# Supplementary material for: The Chronic Effects of a Single Low-Intensity Blast Exposure on Phosphoproteome Networks and Cognitive Function Influenced by Mutant Tau Overexpression
Source: Int J Mol Sci. 2024 Mar 15;25(6):3338. doi: 10.3390/ijms25063338 (PMC10970429; doi:10.3390/ijms25063338)
Supplement: Supplementary file 1 [file ijms-25-03338-s001.zip › Supplementary File S3- WpCNA code in RMarkdown.pdf]

# Supplementary File S3. Weight peptide coexpression-network analysis of 20 mice cortical phosphopeptides

Marcus Jackson

2023-10-18

## Step 1.

- Import or install all required packages:

```
library(readxl)
library(WGCNA)
```

```
## Loading required package: dynamicTreeCut

## Loading required package: fastcluster

##
## Attaching package: 'fastcluster'

## The following object is masked from 'package:stats':
##
##     hclust

##

##
## Attaching package: 'WGCNA'

## The following object is masked from 'package:stats':
##
##     cor
```

```
library(textshape)
library(AnnotationDbi)
```

```
## Loading required package: stats4

## Loading required package: BiocGenerics

##
## Attaching package: 'BiocGenerics'
```

```
## The following object is masked from 'package:textshape':
##
##     combine

## The following objects are masked from 'package:stats':
##
##     IQR, mad, sd, var, xtabs

## The following objects are masked from 'package:base':
##
##     anyDuplicated, aperm, append, as.data.frame, basename, cbind,
##     colnames, dirname, do.call, duplicated, eval, evalq, Filter, Find,
##     get, grep, grepl, intersect, is.unsorted, lapply, Map, mapply,
##     match, mget, order, paste, pmax, pmax.int, pmin, pmin.int,
##     Position, rank, rbind, Reduce, rownames, sapply, setdiff, sort,
##     table, tapply, union, unique, unsplit, which.max, which.min

## Loading required package: Biobase

## Welcome to Bioconductor
##
##     Vignettes contain introductory material; view with
##     'browseVignettes()'. To cite Bioconductor, see
##     'citation("Biobase")', and for packages 'citation("pkgname")'.

## Loading required package: IRanges

## Loading required package: S4Vectors

##
## Attaching package: 'S4Vectors'

## The following objects are masked from 'package:base':
##
##     expand.grid, I, unname

##
## Attaching package: 'IRanges'

## The following object is masked from 'package:grDevices':
##
##     windows
```

```r
library(org.Mm.eg.db)
```

```
##
```

```r
library(GO.db)
library(graphics)
library(cluster)
```

### Step 2.

- Read in the proteomics expression data (Log2FC Intensities only!!!)
- Convert first column "PhosName" to rownames
- Check for genes and samples with too many missing values, if true move on.
- Note: In this dataset we will not remove outliers, they are biologically relevant

```
# The following setting is important, do not omit.
options(stringsAsFactors = FALSE);


df1_expr <- read_excel(path = "C:/Users/Marcu/OneDrive/Documents/Proteomic dataset and metadataset_Marcu
                       sheet = "p-40DPI expression data");
df1_expr <- as.data.frame(df1_expr)

df1_expr <- column_to_rownames(df1_expr, "PhosName")

df1_test = goodSamplesGenes(df1_expr, verbose = 3);
```

```
##  Flagging genes and samples with too many missing values...
##   ..step 1
```

```
df1_test$allOK
```

```
## [1] TRUE
```

```
head(df1_expr)
```

```
##                                                                  Blast rTg4510 3
## _QAS[Phospho (STY)]LDGLQQLR_.2                                           9.526490
## _GQSQLS[Phospho (STY)]NPTDDSWK_.2                                        9.851300
## _ERGS[Phospho (STY)]PVSGR_.2                                             4.013213
## _PQSPVIQATAGS[Phospho (STY)]PK_.2                                        6.751435
## _QSLS[Phospho (STY)]SADNLEPDVQGHQVAAR_.3                                 3.822566
## _ELEKPM[Oxidation (M)]QSKPQS[Phospho (STY)]PVIQAT[Phospho (STY)]AGSPK_.3  5.324073
##                                                                  Blast rTg4510 4
## _QAS[Phospho (STY)]LDGLQQLR_.2                                           9.530376
## _GQSQLS[Phospho (STY)]NPTDDSWK_.2                                        9.539574
## _ERGS[Phospho (STY)]PVSGR_.2                                             9.209353
## _PQSPVIQATAGS[Phospho (STY)]PK_.2                                        5.927391
## _QSLS[Phospho (STY)]SADNLEPDVQGHQVAAR_.3                                 3.574952
## _ELEKPM[Oxidation (M)]QSKPQS[Phospho (STY)]PVIQAT[Phospho (STY)]AGSPK_.3  5.323749
##                                                                  Blast rTg4510 1
## _QAS[Phospho (STY)]LDGLQQLR_.2                                           9.561561
## _GQSQLS[Phospho (STY)]NPTDDSWK_.2                                        9.405308
## _ERGS[Phospho (STY)]PVSGR_.2                                             8.782871
## _PQSPVIQATAGS[Phospho (STY)]PK_.2                                        6.760365
## _QSLS[Phospho (STY)]SADNLEPDVQGHQVAAR_.3                                 6.333914
## _ELEKPM[Oxidation (M)]QSKPQS[Phospho (STY)]PVIQAT[Phospho (STY)]AGSPK_.3  4.105218
##                                                                  Blast rTg4510 2
## _QAS[Phospho (STY)]LDGLQQLR_.2                                           9.310794
## _GQSQLS[Phospho (STY)]NPTDDSWK_.2                                        9.712202
```

```
## _ERGS[Phospho (STY)]PVSGR_.2                                          8.880438
## _PQSPVIQATAGS[Phospho (STY)]PK_.2                                     6.777097
## _QSLS[Phospho (STY)]SADNLEPDVQGHQVAAR_.3                              3.817567
## _ELEKPM[Oxidation (M)]QSKPQS[Phospho (STY)]PVIQAT[Phospho (STY)]AGSPK_.3      6.646825
##                                                                Blast rTg4510 5
## _QAS[Phospho (STY)]LDGLQQLR_.2                                        3.115400
## _GQSQLS[Phospho (STY)]NPTDDSWK_.2                                     8.281087
## _ERGS[Phospho (STY)]PVSGR_.2                                          8.729670
## _PQSPVIQATAGS[Phospho (STY)]PK_.2                                     5.821003
## _QSLS[Phospho (STY)]SADNLEPDVQGHQVAAR_.3                              4.029503
## _ELEKPM[Oxidation (M)]QSKPQS[Phospho (STY)]PVIQAT[Phospho (STY)]AGSPK_.3      3.541612
##                                                                Sham rTg4510 3
## _QAS[Phospho (STY)]LDGLQQLR_.2                                       9.128237
## _GQSQLS[Phospho (STY)]NPTDDSWK_.2                                    8.488968
## _ERGS[Phospho (STY)]PVSGR_.2                                         8.712071
## _PQSPVIQATAGS[Phospho (STY)]PK_.2                                    6.335705
## _QSLS[Phospho (STY)]SADNLEPDVQGHQVAAR_.3                             4.409535
## _ELEKPM[Oxidation (M)]QSKPQS[Phospho (STY)]PVIQAT[Phospho (STY)]AGSPK_.3     3.130726
##                                                                Sham rTg4510 4
## _QAS[Phospho (STY)]LDGLQQLR_.2                                       8.832007
## _GQSQLS[Phospho (STY)]NPTDDSWK_.2                                    9.166122
## _ERGS[Phospho (STY)]PVSGR_.2                                         9.143115
## _PQSPVIQATAGS[Phospho (STY)]PK_.2                                    5.972878
## _QSLS[Phospho (STY)]SADNLEPDVQGHQVAAR_.3                             6.631398
## _ELEKPM[Oxidation (M)]QSKPQS[Phospho (STY)]PVIQAT[Phospho (STY)]AGSPK_.3     4.770406
##                                                                Sham rTg4510 5
## _QAS[Phospho (STY)]LDGLQQLR_.2                                       8.754462
## _GQSQLS[Phospho (STY)]NPTDDSWK_.2                                    8.739547
## _ERGS[Phospho (STY)]PVSGR_.2                                         9.223609
## _PQSPVIQATAGS[Phospho (STY)]PK_.2                                    6.107640
## _QSLS[Phospho (STY)]SADNLEPDVQGHQVAAR_.3                             3.837163
## _ELEKPM[Oxidation (M)]QSKPQS[Phospho (STY)]PVIQAT[Phospho (STY)]AGSPK_.3     3.258363
##                                                                Sham rTg4510 1
## _QAS[Phospho (STY)]LDGLQQLR_.2                                       8.803521
## _GQSQLS[Phospho (STY)]NPTDDSWK_.2                                    8.562182
## _ERGS[Phospho (STY)]PVSGR_.2                                         9.012200
## _PQSPVIQATAGS[Phospho (STY)]PK_.2                                    6.324103
## _QSLS[Phospho (STY)]SADNLEPDVQGHQVAAR_.3                             3.805728
## _ELEKPM[Oxidation (M)]QSKPQS[Phospho (STY)]PVIQAT[Phospho (STY)]AGSPK_.3     3.201110
##                                                                Sham rTg4510 2
## _QAS[Phospho (STY)]LDGLQQLR_.2                                       9.436497
## _GQSQLS[Phospho (STY)]NPTDDSWK_.2                                    9.636452
## _ERGS[Phospho (STY)]PVSGR_.2                                         8.581217
## _PQSPVIQATAGS[Phospho (STY)]PK_.2                                    6.498198
## _QSLS[Phospho (STY)]SADNLEPDVQGHQVAAR_.3                             3.775299
## _ELEKPM[Oxidation (M)]QSKPQS[Phospho (STY)]PVIQAT[Phospho (STY)]AGSPK_.3     4.757065
##                                                                Blast NC 3
## _QAS[Phospho (STY)]LDGLQQLR_.2                                         8.958440
## _GQSQLS[Phospho (STY)]NPTDDSWK_.2                                      8.567541
## _ERGS[Phospho (STY)]PVSGR_.2                                           8.638906
## _PQSPVIQATAGS[Phospho (STY)]PK_.2                                      6.061009
## _QSLS[Phospho (STY)]SADNLEPDVQGHQVAAR_.3                               6.410237
## _ELEKPM[Oxidation (M)]QSKPQS[Phospho (STY)]PVIQAT[Phospho (STY)]AGSPK_.3   5.728836
##                                                                Blast NC 4
```

```
## _QAS[Phospho (STY)]LDGLQQLR_.2                                              8.959951
## _GQSQLS[Phospho (STY)]NPTDDSWK_.2                                           8.494754
## _ERGS[Phospho (STY)]PVSGR_.2                                                8.497609
## _PQSPVIQATAGS[Phospho (STY)]PK_.2                                           5.481435
## _QSLS[Phospho (STY)]SADNLEPDVQGHQVAAR_.3                                    4.070464
## _ELEKPM[Oxidation (M)]QSKPQS[Phospho (STY)]PVIQAT[Phospho (STY)]AGSPK_.3   4.865707
##                                                                           Blast NC 5
## _QAS[Phospho (STY)]LDGLQQLR_.2                                              9.478251
## _GQSQLS[Phospho (STY)]NPTDDSWK_.2                                           8.846596
## _ERGS[Phospho (STY)]PVSGR_.2                                                9.106709
## _PQSPVIQATAGS[Phospho (STY)]PK_.2                                           5.688370
## _QSLS[Phospho (STY)]SADNLEPDVQGHQVAAR_.3                                    3.950248
## _ELEKPM[Oxidation (M)]QSKPQS[Phospho (STY)]PVIQAT[Phospho (STY)]AGSPK_.3   5.152731
##                                                                           Blast NC 6
## _QAS[Phospho (STY)]LDGLQQLR_.2                                              9.231628
## _GQSQLS[Phospho (STY)]NPTDDSWK_.2                                           8.337931
## _ERGS[Phospho (STY)]PVSGR_.2                                                8.130642
## _PQSPVIQATAGS[Phospho (STY)]PK_.2                                           5.878283
## _QSLS[Phospho (STY)]SADNLEPDVQGHQVAAR_.3                                    4.038407
## _ELEKPM[Oxidation (M)]QSKPQS[Phospho (STY)]PVIQAT[Phospho (STY)]AGSPK_.3   6.243723
##                                                                           Blast NC 1
## _QAS[Phospho (STY)]LDGLQQLR_.2                                              8.347308
## _GQSQLS[Phospho (STY)]NPTDDSWK_.2                                           5.893559
## _ERGS[Phospho (STY)]PVSGR_.2                                                2.853323
## _PQSPVIQATAGS[Phospho (STY)]PK_.2                                           4.868358
## _QSLS[Phospho (STY)]SADNLEPDVQGHQVAAR_.3                                    3.891793
## _ELEKPM[Oxidation (M)]QSKPQS[Phospho (STY)]PVIQAT[Phospho (STY)]AGSPK_.3   3.830884
##                                                                           Blast NC 2
## _QAS[Phospho (STY)]LDGLQQLR_.2                                              9.066606
## _GQSQLS[Phospho (STY)]NPTDDSWK_.2                                           8.568276
## _ERGS[Phospho (STY)]PVSGR_.2                                                7.917190
## _PQSPVIQATAGS[Phospho (STY)]PK_.2                                           5.660179
## _QSLS[Phospho (STY)]SADNLEPDVQGHQVAAR_.3                                    6.281934
## _ELEKPM[Oxidation (M)]QSKPQS[Phospho (STY)]PVIQAT[Phospho (STY)]AGSPK_.3   4.714183
##                                                                          Sham NC 3
## _QAS[Phospho (STY)]LDGLQQLR_.2                                             9.238355
## _GQSQLS[Phospho (STY)]NPTDDSWK_.2                                          9.093485
## _ERGS[Phospho (STY)]PVSGR_.2                                               9.019227
## _PQSPVIQATAGS[Phospho (STY)]PK_.2                                          6.536391
## _QSLS[Phospho (STY)]SADNLEPDVQGHQVAAR_.3                                   3.999680
## _ELEKPM[Oxidation (M)]QSKPQS[Phospho (STY)]PVIQAT[Phospho (STY)]AGSPK_.3  6.865086
##                                                                          Sham NC 4
## _QAS[Phospho (STY)]LDGLQQLR_.2                                             8.851384
## _GQSQLS[Phospho (STY)]NPTDDSWK_.2                                          8.686150
## _ERGS[Phospho (STY)]PVSGR_.2                                               9.052475
## _PQSPVIQATAGS[Phospho (STY)]PK_.2                                          5.935714
## _QSLS[Phospho (STY)]SADNLEPDVQGHQVAAR_.3                                   3.683997
## _ELEKPM[Oxidation (M)]QSKPQS[Phospho (STY)]PVIQAT[Phospho (STY)]AGSPK_.3  5.175933
##                                                                          Sham NC 1
## _QAS[Phospho (STY)]LDGLQQLR_.2                                             9.026282
## _GQSQLS[Phospho (STY)]NPTDDSWK_.2                                          8.437126
## _ERGS[Phospho (STY)]PVSGR_.2                                               8.218046
## _PQSPVIQATAGS[Phospho (STY)]PK_.2                                          5.841424
## _QSLS[Phospho (STY)]SADNLEPDVQGHQVAAR_.3                                   3.765512
```

```
## _ELEKPM[Oxidation (M)]QSKPQS[Phospho (STY)]PVIQAT[Phospho (STY)]AGSPK_.3  4.804185
##                                                                          Sham NC 2
## _QAS[Phospho (STY)]LDGLQQLR_.2                                            8.979440
## _GQSQLS[Phospho (STY)]NPTDDSWK_.2                                         8.454514
## _ERGS[Phospho (STY)]PVSGR_.2                                              8.299261
## _PQSPVIQATAGS[Phospho (STY)]PK_.2                                         6.011762
## _QSLS[Phospho (STY)]SADNLEPDVQGHQVAAR_.3                                  3.809294
## _ELEKPM[Oxidation (M)]QSKPQS[Phospho (STY)]PVIQAT[Phospho (STY)]AGSPK_.3  4.582183
```

### Step 3.

- Import trait/clinical/phenotype dataset
- In df2_trait convert first column to rownames
- visualize how the clinical traits relate to the sample dendrogram.
- Convert traits to a color representation (i.e., white means low, red means high, grey means missing entry)
- Plot the sample dendrogram and the colors underneath:

```r
df2_trait <- as.data.frame(read_excel("C:/Users/Marcu/OneDrive/Documents/cogwall_df for WGCNA.xlsx", she

df2_trait <- column_to_rownames(df2_trait, "Animal_ID");

sampleTree2 = hclust(dist(df2_trait), method = "average");

traitColors = numbers2colors(df2_trait$`Learning Index`, signed = FALSE);

dend_trait <- plotDendroAndColors(sampleTree2, traitColors,
                                  groupLabels = names(df2_trait),
                                  main = "Sample Dendrogram and Learning Index Heatmap", cex.dendroLabel
```

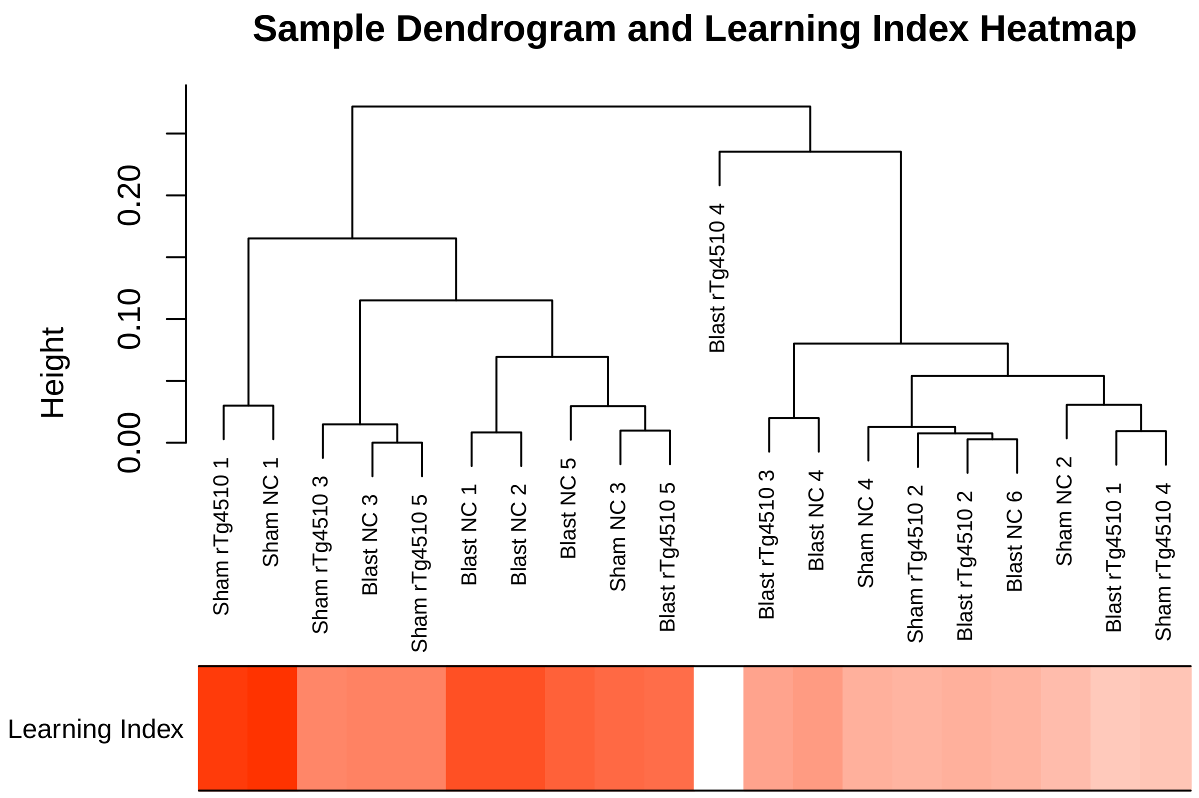


### Step 4.

- Standard peptide screening based on marginal correlation (i.e., Network terminology: GS1 will be referred to as signed peptide significance measure)

```
## Transpose proteomic/expression data to have genes in columns and samples as rows
df1_expr_transposed <- data.frame(t(df1_expr))

GS1= as.numeric(cor(df2_trait, df1_expr_transposed, use="p"));
p.Standard=corPvalueFisher(GS1, nSamples =length(df2_trait));

## Since the q-value function has problems with missing data, we use the following trick
p.Standard2=p.Standard
p.Standard2[is.na(p.Standard)]=1
q.Standard=qvalue(p.Standard2)$qvalues;

# Form a data frame to hold the results
PhosPepNames <- row.names(df1_expr)
StandardPeptideScreeningResults=data.frame(PhosPepNames,PearsonCorrelation=GS1,
                                        p.Standard, q.Standard)

head(StandardPeptideScreeningResults)
```

```
##                                                               PhosPepNames
```

```
## 1                                          _QAS[Phospho (STY)]LDGLQQLR_.2
## 2                                       _GQSQLS[Phospho (STY)]NPTDDSWK_.2
## 3                                            _ERGS[Phospho (STY)]PVSGR_.2
## 4                                       _PQSPVIQATAGS[Phospho (STY)]PK_.2
## 5                                _QSLS[Phospho (STY)]SADNLEPDVQGHQVAAR_.3
## 6 _ELEKPM[Oxidation (M)]QSKPQS[Phospho (STY)]PVIQAT[Phospho (STY)]AGSPK_.3
##   PearsonCorrelation p.Standard q.Standard
## 1        -0.43553296        NaN          1
## 2        -0.25315597        NaN          1
## 3         0.03173034        NaN          1
## 4        -0.05579011        NaN          1
## 5         0.26961245        NaN          1
## 6        -0.15698126        NaN          1
```

### Step 5.

- Now export "StandardGeneScreeningResults" to filter in excel if abs(r) < 0.5.
- Import nw data frame (df3_expr)
- Transpose for network construction

```
# First we need to save the results as a csv
write.csv(StandardPeptideScreeningResults, "C:/Users/Marcu/OneDrive/Documents/WGCNA_StandardGeneScreenin
# Note: due to the small n number for the study, no pvalues were attributable, only pearson r therefore

## Import new filtered dataset
df3_expr <- read_excel("C:/Users/Marcu/OneDrive/Documents/Proteomic dataset and metadataset_Marcus.xlsx
                      sheet = "p-40DPI expression filtered df");

## Transpose proteomic/expression of filtered data to have genes in columns and samples as rows
df3_expr <- column_to_rownames(df3_expr, "PhosName");
df3_expr_transposed <- data.frame(t(df3_expr))

head(df3_expr)
```

```
##                                                        Blast rTg4510 3
## _RKPGAGGS[Phospho (STY)]PALAR_.3                              8.238760
## _IVHSES[Phospho (STY)]QPEKESR_.2                              6.232174
## _TDPGS[Phospho (STY)]IENLC[Carbamidomethyl (C)]PGK_.2         8.986573
## _TGGEGS[Phospho (STY)]LDEEAEGSKK_.2                           4.265219
## _S[Phospho (STY)]MDSLC[Carbamidomethyl (C)]SVPVEGK_.2         4.289535
## _SNS[Phospho (STY)]QENVEASHPSQDGKR_.3                         7.910812
##                                                        Blast rTg4510 4
## _RKPGAGGS[Phospho (STY)]PALAR_.3                              8.928654
## _IVHSES[Phospho (STY)]QPEKESR_.2                              6.598271
## _TDPGS[Phospho (STY)]IENLC[Carbamidomethyl (C)]PGK_.2         3.758421
## _TGGEGS[Phospho (STY)]LDEEAEGSKK_.2                           3.737585
## _S[Phospho (STY)]MDSLC[Carbamidomethyl (C)]SVPVEGK_.2         4.530391
## _SNS[Phospho (STY)]QENVEASHPSQDGKR_.3                         8.126560
##                                                        Blast rTg4510 1
## _RKPGAGGS[Phospho (STY)]PALAR_.3                              7.615425
## _IVHSES[Phospho (STY)]QPEKESR_.2                              5.575043
## _TDPGS[Phospho (STY)]IENLC[Carbamidomethyl (C)]PGK_.2         4.598085
## _TGGEGS[Phospho (STY)]LDEEAEGSKK_.2                           3.300677
```

```
## _S[Phospho (STY)]MDSLC[Carbamidomethyl (C)]SVPVEGK_.2        3.826115
## _SNS[Phospho (STY)]QENVEASHPSQDGKR_.3                        7.609041
##                                                        Blast rTg4510 2
## _RKPGAGGS[Phospho (STY)]PALAR_.3                              8.154468
## _IVHSES[Phospho (STY)]QPEKESR_.2                              3.822685
## _TDPGS[Phospho (STY)]IENLC[Carbamidomethyl (C)]PGK_.2        4.023560
## _TGGEGS[Phospho (STY)]LDEEAEGSKK_.2                           3.424170
## _S[Phospho (STY)]MDSLC[Carbamidomethyl (C)]SVPVEGK_.2        3.294690
## _SNS[Phospho (STY)]QENVEASHPSQDGKR_.3                        7.948428
##                                                        Blast rTg4510 5
## _RKPGAGGS[Phospho (STY)]PALAR_.3                              7.255080
## _IVHSES[Phospho (STY)]QPEKESR_.2                              5.815416
## _TDPGS[Phospho (STY)]IENLC[Carbamidomethyl (C)]PGK_.2        3.767603
## _TGGEGS[Phospho (STY)]LDEEAEGSKK_.2                           3.602941
## _S[Phospho (STY)]MDSLC[Carbamidomethyl (C)]SVPVEGK_.2        2.995633
## _SNS[Phospho (STY)]QENVEASHPSQDGKR_.3                        7.238229
##                                                        Sham rTg4510 3
## _RKPGAGGS[Phospho (STY)]PALAR_.3                             7.559636
## _IVHSES[Phospho (STY)]QPEKESR_.2                             4.509915
## _TDPGS[Phospho (STY)]IENLC[Carbamidomethyl (C)]PGK_.2       3.800942
## _TGGEGS[Phospho (STY)]LDEEAEGSKK_.2                          3.827025
## _S[Phospho (STY)]MDSLC[Carbamidomethyl (C)]SVPVEGK_.2       3.028972
## _SNS[Phospho (STY)]QENVEASHPSQDGKR_.3                       7.742838
##                                                        Sham rTg4510 4
## _RKPGAGGS[Phospho (STY)]PALAR_.3                             8.641213
## _IVHSES[Phospho (STY)]QPEKESR_.2                             5.366398
## _TDPGS[Phospho (STY)]IENLC[Carbamidomethyl (C)]PGK_.2       3.280498
## _TGGEGS[Phospho (STY)]LDEEAEGSKK_.2                          3.938133
## _S[Phospho (STY)]MDSLC[Carbamidomethyl (C)]SVPVEGK_.2       4.052468
## _SNS[Phospho (STY)]QENVEASHPSQDGKR_.3                       7.715931
##                                                        Sham rTg4510 5
## _RKPGAGGS[Phospho (STY)]PALAR_.3                             8.533593
## _IVHSES[Phospho (STY)]QPEKESR_.2                             5.639189
## _TDPGS[Phospho (STY)]IENLC[Carbamidomethyl (C)]PGK_.2       3.786346
## _TGGEGS[Phospho (STY)]LDEEAEGSKK_.2                          4.300083
## _S[Phospho (STY)]MDSLC[Carbamidomethyl (C)]SVPVEGK_.2       4.515215
## _SNS[Phospho (STY)]QENVEASHPSQDGKR_.3                       7.794398
##                                                        Sham rTg4510 1
## _RKPGAGGS[Phospho (STY)]PALAR_.3                             7.604037
## _IVHSES[Phospho (STY)]QPEKESR_.2                             4.530955
## _TDPGS[Phospho (STY)]IENLC[Carbamidomethyl (C)]PGK_.2       3.200704
## _TGGEGS[Phospho (STY)]LDEEAEGSKK_.2                          4.169271
## _S[Phospho (STY)]MDSLC[Carbamidomethyl (C)]SVPVEGK_.2       3.972674
## _SNS[Phospho (STY)]QENVEASHPSQDGKR_.3                       7.446234
##                                                        Sham rTg4510 2 Blast NC 3
## _RKPGAGGS[Phospho (STY)]PALAR_.3                             8.792485   7.930930
## _IVHSES[Phospho (STY)]QPEKESR_.2                             7.393740   5.324989
## _TDPGS[Phospho (STY)]IENLC[Carbamidomethyl (C)]PGK_.2       9.014376   8.701669
## _TGGEGS[Phospho (STY)]LDEEAEGSKK_.2                          4.471540   4.371601
## _S[Phospho (STY)]MDSLC[Carbamidomethyl (C)]SVPVEGK_.2       4.100130   3.451314
## _SNS[Phospho (STY)]QENVEASHPSQDGKR_.3                       8.177623   7.695513
##                                                        Blast NC 4 Blast NC 5
## _RKPGAGGS[Phospho (STY)]PALAR_.3                         8.294293   8.677245
## _IVHSES[Phospho (STY)]QPEKESR_.2                         5.432989   5.951327
```

```
## _TDPGS[Phospho (STY)]IENLC[Carbamidomethyl (C)]PGK_.2   3.484541   3.591541
## _TGGEGS[Phospho (STY)]LDEEAEGSKK_.2                    3.627032   4.105278
## _S[Phospho (STY)]MDSLC[Carbamidomethyl (C)]SVPVEGK_.2   4.256511   4.138116
## _SNS[Phospho (STY)]QENVEASHPSQDGKR_.3                   7.447561   8.318089
##                                                       Blast NC 6 Blast NC 1
## _RKPGAGGS[Phospho (STY)]PALAR_.3                        8.674047   7.550161
## _IVHSES[Phospho (STY)]QPEKESR_.2                        5.900654   4.160454
## _TDPGS[Phospho (STY)]IENLC[Carbamidomethyl (C)]PGK_.2   3.135309   3.305870
## _TGGEGS[Phospho (STY)]LDEEAEGSKK_.2                    3.649046   3.515330
## _S[Phospho (STY)]MDSLC[Carbamidomethyl (C)]SVPVEGK_.2   3.864178   4.077840
## _SNS[Phospho (STY)]QENVEASHPSQDGKR_.3                   8.093542   7.609236
##                                                       Blast NC 2 Sham NC 3
## _RKPGAGGS[Phospho (STY)]PALAR_.3                        7.970520  7.815962
## _IVHSES[Phospho (STY)]QPEKESR_.2                        6.097862  5.464425
## _TDPGS[Phospho (STY)]IENLC[Carbamidomethyl (C)]PGK_.2   4.423620  3.283576
## _TGGEGS[Phospho (STY)]LDEEAEGSKK_.2                    3.109278  3.797314
## _S[Phospho (STY)]MDSLC[Carbamidomethyl (C)]SVPVEGK_.2   3.651650  4.012446
## _SNS[Phospho (STY)]QENVEASHPSQDGKR_.3                   7.830169  7.668289
##                                                       Sham NC 4 Sham NC 1
## _RKPGAGGS[Phospho (STY)]PALAR_.3                       8.554644  7.960728
## _IVHSES[Phospho (STY)]QPEKESR_.2                       6.053357  5.689051
## _TDPGS[Phospho (STY)]IENLC[Carbamidomethyl (C)]PGK_.2  3.865864  4.164917
## _TGGEGS[Phospho (STY)]LDEEAEGSKK_.2                   3.352127  3.651180
## _S[Phospho (STY)]MDSLC[Carbamidomethyl (C)]SVPVEGK_.2  3.136995  3.436048
## _SNS[Phospho (STY)]QENVEASHPSQDGKR_.3                  7.898524  8.032992
##                                                       Sham NC 2
## _RKPGAGGS[Phospho (STY)]PALAR_.3                       7.693437
## _IVHSES[Phospho (STY)]QPEKESR_.2                       5.986509
## _TDPGS[Phospho (STY)]IENLC[Carbamidomethyl (C)]PGK_.2  4.313431
## _TGGEGS[Phospho (STY)]LDEEAEGSKK_.2                   3.326329
## _S[Phospho (STY)]MDSLC[Carbamidomethyl (C)]SVPVEGK_.2  3.541460
## _SNS[Phospho (STY)]QENVEASHPSQDGKR_.3                  7.835881
```

### Step 6.

- Construction of a weighted peptide co-expression network and network modules. Note: The adjacency matrix, also called the connection matrix, is a matrix containing rows and columns which is used to represent a simple labelled graph, with 0 or 1 in the position of (Vi , Vj) according to the condition whether Vi and Vj are adjacent or not. Defining a weighted peptide co-expression network here we define the adjacency matrix using soft thresholding with beta=8 (unsigned = 6, signed = 12).
- Define k-connectivity measure
- Test adjacency network set to beta = 8 for scale free-topology
- Plot results (Histogram and Scatter plot with trend line)

```
ADJ1=abs(cor(df3_expr_transposed,use="p"))^8

# When you have relatively few genes (<5000) use the following code:
k=as.vector(apply(ADJ1,2,sum, na.rm=T))
# When you have a lot of genes use the following code: "k=softConnectivity(datE=datExpr,power=6); this

# Plot a histogram of k and a scale free topology plot
sizeGrWindow(10,5)
par(mfrow=c(1,2))
```

```r
k_histogram <- hist(k)
scale_free_plot <- scaleFreePlot(k, main="Check scale free topology\n")
plot(scale_free_plot)

## Note: k-Histogram and scale free toplogy explained:  The approximate straight line relationship (r^2
```

Figure. Histogram and scatter plot to assess network for scale-free topology Impression: Beta = 8 was the only beta that created scale free topology in data

## Step 7.

- Convert adjacency similarity matrix to a dissimilarity matrix (dissADJ)
- Convert topological overlap matrix to a dissimilarity matrix (dissTOM)
- Define modules using topological overlap dissimilarity
- Plot results.

```r
# Comparing various module detection methods
# Definition of clustering dissimilarity from adjacency
# Many clustering procedures require a dissimilarity matrix as input.
# We define a dissimilarity based on adjacency:
# Turn adjacency into a measure of dissimilarity:
dissADJ=1-ADJ1


# Use of topological overlap to define dissimilarity
# Adjacency can be used to define a separate measure of similarity,
# the Topological Overlap Matrix(TOM).
# We will convert the TOM matrix to a dissimilarity matrix as well (dissTOM)
dissTOM=TOMdist(ADJ1)
```

```
## ..connectivity..
## ..matrix multiplication (system BLAS)..
## ..normalization..
## ..done.
```

```r
simTOM=1-(TOMdist(ADJ1))
```

```
## ..connectivity..
## ..matrix multiplication (system BLAS)..
## ..normalization..
## ..done.
```

```r
collectGarbage()


# Module definition using the topological overlap based dissimilarity.
# We now use the topological overlap based dissimilarity as input to the
# clustering methods.
# Calculate the dendrogram:
hierTOM = hclust(as.dist(dissTOM),method="average");
```

```
# We will compare three different tree-cutting methods:
colorStaticTOM = as.character(cutreeStaticColor(hierTOM, cutHeight=.99, minSize=20))
colorDynamicTOM = labels2colors (cutreeDynamic(hierTOM,method="tree"))
colorDynamicHybridTOM = labels2colors(cutreeDynamic(hierTOM, distM= dissTOM , cutHeight = 0.998,
                                                  deepSplit=2, pamRespectsDendro = FALSE))
```

```
##  ..done.
```

```
# Now we plot the results
sizeGrWindow(10,5)
plotDendroAndColors(hierTOM,
                    colors=data.frame(colorStaticTOM,
                                      colorDynamicTOM, colorDynamicHybridTOM),
                    dendroLabels = FALSE, marAll = c(1, 8, 3, 1),
                    main = "Gene dendrogram and module colors, TOM dissimilarity")
```

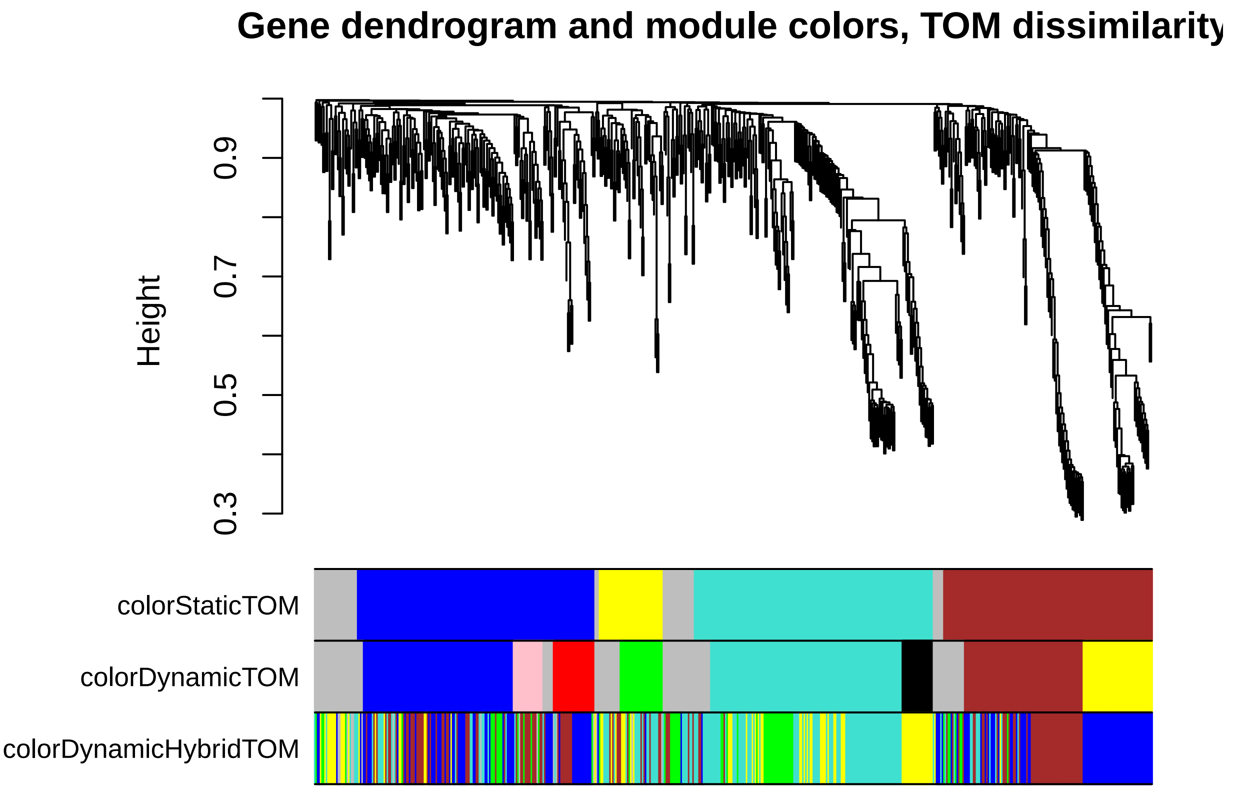


Figure. Weighted peptide coexpression-network

### Step 8.

- Compile the module assignment (colors) into a single list (colorsh1)
- Compute module eigenpeptides for animal's phosphopeptidome
- Relating modules and module-eigenpeptides to learning index
- Calculate Peptide significance (i.e., analogous to gene significance)

```r
# Since the "colorDynamicTOM" tree cutting method captures most of the data
# and has very similiar results to other tree cutting methods,
# will use this method for defining modules (colors at bottom of dendrogram)
# We define a shorter name for the module assignment of choice,
# remove unneeded variables and save the results for use in subsequent
# sessions.
colorh1= colorDynamicTOM


# remove the dissimilarities, adjacency matrices etc to free up space
rm(ADJ1); rm(dissADJ);
collectGarbage()


# Representing modules by eigenpeptides and relating eigenpeptides to one another
# to get a sense of how related the modules are one can summarize each module by # its eigenpeptides (f
datME=moduleEigengenes(df3_expr_transposed, colorh1)$eigengenes
signif(cor(datME, use="p"), 2)
```

```
##             MEblack MEblue MEbrown MEgreen MEgrey MEpink MEred MEturquoise
## MEblack        1.00   0.34    0.55    0.47   0.60   0.23  0.20        0.86
## MEblue         0.34   1.00    0.70    0.58   0.81   0.83  0.68        0.55
## MEbrown        0.55   0.70    1.00    0.59   0.71   0.53  0.51        0.69
## MEgreen        0.47   0.58    0.59    1.00   0.71   0.48  0.43        0.55
## MEgrey         0.60   0.81    0.71    0.71   1.00   0.65  0.64        0.74
## MEpink         0.23   0.83    0.53    0.48   0.65   1.00  0.62        0.44
## MEred          0.20   0.68    0.51    0.43   0.64   0.62  1.00        0.37
## MEturquoise    0.86   0.55    0.69    0.55   0.74   0.44  0.37        1.00
## MEyellow       0.29   0.54    0.82    0.35   0.55   0.35  0.49        0.43
##             MEyellow
## MEblack         0.29
## MEblue          0.54
## MEbrown         0.82
## MEgreen         0.35
## MEgrey          0.55
## MEpink          0.35
## MEred           0.49
## MEturquoise     0.43
## MEyellow        1.00
```

```r
# We define a dissimilarity measure between the module eigenpeptides
# that keeps track of the sign of the correlation between the module
# eigenpeptides, and use # it to cluster the eigenpeptide:
dissimME=(1-t(cor(datME, method="p")))/2
hclustdatME=hclust(as.dist(dissimME), method="average" )


# Plot the eigenpeptide dendrogram
par(mfrow=c(1,1))
plot(hclustdatME, main="Clustering tree based of the module           eigengenes")
```

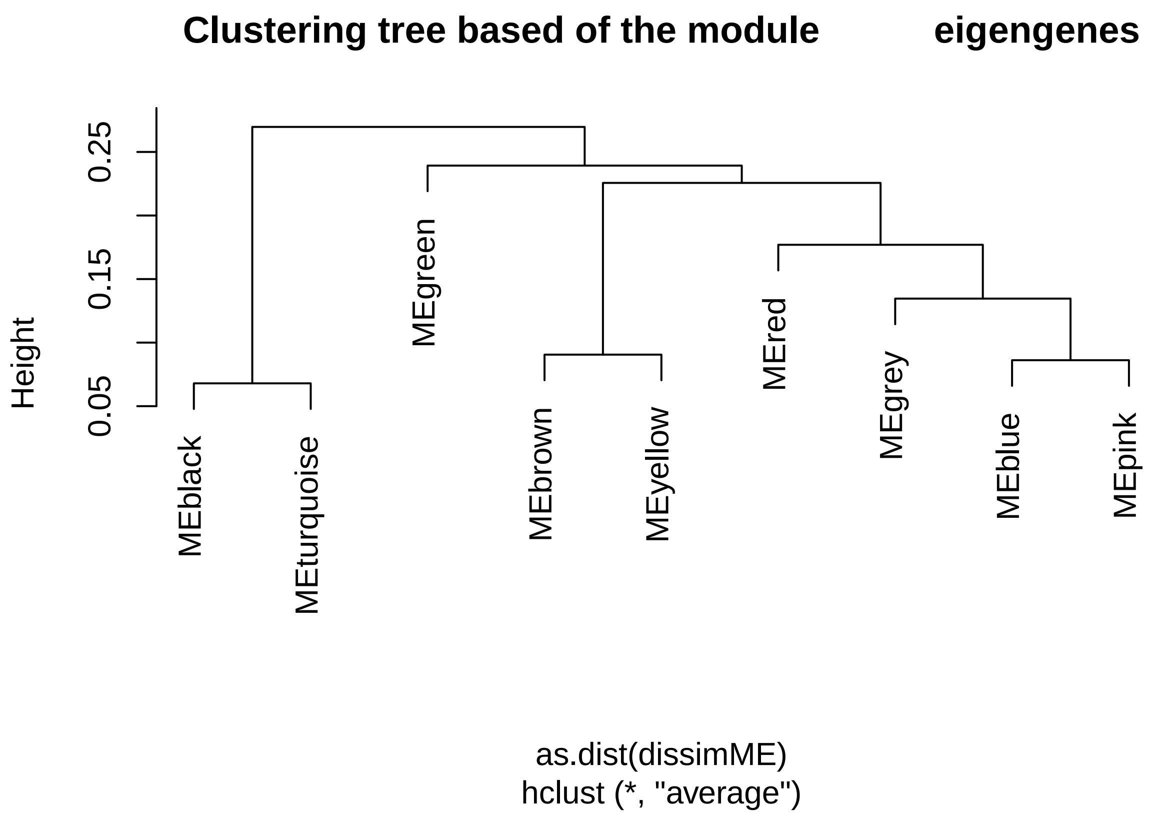


```r
# Impression:  The (I) blue and yellow eigengenes,
# and the (II) brown and turqouise eigenpeptide are highly related, as
# evidenced by their elevated merging height.


# Pairwise scatter plots of mice (n = 20) phenotypes (learning index) # and module eigenpeptides.
PW_scatt_initLL <- plotMEpairs(datME,y=df2_trait$`Learning Index`, )
```

Relationship between module eigengenes

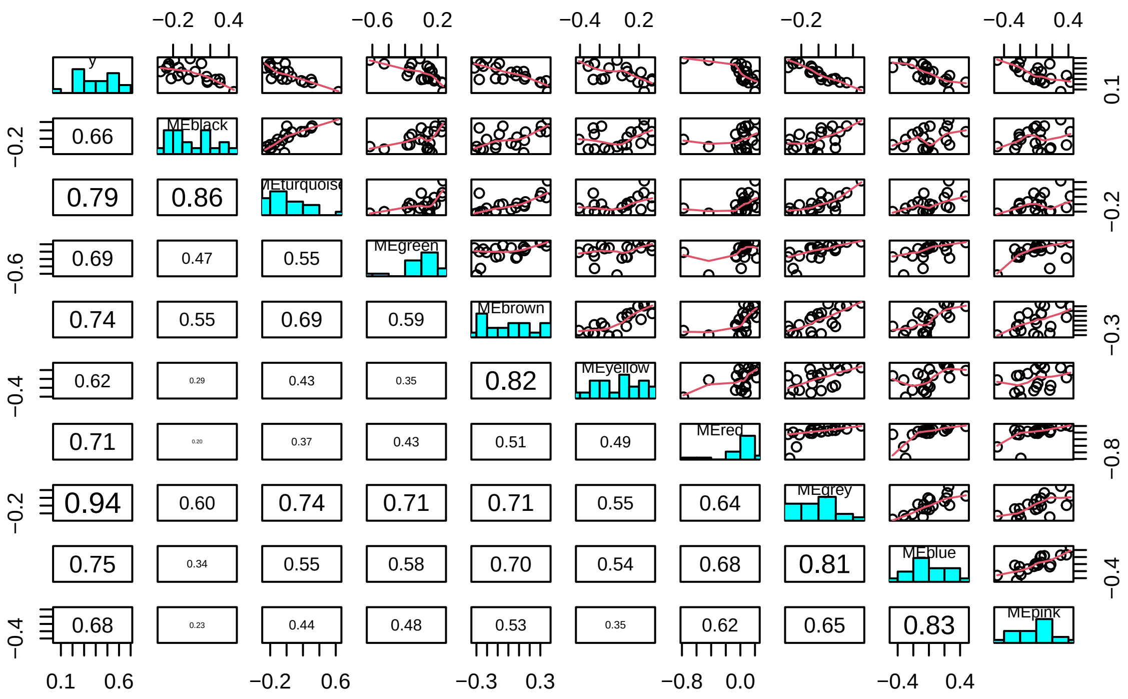


```
# The module eigenpeptides (first PC) of different modules may be
# highly correlated. WpCNA can be interpreted as a biologically
# motivated data reduction scheme that allows for dependency between
# the resulting components. In contrast, principal component analysis # imposes orthogonality between t
# Since modules may represent biological pathways there is no
# biological reason why modules should be orthogonal to each other.
GS1=as.numeric(cor(df2_trait$`Learning Index`,df3_expr_transposed, use="p"))
GeneSignificance=abs(GS1)


# Next module significance is defined as average gene significance.
ModuleSignificance=tapply(GeneSignificance, colorh1, mean, na.rm=T)


# To plot module significance, one can use the following code:
par(mfrow = c(1,1))
plotModuleSignificance(GeneSignificance,colorh1, boxplot = TRUE)
```

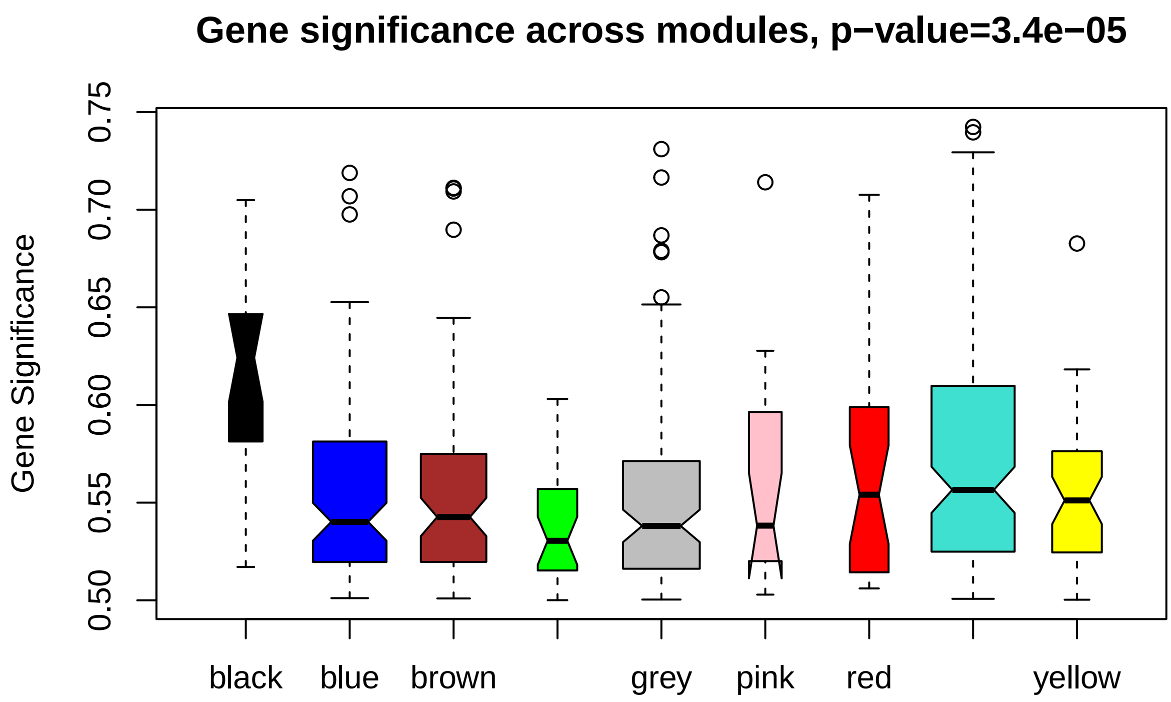


### Step 9.

- Compute intramodular connectivity
- Evaluate relationship between peptide significance and intramodular connectivity
- Compute module membership for each module
- Evaluate relationship between module membership and intramodular connectivity

```
# Module membership and intramodular connectivity: We begin by calculating the
# intramodular connectivity for each peptide. (In network literature,
# connectivity is often referred to as "degree".) The function
# intramodularConnectivity computes the whole network connectivity kTotal, the
# within module connectivity kWithin, kOut=kTotal-kWithin, and
# kDiff=kIn-kOut=2*kIN-kTotal.
ADJ1=abs(cor(df3_expr_transposed,use="p"))^6
Alldegrees1=intramodularConnectivity(ADJ1, colorh1)
head(Alldegrees1)
```

```
##                                                           kTotal   kWithin
## X_RKPGAGGS.Phospho..STY..PALAR_.3                      13.169946  7.829837
## X_IVHSES.Phospho..STY..QPEKESR_.2                       5.157729  1.661596
## X_TDPGS.Phospho..STY..IENLC.Carbamidomethyl..C..PGK_.2  6.854629  4.934478
## X_TGGEGS.Phospho..STY..LDEEAEGSKK_.2                   33.558267 23.776103
## X_S.Phospho..STY..MDSLC.Carbamidomethyl..C..SVPVEGK_.2 27.156917 22.248421
## X_SNS.Phospho..STY..QENVEASHPSQDGKR_.3                 10.217091  5.959858
```

```
##                                                            kOut     kDiff
## X_RKPGAGGS.Phospho..STY..PALAR_.3                      5.340109  2.489728
## X_IVHSES.Phospho..STY..QPEKESR_.2                      3.496133 -1.834537
## X_TDPGS.Phospho..STY..IENLC.Carbamidomethyl..C..PGK_.2 1.920151  3.014327
## X_TGGEGS.Phospho..STY..LDEEAEGSKK_.2                   9.782164 13.993940
## X_S.Phospho..STY..MDSLC.Carbamidomethyl..C..SVPVEGK_.2 4.908496 17.339925
## X_SNS.Phospho..STY..QENVEASHPSQDGKR_.3                 4.257233  1.702625
```

```
# Relationship between peptide significance and intramodular connectivity:
# We plot the peptide significance against intramodular connectivity.
colorlevels=unique(colorh1)
sizeGrWindow(9,6)
par(mfrow=c(2,as.integer(0.5+length(colorlevels)/2)))
par(mar = c(4,5,3,1))
for (i in c(1:length(colorlevels)))
{
  whichmodule=colorlevels[[i]];
  restrict1 = (colorh1==whichmodule);
  verboseScatterplot(Alldegrees1$kWithin[restrict1],
                     GeneSignificance[restrict1], col=colorh1[restrict1],
                     main=whichmodule,
                     xlab = "Connectivity", ylab = "Peptide Significance", abline = TRUE)
}
```

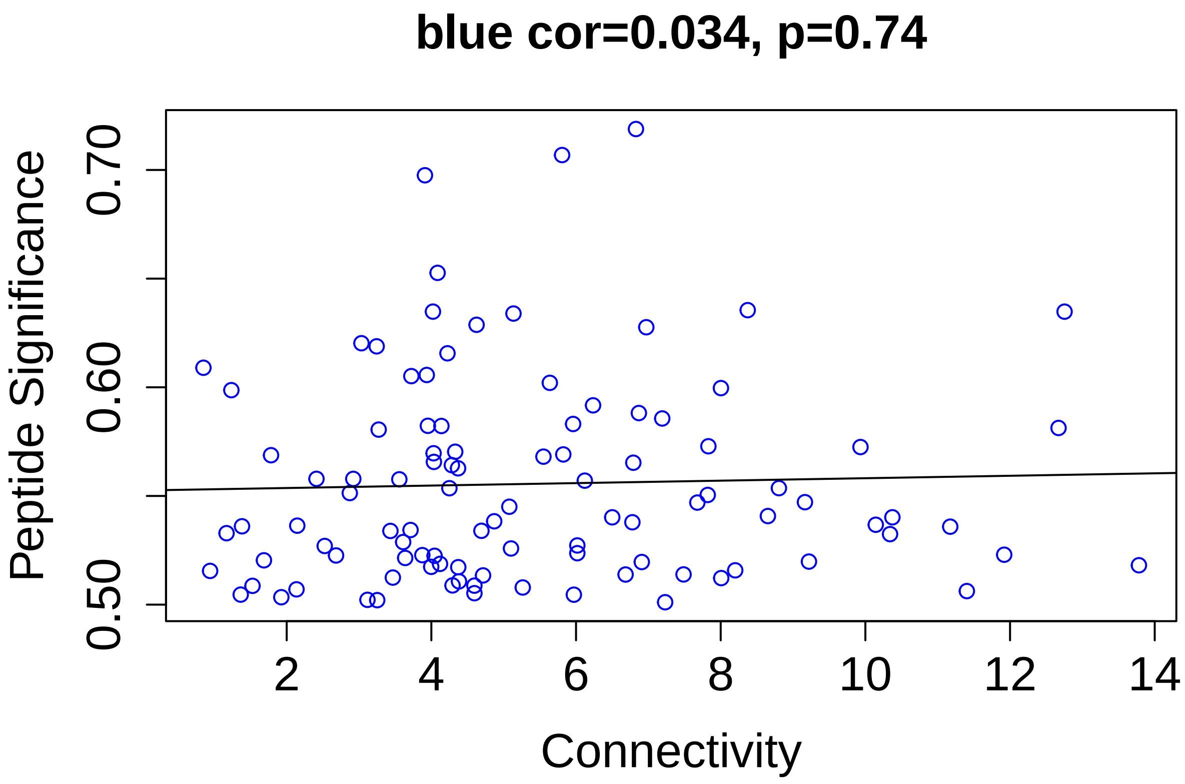

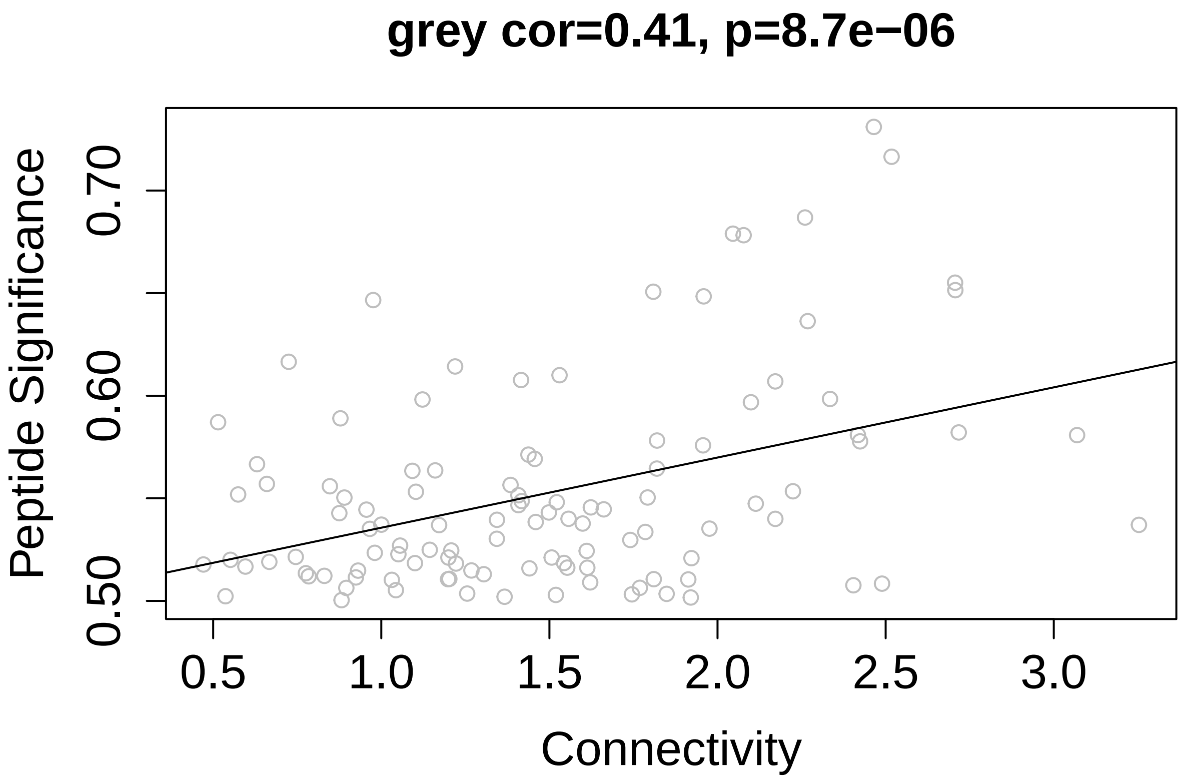
grey cor=0.41, p=8.7e−06
Peptide Significance
0.50
0.60
0.70
0.5
1.0
1.5
2.0
2.5
3.0
Connectivity

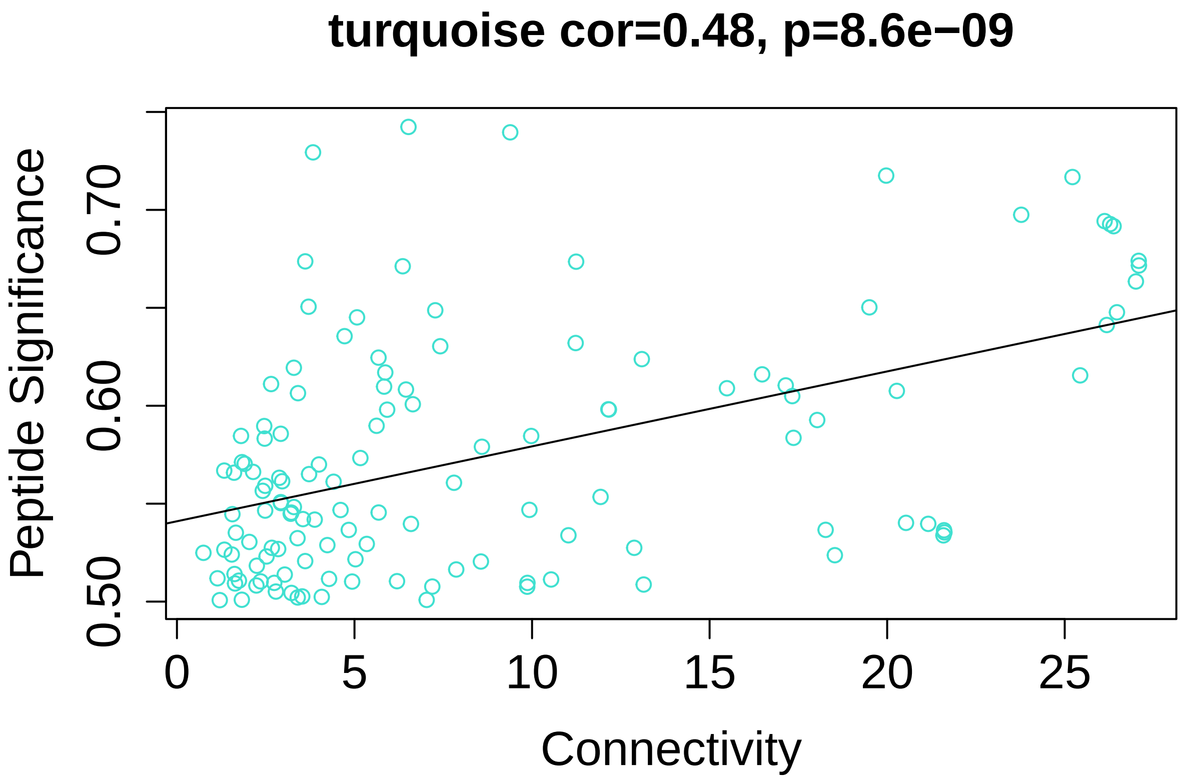
turquoise cor=0.48, p=8.6e−09
Peptide Significance
0.50
0.60
0.70
0
5
10
15
20
25
Connectivity

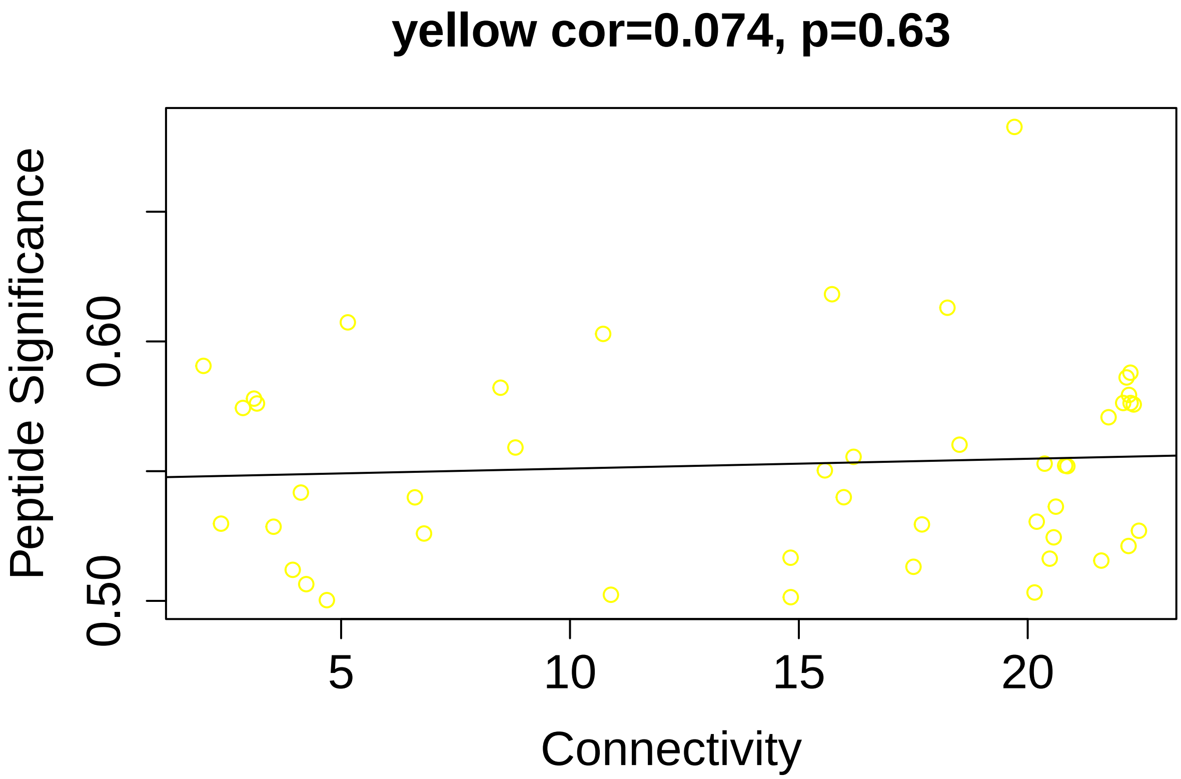

yellow cor=0.074, p=0.63
Peptide Significance
0.50
0.60
5
10
15
20
Connectivity

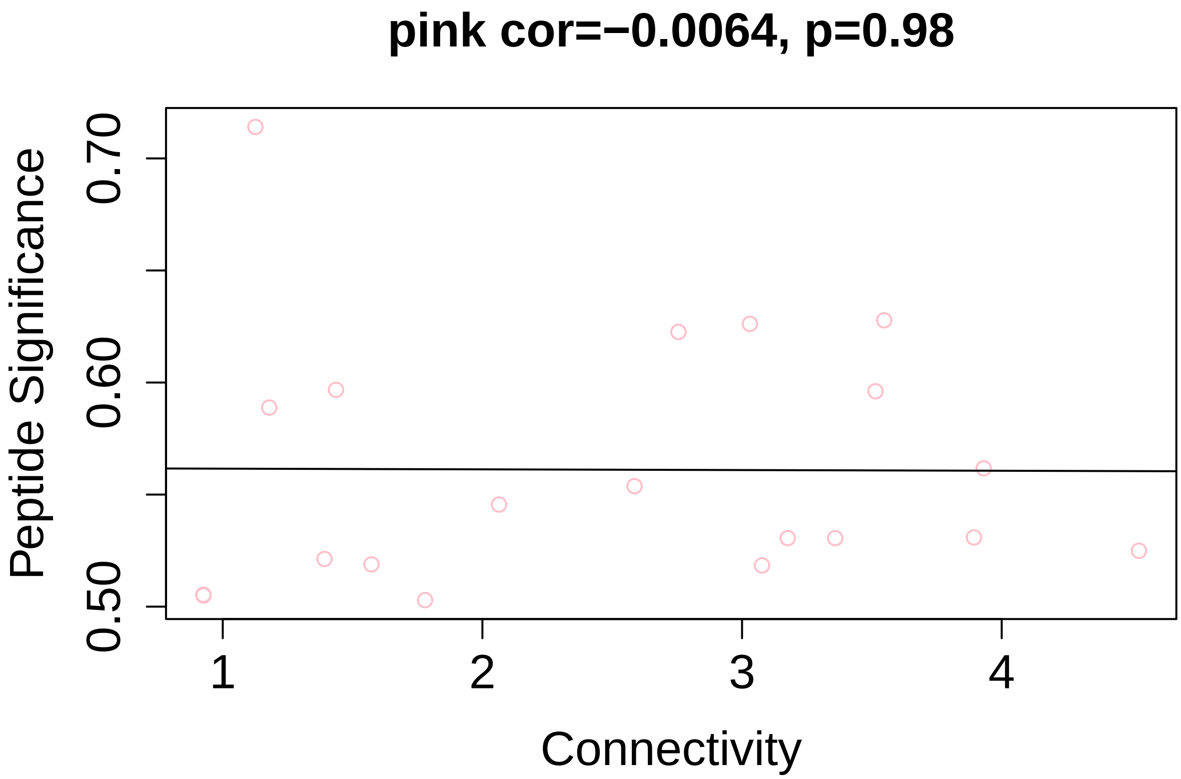
pink cor=−0.0064, p=0.98
Peptide Significance
0.50
0.60
0.70
1
2
3
4
Connectivity

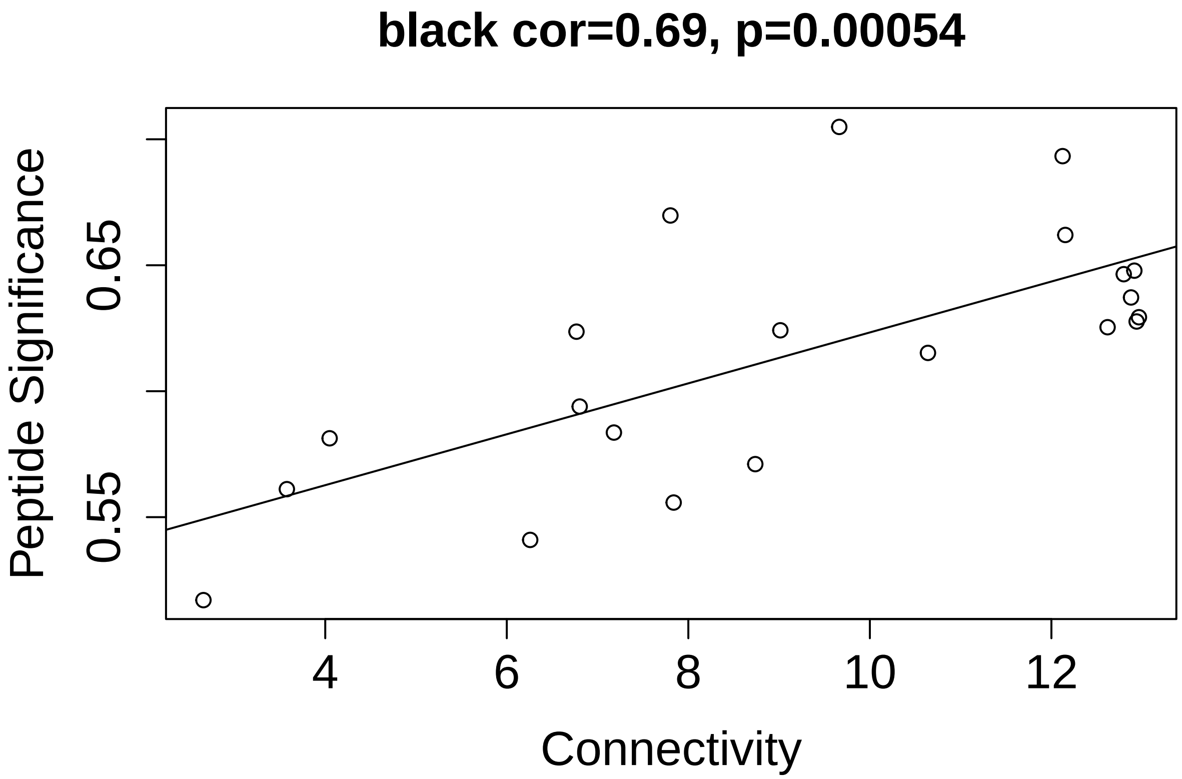
black cor=0.69, p=0.00054
Peptide Significance
0.55
0.65
4
6
8
10
12
Connectivity

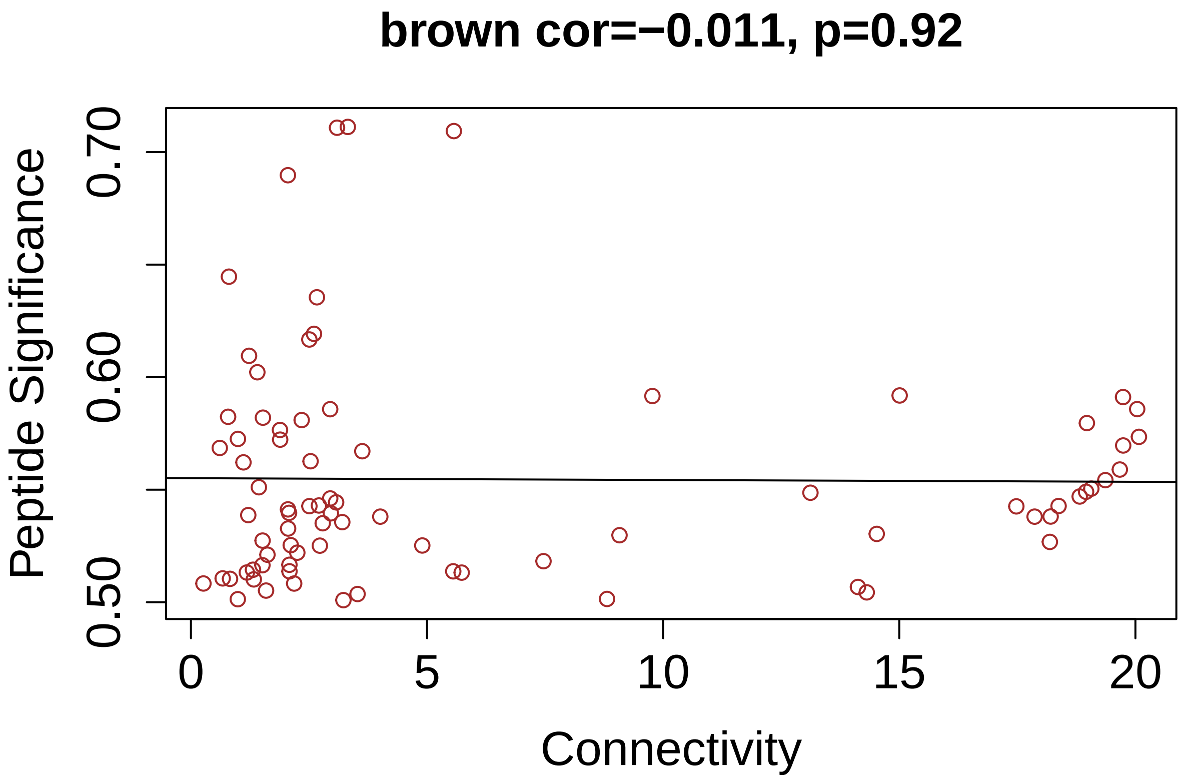
brown cor=−0.011, p=0.92
Peptide Significance
0.50
0.60
0.70
0
5
10
15
20
Connectivity

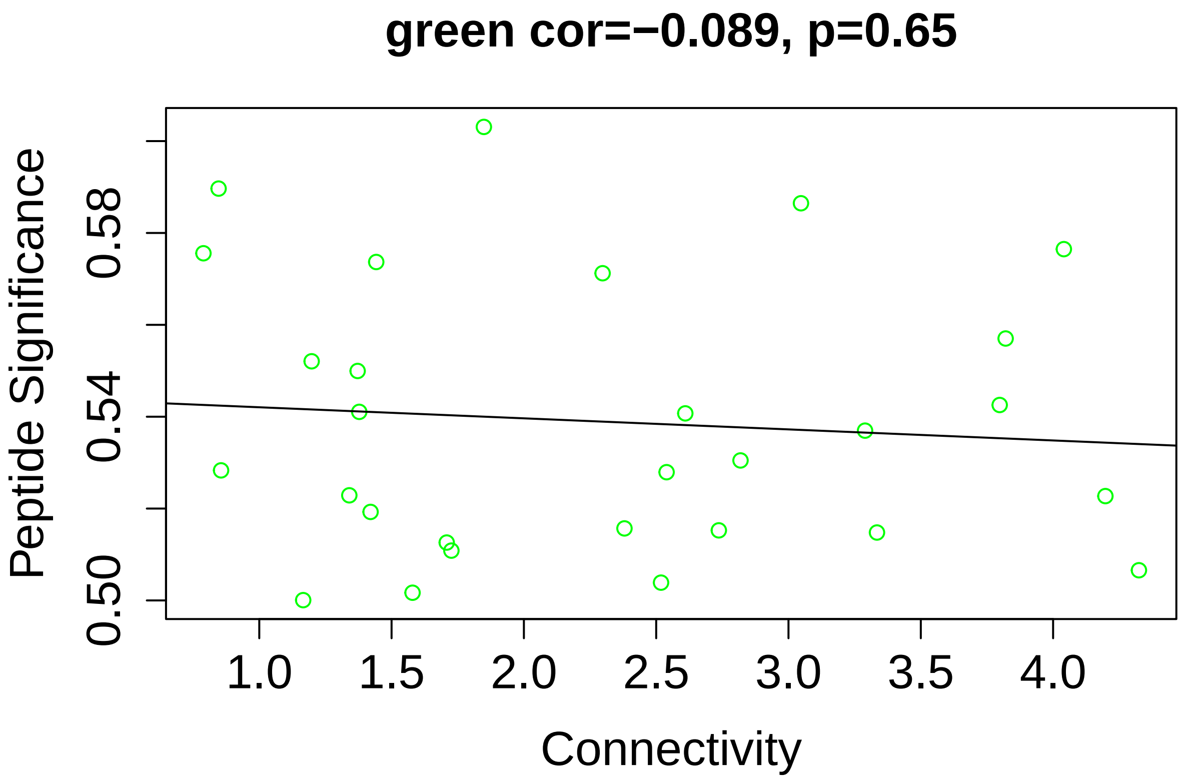
green cor=−0.089, p=0.65
Peptide Significance
0.50
0.54
0.58
1.0
1.5
2.0
2.5
3.0
3.5
4.0
Connectivity

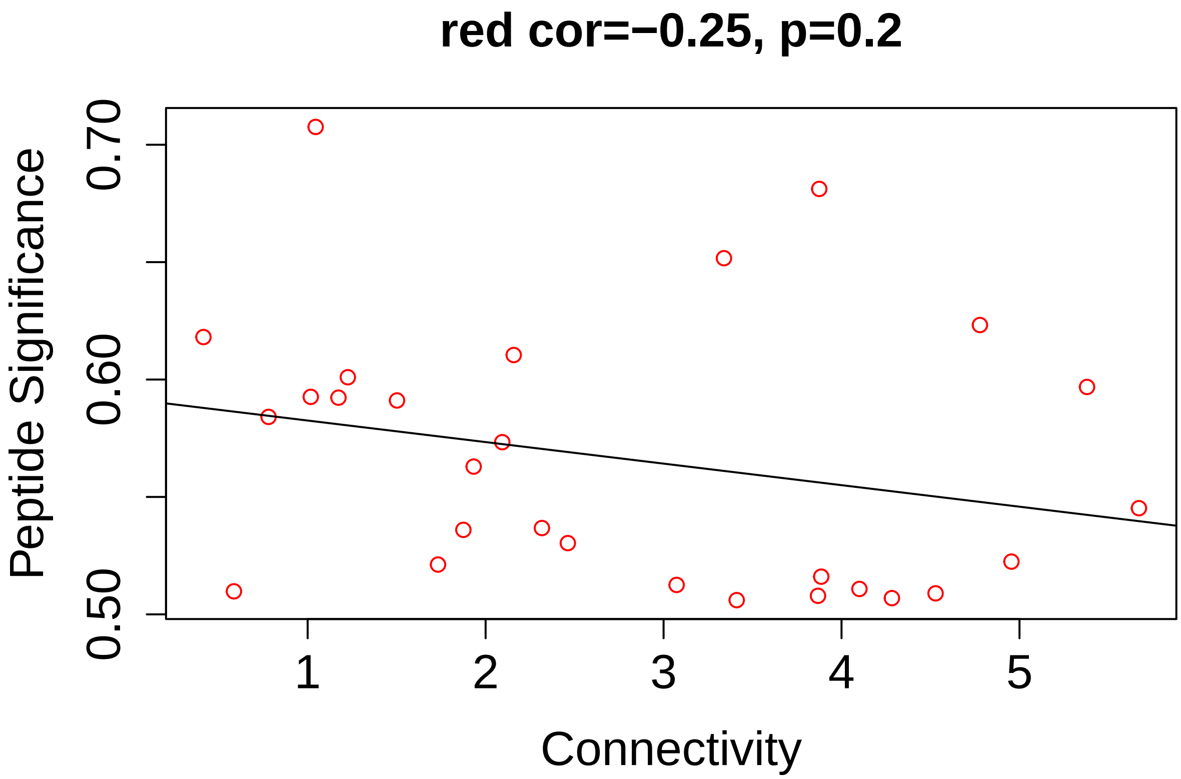


Figure. Peptide significance vs intramodular connectivity

```r
# Define a module eigenpeptide-based connectivity measure for each peptide as the
# correlation between a the peptide expression and the module eigenpeptide.
# Specifically, kMEbrown(i) = cor(xi, MEbrown), where xi is the peptide
# expression profile of peptide i and MEbrown is the module eigenpeptide of the
# brown module. Note that the definition does not require that peptide i is a
# member of the brown module. We define a data frame containing the module
# membership (MM) values for each module. In the past, we called the module
# membership values kME.
datKME=signedKME(df3_expr_transposed, datME, outputColumnName="MM.")
head(datKME)
```

```
##                                                      MM.black   MM.blue
## X_RKPGAGGS.Phospho..STY..PALAR_.3                   0.2151763 0.7867407
## X_IVHSES.Phospho..STY..QPEKESR_.2                   0.2829994 0.6062450
## X_TDPGS.Phospho..STY..IENLC.Carbamidomethyl..C..PGK_.2 0.5995808 0.2356841
## X_TGGEGS.Phospho..STY..LDEEAEGSKK_.2                0.8297820 0.4553535
## X_S.Phospho..STY..MDSLC.Carbamidomethyl..C..SVPVEGK_.2 0.2052835 0.4864796
## X_SNS.Phospho..STY..QENVEASHPSQDGKR_.3              0.2465135 0.7563672
##                                                      MM.brown  MM.green
## X_RKPGAGGS.Phospho..STY..PALAR_.3                   0.5416833 0.2871711
## X_IVHSES.Phospho..STY..QPEKESR_.2                   0.3798168 0.5246010
## X_TDPGS.Phospho..STY..IENLC.Carbamidomethyl..C..PGK_.2 0.1569356 0.2775329
## X_TGGEGS.Phospho..STY..LDEEAEGSKK_.2                0.7307644 0.4792520
## X_S.Phospho..STY..MDSLC.Carbamidomethyl..C..SVPVEGK_.2 0.7296602 0.2922138
## X_SNS.Phospho..STY..QENVEASHPSQDGKR_.3              0.3785909 0.2517185
```

```
##                                                         MM.grey   MM.pink
## X_RKPGAGGS.Phospho..STY..PALAR_.3                     0.5738522 0.5283085
## X_IVHSES.Phospho..STY..QPEKESR_.2                     0.7160377 0.3604438
## X_TDPGS.Phospho..STY..IENLC.Carbamidomethyl..C..PGK_.2 0.5290195 0.1945320
## X_TGGEGS.Phospho..STY..LDEEAEGSKK_.2                  0.5858764 0.3272709
## X_S.Phospho..STY..MDSLC.Carbamidomethyl..C..SVPVEGK_.2 0.4818338 0.3001532
## X_SNS.Phospho..STY..QENVEASHPSQDGKR_.3                0.6404675 0.6769100
##                                                          MM.red MM.turquoise
## X_RKPGAGGS.Phospho..STY..PALAR_.3                     0.6800069    0.3882324
## X_IVHSES.Phospho..STY..QPEKESR_.2                     0.1919280    0.4315670
## X_TDPGS.Phospho..STY..IENLC.Carbamidomethyl..C..PGK_.2 0.1427152    0.7129611
## X_TGGEGS.Phospho..STY..LDEEAEGSKK_.2                  0.3332141    0.9276358
## X_S.Phospho..STY..MDSLC.Carbamidomethyl..C..SVPVEGK_.2 0.4523361    0.3442287
## X_SNS.Phospho..STY..QENVEASHPSQDGKR_.3                0.7186990    0.3616790
##                                                        MM.yellow
## X_RKPGAGGS.Phospho..STY..PALAR_.3                     0.55823609
## X_IVHSES.Phospho..STY..QPEKESR_.2                     0.32494908
## X_TDPGS.Phospho..STY..IENLC.Carbamidomethyl..C..PGK_.2 0.08284898
## X_TGGEGS.Phospho..STY..LDEEAEGSKK_.2                  0.47654669
## X_S.Phospho..STY..MDSLC.Carbamidomethyl..C..SVPVEGK_.2 0.97617547
## X_SNS.Phospho..STY..QENVEASHPSQDGKR_.3                0.29395250
```

```r
# We now have a module membership value for each peptide in each module.
```

```r
# Relationship between the module membership measures (e.g. MM.turquoise) and
# intramodular connectivity. We now explore the relationship between the module
# membership measures (e.g. MM.turquoise) and intramodular connectivity.
# We choose 2 modules to plot: turquoise, black
# For simplicity we write the code out explicitly for each module.
which.color="turquoise"
restrictGenes=colorh1==which.color
verboseScatterplot(Alldegrees1$kWithin[ restrictGenes],
                   (datKME[restrictGenes, paste("MM.", which.color, sep="")])^8,
                   col=which.color,
                   xlab="Intramodular Connectivity",
                   ylab="(Module Membership)^8")
```

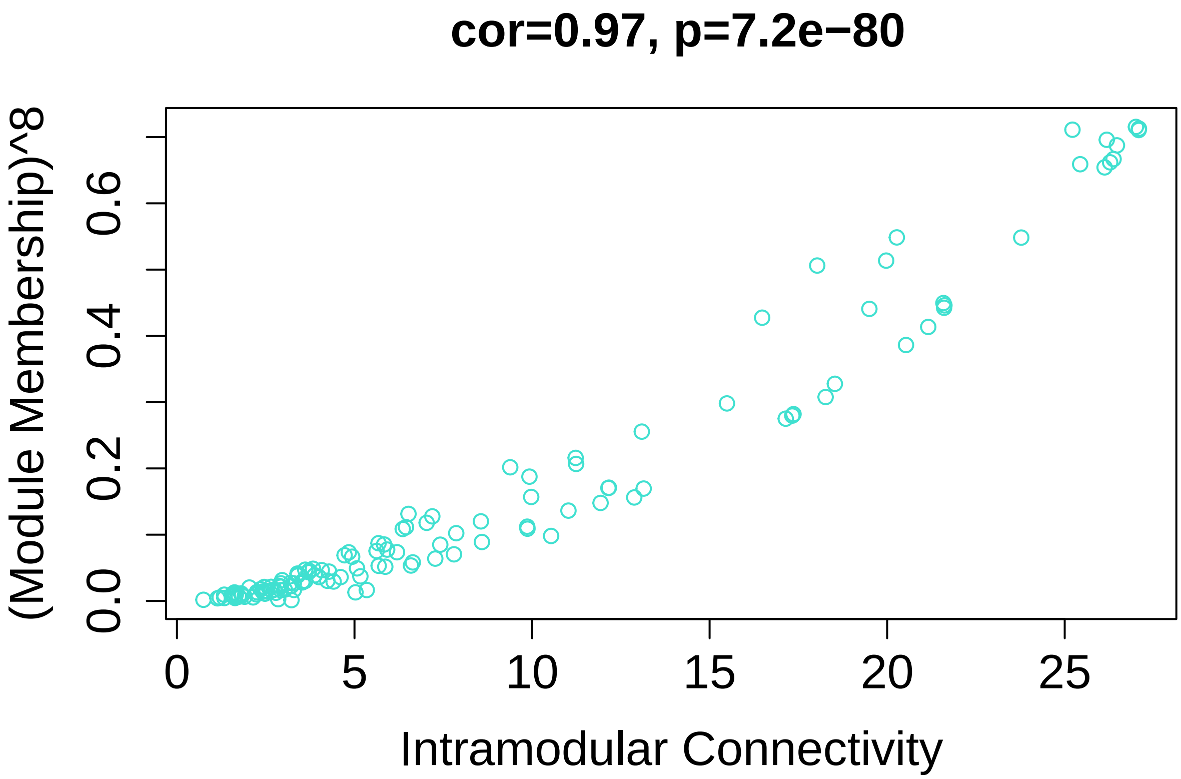


```
which.color="black"
restrictGenes=colorh1==which.color
verboseScatterplot(Alldegrees1$kWithin[ restrictGenes],
                   (datKME[restrictGenes, paste("MM.", which.color, sep="")])^8,
                   col=which.color,
                   xlab="Intramodular Connectivity",
                   ylab="(Module Membership)^8")
```

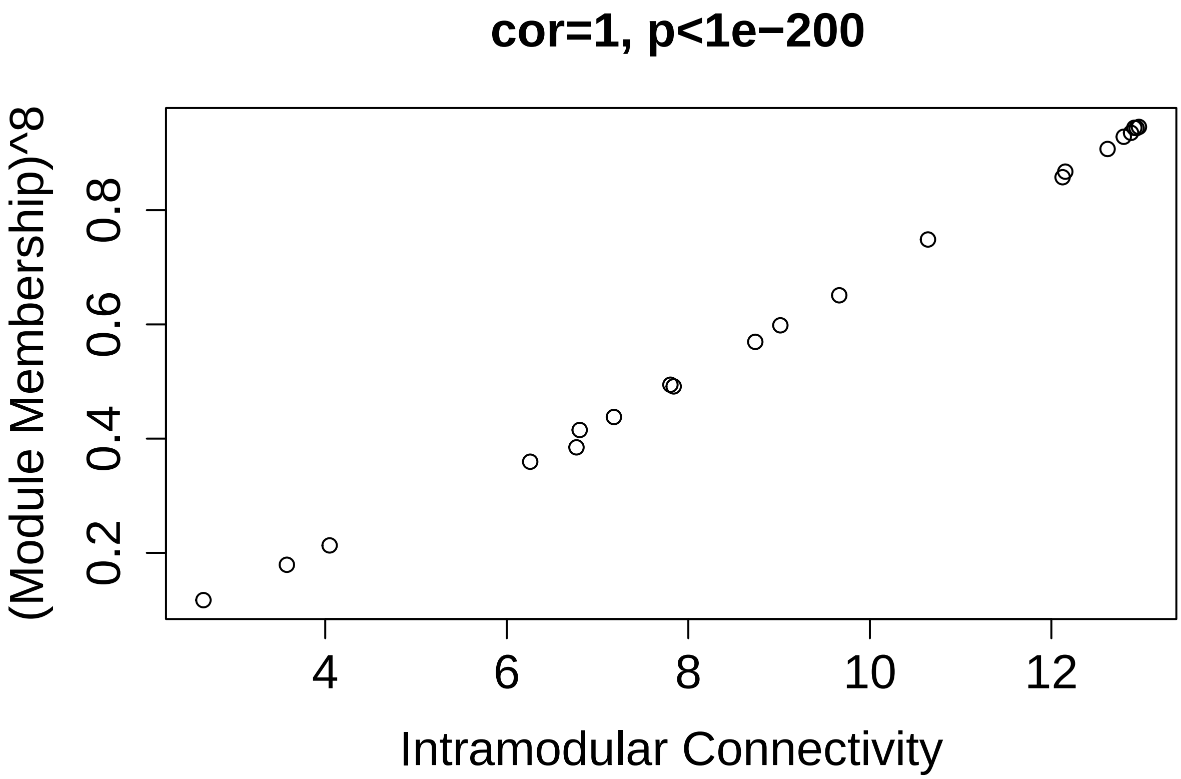


Figure. Intramodular Connectivity vs Module Membership for turquoise and black modules Impression: Module membership raised to Beta-power 8 (y-axis) vs. intramodular connectivity (x-axis) plotted separately for selected modules in the dataset. After raising the module membership to a power of 8, it is highly correlated with the intramodular connectivity (kWithin).

### Step 10.

- Multi-dimensional scaling (MDS) plots
- Visualize topological overlap matrix

```
# In this section we visualize peptide co-expression networks. We begin with a multi-dimensional scalin
cmd1=cmdscale(as.dist(dissTOM),2)


# We now create a TOM plot, a heatmap plot depicting the topological overlap
# matrix supplemented by hierarchical clustering dendrograms and the module
# colors. For large peptide sets, say more than 2000 peptides, this will take a
# long time. We will remove the "grey" module (un-clustered phosphopeptides) to
# save computation time.
power=8
color1=colorDynamicTOM
restGenes= (color1 != "grey")
diss1=1-TOMsimilarityFromExpr( df3_expr_transposed[, restGenes], power = 8)
```

```
## TOM calculation: adjacency..
```

```
## ..will not use multithreading.
##  Fraction of slow calculations: 0.000000
## ..connectivity..
## ..matrix multiplication (system BLAS)..
## ..normalization..
## ..done.
```

```r
hier1=hclust(as.dist(diss1), method="average")
diag(diss1) = NA
```

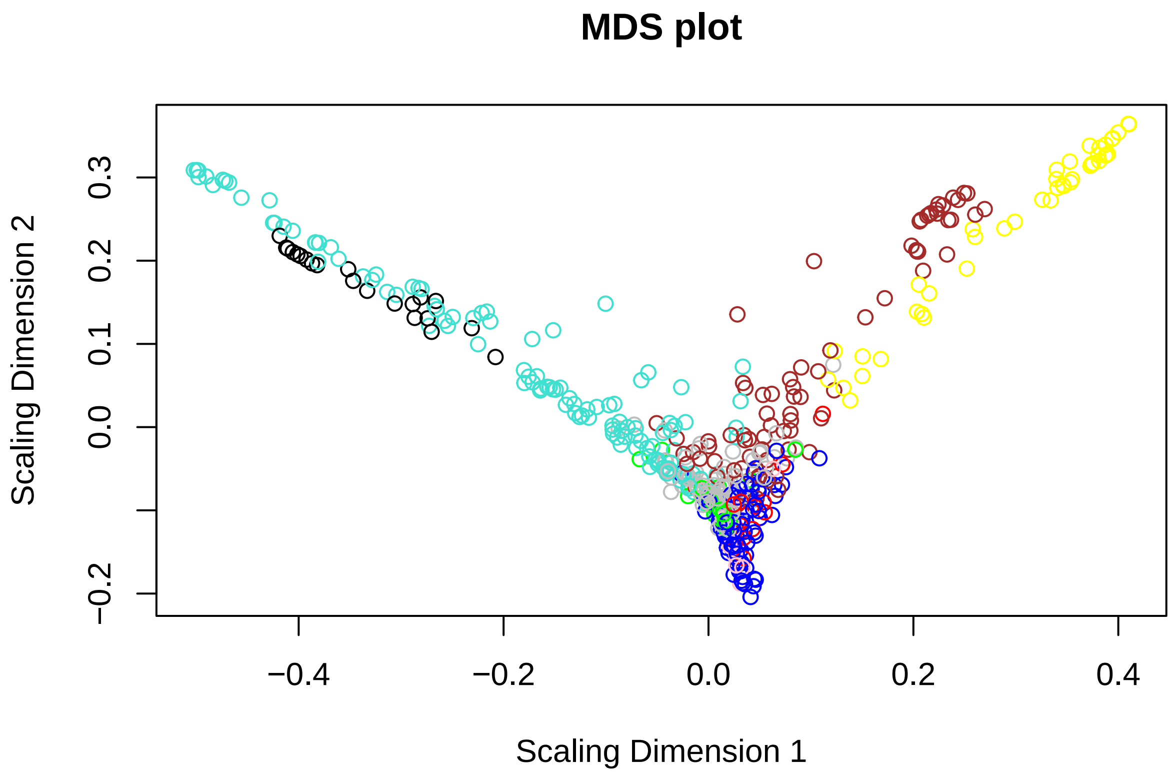


Figure. Multi-dimensional scaling plot

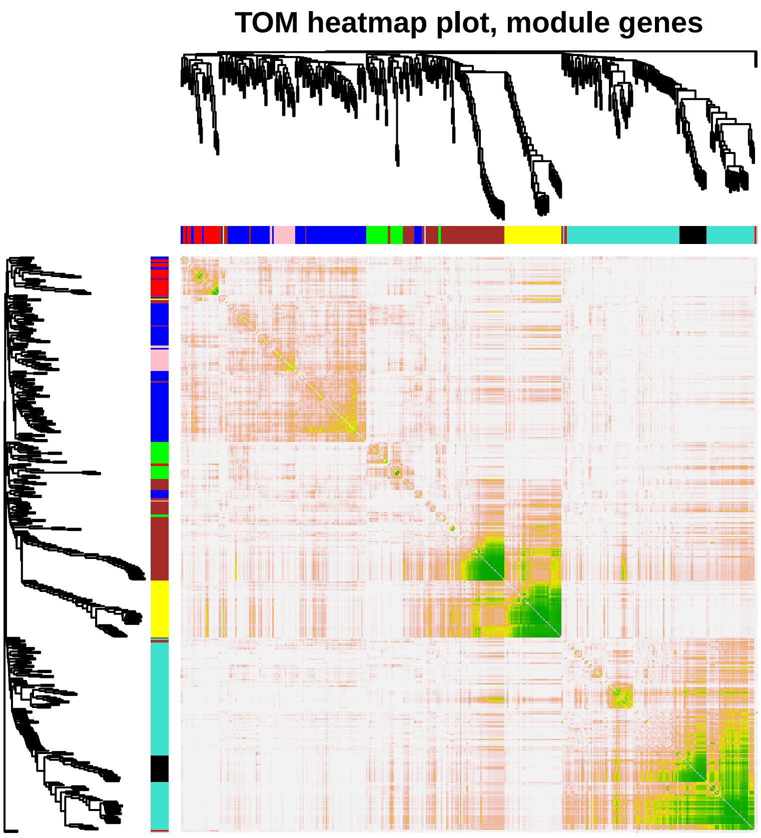


Figure. Topological overlap matrix
