# Supplementary material for: The Chronic Effects of a Single Low-Intensity Blast Exposure on Phosphoproteome Networks and Cognitive Function Influenced by Mutant Tau Overexpression
Source: Int J Mol Sci. 2024 Mar 15;25(6):3338. doi: 10.3390/ijms25063338 (PMC10970429; doi:10.3390/ijms25063338)
Supplement: Supplementary file 1 [file ijms-25-03338-s001.zip › Supplementary File S9- Blast experiment result.pdf]

Supplementary file S9- Blast experiment results

Cohort 1

Table 1. Pressure data

	Stand One			Stand Two			Stand Three		
	Pencil	Pencil'	Flush	Pencil	Pencil'	Flush	Pencil	Pencil'	Flush
Time of Arrival (ms)	*	4.53	4.55	4.51	4.51	4.52	4.47	4.47	4.47
Peak Pressure (psi)	*	6.78	23.6	6.78	6.58	23.2	6.67	6.79	24.7
Postive Phase Threshold (psi)	*	0.2	0.2	0.2	0.2	0.2	0.2	0.2	0.2
Time of Peak Pressure (ms)	*	4.58	4.55	4.53	4.56	4.53	4.52	4.52	4.47
Time of Positive Phase End (ms)	*	7.51	7.3	7.45	7.39	7.21	7.38	7.3	7.17
Rise Time (ms)	*	0.05	0.0	0.02	0.05	0.01	0.05	0.05	0.0
Positive Phase Duration (ms)	*	2.98	2.75	2.94	2.88	2.69	2.91	2.83	2.7
Incident Impulse (psi·ms)	*	8.54	9.16	8.4	8.45	8.97	8.92	8.39	9.02

* Error in Pencil 1 sensor data

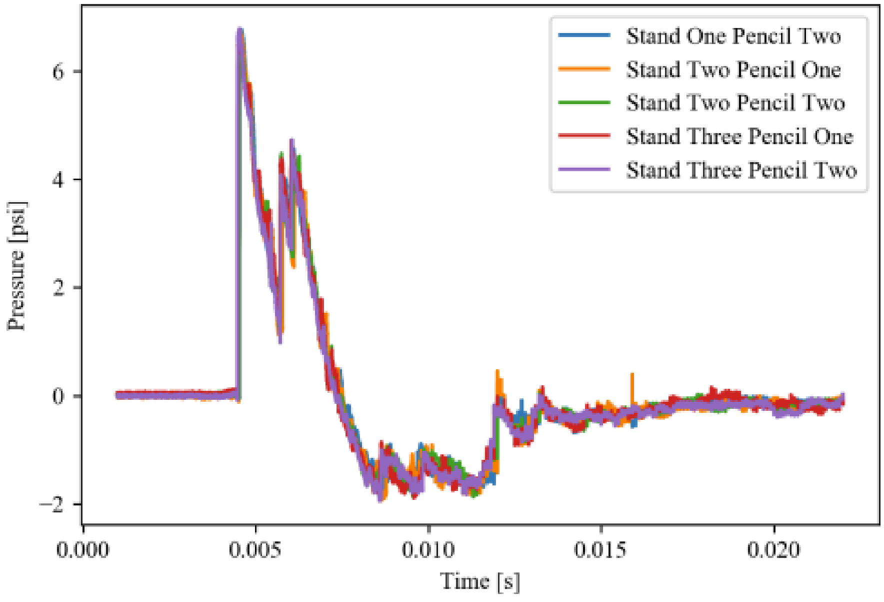

Figure 1. Pressure vs time gathered by pencil sensors

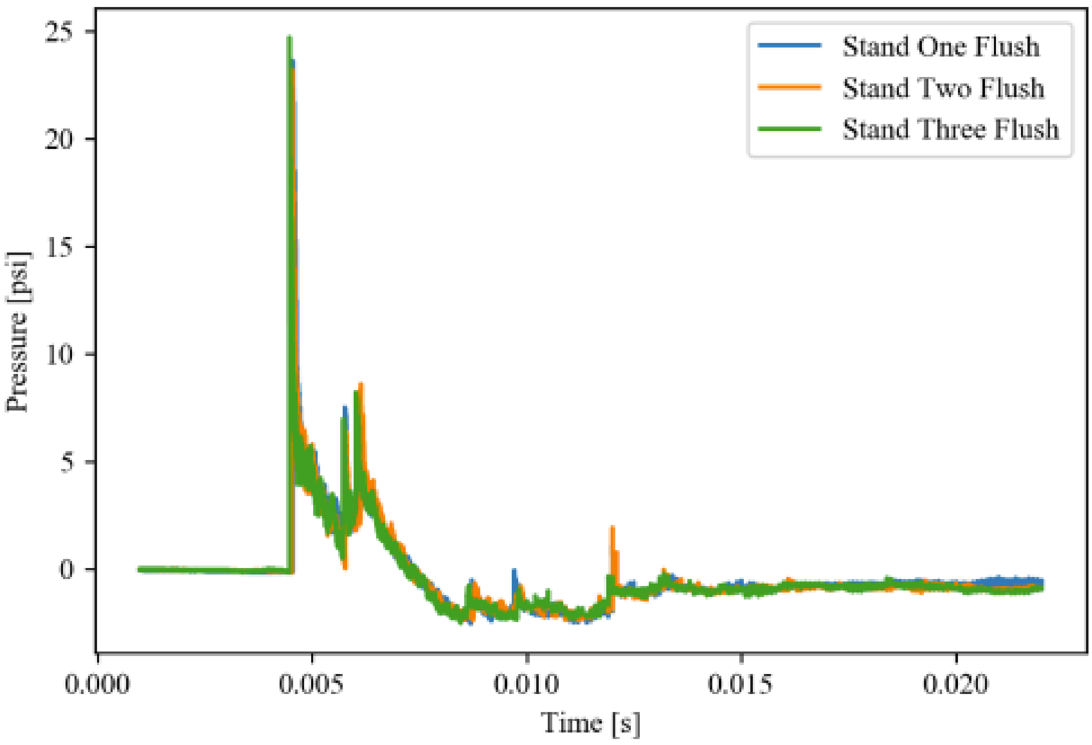

Figure 2. Pressure vs time gathered by flush sensors

Cohort 2

Table 1. Pressure data

	Stand One			Stand Two			Stand Three		
	Pencil	Pencil'	Flush	Pencil	Pencil'	Flush	Pencil	Pencil'	Flush
Time of Arrival (ms)	*	4.53	4.55	4.11	4.54	4.52	4.54	4.57	4.56
Peak Pressure (psi)	*	6.82	21.45	6.97	6.75	21.95	7.43	7.1	30.44
Postive Phase Threshold (psi)	*	0.2	0.2	0.2	0.2	0.2	0.2	0.2	0.2
Time of Peak Pressure (ms)	*	4.54	4.56	4.56	4.6	4.53	4.59	4.6	4.56
Time of Positive Phase End (ms)	*	7.39	7.07	7.35	7.25	6.98	7.4	7.24	7.01
Rise Time (ms)	*	0.01	0.01	0.45	0.06	0.01	0.05	0.03	0.0
Positive Phase Duration (ms)	*	2.86	2.52	3.24	2.71	2.46	2.86	2.67	2.45
Incident Impulse (psi·ms)	*	8.42	9.34	8.65	8.39	9.13	9.1	8.39	9.32

* Pencil sensor 1 was not used

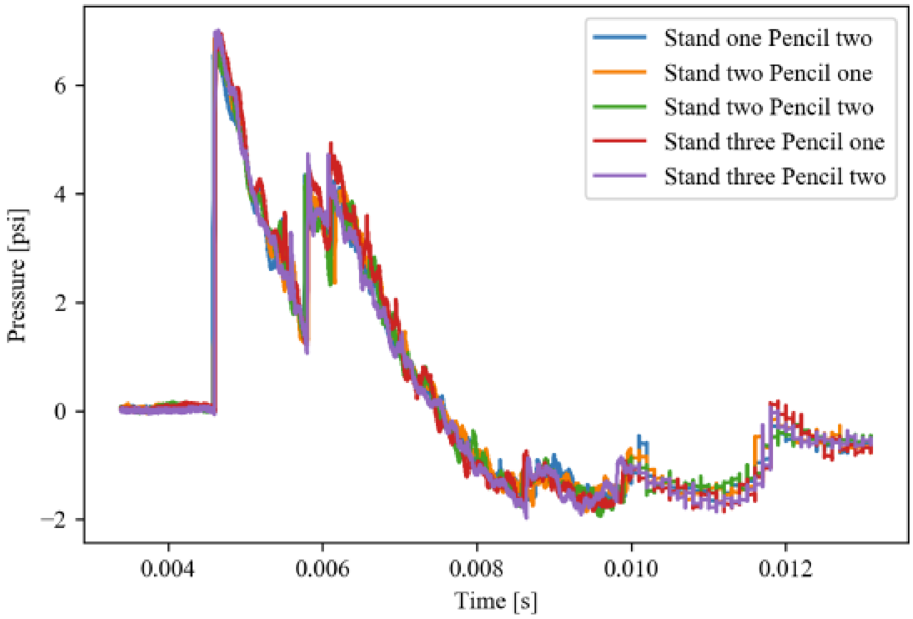

Figure 1. Pressure vs time gathered by pencil sensors

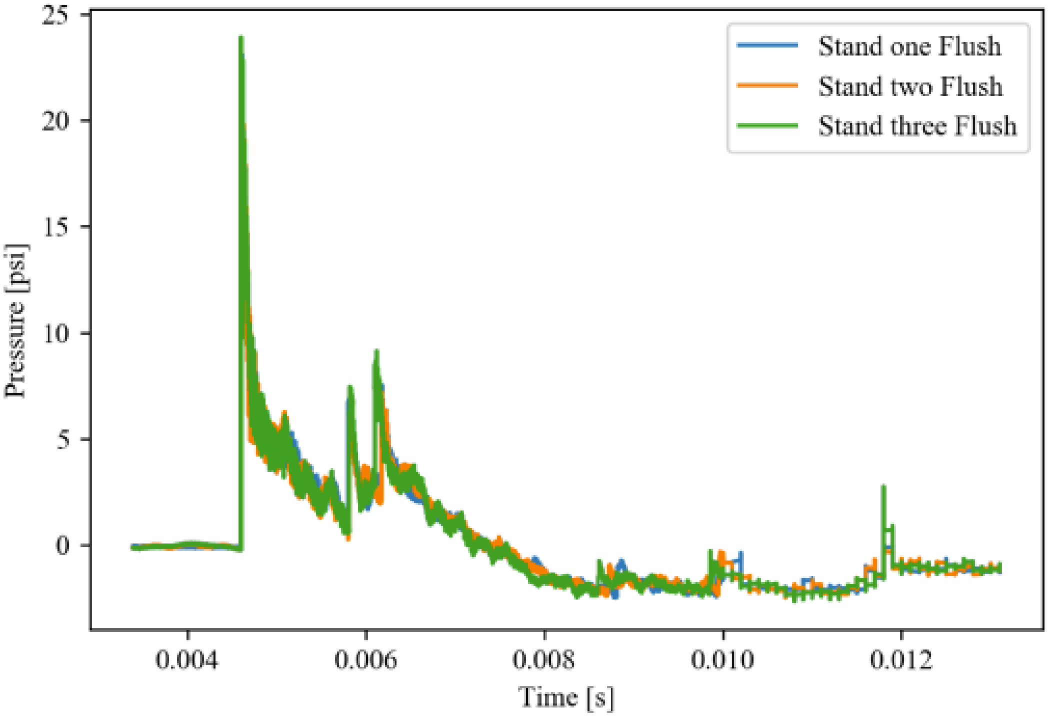

Figure 2. Pressure vs time gathered by flush sensors
