# Supplementary material for: The Chronic Effects of a Single Low-Intensity Blast Exposure on Phosphoproteome Networks and Cognitive Function Influenced by Mutant Tau Overexpression
Source: Int J Mol Sci. 2024 Mar 15;25(6):3338. doi: 10.3390/ijms25063338 (PMC10970429; doi:10.3390/ijms25063338)
Supplement: Supplementary file 1 [file ijms-25-03338-s001.zip › Supplementary Table S1a- MS2 raw data.pdf]

Supplementary Table S1a- MS2 raw data (sheet = 'MS2 raw data')

PG.Genes	PhosName	Phos site	Blast rTg4510 3	Blast rTg4510 4	Blast rTg4510 1	Blast rTg4510 2	Blast rTg4510 5	Sham rTg4510 3	Sham rTg4510 4	Sham rTg4510 5	Sham rTg4510 1	Sham rTg4510 2	Blast NC 3	Blast NC 4	Blast NC 5	Blast NC 6	Blast NC 1	Blast NC 2	Sham NC 3	Sham NC 4	Sham NC 1	Sham NC 2
Tbc1d25	_QAS[Phospho (STY)]LDGLQQLR_.2	A1A5B6_S560_M1_Tbc1d25	9.5265	9.5304	9.5616	9.3108	3.1154	9.1282	8.8320	8.7545	8.8035	9.4365	8.9584	8.9600	9.4783	9.2316	8.3473	9.0666	9.2384	8.8514	9.0263	8.9794
Arfgef2	_GQSQLS[Phospho (STY)]NPTDDSWK_.2	A2A5R2_S1535_M1_Arfgef2	9.8513	9.5396	9.4053	9.7122	8.2811	8.4890	9.1661	8.7395	8.5622	9.6365	8.5675	8.4948	8.8466	8.3379	5.8936	8.5683	9.0935	8.6862	8.4371	8.4545
Arfgef2	_ERGS[Phospho (STY)]PVSGR_.2	A2A5R2_S276_M1_Arfgef2	4.0132	9.2094	8.7829	8.8804	8.7297	8.7121	9.1431	9.2236	9.0122	8.5812	8.6389	8.4976	9.1067	8.1306	2.8533	7.9172	9.0192	9.0525	8.2180	8.2993
Arfgef2	_PQSPVIQATAGS[Phospho (STY)]PK_.2	#N/A	6.7514	5.9274	6.7604	6.7771	5.8210	6.3357	5.9729	6.1076	6.3241	6.4982	6.0610	5.4814	5.6884	5.8783	4.8684	5.6602	6.5364	5.9357	5.8414	6.0118
Arfgef2	_QSLS[Phospho (STY)]SADNLEPDVQGHQVAAR_.3	A2A5R2_S355_M1_Arfgef2	3.8226	3.5750	6.3339	3.8176	4.0295	4.4095	6.6314	3.8372	3.8057	3.7753	6.4102	4.0705	3.9502	4.0384	3.8918	6.2819	3.9997	3.6840	3.7655	3.8093
Arfgef2	_ELEKPM[Oxidation (M)]QSKPQS[Phospho (STY)]PVIQAT[Phospho (STY)]AGSPK_.3	A2A5R2_T224_M2_Arfgef2	5.3241	5.3237	4.1052	6.6468	3.5416	3.1307	4.7704	3.2584	3.2011	4.7571	5.7288	4.8657	5.1527	6.2437	3.8309	4.7142	6.8651	5.1759	4.8042	4.5822
Arfgef2	_HLDVDLDRQS[Phospho (STY)]LSSIDR_.3	A2A5R2_S1518_M1_Arfgef2	3.8670	3.4521	3.4036	3.7172	4.0739	4.1073	4.4750	4.0927	4.3952	3.6776	3.8739	3.1782	3.8979	3.4416	2.9995	3.2291	3.5899	3.5595	3.8586	3.1189
Tanc2	_FS[Phospho (STY)]PPPVGGQSK_.2	A2A690_S1521_M1_Tanc2	10.0605	9.3238	9.4203	8.6983	8.9753	9.5648	9.5244	3.7465	9.5652	9.5742	9.8002	9.3381	9.3071	3.8682	3.7345	9.1769	10.2135	9.6977	9.5116	9.3697
Tanc2	_QGQTS[Phospho (STY)]PIKPK_.2	A2A690_S1981_M1_Tanc2	4.0037	3.3154	6.3676	3.5806	5.7754	3.8978	4.3383	6.3174	3.9265	4.5423	4.3009	3.0415	4.0346	6.3636	4.2184	3.3658	3.7266	3.4229	3.7219	3.2556
Tanc2	_SC[Carbamidomethyl (C)]DELSPVS[Phospho (STY)]PTQGGYPSEPTR_.2	A2A690_S1831_M1_Tanc2	3.7640	6.1754	6.6273	6.4540	5.6566	3.6614	6.1174	3.9209	5.8032	6.8390	2.9332	6.1858	6.0444	5.6663	5.2256	5.9082	6.4362	6.2120	3.6167	6.2782
Tanc2	_EYPS[Phospho (STY)]PPPSPLR_.2	A2A690_S1534_M1_Tanc2	11.0992	11.0283	10.8588	7.4167	10.5361	6.0930	10.5967	11.0278	10.5939	10.7192	10.7981	10.6644	4.0266	10.5299	9.2770	10.6189	11.4241	10.7681	10.5462	10.6853
Tanc2	_AS[Phospho (STY)]PPAESMSVYR_.2	A2A690_S1549_M1_Tanc2	3.1879	9.5036	10.4562	10.2891	4.0264	8.8424	9.3617	4.3891	3.5737	8.2605	9.7430	3.2035	3.6818	9.8563	3.0918	3.5328	8.6278	7.2940	7.0406	4.4037
Tanc2	_QIASDS[Phospho (STY)]PHAS[Phospho (STY)]PK_.2	A2A690_S400_M2_Tanc2	8.6273	8.4359	8.0833	8.1869	7.8122	8.0820	8.2351	8.2567	7.9285	7.9179	7.7871	7.5338	8.3217	7.6900	6.4591	7.3309	8.1265	8.2614	8.0020	7.6722
Tanc2	_DC[Carbamidomethyl (C)]SYGAVTS[Phospho (STY)]PTSTLESR_.2	A2A690_S169_M1_Tanc2	4.8390	10.7460	10.8213	10.7513	9.9863	10.1587	10.3807	10.4394	10.2900	10.7320	10.3597	10.2530	10.7540	10.3549	3.9047	10.1013	10.7762	10.3510	3.3069	10.0826
Tanc2	_SQS[Phospho (STY)]ASYYPVC[Carbamidomethyl (C)]HSK_.2	A2A690_S1726_M1_Tanc2	6.5778	6.4631	7.0144	6.4866	5.8452	5.7330	6.0642	5.9029	5.1413	5.6938	5.8179	3.8045	3.9916	6.0664	3.6727	4.8121	6.0123	3.7661	4.9443	3.7360
Tanc2	_SNSSVGS[Phospho (STY)]PTR_.2	A2A690_S1480_M1_Tanc2	9.7513	9.7458	9.5486	9.3532	9.2787	9.2029	3.7730	9.5922	9.1769	9.5454	9.3448	9.1354	9.5684	3.4529	3.3502	9.1198	9.5525	3.5173	9.1474	9.0411
Tanc2	_SSS[Phospho (STY)]QLGSPDVSHLIR_.2	A2A690_S1744_M1_Tanc2	7.5887	7.5503	6.7233	3.5652	3.6583	5.2037	6.6567	4.2447	3.9419	6.1715	5.6003	6.3894	4.0499	6.9786	3.4600	4.9720	3.7420	6.5076	6.1604	3.2710
Tanc2	_PSQGLPVIQS[Phospho (STY)]PPSS[Phospho (STY)]PAHR_.3	#N/A	3.2825	6.6221	3.7032	4.2057	4.1211	3.9456	3.9360	6.0148	4.7778	3.3615	4.1279	5.5054	3.5043	6.8161	3.9697	4.7966	3.5381	4.1346	5.4118	5.2777
Tanc2	_[Acetyl (Protein N-term)]MFRNS[Phospho (STY)]LK_.2	A2A690_S5_M1_Tanc2	4.4150	4.1819	3.9516	5.3522	3.1211	2.0495	3.8758	3.1399	4.3378	4.1309	3.2114	4.1310	4.4459	3.9897	3.9524	3.7771	4.1379	3.0115	3.3106	3.6669
Fam171a2	_S[Phospho (STY)]VEGPGGLEPSLDEYR_.2	#N/A	4.4034	7.6314	7.2460	7.3453	3.1095	6.6498	7.3508	3.1283	6.7841	7.1733	3.1998	4.1426	4.4343	7.3000	3.8443	5.9759	4.1263	6.9742	7.2118	3.6553
Fam171a2	_S[Phospho (STY)]VEGPGGLEPSLDEYRR_.3	#N/A	4.4685	7.1957	6.3723	6.9404	3.5372	6.2719	6.8513	3.9859	6.0953	6.4075	3.7736	5.4633	4.3062	6.3417	3.6490	6.3578	6.5094	6.4610	6.6912	6.5408
Fam171a2	_TGLC[Carbamidomethyl (C)]SPEDNS[Phospho (STY)]LTPLLDEVVAPEGR_.3	A2A699_S744_M1_Fam171a2	3.0945	3.4074	3.6308	3.0940	3.9331	4.0518	4.8172	4.2957	4.4506	3.3008	3.9293	3.2969	3.1152	3.6179	3.1852	3.4394	3.3326	2.9997	4.1492	3.5849
Fam171a2	_RDS[Phospho (STY)]LTSPEDELGAEVGDEAGDKK_.3	#N/A	4.1419	3.9781	3.6570	3.8027	3.9593	3.7037	5.3582	3.9358	3.7080	3.8929	4.0823	3.8676	3.8609	3.7275	3.7559	4.5483	3.7122	5.6597	4.5657	3.5315
Fam171a2	_RDS[Phospho (STY)]LTSPEDELGAEVGDEAGDK_.3	#N/A	3.4665	4.5363	4.0028	4.2229	4.3051	3.0283	3.2360	3.1669	3.2951	3.6728	3.6695	4.4257	3.4031	4.4477	2.8132	3.8114	3.7046	4.0543	4.3533	4.1250
Fam171a2	_DS[Phospho (STY)]LTSPEDELGAEVGDEAGDKK_.3	#N/A	3.3379	4.3433	4.1181	4.0779	3.9296	3.2403	3.4620	3.5084	3.4422	4.1392	3.3872	4.1634	3.5824	4.3454	3.3158	3.9357	3.5949	3.9714	4.0064	4.1669
Gpatch8	_IYRS[Phospho (STY)]QS[Phospho (STY)]PHYFQSGR_.3	A2A6A1_S1037_M2_Gpatch8	3.4757	4.5724	3.9667	4.2590	3.6826	3.7159	3.3655	3.1308	3.2857	5.1751	4.2652	3.5695	3.4511	3.9538	3.3909	4.3386	3.6685	4.1647	3.8133	3.9208
Gpatch8	_SEPPGSGS[Phospho (STY)]PAPPR_.2	A2A6A1_S735_M1_Gpatch8	8.0347	10.1152	6.8708	4.1280	7.7065	9.2779	8.4461	8.8984	7.9825	7.9331	7.7569	8.4479	4.1049	4.3783	7.6079	4.1851	4.2979	9.8876	9.4158	3.9733
Gpatch8	_SQS[Phospho (STY)]PHYFQSGR_.2	#N/A	7.9447	8.0870	7.7990	8.0884	7.4578	7.8595	7.6669	7.9784	7.7424	7.7745	7.4257	7.6571	8.0181	8.0143	7.2241	7.2252	8.1066	7.7838	7.4469	7.4588
Gpatch8	_KGS[Phospho (STY)]WGHES[Phospho (STY)]PEERR_.3	A2A6A1_S1013_M2_Gpatch8	3.8373	3.0177	4.3736	3.8521	3.1750	3.3991	4.3661	3.5377	4.4252	4.0436	3.2987	4.0550	4.5332	4.3607	3.9433	4.1822	4.0753	3.7578	3.4064	4.3277
Cdr2l	_SC[Carbamidomethyl (C)]S[Phospho (STY)]DTALNAIVAK_.2	A2A6T1_S318_M1_Cdr2l	3.7142	6.9680	3.7282	6.9654	3.9211	6.5053	3.1270	4.3932	6.0763	3.5247	4.0267	3.1995	3.2127	7.3691	3.1524	3.0763	6.7927	4.4032	6.2463	6.4978
Cdr2l	_DLLGGEES[Phospho (STY)]PGEGK_.2	A2A6T1_S407_M1_Cdr2l	3.0938	6.8039	6.4135	7.0547	3.9324	6.5000	6.0063	4.2950	6.6591	6.8401	4.0422	6.6549	3.7758	6.9316	6.0446	6.8828	7.1759	6.5723	6.1005	6.6086
Zmym4	_AAHQES[Phospho (STY)]DNENEIQIQNQLKK_.3	#N/A	5.2739	6.1669	3.9035	5.2043	3.4942	5.0082	5.4144	3.7374	5.3095	4.4700	4.0002	5.0537	4.6440	5.6004	4.4326	4.6454	5.0792	5.0244	4.8614	5.1167
Zmym4	_ENS[Phospho (STY)]KETFSGK_.2	A2A791_S161_M1_Zmym4	5.9146	4.8838	4.5237	5.1000	3.3251	3.5492	5.1543	3.0771	4.2750	4.1937	5.0220	3.9049	5.4677	4.0496	4.8341	4.3323	4.0751	3.0743	3.3734	5.3856
Zmym4	_AAHQES[Phospho (STY)]DNENEIQIQNQLK_.3	#N/A	3.9634	5.0139	5.6419	5.2595	3.9517	4.7205	5.1075	4.3207	4.4933	4.7515	4.1640	4.2622	3.6587	4.5075	3.9097	4.1375	3.5789	5.2060	4.6590	4.6842
Kiaa1522	_GS[Phospho (STY)]PSGGSTAETSDTASIR_.2	A2A7S8_S402_M1_Kiaa1522	3.8666	6.5492	3.4032	3.7176	4.0736	4.0348	3.7304	4.0923	3.7895	3.1785	6.5737	6.1312	3.8975	7.0939	3.3076	3.2288	6.8010	3.5599	3.8589	3.1186
Kiaa1522	_MGS[Phospho (STY)]PGSDPQKK_.2	#N/A	6.2579	6.0390	4.3894	5.4577	4.0772	5.2890	4.7158	6.0189	4.4996	5.3713	3.7447	3.1690	6.2206	5.6176	3.2737	3.4075	6.3304	5.7197	3.9427	5.0019
Kiaa1522	_GLAVAPAS[Phospho (STY)]PGK_.2	A2A7S8_S543_M1_Kiaa1522	4.9174	5.2789	5.1088	3.8020	4.7174	4.7036	3.7293	3.8870	2.7761	4.8427	3.0792	3.4207	3.2655	3.2360	3.2052	4.9074	3.3842	4.3679	4.2836	4.4916
Kiaa1522	_LQLERPVS[Phospho (STY)]PEAQADLQR_.3	A2A7S8_S909_M1_Kiaa1522	4.1591	5.4891	5.2045	5.5824	3.7334	4.4528	5.2593	4.0960	4.6128	5.4395	4.4090	4.6156	4.4164	3.4182	3.3849	3.2397	3.1329	4.8927	4.5833	4.6480
Kiaa1522	_M[Oxidation (M)]GS[Phospho (STY)]PGSDPQK_.2	#N/A	3.6278	3.6913	3.1644	3.9564	3.8347	4.2736	3.2134	3.8535	3.5506	3.4384	3.9250	3.4174	3.6587	3.2024	3.2388	2.9899	3.3507	3.7988	4.0978	4.3805
Hivep3	_GSYS[Phospho (STY)]FDDHVADPEVPSR_.2	#N/A	3.8952	3.8007	3.7463	3.8069	3.5563	3.7845	3.4430	3.9415	3.5664	3.8089	3.3715	3.6104	3.7057	4.7701	3.6692	3.4503	3.7371	3.9848	4.2763	4.2790
Hivep3	_RSS[Phospho (STY)]VESPK_.2	A2A884_S489_M1_Hivep3	4.1622	3.5941	3.6988	3.4220	2.8683	4.4025	4.1798	4.3879	4.1000	3.9728	3.5786	4.3838	4.1931	3.7369	4.2052	3.5243	3.8851	3.2643	3.5634	3.4141
Kiaa1109	_VVFENEQDNNS[Phospho (STY)]LTK_.2	A2AAE1_S2755_M1_Kiaa1109	9.0149	8.7523	8.6695	8.8450	8.1569	8.4058	8.5363	8.7977	8.3550	8.5111	8.4093	8.3601	9.0069	8.3559	6.8427	8.0862	9.0025	8.4516	8.4025	8.1890
Kiaa1109	_SDENVLDS[Phospho (STY)]PK_.2	#N/A	7.9075	7.7384	7.1555	7.5559	7.2609	7.0831	7.4160	7.6187	6.8349	7.7643	7.1435	6.8616	7.4928	7.0079	5.1009	6.8274	7.5410	6.9799	6.3634	6.9452
Kiaa1109	_TLKTES[Phospho (STY)]PNASR_.2	A2AAE1_S3894_M1_Kiaa1109	7.4368	7.7924	4.1444	4.3535	2.9458	7.2117	3.0944	3.4564	3.1535	6.9768	6.9155	4.2841	7.6270	4.3062	4.1724	3.9530	7.1909	4.1959	2.9941	3.9834
Kiaa1109	_ALETIPIT[Phospho (STY)]PIER_.2	A2AAE1_T3996_M1_Kiaa1109	4.0794	3.6752	3.3630	3.3618	4.2863	4.3196	4.2626	4.0280	4.1829	3.8899	3.6615	3.5647	4.3483	3.3501	2.7872	3.4415	3.0648	3.2675	4.4169	3.3171
Kiaa1109	_YTAGSAS[Phospho (STY)]PTPTFK_.2	A2AAE1_S2603_M1_Kiaa1109	8.1010	3.3665	7.5820	7.5981	7.6575	7.6146	3.3810	7.9673	7.1521	7.5494	7.5463	7.4177	8.2006	6.9835	5.5678	6.9470	3.3747	8.3838	8.1230	3.3703
Kiaa1109	_SLDQDS[Phospho (STY)]PSKK_.2	A2AAE1_S3835_M1_Kiaa1109	3.9629	3.3562	3.4995	3.6214	4.1698	4.8190	4.3791	4.1886	4.2993	3.7735	3.7780	3.0823	4.4648	3.5375	2.9037	3.3250	3.6858	3.1510	4.3005	3.4336
Kiaa1109	_SSETFGPAGVRS[Phospho (STY)]PTEPTC[Carbamidomethyl (C)]R_.3	#N/A	3.4502	6.4514	6.1992	4.2335	3.6572	6.4307	6.2236	3.1562	3.3111	5.8961	6.2630	5.7464	6.6138	6.2016	3.4163	4.3132	5.9110	6.2625	3.7878	6.2811
Kiaa1109	_AS[Phospho (STY)]PGSSSYLK_.2	A2AAE1_S3196_M1_Kiaa1109	3.1948	4.1243	4.2322	6.5950	6.3386	6.6014	6.5152	6.4205	6.3037	4.5062	3.0453	5.9678	3.7321	6.1030	3.6718	5.7556	6.2205	6.5580	6.5001	4.2017
Kiaa1109	_S[Phospho (STY)]PTEPTC[Carbamidomethyl (C)]R_.2	#N/A	6.2090	3.6582	3.3800	3.3448	5.7424	6.4231	3.8588	4.0449	6.2377	6.4668	6.2231	3.5477	4.3653	6.1893	3.4360	5.2287	6.5130	5.6121	4.4000	3.3341
Kiaa1109	_SDENVLDS[Phospho (STY)]PKQR_.2	#N/A	7.6825	7.4282	7.4809	6.9327	6.6453	6.8349	7.4458	7.6048	6.9768	7.0973	6.3750	7.1249	7.2110	7.1989	5.7553	6.7225	7.2582	7.5775	6.6980	6.6912
Kiaa1109	_MVPETGIPAEC[Carbamidomethyl (C)]QS[Phospho (STY)]GQK_.2	A2AAE1_S628_M1_Kiaa1109	3.9626	3.3565	3.4992	3.6216	4.1695	3.9389	8.6188	4.1883	4.2996	7.7605	3.7783	3.0826	3.9935	8.7380	2.9040	3.3247	3.6855	3.4640	3.7630	3.2145
Kiaa1109	_RDDGQASVPTEISGTS[Phospho (STY)]PVS[Phospho (STY)]PNTQDK_.3	A2AAE1_S1287_M2_Kiaa1109	3.5903	3.7288	3.1268	3.9940	3.7972	4.3112	4.4830	3.8159	3.1711	3.4009	4.1506	3.4549	3.6211	3.1649	3.2763	2.9524	3.3132	3.8363	4.1353	4.3430
Sh3pxd2b	_KES[Phospho (STY)]IIKSEEELLER_.3	A2AAY5_S528_M1_Sh3pxd2b	4.5240	3.4788	4.4669	3.1654	3.8617	3.2942	3.1146	4.2243	4.3792	3.6936	3.8579	4.1456	4.5447	4.3362	2.9836	3.9642	3.2612	4.2669	4.2206	3.0571
Sh3pxd2b	_LRPAKS[Phospho (STY)]QEK_.2	A2AAY5_S672_M1_Sh3pxd2b	4.4075	4.4125	3.9440	3.6899	3.1136	3.1469	3.9345	3.6998	3.8547	4.2181	3.3334	4.1386	4.0202	4.5228	3.9599	3.7696	4.2375	3.5956	1.4932	2.9890
Arhgap27	_AES[Phospho (STY)]PKQVDDPPEPVYANVER_.3	A2AB59_S215_M1_Arhgap27	5.8388	6.2805	4.0583	5.9186	3.2278	3.2612	3.8203	3.2466	3.7405	4.3323	3.2191	4.0243	3.9059	4.4086	3.8456	3.8839	6.3067	3.7099	3.3585	4.3756
Arhgap27	_KSS[Phospho (STY)]QDS[Phospho (STY)]DTPAQASPPEEK_.2	A2AB59_S462_M2_Arhgap27	3.2134	3.2948	3.7497	5.0217	4.0519	3.9883	3.4675	4.4146	3.3194	4.6593	4.2852	3.1843	3.4610	3.7368	3.1901	3.5583	3.4514	4.0094	4.0365	3.7038
Ddi2	_TQAQHSSPGEM[Oxidation (M)]ASS[Phospho (STY)]PQGLDNPALLR_.3	#N/A	5.5685	5.5973	3.9082	4.7265	3.9783	4.0065	3.7611	5.4514	3.7239	3.5543	4.0005	5.6251	3.7058	5.4411	3.6676	5.0913	3.9232	5.0673	5.3762	5.3421
Ccdc120	_SNS[Phospho (STY)]SEALLVDR_.2	A2AEV7_S357_M1_Ccdc120	9.1820	3.6142	3.4241	9.1055	3.7264	3.9504	8.3147	4.1767	3.8738	9.2618	9.0321	8.4479	9.0231	8.7397	7.9141	8.6688	8.7882	8.3940	8.6549	8.8243
Tmem185a;Tmem185b	_VVITQS[Phospho (STY)]PGK_.2	A2AF53_S333_M1_Tmem185a;Tmem185b	7.9601	8.1482	8.1474	7.5911	7.2332	8.1045	7.7962	7.6478	4.1068	4.3620	7.7450	7.1800	4.2149	7.5867	3.6249	2.9997	4.3937	7.9685	7.7096	3.4359
Map7d2	_EES[Phospho (STY)]LEKR_.2	#N/A	7.7584	7.6707	7.7218	8.1310	7.1541	7.6655	7.5534	6.9843	7.5639	8.1101	7.3636	7.3331	7.6860	7.9785	7.1294	7.2927	7.5311	7.4952	7.6151	7.7443
Map7d2	_RLSS[Phospho (STY)]PVK_.2	A2AG50_S332_M1_Map7d2	7.5011	3.8829	7.5322	7.5407	3.6431	4.4653	3.4050	3.6618	3.3590	8.1845	4.3047	3.6090	3.4670	4.5116	3.4304	4.2991	6.5683	7.0942	4.2894	7.3063
Map7d2	_VATSAASGGHGS[Phospho (STY)]PLR_.2	A2AG50_S315_M1_Map7d2	6.2849	5.8497	6.5390	6.3270	3.7299	4.6984	5.0011	4.4790	3.2384	6.7537	5.4040	5.6584	5.1384	5.4620	3.3436	5.4315	5.2266	4.8774	5.3677	5.4432
Map7d2	_EGAPGQQSTVLPREES[Phospho (STY)]LEKR_.3	#N/A	3.3373	3.9818	4.3747	4.2469	3.5442	3.0633	3.2010	3.5630	3.2601	7.9772	3.6345	3.7079	8.8031	4.4128	4.2791	3.8464	4.5610	4.0892	4.3883	4.0901
Map7d2	_SSYKSS[Phospho (STY)]PTR_.2	A2AG50_S264_M1_Map7d2	3.4830	3.9633	4.3932	4.2285	6.2949	3.0448	5.3503	5.9477	3.2786	6.6080	5.5922	6.0678	6.6281	4.4313	2.7967	3.8279	3.0787	4.0708	4.3698	4.1085
Map7d2	_S[Phospho (STY)]NSLDDSTEDVQSM[Oxidation (M)]DVSPVSK_.2	#N/A	7.7017	7.3468	7.5548	7.2154	4.2161	6.8213	6.5775	4.5476	4.4033	5.2642	3.9955	7.0669	4.1494	7.2736	6.1991	7.1436	5.2909	3.2481	7.1116	7.5173
Map7d2	_NIEGFNS[Phospho (STY)]PGQETTLNTFC[Carbamidomethyl (C)]_.2	#N/A	5.1418	5.1270	4.3744	5.1084	3.1778	4.4488	5.3257	4.9744	3.6834	5.0009	4.7792	4.8363	4.9687	5.1726	3.8517	4.3149	5.3277	4.8454	3.8293	4.0962
Map7d2	_TAKPTYLGS[Phospho (STY)]PVK_.2	A2AG50_S357_M1_Map7d2	10.1373	9.9415	10.1698	10.2593	9.5757	8.8552	9.4548	9.3037	9.0309	10.3251	9.6379	9.6652	9.7648	10.2519	8.3457	9.5338	9.6272	9.4714	9.2732	9.6186
Map7d2	_HLSSSTVAISYS[Phospho (STY)]PDR_.2	A2AG50_S248_M1_Map7d2	3.4573	6.5371	3.9936	4.2321	4.2959	3.6891	3.2452	3.1577	3.3126	3.6636	4.2921	2.9341	3.4780	3.9807	2.8224	3.8022	3.6954	6.3876	3.7864	3.9477
Bclaf3	_SYVNRS[Phospho (STY)]PS[Phospho (STY)]PNVKPVEK_.3	#N/A	6.6045	7.3816	7.4986	6.9943	6.7796	7.0946	6.5704	6.9207	6.9390	7.4226	6.8173	7.1007	6.9599	7.6778	6.6115	7.0315	6.9561	7.0230	6.9493	7.2032
Med12	_EVKPPAKEK_.2	#N/A	10.9778	11.7497	11.2095	11.9841	10.1777	11.6609	10.8857	8.5739	11.8370	11.9783	11.8141	11.4788	11.9209	11.8250	12.0274	11.8257	11.0835	11.0822	12.3209	11.8507
Ambra1	_TSS[Phospho (STY)]PQPSTSR_.2	A2AH22_S1206_M1_Ambra1	7.4308	7.9045	7.1540	3.7643	7.6109	7.5168	7.3233	4.0456	3.7427	7.7288	7.1662	7.3245	3.8508	3.3945	6.0854	3.1820	3.5428	7.4284	3.9057	6.8638
Camsap1	_S[Phospho (STY)]PSDPGGELPEK_.2	A2AHC3_S1133_M1_Camsap1	3.9148	4.1706	4.4511	4.2049	3.2525	3.2803	4.1650	3.4796	4.1668	4.1361	3.3138	3.9817	4.2981	4.3139	3.9493	4.2597	4.2291	4.0698	3.2361	7.7308
Camsap1	_VES[Phospho (STY)]LEALPILSR_.2	A2AHC3_S1407_M1_Camsap1	5.8478	6.9905	3.2292	6.4779	3.5315	4.4534	6.9334	3.8941	5.7912	6.7430	3.5277	3.6985	4.2145	6.9817	3.5868	5.8623	5.7318	6.7252	6.1527	6.2169
Camsap1	_TDVS[Phospho (STY)]PPSPQMPR_.2	A2AHC3_S550_M1_Camsap1	3.8887	3.8783	7.7851	7.9077	3.7777	5.7946	7.2565	3.8707	6.0369	4.6593	6.4393	3.6430	3.6380	7.7031	3.4243	3.5253	3.6826	3.9993	3.8770	3.7637
Camsap1	_SVHREES[Phospho (STY)]YSDSGTK_.3	A2AHC3_S1359_M1_Camsap1	4.4607	4.0565	4.4826	3.7431	3.1668	3.2001	3.8813	3.6466	3.8015	4.2713	3.2802	3.9460	3.9670	4.4696	3.9067	3.8228	4.1843	3.6488	3.2974	4.4366
Lzts3	_LERIES[Phospho (STY)]TEI_.2	A2AHG0_S697_M1_Lzts3	4.1717	3.1474	3.7083	3.4125	2.8778	3.7297	4.0354	4.3974	4.0945	4.3742	5.4311	4.3743	4.2026	6.9203	3.6126	6.6880	3.8946	6.2654	6.8824	3.4236
Ano3	_VHHSGS[Phospho (STY)]IQS[Phospho (STY)]FKQQK_.3	A2AHL1_S10_M2_Ano3	3.5696	4.4332	4.1059	4.1198	2.9073	3.1314	3.1329	3.2700	3.1920	4.2325	4.3685	4.3227	5.0130	4.0930	4.2110	3.9145	3.8076	4.0255	4.0023	4.0220
Ano3	_S[Phospho (STY)]LPC[Carbamidomethyl (C)]LAQSYAHSK_.2	#N/A	9.2769	9.2747	8.5372	8.6803	8.0918	8.2716	8.7775	9.0308	8.3002	8.5952	8.4752	8.5815	8.9266	8.6046	6.7213	7.9036	8.9071	8.6742	8.4646	8.1506
Ano3	_VHHSGS[Phospho (STY)]IQSFK_.3	A2AHL1_S7_M1_Ano3	4.4969	4.3230	4.0335	3.7527	3.2030	3.4045	4.3606	3.2217	4.4197	4.0491	3.2932	4.0492	4.5278	4.0715	3.9378	3.8590	4.2198	4.4305	3.2287	3.7488
Virma	_SFLSEPSS[Phospho (STY)]PGR_.2	A2AIV2_S1578_M1_Virma	8.9089	8.7722	8.8213	8.5725	8.1256	3.4431	8.3861	8.8252	4.3818	8.7069	8.5978	4.0195	9.0951	8.2650	7.2604	7.9960	8.9798	8.4236	8.3261	8.1154
Virma	_HADGEKEDQFNGS[Phospho (STY)]PPRPQPR_.4	A2AIV2_S173_M1_Virma	3.9899	3.3292	3.5264	3.5944	4.1968	6.2391	4.3521	4.2155	6.1855	3.8005	3.7510	3.0553	4.4378	3.5645	5.4905	3.3520	3.7128	5.7431	3.7357	3.2418
Pnpla7	_SC[Carbamidomethyl (C)]S[Phospho (STY)]VPLPSNHGEVDELR_.3	A2AJ88_S379_M1_Pnpla7	3.3267	3.9924	4.3641	4.2575	3.5337	3.0739	3.5144	3.5524	3.2495	3.7184	3.6239	3.7185	3.3576	4.4022	3.5398	4.1897	4.5505	5.8029	4.3989	4.0795
Ajm1	_S[Phospho (STY)]PAQAPEPAAEGLGR_.2	A2AJA9_S593_M1_Ajm1	10.2080	9.9574	10.2940	10.3393	9.8339	9.9923	9.5852	10.0269	9.9129	10.0182	10.0236	9.5877	9.9652	10.1084	8.9678	9.6698	10.2135	9.7681	9.8626	9.8765
Ajm1	_PYLSEEPPRPS[Phospho (STY)]PR_.2	#N/A	8.2753	8.0120	8.3862	8.2254	8.0865	8.1051	7.8189	8.1666	7.8707	8.4082	8.4643	7.8398	8.2041	7.8477	7.1271	8.1302	7.8778	7.2224	7.9918	7.9408
Ajm1	_ELATHAAPLGS[Phospho (STY)]YAR_.3	#N/A	6.2262	6.3811	3.5311	7.3896	7.0392	7.0638	6.0920	6.4242	5.8458	7.1357	7.3553	7.1531	7.6817	3.2602	6.4716	7.2657	7.5195	7.4291	6.7064	6.9922
Ajm1	_GRS[Phospho (STY)]YENLLGR_.2	#N/A	7.9951	7.7837	4.5559	7.6150	7.2631	7.2118	7.0452	7.4031	4.2428	4.2260	7.2173	7.2015	7.9122	7.4473	6.1109	7.1472	7.7791	7.0614	7.3853	6.6839
Ajm1	_S[Phospho (STY)]WDNILAPGPR_.2	A2AJA9_S448_M1_Ajm1	10.2387	10.4635	10.0371	10.2339	3.8353	10.0845	10.2194	5.9576	9.9820	10.5931	7.6168	9.6928	9.0626	10.6261	9.0379	9.8441	10.0118	10.3246	9.9327	9.9807
Ajm1	_S[Phospho (STY)]YENLLGR_.2	#N/A	9.0709	9.1994	8.9711	9.3363	4.1790	8.7396	8.6424	8.0735	8.7316	9.0777	8.8599	8.5676	8.7947	9.2131	7.7805	8.6624	8.9948	8.8493	8.6963	8.5816
Ajm1	_ASQGLATAPAS[Phospho (STY)]PPVLQR_.2	A2AJA9_S109_M1_Ajm1	10.4998	10.5540	9.7069	9.0269	7.9849	8.7212	10.4991	11.5798	8.7787	9.5433	8.8798	10.4566	10.7692	11.4913	8.1039	8.7158	10.4034	10.3340	9.7561	8.7422
Ajm1	_YAALSLSETSLT[Phospho (STY)]EK_.2	A2AJA9_T514_M1_Ajm1	5.2506	5.8923	5.1895	3.8137	3.6290	5.4089	5.6625	3.8605	5.7359	5.2596	3.4057	4.4505	3.9607	4.3360	4.5778	3.9604	3.9848	5.7613	4.5024	5.6841
Ajm1	_S[Phospho (STY)]SPPWADWGPR_.2	A2AJA9_S397_M1_Ajm1	3.6976	8.1915	4.4419	3.8057	3.4034	3.2627	7.8282	3.6060	3.9137	4.3942	3.0570	4.0086	3.9263	8.3134	3.6926	4.2465	4.1437	8.0990	3.3600	3.7453
Ajm1	_RYAALSLSET[Phospho (STY)]S[Phospho (STY)]LTEK_.2	#N/A	4.2313	3.4508	3.5030	3.4288	4.0065	4.1234	4.0828	4.1804	4.0608	3.5152	3.9924	3.2684	4.2020	3.5375	3.1286	3.3204	3.4744	3.4652	3.7643	3.2148
Ajm1	_VALAAGS[Phospho (STY)]PTRPPPAR_.2	A2AJA9_S854_M1_Ajm1	4.5262	4.1221	4.4170	6.8914	3.2323	3.2657	3.8158	10.3644	3.7360	4.3368	3.2146	7.5653	8.8143	10.2166	3.8411	3.8883	8.6557	3.7144	3.3630	4.3711
Ajm1	VALS[Phospho (STY)]RT[Phospho (STY)]PT[Phospho (STY)]PSDLYC[Carbamidomethyl (C)]TDPR	A2AJA9_S236_M3_Ajm1	3.1114	3.5917	3.2640	3.8568	3.9499	4.1740	3.5911	3.9531	3.6502	3.3177	4.0246	3.2800	3.7583	3.3020	3.1683	3.4563	3.4503	3.6991	3.9982	2.9793
Map7d1	_RSS[Phospho (STY)]QPS[Phospho (STY)]PTTVPASDSPPAK_.2	#N/A	8.4178	8.6888	4.0953	4.1947	8.6047	8.8598	8.2166	8.8482	6.5316	9.7074	8.7442	9.0621	6.5695	7.0146	8.2488	9.2748	8.4858	9.4119	8.2240	9.4245
Map7d1	_SSQPS[Phospho (STY)]PTTVPASDSPPAK_.2	A2AJI0_S118_M1_Map7d1	8.4768	8.5522	3.6968	7.7794	6.0956	3.8731	7.4366	7.7806	3.5026	7.9639	7.7505	7.7488	8.1514	8.1830	8.2754	7.6740	8.2182	8.2229	3.7645	6.7968
Map7d1	_SSATLWNS[Phospho (STY)]PSR_.2	A2AJI0_S282_M1_Map7d1	3.7542	7.1919	8.3974	4.1742	7.0305	6.4281	7.4655	3.9462	3.5745	8.3262	3.6116	7.0713	7.7488	6.7622	6.9379	7.9278	7.9505	7.1156	7.5862	7.8786
Map7d1	_RKPGAGGS[Phospho (STY)]PALAR_.3	#N/A	8.2388	8.9287	7.6154	8.1545	7.2551	7.5596	8.6412	8.5336	7.6040	8.7925	7.9309	8.2943	8.6772	8.6740	7.5502	7.9705	7.8160	8.5546	7.9607	7.6934
Map7d1	_KPGAGGS[Phospho (STY)]PALAR_.2	#N/A	8.1234	8.3396	8.1969	8.1160	7.5777	7.8515	7.7764	8.0848	7.6581	8.1703	7.4064	7.8871	7.9494	8.1259	7.2132	7.8664	8.3073	7.9643	7.2680	7.8475
Map7d1	_SAS[Phospho (STY)]ASPLTPC[Carbamidomethyl (C)]SAPR_.2	A2AJI0_S368_M1_Map7d1	7.8216	7.5397	5.4689	7.7281	7.4878	7.1626	7.0633	7.2210	7.1128	7.7614	7.2405	7.0009	7.9871	7.5001	6.4262	7.4743	7.5177	6.9838	6.9639	7.0437
Map7d1	_S[Phospho (STY)]RGPT[Phospho (STY)]PTATGPR_.2	A2AJI0_S95_M2_Map7d1	7.7721	7.6082	8.0564	8.3635	3.3584	7.3443	6.2106	7.1825	7.2959	8.1500	7.3881	7.2156	7.0878	4.3486	6.2286	7.4516	7.2548	7.1663	7.2193	7.5529
Map7d1	_AAEEKEPAAPAS[Phospho (STY)]PAPSPVPSPTPAQPQK_.3	A2AJI0_S544_M1_Map7d1	6.2140	6.4244	4.9058	5.4315	3.2525	5.7746	3.8414	3.9296	4.9798	5.6087	3.8060	5.9400	3.9408	6.4835	4.8371	4.8907	4.4540	5.4424	6.0565	5.0660
Map7d1	_GPT[Phospho (STY)]PTATGPR_.2	#N/A	3.7629	6.7992	4.2992	3.9265	3.1006	6.7622	6.7873	3.4632	3.6181	4.4547	3.0968	4.1294	3.7836	4.2862	3.7233	4.1078	4.0009	3.8322	3.4808	4.2532
Map7d1	_LSTGSELS[Phospho (STY)]PK_.2	A2AJI0_S460_M1_Map7d1	4.0175	3.3016	3.5541	3.5667	4.2245	4.2578	4.3245	4.2432	4.2447	3.8281	3.7233	3.0277	4.9478	3.5922	2.8490	3.3797	3.7404	3.2057	4.3551	3.3789
Pnisr	_QRS[Phospho (STY)]PIALPVK_.2	A2AJT4_S211_M1_Pnisr	5.8496	6.0869	6.0583	5.8810	5.1755	5.8046	5.8394	6.5803	5.0839	5.8651	5.6280	4.9723	5.3550	6.3403	3.9651	5.0721	5.9842	5.6952	4.7429	4.9358
Pnisr	_SNRSS[Phospho (STY)]IER_.2	A2AJT4_S601_M1_Pnisr	4.0018	5.1736	5.5212	3.6876	5.3539	3.1445	5.8886	3.7022	5.0098	5.1046	5.0836	3.8904	5.4984	4.5252	3.7787	4.3467	4.2399	5.6515	5.1785	4.2896
Ptpn3	_SSSSVSPSSNAPGS[Phospho (STY)]C[Carbamidomethyl (C)]SPDGVDQR_.2	A2ALK8_S467_M1_Ptpn3	4.0234	3.9271	4.4312	4.1923	3.6007	3.6532	3.4492	3.6195	3.3940	3.2044	3.3810	3.6533	3.5316	4.4693	3.4746	4.2567	3.1167	4.0346	4.3336	4.1466
Ptpn3	_S[Phospho (STY)]LS[Phospho (STY)]VERLETK_.2	#N/A	3.3713	5.8581	5.8333	4.2946	3.9210	5.9425	5.1684	3.6801	4.8645	3.5923	3.7516	4.1232	3.4853	5.3850	4.9818	6.0335	3.1773	4.2372	5.6765	4.2072
Ptpn3	_RS[Phospho (STY)]LS[Phospho (STY)]VER_.2	#N/A	3.8187	3.4145	5.7119	3.1011	4.3812	5.1762	7.1872	4.5856	5.4956	3.6293	5.4121	4.3814	3.1081	5.4749	4.4885	3.1808	5.5716	3.0068	5.5241	3.5778
Ptpn3	_RS[Phospho (STY)]LS[Phospho (STY)]VERLETK_.2	#N/A	3.9059	3.6685	3.7705	4.3234	4.0588	3.5762	3.5399	3.9018	3.5990	3.4265	3.9733	3.9069	3.7070	3.7687	3.6851	3.5651	4.3715	3.8080	4.1070	3.6463
Rap1gap	_RES[Phospho (STY)]PPAGQK_.2	#N/A	9.9328	9.9309	9.8909	9.7488	9.5761	9.5961	9.6648	10.1390	9.7359	9.7455	10.1122	10.0074	10.1182	10.4890	8.5755	9.4801	10.2639	10.0604	9.2313	10.0586
Rap1gap	_DDVPFFGPPLPDPAVFR_.2	#N/A	4.1313	4.6424	3.8144	5.5989	5.3536	5.0234	5.6410	5.2234	5.0114	5.0794	5.9173	4.8792	4.1070	5.6419	5.2539	5.4582	5.4621	5.4232	5.5389	5.5839
Rap1gap	_RSS[Phospho (STY)]AIGIENIQEVQEK_.2	#N/A	3.8335	6.0627	3.7986	6.0608	3.6581	5.1844	5.4302	4.4753	3.6662	5.4864	3.8614	3.8357	3.8316	5.4086	4.8874	3.6155	3.7561	5.7741	5.7770	3.6416
Rap1gap	_TEVLQGFS[Phospho (STY)]R_.2	A2ALS5_S566_M1_Rap1gap	6.8518	6.8441	7.3064	7.0714	3.8687	6.4949	6.1897	4.2314	6.6175	3.6865	6.8984	3.3612	4.5517	7.0646	5.6255	7.0277	7.2955	6.3919	7.1781	6.9387
Rap1gap	_SENSSTQS[Phospho (STY)]S[Phospho (STY)]PEM[Oxidation (M)]PTTK_.2	#N/A	10.4980	10.9964	11.3830	11.2538	10.4326	10.4389	10.5255	10.5172	10.5413	10.3911	10.8562	10.7310	10.7253	12.0610	10.5995	10.5928	11.0297	10.6233	10.6306	10.8694
Rap1gap	_S[Phospho (STY)]SAIGIENIQEVQEK_.2	A2ALS5_S498_M1_Rap1gap	3.1525	3.3494	3.6888	4.5369	3.9911	4.2152	3.5500	3.9119	3.6091	3.3589	3.9834	3.2389	3.7171	3.2609	3.1272	3.4974	3.4092	3.7403	4.0393	4.4390
Rap1gap	_LPYTEGDAQQLQR_.2	#N/A	3.5085	4.2444	4.5460	4.0757	3.7155	3.8504	3.8293	4.0996	3.8518	4.0638	3.8356	4.0435	3.4183	4.5782	3.2288	4.3255	3.2315	4.3809	3.3508	4.3275
Rap1gap	_SSAIGIENIQEVQEKRES[Phospho (STY)]PPAGQK_.3	#N/A	7.5591	8.5147	6.8237	7.2789	6.4935	6.8324	7.6448	7.8286	7.2095	7.7352	6.4067	8.0145	9.1218	8.7764	7.5233	7.7757	8.0317	8.4828	7.8100	7.4938
Rap1gap	_TPDS[Phospho (STY)]GHVSQEPK_.2	A2ALS5_S525_M1_Rap1gap	3.4754	3.8437	5.3724	4.1088	5.1049	5.5614	3.3657	3.7011	5.7742	6.1852	4.2654	5.8053	3.5063	4.9866	3.3911	4.3384	3.1983	3.9511	4.2501	4.2282
Rap1gap	_SQS[Phospho (STY)]M[Oxidation (M)]DAM[Oxidation (M)]GLSNK_.2	#N/A	7.0881	6.3352	8.2027	6.5903	6.5465	7.2671	7.0418	6.7010	6.7235	6.9771	6.7027	6.8425	6.7642	7.6595	3.1870	6.9325	6.6219	6.5383	6.9497	6.7943
Rap1gap	_RES[Phospho (STY)]PPAGQKT[Phospho (STY)]PDSGHVSQEPK_.3	A2ALS5_S515_M2_Rap1gap	5.6294	5.9987	5.9205	4.5848	4.9879	4.1673	4.9299	4.3058	4.2514	5.7159	4.9910	4.2910	3.7650	3.6280	4.4036	3.4495	3.3427	5.3621	4.1391	3.5950
Shroom2	_DSC[Carbamidomethyl (C)]SS[Phospho (STY)]PPSLNYGK_.2	A2ALU4_S1165_M1_Shroom2	13.3509	13.3870	13.2385	13.3920	13.2416	13.2658	12.9797	13.4047	13.0585	13.3268	13.3082	13.3565	13.8651	14.0161	13.2100	13.3666	13.7729	13.2772	13.3341	13.4417
Shroom2	_IVHSES[Phospho (STY)]QPEKESR_.2	#N/A	6.2322	6.5983	5.5750	3.8227	5.8154	4.5099	5.3664	5.6392	4.5310	7.3937	5.3250	5.4330	5.9513	5.9007	4.1605	6.0979	5.4644	6.0534	5.6891	5.9865
Shroom2	_VENALNNLDDNPSPGDR_.2	#N/A	6.8268	7.4175	6.7432	7.2843	6.6820	7.0173	6.7745	7.0114	6.9621	7.2945	7.0123	7.0560	7.3869	7.7605	6.9108	7.1670	7.7008	6.9372	7.1441	7.4237
Shroom2	_SPS[Phospho (STY)]PQFAPQK_.2	A2ALU4_S1043_M1_Shroom2	10.7451	3.8594	11.1527	11.0527	10.5653	10.5898	10.4155	10.7283	10.4578	11.0681	10.7037	10.8571	11.0674	11.3907	10.4459	10.9138	10.8118	10.5654	10.7144	10.9223
Ubr4	_HVTLPSS[Phospho (STY)]PR_.2	A2AN08_S2716_M1_Ubr4	11.4449	11.3547	10.9179	11.0876	10.9089	11.1821	11.2220	11.4068	11.0898	10.8288	10.9185	10.8637	11.3846	3.5896	9.5034	10.5822	11.3629	11.2128	10.9737	10.7022
Ubr4	_TKEGVGS[Phospho (STY)]PK_.2	A2AN08_S457_M1_Ubr4	7.1540	6.8785	6.9643	3.4571	6.6018	6.6241	3.9908	7.0018	6.3754	6.8694	6.4829	6.5141	6.8892	5.8975	4.4236	5.7913	7.0167	6.7625	6.1187	6.0383
Ubr4	_TLSDVEDQKELAS[Phospho (STY)]PVSPELR_.3	#N/A	6.3170	6.8527	6.2248	6.1077	4.8439	6.2357	6.2755	5.7027	6.4102	6.1079	6.0780	5.8463	6.4903	5.9679	4.7940	5.4000	6.6037	6.8775	5.8399	5.8830
Ubr4	_T[Phospho (STY)]GSTSSKEDDYESDAATIVQK_.3	A2AN08_T360_M1_Ubr4	5.1410	3.8237	4.5328	4.0889	4.1820	4.4061	3.3591	3.7210	3.4182	3.5498	3.7925	3.5499	3.5262	4.5708	2.9363	3.6883	3.2183	3.9312	4.2302	4.2481
Plch2	_GTKPGS[Phospho (STY)]LDSHAAGQPLPR_.3	A2AP18_S987_M1_Plch2	6.9743	7.1451	6.3037	6.0987	6.1848	6.7610	6.6325	6.4296	5.9721	6.7199	6.3363	6.2721	6.5911	7.0346	5.2400	6.0471	6.1661	6.3742	6.1305	5.6854
Plch2	_VAS[Phospho (STY)]VEEGDETLDSPGSQSR_.2	#N/A	10.1644	10.2272	10.0080	10.1384	8.5202	9.8652	9.7853	9.7284	9.5848	10.1018	9.1094	9.6439	9.9499	9.9362	8.5063	9.4580	9.9865	9.6380	9.4814	9.4429
Plch2	_KVAS[Phospho (STY)]VEEGDETLDSPGSQSR_.3	#N/A	6.5968	7.8917	6.7999	7.9046	5.7285	7.4065	6.7926	6.1489	7.2770	7.8547	6.3102	6.3589	6.8376	7.3604	5.4943	7.2512	6.2678	7.2424	7.2861	7.3351
Plch2	_IRDC[Carbamidomethyl (C)]EDPNDFSVSTLS[Phospho (STY)]PSGK_.3	A2AP18_S619_M1_Plch2	3.9519	3.3671	3.4885	3.6323	4.1589	4.1922	4.3901	4.1776	4.3103	3.7625	3.7889	3.0933	4.4757	3.4775	2.9146	3.3141	3.6748	3.1401	4.2895	3.4445
Plch2	SEDAEEGEVS[Phospho (STY)]DEDS[Phospho (STY)]ADEM[Oxidation (M)]EDDC[Carbamidometh	A2AP18_S561_M2_Plch2	3.1000	3.5803	3.2754	3.8454	3.9385	4.1626	3.6025	3.9645	3.6616	3.3063	4.0360	3.3064	3.7697	3.3134	3.1797	3.4449	3.4617	3.6877	3.9868	2.9907
Fmnl2	_S[Phospho (STY)]IEDLHR_.2	A2APV2_S171_M1_Fmnl2	11.7366	11.7701	11.2107	11.4997	11.1825	11.4481	11.5580	11.6340	11.1382	11.5876	11.2913	11.1179	11.7389	11.2720	9.7226	11.0278	11.4328	11.3794	11.2371	11.0851
Fmnl2	_LLEQEALM[Oxidation (M)]EQQDAKS[Phospho (STY)]PSHK_.3	#N/A	6.6644	6.4642	6.5156	6.2509	3.5900	5.5369	5.9467	5.9387	3.9732	6.3313	3.2705	5.6426	6.4886	5.2663	3.9017	3.6154	4.1684	5.5045	3.3336	5.6661
Scn1a	_QAS[Phospho (STY)]FTYNK_.2	A2APX8_S1939_M1_Scn1a	6.5897	5.5995	5.9615	6.4273	5.1314	3.5442	3.5372	3.3025	4.9280	3.1146	5.3838	5.4982	3.6228	4.1255	3.1533	5.4562	5.1206	5.6755	5.1573	5.7955
Scn1a	_FS[Phospho (STY)]IEGNR_.2	A2APX8_S537_M1_Scn1a	5.1468	5.1579	6.1334	5.3966	4.1448	3.3744	4.5928	3.4723	4.3366	5.5548	4.6056	4.6616	3.7927	3.8375	3.7324	4.3829	4.5023	4.6899	4.4563	2.8954
Scn1a	_NSNLS[Phospho (STY)]QTSR_.2	A2APX8_S623_M1_Scn1a	4.0549	4.5353	3.8213	3.2996	3.3927	4.7567	4.1484	4.5104	4.2075	4.2613	3.0810	4.2614	4.3155	3.8593	3.7256	4.3998	5.5263	3.1419	3.4409	3.5366
Scn1a	_RNS[Phospho (STY)]NLSQTSR_.2	A2APX8_S620_M1_Scn1a	4.1665	4.0916	4.2649	4.3567	4.2097	4.2279	3.6137	3.7586	4.5588	4.5389	3.0125	3.8177	3.6993	4.3030	3.6390	4.0905	4.4512	3.9165	3.5651	3.8591
Scn1a	_RDS[Phospho (STY)]LFVPR_.2	A2APX8_S607_M1_Scn1a	4.2632	5.1349	3.6130	4.3798	3.6009	5.0303	3.9402	4.3021	4.5665	4.4695	4.8594	4.7694	4.1073	3.6511	3.6932	3.2995	4.2899	3.3501	3.6491	3.3284
Scn1a	_QQEAAQQAAATTAS[Phospho (STY)]EHSR_.3	A2APX8_S467_M1_Scn1a	3.9025	4.3828	3.9737	3.1471	3.2402	3.4643	4.3008	3.1620	4.3599	4.1088	3.2335	4.1089	4.4680	4.0118	3.8780	4.2474	4.1600	2.9894	3.2885	3.6890
Scn1a	_RYS[Phospho (STY)]S[Phospho (STY)]PHQSLLSIR_.3	A2APX8_S550_M2_Scn1a	4.4798	3.5230	3.5153	3.2096	3.8175	4.1674	4.4149	4.1802	4.3351	3.7377	3.8137	3.4124	4.5005	3.5023	2.9394	3.2892	3.2170	3.1153	4.2647	3.4693
Skt	_VSS[Phospho (STY)]EDPGPTPQTR_.2	A2AQ25_S1087_M1_Skt	10.8067	10.6063	11.1515	10.7989	10.0370	9.8973	10.1260	10.0947	9.9237	10.5963	9.9334	9.5789	10.0922	10.2011	9.4623	10.0274	9.8405	9.8090	10.2097	9.9826

Skt	_NRPGS[Phospho (STY)]LDK_.2	A2AQ25_S1762_M1_Skt	9.9956	9.9817	9.7425	9.7273	9.1782	8.8706	9.2284	9.0831	8.4905	10.3762	9.2177	9.2986	9.6139	9.9710	8.4112	9.2770	9.0560	9.4293	9.2734	9.0638
Skt	_IPALS[Phospho (STY)]PSSGK_.2	A2AQ25_S1808_M1_Skt	8.3177	8.0643	8.5472	8.5813	7.2462	6.8618	3.7200	6.8926	6.6447	8.8669	7.6502	7.8359	3.8866	3.6598	3.6034	7.4000	6.6795	7.2068	3.4544	7.6099
Skt	_NVY[Phospho (STY)]YELNDVR_.2	A2AQ25_Y244_M1_Skt	4.1898	7.2330	7.7615	7.4492	2.8959	3.7116	4.1522	4.4155	4.1126	4.0004	2.9862	7.4671	7.2826	3.7644	7.0733	7.4665	3.9127	3.2367	3.5358	7.8382
Skt	_IGGKS[Phospho (STY)]PPPPPPPPR_.3	#N/A	7.3424	6.5292	6.8794	6.8376	5.8144	6.5145	6.6740	6.9804	5.6168	6.4661	7.2386	7.0608	6.7406	8.2701	5.0039	5.6349	7.1510	7.3447	6.2807	6.3161
Skt	_KYPDSHLPTLGSKT[Phospho (STY)]PPAS[Phospho (STY)]PHR_.4	A2AQ25_S474_M2_Skt	4.3922	7.5219	3.9287	7.1627	3.0982	6.4222	6.7211	6.7768	6.2282	7.2256	6.3044	7.0012	6.6685	7.1984	6.6777	7.1422	6.2703	6.4306	6.8946	3.6441
Skt	_RQEQLS[Phospho (STY)]PEGTLPASR_.2	#N/A	4.6634	4.0318	4.5073	5.9109	5.0298	4.8957	5.6273	3.6714	4.0861	5.8825	5.4525	5.4639	6.7548	5.8261	5.7486	5.4998	4.7969	5.7348	5.1872	6.6403
Skt	_HQEETAHAPGQPLHC[Carbamidomethyl (C)]STGS[Phospho (STY)]PGDVK_.4	#N/A	3.6929	3.7573	3.9533	4.0224	3.5774	4.6744	3.0918	3.8355	3.9436	3.6921	7.6316	6.9251	4.0916	7.2428	3.7709	6.6832	4.1396	3.8647	4.1638	3.6687
Skt	_KAGGDC[Carbamidomethyl (C)]KPTS[Phospho (STY)]PSLPASK_.3	A2AQ25_S1796_M1_Skt	4.4562	4.7309	3.9928	3.1280	3.1623	3.1956	3.8858	3.1810	3.8060	4.2668	3.2847	4.0899	4.4871	4.0308	3.9112	3.8183	4.1791	2.9703	3.2694	3.7081
Skt	_AAPTTSSSSSPPS[Phospho (STY)]PAS[Phospho (STY)]PTSLNQGAR_.3	A2AQ25_S1902_M2_Skt	4.3804	4.4395	3.9170	3.2038	3.0865	3.1199	3.9616	3.1053	3.8818	4.1910	3.3604	6.2663	4.0472	3.9551	5.4614	3.7425	4.1033	3.0461	6.7720	3.6324
Skt	_T[Phospho (STY)]PPASPHR_.2	A2AQ25_T470_M1_Skt	4.2129	3.8088	6.3768	3.4953	5.7505	5.4129	6.7288	5.8748	5.4499	6.1789	6.7799	4.8085	4.2148	6.8772	4.1545	6.3530	7.0865	3.4011	3.0496	3.1836
Skt	_SIS[Phospho (STY)]PSPSAILER_.2	A2AQ25_S361_M1_Skt	9.9568	9.7020	9.3534	8.3373	3.9379	8.7694	8.9947	9.3997	3.0304	9.7833	9.0040	9.3908	9.3464	3.6985	8.2129	3.0931	9.3640	8.9732	4.0686	9.0927
Skt	_S[Phospho (STY)]PPPPPPPPR_.2	#N/A	6.4267	6.0369	4.0300	6.7659	5.9410	5.8652	5.8931	5.8233	5.5505	6.8140	6.4551	6.0825	4.2578	6.7367	5.8631	6.3832	6.0763	5.8837	5.5359	3.4789
Skt	_FQS[Phospho (STY)]PPHAGK_.2	A2AQ25_S1868_M1_Skt	4.2142	3.7886	3.2496	3.4752	3.5519	6.2718	3.9892	3.9145	4.0694	4.4205	3.5481	3.6781	4.2349	6.2308	6.7194	3.0582	4.4522	3.3809	3.0295	3.2037
Skt	_GDAAGDKLELSEDS[Phospho (STY)]PNS[Phospho (STY)]EQELDK_.3	A2AQ25_S1035_M2_Skt	3.0855	3.4164	3.6218	3.8310	3.9241	4.1482	3.6170	3.9789	3.6761	3.2918	4.0505	3.3059	3.7841	3.3279	3.4228	3.4304	3.4762	3.6733	3.9723	3.0052
Skt	_NEGFYADPYLYHEGR_.3	#N/A	4.3092	3.6936	3.3447	3.3802	3.6469	4.3380	3.8942	4.7281	4.1645	3.9083	3.6431	3.5831	4.3299	3.3317	3.4714	3.1533	4.5473	3.2859	4.4353	3.2987
Ppig	_ADREQS[Phospho (STY)]PVSK_.2	#N/A	4.5156	6.2297	3.5510	3.1738	3.8533	4.1317	3.6878	4.2159	4.3708	3.7020	3.8495	3.3767	6.0881	3.5381	3.2650	3.3596	3.2528	3.0795	4.2290	3.5051
Ppig	_FRRS[Phospho (STY)]ET[Phospho (STY)]PPHWR_.3	#N/A	11.1015	11.2109	10.8898	10.9807	9.7671	10.9742	10.9319	10.9641	10.8864	10.8456	10.5099	10.6495	10.8615	10.8675	9.9738	10.5815	11.3683	10.8309	10.7138	10.4759
Ppig	_S[Phospho (STY)]ET[Phospho (STY)]PPHWR_.2	#N/A	8.4760	6.5152	8.4316	8.4164	7.8422	8.7490	8.4279	8.0503	8.1248	8.6220	8.6709	7.3053	3.9072	8.0713	7.2168	8.1148	8.6403	7.9537	8.0936	8.5170
Ppig	_SELNEIKENQRS[Phospho (STY)]PVR_.3	#N/A	7.5562	7.7812	7.4604	7.3183	7.0095	7.5154	7.5479	7.3903	7.1745	7.5158	7.3060	7.0586	7.8121	7.7249	6.2224	6.8332	7.2568	7.4433	7.0070	7.0984
Ppig	_HM[Oxidation (M)]S[Phospho (STY)]ES[Phospho (STY)]PNRKVEK_.3	#N/A	4.0661	4.7418	4.0781	4.3594	3.1786	3.8711	4.3554	3.6605	4.2966	4.8840	4.1911	4.0140	4.3776	4.9112	4.7099	4.3423	3.8270	4.2207	3.6473	3.7591
Ppig	_HMS[Phospho (STY)]ES[Phospho (STY)]PNR_.2	#N/A	4.0692	4.8255	5.1221	6.2238	3.9349	4.6583	4.8091	6.6612	4.1867	4.0791	5.0432	3.7960	3.9287	5.3511	3.5546	3.5008	3.7336	3.6859	3.9672	4.1054
Ppig	_KADREQS[Phospho (STY)]PVSK_.2	#N/A	3.0965	3.4055	3.6328	4.5929	3.9350	5.2009	3.6060	4.2977	4.4526	3.3028	5.4030	6.0845	3.1172	5.1570	4.7961	3.4414	3.3345	6.3922	4.1472	5.8137
Ppig	_ENQRS[Phospho (STY)]PVR_.2	#N/A	5.9457	5.3277	4.8620	3.5292	4.2620	4.8623	4.7425	4.2807	3.9778	4.9057	5.1317	2.9902	4.0859	4.5064	2.8115	3.4171	4.8391	3.3715	3.6706	3.3069
Ppig	_RS[Phospho (STY)]ET[Phospho (STY)]PPHWR_.2	#N/A	9.9131	9.7004	10.0422	9.5974	9.2328	9.8566	9.5436	9.9115	9.6404	9.7227	9.5046	9.3516	9.7182	9.8089	8.6663	9.2695	9.8695	9.4996	9.7139	9.4279
Ppig	_S[Phospho (STY)]PPKADDKER_.2	A2AR02_S288_M1_Ppig	3.8256	6.5704	5.4861	5.2427	5.7462	5.9693	5.9936	6.0205	5.9522	5.7165	5.9013	5.5595	5.8641	6.0086	3.9549	4.1705	5.9216	5.5064	3.2116	5.0233
Ppig	_T[Phospho (STY)]RSPVEKENQK_.2	A2AR02_T712_M1_Ppig	2.6853	4.2471	4.2920	3.9337	3.0935	3.3906	3.6908	3.4561	3.6110	4.4618	3.0896	4.1365	3.7765	4.2791	3.7162	4.1006	3.9938	3.8394	3.4880	4.2461
Ppig	_EQS[Phospho (STY)]PVSK_.2	#N/A	4.3207	1.8257	3.3562	3.3687	3.6584	4.3265	3.8826	4.0211	4.1760	4.5270	3.6546	3.5715	4.3414	3.3432	3.4598	3.1648	4.5588	3.2744	4.4238	3.3102
Ppip5k1	_ALQTS[Phospho (STY)]PQPVEGTGLPR_.2	A2ARP1_S959_M1_Ppip5k1	9.1774	3.7588	4.5977	4.0240	7.9194	7.8246	8.1500	8.4080	8.0592	8.8310	8.1488	8.1128	8.7752	3.1349	6.1463	8.0858	8.3217	8.2762	7.8536	4.3130
Ppip5k1	_TDPGS[Phospho (STY)]IENLC[Carbamidomethyl (C)]PGK_.2	A2ARP1_S939_M1_Ppip5k1	8.9866	3.7584	4.5981	4.0236	3.7676	3.8009	3.2805	3.7863	3.2007	9.0144	8.7017	3.4845	3.5915	3.1353	3.3059	4.4236	3.2836	3.8659	4.1649	4.3134
Ppip5k1	_TLHS[Phospho (STY)]PPLQLR_.2	A2ARP1_S1147_M1_Ppip5k1	3.8829	3.4362	3.4195	3.7013	4.0899	7.3876	3.7466	4.1086	7.4756	3.1622	4.1801	3.1623	3.9138	9.5191	2.9836	3.2450	3.6058	3.5436	3.8427	3.1348
Ankrd63	_GSNSDS[Phospho (STY)]PPGHPAPAPS[Phospho (STY)]PERR_.3	#N/A	6.4833	7.3424	6.7603	6.3802	6.2513	6.4483	6.3607	6.1294	5.7184	7.1411	7.0430	7.0306	6.0299	7.2300	6.5483	7.3064	7.1216	7.2862	6.8377	6.8530
Ankrd63	_GSNSDS[Phospho (STY)]PPGHPAPAPSPER_.2	#N/A	8.7268	9.0564	7.0664	8.1725	8.5400	8.8980	8.7203	7.2154	8.8428	9.0695	9.6126	9.3791	10.0744	8.4462	9.1815	9.5798	9.5122	9.7098	9.6941	9.5703
Rabgap1	_KTTAS[Phospho (STY)]PSVR_.2	#N/A	4.2233	3.8192	3.2191	6.3767	5.8945	6.1009	6.7577	3.8840	4.0389	4.0339	3.5175	3.7086	7.0199	5.9256	4.1441	3.5854	4.4217	3.4115	5.7463	5.9949
Rabgap1	_GVSDEDT[Phospho (STY)]DEEKETLK_.2	A2AWA9_T991_M1_Rabgap1	4.0702	4.8357	4.1129	4.3371	4.3734	4.1353	3.6468	4.1925	3.7054	4.1608	4.0632	3.0164	4.2540	4.6421	4.0056	4.4035	4.7710	4.0085	3.7367	3.6224
Rabgap1	_QGDETPSTNNGS[Phospho (STY)]DDEKTGLK_.2	#N/A	4.0423	3.9353	4.1236	3.6907	3.3800	3.7441	4.6825	3.6845	4.3379	4.1426	3.6947	3.8426	4.4828	4.3652	3.8487	4.2198	4.6169	4.2347	3.4316	3.6296
Rabgap1	_TTAS[Phospho (STY)]PSVR_.2	#N/A	4.5804	4.2395	4.1170	4.5046	3.0969	3.3210	4.4442	3.3053	3.0024	3.9655	3.3768	3.9656	3.1105	4.1551	4.0214	4.1041	4.3034	4.3469	3.1451	3.8324
Atxn7l3	_LQWDGSSDLS[Phospho (STY)]PSDSGSSK_.2	A2AWT3_S281_M1_Atxn7l3	3.8004	3.6318	4.0714	3.8969	3.2409	5.4197	5.7995	4.0261	3.8085	5.5957	3.2872	4.0924	3.9740	6.2952	3.9137	5.7414	4.2577	3.7393	4.0383	3.7092
Arid1a	_PYVPC[Carbamidomethyl (C)]PTPPRK_.2	#N/A	11.7838	11.5666	10.8344	10.6846	11.5934	11.2051	11.0431	12.0033	10.9328	11.1916	10.4248	11.3359	11.5233	10.4786	11.0066	11.0356	11.3734	10.9618	10.8954	10.9059
Arid1a	_IELLPS[Phospho (STY)]R_.2	A2BH40_S1866_M1_Arid1a	10.3783	10.9165	10.7024	10.7730	9.9377	10.6632	10.2385	9.8136	10.3973	10.9881	10.9979	10.6269	11.0896	11.3273	10.6427	11.1178	10.8341	10.4835	10.9453	11.1793
Arid1a	_SNS[Phospho (STY)]VGIQDAFPDGSDPTFQK_.2	A2BH40_S1185_M1_Arid1a	3.1428	6.6868	6.8723	3.8882	3.9813	6.2044	6.2933	6.2803	3.6188	3.3491	3.9932	3.2486	3.7269	3.2706	3.1369	3.4877	6.7005	6.4379	4.0296	5.7448
Arid1a	_SHHAPM[Oxidation (M)]S[Phospho (STY)]PGSSGGGGQPLAR_.3	#N/A	5.8358	6.2249	4.7018	4.9129	4.9854	5.2791	5.7531	6.2481	5.2889	5.8830	4.6107	5.8763	3.8910	6.7967	4.8242	5.7378	5.7392	5.7749	5.7541	3.7343
Arid1a	_GPS[Phospho (STY)]PSPVGSPASVAQSR_.2	A2BH40_S697_M1_Arid1a	4.0048	6.7591	6.7848	7.0829	5.8871	7.0077	7.5000	7.2485	6.2165	7.6630	5.9699	6.7593	6.8930	4.1782	5.7521	6.2583	6.9437	6.6586	3.3354	7.1482
Arid1a	_NPQMPQYTSPQPGS[Phospho (STY)]ALSPR_.2	A2BH40_S770_M1_Arid1a	3.0848	4.2343	4.1222	3.8681	3.2918	3.3251	3.7563	3.5216	3.6765	4.3963	3.1552	3.9604	3.8420	6.2724	3.7817	3.9478	4.0593	3.7738	3.4224	4.3116
Kalrn	_SC[Carbamidomethyl (C)]S[Phospho (STY)]WHTLR_.2	A2CG49_S2374_M1_Kalrn	6.8273	4.2891	4.2500	3.9757	3.0514	3.4327	5.8258	3.4141	5.5610	4.5038	3.0476	5.7097	3.7344	4.2371	3.6741	4.3553	3.9517	3.8814	3.5300	4.2041
Kalrn	_ATAAAESSDGS[Phospho (STY)]IKK_.2	A2CG49_S2368_M1_Kalrn	3.3425	3.1536	4.4490	4.4156	4.1810	4.4051	2.0041	4.5437	3.5857	4.0564	3.6024	3.0489	3.5272	4.5718	2.9372	4.2437	4.0062	3.8859	1.0885	4.2491
Arid4b	_TVSKS[Phospho (STY)]PER_.2	A2CG63_S778_M1_Arid4b	3.1073	3.3946	3.6436	4.5820	3.9459	4.1700	3.5952	4.3086	3.6543	3.3137	4.0286	3.2841	3.7623	3.6307	3.1724	3.4522	3.3454	2.9869	4.1363	3.5977
Uhrf1bp1l	_SAS[Phospho (STY)]DTNLQK_.2	A2RSJ4_S952_M1_Uhrf1bp1l	3.7575	6.1980	4.2938	3.9318	3.0953	5.6169	6.0089	3.4579	3.6128	5.9319	6.1002	5.5342	5.9986	4.2809	3.7180	4.1024	3.9956	5.7800	3.4861	4.2479
Uhrf1bp1l	_SS[Phospho (STY)]PEISLR_.2	A2RSJ4_S1250_M1_Uhrf1bp1l	3.9641	3.3550	3.5007	3.6201	4.1710	5.3697	4.3779	4.1898	5.3331	4.5819	4.2613	3.0811	3.9950	3.5387	2.9025	3.3262	3.6870	5.4751	3.7615	3.2160
Uhrf1bp1l	_DVASTDS[Phospho (STY)]DSVLNYR_.2	A2RSJ4_S1006_M1_Uhrf1bp1l	3.8799	4.1230	4.4161	3.8095	3.2176	3.4417	4.3235	3.5802	4.3826	4.0862	3.2561	4.0124	4.4906	4.4032	3.9007	4.2247	4.1179	3.7153	3.3638	4.3702
Uhrf1bp1l	_SGS[Phospho (STY)]GEMTSEGSHTK_.2	A2RSJ4_S988_M1_Uhrf1bp1l	3.8567	4.6627	5.3339	5.4801	3.5169	4.6424	5.1170	3.8298	3.7163	3.8987	3.7267	3.7564	3.8823	5.7547	3.6438	3.8170	4.1686	3.6561	3.6644	4.2749
Uhrf1bp1l	_NLGS[Phospho (STY)]PPKSPT[Phospho (STY)]HASPQHTQTEK_.3	A2RSJ4_T420_M2_Uhrf1bp1l	4.1859	3.8634	3.1751	3.7023	3.2283	4.5040	4.0882	3.8400	3.9949	4.0782	3.4735	3.7529	4.1603	3.1622	3.9076	3.3235	4.3777	4.3761	4.8464	3.1292
Dennd5b	_EEKQEHLTDS[Phospho (STY)]PVALGPER_.3	#N/A	3.2861	3.2159	3.8224	4.4033	4.1246	3.8603	3.4164	4.4873	3.1413	3.4306	4.1208	5.0340	3.3068	3.8094	2.9936	3.6310	3.5241	4.3090	3.9576	3.7764
Dennd5b	_QEHLTDS[Phospho (STY)]PVALGPER_.2	#N/A	3.6481	3.5474	4.0779	5.6093	3.5725	3.6582	4.8423	3.6894	3.7480	4.9721	3.4827	4.6930	3.7832	4.0946	3.5804	3.8962	4.0786	4.9168	3.5034	4.0453
Dennd5b	_QC[Carbamidomethyl (C)]RT[Phospho (STY)]PPQQKS[Phospho (STY)]PTTTR_.3	A2RSQ0_S1068_M2_Dennd5b	4.9925	4.6427	4.1765	4.0492	3.6444	3.5062	3.2468	3.3406	3.4955	4.5774	2.9741	4.2521	3.6609	4.1636	3.6006	4.1289	3.8782	4.2316	3.9208	4.1305
Dennd5b	_LQSDVLATGPANNNR_.2	#N/A	3.9822	4.1938	4.5194	3.7063	3.3208	3.6471	4.2202	3.6835	4.2793	4.2216	3.1529	3.9091	4.3874	3.9510	3.7974	3.7342	4.9279	2.8061	4.2096	3.6084
Kansl3	_GSRPAS[Phospho (STY)]PAAR_.2	A2RSY1_S515_M1_Kansl3	4.1669	3.1521	3.7035	3.4173	2.8730	4.4072	4.1751	4.3926	4.0953	3.9775	3.5739	4.3791	4.1978	3.7416	4.2004	3.5291	3.8899	3.2596	3.5586	3.4189
Palm3	_TSGAKDDVS[Phospho (STY)]PEEQGK_.2	A2TJV2_S601_M1_Palm3	3.8255	3.4936	3.3621	3.7587	4.0324	4.0658	3.0157	4.2818	5.4417	3.6361	3.9153	3.2197	4.6021	6.2657	3.0410	3.1876	3.3186	5.4969	4.1631	6.1796
Palm3	_TGGEGS[Phospho (STY)]LDEEAEGSKK_.2	A2TJV2_S565_M1_Palm3	4.2652	3.7376	3.3007	3.4242	3.6029	3.8270	3.9381	4.3001	4.1693	4.4715	4.3716	3.6270	4.1053	3.6490	3.5153	3.1093	3.7973	3.3521	3.6512	3.3263
Palm3	_QGTSS[Phospho (STY)]PELPTWVK_.2	A2TJV2_S270_M1_Palm3	3.2746	8.4555	4.6016	4.0200	4.1131	4.3372	3.4280	3.7899	3.4870	3.4809	3.8614	3.1169	3.5951	3.1389	3.0052	3.6195	3.2871	3.8623	4.1614	4.3170
Ralgapa2	_RSS[Phospho (STY)]S[Phospho (STY)]PAELELKDDLQQAHGR_.4	A3KGS3_S819_M2_Ralgapa2	4.0240	3.6199	3.4184	3.3065	4.2310	4.2643	4.3180	4.0833	4.2382	3.8346	3.7168	3.0212	4.4036	4.9771	2.8425	3.3861	3.1201	3.2122	4.3616	3.3724
Exoc6b	_DVYTIFDAEVESTS[Phospho (STY)]PK_.2	A6H5Z3_S261_M1_Exoc6b	4.4670	4.0629	4.4762	3.7495	3.1731	3.2065	3.8750	3.6403	3.7952	4.2776	3.2738	3.9523	3.9606	4.4633	3.9003	3.8291	4.1780	3.6552	3.3038	4.4303
Phrf1	_EVS[Phospho (STY)]PAPATQGESR_.2	A6H619_S1201_M1_Phrf1	10.0542	9.9302	9.5467	9.7997	9.4925	9.7597	9.6484	9.8534	9.5021	9.6089	9.5068	9.2042	10.2017	10.0638	9.1302	9.2187	10.0777	9.6462	9.5898	9.4317
Phrf1	_S[Phospho (STY)]RS[Phospho (STY)]PS[Phospho (STY)]LEHRPR_.3	A6H619_S1169_M3_Phrf1	3.6307	3.6884	3.1673	3.9535	3.8376	4.2707	3.2105	3.8564	3.5535	3.4144	3.9279	3.4145	3.6616	3.2053	3.2358	2.9928	3.3536	3.7958	4.0949	4.3835
Armt1	_[Acetyl (Protein N-term)]AES[Phospho (STY)]PAFLSAK_.2	A6H630_S4_M1_Armt1	3.6851	3.2809	3.7573	3.8397	3.8920	3.9254	3.1561	4.4222	3.4282	3.4957	4.0558	3.4775	3.2417	3.7444	3.1815	3.0472	3.4591	4.1831	4.0227	4.8688
Kcnb2	_DSVDDNHLS[Phospho (STY)]PSR_.2	A6H8H5_S488_M1_Kcnb2	7.9677	8.1189	3.6789	7.2494	7.3172	7.5481	7.7324	7.3638	7.3902	3.5740	7.3852	7.2133	7.5827	3.6660	6.1606	3.1256	8.1697	7.6978	7.3450	7.0682
Kcnb2	_S[Phospho (STY)]M[Oxidation (M)]ELIDVAVEK_.2	#N/A	6.8606	7.0355	6.6034	6.3185	3.8159	6.3360	6.2199	4.1786	6.4247	7.0435	3.8121	5.7514	4.4989	6.0620	5.0606	6.0211	6.1773	6.6794	6.4294	6.3981
Arhgap31	_RNS[Phospho (STY)]APVSVSAVR_.2	A6X8Z5_S1163_M1_Arhgap31	3.9676	3.3515	3.5042	3.6166	4.1745	3.9338	3.8313	5.6876	3.8904	4.5783	4.2648	3.0776	3.9985	3.5423	2.8989	3.3297	3.6905	3.4589	3.7580	3.2195
Arhgap31	_S[Phospho (STY)]MDSLC[Carbamidomethyl (C)]SVPVEGK_.2	A6X8Z5_S343_M1_Arhgap31	4.2895	4.5304	3.8261	3.2947	2.9956	3.0290	4.0525	4.5152	3.9727	4.1001	3.4513	4.2565	4.1381	3.8642	4.0778	3.6516	4.0124	3.1370	3.4360	3.5415
Dpy19l1	_SS[Phospho (STY)]PPPLSGASEVDAGELGSER_.2	A6X919_S23_M1_Dpy19l1	3.4003	8.7918	3.9366	4.3488	3.6124	8.4242	8.4646	4.0765	3.3729	8.2723	8.2593	8.4848	3.3162	8.5792	3.7654	3.7452	4.1120	8.6839	8.3270	3.9201
Vwa5b1	_ETS[Phospho (STY)]LDLEPMAESEEQANPC[Carbamidomethyl (C)]R_.2	A9Z1V5_S782_M1_Vwa5b1	4.5301	3.5095	5.2589	3.7747	3.8678	4.0919	3.6733	4.0352	3.7324	3.2355	4.1068	3.3622	3.8404	3.3842	3.2505	3.3741	3.5325	3.6170	3.9160	3.0615
Ptprs	_SPQGLGAFTAVVR_.2	#N/A	3.8963	4.3527	3.2181	3.7931	3.9900	3.4727	3.8314	3.5967	3.7516	4.3212	3.2302	3.9959	3.9170	4.9418	4.2850	4.0417	4.1344	3.6988	3.3474	4.3867
Uprt	_QVNSTSSPS[Phospho (STY)]PEHLLAEDR_.3	B1AVZ0_S25_M1_Uprt	4.1294	4.1987	3.8019	3.1975	3.3990	3.5255	3.8822	3.8209	3.8430	3.9255	3.6549	3.9586	4.2646	4.7403	4.4273	3.6225	4.0124	3.3943	4.0733	3.2334
Scn2a	_SAS[Phospho (STY)]EDS[Phospho (STY)]IRKK_.2	#N/A	7.6566	7.6235	7.5364	7.5404	6.6678	7.1660	7.4183	7.3254	7.1496	7.4774	6.9850	7.2688	7.5648	6.9206	6.3225	7.2593	7.4943	7.7211	7.4360	6.8127
Scn2a	_KVSS[Phospho (STY)]IYKK_.2	B1AWN6_S1931_M1_Scn2a	6.5097	4.3673	3.9892	3.1316	3.1587	3.1920	3.8894	3.1774	3.8096	4.2632	3.2882	4.0934	3.9751	4.0272	3.9148	3.8147	4.1755	2.9739	3.2730	3.7045
Scn2a	_KSAS[Phospho (STY)]EDSIR_.2	#N/A	7.7662	7.4881	7.1803	6.1318	7.0495	8.0999	7.9539	7.9535	7.7557	6.7602	7.9327	7.8155	8.3764	5.9726	6.8501	7.3391	8.1010	8.0783	7.5351	7.3509
Scn2a	_FS[Phospho (STY)]LEGSR_.2	#N/A	8.1101	7.6395	7.3200	7.5268	6.4078	6.9778	7.3141	6.6778	6.8166	7.6700	7.5877	6.9202	7.0939	6.9703	2.7933	6.3947	7.2471	7.1338	6.9797	6.6918
Scn2a	_S[Phospho (STY)]AS[Phospho (STY)]EDSIRK_.2	#N/A	4.9240	6.4078	4.0594	5.9584	3.7596	6.4482	6.0248	4.8085	5.2231	5.4619	5.9379	4.6984	6.7287	5.8419	5.5076	6.0666	7.3680	7.1054	6.5443	5.5448
Scn2a	_QQEEAQAAAAAASAES[Phospho (STY)]R_.2	#N/A	8.1557	7.9372	6.9616	7.9958	7.4530	7.2134	7.6748	6.9134	7.6093	6.1973	7.8210	7.6178	7.0851	7.7923	5.4086	7.5234	8.1061	6.8608	6.7059	6.7640
Scn2a	_LSSKS[Phospho (STY)]EKELK_.2	B1AWN6_S497_M1_Scn2a	6.4326	6.4181	5.9121	6.0206	5.6893	6.3956	6.4054	6.8507	6.1258	5.3509	6.4535	6.5232	6.9723	6.2658	5.4965	6.0443	6.4987	6.8710	6.2896	6.2525
Scn2a	_GSLFS[Phospho (STY)]PR_.2	B1AWN6_S568_M1_Scn2a	4.2312	4.5887	3.7678	3.5137	2.9373	4.4715	4.1108	3.8761	4.0310	4.0418	3.5096	4.3148	5.0408	3.1982	4.1362	3.5933	4.4138	3.4194	3.0680	3.1652
Scn2a	_[Acetyl (Protein N-term)]AQSVLVPPGPDSFR_.2	#N/A	3.9968	5.8563	6.0442	5.4353	4.2038	6.2464	5.8790	6.3526	3.9197	4.5491	4.2940	3.0484	4.0277	3.5715	5.9877	3.3590	3.7198	6.1671	3.7287	5.0762
Scn2a	_KQQEEAQAAAAAASAES[Phospho (STY)]R_.3	#N/A	3.9667	5.1069	4.0005	3.7261	4.9726	3.4917	4.4310	3.9987	3.6931	4.3040	4.0753	4.1508	3.8011	4.0091	4.1306	3.8162	4.6145	3.6071	4.4321	3.8598
Scn2a	_GFRFS[Phospho (STY)]LEGSR_.2	#N/A	3.8073	3.5118	3.3439	4.5906	4.0143	4.0476	3.0338	4.3000	4.4549	3.6179	3.9335	5.4109	3.1195	3.6221	3.0592	3.1694	3.3368	4.1451	4.1449	3.5891
Scn2a	_VKDIGS[Phospho (STY)]ENDFADDEHSTFEDNDSRR_.4	B1AWN6_S589_M1_Scn2a	4.2271	3.7757	3.2626	3.4623	3.5648	4.0372	5.6458	3.9275	4.0353	4.4334	2.9089	3.5152	4.1434	3.2496	3.5535	3.0712	4.4652	5.1905	3.6131	3.3644
Scn2a	_KSAS[Phospho (STY)]EDS[Phospho (STY)]IRK_.2	#N/A	5.0328	5.4624	4.7081	5.0608	5.0201	5.1687	4.7163	4.1539	5.5494	4.9241	6.4145	5.1023	5.9020	5.7068	4.0861	4.8828	5.6044	5.3273	4.8968	3.9640
Scn2a	_DFSGAGGIGVFS[Phospho (STY)]ESSSVASK_.2	B1AWN6_S484_M1_Scn2a	5.2815	4.0759	4.4632	3.7625	3.2647	3.4887	4.2764	3.1375	4.8197	4.1333	3.2091	3.9653	4.4436	3.9873	3.8536	4.2127	5.6791	3.0138	5.4934	3.6646
Scn2a	Oxidation (M)]SM[Oxidation (M)]ASILT[Phospho (STY)]NT[Phospho (STY)]M[Oxidation (M)]EELI	B1AWN6_T713_M2_Scn2a	4.2169	6.3931	3.2523	3.4725	3.5546	6.0180	5.6550	3.9173	6.4838	4.4232	2.9191	3.6754	4.1536	3.2394	3.5637	3.0609	4.4549	7.0087	5.9138	5.8215
Scn2a	_RPS[Phospho (STY)]NVSQASR_.2	B1AWN6_S623_M1_Scn2a	3.7254	4.2774	4.2617	3.9640	3.0632	3.4209	4.4779	3.4258	3.5807	3.9318	3.0593	4.1668	3.7461	4.2488	4.0551	4.0703	3.9635	3.8697	3.5183	4.2158
Scn2a	_SSSYHVS[Phospho (STY)]M[Oxidation (M)]DLLEDPTSR_.3	#N/A	3.9714	3.4492	3.5902	3.1358	3.8925	4.1458	4.0882	4.2551	4.0598	3.5168	3.9474	3.3387	4.2036	3.4312	3.0827	3.3988	3.2920	3.4132	4.1119	3.2714
Scn2a	_RFS[Phospho (STY)]S[Phospho (STY)]PHQSLLSIR_.3	B1AWN6_S553_M2_Scn2a	3.7400	3.5791	3.2766	4.5233	3.9469	3.9803	3.1012	4.3673	3.0214	3.5506	4.0009	3.3052	3.1868	4.7810	3.1265	3.1021	3.4042	3.8560	4.0776	3.6565
Akirin2	_TLDFDPLLSPAS[Phospho (STY)]PK_.2	B1AXD8_S21_M1_Akirin2	4.2048	5.7008	5.9962	5.5903	2.9109	4.4451	4.6407	4.4305	5.1815	5.1300	3.5360	4.9413	5.7454	3.7795	4.9693	5.1363	4.4992	4.7164	3.5208	5.0279
Nhsl2	_IIAQFS[Phospho (STY)]KDYEPTDNPST_.2	#N/A	3.5123	7.7259	8.5352	4.2531	6.6122	8.5541	7.3196	6.5806	7.5900	8.2568	7.3216	6.5557	7.6614	8.0652	3.6155	7.7804	7.6476	7.4878	7.7972	7.9688
Nhsl2	_SKS[Phospho (STY)]IS[Phospho (STY)]LKK_.2	B1AXH1_S557_M2_Nhsl2	5.9502	3.5045	6.4270	7.2725	3.3706	6.0218	5.2700	6.0643	5.0263	5.8499	4.8771	5.6433	5.9008	5.8565	5.7360	6.3950	6.2929	5.3828	5.9555	3.9164
Nhsl2	_ATTPSQLS[Phospho (STY)]IEVEAR_.2	B1AXH1_S690_M1_Nhsl2	7.7787	3.2175	3.8208	4.4049	3.8285	3.8619	7.1821	4.4857	3.1398	3.4322	4.1192	3.1069	3.3052	7.7425	3.2449	7.3730	3.5225	4.3106	3.9592	7.7406
Nhsl2	_RIIAQFS[Phospho (STY)]KDYEPTDNPST_.3	#N/A	4.5533	4.8185	3.6794	5.0584	3.4334	3.7884	4.3012	3.5765	3.7482	3.5728	3.7868	3.8303	3.5205	5.8161	3.9248	5.1731	3.9037	4.7437	4.3286	4.2424
Nhsl2	_S[Phospho (STY)]LSVPTDSGVTSVDYDEEQK_.2	B1AXH1_S497_M1_Nhsl2	3.6580	3.7989	3.9073	4.0640	3.8649	3.8578	4.0337	3.8837	3.7569	4.1813	3.5757	3.5250	3.9049	3.8611	3.3463	3.7328	4.0936	4.0575	3.8346	3.6498
Nhsl2	_DQGHSSS[Phospho (STY)]PTGSLAR_.2	B1AXH1_S301_M1_Nhsl2	8.0712	4.1412	7.8042	8.0958	7.6736	7.5074	7.3098	3.6921	7.4489	7.9831	4.0449	7.7251	8.0259	8.0536	7.3662	7.4338	7.8200	7.6202	4.1014	7.7490
Frrs1l	_NPARDEEGVFENNR_.3	#N/A	4.9875	3.3132	5.7442	5.8060	5.8536	5.7465	5.6825	5.6272	5.4915	5.7647	5.2673	5.4361	5.5620	5.6977	4.6336	5.5770	5.7619	6.0432	5.7645	6.1163
Nfx1	_S[Phospho (STY)]PQGFFQSSNK_.2	B1AY10_S81_M1_Nfx1	7.4000	7.2850	7.5561	7.6467	6.7853	6.5850	6.4255	5.7608	6.6239	7.8510	7.2977	6.5920	6.6651	7.1606	6.0477	6.7779	7.0751	6.1072	6.4997	6.9317
Nfx1	_EHS[Phospho (STY)]PSESEKEVVIADPR_.3	B1AY10_S147_M1_Nfx1	3.4309	4.2509	4.2152	4.6934	3.2263	3.3388	3.3629	3.2938	3.3442	3.4718	3.2965	4.0723	3.4840	4.2514	3.9340	4.0329	3.8315	4.0652	3.3246	4.0706
Nfx1	_AQGLSEQT[Phospho (STY)]SDTSSLESVAR_.2	B1AY10_T125_M1_Nfx1	4.1888	3.8987	3.9869	3.5853	3.7068	3.8071	4.4205	3.8892	4.4112	3.8676	3.6069	3.7881	4.5471	3.9740	3.5610	3.7702	3.6886	3.8789	3.9022	3.6500
Nfx1	_NYSSS[Phospho (STY)]PPC[Carbamidomethyl (C)]HLPR_.2	B1AY10_S51_M1_Nfx1	4.4752	4.3447	4.0118	3.1090	3.1813	8.0348	3.8668	3.6321	3.7870	4.2858	8.3674	4.0708	3.9524	4.4551	3.8921	3.8373	4.1698	3.6634	3.3120	4.4221
Usp24	_VSDQNS[Phospho (STY)]PVLPK_.2	#N/A	11.5303	11.3039	11.2958	11.1264	10.8339	11.1315	11.0646	11.4266	10.9705	11.0822	10.9816	10.8012	11.4369	10.9645	9.5957	10.5485	11.6124	11.0973	10.7645	10.7476
Usp24	_NMDQGGGGS[Phospho (STY)]PR_.2	B1AY13_S1940_M1_Usp24	3.0825	6.8265	6.9794	7.6763	5.1515	6.4013	7.1387	4.2837	5.8707	3.6342	7.3331	5.1947	4.6040	7.3798	3.1972	3.4274	6.2745	3.9443	4.7514	6.1685
Usp24	_TIS[Phospho (STY)]AQDTLAYATALLNEK_.2	B1AY13_S2558_M1_Usp24	4.2910	4.9473	3.9279	3.4049	3.6287	4.2240	4.4894	3.9914	3.9642	3.6333	3.9336	3.6078	4.1029	3.3135	3.8223	3.1351	3.9185	4.7246	3.8495	3.2805
Usp24	_VSDQNS[Phospho (STY)]PVLPKK_.2	#N/A	9.0703	9.5765	8.5991	9.1772	8.8207	8.9630	9.3173	9.7570	9.1299	8.6927	8.7138	8.9779	9.6214	8.6073	7.2846	8.4574	9.2946	9.0244	8.8367	8.5016
Tmem245	_SSPSSPS[Phospho (STY)]PTLGR_.2	B1AZA5_S329_M1_Tmem245	8.6956	7.0310	8.3180	8.8698	3.4490	7.0077	8.3286	3.8748	7.1761	8.4365	7.9446	8.0079	8.5739	8.0953	6.9651	7.6403	7.1208	8.4020	7.9034	7.6433
Tmem245	_GGPAEAPS[Phospho (STY)]PR_.2	B1AZA5_S12_M1_Tmem245	9.2159	9.5482	9.3454	9.2364	8.6952	9.4049	9.2363	8.8196	9.0297	9.1933	9.0923	8.8555	8.9260	8.4745	7.3831	8.1939	9.5864	8.9648	8.4940	8.6496
Tmem245	_GGPAEAPS[Phospho (STY)]PRGS[Phospho (STY)]PRPESR_.3	#N/A	8.0979	8.1145	7.5838	8.1730	7.4332	7.5336	7.6927	7.8168	7.5617	7.7909	7.3727	7.1515	7.9107	6.9294	6.3767	7.4075	7.8355	7.8347	7.2386	6.7254
Thoc2	_KIDSHPS[Phospho (STY)]PSHSSTVK_.3	B1AZI6_S1417_M1_Thoc2	3.3506	3.2353	4.3880	4.2336	3.5575	3.5909	3.4906	3.5763	3.4108	3.1612	4.3902	3.6946	3.5762	4.0789	3.5159	4.2136	3.0735	4.0396	3.6882	4.0459
Dlgap4	_ATQQS[Phospho (STY)]LGEQSNPR_.2	#N/A	10.7417	10.8697	11.5466	11.0285	10.5243	10.7858	10.4037	10.6808	10.4992	10.8625	10.4306	10.1022	10.5518	10.8584	9.9329	10.4516	10.5852	10.3605	10.6164	10.6218
Dlgap4	_QNS[Phospho (STY)]ATESADSIEIYVPEAQTR_.2	#N/A	8.7530	8.9348	6.6058	9.1000	7.5134	8.3700	8.7563	10.2635	8.4062	9.3911	8.2161	8.0102	4.2463	8.9664	7.9817	8.4109	10.9604	8.4047	8.3338	8.4749
Dlgap4	_ANSWQLVET[Phospho (STY)]PEK_.2	#N/A	7.9826	8.6833	8.7182	8.1563	4.1818	8.5764	8.1644	7.6900	8.4724	8.3987	7.5758	7.8790	7.8472	9.1003	7.7773	8.2359	8.7389	8.3036	8.4814	8.5887
Dlgap4	_SNISGWWS[Phospho (STY)]SDDNLDGEGGAFR_.2	B1AZP2_S206_M1_Dlgap4	4.1594	7.2784	3.4444	3.5458	3.9575	3.8630	3.9024	4.2643	6.7049	4.5075	4.3359	6.8521	4.0695	7.4740	3.4796	3.2622	6.6694	6.9287	3.6871	6.1529
Dlgap4	_ANSWQLVET[Phospho (STY)]PEKR_.2	#N/A	7.6470	7.9219	7.3950	7.7464	6.6939	7.6991	7.3133	7.6810	7.4753	7.7916	7.0549	7.4446	7.9022	8.2449	6.9073	7.5109	8.0607	7.7948	7.6761	7.5130
Dlgap4	_APS[Phospho (STY)]M[Oxidation (M)]EGTAGK_.2	#N/A	5.8104	6.4415	7.0735	7.3290	6.0744	6.1894	6.5701	5.8711	6.3917	7.1442	4.5093	6.5924	4.0026	7.7035	6.0794	5.9651	6.5615	6.2053	6.1815	6.3382
Dlgap4	_ATQQS[Phospho (STY)]LGEQS[Phospho (STY)]NPRR_.2	B1AZP2_S405_M2_Dlgap4	4.5753	6.0808	4.9747	5.1617	4.7500	4.6730	5.7803	4.8070	5.0352	6.0157	4.4893	5.3918	5.1496	3.9478	5.1310	5.0781	5.0609	5.1327	5.7801	4.7025
Dlgap4	_SLPDC[Carbamidomethyl (C)]TSHPNSIS[Phospho (STY)]IDAGPR_.3	#N/A	6.3606	6.8319	6.6715	6.6664	5.1216	6.5170	6.1792	3.8052	4.1468	6.5056	5.4349	5.7308	4.3122	6.8817	5.7695	3.4223	5.2756	6.3687	5.1619	6.2384
Dlgap4	_AM[Oxidation (M)]GDEDS[Phospho (STY)]DESGGGS[Phospho (STY)]PKPSPK_.2	#N/A	4.2386	4.8854	3.8451	4.8145	5.3335	4.6148	5.3096	5.0343	4.6394	5.5175	4.9839	5.4937	5.1884	5.1183	4.6436	5.4006	5.9413	4.8133	5.6794	5.2275
Dlgap4	_S[Phospho (STY)]LDRLDSVDM[Oxidation (M)]LLPSK_.2	#N/A	6.7784	7.4719	7.1757	5.5465	5.2497	4.0259	7.0615	6.6846	7.3129	7.1079	4.1120	6.3746	6.4433	7.7499	7.3436	5.9594	5.7691	7.8486	6.2206	5.6802
Dlgap4	_RS[Phospho (STY)]LDRLDS[Phospho (STY)]VDM[Oxidation (M)]LLPSK_.3	#N/A	3.5650	5.3114	4.6025	4.0192	3.7720	4.3364	3.4288	3.7907	4.5870	3.4801	3.8622	3.4802	3.5959	6.0930	4.8933	4.4280	3.2879	6.4837	4.9463	4.3178
Dlgap4	_RDT[Phospho (STY)]DS[Phospho (STY)]DTQDANDSSC[Carbamidomethyl (C)]K_.2	B1AZP2_S716_M2_Dlgap4	4.3721	4.4478	3.9087	3.2121	3.0782	3.5293	4.2359	3.0970	4.2949	4.1738	3.1685	4.1739	4.4030	3.9468	3.8130	4.3124	4.0950	3.0544	3.3535	3.6240
Cad	_IHRAS[Phospho (STY)]DPGLPAEEPK_.3	B2RQC6_S1859_M1_Cad	7.8042	8.0140	7.7831	7.6451	6.9716	7.4512	7.9415	7.4978	7.7024	8.3098	7.5961	7.2058	7.7295	7.5663	6.3176	7.0628	7.4060	7.8822	7.5989	7.5200
Ythdc2	_ADGIPNDSS[Phospho (STY)]DSEM[Oxidation (M)]EDR_.2	#N/A	3.6398	3.8079	8.5842	7.8051	7.9144	8.1009	8.0560	3.8655	7.9549	3.8967	7.7902	7.9742	3.7071	7.4008	3.9599	3.9644	3.8863	8.3035	8.1038	7.9685
Ythdc2	_STDSSSYPSPC[Carbamidomethyl (C)]AS[Phospho (STY)]PS[Phospho (STY)]PPSSGK_.2	B2RR83_S1282_M2_Ythdc2	3.2382	4.0809	4.2756	4.3460	3.4451	4.1594	3.6030	3.4639	3.1610	4.5496	3.5354	3.8070	3.2691	4.1355	3.6284	4.1011	4.4619	4.3539	2.9866	4.8946
Otud4	_TAADVVS[Phospho (STY)]PGANSVDR_.2	B2RRE7_S1000_M1_Otud4	7.5925	4.5712	7.3255	7.5728	7.4262	7.1858	6.8877	7.2891	7.1154	8.0139	7.1707	7.3674	7.8637	7.8716	6.5693	7.3239	7.6602	3.1778	7.4850	7.3303
Otud4	_EES[Phospho (STY)]S[Phospho (STY)]EDENEVSNILR_.2	#N/A	4.2111	4.6088	3.7477	3.3731	2.9172	3.6903	4.1309	4.4368	4.1339	4.3348	3.0075	4.3349	4.2420	8.7471	4.1563	3.5732	3.9340	7.3080	3.5145	3.4630
Otud4	_LQRPKEES[Phospho (STY)]S[Phospho (STY)]EDENEVSNILR_.3	#N/A	3.8834	3.4356	3.4200	3.7008	4.0904	4.1237	4.4586	4.2239	4.3788	3.6940	3.8574	3.1618	4.5442	3.5936	2.9831	3.2456	3.6064	3.0716	4.2210	3.5130
Rrn3	_LQDPNNPAIIR_.3	#N/A	5.9238	5.7444	5.6684	5.8294	6.1160	5.6651	6.3283	3.8194	6.1439	7.0112	6.6560	6.5589	6.2187	6.8317	6.5805	6.6035	5.5153	6.6327	6.7088	6.4657
Gnai1	_DSGVQAC[Carbamidomethyl (C)]FNR_.2	#N/A	8.5881	8.5703	8.7952	8.7616	8.5936	8.6669	8.4866	8.6766	8.5305	8.8026	8.7651	8.4475	8.5906	8.3761	7.7647	8.3226	8.9366	8.4914	8.4326	8.5851
Gnai1	_IAQPNYIPTQQDVLR_.2	#N/A	8.9928	8.7471	8.6885	8.9546	8.4381	8.6314	8.7975	9.1221	8.7686	8.6692	8.7876	8.5865	8.9803	8.5654	8.1609	8.5157	8.7064	8.6035	8.6276	8.4650
Gnai1	_EYQLNDSAAYYLNDLDR_.2	#N/A	7.4230	6.5948	6.7211	7.2182	6.4050	7.0939	7.0609	6.7554	7.0358	6.7673	6.9271	6.8983	7.0638	6.5054	5.9043	6.5103	6.8676	6.7545	6.4993	6.2734
Gnai1	_IDFGDSAR_.2	#N/A	5.3582	5.7040	5.8705	6.7305	6.1814	5.9135	6.7379	6.0301	6.5837	6.8224	6.7440	6.3064	6.1839	5.9972	5.6695	5.9623	5.8031	6.3287	6.7698	6.2009
Gnai1	_SREYQLNDSAAYYLNDLDR_.3	#N/A	6.9186	6.5806	5.9400	6.8881	6.3238	6.2094	6.0041	6.5136	6.1434	6.6332	6.1989	5.3157	6.7836	6.3800	5.3260	5.8934	6.7293	6.2604	6.1223	6.1200
Gnai1	_AVVYSNTIQSIIAIIR_.2	#N/A	3.8817	3.8910	3.5512	4.0366	3.5990	3.6944	3.7844	3.7537	3.7472	3.8768	3.5739	3.7299	3.8947	3.8694	3.5986	3.7517	4.1209	2.6076	3.6539	3.6555
Apba1	_VVGT[Phospho (STY)]PGGSEVGQR_.2	B2RUJ5_T333_M1_Apba1	8.1125	7.8202	7.9056	8.1570	7.3703	6.6774	7.2564	3.9884	6.8300	8.7823	7.4222	7.4126	7.3834	7.4244	4.2484	7.7327	4.5261	7.0091	7.2485	7.4343
Apba1	_QDIS[Phospho (STY)]PTR_.2	B2RUJ5_S391_M1_Apba1	4.4744	3.5284	3.5098	3.2150	3.8121	4.0362	3.7290	4.0909	3.7881	3.1798	7.0169	3.4179	3.8961	3.4399	3.3062	3.3184	3.5882	3.5613	6.0755	3.1172
Apba1	_APT[Phospho (STY)]PGGGHPDS[Phospho (STY)]PGLPAPAGQQQR_.3	#N/A	10.0368	9.7985	10.3237	10.2530	7.9443	9.6310	8.9791	9.3690	9.5612	10.0495	9.8928	9.4289	9.3078	10.2505	9.2004	9.6311	9.9961	9.3647	9.6747	9.7041
Apba1	_M[Oxidation (M)]DS[Phospho (STY)]YEQEEDIDQIVAEVK_.2	#N/A	4.1075	3.9571	8.0171	7.7441	3.4894	3.6766	3.7549	3.7061	3.7137	4.0477	3.5116	3.8015	3.8629	4.1550	7.9799	3.8596	7.0559	3.7168	3.6379	4.0220
Apba1	_QSM[Oxidation (M)]SSQS[Phospho (STY)]LDK_.2	#N/A	5.3911	5.2964	6.6622	6.3146	5.9201	6.0258	5.7238	6.6973	6.4878	6.3608	6.1512	6.4668	6.1800	6.4283	5.9785	5.6529	6.1664	6.2782	5.9936	6.8218
Apba1	_SNS[Phospho (STY)]QENVEASHPSQDGKR_.3	#N/A	7.9108	8.1266	7.6090	7.9484	7.2382	7.7428	7.7159	7.7944	7.4462	8.1776	7.6955	7.4476	8.3181	8.0935	7.6092	7.8302	7.6683	7.8985	8.0330	7.8359
Apba1	_SNS[Phospho (STY)]QENVEASHPSQDGK_.2	#N/A	7.3649	6.9182	3.5961	7.3851	6.8113	6.9888	7.0209	7.1584	6.6059	7.6037	7.1166	6.8576	6.6266	7.4695	6.4707	7.2777	7.1700	7.1186	7.1177	6.9945
Apba1	_SDGES[Phospho (STY)]DSPEKEAEFAPYPR_.3	#N/A	3.7849	5.4491	5.3787	3.9673	3.8909	5.1437	6.0080	3.6460	5.4349	5.4170	3.9726	4.6155	3.7062	5.7579	3.2734	4.8078	3.9018	3.8126	5.9179	6.1589
Apba1	_SAS[Phospho (STY)]TESGFHNHTDTAEGDVLAAAR_.3	B2RUJ5_S84_M1_Apba1	6.7344	3.8208	5.8368	6.6706	5.7759	5.9973	3.1542	4.1825	5.6712	3.5936	6.1698	3.6666	6.7782	3.9768	5.5022	3.3975	6.4037	6.3528	6.2347	3.8271
Apba1	_LHHY[Phospho (STY)]DERSDGESDS[Phospho (STY)]PEKEAEFAPYPR_.4	#N/A	4.3720	3.4215	3.5728	3.2907	3.5519	4.0114	4.0649	4.2467	6.1715	3.8605	3.7066	3.8146	4.3957	3.5789	5.3918	4.9403	3.4727	3.1739	4.0790	3.4433
Apba1	EDM[Oxidation (M)]PEAEQDLERAPT[Phospho (STY)]PGGGHPDS[Phospho (STY)]PGLPAPAGQQC	#N/A	3.2947	8.0968	8.2706	6.5781	3.5016	8.0820	7.8134	3.5203	7.9110	8.3232	3.5919	7.0027	6.6454	7.7533	7.6843	7.8799	6.8317	8.4045	8.2526	8.1525
Apba1	_LHHYDERS[Phospho (STY)]DGESDSPEK_.3	#N/A	4.0873	3.2318	3.6239	3.4969	4.2943	4.3276	4.2547	4.0200	4.1749	3.8979	3.6535	2.9579	4.3403	3.3422	4.2801	3.4494	4.5577	3.2755	4.4249	3.3092
Cspp1	_[Acetyl (Protein N-term)]ADS[Phospho (STY)]LDEFIEEQK_.2	B2RX88_S4_M1_Cspp1	3.9148	4.0880	4.4511	3.7746	3.2525	3.2316	3.8499	3.6152	3.7701	4.3027	3.2487	3.9775	3.9355	4.4382	4.0185	4.2597	4.1528	3.6803	3.3289	4.4052
Dock5	_S[Phospho (STY)]LQLVDSR_.2	B2RY04_S1765_M1_Dock5	5.6968	6.2959	4.4198	4.5369	3.2542	5.7408	6.3322	3.2730	5.0340	6.0824	3.3445	5.4897	4.5790	5.7582	3.9891	3.9102	4.2710	5.6126	5.0046	3.8001

Rbm25	_LGASNS[Phospho (STY)]PGQPNSVK_.2	#N/A	10.7257	10.7800	10.7777	10.7896	10.4058	10.5769	10.6191	10.6039	10.6725	10.6881	10.7133	10.7882	11.1195	11.2071	9.4864	10.4243	11.0542	10.7875	10.5078	10.5859
Rbm25	_LGASNS[Phospho (STY)]PGQPNSVKR_.2	#N/A	8.3127	8.3723	7.7915	8.2129	7.9265	7.9261	8.0477	8.2978	7.8565	8.0137	7.7014	7.8441	8.4991	8.2005	7.0645	7.8147	8.1835	8.0507	7.7494	7.7878
Ildr2	_GWTYS[Phospho (STY)]PAPAR_.2	B5TVM2_S511_M1_Ildr2	9.0563	9.0482	4.5547	8.7376	3.7242	4.3841	3.3239	3.7430	3.2441	3.3279	8.4731	8.0206	3.5482	4.5928	3.3492	4.3802	3.2402	3.9092	4.2083	4.2701
Ildr2	_GGS[Phospho (STY)]LETPSK_.2	B5TVM2_S562_M1_Ildr2	3.1110	4.2077	4.3314	6.8753	3.3180	3.3513	3.7301	3.4955	3.6504	6.8913	3.1290	5.9433	6.6204	4.3184	3.7555	3.9740	4.0331	3.8000	3.4486	4.2854
Ildr2	_AHGAFYQDGS[Phospho (STY)]LDEYYGR_.2	#N/A	4.1188	6.6837	3.7902	3.6470	3.4566	3.6807	4.3176	3.9372	4.7423	4.3252	4.1063	3.6597	3.8400	3.7923	3.7012	3.5249	4.1213	4.3832	3.2998	3.5122
Clip3	_SPSS[Phospho (STY)]PSLGSLQQR_.2	B9EHT4_S402_M1_Clip3	7.6474	7.0135	7.3697	7.0623	3.7548	6.7335	3.8567	7.0998	3.7769	3.6571	3.9284	3.4884	3.9423	6.7380	5.5749	6.5445	7.2200	6.4264	7.3486	6.7301
Clip3	_KKS[Phospho (STY)]PS[Phospho (STY)]SPSLGSLQQR_.3	#N/A	4.1779	5.9327	5.4241	3.6456	4.6124	5.3380	6.7693	3.7928	3.6807	3.7214	5.6620	3.6072	3.8119	4.0608	3.4617	5.4258	3.7013	5.3026	3.6776	5.6631
Pdzd8	_VQTELKEETQPLSHS[Phospho (STY)]PK_.3	#N/A	4.0127	7.9351	8.2784	3.2951	6.8348	7.3659	7.3743	7.5538	7.4092	7.7760	7.3517	7.1380	7.0899	7.4129	2.8539	7.3484	7.7973	7.3854	7.5309	7.2746
Pdzd8	_VQTELKEETQPLSHS[Phospho (STY)]PKR_.4	#N/A	3.1441	4.1922	4.1815	4.4401	3.3510	3.2565	3.6971	3.3698	3.0669	4.4555	3.4413	3.6131	3.1750	4.2196	3.7224	4.0070	4.3678	3.8492	3.0807	2.2129
Osbpl8	_KES[Phospho (STY)]LKVQK_.2	B9EJ86_S120_M1_Osbpl8	7.0409	6.8947	6.8521	6.3183	6.2507	6.3277	6.5007	6.9470	6.1543	6.2694	5.3210	6.1742	6.9261	5.4734	4.8476	5.3967	5.9911	6.3996	5.8127	5.5625
Osbpl8	_GYSSPEPDIQDS[Phospho (STY)]SGSEAQSVKPSTR_.3	B9EJ86_S807_M1_Osbpl8	3.7335	4.8412	4.1689	5.4555	3.4006	3.4882	5.3700	3.8951	3.3500	3.8282	3.6467	4.5137	3.6533	4.2596	5.0518	5.3255	5.0686	6.0396	3.6548	4.1230
Cttnbp2	_DVS[Phospho (STY)]PTSRDNLVAK_.2	#N/A	3.3257	7.6194	3.8620	7.8426	4.1642	3.8207	6.9139	4.5269	3.4359	3.5320	3.8103	7.7037	8.7856	3.8490	7.0741	7.8454	3.5637	4.2694	3.9180	7.2584
Cttnbp2	_DVS[Phospho (STY)]PTSR_.2	#N/A	4.2789	3.8748	3.8155	3.5613	2.9850	6.7413	4.0631	6.3468	3.9833	4.0895	3.4620	7.0974	4.1488	3.1506	4.0885	3.6410	6.7196	3.4671	3.1156	3.1176
Cttnbp2	_QSSHS[Phospho (STY)]DLGPSPPTALPSSANR_.3	B9EJA2_S362_M1_Cttnbp2	3.8258	3.8848	3.9432	3.8463	3.5500	3.6782	3.7771	7.4202	3.7826	6.7367	3.8171	3.6928	3.9124	4.1597	3.5491	3.7637	6.4638	3.7254	3.9876	3.7447
Cttnbp2	_LSLGS[Phospho (STY)]DDEADLVK_.2	B9EJA2_S1510_M1_Cttnbp2	4.5497	3.4531	3.5851	3.1397	3.8874	5.7473	3.6537	4.2500	5.8974	3.6679	3.8836	3.3426	4.5704	3.5722	3.2309	3.3937	3.2869	3.0454	4.1949	3.5392
Cttnbp2	_FQGNANDPDQNGNNTQS[Phospho (STY)]PPSR_.2	B9EJA2_S465_M1_Cttnbp2	3.9282	4.2380	4.6365	3.7433	3.3942	3.7153	3.7627	4.1539	3.7724	3.9804	3.7417	3.6072	3.9005	4.2618	3.5205	3.8414	3.6511	3.9848	3.6876	3.9590
Cttnbp2	_LASISVATEGPVTR_.2	#N/A	4.3923	4.0330	3.4839	3.6369	3.7301	3.6154	3.8110	4.1730	4.0487	3.0978	4.2445	3.0979	4.3046	3.5219	3.9883	3.2364	3.7505	4.6670	3.7783	3.8255
ATp6v1fnb	_YQY[Phospho (STY)]LNTR_.2	B9EJX3_Y114_M1_ATp6v1fnb	3.7743	5.3463	6.6716	5.5628	5.1145	3.3361	4.5443	5.5987	5.1250	5.3063	3.3617	4.1180	6.4067	5.3104	5.7777	5.9672	4.2882	4.3620	5.5706	3.8173
Tmf1	_SVSEINS[Phospho (STY)]DDELPGK_.2	B9EKI3_S340_M1_Tmf1	5.7977	9.1845	9.6595	9.4072	3.8622	9.3736	8.8143	4.2249	9.0996	9.4739	4.1123	9.1192	4.5452	9.8887	8.9033	9.2524	9.8583	9.1854	9.2427	9.5352
Ptprz1	_EVSADVS[Phospho (STY)]EEENFLTDFK_.2	B9EKR1_S576_M1_Ptprz1	12.0530	11.8346	12.2501	11.9159	11.2740	12.1391	11.5914	11.1251	12.0857	12.3181	11.7956	11.8139	12.1736	12.6500	12.1050	12.3520	12.2121	11.6887	12.0608	12.1609
Ptprz1	_FAVLYQPLAGNDQAK_.2	#N/A	8.6013	8.8388	8.6657	8.9122	8.7402	8.7675	8.5148	8.4506	8.7308	8.9775	8.7068	8.5966	9.1050	9.0485	8.6095	8.9649	8.9025	8.3093	8.7167	8.8084
Ptprz1	_FSSFEEAVK_.2	#N/A	6.6676	6.4977	6.4713	6.3307	6.5771	6.6755	6.5286	6.1537	6.4852	6.8461	6.9959	6.8177	6.5637	6.7280	6.7271	6.1482	6.4457	6.3103	6.1307	6.7655
Ptprz1	_FSEQGLLK_.2	#N/A	6.0477	5.3443	5.8097	5.1336	5.1270	5.9360	5.1010	6.0830	5.5387	5.0540	5.4809	5.4857	4.6827	6.2081	5.7711	6.1401	5.3592	6.0553	5.7784	5.7730
Ptprz1	_QSPINIDEDLTQVNVNLKK_.3	#N/A	5.8639	5.8239	5.6774	5.5132	5.3942	5.1182	4.8881	5.4930	5.1311	5.5109	4.9529	5.3358	6.3162	5.6235	5.9473	5.8525	4.7571	5.5965	5.8975	5.8187
Ptprz1	_QSPINIDEDLTQVNVNLK_.2	#N/A	5.0235	5.2255	5.4321	5.3561	5.0116	4.0547	4.9858	4.6282	5.0211	4.3508	5.2661	4.8248	5.5419	5.9080	4.8915	5.1940	4.9993	5.5241	4.6702	4.4163
Ptprz1	_DTDGVLETDDTGIAPGSPR_.2	#N/A	6.3697	6.6083	7.1826	6.6810	6.5244	6.9603	6.6463	6.4707	7.4252	6.7308	6.5407	6.6218	7.0010	7.6276	7.1176	7.0823	7.0078	6.9649	6.6427	6.6344
Ptprz1	_ASLENTFIHNTGK_.2	#N/A	6.4911	6.5747	6.0460	5.5181	6.5779	6.7560	6.3591	6.6588	5.7587	6.9428	5.6489	6.2718	6.9961	7.2247	6.4949	6.3377	6.9089	6.0121	6.1636	6.6043
Ptprz1	_C[Carbamidomethyl (C)]MSC[Carbamidomethyl (C)]LPYRES[Phospho (STY)]R_.2	#N/A	4.1597	5.7808	6.1923	6.0534	4.8188	5.5027	5.7301	4.0850	5.1928	4.0208	6.1295	5.2968	4.0344	7.0798	4.9678	3.3738	3.9144	3.4995	4.7831	5.8098
Ptprz1	_C[Carbamidomethyl (C)]MSC[Carbamidomethyl (C)]LPYRES[Phospho (STY)]REK_.3	#N/A	4.1558	4.7894	3.6924	3.4284	3.5215	3.7456	4.0195	4.3815	4.0786	4.3901	2.9522	4.3902	4.1867	6.2038	3.5967	3.0279	3.8787	3.2707	3.5698	3.4077
Mfap1a;Mfap1b	_IVEPEVVGES[Phospho (STY)]DSEVEGDAWR_.3	C0HKD8_S116_M1_Mfap1a;Mfap1b	3.9363	4.4151	4.0376	3.7052	3.3076	4.6051	3.7701	3.2129	3.9450	3.7469	3.0662	3.7285	4.0739	4.0484	3.7955	3.9636	3.9824	6.2168	4.4790	3.8868
Mfap1a;Mfap1b	_SLAALDALNTDDENDEEEY[Phospho (STY)]EAWK_.3	C0HKD8_Y276_M1_Mfap1a;Mfap1b	4.2299	4.0093	3.7664	3.4736	2.9360	3.8883	4.1131	4.1570	4.0334	4.0405	3.5120	4.3172	4.1988	3.4953	4.1385	3.5920	4.1643	3.3497	3.3353	3.1676
Mfap1a;Mfap1b	_KIVEPEVVGES[Phospho (STY)]DS[Phospho (STY)]EVEGDAWR_.3	#N/A	4.3044	3.6984	3.3399	3.3850	3.6421	4.3428	3.8990	4.0048	3.9580	4.5107	4.3324	3.5879	4.3251	3.3269	3.4762	3.1485	4.5425	3.2907	4.4401	3.2939
Mfap1a;Mfap1b	_RPDYAPM[Oxidation (M)]ES[Phospho (STY)]S[Phospho (STY)]DEEDEEFQFIK_.3	C0HKD8_S52_M2_Mfap1a;Mfap1b	4.0484	3.9544	4.5847	3.6410	3.3861	3.6102	4.1550	3.7488	4.2140	4.2547	3.0876	3.8439	4.0691	4.5718	3.7322	4.3933	4.2864	3.5467	3.1953	3.0379
H2ac4;H2ac6;H2ac7;H2ac8;H2ac11;	_NDEELNKLLGR_.2	#N/A	6.8351	7.1442	6.2594	6.7895	6.7377	6.1810	6.7206	7.2710	6.6345	6.7849	6.3868	7.0851	7.8793	6.8280	6.7741	6.9857	6.5442	6.9233	6.3749	6.7505
Slc35g2	_REDYQEILDS[Phospho (STY)]PIK_.2	#N/A	6.4555	7.8149	7.4956	6.9276	4.0711	7.8244	7.4511	6.0083	7.8341	6.6784	4.0672	7.2888	7.5886	7.5538	6.3500	7.3848	7.7060	7.6477	7.6143	7.0188
Slc35g2	_EDYQEILDS[Phospho (STY)]PIK_.2	#N/A	7.8964	8.8580	8.7063	8.3322	3.4457	9.0472	8.8239	7.2438	9.0507	7.7244	3.0280	8.0744	7.7588	8.9239	7.5753	8.3221	8.7262	9.0975	8.6004	8.3333
Akap5	_GPVAAS[Phospho (STY)]PQK_.2	#N/A	9.1328	9.1383	9.6069	9.6581	8.9483	9.4817	9.2076	8.6326	9.1729	9.5057	9.9421	9.1732	8.8120	9.8812	8.9522	9.3397	9.2137	9.4295	9.5253	9.8555
Akap5	_VTVDHAEEATVGQAEEATVGQAEK_.3	#N/A	9.8750	9.1541	7.9189	8.8400	8.7344	8.1014	8.2927	9.0673	8.2177	8.6949	9.2418	9.4484	9.8008	10.0198	7.7766	8.7787	9.4187	8.7003	8.7311	9.0319
Akap5	_VTVDQAEEATVGQAEEATVGHTEK_.3	#N/A	7.6500	7.4265	7.2152	7.3025	7.3648	7.1200	6.7724	7.2567	7.5889	7.3147	7.5359	7.3141	8.0903	7.7901	6.9190	7.4476	7.8943	7.1165	7.1968	7.6525
Akap5	_VQGEADDLEIK_.2	#N/A	6.4735	6.5446	6.6810	5.9909	6.7865	6.3685	6.4642	6.7350	6.3855	6.0803	6.7690	6.2707	6.3924	7.1115	6.9542	6.5892	6.7288	6.5955	6.4550	6.8412
Akap5	_LSQAEETTVAQAK_.2	#N/A	5.3291	5.4071	5.6572	4.8156	4.9891	5.3464	5.6611	5.0599	4.4305	5.3570	5.7398	5.6483	5.8528	6.0643	5.9125	6.2849	5.3543	6.0488	5.7260	6.0932
Akap5	_ASLLESSAAGS[Phospho (STY)]PR_.2	D3YVF0_S251_M1_Akap5	13.7354	13.4657	13.8146	13.8576	13.3972	12.9137	12.9598	13.5306	13.2054	13.8095	13.3581	13.4088	14.0096	13.6564	12.8729	13.5381	13.6942	13.0656	13.2665	13.5065
Akap5	_ALSQAGEATAGHPEEATVIQAESQAK_.3	#N/A	3.5698	5.1724	5.2254	3.2971	4.4935	3.5978	5.1295	4.3520	4.4405	4.9992	4.2770	4.6315	3.9782	3.4148	4.1848	4.2219	3.9154	4.2957	3.6459	4.6820
Akap5	_QPAGAWAS[Phospho (STY)]IK_.2	D3YVF0_S79_M1_Akap5	9.2097	8.7381	9.4256	8.7438	8.0752	8.0655	7.6872	7.6552	7.3308	9.5566	8.2836	7.8976	8.3988	8.2914	7.1287	8.4651	8.0016	8.4077	8.4118	8.5282
Akap5	_SSAIQM[Oxidation (M)]GT[Phospho (STY)]PELEKETK_.2	#N/A	9.3180	9.3839	8.2109	9.6311	9.1055	8.6226	8.8557	9.3764	8.8040	9.5784	9.1122	9.2745	10.0875	9.7198	8.3757	9.3089	9.3638	9.0500	9.0087	9.2898
Akap5	_DEKGPVAAS[Phospho (STY)]PQKER_.2	#N/A	10.3371	10.3325	10.1965	10.5298	10.0619	10.0749	10.1128	10.4357	10.1038	10.4885	10.2054	10.3891	10.8944	10.9552	10.3284	10.4603	10.4731	10.3885	10.4125	10.5506
Akap5	_AQTQPDDQAIQAGS[Phospho (STY)]TQGLQEGVLVR_.3	D3YVF0_S171_M1_Akap5	6.5367	4.1902	6.2803	6.6657	5.6628	5.1319	5.6046	5.4703	5.3383	3.8756	5.5792	5.8974	6.2878	6.7417	5.0699	5.8775	5.8271	5.6287	5.1859	5.3638
Akap5	_GPVAAS[Phospho (STY)]PQKER_.2	#N/A	11.8047	11.7981	11.7648	11.9303	11.6387	11.6284	11.4484	11.8633	11.5696	11.8065	11.7720	11.8775	12.0981	12.4338	11.6641	11.9019	12.0246	11.9011	11.7883	11.9804
Akap5	_KATVGQAEEATIDHTEK_.3	#N/A	4.3037	4.5162	3.8403	3.2805	4.7986	5.2065	4.1675	4.5294	4.6744	4.2422	4.7828	4.1583	4.7759	3.8784	5.6198	4.7274	4.0266	3.1228	3.4218	3.5557
Akap5	_TSEQYETLLIETASSLVK_.2	#N/A	4.9339	5.1037	5.0467	4.6841	4.2561	4.9475	4.4247	5.2736	4.6425	5.2557	5.0443	5.4103	4.7684	6.2727	4.8860	4.8436	4.8944	4.5875	4.3313	5.6797
Akap5	_DEKGPVAAS[Phospho (STY)]PQK_.2	#N/A	10.1678	10.2593	10.5837	10.3448	10.0930	10.0989	9.8392	10.0761	9.9427	10.3785	10.4011	9.9815	10.0622	10.8338	9.8667	10.1983	10.5286	10.0733	10.2164	10.4254
Akap5	_SSAIQMGT[Phospho (STY)]PELEK_.2	#N/A	3.5347	8.7814	9.8418	9.8561	7.2228	7.8426	8.4509	3.7604	3.3895	7.9328	9.1748	7.2922	3.5242	10.1268	7.3898	3.8136	7.9287	6.2781	6.5557	7.6957
Akap5	_HTPEAGGS[Phospho (STY)]GQR_.2	D3YVF0_S68_M1_Akap5	4.4656	5.2572	6.4191	3.1186	3.1717	3.4358	4.3293	3.1905	4.3884	4.0803	6.0273	5.4239	4.4965	5.3857	3.9065	3.8277	4.1885	5.7050	3.2600	3.7175
Akap5	_EATVVGQAEEATVDRAEEATVGQAEEATVGHTEK_.3	#N/A	6.2103	3.7024	3.1532	3.9676	3.8236	3.8569	3.2245	4.6637	3.1447	3.4272	4.1242	5.1513	5.8254	8.2909	3.2499	2.9788	5.7818	8.2218	4.1089	4.2385
Akap5	_METS[Phospho (STY)]VSEIQVETK_.2	D3YVF0_S4_M1_Akap5	4.0515	6.0473	3.5881	6.5182	4.2584	3.8499	4.2905	4.2772	4.2107	3.8621	3.6893	2.9937	4.0824	3.6262	2.8150	3.4136	3.7744	3.3750	3.6741	3.3034
Akap5	_TAEETKKHT[Phospho (STY)]PEAGGSGQR_.3	D3YVF0_T62_M1_Akap5	4.4669	4.3530	4.0035	3.1173	3.1730	3.2063	3.8751	3.6404	2.3047	4.2775	3.2740	4.0792	3.9608	4.4634	3.9005	3.8290	4.1781	3.6550	3.3036	4.4304
Akap5	_VTVDQAEETTVGQAEEATVGQAGEAILSQAK_.3	#N/A	3.8481	3.4710	3.3847	2.1097	4.0550	4.0533	2.9931	1.8990	3.7709	3.1970	4.1453	3.1971	3.8790	3.4227	3.0184	3.2102	3.5710	3.5784	3.8775	3.1000
Akap5	_METSVSEIQVETKDEKGPVAAS[Phospho (STY)]PQK_.3	#N/A	3.4947	3.9750	5.8835	6.3729	4.3333	5.0520	4.1029	3.5697	4.8783	4.8187	3.6413	4.3975	3.3749	4.4195	4.7043	3.8396	3.0670	4.0825	4.3815	4.0968
Akap5	_SGETALGQAEEAAVGQADKR_.2	#N/A	4.4070	3.5958	3.4425	3.2823	2.3972	4.2402	4.3421	4.1074	4.2623	3.8105	3.7410	3.4852	4.4278	3.4296	0.4072	3.2511	3.1443	0.8036	4.3375	1.0043
Akap5	_QFLISM[Oxidation (M)]ENEQVGVFANDSDFEGR_.3	#N/A	3.4565	3.8626	4.4939	4.1278	3.6634	3.6967	3.3847	2.1381	3.3049	3.2671	4.2844	3.5887	3.4873	4.5319	3.4101	4.3194	3.3104	3.9701	4.2691	4.2092
Dgki	_VKKPQNS[Phospho (STY)]LK_.2	D3YWQ0_S328_M1_Dgki	6.8242	6.8226	6.6927	6.5895	6.1435	5.9013	6.2844	6.3034	5.2804	6.7546	5.8740	6.4815	6.5935	6.6344	5.5754	5.8991	5.9081	6.2755	6.4381	5.6182
Dgkh	_VPLHLES[Phospho (STY)]PHEER_.3	D3YXJ0_S1067_M1_Dgkh	7.7979	8.4337	6.9110	7.4434	7.5007	8.0498	7.9471	7.8450	7.7712	7.9209	8.0795	7.9426	8.2699	8.1051	7.3784	7.8691	7.5288	8.1723	7.8474	7.8563
Dgkh	_KVS[Phospho (STY)]TSGQMR_.2	D3YXJ0_S53_M1_Dgkh	3.6915	3.6275	3.2281	3.8927	3.8985	4.2099	5.2276	3.9172	3.6144	3.5021	3.9887	3.3537	3.7224	3.2662	3.1750	3.0537	3.4145	3.7350	4.0340	4.4443
Samd1	_AAAATAPPS[Phospho (STY)]PGPAQPGPR_.2	D3YXK1_S150_M1_Samd1	4.5734	4.2466	4.1100	4.5117	3.2795	3.3128	3.7686	3.2982	3.6888	4.3840	3.1675	3.9727	3.8543	4.3569	3.7940	3.9355	4.2963	3.7615	3.4101	7.0486
Samd1	_YLGGS[Phospho (STY)]GGASGR_.2	D3YXK1_S242_M1_Samd1	4.5741	3.4287	3.6096	3.8187	3.9119	4.1359	3.6292	5.4925	3.6883	3.2796	4.0627	3.3181	3.7964	3.3401	5.8123	3.4182	3.4884	3.6610	3.9601	3.0174
Safb	_APTAALS[Phospho (STY)]PEPQDSK_.2	#N/A	10.0124	9.6401	10.4215	9.8935	9.6419	9.4764	9.1432	9.9537	9.7446	9.8810	9.6856	9.6410	9.6917	10.5039	9.4132	9.5438	10.1052	9.5515	9.6494	9.6755
Safb	_APTAALS[Phospho (STY)]PEPQDSKEDVK_.2	#N/A	7.9354	7.9840	8.0855	8.0771	7.7754	7.9353	7.7362	8.2803	7.7275	8.1856	7.9860	7.8991	8.4503	8.3825	7.6386	7.9080	8.2495	7.7403	7.9628	8.0277
Safb	_SVVS[Phospho (STY)]FDKVK_.2	D3YXK2_S626_M1_Safb	9.3083	8.8838	9.4056	9.5088	9.0357	8.1719	8.3792	3.2643	8.4722	9.1521	8.4315	9.1375	9.4680	8.9913	8.0241	8.8725	8.7863	8.3927	8.2066	8.3688
Safb	_APTAALS[Phospho (STY)]PEPQDSKEDVKK_.3	#N/A	10.4623	10.7530	10.5927	10.4646	10.2059	10.2871	10.3430	10.7259	10.4305	10.6476	10.1685	10.4911	11.2994	11.0128	10.4266	10.4620	10.7270	10.5397	10.5009	10.5131
Safb	_[Acetyl (Protein N-term)]AETLSGLGDASAAGAAAVSSAAS[Phospho (STY)]ETGTRR_.3	#N/A	3.8849	3.4926	3.4727	4.1898	3.8168	3.6974	3.7244	3.6594	3.3362	3.0505	3.7418	4.9165	4.2370	3.1052	3.3016	3.4720	3.0666	2.9850	3.4208	3.2742
Safb	_[Acetyl (Protein N-term)]AETLSGLGDASAAGAAAVSSAASETGTR_.3	#N/A	3.2518	3.2133	4.4553	3.9400	3.7437	3.0008	3.3262	3.6436	3.3141	3.8149	4.0229	3.2737	3.4488	4.5589	2.3172	3.8648	3.6787	4.0091	3.7994	3.5197
Safb	_AIEDEGGNPDEIEVTSEC[Carbamidomethyl (C)]NK_.2	#N/A	3.8526	3.4665	3.3892	3.7316	4.0596	4.0488	3.7164	4.0783	4.1167	3.1925	4.1498	3.1926	3.8835	3.4273	3.2936	5.4317	3.5755	3.5739	5.7732	3.1046
Safb	_MSS[Phospho (STY)]VEEDSDTKR_.2	D3YXK2_S406_M1_Safb	4.3745	3.6283	3.4100	3.3149	3.7122	3.9363	3.8288	4.0749	5.1456	4.5808	4.2623	3.5177	4.3952	3.3971	3.4060	3.2186	3.1117	3.2206	4.3700	3.3641
Safb	_SEPVKEEGS[Phospho (STY)]ELEQPFAQATSSVGPDR_.3	D3YXK2_S235_M1_Safb	4.3384	4.3868	3.3738	3.3510	3.6761	4.3088	4.8888	4.0387	4.1936	3.8792	3.6723	3.5539	4.3591	3.3609	3.4422	3.1824	3.0756	4.2640	4.4062	3.3279
Safb	_RPYEVDGR_.2	#N/A	3.5158	4.4870	4.0521	4.1735	2.8536	3.6305	3.4509	3.2162	3.3711	3.2008	4.3506	4.3764	3.5366	4.0392	3.4763	3.8607	3.7539	4.0793	3.7278	4.0062
Ccdc6	_SSS[Phospho (STY)]PDKFK_.2	#N/A	8.0597	8.1503	7.8846	8.1079	7.9218	4.4627	8.0014	7.9110	7.7633	8.1100	8.2217	7.9085	8.1538	8.5360	4.1450	8.2007	8.0636	8.0655	7.9077	8.3904
Ccdc6	_RSSS[Phospho (STY)]PDKFK_.2	#N/A	6.6867	6.7670	3.5963	3.7269	3.8986	4.1339	4.4498	6.4868	6.2814	4.0428	3.7014	6.6513	6.9463	7.1176	3.2673	6.4523	3.8975	6.5502	4.2312	3.5504
Ccdc6	_LDQPVSAPPS[Phospho (STY)]PR_.2	D3YZP9_S237_M1_Ccdc6	10.8233	10.8197	11.0154	10.3745	9.4823	10.2293	10.2023	10.3256	10.1498	11.3275	10.3526	10.6017	10.7240	10.9503	9.7200	10.6543	10.6212	8.8069	10.3066	10.6691
Ccdc6	_QLS[Phospho (STY)]ESESSLEM[Oxidation (M)]DDER_.2	#N/A	6.3588	5.6886	7.0353	6.2760	5.4600	5.3451	5.5039	5.6700	5.6711	6.4795	5.4181	6.0101	3.3117	6.2066	4.9846	6.0320	5.9537	5.6307	5.7953	6.2909
Shank1	_EQDVVPFQES[Phospho (STY)]PK_.2	#N/A	12.6961	12.8511	13.1768	12.8322	11.3845	12.9268	12.5614	11.7581	12.6594	12.8777	12.5969	12.3247	12.4964	13.0668	11.9090	12.5065	13.1898	12.5575	12.7775	12.6634
Shank1	_S[Phospho (STY)]IDEGM[Oxidation (M)]FSAEPYLR_.2	#N/A	12.1092	12.3303	12.2173	11.8263	10.5278	11.6345	11.6003	11.1590	11.6034	11.9024	11.4673	10.9978	11.6560	11.4322	10.9145	11.4485	11.6641	10.6726	11.4745	11.3428
Shank1	_GSST[Phospho (STY)]EDGPGVPPPSPR_.2	D3YZU1_T1518_M1_Shank1	3.3285	3.4724	10.3891	4.3551	13.2251	11.8857	3.3613	4.2186	11.6461	7.5013	3.9417	3.2983	12.7627	3.5475	10.1655	11.8280	3.3845	4.2445	11.5439	9.9319
Shank1	_EQDVVPFQES[Phospho (STY)]PKYAAR_.2	#N/A	8.9087	9.7725	3.8886	3.8789	3.5873	9.1615	9.3872	9.2372	9.3754	8.6309	3.7175	8.6725	10.6421	9.8022	8.9283	8.5766	9.2954	9.6028	8.7898	9.4577
Shank1	_DGPAGGTGGSGGPGGS[Phospho (STY)]LGSR_.2	D3YZU1_S540_M1_Shank1	6.9720	6.9018	7.4716	3.7758	5.9196	3.4754	6.2622	3.1509	6.4670	6.5593	6.5223	6.1989	6.9025	6.9981	3.8670	6.2189	6.6578	5.7263	5.9525	6.4001
Shank1	_NHREQDVVPFQES[Phospho (STY)]PK_.3	#N/A	6.9106	7.2995	5.1608	6.1660	5.2607	5.8741	6.7768	6.8980	6.7337	6.6568	3.7403	6.6324	8.3831	7.1577	6.3751	7.3944	6.9151	7.1571	6.4670	6.6557
Shank1	_GFFATESS[Phospho (STY)]FDPHHR_.2	#N/A	3.7988	3.9263	6.7930	3.6129	3.6561	3.8044	4.4756	3.8899	3.8694	3.8111	3.6599	3.7546	4.2102	3.9466	3.6208	3.7111	3.6612	5.7900	5.5528	3.9136
Shank1	_ALDPASPLGLALAAR_.2	#N/A	4.9481	5.0964	4.3303	4.7324	3.1931	5.2721	4.4887	4.4956	4.7566	5.2767	4.2983	4.6756	4.5151	4.3862	4.0222	4.4355	5.2528	4.8611	4.5406	5.1565
Shank1	_QKS[Phospho (STY)]IGAAEDDRPYLAPPAMK_.3	#N/A	3.3039	3.7842	7.6423	6.9863	4.1424	4.3665	3.3986	3.7606	3.4577	3.5102	6.9370	3.0875	3.5658	7.8862	2.9758	3.6488	3.2578	3.8916	4.1907	4.2877
Shank1	_AGLGS[Phospho (STY)]QEK_.2	D3YZU1_S1434_M1_Shank1	6.6911	7.0318	6.7003	6.7413	6.5158	6.3944	6.7182	4.2053	6.1566	7.0729	7.0133	5.6934	6.6792	6.5571	3.2756	6.1614	3.2422	6.8780	7.2479	6.8478
Shank1	_GGSGGS[Phospho (STY)]TDSHHGGASYIPER_.3	D3YZU1_S1907_M1_Shank1	7.1394	7.5648	4.0504	7.1741	6.5202	6.4790	4.3136	5.7146	6.2875	3.9711	7.1753	3.6025	7.1493	3.8603	5.4211	6.8104	6.1597	7.0182	7.0541	3.5375
Shank1	_GRPSS[Phospho (STY)]SGTPR_.2	D3YZU1_S519_M1_Shank1	4.5996	3.2538	3.7844	4.4413	4.2684	3.8982	3.4544	4.4493	4.3759	3.4686	4.0829	3.1433	3.2688	3.7715	3.0316	3.5930	3.4862	4.3470	3.9956	3.7385
Shank1	_S[Phospho (STY)]IGAAEDDRPYLAPPAM[Oxidation (M)]K_.3	#N/A	7.7338	7.6870	7.6525	7.4690	6.5094	7.1290	6.8662	6.3090	6.5488	7.3417	6.7335	6.3069	6.0846	7.0335	6.0225	6.8287	6.5051	6.8339	7.1164	6.9864
Shank1	_THS[Phospho (STY)]PATS[Phospho (STY)]EDEER_.2	D3YZU1_S4_M2_Shank1	3.2807	3.2212	3.8170	4.4087	4.1193	4.3434	3.4218	4.4819	3.4809	3.4870	3.8553	3.1107	3.3014	3.8041	2.9990	3.6256	3.5188	4.3144	3.9630	3.7711
Shank1	_HS[Phospho (STY)]KSIDEGMFSAEPYLR_.3	D3YZU1_S1289_M1_Shank1	4.0070	5.3462	4.0825	4.1731	3.6924	3.9686	3.6492	3.7111	3.6569	3.7573	3.7696	3.6397	3.7858	6.1156	3.4554	3.3691	3.7299	4.1635	3.8268	3.7979
Shank1	_VGWFPSDC[Carbamidomethyl (C)]LEEVANRS[Phospho (STY)]QEGR_.3	D3YZU1_S615_M1_Shank1	3.7410	6.5404	3.6825	3.8993	4.2268	4.3578	6.3825	3.6601	3.8687	3.7183	3.7347	3.2886	3.9804	3.6696	2.8696	3.5666	3.3843	6.2081	4.1409	3.6366
Shank1	_LESGGS[Phospho (STY)]SGGYGAYAAGSR_.2	D3YZU1_S1310_M1_Shank1	3.9269	3.3922	3.4635	3.6573	4.1338	3.9745	3.7906	4.1526	3.8497	3.1182	4.2241	3.1183	3.9578	3.5015	3.3678	3.2890	3.6498	3.4996	3.7987	3.1788
Selenon	_RPHS[Phospho (STY)]PDPGAQPAPPR_.3	D3Z2R5_S13_M1_Selenon	6.7626	6.2493	6.6165	5.5634	6.6852	5.9565	6.2556	6.4922	6.5279	6.6446	6.5275	6.1630	6.3883	6.1905	5.4388	6.5945	6.7271	6.1285	5.6839	6.2755
Bin2	ASGSGSC[Carbamidomethyl (C)]NAPGS[Phospho (STY)]PEGSSQLC[Carbamidomethyl (C)]SPR	D3Z6Q9_S430_M1_Bin2	5.1416	5.0709	5.5388	3.3254	4.2658	5.1905	5.3329	4.2846	4.2033	4.8603	3.6820	2.9863	4.0897	3.6335	5.0666	3.4210	3.7818	5.0041	3.6667	4.7969
Bin2	_AS[Phospho (STY)]PDASSNPEPAETR_.2	D3Z6Q9_S443_M1_Bin2	6.7212	6.1230	6.4428	4.3139	5.9020	6.7333	6.0737	6.2317	6.4117	3.3412	6.3510	5.9415	3.3962	3.8988	5.8132	5.5511	6.1912	6.1878	6.2263	6.0532
Gsg1l	_IEKGDVS[Phospho (STY)]EEEDFR_.2	D3Z7H4_S274_M1_Gsg1l	6.5629	6.4787	5.5031	6.7712	3.8094	5.8433	5.8101	6.0857	3.5253	3.4130	6.2372	6.3018	6.3874	6.2469	3.2641	5.8439	5.9421	5.7639	6.3397	5.6308
Gls	_VADYIPQLAK_.2	#N/A	8.2446	7.9255	8.3389	8.4659	8.0584	7.7795	7.9833	8.1239	8.0568	8.2390	8.2094	8.0834	7.6916	7.5635	8.4271	8.1490	8.1537	8.0563	8.2546	8.1509
Gls	_VLSPEAVR_.2	#N/A	7.5620	8.0213	7.6413	7.7905	7.9996	7.9971	8.3090	7.8377	7.5499	7.7456	7.8055	8.1473	7.7799	7.5063	8.0683	7.7199	7.2243	8.0567	7.9120	7.6972
Gls	_LTLQTTSDGVM[Oxidation (M)]LDKDLFK_.3	#N/A	6.1185	5.7707	6.1897	5.8714	6.0975	6.1107	5.9590	5.9673	6.0914	6.6802	5.7027	6.0457	6.1718	5.4945	5.9775	6.0504	6.2023	6.0885	5.7853	5.9274
Gls	_DSPGETDAFGNSEGK_.2	#N/A	8.1519	8.2733	8.5641	8.5071	8.1656	8.3482	8.3423	8.3247	8.4151	8.3713	8.0331	8.3174	4.0209	8.5178	8.3128	8.1900	8.6437	8.3445	8.3001	8.2944
Gls	_C[Carbamidomethyl (C)]VQSNIVLLTQAFR_.2	#N/A	3.7195	4.0237	3.5181	4.0807	3.4379	4.2107	3.0147	3.6779	4.2807	2.4189	3.4329	3.2243	4.4461	4.0660	3.5459	4.0792	3.5832	4.0762	3.9322	3.7457
Gls	_YAIAVNDLGTEYVHR_.2	#N/A	5.8316	5.2530	5.2911	5.7510	5.4444	5.3552	5.5260	6.0599	5.6361	6.0986	5.8511	4.9814	6.3206	5.2270	5.1768	5.6580	6.0564	5.0294	5.4159	5.4209
Gls	_M[Oxidation (M)]AGNEYVGFSNATFQSER_.2	#N/A	4.8192	4.2699	3.2185	3.8955	4.4038	4.0190	4.5324	5.2420	4.4216	4.8030	3.6646	4.5182	5.0254	3.2312	3.7070	4.4084	4.8297	4.7933	4.4439	4.3294
Gls	_ILQEYQVQYTPQGDSDDGKGNQTVHK_.3	#N/A	3.6163	3.6370	3.8681	4.8125	3.8233	3.8565	4.3090	3.9002	4.0884	4.1101	3.8147	3.4066	3.2970	4.0241	4.1080	4.6177	3.6441	4.5508	3.8393	4.2160
Gls	_TALHVAAAEGHVEVVK_.2	#N/A	3.6929	3.4929	4.2116	3.7581	3.6430	4.3914	3.8315	4.1129	3.8050	3.8902	2.9711	3.7887	3.9480	4.2497	3.7047	2.8967	3.6196	2.8215	4.4944	3.8530
Gls	_FALSAMDMEQR_.2	#N/A	4.1899	3.8129	3.2253	4.1059	3.5276	3.7517	4.0135	4.3754	4.0726	4.3962	2.9461	3.7024	4.1806	3.7244	3.5907	3.0339	3.8727	3.2768	3.5758	3.4017
Gls	_SVINLLFAAYTGDVSALR_.3	#N/A	3.8409	4.2474	3.6388	3.9340	3.7146	4.4406	3.7965	3.8029	3.7676	3.8101	3.6521	3.3665	3.9117	4.0170	3.6782	3.6461	4.1109	3.7101	3.7865	3.7857
Gls	_ILQEYQVQYTPQGDSDDGK_.2	#N/A	4.1899	3.1292	3.7265	3.3943	2.8960	4.4302	4.1521	4.4156	4.0723	4.0005	3.5510	4.3562	4.2378	3.7645	4.1775	3.5520	3.9128	3.3780	3.0266	3.2066
Prrt2	_AS[Phospho (STY)]PSPSPSEAR_.2	#N/A	10.7566	10.6949	10.7971	10.9859	10.3555	10.6115	10.4311	10.7782	10.4679	10.2848	10.6345	10.4949	10.1956	10.7910	9.0860	10.1706	10.7687	10.8157	10.5022	10.3383
Prrt2	_AHGGHPGS[Phospho (STY)]PRGS[Phospho (STY)]LSR_.3	#N/A	9.5088	9.3854	9.2896	9.4917	8.3357	8.1973	9.1457	8.5084	8.5654	9.7763	8.6776	8.5828	8.8747	8.4741	7.0115	8.3638	8.5127	8.7735	8.7077	8.5740
Prrt2	_HPS[Phospho (STY)]SQLAGPGVEGGEGTQKPR_.3	E9PUL5_S254_M1_Prrt2	6.5831	6.5793	3.6789	4.4301	3.5904	4.2459	5.0860	4.9631	4.5120	5.1810	4.5230	5.2538	4.7483	4.2025	3.6230	3.6778	4.3652	4.2837	4.3939	4.3493
Prrt2	_T[Phospho (STY)]PPANGAPPR_.2	E9PUL5_T214_M1_Prrt2	4.2397	9.8034	3.2026	3.5222	7.1873	9.6809	4.1023	7.2686	9.5106	8.2995	3.5011	3.7251	6.4899	3.1897	5.2006	9.5059	4.4052	9.6542	3.0765	3.1567
Prrt2	_QENGAVVPLQAGDGEEGPAPQPHS[Phospho (STY)]PPSTK_.3	E9PUL5_S208_M1_Prrt2	4.5138	8.0248	4.0504	4.5712	3.2199	3.3876	4.3776	7.9752	4.4367	4.0321	3.3102	6.9155	7.8233	8.4437	5.1830	3.8760	7.3559	4.4135	3.2117	3.7658
Prrt2	_[Acetyl (Protein N-term)]AASS[Phospho (STY)]SQVSEMK_.2	E9PUL5_S5_M1_Prrt2	3.5398	4.0717	3.9600	4.1052	3.9835	3.5928	3.6977	3.7732	3.2462	3.4911	3.5735	3.8734	3.3925	5.4198	3.7271	3.7796	3.9666	4.2328	4.1141	3.9418
Prrt2	_AELAPETTETPVETPETVQATDLSLNPEEGSKAS[Phospho (STY)]PSPSPSEAR_.4	#N/A	9.5883	9.4888	8.6138	9.1417	8.4137	8.8951	9.0327	9.0022	8.4596	8.9409	8.6924	3.9008	9.4700	9.4698	7.9392	8.7011	9.2002	9.3295	8.8019	8.6441
Prrt2	_AHGGHPGS[Phospho (STY)]PR_.2	E9PUL5_S244_M1_Prrt2	4.3592	5.5434	3.3947	3.3302	3.6969	4.2880	4.2943	4.0596	4.2145	3.8583	3.6931	3.5330	4.3799	3.3818	2.8188	3.2033	3.0964	4.8054	4.3853	3.3487
Prrt2	_AELAPETTETPVETPETVQATDLSLNPEEGSK_.3	#N/A	3.3264	3.0399	2.6030	2.9102	2.8325	4.2116	4.1073	3.7977	4.1664	4.8911	2.5514	2.9835	4.2744	4.0367	3.6845	3.8582	5.3388	3.0470	3.1476	3.9123
Fam53a	_SLS[Phospho (STY)]EPEELAR_.2	E9PV82_S119_M1_Fam53a	4.4899	4.3300	4.0265	3.0943	3.1960	7.7362	3.8521	3.6174	3.7723	4.3005	3.2509	4.0561	3.9377	4.4404	3.8775	3.8520	4.1551	7.6737	3.3267	4.4074
Mctp1	_AAS[Phospho (STY)]PSFQAR_.2	E9PV86_S16_M1_Mctp1	8.3595	7.8933	7.8422	4.0206	7.2389	3.8039	3.2775	3.7894	6.7537	3.3743	7.0140	6.6132	6.9215	6.4927	3.3029	4.4266	6.5989	3.8629	4.1619	4.3164
Mctp1	_GSSTATGTGGVT[Phospho (STY)]PPGGR_.2	E9PV86_T145_M1_Mctp1	3.9118	4.0911	4.4480	3.7776	3.2495	3.2346	4.2916	3.6121	3.7670	4.1181	3.2457	3.9805	3.9325	4.4351	3.8688	4.2567	4.1498	3.6834	3.3319	4.4021
Setd1a	_S[Phospho (STY)]GS[Phospho (STY)]PAPETTNESVPFAQHSSLDSR_.3	E9PYH6_S481_M2_Setd1a	3.7112	3.6079	5.0659	3.8731	3.9181	4.1903	3.1300	3.9369	3.6340	3.5218	4.0084	3.3340	3.7420	4.3075	3.1554	3.0733	3.4341	3.7154	4.0144	2.9631
Prr12	_TSS[Phospho (STY)]FHLLR_.2	E9PYL2_S1070_M1_Prr12	7.8575	7.7712	3.3759	7.3896	3.8380	6.5182	6.7572	7.1405	6.8185	3.2058	6.9217	3.2059	3.8702	7.2271	3.2802	3.3444	7.0293	3.5872	6.8249	7.0379
Prr12	_TEDEEFLIQHLLQAPS[Phospho (STY)]PPR_.3	E9PYL2_S648_M1_Prr12	5.0674	5.2641	4.6750	4.4306	4.3467	4.1506	5.6029	4.7666	4.8152	3.5209	3.8214	4.2738	4.2023	5.1224	3.9748	3.4869	3.2471	5.3102	3.9456	3.9956
Prr12	_ASLAC[Carbamidomethyl (C)]SPLGGGEPS[Phospho (STY)]PGAGEPSK_.2	E9PYL2_S338_M1_Prr12	4.0773	4.7386	4.0564	3.7722	3.7416	4.4916	4.2296	3.9603	4.1413	3.4478	3.7806	4.3980	4.1012	4.0763	3.2698	3.8759	3.1944	4.2161	4.2759	3.8624
Prr12	_NLETLPSFS[Phospho (STY)]S[Phospho (STY)]DEEDSVAK_.2	E9PYL2_S1372_M2_Prr12	3.7127	3.6064	3.2493	3.8715	3.9196	3.9530	3.1285	4.3946	3.0487	3.5233	4.0282	3.3325	3.2141	3.7168	3.1538	3.0748	3.4315	4.4017	4.0503	3.6838
Igsf9b	_TGS[Phospho (STY)]PELAAR_.2	E9PZ19_S1215_M1_Igsf9b	7.5548	8.0087	3.6646	8.1197	7.7126	7.7091	7.9078	7.3515	7.5252	7.9819	7.9512	7.4792	4.2996	7.2929	6.6622	7.3871	3.8509	8.0515	7.5496	3.3799
Igsf9b	_AEAEAEATT[Phospho (STY)]PIELISR_.2	E9PZ19_T845_M1_Igsf9b	3.2995	3.2025	3.8358	4.3899	3.8136	7.3289	3.2345	4.5007	6.8666	3.4172	4.1342	3.0919	3.3202	7.2993	5.6732	2.9687	7.0435	7.2442	3.9442	6.8024
Igsf9b	_SLES[Phospho (STY)]PLSSGKVS[Phospho (STY)]PESIR_.2	E9PZ19_S775_M2_Igsf9b	4.2041	4.6159	3.7406	3.3802	2.9102	4.4443	4.1379	4.4297	4.0582	4.0147	3.5368	4.3420	4.2236	5.9275	4.1633	3.5662	3.9270	3.2225	3.5215	5.4834
Igsf9b	_APS[Phospho (STY)]ESS[Phospho (STY)]DDQGQPAAK_.2	E9PZ19_S794_M2_Igsf9b	4.3281	3.6747	3.3635	3.3613	3.6658	3.8899	3.8753	4.0285	3.9344	4.5344	4.3087	3.5642	4.0424	3.3506	3.4525	3.1721	3.0653	3.2670	4.4164	3.3176
Chd2	_LKDEHGLEPAS[Phospho (STY)]PR_.3	E9PZM4_S1365_M1_Chd2	6.4128	6.9650	5.8911	6.3448	5.0090	6.6074	6.1740	5.4910	6.1449	6.8471	6.5389	5.3061	6.4454	5.8639	4.8222	5.6722	6.4524	6.2972	6.4039	5.4927
Chd2	_KQES[Phospho (STY)]S[Phospho (STY)]DDDDDDEAPKR_.2	E9PZM4_S207_M2_Chd2	3.9238	4.0790	4.4601	3.7656	3.2615	3.2226	1.9699	3.6242	2.4340	4.1301	3.2577	3.9685	3.9445	4.4472	3.8568	4.2687	2.0567	3.6713	3.3199	4.4142
Chd2	_SPLDHRS[Phospho (STY)]PLER_.3	E9PZM4_S1806_M1_Chd2	4.1613	3.1578	3.6979	3.4229	2.8674	4.4016	4.1807	4.3870	4.1009	3.9719	3.5796	4.3848	4.2664	3.2682	4.2061	3.5234	3.8842	3.3494	2.9980	3.2352
Irf2bp2	_LEEPPELNRQS[Phospho (STY)]PNPR_.3	#N/A	8.8885	9.0537	9.0213	9.3448	8.8641	8.8111	8.5869	8.6577	8.7544	9.3109	8.9434	8.9126	9.1122	9.5064	8.7466	9.1057	8.9857	8.5590	8.8744	9.2254
Irf2bp2	_S[Phospho (STY)]PTGAQPAAAKPPPLSAK_.2	E9Q1P8_S71_M1_Irf2bp2	7.7312	7.6153	7.0576	3.5848	7.0612	7.4690	7.1769	7.9521	6.4974	7.5110	7.3308	7.7910	7.9788	8.6043	4.0650	7.5343	7.8322	7.5438	7.4680	7.5937
Irf2bp2	_RKPS[Phospho (STY)]PEPEGEVGPPK_.3	E9Q1P8_S343_M1_Irf2bp2	5.4912	6.0521	5.5673	5.6829	5.9376	6.2679	5.3743	4.6852	5.0254	6.3036	5.9679	5.5601	5.5060	6.5525	5.7508	5.9328	4.1109	5.4587	6.0567	5.8010
Irf2bp2	_S[Phospho (STY)]PADSLSSAAGASELSAEGAGK_.2	E9Q1P8_S250_M1_Irf2bp2	4.1196	4.5999	3.7566	3.3642	3.4573	3.7730	4.0838	4.4457	3.6864	4.3259	3.0164	4.3260	4.2509	4.9458	3.6610	2.9637	3.9429	3.2065	4.7073	3.4720
Irf2bp2	_DTLLALHQHGHS[Phospho (STY)]GPFESK_.3	E9Q1P8_S300_M1_Irf2bp2	4.1402	3.8626	3.1757	3.5492	3.4779	3.0062	4.0753	5.4454	4.2050	4.0773	3.4741	6.2960	4.1609	3.1627	4.1007	2.9843	4.3783	3.4549	3.1035	3.1297
Irf2bp2	_NSSS[Phospho (STY)]PPSPSSMNQR_.2	E9Q1P8_S440_M1_Irf2bp2	3.9244	4.0784	4.4607	3.7650	3.2621	3.2220	3.8594	3.6247	3.7796	4.2932	5.8185	3.9679	3.9451	4.4477	3.8848	4.2693	4.1624	3.6707	3.3193	4.4147
Gpr137c	_RYDS[Phospho (STY)]DDDLPR_.2	E9Q343_S351_M1_Gpr137c	3.6291	4.3737	4.1654	4.0602	7.2936	3.1909	3.0734	3.3295	3.1325	3.8355	6.7632	6.7407	3.2405	7.0461	4.1514	6.7000	3.8672	3.9660	6.7361	6.7329
Pi4ka	_TSSVSSISQVS[Phospho (STY)]PER_.2	#N/A	12.6183	12.3413	12.4741	12.2969	11.9480	11.7990	12.0574	12.2242	11.8876	12.1103	12.0252	11.8288	12.5571	11.6455	10.2541	11.6289	12.3298	11.9641	11.7081	11.7074
Pi4ka	_KTSSVSSISQVS[Phospho (STY)]PER_.2	#N/A	6.3571	6.5822	6.1296	6.3868	3.6040	5.9935	6.2277	6.4148	3.7755	5.9764	3.7152	6.0281	6.2747	6.0577	3.6525	6.0127	5.9988	6.0555	3.6803	3.8952
Ryr2	_EAAVPEEEGGT[Phospho (STY)]PEKEISIEDAK_.3	#N/A	3.1730	8.6348	4.2104	4.4112	3.3799	8.8275	8.4689	8.0200	8.4669	4.4844	3.0670	3.8722	8.4083	4.2565	3.6935	4.0360	4.3968	3.8620	3.5106	4.2235
Ryr2	_EAAVPEEEGGT[Phospho (STY)]PEK_.2	#N/A	8.2646	7.6207	7.6264	7.5403	7.4700	8.1248	7.4639	8.1804	7.7654	6.6582	7.5180	7.4498	7.5427	7.3112	6.2324	6.6829	8.2476	7.3296	7.2465	7.3968
Ryr2	idomethyl (C)]YM[Oxidation (M)]VC[Carbamidomethyl (C)]AGESM[Oxidation (M)]S[Phospho (	#N/A	6.7366	7.4425	5.6651	7.2201	5.7361	7.0718	7.3068	6.0897	6.3824	6.1760	6.1329	5.9116	5.5045	6.6745	5.0629	5.5527	6.7218	6.5095	6.2580	5.7001
Ryr2	_IAEDPSRDGPS[Phospho (STY)]PTSGSSK_.2	#N/A	6.5047	4.1706	4.2934	4.1950	5.5284	6.0854	4.3436	6.1811	5.9203	5.1084	4.5141	3.9750	5.9615	4.3028	3.9208	4.1094	5.4525	4.0609	3.2420	4.1487

Ryr2	_DGPS[Phospho (STY)]PTSGSK_.2	#N/A	3.8805	4.1613	5.3793	3.8479	3.2183	5.6669	5.8754	4.0052	3.7402	4.0869	3.2945	4.0508	3.8482	3.9903	3.9391	3.5446	3.9132	3.6319	3.5544	3.8361
Ryr2	_ISQTSQVS[Phospho (STY)]DAAHGYSPR_.3	E9Q401_S2813_M1_Ryr2	4.2296	4.5903	3.7662	3.3546	2.9357	3.6719	4.1124	4.4553	4.1524	4.3164	3.0259	4.3165	4.2605	3.8042	4.1378	3.5917	3.9525	5.3670	3.4960	3.4815
Arid1b	_APS[Phospho (STY)]PASFQR_.2	E9QJN7_S1550_M1_Arid1b	8.2110	4.0838	4.2727	8.1058	3.4422	3.1653	8.8589	8.9228	8.0905	3.8098	8.0091	3.8099	9.2477	4.3108	4.1771	8.1904	8.8340	4.1912	8.0193	8.4157
Arid1b	_SS[Phospho (STY)]PALAAPDASVDPK_.2	E9QJN7_S1757_M1_Arid1b	3.8085	7.1743	3.7530	3.9267	3.1462	3.3837	6.5343	6.7615	6.5627	6.6656	6.9052	4.1295	6.6817	4.3319	4.0178	4.1534	4.0465	6.4988	6.3711	6.3646
Pde8b	_RLS[Phospho (STY)]GNEYVFTK_.2	E9Q4S1_S497_M1_Pde8b	3.1118	4.2085	4.3306	3.8951	3.3187	3.3521	3.7294	4.7328	4.8060	4.4232	4.9251	5.4049	3.8150	4.3177	3.7547	5.5056	4.0324	5.7950	5.1651	4.7997
Pde8b	_IHRDS[Phospho (STY)]GGNSQTEPHSFR_.3	#N/A	3.3904	5.8330	3.7807	5.4415	5.3389	3.7630	3.3574	3.6727	5.5983	5.6563	3.7909	3.8571	5.9712	6.2207	3.7454	3.7353	3.6708	3.8415	3.8213	3.6557
Pde8b	VNSINKPLAAESEGS[Phospho (STY)]DC[Carbamidomethyl (C)]EC[Carbamidomethyl (C)]NPTGK	E9Q4S1_S734_M1_Pde8b	3.7403	4.2206	4.1359	4.7481	3.0780	3.9046	4.4631	3.3242	3.0213	3.9466	3.3957	4.8197	3.3294	4.1740	4.0403	4.0852	4.6630	5.5459	3.1263	3.8513
Nolc1	_ASS[Phospho (STY)]PFRR_.2	E9Q5C9_S626_M1_Nolc1	3.1045	4.2146	7.0287	7.6572	3.3115	3.3448	3.7366	3.3302	3.7829	4.4160	3.1355	7.6481	3.8223	7.5256	3.7620	3.9675	4.3283	3.7935	3.1202	4.2919
Nolc1	_GGSISVQVNSVKFDS[Phospho (STY)]E_.2	E9Q5C9_S701_M1_Nolc1	4.1564	5.9721	3.6930	3.4278	2.8625	5.1563	4.1856	3.9509	4.1058	3.9670	3.5844	4.3896	4.2712	3.2731	4.2110	3.5185	4.4886	5.5548	2.9932	3.2401
Nolc1	_AAKES[Phospho (STY)]EEEEEEEEEEK_.2	#N/A	4.1557	3.7497	4.4181	4.0147	3.5433	2.9368	4.1647	4.4427	4.3447	4.0591	3.2552	4.2367	4.1366	4.2943	3.2542	3.9632	3.6750	4.3794	3.6862	3.6255
Setd2	_TGS[Phospho (STY)]PTSNQLNDSPTTK_.2	E9Q5F9_S614_M1_Setd2	4.9224	6.4782	4.4990	4.4079	3.7125	3.4555	3.2468	3.3191	4.3946	3.5967	3.9685	4.5401	4.8440	4.1421	3.8097	4.1508	5.4732	4.4982	4.3231	4.1091
Setd2	_RGS[Phospho (STY)]LS[Phospho (STY)]PPSSAYER_.2	#N/A	3.9998	5.3956	8.3087	3.2822	4.2067	8.5144	8.2601	7.1425	7.8390	3.8104	3.7411	8.3802	4.4279	3.4297	6.3521	8.0045	8.7406	7.0159	4.3374	6.9971
Setd2	_RRGS[Phospho (STY)]LS[Phospho (STY)]PPSSAYER_.3	#N/A	3.9368	3.3822	3.4734	3.2193	4.1438	4.1771	4.4052	4.1705	4.3254	3.7474	3.8040	3.1084	4.4908	3.4926	2.9297	3.2990	3.0246	3.1250	4.2744	3.4596
Ythdc1	_AKS[Phospho (STY)]PT[Phospho (STY)]PDGSER_.2	#N/A	6.9891	6.6913	4.9997	4.6610	5.6021	6.3029	6.9192	6.1949	6.4413	5.2034	6.3265	6.2349	6.5768	6.1455	3.2858	3.7941	6.6804	6.9395	4.6688	4.3236
Ythdc1	_RAKS[Phospho (STY)]PT[Phospho (STY)]PDGSER_.2	#N/A	4.0368	6.8067	4.5731	3.6526	3.3745	6.0925	4.1665	6.1720	3.8921	4.2431	5.7774	3.8554	4.0575	5.3836	3.7437	4.3817	5.5495	6.3938	5.8402	5.2274
Ythdc1	_GIS[Phospho (STY)]PIVFDR_.2	E9Q5K9_S309_M1_Ythdc1	4.0115	3.9913	4.5478	3.6778	3.3493	3.5734	4.1918	3.7119	4.2509	4.2179	3.1244	3.8807	4.3589	4.5349	3.7690	4.3564	4.2496	3.0985	3.2321	3.5800
Ccdc8c	_S[Phospho (STY)]LDDLSAPPHHR_.2	#N/A	8.8463	9.4822	3.5749	9.2157	5.0835	8.5659	8.8708	7.9597	7.6297	9.6666	6.0810	9.1298	3.5810	9.6246	4.9593	4.1061	8.0172	8.6725	8.7217	9.2168
Bod1l	_EVS[Phospho (STY)]PPGAR_.2	E9Q6J5_S3010_M1_Bod1l	8.4011	8.0502	8.4732	8.2257	7.9311	8.4236	8.5897	8.0507	7.8414	8.3595	8.4050	8.4441	8.3523	8.2823	7.0318	7.9499	8.4605	8.1512	8.0261	7.6577
Bod1l	_TETDITTVEQSS[Phospho (STY)]PSGK_.2	E9Q6J5_S2841_M1_Bod1l	5.0233	3.9199	3.7865	4.1851	3.6672	3.7005	3.4420	5.3095	3.3622	5.7637	3.6305	3.6461	3.5277	5.8208	3.4674	3.6120	5.7096	4.1493	4.4967	3.9973
Bod1l	_YYS[Phospho (STY)]DSDDELTVEQR_.2	E9Q6J5_S480_M1_Bod1l	4.3599	4.4600	3.8965	5.3736	3.0660	5.9452	3.9821	3.0847	5.6632	4.1705	3.3810	4.1862	4.0678	3.9345	4.0075	3.7220	4.0828	3.0666	3.3657	3.6118
Bod1l	_AQLS[Phospho (STY)]PSVKR_.2	E9Q6J5_S3000_M1_Bod1l	3.8611	4.1417	4.3974	3.8282	3.1989	3.4229	4.3422	3.5615	4.4013	7.4876	3.2748	7.3530	3.8819	4.3845	7.2562	7.5456	7.5850	7.3740	7.4857	7.3663
Bod1l	_KLS[Phospho (STY)]SQPSTDVSTDKER_.3	E9Q6J5_S236_M1_Bod1l	3.5506	4.0374	4.0869	4.2019	3.4050	3.5906	3.7920	3.7833	4.1316	6.5802	3.6051	3.9268	3.7976	4.0739	3.8151	3.8955	3.7886	3.6297	3.7562	4.8878
Bod1l	_GSDDVLVSGEVPEC[Carbamidomethyl (C)]EVGHM[Oxidation (M)]S[Phospho (STY)]PR_.3	#N/A	3.9410	6.0293	5.3957	5.7373	3.3585	3.8949	5.2866	5.1577	5.6681	3.7259	3.6242	5.3535	5.7646	5.7683	3.7986	5.5227	5.3824	3.7319	5.7960	5.2908
Ttc7b	_LPVSSS[Phospho (STY)]TSNLHVDR_.2	#N/A	7.7330	7.9261	5.4526	7.0929	7.0583	7.4940	6.9984	7.8947	6.0485	6.2786	6.9464	4.9857	3.7710	7.3898	3.2108	3.2262	7.0293	7.4452	7.2288	3.9020
Ttc7b	_SC[Carbamidomethyl (C)]YNLTNPSDSGRGS[Phospho (STY)]SLLDR_.3	#N/A	3.4725	5.8575	4.3054	4.2283	3.6337	3.8070	6.3657	3.4858	3.8860	4.0704	3.4305	3.8297	4.0098	6.4508	3.5883	5.3164	3.8687	6.2806	3.7055	5.6250
Ttc7b	_GS[Phospho (STY)]SLLDR_.2	#N/A	3.7922	4.0358	3.8542	3.1693	3.6793	3.9060	3.8799	3.7891	3.9440	3.4138	3.4226	3.8081	4.1094	3.8379	4.7360	3.6694	3.5526	3.6281	4.0033	3.8049
Ttc7b	_VEQALSEVASS[Phospho (STY)]LQSSAPK_.2	E9Q6P5_S678_M1_Ttc7b	9.2156	10.3887	9.1726	8.8042	7.8697	9.8386	10.0082	9.2446	8.0872	8.9710	8.1698	8.7326	9.4285	10.3087	8.1448	8.3512	9.3107	10.3313	9.1800	8.4590
Cfap251	_[Acetyl (Protein N-term)]S[Phospho (STY)]DTEENPLEESR_.2	E9Q743_S2_M1_Cfap251	3.1678	8.2297	8.1156	8.4987	3.7385	7.9192	7.9219	3.9829	7.9303	8.5859	3.6745	7.7489	6.6568	7.9885	7.3438	7.8953	8.2685	7.4592	7.8991	4.1301
Zc3h13	_NTEEPSS[Phospho (STY)]PVRK_.2	E9Q784_S110_M1_Zc3h13	8.7337	8.5803	8.4860	8.8130	8.3244	8.3487	8.3812	8.6740	8.3132	8.4023	8.2806	8.3508	8.9578	8.9740	8.0196	8.3141	8.5631	8.6371	8.3440	8.3228
Zc3h13	_SKLS[Phospho (STY)]PS[Phospho (STY)]PSLR_.2	#N/A	9.7275	9.1943	9.6861	9.6206	7.9805	9.0623	8.5545	9.3970	9.3572	4.4090	9.1649	9.2027	9.6570	9.7075	3.7690	9.1487	4.0466	9.1043	9.4107	4.2989
Zc3h13	_S[Phospho (STY)]PERPTGDLR_.2	#N/A	9.3279	9.4107	9.1721	9.2649	9.1002	8.9991	9.1794	9.0657	8.8579	9.1418	8.6665	8.7479	8.9962	9.5610	8.3905	8.9877	9.0436	8.8844	8.7713	8.6875
Zc3h13	_EVS[Phospho (STY)]PEVVR_.2	E9Q784_S198_M1_Zc3h13	7.1719	7.8796	7.5315	7.9802	7.3680	7.8407	7.3549	7.5131	7.5098	7.9821	7.4036	7.5308	7.9380	7.7291	6.9100	7.8400	7.5360	7.4839	7.4670	7.7038
Zc3h13	_DQRPSS[Phospho (STY)]PIR_.2	E9Q784_S704_M1_Zc3h13	7.1442	7.5029	4.2553	6.9396	6.9792	7.1011	7.1310	3.4193	3.8933	7.2908	3.4787	7.0088	7.3340	4.2423	6.6600	4.0639	3.9570	7.2001	3.5865	7.3168
Zc3h13	_AAVVAS[Phospho (STY)]PLLDQDR_.2	E9Q784_S242_M1_Zc3h13	6.2446	5.5235	5.2194	5.9604	5.3481	5.4340	4.7364	5.1311	5.9869	5.4238	5.4475	4.2069	5.5061	5.7794	5.2413	5.7178	6.2955	5.5107	5.4523	5.2742
Zc3h13	_SLS[Phospho (STY)]PSHLTEDR_.2	E9Q784_S953_M1_Zc3h13	8.6480	8.9265	8.6992	8.6693	8.4399	8.7609	8.6199	8.8296	8.6938	8.5816	8.2407	8.5562	9.1404	9.1470	8.0251	8.5707	8.9449	8.5704	8.5688	8.5315
Zc3h13	_S[Phospho (STY)]KGDS[Phospho (STY)]DVS[Phospho (STY)]DEEAAPQNK_.2	E9Q784_S1086_M3_Zc3h13	4.8482	5.2188	5.0595	4.0160	4.8096	5.1182	4.7455	5.3780	3.9757	5.4007	4.7782	4.4730	4.9958	4.4679	4.7200	4.5795	5.1241	4.8095	3.4110	4.0337
Zc3h13	_RS[Phospho (STY)]PERPTGDLR_.3	#N/A	3.6339	4.7527	3.6815	4.0555	2.9717	4.7995	4.2691	3.3343	4.1862	3.8403	5.2108	4.1972	5.8332	3.5990	3.9698	3.9788	3.8720	4.3771	4.5860	3.9576
Zc3h13	_SSS[Phospho (STY)]PGQHHS[Phospho (STY)]PLSSR_.2	#N/A	6.2116	6.7236	3.4932	3.4399	5.8656	6.5450	4.1293	4.1705	6.7889	6.5123	6.2319	3.0644	5.3903	3.5065	6.2048	3.2711	3.4622	4.0719	6.3603	2.6282
Zc3h13	_SKLS[Phospho (STY)]PS[Phospho (STY)]PSLRK_.2	#N/A	4.0383	3.9645	7.2030	3.6510	3.3761	7.5541	7.3355	7.6979	7.4758	7.6623	7.1145	7.4636	7.5670	4.5617	3.9988	7.4318	4.2764	7.4053	7.6085	3.0279
Zc3h13	_LRS[Phospho (STY)]PSNDSAHR_.2	#N/A	3.9913	4.7049	4.7499	4.5073	4.6769	5.1224	4.5055	4.6493	4.5301	5.1874	4.7886	4.6660	4.5115	5.1560	4.1169	4.4164	4.2580	4.9139	4.7882	4.5114
Zc3h13	S[Phospho (STY)]AS[Phospho (STY)]PYPTHC[Carbamidomethyl (C)]LSS[Phospho (STY)]PQR_..	E9Q784_S369_M3_Zc3h13	4.1856	4.2588	4.2645	3.4677	3.0356	3.0689	4.0129	4.1948	3.9331	4.1401	3.4117	4.0530	4.0985	4.2708	4.0383	3.6916	4.1666	3.3460	3.3091	3.0674
Rnf169	_SQS[Phospho (STY)]C[Carbamidomethyl (C)]SDTVQDR_.2	E9Q7F2_S279_M1_Rnf169	3.1609	5.5563	5.6040	4.4233	5.0138	4.8905	3.6803	3.3866	5.1998	5.7515	5.2391	4.3550	5.1189	4.2364	4.5710	4.0238	4.3846	4.8968	3.0639	4.2356
Numa1	_TQPDGTSVPGEPAS[Phospho (STY)]PISQR_.2	E9Q7G0_S1739_M1_Numa1	10.4818	10.5681	10.1738	10.1748	9.7709	10.1767	4.1392	10.5063	10.2341	4.2705	9.9929	10.1327	10.7810	10.3287	9.1895	10.0461	10.7436	10.2385	10.2063	9.8847
Numa1	_VSSETHQGPGT[Phospho (STY)]PESK_.2	E9Q7G0_T1982_M1_Numa1	4.2362	4.9665	3.7728	3.3480	3.6089	3.6652	3.9408	4.4619	3.1760	4.3097	3.0326	4.3098	4.2671	3.8109	3.6772	2.9474	3.9591	3.1903	3.4893	3.4882
Numa1	_LQES[Phospho (STY)]PAPEKGEVLGDALQLDTLK_.3	E9Q7G0_S398_M1_Numa1	3.7376	4.5297	4.1386	4.4831	3.0754	3.2994	4.4657	4.3970	4.5149	4.6955	3.3983	3.9441	3.1320	5.1388	4.0429	4.0825	4.3249	4.3185	3.1236	3.8539
Dnajc21	_YEKEFGDGS[Phospho (STY)]DENEVDQEPR_.3	E9QBD0_S283_M1_Dnajc21	4.1617	5.5026	3.6983	3.4442	2.8678	4.4020	5.1182	3.9456	4.1005	3.9723	3.5791	4.3843	4.2659	5.0743	4.7530	4.5214	4.1514	3.3499	2.9985	3.2347
Fry	_SFS[Phospho (STY)]VPKK_.2	E9QBI9_S1991_M1_Fry	8.4452	8.5119	8.3558	8.2629	8.0197	7.5869	8.1094	8.2405	4.1966	8.0210	7.9021	7.9635	8.2885	7.2422	2.8010	7.5497	8.1638	8.1295	7.6051	7.5315
Fry	_NSDLFTVLS[Phospho (STY)]R_.2	E9QBI9_S1936_M1_Fry	6.8117	6.7601	6.3020	6.6321	5.4667	6.3981	6.4479	5.7511	5.4561	6.6947	5.8122	5.4591	5.5668	5.7684	3.2651	3.3595	5.8510	6.2305	5.2171	5.9109
Fry	_AT[Phospho (STY)]FAGGSRDGVTC[Carbamidomethyl (C)]QPGDSEEK_.3	E9QBI9_T2810_M1_Fry	3.4726	6.2764	5.4465	5.5085	5.2886	5.2965	5.1840	5.1730	5.5669	5.1627	5.1074	4.4196	5.8734	5.2106	2.8071	4.4562	5.6666	5.5656	5.5836	5.2658
Fry	_LLLPGSSPS[Phospho (STY)]S[Phospho (STY)]PEDEVKDR_.2	E9QBI9_S1382_M2_Fry	3.3981	4.3048	4.1840	4.1814	3.6051	3.6384	3.4463	3.2116	3.3665	3.2087	3.7493	3.3845	3.5320	4.0346	3.4717	4.2611	3.7493	5.1407	3.7357	4.0016
Fry	_SSS[Phospho (STY)]PDLSSSSK_.2	E9QBI9_S1940_M1_Fry	9.9385	9.9509	9.5159	9.5898	9.0504	9.6089	9.8062	9.7371	9.6463	9.4516	9.3300	3.0768	9.7067	8.9742	3.4093	3.2153	9.7165	9.8242	9.0082	3.2203
Fry	_S[Phospho (STY)]LDSLDKC[Carbamidomethyl (C)]DM[Oxidation (M)]QILEER_.3	#N/A	4.1676	4.0318	3.9660	4.1614	3.2680	5.1279	3.6765	3.7636	4.6637	4.2177	4.1530	3.8041	3.8143	4.0738	3.8976	3.6698	4.2220	5.3983	4.9898	3.7510
Fry	_EQENMDDTNS[Phospho (STY)]EQQFR_.2	E9QBI9_S2459_M1_Fry	3.4820	3.8371	4.3469	4.8788	4.1954	4.4194	3.1841	3.7077	3.4048	3.5631	3.7792	3.5632	3.5129	4.5575	2.9229	3.7017	3.2049	3.9445	4.2436	4.2348
Rabl2	_NFS[Phospho (STY)]FAKK_.2	E9Q9D5_S147_M1_Rabl2	4.6410	5.2386	5.0813	5.2260	5.1760	4.9130	5.1240	5.5397	5.5661	4.3000	5.1008	4.7400	6.4546	5.4328	5.3050	5.5086	5.5431	5.5646	4.3606	4.2947
Sec16a	_STHS[Phospho (STY)]LPSR_.2	E9QAT4_S1377_M1_Sec16a	3.7721	7.4489	7.6822	7.4633	6.9434	6.6152	7.3523	3.2924	2.9895	3.9784	7.0211	3.9785	4.5984	7.5035	6.6151	7.3446	6.9336	4.3598	7.3957	7.3300
Sec16a	_APS[Phospho (STY)]LTSDSEGKKPAQAVK_.3	E9QAT4_S2101_M1_Sec16a	3.9343	3.3848	3.4709	3.6499	8.0429	3.9671	4.4077	7.4254	4.3279	3.7449	3.8066	8.4541	8.5660	8.8940	2.9323	3.2964	3.6572	3.4923	3.7913	3.1862
Sec16a	_ASHYS[Phospho (STY)]DQLAPR_.2	E9QAT4_S1245_M1_Sec16a	7.4095	3.6065	3.4318	3.6409	3.7341	7.3316	6.9035	4.1690	6.7936	3.1018	6.7049	3.4959	3.9742	3.5179	3.3842	3.2404	7.2721	6.4533	3.7823	3.1952
Sec16a	_S[Phospho (STY)]IHS[Phospho (STY)]EHSAR_.2	E9QAT4_S1362_M2_Sec16a	5.4583	5.3928	5.1985	5.7850	3.4854	4.1629	3.5627	4.9472	4.6361	4.5727	4.5441	3.7667	3.3093	4.4966	3.5881	4.1414	4.9574	3.7486	2.9463	4.0312
Sec16a	_FTGS[Phospho (STY)]FDDDAEHRDPYGEEADRR_.4	E9QAT4_S1342_M1_Sec16a	4.2447	3.8405	3.1977	3.5271	2.9508	2.9841	4.0973	3.8626	4.0175	4.0553	3.4962	4.3014	4.1830	3.1848	4.1227	3.6068	4.4003	3.4328	3.0814	3.1518
Sec16a	_RSS[Phospho (STY)]LS[Phospho (STY)]SHSHQSQYR_.3	E9QAT4_S1384_M2_Sec16a	3.4499	4.5529	3.9862	4.2395	4.2884	3.0117	3.2526	3.1503	3.3117	3.6562	3.6861	2.9415	3.4198	3.9733	2.8298	3.7948	3.6879	4.1452	3.7938	4.1417
Syngap1	_LPS[Phospho (STY)]PTKEKPPPPPPGGGK_.3	F6SEU4_S788_M1_Syngap1	4.1895	3.8133	9.6639	3.4341	3.5272	3.7513	11.5959	4.3758	4.0730	4.3958	11.1021	3.7028	4.1810	3.7248	3.5911	3.0335	11.6613	3.2764	3.5754	3.4021
Syngap1	_S[Phospho (STY)]QPKLDR_.2	#N/A	10.6094	10.7313	10.7424	10.4787	9.9803	9.8683	10.4026	10.0672	10.0374	10.4737	10.2846	9.7750	10.4107	10.0313	8.7813	9.9861	9.7037	10.4396	10.3850	10.0220
Syngap1	_TKS[Phospho (STY)]QPKLDR_.2	#N/A	10.7506	10.8705	10.6493	10.5791	4.3043	10.0351	10.4246	3.1662	3.3211	10.4414	10.1156	9.8236	10.6747	10.2487	8.7457	9.9197	9.9086	10.6038	10.3783	10.0104
Syngap1	_FRS[Phospho (STY)]ADHDR_.2	F6SEU4_S165_M1_Syngap1	8.4310	8.0682	7.9505	8.1063	7.5267	7.3953	7.5387	7.4427	6.7486	8.0730	7.7174	7.7020	8.2103	8.2201	5.6807	7.2245	6.7882	8.1764	7.5707	7.3928
Syngap1	_LLNDISTALR_.2	#N/A	7.3725	7.1782	6.3754	7.3561	7.1213	7.0093	7.3222	6.9262	6.8496	6.2207	6.8486	6.9305	6.5612	6.4875	6.5054	6.7732	7.4630	7.3272	6.7082	6.6978
Syngap1	_ALYESEENC[Carbamidomethyl (C)]EVDPIK_.2	#N/A	5.8555	6.2784	6.3773	5.3432	4.7454	5.7984	6.4049	4.8536	5.5143	5.5536	5.2994	5.2561	5.5942	6.0081	5.6362	5.7782	6.3468	5.6315	5.9918	5.9331
Syngap1	_GKS[Phospho (STY)]QQLTVSAAQKPR_.3	#N/A	6.6451	7.5168	7.0766	4.3344	4.3568	3.1508	7.4163	6.9622	5.8569	4.5612	6.0166	5.5056	6.6431	7.7727	3.6167	4.9837	4.4736	6.8314	7.0596	5.5231
Syngap1	_EFAEYVTNHYR_.2	#N/A	5.8120	5.4586	5.4764	5.8136	5.1967	5.0262	5.0838	5.2744	5.7132	5.3788	5.2602	4.9700	5.6176	5.7387	4.4687	5.3744	5.8609	5.8304	5.4114	5.2740
Syngap1	_QQS[Phospho (STY)]LSKEGS[Phospho (STY)]IGGSGGSGGGGGGGLKPSITK_.3	F6SEU4_S1111_M2_Syngap1	4.7164	5.4553	4.4562	3.9743	3.4665	4.6136	4.7910	3.6333	4.5249	5.4036	3.9357	4.2465	4.0949	4.9900	3.6911	5.0959	3.8809	5.5299	4.8778	4.2872
Syngap1	_EYEEEHS[Phospho (STY)]LKER_.2	#N/A	5.8775	5.3811	5.7295	5.4301	5.3831	5.5854	5.1743	5.4068	5.4972	6.1217	5.6060	5.3339	6.4540	5.5632	3.6573	4.8843	6.0387	5.3167	5.3426	5.6633
Syngap1	_SSPAYC[Carbamidomethyl (C)]TSSS[Phospho (STY)]DTEPEQK_.2	F6SEU4_S825_M1_Syngap1	11.5702	11.4017	11.8225	10.3583	11.8823	10.1734	3.6216	3.9011	9.5997	11.2513	10.3027	3.4488	10.4717	11.2217	9.3414	9.8792	11.3096	11.1415	3.6659	10.8184
Syngap1	_TVS[Phospho (STY)]PVEGR_.2	#N/A	7.4743	4.4702	3.2083	3.7218	6.8616	7.6507	4.8364	4.6213	4.4755	5.3644	4.3402	6.3474	3.9567	5.0246	5.7111	6.5033	4.5217	6.9851	4.5327	4.5786
Syngap1	_DLNSS[Phospho (STY)]DDLQSFM[Oxidation (M)]AR_.2	#N/A	8.8556	9.0539	8.6959	8.0739	6.2359	7.3815	8.3827	8.4860	7.4153	9.1228	7.2392	7.7763	8.5273	9.0110	6.1789	3.0634	7.3218	8.6795	3.6487	7.1313
Syngap1	_ILM[Oxidation (M)]QYQAR_.2	#N/A	6.2672	5.5987	3.2673	5.0609	5.3215	6.0519	3.1104	5.9838	6.3922	5.6572	5.2495	5.5480	5.8525	5.6491	5.8465	5.2496	6.3509	6.5672	5.5651	4.8393
Syngap1	_EGS[Phospho (STY)]GGSGGSGGGGGGGLKPSITK_.3	#N/A	7.5604	8.4806	8.3993	8.2340	7.0348	7.7548	8.0226	6.3885	7.8037	7.6520	7.8609	6.6434	8.2288	7.5929	6.5576	7.5030	7.0949	7.8651	7.6349	7.7642
Syngap1	_GKS[Phospho (STY)]QQLTVSAAQK_.2	#N/A	4.7101	5.3933	5.3218	4.1944	4.9856	4.0630	3.0184	4.0484	4.4395	3.6333	4.8567	3.2225	4.5652	3.3974	3.0438	5.1787	4.3084	3.6038	4.6159	3.5737
Syngap1	_QY[Phospho (STY)]SMEAAPAAPFR_.2	F6SEU4_Y116_M1_Syngap1	3.8358	7.7282	3.9278	4.0079	3.5357	3.5691	3.5989	4.2823	3.5191	3.6464	3.9961	3.8029	3.6845	5.9907	3.6242	3.7533	3.6185	3.8782	4.7186	3.8744
Syngap1	_VKEYEEHS[Phospho (STY)]LKER_.3	#N/A	8.1224	4.4490	8.2942	8.2271	7.2080	7.6912	7.6228	8.0629	7.7626	7.3294	7.4602	7.5598	8.1344	8.1228	7.0606	7.5293	7.8564	7.8821	7.7433	7.3022
Syngap1	_LSQM[Oxidation (M)]GVTTDGVPAQQLR_.2	#N/A	6.2372	6.6300	6.4778	5.2937	5.6887	5.8573	6.4006	6.8688	6.2809	3.4253	5.9716	6.2370	6.8509	5.8879	6.1559	6.3726	6.3552	6.4305	6.1850	5.9920
Syngap1	_S[Phospho (STY)]VSM[Oxidation (M)]LDLQDGPGGR_.2	#N/A	7.0655	6.2768	4.0155	3.7124	3.9086	6.5270	3.9947	3.9674	3.9149	4.2054	3.3935	3.5056	4.0803	3.8899	4.0200	3.7569	6.3500	6.4420	6.6472	3.8569
Syngap1	_DAIGEFIR_.2	#N/A	4.5158	4.0820	4.2745	4.8973	3.4440	4.2381	5.0868	3.4628	4.7072	5.5922	4.7790	4.8394	3.2680	4.3126	1.0007	4.1000	4.4608	4.1894	4.6981	5.1341
Syngap1	_RLLSQEEQTSK_.2	#N/A	3.0684	2.1902	3.5508	3.5700	2.6523	2.9322	4.0841	4.2399	3.6190	3.8248	2.5304	2.7748	4.0451	3.6871	2.9831	4.0829	2.8927	3.4123	3.7114	2.7797
Syngap1	_GPS[Phospho (STY)]AEM[Oxidation (M)]QDYMMR_.2	#N/A	3.7306	7.4490	9.4168	9.1338	3.3764	6.5993	6.3439	3.9563	3.5064	4.8436	7.5649	3.9223	3.6575	7.9873	3.7436	4.0324	4.8191	3.9091	3.4422	3.8302
Syngap1	_GLNSS[Phospho (STY)]M[Oxidation (M)]DMAR_.2	#N/A	4.3831	6.4382	8.5074	8.5526	3.0892	4.9880	3.9625	4.1609	5.2222	5.4214	5.6800	4.1665	4.1690	5.8572	3.8310	3.7452	4.6179	4.4028	4.6648	4.0693
Syngap1	_C[Carbamidomethyl (C)]TAS[Phospho (STY)]SLAEHQANLR_.2	F6SEU4_S534_M1_Syngap1	3.3113	4.1302	4.2829	4.2483	3.5182	3.5516	3.5382	3.2908	3.4585	3.7580	3.5730	3.9509	3.6239	4.1266	3.5636	4.1742	4.1681	4.0003	3.6489	4.0936
Syngap1	_QHS[Phospho (STY)]QTPSTLNPTM[Oxidation (M)]PASER_.3	#N/A	3.7254	3.6072	3.8751	3.8723	3.9323	4.1895	3.3901	3.9511	3.6482	3.3332	4.0226	3.3333	6.4889	3.9132	3.2247	3.7007	3.4483	3.7146	3.6130	5.9485
Syngap1	_S[Phospho (STY)]MDESRLDR_.2	#N/A	3.9096	8.7971	9.5485	9.0857	6.8142	7.7871	8.5615	4.1977	7.4885	3.7202	8.3178	7.4077	4.5180	9.2881	6.3928	3.2717	7.5668	6.5129	6.7548	7.3946
Syngap1	_TVS[Phospho (STY)]PVEGRPHGEHEYHLGR_.4	#N/A	4.2602	3.7426	3.2957	3.4292	2.8528	4.3870	4.1953	3.9606	4.1155	3.9573	3.5941	3.6321	4.2809	3.2827	4.2206	3.5089	4.4983	3.3349	2.9835	3.6055
Syngap1	_EYSKS[Phospho (STY)]M[Oxidation (M)]DESRLDR_.3	#N/A	3.2917	4.8986	4.1086	4.3095	4.1302	4.3543	5.0867	3.8245	3.4763	4.4980	3.8507	3.8920	3.5844	4.1192	2.9944	3.6366	3.4324	4.1255	4.1495	4.0681
Syngap1	_EYSKS[Phospho (STY)]MDESR_.2	#N/A	4.1662	3.7621	3.2762	3.4486	2.8723	4.4065	5.6038	3.9411	4.0960	3.9768	3.5747	3.6515	4.2615	6.1560	4.2012	3.5283	4.4788	3.3544	3.0029	3.2303
Syngap1	_LS[Phospho (STY)]QGSGSSITAAGMR_.2	F6SEU4_S892_M1_Syngap1	3.9844	3.8745	4.4904	6.3960	3.6242	5.3241	5.8042	3.8531	3.6990	4.0280	3.6433	3.7214	3.8644	6.2499	3.5933	3.5205	3.5454	3.9304	4.0261	3.7783
Syngap1	_VKEYEEHS[Phospho (STY)]LK_.3	#N/A	4.1629	3.8523	4.0705	3.5389	3.5006	4.0218	3.5864	4.2928	4.5002	4.5325	3.8456	3.7417	4.3295	3.7132	4.0253	3.8111	4.3047	3.2105	4.1660	4.3090
Syngap1	_TVAWVSNM[Oxidation (M)]PHLS[Phospho (STY)]ADIESAHIER_.3	F6SEU4_S1162_M1_Syngap1	6.1708	6.1724	5.5875	5.5230	3.0196	3.9513	5.8479	6.1235	3.9532	5.2855	4.3319	5.3076	4.1187	6.5899	4.0584	3.6756	4.1110	5.7951	5.1951	3.0875
Syngap1	_S[Phospho (STY)]QPMVLR_.2	F6SEU4_S743_M1_Syngap1	3.3168	3.1852	3.8531	4.3726	4.1553	4.3794	3.3857	4.5180	3.3448	3.5231	3.8192	3.0746	3.5529	3.8401	2.9629	3.6617	3.5548	4.2783	3.9269	4.2748
Tpr	_QTPQAPQS[Phospho (STY)]PR_.2	F6ZDS4_S2141_M1_Tpr	11.9514	12.1259	12.1744	12.1768	11.9295	11.9250	11.7525	12.0616	11.9269	12.1600	11.7125	11.9380	12.2989	12.4385	11.7733	12.1742	12.1719	11.6993	11.9200	12.1018
Tpr	_TDGFAEAIHS[Phospho (STY)]PQVAGVPR_.3	F6ZDS4_S2223_M1_Tpr	5.5744	6.1401	4.5226	3.8861	5.3287	4.2033	5.0667	4.1548	5.2945	5.2104	3.9954	5.2081	3.7290	6.3770	3.1684	3.5520	4.8619	4.8299	4.0274	5.6730
Tpr	_GAILSEEELAAM[Oxidation (M)]S[Phospho (STY)]PTAAAVAK_.2	#N/A	6.1413	5.9725	5.4328	5.5727	5.1558	5.6051	5.5395	6.4425	3.8338	4.0357	5.3882	5.8699	6.7577	6.7098	5.3917	5.7675	6.6579	6.2170	5.4390	5.5133
Farp1	_LGAPENSGIST[Phospho (STY)]LER_.2	F8VPU2_T24_M1_Farp1	10.6439	10.5980	10.3686	10.7134	10.2536	10.3539	10.4076	10.3767	10.1935	10.6911	10.1253	9.7108	10.5247	10.0493	8.0087	9.6217	10.5809	10.2546	9.8609	9.9615
Farp1	_AC[Carbamidomethyl (C)]TLELGPHQS[Phospho (STY)]PALPK_.2	#N/A	7.1036	7.3715	6.0463	6.3752	6.2130	6.0007	6.8626	6.8192	6.2493	6.9382	6.1018	6.3304	6.9982	6.7943	5.0380	6.3029	7.0095	6.8904	6.1315	5.9568
Farp1	_SPDEATAADQES[Phospho (STY)]EDDLSASR_.2	F8VPU2_S892_M1_Farp1	7.5577	7.2583	7.4376	7.0198	6.3885	7.0260	6.9056	7.5117	6.6765	6.9378	6.4497	6.3487	6.2546	6.2667	5.5123	5.9302	7.5466	7.0799	7.0237	5.6973
Farp1	_QSPQSASLTFGEGTES[Phospho (STY)]PGGQSC[Carbamidomethyl (C)]QQAK_.3	F8VPU2_S403_M1_Farp1	4.0251	6.1125	4.4223	3.6913	3.3629	5.6547	3.8767	3.7255	5.1383	4.2194	3.3591	5.2391	4.0459	5.3099	3.7243	3.7095	5.3586	5.3667	5.0519	4.4644
Farp1	_SNGPTPELLASS[Phospho (STY)]PPDNK_.2	F8VPU2_S875_M1_Farp1	4.1798	5.9706	7.4036	7.1475	3.5175	3.7416	7.2438	4.3855	4.0827	4.3861	2.9562	6.5263	4.1907	3.7345	3.6008	3.0238	3.8828	6.9785	6.5538	3.4118
Farp1	_AC[Carbamidomethyl (C)]TLELGPHQS[Phospho (STY)]PALPKS[Phospho (STY)]PPGSK_.3	F8VPU2_S427_M2_Farp1	8.7006	8.6896	7.4697	8.0477	5.7715	6.4046	8.5250	7.3366	6.8525	8.0835	7.0704	8.2397	8.2080	8.6196	5.6190	7.4909	7.4876	8.1810	7.2145	6.4491
Bicra	_GAGS[Phospho (STY)]PTPLPTK_.2	F8VPZ9_S1427_M1_Bicra	6.8205	6.8654	6.9608	5.7994	6.3227	6.5843	4.1868	4.3809	6.1448	6.5717	6.5394	4.3909	6.7289	7.0620	6.3443	6.2568	6.5935	6.2071	6.2222	6.4186
Sipa1l3	_TLS[Phospho (STY)]DESLC[Carbamidomethyl (C)]SGR_.2	G3X9J0_S1538_M1_Sipa1l3	9.0590	9.0026	8.6909	9.0258	8.2112	8.5275	8.3907	8.6003	8.3661	9.0743	8.3914	8.4229	8.6907	8.9849	7.8962	8.3733	8.6740	8.4352	8.4692	8.6622
Sipa1l3	_SSS[Phospho (STY)]EITLSEC[Carbamidomethyl (C)]DVEEPGDPR_.2	G3X9J0_S166_M1_Sipa1l3	4.2228	3.7800	3.2583	3.4674	3.5606	3.7846	3.9805	4.3425	4.0396	4.4292	2.9131	3.6694	4.1477	3.5577	3.0914	3.0669	4.3124	3.3097	3.6088	3.3687
Sipa1l3	_EQSNPS[Phospho (STY)]PSQDTDGVK_.2	G3X9J0_S94_M1_Sipa1l3	4.5039	4.3160	4.0405	6.6564	5.6890	3.3975	4.3676	3.2287	4.4267	6.2395	3.3002	4.0422	4.5348	4.0785	3.9448	3.8660	4.2268	5.8864	3.2217	5.6890
Sipa1l3	_S[Phospho (STY)]KDVEFQDGWPR_.2	G3X9J0_S140_M1_Sipa1l3	6.6007	7.5966	7.7722	6.8355	7.5501	6.9525	7.2076	4.1529	6.9552	7.2343	6.5329	3.1179	3.9581	7.4810	5.5918	6.8579	3.6502	6.0320	6.9667	6.9128
Sipa1l3	_QDAAGKDS[Phospho (STY)]PNR_.2	G3X9J0_S1248_M1_Sipa1l3	3.3296	2.2056	3.8659	4.3598	4.1682	4.3922	3.3729	4.5308	3.4320	3.5359	3.8064	3.0618	3.3503	3.8530	2.9501	3.6745	3.5677	4.2655	3.9141	3.8200
Arfgef1	_LSVSS[Phospho (STY)]NDTQESGNSSGPSPGAK_.2	G3X9K3_S394_M1_Arfgef1	4.3991	3.6037	3.4346	3.2903	3.7368	4.2481	3.8043	4.0995	4.2544	3.8184	3.7330	3.4932	4.4198	3.4216	3.3815	3.2432	3.1363	3.1960	4.3454	3.3886
Arfgef1	_GREGS[Phospho (STY)]LTGTK_.2	G3X9K3_S1076_M1_Arfgef1	6.1155	5.5354	3.5028	3.2220	3.8051	5.7597	3.7360	4.1678	3.7951	3.1729	5.7999	3.4249	4.4881	3.4899	3.3132	3.3114	4.9175	3.1277	4.2772	3.4569
Arfgef1	_S[Phospho (STY)]VDHDSIQFR_.2	G3X9K3_S1566_M1_Arfgef1	8.5510	8.3437	3.6045	8.0761	3.6094	7.1240	7.4561	4.2936	6.2040	8.6854	4.3652	2.9772	4.0988	3.6426	3.5089	7.0243	3.7909	3.3586	7.0864	3.3199
Arfgef1	_QQQHLLQS[Phospho (STY)]PVSHHEPESPHLR_.5	G3X9K3_S234_M1_Arfgef1	3.2232	3.2787	4.7405	4.4662	4.0618	4.0080	4.5260	4.4244	4.4509	4.4935	4.6949	3.1682	3.2439	3.7466	3.0565	3.5681	3.4613	4.3719	4.0205	3.7136
Rab11fip2	_TLS[Phospho (STY)]PFDTSK_.2	G3XA57_S277_M1_Rab11fip2	4.1738	3.8290	3.2092	6.8155	3.5115	5.4501	6.0609	8.8741	6.2692	7.2477	6.0360	3.7185	4.1967	6.7361	5.6749	3.0178	3.8477	6.4009	6.9506	3.1633
Atp2b1	_NSS[Phospho (STY)]PPPSPNK_.2	G5E829_S1178_M1_Atp2b1	3.9537	11.7673	11.5439	3.9758	11.3291	11.6662	11.5844	11.9366	11.5873	11.2473	11.6867	11.6069	12.0028	11.6600	10.0504	11.3250	3.8477	11.9359	11.6390	11.4420
Atp2b1	_SATSSSPGS[Phospho (STY)]PLHSLETSL_.2	G5E829_S1211_M1_Atp2b1	12.6083	12.3469	12.1663	12.2046	11.7356	11.6894	11.9372	12.1926	11.8964	12.2234	11.9535	11.6394	12.5569	11.7573	10.3235	11.7845	12.3551	11.9221	11.7805	11.5852
Atp2b1	_EASDIILTDDNFTSIVK_.2	#N/A	9.0246	8.7269	9.0410	8.5618	7.9353	8.3685	8.5160	8.7615	8.3465	8.4414	8.2321	8.3480	9.0009	8.7663	7.1811	8.8143	8.9441	8.5566	8.1189	8.3247
Atp2b1	_VYTFNSVR_.2	#N/A	8.0431	8.2004	8.2633	8.4981	8.1596	8.0141	8.6750	8.3960	8.1851	8.3039	8.3760	8.2168	8.5262	7.2256	8.0143	7.6125	8.3811	8.6576	8.3453	7.8083
Atp2b1	_TSPNEGLSGNPADLER_.2	#N/A	8.6248	8.0882	8.2063	7.9136	8.0204	8.0958	8.1401	8.2454	8.2193	7.6458	8.3297	7.8382	7.7237	7.5151	7.5006	7.5762	8.1321	8.2221	8.0704	7.6019
Atp2b1	_GIIDSTVSEQR_.2	#N/A	7.9975	8.1736	8.2847	7.6770	7.5799	7.7244	7.8367	8.2463	7.8272	7.6406	7.8715	7.6583	8.4225	7.8348	7.5167	7.5028	8.1018	8.1101	7.6200	7.4741
Atp2b1	_QVVAVTGDGTNDGPALK_.2	#N/A	7.9681	7.7741	8.1190	7.7695	7.3418	7.6433	7.7348	8.1694	7.8862	7.5454	7.2245	7.4052	8.0554	7.5850	6.8828	7.3618	7.6767	7.8975	7.7289	7.3869
Atp2b1	_YGDLLPADGILIQGNDLK_.2	#N/A	8.2522	8.0300	7.0840	7.6850	7.2357	7.3530	8.1795	8.1569	7.8151	7.2975	7.4077	7.5156	7.8475	7.7736	6.8170	6.8713	7.7472	8.0279	7.3375	7.1096
Atp2b1	_IDESSLTGESDHVKK_.3	#N/A	5.5544	5.6775	5.3459	4.4102	5.4399	5.5775	5.5945	5.2997	5.5611	5.2084	5.6457	5.3478	5.5018	4.8030	4.8569	5.4612	5.6480	5.4438	5.4811	5.2632
Atp2b1	_ADVGFAM[Oxidation (M)]GIAGTDVAK_.2	#N/A	6.7677	6.3240	6.1219	5.7293	5.9043	5.9189	6.1995	6.4152	6.4135	6.1509	5.9456	6.2350	6.1441	5.2335	5.6147	6.0801	6.5839	6.9126	6.1611	6.2170
Atp2b1	_EANHDGDFGITLTELR_.3	#N/A	6.3273	6.1572	6.2669	6.2888	5.5985	5.9758	6.0684	5.0809	5.8480	6.0148	6.0136	5.6423	5.9257	5.5581	5.1401	5.6447	6.4294	5.8057	5.5799	6.0991
Atp2b1	_KQDGAIENR_.2	#N/A	5.7681	5.7723	5.9163	5.4864	4.8404	5.6095	5.7642	6.3929	5.6604	5.2328	5.6464	5.1388	5.5432	4.5947	6.0408	5.5103	5.7054	6.0978	5.8894	5.4010
Atp2b1	_NS[Phospho (STY)]LKEANHDGDFGITLTELR_.3	#N/A	6.3597	6.9926	6.4188	3.3189	3.7082	5.6420	6.0519	6.1416	3.8920	6.4543	4.2663	3.5218	5.0553	3.3930	4.2805	4.9442	5.5859	6.4175	4.3740	3.2211
Atp2b1	_TIC[Carbamidomethyl (C)]IAFR_.2	#N/A	3.9794	5.4948	3.9139	5.5939	4.5021	5.7201	5.6875	4.4726	5.6825	2.2771	5.3913	3.2210	5.1654	5.3967	5.1669	5.0044	3.8979	6.4249	5.6438	5.2859
Atp2b1	_M[Oxidation (M)]VTGDNINTAR_.2	#N/A	7.3822	7.3959	7.3579	7.4073	6.8674	7.1225	7.6361	7.1377	8.1251	6.8760	7.1513	7.0414	7.0590	6.9312	7.0602	6.5761	7.3624	7.3302	7.0645	7.4405
Atp2b1	_TVIEPM[Oxidation (M)]ASEGLR_.2	#N/A	7.6643	7.7650	7.6096	7.5172	7.4559	7.3248	7.4847	7.7135	7.2324	6.5895	7.3381	7.4815	7.4964	6.8901	7.1051	6.8004	7.4833	7.6812	7.2213	7.0929
Atp2b1	_TGTLTMNR_.2	#N/A	6.6133	6.1434	7.0742	6.6729	6.5935	6.6588	6.5738	6.5552	6.0889	6.8975	4.3889	7.0143	4.1226	7.3415	6.0010	3.4538	3.8146	6.3829	6.9153	6.5538
Atp2b1	_KADVGFAM[Oxidation (M)]GIAGTDVAK_.3	#N/A	5.6470	5.5813	4.2693	5.7428	5.6280	5.8331	5.8541	5.2966	3.5883	5.7791	5.4724	5.2885	6.1103	5.1872	5.4211	5.7033	5.2993	5.8869	5.9446	5.7640
Atp2b1	_FFDIDSGR_.2	#N/A	5.2386	3.9929	5.0024	5.2674	5.1029	5.0382	4.9644	4.3719	5.1487	4.6407	2.9138	5.4865	3.6006	4.4017	4.2307	3.8446	4.3288	4.7434	5.0434	4.2110
Atp2b1	_QVVAVTGDGTNDGPALKK_.2	#N/A	4.4097	4.1462	4.6836	4.0714	3.9175	3.9038	4.6533	4.4017	4.3563	3.3979	4.3028	3.3875	4.2472	3.8894	4.0475	3.7512	4.7835	4.2408	4.1799	3.5054
Atp2b1	_IQESYGDVYGIC[Carbamidomethyl (C)]TK_.2	#N/A	4.5734	4.5657	5.3358	4.6261	3.9111	4.5387	5.1886	3.9919	5.8979	3.2789	5.3438	3.3189	4.7691	3.3409	3.2072	5.6274	3.4891	3.6603	3.9594	5.0790
Atp2b1	_SSLYEGLEKPES[Phospho (STY)]R_.2	#N/A	6.2559	6.2846	6.4387	5.7108	4.8846	6.1115	5.6369	4.9902	5.3784	7.1864	5.8866	5.8551	5.3485	6.2201	4.6480	5.7632	6.7404	6.0673	6.2891	5.7101
Atp2b1	_EEIPEEELAEDVEEIDHAER_.3	#N/A	6.8592	6.4494	4.5058	4.1159	4.2090	6.7264	6.7890	3.6941	7.1390	3.5767	3.7656	6.6560	3.4993	6.0085	6.5596	6.1714	6.7444	6.7824	6.5605	6.6426
Atp2b1	_SSPTDKHTLVK_.2	#N/A	6.2752	6.4739	6.3640	5.8104	6.0275	5.9580	6.9801	6.5870	5.9707	5.7601	5.8951	6.2094	5.9651	5.8468	6.1147	5.5823	6.3999	6.3946	5.8324	5.7582
Atp2b1	_NVYDSISK_.2	#N/A	3.8514	3.4677	3.3879	3.7329	2.3929	4.0501	3.8093	4.8128	3.7742	3.1937	3.8892	3.1938	3.8822	3.4260	2.8243	2.9617	3.5743	3.5752	3.4844	3.1033
Atp2b1	_[Acetyl (Protein N-term)]GDMANNSVAYS[Phospho (STY)]GVKNSLK_.2	G5E829_S12_M1_Atp2b1	4.0126	4.3739	4.1704	3.8704	4.0200	4.3258	3.5533	3.7129	3.9158	3.3878	3.7694	3.2422	4.0617	3.7048	3.1305	3.5264	3.4195	3.7761	4.0944	3.8758
Atp2b1	_HLDAC[Carbamidomethyl (C)]ETM[Oxidation (M)]GNATAIC[Carbamidomethyl (C)]SDK_.3	#N/A	4.3319	3.2203	3.6354	3.4854	4.5479	4.3391	4.2432	4.0085	5.1459	3.9094	3.6421	2.9464	4.3289	3.3307	4.6489	3.4609	4.1895	3.2870	4.8013	4.8636
Atp2b1	_MTVVQAYINEK_.2	#N/A	4.5439	4.1398	4.3993	3.8264	3.2500	3.8734	3.7981	3.5634	3.7183	4.3545	3.1969	3.5765	3.8837	4.3864	3.8235	3.9060	4.1011	3.7321	3.3807	4.3534
Atp2b1	_IEDS[Phospho (STY)]EPHIPLIDDTDAEDDAPTKR_.3	G5E829_S1155_M1_Atp2b1	3.6731	3.6763	4.2094	4.1270	3.7475	3.9716	3.8768	4.1102	3.9359	3.8794	3.5736	3.5657	3.8984	4.1965	3.4540	4.0180	3.9111	4.0327	3.6813	3.3993
Atp2b1	_AQDGAAMEMQPLKS[Phospho (STY)]EEGGDGDEKDK_.3	G5E829_S338_M1_Atp2b1	3.7092	4.2936	4.2455	3.9802	3.0469	4.3372	3.6443	3.4096	3.5645	4.5083	3.0431	4.1831	3.7299	4.2326	3.6696	4.0541	3.9472	3.8859	3.5345	4.1996
Trip12	_SES[Phospho (STY)]PPAELPSLR_.2	G5E870_S312_M1_Trip12	11.7444	11.3759	11.5809	11.2262	10.4744	11.2901	11.1125	11.2761	11.1171	4.5848	11.2571	10.7696	11.4771	4.1561	3.5932	10.7703	11.6517	11.0768	10.8774	10.8898
Trip12	_AQTAPTKTS[Phospho (STY)]PR_.2	G5E870_S1460_M1_Trip12	8.0447	7.9077	7.6905	7.9518	7.7453	7.8431	8.1334	8.2009	7.6049	7.4551	7.7075	7.6115	8.1291	7.3523	6.2487	7.6961	8.0478	8.0201	7.7822	7.5130
Trip12	_DDSLDLS[Phospho (STY)]PQGR_.2	G5E870_S1024_M1_Trip12	3.6826	3.6365	8.4819	8.5148	3.8895	8.2521	8.3141	3.9083	7.9800	3.4932	4.0583	7.8252	3.2442	3.7469	6.7081	7.9358	8.5494	8.0635	4.0202	7.6479
Trip12	_AAS[Phospho (STY)]KDTISNNR_.2	G5E870_S1111_M1_Trip12	3.9935	3.5894	3.4489	5.9720	4.9242	4.2338	6.2553	5.0978	4.2687	3.8041	3.7473	4.6646	4.4341	3.4359	2.8730	3.3556	3.1506	3.1817	4.3311	3.4029
Trip12	_SAS[Phospho (STY)]PDYNR_.2	G5E870_S77_M1_Trip12	4.2272	3.7756	3.2626	3.4622	3.5649	4.4200	4.1250	3.9276	4.0825	3.9903	3.5611	3.6651	4.2479	3.2497	3.5534	3.0712	4.4652	5.4172	3.0165	3.2167

Ank3	_DFPQYFAVVSR_.2	#N/A	7.9266	7.9513	7.6843	7.8211	8.0762	7.7860	7.9536	8.2347	7.9792	7.8711	7.8731	8.0230	7.9719	7.9875	7.3605	7.3894	7.9918	7.9895	7.6786	7.6559
Ank3	_EFDS[Phospho (STY)]DSLR_.2	G5E8K5_S958_M1_Ank3	6.7717	3.6772	3.1785	4.4252	3.8489	6.2743	6.7158	4.4654	3.1194	7.2304	4.0989	3.4033	3.2849	3.7875	3.2246	7.0694	6.0617	4.3309	6.5999	6.9894
Ank3	_TEEPVS[Phospho (STY)]PLTAYQK_.2	G5E8K5_S1883_M1_Ank3	8.8731	8.3923	8.8045	8.8250	7.5666	7.3456	4.0970	3.8067	4.1561	4.3126	7.9964	7.9069	4.2642	3.1288	3.6742	8.1528	4.3444	7.2385	7.9258	7.9188
Ank3	_NEDLAELLNGM[Oxidation (M)]DEELDS[Phospho (STY)]PEELGTKR_.3	#N/A	3.4796	3.8395	4.5170	4.2629	3.6865	3.7199	3.3616	3.1269	3.2818	3.2902	4.2612	3.5656	6.0458	3.9499	3.3869	4.3426	3.6646	4.1686	5.3101	3.9169
Ank3	_YSYLTEPSMS[Phospho (STY)]PQSPC[Carbamidomethyl (C)]ER_.2	G5E8K5_S1470_M1_Ank3	3.7925	3.5266	3.3291	3.7917	3.9995	4.0328	3.0486	4.0182	4.4697	3.6031	3.9483	3.2527	3.8234	3.3672	3.0740	3.1546	3.5154	3.6340	3.9331	3.0445
Ank3	_IRDTS[Phospho (STY)]QEPC[Carbamidomethyl (C)]GR_.2	G5E8K5_S1406_M1_Ank3	3.8282	6.4137	3.3648	3.7560	4.0351	4.0685	3.0130	4.0539	4.4340	3.6388	3.9127	3.2170	4.5995	3.6013	3.0383	3.1903	3.5511	3.0164	4.1658	3.5683
Ank3	_NEDLAELLNGM[Oxidation (M)]DEELDS[Phospho (STY)]PEELGTK_.3	#N/A	3.1710	3.3309	3.7073	4.5184	4.0096	3.9754	3.5315	4.3722	3.0263	3.3773	3.9650	3.2204	3.1917	3.6944	3.1087	3.5159	3.4091	4.4241	4.0727	3.6614
Ank3	_GETALHM[Oxidation (M)]AAR_.2	#N/A	3.7331	4.3644	3.5029	3.9747	4.2101	5.4247	5.3352	4.4765	3.8761	3.9394	3.5161	3.5059	3.8733	3.8122	3.3942	4.5832	3.7627	5.2002	3.7436	3.6397
Ank3	_RQS[Phospho (STY)]FASLALR_.2	G5E8K5_S1451_M1_Ank3	3.3242	3.9949	4.3616	4.2601	3.5311	3.0764	3.1879	3.5498	3.2470	3.7209	3.6214	3.7210	3.3550	6.1980	3.5424	4.1871	4.5479	4.1024	4.4014	4.0769
Ank3	_ADIVQQLLQQGASPNAATTSGYTPLHLAAR_.3	#N/A	3.3533	3.9658	4.3907	4.2309	3.5603	3.0473	3.2171	3.5790	3.2761	3.6918	3.6505	3.6919	3.3842	4.4288	2.7943	4.2163	3.0762	4.0732	4.3723	4.1061
Ank3	_VALLLLDQGAS[Phospho (STY)]PHAAAK_.2	G5E8K5_S606_M1_Ank3	3.9112	4.0916	4.4475	3.7782	3.2489	3.4730	4.2921	3.6116	4.3512	4.1175	3.2248	3.9810	4.4593	4.0030	3.8693	4.2561	4.1493	1.5375	3.2972	3.6803
Brd1	_VHGEPAS[Phospho (STY)]DLS[Phospho (STY)]DID_.2	G5E8P1_S1052_M2_Brd1	4.1032	8.9077	8.7277	4.0349	3.8739	8.6778	8.5368	3.5799	8.6637	4.0536	3.4511	4.2281	3.6631	8.6127	7.5473	3.8602	3.6843	8.3959	8.5370	8.1996
Cacna2d1	_FVVTDGGITR_.2	#N/A	6.8859	6.7098	6.6635	6.9261	7.0466	6.6596	7.1412	7.0612	6.7054	6.7416	6.8146	6.4518	6.7591	6.9293	6.3351	6.2980	6.2580	7.1415	6.7549	6.4900
Cacna2d1	_SYDYQSVC[Carbamidomethyl (C)]DPGAAPK_.2	#N/A	4.4118	4.2453	5.9636	6.1596	5.0598	5.2875	4.5739	5.0075	5.6091	3.9713	4.9753	4.6817	4.9317	4.9869	5.0412	4.8955	4.2976	5.2339	3.1509	5.1417
Cacna2d1	_IIM[Oxidation (M)]LFTDGGEER_.2	#N/A	3.5081	3.8109	4.5456	4.2914	3.7151	4.5747	4.6323	3.0983	5.0254	3.3187	4.6909	4.5330	3.4187	4.0751	4.9489	4.6810	3.9904	4.2824	3.8457	4.9675
Cacna2d1	_QLVEIAAR_.2	#N/A	3.8328	3.7168	4.1624	4.0632	3.4516	2.7164	1.6247	6.3025	4.8081	3.8325	3.4246	4.2661	3.2435	4.2881	2.0805	3.9710	4.4364	4.6083	3.7900	3.9654
Cacna2d1	_VFTFSVGQHNYDR_.3	#N/A	4.1915	3.5835	3.4172	3.5242	3.3281	3.4652	4.0140	4.0962	4.0021	4.2806	3.0027	3.4113	4.1388	3.4340	3.5912	2.2589	4.1201	3.3993	2.6099	3.2379
Bin1	_VNHEPEPASGAS[Phospho (STY)]PGATIPK_.2	#N/A	8.9166	9.1372	9.2699	9.3640	8.7808	8.7197	8.5614	8.8706	8.5559	9.2936	8.5874	8.9062	9.2760	9.1959	8.7085	8.8725	8.7847	8.6687	8.5209	8.8006
Bin1	_GVFPENFTER_.2	#N/A	8.2988	8.4846	8.4593	8.4552	8.7584	8.1208	8.3617	8.7558	7.9222	8.3857	8.2766	8.6268	8.4541	8.5828	8.7869	8.7485	8.4644	8.4850	8.2336	8.5605
Bin1	_LNQNLNDVLVSLEK_.2	#N/A	5.8918	5.7553	6.1115	6.4403	5.7137	5.7566	5.2976	6.0430	6.2689	5.9125	5.3206	6.1270	6.3279	6.1805	6.0128	5.8930	5.4519	5.7579	5.7337	6.1703
Bin1	_VQAQHDYTATDTDELQLK_.3	#N/A	5.9429	6.0809	5.5682	5.2606	5.4897	5.2314	5.5785	5.0270	5.8264	5.5064	5.3084	5.3088	6.4106	6.1163	5.5538	4.9676	5.6745	5.8482	5.4017	5.4825
Bin1	_GNKS[Phospho (STY)]PS[Phospho (STY)]PPPDGSPAATPEIR_.3	#N/A	13.0605	13.2171	13.6349	13.5580	10.3986	13.3040	12.9163	10.9046	13.1896	13.5585	12.4848	13.0937	12.5310	13.8032	13.2354	13.3188	13.4555	12.9660	13.3057	13.4923
Bin1	_NQAEEELIK_.2	#N/A	7.2999	7.2813	7.2280	7.2364	7.5585	7.2524	7.3376	7.5402	7.3359	8.0771	7.2018	7.0342	4.3995	7.5807	7.5372	7.6456	7.3947	7.1383	6.6171	7.6237
Bin1	_ADETKDEQFEQC[Carbamidomethyl (C)]VQNFNK_.3	#N/A	8.4723	8.5487	8.8589	8.8143	8.4478	8.5145	8.1997	8.8672	8.5297	8.7647	8.5852	8.4735	9.2404	9.2173	8.3552	8.4916	9.0376	8.2536	8.4849	8.6670
Bin1	_SPS[Phospho (STY)]PPPDGSPAATPEIR_.2	#N/A	13.7877	13.8023	13.9817	13.9233	13.5162	13.7555	13.2772	13.8516	13.6685	13.7977	13.5943	13.6028	14.0283	14.1634	13.5447	13.6406	14.1456	13.3738	13.6292	13.7200
Bin1	_LVDQALLTM[Oxidation (M)]DTYLGQFPDIK_.3	#N/A	5.0369	5.0845	4.4115	4.7195	4.5264	4.7895	3.5958	4.0102	4.5014	5.2549	5.2542	4.2701	4.7345	4.7323	4.7495	5.8013	4.5107	4.7309	5.2691	4.7109
Bin1	_VNHEPEPASGASPGATIPKS[Phospho (STY)]PSQLR_.3	O08539_S332_M1_Bin1	6.0032	4.1731	4.1834	4.4382	3.3530	3.3863	3.6951	7.0814	3.6154	4.4574	3.0940	3.8992	3.1769	4.2215	3.7205	4.0090	4.3698	4.2805	5.1453	3.8988
Bin1	_AGDVVLVIPFQNPEEQDEGWLM[Oxidation (M)]GVK_.3	#N/A	4.9864	4.3928	4.7264	4.6831	4.1954	4.4645	4.1572	4.1189	4.8482	3.7990	3.7524	4.1073	4.4392	3.4411	2.8781	3.3249	5.1389	3.9823	3.3011	4.2630
Bin1	_AQPSDNAPEKGNKS[Phospho (STY)]PS[Phospho (STY)]PPPDGSPAATPEIR_.4	#N/A	8.8779	10.1106	8.5151	9.8455	7.3014	9.3852	10.0344	9.4213	8.3685	9.2447	9.3485	9.9657	10.1199	10.5859	9.5559	9.3303	8.4340	10.1502	8.2354	9.6716
Bin1	_HHYESLQTAK_.2	#N/A	6.0685	5.3430	5.9356	5.9535	5.8983	5.6543	5.8756	5.9223	6.1512	6.0354	5.8110	5.8084	5.6600	6.1337	6.4498	6.6404	6.4848	3.2999	5.4213	5.9588
Bin1	_AAPQWC[Carbamidomethyl (C)]QGK_.2	#N/A	5.7825	4.5323	4.0068	4.8391	5.8292	3.0323	5.6692	5.1802	5.2208	5.5252	3.6655	4.4217	3.3992	4.4438	6.2735	3.8154	4.6576	5.5161	5.3967	4.1210
Bin1	_IAENNDLLWM[Oxidation (M)]DYHQK_.3	#N/A	4.3868	4.2415	4.5659	4.5244	3.8369	3.7582	4.0246	3.4048	3.9157	4.1866	3.4619	4.2722	4.4736	4.2393	4.2222	4.0462	4.1568	3.3895	4.2378	4.4450
Bin1	_QHGSNTFTVK_.2	#N/A	3.3570	4.4223	4.3945	4.2272	3.5640	4.2369	4.3818	3.5827	4.4901	3.6881	3.6542	3.6882	3.3879	4.4325	4.5345	4.2200	3.0800	4.0695	4.7508	4.3447
Bin1	_LQAHLVAQTNLLR_.3	#N/A	3.9980	4.1437	3.2880	3.6433	3.8046	3.9147	3.7809	3.6552	3.9405	4.2971	3.2761	3.5097	4.5828	3.6524	3.3722	4.7379	3.8007	3.1329	3.7864	3.4996
Bin1	_ESDWNQHKELEK_.3	#N/A	4.1313	3.1878	3.6678	3.4530	4.3382	3.7702	3.9950	4.3569	3.5651	4.4147	2.9276	3.4756	4.1621	3.7059	4.2361	3.4934	3.8542	3.8941	3.5943	3.3832
Bin1	_VGFYVNTFQSIAGLEENFHK_.3	#N/A	3.7154	4.2874	4.2517	3.9740	3.0531	3.4310	0.7254	3.4157	3.0462	3.9217	3.4206	4.1769	3.7361	4.2387	4.0652	4.0603	3.9534	3.8797	1.9944	4.2057
Sec22b	_NLGS[Phospho (STY)]INTELQDVQR_.2	O08547_S137_M1_Sec22b	10.3243	10.1402	10.4364	10.4294	9.1869	9.7516	9.7985	9.5010	9.8080	10.3155	9.6589	9.0786	9.8108	9.7422	8.1728	9.4793	9.7687	9.5412	9.7514	9.4776
Sec22b	_DLQQYQSQAK_.2	#N/A	4.2667	6.2100	6.0991	6.4027	6.2473	5.7276	5.5374	6.3407	5.7681	4.4730	5.6589	6.1467	4.2874	5.9319	5.7706	6.0517	5.9294	6.3668	5.5159	5.6424
Dpysl2	_GSPLVVISQGK_.2	#N/A	11.8243	11.9254	11.9818	12.0984	11.9939	11.9069	11.8884	12.1057	11.7206	12.0939	11.4550	11.6377	11.6812	11.7172	11.4028	11.5199	11.4948	11.3523	11.3921	11.5068
Dpysl2	_M[Oxidation (M)]VIPGGIDVHTR_.2	#N/A	11.2641	11.3842	11.1721	10.9802	11.3268	11.2387	11.1171	11.5833	11.0352	11.2629	10.5249	10.9339	11.1663	10.8472	10.8976	10.8287	10.8532	10.7658	10.7325	10.8250
Dpysl2	_SAAEVIAQAR_.2	#N/A	12.4322	12.7569	12.7328	12.7678	12.6019	12.6134	12.7053	12.6579	12.5508	12.6689	11.8538	12.3772	12.2340	12.5495	12.4380	12.0339	12.2098	12.1789	12.2538	12.2210
Dpysl2	_QIGENLIVPGGVK_.2	#N/A	12.5222	12.5606	12.6531	12.6812	12.5681	12.4784	12.5802	12.6657	12.4839	12.6586	11.9634	12.2863	12.4176	12.3983	12.4260	12.1407	12.1810	12.1418	12.1546	12.2030
Dpysl2	_VFNLYPR_.2	#N/A	11.3034	11.3510	11.4825	11.8989	11.5785	11.5091	11.3840	11.0936	11.3368	11.5656	11.0445	11.2047	10.9714	10.5140	11.0580	11.0906	10.7712	10.9385	11.1953	11.2545
Dpysl2	_KPFPDFVYK_.2	#N/A	11.0212	11.0769	11.0407	11.1088	11.1934	11.0098	10.9649	11.1885	10.6160	11.1415	10.6591	10.7557	10.5417	10.7213	10.6422	10.4850	10.5629	10.5329	10.5507	10.6940
Dpysl2	_IVLEDGTLHVTEGSGR_.3	#N/A	9.1705	9.4555	9.8129	9.5411	9.2546	9.3008	9.3804	9.1495	9.3111	9.5213	9.0247	8.8536	9.2892	9.3163	9.1671	9.0714	8.8155	8.7466	9.0444	9.0959
Dpysl2	_ILDLGITGPEGHVLSRPEEVEAEAVNR_.4	#N/A	9.2358	9.6337	8.9497	9.4033	8.9773	9.1295	9.3177	9.2749	9.1800	9.2231	8.5599	8.8304	8.9099	9.5409	8.7727	8.7505	8.7731	8.9010	8.8416	9.0130
Dpysl2	_GTVVYGEPITASLGTDGSHYWSK_.3	#N/A	9.8042	9.9042	10.5750	10.2997	9.7337	9.9454	9.7243	9.5979	10.3195	10.0794	9.5293	9.2853	9.3689	9.5735	9.6090	9.6454	9.5345	8.9338	9.4324	9.8041
Dpysl2	_SITIANQTNC[Carbamidomethyl (C)]PLYVTK_.2	#N/A	9.5080	10.0387	9.5321	10.1398	9.4655	9.8513	10.0142	10.1749	9.6917	10.2795	8.6866	9.7249	9.2931	9.6757	9.3643	9.5184	9.4829	9.3225	8.9692	9.1053
Dpysl2	_AVGKDNFTLIPEGTNGTEER_.3	#N/A	8.8967	9.1440	7.7959	9.1031	9.0938	8.6575	8.6630	9.1820	8.3053	9.5191	8.0804	8.6138	9.4318	8.4380	8.0696	8.9759	8.6400	8.4292	8.4221	8.4996
Dpysl2	_TIEAHSR_.2	#N/A	8.4574	7.8798	8.6138	8.1256	8.4084	8.1357	8.5793	7.3414	8.1942	8.6185	8.2394	7.5014	6.9985	6.7049	8.4372	7.3810	7.8603	7.3279	7.2537	8.0382
Dpysl2	_ITS[Phospho (STY)]DRLLIK_.2	O08553_S14_M1_Dpysl2	8.3823	8.7547	8.3009	8.8035	3.0412	8.5011	8.2487	8.6226	8.4917	8.5353	7.6959	8.0718	3.7242	8.6048	7.5994	7.9764	8.1204	7.9489	7.5978	8.0435
Dpysl2	_NIPRIT[Phospho (STY)]SDR_.2	O08553_T13_M1_Dpysl2	7.7559	8.1367	3.1757	3.3849	7.2192	7.5986	7.7951	7.8270	7.3530	7.9956	6.5992	7.3267	4.2302	3.7740	3.6403	2.9843	7.2771	7.0442	3.5262	7.0166
Dpysl2	_TS[Phospho (STY)]PAKQQAPPVR_.2	#N/A	12.8597	13.2417	12.2022	13.2015	12.8024	12.4884	12.7966	13.4356	12.8202	13.8008	12.0697	13.0298	13.9460	13.1749	12.7743	13.0395	3.6274	12.8480	12.7177	12.7794
Dpysl2	_DNFTLIPEGTNGTEER_.2	#N/A	13.1389	13.0627	13.3264	13.3742	3.5193	13.2890	12.8131	13.0075	13.2497	13.3136	7.5493	12.5580	12.5706	13.0454	12.6634	12.8070	12.9732	12.3885	12.6064	12.9019
Dpysl2	_ISVGSDADLVIWDPDSVK_.2	#N/A	9.9269	9.6430	10.4933	10.1973	9.7070	9.9328	9.7653	9.6964	10.2473	10.3155	9.1054	9.4605	9.5270	9.4656	9.6977	9.7520	9.7325	9.3402	9.5288	9.7250
Dpysl2	_M[Oxidation (M)]SVIWDK_.2	#N/A	5.8732	7.6030	7.9277	8.6028	7.7149	7.7614	7.9901	5.4598	7.8536	8.1503	7.2099	7.1812	6.3342	7.6857	7.5792	6.5311	5.0225	6.7434	7.4469	7.0952
Dpysl2	_GIQEEM[Oxidation (M)]EALVK_.2	#N/A	7.5765	8.2004	8.1051	7.9494	7.5995	7.7586	7.6145	7.5670	7.9971	7.5515	7.2240	7.2316	6.7201	7.7341	7.6126	7.2470	7.5274	7.5313	7.1190	7.6845
Dpysl2	_TVT[Phospho (STY)]PASS[Phospho (STY)]AKTS[Phospho (STY)]PAK_.2	#N/A	14.3073	14.1996	14.5498	14.5125	13.5445	13.7850	13.8937	13.8226	13.2190	14.2909	12.3101	13.7280	14.0251	13.4045	13.2512	13.6004	13.6576	13.3479	13.3283	13.6284
Dpysl2	_TVT[Phospho (STY)]PASS[Phospho (STY)]AK_.2	#N/A	11.7270	11.9527	12.1386	12.1386	11.4699	11.6503	11.5384	11.5473	11.7465	12.0752	10.9995	10.9843	11.1561	11.2299	9.7553	10.4081	11.0092	10.8553	10.6145	11.1745
Dpysl2	_TVT[Phospho (STY)]PASS[Phospho (STY)]AKTS[Phospho (STY)]PAKQQAPPVR_.3	#N/A	3.6604	12.1902	10.8366	12.0974	11.4029	10.4614	11.5649	11.2785	10.9980	11.6801	9.8575	10.9701	11.8250	11.6662	11.3911	11.0398	11.3935	11.2534	11.2604	10.7515
Dpysl2	_FQM[Oxidation (M)]PDQGM[Oxidation (M)]TSADDFFQGTK_.2	#N/A	8.6753	8.2480	8.3235	7.6534	8.4821	8.2766	8.0164	8.9375	8.4991	8.7811	7.4751	8.1441	8.5886	7.2417	8.4665	8.5029	8.2867	8.0264	8.0789	8.0656
Dpysl2	_DHGVNSFLVYM[Oxidation (M)]AFK_.2	#N/A	6.4805	6.1002	6.8834	6.2097	6.6115	6.4901	6.0823	6.3207	6.6181	7.0598	5.9982	5.7723	6.3121	5.8420	5.9645	6.2753	6.1822	5.9840	6.0497	6.0550
Dpysl2	_NLHQSGFSLSGAQIDDNIPR_.3	#N/A	9.4739	9.8579	9.6933	9.5553	8.8011	8.7709	9.7058	9.8474	9.1433	9.5893	8.5130	8.9606	9.3503	9.8875	8.7641	8.7940	8.9135	8.8242	8.8619	8.7507
Dpysl2	_THNSALEYNIFEGM[Oxidation (M)]EC[Carbamidomethyl (C)]R_.3	#N/A	9.1313	8.9871	9.5670	9.4631	8.9447	8.9756	9.0052	9.1182	9.1695	9.3176	8.6658	8.7155	9.3118	8.9592	8.6848	8.6344	8.7561	8.2920	8.7063	8.9083
Dpysl2	_FQLTDSQIYEVLSVIR_.3	#N/A	5.6569	5.7061	6.1567	5.6124	5.4506	5.7111	5.3991	5.8140	5.4235	5.9815	5.7011	5.4901	6.7395	6.2965	5.3348	5.5428	5.1550	5.2921	5.7712	5.8816
Dpysl2	_M[Oxidation (M)]DENQFVAVTSTNAAK_.2	#N/A	10.3378	10.7702	10.8901	10.6411	10.2078	10.5527	10.5311	10.4937	10.7005	10.6775	9.7116	10.1339	10.1926	10.3586	10.2193	10.2319	10.0780	9.9822	10.1541	10.2508
Dpysl2	_IVNDDQSFYADIYM[Oxidation (M)]EDGLIK_.2	#N/A	8.1979	8.1428	7.8232	7.5896	7.5305	7.6622	7.7336	8.3092	7.7881	8.3590	7.3507	7.8773	8.2640	8.3819	7.4742	7.8634	7.6150	7.6264	7.2626	7.6585
Dpysl2	_NLHQSGFSLSGAQIDDNIPRR_.4	#N/A	7.4352	7.4058	7.1645	7.3732	7.7061	6.5339	7.3695	7.6942	6.8754	7.9561	7.3093	7.5940	8.1928	6.9044	7.3004	7.4510	7.2958	6.3698	6.7215	7.3295
Dpysl2	_GLYDGPVC[Carbamidomethyl (C)]EVSVT[Phospho (STY)]PK_.2	#N/A	10.2839	13.5178	11.3343	10.5782	10.0848	11.1728	13.0740	10.3450	11.3291	10.8101	9.7579	9.8714	10.0843	11.0986	12.5638	9.9243	13.1528	10.3866	10.2587	12.9558
Dpysl2	_GLYDGPVC[Carbamidomethyl (C)]EVSVTPKT[Phospho (STY)]VT[Phospho (STY)]PASSAK_.3	#N/A	3.7499	10.2552	7.4661	3.9254	3.9337	9.3567	9.8485	4.9343	8.8969	3.8254	3.6234	3.6567	4.2389	10.3311	8.5701	5.7348	3.6645	9.5636	5.5092	5.2510
Dpysl2	_DIGAIAQVHAENGDIIAEEQQR_.3	#N/A	9.8841	9.9420	9.2306	9.6913	9.2167	9.0260	9.1028	9.5995	9.5672	9.9973	9.0218	9.0258	8.6823	9.3508	8.3874	8.7144	9.2989	8.0294	8.7058	9.1476
Dpysl2	_DRFQLTDSQIYEVLSVIR_.3	#N/A	4.4446	4.4703	3.4800	4.2789	3.4439	4.5937	3.7588	4.1207	4.7815	5.2104	4.1922	3.4477	3.9259	3.4697	3.7376	3.2886	3.4967	3.1796	3.8305	3.1470
Dpysl2	_SC[Carbamidomethyl (C)]C[Carbamidomethyl (C)]DYSLHVDITEWHK_.3	#N/A	7.6908	7.5843	7.0143	7.7075	7.2875	6.1720	7.1827	8.3524	6.4910	8.1224	6.6940	6.8769	7.7752	8.3852	4.8592	7.1747	7.5545	5.4620	6.7553	7.3455
Dpysl2	ethyl (C)]EVSVT[Phospho (STY)]PKT[Phospho (STY)]VT[Phospho (STY)]PAS[Phospho (STY)]S[P	#N/A	3.5438	4.0404	3.9787	4.1326	3.6419	3.8111	3.7905	4.1952	3.8117	3.6498	3.4251	3.8020	3.8570	4.0031	3.5088	4.0534	4.0488	4.0034	4.0424	4.0620
Dpysl2	_GRIS[Phospho (STY)]VGSDADLVIWDPDSVK_.3	#N/A	4.0596	3.2595	3.5962	3.5246	4.2665	4.2999	4.2824	4.2853	4.2026	3.8702	3.6813	2.9856	4.3681	3.3699	2.8070	3.4217	3.7825	3.2477	4.3972	3.3369
Dpysl2	_AALAGGTTM[Oxidation (M)]IIDHVVPEPGTSLLAAFDQWR_.3	#N/A	4.4564	4.3636	3.9929	3.1279	3.1625	1.4196	3.8856	3.1812	3.8059	4.2669	3.2845	4.0897	3.9713	2.2454	3.9110	3.8185	4.1793	2.9702	3.2692	3.7083
Dpysl2	FVTSPPLSPDPTTPDFLNSLLSC[Carbamidomethyl (C)]GDLQVTGSAHC[Carbamidomethyl (C)]TFNT.	#N/A	3.8495	4.3298	4.0267	3.0941	3.1872	3.4113	4.3539	3.2150	4.4130	4.0558	3.2865	4.0559	4.5210	4.0648	3.9311	4.1944	4.2131	4.4372	3.2354	3.7421
Rundc3a	_FTQSYDYLT[Phospho (STY)]DEEER_.2	O08576_T215_M1_Rundc3a	3.7361	7.2915	7.7008	5.5565	3.0738	7.7872	6.9841	3.4364	7.1730	5.9828	3.3999	5.1682	3.1336	7.0091	5.6348	4.0810	5.0906	6.8554	6.8276	6.6785
Rundc3a	_SNEC[Carbamidomethyl (C)]LVSDSPEGSPALS[Phospho (STY)]PS_.2	O08576_S444_M1_Rundc3a	3.2144	9.0414	7.2168	4.4084	7.5752	7.5756	8.7707	3.8657	8.8500	8.0459	7.5305	7.8828	3.2417	8.7040	6.2881	3.9139	7.3157	8.3655	8.7186	3.8793
Emd	_DDIFSS[Phospho (STY)]LEEEGKDR_.2	O08579_S142_M1_Emd	3.1191	3.3829	5.4376	4.5703	3.9576	4.0273	3.0541	4.3203	2.9744	3.5976	3.9538	3.2723	3.1398	3.6425	3.1606	3.4640	3.3571	2.9752	4.1246	3.6095
Kcnk1	_QSEPFVASQSPPY[Phospho (STY)]EDGSADH_.2	O08581_Y329_M1_Kcnk1	7.7424	8.3956	8.5107	7.1367	3.8726	8.0370	3.5875	6.4730	8.8869	8.0651	7.0680	7.0991	3.7294	7.1916	3.3216	3.6914	9.3649	8.1576	7.5011	7.1388
Gtpbp1	_LVLVS[Phospho (STY)]PTSEQYDSLLR_.2	O08582_S69_M1_Gtpbp1	5.4212	5.7068	4.1934	3.1379	3.8892	4.0958	5.6611	5.7474	4.1722	3.2569	3.8854	4.7742	5.1188	5.6295	3.5439	3.3955	4.6643	5.3747	4.1931	5.0506
Gtpbp1	_SRS[Phospho (STY)]PVDSPVPASM[Oxidation (M)]FAPEPSS[Phospho (STY)]PGAAR_.3	O08582_S25_M2_Gtpbp1	4.4065	4.4134	3.9431	3.1777	3.1126	3.1459	6.9819	3.1313	3.8557	4.2171	3.3344	4.1396	4.0212	3.9811	3.9609	3.7686	4.1294	3.0200	3.3191	3.6584
Gtpbp1	_SPVDSPVPASM[Oxidation (M)]FAPEPSS[Phospho (STY)]PGAAR_.3	O08582_S25_M1_Gtpbp1	4.0420	3.2771	3.5786	3.5422	4.2490	4.2823	4.3000	4.0653	4.2202	3.8526	3.6988	3.0032	4.3856	6.8819	2.8245	3.4041	3.1021	3.2302	4.3796	3.3544
Gtpbp1	_LLQTTNNS[Phospho (STY)]PMNSKPQQIK_.3	O08582_S580_M1_Gtpbp1	3.1891	4.1300	4.2265	4.3952	3.3960	3.2115	3.6521	3.4147	3.5723	4.5005	3.0510	3.8561	3.2199	5.9579	3.6775	4.0520	4.4128	4.2375	3.0357	3.9418
Alyref	_QQLS[Phospho (STY)]AEELDAQLDAYNAR_.2	#N/A	3.2574	6.1189	6.2438	4.6678	3.7008	5.0327	4.7342	4.0676	5.5288	5.1065	4.1813	5.7988	5.2103	6.6201	5.2698	5.8350	5.3477	6.0758	5.7337	5.9836
Clta	_LQS[Phospho (STY)]EPESIR_.2	#N/A	9.5259	9.4607	9.5366	9.7453	9.4097	9.5095	9.1144	9.4649	9.4296	9.5590	9.3605	9.2549	9.2286	9.8387	9.5132	9.4256	9.4398	9.0512	9.2417	9.4188
Clta	_LEALDANSR_.2	#N/A	9.9735	10.1071	10.3174	10.0700	10.0111	10.0020	10.0345	10.0607	10.2015	10.1318	9.9447	10.2029	10.3305	10.3556	10.6648	10.1297	9.9802	9.9481	10.0840	10.1405
Clta	_ELEEWYAR_.2	#N/A	8.5799	8.3835	8.9801	9.0113	8.5113	8.4823	8.5336	8.4262	8.9285	8.8620	8.8376	8.1691	8.9036	8.7535	9.0396	8.8461	8.0573	8.5227	8.7492	8.8846
Clta	_VADEAFYK_.2	#N/A	6.2012	6.7789	6.6950	6.7985	6.7696	6.5222	7.4108	6.1193	7.2110	7.0464	6.7885	6.5498	7.0333	6.8769	7.5461	7.0984	5.5334	7.0481	7.3862	7.4405
Clta	_LC[Carbamidomethyl (C)]DFNPK_.2	#N/A	5.8692	6.7698	7.0784	7.2896	7.1065	6.7383	7.0053	6.2139	6.6207	6.1895	6.1685	7.2260	6.7446	7.1218	7.3649	6.3893	6.1320	6.5109	6.9144	6.3736
Clta	_KQEAEWK_.2	#N/A	6.3492	5.4696	6.6404	6.0657	6.0707	6.7246	6.2969	5.5731	6.3604	6.4971	6.6285	5.5769	6.2757	5.9518	6.9023	6.3026	6.2115	5.5962	6.0103	6.5313
Clta	_QDEQLQK_.2	#N/A	4.8997	5.5015	5.5732	4.4545	4.9596	4.7297	5.2260	3.5821	5.2523	5.5534	4.7862	4.3689	5.2548	5.7701	6.2129	5.3917	4.4229	5.4810	5.5986	5.2058
Clta	_WREEQTER_.2	#N/A	5.7599	5.9619	5.1979	4.8092	4.4048	5.3228	5.3393	5.3883	5.2445	5.2910	5.2076	5.5707	4.0579	5.4808	5.8836	5.6952	4.7374	5.2715	4.8332	5.4722
Clta	_AIKELEEWYAR_.2	#N/A	4.4202	6.0804	3.4556	6.1430	6.5473	5.5382	5.3878	7.0126	5.9084	5.9996	4.2166	6.9237	7.4244	6.4333	6.4140	6.8717	6.5178	2.3349	6.0754	6.0045
Clta	_LQS[Phospho (STY)]EPESIRK_.2	#N/A	9.9298	10.4032	10.2009	10.1500	9.8255	10.2933	10.1109	10.0510	10.2327	10.3617	9.9846	10.0950	10.4332	10.6195	10.3576	10.2516	10.2029	10.0469	10.1850	10.3584
Pten	_NNIDDVVR_.2	#N/A	3.2407	3.3715	4.3229	4.3435	3.4477	4.4328	3.9823	3.4664	4.0621	4.9743	3.9025	4.7789	3.2716	5.3279	4.5027	4.1037	4.9486	4.4143	4.9477	4.7160
Pten	_VENGS[Phospho (STY)]LC[Carbamidomethyl (C)]DQEIDSIC[Carbamidomethyl (C)]SIER_.2	O08586_S294_M1_Pten	3.9598	4.0430	4.4961	3.7296	3.2975	3.5216	4.2435	3.1046	4.3026	4.1661	3.1762	3.9324	4.4107	3.9544	3.8207	4.3047	4.1027	3.0467	3.3458	3.6317
Stxbp1	_KLNKTDEEISS[Phospho (STY)]_.2	#N/A	7.3667	11.6771	11.5245	11.7742	11.3479	7.2357	11.3903	7.4062	11.4638	11.5127	7.0925	11.5960	12.3772	6.7740	7.0361	7.4656	11.8818	6.9332	11.7126	11.5677
Stxbp1	_SSAS[Phospho (STY)]FSTTAVSAR_.2	O08599_S509_M1_Stxbp1	10.7504	11.1793	9.5647	9.4746	10.1837	9.2366	10.6852	10.5866	9.3295	10.6939	10.2878	9.4404	10.1614	9.5149	9.5437	9.3877	10.6476	9.3899	9.3770	9.3175
Stxbp1	_LNKTDEEISS[Phospho (STY)]_.2	#N/A	12.4585	8.6567	7.5944	9.3490	8.7555	7.2489	8.3573	12.4020	8.1362	12.2289	7.6797	7.4076	7.0701	8.4924	7.7232	12.1835	7.8220	7.1477	7.6239	8.7602
Stxbp1	_SQLLILDR_.2	#N/A	10.5722	10.5545	10.5442	10.7291	10.5008	10.3387	10.8465	10.3981	10.4604	10.6529	10.7390	10.6063	10.9058	10.5541	10.0858	10.6053	10.3061	10.6017	10.7580	10.6239
Stxbp1	_HYPYISTR_.2	#N/A	10.5207	10.5433	10.2610	10.1715	10.5579	10.4074	10.6904	10.7984	10.3356	10.2364	10.3667	10.3821	10.6197	10.2335	10.4587	10.3096	10.3755	10.5319	10.0641	10.3371
Stxbp1	_ISEQTYQLSR_.2	#N/A	10.3559	10.3210	10.3687	10.3450	10.0800	10.2395	10.4394	10.4715	10.2964	10.1657	10.2427	10.2466	10.5994	10.2015	10.1178	10.0973	10.3679	10.2932	10.3149	10.0341
Stxbp1	_NGITEENLNK_.2	#N/A	9.7380	9.7571	10.0260	9.7530	9.5313	9.8096	9.7732	9.9418	9.5995	9.6748	9.6405	9.5139	9.4889	10.0000	8.7870	9.1991	9.8429	9.5819	9.5526	9.5793
Stxbp1	_YETSGIGEAR_.2	#N/A	9.1885	8.9623	9.2680	8.8992	8.7789	9.0657	9.2215	9.0433	9.1697	8.8286	8.9451	8.7953	8.7782	9.0657	9.2170	8.8344	8.6795	9.0314	9.0124	8.9126
Stxbp1	_HIAEVSQEVTR_.2	#N/A	8.5533	8.8662	9.1480	8.9659	8.5416	8.7603	8.8016	8.6794	8.9438	8.4579	8.4071	8.7503	8.2536	8.7311	8.6268	8.6997	8.6443	8.7307	8.7538	8.4101
Stxbp1	_DNALLAQLIQDK_.2	#N/A	8.6575	8.1901	9.2831	8.6507	8.6208	9.0006	8.6275	8.6564	8.4607	8.3021	8.8124	8.3698	8.0631	8.8448	8.2786	8.1008	8.8446	8.3394	8.6019	8.4348
Stxbp1	_AIVPILLDANVSTYDK_.2	#N/A	8.3908	7.9829	8.5522	8.5107	8.0647	8.0662	8.2864	8.0469	8.3111	8.3934	8.1496	8.0839	7.5954	7.9870	7.5802	7.2439	8.1833	7.9231	8.0500	8.2822
Stxbp1	_NKAPGEYR_.2	#N/A	8.0762	7.9908	8.1862	8.1419	7.9893	8.1634	8.3656	8.2663	8.2053	8.2450	8.1154	7.9194	8.2876	7.9469	8.5229	7.8957	7.5549	8.3772	8.0804	7.9082
Stxbp1	_LLDTLKK_.2	#N/A	7.7926	7.4381	7.8901	7.7936	7.9540	7.7635	7.8882	8.0379	7.8069	7.3090	7.5360	7.9851	7.7740	7.4338	8.2110	7.7750	7.7477	7.7939	7.9623	7.6507
Stxbp1	_EPLPSLEAVYLITPSEK_.2	#N/A	7.4293	7.5726	7.5270	7.0879	7.1142	7.5348	7.7974	7.7192	7.6667	7.3907	7.5698	7.5032	7.8091	7.1191	7.7840	7.6207	7.8496	7.5074	7.5206	7.3397
Stxbp1	_ADDPTM[Oxidation (M)]GEGPDK_.2	#N/A	7.4479	7.0965	6.8295	6.2608	6.6731	6.9106	6.7817	7.4676	7.2203	7.2540	6.6858	6.8577	6.3697	6.4028	7.2200	6.8867	6.7329	6.9150	6.5735	6.8069
Stxbp1	_EVLLDEDDDLWIALR_.2	#N/A	6.3355	4.5933	6.8236	6.3807	6.8971	6.7223	6.6622	6.6433	6.7102	6.1376	6.6568	6.3924	6.3366	7.0715	5.5094	6.5079	6.8982	6.3579	6.8818	6.9924
Stxbp1	_IM[Oxidation (M)]HDVIKK_.2	#N/A	6.6986	7.0723	4.8639	5.5859	6.8262	6.9623	7.1294	6.7303	6.6483	7.0138	6.4210	7.2174	6.8798	6.0213	7.2023	6.8792	7.0054	7.0667	6.9818	6.8101
Stxbp1	_SLKDFSSSK_.2	#N/A	6.0521	6.2119	6.4882	6.0853	5.9832	6.1914	6.2373	6.3644	6.3085	6.4686	6.3178	5.9108	6.7027	6.0041	6.4044	6.2995	5.5816	6.4198	6.1080	6.1398
Stxbp1	_LAEQIATLC[Carbamidomethyl (C)]ATLK_.2	#N/A	6.4401	5.9755	6.2355	6.3216	6.7725	6.3541	6.0922	6.8332	6.0850	6.4261	5.9238	6.2474	5.7393	5.3490	5.5168	5.5288	5.8709	6.0395	6.3097	5.9572
Stxbp1	_DNALLAQLIQDKLDAYK_.2	#N/A	6.4895	5.8315	5.5726	5.7605	5.5131	5.8195	5.4406	5.6765	5.8091	5.4877	5.8607	6.1756	5.4457	6.3430	5.1233	6.1537	5.9839	6.0277	6.1589	5.4698
Stxbp1	_AAHVFFTDSC[Carbamidomethyl (C)]PDALFNELVK_.3	#N/A	7.3250	7.4678	7.3358	7.4440	7.0582	7.3057	7.4231	7.5080	7.3277	7.3226	7.0601	7.1131	7.6118	7.5679	6.7780	7.2723	7.2523	7.5450	7.4682	7.3052
Stxbp1	_HYQGTVDK_.2	#N/A	4.7791	4.6313	3.7771	4.9486	4.3509	3.9337	5.1811	4.2635	4.6284	3.9434	5.3538	4.3109	4.0609	4.7940	5.6533	5.3184	4.6217	4.5436	4.7737	5.0786
Stxbp1	_VLVVDQLSM[Oxidation (M)]R_.2	#N/A	9.8536	9.6825	9.7029	9.5968	9.6249	9.5941	9.7331	10.0896	9.5287	9.7193	9.4705	9.7145	10.1236	9.4526	8.8930	9.5410	9.8011	9.7201	9.6029	9.4678
Stxbp1	_REPLPSLEAVYLITPSEK_.3	#N/A	7.6729	7.3763	7.5194	7.1489	7.3040	7.6689	7.2296	7.4651	7.4600	7.1580	7.1029	6.8109	7.5521	7.3819	6.6565	7.0512	7.5177	7.5082	7.6154	7.3148
Stxbp1	_VEQDLAM[Oxidation (M)]GTDAEGEK_.2	#N/A	8.1227	8.0217	8.2584	7.9588	7.5775	8.0671	8.2087	8.0473	8.0844	8.1602	7.9359	7.7703	7.9595	7.9993	7.8625	7.9274	8.2940	8.1525	8.1274	8.1053
Stxbp1	_SVHSLISDFKDPPTAK_.3	#N/A	9.0473	8.8593	8.6359	8.5410	8.6847	8.6457	8.7060	9.1416	8.6463	8.7011	8.6660	8.7800	9.0882	8.7234	8.4647	8.6917	9.0221	8.7352	8.7886	8.5581
Stxbp1	_M[Oxidation (M)]LSSC[Carbamidomethyl (C)]C[Carbamidomethyl (C)]K_.2	#N/A	4.5851	4.9909	6.0946	6.0408	4.8353	4.8756	6.3529	4.8380	4.6078	4.9496	5.5019	4.4771	4.6507	5.6070	5.9818	4.5621	4.6591	4.4551	4.7469	4.5396
Stxbp1	_DFSSSKR_.2	#N/A	4.8997	5.2163	4.3597	4.2242	5.5378	5.8792	5.9252	5.0284	5.5038	5.0884	6.2485	5.7681	4.5471	5.0331	3.9571	5.0493	4.3785	5.4699	3.2094	5.7689
Stxbp1	_KM[Oxidation (M)]PQYQK_.2	#N/A	5.3677	5.8945	6.7621	6.3009	5.3213	5.0926	6.3963	5.3169	5.5543	4.9155	6.2751	4.9485	3.8131	6.7125	6.1029	5.7030	5.3704	5.8898	5.0303	5.2736
Stxbp1	_IM[Oxidation (M)]HDVIK_.2	#N/A	5.9825	6.0292	6.0122	6.0903	6.0248	5.9764	6.6514	6.3665	5.8796	5.8427	6.0710	5.9041	5.6048	6.1794	6.1169	5.8599	5.7509	6.1657	6.1635	5.5348
Stxbp1	_C[Carbamidomethyl (C)]AYEVTQANGK_.2	#N/A	5.9448	4.5607	5.4520	5.6249	5.5793	5.3679	5.6846	7.8967	5.0407	4.1609	5.4517	5.3614	4.4159	5.0994	3.8260	5.3713	5.4015	5.5346	3.3405	5.5314
Stxbp1	_M[Oxidation (M)]TDIM[Oxidation (M)]TEGITIVEDINKR_.3	#N/A	7.9477	7.5925	7.2310	7.5475	6.6736	7.0700	6.7239	7.8492	6.9931	7.7365	7.1683	7.3890	8.4485	7.5603	6.5623	7.5834	7.3403	7.5716	7.5886	7.1846
Stxbp1	_WEVLIGSTHILTPQK_.2	#N/A	7.2794	6.9181	7.1886	6.4336	6.2122	6.5875	6.8270	7.0341	6.5477	6.5090	6.8718	6.4026	6.6742	6.5566	5.8754	6.4579	6.9193	6.4828	6.7250	6.7551
Stxbp1	_M[Oxidation (M)]TDIM[Oxidation (M)]TEGITIVEDINK_.2	#N/A	6.5853	5.6289	6.0637	5.8923	5.7229	5.4755	5.5882	6.4347	5.8022	6.3808	6.1010	5.7670	6.4564	6.2382	5.5849	6.3545	6.1199	5.8346	5.8173	5.9954
Stxbp1	_DIM[Oxidation (M)]EDTIEDKLDTK_.2	#N/A	7.7051	7.6095	8.2226	7.8680	7.4839	7.5849	7.4976	7.7681	7.7651	7.6716	7.6600	7.4626	7.6731	7.6210	7.1745	7.4147	7.8809	7.2468	7.4337	7.6935
Stxbp1	_DIMEDTIEDK_.2	#N/A	3.6071	3.7120	6.6031	6.6826	5.1096	5.1391	3.2340	3.8328	3.1542	3.4177	5.6890	3.4381	3.3197	6.4496	5.7159	2.9692	3.3300	3.8194	4.1185	4.3599
Stxbp1	_YSTHLHLAEDC[Carbamidomethyl (C)]M[Oxidation (M)]K_.3	#N/A	4.6604	6.1158	4.7895	3.7592	3.7413	4.5148	4.8070	4.3824	4.2711	3.9142	4.2700	4.7669	4.0108	5.0584	4.8927	4.6076	3.8162	5.4037	4.7756	3.7781
Stxbp1	_LIQHAQIPPEDSEIITNMAHLGVPIVTDSTLR_.4	#N/A	3.8634	3.4234	3.5923	4.0180	4.8296	4.0386	5.1023	4.7374	3.5384	3.3149	4.1003	3.4758	4.2406	4.8697	2.7848	5.1891	3.5181	3.8035	3.9445	3.3449
Stxbp1	_VEQDLAMGTDAEGEKIKDPMR_.3	#N/A	3.1335	3.6138	4.9876	4.9758	3.9720	4.1961	3.5690	3.9310	3.6281	3.3398	1.4262	3.3399	3.7362	4.2439	3.1462	3.4784	3.4282	3.7212	4.0203	2.9572
Stxbp1	_GFDPSSPVLHELTFQAM[Oxidation (M)]SYDLLPIENDVYK_.3	#N/A	4.3104	4.5095	3.8470	3.2738	3.0165	3.5910	4.0316	4.5361	4.2332	4.1210	3.1067	0.6207	4.3413	3.8850	4.0570	3.6725	4.0333	3.1161	3.4152	3.5623
Stxbp1	_LDAYKADDPTMGEGPDK_.3	#N/A	3.3276	3.9915	4.3650	4.2567	3.5345	3.0730	3.5136	3.5532	3.4338	3.1381	2.9125	3.7177	3.3584	4.4030	3.5390	4.1905	4.5513	4.0990	4.3980	4.0803
Stxbp1	_DLS[Phospho (STY)]QMLKK_.2	O08599_S328_M1_Stxbp1	4.0107	3.3084	3.5472	3.5736	4.2176	4.2509	2.3139	4.2363	4.2516	3.8212	3.7302	3.0346	4.4170	3.5853	2.8559	3.3728	3.7336	3.1988	3.7149	3.3858
Myh11	_VIENTDGS[Phospho (STY)]EEEM[Oxidation (M)]DAR_.2	#N/A	8.8877	4.1986	8.9650	4.2137	3.2362	3.3103	8.1119	3.4324	8.3544	4.1806	8.4610	8.2473	8.6405	8.4578	3.8615	4.0737	8.5669	8.9119	3.2754	8.2220
Ephb6	_GTGYTEQLQQYSS[Phospho (STY)]PGLGVK_.2	O08644_S630_M1_Ephb6	4.8721	5.3443	4.2904	4.6105	3.8389	4.2980	4.0974	4.5568	3.3182	4.2551	3.2210	4.6137	5.0517	4.2775	3.4997	4.0990	4.7260	3.8413	4.6164	4.5737
Map3k4	_ELESGTEES[Phospho (STY)]DEEPTPSPR_.2	O08648_S454_M1_Map3k4	3.9655	6.0363	6.3643	5.6436	3.6403	5.9248	6.0431	6.6304	6.3540	4.0571	3.4299	5.7482	4.4467	5.8155	5.2763	5.3471	6.5745	5.8153	5.9095	5.5616
Map3k4	_LES[Phospho (STY)]EEDSIGWGTADC[Carbamidomethyl (C)]GPEASR_.2	O08648_S492_M1_Map3k4	3.4887	4.5854	5.5393	4.2720	3.6957	5.2913	4.5345	3.1178	4.7870	3.2993	4.2521	2.9740	3.4381	5.0065	3.3778	3.6742	3.6554	4.1777	3.8263	4.1770
Kifc2	_AGVAS[Phospho (STY)]PVQR_.2	O08672_S643_M1_Kifc2	8.5704	8.2304	8.0738	8.1904	7.7525	7.8085	8.5704	7.8833	7.9836	8.1768	7.9782	7.9027	8.3076	7.5990	6.6223	7.2525	8.2333	8.0838	7.6072	7.7249
Prdx6	_LSILYPATTGR_.2	#N/A	10.3129	10.3611	10.3440	10.3634	10.3337	10.2160	10.2717	10.2606	9.9283	10.2972	9.8007	10.2212	10.4229	10.3237	10.3009	10.1052	10.1006	10.1589	10.0873	10.0539
Prdx6	_LPFPIIDDK_.2	#N/A	8.5620	8.8800	9.0061	9.0625	8.9836	8.9820	8.6817	8.5715	8.3272	8.8662	8.5375	8.7174	8.1480	9.0079	9.1299	8.4214	8.5724	8.6940	8.6168	8.5629
Prdx6	_VVFIFGPDKK_.2	#N/A	8.6079	8.5889	8.2206	8.7443	8.5323	8.3927	8.3596	8.5443	8.5467	8.5753	8.0654	8.6110	8.6381	8.8932	8.6684	8.3215	8.3712	8.5359	8.3801	8.5328
Prdx6	_DINAYNGETPTEK_.2	#N/A	8.4276	8.0524	8.4295	8.2746	8.2292	8.2744	8.0672	8.2751	8.4782	8.3564	7.9732	8.1144	7.8011	8.3685	8.6747	8.0618	7.8663	7.7968	8.0321	8.1506

Prdx6	_VVDSLQLTGTKPVATPVDWK_.3	#N/A	6.2834	6.6539	6.1638	7.1187	6.1996	6.7324	6.3112	6.0405	6.9175	6.7665	6.5019	6.4124	6.9053	6.8114	6.8704	6.6295	5.9975	6.3190	6.6552	6.5281
Prdx6	_DLAILLGM[Oxidation (M)]LDPVEK_.2	#N/A	7.0070	7.1443	7.1024	6.6556	7.0951	7.0892	6.7051	6.8729	7.1292	6.7138	6.6637	6.9359	6.1755	5.9234	7.4346	6.5027	7.0959	6.8802	6.8777	7.4795
Prdx6	_NFDEILR_.2	#N/A	5.3554	5.9888	6.8581	6.7641	5.4701	5.9423	6.9964	5.5286	7.0684	5.6337	6.1885	6.2235	7.0166	6.7586	7.0307	5.8936	4.5404	7.1442	6.4689	6.3171
Prdx6	_DFTPVC[Carbamidomethyl (C)]TTELGR_.2	#N/A	7.6319	8.3131	7.4081	7.4583	7.1622	7.9430	7.8529	7.7722	7.8686	8.0086	7.5724	7.0853	7.2427	8.0711	7.8639	6.6436	8.0451	7.2417	7.0284	7.1689
Prdx6	_PGGLLLGDEAPNFEANTTIGR_.2	#N/A	5.4815	6.2625	6.0330	6.0662	5.5872	5.7321	5.9573	5.1347	6.3521	6.1334	5.9287	5.9007	6.2718	6.3157	6.4360	6.0663	5.3593	5.7913	5.6181	5.9419
Prdx6	_VVFIFGPDK_.2	#N/A	4.5605	5.6700	5.3359	6.3050	4.7830	5.7685	4.5022	3.8068	5.7750	5.3268	5.5337	5.2776	5.7841	5.9339	5.8632	6.0819	4.9494	5.0315	5.4457	5.7051
Prdx6	_LIALSIDSVEDHLAWSK_.2	#N/A	3.8714	5.0086	5.0305	4.8827	3.9191	4.6378	4.2815	4.7451	4.7522	4.9917	4.2373	4.6286	4.1354	5.6053	4.6500	4.2609	4.5041	5.0843	4.5677	5.5546
Prdx6	_KGESVM[Oxidation (M)]VVPTLSEEEAK_.3	#N/A	7.2509	7.4264	7.3640	7.5087	6.8900	6.9057	7.2840	7.3989	7.2132	7.0572	6.9873	6.9431	7.3552	7.3997	7.2466	6.8377	6.5220	7.1462	7.0554	7.1276
Prdx6	_LPFPIIDDKGR_.2	#N/A	6.9208	7.0743	6.0537	6.7810	6.9369	6.7669	6.7573	7.2547	6.5240	6.9483	6.5260	6.7858	7.4187	7.0950	7.0843	7.0583	6.9327	6.9301	6.7670	7.0303
Prdx6	_FHDFLGDSWGILFSHPR_.3	#N/A	3.7119	4.2909	4.2482	3.9775	3.0497	3.4344	3.6470	3.4123	3.5672	4.5056	3.0458	4.1803	3.7326	4.2353	3.6724	4.0571	3.9500	3.8832	3.5318	4.2023
Akap1	_RRS[Phospho (STY)]ESSGNLPSVADTR_.3	#N/A	8.8822	9.3891	9.0382	8.9176	8.4385	8.7411	8.8943	4.1038	8.4800	8.9441	8.7867	8.3869	9.1896	8.8508	8.0015	8.4784	8.2756	8.4586	8.6698	3.1300
Akap1	_S[Phospho (STY)]ESSGNLPSVADTR_.2	#N/A	3.3872	4.4839	4.0552	4.1705	3.5942	3.6275	3.4539	3.2192	3.3741	3.1978	7.4203	4.3734	3.5396	4.0422	3.4793	4.2502	3.7569	4.0762	3.7248	4.0092
Dld	_NETLGGTC[Carbamidomethyl (C)]LNVGC[Carbamidomethyl (C)]IPSK_.2	#N/A	8.1144	8.5297	8.8476	8.6247	8.3283	8.5815	8.2892	8.2668	8.3925	8.7482	8.3498	8.5803	8.5553	8.7122	8.5432	8.3959	8.5777	8.2281	8.3156	8.6882
Dld	_GIEIPEVR_.2	#N/A	7.3818	7.6405	7.7973	8.7709	8.1946	7.7622	8.1940	7.2908	8.2535	8.4320	8.5495	7.8440	7.8491	7.6707	8.1629	7.5508	7.0065	8.0448	8.7854	8.3179
Dld	_IPNIYAIGDVVAGPM[Oxidation (M)]LAHK_.3	#N/A	6.8718	7.1376	6.7463	6.9752	6.7592	7.1093	7.0531	6.9597	7.0093	7.2356	6.8664	7.2204	6.8017	7.1360	6.9609	7.1825	6.2688	7.4583	7.2645	7.0224
Dld	_ADGSTQVIDTK_.2	#N/A	7.8393	7.2255	7.7619	7.5692	7.6472	7.1746	6.7799	7.7612	7.6306	7.1291	7.6181	7.8030	7.6445	7.8800	7.9411	7.7255	7.4556	7.5616	7.7037	7.8530
Dld	_EANLAAAFGKPINF_.2	#N/A	7.2795	7.0766	7.0948	7.4250	7.2988	7.1713	7.3674	7.4097	7.4972	7.5070	7.1817	7.2688	7.4251	7.4931	7.5439	7.6158	7.4756	7.3033	7.4042	7.4282
Dld	_IDVSVEAASGGK_.2	#N/A	5.9587	5.5694	4.3503	6.1531	5.7987	6.2317	5.4922	3.5743	6.1725	6.2985	5.4830	5.9876	5.0356	6.5069	6.0536	4.1759	4.5366	5.9363	6.0408	5.9058
Dld	_VC[Carbamidomethyl (C)]HAHPTLSEAFR_.3	#N/A	6.5713	6.7593	6.7062	6.6017	7.0458	7.0035	5.8436	6.6242	6.7580	6.4412	6.7515	5.4649	6.7659	7.2852	7.0639	6.8053	6.9107	6.4390	6.5689	6.5879
Dld	_ALTGGIAHLFK_.2	#N/A	3.8336	3.4294	3.7517	3.1160	4.0785	2.1756	1.3025	4.2737	4.4286	2.0764	3.9073	3.3189	4.5941	1.9133	2.1693	3.1957	3.3106	3.0217	4.1712	2.4903
Dld	_SEEQLKEEGIEFK_.2	#N/A	5.7877	5.7403	5.8713	5.3198	5.7546	5.8691	5.3446	5.9862	5.8478	6.1779	5.9982	5.3385	5.7347	6.2474	5.6002	5.9258	5.5656	5.7909	5.4914	5.8343
Dld	_RPFTQNLGLEELGIELDPK_.3	#N/A	4.2916	4.5284	3.8281	3.2927	2.9976	3.6099	4.1553	4.5172	4.2144	4.2801	3.0879	4.2545	4.3224	3.5734	3.7164	3.6537	4.0145	4.4684	4.3440	3.5435
Dld	_LGADVTAVEFLGHVGGIGIDM[Oxidation (M)]EISK_.3	#N/A	4.0488	3.2703	3.5854	3.5354	4.2558	4.2891	4.2932	4.2745	4.2134	3.8594	3.6920	2.9964	4.3788	3.3806	2.8177	5.5070	3.7717	3.2370	4.3864	3.3476
Dld	_AEVITC[Carbamidomethyl (C)]DVLLVC[Carbamidomethyl (C)]IGR_.2	#N/A	3.8524	3.4666	3.3890	3.7318	3.8249	4.0490	3.7162	4.0781	3.7753	3.1927	4.1496	3.1928	3.8833	3.4271	3.2934	3.3312	3.5754	3.5741	3.8731	3.1044
Dld	_VLGAHILGPGAGEM[Oxidation (M)]VNEAALALEYGASC[Carbamidomethyl (C)]EDIAR_.3	#N/A	3.7268	4.2760	4.2631	3.9625	3.0646	3.2887	4.4765	3.4272	3.0348	3.9332	3.4091	4.1654	3.1428	4.2502	4.0537	4.0717	3.9649	3.8683	3.5168	3.8647
Hsd17b10	_NFLASQVPFPSR_.2	#N/A	7.2163	6.9387	7.3742	7.1002	7.2524	7.0618	7.2025	7.3718	6.7908	7.0204	7.0426	7.2074	6.7288	7.4791	6.8867	7.2071	7.1410	6.9425	7.2442	7.0474
Hsd17b10	_VVTIAPGLFATPLLTTLPEK_.2	#N/A	5.5146	5.2454	5.4935	4.9253	5.5414	5.7020	5.1694	5.1134	4.5166	5.9034	5.5818	5.3289	6.0629	5.2994	4.8945	5.5358	5.2934	4.6606	5.3255	5.1788
Hsd17b10	_LVAGEM[Oxidation (M)]GQNEPDQGGQR_.2	#N/A	5.4197	6.4758	5.2775	6.9186	5.7998	5.2545	6.2749	6.2614	5.7034	5.8123	5.9333	5.6568	5.9835	6.9134	5.3679	5.8504	5.6783	5.8033	5.6079	5.6525
Hsd17b10	_GVIINTASVAAFEGQVGQAAYSASK_.3	#N/A	4.2921	4.0908	4.2248	4.3741	3.6482	3.5099	3.2432	3.5123	3.3011	4.9780	3.0611	3.3144	3.1811	4.2119	1.9566	4.0334	3.4811	1.5531	3.2607	4.1892
Ube3a	_S[Phospho (STY)]PGESQSEDIEASR_.2	O08759_S8_M1_Ube3a	7.9527	7.8210	8.0041	8.1627	7.7313	7.6605	7.8688	8.3346	7.5814	7.7171	7.7227	7.6639	7.9570	7.9500	6.9810	7.9281	8.0924	7.7974	7.7563	7.7799
Ube3a	_NLDFQALEETTEYDGGYTR_.2	#N/A	3.1502	4.1689	4.1876	4.5748	3.6211	3.3905	3.6910	3.3759	3.9430	4.4616	3.0898	3.8950	3.7766	4.2257	3.7164	4.0131	4.3739	4.2764	3.0746	4.2463
Tcof1	_AASAPAKES[Phospho (STY)]PK_.2	#N/A	8.0596	7.9486	7.5753	7.1054	7.3586	7.1851	7.7408	7.9443	7.5495	7.2856	7.3511	7.3660	7.7056	7.6034	5.7022	6.7172	7.9060	7.6455	6.7165	7.2357
Tcof1	_NSS[Phospho (STY)]PAVPAPTPEGVQAVNTTK_.2	O08784_S853_M1_Tcof1	3.5773	8.9062	4.1136	4.1121	2.9150	3.1391	8.5982	3.4872	7.9255	3.7836	7.8757	8.2291	3.2924	4.3370	4.2033	3.9222	4.4853	8.3209	2.9632	4.0143
Tcof1	_KLS[Phospho (STY)]GDLEAGAPK_.2	O08784_S1191_M1_Tcof1	4.6449	5.3074	5.1412	4.9941	4.8957	5.5740	5.1459	5.3594	4.6803	5.2858	4.8269	3.6445	4.1918	5.4620	4.2251	4.7329	5.3472	5.0768	4.6998	4.9192
Tcof1	_AASAPAKES[Phospho (STY)]PKK_.2	#N/A	4.5864	3.8709	3.9906	4.6963	3.5878	3.8217	3.9053	3.0841	4.8210	3.3330	3.1887	4.2559	4.9871	4.2653	3.3442	4.3853	4.5694	3.7886	3.8600	3.8209
Tcof1	_DSAS[Phospho (STY)]PIQKK_.2	O08784_S1303_M1_Tcof1	3.8798	4.1230	5.0650	3.8095	4.1957	3.4416	4.3235	3.5802	4.3826	4.0862	5.0329	4.0124	4.4907	4.0344	3.9007	4.2247	4.1179	4.4676	3.2658	3.7117
Tcof1	_SAEPLANTVLASET[Phospho (STY)]EEEGNAQALGPTAK_.3	O08784_T171_M1_Tcof1	3.8954	4.9384	4.1709	3.7971	3.5482	4.1143	3.7929	4.1211	3.8182	3.8744	3.4307	3.9970	3.9263	4.2090	3.5573	3.7805	3.6183	3.6394	3.9385	3.8863
Tcof1	_ASAVS[Phospho (STY)]PEKAPMTSK_.2	O08784_S1216_M1_Tcof1	3.4852	5.3430	4.5226	5.4011	3.6921	3.7255	5.4064	3.7109	3.2762	3.2958	4.2557	3.5600	3.4416	5.8552	3.3814	4.3481	3.2081	3.9413	4.2404	4.2379
Dctn1	_LDETQTLLR_.2	#N/A	5.4904	5.8055	6.2632	6.3717	5.7478	5.6403	5.6681	5.9278	5.7801	5.5545	5.5232	5.8882	5.4931	5.0650	5.5389	5.8141	4.8054	5.6804	6.1367	5.5455
Dctn1	_ASEQIYGSPSSSPYEC[Carbamidomethyl (C)]LR_.2	#N/A	4.1665	4.6381	5.0200	4.3435	3.5796	4.3273	4.1388	4.5577	4.6534	4.1031	4.5869	4.4109	3.8126	5.6162	3.0909	3.7895	4.1434	3.9363	4.9053	3.9697
Dctn1	_QLTAHLQDVNR_.3	#N/A	3.4048	4.5015	4.0376	2.2763	3.6118	3.5146	3.8206	2.6411	3.3565	3.2154	1.9938	4.3910	3.0915	2.5176	3.3106	4.2678	3.7393	3.1616	3.2522	0.3338
Dctn1	_QSQIQVFEDGADTTS[Phospho (STY)]PETPDSSASK_.3	O08788_S105_M1_Dctn1	7.3852	7.9802	3.9079	7.4854	3.9527	7.5164	7.5110	3.4576	3.3527	3.4242	4.0441	3.0210	7.2959	8.0624	6.9925	7.7042	7.5097	7.7939	7.4309	7.2762
Dctn1	_AFLQGGQEATDIALLLR_.2	#N/A	4.1789	3.1402	3.7154	3.4054	2.8849	4.4191	4.1632	4.4045	3.1044	2.7750	3.5620	4.3672	3.9048	3.7535	4.1885	3.5410	3.3333	3.6711	3.5467	3.4348
Dctn1	_LVLTQEQLHQLHSR_.3	#N/A	4.0269	3.2964	3.5635	3.5616	4.0576	4.1623	3.6781	3.4919	4.0214	3.5630	3.7272	3.0226	4.1613	3.6015	3.8914	3.3890	3.3580	3.4584	4.1476	3.9353
Dctn1	_LHISQLQHENSILR_.3	#N/A	5.1536	3.5705	3.4678	3.2571	3.7701	3.9941	3.7710	4.9681	3.8301	3.1378	5.8998	3.4599	3.9382	3.4549	3.3482	3.2764	3.1696	4.3945	4.3122	3.1592
Dctn1	_LNQLSTHTHVVDITR_.3	#N/A	4.1657	3.1534	3.7022	3.4186	2.8718	4.4059	4.1763	4.3913	4.0966	3.3663	3.5752	4.3804	4.1965	3.7403	4.2017	3.5278	3.8886	3.2609	3.5599	3.4176
Dctn1	_VTFSC[Carbamidomethyl (C)]AAGLGQR_.2	#N/A	3.5349	3.7842	4.5723	4.0494	4.1425	4.3666	3.3986	3.7605	3.4577	3.5103	3.8321	3.5104	3.5657	4.6103	2.9758	3.6488	3.2578	3.8917	4.1907	4.2876
Dctn1	_VEAAQETVADYQQTIK_.2	#N/A	4.4649	4.0607	4.0015	3.7473	3.1710	3.2043	3.8771	3.6424	3.7973	4.2755	3.2760	4.0812	3.9628	4.4654	3.9025	3.8270	4.1801	3.6530	3.3016	4.4324
Prkcsh	_SLEDQVETLR_.2	#N/A	6.0341	6.1611	6.0926	5.9556	6.0571	6.0745	5.8229	5.4195	6.5208	6.1777	6.7264	5.9328	6.1032	6.3042	6.5415	6.2089	6.0361	5.4469	5.8960	6.4533
Prkcsh	_YEQGTGC[Carbamidomethyl (C)]WQGPNR_.2	#N/A	3.5466	6.3166	5.6849	6.1669	5.9644	3.7869	5.9153	5.9981	5.1193	6.1087	5.3838	5.9918	3.3802	6.5930	6.0636	5.8399	6.6600	4.2356	6.1794	5.5312
Eef2k	_RHES[Phospho (STY)]DEDS[Phospho (STY)]LGSSGR_.2	O08796_S469_M2_Eef2k	4.3644	3.6384	3.3999	3.3249	3.7022	2.2309	2.0645	4.0648	4.2197	3.8531	3.6984	3.5278	4.3852	3.3870	2.6479	3.2085	3.1017	3.2307	4.3801	1.7386
Aip	_VES[Phospho (STY)]PGTYQQDPWAMTDEEK_.2	O08915_S159_M1_Aip	4.1201	3.8827	3.1556	3.5693	3.4578	3.2479	4.0832	3.8205	3.9754	4.3264	3.4540	3.7721	4.1408	5.0078	3.6604	2.9642	4.3582	3.4750	3.1236	3.1096
Flot1	_VTGEVLDILSR_.2	#N/A	5.8153	6.3483	4.9859	6.0246	5.4960	6.0475	5.9311	5.9730	5.4330	5.5958	5.0585	5.6465	5.5429	5.6871	5.1358	5.4547	5.3699	5.5046	5.2611	5.2900
Flot1	_DIHDDQDYLHSLGK_.3	#N/A	4.8903	4.4101	5.1037	5.2863	4.8414	4.9029	4.9028	3.8889	5.7268	5.5448	5.9827	4.2240	3.6941	5.4973	4.8922	5.3769	3.9281	4.3923	4.9445	5.6877
Flot1	_VASSDLVNM[Oxidation (M)]GISVVSYTLK_.2	#N/A	6.0767	6.1299	5.8846	5.7507	5.5820	6.5696	3.4823	3.8442	3.5414	6.9698	3.9157	5.7275	6.2212	3.1932	3.0595	6.3303	5.8341	6.0794	5.6119	7.2282
Flot1	_S[Phospho (STY)]PPVMVAGGR_.2	O08917_S19_M1_Flot1	3.6148	4.3880	4.1511	4.0746	2.9525	3.1766	3.0877	3.4497	3.1468	3.8211	6.0081	4.2774	3.2549	6.5692	4.1657	3.9597	3.8529	4.9365	3.0008	3.9767
Flot1	_ITLVSSGSGTM[Oxidation (M)]GAAK_.2	#N/A	3.8018	2.8837	3.3384	3.7824	4.0088	4.0421	3.0393	4.0275	4.4604	3.6124	3.9390	3.2434	3.1250	3.3765	5.9691	3.1639	3.5247	3.6247	3.9238	3.0537
Numbl	_RTPS[Phospho (STY)]EAER_.2	#N/A	3.6916	8.4340	4.2279	8.4597	7.8474	7.8030	7.9432	3.3920	7.5468	3.8980	7.6336	7.9992	7.9536	4.2226	6.2373	4.0365	7.7368	7.7001	3.0776	3.8999
Numbl	AAAAPAVAPGPAQPGHVS[Phospho (STY)]PTPATTS[Phospho (STY)]PGEKGEAGTPVAAGTTAAAIP	#N/A	7.4370	3.4777	6.0524	5.7510	4.1572	4.2228	3.6877	4.1688	5.6712	3.6484	4.9717	3.2540	3.7958	7.9931	2.9816	3.5470	3.5030	8.6710	3.9916	3.8170
Numbl	_SVLWVSADGLR_.2	#N/A	3.9929	5.8127	6.5531	6.0280	5.7306	6.1718	6.2892	4.1144	5.5476	5.6201	5.7926	5.9541	4.4348	5.5661	5.5895	5.2144	5.0916	5.7178	4.3305	6.0428
Numbl	_KAEAAAAPAVAPGPAQPGHVS[Phospho (STY)]PT[Phospho (STY)]PATTSPGEK_.4	#N/A	4.0736	3.3530	3.7135	3.8777	3.7739	3.9075	3.5377	3.6759	3.5395	3.2623	4.1875	3.2425	3.6709	3.6869	3.3222	6.3237	3.6020	3.9291	3.8632	3.5378
Numbl	EAAAAPAVAPGPAQPGHVS[Phospho (STY)]PT[Phospho (STY)]PATTSPGEKGEAGTPVAAGTTAAAI	#N/A	4.5779	4.1738	4.3653	3.8604	3.2840	3.3174	3.7641	3.5294	3.6843	4.3885	3.1629	4.7352	3.8497	4.3524	3.7894	3.9401	4.0671	3.7661	3.4147	4.3194
Numbl	_TPS[Phospho (STY)]EAER_.2	#N/A	4.0615	3.9413	4.5978	3.6279	3.3992	3.6233	4.1419	3.7619	4.2010	4.2678	5.4254	3.8308	4.0822	4.5848	3.7191	4.4064	4.2995	4.6068	3.1822	4.8611
Numbl	_EGS[Phospho (STY)]FRLS[Phospho (STY)]GGGRPAER_.3	O08919_S224_M2_Numbl	3.8455	3.4736	3.3821	3.7387	4.0524	4.0858	2.9957	1.2927	4.4167	3.6561	3.8953	3.1997	4.5821	3.4201	3.0210	3.2076	3.5684	3.5810	3.8801	3.0974
Sdcbp	_[Acetyl (Protein N-term)]SLYPS[Phospho (STY)]LEDLKVDK_.2	O08992_S6_M1_Sdcbp	3.3458	8.2917	3.8821	4.3435	4.1844	3.8005	3.3567	3.0462	3.2011	3.5522	4.1806	3.0456	3.3666	3.8692	2.9339	3.6907	7.2430	7.9731	3.8978	3.8362
Atox1	_AVSYLGPK_.2	#N/A	3.1297	4.9993	3.0892	5.2727	4.0496	4.9667	5.2212	3.5809	4.6240	4.7101	4.8830	5.0150	6.1068	4.1895	6.3390	5.1747	3.9400	4.7647	5.4291	4.8044
Atox1	_VC[Carbamidomethyl (C)]IDSEHSSDTLLATLNK_.3	#N/A	3.8772	4.5830	3.8705	3.8708	3.7602	3.9003	3.7607	3.5759	3.7312	3.8482	3.6116	3.8041	3.8755	3.6487	3.6442	3.6868	4.1136	3.8158	3.4644	3.6157
Sh2b3	_S[Phospho (STY)]AGELPGATSDTNDIDTTAASRPGPAR_.3	O09039_S129_M1_Sh2b3	3.6592	3.6599	3.1958	3.9250	3.8661	4.2422	3.1820	3.8849	3.5820	3.4698	3.9564	3.3860	3.6901	7.3997	3.2074	3.0213	3.3821	3.7673	4.0664	7.1126
Snap23	_ATWGDGGDNS[Phospho (STY)]PSNVVSK_.2	O09044_S110_M1_Snap23	10.6216	10.5343	10.2947	10.1968	9.6318	10.1887	10.0150	10.1367	10.0862	10.2156	9.4348	9.8206	10.3525	9.8870	8.9503	9.6361	3.1084	4.0745	9.8436	9.6480
Psmb1	_NM[Oxidation (M)]QNVEHVPLTLDR_.3	#N/A	6.7649	6.4426	6.5734	6.6729	7.0348	6.3577	6.5495	7.0524	6.3762	7.3826	6.9459	6.9331	6.6985	6.0053	7.1040	7.2335	6.4832	6.8304	7.1967	6.8123
Psmb1	_GAVYSFDPVGSYQR_.2	#N/A	6.6041	6.0332	6.4454	6.7911	6.0726	6.5585	5.8936	5.9538	6.1816	5.9575	6.2956	6.1439	5.9547	5.8743	6.0449	5.8874	6.6232	6.3508	6.3498	6.3288
Psmb1	_AGGSASAM[Oxidation (M)]LQPLLDNQVGFK_.2	#N/A	4.9160	4.8284	4.0820	3.1308	5.1185	5.2125	5.0168	3.1783	3.9674	4.8180	4.4640	4.7294	4.4843	4.0281	4.0478	4.5531	4.1763	4.7774	3.2722	4.4296
Psmb1	_DVYTGDALR_.2	#N/A	4.1721	3.1470	7.4187	7.2037	2.8782	5.9129	6.1962	4.9568	7.4837	6.5311	5.9812	6.5530	4.2030	5.8397	7.3649	6.9357	3.8950	6.7884	6.7369	6.0041
Psmb1	_DVFISAAER_.2	#N/A	4.2117	4.8899	5.0555	5.4106	3.5494	3.7713	5.1776	3.9121	5.7596	5.7062	2.9243	3.6806	4.2324	4.8493	4.8269	5.1063	4.4497	5.7952	5.7744	5.3524
Psmb1	_LSEGFSIHTR_.2	#N/A	4.5615	3.4413	3.5970	3.1279	3.8992	4.1233	3.6418	4.2619	3.7009	3.2670	4.0753	3.3307	4.5822	3.5840	3.2190	3.4056	3.2987	3.0336	4.1830	3.5510
Hdac1	_IAC[Carbamidomethyl (C)]EEEFS[Phospho (STY)]DS[Phospho (STY)]DEEGEGGRK_.2	#N/A	5.6749	6.3104	5.8875	6.3968	3.7374	6.0025	6.1337	5.6150	5.9271	6.4128	5.1168	5.4948	4.4916	6.1738	5.3965	6.0706	6.0983	6.1145	6.0272	5.7968
Hdac1	_MLPHAPGVQM[Oxidation (M)]QAIPEDAIPEES[Phospho (STY)]GDEDEEDPDKR_.4	#N/A	4.2351	6.8312	5.6067	3.2759	3.6983	6.0750	6.0096	4.1371	4.2920	3.8040	3.7706	3.4441	4.4574	8.0110	4.5426	3.4056	3.7647	3.1816	3.9050	3.4262
Ndufb11	_AVIAPSGVEK_.2	#N/A	8.0990	7.8643	7.6116	7.9772	8.2240	7.9593	8.3554	8.0686	8.3249	7.7585	7.9923	8.0243	8.1932	7.9818	8.2339	7.8995	7.7827	8.3128	8.1913	8.0627
Ndufb11	_QREPTMQWQEDPEPEDENVYAK_.3	#N/A	3.4892	3.8298	4.5267	2.5109	3.6962	3.7295	2.2867	3.7149	3.2721	3.2998	2.7481	3.5560	3.4376	3.9402	3.3773	4.3522	3.2122	4.1783	3.8268	3.9072
Ptgds	_AQGLTEEDIVFLPQPDK_.2	#N/A	6.5968	6.3650	7.1113	7.2557	7.1278	6.8253	6.6954	6.3621	6.6880	6.7609	6.9781	6.5269	6.2369	6.8425	6.8070	6.9624	6.4290	6.4204	6.5783	6.2225
Sema4d	_SDFS[Phospho (STY)]DLEQSVK_.2	O09126_S782_M1_Sema4d	7.6238	9.8183	9.6751	8.9596	4.2231	9.7461	9.5427	3.6799	9.7203	8.8328	6.7580	8.7585	3.4851	9.7000	8.4197	8.6706	9.2671	9.7402	9.3367	9.1857
Rpl21	_VYNVTQHAVGIIVNK_.3	#N/A	3.7238	3.3803	3.9652	4.0456	4.0649	2.9815	3.8264	2.8797	3.9860	3.9301	3.7404	3.5691	4.7880	4.3348	3.4036	4.0687	3.6283	3.5451	3.3892	3.4810
Gclm	_TLNEWSSQISPDLVR_.2	#N/A	4.1660	7.1144	3.9608	4.1042	3.4552	4.2897	6.7382	4.2149	4.3252	4.0770	4.2580	4.0321	4.4601	7.5267	3.8620	4.4442	3.8411	6.7067	4.1568	4.2207
Kif1c	_LYADS[Phospho (STY)]DS[Phospho (STY)]GEDSDKR_.2	O35071_S674_M2_Kif1c	5.4361	5.8957	6.2002	6.1433	5.4224	5.8399	5.4512	5.5136	5.5720	5.6868	5.8731	5.4733	3.3186	6.0710	5.3016	5.3034	5.8066	5.6315	5.7930	5.4978
Kif1c	_RNS[Phospho (STY)]LDGGSR_.2	O35071_S1031_M1_Kif1c	4.2987	3.7041	3.3342	3.3907	3.6364	3.8605	5.2885	3.9991	3.9637	4.5050	4.3381	3.5935	4.0718	3.3212	3.4818	3.1428	4.5368	3.2964	2.9450	4.8353
Dpysl4	_VVLEDGNLLVTPGAGR_.2	#N/A	8.2513	8.5980	8.8445	8.8235	8.0362	8.5280	8.3711	8.3637	8.8059	8.7349	8.0735	8.0023	8.4183	8.5549	8.1473	8.4079	8.0199	8.1185	8.0409	8.0559
Dpysl4	_GVPTVVISQGR_.2	#N/A	7.7279	7.8415	7.6007	7.8265	7.8529	8.0923	7.4959	7.6500	7.3691	7.8351	7.2188	7.1227	7.7806	7.7135	7.4816	7.2742	7.1660	7.0620	7.2241	7.2284
Dpysl4	_QIGENLIVPGGIK_.2	#N/A	6.7711	7.0860	7.0201	6.7407	7.2542	6.7145	7.0076	6.9861	6.8794	7.1264	6.3578	6.7142	6.1435	6.5043	6.8590	6.2148	6.3332	6.7994	6.6184	6.6080
Dpysl4	_M[Oxidation (M)]DENEFVAVTSTNAAK_.2	#N/A	10.4026	10.9210	11.1644	10.8919	10.2744	10.6985	10.7417	10.5685	10.7945	8.7655	9.8284	10.1281	3.8995	10.6388	10.2939	10.2092	10.1874	10.0281	7.9571	10.3463
Dpysl4	_QANC[Carbamidomethyl (C)]PLYVTK_.2	#N/A	4.2237	5.4290	5.2138	4.8460	5.3326	5.1910	5.2261	6.0125	4.5332	5.3569	4.9901	5.1133	4.0421	3.9238	5.3385	4.5750	4.9970	4.4419	4.3600	4.8211
Dpysl4	_VAVGSDADLVIWNPR_.2	#N/A	6.7795	6.8472	7.0782	6.8241	6.3890	6.6933	6.5926	6.5362	7.1297	6.8286	6.4168	6.2864	4.0998	3.6436	6.7203	6.2513	6.4562	6.0767	5.8964	6.5273
Dpysl4	_DLGAVAQVHAENGDIVEEEQKR_.3	#N/A	7.6566	6.3766	6.0322	6.6699	6.7189	5.7681	6.0428	6.2576	6.3104	6.7849	6.1365	5.8011	7.1801	4.1386	5.5645	5.5902	6.9241	3.9799	6.1210	6.1105
Dpysl4	_TIDAHGLM[Oxidation (M)]VLPGGVDVHTR_.3	#N/A	6.6283	6.3338	6.1644	6.0044	6.2701	6.2559	5.9231	6.6373	6.2171	7.0683	5.2223	5.3807	6.3997	5.5657	6.0809	6.0442	6.0894	5.7850	6.0933	6.0160
Dpysl4	_NLHQSGFSLSGS[Phospho (STY)]QADDHIAR_.3	O35098_S544_M1_Dpysl4	6.3484	6.9881	5.6864	6.2482	3.2373	7.5125	6.4830	6.5394	7.0626	6.2141	3.7336	5.5520	5.9607	6.7390	4.4381	4.6866	4.0658	5.8961	3.4692	4.7678
Dpysl4	_GAADMVAQAK_.2	#N/A	3.3908	4.6142	5.4430	5.0788	4.8006	3.6310	5.0017	3.6164	3.3706	3.2014	5.1665	3.6544	3.5360	4.9365	4.4650	4.2537	4.4942	4.0798	4.3348	4.0057
Dpysl4	_[Acetyl (Protein N-term)]S[Phospho (STY)]FQGKKS[Phospho (STY)]IPR_.2	O35098_S2_M2_Dpysl4	3.5980	3.8440	3.5603	3.3561	2.8881	4.1224	3.2917	4.1432	3.4040	3.2712	3.8587	2.7532	4.4696	3.5474	2.7275	2.8188	3.2620	3.0703	4.2197	2.5301
Dpysl4	_C[Carbamidomethyl (C)]QC[Carbamidomethyl (C)]TDGQIYEIFSLIR_.2	#N/A	3.3305	3.1714	3.1643	2.5751	2.9947	3.8158	3.3720	4.5317	3.4311	3.8876	2.2735	3.0609	3.3512	3.6935	2.9492	3.6754	3.5686	4.2646	3.9132	2.7911
Dpysl4	_SHNLNVEYNIFEGVEC[Carbamidomethyl (C)]R_.3	#N/A	3.7906	4.2122	4.3269	3.8988	3.1284	3.3524	4.4127	3.2738	4.1421	5.5130	3.3453	4.1016	4.5799	3.3639	4.4504	4.1355	4.2719	4.2763	3.1766	3.8009
Map3k5	_TLFLGIPDENFEDHS[Phospho (STY)]APPSPEEK_.3	O35099_S1036_M1_Map3k5	4.9304	5.2093	4.2135	4.0527	3.8327	4.4248	5.0117	4.3371	4.5089	3.8978	3.8289	4.8339	3.6979	5.0455	3.4466	4.0221	4.3141	5.0614	4.6907	4.1676
Atn1	_TAS[Phospho (STY)]PPGPPQYSK_.2	O35126_S617_M1_Atn1	3.9855	9.1029	3.5221	9.8757	9.0271	10.1639	9.6959	9.9265	9.5049	10.2150	9.2144	10.0105	4.0164	3.5601	2.8811	3.3476	3.7084	9.8190	9.9116	10.1643
Atn1	_RAPS[Phospho (STY)]PGSYK_.2	O35126_S630_M1_Atn1	3.3906	4.6122	3.9269	4.2987	4.2292	4.4533	3.3119	3.0910	3.3710	3.5970	3.7453	3.0008	3.4790	4.5236	6.5604	6.5395	3.6287	3.9784	4.2774	4.2009
Atn1	_SEEIS[Phospho (STY)]ESESEETSAPK_.2	O35126_S77_M1_Atn1	5.2669	5.3132	6.0054	4.4539	5.6669	5.6422	5.2504	5.6890	5.5033	4.4418	4.9848	5.9505	5.5145	6.1104	5.1262	3.9933	5.6933	5.5707	5.5857	5.2786
Atn1	_GRAS[Phospho (STY)]PGGVSTSSSDGKAEK_.3	#N/A	4.2790	4.8179	3.8156	3.3052	2.9851	4.8269	4.4609	4.5047	3.0796	4.0896	3.0754	4.2670	4.2380	3.8537	4.8378	3.6411	3.9340	4.3400	4.5924	3.5310
Atn1	_TEQELPRPQS[Phospho (STY)]PSDLDSLDGR_.3	O35126_S101_M1_Atn1	3.2623	4.0568	4.2997	6.0181	3.4692	6.3789	3.5789	3.4879	3.4991	4.5737	2.9778	3.7830	3.6646	7.3552	5.8389	4.1252	4.4860	6.9763	6.1042	6.4869
Atn1	_TGT[Phospho (STY)]PPGYR_.2	O35126_T654_M1_Atn1	3.4105	4.5923	5.7478	4.2789	4.2491	4.4732	5.8403	3.1109	3.3511	3.6168	5.7619	2.9809	3.4592	3.9339	2.8692	3.7554	3.6486	3.9983	3.8332	6.1838
Atn1	_GRAS[Phospho (STY)]PGGVSTSSSDGK_.2	#N/A	5.8887	5.2989	5.9686	6.1606	5.9077	6.3033	5.4629	5.9576	3.5894	6.1841	6.0970	3.8733	6.3157	6.3352	5.3985	5.9322	6.2165	5.8405	5.6611	5.5727
Atn1	_PHVMS[Phospho (STY)]PGNR_.2	O35126_S881_M1_Atn1	3.3310	3.1710	3.8673	6.1546	4.1696	4.3936	3.3715	4.5322	3.4306	3.5373	5.9027	3.0604	3.5387	6.6339	2.9487	3.6759	3.5691	4.2641	3.9127	3.8214
Atn1	_QEPAEEYEPPES[Phospho (STY)]PVPPARS[Phospho (STY)]PS[Phospho (STY)]PPPK_.3	O35126_S724_M3_Atn1	4.1286	3.8742	3.1641	3.5608	3.4663	3.6904	4.0748	3.8290	4.1338	4.3349	3.0074	3.7637	4.2419	3.1511	3.6520	2.9727	4.3667	3.2155	3.5146	3.4630
Phb2	_VLPSIVNEVLK_.2	#N/A	7.8387	8.0501	7.7157	8.0158	7.9452	7.7500	8.1582	8.1581	7.6518	7.8594	7.5513	7.9764	7.9200	7.8542	7.2824	8.0303	7.7958	8.1686	7.8480	7.6776
Phb2	_ESVFTVEGGHR_.2	#N/A	7.4180	7.4253	7.7510	7.5105	7.6279	7.5401	7.8538	7.4614	7.3564	7.3759	7.6154	7.1448	7.0131	7.6295	7.1562	7.4084	7.1559	7.4676	7.1740	7.4683
Phb2	_FNASQLITQR_.2	#N/A	7.5028	7.4559	7.5767	7.4880	7.4060	7.3032	7.3982	7.8371	7.3161	7.6344	7.2106	7.1740	7.5672	7.4475	7.3649	7.2221	7.6731	7.3637	7.4534	6.9007
Phb2	_LGLDYEER_.2	#N/A	6.7427	6.4401	6.3686	6.4318	6.9236	6.3648	6.5934	6.8264	6.2917	6.5391	6.8460	6.7021	6.1542	6.6248	6.1070	6.3650	6.7865	6.6981	6.3501	6.5147
Phb2	_LLLGAGAVAYGVR_.2	#N/A	5.6758	5.9072	5.1138	5.8670	5.6703	4.9353	5.6020	5.7173	5.7653	5.9910	5.6253	5.5720	5.6345	4.9991	5.0626	4.8585	4.6014	5.5987	5.2033	4.9849
Phb2	_IGGVQQDTILAEGLHFR_.3	#N/A	6.5146	5.5469	5.4403	5.4909	5.8476	5.6207	6.0130	6.2349	5.5339	6.4999	5.8151	5.9170	6.2224	5.5119	5.1746	5.6183	5.4992	5.4784	5.2389	5.2213
Phb2	_DLQM[Oxidation (M)]VNISLR_.2	#N/A	5.9689	6.2664	6.6633	6.2505	6.9065	6.7889	5.7438	6.3192	6.6457	7.2567	5.1704	5.9400	6.5701	3.9962	6.6802	7.4746	5.7631	7.1974	6.7948	7.3072
Phb2	_QVAQQEAQR_.2	#N/A	3.8174	4.5096	4.7649	4.7935	3.8061	4.9388	4.5765	4.6536	4.8518	4.7648	4.6540	5.0518	4.3690	4.3778	4.8027	4.8513	5.0852	5.3759	3.8085	3.7587
Phb2	_AQVSLLIR_.2	#N/A	3.6846	3.4294	3.7452	4.0048	3.0224	3.5025	4.2302	3.3850	3.0770	3.8910	4.6260	4.2076	4.9991	4.2080	4.0959	4.0295	3.9227	4.3296	4.6460	4.6367
Phb2	_IYLTADNLVLNLQDESFTR_.2	#N/A	3.8737	3.1212	4.1686	4.3962	3.8990	4.6757	3.7395	3.9249	4.0699	4.0193	3.8720	4.0306	4.4749	5.1464	3.7956	4.2850	4.5332	3.9614	4.8400	4.2408
Phb2	_IPWFQYPIIYDIR_.2	#N/A	4.2138	3.4645	3.7504	3.3704	3.4928	3.1151	3.3734	4.1585	4.0484	2.2958	3.0477	4.0148	4.2447	3.3393	3.9784	3.5760	3.9368	3.2127	3.5117	3.4072
Phb2	_AQFLVEK_.2	#N/A	4.1783	4.0623	3.2137	3.5626	3.0027	3.1473	4.4595	2.2560	4.0842	4.3846	3.2111	3.7140	2.5448	3.7360	3.6023	3.0223	4.4163	4.2625	3.5642	3.6750
Ncam2	_ASGSPDPTISWFR_.2	#N/A	5.6565	5.4941	6.0074	4.8286	5.4554	5.5149	5.1727	5.7418	5.8426	5.7814	5.3938	5.7172	5.8969	4.5533	5.1642	5.7783	6.4349	6.0379	5.5903	5.2123
Ncam2	_AAYLKDGS[Phospho (STY)]KEPIVEM[Oxidation (M)]R_.3	#N/A	9.8429	9.2863	9.0738	9.7121	8.6209	8.7791	8.9517	9.0726	9.3447	9.1348	9.2189	9.1991	9.9318	9.2858	8.4110	9.4237	9.8856	9.2783	9.6394	9.3954
Ncam2	_IEIFQTLPVR_.2	#N/A	5.0987	3.6920	3.1637	3.9443	4.4855	4.2743	4.9981	3.8528	3.7621	4.2246	5.1599	4.1387	3.6580	4.2865	4.5559	3.5566	5.4445	5.1251	4.3221	4.6750
Ncam2	_ITNHEDGS[Phospho (STY)]PVNEPNETTPLTEPEKLPLKEENGK_.4	#N/A	4.3335	7.3317	6.8923	3.3559	4.2804	7.1659	4.2685	4.0338	4.1887	3.8841	3.6674	3.5588	4.3542	3.3560	2.7931	3.4356	7.3229	3.2616	7.0684	3.3230
Ncam2	_DGS[Phospho (STY)]KEPIVEMR_.2	#N/A	3.4658	9.4477	10.4268	10.2476	7.4836	9.0969	9.4171	3.5987	8.2465	8.0765	10.0865	7.9705	3.4038	10.7071	7.0840	3.8107	8.9918	7.4786	7.5473	8.2686
Ncam2	_LTIYNANIEDAGIYR_.2	#N/A	4.5256	5.5029	3.5611	6.3681	3.8633	4.0874	4.9797	4.2260	3.7368	3.2311	6.9469	3.3666	3.8449	3.5482	4.1429	3.3697	3.2628	3.0695	5.0973	3.5152
Ncam2	_ITNHEDGS[Phospho (STY)]PVNEPNETTPLTEPEK_.3	#N/A	3.6581	3.2967	3.6584	4.0790	4.1381	4.2673	3.9810	4.3343	3.9917	3.6318	3.8246	3.1038	3.8188	3.6687	2.9597	3.4747	3.5986	3.6650	3.8744	3.8813
Ncam2	_GSNTELTVR_.2	#N/A	3.0918	3.4101	3.6281	3.0967	3.9304	6.4476	3.6107	4.2930	3.6698	3.2981	4.0442	3.2996	3.7778	3.6152	3.1879	5.8190	3.3299	3.6796	4.1519	5.7248
Atp5if1	_TKEQLAALR_.2	#N/A	9.1889	9.6516	9.8394	9.4125	9.6610	9.3485	9.6688	9.6251	9.5017	9.3813	9.2344	9.9851	9.7140	9.9717	10.6843	9.7327	9.3990	9.7897	9.7649	9.6753
Atp5if1	_EAGGAFGKR_.2	#N/A	7.0328	7.9408	7.2633	7.8093	7.6533	7.1814	7.7125	7.8384	7.6214	7.8343	7.5896	7.9969	8.2152	7.5026	8.6542	8.1711	7.7257	7.9981	8.1047	7.9399
Atp5if1	_EQLAALR_.2	#N/A	4.9675	6.0265	5.6142	6.4440	5.9111	5.9394	6.5174	5.4988	5.7323	6.2203	5.7284	6.1988	6.6156	5.9574	7.2945	6.2076	4.0522	6.7173	6.3296	6.0812
Atp5if1	_EKAEEDRYFR_.3	#N/A	5.4571	5.0131	5.6685	5.6389	5.3989	5.2510	6.0927	5.8502	5.7675	6.0706	6.0485	5.9013	6.4459	6.2314	7.1063	6.8617	5.6682	5.7863	6.1914	6.2921
Atp5if1	_AEEDRYFR_.2	#N/A	4.8259	3.1516	3.8866	4.3391	4.2174	4.1687	3.2951	4.0547	4.3568	3.3664	4.7843	3.0411	3.9065	3.8737	4.8312	4.7768	3.6594	4.2448	3.8934	3.8407
Atp5if1	_KHHEDEIDHHSKEIER_.4	#N/A	8.3895	7.6480	4.0932	11.1598	10.6841	9.4124	7.4325	11.4473	11.1037	11.3562	9.8729	7.7960	10.1685	8.8302	12.1845	11.4137	11.1308	7.9960	11.2616	10.8707
Atp5if1	_KHHEDEIDHHSK_.3	#N/A	6.6114	5.6774	3.3420	8.2444	8.2709	10.2444	6.1374	9.2152	8.7376	9.2500	10.9417	5.5365	8.1126	7.0337	3.4775	8.0768	11.2997	5.5117	7.7583	9.8524
Atp5if1	_HHEDEIDHHSK_.3	#N/A	3.2079	4.3046	4.2345	4.9706	3.4149	3.4482	3.6332	3.3985	5.1125	4.7453	3.0321	4.1941	3.7189	4.2215	3.6586	4.0709	3.9362	3.8969	3.5455	4.1885
Terf2	_NS[Phospho (STY)]PMTISR_.2	O35144_S399_M1_Terf2	3.8435	3.4394	8.1711	8.0916	4.0504	6.9893	2.9977	4.2638	6.3185	5.6814	7.2673	3.3288	4.5842	8.4913	3.0230	3.2056	3.3007	3.0317	4.1811	6.0762
Terf2	_DLVLANLASPSS[Phospho (STY)]PAHK_.2	#N/A	4.0046	6.2282	3.8861	3.7050	3.4424	5.4308	5.9386	6.5589	3.9924	5.6594	5.2561	5.8444	6.0973	6.3275	3.6672	5.3915	4.2039	5.5780	5.6201	5.3657
Cx3cl1	_SLGS[Phospho (STY)]EEINPVHTDNFQER_.3	#N/A	7.6516	7.4898	7.9475	8.2424	7.3727	7.6193	6.8487	6.4928	8.0381	8.2532	8.1452	7.2630	7.3982	8.0322	7.9087	8.3549	6.4610	7.2471	8.4479	8.6738
Ddt	_IPAGLENR_.2	#N/A	7.9481	8.7584	8.7149	8.4448	8.7297	8.4303	8.8156	8.3550	8.6347	8.4645	7.9711	8.5941	8.8348	8.5912	9.3593	8.6289	8.3191	8.5147	8.5884	8.6425
Ddt	_FFPLEAWQIGK_.2	#N/A	7.2511	7.2742	7.0404	7.6137	7.5563	7.4640	7.3019	7.0837	7.2574	6.8295	7.0161	7.8462	7.2359	7.2608	7.3789	7.3571	7.2099	7.1696	7.0937	7.3990
Ddt	_PFVELETNLPASR_.2	#N/A	6.9270	7.1495	7.0692	6.9595	6.4988	6.9255	6.4744	6.5648	6.9375	7.2740	6.0801	6.3148	6.5791	6.3097	7.1699	6.8743	6.8784	6.6623	6.7181	6.8569
Ddt	_STEPC[Carbamidomethyl (C)]AHLLVSSIGVVGTAEQNR_.3	#N/A	6.4529	6.4609	6.1177	6.7942	6.4724	6.0614	5.8974	6.3234	6.2651	6.8014	6.2140	6.2512	6.9167	6.4562	5.7599	6.6769	6.0972	5.4702	5.9592	6.3229
Ddt	_THSASFFK_.2	#N/A	4.8535	5.4531	6.0490	5.3386	5.9103	5.6909	5.5066	5.2338	5.8304	5.1370	4.7797	5.7646	5.9421	5.6048	6.7021	5.7761	5.7892	5.1984	6.0556	6.1210
Ddt	_LC[Carbamidomethyl (C)]AATATILDKPEDR_.3	#N/A	3.1280	4.4763	4.8336	4.2116	3.2922	4.5657	4.6984	3.3109	3.6761	4.8075	4.6778	3.5928	5.5320	4.3442	5.1378	4.9526	4.3090	4.7843	4.3289	4.1750
Ddt	_FLTEELSLDQDR_.2	#N/A	6.0934	6.2632	6.9612	6.3326	5.7335	6.2472	6.2257	5.3893	6.2624	6.2569	6.0901	6.2165	6.3837	6.7123	6.8672	6.2750	6.2245	6.3541	6.0576	6.5621
Kcnh2	_SGLLNSTS[Phospho (STY)]DSDLVR_.2	O35219_S322_M1_Kcnh2	3.4540	4.2376	4.2116	4.2330	3.2031	5.7071	3.3933	3.3875	5.9003	4.4697	3.0823	4.0504	3.7691	4.2236	3.9085	4.0284	5.3200	3.8473	3.3260	5.0896
Kcnh2	_DTNM[Oxidation (M)]IPGS[Phospho (STY)]PGS[Phospho (STY)]AELESGFNR_.2	#N/A	4.1820	5.1561	3.5201	3.4979	4.0702	4.6248	3.5793	4.2006	4.3681	3.7121	3.8467	3.1874	4.1618	6.1922	2.9724	3.3396	3.5523	3.0897	4.1651	3.5023
Kcnh2	_RAS[Phospho (STY)]S[Phospho (STY)]ADDIEAMR_.2	O35219_S285_M2_Kcnh2	3.5239	4.4789	4.0602	4.1655	2.8616	3.0857	3.1786	3.5406	3.2377	3.7302	3.6121	4.3683	3.3457	4.3904	4.2566	3.8688	4.5386	4.1117	4.4107	4.0676

Psmd4	_VNVDIINFGEEEVNTEK_.2	#N/A	5.1357	4.8705	4.9854	3.9492	4.0913	4.1675	4.2145	4.0698	3.2887	4.7929	4.4568	4.5849	5.1179	4.3802	4.3034	4.1853	4.8062	3.7006	4.6963	3.8813
Psmd4	_EKVNVDIINFGEEEVNTEK_.3	#N/A	5.7211	4.9889	4.7859	5.1093	5.0536	3.8658	4.2860	5.4372	4.3182	5.3035	4.7687	5.5111	5.9711	5.5914	4.1794	4.7392	5.2906	4.7023	4.9871	4.7768
Psmd4	_AAAASAAEAGIAT[Phospho (STY)]PGTEDSDDALLK_.2	#N/A	3.3261	6.3176	6.3488	4.2604	3.5331	5.9867	5.9930	3.4796	5.2911	5.9889	3.3688	5.2124	3.5414	7.1991	6.0459	4.1891	6.0828	4.0011	5.8000	5.7703
Psmd4	_RAAAASAAEAGIAT[Phospho (STY)]PGTEDSDDALLK_.3	#N/A	3.4162	4.5129	4.0262	4.1995	3.6231	3.6565	5.0970	3.1903	3.3452	3.2268	4.3247	4.4024	3.5106	5.8026	3.4503	4.2791	3.7280	5.4815	3.7538	3.9803
Pkd2	_SLDDS[Phospho (STY)]EEEDDEDSGHSSR_.2	O35245_S810_M1_Pkd2	3.7161	4.0495	4.2524	3.9733	3.0538	2.5890	3.6512	3.4165	0.9320	3.4706	2.0045	3.1767	3.7368	4.0765	1.9393	3.0353	3.9542	3.4374	3.0705	2.5157
Exoc7	_QYS[Phospho (STY)]QHGLDGK_.2	O35250_S283_M1_Exoc7	5.8382	3.9425	6.9671	3.6291	6.3378	3.6221	4.1431	4.5050	4.2022	6.5262	6.0186	3.8320	4.3102	6.3550	4.8456	6.0176	4.2983	5.7558	3.4462	5.6958
Exoc7	_AVEYFQDNSPDS[Phospho (STY)]PELNK_.2	O35250_S133_M1_Exoc7	3.6378	3.6813	5.3889	6.3176	3.8447	5.5766	5.5293	3.8635	5.6669	5.2101	4.1030	3.4074	3.2890	5.5158	4.6866	5.5599	3.3607	5.4757	5.6655	5.1581
Purb	_GGGSGGGDES[Phospho (STY)]EGEEVDED_.2	#N/A	11.5135	11.7330	11.8511	11.7640	11.7928	11.5566	11.2593	11.7163	11.3938	11.6817	11.5133	11.6700	11.8260	12.0748	11.1817	11.7308	11.9084	11.6647	11.4053	11.8114
Purb	_VSEVKPSYR_.2	#N/A	6.9460	7.0244	7.3879	6.3701	7.1225	6.9781	7.1389	7.3388	7.0041	6.9885	6.9796	7.1110	6.5593	7.2356	7.7799	7.3482	7.0718	7.0155	6.9415	6.9007
Purb	_RGGGSGGGDES[Phospho (STY)]EGEEVDED_.2	#N/A	12.4189	12.1308	12.4817	12.4433	12.0468	11.7365	11.8362	12.3136	11.8554	12.2267	11.8313	12.0060	12.3355	12.3129	11.5784	12.1749	12.1760	11.8057	11.9764	11.9924
Purb	_GGGGGGGGGPGGFQPAPR_.2	#N/A	5.9491	6.2400	6.7682	6.2636	6.6801	6.0353	6.4482	6.3496	6.3121	6.8064	6.2681	6.6463	6.9339	6.5735	6.9250	6.6104	6.1290	6.2654	6.5621	6.5558
Purb	_DSLGDFIEHYAQLGPSS[Phospho (STY)]PEQLAAGAEEGGGPRR_.4	#N/A	3.6749	3.6442	3.2115	3.9093	3.8819	4.5452	5.8328	3.9006	3.5977	3.3702	3.9721	3.3703	3.7058	3.2496	5.6717	3.0370	3.3978	3.7516	4.0507	4.4277
Purb	_[Acetyl (Protein N-term)]ADGDSGS[Phospho (STY)]ERGGGGGGGGGPGGFQPAPR_.3	O35295_S8_M1_Purb	8.4791	5.5091	4.8751	8.6407	3.5415	7.0115	7.7909	3.8544	9.1331	4.9673	3.8777	8.6402	4.1221	9.1286	4.8603	4.8187	9.2403	8.1670	8.5493	8.4818
Purb	_DSLGDFIEHYAQLGPSS[Phospho (STY)]PEQLAAGAEEGGGPR_.4	#N/A	3.4894	7.4939	4.5268	6.8061	3.6963	3.7296	3.3518	6.9828	7.3784	7.1968	4.2515	3.5558	7.5078	7.9369	3.3772	4.3523	6.8747	3.9372	7.4272	7.2886
Purb	_GGGGGGGGPGGEQETQELASK_.2	#N/A	4.4845	3.5183	3.5200	7.7851	3.8222	4.1880	4.5503	4.1849	4.3398	4.6842	3.8184	7.3542	4.5052	3.5070	3.7973	3.2846	3.2217	3.8642	4.2600	4.3661
Purb	_[Acetyl (Protein N-term)]ADGDS[Phospho (STY)]GSER_.2	#N/A	4.5353	6.4317	3.5708	3.1541	3.8730	4.0971	3.6680	4.2357	3.7271	3.2408	4.1015	3.3569	4.5560	3.5579	3.2452	3.3794	3.2725	3.0598	4.2092	3.5248
Srsf5	_S[Phospho (STY)]GS[Phospho (STY)]RS[Phospho (STY)]PVPEK_.2	O35326_S226_M3_Srsf5	10.1167	9.7013	10.2240	10.1951	9.3029	9.7933	9.9037	9.8123	9.9572	9.9424	9.7665	8.9892	9.0831	9.4003	7.7254	8.8663	9.9099	9.5247	9.3788	9.0874
Srsf5	_SKS[Phospho (STY)]PASVDR_.2	O35326_S247_M1_Srsf5	4.3692	3.6336	3.4047	3.3202	5.6985	3.9310	6.1641	4.0696	3.8932	4.5755	4.2676	3.5230	4.0013	6.5889	3.6088	3.2133	3.1064	6.3209	3.7552	5.5908
Srsf5	_VS[Phospho (STY)]WQDLK_.2	O35326_S120_M1_Srsf5	3.4510	4.5518	3.2901	4.2383	4.2896	3.0827	3.2515	3.1514	3.2085	3.6574	4.2858	2.9404	3.4718	3.9744	3.4400	3.7959	3.2910	3.5644	0.9523	3.9414
Kpna4	_NVPQEDIC[Carbamidomethyl (C)]EDS[Phospho (STY)]DIDGDYR_.2	O35343_S60_M1_Kpna4	3.7928	7.4837	3.3293	3.7915	3.9997	7.0596	3.0484	4.0184	4.4695	3.6033	3.9481	3.2525	3.1341	3.3674	3.0738	3.1549	3.5157	3.6338	3.9328	3.6037
Kpna4	_EAAWAISNLTISGR_.2	#N/A	3.9223	3.1430	4.5779	4.6679	2.6917	3.9792	4.4197	4.1978	3.8451	3.1228	4.2195	3.1229	3.9531	3.4969	2.9443	3.2844	3.6452	3.5043	3.8033	3.1742
Kpna4	_VQNTSLEAIVQNASSDNQGIQLSAVQAAR_.3	#N/A	4.5783	4.2416	4.1149	4.5067	3.2844	3.3178	3.7637	3.3032	3.6839	4.3889	3.1625	3.9677	3.8493	4.1530	3.7891	3.9404	4.3012	4.3491	3.1473	3.8302
Kpna3	_NVPQEESLEDS[Phospho (STY)]DVDADFK_.2	O35344_S60_M1_Kpna3	4.0903	8.3199	3.7215	3.8957	3.6156	7.6440	7.5804	3.9528	10.9714	3.8733	3.7704	3.5534	4.0965	10.7008	5.7626	3.5610	8.6708	12.0921	3.9037	3.7100
Kpna6	[Acetyl (Protein N-term)]M[Oxidation (M)]ETM[Oxidation (M)]AS[Phospho (STY)]PGKDNYR .	#N/A	6.0310	6.2554	5.2715	5.7340	4.9609	4.1041	5.2882	5.9614	5.1346	5.3081	5.5181	5.3500	5.6202	5.4991	3.1235	5.2118	5.3569	5.2425	5.3171	5.0748
Abcc1	_GSS[Phospho (STY)]QLDVNEEVEALIVK_.2	O35379_S290_M1_Abcc1	3.1084	6.3281	4.1459	5.6800	3.3154	6.2771	3.7327	3.4980	5.5994	5.5505	5.7213	5.8287	6.4985	5.7359	3.7581	5.6574	4.0357	6.4758	3.4460	4.2880
Anp32a	_KLELSENR_.2	#N/A	7.8672	7.9185	8.3175	7.5189	7.7827	8.0310	8.1473	7.6939	7.9512	8.3043	7.9259	7.9578	7.9469	8.3140	7.9692	7.9493	7.8609	7.6446	8.0130	7.8745
Anp32a	_ELVLDNC[Carbamidomethyl (C)]K_.2	#N/A	5.6994	6.1924	6.3192	6.2150	5.8607	6.2348	6.6197	5.6593	6.0178	6.2535	6.3409	6.2383	6.5129	5.9285	6.5201	5.7927	6.2778	6.4179	5.9051	6.1214
Anp32a	_NRT[Phospho (STY)]PSDVK_.2	O35381_T15_M1_Anp32a	3.7432	4.2596	4.2795	3.9462	3.0809	7.4480	4.4602	7.5918	3.0184	3.9495	3.3928	4.1491	3.1265	4.2666	4.0374	4.0881	7.4088	7.3163	3.1292	7.0492
Anp32a	_SLDLFNC[Carbamidomethyl (C)]EVTNLNAYR_.2	#N/A	5.3245	5.7720	5.7213	6.1285	5.4716	5.1876	5.8104	6.2380	5.3871	5.3336	4.6578	5.3345	6.3435	6.0293	5.2868	5.7527	5.8960	5.1446	4.8483	4.7653
Anp32a	_ISGDLEVLAEK_.2	#N/A	3.8663	5.3814	4.4026	5.2066	5.2963	5.4853	6.8007	5.3172	5.2602	5.4956	5.2802	4.2339	5.5111	5.8188	5.7159	4.1152	5.2612	5.1643	5.6313	4.8774
Anp32a	_HLNLSGNK_.2	#N/A	5.4855	4.5874	4.3501	5.2762	5.2502	5.2103	5.1043	4.5771	5.0702	5.6571	4.8361	4.9547	4.6687	5.4209	5.8479	5.6033	4.3439	5.7479	5.1736	5.4331
Anp32a	_LLPQVM[Oxidation (M)]YLDGYDR_.2	#N/A	6.3422	5.1428	4.1916	4.0341	5.4123	3.4911	3.3913	6.1426	3.5106	4.5622	2.9892	4.2370	3.6760	4.1787	4.9819	4.1138	6.4745	5.2839	5.2148	4.1457
Anp32a	_KREPDDEGEEDD_.2	#N/A	4.3661	3.6367	3.4015	3.3233	4.2354	5.7996	3.8373	5.4045	3.8964	5.1524	4.2708	3.5262	4.0044	3.5482	5.8258	3.2101	5.2714	4.7431	3.7520	3.2255
Exoc4	_S[Phospho (STY)]GELQGGPDDNLIEGGGTK_.2	O35382_S469_M1_Exoc4	6.6635	6.4995	6.9078	6.9333	4.3251	6.1068	6.4616	3.1869	5.8068	6.9246	3.6495	5.7275	6.0658	6.2028	4.5852	5.4513	7.1622	6.6004	6.2218	5.3323
Efnb3	_GGS[Phospho (STY)]LGLGGGGGMGPR_.2	O35393_S274_M1_Efnb3	3.2846	7.0498	8.4827	7.8971	4.1232	6.4776	6.4324	3.7798	5.7999	3.4909	3.8514	3.4910	3.5850	7.3350	6.2366	3.6295	6.5793	3.8724	4.1714	4.3069
Pld3	_LFVVPTDESQAR_.2	#N/A	8.3600	7.6024	7.4806	7.7649	7.8723	7.8986	7.6588	8.0969	7.6898	8.1813	7.8620	7.6749	7.9516	7.6710	7.3575	7.7568	8.0973	7.5826	7.7139	7.3972
Pld3	_ALLNVVDSAR_.2	#N/A	7.0497	6.6526	6.6889	6.9071	6.8579	7.0691	6.7594	6.9135	5.9362	7.0395	6.6094	6.9561	7.1676	6.6150	6.2000	6.6232	6.7850	6.4380	6.6636	6.7914
Pld3	_SQLEAVFLR_.2	#N/A	4.8517	5.7667	5.2421	5.3858	5.5675	5.2998	5.1397	4.3220	5.0752	5.5194	5.6071	5.7283	3.6765	5.9150	5.4767	4.6316	4.4740	5.8882	5.3600	5.6033
Pdyn	_S[Phospho (STY)]QENPNTYSEDLDV_.2	O35417_S235_M1_Pdyn	3.3469	5.8612	5.9776	4.3425	4.1854	5.9952	5.9125	3.0473	6.5324	5.7355	4.1816	3.0445	3.3676	6.6490	6.5697	6.5060	5.6463	6.2364	6.4487	6.5893
Clcn6	_SYPS[Phospho (STY)]SELR_.2	O35454_S684_M1_Clcn6	3.9456	4.0572	4.4819	3.7438	3.2833	3.2008	4.2578	3.6460	3.8009	4.2719	3.2795	3.9467	3.9663	4.4690	3.8350	4.2905	4.1836	3.6495	3.2981	4.4360
Clcn6	_ES[Phospho (STY)]RTPEELTILGETQEEEDEILPR_.3	O35454_S23_M1_Clcn6	4.3555	3.6473	3.3909	3.3339	3.6932	3.9173	3.8479	4.0559	3.9070	4.5618	4.2813	3.5368	4.0150	3.3780	3.4251	3.1995	3.0927	3.4424	4.3890	3.2361
Fkbp8	_VLAQQGEYSEAIPILR_.2	#N/A	5.8465	5.8174	5.3221	5.5934	4.9534	5.4795	5.5445	5.4338	5.5552	4.6847	4.5182	4.9858	3.4023	4.6000	4.9720	4.3875	5.5108	5.2534	4.7680	4.7262
Clk3	_S[Phospho (STY)]RS[Phospho (STY)]HDRIPYQR_.3	O35492_S197_M2_Clk3	5.1001	3.6570	3.3812	4.3229	4.2681	4.8876	4.5090	4.0461	4.2010	3.8718	3.7128	2.9840	4.3665	3.3683	2.8054	3.4233	3.0830	3.9530	4.3988	3.3353
Clk3	_YRS[Phospho (STY)]PEPDPYLSYR_.3	O35492_S157_M1_Clk3	3.6774	4.3254	4.2137	4.0119	3.0152	3.2393	3.0251	3.3778	3.0842	3.8838	3.4585	4.2148	3.1922	4.2008	4.1031	4.0223	3.9155	3.9177	3.5662	4.1678
Cdk14	_ADS[Phospho (STY)]YEKLEK_.2	#N/A	10.8241	10.9893	10.7589	10.7537	10.5447	10.6434	10.6571	11.0052	10.5437	10.8307	10.6111	10.5411	11.0248	10.8788	9.9231	10.5058	10.9024	10.7001	10.6512	10.6082
Cdk14	_SSSAGKES[Phospho (STY)]PKVR_.2	#N/A	5.2330	5.3990	4.8577	5.2651	4.2721	4.9661	5.3726	5.3274	4.9958	4.8202	4.5904	4.8015	5.8591	4.7935	3.6206	4.8478	4.5020	5.1225	4.5442	4.7938
Cdk14	_HSSPSS[Phospho (STY)]PTS[Phospho (STY)]PK_.2	#N/A	4.6493	5.3070	4.0553	6.2884	4.3129	3.6882	4.8376	5.3433	5.8902	3.8774	5.7570	3.7190	4.8096	4.9396	3.5548	3.8759	4.1163	4.0403	5.0876	3.6696
Cdk14	_RVHS[Phospho (STY)]ENNAC[Carbamidomethyl (C)]INFK_.3	#N/A	5.9419	6.3882	6.1017	6.1116	4.8674	5.5869	5.9749	5.3626	5.6952	6.1218	5.0621	5.4784	5.6141	6.3838	4.6593	5.9601	5.6538	5.9208	6.0922	5.3730
Cdk14	_VHS[Phospho (STY)]ENNAC[Carbamidomethyl (C)]INFK_.2	#N/A	8.4189	8.1359	8.0762	8.1001	7.3528	7.6727	7.6791	7.9966	7.5945	8.1562	7.8444	7.7714	8.1701	8.3376	6.8913	7.8839	7.9215	7.5495	7.9228	7.8737
Cdk14	_FGKADS[Phospho (STY)]YEKLEK_.2	#N/A	5.0448	4.3632	4.5255	4.0962	3.6950	4.4134	3.3531	3.7137	3.4109	3.2986	3.7852	3.5572	3.5189	4.5635	3.3785	4.3510	3.2110	3.9385	4.1000	4.2408
Cdk14	_RHS[Phospho (STY)]SPSS[Phospho (STY)]PTSPK_.2	#N/A	4.1849	3.7683	3.7597	3.2711	3.4715	4.0606	4.3074	3.8317	5.3770	3.9468	3.5682	4.0054	4.4205	3.6319	3.7606	3.5470	4.0398	5.4981	3.6094	3.4809
Cdk14	_TLS[Phospho (STY)]ESFSR_.2	O35495_S24_M1_Cdk14	3.8657	3.4534	3.4023	3.7185	4.0726	4.1060	4.4763	4.0914	4.3965	3.6763	3.8752	3.1795	4.5620	3.4403	3.0008	3.2278	3.5886	3.7556	3.8599	3.5308
Cdk14	_HSSPSSPT[Phospho (STY)]S[Phospho (STY)]PKFGK_.3	#N/A	3.3797	3.9393	4.4172	4.2045	3.5867	3.6200	3.4614	3.6054	3.3816	3.1903	4.3611	3.6655	3.4106	4.4552	3.4868	4.2427	3.1027	4.0468	4.3458	4.1325
Stx1a	_HSAILAS[Phospho (STY)]PNPDEK_.2	#N/A	10.3012	9.9276	9.8287	9.7116	9.4524	9.9765	9.7021	10.0488	9.6672	9.6714	10.0631	9.7185	10.0597	9.8340	8.5268	9.3614	10.3974	10.0961	9.7667	9.6966
Stx1a	_QALSEIETR_.2	#N/A	7.8744	7.1557	7.6767	7.0219	6.9751	7.1132	7.3308	7.5278	7.6433	7.1107	7.7115	6.8873	7.4533	7.0494	6.7884	6.9200	7.6635	7.6137	7.1174	6.8790
Stx1a	_IEYNVEHAVDYVER_.2	#N/A	6.3317	6.2166	6.2040	6.1091	5.8531	6.2359	5.9304	5.1731	5.9418	6.1397	6.5074	6.1959	6.1321	4.6988	4.3700	4.7793	6.0269	5.9134	5.6125	6.2426
Stx1a	_FM[Oxidation (M)]DEFFEQVEEIR_.2	#N/A	7.5530	7.0533	7.4794	7.0529	6.2692	7.0000	6.8270	6.7475	7.0303	6.9582	6.8812	6.6361	7.4480	7.2586	6.2379	6.9077	7.3226	7.0582	6.8789	6.8088
Stx1a	_TAKDS[Phospho (STY)]DDDDDVTVTVDR_.2	#N/A	11.3197	10.9711	11.2432	10.9108	7.8889	10.8761	10.8075	10.7431	10.6350	10.8304	10.6802	10.6371	10.2029	11.1705	9.4718	10.5702	11.1834	10.8519	10.7766	10.6509
Stx1a	_KHSAILAS[Phospho (STY)]PNPDEK_.3	#N/A	8.5047	8.2981	7.8660	8.1241	7.7116	8.3368	8.1852	7.9921	7.3053	7.5273	8.4941	7.9298	8.3712	8.1266	6.6473	6.9825	8.5662	8.5418	8.3413	7.8784
Stx1a	_TAKDS[Phospho (STY)]DDDDDVTVTVDRDR_.3	#N/A	11.6972	12.0168	11.4597	11.7370	10.5077	11.5097	11.7251	11.3870	11.4256	11.4053	11.1248	11.4642	11.5387	12.1760	10.6627	11.4252	11.7404	12.0353	11.5240	11.4620
Stx1a	_SIEQSIEQEEGLNR_.2	#N/A	10.1199	9.8333	9.8860	9.7823	9.4483	9.7252	9.6073	9.9310	9.6994	9.4245	9.8819	9.4883	10.4526	9.5645	8.6771	9.5236	10.4420	9.9363	9.7442	9.6441
Stx1a	_DS[Phospho (STY)]DDDDDVTVTVDRDR_.2	#N/A	3.9404	5.8515	4.8004	3.7490	3.2782	3.5022	4.9878	3.1240	4.3220	4.1467	3.1956	5.5494	4.4301	5.3807	3.8401	4.2853	4.1221	5.7163	3.3264	5.0010
Stx1a	_TKEELEELMSDIKK_.3	#N/A	4.4273	5.7781	6.9503	6.5691	3.1334	5.6336	6.0887	3.1521	4.4994	4.2379	6.1922	4.5701	4.4581	6.5691	3.9401	3.7894	4.9738	2.9993	4.2991	4.8171
Stx1a	_LKS[Phospho (STY)]IEQSIEQEEGLNR_.3	O35526_S95_M1_Stx1a	4.0086	4.1234	3.9701	3.9735	3.7196	3.4837	3.3923	3.5817	3.3524	3.9273	4.3844	3.9748	4.5648	4.4203	3.7945	3.6817	3.7793	4.4854	3.7455	4.2573
Stx1a	_KFVEVM[Oxidation (M)]SEYNATQSDYR_.3	#N/A	5.0303	5.0400	5.4220	3.9076	3.9525	4.9486	4.2975	5.2637	3.7099	4.4397	4.4199	3.9938	5.1368	3.8845	3.8151	4.1886	4.7587	4.4698	4.4497	3.7199
Stx1a	_FVEVM[Oxidation (M)]SEYNATQSDYR_.2	#N/A	3.3753	3.5643	3.4206	4.3052	3.8836	4.0215	3.4221	4.4335	3.4209	7.0383	4.5606	3.3640	3.5535	3.7556	3.2168	3.2361	3.4703	5.1287	4.0177	3.4485
Rabep1	_AQS[Phospho (STY)]TDSLGTSSSLQSK_.2	#N/A	9.3607	9.3619	9.6495	8.6319	8.6103	8.7501	9.0327	8.8000	8.7083	10.2212	9.0675	9.0160	9.1446	9.6830	7.9090	9.2149	9.0980	8.7827	9.0486	8.8502
Rabep1	_RAQS[Phospho (STY)]TDS[Phospho (STY)]LGTSSSLQSK_.2	#N/A	4.7034	4.2801	4.2256	4.1422	4.4725	4.0108	3.2446	3.7398	3.2517	3.6149	4.4666	4.1285	4.4623	4.8399	4.0138	4.0407	4.1470	4.0372	3.8397	4.0786
Rabep1	_QNAVLQAAQDDLGHLR_.3	#N/A	3.2871	4.0320	4.3245	4.2971	3.4941	3.5274	3.5540	3.5128	3.4743	3.0977	2.9529	3.7581	3.6397	4.3626	3.5794	4.1501	4.5109	4.1394	4.4385	4.1094
Fyb1	_VAGQSS[Phospho (STY)]PSGIQSR_.2	O35601_S28_M1_Fyb1	3.4514	4.5515	3.9876	6.8765	4.2899	7.1444	7.1814	7.1948	7.0572	7.7962	6.8605	7.1701	7.5683	4.4629	2.8284	7.1808	3.6894	7.1827	4.3382	7.2787
Fyb1	_TTAVEIDYDS[Phospho (STY)]LKR_.2	O35601_S561_M1_Fyb1	3.1172	4.2019	4.2033	3.7213	3.3242	3.3874	4.1648	3.4892	3.8868	4.4287	3.1228	2.6417	3.8096	4.7087	3.5085	3.9802	4.0269	4.9861	3.8843	3.8894
Bmpr2	_LNINSS[Phospho (STY)]PDEHEPLLR_.2	#N/A	6.7510	7.1430	7.3805	3.5468	5.1067	6.8916	5.4217	6.3750	3.7805	3.8939	3.7494	6.7635	3.9460	6.9542	4.8733	6.1625	3.6779	6.6389	6.2949	3.9302
Bmpr2	_LNINSS[Phospho (STY)]PDEHEPLLRR_.3	#N/A	6.3801	4.1476	3.5553	4.0620	3.4856	3.5927	3.9679	4.1750	5.5689	3.8293	3.8085	3.8737	3.7553	4.2580	5.3200	3.3808	6.4528	4.0002	2.6212	3.5432
Bmpr2	_SVS[Phospho (STY)]PTVNPMSTAMQNER_.2	O35607_S515_M1_Bmpr2	3.3165	5.6203	4.3539	4.2677	3.5234	3.5568	3.5247	3.5422	3.4449	3.1271	2.9235	3.7287	3.6103	7.7537	3.5501	4.1794	4.5402	4.0055	4.4091	4.0800
Bmpr2	_NLKES[Phospho (STY)]SDENLMEHSLK_.3	O35607_S680_M1_Bmpr2	3.5773	4.0576	4.5900	4.3227	2.9150	3.1391	3.1252	3.4872	3.1843	3.7836	3.5587	3.7837	3.2924	4.3370	4.2033	3.9222	4.4853	4.1650	2.9632	4.0143
Bmpr2	_EVDKNLKES[Phospho (STY)]S[Phospho (STY)]DENLMEHSLK_.3	O35607_S680_M2_Bmpr2	3.8221	3.4970	3.3587	3.7621	4.0290	4.0624	3.0191	4.0478	4.4401	3.6327	3.9188	3.2231	4.6056	3.6074	3.0445	3.1842	3.5450	3.0103	4.1597	3.5744
Scamp3	_KLS[Phospho (STY)]PTEPR_.2	#N/A	10.9120	10.7123	10.5817	10.9730	9.9498	10.2159	10.4621	10.4519	10.3409	9.9904	9.9693	10.0764	10.7453	9.9186	8.7652	9.5539	10.3180	10.5394	9.9002	9.7875
Scamp3	_LS[Phospho (STY)]PTEPR_.2	#N/A	5.5506	6.5008	3.4227	3.6981	4.0931	4.1264	4.4559	4.1118	4.3761	3.6967	6.0655	3.1591	5.8700	6.1396	2.9804	6.4006	3.6090	3.5404	3.8394	3.1381
Scamp3	_TAAANAAAGAAENAFR_.2	#N/A	3.0936	4.2255	4.1310	4.4906	3.3005	5.3372	3.7476	5.5680	5.5833	4.4050	3.1464	3.9516	3.1245	4.1691	3.7729	3.9566	4.3174	4.3329	5.5925	3.8464
Zfpm1	_AGS[Phospho (STY)]PVSATEEKPK_.2	O35615_S286_M1_Zfpm1	3.5806	3.7385	3.1172	4.0036	5.3687	3.8209	3.2606	4.5267	6.1466	3.3912	4.1602	3.4646	3.3462	3.8489	3.2859	2.9427	3.5636	4.2696	5.9193	3.8159
Zfpm1	_GSEGSQS[Phospho (STY)]PGSSVDDAEDDPSR_.2	O35615_S681_M1_Zfpm1	3.5148	4.4880	4.0511	4.7015	2.8525	3.0766	3.6959	3.2151	3.2468	3.7211	3.6212	4.2640	3.3549	4.0381	4.2658	4.3889	3.7528	4.0803	3.7289	4.0051
Zfpm1	_APRS[Phospho (STY)]PS[Phospho (STY)]PAPENTPSDPADQGAR_.3	O35615_S925_M2_Zfpm1	4.0298	5.6098	5.4522	5.3381	4.5285	5.1177	5.5331	5.7743	5.5342	5.5079	4.7397	5.7777	5.6249	5.9576	3.6841	3.3919	5.6246	5.0015	5.3765	5.5827
Mdm4	_TIS[Phospho (STY)]APVVRPK_.2	O35618_S367_M1_Mdm4	6.6347	3.6765	3.1792	6.1463	6.5168	6.4912	6.0446	3.8683	6.2482	3.4532	4.0982	6.7848	6.7561	3.2172	5.7831	3.0047	3.3655	6.5492	6.6698	4.3954
Pmm1	_TVGHSVVS[Phospho (STY)]PQDTVQR_.2	#N/A	8.2679	8.7224	7.4971	8.6103	7.8679	8.1180	7.5896	8.2839	8.1097	8.2353	8.3708	8.4033	8.9870	9.1177	8.0166	8.4302	8.6567	8.5153	8.4034	8.4582
Bet1	_SLS[Phospho (STY)]IEIGHEVK_.2	O35623_S50_M1_Bet1	6.6780	6.6962	6.6357	6.4163	3.2159	5.3661	3.8322	3.2346	3.7525	7.1488	3.2311	5.3321	3.9179	6.3404	3.8576	3.8719	5.6580	6.1777	5.9935	5.2804
Slc32a1	_GGAPLPPSGS[Phospho (STY)]KDQAVGAGGEFGGHDKPK_.3	#N/A	3.8336	3.7487	8.9642	7.6181	7.8842	8.4628	4.0068	7.8045	7.0731	9.1099	3.8482	7.5251	9.5672	4.0952	5.9600	3.8684	9.5078	3.5055	8.4022	9.1524
Slc32a1	_SLFQEGSR_.2	#N/A	6.5815	6.2395	5.8375	5.8388	5.8056	6.5962	6.7432	6.8651	5.7424	6.2922	6.4360	6.4038	5.9237	4.9001	5.6843	6.1253	6.0391	6.2331	5.7371	5.8941
Slc32a1	_GGAPLPPSGS[Phospho (STY)]K_.2	#N/A	9.1972	4.4554	4.0837	4.1420	8.9003	3.5990	3.1552	3.2477	9.0739	3.7537	4.3821	8.5630	3.5681	7.9177	4.2332	3.8923	3.7854	4.0477	3.6963	8.0320
Slc32a1	_LTNVATSVSNK_.2	#N/A	5.0375	5.9208	5.8176	4.5586	4.9528	6.0862	6.1688	5.3742	6.0326	6.5420	4.5515	5.3346	4.5574	4.1011	5.1724	4.1580	4.9413	5.5819	5.5008	4.7941
Slc32a1	_ILIAC[Carbamidomethyl (C)]LYEENEDGEVVR_.2	#N/A	5.1572	4.4011	3.8049	4.1749	3.7305	2.7909	3.2422	4.3768	3.4464	3.5216	4.1348	4.3158	5.0421	3.9360	2.9645	4.2105	3.9339	4.6830	4.2020	3.7874
Ap1b1	_DC[Carbamidomethyl (C)]EDPNPLIR_.2	#N/A	9.6439	9.4453	9.8817	9.5588	9.6772	9.6085	9.6649	9.6861	9.7328	9.4946	9.6800	9.2866	9.4771	9.2455	9.1053	9.1026	9.6123	9.4742	9.3386	9.2978
Ap1b1	_DC[Carbamidomethyl (C)]PLNTEAASNK_.2	#N/A	8.9526	8.8048	9.1533	9.1229	8.9130	8.9732	8.9848	9.2898	8.7488	8.9392	8.7894	8.8091	8.4623	8.7321	7.9137	8.3816	9.0732	8.8064	8.6787	8.7168
Ap1b1	_VDKITEYLC[Carbamidomethyl (C)]EPLRK_.3	#N/A	7.7947	7.3917	7.3865	7.5931	7.6102	7.6236	7.7571	8.1461	7.3411	7.6769	7.5379	7.0619	7.3837	6.8981	6.7420	7.5708	7.8187	7.3903	7.4786	7.0479
Ap1b1	_YNDPIYVK_.2	#N/A	7.2342	7.2660	7.3527	6.9436	7.3089	7.2660	7.2353	7.0712	7.5664	7.5120	7.1500	6.7595	6.8307	6.9944	6.8944	7.2484	6.1966	7.2632	7.3953	7.1943
Ap1b1	_KGEIFELK_.2	#N/A	6.7038	6.8539	6.0249	6.7887	6.1881	6.5979	6.8366	7.4664	6.3672	6.5191	6.5145	6.6835	6.0950	6.3222	5.7272	5.9326	7.0556	6.4754	6.2716	6.4300
Ap1b1	_LASQANIAQVLAELK_.2	#N/A	6.4626	6.3678	6.6607	6.6167	6.7579	6.4306	6.2808	5.8664	6.3988	6.4380	6.2191	5.8509	5.5099	5.0283	5.4204	5.1961	6.2553	5.8870	5.9930	6.0451
Ap1b1	_VIASMTVGK_.2	#N/A	6.0149	6.0490	5.4228	3.5485	6.1989	6.3971	6.4355	6.6481	5.8067	6.3951	5.8032	5.8274	4.0666	5.9669	6.1724	6.2056	5.7696	6.3088	6.7609	6.4751
Ap1b1	_NINLIVQK_.2	#N/A	3.8121	4.4809	4.0944	5.5133	3.4516	4.4733	5.5716	3.3821	4.9927	5.1819	5.6737	4.7446	5.4769	3.3495	3.6417	3.9356	3.0094	5.6219	4.5805	5.0443
Ap1b1	_EYATEVDVDFVR_.2	#N/A	5.7182	4.4545	6.1397	5.1715	5.0835	5.3843	4.7490	3.4705	5.5238	5.6274	5.3749	3.9092	3.7908	4.8647	4.5505	5.4111	5.3769	5.1673	4.4472	4.9590
Ap1b1	_LSHANSAVVLSAVK_.2	#N/A	5.3036	5.6104	5.7844	4.8768	4.7938	5.5509	5.5108	5.1350	4.7009	4.5905	4.7651	4.9587	3.6814	5.1813	4.8646	5.2279	4.9784	5.0652	5.1114	4.8778
Ap1b1	_LQSSNIFTVAK_.2	#N/A	4.9639	5.7996	5.5451	5.4225	5.0758	5.0138	5.2583	5.3935	4.9077	4.6432	3.6310	4.9703	3.3068	4.2451	4.6500	2.9821	3.5242	5.9413	4.2524	4.6053
Ap1b1	_LVYLYLM[Oxidation (M)]NYAK_.2	#N/A	5.6053	3.4545	5.4448	5.0309	5.1536	5.1252	4.9653	6.4358	5.5080	4.8102	4.5370	3.1806	5.1488	5.0830	3.3055	3.3191	4.7453	5.4976	5.1534	5.1548
Ap1b1	_AAM[Oxidation (M)]IWVGEYAER_.2	#N/A	7.0857	6.8619	7.1559	6.3223	6.6441	6.3317	6.4550	6.5766	6.6689	6.2235	6.5312	3.2862	7.2047	3.3082	6.0338	6.7260	6.8671	3.6930	6.3448	6.5920
Ap1b1	_NVEGQDM[Oxidation (M)]LYQSLK_.2	#N/A	4.2491	5.7366	4.9336	5.4585	4.7229	5.4826	5.5537	5.4770	4.3630	4.9665	4.6289	4.5992	5.2370	5.9326	4.8284	4.7411	4.7669	4.8731	5.1891	4.6265
Ap1b1	_APEVSQHVYQAYETILKN_.3	#N/A	4.4583	4.3497	2.4315	3.8287	3.8966	4.1253	2.7490	3.9031	3.1085	3.7263	3.4828	4.2391	4.3773	3.9304	4.1274	3.0757	4.4094	3.2219	3.6904	3.9384
Ap1b1	_ITEYLC[Carbamidomethyl (C)]EPLRK_.2	#N/A	5.9504	5.8164	6.1027	5.8059	5.8481	5.8084	5.7362	6.1091	5.4099	5.2987	5.8233	5.7246	5.1613	5.6726	5.0944	5.0173	6.1237	5.8799	5.6070	5.5555
Ap1b1	_TAAVC[Carbamidomethyl (C)]VAK_.2	#N/A	2.6997	4.3169	3.9897	2.3674	3.1592	3.4974	4.9243	3.6542	3.2759	4.4075	3.5356	4.0929	3.9745	4.4772	3.2660	3.7028	4.1918	4.4186	3.2899	4.4442
Ap1b1	_DDREAQSIC[Carbamidomethyl (C)]ER_.3	#N/A	3.9816	3.7141	3.2346	3.7138	3.6283	3.7326	4.6202	5.3023	6.0709	4.3914	4.0521	3.5256	4.3388	3.7267	3.6537	3.0513	4.0912	3.8787	4.1608	3.5960
Ap1b1	_C[Carbamidomethyl (C)]LKDEDPYVRK_.3	#N/A	1.7283	3.4380	3.4176	3.7032	4.0880	4.1213	4.4609	4.1067	4.3812	3.6917	3.8598	3.1641	4.5466	3.5484	2.9855	3.2432	3.6040	3.0692	4.2187	0.3450
Ap1b1	_KPTETQELVQQVLSLATQDSDNPDLR_.3	#N/A	3.6131	3.7060	3.1497	1.7687	3.8201	3.8534	3.2280	3.8388	3.1482	3.4237	4.1277	3.4321	3.3137	3.8163	3.2534	2.9752	3.3360	2.5756	3.9507	3.7833
Ap1b1	_C[Carbamidomethyl (C)]LKDEDPYVR_.3	#N/A	3.6064	3.7127	3.1430	3.9778	3.8133	3.8467	3.2348	3.8321	3.1550	3.4170	4.1345	3.4388	3.3204	3.8231	3.2602	2.9685	3.3293	4.2954	3.9440	3.7901
C1qbp	_AFVEFLTDEIKEEK_.2	#N/A	4.1189	4.1174	4.6325	4.5536	4.1378	4.8041	3.4588	4.2871	4.0085	4.0117	4.7334	3.8034	3.8832	4.9018	3.5233	4.1125	4.7325	4.4354	4.4293	4.2009
Hap1	_ATHS[Phospho (STY)]PSAREEEGPSGAT_.2	O35668_S615_M1_Hap1	4.9111	5.1579	3.4003	3.3246	4.9762	4.9014	5.7584	6.0159	5.7093	3.8527	4.8727	6.6012	4.3855	6.0381	5.2985	5.9691	5.4596	3.2303	5.3198	3.3544
Mgll	_NKS[Phospho (STY)]EVDLYNSDPLVC[Carbamidomethyl (C)]R_.2	#N/A	7.1546	7.8944	7.1475	7.7450	6.4610	7.6181	3.7545	7.2346	6.8056	7.8363	7.2804	7.0840	7.2563	7.9546	6.4537	7.3928	7.4920	7.3310	7.6988	7.6037
Mgll	_LTLPFLLLQGSADR_.2	#N/A	4.4385	4.6482	5.0198	5.1975	4.8871	5.0792	4.8728	3.8684	4.5465	4.3743	4.5838	5.3161	4.1887	4.8758	3.8396	4.5558	4.3622	4.3482	4.8307	4.3584
Mgll	_IDSSVLSR_.2	#N/A	3.8041	4.8632	3.3407	5.0975	4.3543	4.0444	5.3753	3.4032	4.2417	5.0528	5.5440	5.1009	3.6806	2.4944	5.0072	4.7596	3.3401	5.4476	4.8960	5.1411
Mgll	_MPEASS[Phospho (STY)]PR_.2	O35678_S6_M1_Mgll	4.3747	3.6281	3.4101	3.3147	3.7124	3.9365	3.8287	4.0751	3.8878	5.5265	4.2621	3.5176	3.9958	6.4534	3.4059	3.2187	3.1119	3.4616	3.7606	3.2169
Mgll	_VC[Carbamidomethyl (C)]FGIQLLNAVAR_.2	#N/A	4.5181	3.4847	3.7714	3.1713	3.8558	3.9779	3.6852	4.2185	3.7443	3.2236	4.1187	3.3741	4.5388	3.5407	3.2624	3.5160	3.2553	3.0770	3.8097	3.5077
Mgll	_ALIFVSHGAGEHC[Carbamidomethyl (C)]GR_.3	#N/A	3.3740	3.1280	3.9103	4.3154	4.2126	3.7724	3.3091	3.0744	3.2293	2.2011	4.2088	3.0174	3.3947	3.8974	2.9057	3.7189	3.6121	4.2211	3.8697	3.8644
Syt3	_GLSEKENS[Phospho (STY)]E_.2	O35681_S586_M1_Syt3	9.6574	10.0366	9.3125	9.5149	9.2944	9.6475	9.8797	9.5875	9.7535	9.4875	9.6046	9.4208	10.1768	9.3851	8.7510	9.2833	9.7681	9.8967	9.6412	9.5585
Syt3	_KLHFS[Phospho (STY)]VYDFDR_.3	#N/A	3.2775	3.2245	3.8138	4.4119	4.1161	6.8027	6.7994	4.4787	6.7416	3.4838	6.3384	6.2686	3.5922	7.1827	3.0022	3.6224	3.5156	7.4514	7.1408	3.7679
Syt3	_LHFS[Phospho (STY)]VYDFDR_.2	#N/A	3.0958	6.5698	3.6321	5.6196	3.9344	6.1317	6.3995	4.2970	4.4519	3.6209	3.9306	3.2956	5.6308	5.7404	4.9741	3.1724	6.3353	2.9984	4.1479	3.5862
Syt3	_GGGS[Phospho (STY)]GEAGAPC[Carbamidomethyl (C)]GR_.2	O35681_S290_M1_Syt3	3.8033	6.0270	4.3396	5.6719	3.1410	5.4829	5.3511	5.0289	6.0219	6.1896	5.7599	5.2895	3.8240	5.4276	3.7637	6.8797	5.5156	6.2259	3.4404	5.7089
Nudc	_LQLEIDQK_.2	#N/A	3.2847	4.0344	4.2689	4.2995	3.4916	4.2846	3.5565	3.3218	4.2685	3.8655	2.9553	3.7605	3.6421	4.8450	3.9427	3.7539	3.8595	4.6435	3.6223	4.9138
Nudc	_WTQTLAELDLAVPFR_.2	#N/A	3.8988	3.4203	3.4354	3.6854	4.4421	3.4412	4.4432	3.7767	3.8216	3.7094	4.1194	2.5431	3.9297	4.3322	4.2856	4.2241	3.6217	3.5277	3.2163	3.8842
Pnn	_GFS[Phospho (STY)]DSGGGPPAK_.2	#N/A	3.9078	4.3881	3.9684	3.1524	3.2455	3.4696	4.2956	3.1567	4.3546	4.1141	3.2282	4.1142	4.4627	4.0065	3.8727	4.2527	4.1547	2.9947	3.2938	3.6837
Pnn	_RGFS[Phospho (STY)]DSGGGPPAK_.2	#N/A	3.4680	4.5647	3.9744	4.2513	3.6749	3.7083	3.3732	3.1385	3.2934	3.2786	4.2729	2.9533	4.4588	3.9615	3.3985	4.3309	6.6459	4.1570	3.8056	3.9285
Pnn	_SLS[Phospho (STY)]PGKENINSQEVEK_.2	#N/A	6.8263	6.3199	6.7069	6.8222	5.8746	6.7424	6.0287	6.4403	3.6220	4.0277	6.0576	5.8775	3.5410	6.6316	5.8030	5.7185	6.7155	6.3182	3.3275	5.8716
Pnn	_QES[Phospho (STY)]DPEDDDVKKPALQSSVVATSK_.3	#N/A	4.3638	5.4455	4.0598	4.7463	3.8166	5.3005	4.4884	3.8353	4.6931	3.9697	3.9742	4.7764	3.4385	5.5645	3.9693	4.6592	4.7273	4.7830	4.5163	4.6191
Ppfibp2	_C[Carbamidomethyl (C)]VDGNQLS[Phospho (STY)]PVGEPK_.2	O35711_S405_M1_Ppfibp2	5.0368	4.3455	5.7273	3.1098	5.5368	5.0038	3.8676	5.0276	3.7878	5.6465	5.0146	5.7801	3.9532	4.8074	3.8930	4.4072	5.3888	4.5826	3.2512	4.4229
Ppfibp2	_T[Phospho (STY)]QSGNFNTDAPGM[Oxidation (M)]AEFR_.2	O35711_T516_M1_Ppfibp2	3.4709	3.8995	4.0072	4.2442	3.6513	3.8754	3.2574	4.0140	3.3165	3.6772	4.5269	3.7890	3.4245	3.7506	3.6773	3.8158	3.7089	3.8993	3.8993	4.1464
Hnrnph1	_VHIEIGPDGR_.2	#N/A	8.3158	8.6124	8.6095	8.4247	8.3664	8.5495	8.3781	8.5213	8.4209	8.2671	8.3074	8.2994	8.4527	8.7338	8.5261	8.3345	8.4855	8.3552	8.4736	8.2870
Hnrnph1	_GLPWSC[Carbamidomethyl (C)]SADEVQR_.2	#N/A	8.5472	8.1466	8.5341	8.5076	8.1188	8.1912	8.1769	8.3791	8.1918	8.2041	8.2764	8.1838	8.5104	8.5086	8.4041	8.3040	8.2991	8.0433	8.3076	8.1763
Hnrnph1	_STGEAFVQFASQEIAEK_.2	#N/A	7.5394	7.2612	7.4300	7.6250	7.0616	7.0743	6.4848	7.6243	6.9701	7.7316	7.2458	7.2530	8.3231	8.1217	6.9472	7.5513	7.5634	6.9063	7.1180	7.8032
Hnrnph1	_ATENDIYNFFSPLNPVR_.2	#N/A	5.9258	5.5847	6.0876	6.0158	5.8382	5.6739	5.5518	5.5096	4.9658	5.9889	5.9273	5.6398	5.9436	5.8100	5.9547	5.8410	6.0383	5.2056	5.2879	5.9128
Hnrnph1	_HTGPNS[Phospho (STY)]PDTANDGFVR_.2	#N/A	9.5112	9.8014	9.3545	9.7378	9.0597	9.1716	9.4812	9.7872	9.4642	9.8421	9.4193	9.6978	10.3475	10.3712	9.5484	9.7380	9.8100	9.6597	9.7415	9.8076
Hnrnph1	_THYDPPRK_.2	#N/A	3.7681	3.5510	6.8891	6.1545	5.6590	4.1333	5.8229	5.8693	5.7196	3.5787	6.4830	5.4742	3.7990	5.2643	6.3581	3.1302	6.2161	3.6584	4.5651	6.2920
Hnrnph1	_THYDPPR_.2	#N/A	4.1337	3.8691	4.2844	3.5557	4.8650	4.2337	4.0696	3.8341	5.4329	5.6209	5.3367	3.7585	4.1544	3.5994	5.2308	2.9778	3.4197	4.1166	4.5774	5.4413
Hnrnph1	_DLNYC[Carbamidomethyl (C)]FSGM[Oxidation (M)]SDHR_.2	#N/A	3.6203	5.5678	5.3887	6.1742	3.8503	4.9214	4.0806	5.3367	5.0871	4.2814	3.9446	5.2722	3.3433	5.5506	4.4993	5.1190	6.5130	5.1028	5.2844	3.9279
Hnrnph1	_EGRPSGEAFVELES[Phospho (STY)]EDEVK_.3	O35737_S63_M1_Hnrnph1	3.3533	4.4500	4.3907	4.1366	3.5603	3.5936	3.4878	3.2532	3.4080	3.1639	4.3875	3.6919	3.5735	4.0761	3.5132	4.2163	3.7908	4.0423	3.6909	4.0431
Api5	_TSEDTSSGS[Phospho (STY)]PPK_.2	#N/A	8.3909	8.3067	7.7647	8.2124	3.8967	8.3799	3.1514	3.9154	7.9385	8.1662	8.1691	7.6564	7.8864	7.7199	3.1768	7.7610	8.6123	8.0635	8.1975	7.8748
Api5	_TSEDTSSGS[Phospho (STY)]PPKK_.2	#N/A	7.5907	7.2277	7.3133	7.0560	2.9338	7.0097	7.1043	7.3739	6.8869	6.7235	6.9131	4.8937	7.2431	7.2885	5.6364	6.2993	7.3477	7.4113	7.1898	6.2750
Api5	_TSEDTSSGS[Phospho (STY)]PPKKSPGGPK_.3	#N/A	3.9805	3.3386	3.5171	3.6037	4.1874	4.2208	2.6403	4.2062	4.2817	3.7911	3.7604	3.0647	4.4472	3.5551	2.8860	3.3426	3.7034	3.4460	3.6188	3.2324
Slc1a4	_SEEETS[Phospho (STY)]PLVTHQNPAGPVAIAPELESK_.3	O35874_S507_M1_Slc1a4	8.8544	8.8394	8.2522	8.3805	7.7723	8.2932	8.5218	8.4705	8.1314	8.2476	7.9688	7.9258	8.5959	8.6603	6.7076	7.8949	8.6974	8.5936	7.9097	7.5668
Calu	_EQFVEFR_.2	#N/A	4.7358	4.5842	5.0032	4.8609	4.7512	4.9984	4.9375	3.6647	5.0821	5.8180	4.5532	4.8745	5.6483	5.6016	5.7037	5.5268	3.9587	5.1008	5.3660	5.6444
Ctnnd2	_GGSPLTTTQGGS[Phospho (STY)]PTK_.2	O35927_S273_M1_Ctnnd2	12.7299	12.4613	12.7592	12.7235	12.3663	12.2293	12.1190	12.7460	12.0230	12.4072	12.2310	12.3910	12.7699	12.7367	11.8111	12.3067	12.7678	12.3899	12.2947	12.3665
Ctnnd2	_GGS[Phospho (STY)]APEGAAYAAPR_.2	O35927_S282_M1_Ctnnd2	10.8256	10.6139	10.5825	10.6338	10.0569	9.9297	10.1728	10.4156	9.9134	11.0176	10.3136	10.2338	10.7238	10.7726	2.8422	10.1956	10.5943	10.3497	10.3851	10.2294
Ctnnd2	_SPS[Phospho (STY)]IDSIQK_.2	#N/A	10.2255	9.8713	10.3805	4.4146	9.5327	4.3374	9.6026	4.4760	9.9414	10.2145	10.3687	9.5159	3.5949	10.1108	8.3816	3.6197	10.0034	9.8111	9.7770	4.3168
Ctnnd2	_SASAPAS[Phospho (STY)]PR_.2	O35927_S1098_M1_Ctnnd2	11.0268	11.1636	10.8633	10.5561	10.7583	10.8538	10.8760	11.0652	10.3903	11.0124	10.8684	10.9556	11.2774	11.0726	9.7705	10.7748	11.1161	11.0200	10.8334	10.8977

Ctnnd2	_ALQS[Phospho (STY)]PEHHIDPIYEDR_.3	#N/A	11.4820	11.2676	11.2980	11.3857	10.6598	10.7463	10.8839	11.2050	10.9166	11.3717	10.9511	10.8315	11.5737	11.2731	10.1779	10.9360	11.2900	10.9809	11.0444	10.8980
Ctnnd2	_T[Phospho (STY)]GSQHGPQNAAAATFQR_.3	O35927_T469_M1_Ctnnd2	6.8340	3.6277	6.2260	3.5810	3.5958	3.8199	7.6217	4.0303	4.0133	4.4644	6.8581	6.9513	4.1214	3.3676	3.5314	3.1021	5.9982	7.1672	3.4647	3.1777
Ctnnd2	_TSTAPSS[Phospho (STY)]PGVDSVPLQR_.2	O35927_S458_M1_Ctnnd2	12.5657	12.3746	12.1993	12.1612	11.5808	11.6694	11.9018	12.4719	11.8714	12.3373	11.8892	11.9818	12.7168	12.2352	10.9599	11.7341	12.4276	11.8748	11.7646	11.6899
Ctnnd2	_NS[Phospho (STY)]YGAPAEDIK_.2	O35927_S1149_M1_Ctnnd2	3.1983	3.3037	3.7346	4.4911	4.0369	4.2610	3.5042	4.3995	3.5633	3.4046	3.9377	3.1931	3.6714	3.2151	3.0814	3.5432	3.4364	3.7861	4.0851	4.3932
Ctnnd2	_TPSIS[Phospho (STY)]PVR_.2	O35927_S1082_M1_Ctnnd2	8.9198	9.1510	8.6238	8.9548	8.7035	8.8353	5.5334	8.9129	8.7801	9.1364	8.9412	8.8328	9.4253	9.1373	8.2412	8.9671	9.3802	9.2717	9.1303	9.2834
Ctnnd2	_NSSLS[Phospho (STY)]PGLNTSNGDGSETETTSAILASVK_.3	O35927_S32_M1_Ctnnd2	5.8125	6.2403	3.7769	4.1979	3.6436	3.6770	3.4548	3.6624	3.5354	5.0794	3.6574	3.6589	3.5405	4.0011	3.4149	3.6025	3.9633	4.3288	3.6261	4.0101
Ctnnd2	_TPSIS[Phospho (STY)]PVRVS[Phospho (STY)]PNNR_.2	#N/A	8.6764	8.8596	8.5240	9.1227	8.0670	8.6116	8.6740	3.9887	8.5625	8.6431	8.7067	8.7655	8.8922	9.1491	8.1205	8.8321	9.1483	8.4830	8.7225	8.8782
Ctnnd2	_QLQYC[Carbamidomethyl (C)]ASVDS[Phospho (STY)]PYSK_.2	O35927_S511_M1_Ctnnd2	4.2081	7.4744	7.8390	6.1403	5.7779	3.8296	6.6925	3.9084	6.4165	7.0843	3.6398	3.8122	6.6431	6.7371	3.7005	6.5357	6.9464	5.1585	5.5418	6.3634
Ctnnd2	_SLSQS[Phospho (STY)]QGDPLPPAHTGTFR_.2	O35927_S437_M1_Ctnnd2	3.6956	3.6465	3.9571	4.0102	3.8453	3.7129	5.6120	4.0930	3.4549	3.8281	4.7976	3.4865	3.5350	3.9551	3.3556	3.7693	4.5870	3.8975	3.7634	4.0046
Ctnnd2	_SPS[Phospho (STY)]IDSIQKDPR_.2	#N/A	9.9086	9.7921	9.2729	9.3635	9.0294	9.3541	9.3698	9.5809	9.3841	9.9740	9.8182	9.3527	9.8613	9.8854	8.2272	9.3435	10.2498	9.6160	9.5441	9.7372
Ctnnd2	_SGPALPPEGTLARS[Phospho (STY)]PS[Phospho (STY)]IDSIQK_.3	#N/A	3.5521	8.0851	4.0349	4.1738	3.7590	3.7384	5.6965	3.7777	4.5224	4.3089	3.4252	3.6348	3.8462	9.1256	3.9140	3.8604	4.2212	7.9527	3.2722	3.7502
Ctnnd2	_AS[Phospho (STY)]YSSQHGHLAPELR_.3	O35927_S395_M1_Ctnnd2	3.7180	3.5607	4.7208	3.8894	3.9249	4.2591	4.0161	3.9437	3.9827	3.6136	3.8362	3.3504	4.1286	4.0364	3.1717	3.9278	3.6499	3.7317	4.1559	3.9423
Ctnnd2	_SGPALPPEGTLARS[Phospho (STY)]PS[Phospho (STY)]IDSIQKDPR_.3	#N/A	3.7568	6.9228	4.4651	4.0746	3.4131	7.2250	9.2940	3.6361	7.4280	3.8558	3.6488	3.7693	3.7498	8.1795	6.6675	3.8712	4.0376	10.0670	5.9882	3.8486
Ctnnd2	_AAGQSFSQGTTGR_.2	#N/A	4.4505	3.5523	3.4859	3.2389	3.7882	5.1123	3.7529	4.1508	3.8120	3.1559	5.2866	3.8433	5.8743	3.4730	5.3201	3.2945	3.1877	3.1446	5.1604	5.3387
Pitpnm1	_C[Carbamidomethyl (C)]NTGSEGPEAQT[Phospho (STY)]PGK_.2	O35954_T270_M1_Pitpnm1	5.0838	5.5139	6.0021	5.7719	5.4431	4.5682	4.8174	3.7863	5.0474	5.4981	5.6465	3.8063	5.0527	4.6093	4.0464	4.6018	5.9067	3.9752	5.3775	4.6333
Pitpnm1	_PGTSTAGT[Phospho (STY)]PDGPEAPPGPDASPDAS[Phospho (STY)]FGK_.3	O35954_S304_M2_Pitpnm1	3.8964	3.4035	3.5420	4.0827	3.6686	3.9018	5.6591	4.2224	3.4730	3.8813	3.7331	3.7634	3.6094	3.5619	3.5800	3.2585	3.5720	3.3608	3.9292	3.8929
Pitpnm1	_SSYSSQHGGGVS[Phospho (STY)]PQS[Phospho (STY)]LSEWR_.3	O35954_S326_M2_Pitpnm1	4.1765	3.1426	3.7131	3.4077	2.8826	3.7249	4.0402	4.4022	4.0993	4.3695	2.9728	4.3696	4.2074	3.2142	4.1909	3.5386	3.8994	4.9905	3.5491	3.4284
Pitpnm1	_RGS[Phospho (STY)]M[Oxidation (M)]NNEM[Oxidation (M)]LSPEVGPVRDPLADGVEVLGR_.4	O35954_S593_M1_Pitpnm1	4.5560	3.4468	3.5914	3.1334	3.8937	4.0912	3.6474	4.2564	4.4113	3.6615	3.8899	3.3363	4.5767	3.5785	3.2246	3.4000	3.2932	3.0391	4.1885	3.5455
Rab33b	_S[Phospho (STY)]M[Oxidation (M)]VQHYYR_.2	#N/A	5.5203	5.9303	6.0435	5.3681	4.3495	5.7057	5.2758	4.7672	3.4259	5.8511	4.3225	4.3086	5.5686	5.1696	3.9686	4.5552	4.3541	5.1708	4.5805	4.9996
Gamt	[Acetyl (Protein N-term)]SSSAAS[Phospho (STY)]PLFAPGEDC[Carbamidomethyl (C)]GPAWR_.	O35969_S7_M1_Gamt	4.5441	4.2758	4.0807	3.8266	3.2502	3.2836	3.7979	3.5632	3.7181	4.3547	3.1967	4.0019	3.8835	4.3862	3.8233	3.9062	4.1009	3.7323	4.8298	4.3532
Pbx2	_TAVSVAQGGHS[Phospho (STY)]R_.2	O35984_S330_M1_Pbx2	6.0219	5.7726	5.3468	6.3209	4.9266	4.2474	4.1312	4.1565	5.0626	5.5934	4.9352	5.0972	5.4645	5.0790	4.1228	4.4968	4.6495	5.1083	5.3044	3.0617
Cavin1	_ATEEPSGTGS[Phospho (STY)]DELIK_.2	O54724_S42_M1_Cavin1	7.8655	7.6992	8.4812	7.7179	3.7891	7.8906	7.1843	7.3049	7.8351	3.1569	7.3485	7.7771	3.9203	8.5834	7.3590	7.6481	8.3637	7.6632	7.5982	7.8141
Cavin1	_IIGAVDQIQLTQAQLEER_.2	#N/A	3.2281	3.2738	3.7644	2.0367	4.0667	3.9182	3.4744	4.4294	3.1458	3.4345	4.0629	2.8723	3.2489	3.7515	3.0516	3.5730	3.4662	3.0389	4.0155	2.6942
Ddost	_TLVLLDNLNVR_.2	#N/A	4.5302	3.4501	4.0745	4.4830	4.7920	5.3430	4.7477	4.2531	5.6122	5.3430	3.8867	4.7638	5.0209	4.6590	4.4347	4.3902	4.9680	4.5084	4.7280	4.1183
Ap3d1	_MVKGS[Phospho (STY)]IDR_.2	#N/A	9.8457	10.3635	10.0376	10.5947	8.8516	8.6124	9.9184	8.3111	8.3821	10.2092	9.2832	8.6773	8.6273	9.5042	5.9261	8.2412	8.5260	8.7694	8.5360	8.5342
Ap3d1	_NAEAVKS[Phospho (STY)]PEKEGVLGVEK_.3	#N/A	8.9155	8.3267	8.5575	8.5951	7.9126	8.1238	8.1425	8.2645	7.8387	8.3145	8.2538	7.7584	7.8566	8.1500	6.5110	7.8449	8.0551	8.1620	8.0806	7.6475
Ap3d1	_RHSSLPTES[Phospho (STY)]DEDIAPAQR_.3	#N/A	9.6413	9.3916	9.6595	9.5955	3.3988	9.4528	9.4945	9.2359	9.3740	9.2804	8.5230	8.8985	9.1228	8.7137	8.1556	9.4123	9.7161	9.4341	9.5412	9.0737
Ap3d1	_HSSLPTES[Phospho (STY)]DEDIAPAQR_.3	#N/A	11.6363	11.8356	11.4752	11.7157	9.5072	11.5998	11.8104	11.2451	11.6733	11.7534	11.3297	11.1896	11.9951	11.3957	10.2398	11.3577	11.5812	11.8263	11.6231	11.4202
Ap3d1	_NAEAVKS[Phospho (STY)]PEK_.2	#N/A	6.7531	5.4656	5.1448	4.6788	5.1752	5.2862	5.6500	3.8016	3.4988	5.2529	5.3506	3.9838	3.6068	5.7550	3.2906	2.9381	5.5167	5.1566	5.3667	4.9840
Ap3d1	_DLILQC[Carbamidomethyl (C)]LDDKDESIR_.3	#N/A	5.6444	5.1381	3.7361	6.1164	5.1985	6.0012	5.8792	5.0146	5.5399	3.4062	5.6914	4.8826	4.6082	3.2136	4.5806	3.5447	5.3392	5.5370	4.7343	4.3917
Ap3d1	_EQAADTEAAQEVTQLLVER_.2	#N/A	4.6612	4.5515	4.1996	4.4946	3.7910	4.3484	4.8113	4.8476	4.0576	4.1067	3.8591	4.0971	4.5934	4.6388	3.6141	4.5435	4.3998	4.3653	4.4489	3.7955
Ap3d1	_NLQDLVR_.2	#N/A	3.9769	2.3590	4.5132	3.7125	3.3146	3.5387	4.1751	3.6773	4.2856	4.1832	3.1591	3.9154	3.9976	4.5003	3.8037	3.5646	4.2149	3.6182	3.2668	2.9664
Ap3d1	_LEDPDPGVQSAAVNVIC[Carbamidomethyl (C)]ELAR_.2	#N/A	3.2466	4.3433	4.1958	4.0299	3.4535	3.4869	1.9413	3.3599	3.5148	4.5580	2.9934	4.2328	3.6802	4.1829	3.6199	4.1096	3.8975	1.4043	3.5842	4.1499
Ap3d1	_VDIITEEMPENALPS[Phospho (STY)]DEDDKDPNDPYR_.4	O54774_S784_M1_Ap3d1	3.3812	3.1208	3.9175	4.3082	4.2198	4.4438	3.3213	3.6833	3.3804	3.5875	3.7548	3.0102	3.4885	4.5331	2.8985	3.7261	3.6193	3.9689	4.2680	4.2103
Srpk2	_SSS[Phospho (STY)]SERPEPQQK_.2	O54781_S10_M1_Srpk2	7.7691	7.6111	7.3294	7.6779	3.0799	3.5650	7.4179	7.8408	4.2691	7.7589	7.2026	4.2096	4.3771	7.7465	6.8567	7.5561	7.3976	7.4994	7.5215	7.2826
Srpk2	_TVSASS[Phospho (STY)]TGDLPK_.2	O54781_S490_M1_Srpk2	5.0695	3.5029	6.9624	6.7561	4.0279	6.2640	3.3629	6.7383	6.3588	6.6570	4.0705	3.2290	3.6827	6.8939	5.0041	6.3764	3.5439	3.9285	4.6629	4.3921
Srpk2	_AADLEDT[Phospho (STY)]T[Phospho (STY)]EEETAK_.2	O54781_T331_M2_Srpk2	5.2823	3.8015	5.5474	4.4720	5.1189	5.6000	5.1407	4.4351	5.1034	5.4237	5.5927	4.8299	4.7477	4.9938	4.4058	4.8533	5.2579	4.3429	4.6585	3.6048
Srpk2	_[Acetyl (Protein N-term)]S[Phospho (STY)]VNS[Phospho (STY)]EKSSSSERPEPQQK_.2	O54781_S2_M2_Srpk2	3.8453	3.5178	3.5560	4.0299	3.8583	3.8780	3.7184	4.2210	3.6049	4.0516	4.2201	3.4073	3.8660	3.5431	3.2956	3.3646	3.6810	3.9356	3.5842	3.5101
Dapk3	_NTSYAS[Phospho (STY)]FER_.2	O54784_S316_M1_Dapk3	3.5093	8.3672	4.3669	4.2547	8.0192	7.9054	8.2307	3.5552	3.2523	8.2607	3.6267	3.7157	8.5479	4.4050	4.2712	3.8542	8.2277	7.9950	8.0150	7.9964
Dffa	_RS[Phospho (STY)]PLPGEPQRPK_.3	O54786_S314_M1_Dffa	5.8361	3.8093	6.0930	4.9408	5.6784	5.2275	6.1065	5.4499	6.0122	6.5927	6.2098	4.9915	7.0862	6.6711	3.3567	5.9364	5.6444	5.8719	6.2162	6.3393
Tpd52l1	_S[Phospho (STY)]WHDM[Oxidation (M)]QTTTAYK_.2	#N/A	6.7268	4.0228	4.3670	4.4162	3.5366	7.1709	3.5449	3.2014	5.6116	3.9694	6.0474	5.7513	6.4288	4.3263	3.5703	6.8156	4.0068	6.0707	3.5478	6.3671
Tpd52l1	_S[Phospho (STY)]WHDM[Oxidation (M)]QTTTAYKK_.3	#N/A	4.1139	5.5415	4.0637	5.1803	3.0616	3.1810	4.1734	3.2400	4.7487	6.1030	5.1614	5.2258	4.0797	4.8240	4.7724	3.8809	4.4912	4.2429	3.4814	4.9596
Tpd52l1	_NSSTFKS[Phospho (STY)]FEER_.2	O54818_S149_M1_Tpd52l1	9.2045	8.8377	9.1248	9.0497	8.1183	8.5470	8.5375	8.3862	8.4025	8.8071	8.6026	8.5217	8.6543	9.0190	7.9466	8.8403	8.8888	8.3813	8.6807	8.7490
Rgs9	_GC[Carbamidomethyl (C)]LAS[Phospho (STY)]PVFAR_.2	O54828_S588_M1_Rgs9	8.6524	8.7063	8.1631	8.7355	3.8200	8.0989	8.0033	9.0811	8.2826	8.5983	9.6623	9.4304	10.1033	9.7349	8.1501	9.2181	9.8453	9.3389	9.0124	9.2805
Rgs7	_FLESEFSSENLR_.2	#N/A	6.6466	6.3546	7.1700	6.8935	6.1424	6.4231	6.7466	6.8099	6.6273	6.3262	6.6925	6.4557	7.0673	7.0101	6.6684	6.4127	6.7718	6.2799	6.2157	6.5495
Rgs7	_SHS[Phospho (STY)]PTHTPTPETKPPTEDELHQQIK_.4	#N/A	3.9392	11.0602	5.5200	11.6095	7.5003	8.2028	10.4309	7.8257	11.2138	4.1022	8.3101	11.0158	12.0172	11.4083	8.4968	11.1256	3.8493	7.7147	11.3321	11.2323
Rgs7	_SSAYQELLQAK_.2	#N/A	3.9749	5.7877	3.0973	5.7696	5.8921	5.4425	5.4652	3.0895	5.2093	5.5278	4.9897	5.9600	6.3577	6.0691	5.8201	5.4707	5.7404	5.6295	5.6754	5.1509
Rgs7	_YTFEDAQEHIYK_.2	#N/A	5.2397	4.9253	5.0739	3.9451	4.9676	4.8076	4.0045	5.1693	4.4709	4.5416	4.6622	4.1375	4.4775	5.2529	4.4494	4.0878	5.0966	3.8151	4.9171	4.5262
Rgs7	_S[Phospho (STY)]VYGLQNDIR_.2	O54829_S229_M1_Rgs7	3.6930	3.6261	4.2545	3.8912	3.9000	3.9333	4.2495	3.9187	3.0683	3.5036	4.0478	3.3522	3.2338	3.7364	3.1735	3.0551	3.4159	4.3820	4.6770	3.7034
Cd200	_EVIC[Carbamidomethyl (C)]QVLYLGNVIDYK_.2	#N/A	4.3392	4.6609	3.3696	3.5838	3.6770	3.7100	3.8641	4.2261	4.3213	4.5456	4.2976	3.0448	4.0313	3.5750	3.4413	3.1833	4.3524	3.6152	3.7252	4.3928
Reps1	_RQS[Phospho (STY)]SSYEDPWKITDEQR_.3	#N/A	3.9826	6.1476	4.1285	4.4994	4.1847	3.3157	3.8463	3.3167	3.9054	5.3768	3.3883	3.9603	4.0135	4.1665	5.2783	6.0936	4.3148	5.7729	5.9268	4.4375
Reps1	_RQS[Phospho (STY)]SSYEDPWK_.2	#N/A	8.0973	7.9615	8.6483	8.6998	7.6567	6.7026	7.5038	7.5093	8.0274	8.9455	3.6477	8.1989	7.9290	9.0195	6.9240	7.6754	8.1689	7.7423	8.4090	7.6006
Reps1	_SSS[Phospho (STY)]LDMNR_.2	O54916_S561_M1_Reps1	3.2921	3.2098	3.8284	8.6016	5.4404	6.8220	7.7401	3.7723	3.4695	6.9329	3.8439	3.0993	3.5775	8.5973	2.9876	3.6370	3.2696	6.3456	6.1724	4.2994
Reps1	_LKS[Phospho (STY)]EDELRPDVDEHTQK_.3	#N/A	5.4086	5.7663	5.7364	5.4215	4.6959	5.0184	5.4132	4.8155	4.7621	5.7420	5.0507	5.2745	5.9967	5.6270	4.2853	5.2191	5.5181	5.2490	5.0248	5.4063
Reps1	_KGPGS[Phospho (STY)]HDAVQPR_.3	O54916_S145_M1_Reps1	4.1986	4.2334	4.2220	3.4908	3.5363	2.8144	3.6922	3.8990	3.3678	4.2060	2.6734	3.6937	4.2193	3.3972	3.5820	3.0426	4.4366	3.8184	3.8824	2.8816
Reps1	_LIDLEDSADVGEQPGEVGY[Phospho (STY)]SGSPAEAPPSK_.3	O54916_Y389_M1_Reps1	3.3063	4.0128	4.3437	4.2780	3.5132	3.5465	3.5349	3.5319	3.4551	3.1168	2.9338	3.7390	3.6206	4.3817	3.5603	4.1692	4.5300	3.9953	4.4193	4.0902
Akap2	_TLS[Phospho (STY)]M[Oxidation (M)]IEEEIR_.2	#N/A	3.6082	6.2975	6.0953	5.2924	3.4210	3.5298	4.6725	3.2433	3.1579	3.6413	3.8831	3.9029	3.2965	6.6281	3.7659	4.2207	3.7810	5.6283	3.7169	4.0333
Bnip2	_KGS[Phospho (STY)]ITEYTATEEK_.2	O54940_S114_M1_Bnip2	5.1806	5.4519	3.5552	3.5656	4.2256	4.2589	5.5735	4.2443	5.5088	5.7567	5.1877	3.0266	4.4090	5.6541	2.8479	3.3807	5.0830	5.3791	4.6118	4.9292
Dnajb6;Dnajb7	_VDYYEVLGVQR_.2	#N/A	4.8249	5.6859	5.5968	6.4330	5.0223	4.7700	5.5981	5.7514	5.2782	6.0413	5.6003	5.2567	5.6542	5.7291	5.0075	5.2230	6.1923	5.6043	5.3937	5.3770
Banf1	_DC[Carbamidomethyl (C)]FGC[Carbamidomethyl (C)]LR_.2	#N/A	3.7897	5.3326	5.6140	4.6748	5.3432	5.1957	3.8747	4.0154	3.7125	5.2758	4.0869	5.0572	5.6726	5.2910	4.6954	4.1559	3.5126	4.5465	4.8370	4.4780
Crym	_SSSLLIPPLEAALANFSK_.2	#N/A	7.8092	7.5837	7.7514	7.6966	8.0830	8.0601	7.3962	7.6829	7.4573	7.9705	7.5921	7.5270	7.2510	7.3446	7.8055	7.4257	6.9360	7.0104	7.5339	7.5316
Crym	_LVYDSWSSGK_.2	#N/A	7.8151	7.5209	7.9557	8.2360	7.8973	7.8671	7.8242	8.0907	7.8559	8.1078	7.6503	7.6571	8.0038	7.5830	7.6454	7.5821	7.3981	7.3903	7.6614	7.3614
Crym	_QAVLYVDSR_.2	#N/A	7.1742	7.1569	7.3381	6.7024	7.2707	7.4311	7.2636	7.3703	7.2458	7.0688	6.8508	7.0214	7.3682	7.1332	6.9646	7.1251	6.9520	6.4794	6.9561	6.9552
Crym	_FASTVQGDVR_.2	#N/A	6.2243	6.9447	6.7680	7.1370	6.8842	7.0587	7.4152	7.1638	7.4166	6.7547	5.8179	6.6603	6.4340	7.0737	7.0669	6.9343	6.5116	6.3996	6.7747	6.5633
Crym	_SLGM[Oxidation (M)]AVEDLVAAK_.2	#N/A	7.1091	6.9458	6.5217	6.6278	7.2632	7.4119	7.0639	7.2497	6.4865	6.8036	6.6158	6.9329	7.6653	6.5462	6.3923	7.1650	6.8714	7.1188	7.1713	6.8203
Crym	_GFLGVM[Oxidation (M)]PAYSAAEDALTTK_.2	#N/A	6.8900	6.3391	5.8399	6.5707	6.5212	7.1615	6.7649	6.8410	6.6508	6.7608	6.0931	6.6085	6.3143	6.3119	6.1779	6.8147	6.2136	6.6295	6.3236	6.4316
Crym	_TAAVSAIATK_.2	#N/A	5.7138	5.5647	5.7843	6.7632	6.3106	6.3904	6.3298	5.2417	5.7594	6.4031	6.1019	6.0436	5.0752	5.5753	6.2944	6.3840	5.1825	5.7535	6.4152	5.6476
Crym	_RAPAFLSAEEVQDHLR_.3	#N/A	7.4787	7.9071	7.1507	7.2774	7.6923	7.7127	7.5410	7.9813	7.3299	7.5657	7.1148	7.1827	7.4368	7.2383	7.0328	7.1984	7.0311	6.9004	7.5707	7.1208
Crym	_GPDGGVM[Oxidation (M)]QPVR_.2	#N/A	3.9978	6.4022	6.2665	6.9258	6.0958	6.5161	6.5951	6.4585	6.4328	6.2617	6.0165	6.0115	6.0747	6.4259	6.6550	5.9948	6.0213	6.2413	6.0057	6.1169
Crym	_APAFLSAEEVQDHLR_.2	#N/A	5.3595	4.1801	5.3832	5.5039	5.0971	5.6441	4.7532	5.3542	5.3404	5.4898	5.3922	4.3630	5.4178	4.6506	4.6364	4.6626	5.4117	4.6914	5.4192	5.3480
Crym	_ELDDELMR_.2	#N/A	3.7533	3.5658	5.5217	6.3659	3.9602	3.9936	5.6627	3.9790	3.8770	4.2035	4.8137	3.8857	3.1735	5.7237	4.7982	3.1154	3.4762	4.4423	4.0909	3.6432
Get3	_LEETLPVIR_.2	#N/A	5.6387	5.7712	6.1536	5.9988	6.4211	6.0981	5.9677	6.1712	6.3072	5.7756	6.0748	6.2248	5.8359	5.7957	6.4092	6.1554	6.2425	5.3842	6.3578	5.8744
Get3	_LLNFPTIVER_.2	#N/A	6.7211	6.6354	6.3184	6.6299	6.8504	6.8259	6.2811	6.8159	6.9075	6.5493	6.6965	6.4529	6.3704	6.8851	6.3857	6.4474	4.4844	6.4901	6.2703	6.5771
Get3	_ESVLIISTDPAHNISDAFDQK_.3	#N/A	3.4760	4.5268	5.0600	5.5139	4.5723	4.6127	4.9064	3.5884	4.1511	4.7111	5.3660	4.1811	3.3936	4.4382	4.1510	3.8209	3.0857	4.6767	5.1546	5.2256
Slk	_VITSDRSS[Phospho (STY)]EVGTDEALDDTQK_.3	O54988_S543_M1_Slk	3.3892	4.6136	3.9255	4.3002	4.2277	4.4518	3.3134	3.0895	3.3724	6.2453	3.7468	3.0023	3.4805	6.0470	2.8906	5.9949	3.6272	4.2059	3.8545	3.8795
Slk	_ATLEQPETDEVEQVSESNS[Phospho (STY)]IEELER_.3	O54988_S647_M1_Slk	6.4412	7.3600	6.7326	6.1403	3.5677	3.6011	6.9851	3.5865	6.5586	6.4614	4.3801	6.6556	3.5660	4.0687	6.1884	4.2237	3.0837	4.0498	6.6510	5.8859
Slk	_LENLPDTQDQQTVDVNSVSEENENNR_.3	#N/A	6.5918	7.1140	4.2567	3.9689	5.1789	6.8121	6.2380	3.4208	3.5757	4.4971	3.0544	4.1718	3.7412	6.9731	3.6809	5.5692	5.9755	6.3581	3.5232	4.2108
Cntnap1	_DQNLPQILEESRS[Phospho (STY)]E_.2	O54991_S1384_M1_Cntnap1	13.2423	13.2807	13.3939	13.1628	10.1656	13.1735	12.9320	12.6445	13.1366	12.5727	12.1773	12.5295	12.9586	12.9788	11.7242	12.5521	13.2222	12.9358	12.8736	12.6681
Cntnap1	_AVATQGAFNSWDWVTR_.2	#N/A	3.6312	4.1115	4.2450	4.3766	2.9689	3.1930	3.0713	3.4333	3.1304	3.8375	3.5048	3.8376	3.2385	4.2831	4.1494	3.9761	4.4313	4.2189	3.0171	3.9604
Bnip3	_NSTLS[Phospho (STY)]EEDYIER_.2	O55003_S88_M1_Bnip3	9.9242	9.8691	9.7449	9.9202	8.8254	9.5186	9.4707	9.2430	9.2311	9.7075	9.3837	9.4983	9.7889	10.0130	8.6175	9.8544	10.0294	9.6279	9.5462	9.5207
Bnip3	_SSHC[Carbamidomethyl (C)]DS[Phospho (STY)]PPR_.2	O55003_S60_M1_Bnip3	3.7147	8.3357	7.7981	7.4972	6.7560	8.2877	2.9878	8.1257	3.5700	8.0747	8.2967	8.0191	3.7354	8.1333	7.4590	6.8404	8.6887	8.1027	8.4509	8.1646
Bnip3	_AEIDSHS[Phospho (STY)]FGEK_.2	O55003_S79_M1_Bnip3	6.8634	3.9417	4.5974	6.7304	6.6516	3.0853	6.1032	6.9301	3.9164	4.1564	5.8091	6.0474	6.4936	6.3546	4.0215	6.6170	4.2992	5.7361	5.5790	5.8306
Bnip3	_SQT[Phospho (STY)]PQDTNRAEIDSHSFGEK_.3	O55003_T66_M1_Bnip3	5.4839	4.5031	3.8534	5.0163	5.7938	3.5846	5.6042	4.5425	4.2396	4.1274	3.1131	5.0625	5.2486	3.8914	4.4495	5.5078	5.7876	3.1097	3.4088	5.8504
Bnip3	_ILLDAQHES[Phospho (STY)]GR_.2	O55003_S48_M1_Bnip3	7.3866	7.0972	7.2172	7.1742	6.8559	6.6814	6.3320	7.4922	6.3815	6.5632	7.4720	6.8018	7.1949	7.3412	6.1467	3.8970	6.9622	6.5325	7.0978	7.1906
Cacna1b	_ESLS[Phospho (STY)]WGTQR_.2	O55017_S1929_M1_Cacna1b	8.1162	7.9682	7.5634	7.6993	3.3617	7.6839	4.1794	4.5413	4.2385	4.2303	7.6509	7.1138	4.3465	3.8903	4.8169	7.0709	4.0386	3.1109	6.6921	3.5676
Cacna1b	_SHS[Phospho (STY)]KETPGADTQVR_.3	#N/A	5.8792	5.7786	5.4174	5.7375	5.0324	4.5725	6.0609	4.8579	4.9527	6.1130	5.4594	5.2947	5.4237	5.5785	3.5413	5.1423	4.7760	5.2078	5.3600	5.0335
Cacna1b	_AS[Phospho (STY)]C[Carbamidomethyl (C)]EALYSEM[Oxidation (M)]DPEER_.2	#N/A	8.8676	8.9547	9.1289	8.9031	7.4835	8.8625	8.7904	8.4595	8.2959	8.2418	8.4447	8.0834	7.9201	7.7343	6.4147	7.7965	8.6440	8.6969	8.2147	8.0800
Cacna1b	_GLS[Phospho (STY)]EHNALLQK_.2	O55017_S2221_M1_Cacna1b	7.6597	7.1657	7.6902	6.7687	6.2940	6.5815	6.4809	3.3664	6.5200	7.2389	6.9219	5.4953	5.9999	5.3698	4.1145	5.9163	6.6787	6.3514	6.0120	5.5904
Cacna1b	_RGS[Phospho (STY)]PEEATER_.2	#N/A	5.9896	3.3961	3.4596	3.6612	5.2787	3.9784	6.1037	5.3909	5.4882	3.1221	5.9413	4.5460	3.9539	4.2038	3.3639	3.2607	6.2674	6.0579	5.7603	3.1749
Cacna1b	_IGS[Phospho (STY)]DPYLGQR_.2	O55017_S2244_M1_Cacna1b	9.7005	9.6745	9.2536	9.4824	8.6647	8.7815	9.2401	8.7137	8.7517	9.5337	9.0519	8.7365	9.4374	8.7693	6.8893	8.7225	9.2911	9.2664	8.8708	8.6617
Cacna1b	_RGS[Phospho (STY)]PEEATEREPR_.3	#N/A	6.8190	6.8308	6.3723	6.6005	3.5385	6.7035	6.9626	3.8001	3.8600	3.9424	6.7489	6.0215	4.4189	5.7741	5.2448	5.8971	6.5267	6.5144	6.5822	6.3559
Cacna1b	_RSHS[Phospho (STY)]KETPGADTQVR_.3	#N/A	3.5041	6.6849	4.0704	6.5093	3.6992	3.9233	4.0303	4.0251	3.2619	6.5229	6.0515	6.3139	6.9393	6.1722	3.6075	5.5158	3.9805	6.5829	2.5706	3.8180
Cacna1b	_S[Phospho (STY)]LEKGPSLSVDPEGAPSTAAGPGLPHGEGSTAC[Carbamidomethyl (C)]R_.4	O55017_S2056_M1_Cacna1b	3.5313	4.4715	4.0676	4.1581	2.8690	4.4159	6.1589	3.2317	3.3866	3.1854	4.3660	4.3610	3.5520	6.5040	4.2493	3.8762	3.7693	5.9972	3.7124	4.0216
Cacna1b	_EVAEVS[Phospho (STY)]PM[Oxidation (M)]S[Phospho (STY)]AANISIAAR_.2	#N/A	3.5893	3.7638	4.1256	4.1598	3.7185	4.7249	5.9515	4.0811	3.4446	3.7956	3.7147	3.6533	3.6100	7.0840	3.5416	3.9342	3.8273	7.2316	3.7141	3.4512
Cacna1b	_LQNLRAS[Phospho (STY)]C[Carbamidomethyl (C)]EALYSEM[Oxidation (M)]DPEER_.3	#N/A	4.2497	2.7816	3.2852	3.4397	3.5874	3.8115	3.9537	3.9501	4.0127	4.4560	4.3871	3.6426	4.2704	3.2722	3.5309	3.0938	4.4878	2.3820	2.9940	3.2392
Pgrmc1	_GDQPGASGDNDDDEPPPLPR_.2	#N/A	7.8951	8.4224	8.4033	8.5507	7.8652	8.9358	8.3893	8.7979	8.5627	8.6539	8.5560	8.0295	7.7504	8.9514	8.5285	8.6937	8.9232	7.9138	8.4730	8.3198
Pgrmc1	_RFDGVQDPR_.2	#N/A	7.8306	7.8929	7.7421	7.8515	7.7155	7.9500	8.2998	8.1126	7.9825	7.9454	7.6113	7.7477	7.9013	7.6164	8.3973	7.9907	7.6401	7.9060	7.8451	7.4500
Pgrmc1	_FYGPEGPYGVFAGR_.2	#N/A	6.9791	6.9085	6.7993	6.7672	7.4890	7.5519	7.1845	7.2194	6.8188	7.0034	7.0175	7.1605	6.8716	7.0795	7.0782	7.0441	7.3594	7.6260	7.0333	7.1987
Pgrmc1	_EGEEPTVYS[Phospho (STY)]DDEEPKDETAR_.3	#N/A	11.5331	11.6398	11.5407	11.7713	9.7074	11.7109	11.6335	11.0706	11.4683	11.3613	11.6277	11.3913	11.7904	12.0218	11.0030	11.4171	11.3794	11.5384	11.1949	11.1582
Pgrmc1	_LLKEGEEPTVYSDDEEPKDETAR_.4	#N/A	6.7897	9.6834	8.6072	9.8495	6.5083	9.2654	9.3519	6.3070	9.3635	9.6083	6.6450	9.5589	7.4186	9.7908	9.4053	9.8342	6.7780	9.6417	9.6144	9.7775
Pgrmc1	_DFTPAELR_.2	#N/A	3.0861	4.0275	3.3963	3.8315	3.9247	4.2045	5.1395	4.0919	4.7588	3.2924	5.4704	3.2925	3.7836	5.2116	5.0060	3.4310	3.4756	5.1070	4.4224	5.0617
Pgrmc1	_EGEEPTVYS[Phospho (STY)]DDEEPKDETARK_.4	#N/A	4.3463	3.6565	3.3818	3.3431	3.6840	3.9081	3.8570	4.2190	3.9161	4.5526	4.2905	3.5459	4.0242	3.5679	3.4342	3.1904	3.7162	3.4332	3.7323	3.2452
Impa1	_SLLVTELGSSR_.2	#N/A	7.9841	8.0837	8.2028	8.4739	8.0137	7.8906	8.0306	8.2898	7.8194	8.0683	7.6723	8.1217	8.2481	8.3932	7.9657	7.8220	8.0789	7.8159	7.8597	8.0587
Impa1	_SS[Phospho (STY)]PADLVTVTDQKVEK_.2	#N/A	6.1111	6.3807	6.2818	6.4805	5.0546	6.5240	6.0532	5.6671	5.0337	6.5649	5.9018	5.9985	5.4018	4.9705	6.0033	6.3506	6.6511	6.0548	6.3273	6.3637
Impa1	_EIEIIPLQR_.2	#N/A	7.3843	7.2971	7.6015	7.4638	7.6629	7.6341	7.5620	7.5196	7.2095	7.4055	7.5667	7.2518	6.9230	8.1057	7.4111	7.3240	7.5083	7.7348	7.3659	7.4624
Impa1	_LC[Carbamidomethyl (C)]SIPIHGIR_.2	#N/A	7.3034	7.6537	7.0242	7.2332	7.4023	7.2590	7.2356	7.5356	6.6863	7.3818	7.4388	7.4617	7.2116	7.2340	7.3231	6.7576	7.7672	7.1725	7.2391	6.3996
Impa1	_LQVSQQEDITK_.2	#N/A	4.4599	5.2118	3.7502	6.2005	5.4033	5.4601	4.9162	4.6021	5.4115	5.3963	4.9214	5.3546	4.2445	5.2095	5.5052	5.4529	4.8042	5.4933	5.0632	5.1618
Impa1	_S[Phospho (STY)]SPADLVTVTDQK_.2	#N/A	6.7274	6.9750	8.2004	6.7419	5.8234	5.8660	5.6453	5.8861	7.4727	7.7559	5.9080	6.3708	4.2986	5.7737	5.7748	6.7515	6.6210	5.0176	5.6834	7.3215
Impa1	_IIAANSITLAK_.2	#N/A	3.2367	5.6972	5.7088	5.7399	5.5101	5.6467	5.5275	4.2331	3.5841	5.0090	4.6489	3.1548	4.5146	5.7295	5.8844	5.5011	4.2703	5.8535	4.4095	5.0536
Impa1	_[Acetyl (Protein N-term)]ADPWQEC[Carbamidomethyl (C)]M[Oxidation (M)]DYAVILAR_.2	#N/A	3.5449	3.7742	4.5823	4.0394	4.4515	4.2000	4.0467	3.7705	3.2165	3.3555	4.2566	5.1163	3.3820	3.8846	4.2934	4.2335	3.2678	3.0092	4.3291	4.9390
Bckdk	_S[Phospho (STY)]TSATDTHHVELAR_.3	O55028_S31_M1_Bckdk	3.6953	3.4172	4.0846	4.0046	7.6207	8.1292	3.7043	3.9650	8.1760	3.6197	7.8623	3.1909	8.0232	4.1017	7.8029	3.9052	7.7971	3.8715	8.3989	3.8688
Copb2	_DNNQFASASLDR_.2	#N/A	7.5056	7.2564	7.2289	7.1453	6.9676	7.4951	7.0778	7.3947	7.1897	7.1384	7.0153	6.9360	7.0332	7.1509	6.3604	6.6191	7.1315	7.0317	6.9903	6.5790
Nck2	_DSESS[Phospho (STY)]PSDFSVSLK_.2	O55033_S316_M1_Nck2	3.5996	4.4032	4.1359	4.0898	2.9373	3.1614	3.1029	3.3000	5.7380	3.8059	3.5364	4.2926	3.6203	4.1230	4.1809	3.9445	3.8377	3.9955	3.6441	5.6175
Nck2	_DAS[Phospho (STY)]PTPSTDAEYPANGSGADR_.2	O55033_S90_M1_Nck2	4.1796	5.5259	3.2151	3.5098	3.5173	4.4676	4.1147	3.8800	4.0349	4.0379	3.5135	3.7126	4.2003	3.2021	5.0080	3.0237	4.4177	3.4155	3.0641	3.1691
Snca	_EQVTNVGGAVVTGVTAVAQK_.2	#N/A	8.7798	8.5136	8.9021	9.0840	8.4256	8.0627	7.9357	8.6742	8.5182	8.8190	8.6941	8.6482	9.0816	9.1800	8.4877	8.8233	8.9066	8.2103	8.7096	8.8691
Snca	_AKEGVVAAAEK_.2	#N/A	8.7170	8.8498	8.9207	8.9395	8.9417	8.7990	9.0399	9.1220	9.0420	8.9102	8.5664	8.7611	9.2302	8.9505	9.5421	9.3328	8.8040	8.7996	9.0058	9.0594
Snca	_TVEGAGNIAAATGFVK_.2	#N/A	8.6835	8.5933	9.0768	8.7291	8.6169	8.4841	8.6650	8.4758	8.7232	8.7332	8.4148	8.3565	8.9930	9.1402	9.6023	8.8632	8.3594	8.7061	8.8283	8.8531
Snca	_EGVLYVGSKT[Phospho (STY)]K_.2	O55042_T44_M1_Snca	7.1393	3.2287	3.8096	7.0628	6.7473	4.3359	3.4292	4.4745	3.4883	3.4796	3.8627	3.1181	3.5964	3.1402	3.0064	7.2351	3.5113	3.8610	6.6512	4.3183
Snca	_TKEGVVHGVTTVAEK_.3	#N/A	9.8367	10.0061	6.8747	6.7360	8.3819	10.0834	9.8104	10.2910	6.6942	6.4320	10.0378	9.8945	10.6769	9.9964	7.9978	7.3655	10.1898	10.0352	7.6205	6.8140
Snca	_TKEQVTNVGGAVVTGVTAVAQK_.3	#N/A	9.5105	9.0846	8.7514	9.1897	9.0698	8.8841	8.6317	9.5324	9.0944	9.4038	9.1858	9.3829	10.1392	9.6541	8.9385	9.1305	9.3721	8.7746	9.4073	9.5483
Snca	_TVEGAGNIAAATGFVKK_.2	#N/A	5.8527	5.3690	5.3210	5.8236	5.3657	5.2164	5.7091	5.9320	5.5717	5.7134	5.0936	5.8131	5.9418	6.1532	6.2026	6.0938	5.6659	5.3979	5.9811	6.0077
Snca	_EGVVHGVTTVAEK_.2	#N/A	6.4074	6.9095	7.1067	7.2364	6.4763	6.9791	6.5206	6.7644	6.9928	6.7903	6.7313	6.9952	6.8249	7.3788	6.9664	6.5222	6.8324	6.6855	6.5659	6.8709
Tpmt	_[Acetyl (Protein N-term)]S[Phospho (STY)]LDM[Oxidation (M)]KEHPDAEVQK_.2	#N/A	5.1686	5.4778	5.7063	4.6989	4.9254	4.6658	4.7284	5.6738	4.6716	5.0172	4.3313	4.2772	5.0290	5.2629	4.4847	4.9196	5.0238	4.8673	4.9169	4.3864
Akap7	_AVQQYLEETQNK_.2	#N/A	7.5314	7.9417	5.7412	7.2260	7.3310	7.8342	7.4999	7.7856	7.0309	7.1478	7.3562	7.6223	7.8409	7.4583	7.0657	7.7111	8.0017	7.7142	7.6420	7.6593
Impact	_TEEVEVES[Phospho (STY)]EEDPILEHPPENPVK_.3	#N/A	4.4738	9.8642	8.9048	3.1105	3.1799	3.2132	9.4210	3.1986	9.0927	9.1324	3.2671	4.0723	3.9539	10.3709	3.8936	8.8851	8.4298	9.6682	9.1535	8.7709
Impact	_KKTEEVEVES[Phospho (STY)]EEDPILEHPPENPVK_.4	#N/A	3.7762	10.0223	9.0211	8.6901	3.1139	3.3380	9.8001	3.2882	2.9854	3.9825	3.3598	8.7395	9.4915	10.5446	8.6964	4.1211	4.2863	9.8680	8.7507	8.0388
Impact	_KTEEVEVES[Phospho (STY)]EEDPILEHPPENPVK_.3	#N/A	4.3370	3.6660	3.3724	3.3526	3.6747	4.1048	5.8960	4.0373	4.0794	4.2353	4.0338	3.5554	4.2190	3.3595	3.1704	3.1810	4.0047	3.3290	4.4077	3.3053
Syngr1	_AGGAFDPYTLVR_.2	#N/A	9.1245	9.0176	9.0622	8.8497	8.9051	8.6929	9.1412	9.0928	8.6548	8.8294	9.0002	8.6502	8.5383	8.5572	8.1526	8.2691	9.0560	8.7856	8.5402	8.6600
Strn	_LPEQELSR_.2	#N/A	5.0057	5.1098	3.5133	5.4937	5.9292	5.6170	5.4316	5.9310	5.2589	4.9495	5.8572	6.1103	4.9629	5.5724	6.4100	5.9825	5.0061	5.8117	5.3519	5.4707
Strn	_FLESAAADVS[Phospho (STY)]DEDEDEDTDGR_.2	O55106_S245_M1_Strn	4.3619	7.7131	6.9225	6.1143	3.0680	7.4399	7.1810	3.6077	6.8859	4.0258	3.4260	5.5376	4.3902	9.0250	6.7504	5.9774	4.3405	8.2173	6.8314	7.2010
Strn	_EALKEFDFLVTSEEGDNESR_.3	#N/A	4.7269	4.4712	4.3723	4.8689	3.9456	4.1077	4.1283	4.3636	4.1331	3.9770	4.7470	4.6881	4.2458	4.6588	3.7691	4.0911	4.3751	4.5380	4.4237	4.5968
Strn	_FYDNNTGK_.2	#N/A	3.7650	3.3609	3.6774	4.5483	3.9719	4.0053	3.0762	4.3423	2.9964	3.5756	3.9759	4.7965	3.1618	3.6645	3.1016	3.1271	3.3792	4.4540	4.1026	3.6315
Strn	_VISHPTLPISITAHEDR_.3	#N/A	3.8932	4.3735	3.9830	3.1378	3.2309	3.4550	4.3102	3.1713	4.3693	4.0995	3.2428	4.0996	4.4773	4.0211	5.1631	4.2381	4.1694	2.9801	3.2791	3.6984
Nipsnap1	_AGPNIYELR_.2	#N/A	8.8052	8.5605	8.5819	8.5045	8.6345	8.5702	8.6602	8.5393	8.5077	8.6357	8.5311	8.7888	8.7317	8.5466	8.4877	8.1195	8.8623	8.6066	8.5872	8.3522
Nipsnap1	_GWDENVYYTVPLVR_.2	#N/A	7.9936	8.0802	8.2031	8.2028	7.8144	7.9322	8.0402	7.9585	7.8159	8.0229	7.9818	7.9074	7.9191	7.9557	8.0374	8.3041	8.2172	8.0085	8.0750	7.9575
Nipsnap1	_LKPGTM[Oxidation (M)]IEWGNNWAR_.3	#N/A	6.8817	7.5447	6.4259	6.9318	7.4877	7.4817	6.7897	7.4089	6.7941	6.8395	6.3138	7.5448	7.4310	6.1442	7.5147	7.6679	6.9975	7.2577	7.2068	6.9222
Nipsnap1	_KETSNLYK_.2	#N/A	4.3394	4.4688	3.1996	5.2262	4.8673	5.3213	5.1708	3.8645	4.7808	5.2886	4.5844	4.4853	4.2063	5.4875	4.6758	5.5531	4.6486	4.6761	5.4916	5.2184
Nipsnap1	_DSEGSWFR_.2	#N/A	5.5488	4.4365	6.0568	5.3683	5.5226	5.5458	5.3939	4.5308	5.6564	5.3402	5.9888	5.1365	3.5870	5.7991	6.1297	3.9112	3.8043	5.8900	5.1379	5.6488
Nipsnap1	_IQFHNVKPEC[Carbamidomethyl (C)]LDAYNSLTEAVLPK_.4	#N/A	4.0164	3.3027	3.5530	3.5678	4.2233	4.2567	4.3256	4.2421	4.2458	3.8270	3.2907	3.0288	4.4113	3.4131	2.8501	3.3785	3.7393	3.2046	4.3540	4.5574
Nipsnap2	_PGPNIYELR_.2	#N/A	8.2385	7.7993	7.6563	7.7021	7.8640	7.7556	7.9639	7.9058	7.7030	7.8326	8.0624	8.2148	7.9658	7.8823	7.8530	7.7313	7.9510	8.0635	7.9332	7.8278
Nipsnap2	_LKENQEFVNFR_.3	#N/A	5.3793	4.7976	3.9544	5.8392	5.9331	5.2261	5.2229	4.8345	5.8312	5.5758	6.1384	4.7559	6.0687	5.5154	5.8371	5.8768	4.8750	5.6643	5.2321	5.7767
Nipsnap2	_IC[Carbamidomethyl (C)]QEVLPK_.2	#N/A	5.1881	5.5794	6.2786	5.5448	5.9047	6.1362	5.4323	5.4873	5.8860	5.9988	5.3067	5.4460	5.2539	5.6543	6.2254	5.8742	4.5878	5.4449	5.6662	6.3192
Nipsnap2	_KETSSLYK_.2	#N/A	4.3121	5.2948	5.5302	5.2809	4.7110	5.4339	5.2218	4.9036	5.1431	5.6177	5.5914	5.1185	4.7511	5.4446	5.6086	3.2555	3.2508	5.2267	5.3223	5.3158
Nipsnap2	_LQFHNVKPEC[Carbamidomethyl (C)]LDAYNK_.3	#N/A	4.8991	5.1807	3.6570	4.5687	5.3504	5.4838	5.0136	5.6025	5.1026	4.8196	5.1616	6.1719	5.7011	3.6441	5.5908	4.9261	5.7020	5.6132	5.7616	5.3985
Nipsnap2	_SDMLLSR_.2	#N/A	3.1742	3.3277	3.7105	4.5151	4.0128	3.9721	3.1093	4.3755	3.0295	3.5425	4.0090	2.0105	3.1950	3.6976	3.1347	3.5191	3.4123	4.4209	4.0694	3.6646
Septin7	_ILEQQNSSRT[Phospho (STY)]LEK_.2	#N/A	8.6873	8.7648	8.6370	9.4514	8.3739	8.2069	8.2503	8.5223	8.1250	9.1286	8.7109	8.6421	8.8287	9.1454	7.3614	8.6201	8.6451	8.2603	8.6611	8.9047
Septin7	_LKDSEAELQR_.2	#N/A	8.9246	9.0666	9.4189	8.9950	8.9875	8.9946	9.2335	8.9058	9.2900	9.3543	9.1539	9.1381	9.0190	9.2535	9.1603	9.1546	9.1181	9.0154	9.2093	9.2216
Septin7	_FEDYLNAESR_.2	#N/A	8.8559	8.5101	9.1526	8.8014	8.6693	8.6404	8.5016	8.7271	8.9170	8.8443	8.8254	8.2076	8.2858	8.8925	8.8209	8.6512	8.7734	8.5102	8.5479	8.5226
Septin7	_NLEGYVGFANLPNQVYR_.2	#N/A	8.8692	8.6657	8.6134	8.7580	8.5318	8.4205	8.5418	8.6198	8.3898	8.6709	8.6147	8.4454	8.5413	8.8411	8.4820	8.8110	8.5105	8.4808	8.7244	8.5850

Septin7	_ANWEAQQR_.2	#N/A	8.1412	8.0362	8.8686	8.5322	8.2795	8.3904	8.5096	8.3757	8.5094	8.4863	8.2541	8.1866	8.3046	8.4701	8.6089	8.3643	8.3296	8.2220	8.3884	8.3749
Septin7	_ADTLTPEEC[Carbamidomethyl (C)]QQFK_.2	#N/A	7.9139	7.7415	8.4146	7.9844	7.6304	7.8437	7.8888	8.0738	7.8185	7.9086	8.1547	7.6706	7.4893	7.8295	7.9637	7.7853	7.9162	7.8148	7.5345	7.6784
Septin7	_ADTLTPEEC[Carbamidomethyl (C)]QQFKK_.2	#N/A	7.6756	7.3584	7.4049	7.8684	8.0386	7.4671	7.1378	7.6354	7.5418	7.4896	7.2978	7.5008	7.6897	7.2671	7.7680	7.7106	7.6387	7.1201	7.1249	7.7300
Septin7	_LAAVTYNGVDNNK_.2	#N/A	6.8666	6.8613	7.0403	6.9446	6.5968	7.3239	6.3169	7.0497	6.8794	7.0760	6.8236	6.7602	7.1646	7.0013	6.5056	6.5827	7.1155	6.3976	7.0297	6.9873
Septin7	_STLINSLFLTDLYSPEYPGPSHR_.3	#N/A	5.2097	6.7182	5.7179	5.9418	5.3797	5.7803	6.4216	5.1683	5.4093	5.4592	4.7643	6.3777	6.5058	7.7636	6.1616	5.5475	5.4188	6.3818	5.4722	5.9695
Septin7	_IYEFPETDDEEENKLVK_.3	#N/A	6.4888	5.7429	5.9453	5.5091	5.9733	5.5220	5.8603	6.3725	5.4759	6.1452	6.0672	5.8878	6.4504	6.4760	6.8193	6.2794	5.8744	5.7318	5.8561	6.2718
Septin7	_DVTNNVHYENYR_.2	#N/A	7.0780	7.0525	7.5559	7.1836	6.8736	7.1701	7.1228	7.0175	7.3196	7.0378	6.9587	6.8458	7.0964	7.1471	7.1746	7.1388	6.8072	6.7117	7.2633	7.2639
Septin7	_GQLTKS[Phospho (STY)]PLAQM[Oxidation (M)]EEERR_.3	#N/A	6.8419	6.8590	3.4666	6.3547	6.3153	6.2040	6.3705	6.6744	6.2848	6.5057	5.9800	7.0594	7.8003	3.4755	5.9379	6.6120	6.3082	6.8203	6.6024	6.5031
Septin7	_IYEFPETDDEEENK_.2	#N/A	6.5718	6.4728	7.2134	7.3230	3.8738	6.3902	6.4078	5.7559	6.5638	7.3007	6.6312	6.1021	4.1389	9.0034	6.4056	6.0074	6.7226	5.9583	6.2885	6.7310
Septin7	_S[Phospho (STY)]PLAQM[Oxidation (M)]EEER_.2	#N/A	8.6231	8.4628	9.1281	9.0451	8.2868	8.5759	7.7164	7.7577	8.1013	8.6323	9.0116	7.9937	7.5165	8.8223	7.6699	8.1179	8.1200	7.9747	8.4109	8.1246
Septin7	_NLEAQHKELEEK_.2	#N/A	4.4640	3.9133	4.7478	4.9839	4.1435	5.0201	5.3523	5.1750	4.6172	5.7839	4.7883	5.4262	5.1037	4.2905	4.2669	4.9863	4.1070	4.7908	4.0019	5.3939
Septin7	_KTVQVEQSK_.2	#N/A	4.0708	2.5680	4.5990	4.0226	2.8575	4.2140	3.2796	2.4274	2.9972	4.1059	3.1994	1.9263	3.5925	2.5508	3.6332	3.7683	3.2845	3.6990	3.5123	3.6946
Septin7	_THMQDLK_.2	#N/A	4.4781	4.3778	5.4480	5.3389	3.8158	4.1443	4.9944	4.1784	4.2930	3.1835	4.1438	3.3370	3.8924	5.4026	4.7548	3.3221	3.2153	3.1170	4.2665	3.9014
Septin7	_S[Phospho (STY)]PLAQM[Oxidation (M)]EEERR_.2	#N/A	10.3006	10.5819	10.6760	10.7751	10.0723	10.3068	10.1414	9.9055	9.9560	10.4380	10.5858	10.0524	9.7924	11.1053	9.5364	9.8527	10.3507	10.0369	10.0766	10.2073
Septin7	_ILEQQNSSR_.2	#N/A	7.2060	7.0686	7.2258	7.2254	6.9801	6.9808	7.1413	7.1722	7.2296	7.0921	6.8522	7.1607	7.1715	7.2769	7.3877	7.2754	6.9748	7.0613	6.9079	6.8185
Septin7	_QFEEKANWEAQQR_.2	#N/A	3.9263	3.7584	3.1920	3.7434	3.6910	3.9986	3.8146	3.8691	3.1228	3.9492	3.5541	3.5741	3.9817	3.2049	3.3918	3.0091	3.9716	3.5237	3.6584	4.0097
Acot1	_YRADS[Phospho (STY)]HGELDLAR_.3	O55137_S56_M1_Acot1	5.8435	3.7099	3.3284	6.7110	3.6307	4.3543	5.7623	3.9933	4.1482	5.8974	3.6268	3.5993	4.3136	3.3155	4.2534	3.1370	5.9900	3.3022	2.9508	3.2825
Acot1	_QLGSEC[Carbamidomethyl (C)]LHVS[Phospho (STY)]PK_.2	#N/A	5.6739	5.2562	4.6572	5.3255	4.6823	5.4890	4.0213	5.0314	4.7770	4.8408	4.9308	4.6228	3.8435	5.1171	4.1163	4.9740	4.9148	4.6630	5.1015	4.5514
Rpl35a	_IEGVYAR_.2	#N/A	4.8324	3.3551	3.6832	5.1992	4.3345	5.3067	5.4596	4.3481	5.6095	5.5776	5.1865	5.2620	4.3490	5.2832	4.4669	4.9514	4.1729	5.8393	5.0164	5.2506
Rpl35a	_NNTVTPGGKPNK_.2	#N/A	5.2762	5.6737	6.1796	5.3685	5.0920	5.5409	5.4989	5.7844	5.9398	5.4779	5.2920	5.7875	5.1297	5.3563	5.9843	5.3127	5.6597	5.3383	5.0311	5.0633
Atp2a2	_EFDELSPS[Phospho (STY)]AQR_.2	O55143_S663_M1_Atp2a2	12.5000	12.1801	12.0261	12.1390	11.9833	12.0508	11.9991	12.4870	12.1501	11.9653	12.2176	12.0924	12.6725	11.9788	11.1335	11.8866	12.7909	12.2053	12.1555	11.9503
Atp2a2	_WGS[Phospho (STY)]NELPAEEGK_.2	O55143_S38_M1_Atp2a2	8.4219	8.2037	8.3624	7.8242	7.5210	7.9344	7.8373	8.3150	7.9870	7.7649	8.2114	7.8609	8.1931	7.8634	7.0975	7.9228	8.5805	7.9188	8.0677	7.9907
Atp2a2	_VGEATETALTC[Carbamidomethyl (C)]LVEK_.2	#N/A	6.7928	6.8437	7.2899	6.6142	6.3792	6.9239	6.5406	6.8848	6.6388	6.3568	6.7258	6.0947	6.7929	5.9400	6.4280	6.2426	6.8104	6.5504	6.9669	6.1568
Atp2a2	_VDQSILTGESVSVIK_.2	#N/A	6.5133	6.3388	6.6959	6.7343	5.4402	5.4906	5.8064	6.5128	6.3466	5.3040	6.5091	6.0510	5.6249	6.1612	5.1997	5.6927	5.9363	6.1398	6.0033	6.0437
Atp2a2	_NM[Oxidation (M)]LFSGTNIAAGK_.2	#N/A	6.5081	6.6606	5.1320	4.7892	5.9719	5.9461	5.6530	6.2060	6.2072	5.9677	6.0655	6.4199	6.7073	4.3279	4.6075	5.9814	5.5914	6.5832	6.7074	6.1201
Atp2a2	_EWGS[Phospho (STY)]GSDTLR_.2	O55143_S553_M1_Atp2a2	7.6123	8.0197	7.2931	3.4456	6.3495	4.4034	7.1646	3.9442	6.7436	3.9737	3.5777	3.6484	4.2645	3.2664	4.2043	6.9221	4.4819	3.3513	2.9999	7.5230
Atp2a2	_VIM[Oxidation (M)]ITGDNK_.2	#N/A	5.2459	5.6785	5.2773	4.8881	5.6583	6.0353	5.6839	5.4630	5.4496	5.8445	5.9268	5.3303	5.9377	5.1448	4.7575	6.0719	5.8597	5.7959	5.6989	5.2678
Atp2a2	_SLPSVETLGC[Carbamidomethyl (C)]TSVIC[Carbamidomethyl (C)]SDK_.2	#N/A	6.8368	6.2864	7.5791	6.6900	6.2624	6.4341	6.3338	6.4868	6.8765	6.3781	6.5307	6.6515	3.8955	6.2502	6.1428	6.2980	7.0697	6.1799	6.5927	6.3255
Atp2a2	_ANAC[Carbamidomethyl (C)]NSVIK_.2	#N/A	6.0092	6.4066	5.8734	5.9020	6.3989	6.3639	6.9693	6.1269	6.7700	6.0237	6.0398	6.3691	5.6882	3.4673	6.2009	5.9336	6.1931	6.9399	6.5861	6.6594
Atp2a2	_IGIFGQDEDVTSK_.2	#N/A	6.9470	6.2633	6.4257	5.9978	5.7786	6.3697	6.5839	6.1006	6.1623	6.0480	6.3757	5.7876	3.1954	6.4305	4.7290	5.3358	6.7974	6.6416	6.0584	6.1222
Atp2a2	_DIVPGDIVEIAVGDKVPADIR_.3	#N/A	4.7459	5.5792	5.0453	4.8471	4.1977	5.6547	5.7131	3.0595	6.0616	4.3027	5.9060	5.6179	5.6636	4.4760	4.8582	5.7330	6.0340	5.2923	4.7017	5.0959
Atp2a2	_IEVASSVK_.2	#N/A	3.7061	3.8654	3.3873	4.5045	3.2866	4.6488	5.1430	4.0761	4.8497	3.6912	4.9213	4.0743	3.8471	4.3898	4.0566	4.1124	4.3034	4.5611	4.4170	4.4012
Atp2a2	_MNVFDTELK_.2	#N/A	3.8670	4.1202	3.5820	4.4227	3.1581	4.1185	5.0988	3.5674	4.5249	2.9907	3.8591	3.6643	3.8877	4.8579	3.7575	4.2119	4.1761	3.3092	3.3767	4.3573
Atp2a2	_NYLEQPGKEC[Carbamidomethyl (C)]VQPATK_.3	#N/A	4.7859	5.0529	3.6229	4.3642	4.0585	4.2172	5.1798	5.0655	4.7508	4.3779	5.2111	5.0920	3.9994	5.0289	4.7112	4.7927	4.6921	5.4724	4.8570	4.1107
Atp2a2	_GTAVAIC[Carbamidomethyl (C)]R_.2	#N/A	4.4988	4.4117	3.6553	4.6259	3.9677	4.0950	4.8682	4.3444	4.3347	3.7443	2.9151	4.0027	4.0179	3.6934	3.5597	4.1235	3.3219	4.1685	4.0674	3.9311
Atp2a2	_TASEM[Oxidation (M)]VLADDNFSTIVAAVEEGR_.3	#N/A	6.4204	5.9116	6.2229	5.9487	5.4475	5.5540	5.0475	5.7492	5.5584	5.6414	5.5345	5.5671	6.3412	5.9564	4.5627	6.0977	5.5508	5.5534	5.7953	5.6298
Atp2a2	_TGTLTTNQMSVC[Carbamidomethyl (C)]R_.2	#N/A	4.1844	7.5159	7.4208	7.5321	7.0727	7.5710	6.0427	7.8590	7.2168	7.6777	3.2709	7.6895	8.3535	8.2360	7.3537	7.6358	7.8617	7.5594	7.4270	7.6973
Atp2a2	_IVEFLQSFDEITAM[Oxidation (M)]TGDGVNDAPALK_.3	#N/A	3.5731	4.4297	3.7768	4.1163	2.9108	2.7721	3.1294	3.2735	3.1885	3.7794	3.5629	4.3191	3.2965	4.0965	4.2074	3.9180	3.8112	4.0220	3.6706	4.0635
Atp2a2	_IVEFLQSFDEITAM[Oxidation (M)]TGDGVNDAPALKK_.3	#N/A	4.2857	4.5342	3.8223	3.2985	2.9918	3.0251	4.0563	4.5114	3.9765	4.0963	3.4552	4.2604	4.3166	3.8603	4.0817	3.6478	4.0086	3.1408	3.4399	3.5376
Atp2a2	_YETNLTFVGC[Carbamidomethyl (C)]VGM[Oxidation (M)]LDPPR_.2	#N/A	3.2924	4.3891	4.1500	4.0757	3.4994	5.5194	3.5487	3.3140	3.4689	3.1030	2.9476	4.2786	3.6344	4.1370	3.5741	4.1554	3.8517	3.9814	3.6300	4.1040
Atp2a2	_IRDEMVATEQER_.3	#N/A	4.2333	3.7695	3.2687	4.1557	3.5710	3.7951	3.9701	3.9336	4.0292	4.4396	2.9027	3.6590	4.1372	3.2558	3.5473	3.0773	4.4713	3.3618	3.0104	3.2228
Pja1	_SRS[Phospho (STY)]PFASTR_.2	O55176_S32_M1_Pja1	3.2767	7.5426	4.3141	7.0121	3.4836	7.1910	7.1776	3.5024	6.5757	7.6781	6.9584	7.1567	6.7995	7.1402	3.5899	4.1396	7.0778	6.9832	7.0058	6.5688
Pja1	_EQPQSSS[Phospho (STY)]GESWELLPGKEELER_.3	O55176_S341_M1_Pja1	3.6854	3.6337	3.2219	3.8989	3.8923	4.2161	3.5491	3.9110	3.6082	3.3597	3.9826	3.3598	3.7162	3.2600	3.1263	3.0475	3.4083	3.7412	4.0402	4.4381
Pja1	_FTSVNHDAKEEC[Carbamidomethyl (C)]GKVES[Phospho (STY)]PPAAR_.4	O55176_S137_M1_Pja1	4.5409	7.6039	4.0775	8.1701	6.1115	3.3605	6.6648	6.6271	4.4637	4.0051	3.3372	6.6536	4.5718	7.7502	7.0531	7.3170	6.5079	7.0890	5.4684	7.1253
Pja1	_VFFDT[Phospho (STY)]DDDDDVPHSTSR_.2	#N/A	3.8274	7.6768	5.7297	3.5045	3.6497	7.3844	7.1867	3.6522	6.4427	3.7320	3.7237	3.6901	4.1915	8.3740	5.9971	3.7792	3.8836	7.4085	6.8521	6.7654
Pja1	_YYEEDS[Phospho (STY)]DSDKEWM[Oxidation (M)]AALR_.3	O55176_S317_M1_Pja1	4.4162	3.5866	3.4517	3.2732	3.7540	3.9780	3.7871	4.1166	3.8462	3.1217	4.2206	3.4760	3.9543	3.4388	3.3643	3.2603	3.1534	3.1789	4.3283	3.1753
Pja1	_GNNS[Phospho (STY)]LER_.2	O55176_S175_M1_Pja1	3.7652	4.2376	4.3015	3.9242	3.1029	3.3812	4.4382	3.4656	3.6205	3.9715	3.0991	4.1271	3.7859	4.2886	4.0154	4.3172	4.0032	3.8299	3.4785	4.2556
Psmb5	_GPGLYYVDSEGNR_.2	#N/A	7.7117	7.6665	7.8301	7.9058	7.6242	7.8950	7.6119	8.0901	7.8524	7.9146	7.7982	7.9051	8.1633	7.6265	7.0185	7.6972	8.0024	7.4009	7.9094	7.7982
Psmb5	_RGPGLYYVDSEGNR_.3	#N/A	6.3572	7.0378	7.1783	6.9942	7.0157	7.2643	6.6110	6.8114	7.1538	7.1715	7.3648	7.0203	7.7283	6.7316	5.9688	7.1787	6.9554	7.3335	7.3145	6.8857
Psmb5	_AIYQATYR_.2	#N/A	5.6247	5.4541	6.0611	5.7706	5.8853	5.5726	5.5167	3.5531	5.8529	5.5182	5.4262	5.2835	3.3583	4.9265	5.7223	6.0243	6.1289	5.8124	5.8806	5.9004
Psmb5	_ATAGAYIASQTVK_.2	#N/A	4.8185	5.8208	5.2047	5.7324	5.2713	5.0691	5.1649	5.4097	5.4740	4.8472	5.0267	5.2941	3.5113	5.5985	5.1648	4.8392	4.7892	5.3988	5.5869	5.6091
Psmb5	_VEEAYDLAR_.2	#N/A	2.6626	5.1644	5.8134	5.0850	4.7765	4.6766	5.5237	3.4535	5.3626	5.2287	5.0262	4.5661	3.7738	4.2563	5.2837	5.4648	3.9911	5.4601	5.2751	4.4456
Psmb5	_DAYSGGAVNLYHVR_.2	#N/A	4.7963	3.9864	5.0412	4.2695	4.5422	4.0010	1.9544	3.5584	4.4628	3.9795	4.0557	3.7125	3.3636	4.4082	3.3330	4.2504	3.6681	4.0938	4.3929	4.7977
Psmb5	_FLHGVIVAADSR_.3	#N/A	3.3243	3.9948	4.3617	4.1076	3.5312	3.5646	3.5169	3.2822	3.4371	3.1349	2.9157	3.7209	3.6025	4.1052	3.5422	4.1873	3.8198	4.0133	3.5285	3.8137
Dhx9	_AEENNSGVESSGYGS[Phospho (STY)]PGPTWDR_.2	O70133_S136_M1_Dhx9	3.1998	3.3022	3.7361	5.7998	5.9699	4.2624	6.3156	4.4010	3.5618	3.4061	3.9362	3.1916	3.2205	3.7231	3.0799	3.5447	3.4378	6.6590	4.0439	3.6901
Dhx9	_DFVNYLVR_.2	#N/A	3.1012	4.6382	3.6375	4.0421	3.9398	3.6686	3.9710	4.3025	3.6604	4.0737	4.4557	3.2902	3.7684	3.6470	3.1785	3.4461	3.3393	4.1617	4.1425	3.5916
Dhx9	_YTQVGPDHNR_.2	#N/A	3.5438	3.1397	4.7491	4.3271	3.7507	3.7841	5.9589	3.0627	5.1862	3.3544	4.9401	5.4358	3.3830	3.8857	3.3227	4.4067	3.6004	5.4947	4.7582	4.6464
Tesk1	_S[Phospho (STY)]LPSSPELPR_.2	O70146_S439_M1_Tesk1	4.0769	3.6727	3.3655	3.3593	4.2838	4.3171	7.0733	4.0305	4.1854	3.8875	3.6640	2.9683	4.3508	3.3526	2.7897	3.4390	3.0673	7.4068	7.5305	7.6419
Pip5k1c	_SSSLKSS[Phospho (STY)]PSK_.2	O70161_S453_M1_Pip5k1c	6.5558	5.2774	5.4667	3.6944	5.5283	5.3068	6.3047	5.7521	5.0823	6.0153	3.4548	3.6124	6.4160	6.0665	3.4793	5.5654	6.3031	5.9601	5.5249	3.9231
Pip5k1c	_SPS[Phospho (STY)]DTSEQPR_.2	O70161_S540_M1_Pip5k1c	5.7821	3.2912	7.0987	6.5121	4.2349	4.2682	5.4717	4.2536	5.4929	6.4908	6.5128	5.8904	4.3997	6.0671	4.8945	3.3900	3.7508	5.7830	6.1014	6.1017
Pip5k1c	_ITVQVEPVC[Carbamidomethyl (C)]GVGVVPK_.2	#N/A	5.6932	5.1283	3.5605	5.2696	4.7474	4.5267	5.1244	5.8072	5.6607	5.7008	5.7214	5.4115	5.3525	6.0370	5.3275	5.8536	5.2836	5.4992	5.2751	5.1494
Pip5k1c	_RTQS[Phospho (STY)]SGQDGRPQEEPHAEDLQK_.4	#N/A	3.7029	8.2885	8.3303	8.3932	3.5562	3.7715	8.1478	3.6999	3.2728	8.8219	7.7702	3.8455	8.4066	8.2826	7.3536	8.2335	3.6962	3.8159	7.8754	3.6799
Pip5k1c	_SSS[Phospho (STY)]LKSS[Phospho (STY)]PSKK_.2	#N/A	4.4966	4.3234	4.0331	4.5885	3.2027	3.4049	4.3603	3.2214	4.4194	4.7416	3.2929	4.0495	4.5274	4.0712	3.8708	3.8587	4.2195	3.3805	3.2290	3.7485
Pip5k1c	_TQS[Phospho (STY)]SGQDGRPQEEPHAEDLQK_.3	#N/A	3.6389	7.7279	4.5927	6.7219	3.7968	6.4968	7.0005	4.7153	6.0351	7.8103	6.8720	6.3433	7.8189	7.2244	6.1121	7.0290	6.5148	7.1151	7.0848	6.4238
Pip5k1c	_DC[Carbamidomethyl (C)]LVLESFK_.2	#N/A	3.0992	3.4028	4.1497	4.1571	3.9377	4.0472	3.0342	4.3004	2.6392	3.6175	4.8661	5.0658	3.1199	3.6225	3.1806	3.4441	3.3372	3.7898	4.3623	4.5409
Pip5k1c	_IM[Oxidation (M)]DYSLLLGVHNIDQQER_.3	#N/A	4.2033	4.6167	3.7398	3.3810	2.9094	4.4435	4.1387	4.4289	4.0589	4.9845	3.5376	4.3428	4.2244	3.7779	4.1641	3.5654	3.9262	3.2233	3.5223	3.4552
Pip5k1c	_SWVYSPLHYSARPASDGESDT_.3	#N/A	3.5273	4.0693	3.6181	4.5442	4.0060	3.7860	3.2475	4.4005	3.5097	4.2389	4.1986	3.2130	3.3846	5.8005	3.9739	4.1981	4.4986	4.2348	4.5597	4.2046
Stmn3	_DAS[Phospho (STY)]LEELQKR_.2	#N/A	12.2382	12.3499	11.8423	12.1436	11.6469	11.9733	11.8623	12.2692	11.9195	12.0427	11.8231	12.0889	12.6402	12.6030	11.5431	11.9130	12.4436	12.1596	11.9872	12.0412
Stmn3	_DAS[Phospho (STY)]LEELQK_.2	#N/A	9.2428	9.9412	8.9237	8.9988	8.1275	9.4072	9.7321	9.6479	8.7551	8.9033	8.8809	9.7961	10.1131	10.1362	8.9966	8.6059	8.9539	9.7417	8.6876	8.7363
Stmn3	_ALEENNNFSR_.2	#N/A	6.6961	6.6567	6.2243	6.6624	6.0472	6.7769	6.3556	6.5207	6.2851	6.9718	6.5932	6.4563	7.2648	6.3658	6.3954	6.6343	6.4984	6.1622	6.6523	6.4175
Stmn3	_KDAS[Phospho (STY)]LEELQK_.2	#N/A	5.5384	3.4876	4.3492	5.4349	4.5072	4.6835	4.5682	5.3639	4.0611	4.9632	5.3107	4.6141	4.7316	5.2281	4.1946	5.0233	4.9865	5.1246	4.3297	4.7209
Stmn3	_SPSDLSPES[Phospho (STY)]PVLSSPPK_.2	O70166_S68_M1_Stmn3	9.3405	8.7660	8.5559	8.3275	8.1685	8.7986	8.7776	8.7593	8.7119	8.1613	8.4925	9.1364	9.7957	9.3849	8.8363	9.1357	9.2127	8.7855	8.7210	9.1135
Stmn3	_ASGQS[Phospho (STY)]FEVILK_.2	O70166_S53_M1_Stmn3	3.9238	4.7282	4.4647	3.7111	3.8730	3.9538	3.9651	4.1621	4.3783	3.8903	3.6767	3.4526	3.9822	5.2772	3.3037	4.3044	4.2118	5.4417	3.8849	4.4942
Stmn3	_RAS[Phospho (STY)]GQS[Phospho (STY)]FEVILK_.2	#N/A	3.5346	3.8215	4.0709	4.2050	3.6762	4.0919	4.0220	3.6627	3.5795	4.0256	3.5574	3.7109	3.7176	4.7486	3.5992	3.8795	3.7113	4.7803	3.8329	4.3115
Stmn3	_RKDAS[Phospho (STY)]LEELQK_.2	#N/A	3.3801	4.8890	4.3792	4.6249	4.4393	4.3941	4.4182	4.7718	3.2160	3.8977	4.3109	4.6030	4.6856	4.4939	4.4759	3.8919	4.3537	5.0308	3.3416	4.1193
Stmn3	_KDAS[Phospho (STY)]LEELQKR_.3	#N/A	3.2072	4.1119	4.2446	4.3771	3.4141	3.4474	3.3442	3.4329	3.5542	3.8123	4.0210	3.8380	3.4640	4.2827	3.6594	4.0701	4.4309	2.9892	3.4072	4.1782
Stmn3	_NKEQREEMS[Phospho (STY)]G_.2	O70166_S179_M1_Stmn3	4.3595	4.4604	3.8961	3.2247	3.0656	3.5419	3.9825	3.0844	3.9027	4.1701	3.3813	4.1865	4.3904	3.9342	4.0079	3.7216	4.0824	3.0670	3.3661	3.6114
Pip4k2a	_IYIDDNNKK_.2	#N/A	7.0864	6.6704	6.4727	6.9041	7.2703	6.8750	6.6362	7.2074	6.0238	6.8489	6.5760	6.4527	6.4374	7.0826	6.4857	6.5602	6.9170	6.9888	6.4560	6.8363
Pip4k2a	_DVEFLAQLK_.2	#N/A	3.9590	3.3601	4.5754	3.2415	4.1660	4.3212	4.4813	3.9283	4.3032	4.4813	3.9282	3.0862	4.4686	3.4704	4.1164	4.1757	3.1851	4.4546	4.6006	4.9279
Pip4k2a	_FGIDDQDFQNSLTR_.2	#N/A	3.2155	5.7151	4.2529	4.3688	3.4224	5.1660	5.2882	3.4411	4.8460	4.5269	3.0246	5.4596	3.7114	4.2909	5.0750	4.7406	5.4677	5.2802	5.2133	4.1810
Chrna4	Carbamidomethyl (C)]TC[Carbamidomethyl (C)]KEPS[Phospho (STY)]PVS[Phospho (STY)]PITVL	O70174_S540_M2_Chrna4	3.5196	4.4832	4.0559	4.1698	2.8573	3.6268	3.4547	3.2200	3.3749	3.1971	4.3544	4.3726	3.5403	4.0430	4.2609	3.8645	3.7577	4.0755	3.7241	4.0100
Eif3d	_IFHTVTTTDDPVIR_.3	#N/A	5.5898	4.7805	3.3672	4.9255	4.9607	5.2413	4.6465	6.1848	5.5667	5.2083	5.1152	4.7267	4.0388	3.3542	4.5229	3.1758	4.2207	4.4520	5.0438	4.7928
Eef1b	_TPAGLQVLNDYLADK_.2	#N/A	5.8503	4.8810	6.0129	6.0867	6.5268	6.2627	5.9178	6.4713	5.8852	6.4276	5.8797	6.0685	5.8737	6.2794	6.6386	6.2981	6.4242	6.2193	6.3628	6.2474
Eef1b	_SIQADGLVWGSSK_.2	#N/A	4.1574	4.2490	4.8799	3.4399	2.8635	5.0742	4.7339	4.8013	4.1048	5.3604	3.5834	5.2512	5.5796	4.2426	5.7317	4.7781	4.4876	4.0417	2.9942	3.2390
Eef1b	_DDDDIDLFGS[Phospho (STY)]DDEEESEEAKK_.3	#N/A	3.5477	6.4965	4.0840	4.1417	2.8854	6.8793	3.4827	3.2480	3.4029	3.1690	4.3824	4.3446	3.5684	9.5124	5.8975	3.8926	3.7857	8.0697	3.6960	4.0380
Eef1b	_YGPSS[Phospho (STY)]VEDTTGSGAADAK_.2	O70251_S83_M1_Eef1b	3.7371	3.2277	3.7228	4.0233	4.2079	4.1098	3.8323	4.3990	3.7525	3.6801	3.9078	3.0373	3.9179	3.7338	3.0335	5.3593	3.6701	5.8407	3.8104	3.5708
Eef1b	_YGPSSVEDTTGSGAADAKDDDDIDLFGS[Phospho (STY)]DDEEESEEAK_.4	#N/A	4.0826	3.2364	3.6192	3.5016	4.2896	3.8188	3.9464	4.3083	4.0055	4.4633	4.3798	2.9626	4.1135	3.6573	3.5236	3.1010	3.8056	3.3439	3.6429	3.3346
Eef1b	_DDDDIDLFGS[Phospho (STY)]DDEEESEEAK_.2	#N/A	3.6031	4.3997	4.1394	4.0863	2.9408	3.1649	3.0994	3.4614	3.1585	3.8094	3.5329	4.2892	3.2666	9.3351	4.1775	3.9480	4.4595	9.0104	2.9890	3.9885
Grk6	omethyl (C)]C[Carbamidomethyl (C)]GNC[Carbamidomethyl (C)]S[Phospho (STY)]DS[Phospho (	O70293_S566_M2_Grk6	3.3380	3.1640	3.8743	4.3514	4.1765	3.8084	3.3645	4.5392	3.1933	3.5443	4.1727	3.0534	3.3587	3.8614	2.9417	3.6829	3.5760	4.2571	3.9057	3.8283
Grk6	_DVLDIEQFS[Phospho (STY)]TVK_.2	O70293_S484_M1_Grk6	3.9108	4.3911	3.9654	3.1554	3.2485	3.4726	4.2926	3.1537	4.3517	4.1171	3.2252	4.1172	4.4597	4.0035	3.8698	4.2557	4.1518	2.9977	3.2967	3.6808
Atxn2	_DNS[Phospho (STY)]EEFLKR_.2	O70305_S400_M1_Atxn2	9.8875	9.9322	9.8171	9.7210	9.3315	9.4068	9.4135	9.5169	9.1852	10.1386	9.5986	9.4772	9.7870	10.2941	8.4958	9.5007	9.8444	9.5169	9.3838	9.7631
Atxn2	_VALENDDRS[Phospho (STY)]EEEKYTAVQR_.3	O70305_S435_M1_Atxn2	4.2863	4.7651	3.8229	4.0917	2.9924	3.0257	4.0557	4.5522	3.9759	4.0969	3.7895	4.2598	4.1414	3.1432	4.0811	4.3355	4.3587	4.7371	3.1230	3.1102
Atxn2	_ASETS[Phospho (STY)]PSFSK_.2	O70305_S741_M1_Atxn2	3.2751	4.0440	4.3125	6.2662	3.4820	3.1255	6.3477	5.5593	4.5465	6.6879	6.0586	3.7701	3.3060	4.3506	4.2169	4.1380	4.4988	5.4583	6.0610	4.0278
Atxn2	_QNS[Phospho (STY)]PAGSKENVK_.2	O70305_S727_M1_Atxn2	3.1066	5.4293	3.6429	5.0997	5.5391	4.1693	4.5340	5.1043	3.6550	3.3129	5.3861	3.2848	3.7630	4.4200	3.1731	3.4515	3.4551	3.6944	4.7759	2.9841
Atxn2	_TNS[Phospho (STY)]PSIS[Phospho (STY)]PSMLSNAEHKR_.3	#N/A	4.3651	3.6377	3.4006	3.3242	3.7029	3.6254	3.8382	4.0655	3.8973	4.5715	4.2717	3.5271	4.0053	3.3877	3.4154	3.2092	3.1024	3.2300	4.3794	3.3547
Atxn2	_TNS[Phospho (STY)]PSIS[Phospho (STY)]PSMLSNAEHK_.2	#N/A	3.8001	4.2774	3.7404	3.8206	3.7159	3.8188	4.1348	3.9946	3.7893	3.5382	3.9149	3.3740	3.9114	5.4330	3.4182	4.0657	3.7525	3.6912	3.7315	3.7080
Atxn2	_MSSEGPPRM[Oxidation (M)]S[Phospho (STY)]PK_.2	O70305_S593_M1_Atxn2	3.5017	4.6783	4.5391	4.8610	3.7086	3.7420	3.3395	3.7274	3.2597	3.3123	4.2392	3.5435	3.4251	4.5772	3.3649	4.3646	3.2246	4.1907	4.2239	3.8948
Atxn2	_DSFIDSSSSSSNC[Carbamidomethyl (C)]TSGSSK_.2	#N/A	3.3109	3.1911	3.8472	4.0563	4.1495	4.3735	3.3916	4.2622	3.4507	3.5172	3.8251	3.0805	3.5588	4.4209	2.9688	3.6558	3.2508	3.8986	4.1977	4.2806
Atxn2	_GM[Oxidation (M)]S[Phospho (STY)]PVVSEHRK_.3	O70305_S753_M1_Atxn2	3.7797	2.8829	4.3160	3.9097	3.1175	3.3415	2.7347	3.4801	2.9819	3.9861	3.3563	4.1125	4.5908	4.1345	4.0008	4.1246	4.0178	4.3675	3.1657	3.8118
Nmt1	_SGLS[Phospho (STY)]PANDTGAK_.2	#N/A	11.2343	11.0964	10.9241	11.2015	10.5971	10.4859	10.6028	10.9205	10.4573	10.9044	10.4469	10.6201	10.9565	11.0749	9.2975	10.5796	10.6986	10.6946	10.3395	10.5176
Nmt1	_SGLS[Phospho (STY)]PANDTGAKK_.2	#N/A	7.2602	7.1721	6.9722	3.3306	6.7202	3.6478	6.9052	7.1658	6.4010	7.2244	6.4042	6.5999	7.5438	6.6822	5.9926	3.6157	7.2228	3.1729	6.3754	6.7156
Nmt2	_GS[Phospho (STY)]PGGDLGAK_.2	O70311_S38_M1_Nmt2	7.3848	7.6021	7.7876	7.6567	7.0083	7.7376	7.6755	7.3744	7.1721	7.8307	7.7682	6.8675	6.7699	7.7706	7.1699	6.6364	7.4792	3.1206	7.2928	7.1620
Nmt2	_EKPNSGGTKS[Phospho (STY)]DS[Phospho (STY)]AS[Phospho (STY)]DSQEIK_.2	O70311_S66_M3_Nmt2	3.8693	4.7680	5.5357	5.1376	3.7672	3.5512	4.9092	5.3103	3.6214	5.0514	3.9958	3.7523	3.7295	3.9256	3.6213	3.3659	3.8344	5.2590	4.2550	3.7804
Epb41l2	_QRS[Phospho (STY)]YNLVVAK_.2	#N/A	4.0459	3.9569	8.0085	3.6435	3.3836	3.1005	6.5675	3.7462	6.5408	4.1717	6.8537	3.8464	4.0666	4.5692	3.7347	4.3908	4.2839	3.5492	3.1978	3.0354
Epb41l2	_[Acetyl (Protein N-term)]TTEVGS[Phospho (STY)]ASEVKK_.2	O70318_S7_M1_Epb41l2	6.7919	4.3025	6.2471	3.9891	5.8741	6.2735	3.0022	6.3268	3.5556	6.5147	5.6786	6.0861	3.7210	4.2237	4.0802	6.1311	5.8570	6.3571	6.2664	4.1907
Epb41l2	_LVS[Phospho (STY)]PEQPPK_.2	O70318_S492_M1_Epb41l2	6.4655	6.4834	6.5662	6.3187	6.2798	6.7920	5.9253	3.3591	5.8882	6.7559	6.3556	5.8055	3.7934	6.0728	3.7331	5.3327	6.5327	5.6193	5.8721	6.1837
Epb41l2	_EVQTSELKAEVAS[Phospho (STY)]QK_.2	#N/A	6.5516	5.9926	5.1595	6.0862	5.9300	6.1938	6.2151	5.5976	6.1538	5.5431	4.9804	6.2093	5.8704	5.7329	5.6850	6.4431	6.4298	6.0611	5.5353	5.8356
Epb41l2	_EM[Oxidation (M)]S[Phospho (STY)]PGHGPGETR_.2	#N/A	6.1467	5.5489	4.8432	6.5076	5.7533	5.8928	5.7269	5.9302	5.5490	5.6685	6.4730	5.5489	6.2349	6.9475	5.0813	5.8182	5.7868	5.3897	5.9234	5.4628
Epb41l2	_EPTQADVEDQILGKEES[Phospho (STY)]LPEEESR_.3	#N/A	3.2525	5.3353	4.9947	4.3317	3.4594	4.8410	4.4361	3.4782	3.5089	4.5639	2.9875	3.7927	3.6743	5.2116	3.6140	4.1154	4.4762	4.8030	3.5901	4.1440
Epb41l2	_S[Phospho (STY)]YNLVVAK_.2	#N/A	3.7900	3.3859	3.6524	4.5733	3.9969	4.0303	3.0512	4.3173	2.9714	3.6006	3.9509	3.2753	3.1368	3.6395	3.0765	3.1521	3.3542	2.9782	4.1276	3.6065
Epb41l2	_IVITGDAALDHDQALAQAIR_.3	#N/A	4.5229	3.5357	4.5360	5.4970	4.6547	4.3540	4.2589	4.0091	4.5135	4.1175	4.8323	3.2618	3.1434	3.3581	2.6138	3.1455	3.5063	3.6431	4.2274	3.6130
Epb41l2	_AKEVENEQTPVS[Phospho (STY)]EPEEEKGSQPGPPVER_.4	O70318_S38_M1_Epb41l2	3.6693	4.3335	4.2056	4.4148	3.0071	3.2311	3.0332	3.3951	3.0923	3.8757	3.4666	4.2229	3.2003	4.2449	4.1112	4.0142	4.3932	4.2571	3.0553	3.9222
Epb41l2	_GLSPAQADSQFLENAK_.2	#N/A	3.4573	4.2263	5.8763	6.2072	6.1516	6.3535	3.3299	6.4222	6.4536	3.4943	3.2740	6.4647	3.3400	7.0427	3.9116	6.0508	5.7977	4.1181	6.2076	6.0020
Epb41l2	_VTEGTIREEQEEC[Carbamidomethyl (C)]DEELEEEPGQGAK_.3	#N/A	4.1393	3.1187	3.7369	3.3839	3.4770	3.7011	4.0641	4.4260	4.1232	4.3456	2.9967	3.7530	4.2312	3.7750	3.6413	2.9833	3.9233	3.2262	3.5252	3.4523
Epb41l2	_KEPTQADVEDQILGKEES[Phospho (STY)]LPEEESR_.4	#N/A	4.4771	3.5258	3.5125	3.2123	3.8148	4.1702	3.7263	4.1774	4.3323	3.1825	3.8110	3.4152	4.4978	3.4996	3.3035	3.3211	3.2143	3.1180	4.2675	3.4666
Gpx4	_ILAFPC[Carbamidomethyl (C)]NQFGR_.2	#N/A	6.8463	6.5426	5.6861	6.4750	6.5651	6.3194	7.3529	6.8419	6.7385	7.0133	6.3990	6.3933	6.6651	6.3824	6.4503	6.6162	6.2786	6.9493	6.0390	6.3490
Gpx4	_YAEC[Carbamidomethyl (C)]GLR_.2	#N/A	4.8299	5.6472	3.5636	5.5793	4.6067	5.5440	5.8322	5.6053	5.2990	4.7577	5.4148	4.9274	4.8154	4.7726	5.1783	4.4209	4.4022	5.5498	4.5067	4.4893
Gpx4	_IC[Carbamidomethyl (C)]VNGDDAHPLWK_.3	#N/A	4.2147	3.7881	4.0893	4.4869	3.5524	3.7765	3.9887	3.9151	4.0478	3.5758	3.3775	4.5661	4.2354	3.2372	3.6933	3.0587	4.4527	3.7124	3.0290	3.8135
Gpx4	_TDVNYTQLVDLHAR_.2	#N/A	4.3365	3.3607	4.0441	3.7506	3.4482	3.6723	3.7290	3.2594	3.4751	3.9623	3.0301	3.7863	4.2782	3.5887	3.6746	3.3985	3.9131	3.2673	3.2448	3.3845
Cbfa2t2	_QHS[Phospho (STY)]PGSTDSLSNDSQR_.2	O70374_S400_M1_Cbfa2t2	4.0032	3.9996	4.5395	3.6862	3.3409	3.1431	3.9383	3.7036	3.8585	4.2143	3.3371	3.8890	4.0239	4.5266	1.8146	4.3481	4.2413	3.5919	3.2405	2.9928
Ulk1	_IEQNLQS[Phospho (STY)]PTQQTAR_.2	O70405_S450_M1_Ulk1	8.0893	7.9800	7.5055	7.6686	6.9101	7.3012	3.8609	7.7362	7.2080	4.2916	3.2598	7.4309	7.3382	7.3238	5.3294	3.8432	7.6453	7.6361	3.3178	6.8605
Ulk1	_VPS[Phospho (STY)]PQGADVR_.2	O70405_S521_M1_Ulk1	4.9190	4.8178	3.9245	4.3012	4.2268	4.9806	5.6730	3.0886	3.3734	3.5945	3.7478	3.0032	3.4814	4.5261	2.8915	3.7331	3.6263	3.9760	4.2750	4.6147
Psma3	_DIREEAEK_.2	#N/A	5.4112	5.2862	5.4668	5.6383	5.8337	6.0139	5.6622	5.6672	6.1394	5.9855	5.7141	5.7815	5.9124	6.1377	6.0445	5.6929	5.3866	5.7500	5.2876	5.5648
Psma3	_SNFGYNIPLK_.2	#N/A	6.1962	6.7790	6.1082	6.7921	6.5960	6.3973	6.0735	6.7894	6.4980	5.6552	5.9834	6.7806	6.6690	6.4978	6.8840	6.5616	6.3692	6.6071	6.7487	6.4827
Psma3	_[Acetyl (Protein N-term)]SSIGTGYDLSASTFSPDGR_.2	#N/A	5.5473	3.4428	5.6112	5.8322	4.8393	5.1395	4.4199	4.9593	4.6410	5.8304	5.0831	5.3343	3.8105	4.6022	5.5073	5.8716	5.4559	4.3791	4.6941	4.9593
Psma3	_AVENSSTAIGIR_.2	#N/A	5.0753	5.1159	4.8372	4.4639	3.5558	3.6461	4.9491	4.0106	4.0877	4.6198	3.6441	4.7625	4.3309	4.9298	5.1499	4.1506	3.6049	3.2849	3.9008	4.2694
Psma3	_ESLKEEDES[Phospho (STY)]DDDNM[Oxidation (M)]_.2	#N/A	12.4736	12.4235	12.8703	12.9441	12.2017	12.1621	12.1822	12.4504	12.2318	12.3971	12.1506	12.2440	12.6894	12.8730	12.2021	12.4247	12.4828	12.0563	12.2228	12.2891
Psma3	_YAKESLKEEDES[Phospho (STY)]DDDNM[Oxidation (M)]_.2	#N/A	4.9722	5.1342	3.6460	4.0062	4.4889	4.8222	4.5469	4.1664	3.9690	4.6967	4.1377	5.0967	5.1163	3.9903	4.3593	4.4082	4.9942	4.2497	4.4425	3.1987
Stx7	_EFGSLPTT[Phospho (STY)]PSEQR_.2	O70439_T79_M1_Stx7	11.2466	11.0581	11.1350	10.8640	10.6342	10.7782	10.5410	10.9402	10.8839	10.8804	10.7139	10.4534	11.1909	10.5643	9.8196	10.6214	11.2733	10.6486	10.7741	10.5270
Stx7	_TLNQLGT[Phospho (STY)]PQDSPELR_.2	O70439_T41_M1_Stx7	8.1999	8.2618	7.5543	7.9289	7.1210	7.4405	7.2862	7.6480	8.3622	7.6177	4.7962	7.2650	7.5506	7.5043	6.6395	7.2692	7.6277	7.2897	7.7030	6.5990
Stx7	_[Acetyl (Protein N-term)]S[Phospho (STY)]YTPGIGGDSAQLAQR_.2	O70439_S2_M1_Stx7	5.2915	5.0191	4.5945	4.6786	4.8715	5.0530	5.1254	5.1587	6.9013	7.0245	4.9319	4.9920	5.0148	4.9371	4.8999	4.6303	5.2746	4.7896	4.7556	6.2569
Stx7	_ITQC[Carbamidomethyl (C)]SVEIQR_.2	#N/A	3.6790	4.3238	4.2153	4.0103	3.0168	3.2409	3.0235	3.3854	3.0826	3.8854	3.4569	4.2132	3.1906	4.2352	4.1015	4.0239	4.3835	4.2668	3.0650	3.9125
Gnaz	_SSEYHLEDNAAYYLNDLER_.3	#N/A	8.0235	7.6481	6.6978	7.5524	7.1354	6.9819	7.1975	7.4292	7.1207	7.5126	7.4403	7.2822	7.9536	7.1758	5.9419	6.9171	7.6161	7.1647	7.0102	6.8190
Gnaz	_QFEDLNR_.2	#N/A	5.6697	5.4412	6.0671	5.8666	5.4507	5.9117	5.9697	5.7486	5.6117	6.2224	6.0184	5.8698	5.5884	5.6618	5.6605	5.4955	6.4704	5.6120	6.0592	5.7503
Gnaz	_IAAPDYIPTVEDILR_.2	#N/A	7.1358	7.1280	6.7576	6.9425	6.8178	6.9293	6.9097	7.1853	6.5782	6.8148	7.1478	6.4304	6.4917	3.9291	6.1255	6.6117	6.9373	6.9352	6.8653	6.6965
Gnaz	_GQNTYEEAAVYIQR_.2	#N/A	6.0479	4.4583	4.0808	4.4528	4.7264	3.6018	3.1580	5.2845	3.3998	5.0770	5.1324	4.3477	6.0974	4.0679	4.2360	3.8894	5.5443	4.0506	3.6992	4.0349
Gnaz	_LWADPGAQAC[Carbamidomethyl (C)]FGR_.2	#N/A	4.3827	4.4372	3.9193	3.2015	3.0888	3.1222	3.9593	3.1076	3.8795	4.1933	3.3581	4.1633	4.0449	3.9574	3.9846	3.7448	4.1056	3.0438	3.3429	3.0137
Slc16a7	_VGS[Phospho (STY)]RHDS[Phospho (STY)]TLKK_.2	#N/A	6.1901	6.2025	6.7435	6.1304	5.6305	4.0097	5.4151	5.1274	3.9035	5.6618	5.3832	5.8328	5.7122	5.5123	3.0995	5.6662	5.4934	5.5561	4.1070	3.9774
Slc16a7	_VGS[Phospho (STY)]RHDS[Phospho (STY)]TLK_.2	#N/A	3.2457	4.0734	4.2831	4.3385	3.4527	3.1549	3.5954	3.4714	3.5157	4.5572	2.9943	3.7995	3.2766	4.3212	3.6208	4.1087	4.4695	4.1808	3.9324	3.9985
Vamp4	_NLLEDDS[Phospho (STY)]DEEEDFFLR_.2	#N/A	8.9286	8.7521	8.5452	7.6119	5.5722	8.5321	7.6050	5.6371	8.8022	7.2689	6.2770	6.9619	7.4532	7.4393	7.9191	6.3066	8.7731	8.6857	8.3102	6.1513
Vamp4	_RNLLEDDS[Phospho (STY)]DEEEDFFLR_.3	#N/A	4.0252	4.7842	3.5618	3.5988	3.7272	3.8414	5.2702	4.2509	3.6980	3.9833	3.5829	3.8788	3.8240	3.5998	3.4557	3.0360	3.7481	4.7887	3.1847	3.5658
Ubr1	_MDVS[Phospho (STY)]PEPPLAPQR_.2	O70481_S15_M1_Ubr1	4.5718	8.1809	8.9602	8.3553	3.2779	7.8896	8.0588	3.2967	2.9938	4.3824	3.3682	3.9742	4.6027	4.1465	3.7955	3.9339	4.2947	4.3555	6.2926	3.8238
Snx3	_FTTYEIR_.2	#N/A	4.4029	5.1954	5.3181	5.5315	5.7300	5.0071	5.7827	3.2949	5.6583	6.1162	5.6379	4.5060	5.4901	5.2345	4.9636	5.2122	4.9252	5.3917	4.6881	5.0104
Snx3	_YSDFEWLR_.2	#N/A	3.9353	4.7409	4.3817	4.1948	4.4960	4.5013	4.5430	4.0516	4.0409	4.3049	4.3929	4.5506	3.8568	4.9920	4.4877	4.3794	3.5488	4.7025	4.7355	4.6597
Snx3	_[Acetyl (Protein N-term)]AETVADTRR_.2	#N/A	5.0068	3.9109	4.2465	3.9791	4.6882	4.9479	4.3236	3.4106	5.0071	4.0529	4.2908	4.5112	5.1649	3.9651	5.5474	4.0551	3.9483	3.8868	4.2384	4.1587
Snx12	_[Acetyl (Protein N-term)]S[Phospho (STY)]DTAVADTRR_.2	O70493_S2_M1_Snx12	3.5207	4.4821	7.2885	7.7994	7.1330	3.0825	7.2999	3.2211	7.1063	7.0772	3.6152	4.3715	3.3489	4.0441	6.6131	7.2470	7.2378	6.9144	6.5398	4.0111
Snx12	_GDEGIFEESFIEER_.2	#N/A	6.1944	5.5583	5.8006	5.5821	5.4618	6.0358	4.7720	6.2242	5.8601	6.4254	5.2653	5.6354	6.6309	6.9862	5.1659	6.0603	6.1452	4.6064	5.6441	5.1685
Snx12	_FTTYEVR_.2	#N/A	3.7501	4.9075	4.3158	5.9962	5.1693	4.7484	5.0761	3.4798	5.5366	5.5319	5.1442	5.6574	4.5910	5.5235	5.7066	6.0698	3.7252	5.3798	5.4979	5.9559
Snx12	_RYS[Phospho (STY)]DFEWLK_.2	O70493_S73_M1_Snx12	4.3337	3.6691	3.3692	3.3557	3.6714	5.4002	4.2688	4.0341	4.6300	3.8838	3.6676	3.5585	4.3544	3.3562	3.4468	3.1778	3.0709	5.6523	4.4108	3.3232

Snx12	_IAGHPLAQNER_.3	#N/A	3.1132	4.2059	4.1506	3.8965	3.3202	3.3535	3.7279	3.4932	3.6481	4.4247	3.1268	3.9320	3.8136	4.3162	3.7533	3.9762	4.0309	3.8022	3.4508	4.2832
Hcn4	_S[Phospho (STY)]PQAAQPPPPLPGAR_.2	O70507_S921_M1_Hcn4	10.2578	9.8286	9.2311	9.9487	8.4892	8.8577	9.2170	7.4832	8.4756	10.0438	8.8582	8.9102	7.3139	8.9362	6.3707	8.5690	9.3187	9.0319	8.3863	8.5857
Hcn4	_GGLS[Phospho (STY)]PPGHSPGPPR_.2	#N/A	3.6715	4.3093	4.2564	3.9959	3.4329	3.5528	3.9354	7.0587	7.1615	3.7273	3.8938	6.8575	4.0740	4.0227	3.6886	4.2532	7.6801	3.6386	3.5281	3.8532
Hcn4	_RAS[Phospho (STY)]PHS[Phospho (STY)]S[Phospho (STY)]GESVAAFSLYPR_.3	O70507_S1090_M3_Hcn4	4.4694	3.5334	3.5049	3.2200	3.8071	4.0312	3.7339	4.1698	3.7930	3.1749	4.1674	3.4228	3.9011	3.4920	3.3111	3.3135	3.2066	3.1257	4.2751	3.4590
Srpk1	_ADT[Phospho (STY)]PS[Phospho (STY)]GDEQEPNGALDSK_.2	O70551_S450_M2_Srpk1	6.1648	3.5567	7.0481	6.1030	6.2974	5.9043	5.0424	6.5722	5.7782	6.0925	5.8768	6.1697	3.6829	3.3566	3.3229	4.8214	6.0726	5.3509	5.7690	4.9374
Cask	_EINGISVANQTVEQLQK_.2	#N/A	4.3937	3.6778	3.9303	3.2349	3.5044	3.3162	4.1356	3.4925	4.5672	4.1607	3.1901	3.6947	4.4246	3.9684	3.1778	3.8010	3.6137	3.0534	2.9788	3.6549
Cask	_TQSSSC[Carbamidomethyl (C)]ERDS[Phospho (STY)]PSTSR_.2	O70589_S582_M1_Cask	5.4017	3.1616	3.8767	4.3490	3.7727	4.7357	5.1796	4.5416	4.3015	5.6982	5.0657	4.7201	5.5673	3.8637	4.1990	4.4287	5.3720	5.3970	5.5397	4.8365
Pfdn2	_GAVSAEQVIAGFNR_.2	#N/A	5.6280	5.8903	6.1955	5.8568	5.2945	5.3301	5.2724	6.0882	6.0212	5.8449	5.9132	5.7831	6.4495	4.5772	6.2111	5.4278	6.1330	5.2617	6.0440	5.6479
Pfdn2	_IIETLSQQLQAK_.2	#N/A	4.4755	2.8311	4.8361	3.4504	4.3293	4.6291	3.2975	4.1661	4.0090	3.9785	3.4421	3.6533	3.2932	3.8319	4.2347	3.5301	4.4770	3.2353	4.3801	3.2285
Rtn2	_EFS[Phospho (STY)]EDEEEETTSQDWGTPR_.2	O70622_S44_M1_Rtn2	3.5861	6.2031	5.8929	4.3316	2.9238	5.6943	5.8780	3.4783	5.4394	3.7924	3.5499	3.7925	3.2835	6.0661	4.3960	3.9310	4.4764	6.1919	4.7690	4.6759
Ttc3	_SSQS[Phospho (STY)]PPLAAAGPSAR_.2	O88196_S1903_M1_Ttc3	3.3669	9.8282	9.7166	9.6759	9.1270	9.7405	9.5160	10.0401	9.3888	9.5136	9.2414	9.4718	9.7846	9.7196	8.7780	9.2729	9.9940	9.4347	9.2500	9.1252
Ttc3	_RNSDS[Phospho (STY)]AGPFAVPDHLR_.3	O88196_S1062_M1_Ttc3	6.3273	3.4245	3.5324	7.7651	3.6952	7.8695	8.4676	4.2100	4.1419	7.3771	4.0906	3.2433	4.2820	8.8247	6.2240	3.2015	3.5074	3.2695	7.6949	3.3667
Ttc3	_GASQVS[Phospho (STY)]PSEQS[Phospho (STY)]PEADEK_.2	O88196_S1794_M2_Ttc3	4.4083	4.8892	5.2609	3.6863	3.7460	3.9701	3.8037	4.2446	5.0659	3.9970	4.4452	3.1998	3.9708	3.5805	3.3809	3.2523	3.5344	4.6631	3.9799	4.5233
Ttc3	_KQDS[Phospho (STY)]DEM[Oxidation (M)]PFGC[Carbamidomethyl (C)]IK_.2	#N/A	6.4939	7.2542	6.8353	6.4573	3.4331	7.1422	6.9949	6.2429	7.0716	7.0847	4.7394	7.1721	7.0075	6.5993	5.9833	6.9969	7.7965	7.4637	7.1116	6.8760
Ttc3	_IIIYSSGGQVK_.2	#N/A	3.9707	3.3484	3.5073	3.6135	4.1776	3.9307	3.8344	4.1964	3.8935	4.5753	4.2679	3.0745	4.0016	3.5453	2.8959	3.3328	3.6936	3.4558	3.7549	3.2226
Grem2	_EVLASS[Phospho (STY)]QEALVVTER_.2	O88273_S56_M1_Grem2	7.9267	8.0559	7.9783	7.7336	3.8439	7.6456	7.5264	7.5073	7.8856	8.0436	7.5507	7.6833	8.0476	4.2551	7.4605	7.5725	7.6599	7.6375	7.5519	7.4189
Znf326	_MNLSKS[Phospho (STY)]PTK_.2	#N/A	4.0508	5.8147	4.5871	3.6386	3.3885	3.6126	4.1526	3.7511	4.2117	5.2251	3.0852	3.8415	4.3197	3.8635	3.7298	4.3957	4.7173	3.1377	3.4367	5.3454
Znf326	_S[Phospho (STY)]PTKTDPKNEEEEK_.2	#N/A	3.8029	4.3822	4.0029	3.9839	3.1724	3.2057	3.9043	4.0518	3.8245	4.2769	3.3031	4.1083	3.9899	4.0666	3.9297	3.8284	3.9805	3.5488	3.4846	3.9183
Znf326	_M[Oxidation (M)]NLSKS[Phospho (STY)]PTKTDPK_.2	#N/A	3.3380	4.3779	4.7948	4.3218	3.7481	3.8529	4.8863	4.0814	3.1593	3.4232	4.1388	3.3988	3.3248	5.7839	3.2645	3.8521	3.3139	4.2007	4.1575	3.7944
Ctnnal1	_S[Phospho (STY)]AAEELELTVLK_.2	O88327_S373_M1_Ctnnal1	4.3262	3.6766	3.3616	3.3632	3.6639	3.8880	3.8772	4.0265	3.9363	4.5325	4.3107	3.5661	4.0443	4.2999	3.4544	3.1702	3.0634	3.4131	3.7121	3.2654
Wdr1	_VFASLPQVER_.2	#N/A	8.2321	8.2320	7.9474	8.1637	8.3617	7.9233	8.1661	8.6549	8.0213	8.1752	8.2448	8.4821	8.2759	8.3180	8.4136	8.1564	7.9214	8.2267	8.1366	8.0481
Wdr1	_YAPSGFYIASGDISGK_.2	#N/A	8.0756	8.1428	8.4022	8.4683	7.9095	7.8857	7.6777	8.1172	8.0385	8.1669	7.9192	7.5188	8.4908	7.7341	7.7088	8.0579	8.1502	7.8598	7.9001	7.8962
Wdr1	_YEYQPFAGK_.2	#N/A	6.7953	7.2133	7.3063	7.5566	7.3657	6.8864	7.3566	7.4867	7.2072	7.3922	7.1135	7.3551	7.5338	7.0038	7.4796	7.2062	7.2365	7.4166	7.3772	7.1988
Wdr1	_IAVVGEGR_.2	#N/A	6.5123	6.5746	6.6432	6.8350	6.5919	6.7019	7.3217	6.8101	6.7167	6.6586	6.5902	6.5917	6.2842	6.3937	7.1657	6.6811	6.4364	6.5759	6.7377	6.9956
Wdr1	_SIQC[Carbamidomethyl (C)]LTVHR_.2	#N/A	6.2544	6.5919	6.6772	6.4283	6.6414	6.3732	6.7235	6.5169	6.5219	6.2406	6.3684	6.5421	6.4213	6.7026	6.3565	6.4871	6.2301	6.6166	6.6958	6.6295
Wdr1	_DYSGQGVVK_.2	#N/A	5.8287	6.3895	6.3187	6.2371	6.0381	6.6720	6.9980	5.6487	6.7352	6.8786	6.8746	6.6472	7.0006	6.3519	6.9655	6.5252	5.7430	6.5880	6.5623	6.4508
Wdr1	_DIAWTEDSKR_.2	#N/A	6.4473	6.1044	6.2101	6.0607	6.0703	6.2540	6.2562	5.6718	6.2267	6.2972	6.4770	6.0542	5.5204	6.3744	6.0973	6.4288	6.2272	6.0470	6.2106	6.2786
Wdr1	_GPVTDVAYSHDGAFLAVC[Carbamidomethyl (C)]DASK_.3	#N/A	7.3619	6.2563	7.0072	7.0476	6.5342	6.6928	6.3627	7.5205	6.6324	6.9158	6.4697	6.5301	7.8240	7.3447	5.7069	6.5334	7.4120	5.9196	6.6170	6.3445
Wdr1	_VINSVDIK_.2	#N/A	5.1677	5.0599	5.5428	5.1828	4.7196	4.8559	5.1796	2.7823	5.6320	4.5156	5.2164	4.9319	4.7824	3.9129	5.5825	4.7948	3.1610	5.0966	5.2126	4.6752
Wdr1	_LATGSDDNC[Carbamidomethyl (C)]AAFFEGPPFK_.2	#N/A	3.1753	5.2988	5.8831	5.7542	3.3822	5.7210	5.5095	6.3594	5.9228	6.4032	5.7910	5.9363	6.1247	6.1149	5.6116	4.0383	6.0496	4.2512	6.1603	5.5605
Wdr1	_VC[Carbamidomethyl (C)]ALGESK_.2	#N/A	4.0746	3.9526	4.5865	4.9283	3.3879	4.5020	4.9519	3.6965	4.8896	4.6845	4.5232	4.5996	5.2268	4.5736	5.6850	5.2929	4.2883	4.7795	4.6601	4.5547
Wdr1	_VYSILASTLKDEGK_.2	#N/A	6.0708	6.1995	6.0643	6.2202	6.1046	6.0300	5.9327	6.6819	6.1401	5.9253	5.7557	6.1847	6.2210	6.4609	5.8325	6.2529	6.1670	6.0920	5.9745	5.8779
Wdr1	_AHDGGIYAISWSPDSTHLLSASGDK_.3	#N/A	4.0044	3.5388	3.6330	5.0899	3.3833	3.6056	3.9309	3.2551	4.4322	4.5949	3.5114	3.2398	4.0298	3.5568	4.3492	4.3995	4.2557	3.5111	4.5827	4.4924
Wdr1	_VVTVFSVADGYSENNVFYGHHAK_.3	#N/A	4.4163	3.7742	4.0768	3.9450	3.8039	3.2054	3.5168	4.4039	3.8627	4.4112	4.4908	4.0205	5.3082	4.5107	3.5187	4.2476	4.1145	3.8928	4.1807	4.4956
Wdr1	_GDHFLYTNGK_.2	#N/A	5.4822	4.1006	4.2559	4.3657	3.4254	4.9954	5.4935	3.4442	3.1413	3.8266	4.2446	3.8267	3.2494	4.2940	3.9871	3.9652	4.4422	4.4516	3.0063	4.8452
Wdr1	_M[Oxidation (M)]TVNESEQLVSC[Carbamidomethyl (C)]SMDDTVR_.2	#N/A	4.1413	3.8242	4.2193	4.2518	3.7996	3.9822	3.8477	4.0208	4.1396	4.2395	3.7844	3.5149	4.2689	4.7230	3.3873	3.7495	4.0269	3.1813	4.1082	3.4007
Wdr1	_SYIYSGSHDGHINYWDSETGENDSFSGK_.4	#N/A	6.2060	4.5937	5.4274	3.3580	5.1891	4.4665	4.1158	5.5729	5.6080	5.6513	5.5449	4.6696	6.1612	5.1399	4.5553	3.5113	5.2491	5.8065	5.0344	3.1703
Wdr1	_NIDNPAIADIYTEHAHQVVVAK_.3	#N/A	6.3477	5.5826	3.5977	6.0653	6.0024	5.6333	5.7052	4.2626	6.1241	6.2966	3.8962	5.8872	4.5830	3.5848	5.7472	5.4326	5.7782	3.0329	5.9030	5.7844
Slc4a4	_GSLDS[Phospho (STY)]DNDDSDC[Carbamidomethyl (C)]PYSEK_.2	O88343_S1029_M1_Slc4a4	6.1482	7.1695	6.5899	6.2508	5.4850	6.1869	6.5840	3.4062	3.1034	4.4920	6.3860	5.9402	6.1560	6.2899	4.6232	5.9558	7.0555	6.4156	3.0442	6.1382
Slc4a4	_NLTSSS[Phospho (STY)]LNDISDKPEKDQLK_.3	#N/A	11.3895	11.4766	10.9646	11.1349	9.8668	11.0431	11.0062	10.6329	10.7802	10.7584	10.1344	10.7837	10.8349	10.6843	9.0846	10.6000	11.2719	10.9104	10.5946	10.5894
Slc4a4	_LQQAVM[Oxidation (M)]LGALTEVPVPTR_.2	#N/A	4.5694	5.0135	2.9049	4.3180	4.4123	5.6490	5.0521	3.4919	4.5009	4.5777	4.3645	4.2516	4.9913	4.4966	1.8338	3.7432	4.7022	4.9002	3.6038	4.4602
Slc4a4	_NLTSSS[Phospho (STY)]LNDISDKPEK_.2	#N/A	11.9543	11.6812	11.7445	11.3093	10.9047	11.4063	11.1940	3.8661	11.0070	11.0988	10.9259	10.7434	3.6713	11.0856	3.3906	10.5866	11.6093	11.1292	10.6087	10.5348
Slc4a4	_LADYYPINSDFK_.2	#N/A	6.4717	6.3416	5.4974	6.1413	6.5203	6.2019	6.8456	3.8067	5.5043	5.8178	3.0296	6.3111	4.2641	6.1746	3.6742	5.3462	6.6874	6.4444	6.2520	6.0484
Slc4a4	_MFSNPDNGS[Phospho (STY)]PAM[Oxidation (M)]THR_.2	#N/A	5.0325	8.7169	8.7726	9.0349	7.0609	8.0897	8.6910	6.2246	7.3383	6.8102	7.5429	6.7369	5.6957	7.9719	5.8008	4.6752	7.5188	6.8891	4.5704	6.6636
Slc4a4	_ISENYSDKS[Phospho (STY)]DVENADESSSSILKPLISPAAER_.4	O88343_S68_M1_Slc4a4	3.8940	3.9622	3.8909	3.8402	3.2317	3.4558	3.6570	3.9172	3.7160	4.1003	3.3157	4.3768	3.8241	3.9004	3.9603	3.5127	3.8239	3.7556	3.6440	3.7713
Slc4a4	_KGS[Phospho (STY)]LDS[Phospho (STY)]DNDDSDC[Carbamidomethyl (C)]PYSEK_.2	#N/A	3.5922	3.7268	3.1288	3.9920	3.7992	4.3107	3.2489	3.8179	3.1691	3.4028	4.4680	3.4530	3.3346	3.1669	3.2743	2.9544	3.3152	3.8343	3.5711	3.8042
Slc4a4	_KKGS[Phospho (STY)]LDS[Phospho (STY)]DNDDSDC[Carbamidomethyl (C)]PYSEK_.3	#N/A	4.1002	3.9026	3.1357	3.5892	3.4380	3.6620	4.1031	3.8006	4.1622	4.3066	3.0357	3.7920	4.2703	4.6236	3.6803	2.9443	4.3383	3.4949	3.1435	3.0898
Mtx2	_TLDQVLEDVDQC[Carbamidomethyl (C)]C[Carbamidomethyl (C)]QALSQR_.3	#N/A	4.9830	5.0816	5.1314	5.3537	5.0449	4.8717	5.1820	5.2323	4.5305	5.5237	4.6979	5.0765	3.9810	5.9561	4.9556	4.7512	5.3923	4.8885	5.1782	5.3196
Mtx2	_[Acetyl (Protein N-term)]SLVAEAFVSQIAATEPWPENATLYQQLR_.3	#N/A	4.1687	3.7646	3.2737	3.4512	2.8748	4.4090	4.1733	3.9386	4.0935	3.9793	3.5721	4.3773	4.2589	3.2607	4.1987	3.5308	4.4763	3.3569	3.0055	3.2277
Klc1	_VDS[Phospho (STY)]PTVTTTLK_.2	#N/A	10.4266	10.4872	10.7428	10.5088	10.2204	10.3933	10.0047	10.4654	10.0872	10.6993	10.1141	9.9825	10.1577	10.3661	9.4491	10.0142	10.5233	9.9573	9.9935	10.2226
Klc1	_DAANLLNDALAIR_.2	#N/A	5.5684	5.5296	5.1488	5.2806	5.9040	5.8469	5.6938	4.9795	5.2959	5.5602	5.5639	5.0245	4.3386	5.6203	5.5753	4.8027	4.0121	5.4555	5.6515	5.6728
Klc1	_S[Phospho (STY)]RESLNM[Oxidation (M)]DVVK_.2	#N/A	8.4836	8.5594	8.5700	8.9041	8.1133	8.2661	8.1242	8.1308	8.0615	8.7462	8.1070	7.7903	8.2620	8.7993	7.2394	8.1371	8.3059	7.8950	7.8460	8.3392
Klc1	_AC[Carbamidomethyl (C)]KVDS[Phospho (STY)]PTVTTTLK_.2	#N/A	4.0754	3.2437	3.6120	8.3259	7.8008	7.9401	4.2666	4.3011	8.0096	7.9144	4.3726	8.1304	8.3685	8.4683	2.7911	3.4375	8.5128	3.3511	7.6400	8.0956
Klc1	_S[Phospho (STY)]RES[Phospho (STY)]LNMDVVKYESGPDGGEEA_.3	#N/A	3.1506	4.2473	4.2918	3.9339	3.3576	3.3909	3.6905	3.4558	3.6107	4.4621	3.0894	4.1368	3.7762	4.2788	3.7159	4.0136	3.9935	3.8396	3.4882	4.2458
Klc1	_RS[Phospho (STY)]RES[Phospho (STY)]LNMDVVK_.3	#N/A	4.1921	3.1270	3.7287	3.3921	2.8982	3.7093	4.0558	4.4178	4.1149	4.3538	2.9885	4.3539	4.2230	3.7667	3.6330	2.9916	3.9150	3.2344	3.5335	3.4440
Klc2	_VDS[Phospho (STY)]PTVNTTLR_.2	#N/A	10.6780	10.4025	10.7407	10.6965	9.7199	9.1027	9.9333	8.9815	9.0108	10.8435	9.7952	9.6910	4.4324	10.0159	6.6180	9.6887	9.1787	9.2135	9.3674	9.6679
Klc2	_ASS[Phospho (STY)]LNFLNK_.2	O88448_S575_M1_Klc2	9.7519	10.1153	10.2487	10.4002	4.0413	9.1347	9.4094	9.0816	9.7351	10.4185	9.7473	9.0976	9.8404	9.9145	9.0258	9.5413	9.0842	9.2878	9.6900	9.6751
Klc2	_YEVAVPLC[Carbamidomethyl (C)]K_.2	#N/A	5.3804	6.0631	5.4384	6.3683	6.0145	5.5709	6.2818	6.0794	5.7742	6.3375	5.8302	5.5069	5.8524	5.7793	6.2039	5.5467	4.9355	5.8357	6.0955	5.9249
Klc2	_S[Phospho (STY)]GS[Phospho (STY)]FGKLR_.2	O88448_S537_M2_Klc2	5.7036	6.8424	4.7815	4.9027	5.6374	5.6351	5.6980	6.4891	5.3689	6.5861	5.1633	5.1352	6.3812	5.2839	4.3564	6.0138	4.7541	4.8413	4.1987	5.4921
Klc2	_AC[Carbamidomethyl (C)]KVDS[Phospho (STY)]PTVNTTLR_.2	#N/A	7.8165	4.2644	4.0921	7.9524	6.7739	3.3459	7.1998	6.3298	2.9775	3.9904	3.3519	3.9905	7.6468	7.6189	3.9965	4.1290	6.0150	4.3718	6.7534	3.8075
Klc2	_TLHNLVIQYASQGR_.2	#N/A	4.8843	3.9288	3.4904	3.7327	5.1723	5.2257	3.9887	5.1590	5.1874	5.2617	4.6852	4.7392	5.0049	3.8330	3.6993	5.3102	5.1389	4.3819	5.1108	5.8686
Klc2	_RSS[Phospho (STY)]EMLVR_.2	O88448_S551_M1_Klc2	3.3216	3.9975	4.3590	4.2626	3.5285	3.5619	3.5196	3.5473	3.4398	3.1322	2.9184	3.7236	3.6052	4.1079	3.5449	4.1846	4.5454	4.0106	3.6592	4.0749
Capns1	_YADESGNM[Oxidation (M)]DFDNFISC[Carbamidomethyl (C)]LVR_.2	#N/A	3.0159	3.3127	4.1240	4.1875	2.9255	3.5586	1.6991	3.3334	3.4430	2.8908	2.9216	4.3045	3.6084	4.1111	4.1928	1.7600	3.8258	1.3896	3.6560	3.5647
Capns1	_ILGGVISAISEAAAQYNPEPPPPR_.3	#N/A	4.0842	3.8280	3.5303	3.5905	4.2006	4.0987	3.7631	4.2194	3.9165	4.5523	4.2909	3.0515	4.0246	3.5683	4.0336	3.1900	3.7166	3.4328	3.6772	3.2456
Znf106	_ATHVPENS[Phospho (STY)]DTEQDVFTAKPAR_.3	O88466_S1381_M1_Znf106	3.7575	3.5616	6.6107	3.8267	3.9644	3.9978	3.0837	4.3498	3.0039	3.5681	3.9834	3.2877	3.1693	3.6720	3.1090	3.1196	3.3867	4.4465	4.0951	3.6390
Dync1i1	_IGHDS[Phospho (STY)]ELENQEK_.2	O88485_S180_M1_Dync1i1	8.0742	8.6614	8.3566	8.5271	7.9443	8.3124	8.3926	8.0714	8.5425	7.9224	8.0865	8.1068	8.7179	8.8930	7.7385	8.1998	8.4390	8.2653	8.2083	8.4960
Dync1i1	_VTQVDFLPR_.2	#N/A	6.2751	6.9298	6.5347	6.1015	6.7623	6.3806	6.5784	6.5909	6.2671	6.5970	6.5233	6.6542	6.3250	7.0213	6.5777	6.1792	6.1285	6.1527	6.5794	6.4153
Dync1i1	_ANRADS[Phospho (STY)]EEEGAVELAA_.2	O88485_S618_M1_Dync1i1	11.0361	12.5250	12.3393	12.3903	4.3224	12.1204	11.9665	10.1533	12.2186	12.3132	9.9798	12.1249	11.9880	12.5946	11.9378	12.3388	12.2047	12.1011	12.1842	12.3476
Dync1i1	_KEPVQDDS[Phospho (STY)]DLDR_.2	O88485_S50_M1_Dync1i1	7.3595	6.8082	7.6112	7.6315	6.4738	6.9401	6.9581	7.6281	6.9739	7.2633	7.1070	7.2413	7.0548	7.9919	6.7738	7.7118	7.8841	7.5803	6.7987	7.3805
Dync1i1	_ALAEDSDIFFDYSGR_.2	#N/A	5.9732	5.3839	6.5109	5.9643	4.3083	5.4357	4.2406	5.6395	5.7014	3.9120	6.1335	2.9438	6.9793	5.8761	6.1510	5.8903	3.8243	3.2896	5.4482	5.7599
Dync1i1	_ETQTPLATHQS[Phospho (STY)]EEDEEDEEMVEPK_.3	#N/A	5.2458	7.3868	3.8393	3.6046	3.7830	3.8760	5.1287	4.0096	6.0700	3.7582	3.8635	3.5912	5.7325	8.0135	5.2532	7.0785	3.6817	6.0322	3.8396	5.7045
Dync1i1	_SVSTPSDAGS[Phospho (STY)]QDSGDLGPLTR_.2	O88485_S94_M1_Dync1i1	6.7963	7.3875	3.6346	3.9854	5.3503	3.6515	6.7098	3.7396	3.7314	4.0613	6.3307	6.8377	7.2905	7.7079	6.1690	7.3203	7.1944	7.0132	6.8888	7.3599
Dync1i1	_[Acetyl (Protein N-term)]SDKSDLKAELER_.3	#N/A	4.4625	3.2870	5.5754	4.4744	4.0535	4.9581	5.6714	4.4955	5.5465	5.9952	5.4822	5.4663	5.4858	4.8509	5.1150	3.5599	3.4530	5.5584	4.0287	5.3342
Dync1i1	_TLQWDTDPSVLQLQSDSELGR_.2	#N/A	3.7872	4.0950	3.4874	3.9344	3.3409	3.8437	3.6468	3.8728	3.5628	4.2442	3.3981	4.0382	3.9052	4.3249	3.9061	3.8135	4.2578	3.9018	3.2177	3.7868
Dync1i1	_LDLWNLNSDTEVPTASVAIEGASALNR_.3	#N/A	4.5780	2.8373	4.1145	4.5071	3.2840	3.3174	3.7641	3.3028	4.0566	4.1935	3.1629	3.9681	3.8497	4.1526	3.7894	3.2635	4.3009	3.0900	3.1476	3.8299
Dync1i2	_SVSTPSEAGS[Phospho (STY)]QDSGDGAVGSR_.2	O88487_S95_M1_Dync1i2	8.0055	7.8145	6.2916	6.1520	5.1062	7.7432	7.4154	6.7603	8.3204	8.0299	7.4602	7.8098	6.8732	8.5369	6.4559	7.7592	8.2880	7.9685	6.1419	6.0355
Dync1i2	_TLAEINANR_.2	#N/A	6.9303	6.8387	6.4943	6.9822	4.0251	6.8637	6.7730	6.9220	6.9080	6.8820	6.5744	6.5988	6.9724	6.8060	6.6929	3.1803	6.6082	6.2822	7.0713	7.0132
Dync1i2	_EIAVGDSEGQIVIYDVGEQIAVPR_.3	#N/A	5.6190	5.5958	4.8025	5.6039	5.0149	4.9090	5.1386	6.1604	5.3927	5.4722	5.2494	5.4447	5.8830	6.3841	4.2149	5.4016	5.7962	4.9800	5.1409	5.6389
Dync1i2	_DLEDKEGEIQAGAK_.2	#N/A	3.2869	4.0527	5.0850	3.0220	4.4164	4.4207	4.1297	3.5126	3.2098	4.3452	4.8513	4.5258	5.5666	4.8261	4.8849	4.2072	5.0858	4.3835	4.1495	4.0667
Dync1i2	_EAEALLQSM[Oxidation (M)]GLTTDSPIVPPPM[Oxidation (M)]S[Phospho (STY)]PSSK_.3	#N/A	3.7489	4.3818	3.9428	6.1417	3.9559	3.9384	3.4108	4.0603	3.9227	3.3742	4.1298	3.3473	4.0705	3.9675	3.1399	3.7047	3.3955	3.5411	4.0858	6.1253
Nsd1	_AAS[Phospho (STY)]PQEVTPQADEK_.2	O88491_S2369_M1_Nsd1	4.2848	8.7523	8.2722	3.5672	2.9909	3.0242	8.5590	8.8483	8.3744	8.7137	8.3502	4.2613	4.1429	3.1447	8.0460	3.6469	8.7966	8.3705	3.1215	3.1117
Pde8a	_RFS[Phospho (STY)]GNEYILATK_.2	O88502_S452_M1_Pde8a	3.3049	4.0141	4.3424	4.2793	3.5119	3.5452	3.5362	3.3015	3.4564	3.1155	2.9351	3.7403	3.6219	4.1245	3.5616	4.1679	3.8392	3.9940	3.6425	4.0915
Dnmt3a	_SEPQPEEGS[Phospho (STY)]PAAGQK_.2	O88508_S102_M1_Dnmt3a	9.5845	9.2707	9.4427	9.1831	9.0768	9.2356	9.0850	9.5721	9.2903	9.1072	9.0766	2.9517	9.3707	9.4141	9.3834	9.2691	9.6069	9.1252	9.3473	9.0889
Ikbkg	_S[Phospho (STY)]PPEEPPDFC[Carbamidomethyl (C)]C[Carbamidomethyl (C)]PK_.2	#N/A	9.0041	9.8066	9.3116	9.3324	7.0525	9.4729	8.3379	9.2039	9.1128	8.7152	9.4675	9.3930	9.0500	10.0842	9.1356	9.0680	10.1141	9.2591	9.3043	9.5235
Ikbkg	_RS[Phospho (STY)]PPEEPPDFC[Carbamidomethyl (C)]C[Carbamidomethyl (C)]PK_.2	#N/A	3.7992	3.3951	3.6432	4.5825	4.0061	4.0395	3.0420	4.3081	4.4630	3.6098	3.9417	3.2460	3.1276	3.6303	3.0673	3.1613	3.3450	2.9874	4.1368	3.5973
Ppt1	_ETIPLQESTLYTEDR_.2	#N/A	7.0770	6.8092	7.2240	7.2273	7.2609	6.6982	6.6389	7.1035	7.1738	7.0496	7.2018	7.0769	7.5657	7.2010	7.1716	7.3863	7.2155	6.5118	7.2663	7.1751
Zfr	_RDS[Phospho (STY)]DGVDGFEAEGKK_.3	#N/A	6.5707	7.4257	7.0637	6.5979	6.3834	7.4674	7.3466	6.8872	7.0017	7.2889	7.0107	6.8081	7.7368	7.1703	6.7326	6.9220	7.4305	6.8517	7.2239	7.0587
Zfr	_RRDS[Phospho (STY)]DGVDGFEAEGK_.3	#N/A	5.1306	5.3436	6.3800	4.5872	3.1575	4.7355	5.3673	6.5937	5.2101	5.5968	5.6322	5.1343	5.8475	5.3691	4.8401	5.1361	6.3504	6.4162	5.4132	6.0904
Zfr	_RDS[Phospho (STY)]DGVDGFEAEGK_.2	#N/A	3.4215	7.3508	4.4589	4.1627	3.6284	6.4704	6.4148	6.7499	3.3443	5.7623	3.7187	3.6237	3.4524	4.4970	5.7578	4.2845	3.1444	7.0071	5.6394	4.1743
Zfr	_DS[Phospho (STY)]DGVDGFEAEGK_.2	#N/A	4.4860	3.5168	6.7084	6.4946	3.8237	4.1612	3.7174	4.1864	4.3412	6.2708	3.8199	3.4063	4.5067	3.5085	3.2946	3.3300	6.7926	6.3830	5.9841	6.2925
Cops3	_[Acetyl (Protein N-term)]ASALEQFVNSVR_.2	#N/A	5.9032	5.1602	5.5604	5.4891	5.0870	5.7463	5.5596	5.6067	6.1216	4.5194	5.5346	5.7142	4.6938	4.9463	4.7633	4.7911	4.8702	5.4386	5.4219	5.5679
Cops3	_YTSQIVGR_.2	#N/A	3.8450	5.2450	5.3129	3.1275	5.5084	4.0853	5.4424	5.2821	5.4093	3.6556	4.9145	3.2002	4.7439	5.0777	4.2915	5.6936	5.1438	4.9793	4.6388	3.5515
Cops3	_SM[Oxidation (M)]GS[Phospho (STY)]QEDDSGNKPSSYS_.2	#N/A	7.2121	7.2393	7.1242	7.4753	6.4142	6.9314	6.8490	7.2487	6.5251	7.2705	7.1634	6.8888	7.6386	7.1876	6.2056	7.0109	7.3035	7.3618	7.2136	6.9516
Cops3	_YATDTFAGLC[Carbamidomethyl (C)]HQLTNALVER_.3	#N/A	4.5192	4.3008	4.0557	3.5479	3.2252	3.2586	2.4524	3.2440	2.7376	4.3297	3.2217	4.0269	3.9085	4.0938	3.8482	3.8813	4.2421	4.4082	3.2064	3.7711
Cops4	_NAAQVLVGIPLETGQK_.2	#N/A	5.7827	5.6694	6.5068	6.1593	5.9571	6.3651	5.4019	5.8732	6.2605	6.1937	5.9000	5.7465	6.1490	6.0526	5.6555	6.3987	6.2638	5.9240	6.2011	6.0136
Cops4	_ATTADGSSILDR_.2	#N/A	6.0374	5.9248	6.5096	6.1976	6.2805	6.2171	6.6174	6.5339	6.6239	6.5132	6.4935	6.1306	4.1004	6.5634	6.6062	6.5024	5.9227	6.2629	5.6686	6.2841
Cops4	_LYLEDDDPVQAEAYINR_.2	#N/A	5.1215	4.7140	5.3876	5.2121	4.6671	5.4855	5.1549	4.9509	5.2544	4.3590	5.3762	4.4332	4.4454	4.6628	4.1164	4.8975	4.4685	4.9672	4.6009	4.4876
Cops4	_VISFEEQVASIR_.2	#N/A	4.8423	3.2271	5.5887	5.4393	4.1134	4.8893	5.0139	5.0376	4.8945	5.4217	3.8611	4.9389	5.4532	5.2840	5.2605	3.6198	5.2939	4.8909	5.5277	4.5907
Cops4	_ISQTAPEWTAQAM[Oxidation (M)]EAQM[Oxidation (M)]AQ_.2	#N/A	4.9616	3.5134	3.5248	3.2000	4.5560	5.0548	5.7625	6.0739	5.1845	5.0813	4.1475	4.8475	4.8133	5.1411	4.9919	4.9726	5.1576	5.3529	4.9790	4.9784
Cops4	_IASQMITEGR_.2	#N/A	3.1879	3.3141	4.8999	4.5015	4.0264	3.2521	3.5147	4.3891	3.5737	3.3942	3.9481	3.2036	3.6818	3.7112	3.9743	3.5328	3.4259	4.4072	4.0558	4.4037
Hnrnpa2b1	_GGGGNFGPGPGSNFR_.2	#N/A	9.2273	8.6795	9.7624	9.3661	9.2956	8.5330	8.9114	9.4871	9.2131	8.8500	9.1172	9.4002	9.7872	9.5823	9.6528	9.1056	9.2798	9.0217	9.0994	8.8979
Hnrnpa2b1	_GGNFGFGDSR_.2	#N/A	8.8599	9.0147	8.9475	9.1384	9.1695	9.1037	8.9505	9.3637	9.1081	9.0724	9.0503	9.1077	9.5078	9.1501	9.2489	8.9606	8.8156	8.9536	8.9729	8.9861
Hnrnpa2b1	_IDTIEIITDR_.2	#N/A	8.8694	9.0445	9.3414	8.9218	8.5838	8.6163	8.6776	8.6946	8.6410	8.7328	8.7801	8.6542	8.6000	9.1184	8.4937	8.5820	8.7475	8.5146	8.4562	8.6375
Hnrnpa2b1	_EDTEEHHLR_.2	#N/A	6.7364	6.4054	7.7046	6.5029	6.3965	7.0025	6.7156	6.8395	6.7175	6.7017	7.0832	6.7010	5.7866	7.1780	7.6331	7.1345	7.2421	7.0492	6.2545	6.9754
Hnrnpa2b1	_GFGFVTFDDHDPVDK_.2	#N/A	6.3893	6.4664	7.0696	6.5360	6.2170	6.6639	6.6148	6.2232	6.3771	6.7449	6.7639	6.5631	6.8626	7.2184	6.3602	6.4034	6.6321	6.1767	6.3693	6.6844
Hnrnpa2b1	_NYYEQWGK_.2	#N/A	6.8127	7.4274	7.2038	7.4504	7.3196	7.5027	7.6519	7.1576	6.9091	7.8349	7.6835	7.5270	7.4598	7.4183	7.9029	7.5484	5.9702	7.4402	7.8391	7.9428
Hnrnpa2b1	_PHS[Phospho (STY)]IDGR_.2	O88569_S85_M1_Hnrnpa2b1	5.0584	4.1229	4.2197	5.5711	5.5291	4.2598	4.4431	5.5376	5.0343	5.6832	4.2811	4.6225	4.3194	5.7307	3.4818	5.1431	5.2684	4.3891	4.6416	5.2991
Hnrnpa2b1	_YHTINGHNAEVR_.3	#N/A	3.8975	5.3095	6.2011	6.1944	5.5631	5.6649	6.0848	3.1670	6.1772	6.0811	5.9466	5.3259	4.4730	4.0168	6.3394	5.9884	5.2185	5.0109	5.6827	5.0577
Hnrnpa2b1	_QEMQEVQSSR_.2	#N/A	3.4135	6.8594	7.5870	7.6503	5.5696	6.4776	6.9591	3.6392	6.5141	6.0719	6.9366	5.5361	3.5133	7.7990	6.6587	4.2765	3.1364	5.4292	4.6656	5.3358
Hnrnpa2b1	_LTDC[Carbamidomethyl (C)]VVMR_.2	#N/A	4.2672	3.9494	5.5390	5.7123	3.0929	4.2176	4.7466	4.4929	3.9950	4.0778	4.5187	4.2788	4.2981	5.3542	4.3552	3.6293	3.9901	3.1593	3.4584	3.5191
Hnrnpa2b1	_DYFEEYGKIDTIEIITDR_.3	#N/A	7.2901	7.3787	7.0819	7.5824	6.5678	6.1396	6.7495	7.6353	6.4047	7.6159	6.4705	7.2864	8.3907	8.0432	7.1315	7.6378	6.6203	6.7358	6.4461	6.9246
Hnrnpa2b1	_DYFEEYGK_.2	#N/A	3.9474	4.0555	4.4837	4.5599	3.4204	3.6496	4.3461	3.6477	5.1092	4.2799	3.9849	3.9449	4.4231	4.5271	3.8747	4.5651	4.1854	3.9715	3.3333	3.6442
Hnrnpa2b1	_EES[Phospho (STY)]GKPGAHVTVK_.2	O88569_S102_M1_Hnrnpa2b1	4.0678	3.3847	7.0158	3.3284	3.8362	7.1274	2.4923	4.2868	3.8453	7.5809	6.9366	7.9957	8.5332	7.9669	7.4091	7.9785	8.1043	3.0228	3.8494	3.7149
Hnrnpa2b1	_LFIGGLSFETTEESLR_.2	#N/A	5.0068	4.2445	4.4798	5.0107	4.7981	4.2447	4.4071	4.9955	4.7025	4.7998	4.3453	4.1893	5.0870	5.1437	3.5107	4.6654	4.9562	4.0171	4.6221	4.8426
Hnrnpa2b1	_EESGKPGAHVTVKK_.3	#N/A	3.4258	3.8932	4.4633	4.1584	3.6328	3.6661	3.3753	3.6515	3.6454	3.8512	4.3150	2.8065	4.4728	4.5013	4.7447	4.2888	3.1487	4.0007	5.3827	4.1014
Hnrnpa2b1	_GFGDGYNGYGGGPGGGNFGGS[Phospho (STY)]PGYGGGR_.3	O88569_S259_M1_Hnrnpa2b1	3.3993	3.9198	4.4367	4.1849	4.2780	3.0013	3.2630	3.6250	3.3221	3.6458	3.6965	3.6459	3.4302	4.4748	2.8402	3.7844	3.1222	4.0272	4.3263	4.1521
Hnrnpa2b1	_NM[Oxidation (M)]GGPYGGGNYGPGGSGGS[Phospho (STY)]GGYGGR_.3	#N/A	5.3120	4.0390	3.9091	3.8488	3.6456	3.6798	4.2332	5.2301	3.7660	5.0565	3.4697	3.7137	3.9204	4.5958	3.6046	3.7347	4.1015	3.8535	3.3959	3.9486
Hnrnpa2b1	_GGSDGYGSGR_.2	#N/A	3.6708	3.6483	3.2073	3.9135	3.8777	2.7009	3.1704	3.8964	3.5936	3.4813	3.9680	3.3745	3.7016	3.2454	3.1958	3.0329	3.3937	3.7558	4.0548	4.4235
Comt	_AVYQGPGSS[Phospho (STY)]PVKS_.2	O88587_S261_M1_Comt	12.2463	12.2458	12.0866	12.1501	11.8409	11.9662	11.8376	12.3270	11.9237	12.0445	11.7758	11.9327	12.5741	12.1576	11.3748	11.8272	12.3752	11.9356	11.8870	11.8555
Cacng2	_DAS[Phospho (STY)]PVGVK_.2	O88602_S253_M1_Cacng2	6.8411	7.3247	7.3946	8.0173	7.1432	7.8866	8.4939	6.3086	7.9008	7.9749	8.4610	7.3813	7.7348	7.4636	6.7136	7.4454	6.8939	3.7890	8.1504	8.0795
Cacng2	_STEPSHS[Phospho (STY)]RDAS[Phospho (STY)]PVGVK_.3	O88602_S249_M2_Cacng2	4.3793	5.5800	3.9159	3.2049	3.0854	3.1188	3.9627	3.7280	3.8829	4.1899	4.8092	4.1667	5.6914	4.5510	3.9880	3.7414	4.2657	5.9869	4.7954	3.0171
Pak1	_LLQTSNITK_.2	#N/A	7.1040	7.0237	7.3540	7.3162	7.3918	7.1661	7.3390	7.2346	7.7045	7.3040	7.4110	7.3897	7.6764	7.5489	7.8028	7.3241	7.3022	7.2382	7.4559	7.0019
Pak1	_STM[Oxidation (M)]VGTPYWM[Oxidation (M)]APEVVTR_.2	#N/A	7.6394	7.0154	6.2674	6.1733	6.9546	6.8886	7.0006	7.7334	7.1677	7.3836	7.0704	7.5722	7.5297	7.0372	7.2819	7.6052	7.2618	7.4466	7.2574	7.0584
Pak1	_ALYLIATNGTPELQNPEK_.2	#N/A	5.7329	5.8275	6.1992	6.4840	6.3067	5.7422	6.2841	6.3639	6.5463	6.6801	5.7763	5.5204	6.2295	6.2742	6.3124	6.3165	6.0081	6.0260	5.8671	6.0303
Pak1	_EC[Carbamidomethyl (C)]LQALEFLHSNQVIHR_.3	#N/A	6.3569	6.4115	5.2014	6.5042	6.3613	5.5041	5.9835	6.4828	5.8582	6.1428	6.3248	6.0993	6.5556	6.3707	5.8365	5.9011	6.3009	5.7441	5.8631	6.4798
Pak1	_SAEDYNS[Phospho (STY)]SNTLNVK_.2	O88643_S155_M1_Pak1	6.8438	6.5672	6.3246	3.7416	4.1689	6.1237	4.2609	6.0580	6.1852	6.5976	4.5119	6.2681	5.9350	3.8259	5.8705	4.5870	4.2703	4.1409	4.1619	4.2282
Pak1	_DVATSPIS[Phospho (STY)]PTENNTTPPDALTR_.2	#N/A	13.4239	13.5795	13.4144	13.1989	12.7264	13.0048	12.8374	13.2669	12.9075	13.3174	9.5005	13.3047	13.8696	13.9094	12.5771	13.2727	13.4996	13.0331	13.1162	13.0736
Pak1	_MSDEEILEK_.2	#N/A	3.2558	4.0917	4.9727	5.3331	4.5029	4.8572	4.5060	3.4814	4.6349	4.5672	4.4084	4.7126	3.6710	5.2205	4.6960	4.1187	4.4795	4.1708	2.9792	4.0085
Pak1	_ELIINEILVM[Oxidation (M)]R_.2	#N/A	4.5168	4.1914	5.7376	5.1281	4.4607	4.2213	4.3805	4.4828	4.9437	4.0291	3.3132	4.5667	4.5477	4.8127	4.3050	3.8789	4.9653	4.5399	4.4595	3.9920
Pak1	_SDNILLGM[Oxidation (M)]DGSVK_.2	#N/A	6.2422	3.3281	3.7102	4.5155	4.0125	7.0913	3.5286	6.6676	6.6550	3.3802	3.9621	6.8958	6.1495	3.6973	6.8106	6.1344	6.8071	6.3524	7.1119	6.6696
Pak1	_LTDFGFC[Carbamidomethyl (C)]AQITPEQSK_.2	#N/A	4.3001	4.5198	6.5386	6.8558	6.1502	4.7195	4.0419	3.8072	6.4332	4.1107	3.4407	4.2459	4.1275	3.1293	5.8655	3.6623	4.3448	3.4883	3.1369	3.0963
Pak1	_SVIEPLPVTPTR_.2	#N/A	8.3843	8.5795	8.7506	8.2807	8.5810	8.4464	8.2899	8.7502	8.4917	8.4741	8.3871	8.3011	8.6251	8.5369	8.6508	8.3223	8.0618	8.5562	8.4010	8.4957
Pak1	_LLQTSNITKS[Phospho (STY)]EQK_.2	O88643_S115_M1_Pak1	7.0190	7.1254	6.9839	7.2842	6.4623	6.7585	6.2619	7.0882	6.9076	6.9107	6.5028	6.5692	7.3899	7.4145	6.8818	6.8687	7.1959	6.7798	6.8327	7.0832
Pak1	_[Acetyl (Protein N-term)]S[Phospho (STY)]NNGVDIQDKPPAPPMR_.2	O88643_S2_M1_Pak1	4.2795	5.1532	3.8161	4.8980	2.9856	3.6219	4.2654	4.5052	4.2023	4.2664	3.0759	4.2665	4.3104	6.4244	2.9193	3.6416	5.4306	3.1470	3.4461	3.5314
Pak1	_IGQGASGTVYTAMDVATGQEVAIK_.3	#N/A	2.3177	4.1157	4.2408	4.3809	3.4103	3.4436	4.2308	3.4290	3.5580	4.5148	3.0367	3.8419	3.2342	4.2788	3.6632	4.0663	4.4271	4.2232	3.0214	3.9561
Psmc3	_VDILDPALLR_.2	#N/A	6.7342	6.5309	6.7651	7.0784	6.9767	6.7332	6.9904	6.9092	6.2756	6.0686	6.5462	6.4838	6.0381	7.0358	6.7601	6.0829	6.2818	7.0296	6.7411	6.5475
Psmc3	_DSYLILETLPTEYDSR_.2	#N/A	6.3460	6.2485	6.6784	6.4338	6.4401	6.6333	6.1250	5.8778	6.7782	6.8596	7.0006	5.8925	6.4669	6.1991	6.7574	6.4747	6.6224	6.1096	6.1410	7.0045
Psmc3	_QTYFLPVIGLVDAEK_.2	#N/A	4.1527	3.1664	5.8537	5.2523	4.8302	3.7487	4.0164	5.1239	4.0755	4.3933	5.4553	4.8928	4.1836	5.5810	5.3765	4.7919	5.2075	4.8289	4.1766	5.7749
Psmc3	_M[Oxidation (M)]ATVWDEAEQDGIGEEVLK_.2	#N/A	5.4122	5.4412	4.4479	4.8173	5.2803	4.3644	4.8543	5.6766	4.1255	4.4212	4.3493	5.2613	6.0869	6.1678	4.0755	5.3947	5.4028	5.1799	5.1741	4.5186
Psmc3	_[Acetyl (Protein N-term)]M[Oxidation (M)]QEMNLLPTPES[Phospho (STY)]PVTR_.2	#N/A	3.3578	8.2310	7.6058	4.0763	3.9865	7.1523	8.1237	3.6530	3.3811	3.3380	7.3235	3.2764	3.5237	9.3587	3.2773	3.9230	3.4568	6.8746	4.0150	3.6199
Hcn2	_TSPYGVPGS[Phospho (STY)]PATR_.2	O88703_S771_M1_Hcn2	11.1362	10.4899	10.6310	11.2738	3.4544	9.9546	10.8614	10.4330	9.4445	10.3225	10.2909	9.3380	9.5097	10.0341	7.5736	3.9211	11.0213	10.2502	9.7744	10.5232
Hcn2	_GEPQC[Carbamidomethyl (C)]S[Phospho (STY)]PEGPAR_.2	O88703_S90_M1_Hcn2	12.2867	12.2750	12.1861	12.0674	11.9069	11.9470	12.0731	12.2511	11.8364	11.8593	11.9864	11.6529	11.7096	11.8967	10.6025	11.4381	12.4036	11.9400	11.8058	11.6311
Hcn2	_PLVGPLALGS[Phospho (STY)]PR_.2	O88703_S726_M1_Hcn2	7.4772	7.8642	6.3662	6.2936	3.9306	4.8466	8.2369	8.5520	4.3866	5.5867	5.2905	7.6889	5.9885	8.5723	3.8655	4.6349	6.5495	7.1833	5.6170	4.4521
Hcn2	_GPKVS[Phospho (STY)]FSC[Carbamidomethyl (C)]R_.2	O88703_S101_M1_Hcn2	3.7157	4.2871	4.2520	3.9737	6.3604	3.4307	3.6508	3.4161	3.5710	4.5018	3.0496	4.1765	3.7364	6.4082	3.6762	4.0606	3.9538	3.8794	3.5280	4.2061
Hcn2	_GSQAS[Phospho (STY)]FLQR_.2	O88703_S134_M1_Hcn2	7.7722	7.7164	8.1684	7.9651	7.1722	6.4957	6.3472	7.6297	7.9202	8.2706	8.0894	7.6403	7.6560	6.2291	4.6183	7.4565	8.3604	8.2747	8.2328	8.0376
Hcn2	_TAAPS[Phospho (STY)]PDRR_.2	O88703_S834_M1_Hcn2	8.3026	8.1855	4.1816	7.8821	7.8960	8.0763	7.8612	3.4623	7.8913	7.9363	7.5071	7.5125	7.9501	7.6769	6.1854	7.4113	8.0643	7.9272	7.7751	7.4373
Hcn2	_DSAS[Phospho (STY)]PGAASGLDPLDSAR_.2	#N/A	11.1914	11.0790	11.0575	10.8885	3.9139	10.6657	10.6464	10.7876	10.6868	10.8182	10.6065	10.4433	11.2145	10.5582	9.3241	10.3952	11.2015	10.5684	10.4790	10.3851
Hcn2	[Carbamidomethyl (C)]S[Phospho (STY)]RDS[Phospho (STY)]AC[Carbamidomethyl (C)]TPGAAI	O88703_S64_M2_Hcn2	6.4975	6.0263	5.4971	6.2669	5.9621	5.5101	5.6481	5.8810	4.8018	5.8189	5.8904	3.9724	5.6978	5.7045	4.2087	5.1469	3.8513	3.4763	3.8304	5.5645
Hcn2	_GAASGPSAAEEAGS[Phospho (STY)]EEAGPAGEPR_.2	O88703_S119_M1_Hcn2	9.3436	9.2336	9.0518	9.1271	8.5544	8.8983	9.0053	9.1750	8.6557	4.0506	8.6231	8.6508	9.1811	8.6917	7.4783	8.4969	9.3528	8.8461	8.6327	8.4099

Hcn2	_RDS[Phospho (STY)]ASPGAASGLDPLDSAR_.3	O88703_S840_M1_Hcn2	3.7195	3.2662	3.6823	3.9822	4.1760	4.2980	3.7257	4.3591	3.8942	3.6787	3.8022	3.0795	3.8635	3.5233	3.7908	3.4992	3.6410	3.9629	3.6115	3.4903
Hcn2	_TAAPS[Phospho (STY)]PDRRDS[Phospho (STY)]AS[Phospho (STY)]PGAASGLDPLDSAR_.3	O88703_S834_M3_Hcn2	3.7274	3.9854	4.4695	3.9989	3.4493	3.3905	3.8759	3.6445	3.7889	4.0350	3.3527	3.7930	3.8970	3.6398	3.6491	4.2857	4.4110	3.7262	4.0252	3.8083
Hcn2	_RAPPGPLPPAAS[Phospho (STY)]PGPPAAS[Phospho (STY)]PPAAPS[Phospho (STY)]SPR_.3	O88703_S743_M3_Hcn2	3.2443	3.2576	3.7806	4.4451	4.0829	3.9021	6.5823	4.4455	3.5173	3.4506	3.8917	3.1471	3.2650	3.7677	3.0354	3.5892	3.4824	6.8306	3.9994	3.7347
Hcn1	_LGT[Phospho (STY)]PPGGGAAGK_.2	#N/A	11.8739	11.6578	11.3569	11.3957	11.4880	11.5043	11.3635	11.7534	11.3219	11.3100	11.4549	11.2703	11.6757	11.3431	10.2648	10.8158	11.7445	11.6035	11.2767	11.1434
Hcn1	_RLGT[Phospho (STY)]PPGGGAAGK_.2	#N/A	8.5343	8.4334	8.7988	8.2994	8.7069	8.5149	8.8651	8.9525	8.5265	8.8781	8.7290	8.7777	8.8083	9.0156	7.9186	8.6068	9.2895	8.9095	8.6130	8.6392
Hcn1	_VDGGGGEEPAGS[Phospho (STY)]FEDAEGPR_.2	#N/A	7.2964	7.3370	7.2199	6.9790	3.7936	7.0272	7.1302	6.7947	7.2545	6.9022	6.0008	7.1713	7.7335	7.1765	5.6918	7.0235	8.0041	7.5353	7.2412	6.8973
Hcn1	_KNS[Phospho (STY)]ILLQK_.2	O88704_S588_M1_Hcn1	5.2623	5.4030	4.3107	4.3274	4.6936	5.4015	5.1286	4.8926	4.5882	4.4215	4.9655	4.0536	4.2600	3.7737	2.4948	3.9913	5.2987	4.9805	4.6749	4.5198
Hcn1	_VDGGGGEEPAGS[Phospho (STY)]FEDAEGPRR_.3	#N/A	4.5986	5.3096	4.1846	4.2793	3.1293	3.4833	4.4918	3.3598	3.8950	4.6028	3.7951	4.7842	4.2192	5.2614	3.9894	4.4118	5.0054	5.3562	5.4318	4.6100
Hcn1	_QMSS[Phospho (STY)]GAIPPNR_.2	O88704_S868_M1_Hcn1	3.8599	3.4558	3.5825	3.1424	4.0668	4.1002	2.9813	4.2474	4.4023	3.6705	3.8809	3.1853	4.5677	3.5696	3.0066	3.2220	3.2842	3.0481	4.1975	3.5366
Hcn3	_KRS[Phospho (STY)]EPS[Phospho (STY)]PGSSGGVM[Oxidation (M)]EQHLVQHDR_.4	#N/A	4.1085	5.0123	4.3985	3.3168	3.7346	3.6340	4.1340	4.9969	4.1931	4.2786	3.0666	4.2787	4.3012	3.8449	3.8572	4.0113	4.9833	3.1591	3.4582	3.5222
Ctbp1	_PLVALLDGR_.2	#N/A	7.3127	7.5693	7.5124	7.6709	7.7683	7.7872	7.6296	7.5615	7.7126	7.6357	7.6145	7.6466	7.3513	7.7954	7.4895	7.4766	7.3218	7.6671	7.7919	7.5871
Ctbp1	_IGSGFDNIDIK_.2	#N/A	4.9328	4.8921	5.1042	5.2306	4.9494	5.3995	5.3595	5.1587	5.7416	5.2729	5.1878	5.7224	5.7806	4.7124	4.4578	4.0902	5.0776	5.3174	4.9507	5.0601
Ctbp1	_GAALDVHESEPFSFSQGPLK_.3	#N/A	3.2328	5.5624	6.3262	5.6189	5.2850	5.0131	4.9730	4.2754	5.6095	5.5712	5.6871	5.3932	5.8704	5.8028	5.7750	4.5569	5.2138	4.9900	5.6131	5.0210
Ctbp1	_VGQAVALR_.2	#N/A	4.5332	4.9119	4.3435	5.2244	5.0610	4.6360	5.3903	4.8297	5.2858	5.3115	5.2885	5.0545	5.5751	5.2621	5.4285	4.8699	4.7434	5.2479	5.4872	5.2225
Ctbp1	_TTWLHQALR_.2	#N/A	6.7668	6.9217	3.8941	7.1436	7.1553	7.0287	6.8362	7.0067	6.7178	6.8729	6.2086	6.9788	6.9287	6.5529	6.7517	6.6631	7.3883	6.9131	6.9376	6.5020
Ctbp1	_VQSVEQIR_.2	#N/A	6.3797	6.3954	6.5844	6.4591	6.7538	6.5546	6.8003	6.7221	6.7739	6.6766	6.7654	6.7602	6.8244	7.1255	6.9224	6.6964	6.6221	6.6782	6.8811	6.7850
Ctbp1	_DVATVAFC[Carbamidomethyl (C)]DAQSTQEIHEK_.3	#N/A	7.4158	7.3502	6.9017	7.2268	6.9113	6.5932	6.6451	7.5963	6.8271	7.1345	7.1584	6.8382	6.1853	7.8206	6.1338	7.0168	7.3303	6.5949	6.5993	6.7300
Ctbp1	_VLNEAVGALM[Oxidation (M)]YHTITLTR_.2	#N/A	2.7824	4.2677	3.9970	3.7511	2.7686	2.7552	3.0119	3.2475	3.3500	2.6530	3.3166	3.0501	3.1173	4.0705	3.0532	3.4523	3.7852	4.0490	2.8832	4.0375
Map7	_LSSSSATLNS[Phospho (STY)]PDR_.2	O88735_S209_M1_Map7	10.4300	10.2544	10.2920	10.6270	9.9013	9.8286	9.7950	9.9116	9.5200	10.3907	10.2649	9.9692	10.4756	10.7407	9.4296	10.1575	10.4887	9.9587	9.8935	10.3152
Bsn	_SLS[Phospho (STY)]DPKPLS[Phospho (STY)]PTAEESAKER_.3	#N/A	8.2419	9.0482	8.0599	9.0496	6.4731	7.4378	7.3280	7.0653	7.7117	9.0763	6.3251	8.5455	8.1678	8.5003	7.5010	8.6977	8.1821	7.8736	7.9086	8.6482
Bsn	_GEEES[Phospho (STY)]EEDSYDPR_.2	O88737_S3301_M1_Bsn	10.6971	10.3655	9.9996	10.6742	10.2276	10.1863	10.1480	10.6874	9.9401	10.8342	10.4232	10.5324	10.9606	11.3352	9.7988	10.6358	10.7462	10.6136	10.4911	10.7857
Bsn	_DYLS[Phospho (STY)]DSELNQLR_.2	#N/A	8.9979	10.2742	10.0442	9.7469	3.8561	9.4175	9.5382	4.2187	9.2842	10.3511	3.8523	8.5950	4.5391	11.1581	8.5512	9.4155	3.2556	10.0938	9.2499	9.6937
Bsn	_DAC[Carbamidomethyl (C)]EPES[Phospho (STY)]GPDPSTVR_.2	#N/A	9.5333	9.6276	9.7685	9.9996	9.3371	9.3666	9.2234	9.2972	9.2617	9.9268	9.4514	8.9790	9.1810	10.2052	8.7684	9.5005	9.6884	9.1068	9.3985	9.3939
Bsn	_DST[Phospho (STY)]PTASESQR_.2	O88737_T3254_M1_Bsn	9.3798	9.2394	9.1580	9.4318	8.7023	8.5056	9.0773	8.9462	8.8498	3.8105	3.7410	3.4852	4.4278	9.6339	2.8666	8.5270	3.1443	9.0754	9.0085	9.0841
Bsn	_ATSVPGPTQATAPPEVGR_.2	#N/A	8.2334	8.2434	8.3896	8.5920	8.1123	8.1326	8.1568	8.3261	8.1780	8.2470	8.0229	8.4291	8.9584	8.7093	8.6590	8.4523	8.6422	8.2487	8.4324	8.3186
Bsn	_DPEPPEPLTFR_.2	#N/A	6.7157	6.8812	7.0862	6.9938	7.3227	6.8277	7.0070	6.7133	7.0385	7.0392	7.3640	7.1296	7.0541	7.2038	7.0832	7.0977	7.1492	7.0354	6.9841	7.6920
Bsn	_AS[Phospho (STY)]PQAKPLR_.2	#N/A	6.5880	6.8439	6.3235	6.7010	5.7962	5.9394	5.5546	6.7546	5.8400	6.9981	6.5782	6.2371	6.8594	6.7062	5.9745	6.7425	6.5620	6.5256	6.5720	6.2389
Bsn	_ELQELQTIK_.2	#N/A	5.9667	5.6048	5.6706	5.9002	6.0091	5.6267	5.8935	5.6810	5.9318	5.8490	5.5981	6.4090	6.5950	6.1203	6.1576	5.6603	5.1647	5.4740	5.9208	5.9729
Bsn	_AQSPSGQEAES[Phospho (STY)]PR_.2	#N/A	5.7206	6.2141	3.7635	6.5556	6.0874	5.6231	6.0313	4.4284	5.1224	6.1532	5.5377	6.0789	5.9484	6.5546	3.0525	6.2601	5.9005	5.7843	5.5311	6.2337
Bsn	_YLELGITQR_.2	#N/A	4.1159	4.4522	6.1611	4.8108	4.9950	4.7799	5.7652	5.0413	5.9695	5.2920	4.9723	5.7188	5.6812	5.6481	4.9950	5.4730	5.4939	5.4466	5.3178	5.3869
Bsn	_SQASEEES[Phospho (STY)]PVS[Phospho (STY)]PLGRPR_.2	#N/A	8.6393	9.4890	8.9607	9.5782	8.2577	9.2270	9.3947	8.8736	9.1997	9.5727	8.9323	9.5279	9.8516	10.2336	9.4117	9.7096	9.7726	9.5262	9.7310	9.7175
Bsn	_QTS[Phospho (STY)]LADLEQK_.2	O88737_S3158_M1_Bsn	8.3084	8.1544	9.8332	3.3995	2.8908	3.7167	4.1573	4.4104	4.1075	4.3612	2.9811	4.3613	4.2156	8.4409	4.1827	3.5468	3.9076	3.2418	3.5409	3.4366
Bsn	_SHGPLLPTIEDSS[Phospho (STY)]EEEELREEEELLREQEK_.4	#N/A	3.6138	4.0725	4.3778	4.0604	3.4089	3.1871	3.5200	3.5540	3.4973	6.4756	3.4238	3.8873	3.6387	4.3905	5.9210	4.0649	4.3409	3.9303	3.9782	4.2180
Bsn	_LRDAC[Carbamidomethyl (C)]EPES[Phospho (STY)]GPDPSTVR_.3	#N/A	4.1518	3.2541	3.6019	5.8619	3.9577	5.2681	4.1351	4.1937	4.1181	4.1879	4.0813	2.9802	5.4460	3.5304	3.2182	5.3247	4.6060	3.2865	4.0570	5.3580
Bsn	_SQAS[Phospho (STY)]EEESPVSPLGR_.2	#N/A	12.7492	12.6121	12.6091	12.6034	12.0823	12.1477	12.0145	12.3609	12.0265	12.5621	12.1586	12.0561	12.6443	12.7590	11.6427	12.2531	12.5375	11.9312	12.1509	12.3502
Bsn	_IADSSVQT[Phospho (STY)]DDEEGEGR_.2	#N/A	9.9242	10.0269	9.6199	9.9890	9.4084	9.5236	9.7558	9.7037	9.5282	10.2085	9.3918	9.7547	10.0452	10.6012	9.0538	9.7567	9.8586	9.6441	9.4975	9.7771
Bsn	_EKPLSGQDSEVGAPQPSR_.3	#N/A	6.5748	6.8683	4.2504	6.5038	6.4166	6.6243	6.7115	3.4387	6.9249	6.6837	7.0014	6.9220	6.8045	7.2758	7.0854	6.8090	6.2662	6.8647	6.8119	6.5486
Bsn	_ASPQTTKAS[Phospho (STY)]PQAK_.2	#N/A	3.7937	5.7596	4.3300	3.8956	5.3648	4.8976	5.4943	3.9441	4.4687	5.5586	5.2035	4.0985	3.8145	6.2303	3.9868	4.1386	5.3078	3.8014	3.4499	5.8390
Bsn	_SLS[Phospho (STY)]DPKPLSPTAEESAK_.2	#N/A	13.6112	13.4525	13.8239	13.5828	12.9777	12.8795	12.9431	13.3318	13.0192	13.5228	13.1305	13.2754	13.8438	13.7764	12.6337	13.3149	13.6239	13.0601	13.2207	13.4276
Bsn	_SADC[Carbamidomethyl (C)]SVQT[Phospho (STY)]DDEDNADWEQPVRR_.3	O88737_T2622_M1_Bsn	3.4284	3.8907	4.4658	4.1558	3.6354	7.0176	3.2922	3.6541	3.3512	3.6167	3.7256	3.6168	3.4593	7.6901	2.8693	4.2914	3.1513	3.9981	4.2972	7.2487
Bsn	_AQSPSGQEAES[Phospho (STY)]PRR_.2	#N/A	8.1741	8.1648	8.0310	8.3388	7.5721	7.6217	7.7371	7.8159	7.4532	8.2300	7.4976	7.9907	8.3197	8.3684	7.6304	8.0588	7.9280	7.8977	7.8769	7.9314
Bsn	_YLGQGLQYGS[Phospho (STY)]TDLR_.2	O88737_S2046_M1_Bsn	4.2686	4.1053	5.3402	5.0255	4.7643	4.7044	5.0142	4.0391	5.0163	4.3774	4.3164	3.7884	4.0501	4.4545	3.7060	4.0910	4.5354	4.9287	4.8366	4.8586
Bsn	_QVEQAVQTAPYR_.2	#N/A	4.3295	5.6200	5.8636	4.9437	5.8223	4.6272	5.2802	4.6864	5.3434	5.8683	4.5388	5.1052	4.0410	5.5848	4.5968	5.1409	5.4635	5.3679	5.6040	4.7608
Bsn	_GLGEHRDYLS[Phospho (STY)]DSELNQLR_.3	#N/A	3.7569	6.0518	4.2932	4.5416	3.0947	3.3188	4.4464	3.4573	3.0047	3.9633	3.3790	4.0687	3.1127	7.0456	4.0236	4.1018	3.9950	6.2532	4.6058	3.8346
Bsn	_RIADSS[Phospho (STY)]VQTDDEEGEGR_.2	O88737_S2592_M1_Bsn	3.7455	3.3676	3.6112	3.9592	3.7245	4.3800	3.7094	3.9988	3.7058	5.4260	3.7526	3.7376	5.5707	5.8255	4.0992	5.2378	4.7778	4.9076	3.8939	5.0824
Bsn	_EQQDTAESS[Phospho (STY)]DDFGSQLR_.2	#N/A	3.5920	5.8611	4.3225	4.9743	4.0590	3.9590	4.0452	4.6366	5.3431	3.5577	4.4256	3.7484	4.1610	4.3304	3.9831	3.8544	3.9208	3.4678	4.5481	3.9403
Bsn	_LLEGSLGEPAPLPLPTPQQPPAGVPHR_.3	#N/A	3.1416	3.8206	3.8685	5.2333	4.3967	4.2358	3.6931	3.3673	4.6353	4.4691	4.6799	3.9036	3.1725	4.1343	3.6754	4.2545	4.3653	4.1652	4.2949	5.3313
Bsn	_SQQQLHS[Phospho (STY)]PALS[Phospho (STY)]PAHSPAK_.3	#N/A	6.1964	5.4477	5.9573	6.2426	4.5101	4.2975	4.5022	4.7221	4.4649	7.1334	4.9742	5.2020	3.9929	4.7412	3.4911	4.4918	3.9956	4.9469	5.0173	5.4827
Bsn	_SSC[Carbamidomethyl (C)]SEYS[Phospho (STY)]PSPSLDSEAETLDGGPTR_.3	O88737_S1137_M1_Bsn	8.7902	8.8084	4.3176	4.0635	3.4871	3.5205	3.5610	3.3263	3.4812	3.0908	7.8925	3.7650	3.6466	4.1493	3.5864	4.1431	3.8640	3.9692	3.6178	8.2199
Bsn	_EPELEM[Oxidation (M)]ESLTGS[Phospho (STY)]PEDR_.2	#N/A	6.5521	7.9701	8.3903	7.8209	3.8177	8.0842	7.7352	3.2812	8.1115	8.2522	4.6763	7.6720	6.3691	8.1139	7.4404	7.9050	8.2393	8.0075	8.4028	8.3829
Bsn	_SQGS[Phospho (STY)]FEYQDTQDHDYGGR_.3	#N/A	8.4873	8.4714	9.1421	8.9136	6.9594	7.5045	8.0260	7.0997	7.3165	9.2791	8.2970	7.6866	8.1489	8.5187	6.6123	8.3616	7.0503	7.8181	8.2167	8.3074
Bsn	_SHGPLLPTIEDSS[Phospho (STY)]EEEELREEEELLR_.4	#N/A	3.7570	3.5958	4.8075	3.6222	3.9640	3.9973	3.9686	4.3627	3.8888	4.7440	4.5775	3.3996	4.0543	3.6649	3.1219	3.1192	3.3995	3.8173	4.0946	3.6519
Bsn	_SSS[Phospho (STY)]SAVEDQKR_.2	O88737_S767_M1_Bsn	4.1093	4.3355	3.9172	3.6413	3.6071	3.7098	3.5201	4.0085	4.0815	3.9572	4.5780	3.4891	3.8796	3.6304	4.5178	3.8464	4.7018	3.2902	3.8598	3.0944
Bsn	_MASAYS[Phospho (STY)]GEK_.2	O88737_S3460_M1_Bsn	4.2211	5.8979	6.0248	3.4683	3.5589	4.0353	5.2625	3.9215	4.0764	3.9964	3.5550	3.6711	4.2419	3.2437	3.5594	3.0652	4.4592	3.3740	3.0226	3.2107
Bsn	_LQPQSQPTTR_.2	#N/A	3.7367	4.2661	5.7502	4.4822	5.5826	3.2985	6.2001	6.5127	3.0249	6.0684	3.3993	6.1121	3.1329	6.5392	6.9013	5.4386	5.8550	5.9562	5.6835	5.7762
Bsn	_QASATAPGRES[Phospho (STY)]PR_.2	O88737_S105_M1_Bsn	8.3067	7.8502	8.2149	8.0290	7.5765	7.8741	7.6656	7.7783	7.3426	8.3633	7.7698	7.8067	7.6520	8.3158	7.3584	7.7069	8.2045	7.6453	7.7262	7.8621
Bsn	_ASPQATKAS[Phospho (STY)]PQTTK_.2	O88737_S593_M1_Bsn	7.6198	7.8553	7.5211	8.0402	6.9242	6.8454	7.3047	7.3108	7.5364	7.8219	7.1442	7.6855	7.3160	8.2226	7.0480	7.7937	7.4010	7.4502	7.5600	7.6300
Bsn	_GYM[Oxidation (M)]TPT[Phospho (STY)]SPAGSER_.2	#N/A	3.5282	4.1138	3.9333	7.2673	3.7352	3.8549	5.5113	3.5827	3.2500	3.3388	3.8292	6.4951	3.5296	3.9544	3.3551	3.9827	4.2850	4.0047	4.0914	3.7335
Bsn	_LLDTSFAS[Phospho (STY)]SER_.2	O88737_S2822_M1_Bsn	9.4490	9.1209	9.1202	9.4116	5.0451	4.7648	8.7249	8.4399	8.6923	9.6125	8.9684	8.8455	4.9249	9.2424	7.9627	8.9159	9.0769	8.4766	9.0175	9.0368
Bsn	_GPGATQSGPR_.2	#N/A	3.9838	3.3353	3.5204	4.8977	5.2721	3.9177	5.2408	4.2095	4.2784	3.7944	5.3883	3.0614	4.0147	5.8202	6.6565	4.5570	3.7067	5.2073	5.5111	5.7357
Bsn	_DSAVDLSSLK_.2	#N/A	4.3403	3.8888	3.2897	4.1539	3.6373	3.3050	3.4108	3.6560	4.0208	3.2409	5.0637	4.3232	3.4612	4.5058	5.2590	4.7440	3.1532	4.3660	5.1639	5.7372
Bsn	_SKTPPSNLSPIEDAS[Phospho (STY)]PTEELR_.3	O88737_S1114_M1_Bsn	3.9629	7.6068	5.2322	6.9604	4.0209	7.4566	7.4607	5.5912	8.1711	6.6096	7.0123	7.5993	7.5601	7.6961	8.1390	7.9100	6.8831	8.1870	8.3502	8.4088
Bsn	_DAC[Carbamidomethyl (C)]EPES[Phospho (STY)]GPDPSTVRR_.3	#N/A	4.1280	3.7239	3.3144	4.7351	4.3350	4.3683	4.2140	3.9793	4.1342	3.9386	3.6128	3.6133	4.2996	3.3015	4.2394	3.4901	4.5170	3.3162	2.9648	4.5465
Bsn	_LHSS[Phospho (STY)]PVSSTLTSK_.2	O88737_S1370_M1_Bsn	3.1854	4.2821	4.2570	3.9687	3.3923	3.4257	3.6558	3.4211	3.5760	4.4968	3.0546	3.6128	3.7414	4.2441	3.6812	4.0483	3.9588	3.8744	3.5230	4.2111
Bsn	_HSYS[Phospho (STY)]LGFADGR_.2	O88737_S2029_M1_Bsn	3.5437	7.1552	4.0800	7.1244	3.7443	5.9802	3.1749	3.5424	3.2340	3.7500	3.6084	3.7267	3.3421	4.8404	4.1860	3.8886	3.6003	6.5077	3.9565	4.1936
Bsn	_QPQTQQQQQQQQQQQGLGQQAPQQAPSQAR_.3	#N/A	4.2274	3.7548	4.0676	3.6912	3.7440	3.8390	4.1601	3.9336	3.6860	4.6122	3.5590	3.5838	4.0591	4.9699	4.4951	3.9936	4.1848	3.4732	4.3276	4.0251
Bsn	_SPQVLYS[Phospho (STY)]PVS[Phospho (STY)]PLSPHR_.2	#N/A	3.6598	6.8465	4.3533	5.1669	3.2952	4.4340	6.2076	3.4175	5.4746	5.1092	3.7706	3.9803	3.8576	7.4902	4.9900	4.5433	3.8215	6.0585	4.6877	5.2699
Bsn	_QAAPGPQQSQPPSSR_.2	#N/A	3.5439	4.0242	4.3323	4.2893	2.8816	6.2856	3.1587	3.5206	6.5380	3.7502	6.4957	6.7175	6.7543	7.2409	6.9911	3.8888	6.8476	6.8008	4.4307	6.0477
Bsn	_YSS[Phospho (STY)]VSNHYSDHR_.2	O88737_S2068_M1_Bsn	6.4967	6.9541	7.4872	7.1175	6.7222	6.5983	5.9985	6.6175	6.2281	6.0135	6.2024	3.9529	4.6116	6.5421	6.0834	4.9279	3.8257	4.4403	6.0999	6.5858
Bsn	_AS[Phospho (STY)]GAGGPPR_.2	O88737_S2295_M1_Bsn	3.1725	4.0448	3.7088	5.2704	3.6096	3.9739	4.0711	4.3737	3.0278	4.6466	4.2197	3.2189	3.1932	3.6959	3.1330	3.9787	3.4106	4.4226	4.0712	3.6629
Bsn	_LQLEQIQQLQQQLQLEEQK_.3	#N/A	4.1885	3.1306	3.7251	3.5696	2.8946	4.4288	4.1535	3.1337	4.0737	4.0107	3.5523	2.7034	4.2391	2.8371	2.2642	3.8292	3.4067	3.3767	3.4610	3.2079
Bsn	_ISKFS[Phospho (STY)]PIEEAK_.2	#N/A	9.1437	3.8616	9.6507	9.2316	3.4789	8.6562	8.3218	3.8416	8.3174	9.1380	8.9294	3.7510	8.5833	9.2536	7.9840	8.7359	9.0258	8.4364	8.7956	9.0054
Bsn	_ASPQATKASS[Phospho (STY)]PQATK_.2	O88737_S579_M1_Bsn	10.6386	10.5776	10.7132	10.7568	10.1778	9.8437	10.2253	10.4278	9.9628	10.7337	10.0866	10.5797	10.9614	10.9619	9.8766	10.5727	10.4842	10.3368	10.1690	10.4203
Bsn	_QREQQDTAESS[Phospho (STY)]DDFGSQLR_.3	#N/A	3.9701	3.8139	4.0171	4.0422	2.9786	3.2773	3.2635	3.9611	3.6582	4.1730	2.5353	4.2696	3.7663	3.9799	3.3434	4.6799	3.5635	3.8845	3.3526	3.6572
Bsn	_LYSSM[Oxidation (M)]SDT[Phospho (STY)]NLAEAGLNYHAQR_.3	O88737_T1992_M1_Bsn	4.4795	4.3404	4.0161	3.1047	3.1856	3.2189	3.8625	3.2043	3.7827	4.2901	3.2614	4.0666	3.9482	4.4508	3.8879	3.8416	4.2024	3.6677	3.3162	4.4178
Bsn	_HREEEQLLVQR_.3	#N/A	3.7391	3.6114	4.2753	4.8065	4.6950	4.5570	4.4322	3.4394	3.6563	4.0753	4.0932	3.5008	3.7983	4.5453	4.1312	4.0839	4.7762	3.7786	4.8999	3.7378
Bsn	_GYSYFTGSS[Phospho (STY)]PPLSPSTPS[Phospho (STY)]ESPTFSPGK_.3	#N/A	4.2315	4.3451	4.0946	3.4463	3.1261	3.2895	3.8940	3.2731	4.0222	4.2645	3.2747	4.1212	4.1636	7.5310	3.9194	3.9133	4.1037	3.3169	3.3576	3.9114
Bsn	_VTQHFAK_.2	#N/A	3.3353	3.9838	4.3727	4.2489	3.5423	3.5756	3.5058	3.5610	3.4260	3.1459	2.9047	3.7099	3.5915	4.0941	4.5725	3.8983	4.5591	3.3863	3.6729	4.0611
Bsn	_SQQQLHS[Phospho (STY)]PALS[Phospho (STY)]PAHS[Phospho (STY)]PAKQPLGKPEQER_.4	O88737_S241_M3_Bsn	4.1571	3.7432	4.1343	4.3115	3.0595	3.6889	3.0682	3.3101	4.0656	4.2020	3.1734	4.1742	4.1933	4.1462	4.0625	4.0667	4.0902	4.0716	4.3028	3.9780
Bsn	_SPSVS[Phospho (STY)]PDR_.2	O88737_S142_M1_Bsn	4.0456	3.9573	4.5818	3.6438	3.3833	3.6074	4.1578	3.7459	4.2169	4.2519	4.8236	3.8467	4.3249	4.5689	3.7350	4.3904	4.2836	3.5496	3.1981	3.0351
Bsn	_SSVSQSPAPTYPSDSHYTSLEQNVPR_.3	#N/A	3.3474	3.9717	4.3848	4.2368	3.5543	3.5877	3.4938	3.5731	3.1999	3.1580	3.4499	3.6978	5.0578	4.4229	3.4061	4.2103	3.0703	2.4856	4.3782	4.1002
Bsn	_HDY[Phospho (STY)]VEDSS[Phospho (STY)]EGGLSPLPPQPPAR_.3	O88737_Y829_M2_Bsn	4.0404	6.1795	4.3171	3.9636	3.6607	3.8400	3.8255	3.6694	3.8398	4.0762	3.4023	3.6977	3.8338	4.5016	3.5677	4.0092	3.9291	3.7542	3.6866	3.9872
Bsn	_GEHSSTLPASTPSYTSGTSPTLSSLEEDSDSSPS[Phospho (STY)]RR_.4	O88737_S1021_M1_Bsn	3.2590	5.5167	4.2965	4.3252	3.4660	3.1416	3.5821	3.4847	3.1819	3.7861	3.5562	3.7862	3.2899	4.3345	3.6075	4.1220	4.4828	4.1675	2.9657	4.0118
Bsn	_FS[Phospho (STY)]PIEEAK_.2	#N/A	3.8077	4.1951	4.3440	3.8817	3.1454	3.3695	4.3957	3.5081	4.4548	4.0140	3.3283	4.0846	4.5628	3.0597	3.9729	4.1526	4.0457	4.3954	3.1936	3.7839
Bsn	_ASQPVAESTPAGLGAAVYEELQTSQSIAR_.3	#N/A	3.3845	4.6183	3.9208	4.3049	4.2231	4.4472	3.3180	3.0849	3.3771	3.5908	3.7515	3.0069	3.4851	3.9079	2.8952	3.7294	3.6226	4.2106	3.8592	3.8749
Bsn	_GPANYNTC[Carbamidomethyl (C)]TAC[Carbamidomethyl (C)]K_.2	#N/A	3.8893	4.1135	4.4256	6.9821	3.2270	3.4511	4.3141	3.1752	4.3732	4.0956	3.2467	4.0030	4.4812	4.0250	3.8913	4.2342	4.1733	2.9762	3.2752	3.7023
Bsn	_S[Phospho (STY)]KSQQQLHSPALSPAHSPAK_.4	O88737_S233_M1_Bsn	3.2143	4.1047	4.2518	3.9976	3.4213	3.4546	4.5656	3.3921	3.5470	4.4258	3.0257	3.8309	3.7125	4.2151	3.6522	4.0773	3.9298	3.9033	3.5519	4.1821
Bsn	_ADTQAQPQMQGR_.2	#N/A	4.5095	3.4933	3.5449	3.1799	3.8472	4.0713	4.1312	4.2098	3.7530	3.2149	4.1274	3.3828	3.8610	5.4642	3.2711	3.3535	3.2467	3.0856	4.2351	3.0821
Birc6	_SDS[Phospho (STY)]VTGHTSQR_.2	O88738_S493_M1_Birc6	5.8547	5.7865	6.3237	5.1254	2.9699	3.0032	5.5023	3.8435	5.7822	4.0744	6.0877	4.9628	5.4657	5.2146	4.1036	3.6259	6.1328	6.2049	3.1005	5.6932
Scrg1	_ISFVPC[Carbamidomethyl (C)]NNH_.2	#N/A	6.8499	7.3142	7.1277	6.6461	6.5599	6.9877	7.0152	7.9130	6.9109	6.9768	6.6591	7.4104	8.0256	8.6499	6.9750	6.5705	7.1969	6.7689	6.8200	7.5577
Tom1	_QQS[Phospho (STY)]TGAIPATQAR_.2	O88746_S405_M1_Tom1	7.7222	7.7995	4.5571	4.3029	3.7266	7.1122	7.1591	3.0868	7.2930	3.3302	4.2212	7.5156	7.6619	3.9098	3.3469	4.3826	3.6245	4.2086	7.3924	3.8768
Tom1	_QNS[Phospho (STY)]VSSNTSQR_.2	O88746_S180_M1_Tom1	3.2119	8.4966	3.7482	4.4774	7.7076	7.6122	3.1470	7.4070	7.5489	9.1598	7.0260	7.8432	7.7214	8.2735	3.1724	3.5568	3.4500	7.7309	4.0317	7.7023
Tom1	_GSS[Phospho (STY)]LADQRK_.2	O88746_S376_M1_Tom1	3.1151	4.2118	4.3273	3.8984	3.3220	4.3234	3.7261	3.4914	3.6463	4.4265	3.1249	3.9301	3.8117	4.3144	3.7515	3.9780	4.0291	3.8041	4.4311	4.2814
Tom1	_AADRLPNLAS[Phospho (STY)]PSAEGPPRPSPGTAPR_.4	O88746_S462_M1_Tom1	3.2117	3.2902	3.7480	4.4777	4.0503	4.2744	4.4908	4.4130	3.5499	4.4181	3.9242	3.1797	3.6579	7.0289	3.0680	3.5566	3.4498	3.5428	4.0320	4.3798
Dcx	_SKQS[Phospho (STY)]PISTPTS[Phospho (STY)]PGSLRK_.3	O88809_S332_M2_Dcx	4.2059	3.7969	3.2413	3.4835	3.5436	4.4414	4.1409	3.9062	4.0611	4.0117	3.5398	3.6864	4.2266	3.2284	3.5747	3.0499	4.4439	3.3892	3.0378	3.1954
Dcx	_GNPSAAAGPKAS[Phospho (STY)]PTPQK_.2	O88809_S287_M1_Dcx	3.9006	3.4185	3.4372	3.1831	4.1075	4.1409	4.4414	4.2067	4.3616	3.7112	3.8403	3.1446	4.5271	3.5289	2.9659	3.2627	3.2436	3.0888	4.2382	3.4959
Idh1	_ATDFVVPGPGK_.2	#N/A	7.4308	7.0420	7.2868	7.1229	7.0227	7.2113	6.8892	6.8988	7.3354	7.2040	6.5729	6.9855	6.2224	7.1054	7.3231	7.0544	6.4195	6.8634	6.8315	6.9599
Idh1	_ALEDVC[Carbamidomethyl (C)]IETIEAGFM[Oxidation (M)]TK_.2	#N/A	6.2817	5.3248	5.5517	5.5716	3.6249	4.8505	5.1165	6.0239	4.8453	5.6077	4.7108	5.2850	5.8699	6.1815	4.9372	5.6840	5.0957	5.5560	5.1872	5.0891
Idh1	_TVEAEAAHGTVTR_.2	#N/A	4.1858	3.8412	4.3477	3.6720	4.4862	4.5124	4.2468	4.1817	4.7078	4.1620	4.2814	4.0885	4.1875	4.0844	4.3473	3.9578	4.1701	4.0290	3.9649	3.8802
Idh1	_HAYGDQYR_.2	#N/A	3.1344	5.0571	4.1718	4.4499	5.5308	3.3746	3.7068	3.4721	3.6270	4.4458	5.2067	4.5829	3.7924	5.0566	5.7953	3.9973	4.3581	4.2450	3.4720	4.6552
Akap10	_QSSLAEPVS[Phospho (STY)]PSKR_.2	O88845_S187_M1_Akap10	4.1333	3.1858	3.6698	7.0778	4.3402	4.3735	4.2087	4.3589	4.1290	7.5758	3.6076	4.4128	4.2944	3.2962	4.2341	6.7649	3.8562	3.3214	2.9700	6.3565
Akap10	_ASVAVHS[Phospho (STY)]PQK_.2	O88845_S52_M1_Akap10	7.3960	7.1828	3.7412	4.4845	6.7465	3.9414	4.4976	4.4061	6.0493	7.1771	6.6058	6.5224	3.2256	3.7283	5.4696	3.5498	6.2281	4.3902	4.0388	3.6953
Akap10	_NSC[Carbamidomethyl (C)]SS[Phospho (STY)]PLRELSEK_.2	O88845_S281_M1_Akap10	3.3444	3.9747	4.3818	4.1277	3.5514	3.5847	3.4967	3.2620	3.4169	4.3179	2.8956	3.7008	3.5824	4.0850	3.5221	4.2074	3.7997	4.0334	3.6820	4.0520
Tspyl1	_[Acetyl (Protein N-term)]SS[Phospho (STY)]PERDEGTPVPDSR_.2	O88852_S3_M1_Tspyl1	4.1915	3.1276	6.0094	6.1063	2.8976	4.4318	4.1505	4.4172	6.1645	4.0021	5.3493	4.3545	4.2361	3.7661	4.1759	3.5536	3.9144	6.1528	3.5341	6.6587
Ccnk	_KPS[Phospho (STY)]PQPS[Phospho (STY)]PPR_.2	O88874_S325_M2_Ccnk	9.9836	10.1874	9.7798	9.8588	9.4501	9.6878	9.7699	9.9077	9.8447	9.7611	9.4739	9.5617	10.0494	10.0123	8.9025	9.7018	9.8151	10.0152	10.0256	9.4261
Ccnk	_AVVVS[Phospho (STY)]PKEENK_.2	O88874_S341_M1_Ccnk	6.8815	6.9038	6.5336	5.7908	6.6116	6.2536	6.4543	5.9377	5.7177	6.8885	5.6634	5.7603	5.9906	5.8338	5.4298	5.6272	6.2299	6.0728	6.0600	6.0536
Zfand5	_M[Oxidation (M)]S[Phospho (STY)]PMGTASGSNSPTSDSASVQR_.2	#N/A	3.7919	3.9102	3.9118	3.8460	3.6300	3.7761	3.8874	3.7795	3.3942	3.6689	3.7686	3.7394	4.2541	4.1487	3.6042	3.7261	3.7945	4.0503	3.6003	3.4751
Mpp3	_TTGTLPS[Phospho (STY)]PQNFK_.2	O88910_S307_M1_Mpp3	12.5471	12.3161	12.0968	12.3981	12.1574	11.8959	11.8501	12.2717	11.8070	12.3493	12.4398	12.3490	12.7708	12.9848	11.6402	12.4159	12.8204	12.0779	12.1734	12.4504
Syn1	_QAS[Phospho (STY)]SGPAPTK_.2	O88935_S568_M1_Syn1	12.7315	12.4194	12.5601	12.9111	12.3040	12.1418	11.8960	12.2110	11.9820	12.9099	12.5073	11.7252	12.4779	12.8166	11.6144	12.4583	12.0395	11.6247	12.1069	11.9287
Syn1	_SLKPDFVLIR_.2	#N/A	11.5510	11.4628	11.2930	11.3584	11.5003	11.3178	11.4786	11.7269	11.0295	11.4352	11.2856	11.3783	11.5917	11.7827	11.1499	11.3461	11.4356	11.3587	11.2680	11.3540
Syn1	_TYATAEPFIDAK_.2	#N/A	11.2511	10.9217	11.2861	11.1517	11.1138	10.8589	11.0080	11.1086	11.2179	11.1553	11.0154	11.0538	11.1929	11.1389	11.1556	11.0689	11.0542	10.9267	10.8828	11.0782
Syn1	_ASGAPPGGQQR_.2	#N/A	9.7042	9.6247	9.8475	9.9028	9.9988	9.9483	9.5522	10.0752	9.9596	9.9148	9.8663	10.0476	9.5665	10.1840	10.8963	9.8743	9.6975	9.8753	9.6422	10.0325
Syn1	_VLLVIDEPHTDWAK_.2	#N/A	8.9850	8.9513	8.8905	8.9804	8.6440	8.5481	8.9119	9.1153	8.5834	8.7501	8.5472	8.7237	9.0408	9.3713	8.8575	8.6492	8.9695	8.5686	8.7081	8.5948
Syn1	_TGPPTTQQPR_.2	#N/A	9.4478	9.5657	10.0031	9.5839	9.4911	9.7698	10.1529	9.5914	9.9406	9.7266	9.9012	9.5931	9.4931	9.9299	10.3077	9.7107	9.6254	9.8665	9.6677	9.7605
Syn1	_VDNQHDFQDIASVVALTK_.3	#N/A	7.7588	8.1598	8.4203	7.6684	8.0587	8.2596	7.9509	7.8893	7.7184	8.2148	7.9306	7.6196	7.9185	8.4622	7.6463	7.8771	7.8617	7.7193	7.9121	8.0317
Syn1	_KLGTEEFPLIDQTFYPNHK_.3	#N/A	8.4620	8.5611	8.3054	8.3354	8.4362	8.2081	8.1668	8.4364	8.3288	8.4135	8.3492	8.2280	8.3898	8.3973	8.2132	8.6470	8.4015	8.2615	8.1258	8.3772
Syn1	_IHGEIDIK_.2	#N/A	7.1120	7.5073	7.8922	7.4604	7.1624	7.3691	7.1660	7.0543	7.4850	7.5344	7.8392	6.6841	7.2372	8.1235	7.1939	7.4833	7.5687	7.6155	7.7498	7.7940
Syn1	_QGPPQKPPGPAGPTR_.3	#N/A	5.9246	7.0668	7.4011	7.0828	7.1906	6.9465	7.8148	6.7222	7.4745	7.4948	7.1802	6.7997	7.4133	6.7585	8.1371	7.3623	7.0924	7.0285	7.4885	7.0763
Syn1	_EM[Oxidation (M)]LSSTTYPVVVK_.2	#N/A	9.3010	9.1897	9.3232	9.3528	9.2350	8.9668	9.2361	9.3324	9.1727	9.2861	9.1611	9.1147	9.4971	9.3304	9.2353	9.3036	9.1690	9.0349	9.1677	9.1191
Syn1	_QAGAPQATR_.2	#N/A	6.6712	6.7705	7.3718	7.0943	7.0680	6.4130	7.0613	6.9624	7.3855	7.0122	6.8067	7.3184	7.2084	7.0928	7.9394	7.2924	6.6246	7.3492	7.1570	6.9064
Syn1	_M[Oxidation (M)]TQALPR_.2	#N/A	7.5286	8.3297	8.1255	8.5918	8.0950	8.0977	8.7761	8.0541	8.3031	8.1926	7.9914	8.2938	8.2234	8.3751	8.7354	8.0258	7.6715	8.3835	8.4250	8.2951
Syn1	_QPQRDAS[Phospho (STY)]PGR_.2	#N/A	8.1316	7.7665	7.6760	7.9777	7.3585	7.4075	7.5507	7.7704	7.1809	7.8538	7.3914	7.6111	7.2232	7.5813	3.9672	6.4942	7.5383	7.1190	6.8198	7.1519
Syn1	_LGTEEFPLIDQTFYPNHK_.3	#N/A	3.7582	7.3153	4.2945	7.6653	3.0959	7.2734	7.5044	7.0468	7.4314	7.1980	7.2286	7.2192	7.3862	7.4236	7.0609	7.2173	6.8335	7.2787	7.2946	7.5788
Syn1	_ASTAAPVASPAAPSPGSSGGGGFFSSLSNAVK_.3	#N/A	6.7384	6.7388	6.7359	6.0144	5.2979	5.2034	7.3418	7.6876	5.2716	5.4329	5.3650	6.9196	6.8360	8.3815	5.5370	4.8643	5.2728	6.3833	5.0129	5.2915
Syn1	_DAS[Phospho (STY)]PGRSHSQSSSPGALTLGR_.3	#N/A	9.7614	10.0453	8.7946	9.3384	8.0694	8.3151	9.7270	10.6490	8.6542	9.3079	8.1115	9.7470	10.7247	10.6141	8.4662	8.9865	9.4266	9.5475	9.0271	8.3477
Syn1	_VKVDNQHDFQDIASVVALTK_.3	#N/A	7.5959	7.7239	7.2860	7.6577	7.5882	7.3964	7.3025	7.7972	7.3944	7.9799	7.5937	7.7571	8.3561	8.0138	7.2239	7.6126	7.7173	7.2812	7.6520	8.1168
Syn1	_QLIVELVVNK_.2	#N/A	3.0997	4.1964	5.6139	4.9644	4.1269	4.2416	4.4727	4.4983	4.4470	5.4302	4.8485	4.5056	3.8271	4.9096	3.8047	3.9626	3.4993	4.0820	4.4708	5.0823
Syn1	_TSVSGNWK_.2	#N/A	7.0774	6.7306	7.8280	7.7656	7.3020	6.8429	7.4826	7.3866	7.8492	7.7996	7.5598	7.8354	7.7356	7.7177	8.2819	7.8150	7.0312	7.7375	7.8996	7.8186
Syn1	_GSHSQSSS[Phospho (STY)]PGALTLGR_.2	#N/A	10.9619	10.8754	10.7550	9.2680	10.3985	10.3616	10.5595	10.7549	10.5029	10.6973	10.4830	10.4804	11.1335	11.0190	10.1750	10.4336	10.7186	10.1915	8.7567	10.5541
Syn1	_KIHGEIDIK_.2	#N/A	6.0864	5.3775	4.8105	5.8089	5.8894	5.2356	3.3762	5.8956	3.2964	4.9632	6.7608	5.5803	6.2750	3.9645	5.8864	5.9926	4.3600	4.7143	5.9749	4.9315
Syn1	_SQSLTNAFNLPEPAPPRPSLSQDEVKAETIR_.4	#N/A	3.8654	3.5586	3.4020	4.0815	3.1868	4.0353	4.2942	4.0911	3.0955	4.8884	3.8060	3.3903	4.6783	3.9895	3.6527	4.5341	3.5884	4.3106	3.9906	2.9856
Syn1	_QTTAAAAATFSEQVGGGSGGAGR_.2	#N/A	6.9681	5.8522	7.2481	7.3561	7.0367	6.1645	6.4366	7.2056	5.9753	7.0726	7.1742	6.5067	7.9636	6.7837	6.2158	6.7105	6.8059	5.8907	6.7962	6.4890
Syn1	_PQPPPPPPSAAS[Phospho (STY)]PGATPGSATASAER_.3	O88935_S39_M1_Syn1	10.9990	11.0590	10.6475	10.2910	9.1895	9.4158	10.7261	10.8609	9.2616	10.6709	9.7240	10.5340	10.2498	11.3573	8.4133	9.6248	9.8307	10.1433	9.6530	9.4359
Syn1	_QAS[Phospho (STY)]QAGPGPR_.2	#N/A	10.9594	11.3117	11.4649	11.2593	10.6818	10.1285	10.5020	10.0758	10.0060	10.9746	10.6155	9.5168	10.6132	10.6792	9.5147	10.8069	9.6428	10.0450	10.7929	10.4663
Syn1	_LPSPT[Phospho (STY)]AAPQQSASQATPVTQGQGR_.3	O88935_T512_M1_Syn1	7.1927	7.4041	3.9217	5.6023	3.3189	3.4573	5.3181	8.9777	3.1247	3.7295	7.3364	4.0169	5.9703	6.5929	3.8904	3.7335	5.8792	6.5459	4.6278	3.8907
Syn1	_QHAFSMAR_.2	#N/A	3.3275	4.2034	4.9724	5.3493	4.5020	3.5677	3.5137	3.5531	4.1473	3.1381	3.7471	5.9781	3.5993	4.9500	4.6410	5.1077	4.5512	3.7491	4.3981	4.7689
Syn1	_SQSLTNAFNLPEPAPPRPSLSQDEVK_.3	#N/A	5.0160	5.2541	5.8262	5.8043	5.4109	5.3091	6.9559	5.2547	5.5773	5.3838	5.0215	5.0793	5.4322	8.7212	5.7481	5.6942	5.9374	6.9220	5.5483	5.5413
Syn1	_RLS[Phospho (STY)]DSNFM[Oxidation (M)]ANLPNGYM[Oxidation (M)]TDLQR_.3	#N/A	7.3868	3.9567	7.9502	6.6803	5.6957	6.3105	8.0094	8.8912	3.6672	7.9031	3.6402	7.2144	3.8221	8.7728	5.8300	6.6778	5.6779	8.3525	7.1221	4.0678
Syn1	_PVAGGPGAPPAARPPASPS[Phospho (STY)]PQR_.3	#N/A	6.2402	6.7763	5.4361	5.9929	3.5152	4.6996	5.0159	3.6043	3.8117	6.8301	3.6758	5.5736	3.9197	8.3179	3.8064	3.8967	4.0883	5.2236	4.6354	3.6174
Syn1	_QSRPVAGGPGAPPAARPPASPS[Phospho (STY)]PQR_.4	#N/A	3.7375	6.0783	3.9171	4.0018	3.9423	4.1495	3.7605	6.4437	3.7173	4.0892	3.4998	4.0242	6.3580	4.1900	3.6847	4.9857	3.7720	3.7582	3.1560	3.9680
Syn1	_DHIEVVGSSM[Oxidation (M)]PLIGDHQDEK_.3	#N/A	5.5482	4.4538	4.7590	4.8199	4.2264	4.3462	4.7934	4.8901	4.2864	5.1943	4.5515	4.6532	4.8443	5.0343	4.9303	5.1037	5.0748	5.1488	4.6250	5.0647
Syn1	_VEQAEFSDLNLVAHANGGFSVDM[Oxidation (M)]EVLR_.3	#N/A	3.8427	4.3230	4.0335	4.5882	3.3544	3.4045	3.0678	3.2217	4.4197	4.0491	3.2932	4.0495	4.5278	5.0316	3.9378	4.1876	4.2198	4.4305	3.2287	3.7488
Syn1	_LWVDTC[Carbamidomethyl (C)]SEIFGGLDIC[Carbamidomethyl (C)]AVEALHGK_.3	#N/A	3.5315	3.7876	4.5689	4.0528	3.7384	3.7717	3.3097	3.7571	3.2299	3.3421	4.2094	3.5137	3.3954	4.6069	3.3351	4.3944	3.2544	1.9095	4.1941	3.8650
Zbtb7a	_HFKDEEDEEEAS[Phospho (STY)]PDGSGR_.3	O88939_S537_M1_Zbtb7a	3.3913	3.9278	4.4287	4.1930	3.5982	3.6315	3.4499	3.6169	7.8194	3.2019	4.3496	3.6539	8.6492	8.0836	3.4753	7.8861	7.4989	8.2254	3.7289	7.6855
Lin7b	_[Acetyl (Protein N-term)]AALVEPLGLER_.2	#N/A	6.0135	6.2969	6.7267	6.1158	6.1718	5.7797	6.4177	6.0949	6.3688	6.1547	5.8908	6.5464	6.1506	6.1131	6.6621	6.1888	6.2770	6.2049	6.0529	6.1208
Lin7b	_QQHHSYTS[Phospho (STY)]LESRG_.3	O88951_S202_M1_Lin7b	4.3069	5.2037	3.9761	4.9741	4.3534	3.6145	3.3748	4.0008	3.2950	4.5824	4.5197	3.9263	4.9438	3.9631	4.3591	3.7061	3.6778	4.1724	4.8958	3.9301
Lin7b	_QQHHSY[Phospho (STY)]TSLESR_.3	O88951_Y200_M1_Lin7b	4.4290	4.3910	3.9655	3.1553	3.1351	3.1684	3.9130	3.1538	3.8332	4.2396	3.3119	4.1171	3.9987	4.0036	3.9384	3.7911	4.1519	2.9976	3.2966	3.6809
Lin7c	_VLQSEFC[Carbamidomethyl (C)]NAVR_.2	#N/A	5.2183	5.3247	5.5000	5.6956	5.2564	5.0867	5.1171	4.7280	6.6383	5.3366	3.4518	5.2907	5.3797	5.4285	5.5877	4.6556	7.5910	4.8011	5.1593	5.2584
Cpd	_KSLLSHEFQDET[Phospho (STY)]DT[Phospho (STY)]EEETLYSSKH_.4	#N/A	3.9023	4.1005	4.4386	3.7871	3.2400	3.4641	4.3010	3.6027	4.3601	4.1086	3.2337	3.9899	4.4682	4.4257	3.8782	4.2472	4.1404	2.9892	3.3414	3.6892

Cpd	_SLLSHEFQDET[Phospho (STY)]DT[Phospho (STY)]EEETLYSSKH_.3	#N/A	3.7790	4.2593	4.0972	4.5244	3.1167	3.3408	4.4244	3.2855	2.9826	3.9853	3.3570	3.9854	4.5915	4.1353	4.0016	4.1239	4.2835	4.3667	3.1650	3.8126
Cpd	_SLLSHEFQDET[Phospho (STY)]DT[Phospho (STY)]EEETLYSSK_.3	#N/A	4.4708	3.5320	3.5062	3.2186	3.8085	4.0326	3.7326	4.1711	3.7917	3.1762	4.1661	3.4215	3.8997	3.4435	3.3098	3.3148	3.2080	3.5577	3.8567	3.1208
Lgmn	_DYTGEDVTPENFLAVLR_.2	#N/A	3.7721	3.5469	4.1588	3.8121	3.9791	3.9329	3.0690	3.9978	3.6950	3.5827	2.8138	3.2731	3.8030	3.3468	3.1071	4.3752	3.4950	3.6544	3.5461	3.6952
Sh3pxd2a	_AAS[Phospho (STY)]QGSESPLLPTQR_.2	O89032_S1029_M1_Sh3pxd2a	10.9907	10.5978	10.4380	10.6134	9.8925	9.8709	10.0060	10.0118	9.7324	10.6717	10.1914	10.0594	10.2819	3.4009	3.4022	10.0697	10.3254	10.1978	10.1359	10.1186
Sh3pxd2a	_EAEENPVGAC[Carbamidomethyl (C)]ESQGS[Phospho (STY)]PLK_.2	O89032_S546_M1_Sh3pxd2a	7.5170	7.8082	7.6593	7.2464	7.1328	7.5568	4.3294	4.2383	7.6019	4.5333	7.9301	7.0265	7.7385	3.5873	6.6693	3.3747	7.8734	7.5016	3.7130	7.1591
Sh3pxd2a	_GSSGDSDS[Phospho (STY)]PGSSSLSLAVK_.2	O89032_S643_M1_Sh3pxd2a	3.5181	5.0850	4.5542	4.1713	2.8558	5.1601	5.0554	3.2185	5.2991	3.7244	4.3529	4.3741	3.5388	6.0196	4.2624	4.2108	3.7562	4.6881	3.7256	5.0026
Sh3pxd2a	_SAS[Phospho (STY)]DAGIRDTPK_.2	O89032_S721_M1_Sh3pxd2a	3.4793	4.5235	4.0156	4.2101	4.3179	3.6670	3.4144	3.1797	3.3346	4.1796	4.3141	4.6485	3.5000	4.0027	3.4398	3.8242	2.6895	4.1158	3.7644	3.9697
Sh3pxd2a	_RIS[Phospho (STY)]PASSLQR_.2	O89032_S592_M1_Sh3pxd2a	4.0489	3.9539	4.5852	3.6405	3.3866	3.0975	6.4105	3.7493	3.9042	4.2552	5.0133	3.8434	4.0696	4.5723	3.7317	4.3938	4.2869	6.1017	3.1948	3.0384
Sh3pxd2a	_NSS[Phospho (STY)]FSTAR_.2	O89032_S1008_M1_Sh3pxd2a	4.5198	3.4830	3.5553	3.1696	3.8575	4.0816	3.6836	4.2202	3.7426	3.2253	4.1170	3.3725	3.8507	3.3945	3.2608	3.3639	3.2570	3.6067	3.9058	3.0718
Sh3pxd2a	_PEDVNPPK_.2	#N/A	4.1376	3.8652	3.1731	3.5518	3.4753	3.0088	4.0657	3.8380	3.9929	4.3439	3.4715	3.7546	4.1583	3.1601	3.6429	2.9817	4.3757	3.4575	3.1061	3.1271
Coro1a	_ATPEPSGTPSSDTVSR_.2	#N/A	9.0316	8.8148	9.7796	9.1970	9.1786	8.9486	8.9845	9.0427	9.1521	9.2803	8.8971	8.7027	8.8888	9.2351	9.1904	9.0496	8.7728	8.7724	8.7428	8.9386
Coro1a	_DGALIC[Carbamidomethyl (C)]TSC[Carbamidomethyl (C)]R_.2	#N/A	7.5273	7.5044	8.0409	7.7909	7.2850	7.4897	7.8798	8.0777	7.9179	7.7855	7.7411	7.3051	7.5575	7.8292	7.5637	7.4859	7.4658	7.3989	7.6409	7.7136
Coro1a	_ILTTGFSR_.2	#N/A	6.5555	7.1223	6.8279	7.3419	7.3892	6.8532	7.2226	6.9489	7.0355	7.2916	6.8208	7.1633	6.9764	6.3543	7.3698	7.0196	6.4716	7.4323	7.3730	7.1300
Coro1a	_QVALWDTK_.2	#N/A	6.8676	7.1811	7.2800	7.1708	7.0620	6.8710	6.5945	6.7844	6.8321	6.9329	6.8026	6.3818	7.0879	7.0172	7.1268	6.7571	6.7128	6.7879	7.0547	6.8054
Coro1a	_DAGPLLISLK_.2	#N/A	5.7478	6.1637	6.0409	5.7764	5.9509	6.5985	6.2032	4.6889	6.2322	5.8860	5.2971	6.3212	5.7702	6.0906	5.9627	6.4049	6.0514	6.5563	6.1395	5.5874
Coro1a	_HVFGQPAK_.2	#N/A	5.1342	6.3130	6.2732	6.7994	6.0287	6.3863	6.6615	6.2846	6.6390	5.8128	6.2813	6.3669	5.2107	6.0033	6.8577	5.3098	5.9155	6.6224	6.3982	6.0718
Coro1a	_VSQTTWDSGFC[Carbamidomethyl (C)]AVNPK_.2	#N/A	5.5162	4.6821	5.6164	5.4026	5.2868	5.6045	4.9578	5.4775	5.2545	4.8254	4.0307	4.3067	3.2166	3.7193	5.2390	5.0202	4.8560	4.2402	4.9823	5.6407
Coro1a	_LDRLEETVQAK_.2	#N/A	4.0436	4.2061	4.4427	3.0622	4.5278	4.8231	4.2691	4.3778	3.4336	3.5680	4.2694	3.7156	3.9350	4.0507	4.3443	4.3875	4.1008	4.1828	4.1491	4.1168
Coro1a	_NLNAIVQK_.2	#N/A	4.0585	3.9443	4.1379	3.2261	3.3962	3.4150	1.4477	4.5068	4.2039	4.2648	3.2655	2.4331	4.3120	2.5512	3.8314	4.4034	4.0040	3.1454	4.3951	1.1844
Coro1a	_KSDLFQEDLYPPTAGPDPALTAEEWLGGR_.3	#N/A	3.7484	4.2544	4.2847	3.9410	3.0861	3.3102	4.4549	3.4488	3.0132	3.9547	3.3876	4.1438	3.1212	4.2718	4.0321	4.0933	3.9865	4.3362	3.4953	3.8431
Cope	_APPVPGAVSGGS[Phospho (STY)]GEVDELFDVK_.2	#N/A	3.8211	6.5120	7.9220	4.1195	3.5432	7.8037	6.2453	3.9210	7.3416	7.3399	3.9906	6.2602	7.6062	6.8464	5.9832	6.4277	3.9185	9.0114	8.2454	3.6462
Pde4a	_S[Phospho (STY)]PLGGPPSVC[Carbamidomethyl (C)]K_.2	O89084_S204_M1_Pde4a	9.0059	8.5728	9.1281	8.9426	7.6844	7.6845	7.9034	7.4990	7.6221	9.4879	8.5561	7.9433	8.0214	8.5172	6.7659	8.2014	8.3642	7.9711	8.3281	8.2261
Pde4a	_GYS[Phospho (STY)]DSAER_.2	O89084_S49_M1_Pde4a	3.8635	4.1393	4.3998	3.8259	3.2012	3.4253	4.3398	3.2009	4.3989	4.0698	3.2725	4.0287	4.5070	4.0508	3.9170	4.2084	4.1016	4.4513	3.2495	3.7280
Pde4a	_SLS[Phospho (STY)]LSLPGPR_.2	O89084_S13_M1_Pde4a	3.6886	4.3142	7.4529	4.0008	3.0263	3.2504	3.0139	3.3890	3.0730	3.8949	3.4474	4.2037	3.1811	8.3462	4.0920	4.0335	3.9266	7.3687	3.0745	3.9030
Pde4a	_QS[Phospho (STY)]PSPTLEEEPGVLSDPALPDK_.2	O89084_S672_M1_Pde4a	3.4001	4.4968	4.0423	4.1834	3.6071	3.6404	3.4410	3.2063	3.3612	3.2107	4.3407	4.3863	3.5267	4.0293	3.4664	4.2631	3.7440	4.0891	3.7377	3.9963
Pde4a	_S[Phospho (STY)]GNQVSEYISNTFLDK_.2	O89084_S274_M1_Pde4a	3.6071	3.7120	5.6390	3.9771	3.8141	3.8474	3.2340	3.8328	3.1542	3.4177	4.1337	3.4381	3.3197	3.1818	3.2594	2.9693	3.3300	3.8194	4.1184	3.7893
Pde4a	_TLSPS[Phospho (STY)]EEAQGLLGLPSM[Oxidation (M)]AAEVEAPR_.3	O89084_S788_M1_Pde4a	5.5753	3.4493	3.4063	3.7145	3.8076	4.0317	3.7335	4.0954	3.7926	6.0012	4.1669	3.1755	3.9006	3.4444	3.3107	3.3139	3.5927	5.4496	3.8558	3.1217
Pde4a	_SDS[Phospho (STY)]DYDMSPK_.2	O89084_S147_M1_Pde4a	4.5668	3.4360	3.6023	3.1226	3.9045	4.1286	3.6365	4.2672	3.6956	3.2723	4.0700	3.3254	3.8037	3.5893	3.2137	3.4109	3.3040	3.6537	4.1777	3.0247
Rbm3	_YDSRPGGYGYGYGR_.3	#N/A	5.0023	5.9097	5.6404	6.4136	5.5158	5.4124	5.4958	4.0718	5.2919	6.1772	5.5769	4.9645	6.0996	5.6209	6.5156	5.2613	4.0612	5.2967	5.4644	5.8811
Rbm3	_YSGGNYRDNYDN_.2	#N/A	5.4132	5.9311	4.2539	6.2739	5.8944	6.3251	5.7047	6.0748	5.9905	5.4035	5.6160	6.3666	7.1876	6.0396	6.1047	5.9310	6.0981	5.5717	6.1539	5.2562
Lancl1	_SLAENYFDSTGR_.2	#N/A	6.2306	5.9133	4.0330	4.1927	5.9444	5.9047	5.8929	5.8952	5.8983	6.0143	5.7173	5.4025	6.5752	5.2681	5.1287	4.9348	6.3317	5.7240	6.0501	3.9871
Lancl1	_IPQSHIQQIC[Carbamidomethyl (C)]ENILTSGENLSR_.3	#N/A	5.6517	5.6998	5.7314	4.1466	2.8805	6.1155	5.7571	5.8197	5.2069	5.3409	4.5313	5.3061	3.3269	5.6166	3.9916	5.3737	3.7808	5.8709	4.4641	5.0954
Mt-Cyb	_DVNYGWLIR_.2	#N/A	5.9538	4.9393	5.5478	5.1504	5.1081	5.0365	5.8948	4.9700	5.3200	5.4060	4.9496	2.8694	4.9123	5.0078	4.5117	4.8793	5.5296	4.7568	4.8794	4.9669
Mtco2	_VVLPM[Oxidation (M)]ELPIR_.2	#N/A	10.3035	10.3308	9.7755	9.9499	9.2173	10.0268	9.6271	10.3977	9.7693	8.9932	9.2208	10.1823	9.2461	9.6686	9.3167	9.7339	10.0304	9.4624	9.0508	9.8710
Mtco2	_LLEVDNR_.2	#N/A	10.2775	10.4297	10.2200	10.2711	10.1187	10.4066	10.7407	10.3056	10.4652	10.3510	10.5049	10.4991	10.7957	10.1836	10.0195	10.1505	10.2382	10.6884	10.5279	10.4030
Mtco2	_ILYM[Oxidation (M)]M[Oxidation (M)]DEINNPVLTVK_.2	#N/A	7.3623	7.0354	5.6227	5.4558	7.0509	6.6693	6.7014	7.5814	6.3793	6.9493	6.3554	6.7637	7.0026	6.1194	6.1994	6.8619	7.0379	7.2116	6.7671	6.5821
Mtco2	_M[Oxidation (M)]LISSEDVLHSWAVPSLGLK_.3	#N/A	6.4885	5.4751	5.6026	6.4920	6.2051	6.2249	6.3652	6.8496	5.7808	6.9070	6.2480	6.4299	6.6050	5.8113	5.2542	6.4508	6.0884	6.8346	6.5912	6.2659
Hprt1	_VIGGDDLSTLTGK_.2	#N/A	6.6400	6.8901	7.4606	7.4326	6.8463	7.2132	7.2018	7.2827	6.6455	6.7000	6.9598	7.1403	7.1302	6.9503	7.0205	6.7231	6.5841	7.1540	6.8790	6.7463
Hprt1	_NVLIVEDIIDTGK_.2	#N/A	5.8032	5.8256	6.2129	6.3858	5.7145	5.8316	5.8142	5.7462	6.1294	6.1086	5.4819	6.2043	6.0199	6.1238	5.9276	6.4305	6.4063	5.7977	6.1759	6.1111
Hprt1	_SIPM[Oxidation (M)]TVDFIR_.2	#N/A	6.4759	5.5536	5.1875	5.1499	6.4097	6.3293	5.4407	6.4415	6.0083	6.2298	5.6914	6.3835	4.2029	3.7466	5.1934	6.4752	6.7073	6.1849	5.4547	6.1899
Hprt1	_EM[Oxidation (M)]GGHHIVALC[Carbamidomethyl (C)]VLK_.3	#N/A	3.5875	3.9894	4.0351	4.5865	3.1788	3.4029	4.3623	3.2234	3.8922	4.0474	2.7569	4.0475	4.4676	4.0732	4.1506	4.1860	4.2214	2.7552	3.2271	3.7505
Hprt1	_DLNHVC[Carbamidomethyl (C)]VISETGK_.2	#N/A	3.3825	4.0411	4.4199	4.0260	5.1649	3.7647	5.0232	5.6367	3.3446	4.9493	4.0383	3.0097	3.4032	3.9059	4.1034	4.9046	3.6206	4.2134	3.6686	3.8729
Abl1	...C[Carbamidomethyl (C)]S[Phospho (STY)]ASC[Carbamidomethyl (C)]M[Oxidation (M)]PHGAR	#N/A	4.7932	4.8405	3.6465	4.7197	4.3496	3.5614	4.0092	3.8778	3.5021	3.9205	3.7695	3.7859	3.6675	4.1702	3.6072	3.4720	3.7936	4.0101	3.6587	4.3046
Ca2	_DFPIANGDR_.2	#N/A	9.6540	9.6479	9.9942	9.8724	9.7313	9.7986	9.6146	9.6122	9.7488	9.7847	9.7881	9.5867	9.7421	10.0332	10.1209	9.8200	9.5845	9.5127	9.6938	10.0616
Ca2	_GGPLSDSYR_.2	#N/A	8.6654	8.5934	8.7675	8.4172	8.3466	8.6587	8.6693	8.4246	8.4772	8.4645	8.0886	8.4144	8.6754	8.5724	9.0164	8.3951	8.0923	8.3634	8.4007	8.4998
Ca2	_VLEALHSIK_.2	#N/A	8.8856	9.0647	8.8925	9.0274	9.1269	8.7929	8.9952	9.4470	8.5885	8.7604	8.5144	9.1154	9.1083	8.9463	9.5430	8.8455	8.9180	8.8639	8.9449	8.8994
Ca2	_[Acetyl (Protein N-term)]S[Phospho (STY)]HHWGYSK_.2	P00920_S2_M1_Ca2	7.4579	7.7457	7.1035	7.1595	7.2548	7.4326	7.3159	7.4579	7.2577	7.4490	7.0505	7.4864	7.8747	7.5605	7.5297	7.2058	7.3304	7.0942	7.3012	7.3688
Ca2	_AVQQPDGLAVLGIFLK_.2	#N/A	7.4343	7.2309	7.6631	7.3822	7.3801	7.0612	6.9305	7.2272	7.4210	7.4634	7.2592	7.3876	7.0829	6.9279	7.7224	7.5581	7.0807	7.1756	7.3675	7.5194
Ca2	_IGPASQGLQK_.2	#N/A	6.9362	6.8868	6.4915	6.3134	6.5895	6.7469	6.8163	6.6325	6.0722	6.0112	5.7807	6.5741	6.7125	6.9172	6.9716	6.1055	6.2415	6.7129	6.5779	6.4353
Ca2	_SIVNNGHSFNVEFDDSQDNAVLK_.3	#N/A	6.9049	6.6878	6.6132	6.7136	6.1903	6.5092	6.4944	6.7564	6.5091	6.5241	6.2113	6.5003	6.7537	6.7984	6.4924	6.2845	6.7642	6.2935	6.3440	6.6521
Ca2	_QSPVDIDTATAQHDPALQPLLISYDK_.3	#N/A	4.8732	5.7394	5.3717	5.8296	5.6398	6.1153	5.8841	5.9971	5.4421	5.3874	5.1086	5.5318	5.5005	6.3198	6.0771	6.0249	6.2502	5.6850	5.6582	5.6838
Ca2	_YAAELHLVHWNTK_.3	#N/A	5.9538	5.2266	6.4624	5.8095	5.4625	5.2231	5.2753	5.9869	4.9353	5.6830	5.1278	5.1201	6.0839	5.1983	5.8077	5.5324	5.2651	3.9154	4.3953	6.1377
Ca2	_TLNFNEEGDAEEAM[Oxidation (M)]VDNWRPAQPLK_.3	#N/A	4.5738	4.7560	3.8553	4.3704	3.9832	4.9719	3.7480	5.3047	5.0684	4.0610	4.1537	4.5262	5.8817	4.4268	4.3209	4.7210	5.5040	3.7497	4.5222	4.8765
Ca2	_EPITVSSEQM[Oxidation (M)]SHFR_.2	#N/A	4.1802	4.5802	4.0403	4.7627	3.7631	3.2635	4.6653	3.9812	3.9950	3.9425	3.9987	3.8534	4.3578	4.7371	4.5866	4.4253	4.0021	4.7104	3.8048	4.2717
Ca2	_HNGPENWHKDFPIANGDR_.4	#N/A	4.4387	6.2843	3.4742	3.2507	3.7765	5.3117	3.7646	7.1108	6.1794	3.1442	4.1981	3.4535	3.9318	4.9932	7.1697	6.6264	3.6238	6.0919	5.8344	6.3744
Thy1	_VTLSNQPYIK_.2	#N/A	9.2152	8.4792	8.9580	8.5981	9.1366	8.9738	9.0151	9.2882	8.8813	8.4059	8.9259	9.0160	9.1447	9.0105	8.6393	8.5672	8.9660	9.3846	8.8570	8.7639
Thy1	_DNSIQHEFSLTR_.2	#N/A	6.3635	6.4734	6.5098	6.3512	5.9282	6.6435	6.8862	5.7794	6.0788	6.2017	6.2385	6.0304	5.9416	6.8609	5.8570	5.8631	6.3396	6.9658	6.0928	6.2093
Thy1	_HVLSGTLGIPEHTYR_.3	#N/A	9.6213	9.6234	9.3856	9.4360	9.4418	9.5241	9.7636	9.7002	9.4456	9.3079	9.4622	9.3910	9.8939	9.7052	9.2266	9.3271	9.5508	9.7399	9.3466	9.3895
Thy1	_VTSLTAC[Carbamidomethyl (C)]LVNQNLR_.2	#N/A	7.8829	7.9163	8.2819	7.9176	7.6575	7.9372	8.1752	8.0605	7.9291	7.3282	7.7270	7.5217	7.9826	7.9333	7.6094	7.5806	8.1417	7.9061	7.9033	7.8728
Hba	_M[Oxidation (M)]FASFPTTK_.2	#N/A	7.3843	6.8926	7.8406	6.9119	7.4793	7.3074	7.3009	8.0501	6.7411	4.2511	6.7737	6.8578	7.5786	7.0508	8.4247	6.5012	7.2632	7.4026	6.4022	6.4834
Hba	_FLASVSTVLTSK_.2	#N/A	7.3753	6.1407	7.4984	6.0928	6.6693	6.2846	5.3563	6.8530	6.0574	3.2240	6.1574	5.4502	6.6470	5.9113	7.4055	5.8725	6.8221	6.1736	5.5019	5.7400
Hba	_VDPVNFK_.2	#N/A	5.9646	5.0970	6.6831	6.0851	6.7427	6.0640	5.9601	6.4856	5.8900	3.2626	5.4376	5.9428	6.6150	5.0817	7.8103	5.4669	5.6083	5.8296	5.7097	5.9250
Hba	_VLSGEDKSNIK_.2	#N/A	5.6915	5.7812	4.9178	4.8528	5.5822	5.4586	5.1226	6.4990	5.2020	4.0031	4.7044	5.5670	6.9190	4.9582	6.5120	5.2586	5.9389	5.4530	5.1427	4.4994
Hba	_IGGHGAEYGAEALER_.3	#N/A	5.7273	5.6487	6.3786	5.2739	5.4164	5.8613	5.6437	6.2179	5.1286	3.5336	4.8873	5.4868	6.3274	5.1670	6.3432	4.8488	5.3783	6.1288	4.6322	5.1397
Hba	_TYFPHFDVSHGSAQVK_.3	#N/A	7.6232	6.6034	8.0189	6.3338	7.2140	6.7835	6.6033	7.5520	6.8559	4.3184	6.6743	6.6447	7.5816	6.0027	7.9490	6.5109	7.1065	6.9069	6.2597	6.4456
Hbb-b1	_LLVVYPWTQR_.2	#N/A	10.6780	10.1968	10.5285	9.5490	10.3628	10.3033	9.7479	10.9882	9.4427	6.9182	9.4886	9.8270	10.7103	9.7108	10.6669	9.6862	10.4803	10.4161	9.4189	9.6151
Hbb-b1	_LHVDPENFR_.2	#N/A	10.0904	9.2204	10.1893	8.6690	9.8053	9.4549	9.0320	10.3642	8.6411	6.1354	8.9019	8.9976	9.4186	9.1270	10.4746	8.6842	9.8245	9.7165	8.7416	8.5516
Hbb-b1	_DFTPAAQAAFQK_.2	#N/A	9.3269	8.4608	9.1072	8.1327	8.5314	8.4755	8.1941	9.3965	8.0474	3.4470	7.8647	8.1478	8.7697	8.1792	9.5301	7.6800	9.0352	8.5609	7.9400	7.9189
Hbb-b1	_VHLTDAEK_.2	#N/A	8.8991	7.9989	8.8467	7.3234	8.1948	8.3204	7.8142	8.8710	7.6960	4.9338	7.7766	7.9314	8.6705	7.5803	9.1999	7.8666	8.1006	8.5334	7.8141	7.6949
Hbb-b1	_VNSDEVGGEALGR_.2	#N/A	8.9416	8.1128	9.0773	8.0159	8.5677	8.2400	8.0245	9.3440	8.3532	3.5299	7.7565	7.8581	8.6997	8.0729	9.2275	7.9773	8.6984	8.1925	8.0001	7.7330
Hbb-b1	_VITAFNDGLNHLDSLK_.3	#N/A	6.6240	6.4616	6.9407	5.7885	6.4270	6.4534	5.8112	7.1232	5.8841	4.4543	5.5907	5.9318	6.4804	6.2727	7.1159	6.1177	6.6528	6.4956	5.1356	5.5267
Hbb-b1	_GTFASLSELHC[Carbamidomethyl (C)]DK_.2	#N/A	7.6894	6.9434	8.0313	6.4649	7.1815	6.8530	6.1853	7.6464	5.9007	4.5527	6.6042	6.7127	6.5788	6.4761	7.9218	5.6554	6.7041	6.7546	6.5373	6.3542
Hbb-b1	_YFDSFGDLSSASAIM[Oxidation (M)]GNAK_.2	#N/A	5.8939	4.9887	6.1208	5.2207	4.6160	4.7821	4.3359	5.5978	4.5395	3.9113	3.9129	4.3150	6.0363	4.7605	5.4324	4.2852	5.4600	4.6613	4.5040	4.6361
Hbb-b2	_VNPDEVGGEALGR_.2	#N/A	8.8133	7.7714	8.5482	7.7718	8.4743	8.1734	7.7454	8.9292	8.3577	4.0553	7.8170	7.9471	8.8133	7.9331	9.0816	8.2488	8.5384	8.1497	8.1686	7.9157
H3-5;H3-3a	_YRPGTVALR_.2	#N/A	10.4327	10.8252	10.4257	10.3737	10.2160	10.6744	10.8827	10.5033	10.4886	10.4176	10.0443	10.4987	10.6065	10.4188	10.7767	10.2027	10.3955	10.7786	10.5108	10.1767
H3-5;H3-3a	_RVTIM[Oxidation (M)]PK_.2	#N/A	8.9521	9.0946	8.2858	7.8283	8.8961	8.9340	9.1870	8.2741	8.9748	9.0199	7.9136	8.4028	8.7346	7.8146	9.4405	8.6249	8.6682	9.4885	8.8984	8.7695
H3-5;H3-3a	_STELLIR_.2	#N/A	6.1388	6.5731	7.2232	7.4533	6.9041	6.9058	8.4972	4.5737	8.2173	8.1561	7.8542	6.9962	8.6620	7.5953	7.6974	8.1441	5.1356	8.4628	8.6133	8.1153
H3-5;H3-3a	_EIAQDFK_.2	#N/A	4.4699	5.8024	5.8051	6.2409	6.1649	6.5566	6.9281	4.3427	6.1582	6.7740	5.7021	5.9809	6.0679	5.2945	6.3837	6.1526	3.0290	6.8353	5.9775	6.5673
H3-5;H3-3a	_EIAQDFKTDLR_.2	#N/A	4.9869	5.5411	5.1634	5.0439	5.0370	5.2133	5.2092	5.0093	5.3544	4.3191	3.9332	5.4498	5.4743	4.2064	5.4600	5.7187	4.0814	5.2830	4.8514	4.3766
H3-5;H3-3a	_FQSAAIGALQEASEAYLVGLFEDTNLC[Carbamidomethyl (C)]AIHAK_.3	#N/A	4.2889	4.5311	3.8254	3.2954	2.9950	3.0283	4.0531	4.5145	3.9733	4.0995	3.4520	4.2572	4.1388	3.8635	4.0785	3.6510	4.0118	3.4770	3.1256	3.1076
Mt1	...yl (C)]C[Carbamidomethyl (C)]SC[Carbamidomethyl (C)]C[Carbamidomethyl (C)]PVGC[Carba...	#N/A	3.6478	4.3550	5.5542	4.0416	2.9855	3.4986	5.2711	3.3482	4.6471	4.5698	2.9817	4.2445	3.6685	4.1712	6.3316	3.9927	3.8858	3.9473	4.5480	4.1382
Mtnd3	_ANPYEC[Carbamidomethyl (C)]GFDPTSSAR_.2	#N/A	4.5629	8.0981	4.0995	4.5221	3.1144	3.3385	7.4353	3.2878	7.7218	3.9830	7.4389	7.6567	4.5938	4.1376	4.0038	4.1216	8.4730	8.0026	7.7392	7.2130
Mtnd4	_DNNVLQK_.2	#N/A	4.5211	4.1990	4.0577	4.5640	3.2272	3.3803	4.3848	3.2459	3.8888	4.0248	3.3175	4.0249	3.0380	4.4454	3.8463	3.8832	4.2440	4.4063	3.8658	3.7730
Mtatp8	_IYLPHSLPQQ_.2	#N/A	7.7752	7.9622	7.7100	7.4133	7.7351	7.6768	7.6482	7.9298	7.4201	7.1598	7.8247	7.5787	8.0845	7.3630	7.3247	7.3133	7.7727	8.0899	7.9723	7.4806
Gfap	_FADLTDAASR_.2	#N/A	8.2842	8.8380	9.1561	8.6482	8.5496	8.8989	8.7232	8.6988	8.8583	8.6338	7.6188	8.2652	8.3520	8.3669	8.5401	7.7091	8.0486	8.2285	8.0341	8.0334
Gfap	_LADVYQAELR_.2	#N/A	8.2600	8.8019	8.8520	8.6633	8.3238	8.7368	8.4402	8.5326	8.8127	8.5150	7.2023	7.9137	7.7553	7.9856	7.9801	7.2437	7.4871	8.0421	7.7629	7.8462
Gfap	_ITIPVQTFSNLQIR_.2	#N/A	8.0687	8.2136	8.7433	8.4363	8.0698	8.3861	7.9141	7.6809	8.3614	8.4026	7.5023	7.6118	7.8400	7.5959	7.7880	7.2283	7.2823	7.5943	7.6701	7.6842
Gfap	_ESASYQEALAR_.2	#N/A	7.6006	7.6981	8.0709	8.2484	7.8277	8.0492	7.4606	7.5301	8.3140	7.9213	6.9037	6.9390	7.2818	7.5477	7.7115	7.3333	7.1876	7.2035	7.1092	7.6877
Gfap	_FLEQQNK_.2	#N/A	7.1077	7.9812	7.9244	7.5803	7.7289	8.0887	7.2556	7.4821	8.2006	7.6370	6.7737	7.7832	7.6210	7.3427	7.8915	6.7212	6.8913	7.5853	7.4608	7.4741
Gfap	_KLLEGEENR_.2	#N/A	7.3913	7.9139	7.9932	7.8040	7.8090	8.1919	7.4818	7.7018	7.9591	7.7844	6.7357	6.9586	7.0063	7.1196	7.1771	7.2282	6.8676	7.1058	6.7728	7.2544
Gfap	_ALAAELNQLR_.2	#N/A	6.5809	7.4106	6.8576	7.1468	7.2744	7.5944	6.9169	7.0516	6.8176	7.3920	6.1827	6.6800	7.0096	6.1625	7.1177	6.4975	6.2703	6.9141	6.2090	6.4735
Gfap	_QEADEATLAR_.2	#N/A	5.8827	6.8581	7.2135	6.4454	6.5559	7.3419	6.9320	6.4740	7.1658	7.2582	5.8358	5.8391	5.1072	6.4154	7.0227	6.3182	6.0362	6.2937	6.1764	5.9775
Gfap	_HLQEYQDLLNVK_.2	#N/A	5.7112	6.2478	6.2535	6.1448	5.9049	5.7249	5.0722	5.0640	5.9107	6.4025	4.7879	4.9449	5.5457	5.2130	4.9075	4.9629	4.0815	4.9146	5.5028	4.7425
Gfap	_KVESLEEEIQFLR_.3	#N/A	6.0619	6.6014	6.2097	5.8163	6.0946	5.9745	6.2104	5.8303	5.8257	6.4439	5.6597	5.8438	5.6955	6.1286	5.7471	5.2282	5.6466	5.4713	5.0820	4.8851
Gfap	_LEAENNLAAYR_.2	#N/A	6.9869	7.0208	7.8295	7.3539	7.0140	7.5257	6.8959	5.7592	7.4371	7.1074	6.3996	6.1722	3.2654	6.0605	7.0015	6.6584	6.4196	6.6500	6.3628	6.8201
Gfap	_S[Phospho (STY)]YASETVVR_.2	P03995_S12_M1_Gfap	3.2286	7.8130	8.4439	8.0085	5.9987	6.4119	6.9901	7.1884	7.8838	7.8552	8.6111	5.5443	6.6722	5.6890	7.2348	7.0623	3.4667	6.1471	9.0510	3.7190
Gfap	_QLQALTC[Carbamidomethyl (C)]DLESLR_.2	#N/A	4.4308	5.3993	6.1928	5.2200	5.4459	5.6229	5.3733	5.7476	5.9453	5.4996	4.0520	4.4386	3.9397	5.5839	6.0246	4.8667	5.0303	5.2404	4.7297	4.9293
Gfap	_DGEVIKDSK_.2	#N/A	4.3326	5.8116	5.5841	5.4834	4.6365	5.8135	4.1165	3.3368	5.4646	5.4154	3.9238	4.1940	3.6572	4.9772	5.0985	3.4078	5.3399	4.7516	4.6533	4.0623
Gfap	_QKLQDETNLR_.2	#N/A	3.8295	5.1246	4.3658	5.5037	4.8708	4.8959	5.1753	5.4422	5.1179	5.0805	4.8408	4.6975	3.8503	4.5928	5.0221	4.1744	4.6542	3.9645	5.2404	4.6375
Gfap	_MTPPLPAR_.2	#N/A	4.0036	7.8231	8.4791	7.7559	4.4544	6.8262	6.2364	4.1004	6.4079	5.1378	5.2048	4.7579	4.1099	7.3301	6.6150	3.7384	5.8097	5.0476	4.7993	4.3755
Gfap	_DNFAQDLGTLR_.2	#N/A	6.7232	7.7692	7.6619	6.9498	7.2061	7.5241	7.1458	7.1363	7.4718	7.8828	6.4732	6.4258	6.5330	7.3116	6.3656	6.5959	6.7003	6.8711	6.8296	6.4914
Gfap	_LRLDQLTANSAR_.2	#N/A	3.9083	4.8663	5.2063	4.9189	4.3160	5.1991	4.5099	3.6087	4.3541	4.3937	3.2276	3.4323	4.4622	4.4317	4.6775	3.9875	4.4617	3.5381	2.9440	3.6832
Gfap	_LEVERDNFAQDLGTLR_.3	#N/A	3.8169	5.1399	4.0593	4.5624	4.0260	4.0717	3.8664	5.4759	5.1855	5.0308	3.3190	4.6492	4.8821	4.1765	5.0189	3.8321	4.3166	4.5825	4.7778	3.7746
Gfap	_FASYIEK_.2	#N/A	3.6121	4.4067	3.1487	4.9443	3.8191	4.1468	4.7499	3.8378	4.5364	4.6223	3.9093	3.3520	3.6430	3.1868	4.0375	2.1080	3.3350	3.8144	3.6983	4.0225
Gfap	_TQYEAVATSNM[Oxidation (M)]QETEEWYR_.2	#N/A	8.1401	7.9982	8.2506	7.8759	7.0751	7.0582	6.8260	8.0750	6.8348	7.9994	6.2338	6.8678	8.6993	8.3805	6.8729	7.2362	6.7708	6.6690	6.3188	6.4210
Gfap	_EQLAQQQVHVEM[Oxidation (M)]DVAKPDLTAALR_.3	#N/A	5.4253	5.8614	5.9328	4.7845	3.7237	5.5967	5.9300	4.4562	4.9469	5.8442	4.1111	4.3359	5.1837	3.5482	4.6503	3.8807	5.1003	3.6126	5.0176	3.0659
Gfap	_AEMMELNDR_.2	#N/A	3.3199	3.9992	6.8900	6.9021	3.5268	6.7458	4.3810	3.5455	3.2427	3.7253	5.3109	3.7254	3.3507	6.5182	4.4159	4.1828	4.5436	4.1067	4.4057	4.0726
Gfap	_LEEEGQSLKEEMAR_.2	#N/A	3.6982	4.6428	4.3909	4.6842	3.7647	3.5675	4.1534	4.1274	3.2981	3.8291	3.7609	3.5413	3.7189	3.7012	3.4748	4.0431	3.9363	4.0162	3.6648	3.4165
Gfap	_LALDIEIATYR_.2	#N/A	3.3518	3.1502	3.8881	4.3957	2.7879	4.4144	3.3507	3.7127	3.4098	3.5581	3.7842	3.0396	3.5179	4.5625	4.3046	3.6967	3.2099	3.9395	4.2386	4.2398
Gfap	_GTNES[Phospho (STY)]LER_.2	P03995_S302_M1_Gfap	3.3055	4.0136	4.3429	4.2787	3.5124	3.0951	3.5357	3.5312	3.2283	3.1161	3.6027	3.7397	3.3364	4.3810	3.5610	4.1684	4.5292	4.1210	4.4201	4.0583
Mbp	_FFS[Phospho (STY)]GDRGAPK_.2	P04370_S178_M1_Mbp	9.2144	7.1817	7.8922	7.4149	6.9685	6.7074	6.8144	7.2806	6.6735	8.0488	7.0866	7.8027	7.9607	7.6802	6.8974	7.1145	7.9226	6.8182	7.1270	8.6046
Mbp	_TQDENPVVHFFK_.2	#N/A	12.6164	12.6255	12.5578	12.5680	3.0919	12.6747	12.3045	12.6521	12.4219	12.4142	6.3003	12.5144	12.7552	12.7997	12.6866	12.5117	12.5913	12.2340	12.3141	12.4640
Mbp	_LFS[Phospho (STY)]RDAPGR_.2	P04370_S96_M1_Mbp	4.5049	5.2761	3.5981	3.0587	3.7258	4.2280	3.1172	4.0604	4.1051	4.7580	3.4002	4.0406	5.2308	4.7779	4.9940	4.6375	4.7229	3.6314	4.5481	3.8973
Mbp	_NIVTPRT[Phospho (STY)]PPPSQGK_.2	#N/A	12.2015	12.8738	11.3816	12.2540	12.2408	11.8425	11.9458	13.1953	11.9078	11.9039	11.1941	12.7246	13.7178	12.7265	12.5430	12.4849	12.5160	12.3314	12.1015	12.0232
Mbp	_DTGILDS[Phospho (STY)]IGR_.2	#N/A	3.9277	9.0294	11.7569	11.6820	3.5878	11.6294	11.3223	4.7939	11.5469	11.5681	3.7308	11.2770	3.6448	9.2282	8.8675	11.4021	11.0723	11.1233	11.3413	8.7582
Mbp	_TTHYGS[Phospho (STY)]LPQK_.2	P04370_S201_M1_Mbp	13.1020	13.2804	13.3724	13.3085	12.9748	13.2733	12.9572	13.2298	13.2462	13.0495	12.7307	12.9397	13.3709	13.3916	13.3217	13.0060	13.0727	12.7429	12.8678	12.9387
Mbp	_HRDTGILDSIGR_.3	#N/A	11.8038	11.9487	11.9779	12.1280	11.6534	12.0147	11.8428	11.9052	11.9030	11.7880	11.5327	12.0577	12.4725	12.0830	12.3550	12.0842	11.8222	11.7587	11.7674	11.8560
Mbp	_TTHYGSLPQKS[Phospho (STY)]QHGR_.3	#N/A	6.9936	6.7929	7.8554	6.3974	6.4245	6.9092	5.9851	6.6444	6.3501	6.4963	6.2032	5.8499	7.1975	7.0835	6.7841	6.2296	6.6303	5.6841	5.9085	6.7968
Mbp	_DSRSGS[Phospho (STY)]PMAR_.2	#N/A	4.0807	3.7664	4.0524	3.6829	3.4184	5.2369	4.1524	3.9715	3.8580	4.2870	3.8937	3.6007	3.9923	4.0584	3.7296	3.7601	3.9510	3.5607	3.5288	3.9236
Mbp	_S[Phospho (STY)]KYLATASTM[Oxidation (M)]DHAR_.2	#N/A	6.9996	7.5834	8.5490	7.7362	7.0328	7.8053	7.0954	7.5494	7.7253	6.7256	7.2896	7.3941	6.9204	7.9626	8.1376	7.1981	7.7868	6.6855	6.7515	6.7599
Mbp	_YLATASTM[Oxidation (M)]DHAR_.2	#N/A	10.4596	10.7532	11.0670	10.9351	10.5496	10.8927	10.8264	10.5969	10.8340	10.5556	10.3969	10.6773	10.2836	10.9663	11.3109	10.6791	10.3722	10.4803	10.6087	10.4710
Mbp	_T[Phospho (STY)]PPPSQGK_.2	#N/A	4.2283	3.8242	3.2140	2.7944	2.9344	3.7002	4.1137	3.8790	4.0339	4.0389	3.5125	4.2128	4.1993	3.2011	4.1390	3.5905	4.4166	3.4165	3.0651	3.1681
Mbp	_S[Phospho (STY)]QHGRTQDENPVVHFFK_.4	#N/A	3.1864	4.1327	4.2238	4.3978	3.3933	3.2142	3.0501	3.4121	3.1092	3.8587	3.4836	3.8588	3.2173	5.9152	3.6801	4.0493	4.4101	4.2401	3.0384	3.9392
Mbp	_SGS[Phospho (STY)]PM[Oxidation (M)]AR_.2	#N/A	4.1427	3.8601	3.1781	3.5467	3.4804	3.0037	4.0777	3.8430	3.9979	4.0749	3.4766	3.7496	4.1634	3.1652	3.6379	2.9867	4.3807	3.4524	3.1010	3.1322
Prnp	_YPGQGSPGGNR_.2	#N/A	4.3545	7.3713	6.9936	7.3001	3.6922	7.2725	3.8488	7.2870	6.9889	7.1658	7.2885	6.9330	4.0160	6.9808	6.8318	7.0689	6.8281	7.0803	7.2615	6.8930
Prnp	_VVEQM[Oxidation (M)]C[Carbamidomethyl (C)]VTQYQK_.2	#N/A	6.0655	6.2825	5.7262	4.1210	6.6972	6.5399	6.6971	5.9899	6.3742	5.6945	5.9115	6.4452	6.4678	4.0918	6.2630	6.5428	6.7272	6.3315	6.1840	6.3552
Prnp	_PM[Oxidation (M)]IHFGNDWEDR_.3	#N/A	4.4079	3.6397	4.2456	4.0072	4.9872	5.2340	5.3369	5.1308	4.9874	4.8822	4.9527	3.5292	4.0074	4.0090	4.4966	4.9380	4.2464	5.3565	5.1062	3.9853
Prnp	_ESQAYYDGRR_.2	#N/A	4.5145	3.4883	3.5500	3.1749	3.8522	4.1327	6.6993	4.2149	4.3698	3.7030	3.8484	3.3778	4.5352	3.5370	3.2660	3.3586	3.2517	3.0806	4.2300	3.5040
Prnp	_HVAGAAAAGAVVGGLGGYM[Oxidation (M)]LGSAM[Oxidation (M)]SR_.3	#N/A	4.0986	3.9042	3.1341	3.5908	3.4364	3.6604	4.1047	3.7990	4.1638	4.3050	3.0374	3.7936	4.2719	4.6220	3.6819	2.9427	4.3367	3.4965	3.1451	3.4929
Aldoc	_DDNGVPFVR_.2	#N/A	9.5561	9.5314	10.1546	10.1621	9.9331	9.8743	9.6131	9.4332	9.9752	10.1070	10.1361	9.5478	9.6815	9.8202	9.9295	9.8309	9.4201	9.7002	9.9696	10.0869
Aldoc	_QVLFSADDR_.2	#N/A	9.4047	9.1590	9.7353	9.5389	9.6106	9.2161	9.4121	9.4309	9.4029	9.5337	9.1607	9.2417	9.3658	9.3467	9.3934	9.2740	9.2693	9.0409	9.2851	9.3949
Aldoc	_ELSDIALR_.2	#N/A	9.2076	9.0922	9.2974	9.3474	9.4534	9.4273	9.5291	8.9971	9.5417	9.5776	9.1939	9.2377	9.6941	9.1985	9.6661	9.4058	8.4869	9.4345	9.5115	9.4913
Aldoc	_ALQASALNAWR_.2	#N/A	8.4632	8.8762	8.9805	9.2297	8.7060	8.9912	8.7817	8.6674	8.8364	8.6867	8.5509	8.7195	8.4454	8.7849	9.0437	8.7225	8.5093	9.3492	8.5952	8.7085
Aldoc	_DNAGAATEEFIK_.2	#N/A	8.4589	8.0056	9.0695	8.8239	8.4907	8.5585	7.9833	8.4008	8.6870	8.7396	8.2959	8.1824	8.0267	8.3771	8.4310	8.4326	8.8194	7.9211	8.2003	8.6451
Aldoc	_PHSYPALSAEQKK_.3	#N/A	7.9616	8.5010	7.8604	7.7657	8.2639	8.1161	8.4686	8.3857	8.5109	8.1735	8.0621	8.0910	8.2966	8.4945	8.9641	8.4071	8.4287	8.0507	8.2917	8.1546
Aldoc	_LSQIGVENTEENR_.2	#N/A	8.2737	7.9084	8.8732	8.6797	8.1824	8.6946	8.0768	8.1681	8.3317	8.5134	8.2190	8.0239	7.8752	8.3122	7.8318	7.9906	8.5157	7.7484	8.1787	8.2499
Aldoc	_VDKGVVPLAGTDGETTTQGLDGLLER_.3	#N/A	6.0490	7.1863	5.6786	7.2547	6.6890	6.6960	6.6343	7.0009	6.9761	6.4290	6.3432	6.4341	6.7651	7.1773	6.7927	6.6869	6.3157	6.1295	6.6890	6.2871
Aldoc	_ISDRTPSALAILENANVLAR_.3	#N/A	6.4502	7.1424	4.8768	6.2133	6.6270	6.3729	6.4559	6.8414	6.1978	6.4398	6.1259	7.0728	6.7129	6.6954	6.4856	6.9978	6.4013	6.3474	6.2507	6.4770
Aldoc	_YEGSGDGGAAAQSLYIANHAY_.2	#N/A	6.6059	6.0662	6.5962	6.3646	6.2246	6.1753	6.1958	6.3870	6.2709	6.2786	5.9032	6.1829	6.3965	6.0599	6.0786	6.3429	6.2555	5.7455	6.0342	5.7887
Aldoc	_TPSALAILENANVLAR_.2	#N/A	5.4988	5.9097	5.2957	5.1079	5.8257	5.3416	5.9398	4.8080	5.3426	4.6450	5.7630	5.7896	4.9881	5.6013	5.1966	4.1486	4.9161	5.3519	5.3473	6.0263
Aldoc	_PHSYPALSAEQK_.2	#N/A	7.3024	7.3052	7.3578	7.0967	7.2756	7.5722	7.3757	6.8131	7.5403	7.2718	7.4372	7.4128	6.6384	7.6686	7.7501	7.4326	7.1243	7.4927	7.2605	7.4795
Aldoc	_TVPPAVPGVTFLSGGQSEEEASLNLNAINR_.3	#N/A	6.6401	6.8351	4.1273	6.5796	5.8584	5.9105	6.1180	6.3595	6.0770	6.7105	5.7574	5.7784	6.1582	6.7972	5.4068	6.4146	6.8227	6.1324	5.9030	6.7111
Aldoc	_GILAADESVGS[Phospho (STY)]M[Oxidation (M)]AK_.2	#N/A	9.0753	9.0623	8.9103	9.1612	8.5188	8.9854	8.5359	8.6451	9.0636	9.1485	9.1060	8.6707	8.9248	8.6144	9.3994	9.8470	7.6312	8.6904	8.9085	8.8791
Aldoc	_GVVPLAGTDGETTTQGLDGLLER_.2	#N/A	6.6182	7.2432	6.7373	7.2354	7.1292	7.0606	6.8031	6.9493	7.3574	6.9383	6.5763	6.9344	6.8620	7.2444	7.2569	7.1522	7.1820	6.7861	7.0471	7.1048
Aldoc	_YSPEEIAM[Oxidation (M)]ATVTALR_.2	#N/A	8.8427	8.8218	8.8019	8.8900	8.7016	8.7883	8.5109	8.7984	8.6752	8.6797	8.5849	8.4409	8.5221	8.5776	8.7194	8.5390	8.7384	8.5569	8.6024	8.6078
Aldoc	_AEM[Oxidation (M)]NGLAAQGR_.2	#N/A	6.6735	7.1528	7.5236	6.9974	6.7618	7.3010	7.1927	7.2382	6.9468	6.6805	6.6400	6.8825	6.6752	7.0187	7.5655	6.7745	6.6145	6.5942	6.8049	6.8068
Aldoc	_LSQIGVENTEENRR_.3	#N/A	5.0315	5.4265	5.0404	5.5998	5.8057	5.5797	5.8017	3.5175	5.8647	6.2280	5.2557	5.2362	6.1681	5.3357	6.0490	5.9675	4.5514	5.2290	5.8725	5.8912
Aldoc	_RLS[Phospho (STY)]QIGVENTEENRR_.3	P05063_S45_M1_Aldoc	5.3166	5.5701	4.3411	6.1199	4.9007	5.4183	5.7290	4.7310	5.8602	5.5144	5.3711	5.1530	5.8818	5.9310	5.3380	5.4009	4.5931	5.4444	5.6062	5.6854
Aldoc	_DNAGAATEEFIKR_.2	#N/A	8.8404	9.1356	9.2100	9.3444	9.1134	8.8577	9.0248	9.3373	9.3352	8.8849	8.6873	9.1312	9.2492	9.2863	9.1845	9.2338	8.8959	8.9048	9.0165	9.0663

Aldoc	_GILAADESVGS[Phospho (STY)]M[Oxidation (M)]AKR_.2	#N/A	5.6922	6.6827	5.9957	6.1684	5.3585	6.0221	6.1201	6.2468	5.9053	5.9327	4.7845	5.5981	6.4997	6.4252	5.5475	6.0877	5.6140	5.6138	5.3073	5.7584
Aldoa	_GILAADESTGS[Phospho (STY)]IAK_.2	#N/A	10.6547	11.5450	11.7573	11.4974	10.9682	11.4544	11.0886	10.5536	11.4461	11.4629	11.1150	11.1918	10.8321	11.8369	11.2833	11.4708	11.6568	11.2547	11.4044	11.5911
Aldoa	_ELSDIAHR_.2	#N/A	11.3035	11.4079	11.5684	11.2878	11.4121	11.4699	11.4432	11.5015	11.5329	11.3988	11.3679	11.4695	11.3989	11.5649	11.7814	11.4611	11.2754	11.3808	11.3106	11.4717
Aldoa	_ALANSLAC[Carbamidomethyl (C)]QGK_.2	#N/A	10.8350	11.1111	11.0834	11.0058	10.9302	10.9394	11.2699	11.0562	10.9301	10.8793	10.7869	11.1911	11.1548	11.2139	11.6510	10.8242	10.8257	11.0334	11.0994	10.9367
Aldoa	_PHPYPALTPEQK_.2	#N/A	10.0243	10.0003	10.1961	9.8007	10.0025	10.0150	10.0218	10.0122	10.0241	10.0813	10.0519	10.1560	9.9574	10.2300	10.3704	9.9884	10.1021	10.1369	10.0424	10.1203
Aldoa	_DGADFAKWR_.2	#N/A	10.9685	11.2979	10.8992	10.9420	11.0822	11.1491	10.7360	10.9823	10.6370	10.9166	11.0569	10.9413	11.5774	11.3285	10.2548	10.3471	11.5955	10.8372	10.5860	11.0269
Aldoa	_AAQEEYIKR_.2	#N/A	10.7773	10.7771	10.8887	10.8440	10.8170	10.7890	10.9131	10.9013	11.0914	10.8560	10.6264	10.7813	11.0773	10.9642	11.1136	10.9805	10.6609	10.7431	10.7127	10.8889
Aldoa	_LQSIGTENTEENR_.2	#N/A	8.5662	8.4443	8.7918	8.6859	8.4752	8.7656	8.2470	8.3833	8.5280	8.6328	8.6885	8.3272	8.3013	9.0779	8.2921	8.6264	8.8767	8.3507	8.4338	8.7811
Aldoa	_GVVPLAGTNGETTTQGLDGLSER_.2	#N/A	8.4412	8.6700	8.6745	8.7317	8.4057	8.5537	8.6207	8.5027	8.5393	8.6614	8.6542	8.5092	8.2578	9.0538	9.0132	8.5502	8.7145	8.5429	8.4759	8.4250
Aldoa	_YASIC[Carbamidomethyl (C)]QQNGIVPIVEPEILPDGDHDLKR_.4	#N/A	9.0506	9.4775	9.5518	9.5307	9.0628	9.3148	9.4500	9.3712	9.4846	9.2595	8.9833	9.3399	9.1928	9.8114	9.6945	9.1569	9.0941	9.4591	9.4502	9.3122
Aldoa	_PHPYPALTPEQKK_.3	#N/A	9.0074	9.2483	8.3122	9.0632	9.2897	9.0481	9.1775	9.5585	8.9769	9.1937	8.7107	9.6243	9.7160	9.3322	9.6358	9.3045	9.2146	9.2223	9.2926	9.2502
Aldoa	_IGEHTPSALAIM[Oxidation (M)]ENANVLAR_.3	#N/A	8.1347	8.1218	8.4340	8.8105	8.0311	8.3185	8.0868	8.1148	7.6030	8.3629	8.4340	8.0691	8.3778	9.0669	8.0110	7.7040	8.3813	8.5017	7.9323	8.3018
Aldoa	_KELSDIAHR_.2	#N/A	7.4416	8.5415	8.4780	8.0409	7.8579	8.3325	8.5421	8.1443	8.0255	8.5463	8.4094	8.1945	8.2758	8.2751	8.7469	8.3201	8.3487	8.1937	8.4677	8.3561
Aldoa	_VDKGVVPLAGTNGETTTQGLDGLSER_.3	#N/A	8.0538	8.3962	8.0298	8.5914	8.1905	8.2225	8.2809	8.7185	8.2519	8.3491	8.1297	8.6377	8.9460	8.3666	8.8222	8.9480	8.4163	8.2436	8.2102	8.2621
Aldoa	_KDGADFAK_.2	#N/A	7.3191	6.9180	7.4104	6.9041	7.4110	7.6048	7.3377	7.1542	7.4320	7.6467	7.6817	7.1413	7.2670	7.3685	7.6771	7.5172	7.4430	7.1965	7.4602	7.4602
Aldoa	_AAQEEYIK_.2	#N/A	6.3899	6.4738	7.4918	6.8353	6.7674	7.0014	7.0083	6.8548	7.7520	7.1264	7.4981	7.2272	6.6144	7.5781	8.0335	7.0838	6.7807	6.9095	6.9130	7.4295
Aldoa	_GGVVGIKVDK_.2	#N/A	6.5846	7.5738	6.3041	6.8342	5.9743	6.3678	6.9048	7.0082	7.3391	6.8313	6.4940	6.8923	7.5641	7.4409	6.7357	7.0422	6.8641	6.5537	7.0175	6.9625
Aldoa	_QLLLTADDR_.2	#N/A	10.8745	10.5730	11.0469	10.7296	10.8343	10.8460	10.6193	3.4938	10.8341	10.8194	10.9353	10.5742	10.7051	10.8956	10.7053	10.6525	10.8746	10.5437	10.6133	10.9195
Aldoa	_LQSIGTENTEENRR_.3	#N/A	7.5334	7.7191	7.6451	7.4665	7.3112	8.0044	7.7672	6.8040	7.9668	7.9700	7.9607	7.6345	7.9279	7.6732	8.0823	7.5795	7.1465	7.3838	7.7072	7.9495
Aldoa	_SKGGVVGIK_.2	#N/A	7.3271	7.2262	6.8270	7.1593	7.3015	7.2857	7.1097	7.4481	7.1761	7.0383	7.1874	7.2638	6.7880	7.5011	7.6359	7.2014	7.2517	7.3674	7.3976	7.2822
Aldoa	_ALQASALK_.2	#N/A	4.1147	5.6211	4.6823	6.5098	4.6675	5.7793	6.4974	3.4682	6.1896	6.2672	6.1959	6.6099	6.7004	5.3256	6.9016	5.6761	3.8946	6.2107	6.6399	6.6354
Aldoa	_ADDGRPFPQVIK_.2	#N/A	9.1838	9.1286	9.2352	9.0725	9.1958	9.0029	9.0186	9.2229	8.9549	9.0297	8.9013	9.1584	9.0167	9.3194	9.3589	9.0872	9.2313	9.0132	8.9377	8.9766
Aldoa	_GILAADESTGS[Phospho (STY)]IAKR_.2	#N/A	10.5981	10.5710	10.8836	10.9385	10.5190	10.3405	10.3289	10.6246	10.6174	10.7041	10.3824	10.6463	11.2513	11.0366	10.4107	10.7544	10.8113	10.5757	10.6285	10.7282
Aldoa	_ALSDHHVYLEGTLLKPNM[Oxidation (M)]VTPGHAC[Carbamidomethyl (C)]TQK_.5	#N/A	7.8743	6.4347	8.1680	7.5636	7.8910	7.9466	7.2936	8.3432	8.2463	8.5898	7.7206	8.3882	8.5545	8.2666	8.3668	8.2995	8.3791	8.3437	7.5971	8.2615
Aldoa	_FSNEEIAM[Oxidation (M)]ATVTALR_.2	#N/A	7.5471	6.9140	7.1500	7.0093	7.3880	7.5152	6.7945	7.4393	7.2372	7.4950	7.3936	7.1481	7.4827	7.5151	7.0308	7.5218	7.4654	6.8842	7.6075	7.1704
Aldoa	_C[Carbamidomethyl (C)]QYVTEK_.2	#N/A	4.4731	7.5888	4.0097	3.1111	3.1792	7.9271	7.5038	3.6342	8.0713	7.2923	3.2677	4.0729	3.9545	4.4572	8.0644	7.8794	7.6391	7.9130	7.2291	8.1532
Aldoa	_RLQSIGTENTEENRR_.3	#N/A	4.0948	5.4465	4.7174	4.4884	3.0852	4.7302	5.2456	3.7933	5.0581	5.2864	4.6303	4.6407	3.8924	5.2652	5.3061	5.5352	5.1415	4.5014	4.8282	5.0753
Aldoa	_YTPSGQSGAAASESLFISNHAY_.2	#N/A	7.2731	7.4684	6.9745	7.2039	6.6155	7.1746	6.7248	7.2974	7.2077	7.3188	6.7953	6.9181	7.0493	7.9638	7.2790	7.3252	7.1502	7.2152	6.8204	7.2187
Aldoa	_VNPC[Carbamidomethyl (C)]IGGVILFHETLYQK_.2	#N/A	4.2391	3.9461	4.3001	3.6332	4.5746	4.4856	3.6129	4.7475	4.5006	4.3115	4.1853	4.0455	3.8638	4.1259	4.3201	3.8735	4.2922	4.2229	4.6667	4.7555
Aldoa	_TVPPAVTGVTFLSGGQSEEEASINLNAINK_.3	#N/A	5.8777	6.1222	3.5684	5.5109	4.0207	4.5674	5.0164	6.3847	5.6617	5.0074	4.1503	5.2620	6.0964	6.2525	5.4905	3.8745	5.8753	5.5080	5.4681	4.3082
Aldoa	_FSNEEIAMATVTALRR_.3	#N/A	3.3599	3.1421	3.8962	4.3295	4.1985	4.4226	3.2210	3.0603	3.4017	3.5662	3.7761	3.0315	3.3806	5.4363	2.9269	3.7048	3.5980	4.2352	3.8838	3.5575
Prkaca	_FKGPGDTSNFDDYEEEEIRVS[Phospho (STY)]INEK_.4	P05132_S339_M1_Prkaca	6.9078	6.8627	7.0307	6.9825	5.7902	5.6069	5.4323	7.0910	3.8635	4.2093	6.9389	4.1473	4.0289	4.5316	4.6131	4.5373	6.8809	3.5869	6.4354	2.9977
Prkaca	_WETPSQNTAQLDQFDR_.2	#N/A	5.7586	4.1100	4.2465	4.3752	4.9568	3.1915	3.0728	3.4347	3.1319	3.8361	5.1711	3.8362	3.2399	4.2845	3.8354	4.0720	4.4328	5.3158	3.0157	3.9618
Got1	_HIYLLPSGR_.2	#N/A	9.8410	9.8853	9.9343	9.7501	9.8441	9.6320	9.9432	10.2705	9.6862	9.8642	9.6870	9.7671	10.0879	9.7923	9.6017	9.6805	9.7260	9.6900	9.7929	9.6296
Got1	_ITWSNPPAQGAR_.2	#N/A	9.6078	9.5598	9.6474	9.4789	9.4129	9.4786	9.5689	9.7158	9.4165	9.5510	9.3423	9.3391	9.5115	9.5576	9.3007	9.3012	9.5228	9.1733	9.3161	9.3631
Got1	_NFGLYNER_.2	#N/A	9.7139	9.5645	9.2744	9.5260	9.6585	9.4813	9.6079	9.9809	9.0420	9.4513	9.2912	9.6612	9.8156	9.3964	8.9769	9.2422	9.4813	9.5193	9.5426	9.4527
Got1	_VGGVQSLGGTGALR_.2	#N/A	8.7830	9.1370	9.1797	8.9556	8.6650	8.9290	8.9329	9.0139	8.9711	8.7512	8.3345	8.7151	8.6207	9.0080	8.8365	8.7525	8.4864	8.9924	8.7409	8.7158
Got1	_IVAATLSDPELFK_.2	#N/A	8.9050	8.9684	9.2206	9.0996	8.7717	9.0024	8.8622	8.7685	8.7960	8.8449	8.6344	8.5164	8.7173	8.7744	8.6745	8.7140	8.7724	8.7875	8.8349	8.5834
Got1	_LVLGDNSLAIR_.2	#N/A	8.9806	9.0569	9.0214	9.2810	8.8712	9.0800	9.1582	9.3272	8.7271	8.9027	8.6527	9.0581	8.9531	8.8511	8.9001	8.7606	8.7969	8.9195	8.8729	8.8444
Got1	_DIRPYC[Carbamidomethyl (C)]YWDAEKR_.3	#N/A	7.9043	8.3618	8.6307	7.8605	8.2280	8.1494	8.2525	8.5261	8.1971	8.2634	7.9535	8.2261	8.5016	8.3480	8.0412	8.2807	8.2535	7.9945	8.1760	8.0376
Got1	_QVEYLVNEK_.2	#N/A	8.2241	7.9727	8.2994	8.3202	8.2835	8.2017	8.3719	8.4281	8.3596	8.4230	8.0123	8.0219	8.3972	8.1104	8.3555	8.2833	7.8818	7.8627	8.3206	8.2829
Got1	_APPSVFAQVPQAPPVLVFK_.2	#N/A	7.1438	7.0661	6.8989	6.9621	6.6999	7.1443	7.0994	6.5648	6.8573	6.7967	6.9271	6.8186	6.9634	7.3142	7.1399	6.7218	6.8426	7.1083	6.8692	7.1665
Got1	_NTPIYVSSPTWENHNAVFSAAGFK_.3	#N/A	7.9839	8.1480	7.3539	7.9030	7.7520	7.8371	7.3024	8.1944	7.7100	7.5595	7.8374	7.6268	7.7782	7.6261	7.4914	7.2917	7.7526	7.5940	7.5539	7.6297
Got1	_IGADFLGR_.2	#N/A	6.8016	7.1149	6.8949	7.0605	7.1335	7.1344	7.3402	7.2404	6.9923	7.1131	7.1615	7.2225	7.4997	6.8399	6.5498	6.8386	7.0246	7.2622	6.8439	7.1280
Got1	_VNLGVGAYR_.2	#N/A	4.7547	5.9190	5.1396	5.9154	6.3199	5.8514	6.2107	5.4112	5.7557	6.0512	5.3569	6.3990	5.2104	5.7858	6.3785	5.9161	5.6078	6.0685	5.8636	5.5287
Got1	_VGNLTVVGK_.2	#N/A	4.9566	5.7603	4.8375	5.6726	5.6059	6.0232	6.1630	4.3282	6.1015	5.6957	5.9532	5.8693	6.3452	5.3232	5.5912	4.9705	3.9510	5.9828	5.5733	5.6766
Got1	_IANDNSLNHEYLPILGLAEFR_.3	#N/A	5.3937	3.7516	5.1818	4.6284	5.7734	4.8364	3.7405	5.0032	5.4129	4.5218	5.4528	5.0398	3.9021	5.4270	5.0331	5.3968	4.7481	4.5227	5.3312	4.8037
Got1	_IVAATLSDPELFKEWK_.3	#N/A	2.8092	4.5350	4.5006	5.1829	4.6983	3.8129	4.6939	3.9966	4.2369	4.8622	3.6157	4.4904	5.0245	4.1835	3.9489	4.7984	3.4288	5.0807	4.5540	4.7939
Got1	_QIAAVM[Oxidation (M)]QR_.2	#N/A	5.8547	6.6268	7.2274	7.3096	6.8393	6.9883	7.1913	7.0014	6.2551	5.9991	6.9246	6.4718	6.5431	7.3945	7.2723	6.7553	5.9800	6.8353	7.0349	6.6184
Got1	_INM[Oxidation (M)]C[Carbamidomethyl (C)]GLTTK_.2	#N/A	5.8004	6.1280	6.1090	6.3868	6.1082	5.9650	6.8757	6.0258	5.7920	5.9822	5.8002	6.2055	6.2755	6.1376	6.9342	5.7090	5.8826	6.2463	6.3948	6.0848
Got1	_TPGTWSHITEQIGM[Oxidation (M)]FSFTGLNPK_.3	#N/A	6.0272	6.3465	5.9977	6.9142	6.2163	6.5706	6.2250	7.2665	3.9131	7.4011	6.6055	6.6674	7.7107	7.2696	6.0184	6.6950	6.9470	5.6857	6.3426	7.4139
Got1	_TDESQPWVLPVVR_.2	#N/A	8.8767	8.9181	9.0326	9.0165	8.7017	8.9126	8.8284	8.8604	9.0408	8.7739	8.6450	8.7444	8.9135	8.9159	8.9255	8.9384	8.8832	8.7893	8.7469	8.6101
Got1	_FLFPFFDSAYQGFASGDLEK_.2	#N/A	4.6499	4.7529	5.6314	5.0465	3.0587	4.3758	2.6479	4.9914	3.2751	4.3975	3.8199	4.2467	3.4405	4.6937	2.5053	4.1599	5.2523	3.8897	4.5223	4.7353
Got1	_LTADFRDDPDPR_.2	#N/A	7.7344	7.0051	7.3630	7.3354	7.3165	6.8763	6.6742	7.5178	7.8295	7.6575	6.7110	6.8384	6.9783	6.7261	7.5333	7.9527	7.4357	6.9831	7.3907	7.4917
Got1	_DIRPYC[Carbamidomethyl (C)]YWDAEK_.3	#N/A	4.3577	3.6451	3.3932	3.3317	3.4982	4.6525	4.7026	4.0581	3.8156	3.8598	3.6916	3.4073	4.3784	4.5521	4.4953	4.1102	2.6431	3.2374	4.4714	3.3472
Got1	_NLDYVATSIHEAVTK_.2	#N/A	7.6525	7.7568	7.9907	7.2208	7.4782	7.4310	7.3525	7.9369	7.3050	7.7476	7.2148	7.4099	8.1573	7.6715	6.9234	7.5634	7.5896	7.3691	7.5765	7.5150
Got1	_WYNGTDNK_.2	#N/A	3.5697	3.7494	4.6071	4.0146	3.7766	3.8099	5.2987	3.7953	5.4762	5.4596	4.1712	4.9451	3.3572	4.5495	5.3727	4.4326	3.2926	4.6540	4.1559	3.8268
Got1	_VLSQMEK_.2	#N/A	3.4073	3.2708	3.9436	3.9315	4.2458	3.3017	3.2952	3.1077	3.2626	3.6136	4.2420	2.9841	3.4280	4.2210	3.3423	3.7522	3.6453	4.1878	3.8364	3.5500
Got1	_YFVSEGFELFC[Carbamidomethyl (C)]AQSFSK_.2	#N/A	3.5307	4.4721	4.0670	4.1587	2.8685	3.0925	3.1718	3.5337	3.2309	3.7371	3.6053	4.3615	3.3389	4.3835	4.2498	3.8756	3.7688	4.1185	4.4175	4.0608
Got2	_IAATILTSPDLR_.2	#N/A	10.7010	10.8652	11.0298	10.9735	10.5308	10.7108	10.7995	10.8104	10.7352	10.6882	10.5640	10.6937	10.8028	10.9839	10.6145	10.8795	10.7702	10.8573	10.9098	10.6111
Got2	_ASAELALGENNEVLK_.2	#N/A	9.7914	9.8452	9.8320	9.7817	9.5454	9.4230	9.4226	9.8045	9.7829	9.8184	9.6642	9.4990	9.9820	10.0903	9.5174	9.7222	9.8426	9.7055	9.7447	9.6504
Got2	_TQLVSNLKK_.2	#N/A	8.2646	8.7366	8.7959	8.9023	8.7296	8.5946	8.8688	8.8401	8.7486	8.6468	8.5292	8.9458	8.9519	8.9401	8.9129	8.7511	8.4548	8.8957	8.9250	8.7200
Got2	_DDNGKPYVLPSVR_.2	#N/A	8.7048	8.4804	8.9115	8.7758	8.5591	8.5572	8.5139	8.4813	8.7341	8.8151	8.9919	8.5584	8.5883	8.8567	8.7533	8.6494	8.6642	8.4429	8.8179	8.6885
Got2	_PSWGNHTPIFR_.3	#N/A	8.6910	8.6469	8.3280	8.5619	8.7261	8.4653	8.5924	8.5354	8.0563	8.4620	8.4889	8.5962	8.6963	8.7831	8.6180	8.5684	8.3659	8.6728	8.5335	8.5399
Got2	_FVTVQTISGTGALR_.2	#N/A	9.3005	9.3376	9.3813	9.4500	9.2328	9.0471	9.1637	9.3843	9.2427	9.2615	9.2551	9.0368	9.7737	9.1894	9.2082	9.2923	8.9774	9.0740	9.4185	9.2520
Got2	_VGAFTVVC[Carbamidomethyl (C)]K_.2	#N/A	9.1575	9.2094	9.3443	9.2914	9.0712	9.0488	9.4068	9.4062	9.0078	9.1718	9.1105	9.3368	9.1889	9.3281	8.7809	8.8976	9.1073	9.2338	8.9969	8.9129
Got2	_VGASFLQR_.2	#N/A	9.2467	9.1398	9.1484	9.1642	9.2927	8.9111	9.2559	9.4716	8.9468	9.0188	8.8960	9.0353	9.3125	8.9682	9.0035	8.8444	9.0527	9.1567	9.1437	9.0082
Got2	_NLDKEYLPIGGLAEFC[Carbamidomethyl (C)]K_.3	#N/A	7.3662	7.8199	7.8906	7.8110	7.2575	7.3662	7.7548	7.4033	7.7214	7.7148	7.4266	7.5221	7.9060	8.0269	7.7798	7.6769	7.7243	7.7321	7.6133	7.8637
Got2	_KQWLQEVK_.2	#N/A	8.7382	8.2919	8.4076	8.2364	8.2164	8.2981	8.4102	8.6144	8.3104	8.4738	8.2462	8.4649	8.2039	8.6154	8.5067	8.3933	8.6971	8.5204	8.7998	8.5413
Got2	_KAEAQIAAK_.2	#N/A	7.8620	7.7806	8.0778	8.0620	7.8184	7.9157	7.7704	7.8800	8.2048	7.8316	8.0622	7.5067	7.5862	7.5284	8.2076	8.0308	7.7966	7.8089	7.9194	7.5885
Got2	_QWLQEVK_.2	#N/A	7.2191	6.6277	6.2690	7.1446	7.3661	6.8910	7.2161	7.0741	6.7965	6.8970	7.0862	7.1102	7.1854	7.1285	7.1325	6.8960	7.0201	7.4413	7.2159	7.2893
Got2	_TQLVSNLK_.2	#N/A	6.1145	7.0450	7.3486	7.3422	7.2470	6.8751	7.1038	6.5974	6.9306	7.1811	7.3140	6.8729	6.7641	7.0031	7.7287	7.0656	6.0815	6.9171	7.3850	7.1548
Got2	_TC[Carbamidomethyl (C)]GFDFSGALEDISK_.2	#N/A	6.0220	6.2657	7.4897	6.3324	6.5277	6.4016	5.9765	5.8853	5.9811	6.8316	6.3652	6.3427	6.3733	6.5353	6.7723	6.6250	6.9572	6.0385	6.3314	6.5841
Got2	_M[Oxidation (M)]NLGVGAYR_.2	#N/A	7.2510	6.5544	6.6410	6.6854	6.7955	6.8029	6.8870	6.7161	6.5540	7.0842	6.8072	6.7409	6.4649	6.4577	6.7604	7.2049	7.0019	6.9685	6.9040	6.6901
Got2	_DAGM[Oxidation (M)]QLQGYR_.2	#N/A	9.3690	9.2190	9.4924	9.2997	9.2644	9.2392	9.3954	9.3968	9.4942	9.2779	9.2886	9.4817	9.5886	9.5546	9.5402	9.2426	9.3885	9.3465	9.3787	9.3177
Got2	_IAATILTSPDLRK_.2	#N/A	6.3035	7.2343	6.7302	7.0641	6.6709	6.4733	6.7937	6.8021	6.6189	6.6833	5.9938	7.0157	7.0733	7.1145	6.7716	6.9723	7.0627	7.1773	6.8208	7.1645
Got2	_NM[Oxidation (M)]GLYGER_.2	#N/A	7.5517	8.1198	8.3520	8.0567	8.8485	8.3443	8.6647	8.1567	8.5620	8.3208	8.2657	9.2623	8.1610	8.5469	8.3558	8.1382	7.8173	8.3352	8.1965	8.1485
Got2	_EYLPIGGLAEFC[Carbamidomethyl (C)]K_.2	#N/A	3.3283	5.4186	6.6324	5.9719	5.7668	5.7818	5.4790	4.8571	5.0399	5.5041	5.2606	5.5299	3.3591	5.6007	5.2426	5.5160	5.9063	4.9955	5.5416	5.3632
Got2	_EFSVYMTK_.2	#N/A	4.2696	5.0207	6.5350	6.8600	5.2884	5.6913	5.8994	4.2957	5.5345	4.4759	6.5489	4.6580	4.1009	7.0532	5.7293	3.1136	3.7929	4.8226	3.6555	5.1586
Got2	_IPEQSVLLLHAC[Carbamidomethyl (C)]AHNPTGVDPRPEQWK_.5	#N/A	3.5440	6.2681	4.0803	6.7638	5.1681	5.7206	5.2172	3.2444	6.0147	3.7503	6.4891	5.7454	5.8011	6.0360	6.1417	3.8889	3.7821	6.3081	6.3459	5.9261
Got2	_ISVAGVTSGNVGYLAHAIHQVTK_.3	#N/A	5.5598	5.2531	5.6703	5.6045	5.4355	5.0496	4.4112	5.5191	5.5289	5.2865	5.4223	5.3383	6.4278	5.7674	5.3454	5.3551	5.6583	5.2134	5.2765	5.9283
Tuba1b	_EIIDLVLDR_.2	#N/A	14.4805	14.6979	14.8876	14.7328	14.3116	14.5731	14.6724	14.3596	14.5637	14.8185	13.9241	13.8640	13.9029	14.0083	13.7429	13.9567	13.8003	13.8569	13.9395	13.8221
Tuba1b	_VGINYQPPTVVPGGDLAK_.2	#N/A	14.2755	14.3236	14.3097	14.2830	8.0969	14.1893	14.2629	14.3338	14.2292	14.3998	13.5861	13.5163	13.6964	13.6863	13.1149	13.3517	13.6239	13.4846	13.3111	13.3770
Tuba1b	_IHFPLATYAPVISAEK_.2	#N/A	12.7253	12.8401	12.4820	12.5793	12.5827	12.6042	12.7621	12.8327	12.3380	12.8366	11.9568	12.0711	12.1844	12.2778	11.6884	11.8745	12.0020	12.0983	11.9713	11.9605
Tuba1b	_LSVDYGKK_.2	#N/A	12.2660	12.5063	12.3826	12.1169	12.2689	12.3417	12.4235	12.5745	12.5249	12.4882	11.5471	11.7184	11.9436	11.7549	11.9001	11.7020	11.5898	11.7134	11.5513	11.6830
Tuba1b	_DVNAAIATIK_.2	#N/A	12.9216	13.0312	13.2221	13.3969	13.0805	13.0954	13.2869	12.9837	13.1341	13.4810	12.5266	12.4470	12.8931	12.4720	12.5266	12.6203	11.8031	12.5319	12.6654	12.5156
Tuba1b	_QLFHPEQLITGK_.2	#N/A	11.5496	11.4636	11.7858	11.3484	11.3897	11.5018	11.4686	11.6244	11.2015	11.6488	10.7561	10.6650	10.7759	10.9002	10.9108	10.5935	10.9027	10.8024	10.8400	10.8317
Tuba1b	_SIQFVDWC[Carbamidomethyl (C)]PTGFK_.2	#N/A	9.8030	10.7573	10.1830	10.5056	9.8366	10.5196	10.8046	9.9333	10.2809	10.9705	8.6489	9.2905	9.7839	10.2213	9.9689	9.9600	9.7329	8.8838	9.5274	8.9812
Tuba1b	_QLFHPEQLITGKEDAANNYAR_.4	#N/A	10.1691	10.2850	9.9477	10.2768	10.1515	10.1123	10.3738	10.4499	10.1876	10.6193	9.4318	9.7509	10.3456	9.6351	9.6445	9.7881	9.6783	9.3982	9.6879	9.7617
Tuba1b	_EDAANNYAR_.2	#N/A	10.5924	10.9907	11.2389	11.1680	10.6924	11.2915	11.4870	10.2578	11.4575	11.4723	10.6657	10.3683	10.2908	10.3211	10.9003	10.3790	9.7644	10.4951	10.7295	10.5466
Tuba1b	_RNLDIERPTYTNLNR_.3	#N/A	8.8673	8.9387	8.7690	8.8519	8.8690	8.6020	8.7498	9.2182	8.6309	9.0390	8.0730	7.6746	8.5307	8.4452	7.8943	8.1064	7.9092	8.0100	8.0938	7.6284
Tuba1b	_DVNAAIATIKTK_.2	#N/A	7.2654	7.5863	6.8952	7.2870	7.1398	7.7932	7.3730	7.5047	6.9778	7.4399	6.5036	6.4080	6.3905	7.6009	5.6158	6.2460	6.8720	6.6123	6.6083	6.3940
Tuba1b	_YM[Oxidation (M)]AC[Carbamidomethyl (C)]C[Carbamidomethyl (C)]LLYR_.2	#N/A	11.6440	11.7997	11.6200	11.5723	11.5925	11.5498	11.7763	11.8838	11.4459	11.8163	10.6421	11.1726	11.2900	10.6851	10.7665	10.7334	10.9347	11.1076	11.0423	10.8726
Tuba1b	_AYHEQLSVAEITNAC[Carbamidomethyl (C)]FEPANQM[Oxidation (M)]VK_.3	#N/A	10.9095	11.1875	11.2824	11.0755	9.3145	10.7651	10.7192	10.9773	10.7418	11.1298	10.0237	9.9657	10.7497	10.7251	9.7898	10.1103	10.3089	9.8748	10.1867	10.5357
Tuba1b	_DYEEVGVDSVEGEGEEEGEEY_.2	#N/A	9.4597	9.0138	9.8835	9.5301	7.0116	8.8937	8.2547	9.1253	8.6635	9.3970	6.8470	7.9550	7.6305	9.4942	7.5446	8.5359	8.7958	7.3190	6.9574	7.3860
Tuba1b	_FDGALNVDLTEFQTNLVPYPR_.3	#N/A	8.3636	8.4721	8.2531	8.2654	7.9192	8.4440	8.2713	8.3555	8.0956	8.0496	7.3991	7.8127	7.8143	8.1985	7.4972	7.6723	7.8285	7.6865	7.6827	7.8535
Tuba1b	_LDHKFDLM[Oxidation (M)]YAK_.2	#N/A	8.8676	9.6367	8.9772	9.1295	8.8061	9.1762	9.1599	9.4201	8.8953	9.1341	7.7467	8.7169	8.7381	8.8180	8.5882	8.5734	8.4104	8.7632	8.4613	8.3181
Tuba1b	_NLDIERPTYTNLNR_.3	#N/A	13.6270	13.5255	14.0288	13.6915	13.2509	13.3921	13.3362	13.8283	13.4805	13.9462	13.0461	12.9344	13.4751	13.3712	12.8373	13.0537	13.2236	12.7231	13.0048	13.1179
Tuba1b	_AVFVDLEPTVIDEVR_.2	#N/A	13.5881	13.6623	13.9203	13.7553	13.3974	13.5716	13.5611	13.6711	13.7262	13.8076	12.8912	12.7768	13.2443	12.9189	12.8820	13.0467	13.0298	12.7526	12.9805	12.8838
Tuba1b	_EDMAALEK_.2	#N/A	3.0958	8.7561	9.5571	10.1158	7.2525	8.6042	9.4478	4.8192	8.3002	8.0603	8.5983	7.0421	3.1165	8.7999	8.3358	3.4407	6.1976	6.7276	7.1454	7.5915
Tuba1b	_TIGGGDDS[Phospho (STY)]FNTFFSETGAGK_.2	P05213_S48_M1_Tuba1b	6.2584	9.0046	9.1207	6.5807	5.7902	6.5545	8.7132	6.2482	6.6270	6.8712	6.0316	6.0405	5.5151	8.6767	5.7513	4.3078	6.2015	8.2860	6.1918	5.6714
Tuba1b	_LISQIVSSITASLR_.2	#N/A	7.9835	7.7458	8.2026	8.7554	8.4284	8.8928	7.8957	7.9740	8.8692	9.4655	7.4931	7.3927	8.0923	6.3849	8.1027	8.3059	8.2997	7.1993	8.1937	7.6532
Tuba1b	_FDLM[Oxidation (M)]YAK_.2	#N/A	9.1512	8.8805	9.3126	9.4958	9.2728	9.2844	9.5805	8.7874	9.3050	9.8715	8.8956	8.7533	9.0248	8.1650	8.7346	8.7468	7.7224	9.0856	9.0774	9.1417
Tuba1b	_AFVHWYVGEGM[Oxidation (M)]EEGEFSEAR_.3	#N/A	12.3809	12.7958	12.2018	12.2113	10.8202	11.5572	11.8609	12.6266	11.6565	12.0317	10.5391	11.3740	12.0678	12.7908	10.9341	11.3039	11.3866	11.3480	11.3159	11.0602
Tuba1b	_AVC[Carbamidomethyl (C)]M[Oxidation (M)]LSNTTAIAEAWAR_.3	#N/A	9.5071	10.1471	10.7382	10.7276	9.3720	9.7493	9.6944	9.2936	9.2691	10.4967	9.3121	8.9468	9.8791	10.2719	8.9064	9.0853	9.1067	8.6348	8.9034	9.6068
Tuba1b	_GDVVPKDVNAAIATIK_.2	#N/A	5.7628	6.2595	6.2358	6.1053	5.9422	6.5923	6.0092	6.7641	5.9692	5.7964	4.8933	5.3289	5.9924	5.9561	5.0478	3.8286	5.4445	5.4462	5.6334	5.3204
Tuba1b	_AFVHWYVGEGM[Oxidation (M)]EEGEFSEAREDM[Oxidation (M)]AALEK_.4	#N/A	7.2393	7.7894	7.6314	7.7257	6.7096	7.7167	7.0210	7.7319	6.5393	7.2138	5.8433	7.6838	8.1976	7.4611	5.6902	6.7582	6.0377	6.8179	6.8737	7.3347
Tuba1b	_LS[Phospho (STY)]VDYGK_.2	#N/A	3.1659	3.6462	3.2094	3.9114	4.0045	4.2286	3.5366	3.8985	3.5957	3.3723	5.5982	3.3724	3.7037	3.2475	3.1138	3.5108	3.3958	3.7537	4.0527	4.4256
Tuba1b	_LADQC[Carbamidomethyl (C)]TGLQGFLVFHSFGGGTGSGFTSLLM[Oxidation (M)]ER_.3	#N/A	3.3965	3.9225	4.4340	4.1877	3.6035	3.6368	3.4446	3.6222	3.3648	3.2071	4.3443	3.6487	3.5303	4.4720	3.4700	4.2595	3.1195	4.0855	4.3290	3.9999
Tuba1b	_EDM[Oxidation (M)]AALEKDYEEVGVDSVEGEGEEEGEEY_.3	#N/A	3.2344	3.7147	3.1410	3.9798	4.0729	4.2970	3.4682	3.8301	3.5272	3.4407	3.9016	3.4408	3.6353	3.1791	3.0454	3.5793	3.3273	3.8221	4.1212	4.3572
Tuba1b	_RAFVHWYVGEGMEEGEFSEAR_.3	#N/A	4.0078	3.3113	3.5444	3.5764	4.2147	4.2481	4.3342	4.2335	4.2544	3.8184	3.7331	3.0374	4.4199	3.5824	2.8587	3.3699	3.7307	3.4187	3.7178	3.3887
Tuba3a	_TIQFVDWC[Carbamidomethyl (C)]PTGFK_.2	#N/A	11.1619	11.3826	11.7240	11.5142	11.2274	11.4528	11.3364	11.2333	11.3488	11.5380	10.4568	10.4638	10.7791	10.5237	10.5818	10.4366	10.5557	10.4198	10.6553	10.4305
Tuba3a	_LIGQIVSSITASLR_.2	#N/A	8.3493	8.9619	8.3660	8.7865	8.4354	8.9298	8.9665	7.7587	8.7892	8.2671	7.2962	7.4557	7.5628	7.3742	6.6672	6.8852	7.7809	8.3551	7.9436	7.3209
Src	_S[Phospho (STY)]LEPSENVHGAGGAFPASQTPSKPASADGHR_.4	P05480_S17_M1_Src	10.1488	10.7735	9.8369	9.4620	2.8867	9.2173	10.4013	10.4155	8.6185	3.1677	4.3838	9.9849	9.8282	11.1876	4.2315	8.9958	9.4711	10.2948	9.8077	8.7374
Src	_LFGGFNSSDTVTS[Phospho (STY)]PQR_.2	P05480_S74_M1_Src	7.9337	8.6949	7.8369	7.6924	3.8094	4.2990	8.0896	8.0483	6.8011	8.1657	3.8996	7.6755	8.1337	9.0277	5.6461	7.1897	7.0888	8.2399	7.4381	6.9571
Kit	_VNS[Phospho (STY)]VGSSASSTQPLLVHEDA_.2	P05532_S962_M1_Kit	3.9557	3.3634	3.4923	3.6285	4.1627	4.1960	3.9673	4.1814	4.3065	3.7663	3.7851	3.0895	3.9866	3.5304	2.9108	3.3178	3.6786	3.4708	3.7699	4.1291
Jun;Jund	_LAS[Phospho (STY)]PELER_.2	P05627_S73_M1_Jun;Jund	5.9356	7.0695	6.3082	6.5514	6.3846	6.8661	6.1672	6.1263	6.2583	7.4507	6.8580	5.9242	7.3552	6.5847	5.4639	6.7785	6.3471	6.3849	6.4881	6.5472
Ldha	_VTLTPEEEAR_.2	#N/A	9.9322	9.7515	9.9649	9.7996	9.7439	9.8708	9.8824	9.9928	9.7615	9.7331	9.6708	9.5499	9.9549	9.6747	9.5883	9.6590	9.6837	9.7855	9.6417	9.6386
Ldha	_LVIITAGAR_.2	#N/A	8.8972	9.2299	9.3590	9.2319	8.4013	8.8964	9.1309	8.9725	9.1312	8.9793	8.7609	8.5740	8.9552	9.1847	8.7130	8.9339	8.4020	9.4509	9.0979	9.0707
Ldha	_SADTLWGIQK_.2	#N/A	9.0170	8.9767	8.8427	8.9373	8.8212	8.8198	8.6364	9.1371	8.9945	8.8238	8.8196	8.6741	9.0059	8.7980	8.7206	8.8052	8.9677	8.8375	8.7476	8.5695
Ldha	_VIGSGC[Carbamidomethyl (C)]NLDSAR_.2	#N/A	8.5633	8.9660	8.8108	8.6865	8.4309	8.4524	8.9504	9.1761	8.7887	8.7563	8.4961	8.7013	8.7713	8.5809	9.1008	8.4153	8.6569	8.7916	8.6247	8.3585
Ldha	_QVVDSAYEVIK_.2	#N/A	8.0490	8.0418	7.9844	7.8140	7.6857	7.4641	7.8075	8.1316	8.3001	8.0574	7.8904	7.3557	7.2776	7.7392	7.6646	7.8153	7.6106	7.6380	7.8734	7.5544
Ldha	_DQLIVNLLK_.2	#N/A	6.9695	7.3006	7.7915	7.3684	7.2640	7.3263	7.3105	6.8453	7.1297	7.1649	7.6880	6.9270	6.4301	7.1348	6.8454	7.0986	6.5865	7.3036	7.0996	7.3143
Ldha	_DYC[Carbamidomethyl (C)]VTANSK_.2	#N/A	7.3982	7.1355	7.2579	7.3431	7.0146	6.9183	7.2627	7.3223	7.0577	7.0053	7.1932	6.5725	6.7556	7.0483	7.3558	6.4787	6.8030	6.9877	6.7343	7.0797
Ldha	_DQLIVNLLKEEQAPQNK_.3	#N/A	7.2632	7.7445	7.1441	7.3178	7.2713	7.2578	7.4186	7.3055	7.2058	7.5568	6.9103	7.2048	7.7343	7.6386	6.3063	7.4335	7.1664	7.5587	7.0563	7.4882
Ldha	_DLADELALVDVM[Oxidation (M)]EDK_.2	#N/A	7.4603	6.8596	7.9756	7.1953	7.3303	7.2252	7.0301	6.8569	7.3879	7.3072	7.3658	6.7056	6.9364	6.6445	6.6793	7.1643	7.1553	6.8162	7.1234	7.2952
Ldha	_GEM[Oxidation (M)]M[Oxidation (M)]DLQHGSLFLK_.3	#N/A	4.4088	4.8011	4.1089	4.3826	4.8375	5.5474	4.4109	4.1047	4.0442	4.4236	3.3511	4.3775	3.3237	3.6425	4.2389	5.3137	3.0293	5.1115	4.4709	4.5024
Ldha	_SLNPELGTDADKEQWK_.2	#N/A	7.7659	7.2133	7.5817	7.5165	7.3118	7.6526	7.5295	7.5672	7.6540	7.5218	7.4634	6.9377	7.1598	7.2303	7.1572	7.3928	7.6861	7.3532	7.4974	7.3612
Ldha	_DLADELALVDVM[Oxidation (M)]EDKLK_.2	#N/A	4.8497	5.0289	4.8880	4.6559	4.9944	5.2264	4.9695	5.2110	5.0595	5.3992	4.8229	5.0160	4.5783	4.5239	4.3138	4.7453	5.0058	5.0762	5.4009	4.8812
Ldha	_LLIVSNPVDILTYVAWK_.2	#N/A	3.3802	3.1218	3.7094	4.1256	1.3226	2.5017	3.3223	1.4315	3.3814	3.5865	3.7558	3.0112	3.4895	4.5341	2.1193	2.6130	3.1815	1.7428	2.2604	4.2114
Gpi	_SNT[Phospho (STY)]PIKVDGK_.2	#N/A	8.8076	8.9199	8.3996	8.6426	8.2310	8.6416	8.7641	8.7157	7.6307	8.8544	8.6860	8.4575	8.8147	8.9418	8.4512	8.6746	8.7856	8.7471	8.7317	8.6326
Gpi	_ELFEADPER_.2	#N/A	10.0408	9.1263	9.6640	9.4649	9.6205	9.6003	9.4164	10.0101	9.5119	9.8267	9.6984	9.3549	9.4286	9.4271	9.0657	9.1287	9.5597	9.5506	9.1782	9.1911
Gpi	_INYTENR_.2	#N/A	8.9538	8.8210	8.9911	9.0062	8.5192	8.8113	8.9894	8.6110	9.1038	8.7653	8.7771	8.6763	9.1462	8.5710	8.7422	8.6944	8.5727	9.0268	8.7399	8.7511
Gpi	_AVLHVALR_.2	#N/A	8.7370	8.9139	8.6272	8.2945	8.5157	8.8245	8.4708	8.6398	8.4334	8.2983	8.1546	8.4930	8.2805	8.4382	8.2202	8.0202	8.3231	8.8618	8.3629	8.1995
Gpi	_VFEGNRPTNSIVFTK_.3	#N/A	8.2095	7.6923	8.4459	8.2822	7.8722	8.1124	8.4231	7.7862	8.4930	8.6594	8.4284	7.7892	8.2651	7.9596	8.2216	8.5299	7.1399	8.0932	8.5329	8.3731
Gpi	_GKLPEEARK_.2	#N/A	8.1454	8.3246	8.2362	7.8717	8.1153	8.2108	8.0204	8.6923	8.1420	8.3708	8.3252	8.0302	8.2859	7.8746	7.5536	8.3292	8.2059	8.0855	7.9370	8.0256
Gpi	_HFVALSTNTAK_.2	#N/A	7.5056	8.0213	7.9411	7.6584	7.8639	7.6997	7.8701	7.8952	7.9115	7.5552	6.9607	7.3558	7.2017	7.4758	7.4938	7.7019	7.6472	7.4814	7.6186	7.3381
Gpi	_TLASLSPETSLFIIASK_.2	#N/A	7.9216	7.8644	7.7401	7.5548	7.4825	7.9118	7.7064	7.4621	7.5470	7.9792	7.5964	7.4204	7.4330	6.9447	7.2090	7.2272	7.3578	7.7693	7.7750	7.5193
Gpi	_EWFLEAAKDPSAVAK_.2	#N/A	6.5215	6.6893	6.7508	6.3688	6.3607	6.7424	6.3752	6.6978	6.3991	6.3395	6.4119	5.6205	6.4836	5.7811	5.8806	6.3768	6.5288	6.1763	5.6612	5.8929
Gpi	_NRS[Phospho (STY)]NTPIK_.2	#N/A	4.0936	3.9092	3.1291	3.5958	6.4703	3.0528	4.1097	5.7042	3.9489	4.2999	3.4275	3.7986	4.1143	4.6170	3.6869	2.9377	4.3317	3.5015	3.1501	3.0831
Gpi	_LRELFEADPER_.2	#N/A	7.1568	7.0319	4.8147	6.9857	6.6723	7.2367	7.1272	7.0730	7.1811	7.0175	6.4871	7.0633	7.2432	6.8279	6.8514	7.0580	6.6490	7.1238	6.6014	6.6741
Gpi	_NRS[Phospho (STY)]NTPIKVDGK_.3	#N/A	4.7502	4.8761	4.0751	3.8890	3.4302	4.8251	4.9021	4.0284	5.1444	5.0067	4.8410	4.5234	3.8413	5.0355	4.5969	4.7275	3.8752	5.3382	4.7164	4.5496
Gpi	_EWFLEAAK_.2	#N/A	4.8390	4.3566	5.5035	4.4124	4.8611	5.3226	4.8704	3.2898	5.2967	5.7588	4.8823	5.2318	3.1082	4.5090	4.7342	4.7236	2.6865	4.9907	5.5274	5.5623
Gpi	_DVM[Oxidation (M)]PEVNR_.2	#N/A	8.1661	8.6461	9.2684	9.1063	8.8699	8.7152	8.7889	8.4225	9.3179	8.2330	8.6173	8.2718	9.0858	8.7940	8.2541	8.7385	8.3062	8.4410	7.9963	8.2298
Gpi	_TFTTQETITNAETAK_.2	#N/A	6.2360	6.8934	6.8853	6.6383	5.6976	6.4188	6.1616	5.6458	6.5850	5.6683	5.8899	5.9218	5.9424	6.5974	5.8863	5.9423	6.1161	6.5199	6.0107	6.0915
Gpi	_TFTTQETITNAETAKEWFLEAAK_.3	#N/A	5.5674	4.7336	4.0182	5.5791	2.9656	4.4433	3.8722	5.0880	4.8833	5.5619	4.7641	4.1211	5.2373	5.3285	4.2466	4.1477	4.5023	3.9046	5.0688	5.2108
Gpi	_VWFVSNIDGTHIAK_.2	#N/A	7.6065	7.4785	7.4863	7.1771	7.1322	7.3056	7.4969	7.4424	7.2079	7.3358	7.1462	7.1983	7.3710	7.1546	6.4962	7.0577	7.4256	7.0624	7.1372	7.0435
Gpi	_M[Oxidation (M)]KS[Phospho (STY)]FC[Carbamidomethyl (C)]QR_.2	#N/A	6.2400	6.9207	6.4279	6.9089	6.1641	6.8482	6.5162	6.6062	6.3787	5.9848	6.4187	5.8858	4.1359	6.5849	6.2231	5.7806	6.6293	6.4243	6.3636	6.2674
Gpi	_KIEPELEGSSAVTSHDSSTNGLISFIK_.4	#N/A	4.9779	5.9885	5.8589	5.9445	3.0119	5.5933	5.1408	3.3745	5.7833	5.4050	5.5151	5.8911	6.2430	6.4410	5.8272	5.3788	5.2944	5.5623	5.8112	5.9118

Gpi	_EVM[Oxidation (M)]QM[Oxidation (M)]LVELAK_.2	#N/A	5.8565	5.8074	4.9417	5.7222	5.2151	5.2013	5.2600	5.4228	5.1441	5.2314	5.6574	5.0557	5.5972	4.9259	4.7423	4.9896	4.8697	5.4169	5.5062	5.3241
Gpi	_ILLANFLAQTEALM[Oxidation (M)]K_.2	#N/A	5.3667	5.4673	5.3178	5.0839	5.7099	5.6291	4.9914	5.8698	5.4956	4.7947	4.8581	5.3245	4.9106	4.9850	5.1842	5.0519	5.7146	5.7579	5.6744	4.8081
Gpi	_FAAYFQQGDMESNGK_.2	#N/A	3.6853	5.3079	6.0936	5.6939	4.3833	5.6214	5.3226	5.4134	5.7602	3.5569	5.6992	3.3712	4.5651	5.9920	4.8230	3.9226	3.4082	5.4047	4.0516	4.7105
Gpi	_SPEDLEK_.2	#N/A	4.4394	4.0353	3.3343	3.7218	3.1455	2.5618	4.3973	3.6679	3.8228	3.7957	3.3015	4.1067	3.9883	4.4909	3.5249	3.8015	4.2056	2.8859	4.3299	3.2542
Gpi	_DNM[Oxidation (M)]FSGSK_.2	#N/A	4.0149	4.1088	3.7778	3.7953	3.3526	3.3100	3.5359	3.7153	4.2402	3.5660	3.9196	4.2970	3.7030	3.7648	4.0225	3.7741	3.4795	4.2272	4.0770	3.6763
Gpi	_ELQAAGKS[Phospho (STY)]PEDLEK_.2	P06745_S455_M1_Gpi	4.0473	7.6231	7.8361	3.6421	7.0681	3.6091	5.9826	3.7477	4.2152	4.2536	6.6300	6.6449	4.3232	7.0446	3.7333	4.3922	4.2853	3.5478	3.1964	3.5443
Ptprc	_NRNS[Phospho (STY)]NVVPYDFNR_.2	#N/A	6.9701	5.6433	3.7565	6.8877	4.7894	5.8468	7.1236	5.1667	5.7213	6.3037	6.8750	6.1835	7.5089	5.4093	3.8738	5.2532	7.1623	7.3931	5.5334	6.6777
Me1	_AIFASGSPFDPVTLPDGR_.2	#N/A	5.6873	5.8385	5.9502	5.4212	5.8950	5.7510	5.6285	5.9138	6.0309	5.9496	5.2670	5.1596	5.6941	5.8597	5.5640	4.9724	5.4888	5.2230	5.3152	4.8381
Gap43	_EGDGSATTDAAPATS[Phospho (STY)]PK_.2	#N/A	13.2543	13.0491	13.3685	13.3706	12.7449	13.0910	12.8160	13.1729	12.9926	13.1387	12.9492	12.9172	13.1247	13.4250	9.5195	12.7992	13.2752	12.8702	12.8363	13.0540
Gap43	_EKDDAPVADGVEKK_.3	#N/A	6.6737	6.9426	6.9678	6.8613	6.7519	7.0213	6.9758	6.9985	7.4385	7.2284	6.9147	7.3164	7.5094	7.1577	7.7826	7.4536	6.7931	6.4476	7.2742	7.4416
Gap43	_AEDGPAKEEPK_.2	#N/A	7.1010	6.6442	7.5416	7.0744	6.7469	7.4320	6.8371	7.2329	7.4416	7.0493	7.0947	7.4522	7.4868	7.6799	8.1332	7.7602	7.3018	7.2875	7.1094	7.1857
Gap43	_KGDAPAAEAEAK_.2	#N/A	6.7260	6.8161	7.1439	7.1830	6.6954	7.2246	7.2314	6.8487	7.2829	6.6494	7.1059	7.3401	7.0312	6.9331	7.3530	7.0908	7.0592	7.2261	6.6635	7.0205
Gap43	_KGEGDAAPSEEK_.2	#N/A	5.0630	5.3972	5.5325	5.7854	5.6369	5.4376	5.2613	5.8484	6.3383	6.0410	5.8629	6.0106	5.1418	5.7469	7.4693	6.2076	5.4887	6.0936	5.3275	5.9832
Gap43	_EGDGSATTDAAPATS[Phospho (STY)]PKAEEPSK_.3	#N/A	11.8585	11.8023	12.0968	11.0083	11.7768	11.8253	11.6248	12.8975	10.8671	11.9015	11.8132	11.9759	12.4890	11.5583	11.6285	11.8906	12.0947	11.8989	11.6907	12.1962
Gap43	_KGEGDAAPSEEKAGS[Phospho (STY)]AETESAAK_.2	#N/A	5.8996	6.2764	4.5984	5.9636	5.7053	6.2919	6.1771	5.6525	5.9424	6.1568	6.1321	6.2614	6.4802	6.8347	6.2878	6.3810	6.7196	6.3721	6.4514	6.1347
Gap43	_QADVPAAVTDAAATT[Phospho (STY)]PAAEDAATK_.2	#N/A	13.4665	13.4105	13.5971	13.4579	12.8459	13.0787	12.9585	13.3509	13.2533	13.4815	13.1697	13.5047	14.1377	13.8668	13.0964	13.4472	13.7281	13.1803	13.2874	13.4661
Gap43	_NDEDQKIEQDGVKPEDK_.3	#N/A	5.9316	6.3285	6.5910	6.0243	5.7363	6.5027	6.7133	4.2640	5.1129	7.0779	6.6909	6.3099	6.5331	6.7349	7.0427	6.2943	6.0925	6.1512	6.4592	6.3792
Gap43	_KEGDGSATTDAAPATS[Phospho (STY)]PK_.2	#N/A	4.2050	6.9127	7.6292	7.2568	3.5427	7.4602	7.2765	3.8324	7.0435	7.3896	7.3043	3.7674	6.8052	7.6171	6.6866	7.0428	4.2034	6.7154	7.2770	7.2275
Gap43	_ATTDNS[Phospho (STY)]PSSKAEDGPAKEEPK_.3	#N/A	11.3087	11.6378	11.2276	11.5983	11.1113	11.0793	11.2840	11.6104	11.3704	11.4021	11.0301	11.6592	12.4748	12.0870	11.8027	11.6985	11.6819	11.5694	8.7549	11.4898
Gap43	_QDEGKEDPEADQEHA_.2	#N/A	3.9358	4.0670	4.4721	3.7535	3.2736	5.5412	5.0311	3.6362	5.1841	5.0932	4.7648	5.3331	4.4347	4.4592	5.9225	5.0650	4.1739	4.5251	4.7622	4.9202
Gap43	_AGS[Phospho (STY)]AETESAAK_.2	#N/A	4.2561	8.3162	8.5746	8.4120	8.1992	8.5939	8.1355	8.0367	8.0833	8.4725	8.6845	8.4732	8.3491	8.8623	8.3822	7.8486	8.5053	7.6148	3.5226	8.8552
Gap43	_AEDGPAKEEPKQADVPAAVTDAAATT[Phospho (STY)]PAAEDAATK_.4	#N/A	3.8257	3.4934	3.3623	3.7585	4.0326	3.3871	3.0155	4.0514	4.4365	3.6363	3.9151	3.2245	4.6019	3.4004	3.0408	3.1878	3.5486	3.6008	3.8999	3.0776
Gap43	_AAQPPTETAESSQAEEEKDAVDEAKPK_.4	#N/A	8.7111	9.6134	8.9407	9.8993	8.5373	10.0975	9.2474	9.4258	8.2373	9.6945	8.6826	10.4322	9.1230	10.2855	10.1456	8.7451	9.5094	10.3117	9.3297	10.1518
Gap43	_EGDGSATTDAAPATS[Phospho (STY)]PKAEEPSKAGDAPSEEK_.4	#N/A	7.4906	8.5746	3.3189	4.0725	3.8298	8.0989	8.3481	7.7143	8.0488	8.4994	8.3083	8.2120	9.4071	4.1773	8.3367	4.3960	4.3326	8.4785	8.4876	4.8862
Gap43	_AGDAPS[Phospho (STY)]EEKKGEGDAAPSEEK_.3	#N/A	4.3047	3.3800	3.5704	3.3650	4.0694	4.2031	4.0316	4.2475	3.5805	3.7734	3.8957	2.9032	4.4694	3.5833	3.0550	2.7459	3.5360	3.2819	3.1912	3.4094
Gap43	_KEGDGSATTDAAPATS[Phospho (STY)]PKAEEPSK_.3	#N/A	3.3829	5.4183	4.0985	4.4129	3.5898	3.4844	3.3753	3.8422	4.5573	5.0351	3.7118	4.3172	5.9642	4.9018	6.3352	5.4638	5.3414	5.4479	3.9534	5.1279
Gap43	_ATTDNS[Phospho (STY)]PSSKAEDGPAK_.2	#N/A	7.8113	7.8884	7.5627	8.0032	7.5766	8.1802	7.8434	7.8910	7.6189	7.8312	7.9461	7.9769	7.6390	8.7822	8.2429	8.2739	8.3068	7.9894	7.9463	7.9743
Gap43	_AGSAETESAAKATTDNS[Phospho (STY)]PSSK_.2	#N/A	4.5876	4.5104	4.3697	4.2837	3.1712	3.1685	5.0004	3.5338	3.5408	4.0398	3.4230	4.0814	3.6850	4.9572	3.9710	4.1783	4.0715	3.9403	4.0889	3.5547
Gap43	_GEGDAAPS[Phospho (STY)]EEKAGSAETESAAK_.2	P06837_S122_M1_Gap43	3.9667	3.3523	3.5033	3.6175	4.1737	3.9347	3.8305	4.1924	3.8896	1.6615	4.2639	3.0785	3.9976	3.5414	3.4077	3.2169	3.6897	3.4598	3.7588	3.2187
Gap43	_AGDAPSEEK_.2	#N/A	3.1868	3.3151	4.0489	3.9323	4.0254	4.2495	3.0519	3.6040	3.3894	3.3932	3.3417	3.2046	3.6828	3.2266	3.0929	3.5317	3.3749	3.7746	4.0736	3.5579
Gap43	_AEEPSKAGDAPSEEK_.3	#N/A	3.8833	4.1195	4.4196	3.8061	3.2210	3.4451	4.3201	3.5836	4.3792	4.0896	3.2527	4.0090	4.4872	4.4066	5.2598	4.2282	4.1213	3.8075	3.3604	4.3736
Gap43	_ESARQDEGKEDPEADQEHA_.3	#N/A	4.1846	3.8182	3.2201	3.5047	3.5224	4.4626	4.1197	3.8850	4.0399	4.0329	3.5186	3.7076	4.2054	3.2072	4.1451	3.0287	4.4227	3.4105	3.0590	3.1742
Alb	_LVQEVTDFAK_.2	#N/A	4.6753	3.7957	5.4963	4.8751	5.0799	4.9368	4.3276	5.6491	4.8406	3.1341	3.9310	4.8604	5.1683	5.4187	5.0419	3.7485	5.0764	3.8961	4.9435	4.1399
Alb	_APQVSTPTLVEAAR_.2	#N/A	6.4248	5.7868	5.2763	6.1535	5.9575	6.1976	5.5436	6.2615	5.8373	4.6112	2.9576	5.4823	6.5804	5.2417	6.0960	5.0343	6.0461	5.6868	5.8151	5.2015
Alb	_QTALAELVK_.2	#N/A	4.4643	3.4461	3.6929	3.7232	3.9271	4.3635	3.4670	3.2070	4.3218	3.5719	4.3414	3.6447	3.5274	2.6208	3.1794	3.8515	4.1017	0.3383	3.7370	3.9970
Alb	_TC[Carbamidomethyl (C)]VADESAANC[Carbamidomethyl (C)]DK_.2	#N/A	3.4683	4.5645	5.6575	5.0476	4.8574	5.0292	3.3734	5.4015	4.4963	3.2784	4.2731	2.9531	3.4590	3.9617	4.4958	3.5446	5.2816	4.4957	3.8054	4.7274
Hsp90aa1	_DQVANSAFVER_.2	#N/A	9.3538	9.3105	9.9131	9.3894	9.4921	9.3918	9.3526	9.5813	9.5353	9.4571	9.3415	9.2907	9.6330	9.2770	9.3241	9.5063	9.2491	9.3296	9.2329	9.2101
Hsp90aa1	_NPDDITNEEYGEFYK_.2	#N/A	9.1265	8.8689	9.2313	8.9426	8.8185	8.8689	8.8130	9.1347	9.1138	9.0667	8.6098	8.4455	9.3172	9.2088	8.5777	8.9721	9.0673	8.8363	8.5829	8.7532
Hsp90aa1	_LGIHEDSQNR_.2	#N/A	8.1392	7.8262	8.0922	7.9923	7.8335	7.9237	8.0447	8.0763	8.1684	7.7715	7.6342	7.8700	7.7962	7.9256	7.2811	7.7272	8.2038	7.8278	7.7077	7.6751
Hsp90aa1	_ELISNSSDALDK_.2	#N/A	8.7533	8.5291	9.2473	8.8164	8.6828	8.4757	8.6913	8.9506	8.7908	8.4444	8.9322	8.6506	8.0760	8.9677	8.5969	8.6176	8.8184	8.6101	8.6096	8.6025
Hsp90aa1	_DKEVS[Phospho (STY)]DDEAEEK_.2	#N/A	8.6800	8.5395	8.5121	8.2532	8.1547	8.5349	8.2657	8.4019	8.1845	8.7358	8.3881	7.9722	8.3184	8.8683	7.7740	8.1632	8.4849	8.4475	8.3260	7.9258
Hsp90aa1	_HIYFITGETK_.2	#N/A	7.6972	8.2657	7.6543	7.5106	7.9871	8.0534	8.1499	8.1511	7.8533	7.6492	7.8711	7.8318	8.1074	8.1545	7.6797	8.0380	7.9020	7.9775	7.6821	7.7589
Hsp90aa1	_ELHINLIPSK_.2	#N/A	7.8522	7.8729	8.3407	7.6420	7.8687	8.0094	7.7659	8.1407	7.8803	7.3807	7.9986	7.3366	7.2358	8.0332	7.9040	7.9534	8.2456	7.9152	8.0983	7.6113
Hsp90aa1	_ELHINLIPSKQDR_.3	#N/A	6.9654	7.7940	7.2988	8.1801	7.8687	7.8940	8.0511	7.2716	7.6290	8.0950	7.8759	7.7301	8.0068	7.9520	7.9135	8.3409	7.5125	7.9465	8.2170	8.2199
Hsp90aa1	_APFDLFENR_.2	#N/A	5.9834	6.4241	6.1588	5.9720	6.3138	6.0606	6.1225	5.9675	5.9157	6.5218	6.2854	6.1381	6.2103	6.3911	6.2380	6.0643	5.9297	5.8911	6.4435	5.7893
Hsp90aa1	_RAPFDLFENR_.2	#N/A	8.0397	8.0018	7.6608	7.8693	8.0151	7.6761	8.1186	7.8276	3.7085	8.1487	7.9815	8.0919	7.4362	7.7735	7.3411	7.5752	7.7380	7.9251	7.9736	8.0490
Hsp90aa1	_ESDDKPEIEDVGS[Phospho (STY)]DEEEEEK_.2	#N/A	5.8116	5.2539	7.8966	7.0401	3.8110	5.6375	5.6256	3.7032	6.4407	5.7310	3.3368	4.9694	4.0236	6.6502	6.0296	5.2809	6.1393	5.4767	6.1238	5.2161
Hsp90aa1	_ESDDKPEIEDVGS[Phospho (STY)]DEEEEKK_.3	#N/A	8.7099	8.5789	9.2168	8.5984	3.4974	8.6089	8.4462	8.3967	8.3660	8.7794	8.5287	8.1834	7.6206	9.1993	7.9260	8.2961	8.7298	8.4998	8.4248	8.5648
Hsp90aa1	_ELISNSSDALDKIR_.2	#N/A	6.4460	5.9466	5.1055	5.1774	5.8781	5.4068	5.7579	6.2883	5.5525	5.6245	5.6403	5.6588	6.3056	5.8177	5.7897	5.5833	5.8121	5.7459	5.6038	5.4013
Hsp90aa1	_HGLEVIYM[Oxidation (M)]IEPIDEYC[Carbamidomethyl (C)]VQQLK_.3	#N/A	3.4523	3.8668	4.4897	4.7481	3.7769	5.0981	5.1395	5.3547	5.5672	4.8320	3.7015	3.6711	4.9228	4.7508	4.0530	4.3153	5.0342	4.5022	4.6832	4.4387
Hsp90aa1	_DKEVS[Phospho (STY)]DDEAEKEEK_.2	#N/A	4.9605	5.4497	5.9419	5.0652	5.4728	5.1290	5.5399	5.0515	5.7369	5.8036	5.4749	5.4378	4.9533	6.4960	4.6782	4.9395	5.6476	5.3938	5.6302	5.4941
Hsp90aa1	_FYEQFSK_.2	#N/A	4.4325	3.5703	3.6145	3.2569	2.9875	3.1255	4.2293	4.1329	4.4685	3.8666	5.0247	4.2838	3.9380	3.8074	4.3820	4.3553	3.1697	4.2323	3.9799	4.2060
Hsp90aa1	_SLTNDWEEHLAVK_.2	#N/A	6.4644	5.6778	6.1563	5.2850	5.7383	5.8316	5.2277	5.6187	5.2898	5.3074	5.0591	5.6012	6.0529	5.1209	5.8339	5.6562	5.6432	5.6032	4.9134	4.8238
Hsp90aa1	_HLEINPDHSIIETLR_.3	#N/A	6.8671	6.9106	6.5728	7.0225	6.5401	7.0297	6.8379	6.9370	6.4507	6.8912	6.7200	6.8157	6.7562	6.6504	6.5085	7.2608	6.7913	6.2735	7.0020	6.6206
Hsp90aa1	_DNSTM[Oxidation (M)]GYMAAK_.2	#N/A	3.5659	3.8717	5.5164	5.7295	3.7728	5.2768	3.9023	3.8539	3.8612	3.8050	3.6596	3.5751	4.0103	5.6344	5.3625	3.6792	3.9155	5.4843	3.7664	3.7142
Hsp90aa1	LVTSPC[Carbamidomethyl (C)]C[Carbamidomethyl (C)]IVTSTYGWTANM[Oxidation (M)]ER_.3	#N/A	3.5890	3.7301	3.1256	3.9952	3.3815	4.3124	3.4527	3.8147	3.5118	4.9247	3.8862	3.4562	3.6199	4.6596	3.0299	3.5947	3.3119	3.5323	4.1366	4.3418
Hsp90aa1	_KHLEINPDHSIIETLR_.4	#N/A	3.7222	4.2806	4.2585	4.7458	3.0600	3.7585	2.9803	3.5119	4.5791	5.0007	3.4137	4.1700	3.1474	4.1920	4.1964	4.0671	4.3403	4.3100	3.1082	4.2664
Hsp90aa1	_ERDKEVS[Phospho (STY)]DDEAEEK_.2	#N/A	3.7042	3.1741	3.5574	2.9136	3.3342	3.8851	3.3996	4.2360	3.3410	3.5532	3.9765	4.0133	4.1421	3.1377	3.8595	3.0284	3.5161	3.3982	3.0699	2.6607
Hsp90aa1	_LGLGIDEDDPTVDDTSAAVTEEMPPLEGDDDTSR_.3	#N/A	3.2984	3.7787	3.8347	4.0438	4.1370	4.3610	3.4041	3.7661	3.4632	3.5047	3.8376	3.0930	3.5713	4.6159	2.9813	3.6433	3.2633	3.8861	4.1852	4.2931
Hsp90aa1	_LGIHEDSQNRK_.3	#N/A	4.1187	3.2004	3.6553	3.4655	4.3257	3.7827	4.2233	4.3444	4.0415	3.9293	2.9151	2.9265	4.1496	3.6934	4.2486	3.4808	3.8416	3.3078	3.6069	3.2426
Hsp90b1	_SILFVPTSAPR_.2	#N/A	8.3859	8.6032	8.3899	8.3141	8.3708	8.4760	8.3813	8.6365	8.2142	8.5005	8.0608	8.4472	8.6292	8.9055	8.3315	8.5527	8.5036	8.3236	8.1487	8.4745
Hsp90b1	_TFEINPR_.2	#N/A	6.6953	7.1735	6.8728	7.0346	7.2973	6.8933	6.6846	7.1766	6.9130	6.9829	6.8921	7.1858	6.5398	6.7884	7.2441	6.5820	6.7665	7.0777	6.5697	7.2421
Hsp90b1	_FAFQAEVNR_.2	#N/A	6.3934	6.7829	7.2133	6.5523	6.8304	5.5996	6.8328	6.1508	6.3803	6.7207	6.4245	6.6526	6.2741	6.6079	6.4995	6.3105	6.7689	6.6706	6.8028	6.3857
Hsp90b1	_GLFDEYGSK_.2	#N/A	5.9432	6.1358	6.7882	6.4215	6.7462	6.9362	6.7853	5.9610	6.6100	6.6519	6.9162	6.4670	6.5161	7.0811	6.6089	6.9986	6.4669	6.5523	6.4169	7.0128
Hsp90b1	_TDDEVVQREEEAIQLDGLNASQIR_.3	#N/A	5.9596	6.0006	5.3045	5.1334	5.5229	5.1042	5.5430	5.8145	5.2980	5.5094	5.4611	4.6480	5.8251	5.9381	5.1653	4.7775	6.0735	5.2644	4.3169	5.9331
Hsp90b1	_GVVDSDDLPLNVSR_.2	#N/A	4.0525	7.9006	8.0767	7.3964	6.2115	7.6294	7.6922	7.2372	7.6680	7.2330	6.4444	7.6695	7.5712	8.2402	7.8863	7.5022	7.6989	7.7131	7.5825	7.6267
Hsp90b1	_SGTSEFLNK_.2	#N/A	3.7295	5.2330	5.3111	4.9548	4.9566	5.4233	5.5698	4.4500	4.7848	5.1889	5.4145	4.4471	5.5303	5.2442	5.0065	4.4423	3.6806	5.4309	4.3982	5.5535
Hsp90b1	_DISTNYYASQK_.2	#N/A	5.3617	4.4748	5.5696	5.4515	5.6763	5.3420	4.9106	5.9156	5.4064	5.7675	5.8927	5.4018	6.3379	6.2323	5.5998	5.9044	4.5346	5.4318	5.1392	6.0021
Hsp90b1	_SGYLLPDTK_.2	#N/A	3.8618	5.5967	4.7485	5.0871	4.3499	5.0388	5.5983	3.2027	4.8879	4.9694	4.8657	4.9047	5.0634	5.2215	3.9188	4.2067	4.2007	4.9074	4.9466	4.5335
Hsp90b1	_NLLHVTDTGVGM[Oxidation (M)]TR_.2	#N/A	3.8933	4.7016	4.4296	3.7961	4.5424	3.9570	4.3832	5.5812	3.7485	4.9536	4.0773	4.7290	4.8484	4.4166	3.5804	4.0713	4.9799	4.6363	4.6140	4.1213
Hsp90b1	_FQSSHHSTDITSLDQYVER_.3	#N/A	5.2260	5.4217	5.6023	5.2914	5.6586	5.2836	4.8594	5.9121	5.4773	6.4509	5.7052	5.6167	6.1846	5.4487	5.5328	5.7517	5.6066	4.0700	5.3457	5.2431
Hsp90b1	_LISLTDENALAGNEELTVK_.2	#N/A	4.3961	5.3016	4.9107	4.9554	5.2265	4.3862	3.8305	3.7112	3.8661	4.2067	4.9060	5.1286	5.6223	4.5342	3.9713	4.4854	4.2489	3.5842	5.1061	5.0840
Apoe	_ELEEQLGPVAEETR_.2	#N/A	7.9272	7.9721	8.0395	7.5032	7.1779	7.7590	7.8147	7.8523	8.0760	7.6316	7.4680	7.9833	7.9949	8.1821	7.6978	8.1664	7.6987	7.9174	7.9517	7.9538
Apoe	_LQAEIFQAR_.2	#N/A	7.2569	7.0046	7.4473	7.2927	7.5806	7.7229	7.4673	7.4928	7.2612	7.3826	7.0735	7.3783	7.5537	7.4076	7.3118	7.3127	7.5311	7.5273	7.2437	7.1066
Apoe	_LGPLVEQGR_.2	#N/A	7.2409	7.3971	7.0361	7.1380	7.5993	7.1364	7.4925	7.7214	7.0726	6.8333	7.0740	7.4471	7.7479	7.1785	7.1300	6.8963	7.2008	7.4733	7.1934	7.2333
Apoe	_DRLEEVR_.2	#N/A	6.3222	6.2798	6.4139	5.7764	5.8007	6.2681	6.3754	5.4430	5.9961	6.7147	6.4748	5.7252	5.5935	6.0799	6.5299	5.5086	5.6801	6.1405	6.4311	5.3917
Apoe	_IQASVATNPIITPVAQENQ_.2	#N/A	8.7793	9.0929	8.9769	8.6173	3.3335	8.5503	8.9885	9.2785	8.4884	8.9397	8.5541	8.9189	9.0573	9.9373	8.7218	8.4770	8.9867	9.0129	8.6827	8.5618
Apoe	_NEVHTM[Oxidation (M)]LGQS[Phospho (STY)]TEEIR_.2	#N/A	4.4068	5.5563	4.1118	4.2166	5.2301	4.5069	5.5211	5.1929	5.1139	5.3334	5.2103	5.1059	5.4630	5.8201	5.0782	4.3755	3.9183	4.4745	4.1783	4.5572
Apoe	_LGADMEDLR_.2	#N/A	3.3632	3.9066	4.9467	4.9632	4.2018	3.8203	3.8856	3.0636	3.3984	3.3993	3.7805	3.0282	3.5064	4.1628	4.5018	3.7081	3.6013	2.0440	3.8804	3.8536
Apoe	_DAEDLQKR_.2	#N/A	3.4887	4.5141	4.0250	4.2007	4.3273	3.0505	3.2138	3.1891	3.2729	3.6951	3.6473	4.4035	3.3809	4.0121	2.7910	3.8336	3.7268	4.1220	3.7550	3.9791
Apoe	_SKMEEQTQQIR_.2	#N/A	4.0777	3.2414	3.6143	3.2931	4.2846	3.8237	2.7590	4.3034	4.1845	3.8883	3.6632	2.9675	4.1086	3.6523	2.7889	3.4398	3.8006	3.3488	3.6479	3.3296
Sod1	_DGVANVSIEDR_.2	#N/A	9.5865	9.6178	9.6908	9.6286	9.1437	9.4492	9.3491	9.2595	9.7019	9.5513	9.2411	9.4640	9.5329	9.8061	9.3145	9.5326	9.6688	9.3068	9.2397	9.5711
Sod1	_HVGDLGNVTAGK_.2	#N/A	8.8586	9.1764	9.5151	9.1726	9.2598	9.1583	9.0953	9.3981	9.4193	9.1698	8.8497	9.4229	9.2848	9.3987	10.1541	9.4995	9.2240	9.0340	9.2570	9.2046
Sod1	_VISLSGEHSIIGR_.2	#N/A	6.1082	6.7293	8.2415	8.2070	6.9998	7.1152	6.7708	6.2949	8.2752	7.8251	6.7514	6.7285	7.2534	6.9652	8.7903	8.2496	7.5425	7.0691	7.8765	7.9288
Sod1	_TM[Oxidation (M)]VVHEK_.2	#N/A	5.9129	6.1231	6.6968	6.3178	6.2895	6.2616	6.2277	6.3771	6.3945	6.0350	6.4017	4.2596	6.3294	6.4332	8.0852	6.8846	5.9264	6.0346	6.6187	6.2600
Sod1	_LAC[Carbamidomethyl (C)]GVIGIAQ_.2	#N/A	4.2189	5.3286	5.4737	5.8765	4.4775	4.7286	4.8529	3.6205	5.0825	5.3273	4.9524	4.8788	4.8309	4.6494	5.1847	5.3158	4.1582	5.3054	4.6281	5.0080
Sod1	_GDGPVQGTIHFEQK_.2	#N/A	7.6024	7.8537	8.3605	7.8477	7.5375	8.0127	7.5159	7.7441	8.1523	7.7022	7.3539	7.8390	7.7276	8.4569	8.5853	8.1577	7.8530	8.0012	8.0348	7.9616
Sod1	_KHGGPADEER_.2	#N/A	5.4495	4.5579	6.4653	6.1351	5.4090	5.3910	4.6499	3.1453	4.6867	5.9007	5.8279	2.9465	3.4656	3.9683	3.4053	5.4511	6.6245	4.8023	5.1877	3.9353
Sod1	_QDDLGKGGNEESTK_.2	#N/A	5.5780	5.4347	4.9726	5.1913	5.4557	5.1741	5.8195	5.6578	5.9235	5.1176	4.5948	5.3498	6.3560	5.1896	6.2975	6.2823	5.2295	5.2513	5.5156	4.6511
Mdh2	_GC[Carbamidomethyl (C)]DVVVIPAGVPR_.2	#N/A	12.5419	12.6497	12.7269	12.9224	12.7085	12.5680	12.5306	12.8206	12.6658	12.7130	12.7725	12.7082	12.8435	13.1242	12.8884	12.8174	12.8239	12.4845	12.6431	12.7204
Mdh2	_VDFPQDQLATLTGR_.2	#N/A	12.2391	12.1680	12.5835	12.4476	12.3888	12.2930	12.2638	12.4376	12.3896	12.3426	12.2905	12.3528	12.7091	12.6342	12.7648	12.4153	12.4534	12.3091	12.3639	12.4911
Mdh2	_GYLGPEQLPDC[Carbamidomethyl (C)]LK_.2	#N/A	11.6215	11.6757	11.9239	11.8861	11.6367	11.6577	11.5885	11.6843	11.6444	11.5002	11.6265	11.7003	11.7871	11.9189	12.0062	11.6605	11.7449	11.6759	11.6162	11.6894
Mdh2	_IFGVTTLDIVR_.2	#N/A	11.4240	11.5154	11.6235	11.6541	11.5488	11.5594	11.4528	11.5917	11.3373	11.6501	11.5097	11.6704	11.7384	11.7288	11.6184	11.6785	11.4922	11.4833	11.5457	11.5915
Mdh2	_M[Oxidation (M)]IAEAIPELK_.2	#N/A	10.3192	10.4349	10.3120	10.5654	10.5755	10.3331	10.4806	10.5276	10.1903	10.3919	10.3397	10.6949	10.6551	10.5636	10.7132	10.4488	10.3647	10.4459	10.3722	10.4926
Mdh2	_TIIPLISQC[Carbamidomethyl (C)]TPK_.2	#N/A	10.8868	10.9112	11.1402	10.9885	10.9351	10.8793	11.0203	11.0142	10.7425	10.7932	10.7233	10.9988	11.0421	11.1531	11.1393	10.7706	10.9880	11.0490	10.8623	10.9819
Mdh2	_IQEAGTEVVK_.2	#N/A	9.7363	9.7130	9.9708	10.0007	9.8730	9.7938	9.7438	9.8865	10.0506	10.0788	9.8111	9.8452	9.9801	10.0270	10.2766	10.0502	9.7215	9.7714	9.8145	10.0195
Mdh2	_ETEC[Carbamidomethyl (C)]TYFSTPLLLGK_.2	#N/A	10.5103	10.4282	10.9305	10.9182	10.3970	10.5392	10.4751	10.4412	10.7341	10.6764	10.6093	10.5194	10.8223	10.6353	10.8613	10.6770	10.6198	10.2512	10.5198	10.6314
Mdh2	_VNVPVIGGHAGK_.2	#N/A	10.6173	9.6866	8.6612	9.7539	10.9300	10.2861	9.7081	10.6767	9.7171	10.4030	10.5532	10.7639	10.5679	9.8710	10.9432	10.5975	10.7651	9.9223	10.3576	10.6500
Mdh2	_EGVVEC[Carbamidomethyl (C)]SFVQSK_.2	#N/A	9.3211	8.8531	9.8518	9.5659	9.1010	8.9949	9.0541	9.2449	9.5505	9.4542	9.0909	9.2203	9.2226	9.7715	9.0362	9.0183	9.4481	9.2120	9.0949	9.5092
Mdh2	_VAVLGASGGIGQPLSLLLK_.2	#N/A	8.9822	8.9871	8.7834	9.1121	8.8418	8.8885	8.9257	8.7706	8.9432	8.9545	8.9750	8.9138	9.0836	8.9894	9.1701	9.1516	8.8154	9.0376	9.0652	9.2065
Mdh2	_ANTFVAELK_.2	#N/A	8.3017	8.3773	8.5958	9.1576	8.3927	8.7213	8.7180	7.6782	8.7165	8.8844	8.7142	8.8129	8.5344	8.8997	8.9286	9.1286	7.0951	8.7385	8.9196	9.0836
Mdh2	_KGEDFVK_.2	#N/A	8.0258	8.1822	8.2197	8.1824	7.8308	8.1895	8.3791	7.9333	8.2333	8.3942	8.5562	8.1775	8.3062	8.4984	8.4918	8.1346	7.9747	8.1088	8.2208	8.5679
Mdh2	_LTLYDIAHTPGVAADLSHIETR_.3	#N/A	7.2263	7.1813	7.3811	7.4467	6.9940	7.0622	6.9426	7.4221	7.1347	7.1811	7.0194	7.3063	7.5652	7.7446	7.3869	7.2581	7.1070	7.2651	7.2103	7.5683
Mdh2	_HGVYNPNK_.2	#N/A	7.8692	8.1138	8.2222	7.5300	7.7234	8.2706	8.2688	8.1428	8.0882	8.4348	8.5639	7.9947	8.1377	8.0117	8.3439	7.8292	8.0573	8.2471	3.9196	8.3064
Mdh2	_NSPLVS[Phospho (STY)]R_.2	P08249_S51_M1_Mdh2	3.7996	7.5903	4.3359	7.7319	7.6813	7.5560	7.5029	7.3329	7.5806	4.0059	7.8439	7.5213	4.5709	8.1492	7.4620	7.8712	7.4951	7.5894	7.9551	7.9774
Mdh2	_FVFSLVDAM[Oxidation (M)]NGK_.2	#N/A	8.6456	8.7466	9.0224	8.9991	8.6542	8.7487	8.6486	8.8656	8.4232	8.8922	8.6340	8.7776	8.4959	8.9362	8.8814	8.7439	8.7324	8.6946	8.7194	8.9449
Mdh2	_AGAGSATLSM[Oxidation (M)]AYAGAR_.2	#N/A	7.8457	7.3014	7.5631	7.3478	7.7562	7.3417	7.5390	7.9676	7.5467	7.6545	7.4552	7.7324	7.1777	7.4687	7.2660	7.4018	7.6664	7.7397	7.4732	7.6963
Mdh2	_ITPFEEK_.2	#N/A	10.4033	10.4368	10.5272	4.1755	4.3525	3.0757	10.5974	10.5928	10.6805	3.7202	3.6221	4.3783	3.3558	4.4004	4.2666	3.8588	10.2275	4.1017	4.4007	4.0776
Camk4	_ASS[Phospho (STY)]DPPSTQDAK_.2	P08414_S353_M1_Camk4	3.2153	3.2867	6.7953	4.4741	4.0538	4.2779	3.4872	3.8492	3.5463	3.4216	3.9207	3.1761	3.6544	3.1981	3.0644	3.5602	3.3464	3.8030	4.1021	4.3763
Camk4	_DSTDLLGKK_.2	#N/A	3.4651	4.2130	4.1262	4.2724	4.2644	3.3751	3.7918	3.8989	3.7837	3.5046	4.0853	4.1742	4.6523	4.0369	5.2580	4.4882	4.0927	4.2960	3.6131	4.1433
Camk4	_RDS[Phospho (STY)]EEKLK_.2	P08414_S433_M1_Camk4	4.3230	3.7691	4.1030	4.0454	3.6901	3.9378	3.2963	3.8994	4.4819	3.7329	4.2944	3.6852	4.7657	3.6621	3.1384	3.4884	4.7873	4.5193	4.2599	3.1396
Camk4	_TEIGVLLR_.2	#N/A	3.5927	3.3176	3.4408	4.4457	3.7996	4.4815	3.7515	3.8183	3.5155	4.7543	3.8432	3.6205	3.8482	3.9597	3.5636	2.9548	3.3156	4.6407	4.1126	2.9318
Camk4	_LGS[Phospho (STY)]ASSSHTSIQENHK_.3	P08414_S337_M1_Camk4	3.8881	4.1147	4.4244	3.1327	3.2259	3.4500	4.3152	3.1763	4.3743	4.0945	3.2478	4.0041	4.4824	4.0261	3.8924	4.2330	4.1744	2.9751	3.2741	3.7034
Nefl	_AQLQDLNDR_.2	#N/A	11.7604	12.1178	12.1039	12.0696	12.0495	12.2235	11.6210	12.2237	11.9553	12.0337	12.1994	12.4200	12.7134	12.8676	12.4626	12.3133	12.4888	12.2060	12.3703	12.4788
Nefl	_EYQDLLNVK_.2	#N/A	10.5073	10.1866	10.7868	10.7583	10.5572	10.3301	10.0338	10.4480	10.4879	10.5893	10.6888	10.7469	10.8794	10.9509	10.8985	10.7723	10.7118	10.2982	10.6071	10.8500
Nefl	_KLLEGEETR_.2	#N/A	9.7436	9.8855	8.8796	8.7840	9.9869	9.9260	9.7260	10.1390	9.9200	9.8759	9.9388	10.2661	10.3767	10.3816	10.4703	10.1961	10.2277	9.8412	9.9914	10.2941
Nefl	_VHELEQQNK_.2	#N/A	8.9192	9.0712	9.1791	9.3376	9.2113	9.2788	8.9071	9.1507	9.2748	9.1920	9.5440	9.6937	9.7694	9.8621	9.9776	9.5766	9.6515	9.4134	9.5523	9.6963
Nefl	_YEEEVLSR_.2	#N/A	8.9963	8.6532	9.3234	9.2665	8.9264	9.0328	8.9369	8.9395	8.8316	8.9601	9.2285	9.3427	8.9982	9.6174	9.7150	9.2359	9.2253	9.1806	9.1704	9.4847
Nefl	_ALYEQEIR_.2	#N/A	8.9200	8.8342	8.8840	9.1895	9.0526	9.1946	9.2658	8.7112	8.7996	9.1707	9.5677	9.4933	9.7113	9.4585	9.5454	9.0721	8.7044	9.3137	9.0970	9.3637
Nefl	_FTVLTESAAK_.2	#N/A	8.2435	8.3730	8.8953	8.9613	8.4928	8.5015	8.4946	8.2911	8.4854	8.6925	8.5761	8.9539	9.0542	9.3713	9.1740	8.8883	8.5372	8.5747	8.9091	8.9369
Nefl	_TLEIEAC[Carbamidomethyl (C)]R_.2	#N/A	8.0409	8.2535	8.4411	8.2773	8.3043	8.4300	8.1009	8.2141	8.2117	8.4040	8.3985	8.5226	8.7132	8.9261	8.6695	8.4594	8.2232	8.1991	8.1903	8.6847
Nefl	_VLEAELLVLR_.2	#N/A	7.9533	8.5003	8.7031	8.6149	8.1702	8.3301	7.9987	8.2070	8.3509	8.0862	8.2594	8.7471	8.6101	8.5975	8.8292	8.5265	8.7033	8.4588	8.5355	8.6433
Nefl	_KGADEAALAR_.2	#N/A	7.4818	7.5304	7.6571	7.8807	7.4516	7.9199	7.7609	7.7996	7.8968	7.5357	8.1684	8.0573	7.9291	8.0920	7.8914	7.7847	8.1041	7.6070	7.5326	7.9232
Nefl	_[Acetyl (Protein N-term)]SSFGYDPYFSTSYK_.2	#N/A	7.3094	6.8866	7.5887	6.9924	7.2942	7.4485	6.6061	7.1023	7.3470	7.1137	7.4326	7.6188	7.6648	7.4005	7.4544	7.5024	7.6845	7.0934	7.3336	7.7030
Nefl	_FASFIER_.2	#N/A	6.5873	6.5007	6.8534	7.9399	7.0044	7.2499	7.2099	5.9729	6.8406	7.4848	7.5164	7.5419	7.1432	7.3063	7.9165	7.8370	5.6641	7.7184	7.7974	7.9446
Nefl	_AAKDEVSESR_.2	#N/A	6.6221	6.4113	7.3080	7.4445	6.7864	6.9902	6.6740	6.6526	6.8273	6.8109	7.3097	7.2628	7.3495	7.4296	8.3382	7.7468	6.8412	6.6440	7.4530	6.8241
Nefl	_EGLEETLR_.2	#N/A	6.8093	6.4184	7.1806	7.0244	7.1212	7.5682	6.6532	6.6321	6.8488	6.9527	7.5866	6.9544	6.4303	7.6586	7.2741	7.1996	6.8454	7.3819	7.2752	7.4955
Nefl	_KEES[Phospho (STY)]AGEEQVAK_.2	#N/A	6.9664	6.3288	7.1647	6.8606	6.6951	7.0021	6.1584	7.1358	6.5256	6.7247	7.1509	6.9987	6.8392	7.7056	6.6778	6.7079	7.3079	6.6037	6.8199	7.0979
Nefl	_YLKEYQDLLNVK_.2	#N/A	6.4903	6.6544	5.2173	6.1032	6.8335	5.9491	5.6176	7.2010	6.2530	6.8226	6.5587	7.0070	7.6223	7.2983	7.1561	6.7197	6.4529	6.6529	6.5092	7.1306
Nefl	_AEEAKDEPPS[Phospho (STY)]EGEAEEEEK_.2	#N/A	7.2686	7.2754	7.9155	7.3210	6.4464	7.5000	6.5481	7.0576	7.1180	7.3885	7.9800	5.4318	6.8365	8.4562	6.8165	7.0331	7.8161	7.0891	7.6613	7.4091
Nefl	_LLEGEETR_.2	#N/A	4.2438	7.7527	7.5267	7.9206	7.4068	8.0161	7.8545	7.1331	8.0700	8.0352	8.0417	8.2202	8.0662	8.5448	8.8031	8.3395	7.9726	7.9159	8.0950	8.2285
Nefl	_SFPAYYTSHVQEEQTEVEETIEATK_.3	#N/A	8.2441	8.7317	6.9142	7.9311	7.4730	7.8654	7.5790	8.5220	6.8268	7.6719	8.0572	8.7422	8.8278	10.6662	7.4884	7.8707	8.4350	8.5572	7.5967	8.2191
Nefl	_EES[Phospho (STY)]AGEEQVAK_.2	#N/A	5.3053	6.1428	5.8261	5.9506	5.4822	6.1597	5.3525	4.9248	5.2956	5.9893	6.0837	6.0345	3.2978	6.5801	5.5326	6.1693	6.3010	5.8995	6.2928	5.6071
Nefl	_IDSLM[Oxidation (M)]DEIAFLKK_.3	#N/A	5.4570	3.9711	4.2178	5.6063	4.3081	4.9422	4.4129	4.4660	4.1164	4.7069	5.3551	5.4971	5.4391	4.3159	4.4663	4.4713	4.1185	4.4274	4.6917	5.1445
Nefl	_QALQGEREGLEETLR_.3	#N/A	6.0059	6.5314	4.8482	5.9818	6.1951	5.0196	6.6285	6.6086	6.6523	5.7819	5.9908	6.9211	7.7675	7.3472	7.5050	6.9895	6.6289	6.5646	6.9874	6.7862
Nefl	_RYVETPR_.2	#N/A	3.6974	3.8210	4.6740	4.7027	4.7438	4.9491	4.3500	4.8795	4.5092	5.0730	5.0014	5.0756	3.7052	3.6965	4.9658	4.6409	4.3418	3.8415	4.7917	4.7019
Nefl	_NM[Oxidation (M)]QNAEEWFK_.2	#N/A	6.9016	6.4751	6.2445	6.5401	6.7112	6.6500	6.0219	6.8793	6.3854	6.3564	6.9291	7.1392	7.1970	7.1197	6.9553	6.9301	7.2711	6.9327	6.8289	7.0686
Nefl	_ESEEEEKKEES[Phospho (STY)]AGEEQVAK_.3	#N/A	4.2505	3.5032	3.8818	4.0052	3.6320	3.9041	2.9973	3.2722	3.8076	4.0398	4.4982	4.0019	3.2834	4.5443	3.6978	3.7458	4.0821	3.6628	4.9449	4.3328
Nefl	_IDSLM[Oxidation (M)]DEIAFLK_.2	#N/A	4.7945	5.0744	5.3578	4.7547	4.8991	4.8326	4.6990	4.8594	4.8042	5.5955	4.8986	5.2443	4.9598	5.3983	5.0106	5.2032	4.7628	5.0796	4.9044	5.0068
Nefl	_SAYSGLQSSSYLM[Oxidation (M)]SAR_.2	#N/A	7.1788	6.8412	7.3775	7.1457	6.5096	7.2473	6.5691	7.1609	6.9062	6.9799	6.9303	6.9787	7.4033	7.7417	7.1299	7.2909	6.8711	7.1222	7.0950	7.2083
Nefl	_M[Oxidation (M)]ALDIEIAAYR_.2	#N/A	7.2558	6.5513	7.1829	7.5544	7.0642	6.9568	6.4844	6.9706	7.5594	7.1842	6.9229	7.2005	7.1621	7.2635	7.2810	7.2713	7.4795	7.1198	7.2486	7.3235
Nefl	_AAKDEVSESRR_.3	#N/A	3.9080	4.0948	4.4443	3.7813	3.2458	4.0458	3.8431	3.6084	4.5097	4.3802	4.3808	3.2293	3.9288	5.5054	5.6981	5.1606	4.1461	3.7962	4.6436	4.3984
Nefl	_SAYSSYSAPVSSSLSVR_.2	#N/A	7.3542	7.2538	7.2566	7.5085	7.2934	7.2458	6.8975	7.4540	7.3943	7.3617	7.0689	7.6622	7.7226	7.9239	7.7116	7.4198	7.5656	7.1570	7.3712	7.3133
Nefl	_LSFTSVGSITSGYSQSSQVFGR_.2	#N/A	4.8149	5.1848	4.9818	5.0127	3.9960	4.7988	5.0136	5.3309	4.2577	5.1710	4.9479	5.1742	6.0750	6.5129	4.6439	5.4972	5.3175	4.7965	5.0096	4.9268
Nefl	_SYSSSGSLM[Oxidation (M)]PSLENLDLSQVAAISNDLK_.3	#N/A	4.0234	4.5037	3.8528	3.2680	3.3611	3.5852	4.1799	4.5419	4.2390	4.2297	3.1126	4.2298	4.3471	3.8909	3.7571	4.3683	4.0391	3.1103	3.4094	3.5681
Nefl	_TQEKAQLQDLNDR_.3	#N/A	3.7572	3.3531	3.6852	4.5405	3.9641	4.9107	3.0840	4.3501	4.2247	4.5348	4.4473	4.2527	3.1696	3.6723	4.9492	4.9523	3.3870	4.4462	5.3198	4.9424
Nefl	_YEEEVLSREDAEGR_.3	#N/A	3.5902	4.5221	3.1268	3.9940	3.7972	4.3112	3.2509	3.8159	3.5131	3.4008	3.8874	4.0859	3.6211	3.1649	4.4352	5.1208	3.3131	3.8363	5.1245	4.8078
Nefl	_AEEAKDEPPS[Phospho (STY)]EGEAEEEEKEK_.3	#N/A	4.1362	3.8666	7.9612	3.5532	3.4739	3.6980	4.0672	3.8366	7.1748	7.4953	2.9998	3.7561	4.2343	8.6347	7.4005	2.9803	4.3743	3.4589	3.1075	7.9771
Nefm	_S[Phospho (STY)]PVEEVKPKPEAK_.3	#N/A	12.7200	12.8007	12.9368	13.2054	12.3886	13.0271	12.4673	11.9923	12.8142	13.1822	13.1248	12.7703	12.9273	13.5524	12.9537	13.0459	12.8866	12.7123	13.1432	13.1531

Nefm	_GVVTNGLDVS[Phospho (STY)]PAEEK_.2	#N/A	10.6355	11.0352	11.5478	11.1357	9.8440	11.0679	10.3527	10.5480	10.8573	11.0209	11.0344	10.9928	10.9433	11.8895	10.7555	11.1485	11.6718	10.7922	11.1198	11.3078
Nefm	_AES[Phospho (STY)]PVKEK_.2	#N/A	10.2110	9.7935	9.9307	10.1160	9.9348	9.9065	9.6280	9.6282	9.6495	10.0133	10.2105	10.0298	10.3544	10.0542	9.6196	9.8053	10.2545	9.8464	9.9118	10.2946
Nefm	_QLS[Phospho (STY)]DIEER_.2	P08553_S344_M1_Nefm	8.0763	8.1074	8.2222	8.2987	8.1441	8.2778	7.8433	8.3470	8.1856	8.2327	8.5865	8.7644	9.0893	9.1544	8.8248	8.7435	8.8473	8.4615	8.5993	8.9338
Nefm	_VHYLEQQNK_.2	#N/A	8.4713	8.1756	8.7270	8.3674	8.5407	8.5844	8.5146	8.3952	8.5831	8.6076	8.6784	8.8186	8.0713	9.4207	9.0033	8.8005	8.6416	8.6679	8.5726	8.8256
Nefm	_EIEAEIQALR_.2	#N/A	7.8506	7.9169	8.4315	8.3536	7.7380	7.8337	7.5527	7.5623	8.1150	7.7047	8.3431	8.0899	8.1999	8.7477	8.6017	8.1550	8.1658	8.2254	8.3957	8.5329
Nefm	_[Acetyl (Protein N-term)]SYTLDSLGNPSAYR_.2	#N/A	7.8831	7.8146	8.3617	8.1948	7.9973	8.0188	7.5776	8.0075	7.9045	7.5327	8.2697	8.3192	8.4047	8.4797	8.7548	8.3900	8.6386	7.8235	7.9781	8.1440
Nefm	_VQSLQDEVAFLR_.2	#N/A	7.5445	7.4599	7.6615	7.9806	7.6130	7.6945	7.3358	7.7027	7.4263	7.3908	7.4758	8.0569	8.1270	8.2207	8.4388	7.8791	8.1051	7.8027	7.9082	7.8308
Nefm	_FSTFSGSITGPLYTHR_.2	#N/A	6.4832	5.8954	6.4298	6.5968	6.3083	6.2297	5.7809	6.3342	6.1741	6.4332	6.8199	6.7480	6.8080	6.7136	7.0690	6.7991	6.6615	5.9723	6.6308	6.9561
Nefm	_LTEAAEQNK_.2	#N/A	7.2179	7.0742	7.8001	7.6565	7.5777	7.4667	6.9069	7.4766	7.0463	7.4701	7.4879	7.7625	7.6543	7.9313	7.9651	7.7590	7.7461	7.5469	7.4528	7.9232
Nefm	_SAKEEIAEYR_.2	#N/A	6.7534	6.6357	7.1838	6.8583	6.7958	7.0873	6.8999	6.6216	6.5036	6.8736	7.2784	6.9675	7.1022	7.4586	7.7299	7.5125	6.5237	6.8971	7.0987	7.2386
Nefm	_AVEEVITISK_.2	#N/A	5.6151	6.6403	6.9067	6.8965	6.4283	6.4097	6.0548	6.0883	6.3189	5.8187	5.8252	6.5438	6.2912	7.7100	7.2810	6.6696	6.7077	6.5955	6.8422	6.6938
Nefm	_SAKEEIAEYRR_.3	#N/A	6.2493	6.6211	5.8279	6.9039	6.2705	6.4750	6.2446	6.0108	6.7456	7.4142	7.0111	6.8090	6.3151	6.3018	7.5210	7.4302	6.7165	6.5620	7.0010	7.3171
Nefm	_VTSHAIVK_.2	#N/A	5.7578	5.2643	6.3613	5.6307	6.1417	6.0739	6.3748	6.2620	6.0374	5.7878	5.6490	7.0674	5.9553	6.8115	7.0746	6.5917	6.3265	6.5757	6.5895	6.4543
Nefm	_LRDDTEAAIR_.2	#N/A	5.9235	6.0931	7.0506	5.7703	5.6790	6.4268	5.7954	5.7047	5.9967	6.0663	6.0244	6.2821	5.9610	6.2002	6.6393	6.7832	6.4064	5.8003	6.3901	6.1191
Nefm	_SNHEEEVADLLAQIQASHITVER_.3	#N/A	5.7348	5.0486	6.3020	5.3810	5.7598	4.8084	5.0742	5.2583	5.4809	5.5224	5.8829	5.8159	5.7187	6.4680	6.1678	5.9321	5.8372	4.6696	5.7854	6.3584
Nefm	_GS[Phospho (STY)]PSTVSSSYKR_.2	#N/A	8.2269	8.3624	7.7559	8.4133	8.4847	8.0831	7.8158	8.4484	7.9178	3.8085	8.2659	8.8110	9.0804	8.9411	8.5690	8.6463	8.6874	8.3726	8.3995	8.6983
Nefm	_KGEDS[Phospho (STY)]SDDKVVVTK_.2	#N/A	6.6644	6.5969	6.4695	7.0268	6.2359	6.6544	5.9446	6.4077	6.5225	6.1238	6.0414	6.8555	7.3516	7.4472	5.6347	6.8615	7.2031	6.9285	6.7135	7.0451
Nefm	_KAES[Phospho (STY)]PVKEK_.2	#N/A	8.7476	8.1582	8.5011	8.7342	8.8314	8.4233	7.6909	7.7930	8.3222	8.2921	8.6049	3.8588	8.6329	8.3761	8.7898	8.5704	8.8896	8.2256	8.3795	8.9191
Nefm	_EEEPEAEKS[Phospho (STY)]PVKS[Phospho (STY)]PEAK_.3	#N/A	5.1349	5.5455	5.8632	5.8165	4.8516	6.3084	5.0851	4.7899	5.4836	5.8461	6.1529	5.6198	5.5565	6.3613	5.1081	5.5376	5.6978	5.3100	5.6079	5.6427
Nefm	_HNHDLSSYQDTIQQLENELR_.3	#N/A	5.2132	5.4581	5.4099	5.8877	5.7306	5.9055	5.5574	5.8786	5.1970	4.4880	5.9856	6.0746	5.2480	6.6018	5.1568	5.4560	5.8753	5.5380	5.9585	6.0732
Nefm	_VSGS[Phospho (STY)]PSSGFR_.2	P08553_S30_M1_Nefm	11.0034	10.9998	10.9815	11.4675	11.1287	11.1505	10.7790	11.0061	10.8105	11.2893	11.4175	11.5093	11.7964	11.8630	11.4327	11.5496	11.5596	11.2535	11.3636	11.7386
Nefm	_SIELESVR_.2	#N/A	4.7620	5.3927	5.1059	5.2887	4.9236	5.9475	5.4096	4.3101	6.6160	6.5652	6.5096	5.3477	6.5043	6.9368	7.0745	7.1159	5.5298	6.8752	7.4035	7.5906
Nefm	_VKEKAEEEGGS[Phospho (STY)]EEEGSDR_.3	#N/A	3.9383	3.1699	4.4746	4.2089	4.2532	4.2560	3.6389	3.4765	3.9824	5.1416	4.7726	4.6916	5.7858	5.7693	5.8845	5.0377	4.6147	4.6536	5.1273	4.8924
Nefm	_ATLEMVNHEK_.2	#N/A	4.3344	5.4130	7.0727	6.1421	4.7346	5.4434	6.0586	4.0348	4.5910	4.9054	5.8607	5.1547	4.3552	7.4189	6.0341	3.1785	5.1156	4.3651	4.7987	5.3662
Nefm	_S[Phospho (STY)]PVEEVKPK_.2	#N/A	6.0860	5.3434	5.9720	6.2912	4.9350	6.0063	5.5206	5.4675	5.4724	5.8892	6.0692	5.5028	3.4175	6.2241	5.6858	5.5719	5.8648	5.5797	6.0651	5.8034
Nefm	_SNEKEQLQGLNDR_.2	#N/A	7.5922	8.2422	7.8909	8.3672	7.9729	8.2595	7.7163	8.3248	8.1835	8.3960	8.2294	8.6903	9.0706	9.1633	8.6493	8.6243	8.5562	8.4090	8.6432	8.7349
Nefm	_EEEKGVVTNGLDVS[Phospho (STY)]PAEEK_.3	#N/A	5.5652	7.1269	4.1463	7.8771	3.1432	7.4774	7.2169	3.3935	7.6020	7.4021	3.3418	7.4354	3.5661	8.8470	7.9489	8.2969	6.7438	7.9524	8.2677	8.6004
Nefm	_GS[Phospho (STY)]PSTVSSSYK_.2	#N/A	8.2914	8.0830	8.5532	8.3783	8.1758	8.5316	7.7781	8.0560	8.2369	8.2336	8.5281	8.4481	8.5874	8.9874	8.6228	8.6868	8.7164	8.2206	8.3631	8.7192
Nefm	_AEEEGGS[Phospho (STY)]EEEGSDRSPQESK_.3	#N/A	5.5626	4.1360	3.8183	5.1528	5.2263	5.2087	5.6467	4.7090	4.0926	3.9604	4.8311	5.6171	5.7850	7.0640	5.5788	5.5103	5.6152	5.7412	5.8915	5.6024
Nefm	_EEIAEYRR_.2	#N/A	4.8731	3.6453	5.4586	3.3319	5.1594	4.5773	3.8458	4.8470	4.2128	3.8600	5.0228	5.7319	5.3278	5.7260	5.5192	5.5476	3.0947	3.2376	5.3162	4.9073
Nefm	_GTKES[Phospho (STY)]LER_.2	P08553_S338_M1_Nefm	3.1870	3.3150	3.7233	6.0174	5.9886	3.9594	3.1221	4.3882	3.0423	3.5297	4.0217	6.0768	3.2077	3.7104	3.0927	3.5319	5.9685	4.4081	4.0567	3.6774
Nefm	_ITS[Phospho (STY)]EGGDGATK_.2	P08553_S795_M1_Nefm	4.5159	3.4812	3.5570	3.1678	3.8593	4.5752	3.6818	4.0437	3.7409	3.2271	4.1152	3.3707	4.8392	3.3927	3.2590	3.3656	5.3242	3.6085	3.9075	3.0700
Nefm	_QASHAQLGDAYDQEIR_.3	#N/A	5.3180	4.8842	5.9695	5.4845	5.7083	5.4939	5.0618	5.1609	5.4036	5.0578	5.1646	5.2162	4.9746	6.0447	5.9902	6.1447	4.7924	4.8348	5.6928	5.6388
Nefm	_EEKPQPQEK_.2	#N/A	4.4870	4.4641	4.4048	4.1507	5.2519	4.9378	4.8137	3.2391	4.7328	4.4849	4.4075	4.4550	3.5595	4.6226	4.8199	4.2303	4.9530	4.0564	3.7050	4.9443
Nefm	_AQVQLDSDHLEEDIHR_.3	#N/A	7.9582	7.9291	8.1739	7.6771	7.9453	7.8206	7.1899	8.1107	7.5278	7.9478	8.1761	8.2956	8.4829	8.8461	8.2337	8.2526	8.3059	7.6943	7.8829	8.2840
Nefm	_FAGYIEK_.2	#N/A	4.5548	3.6660	3.5902	3.1346	3.6988	4.0648	4.7490	4.2551	4.8967	3.7742	4.1944	4.5923	4.9198	3.5773	4.9518	4.6201	3.2920	5.1370	4.5644	4.2845
Nefm	_LTEAAEQNKEAIR_.2	#N/A	3.2112	3.2969	3.9172	3.9945	3.4182	3.4515	2.8766	3.3952	3.5147	2.0769	3.0288	4.0982	3.9788	4.4664	3.9744	5.0364	3.9388	2.5811	3.5488	3.9676
Nefm	_LS[Phospho (STY)]RSNEKEQLQGLNDR_.3	P08553_S95_M1_Nefm	4.5413	3.5208	3.3349	3.7859	3.8790	4.1031	3.6621	4.0240	3.7211	3.2468	4.0955	3.2469	3.8292	3.3730	3.2393	3.3854	3.5212	3.6282	3.9273	3.0503
Nefm	_TDISTALK_.2	#N/A	4.4460	4.3739	3.9826	3.1382	3.1521	3.1855	3.8960	3.6613	3.8162	4.2566	3.2948	4.1000	2.6958	4.4843	3.9818	2.4825	4.1989	3.6449	4.1166	2.7808
Nefm	_EVTKES[Phospho (STY)]PKEEK_.2	#N/A	6.7770	3.7981	7.0066	6.2347	6.7143	7.1394	6.5510	6.2444	6.5883	6.8028	7.0654	6.9705	4.4116	7.9556	3.8216	6.4524	7.2756	6.6994	6.7676	7.2224
Nefm	_AEEEGGS[Phospho (STY)]EEEGSDR_.2	#N/A	5.6831	6.3133	6.6426	5.8168	5.8585	6.6597	5.0272	5.7515	4.9849	5.9310	6.8173	6.0670	6.1453	7.3889	7.2941	6.3154	7.1795	6.3441	6.7390	6.8660
Nefm	_VEEHEETFEEK_.2	#N/A	5.8307	5.4114	6.2105	5.7544	5.8839	5.6987	5.4479	5.9068	6.0405	5.7830	5.6744	6.1204	5.3275	5.8000	6.5772	6.4752	6.0800	5.8132	5.7336	5.9790
Nefm	_SPVKS[Phospho (STY)]PEAK_.2	P08553_S506_M1_Nefm	4.4456	3.5572	6.4328	3.2437	5.6791	6.3478	6.0384	6.2026	3.8168	6.4726	6.8422	6.8983	3.9248	7.5668	5.4714	3.2897	6.9550	3.1495	6.8017	3.4352
Nefm	_EKAEEEGGS[Phospho (STY)]EEEGSDR_.2	#N/A	4.4499	4.3700	3.9865	4.8352	3.1560	3.1894	3.8921	3.8621	3.8123	4.2605	3.2909	4.5561	3.9777	6.1818	5.9589	3.8121	5.3537	5.1560	3.2867	4.4474
Nefm	_QPSVTISSK_.2	#N/A	3.2338	3.2682	3.7701	4.7216	4.0723	2.9021	3.4688	3.8307	4.7655	3.4401	3.9022	4.9238	3.6359	3.1797	5.1078	4.1043	3.3279	3.8215	5.1998	5.2316
Nefm	_AKS[Phospho (STY)]PMPK_.2	P08553_S605_M1_Nefm	3.5739	4.4289	6.1269	5.8478	2.9116	3.1357	3.1286	3.4906	3.1877	4.3404	5.7828	4.3184	3.2958	6.5980	4.2067	3.9188	4.4887	4.1616	2.9598	4.0177
Nefm	_EGSS[Phospho (STY)]EKDEGEQEEEEGETEAEGEGEEAEAKEEK_.4	P08553_S551_M1_Nefm	4.0139	4.0599	3.8294	3.7465	3.6856	3.8257	3.6195	3.7843	3.6166	3.1658	4.2854	3.5674	4.1046	4.9271	3.4195	3.8301	3.5311	3.6522	4.0108	3.7834
Nefm	_SVTVTQKVEEHEETFEEK_.3	#N/A	8.2096	6.5290	7.1425	7.9311	7.8247	3.5049	4.2603	8.5389	4.3194	6.8785	7.5389	8.1631	9.8360	5.0141	8.0262	8.0111	7.8301	5.0761	7.1152	7.2465
Nefm	_AEEEGGS[Phospho (STY)]EEEGSDRSPQESKK_.3	#N/A	3.4541	3.9477	3.8743	3.8837	3.5627	3.7868	4.0964	3.7418	3.3846	3.6604	3.7590	3.7769	3.4926	3.4010	3.6736	3.7989	3.7569	3.2478	3.9974	3.4846
Nefm	_EKAEEEGGS[Phospho (STY)]EEEGSDRSPQESK_.3	#N/A	4.0215	4.1845	4.1830	4.4497	3.3525	3.4480	4.0318	3.2906	4.0418	4.3303	2.7194	3.9107	4.1697	4.1266	3.7320	4.0086	4.3693	3.5550	3.4209	3.9597
Nefm	_SQLEC[Carbamidomethyl (C)]HSDQNMHQAEEWFK_.3	#N/A	4.2708	4.2185	4.4937	3.3676	3.3410	4.0376	4.2054	4.2458	4.1587	3.9502	3.6473	4.0025	4.2963	3.5832	3.4174	3.3853	3.5618	4.0584	4.0331	3.3915
Nefm	_DTKEEKPQPQEK_.3	#N/A	4.3471	3.6557	3.3826	3.3422	4.2667	4.3001	4.2822	4.0475	4.2024	3.8704	3.6811	3.5451	4.3679	3.3697	2.8067	3.4219	3.0844	3.2480	4.3974	3.3367
Gnai2	_LFDSIC[Carbamidomethyl (C)]NNK_.2	#N/A	8.8929	8.1522	9.0721	8.8932	8.7356	8.6324	8.8825	9.3263	8.5270	8.3049	8.5569	8.8507	8.7793	8.3256	8.0986	8.0641	9.1734	8.5130	8.2047	8.4040
Gnai2	_IAQSDYIPTQQDVLR_.2	#N/A	8.6394	8.2115	8.6479	8.2592	8.1387	8.2220	8.3953	8.4396	8.5049	8.3264	7.9961	8.1085	8.2059	7.8184	7.5033	7.9841	8.2412	7.8100	7.9208	8.0200
Gnai2	_EYQLNDSAAYYLNDLER_.2	#N/A	6.8420	6.7802	6.5341	6.2492	5.9822	6.0355	6.2720	5.9377	6.1586	6.1412	5.5559	5.2679	5.8677	5.9383	4.6884	5.7269	5.9926	6.2393	5.6720	5.6634
Gnai2	_AVVYSNTIQSIM[Oxidation (M)]AIVK_.2	#N/A	5.0542	4.7366	3.5001	5.6464	4.2361	3.5909	4.5891	4.1320	3.1124	4.9102	4.9363	3.3659	5.6087	4.6532	2.3908	4.0187	4.1699	4.3502	3.9321	4.2591
Gnai2	_NLREDGEK_.2	#N/A	6.2481	5.9161	6.1257	5.3436	5.3057	5.5912	5.7483	4.9539	5.9048	6.1403	5.6363	5.6948	4.7171	5.7405	6.6264	5.5491	5.5342	5.9767	5.1598	5.0388
Gnai2	_NNLKDC[Carbamidomethyl (C)]GLF_.2	#N/A	4.1724	5.6825	5.1845	5.5658	4.7957	4.9803	4.9730	6.0686	4.9394	4.9062	5.6955	5.3222	5.6495	4.9865	4.5292	5.2052	5.3612	5.1109	4.9661	4.8500
Gnai2	_M[Oxidation (M)]FDVGGQR_.2	#N/A	5.9781	7.0355	6.9321	7.0398	5.7177	6.7694	7.4000	6.6141	6.3573	6.4872	6.5675	6.5110	5.8067	6.7208	5.7940	4.9320	6.1053	6.3307	6.2621	6.1912
Gnai2	_YDEAASYIQSK_.2	#N/A	4.3120	4.2739	6.3540	5.8462	4.7865	5.3534	4.9764	4.4263	5.4363	5.2096	5.4520	3.5803	5.3818	3.3345	5.6118	5.6781	5.4241	5.3374	5.1997	5.3442
Gnai2	_SREYQLNDSAAYYLNDLER_.3	#N/A	4.9849	6.1312	3.1727	5.4972	4.7490	4.5023	4.8918	5.4670	5.5465	5.1778	5.2541	5.3673	5.3295	3.2108	4.9308	3.5475	5.9105	5.5474	4.0894	4.6607
Gnai2	_IIHEDGYSEEEC[Carbamidomethyl (C)]R_.3	#N/A	3.9771	3.6487	3.6801	3.6071	3.0216	3.9243	4.4862	3.7212	3.9292	3.7877	4.8253	3.4493	4.0080	3.5517	2.5456	3.3392	3.1100	4.3834	2.0347	3.9981
Gnai2	_TTGIVETHFTFK_.2	#N/A	7.4165	6.9167	7.0901	7.3557	7.2688	6.5863	6.3120	7.2230	7.0056	7.0296	7.2903	6.9176	7.2765	6.3717	6.7911	6.8595	6.8822	6.8707	6.9153	6.7463
Gnai2	_MFDVGGQRS[Phospho (STY)]ER_.2	P08752_S207_M1_Gnai2	3.5211	5.4351	4.0574	4.1683	2.8588	4.3010	5.1558	3.2215	3.2405	3.7274	5.0597	4.3711	3.3485	4.0445	4.2594	3.8660	3.7592	4.0740	3.7226	4.0115
Gnai2	_QLFALSC[Carbamidomethyl (C)]AAEEQGM[Oxidation (M)]LPEDLSGVIR_.3	#N/A	3.3437	3.1582	3.8800	4.3456	4.1823	3.8026	3.2788	3.0441	3.1990	2.3329	1.8542	3.0477	4.4536	3.8671	2.9360	3.6886	3.5818	4.2514	3.8999	3.8341
Gnai2	_LWADHGVQAC[Carbamidomethyl (C)]FGR_.3	#N/A	4.3351	3.3461	3.6149	3.3148	3.4255	4.2877	3.9136	4.2927	4.2252	3.6365	3.6590	4.3480	4.3906	3.6292	4.0633	3.4325	3.5939	3.1868	3.9506	3.5595
Gnai2	_EIYTHFTC[Carbamidomethyl (C)]ATDTK_.3	#N/A	3.5719	3.7816	4.1082	4.1759	3.7027	3.9268	3.9821	4.0654	4.0412	3.7782	3.6225	3.6710	3.7412	3.8800	3.5593	3.9168	3.8099	3.8122	3.8196	4.3322
Gnai2	_AMGNLQIDFADPQR_.2	#N/A	3.8987	4.1041	4.4350	3.7907	3.2365	3.4605	4.3046	3.5991	4.3637	4.1051	3.2372	3.9935	4.4718	4.4221	3.8818	4.2436	4.1368	3.6964	3.3450	3.6928
Gnai2	_NVQFVFDAVTDVIIK_.2	#N/A	3.7560	4.2363	4.1202	4.5014	3.0937	3.3178	4.4474	3.3085	3.0056	3.9623	3.3800	3.9624	3.1137	4.1583	4.0245	4.1009	4.3065	4.3437	3.1420	3.8355
Polr2a	_YSPTS[Phospho (STY)]PTYSPTTPK_.2	P08775_S1878_M1_Polr2a	3.1799	3.3221	3.7162	4.5095	4.0185	4.2425	3.5226	8.8877	3.5817	3.3862	3.9561	3.2115	3.6898	3.2335	3.0998	3.5248	3.3818	3.7676	4.0667	4.4117
Polr2a	_YSPTS[Phospho (STY)]PTYSPTSPK_.2	P08775_S1913_M1_Polr2a	7.0838	3.9967	7.1154	7.3808	6.9857	3.5359	7.0868	7.7399	7.0200	7.3568	4.0089	7.2905	7.7497	4.0435	6.2042	6.0931	4.1918	7.1713	4.1037	3.7208
Polr2a	_YTPTSPSYSPSS[Phospho (STY)]PEYTPASPK_.2	P08775_S1850_M1_Polr2a	3.7879	5.2576	4.3242	3.9014	3.1257	3.3584	4.4154	3.4883	3.6432	3.9943	3.1219	4.1043	3.8087	4.3113	3.9926	4.1328	4.0260	3.8072	3.4557	4.2783
P4hb	_EADDIVNWLK_.2	#N/A	5.7198	5.4616	5.8904	5.8933	5.7971	6.0247	5.6706	4.9625	5.5905	5.8097	5.9567	6.0496	5.4730	6.1611	6.3089	5.9648	5.8366	6.5354	6.1850	5.8507
P4hb	_NNFEGEITK_.2	#N/A	6.3869	5.5462	6.5472	6.2691	6.2399	6.6162	6.2198	3.6546	6.4334	6.7791	6.1967	5.9193	6.4090	6.6276	6.2175	6.2303	6.1606	6.1608	6.5791	6.6856
P4hb	_TGPAATTLSDTAAAESLVDSSEVTVIGFFK_.3	#N/A	3.9786	4.1584	4.9787	2.9698	2.4189	4.4794	4.2576	3.8939	1.9035	2.9881	3.2077	3.7220	4.7602	5.3370	2.4944	4.4230	3.2272	3.6667	3.3228	3.7645
P4hb	_TVIDYNGER_.2	#N/A	6.3062	6.4866	7.1255	6.7196	6.4467	3.5732	6.6952	3.5586	6.3127	6.5304	5.1993	6.6394	6.7176	7.0541	7.0370	4.9473	6.8474	6.1137	6.0556	6.5183
P4hb	_ILFIFIDSDHTDNQR_.3	#N/A	3.8660	4.1368	4.9656	3.8234	4.1299	3.2804	3.8010	3.5663	4.9272	4.3516	3.1999	4.0263	3.8867	5.0898	3.9146	4.2109	4.1040	3.7291	3.3777	4.3563
Cck	_NLQSLDPSHR_.2	#N/A	6.7272	4.0453	6.3269	4.3104	6.8440	6.5187	6.7246	6.7751	4.9760	6.8033	6.4551	6.7597	6.8385	4.3493	7.3398	6.7674	6.0944	6.2322	6.5168	6.8776
Alpl	_TYNTNAQVPDS[Phospho (STY)]AGTATAYLC[Carbamidomethyl (C)]GVK_.3	P09242_S110_M1_Alpl	4.1222	3.2478	3.6097	3.5129	3.9302	4.0401	4.1634	4.2988	4.0034	4.8174	3.3743	3.3864	4.2857	5.3181	3.2490	3.2310	3.7960	3.2572	4.0297	3.3751
Ncl	_NDLAVVDVR_.2	#N/A	5.1607	5.2580	5.7109	6.1935	5.1961	5.4497	5.8857	5.1550	6.1315	5.4755	5.4015	4.8067	6.1008	4.8128	5.2976	5.9973	5.1346	5.3894	5.8886	5.3759
Ncl	_LELQGSNSR_.2	#N/A	6.3378	5.6663	5.9656	5.7290	5.9991	5.5726	6.5431	5.3608	6.0404	6.3285	6.2434	6.3218	5.8580	6.1841	6.3086	5.3382	6.0277	6.2933	6.3524	5.6624
Ncl	_ETLEEVFEK_.2	#N/A	5.8952	5.3990	5.3171	5.8890	5.7272	5.7546	6.0307	4.8166	5.8058	5.6358	5.8970	5.1993	3.6314	5.7382	5.5650	5.5496	5.7831	5.7179	5.7674	5.0135
Ncl	_VEGSEPTTPFNLFIGNLNPNK_.2	#N/A	2.9971	3.3189	3.8122	4.2657	4.0715	3.8715	4.3964	3.8113	4.1208	3.9053	3.4276	3.6137	4.9513	4.2643	3.3369	2.9857	4.1700	3.4526	2.2199	3.4499
Ncl	_NLS[Phospho (STY)]FNITEDELK_.2	P09405_S403_M1_Ncl	4.8189	3.3168	5.3130	3.5819	4.2092	6.1902	4.3397	4.2280	5.8777	3.8129	3.7386	3.0429	4.0332	3.5769	2.8643	3.3644	6.6033	5.6791	3.7233	3.2542
Ncl	_GLS[Phospho (STY)]EDTTEETLK_.2	P09405_S577_M1_Ncl	3.1493	3.3527	3.6856	4.5401	3.9878	4.2119	3.5532	4.3505	3.6123	3.3556	3.9867	3.2421	3.7204	3.6726	3.1304	3.4942	7.1654	4.4458	6.1830	3.6396
Pgk1	_NNQITNNQR_.2	#N/A	11.5065	12.0344	12.0072	12.1130	11.8450	12.0218	11.7857	12.2538	11.7688	12.0733	11.6793	12.1436	12.0743	12.9317	12.3335	12.1120	12.0703	12.0030	12.1152	12.0389
Pgk1	_LTLDKLDVK_.2	#N/A	8.6357	8.7064	8.6434	8.7877	8.8673	8.4658	8.5077	8.9781	8.6400	8.7734	8.2153	8.9488	8.8369	8.9785	8.8694	8.6581	8.6885	8.4873	8.4124	8.5978
Pgk1	_FHVEEEGKGK_.2	#N/A	8.1014	7.8712	9.0026	8.1612	8.1467	8.5318	8.2477	8.3250	8.6731	8.3699	8.5327	7.7703	7.9591	7.8817	8.4969	8.6446	8.3260	8.0283	8.3985	8.6496
Pgk1	_ITLPVDFVTADKFDENAK_.2	#N/A	7.6757	7.8127	7.4005	7.5289	7.4679	7.6120	7.5179	7.6347	7.4930	7.7288	7.0801	7.4762	8.0472	7.9557	7.5254	7.5702	8.0512	7.3571	7.4728	7.2774
Pgk1	_YSLEPVAAELK_.2	#N/A	8.8332	8.4479	8.6415	8.4584	8.5158	8.5249	8.4878	8.5050	8.4937	8.3241	8.8124	8.3399	8.0247	8.6829	8.5536	8.3176	8.6410	8.4643	8.3552	8.6133
Pgk1	GC[Carbamidomethyl (C)]ITIIGGGDTATC[Carbamidomethyl (C)]C[Carbamidomethyl (C)]AK_.	#N/A	7.9186	8.7382	8.5654	8.3432	7.6154	8.0276	8.3370	7.9382	7.8372	8.0771	7.7417	8.0930	8.1364	9.1533	7.9303	7.5253	7.7210	8.1138	7.8029	7.8129
Pgk1	_LGDVYVNDAFGTAHR_.3	#N/A	7.0780	7.1045	7.2177	6.9955	6.9788	6.7990	6.7522	6.5443	7.2382	7.2916	7.3991	6.8891	5.9677	6.8722	6.8953	6.7849	7.1640	6.7788	6.9617	6.9602
Pgk1	_FC[Carbamidomethyl (C)]LDNGAK_.2	#N/A	7.5888	7.8994	7.9886	7.9614	7.6222	7.8304	8.1332	7.7308	7.9725	7.9171	7.8619	7.9142	7.9290	7.9679	8.3888	7.6904	7.2769	7.9180	7.7675	7.9674
Pgk1	_FHVEEEGK_.2	#N/A	7.6582	7.6385	7.6213	7.5499	7.5302	7.9879	7.6332	7.6495	7.9958	7.9188	7.8948	7.3870	6.9265	7.5573	7.9844	7.6277	7.6843	7.6906	7.7160	7.5911
Pgk1	_KYAEAVGR_.2	#N/A	6.9895	7.2246	6.8447	7.0878	7.3572	7.1849	7.1637	7.1577	7.3347	7.1707	7.3218	6.8880	7.2443	6.9700	7.5019	7.1515	7.2482	6.7830	6.9486	7.1561
Pgk1	_IQLINNM[Oxidation (M)]LDK_.2	#N/A	7.1223	6.5727	7.0681	6.5414	7.1082	6.9297	6.4823	7.2199	6.4722	6.6689	6.4710	6.9750	6.3460	6.5993	6.8745	6.5510	6.9962	6.9714	7.1298	6.7770
Pgk1	_ELNYFAK_.2	#N/A	5.4093	5.6158	4.7352	5.4618	5.2753	5.4390	5.9117	4.1055	5.6854	6.0570	5.9788	5.0983	7.1124	5.3085	5.9757	5.6162	4.1722	6.7151	6.4483	6.1088
Pgk1	_VNEM[Oxidation (M)]IIGGGM[Oxidation (M)]AFTFLK_.2	#N/A	6.7595	6.0480	7.6752	6.6317	7.1893	7.3265	5.7120	6.2748	6.8530	7.0863	6.8294	6.0544	4.3338	5.9731	6.7597	6.7274	6.9837	6.4246	6.3238	7.0268
Pgk1	_AEPAKIDAFR_.2	#N/A	6.2401	6.5262	5.4549	6.1532	6.5602	5.6586	6.4050	7.0081	5.3372	6.3423	5.7509	6.5450	7.5143	6.3272	6.1427	6.5943	6.8993	5.2975	6.2183	5.6914
Pgk1	_VLNNM[Oxidation (M)]EIGTSLYDEEGAK_.2	#N/A	6.0900	6.5875	7.1702	6.9094	6.1610	6.4111	6.2953	6.4232	6.3939	6.5288	6.4776	6.2137	6.2555	6.8039	6.4534	5.9004	6.5822	5.9330	6.0981	6.3042
Pgk1	_SLM[Oxidation (M)]DEVVK_.2	#N/A	6.6118	6.4030	6.7867	6.9053	6.9214	7.1024	7.2327	6.4049	6.4184	6.9563	7.0897	6.5120	6.6543	7.2426	7.1001	6.7982	5.7979	6.9138	6.8046	6.9239
Pgk1	_VDFNVPMK_.2	#N/A	3.6700	5.9688	6.7578	7.4960	4.0453	5.9300	6.7571	4.4373	5.5617	5.0397	6.6998	4.6632	3.2568	7.1962	6.4246	3.0321	3.4741	3.4901	4.3535	5.1854
Pgk1	_ALESPERPFLAILGGAK_.3	#N/A	6.9715	7.4491	7.5005	6.8122	7.3889	7.4863	7.4517	6.9571	7.0316	7.2941	6.6743	7.2605	7.5739	7.8124	7.2094	7.0404	7.1799	7.4948	7.3485	7.5731
Pgk1	_AHSSM[Oxidation (M)]VGVNLPQK_.2	#N/A	6.7046	6.9750	7.3373	7.1753	6.9564	7.1953	7.0782	6.8788	6.9797	7.0287	6.8977	6.8886	6.9969	7.1284	7.2516	7.2679	6.7082	6.7539	7.0225	7.1760
Pgk1	_DC[Carbamidomethyl (C)]VGPEVENAC[Carbamidomethyl (C)]ANPAAGTVILLENLR_.3	#N/A	4.0508	3.6467	5.7167	4.6298	4.2578	4.2911	4.2912	3.6994	4.7604	4.3035	4.5052	3.3422	4.3768	4.6918	5.3956	3.4129	3.0933	3.2390	4.3884	3.3456
Pgk1	_VSHVSTGGGASLELLEGK_.3	#N/A	6.6896	4.1754	6.1017	4.8617	6.4727	5.5413	4.6982	3.5579	6.2346	5.8254	5.5655	4.6442	3.8783	5.3496	3.8180	5.1046	5.4603	3.7376	3.5787	5.5543
Pgk1	_WNTEDKVSHVSTGGGASLELLEGK_.3	#N/A	3.3418	4.2833	4.0732	4.0476	3.3295	3.3648	4.4004	3.2615	4.4595	4.4602	3.3330	4.0094	4.5675	4.1113	3.9776	4.1479	4.2596	4.3907	3.1889	3.2954
Pgk1	_QIVWNGPVGVFEWEAFAR_.2	#N/A	3.9306	4.0722	4.4669	3.7588	3.2683	3.2158	3.8656	3.6310	3.7859	4.2869	3.2645	3.9617	3.9513	4.4540	3.8910	4.2755	4.1686	3.6645	3.3131	4.4210
Fth1	_[Acetyl (Protein N-term)]TTAS[Phospho (STY)]PSQVR_.2	P09528_S5_M1_Fth1	11.3362	11.2511	11.7889	11.4132	10.9612	11.1646	10.8581	11.3875	11.1080	11.2850	11.1216	11.3438	11.3896	11.7922	11.2010	11.1980	11.4745	10.9850	11.1419	11.2678
Fth1	_QNYHQDAEAAINR_.3	#N/A	3.2371	3.2649	3.7734	3.9825	4.0756	4.2997	2.4866	3.8274	3.5245	3.4434	3.8989	3.1544	3.6326	3.1764	3.0427	3.5820	3.3246	3.8248	4.1239	4.3545
Sod2	_GELLEAIKR_.2	#N/A	9.9072	10.2338	10.1968	10.1328	10.2363	10.1021	9.9750	10.2598	9.8770	10.0093	9.9577	10.3127	10.4428	10.4956	10.4657	10.3174	10.1358	10.1068	10.0112	10.1205
Sod2	_NVRPDYLK_.2	#N/A	9.2798	9.6414	9.7632	9.3535	9.4564	9.3143	9.6781	9.5269	9.3690	9.4394	9.2416	9.7094	9.5202	9.6979	10.2001	9.4444	9.3508	9.5124	9.4171	9.5440
Sod2	_GDVTTQVALQPALK_.2	#N/A	8.3439	8.7472	8.9371	8.9139	8.1964	8.7553	8.7656	8.2471	9.0197	8.4085	8.0693	8.6204	8.4770	9.3711	9.3486	9.0336	8.4148	8.7227	8.6834	8.7452
Sod2	_AIWNVINWENVTER_.2	#N/A	8.4181	8.1647	8.8373	8.7009	8.3711	8.2797	8.1798	8.4829	8.5599	9.0265	8.4623	8.3548	8.7106	8.6427	8.6431	8.6273	8.6238	8.1616	8.4744	8.5439
Sod2	_YHEALAK_.2	#N/A	5.7258	5.5270	6.1557	5.6605	5.5670	5.3688	5.6527	5.4552	5.8425	6.2316	5.7241	5.8444	4.6490	5.5343	6.2223	5.6230	5.0832	6.1629	5.6217	5.1981
Sod2	_GELLEAIK_.2	#N/A	4.0202	4.1597	4.1144	5.3914	4.5956	4.7153	4.3564	4.5146	3.8429	4.7658	5.8410	4.4247	4.1857	4.7797	4.5009	5.3965	3.0432	4.6670	4.4442	5.0425
Sod2	_HHAAYVNNLNATEEK_.3	#N/A	3.7489	4.2539	6.0620	6.4799	3.0867	7.0392	6.6669	3.3155	6.8277	5.9275	6.2198	5.9002	3.1207	4.1653	7.4774	6.4871	4.3136	5.7106	3.1349	6.7082
Sod2	_FNGGGHINHTIFWTNLSPK_.3	#N/A	4.0511	5.2193	3.3547	3.2957	3.3889	4.1838	4.7678	5.1321	3.9878	4.2575	3.0848	4.9232	4.3194	5.0267	4.8285	4.3960	4.0114	4.9920	3.4371	3.5404
Sod2	_DFGSFEK_.2	#N/A	4.0312	3.2879	3.9201	3.5530	4.2381	3.8702	3.8949	4.2569	3.9540	4.5147	4.2167	3.0140	4.0621	3.6058	3.4721	3.9793	3.7541	3.3953	3.6944	3.9148
Nudt10;Nudt11	_LGGS[Phospho (STY)]PTNGNSAAPSPPESEP_.2	P0C027_S148_M1_Nudt10;Nudt11	4.3885	11.0861	3.9250	3.1958	3.0946	3.5130	4.2522	3.1133	4.3113	11.3150	3.1848	11.0787	4.4193	11.3985	3.8294	10.9238	4.1114	10.8906	10.9243	3.6404
Nudt10;Nudt11	_LKLGGSPT[Phospho (STY)]NGNSAAPSPPESEP_.2	P0C027_T150_M1_Nudt10;Nudt11	4.3242	4.4958	3.8608	3.6066	3.0303	3.0636	4.0178	10.1989	3.9380	4.1348	3.4167	4.2219	4.1035	4.6061	9.9853	3.6863	4.3208	3.5123	7.5659	3.0723
Nudt10;Nudt11	_SEREDEVLLVSSSR_.3	#N/A	3.9154	3.4037	3.4520	3.6688	4.1224	3.9860	4.4266	4.1411	3.8382	3.7260	4.2126	3.1298	3.9463	3.4901	2.9511	3.2775	3.6383	3.5111	3.8102	3.1673
Rc3h2	_T[Phospho (STY)]GDPANLNRLR_.2	P0C090_T341_M1_Rc3h2	3.2011	3.3009	3.7374	4.4883	4.0397	4.2638	3.5014	4.4023	3.5605	3.4074	3.9349	3.1903	3.6686	7.8488	3.0786	3.5460	3.4392	3.7889	4.0879	4.3904
Chmp6	_MHQVM[Oxidation (M)]S[Phospho (STY)]IEEVER_.2	#N/A	4.0759	4.9074	5.5734	5.4676	4.7133	4.5384	4.8828	3.9849	4.7639	4.9212	5.5687	3.7577	4.1175	5.9322	4.3661	4.5594	4.6038	4.6385	4.8996	5.0690
H2az1;H2az2	_ATIAGGGVIPHIHK_.3	#N/A	6.0616	6.0201	6.1666	6.4143	6.5402	6.7564	6.0232	5.5069	6.3156	6.9196	6.8656	5.8267	7.0587	5.8125	6.9435	6.3678	5.2217	6.3163	6.4251	6.2659
H2az1;H2az2	_GDEELDSLIK_.2	#N/A	4.2770	4.5429	3.8136	3.3072	2.9831	3.0164	4.0650	4.5027	3.9852	4.0876	3.4639	4.2691	4.1507	3.8516	4.0904	3.6391	3.9999	3.1495	3.4486	3.5289
Lrrc4b	_SGS[Phospho (STY)]KENVQETQI_.2	P0C192_S700_M1_Lrrc4b	10.6782	10.5969	10.5715	10.6711	10.2509	10.0486	10.2515	10.6009	3.5583	10.4972	10.0747	10.0564	10.8888	10.5548	9.2502	3.8876	10.5768	10.2343	3.2605	10.1307
Gpr52	_RRANS[Phospho (STY)]C[Carbamidomethyl (C)]S[Phospho (STY)]I_.2	P0C5J4_S358_M2_Gpr52	6.0636	6.4706	4.4165	6.3103	6.2068	5.7686	6.4298	5.8031	6.0108	5.0058	5.9462	6.3168	7.1874	6.6446	5.4365	6.1783	6.9553	6.9605	6.5866	6.0640
Igsf11	_GSS[Phospho (STY)]PQVLPR_.2	P0C673_S378_M1_Igsf11	10.6837	10.6357	10.2923	10.5161	3.4403	10.2705	3.6078	10.4166	10.0743	10.5564	9.9322	10.1707	10.6730	10.1147	9.1665	9.9254	10.5025	10.1218	9.8449	9.9502
Wipf3	_AQLVS[Phospho (STY)]PPAPPTK_.2	P0C7L0_S211_M1_Wipf3	13.1124	13.1991	12.7792	13.1136	13.0166	12.9186	12.6114	13.3166	12.7206	12.9140	12.7930	13.0025	13.4765	13.3680	12.4844	12.8472	13.2971	12.7209	12.6768	12.9873
Wipf3	_TAVAPPPLPGANNSGSET[Phospho (STY)]PPPLPPK_.3	P0C7L0_T338_M1_Wipf3	3.8404	4.8007	4.3767	3.8490	3.1781	3.4022	4.3630	3.5407	4.4221	4.0467	3.2956	4.0519	4.5301	4.3637	3.9402	4.1853	4.0784	3.7547	3.4033	4.3307
Clec2l	_SGS[Phospho (STY)]GYEGSTSWK_.2	P0C7M9_S47_M1_Clec2l	8.3796	7.9096	7.0696	8.4202	3.6922	3.7892	8.6202	8.5238	8.2805	8.3609	7.9928	7.1109	8.4211	8.4096	6.1427	6.8259	8.8285	8.4251	7.0974	7.2082
Clec2l	_S[Phospho (STY)]PAEAEAR_.2	#N/A	10.2084	10.0482	9.6682	9.6282	9.2707	9.8445	9.7877	9.3215	9.6387	9.5936	9.9249	9.3917	9.6155	9.4095	8.3844	8.8881	9.8975	9.8324	8.9917	9.5236
Clec2l	_PRS[Phospho (STY)]PAEAEAR_.2	#N/A	8.3552	7.8924	8.1494	7.7852	7.8947	7.9201	7.6852	7.6505	7.7384	7.5882	7.6497	7.7705	7.8919	7.5748	7.0443	7.6509	8.4293	7.9369	8.0126	7.6729
Clec2l	_PAPAAPRPRS[Phospho (STY)]PAEAEAR_.3	#N/A	8.8250	9.4325	8.0736	8.8243	8.3327	8.2040	9.0077	9.3676	8.3366	8.1179	8.0798	8.8299	10.2807	8.5283	7.9192	8.7743	8.8779	9.0778	8.4310	8.4824
	DTTEDS[Phospho (STY)]ITEDDKRR.3	#N/A	3.6816	7.3199	6.9597	7.2891	6.6419	5.2833	4.0231	5.3763	5.1329	7.5437	7.4008	5.1205	7.7567	7.3833	7.4353	5.1168	6.6812	5.3831	7.2894	7.0850
	DTTEDS[Phospho (STY)]ITEDDKR.2	#N/A	3.2911	4.0279	4.3286	4.2931	3.4981	3.1094	3.1549	3.5168	3.2140	3.7540	3.5883	3.7541	3.3220	4.3666	4.2329	4.1541	4.5149	4.1354	4.4344	4.0439
Ubb;Ubc;Rps27a;Uba52	_TLSDYNIQK_.2	#N/A	9.7066	9.7940	10.0638	9.6079	9.1608	9.8182	9.5944	9.6436	9.7827	9.6481	9.6378	9.6343	9.6990	9.9010	10.1502	9.8688	9.8763	9.7357	9.7807	9.7354
Ubb;Ubc;Rps27a;Uba52	_ESTLHLVLR_.2	#N/A	9.9396	10.2405	10.6874	9.9683	9.8281	9.9967	9.8507	10.0453	9.9830	9.7501	9.6879	9.7428	10.1587	9.9675	10.3636	10.0543	10.1212	10.0176	10.3104	9.9340
Ubb;Ubc;Rps27a;Uba52	_TITLEVEPSDTIENVK_.2	#N/A	9.3011	9.4975	9.6686	9.3211	9.1021	9.3921	9.1641	9.3550	9.5070	9.1787	9.1743	9.1189	9.4874	9.5252	9.7161	9.4730	9.4424	9.2143	9.2861	9.2001
Ubb;Ubc;Rps27a;Uba52	_QLEDGRTLS[Phospho (STY)]DYNIQK_.3	#N/A	3.8549	3.8687	3.4267	3.5865	3.9431	4.2497	3.9941	3.9627	3.9979	4.1371	3.6176	3.3747	4.1284	3.2938	3.6437	3.3143	4.1154	3.4220	4.3533	3.2547
Nrxn1	_FNVGTDDIAIEESNAIINDGK_.2	#N/A	5.4415	3.8768	4.3284	3.5634	3.4637	4.2075	4.6989	5.6935	3.9813	3.1838	3.4599	5.8355	4.1467	3.1485	3.6546	2.9700	4.3640	4.2204	3.1177	5.1690
Nrxn1	_DEGS[Phospho (STY)]YHVDESR_.2	P0DI97_S423_M1_Nrxn1	3.6618	4.3410	4.1981	4.0276	2.9995	5.8322	3.0407	3.3622	3.0998	3.8681	3.4742	4.2305	3.2079	4.2525	4.1188	4.0067	3.8998	4.2495	3.0477	3.9298
Nrxn1	_YPAGNNDNER_.2	#N/A	4.0004	4.5624	4.0202	4.2054	4.3225	4.9648	3.1260	3.1843	3.3392	3.2327	4.3187	4.4083	4.6190	4.0073	2.7958	3.8288	4.5986	4.4977	3.7597	4.8110
Nrxn1	_NYIS[Phospho (STY)]NSAQSNGAVVK_.2	P0DI97_S434_M1_Nrxn1	3.3942	4.2829	4.4490	4.6357	3.8919	3.9689	3.1174	4.3836	4.6079	4.3660	4.0171	3.3508	3.2031	3.7058	3.1428	4.1937	4.2294	3.8133	4.0663	3.8065
Ndufb1	_TLFQLLR_.2	#N/A	6.7250	7.0769	6.9124	6.9044	6.3266	6.8638	6.8073	6.1684	6.7016	7.0733	7.1186	7.4870	7.3052	6.8065	6.9284	7.3748	6.7969	7.4075	7.0057	7.1037
Ndufb1	_ELRPNEEVTWK_.2	#N/A	5.5100	4.4934	5.4920	4.6650	4.8012	4.9794	5.3780	5.7009	5.0366	5.0782	4.7999	4.8775	5.1726	5.2061	5.7084	5.1174	4.6271	5.0453	4.7301	5.2113
Calm1;Calm2;Calm3	_EAFSLFDK_.2	#N/A	5.9879	5.0942	7.1855	6.4745	5.8315	5.9865	5.7982	5.3671	6.4491	6.3005	6.7835	6.3606	4.3376	6.1725	7.1594	5.8276	5.7185	5.8336	6.5815	6.3149
Calm1;Calm2;Calm3	_EAFSLFDKDGDGTITTK_.3	#N/A	10.3134	10.1080	11.2025	10.6452	3.7514	10.3615	10.0957	9.5911	10.6797	10.5668	10.5984	10.3415	10.3234	10.8804	11.6011	11.1057	10.5990	10.2062	10.9187	10.9660

Calm1;Calm2;Calm3	_ELGTVMR_.2	#N/A	3.5485	7.5471	8.6477	9.0721	6.1313	7.2816	8.4253	3.7742	7.2510	6.6518	8.4477	6.9704	3.5794	9.0751	8.8166	4.4115	5.3629	6.3599	6.9805	7.3561
Calm1;Calm2;Calm3	_VFDKDGNGYISAAELR_.3	#N/A	8.5367	8.1471	8.7915	8.6502	8.3471	8.2964	8.1987	8.2198	8.5041	8.5222	8.6940	8.5475	8.4445	9.0541	9.3916	8.8476	8.7981	8.6094	8.7004	8.7384
Calm1;Calm2;Calm3	_DGNGYISAAELR_.2	#N/A	4.4761	5.8526	6.6585	6.1350	3.8138	5.9949	4.9591	4.1765	6.4136	6.7690	6.6028	3.3860	3.8944	6.4409	4.9281	5.1376	6.5336	6.2487	6.5624	7.1808
Calm1;Calm2;Calm3	_M[Oxidation (M)]KDTDSEEEIREAFR_.3	#N/A	7.5247	8.4907	9.2859	8.5605	7.7959	7.9380	8.1503	7.9094	8.5735	8.1367	8.2193	8.3552	8.2761	9.2797	9.1044	8.2993	8.1506	8.2362	8.4529	8.5326
Calm1;Calm2;Calm3	_DTDSEEEIREAFR_.2	#N/A	5.6279	5.3125	6.3796	5.9684	4.8186	5.5901	5.6535	5.5118	6.1902	5.3536	4.9684	5.6167	5.1424	6.1386	6.5625	6.1758	6.0563	6.0256	6.0931	6.4235
Calm1;Calm2;Calm3	_M[Oxidation (M)]KDTDSEEEIR_.2	#N/A	4.6532	4.4530	5.8639	4.9748	4.5466	4.9275	5.2456	4.7392	5.2961	4.7291	5.6643	4.8585	4.8326	6.1705	5.8048	4.8358	4.6048	4.7289	4.7810	5.1122
Calm1;Calm2;Calm3	_[Acetyl (Protein N-term)]ADQLTEEQIAEFKEAFSLFDKDGDGTITTK_.3	#N/A	3.5324	2.5153	4.0687	4.1570	1.8242	1.9755	3.3901	4.3346	3.6498	3.7387	3.6036	3.2001	3.3373	5.2866	3.2347	1.6606	3.7704	2.7812	3.7113	2.2194
Calm1;Calm2;Calm3	_[Acetyl (Protein N-term)]ADQLTEEQIAEFK_.2	#N/A	3.9650	4.6038	4.5013	3.7243	3.3028	3.4237	3.9001	3.6654	3.8203	4.2525	4.6794	3.9272	3.9858	12.1123	3.9255	4.3099	4.2031	11.5594	3.2786	2.9546
Calm1;Calm2;Calm3	_DTDSEEEIR_.2	#N/A	4.1550	3.8478	3.1905	3.5344	3.4927	3.7168	4.0484	3.8554	5.5323	4.3613	2.9810	3.7373	4.2155	3.1775	3.6256	2.9991	4.3930	3.4401	3.0887	5.2184
Eef1a1	_IGGIGTVPVGR_.2	#N/A	10.7752	10.8576	10.7644	10.8524	10.8944	10.6992	10.8713	11.1640	10.7741	10.7655	10.5656	10.6423	10.8153	10.3027	10.5158	10.3522	10.5339	10.7363	10.6198	10.5965
Eef1a1	_RYEEIVK_.2	#N/A	9.0461	8.9730	9.1968	8.6711	8.9259	9.0943	9.0557	8.9425	9.0657	9.1215	8.9030	8.8378	8.9087	8.8893	9.0096	8.9901	8.7249	8.9457	8.8505	8.8407
Eef1a1	_LPLQDVYK_.2	#N/A	8.9930	8.8399	9.1007	8.4469	9.2576	9.0462	9.0636	9.1611	8.7557	8.8651	8.9691	8.8623	8.5126	8.5220	8.9360	8.4828	8.5930	8.8235	8.7871	8.7429
Eef1a1	_QLIVGVNK_.2	#N/A	8.7502	9.0603	9.0261	8.6046	8.9913	8.9979	9.0622	9.1341	8.7684	8.9534	8.5651	8.8980	8.8730	8.9639	8.4787	8.5671	8.5753	8.9376	8.8588	8.5362
Eef1a1	_QTVAVGVIK_.2	#N/A	8.6134	8.6487	8.9114	8.8324	8.8635	8.9192	8.7271	8.6128	9.0253	8.8298	8.8199	8.5874	8.5361	8.6216	8.6967	8.8428	8.2497	8.6036	8.8257	8.7207
Eef1a1	_STTTGHLIYK_.2	#N/A	8.6613	8.6160	8.8291	8.8436	8.6699	8.7065	8.6229	8.8953	8.7684	8.9256	8.0521	8.6917	8.5239	8.6766	8.9531	8.5767	8.3941	8.7156	8.8644	8.3963
Eef1a1	_TIEKFEK_.2	#N/A	7.7402	7.8031	7.4013	7.5645	7.9136	7.8334	7.7674	7.8078	7.7455	7.6205	7.4823	7.5650	7.6491	7.7081	7.8768	7.6905	7.2955	7.6850	7.6769	7.3855
Eef1a1	_EHALLAYTLGVK_.2	#N/A	6.8601	6.5294	6.8012	7.1225	6.9468	6.7979	6.7108	6.9659	6.9626	7.1847	6.7695	6.8598	7.4679	6.4868	6.5665	6.9716	6.3222	6.4518	6.9116	6.6464
Eef1a1	_M[Oxidation (M)]DSTEPPYSQK_.2	#N/A	6.9003	7.6778	8.3223	8.1935	7.5690	7.9510	7.7818	6.7891	7.7086	7.6091	7.6942	7.4667	7.3919	7.9123	7.6631	7.8581	7.3462	7.4495	7.1808	7.3883
Eef1a1	_VETGVLKPGM[Oxidation (M)]VVTFAPVNVTTEVK_.3	#N/A	5.9025	6.8601	6.6963	6.7410	6.2115	6.4692	6.7689	6.6676	6.4219	6.0347	6.1352	5.9038	6.5488	6.6927	6.3212	6.1862	5.9555	6.5206	6.4552	5.9019
Eef1a1	_YYVTIIDAPGHR_.2	#N/A	8.5319	8.7823	8.4767	8.4444	8.4689	8.6800	8.5792	8.7724	8.5679	8.6295	8.1327	8.5430	8.5809	8.6119	8.0370	8.6210	8.5337	8.4575	8.4710	8.2469
Eef1a1	_DGSASGTTLLEALDC[Carbamidomethyl (C)]ILPPTRPTDKPLR_.3	#N/A	4.3217	2.6740	2.4965	4.3386	3.0048	3.4577	3.5027	3.3716	3.3823	3.7916	4.2663	3.7996	3.2765	4.1669	3.4257	2.9806	4.1667	3.7094	1.5996	3.9984
Eef1a1	_NMITGTSQADC[Carbamidomethyl (C)]AVLIVAAGVGEFEAGISK_.3	#N/A	4.3205	3.3607	2.9986	3.3294	3.4110	4.2235	3.9795	4.2781	3.9598	3.6219	3.9056	3.9888	4.1000	3.6146	3.8330	3.4179	3.5793	3.2014	3.9651	3.4326
Eef1a1	_THINIVVIGHVDSGK_.3	#N/A	3.4026	3.8829	4.4736	4.1480	4.2412	4.4652	3.2999	3.6619	3.3590	3.6089	3.7334	3.6090	3.4671	4.5117	2.8771	3.7475	3.1591	3.9903	4.2894	4.1889
Alad	_DAAQSS[Phospho (STY)]PAFGDR_.2	#N/A	10.3839	10.3370	10.5049	10.6740	10.1377	10.1358	10.0036	10.3147	10.1662	10.4163	10.1417	10.1883	10.4653	10.8431	3.2613	10.2008	10.5584	10.0880	10.2316	10.2429
Alad	_DAAQSS[Phospho (STY)]PAFGDRR_.2	#N/A	7.7854	7.7046	7.9852	8.0999	7.6670	7.9549	8.1147	8.2047	7.4422	8.3057	8.0288	8.3108	8.6484	8.6686	8.0075	8.3356	8.2882	8.1591	8.1305	8.0923
Ctsb	_EQWSNC[Carbamidomethyl (C)]PTIGQIR_.2	#N/A	7.0859	7.3750	7.5365	7.7419	7.7738	7.6627	7.5326	7.6265	7.7978	7.6899	7.6166	7.5913	7.1199	7.8964	7.8267	7.6429	7.3932	7.1045	7.1855	7.5996
Ctsb	_GENHC[Carbamidomethyl (C)]GIESEIVAGIPR_.3	#N/A	4.5343	6.0700	7.6690	7.2818	7.2224	7.0877	6.4912	6.1352	6.9176	6.8950	6.9498	6.3485	5.7073	7.0891	7.3149	7.0336	6.7793	7.0657	6.4437	7.6623
Ctsb	_SC[Carbamidomethyl (C)]EAGYSPSYKEDK_.2	#N/A	4.0272	3.8055	4.3052	3.8396	3.9883	4.2165	4.7629	1.7909	3.0580	4.5730	4.0708	3.9100	3.1661	4.7550	4.9103	4.1658	3.7319	4.8578	4.0029	3.9878
Ctsb	_DQGSC[Carbamidomethyl (C)]GSC[Carbamidomethyl (C)]WAFGAVEAISDR_.2	#N/A	5.5295	4.6205	5.7779	4.9586	4.9299	4.6358	4.9223	5.7695	5.1172	5.6599	4.6537	5.4262	5.4412	6.1601	4.9322	5.1443	5.3519	4.8479	5.2364	4.8505
Ctsb	_NGPVEGAFTVFSDFLTYK_.2	#N/A	3.3894	4.6134	3.9257	4.3000	4.2279	4.4520	3.3131	3.6751	5.4898	3.5957	3.7466	3.0020	3.4803	2.8966	2.8903	3.7343	3.1723	3.9771	4.2762	5.2957
Ctsb	_EIMAEIYK_.2	#N/A	3.0922	4.2269	4.1296	3.8755	3.2991	3.3325	3.7490	3.5143	3.6692	4.4036	3.1478	3.9530	3.8346	4.3373	3.7744	3.9551	4.0520	1.1239	3.4298	4.3043
Eif4a2	_LQAEAPHIVVGTPGR_.3	#N/A	6.1157	5.9974	6.1225	6.2489	5.8332	6.0222	6.4649	6.3153	6.0327	6.2640	6.2036	5.0800	6.4103	6.0463	6.1412	6.5072	5.1294	6.0849	6.4667	5.5730
Eif4a2	_ETQALVLAPTR_.2	#N/A	6.2030	5.7742	5.1952	5.0680	5.8001	5.7140	5.4576	6.1193	5.6140	5.6130	5.0338	5.5015	4.2347	6.1146	4.2121	4.8191	4.8806	5.7899	5.1485	5.5226
MAPT	_SGYSSPGS[Phospho (STY)]PGTPGSR_.2	#N/A	16.7214	16.2339	17.0335	17.0945	16.3582	15.8049	16.0292	16.8976	15.8759	16.2637	14.5840	14.8898	15.5797	15.4325	14.6664	14.8157	15.3150	14.6830	14.5246	14.9746
MAPT	_SKIGS[Phospho (STY)]TENLK_.2	#N/A	12.8080	12.5899	12.6213	13.0079	11.7450	10.9552	12.0526	11.6658	11.2136	8.6076	9.5329	9.8645	9.9427	10.3526	9.1282	9.8960	9.4876	9.6684	9.6567	10.1364
MAPT	_TDHGAEIVYK_.2	#N/A	10.4598	10.6606	10.9499	10.2633	10.4721	10.8517	10.5443	10.6695	10.9185	10.6938	7.4549	7.4704	7.5006	7.5559	8.1970	7.7003	7.5166	7.3994	7.1673	7.6333
MAPT	_LDLSNVQSK_.2	#N/A	10.5812	10.9378	10.8348	10.5720	10.5387	10.6798	10.6934	10.6702	10.7167	10.9144	7.4460	7.5619	7.6820	7.9077	8.1559	7.7331	7.7101	7.2750	7.5860	7.9432
MAPT	_SEKLDFKDR_.2	#N/A	10.3770	10.7139	10.5793	10.2601	10.4760	10.3187	10.3467	11.0066	10.5579	10.9714	7.5489	7.7220	7.9684	7.8523	8.1390	8.1782	7.7622	7.7838	7.4122	7.5252
MAPT	_HVPGGGSVQIVYKPVDLSK_.3	#N/A	9.2570	9.0031	9.8229	9.4228	9.1881	9.2795	9.0744	9.4219	9.3841	8.9660	8.8430	9.1139	9.5888	9.4215	9.4234	8.9175	9.1151	8.9218	9.4478	9.2768
MAPT	_SEKLDFK_.2	#N/A	9.0120	9.3113	9.5388	8.9807	8.8716	9.2733	9.0347	8.8463	8.9531	9.5365	6.2690	5.9122	6.0812	6.3342	6.7408	6.4666	5.8871	6.0703	5.5049	6.4032
MAPT	_VQIINKK_.2	#N/A	7.6494	8.4407	8.1423	7.9786	8.0185	7.9376	8.2117	8.1296	7.9188	8.2109	4.9165	5.1082	4.2898	5.3804	5.5121	4.8544	4.0860	5.2794	4.4875	5.2612
MAPT	_LQTAPVPM[Oxidation (M)]PDLK_.2	#N/A	11.5422	12.0938	12.0135	11.9097	11.6932	11.8227	11.8881	11.7778	11.6683	12.0862	9.0415	9.0945	8.5902	9.2683	9.4398	8.6462	8.8108	8.9747	8.1988	8.3390
MAPT	_T[Phospho (STY)]PPKSPSSAK_.2	#N/A	12.9575	13.0279	14.1126	13.1765	13.1012	13.1114	13.2759	12.9865	13.0032	13.3603	10.0973	9.8324	10.7880	11.5780	9.3849	9.9071	10.0410	10.8485	10.3747	11.1053
MAPT	_IGS[Phospho (STY)]LDNITHVPGGGNK_.2	#N/A	13.5627	13.4304	12.9434	13.1595	12.0067	12.0220	12.6298	12.1129	12.0116	14.1356	9.4625	9.5276	9.7899	9.8449	8.2841	9.2345	9.0630	8.9558	9.0633	9.9008
MAPT	_IGSTENLK_.2	#N/A	10.2514	10.4119	10.6185	10.5135	9.8564	10.2272	10.4954	9.8743	10.2849	11.2292	7.3513	7.4359	7.2937	7.2079	7.6561	7.1972	7.0818	7.2702	6.9919	7.8174
MAPT	_VAVVRT[Phospho (STY)]PPKS[Phospho (STY)]PSSAK_.2	#N/A	12.9643	12.9711	13.3632	13.1982	11.9766	11.5710	12.2899	11.6502	11.8043	13.5568	10.7106	10.7830	10.8977	11.4364	9.7050	10.9945	10.2280	10.3910	10.6013	10.7625
MAPT	_TPS[Phospho (STY)]LPTPPTR_.2	#N/A	13.9918	13.9484	14.3462	13.7186	13.2247	13.3636	13.3690	13.2371	12.9885	14.2020	10.9829	10.9920	10.6444	11.4914	10.2526	10.5289	10.7425	10.3595	10.6400	10.7085
MAPT	_IGS[Phospho (STY)]LDNITHVPGGGNKK_.3	#N/A	11.0483	11.3745	10.4623	10.7062	9.4624	9.4883	10.5969	10.0899	9.3802	11.9082	6.2395	6.6623	7.2228	7.2092	6.3050	5.6382	6.5419	6.9363	6.1622	7.0017
MAPT	_SRT[Phospho (STY)]PS[Phospho (STY)]LPTPPTREPK_.3	#N/A	3.4602	13.2082	11.1558	12.6876	9.0170	10.5916	12.8298	12.3288	12.1802	11.2740	3.7019	8.3615	10.3275	9.7733	7.1417	9.8275	9.5358	9.6041	9.1108	9.9146
MAPT	_AKTDHGAEIVYKS[Phospho (STY)]PVVS[Phospho (STY)]GDTSPR_.4	#N/A	10.9865	11.3615	11.8912	11.5285	7.4512	10.3356	10.0596	9.2154	10.3866	10.8068	7.5079	9.8005	8.3852	10.0005	8.9788	9.7745	6.9479	9.6565	8.7011	9.4091
MAPT	_IGS[Phospho (STY)]TENLKHQPGGGK_.3	#N/A	3.8386	9.6048	6.2143	9.2121	3.4470	5.1934	8.8495	3.5549	3.7998	10.3444	3.6526	3.8054	3.9408	4.1140	3.6809	3.7928	4.0926	3.7053	3.5328	4.0078
MAPT	_SPVVS[Phospho (STY)]GDTS[Phospho (STY)]PR_.2	#N/A	15.7745	15.6456	15.7140	15.9361	15.6695	15.4961	15.3370	15.8467	15.8095	15.8672	13.9636	14.3335	14.9791	14.5773	13.9609	14.2428	14.5956	14.0425	14.1148	14.3742
MAPT	_TDHGAEIVYKS[Phospho (STY)]PVVSGDTS[Phospho (STY)]PR_.3	#N/A	11.6633	12.5672	13.0372	12.9112	10.8534	12.2365	12.1210	4.2497	11.5792	13.6002	10.0876	10.6189	9.9949	11.0432	9.9760	10.8364	10.6777	10.1721	10.5849	10.8721
MAPT	_TPS[Phospho (STY)]LPTPPTREPK_.2	#N/A	13.6521	13.7871	13.6801	13.6091	12.8146	12.8452	13.0037	13.1361	12.7927	13.9761	10.2839	10.8451	11.1820	11.1215	10.2926	10.6026	10.9515	10.3275	10.3213	10.4464
MAPT	_AKTDHGAEIVYK_.2	#N/A	6.9379	7.7110	8.0063	6.9131	6.8525	7.7167	7.5524	6.6390	8.0872	7.5836	4.4478	3.7884	4.1958	4.5146	5.3572	4.9911	3.8878	4.4185	3.8667	4.8103
MAPT	_SRT[Phospho (STY)]PS[Phospho (STY)]LPTPPTR_.2	#N/A	11.6565	4.1370	12.3837	11.9752	10.8098	12.0815	12.6451	10.7993	12.6923	12.7072	10.2939	3.9202	4.0203	4.1507	7.6505	10.2456	8.4630	8.2255	3.4881	8.6933
MAPT	_KLDLSNVQSK_.2	#N/A	9.1627	9.7419	9.5780	9.3404	9.2511	9.3749	9.5552	9.4004	9.4403	9.7746	6.4968	6.5098	6.9842	7.0204	7.1532	6.4240	6.8088	6.7029	6.4790	6.6137
MAPT	_IPAKT[Phospho (STY)]PPAPK_.2	#N/A	10.9532	10.7144	11.4887	11.5370	10.5067	10.0637	10.1675	10.1197	10.0045	11.3163	3.2345	1.6289	4.4690	4.4249	3.8791	4.2464	4.1395	3.6936	3.3422	5.5239
MAPT	_T[Phospho (STY)]PPSSGEPPK_.2	#N/A	13.9618	13.9544	14.5779	14.0970	13.4729	14.0291	13.6697	13.2149	13.7909	14.3709	3.8656	3.4837	3.5993	4.0319	3.0613	3.4140	3.2913	3.8651	4.1641	4.3211
MAPT	_TPPAPKT[Phospho (STY)]PPSSGEPPK_.2	#N/A	13.8008	14.0887	14.3673	14.0446	13.5071	13.7623	13.6675	13.7917	13.7658	14.3080	6.3606	5.1306	5.9670	5.9803	3.5424	3.0428	6.2365	5.6443	3.7737	7.3292
MAPT	_SKIGS[Phospho (STY)]TENLKHQPGGGK_.3	#N/A	9.1109	9.7920	3.3554	9.6410	7.8307	4.0826	8.8312	4.0445	3.7416	10.4302	4.1160	3.2264	3.8497	6.5815	4.8535	3.3649	3.5417	3.6077	3.9068	3.0707
MAPT	_DQGGYTM[Oxidation (M)]HQDQEGDTDAGLK_.3	#N/A	6.3632	5.5001	6.3524	5.9368	6.2451	6.8630	6.0615	5.6713	6.3660	6.9395	4.2517	3.5072	3.9854	3.5292	3.3955	3.2291	3.6774	3.4720	3.7711	3.2065
MAPT	_QEFEVM[Oxidation (M)]EDHAGTYGLGDR_.3	#N/A	8.0256	7.2448	8.3347	7.9233	7.7995	7.3269	7.3077	7.4995	7.3247	8.3607	3.7739	3.5127	4.0363	4.1358	2.9091	3.5244	3.1890	3.5896	4.2827	3.9052
MAPT	_VQS[Phospho (STY)]KIGS[Phospho (STY)]LDNITHVPGGGNK_.3	P10636_S669_M2_MAPT	3.4093	9.2478	8.4237	7.3731	4.2478	8.1696	8.8313	3.1097	8.3361	8.6721	3.7267	2.9821	3.4300	3.9326	2.8704	3.7542	3.6473	4.1858	3.8344	5.9045
MAPT	_SGDRSGYSSPGS[Phospho (STY)]PGTPGSR_.3	#N/A	6.8948	7.5286	5.0243	5.2404	4.5493	5.0398	5.0014	4.7293	5.2015	5.3879	3.9203	3.6248	3.6961	3.9351	3.4648	3.9533	3.9442	4.0085	3.6185	3.7217
MAPT	_IPAKT[Phospho (STY)]PPAPKT[Phospho (STY)]PPSSGEPPK_.3	#N/A	12.6962	12.7768	12.8518	12.8261	11.5655	11.5441	12.1267	11.2372	11.2351	13.2100	3.8621	3.1378	3.2855	3.7882	2.9961	3.6097	3.5028	4.3415	3.9901	4.7338
MAPT	_TPPAPKT[Phospho (STY)]PPSSGEPPKSGDR_.3	#N/A	10.3860	10.9891	9.4680	10.9113	10.4005	10.6090	10.1931	10.8786	10.2107	9.5558	3.7384	3.5949	3.9930	3.8379	3.3718	3.6038	3.6652	3.8400	3.8906	4.3773
MAPT	_C[Carbamidomethyl (C)]GSLGNIHHKPGGGQVEVK_.3	#N/A	7.1702	7.4470	7.3766	6.1726	7.0595	7.7502	7.4385	7.6537	7.1438	7.5185	3.4162	4.5457	4.4344	5.6182	5.3273	5.1432	5.1864	4.4392	3.6728	5.4057
MAPT	_T[Phospho (STY)]PPSSGEPPKSGDR_.2	#N/A	12.3955	8.0258	12.2674	12.3493	11.8196	12.0667	12.0253	12.0198	12.0511	12.4716	3.2702	4.0817	4.0872	4.2666	3.8967	4.0863	4.1100	4.4901	3.3742	3.6907
MAPT	_HLSNVSSTGS[Phospho (STY)]IDM[Oxidation (M)]VDSPQLATLADEVSASLAK_.4	#N/A	3.5822	8.6110	9.0857	8.0101	3.7146	3.8125	7.1847	9.1609	3.5436	3.6739	3.8578	3.5933	3.6901	6.6018	5.3322	4.1130	3.7815	5.7850	3.7645	4.2470
MAPT	_QEFEVMEDHAGTYGLGDRK_.3	#N/A	3.6580	3.9323	3.6736	4.3330	3.8104	4.0345	3.9019	3.4491	3.9609	3.8643	3.5206	3.5908	4.0690	4.2989	3.4791	4.0029	3.7610	3.5596	3.8586	3.9762
Mapt	_IPAKTT[Phospho (STY)]PS[Phospho (STY)]PK_.2	#N/A	11.0227	11.2053	11.2155	11.5701	10.5911	10.8387	10.8272	10.9702	11.1573	11.2736	3.9289	10.5655	10.8449	11.1036	10.6229	3.1741	10.6634	10.5216	10.5692	10.5647
Mapt	_IPAKTT[Phospho (STY)]PS[Phospho (STY)]PKT[Phospho (STY)]PPGSGEPPK_.2	#N/A	8.4918	8.2656	8.2518	4.3540	8.0248	3.7280	7.9237	6.7151	3.2367	8.1183	5.5295	3.6084	6.4124	4.3894	3.5511	7.5250	3.6405	4.1312	6.5885	5.6795
Mapt	_T[Phospho (STY)]PPKSPSASK_.2	#N/A	12.7894	14.0165	13.5441	14.1487	13.2750	13.3214	13.4232	13.1713	13.2248	13.5413	11.1539	10.0484	10.8540	11.6892	9.5793	10.1484	10.2775	10.8994	10.5818	11.2161
Mapt	_ESPPQPPADDGAEEPGS[Phospho (STY)]ETSDAK_.2	#N/A	8.9077	8.9595	8.9262	9.2653	8.5375	8.6313	8.7842	8.9835	8.5727	8.8020	8.6793	9.1257	9.5120	9.8614	8.8186	9.2618	9.4759	8.6955	8.7093	8.9417
Mapt	_VAVVRT[Phospho (STY)]PPKS[Phospho (STY)]PSASK_.2	#N/A	12.6360	12.7201	13.1040	12.9924	11.6204	11.3114	12.0128	11.2471	11.5299	13.3496	10.5527	10.6436	10.7121	11.1933	9.5494	10.8312	10.0755	10.1376	10.4115	10.6116
Mapt	_TTPS[Phospho (STY)]PKT[Phospho (STY)]PPGSGEPPKSGER_.3	#N/A	7.9144	10.0152	6.8042	8.8124	7.6140	8.2238	8.2216	8.5271	9.4996	3.8080	8.5750	6.8016	9.3333	9.1595	8.9001	9.3830	9.2519	8.8674	7.0785	8.3447
Mapt	_QEFDTM[Oxidation (M)]EDHAGDYTLLQDQEGDM[Oxidation (M)]DHGLK_.4	#N/A	5.3784	3.2873	4.2271	3.4574	4.5500	4.6077	4.2207	4.3333	4.6430	4.6727	4.3392	4.6815	4.1385	3.3003	3.8975	5.1006	3.3543	3.4553	4.4003	5.2726
Mapt	_TTPS[Phospho (STY)]PKT[Phospho (STY)]PPGSGEPPK_.2	#N/A	12.5006	12.5350	12.8694	12.2162	12.3337	12.3587	11.4548	12.5140	12.3648	12.6001	12.0422	12.2648	12.5913	12.8910	11.5574	12.2992	12.5364	12.0979	12.1453	12.3322
Mapt	_ST[Phospho (STY)]PTAEDVTAPLVDER_.2	P10637_T58_M1_Mapt	9.8019	9.8705	10.1672	10.0074	8.8895	9.9447	9.4163	9.4505	9.7403	9.6259	9.5682	9.5354	3.8953	10.2993	9.4129	9.7500	10.1046	9.3457	9.7228	9.8995
Mapt	_T[Phospho (STY)]PPGSGEPPK_.2	#N/A	11.5383	11.3214	12.0436	11.8483	11.4656	11.7426	11.1244	10.8713	11.4444	11.7746	11.5996	10.1460	9.4560	11.6561	11.3377	11.1740	11.3916	10.8738	11.2310	11.5160
Mapt	_SGERS[Phospho (STY)]GYSS[Phospho (STY)]PGSPGTPGSR_.2	P10637_S487_M2_Mapt	3.9380	3.9065	3.6919	3.7458	3.6340	3.7686	3.9269	3.8495	3.9246	4.0212	3.4474	3.7605	5.0346	3.7259	3.6352	3.5055	4.1930	3.7138	5.3315	3.5651
Mapt	_T[Phospho (STY)]PPGSGEPPKSGER_.2	#N/A	3.9299	9.8271	3.6375	9.3827	9.1516	9.5556	8.7681	10.5486	9.5834	9.0243	9.1498	8.9656	8.6433	10.8446	9.7190	10.4742	10.4367	3.3662	3.6653	10.3529
Txn	_EAFQEALAAAGDK_.2	#N/A	8.9823	8.9783	9.7236	9.3416	8.6879	9.1136	8.9799	9.0002	9.3955	9.2731	8.9934	8.8409	8.8975	9.2962	9.5258	8.9776	9.1372	8.9672	9.2038	9.1780
Txn	_VGEFSGANK_.2	#N/A	8.0649	7.9093	8.2858	8.2607	8.1023	8.1866	8.1343	8.1485	8.1396	7.7638	7.8126	7.8892	7.8228	8.0525	8.5293	7.8185	7.6713	7.9905	7.6641	8.1127
Txn	_VKLIESK_.2	#N/A	6.5998	7.0593	6.0782	6.7563	6.6535	6.5547	6.5663	7.0080	6.4009	6.2589	6.5843	7.0926	7.8604	6.7941	6.8399	6.7049	7.0211	7.0235	6.6646	7.0968
Txn	_VGEFSGANKEK_.2	#N/A	4.3352	5.4954	4.9850	5.4519	4.8232	5.1639	4.6064	4.9435	5.3910	5.6350	5.0531	5.6101	5.6074	4.6745	5.6008	5.5250	5.4100	5.0127	4.9335	5.3746
Gstm1	_ITQSNAILR_.2	#N/A	9.8960	10.0567	10.0064	10.0224	9.8160	10.0476	10.0738	10.1729	10.0789	10.0962	9.2725	9.6575	9.8722	9.7082	9.8040	9.5276	9.5070	9.6461	9.6848	9.3977
Gstm1	_YIATPIFSK_.2	#N/A	9.2640	9.2186	9.2542	9.4335	9.2282	9.1821	9.1518	9.0849	8.9427	9.0125	8.7145	9.0763	8.8391	8.7516	9.0192	8.7871	8.6639	8.9538	9.0601	8.8709
Gstm1	_PM[Oxidation (M)]ILGYWNVR_.2	#N/A	8.2372	8.3901	8.1152	8.2555	8.6755	8.4501	8.1764	8.6522	8.2677	8.5102	7.8625	8.1904	8.1323	7.8774	8.0524	8.1157	8.0807	8.2581	8.2975	8.2020
Gstm1	_SQWLNEK_.2	#N/A	8.0522	7.9909	7.9226	8.3906	8.1298	8.3061	8.4756	8.1804	8.2045	8.5800	7.9421	7.9134	8.0561	7.9653	8.1722	7.8810	7.1482	8.0088	7.9936	8.1987
Gstm1	_C[Carbamidomethyl (C)]LDAFPNLR_.2	#N/A	7.9640	8.2501	8.3791	7.9513	8.1297	8.1339	8.1938	7.9818	8.0316	8.1408	7.4743	8.0040	7.7204	7.6280	8.0095	7.7815	7.7668	7.6330	7.8986	7.3755
Gstm1	_RPWFAGDK_.2	#N/A	7.6455	8.0286	8.1434	7.8189	8.0092	7.9024	8.2574	7.7322	7.5689	7.5119	7.3923	7.6066	6.9642	7.8575	7.8636	7.3061	7.5554	7.6825	7.0346	7.3540
Gstm1	_LYSEFLGK_.2	#N/A	6.8557	7.0388	7.1137	7.5992	7.1111	7.3010	7.7441	6.1105	7.5028	7.2809	6.7259	7.1569	7.1501	6.8554	7.2209	7.1797	5.4975	7.0716	7.6867	7.1984
Gstm1	_YTM[Oxidation (M)]GDAPDFDR_.2	#N/A	9.1660	9.0756	9.4592	9.2404	9.0611	9.1936	8.9563	9.0431	9.1850	9.1721	8.7407	8.6990	8.8030	8.7539	8.7293	8.7263	8.7227	8.6157	8.6195	8.7969
Gstm1	_ADIVENQVM[Oxidation (M)]DTR_.2	#N/A	8.4533	8.5382	8.7083	8.6379	8.5340	8.7208	8.4378	8.6175	8.6660	8.5788	8.1224	8.1912	8.2083	8.4750	8.4641	8.1041	8.1288	8.1568	8.1346	8.3418
Gstm1	_LGLDFPNLPYLIDGSHK_.3	#N/A	6.8027	6.3267	6.4981	6.7364	6.3284	6.5329	6.2947	6.1518	6.7129	6.7732	6.1123	6.1259	5.7842	5.9328	5.6697	5.8668	6.5045	6.1130	6.1145	6.0355
Gstm1	_MLLEYTDSSYDEK_.2	#N/A	3.8925	4.9822	5.4469	5.5326	4.2368	4.7122	4.7494	4.1182	4.6660	3.7031	3.8484	3.1527	4.5352	5.7727	3.6441	3.2546	3.6154	3.0505	3.1414	3.5040
Gstm1	_M[Oxidation (M)]LLEYTDSSYDEKR_.2	#N/A	6.0189	5.9293	6.1530	6.0826	6.4363	6.2471	5.9132	6.0919	6.0256	5.5078	5.0530	5.7430	5.9695	5.9991	6.0955	5.9921	5.6879	5.5048	5.5702	5.8486
Gstm1	_IRADIVENQVM[Oxidation (M)]DTR_.3	#N/A	4.4551	5.8442	4.0674	4.8947	4.5870	4.9840	5.6758	5.4212	5.0937	5.0684	3.5247	5.1752	6.0058	4.7811	5.5528	5.5069	4.7659	5.3129	5.0093	5.0877
Gstm1	_VTYVDFLAYDILDQYR_.2	#N/A	3.4422	3.4057	4.1843	2.8976	4.0512	2.3476	3.4898	4.4139	3.5489	3.4190	3.9233	3.1787	3.6570	2.9982	1.8323	3.5576	3.4507	4.3824	2.2879	3.7030
Tcea1	_EPAISSQNS[Phospho (STY)]PEAR_.2	#N/A	9.3694	3.6738	9.5630	9.3454	9.3353	9.2089	8.7490	8.9187	9.0196	9.3149	9.4308	9.1973	3.2815	9.9699	9.1109	9.4398	9.6399	8.8690	9.3586	9.5131
Tcea1	_KKEPAISSQNS[Phospho (STY)]PEAR_.3	#N/A	8.9577	10.2717	9.6234	9.7937	9.7177	9.9265	9.8144	9.1540	9.7581	10.2415	9.6788	8.7104	10.2024	10.3830	10.1321	10.0628	10.0041	9.9610	9.4484	9.9722
Tcea1	_DTYVSSFPR_.2	#N/A	3.6165	4.3863	4.1528	4.0728	3.4965	3.5298	4.7997	3.3169	3.4718	3.1001	2.9505	4.2757	3.6373	4.1399	3.5770	4.1525	3.8546	3.9786	3.6271	4.1069
Slc3a2	_LLLSTDSAR_.2	#N/A	7.4481	7.4978	7.1605	7.3415	7.4418	7.3076	7.8017	7.6720	7.5204	7.6103	7.5919	7.4582	7.6013	7.0895	7.1156	7.4304	7.1637	7.7806	7.7469	7.2021
Slc3a2	_GQNEDPGSLLTQFR_.2	#N/A	7.4603	7.4530	7.4955	6.9063	6.9504	7.2742	6.8570	7.5931	7.2680	7.1672	7.2213	6.9169	6.9376	7.1645	6.8076	6.9710	7.4338	7.1002	6.9653	7.0884
Slc3a2	_LGASNLPAGISLPASAK_.2	#N/A	7.2627	6.8711	7.0484	7.1182	6.8047	7.0062	7.1549	7.2450	7.0943	6.6394	7.0252	6.7252	6.2952	7.0954	6.3664	6.0861	6.6619	7.4207	5.9243	6.7864
Slc3a2	_IGDLQAFVGR_.2	#N/A	7.0661	7.0043	6.7341	6.6630	6.7489	6.4420	6.9183	6.8542	6.3564	5.9854	6.7124	6.6978	6.3548	6.0182	6.0729	5.8753	6.5272	6.8946	6.2830	6.3011
Slc3a2	_QINPTLGSQEDFKDLLQSAK_.3	#N/A	6.2428	5.9773	5.7435	6.1377	5.6475	5.8562	5.3490	5.9245	5.9203	5.0552	5.8971	5.3272	5.6915	5.6392	5.8211	5.3194	5.9061	6.1085	6.2350	5.4815
Slc3a2	_EALSSWLQDGVDGFQFR_.2	#N/A	5.3321	5.0067	4.3790	5.1245	5.3631	4.2327	5.0393	4.4467	5.2371	5.2444	4.8489	4.9943	4.6117	4.7738	4.8245	4.7567	5.4896	4.9741	4.6465	4.0578
Slc3a2	_SHLEYLSTLK_.2	#N/A	4.9898	4.0025	4.4702	5.3088	4.9189	4.5359	5.3128	4.3554	3.8603	5.0366	4.8032	4.6864	5.5478	4.8116	5.0361	4.8478	4.3044	4.5668	4.6691	4.7140
Slc3a2	_HWDQNER_.2	#N/A	5.0777	5.8380	5.6782	4.1736	5.1362	5.5114	5.7620	5.7446	5.4592	5.4245	5.5156	4.8505	3.4415	4.4861	2.8515	5.0431	3.1335	4.0159	5.4291	5.1706
Slc3a2	_DAGGIAGLK_.2	#N/A	3.6272	3.6918	3.1638	3.9570	3.8342	4.2742	2.1524	3.8529	3.5501	3.4456	3.9244	3.4180	3.6581	3.2019	2.5423	2.9894	3.3502	2.6380	4.0983	1.6716
Slc3a2	_NQKDEINETDLK_.3	#N/A	3.3396	3.1624	3.8759	4.3498	4.1781	4.4022	2.2285	4.5408	3.4220	3.5459	3.7964	3.0518	3.5301	3.8630	2.9401	3.6845	3.5776	4.2555	3.9041	3.8300
H2bc7;H2bc14;H2bc3;H2bc9;Hist2h	_LLLPGELAK_.2	#N/A	12.0190	12.0130	12.1473	12.3333	12.1168	12.1660	12.1254	11.7629	12.0601	12.2618	11.8504	12.1697	11.9875	11.6559	12.2161	11.9933	11.7994	11.9512	12.1737	12.0438
H2bc7;H2bc14;H2bc3;H2bc9;Hist2h	_ESYSVYVYK_.2	#N/A	6.8203	7.4838	8.1020	8.1808	7.2277	7.7775	8.3663	6.1100	8.5283	8.3295	8.0023	7.5065	8.0621	8.4650	8.1962	8.3475	6.0303	8.3866	8.2450	8.3331
H2bc7;H2bc14;H2bc3;H2bc9;Hist2h	_EIQTAVR_.2	#N/A	6.2620	7.2832	6.8837	7.5853	6.5744	7.4977	7.9124	6.0194	8.0698	7.9619	7.4531	7.2484	7.6902	6.8500	8.2459	7.4790	5.6903	7.5971	7.7633	7.6580
H2bc7;H2bc14;H2bc3;H2bc9;Hist2h	_KESYSVYVYK_.2	#N/A	7.4562	7.6472	7.5264	7.3354	6.8936	7.3932	7.6323	7.2381	7.4879	7.5198	6.9531	6.7446	7.7350	7.5635	7.2153	7.5010	7.1498	7.5052	7.2209	7.5335
H2bc7;H2bc14;H2bc3;H2bc9;Hist2h	_AM[Oxidation (M)]GIM[Oxidation (M)]NSFVNDIFER_.2	#N/A	8.8000	8.6830	9.1744	8.8801	8.3564	8.6508	8.4465	8.7185	8.6807	8.8577	8.5308	8.6331	8.6956	8.0154	8.3265	8.5893	8.4665	8.4264	8.5418	8.3277
H2bc7:H2bc14:H2bc3:H2bc9:Hist2h	_QVHPDTGISSK_.2	#N/A	10.9737	10.9209	11.1338	10.8300	10.8953	10.9391	10.9238	11.0715	11.1823	10.9582	10.5309	10.8089	10.9623	10.7904	11.1962	10.9192	10.6355	10.7849	10.7246	10.8174
H1-0	_VGENADSQIK_.2	#N/A	6.7505	6.2677	6.3495	5.4902	6.1717	6.4228	6.6881	6.1602	6.9591	5.8471	6.0507	5.8880	3.9026	5.7604	6.5134	5.1980	6.2341	6.3469	5.7471	6.0746
H1-0	_[Acetyl (Protein N-term)]TENS[Phospho (STY)]TSAPAAKPK_.2	#N/A	3.9724	4.3490	3.7016	3.7894	4.3001	4.8052	5.2792	6.3600	3.6809	3.9846	4.4933	5.3936	4.7669	3.5245	4.1389	3.8747	3.2900	4.7101	4.3708	4.2029
H1-0	_GVGASGSFR_.2	#N/A	4.1072	3.2119	3.6438	3.4770	4.3141	4.3475	1.8553	4.3329	4.1550	3.9178	4.8861	5.6692	4.3204	4.8299	6.3312	4.9840	3.8301	5.7755	5.4648	4.9843
Sub1	_EQISDIDDAVR_.2	#N/A	7.1016	6.4845	6.8210	6.8449	6.3051	7.1329	6.9991	6.3367	6.4076	6.9869	6.5434	6.5826	6.2078	6.5206	6.3357	6.0562	6.7361	6.2860	6.2562	6.2389
Sub1	_QSS[Phospho (STY)]SSRDDNMFQIGK_.3	#N/A	4.0365	3.8549	3.9363	6.0816	3.7203	3.6350	3.5135	4.0612	3.4337	3.9877	3.6948	3.6375	3.5992	4.1018	3.4832	3.7543	3.8165	4.0837	3.7323	4.0688
Gpx1	_TIDIEPDIETLLSQQSGNS_.2	#N/A	9.4581	9.7681	8.9483	9.0935	8.4346	8.7773	9.4761	10.3356	8.8224	9.0528	8.6880	9.7170	9.9897	10.2730	9.1530	8.9239	9.6069	9.3770	8.9784	8.8354
Fabp3	_SLGVGFATR_.2	#N/A	7.4469	8.2280	8.3222	8.2094	7.8236	8.1135	8.1843	7.9942	8.0936	8.2101	7.5389	8.1408	8.0167	8.2887	8.3722	7.8845	7.8307	8.3551	8.2444	8.0062
Fabp3	_[Acetyl (Protein N-term)]ADAFVGTWK_.2	#N/A	7.4930	7.9425	7.9730	7.8093	7.7794	7.7012	7.7958	7.6209	7.6578	7.9365	7.5743	7.7488	7.3811	7.4897	7.9155	8.0734	7.7130	7.8217	7.9424	7.4878
Fabp3	_SLVTLDGGK_.2	#N/A	5.8827	6.9894	6.6799	7.0483	6.9730	6.4383	7.2325	6.3940	7.5979	6.7386	6.8689	6.4736	7.2843	6.2093	7.6517	7.0119	5.6148	7.2044	7.3775	7.4625
Fabp3	_WNGQETTLTR_.2	#N/A	6.3085	5.6904	6.7392	6.8380	6.2551	6.1502	6.5109	6.8259	5.9510	6.3423	6.4525	6.2020	6.6983	6.1684	7.2523	6.4354	6.1140	6.5444	6.3126	5.6351
Fabp3	_LILTLTHGSVVSTR_.2	#N/A	4.0084	3.6764	3.2992	3.4018	2.2686	3.9263	4.2772	4.0152	4.2605	3.4750	2.4005	3.5001	3.7326	4.1773	3.3627	3.1172	3.8920	3.3075	3.7960	3.4889
Fabp3	_NGDTITIK_.2	#N/A	3.3444	3.9747	4.3818	4.1277	3.5513	3.5846	3.4968	3.2621	3.4170	3.1550	2.8956	3.7008	3.5824	4.0851	3.5222	4.2073	3.7998	4.0334	3.6820	3.4463
Hsp90ab1	_IEDVGS[Phospho (STY)]DEEDDSGK_.2	#N/A	11.7055	11.0397	12.1314	11.8436	11.4432	11.4918	10.8452	11.4028	10.7756	11.5313	12.2660	11.9205	11.6824	12.8398	11.4881	11.9515	12.3851	11.9922	12.0052	12.1609
Hsp90ab1	_TKPIWTR_.2	#N/A	10.2343	10.4779	10.9335	10.5602	10.5358	10.3001	10.6486	10.6803	10.3246	10.4158	10.0879	10.6107	10.6214	10.4701	10.7734	10.1755	10.3011	10.6601	10.5280	10.2536
Hsp90ab1	_ADLINNLGTIAK_.2	#N/A	10.4890	10.5154	10.6344	10.3908	10.4857	10.4423	10.5694	10.5715	10.4238	10.3013	10.3684	10.4748	10.5663	10.7182	10.5910	10.3086	10.3889	10.6266	10.3555	10.3001
Hsp90ab1	_IDIIPNPQER_.2	#N/A	10.2696	10.2234	10.5254	10.1995	10.4438	10.0933	10.2205	10.0971	10.1442	10.1366	10.1778	10.2288	10.2592	10.2501	10.1491	10.1248	10.3599	10.2633	10.1924	10.0099
Hsp90ab1	_YIDQEELNK_.2	#N/A	9.9741	9.9899	10.2893	10.1007	10.0786	10.0412	10.0025	10.2904	9.9289	9.8498	9.8129	9.9772	10.1884	10.1989	10.0403	9.9346	9.9750	9.9612	9.7806	9.8642
Hsp90ab1	_EDQTEYLEER_.2	#N/A	9.7286	9.2445	10.0530	9.6088	9.4867	9.5392	9.1957	9.5294	9.5983	9.5592	9.6293	9.2241	9.2803	9.7129	9.3155	9.2442	9.5345	9.2047	9.1296	9.4354
Hsp90ab1	_ELISNASDALDK_.2	#N/A	9.5554	9.2526	9.7245	9.1243	9.2211	9.2637	9.2586	9.5950	9.3946	9.4635	9.5094	9.1657	9.1798	9.5922	9.0887	9.0976	9.3425	9.2751	9.1463	9.1867
Hsp90ab1	_SIYYITGESK_.2	#N/A	8.8611	8.7870	9.2048	8.9215	8.8389	8.8512	8.8450	8.8678	9.0545	8.8854	8.8957	8.6079	9.0201	9.0896	8.3841	8.8642	8.6402	8.8031	8.7006	8.9179
Hsp90ab1	_EGLELPEDEEEKKK_.3	#N/A	8.3440	8.2523	8.9419	8.4100	8.4040	8.7036	8.5646	8.3822	8.6997	8.5514	8.6575	8.2200	8.3027	8.3900	8.3202	8.2410	8.0456	8.4642	8.1433	8.5910
Hsp90ab1	_NPDDITQEEYGEFYK_.2	#N/A	8.0275	8.1276	8.5559	8.2081	8.0649	8.2586	7.8993	8.1611	8.3931	8.3099	8.2916	8.1340	7.7506	8.2199	7.9826	8.4544	8.4458	8.2516	7.9321	8.2102
Hsp90ab1	_ADHGEPIGR_.2	#N/A	8.4065	8.1207	8.6286	7.6838	7.9888	8.5062	8.4507	8.3303	8.4975	8.1723	8.5119	7.9670	8.3611	8.1210	8.3319	8.1565	7.8883	8.3580	8.1973	8.1707
Hsp90ab1	_EQVANSAFVER_.2	#N/A	7.7202	7.8746	8.3564	7.9569	7.7260	7.6068	7.5547	8.0629	7.9814	7.6412	7.3913	7.6210	8.1192	7.8537	7.7910	7.9244	7.6511	7.5937	7.9833	7.8956
Hsp90ab1	_ALLFIPR_.2	#N/A	7.1377	7.4526	7.8096	8.1644	7.4180	7.1786	7.9828	6.5244	7.5035	7.6414	7.1228	7.9299	7.4205	7.1158	7.3884	7.5990	6.4610	7.9156	7.7778	7.2141
Hsp90ab1	_EIS[Phospho (STY)]DDEAEEEK_.2	#N/A	7.7653	7.5974	8.2233	7.3887	7.5360	7.6438	7.2636	7.5427	7.4976	7.7451	7.3882	6.8959	7.0496	7.6969	6.5742	7.3135	7.6484	7.2028	6.8881	7.2391
Hsp90ab1	_YESLTDPSK_.2	#N/A	6.6620	7.3579	7.1121	7.1427	6.6186	7.2874	7.7140	6.2827	7.4363	7.6888	7.5114	6.7849	6.6619	7.3590	7.4489	7.2368	6.2186	7.2913	7.3124	7.5321
Hsp90ab1	_EKEIS[Phospho (STY)]DDEAEEEK_.2	#N/A	7.1117	7.2914	7.1125	7.3341	6.7970	7.1007	6.1845	7.3249	6.9551	7.4773	6.9099	6.8497	6.2997	7.6299	6.5693	6.5789	7.1092	6.9609	6.4313	6.7464
Hsp90ab1	_EKYIDQEELNK_.2	#N/A	6.1625	6.3420	6.7121	5.5657	6.5593	6.2054	6.3905	7.1055	6.6928	6.1406	6.8395	6.2918	6.5733	6.7058	6.1144	6.2811	6.7055	6.6545	6.4510	6.0933

Hsp90ab1	_VFIM[Oxidation (M)]DSC[Carbamidomethyl (C)]DELIPEYLNFIR_.2	#N/A	5.9004	6.1169	5.7161	5.6014	5.3474	6.1242	6.2765	6.3902	5.7897	5.8990	5.7985	6.2929	6.6749	6.3579	5.0735	5.2360	4.9915	5.7674	5.7283	5.6806
Hsp90ab1	_GVVDSEDLPLNIS[Phospho (STY)]R_.2	P11499_S391_M1_Hsp90ab1	6.7923	7.6395	7.4049	6.9200	4.0101	6.8802	7.0264	6.3917	7.0106	6.5749	6.9487	6.5152	3.6982	7.7949	6.6599	6.8598	6.9008	7.3594	7.1200	6.7477
Hsp90ab1	_APFDLFENK_.2	#N/A	4.9708	3.9579	4.3440	5.0583	4.9993	5.0528	5.2453	5.4049	5.0309	3.6957	5.0915	5.0008	4.1651	3.7262	4.2493	4.8340	4.8450	5.0446	5.0894	5.3992
Hsp90ab1	_LGIHEDSTNR_.2	#N/A	7.4575	6.8121	7.3434	6.9476	7.0872	6.6726	7.2376	7.3323	7.2751	6.7241	7.0557	7.4160	6.7368	7.0256	6.2463	6.6464	7.1256	7.0350	6.9372	6.9144
Hsp90ab1	_ELISNASDALDKIR_.2	#N/A	6.1029	5.9130	5.2754	6.2943	6.0911	6.0050	6.0782	6.2160	6.1204	6.2171	5.7992	6.2351	6.3742	6.1036	6.0665	6.3638	6.1237	5.8739	6.1509	5.9349
Hsp90ab1	_EKEIS[Phospho (STY)]DDEAEEEKGEK_.2	#N/A	5.0631	5.0136	5.3747	4.5125	5.0689	4.7201	5.4670	4.7259	5.2275	5.9784	5.4389	4.6627	5.4161	5.8775	4.3630	4.9734	4.9874	5.8110	5.4219	5.1171
Hsp90ab1	_EIS[Phospho (STY)]DDEAEEEKGEK_.2	#N/A	4.8204	4.2318	4.9437	4.3815	4.0380	4.4782	5.1927	6.1087	5.2224	4.7431	5.1188	3.9645	3.5618	4.7426	3.8475	3.8452	4.8388	4.2872	4.1979	3.8583
Hsp90ab1	_AKFENLC[Carbamidomethyl (C)]K_.2	#N/A	7.4173	7.6471	3.4617	7.1149	7.2998	7.5775	7.5186	7.3447	7.1707	7.6511	7.4486	7.4241	8.0412	7.5460	7.4960	7.3100	7.5575	7.5223	7.6175	7.6233
Hsp90ab1	_IEDVGS[Phospho (STY)]DEEDDSGKDK_.2	#N/A	11.4546	11.2450	11.5495	11.4165	10.8679	11.0541	11.0508	11.3735	11.0595	11.2552	10.9431	11.0258	11.4497	11.6810	10.5962	10.9159	11.3081	11.0404	10.9206	10.9700
Hsp90ab1	_FYEAFSK_.2	#N/A	3.3887	3.8865	4.4378	4.1341	4.1926	3.8267	5.0486	3.6758	4.8257	5.0182	3.7473	3.0027	4.9217	2.2650	5.2405	5.0702	3.1730	5.6455	5.3822	4.4462
Hsp90ab1	_IEDVGS[Phospho (STY)]DEEDDSGKDKK_.3	#N/A	12.8925	12.9999	13.1982	12.9086	12.4991	12.9956	12.8481	12.5640	12.9793	13.3320	12.9984	12.6342	13.2042	13.0155	12.8589	12.8652	12.8343	12.7928	13.0644	12.7513
Hsp90ab1	_EM[Oxidation (M)]LQQSK_.2	#N/A	5.3303	5.9288	5.2792	4.1645	5.5357	5.3706	5.9708	5.6879	4.7009	5.4627	5.3645	5.2304	5.0455	5.4015	6.9878	6.2308	5.1668	5.7526	5.8430	5.4515
Hsp90ab1	_YESLTDPSKLDSGK_.2	#N/A	7.8306	7.7187	7.8037	7.6178	7.5451	7.6221	7.7399	7.8477	7.7535	7.5213	7.6205	7.6300	7.8388	7.9735	7.5588	7.7773	7.6456	7.6478	7.3030	7.4044
Hsp90ab1	_SLTNDWEDHLAVK_.2	#N/A	6.1100	5.8287	6.8687	5.4207	6.0029	5.7348	5.7954	6.2459	5.9761	5.7139	5.9618	5.8932	5.7220	5.4530	5.9336	5.4214	6.3749	5.4491	6.1058	5.5790
Hsp90ab1	_HLEINPDHPIVETLR_.3	#N/A	7.0663	6.5410	6.3190	6.5292	7.1886	6.6793	6.6011	6.9660	6.7449	6.8316	6.7619	7.2974	4.2356	6.6885	6.1328	6.6538	7.1988	6.7274	6.7524	6.7566
Hsp90ab1	_YHTSQSGDEM[Oxidation (M)]TSLSEYVSR_.3	#N/A	6.4751	6.3323	6.3737	5.6349	6.1670	5.5661	4.8978	6.5719	3.3516	6.4402	6.4755	6.2555	6.1302	6.5145	5.3199	5.2439	5.9501	4.0682	5.6545	4.8330
Hsp90ab1	_HNDDEQYAWESSAGGSFTVR_.3	#N/A	6.6679	3.6899	3.2606	4.8880	5.2672	4.5237	4.5625	6.4996	4.9472	5.5331	5.5992	3.5056	3.9722	6.3350	4.8717	5.9227	6.4055	5.6894	5.5341	5.3712
Hsp90ab1	_EGLELPEDEEEKK_.3	#N/A	3.5456	4.0259	4.3306	4.2911	2.8834	3.1074	1.7419	3.5188	4.2623	4.5296	3.5903	3.7521	3.3240	4.3686	4.2349	3.9611	4.5169	4.1334	3.2040	4.0459
Hsp90ab1	_VILHLKEDQTEYLEER_.3	#N/A	7.0141	3.7534	4.6031	4.2173	5.7105	3.8060	5.5370	5.9798	3.1957	3.3763	5.1842	5.9485	6.4294	3.8638	3.3009	5.7972	5.2407	4.2547	3.9033	5.3531
Hsp90ab1	_KHLEINPDHPIVETLR_.4	#N/A	3.1896	3.3123	3.7259	4.8206	4.0282	4.2523	3.5129	3.8748	3.5720	3.3960	4.9416	3.2018	3.6800	3.2238	3.0901	3.5345	3.3721	3.7774	5.4960	4.4019
Hsp90ab1	_LGIHEDSTNRR_.3	#N/A	3.7619	4.2409	4.2982	3.9275	3.0996	3.3237	4.4414	3.4623	2.9997	3.9682	3.3741	4.1303	3.1077	4.2853	4.0186	4.1068	4.0000	3.8332	3.4818	4.2523
Hsp90ab1	_LGLGIDEDEVTAEEPSAAVPDEIPPLEGDEDASR_.3	#N/A	3.7372	2.4077	4.2735	3.9522	3.0749	3.4092	4.4662	3.4376	3.0244	3.9435	3.3988	4.1551	3.7579	4.2606	4.0434	4.0821	3.9752	3.8579	3.5065	4.2276
Dmd	_SDSS[Phospho (STY)]QPMLLR_.2	P11531_S3617_M1_Dmd	4.3324	4.1089	8.4139	4.1454	3.3354	3.1918	3.9715	3.5447	3.8180	4.3210	3.2966	3.9097	3.9834	4.4861	3.7630	4.1863	4.2923	3.6405	5.3002	3.8370
Dmd	_NAPGKPMREDT[Phospho (STY)]M_.2	P11531_T3677_M1_Dmd	4.1276	3.1915	9.1550	3.4566	4.3345	4.3679	4.2144	3.9797	4.1346	3.9382	5.9910	4.4185	4.3001	3.3019	4.2398	3.4897	4.5174	3.3158	2.9643	3.2689
L1cam	_SLES[Phospho (STY)]DNEEK_.2	P11627_S1184_M1_L1cam	11.1289	11.0522	11.0323	11.0099	10.4551	11.1123	10.9431	10.7319	10.8994	10.4644	10.9620	10.6834	10.7251	11.0524	9.8418	10.3838	11.1120	11.0151	10.7263	10.6849
L1cam	_EKEAAGGNDSSGAT[Phospho (STY)]SPINPAVALE_.2	P11627_T1250_M1_L1cam	4.2728	4.1690	3.6068	3.3872	3.2953	3.5796	4.1917	3.9443	4.2508	4.2241	3.1244	3.9649	4.3589	3.9027	5.4178	6.4656	6.7730	3.1047	3.4038	3.5799
L1cam	_TLQLLNVGEEDDGEYTC[Carbamidomethyl (C)]LAENSLGSAR_.3	#N/A	5.5948	5.5571	5.6178	5.2441	4.8976	5.1763	5.2362	5.2008	5.5847	5.2195	5.0994	5.1873	5.6325	6.4896	5.1466	5.7921	5.5933	5.0052	5.2164	5.5236
L1cam	_AQLLVVGSPGPVPHLELSDR_.3	#N/A	4.2468	3.7560	3.2822	5.2645	4.8577	5.2668	5.2392	4.9473	3.9455	5.2834	5.2399	4.7848	4.1237	5.4899	5.2509	3.0908	5.4625	5.5246	5.6222	5.0350
L1cam	_EAAGGNDSSGATS[Phospho (STY)]PINPAVALE_.2	#N/A	8.1023	9.0432	8.3265	8.0426	7.4656	9.5344	8.8882	7.9747	8.9792	7.7383	8.0775	7.6923	8.2157	8.2066	8.1433	7.8783	8.5079	9.3903	8.4839	8.1691
L1cam	_DETFGEYRS[Phospho (STY)]LES[Phospho (STY)]DNEEK_.2	#N/A	4.1563	5.3243	5.5727	4.1225	3.0572	5.2269	3.9261	3.3057	3.9388	3.9901	3.4404	3.9901	4.0656	4.1555	4.4092	3.8240	4.3038	5.3010	4.8544	5.3146
L1cam	_PM[Oxidation (M)]KDETFGEY[Phospho (STY)]RSLES[Phospho (STY)]DNEEK_.3	#N/A	4.2080	5.2929	5.1984	3.7734	3.5457	5.2566	4.8172	4.2621	5.0078	5.3069	3.6551	5.0085	4.1242	4.1944	3.9375	4.6743	4.3858	5.1881	5.3823	5.0330
L1cam	_DKEDTQVDS[Phospho (STY)]EARPMK_.3	P11627_S1166_M1_L1cam	3.6983	3.6208	3.2349	3.8859	3.9052	4.2031	3.5620	3.9240	3.6211	3.3468	3.9955	3.3469	3.7292	3.2729	3.1682	3.0604	3.4212	3.7282	4.0273	4.4511
L1cam	_LVALQGQSLILEC[Carbamidomethyl (C)]IAEGFPTPTIK_.3	#N/A	4.5271	4.2928	4.0637	4.5579	3.2332	3.0714	3.8149	3.2520	3.7351	4.3377	3.2137	4.0189	4.5580	4.1018	3.8402	3.8893	4.2500	4.4002	3.1984	3.7791
Camk2a	_ITQYLDAGGIPR_.2	#N/A	11.5982	11.5452	11.5463	11.5591	11.3063	11.4178	11.5202	11.7586	11.3463	11.3116	11.3658	11.3895	11.7050	11.3266	11.1842	11.2413	11.6615	11.4671	11.3420	11.0616
Camk2a	_HPWIS[Phospho (STY)]HR_.2	P11798_S272_M1_Camk2a	9.4616	9.3095	9.8798	8.8964	9.7043	10.1046	9.7114	10.4407	10.0030	8.8742	9.4177	9.5061	9.8567	9.5111	9.5300	9.3094	10.1192	10.0538	9.8790	9.4965
Camk2a	_FTEEYQLFEELGK_.2	#N/A	9.9511	9.5791	10.1960	9.8716	9.3839	9.5132	9.5503	9.6255	9.6813	9.5853	9.6980	9.4251	9.9056	9.3540	9.0811	9.4800	9.7517	9.3526	9.6240	9.5852
Camk2a	_VLAGQEYAAK_.2	#N/A	9.6121	9.4752	9.4061	9.4971	9.3400	9.5507	9.8488	9.6677	9.6172	9.4846	9.4268	9.6110	9.5658	9.2668	9.5292	9.2556	9.4423	9.9343	9.4859	9.3417
Camk2a	_WQIVHFHR_.2	#N/A	9.3161	9.1154	8.8726	9.0889	8.7560	8.7354	8.8236	9.6639	8.6716	8.8143	8.1442	8.9350	9.0479	8.7906	8.0121	8.9507	9.1627	8.7151	8.6775	8.1720
Camk2a	_FYFENLWSR_.2	#N/A	9.3910	9.2370	8.9183	9.0791	8.8119	8.9912	9.0898	9.3524	8.8300	9.0798	8.6949	9.2476	8.9415	9.1934	8.5280	8.6339	9.3121	9.2052	9.1246	8.7435
Camk2a	_ITAAEALK_.2	#N/A	5.4212	6.4456	5.9956	6.6523	6.2226	6.5672	7.3050	4.2470	6.4892	6.4777	6.7985	6.8978	6.8775	5.8309	6.9010	6.9763	4.7187	7.0012	7.0061	7.1950
Camk2a	_ESSES[Phospho (STY)]TNT[Phospho (STY)]T[Phospho (STY)]IEDEDTKVR_.2	#N/A	8.4950	11.3266	11.0902	10.1575	9.6307	10.8449	10.7196	11.1290	11.2755	11.3461	10.7444	11.1939	4.0298	8.7838	7.8384	11.1742	11.3703	11.2557	10.8753	8.2886
Camk2a	_M[Oxidation (M)]C[Carbamidomethyl (C)]DPGM[Oxidation (M)]TAFEPEALGNLVEGLDFHR_.3	#N/A	4.6522	4.1341	5.3721	3.7397	4.3148	4.8535	4.2211	4.3497	4.7350	4.5916	4.7136	4.0016	5.2332	4.0225	4.5766	4.3498	4.5108	4.3319	4.4467	4.3569
Camk2a	_ESS[Phospho (STY)]ESTNTTIEDEDTK_.2	#N/A	11.5391	11.4590	12.2124	11.4872	10.9299	11.4629	10.9754	11.4934	11.1337	11.3187	11.6467	11.3086	11.2422	11.6532	10.6939	10.9227	11.8362	11.1300	11.4098	11.5414
Camk2a	_NFS[Phospho (STY)]GGKSGGNK_.2	P11798_S314_M1_Camk2a	3.7133	5.8677	5.5533	3.8710	3.9202	6.7582	6.2178	4.8041	5.5059	3.5239	4.0276	3.3319	6.1618	3.7162	3.1533	3.0754	5.8706	6.2578	6.1020	3.6832
Camk2a	_[Acetyl (Protein N-term)]ATITC[Carbamidomethyl (C)]TR_.2	#N/A	4.5585	4.1911	4.8110	4.9493	5.0512	4.5894	5.6838	4.9802	4.9235	5.3646	4.7559	5.2506	3.1588	4.8998	4.8019	3.9909	3.9516	4.2986	4.9206	3.8807
Camk2a	_S[Phospho (STY)]TVASC[Carbamidomethyl (C)]M[Oxidation (M)]HR_.2	#N/A	8.2376	9.7529	10.3957	9.7581	9.1762	10.0416	9.7482	9.6753	9.7475	8.4835	9.4275	8.9638	9.0118	10.0124	10.0001	8.7256	9.7372	9.1557	9.0310	9.1095
Camk2a	_NDGVKES[Phospho (STY)]SESTNTTIEDEDTK_.2	#N/A	5.3031	4.6297	4.4187	3.9522	3.3655	4.7418	4.3732	4.5814	3.8242	5.0768	4.8755	4.9482	3.9482	4.2789	3.8808	5.4371	3.8263	4.4357	5.1514	5.0055
Camk2a	_VTEQLIEAISNGDFESYTK_.2	#N/A	9.3983	8.9755	9.0390	9.1848	8.5285	8.7735	8.8980	9.5388	8.6076	9.0010	8.8556	8.8936	9.7279	9.7064	8.3062	8.9490	9.3203	8.8961	8.7650	9.1671
Camk2a	_QETVDC[Carbamidomethyl (C)]LKK_.2	#N/A	5.7755	4.9626	6.7767	5.8978	5.3076	6.0286	5.6361	5.2577	5.7177	5.4171	6.0396	4.7029	4.9497	5.5850	5.2164	5.3279	5.7427	5.2428	5.0493	4.7297
Camk2a	_QET[Phospho (STY)]VDC[Carbamidomethyl (C)]LK_.2	#N/A	6.5160	6.1379	7.5709	6.3392	5.4287	5.8027	2.9877	3.3496	3.0468	3.9212	5.7957	4.1774	3.1548	4.1994	4.2538	4.0597	4.3477	4.3026	3.1008	5.4506
Camk2a	_SGAPSVLPH_.2	#N/A	7.6316	7.9525	7.6580	8.0162	7.1584	7.4961	8.1249	8.1018	8.0388	7.4301	7.9322	7.8814	7.7404	7.4907	7.6761	7.1834	7.6896	8.1479	7.3219	7.4230
Camk2a	_NDGVKESS[Phospho (STY)]ESTNTTIEDEDTKVR_.3	#N/A	5.3115	5.9665	5.1448	3.6560	4.2094	3.8439	5.5463	4.0267	4.8439	3.8481	3.8349	5.4491	4.1534	3.7867	4.9340	5.8699	5.9084	6.4684	5.9583	3.6687
Camk2a	_KNDGVKESS[Phospho (STY)]ESTNTTIEDEDTK_.3	#N/A	3.8064	3.8714	3.7380	3.7519	3.5660	3.6674	3.7036	3.7990	3.6815	4.1334	3.0622	3.7152	3.8227	3.8556	3.5644	3.7888	4.0512	3.6168	3.7163	3.8930
Gas2	_VDGKTS[Phospho (STY)]PVQSK_.2	P11862_S283_M1_Gas2	4.1971	6.6799	6.3578	3.3872	6.0869	5.9741	6.0807	4.4227	4.1199	5.6211	5.9206	6.5858	4.2279	5.9887	4.1703	3.5592	3.9200	3.2295	3.5285	6.1038
Tcp1	_SLLVIPNTLAVNAAQDSTDLVAK_.2	#N/A	7.2098	7.3142	7.1066	7.1860	6.7916	7.1618	7.4624	7.5017	7.3549	7.3273	6.8380	7.2895	7.6982	6.8704	6.9169	7.0460	7.3624	7.5760	7.0352	7.2750
Tcp1	_SSFGPVGLDK_.2	#N/A	6.8101	6.5774	6.5923	6.5139	6.3417	6.2373	6.9709	6.6779	6.1136	6.3541	6.3435	6.6746	6.7373	6.2344	6.4437	6.1858	5.5396	6.7023	6.5137	5.9826
Tcp1	_LLEVEHPAAK_.2	#N/A	6.3329	6.4855	6.6038	6.0526	6.4430	6.7468	6.6411	7.1357	6.3029	6.8118	6.3482	6.7906	6.2102	5.7418	6.4907	5.9838	6.3860	6.4953	6.2554	6.0638
Tcp1	_ILATGANVILTTGGIDDM[Oxidation (M)]YLK_.2	#N/A	4.5456	5.0420	4.3103	4.9407	5.3048	4.9398	4.7483	4.6404	5.1940	4.7620	5.1080	3.9598	5.3065	4.1793	4.3517	4.4927	3.2476	4.6783	3.9624	5.1205
Tcp1	_YINENLIINTDELGR_.2	#N/A	3.1508	6.4545	6.5959	6.2859	5.7889	6.4815	6.2710	6.3056	6.4122	6.4165	6.5748	6.1213	5.8271	6.4962	5.6285	6.0784	6.5614	5.9720	6.3042	6.4058
Tcp1	_FATEAAITILR_.2	#N/A	5.7235	5.7448	6.5204	6.0525	5.6055	5.7379	4.8647	5.2651	5.8904	6.1988	5.5849	5.3025	5.3308	5.6606	5.2329	4.7072	5.8748	3.2712	5.5663	5.4675
Tcp1	_IC[Carbamidomethyl (C)]DDELILIK_.2	#N/A	5.1528	4.6573	4.9823	5.3372	5.2547	4.5854	4.4845	4.8682	5.2984	4.5801	4.9025	5.2268	3.8500	4.6902	3.1045	4.7906	4.6006	4.9477	3.9008	4.2439
Tcp1	_DDKHGS[Phospho (STY)]YENAVHSGALDD_.3	#N/A	6.8927	6.9156	6.2169	6.6334	6.1622	6.5388	6.7817	6.5638	6.6541	6.6895	6.6498	6.6482	7.0223	6.6983	6.1636	6.6753	6.2272	6.5932	6.8413	6.5311
Tcp1	_M[Oxidation (M)]LVDDIGDVTITNDGATILK_.2	#N/A	6.1411	5.6801	5.2792	5.4024	5.5047	5.3038	5.2836	5.9147	5.5673	5.4914	5.7456	5.9348	6.0346	6.1892	4.8331	5.3593	5.3880	5.2804	5.3058	5.7087
Tcp1	_HGSYENAVHS[Phospho (STY)]GALDD_.2	#N/A	5.8608	5.6731	5.9330	4.8401	4.8730	5.1046	5.5547	4.1862	5.4909	5.4646	5.5139	4.6929	4.6052	4.7913	4.0572	4.7954	5.1356	4.9606	4.3432	5.0187
Tcp1	_DC[Carbamidomethyl (C)]LINAAK_.2	#N/A	5.0714	3.9529	4.5221	4.3914	4.1056	4.7682	4.7122	3.7104	3.4075	5.1480	4.2179	4.3879	3.5156	4.6497	5.1969	4.3477	3.2076	4.3847	0.8686	2.1606
Tcp1	_SLHDALC[Carbamidomethyl (C)]VVK_.2	#N/A	4.2639	3.9075	3.6466	3.4255	4.8748	3.8257	5.3917	5.5046	5.0929	4.4702	3.5544	5.3074	4.7349	5.4118	4.6042	4.4880	4.5019	4.9412	5.0591	4.5190
Tcp1	_IAC[Carbamidomethyl (C)]LDFSLQK_.2	#N/A	4.0754	4.3466	4.0099	4.0334	3.1795	3.2128	3.7172	4.3165	3.7459	4.2840	3.2675	4.0727	3.9543	4.5771	3.8940	3.8355	4.1963	3.6546	4.0792	3.7253
Tcp1	_SQNVM[Oxidation (M)]AAASIANIVK_.2	#N/A	3.6594	4.7018	3.6986	3.4222	4.0899	3.7330	4.0257	4.3877	4.9383	4.5680	2.9584	3.0522	4.1929	3.7367	3.1736	3.0217	3.8849	3.6601	4.0016	3.4139
App	_VES[Phospho (STY)]LEQEAANER_.2	P12023_S441_M1_App	7.9213	7.8546	8.0878	8.0373	7.6152	7.9632	7.1737	7.5930	7.4591	7.8622	8.1671	7.5514	7.6073	7.9822	7.3445	7.6179	8.0805	7.1502	7.4758	7.6948
App	_C[Carbamidomethyl (C)]LVGEFVSDALLVPDK_.2	#N/A	3.1916	3.3928	4.7350	4.7231	3.8217	4.2607	3.0442	3.9515	4.8582	3.6075	3.9730	4.2591	3.1299	4.4959	4.2453	3.4541	3.3472	2.9851	4.1345	4.6274
App	_QYTSIHHGVVEVDAAVT[Phospho (STY)]PEER_.3	P12023_T743_M1_App	4.9303	3.8076	4.5165	3.4942	3.5329	4.4520	4.1302	3.8956	4.0505	5.0988	3.5291	3.6356	5.4104	3.2177	3.5854	3.0392	4.4332	3.3999	3.0485	3.1847
Prkar2a	_TDEHVIDQGDDGDNFYVIER_.3	#N/A	8.4692	8.5444	8.2882	8.3496	7.8995	8.1595	7.6307	7.8927	8.0357	8.1904	8.0087	8.0293	8.4588	8.8261	7.3057	8.2420	8.1585	7.9835	8.1428	8.4022
Prkar2a	_SVGQYDNR_.2	#N/A	6.3998	6.4988	6.7374	6.5267	6.4200	6.7289	6.2426	6.4147	6.6644	6.6767	6.1778	6.8166	6.4968	6.1467	6.8413	6.7032	6.2549	6.5133	6.3849	6.3260
Prkar2a	_AATIIATSEGSLWGLDR_.2	#N/A	5.9482	6.1536	6.3529	6.5778	6.1609	5.9244	5.4333	5.9842	6.3646	6.6685	6.0909	5.9245	6.9796	6.0598	5.1132	6.3471	6.2576	5.9430	5.3467	6.4489
Prkar2a	_GTYDILVTK_.2	#N/A	4.8697	4.8397	5.8632	4.8601	5.2730	4.5649	5.3449	4.4700	5.6328	5.6168	4.7858	5.1464	5.2874	5.5195	5.1815	4.9405	4.3325	4.3432	5.0981	5.4229
Prkar2a	_M[Oxidation (M)]FGS[Phospho (STY)]NLDLM[Oxidation (M)]DPGQ_.2	#N/A	7.9632	8.1108	8.2874	8.0797	7.5017	7.7634	7.5272	7.2402	8.1056	8.0515	7.4541	7.3745	7.9980	7.6911	7.3691	8.1457	7.8843	7.8012	8.0842	7.9360
Prkar2a	_NISHYEEQLVK_.2	#N/A	4.3982	4.7279	5.2655	5.4838	4.8687	5.6474	4.9469	5.0518	5.2658	5.5427	4.2720	4.6961	5.2746	5.0868	4.6913	5.5566	5.5374	5.0944	5.3111	5.2581
Prkar2a	_RVS[Phospho (STY)]VC[Carbamidomethyl (C)]AETFNPDEEEEDNDPR_.3	P12367_S96_M1_Prkar2a	6.2877	7.4548	6.2457	6.6360	3.8249	6.4396	7.4633	5.8107	6.6189	4.3776	3.9231	5.9967	4.0352	8.4155	5.7573	6.1041	5.6476	7.6691	6.4684	5.2434
Prkar2a	_VSVC[Carbamidomethyl (C)]AETFNPDEEEEDNDPR_.2	#N/A	4.3339	3.6689	3.3693	2.6782	3.6716	3.5334	3.8695	4.0342	3.9286	4.5402	4.3030	3.5584	4.0366	3.3564	3.4467	3.1779	3.0711	3.2612	4.4107	3.3234
Pfkl	_RTLS[Phospho (STY)]IDKGF_.2	#N/A	12.6341	12.3189	3.1616	3.5633	11.8080	11.9181	11.9563	12.3733	11.6766	12.2456	12.1092	11.8140	12.6657	11.9813	10.3404	11.9490	12.4709	12.0317	11.9545	11.8927
Pfkl	_RTLS[Phospho (STY)]IDK_.2	#N/A	10.3761	10.0078	9.7323	10.0398	9.4225	9.9665	9.8986	9.8387	9.4125	10.1124	9.8842	9.2319	9.5396	9.9207	8.0999	9.2557	9.8136	9.7032	9.5515	9.3290
Pfkl	_GGTPSAFDR_.2	#N/A	8.9483	9.0158	7.7420	8.9431	8.6198	8.8348	8.9166	9.0240	7.9187	8.6255	8.7245	7.2927	8.9247	8.8329	8.2298	7.3165	8.8628	7.6984	8.7476	8.7030
Pfkl	_VTVLGHVQR_.2	#N/A	7.7365	8.1664	7.7172	7.2658	7.7332	7.6419	8.0858	7.8042	7.4426	7.6462	7.0735	7.4872	7.4155	7.4877	7.2232	7.0933	7.0159	7.7744	7.6381	7.2914
Pfkl	_TNVLGHLQQGGAPTPFDR_.3	#N/A	7.2770	6.5765	6.1488	5.6821	6.5723	5.8676	7.0292	6.8257	6.1471	6.6754	6.7097	6.0568	6.6097	6.7285	5.7787	6.2733	6.5357	6.4647	6.3266	6.4186
Pfkl	_SFENNWK_.2	#N/A	6.0346	6.3277	5.9396	5.8416	5.8440	5.8801	6.3398	6.3489	5.3367	5.4391	6.6142	5.5802	5.4941	6.1951	4.3264	5.5412	6.1454	6.6430	5.5709	5.2647
Pfkl	_VFANAPDSAC[Carbamidomethyl (C)]VIGLR_.2	#N/A	6.1895	4.8468	5.8037	5.1877	5.1323	5.3521	5.4329	5.2107	5.2185	5.1709	5.3862	4.8141	5.4163	5.2617	4.9962	4.1988	5.6023	4.7355	5.7863	5.2045
Pfkl	_TLS[Phospho (STY)]IDKGF_.2	#N/A	7.2726	7.8890	3.6865	8.8686	3.5275	3.7516	8.7412	6.3983	8.0720	8.9420	2.9462	7.9091	8.6184	7.8812	3.5908	8.3022	3.8728	8.1877	8.8272	8.8323
Pfkl	_TFVLEVM[Oxidation (M)]GR_.2	#N/A	6.5494	6.1669	5.3294	6.5083	6.0016	5.8239	6.4063	6.0719	6.3703	5.5981	5.6959	5.4244	5.9014	6.1805	5.2257	5.1691	4.0668	6.3233	5.6981	5.4214
Pfkl	_NEWGSLLEELVK_.2	#N/A	4.0739	5.1615	4.1861	3.5104	4.5705	3.9741	4.4314	4.2995	4.0585	4.4721	4.5939	3.9002	4.3624	3.5278	3.8391	2.5949	3.7968	3.9859	3.6517	3.3258
Pfkl	_AIGVLTSGGDAQGM[Oxidation (M)]NAAVR_.2	#N/A	5.8271	5.7176	5.4926	5.4163	5.6183	5.5264	4.9806	5.8433	5.6833	5.6608	5.0702	5.1433	5.5644	4.6650	5.2845	5.4174	5.4448	5.9534	5.0245	5.4598
Pfkl	_IM[Oxidation (M)]EVIDAITTTAQSHQR_.2	#N/A	4.3738	4.9048	4.3679	4.0653	4.4608	3.6294	4.6466	5.4941	4.7942	5.0286	3.3717	4.7914	5.0904	4.0946	3.6501	4.3315	5.4716	4.7313	4.1791	4.3411
Pfkl	_TGISEGHTVYIVHDGFEGLAK_.3	#N/A	3.5598	2.7967	4.0012	4.2245	4.3035	3.2457	3.2376	3.1653	3.3202	3.6712	4.2997	2.9265	3.4856	3.9883	2.8148	3.8098	4.4927	4.1302	3.7788	3.9553
Chrm1	_SQPGAEGSPES[Phospho (STY)]PPGR_.2	P12657_S254_M1_Chrm1	10.7769	10.7430	9.9498	10.3476	9.9549	10.2673	10.3537	10.6245	10.1450	10.0465	10.4920	10.5310	11.3116	10.3285	9.1056	10.1605	11.0480	10.8792	10.5476	10.2301
Chrm1	_RPGS[Phospho (STY)]VHRT[Phospho (STY)]PSR_.3	#N/A	5.8381	6.3095	4.9881	5.4101	3.8856	4.6438	3.5482	4.9932	4.2678	4.0490	3.4453	5.6383	5.2180	5.2769	4.0821	5.1741	3.9474	3.9123	6.1039	4.1997
Calb1	_TDLALILSAGDN_.2	#N/A	8.3374	8.8799	8.4048	8.4447	7.6749	8.3821	8.7089	9.2093	8.5119	8.5457	8.7981	8.9861	8.8586	9.6085	9.0622	8.9023	9.1372	9.1235	9.2474	8.7702
Calb1	_SFVDQYGQR_.2	#N/A	6.3952	6.5872	7.0871	5.6379	6.7631	6.2296	6.5211	5.5225	6.8188	6.4025	7.0142	6.5376	6.1339	6.7859	7.5184	7.0172	7.1265	6.8886	7.0010	7.0970
Calb1	_ELQNLIQELLQAR_.2	#N/A	5.0932	4.2606	5.2901	4.8428	4.5450	4.9546	4.7121	4.2219	5.1495	5.0338	4.9667	5.3232	4.0094	4.8389	4.6429	4.8099	5.4306	5.0361	5.3158	4.6610
Calb1	_NIMALSDGGK_.2	#N/A	3.1388	5.6367	3.6751	6.0422	3.9774	4.0538	3.5637	4.3401	3.6228	3.3452	6.0340	3.2526	3.7308	5.9674	3.1409	3.4837	3.3769	3.7266	4.0256	2.9519
Cox5a	_LNDFASAVR_.2	#N/A	9.5706	9.9247	9.8465	9.7761	9.9116	9.5425	9.7260	9.9126	9.7152	9.7913	9.7093	10.0385	9.9978	10.0590	10.6479	9.8809	9.8108	9.9615	9.9334	9.9027
Cox5a	_ILEVVKDK_.2	#N/A	8.6351	9.0758	8.9314	9.0380	9.0046	8.7232	9.0877	9.1259	8.9409	8.7645	8.5839	9.4821	9.8337	9.5540	9.8052	9.3958	9.0807	9.0334	9.2879	9.0466
Cox5a	_GM[Oxidation (M)]NTLVGYDLVPEPK_.2	#N/A	7.8893	8.3833	9.1036	8.7560	8.3088	8.3284	8.3956	7.9986	8.2424	8.2435	8.2898	8.4947	9.3745	8.9717	8.7289	9.3975	8.3506	8.1753	8.1951	8.1563
Cox5a	_RLNDFASAVR_.2	#N/A	5.8515	6.3298	6.2667	6.7902	3.3203	6.3552	6.6157	6.7110	5.8516	6.5401	6.0497	7.0026	6.8493	6.8670	7.2277	7.1299	6.6652	6.3038	6.5603	6.2698
Nr4a1;Nr4a2;Nr4a3	_TDS[Phospho (STY)]LKGR_.2	P12813_S344_M1_Nr4a1;Nr4a2;Nr4a3	4.0021	6.7952	6.7362	6.4874	6.1270	6.8521	6.6185	3.2954	3.3944	7.1187	3.2823	3.9755	3.5383	4.1452	3.9185	3.9327	6.0235	4.3569	6.0871	3.8225
Pdcd6	_LSDQFHDILIR_.2	#N/A	3.9918	4.1648	4.7721	5.0099	4.8607	4.5325	4.3072	4.8059	3.9010	3.3520	4.3260	4.5421	4.8325	3.4830	4.5925	4.0041	4.6049	3.9490	4.5551	3.5740
Pdcd6	_YITDWQNVFR_.2	#N/A	3.3067	5.0268	5.4502	4.2775	4.5626	4.7541	5.4145	5.1942	3.2295	3.7384	3.6039	5.1036	3.3376	6.0387	4.6912	4.1697	5.1225	4.1198	4.6947	4.0595
Prkar1b	_NVLFSHLDDNER_.3	#N/A	4.7219	4.3914	5.4653	5.1882	5.0135	5.0739	5.0460	5.5632	5.4078	5.8916	5.6542	5.0649	4.1594	5.1826	5.0051	5.4071	5.3672	5.0985	4.8945	5.0648
Prkar1b	_S[Phospho (STY)]PNEEYVEVGR_.2	P12849_S307_M1_Prkar1b	6.0371	6.7318	3.4715	3.6493	3.7424	3.9665	5.9577	4.1606	3.8577	3.1102	6.3205	3.1103	3.9658	6.7493	3.3759	3.2488	3.6578	6.6572	3.7907	3.1869
Prkar1b	_[Acetyl (Protein N-term)]ASPS[Phospho (STY)]C[Carbamidomethyl (C)]FHSEDEDSLK_.2	P12849_S5_M1_Prkar1b	3.4641	3.9246	4.0004	4.2365	3.6774	5.3330	3.2496	4.0400	3.3087	3.6704	3.6830	3.8140	3.4167	3.9875	3.7023	3.8090	4.6989	4.1422	3.7908	3.9545
Prkar1b	_SNSQC[Carbamidomethyl (C)]DSHDEEIS[Phospho (STY)]PTPPNPVVK_.3	P12849_S83_M1_Prkar1b	6.4200	7.2623	7.0289	6.4287	4.5091	6.9591	7.0662	6.0308	7.1179	6.4395	6.1782	6.0453	7.0199	7.7367	6.5839	6.9565	6.7481	7.1789	7.2956	6.0643
Prkar1b	_GGVSAEVYTEEDAVSYVR_.2	#N/A	6.4086	4.2802	4.6675	4.5453	4.3587	3.2792	5.1505	5.9584	3.7224	5.5782	5.6248	5.7812	6.6624	4.1144	3.8276	3.9019	4.2627	4.3876	3.1858	3.7917
Prkar1b	_IVVQGEPGDDFYIITEGTASVLQR_.3	#N/A	3.1182	4.2009	4.1556	4.4661	3.3251	3.3584	3.7230	3.3438	3.6432	2.1308	3.1218	3.9270	3.8087	4.1936	3.7484	3.9811	4.3419	4.3084	3.1066	3.8709
Cntn1	_NDGGIYTC[Carbamidomethyl (C)]FAENNR_.2	#N/A	11.4956	11.8872	11.6479	11.7636	10.6127	11.4281	11.3637	11.5415	11.3748	11.1393	10.9907	10.9571	11.6426	12.1997	10.5209	11.0766	11.4403	11.5923	11.3209	11.1896
Cntn1	_M[Oxidation (M)]NNGDVDLTNDR_.2	#N/A	9.3606	9.4180	9.6076	9.6571	9.2769	9.3236	9.2460	9.4957	9.3682	9.3327	9.4027	9.1579	9.5324	9.8017	8.6980	9.0583	9.6648	9.2917	9.2929	9.3142
Cntn1	_GPPGPPGGLR_.2	#N/A	8.4702	8.8721	8.6151	8.7452	8.6592	8.7157	9.1191	8.5302	8.4793	8.2880	8.4274	8.8726	8.7530	8.4735	8.7523	8.3377	8.4852	8.9258	8.4710	8.5522
Cntn1	_IKTDGAAPNVAPSDVGGGGGTNR_.3	#N/A	8.4582	8.7187	8.6886	8.7673	8.3066	8.4046	8.1737	8.7262	8.4839	8.5464	8.2104	8.3889	8.7696	8.4615	8.0797	8.3712	8.4457	8.5788	8.3550	8.3169
Cntn1	_VTVTNPDTGR_.2	#N/A	8.2188	7.9061	8.2189	8.0957	8.1347	7.9287	7.9716	8.2663	8.2806	7.3981	7.8576	7.9016	8.2132	8.1388	8.1793	7.9283	7.9556	8.0781	8.0052	8.3109
Cntn1	_FIPLIPIPER_.2	#N/A	7.6836	8.0799	7.7859	7.8849	8.3118	8.3296	8.2776	8.3048	7.5693	8.2529	8.0857	8.2184	8.1130	7.7033	7.8052	7.9485	8.2786	8.3930	7.7992	7.9973
Cntn1	_IVESYQIR_.2	#N/A	7.9355	8.0562	8.0517	8.1153	8.0630	8.1683	8.4733	8.0688	8.3303	8.0640	8.0655	7.9931	8.3243	8.0784	7.8687	8.1256	7.7935	8.2681	8.0513	7.7395
Cntn1	_ELTITWAPLSR_.2	#N/A	7.8142	7.6804	7.5193	7.9089	7.6244	7.7448	7.8938	7.4673	7.9323	7.4951	7.8144	7.7188	7.7034	7.8421	7.4889	7.6880	8.0038	7.8831	8.0373	7.7346
Cntn1	_VQVTSQEYSAR_.2	#N/A	7.5546	7.0530	7.7504	7.4365	7.3074	7.4566	7.3969	7.2313	7.6762	7.6137	7.1804	7.1429	7.6999	7.0355	7.3626	7.2845	7.4002	7.1168	7.4977	7.5692
Cntn1	_TDGAAPNVAPSDVGGGGGTNR_.2	#N/A	7.5409	7.5241	7.3302	7.4163	7.0218	7.4196	6.9738	7.3708	7.8283	6.7344	7.1575	7.2039	7.2722	7.5854	6.7240	7.0598	7.9326	7.3278	7.4712	6.9325
Cntn1	_DGEYVVEVR_.2	#N/A	7.4164	7.3522	6.9970	6.8066	6.7800	7.4732	7.5159	6.8711	7.4905	7.0950	7.4674	7.3714	7.1000	7.5735	6.1771	7.0405	7.0966	7.6324	7.0204	7.4158
Cntn1	_GM[Oxidation (M)]VLLC[Carbamidomethyl (C)]DPPYHFPDDLSYR_.3	#N/A	6.9466	7.4511	6.5247	6.4537	6.8785	6.8745	6.7370	6.9398	6.7132	6.7312	6.3780	6.5704	6.6114	6.5613	6.5767	6.7481	6.8480	7.1270	6.7675	6.4542
Cntn1	_HSIEVPIPR_.2	#N/A	6.9700	6.8665	6.3847	6.3207	6.5757	6.5845	7.0319	6.9797	6.7735	6.5197	6.3071	6.9496	6.4663	6.8467	6.4439	6.1775	6.6549	6.9767	6.3149	6.9008
Cntn1	_ILYRPDGQHDGK_.3	#N/A	5.6467	6.1192	5.0767	5.4781	5.4526	6.2220	6.3492	5.3028	6.2884	6.0022	5.7812	5.2752	5.6455	5.5868	6.4695	5.7671	5.2328	6.0922	6.0031	5.9692
Cntn1	_AHSDGGDGVVSQVK_.2	#N/A	5.5955	6.1939	5.3895	5.4945	5.1444	6.2666	5.7635	5.3098	5.7552	5.9295	5.9148	5.4870	5.9396	6.2697	5.1283	6.0515	5.9169	5.9062	5.7183	5.8481
Cntn1	_VVATNTLGTGEPSIPSNR_.2	#N/A	9.0944	8.6056	8.6366	9.0482	8.7882	8.8086	8.5923	8.8092	9.0090	8.9012	8.2503	8.6859	4.2217	8.6353	8.8024	8.8375	9.1075	9.1306	8.2501	8.4013
Cntn1	_ILALAPTFEM[Oxidation (M)]NPM[Oxidation (M)]K_.2	#N/A	7.7786	7.0478	6.8537	7.0235	7.3751	7.2758	6.8674	7.6079	7.3455	6.9693	6.7289	7.6195	7.2958	6.3580	6.7698	7.5693	7.3983	7.8216	7.5162	7.1297
Cntn1	_GSDNHSPISK_.2	#N/A	4.9730	5.1748	3.5601	4.6397	5.6984	5.4035	4.9925	5.6474	5.5880	5.5537	5.0918	4.4350	5.5019	5.5019	5.6670	5.5610	5.6141	5.5766	5.5388	5.1027
Cntn1	_STEATLSFGYLDPFPPEERPEVK_.3	#N/A	5.6363	5.3160	3.9011	4.5605	4.7744	5.1796	5.4417	5.3936	5.3437	5.0682	3.8056	5.3879	4.4598	5.4900	5.1110	5.3453	5.0417	5.1345	4.5881	5.2290
Cntn1	_VLEPM[Oxidation (M)]PSTAEISTSGAVLK_.2	#N/A	6.2995	6.3082	6.0367	6.0785	5.5934	6.0542	6.3494	5.9328	5.7565	5.8647	5.8971	6.3941	6.3855	6.2112	5.6800	5.8353	5.9096	6.0600	6.0482	5.9520
Cntn1	_TDPPIIEGNMESAK_.2	#N/A	3.1882	7.7530	8.3877	8.0284	6.5347	6.8109	7.7556	4.3894	6.7045	7.2223	7.6624	7.3219	3.6814	7.9116	7.0769	3.5331	3.4263	5.7747	4.0750	6.4148
Cntn1	_KVTVTNPDTGR_.2	#N/A	4.6274	5.5150	3.6016	5.3769	3.9039	4.8865	4.6791	4.2665	4.4214	4.9328	4.7227	3.4904	4.7277	3.5887	4.3742	3.8280	3.3034	5.0502	3.8473	3.5557
Cntn1	_IFNIQLEDEGLYEC[Carbamidomethyl (C)]EAENIR_.3	#N/A	5.7355	5.6606	4.6521	5.2908	5.2815	5.2492	5.4976	5.6959	5.2284	5.3432	4.1904	5.3642	3.3763	3.8790	2.9241	4.4890	5.3741	5.3554	4.1124	5.0841
Cntn1	_FVSQTNGNLYIANVESSDR_.2	#N/A	4.4858	4.0817	4.4574	3.7683	3.1919	3.2252	3.7313	3.6215	3.7764	4.2964	3.5238	3.9711	3.9418	4.4445	3.8816	3.8479	4.1592	3.6740	3.3226	4.4115
Cntn1	_VLSSSEISVHWK_.2	#N/A	3.2238	4.0953	4.2878	4.1787	3.4308	1.7299	3.6173	4.0664	3.5375	4.5353	3.0162	3.8214	3.7030	4.2056	3.6427	3.7769	3.9203	3.9128	3.5614	4.1726
Cntn1	_DAGVYYC[Carbamidomethyl (C)]LASNNYGM[Oxidation (M)]VR_.2	#N/A	4.4023	3.9981	4.5410	3.6847	5.2627	3.1417	3.9397	3.7051	3.8599	5.9100	5.3571	3.8876	5.3131	5.0717	3.9651	3.7644	4.2427	3.5904	4.8098	5.2553
Scg5	_TADDGC[Carbamidomethyl (C)]LENAPDT[Phospho (STY)]AEFSR_.2	P12961_T137_M1_Scg5	6.7014	7.0684	7.6125	7.3452	5.2188	7.5424	6.8363	3.9322	7.4127	7.1769	5.0742	7.0694	6.3490	7.6540	7.2538	7.0167	7.6390	6.6429	7.2109	7.0952
Scg5	_KSVPHFS[Phospho (STY)]EEEKEAE_.3	#N/A	4.1521	3.4391	3.1876	4.1030	3.4898	2.9943	4.0872	3.8525	4.0074	4.2163	3.4860	3.4960	4.1728	3.1746	3.6284	3.6477	4.3902	3.5446	4.4950	3.1416
Scg5	_SVPHFS[Phospho (STY)]EEEKEAE_.2	#N/A	8.8722	8.6684	8.6531	8.6665	8.3136	8.4072	3.0590	8.5546	8.8816	8.6450	8.3113	8.7724	9.4317	9.2486	9.3781	9.1437	9.3402	8.5717	9.3290	8.9794
Scg5	_DFSEDQGYPDPPNPC[Carbamidomethyl (C)]PLGK_.2	#N/A	3.9558	4.4361	3.9204	3.2004	3.2935	4.7293	4.2476	3.1087	4.3066	4.1621	5.4455	5.1264	4.4147	5.4658	5.0669	4.3007	4.1067	4.6597	4.5141	3.6358
Rpl7a	_QTATQLLK_.2	#N/A	4.5330	1.5483	6.1375	5.8766	3.5048	2.1839	5.8311	3.5743	3.3006	6.1738	6.2318	2.7530	3.9113	2.5856	4.0386	2.6569	4.1120	3.1803	3.1837	2.3109
Rpl7a	_NFGIGQDIQPK_.2	#N/A	6.3383	6.2089	5.2337	5.6164	5.7325	5.7788	6.5089	6.3793	6.4897	4.4741	3.0774	4.1488	6.2038	4.2668	6.0807	6.2131	6.4692	6.2782	5.8406	6.1397
Gsn	_HVVPNEVVVQR_.2	#N/A	5.7445	5.6701	5.0914	5.2094	5.7705	5.5200	5.5861	5.5576	5.3281	5.7557	5.8598	5.0557	5.0453	5.7889	5.8257	4.8610	5.7157	5.3978	5.1700	5.6536
Gsn	_DSQEEEKTEALTSAK_.2	#N/A	3.7412	4.2616	4.2775	3.9482	3.0789	3.4052	3.6762	3.4416	3.5965	4.4763	3.9016	3.7455	3.7619	4.2646	3.3653	4.0861	3.5465	3.6494	3.5025	3.5036
Ncam1	_NPPEAATAPAS[Phospho (STY)]PK_.2	#N/A	14.7492	14.8021	14.8165	14.5379	13.8063	14.1998	14.4628	14.9413	14.6490	14.1955	14.4856	14.6933	15.3196	14.9451	14.0003	14.5969	15.1388	14.8191	14.6494	14.5802
Ncam1	_GVTASSSS[Phospho (STY)]PASAPK_.2	P13595_S887_M1_Ncam1	12.4389	12.2943	12.2301	12.1736	11.7730	12.0214	12.0790	12.3148	12.1108	11.9676	11.8316	11.8577	12.4327	11.8849	10.9058	11.7371	12.3270	12.1094	11.9112	11.7712
Ncam1	_FFLC[Carbamidomethyl (C)]QVAGDAK_.2	#N/A	8.3724	8.5683	8.8497	8.5520	8.3809	8.5801	8.5181	8.6358	8.2638	8.4465	8.4185	8.2449	8.1503	8.3059	7.9956	7.8098	8.3611	8.3258	8.2193	8.1089
Ncam1	_GLGEISAATEFK_.2	#N/A	7.9004	8.2622	8.1077	8.0901	7.2491	8.0253	7.8991	7.7722	7.9311	7.6183	7.3871	7.3956	7.6197	7.1378	7.5977	7.7153	7.6062	8.0896	7.7557	7.3480

Ncam1	_ALASEWKPEIR_.2	#N/A	8.3281	8.2592	7.9504	7.9133	8.0211	8.1265	7.9891	8.3887	8.0499	7.6325	7.8384	7.8091	7.9640	7.7550	7.6818	7.5475	8.1597	7.9811	7.6680	7.6645
Ncam1	_NAPTPQEFK_.2	#N/A	7.7160	7.4849	7.8304	7.2349	7.2220	7.5276	7.6544	7.4564	7.4696	7.2357	7.8070	7.1829	7.3102	7.2631	7.2524	6.8732	7.3917	7.1686	6.9972	7.4540
Ncam1	_AGEQDASIHLK_.2	#N/A	6.7786	7.0062	6.7112	6.6302	6.8099	6.7018	7.0093	6.7607	7.1662	6.8871	6.8263	6.3889	6.0079	6.7383	6.0736	6.0235	6.0159	6.8906	6.2283	6.0969
Ncam1	_FIVLSNNYLQIR_.2	#N/A	6.7780	6.6390	6.6819	6.5806	6.6306	6.5833	6.1773	6.4320	6.8067	6.8928	6.1703	6.1429	6.7270	6.2452	5.6065	5.7830	7.0509	6.4998	6.3783	6.3370
Ncam1	_NPPEAATAPAS[Phospho (STY)]PKSK_.2	#N/A	4.1627	3.1564	7.0296	3.4215	7.7793	3.7387	7.8364	8.4689	4.0855	4.3832	7.8079	4.3833	8.7271	3.7373	6.5547	3.0210	3.8856	7.9428	8.0172	3.4146
Ncam1	_VEPYSSTAQVQFDEPEATGGVPILK_.3	#N/A	5.4635	6.0990	6.2489	6.0996	4.6230	5.6026	5.0453	5.0824	6.0906	4.9586	5.6676	5.3221	5.3243	4.3857	5.6651	4.7059	5.5096	5.0012	5.0575	5.3027
Ncam1	_NVDKNDEAEYVC[Carbamidomethyl (C)]IAENK_.3	#N/A	7.7343	7.5480	8.4804	7.8975	7.4034	7.8172	7.5452	7.7995	7.8484	7.9383	7.5720	7.1956	8.0855	7.9179	6.8768	7.4354	7.3873	7.7670	7.6028	7.5391
Ncam1	_AAHFVFR_.2	#N/A	4.1878	4.8201	4.6322	4.3730	4.9775	5.8921	4.8988	5.0168	5.1069	3.2984	5.0513	5.1920	4.2850	4.7868	4.7465	4.8390	5.0264	5.5937	5.2603	3.5633
Ncam1	_AAFSKDES[Phospho (STY)]KEPIVEVR_.3	#N/A	12.7464	12.7268	12.5688	12.5461	11.5572	12.4339	12.3396	12.7559	12.4477	12.4181	12.1176	12.3327	12.9295	12.4540	11.2528	12.2115	12.8983	12.6033	12.4715	12.1947
Ncam1	_GPDPEPTQPGTVKNPPEAATAPAS[Phospho (STY)]PK_.3	#N/A	3.4584	7.8802	5.5660	6.9523	5.5009	4.5908	7.3485	7.6119	3.3030	3.2690	4.2825	6.9623	9.4810	6.5673	3.4150	5.5754	5.7514	7.2089	3.7960	5.2131
Ncam1	_DES[Phospho (STY)]KEPIVEVR_.2	#N/A	12.3466	12.1015	12.1875	11.8154	11.3600	11.8983	11.7797	11.7789	11.6889	11.8062	11.7195	11.3057	11.8491	11.8338	10.2905	11.1652	12.0884	11.7328	11.5049	11.3910
Ncam1	_EANM[Oxidation (M)]EGIVTIM[Oxidation (M)]GLKPETR_.3	#N/A	5.6033	5.5817	4.7399	4.3237	5.0155	5.3894	5.4286	5.0402	4.3529	6.0364	5.5518	5.1626	5.6422	4.4485	4.2907	5.1169	4.9487	5.6123	5.2332	5.1979
Ncam1	_C[Carbamidomethyl (C)]VVTAEDGTQSEATVNVK_.2	#N/A	7.2141	7.1246	3.8405	3.2803	5.9121	4.8324	5.3329	6.7093	3.9583	5.5603	5.6600	4.2421	6.4683	6.9576	4.0634	3.6661	6.0267	6.0291	3.1407	3.0925
Ncam1	_LEGQM[Oxidation (M)]GEDGNSIK_.2	#N/A	6.6400	6.0589	7.0260	5.5730	6.4118	6.2686	6.3939	6.5935	6.5872	6.1711	6.0115	6.1106	6.4485	5.4111	6.0868	6.0554	6.3116	6.3178	6.0928	6.1783
Ncam1	_ASPAPT[Phospho (STY)]PTPAGAASPLAAVAAPATDAPQAK_.3	P13595_T950_M1_Ncam1	7.1946	7.4443	6.7382	5.3256	3.1950	3.3761	6.7219	8.1190	3.9345	5.7170	3.1977	6.6296	7.2182	7.5930	4.2717	4.1037	6.3012	6.3111	5.8843	4.1722
Ncam1	_DGEPIENEEEDDEKHIFSDDSSELTIR_.4	#N/A	9.8876	9.5096	9.2941	9.1038	8.5566	9.1551	9.1773	8.5648	9.3716	9.1954	9.3489	9.2300	9.2270	9.6144	7.9560	8.7647	9.4104	9.0731	8.9060	9.5342
Ncam1	_SLGEESWHFK_.2	#N/A	7.3444	7.1922	7.1351	6.8895	7.0710	7.0587	7.2010	7.1910	7.2047	6.7499	6.9039	7.0277	6.9811	7.0163	6.3912	6.9608	7.0907	6.8716	6.8121	6.6044
Ncam1	_IGQESLEFILVQADTPSSPSIDR_.3	#N/A	3.5226	4.1235	3.7883	4.1668	2.8604	4.3626	4.2936	3.4377	4.4364	3.7290	3.6133	3.2854	3.3470	4.3916	4.2579	3.8675	4.5399	4.0370	3.1611	3.2279
Ncam1	_LPSGSDHVMLK_.2	#N/A	3.9676	6.6047	6.8822	6.7311	4.1746	6.0630	5.9813	4.1933	3.8904	4.5783	5.3566	3.0776	3.9985	6.5768	3.4085	3.3297	3.6905	3.4589	3.7580	3.2195
Ncam1	_TEEERT[Phospho (STY)]PNHDGGK_.2	P13595_T788_M1_Ncam1	6.0541	4.4638	4.0753	4.1503	2.8768	3.6073	5.9122	3.2394	3.3943	3.1776	4.3738	5.4776	3.5598	4.0624	3.4995	4.2300	3.7771	6.0242	3.7046	4.0294
Gpd1	_LISEVIGER_.2	#N/A	6.5599	6.8739	7.0475	6.6620	6.9168	7.0338	7.1490	7.0446	7.0908	7.2809	7.0529	6.8044	7.2352	6.9406	6.8541	6.8188	7.4654	6.7761	7.4856	6.8954
Gpd1	_DLM[Oxidation (M)]QTPNFR_.2	#N/A	6.6090	5.8450	3.8924	4.3333	6.1289	6.2066	3.3465	6.1718	3.2113	5.5792	5.8488	6.3995	3.3768	3.8794	6.2833	6.0777	3.5941	6.3719	6.1301	5.8284
Dnmt1	_EADDDEEADDDVSEM[Oxidation (M)]PS[Phospho (STY)]PK_.2	#N/A	5.3391	4.2225	4.9971	3.7190	5.4689	5.1491	4.9876	4.8253	4.8887	3.9485	4.9678	5.1324	5.2684	4.6860	3.5058	5.4862	5.4532	4.9368	3.8604	5.1437
Dnmt1	_S[Phospho (STY)]KSDSDTLSVETSPSSVATR_.3	P13864_S138_M1_Dnmt1	4.3168	4.5031	3.8534	3.2674	3.0229	3.0562	4.0252	4.5425	3.9454	4.1274	3.4241	4.2293	4.1109	4.6135	4.0506	3.6789	4.0397	3.5050	3.1535	3.0797
Atp1b1	_YNPNVLPVQC[Carbamidomethyl (C)]TGK_.2	#N/A	11.5283	11.3851	11.5484	11.3057	11.1314	11.2913	11.5414	11.5770	11.2939	11.1417	11.2504	11.1104	11.2579	11.0985	11.2067	10.7637	11.5628	11.2791	11.0470	10.8127
Atp1b1	_SYEAYVLNIIR_.2	#N/A	10.4783	10.2762	10.3951	10.3117	9.9785	10.2847	10.3473	10.3447	10.2759	9.9280	10.0109	9.9030	10.1551	9.7996	9.6938	9.9999	10.3178	10.2031	10.1879	9.8478
Atp1b1	_AYGENIGYSEK_.2	#N/A	9.5023	9.2721	9.3844	9.2895	9.1573	9.4779	9.4832	9.4353	9.5106	9.2849	9.3714	9.0092	9.4338	9.2609	9.2985	9.0197	9.3622	9.2801	9.1414	9.2668
Atp1b1	_AKEEGSWKK_.2	#N/A	7.8680	7.9699	8.4128	7.5440	7.6840	7.6910	7.7695	8.3351	7.9940	7.8535	8.0231	7.7163	8.3165	7.6439	7.6385	7.9456	8.3243	8.0324	7.8428	7.6803
Atp1b1	_EGKPC[Carbamidomethyl (C)]IIIK_.2	#N/A	8.8461	8.8824	8.9466	8.9182	8.4364	8.6755	9.1756	9.1660	8.8619	8.5069	8.5623	8.7434	8.8234	8.5780	8.0099	7.9630	8.8956	8.6707	8.2872	8.3593
Atp1b1	_FIWNSEK_.2	#N/A	8.1771	8.4967	8.4853	8.9769	8.3601	8.5399	9.2309	7.8839	8.7405	8.5996	9.0251	8.5301	8.8529	8.4320	8.5908	8.3061	7.3607	9.0795	8.9238	8.7165
Atp1b1	_AKEEGSWK_.2	#N/A	7.6240	7.5658	7.5219	7.4798	7.5407	7.8838	7.8185	7.8808	7.8317	7.8510	8.0222	7.3543	7.6309	7.3665	7.8534	7.6375	7.5621	7.8777	7.8178	7.5228
Atp1b1	_VAPPGLTQIPQIQK_.2	#N/A	12.1507	12.1366	12.0230	11.9950	11.8767	11.9448	12.1923	12.2829	12.0370	11.7276	11.8837	11.8213	12.1844	11.7684	11.7713	11.6749	12.1323	12.0421	11.8739	11.6381
Atp1b1	_AYGENIGYSEKDR_.2	#N/A	8.8540	8.5482	8.9264	8.7934	8.5676	8.5834	8.8237	9.1784	9.0347	8.5532	8.6160	8.5204	9.0655	8.3705	8.5165	8.7796	8.8129	8.5983	8.6699	8.5476
Atp1b1	_KFIWNSEK_.2	#N/A	3.2799	9.2686	6.2083	6.4014	3.4868	9.1788	9.0316	6.0516	6.3820	7.7164	3.5771	6.1425	9.8317	7.5159	6.1318	5.3388	7.0356	7.7947	6.3945	4.0326
Atp1b1	_YKDSAQKDDM[Oxidation (M)]IFEDC[Carbamidomethyl (C)]GNVPSEPK_.4	#N/A	8.8601	8.2184	8.4493	8.6989	8.1630	8.2988	8.2189	8.7138	8.7672	8.0547	8.7145	8.0866	8.9816	8.4826	7.9918	8.2577	8.2068	8.3198	8.4803	8.5900
Atp1b1	_DDM[Oxidation (M)]IFEDC[Carbamidomethyl (C)]GNVPSEPK_.2	#N/A	8.6935	8.0118	8.7427	8.0509	7.9860	8.4079	8.3067	8.4020	8.4272	7.8511	8.0954	7.7778	8.0021	8.0112	7.6165	7.9045	8.5497	8.1956	8.2338	7.9019
Atp1b1	_DSAQKDDM[Oxidation (M)]IFEDC[Carbamidomethyl (C)]GNVPSEPK_.3	#N/A	8.1203	7.4239	7.5987	7.2050	7.5834	7.3888	7.5124	7.5340	7.7191	7.5753	7.8326	7.4584	7.4493	7.8003	6.6270	7.2915	8.0468	7.5916	7.4146	7.2955
Atp1b1	_TEISFRPNDPK_.2	#N/A	9.6160	9.5799	9.5059	9.1846	9.1839	9.4353	9.7439	9.8085	9.4601	9.0363	9.2515	9.3572	9.5918	9.3748	9.2327	9.0708	9.5615	9.5621	9.2179	9.1058
Atp1b1	_GDINHER_.2	#N/A	4.5339	3.4689	3.5694	3.1555	3.8716	4.1133	3.6694	4.2343	4.3892	3.2394	3.8678	3.3583	4.5546	3.5564	3.2466	3.3780	3.2711	3.0612	4.2106	3.5234
Rpl27a	_TGVAPIIDVVR_.2	#N/A	6.7966	6.6934	6.6974	6.4394	6.6391	6.6102	7.2863	7.1813	6.6148	6.3485	6.8326	6.8337	6.9771	6.7230	6.1506	6.2698	6.8503	6.9434	6.3163	6.7807
Rpl27a	_LWTLVSEQTR_.2	#N/A	3.8774	6.6469	5.9815	6.7196	4.0843	6.4513	6.4316	4.1030	6.5653	3.6880	7.1542	6.2059	3.9082	6.2845	6.1137	6.6386	3.6003	6.5893	3.8482	3.1293
Rps16	_LLEPVLLLGK_.2	#N/A	6.4284	6.0010	5.7807	5.5030	5.9623	5.2405	6.5557	5.9856	5.9408	5.8583	6.4660	5.7999	5.9058	5.7363	4.7765	4.9246	5.0489	5.8675	5.4516	5.5065
Rps16	_DILIQYDR_.2	#N/A	4.2759	5.8797	5.4665	4.7948	5.8667	6.2080	5.8827	5.2841	5.4875	5.1822	5.6037	4.7532	4.3068	3.8672	5.5383	5.2850	4.6102	6.0532	6.7180	4.5945
Rps16	_TATAVAHC[Carbamidomethyl (C)]K_.2	#N/A	3.2311	3.9884	3.7674	4.3984	4.0697	4.6063	4.0719	3.8152	4.1951	3.4375	3.9048	3.1603	3.6385	3.7545	4.9666	3.5760	3.4692	3.3090	4.0125	4.3604
Rps16	_EIKDILIQYDR_.3	#N/A	3.1089	4.2102	4.1463	4.4753	3.3158	3.3492	3.7323	3.3346	3.6525	2.1756	3.1311	3.9363	3.8179	4.1844	1.6732	3.9718	4.3326	4.3176	1.7905	4.2876
Mdh1	_FVEGLPINDFSR_.2	#N/A	11.6428	11.7758	11.5876	11.6537	11.5919	11.6406	11.6889	11.7088	11.5006	11.5831	11.3614	11.5961	11.6733	11.7941	11.8228	11.5545	11.6586	11.6738	11.5928	11.5240
Mdh1	_GEFITTVQQR_.2	#N/A	11.3817	11.5209	11.8557	11.4856	11.3708	11.4371	11.4489	11.5639	11.4112	11.4679	11.2742	11.2827	11.3117	11.6146	11.3860	11.3178	11.4242	11.2811	11.3964	11.2692
Mdh1	_VIVVGNPANTNC[Carbamidomethyl (C)]LTASK_.2	#N/A	10.7884	10.7089	10.9824	10.9514	10.5607	10.5807	10.7024	10.8658	10.8541	10.8128	10.5741	10.6159	11.0203	10.9761	10.7061	10.7024	10.7616	10.5875	10.7376	10.5130
Mdh1	_ENFSC[Carbamidomethyl (C)]LTR_.2	#N/A	10.0760	10.1334	10.4472	10.3645	10.2606	10.1436	10.2891	10.3992	10.2319	10.1860	10.1176	10.2734	10.2120	10.4240	10.6208	10.1525	10.0934	10.2083	10.2490	10.1685
Mdh1	_DLDVAVLVGSM[Oxidation (M)]PR_.2	#N/A	8.6300	8.9022	9.6476	9.0937	8.8795	8.7820	8.7494	8.7441	9.0034	8.9725	8.6677	8.7537	8.7129	8.9201	9.0669	8.8433	8.9942	8.8852	9.0445	9.0143
Mdh1	_LGVTADDVK_.2	#N/A	9.5030	9.4724	9.6945	9.5508	9.3566	9.4793	9.7447	9.5240	9.5734	9.5455	9.3682	9.4662	9.3521	9.4655	9.3577	9.0232	9.1343	9.4816	9.2782	9.4657
Mdh1	_EVGVYEALKDDSWLK_.2	#N/A	7.0217	7.1603	7.4211	7.3136	7.0169	7.0747	7.2059	7.2248	7.3610	7.3359	7.2638	7.3138	6.7650	7.5839	7.0961	7.4042	7.3444	6.7439	7.2427	7.2845
Mdh1	_SQGTALEK_.2	#N/A	5.1379	6.5112	6.2197	6.6917	6.3231	6.6764	6.9804	5.2267	6.7953	6.8078	6.5648	6.7848	6.4461	6.4826	7.0867	6.7595	5.8279	7.0810	6.9265	6.7712
Mdh1	_ELTEEKETAFEFLSSA_.2	#N/A	11.2975	11.0413	11.2594	11.3590	10.3786	10.4850	10.3734	11.5156	10.5104	11.1683	10.2733	10.8215	12.1322	11.8609	10.6042	11.2522	11.0501	10.4525	10.5985	10.7679
Mdh1	_NVIIWGNHSSTQYPDVNHAK_.3	#N/A	9.2018	9.1847	9.5729	9.3220	8.9958	9.0266	8.9045	9.2166	9.1298	9.3167	8.8658	8.9106	9.2852	9.2794	9.0136	9.0839	9.2047	9.0647	9.2190	9.1017
Mdh1	_KLS[Phospho (STY)]SAM[Oxidation (M)]SAAK_.2	#N/A	8.6589	9.2568	9.2048	9.2112	8.7094	8.8226	8.8746	8.7855	8.5173	8.5509	8.8550	8.7339	9.0991	9.6639	8.6541	8.4952	9.0496	8.8218	8.7256	8.6310
Mdh1	_EKM[Oxidation (M)]DLTAK_.2	#N/A	6.4641	6.6333	7.4928	7.0194	6.4876	6.6751	6.9482	6.7232	6.5727	6.9299	7.2593	6.0500	6.2699	7.2437	7.4428	6.6935	5.9456	6.7430	6.7035	6.4410
Mdh1	_IFKS[Phospho (STY)]QGTALEK_.2	P14152_S111_M1_Mdh1	3.5390	3.7801	4.5764	4.0452	5.5132	5.7625	3.3022	3.7647	4.3759	3.3496	4.2018	5.0566	5.8503	4.6145	5.1400	5.4081	3.2619	5.2683	5.0767	3.8575
Mdh1	_LSSAMSAAK_.2	#N/A	3.8537	4.1491	4.3900	4.2797	3.1914	3.4155	3.5404	3.5541	4.4087	4.0600	3.2823	4.0385	4.5168	4.3771	3.9268	4.1986	4.0918	3.7414	3.3900	4.3441
Mdh1	_VLVTGAAGQIAYSLLYSIGNGSVFGK_.3	#N/A	3.7556	3.5635	3.2922	3.8286	3.9625	3.9959	3.0856	3.9813	3.0058	3.5662	3.9853	3.2896	3.1712	3.6739	3.1110	3.1177	3.4785	4.4446	4.0932	3.6409
Rpsa	_YVDIAIPC[Carbamidomethyl (C)]NNK_.2	#N/A	7.8402	8.1077	8.5503	8.5887	8.2795	8.1302	8.1276	8.7465	8.3696	8.3024	8.0825	8.3400	8.2805	8.6449	8.3379	8.1009	8.3141	7.8746	8.1428	8.2907
Rpsa	_FAAATGATPIAGR_.2	#N/A	7.9308	7.8841	8.0815	7.5096	8.0298	7.8277	7.8534	8.1197	7.6731	7.9705	7.8147	7.6106	7.5441	7.8354	8.2406	7.8185	7.7864	7.5769	7.9314	7.7735
Rpsa	_AIVAIENPADVSVISSR_.2	#N/A	6.9445	6.6341	7.9228	7.3504	7.1639	7.0678	6.9513	6.9456	7.2056	7.4556	6.7760	6.5509	6.4403	7.2649	7.0018	7.2211	6.8443	6.4026	6.9537	7.1451
Rpsa	_LLVVTDPR_.2	#N/A	5.8544	6.3438	6.2524	6.3526	6.1040	6.1356	6.7857	6.4269	6.1621	6.6999	6.2506	6.4955	6.5543	6.6643	6.4244	6.7633	5.5522	6.4278	6.1139	5.9763
Rpsa	_[Acetyl (Protein N-term)]SGALDVLQM[Oxidation (M)]KEEDVLK_.2	#N/A	4.0945	4.5182	4.6428	4.6023	4.4089	4.3650	4.5585	4.5702	4.9639	4.7345	4.1457	4.5458	5.0930	4.9924	4.4359	4.5322	3.8990	5.6293	4.3508	4.6704
Rpsa	_ADHQPLTEASYVNLPTIALC[Carbamidomethyl (C)]NTDSPLR_.3	#N/A	3.2006	4.1185	4.2380	4.3836	3.4075	3.1537	3.6406	3.4263	3.3064	4.5120	3.0394	4.3374	3.7262	4.2761	4.1333	3.5409	4.4244	4.2259	3.9940	3.9534
Calr	_EQFLDGDAWTNR_.2	#N/A	8.8591	8.8623	8.7287	8.8630	8.8979	8.9542	8.6673	8.8174	8.7283	8.9050	8.9715	8.9909	9.1193	9.1341	9.0806	9.0433	8.9070	8.8683	8.7568	9.1772
Calr	_LFPSGLDQK_.2	#N/A	8.5796	8.6678	7.5165	7.9043	9.0300	8.3218	8.6484	8.3509	8.1659	8.4867	8.4803	8.7245	8.9201	8.7750	9.1767	8.2338	8.3358	8.8192	8.5536	8.7963
Calr	_FYGDLEKDK_.2	#N/A	8.1840	7.9726	8.1092	8.0186	8.3064	7.8339	7.8300	8.3308	8.0333	8.2608	8.0327	8.4078	8.3271	8.4418	8.7601	8.1801	8.0728	8.2906	7.9447	8.2365
Calr	_QIDNPDYK_.2	#N/A	5.6497	4.9794	5.9219	5.4431	5.7756	5.9831	5.5398	4.7137	5.2912	6.0278	5.6708	5.7976	6.0446	5.6960	6.9379	6.1482	5.5641	5.6574	5.5589	6.2049
Calr	_FEPFSNK_.2	#N/A	6.9318	7.1682	6.6930	7.3557	8.2839	6.9037	6.6204	7.4553	7.5817	6.9073	7.1437	8.0076	6.6865	7.4029	8.2110	7.7161	7.7077	8.1855	7.6742	8.2081
Calr	_IDDPTDSKPEDWDKPEHIPDPDAK_.4	#N/A	5.6416	6.2920	6.5373	6.7477	6.1966	6.6503	6.3209	5.4670	6.7999	7.1012	6.7414	6.6011	6.6540	7.1832	7.4477	7.1458	6.5320	6.4020	6.7685	7.1356
Calr	_DKGLQTSQDAR_.2	#N/A	4.2368	3.9022	4.4543	5.4811	4.3247	5.0896	5.0620	5.8841	4.8395	5.9933	5.4237	4.4036	4.8290	5.5309	5.9315	5.5656	5.0724	5.5166	5.5800	4.9824
Calr	_VHVIFNYK_.2	#N/A	4.9897	4.6126	4.1443	4.0814	5.2096	5.2515	4.8739	5.7005	5.0359	5.3860	5.0266	4.2842	5.0760	5.6136	5.4179	4.1610	6.2454	5.6635	5.3006	5.1745
Calr	_HEQNIDC[Carbamidomethyl (C)]GGGYVK_.2	#N/A	3.1903	4.5783	5.5626	4.3939	5.1568	3.4306	4.5047	5.4018	3.9994	4.5017	4.8502	4.8783	3.7365	5.1481	3.6762	5.4461	3.9539	4.8503	3.0564	4.6939
Calr	_IKDPDAAKPEDWDER_.3	#N/A	6.0173	6.8831	6.7423	6.4000	6.6658	6.6086	6.4922	6.5392	6.8759	6.4594	6.9780	6.8405	7.3185	7.1149	7.3075	6.9067	6.5173	6.6595	6.7424	6.9575
Calr	_LKEEEEDKK_.2	#N/A	3.3020	4.0171	3.2008	4.2822	1.7172	3.0986	3.1657	3.5277	3.0657	3.7431	2.7176	3.7432	3.3329	4.3775	3.5645	4.1650	4.5258	4.1245	4.4236	3.2949
Calr	_DM[Oxidation (M)]HGDSEYNIM[Oxidation (M)]FGPDIC[Carbamidomethyl (C)]GPGTK_.3	#N/A	4.5602	3.4426	3.5956	3.1292	3.8979	4.1220	3.6432	4.2606	3.7023	3.2657	4.0766	3.3321	3.8103	3.5827	3.2204	3.4042	3.2974	3.0349	4.1843	3.5497
Atp1b2	_FLNVTPNVEVNVEC[Carbamidomethyl (C)]R_.2	#N/A	7.0418	7.2230	6.5291	6.5911	6.1606	6.7296	7.2607	7.3791	6.9068	6.7147	6.5686	6.3160	7.0398	6.6886	6.2585	6.3086	6.9758	6.7629	6.5698	6.0998
Atp1b2	_SC[Carbamidomethyl (C)]GQVVEEWK_.2	#N/A	6.6668	5.9187	7.6423	6.4785	6.7977	6.8286	5.9110	6.6521	6.4432	6.2454	6.7889	5.7073	6.8396	6.0421	6.3421	5.7544	6.1574	6.1622	5.5885	6.3874
Atp1b2	_NDVC[Carbamidomethyl (C)]RPGR_.2	#N/A	5.9175	6.2024	5.7747	5.4796	5.6641	6.1454	6.7675	5.3488	5.9684	5.7477	5.6326	5.0413	5.8535	5.8861	6.0661	5.3933	6.2046	6.3636	6.3053	5.7128
Atp1b2	_INAANIATDDERDK_.2	#N/A	6.1500	5.8096	5.9404	5.8780	5.9499	6.0064	5.8026	5.5400	5.9603	5.4153	6.0868	5.6102	5.4125	5.8674	5.5693	5.3827	5.9178	5.8288	5.2675	5.6004
Atp1b2	_EFVWNPR_.2	#N/A	6.4493	6.0542	5.3898	5.7857	5.7931	5.8479	5.9475	5.8855	6.0541	5.3460	5.8380	5.6424	5.0633	6.0062	5.1962	5.2352	5.4593	5.7209	5.9126	5.5188
Atp1b2	_YYEQPDNGVLNYPK_.2	#N/A	4.4458	6.6907	6.4698	6.5344	6.4128	6.3142	6.6126	8.0488	6.9673	6.4502	5.7809	5.5147	4.4767	5.3950	5.0798	4.2387	6.3089	5.7365	5.0275	5.2012
Atp1b2	_SC[Carbamidomethyl (C)]GQVVEEWKEFVWNPR_.3	#N/A	5.0056	5.0934	3.7799	6.0377	2.9494	4.3692	5.3794	5.2523	4.8030	5.2678	4.7777	4.7622	4.9048	5.6754	4.7455	3.6054	4.3800	4.6057	5.0288	5.1233
Atp1b2	_INAANIATDDERDKFAGR_.3	#N/A	8.2318	8.1910	7.9418	7.9084	7.9232	7.8769	8.1364	8.3902	8.0419	7.7760	7.4658	7.8618	8.3632	7.8807	7.7582	7.6414	8.0474	7.8392	7.7767	7.6354
Atp1b2	_TQLGDC[Carbamidomethyl (C)]SGIGDPTHYGYSTGQPC[Carbamidomethyl (C)]VFIK_.3	#N/A	3.0385	3.6543	3.7042	3.4166	2.2676	1.4960	3.3108	1.7162	4.0904	4.3784	2.1850	2.7903	4.1985	3.6979	3.6085	3.0161	1.9409	3.2589	3.5580	3.4195
Hspb1	_QLS[Phospho (STY)]SGVSEIR_.2	P14602_S86_M1_Hspb1	9.3059	9.5963	10.4038	9.3949	9.6657	9.5781	8.8559	9.6756	9.4860	9.3672	9.7332	9.7293	10.3468	10.3867	9.8962	9.7502	9.9416	9.3811	9.8530	10.1176
Hspb1	_SPS[Phospho (STY)]WEPFR_.2	P14602_S15_M1_Hspb1	6.8178	4.3603	9.0607	7.5464	3.1657	3.1990	3.8824	3.6477	8.5130	7.8953	3.2813	4.0865	3.9681	9.2076	8.1388	3.8217	8.1890	8.5040	3.2963	8.3360
Psmd3	_SVFPEQANNNEWAR_.2	#N/A	8.0882	8.3632	4.6036	4.0180	7.0624	7.6874	7.9509	8.6369	7.8957	8.0082	7.6173	3.4790	8.1695	8.2238	3.3003	7.5222	8.2326	8.0060	7.7414	7.3987
Psmd3	_LQLDS[Phospho (STY)]PEDAEFIVAK_.2	P14685_S426_M1_Psmd3	3.8660	3.4618	3.4026	3.1484	4.0729	4.1063	4.4760	4.2413	4.3962	3.6766	3.8749	3.1792	4.5617	3.5635	3.0006	3.2281	3.2782	3.0541	4.2036	3.5305
Lmnb1	_[Acetyl (Protein N-term)]ATAT[Phospho (STY)]PVQQQR_.2	P14733_T5_M1_Lmnb1	9.1044	9.4070	9.3242	9.5444	9.4055	9.0250	8.8412	9.5913	8.9841	4.3387	9.0744	9.1895	9.6450	9.5265	8.6229	9.2631	9.0316	9.1152	8.9753	9.0700
Lmnb1	_ASAPATPLS[Phospho (STY)]PTR_.2	P14733_S24_M1_Lmnb1	7.8453	3.9693	4.5698	3.6559	7.5994	3.1129	7.5640	3.7338	7.3078	7.6656	7.6731	3.8588	8.6159	4.5568	7.7605	4.3784	4.2715	3.5616	7.9837	7.6899
Lmnb1	_LKLS[Phospho (STY)]PSPSSR_.2	P14733_S392_M1_Lmnb1	8.2114	8.2744	7.3984	8.0668	7.6791	6.0941	5.4753	7.8360	7.8825	8.2784	5.8655	7.7939	6.3181	8.1386	7.3940	8.2350	6.3755	7.9806	7.7792	6.0764
Lmnb1	_LSPS[Phospho (STY)]PSSR_.2	P14733_S394_M1_Lmnb1	4.5911	5.0041	3.6301	8.7152	3.7624	8.3534	8.4362	8.7770	8.3837	8.5345	8.1670	8.4956	8.8958	8.6967	8.4043	8.6057	8.6130	8.5251	8.5236	8.4715
Lmnb1	_NQNSWGTGEDVK_.2	#N/A	3.8336	5.1693	5.6060	3.7506	4.0405	5.1053	5.4289	6.1335	4.9996	3.6442	5.2635	5.2373	4.5941	6.2122	5.1856	3.1957	5.1914	5.6239	5.6821	5.5843
Lmnb1	_AGSRAS[Phospho (STY)]APAT[Phospho (STY)]PLSPTR_.2	P14733_S17_M2_Lmnb1	5.8126	5.5727	5.5898	5.7035	5.6432	5.8780	4.0387	6.1091	5.8272	6.1330	5.1679	3.5589	4.0543	6.1436	4.8893	5.7954	6.0823	5.4049	5.5864	5.6499
Anxa6	_SEIDLLNIR_.2	#N/A	7.0913	6.8586	6.8723	6.7506	6.7633	6.7869	6.7829	6.6082	6.0924	6.5398	7.1535	6.8085	6.1767	7.0488	6.5810	6.6635	6.4176	6.7540	6.6667	6.6931
Anxa6	_DLESDIIGDTSGHFQK_.2	#N/A	6.5983	6.8111	6.5744	6.8370	6.3418	6.0555	6.7724	6.5887	6.2932	6.5546	6.9760	6.4567	6.9366	6.9476	6.4198	6.7111	6.4767	6.6887	6.6381	5.9410
Anxa6	_TLIEILATR_.2	#N/A	6.1431	6.3395	6.7909	6.4406	6.3306	5.9581	6.1202	5.3626	6.0720	6.4320	6.2290	5.9763	3.4584	5.3028	6.1274	6.3567	6.1548	6.2472	6.4048	6.5209
Anxa6	_SLEDALSSDTSGHFR_.2	#N/A	5.6277	5.4587	5.5678	5.7552	4.7955	5.4015	5.1844	4.6694	5.4627	4.7435	5.5102	5.1752	5.5779	5.2865	4.5119	5.3384	5.3151	5.0819	5.0102	4.5889
Anxa6	_M[Oxidation (M)]LVVLLQGTR_.2	#N/A	4.8387	4.5012	4.9886	4.0428	4.8059	4.4143	5.0383	5.0498	5.2035	5.6673	4.4152	4.5057	3.3513	4.9262	4.5872	5.2755	5.0189	5.4482	4.9530	5.4555
Anxa6	_ENDDVVSEDLVQQDVQDLYEAGELK_.3	#N/A	4.5860	4.4675	4.6514	3.7043	3.7348	3.6867	2.8988	4.1955	4.6674	5.6225	4.8836	5.0910	4.0096	5.1613	4.7842	4.3168	4.4849	4.8738	3.1376	5.1396
Anxa6	_GIGTDEATIIDIVTHR_.2	#N/A	4.2118	3.8457	3.9291	3.8149	3.7012	3.6841	3.4320	4.6730	3.8337	4.3183	4.2319	3.7881	3.2048	4.4098	4.1021	3.6300	4.5458	3.8116	4.8733	3.9803
Anxa6	_WGTDEAQFIYILGNR_.2	#N/A	4.9232	4.0765	4.6242	3.7631	4.5075	4.0646	4.2770	3.6267	5.1632	5.2519	3.2097	4.1137	3.9470	4.4497	3.8542	3.9787	4.5145	3.6688	3.3174	4.4167
Anxa6	_ILISLATGNR_.2	#N/A	3.6997	4.1800	4.1765	4.4452	3.0374	5.6321	7.4845	7.6064	3.0619	3.9060	7.8677	3.9061	3.1699	7.3047	4.0808	4.0446	4.3628	3.9636	3.0857	3.8918
Anxa6	_AINEAYKEDYHK_.3	#N/A	4.5052	2.9706	4.0418	4.5799	3.1722	2.9000	3.6607	3.2300	4.4280	4.0408	3.3015	4.0409	4.5361	1.3350	3.2979	4.1407	4.2281	1.4614	2.6281	3.7571
Anxa6	_IAQGAMYR_.2	#N/A	3.4491	3.8700	4.4865	4.1351	3.6560	3.6894	3.3921	3.6748	3.3123	3.2597	4.2918	3.5961	3.4777	4.5246	3.4175	4.3120	3.1720	4.1381	4.2765	3.9474
Rplp0	_IIQLLDDYPK_.2	#N/A	6.1877	6.6123	6.4008	6.4863	6.2225	6.5370	6.7376	6.4854	5.8003	6.1053	6.2679	6.3717	6.2759	6.4460	6.2399	6.1327	6.1811	6.5698	6.5191	5.6862
Rplp0	_GHLENNPALEK_.2	#N/A	6.4675	4.9543	5.9815	6.0610	6.3360	5.9543	5.9183	5.9406	5.4192	6.3830	6.1489	5.7354	5.1604	6.1070	6.0379	6.3175	5.8701	5.8696	5.9047	6.2877
Rplp0	_TSFFQALGITTK_.2	#N/A	4.1044	5.5563	5.5945	5.6022	5.5265	4.7239	5.3900	5.3296	5.5268	5.9916	5.4448	5.3850	5.6650	5.9602	5.2496	5.4363	5.1673	5.1632	5.3496	5.6985
Rplp0	_C[Carbamidomethyl (C)]FIVGADNVGSK_.2	#N/A	5.3378	3.3513	6.2183	6.2012	6.3218	6.0628	5.9946	4.3519	6.2484	6.0382	6.3767	5.9868	6.1486	5.2578	6.2625	4.2793	5.6414	5.6369	6.2378	6.1081
Rplp0	_AFLADPSAFAAAAPAAAATTAAPAAAAAPAK_.3	#N/A	4.0701	4.2599	4.6354	3.8094	4.3696	4.1599	4.0261	2.4362	3.3959	3.7895	4.4200	4.2100	3.2865	4.0000	4.2166	3.5310	3.9831	3.7739	3.5558	4.4054
Rplp0	_EDLTEIR_.2	#N/A	3.5688	3.7503	4.6062	3.0694	4.1086	4.2900	3.4325	3.7944	3.1799	3.8581	3.8660	3.4765	3.5996	3.1434	3.5465	3.6327	3.2917	4.7049	2.7763	3.7412
Rplp0	_AEAKEES[Phospho (STY)]EESDEDM[Oxidation (M)]GFGLFD_.2	P14869_S304_M1_Rplp0	3.8220	4.1181	3.8334	3.9428	3.4621	4.5616	4.5147	3.4808	4.2047	4.1074	3.5896	3.8443	3.8057	4.6806	4.6013	3.6589	4.0197	5.0995	3.8457	4.1067
Map1b	_DLTGQVPT[Phospho (STY)]PPVK_.2	P14873_T527_M1_Map1b	12.6173	12.4765	12.3418	12.5505	12.2167	12.3326	12.0091	12.6357	12.2709	12.6115	12.2757	11.9935	12.8313	12.5115	11.6024	12.2548	12.7521	12.1885	12.1947	12.1992
Map1b	_VLS[Phospho (STY)]PLRS[Phospho (STY)]PPLLGSESPYEDFLSADSK_.3	P14873_S1391_M2_Map1b	10.0864	10.8266	10.2445	9.8837	8.3083	9.4943	10.6076	10.3963	9.7543	10.2864	8.8964	10.3081	10.7741	11.1085	9.6624	9.6634	9.6722	10.3270	10.1461	9.5664
Map1b	_SKPLAAS[Phospho (STY)]PKPGALK_.2	#N/A	9.5188	9.6466	8.6281	9.1345	8.7110	8.7196	8.9933	9.1227	8.5670	9.4484	8.6550	9.1784	8.7576	9.9463	7.9924	8.4857	9.0553	9.2334	8.9513	8.4484
Map1b	_T[Phospho (STY)]PEEGGYSYEISEK_.2	#N/A	9.6167	9.8544	10.2263	10.0628	7.9949	9.6769	9.4987	8.6255	9.6204	9.7290	8.9986	9.4120	8.7735	10.4057	8.6874	9.6490	9.7536	9.6586	9.6760	9.9204
Map1b	_QPGIQS[Phospho (STY)]PSR_.2	P14873_S1073_M1_Map1b	9.9138	4.1958	4.3433	9.8858	9.4974	9.9239	9.5506	3.5074	9.5779	4.4105	9.6679	9.3046	9.1560	4.3304	8.6178	4.1519	9.6925	3.7881	9.7022	9.6350
Map1b	_C[Carbamidomethyl (C)]YTTEKKS[Phospho (STY)]PSEAR_.3	#N/A	7.0621	8.3227	7.7688	7.0471	7.5034	7.8206	7.4201	8.1545	8.1040	8.2175	7.8678	7.9339	8.3850	8.2739	7.8845	7.9362	8.2421	8.0516	8.3165	7.9365
Map1b	_SSYYVVSGNDPAAEEPSR_.2	#N/A	8.1497	8.2432	8.2388	8.1383	8.1203	8.3483	8.0128	7.8995	8.4302	8.4125	8.0553	8.1203	8.2187	8.6274	8.2173	8.6092	8.5211	7.9359	8.2798	8.4419
Map1b	_AVLDALLEGK_.2	#N/A	7.0873	7.6406	7.7323	7.9683	7.5408	7.2926	7.5816	7.3838	7.0585	7.9880	7.6175	7.8957	7.7077	7.9894	8.1216	7.9551	7.3654	7.9684	7.8689	8.0540
Map1b	_VLFPGNSTQYNILEGLEK_.2	#N/A	7.6692	7.9393	6.9154	7.9505	7.2172	7.2961	7.9043	7.8985	7.2436	7.4004	7.6792	7.7536	7.9972	7.5800	7.6374	7.3997	7.4554	7.7402	7.1829	7.4990
Map1b	_FSPEVPGQK_.2	#N/A	7.2468	7.2144	7.5566	7.3029	7.3634	7.4630	7.2246	7.6959	7.0525	7.2322	7.0067	7.2112	7.0680	7.1142	7.4870	7.3742	7.1520	7.4180	7.2102	7.2676
Map1b	_T[Phospho (STY)]PEDGGYTC[Carbamidomethyl (C)]EITEK_.2	#N/A	7.5597	7.3766	7.2951	7.2063	5.9154	6.8927	6.7206	6.2863	6.8253	7.2986	6.7012	6.4639	6.0927	7.6984	5.7476	6.1844	7.3620	5.9907	6.6917	6.8319
Map1b	_AIGNIELGIR_.2	#N/A	5.7931	6.8630	6.6165	6.2341	6.7180	6.3315	6.5229	6.4236	6.4280	6.3089	6.2462	6.4671	7.0768	6.3434	6.4045	6.4941	6.4562	6.5430	6.4187	6.1702
Map1b	_SVGNTIEPVILFQK_.2	#N/A	6.3676	6.5285	6.7921	6.3503	6.3083	6.7269	6.1162	6.5375	6.1484	6.5358	6.3566	6.1778	7.0093	6.2802	5.9901	6.7180	6.4786	6.2262	6.7390	6.2721
Map1b	_DVSDERLS[Phospho (STY)]PAK_.2	#N/A	6.8179	6.3867	6.9754	6.8175	6.4429	6.8620	6.4416	6.3772	6.4519	6.6826	6.6513	6.0420	5.6271	6.8921	5.1792	5.4515	6.6587	6.0774	6.0933	5.8646
Map1b	_TPEVSGYTYEK_.2	#N/A	6.7221	7.0071	6.6402	6.6155	5.9551	6.6421	6.1901	6.4488	6.8027	5.9569	6.4933	6.5537	6.1888	7.0734	6.1754	6.5197	6.6698	6.4601	6.3538	6.5449
Map1b	_QGFPDRES[Phospho (STY)]PVSDLTSTGLYQDK_.3	#N/A	7.1969	4.3881	7.1173	7.4120	6.0465	6.4550	7.2553	3.3151	6.9216	3.1020	7.2808	3.7538	3.6354	7.9369	5.9041	6.4861	7.1060	3.9804	6.7300	6.4790
Map1b	_TTEAAATAVGTAATTAAVVAAAGIAASGPVK_.3	#N/A	6.0611	6.2433	6.2271	6.6703	5.8206	6.2426	5.4857	6.1413	6.2500	6.2626	6.4822	6.3210	6.9696	7.4474	5.5118	6.0307	6.7291	6.0265	6.2896	7.2886
Map1b	_SWDTNLIEC[Carbamidomethyl (C)]NLDQELK_.2	#N/A	5.7284	5.9929	6.5314	6.3779	6.1126	5.6750	6.1443	6.4403	5.8834	5.9271	6.2291	5.9465	5.6835	6.0304	5.7920	6.0957	5.8320	5.4237	6.1324	5.7170
Map1b	_SVNFSLTPNEIK_.2	#N/A	5.1444	6.3106	6.0734	5.7281	5.7265	6.3676	5.7968	6.0177	6.2190	6.1108	5.3644	5.6801	6.1713	5.7478	5.9105	5.5874	5.9822	5.9724	5.8455	6.0395
Map1b	_DAYC[Carbamidomethyl (C)]S[Phospho (STY)]EEKELK_.2	P14873_S1225_M1_Map1b	4.5093	4.3106	4.0459	4.5757	3.2154	3.2488	6.7270	3.2342	3.7529	4.3199	3.2315	4.0367	3.9183	7.5432	3.8580	3.8715	4.2323	3.6975	3.3461	4.3880
Map1b	_SPSLSPSPPS[Phospho (STY)]PIEK_.2	#N/A	13.5929	13.6174	13.6878	13.5018	12.7378	13.2493	13.0598	13.1766	13.1914	13.5939	12.9357	13.2123	13.6997	13.6530	12.4118	13.3033	13.7045	13.1547	13.2568	13.3362
Map1b	_SDIS[Phospho (STY)]PLTPR_.2	P14873_S1781_M1_Map1b	10.7503	10.9300	10.5442	10.9828	10.3568	10.5696	10.5056	10.4550	10.3621	11.0754	10.4422	10.7624	11.0569	10.9045	9.8772	10.7170	11.0507	10.6695	10.7258	10.7534
Map1b	_S[Phospho (STY)]PPLLGSESPYEDFLSADSK_.2	#N/A	9.7108	9.9617	10.0604	9.5789	8.9152	10.1586	9.7250	9.3698	9.8852	9.7403	9.9112	9.3837	9.2556	10.2122	9.3883	9.6394	10.2503	9.7533	9.8567	9.6414
Map1b	_TIKS[Phospho (STY)]PC[Carbamidomethyl (C)]DSGYSYETIEK_.2	#N/A	7.4057	8.1550	7.7681	8.2022	3.8010	7.6975	7.8385	7.0809	5.8800	8.7197	7.2396	7.4298	7.0504	8.3111	5.7061	7.7859	7.2707	7.6387	6.9819	7.7718
Map1b	_ESVVS[Phospho (STY)]GDDR_.2	#N/A	3.5237	3.8777	3.8254	8.6775	3.6179	8.5220	8.0244	8.0940	8.2412	8.2240	8.4100	7.9562	7.9480	3.8635	3.5183	7.9233	4.0117	8.0880	8.3233	8.3407
Map1b	_ADS[Phospho (STY)]RESLKPATK_.3	#N/A	6.3413	5.8147	5.1368	5.7556	5.8742	6.4482	5.9593	5.9416	5.9570	5.5077	6.1806	6.1521	3.7734	6.3628	4.9368	5.8130	6.2506	6.0121	5.8731	5.5645
Map1b	_TIKT[Phospho (STY)]PEDGGYTC[Carbamidomethyl (C)]EITEK_.2	#N/A	5.9778	6.2686	6.3457	6.3773	3.8853	3.9680	5.8767	4.0734	3.9010	6.7327	4.8787	5.6877	6.2011	6.0016	5.4274	5.7225	6.1355	6.2425	5.4387	6.1387
Map1b	_ASLTLFC[Carbamidomethyl (C)]PEEGDWK_.2	#N/A	6.4850	6.6048	3.8117	6.3478	6.2225	6.3306	6.1240	6.2315	6.6528	6.2065	6.0861	5.9545	6.3030	6.3328	6.0395	5.8429	6.6064	5.6414	5.9181	5.5521
Map1b	_KLGGDVS[Phospho (STY)]PTQIDVSQFGSFK_.3	#N/A	6.2685	7.4800	6.1047	5.8335	3.2236	4.8494	7.2119	6.5640	3.7412	4.3316	3.2198	7.1389	3.9066	8.4828	5.1346	4.2308	4.1240	7.3859	6.2759	4.2741
Map1b	_THDVGGYYYEK_.2	#N/A	5.6667	4.4693	4.4851	4.9669	5.2355	5.8280	4.5103	5.5189	5.6339	5.1263	5.5198	5.0236	5.8389	3.4862	5.5169	5.2547	5.7435	5.4184	4.7195	5.7204
Map1b	_SVSPGVTQAVVEEHC[Carbamidomethyl (C)]AS[Phospho (STY)]PEEK_.3	#N/A	12.3347	11.1794	11.9393	11.3876	11.7670	11.0262	11.0069	12.1789	11.8006	12.5114	12.3565	11.4551	12.9182	11.9059	11.8039	11.3478	12.6154	11.6026	12.0008	11.1070
Map1b	_FEDEGAGFEESS[Phospho (STY)]ETGDYEEK_.2	P14873_S934_M1_Map1b	5.2535	5.4524	6.8123	6.0142	3.4130	6.0842	3.6860	3.1444	3.9098	5.8264	3.8466	3.8900	4.0370	3.9808	3.7114	5.2048	3.7320	5.3586	3.5722	5.7064
Map1b	_SPC[Carbamidomethyl (C)]DSGYSYETIEK_.2	#N/A	6.1774	6.0514	5.8315	6.0889	4.9183	5.7433	5.6831	4.8594	6.3564	6.6200	4.9042	5.6228	5.1139	6.3153	5.6848	5.6732	6.0055	5.9532	5.7261	5.5539
Map1b	_AAEAGVTEEQYGYLGTSAK_.2	#N/A	7.3418	5.9112	5.2132	5.3575	6.0549	3.8629	5.6794	6.4602	5.6731	5.8008	5.5417	5.2521	6.7323	6.7690	3.4680	5.4715	5.7025	4.7857	4.7625	4.8845

Map1b	_HNLQDFINIK_.2	#N/A	5.0412	5.2174	5.4729	5.5125	5.0331	5.8574	4.8926	5.1299	5.8341	4.2128	4.9754	5.0363	5.3040	5.2161	4.7799	5.1843	5.1642	4.9469	5.5708	6.0604
Map1b	_LSPAKS[Phospho (STY)]PS[Phospho (STY)]LSPSPPSPIEK_.2	#N/A	4.0416	3.5901	3.9176	3.6987	3.9102	4.1727	3.6286	3.9906	6.5558	3.9138	5.3075	3.3690	3.7958	3.9106	3.2058	3.7388	3.3900	3.6978	6.0417	3.5878
Map1b	_TTRT[Phospho (STY)]PEEGGYSYEISEK_.3	#N/A	3.7555	6.6789	5.2081	3.9457	4.6681	3.8218	4.5938	3.4644	3.8473	4.1478	5.9709	4.7782	5.0106	7.1077	3.5805	5.1633	5.5656	6.4563	6.1816	4.0798
Map1b	_EVSSKEEQS[Phospho (STY)]PVK_.2	#N/A	4.1951	3.8077	6.5116	5.8961	5.7530	6.4362	6.0319	5.6101	4.0673	6.3712	2.9409	5.6404	5.4519	3.2176	4.9491	3.0392	4.4331	5.9715	6.0461	6.7117
Map1b	_ESS[Phospho (STY)]PLYSPGFSDSTSAAK_.2	P14873_S1789_M1_Map1b	12.2918	12.2486	12.2984	12.2747	11.4931	11.7741	11.6536	11.8040	11.8185	12.0907	11.7394	11.7842	12.3663	12.1997	11.0408	11.9316	12.2573	11.7431	11.8912	11.8882
Map1b	_ETAAAHQASS[Phospho (STY)]SPPIDAATAEPYGFR_.3	P14873_S1814_M1_Map1b	4.6564	6.7076	3.7849	4.4899	4.2918	5.8793	5.8475	6.5317	6.0168	4.8826	4.5542	6.1836	3.4810	6.8028	4.3301	4.6706	4.9678	6.1425	4.5048	4.3570
Map1b	_TTRS[Phospho (STY)]PDTSAYC[Carbamidomethyl (C)]YETM[Oxidation (M)]EK_.2	#N/A	5.9899	6.0884	3.9503	3.7651	5.4593	4.2131	5.0435	6.2679	3.9046	4.0237	5.6413	5.5721	6.0977	3.9884	5.1589	5.6965	5.6313	3.6074	3.9064	3.6657
Map1b	_RES[Phospho (STY)]VVS[Phospho (STY)]GDDR_.2	#N/A	6.0671	6.1261	6.2051	5.5549	5.3450	6.0833	5.7983	5.3824	5.3928	5.9506	5.4809	5.3325	4.9139	6.1976	4.9341	5.6214	6.0659	5.5278	5.9314	5.4911
Map1b	_GEAEQS[Phospho (STY)]EEEGEEEDKAEDAR_.3	#N/A	3.8030	5.6604	5.0852	6.1501	4.2911	5.8976	5.2105	4.1260	5.6982	5.9785	5.8662	4.7577	3.8509	5.6498	4.8098	4.6700	4.1175	5.0101	5.0628	5.4422
Map1b	_VDSILLTHGDDNLPGINSM[Oxidation (M)]LQR_.3	#N/A	5.5332	5.5331	3.5950	3.5258	4.9430	5.6367	3.9221	5.1845	5.2186	5.5391	4.3881	5.3757	4.0893	4.7387	4.1050	5.2354	5.6204	4.6458	5.7091	4.6790
Map1b	_ASLSPM[Oxidation (M)]DEPVPDSES[Phospho (STY)]PVEK_.2	#N/A	12.1770	11.5931	11.1470	11.0106	10.7527	11.5719	11.0459	11.8884	11.5397	11.5666	11.2830	11.5367	12.5337	11.3262	9.9899	11.6979	12.0632	11.6055	11.6562	11.5942
Map1b	_SQGSTSNS[Phospho (STY)]DWM[Oxidation (M)]K_.2	#N/A	3.8314	6.3022	7.1363	7.5455	8.8093	6.2900	5.8842	4.7163	5.4410	5.0389	6.7323	3.8819	3.8556	7.5120	5.4710	3.9105	5.9883	4.5045	4.6007	5.5775
Map1b	_DLTTSS[Phospho (STY)]VEKDSGGK_.2	P14873_S1851_M1_Map1b	4.4104	4.4095	6.6280	6.5392	4.5741	3.1498	3.9316	5.0969	5.4933	7.2204	5.6242	5.3153	4.0172	5.3750	4.5025	3.7725	5.3240	3.0161	5.5046	5.9156
Map1b	_TPQASTYSYETSDR_.2	#N/A	6.6070	6.8076	4.0303	4.5913	6.2652	6.2299	5.7428	6.6564	6.2832	6.5886	5.5528	6.3527	3.9339	6.6763	6.0343	6.0349	4.2167	6.4019	6.0224	6.5655
Map1b	_TTEAAATAVGTAATTAAVVAAAGIAASGPVKELEAER_.3	#N/A	4.4405	4.5889	4.0444	2.5952	3.8561	3.0399	3.9913	3.3001	3.3941	4.3809	3.9750	4.3296	3.9486	4.0459	3.9777	4.2795	4.3548	4.5153	3.8850	4.4155
Map1b	_[Acetyl (Protein N-term)]ATVVVEATEPEPSGSIGNPAASTS[Phospho (STY)]PSLSHR_.3	#N/A	9.7520	10.7363	4.8886	4.9747	4.7849	8.4039	10.1081	10.4049	5.0345	5.2467	4.8342	9.6601	9.8737	11.2252	7.7821	5.9900	5.5312	10.1059	9.1367	4.6443
Map1b	_RSES[Phospho (STY)]PFEGK_.2	P14873_S1422_M1_Map1b	8.1700	8.2164	7.5432	4.8509	7.0280	7.1507	7.5879	7.4847	7.1537	8.6258	6.9032	7.5198	7.7220	7.5826	6.5592	7.4219	7.5518	7.0832	7.4633	7.1800
Map1b	_TESKPS[Phospho (STY)]VTEK_.2	P14873_S601_M1_Map1b	3.5092	3.8098	4.5467	4.0750	3.7162	4.6727	3.3319	3.7349	3.2521	4.7643	5.6315	4.9993	3.4176	5.1667	3.3573	4.5718	3.2322	4.8275	4.2163	3.8872
Map1b	_SLM[Oxidation (M)]S[Phospho (STY)]S[Phospho (STY)]PEDLTK_.2	#N/A	7.7825	11.2177	11.3030	10.9662	3.5298	11.5155	10.8451	3.5605	11.1148	12.0802	3.5420	10.9762	3.3475	11.7912	10.5533	11.0164	12.2043	10.9481	11.1267	11.0821
Map1b	_SPSLSPSPPS[Phospho (STY)]PIEKTPLGER_.3	#N/A	4.2439	6.7295	3.5007	3.4430	3.9931	4.5559	6.0228	7.3833	4.0781	4.6361	3.9830	6.3733	4.2209	6.0345	3.1187	3.3175	5.4359	4.8080	6.1831	4.6628
Map1b	_LGGDVS[Phospho (STY)]PTQIDVSQFGSFK_.2	#N/A	3.6789	3.2748	3.7635	4.4622	3.8859	3.9192	6.6857	4.6610	3.0824	5.5979	4.0619	6.5807	3.2479	7.5275	3.1876	3.0410	3.4652	6.3611	4.0165	3.7175
Map1b	_KES[Phospho (STY)]KEETPEVTK_.2	#N/A	9.0764	9.1618	8.7880	9.2384	8.8022	8.9047	8.9591	8.9754	8.7171	9.2610	9.1044	9.3712	9.7411	9.4990	8.8689	9.1159	9.2880	9.2619	9.4231	9.1661
Map1b	_MSISEGTVS[Phospho (STY)]DK_.2	P14873_S1523_M1_Map1b	3.2508	7.1344	7.9347	7.4598	4.0893	7.0255	7.0752	5.3904	6.3850	5.5339	7.3554	6.1629	3.6329	8.2780	6.3423	3.5956	6.7711	5.3395	5.7625	6.6447
Map1b	_VQSLEGEKLS[Phospho (STY)]PK_.2	#N/A	11.8205	11.9352	11.7478	11.6673	11.4151	11.6584	11.4170	11.9711	11.4776	11.3390	11.4455	11.6399	12.2468	12.0579	11.0563	11.6175	12.0354	11.7173	11.5925	11.6403
Map1b	_EKRES[Phospho (STY)]VVSGDDR_.2	#N/A	4.2169	5.2839	4.4638	4.5095	4.3582	5.7093	4.7781	4.2861	5.2329	4.2831	5.7784	4.1743	4.0744	5.9200	3.8068	4.8570	4.4985	4.5472	5.2444	4.9494
Map1b	_HM[Oxidation (M)]DPPPAPMQDRS[Phospho (STY)]PS[Phospho (STY)]PR_.3	#N/A	4.0035	7.2190	7.0730	7.5135	3.3895	5.6305	6.1883	4.0349	4.6791	4.3910	6.0562	3.9989	4.0949	7.2214	4.6201	3.6884	5.4314	3.5443	4.4913	4.6025
Map1b	_SSHLPTEVTEKPQAVPVSPEFSEAKDENER_.4	#N/A	5.6706	7.5889	5.8957	6.0357	5.5623	5.2585	5.3876	5.9518	5.7178	5.8807	5.1353	5.6369	6.0028	5.9773	5.4419	5.5525	6.1307	5.6360	5.1174	5.4327
Map1b	_EVSSKEEQS[Phospho (STY)]PVKAEVAEK_.2	#N/A	8.8294	9.2682	9.0678	9.1173	8.8029	9.1915	9.0346	8.8679	9.0275	9.1859	8.8563	8.7572	9.2252	9.3277	8.6955	8.9467	9.1433	9.1885	9.1341	8.9348
Map1b	_SRT[Phospho (STY)]PVQDHR_.2	#N/A	5.9655	6.3093	6.1651	6.5916	5.8621	5.5533	5.8178	4.1378	5.4860	6.8581	5.8097	6.1000	6.2094	6.1523	5.3523	6.1501	5.9204	6.2204	5.6218	5.8303
Map1b	_LM[Oxidation (M)]TDAAR_.2	#N/A	5.2369	5.3085	4.6764	4.8986	5.1520	5.0653	5.1761	5.4583	5.2074	5.3276	4.7690	5.6220	5.1369	4.5802	5.3913	4.6138	5.2907	5.0638	5.3348	5.5744
Map1b	_LEMYVLNPVK_.2	#N/A	3.7582	4.5572	4.6149	5.2109	3.9651	3.1717	4.2887	3.9839	3.6810	3.2869	4.5639	3.2870	3.7891	4.6211	3.8453	3.1203	3.4811	3.6683	3.9674	3.0101
Map1b	_KLGGDVS[Phospho (STY)]PTQIDVSQFGSFKEDTK_.3	#N/A	4.0033	5.4618	3.5399	3.5809	4.2102	3.5545	4.3387	4.2290	4.2589	3.8139	7.3376	4.7898	5.7742	6.1250	2.8632	3.3654	7.3262	3.1915	4.1653	3.3932
Map1b	_T[Phospho (STY)]PVQDHR_.2	#N/A	5.6018	5.1332	4.3028	5.1167	3.1043	4.2817	3.7016	3.4669	3.9512	5.2796	4.8738	4.4544	3.7873	4.6419	3.7270	4.1114	4.8669	3.8286	4.6369	2.4475
Map1b	_NVDVEFFK_.2	#N/A	3.0835	3.4185	2.1611	4.2308	2.3457	4.0629	2.6193	4.2847	4.4396	3.6332	3.9182	3.3915	4.6050	2.5632	3.0439	3.4284	3.3215	1.3826	3.8711	4.0174
Map1b	_ITSFPESESYSYETSTK_.2	#N/A	10.6191	7.4088	10.2698	8.1816	8.0774	10.3099	7.4519	8.8048	8.3233	11.3773	7.5655	3.5516	3.7162	4.0327	7.6617	7.6152	7.6091	7.7789	7.5213	7.5948
Map1b	_VQSLEGEKLS[Phospho (STY)]PKS[Phospho (STY)]DISPLTPR_.3	P14873_S1778_M2_Map1b	3.5099	8.1263	5.4983	4.5605	3.6965	6.5287	6.6719	3.3952	6.2869	4.6828	3.8205	3.6794	3.5835	9.8440	5.8137	4.0369	3.6063	9.1728	5.2063	4.2153
Map1b	_EEQS[Phospho (STY)]PVKAEVAEK_.2	#N/A	10.5015	10.5199	10.5432	10.3494	9.8452	10.7099	9.9616	10.3106	10.0660	10.2542	10.1166	9.8764	9.6914	10.8811	9.7876	9.8121	10.6463	10.2194	10.2671	10.2746
Map1b	_S[Phospho (STY)]PDTSAYC[Carbamidomethyl (C)]YETMEK_.2	#N/A	4.5161	8.3889	9.1190	9.2064	6.3950	7.6186	8.0173	5.1431	6.8185	6.9716	8.3001	6.5788	5.0397	9.2651	6.4853	4.5917	7.6510	6.0129	6.1534	6.9362
Map1b	_TSDVETM[Oxidation (M)]SSQSALALDER_.2	#N/A	5.9717	6.4748	5.4562	5.9091	5.2162	5.5869	5.6627	5.8404	5.7175	5.6637	4.9395	5.4541	5.6921	6.8489	4.9616	5.4708	5.5120	6.1144	5.7542	5.8441
Map1b	_EWYQETHEK_.2	#N/A	5.4039	6.1374	6.1024	6.3274	5.9508	6.2028	6.2129	6.0113	5.7848	6.1876	5.8035	5.0019	5.2605	6.2181	6.5702	6.1236	5.4289	5.6344	6.6437	6.3973
Map1b	_SDVLETVVLINPSDEAVSTEVR_.2	#N/A	5.6510	4.9859	4.9447	5.5624	3.9669	5.2067	3.8637	5.2288	4.6677	5.1336	4.7219	5.3149	5.4043	5.3848	4.3773	5.3402	5.0663	5.4548	4.8181	5.2014
Map1b	_ESVVS[Phospho (STY)]GDDRAEEDM[Oxidation (M)]DDVLEK_.3	#N/A	3.9615	4.0413	4.4978	6.5110	3.2992	7.4137	4.2418	3.6619	6.5999	6.4079	3.1745	5.6691	4.4090	7.1426	6.4430	4.3064	4.1996	7.1152	7.0530	2.9511
Map1b	_AEEDM[Oxidation (M)]DDVLEKGEAEQS[Phospho (STY)]EEEGEEEDKAEDAR_.4	#N/A	3.7567	5.6389	5.3852	4.5396	3.7480	5.8132	5.0958	4.3171	4.8881	4.7971	5.3963	3.9695	3.9836	4.6653	3.2837	4.6332	3.9948	4.3291	4.5869	4.0283
Map1b	_PMSIS[Phospho (STY)]PPDFS[Phospho (STY)]PK_.2	P14873_S1616_M2_Map1b	4.1309	5.0236	6.8249	3.4133	3.6764	3.7097	5.9210	4.0039	4.1588	3.9415	3.6375	3.8746	4.3243	7.6616	5.1675	3.4930	3.8802	3.8783	3.8070	3.2931
Map1b	_QGFPDRES[Phospho (STY)]PVSDLTSTGLYQDKQEEK_.4	#N/A	3.2287	3.7338	4.0405	4.4100	3.7817	3.9327	5.5441	4.0169	3.5330	4.0979	3.5337	3.5223	3.6696	4.0580	3.3704	3.8592	4.0485	4.2859	3.6003	3.8580
Map1b	_AETEEAEEPEEDGEDNASGSASK_.2	#N/A	3.7598	2.2128	4.6280	1.6817	3.8470	4.5175	3.4778	4.1663	3.8634	4.2663	4.2378	3.1046	3.7866	1.6016	2.9798	3.8681	4.3330	3.4859	3.8534	3.1925
Map1b	_LGGDVS[Phospho (STY)]PTQIDVSQFGSFKEDTK_.3	#N/A	3.4199	5.7498	3.9562	4.1654	4.2585	2.9817	3.2826	3.6445	3.3417	3.6263	3.7160	2.9715	3.4497	5.6689	2.8598	3.7648	3.1418	4.0077	4.3067	4.1716
Map1b	_DYNASASTIS[Phospho (STY)]PPSSMEEDKFSK_.3	#N/A	3.6233	5.3110	4.3464	5.6947	3.5094	3.4484	3.7470	3.8881	3.6934	3.6217	4.9559	3.7419	3.8170	4.1329	3.5879	4.2805	3.6727	3.8251	3.8987	3.9069
Map1b	_DVM[Oxidation (M)]SDETNNEETES[Phospho (STY)]PSQEFVNITK_.3	#N/A	9.2781	9.7361	8.8931	8.8041	7.8392	8.9893	9.0544	8.6184	9.2413	9.2072	8.4682	9.1451	9.7418	9.6239	8.6651	9.3041	9.2268	9.3801	9.3472	9.1058
Map1b	_IAELEEERS[Phospho (STY)]QGSTSNSDWM[Oxidation (M)]K_.3	#N/A	7.5001	7.5534	8.2160	7.9609	6.5990	7.7573	7.1211	6.8541	7.2197	7.7880	7.3045	7.1683	7.7956	8.1541	7.1147	7.5413	7.5377	7.4742	7.4540	7.7518
Map1b	_SSM[Oxidation (M)]LFDTM[Oxidation (M)]QHHLALNR_.3	#N/A	6.0086	5.1532	3.7118	4.5139	4.0141	4.8719	4.9891	6.3148	3.5861	3.3818	3.9605	4.2237	3.6942	3.2379	3.1042	3.5204	6.0865	4.9089	5.2959	4.4160
Map1b	_ASAELDIKDVS[Phospho (STY)]DER_.2	#N/A	3.3803	6.4905	6.0852	6.2704	3.5873	6.6006	5.8701	3.6060	5.6306	6.0379	4.3605	5.5186	3.5465	6.3741	3.4862	6.0094	5.1145	6.6365	5.9938	5.9973
Map1b	_GEAEQS[Phospho (STY)]EEEGEEEDK_.2	#N/A	3.1969	3.3051	6.3829	4.4925	4.0354	5.8246	3.5057	4.3981	3.0521	3.4032	4.0316	3.1946	3.2176	2.6281	3.0829	3.5417	4.3349	4.3983	4.0468	3.6872
Map1b	_TAKS[Phospho (STY)]RT[Phospho (STY)]PVQDHR_.3	#N/A	4.1859	3.8169	3.2214	3.5034	3.5237	4.4613	4.0174	3.8863	4.0412	4.0316	3.5199	3.7063	4.2067	3.2085	3.5946	3.0300	4.4240	3.4092	3.0577	2.3035
Map1b	_ASAELDIKDVS[Phospho (STY)]DERLSPAK_.3	#N/A	4.2162	3.4778	3.4708	3.4763	3.9707	4.4944	4.0104	4.2465	4.0989	3.4740	4.0092	3.3612	4.2414	4.8879	3.1450	4.0309	3.4250	3.3450	4.0652	3.3154
Map1b	_DENERAS[Phospho (STY)]LS[Phospho (STY)]PM[Oxidation (M)]DEPVPDSESPVEK_.3	P14873_S1371_M2_Map1b	3.8463	3.4728	3.3829	3.7379	4.0532	4.0866	2.9949	4.2610	4.4159	3.6569	3.8945	3.1989	4.5814	3.5832	3.0202	3.2084	3.2979	3.0345	4.1839	3.5502
Map1b	_ELEAERS[Phospho (STY)]LM[Oxidation (M)]S[Phospho (STY)]S[Phospho (STY)]PEDLTK_.2	P14873_S825_M3_Map1b	3.7332	9.5428	4.2695	3.9562	3.0709	8.4864	9.2893	4.3335	3.0284	3.9395	3.4028	4.1591	3.1365	10.0890	4.0474	4.0781	3.9712	9.5610	3.5105	4.2235
Map1b	_TDATDGKDYNASASTIS[Phospho (STY)]PPSSM[Oxidation (M)]EEDKFSK_.4	#N/A	4.0520	3.5553	3.9534	3.6005	4.1162	4.2884	4.1040	4.0070	4.0525	3.7160	3.7718	3.3354	4.2060	6.2884	2.9035	3.5034	3.1826	3.4764	4.2911	3.7450
Map1b	QDDLNIM[Oxidation (M)]VLASSSTVVM[Oxidation (M)]QDESFPAC[Carbamidomethyl (C)]K .3	#N/A	3.3428	3.1592	3.8791	3.4466	4.1813	4.4054	3.3597	3.7217	4.1888	3.5491	3.7932	3.0486	3.5269	4.5715	4.1095	5.0673	3.2189	4.2373	1.8440	4.2488
Map1b	_SEQSSM[Oxidation (M)]SIEFGQES[Phospho (STY)]PEHSLAMDFSR_.3	#N/A	3.4731	3.4687	3.5047	4.2222	3.9727	4.2181	3.5477	4.1798	3.6067	3.3618	3.9811	3.2706	3.7148	4.4475	3.1243	3.3204	3.4296	4.1034	4.0358	3.3635
Map1b	_KIAELEERS[Phospho (STY)]QGSTSNSDWM[Oxidation (M)]K_.3	#N/A	3.6320	3.6831	3.8240	4.1982	3.2704	3.3412	4.1799	4.1382	3.5717	3.6547	3.9460	3.8986	3.5461	4.9324	3.4189	3.6372	3.9317	4.4809	3.7413	3.7265
Map1b	_RES[Phospho (STY)]VVS[Phospho (STY)]GDDRAEEDMDDVLEK_.3	#N/A	3.1764	2.8576	3.7127	3.6457	4.0150	3.5405	3.9694	3.8900	3.5871	3.3828	3.9615	2.9357	3.6952	3.8105	2.7207	3.5213	3.4442	3.0928	3.8685	3.8278
Map1b	_DIKPQLEIIEDEEK_.3	#N/A	4.4222	3.5806	3.4576	3.2672	3.7599	3.5202	4.3572	4.1225	4.2774	3.7954	3.7561	3.4701	4.4429	3.4447	2.8818	3.3469	3.1594	3.1729	3.2158	3.4117
Map1b	_NLISPDLGVVFLNVPENLKDPEPNIK_.3	#N/A	4.5170	4.1128	4.4263	3.7994	3.2231	3.2564	3.8250	3.5903	3.7452	4.3276	3.2239	4.0023	3.9107	4.4133	3.8504	3.4790	4.1280	3.7051	3.3537	4.3803
Map1b	_AQWGSNM[Oxidation (M)]QVTLIPTHDSEVM[Oxidation (M)]R_.3	#N/A	6.5014	4.2742	4.2649	3.9608	3.0663	7.2629	4.4748	3.4290	7.1650	3.9349	3.4074	4.1637	3.1411	4.2520	7.0703	4.0735	6.8527	3.8665	7.2906	4.2190
Map1b	_EEC[Carbamidomethyl (C)]PRPMSIS[Phospho (STY)]PPDFSPK_.3	P14873_S1616_M1_Map1b	4.1234	3.1957	3.6600	3.4608	4.3303	4.3637	4.2186	4.3491	4.1388	3.9340	3.6175	4.4227	4.3043	3.6980	4.2440	3.4855	3.8463	3.3031	3.6022	3.2731
Map1b	_TLEVVSPSQSVTGSAGHTPYYQSPTDEK_.3	#N/A	3.4712	4.5316	4.0075	4.2182	4.3098	2.7657	3.2313	3.1716	3.3265	3.6775	4.3060	4.4210	3.4919	3.9946	2.8085	3.8161	3.7093	4.1239	3.7725	3.9616
Map1b	_SDISPLTPRES[Phospho (STY)]S[Phospho (STY)]PLYSPGFSDSTSAAK_.3	#N/A	3.0988	4.2203	4.1362	4.4854	3.3057	3.3391	3.7424	3.5077	3.6626	4.4102	3.1412	3.9464	3.8280	4.3307	3.7677	3.9618	4.3226	3.7878	4.3364	4.2977
Map1b	_HSPTEDDES[Phospho (STY)]AKAEADVHLK_.3	P14873_S974_M1_Map1b	3.1135	3.3884	3.6498	4.5759	3.9521	4.1762	3.5890	4.3147	3.6481	3.3198	4.0225	3.2779	3.7561	3.6369	3.1662	3.4584	3.3516	2.9807	4.1302	2.9772
Glul	_DIVEAHYR_.2	#N/A	10.8556	11.1560	11.2335	11.0474	11.0546	11.0786	11.1303	11.1115	11.1918	11.1569	10.8939	10.8311	11.0313	11.0139	11.2157	11.0260	10.7159	10.8043	10.9560	10.9615
Glul	_[Acetyl (Protein N-term)]ATSASSHLNK_.2	#N/A	10.6609	10.6430	10.8585	10.8452	10.5950	10.5368	10.8336	10.8005	10.8789	10.6285	10.1399	10.5697	10.7066	10.6049	10.9750	10.3956	10.3813	10.4866	10.4591	10.4467
Glul	_KPAETNLR_.2	#N/A	10.3415	10.4059	10.7514	10.4462	10.3854	10.3993	10.5023	10.4226	10.4438	10.4703	10.4197	10.1607	10.1858	10.4204	10.7751	10.2682	10.1655	10.1720	10.2623	10.3469
Glul	_TC[Carbamidomethyl (C)]LLNETGDEPFQYKN_.2	#N/A	10.5494	10.5837	10.6117	10.6681	10.3617	10.2875	10.3940	10.5988	10.6156	10.6118	10.2913	10.1229	10.7064	10.5655	10.3814	10.3518	10.6629	10.0999	10.3484	10.3542
Glul	_LVLC[Carbamidomethyl (C)]EVFK_.2	#N/A	7.3528	8.1506	8.6346	8.4970	8.0130	8.0531	7.9674	7.0010	8.2656	8.2842	7.8978	7.8787	7.2052	7.8377	8.0248	7.8750	7.2157	7.8175	8.0442	8.0669
Glul	_TLDC[Carbamidomethyl (C)]EPK_.2	#N/A	7.3505	7.5919	7.8125	7.8318	7.7378	7.5497	8.2132	7.0194	7.8592	7.9479	7.0164	7.7403	7.6404	7.1959	8.2048	7.3710	6.9245	7.7522	7.6741	7.8349
Glul	_TC[Carbamidomethyl (C)]LLNETGDEPFQYK_.2	#N/A	8.0564	7.8347	8.3008	7.8533	7.5308	7.8047	7.7216	7.7864	8.1248	7.9695	7.6633	7.1965	7.2526	7.9950	7.6699	7.4571	7.8519	7.2101	7.2499	7.7890
Glul	_KGYFEDR_.2	#N/A	7.5997	7.9099	7.8514	7.7592	7.6417	7.4948	7.6135	7.9775	7.9169	7.4917	7.5702	7.5769	7.7278	7.4863	7.5066	7.2990	7.6401	7.4861	7.0882	7.7245
Glul	_AC[Carbamidomethyl (C)]LYAGVK_.2	#N/A	5.3823	7.2438	6.8891	6.9830	6.7389	7.1604	7.5460	5.8120	7.3443	6.8999	6.9472	6.9819	6.7675	7.1420	7.4967	7.2133	5.5756	6.9584	7.3578	7.3083
Glul	_C[Carbamidomethyl (C)]IEEAIDK_.2	#N/A	6.2224	6.2652	6.4005	6.8437	5.5634	6.1194	6.8010	4.8998	7.1632	7.0362	6.0486	5.9335	6.1437	6.1904	7.3179	6.2457	5.3151	6.2850	6.5519	6.7348
Glul	_M[Oxidation (M)]GDHLWIAR_.2	#N/A	8.5309	8.2499	7.7667	7.5339	8.3813	8.0708	8.0689	8.5380	7.9229	7.9170	7.5371	8.0682	8.3501	7.7804	7.4189	7.5710	7.7333	7.7372	7.5766	7.8052
Glul	_VQAM[Oxidation (M)]YIWVDGTGEGLR_.2	#N/A	8.2365	7.9692	8.4583	8.2914	7.9564	7.9037	7.7850	8.0214	8.1024	8.4472	7.6976	7.5174	8.0874	7.7426	7.6932	7.8167	8.1841	7.4525	7.8200	8.0100
Glul	_ITGTNAEVM[Oxidation (M)]PAQWEFQIGPC[Carbamidomethyl (C)]EGIR_.3	#N/A	7.7820	8.1820	7.8166	8.1470	7.5602	7.6348	7.9447	8.0930	7.8408	8.9342	7.5519	7.6297	7.8093	8.3130	7.7823	8.0477	7.9158	7.6799	8.5684	7.7218
Glul	_QM[Oxidation (M)]YM[Oxidation (M)]SLPQGEK_.2	#N/A	8.0772	7.6571	6.8851	8.0116	8.1011	8.5267	7.6213	9.0709	8.9037	8.7728	7.3664	8.0349	8.9090	8.6199	8.0568	8.1402	8.4786	8.1065	7.8855	7.7291
Glul	_Y[Phospho (STY)]NRKPAETNLR_.3	#N/A	4.1987	4.5675	3.6403	4.3844	4.2849	4.6871	4.8846	4.5543	4.5673	4.6712	3.7815	3.6512	4.9599	3.8583	4.4981	3.8084	3.9864	4.3134	4.5443	4.4554
Glul	_RPSANC[Carbamidomethyl (C)]DPYAVTEAIVR_.3	#N/A	6.2677	6.6864	7.2685	7.0338	6.0656	6.9192	7.0779	6.1400	6.8831	6.4935	6.1854	5.9662	5.8830	7.0025	6.9502	6.4185	6.1890	6.8140	6.8782	6.9711
Glul	_AMREENGLK_.2	#N/A	4.2825	4.5375	4.1292	3.6774	2.9886	3.0219	3.6992	3.8249	0.8201	4.0931	4.0082	4.2636	4.1452	3.8097	2.9930	3.6446	1.6947	3.4706	3.1192	3.1140
Glul	_C[Carbamidomethyl (C)]IEEAIDKLSK_.2	#N/A	5.9795	5.9832	5.3444	6.2818	6.2445	5.8112	5.7912	6.2380	6.3068	5.9041	5.1727	6.0421	6.6343	5.9292	6.4797	5.8025	5.7037	5.5877	6.1045	5.8376
Glul	_RLTGFHETSNINDFSAGVANR_.4	#N/A	6.8105	3.7461	5.4701	7.1021	6.8949	5.9706	5.5187	6.6553	4.0057	6.6550	6.7589	5.8123	6.5785	3.6575	5.1432	6.5884	6.6026	3.4180	5.4546	5.5520
Glul	_LTGFHETSNINDFSAGVANR_.3	#N/A	3.4241	3.1753	3.8638	4.3627	4.4264	3.8197	3.7438	3.9487	3.6986	3.3900	4.1622	3.0648	3.3482	3.8508	2.9144	4.0733	3.7428	4.2685	3.9170	3.9308
Glul	_VC[Carbamidomethyl (C)]EDFGVIATFDPK_.2	#N/A	4.5117	4.3911	3.5472	3.7563	3.7539	4.0735	3.6916	4.0536	4.9404	3.2172	4.1251	3.3805	3.8588	3.4025	3.2688	3.3558	3.5508	3.9063	3.8977	3.0798
Cdh2	_SAAPHPGDGDFINEGLK_.3	#N/A	3.6018	4.6662	4.1381	4.0876	4.2594	3.5886	3.5511	4.5496	3.4571	3.8082	4.3603	4.2643	4.7388	3.9256	3.2739	3.9467	3.3543	4.2560	4.5818	4.0421
Ntrk2	_IWC[Carbamidomethyl (C)]TEPSPGIVAFPR_.2	#N/A	5.7661	6.0802	5.9007	5.9207	5.0207	5.8414	5.6118	6.0828	5.4295	5.3371	5.9733	5.4547	5.4807	5.5808	5.4167	5.1949	4.9822	5.9767	5.8594	5.3161
Prph	_LLGSGS[Phospho (STY)]PSSSAR_.2	P15331_S57_M1_Prph	3.1610	4.2577	4.2814	3.9443	5.1362	3.4012	3.6802	3.4455	3.6004	4.4724	3.0790	4.1471	3.7659	4.2685	3.7056	4.0239	3.9832	3.8500	3.4986	4.2355
Kcnc1	_LAWLAEPDAHS[Phospho (STY)]HFDYDPR_.3	P15388_S44_M1_Kcnc1	4.8035	7.0907	4.5011	3.4074	3.6197	3.8438	3.9213	3.9824	5.0541	5.1688	4.3548	3.6102	4.0885	6.5483	3.4985	3.1261	4.5201	6.1856	2.9617	3.2716
Kcnc1	_HIPRPPQLGS[Phospho (STY)]PNYC[Carbamidomethyl (C)]K_.3	P15388_S468_M1_Kcnc1	7.2564	3.9234	4.4331	4.1886	3.6026	3.6359	3.4455	3.6213	3.3657	3.2062	4.3452	3.6496	3.5312	4.0338	3.4709	4.2586	3.1186	4.0847	3.7332	4.0008
Sptb	_ILS[Phospho (STY)]PVDSGNK_.2	P15508_S1218_M1_Sptb	6.7259	6.4277	5.9878	5.9992	6.3718	6.2719	6.2828	6.1790	6.0106	6.0023	5.9974	6.2004	6.3968	5.4179	2.8069	5.3828	6.6170	6.2612	3.7709	5.4280
Sptb	_LLTS[Phospho (STY)]PDVSYDEAR_.2	P15508_S1289_M1_Sptb	3.8628	6.3562	4.3991	3.8266	3.2005	6.0248	5.7421	3.5632	3.7181	4.0691	5.6146	5.1358	6.0777	4.3862	4.8739	5.5643	6.5298	6.2011	3.3809	4.3532
Nme1	_NIIHGSDSVK_.2	#N/A	7.4780	7.3084	4.4462	6.6153	7.4105	7.6168	7.3884	7.9066	7.5130	7.6148	7.4491	7.5804	7.7898	7.8730	7.9329	7.5753	7.5126	7.5306	7.5736	7.6950
Nme1	_FLQASEDLLK_.2	#N/A	7.7589	7.7464	7.8851	8.0115	7.7412	8.0656	7.9194	7.8702	7.7546	7.8979	8.1378	7.9056	7.3935	8.1839	8.2690	7.9751	7.7524	8.0179	8.0698	8.3307
Nme1	_YM[Oxidation (M)]HSGPVVAM[Oxidation (M)]VWEGLNVVK_.3	#N/A	8.1706	8.4401	7.9238	7.8979	8.2456	8.1184	7.5378	8.2588	7.9074	8.3019	7.9639	8.0273	7.9854	7.7205	8.0013	8.2716	8.1569	8.1853	8.5015	8.0036
Nme1	_FLQASEDLLKEHYTDLK_.3	#N/A	7.1854	7.5541	7.0076	6.8676	7.2994	7.4161	7.0791	7.5723	6.9935	6.7241	6.9718	7.0137	7.3159	7.4559	7.6141	6.7987	7.1826	7.1481	7.7104	7.6076
Nme1	_EISLWFQPEELVEYK_.2	#N/A	6.4157	6.4808	6.6316	6.8898	5.8751	6.5819	6.7384	6.0189	6.6255	6.5140	6.3004	6.1684	6.6073	6.9778	6.2378	6.6862	6.5993	5.9321	6.4803	6.7535
Nme1	_SC[Carbamidomethyl (C)]AQNWIYE_.2	#N/A	3.6834	3.3824	4.5351	4.3483	3.3365	4.6747	4.3855	3.6992	4.9311	5.0484	3.1372	4.5496	4.3717	4.5222	4.7703	4.3838	4.3195	4.1428	4.5818	3.5928
H1-2	_[Acetyl (Protein N-term)]S[Phospho (STY)]EAAPAAPAAPPAEK_.2	#N/A	10.5523	9.9459	10.2815	10.4894	9.7448	10.0998	9.9283	10.1439	10.5551	10.0387	10.0865	9.8123	10.4588	10.3864	10.0604	10.0082	10.7468	10.0951	9.8396	10.2318
H1-2	_ALAAAGYDVEK_.2	#N/A	7.8401	8.2677	8.1547	7.7564	7.3410	7.9124	8.3297	7.8252	8.3635	7.4711	7.5530	7.4612	7.9810	7.9663	8.0968	7.5667	7.6530	8.1206	7.3929	7.6276
H1-2	_GTGASGSFK_.2	#N/A	6.1840	6.5173	5.9457	6.3222	5.4204	6.3547	6.7665	5.8280	7.0842	5.9268	6.1542	5.7447	6.5404	6.7133	6.6560	6.5672	5.7530	6.0577	6.0924	6.2508
H1-2	_ASGPPVSELITK_.2	#N/A	5.9634	3.3236	5.7235	4.5110	5.7917	6.1344	6.7249	5.9218	6.3076	6.0975	6.0858	5.6697	6.5530	6.4734	5.5507	6.0359	5.7580	6.4755	5.4570	5.7180
H1-2	_[Acetyl (Protein N-term)]S[Phospho (STY)]EAAPAAPAAPPAEKAPAK_.2	#N/A	5.8937	6.4177	4.0674	5.4559	4.5677	4.9129	5.5534	6.3393	5.6834	5.8189	4.4330	5.8812	7.2980	5.7131	5.6521	5.8731	6.1697	5.5923	5.6988	5.2061
H1-2	_ALAAAGYDVEKNNSR_.2	#N/A	3.3991	3.9644	3.7477	4.0690	3.6060	3.6045	3.1030	3.6248	2.9796	3.2387	3.6928	3.6905	3.2268	4.0125	3.7091	2.9235	2.9329	4.3116	3.5991	2.9431
Atp6v0a2	_KDS[Phospho (STY)]EEEVSLLGNQDIEEGNSR_.3	P15920_S695_M1_Atp6v0a2	4.1150	3.2041	3.6516	3.4692	4.3220	3.7864	4.2270	3.4407	4.0378	4.4309	2.9114	2.9302	4.1459	3.6897	4.2524	3.4771	3.8379	5.4755	3.6106	3.3670
Chgb	_IYHS[Phospho (STY)]EER_.2	P16014_S155_M1_Chgb	11.3315	11.7882	11.3773	11.4500	11.3687	11.5685	11.4177	11.5791	11.4288	11.7177	11.1903	11.7133	12.1524	12.0183	12.6141	12.0794	11.6508	11.7149	11.9569	11.6995
Chgb	_WASS[Phospho (STY)]REDAGAPVEDSQGQTK_.3	#N/A	3.9507	8.7355	8.3611	5.1462	8.1341	8.6171	4.6138	7.8612	8.7608	9.0734	8.7334	8.5393	9.4108	9.0248	9.3806	9.1308	5.4907	8.5196	9.2704	3.6198
Chgb	_SSYEGHPLS[Phospho (STY)]EER_.2	#N/A	9.1813	9.4849	9.6018	9.5631	9.2401	9.3579	8.9224	9.3975	9.3778	9.3491	9.1286	9.0934	9.2588	9.8014	9.6052	9.4770	9.3748	9.0436	9.4596	9.5612
Chgb	_WWQQEEQLGPEES[Phospho (STY)]REEVRFPDR_.4	#N/A	6.1551	6.7388	7.1655	6.4982	5.5717	5.9737	6.4387	6.1184	7.1174	6.8472	6.2678	6.7617	6.4683	7.7402	7.7721	6.7521	6.0298	7.4497	7.3385	6.6716
Chgb	_ES[Phospho (STY)]KEADVATVR_.2	P16014_S318_M1_Chgb	7.2626	7.2529	7.9766	7.7346	7.3713	7.7545	7.2664	7.3744	7.8319	7.7667	7.3745	7.3811	7.8569	8.0361	7.6682	7.5620	7.5095	7.3807	7.5600	7.4805
Chgb	_EGVDDQESLRPSNQQAS[Phospho (STY)]KEAK_.3	#N/A	4.0898	5.7726	3.6264	3.5185	3.6417	4.8868	5.3366	4.3155	5.2289	4.7405	4.9382	3.8253	5.5005	5.6355	5.4061	5.2550	5.2639	5.4965	5.2322	5.4540
Chgb	_AS[Phospho (STY)]EEEPEYGEESR_.2	#N/A	9.9605	9.7112	9.6329	9.6655	9.8213	9.6013	9.1979	9.8030	9.5895	9.4179	10.0436	10.3335	10.0999	10.9140	10.4319	10.6860	10.3960	10.0217	9.9115	10.5266
Chgb	_WTEGGGHS[Phospho (STY)]R_.2	#N/A	10.0971	10.1325	10.2307	10.2312	9.7764	10.1436	9.7263	9.9350	10.0777	10.2806	10.0188	9.9244	9.8908	10.4748	10.4812	10.2524	10.1735	9.9126	10.3373	10.0785
Chgb	_EGVDDQESLRPSNQQAS[Phospho (STY)]K_.3	#N/A	4.0340	3.9688	4.5703	3.5132	3.3717	3.5958	4.1693	4.5313	4.2284	5.1542	4.9444	3.8582	4.3365	3.8803	5.1265	4.3709	4.0285	4.5740	3.4200	5.3941
Chgb	_KHIEDS[Phospho (STY)]GEKPNTFSNK_.3	#N/A	4.2097	4.6102	4.8873	3.4922	2.9158	4.4500	4.1323	3.8976	4.3923	5.0560	3.5311	4.3363	4.9170	5.3096	5.3389	4.0840	4.5803	3.3979	5.1730	4.5499
Chgb	_WWQQEEQLGPEES[Phospho (STY)]REEVR_.3	#N/A	3.1142	5.8415	5.3133	3.8597	3.9528	5.0873	5.4371	3.9502	4.8772	3.3206	4.0217	3.2772	3.7554	6.5411	5.5448	3.4591	3.4475	5.0677	5.9122	5.3325
Chgb	_SGSNKSS[Phospho (STY)]YEGHPLSEER_.3	P16014_S301_M1_Chgb	3.5126	4.0882	5.0309	4.1937	3.3803	3.2181	5.3995	3.5185	3.5854	4.1691	3.3504	5.3824	3.7365	5.4523	3.7127	4.9292	4.3607	3.8730	3.2356	3.8609
Chgb	_S[Phospho (STY)]HHLAHYRASEEEPEYGEESR_.4	P16014_S333_M1_Chgb	3.8284	3.6399	3.4461	3.7461	7.6509	7.0946	3.8583	7.1728	5.0922	3.5388	7.9098	3.3873	8.6517	8.2516	3.0258	3.4614	3.3064	3.6036	4.1608	6.8238
Chgb	_GRGS[Phospho (STY)]EEDRAPR_.2	#N/A	4.3622	5.0610	4.1516	5.2773	5.0822	5.5654	4.9939	5.0165	4.3845	4.7384	7.6684	4.9821	5.0932	5.4890	6.1481	5.7081	5.2274	5.3505	5.7761	5.2991
Chgb	_EDAGAPVEDSQGQTK_.2	#N/A	3.7955	4.2073	4.3318	3.8939	3.1332	5.1917	7.9515	3.4959	4.0325	7.8099	7.8624	4.0967	3.8162	4.3189	4.9268	5.2849	4.0336	3.7996	5.0821	4.2859
Chgb	_WTEGGGHS[Phospho (STY)]REGVDDQESLRPSNQQASK_.4	#N/A	7.7035	8.3061	7.6586	8.3550	6.8927	8.0875	7.6931	6.8703	8.1132	7.5459	7.3781	8.1988	8.3178	8.0841	7.3847	8.5859	3.6667	8.1421	8.0925	8.0104
Chgb	_SSYEGHPLS[Phospho (STY)]EERRPSPK_.3	#N/A	7.0963	4.1902	7.0814	3.8768	6.7576	7.4180	4.3908	3.5129	4.4449	4.0189	7.0438	4.0797	8.0862	7.9006	7.9637	7.9014	7.7975	7.7716	3.4311	4.3029
Chgb	_ADAHS[Phospho (STY)]M[Oxidation (M)]ELEEK_.2	#N/A	9.7173	9.6413	9.7414	9.7863	9.2266	9.6913	9.5075	9.4260	9.3841	8.7583	9.2454	9.4368	9.4499	9.9475	9.4013	8.3443	9.5816	9.2896	9.3017	9.2492
Chgb	_SEES[Phospho (STY)]QEREYKR_.3	P16014_S378_M1_Chgb	3.8792	4.1236	4.4155	3.8102	3.2169	3.4410	4.3242	3.5796	4.3832	4.0855	3.2568	4.0130	4.4913	4.4026	3.9013	4.2241	4.1173	3.7159	3.3645	3.7123
Chgb	_ADAHS[Phospho (STY)]MELEEKTHS[Phospho (STY)]R_.2	P16014_S220_M2_Chgb	4.2283	3.2078	3.6479	3.4729	3.5661	3.7901	3.9750	4.3370	4.0341	4.4347	2.9076	2.9339	4.1422	3.6859	3.5522	3.0724	3.8342	3.3152	3.6143	3.3632
Chgb	_HIEDS[Phospho (STY)]GEKPNTFSNK_.3	#N/A	3.7502	4.2527	4.2865	3.9392	3.0879	3.3120	4.4532	3.4505	3.0114	3.9565	3.3858	4.1421	3.1195	4.2735	4.0304	4.0951	3.9882	3.8450	3.4935	4.7626
Chgb	_ADEFPDFY[Phospho (STY)]DS[Phospho (STY)]EEQM[Oxidation (M)]GPHQEANDEK_.3	P16014_S626_M2_Chgb	3.3728	3.9463	4.4102	4.2114	3.5797	3.6131	3.4684	3.2337	3.3886	3.1834	4.3680	3.6724	3.5540	4.0567	3.4937	4.2358	3.7713	4.0618	3.7104	4.0237
Prkce	_LAAGAESPQPASGNS[Phospho (STY)]PSEDDR_.2	#N/A	10.3694	10.2221	10.5218	10.3713	9.8974	10.2444	10.0324	10.1855	9.9886	10.2390	10.0585	9.8186	9.9611	10.4735	9.1091	9.7577	10.3384	10.0495	9.9690	9.8532
Prkce	_SAPTS[Phospho (STY)]PC[Carbamidomethyl (C)]DQELK_.2	#N/A	9.4665	3.7772	9.6702	10.3209	9.2290	10.4584	10.0593	3.5053	3.5221	4.0512	9.9365	9.9882	3.6302	9.3625	9.0332	8.2325	11.0562	10.1669	9.5000	3.8433
Prkce	_ALS[Phospho (STY)]FDNRGEEHR_.3	#N/A	9.2650	9.2704	9.4627	9.3553	8.5270	8.8382	8.9118	8.8430	8.6961	9.3979	8.8606	8.7414	9.5866	9.1974	8.2098	8.8935	8.8608	9.0032	9.0504	8.9424
Prkce	_KALS[Phospho (STY)]FDNR_.2	#N/A	9.5563	9.7549	9.8118	9.5382	9.1098	9.4431	9.2718	9.0287	9.0634	9.6370	9.4413	9.0376	9.3961	9.8178	7.9325	9.0523	9.3126	9.3049	9.2678	8.8795
Prkce	_ASSATDGQLAS[Phospho (STY)]PGENGEVRPGQAK_.3	#N/A	9.2630	9.4680	8.8666	9.0899	8.1725	8.8497	9.1986	9.1392	8.6490	8.8604	8.6067	8.9816	9.1798	9.7710	8.1898	8.9555	9.1479	9.2245	9.1000	8.4458
Prkce	_KQET[Phospho (STY)]PDEVGSQR_.2	P16054_T228_M1_Prkce	7.8801	7.8953	7.2837	7.8128	3.9541	7.8117	7.7312	7.8400	7.6791	7.5621	7.5024	3.2981	8.3531	7.9768	7.1021	7.4520	7.7964	7.6841	7.6296	2.9991
Prkce	_SAPTS[Phospho (STY)]PC[Carbamidomethyl (C)]DQELKELENNIRK_.3	#N/A	3.7159	4.2907	4.2522	3.9773	3.0536	6.8349	6.9893	3.4163	7.1427	3.9222	3.4239	4.1801	4.0593	4.2393	6.9886	4.0608	3.9539	4.0330	7.0998	4.1239
Prkce	_ALS[Phospho (STY)]FDNR_.2	#N/A	7.9386	7.5409	7.6567	7.9634	3.2266	3.3809	7.5869	6.5757	7.3083	8.3815	7.8868	4.0255	7.1980	4.0952	6.3847	7.6458	7.1772	4.4069	7.7222	7.6077
Prkce	_SKS[Phospho (STY)]APTS[Phospho (STY)]PC[Carbamidomethyl (C)]DQELK_.2	#N/A	9.6124	9.3538	9.8474	9.7025	8.9022	9.2325	9.2573	9.3365	9.1447	9.4921	9.3278	7.0007	9.1425	9.8309	8.1108	8.8707	7.4300	9.3306	7.1560	9.1406
Prkce	_KLAAGAESPQPASGNS[Phospho (STY)]PSEDDR_.3	#N/A	5.8911	6.7512	5.9377	6.4593	6.4915	6.8022	6.4325	6.4428	6.4651	6.6012	6.4470	5.5377	5.7478	6.7504	5.6436	6.1648	6.3380	6.5709	6.6510	5.8208
Prkce	_LAAGAESPQPASGNS[Phospho (STY)]PSEDDRSK_.3	#N/A	7.7540	8.4847	7.4162	7.7777	7.0381	8.2686	8.0996	7.9353	7.4776	7.9770	7.7810	7.9792	8.6897	8.4076	7.7151	8.0193	8.1393	8.2891	8.0330	8.0139
Prkce	_KALS[Phospho (STY)]FDNRGEEHR_.3	#N/A	7.4856	7.8381	7.6670	7.2896	7.2014	7.1542	7.0604	7.3047	7.3570	7.2337	7.1678	6.7914	7.7572	7.3319	6.7024	7.2606	6.9979	7.2285	7.5108	7.1284
Prkce	_SAPTS[Phospho (STY)]PC[Carbamidomethyl (C)]DQELKELENNIR_.3	#N/A	3.9343	5.7503	3.8915	3.7394	3.8711	3.9949	3.9378	3.9835	3.9009	3.9656	3.2963	3.4636	4.0090	3.9195	3.8128	3.7136	3.8737	3.6008	3.7295	3.7518
Prkce	_ASSATDGQLAS[Phospho (STY)]PGENGEVRPGQAKR_.3	#N/A	3.9217	4.0811	4.4580	3.7677	3.2594	3.4835	4.2817	3.6221	4.3408	4.1280	3.2143	3.9706	4.4488	4.4450	3.8589	3.7851	4.1597	3.6734	3.3220	4.4120
Prkce	_DVILQDDDVDC[Carbamidomethyl (C)]TMTEK_.2	#N/A	4.3132	3.6896	4.5812	3.3762	3.6509	3.8750	3.8902	4.0136	3.9492	4.5195	4.3236	3.5791	4.0573	3.3357	4.3674	3.1572	4.5512	3.4001	4.4313	3.2784
Prkce	_SKS[Phospho (STY)]APTS[Phospho (STY)]PC[Carbamidomethyl (C)]DQELKELENNIR_.3	#N/A	3.3965	4.6063	3.9328	4.2929	4.2351	3.7499	3.3060	3.0969	3.2518	3.3202	4.2312	2.9949	3.4172	3.9199	2.8832	3.7414	3.6345	3.2021	3.8472	3.8869
Ldhb	_LIASVADDEAAVPNNK_.2	#N/A	11.8270	11.7758	12.0979	11.9787	11.5654	11.8241	11.5188	11.9725	11.8841	11.8500	11.6549	11.6844	12.0609	12.0778	11.4444	11.7916	12.1265	11.6070	11.7714	11.8474

Ldhb	_FIIPQIVK_.2	#N/A	10.1274	10.5445	10.2306	10.4987	10.4929	10.2923	10.5989	10.3198	9.9677	10.3908	10.1153	10.3526	10.4415	10.1696	10.1488	10.3176	10.1224	10.4986	10.4320	10.3946
Ldhb	_LKDDEVAQLR_.2	#N/A	10.0128	9.8408	10.4120	9.8411	9.6705	9.9551	9.9245	10.0524	10.0009	9.8841	9.8417	9.6432	9.8456	9.9256	9.9197	9.8078	9.9114	9.6262	9.7295	9.8277
Ldhb	_IVVVTAGVR_.2	#N/A	9.0892	9.9595	9.9090	9.7927	9.3703	9.5392	9.6086	9.0415	9.8058	9.6156	9.1668	9.4157	9.2439	9.7154	9.6772	9.7926	9.1564	9.6884	9.6295	9.5728
Ldhb	_SADTLWDIQK_.2	#N/A	9.6602	9.6579	9.7208	9.7186	9.4495	9.4795	9.4098	9.8603	9.6831	9.6160	9.4789	9.4143	9.8059	9.4601	9.4449	9.2955	9.5646	9.3294	9.4730	9.4183
Ldhb	_GLTSVINQK_.2	#N/A	8.7841	8.4596	8.9574	8.9996	9.0168	8.4748	8.5265	8.7934	8.9836	8.9413	8.9071	8.7218	8.8515	8.7698	9.0649	8.7713	8.2999	8.7664	8.9107	8.8267
Ldhb	_LKDDEVAQLRK_.3	#N/A	7.0677	7.3329	6.8508	6.6528	7.2581	7.1033	7.1935	7.4453	7.1996	7.3208	7.0654	7.2381	7.6159	6.9935	7.4552	7.2153	7.1288	7.1693	7.3696	7.3431
Ldhb	_SLADELALVDVLEDK_.2	#N/A	7.8235	7.3806	8.4941	8.1804	7.3661	7.8510	7.8551	7.4927	7.8282	7.9345	8.0252	7.8202	7.7253	7.6870	7.7097	7.6955	8.0502	7.4500	7.5237	8.0040
Ldhb	_EKLIASVADDEAAVPNNK_.3	#N/A	7.2207	6.9703	7.0786	7.2518	6.7168	6.7535	6.7843	6.9852	6.8850	7.1540	6.5092	6.6011	7.0685	6.9905	6.6165	6.5465	6.9776	6.8871	6.9580	6.9036
Ldhb	_KSADTLWDIQK_.2	#N/A	6.9459	6.4477	6.6449	6.2228	6.1141	6.0051	6.2576	6.5929	6.0676	6.3884	5.9204	6.5459	6.0972	6.8082	6.3301	6.4084	5.9083	6.2338	6.6196	6.4452
Ldhb	_M[Oxidation (M)]VVDSAYEVIK_.2	#N/A	9.1258	8.9025	9.0093	8.8893	8.8874	8.7345	8.8470	9.0278	8.9711	8.9589	8.6412	8.8730	10.1077	8.8209	8.6599	10.0435	8.9332	8.9133	8.9234	8.8296
Ldhb	_IHPVSTM[Oxidation (M)]VK_.2	#N/A	7.9602	8.0302	7.0171	7.6777	8.1826	7.9170	8.2969	8.1317	8.0468	7.9133	7.3675	8.1652	7.9488	7.4817	8.2501	7.8359	7.6479	8.2366	8.0818	7.6808
Ldhb	_IVADKDYSVTANSK_.2	#N/A	7.2968	7.4747	7.8072	7.7291	7.4045	7.3031	7.8078	7.2713	7.8853	7.8808	7.4881	7.2286	7.5586	7.5009	7.7939	7.6601	7.3823	7.5206	7.7736	7.6935
Ldhb	_SLADELALVDVLEDKLK_.2	#N/A	4.7803	4.9291	4.6519	4.0100	4.6894	4.3230	4.8484	4.8899	3.6385	4.5228	4.6620	4.6068	4.2385	4.9461	4.4900	4.4359	4.7059	4.6962	4.3362	4.4556
Ldhb	_ITVVGVGQVGM[Oxidation (M)]AC[Carbamidomethyl (C)]AISILGK_.2	#N/A	5.8624	5.9535	5.0559	5.2751	4.6111	5.2405	5.2239	5.5019	4.4545	5.1496	5.1644	4.9925	5.4336	5.8482	4.5993	5.1155	5.7895	5.5922	4.8832	5.1032
Ldhb	_GEM[Oxidation (M)]M[Oxidation (M)]DLQHGSLFLQTPK_.3	#N/A	4.9995	5.4749	5.6904	3.3991	5.3752	5.7582	4.2253	5.2863	6.2650	6.2230	4.9651	4.8742	6.5192	3.3128	6.2521	6.1436	5.0372	5.8884	5.7555	5.9222
Ldhb	_GM[Oxidation (M)]YGIENEVFLSLPC[Carbamidomethyl (C)]ILNAR_.3	#N/A	3.3990	4.6038	3.9353	4.1445	4.2376	4.4617	3.3035	3.6654	3.3626	3.6054	3.7369	2.9924	3.4706	4.5152	2.8807	3.7439	3.1627	3.9868	4.2858	4.1925
Srp14	_KSS[Phospho (STY)]VEGLEPAENK_.2	P16254_S45_M1_Srp14	3.3605	8.5586	8.6500	4.3289	4.1990	4.4231	7.9322	8.4509	3.4011	8.4938	8.4747	3.0309	8.8898	3.3838	8.0809	8.3737	3.5985	3.9482	8.6148	8.5690
Slc4a3	_ALPSVGLQSDQS[Phospho (STY)]PQR_.2	P16283_S198_M1_Slc4a3	11.4683	11.4266	11.1986	11.1254	10.1186	10.9298	11.0136	10.7971	11.0278	10.8208	10.8730	10.8546	11.2934	11.0782	9.5284	10.7541	11.4804	11.2406	10.9515	10.8182
Slc4a3	_SYS[Phospho (STY)]ERDFEFHR_.3	P16283_S67_M1_Slc4a3	3.2479	4.0712	4.2853	4.3363	3.4549	6.9445	3.5932	3.4736	3.5135	4.5594	2.9921	3.7973	3.6789	4.3234	3.6186	4.1109	4.4717	4.1786	2.9768	4.0007
Slc4a3	_FSIGS[Phospho (STY)]DEDDSPGLPVK_.2	#N/A	9.3911	9.9999	9.7641	9.4335	9.1637	9.8457	9.7559	9.5707	9.8464	9.4329	9.1187	9.4549	9.2896	10.4874	8.9311	9.3737	10.0574	10.2903	9.6145	9.5081
Slc4a3	_AKFSIGS[Phospho (STY)]DEDDSPGLPVK_.2	#N/A	3.7500	5.7754	3.7472	3.9918	4.2280	4.3567	3.8885	4.4232	3.8584	3.7225	3.7353	3.0144	4.0029	6.8508	4.7507	3.5635	3.6889	5.7276	4.1770	3.8623
Slc4a3	_SRPWS[Phospho (STY)]PSASYDLR_.2	P16283_S224_M1_Slc4a3	4.1944	3.1247	3.7310	3.3898	2.9005	4.3347	4.1476	6.5364	4.0678	4.0050	3.5464	4.3516	4.2253	3.7691	4.1730	3.5565	3.9173	6.3450	3.5312	3.4464
Slc4a3	_GS[Phospho (STY)]PSGLAPILR_.2	#N/A	3.1926	3.3093	3.7289	4.4968	4.0312	4.2553	3.5099	3.8718	3.5690	3.3990	3.9434	3.1988	3.6770	3.1353	3.0871	3.5375	3.3691	3.7804	4.0794	4.3989
Slc4a3	_IQGGRGS[Phospho (STY)]PSGLAPILR_.3	#N/A	4.3371	4.9519	3.8737	3.2471	3.0432	3.0765	4.0049	5.4235	3.9251	4.1477	3.4038	4.2090	4.0906	4.5932	4.0303	3.6992	4.0600	3.5252	3.1738	3.0594
Cnp	_NQWQLS[Phospho (STY)]ADDLKK_.2	#N/A	9.4619	9.6936	9.4303	9.3497	8.4183	9.4177	9.1940	9.2360	9.3894	9.0328	9.0163	9.1853	9.3219	9.6890	8.7133	9.0417	9.7915	9.3066	9.2047	9.1906
Cnp	_HFISGDEPK_.2	#N/A	8.6198	8.5504	8.7473	8.4004	8.1748	8.7184	8.4453	8.3275	8.4273	8.2330	8.4939	8.1528	7.9361	8.2791	8.2224	7.9895	8.6459	8.3279	8.1378	8.1976
Cnp	_KS[Phospho (STY)]HTFLPK_.2	#N/A	9.7901	10.0675	9.7311	9.7440	9.3583	9.7546	9.3452	10.0269	9.5325	9.6152	9.1855	9.5083	9.5626	9.5176	8.7519	9.0436	9.9260	9.2786	9.1852	9.1613
Cnp	_LDEDLAGYC[Carbamidomethyl (C)]R_.2	#N/A	9.4313	9.3096	9.5806	9.4638	9.0018	9.3193	9.1253	9.5538	9.3306	9.0488	9.0771	9.0267	9.2080	9.0455	9.0076	8.7270	9.2218	8.9773	9.0751	8.9227
Cnp	_ADFSEAYKR_.2	#N/A	9.1898	9.2417	9.2663	8.9609	8.9769	9.2175	9.1241	9.1400	9.2935	8.7996	8.9485	8.7993	8.9566	8.7069	8.7831	8.7763	8.7969	8.8791	8.7482	8.8106
Cnp	_LSISALFVTPK_.2	#N/A	8.3371	8.6651	8.6142	8.5825	8.5180	8.4618	8.3677	8.0494	8.5176	8.1863	8.2615	8.2355	8.3218	8.0804	7.9701	7.9983	8.2104	8.2812	8.2163	8.3138
Cnp	_AAGAEEYAQQEVVK_.2	#N/A	8.5496	8.3708	8.6131	8.5830	8.1972	8.5457	8.2800	8.4777	8.8092	8.1634	8.2356	8.1690	8.1878	8.2273	7.7424	8.1154	8.4585	7.7584	7.6430	8.3633
Cnp	_AAGAEEYAQQEVVKR_.3	#N/A	7.1503	7.4741	7.1216	7.0832	6.6503	6.9863	6.8337	6.6576	7.0313	6.5379	6.6402	6.8762	7.2374	7.1175	6.3744	6.5641	6.8379	6.8860	6.7614	6.7436
Cnp	_KAGQVFLEELGNHK_.3	#N/A	8.0204	7.8508	7.3396	7.7643	7.9332	7.9474	7.5296	7.9697	7.6457	7.7333	7.9036	7.5919	7.4681	7.2301	7.2191	7.6102	7.9736	7.7484	7.7933	8.0282
Cnp	_AIFTGYYGK_.2	#N/A	7.5844	7.7426	7.6392	7.9548	7.3664	7.6600	8.2308	7.6975	7.9544	7.7532	7.9456	7.4254	7.9448	7.1580	7.0246	6.8605	6.2272	8.1414	7.4663	7.7640
Cnp	_RPPGVLHC[Carbamidomethyl (C)]TTK_.3	#N/A	6.6075	7.3090	6.5749	7.2202	7.1007	7.8585	7.8095	6.0241	6.8900	6.4234	7.3888	7.3904	7.3240	5.7812	7.4817	7.2072	6.4022	7.6234	7.1491	6.9800
Cnp	_EKPELQFPFLQDEDTVATLHEC[Carbamidomethyl (C)]K_.4	#N/A	6.9661	7.6886	6.7416	6.9256	6.3830	7.1990	7.1033	6.8482	7.1730	6.2831	7.2182	6.9890	6.9392	7.2295	6.2924	6.4451	6.0243	6.8624	7.0216	6.9166
Cnp	_LDC[Carbamidomethyl (C)]AQLK_.2	#N/A	5.6745	5.7485	5.1128	5.6234	5.4106	5.1659	5.7057	5.1931	4.7305	5.5129	5.0571	4.8654	4.7947	3.6196	5.5194	5.2045	4.1816	5.1969	4.7258	4.9663
Cnp	_GKLY[Phospho (STY)]SLGK_.2	P16330_Y372_M1_Cnp	3.1799	4.1391	4.2174	6.5187	3.3869	3.4202	3.6612	3.4265	3.5814	4.4914	3.0601	6.4218	3.7469	4.2495	3.6866	4.0429	3.9642	6.1982	3.5175	4.2165
Cnp	_GGSQGEAVGELPR_.2	#N/A	10.8630	10.6496	10.8094	10.7564	10.3706	10.7004	10.5414	10.9671	10.7776	10.4826	10.5242	10.5111	10.8755	10.3921	10.1880	10.3035	10.7143	10.4186	10.2833	10.3828
Cnp	_VLVLDDTNHER_.2	#N/A	10.4126	10.1575	10.3022	9.9559	10.0056	10.2788	10.1186	10.4052	10.2170	9.6812	10.1103	9.7397	9.9650	9.7660	9.6334	9.7841	10.1359	9.7891	9.7476	9.7840
Cnp	_[Acetyl (Protein N-term)]M[Oxidation (M)]NTS[Phospho (STY)]FTRK_.2	#N/A	9.6653	9.4427	8.8303	9.5672	8.8502	7.3050	8.9123	10.0437	10.0872	8.9878	8.6500	9.6725	3.9980	4.0850	9.3698	8.8640	8.9356	9.2025	9.1690	9.2822
Cnp	_NQWQLSADDLK_.2	#N/A	7.9197	8.5688	8.1674	8.1496	7.1317	8.6357	8.1822	7.6856	8.1967	7.7828	7.5614	7.9669	7.6485	8.2756	7.5150	7.7207	8.1973	8.1996	7.9775	8.0392
Cnp	_EKNQWQLSADDLKK_.3	#N/A	6.7869	6.9864	7.2524	7.1532	5.9353	6.5209	7.0808	6.5677	6.9085	6.4733	6.2238	6.6680	6.5431	7.0717	6.5667	6.7342	6.6833	6.9354	6.9364	6.6883
Cnp	_S[Phospho (STY)]HTFLPK_.2	#N/A	4.3542	11.9389	11.7454	11.8182	11.2778	11.7695	11.2967	11.8582	11.7165	11.4648	11.3666	11.4686	11.8290	11.5164	10.6583	10.7411	11.8192	11.2613	10.7779	3.6062
Cnp	_RLDEDLAGYC[Carbamidomethyl (C)]R_.3	#N/A	7.7314	8.0692	7.9748	8.3381	7.8222	8.1702	8.0852	7.6742	8.3613	7.8336	8.1619	7.5571	7.8144	7.7888	7.9641	7.4588	7.6682	7.6702	7.6633	7.3967
Cnp	_AGQVFLEELGNHK_.2	#N/A	7.4527	7.0844	7.0751	7.4894	7.2070	7.2555	7.0400	7.7304	7.3974	6.8911	6.6071	6.8375	7.1855	6.5744	6.2945	6.3628	7.0068	6.9709	7.0270	6.7399
Cnp	_AHVTLGC[Carbamidomethyl (C)]AADVQPVQTGLDLLDILQQVK_.3	#N/A	4.0668	5.1817	3.4821	1.9563	3.6534	4.1430	3.5468	4.7598	3.0550	3.2234	4.4054	3.0396	3.8526	4.6578	2.8931	3.0104	4.7655	4.2606	3.9738	2.8811
Cnp	_EKLELVSYFGK_.2	#N/A	6.5183	6.7464	6.4865	6.7344	6.0764	6.5584	5.8416	6.5745	6.4361	6.2509	5.9657	5.7573	6.0998	5.9976	6.0432	6.0836	6.3712	6.1828	6.2059	6.3293
Cnp	_ADFSEAYK_.2	#N/A	3.2102	6.0552	4.2476	5.8691	4.8452	5.3177	6.4875	3.3963	6.7805	5.7711	6.0854	5.8746	5.9626	4.4496	6.0192	6.1234	5.9340	6.2018	5.4060	6.0278
Cnp	_SSETLRK_.2	#N/A	5.3266	4.0091	5.9106	4.0009	5.0803	5.0957	4.6582	3.3889	4.6521	5.3516	6.0914	4.6189	4.0048	4.2119	4.8478	4.1370	5.0323	4.9918	4.2545	5.6261
Cnp	_VLVLDDTNHERER_.3	#N/A	7.2041	8.0125	7.3576	7.5796	7.3097	7.2406	7.8447	7.6967	7.8634	7.4286	6.9971	7.9047	8.4334	7.7716	7.5820	7.9712	7.7445	7.8124	7.8083	7.6988
Cnp	_HFISGDEPKEK_.3	#N/A	6.3212	6.0638	5.6095	6.1405	6.1523	6.3115	6.3493	6.7377	6.5775	5.8447	5.8230	5.8856	5.8971	5.5625	5.9274	6.3523	6.2985	5.8530	5.7595	5.5651
Cnp	_M[Oxidation (M)]VSADAYK_.2	#N/A	4.9828	5.3375	4.9489	5.0695	4.6111	5.4604	5.5545	5.4191	5.7226	5.6773	4.7239	4.6883	5.9235	4.9370	5.4396	5.4067	4.9936	5.5311	4.7900	4.3247
Cnp	_LELVSYFGK_.2	#N/A	8.0492	8.5739	8.7167	8.7589	8.1058	8.5827	8.3634	7.9925	8.3761	8.1877	8.1121	8.3884	8.0209	8.1208	7.8279	8.0801	7.7822	8.3792	8.3410	8.0529
Cnp	_LDQLFEM[Oxidation (M)]ADQYQYQVVLVEPK_.3	#N/A	3.8240	4.1000	4.2619	4.6072	2.1820	3.4215	2.1765	4.0497	3.7468	4.2940	4.1212	2.9331	3.8549	3.3986	2.1293	3.1861	4.0007	2.9974	3.9016	3.4301
Cnp	_[Acetyl (Protein N-term)]M[Oxidation (M)]NTS[Phospho (STY)]FTR_.2	#N/A	4.0397	3.6584	4.0612	3.9235	7.0539	7.7411	6.9514	3.9177	3.5778	7.3232	7.1417	7.8458	3.7097	4.0993	7.5952	3.9008	8.0910	3.8774	4.0649	3.5907
Cnp	_EKNQWQLSADDLK_.3	#N/A	3.4773	3.8418	4.5147	4.1070	3.6842	3.7175	3.3639	3.7029	3.2841	3.2879	3.6392	3.5680	3.4496	4.4527	3.1071	4.3402	3.2002	3.9493	4.2483	4.2300
Cnp	_ERLDQLFEM[Oxidation (M)]ADQYQYQVVLVEPK_.3	#N/A	4.2805	3.7223	3.3160	3.4089	3.6182	3.8423	3.9228	3.9809	3.9819	4.4868	4.3563	3.6117	4.0900	3.6337	3.5000	3.1246	4.5186	3.3674	3.6665	3.3110
Ass1	_IDIVENR_.2	#N/A	5.2242	6.0149	6.3277	6.1211	6.2700	6.2658	6.6460	5.9002	6.6110	6.2435	6.3919	6.1839	6.0387	6.0572	6.1875	6.4971	5.3553	6.2482	5.9865	6.4057
Ass1	_RQVEIAQR_.2	#N/A	3.2544	3.2476	6.2367	4.4350	5.2886	4.3170	3.4481	4.4556	3.5072	3.4607	4.3193	5.7800	3.2751	7.1165	5.2993	4.5449	3.4925	7.3867	3.9893	3.7448
Ass1	_TTSLELFM[Oxidation (M)]YLNEVAGK_.2	#N/A	3.6070	3.7120	3.1436	3.9772	3.8140	3.8473	3.2341	3.8327	3.7557	3.4176	4.1338	3.4382	3.6379	3.1817	3.2595	2.9692	3.3300	3.8195	3.8967	3.3915
Sptan1	_S[Phospho (STY)]LQQLAEER_.2	#N/A	11.7073	11.5116	11.8622	11.6856	11.3711	11.5445	11.1800	11.6758	11.4871	11.6124	11.6799	11.3927	11.7595	12.0256	11.1954	11.5070	11.8636	11.3096	11.5599	11.5119
Sptan1	_KLDPAQSAS[Phospho (STY)]R_.2	#N/A	7.4862	9.6495	9.9550	9.7392	9.1857	9.5006	9.5334	9.4259	9.3703	9.9780	9.6909	9.2808	9.0573	9.7455	9.0592	9.4778	9.4919	9.3454	9.4185	9.4774
Sptan1	_EANQQQQFNR_.2	#N/A	9.4947	9.6538	9.9001	9.8226	9.5294	9.7738	9.6097	9.8653	9.8180	9.7826	9.5109	9.6930	10.0886	10.0196	9.5081	9.6120	9.7166	9.6923	9.7740	9.7085
Sptan1	_EKEPIAASTNR_.2	#N/A	9.5233	9.5781	9.7376	9.6340	9.5151	9.5643	9.5627	9.8574	9.8691	9.4978	9.3759	9.4851	9.8389	9.7095	9.6824	9.6718	9.5301	9.6604	9.5454	9.6687
Sptan1	_DLTGVQNLR_.2	#N/A	9.1470	9.0615	9.4181	9.2737	9.2242	9.1515	9.2481	9.2686	9.2071	9.1252	9.2133	9.2706	9.2298	9.2743	9.5557	8.8908	9.1756	9.1522	9.2279	9.1301
Sptan1	_LSDDNTIGQEEIQQR_.2	#N/A	9.1795	9.8691	9.2902	9.1509	8.9217	8.9632	9.0647	9.3544	9.1249	9.2664	8.9003	9.1008	9.4054	9.3815	8.9321	9.1695	9.4339	9.0606	9.2599	9.1803
Sptan1	_SSEEIESAFR_.2	#N/A	8.8291	8.8866	9.3747	9.0180	8.7319	8.8670	8.8110	9.3031	8.9957	8.6113	8.8031	8.6814	9.1782	9.0846	9.0036	8.5257	8.6614	8.9190	8.6787	8.8498
Sptan1	_ELPTAFDYVEFTR_.2	#N/A	8.5034	8.5757	9.1635	8.8082	8.5592	8.7550	8.7023	8.7291	8.7263	8.7990	8.6349	8.6651	9.0211	8.7717	8.9547	8.9232	8.9745	8.6276	8.8924	8.6757
Sptan1	_LFGAAEVQR_.2	#N/A	8.9438	8.8234	8.9346	8.9067	8.8465	8.6467	8.9540	9.1837	8.5345	8.8807	8.6310	8.9874	8.7643	8.8614	8.7999	8.5643	8.9413	8.9243	8.6207	8.7297
Sptan1	_LLVSSEDYGR_.2	#N/A	9.0204	8.5687	8.7566	8.8270	8.7977	8.8872	8.7855	9.2024	8.9341	8.9218	8.8803	8.7753	8.9217	9.1117	8.7967	8.6738	8.7927	8.6382	8.7902	8.8743
Sptan1	_EELYQNLTR_.2	#N/A	8.8586	8.7325	9.0891	8.7590	8.8541	8.8129	8.6899	8.9419	8.7605	8.8238	9.0709	8.5935	8.4808	8.9864	8.8396	8.7130	8.6958	8.5932	8.8445	8.8427
Sptan1	_LIQEQHPEEELIK_.2	#N/A	7.5228	7.2843	7.7786	7.7386	7.7854	7.5280	7.5454	7.7888	7.6934	7.3813	7.7393	7.3840	7.9530	7.4598	7.4449	7.6673	7.4057	7.3577	7.5889	7.6366
Sptan1	_ADVVESWIGEK_.2	#N/A	8.7361	8.2943	8.8345	8.6914	8.2966	8.6236	8.0761	8.5723	8.3706	8.3963	8.7882	8.0845	8.4889	8.6256	8.4300	8.6336	8.8662	8.1816	8.2892	8.6102
Sptan1	_WTQLLANSATR_.2	#N/A	8.6406	8.4318	8.6099	8.6204	8.3037	8.4314	8.4999	8.8641	8.4226	8.4943	8.2770	8.4988	8.6296	8.4346	8.4045	8.7488	8.3884	8.4858	8.5692	8.4017
Sptan1	_EANELQQWITEK_.2	#N/A	8.5513	8.5495	8.6923	8.7886	8.5255	8.4378	8.3495	8.3185	8.5781	8.4263	8.4349	8.3130	8.5569	8.7241	8.2198	8.5343	8.6964	8.4516	8.5041	8.7210
Sptan1	_DLAALEDKVK_.2	#N/A	8.5480	8.5845	8.4377	8.6693	8.7557	8.3330	8.5392	8.8942	8.4754	8.4559	8.0293	8.6909	8.9825	8.4268	8.6417	8.7515	8.3747	8.7107	8.5504	8.7214
Sptan1	_DVEDEETWIR_.2	#N/A	8.6427	8.4242	8.5571	8.3482	8.5468	8.6152	8.5530	8.7387	8.4802	8.7997	8.7382	8.6404	8.8955	8.8503	8.0503	8.8660	8.6797	8.5435	8.6474	8.6937
Sptan1	_DLASVQALLR_.2	#N/A	8.2616	8.1748	8.3837	8.3457	8.5255	8.5874	8.5956	8.5254	7.9543	8.3053	8.6745	8.2255	7.7822	8.4582	8.2707	8.2175	8.3701	8.6032	8.5229	8.5136
Sptan1	_DLSSVQTLLTK_.2	#N/A	8.3037	8.3526	8.7846	8.5152	8.3588	8.3732	8.3324	8.7406	8.2948	8.4544	8.5131	8.3223	8.4117	8.5075	8.4784	8.1178	8.5882	8.3158	8.4257	8.5053
Sptan1	_ITALDEFATK_.2	#N/A	8.6456	8.5034	8.3492	8.5252	8.6061	8.4020	8.6635	8.5089	8.3552	8.6050	8.5063	8.7160	8.4620	8.5210	8.2992	8.5122	8.5024	8.7183	8.3507	8.5558
Sptan1	_VLETAEDIQER_.2	#N/A	8.2449	8.3078	8.6477	8.3162	8.1255	8.2375	8.2331	8.4873	8.4602	8.2139	8.1154	8.1011	8.1570	8.3799	8.2069	8.4859	8.2259	8.1470	8.3387	8.4501
Sptan1	_WRS[Phospho (STY)]LQQLAEER_.3	#N/A	7.3562	7.5720	7.1539	7.4404	6.8612	7.0214	7.0490	7.1765	6.9693	7.3346	7.2922	7.1174	8.0610	7.8886	6.3971	7.5836	7.1746	7.6183	7.4441	7.2859
Sptan1	_TATDEAYKDPSNLQGK_.2	#N/A	7.0162	6.8771	7.1053	6.9014	6.8376	6.9135	7.2693	7.0287	7.3942	7.1958	7.0142	7.1734	7.0788	7.2860	7.4923	7.3489	6.4500	7.1306	7.2039	7.1865
Sptan1	_GLVSSDELAK_.2	#N/A	7.7869	7.2770	8.4883	7.9988	7.9714	8.1580	8.1738	8.0031	8.0366	8.0524	8.2921	8.0269	7.6268	7.7675	8.1626	7.8784	7.7094	7.9887	7.5157	8.1998
Sptan1	_LLEAQSHFR_.2	#N/A	7.1600	7.3400	6.9397	6.8720	7.0855	7.0016	7.0961	7.4384	6.2132	6.6683	6.6571	6.8560	7.1258	7.3120	6.9718	6.6647	7.4126	7.1462	6.7032	7.0237
Sptan1	_KFEEFQTDLAAHEER_.3	#N/A	8.1582	8.0014	8.6045	7.9406	7.6336	7.8206	7.4539	8.2517	7.6727	8.2056	7.7817	7.6123	8.2051	8.4679	7.7745	7.8348	7.8797	8.0534	7.9751	7.9183
Sptan1	_IDGITIQAR_.2	#N/A	7.9242	7.8902	8.3839	7.7635	7.8058	8.0482	7.7359	7.5531	7.8742	7.4847	7.8726	7.6477	7.1320	7.3842	7.5639	7.6491	7.7707	7.6850	7.8237	7.9301
Sptan1	_LIDVNHYAK_.2	#N/A	7.7155	7.7844	8.0903	7.5195	8.0403	7.7397	7.7736	8.0473	7.7658	7.6960	7.5593	8.0767	7.6678	7.7456	7.9057	7.5734	7.9150	7.8230	8.0453	7.6141
Sptan1	_VNEVSQFAAK_.2	#N/A	7.6313	7.8140	8.0391	7.7726	7.7456	7.9043	7.8644	7.5077	7.3299	7.4226	7.5216	7.6562	7.7257	7.7495	7.7168	7.5676	7.5205	7.6402	7.5963	7.3916
Sptan1	_REELITNWEQIR_.3	#N/A	7.5525	7.4188	6.9401	8.0181	7.8202	7.4953	7.7661	7.3380	7.6813	7.9262	8.1369	7.7135	8.2451	7.7460	6.6780	7.6948	7.6072	7.1436	8.0498	7.9978
Sptan1	_VNSLGETAQR_.2	#N/A	7.9259	7.1527	8.1354	7.8371	7.4288	7.6571	7.8832	7.9066	7.6817	7.6351	7.7073	7.5847	7.4647	7.7661	7.6525	7.3028	7.7798	7.4926	7.7294	7.5601
Sptan1	_QKLEDSYR_.2	#N/A	7.5406	7.5211	7.7069	7.0318	7.6552	7.7124	7.8589	7.8032	7.8335	7.7543	7.8519	7.4659	7.7277	7.7616	8.0254	7.5960	7.3407	7.6609	7.6180	7.3457
Sptan1	_LAQFVEHWK_.2	#N/A	7.7070	7.8367	7.7041	7.8026	7.5321	7.7918	7.6739	7.6900	7.6250	7.5468	7.5044	7.7165	7.8721	7.8537	7.6602	7.7185	8.0298	7.6091	7.6315	7.7873
Sptan1	_DVDIEIAWISEK_.2	#N/A	7.7772	7.5991	7.9419	7.8412	7.3628	7.7126	7.2937	7.5418	7.8486	7.3912	7.5644	6.9342	7.8424	7.6179	7.6108	7.8635	7.9643	7.3357	7.7093	7.6301
Sptan1	_LDPAQSAS[Phospho (STY)]RENLLEEQGSIALR_.3	#N/A	8.2563	8.5483	8.1967	8.3587	7.1220	7.2751	8.1462	7.9440	7.1532	3.6802	7.6768	8.0204	8.1911	8.4867	7.0802	7.4135	8.0127	7.9836	7.3737	7.4995
Sptan1	_DVDETIGWIK_.2	#N/A	7.4160	7.5756	7.6983	7.8633	6.9703	7.4796	7.1468	6.8569	7.8368	7.4103	7.7385	6.9379	7.1003	7.8277	7.3176	7.3517	6.9635	7.4247	7.3619	7.7552
Sptan1	_NTTGVTEEALK_.2	#N/A	7.5740	7.4746	8.0062	7.4187	7.4975	7.3819	7.3247	7.5580	7.5770	7.3788	7.7961	7.1122	7.0642	7.4447	7.5740	7.2934	7.1226	6.9556	7.3793	7.4257
Sptan1	_M[Oxidation (M)]NEVISLWK_.2	#N/A	7.8214	6.7983	6.3965	6.3161	7.6246	6.8889	6.9465	7.3983	6.7917	7.4678	6.9031	7.1781	7.4529	7.1092	7.3031	7.7417	7.2241	7.5254	7.4440	7.4588
Sptan1	_LSILSEER_.2	#N/A	7.3121	6.9216	6.8879	7.3347	7.3394	7.2075	7.1432	8.0050	7.1009	7.0106	7.1628	7.3008	7.5271	7.2861	7.1102	7.6923	6.6146	7.4176	7.5344	7.5071
Sptan1	_LGESQTLQQFSR_.2	#N/A	7.1690	7.1476	6.8111	7.1758	7.1890	6.8782	7.3096	7.2187	7.4337	7.3356	7.1369	7.3091	7.0701	7.5896	6.9941	6.6158	7.1795	6.8981	7.0057	6.9867
Sptan1	_LDENSAFLQFNWK_.2	#N/A	6.8845	6.6149	7.2129	7.0236	6.8454	6.7975	6.8252	6.9294	7.2047	7.1336	6.6591	6.6953	7.2079	6.4673	6.7350	6.7253	6.4871	6.4039	7.0815	6.9052
Sptan1	_QDEVNAAWQR_.2	#N/A	6.5538	5.8840	6.9455	7.2638	6.9641	7.0996	6.1893	6.1581	6.7287	6.8674	6.5240	6.4668	6.1173	5.4060	6.5613	7.0991	6.8537	5.8797	6.6180	7.0626
Sptan1	_DLTNVQNLQK_.2	#N/A	7.7689	6.6277	7.0506	6.9693	6.6481	7.0383	6.3683	7.1925	6.9521	6.7869	6.8726	6.4804	6.6430	6.9885	6.6852	6.5816	6.7255	6.6807	6.6337	6.6726
Sptan1	_KLEATELK_.2	#N/A	6.4163	6.7187	6.5962	6.8164	7.0337	6.6463	6.3515	6.0663	6.3803	6.6232	6.7567	6.4842	6.3109	6.8065	6.7761	6.7973	6.6217	6.1648	6.3669	6.5011
Sptan1	_EELITNWEQIR_.2	#N/A	6.7209	6.6285	6.8946	6.1562	6.3865	6.4110	6.3471	6.2026	6.3976	6.3075	6.3306	6.2233	6.5186	6.6028	6.6404	6.9350	6.5831	6.3911	6.9104	6.4178
Sptan1	_KVEDLFLTFAK_.2	#N/A	6.8339	6.5404	6.7763	6.3772	6.5324	6.4092	6.3029	6.6333	6.0290	6.6243	6.0027	6.4227	6.6929	6.8904	6.2016	6.4213	6.1784	6.5275	6.4388	6.3833
Sptan1	_LGDSHDLQR_.2	#N/A	6.6156	6.2038	6.5517	6.3614	6.6077	6.9739	6.8725	6.6778	6.6118	6.4250	6.9396	6.3565	6.1827	5.8746	6.5873	6.3922	6.4580	6.5958	6.5879	6.6090
Sptan1	_KFDDFQK_.2	#N/A	5.9052	6.2827	6.6481	5.9193	6.3137	5.7646	6.2286	6.8140	6.3980	6.2714	6.4741	6.5117	5.7226	6.4966	7.0092	6.5036	6.2481	6.4572	6.4031	6.1489
Sptan1	_LLEATELK_.2	#N/A	5.5592	5.3036	5.5347	6.1932	6.1415	5.9595	6.3069	5.8628	6.2305	6.4838	6.3564	6.1561	7.1751	5.8490	5.5232	6.0039	4.8536	6.9208	6.1580	6.3507
Sptan1	_QWELLLEK_.2	#N/A	5.5946	5.7334	6.7984	6.5482	6.0711	5.7141	6.2831	6.6085	6.2426	6.0730	6.3159	5.9206	5.9866	6.7380	6.1933	6.2669	6.2003	6.4168	6.1681	6.3629
Sptan1	_VNDVC[Carbamidomethyl (C)]TNGQDLIK_.2	#N/A	6.3639	5.3928	5.8085	6.2437	6.0966	6.5456	5.8103	6.2212	6.1429	6.2532	6.2530	5.9914	6.0290	5.9372	6.7222	6.3721	6.5262	6.1057	6.2352	6.1376
Sptan1	_DLM[Oxidation (M)]SWINGIR_.2	#N/A	6.0999	5.7294	6.1697	6.0445	6.4450	6.5148	5.9438	6.1110	6.0942	6.5953	6.3375	6.4709	5.5485	6.4828	6.0300	6.0150	6.1571	5.9993	6.4912	6.2861
Sptan1	_C[Carbamidomethyl (C)]TELNQAWTSLGK_.2	#N/A	5.6259	5.2800	6.4734	5.5607	5.7552	6.1771	5.8246	6.1120	5.9529	5.6817	5.3812	4.9620	6.2191	5.8437	6.1560	5.4451	5.5186	5.7455	5.4212	5.5600
Sptan1	_LAALADQWQFLVQK_.2	#N/A	6.0665	5.9955	6.2817	6.1761	6.0198	5.6706	6.2463	6.4088	5.8850	5.7495	6.2210	6.5303	6.5461	6.3067	5.9455	6.2091	6.4191	5.6339	6.0864	6.4251
Sptan1	_IAALQAFADQLIAVDHYAK_.3	#N/A	4.8711	5.5578	5.3018	4.8983	5.0212	5.3085	4.9245	5.2080	4.0326	5.1445	4.8113	5.0846	5.2292	5.9443	4.7361	5.2288	5.5332	5.0860	4.9366	4.8531
Sptan1	_KLSDDNTIGQEEIQQR_.3	#N/A	5.1964	6.0030	5.7500	6.8349	6.2242	5.7871	6.1999	5.6009	6.2111	6.2304	5.9620	5.8570	6.8039	6.3046	6.2373	5.9182	5.3790	5.8701	5.9174	6.2370
Sptan1	_QVEELYQSLLELGEK_.2	#N/A	5.6740	5.6542	6.4418	6.1891	5.6792	5.7967	5.9968	5.9052	5.9242	5.0833	5.7835	5.6426	6.0091	6.1228	5.7108	5.4795	5.2666	5.7136	6.0287	5.4791
Sptan1	_DVTGAEALLER_.2	#N/A	8.9571	8.5812	9.0706	8.6945	4.2791	8.8270	8.5729	8.8959	8.6257	8.8688	9.1210	8.6243	8.6969	8.9325	8.5376	8.3100	9.1570	8.8265	8.5118	8.7717
Sptan1	_EAIVTSEELGQDLEHVEVLQK_.3	#N/A	7.6538	7.9075	7.1496	7.4333	6.7080	7.2730	6.9241	7.1483	7.1485	6.9487	7.3874	7.1150	7.2818	8.3147	6.3232	7.2123	7.5198	7.2344	7.0610	7.4532
Sptan1	_HQALQAEIAGHEPR_.3	#N/A	6.4363	7.3122	6.2650	7.6911	6.7429	7.0201	6.8728	6.5955	6.8477	6.8362	6.7260	6.4199	3.3835	7.5147	7.5234	7.1665	5.7253	7.1717	7.2108	6.8069
Sptan1	_LQQSHPLSASQIQVK_.2	#N/A	5.7776	5.5353	6.1589	6.2748	5.9657	6.1213	5.9561	5.7989	6.1808	5.9110	6.2140	5.8997	4.4652	6.1813	6.2281	6.0474	6.0215	5.9157	6.1770	6.0238
Sptan1	_ENLLEEQGSIALR_.2	#N/A	6.3598	6.9107	6.6746	6.5917	6.6080	6.5407	7.0687	5.8970	6.7005	6.7549	6.3947	6.4356	3.1323	6.5094	6.7528	6.2348	6.0613	6.4497	7.1412	6.8159
Sptan1	_EAHDAFR_.2	#N/A	6.4172	6.3326	5.8704	6.2017	5.9892	6.3053	6.4410	6.1523	6.1173	6.2599	6.4224	6.5393	3.5670	6.1876	6.6380	6.1394	6.1295	6.0005	6.2183	6.3065
Sptan1	_TALLELWELR_.2	#N/A	6.1629	6.0462	6.1438	6.3245	6.0257	6.1847	6.1311	5.3731	5.8840	6.2802	6.4074	5.5048	3.3099	5.9572	5.6679	5.3759	6.3506	5.7437	6.0463	5.9786
Sptan1	_RQQVLDR_.2	#N/A	6.1184	5.3159	5.7768	5.9019	5.9736	5.3109	5.9772	6.1634	5.3975	5.3236	6.1760	6.0986	6.5130	5.3026	6.0653	6.0621	5.9585	5.3929	5.8272	5.8087
Sptan1	_TALLELWELRR_.2	#N/A	3.8788	4.5614	4.8043	5.0849	4.9112	3.8036	4.7338	3.9075	4.8685	4.9693	4.4646	5.0023	4.1245	3.6788	4.9682	4.5948	4.4711	5.1972	5.4403	5.2639
Sptan1	_LKDLNSQADSLM[Oxidation (M)]TSSAFDTSQVK_.3	#N/A	6.4228	5.4153	5.3158	3.9845	5.5748	5.3335	5.4733	6.0065	5.3272	5.3332	5.3000	6.3515	5.9064	5.2698	4.7048	5.0455	6.2949	5.2085	4.6711	5.7583
Sptan1	_DAEELEKWIQEK_.2	#N/A	5.4740	5.5494	4.2304	5.4540	5.2728	4.6119	5.4310	5.8468	5.3348	4.4816	3.3282	6.2419	7.0379	5.4485	5.5092	5.6944	5.3546	5.7435	5.1346	5.2067
Sptan1	_C[Carbamidomethyl (C)]NSLEEIK_.2	#N/A	4.6913	3.4431	4.9070	4.5500	4.5905	4.4292	4.5166	4.2700	5.0687	4.6727	4.2622	4.7770	4.6590	4.2995	5.0138	4.7172	4.0671	4.6591	4.6666	4.1307
Sptan1	_KHEGFER_.2	#N/A	8.2980	8.0780	9.6949	8.0977	8.2673	8.5992	7.5635	9.1674	8.3085	9.1610	9.3667	7.6638	8.0072	8.2635	8.7874	8.7973	9.2601	7.8014	8.7400	8.5438
Sptan1	_VAEDLESEGLM[Oxidation (M)]AEEVQAVQQQEVYGAM[Oxidation (M)]PR_.3	#N/A	7.9673	7.0955	5.8285	5.6664	7.0388	6.6395	6.7088	8.1393	6.6526	7.5708	6.4993	7.5277	7.9831	7.5536	6.0121	7.3389	7.6791	7.2956	7.2292	7.6052
Sptan1	_ALINADELANDVAGAEALLDR_.2	#N/A	5.6662	5.8286	5.7745	5.8099	4.4901	5.7486	6.0554	5.5734	5.6177	5.8501	5.5498	5.9484	5.1048	6.1539	5.2462	5.1643	5.2724	5.9723	6.0461	5.7783
Sptan1	_LNHQEFK_.2	#N/A	5.6307	5.7356	6.2569	6.2856	6.0833	5.7983	6.1534	5.7700	5.7248	6.4311	5.9679	5.5550	5.6238	5.7736	6.3793	5.6844	5.8507	6.0329	5.9988	5.6506
Sptan1	_M[Oxidation (M)]M[Oxidation (M)]LDHC[Carbamidomethyl (C)]LELQLFHR_.3	#N/A	6.8240	6.3868	4.8705	5.6112	6.4231	6.0593	6.6599	6.5924	6.0386	6.3199	6.2559	5.2455	6.2634	5.5290	6.0133	6.1523	6.1773	6.6631	6.7059	6.0177
Sptan1	_GEIDAHEDSFK_.2	#N/A	4.9728	4.5742	5.7364	4.9278	4.3437	5.8570	5.5643	5.6144	4.8117	5.3014	5.2773	4.8759	4.4219	4.9622	5.5742	5.2306	5.4367	5.4311	5.5004	5.3023
Sptan1	_DTEQVDNWM[Oxidation (M)]SK_.2	#N/A	7.8950	7.8720	8.2751	7.9760	7.6909	7.8746	7.5554	7.9841	7.8688	8.0952	7.7991	7.8738	7.9848	8.0794	7.6796	7.6789	7.9948	7.5606	7.7840	7.9449
Sptan1	_GVIDM[Oxidation (M)]GNSLIER_.2	#N/A	6.8794	7.1298	7.3448	7.4270	6.8835	6.9201	7.2623	7.1145	6.6277	6.9415	6.9844	6.8520	6.8644	7.4430	7.0483	6.7435	6.9816	6.8158	6.5370	6.7525
Sptan1	_DLAALGDKVNSLGETAQR_.3	#N/A	5.8509	6.1079	5.8168	6.4115	6.4082	6.0168	6.3887	6.2275	5.9715	6.3719	5.9501	6.1923	6.4053	6.0401	6.2246	6.3382	5.9210	6.3455	6.1169	6.5941
Sptan1	_AQLADSFHLQQFFR_.3	#N/A	4.7632	4.9503	5.4661	4.7599	5.0525	4.7880	4.5642	4.6601	5.1611	4.3263	5.2212	4.9371	5.5968	5.1377	4.9304	5.1564	5.0523	4.9239	4.8741	5.4873
Sptan1	_QQVAPM[Oxidation (M)]DDETGKELVLALYDYQEKS[Phospho (STY)]PR_.3	#N/A	4.4274	3.8692	3.9726	3.7547	2.9407	4.7650	3.7063	3.6612	4.3910	4.4551	3.2948	4.3022	3.9816	5.4859	5.3774	4.5935	4.1989	5.7393	3.3389	4.6516
Sptan1	_SADESGQALLAASHYASDEVR_.3	#N/A	6.0160	5.6293	5.8310	5.8505	5.7100	5.6063	5.6744	5.4931	5.3667	6.2418	5.6661	5.5016	5.7360	6.4166	4.8829	5.8618	5.8546	5.4621	5.3855	5.9975
Sptan1	_DLIGVQNLLK_.2	#N/A	3.2265	3.2755	5.7335	6.0984	5.0134	5.7203	5.7066	4.4277	4.9337	5.3391	5.2183	5.0459	3.2472	5.1096	5.7055	5.0402	5.0477	4.8575	5.5974	5.8049
Sptan1	_DC[Carbamidomethyl (C)]EQAENWM[Oxidation (M)]AAR_.2	#N/A	5.5655	6.9251	7.3800	7.4659	6.9309	6.9454	6.9076	6.8444	7.0092	6.9494	7.2370	6.7070	6.8495	7.1019	7.1149	6.5603	7.1984	6.8799	6.8343	7.2060
Sptan1	_DSDELKSWVNEK_.2	#N/A	5.7462	5.8836	4.4399	5.8327	4.5562	5.5060	5.4281	5.8344	5.8438	5.3852	5.3971	6.1127	6.1003	5.6910	5.8055	6.3963	5.7036	5.7767	6.0162	6.2898
Sptan1	_AINVQEEK_.2	#N/A	6.6954	5.9536	6.2335	6.4492	6.1516	6.4627	6.6587	5.3039	6.3618	5.7669	7.0750	6.1864	5.6927	6.2288	6.8766	6.8068	4.5020	5.8738	6.6011	6.6750
Sptan1	_SSLSSAQADFNQLAELDR_.2	#N/A	6.8351	6.9224	7.1342	6.9081	6.9536	6.9315	6.7929	7.1445	6.9739	7.2365	7.2506	7.1899	7.3643	7.2650	7.0338	7.1590	7.0603	6.9846	7.0886	6.8978
Sptan1	_GLVSSDELAKDVTGAEALLER_.3	#N/A	3.9539	4.2299	4.3904	3.5917	2.9761	4.1881	3.5820	3.8101	3.8813	4.2710	3.1820	3.6739	4.6774	2.6875	3.3251	3.9563	4.1920	3.0408	4.1722	4.3963
Sptan1	_ASAFNSWFENAEEDLTDPVR_.2	#N/A	4.3099	3.8958	3.1948	3.6764	3.9833	3.3588	3.0798	3.1223	3.3281	3.6802	3.8858	3.1114	3.1654	4.1569	3.3958	1.2319	4.4030	3.8810	3.2730	2.9813

Sptan1	_LIQSHPESAEDLKEK_.3	#N/A	6.1006	5.8271	6.5649	6.0732	6.1225	5.9934	6.2383	6.3622	6.1756	6.0643	5.9549	6.0409	6.3029	6.3142	6.6343	6.3307	6.0609	6.1931	6.1248	6.0841
Sptan1	_DM[Oxidation (M)]DDEESWIK_.2	#N/A	6.8039	6.3770	7.0150	6.6371	6.2636	7.0091	6.2889	6.3957	5.9828	6.6107	5.6881	6.5467	5.9276	6.6525	6.2081	6.4787	6.5269	6.4935	6.4592	6.8186
Sptan1	_EKLDILDQERTDLEK_.3	#N/A	6.3707	4.2187	6.8987	4.4839	5.7958	3.3002	6.1389	6.2977	6.8951	6.2901	6.0687	5.6828	7.1650	6.4622	6.3912	5.7359	6.3418	5.8107	5.8256	3.8531
Sptan1	_M[Oxidation (M)]QHNLEQQIQAR_.3	#N/A	5.5083	5.3424	5.6159	5.0864	5.6422	5.2265	5.1117	5.2986	5.2008	5.1194	5.2072	5.3739	6.0611	5.1445	5.6494	5.9972	4.5300	5.3911	5.7936	5.4725
Sptan1	_DLNSQADSLM[Oxidation (M)]TSSAFDTSQVK_.2	#N/A	4.5750	5.4168	4.9861	4.0646	4.3006	4.2374	4.5427	4.9860	5.2342	4.6142	4.1867	4.8868	5.0050	5.9502	4.5776	4.9075	4.9033	4.7185	4.2213	4.7007
Sptan1	_DLTSWVTEMK_.2	#N/A	4.0803	5.0063	5.8872	6.0405	4.2550	4.4822	5.3318	3.7807	4.1518	2.9474	5.6778	4.0785	4.2902	6.2586	4.3218	4.4252	4.0321	3.5148	3.1633	3.5911
Sptan1	_M[Oxidation (M)]TLVASEDYGDTLAAIQGLLK_.2	#N/A	5.4924	5.6029	4.8758	5.0792	5.0822	4.9990	5.3411	5.0723	5.4193	5.6950	4.6907	5.4519	5.9053	5.6784	5.1888	5.5780	5.4635	5.4991	5.2834	5.3446
Sptan1	_ADVVESWIGEKENSLK_.2	#N/A	3.6731	4.4037	4.2438	4.5408	3.8132	4.2869	3.1324	4.3059	4.2346	3.7517	4.2919	3.6605	4.7178	4.3896	4.1923	4.6169	4.4148	4.4103	4.6608	4.7176
Sptan1	_QQNFNTGIK_.2	#N/A	4.4473	4.1910	3.4827	5.7400	4.8391	5.0616	5.1618	4.1476	5.2428	3.1527	3.8803	3.9911	3.9232	4.7848	6.3154	4.1263	3.1845	4.7526	5.3602	3.4368
Sptan1	_YTEHSTVGLAQQWDQLDQLGM[Oxidation (M)]R_.3	#N/A	5.5535	5.7487	4.5675	4.6032	3.7370	5.1323	3.3111	5.3896	5.0346	5.4353	5.6843	5.2255	5.7298	5.2149	4.1249	4.1325	5.6768	5.4070	4.9164	4.7513
Sptan1	_LDETGNLM[Oxidation (M)]ISEGHFASETIR_.3	#N/A	6.4435	6.0660	6.5244	6.6729	5.5252	5.6971	5.7976	6.0881	5.1113	6.2329	6.0972	5.9197	6.6977	7.0420	5.1046	5.7293	5.7952	5.5570	5.9067	5.8993
Sptan1	_RNEVLDR_.2	#N/A	4.5352	4.3173	3.9933	4.2324	4.2956	4.7589	4.3502	5.1032	3.3046	4.5650	5.2069	4.4080	3.4126	4.6704	5.9085	5.1391	5.7696	4.0283	4.7601	4.1345
Sptan1	_KLLVSSEDYGR_.2	#N/A	6.4129	6.6536	6.3900	6.2607	6.4412	6.2509	6.2489	6.5288	6.2710	6.1490	6.1540	6.2089	6.1801	6.3737	6.3395	6.4370	6.5457	6.0342	6.3839	5.9262
Sptan1	_EC[Carbamidomethyl (C)]EDVM[Oxidation (M)]DWINDK_.2	#N/A	6.2484	5.8561	5.5458	5.0261	6.1198	6.2575	5.7399	6.0568	5.7654	6.0795	6.0604	5.4345	5.6489	6.4372	4.2057	5.4069	6.0369	5.4493	5.7197	5.9886
Sptan1	_EAFLNTEDKGDSLDSVEALIK_.3	#N/A	5.2961	5.2339	5.2918	4.6913	5.7935	5.2844	5.7750	5.2395	4.7995	5.0903	5.5305	5.6059	5.8310	6.0233	4.5742	5.6080	5.5184	5.4545	5.3707	5.6669
Sptan1	_KLDPAQSAS[Phospho (STY)]RENLLEEQGSIALR_.3	#N/A	3.7384	6.3124	4.2747	4.0366	3.8377	5.1217	3.8114	7.4552	3.7933	6.3172	3.7328	7.2269	3.9206	3.8657	4.6293	4.0833	3.8133	3.7997	3.6868	3.6951
Sptan1	_ELVLALYDYQEKS[Phospho (STY)]PR_.2	#N/A	3.1812	4.5778	5.5115	4.7271	3.7226	3.8703	3.5925	3.7372	3.5840	4.0695	3.6005	3.5734	3.7195	3.9052	3.4107	3.8210	4.8605	3.9547	3.5719	4.1568
Sptan1	_SLGYDLPM[Oxidation (M)]VEEGEPDPEFEAILDTVDPNR_.3	#N/A	3.1717	3.3302	3.7080	4.5177	3.9413	3.9747	3.1068	4.3729	3.0270	3.5450	4.0065	3.2197	3.1924	3.6951	3.1322	3.0965	1.5873	4.4234	1.2600	3.6621
Sptan1	_DEADS[Phospho (STY)]KTAS[Phospho (STY)]PWK_.2	P16546_S1186_M2_Sptan1	9.3699	8.6909	8.2261	9.4169	8.8510	8.0507	8.1689	9.1403	8.3853	8.9757	8.6646	8.9331	9.6279	9.2544	7.6716	8.9198	9.0323	8.2690	8.6196	8.8499
Sptan1	_DLAALEDK_.2	#N/A	4.0375	3.2816	3.5741	5.3292	4.3525	4.0246	3.8188	4.2632	4.4573	3.8480	5.5804	3.0077	4.0684	3.6122	5.1001	4.9326	3.7604	4.3740	4.8293	4.9245
Sptan1	_LQVASDENYKDPTNLQGK_.3	#N/A	6.2528	6.2479	6.8255	6.4242	5.7441	6.2618	6.3589	6.0299	6.3804	6.1788	5.8474	6.0172	6.4405	6.7442	6.3584	6.4359	6.0141	6.2697	6.1041	6.3772
Sptan1	_YEALKEPM[Oxidation (M)]VAR_.2	#N/A	6.3632	5.4433	6.7530	6.5201	5.8827	6.1784	5.6163	6.2255	5.5609	6.3143	6.2845	5.8228	5.6773	6.4478	6.2439	6.2682	6.3722	5.7172	5.9013	6.0915
Sptan1	_EAALTNEEVGADLEQVEVLQK_.2	#N/A	3.3225	8.6628	7.8192	4.3398	3.9157	3.1562	7.9681	3.4780	3.1752	3.8007	3.5495	3.8008	3.2832	8.9449	3.3343	3.9190	3.8606	8.2231	3.8657	4.0051
Sptan1	_SQLLGSAHEVQR_.2	#N/A	7.2624	7.1431	7.6066	7.4165	7.3294	7.3811	7.4145	7.6966	7.3719	7.1906	7.3007	7.2547	7.2402	7.3496	7.8816	7.4594	7.6555	7.5393	7.5016	7.4849
Sptan1	_EKEPIVGSTDYGKDEDSAEALLK_.3	#N/A	6.9085	6.4880	6.7950	6.7401	6.4956	6.5320	6.4409	7.1016	6.1929	6.5960	6.6741	6.4490	6.7244	7.2947	6.4350	6.5079	6.9281	6.5940	6.0354	6.6520
Sptan1	_QQVAPM[Oxidation (M)]DDETGKELVLALYDYQEK_.3	#N/A	5.0000	5.3248	4.8409	3.4966	4.2532	4.1965	4.7299	5.1660	4.6815	5.3407	3.7967	5.2232	5.7113	5.5032	4.6100	4.3503	5.6236	4.7035	4.5204	4.5246
Sptan1	_TYLLDGSC[Carbamidomethyl (C)]MVEESGTLESQLEATK_.3	#N/A	3.4006	4.2012	4.9585	5.2373	3.6076	3.6409	3.4405	3.2058	3.3607	3.2112	4.3402	4.3868	3.5262	5.7011	3.4659	4.2636	3.7435	3.2423	3.7382	3.9958
Sptan1	_NQALNTDNYGHDLASVQALQR_.3	#N/A	6.6984	6.5547	6.7405	6.7036	6.3948	6.5498	6.5342	6.9058	6.6517	6.8112	6.8298	6.6043	6.9437	7.0400	6.7122	6.3826	7.0667	6.5615	6.8009	6.7932
Sptan1	_NNHHEENISSK_.2	#N/A	4.1130	3.8704	4.1145	4.4891	4.0791	4.4192	3.2686	5.1899	3.8189	4.6502	4.7577	4.2487	4.3591	3.9337	4.1146	4.1361	4.9682	4.0431	4.3099	4.3417
Sptan1	_EKLDILDQER_.2	#N/A	7.8970	7.1157	7.8518	7.3439	7.3855	7.4572	7.1303	7.7332	7.4111	7.4814	7.6286	7.3388	7.4841	7.1760	7.3947	7.1184	7.7167	6.8892	7.2603	7.4413
Sptan1	_DADETKEWIEEK_.2	#N/A	5.5625	5.8409	5.7454	6.1161	6.1321	6.1213	5.5326	5.8771	6.1905	6.4325	6.0465	5.9106	5.7235	6.2469	6.0534	6.1043	6.4149	5.9289	6.2299	5.9518
Sptan1	_HQEHKGEIDAHEDSFK_.4	#N/A	3.3993	4.3433	4.1991	4.0298	3.1744	3.4868	3.5979	3.3632	3.5181	4.4454	5.5904	4.2327	3.6835	4.1862	5.3259	4.0312	3.5831	4.0955	5.7297	4.1532
Sptan1	_QFQDAGHFDAENIKK_.3	#N/A	5.1868	5.9305	5.6046	5.4959	4.9212	4.8206	6.0619	6.1730	5.7716	5.4475	5.1040	5.9248	5.0811	5.5058	4.9671	5.3578	5.3227	6.1847	5.4499	4.8174
Sptan1	_KIEDLGAAM[Oxidation (M)]EEALILDNK_.3	#N/A	5.9460	3.8941	3.6817	4.8342	5.1256	5.0315	4.6024	4.7413	4.3243	4.7799	4.4312	4.9824	5.0936	4.4901	4.2723	4.1161	5.2745	4.0397	5.4024	4.8874
Sptan1	_TKQDEVNAAWQR_.3	#N/A	3.8100	3.8555	3.3466	3.7742	4.0169	4.4532	4.7044	4.2973	5.7244	5.3468	4.0299	4.3574	3.1168	3.6195	4.7767	3.1721	3.3341	4.4555	4.1476	5.0413
Sptan1	_QEFAQHANAFHQWIQETR_.3	#N/A	6.3538	5.8942	4.3818	6.4019	6.2368	6.0077	5.8521	6.2906	5.6528	6.2113	6.4510	5.7096	6.5923	6.8756	6.2796	5.8147	6.8496	6.3300	6.3448	6.5884
Sptan1	_EKLSILSEER_.2	#N/A	5.6594	5.8165	5.7280	4.5732	3.9547	5.7208	6.0724	6.4128	3.6454	6.4301	4.0198	3.2752	3.7535	5.8097	5.1953	3.4611	3.3542	5.4760	5.9071	2.9745
Sptan1	_SADESGQALLAASHYASDEVREK_.3	#N/A	5.9002	5.7571	4.3740	5.8625	5.7526	5.5049	5.3679	6.4627	5.8016	6.1306	4.8040	6.0052	7.1456	5.8980	5.7576	6.7849	6.0057	5.2833	5.8464	6.1820
Sptan1	_GNAM[Oxidation (M)]VEEGHFAAEDVK_.2	#N/A	4.5040	4.4054	4.0158	3.2044	3.9179	3.9848	3.8335	4.3260	3.9867	4.3077	3.3962	4.2902	3.8128	4.6418	4.1853	4.5202	4.4473	4.4854	3.8381	4.5009
Sptan1	_EQLMASDDFGR_.2	#N/A	4.1450	3.1741	6.3767	5.6895	4.3519	4.3852	5.6371	3.9623	4.1172	3.9556	5.3065	4.4011	4.2827	3.2845	4.2224	3.5071	4.5000	3.3331	2.9817	3.2515
Sptan1	_QETFDAGLQAFQQEGIANITALK_.3	#N/A	3.3416	3.1604	3.8779	4.3478	4.1801	3.6171	3.3610	4.5428	3.0331	3.3751	3.4801	3.0499	3.3623	4.6967	2.9382	3.6865	3.5796	4.2535	3.2759	4.0583
Sptan1	_HALLEADVAAHQDR_.3	#N/A	4.6423	4.1339	4.4052	3.5541	3.2066	3.4307	4.3345	4.2467	4.3936	4.0752	4.5976	4.0234	4.5016	4.3922	3.9117	4.2138	4.1069	3.7262	3.3748	3.7227
Sptan1	_HQLLEADISAHEDR_.3	#N/A	4.2297	3.7731	3.2651	5.0193	4.8609	3.7915	3.9737	5.1693	4.0328	5.5226	5.1143	3.6626	4.1408	3.6846	4.5107	3.0737	4.6375	3.3166	4.7455	4.5058
Sptan1	_LIQNNHYAM[Oxidation (M)]EDVATR_.3	#N/A	3.5644	3.8670	4.2068	4.1716	3.5513	5.6945	3.5184	3.2282	5.1834	3.8555	3.7439	3.7441	3.3790	4.0611	4.0823	3.7721	3.9534	4.0058	3.5953	3.9266
Sptan1	_KHEAFETDFTVHK_.3	#N/A	3.7951	4.9259	3.3317	3.7891	4.0021	4.6751	3.0460	4.0208	5.8846	3.6057	4.9715	3.3419	3.1317	3.6343	3.0714	3.1572	3.5180	2.9833	4.1327	3.6013
Sptan1	_AKLGDSHDLQR_.3	#N/A	4.0901	2.6361	3.6267	3.0242	4.2971	3.8113	3.4025	4.3158	4.0130	2.9193	4.3873	3.4018	4.1210	3.6648	4.2772	3.4522	3.8130	3.3364	4.3487	3.3421
Sptan1	_DMDDEESWIKEK_.2	#N/A	3.6484	3.4903	4.1847	4.7001	3.7448	3.7253	3.3230	4.1075	3.2515	3.8623	3.6965	3.5901	3.9466	4.8950	3.5889	3.9933	3.6856	4.0351	3.6837	3.3966
Sptan1	_LM[Oxidation (M)]VHTVATFNSIK_.2	#N/A	3.1313	3.5520	3.6676	4.5581	4.6737	4.1940	3.5712	4.3229	3.6303	2.2576	4.0047	1.3753	3.7383	3.2821	3.1484	3.5847	3.3694	3.7191	4.0181	2.9594
Sptan1	_AGTFQAFEQFGQQLLAHGHYASPEIK_.3	#N/A	3.5624	1.9975	3.8800	4.3457	3.7693	1.0913	3.1049	3.4532	3.1990	3.3730	4.1785	3.1904	3.3644	3.8671	3.3041	4.4254	1.4793	3.7415	0.9639	1.7129
Sptan1	_HQAFEAELHANADR_.3	#N/A	4.0015	3.3176	3.5381	3.5827	5.8855	4.2418	4.3405	4.2272	4.2607	3.8121	5.2847	3.0437	4.4261	3.5762	2.8650	3.3636	3.7244	3.1897	3.7241	3.3950
Sptan1	_LIQNNHYAMEDVATRR_.3	#N/A	4.1255	3.1936	3.6621	3.4587	4.3324	4.3658	3.5653	4.3512	4.1367	3.9361	3.6154	4.4206	4.3022	5.4094	4.2419	3.4876	3.8484	3.3136	3.6001	3.2710
Sptan1	_HEAFETDFTVHK_.3	#N/A	3.7697	3.5494	3.3062	3.8146	3.9766	4.1318	3.0715	3.9953	3.6925	3.5803	3.4578	3.2755	3.8005	3.3443	3.0969	3.1318	3.4926	3.6569	3.9559	3.0216
Sptan1	_QETFDAGLQAFQQEGIANITALKDQLLAAK_.3	#N/A	3.1296	4.1895	4.1670	4.4547	3.3365	3.3698	3.7116	3.3553	3.6318	4.4410	3.1104	3.9156	3.1604	4.2051	3.7370	3.9925	4.3533	4.2970	3.0952	3.8823
Sptan1	_EKEQLMASDDFGR_.3	#N/A	4.2494	3.2289	3.6268	3.4940	3.5871	3.8112	3.9539	4.3159	4.0130	3.2072	4.3874	3.6428	4.1211	3.6648	3.5311	3.0935	3.8131	3.3363	3.6354	3.3421
Sptan1	_IEDLGAAMEEALILDNK_.2	#N/A	3.1562	3.3458	3.8059	4.5332	3.9947	3.9902	3.0912	4.3574	3.0114	3.5605	3.9909	3.2353	3.1769	3.6795	3.1166	3.5011	3.3942	4.4389	4.0875	3.6465
Sptan1	_RQDLEDSLQAQQYFADANEAESWM[Oxidation (M)]R_.3	#N/A	4.3194	4.5005	3.8560	3.2648	3.0255	3.0588	4.0226	3.7879	3.9428	4.1300	3.4215	4.2267	4.1083	4.6109	4.0480	3.6815	4.3256	3.5076	3.1561	3.0771
Sptan1	_KHEAFETDFTVHKDR_.4	#N/A	4.2588	4.5612	3.7953	3.9909	2.9649	3.6427	4.0832	4.4844	4.1816	4.2872	3.0551	4.2873	4.2896	3.8334	4.1086	3.6209	3.9817	3.1678	3.4668	3.5107
Gapdh	_GAAQNIIPASTGAAK_.2	#N/A	13.1285	12.8874	13.2474	12.6884	13.0459	13.0428	12.8985	12.9532	12.6717	13.0744	12.9012	13.0132	13.4463	13.0267	13.2460	12.9031	13.1720	12.8720	13.1715	12.8452
Gapdh	_VPTPNVSVVDLTC[Carbamidomethyl (C)]R_.2	#N/A	10.7643	10.7978	11.0121	11.0311	10.6920	10.8371	10.7455	10.8679	10.8314	10.8660	10.6705	10.6767	10.8709	11.0485	10.8051	10.7300	10.8554	10.4803	10.7612	10.6194
Gapdh	_VIPELNGK_.2	#N/A	10.2846	10.7365	10.6474	11.0070	10.7616	10.6606	10.9559	10.3045	10.8301	10.8427	10.7416	10.8409	10.7273	10.5126	11.2720	10.5609	9.5376	10.8172	10.8136	10.8462
Gapdh	_LTGM[Oxidation (M)]AFR_.2	#N/A	10.4757	10.6946	10.1844	10.2356	10.8411	10.6972	10.7921	10.6576	10.5700	10.8245	10.1535	10.9001	10.7639	9.8632	11.0173	10.6627	10.2452	11.0055	10.9352	10.7112
Gapdh	_VGVNGFGR_.2	#N/A	9.9961	9.8489	9.9487	9.8862	9.8382	9.8368	10.1707	9.9229	9.7862	9.8755	9.5758	9.8844	9.6053	9.5879	10.1851	9.3633	9.5766	9.9563	9.6717	9.7558
Gapdh	_LVINGKPITIFQER_.3	#N/A	8.9264	8.9226	9.0621	8.9246	8.8013	8.9775	8.8073	8.5701	8.5482	8.9735	8.9613	8.6733	8.5089	9.0940	8.6162	8.5854	8.7837	8.8040	8.6956	9.0183
Gapdh	_PITIFQER_.2	#N/A	8.6866	8.6204	9.5240	8.8819	8.8538	9.0340	8.6284	8.5223	8.5629	8.9996	9.0213	8.4576	7.8963	8.9145	8.4980	8.7432	8.6768	8.5138	8.8151	8.6906
Gapdh	_VVKQASEGPLK_.2	#N/A	7.0963	7.0648	7.4986	7.5703	7.1616	7.4228	7.1944	7.3660	7.3542	7.0792	7.5238	7.1538	7.3163	7.2588	7.5856	7.0445	7.5902	7.2481	6.9609	7.2811
Gapdh	_VIISAPSADAPM[Oxidation (M)]FVM[Oxidation (M)]GVNHEKYDNSLK_.4	#N/A	7.3771	6.9388	3.6773	6.8166	7.2293	6.8849	7.2609	7.3268	7.4658	7.9747	6.9600	7.7759	8.8448	6.2256	7.0853	8.4788	7.1744	7.6570	7.9407	7.6569
Gapdh	_PITIFQERDPTNIK_.3	#N/A	7.4386	7.6394	7.7240	7.8616	7.4793	7.7162	7.3984	7.8758	7.4769	7.7523	7.7029	7.5178	7.5852	8.0460	7.5191	7.7162	7.5415	7.5039	7.5835	7.6242
Gapdh	_WGEAGAEYVVESTGVFTTM[Oxidation (M)]EK_.2	#N/A	9.6217	9.2235	9.6620	9.6270	8.5468	8.7214	8.6077	9.8635	8.7321	9.6418	8.5813	8.9440	10.0467	10.0754	8.6166	9.4332	9.1004	8.9494	8.6465	8.9020
Gapdh	_VIISAPSADAPM[Oxidation (M)]FVM[Oxidation (M)]GVNHEK_.3	#N/A	8.2418	7.0495	8.2552	8.3884	8.2022	8.1220	8.0393	8.2247	7.6950	8.2199	8.3489	7.8000	7.4498	8.5987	7.8208	7.7860	7.4430	7.7575	7.2114	7.9054
Gapdh	_RVIISAPSADAPM[Oxidation (M)]FVM[Oxidation (M)]GVNHEK_.3	#N/A	8.6928	8.3543	8.1239	8.2268	8.5136	8.5823	8.2366	9.2446	8.8869	8.8175	7.9324	8.5523	9.3912	8.2290	8.6215	8.9633	8.4260	8.7460	8.8250	9.0815
Gapdh	_LISWYDNEYGYSNR_.2	#N/A	10.7707	10.6624	10.8807	10.8850	7.2820	10.5491	10.3826	10.7907	10.5897	10.8652	10.5877	10.4349	11.1230	10.6050	10.2385	10.6417	10.7735	10.1679	10.5669	10.5771
Gapdh	_DPTNIKWGEAGAEYVVESTGVFTTM[Oxidation (M)]EK_.3	#N/A	5.8163	5.4547	5.7640	5.9901	5.1294	5.2958	4.1147	6.3087	4.7185	6.1174	5.1439	4.8676	6.8277	5.2174	4.3515	5.7609	5.3699	4.5578	5.3844	5.3772
Gapdh	_VVDLM[Oxidation (M)]AYM[Oxidation (M)]ASK_.2	#N/A	7.8992	7.6174	8.1418	7.6906	8.2017	7.9070	7.4997	7.9759	7.8437	7.7004	7.5324	7.8607	7.9362	7.1822	7.8849	8.0027	7.6975	7.6133	8.0153	8.1582
Gapdh	_VVDLM[Oxidation (M)]AYM[Oxidation (M)]ASKE_.2	#N/A	8.4211	8.3725	8.0810	7.7610	8.1118	8.4424	8.0768	8.4534	8.2514	7.9545	7.1458	8.5067	8.4190	8.3508	8.4740	8.4272	8.3111	8.8379	8.5343	8.4303
Gapdh	_QASEGPLK_.2	#N/A	10.4117	10.4658	10.7909	10.3625	10.4523	10.4317	3.5554	10.5803	3.4756	10.6622	10.2872	10.5981	10.4048	10.4406	3.5808	4.1487	3.8584	3.9747	3.6233	10.5641
Gapdh	_LEKPAKYDDIK_.3	#N/A	4.1333	3.7291	3.3091	3.4157	4.3402	4.0520	5.3927	3.9741	5.1451	4.7863	4.5608	3.6186	4.2944	4.2203	5.2808	3.4954	2.6841	4.9234	5.1546	4.8733
Gapdh	_IVSNASC[Carbamidomethyl (C)]TTNC[Carbamidomethyl (C)]LAPLAK_.2	#N/A	10.9905	11.0573	11.5071	11.3584	11.0170	10.9701	11.1531	11.3553	11.2368	11.2137	10.8743	10.9210	11.1796	11.3508	11.3452	10.9329	11.0092	10.7727	10.9797	10.9523
Gapdh	_TVDGPSGKLWR_.2	#N/A	6.3270	6.6452	6.2475	5.6455	6.6387	6.8502	6.2636	6.6262	6.2361	6.5389	7.0793	6.2737	6.5861	7.1069	6.5057	6.2692	7.2516	6.2820	6.3051	6.7747
Gapdh	_VIHDNFGIVEGLM[Oxidation (M)]TTVHAITATQK_.4	#N/A	7.7684	8.3723	8.4186	8.6694	7.4222	8.2190	7.9203	7.5858	8.2192	8.6919	8.1204	8.2568	8.3734	8.2682	7.9686	8.5799	7.0222	8.1281	8.5642	8.3852
Gapdh	_FNGTVKAENGK_.2	#N/A	3.5527	3.7664	4.5901	4.0315	3.7596	4.3487	3.2885	3.7784	3.4755	3.3633	3.8499	3.4925	3.5836	4.6282	2.4954	4.4156	3.2756	3.8738	4.1729	3.1549
Gapdh	_VEIVAINDPFIDLNYMVYMFQYDSTHGK_.3	#N/A	3.6957	4.0259	4.4331	4.0448	3.4094	3.2905	3.9098	3.6079	3.7345	4.4024	3.2132	3.8318	3.9000	4.4026	3.6872	4.2492	4.3699	3.7392	3.3878	3.7160
Atf2	_NDSVIVADQTPT[Phospho (STY)]PTR_.2	P16951_T53_M1_Atf2	7.1684	7.0644	6.9707	7.0061	6.6533	6.5456	6.6323	6.9755	6.9967	6.6568	6.4573	6.9404	7.0893	7.6292	3.3533	7.0595	7.4267	7.0400	6.5198	6.8039
Atf2	_SKIEEPSVVETTHQDS[Phospho (STY)]PLPHPESTTSDEK_.4	P16951_S118_M1_Atf2	3.8676	3.7416	3.5748	3.1500	4.0745	4.1079	6.8603	4.2397	4.3946	3.6782	3.8733	3.3529	4.5601	3.5619	2.9990	3.2297	3.2766	3.0557	4.2052	3.5289
Hmga1	_[Acetyl (Protein N-term)]SESGS[Phospho (STY)]KSSQPLASK_.2	P17095_S6_M1_Hmga1	5.0904	3.5581	3.2976	3.8232	3.9679	3.8318	3.7074	5.0017	3.6838	3.2841	4.0174	3.2842	3.7919	3.3356	3.1056	4.5517	4.2724	3.6655	3.9903	3.0129
Hmga1	_KLEKEEEEGISQES[Phospho (STY)]S[Phospho (STY)]EEEQ_.2	#N/A	7.6095	7.8417	7.7521	7.5667	7.2537	7.5166	7.5446	7.7183	7.8364	7.6651	7.2594	7.4898	7.9114	7.7922	7.2920	7.2346	8.0881	7.7683	7.2688	7.2885
Zfp37	_IHSEEQS[Phospho (STY)]EEED_.2	P17141_S590_M1_Zfp37	8.2221	8.7802	8.1280	8.0318	3.8296	8.2544	8.4734	8.2779	8.3140	7.9331	7.7748	3.4226	8.9535	8.3937	8.3568	7.7260	3.3456	8.2880	8.0197	8.4063
Hspa2	_DAGTITGLNVLR_.2	#N/A	6.6337	6.7817	6.3734	6.9314	6.3382	6.8960	6.6814	6.2336	6.2904	6.4965	6.4518	6.5792	6.5194	6.9474	6.5673	6.8018	6.5082	6.1952	6.5612	6.5945
Hspa2	_LYQGGPGGGGSSGGPTIEEVD_.2	#N/A	4.6130	4.9297	3.3431	6.0769	4.9248	4.9804	4.7749	4.0192	4.6518	4.2341	4.6292	4.7073	4.0176	4.4546	4.9470	3.6110	4.4451	4.5628	5.3388	4.2765
Hspa2	_QTQTFTTYSDNQSSVLVQVYEGER_.3	#N/A	3.8731	3.4460	3.4097	3.7111	4.0801	4.1134	4.4689	4.0988	4.3891	3.6837	3.8677	3.1721	4.5545	3.4478	2.9934	6.8030	3.5960	3.5534	3.8525	3.5233
Eno1	_YITPDQLADLYK_.2	#N/A	11.5946	11.7411	11.6609	11.7022	11.6172	11.7089	11.5526	11.6590	11.5192	11.7866	11.4682	11.6888	11.6456	11.6546	11.7386	11.6752	11.6548	11.5568	11.4737	11.7265
Eno1	_GVSQAVEHINK_.2	#N/A	11.0500	11.3570	11.3913	11.1150	11.1837	11.1268	11.1945	11.2748	11.2512	11.2380	10.8197	11.2462	11.1267	11.4002	11.6749	11.1256	11.0200	11.0919	11.1061	11.0277
Eno1	_GNPTVEVDLYTAK_.2	#N/A	10.8480	11.1684	11.2359	11.2340	10.8417	10.8282	10.8300	10.9778	11.1885	11.0868	10.7930	10.9452	11.3753	11.3961	10.9850	11.0188	10.7083	10.9706	10.6923	11.1505
Eno1	_TIAPALVSK_.2	#N/A	8.1458	8.4502	8.6195	8.8657	8.5796	8.4687	8.6214	8.0197	8.5259	8.7708	8.4075	8.4584	8.6260	8.6633	8.9280	8.6843	7.8508	8.4204	8.7868	8.8635
Eno1	_SC[Carbamidomethyl (C)]NC[Carbamidomethyl (C)]LLLK_.2	#N/A	9.0117	9.3484	9.6587	9.5270	9.6379	9.3819	9.4206	9.6063	9.1069	9.5719	9.3170	9.5040	9.4748	9.5102	9.6187	9.0799	9.2458	9.3384	9.2895	9.3057
Eno1	_IGAEVYHNLK_.2	#N/A	9.0360	9.3623	9.0734	9.2757	8.9924	9.1528	9.1833	9.2510	9.0638	9.1233	8.7935	9.2843	8.5705	9.3561	8.8716	8.7352	9.0356	9.1950	8.7755	8.9724
Eno1	_KVNVVEQEK_.2	#N/A	8.8670	8.8411	9.4339	8.9432	8.9147	8.8454	9.0372	8.9448	9.0198	9.1303	8.8880	8.8814	8.6568	9.1261	9.5088	9.0144	8.6383	8.6376	8.8924	8.9927
Eno1	_IEEELGSK_.2	#N/A	6.2676	6.8462	6.7979	7.7245	6.3494	6.9008	7.4075	5.1929	8.2064	7.5882	8.1065	7.5967	8.1491	7.2993	8.6773	7.9843	5.9570	7.8568	8.2553	8.3544
Eno1	_DATNVGDEGGFAPNILENKEALELLK_.3	#N/A	7.0838	7.6717	6.2481	7.8116	7.1699	7.1170	7.2418	7.7725	7.5701	7.5349	6.5619	7.7660	8.5464	7.4542	7.8286	7.8299	7.5338	7.3604	7.3666	7.5130
Eno1	_VNVVEQEK_.2	#N/A	6.6725	6.0034	6.4486	6.3509	6.3682	6.7829	6.5122	5.9798	6.1995	6.3991	6.8474	6.3569	5.6601	5.9863	6.8375	6.6268	5.8757	5.9142	6.5982	6.7686
Eno1	_HIADLAGNPEVILPVPAFNVINGGSHAGNK_.4	#N/A	7.2918	7.5941	8.0473	7.9496	7.4897	7.4428	7.1566	6.9808	8.0846	8.0700	6.9495	6.5690	7.6950	7.9131	8.1679	7.7340	7.3041	6.9864	7.6450	8.0177
Eno1	_AAVPSGASTGIYEALELRDNDK_.3	#N/A	6.9360	8.2145	7.4088	7.5443	7.1148	6.6474	7.5608	8.2015	7.4301	7.4413	6.9769	7.9745	8.5686	8.2740	7.7376	7.2221	7.3120	7.6988	7.5763	7.2119
Eno1	_SGKYDLDFK_.2	#N/A	8.3225	8.5250	8.3536	8.6159	8.1975	8.4321	8.1287	8.0963	8.1052	8.2689	8.0237	8.0968	8.1103	8.6625	8.1877	7.9166	8.2658	8.0523	7.5850	8.6045
Eno1	_KVNVVEQEKIDK_.2	#N/A	4.3660	5.4854	5.1548	5.3917	5.4559	4.9980	5.3446	5.4317	5.6681	5.1988	4.7984	4.9323	5.4926	4.2887	5.8886	5.7822	5.4585	4.9919	5.4415	5.0114
Eno1	_IEEELGSKAK_.2	#N/A	8.3157	5.2751	8.3868	8.5980	8.1429	7.0039	4.9224	8.5901	7.7353	5.3816	5.3669	8.3438	7.3658	8.6504	7.3867	8.1757	8.3002	7.7760	5.1511	7.7120
Eno1	_DATNVGDEGGFAPNILENK_.2	#N/A	11.6652	11.4753	11.8050	11.7328	3.8641	11.5922	11.3008	11.5609	11.6113	11.7373	11.6144	11.4751	11.6544	11.9924	11.3813	11.5769	11.8794	11.2388	11.3649	11.6577
Eno1	_LAM[Oxidation (M)]QEFM[Oxidation (M)]ILPVGASSFR_.2	#N/A	9.6092	9.4236	9.1495	9.0828	9.5113	9.6176	9.4671	9.7000	9.3925	9.7551	9.2426	9.5277	9.5821	8.9842	9.5832	9.7395	9.3149	9.4887	9.5263	9.6354
Eno1	_YGKDATNVGDEGGFAPNILENK_.3	#N/A	8.9333	8.7297	8.8404	9.2146	8.8765	9.0902	8.4896	8.5041	8.6720	9.1112	9.1272	8.6960	9.2555	9.3441	8.5521	8.6732	9.4205	8.2203	8.5780	8.9334
Eno1	_FGANAILGVSLAVC[Carbamidomethyl (C)]K_.2	#N/A	3.3923	5.8462	4.8200	6.1123	6.5620	6.1057	5.5802	6.5307	5.5492	3.6528	6.4138	6.0907	3.4232	4.4678	5.6008	5.7656	4.9615	4.1037	5.4626	6.8055
Eno1	_SGKYDLDFKS[Phospho (STY)]PDDPSR_.3	#N/A	10.5185	10.9486	10.5758	10.9985	8.5678	10.7386	10.8432	9.9046	10.7211	11.2103	10.1418	10.8005	11.1808	11.3537	9.9520	10.7623	10.9230	10.8950	10.7143	10.9439
Eno1	_LM[Oxidation (M)]IEM[Oxidation (M)]DGTENK_.2	#N/A	7.9128	7.8791	6.5908	6.8355	8.3660	7.8361	7.2003	7.8557	7.7799	8.0004	7.4642	7.6052	7.8662	7.5161	7.5627	7.8842	7.6317	7.7669	7.8719	8.5251
Eno1	_AAVPSGASTGIYEALELR_.2	#N/A	10.6926	10.8587	10.7832	10.6929	10.4859	10.6127	10.6398	10.5034	10.6540	10.5490	10.4446	10.7040	10.9852	10.7008	10.6481	10.5053	10.3562	10.1907	10.5493	10.5058
Eno1	_YDLDFKS[Phospho (STY)]PDDPSR_.2	#N/A	9.5479	10.7126	10.8973	10.2219	7.5437	10.6767	10.2364	7.6293	10.4408	10.3999	8.7148	10.0418	9.2282	11.0621	10.1196	10.1712	10.3322	10.2336	10.3871	10.5342
Eno1	_SGETEDTFIADLVVGLC[Carbamidomethyl (C)]TGQIK_.2	#N/A	4.3117	4.0660	4.3046	4.1498	4.3153	4.6016	4.3944	4.9110	4.0606	3.8768	3.9530	4.5022	4.9846	5.4800	3.5821	4.2927	4.9499	3.8574	3.8802	4.0348
Eno1	_FTASAGIQVVGDDLTVTNPK_.2	#N/A	8.1978	8.1011	7.5514	7.8553	7.3667	7.7036	7.4855	7.8719	7.7896	7.7783	7.5043	7.7994	7.8697	8.8849	7.1938	7.5660	7.6918	7.8277	7.6995	8.0675
Eno1	_SFVQNYPVVSIEDPFDQDDWGAWQK_.3	#N/A	3.8340	4.0312	3.6084	3.1165	4.0409	2.8225	1.7506	4.0982	4.4282	3.6446	3.9068	2.6888	4.5936	3.1807	3.0325	3.1961	3.3101	3.5128	2.5237	3.5625
Eno1	_VNVVEQEKIDK_.2	#N/A	5.9768	5.7116	6.1468	5.8836	5.4850	6.4434	5.5177	3.3247	6.1588	5.7822	5.4526	6.1188	3.2453	6.3837	6.1527	5.9723	5.3184	5.9572	5.3920	5.8023
Eno1	_LAQSNGWGVM[Oxidation (M)]VSHR_.2	#N/A	4.7865	5.1843	5.5843	4.5028	4.6538	5.5126	4.2518	4.0108	5.2438	4.8705	5.0838	5.0707	4.4541	7.5072	4.6960	4.2335	5.9402	5.6228	5.4085	4.7111
Eno1	_FTASAGIQVVGDDLTVTNPKR_.3	#N/A	4.1074	4.5368	3.7688	5.5905	4.6847	3.6692	4.0959	4.4579	4.3249	4.3137	3.0286	4.1958	4.7281	3.8069	3.6731	3.3573	3.3917	5.6549	5.7185	5.5668
Eno1	_AGYTDQVVIGM[Oxidation (M)]DVAASEFYR_.2	#N/A	6.7400	7.8481	7.5955	7.0526	6.6651	7.4321	6.9855	7.4954	6.4485	7.4678	6.3687	7.0552	7.6526	8.4543	6.2422	7.1454	7.7778	7.2229	6.5112	7.0819
Eno1	_IDKLMIEM[Oxidation (M)]DGTENK_.3	#N/A	3.1706	3.4619	3.5155	4.2108	4.0092	3.7643	3.5329	4.1905	3.5920	3.3769	3.9664	3.2630	3.5055	3.5239	3.1101	3.5155	3.4379	4.0917	4.0273	3.3755
Eno1	_EALELLK_.2	#N/A	3.5972	3.7219	3.1338	3.9870	3.8041	4.3042	3.2440	3.8229	3.5200	3.4078	3.8944	3.4480	3.6281	3.1718	3.2694	2.9593	3.3201	3.8293	1.6235	4.3499
Eno2	_IEEELGDEAR_.2	#N/A	11.6880	11.6130	12.0244	11.8712	11.7785	11.7245	11.7254	11.9176	12.0480	11.8589	11.6865	11.9035	11.9066	12.0979	12.3624	12.1045	11.6609	11.6072	11.6721	11.9642
Eno2	_VNQIGSVTEAIQAC[Carbamidomethyl (C)]K_.2	#N/A	10.5798	10.9604	11.2598	11.0132	10.5432	10.6934	10.6860	10.8245	10.8721	10.8621	10.5748	10.6496	11.1243	11.1196	11.0550	10.7679	10.7260	10.5691	10.6058	10.7927
Eno2	_AVDHINSR_.2	#N/A	9.8970	10.1384	10.4779	10.1866	10.0998	10.1670	10.1892	10.2100	10.2346	10.1784	10.1881	10.2941	10.3164	10.3840	11.0368	10.3387	9.9659	10.0018	10.1849	10.2685
Eno2	_YITGDQLGALYQDFVR_.2	#N/A	9.3964	9.5929	9.5327	9.6367	9.3140	9.3717	9.3874	9.7169	9.5150	9.4576	9.3773	9.6565	9.9313	9.8993	9.6568	9.6533	9.4974	9.3760	9.4207	9.5704
Eno2	_DGKYDLDFK_.2	#N/A	9.2133	8.9429	9.0774	9.0766	9.1776	8.9054	9.1106	9.1231	9.1698	9.2438	9.0389	9.2322	9.2430	9.2923	8.9085	9.0376	9.3806	8.8337	8.8379	9.2151
Eno2	_AC[Carbamidomethyl (C)]NC[Carbamidomethyl (C)]LLLK_.2	#N/A	8.5542	8.5881	8.9573	9.1244	9.1858	8.9152	8.8312	8.8845	8.7302	8.7891	8.6709	9.1538	9.3659	9.0742	9.4180	8.7772	8.7514	8.7504	8.6578	8.7534
Eno2	_LGAEVYHTLK_.2	#N/A	8.3633	7.2288	8.6868	8.5452	8.6067	8.3360	8.0052	8.3327	8.3440	8.5851	8.1976	8.1872	8.4354	7.4783	8.5692	8.2126	8.3221	7.3502	8.3320	8.6875
Eno2	_DATNVGDEGGFAPNILENSEALELVK_.3	#N/A	7.9656	7.7558	8.4387	8.3167	7.6491	7.5836	7.5859	7.7596	8.4050	8.2719	7.7272	7.9754	8.3143	8.2196	8.3913	7.9463	8.2496	7.7564	7.8076	8.3142
Eno2	_FAGHNFR_.2	#N/A	7.3598	7.7572	7.1098	7.8286	7.8136	7.6432	7.9733	7.7281	7.2840	7.6573	6.9695	8.1339	7.3956	7.5271	8.5056	7.9657	7.4743	7.5900	7.6478	7.4904
Eno2	_LDNLM[Oxidation (M)]LELDGTENK_.2	#N/A	6.9667	6.7188	7.2164	6.9176	6.9206	7.3514	6.3445	6.7251	7.2437	7.5282	6.9426	6.9751	7.1181	7.3271	7.3802	7.4270	7.4221	7.1682	7.3245	7.1844
Eno2	_DKYGKDATNVGDEGGFAPNILENSEALELVK_.4	#N/A	5.4096	5.9627	5.8767	7.0744	5.7181	6.0270	5.5224	6.1818	5.7992	6.6053	6.3257	5.8680	6.4377	6.5327	7.1192	6.6907	6.9049	5.3211	6.5087	6.5007
Eno2	_[Acetyl (Protein N-term)]S[Phospho (STY)]IEKIWAR_.2	P17183_S2_M1_Eno2	6.0927	6.9235	4.6145	6.1316	6.1178	5.3426	5.6604	5.7577	5.2358	5.6245	5.1848	6.8049	6.7250	6.0352	6.4355	6.1794	5.7865	6.2503	6.0912	6.3036
Eno2	_DGKYDLDFKS[Phospho (STY)]PADPSR_.3	#N/A	8.1442	9.3077	8.6866	9.1926	6.9617	8.5675	8.7822	7.4126	8.3228	8.9461	7.7346	8.7836	8.8316	9.5575	8.1689	8.2835	8.9681	8.6171	8.4599	8.7928
Eno2	_IAPALISSGISVVEQEK_.2	#N/A	6.9650	6.4012	7.3350	6.7418	6.3521	6.6466	6.2771	6.5019	6.8757	6.7615	6.9621	6.3395	5.9356	7.2526	6.9683	6.8361	6.7595	5.7243	6.3633	7.0294
Eno2	_HIAQLAGNSDLILPVPAFNVINGGSHAGNK_.3	#N/A	4.8079	6.2625	7.2633	4.9949	5.7416	6.1486	5.4635	6.1830	7.2589	5.8113	4.9736	5.5392	6.3806	6.6063	7.6394	6.1187	5.2945	6.2910	4.8653	7.2001
Eno2	_M[Oxidation (M)]VIGM[Oxidation (M)]DVAASEFYR_.2	#N/A	8.1497	7.7628	7.7450	7.9409	7.7134	7.4476	7.3708	8.1601	7.9183	8.1498	7.6142	7.8986	8.6622	7.5402	7.9321	8.1655	7.9162	7.8441	7.8157	7.8262
Eno2	_LAM[Oxidation (M)]QEFM[Oxidation (M)]ILPVGAESFR_.3	#N/A	7.1832	7.5526	7.5214	6.9092	7.3416	7.0582	6.9040	7.0618	7.1775	7.5023	7.3060	7.4027	7.4990	6.6588	7.5002	7.7939	6.7846	7.3683	7.4106	7.3569
Eno2	_YDLDFKS[Phospho (STY)]PADPSR_.2	#N/A	5.9889	6.8777	7.3423	6.6271	3.6959	6.9378	6.6237	5.2708	6.6332	7.1842	6.0415	6.7215	6.3314	7.5962	6.4052	6.5028	6.7201	6.4871	6.6261	6.8959
Eno2	_IAPALISSGISVVEQEKLDNLM[Oxidation (M)]LELDGTENK_.4	#N/A	5.1988	5.8708	5.5731	4.8167	5.2290	5.1316	5.6690	5.8062	5.5023	5.3038	5.2273	6.0189	6.3725	6.5731	5.5030	5.9071	5.8761	5.8318	5.8391	5.5257
Eno2	_FTANVGIQIVGDDLTVTNPK_.2	#N/A	6.8026	7.1851	5.8220	6.3310	6.6437	6.6423	6.9709	7.3231	6.3673	6.9307	6.2902	6.7818	6.9810	8.5618	5.7586	6.4781	6.9485	7.1776	6.5895	6.7888
Eno2	_LAQENGWGVM[Oxidation (M)]VSHR_.3	#N/A	5.7051	3.5945	3.9398	3.6958	4.5015	4.9169	5.2346	5.3513	4.9362	4.4800	5.5251	5.0409	4.2720	3.3554	5.2707	4.8464	3.3620	3.5539	6.1255	6.1726
Eno2	_NYPVVSIEDPFDQDDWAAWSK_.3	#N/A	3.4729	4.5299	4.0092	4.2165	4.3115	3.0347	3.2296	3.1733	3.2887	2.4375	3.6631	4.4193	3.3967	3.9963	2.8068	3.8178	3.7110	4.0607	3.7708	4.1186
Ap2a1	_NADVELQQR_.2	#N/A	9.1702	8.0114	8.8666	8.7023	8.6653	8.0927	8.0461	8.8587	9.0481	7.9444	8.4571	8.7245	8.7472	8.1078	7.9017	8.4494	8.7128	8.7446	8.4394	8.3490
Ap2a1	_DFLTPPLLSVR_.2	#N/A	8.8673	8.6680	8.5907	8.8477	8.5215	8.6861	9.0746	8.6582	8.5007	8.2728	8.4257	8.7066	8.3411	8.0706	7.8141	7.7855	8.8908	8.7247	8.6123	8.3613
Ap2a1	_LLQC[Carbamidomethyl (C)]YPPPEDAAVK_.2	#N/A	8.6244	8.3353	8.2330	8.2438	8.1008	8.2124	8.5090	8.5578	8.5154	8.0566	8.2273	8.2354	7.9957	8.0925	8.0346	7.3475	8.4587	8.4006	8.1825	7.8901
Ap2a1	_GLAVFISDIR_.2	#N/A	7.8495	8.0405	8.2743	7.9255	7.6318	7.8035	8.0381	7.9324	8.0215	7.9094	7.9604	7.6405	8.0254	7.1989	6.6470	7.5548	7.7545	7.9259	7.7819	7.4143
Ap2a1	_QLSLPLQEAQK_.2	#N/A	7.7075	7.5683	7.5828	7.0865	7.4896	7.6587	7.9739	7.7335	7.3038	7.3155	7.3747	7.4499	6.2893	7.1788	7.2928	6.9651	7.7794	7.5251	7.4705	7.2047
Ap2a1	_HLC[Carbamidomethyl (C)]ELLAQQF_.2	#N/A	7.3292	7.2059	7.5695	7.1554	6.8731	7.0340	7.4187	7.6118	6.9731	6.7504	6.6752	7.0472	7.0015	6.6808	6.3312	6.7207	7.1229	6.8788	6.7446	6.7209

Ap2a1	_QSAALC[Carbamidomethyl (C)]LLR_.2	#N/A	6.9856	7.2499	6.5683	7.4916	7.1952	7.0977	7.4410	7.2144	6.9381	6.5777	6.9187	6.8906	6.9842	5.7982	6.7021	6.3962	7.3509	7.2221	6.8061	6.6024
Ap2a1	_ATIQGVLR_.2	#N/A	6.5878	6.6080	6.8112	6.6182	7.2176	6.6885	7.4045	7.0360	7.0271	6.8104	6.9321	6.8994	6.7776	6.6062	6.3437	5.9308	6.8486	7.3684	6.7310	6.6537
Ap2a1	_LVEC[Carbamidomethyl (C)]LETVLNK_.2	#N/A	6.5725	6.8281	6.6965	6.5463	6.3098	6.4475	6.6852	6.8753	6.3744	6.8035	6.3191	6.3885	5.9193	6.2079	5.9667	6.3350	6.6037	6.6251	6.3156	6.0679
Ap2a1	_NSGVLFENQLLQIGVK_.2	#N/A	7.0185	6.2311	6.1560	6.8340	5.9010	5.8356	6.3297	6.1316	6.1257	6.6804	6.1902	6.2235	6.3079	5.7508	5.6707	6.2432	6.3853	6.5153	6.0553	6.4236
Ap2a1	_THIDTVINALK_.2	#N/A	6.2344	6.2942	6.0884	6.7924	6.2836	6.3858	6.3495	6.5273	5.3594	6.4103	6.5237	6.0121	6.5381	6.5215	4.5391	5.7431	6.7915	6.4646	5.7840	6.6040
Ap2a1	_YGGTAQSLTLK_.2	#N/A	6.4699	5.5795	5.7053	6.2414	5.7414	5.7529	5.8853	5.5835	5.8908	5.0604	5.1116	4.9708	5.4687	5.6669	5.5183	4.8140	5.2709	6.0547	5.4404	5.4164
Ap2a1	_IAGDYVSEEVWYR_.2	#N/A	6.6185	6.5259	6.4509	6.4362	6.0364	6.3246	5.9375	6.7015	6.3598	5.6759	6.0779	5.5018	7.0285	4.0012	4.3075	5.3982	6.4412	5.1540	5.7517	5.6969
Ap2a1	_TSKEPVSR_.2	#N/A	5.6441	4.3095	4.3337	5.0431	5.2110	5.1007	4.9433	4.6495	5.5366	5.4059	5.3884	4.3711	5.1961	3.8438	5.0979	5.1555	4.1166	4.0939	4.3669	5.4966
Ap2a1	_LLGFGSALLDNVDPNPENFVGAGIIQTK_.3	#N/A	3.5071	4.7786	5.2615	3.6119	4.4239	4.5721	4.3563	4.1154	4.1706	4.0859	4.3561	4.5966	4.9177	4.2968	4.1869	4.7143	4.7173	4.9058	5.4178	3.6294
Ap2a1	_AC[Carbamidomethyl (C)]NQLGQFLQHR_.3	#N/A	7.0242	6.2391	6.7373	6.5837	6.9245	6.7581	7.2158	7.0112	6.3606	6.8370	6.9126	6.6293	6.4942	6.3043	6.0771	5.9476	6.3870	6.5501	6.0878	6.4298
Ap2a1	_VGGYILGEFGNLIAGDPR_.2	#N/A	5.6191	4.6575	4.4734	4.9964	4.5615	4.7579	4.8447	4.8085	4.7662	4.0520	5.2480	4.3364	3.9578	4.4970	4.0049	4.2361	4.9133	5.1291	4.6819	3.6747
Ap2a1	_AADLLYAM[Oxidation (M)]C[Carbamidomethyl (C)]DR_.2	#N/A	6.8853	6.3540	6.0450	6.2290	6.2194	6.4938	6.5884	6.8842	6.3931	6.2539	6.2028	6.1908	6.2996	6.0377	5.6740	5.8973	6.7846	6.7352	6.3638	6.3914
Ap2a1	_FFQPTEM[Oxidation (M)]AAQDFFQR_.2	#N/A	7.2787	6.9374	7.0131	6.8803	6.6844	6.9106	6.8257	7.1631	6.3672	6.6249	6.8889	6.4759	6.9072	6.0595	6.0675	6.3360	7.0986	6.8557	6.4225	6.4395
Ap2a1	_ILVAGDSMDSVK_.2	#N/A	4.4078	5.3557	5.7867	5.1251	3.8117	4.0531	5.3436	4.1575	3.4535	3.1133	5.4562	3.4844	3.9627	5.6463	2.9750	3.2519	3.6547	3.4947	3.7938	3.1837
Ap2a1	_ANHPMDAEVTK_.2	#N/A	3.1548	6.4651	7.0856	7.2326	4.5920	6.3153	7.2440	3.9097	5.4649	3.3611	5.9548	3.3612	3.7149	6.3436	5.1609	3.4997	3.4069	3.7425	4.0416	4.4368
Ap2a1	_YLALESM[Oxidation (M)]C[Carbamidomethyl (C)]TLASSEFSHEAVK_.3	#N/A	6.2208	4.7120	5.3631	4.6781	4.9333	4.1154	4.6618	5.2012	4.6977	5.1674	4.8495	4.5163	6.2576	5.1528	3.5335	4.3976	4.7951	4.4171	4.5656	5.3636
Ap2a1	_TSVQFQNFLPTVVHPGDLQTQLAVQTK_.3	#N/A	2.7601	3.7116	3.7676	3.9767	4.0699	4.2939	3.7636	3.8087	1.9531	3.4376	3.4570	3.4038	3.6384	3.1821	3.0484	3.5762	3.4581	2.1989	3.5842	4.3603
Ap2a1	_ASPDLVPMGEWTAR_.2	#N/A	3.4564	3.8627	6.8651	6.4474	4.6744	5.3754	3.3202	3.6821	4.9119	3.5887	5.5845	3.5888	3.4873	6.2707	3.4101	4.3194	3.1793	3.9701	4.2692	4.2092
Ap2a1	_VAAQVDGGAQVQQVLNIEC[Carbamidomethyl (C)]LR_.2	#N/A	3.7021	4.2014	4.1551	4.4666	3.3485	3.0960	4.0286	3.6747	3.5743	4.2086	3.6766	3.9276	3.5077	4.2683	3.5883	3.6868	3.9756	4.3089	2.9319	3.9420
Ap2a1	_LEPNAQAQMYR_.2	#N/A	3.1880	4.2847	6.9927	6.8227	3.3949	3.4282	6.7710	3.4185	3.5734	4.4994	5.6733	4.1741	3.7389	4.2415	3.6786	4.0509	3.9562	3.8770	3.5255	4.2085
Ap2a1	_AVEYLTLSSVASTDVLATVLEEM[Oxidation (M)]PPFPER_.3	#N/A	4.1346	3.8682	3.1700	3.3792	3.4723	3.6964	4.0688	4.4307	4.1279	4.3409	3.0014	2.3392	4.2359	3.7797	3.6460	2.9786	3.9280	3.2215	3.5205	3.4570
Ap2a2	_TTQIGC[Carbamidomethyl (C)]LLR_.2	#N/A	7.6576	7.4868	7.2837	7.6802	7.5382	7.6016	7.8696	7.8974	7.6936	7.1648	7.2975	7.3030	7.0209	6.5180	7.2081	6.9379	7.3960	7.5923	7.3476	6.9010
Ap2a2	_LLQC[Carbamidomethyl (C)]YPPPDPAVR_.2	#N/A	7.4862	7.2274	7.3291	6.9185	7.5219	7.7415	7.6553	7.4218	7.3081	7.0165	7.4854	7.0987	6.5049	6.9379	6.4897	6.4573	7.2109	7.3412	6.9630	6.4852
Ap2a2	_ATIQDVLR_.2	#N/A	6.6664	6.5111	7.0235	7.1510	6.5286	7.2234	7.3971	6.9543	6.9595	7.2761	7.1178	6.7231	7.0873	6.3278	6.5110	6.7723	6.6131	7.3904	6.4831	6.4263
Ap2a2	_QLSNPQQEVQNIFK_.2	#N/A	6.3480	6.3031	6.4841	6.3560	6.4115	6.6482	6.6725	6.8423	6.7326	5.7603	6.8155	6.7043	6.8750	5.4077	5.9170	6.1054	6.5321	6.5690	6.3582	6.1273
Ap2a2	_SSPLIQFNLLHSK_.2	#N/A	6.7548	6.5872	6.9586	6.0140	6.2428	6.0600	6.8537	6.7795	6.4975	5.9705	6.1353	6.1222	6.5087	5.5863	5.6486	5.7108	6.4002	6.4726	5.5231	6.5402
Ap2a2	_KGPSTVTDLEETKR_.3	#N/A	6.4255	5.8546	6.4056	6.2468	5.8953	6.2446	6.8329	6.5655	6.5374	6.0860	6.4353	6.2631	6.3271	5.8940	6.2647	5.4372	6.3101	5.9446	6.0625	5.9167
Ap2a2	_LTEC[Carbamidomethyl (C)]LETILNK_.2	#N/A	5.1141	5.5998	5.2731	5.1814	5.4916	5.8186	5.6369	5.1716	5.9983	5.6748	5.3286	4.9513	6.3311	4.3293	4.3677	5.1651	5.5755	5.1733	5.1574	4.8395
Ap2a2	_FFQPTEMASQDFFQR_.2	#N/A	7.5939	7.1763	6.2134	6.6311	6.9048	7.3448	6.7089	7.8163	7.2383	7.2120	7.0051	6.9858	6.1662	6.5953	6.3853	6.5977	7.3424	7.2595	6.9482	6.5489
Ap2a2	_THIETVINALK_.2	#N/A	6.0469	5.4633	5.6442	5.6611	5.2499	5.5295	5.7390	6.3556	5.7045	5.3911	5.8843	5.2427	4.3735	3.9519	4.3593	5.2087	5.0780	6.1441	3.8152	3.9678
Ap2a2	_YGGTFQNVSVK_.2	#N/A	6.2354	4.2362	5.6179	5.0775	4.5761	4.6089	5.6092	5.1915	5.6016	5.5250	4.8923	3.0310	4.8455	3.5888	3.9517	4.8490	5.7657	5.4104	5.6468	4.9612
Ap2a2	_LSTVASTDILATVLEEM[Oxidation (M)]PPFPER_.3	#N/A	5.0936	5.5270	4.9400	4.8740	4.9886	4.1549	4.9125	4.5009	4.6760	4.1210	3.0716	4.2172	5.2192	4.5611	3.7427	4.7836	5.5353	4.6553	5.0092	4.2940
Ap2a2	_TVFEALQAPAC[Carbamidomethyl (C)]HENLVK_.3	#N/A	3.1572	4.8175	3.3343	4.4700	3.5493	4.2241	4.4360	3.9992	4.4327	4.5052	4.3340	4.0707	4.3196	3.3214	3.4284	3.1429	4.5369	4.0907	5.1791	3.2884
Ap2a2	_VIQIVINR_.2	#N/A	4.4958	4.3766	3.9799	3.1409	3.1494	4.4603	4.5905	3.1399	4.6331	4.2539	3.2397	4.1027	4.4742	3.7242	3.9241	3.8054	4.1662	4.6422	3.2823	4.4026
Ap2a2	_AVDLLYAMC[Carbamidomethyl (C)]DR_.2	#N/A	3.8653	4.0685	5.4942	3.7189	4.0723	4.3785	5.1274	4.0910	4.1585	3.6759	3.8755	3.1799	3.8962	5.3290	3.0012	3.2275	3.5883	3.5612	3.4784	2.6706
Ap2a2	_TSPDLVPMGDWTSR_.2	#N/A	3.6463	5.9151	6.8661	6.2243	3.8533	4.2551	6.1329	3.8720	3.5692	3.3988	6.3665	3.3989	3.6772	3.2210	3.2202	3.0085	3.3693	3.7802	4.0792	4.3991
Ap2a2	_ILVAGDTMDSVK_.2	#N/A	3.5839	4.0642	4.1071	4.5914	2.9216	3.1457	3.1186	3.4806	3.1777	3.7902	3.5521	3.7903	3.2858	4.3304	1.9559	3.9288	4.4787	4.1716	2.9698	4.0077
Cox7c	_SHYEEGPGK_.2	#N/A	7.8732	7.7512	7.6976	7.4509	7.7542	7.5276	7.6223	8.0808	7.7714	7.9546	8.0206	7.4161	7.7748	7.5989	8.5953	8.0510	7.8718	7.7207	7.8893	7.5626
Cox7c	_NLPFSVENK_.2	#N/A	7.2372	6.0595	6.5948	6.9745	6.5401	6.8404	7.1831	6.7310	6.9127	6.5921	6.4445	6.7366	7.1477	6.0742	7.1159	5.9225	6.5494	7.0534	6.8124	6.7542
Hk1	_FLSQIESDR_.2	#N/A	9.3063	9.1563	9.1086	9.1345	9.0299	9.1468	9.1725	9.5536	8.9375	9.0588	9.1418	9.0393	9.1273	8.9219	8.2436	8.5558	9.3657	8.9938	9.0359	8.7558
Hk1	_ITPELLTR_.2	#N/A	8.9923	8.8912	8.7957	9.1064	9.0471	8.9273	9.2374	8.9641	8.7347	8.8478	8.9107	8.9834	8.9978	8.1266	8.4112	8.4732	8.6120	9.0841	8.9167	8.7814
Hk1	_ESLLFEGR_.2	#N/A	8.9285	8.9128	9.1713	9.0499	8.7057	8.7091	9.0666	8.8989	8.8207	8.6523	8.9962	8.8071	8.9307	8.4586	8.0164	8.3766	8.8268	8.8827	8.7111	8.6725
Hk1	_HIDLVEGDEGR_.2	#N/A	8.9530	8.6796	8.6843	8.6492	8.5302	8.7453	8.7245	9.0164	8.7304	8.7046	8.7818	8.4101	8.9128	8.4239	8.1742	8.6255	8.9689	8.6279	8.6176	8.3522
Hk1	_ASGVEGADVVK_.2	#N/A	8.8880	8.7069	8.8545	8.5461	8.5392	8.7478	8.8831	8.8263	8.8009	8.6774	8.8411	8.2249	8.6886	8.3822	8.4546	8.2056	8.5218	8.5568	8.6120	8.5289
Hk1	_LVPDSDVR_.2	#N/A	8.5013	8.4572	8.4760	8.3690	8.3829	8.6259	9.0124	8.6345	8.5987	8.4732	8.5175	8.4011	8.2616	8.2494	8.3894	8.3187	7.9023	8.6234	8.2849	8.2324
Hk1	_GAALITAVGVR_.2	#N/A	8.2505	8.5435	8.4316	8.5900	7.8990	8.1180	8.6490	8.3869	8.2809	7.7324	7.6467	7.8987	8.3348	8.2105	7.6529	7.9216	7.7395	8.5383	8.4595	8.4031
Hk1	_SIPDGTEHGDFLALDLGGTNFR_.3	#N/A	8.3830	8.5861	8.2323	8.6126	7.9761	8.2547	8.3951	8.4471	8.3965	8.3148	8.1806	8.2164	8.1850	8.3256	7.2194	8.0232	8.3298	8.3301	7.8906	8.1289
Hk1	_ATDC[Carbamidomethyl (C)]VGHDVATLLR_.3	#N/A	7.0577	6.9402	7.5781	6.7902	6.6368	6.8555	7.2271	6.8882	6.9924	7.3389	6.5882	6.8948	6.6347	7.1863	6.3397	6.7959	6.9567	6.8815	6.8400	6.1384
Hk1	_TTVGVDGSLYK_.2	#N/A	7.9258	7.8644	7.9812	7.7486	7.5205	8.0776	7.9735	7.5627	7.5983	7.8094	7.2947	7.6435	7.6999	7.5948	7.6608	7.6048	7.4563	8.1801	7.5836	7.3784
Hk1	_GDFIALDLGGSSFR_.2	#N/A	8.0311	7.7082	8.3499	8.0017	7.7455	7.8237	7.6539	7.7383	8.1364	7.7631	7.5468	7.2783	7.9557	7.3727	7.4588	7.6919	7.9576	7.3371	7.7162	7.4043
Hk1	_LSDEILIDILTR_.2	#N/A	7.8749	8.0313	8.0097	7.6352	7.5615	7.6427	7.7968	7.7406	7.5312	7.5914	7.5148	7.7013	7.6067	7.7357	6.8598	7.3594	7.9502	7.7931	7.5767	7.5205
Hk1	_VVDEYSLNSGK_.2	#N/A	7.1251	7.1200	7.3587	7.1860	7.0761	7.2457	7.4973	6.7080	6.7459	6.9320	7.2807	6.9777	7.1455	6.9568	7.0255	6.8735	7.1415	7.2174	6.8830	7.1429
Hk1	_RLVPDSDVR_.2	#N/A	6.7584	7.2070	7.1510	6.8701	7.1768	7.4716	7.3811	7.3197	7.1632	6.7711	7.2823	7.0069	7.2360	7.1860	6.9588	7.1548	7.3483	7.1822	7.1347	7.1293
Hk1	_IDEAVLITWTK_.2	#N/A	6.8619	6.8386	7.1418	7.0425	6.6429	6.9917	7.1987	6.9946	6.9428	6.8246	6.8614	6.6758	6.3402	6.6846	6.4829	6.0954	6.9394	7.1966	7.1694	6.6708
Hk1	_GQISEPLK_.2	#N/A	5.8996	6.5104	6.5515	7.0363	6.1862	6.7222	7.3179	5.9414	6.7450	6.7038	6.7098	6.9802	6.1422	5.7454	6.3417	6.3808	5.7295	6.7866	6.7022	7.0659
Hk1	_AILQQLGLNSTC[Carbamidomethyl (C)]DDSILVK_.2	#N/A	7.1364	7.1950	6.4109	6.9231	6.6555	6.9373	7.3030	6.8511	6.6254	6.4855	6.7472	6.7897	7.1659	7.2750	5.3770	6.5116	4.6608	6.4875	6.4266	6.3995
Hk1	_NILIDFTK_.2	#N/A	5.4163	6.4422	6.4808	7.2402	6.1878	6.1258	6.4940	5.3287	6.8227	6.6275	6.4820	6.5913	6.2186	6.2062	5.7700	5.9037	5.5671	6.8009	6.6600	6.4323
Hk1	_M[Oxidation (M)]HPQYSR_.2	#N/A	7.5779	6.9680	6.2710	6.6388	7.0543	7.2977	7.2131	7.7487	6.9492	7.2701	6.9847	3.2720	7.4260	6.7071	8.3348	7.5302	6.8609	7.4532	7.4385	7.3836
Hk1	_M[Oxidation (M)]LPTFVR_.2	#N/A	7.8201	7.5708	7.4845	7.8913	7.8722	7.7158	8.2602	8.3910	7.6216	7.3244	7.3442	8.0224	7.9940	6.3997	7.4137	7.6022	7.3453	8.2439	8.8424	7.6656
Hk1	_TDFDKVVDEYSLNSGK_.3	#N/A	6.3648	6.7294	7.2772	4.2361	4.3292	6.2220	6.5978	6.4585	7.4464	6.3489	5.7974	6.1129	6.6204	6.2451	6.3935	6.8917	6.8142	6.1525	6.5650	6.4775
Hk1	_M[Oxidation (M)]ISGM[Oxidation (M)]YLGEIVR_.2	#N/A	6.3056	5.4589	5.0278	5.5430	5.7073	5.8451	5.5350	6.1984	5.4726	5.6336	5.5429	5.4799	6.1170	5.3016	5.1717	5.3857	5.7816	5.9555	5.8799	5.8529
Hk1	_IM[Oxidation (M)]HQTVK_.2	#N/A	5.7608	6.2811	4.5835	5.3677	5.5524	5.8809	6.4411	5.8188	5.5000	5.9197	4.4516	2.9854	5.9578	5.0181	7.0214	6.0148	5.8093	6.4297	6.4327	5.9633
Hk1	_GAAM[Oxidation (M)]VTAVAYR_.2	#N/A	6.5260	6.2545	6.7976	6.9254	6.2587	6.2282	6.2362	6.3805	6.0724	5.4447	5.6269	6.5154	6.9416	6.3997	5.6474	6.1982	6.2500	6.7379	6.2012	6.0032
Hk1	_LPVGFTFSFPC[Carbamidomethyl (C)]R_.2	#N/A	5.7978	4.4848	4.9781	4.6905	5.4995	4.7753	5.4990	3.2184	5.3130	5.2090	3.6180	4.3742	4.8454	5.3716	4.2625	3.8629	3.7561	5.7099	4.8124	4.9452
Hk1	_DYNPTASVK_.2	#N/A	3.5119	3.8072	5.1181	5.8803	3.7188	5.6752	6.6890	3.7376	6.0023	5.6355	6.0485	5.8082	3.4149	5.2663	5.3425	4.9772	3.2348	5.6803	3.8495	3.8846
Hk1	_C[Carbamidomethyl (C)]TVSFLLSEDGSGK_.2	#N/A	4.6954	4.9854	5.3120	5.1523	4.2493	4.1390	4.4829	5.4066	3.3509	3.6170	5.8421	4.8329	3.4589	3.9341	2.8690	3.7556	5.1431	3.9985	5.4676	5.1908
Hk1	_QIEETLSHFR_.2	#N/A	6.9981	7.1868	7.1649	6.7567	6.9525	7.0055	7.1913	7.4580	7.1131	6.8784	6.9349	7.1183	7.0134	6.8851	6.4918	6.6115	7.4178	7.1439	6.6585	6.7511
Hk1	_FTTSDVAAIETDKEGVQNAK_.3	#N/A	5.4251	5.7849	6.3458	6.0791	5.4141	5.3086	5.8622	5.7901	5.8343	5.1520	5.0741	5.1749	5.5486	5.8973	5.6180	5.3844	5.4668	5.4337	6.0273	5.2105
Hk1	_SANLVAATLGAILNR_.2	#N/A	4.3444	2.9045	4.0568	3.3476	4.7129	3.2542	2.7745	4.2451	3.9423	3.9010	4.3431	3.8070	4.0503	3.2454	3.4604	3.0514	3.8511	2.6138	3.7251	4.0626
Hk1	_M[Oxidation (M)]C[Carbamidomethyl (C)]INTEWGAFGDDGSLEDIR_.2	#N/A	3.5242	4.1221	4.1085	3.7613	3.8725	4.2418	3.8531	4.2022	3.2561	4.2084	3.3346	4.1741	4.1090	4.6174	3.2767	3.8170	3.6480	3.5860	3.0617	4.3337
Hk1	_FLLSESGSGK_.2	#N/A	7.5203	7.7574	7.4098	7.7657	7.4562	7.3905	8.1907	6.9780	7.5980	7.8237	7.3986	7.4229	7.7599	7.5782	7.0611	7.6333	7.1969	7.8961	7.8714	7.6868
Hk1	_LGVEPSHDDC[Carbamidomethyl (C)]VSVQHVC[Carbamidomethyl (C)]TIVSFR_.4	#N/A	4.9986	5.2937	4.7476	5.1584	3.3095	3.8182	4.6193	4.8431	3.6588	3.1557	3.1374	5.2133	4.3804	4.3269	3.7640	3.9655	4.5243	3.7916	4.3982	4.2939
Hk1	_QALMEVK_.2	#N/A	3.6541	4.3487	4.1904	4.0353	2.9918	3.2159	3.5963	3.3544	3.1075	3.8604	3.4819	4.2382	3.2156	4.2602	1.3968	3.9990	3.8921	4.2418	3.0400	2.2640
Hk1	_AAQLC[Carbamidomethyl (C)]GAGMAAVVEK_.2	#N/A	3.1110	4.2081	4.1484	4.0296	3.3180	4.0178	3.7301	3.3367	3.6503	4.4225	3.1290	3.9342	3.8158	5.5029	3.1390	3.9740	4.3348	4.3155	3.1137	3.8638
Hk1	_TVC[Carbamidomethyl (C)]GVVSK_.2	#N/A	3.9394	4.0634	4.4757	3.7500	3.2771	3.5012	4.2640	3.6398	4.3231	4.1457	3.1966	3.9529	4.4311	4.4628	4.4848	4.2843	4.1774	3.6557	4.8580	3.6522
Hk1	_SEMEMGLR_.2	#N/A	4.4328	3.5700	3.4683	3.2565	3.7706	4.2144	4.3679	4.1332	4.2881	3.7847	3.7668	3.4594	4.4536	3.4554	2.8924	3.2769	3.1701	3.1623	4.3117	3.4224
Ppia	_SIYGEKFEDENFILK_.3	#N/A	9.9570	10.4139	10.3179	10.3211	9.9565	10.0378	9.9469	9.9980	10.0217	10.2895	9.9535	10.3205	10.6756	10.6555	10.1776	10.4084	9.8571	10.0222	10.1281	10.4066
Ppia	_VSFELFADK_.2	#N/A	7.9578	8.4381	8.8580	8.8033	8.0439	8.3957	8.4464	7.2661	8.3847	8.5617	8.4734	8.4775	8.1672	8.6674	8.7934	8.3307	7.8695	8.3754	8.4278	8.5097
Ppia	_KITISDC[Carbamidomethyl (C)]GQL_.2	#N/A	8.1151	8.1388	8.6174	8.0137	7.8421	8.0388	8.1410	8.2550	8.1264	8.2314	7.8762	8.0504	7.5985	8.6903	8.4128	7.6585	8.0964	8.2372	7.6422	7.9465
Ppia	_VNPTVFFDITADDEPLGR_.2	#N/A	9.3049	9.5024	9.4596	9.4089	9.1814	9.3066	9.3201	9.1966	9.5500	9.5201	9.0207	9.3621	9.5822	9.5437	9.5497	9.4970	9.4202	9.1868	9.4554	9.4565
Ppia	_VSFELFADKVPK_.2	#N/A	9.5296	9.7596	9.7091	9.7238	9.6222	9.4780	9.4265	9.8867	9.7041	9.7082	9.2231	9.4606	10.0390	9.6044	9.7706	9.6546	9.5327	9.4237	9.7173	9.6998
Ppia	_IIPGFMC[Carbamidomethyl (C)]QGGDFTR_.2	#N/A	3.3489	9.4068	10.6951	10.5467	8.0706	9.1375	9.6913	7.0148	8.6597	8.3117	9.4655	8.1714	3.5208	10.4148	8.9765	3.6938	8.5271	7.5036	7.7830	8.5618
Ppia	_HTGPGILSM[Oxidation (M)]ANAGPNTNGSQFFIC[Carbamidomethyl (C)]TAK_.3	#N/A	6.8879	8.1475	6.7928	7.2991	6.8861	6.9243	7.1347	6.9478	7.3148	8.3364	6.4337	6.8582	7.3094	7.4245	7.2833	7.0097	7.0130	7.0114	6.9730	7.2203
Ppia	_FEDENFILK_.2	#N/A	9.7023	9.4523	10.0730	9.5144	3.6031	9.5490	9.5234	3.6219	9.4269	9.5614	4.3447	9.0223	9.3392	9.7358	9.6889	9.2063	9.4149	9.3307	9.1663	9.2277
Ppia	_EGM[Oxidation (M)]NIVEAMER_.2	#N/A	3.5063	6.9931	8.5294	8.1921	5.2788	5.8621	6.2681	3.7872	5.6888	3.9951	5.6338	3.3870	3.9030	8.1654	5.4154	3.7124	6.5157	3.7948	3.9052	5.4808
Ppia	_VKEGM[Oxidation (M)]NIVEAM[Oxidation (M)]ER_.3	#N/A	7.1322	7.0856	5.9965	6.9882	6.5451	7.3215	7.6225	7.4002	7.5033	7.4050	6.7624	7.0050	7.6163	7.1699	7.7782	7.7545	6.3230	7.2024	7.4744	7.6983
Ppia	_TEWLDGK_.2	#N/A	4.4250	3.5778	4.9866	6.2851	3.7627	3.9868	5.2044	4.6351	5.9335	5.1324	4.2118	3.4672	3.9455	4.4705	3.3555	5.8824	3.6767	3.5119	3.8110	6.0510
Ppia	_[Acetyl (Protein N-term)]M[Oxidation (M)]VNPTVFFDITADDEPLGR_.2	#N/A	3.6195	4.3833	4.1558	4.0699	2.9572	3.1813	3.0830	3.3199	3.1421	3.8258	3.5165	4.2727	3.2501	4.2947	1.4367	3.9644	3.8576	4.2073	3.0055	3.9720
Tpi1	_VVLAYEPVWAIGTGK_.2	#N/A	10.8001	10.7869	11.2303	10.9501	10.9000	10.7600	10.6424	10.7394	10.9287	10.9854	10.7375	10.6616	11.0479	10.8481	11.0383	10.9173	10.8617	10.6153	10.8381	10.9083
Tpi1	_IIYGGSVTGATC[Carbamidomethyl (C)]K_.2	#N/A	8.9584	8.1612	9.4361	9.2352	8.7045	9.1201	9.0006	8.9799	8.9354	9.0431	8.7437	8.9825	9.0469	9.4451	9.1777	8.9612	9.1511	8.9453	7.7176	9.1511
Tpi1	_SNVNDGVAQSTR_.2	#N/A	9.9497	10.1863	10.1521	10.2908	9.9680	10.1358	10.2017	10.1875	10.3579	10.1151	9.9145	10.1177	10.2179	10.2936	10.5856	10.2457	9.8068	10.0180	9.9935	10.2672
Tpi1	_FFVGGNWK_.2	#N/A	9.4587	9.4518	9.4786	9.7053	9.2847	9.3467	9.5168	9.4975	9.3229	9.5708	9.4486	9.6963	9.4755	9.3716	9.3914	9.3452	9.1513	9.5969	9.5274	9.6818
Tpi1	_IAVAAQNC[Carbamidomethyl (C)]YK_.2	#N/A	8.0967	8.7669	8.7316	8.8813	8.5931	8.4312	8.7032	8.2111	8.7835	8.4579	8.0930	8.5221	8.0523	8.7552	9.2807	8.5314	8.5045	8.5519	8.4476	8.7593
Tpi1	_VSHALAEGLGVIAC[Carbamidomethyl (C)]IGEK_.3	#N/A	6.8457	6.6887	7.0894	6.8955	6.8278	7.1949	6.4008	6.5414	6.9234	7.0235	6.7681	6.7796	7.2994	6.8947	6.7210	6.5865	7.0758	6.3454	6.7791	7.1203
Tpi1	_VIADNVKDWSK_.2	#N/A	7.6994	8.1633	7.0830	8.0058	8.1524	7.8314	7.9851	8.7023	7.7048	7.7729	7.2748	8.3974	8.8785	7.8904	8.3878	8.0949	8.1853	8.1272	7.9511	8.0245
Tpi1	_DLGATWVVLGHSER_.2	#N/A	7.5623	8.0871	9.3858	8.8216	7.8879	8.2433	7.9312	8.0130	8.5427	8.8138	8.6054	8.3191	7.1460	8.3108	8.6432	8.5428	7.9657	8.0256	8.2373	8.7590
Tpi1	_VVFEQTK_.2	#N/A	5.1255	6.2996	5.8482	7.2299	5.6940	6.7182	7.4891	4.9100	7.0541	7.0947	7.1575	7.0231	7.3286	6.5770	7.5040	7.5490	5.4518	7.4080	7.4072	7.3727
Tpi1	_VTNGAFTGEISPGM[Oxidation (M)]IK_.2	#N/A	7.8826	7.7772	8.6003	7.8392	7.2402	8.1835	8.0472	8.0059	8.3989	8.3438	7.9590	8.0523	7.1495	8.1567	8.7205	8.3825	8.2231	8.1386	8.2082	7.8419
Tpi1	_TATPQQAQEVHEK_.2	#N/A	10.1138	9.9772	10.3943	10.2379	10.1740	10.1304	10.1687	10.4181	10.3080	9.7651	9.9240	10.2062	10.1725	10.2016	10.5873	10.2882	9.9970	10.1010	9.8928	10.2203
Tpi1	_HVFGESDELIGQK_.2	#N/A	6.7496	7.1508	7.2328	6.9391	6.9297	6.5954	6.8279	6.8936	7.0539	6.9774	6.5903	6.9520	7.0839	7.5938	7.1367	7.3479	7.0849	7.0052	6.9981	7.3645
Tpi1	_ELASQPDVDGFLVGGASLKPEFVDIINAK_.3	#N/A	3.8800	4.1067	4.0775	3.6206	1.4782	4.5750	4.3569	5.2612	4.6067	4.2751	3.0672	4.9522	5.4282	6.3235	5.3770	3.6758	5.4829	5.0766	5.0045	4.4248
Tpi1	idomethyl (C)]LGELIC[Carbamidomethyl (C)]TLNAANVPAGTEVVC[Carbamidomethyl (C)]APPT.	#N/A	3.9406	3.6408	4.9246	3.7488	2.0905	2.6392	4.2628	3.6409	2.3555	4.1469	3.0940	3.5642	4.4299	4.4639	3.7071	4.2854	4.3484	3.3880	3.3487	3.6510
Tpi1	_RHVFGESDELIGQK_.3	#N/A	3.6329	4.4291	5.1084	5.3000	5.2996	5.1704	4.7315	5.2280	3.3805	5.2083	5.5681	5.4618	3.7816	6.1000	6.0001	5.0116	4.0759	5.7916	5.4382	5.7022
Slc2a1	_TFDEIASGFR_.2	#N/A	6.6021	7.0637	6.5637	6.6321	6.4898	6.6358	7.1628	6.6893	6.1475	6.3224	6.4763	6.7161	6.6976	5.9099	5.8573	5.8312	6.3359	7.0047	6.1288	6.1567
Slc2a1	_AKS[Phospho (STY)]VLKK_.2	P17809_S226_M1_Slc2a1	5.4602	5.4247	5.3669	5.5761	5.6450	5.4751	5.3698	5.2273	5.4519	5.1289	5.1131	4.6448	6.1404	4.4108	1.8550	4.2361	6.2464	4.6839	4.5322	5.1101
Slc2a1	_VTILELFR_.2	#N/A	3.8922	5.1179	4.3017	4.7916	4.5894	4.6467	4.9675	3.7418	5.2780	3.6422	4.9972	4.9393	5.3398	4.3397	3.6161	3.7071	4.4880	4.6859	4.4133	4.0170
Slc2a1	_QGGASQSDKT[Phospho (STY)]PEELFHPLGADSQV_.3	P17809_T478_M1_Slc2a1	3.8052	3.5138	3.3418	3.7790	4.0122	9.0503	3.6690	4.0309	3.7281	3.2399	4.1024	3.2400	3.8361	3.3799	3.0613	3.1674	3.5282	9.5676	3.9203	8.3764
Hspa1b;Hspa1a	_IINEPTAAAIAYGLDR_.2	#N/A	6.1091	4.9518	5.2263	5.5598	6.0271	5.1532	5.0490	5.4353	4.8579	4.7261	4.9487	5.0629	4.9355	5.4264	4.5063	4.7760	5.4524	4.9555	5.4980	4.4094
Hspa1b;Hspa1a	_NQVALNPQNTVFDAK_.2	#N/A	6.1101	6.1853	6.7076	6.8042	6.2917	6.2602	6.3912	6.5156	6.5172	6.3260	6.2682	6.3693	6.1770	6.4890	6.3084	6.2846	6.6359	6.3242	6.3176	5.9939
Hspa1b;Hspa1a	_ATAGDTHLGGEDFDNR_.3	#N/A	4.2335	10.9178	4.0966	5.9010	3.5713	8.9369	5.6451	9.4647	4.6620	6.1501	3.7644	3.6930	4.1713	4.0837	3.5813	9.0542	3.7984	8.7211	3.8721	10.9008
Ptpra	_QAGSHSNS[Phospho (STY)]FR_.2	P18052_S180_M1_Ptpra	5.4656	5.5971	6.2097	5.6485	5.9036	6.3796	6.0099	6.2016	6.1859	6.1101	4.9812	5.2408	3.6079	5.1624	5.2148	5.5949	5.8431	6.5021	3.4957	5.7186
Ptpra	_VVQEYIDAFSDY[Phospho (STY)]ANFK_.2	P18052_Y825_M1_Ptpra	7.4116	8.1554	7.0587	6.6600	5.2975	6.8030	7.5667	7.0255	5.9369	7.3878	6.4517	7.4912	7.6110	7.5723	4.7982	6.3307	6.5928	7.3328	6.5364	6.5043
Ctsd	_VSSLPTVYLK_.2	#N/A	7.3534	7.3792	7.2258	7.5802	7.7022	7.2177	7.4245	7.0894	6.8928	7.5416	7.3028	7.5868	7.5722	7.5596	7.5623	7.5344	7.4431	7.4853	7.0745	7.5129
Ctsd	_TTEPVSELLK_.2	#N/A	6.6059	6.5272	7.2287	6.8246	6.9544	6.6186	6.7497	6.6812	6.8254	7.0331	6.9073	6.7789	6.4694	6.9469	6.9264	7.0275	6.8118	6.3354	6.8712	7.3585
Ctsd	_QPGIVFVAAK_.2	#N/A	5.8908	5.5420	5.9276	6.0298	6.0182	6.2352	5.9729	5.9257	5.9684	5.7208	6.0391	5.8053	5.6259	5.7147	6.5463	5.7344	5.8802	5.6894	5.7534	6.1865
Ctsd	_QIFGEATK_.2	#N/A	4.6087	4.3584	5.3000	4.1538	4.8792	5.2800	4.7184	4.5250	5.0758	5.1916	4.9290	4.9335	4.7278	5.5607	5.9934	5.7016	4.2220	4.8869	4.9301	5.3032
Ctsd	_ILDIAC[Carbamidomethyl (C)]WVHHK_.3	#N/A	3.0307	4.3276	5.0867	4.5456	4.3303	3.5366	3.0118	2.4137	3.7148	4.3029	3.6437	3.8639	3.9354	3.4080	3.3173	3.1891	4.2152	3.1835	3.3290	4.4695
Ctsd	_AIGAVPLIQGEYMIPC[Carbamidomethyl (C)]EK_.2	#N/A	3.6395	4.9624	5.3069	5.7988	1.9009	3.5069	5.1302	3.3399	2.8450	3.8458	4.1532	3.4030	3.6602	6.1833	4.4654	3.9844	3.8776	3.9556	3.6042	3.9837
Bsg	_YIVDADDR_.2	#N/A	6.7234	6.7424	6.8280	7.1640	6.6735	6.9322	6.8203	6.8107	6.9379	6.9975	6.9524	7.1887	6.8756	7.1843	6.7277	6.5250	6.7541	6.9396	6.8208	7.0225
Bsg	_SEINVEGPPR_.2	#N/A	7.0018	6.6905	6.6108	6.6894	6.5292	6.6338	6.9213	6.9696	7.0961	6.5780	6.7389	6.9375	6.9784	6.9247	6.7691	6.8700	6.4268	6.7741	6.5431	6.5420
Bsg	_SGEYSC[Carbamidomethyl (C)]IFLPEPVGR_.2	#N/A	6.6709	6.7089	7.2311	6.9315	6.4914	6.9191	6.8231	6.7073	6.1753	6.7400	6.7204	6.9311	6.5823	6.8013	6.4181	6.4777	6.9147	6.8075	6.4018	6.5889
Bsg	_YVVVSTPEK_.2	#N/A	6.2410	6.5968	6.5830	6.5966	6.6848	6.6221	6.9050	6.2816	6.5815	6.8658	6.8450	6.2966	6.9892	6.9738	6.8572	6.4144	6.3611	6.7780	6.6253	6.1437
Bsg	_VLQEDTLPDLHTK_.3	#N/A	3.7450	3.5740	3.2816	4.5283	3.3642	3.9853	3.0961	4.3623	3.0163	4.6070	5.0314	3.3002	3.1818	3.6844	4.4668	3.1072	3.3991	4.4340	4.0826	4.3288
Slc7a2	_NLS[Phospho (STY)]LPFILHEK_.2	P18581_S645_M1_Slc7a2	7.9233	8.5110	6.0711	6.8423	5.4163	6.3971	8.0740	8.2846	5.7466	6.8773	5.1456	7.2343	7.1907	7.7068	4.5565	5.1459	6.5996	7.7613	6.4333	5.3419
Rps6ka3	_NQS[Phospho (STY)]PVLEPVGR_.2	P18654_S715_M1_Rps6ka3	9.0204	8.7889	8.8473	8.7962	8.3820	8.4798	8.3757	8.7069	8.2198	8.9259	8.6646	8.5138	8.9965	8.6958	7.6932	8.5504	8.7773	8.5876	8.6146	8.5919
Rps6ka3	_DLKPENILLDEEGHIK_.3	#N/A	3.6858	4.4248	3.7566	4.4691	3.8928	4.6718	4.7321	4.8843	3.0755	4.5811	4.0550	4.8788	3.2410	4.8723	3.9993	4.5039	4.7635	4.3193	4.2516	4.0339
Cfl1	_[Acetyl (Protein N-term)]AS[Phospho (STY)]GVAVSDGVIK_.2	P18760_S3_M1_Cfl1	14.5102	14.6223	14.4038	14.7618	14.4672	14.2672	14.0943	14.5387	14.4635	14.9594	14.3716	14.2551	15.1361	14.7036	14.1076	14.6555	14.8877	14.2171	14.4002	14.4701
Cfl1	_YALYDATYETK_.2	#N/A	10.7895	10.5905	11.0077	10.7595	10.7158	10.6173	10.3302	10.7654	10.8662	10.7911	10.5639	10.5577	10.7977	10.9035	10.7943	10.9315	10.6523	10.4491	10.5974	10.7497
Cfl1	_KSSTPEEVK_.2	#N/A	7.1257	6.6861	6.9429	6.5822	7.3087	7.3908	7.5453	6.5068	6.7245	6.7137	7.3495	7.2695	7.1244	7.6547	7.4026	6.8114	6.5993	7.4719	7.5797	7.4886
Cfl1	_LGGSAVISLEGKPL_.2	#N/A	8.3249	7.8590	7.7562	7.8060	8.2428	8.1514	8.0349	7.8648	8.2118	8.1828	8.0513	7.9180	7.6063	7.8950	7.8095	7.7964	8.2947	7.9483	7.9251	8.3900
Cfl1	_SSTPEEVKK_.2	#N/A	6.1506	6.4986	6.8531	6.6246	6.5398	6.6541	6.5770	6.8986	7.1017	6.7880	6.2473	6.4777	6.1769	6.8286	7.1918	7.0881	6.6052	6.1905	6.7512	6.5830
Cfl1	_EIILVGDVGQTVDDPYTTFVK_.2	#N/A	6.6656	6.1917	7.1024	6.3815	5.9782	6.9447	6.4831	6.0731	6.6779	6.3766	6.7146	6.1841	6.1675	6.7239	7.1861	6.5387	6.6426	6.9017	6.3238	6.6422
Cfl1	_M[Oxidation (M)]LPDKDC[Carbamidomethyl (C)]R_.2	#N/A	8.1704	8.7167	9.1457	8.6167	8.3523	8.7190	8.9424	8.2428	8.4635	8.4728	8.6417	8.7125	7.9560	9.2031	9.4803	8.4133	8.4843	8.6802	8.5991	8.5943
Cfl1	_NIILEEGKEILVGDVGQTVDDPYTTFVK_.3	#N/A	5.2177	6.0429	3.7785	2.9089	4.0807	3.2621	5.4770	6.5173	4.5075	3.4586	2.7506	5.6947	6.3628	7.4964	3.2118	4.0435	4.8847	5.1835	4.4526	1.7331
Cfl1	_NIILEEGK_.2	#N/A	4.4815	4.5439	5.2186	5.6626	4.9394	5.3237	5.6437	3.7981	5.1004	5.5105	6.3045	5.8944	5.3998	5.4423	6.3477	5.2885	3.2187	5.8017	5.7954	6.1212
Cfl1	_AVLFC[Carbamidomethyl (C)]LSEDKK_.2	#N/A	6.7325	7.2652	7.1840	6.9499	5.9036	6.7748	6.7036	6.6370	6.7199	6.7391	6.4891	6.9262	6.9182	7.7076	7.1205	7.0239	7.0159	7.2443	7.0043	7.2000
Cfl1	_[Acetyl (Protein N-term)]ASGVAVSDGVIKVFNDM[Oxidation (M)]K_.2	#N/A	3.4254	2.6956	4.4628	4.1496	2.8939	4.0026	2.8816	5.2610	3.6777	4.8752	4.0031	3.5521	5.0528	4.0840	3.9914	4.4733	3.6338	3.3082	2.1914	2.5163
Cfl1	_KEDLVFIFWAPENAPLK_.3	#N/A	5.1041	5.4529	5.8720	5.0173	5.2997	5.3936	5.2187	5.2797	5.3220	5.2046	5.5002	5.4228	5.3388	5.4619	5.2347	4.8879	5.5062	5.4874	5.7123	4.9405
Cfl1	_HELQANC[Carbamidomethyl (C)]YEEVKDR_.3	#N/A	5.4716	5.8288	6.4140	5.7025	5.5381	6.2086	5.7746	4.4379	5.9781	5.8954	6.0286	5.3782	4.3402	6.4852	7.0430	6.4033	5.0086	6.1739	6.6660	5.9913
Cfl1	_SST[Phospho (STY)]PEEVKKR_.2	#N/A	3.4884	3.8307	4.5258	4.0958	6.4732	4.4130	3.3528	3.7141	3.4112	3.2990	3.7856	3.5568	3.5193	4.5639	3.3781	4.3514	3.2113	3.9381	4.2372	4.2412
Cfl1	_KSST[Phospho (STY)]PEEVKK_.2	#N/A	4.8568	3.9309	6.0919	4.9190	6.5208	6.0911	6.0864	4.9189	6.5186	6.4899	6.2366	4.9436	5.4906	6.0466	4.8280	4.8552	6.3250	4.2237	6.1403	6.0520
Cfl1	_LTGIKHELQANC[Carbamidomethyl (C)]YEEVKDR_.4	#N/A	3.5543	8.1303	4.0906	5.1499	2.8920	3.5921	3.1482	6.2549	3.4096	3.7606	5.7175	4.3380	3.5750	4.0777	6.2192	5.0062	3.7923	3.9131	3.6894	4.0447
Cfl1	_MIYASSK_.2	#N/A	3.5284	3.4253	3.5307	4.2057	4.0096	4.1338	3.7170	3.9964	3.6935	3.2775	4.0679	3.2320	3.8016	3.3453	3.2785	3.3469	3.4936	3.6589	3.9042	3.0226
Gnao1	_IGAGDYQPTEQDILR_.2	#N/A	11.4165	11.0890	11.1407	11.1638	10.8362	10.9906	11.1091	11.3223	11.1876	11.1549	11.0458	10.8904	11.0852	11.0718	10.3190	10.6802	11.2169	10.8570	10.6838	10.7214
Gnao1	_LFDVGGQR_.2	#N/A	10.6283	10.5874	10.3353	10.4773	10.6655	10.3948	10.6998	10.8641	10.3269	10.4862	10.3389	10.6818	10.7110	10.1539	10.1191	9.9414	10.4731	10.5949	10.4037	10.1502
Gnao1	_NLKEDGISAAK_.2	#N/A	8.8995	8.4927	8.5548	8.3485	8.4196	8.5245	8.7086	8.8878	8.7335	8.4927	8.3609	8.4069	8.3544	8.5828	8.3008	8.5879	8.5982	8.5157	8.4665	8.5091
Gnao1	_LWGDSGIQEC[Carbamidomethyl (C)]FNR_.2	#N/A	8.8963	8.8961	8.7958	9.0981	8.3708	8.7539	8.9498	8.9548	8.7385	8.8170	8.6053	8.4925	9.1123	8.7655	8.5682	8.3151	8.5029	8.4701	8.6039	8.3806
Gnao1	_EYQLNDSAK_.2	#N/A	8.5816	6.5658	8.8149	8.7631	8.3070	6.1796	7.5232	8.3387	9.0186	8.5189	8.7920	8.4304	8.8444	8.4935	8.7546	8.5614	8.5150	8.8733	8.6670	8.8431
Gnao1	_KDLFGEK_.2	#N/A	6.2593	5.7472	5.6524	5.0075	6.1929	5.5618	6.1585	5.7983	5.6082	6.0270	6.0244	5.3449	5.2109	5.0994	5.9712	5.7123	5.1011	5.6089	5.5555	4.9699

Gnao1	_YYLDSLDR_.2	#N/A	10.0327	9.8171	9.8621	9.9393	9.7119	9.8307	10.0475	9.9972	9.8258	9.8057	9.8620	9.6768	10.1157	9.5613	9.4252	9.5321	9.3948	9.8670	9.5978	9.7172
Gnao1	_M[Oxidation (M)]VC[Carbamidomethyl (C)]DVVSR_.2	#N/A	8.4763	8.6736	8.9442	8.9825	8.4721	8.3578	9.1559	8.8174	8.4839	8.5516	8.4297	8.5696	8.3980	8.4922	8.8811	8.2171	8.3420	8.6263	8.5701	8.3734
Gnao1	_IIHEDGFSGEDVK_.3	#N/A	8.3838	8.4488	8.9376	8.8471	8.2348	8.6830	8.7652	8.2223	9.0733	8.8786	8.8254	8.4566	8.7715	8.6048	8.7447	8.6906	7.9802	8.6785	8.8383	8.7495
Gnao1	_QYKPVVYSNTIQSLAAIVR_.3	#N/A	7.3984	7.4977	7.5016	7.3293	7.2798	7.1447	7.3915	7.5018	7.1843	7.3968	7.4119	7.2203	7.7485	7.3942	6.7248	7.1872	7.2520	7.4199	7.3110	7.6651
Gnao1	_LFDVGGQRS[Phospho (STY)]ER_.2	#N/A	9.1754	9.1623	8.8874	9.0772	8.8450	8.8415	8.8207	9.2107	8.7301	8.8911	8.8942	8.8473	9.7064	9.3713	8.3453	8.9954	9.4023	9.1231	8.9149	9.0765
Gnao1	_M[Oxidation (M)]HESLM[Oxidation (M)]LFDSIC[Carbamidomethyl (C)]NNK_.3	#N/A	6.6436	6.2059	6.5934	6.4642	6.3231	5.7896	5.5948	6.7239	6.1789	6.7622	6.1830	6.1681	6.6788	6.3140	5.1967	6.1556	6.4073	6.0512	6.1388	6.5425
Gnao1	_M[Oxidation (M)]EDTEPFSAELLSAM[Oxidation (M)]MR_.2	#N/A	3.5569	3.8987	5.7085	6.2275	3.8695	5.7652	6.0235	4.2421	3.7963	3.7632	5.8440	4.6758	3.9044	6.7962	3.2910	3.9018	3.6738	3.6467	3.8562	4.1139
Gnao1	_AM[Oxidation (M)]DTLGVEYGDKER_.2	#N/A	7.1277	6.9389	7.7432	7.3149	6.8366	7.1424	7.0705	7.2508	7.6119	6.9788	7.7764	6.9288	6.5492	7.7914	6.8591	7.2003	6.6948	7.1108	6.8613	7.1660
Gnao1	_NLKEDGIS[Phospho (STY)]AAKDVK_.3	#N/A	4.2425	4.6798	2.9925	4.1575	3.2837	4.4047	4.6285	4.3589	4.2158	3.9750	4.7185	4.7977	5.4934	5.0017	2.9058	4.2403	4.2575	4.5149	4.7219	3.8467
Gnao1	_SREYQLNDSAK_.2	#N/A	6.3080	5.7618	5.6053	6.0245	5.7892	5.9882	5.7256	6.1660	5.6767	5.9948	5.6727	6.1071	6.0467	5.8282	5.4198	6.1560	6.2140	5.4074	5.6583	6.1920
Gnao1	_SPLTIC[Carbamidomethyl (C)]FPEYPGSNTYEDAAAYIQTQFESK_.3	#N/A	4.0928	3.0261	4.5044	4.1173	3.6739	4.4345	3.3742	3.6926	3.3898	3.2775	3.7641	3.5783	3.4978	4.1811	3.3996	4.3299	3.1898	3.9596	4.2586	4.2197
Gnao1	_PVVYSNTIQSLAAIVR_.2	#N/A	3.4324	4.5704	3.9687	4.2569	4.2710	2.9942	3.2701	3.1328	3.3292	3.6388	3.7035	2.9590	3.4272	3.9558	2.8473	3.7773	3.6705	4.1627	3.8112	3.9228
Fasn	_SRS[Phospho (STY)]DTSLK_.2	P19096_S2201_M1_Fasn	7.0879	7.0390	6.8953	6.8660	6.5373	6.6617	6.9443	6.8813	6.6832	6.4579	6.7480	6.4924	6.0778	6.1675	6.7455	5.3800	7.9203	6.7179	6.4958	6.3338
Fasn	_VQEVQQVSTNK_.2	#N/A	6.4538	5.9164	6.1751	6.0229	6.0878	5.8872	6.1434	6.1254	6.5420	5.7573	6.1724	5.4791	5.8351	5.4322	5.4032	5.9878	6.2967	5.8954	5.7025	5.5957
Fasn	_VTAIYIDPATHR_.2	#N/A	6.6664	5.6694	6.0884	6.1298	6.2693	6.1818	6.4297	6.3397	5.8196	6.0646	5.3968	6.0788	4.7229	5.0889	4.6924	5.2307	6.1240	6.1435	5.6790	4.7406
Fasn	_TLEAVQDLLEQGR_.2	#N/A	5.4725	4.2251	4.8294	5.2809	4.9889	5.4395	5.6473	5.2670	5.8211	5.9748	4.0741	5.0778	4.5035	4.3011	5.2719	5.2935	5.7146	5.8695	5.2456	5.5610
Fasn	_GYTVLGVEGR_.2	#N/A	5.9021	5.9082	5.9269	6.3119	6.2590	5.7764	6.2118	4.0385	5.7514	5.8639	5.4719	5.2794	3.8437	3.3875	5.5447	6.6209	5.7467	5.9934	5.4088	5.6438
Fasn	_LFDHPEVPTPPESASVSR_.3	#N/A	3.5674	6.1676	6.0311	4.3507	6.2924	5.9473	6.3560	4.5399	6.6267	6.0676	4.1734	3.4778	3.3594	5.1571	5.9389	5.7060	6.3717	5.9485	5.7960	3.8291
Gstp1	_PPYTIVYFPVR_.2	#N/A	6.3962	7.1309	7.0235	7.0248	6.9244	7.0436	7.0349	6.8873	6.7864	6.3520	6.1034	6.8170	6.8625	6.7905	7.1979	6.9685	6.7033	6.9733	6.5969	6.7409
Gstp1	_YVTLIYTNYENGK_.2	#N/A	6.3675	6.2635	7.0577	7.0370	6.3721	7.0152	5.9079	6.3102	6.9316	6.9778	6.7296	6.5317	6.2486	6.8693	6.8052	6.3343	6.7801	6.3428	6.1698	6.7728
Gstp1	_MLLADQGQSWK_.2	#N/A	4.2377	6.4496	6.7726	6.2905	5.1645	6.2603	6.4627	4.3276	5.8804	4.8121	5.5886	3.6545	4.1328	6.7818	6.4579	5.3696	5.4346	4.9304	5.0961	5.0815
Gstp1	_EAAQM[Oxidation (M)]DM[Oxidation (M)]VNDGVEDLR_.2	#N/A	6.7654	6.3764	5.5369	7.2497	5.9967	6.4531	5.9085	6.8577	6.5852	6.0852	5.8462	6.3529	6.8154	5.7288	6.5442	6.5314	6.2556	6.1245	6.3855	6.2467
Gstp1	_ALPGHLKPFETLLSQNQGGK_.3	#N/A	8.2813	8.5460	7.9543	8.1989	8.3241	8.3659	8.2269	8.7261	8.0696	8.4509	8.0635	8.2431	8.4422	8.7679	8.2909	8.3498	8.3691	8.2201	8.1286	8.5832
Gstp1	_FEDGDLTLYQSNAILR_.2	#N/A	8.5687	8.5422	8.9066	8.4948	7.9953	8.4049	8.4503	8.3991	8.3635	8.5613	8.0961	8.3779	8.7379	8.6315	8.5298	8.3704	8.3957	8.0868	8.3237	8.3550
Gstp1	_EAAQMDMVNDGVEDLRGK_.3	#N/A	3.1706	3.3314	3.7069	4.4326	3.3243	3.9758	3.1057	4.3718	3.0259	3.5461	4.0053	3.2208	3.1913	4.5153	3.1091	3.5155	3.4086	4.4245	4.0731	3.3610
Nefh	_AKPLDVKS[Phospho (STY)]PEAQTPVQEEAK_.3	#N/A	10.9874	10.9689	11.4844	11.2206	10.6216	11.0222	10.3360	10.9034	10.6810	10.9340	11.0947	10.9506	11.2466	11.6719	10.9374	10.8925	11.3096	10.6846	10.9106	11.0159
Nefh	_S[Phospho (STY)]PGEAKS[Phospho (STY)]PAEAK_.2	#N/A	10.0257	10.0272	10.6814	10.4539	10.0452	10.3241	9.7120	9.7498	10.1003	10.2230	10.2749	10.0736	9.8707	10.7054	9.4061	10.0263	10.4937	9.8084	10.1212	10.4172
Nefh	_SPAEAKS[Phospho (STY)]PIEVK_.2	#N/A	10.0586	10.0356	10.2628	10.2629	9.8948	10.0519	9.6623	9.8183	9.9150	10.0241	10.0628	10.2186	10.1305	10.8260	10.0239	10.1335	10.5945	9.9325	10.0239	10.1850
Nefh	_S[Phospho (STY)]PAEAKS[Phospho (STY)]PAAVK_.2	#N/A	9.3750	9.4001	9.7962	9.7116	9.4511	9.5831	9.0571	9.2594	9.3708	9.5483	9.4164	9.4649	9.4086	9.9644	9.4208	9.5189	9.8134	9.2727	9.5864	9.6697
Nefh	_S[Phospho (STY)]PGEAKS[Phospho (STY)]PAAVK_.2	#N/A	9.4314	9.4130	9.8561	9.6546	9.3269	9.5423	8.9487	9.1498	9.3365	9.5536	9.5132	9.1852	9.2807	9.8847	9.1953	9.3648	9.5511	9.1217	9.3684	9.5884
Nefh	_TSVSSVSAS[Phospho (STY)]PSR_.2	P19246_S61_M1_Nefh	9.4562	9.3286	9.4884	9.5906	9.2355	8.6602	8.4642	8.7767	8.5918	9.9817	8.5758	8.8354	9.5553	9.3152	8.7039	8.8083	9.7090	8.5142	8.6540	8.8373
Nefh	_S[Phospho (STY)]PAEAKS[Phospho (STY)]PGEAK_.2	#N/A	8.7521	8.8276	9.4307	9.1932	8.9272	9.0248	8.6034	8.6046	8.8389	9.0121	9.0136	8.9296	8.6963	9.6067	8.1124	8.7487	9.3042	8.6636	8.9164	9.1845
Nefh	_SPIEVKS[Phospho (STY)]PEK_.2	#N/A	8.8069	8.6496	9.0958	8.8738	8.6624	8.8647	8.2878	8.5145	8.4033	8.8062	8.8851	8.7515	8.6714	9.4626	8.7101	8.6894	9.3901	8.4629	8.8400	9.0282
Nefh	_SEKEQLQALNDR_.2	#N/A	8.2296	8.5473	8.3097	8.7522	8.7737	8.6333	8.1455	8.8016	8.3142	8.6030	8.4351	8.8478	8.9933	9.2116	8.7012	8.8043	9.0518	8.4330	8.6488	8.8788
Nefh	_S[Phospho (STY)]PGEAKS[Phospho (STY)]PAEPK_.2	#N/A	8.2346	8.0517	8.8986	8.5986	8.0270	8.3844	7.6737	8.0698	8.0787	8.5221	8.6213	8.3202	8.0995	8.8091	8.2893	8.3104	8.7895	8.0779	8.4966	8.4374
Nefh	_S[Phospho (STY)]PSEAKS[Phospho (STY)]PAEPK_.2	#N/A	7.6761	7.4518	7.9647	6.8006	7.6381	7.7288	6.1432	7.3813	7.4743	7.8368	7.8244	7.7466	7.7855	8.4918	7.2642	7.8644	8.0734	7.4536	7.6686	7.8812
Nefh	_S[Phospho (STY)]PVKEDIKPPAEAK_.3	P19246_S795_M1_Nefh	7.7975	8.1703	8.6227	8.7794	8.1056	8.8166	8.0103	7.3517	7.8826	8.7750	8.7072	8.3557	8.3503	8.8836	8.7134	8.6418	8.1382	8.0337	8.6916	8.7098
Nefh	_S[Phospho (STY)]PAEAKS[Phospho (STY)]PAEPK_.2	#N/A	7.7231	7.2812	7.7117	7.6735	7.4069	7.7623	6.8328	6.9572	7.3079	7.8684	7.5528	7.4853	7.2302	8.5155	7.9716	7.5872	7.9448	7.0017	7.3470	7.7506
Nefh	_EQLQALNDR_.2	#N/A	6.5127	6.9697	7.4598	7.4514	7.2899	7.4595	6.8130	7.1141	6.9945	6.8699	7.4124	7.2911	7.2952	8.0826	7.2602	6.9560	7.6143	6.9405	7.4489	7.0950
Nefh	_AVAS[Phospho (STY)]EEETPAK_.2	P19246_S965_M1_Nefh	7.3586	3.2619	3.7764	7.8365	7.0835	7.3375	6.9745	4.4413	6.6352	7.3422	6.9456	7.6475	8.1556	8.0256	7.4075	7.1906	3.4781	4.3550	7.2368	3.7304
Nefh	_S[Phospho (STY)]PVKEEVK_.2	#N/A	5.6193	5.5869	6.9556	6.4659	5.8930	6.0498	5.7317	5.7641	5.7451	6.1025	6.7191	5.6500	5.6084	6.6598	6.0832	5.7517	5.9492	6.1994	6.5780	6.5045
Nefh	_IPSISTHIK_.2	#N/A	6.0734	5.8932	5.5496	5.4900	6.1570	5.3149	5.2916	5.0099	5.5721	5.5668	5.9277	6.1251	5.1048	5.4894	6.0532	5.9255	5.2429	5.3331	6.1442	5.8024
Nefh	_HQADIASYQDAIQQLDSELR_.3	#N/A	5.1947	5.5722	5.1744	5.6556	5.4894	5.5832	5.8995	5.5986	5.7469	5.6025	5.5010	6.2241	6.3331	6.3402	6.1296	5.2126	5.4453	5.3853	5.8201	5.7998
Nefh	_S[Phospho (STY)]PEAQTPVQEEAK_.2	#N/A	8.4685	8.2051	9.0744	8.6389	8.2880	8.5815	8.4692	7.9075	8.2938	8.5533	8.7454	8.1155	6.4501	9.1144	7.8956	8.1942	8.7120	8.0251	8.1478	8.5681
Nefh	_SPIEVKS[Phospho (STY)]PEKAK_.2	#N/A	6.7863	6.6056	6.7477	6.9184	6.9809	6.6572	6.4048	7.2195	6.7814	6.1835	6.5611	7.3419	8.1886	7.7718	7.2839	7.2794	7.5061	7.2380	7.1467	7.1158
Nefh	_S[Phospho (STY)]PAEAKS[Phospho (STY)]PEK_.2	#N/A	7.9131	7.6267	8.0409	7.8916	7.2572	7.5906	7.6103	7.2920	7.2516	7.7615	7.7849	7.3541	7.1446	8.0900	6.5183	6.8481	7.8874	7.3953	7.1254	7.3274
Nefh	_C[Carbamidomethyl (C)]DVTSALR_.2	#N/A	4.0789	4.5287	4.2809	5.4296	4.2383	4.9877	4.4620	4.3297	4.0820	4.3486	5.3351	5.3738	4.7488	3.6787	5.5231	5.2736	4.8896	4.4536	3.2590	4.4748
Nefh	_SPAEAKS[Phospho (STY)]PAEAKS[Phospho (STY)]PIEVK_.3	#N/A	8.7414	8.9370	9.2295	9.2440	8.5988	8.9198	8.4179	8.6146	8.9035	9.2291	9.0090	9.1233	9.1739	9.6737	9.1894	9.2295	9.4271	8.8539	9.1902	9.2886
Nefh	_S[Phospho (STY)]LEGEAAALR_.2	P19246_S122_M1_Nefh	3.5960	5.3793	3.1326	3.9882	3.8029	5.4723	5.0426	5.1709	4.8356	5.8321	6.3788	5.7298	6.3497	6.4162	5.7341	6.4035	6.6104	5.7704	6.3626	6.4413
Nefh	_S[Phospho (STY)]PEKAKS[Phospho (STY)]PVK_.2	P19246_S795_M2_Nefh	5.1125	3.6590	5.7920	3.9276	4.4199	4.6538	3.6255	4.6759	4.3371	4.2187	5.0370	3.7257	4.3062	3.6394	4.2459	3.4836	4.8603	3.2791	3.6041	3.5912
Nefh	_VKEEAKS[Phospho (STY)]PGEAK_.2	#N/A	8.4114	8.6385	8.4755	8.5197	8.4302	8.4507	8.1291	8.7929	8.2485	8.5466	8.3371	8.7879	8.7506	9.1851	8.3322	8.9968	8.7222	8.4664	8.7155	8.7469
Nefh	_SPAAVKS[Phospho (STY)]PAEAKS[Phospho (STY)]PAAVK_.2	#N/A	8.6300	8.5806	8.8581	8.7687	8.2080	8.4689	7.9078	8.2487	8.2616	8.8694	8.4421	8.5249	8.5870	9.2355	8.1916	8.6645	8.8334	8.3128	8.3983	8.3661
Nefh	_SPAEPKS[Phospho (STY)]PAEAKS[Phospho (STY)]PAEVK_.2	#N/A	7.6347	7.6360	7.8431	7.8056	7.4021	7.5498	7.3276	7.1920	7.4428	7.8967	7.6970	7.8281	7.9125	8.3985	7.9626	7.8973	8.0666	7.4681	7.7879	7.7261
Nefh	_SPAEAKS[Phospho (STY)]PAEVKS[Phospho (STY)]PAEAK_.2	#N/A	7.0373	7.0005	7.2675	7.1278	6.5361	6.8909	6.3690	7.1522	6.8649	7.1835	6.9208	7.0501	7.0405	7.5466	6.9078	7.1930	7.3570	6.7944	6.8955	6.9041
Nefh	_ETVIVEGQTEEIR_.2	#N/A	5.7686	3.5052	6.4081	3.7703	6.1391	5.2925	3.6777	4.0396	5.3168	3.2312	5.3463	5.5280	5.9348	6.8980	3.2549	4.8492	6.0182	2.4822	4.5961	6.0248
Nefh	_SPAEAKS[Phospho (STY)]PIEVKS[Phospho (STY)]PEK_.2	#N/A	8.0809	7.9430	8.4755	8.1983	7.7080	8.1454	7.7073	7.8054	7.9022	8.3220	8.2697	7.9618	7.4790	8.6564	7.8672	8.0347	8.5429	7.8555	7.9721	8.2159
Nefh	_SPAEPKS[Phospho (STY)]PAEAKS[Phospho (STY)]PAEPK_.2	#N/A	7.5137	7.6464	7.7763	7.8576	7.5576	7.7276	7.0664	7.4888	7.3507	7.5647	7.3343	7.8296	7.9663	8.0634	8.0687	7.5114	8.2084	7.5253	7.7111	7.4295
Nefh	_SPAEAKS[Phospho (STY)]PGEAKS[Phospho (STY)]PAEVK_.2	#N/A	7.1151	7.0890	6.9781	7.2198	7.0519	6.9526	6.7416	6.7122	6.7700	7.1237	7.1455	7.0897	7.1537	7.7466	7.1506	7.1107	7.4466	6.8344	6.8735	7.1310
Nefh	_S[Phospho (STY)]PAEAKS[Phospho (STY)]PATVKS[Phospho (STY)]PGEAK_.2	#N/A	4.8395	5.2642	4.2025	4.0653	5.6440	3.2967	4.8231	4.9452	3.6641	4.5588	4.7560	5.6053	5.6542	5.9181	5.0948	5.8332	6.0795	3.9312	4.7901	5.3089
Nefh	_AQALQEEC[Carbamidomethyl (C)]GYLR_.2	#N/A	3.7796	4.2233	4.9386	4.6896	4.6245	5.1804	4.4231	3.4799	4.7433	3.9859	4.1784	4.6710	3.8003	3.8885	5.4687	5.0523	4.0176	4.7037	4.4792	5.1933
Nefh	_SPGEAKS[Phospho (STY)]PAAVKS[Phospho (STY)]PAEAK_.2	#N/A	8.4306	8.3869	8.9096	8.8864	8.5759	8.6611	7.9443	8.3476	8.2978	8.9085	8.7791	8.5972	8.5854	9.1328	8.2371	8.7613	8.9987	8.3506	8.6170	8.9185
Nefh	_SPGEAKS[Phospho (STY)]PAEPKS[Phospho (STY)]PAEAK_.2	#N/A	8.1072	8.2413	8.6308	8.4525	7.9746	8.1049	7.8950	8.2731	8.1814	8.1919	7.9753	8.4002	8.5983	9.0611	8.4187	8.1610	8.6556	8.2434	8.1923	8.2381
Nefh	_SPAEAKS[Phospho (STY)]PAEAKS[Phospho (STY)]PAEAK_.2	#N/A	7.3512	7.1266	7.7757	7.6935	6.8132	7.4575	6.5621	7.5107	7.3641	7.5798	7.3096	7.6304	7.7691	8.1839	7.4027	7.2375	7.9292	7.4765	7.7200	7.7758
Nefh	_SPAEVKS[Phospho (STY)]PAEAKS[Phospho (STY)]PAEVK_.2	#N/A	7.3572	7.7554	8.1248	7.7158	7.3775	7.8083	7.0632	7.7247	7.5429	7.7944	7.5148	7.6073	7.6780	8.6289	7.7648	7.8203	8.1322	7.6078	7.8834	7.8121
Nefh	_S[Phospho (STY)]PGEAKS[Phospho (STY)]PAEAKS[Phospho (STY)]PAEAK_.2	#N/A	9.9320	9.9013	10.4645	10.1939	9.7705	10.0105	9.5537	9.7407	9.8937	10.0448	9.9297	9.9643	9.8178	10.5285	9.9522	10.0003	10.3405	9.6691	9.9518	10.2069
Nefh	_SPAEAKS[Phospho (STY)]PAEAKS[Phospho (STY)]PAEAK_.2	#N/A	9.2060	9.1675	9.4799	9.3900	9.1821	9.1309	8.9408	9.2742	9.1285	9.2229	9.1709	9.5346	9.6787	9.9039	9.4201	9.5469	9.6667	9.3296	9.3781	9.4922
Nefh	_S[Phospho (STY)]PGEAKS[Phospho (STY)]PAEAKS[Phospho (STY)]PGEAK_.2	#N/A	9.2378	9.1285	9.9264	9.6534	9.1696	9.4975	8.8917	8.9534	9.2676	9.3998	9.2821	9.2787	8.9740	10.0318	9.2108	9.1774	9.6574	8.9147	9.3421	9.4099
Nefh	_SPAEAKS[Phospho (STY)]PAEVKS[Phospho (STY)]PGEAK_.2	#N/A	8.5749	8.6892	8.7244	8.9119	8.4550	8.5463	8.2378	8.5828	8.5313	8.5601	8.5259	9.0241	8.8617	9.3823	8.8127	8.7326	9.0565	8.6044	8.5141	8.6986
Nefh	_S[Phospho (STY)]PGEAKS[Phospho (STY)]PAEPKS[Phospho (STY)]PAEPK_.2	#N/A	7.3984	7.0758	7.7935	7.6754	7.2315	7.5848	7.3008	7.0021	7.2930	7.6998	7.6806	7.4889	7.5784	8.3084	7.7582	7.6953	7.9816	7.3892	7.8443	7.4683
Nefh	_SPAEAKS[Phospho (STY)]PAEAK_.2	#N/A	10.6622	10.6982	10.8022	10.7840	10.5532	10.5578	10.2520	10.8275	10.4580	10.5538	10.5553	10.9273	11.1400	11.3536	10.3923	10.9078	11.0979	10.7074	10.8094	10.8673
Nefh	_S[Phospho (STY)]PGEAKS[Phospho (STY)]PGEAKS[Phospho (STY)]PAEAK_.2	#N/A	9.6215	9.5140	10.1475	9.9410	9.4548	9.8093	9.2793	9.4127	9.5947	9.6641	9.6696	9.7228	9.6345	10.3435	9.7811	9.5718	10.0170	9.3780	9.7653	9.7174
Nefh	_S[Phospho (STY)]PGEAKS[Phospho (STY)]PGEAKS[Phospho (STY)]PGEAK_.2	#N/A	9.2817	9.2721	9.8209	9.6417	9.1646	9.4966	8.9666	9.2275	9.2226	9.3431	9.3438	9.4285	9.2102	10.0000	9.4673	9.2859	9.7017	9.0181	9.4722	9.4380
Nefh	_SPATVKS[Phospho (STY)]PGEAK_.2	P19246_S589_M1_Nefh	8.7657	8.8511	9.0892	8.9766	8.8899	8.7711	8.4718	8.9014	8.7314	8.9418	8.7149	9.2133	9.3997	9.4611	9.1493	8.5666	9.3614	8.5048	9.0479	9.0812
Nefh	_VKS[Phospho (STY)]EEMIK_.2	#N/A	4.1031	6.4045	6.7719	6.8483	3.4408	5.7139	6.1547	3.8035	4.1594	5.3636	6.2737	5.3642	4.2674	7.3671	5.2042	2.9471	5.6034	4.7449	4.8588	5.3202
Nefh	_S[Phospho (STY)]PAEAKS[Phospho (STY)]PATVK_.2	#N/A	8.1718	7.9438	8.6355	8.4622	7.1193	8.3656	7.6613	8.0413	7.8958	8.2968	7.6601	8.1491	7.8181	8.7534	8.0759	8.2037	8.5212	7.9470	8.0903	8.3318
Nefh	_SPGEAKS[Phospho (STY)]PSEAK_.2	#N/A	9.7230	9.8952	10.0233	10.0193	9.7460	9.6737	9.3999	9.8397	9.6601	9.7803	9.6727	10.0877	10.1713	10.5619	9.3966	9.9226	10.1960	9.7919	9.7930	10.1096
Nefh	_SPAEPKS[Phospho (STY)]PAEAKS[Phospho (STY)]PAEAK_.2	#N/A	7.7521	8.0653	8.0263	7.8110	7.7453	7.8474	7.5086	7.9050	7.5350	7.9140	7.8951	8.2201	8.4616	8.8277	8.3624	8.0380	7.0696	7.8099	8.0364	7.8643
Nefh	_SPAEAKS[Phospho (STY)]PAAVKS[Phospho (STY)]PAEAK_.2	#N/A	7.8257	7.7299	7.5649	8.2254	7.8657	7.9093	7.3994	7.8150	7.2196	8.2393	7.5098	8.1724	8.0273	8.4251	7.4021	8.2044	8.2709	8.0122	7.9897	7.9516
Nefh	_SPAEVKS[Phospho (STY)]PGEAKS[Phospho (STY)]PAEPK_.2	#N/A	6.9218	7.4974	7.3702	7.1856	6.7535	7.0099	5.8110	6.6708	7.3076	7.3693	7.2919	7.2045	7.8330	7.6116	7.8024	7.5442	7.3429	7.2656	7.3233	7.5050
Nefh	_TPVKEGAKS[Phospho (STY)]PAEAK_.2	#N/A	4.1217	4.1832	4.0937	4.4552	4.8879	4.1228	3.8665	4.5582	3.8730	3.5310	4.6502	4.4381	4.3926	4.6441	4.7103	3.6171	4.5422	4.1415	3.7254	4.4532
Nefh	_SPAEVKS[Phospho (STY)]PGEAK_.2	P19246_S679_M1_Nefh	11.1783	11.2900	11.2319	11.2059	11.0761	11.1143	10.7231	11.3066	11.0420	11.0055	11.0511	11.4561	11.7857	11.7969	11.3431	11.4056	11.6353	11.1771	11.2732	11.3620
Nefh	_S[Phospho (STY)]PGEAKS[Phospho (STY)]PGEAK_.2	#N/A	10.1181	10.1040	10.4580	10.4433	10.0699	10.1097	9.7793	10.1419	10.0757	10.2399	10.0316	10.3746	10.4069	10.8480	9.7500	10.1852	10.5180	10.0518	10.0255	10.3140
Nefh	_S[Phospho (STY)]PAEAKS[Phospho (STY)]PAEVK_.2	#N/A	10.0794	9.9545	10.4531	10.2977	9.9290	10.1164	9.4733	9.7832	9.9757	10.1767	10.0852	10.1493	9.8675	10.6891	9.9912	10.1169	10.4816	9.8602	10.0037	10.3047
Nefh	_SPAAVKS[Phospho (STY)]PGEAK_.2	P19246_S727_M1_Nefh	9.8573	9.9702	10.2741	10.1800	9.8292	10.0118	9.5336	9.7981	9.8919	9.9226	10.0053	9.9456	10.1125	10.4852	10.0123	9.9575	10.1754	9.8552	9.9617	10.0945
Nefh	_S[Phospho (STY)]PAEVKS[Phospho (STY)]PGEAK_.2	#N/A	9.8160	9.7322	10.1908	10.0718	9.7481	9.8456	9.4048	9.5194	9.7667	9.8326	9.8273	9.9083	9.8596	10.4612	9.7510	9.8923	10.2778	9.6126	9.7729	10.0618
Nefh	_S[Phospho (STY)]PAEAKS[Phospho (STY)]PGEAK_.2	#N/A	9.6822	9.6814	9.8771	9.7733	9.6212	9.7342	9.4746	9.1985	9.5867	9.7882	9.8258	9.9548	10.0860	10.3689	9.8476	9.9011	10.1987	9.6713	9.8416	10.0159
Nefh	_SPAAVKS[Phospho (STY)]PGEAK_.2	P19246_S739_M1_Nefh	9.6647	9.7330	9.8952	9.8897	9.5643	9.7354	9.3716	9.7141	9.6584	9.6480	9.5657	9.7752	9.9565	10.1750	9.8327	9.7573	10.0759	9.5684	9.7032	9.8473
Nefh	_S[Phospho (STY)]PAEPKS[Phospho (STY)]PATVK_.2	#N/A	9.6608	9.5424	10.0877	9.8817	9.4648	9.9635	9.1553	9.8554	9.5728	9.7825	9.8737	9.5736	9.2790	10.2768	9.3386	9.5957	10.0530	9.2607	9.6816	9.8817
Nefh	_SPATVKS[Phospho (STY)]PGEAKS[Phospho (STY)]PSEAK_.2	#N/A	7.1540	7.7372	7.1719	7.5672	7.5585	7.5209	7.2458	7.7187	7.6402	7.7190	7.4327	5.7004	8.2326	8.3146	8.2212	8.1155	8.3065	7.8314	7.9420	7.7568
Nefh	_SPAEVKS[Phospho (STY)]PGEAKS[Phospho (STY)]PAAVK_.2	#N/A	7.3592	7.1176	7.7137	7.6423	7.4674	7.2400	6.6560	7.1553	7.1974	7.1984	7.7685	7.3924	7.5178	8.2721	7.5688	7.5422	7.6078	7.1221	7.1472	7.5428
Nefh	_SPAEAKS[Phospho (STY)]PAEAKS[Phospho (STY)]PATVK_.2	#N/A	7.9830	7.6072	7.9630	8.2350	7.5290	7.7910	7.2292	7.4433	7.4342	8.0097	7.6282	7.8711	7.6594	8.4552	7.5874	7.7430	8.1730	7.4797	7.7838	7.9219
Nefh	_EEVKS[Phospho (STY)]PVKEEVK_.2	#N/A	4.9054	4.9378	4.2540	4.8877	4.6491	5.3127	4.5031	4.8661	4.2764	5.0444	5.1170	4.8067	5.4585	5.3554	4.7291	5.3690	4.8988	4.7594	4.4795	5.2453
Nefh	_AKT[Phospho (STY)]PVKEGAK_.2	P19246_T775_M1_Nefh	3.5247	4.7091	4.4362	4.0595	3.5512	4.1308	3.3164	3.7504	3.2366	3.9288	3.1542	3.4972	3.4021	4.2679	3.3418	4.3877	4.2038	2.9257	4.2009	4.2466
Nefh	_HPTDIRPPEQVKS[Phospho (STY)]PAK_.3	#N/A	7.5733	7.3533	8.2186	6.5797	7.3938	7.8860	6.5797	7.2871	6.9936	7.5236	6.0579	5.6262	4.0005	8.5367	7.4507	7.2867	8.0221	7.6270	7.2979	7.3734
Nefh	_SPAEAKS[Phospho (STY)]PAEPKS[Phospho (STY)]PATVK_.2	#N/A	6.1046	7.4977	7.9307	7.5933	7.4236	7.1911	7.2195	8.5670	7.4067	7.6627	7.4573	7.7664	7.6441	8.4749	7.4063	7.5395	8.0123	7.3095	6.6762	7.6978
Nefh	_S[Phospho (STY)]PGEAKS[Phospho (STY)]PSEAKS[Phospho (STY)]PAEAK_.2	#N/A	7.6851	7.5096	8.0967	7.8277	7.3166	4.2089	8.0129	7.7759	8.3536	7.5328	8.3827	8.6637	8.6524	8.2727	7.2576	4.5491	7.7502	6.9659	7.7897	7.6689
Nefh	_S[Phospho (STY)]PGEAKS[Phospho (STY)]PGEVKS[Phospho (STY)]PGEAK_.2	#N/A	6.0375	6.1854	6.4448	6.4680	5.7531	6.0767	5.8051	5.8721	6.3015	6.2722	6.3700	6.3188	6.5766	7.0389	6.6444	5.7287	7.0281	6.1587	6.1499	6.6517
Nefh	_SPAEPKS[Phospho (STY)]PAEPK_.2	P19246_S565_M1_Nefh	7.7003	7.3071	7.1921	7.6468	7.2230	7.8431	7.3590	7.3060	6.5516	7.4073	7.1124	7.3221	7.6920	7.9109	7.8845	8.0246	7.7437	7.4986	7.7344	8.0949
Nefh	_S[Phospho (STY)]PAEPKS[Phospho (STY)]PATVKS[Phospho (STY)]PGEAK_.2	#N/A	5.3829	4.7462	5.9236	3.8868	3.7411	5.1150	4.1969	3.8589	5.1006	5.5283	5.1740	4.1399	5.1990	5.9109	5.2509	4.6945	4.2044	4.7930	5.5453	4.3322
Nefh	_QREEAEAAAR_.2	#N/A	4.2179	2.8227	1.1729	3.3663	2.9240	4.4582	1.8382	4.4436	4.0443	4.0285	3.5229	4.3281	4.2488	3.7926	3.1473	2.3240	3.9408	3.2086	1.7595	0.6108
Nefh	_EGAKS[Phospho (STY)]PAEAKS[Phospho (STY)]PEK_.2	#N/A	7.7303	7.6230	8.2131	8.0999	7.2219	7.8153	7.2171	7.2378	7.5452	7.6665	7.3931	7.6253	6.8254	8.2999	6.8897	7.4400	7.9694	7.3735	7.4834	7.7003
Nefh	_S[Phospho (STY)]PVKEGAKPPEK_.2	#N/A	6.9749	6.9682	7.2112	7.3530	7.0897	7.3230	6.7927	7.7662	6.6098	7.2153	6.9454	7.0597	6.4289	7.7577	6.9505	7.2214	7.4833	6.7524	6.9504	7.2043
Nefh	_S[Phospho (STY)]PGEAKS[Phospho (STY)]PGEAKS[Phospho (STY)]PAEPK_.2	#N/A	6.0183	6.1916	6.6624	5.9803	6.0363	6.4284	5.9739	5.2159	6.1443	6.3184	5.7373	6.5166	6.1288	7.0486	6.2794	5.9757	6.8155	6.1102	6.1674	5.9113
Nefh	_S[Phospho (STY)]PAEAKS[Phospho (STY)]PEAK_.2	#N/A	5.5800	5.6678	5.2186	5.1786	5.1846	4.8366	5.1170	5.4849	4.8752	5.3028	5.5519	5.3976	5.8387	6.1272	4.6666	5.3459	5.5349	5.5156	5.4491	5.4800
Nefh	_LEQEHLLEDIAHVR_.3	#N/A	6.9263	6.6792	6.4566	6.5011	7.1420	6.7652	6.4823	7.1621	6.0227	6.9333	6.8369	6.8489	6.9620	7.4023	7.1493	6.8650	6.8647	6.5449	6.7076	6.9040
Nefh	_SPAAVKS[Phospho (STY)]PGEAKS[Phospho (STY)]PGEAK_.3	#N/A	6.2481	6.3715	5.4938	6.2529	5.9077	6.2542	6.0453	6.0770	6.3779	6.5798	6.0432	6.4820	6.8003	6.8642	6.7181	6.4945	6.3223	5.9421	6.5165	6.6917
Nefh	_EEAKS[Phospho (STY)]PAEVKS[Phospho (STY)]PGEAK_.2	#N/A	3.2435	3.8779	3.7798	4.4459	4.0821	3.6701	2.3262	4.0663	2.1570	3.5021	3.5862	2.6390	3.6262	2.6160	4.0880	4.4146	4.1897	4.3516	1.7788	3.5398
Nefh	_SAAGSSSGFHSWAR_.2	#N/A	4.5381	5.3474	4.0747	4.5470	5.3967	5.2542	5.1603	3.2629	5.9122	4.0079	5.3498	5.3282	4.5690	5.9537	5.6717	5.4569	5.8995	5.6542	4.6941	3.7900
Nefh	_VKS[Phospho (STY)]EEMIKVEEK_.3	#N/A	4.1921	3.1269	3.7287	3.9321	2.8982	4.4324	4.1499	4.4178	4.0701	4.0027	5.5487	4.3539	4.2355	3.7668	4.1752	5.5543	3.9151	3.3803	3.5334	3.2043
Nefh	_EGAKS[Phospho (STY)]PAEAK_.2	#N/A	3.9481	3.3710	4.8447	3.6861	3.7671	4.8353	6.8618	3.7893	4.3141	3.7587	3.7927	4.8070	4.4795	3.5227	2.9184	3.3102	4.8754	3.7592	4.2857	4.9451
Nefh	_AKS[Phospho (STY)]PEKEEAK_.2	P19246_S867_M1_Nefh	3.4784	2.4845	4.0147	4.2110	3.6346	3.6680	3.4135	3.1788	3.3337	3.2383	4.3131	4.4139	3.4991	4.0018	3.4388	4.2907	3.7165	4.1167	3.7653	3.9688
Nefh	_VTEGVTEEEDKEAQGQGEGEEEEGEEKEEEGAAATS[Phospho (STY)]PPAEEAASPEK_.5	P19246_S500_M1_Nefh	4.3627	4.4573	3.8992	3.2216	3.0688	3.1021	3.9793	3.0875	3.8995	4.1733	3.3782	4.1834	4.0650	3.9373	4.0067	3.7248	4.0856	3.5508	3.1994	3.0038
Nefh	_EDIKPPAEAKS[Phospho (STY)]PEK_.3	#N/A	3.2416	3.2603	3.7779	4.4478	4.0802	4.3043	3.4609	4.4429	3.5200	3.4480	5.9873	3.1498	3.6280	3.1718	3.0381	3.5865	3.4797	3.8294	4.1284	3.4499
Nefh	_SEKETVIVEGQTEEIR_.3	#N/A	4.4113	4.4086	3.9479	3.1729	3.1174	3.1507	3.9307	3.3161	3.8509	4.2219	5.5172	4.1347	4.0163	4.5190	4.3543	3.7734	4.1342	3.5995	3.2481	2.9852
Nefh	_AQLEGHAVQSTLQSEEWFR_.3	#N/A	4.4660	4.0619	4.4772	3.7485	3.1721	3.2055	3.8760	3.6413	3.7962	4.2766	3.2748	3.9514	3.9616	4.4643	3.9013	3.8282	4.1790	3.6542	3.3028	4.4313
Nefh	_PPAEAKS[Phospho (STY)]PEK_.3	#N/A	4.8021	3.4101	3.6282	3.8373	3.9305	4.1545	3.6106	3.9726	3.6697	3.2982	4.0441	3.2905	3.7778	3.3215	3.1878	3.4368	3.4798	3.6796	3.9787	2.9988
Nefh	_SAQEEITEYRR_.3	#N/A	3.4996	4.5632	3.9759	4.2498	4.2781	3.7068	3.2629	3.1400	3.2949	3.6459	4.2743	2.9518	4.3633	3.9630	2.8401	3.7845	3.6776	4.1555	3.8041	3.9300
Rpl13a	_YQAVTATLEEK_.2	#N/A	4.2447	3.9041	3.8293	4.6496	4.2952	4.6435	4.8484	3.1177	5.0539	4.8327	3.3086	4.1343	3.4524	4.6172	3.9734	3.7622	4.9339	5.0785	3.5764	4.3912
Nefe	_SLS[Phospho (STY)]EQPVVDTATATEQAK_.2	P19426_S51_M1_Nefe	8.7759	9.0697	9.1954	9.0452	8.6640	8.6865	8.7104	8.6526	8.7825	9.2057	8.5664	8.8435	9.3980	9.3312	8.3230	9.0848	9.3668	8.6033	8.6878	8.9686
Nefe	_SM[Oxidation (M)]S[Phospho (STY)]ADEDLQEPSR_.2	#N/A	9.2420	9.2782	8.3773	9.5434	8.8307	9.0553	8.9204	9.1908	8.9872	9.5227	8.9830	9.0006	9.3666	9.7837	8.7315	9.1313	9.2081	8.7755	8.9691	9.1185
Cox5b	_EDPNLVPSISNK_.2	#N/A	9.3362	9.2509	10.0771	9.5775	9.6233	9.4384	8.9940	9.2710	9.4944	9.5641	9.8962	9.5487	9.3282	9.8496	10.1034	9.8531	9.7526	9.4052	9.5844	9.7917
Cox5b	_AASGTKEDPNLVPSISNK_.3	#N/A	7.1143	7.3068	7.2172	7.5046	7.7020	7.1905	7.3487	7.4841	7.9001	7.7952	7.5417	7.6641	8.3008	8.1632	8.5872	8.0723	7.4344	7.5121	7.9641	7.9277
Cox5b	_EIM[Oxidation (M)]AAQK_.2	#N/A	6.1868	6.4154	6.8222	7.2195	6.5052	6.9450	7.4689	6.1027	7.3253	7.0948	7.3103	7.2140	7.3462	7.4708	7.9772	8.1164	6.3042	7.0860	7.4058	7.5066
Cox5b	_LVPHQMAH_.2	#N/A	3.4923	6.6096	7.0754	7.5289	5.6958	5.9092	6.7845	7.3677	5.2372	4.3743	6.0482	6.4620	5.3232	7.4257	6.9442	4.3553	5.0812	4.7531	4.5478	5.4832
Cox5b	_AASGTKEDPNLVPSISNK_.3	#N/A	9.4037	9.7728	9.4311	9.6115	9.6818	9.4071	9.6471	9.9840	9.7231	9.6087	9.4556	10.0604	10.5635	10.0842	10.6138	10.1712	9.7158	9.7673	9.8693	9.7149
Cox5b	_EDPNLVPSISNKR_.2	#N/A	9.4710	9.5427	9.8266	9.5297	9.6403	9.5762	9.4078	9.6762	9.4681	9.5700	9.7346	9.7708	9.8144	9.9971	10.4483	9.8500	9.8965	9.5150	9.7131	9.5344
Cox5b	_C[Carbamidomethyl (C)]PNC[Carbamidomethyl (C)]GTHYK_.2	#N/A	3.4734	4.5294	4.0097	4.2159	4.3120	3.0352	3.2291	3.1738	4.4549	3.6798	3.5279	4.4108	3.8183	3.9968	6.2722	3.7115	4.3368	5.2381	4.3602	4.1181
Cox4i1	_SEDYAFPTYADR_.2	#N/A	10.4267	10.0708	10.4193	10.0640	9.9684	10.0863	10.1428	10.1451	10.2710	10.0981	10.3766	9.7713	9.9332	10.1107	9.7036	9.9073	10.3774	9.9559	10.0207	9.9622
Cox4i1	_ANPIQGFSAK_.2	#N/A	9.5756	9.5797	9.4716	9.2302	9.6551	9.2906	9.7178	9.9161	9.2771	9.1564	9.4049	9.5368	9.8673	9.1759	9.5031	9.1055	9.3444	9.8621	9.2282	9.2576
Cox4i1	_ADWSSLSR_.2	#N/A	8.9364	9.1718	9.1188	9.0044	9.0255	9.2234	9.3640	9.0717	8.8427	8.8443	9.1215	9.0611	9.5229	8.9138	8.9627	8.6556	8.7008	9.1239	8.9301	8.9050
Cox4i1	_EKADWSSLSR_.2	#N/A	7.8707	8.2029	7.8913	8.0030	7.7789	7.9009	8.2131	8.3787	8.0110	7.7404	7.9849	8.2745	8.5260	8.1453	8.0295	8.0263	8.5029	8.2126	8.1363	8.0415
Cox4i1	_SEDYAFPTYADR_.3	#N/A	7.3768	6.9714	6.8848	7.1199	6.8483	6.9209	7.3053	7.2259	7.4448	6.9813	7.1456	7.1294	7.4206	7.0795	7.0372	7.3834	6.6718	7.2250	7.1144	7.1109
Cox4i1	_IQFNESFAEM[Oxidation (M)]NR_.2	#N/A	6.8167	6.6712	7.0400	6.7872	6.4209	6.6017	6.5355	6.6487	6.2247	6.5957	6.3062	6.1542	6.6554	6.2894	6.0547	6.1855	6.8124	6.3203	6.7607	6.4169
Cox4i1	_DWVAM[Oxidation (M)]QTK_.2	#N/A	6.6701	6.8344	7.0366	6.6990	6.3251	7.0346	6.9219	6.8387	6.5720	6.5559	6.8206	6.6690	6.6736	6.4914	6.6496	6.3292	6.6937	7.0184	6.6830	6.7775
Cox4i1	_DYPLPDVAHVTM[Oxidation (M)]LSASQK_.3	#N/A	8.1653	7.8828	7.8021	7.8086	7.8441	7.6449	8.0095	7.8238	7.9405	8.3584	7.7820	8.1065	8.3908	7.4072	7.3460	7.9719	7.9238	7.9957	8.2999	7.6967
Lsp1	_QPS[Phospho (STY)]ELPSMAVASTK_.2	P19973_S243_M1_Lsp1	3.5754	3.7437	6.0741	4.0088	3.7824	3.8157	5.7458	3.8011	3.1859	3.3860	4.1654	3.4698	3.3514	3.1501	3.2911	2.9375	3.2983	3.8511	4.1501	3.8210
Hspa5	_ITITNDQNR_.2	#N/A	9.1633	8.6986	9.3550	9.1079	9.2214	8.6442	8.4640	9.2290	9.1155	8.8879	9.1633	9.3745	9.5828	9.6071	9.4837	9.1209	9.0929	9.1786	9.1824	9.4753
Hspa5	_ITPSYVAFTPEGER_.2	#N/A	8.3092	8.2559	8.6575	8.5136	8.5745	8.3486	8.1965	8.6345	8.3178	8.2490	8.1862	8.7201	8.4768	8.6012	8.6708	8.5720	8.7906	8.2720	8.1684	8.4043
Hspa5	_LTPEEIER_.2	#N/A	8.1764	8.2888	8.1244	8.3927	8.3890	8.3346	8.3828	8.2018	8.2289	8.0286	8.1787	8.4382	8.8154	8.5508	8.7651	8.1266	8.1766	8.5565	8.1546	8.5308
Hspa5	_NQLTSNPENTVFDAK_.2	#N/A	7.9183	7.8505	8.3023	8.0809	7.7318	7.6780	7.6346	7.8050	7.7354	8.0552	7.8166	8.0345	8.1772	8.2805	8.2217	8.0024	8.0161	7.6806	7.7608	8.3246
Hspa5	_SQIFSTASDNQPTVTIK_.2	#N/A	6.8849	7.2086	7.7667	7.3064	7.0063	6.8030	6.9860	7.3626	7.5545	7.2238	7.5478	7.2188	7.7317	7.5179	7.9195	7.7441	7.0718	6.9246	7.3528	7.3172
Hspa5	_TWNDPSVQQDIK_.2	#N/A	7.3494	7.3838	7.7349	6.8046	7.4570	7.3695	7.5264	7.5645	7.6849	7.4489	7.5155	7.4006	7.8556	7.8575	7.7970	7.2326	7.1849	7.4177	7.5351	7.6430
Hspa5	_VYEGERPLTK_.2	#N/A	7.1393	6.5486	7.2291	7.1522	7.4861	6.8903	7.1091	7.3314	7.1414	6.8445	7.0238	7.6573	7.2760	7.7570	7.8514	7.2945	7.6343	7.2917	6.9973	7.5298
Hspa5	_NELESYAYSLK_.2	#N/A	6.9670	6.9831	6.9890	7.5200	7.2183	7.0074	6.5967	7.1673	6.7254	7.3414	7.3660	7.2163	7.5112	7.7679	7.3340	7.2184	6.8960	7.0662	6.7560	7.4573
Hspa5	_ELEEIVQPIISK_.2	#N/A	6.6576	6.2193	6.9614	6.0060	6.0441	6.4418	5.9848	6.0322	6.6034	6.8094	6.2700	6.2648	6.3008	6.7476	6.3736	6.7330	6.3069	5.7950	6.5064	6.2823
Hspa5	_NTVVPTKK_.2	#N/A	5.7073	6.9790	6.2700	5.8771	6.3278	5.7371	6.3167	6.3875	5.7759	6.4656	6.2158	6.4149	6.5802	6.1490	7.0610	6.2879	5.7911	6.5430	6.2020	6.6562
Hspa5	_IEIESFFEGEDFSETLTR_.2	#N/A	6.0828	6.3950	6.4704	4.8281	4.8142	4.7514	4.8082	6.8246	5.1443	5.6651	4.8845	5.9035	6.7995	7.4019	6.5513	6.3571	5.3562	5.6297	5.4417	4.9841
Hspa5	_VTHAVVTVPAYFNDAQR_.3	#N/A	6.1260	6.2635	6.1103	5.5223	6.2132	6.3061	5.8447	6.0077	5.8809	6.5574	6.4795	6.3816	6.2632	7.1486	6.3770	6.5945	6.2592	6.4115	6.4848	6.5900
Hspa5	_NQLTSNPENTVFDAKR_.3	#N/A	5.4654	5.8429	5.1158	5.0169	5.7316	4.4590	5.5181	5.1338	4.9107	4.7283	5.4087	5.9694	4.2089	4.8008	5.3114	5.8256	5.2485	4.7905	5.3952	6.0092

Hspa5	_IINEPTAAAIAYGLDKR_.3	#N/A	4.4871	4.8983	5.1557	5.0436	4.9881	4.9846	5.0084	4.5197	4.5385	5.2240	5.2219	5.1274	5.2822	5.4541	5.2101	4.9638	3.0305	4.8889	5.3359	5.5180
Hspa5	_TFAPEEISAM[Oxidation (M)]VLTK_.2	#N/A	5.5737	5.8766	6.6610	6.3125	5.4831	5.6985	5.9945	5.8847	5.1774	4.9253	5.6235	6.0385	5.5507	6.5794	6.1055	5.6153	6.1101	5.5839	5.7594	5.8547
Hspa5	_KSDIDEIVLVGGSTR_.3	#N/A	5.3369	3.1834	5.4543	5.4310	5.2298	5.5149	4.4905	4.3614	5.8844	4.4103	6.2642	4.9054	5.5068	5.9380	5.9035	5.5842	5.4098	4.5477	4.9100	5.5004
Hspa5	_ALSSQHQAR_.2	#N/A	4.4525	3.1090	3.2298	2.3930	4.0486	3.4489	4.1886	3.6859	3.1173	2.5432	3.7705	4.0936	2.8636	3.7250	5.0142	3.8146	3.8511	3.1424	4.1085	4.7469
Hspa5	_IEWLESHQDADIEDFK_.3	#N/A	4.9983	4.5779	5.7588	5.0506	4.8261	5.3369	5.5889	4.1810	5.8984	5.4759	4.8852	5.3424	5.4687	6.1560	4.3771	5.1025	5.1018	4.8956	5.1233	5.8575
Hspa5	_VLEDSDLK_.2	#N/A	3.1941	3.6103	3.7304	4.4953	4.1183	4.2907	4.0034	4.3953	4.8515	3.4005	4.6338	4.1646	3.6755	3.2193	5.3243	3.5390	1.7737	4.1475	4.4884	4.6782
Hspa5	_DAGTIAGLNVMR_.2	#N/A	4.2503	5.5335	6.5570	6.5170	2.9564	2.9897	6.0423	3.8570	4.0119	4.0609	3.4906	4.2958	4.1774	6.8660	5.6999	3.6124	3.9732	3.4384	3.0870	3.6162
Hspa5	_LYGSGGPPPT[Phospho (STY)]GEEDTSEKDEL_.2	P20029_T644_M1_Hspa5	3.8243	4.0825	3.9900	3.7359	3.1596	3.1929	5.2357	3.6664	3.8213	4.2641	3.3000	4.1052	3.9868	4.4894	3.9265	3.8156	4.2041	5.3288	3.2902	3.8272
Hspa5	_AKFEELNM[Oxidation (M)]DLFR_.3	#N/A	4.3436	3.6592	3.3790	3.6112	2.8763	4.4553	3.8598	4.0439	3.9189	4.5499	3.7290	4.0092	4.0269	3.3661	3.4370	4.3776	3.0808	3.4761	3.7295	3.8752
Tmsb4x	_NPLPSKETIEQEK_.3	#N/A	6.7290	6.9295	7.6076	7.4333	6.9105	7.1602	7.0215	6.9491	7.4767	7.6139	6.9510	7.1853	7.2728	7.5985	8.5880	7.6911	7.6820	7.1987	8.0749	7.3721
Tmsb4x	_ETIEQEKQAGES[Phospho (STY)]_.2	P20065_S50_M1_Tmsb4x	7.0687	7.1085	7.2653	7.3367	6.9914	6.8350	6.7119	7.2369	7.1784	7.3746	6.8796	7.3984	7.8103	7.4311	7.9914	7.6272	7.2413	7.1602	7.7003	7.0977
Tmsb4x	_TETQEKNPLPSKETIEQEK_.3	#N/A	5.4282	5.6681	7.0043	7.2385	6.1920	5.9612	5.7885	6.5118	6.6238	7.6208	5.8799	5.9653	6.7173	6.4446	7.3688	6.5253	6.6116	6.1696	7.3256	6.1708
Tmsb4x	_KTETQEKNPLPSK_.3	#N/A	6.1901	5.6521	6.9369	6.6779	6.5945	6.1783	6.5238	5.2653	6.3657	6.8675	6.3708	5.3907	5.6044	6.9134	7.0988	6.9302	5.7985	6.2521	7.0604	5.6908
Tmsb4x	_TET[Phospho (STY)]QEKNPLPSK_.2	#N/A	7.9342	7.7232	8.6781	8.3514	8.0223	7.6152	7.7166	7.6084	7.7782	8.7566	7.9351	7.7411	7.6874	8.2484	8.6030	8.3493	7.8622	7.6465	8.2724	8.0743
Prdx3	_GLFIIDPNGVVK_.2	#N/A	8.5659	8.5375	8.4100	8.6301	8.6872	8.4321	8.4208	8.5503	8.2113	8.5972	8.4808	8.6774	8.5317	8.7330	8.6399	8.6445	8.4330	8.5017	8.5073	8.6407
Prdx3	_HLSVNDLPVGR_.2	#N/A	8.0976	8.0363	8.2184	8.3410	8.5190	7.9413	7.7617	8.0898	8.2815	8.0503	8.0017	8.3642	8.1172	8.2672	8.7105	8.3385	8.2225	7.9695	8.2237	7.9926
Prdx3	_DYGVLLESAGIALR_.2	#N/A	6.8502	7.0332	7.3633	6.4487	6.8196	6.1881	6.7921	6.3036	6.6071	6.4836	6.3443	6.4581	7.1656	6.7323	6.9545	7.0028	6.8520	6.0344	6.2690	7.1547
Prdx3	_GTAVVNGEFK_.2	#N/A	6.4766	6.2554	6.1559	6.6312	6.3320	6.4927	6.2471	5.7902	6.4347	6.4050	6.6196	6.4092	5.7433	6.5752	6.8069	6.6443	6.1418	6.4507	6.3121	6.3481
Prdx3	_ELSLDDFK_.2	#N/A	5.3915	6.4234	6.7252	6.7738	6.4340	6.2947	6.7241	3.9738	6.4238	6.6873	6.7634	6.6969	5.9002	5.5091	6.4612	6.8322	5.6464	6.3153	6.7515	7.0762
Vim	_VELQELNDR_.2	#N/A	7.6169	7.6510	8.4373	7.5101	7.9138	7.5679	6.9237	7.6078	7.5472	7.7760	7.6976	7.3649	7.1809	7.7969	7.1224	7.1192	7.4985	6.7911	7.5565	7.5296
Vim	_FADLSEAANR_.2	#N/A	7.0184	7.2232	7.6152	6.5075	7.4698	7.4982	6.7121	6.7376	7.2200	7.3622	6.7902	7.3240	7.1104	7.1623	7.1731	6.5946	6.8328	6.5695	6.8734	6.9061
Vim	_NLQEAEEWYK_.2	#N/A	5.2994	5.9145	6.4729	5.6183	6.0920	5.6533	5.3842	6.1153	6.1256	6.2723	5.2110	5.7395	6.3786	5.9366	6.2976	5.5029	6.1115	5.5305	6.2901	6.0069
Vim	_QDVDNAS[Phospho (STY)]LAR_.2	P20152_S214_M1_Vim	5.3614	6.2757	5.9733	5.4654	5.5931	5.2425	5.5596	4.8914	5.3257	5.8564	4.7884	5.3754	4.8985	4.2691	4.6057	5.1363	5.1666	4.8001	4.9259	4.9769
Vim	_TNEKVELQELNDR_.3	#N/A	5.6553	6.6773	6.3158	6.1081	3.8651	6.4623	6.5961	5.7932	6.4348	6.8453	6.0900	6.3111	6.5886	7.1216	5.5757	6.0498	5.3996	6.3786	5.7406	6.3042
Vim	_ISLPLPTFSSLNLR_.2	#N/A	4.3222	6.3655	6.9242	5.6454	6.1037	5.0461	5.8541	3.7852	5.6628	5.6243	4.2796	5.7899	6.2116	5.2418	5.6197	5.1219	5.5826	5.8042	5.9467	5.8914
Vim	_ILLAELEQLK_.2	#N/A	3.8399	6.2100	5.4362	5.2042	5.3264	5.6650	5.6941	5.1800	5.5694	5.1150	5.3913	5.2207	4.5878	5.1212	5.4762	4.7093	5.2924	5.4175	5.0733	5.5079
Vim	_SLYSSS[Phospho (STY)]PGGAYVTR_.2	P20152_S56_M1_Vim	4.3181	3.3722	7.8030	3.0881	6.7384	7.2264	6.3281	5.9756	7.3560	7.5414	3.9080	7.0199	7.4575	3.4626	7.1291	5.3608	3.5345	3.5485	3.8475	4.8961
Vim	_QQYESVAAK_.2	#N/A	3.0614	3.6743	5.0010	3.3609	4.1620	4.5167	4.6383	4.3586	4.5423	3.8883	4.3312	3.5638	4.0420	4.5842	5.4502	4.7335	3.0657	4.0032	4.7496	3.8368
Vim	_EM[Oxidation (M)]EENFALEAANYQDTIGR_.2	#N/A	4.9488	4.9509	4.7229	4.4039	4.1411	4.3775	4.5428	4.4184	4.8688	4.7364	3.9845	4.5266	5.2102	5.1453	4.0872	4.5967	4.6743	3.9118	4.0869	4.2781
Vim	_FANYIDK_.2	#N/A	3.1328	4.1863	4.1702	3.1224	3.3397	3.3731	2.5029	3.4737	4.7824	4.4442	3.1072	3.9124	3.7940	3.9823	4.5912	3.9958	4.0114	3.8218	4.0091	4.2637
Vim	_SYVTTSTR_.2	#N/A	3.9768	3.3423	3.5134	3.6074	4.1838	3.4478	4.1409	4.2025	5.0390	4.9197	4.2740	3.6814	4.0077	3.5515	4.5742	3.3389	3.6997	3.7751	3.7488	3.2288
Vim	_DNLAEDIMR_.2	#N/A	4.2732	4.5467	3.8098	4.7331	2.9793	3.0126	3.8553	4.4989	3.9890	4.0838	4.6291	4.2728	4.1545	3.8478	4.0942	3.6353	3.9961	3.1533	3.4524	3.5251
Vim	_ETNLESLPLVDTHSK_.3	#N/A	4.5366	3.4662	3.5721	3.4000	3.8744	4.1106	4.4717	4.2370	4.3919	3.6809	3.8706	3.3556	4.5574	3.5592	3.2439	3.3807	3.2739	3.0585	4.2079	3.5262
Vim	_SVSSSSYR_.2	#N/A	3.9914	3.3277	3.5280	3.5929	4.1983	4.2317	4.3506	4.1159	4.2708	3.8020	3.7495	3.0538	4.4363	3.4381	2.8752	3.3535	3.1528	3.1795	4.3290	3.4051
Map2	_ARVDHGAEIITQS[Phospho (STY)]PSR_.3	#N/A	12.4076	13.0185	12.3928	12.5117	11.7524	12.0205	12.4244	12.3314	12.2315	12.1224	11.7462	12.3955	13.1650	13.0039	11.9688	12.3245	12.3331	12.4822	12.3212	12.3855
Map2	_IST[Phospho (STY)]PERK_.2	#N/A	13.6070	13.8128	13.7030	13.7476	13.4450	13.4176	13.4673	13.6227	13.2942	13.9361	13.3357	13.5393	14.0177	14.0571	13.1066	13.7620	13.6370	13.5983	13.6849	13.7617
Map2	_KSEVQAHS[Phospho (STY)]PSRK_.3	#N/A	9.7133	9.8047	8.8599	9.5557	9.3957	8.3512	9.5247	8.0614	9.4998	8.7269	9.3715	9.7111	9.1343	9.9351	8.4942	8.0151	8.6640	9.4765	9.7656	9.4264
Map2	_GLSS[Phospho (STY)]VPEVAEVEPTTK_.2	P20357_S1023_M1_Map2	9.8778	9.9488	9.9060	9.9635	9.2251	9.6340	8.3145	9.6972	9.7515	9.8606	9.3916	9.7426	8.8983	10.2678	9.6149	9.9715	10.0623	9.6112	9.5676	9.8430
Map2	_SSVAS[Phospho (STY)]PRR_.2	P20357_S1791_M1_Map2	9.5687	10.0303	9.5823	9.5017	9.6917	9.7740	9.6492	10.1681	9.8180	9.4337	9.4117	9.9929	10.6461	10.3287	9.8206	9.9945	9.9618	10.0495	9.9409	10.0023
Map2	_DGS[Phospho (STY)]PDAPATPEK_.2	#N/A	9.9004	9.9376	10.2559	9.9828	9.3593	9.9745	9.5625	9.7875	9.7790	9.9556	9.6939	9.4513	9.5401	10.3518	9.5305	9.5846	10.0633	9.5129	9.4813	9.7229
Map2	_GRIST[Phospho (STY)]PER_.2	#N/A	3.3187	4.0004	4.3561	4.2655	3.5256	3.5590	3.5225	3.5444	3.4427	3.1293	8.8808	3.7265	3.3496	4.3942	3.5478	4.1817	4.5424	4.1078	9.4092	4.0715
Map2	_FAAPAQPEEER_.2	#N/A	8.7721	8.8419	9.3431	9.0483	9.1073	9.2322	9.0073	9.0991	9.0637	9.1477	9.1275	8.8312	8.8786	9.3023	9.2514	9.0733	9.0588	8.8384	9.0561	9.1884
Map2	_LINQPLPDLK_.2	#N/A	8.7322	9.2089	9.2608	9.0650	9.0889	8.9745	9.0593	9.3420	9.0398	9.3129	8.9217	9.2944	9.2143	9.1393	9.3605	8.9203	9.1765	9.3227	9.1275	9.0905
Map2	_NKDDLTLSR_.2	#N/A	8.5942	8.9446	8.9409	8.7178	8.7992	8.6954	8.7154	8.8794	8.9152	8.6561	8.6505	8.8611	8.8927	9.1977	8.9518	8.9112	8.5122	8.7845	8.5692	8.9268
Map2	_AEPSQLDIK_.2	#N/A	8.1799	8.1887	8.5336	8.0566	8.4987	8.0183	8.1285	8.5572	8.1496	8.4257	8.3869	8.3205	8.3791	8.3795	8.5094	8.4338	7.9881	8.3263	8.1086	8.2137
Map2	_ADQGLDFAATK_.2	#N/A	8.1193	8.1199	8.4017	8.1992	8.2463	8.0766	7.9307	8.2972	8.0732	8.2924	8.0088	8.0402	7.8852	8.1829	8.3134	8.3193	8.1044	8.1497	7.8662	8.2376
Map2	_ELITTKDTS[Phospho (STY)]PEK_.2	#N/A	7.4750	8.4439	7.6112	3.1782	8.0992	8.2128	8.0015	8.3460	7.6912	8.0565	7.7986	8.2849	8.6710	8.4612	7.9277	8.1323	8.2701	3.0205	7.7026	8.1375
Map2	_VKDEFTAEK_.2	#N/A	7.5768	7.7815	8.1107	7.4661	7.7665	7.7967	7.7001	7.6728	8.0368	7.8681	7.9262	7.5822	7.7053	7.9460	8.0067	7.9723	7.8592	7.6381	7.6274	7.9990
Map2	_EIQGLFEEK_.2	#N/A	7.9943	7.8247	8.1722	7.6523	7.9482	7.7251	7.6475	7.9041	7.6377	8.5296	7.8216	7.9178	7.8359	8.1524	7.5174	8.0810	7.7992	7.7234	7.9111	8.0927
Map2	_VKIESVK_.2	#N/A	7.5734	7.4569	7.5199	7.1212	7.3974	7.5529	7.5919	7.2699	7.3709	7.2161	7.4035	7.5468	7.5816	7.6452	7.5615	7.4721	7.0903	7.4009	7.2173	7.5683
Map2	_ETSAPSVQEPTLTETEPQTK_.2	#N/A	7.4947	7.1586	7.7261	7.5212	7.1049	7.2620	7.1451	7.5181	7.7788	7.3876	7.2891	7.2112	7.2992	7.9189	7.6296	7.6418	7.6440	7.1851	7.0062	7.3569
Map2	_SDTLQISDLLVSESR_.2	#N/A	7.1671	6.5481	7.8413	7.3667	6.8537	7.0163	6.8266	6.5522	7.4733	7.4131	7.0478	6.8875	7.4507	7.1987	6.9853	7.4479	7.2038	6.4629	6.9785	7.1996
Map2	_GGQVQIVTK_.2	#N/A	6.5294	6.4042	6.4485	6.6193	6.7057	6.1590	6.5832	6.6132	6.9297	6.8238	6.2521	6.2785	6.9130	7.1675	6.8209	6.7959	6.1526	6.2953	6.7528	6.9018
Map2	_SKIGS[Phospho (STY)]TDNIK_.2	P20357_S1680_M1_Map2	3.8592	7.4495	7.6801	7.5946	6.2967	4.0995	6.6678	4.0849	4.4030	8.2922	7.0187	6.8220	6.5557	7.4626	3.0074	6.9801	5.5071	7.0968	7.3147	7.1805
Map2	_VTDGISKS[Phospho (STY)]PEK_.2	#N/A	6.7242	4.0992	4.2573	6.7746	6.7898	7.1837	6.7110	6.5656	6.7425	7.0487	6.7973	6.5620	6.4538	7.1969	3.6467	6.3774	7.1836	6.8072	6.9657	6.5290
Map2	_GSAQESLDTIS[Phospho (STY)]PK_.2	P20357_S608_M1_Map2	12.7617	11.9387	12.4536	12.3181	12.3681	11.9871	11.4823	12.7404	12.0725	12.8436	12.5142	12.4371	13.1592	12.8675	12.2506	12.6195	13.0519	12.2329	12.6066	12.6361
Map2	_TPGTPGT[Phospho (STY)]PSYPR_.2	P20357_T1623_M1_Map2	12.6756	12.2061	12.5821	12.2981	12.1534	10.6690	11.8697	11.4105	10.9482	13.0661	11.2716	11.5677	11.9970	11.7887	7.8696	11.6818	11.0327	11.1190	10.9251	11.4076
Map2	_VAIIRT[Phospho (STY)]PPKS[Phospho (STY)]PATPK_.2	#N/A	10.5051	10.3689	10.1951	9.9120	9.5449	7.8308	8.9233	9.7747	8.1397	10.3258	9.1293	8.4154	10.3843	10.7257	6.1405	9.7297	8.8315	9.7330	8.6760	9.0402
Map2	_LAS[Phospho (STY)]VSADAEVAR_.2	P20357_S823_M1_Map2	6.1824	10.0684	10.6540	10.1335	5.9985	10.3479	9.6810	5.3609	9.8308	5.7969	5.7012	5.3553	10.0061	10.7141	9.6634	9.9777	9.7724	10.3269	10.2353	10.2892
Map2	_T[Phospho (STY)]PPKSPATPK_.2	P20357_T1650_M1_Map2	9.7761	9.8529	9.7086	9.5811	9.5657	9.7033	8.8968	9.3548	9.5641	9.6766	9.7559	8.9895	9.0636	10.3913	9.4619	9.5543	9.6259	9.2695	9.4229	9.3324
Map2	_KETS[Phospho (STY)]PETSLIQDEVALK_.2	#N/A	6.7540	6.9655	6.9097	3.7653	5.8946	3.2888	6.1836	3.8893	6.4709	6.0595	6.3861	6.2339	6.8115	7.2931	5.9853	6.6667	7.1975	3.7376	6.5589	6.7110
Map2	_TTAASGDLAQAPGAFK_.2	#N/A	7.0416	6.8653	7.1683	6.9198	6.9961	7.0422	7.1617	7.4188	7.1680	6.9687	6.8363	6.8792	7.3237	7.6397	7.3804	7.0145	7.0263	6.8741	7.1535	6.8151
Map2	_STVS[Phospho (STY)]IEEAVAK_.2	P20357_S447_M1_Map2	5.4347	5.8794	5.6197	5.9233	4.7391	5.7192	5.4854	6.7411	5.8438	5.4722	5.2304	6.9442	5.7542	7.9592	5.7364	5.7597	6.8708	5.8278	5.3335	7.4654
Map2	_SILTEQLETIPKEER_.2	#N/A	5.8708	5.8121	6.2585	6.1553	6.1374	5.9310	5.6710	6.4667	5.8477	6.1615	5.7877	6.3806	6.0932	6.6275	6.0521	6.5850	6.0340	6.5606	6.1648	6.2496
Map2	_SILTEQLETIPK_.2	#N/A	6.1171	6.4585	6.1057	6.9275	6.0797	6.5040	6.1197	5.2903	6.1192	5.1503	3.4816	6.1163	5.5486	6.3447	5.8958	5.7533	6.0656	5.9552	6.3583	6.3909
Map2	_SGILVPSEK_.2	#N/A	8.6456	7.7882	8.2168	8.8888	7.9127	7.3158	8.1652	7.5210	7.6307	9.3539	7.9652	7.5999	7.8793	7.8823	7.2900	7.7592	7.0714	7.7094	7.1348	7.6601
Map2	_VELFGLGITYDQASTK_.2	#N/A	6.2395	6.0607	6.2404	5.9269	5.7445	5.4125	6.0739	5.4891	5.8891	6.0580	5.2168	5.2922	3.1734	6.1869	5.5083	5.6326	5.8839	5.2137	5.3029	5.8793
Map2	_IDLSHVTSK_.2	#N/A	5.2805	5.4462	5.0755	4.9080	4.8867	5.4178	4.9954	5.6336	5.1471	5.3757	5.4073	5.3939	5.0862	5.7647	5.6489	5.4488	5.1190	5.1872	5.1085	4.4241
Map2	_KSEVQAHS[Phospho (STY)]PSR_.2	#N/A	11.2503	11.5312	11.4720	11.5680	11.1427	11.0402	11.1162	11.3691	11.1288	11.5949	10.7596	11.3867	11.7420	11.7355	11.2812	11.6416	11.1848	11.1899	11.4145	11.2650
Map2	_STELGSDYYELSDSR_.2	#N/A	6.8646	8.9640	9.3187	9.2157	6.8327	7.6761	7.9994	6.6372	7.6117	6.6762	7.2378	6.7451	6.9479	7.2533	6.5738	7.0277	6.7746	6.5211	6.6550	8.8622
Map2	_DLATDLSLIEVK_.2	#N/A	6.3000	6.4227	6.8987	6.3666	6.4940	6.5833	6.4455	6.4672	6.3900	6.8268	6.5017	6.6916	6.7125	6.3431	6.7619	6.6671	6.4697	6.3299	6.3834	6.8028
Map2	_VTS[Phospho (STY)]EPEAVSERR_.2	#N/A	3.4009	6.0409	4.1128	4.1314	3.9690	4.1754	5.4488	4.6257	4.2478	3.5210	3.9526	4.1700	4.5765	6.3203	4.1083	4.5844	5.9415	5.6246	5.6865	5.6406
Map2	_SEVQAHS[Phospho (STY)]PSR_.2	#N/A	12.1626	12.2587	12.2925	12.3693	11.9409	11.9733	11.9987	12.0828	11.7959	12.3029	11.7737	12.0535	12.3496	12.4889	11.9457	12.2730	11.9704	11.9070	12.0869	12.0796
Map2	_ELITTKDTS[Phospho (STY)]PEKTEK_.3	#N/A	9.5239	10.4213	8.8609	9.6413	9.6187	7.5811	9.8263	10.3312	9.8870	10.0706	9.1649	10.2276	11.4056	10.4370	10.0660	9.9639	9.6160	10.1442	10.9415	10.0570
Map2	_DGS[Phospho (STY)]PDAPATPEKEEVAFSEYK_.3	#N/A	11.3995	11.5807	12.0616	11.8320	10.5133	11.5831	11.1529	10.5359	11.7529	11.8273	11.2432	10.9781	11.1578	11.7806	11.2473	11.4707	11.4327	11.2838	11.4863	11.6871
Map2	_VTS[Phospho (STY)]EPEAVSER_.2	#N/A	9.1117	9.1240	9.4233	9.2246	8.6135	8.9220	8.7159	9.2145	3.9071	9.2377	9.3955	9.3044	9.3947	9.9635	9.2850	9.3502	9.6338	9.3499	9.4837	9.6584
Map2	_DWFIEM[Oxidation (M)]PTESK_.2	#N/A	5.9269	5.7180	6.6929	6.0533	5.6437	6.1277	5.9363	5.5488	5.6537	5.9383	5.7253	5.5891	5.5428	6.4896	5.7049	5.8355	6.2614	5.2451	5.5686	6.1235
Map2	_VDHGAEIITQS[Phospho (STY)]PSR_.2	#N/A	14.1913	14.2356	14.5738	14.4117	13.9037	13.9209	13.9217	14.0810	14.1044	14.3060	14.1043	14.0381	14.8426	14.5128	13.7691	14.1310	14.2598	13.9603	14.2059	14.2655
Map2	_VTDGISKS[Phospho (STY)]PEKR_.2	#N/A	10.5432	10.1205	10.4604	10.6144	10.3305	10.4967	10.3529	10.5545	10.2650	10.5991	9.7008	10.3504	10.7049	10.7503	10.1198	10.3813	10.3800	10.4115	10.4032	10.4119
Map2	_VDHGAEIITQSPSRSS[Phospho (STY)]VAS[Phospho (STY)]PR_.3	P20357_S1788_M2_Map2	5.9842	6.0186	5.7208	6.3968	3.5169	6.4245	5.6298	3.6972	5.2873	6.6410	3.5899	5.9641	3.7449	6.2692	4.7598	6.3651	5.2422	3.7972	5.9331	6.0276
Map2	_S[Phospho (STY)]GILVPSEKK_.2	#N/A	8.3525	7.1655	6.7572	8.2468	3.5618	4.4231	6.8341	3.9474	4.0794	8.7271	3.5580	6.2676	4.2448	5.8436	4.1845	6.1439	4.4621	5.6284	5.6663	3.2136
Map2	_ETS[Phospho (STY)]PETSLIQDEVALK_.2	#N/A	4.0406	8.7858	9.2329	3.9435	8.9939	8.7647	8.6531	8.7700	3.8542	9.3455	9.3712	3.8708	9.6964	12.8506	8.6538	4.1663	9.4116	12.0905	3.4978	9.1177
Map2	_EKDVLEDIPR_.2	#N/A	7.5553	7.7556	7.9144	7.6257	8.2158	7.7245	7.5114	7.8606	7.7069	7.7886	7.7536	7.4085	7.4569	7.8426	7.6873	7.7295	7.9239	7.6088	7.6663	7.7955
Map2	_VSDFGQM[Oxidation (M)]ASGM[Oxidation (M)]NVDAGK_.2	#N/A	6.0292	6.1887	6.1099	5.6785	6.3291	5.9027	5.6772	6.8346	6.1586	6.3572	5.7595	6.4805	6.3620	5.6854	6.4821	6.7095	6.1039	6.2380	6.3962	5.9776
Map2	_M[Oxidation (M)]PSKPGEDFEHAALVPDTSK_.4	#N/A	5.9910	5.8411	5.3816	4.8625	5.6046	5.7568	5.8836	5.0464	6.1833	6.5704	6.1105	5.5960	6.8322	6.2215	6.4410	6.0865	4.8165	6.1721	6.3464	6.4214
Map2	_YFETSALKEDM[Oxidation (M)]TR_.2	#N/A	4.7943	5.1737	5.2652	5.6369	4.4079	5.4119	4.8639	4.9992	5.1491	5.0704	5.0759	4.8730	4.4531	6.0536	5.2786	5.2534	4.8713	5.1324	5.1557	5.1750
Map2	_ESS[Phospho (STY)]KDEEPLKDK_.2	#N/A	7.5282	7.5362	7.3727	7.2637	6.8109	6.9655	7.1867	6.8583	6.4597	7.8875	7.1751	5.0143	7.1288	7.8572	6.5788	7.2363	7.2764	7.3940	7.2757	7.3469
Map2	_TGVIQTS[Phospho (STY)]TEQSFSK_.2	#N/A	5.2070	4.4592	4.2073	3.8768	3.7047	4.7426	3.4430	3.8141	4.4071	3.8778	4.6862	3.6140	4.0821	5.6539	3.4553	3.8376	3.6939	4.8409	4.4562	3.7635
Map2	_VGS[Phospho (STY)]LDNAHHVPGGGNVK_.3	#N/A	9.0882	8.9878	8.8166	9.0581	7.8399	7.5432	8.2778	7.4886	7.3909	9.5538	8.4347	8.1486	8.2938	8.7546	6.9796	8.2310	7.5746	8.3562	8.4298	8.6425
Map2	_SEVQAHS[Phospho (STY)]PSRK_.2	#N/A	8.9229	9.0415	9.0187	9.1085	8.8553	8.7049	8.6993	9.0346	8.7794	9.2739	8.5384	9.0267	9.2661	9.3445	8.4548	9.0615	8.7150	3.8625	8.7600	8.9810
Map2	_NQHDEKELQAK_.2	#N/A	5.1147	4.5684	4.1357	4.8267	3.9842	5.0514	4.4000	4.7508	4.3674	4.9086	4.8034	4.4707	4.0088	5.0462	5.4409	5.3894	5.3845	4.6858	4.3638	4.9950
Map2	_GVSGDREENS[Phospho (STY)]FSLNSSISSAR_.3	P20357_S1567_M1_Map2	6.5514	4.1550	4.2191	4.4201	3.3886	3.4830	4.0078	3.6704	4.2659	3.6548	5.0783	4.2920	5.6338	4.0929	3.7599	4.0447	4.0400	5.4659	4.7396	5.5211
Map2	_SGTSTPTTPGSTAITPGT[Phospho (STY)]PPSYSSR_.3	P20357_T1609_M1_Map2	8.7391	9.1443	7.5176	4.3094	3.7946	7.4019	3.4773	10.0433	7.1138	8.2874	3.7749	8.9776	9.5061	10.6216	3.3629	7.5069	8.6778	8.7956	3.7789	3.7512
Map2	_GRIST[Phospho (STY)]PERK_.2	#N/A	9.1861	9.4609	4.2178	9.2752	9.1601	8.4947	9.0725	10.7150	3.5962	9.3872	3.0748	4.0447	3.7616	9.6199	9.2381	9.5867	9.2341	9.4831	9.2297	4.2313
Map2	_AGKS[Phospho (STY)]GTSTPTTPGSTAITPGTPPSY[Phospho (STY)]SSR_.3	P20357_S1592_M2_Map2	3.5249	4.4779	4.0612	4.2704	2.8627	3.0867	3.1776	3.5395	3.2367	3.7313	3.6110	4.3673	3.3447	4.3893	4.2556	3.8698	4.5376	4.1127	4.4117	4.0666
Map2	_VTGGQTIQVETSSESPFPAK_.2	#N/A	4.4482	4.0384	5.1573	4.7160	3.9262	3.8348	3.5294	3.5767	4.4914	3.7615	4.3117	4.4623	4.4602	5.0976	3.5548	4.0921	4.0045	3.9070	4.0979	4.6723
Map2	_MFTIDPK_.2	#N/A	3.1429	4.3240	4.1803	4.4413	4.3669	4.3692	5.2179	3.3686	3.6185	4.2279	3.0971	3.6905	3.7839	5.4014	3.9733	4.0058	3.0131	4.0939	3.4805	3.9546
Map2	_EAS[Phospho (STY)]PPTSADK_.2	#N/A	14.0803	14.0456	14.2343	13.9115	13.8580	13.9542	13.7974	13.9837	13.8103	14.1286	14.0685	13.9246	14.0690	14.7881	13.6937	13.9784	14.1537	14.0447	14.0202	14.1900
Map2	_KANDKLDTVLEK_.3	#N/A	3.3392	4.2063	4.3766	4.2450	3.9653	5.0056	4.8298	3.9389	4.1645	3.8478	2.9008	4.1608	3.5876	4.6481	5.7461	4.4280	3.0621	4.7049	4.3864	4.4950
Map2	_VKDEFTAEKEAS[Phospho (STY)]PPTSADK_.3	#N/A	11.8052	12.1373	11.6215	11.6148	11.6371	11.5868	11.5940	12.1607	11.5759	11.8626	11.5521	12.0378	12.8769	12.8805	11.8921	12.1941	12.1737	12.1740	11.9211	12.0285
Map2	_TGVIQTSTEQS[Phospho (STY)]FSKEDQK_.3	#N/A	5.2601	5.8338	5.8574	5.6676	4.0353	4.9995	5.1495	3.7904	5.1814	4.9613	5.1925	5.2281	4.5735	3.7133	3.6806	4.8888	4.9505	5.1161	4.2374	5.0647
Map2	_DKVTDGISKS[Phospho (STY)]PEK_.2	#N/A	7.7194	7.4990	7.9943	7.6385	7.4468	7.6002	7.2614	7.2813	6.9468	7.6729	7.3534	7.4442	7.5876	8.0968	7.3149	7.3878	7.7109	7.3296	7.3513	7.3058
Map2	_IVQVVTAEAVAVLKGEQEK_.2	#N/A	5.1619	4.7434	3.8680	4.6703	4.8555	4.5248	3.6622	4.7785	4.0990	5.1193	4.7143	4.1806	5.5057	5.4028	3.7606	4.0686	5.6259	4.2439	4.3227	4.5588
Map2	_TPGT[Phospho (STY)]PKS[Phospho (STY)]GILVPSEK_.2	#N/A	6.7679	8.3609	8.5104	9.4917	3.8694	5.7387	7.8185	3.8832	6.2015	9.6479	3.8235	7.5490	3.8805	7.8900	3.7535	8.0726	5.3591	6.9564	7.1951	7.2200
Map2	_APHWTSASLTEAAAHPHS[Phospho (STY)]PEM[Oxidation (M)]K_.3	#N/A	4.0479	9.3815	8.3531	3.8784	6.7420	6.6546	9.0960	9.1135	6.4556	8.6407	7.0857	8.7223	7.7938	10.7063	7.1838	8.0813	7.7895	9.1326	8.6091	8.1742
Map2	_EEFVETC[Carbamidomethyl (C)]PGELK_.2	#N/A	3.6935	3.6255	7.6172	7.3903	7.4907	7.5349	3.5573	7.4239	7.5787	3.3516	3.9907	3.3517	3.7244	7.7461	7.5022	7.3318	3.4165	3.7330	6.9384	4.4463
Map2	_LEGAGSATIAEVEM[Oxidation (M)]PFYEDK_.2	#N/A	3.6468	4.3013	5.3657	4.9809	4.8016	5.0697	5.0839	5.4082	4.7456	5.3912	5.0453	4.8541	3.4858	5.6453	4.8987	3.0089	5.0205	4.3893	4.0390	4.8949
Map2	_DQPAALPLAAEETANLPPS[Phospho (STY)]PPPS[Phospho (STY)]PASEQTATVEEDLLTASK_.4	#N/A	3.8438	6.5706	3.9932	3.8174	3.4270	6.1216	4.1351	3.6331	3.6345	4.1840	3.3588	4.0203	3.7878	3.9803	3.9914	3.8635	4.1708	3.7231	3.9066	3.9473
Map2	_TPGTPGTPSYPRT[Phospho (STY)]PGT[Phospho (STY)]PK_.2	#N/A	7.2560	6.6855	4.0267	7.4755	3.6692	5.0628	6.2169	3.8326	3.7845	7.5085	3.6364	5.5839	3.9096	6.1245	4.3929	6.4810	5.3684	5.6982	3.6664	4.7913
Map2	_ARVDHGAEIITQS[Phospho (STY)]PSRSS[Phospho (STY)]VAS[Phospho (STY)]PR_.3	#N/A	3.5760	3.7702	3.8332	4.1202	3.7390	4.0426	3.8716	3.9861	3.7872	5.4558	3.7595	3.6109	4.0388	3.7510	3.4488	3.6454	3.6529	4.0153	3.8482	6.3213
Map2	_IGSTDNIK_.2	#N/A	2.6695	3.5234	2.5005	4.0957	4.1835	4.0781	3.7997	4.0829	3.4343	3.6678	4.3585	3.4041	3.1657	4.4354	4.8280	3.5652	3.6902	3.7415	4.2118	3.9333
Map2	_YFETSALK_.2	#N/A	3.2051	4.1140	4.2377	4.5960	3.4120	3.4453	3.6361	3.4307	3.5563	4.5165	4.1281	3.1856	3.7218	4.3519	4.3771	3.7917	4.4288	3.8941	4.4241	4.1914
Map2	_GHDLSPLASDILTNTSGSM[Oxidation (M)]DEGDDY[Phospho (STY)]LPPTTPAVEK_.4	#N/A	3.2923	6.0507	4.1847	4.3340	3.7369	3.6924	6.3815	3.9130	4.8752	3.8463	3.8423	3.5679	3.4961	5.3355	3.4060	4.0060	3.7901	4.0915	3.9791	4.0517
Map2	_SM[Oxidation (M)]SINLPM[Oxidation (M)]SC[Carbamidomethyl (C)]LDSIALGFNFGR_.3	#N/A	4.3696	4.1420	4.2145	4.4071	3.8431	4.6895	3.6640	3.4028	3.5842	4.4886	3.0629	3.8681	3.7497	4.2526	3.6894	4.0401	4.4009	4.2494	3.3227	3.9299
Map2	_LSNVSS[Phospho (STY)]SGSINLLESPQLATLAEDVTAALAK_.3	P20357_S1800_M1_Map2	3.6995	5.7602	3.2361	3.8847	3.9064	3.7008	7.1506	3.9252	3.0619	3.5101	4.0413	5.9783	3.2273	3.2741	3.1670	3.0616	3.4224	3.7270	4.0261	2.9514
Map2	_APHWTSASLTEAAAHPHS[Phospho (STY)]PEM[Oxidation (M)]KDQGGAGEGLSR_.5	#N/A	4.5963	3.3627	3.5338	3.6278	3.7996	4.3491	3.9973	6.8274	3.9515	3.7983	3.7152	3.6955	6.5111	3.4450	3.9287	4.8654	4.0079	3.4694	5.6165	3.7877
Map2	_ASQPSPPAQEAGYSTLAQSYTPDHPSELPEEPSS[Phospho (STY)]PQER_.4	P20357_S655_M1_Map2	3.9016	3.3610	3.9259	3.9006	3.5851	3.8367	3.7211	3.7960	3.6182	3.9899	3.9925	3.6616	3.9257	3.9469	3.5146	3.7457	3.9655	5.9258	4.4248	3.7266
Map2	_KDEWGLAAPIS[Phospho (STY)]PGPLTPM[Oxidation (M)]R_.3	P20357_S283_M1_Map2	4.5409	4.8689	4.0986	3.7280	3.5925	3.8166	4.7152	4.4252	4.0547	4.8202	3.7366	4.9677	3.9357	5.6985	3.5728	3.9072	3.9243	4.3645	4.0373	3.0312
Map2	_DTS[Phospho (STY)]PEKTEK_.2	#N/A	4.4814	4.3386	4.0179	3.1029	3.1875	3.4201	4.3451	3.2062	4.4042	4.0646	3.2777	4.0647	4.5122	4.0560	3.9223	3.8435	4.2043	4.4460	3.2442	3.7333
Map2	_ANDKLDTVLEKS[Phospho (STY)]EEHIDSK_.3	P20357_S969_M1_Map2	3.9987	4.0041	4.5350	3.6907	3.3364	3.9226	4.2047	3.6990	3.8539	5.4278	3.3326	3.8936	4.0194	4.5220	3.7819	4.3436	4.2367	3.5964	3.2450	4.8315
Map2	_ESEEM[Oxidation (M)]GGKVELFGLGITYDQASTK_.3	#N/A	3.1875	3.3144	3.7238	4.5019	4.0261	4.2502	3.5150	4.3887	4.1482	3.3939	3.9484	3.2039	3.6821	3.7109	3.0922	5.2989	3.4256	4.4076	4.6795	4.4040
Map2	_YTVPLPSPVQDSENLSGESGSFYEGTDDKVR_.3	#N/A	4.1248	3.8780	3.1602	3.5646	3.4625	3.6866	4.0786	3.8252	4.1377	4.3311	3.0112	3.7675	4.2457	3.7895	3.6558	2.9688	4.3628	3.2117	3.5107	3.4668
Map2	_IVQVVTAEAVAVLK_.2	#N/A	1.3482	4.5770	3.7795	3.3413	2.9490	3.6585	4.0991	4.4686	4.1658	1.5121	3.0393	4.3031	4.2738	3.8176	4.1244	3.6050	3.9658	3.1836	3.4826	3.4949
Prkca	_VIS[Phospho (STY)]PSEDR_.2	#N/A	12.3272	12.0940	12.1189	12.6250	12.0299	12.1425	12.1665	11.2889	11.9585	12.3622	12.0181	11.6648	12.0850	11.9779	10.9896	12.1824	11.8219	11.9756	12.1258	11.9261
Prkca	_[Acetyl (Protein N-term)]ADVYPANDSTASQDVANR_.2	#N/A	8.6279	8.7601	9.0582	7.4074	7.8353	7.6207	8.0790	7.3973	7.2167	8.9410	6.8528	8.1896	8.3850	8.0485	7.5502	8.0712	8.3783	8.1624	7.3367	6.9604
Prkca	_VIS[Phospho (STY)]PSEDRKQPSNNLDR_.3	#N/A	7.6732	8.3809	7.3924	7.9343	7.6832	7.2592	8.0220	8.5251	7.6642	7.7281	6.8716	8.0449	9.1569	7.8116	7.2884	7.8894	7.7402	8.0329	7.5792	7.5403
Prkca	_VIS[Phospho (STY)]PSEDRK_.2	#N/A	4.5594	3.4434	3.5949	3.1300	8.1922	8.0603	8.1465	7.9462	3.7030	3.2649	7.9081	7.8362	3.8111	3.5819	7.5908	3.4035	3.2966	3.0357	7.7738	7.7692
Prkca	_LTDFNFLM[Oxidation (M)]VLGK_.2	#N/A	6.0328	5.3677	5.5753	5.8241	5.9267	5.7680	5.9184	6.0411	5.2933	5.3264	5.7105	5.6616	5.6899	4.6520	5.1454	6.0679	6.0917	5.9721	5.7029	5.3694
Prkca	_DVVIQDDDVEC[Carbamidomethyl (C)]TM[Oxidation (M)]VEK_.2	#N/A	7.1307	7.9362	7.4775	7.5840	6.2271	6.7278	7.3464	7.0899	7.1595	6.8855	6.4595	6.6264	7.0454	8.6444	6.1949	6.7217	6.8449	7.1700	6.3553	6.7062
Prkca	_T[Phospho (STY)]FC[Carbamidomethyl (C)]GTPDYIAPEIIAYQPYGK_.3	P20444_T497_M1_Prkca	3.6329	4.5025	4.7102	5.1316	4.6622	4.9071	5.0839	4.8979	4.2856	6.2148	6.0966	4.1541	4.7974	3.9034	4.1591	6.0697	4.4898	5.7737	4.3349	4.5266
Prkca	_LKPS[Phospho (STY)]DKDR_.2	P20444_S234_M1_Prkca	5.5605	5.5198	5.0341	5.6624	4.7904	3.1207	4.9405	5.3113	3.7537	5.7915	3.5771	3.7653	5.8818	4.3553	4.2216	4.5719	5.4538	5.7635	4.7898	4.0326
Prkca	_LSVEIWDWDR_.2	#N/A	4.8276	4.9064	5.1455	4.0870	4.9342	4.7002	4.9028	4.8907	4.9492	5.0734	4.0765	3.9240	5.3415	4.2186	3.8123	4.3132	4.2063	3.0552	3.2754	3.6233
Prkca	_NLIPM[Oxidation (M)]DPNGLSDPYVK_.2	#N/A	7.4364	7.1606	6.7464	6.3775	7.0444	7.0924	6.9398	7.2623	7.0568	7.1085	6.9769	7.1652	7.1350	6.1586	6.6782	7.0422	6.8040	7.1843	7.2072	6.9721
Prkca	_DVVIQDDDVEC[Carbamidomethyl (C)]TM[Oxidation (M)]VEKR_.3	#N/A	6.0664	6.6474	4.5980	6.0194	5.2778	5.0564	5.6425	5.9307	5.0633	5.7436	4.6235	5.5523	6.3334	6.7962	4.5988	5.4918	5.5534	5.8436	5.0615	5.6591
Prkca	_KDVVIQDDDVEC[Carbamidomethyl (C)]TMVEK_.3	#N/A	4.2867	2.5641	3.3222	3.4027	3.6244	3.8485	3.9166	3.9871	3.9757	4.4930	4.3501	3.6055	4.0838	3.3092	3.4938	3.1308	4.5248	3.3084	2.9570	3.3048
Mag	_LLGDLGLR_.2	#N/A	8.5625	8.2501	7.9144	8.3705	8.2612	8.1916	8.0152	8.0818	7.8319	7.7654	8.1318	8.2712	8.1692	7.8268	7.4643	7.6331	8.2367	8.0111	7.8581	8.1894
Mag	_NLYGTQSLELPFQGAHR_.2	#N/A	4.9688	5.6367	4.3878	5.7778	5.1915	5.6030	5.3834	5.0999	5.3531	5.5857	5.2050	5.1740	4.7761	5.2713	5.6364	4.8362	4.4478	5.2612	5.6963	5.2808
Mag	_TQVVHESFQGR_.2	#N/A	4.8071	5.7811	5.7822	4.3939	5.1698	5.7985	5.6143	4.7486	5.2911	5.0185	5.3466	5.2666	5.2846	4.8268	5.2670	5.4350	5.5094	5.4266	5.2940	5.5015
Mag	_SGLLLTSILTIR_.2	#N/A	3.7594	3.9434	4.8173	4.0396	5.3281	4.6580	4.9833	4.0691	3.8937	5.5736	4.0983	3.5235	4.1486	3.9230	3.4118	3.6930	5.1767	4.4705	5.0751	3.9714
Mag	_GESPELDLSYSHSDLGK_.2	#N/A	3.9388	5.9623	5.7827	5.4204	3.8436	5.5796	5.1827	4.2496	5.5491	4.0552	4.0470	4.8764	4.2694	6.2730	4.6312	4.0901	5.6429	5.7015	5.0026	3.8948
Mag	_NVTESSSFS[Phospho (STY)]GGDNPHVLYSPEFR_.3	P20917_S549_M1_Mag	7.9167	7.7691	4.2567	7.5678	3.1721	6.6575	7.3128	7.2875	6.8244	3.5578	6.5982	7.2618	7.6093	7.9254	5.4107	6.4588	6.9210	7.2772	6.9095	6.2268
Mag	_LREDEGTWVQVSLLHFVPTR_.3	#N/A	4.2390	3.7638	3.2744	3.4504	3.5767	3.8008	3.9644	3.9393	4.0235	4.4453	2.8970	3.6533	4.1315	3.2615	3.5416	3.0830	4.4770	3.3259	2.5639	3.3526
Tpm3	_IQLVEEELDR_.2	#N/A	9.3342	9.2661	9.7735	9.6164	9.2270	9.4448	9.2040	9.2707	9.3847	9.5234	9.6437	9.3730	9.3629	9.8409	9.3893	9.4966	9.4756	9.1986	9.2267	9.6892
Tpm3	_AADAEAEVASLNR_.2	#N/A	8.3553	8.2046	8.8028	8.7055	8.2875	8.2961	7.8606	8.3093	8.3783	8.5437	8.4449	8.2656	8.4063	8.5889	8.6117	8.4180	8.2146	7.9768	8.2846	8.4732
Tpm3	_KYEEVAR_.2	#N/A	7.3127	6.8216	8.6849	7.0172	7.6833	7.5175	7.3793	7.4228	7.4307	7.6401	7.6837	7.3077	7.8711	7.5065	8.5786	7.4798	7.1799	7.3117	7.4098	7.6419
Tpm3	_HIAEEADR_.2	#N/A	7.1086	6.9626	7.6283	7.2486	7.1404	7.5045	6.8470	6.9139	7.5081	7.5049	7.5106	6.9473	7.0773	7.3845	8.3701	7.3011	6.7318	7.0930	7.4727	7.3064
Tpm3	_YSQKEDKYEEEIK_.3	#N/A	6.4017	6.9445	6.8066	5.8708	6.4538	6.5445	6.1070	6.0980	6.3803	6.9587	6.3426	6.7240	6.7854	6.6890	7.1444	6.5096	6.1360	6.4380	6.5994	6.9671
Tpm3	_KLVIIEGDLER_.2	#N/A	5.9057	5.9513	6.2393	6.0196	6.2877	5.7686	5.6425	6.1238	5.4544	5.9052	5.8720	5.7984	5.9282	6.1827	6.4488	5.9904	6.3502	5.5865	6.1713	6.2379
Tpm3	_IQLVEEELDRAQER_.3	#N/A	6.5728	6.2872	6.3477	6.7087	6.6150	6.0481	6.2775	5.8133	6.2284	7.3092	7.0630	6.6386	7.6727	6.7352	6.4503	7.0985	6.4519	6.4802	6.4524	7.2494

Tpm3	_LEEAEKAADESER_.2	#N/A	5.4327	5.1394	4.7492	5.4904	4.9518	5.0713	5.1296	5.2824	5.4469	4.8218	4.7970	5.7994	5.4666	5.6940	5.3655	5.4860	5.6878	5.4743	5.1899	4.7506
Tpm3	_KLVIIEGDLERTEER_.3	#N/A	6.2088	6.3002	4.4828	6.9819	6.1787	4.9974	5.6916	6.5555	6.1293	6.6647	6.2317	6.7090	7.7523	7.0850	6.7094	6.3034	6.3099	6.6090	6.2020	7.2656
Tpm3	_C[Carbamidomethyl (C)]SELEEELKNVTNNLK_.2	#N/A	4.1210	4.2772	2.7396	2.9169	3.6900	3.4594	3.8777	3.7140	3.4698	3.8866	2.9793	4.2094	3.8416	4.2240	3.6249	4.3098	3.8512	3.1223	4.0458	3.4166
Tpm3	_ALKDEEKM[Oxidation (M)]ELQEIQLK_.3	#N/A	4.8957	4.9592	4.2696	4.0129	4.4793	4.7147	4.6226	5.0500	4.7342	4.9452	5.0441	5.2866	5.9199	3.7434	4.6259	5.6331	4.7055	4.6859	4.7440	5.2623
Tpm3	_AADAEAEVASLNRR_.2	#N/A	3.6066	3.8664	3.6859	4.0424	3.8048	3.9709	4.0328	3.3964	3.8826	3.5937	3.7744	2.9661	3.8050	3.4229	3.2655	3.6592	3.4901	3.9590	4.0567	3.8878
Tpm3	_TEERAELAESK_.2	#N/A	3.6620	3.9698	4.1983	4.0274	3.9544	3.2238	3.0405	2.9412	3.0996	3.8683	2.4390	3.5699	3.2077	4.2523	3.3421	3.7414	4.4006	4.2497	3.0479	3.9296
Tpm3	_LVIIEGDLERTEER_.3	#N/A	3.0961	3.4059	3.6324	4.5933	3.9347	4.0503	3.0312	4.2973	4.4522	5.7310	4.7668	5.0278	3.1168	3.6195	5.0465	3.4410	3.3341	2.9982	4.1476	3.5865
Tpm3	_RIQLVEEELDR_.2	#N/A	4.5510	4.1468	4.3923	3.8334	3.9785	3.2904	3.7910	3.5564	3.7112	4.3616	3.1899	4.0363	3.8767	10.1036	3.8164	9.3743	4.0940	3.7391	3.3877	4.3463
Tpm3	_HIAEEADRKYEEVAR_.4	#N/A	3.6626	3.6565	3.1992	3.9216	3.8696	3.9029	3.1785	3.8883	3.0987	3.4732	4.0782	3.3826	3.2642	3.7668	3.2039	3.0247	3.3855	4.3516	3.3428	3.7338
Tpm3	_AISDELDHALNDMTS[Phospho (STY)]I_.2	P21107_S284_M1_Tpm3	3.7316	3.5875	3.2682	3.8526	3.9385	3.9719	3.1096	3.9573	3.0298	3.5422	4.0093	3.3136	3.1952	3.3062	3.1350	3.0937	3.4545	3.6950	3.9940	2.9835
Gna11	_LLLLGTGESGK_.2	#N/A	7.9938	7.8250	7.8087	7.8078	7.6588	7.7981	7.9031	7.9064	7.6864	7.9502	7.6828	7.6307	7.4247	7.3153	6.7637	7.0720	7.4911	7.6363	7.4957	7.5994
Gna11	_IATVGYLPTQQDVLR_.2	#N/A	6.9251	6.5713	7.1630	6.9677	6.9032	6.9887	7.2459	7.0980	6.7974	6.7435	7.0101	6.3105	6.9618	6.9111	6.3272	6.8238	6.7693	6.7150	6.8749	6.4595
Gna11	_DTILQLNLK_.2	#N/A	5.0318	4.9212	5.5087	5.1925	4.7657	4.5948	5.4019	4.9720	5.1606	3.2869	5.0901	3.3108	3.4729	4.7379	4.6767	4.4762	4.6139	5.2298	4.7855	4.6981
Gna11	_MVDVGGQR_.2	#N/A	3.4866	6.1062	5.4724	4.0977	3.6935	5.9317	6.6905	6.4172	5.8748	5.0515	6.0132	5.1541	3.5174	5.5570	6.7142	4.3495	4.7339	4.3628	4.8603	4.2393
Gna11	_TLWSDPGVQEC[Carbamidomethyl (C)]YDR_.2	#N/A	3.6926	3.6265	5.9582	6.0995	3.8995	5.8016	3.5563	3.9183	5.9689	3.3525	3.9898	3.3526	3.7235	3.2672	4.7648	3.4911	3.4155	3.7339	4.0330	5.0195
Gnaq	_VADPSYLPTQQDVLR_.2	#N/A	8.8871	8.5173	8.5415	8.7498	8.3879	8.5004	8.7428	9.0033	8.6974	8.6236	8.4366	8.4326	8.4532	8.6964	8.3689	8.6274	8.6840	8.5966	8.6208	8.1154
Gnaq	_YYLNDLDR_.2	#N/A	7.0430	7.3566	7.4518	7.1509	6.8790	7.1669	7.1821	6.4970	7.5332	6.8980	7.0538	6.6819	7.3875	6.2897	6.5595	7.0656	6.5448	7.2483	6.9756	6.8201
Gnaq	_VSAFENPYVDAIK_.2	#N/A	6.1669	6.5680	6.5251	6.4633	6.4258	6.3554	6.7683	6.7552	6.8034	6.5537	6.6606	6.0981	6.6572	6.3663	6.5688	6.4290	6.3595	6.4642	6.3796	5.6801
Gnaq	[Acetyl (Protein N-term)]TLESIMAC[Carbamidomethyl (C)]C[Carbamidomethyl (C)]LSEEAK_.2	#N/A	3.8903	2.9227	4.7322	3.6939	4.0973	4.1306	3.6387	4.1160	4.3719	2.5132	2.4097	3.1549	4.5373	3.4395	2.9762	3.2524	3.6132	3.0785	4.2279	3.5061
Gnaq	_EVDVEKVS[Phospho (STY)]AFENPYVDAIK_.2	#N/A	3.5253	3.9624	3.8525	3.8909	3.7322	3.8136	3.6484	3.7440	3.6400	4.0150	3.6576	3.5802	3.7756	3.8618	3.4660	3.6781	3.7455	3.7673	3.7493	3.7326
Gnaq	_VPTTGIIEYPFDLQSVIFR_.2	#N/A	3.8534	3.4657	3.3900	3.7309	3.6380	1.9554	3.0219	4.0791	4.4088	2.6101	3.8875	3.1918	4.5743	3.5761	3.0132	3.2155	3.1249	2.9220	4.1910	3.5431
Abcb1a	_S[Phospho (STY)]TRKS[Phospho (STY)]IC[Carbamidomethyl (C)]GPHDQDR_.3	P21447_S663_M2_Abcb1a	3.1678	4.1513	4.2052	4.4164	3.3747	3.4081	3.6734	3.3935	3.5936	4.4792	3.0722	3.8774	3.7590	4.2433	3.6987	4.0308	4.3916	4.2587	3.0569	3.9206
Abcb1a	_S[Phospho (STY)]IC[Carbamidomethyl (C)]GPHDQDR_.2	P21447_S667_M1_Abcb1a	9.0417	9.3823	8.9594	9.0973	8.5918	8.8414	8.9510	9.1434	9.1691	8.5996	8.8263	8.6043	8.4723	8.8063	7.7369	8.3044	8.9731	9.0723	8.7819	8.4960
Abcb1a	_DSGSS[Phospho (STY)]LIR_.2	P21447_S657_M1_Abcb1a	3.9471	4.0557	4.4834	3.7423	3.2848	3.5089	4.2562	3.6475	4.3153	4.1534	3.1889	3.9451	4.4234	4.4705	3.8334	4.2920	4.1852	3.6480	3.2966	4.4375
Cst3	_ALDFAVSEYNK_.2	#N/A	6.6781	7.1418	7.0224	6.9566	6.6730	7.0997	7.0827	6.9364	7.2787	6.6423	6.2994	6.7211	6.5026	7.1202	7.0346	6.9480	7.2803	6.7581	6.7597	7.0089
Lmnb2	_SQT[Phospho (STY)]NWGPGESFR_.2	P21619_T528_M1_Lmnb2	5.9075	4.2748	5.8168	6.1714	6.1244	6.1594	5.5709	4.1181	6.1558	6.2800	3.4175	5.6948	3.9233	6.4464	3.5765	4.3581	3.6153	5.6301	6.2091	3.8731
Lmnb2	_RRLETEDTSGS[Phospho (STY)]PSR_.3	#N/A	4.5233	5.6129	3.9904	5.0459	4.3855	3.6965	3.5222	3.7337	5.1482	3.7258	3.9316	3.7262	5.7661	5.2532	5.5716	5.2012	4.8109	5.3339	5.7011	5.4413
Lmnb2	_RLETEDTSGS[Phospho (STY)]PSR_.2	#N/A	9.7320	9.6330	9.4690	9.7012	9.3996	9.5829	9.3955	9.5923	3.7240	9.6881	9.2918	9.5860	9.9633	9.9586	9.2929	9.5694	9.6494	9.4812	9.5659	9.4841
Lmnb2	_LETEDTSGS[Phospho (STY)]PSR_.2	#N/A	8.6160	4.2781	4.0973	8.6521	8.4912	8.5284	8.4504	8.6876	8.4463	4.8919	8.3823	8.4509	8.7290	4.4232	8.4191	8.7258	8.6455	8.4806	8.5014	8.5969
Lmnb2	_ATSSSSSSS[Phospho (STY)]GVGM[Oxidation (M)]SVGQGR_.2	#N/A	4.0116	4.1082	3.9444	4.0380	3.3494	4.5876	3.8431	5.0973	3.3444	5.4616	3.7188	4.1877	4.0103	4.0179	3.8668	3.8440	3.8441	3.9920	3.5915	4.8534
Pou3f1	_WLEETDSSSGS[Phospho (STY)]PTNLDK_.2	P21952_S324_M1_Pou3f1	3.3429	3.9866	4.3803	4.9752	3.5498	4.0536	4.6891	3.5686	3.4668	4.1313	4.7454	3.7127	3.6055	5.5278	4.2947	4.2058	4.6568	4.6933	3.7700	4.1971
Emb	_SDDS[Phospho (STY)]NGIENNVPR_.2	P21995_S312_M1_Emb	6.8153	3.6014	7.1801	6.7051	3.8350	4.2131	3.4803	6.8923	3.8694	3.3254	3.9802	3.4061	7.0317	3.3701	5.9471	3.1765	3.2938	3.4906	6.3546	6.4489
Penk	_FAESLPSDEEGENYS[Phospho (STY)]K_.2	#N/A	7.2117	7.0779	7.8269	8.4129	6.1730	7.4516	8.2323	4.1162	8.0147	8.5170	8.7179	7.9170	7.3165	8.4482	8.4345	9.3758	8.4302	7.9179	9.3888	8.3964
Penk	_ELLGTGDNR_.2	#N/A	6.9183	7.7277	7.6465	7.7058	5.3536	7.7411	7.5372	8.0383	7.9454	7.4208	8.3989	8.4978	8.6441	8.5978	9.2893	8.6172	8.4893	8.6890	8.6602	8.7359
Penk	_FAESLPSDEEGENY[Phospho (STY)]SKEVPEIEK_.3	#N/A	3.9875	4.0153	4.5238	5.6141	5.2787	6.8464	4.2159	4.8225	4.0823	4.2047	6.3333	3.9048	6.3902	7.3985	6.7600	6.9614	4.1596	6.8295	3.3087	6.9042
Penk	_FAESLPSDEEGENYS[Phospho (STY)]KEVPEIEKR_.3	#N/A	3.8412	3.8416	4.0025	3.8024	3.6420	3.7534	3.7920	3.8059	3.8087	5.7322	3.6639	3.6640	3.9339	4.0049	3.5261	6.7625	3.8659	4.0704	3.7190	3.6582
Penk	_LVRPGDINFLAC[Carbamidomethyl (C)]TLEC[Carbamidomethyl (C)]EGQLPSFK_.3	#N/A	3.1572	4.0620	4.1946	4.5146	3.3642	3.2434	3.1461	4.0912	3.0800	3.8879	3.6572	3.8880	3.1881	4.3057	3.6692	4.5663	4.3810	4.4437	3.9791	3.5441
Penk	_DADEGDTLANS[Phospho (STY)]SDLLK_.2	P22005_S153_M1_Penk	4.2397	4.2571	4.7830	4.4282	3.3993	5.2526	4.9521	4.0201	5.5005	3.8550	3.6708	5.2385	4.1949	5.2041	4.4086	3.8002	5.4256	4.8651	6.4033	3.4160
Gabrg2	_VPEGDVTVILNNLLEGYDNK_.2	#N/A	5.3356	5.7720	3.3896	3.5514	3.0370	3.7704	5.3213	5.5282	4.3075	3.9157	3.4561	4.9701	5.4731	5.4770	3.1237	3.4572	4.1762	5.0176	4.0720	3.1370
Ap1g1	_NLIM[Oxidation (M)]SGYS[Phospho (STY)]PEHDVSGISDPFLQVR_.3	P22892_S232_M1_Ap1g1	3.3746	3.1273	3.9109	4.3147	4.2132	3.7717	3.3097	3.0750	3.2299	3.3420	4.2094	3.0168	3.3954	3.8980	3.3351	3.7195	3.6127	4.8687	3.8690	3.8650
Cbx3	_LTWHSC[Carbamidomethyl (C)]PEDEAQ_.2	#N/A	4.5226	5.7439	4.9122	4.8067	5.4742	5.5231	5.0620	5.2315	5.1874	5.6236	5.2039	5.0628	5.7703	6.0616	5.0642	4.9390	5.7191	4.5876	4.7404	5.8011
Cbx3	_KVEEAEPEEFVVEK_.2	#N/A	5.9945	5.6184	5.8781	4.1600	5.0637	4.4772	5.7140	5.8962	5.9882	5.2119	5.3920	5.7423	5.9797	6.4979	6.1172	5.9499	5.8751	5.2283	4.3014	5.5782
Cbx3	_SLS[Phospho (STY)]DSESDDSKSK_.2	#N/A	7.6068	7.9610	7.2395	7.7973	7.4168	7.4404	7.5498	7.9717	7.5063	7.7866	6.7913	7.6877	8.4731	7.9677	8.4708	7.8982	7.6038	7.7340	7.9773	7.8331
Cbx3	_KSLS[Phospho (STY)]DSESDDSK_.2	#N/A	4.7094	6.1081	3.8873	5.9835	5.4368	5.9093	5.4234	5.6572	5.4705	6.0585	5.7052	5.7161	5.7774	5.9816	6.4611	7.9947	6.0787	5.9592	8.3681	5.7377
Cbx3	_SLS[Phospho (STY)]DSESDDSK_.2	#N/A	3.4524	7.3285	4.4329	6.6913	4.0147	6.3928	6.3174	3.6211	5.7875	3.4777	6.0810	5.4316	5.4262	7.5026	3.1556	4.0752	3.1184	5.7515	5.5143	6.9946
Cbx3	_RKS[Phospho (STY)]LS[Phospho (STY)]DSESDDSK_.2	P23198_S93_M2_Cbx3	4.9109	5.8935	4.9297	5.8708	5.1465	5.3356	5.5966	4.7572	5.7769	5.8138	5.3224	5.7116	6.2217	6.5223	6.8198	6.0415	5.9245	5.8639	5.5012	6.3281
Cbx3	_KSLS[Phospho (STY)]DSESDDSKSK_.2	#N/A	3.4851	4.5177	4.0214	4.2042	4.3237	3.6612	3.2174	3.1855	3.2765	3.6915	3.6508	4.4071	3.5059	4.0085	7.0109	3.8300	3.7232	4.1100	3.7585	3.9755
Cbx3	_C[Carbamidomethyl (C)]PQIVIAFYEER_.2	#N/A	4.3648	3.6380	3.4003	3.3246	3.7025	4.2824	3.8386	4.0652	4.2201	4.5711	3.6987	3.5275	4.3855	3.3873	3.4158	3.2089	3.1020	3.2303	4.3797	3.3543
Gja1	_ASS[Phospho (STY)]RPRPDDLEI_.2	#N/A	11.2727	10.8857	9.9094	10.6629	10.3729	10.5168	10.5634	10.6195	10.1924	10.6795	10.2996	10.1185	11.0191	10.1135	7.8590	10.0298	10.8829	10.3725	10.0049	10.0010
Gja1	_PRPDDLEI_.2	#N/A	5.7230	5.3567	6.1868	4.5372	5.4435	6.0662	4.2789	5.6473	6.0245	4.1308	2.8984	5.1648	5.1492	3.9898	5.2966	3.7773	5.9402	5.8761	5.7429	5.5587
Gja1	_LVTGDRNNS[Phospho (STY)]S[Phospho (STY)]C[Carbamidomethyl (C)]RNYNK_.3	P23242_S296_M2_Gja1	3.9309	4.0719	4.4672	3.7585	3.2686	3.4927	4.2457	3.6312	4.3316	5.3824	3.2051	3.9614	4.0182	4.4542	3.8497	4.2758	4.1689	3.6642	3.3128	4.4212
Gja1	_QASEQNWANYSAEQNR_.2	#N/A	7.5176	7.3271	6.8624	7.2646	7.0091	6.6331	7.1453	6.5844	6.9088	7.3424	6.0196	5.6471	6.8764	6.5215	4.8855	6.5626	6.4838	6.5171	6.6184	5.8575
Gja1	_SDPY[Phospho (STY)]HATTGPLSPSK_.2	P23242_Y247_M1_Gja1	3.1897	3.3122	3.7260	3.9352	4.0283	4.2524	6.3909	3.8747	6.3019	3.3961	3.9462	5.9229	3.6799	3.2237	3.0900	3.5346	3.3720	5.6031	4.0765	4.4018
Gja1	_LVTGDRNNS[Phospho (STY)]SC[Carbamidomethyl (C)]R_.2	P23242_S296_M1_Gja1	3.8974	3.4217	3.4340	6.2984	5.5209	5.3068	6.1430	4.1231	3.8203	5.9164	4.1946	4.3803	5.8943	4.9713	3.3384	5.2337	5.4534	5.2072	4.7976	4.8949
Gja1	_VAAGHELQPLAIVDQRPSS[Phospho (STY)]RAS[Phospho (STY)]S[Phospho (STY)]R_.4	P23242_S365_M3_Gja1	4.0806	3.6765	3.3618	3.3631	4.2875	4.3209	4.2614	4.0267	4.1816	3.8912	3.6602	3.5659	4.3470	7.5483	2.7859	3.4427	3.0635	3.2688	4.4182	3.3159
Gja1	C[Carbamidomethyl (C)]NTQQPGC[Carbamidomethyl (C)]ENVC[Carbamidomethyl (C)]YDK_.2	#N/A	4.2455	4.5744	3.7821	3.3387	2.9516	4.4191	5.4244	4.4712	4.0167	4.0561	3.4953	4.3005	4.1821	3.8202	4.1218	3.6077	3.9685	3.4337	3.4800	3.1509
Xrcc6	_VELS[Phospho (STY)]EEELK_.2	P23475_S558_M1_Xrcc6	3.0963	6.5339	5.5516	4.4879	3.3032	6.3005	5.7218	3.3220	6.1963	4.4077	3.1437	3.9489	3.8305	6.3398	5.8651	5.7726	4.3201	5.5047	6.1540	6.2145
Pcmt1	_ELVDDSITNVK_.2	#N/A	7.2271	7.0215	7.3288	7.3377	6.9148	7.0486	6.8950	7.1257	7.0229	7.5233	7.3964	6.9546	7.1641	6.9102	7.7326	7.1598	6.8133	6.8601	7.0907	7.7330
Pcmt1	_ALDVGSGSGILTAC[Carbamidomethyl (C)]FAR_.2	#N/A	5.8208	5.5590	6.1930	6.6401	5.5940	5.9086	4.4000	5.6513	6.0304	6.0932	5.1191	6.0124	6.9264	6.0507	6.0543	5.9461	5.9000	5.3343	6.2029	6.1925
Pcmt1	_LILPVGPAGGNQM[Oxidation (M)]LEQYDKLQDGSVK_.3	#N/A	6.4056	6.7393	3.6288	6.0347	5.9033	6.2324	6.4296	6.5627	6.2794	6.6000	5.5912	6.5905	6.7785	6.6207	6.8056	5.7087	6.5802	6.4791	6.6097	6.5985
Pcmt1	_VFEVM[Oxidation (M)]LATDR_.2	#N/A	3.1165	5.9110	3.6528	4.5729	4.2684	4.6337	3.5860	4.9762	5.2429	3.9338	3.8414	5.1722	4.6021	5.2323	4.7699	3.4614	3.3546	5.2530	5.5847	5.2038
Pcmt1	_SGGASHSELIHNLR_.3	#N/A	3.6599	3.6592	3.1965	5.4809	5.1992	4.7339	5.2173	4.6195	4.3292	4.6111	4.9250	5.0179	5.4277	4.9580	5.1353	5.1111	5.3053	4.1443	5.0070	5.6238
Pcmt1	_TDKVFEVM[Oxidation (M)]LATDR_.3	#N/A	4.2452	4.5408	3.1358	3.5891	3.4380	3.6621	2.9974	3.8007	4.1621	4.2046	4.2602	3.6433	4.6977	4.0491	3.6803	2.9444	4.1728	4.1400	3.1434	3.0898
Pcmt1	_ELVDDSITNVKK_.3	#N/A	3.5653	3.7538	4.6028	4.2787	3.7723	4.3361	3.4291	3.7910	3.4882	2.5829	3.8625	2.3380	3.5962	3.1400	3.1996	3.9461	3.2882	3.8612	2.6822	1.7354
Gria2	_ADIAIAPLTITLVR_.2	#N/A	5.3941	5.3940	5.2550	5.3695	5.3456	4.6415	5.1964	4.9251	5.4003	4.2037	4.5802	5.0752	4.0346	4.6791	4.8549	3.7457	4.8834	5.0436	3.9999	4.6851
Gria2	_NAQNINPSSSQNS[Phospho (STY)]QNFATYK_.2	P23819_S863_M1_Gria2	6.3123	4.4530	3.9035	3.2173	3.0731	3.1064	3.9750	3.0918	3.8953	4.1776	3.3739	4.1791	4.3978	3.9416	4.0004	3.7291	7.2002	3.0596	3.3586	3.6189
Gria2	_YAYLLESTM[Oxidation (M)]NEYIEQR_.2	#N/A	4.4882	4.3317	4.0248	5.1073	3.1943	3.4132	3.8538	3.2131	4.4110	4.0577	3.2846	4.0578	5.6852	4.0629	3.8792	3.8503	4.2111	3.4999	3.5093	3.7401
Gria2	_YTSALTYDAVQVM[Oxidation (M)]TEAFR_.2	#N/A	4.4458	4.5486	3.9905	4.2352	4.2928	3.6811	3.2483	3.1546	3.3074	3.6605	3.6818	2.9372	3.4155	3.9776	7.9033	4.2304	4.2709	4.0570	3.7895	4.1374
Cryab	_VLGDVIEVHGK_.2	#N/A	5.2942	6.0431	6.0465	5.5120	6.2605	5.7375	5.5539	6.4943	6.0896	6.5614	5.6728	6.0425	5.1450	6.2106	6.4236	5.7723	6.7080	5.3142	5.6182	6.3881
Cryab	_[Acetyl (Protein N-term)]M[Oxidation (M)]DIAIHHPWIR_.3	#N/A	5.6544	5.8438	5.4963	5.0269	6.4909	5.7173	4.9663	6.2640	5.2862	6.1480	5.1129	6.3012	6.0466	5.1942	6.3052	5.6021	5.5173	5.2049	6.2090	6.1551
Cryab	_QDEHGFISR_.2	#N/A	4.9006	4.5693	5.8171	3.3336	5.4942	5.2342	4.9504	4.4763	5.8408	5.9634	3.4903	5.2370	4.1771	5.7033	6.1285	4.4729	4.4354	5.8450	5.1193	3.1459
Cryab	_FSVNLDVK_.2	#N/A	4.3536	4.0553	2.6084	3.2306	3.1689	2.2563	3.8556	3.0784	3.3659	4.1924	3.1499	4.1925	4.3845	3.9282	4.2662	3.7157	3.3012	3.8681	2.3386	3.5168
Cryab	_APSWIDTGLSEMR_.2	#N/A	3.9138	4.0890	4.8325	4.9384	3.2515	5.3287	4.2895	3.6142	3.7691	4.3037	4.2687	3.9784	3.9345	5.4327	4.3128	4.2587	4.1519	3.6813	3.3299	4.1666
Cryab	_HEERQDEHGFISR_.4	#N/A	3.4298	4.5265	4.0126	4.2131	3.6367	3.6701	3.4114	3.1767	3.3316	3.2404	4.3110	4.4160	3.4970	3.9997	3.4367	4.2928	3.7143	4.1188	3.7674	3.9667
Bcat1	_LTDIQYGR_.2	#N/A	5.0518	5.7426	5.1282	5.2446	5.4780	5.1890	5.6234	5.5324	5.4466	5.5714	5.5651	5.7045	3.2067	5.1599	5.8120	5.3655	4.8147	5.5484	5.3227	5.0715
Bcat1	_HLTM[Oxidation (M)]DDLATALEGNR_.3	#N/A	4.0954	4.5106	4.8457	4.1847	4.4215	4.0754	5.0088	4.4988	4.7630	4.4808	4.4904	4.8130	4.1229	5.1470	4.2221	4.5506	4.1511	5.3037	5.0316	4.3610
Bcat1	_QSILELAQQWGEFK_.2	#N/A	4.1306	4.1134	3.9026	3.4570	3.6195	3.5354	4.2298	3.0909	4.2889	4.1799	3.1624	3.9187	4.3969	3.9407	3.8070	4.3185	4.0889	3.3405	3.3596	3.6180
Ppib	_TVDNFVALATGEK_.2	#N/A	7.2289	7.6154	7.3306	6.9064	6.9733	7.1104	6.9988	6.7402	7.0227	7.1355	6.8759	7.1472	7.1662	7.5721	7.1132	7.1885	7.0676	7.1621	7.0441	7.0324
Ppib	_VLEGMDVVR_.2	#N/A	3.8892	3.4298	3.4258	5.9290	4.3522	4.0067	5.3216	4.2181	4.3730	3.6998	5.3936	3.1560	4.5384	6.0763	4.6159	3.2514	3.2549	3.0774	4.2268	3.5072
Ppib	_DTNGSQFFITTVK_.2	#N/A	4.4061	3.5967	3.4415	3.2833	3.7438	3.9679	3.7973	4.1065	3.7567	3.1116	4.2307	3.4862	3.9644	3.4286	4.7087	4.0564	3.1433	3.1890	4.3384	3.3956
Ppib	_VYFDLQIGDESVGR_.2	#N/A	3.7546	4.2482	4.2909	3.9348	3.0923	3.3918	3.6896	3.4549	3.6098	4.4630	5.2251	4.1377	3.7753	4.2779	3.7150	4.0995	3.9926	3.8405	3.4891	4.4585
Gsta4	_FLQPGSQR_.2	#N/A	6.5994	6.8954	6.6262	6.3760	6.5669	6.5465	6.4145	6.5666	6.7774	6.9815	5.8749	7.0599	6.7862	6.4289	7.2138	6.7829	6.4789	6.6615	6.8560	6.7380
Gsta4	_EKEESYDLILSR_.3	#N/A	4.5283	3.4745	3.5638	3.7730	3.8661	4.0902	3.6750	4.0370	3.7341	3.2338	4.1085	3.3639	3.8421	3.3859	3.2522	3.3724	3.5342	3.6153	3.9143	3.0632
Th	_PTPSASS[Phospho (STY)]PQPK_.2	P24529_S8_M1_Th	11.2244	11.1654	11.0424	11.4169	10.9000	10.8597	10.7375	11.3033	10.9110	11.2826	10.8268	10.9693	11.2740	11.0751	10.6601	11.0752	10.8713	3.4047	10.9186	10.9356
Th	_RQS[Phospho (STY)]LIEDARK_.3	#N/A	7.9189	8.4129	8.8350	8.0615	7.3482	7.2022	7.7913	7.8075	7.8022	8.4636	7.6319	8.0404	8.0555	7.7581	7.5512	7.7749	7.9317	7.5740	7.8396	7.6863
Th	_RQS[Phospho (STY)]LIEDAR_.2	#N/A	6.8874	6.5109	7.4237	6.6464	5.8340	6.2510	5.8128	6.3172	6.5403	7.1116	5.9100	6.7838	6.2494	6.2917	5.4443	6.3619	6.4663	5.7750	6.1787	5.5960
Th	_QAEAVTS[Phospho (STY)]PR_.2	P24529_S31_M1_Th	9.8048	10.0181	10.0027	10.0672	9.6422	9.8242	9.4896	10.1771	10.0247	10.0301	10.0109	10.1156	10.2087	10.4230	9.8484	10.0581	10.1264	10.1044	10.0649	10.2247
Th	_RAVS[Phospho (STY)]EQDTK_.2	P24529_S19_M1_Th	4.5400	6.1944	3.5754	3.1494	3.8777	5.2487	3.6634	4.2404	3.7225	3.2455	5.3806	3.3523	4.5607	5.5832	3.2406	3.3840	5.8194	5.5480	4.7297	3.5295
Th	_FEVPSGDLAALLSSVR_.2	#N/A	5.4504	5.0055	4.2121	5.6861	5.3153	5.2369	5.8492	3.4004	5.8045	4.5881	5.0155	4.7375	3.2055	4.8296	4.8322	4.0090	4.8223	4.2844	5.2770	5.6189
Th	_FDPYTLAIDVLDS[Phospho (STY)]PHTIR_.2	#N/A	3.3697	4.3119	4.1533	4.2772	3.9574	3.2767	3.3340	3.5020	3.4061	4.1784	3.3572	4.1331	3.5460	4.3518	3.9947	3.9710	4.5000	4.0750	4.1344	3.7689
Th	_SLEGVQDELHTLTQALSAIS_.2	#N/A	3.6166	3.7025	3.1532	3.9676	3.3784	4.2848	3.2245	3.8423	2.4189	3.4272	3.9138	3.4286	3.6475	3.9065	4.2680	2.9787	3.3395	3.1049	2.5860	4.3694
Th	_VFETFEAK_.2	#N/A	3.3895	3.8698	4.4867	4.1349	4.2280	4.4521	3.3131	3.6750	3.3721	3.5958	3.7465	3.0020	3.4802	4.5248	2.8903	3.7344	3.1722	3.9772	4.2763	4.2021
Impdh2	_GMGS[Phospho (STY)]LDAMDK_.2	P24547_S416_M1_Impdh2	3.4421	3.8770	4.4795	4.9105	3.6490	3.6824	3.3991	3.6678	3.3193	3.2527	4.2988	3.6031	3.4847	4.4526	3.4245	4.3050	3.1650	4.1311	3.7797	3.9544
Aldh1a1	_[Acetyl (Protein N-term)]SS[Phospho (STY)]PAQPAVPAPLADLK_.2	P24549_S3_M1_Aldh1a1	9.8621	9.7375	4.2112	9.5491	9.0301	8.7860	9.1968	9.9482	8.9291	9.4314	9.2159	9.4270	9.7027	10.0281	8.9942	9.3008	3.9130	9.2993	9.1546	9.0429
Aldh1a1	_GFFVQPTVFSNVTDEM[Oxidation (M)]R_.2	#N/A	6.6758	5.7486	5.2563	5.2433	5.3702	5.6047	4.7717	5.1383	6.2338	5.8566	4.8950	6.1675	6.1222	5.3950	6.0009	6.1161	5.9475	5.7429	5.7128	5.7874
Aldh1a1	_IHGQTIPSDGDIFTYTR_.3	#N/A	3.5976	5.2495	5.9057	6.1883	5.6375	5.5145	5.2835	5.4631	6.1927	6.2926	6.1519	5.6994	3.6183	5.2290	6.1088	6.0852	5.9898	5.6516	5.3431	5.9079
Aldh1a1	_VFANAYLSDLGGC[Carbamidomethyl (C)]IK_.2	#N/A	3.7172	5.3273	4.9788	5.7123	5.0426	5.1655	4.6489	4.2685	4.2617	5.0738	5.1615	4.9118	4.9561	4.9053	3.1493	3.0793	5.2617	5.1510	3.5672	4.8961
Aldh1a1	_VTLELGGK_.2	#N/A	4.1049	4.2255	3.1404	3.9584	3.4426	3.9303	4.7287	3.8053	3.7146	4.1470	3.7190	4.3998	4.3984	3.8868	4.3429	5.1883	3.4318	5.0235	4.6288	4.9287
Aldh1a1	_VAFTGSTQVGK_.2	#N/A	3.8158	3.5033	3.3523	5.1839	4.0227	5.1050	3.6795	4.0414	3.7386	3.2294	4.1130	3.2295	3.8466	5.0811	3.2567	3.3679	3.5387	3.6108	3.9098	3.0677
M6pr	_SFESTVGQGSDTYSYIFR_.2	#N/A	3.7943	3.5601	4.3306	4.8900	4.0122	4.1307	3.5645	3.9416	4.4681	4.1645	4.4677	3.3672	4.1922	4.5902	3.9862	4.4084	4.4904	4.3023	2.4307	4.5037
M6pr	_GVGDDQLGEES[Phospho (STY)]EERDDHLLPM[Oxidation (M)]_.3	P24668_S268_M1_M6pr	3.1764	4.1427	4.2138	4.4078	3.3834	3.2242	3.6647	3.4021	3.0992	2.0894	3.0636	1.9783	2.3385	2.9055	1.1304	4.0394	4.4002	4.2501	3.0483	3.9292
Cdk11b	_S[Phospho (STY)]PLRPPR_.2	P24788_S234_M1_Cdk11b	9.5222	9.4801	9.3083	9.1304	8.9528	9.5435	9.4705	9.4028	9.1813	9.4139	9.4551	9.2801	7.7280	9.4299	8.0117	8.7543	9.7398	9.5177	9.1379	8.9131
Cdk11b	_DLLSDLQDIS[Phospho (STY)]DSER_.2	P24788_S270_M1_Cdk11b	3.3273	3.1746	8.0046	4.3621	4.1659	4.3900	6.8950	4.5286	3.4343	3.5337	3.8086	3.0641	3.5423	3.8507	6.7572	3.6722	3.5654	3.9151	3.9164	4.2642
Rps2	_TYSYLTPDLWK_.2	#N/A	6.0113	6.2735	6.4137	5.7233	5.8868	6.1508	6.3674	5.9890	5.8755	5.4089	5.6372	6.0152	6.6159	6.1213	5.8261	6.1037	5.7483	5.7060	5.9941	5.4399
Rps2	_AEDKEWIPVTK_.3	#N/A	3.6479	4.3549	4.1842	3.5231	2.9856	3.2097	4.1822	3.3483	4.5267	4.6075	3.4881	3.4746	4.7108	3.0990	3.9646	2.8171	3.8860	3.9839	3.6253	3.2057
Tln1	_VLVQNAAGS[Phospho (STY)]QEK_.2	P26039_S2040_M1_Tln1	7.1683	6.7053	7.1669	6.2615	6.2952	6.7045	6.7573	7.0774	6.2743	6.9562	6.5048	6.3817	6.7999	6.5872	5.7722	6.1743	6.9651	6.9916	6.4345	6.4361
Ezr	_APDFVFYAPR_.2	#N/A	6.7964	7.4649	7.2240	6.4865	7.4826	7.4014	6.9931	7.3830	6.6127	7.2992	6.6123	6.9245	6.9860	7.4162	6.9060	6.9723	6.6044	7.1858	7.0921	7.3764
Ezr	_IGFPWSEIR_.2	#N/A	7.0023	6.9044	5.8367	6.4973	6.7596	6.5829	6.2766	7.0623	6.2514	6.8025	6.5604	6.8141	5.6427	6.2706	5.8162	5.9896	6.5030	6.5511	5.6081	6.3683
Ezr	_NISFNDKK_.2	#N/A	5.9369	6.1284	6.4031	4.5017	4.0263	6.2277	6.0993	5.9998	6.5059	6.7616	5.9639	5.5395	6.2026	6.5412	6.6722	6.1321	3.4258	5.6764	6.0689	6.2713
Ezr	_QLLTLSNELSQAR_.2	#N/A	3.9447	3.9347	4.5236	4.7771	4.1589	4.4108	3.3550	3.7119	4.0186	3.2968	4.2547	4.4224	3.4406	3.5100	3.3803	4.3491	3.2091	4.2783	4.1334	3.6500
Ezr	_IAQDLEM[Oxidation (M)]YGINYFEIK_.2	#N/A	4.6741	3.6040	3.6805	3.8220	3.6933	3.9848	2.6582	4.0589	3.9063	3.6619	3.7475	3.4656	4.5399	4.0268	3.8541	3.7851	3.3842	3.2770	4.3246	3.1240
Ezr	_RKEDEVEEWQHR_.3	#N/A	4.3316	3.6731	3.3670	4.8522	4.7906	4.2169	4.1340	4.9684	5.2823	5.5447	5.5048	3.5625	4.3523	3.3541	4.5250	5.1030	5.1919	3.2654	3.8266	4.8360
Ezr	_GTDLWLGVDALGLNIYEK_.2	#N/A	3.3405	2.4821	3.8768	4.3489	4.1791	3.8058	3.2756	4.5417	3.1958	3.3762	4.1753	3.0509	3.3612	3.8639	3.3010	4.4285	3.5786	4.2546	3.9032	3.8309
Gabra3	_LLDGYDNR_.2	#N/A	6.6312	7.0191	5.7870	7.0001	6.5478	6.9229	6.8932	6.8707	6.5242	6.7388	6.5521	6.8256	6.9206	6.5619	5.8529	5.9348	6.7888	6.9293	6.5766	6.7429
Gabra3	_SAAAPSASSTPTAIAS[Phospho (STY)]PK_.2	P26049_S433_M1_Gabra3	9.1435	8.9356	8.5264	9.1463	8.3245	9.0152	9.0988	8.9992	8.3542	8.6710	8.6247	8.7496	9.0921	8.3579	6.9771	8.6878	9.3463	9.1385	8.4495	8.6147
Ctnna1	_SRTS[Phospho (STY)]VQTEDDQLIAGQSAR_.3	#N/A	3.9409	8.8884	8.7756	7.1086	4.3507	9.0861	6.8017	5.2476	7.0322	6.9716	3.7867	8.8088	3.9644	9.1518	8.6930	6.9594	8.6820	6.9428	7.0125	9.0014
Ctnna1	_QDLLAYLQR_.2	#N/A	5.3439	5.2430	5.6030	3.9766	3.0505	5.5167	5.2776	4.7923	4.1333	4.3919	5.4931	5.6581	3.1568	5.1294	4.7462	3.1525	3.9509	5.4585	3.0988	5.4738
Ctnna1	_EYAQVFR_.2	#N/A	4.3070	4.1443	4.6196	5.1406	4.4128	4.6908	5.1660	3.8827	4.5871	4.3237	5.2685	4.5928	5.0380	3.2049	4.9873	4.9450	4.8789	4.8366	5.4692	3.1719
Ctnna1	_TS[Phospho (STY)]VQTEDDQLIAGQSAR_.2	#N/A	7.9629	7.6976	8.0493	5.5831	3.9042	9.3053	7.3469	7.6608	7.5856	7.5561	7.5561	7.1721	3.9512	7.9708	6.8711	7.6759	8.3170	7.4752	7.8012	7.3084
Ctnna1	_TPEELDDSDFET[Phospho (STY)]EDFDVR_.2	P26231_T645_M1_Ctnna1	3.7669	6.1317	4.1414	3.9170	3.7386	3.8292	3.8491	3.8382	3.7693	3.9335	3.8113	3.6715	3.8837	6.4258	3.8745	3.9607	3.6821	6.4464	3.8866	3.9398
Ctnna1	_LESIISGAALM[Oxidation (M)]ADSSC[Carbamidomethyl (C)]TR_.2	#N/A	4.2905	3.7123	3.3260	2.9498	3.6283	3.8523	3.9128	4.2748	3.7080	4.4969	4.3463	3.6017	4.0800	3.1584	3.4900	3.1346	3.7720	3.3774	3.6765	3.3010
Chga	_GWRPSS[Phospho (STY)]REDS[Phospho (STY)]VEAR_.2	#N/A	7.1788	7.8274	7.6700	7.8825	7.5441	7.2402	7.1126	7.6933	6.0375	7.6616	7.1788	7.7213	7.5527	8.2025	8.0078	7.7776	5.4821	7.4055	7.7742	7.7408
Chga	_DDGQSDS[Phospho (STY)]QAVDGDGK_.2	P26339_S282_M1_Chga	4.3280	3.3074	3.5482	3.5726	3.6657	3.8898	3.8754	4.2373	3.9345	4.5343	4.3089	3.0335	4.0425	3.5863	3.4526	3.1720	3.7346	3.4149	3.7139	3.2636
Ptma	_[Acetyl (Protein N-term)]SDAAVDTSSEITTK_.2	#N/A	8.2773	8.7208	9.1229	8.9797	6.3777	8.6589	8.3282	7.4583	8.9285	9.0015	6.7075	9.1008	7.8231	9.5089	9.3194	9.0575	8.9057	8.3466	8.9058	8.9788
Ptma	_VAEDDEDDDVDTKK_.2	#N/A	3.3553	4.4931	4.7305	5.0248	4.9603	4.5889	4.2854	4.8016	4.7208	4.6896	3.8261	4.7924	4.1883	3.8846	5.3928	4.6914	4.6281	3.9533	4.8287	3.5592
Ptma	_RVAEDDEDDDVDTKK_.3	#N/A	3.1978	4.1213	5.9011	5.6430	3.4047	4.9781	5.7110	3.4235	5.7289	5.6277	3.4950	5.2382	5.7882	5.8006	6.3987	5.3961	5.2981	5.2987	5.1921	5.0024
U2af2	_GAKEEHGGLIRS[Phospho (STY)]PR_.3	#N/A	9.7253	10.2166	9.5966	9.6031	9.3335	9.6488	9.9469	9.9866	9.5984	10.0379	9.3528	9.6819	10.6698	9.7137	9.3190	9.5643	9.7687	9.7611	9.7919	9.4897
U2af2	_EEHGGLIRS[Phospho (STY)]PR_.3	#N/A	9.4447	9.5161	9.5836	9.1632	9.0361	9.3663	9.0296	9.1388	8.7880	9.3484	9.1593	8.8518	9.3169	9.4851	8.6164	8.9342	9.3351	9.0877	9.0809	9.1487
U2af2	_[Acetyl (Protein N-term)]S[Phospho (STY)]DFDEFER_.2	P26369_S2_M1_U2af2	4.3612	7.6051	3.3966	3.3282	3.6989	7.6536	7.4824	4.0615	7.3560	4.5675	4.2757	3.5311	4.0093	7.8985	6.5757	5.3969	3.0984	6.6871	7.2702	3.2304
Glud1	_NYTDNELEK_.2	#N/A	8.8254	8.7528	9.3910	9.0844	8.9293	8.9146	8.7615	9.0693	9.1659	8.9157	8.6501	8.7907	8.7218	9.0214	8.8650	8.7731	8.9402	8.7448	8.7113	8.7985
Glud1	_TAAYVNAIEK_.2	#N/A	8.0286	8.8002	8.9419	8.9249	8.0807	8.2437	8.7453	8.6563	8.4878	8.0621	8.1291	8.3362	9.0359	8.4521	8.1003	8.1670	7.4174	8.4851	7.6176	7.8659
Glud1	_YSTDVSVDEVK_.2	#N/A	8.4664	8.5230	8.6734	8.6572	8.3879	8.7155	8.6812	8.5966	8.7885	8.6892	8.1551	8.2879	8.3554	8.4974	8.0788	8.6630	7.9494	8.1000	8.2570	8.2821
Glud1	_C[Carbamidomethyl (C)]AVVDVPFGGAK_.2	#N/A	8.2031	7.5918	8.5005	8.1071	8.0507	8.1934	7.9244	7.9902	8.3236	8.3323	7.6871	7.9015	8.0028	7.8319	8.2908	8.0229	7.7317	7.9791	8.0966	7.8797
Glud1	_YNLGLDLR_.2	#N/A	7.7821	8.1163	8.3178	8.5629	7.8428	8.1613	8.4610	7.7234	7.9489	8.0698	7.9992	8.1782	8.1712	7.8076	8.0277	7.9432	7.4535	8.1909	7.8091	7.9432
Glud1	_HGGTIPVVPTAEFQDR_.2	#N/A	7.5997	7.4881	7.3634	7.4030	7.3588	7.2610	7.3747	7.6271	7.1523	7.6838	7.2989	7.3824	7.4657	7.6892	6.6603	7.0919	7.4130	6.9293	6.9813	6.9697
Glud1	_NLNHVSYGR_.2	#N/A	7.5020	8.2069	8.2208	8.0734	7.6602	7.9847	8.0544	7.8420	8.2094	8.1012	7.2325	7.5966	7.5399	7.7719	7.9615	7.1610	7.6192	7.5918	8.0590	7.3732
Glud1	_C[Carbamidomethyl (C)]VGVGESDGSIWNPDGIDPK_.2	#N/A	6.5815	6.4472	6.7419	6.7046	6.1782	6.7741	6.5962	6.2198	6.9258	6.7396	6.2773	6.1599	6.3761	6.9413	6.7726	6.5377	6.6340	5.9412	6.2763	5.7721
Glud1	_RDDGSWEVIEGYR_.3	#N/A	5.6111	5.2817	6.5282	6.6098	6.1559	5.9717	4.8198	3.6997	6.4192	6.1341	4.6856	4.7264	5.1635	3.9150	5.3152	5.7880	5.5563	5.2204	5.9632	5.8871
Glud1	_M[Oxidation (M)]VEGFFDR_.2	#N/A	7.7889	7.7555	7.7096	8.0567	7.5091	7.6457	8.0853	7.7859	7.7575	7.8311	7.3167	7.8282	7.6740	7.6419	7.4970	7.5760	7.2895	7.8507	7.8577	7.4075
Glud1	_GFIGPGIDVPAPDM[Oxidation (M)]STGER_.2	#N/A	8.0194	7.7855	7.8712	7.4957	7.4720	7.4689	7.6839	7.9447	7.4221	7.5426	7.5056	7.4557	7.4138	8.0154	7.2450	7.3443	7.6725	7.4601	7.2063	7.4529
Glud1	_DDGSWEVIEGYR_.2	#N/A	8.7605	8.6688	9.4875	8.8449	8.3804	9.0343	8.4983	8.7624	8.9121	8.8092	8.4990	8.3937	8.7897	9.2871	8.5254	8.6023	8.6738	8.7699	8.8255	8.8959
Glud1	_IIAEGANGPTTPEADKIFLER_.3	#N/A	6.8979	7.5168	7.2214	7.3871	7.3810	7.2664	7.3000	7.5025	7.1536	7.3913	7.4003	7.0827	7.3096	7.4474	6.8657	6.9843	7.1134	7.2779	6.9645	7.0423
Glud1	_TFVVQGFGNVGLHSM[Oxidation (M)]R_.2	#N/A	5.4839	3.2229	5.0487	4.6602	5.5958	5.1313	3.9966	5.8222	5.3810	5.7790	4.0415	4.5766	5.0623	2.5466	4.8963	5.0096	3.3835	3.4846	5.5963	5.4152
Glud1	_IIAEGANGPTTPEADK_.2	#N/A	5.4634	5.1666	5.4789	5.6810	4.6498	5.6044	5.6980	4.3098	5.5614	5.7478	5.7191	3.1667	3.6449	5.1654	5.3241	5.1495	3.4628	4.3704	5.0377	4.9881
Glud1	_GASIVEDKLVEDLK_.2	#N/A	5.6687	6.0248	6.2235	6.0375	5.8686	5.8505	5.2527	6.0522	5.5484	5.7277	5.5909	6.0872	6.2585	5.8418	5.0680	5.7125	6.0184	5.7886	5.6249	5.1852
Glud1	_ALASLMTYK_.2	#N/A	3.0852	4.5791	5.1494	5.0225	3.2922	3.4878	5.0034	3.3109	3.0081	3.5116	3.4235	3.9600	3.1161	5.0745	3.1261	3.9482	4.3090	4.3413	3.5483	2.5560
Glud1	_DSNYHLLM[Oxidation (M)]SVQESLER_.3	#N/A	5.3528	5.4817	5.7275	5.6370	5.2746	4.9398	5.7568	5.4041	5.0450	6.6174	4.9029	5.4313	5.7192	5.4889	4.7986	5.7791	4.7952	5.1977	6.0778	5.1542
Glud1	_VYEGSILEADC[Carbamidomethyl (C)]DILIPAASEK_.2	#N/A	8.3792	8.2899	8.3748	8.3490	7.6615	8.0647	8.0880	8.2390	8.1501	8.2092	7.7458	8.0495	8.4082	8.3789	7.6050	7.9877	7.8778	7.5483	7.7493	8.0358
Glud1	_DIVHSGLAYTM[Oxidation (M)]ER_.3	#N/A	5.2447	5.3662	6.0464	4.8638	5.1871	5.6459	5.3911	5.0869	5.2942	5.4770	5.2611	5.0209	4.5693	5.1967	5.0131	5.2882	5.6654	5.3494	5.1240	4.8704

Glud1	_FTMELAK_.2	#N/A	4.3608	3.9567	4.5824	2.9590	3.0669	3.1002	2.3021	3.7465	3.9014	4.1714	3.3800	3.8461	4.0668	2.7469	1.7826	3.7229	4.2842	3.5490	3.1976	3.0357
Psmd7	_SVVALHNLINNK_.2	#N/A	4.6210	4.6803	3.9572	3.8213	4.6617	4.8957	4.3331	4.3630	4.0722	4.1358	3.4156	4.7477	5.9270	4.1275	3.5183	2.6637	4.0481	4.8051	5.0466	5.4030
Sars1	_LLIDEAIQK_.2	#N/A	6.2219	6.3963	6.6258	5.8561	5.4655	5.7644	5.9608	5.6161	5.7631	5.4355	5.8352	5.9299	5.3131	5.9932	6.4137	5.6578	3.1754	6.3183	6.5199	6.0774
Sars1	_VLDLDLFR_.2	#N/A	4.4638	5.4613	5.7315	5.9145	5.5564	4.9613	5.6502	5.6142	5.8485	5.5155	5.8201	5.8387	5.6696	5.2477	5.4358	5.5927	3.1896	5.5717	5.8034	4.5766
Sars1	_YLIATSEQPIAALHR_.3	#N/A	5.7580	5.4481	5.4553	4.7398	4.8498	5.2441	5.0261	3.0652	5.3287	6.0095	5.1042	5.1730	4.7288	4.3694	5.1225	5.5340	3.4950	5.2079	5.7508	5.5880
Sars1	_TIC[Carbamidomethyl (C)]AILENYQAEK_.2	#N/A	5.4161	4.7087	5.5566	5.1461	5.6716	4.0262	4.5241	5.2870	5.3169	5.3683	4.9411	5.2264	4.2623	4.4145	5.0601	4.5647	5.2998	4.0168	4.9075	4.9288
Sars1	_YAGLSTC[Carbamidomethyl (C)]FR_.2	#N/A	5.1316	5.5589	4.4606	5.3006	5.4468	5.3282	5.5986	3.6247	3.7796	4.7370	4.8131	4.5108	4.1282	5.1854	5.7739	4.6320	4.1623	5.0757	5.3654	5.0519
Sars1	_IEQFVYSSPHDNK_.2	#N/A	6.3015	5.5714	5.8250	5.7066	6.4821	5.4459	5.7456	5.9254	5.8993	6.3579	5.5690	5.7661	5.6579	6.1829	6.4327	6.0143	6.1855	5.6795	6.1265	6.1390
Sars1	_EVPLENQLQS[Phospho (STY)]M[Oxidation (M)]EVTEA_.2	#N/A	6.7937	7.5418	4.3709	6.6327	3.5404	6.0453	6.7163	3.5592	5.6271	8.7559	3.6307	6.6906	3.3644	7.1171	4.0878	6.5926	4.5572	7.1321	6.1895	4.0863
Sars1	_ELVSC[Carbamidomethyl (C)]SNC[Carbamidomethyl (C)]TDYQAR_.2	#N/A	3.2258	3.9111	4.2632	4.3585	4.6105	3.6581	3.6154	3.4514	3.1486	3.8193	3.5230	3.8194	3.2566	4.1900	4.5707	3.0610	4.1714	4.2008	4.2423	3.4961
Sars1	_AKEVPLENQLQS[Phospho (STY)]MEVTEA_.2	#N/A	4.3357	3.6671	3.3711	3.3537	3.6734	4.3115	4.2707	4.0360	4.1909	3.8819	3.6696	3.5566	4.3564	3.3582	3.4449	3.1797	3.0729	3.2594	4.4089	3.3252
Sars1	_EIGNLLHPSVPISNDEDADNKVER_.4	#N/A	3.3946	3.9245	4.4320	4.1896	3.6016	3.6349	3.4465	3.2119	3.3668	3.2052	4.3462	3.6506	3.5322	4.0835	3.4719	4.2576	3.7495	4.0836	3.7322	4.0018
Marcks	_AEDGAAPS[Phospho (STY)]PSSETPK_.2	#N/A	13.8742	14.0839	14.3801	14.3143	13.9282	13.8028	13.6486	14.2498	13.8069	14.2822	14.0336	14.2945	14.7087	13.9131	14.0504	14.3007	14.4121	14.0676	13.8637	14.3347
Marcks	_LSGFS[Phospho (STY)]FKK_.2	#N/A	14.8113	14.8915	14.3437	14.4054	14.2729	14.0337	13.9143	14.7211	13.4894	14.7585	14.1200	14.1506	14.8471	14.7197	13.5893	14.0890	14.3619	14.0733	13.9115	14.2690
Marcks	_GEATAERPGEAAVASS[Phospho (STY)]PSK_.2	#N/A	9.3222	9.9816	10.3277	10.4090	9.2100	10.2181	9.0414	9.5393	10.1080	10.3947	10.2629	9.7286	10.0601	10.4658	10.3971	10.1491	9.9082	9.7035	10.2261	9.4048
Marcks	_VNGDAS[Phospho (STY)]PAAAEPGAK_.2	P26645_S46_M1_Marcks	11.1682	10.9928	11.3678	11.0922	10.7137	11.0725	10.5559	10.8058	10.7381	11.2034	10.7822	10.7075	10.4350	11.4679	10.5578	10.7362	10.8834	10.5700	10.6019	10.9357
Marcks	_FS[Phospho (STY)]FKKS[Phospho (STY)]FK_.2	P26645_S152_M2_Marcks	10.0879	10.4289	10.3855	10.6345	9.9599	9.9041	9.8521	10.1571	9.8142	10.5831	9.6443	10.0720	10.6935	10.3471	9.8239	10.2818	10.1044	9.7229	10.0314	10.2491
Marcks	_LSGFS[Phospho (STY)]FKKS[Phospho (STY)]K_.2	P26645_S163_M2_Marcks	8.5837	8.9861	8.9434	9.1103	8.4937	7.4307	8.0710	7.8095	7.3710	9.6093	7.9589	7.5291	8.0248	8.4985	7.4064	8.2204	8.0880	7.5308	8.1647	8.3574
Marcks	_DEAAAATEPGAGAADKEAAEAEPAEPSS[Phospho (STY)]PAAEAEGASASSTSSPK_.4	#N/A	4.0051	6.9018	3.6305	3.3695	3.3428	6.8456	4.2074	3.5317	6.2315	4.2114	6.2755	3.8963	4.3745	3.6505	6.6630	3.7309	4.2674	3.2487	3.2022	3.4361
Marcks	_ESGEGAEAEGATAEGAK_.2	#N/A	4.8273	4.9556	5.3406	5.5123	5.5084	5.7567	5.3496	5.1630	5.6287	5.4832	5.6342	5.8542	4.4573	5.3604	5.8618	5.3687	4.3401	4.9396	5.4659	6.2347
Marcks	_PGEAAVASS[Phospho (STY)]PSK_.2	#N/A	4.0997	3.2194	3.6362	3.4846	4.3066	3.8018	3.9634	4.3253	4.0225	4.4463	2.8960	5.8890	4.1305	6.7662	4.2677	3.4618	6.4491	3.3269	5.8832	3.3516
Marcks	_SKKES[Phospho (STY)]GEGAEAEGATAEGAK_.3	#N/A	5.7244	6.0845	6.1371	5.8711	5.1589	5.9648	6.0591	5.4352	5.7913	6.5306	6.3582	6.0621	6.5828	6.7543	6.5301	6.1774	6.1606	6.2593	6.6307	4.1051
Marcks	_AEDGAAPS[Phospho (STY)]PSSET[Phospho (STY)]PKKK_.2	#N/A	5.7234	7.9716	7.4425	8.1033	7.5184	7.7213	8.7662	8.1292	8.8848	9.5153	8.4834	9.2770	10.1222	8.4979	8.4102	8.2479	7.9871	9.3372	7.1223	9.4230
Marcks	_EAAEAEPAEPSS[Phospho (STY)]PAAEAEGASASSTSSPK_.3	#N/A	12.1976	11.9607	12.8580	12.3111	11.5227	12.0977	11.4867	11.9240	11.8522	12.3705	12.1611	11.8835	12.0959	12.6404	11.9993	12.1424	12.4967	11.7483	11.9478	12.2270
Marcks	_S[Phospho (STY)]FKLS[Phospho (STY)]GFSFK_.2	P26645_S160_M2_Marcks	3.8688	3.4503	3.4054	3.7155	4.0757	4.1090	5.5537	4.2385	5.1651	3.6794	3.8721	3.1764	4.5589	6.6676	5.6743	3.2309	3.2754	5.8239	4.2064	3.5277
Marcks	_AEDGAAPSPSSET[Phospho (STY)]PKK_.2	#N/A	10.7878	10.3995	10.8902	10.4695	10.6391	10.5852	9.9190	10.3579	10.0486	10.6064	10.5390	10.8773	11.3060	11.3972	10.4512	10.9803	10.8468	10.2922	10.7140	10.8664
Marcks	_GEATAERPGEAAVASS[Phospho (STY)]PSKANGQENGHVK_.4	#N/A	4.0715	8.7519	3.6081	3.5903	3.7995	7.6728	4.1097	3.8183	4.1157	3.7901	8.4770	3.5784	8.6366	3.6462	8.8140	3.4336	3.7944	7.7906	8.8253	3.8985
Marcks	_LSGFS[Phospho (STY)]FK_.2	#N/A	3.8073	9.0911	9.9063	4.5906	4.0142	4.0475	3.0339	4.3000	9.4446	10.6343	3.9336	3.2926	3.1195	3.6222	3.0593	3.1694	3.3369	9.1570	4.1449	3.5892
Marcks	_TAAKGEATAERPGEAAVASS[Phospho (STY)]PSK_.3	#N/A	6.5780	3.7516	5.7909	4.0167	6.2331	6.1796	6.2513	6.4166	6.4535	3.7663	6.0360	6.4348	7.1586	6.8037	6.4943	6.8109	3.9858	6.6810	3.7670	6.5625
Marcks	_KES[Phospho (STY)]GEGAEAEGATAEGAKDEAAAAAGGEGAAAPGEQAGGAGAEGAAGGEPR_.4	#N/A	4.1891	5.3771	3.7002	3.2448	3.5269	4.5439	4.0486	3.6872	4.1077	4.7270	3.8087	3.8634	4.2157	3.7174	3.6258	3.8607	3.7049	5.1763	3.9296	4.4703
Marcks	_ESGEGAEAEGATAEGAKDEAAAAAGGEGAAAPGEQAGGAGAEGAAGGEPR_.4	#N/A	3.6435	4.3593	4.1798	4.0459	2.9812	3.2053	3.0590	3.3439	3.1181	3.8498	3.4925	4.2488	3.2262	4.1669	4.1371	3.9884	3.8815	3.9516	3.6002	4.1339
Marcks	_KES[Phospho (STY)]GEGAEAEGATAEGAK_.2	#N/A	3.5884	3.7307	3.1249	3.9959	3.7953	4.3131	3.4521	3.8140	3.5112	3.4567	3.8856	3.4568	3.6192	5.4411	3.0293	2.9505	3.3113	3.8382	4.1372	4.3411
Fkbp1a	_GVQVETISPGDGR_.2	#N/A	11.0556	11.0473	11.1911	11.1428	10.2056	10.9052	10.7985	11.1826	11.1281	11.0847	10.8937	11.1971	11.6066	11.4672	11.4195	11.3470	11.3802	10.9148	11.1054	11.1846
Fkbp1a	_GWEEGVAQM[Oxidation (M)]SVGQR_.2	#N/A	8.5381	8.3930	8.9078	8.6200	8.5459	8.3012	8.1080	8.8668	8.4939	8.4663	8.2405	8.6108	8.8447	8.6063	8.9376	8.7395	8.4831	8.2461	8.3939	8.4961
Map4	_SKVGS[Phospho (STY)]TENIK_.2	#N/A	10.1550	10.2627	9.7424	3.7466	9.6901	9.4780	9.7402	9.7847	9.3755	10.3293	9.7403	9.7859	10.2805	10.3939	3.9032	9.8857	10.0814	9.9665	9.8754	9.8878
Map4	_SAS[Phospho (STY)]ADLSR_.2	P27546_S828_M1_Map4	8.0209	3.8129	7.3220	7.8747	7.4237	7.1347	6.9363	6.6645	4.0451	4.3962	7.5878	7.9200	7.8342	7.7967	6.3403	7.7732	7.0666	7.4958	7.8015	7.9560
Map4	_LATTVS[Phospho (STY)]APDLK_.2	P27546_S901_M1_Map4	7.7846	8.2835	7.9719	4.5932	3.9348	4.1589	3.6063	7.3323	3.6654	8.2384	4.0398	7.5041	3.7734	8.3145	7.6165	8.2971	3.3343	7.7121	4.1475	8.0522
Map4	_AAVGVTGNDITTPPNKEPPPS[Phospho (STY)]PEK_.3	#N/A	8.8802	4.1379	7.7882	8.0208	8.2052	3.4267	8.3360	8.8310	4.3975	4.0713	8.2432	8.2854	8.4742	4.0493	3.9156	7.9668	8.4500	4.4527	3.2509	8.0320
Map4	_DM[Oxidation (M)]S[Phospho (STY)]PSAETEAPLAK_.2	#N/A	10.8951	11.0546	11.3836	11.0536	10.2398	11.0253	10.4857	10.7909	10.4053	10.8694	10.8180	10.6776	10.9530	11.5014	10.4441	10.6050	11.2079	10.3348	10.7673	10.8445
Map4	_TEFIPLLDGDEK_.2	#N/A	7.3253	7.4065	7.5413	7.6093	7.5091	7.2933	7.3701	7.0229	7.3963	7.2512	7.6884	7.3194	3.6815	7.6133	8.1296	7.8154	7.2109	6.7249	7.4843	7.7028
Map4	_NTT[Phospho (STY)]PTGAAPPAGM[Oxidation (M)]TSTR_.2	#N/A	11.5555	11.6174	11.4093	11.4817	11.3358	11.1297	11.0381	11.5533	11.1104	11.3448	11.0182	11.3427	11.8405	11.9557	10.8851	11.3271	11.5911	11.2125	11.0484	11.1854
Map4	_ATS[Phospho (STY)]PSTLVSTGPSSR_.2	P27546_S785_M1_Map4	9.8461	9.8646	9.2662	9.5071	8.8923	8.7700	9.2467	9.7509	8.6942	9.5732	8.7598	9.4980	9.9409	3.9421	8.3908	9.1691	5.6525	4.9557	9.0815	8.9940
Map4	_DM[Oxidation (M)]S[Phospho (STY)]PLPESEVTLGK_.2	#N/A	8.4699	9.2873	9.3661	8.6962	3.2352	8.7663	7.5734	7.6658	8.3657	8.6387	7.7421	8.2588	7.9623	9.6755	8.2148	8.1994	8.6837	8.1233	8.3268	8.4870
Map4	_GQSTVPPC[Carbamidomethyl (C)]TAS[Phospho (STY)]PEPVK_.2	P27546_S598_M1_Map4	9.4370	8.4751	9.1756	8.7747	8.6222	9.8316	7.1240	9.8956	7.7900	8.5257	8.5795	8.6058	8.9263	10.3152	3.9311	3.7984	4.2087	9.4206	7.8901	2.9602
Map4	_SKVGST[Phospho (STY)]ENIKHQPGGGR_.3	P27546_T915_M1_Map4	4.5163	3.4957	3.3599	3.7609	3.8540	4.0781	3.6871	4.0490	3.7462	3.2217	4.1206	3.2218	3.8542	5.8702	5.1670	3.3603	3.5463	3.6032	3.9022	3.0753
Map4	_TTSASS[Phospho (STY)]VKR_.2	P27546_S841_M1_Map4	4.2275	3.7753	3.2630	3.4619	3.5652	3.7893	3.9759	3.9279	4.0349	4.4338	2.9085	3.6648	4.1430	3.2500	3.5531	3.0716	4.4656	3.3676	3.0162	3.3641
Map4	_VGS[Phospho (STY)]TENIKHQPGGGR_.3	#N/A	4.5017	3.5011	3.5372	3.1877	3.8394	4.0635	3.7016	4.2021	3.7607	3.2072	4.1351	3.3905	3.8688	3.5242	3.2788	3.3458	3.2389	3.5886	4.2428	3.0898
Map4	_DM[Oxidation (M)]S[Phospho (STY)]PLPESEVTLGKDVVILPETK_.3	#N/A	5.6198	6.2065	3.6397	3.4811	4.3100	4.8726	4.7568	4.3288	4.0259	5.0224	2.8994	5.4934	4.9300	6.3006	5.0882	3.4652	3.8260	3.3234	3.6225	3.3550
Map4	_VGS[Phospho (STY)]LDNVGHLPAGGAVK_.2	#N/A	4.7010	5.8903	4.7893	5.2378	5.0214	5.1208	5.6222	5.4654	4.0683	5.2270	4.9869	5.9855	6.1470	7.0151	4.6351	5.5312	5.9870	6.2604	5.8166	5.8939
Map4	_VAEFNNVT[Phospho (STY)]PLS[Phospho (STY)]EEEVTSVK_.2	P27546_S506_M2_Map4	3.3114	4.6606	3.8477	4.3780	4.1499	3.8350	3.2464	4.5126	3.1666	3.4053	4.1461	3.0801	3.3321	3.8347	3.6607	3.6563	3.5494	5.1822	3.9323	3.8017
Map4	_AAVGVTGNDITT[Phospho (STY)]PPNKEPPPS[Phospho (STY)]PEKK_.3	P27546_T658_M2_Map4	10.4602	10.6018	10.3379	10.3938	9.0766	10.1042	10.1760	10.3757	10.0517	10.4428	9.8640	10.5283	10.8751	11.3775	9.8565	10.4297	10.6884	10.4709	10.2376	10.3285
Map4	_DFMAALEAEPYDDIVGETVEK_.2	#N/A	3.9040	3.5482	4.8994	4.0856	4.1109	2.7735	3.1264	4.1297	3.8268	3.1411	4.2012	3.1412	3.9349	3.2795	4.1371	3.2661	3.6269	3.5225	3.8216	3.1559
Map4	_S[Phospho (STY)]PATTLPK_.2	P27546_S798_M1_Map4	3.1757	4.1626	4.2131	4.4085	3.3827	3.2249	3.0395	3.4014	3.0985	3.8694	3.4729	3.8695	3.2066	4.2512	3.6908	3.7010	4.3995	4.2508	3.0490	3.9285
Map4	_VGS[Phospho (STY)]TENIK_.2	#N/A	4.2402	3.7626	3.2756	3.4848	3.5779	3.8020	3.9632	4.3251	4.0223	4.4465	2.8958	3.6521	4.1303	3.6741	3.5404	3.0842	3.8224	3.3271	3.6261	3.3514
Map4	_AAEQM[Oxidation (M)]STLPIDAPS[Phospho (STY)]PLENLEQK_.3	P27546_S617_M1_Map4	3.7428	4.2600	4.2791	3.9466	3.0805	3.4036	3.5430	3.4432	3.5981	3.3530	3.0767	4.1495	3.7635	4.2662	4.0378	4.0877	3.9808	3.8128	3.5009	4.2332
Gna13	_LTESLNIFETIVNNR_.2	#N/A	4.0053	4.3122	4.0969	4.4178	4.7353	3.9857	3.7149	5.0184	4.8006	4.8034	4.1938	3.4631	4.8913	4.0319	4.1786	3.9146	4.3059	4.3524	3.4098	3.5616
Gna13	_ALWEDSGIQNAYDR_.2	#N/A	6.0699	6.0822	6.2758	5.6555	5.3573	6.0656	5.6674	5.7979	6.2986	3.5683	3.9831	5.5383	3.1691	5.1482	5.8392	5.1682	6.1403	5.5303	5.8984	5.3529
Rpl3	_NNASTDYDLSDK_.2	#N/A	6.6081	6.7875	6.8418	7.0183	6.5972	6.8941	7.1328	7.2371	7.2338	6.6735	7.0187	6.4577	6.6567	7.2866	6.3410	6.9519	7.2518	6.8915	6.4217	6.9743
H2ax	_LLGGVTIAQGGVLPNIQAVLLPK_.2	#N/A	4.5261	4.6957	4.8202	3.7088	4.7800	4.1390	4.3979	4.6363	3.8276	3.6983	2.0914	3.3611	3.9248	4.5011	3.9007	4.2471	4.9433	4.5344	4.5600	2.6774
Rasgrf1	_LLYGDAPKS[Phospho (STY)]PR_.2	P27671_S718_M1_Rasgrf1	7.3473	3.4164	6.3391	3.6815	4.1096	5.8681	4.4393	6.3379	4.3595	3.7133	3.8382	3.1425	3.9336	3.4773	2.9638	3.2648	3.6256	6.5064	3.8229	3.1546
Rasgrf1	_AGS[Phospho (STY)]ADDEDSDIKK_.2	P27671_S199_M1_Rasgrf1	3.5600	4.4428	4.0963	4.1293	2.8978	3.5863	3.1425	3.2604	3.2016	3.7664	3.5759	4.3322	3.5808	4.0834	4.2205	3.9049	3.7981	4.0351	3.6836	4.0504
Pdia3	_SEPIPESNEGPVK_.2	#N/A	8.6386	8.4981	8.9718	8.6353	8.6465	8.5527	8.6105	8.5399	8.7216	8.6387	8.5994	8.8064	8.6027	9.0782	8.8632	8.8300	8.5276	8.6596	8.5681	9.0449
Pdia3	_LAPEYEAAATR_.2	#N/A	8.4507	8.6180	8.7742	8.3572	8.7095	8.4359	8.4348	8.6944	8.3487	8.7370	8.2171	8.7919	8.5345	8.5537	8.7953	8.5037	8.2467	8.5601	8.4954	8.6630
Pdia3	_EATNPPIIQEEKPK_.2	#N/A	7.3657	6.8005	7.6037	7.2666	7.4288	7.1905	7.0077	7.5518	7.4222	7.3574	7.0140	7.7049	7.3930	7.8052	7.6766	7.6460	7.3691	7.2787	7.0145	7.6771
Pdia3	_ELNDFISYLQR_.2	#N/A	6.8401	7.1159	7.4896	6.5741	7.0577	7.1861	6.8879	7.2466	7.0116	7.3205	7.0098	7.4170	7.3179	7.3125	7.3990	7.2039	7.2152	7.0661	6.7118	7.2569
Pdia3	_DLFSDGHSEFLK_.2	#N/A	6.8669	7.0423	7.0413	7.0444	6.9297	6.6142	6.9945	7.2958	6.9002	6.9355	6.8542	6.9325	7.5421	7.3936	7.1167	7.0373	7.2723	6.8933	6.8245	6.9455
Pdia3	_TADGIVSHLK_.2	#N/A	5.5966	5.2422	5.8030	5.1268	5.4858	5.0498	5.9078	5.8059	5.6743	4.9181	5.8318	5.4658	5.6317	5.7080	6.3318	5.6776	5.5283	5.6004	5.4228	5.5149
Pdia3	_VDC[Carbamidomethyl (C)]TANTNTC[Carbamidomethyl (C)]NK_.2	#N/A	3.8084	5.3149	4.5607	5.0732	3.8865	4.3793	4.5497	4.3297	4.2878	4.5136	4.3019	3.8141	4.9947	3.6288	4.7177	4.8187	4.2351	3.7650	4.6309	3.7008
Pdia3	_FISDKDASVVGFFR_.3	#N/A	6.0751	6.8187	6.4861	6.6854	6.0402	6.6602	6.5674	6.7713	6.7194	5.6841	6.6573	6.5376	7.0458	7.0029	7.0591	6.5190	6.7859	6.5452	6.8257	7.0476
Pdia3	_DLLTAYYDVDYEK_.2	#N/A	6.2070	3.9014	6.8516	4.1665	5.8540	6.4982	6.0088	5.5293	6.3222	6.4313	6.0560	5.8349	6.5614	6.1573	6.3318	6.7835	6.0330	5.6399	6.0429	6.6616
Pdia3	_TFSHELSDFGLESTTGEVPVVAIR_.3	#N/A	5.9238	5.4466	5.1419	5.4620	5.4967	5.7970	4.7751	5.9539	5.9586	5.4625	5.3132	5.7955	3.1914	6.3983	5.1421	5.5893	5.9096	5.9217	5.8359	5.9246
Pdia3	_QAGPASVPLR_.2	#N/A	4.2640	3.5198	4.7464	4.3539	3.6826	4.6416	4.5230	3.7259	4.3256	4.5508	4.4994	3.5923	3.2612	4.0015	5.4993	4.3488	4.4541	4.7738	4.5488	4.5518
Pdia3	_FVM[Oxidation (M)]QEEFSR_.2	#N/A	3.2773	5.9496	6.3383	5.3271	5.9811	5.9418	5.5650	6.3460	5.4446	5.2344	5.6135	5.7971	6.2126	6.6583	6.3088	5.7171	5.7140	6.1127	5.9193	5.8980
Pdia3	_FAHTNIESLVK_.2	#N/A	5.8907	5.7551	5.9011	5.6791	5.9694	5.5481	5.5119	5.8804	5.1896	5.6737	5.5196	5.6629	5.9213	5.7976	5.7734	5.3416	5.7025	5.4221	5.1933	5.3659
Pdia3	_DGEEAGAYDGPR_.2	#N/A	3.8167	3.5448	3.3533	3.7675	7.5310	4.0570	5.8963	4.0424	4.4455	3.6273	7.6740	7.5729	3.1101	3.3914	7.1761	7.3844	7.2371	7.3609	7.2408	7.8871
Pdia3	_MDATANDVPSPYEVK_.2	#N/A	4.5364	3.4664	6.7940	7.3403	3.8742	4.1108	6.9728	4.2368	4.3917	3.2419	3.8704	5.4054	4.5572	7.6280	6.7070	3.3805	3.2737	3.0587	4.2081	3.5260
Braf	_SAS[Phospho (STY)]EPSLNR_.2	P28028_S714_M1_Braf	12.5772	12.7949	12.6490	12.7725	12.3598	12.7451	12.4652	12.8720	12.7538	12.4948	12.4928	12.3782	13.1082	12.9632	12.1582	12.8333	12.9489	12.7549	12.8778	12.6122
Braf	_NNPKS[Phospho (STY)]PQKPIVR_.3	#N/A	9.8457	9.9286	9.2109	9.4285	8.5178	8.4230	9.4247	8.7022	8.5572	9.7879	9.0006	9.3081	9.4253	9.8572	7.3747	8.9883	8.6761	9.2298	9.1350	9.0289
Braf	_S[Phospho (STY)]PQKPIVR_.2	#N/A	8.2822	8.1217	8.5381	7.8929	7.6176	7.3140	7.3625	7.0792	7.2277	8.6663	8.1323	7.6498	6.7797	7.9354	6.1526	7.4985	7.5265	7.0771	7.6919	7.4253
Braf	_SSS[Phospho (STY)]APNVHINTIEPVNIDDLIR_.3	P28028_S348_M1_Braf	4.1639	3.1434	3.7123	3.4085	3.5016	3.7257	8.4629	8.4858	4.0985	4.3702	2.9721	8.5430	4.2066	9.0173	6.5250	6.9246	7.5265	8.3511	3.5499	3.4277
Braf	_RDS[Phospho (STY)]SDDWEIPDGQITVGQR_.3	#N/A	4.0328	9.1165	3.8854	8.7689	3.4021	8.5455	9.3931	4.0697	9.0111	3.9905	3.4561	6.7552	9.2153	9.1544	8.1721	7.7280	4.3743	9.3976	8.8375	3.6588
Braf	_AGFQTEDFSLYAC[Carbamidomethyl (C)]AS[Phospho (STY)]PK_.2	#N/A	7.5740	7.4480	7.2751	6.9585	6.3991	6.9297	7.1270	6.5372	6.9458	7.0321	7.2230	2.9765	3.4209	7.1143	2.8648	6.4243	7.2886	7.0851	3.8288	6.2245
Braf	_DS[Phospho (STY)]SDDWEIPDGQITVGQR_.2	#N/A	4.2917	4.5282	3.8283	3.2925	2.9978	3.6097	4.1555	4.5174	4.2145	4.2542	3.0881	4.2543	4.3226	3.8664	4.0756	3.6538	4.0146	3.1348	3.4339	3.5437
Mt3	yl (C)]C[Carbamidomethyl (C)]SC[Carbamidomethyl (C)]C[Carbamidomethyl (C)]PAGC[Carbar	#N/A	6.1291	6.9493	6.4434	6.6421	6.2598	6.7283	6.7215	5.8930	6.8108	6.5719	6.2676	6.5508	6.9858	7.1264	7.8409	6.1746	6.2215	6.5882	7.0604	6.3190
Mt3	_GEEGAKAEAEK_.2	#N/A	2.9115	2.7910	3.6400	3.6238	3.9653	2.9726	3.2841	4.3291	3.8310	2.8445	3.9509	2.9418	4.1441	4.3013	4.8346	3.4899	4.0446	3.1149	4.0649	3.3553
Mt3	_DC[Carbamidomethyl (C)]VC[Carbamidomethyl (C)]KGEEGAK_.2	#N/A	5.4503	5.8590	5.8107	5.5575	5.0559	5.3184	5.6242	4.9316	5.4437	5.4636	4.6153	5.5626	5.3406	5.2645	6.2383	5.6525	5.6867	5.5849	4.9928	5.0108
Mt3	lation (M)]DPETC[Carbamidomethyl (C)]PC[Carbamidomethyl (C)]PTGGSC[Carbamidomethyl (	#N/A	6.1393	6.7426	7.2379	7.1205	6.7980	6.5606	6.5727	6.3781	7.1023	6.8234	6.4538	6.6882	6.8096	7.1530	7.3354	6.7219	6.8008	6.5126	6.7048	6.7399
Mt3	_DC[Carbamidomethyl (C)]VC[Carbamidomethyl (C)]KGEEGAKAEAEK_.3	#N/A	4.7328	5.6397	4.9332	4.5996	5.2901	5.0531	5.3286	4.4627	5.2504	4.8599	4.6998	5.3226	5.9455	5.4363	6.0847	5.5945	5.2760	4.9347	5.4492	5.4480
Gjb1	_KGS[Phospho (STY)]GFGHR_.2	P28230_S233_M1_Gjb1	6.0490	6.0070	4.1091	5.8430	5.4573	6.2190	5.6960	5.2060	4.8524	4.2095	5.6370	5.1354	5.8245	4.0962	3.5333	4.3216	6.0060	5.7129	5.3742	5.1933
Aco1	_VILQDFTGVPAVVDFAAM[Oxidation (M)]R_.2	#N/A	3.8843	2.9497	4.3335	4.2882	3.5030	3.1175	3.9808	3.5217	3.8725	3.1200	3.5932	2.8752	3.3269	4.3715	3.5705	4.1590	4.5198	4.1305	1.6061	4.0488
Apex1	_AAADDGEEPKS[Phospho (STY)]EPETKK_.3	P28352_S18_M1_Apex1	3.2391	4.0800	4.2765	5.4110	3.4460	3.4793	5.2545	4.9466	3.5223	6.3882	3.0010	3.8062	3.6878	5.2659	3.6275	4.1020	3.9051	3.9281	3.5766	4.1574
Adh5	_IDPSAPLDK_.2	#N/A	6.3225	6.2036	6.5449	6.4257	6.0773	6.4272	6.7212	6.8295	6.3657	6.1211	6.4418	5.9910	5.4268	5.8284	6.6592	5.9096	6.4641	6.5754	6.3391	6.1897
Adh5	_AAVAWEAGKPLSIEEIEVAPPK_.3	#N/A	4.1268	4.6240	4.2187	3.4574	4.3724	5.5312	4.2141	5.3170	4.3143	4.0216	4.2088	4.4044	4.3009	4.0671	4.9006	4.4678	3.4989	4.3985	4.4981	4.6174
Adh5	_EFGASEC[Carbamidomethyl (C)]ISPQDFS[Phospho (STY)]K_.2	#N/A	3.6632	7.6316	7.4464	4.0262	3.0009	3.4832	7.0680	3.3636	6.9244	7.2759	7.6722	6.3050	3.6839	7.6965	6.1997	6.8944	3.9012	7.0679	6.8265	7.0554
Adh5	_AKEFGASEC[Carbamidomethyl (C)]ISPQDFS[Phospho (STY)]K_.3	#N/A	3.8359	4.1669	4.3722	4.6259	3.1736	3.3977	4.3675	3.5363	4.4175	4.0422	3.3001	4.0564	4.5346	5.6226	3.9447	4.1808	4.0739	5.1819	3.4078	3.7557
Adh5	_LVSEYMSK_.2	#N/A	4.3207	3.6821	3.3562	3.3687	3.6585	4.3265	4.2558	4.0211	4.1760	3.8968	3.6546	3.5715	4.3414	3.3433	3.4598	3.1648	4.5588	3.2744	4.4238	3.3103
Adh5	_SIQEVLVEM[Oxidation (M)]TDGGVDYSFEC[Carbamidomethyl (C)]IGNVK_.3	#N/A	3.2532	3.2487	3.7895	4.4362	4.0918	3.3372	3.4493	4.4544	3.5084	3.4595	3.8828	3.1382	3.6164	3.7766	3.0265	3.5981	3.4913	4.3419	3.9905	3.7436
Slc6a9	_YAPTTTPS[Phospho (STY)]PEDGFEVQPLHPDK_.3	P28571_S659_M1_Slc6a9	4.1989	4.0217	3.7584	3.3630	3.2439	3.3929	4.0748	4.4475	4.0653	4.2138	3.2621	4.3248	4.2527	3.7965	3.8962	3.3342	3.9447	3.2053	3.5043	3.4737
Adss1	_VVDLLATDADIVSR_.2	#N/A	4.9803	4.1690	5.2002	6.0985	5.2132	5.1240	4.9970	5.4451	5.1955	4.8488	3.9150	3.3134	3.7916	5.1940	5.3079	4.4026	3.3161	5.0510	5.1998	4.3060
Adss1	_IC[Carbamidomethyl (C)]DLLSDFDEFSAR_.2	#N/A	3.9871	3.3320	3.5237	3.5971	4.1941	4.2274	4.2046	4.2128	4.6593	3.7977	3.7537	3.0581	4.0180	3.5618	4.0300	3.1357	3.7100	3.4394	3.7384	3.2391
Camk2b	_ESSDS[Phospho (STY)]TNTTIEDEDAK_.2	#N/A	12.1807	11.9322	12.7461	11.3424	11.5957	11.8084	11.0632	12.1826	12.2749	11.1304	10.7724	11.1918	12.3941	12.4352	12.2410	11.7681	12.3065	12.4795	11.9114	11.8572
Camk2b	_FTDEYQLYEDIGK_.2	#N/A	7.8370	7.5823	7.9687	7.6652	7.4607	7.6359	7.4954	7.9496	7.9481	7.5670	7.7236	7.3735	8.1209	7.0499	6.9743	7.9891	7.6135	7.1886	7.2154	7.7495
Camk2b	_HPWVC[Carbamidomethyl (C)]QR_.2	#N/A	7.4710	7.3336	7.2236	7.1015	7.1650	7.6535	8.0775	7.6110	6.7489	7.2651	7.4712	7.3854	6.6121	7.5234	7.8167	7.1190	7.1416	7.7220	7.5259	7.0416
Camk2b	_ITAHEALK_.2	#N/A	5.9456	6.1184	6.5566	6.2191	6.4132	6.6104	6.5827	6.3366	6.1909	6.5806	6.5097	5.9554	5.9875	6.1526	6.5596	6.3030	5.9943	6.7154	6.9359	6.1730
Camk2b	_LC[Carbamidomethyl (C)]TGHEYAAK_.2	#N/A	5.4382	5.6841	5.7259	5.0365	6.0164	5.4432	5.6856	6.2226	5.5661	5.4006	5.9595	5.8010	5.4679	5.7100	6.2732	5.5951	5.3437	6.3484	5.4883	5.4015
Camk2b	_ADGVKPQTNS[Phospho (STY)]TK_.2	#N/A	5.8399	5.1444	4.3751	4.2466	3.5446	3.0630	5.4092	3.5633	3.2605	3.1482	3.6348	3.7076	3.3685	4.4131	3.5289	4.2006	3.0605	4.0889	5.0745	4.0904
Camk2b	_FYFENLLAK_.2	#N/A	4.9799	3.3758	6.0520	5.0554	6.4262	6.1128	3.4716	3.8558	4.8768	5.7602	5.5036	4.4027	3.9301	3.7994	5.3443	4.9103	4.5452	2.6224	4.6147	6.3549
Camk2b	_AGAYDFPSPEWDTVTPEAK_.2	#N/A	9.8555	11.1801	10.8378	9.5641	9.2762	9.5648	10.9489	9.7864	9.6745	9.6941	9.6665	9.6935	9.6323	11.1396	9.0496	9.3373	11.2711	11.1676	9.2629	9.3662
Camk2b	_NLINQM[Oxidation (M)]LTINPAK_.2	#N/A	5.8472	6.0852	6.1229	5.6792	5.7836	6.0299	6.2242	5.9789	5.8803	5.4168	5.8732	5.7433	6.0355	5.6920	5.9675	4.7755	5.7345	6.3274	5.6983	6.0505
Camk2b	_QET[Phospho (STY)]VEC[Carbamidomethyl (C)]LKK_.2	#N/A	5.8142	5.9237	6.5044	6.3827	5.8991	6.0820	5.2892	3.6746	3.3717	5.2310	5.9068	3.5963	3.4798	5.5170	4.4429	5.5758	5.7341	4.9423	4.2767	5.5878
Camk2b	_GAILTTM[Oxidation (M)]LATR_.2	#N/A	6.6970	6.3896	6.7750	6.3987	6.1729	6.9793	6.3634	6.2986	6.6941	6.2748	6.2775	6.2276	5.4391	6.7631	6.3495	5.9552	6.2442	6.8052	6.4204	6.3514
Camk2b	_WQNVHFHC[Carbamidomethyl (C)]SGAPVAPLQ_.3	#N/A	5.5474	5.2214	5.8747	5.4430	4.7771	5.3052	5.7493	4.6464	5.2727	5.5702	5.3331	4.8150	5.0843	5.0318	5.4622	5.8482	5.5303	5.3598	5.0620	5.3192
Camk2b	_LTQYIDGQGR_.2	#N/A	3.3498	6.3492	5.9891	4.2344	3.5568	6.1080	6.0006	6.5672	6.1725	3.1604	6.4286	5.5833	3.3807	4.4253	5.9589	5.7974	6.0199	6.0720	5.7473	5.2030
Camk2b	_QET[Phospho (STY)]VEC[Carbamidomethyl (C)]LK_.2	#N/A	4.0247	4.9228	6.3146	4.7161	5.4374	5.3717	5.1687	3.7250	4.9965	5.0725	5.5878	3.8676	4.3458	3.8896	3.7559	5.5235	5.4313	3.1116	4.6739	3.5669
Camk2b	_ESSDST[Phospho (STY)]NT[Phospho (STY)]T[Phospho (STY)]IEDEDAKAR_.2	#N/A	8.2316	7.9935	4.2448	6.9022	3.2681	3.3877	6.0482	3.7762	7.7987	4.1662	3.2570	3.9871	7.8669	7.5132	6.5181	6.1910	7.5193	3.6493	3.3167	3.6705
Camk2b	_DLKPENLLLASK_.2	#N/A	10.2196	10.3044	10.0719	10.0719	10.0127	10.1389	10.2791	10.4564	9.9205	9.9366	9.9736	10.1006	10.3976	10.0473	9.9273	9.9209	10.2015	10.2142	10.0788	9.8946
Camk2b	_NSSAITS[Phospho (STY)]PK_.2	P28652_S367_M1_Camk2b	13.0188	13.1533	12.9219	13.1027	12.7089	13.0664	13.0718	12.9298	12.9612	13.0635	12.9656	12.9743	13.2773	13.1576	12.3790	12.8694	13.0425	13.1558	13.0466	12.9942
Camk2b	_LTQYIDGQGRPR_.2	#N/A	6.2282	6.3623	6.1231	6.3131	6.2206	6.3272	6.2810	6.3386	6.1384	6.2245	5.9078	6.7673	5.6115	6.7428	6.5737	6.1052	6.4184	6.3948	6.4634	6.0260
Camk2b	_S[Phospho (STY)]TVASMMHR_.2	P28652_S276_M1_Camk2b	3.2184	4.5291	7.0935	6.4057	3.8915	5.5648	5.8662	4.1849	3.2678	3.8180	4.4439	3.4519	3.3942	6.2818	4.8958	3.7421	3.8344	4.3248	3.7399	4.0389
Camk2b	_IC[Carbamidomethyl (C)]DPGLTSFEPEALGNLVEGM[Oxidation (M)]DFHR_.3	#N/A	3.3035	4.0156	4.3409	4.2807	4.2919	3.5438	4.6560	5.2307	3.4579	3.1141	4.3622	4.7884	5.4547	4.1260	4.8295	4.3132	4.5272	3.9925	4.4465	3.4142
Camk2b	_TTEQLIEAVNNGDFEAYAK_.2	#N/A	7.0609	6.8830	6.2370	6.7677	6.5395	6.6576	6.5827	7.2510	6.7252	6.7891	6.7841	6.8080	7.2634	6.8337	5.9831	6.8866	7.0141	6.8937	6.6459	6.7287
Camk2b	_KADGVKPQTNS[Phospho (STY)]TK_.2	#N/A	4.7219	4.4535	3.7786	3.1523	3.6271	3.8131	4.0066	4.7747	4.1937	3.9339	3.6724	3.7981	3.9018	4.4865	3.4037	3.9378	3.7664	4.8134	3.8048	3.6065
Camk2b	_QTTAPATMSTAASGTTM[Oxidation (M)]GLVEQAK_.2	#N/A	3.9656	3.7965	3.8295	3.5965	3.3293	3.4297	3.4584	3.9964	3.6457	4.3779	3.4846	3.9658	4.1338	3.6362	3.6779	4.9859	3.9549	4.5476	4.3211	4.0479
Nap1l1	_LDGLVDTPTGYIESLPK_.2	#N/A	5.7485	5.6610	5.9543	5.8226	5.8123	5.5982	5.5237	5.6318	5.6317	6.0009	5.6835	5.3768	6.0297	5.7527	6.1676	5.9368	5.5905	5.3142	5.7350	5.9007
Nap1l1	_NVDLLSDMVQEHDEPILK_.3	#N/A	4.1836	3.8192	3.2191	3.5058	3.5213	4.4636	4.1187	3.8840	4.0389	4.0339	3.5175	3.7086	4.2043	3.2061	3.5969	3.0277	4.4217	3.4115	3.0601	3.1731
Celf1	_TWS[Phospho (STY)]EKDLR_.2	P28659_S28_M1_Celf1	4.5630	7.5056	3.5984	3.1264	7.4225	7.1454	2.9972	7.6947	4.4182	7.3368	7.0856	7.1076	4.5837	7.7237	3.2176	6.9580	3.3002	7.4676	4.1816	7.7524
Nckap1	_NNNQQLAQLQK_.2	#N/A	4.9645	5.1004	5.9021	5.3481	5.5796	5.5519	6.0799	3.1669	5.6151	5.1719	5.0173	5.4129	3.9856	5.9017	3.5945	5.6082	5.8129	5.0472	5.3545	5.4399
Nckap1	_NLSADQWR_.2	#N/A	6.3661	6.0545	5.8181	5.8025	5.4766	5.8842	5.7903	5.9614	5.9776	5.5565	5.3463	5.6708	3.8165	4.7831	5.9243	5.3452	5.0415	5.4629	5.1467	4.7362
Nckap1	_AAEDLFVNIR_.2	#N/A	5.4052	3.9091	3.7618	4.4092	4.8320	4.2486	3.8090	4.4111	4.3583	4.8625	4.2718	4.5957	4.2024	4.5901	3.9837	3.5293	3.9105	4.4893	4.8151	5.1159
Nckap1	_SVLQPSQQK_.2	#N/A	6.0239	6.5379	3.3631	3.7577	5.8805	6.9810	7.0705	6.7783	6.6112	3.6372	6.3954	6.1991	5.7992	5.9582	5.6670	5.7191	5.8624	6.4997	3.8990	3.0785
Nckap1	_NAYHAVYK_.2	#N/A	4.3565	4.4634	3.8931	3.2277	3.0626	3.7490	5.1909	5.2771	4.2793	4.7838	4.4799	4.1547	4.3874	4.2431	3.7974	3.7186	4.7743	4.6336	3.3691	3.6084
Nckap1	_LVVENVDVLTQM[Oxidation (M)]R_.2	#N/A	4.3903	4.4296	5.0681	3.1939	4.8868	4.6168	3.3231	4.8874	4.8152	4.2009	3.3506	4.1558	4.0374	3.9649	3.9771	3.7530	4.1132	3.9946	4.2685	3.9186
Nckap1	_ASLSLADHR_.2	#N/A	3.5238	3.1196	3.9186	4.3071	3.7307	3.7640	3.3174	3.0827	3.2376	3.3344	4.2171	3.0091	3.4031	3.9057	3.3428	4.3867	3.6204	4.2128	3.4473	3.8727
Septin4	_PQS[Phospho (STY)]PDLC[Carbamidomethyl (C)]DDDVEFR_.2	#N/A	9.3938	9.8139	10.2308	9.5681	6.2433	9.6317	9.3123	7.6683	9.4487	9.8446	8.7135	8.9451	8.7376	9.9811	8.6534	9.1467	9.5969	9.0510	9.2332	9.3801
Septin4	_PRS[Phospho (STY)]PWGK_.2	P28661_S107_M1_Septin4	7.3098	7.8271	7.3515	7.7187	6.9031	7.3776	6.8726	6.8995	6.5795	7.4927	6.6002	7.3137	7.1469	7.4991	6.3680	7.0185	7.7293	6.8554	7.2183	7.1251
Septin4	_ESIPFAVIGSNTVVEAR_.2	#N/A	5.4834	5.9812	6.6704	6.0561	6.3018	6.1777	5.5449	5.6905	6.2305	6.4882	5.3104	5.1551	6.1341	6.0102	5.6537	5.0338	5.3614	5.4695	5.6204	6.3298
Septin4	_VARPQILEPRPQS[Phospho (STY)]PDLC[Carbamidomethyl (C)]DDDVEFR_.4	#N/A	7.0647	8.6668	6.6479	7.4388	4.5116	6.9421	8.4494	7.1284	6.4329	7.0386	6.0690	7.4120	7.5706	9.3883	6.2983	6.4453	6.6324	8.1520	7.4160	6.6888
Septin4	_PQILEPRPQS[Phospho (STY)]PDLC[Carbamidomethyl (C)]DDDVEFR_.3	#N/A	7.5026	8.6449	8.0664	7.9293	3.5717	7.8625	7.5178	6.5315	7.4685	7.4510	7.1010	7.2360	6.6862	9.2370	7.2074	6.9956	7.5973	7.9348	7.5546	7.4189
Septin4	_IYQFPDC[Carbamidomethyl (C)]DS[Phospho (STY)]DEDEDFKLQDQALK_.3	#N/A	3.1270	9.0629	3.6633	3.8725	3.9656	4.1897	3.5755	3.9374	3.6346	3.3334	4.0089	3.2644	3.7426	3.2864	3.1527	3.4719	3.4347	8.6118	4.0138	2.9637
Septin4	_IYQFPDC[Carbamidomethyl (C)]DS[Phospho (STY)]DEDEDFK_.2	#N/A	4.2637	8.6127	3.8003	3.3205	2.9698	6.1764	7.9907	4.4894	3.9985	4.0743	3.4771	4.2823	4.1640	9.9887	4.1037	3.6258	3.9866	8.6233	3.4619	3.1328
Septin4	_STLVNSLFLTDLYR_.2	#N/A	3.6315	4.0817	4.1678	4.0579	4.4260	3.8558	3.0710	3.6719	3.8136	4.3495	2.6703	3.6570	3.6522	4.2102	3.8513	3.7984	3.8695	3.8184	4.0622	4.0518
Septin4	_HFLEDS[Phospho (STY)]SDDAELSK_.2	P28661_S28_M1_Septin4	3.9808	5.9544	5.7098	5.9491	3.7645	5.7155	5.6546	4.2065	5.5194	5.9269	3.8056	5.7005	3.8377	6.2883	5.3031	5.3490	3.7037	5.3054	5.7885	5.4581
Septin4	_LDPY[Phospho (STY)]DSSEDDKEYVGFATLPNQVHR_.3	#N/A	9.6598	10.3077	9.2420	7.7493	3.8712	10.0009	9.3402	8.7640	9.2407	9.7870	3.8496	3.4747	7.6230	10.3410	8.9294	8.4905	9.8317	9.6812	9.4580	9.7354
Napb	_VAAYAAQLEQYQK_.2	#N/A	7.4960	7.6815	7.6985	7.5623	7.5894	7.7017	7.6946	7.4545	7.8945	7.7675	7.2574	7.5450	8.0120	8.1676	7.5405	7.5963	7.3947	8.1149	7.6394	7.4620
Napb	_NWSAAGNAFC[Carbamidomethyl (C)]QAAK_.2	#N/A	6.2459	5.8913	6.0419	5.6317	5.8789	6.2555	6.1720	5.8043	6.4976	6.2245	6.0442	6.1985	5.5517	6.1810	6.0943	6.1676	5.7219	5.7445	6.0762	6.0819
Napb	_KADPQEAINC[Carbamidomethyl (C)]LNAAIDIYTDM[Oxidation (M)]GR_.3	#N/A	5.3809	6.1264	5.5567	5.9562	4.7130	5.8780	5.9630	5.8717	5.6934	5.9669	5.7172	5.3558	5.9499	5.4479	5.1773	6.0471	5.8330	6.1747	6.1387	6.0443
Napb	_YEEM[Oxidation (M)]FPAFTDSR_.2	#N/A	8.4003	8.1672	8.3808	8.2293	8.0215	8.2170	8.1034	8.1743	8.2373	8.3076	8.3779	7.9765	8.0466	8.3772	8.0036	8.1214	8.0721	7.8574	8.0212	8.1480
Napb	_AALC[Carbamidomethyl (C)]HFIVDELNAK_.3	#N/A	5.8333	6.3970	5.6885	5.8420	6.1926	5.8729	5.9134	6.3341	5.3178	6.1998	5.3432	6.1415	6.2034	5.9934	5.9947	5.8685	5.9005	6.1600	5.7745	6.3582
Napb	_SIQGDGEGDGDLK_.2	#N/A	4.5293	7.7940	3.5647	3.1601	5.4672	7.2633	7.2051	4.2296	7.0102	7.6022	7.4411	5.0067	7.8498	7.1566	7.7407	7.6536	7.0731	4.7315	7.5768	7.4567

Napb	_HDSATSFVDAGNAYK_.2	#N/A	5.6615	5.6726	3.5308	5.0484	5.7359	5.9300	5.4893	5.8324	5.7041	5.5519	6.1352	5.3036	5.8472	5.8239	5.7649	5.0017	5.1071	5.2698	5.7632	5.0631
Napb	_LDQWLTTM[Oxidation (M)]LLR_.2	#N/A	8.1852	8.0160	7.9821	8.0807	8.1149	8.1718	7.8221	8.1792	7.7585	8.0549	7.9506	8.1023	7.7655	7.6430	7.8839	7.7696	7.7653	7.9271	8.2078	7.6884
Napb	_LLEAHEEQNSEAYTEAVK_.3	#N/A	6.5691	6.3146	5.5428	5.8752	5.8774	4.9301	5.2916	5.6317	5.1699	5.7901	5.8294	5.9049	6.1911	6.6653	4.3716	5.4774	5.5544	5.7374	3.9664	5.3258
Napb	_ASHS[Phospho (STY)]FLR_.2	P28663_S26_M1_Napb	4.0379	3.2812	3.5745	3.5463	4.2449	4.2782	4.3041	4.2636	4.2243	3.8485	3.7029	3.0073	4.3897	3.6126	2.8286	3.4000	4.6423	3.3886	3.6877	3.2898
Napb	_AIEIYEQVGANTM[Oxidation (M)]DNPLLK_.2	#N/A	4.7703	5.2491	5.1822	5.4465	4.6001	4.5798	5.1964	4.6611	5.2850	5.1909	4.5053	4.8829	5.1970	5.3681	4.1249	4.9314	5.2673	5.3143	4.6419	5.0128
Napb	_LHMQLQSK_.2	#N/A	4.2561	5.0344	6.3096	5.5878	4.1600	3.8249	5.4514	4.5227	3.1767	3.3952	5.3405	3.0700	3.3422	6.5227	5.0229	3.6663	3.5595	4.2737	3.9222	3.8118
Napb	_IEEAC[Carbamidomethyl (C)]EMYTR_.2	#N/A	3.5847	5.1153	6.3409	6.2480	2.9225	5.2430	5.9007	3.2851	3.4400	4.4706	4.7724	4.3075	3.6055	5.9389	4.8502	3.9296	3.8228	3.1415	3.6589	4.0751
Napb	_LLEAHEEQNSEAYTEAVKEFDSISR_.4	#N/A	4.2534	5.1894	4.5248	5.1441	3.4627	3.6207	4.2562	3.7660	4.3509	3.9257	4.1224	4.5281	4.3922	5.0990	4.1088	3.9497	3.7451	4.1793	4.6294	4.1976
Napb	_AIAHYEQSADYYKGEESNSSANK_.3	#N/A	7.1041	8.1213	6.4092	7.1981	6.3831	6.7396	7.2187	6.9847	7.2179	6.6326	6.7567	7.1111	7.3377	8.1660	6.3436	6.7884	6.6860	7.2942	7.0858	7.0932
Napb	_EAVQLMAEAEKR_.2	#N/A	3.4212	4.5816	5.4351	5.1360	4.2598	3.9643	4.2551	3.1216	2.4360	3.6275	4.2325	2.9702	3.4419	4.9732	3.2707	3.7661	3.6593	4.1739	3.8225	3.9116
Napb	_KLLEAHEEQNSEAYTEAVK_.3	#N/A	2.1011	4.4505	3.9060	3.2148	1.6502	3.1089	1.7597	3.0943	3.8928	4.1800	3.3714	2.0764	4.0582	1.2518	3.9979	3.7316	4.0924	3.0571	3.3561	3.6214
Napb	_AIAHYEQSADYYK_.3	#N/A	3.3440	4.4408	4.0983	4.1273	3.5510	3.5843	3.4971	3.2624	3.4173	3.1546	2.8960	3.7012	3.5828	4.0854	3.5225	4.2070	3.8001	4.0331	3.8387	4.0524
Marcksl1	_AAAT[Phospho (STY)]PESQEPQAK_.2	P28667_T148_M1_Marcksl1	11.0057	10.9895	11.0236	11.0161	10.7180	11.0390	10.4848	11.0480	10.8234	10.9197	10.7438	11.0448	11.7055	11.5419	11.5143	11.2521	11.2851	11.1138	11.1428	11.2120
Marcksl1	_GDVTAEEAAGAS[Phospho (STY)]PAK_.2	P28667_S22_M1_Marcksl1	10.4912	10.8815	11.0949	11.2137	10.2952	11.1366	10.7138	10.8417	10.0880	11.7654	11.4061	11.2238	11.5305	11.9932	11.6198	11.7441	11.6224	11.3573	11.7578	11.7265
Marcksl1	_LSGLS[Phospho (STY)]FKR_.2	#N/A	11.3225	11.6226	11.1186	11.0626	10.2218	10.7173	10.8547	11.6279	10.0157	11.2295	10.6817	10.9526	11.7747	11.8605	10.7705	10.6697	11.0616	11.0249	11.0408	10.7318
Marcksl1	_GEVAPKET[Phospho (STY)]PK_.2	#N/A	10.5778	10.5453	10.9825	10.5055	10.3070	10.7908	10.3131	10.6583	10.5627	10.7614	10.6390	10.8067	11.0756	11.2205	11.3148	10.8882	10.9975	10.8660	11.0198	10.9606
Marcksl1	_LSGLS[Phospho (STY)]FK_.2	#N/A	3.9243	4.0785	4.8624	3.7651	3.2620	4.7695	4.2791	3.6247	3.7796	6.1247	3.2582	4.1714	3.9450	4.4743	3.8562	4.2692	4.1624	3.6708	3.3194	5.9153
Marcksl1	_GEVAPKET[Phospho (STY)]PKK_.2	#N/A	6.1256	6.3149	6.4009	5.9725	5.9971	6.4837	5.8451	6.2768	5.9440	6.4760	5.9562	6.0903	7.0134	6.9430	7.3579	6.6991	5.8982	6.1880	6.2841	6.3221
Kif5c	_SVELDS[Phospho (STY)]DDGGGSAAQK_.2	P28738_S820_M1_Kif5c	7.4256	7.4553	7.1823	7.2309	7.1013	7.1827	7.0020	7.0304	6.9704	7.4520	6.3240	6.5096	7.1933	7.4049	6.1626	6.4816	7.2175	6.7136	6.5974	6.6579
Kif5c	_LYLVDLAGSEK_.2	#N/A	6.4919	6.4326	6.5440	6.6524	6.4661	6.5635	6.3478	6.2403	6.8374	6.7848	5.9323	6.0196	6.1917	6.7367	5.6297	6.1195	6.6757	6.2416	6.2917	6.1272
Kif5c	_ILQDSLGGNC[Carbamidomethyl (C)]R_.2	#N/A	3.2381	5.9273	6.8538	5.8692	6.1713	6.5435	6.2214	6.4566	6.2809	5.8542	6.5321	6.2802	5.2000	3.1753	6.3473	5.8708	6.2991	5.8995	5.3650	6.1396
Kif5c	_SLEPC[Carbamidomethyl (C)]DNT[Phospho (STY)]PIIDNITPVVDGISAEK_.3	P28738_T396_M1_Kif5c	5.7882	6.0364	5.9423	5.5637	4.0728	5.1036	5.5067	4.0915	5.1723	5.8755	4.1631	5.3589	5.0930	6.6431	3.3548	4.2329	3.5888	5.6037	3.8597	4.5579
Kif5c	_SLSALGNVISALAEGTK_.2	#N/A	5.1811	4.0414	4.0008	4.8901	5.1000	3.4237	4.1393	4.8930	4.8629	4.2155	4.9836	4.7986	4.6358	4.5652	4.3000	4.7312	3.4638	4.4381	4.4790	4.9167
Kif5c	_ISFLENNLEQLTK_.2	#N/A	4.4414	4.2469	3.9780	4.8507	3.1475	3.9326	4.4719	4.5440	3.8821	4.4382	4.5142	4.1046	3.9862	4.4889	3.2210	4.2037	4.2036	3.6001	3.2782	4.1007
Kif5c	_TTIVIC[Carbamidomethyl (C)]C[Carbamidomethyl (C)]SPSVFNEAETK_.2	#N/A	4.0247	3.2944	4.0135	3.0929	4.2316	3.5311	4.3173	4.2504	4.2375	3.8353	3.7162	3.0205	4.4030	3.5993	3.1681	3.3868	3.7476	3.2128	3.7009	3.3718
Kif5c	_HVAVTNMNEHSSR_.3	#N/A	4.4778	3.5250	3.5133	3.2116	3.8155	4.0396	3.7255	4.1782	3.7846	3.1833	4.1590	3.4144	4.4985	4.1736	3.3027	3.3219	3.2150	3.1173	4.2667	3.4673
Kif2a	_GSLDYRPLTTADPIDEHR_.3	#N/A	6.9551	6.3986	6.7655	6.8596	6.2399	6.2240	6.4449	6.5722	6.1861	6.7753	6.2098	6.0866	6.7985	6.1127	6.3840	6.0526	6.4968	6.1810	5.9591	6.1523
Kif2a	_PSQLPEQSSSAQQNGSVSDIS[Phospho (STY)]PVQAAK_.3	P28740_S139_M1_Kif2a	6.6359	3.8145	5.7052	5.6392	3.7115	5.8323	5.8577	3.7303	5.2180	5.7385	4.2362	5.8200	6.1201	7.2939	3.3619	5.4181	5.8013	6.1348	5.9754	5.7814
Kif2a	_YLENQTFR_.2	#N/A	5.7944	5.6743	4.0371	5.9697	5.8377	5.5824	5.1213	5.7387	5.5030	5.5899	5.6034	5.8768	3.7570	5.9324	6.1020	5.6065	5.6277	5.5215	4.8801	5.4807
Kif2a	_IDILTELR_.2	#N/A	3.4205	3.6373	3.9568	4.6527	4.4809	4.7633	4.5655	3.0105	3.6962	4.2630	4.5633	4.5149	3.4412	2.9253	4.2053	3.4184	3.3672	4.7737	4.6934	4.7164
Kif2a	_LIDIGNSC[Carbamidomethyl (C)]R_.2	#N/A	4.2846	3.7196	3.3187	3.4062	3.6209	4.5592	5.6754	4.9466	4.6973	4.1572	4.7798	4.4970	4.3039	3.3057	4.2584	4.0816	4.9660	4.2863	4.1293	4.4651
Kif2a	_DFRGS[Phospho (STY)]LDYRPLTTADPIDEHR_.4	P28740_S206_M1_Kif2a	3.4642	3.9445	4.4120	4.2096	4.3027	3.0260	3.2383	3.6003	3.2974	3.6705	3.6718	3.6706	3.4055	4.4501	2.8155	4.4038	3.0975	4.0519	4.3510	4.1274
Kif2a	_AVFQESIR_.2	#N/A	4.2164	3.7864	3.2519	3.4730	3.5541	4.4308	3.9870	3.9168	4.5999	4.0011	3.5503	3.6759	4.2371	6.1133	3.5642	3.3549	4.4545	3.3787	2.9308	3.2059
Kif3a	_S[Phospho (STY)]AKPETVIDSLLQ_.2	P28741_S689_M1_Kif3a	5.6694	6.6849	6.8908	4.3267	6.0951	3.7837	3.2978	6.4378	3.2180	3.3540	6.4572	3.5018	6.5764	6.8790	6.1630	4.4064	3.6007	6.5121	6.0142	6.4675
Kif3a	_KQT[Phospho (STY)]PVPDKK_.2	P28741_T635_M1_Kif3a	4.7891	3.7454	2.7590	3.7726	3.6949	3.0271	1.9812	3.9955	3.5940	3.6700	3.0350	3.6789	1.9246	3.0408	3.5672	3.0574	4.4514	2.7482	3.0304	2.6581
Prkcd	_LLAEALNQVTQR_.2	#N/A	3.4201	3.9024	3.9564	5.2060	4.7849	4.3091	4.3127	5.5514	4.5129	4.4863	4.3934	3.5823	3.4408	4.5988	4.5198	3.7650	4.2130	4.3861	4.7021	4.2256
Prkcd	_SPSDYSNFDPEFLNEKPQLS[Phospho (STY)]FSDK_.3	P28867_S643_M1_Prkcd	4.5589	3.4439	3.5944	3.1304	3.8967	2.8329	3.6444	4.2593	4.4142	3.2644	3.8929	3.3333	4.5797	3.5815	3.2216	3.4030	3.2962	3.0362	4.1856	3.5485
Pabpc1	_FSPAGPILSIR_.2	#N/A	7.3068	7.4983	7.2078	7.5532	7.4610	7.5357	7.4194	7.3828	7.2008	7.6639	7.4136	7.2218	7.5561	7.7611	7.3049	7.0740	7.4701	7.3258	7.1517	7.2227
Pabpc1	_SLGYAYVNFQQPADAER_.2	#N/A	3.4383	5.8780	5.9817	5.5277	3.6453	5.9082	5.4082	5.8126	6.1788	5.8948	5.7459	6.2396	6.4122	5.9810	3.4282	5.7667	6.0534	5.6042	5.8686	5.6323
Pabpc1	_ALYDTFSAFGNILSC[Carbamidomethyl (C)]K_.2	#N/A	3.3278	4.4184	4.7357	5.1454	4.2762	3.8154	3.2333	4.5432	4.7145	5.0511	2.9122	4.2747	3.5990	4.1016	4.5412	3.9577	4.2989	4.3050	3.6654	4.0686
Pabpc1	_SKVDEAVAVLQAHQAK_.3	#N/A	5.4186	3.2419	3.7501	4.4293	5.7668	3.8863	3.1951	5.8485	3.1154	6.0641	4.9921	4.5285	3.2808	4.4548	4.8278	5.1207	3.4981	4.9183	3.9836	5.1810
Pabpc1	_NLDDGIDDERLR_.3	#N/A	3.9175	4.3978	3.9587	3.1621	3.2552	3.4793	4.2858	3.1470	4.3449	4.1238	3.2185	4.1239	4.4530	3.9968	3.8630	4.2624	4.1450	3.0044	3.3035	3.6740
Gnb4	_SGVLAGHDNR_.2	#N/A	5.3318	6.0804	3.8012	4.4245	5.2092	10.6192	5.9928	10.9788	6.0500	10.5288	10.6379	3.1265	3.2856	10.4611	5.5971	10.2075	10.5212	10.6128	10.3377	10.4335
Vcam1	_QEFSS[Phospho (STY)]EEM[Oxidation (M)]TK_.2	#N/A	8.5173	8.6901	8.1061	7.9527	8.1211	8.5244	8.2510	8.6188	8.3264	8.6294	8.2946	8.2440	8.8065	9.0136	8.1357	8.2022	8.7472	8.3043	8.2483	3.1796
Nedd8	_EIEIDIEPTDKVER_.2	#N/A	6.5182	6.5414	7.0358	6.8359	5.8726	6.1576	6.2478	6.4639	6.9224	6.6321	5.8102	6.1530	6.5885	6.5798	6.7601	6.6629	6.5330	6.4456	6.5728	6.7762
Ahsg	_VM[Oxidation (M)]HTQC[Carbamidomethyl (C)]HSTPDS[Phospho (STY)]AEDVR_.3	#N/A	3.9356	4.0197	4.3242	3.5764	3.9788	4.3835	3.7961	4.3707	3.4164	3.5103	3.1465	3.8509	4.7193	3.5989	3.9951	3.5629	4.5381	3.6704	4.0748	4.0324
Ahsg	_VM[Oxidation (M)]HTQC[Carbamidomethyl (C)]HSTPDS[Phospho (STY)]AEDVRK_.4	#N/A	4.1467	3.9892	4.0393	3.6966	3.6334	3.7812	3.5965	3.8693	3.9461	3.9402	3.4536	3.5936	4.1115	4.4296	3.4858	3.7095	4.0403	3.5835	3.5515	3.9681
Oat	_LPSDVVTSVR_.2	#N/A	6.6953	6.4480	6.3134	6.9942	6.6391	6.4454	6.2736	6.5173	6.0130	6.2966	6.4717	6.2237	6.2130	6.2549	6.8378	6.5429	6.4033	6.4403	6.2613	6.2765
Oat	_IAIAALEVLEEENLAENADK_.2	#N/A	5.8349	5.8197	5.9018	5.3553	4.6014	4.3076	5.6186	5.8892	5.3304	5.6673	5.3390	5.1886	5.5384	6.1105	5.2148	4.5458	5.3240	5.4146	4.3858	5.2152
Oat	_IVFADGNFWGR_.2	#N/A	5.0307	4.6930	5.8908	4.9284	5.6087	5.1031	4.9398	4.7240	4.8164	5.2818	4.7726	5.0680	3.1588	5.3946	4.5200	5.3338	5.5473	5.2443	5.1292	4.6709
Oat	_AFYNNVLGEYEEYITK_.2	#N/A	4.4998	4.4844	4.7513	4.9491	4.9544	4.4089	4.7900	4.7523	4.3464	4.7312	4.1301	4.6221	4.7185	5.0036	5.1132	4.7400	4.7768	4.3069	3.2209	4.9207
Oat	_KTEQGPPSSEYIFER_.3	#N/A	3.8810	3.4381	3.4176	3.7032	4.0879	5.6591	4.3660	4.1067	3.8038	3.1641	4.1782	5.4285	3.9119	5.2146	5.6522	5.2524	3.6039	5.5221	6.2123	3.1329
Ckmt1	_LYPPSAEYPDLR_.2	#N/A	10.4130	10.3314	10.4588	10.4752	10.4679	10.4385	10.4705	10.5372	10.2796	10.4452	10.6666	10.6123	10.5288	10.6592	10.6365	10.5448	10.5733	10.3859	10.3398	10.5769
Ckmt1	_VVVDALSGLK_.2	#N/A	9.9398	10.0442	10.1570	10.2730	10.0562	9.8653	10.0720	9.7896	9.8880	9.9798	10.0545	10.3955	10.2646	10.3068	10.2668	10.1219	10.0845	10.1641	10.0890	10.1613
Ckmt1	_TTPTGWTLDQC[Carbamidomethyl (C)]IQTGVDNPGHPFIK_.3	#N/A	8.6196	8.4728	8.8411	8.7891	8.2779	8.3114	8.3635	8.5274	8.5107	8.6713	8.6029	8.7243	9.0827	8.8021	8.8937	8.7362	8.5852	8.3554	8.5644	8.6007
Ckmt1	_GWEFM[Oxidation (M)]WNER_.2	#N/A	8.4930	8.5146	8.7690	8.3849	8.7291	8.3628	8.2347	8.4770	8.3936	8.6466	8.4989	8.4269	8.8984	8.3425	8.4425	8.6204	8.6148	8.2804	8.4002	8.7612
Ckmt1	_LYPPSAEYPDLRK_.3	#N/A	7.3901	7.7007	7.6211	7.9156	8.0999	7.8090	8.0494	7.7619	7.8687	7.9546	8.1536	8.0430	8.3886	8.0612	8.5924	8.5642	7.3623	8.0425	8.2884	8.5741
Ckmt1	_TVGM[Oxidation (M)]VAGDEETYEVFAELFDPVIQER_.3	#N/A	7.7028	7.8791	8.2147	7.6392	7.2087	7.6493	7.2330	8.2234	6.9031	7.8975	7.4065	7.6630	8.3459	9.2808	7.9995	8.2275	8.1766	7.4458	7.3921	7.7491
Ckmt1	_HTTDLDASK_.2	#N/A	7.7196	7.7652	7.4477	8.0305	7.7233	7.2483	7.6420	7.5494	7.4871	7.6049	7.9700	7.6747	7.4483	7.8858	8.4858	8.0044	7.5100	7.3199	7.6435	7.9491
Ckmt1	_GIWHNNEK_.2	#N/A	7.4907	7.3975	7.6971	7.4781	7.7004	7.8943	7.6427	7.4096	7.5177	7.6792	7.8268	7.8139	7.6601	7.9609	7.8980	7.7341	7.5587	7.7309	7.5856	7.6831
Ckmt1	_HNGYDPR_.2	#N/A	5.3934	6.0824	6.4020	5.4313	5.7267	5.7503	6.2251	5.9112	6.4796	6.1138	6.3640	5.3247	4.9922	6.0918	6.6053	5.8063	5.6367	6.1573	5.4584	6.0789
Ckmt1	_HTTDLDAS[Phospho (STY)]KIR_.2	P30275_S148_M1_Ckmt1	5.4969	5.1527	4.3719	4.7062	5.2791	5.8778	4.9855	5.4441	5.4007	4.7042	4.9626	5.3957	6.1281	6.0811	5.1453	5.4942	5.8984	5.2442	5.3677	5.7645
Ckmt1	_GLSLPPAC[Carbamidomethyl (C)]TR_.2	#N/A	8.6508	7.5668	9.2466	10.3889	9.3217	7.4609	6.9039	6.8730	6.2003	7.6460	8.6233	8.6622	4.3056	9.6810	8.5510	7.8460	8.3820	9.2221	8.4746	9.2179
Ckmt1	_GLKEVEK_.2	#N/A	6.9148	7.0080	4.2652	6.6572	6.9033	7.1804	7.0607	6.8052	6.5907	6.6226	6.8947	7.2038	7.3515	4.3033	7.2960	7.1366	4.4516	6.8542	6.9549	7.1428
Ckmt1	_HNNC[Carbamidomethyl (C)]M[Oxidation (M)]ASHLTPAVYAR_.3	#N/A	7.9790	7.7520	8.1688	8.1811	8.0529	7.7051	7.9640	8.1291	7.8678	8.1463	7.9102	8.0628	8.5685	8.3377	8.6555	8.0952	7.8863	7.8346	7.9440	8.1609
Ckmt1	_GTGGVDTAATGSVFDISNLDR_.2	#N/A	6.9979	7.7756	7.2129	6.7562	6.7527	7.3613	6.4695	7.1879	7.1248	6.9281	7.3179	7.3477	7.8536	8.3947	7.4212	7.2379	7.2207	7.5549	7.1273	7.2600
Ckmt1	_GWEFMWNERLGYILTC[Carbamidomethyl (C)]PSNLGTGLR_.3	#N/A	4.5885	5.3429	6.0167	4.3998	4.4313	4.8216	4.9191	5.1767	4.0940	4.9373	4.4262	4.6154	3.2153	5.6489	5.0222	4.6823	5.3871	5.3359	4.8971	4.1630
Ckmt1	_SFLIWVNEEDHTR_.2	#N/A	6.8331	6.7440	7.5026	6.9654	6.6541	6.7630	6.6313	6.8752	6.6558	5.9953	7.0385	6.9923	7.1391	7.2494	7.1191	6.9433	7.0245	6.2641	6.9324	7.0502
Ckmt1	_LGYILTC[Carbamidomethyl (C)]PSNLGTGLR_.2	#N/A	6.9105	6.7536	7.1298	6.9082	6.9565	6.6461	6.7238	7.3763	6.5862	7.0298	6.8456	7.2936	7.1149	7.4052	6.8181	6.9076	6.9662	6.4971	6.5987	7.0591
Ckmt1	_VVVDALSGLKGDLAGR_.2	#N/A	3.3876	3.3583	3.6791	3.7549	4.3042	2.9285	2.9913	2.9121	3.4004	3.7992	4.1399	2.9264	4.8156	3.8818	3.3884	4.7882	4.5776	3.7041	4.4225	3.8874
Ckmt1	_SGYFDER_.2	#N/A	4.3445	4.4976	4.3682	5.7589	4.7844	5.2211	5.5961	3.8219	5.2824	5.6386	5.6356	5.1222	5.5586	5.3759	5.4580	5.7087	4.0289	5.3980	5.3639	5.9557
Ckmt1	_IRS[Phospho (STY)]GYFDER_.2	P30275_S152_M1_Ckmt1	7.9209	7.6426	4.0194	7.9366	8.0257	7.8516	7.4134	7.8843	3.2785	7.9205	7.9371	7.9994	8.4623	8.1825	7.8918	8.1201	8.2901	7.6600	8.0295	8.0856
Ckmt1	_RGTGGVDTAATGSVFDISNLDR_.3	#N/A	6.6005	6.5628	6.0950	6.4956	6.5867	6.3069	6.4933	6.4025	5.9784	6.3514	6.6632	6.8279	6.4463	7.1040	6.1251	6.1314	7.0495	6.3948	6.1974	6.6555
Nktr	_TC[Carbamidomethyl (C)]DEGSS[Phospho (STY)]PSKPK_.2	P30415_S1060_M1_Nktr	4.2475	4.5724	3.7841	3.3367	2.9536	3.6539	4.1112	4.4732	4.1703	4.2985	3.0438	4.2986	4.2784	3.8221	3.6884	2.9362	3.9704	3.1790	3.4781	3.4994
Fkbp4	_TQLAVC[Carbamidomethyl (C)]QQR_.2	#N/A	3.1547	4.1644	4.1921	5.8485	3.3616	3.2459	3.0184	4.9324	5.1751	3.8904	3.4519	5.0171	3.1856	4.2302	5.8173	4.0176	4.3784	4.2719	5.4150	4.8730
Sstr1	_AYS[Phospho (STY)]VEDFQPENLESGGVFR_.2	P30873_S364_M1_Sstr1	4.3878	5.7199	3.4233	4.8457	3.7255	4.2594	4.3229	3.9354	4.4251	5.1186	3.7217	3.5044	4.4085	3.4104	3.3927	3.2319	3.1250	4.9646	5.0659	3.3774
Ctnnd1	_VGGSS[Phospho (STY)]VDLHR_.2	P30999_S269_M1_Ctnnd1	6.3798	5.0177	6.2993	5.5295	5.1651	4.8524	5.7398	4.4825	4.9081	6.4004	5.7032	5.3643	4.7903	4.9212	3.9427	5.0388	5.3596	5.6016	6.2379	4.9749
Ctnnd1	_HYEDGYPGGSDNYGS[Phospho (STY)]LSR_.3	#N/A	3.8237	4.0674	3.6356	3.5695	3.6271	3.8867	3.9628	3.5868	5.4938	7.5389	6.2279	6.3220	6.6841	3.6737	3.5400	5.7496	5.2397	6.5109	3.7109	5.3472
Ctnnd1	_GSLAS[Phospho (STY)]LDSLRK_.2	P30999_S349_M1_Ctnnd1	8.4908	8.7800	8.7926	8.2545	7.3893	8.1296	8.1324	7.8780	7.4158	8.0112	7.8294	7.6079	7.9333	8.8628	7.3891	7.8492	8.1321	7.9652	8.2573	7.9563
Ctnnd1	_SQSSHSY[Phospho (STY)]DDSTLPLIDR_.3	P30999_Y865_M1_Ctnnd1	5.7315	5.8583	4.1165	4.1292	3.2860	6.6587	6.5146	3.3047	3.6869	4.3905	3.1656	6.3288	6.0496	7.7310	5.9461	3.9420	4.3028	7.0776	7.2426	4.1880
Ctnnd1	_GS[Phospho (STY)]LASLDSLR_.2	#N/A	3.8956	5.6021	5.1189	3.8253	3.6209	6.2520	5.3252	4.0561	3.7128	3.9667	4.5698	4.0087	3.8425	4.7493	3.4785	3.8361	3.5533	5.4990	3.7127	3.6732
Ctnnd1	S[Phospho (STY)]M[Oxidation (M)]GYDDLDYGM[Oxidation (M)]M[Oxidation (M)]SDYGTAR_.2	P30999_S288_M1_Ctnnd1	3.1036	5.5386	4.8080	4.4806	3.3105	3.3439	3.7376	3.3293	6.0017	4.4150	3.1364	3.9416	3.1345	4.1791	3.7630	3.9665	4.9624	5.4788	5.1338	5.4451
Ctnnd1	_TLDRS[Phospho (STY)]GDLGDMEPLK_.3	P30999_S920_M1_Ctnnd1	4.3712	3.6316	3.4066	3.3182	3.7089	3.9330	3.8322	4.0715	3.8913	4.5775	4.2657	3.5211	3.9993	3.3937	3.4094	3.2152	3.1084	3.2239	4.3734	3.2204
Prkar2b	_AATITATSPGALWGLDR_.2	#N/A	7.1295	6.8124	7.2094	7.4204	7.0377	7.0304	6.8360	6.9097	7.2142	7.5010	7.1107	6.7841	6.9645	7.1222	7.2675	7.4610	7.0927	7.0731	7.1394	7.1146
Prkar2b	_TWGDAGAAAGGGIPSK_.2	#N/A	6.3804	6.8159	7.2343	6.3909	6.5980	6.8524	6.4147	6.3137	6.3645	6.4032	6.6593	6.2618	6.9600	6.9645	6.7479	6.8743	6.5692	6.1794	6.6394	6.4169
Prkar2b	_C[Carbamidomethyl (C)]VGNYDNR_.2	#N/A	4.7635	5.4550	5.3112	5.8556	5.5937	5.3167	5.2342	5.3776	5.2918	5.7251	5.6031	5.1243	4.8337	5.9334	5.6778	5.7381	5.4422	5.3852	5.8867	5.6616
Prkar2b	_ASVC[Carbamidomethyl (C)]AEAYNPDEEEDDAESR_.2	#N/A	5.2168	5.6726	5.9285	4.9841	3.8096	4.5387	4.9511	4.5574	5.5331	5.5799	4.5232	5.1299	3.9520	6.0780	5.1254	5.5783	5.8218	4.6682	5.6710	5.0502
Prkar2b	_AASAHAIGTVK_.2	#N/A	5.0336	5.2845	4.6160	4.8518	4.8338	5.0630	5.1744	3.9348	5.0780	4.9989	4.6624	5.3504	4.2552	4.8752	5.1727	5.1233	4.9705	4.7824	4.8274	5.1145
Prkar2b	_VYNDGEQIIAQGDLADSFFIVESGEVK_.3	#N/A	4.4627	5.3446	5.2644	4.5620	4.5253	4.9095	5.0119	5.9947	4.0988	5.3785	4.1352	5.4330	6.0632	6.5889	4.8559	5.6736	4.4952	4.7372	4.1645	5.3608
Prkar2b	_LQQENER_.2	#N/A	3.9819	6.0393	5.4089	5.1441	4.7956	5.7652	5.6462	4.9119	5.3318	5.3966	5.3852	5.2128	4.3886	5.8463	3.7986	5.1135	5.6364	3.0688	4.7882	6.1349
Prkar2b	_GSFGELALM[Oxidation (M)]YNTPR_.2	#N/A	5.7710	5.3466	6.1731	5.9239	5.4884	5.5080	5.5660	5.7204	5.6632	5.6953	5.5090	5.1140	6.0252	5.8502	5.9327	5.7898	5.3531	5.4853	5.4611	5.2109
Prkar2b	_VVDVIGTK_.2	#N/A	3.3525	3.1494	3.8888	3.2528	4.1911	4.1029	4.6344	3.0529	3.7727	3.5589	3.7985	3.7900	3.5171	3.8759	5.4589	4.9458	3.5906	4.8610	5.1299	4.6521
Prkar2b	_EGEHVIDQGDDGDNFYVIDR_.3	#N/A	7.6564	7.4710	7.5829	7.7355	7.3014	7.4717	7.3843	7.5885	7.6416	7.6541	7.8031	7.4381	7.9106	8.0272	7.0203	7.5428	7.7377	7.6508	7.3375	7.7306
Prkar2b	_GTFDIYVK_.2	#N/A	4.0642	4.5445	2.9485	3.3088	3.4019	3.7269	1.6782	4.5011	4.7351	4.2705	3.0718	3.8280	4.3063	2.3239	3.5124	4.4091	3.9983	3.6645	3.1355	3.5273
Prkar2b	_GVNFAEEPM[Oxidation (M)]RS[Phospho (STY)]DSENGEEEEAAEAGAFNAPVINR_.4	P31324_S83_M1_Prkar2b	3.8600	5.1447	4.4262	3.9805	3.6680	3.7778	4.3586	3.7383	4.3017	3.7874	3.8843	3.6127	3.4945	5.2461	4.4748	3.9809	3.7920	7.3276	3.8104	3.6548
Prkar2b	_HQPADLLEFALQHFTR_.3	#N/A	4.4687	3.7267	3.5042	3.2207	2.8113	4.0305	3.7346	4.1691	3.7937	3.1742	3.7688	3.4235	4.4894	3.4913	3.3118	3.0923	3.2059	3.9532	3.8925	3.4583
Prkar2b	_NLDPEQM[Oxidation (M)]SQVLDAMFEK_.2	#N/A	3.2219	3.2801	3.7582	5.9932	4.0604	4.2845	3.4807	3.8426	3.5397	3.4282	3.9141	3.1696	3.6478	3.1916	3.0578	3.5668	3.3398	3.8096	4.1087	4.3697
Prkar2b	_RAS[Phospho (STY)]VC[Carbamidomethyl (C)]AEAYNPDEEEDDAESR_.3	P31324_S112_M1_Prkar2b	3.6111	4.5171	3.8313	3.4494	3.8180	3.8513	4.4332	4.4963	3.1503	3.4217	4.1298	3.4341	3.3158	6.0791	3.2555	2.9732	5.3331	4.6286	5.4323	3.7854
Prkar2b	_C[Carbamidomethyl (C)]LAMDVQAFER_.2	#N/A	3.1431	4.1760	4.1805	4.4411	3.3500	3.3834	3.6981	3.3688	3.6183	4.4545	3.0969	3.9021	3.7837	4.2186	3.7235	4.0060	4.3668	4.2835	3.0817	4.2534
Pou3f2	_WLEEADSSSGS[Phospho (STY)]PTSIDK_.2	P31360_S343_M1_Pou3f2	6.4261	4.2604	4.1915	4.2685	3.1749	5.9078	5.6252	5.4164	6.1342	6.0797	5.3964	5.5276	4.2306	6.4278	3.8577	5.8637	6.3398	4.1362	3.3414	4.0401
Pou3f3;Pou3f4	_WLEEADSSTGS[Phospho (STY)]PTSIDK_.2	P31361_S390_M1_Pou3f3;Pou3f4	6.5512	6.6498	6.9883	6.9649	6.1509	6.3990	3.2254	3.7281	6.2162	6.8747	6.2149	6.2694	6.3399	6.8940	6.0512	6.9062	3.2254	3.9353	6.8925	6.6162
Slc6a1	_QC[Carbamidomethyl (C)]DNPWNTDR_.2	#N/A	7.6990	7.2195	7.5449	7.7325	7.5624	7.3309	7.6416	7.4415	7.3900	7.4976	7.4841	7.1637	7.6249	7.0205	6.9277	6.7181	7.7914	7.2893	7.4259	7.1007
Slc6a1	_AGDLPDRDTWK_.2	#N/A	6.1851	3.8155	4.5410	5.4400	4.7624	5.5235	4.7003	5.5556	4.7541	5.7117	5.2695	5.7583	5.2091	5.2699	2.9445	3.6801	5.5466	5.1989	4.7858	5.1654
Slc6a1	_VADGQIS[Phospho (STY)]TEVSEAPVASDKPK_.3	#N/A	7.2976	6.1629	5.7337	7.4171	4.8191	7.2252	5.6799	6.2516	7.0596	5.7767	6.5584	6.6787	7.1389	5.2109	5.6171	5.2875	6.9860	7.3932	7.0895	5.6151
Slc6a11	_GS[Phospho (STY)]ETLGGGGGGAAGTR_.2	#N/A	7.7554	7.4242	5.8515	6.0671	6.9772	7.6671	7.7444	7.3635	7.8672	7.5190	5.9048	6.9672	7.1350	7.0256	5.6731	7.1182	7.5205	5.8638	7.1471	7.2404
Slc6a11	_LTVPSADLK_.2	#N/A	7.5581	7.1110	7.0279	6.8662	7.3363	7.2029	7.4819	7.3275	7.8508	6.9714	7.2728	7.3034	7.6676	6.4481	7.1280	7.1163	7.1134	7.4801	7.2817	7.2365
Slc6a11	_AAEEARGS[Phospho (STY)]ETLGGGGGGAAGTR_.3	#N/A	3.8502	3.5168	3.5513	4.0855	5.7070	7.1140	3.5769	4.3422	6.5769	4.0876	4.2009	3.4063	3.7144	3.6772	3.2946	3.3599	3.6166	3.9699	3.8685	3.5121
Slc6a11	_VKGDGTISAITEK_.3	#N/A	4.4907	3.4701	3.3855	3.7353	3.8284	4.0525	3.7127	4.0746	3.7718	3.1962	4.1461	3.1963	3.8798	3.4236	3.2899	3.3347	3.5719	3.5776	3.8766	3.1009
Akt1	_SGS[Phospho (STY)]PSDNSGAEEM[Oxidation (M)]EVSLAKPK_.3	#N/A	3.7513	4.0502	4.0409	3.9064	3.4224	3.7100	3.7287	6.9273	3.7878	7.6944	3.5838	7.4410	4.0346	7.0740	3.7731	3.8526	3.8463	7.6320	3.6855	4.3295
Dbi	_AKWDSWNK_.2	#N/A	7.2042	8.1297	7.8892	8.0064	8.0244	8.1967	8.1222	8.2005	8.2476	8.0945	7.6353	7.9438	8.4174	8.2033	8.6857	8.0086	7.7344	7.4922	7.9568	8.0174
Dbi	_TYVEKVDELKK_.3	#N/A	5.6553	6.0376	6.3223	6.5364	5.5692	6.2221	6.5060	5.8482	6.1609	6.4748	5.4309	6.0812	6.8263	5.7377	7.1385	6.2501	6.1507	5.7452	6.5882	6.6616
Dbi	_[Acetyl (Protein N-term)]SQAEFDKAAEEVKR_.3	#N/A	7.5324	7.6531	7.4594	7.6732	7.7265	7.5582	7.4903	7.9792	7.8933	7.7884	7.2971	7.7120	8.2923	7.9688	8.0109	7.8753	7.5014	7.4961	7.6903	7.5918
Dbi	_TQPTDEEM[Oxidation (M)]LFIYSHFK_.3	#N/A	7.0358	7.0247	7.2103	7.2219	7.0044	7.1977	7.0317	6.4727	7.4093	7.4795	7.1669	7.1106	7.0001	6.9263	7.7634	7.5031	7.0930	6.7682	7.7547	7.4932
Dbi	_QATVGDVNTDRPGLLDLK_.3	#N/A	8.1757	8.0198	8.2930	8.2156	7.9568	8.1360	8.1530	8.2385	8.3792	8.3694	7.9616	8.1406	8.4973	8.3418	8.9131	8.6764	8.0869	8.0529	8.4223	8.0354
Dbi	_[Acetyl (Protein N-term)]SQAEFDKAAEEVK_.2	#N/A	3.3821	4.5872	3.8065	3.0005	4.1966	3.7548	4.1661	5.3219	3.3793	4.2971	2.8142	2.9880	3.5448	4.5952	4.0780	4.6347	3.7621	3.3399	4.0047	4.1067
Dbi	_TYVEKVDELK_.2	#N/A	7.9688	8.3357	8.1287	8.2628	8.3361	8.2522	8.1943	8.5064	8.3547	8.2354	7.8857	8.3376	8.7841	8.3149	8.8230	8.4703	8.0002	8.2238	8.2136	8.1690
Map2k1	_DVKPSNILVNSR_.2	#N/A	8.3364	8.3062	8.5213	8.4680	8.4567	8.3383	8.2714	8.7369	8.1905	8.4821	8.1456	8.4185	8.3111	8.5955	8.7067	8.1854	8.4092	7.8568	8.1446	8.0343
Map2k1	_KLEELELDEQQR_.3	#N/A	6.4143	6.7100	7.4774	7.1277	6.1054	7.0305	6.8486	6.2364	7.0632	7.3923	6.6177	6.8354	6.6288	6.8842	6.8486	7.3219	6.2960	7.0181	7.2574	6.9380
Map2k1	_IPEQILGK_.2	#N/A	4.2082	3.9268	3.1548	3.2902	5.1174	4.6277	5.0760	3.8197	3.5839	4.9421	5.1307	4.8581	2.2775	4.6825	4.6645	5.3378	4.2936	4.1444	4.3421	4.2707
Map2k1	_LEAFLTQK_.2	#N/A	3.0844	4.3926	2.6828	3.8677	4.7095	5.3264	5.8938	3.5221	3.6770	3.3547	5.7836	3.9011	3.8425	4.5530	4.0139	4.0287	4.0598	4.9939	4.1634	4.3121
Map2k1	_ISELGAGNGGVVFK_.2	#N/A	5.9567	5.7631	3.5265	3.5943	5.0582	5.9527	5.4420	5.4282	3.9128	6.0613	5.1662	5.8449	4.0208	5.5838	4.3020	3.3520	3.7128	4.9192	3.7356	5.2189
Map2k1	_LPSGVFSLEFQDFVNK_.2	#N/A	4.0225	3.2965	3.5591	3.5617	4.8368	4.1816	4.3195	4.2482	4.4837	3.8331	5.0689	4.0069	3.9342	3.4069	2.8531	3.3847	3.7454	3.8297	4.8085	3.3739
Map2k1	_LC[Carbamidomethyl (C)]DFGVSGQLIDSM[Oxidation (M)]ANSFVGTR_.2	#N/A	4.7686	5.2540	5.0101	3.8031	4.1742	4.1260	3.6131	4.9555	4.4883	4.7774	3.8516	4.1958	4.5084	3.8839	4.3226	4.6781	4.7184	4.8563	4.7432	4.8342
Map2k1	_KLEELELDEQQRK_.3	#N/A	3.6052	3.3707	4.1415	2.4410	2.9429	3.1670	3.0973	3.3055	3.1564	3.8115	3.6666	4.2871	3.2645	4.1285	2.5609	3.9501	3.8432	4.1929	2.9911	2.3835
Map2k1	_VGELKDDDFEK_.3	#N/A	1.0961	3.7070	3.1486	2.1587	3.8190	4.2894	2.3677	3.8377	0.9602	3.4227	4.1288	3.4331	2.7244	3.1867	2.5273	2.9742	3.3350	3.8145	4.1135	3.2410
Map2k1	_LEELELDEQQR_.2	#N/A	3.4660	3.8531	6.0602	4.1182	3.6729	5.1758	3.3752	3.6917	3.3888	3.2766	3.7632	3.5792	3.4969	4.5415	3.4005	4.3289	3.1889	3.9605	4.2596	4.2188
Map2k1	_LQGTHYSVQSDIWSM[Oxidation (M)]GLSLVEM[Oxidation (M)]AVGR_.3	#N/A	4.1158	3.8870	3.1513	3.5736	3.4535	3.6391	4.0509	3.8162	3.9711	4.1017	3.4497	3.7765	4.1365	3.1383	3.6648	2.9599	4.3539	3.4793	3.1279	3.1053
Scp2	_KLEEEGEQFVKK_.3	#N/A	3.4452	3.8739	4.7932	4.2929	2.7539	3.1270	3.3960	3.1613	3.3162	4.1253	4.2957	3.6000	3.4816	3.9843	4.9281	3.3487	3.6990	4.1342	3.7828	3.4810
Scp2	_GSVLPNSDKK_.2	#N/A	4.4869	3.5159	3.5224	3.2025	3.8246	4.9684	5.6122	4.1873	5.0849	5.3054	4.1499	3.4053	3.8836	3.5095	5.5458	4.9533	3.2241	3.7936	4.9693	3.1046
Scp2	_EATWVVDVK_.2	#N/A	4.1220	3.1970	3.6586	3.4622	4.3290	3.3295	2.7441	2.0852	4.1402	3.9326	3.6188	4.4240	4.1529	2.7816	4.2453	3.4842	3.8449	3.4902	3.6035	3.3740
Slc2a3	_EEDQATEILQR_.2	#N/A	7.5310	7.0483	6.6985	6.6672	6.8687	6.8578	6.7358	7.5885	7.3332	6.5808	7.2260	6.3835	6.9407	6.3134	6.4416	6.4725	7.0842	7.0747	6.6382	6.7545
Slc2a3	_QVTVLELFR_.2	#N/A	6.4796	6.4948	6.1352	6.7755	6.1822	6.7043	6.5649	6.4878	6.5604	6.3190	6.2975	6.2671	6.3808	5.9160	5.6256	6.0354	6.7678	6.6315	6.0733	6.2642
Slc2a3	_LWGTSDVVQEIQEM[Oxidation (M)]KDESVR_.3	#N/A	7.1681	6.5629	7.1162	6.5766	6.6071	7.0045	6.1999	6.7998	7.1077	6.0168	6.4751	6.5211	7.0103	6.8743	6.2569	4.1535	7.3271	6.6904	6.7583	7.0908
Slc2a3	_TFEDIAR_.2	#N/A	3.9075	4.4845	4.4438	4.7347	3.7195	3.8579	5.2808	3.6079	4.3277	4.1138	4.8241	4.7298	3.9282	4.4309	3.8731	4.7461	4.1456	4.3518	4.5567	4.3979
Ssb	_S[Phospho (STY)]PSRPLPEVTDEYKNDVK_.3	P32067_S92_M1_Ssb	3.8665	6.0805	3.4030	3.7178	4.0734	4.1067	4.4756	4.0921	5.5486	6.3396	3.8744	3.1788	4.5612	3.4411	5.8726	3.2286	3.5894	3.5601	5.7810	6.5318
Chrm4	_KFAS[Phospho (STY)]IAR_.2	P32211_S379_M1_Chrm4	10.8816	10.7195	9.9874	10.5352	10.1921	10.3596	10.3287	10.7612	10.3214	10.3942	10.6613	10.5632	11.0903	10.4251	9.3070	10.2169	10.9918	10.7451	10.5028	10.3505
Kras	_LVVVGAGGVGK_.2	#N/A	6.9201	7.2511	7.4980	7.2943	7.3394	7.4971	7.3222	7.1555	7.5854	6.9840	6.9658	7.3945	6.9820	7.3743	7.3743	7.0750	6.9357	7.5699	7.3984	7.2468
Kras	_SYGIPFIETSAK_.2	#N/A	7.1695	7.5338	7.2332	7.6161	7.4714	7.6887	7.7009	7.2038	7.6676	7.3036	7.4023	7.5521	7.4392	7.6894	7.5411	7.2623	7.5550	7.3665	7.4617	6.9457
Kras	_TGEGFLC[Carbamidomethyl (C)]VFAINNTK_.2	#N/A	6.2809	5.0216	5.9359	5.6612	5.3205	5.0504	5.1918	5.3894	5.4631	6.1321	5.2444	4.8588	5.8653	5.6413	4.9782	4.9903	5.7158	4.6982	5.2666	6.2123
Kras	_QVVIDGETC[Carbamidomethyl (C)]LLDILDTAGQEEYSAM[Oxidation (M)]R_.3	#N/A	4.3321	3.9275	3.9434	4.1661	3.7499	3.9673	3.5842	4.3328	3.8569	3.2031	3.1614	3.7684	5.1002	5.1520	3.1866	3.8356	3.6570	3.5129	2.4807	3.7089
Kras	_SFEDIHHYR_.3	#N/A	3.8137	3.7856	3.2527	3.4721	4.3626	4.3531	4.7267	3.9409	4.0725	5.3459	5.1808	4.5724	4.2380	3.2398	4.7069	5.2930	3.3162	5.2579	4.1664	4.7533
Wars1	_ASEDFVDPWTVR_.2	#N/A	6.4201	6.7463	7.2198	6.6560	6.8352	6.6531	6.6788	6.8234	6.6945	6.6229	7.0549	6.6829	6.3820	7.0274	6.8519	6.6690	6.9627	6.9520	6.4380	6.0064
Wars1	_TLIDVLQPLIAEHQAR_.2	#N/A	4.3776	3.9173	3.4131	3.3163	4.0494	4.1819	3.5069	4.4060	3.5468	3.6662	4.2637	4.0300	3.9974	4.0852	3.8211	3.7500	4.2973	3.7030	2.8710	3.3342
Wars1	_DLTLEQAYSYTVENAK_.2	#N/A	3.4194	3.8996	4.4569	4.1648	4.2579	2.9811	3.2832	3.6451	3.3423	3.6257	3.7166	3.6258	3.4503	4.4949	2.8604	5.5933	3.1424	4.0071	4.3061	4.1722
Kif1a	_C[Carbamidomethyl (C)]NAIINEDPNNK_.2	#N/A	6.9956	6.8056	7.0381	7.0683	6.6927	6.4074	6.8271	7.1145	6.7706	6.7891	6.8793	6.1924	6.8844	6.3331	5.5120	6.6092	6.9072	6.8017	6.7391	6.3610
Kif1a	_AASVSS[Phospho (STY)]LHER_.2	P33173_S419_M1_Kif1a	7.6673	7.4360	3.7034	3.4174	3.5105	5.9387	6.7305	6.4546	4.0897	4.3791	2.9632	3.7195	4.1977	3.7415	3.6078	3.0168	3.8898	6.7255	3.5587	5.9607
Kif1a	_NLFGSGS[Phospho (STY)]LR_.2	P33173_S1317_M1_Kif1a	7.2167	6.8645	6.8829	3.3799	4.3044	4.3377	6.2974	5.9205	4.1648	6.6938	3.6434	3.5828	4.3302	3.3320	4.2699	3.4596	4.5475	3.2856	5.4659	3.2990
Kif1a	_DVLS[Phospho (STY)]PLRPSR_.2	P33173_S1093_M1_Kif1a	9.3438	4.4425	8.1956	8.3829	3.0836	7.8191	8.3711	8.4035	7.5869	8.3553	8.4550	8.3347	8.4372	8.4924	5.5337	7.9969	8.3708	8.0490	7.5877	7.8516
Kif1a	_SGTS[Phospho (STY)]QEELR_.2	P33173_S937_M1_Kif1a	9.2738	9.4580	8.8044	9.7387	9.0387	9.2785	9.6345	8.9610	9.2447	9.5847	9.4709	9.1021	9.5236	9.0712	7.8175	8.9350	8.9695	9.7040	9.1815	9.1357

Kif1a	_KTPS[Phospho (STY)]PVR_.2	P33173_S1553_M1_Kif1a	4.9836	9.2320	8.8563	8.7036	8.4082	8.7418	9.0910	3.8892	8.6402	8.7318	8.7316	8.7053	8.9784	4.6359	7.5387	8.3297	9.0177	8.9198	8.6880	4.5083
Kif1a	_ISLVDLAGSER_.2	#N/A	4.1851	8.1688	3.2573	4.2772	8.3672	8.3531	8.2057	3.9222	5.6161	8.5608	4.9722	8.0426	4.2426	7.1677	7.2846	3.5472	5.2493	4.6062	5.0074	7.7299
Kif1a	_FQSESC[Carbamidomethyl (C)]PVVGMS[Phospho (STY)]R_.2	P33173_S932_M1_Kif1a	4.0519	5.2979	5.5656	6.5778	3.3896	3.6137	5.1243	3.7523	3.7179	4.2582	5.4050	3.8403	4.3186	4.9614	3.7286	4.3968	4.2900	3.1388	3.4379	3.5396
Kif1a	_IS[Phospho (STY)]FDDQHFEK_.2	P33173_S912_M1_Kif1a	4.0700	3.2491	3.6066	3.5142	4.2770	4.1545	4.2720	4.0373	4.1922	3.8806	3.6708	3.6922	4.3576	5.1447	3.3312	3.4321	3.8905	5.2333	4.1468	3.3264
Kif1a	_S[Phospho (STY)]DSLILDHQWELEK_.2	P33173_S1373_M1_Kif1a	4.2302	3.9406	3.7667	4.2057	3.7302	7.4684	4.1696	3.7489	3.8032	4.0408	3.8076	3.6447	4.2610	3.8048	3.4880	3.5923	3.9531	4.0480	3.5532	3.4821
Kif1a	_VTGVYELSLC[Carbamidomethyl (C)]HVADAGS[Phospho (STY)]PGM[Oxidation (M)]QR_.3	P33173_S1342_M1_Kif1a	4.4715	4.3485	4.0081	3.7539	3.1776	3.2109	3.8705	3.6358	3.7907	4.2821	3.2694	4.0746	3.9562	4.4588	3.8959	3.8336	4.1735	3.6596	3.3082	4.4258
Kif1a	_TPQPC[Carbamidomethyl (C)]S[Phospho (STY)]RPAS[Phospho (STY)]PEPELLPELDSK_.3	P33173_S1533_M2_Kif1a	3.2156	3.6959	3.1598	3.9610	4.0542	4.2782	3.4869	3.8489	3.5460	3.4219	3.9204	3.4220	3.6541	6.4947	3.0641	3.5605	3.3461	6.3152	4.1024	4.3759
Kif1a	_PASPEPELLPELDSKKT[Phospho (STY)]PS[Phospho (STY)]PVR_.4	#N/A	4.3056	2.7833	3.5797	3.3512	4.0849	4.2009	4.0040	4.2572	3.9828	3.5947	3.9185	3.1943	4.1236	3.5935	3.8622	3.3971	3.5534	3.2004	3.9767	3.3898
Kif1a	_PAS[Phospho (STY)]PEPELLPELDSK_.2	P33173_S1537_M1_Kif1a	3.8198	3.4993	3.3564	3.7644	4.0267	4.0816	3.0214	4.0455	3.7426	3.6304	4.1170	3.7457	3.8507	3.3944	3.0467	3.1819	3.5427	3.6067	3.9058	3.0717
Ranbp1	_FLNAENAQK_.2	#N/A	6.4372	6.5504	6.7993	6.8203	6.7880	6.7666	6.3746	6.9248	5.9445	6.8239	6.4732	6.7209	7.3117	7.0267	7.3619	6.7201	6.6174	6.9917	6.9524	6.5450
Ranbp1	_FASENDLPEWK_.2	#N/A	6.3041	8.3587	8.0503	7.8501	6.4993	8.2459	7.7899	7.0016	7.9327	7.7639	6.4387	7.5985	6.5927	8.7243	7.6297	8.0210	7.8566	8.0220	8.3339	8.3625
Ranbp1	_TLEEDEEELFK_.2	#N/A	5.2237	5.5528	5.9555	5.7585	3.3065	3.5306	5.1501	3.0957	6.2252	6.2794	3.1672	3.9235	5.8587	5.8438	5.8806	4.3137	4.9011	5.5091	3.3547	3.6228
Ranbp1	_VAEKLEALSVR_.3	#N/A	3.4928	2.9452	3.2875	4.4202	3.9948	3.6306	3.3899	3.9252	3.0192	4.6815	3.9064	3.2943	5.0606	3.6673	4.1584	4.7001	3.4738	4.1311	3.9072	4.2183
Ptk2	_YMEDSTY[Phospho (STY)]YK_.2	P34152_Y576_M1_Ptk2	3.6961	4.3067	4.2324	3.9933	3.0338	3.2579	3.0064	3.3965	3.0655	3.9024	5.8749	4.1962	3.1736	5.9628	4.0845	4.0410	3.9341	3.8990	3.5476	4.1865
Ptk2	_LQPQEIS[Phospho (STY)]PPPTANLDR_.2	P34152_S910_M1_Ptk2	13.1851	13.0503	12.5854	12.6423	12.2307	3.8642	12.6478	13.0893	12.3879	12.6527	12.8118	12.6636	13.3519	13.1023	11.5355	12.4682	13.3482	12.9132	12.7205	12.5078
Mif	_LHISPDR_.2	#N/A	9.7169	9.8000	9.8686	9.6617	9.7539	9.8326	10.1212	9.8371	9.7964	9.9102	9.7990	9.9594	10.0990	9.9564	10.5548	9.8889	9.6004	9.8541	9.9882	9.9335
Mif	_LLC[Carbamidomethyl (C)]GLLSDR_.2	#N/A	9.9879	10.3628	9.9388	10.1712	10.2236	10.1210	10.1827	10.2249	9.6159	10.2363	9.9109	10.3060	10.3378	10.2502	10.0727	10.0701	9.9412	10.0983	9.9760	10.1180
Mif	_ASVPEGFLSELTQQLAQATGK_.2	#N/A	8.2867	8.2286	8.5109	7.8696	7.8754	8.3075	7.8594	7.8055	8.2588	8.1122	8.0293	7.9690	7.6448	8.8223	8.2643	8.2793	7.9529	7.9010	8.1461	8.0520
Mif	_PM[Oxidation (M)]FIVNTNVPR_.2	#N/A	9.2062	9.6980	9.8320	9.7962	9.5187	9.6474	9.5322	9.5547	9.6037	9.7277	9.2074	9.5424	9.8974	10.0777	9.7190	9.9229	9.4206	9.4807	9.6089	9.6837
Mif	AVHVVPDQLM[Oxidation (M)]TFSGTNDPC[Carbamidomethyl (C)]ALC[Carbamidomethyl (C)]SL	#N/A	4.4933	3.3120	3.3497	3.4382	3.2628	3.7554	4.0097	4.3717	4.0688	4.3999	3.0162	3.0227	3.9303	3.7206	3.7498	3.0377	3.8689	3.1856	3.5796	3.4729
Mif	_VYINYYDM[Oxidation (M)]NAANVGWNGSTFA_.2	#N/A	4.1432	5.6155	5.3148	3.5470	3.8898	4.0130	3.4596	6.0817	4.1824	3.3910	4.0845	3.3622	3.5969	4.6804	3.2202	3.2153	3.3540	3.0911	4.2405	5.6315
Ptpn11	_IQNTGDY[Phospho (STY)]YDLYGGEK_.2	P35235_Y62_M1_Ptpn11	6.9982	7.5692	7.2080	7.1607	4.2289	7.0999	7.0230	3.9738	6.9027	7.2128	4.4810	7.1611	7.5064	7.4720	3.3988	7.3660	4.9318	7.3983	7.3237	6.8203
Ptpn11	_ESQSHPGDFVLSVR_.3	#N/A	4.1434	3.7393	3.2990	3.4258	4.3503	4.3837	4.1986	3.9639	4.1188	3.9540	3.5975	3.6287	4.2843	3.2861	4.2240	3.5055	4.5016	3.5317	2.9801	3.2531
Rab5c	_FEIWDTAGQER_.2	#N/A	7.3523	6.8457	7.7096	6.9438	6.9398	7.2925	7.0840	6.7051	7.1583	6.9042	7.2129	6.6887	6.5367	7.0118	6.6378	6.3443	7.1357	6.8828	6.7577	7.1478
Rab5c	_GVDLQESNPASR_.2	#N/A	7.5380	7.2809	7.1226	7.1955	7.1045	7.2854	7.2591	7.6182	7.1525	7.2037	7.0848	7.3209	6.9859	7.2512	6.9762	7.2047	7.6499	7.6794	7.2853	7.0833
Rab5c	_LVLLGESAVGK_.2	#N/A	7.4509	7.7244	7.2278	7.3260	6.9960	7.0853	7.7843	7.4141	7.2519	6.7231	6.4659	7.0032	6.9522	7.3433	6.0708	6.4245	7.0231	7.6492	6.8835	6.9485
Rab5c	_QASPNIVIALAGNK_.2	#N/A	5.3008	5.7506	5.1534	4.6719	5.1197	5.6874	5.0320	4.8463	5.0812	5.2949	5.0816	4.6163	5.9221	4.9980	4.0998	5.0894	5.0729	5.2000	5.1515	5.7487
Rab5c	_YHSLAPM[Oxidation (M)]YYR_.3	#N/A	4.2967	4.4638	3.8332	4.7003	5.3344	5.1670	5.7679	4.3636	3.9655	3.6322	4.8144	5.0737	4.9590	4.3435	4.0707	3.6588	4.3483	5.8706	3.1334	3.0998
Rab5c	_GAQAAIVVYDITNTDTFAR_.2	#N/A	6.7634	6.5327	6.5587	6.4243	6.0368	5.8976	6.2273	6.6008	6.1958	6.2613	6.0996	6.2420	6.9705	6.6655	4.6837	6.0669	6.6478	6.1180	6.0847	6.3941
Rab5c	_AVEFQEAQAYADDNSLLFM[Oxidation (M)]ETSAK_.3	#N/A	4.2406	4.4475	4.2503	3.3436	3.7292	4.4809	3.7278	4.4663	4.0216	4.0512	3.5002	4.3054	4.1870	3.8153	4.1267	3.6028	3.9636	3.1859	3.4849	3.4926
Rab6a	_[Acetyl (Protein N-term)]S[Phospho (STY)]AGGDFGNPLRK_.2	P35279_S2_M1_Rab6a	7.9995	7.6666	7.2329	7.6209	6.6641	6.4830	6.9634	7.0994	6.8573	7.9054	6.9324	6.6820	7.1929	6.7480	4.6160	6.9147	7.1061	6.9099	6.4880	6.2917
Rab6a	_TDLADKR_.2	#N/A	5.7403	5.4317	5.5412	3.6348	5.9716	5.4415	5.9623	6.2634	5.4662	4.3371	5.9972	4.7933	4.9202	4.8213	5.1754	6.3375	5.0925	5.5974	5.4910	5.4343
Rab6a	_LVFLGEQSVGK_.2	#N/A	6.2901	6.6669	6.2511	6.2650	5.4031	6.0671	6.5373	6.2095	7.6507	6.3852	6.2548	6.1507	3.9681	6.2052	6.9321	5.8840	5.2060	6.5077	5.1792	5.1164
Rab6a	_SREDMIDIK_.2	#N/A	4.2684	5.1166	3.3038	5.1669	3.6061	4.3789	5.1896	3.9687	4.1236	3.9492	3.3047	1.7651	4.2891	5.0346	4.2288	3.1124	4.5064	3.3267	2.9753	3.2579
Rab6a	_VAAALPGMESTQDR_.2	#N/A	3.8016	3.5175	3.3382	3.7826	3.8757	4.0998	3.6654	4.0273	3.7245	3.2435	4.0988	3.2436	3.8325	3.3763	3.2426	3.3820	3.5245	3.6249	3.9240	3.0536
Rab21	_DSNGAILVYDVTDEDSFQK_.2	#N/A	7.1311	6.2839	3.7877	5.6340	5.0728	5.1064	5.1538	6.0357	6.3081	5.1036	5.1017	5.5907	6.5040	6.1003	4.2168	5.2101	5.9793	5.6028	5.4912	5.1556
Rab21	_VNLAIWDTAGQER_.2	#N/A	4.9629	4.2175	3.8664	4.0755	3.6298	4.0407	4.5755	4.9653	3.4315	4.4769	3.8059	3.0613	4.7080	4.0812	4.2733	3.6750	4.3325	3.6083	3.7072	4.1742
Rab12	_FNS[Phospho (STY)]ITSAYYR_.2	P35283_S105_M1_Rab12	8.8113	8.8046	8.0319	8.2779	8.1149	8.2475	8.2061	8.2683	8.1152	8.4291	8.0562	7.8656	8.4712	4.0927	3.9590	7.5622	8.4735	8.2209	8.1458	7.5865
Rab18	_IIQTPGLWESENQNK_.2	#N/A	7.4491	7.1778	7.0184	6.6400	5.9811	6.5365	6.7040	7.5432	6.5972	6.7903	6.7096	6.7449	7.0864	7.4330	5.7909	6.5279	7.2238	6.9654	6.8720	6.9314
Rab18	_LAIWDTAGQER_.2	#N/A	6.7560	6.4610	6.6579	6.9116	6.7927	6.8513	6.5924	6.4057	6.9665	6.2825	6.6693	6.5138	4.0478	6.4473	6.4365	6.3467	7.0827	6.5791	6.2857	6.8739
Rab18	_TC[Carbamidomethyl (C)]DGVQC[Carbamidomethyl (C)]AFEELVEK_.2	#N/A	5.0871	4.3647	5.3801	4.8669	3.0330	4.5944	4.7995	4.3758	5.2893	4.9179	4.3358	4.4174	3.1744	4.2190	4.0853	4.5775	4.3672	4.7453	4.8115	4.6114
Crhr1;Crhr2	_AMSIPTS[Phospho (STY)]PTR_.2	P35347_S400_M1_Crhr1;Crhr2	3.7949	4.2752	4.0813	4.5403	3.1326	3.3567	4.4085	3.2696	4.4675	4.0012	3.3411	4.0013	4.5756	4.1194	3.9857	4.1398	4.2676	4.3826	3.1809	3.7967
Htr2a	_S[Phospho (STY)]IHREPGSYAGR_.3	P35363_S298_M1_Htr2a	6.2904	6.7157	4.0385	6.1461	3.2081	5.5262	6.5440	5.0413	5.5714	7.0174	6.3561	4.0441	4.5328	4.0766	4.8640	3.8641	4.2249	6.1036	3.2236	5.8265
Grin2a	_QHS[Phospho (STY)]YDNILDKPR_.3	#N/A	6.4828	6.4963	6.0049	6.9974	3.4849	3.6187	6.3441	3.7897	4.9110	5.3218	5.6969	3.8759	3.8491	4.1219	3.6721	4.9201	3.8365	6.3892	3.7468	4.0889
Grin2a	_YLPEEVAHS[Phospho (STY)]DIS[Phospho (STY)]ETSSR_.2	#N/A	3.7476	7.1351	6.0676	5.8925	3.7997	6.0270	7.4547	4.1624	5.9151	3.8022	3.7959	3.4940	3.7684	6.8425	4.9181	5.2804	6.3236	7.5816	7.5370	7.3175
Grin2a	_KMPS[Phospho (STY)]IESDV_.2	P35436_S1459_M1_Grin2a	3.6611	4.3417	6.9426	7.2715	4.4476	5.7207	6.0491	3.3614	3.1005	3.8674	6.6098	4.2312	3.2086	7.1580	4.6464	4.0060	3.8991	4.2488	3.0470	3.9305
Grin2a	_SM[Oxidation (M)]ESLRQDS[Phospho (STY)]LNQNPVSQR_.3	#N/A	7.1491	6.9303	6.9685	6.7821	5.8475	6.2406	6.6261	6.7593	5.9898	6.5759	6.4056	5.8336	6.6277	6.3624	4.5248	6.0997	5.9915	6.3613	5.9901	5.9830
Grin2a	_SIS[Phospho (STY)]LKDR_.2	P35436_S1312_M1_Grin2a	3.7853	4.2175	4.3216	3.9041	3.1230	3.3471	4.4181	3.4857	2.9763	3.9916	3.3507	4.1070	4.5852	4.3087	3.9953	4.1302	4.0234	3.8098	3.4584	4.2757
Grin2a	_DKGS[Phospho (STY)]PHSEGSDRYR_.3	P35436_S1198_M1_Grin2a	4.8307	5.3937	4.0116	4.5529	4.3929	4.5107	4.9648	4.5487	4.6504	5.5222	4.6892	4.6478	5.5991	4.1647	4.7279	3.8372	4.1980	5.4252	5.2693	4.1367
Grin2a	_SPDFNLTGS[Phospho (STY)]QSNM[Oxidation (M)]LK_.2	#N/A	7.0350	7.2714	3.8356	3.9846	5.5475	6.6283	6.6236	6.6402	3.8202	6.5600	3.6708	6.6984	7.1726	5.8526	3.5102	3.9613	7.0453	3.8724	3.7336	3.6669
Grin2a	_QDS[Phospho (STY)]LNQNPVSQR_.2	#N/A	4.2182	4.6018	3.7547	6.8558	2.9243	4.4584	4.1238	4.4438	4.0440	4.0288	3.5227	4.3279	4.2490	6.0018	4.1492	3.5803	6.5320	3.2084	3.5074	3.4701
Grin2a	_KKS[Phospho (STY)]PDFNLTGS[Phospho (STY)]QSNMLK_.3	P35436_S882_M2_Grin2a	4.5285	4.2914	4.0651	4.5565	3.2346	3.3729	4.7893	3.2534	4.4514	4.0174	3.3249	4.0175	4.5594	5.1563	3.8388	3.8907	4.2514	4.3988	3.1970	3.7805
Grin2a	_STASYC[Carbamidomethyl (C)]SRDS[Phospho (STY)]R_.2	P35436_S1416_M1_Grin2a	3.7774	4.2254	4.3137	3.9120	3.1151	3.3690	3.7125	3.4778	3.6327	4.4401	3.5895	4.1148	3.7981	4.3008	3.7379	3.9916	4.0155	4.2180	3.4663	4.2678
Pdha1	_LEEGPPVTTVLTR_.2	#N/A	10.3718	10.1864	10.1564	10.5290	10.3157	10.1999	10.3081	10.4482	10.3673	10.3467	10.3383	10.3795	10.4365	10.6009	10.5288	10.4595	10.3483	10.2098	10.3582	10.3746
Pdha1	_AILAELTGR_.2	#N/A	7.8843	8.1060	8.2707	8.6593	8.4793	8.1858	8.3809	7.7309	8.1886	8.8134	8.4592	8.3426	7.9536	8.3674	8.2914	8.4770	7.6186	8.6193	8.5972	8.8424
Pdha1	_FAAAYC[Carbamidomethyl (C)]R_.2	#N/A	7.0630	7.8468	7.1284	7.7578	7.7970	7.5773	8.1208	7.2089	7.5401	7.7045	7.5729	7.5420	7.8048	7.5726	8.0520	7.2057	7.4606	7.8726	8.0862	7.8074
Pdha1	_TREEIQEVR_.2	#N/A	6.9725	6.8828	6.9643	7.1233	6.7881	7.1794	6.8605	6.8129	7.2069	6.9528	7.1637	7.0858	6.5492	7.3971	7.1473	6.9289	6.8601	6.8323	6.5900	7.1064
Pdha1	_AAASTDYYKR_.2	#N/A	6.0218	5.8178	5.5060	5.8265	5.5087	6.4379	6.1959	5.9997	6.4994	6.4100	6.3268	5.8752	6.2877	6.1208	6.6836	6.1780	5.7308	5.8050	6.2077	6.0735
Pdha1	_VDGM[Oxidation (M)]DILC[Carbamidomethyl (C)]VR_.2	#N/A	6.2471	7.2826	7.1880	7.7888	6.9790	7.1554	7.2669	6.7241	6.8838	6.8824	6.7874	6.6765	6.3578	7.5389	7.0074	6.4892	7.2309	6.4843	6.8973	6.9066
Pdha1	_YGM[Oxidation (M)]GTSVER_.2	#N/A	6.7862	6.1658	6.3331	8.0958	6.4597	6.4020	6.9496	6.6734	7.3336	6.5748	7.0916	6.9205	6.5767	7.2489	6.6657	6.7254	6.6260	6.7982	6.8592	6.9500
Pdha1	_NFYGGNGIVGAQVPLGAGIALAC[Carbamidomethyl (C)]K_.3	#N/A	4.6244	3.9467	5.3335	4.9654	3.7722	4.4672	3.2759	3.7910	4.5847	4.4162	4.2418	3.9183	3.5962	4.3160	3.8084	4.4282	3.1249	3.3589	5.0366	4.1015
Pdha1	_GPILM[Oxidation (M)]ELQTYR_.2	#N/A	6.3566	7.1196	7.3484	7.5666	6.8636	6.7657	6.9416	6.8996	6.5510	6.5338	7.0373	7.0046	6.3831	7.2499	7.0926	6.7492	6.8626	6.4640	6.7628	6.8390
Pdha1	_M[Oxidation (M)]VNSNLASVEELKEIDVEVR_.3	#N/A	7.4765	7.0232	6.6589	6.8267	6.2189	6.5412	6.3010	6.5221	6.3420	6.7383	6.2522	6.4625	7.2599	7.4442	6.2341	7.2127	7.1434	7.2878	6.6184	6.9072
Pdha1	_YHGHS[Phospho (STY)]M[Oxidation (M)]SDPGVSYR_.3	#N/A	10.5015	10.3479	10.6535	10.9682	9.9907	9.9524	9.8934	10.0253	10.3482	10.7454	10.0654	9.5238	10.4800	10.4200	10.1920	10.2563	10.2294	9.6136	10.2939	9.8967
Pdha1	_LPC[Carbamidomethyl (C)]IFIC[Carbamidomethyl (C)]ENNR_.2	#N/A	3.1202	4.2169	4.3222	3.9035	3.3271	3.3605	3.7210	3.4863	3.6412	4.4316	3.1198	4.1064	3.8066	10.6823	3.7463	3.9832	4.0240	3.8092	3.4578	4.2763
Pdha1	_YHGHSMSDPGVS[Phospho (STY)]YRT[Phospho (STY)]REEIQEVR_.4	#N/A	3.8460	4.0395	3.8499	3.7261	3.5234	3.6622	3.7819	3.7232	3.7800	4.5061	3.8365	3.9290	3.9129	5.6820	3.5787	3.6758	4.2609	3.6586	3.2804	3.8136
Pdha1	_M[Oxidation (M)]VNSNLASVEELKEIDVEVRK_.4	#N/A	3.4111	4.5917	3.9474	4.2783	4.2497	4.4738	3.2914	3.1115	3.3505	3.6174	3.7249	3.3472	3.4585	3.9345	3.7784	3.0004	3.6492	3.9989	3.8326	4.1804
Fah	_ASLQNLLSASQAR_.2	#N/A	3.8106	6.3584	5.8285	5.8975	5.6124	6.1546	5.5011	6.5125	5.7014	5.7152	4.8361	5.5278	6.0258	6.0266	6.2923	5.4726	5.5627	6.0412	5.9701	5.7535
Fah	_ASSIVVSGTPIR_.2	#N/A	5.6791	6.3753	6.0208	6.1766	5.1914	5.4396	5.6587	6.2875	5.3448	6.0121	3.3380	5.5544	5.7695	5.9631	5.9742	5.5648	5.9244	6.0036	5.4376	5.5895
Canx	_APVPTGEVYFADSFDR_.2	#N/A	7.6098	7.4978	7.7354	7.3160	6.9812	6.9040	7.2578	6.8764	7.3872	7.0629	7.3011	6.9931	7.1981	7.1456	7.0745	7.1276	6.8592	7.0761	7.4505	7.2730
Canx	_TYFTDKK_.2	#N/A	6.2118	6.4116	5.7641	5.8861	6.0806	5.5896	6.7355	6.1168	6.4565	5.7528	6.4229	6.2125	6.2167	6.0483	5.9483	5.4387	5.4456	6.2350	5.6791	5.9533
Canx	_AEEDEILNRS[Phospho (STY)]PR_.2	#N/A	11.4820	11.3663	11.3700	11.3474	10.9039	11.1060	10.9855	11.2037	11.0390	11.2853	11.0358	10.8861	11.3588	11.0973	10.2080	10.8412	11.3658	10.9275	10.9833	11.0102
Canx	_QKS[Phospho (STY)]DAEEDGVTGS[Phospho (STY)]QDEEDSKPK_.3	P35564_S563_M2_Canx	7.0480	7.0221	7.0666	6.9376	5.9728	6.7786	6.9588	6.8801	7.0463	7.1498	6.7950	6.5906	5.6807	7.0223	5.3809	6.5629	6.6112	6.9216	6.9516	6.5869
Canx	_AEEDEILNR_.2	#N/A	6.4366	6.0189	6.9189	6.7448	6.1788	6.6547	6.1744	6.0266	6.8194	6.4820	6.7832	6.4202	3.4653	6.4546	6.1843	5.8750	6.2379	6.4984	6.1529	5.9478
Canx	_SDAEEDGVTGS[Phospho (STY)]QDEEDSKPK_.3	#N/A	4.9555	4.5073	4.9432	3.8282	4.3040	4.5397	5.8462	4.9011	5.1104	4.4054	5.1383	4.6791	4.4152	5.3496	4.7090	4.2310	4.5776	5.0299	5.4238	3.5636
Canx	_VVDDWANDGWGLK_.2	#N/A	4.4479	5.1722	3.4833	3.6925	3.7856	5.2291	4.7542	4.1174	3.8146	4.4104	4.1889	4.6156	5.2363	3.4664	4.4301	4.7860	5.8039	3.5348	3.8338	4.3683
Irs1	_[Acetyl (Protein N-term)]AS[Phospho (STY)]PPDTDGFSDVRK_.2	#N/A	9.0826	4.2796	4.2595	8.7914	3.0609	8.9258	8.8649	8.6283	8.7234	3.9295	8.0832	8.9518	9.1853	4.2465	8.1585	8.2693	8.9706	8.7714	8.8664	8.3238
Irs1	_[Acetyl (Protein N-term)]AS[Phospho (STY)]PPDTDGFSDVR_.2	#N/A	3.5774	7.5474	7.5594	4.3607	3.7843	6.6166	3.2638	4.5299	7.5615	7.7127	4.1635	6.8276	3.3494	7.5384	6.5578	6.5674	3.5668	7.4060	7.2288	7.6662
Ap1m1	_VLFDNTGR_.2	#N/A	5.0695	3.3672	3.4885	5.3709	4.1589	4.1922	4.6429	4.8235	5.7625	3.7625	4.9456	4.7517	4.4757	4.4153	4.3960	4.9560	3.6748	5.0930	3.7736	4.3989
Prdx1	_IGYPAPNFK_.2	#N/A	8.8303	9.0126	8.1979	8.1106	8.2536	9.0096	9.0273	9.1518	8.8909	8.7512	8.1762	8.7556	8.2322	8.1752	8.5580	8.3373	8.7838	8.5266	8.5553	8.5177
Prdx1	_TIAQDYGVLK_.2	#N/A	8.2437	7.9501	8.5550	8.5135	8.3994	8.1634	8.4210	8.0345	8.3552	8.3783	8.0780	8.2826	8.1751	8.2471	8.7457	8.2514	7.4973	8.3418	8.3485	8.4783
Prdx1	_LVQAFQFTDK_.2	#N/A	7.0190	7.5250	7.7494	8.0416	6.9862	7.6931	7.3627	7.2044	7.2565	7.7861	6.6565	7.1201	7.1253	7.7833	7.4381	7.5729	7.3323	7.1969	7.4399	7.2443
Prdx1	_ADEGISFR_.2	#N/A	7.0808	6.0735	7.3492	7.4559	7.1445	6.8379	6.1297	6.6827	7.5473	7.8954	7.2307	7.1373	6.7802	6.9526	7.9186	7.4418	6.2611	6.7836	7.3790	7.5816
Prdx1	_SKEYFSK_.2	#N/A	5.8840	5.7089	6.7068	6.0897	6.2333	6.2174	6.0993	6.6621	6.4361	6.3412	5.5877	6.2473	6.5041	6.1238	6.8237	6.5338	5.7453	5.7769	6.2483	6.0806
Prdx1	_GLFIIDDK_.2	#N/A	5.2410	5.8066	7.4737	7.3148	6.2071	6.6846	6.7166	4.3583	6.8018	7.2258	6.6488	6.8215	5.9314	6.4581	6.9364	7.0507	5.2666	6.0749	7.0841	6.8615
Prdx1	_ATAVM[Oxidation (M)]PDGQFK_.2	#N/A	8.0843	8.6024	8.4673	8.5121	8.4445	8.4484	8.5833	8.4694	8.2962	8.3079	8.0536	8.5577	7.9825	8.3169	8.9391	8.1274	8.2278	8.2562	8.2390	8.3694
Prdx1	_DISLSEYK_.2	#N/A	3.9589	3.5548	5.6757	3.2413	4.3002	4.6441	5.2489	4.1484	6.1526	6.4539	5.6581	3.4442	4.4688	2.0233	5.2809	6.2357	3.1853	5.4190	6.6068	5.8255
Prdx1	_SVDEIIR_.2	#N/A	3.1495	3.6595	3.5092	5.6297	4.1795	4.3554	5.3316	4.1982	5.9970	5.8053	5.6093	5.5338	4.3729	5.4683	5.3483	4.9609	3.6955	5.5213	5.7765	5.5411
Prdx1	_LNC[Carbamidomethyl (C)]QVIGASVDSHFC[Carbamidomethyl (C)]HLAWINTPK_.3	#N/A	4.0127	5.3469	4.0336	3.7258	4.0729	3.4058	3.7053	3.2093	3.6255	3.8117	3.9396	4.2280	3.7909	5.4085	3.7306	4.0285	3.9892	3.6107	3.8959	3.3184
Cd81	_QFYDQALQQAVM[Oxidation (M)]DDDANNAK_.3	#N/A	9.5131	9.4090	8.7915	8.6619	8.3926	8.8901	9.0150	9.3910	8.8279	8.5292	8.5698	8.9165	10.1544	9.1407	7.5706	8.5359	9.3494	9.2990	8.7405	8.5765
Cd81	_NSLC[Carbamidomethyl (C)]PSGGNILTPLLQQDC[Carbamidomethyl (C)]HQK_.3	#N/A	7.1524	7.0105	7.3912	6.8987	6.3543	7.1045	7.2833	6.4155	6.6189	6.6403	6.5909	6.2939	7.0113	6.4779	6.5683	3.6656	7.2860	6.9590	6.7460	6.7117
Cd81	_TFHETLNC[Carbamidomethyl (C)]C[Carbamidomethyl (C)]GSNALTTLTTTILR_.3	#N/A	5.3452	5.3309	5.0692	5.6247	4.0910	5.4150	4.2625	5.1759	4.2523	5.0507	3.8858	4.5786	5.1791	4.8496	3.6765	3.9205	5.2947	5.2691	5.0873	4.7802
Gpm6a	_QFGIVTIGEEK_.2	#N/A	10.3125	10.1880	10.2511	10.1101	9.7329	10.0420	10.1768	10.0322	10.0562	9.5982	10.0663	9.6952	10.0326	9.9537	9.1628	9.7393	10.1028	9.9983	9.9762	9.6626
Gpm6a	_IC[Carbamidomethyl (C)]TASENFLR_.2	#N/A	10.2693	10.1245	10.2579	9.9292	9.8129	9.9439	10.3149	10.3190	10.0128	9.7400	9.9544	9.8293	10.0666	9.4402	9.6430	9.4152	10.1274	10.0541	9.8589	9.6473
Gpm6a	_KIC[Carbamidomethyl (C)]TASENFLR_.2	#N/A	6.8988	6.5450	6.4397	6.0824	6.3960	6.3951	6.6455	7.0822	6.4069	6.0304	6.1164	6.3866	6.7064	5.9085	5.7450	5.7679	6.4086	6.4272	6.2312	5.9747
Gpm6a	_SKEEQELHDIHSTR_.3	#N/A	8.8289	8.5831	9.5683	8.8134	8.4288	8.8686	8.7388	8.8821	9.0182	8.6686	9.2008	8.1188	8.6400	8.0421	8.6661	8.6730	9.2358	8.4753	8.9247	7.9705
Gpm6a	_M[Oxidation (M)]QKYEDIK_.2	#N/A	6.2345	6.4129	5.2584	4.7415	5.5376	6.0516	6.3692	6.3634	6.1567	6.1663	5.2196	6.2180	6.3196	5.4407	5.6227	5.9352	6.2492	6.5365	6.4983	5.5464
Gpm6a	_QFGIVTIGEEKK_.2	#N/A	5.9602	5.8890	5.5350	5.6800	5.0754	5.9052	6.2288	5.6793	5.2547	4.6778	5.5326	5.6527	6.0175	5.0422	4.8820	5.4316	5.8201	5.5950	5.3918	5.5709
Gpm6a	_DLYGDFK_.2	#N/A	3.3485	8.6711	5.0060	5.0381	4.1164	3.5888	3.9064	3.5742	4.1951	4.6677	5.3863	3.8247	3.5783	4.3825	4.5420	4.4007	3.0714	5.3001	5.9802	4.9910
Gpm6a	_EEQELHDIHSTRS[Phospho (STY)]K_.3	#N/A	5.5277	6.1487	4.3834	4.8930	4.7940	4.6152	5.9677	5.9056	5.2837	5.2043	4.0715	5.5044	7.3131	5.4557	4.7989	5.5410	5.5363	6.0619	3.9292	5.0909
Gpm6a	_S[Phospho (STY)]KERLNAYT_.2	#N/A	6.6173	6.4834	3.6253	3.0995	6.0730	6.4008	3.6135	5.9673	6.2085	3.2954	5.7080	6.2700	6.9458	6.5073	5.7411	6.3351	6.4765	6.7798	6.1452	5.9812
Gpm6a	_SKEEQELHDIHSTRS[Phospho (STY)]K_.3	#N/A	8.8446	7.3963	7.3793	8.7393	8.1332	8.1577	9.4059	9.3490	8.5468	7.2558	7.0962	8.7862	9.2870	8.5504	7.5179	8.4370	9.0165	9.4827	9.0924	7.7475
Gpm6a	_EEQELHDIHSTRS[Phospho (STY)]KER_.3	#N/A	4.1547	4.3041	3.9721	3.6022	3.4924	3.8411	4.3825	6.1476	4.0100	3.9033	3.4886	6.1642	7.7727	5.9478	4.6218	5.3899	3.6738	6.1458	3.9384	4.9785
Gpm6a	_YEDIKS[Phospho (STY)]KEEQELHDIHSTR_.4	#N/A	9.5401	9.4645	9.0936	9.0524	8.5016	9.1786	9.0884	9.1641	9.0231	8.7218	9.1584	9.1191	9.7358	9.2329	7.9873	8.8952	9.4788	9.5894	9.2907	9.2081
Gpm6a	_EEQELHDIHSTR_.3	#N/A	5.0391	5.4464	4.7035	5.4034	5.1856	5.0825	5.9273	3.3650	5.6034	5.2930	5.2519	4.8113	3.9626	3.6998	4.6245	5.0116	4.0712	5.1293	5.0809	4.6692
Gpm6a	_[Acetyl (Protein N-term)]MEENMEEGQTQK_.2	#N/A	4.2035	3.7993	6.7349	6.7433	3.5412	5.3767	4.6383	3.9039	4.0588	4.4098	5.3210	3.6887	4.2242	6.0230	3.5770	3.0476	4.4416	3.3916	3.0402	3.1930
Gpm6b	_AKEEQELQDIQS[Phospho (STY)]R_.2	#N/A	8.3804	8.1763	8.1814	8.2975	7.7275	7.9465	7.8426	8.1313	7.7452	7.8496	7.8525	7.4708	7.9522	7.8086	6.7250	7.5309	8.0705	7.6984	7.9867	7.3895
Gpm6b	_EEQELQDIQS[Phospho (STY)]R_.2	#N/A	9.1911	8.7514	9.4256	9.1563	8.7508	8.7474	8.5384	8.8449	8.3778	9.1101	8.7442	8.0157	8.3041	8.2434	6.9150	8.3745	8.8466	8.2120	8.4457	8.3565
Gpm6b	_QYGIIPWNAFPGK_.2	#N/A	8.1451	7.7604	7.6335	8.0570	7.9195	7.8070	7.8758	8.0508	7.7863	7.7359	7.7753	7.8837	10.9784	6.9878	7.5562	7.2385	7.9827	8.0277	7.5177	7.5172
Gpm6b	_ELHGEFK_.2	#N/A	6.7726	6.8785	6.4456	6.7883	5.9516	6.6239	7.2875	6.6335	6.7729	6.5944	6.9766	6.3673	6.3792	6.2194	6.3711	6.5884	6.8179	6.9937	6.7338	6.6626
Gpm6b	_S[Phospho (STY)]KEQLNSYT_.2	#N/A	11.9609	11.9778	11.9058	12.0117	11.3404	11.5614	11.7133	12.1046	11.8024	11.7225	11.6855	11.6241	12.3681	11.9047	10.8770	11.4392	11.9578	11.8602	11.7748	11.5890
Gpm6b	_MQAYQDIK_.2	#N/A	3.8202	5.0528	5.1570	4.6236	3.7340	4.0813	5.2262	4.0458	3.5387	3.2249	5.1213	3.2250	3.8510	3.5684	3.9761	3.1823	4.7509	3.6064	3.9054	3.0721
Gpm6b	cetyl (Protein N-term)]M[Oxidation (M)]KPAM[Oxidation (M)]ETAAEENTEQS[Phospho (STY)]Q	#N/A	3.4328	3.9131	3.8114	4.1782	3.6139	3.8380	3.2951	4.0170	3.2848	3.0317	3.7286	2.8408	3.4622	5.9757	2.8107	3.7777	3.9977	3.8444	3.6622	4.1841
Ptpn12	_DADVSEES[Phospho (STY)]PPPLPER_.2	P35831_S673_M1_Ptpn12	3.1950	4.1241	4.2324	4.3893	3.4019	3.2056	3.6462	3.4207	3.1178	3.8501	3.4922	3.8502	3.2258	4.2705	3.6716	4.0579	4.4187	7.5112	3.0298	3.9477
Ptpn12	_NLS[Phospho (STY)]FEIKK_.2	#N/A	5.6845	4.9490	4.7399	5.1002	4.7895	4.5832	4.7558	3.8770	4.0319	4.8590	4.0266	4.1616	4.2340	3.8800	3.2141	4.3223	4.6164	4.9911	3.0670	4.8871
Ptpn12	_S[Phospho (STY)]PAEVTDIGFGNR_.2	#N/A	3.9534	5.5757	3.9228	4.8440	3.2911	4.6758	4.2500	3.1111	4.1313	5.6091	3.1826	4.1598	4.4171	3.9609	3.8272	4.2983	5.1482	3.0403	3.3393	3.6382
Ptpn12	_DQITKS[Phospho (STY)]PAEVTDIGFGNR_.3	#N/A	4.2905	3.7123	3.3260	3.3989	3.6282	4.0725	4.7320	3.9909	3.9719	4.4968	4.3463	3.6018	4.0800	3.3130	3.4901	3.1346	4.5286	3.3046	2.9532	3.3011
Ptpn12	_NLS[Phospho (STY)]FEIK_.2	#N/A	4.4967	4.3232	4.0333	4.5883	3.2028	2.4635	3.7849	3.2216	3.7655	4.3073	3.2441	3.7110	3.9309	4.0714	3.8706	3.8589	4.2197	0.7267	3.2288	2.8855
Flt1	_DGFADPHFHSGS[Phospho (STY)]SDDVR_.3	P35969_S1207_M1_Flt1	3.5124	4.4904	4.0487	4.1769	4.3510	3.0743	3.1901	3.2128	3.2492	3.7188	3.6235	4.3798	3.3572	4.4018	4.4558	3.8573	3.7505	4.1002	4.3992	4.0791
Rpl12	_EILGTAQSVGC[Carbamidomethyl (C)]NVDGR_.2	#N/A	6.0399	6.4252	6.4651	6.4335	5.6863	6.6784	6.1044	6.8740	6.3979	6.6921	6.3132	6.1493	5.4216	7.1685	6.8265	6.1383	6.5959	6.7082	6.5429	6.4762
Rpl12	_QAQIEVVPSASALIIK_.2	#N/A	6.0629	5.5006	6.8865	6.8254	6.2783	6.2387	5.6298	5.4915	6.6142	6.3125	6.0896	5.5248	5.9842	5.3361	6.2849	5.8857	5.7952	5.2849	6.4427	6.4691
Rpl12	_HSGNITFDEIVNIAR_.3	#N/A	4.0224	3.2967	3.0046	4.5035	4.0380	3.6621	4.0984	4.2480	3.9140	4.5236	4.3196	3.0228	4.0532	3.5970	3.9543	3.1613	3.7453	4.0651	4.0383	3.2743
Rpl18	_ILTFDQLALESPK_.2	#N/A	3.9791	4.5346	4.6622	3.2237	3.3168	5.0022	4.9973	4.7882	4.7293	4.1854	3.1569	4.6855	4.3914	4.5203	3.6710	5.0388	5.9882	5.4923	5.0827	4.4435
Rpl18	_SQDIYLR_.2	#N/A	3.7418	3.8484	2.8827	3.5350	2.5427	3.6969	4.6767	3.8548	3.8674	1.3467	4.2054	4.2935	4.1751	3.1770	4.1149	3.6363	4.3925	4.4741	4.4989	4.6761
Sar1a;Sar1b	_LVFLGLDNAGK_.2	#N/A	5.1671	5.2787	5.9196	6.0595	4.6393	5.5835	5.8573	5.4690	5.4785	5.3150	5.5221	5.6593	4.0833	5.8406	5.0827	6.0020	4.1151	6.0296	5.7262	5.5909
Cpox	_SPS[Phospho (STY)]PGRREEDGDELAR_.3	P36552_S101_M1_Cpox	4.3121	4.5079	3.8486	3.2722	3.0182	3.0515	4.0299	4.5377	3.9501	4.1227	3.4288	4.2340	4.3429	3.8867	4.0553	3.6742	4.0350	3.1145	3.4135	3.5640
Gnl1	_GLQDGLRS[Phospho (STY)]SS[Phospho (STY)]NSR_.2	P36916_S32_M2_Gnl1	3.8721	3.4470	3.4087	3.7121	4.0790	4.1124	4.4699	4.2352	4.3901	3.6827	5.3087	3.7351	4.5555	3.5574	2.9944	3.2342	3.5950	3.0603	5.5748	3.5244
Gnl1	_REEQTDTS[Phospho (STY)]DGESVTHHIR_.4	#N/A	9.5794	9.0399	9.3939	9.5461	8.7364	9.2554	9.4280	9.0370	9.1822	9.6164	8.9147	8.8802	9.6091	9.1932	7.8760	9.1711	9.1876	9.2658	9.1355	9.0573
Gnl1	_EEQTDTS[Phospho (STY)]DGESVTHHIR_.3	#N/A	8.3506	8.3876	8.2955	8.3541	7.8957	8.4698	8.5288	7.8077	8.1056	8.4268	8.2567	7.7728	7.9765	8.2187	7.1604	7.8880	8.2325	8.1904	7.9661	7.9120
Gnl1	_ERREEQTDT[Phospho (STY)]S[Phospho (STY)]DGESVTHHIR_.4	#N/A	4.3431	4.2731	3.8797	3.2504	3.0492	7.0716	4.0081	6.8923	6.6937	4.4518	3.3830	4.2122	7.9003	7.2752	4.0335	7.3552	6.7818	7.2433	4.1963	3.3954
Gnl1	_EVYQPGSVLDFPR_.2	#N/A	4.4358	3.4153	5.5285	3.6804	3.7735	5.1008	3.7676	5.2874	3.8266	3.1413	5.1641	3.1414	3.9347	3.4785	4.4283	3.2799	3.6267	3.5227	4.9117	3.1557
Hmgcl	_LLEAGDFIC[Carbamidomethyl (C)]QALNR_.2	#N/A	3.4442	3.9245	5.7606	4.1897	5.6457	5.7963	4.8709	3.6202	5.6122	3.6505	3.6918	3.6506	5.1663	5.0418	4.9467	5.1046	5.6881	4.8808	5.5768	5.6800
Hmgcl	_EVSVFGAVSELFTR_.2	#N/A	3.8180	4.0539	3.3546	3.7662	4.0249	4.0583	3.0232	4.0437	4.4442	4.8491	3.9228	3.2272	3.6600	3.6115	3.0485	3.1801	3.5409	3.2402	4.1556	3.5785
Hsf1	_VKQEPPS[Phospho (STY)]PPHS[Phospho (STY)]PR_.2	#N/A	4.1485	7.4444	7.5128	3.5428	3.5947	7.0944	6.7029	7.7281	7.0065	3.7438	6.9226	7.5014	6.8238	8.0629	6.9186	7.3361	7.6609	7.2364	7.4428	7.3597
Hspa9	_DAGQISGLNVLR_.2	#N/A	8.3822	8.5518	8.7032	8.6432	8.7379	8.4458	8.4366	8.5913	8.4273	8.5481	8.5249	8.6111	8.7163	8.9396	8.9006	8.5809	8.4268	8.6205	8.7299	8.5956
Hspa9	_NAVITVPAYFNDSQR_.2	#N/A	8.1228	7.9088	8.2830	8.1227	7.9805	8.0751	7.8969	8.4738	7.7884	7.9955	8.0384	7.8231	8.3633	8.4891	7.6695	7.9745	8.3508	7.8712	7.5334	7.9747
Hspa9	_TTPSVVAFTADGER_.2	#N/A	7.7796	7.6560	8.1611	7.4492	7.8966	7.7325	7.4690	7.8056	7.9858	7.7366	7.9097	7.7580	7.8713	7.7561	7.8025	7.9471	8.0021	7.6786	7.4171	8.0467
Hspa9	_VQQTVQDLFGR_.2	#N/A	7.5695	7.7776	8.0535	7.9818	7.8984	7.6964	7.8090	7.9297	7.8862	8.0029	7.9868	8.0412	8.1167	8.1198	8.0795	8.1107	7.9392	7.5131	7.8209	7.7178
Hspa9	_AQFEGIVTDLIK_.2	#N/A	6.6829	6.6173	7.1149	6.9157	7.0091	6.7154	6.7910	6.4011	6.6739	6.7880	6.8525	6.3861	6.6989	7.1285	6.9678	6.9888	6.1069	6.6397	6.1909	6.8566
Hspa9	_NTTIPTKK_.2	#N/A	6.3809	6.3427	6.6118	5.8861	6.8235	6.4333	6.6121	6.7913	6.8574	6.7496	6.2405	6.6264	7.2285	7.2330	7.3090	6.6017	6.5534	6.7609	6.5365	6.7082
Hspa9	_VINEPTAAALAYGLDK_.2	#N/A	5.2890	5.5680	6.9689	6.4354	6.6608	6.0471	5.5340	5.9460	6.5505	6.7123	6.4074	6.0058	4.8761	6.7669	6.6847	6.2837	6.3280	5.5108	5.9668	6.1400
Hspa9	_QAVTNPNNTFYATK_.2	#N/A	6.0963	5.4488	6.3725	6.0648	6.6119	6.0955	5.6546	6.3875	5.7964	5.4580	5.8960	6.0398	5.9955	6.3855	6.4508	5.8983	6.1287	5.9286	5.7470	5.9398
Hspa9	_QAASSLQQASLK_.2	#N/A	5.3011	5.6750	5.8314	4.8836	6.0802	5.6853	6.4947	5.5922	6.2325	6.2619	6.4321	6.2016	5.6916	6.0138	6.3580	6.4995	5.8764	5.9435	6.2852	5.9166
Hspa9	_STNGDTFLGGEDFDQALLR_.2	#N/A	5.4932	5.5239	5.5179	5.0999	5.3966	4.9154	5.6024	4.9395	4.7848	5.1728	4.9919	5.4375	5.0824	4.9928	5.5685	5.5578	5.6264	4.9521	4.8086	4.7444
Hspa9	_EQQIVIQSSGGLSKDDIENM[Oxidation (M)]VK_.3	#N/A	5.8212	6.1980	4.9232	5.8784	5.9747	4.9311	5.1412	6.1887	4.9927	5.5168	5.6364	5.9845	6.6437	6.2781	4.8235	5.4717	5.4711	5.7042	5.4682	5.9368
Hspa9	_LLGQFTLIGIPPAPR_.2	#N/A	6.5934	6.0582	5.6780	4.1718	6.3948	6.2216	4.6111	7.5394	5.8307	6.6279	5.9732	6.2209	6.1532	6.6570	7.3955	6.8923	6.5827	7.2868	6.6707	7.8225
Hspa9	_ETAENYLGHTAK_.2	#N/A	5.8837	5.3633	5.5375	5.4123	4.9198	5.9563	5.6682	5.5633	5.0854	5.8001	4.7098	4.9573	4.2514	5.4079	5.9705	4.9753	4.7764	3.2060	5.3501	5.5494
Hspa9	_ALLAGKDSETGENIR_.2	#N/A	3.8798	4.3782	2.5626	4.4780	3.6627	3.4657	2.1088	4.8406	4.5248	3.0668	4.0190	4.9446	4.6813	4.0599	3.4945	3.8336	4.0759	3.3209	4.2266	3.5059
Hspa9	_ETGVDLTKDNM[Oxidation (M)]ALQR_.3	#N/A	4.9150	5.5499	3.5094	4.8234	5.3401	5.1692	5.7266	4.1743	5.3346	5.8492	5.2608	5.9156	6.2299	3.4965	5.4226	5.9947	3.2112	5.3284	5.4570	5.9872

Hspa9	_S[Phospho (STY)]DIGEVILVGGMT[Phospho (STY)]R_.2	P38647_S378_M2_Hspa9	11.1100	11.1139	11.1890	11.0036	10.4058	10.7039	10.7736	10.7654	10.8978	10.8227	10.6535	10.8489	11.0389	11.4149	10.4242	11.0057	8.9535	10.8635	10.8899	10.9046
Hspa9	_AM[Oxidation (M)]QDAEVSK_.2	#N/A	4.4442	3.7362	3.1505	3.1400	4.5118	4.4394	4.6731	3.1691	4.8066	4.8564	4.1063	4.5303	4.4751	4.0189	6.4098	4.5972	4.1671	4.7456	3.6726	4.2516
Hspa9	_RYDDPEVQKDTK_.3	#N/A	3.1526	5.2981	3.6889	4.5368	5.0431	5.5517	5.2333	5.3297	3.6090	5.8538	5.3830	3.2388	5.0900	4.9653	6.0756	5.7338	4.8883	5.2423	5.0021	5.0064
Hspa9	_M[Oxidation (M)]EEFKDQLPADEC[Carbamidomethyl (C)]NK_.3	#N/A	5.0368	3.8015	4.8616	4.0666	4.3932	4.4793	3.3236	3.7433	5.1561	4.8494	5.0739	4.3111	3.5485	4.1932	5.2851	4.7838	3.2405	4.5647	4.5530	4.9125
Hspa9	_AM[Oxidation (M)]QDAEVSKSDIGEVILVGGMTR_.3	#N/A	3.0942	3.4078	3.6305	4.9039	3.9328	4.0932	4.5723	3.9703	3.6674	3.3005	4.0418	4.3356	3.7755	6.4855	3.1855	3.4391	3.4675	3.6819	3.9810	2.9965
Hspa9	_ASNGDAWVEAHGK_.2	#N/A	5.1972	4.5375	5.7536	5.4311	5.2477	4.6264	4.9857	5.8527	5.4041	4.3270	5.1610	5.1285	4.5500	5.3049	6.2279	5.5369	5.4810	4.6341	5.6205	5.8486
Hspa9	_VEAVNM[Oxidation (M)]AEGIIHDTETK_.3	#N/A	4.6598	4.0958	4.0124	4.5872	3.3642	3.8175	4.2542	3.6259	3.9864	4.0303	3.7971	3.8405	5.0424	5.1724	4.0581	3.4952	4.3466	3.0148	3.9556	4.2536
Hspa9	_VINEPTAAALAYGLDKSEDK_.3	#N/A	4.1054	4.5857	3.7708	3.3500	3.4431	3.6672	4.0979	4.4599	3.0254	4.5452	3.0306	3.7868	4.2651	3.8088	3.6751	2.9495	3.9571	3.1923	3.4914	3.6682
Hspa9	_TIAPC[Carbamidomethyl (C)]QK_.2	#N/A	4.5766	4.2433	4.1132	4.5084	3.2827	3.3248	4.0328	3.3015	3.6856	4.3872	3.1642	3.9694	3.1067	3.8896	4.3051	3.9387	4.2995	4.3507	3.1490	4.7038
Hspa9	_MKETAENYLGHTAK_.3	#N/A	4.4566	4.3633	3.9932	4.8004	3.1627	3.1960	3.8854	3.1814	3.8056	4.2672	3.2843	4.0895	3.9711	5.2325	4.3952	3.8187	4.1795	2.9699	3.2690	4.4407
Hspa9	_GAVVGIDLGTTNSC[Carbamidomethyl (C)]VAVM[Oxidation (M)]EGK_.2	#N/A	3.5776	4.1236	4.3272	4.3535	3.3127	3.3284	4.0243	4.1833	3.8846	3.9729	3.3790	3.9336	4.3207	4.4752	3.7907	4.1437	4.3056	4.3544	3.1526	3.8346
Hspa9	_SQVFSTAADGQTQVEIK_.2	#N/A	4.0420	3.6379	3.4004	3.3245	4.2490	8.1107	4.3000	8.1296	4.2202	4.0219	3.6988	3.5273	4.3856	3.3874	6.8209	3.4041	3.1021	3.2302	4.3796	3.3544
Dnm1	_S[Phospho (STY)]PTSSPTPQR_.2	#N/A	10.0923	9.1177	9.6877	9.8071	8.4139	9.2534	9.0134	8.8818	9.1188	10.0010	9.4702	9.6096	8.1080	9.8184	8.4900	9.3810	9.5852	8.9978	9.3811	9.1029
Dnm1	_GISPVPINLR_.2	#N/A	10.5920	10.5168	10.6147	10.6596	10.7039	10.5569	10.7509	10.7812	10.3323	10.5709	10.4254	10.6599	10.6994	10.3904	10.5832	10.4811	10.5085	10.6409	10.5992	10.4775
Dnm1	_SSVLENFVGR_.2	#N/A	10.3526	10.4346	10.1581	10.1638	10.2388	10.1339	10.4402	10.5222	9.8266	10.1441	10.1011	10.3269	10.6037	10.3925	10.1075	10.1216	10.2779	10.3142	10.1068	9.9897
Dnm1	_VPVGDQPPDIEFQIR_.2	#N/A	9.3739	9.5239	9.7472	9.6605	9.0860	9.2908	9.4850	9.5456	9.5996	9.4622	9.3405	9.4000	9.7692	9.7207	9.2311	9.4738	9.5197	9.3825	9.4934	9.3035
Dnm1	_DITAALAAER_.2	#N/A	8.9376	9.5108	9.5981	9.4089	8.8569	9.3132	9.2348	8.9856	9.2097	9.2904	9.0997	9.1256	9.0106	9.5174	9.1661	9.0713	9.1448	9.2111	9.0692	9.0771
Dnm1	_VLNQQLTNHIR_.2	#N/A	7.9244	8.0939	8.0017	7.8439	8.0954	7.8304	8.3066	8.2073	7.6655	7.6264	7.7097	7.9643	7.9545	8.0506	8.1357	7.6934	7.7289	8.0931	7.8960	7.8766
Dnm1	_VYSPHVLNLTLVDLPGM[Oxidation (M)]TK_.3	#N/A	9.3502	9.1142	8.4516	8.7838	8.9206	8.7257	8.7560	9.3536	8.9618	8.9946	8.8428	8.8957	9.2225	8.9055	8.6996	9.0644	8.9958	9.1856	9.0345	8.9927
Dnm1	_IVTTHIR_.2	#N/A	8.6818	8.8378	8.9504	8.6480	8.6724	8.6908	9.1995	9.0163	8.5509	8.7040	8.2910	8.8853	8.7288	8.8136	8.9723	8.4750	8.5865	8.7129	8.9179	8.7719
Dnm1	_FFLSHPSYR_.2	#N/A	9.1089	8.8229	8.6316	8.9478	8.9159	8.7892	8.6137	9.1702	8.3272	8.2426	8.6304	8.8376	8.9409	8.6761	8.1417	8.3723	8.6874	8.8035	8.7360	8.7139
Dnm1	_NFRPDDPAR_.2	#N/A	8.3334	7.8726	8.8864	8.1787	8.7144	8.3962	7.9502	8.2169	8.5836	7.7032	8.6676	8.1238	7.7043	8.7076	8.6549	8.1403	8.5329	8.4560	8.2945	8.4480
Dnm1	_GYIGVVNR_.2	#N/A	7.0965	7.9067	7.9191	8.4177	7.7418	8.0948	8.5530	7.0149	8.2522	8.5601	8.4529	7.9896	8.7717	8.2273	8.3659	8.4471	6.5964	8.5198	8.4991	8.2778
Dnm1	_LQDAFSAIGQNADLDLPQIAVVGGQSAGK_.3	#N/A	7.4257	7.2234	7.9340	7.8406	7.4472	7.2661	7.5498	7.6499	7.5122	7.6682	7.4678	7.5070	7.8415	8.0631	7.6962	7.8023	7.8281	7.7157	7.6505	7.6857
Dnm1	_NVYKDYR_.2	#N/A	7.0980	7.6438	7.1089	7.0761	7.3471	7.3654	7.6242	7.3495	7.2618	7.4345	7.4262	7.2525	7.6148	7.3102	7.3369	7.5231	6.9231	7.3892	7.1276	7.3354
Dnm1	_C[Carbamidomethyl (C)]VDM[Oxidation (M)]VISELISTVR_.2	#N/A	5.2839	4.7287	5.7509	5.4088	5.5225	5.0919	4.6237	5.8967	5.4569	6.2187	5.2881	5.1438	5.2813	4.6964	5.7625	5.9824	5.3480	4.9082	5.8197	5.3009
Dnm1	_GM[Oxidation (M)]EDLIPLVNR_.2	#N/A	9.8755	10.0025	9.8566	10.0042	9.9622	9.9651	10.0175	10.1074	9.5518	9.5744	9.8879	9.8267	9.8616	9.9359	9.6208	9.5772	9.9319	10.0173	9.8884	9.9001
Dnm1	_KKTS[Phospho (STY)]GNQDEILVIR_.3	#N/A	3.4628	3.6812	8.6393	7.6144	3.6698	7.3030	8.5868	4.0807	3.3178	7.5914	3.6522	3.4686	3.4832	8.2987	3.4229	7.5477	7.3470	7.9852	4.2183	7.6569
Dnm1	_EVDEYKNFRPDDPAR_.3	#N/A	6.7114	6.4042	7.5356	6.8049	6.3493	6.8549	6.5739	7.1007	7.3648	6.5112	6.6355	6.7127	4.4797	7.1971	7.4740	7.4295	6.6461	6.8228	7.1193	6.8299
Dnm1	_M[Oxidation (M)]GTPYLQK_.2	#N/A	6.9125	7.0916	3.3459	5.9025	6.9290	6.8023	7.1455	7.0614	7.0850	6.9499	6.3210	7.0518	6.9136	6.2611	6.6768	6.6139	6.4071	7.1249	6.2391	7.0068
Dnm1	_FTDFEEVR_.2	#N/A	6.2749	5.9602	6.3481	6.5080	5.7054	6.3353	6.4983	5.5492	6.3401	6.0898	6.8343	6.0655	4.0172	6.3139	6.3593	5.9866	6.2157	6.3954	6.4763	5.9605
Dnm1	_ALLQM[Oxidation (M)]VQQFAVDFEKR_.3	#N/A	4.8659	3.9348	4.4058	4.1731	5.0263	4.5216	3.4446	5.2777	3.2601	4.8788	4.5382	5.0857	4.7236	3.8745	4.3400	4.4855	5.0846	5.1857	3.7997	4.1537
Dnm1	_LDLM[Oxidation (M)]DEGTDAR_.2	#N/A	8.0777	8.1958	8.8211	8.7065	7.8070	8.1517	8.4170	8.0104	8.2100	8.2658	8.3294	7.8733	7.8411	8.6554	8.1385	7.6158	8.3544	8.1149	7.8590	7.8866
Dnm1	_NLVDSYM[Oxidation (M)]AIVNK_.2	#N/A	6.6443	6.6266	6.7189	6.8634	6.6324	6.4371	6.4675	6.8991	6.3589	6.7374	6.8957	6.6596	6.8315	6.8804	6.0828	6.2014	6.4946	6.4507	6.4012	6.8855
Dnm1	_LREEM[Oxidation (M)]ER_.2	#N/A	4.3969	5.4014	5.6973	5.9140	4.6700	5.3652	5.7410	5.0111	4.9620	4.7866	5.8024	4.9484	4.6723	5.8744	5.9044	5.4865	4.5602	4.6519	4.2002	4.6102
Dnm1	_EISYAIK_.2	#N/A	3.8255	6.2931	5.8425	5.7628	5.4815	6.0860	6.3971	4.0469	6.6204	4.0318	6.5211	6.2169	6.6306	5.7852	6.8545	6.3978	4.0636	6.6470	6.4320	7.0912
Dnm1	_KDITAALAAER_.2	#N/A	5.8884	5.4638	5.3658	5.4280	4.9627	5.5999	5.4840	5.2495	5.4176	5.6058	5.5540	3.8327	4.3110	5.0341	5.3120	5.2412	5.8768	4.8362	5.4025	8.1925
Dnm1	_TSGNQDEILVIR_.2	#N/A	7.2850	7.1297	7.9586	8.0057	6.6340	7.9179	7.0831	7.1461	7.5098	7.4881	6.7015	6.9458	6.7897	7.9608	6.8309	7.3380	7.4290	7.5721	7.0185	7.6341
Dnm1	_ENC[Carbamidomethyl (C)]LILAVSPANSDLANSDALK_.2	#N/A	7.0810	7.3123	7.2675	7.1535	6.7180	6.9654	7.5925	7.1553	7.3707	7.0255	7.1022	7.2704	7.2804	7.5966	6.8702	7.2158	7.2654	7.1939	7.1691	7.2436
Dnm1	_RSPTSS[Phospho (STY)]PTPQR_.2	#N/A	7.4299	6.8595	3.9827	7.0735	3.3491	6.5860	6.6536	4.0990	6.5384	7.1812	6.7402	3.9514	7.3399	7.1110	7.4900	6.5703	7.1169	6.7995	3.8084	9.1138
Dnm1	_LEIEAETDRVTGTNK_.2	#N/A	3.9074	4.9949	5.3851	3.9589	3.2693	4.0641	4.8823	3.7064	5.0626	4.3896	4.7535	3.8346	4.2252	4.9107	3.7198	4.6979	3.6988	4.4246	4.4894	4.7741
Dnm1	_S[Phospho (STY)]PTSS[Phospho (STY)]PTPQRR_.2	#N/A	7.3714	6.7174	5.4356	7.3217	6.7600	7.0858	6.9264	5.1055	7.2845	8.0921	6.9993	7.8255	7.8275	3.8039	4.8693	3.4305	7.5393	7.1149	7.1546	7.3195
Dnm1	_IEGSGDQIDTYELSGGAR_.2	#N/A	6.7784	7.1566	6.4006	6.8708	6.1182	6.9584	6.7322	6.3777	6.6384	6.8458	5.8859	6.0593	6.2519	7.3846	6.0165	5.9458	5.9162	6.6923	5.8158	6.4806
Dnm1	_KTSGNQDEILVIR_.3	#N/A	4.8672	4.6714	4.3089	4.7827	4.6144	4.7788	4.5276	3.8129	4.3759	5.0198	4.6611	4.5115	3.7489	3.9781	4.0383	4.4229	4.7212	4.1811	5.2680	4.8919
Dnm1	_RS[Phospho (STY)]PT[Phospho (STY)]SSPTPQRR_.2	#N/A	4.0942	4.1226	3.8529	3.9174	4.1577	4.0851	3.7978	3.8079	3.7612	4.3256	3.4221	3.9834	3.9085	3.6385	4.0119	3.6784	4.1995	4.5135	4.0930	3.5316
Dnm1	_YM[Oxidation (M)]LSVDNLK_.2	#N/A	6.6466	6.6683	6.7393	7.2711	6.8348	6.6245	6.9123	6.6034	7.2179	6.5759	6.7933	6.8058	6.7635	7.0484	6.4554	6.4300	6.4827	7.2745	6.6902	6.5611
Dnm1	_TIM[Oxidation (M)]HLM[Oxidation (M)]INNTK_.2	#N/A	5.3063	4.9526	5.3489	4.6229	5.2255	4.6810	4.9942	5.1147	5.1178	5.3293	4.1716	5.5895	5.4546	3.8649	4.8925	5.6930	4.9796	5.2605	5.2093	5.0812
Dnm1	_LQSQLLSIEKEVDEYK_.3	#N/A	4.2460	4.4499	3.8701	4.2853	3.2954	3.8187	4.0452	4.2385	3.9458	4.4238	3.9156	3.8998	4.4842	4.1734	3.7657	3.3778	4.2438	4.4433	3.6277	4.3063
Dnm1	_LDLM[Oxidation (M)]DEGTDARDVLENK_.3	#N/A	3.6046	5.4083	4.6277	5.4172	4.7921	4.8050	5.0195	5.3913	5.0668	5.2422	5.0221	5.4194	6.0067	5.6680	5.0030	5.5909	4.6166	5.1209	5.5356	5.0705
Dnm1	_ALLQM[Oxidation (M)]VQQFAVDFEK_.2	#N/A	4.6205	5.1790	4.6966	4.7905	5.3279	5.1401	4.7785	5.2901	4.7535	4.9278	5.2672	4.7778	4.9374	4.8722	4.7313	4.9000	5.4632	5.3704	4.0742	4.2044
Dnm1	_HIFALFNTEQR_.2	#N/A	7.0690	7.2883	7.5105	6.9852	6.9730	6.7106	7.0726	7.2483	7.0343	6.8572	7.1125	6.6670	7.4962	7.3698	6.5474	6.8073	7.0522	6.9231	7.1414	6.6968
Dnm1	_RIEGSGDQIDTYELSGGAR_.3	#N/A	4.6718	5.1445	4.8008	5.1999	4.7414	4.4886	5.1126	4.6453	4.8306	5.0874	4.6502	4.9979	5.1381	5.4180	4.5732	4.4006	4.7597	5.0529	4.3341	4.7691
Dnm1	_QLELAC[Carbamidomethyl (C)]ETQEEVDSWK_.2	#N/A	3.8536	4.1492	4.3899	3.8358	3.1914	3.4154	4.3497	3.2108	4.4088	4.0600	3.2824	4.5679	4.5169	8.5493	3.9269	4.1985	4.2089	7.4491	3.2396	3.7379
Dnm1	_RPLVLQLVNSTTEYAEFLHC[Carbamidomethyl (C)]K_.3	#N/A	3.7473	3.5718	3.9579	3.8369	3.9542	4.3481	3.0706	4.5786	3.6701	3.2978	4.0445	3.2979	3.7782	3.6432	3.1192	3.1094	3.4702	3.6792	3.9783	2.9992
Dnm1	_EALSIIGDINTTTVSTPM[Oxidation (M)]PPPVDDSWLQVQSVPAGR_.4	#N/A	3.6890	4.2901	3.2256	3.8952	3.8959	3.9293	3.1522	3.9147	3.0724	3.6128	4.0518	3.3562	3.2378	3.2636	3.1775	3.0511	3.4119	4.3780	2.7729	3.7075
Dnm1	_EFIFSELLANLYSC[Carbamidomethyl (C)]GDQNTLM[Oxidation (M)]EESAEQAQR_.3	#N/A	3.8809	3.4382	3.4174	3.7034	4.0878	4.0206	4.4612	4.1065	3.8037	3.6914	4.1780	3.1644	3.9117	3.4555	2.9857	3.2430	3.6038	3.5457	3.8447	3.1328
Dnm1	_ASETEENGSDSFMHSMDPQLER_.3	#N/A	3.4477	3.8714	4.4851	3.6453	3.6546	4.4537	3.3114	3.6734	3.3705	3.5974	3.7449	3.5975	3.4786	4.5232	2.8886	4.3107	3.1706	3.9788	4.2779	4.2005
Tjp1	_ERS[Phospho (STY)]LS[Phospho (STY)]PR_.2	P39447_S166_M2_Tjp1	9.6913	10.0035	10.2627	10.1951	9.7532	9.8047	9.5898	9.8818	10.0248	9.7641	9.6412	9.6951	10.0439	10.1150	9.4127	9.7213	9.9611	9.6007	9.7158	9.7837
Tjp1	_AVPVS[Phospho (STY)]PSAVEEDEDEDGHTVVATAR_.3	P39447_S1614_M1_Tjp1	9.2656	9.5842	4.3344	4.2872	3.5039	8.7412	8.8570	8.8265	3.4644	9.9730	9.0154	9.2447	3.6298	10.0036	3.5695	9.3899	4.5208	4.1295	9.2152	9.0883
Tjp1	_EIS[Phospho (STY)]QDSLAAR_.2	P39447_S212_M1_Tjp1	7.2999	4.3100	7.6018	7.4722	3.0306	3.4535	3.0097	6.4527	3.5481	8.1297	3.0267	4.1994	3.7135	4.2162	6.1263	4.0377	3.9309	3.9023	3.5509	4.1832
Tjp1	_S[Phospho (STY)]VAS[Phospho (STY)]SQPAKPTK_.2	P39447_S175_M2_Tjp1	8.7218	8.7938	8.8133	8.9351	8.3355	8.5991	8.4233	8.4036	8.4959	8.9064	8.1916	8.3883	8.4531	8.9352	7.6394	8.3234	8.6468	7.7773	8.3801	8.3644
Tjp1	_ADGAT[Phospho (STY)]SDDLDLHDDR_.2	P39447_T809_M1_Tjp1	4.1646	4.0157	5.2570	5.1611	3.6964	4.9045	4.2129	3.7151	4.1331	3.9752	3.6118	3.7418	4.4569	5.4898	3.5632	3.5267	3.8875	3.2974	3.5965	3.4165
Tjp1	_KS[Phospho (STY)]REDLSAQPVQTK_.3	#N/A	4.3730	6.1830	4.8493	4.5699	5.0044	4.3971	3.5830	4.9773	3.6421	3.3258	4.0165	4.2512	6.7609	3.6429	3.1602	5.5155	3.3576	5.5342	5.6068	5.2559
Tjp1	_LSYLSAPGSEYSM[Oxidation (M)]YSTDS[Phospho (STY)]R_.2	P39447_S837_M1_Tjp1	4.5307	3.4721	3.5661	3.1587	3.8684	4.0925	3.6727	4.2311	5.6904	3.2362	4.1061	3.3616	3.8398	3.5532	4.6792	3.3747	3.2679	3.6176	4.2139	3.0609
Tjp1	_DDISEIQSLAS[Phospho (STY)]DHS[Phospho (STY)]GR_.2	P39447_S297_M2_Tjp1	4.1891	4.9431	3.7256	3.3952	2.8952	4.4293	4.1529	4.4147	4.0731	3.9997	3.5518	4.3570	4.2386	6.0216	4.6019	3.5512	3.9120	3.9015	3.5365	3.2074
Tjp1	_SAS[Phospho (STY)]LENKK_.2	P39447_S1277_M1_Tjp1	4.7647	4.8880	3.6042	3.1207	3.9064	3.3926	3.6346	4.2691	3.6937	3.8857	3.6172	3.3235	4.5894	3.5912	3.2118	3.4128	3.3059	3.0264	4.1758	3.5582
Tjp1	_S[Phospho (STY)]REDLSAQPVQTK_.2	#N/A	8.7838	8.5682	8.7439	8.9190	8.4790	8.5411	8.1926	8.7017	8.4373	8.9063	8.3719	8.5474	8.7753	8.9764	8.0734	8.4462	8.7748	8.3803	8.3475	8.6347
Tjp1	_IDS[Phospho (STY)]PGLKPASQQK_.2	#N/A	5.4374	5.7872	5.6491	5.6563	5.5524	5.5539	5.1797	5.2958	5.2869	5.1090	5.0943	5.8475	3.5239	5.6649	5.3347	5.5652	6.1692	5.3006	5.4624	4.4948
Tjp1	_ATLLNVPDLSDSIHS[Phospho (STY)]ANAS[Phospho (STY)]ER_.3	P39447_S284_M2_Tjp1	3.7959	4.3877	3.9995	3.9858	3.1690	4.0361	3.9097	3.6472	3.8299	4.2735	3.3086	4.1138	3.9954	3.8045	3.9351	3.8250	4.3946	3.9080	3.0705	3.7980
Tjp1	_VQIPVSHPDPEPVS[Phospho (STY)]DNEDDSYDEEVHDPR_.4	P39447_S125_M1_Tjp1	3.9359	7.9054	4.4437	3.9807	3.2736	7.1232	7.2194	3.6144	6.3270	4.1422	3.2160	3.8837	4.0404	9.2403	6.6550	4.2808	4.2946	8.8192	4.8486	3.5532
Tjp1	_KVQIPVSHPDPEPVS[Phospho (STY)]DNEDDSY[Phospho (STY)]DEEVHDPR_.4	#N/A	3.7968	4.2060	4.3331	3.8926	3.1345	3.3586	4.4066	3.4972	4.4657	4.0031	3.3392	4.0955	4.5737	4.3202	3.9838	4.1417	4.0348	4.3845	3.4469	3.7948
Tjp1	_QAS[Phospho (STY)]RDLEQPSYR_.2	P39447_S1051_M1_Tjp1	4.3997	4.4202	3.9363	3.1845	3.1058	3.5017	4.2634	6.8866	4.3225	4.1462	3.1961	4.1463	4.4306	3.9744	3.9677	6.3150	4.1226	3.0268	3.3259	5.7664
Fyn	_DGS[Phospho (STY)]LNQSSGYR_.2	#N/A	10.6365	10.5063	10.8478	10.6743	10.1292	10.3167	10.2973	10.1849	10.1531	10.5255	10.1431	9.7928	10.1043	10.4043	9.0640	9.7958	10.0167	10.0722	9.9415	9.9970
Fyn	_GAY[Phospho (STY)]SLSIR_.2	P39688_Y185_M1_Fyn	4.2975	5.0357	3.8341	4.9548	4.4605	3.0369	4.0445	4.5232	4.8307	5.2461	5.4231	5.2505	4.1302	5.2767	4.7317	5.7758	4.0204	3.1290	3.4281	5.5723
Fyn	_WTAPEAALYGR_.2	#N/A	5.6165	5.2333	3.1844	5.2560	5.3976	5.4177	5.3456	6.3996	5.5810	4.6341	5.2654	5.8636	4.3614	3.6475	5.4193	5.3069	5.6494	5.9030	5.4430	5.2151
Fyn	_LT[Phospho (STY)]EERDGSLNQSSGYR_.3	P39688_T15_M1_Fyn	3.9939	4.7976	3.5484	4.0994	3.7262	4.1247	4.5504	3.7488	4.4700	3.8589	4.0113	3.7237	5.1750	3.5719	4.7538	4.0669	4.5011	3.6610	5.0398	4.4441
Cap1	_ALLATASQC[Carbamidomethyl (C)]QQPAGNK_.2	#N/A	7.4403	7.5607	7.9550	7.7820	7.2794	7.4540	7.7069	7.7755	7.7432	7.6672	7.2367	7.8820	7.2202	8.0089	7.7457	7.3921	7.6906	7.4566	7.6042	7.7823
Cap1	_NSLDC[Carbamidomethyl (C)]EIVSAK_.2	#N/A	7.2825	7.1791	7.4038	6.9902	7.2478	7.0399	7.2446	7.7478	7.5290	7.0882	7.2180	6.8692	7.2997	7.6872	7.2209	7.1426	7.2558	7.0245	7.1892	7.4010
Cap1	_VPTISINK_.2	#N/A	6.6373	7.1891	7.0651	7.2289	7.2391	6.8688	7.3153	6.8720	6.9667	7.3089	7.1498	7.0466	7.7841	7.3283	7.5684	7.5134	6.8118	7.5083	7.6086	7.5406
Cap1	_KEPALLELEGKK_.3	#N/A	5.9776	6.1443	5.4905	5.9037	5.3652	5.5606	5.8370	6.2085	5.0885	6.1626	5.7464	5.2158	5.6413	5.7865	6.1528	6.4270	6.3213	5.9604	5.9304	5.9855
Cap1	_TDGC[Carbamidomethyl (C)]HAYLSK_.2	#N/A	5.5610	5.3790	4.9167	5.6736	4.9036	5.6761	5.6441	3.5482	5.1445	5.5321	5.3372	5.0547	5.8582	5.7535	5.7410	5.6002	5.5767	5.1574	5.6603	4.9350
Cap1	_INSITVDNC[Carbamidomethyl (C)]KK_.2	#N/A	4.5879	6.6117	6.0275	5.6096	5.6436	5.6749	6.1395	5.1592	5.5353	5.8620	5.6894	5.0253	3.4947	5.6549	6.0651	4.8595	5.0275	5.5642	4.9169	5.2754
Cap1	_SALFAQINQGESITHALK_.2	#N/A	4.8066	4.8843	4.6571	4.4251	5.5182	4.6501	4.7564	3.7336	5.1954	5.0550	5.1716	5.3829	5.7750	5.2414	4.1224	3.9603	4.8027	4.8397	4.4999	5.1112
Cap1	_LSDLLAPISEQIQEVITFR_.2	#N/A	4.0076	5.1127	4.6089	3.0176	3.6233	5.5446	5.1675	5.5913	4.0520	4.5250	4.6103	4.7647	4.7804	5.4936	3.5701	4.5039	5.6237	5.3804	4.9483	4.0549
Cap1	_TLWNGQK_.2	#N/A	4.0627	3.5996	3.4386	4.4272	4.5171	4.3890	3.1252	4.1035	3.2262	3.5157	3.6541	4.1229	3.9673	2.5699	4.5689	3.2472	3.1723	4.5888	4.1472	3.8993
Cap1	_[Acetyl (Protein N-term)]ADMQNLVER_.2	#N/A	4.4834	6.0407	6.7897	6.7575	3.8211	5.5213	6.1943	4.1837	4.9066	4.4498	6.0884	4.7200	3.8871	6.8841	5.5667	3.3274	3.2206	3.1117	4.2612	4.9776
Cap1	_LEAVSHTSDM[Oxidation (M)]HC[Carbamidomethyl (C)]GYGDS[Phospho (STY)]PSK_.3	#N/A	6.3410	4.6986	5.5789	5.7938	5.1803	4.7863	3.9891	5.2125	6.0250	6.6034	6.2405	6.0235	5.8955	5.2605	5.9021	5.8688	6.1411	5.8303	5.9255	6.0185
Cap1	_C[Carbamidomethyl (C)]VNTTLQIK_.2	#N/A	3.9845	4.5765	4.5208	4.5734	5.1674	4.8930	4.2188	3.6849	4.7107	4.1909	3.1514	3.9731	4.3860	4.7776	4.2892	4.3294	3.8205	3.6106	3.2592	3.6070
Cap1	_VENQENVSNLVIDDTELK_.2	#N/A	7.4624	6.8200	6.9518	6.9856	3.8421	4.0662	6.5479	3.9912	6.8845	6.8025	4.1466	3.4020	7.3516	7.3725	3.2903	7.0387	6.8909	6.8542	6.6299	3.1607
Cap1	_EM[Oxidation (M)]NDAAM[Oxidation (M)]FYTNR_.2	#N/A	3.8951	5.4735	5.2229	4.8697	5.9815	5.7123	5.2594	6.1154	5.7390	5.6453	5.3564	5.8291	5.4664	5.0340	5.7872	6.0525	5.9181	5.9278	5.7655	5.8887
Cap1	_PQTS[Phospho (STY)]PSPKPATK_.2	P40124_S307_M1_Cap1	8.5269	8.4763	8.6298	8.6425	3.4066	3.6307	7.6556	4.4964	7.7792	8.1378	3.0671	7.8960	8.1195	8.7870	7.5731	8.4062	8.4560	8.2820	3.4548	8.3961
Cap1	_EFHTTGLAWSK_.3	#N/A	3.9368	4.0661	5.2531	3.7526	1.8170	3.2096	3.5833	3.6371	3.9654	4.2808	2.0219	3.9555	4.1956	4.4601	3.8972	4.2816	4.1748	3.6584	1.7412	4.4271
Tkt	_VLDPFTIKPLDR_.2	#N/A	7.1842	7.1348	6.8111	7.1355	7.1668	7.1536	6.8021	7.3650	6.4896	7.2087	6.7568	6.8703	7.0803	7.2346	7.1311	6.8313	7.2050	6.9173	6.8044	7.0992
Tkt	_LAVSQVPR_.2	#N/A	7.1384	7.9695	7.5033	7.7143	7.7808	7.9543	7.8941	7.3170	7.7706	7.9668	7.4489	7.8878	6.8975	7.5692	8.1465	7.7171	6.3446	7.9365	7.9860	7.9893
Tkt	_LDNLVAIFDINR_.2	#N/A	7.8475	7.9988	8.2383	8.0289	7.7057	7.5203	7.7139	7.6774	7.8081	7.8246	7.2699	6.9448	7.6946	7.5036	7.8628	7.9321	8.0040	7.7378	7.6457	7.7852
Tkt	_LGQSDPAPLQHQVDIYQK_.3	#N/A	6.8093	7.2271	7.1693	7.1855	7.1463	7.0956	7.0678	7.3685	7.3479	7.0419	6.8206	6.6261	7.6164	7.4699	7.0853	6.8096	7.0320	6.9671	6.7784	7.1527
Tkt	_HQPTAIIAK_.2	#N/A	7.1260	7.3839	7.2347	7.1070	6.9629	7.1112	7.0054	7.0745	6.4421	7.2406	6.6872	7.2212	6.6439	6.5602	7.3002	6.8819	6.7145	6.5880	7.0352	7.1323
Tkt	_IIALDGDTK_.2	#N/A	6.8173	6.1263	6.9717	6.3487	6.8465	6.5712	6.3978	6.5372	6.4640	6.7018	6.6317	6.7666	6.2612	6.4620	6.9212	6.7302	5.5532	6.2330	6.8564	6.4690
Tkt	_NSTFSELFK_.2	#N/A	6.3918	6.0151	6.1240	6.7070	6.3833	6.5850	6.1362	6.3447	6.3383	6.7347	6.5485	6.2586	6.2541	6.7656	6.2455	6.2463	5.5546	6.2019	6.0110	6.3992
Tkt	_NM[Oxidation (M)]AEQIIQEIYSQVQSK_.2	#N/A	5.4976	5.7537	6.0530	5.3302	5.6119	5.7524	5.2261	5.5589	5.6655	6.1599	5.8653	5.2791	4.4817	6.1746	5.6787	6.0396	5.5240	5.8400	5.7493	5.7682
Tkt	_ILATPPQEDAPSVDIANIR_.2	#N/A	7.8979	7.9553	7.9378	8.1298	7.7699	7.8302	7.7725	7.9662	8.0393	7.7785	7.3606	7.6322	7.8290	8.1646	7.7278	8.0536	7.7530	7.6102	7.6057	7.5281
Tkt	_TVPFC[Carbamidomethyl (C)]STFAAFFTR_.2	#N/A	6.1763	5.7090	6.7128	7.1439	5.9645	6.0281	6.0698	5.5941	6.1288	3.7179	3.6244	5.9792	7.2217	6.6060	6.1361	5.7773	6.4419	5.4120	6.1562	5.9544
Tkt	_SVPM[Oxidation (M)]STVFYPSDGVATEK_.2	#N/A	8.3465	8.2625	8.4201	8.0395	8.2700	8.2192	8.1701	8.6248	7.9666	8.3471	8.2156	8.3738	8.2032	8.4931	8.1275	8.4147	8.2217	7.7724	7.7726	8.0683
Tkt	_M[Oxidation (M)]PTPPSYK_.2	#N/A	5.4140	5.8250	4.1468	4.4749	4.7131	4.9907	5.6211	5.0563	4.8662	5.2192	4.7136	5.6817	3.8175	5.0908	5.8402	5.0787	5.0381	5.3958	4.4893	5.7191
Tkt	_TSRPENAIIYSNNEDFQVGQAK_.3	#N/A	5.3561	5.8200	5.6945	3.4265	5.4404	5.3593	4.9981	5.6297	4.5155	5.4097	5.2019	4.7870	5.2658	6.1282	4.9251	4.8764	4.8134	5.1373	5.7208	4.8435
Tkt	_C[Carbamidomethyl (C)]EAFGWHTIIVDGHSVEELC[Carbamidomethyl (C)]K_.3	#N/A	3.6027	4.0830	4.2735	4.3482	2.6945	3.1645	3.0998	3.4617	3.0463	3.8090	3.5333	3.8091	4.2538	4.4698	4.1778	3.9476	4.4598	3.6248	2.9887	2.8861
Tkt	_ISSDLDGHPVPK_.3	#N/A	3.3043	4.0148	4.3417	4.2799	3.5112	3.5446	3.5369	3.3022	3.4571	2.7321	2.9357	1.0214	3.6225	4.1252	2.0148	4.1672	3.8399	3.9933	3.6419	4.0922
Tkt	_ILTVEDHYYEGGIGEAVSAAVVGEPGVTVTR_.3	#N/A	4.1351	3.8677	3.1705	3.5543	3.4728	1.8460	2.0147	4.2887	4.1274	1.4878	3.0009	1.6861	4.2354	3.4813	3.6455	2.9791	4.3731	3.2220	3.5210	3.4565
Tkt	VYC[Carbamidomethyl (C)]M[Oxidation (M)]LGDGEVSEGSVWEAM[Oxidation (M)]AFAGIYK_.	#N/A	4.2241	3.7787	3.2596	3.4687	3.5618	3.5722	3.9792	4.3412	4.0383	4.4304	2.9119	3.6681	4.1464	3.6902	3.5564	3.0682	3.8384	3.3110	3.6101	3.3674
Vps26a	_FES[Phospho (STY)]PDSQASAEQPEM_.2	P40336_S315_M1_Vps26a	3.6849	8.9899	4.2212	9.7246	3.0227	3.2467	8.7931	3.3853	7.4715	3.8913	3.4510	4.2073	3.1847	9.5565	4.0956	4.0298	3.9230	6.4823	3.0709	3.9066
Syt4	_LFTETEKEANS[Phospho (STY)]PESLK_.2	P40749_S135_M1_Syt4	6.3848	6.6311	6.1984	6.7397	5.3634	6.3405	5.9056	4.1250	6.4797	6.6075	3.8416	3.1459	6.5699	6.2916	4.8289	5.6015	7.0325	6.3799	6.0901	5.6621
Rpl28	_QTYSTEPNNLK_.2	#N/A	5.5172	5.5840	5.1252	5.5720	3.0873	5.6173	5.5843	5.6145	5.9914	3.9559	5.4545	5.8995	4.7847	5.3467	5.7934	5.3725	5.2104	5.5854	4.9030	5.1315
Rpl28	_[Acetyl (Protein N-term)]SAHLQWMVVR_.2	#N/A	3.9579	3.5311	4.0884	3.7962	3.6879	4.1134	3.7518	4.0590	3.3177	3.8025	4.1305	3.5565	4.1873	4.0283	3.2742	3.8560	4.4030	3.6385	3.8950	4.5794
Abca2	_VSEEDQS[Phospho (STY)]LENS[Phospho (STY)]EADVK_.2	#N/A	6.6216	6.9279	6.9929	6.7715	5.8082	6.6732	6.3771	6.9885	6.4696	6.6928	6.2352	5.7659	6.4390	5.7957	5.1757	5.8258	6.8174	6.4750	6.0744	5.8164
Abca2	_QPAEPGTSQEPGLASS[Phospho (STY)]PSGC[Carbamidomethyl (C)]PR_.2	P41234_S1238_M1_Abca2	3.2122	4.1068	4.2497	4.3720	3.4192	3.1884	4.6607	3.4379	4.6989	3.8329	3.5094	5.0896	3.2431	4.2877	3.3519	4.0752	4.4360	5.4409	3.0125	3.9650
Abca2	_VSEEDQS[Phospho (STY)]LENS[Phospho (STY)]EADVKESR_.2	#N/A	4.6358	4.5903	3.7640	4.3675	4.4764	4.1428	4.5256	4.9483	4.2389	4.3934	4.2308	3.6650	4.2583	4.5258	3.6568	4.1578	5.2210	5.0257	4.1561	3.8543
Tac1	_DADSS[Phospho (STY)]VEKQVALLK_.2	P41539_S76_M1_Tac1	4.5273	3.4755	3.5627	3.1621	3.8650	5.3743	4.7485	4.2277	5.8410	3.2328	4.1095	6.0697	4.5480	6.8088	6.8973	6.6445	6.4176	3.0678	4.2172	6.3055
Eci1	_EADIQNFTSFISK_.2	#N/A	4.0752	4.6163	3.9228	4.3028	4.2251	3.7598	3.8025	3.4568	3.3751	3.5929	3.7494	3.0049	3.4073	3.9099	3.6369	3.7320	4.2809	3.5163	3.7975	3.4868
Foxk1	_QS[Phospho (STY)]PGPALAR_.2	P42128_S87_M1_Foxk1	8.1118	8.4784	8.7527	7.9365	8.1022	8.4294	8.1113	8.3931	7.6365	8.5253	8.1871	8.0479	8.2226	8.8029	7.9613	8.1759	8.5391	8.0255	8.2195	8.4185
Foxk1	_EGS[Phospho (STY)]PIPHDPDLGSK_.2	#N/A	8.5543	8.5769	8.2998	8.3973	7.9509	8.5210	8.0688	8.5403	8.2477	8.4461	8.2976	8.2019	8.7701	8.8691	7.6815	8.2484	8.6519	8.3196	8.2988	8.3559
Foxk1	_S[Phospho (STY)]APASPT[Phospho (STY)]HPGLM[Oxidation (M)]S[Phospho (STY)]PR_.2	P42128_T408_M3_Foxk1	3.9006	6.7906	8.6259	8.0888	3.6615	4.7211	6.4455	7.0440	3.7958	4.1236	3.3371	3.8213	3.9271	8.0800	4.0546	3.7200	4.0808	3.7120	3.4128	3.5360
Septin2	_LTVVDTPGYGDAINC[Carbamidomethyl (C)]R_.2	#N/A	5.8623	6.2152	5.4771	5.2692	5.7572	5.2793	5.2685	5.5682	5.0214	5.3990	5.4233	5.3469	3.1857	6.1407	5.3787	5.1121	6.0591	5.2538	5.5500	4.3837
Septin2	_TVQIEASTVEIEER_.2	#N/A	7.9284	8.1403	7.1331	6.7720	6.7616	6.7782	6.7233	7.9962	6.9744	7.4405	7.0543	7.6812	8.6094	8.3164	6.7956	7.2982	7.4292	6.6923	6.7598	6.9882
Septin2	_TIISYIDEQFER_.2	#N/A	4.4037	4.9741	4.7834	4.8356	5.2143	4.6928	5.0280	5.5405	5.1241	6.0444	4.7062	4.7003	5.1036	4.1058	4.9801	3.5274	4.3530	5.1742	4.9104	4.4961
Septin2	_IYHLPDAES[Phospho (STY)]DEDEDFKEQTR_.3	#N/A	9.2069	10.8573	10.3394	9.7390	5.2668	10.5033	10.5011	4.2578	10.4246	10.4861	8.1596	9.6779	8.6920	11.8079	10.3699	9.8183	8.9796	10.8948	10.7984	10.5734
Septin2	_STLINSLFLTDLYPER_.2	#N/A	2.9044	3.3614	3.7547	4.1342	3.2677	4.0715	4.9296	3.3466	4.4310	4.8001	3.7074	4.3151	4.3482	3.5983	4.4733	4.1862	3.3130	2.7501	3.5426	4.7587
Septin2	_IYHLPDAES[Phospho (STY)]DEDEDFK_.2	#N/A	3.6549	4.1075	3.7169	4.0105	4.1738	3.8813	3.1315	3.6728	3.9891	3.6529	4.1251	3.9454	3.3110	3.7240	2.9337	3.5321	3.6271	3.8898	3.8419	3.5826
Septin2	_QQPTQFINPETPGYVGFANLPNQVHR_.3	#N/A	4.3492	3.7866	3.8858	3.2350	4.6038	4.8210	5.0917	3.0741	4.2721	4.1967	3.1456	4.9895	4.3801	3.9239	3.7902	4.3353	5.3005	3.8407	4.1153	3.6012
Eps15	_AEITSQESQISSYEEELLK_.2	#N/A	5.5981	6.2463	5.0551	5.3367	5.0530	4.3256	4.8845	5.3673	4.1018	5.0307	5.0618	5.5773	5.9443	6.7129	4.7664	4.9471	6.0399	5.0360	4.1305	5.3181
Eps15	_NITGSS[Phospho (STY)]PVADFSAIK_.2	P42567_S324_M1_Eps15	7.9587	7.6380	7.7129	4.0616	4.1547	7.2919	7.1348	7.3948	6.8870	7.3541	7.3650	3.0752	7.4619	8.2303	6.6061	3.6611	7.7638	7.1851	7.1941	7.2166
Pura	_VSEVKPTYR_.2	#N/A	8.9976	8.8452	9.1885	9.0185	8.8086	8.7723	8.9382	9.1052	9.1097	8.9004	8.7068	8.9475	8.8804	9.2759	9.2795	8.8825	8.6436	9.0401	9.0560	8.8932
Pura	_IAEVGAGGNK_.2	#N/A	7.8713	8.1172	8.1612	8.3411	8.1921	8.4618	8.3527	8.1526	8.6073	8.6184	8.0550	8.5163	8.8309	8.2889	8.8378	8.5533	7.9897	8.4796	8.6136	8.2475
Pura	_FFFDVGSNK_.2	#N/A	7.5270	7.7141	7.8717	8.1969	7.9257	7.7858	7.9618	7.5950	7.8567	7.9912	7.8839	8.0362	7.9523	8.0477	7.9192	7.8912	8.1251	7.9463	7.8911	7.8503
Pura	_LIDDYGVEEEPAELPEGTSLTVDNKR_.3	#N/A	7.3030	7.4723	7.0290	7.3915	7.0401	7.1353	7.1112	7.0810	7.4257	6.8822	7.1435	7.0550	6.7796	7.9603	7.3800	7.5876	7.0544	6.9684	7.1952	6.9206
Pura	_GPGLGSTQGQTIALPAQGLIEFR_.3	#N/A	4.6836	4.9742	5.4646	4.7990	4.9253	5.3152	5.2269	5.4413	5.3055	4.8985	5.0786	5.1927	5.5878	5.7084	5.5579	5.8919	5.1680	5.3009	5.4340	5.3720
Pura	_VDIQNKR_.2	#N/A	5.9323	5.8818	6.0102	5.8596	5.7151	5.8217	6.1603	4.0233	5.7268	6.0547	6.0697	5.5561	5.6547	5.4083	6.4190	5.7266	5.1646	5.9254	6.3964	5.2741
Pura	_NSITVPYK_.2	#N/A	4.4558	4.9139	5.1209	5.9633	4.5857	4.7033	5.4736	3.5399	4.8707	5.3348	4.7263	5.2152	4.8567	4.7356	4.8951	5.1174	4.2872	5.3830	5.4459	5.3697
Pura	_YSEEMKK_.2	#N/A	4.4655	4.3545	5.1821	5.0266	3.1716	3.2049	5.6728	3.6418	3.7967	4.2761	3.2754	4.0806	3.9622	5.9084	4.1011	3.8276	4.1795	3.6536	3.4160	3.5692
Htt	_RHS[Phospho (STY)]LSC[Carbamidomethyl (C)]TK_.2	P42859_S1841_M1_Htt	4.4606	5.0438	4.3380	3.9799	3.8113	4.6176	4.2190	3.8300	5.0086	4.0283	4.1365	4.8129	3.6352	3.1790	3.2622	1.5103	4.8149	5.0701	4.1213	4.0989
Htt	_S[Phospho (STY)]GEEEDSGSAAQLGMC[Carbamidomethyl (C)]NR_.2	#N/A	4.1328	3.1863	6.8708	3.4514	4.3397	4.3731	4.2092	4.3585	4.1294	3.9434	3.6081	4.4133	4.2949	3.2967	4.2346	3.4949	3.8557	3.3210	2.9695	3.2637
Htt	_DEVAEAS[Phospho (STY)]DPESKPC[Carbamidomethyl (C)]R_.2	P42859_S637_M1_Htt	3.2153	3.9305	3.1601	3.9607	3.1984	4.2779	4.1576	3.8492	3.5463	3.4216	3.9207	3.4217	3.6544	3.1982	3.0644	3.5602	3.3464	3.8030	4.1021	4.3763
Htt	_SGS[Phospho (STY)]IVELLAGGGSS[Phospho (STY)]C[Carbamidomethyl (C)]SPVLSR_.2	P42859_S409_M2_Htt	3.9006	3.8206	3.6909	3.9385	3.7745	3.9478	4.1696	4.5246	4.1731	3.7382	3.7502	3.5890	4.1811	3.9444	3.4287	3.5110	3.7379	4.8460	3.8038	3.4876
Htt	_SLNPQKS[Phospho (STY)]GEEEDSGSAAQLGM[Oxidation (M)]C[Carbamidomethyl (C)]NR_.3	#N/A	4.2999	7.3253	3.7089	3.2219	6.8798	7.3748	7.4661	4.3872	4.0044	3.8212	7.2634	6.8405	7.8858	7.0446	5.3542	3.9598	7.1806	7.7315	7.5817	7.1670
Oprm1	_EFC[Carbamidomethyl (C)]IPTSSTIEQQNS[Phospho (STY)]AR_.2	P42866_S363_M1_Oprm1	8.4233	8.6795	8.7965	8.6499	4.3457	8.5589	8.4326	8.0872	8.6619	8.2598	8.3693	7.7680	8.0461	8.2156	7.0970	7.9724	8.1611	8.5400	8.1390	8.1224
Cct8	_KDWDDDQND_.2	#N/A	6.5602	7.0762	6.7421	6.2291	6.8953	7.2505	7.3295	7.4392	6.9247	7.0668	6.9889	7.2539	6.6572	7.0077	6.6300	4.9160	7.2998	7.0701	7.3516	7.0470
Cct8	_DVDEVSSLLR_.2	#N/A	6.7825	6.9159	7.0195	7.0751	7.0358	6.9987	6.7471	7.2311	6.7682	6.9111	6.9883	6.8440	7.0551	7.0401	6.4356	6.6235	6.9136	6.5048	6.8037	6.5475
Cct8	_FAEAFEAIPR_.2	#N/A	6.0933	6.5982	6.1027	6.7238	6.3737	6.6868	6.5290	6.6059	6.3878	6.2153	6.0056	6.4499	5.7694	6.2174	6.0033	6.1323	6.9610	6.2793	6.4006	6.1791
Cct8	_LATNAAVTVLR_.2	#N/A	5.1433	6.3524	5.6019	6.3280	5.7632	6.0488	6.0419	6.1928	5.2730	5.6523	5.2409	5.8256	5.6906	6.1153	5.7443	5.5966	6.1782	6.0969	5.9369	5.8748
Cct8	_AVDDGVNTFK_.2	#N/A	6.0996	6.4495	5.9387	5.8481	5.5894	5.9809	6.4466	6.6183	6.2604	6.4008	5.6421	6.3046	6.3764	6.0755	5.4216	6.1101	5.0768	6.1307	6.0162	5.6660

Cct8	_LFVTNDAATILR_.2	#N/A	6.3887	5.8390	6.1888	5.7130	5.6569	5.8602	6.0291	6.4799	6.0791	6.1908	5.8896	6.3104	6.4205	5.7977	5.6161	5.8651	5.7738	5.8958	6.4837	5.5975
Cct8	_LYSVHQEGNK_.2	#N/A	5.8975	5.5483	6.0359	5.5569	5.7244	5.8596	5.4266	5.6442	5.5465	5.6870	5.0821	5.8081	5.8616	6.0340	5.6590	5.6717	5.7186	5.9531	5.7031	5.7790
Cct8	_EDGAISTIVLR_.2	#N/A	5.7114	6.0273	6.3832	2.5310	5.1416	5.9712	5.1002	4.7753	5.4366	3.2358	4.9194	4.7526	5.5106	5.9565	5.1469	5.5830	5.8372	5.5277	5.0520	3.0612
Cct8	_AIAGTGANVIVTGGK_.2	#N/A	3.7544	4.2484	4.2907	3.9350	3.0922	3.3162	4.1363	3.6695	3.0072	3.4719	3.3816	3.4599	3.1152	4.3378	4.2006	3.9905	3.9925	4.5197	4.0763	3.0821
Cct8	_HEKEDGAISTIVLR_.3	#N/A	3.6630	3.0460	3.1996	3.9212	4.2025	4.0549	2.9173	3.8887	4.8699	3.1808	3.9519	3.3822	3.2638	3.2377	4.6879	2.9124	3.3859	4.6634	3.9133	3.7334
Cct8	_GSTDNLM[Oxidation (M)]DDIER_.2	#N/A	4.2074	4.6289	5.8608	5.5643	5.0740	5.9123	5.5775	5.6490	5.3700	5.8223	4.9123	5.3403	5.4126	5.5702	5.3496	5.0272	5.4749	5.3218	5.1242	4.0496
Cct8	_QYGSETFLAK_.2	#N/A	3.4559	4.5469	5.6226	5.4286	3.6571	3.6905	3.3910	3.1563	3.3112	3.2608	4.2907	5.8652	3.4766	3.9793	5.6157	4.3131	3.6940	4.1392	5.5882	5.0823
Cct8	_HFSGLEEAVYR_.2	#N/A	3.1830	4.6359	3.7193	4.5064	4.0216	3.9634	3.5195	4.3842	4.2565	3.2903	4.0178	3.2084	3.2037	3.7064	3.0967	3.5279	3.4211	4.3905	4.0499	4.8263
Slc1a2	_SADC[Carbamidomethyl (C)]S[Phospho (STY)]VEEEPWK_.2	#N/A	3.9966	7.7337	7.9464	7.6402	3.7583	7.8920	7.5239	3.4689	7.5327	7.6672	6.3161	6.8016	3.7722	7.1423	5.8007	6.8399	8.0520	7.3620	7.3955	3.7391
Slc1a2	_NLFPENLVQAC[Carbamidomethyl (C)]FQQIQTVTK_.2	#N/A	6.7212	6.6621	6.8589	6.4992	6.3513	6.6176	6.4110	6.5991	6.5485	6.2435	6.3666	5.9701	5.9030	6.5343	5.3196	5.5470	6.2490	6.4686	6.2314	6.5126
Slc1a2	_KNDEVSSLDAFLDLIR_.3	#N/A	6.0413	6.3819	6.1146	6.1258	5.6875	6.3739	6.3045	5.2553	6.3109	6.0900	5.9357	5.6362	5.7870	5.8394	4.9568	5.5234	5.8131	6.1391	6.0105	5.8868
Slc1a2	_[Acetyl (Protein N-term)]ASTEGANNM[Oxidation (M)]PK_.2	#N/A	8.0368	9.0844	9.2764	8.4258	8.2642	8.6240	8.4908	8.0491	8.0714	7.4682	8.5670	7.4631	7.6117	8.2957	6.9376	7.0014	8.0096	8.0322	7.4882	7.5208
Slc1a2	_TSVNVVGDSFGAGIVYHLSK_.2	#N/A	7.5713	7.2363	5.7649	6.1180	5.9384	6.5110	6.8094	6.9651	6.1833	6.4750	6.0960	6.0107	6.8310	6.9596	4.8804	5.7528	7.0565	7.1584	6.6586	6.5364
Slc1a2	_SELDTIDSQHR_.2	#N/A	10.2295	9.9631	10.0476	9.7834	9.7284	9.9823	10.1696	10.1891	10.1668	9.7968	9.6716	9.5896	9.7483	9.4552	9.3711	9.3961	9.8075	9.8558	9.5881	9.4760
Slc1a2	_C[Carbamidomethyl (C)]LEDNLGIDKR_.2	#N/A	8.1202	8.0126	8.0249	7.5916	7.8156	7.8507	8.0116	7.9506	8.1969	7.8340	7.6353	7.3156	7.4369	7.1993	7.4774	7.2745	7.7886	7.6497	7.6555	7.4099
Slc1a2	_SADC[Carbamidomethyl (C)]S[Phospho (STY)]VEEEPWKR_.2	#N/A	9.4546	9.5926	9.2675	9.4569	8.5287	9.2946	9.3626	9.0974	9.2133	9.0279	8.8700	8.8782	9.1614	9.1525	8.1137	8.6590	9.4071	9.2450	8.9674	8.5816
Slc1a2	_M[Oxidation (M)]QEDIEMTK_.2	#N/A	4.1075	6.4153	7.9911	8.0963	6.0808	6.9202	7.8633	5.2717	6.6413	6.1795	6.9894	5.6122	3.6669	7.8166	6.4055	3.4696	5.8281	5.5076	5.3626	6.0172
Slc1a2	_TQSIYDDKNHR_.2	#N/A	5.8751	5.3308	5.5600	5.4784	4.5939	5.5326	5.7425	5.2568	5.4264	5.6418	5.2045	4.4468	5.1261	4.5824	5.1919	4.8325	5.0088	5.4831	5.0495	4.4478
Slc1a2	_M[Oxidation (M)]HDSHLS[Phospho (STY)]SDEPK_.2	#N/A	8.0226	8.2466	7.4267	5.9103	7.7106	8.0689	8.0609	6.8692	8.1969	8.2272	6.2029	7.5143	8.0436	7.0444	6.4254	7.6112	7.9832	8.2393	6.7533	6.6883
Slc1a2	ESNS[Phospho (STY)]NQC[Carbamidomethyl (C)]VYAAHNSVVIDEC[Carbamidomethyl (C)]K_.	#N/A	7.5804	8.1626	7.8236	7.6552	6.6004	7.3358	7.7415	5.8670	6.7826	5.5210	7.0874	6.6460	7.1910	8.0667	4.1518	6.0559	7.1042	7.7649	6.9019	6.6909
Slc1a2	_NDEVSSLDAFLDLIR_.2	#N/A	8.1575	8.0586	7.7734	7.8765	7.6519	7.7085	7.9196	7.8182	7.9147	7.7556	7.4549	7.3698	8.0171	7.3690	6.9681	7.3856	7.8176	7.6295	7.7663	7.1772
Slc1a2	_TQSIYDDK_.2	#N/A	7.7096	7.6840	7.5931	7.4429	6.8022	7.6202	7.7577	7.5332	7.7053	7.4363	7.3635	6.6426	7.0540	6.7502	6.9533	6.7866	6.6279	7.5719	7.1899	6.5306
Slc1a2	_M[Oxidation (M)]HDSHLS[Phospho (STY)]S[Phospho (STY)]DEPKHR_.3	#N/A	6.5217	5.6066	4.4783	4.0678	5.9033	5.7411	5.1515	5.8870	6.3878	5.7426	5.0085	5.7185	6.8855	5.0685	4.3318	5.5804	5.9617	6.1723	6.4507	5.1346
Slc1a2	_DLEVVAR_.2	#N/A	3.9614	4.4417	3.9148	3.9987	3.2991	4.2037	3.8738	3.1031	3.7335	4.1677	3.9663	3.9309	4.4091	3.5036	3.8192	4.3063	4.1012	4.1105	3.3473	4.4137
Slc1a2	NHRESNS[Phospho (STY)]NQC[Carbamidomethyl (C)]VYAAHNSVVIDEC[Carbamidomethyl (C)]K	#N/A	3.8509	4.5509	3.9057	3.8311	3.9731	3.6625	3.7953	3.9516	3.8015	3.8769	3.8264	4.3577	3.9311	3.9158	3.5652	3.4794	3.8452	3.7141	3.6273	3.7574
Slc1a2	[Phospho (STY)]DDKNHRESNSNQC[Carbamidomethyl (C)]VYAAHNSVVIDEC[Carbamidomethyl	P43006_Y522_M1_Slc1a2	4.1535	5.8806	3.6901	3.4307	2.8596	3.7479	6.8450	4.3792	4.0763	9.5782	8.9370	4.3925	10.3827	3.7282	3.5944	3.5156	3.8764	3.2730	3.5721	3.4054
Cox6a1	_HEEHERPPFVAYPHLR_.4	#N/A	8.7961	6.4088	7.8701	8.1793	8.8172	7.1211	7.9294	8.3402	6.5380	8.4514	7.9443	8.7959	8.1828	7.7290	8.0476	8.8752	8.8878	7.6412	8.2594	8.9070
Cox6a1	_ALTYFVALPGVGVSM[Oxidation (M)]LNVFLK_.2	#N/A	4.5752	3.4276	3.6106	3.1142	3.9129	4.1370	3.6282	4.2755	3.6873	3.2807	4.0617	3.3171	3.7953	3.5977	3.2054	3.4192	3.3124	3.0199	4.1694	3.5647
H1-4	_[Acetyl (Protein N-term)]S[Phospho (STY)]ETAPAAPAAPAPAEK_.2	#N/A	13.7518	13.3296	13.5975	13.6418	13.1108	13.3953	11.5289	13.7231	13.8136	13.2546	13.1401	13.3665	14.3193	13.6442	13.2897	13.2790	14.0105	13.4226	11.4903	13.3832
H1-4	_TSGPPVSELITK_.2	#N/A	8.8318	8.9600	8.9565	8.6600	8.9123	8.9945	9.0609	8.9172	9.5240	8.2568	8.7289	8.8263	9.3149	8.9822	9.1124	8.7221	9.1656	8.9328	8.6147	8.5964
H1-4	_[Acetyl (Protein N-term)]S[Phospho (STY)]ETAPAAPAAPAPAEKTPVK_.2	#N/A	5.7087	6.6619	6.6549	7.6267	6.3903	7.1104	7.7203	8.0451	7.7328	5.7432	6.5423	7.4861	8.8952	7.4891	7.2109	5.5900	7.8903	7.5611	7.0274	6.8734
H1-5	_[Acetyl (Protein N-term)]S[Phospho (STY)]ETAPAETAAPAPVEK_.2	#N/A	6.8981	11.7947	11.1880	11.4187	10.4143	11.5784	6.3668	11.0802	11.8152	11.2798	6.0631	11.2620	11.5447	11.5570	11.1706	11.2203	12.0314	11.5326	11.2090	11.2737
H1-5	_[Acetyl (Protein N-term)]SETAPAETAAPAPVEKS[Phospho (STY)]PAKK_.3	#N/A	8.1859	8.0886	7.7895	7.8305	7.0572	7.8970	7.8322	8.0408	7.6930	7.2904	7.1822	7.2807	8.4873	7.9271	7.3976	6.9729	8.0639	7.6761	7.4682	7.1185
H1-5	_ALAAGGYDVEK_.2	#N/A	4.6907	4.9381	4.7956	4.6367	4.0789	4.0124	4.8357	4.6645	4.8627	4.1194	4.2010	4.9849	5.0075	4.4700	4.9487	4.2958	4.7724	3.8323	4.0525	4.5654
H1-5	_[Acetyl (Protein N-term)]SETAPAETAAPAPVEKS[Phospho (STY)]PAK_.2	#N/A	8.9733	9.2210	8.5340	8.4945	7.6466	8.4887	8.9709	8.7398	8.8540	8.0954	7.9948	8.2776	9.5822	8.7910	8.1187	8.2045	9.0071	8.5193	8.2418	8.0506
H1-3	_[Acetyl (Protein N-term)]S[Phospho (STY)]ETAPAAPAAPAPVEK_.2	P43277_S2_M1_H1-3	12.6898	12.7707	12.3354	12.4963	11.8732	12.4488	12.4709	12.4743	12.7211	12.3095	12.1714	12.1815	13.0636	12.6091	12.2262	12.2714	12.9852	12.5697	12.2634	12.3272
H1-3	_[Acetyl (Protein N-term)]SETAPAAPAAPAPVEKT[Phospho (STY)]PVK_.2	P43277_T18_M1_H1-3	5.2552	4.8413	4.7725	4.5907	7.5384	8.6349	4.4447	5.0596	4.6234	4.8447	4.4030	4.5684	6.5417	4.9061	4.6582	4.5610	5.3643	5.1030	4.0506	4.4294
Dck	_FC[Carbamidomethyl (C)]PS[Phospho (STY)]PSTSSEGTR_.2	P43346_S11_M1_Dck	8.8719	9.4453	9.2398	4.3656	9.0490	3.4589	3.6226	3.4443	8.3518	4.5300	8.7865	3.8266	3.2495	9.1754	8.6751	9.0774	8.8058	8.5926	8.7196	3.9714
Itgav	_EQLQPHENGEGNS[Phospho (STY)]ET_.2	P43406_S1042_M1_Itgav	4.3077	3.8682	3.8442	3.2766	3.0138	4.7055	5.1393	3.7996	3.4314	4.1183	3.4332	4.2384	4.1200	4.6226	4.0597	3.6698	5.4283	3.4958	3.1444	3.0888
Itgav	_YNNQLATR_.2	#N/A	3.9678	3.3513	3.5044	3.6165	4.1747	4.2080	4.3742	5.2362	4.2944	3.7784	3.7731	3.0774	4.4599	3.5424	2.8988	3.3299	3.6907	3.1559	3.7578	3.4287
Akr1b1	_TIGVSNFNPLQIER_.2	#N/A	6.6176	6.5858	6.7101	6.8384	6.6688	6.7583	6.6176	6.3500	6.2439	6.4980	6.5914	6.2490	6.2498	7.0350	6.5029	6.7911	6.7656	6.5223	6.5001	6.5130
Akr1b1	_[Acetyl (Protein N-term)]ASHLELNNGTK_.2	#N/A	6.1980	6.6502	6.1794	6.6976	6.2350	6.5926	6.1693	6.2332	6.3524	6.1297	6.5434	6.6667	6.5415	6.4175	6.7845	6.2485	6.1272	6.2422	6.3222	6.4752
Akr1b1	_TTAQVLIR_.2	#N/A	5.3852	6.0676	6.2780	5.9257	6.2637	6.2307	5.7043	5.4288	6.1361	5.8673	6.0813	5.9245	5.4960	5.3133	6.0704	6.5046	5.4748	6.2299	6.5425	6.0444
Akr1b1	_VAIDLGYR_.2	#N/A	5.8790	5.9125	6.8528	6.1590	5.9451	6.0954	6.7847	5.5079	6.2643	6.4664	6.1098	6.4398	4.2814	6.0623	6.2961	6.4762	4.9578	6.5107	6.3907	6.3465
Akr1b1	_SPPGQVTEAVK_.2	#N/A	6.5184	7.0528	7.2348	7.4387	6.9993	7.2552	7.2230	6.6213	7.6287	6.8493	6.8890	6.9146	6.8757	7.4050	7.4453	7.3100	6.5493	7.0078	7.2597	7.5153
Akr1b1	_LIEYC[Carbamidomethyl (C)]HSK_.2	#N/A	4.1753	5.4787	5.6510	5.4829	2.8814	5.5584	5.7914	5.2193	5.6268	5.9976	5.3352	5.1510	5.1622	5.5024	6.0190	5.1996	5.2945	4.7821	4.9437	4.6214
Akr1b1	_HIDC[Carbamidomethyl (C)]AQVYQNEK_.2	#N/A	3.4612	4.5416	3.9975	4.2282	4.2997	5.2549	3.3962	3.1615	3.3164	6.6616	5.6291	2.9303	3.4819	3.9845	3.4216	4.3079	3.6992	5.9302	3.7825	3.9515
Cfl2	_[Acetyl (Protein N-term)]AS[Phospho (STY)]GVTVNDEVIK_.2	P45591_S3_M1_Cfl2	9.7796	6.5905	9.4241	10.0701	9.7848	9.7498	9.3000	6.2925	9.6342	10.3179	9.6724	9.2934	10.0520	9.9833	9.1139	9.9874	10.0698	9.3730	9.6096	9.8557
Cfl2	_QIIVEEAK_.2	#N/A	5.7878	6.0051	5.4770	5.7273	5.9264	5.8485	6.1822	5.7466	6.2107	6.2181	6.0884	6.1877	6.8177	5.5283	6.4241	6.2739	5.3476	5.8401	6.2736	5.3540
Cfl2	_QILVGDIGDTVEDPYTSFVK_.2	#N/A	4.5787	5.3709	4.1541	4.7558	4.2115	5.1609	4.6409	4.4665	5.5377	4.4364	4.7322	4.9085	5.2692	5.0790	4.3521	3.5024	4.3492	4.4476	4.7764	4.7151
Cfl2	_LLPLNDC[Carbamidomethyl (C)]R_.2	#N/A	6.6111	7.1879	3.3229	7.0031	7.3927	6.9679	7.0243	7.3115	6.4907	3.5969	7.0234	7.4293	6.9771	6.4712	7.2994	6.7577	7.1519	7.3050	6.9402	6.9411
Cfl2	_HEWQVNGLDDIKDR_.3	#N/A	5.7223	5.8189	6.0591	5.6648	5.9040	5.7009	5.2510	4.9139	5.4093	6.3004	5.4454	5.6752	5.5710	5.7714	6.6711	5.8637	4.3467	5.6171	5.7570	5.4589
Fkbp2	_LVIPSELGYGER_.2	#N/A	6.5375	7.0641	6.9115	6.8544	6.8784	6.4202	6.9062	7.0452	6.9836	7.1198	6.7505	6.9830	6.9963	7.0673	7.3546	6.6294	6.4582	6.8920	6.6432	6.5531
Fkbp2	_IPGGATLVFEVELLK_.2	#N/A	4.0599	3.9429	4.5962	3.6295	3.3976	3.0865	4.1435	3.7602	3.9151	4.2662	3.0761	3.8324	4.0806	4.5832	3.7207	4.4048	4.2979	3.5352	3.1838	3.0494
Rangap1	_GSGEEPAT[Phospho (STY)]PSRK_.2	P46061_T411_M1_Rangap1	3.3225	6.0869	3.8588	4.3669	4.1611	4.3852	3.3800	4.5237	3.4391	6.4059	5.8958	4.9659	3.5471	3.8459	2.9572	3.6674	3.5606	3.9103	3.9212	4.2690
Syt1	_VFVGYNSTGAELR_.2	#N/A	9.4614	9.2548	9.3533	9.1036	8.9911	9.1441	9.3726	9.6115	9.3284	9.0631	8.9151	8.9511	9.4306	8.4743	8.0436	8.5078	9.1934	9.1976	8.8851	8.5487
Syt1	_HDIIGEFK_.2	#N/A	8.4711	8.0564	8.3298	7.9699	8.1827	8.3552	8.4463	8.5192	8.1741	8.1447	8.3533	8.0684	8.3083	7.7582	7.7466	7.6922	8.4532	8.1835	7.9942	7.8232
Syt1	_TLNPVFNEQFTFK_.2	#N/A	8.1999	8.0027	7.8298	7.9916	7.9050	7.8570	8.1863	8.1176	7.9944	7.7765	7.8560	7.8632	7.8116	7.0618	7.1222	7.3480	7.7839	8.1474	7.9732	7.2803
Syt1	_VPYSELGGK_.2	#N/A	7.5624	8.0832	6.9782	7.4898	7.3146	7.9580	8.4493	7.2171	8.2508	7.7309	8.0557	7.7005	8.0327	7.3113	7.2742	7.7421	7.1139	8.1622	7.8790	7.9373
Syt1	_LTVVILEAK_.2	#N/A	6.7505	6.4930	6.9384	6.8264	6.4443	6.3941	6.7558	6.4633	6.9311	6.1288	6.9044	6.6421	6.2362	5.7356	5.2682	6.5847	6.1410	6.5080	6.8700	6.5986
Syt1	_M[Oxidation (M)]DVGGLSDPYVK_.2	#N/A	7.2789	7.7850	7.6707	7.8223	7.2835	7.7305	8.0505	7.4072	7.3196	6.8086	7.5537	7.2659	7.3398	7.8679	6.9112	6.6185	7.6162	7.2803	7.1502	6.8300
Syt1	_DDDAETGLT[Phospho (STY)]DGEEKEEPKEEEK_.3	P46096_T128_M1_Syt1	3.6751	4.3277	4.2114	4.0143	3.0128	3.2369	5.3444	3.3894	3.0865	3.8814	3.4609	4.2172	3.1946	4.2392	4.1055	4.0200	3.9131	4.2628	3.0610	3.9165
Syt1	_HWSDM[Oxidation (M)]LANPR_.2	#N/A	6.0417	6.8117	6.5437	6.8864	5.8942	6.1604	6.7847	5.7907	5.8082	5.8497	6.7025	6.3304	5.2023	6.7946	5.6855	5.5347	6.6107	6.1627	5.5773	5.2837
Syt1	_DLQSAEKEEQEK_.2	#N/A	6.4860	6.8814	6.2352	6.9701	6.3546	6.9102	6.7666	6.5388	6.4525	6.6427	6.8919	6.7047	6.6689	6.5378	5.5058	6.1559	6.9098	6.8688	6.7704	6.8164
Syt1	_VFLLPDK_.2	#N/A	5.7317	5.4312	5.6226	6.1633	5.2792	5.7218	6.5616	4.1102	5.8402	5.9569	6.1385	6.2275	6.2673	5.7157	4.8679	4.8459	3.6074	6.1934	6.0272	5.7028
Syt1	_VQVVVTVLDYDK_.2	#N/A	3.6416	6.9980	6.9405	4.0478	6.5216	3.2034	3.0609	6.8736	7.1773	6.6136	6.6572	6.1615	6.3810	6.4089	5.6322	5.9525	7.1541	6.4978	3.0276	6.6012
Syt1	_TLVM[Oxidation (M)]AVYDFDR_.2	#N/A	8.3167	8.1201	8.0783	8.0254	7.6357	7.9671	8.1087	7.8315	7.8877	7.5168	7.9093	7.6919	8.0209	7.6014	6.8887	7.6481	8.4124	8.1842	7.8703	7.6362
Syt1	_VPM[Oxidation (M)]NTVDFGHVTEEWR_.3	#N/A	8.2709	7.9594	8.0576	7.5829	7.6500	7.6500	8.1156	8.0227	7.6176	7.4757	7.8179	7.5412	7.9881	7.8887	7.0943	7.1409	8.0488	7.8359	7.5337	7.2696
Syt1	_NTLNPYYNESFS[Phospho (STY)]FEVPFEQIQK_.3	P46096_S344_M1_Syt1	3.9067	4.9920	3.4433	3.1892	4.1137	4.1470	4.4353	4.2006	4.3555	3.7173	3.8341	3.1385	4.5209	3.5227	2.9598	3.2689	3.2374	3.0949	4.2443	3.4897
Cdkn1b	_VSNGS[Phospho (STY)]PSLER_.2	P46414_S10_M1_Cdkn1b	9.0517	9.2972	3.5205	9.3936	4.1283	7.9213	8.8099	9.1010	8.9000	9.1839	8.7280	8.8369	4.0148	9.9616	8.9832	9.0771	9.0514	8.7558	8.7510	8.0076
Cdkn1b	_TEENVSDGS[Phospho (STY)]PNAGTVEQTPK_.2	P46414_S178_M1_Cdkn1b	3.5532	6.7080	6.5894	6.6458	3.7601	6.4243	3.3245	3.9927	3.2447	3.3638	6.0102	3.5286	6.5725	6.6560	5.8034	6.3652	6.6879	6.2448	6.2052	3.8602
Cdkn1b	_VLAQES[Phospho (STY)]QDVSGSR_.2	P46414_S106_M1_Cdkn1b	3.5931	3.7260	3.1297	3.9911	3.8001	3.8334	3.2481	4.5142	3.1683	3.4037	4.1477	3.4521	3.3337	3.8364	3.2734	6.7700	3.3160	6.5257	3.9307	3.8034
Nsf	_GILLYGPPGC[Carbamidomethyl (C)]GK_.2	#N/A	9.8582	10.0457	9.9436	9.9607	9.9715	9.8386	9.9679	10.2361	9.5851	9.7497	9.8163	9.7557	10.0503	9.7654	9.6297	9.5546	9.7128	9.8532	9.7725	9.8333
Nsf	_LLDYVPIGPR_.2	#N/A	10.0880	10.0954	9.8898	10.1794	10.0221	9.9704	10.3507	10.3018	9.5607	9.7994	9.6921	10.0806	9.9487	9.5752	9.4397	9.7404	9.8644	10.0919	9.9788	9.6197
Nsf	_YVGESEANIR_.2	#N/A	9.5884	9.3394	9.8125	9.6059	9.5264	9.7139	9.7209	9.7997	9.7133	9.5457	9.3970	9.3720	9.4678	9.4729	9.4773	9.5059	9.3873	9.2730	9.5431	9.3103
Nsf	_VLDDGELLVQQTK_.2	#N/A	9.5756	9.4911	9.6859	9.5338	9.3788	9.4744	9.6328	9.8496	9.5074	9.5098	9.4470	9.3634	9.6460	9.5204	9.1307	9.3383	9.5332	9.2925	9.4734	9.2868
Nsf	_SQLSC[Carbamidomethyl (C)]VVVDDIER_.2	#N/A	9.2731	9.1164	9.5999	9.3855	9.0052	9.2202	9.2365	9.2973	9.1026	9.2638	9.2258	8.9965	9.3584	9.1394	8.6201	8.8809	9.4030	8.9574	9.0339	8.9464
Nsf	_NFSGAELEGLVR_.2	#N/A	8.9609	9.1394	9.1003	8.8184	9.0632	9.0420	9.1611	9.3090	8.8032	8.9522	8.8628	9.0839	9.1759	8.8312	8.7767	8.7933	8.9375	8.7902	8.9227	8.8575
Nsf	_LLIIGTTSR_.2	#N/A	8.8284	8.5689	8.5748	8.4370	8.5549	8.6944	8.8444	8.8114	8.4815	8.6285	8.6488	8.3140	8.6126	8.6708	7.4709	8.6949	8.5367	8.6003	8.4810	8.6602
Nsf	_NIDSNPYDTDK_.2	#N/A	8.5022	8.4152	8.7348	8.5173	8.3902	8.6818	8.4665	8.4947	8.5544	8.4749	8.4651	8.2988	8.2381	8.6698	8.4518	8.5764	8.7445	8.3912	8.4981	8.4812
Nsf	_KLLIIGTTSR_.2	#N/A	8.2461	8.4766	8.7272	8.5852	8.2602	8.3008	8.4874	8.7126	8.4929	8.1752	7.5654	8.2409	8.4333	8.1293	7.9169	8.1927	8.5002	8.2283	8.3220	8.2600
Nsf	_IAEESNFPFIK_.2	#N/A	8.7243	8.4270	8.3694	8.3740	8.5465	8.3619	8.7478	8.7858	8.2864	8.5839	8.4436	8.4252	8.4483	8.2854	8.2386	8.1835	8.3795	8.5222	8.3428	8.0876
Nsf	_WGDPVTR_.2	#N/A	8.5272	8.5468	8.3204	8.0565	8.2066	8.3782	8.8357	8.6675	8.4022	8.4284	8.0215	8.3816	7.9804	8.2286	8.0292	7.7856	7.4774	8.4029	8.0671	7.7746
Nsf	_VVNGPEILNK_.2	#N/A	8.5161	8.1725	8.2821	8.0303	8.3582	8.3042	8.5520	8.5941	7.9721	7.8799	7.9832	8.1588	8.1545	8.0228	8.5559	7.7730	8.2050	8.1904	8.0452	7.7650
Nsf	_AENSSLNLIGK_.2	#N/A	8.0077	7.7051	8.1053	7.9262	7.9511	7.2981	7.7031	8.1346	7.6089	7.6161	7.6525	7.6639	7.7935	7.3579	7.7429	7.3084	7.6766	7.5193	7.7947	7.5446
Nsf	_AESLQVTR_.2	#N/A	6.8928	7.2929	7.7537	7.4644	7.2609	7.7153	7.8685	6.8285	7.9395	7.6093	7.8419	7.2711	7.1334	7.0295	7.4415	7.2295	6.2101	7.3169	7.6146	7.7928
Nsf	_TTIAQQVK_.2	#N/A	6.9254	7.4764	6.9368	6.7639	6.2785	7.2668	7.8670	6.8270	7.2486	7.4148	7.1598	7.3263	7.0733	7.1942	7.5028	7.1447	6.4537	7.5195	7.2548	7.0653
Nsf	_TKENRQS[Phospho (STY)]IINPDWNFEK_.3	#N/A	3.7614	8.1833	4.2977	3.9280	6.5984	7.4301	7.5246	7.7525	7.1440	8.0230	7.1309	7.2375	7.7082	8.1894	3.7219	7.3791	7.1615	7.5294	7.3679	7.2455
Nsf	_YVGESEANIRK_.2	#N/A	6.5121	4.6581	5.7134	5.5810	6.1349	5.4439	5.2446	6.7053	6.6702	4.9771	5.4001	5.8374	6.1665	5.6601	6.2679	5.9189	5.7001	6.0478	5.7103	6.1596
Nsf	_M[Oxidation (M)]GIGGLDKEFSDIFR_.3	#N/A	5.8400	5.3471	6.0299	5.9002	5.7811	5.4681	5.9097	5.8908	5.2103	6.0007	5.7822	5.9488	4.5183	5.6314	4.5570	5.2368	5.8486	5.7475	5.5280	5.6704
Nsf	_LQILHIHTAR_.3	#N/A	7.8401	7.6916	6.9773	7.6914	8.1064	7.3717	7.4564	8.1340	7.2611	7.8485	7.4781	7.5647	7.6735	7.6759	6.8660	7.4236	7.6552	7.5010	7.2516	7.1035
Nsf	_KIFDDAYK_.2	#N/A	6.0923	6.7438	6.3174	6.3804	6.8639	6.6545	6.5694	6.2822	6.3023	6.6410	6.7914	6.8255	5.7485	6.3814	6.2885	6.5332	6.6555	6.6712	6.5743	6.4110
Nsf	_IEVGLVVGNSQVAFEK_.2	#N/A	5.9499	5.7999	7.2449	5.8732	6.1023	5.9562	5.0562	5.7693	5.9373	5.8076	6.0089	4.6953	4.9322	5.2952	4.7384	5.2903	5.4679	5.5754	5.6256	5.8986
Nsf	_QC[Carbamidomethyl (C)]IGTM[Oxidation (M)]TIEIDFLQK_.2	#N/A	3.8268	3.8407	5.2586	5.0691	5.1558	4.2045	4.5161	4.6284	4.0133	5.0929	5.0856	2.9547	4.4677	3.5122	4.0051	4.5414	4.9617	4.2225	4.7286	3.8548
Nsf	_M[Oxidation (M)]EIGLPDEK_.2	#N/A	7.3763	7.5853	7.1136	7.8140	7.0928	7.2872	7.8714	7.0159	7.4720	7.6984	7.3387	7.5103	6.4591	7.3320	7.0623	7.1650	6.8310	7.3174	7.2584	7.3671
Nsf	_DIEAM[Oxidation (M)]DPSILK_.2	#N/A	7.4264	7.3083	7.3703	7.2207	7.3610	7.4850	7.1904	7.2957	7.0685	7.0787	7.3602	6.8236	6.7766	7.1340	7.0186	6.8898	7.0858	7.1219	6.9056	7.0912
Nsf	_TPLVSVLLEGPPHSGK_.2	#N/A	6.9410	7.0480	7.0556	6.5090	6.9867	7.3740	7.0407	6.9374	6.5803	7.0237	6.7752	6.7632	6.6331	6.8189	6.1811	6.7735	7.0012	6.8598	6.8236	7.0312
Nsf	_IDGVEQLNNILVIGM[Oxidation (M)]TNRPDLIDEALLRPGR_.4	#N/A	4.0225	5.2995	5.3987	4.1230	4.5779	5.6245	4.7538	5.8347	4.6010	4.5233	4.9012	4.4967	5.4591	4.7947	4.3244	3.5190	5.7834	5.0130	4.7676	4.0654
Nsf	_KLFADAEEEQR_.3	#N/A	3.9649	4.4936	5.7062	5.6236	5.5605	5.4791	4.9800	3.5715	5.1760	4.8995	5.2296	4.9704	3.6923	4.4909	5.3831	4.6825	3.6747	4.2972	5.2816	5.0571
Nsf	_M[Oxidation (M)]IGFSETAK_.2	#N/A	4.4404	4.2536	5.5404	5.3571	5.1009	5.1869	5.7191	4.7162	5.5654	4.8345	4.7904	4.8383	3.9234	5.0537	4.9552	4.5629	4.0815	4.7975	4.6292	4.3090
Nsf	_AAQSTAMNR_.2	#N/A	3.8752	6.0057	6.6432	7.1422	4.8157	5.5673	6.6886	4.2321	5.3497	3.6858	5.6721	3.1700	4.5524	6.9255	5.6506	3.2373	4.9783	3.0634	4.3763	4.8367
Nsf	_EFSDIFR_.2	#N/A	3.8270	4.1976	4.0902	5.1163	3.9197	4.7910	4.9116	3.2543	3.9232	5.3448	5.5443	4.3121	3.5746	5.3183	3.8196	3.8988	3.3163	4.3545	4.7644	5.2686
Nsf	_GHQLLSADVDIK_.2	#N/A	5.0182	4.5971	5.4316	5.1644	2.9290	4.3382	5.0519	5.2272	4.3383	5.2274	5.5891	5.3179	4.2537	3.7975	5.5411	4.8395	5.3122	5.0942	4.5413	5.5043
Nsf	_ENRQS[Phospho (STY)]IINPDWNFEK_.3	#N/A	6.1786	6.5631	6.6580	6.5687	5.4273	6.4118	6.1435	5.5240	6.2063	6.2580	6.0715	5.6974	5.6495	6.0651	5.1909	6.1919	5.4783	5.7744	6.4659	6.1969
Nsf	_KLFADAEEEQRR_.3	#N/A	5.6861	5.9791	6.2391	6.2838	6.0339	6.4412	6.2806	5.5734	6.0654	6.3580	6.1080	5.7320	5.6841	5.7118	6.2290	6.0084	6.1879	6.3196	6.5164	6.1857
Nsf	_QSIINPDWNFEK_.2	#N/A	6.9074	6.8692	7.5145	7.6035	7.1195	7.2479	7.1413	7.3030	7.2673	7.0988	7.0342	6.9761	7.3424	6.9910	6.9307	7.0584	7.1060	7.0517	6.9861	6.9027
Nsf	_GSM[Oxidation (M)]AGSTGVHDTVVNQLLSK_.3	#N/A	8.2948	8.3073	7.0778	7.9324	8.2340	7.9479	7.9105	8.5444	7.3423	8.2519	7.9818	8.0094	8.8747	7.7603	7.6072	7.8707	7.8845	7.9659	7.9949	8.0377
Nsf	_VEVDM[Oxidation (M)]EKAESLQVTR_.3	#N/A	4.4949	4.5553	3.5304	2.8016	3.8326	3.2433	4.0330	4.1953	3.2094	3.2004	4.1419	3.3477	3.8756	3.3559	3.2856	3.6158	3.2321	3.8260	4.2496	3.0966
Nsf	_C[Carbamidomethyl (C)]PTDELSLSNC[Carbamidomethyl (C)]AVVNEK_.2	#N/A	3.7394	4.5077	4.2447	3.2720	4.7707	3.5892	4.1593	4.5380	4.2351	3.6751	5.0066	4.2338	4.3431	3.8869	3.7532	4.3722	4.1432	4.2579	3.4133	3.5642
Nsf	_KNIDSNPYDTDK_.2	#N/A	4.0710	3.9318	4.6073	3.3156	5.1648	5.0068	5.2463	5.9162	5.9395	4.2773	3.0650	3.8212	4.2995	3.8432	3.7095	5.6133	5.9429	5.5386	6.0292	3.5205
Nsf	_LFADAEEEQRR_.2	#N/A	4.0437	3.6407	3.3100	4.9263	4.0212	4.5453	4.1727	4.1631	3.5766	4.0977	3.8935	3.4565	3.7039	3.4272	3.3159	4.8653	4.2988	3.4401	4.3721	3.6239
Nsf	_FLALM[Oxidation (M)]REEGAS[Phospho (STY)]PLDFD_.2	#N/A	3.6426	3.6765	3.1792	3.9416	3.8495	4.2588	3.5063	3.8683	3.5654	3.4025	3.9398	3.4026	3.6735	3.2172	3.0835	3.1339	3.3655	3.7839	4.0830	4.3953
Nsf	_EEGAS[Phospho (STY)]PLDFD_.1	#N/A	4.4088	4.4111	3.9454	3.1754	3.1149	3.1482	3.9332	3.1336	3.8534	4.2194	3.3320	4.1372	4.0188	3.3205	3.9586	3.7709	4.1317	3.0177	3.3168	3.6607
Nsf	_DFQSGQHVM[Oxidation (M)]VR_.2	#N/A	3.8265	5.1430	4.7102	3.9237	3.7068	3.7469	3.8208	3.8786	3.7246	3.9512	3.7298	3.8541	3.8536	5.3393	6.2608	3.6348	3.7627	3.8072	4.8862	3.6679
Nsf	_VEVDMEK_.2	#N/A	3.4596	4.5432	3.9959	4.2298	4.2981	3.0214	3.2429	3.1599	3.3020	3.6659	3.6764	2.9318	3.4101	3.9829	2.8201	3.8045	3.6976	4.0473	3.7841	4.1320
Vps4b	_GNDS[Phospho (STY)]DGEAESDDPEK_.2	#N/A	3.7644	7.9792	4.3007	3.9249	3.1022	8.9734	4.4389	7.2203	6.2379	8.6081	7.1729	4.1278	7.1980	9.0155	6.4208	7.2917	8.1002	7.7018	3.4792	8.2387
Vps4b	_GNDS[Phospho (STY)]DGEAESDDPEKKK_.2	#N/A	4.8011	5.1810	5.3554	4.9621	4.7506	4.9491	4.9635	5.1523	5.0110	5.0666	4.8932	5.0443	4.6733	5.8726	5.3829	5.1393	5.1215	4.9492	4.7790	5.3896
Psmc2	_TM[Oxidation (M)]LELINQLDGFDPR_.2	#N/A	4.7796	4.9031	5.3087	3.1495	4.5860	4.7302	4.4060	5.4336	4.7525	5.7730	4.5026	4.7765	4.4252	4.0094	4.4780	5.4612	5.2786	4.9868	5.2017	5.0061
Psmc2	_FVVDLSDQVAPTDIEEGM[Oxidation (M)]R_.2	#N/A	3.2331	4.3980	3.6887	3.9786	4.0717	4.6176	3.4694	3.8313	3.5285	3.4395	3.9028	4.5738	3.6365	3.1803	3.9686	4.2060	3.3285	4.8761	4.2155	4.3179
Rab11b	_AQIWDTAGQER_.2	#N/A	7.9555	8.1698	8.0866	7.7126	7.7302	7.9093	7.8122	7.8319	8.1280	7.8463	7.7280	7.7830	8.0136	7.3269	7.1934	7.3779	7.7997	7.7440	7.7175	7.6145
Rab11b	_VVLIGDSGVGK_.2	#N/A	7.5730	7.1757	7.6537	7.3761	7.4833	7.4762	7.4978	7.7745	7.1981	7.6881	7.2065	7.5713	6.7871	7.4486	7.0381	7.2415	7.3787	7.5759	7.4177	7.3634
Rab11b	_[Acetyl (Protein N-term)]GTRDDEYDYLFK_.2	#N/A	6.0026	6.0063	6.3763	5.7960	5.7693	6.0364	5.5857	6.1518	5.8268	6.3594	5.4048	5.4659	6.3100	5.8511	4.9628	6.0518	6.0735	5.7738	5.9563	6.1177
Rab11b	_NNLSFIETSALDSTNVEEAFK_.2	#N/A	6.0503	5.5488	5.7310	5.4023	4.9745	4.8876	5.2523	5.8258	4.1333	5.8490	4.3470	5.1401	5.4537	6.2330	5.2177	5.1456	5.0100	5.3666	4.9927	5.8175
Rab11b	_HLTYENVER_.2	#N/A	7.1460	7.2259	7.2171	6.8360	6.8300	6.7109	7.3082	7.4255	6.8997	7.0925	6.9787	6.5502	7.2556	6.4869	6.7349	6.7748	7.0050	6.7395	6.9694	6.5918
Rab11b	_NILTEIYR_.2	#N/A	5.0054	5.4653	5.1608	5.5956	5.1848	5.4048	5.7007	5.0863	5.8762	5.5545	5.5259	5.5643	5.8494	5.6626	5.1477	4.9826	4.4914	5.5238	5.8137	5.3731
Rab11b	_STIGVEFATR_.2	#N/A	6.1000	6.6116	4.4332	3.7925	6.3046	5.1499	5.8337	5.8294	6.8479	4.1032	6.1430	3.9954	4.4736	5.5029	3.8837	5.6177	4.1349	6.3698	6.0959	3.6947
Rab11b	_AITSAYYR_.2	#N/A	4.3397	4.2356	4.3035	3.9222	3.1049	3.9843	3.7022	3.4676	3.6225	4.4504	4.2124	3.6713	3.7879	4.2906	4.6497	4.1121	4.0052	3.9316	3.1492	4.2575
Rab11b	_NEFNLESK_.2	#N/A	4.4202	4.3997	3.9568	3.1640	3.1263	4.5935	4.4631	3.1450	4.3430	5.6008	4.6092	4.1258	4.4511	3.9948	3.8611	3.7823	4.1431	5.1181	4.8840	5.0641
Rab11b	_AAHDES[Phospho (STY)]PGNNVVDISVPPTTDGQR_.3	P46638_S190_M1_Rab11b	4.4647	4.3552	4.0013	3.1195	3.1708	3.2041	3.8773	3.1895	3.7975	4.2753	3.2762	4.0814	3.9630	4.4656	3.9027	3.8268	4.1876	3.6529	3.3014	4.4326
Ina	_TNEKEQLQGLNDR_.3	#N/A	8.3092	8.6446	8.5209	9.0574	8.8008	8.6586	8.5022	8.8012	8.9785	8.9014	8.7319	9.0423	9.3208	9.2754	9.0342	9.0796	8.9965	8.7995	8.9676	9.3161
Ina	_DGLAEEVQR_.2	#N/A	9.0513	9.1811	9.4704	9.7179	9.2736	9.2180	9.3105	9.3295	9.3905	9.3565	9.4122	9.4953	9.5856	9.6898	9.2926	9.2865	9.3478	9.1696	9.3383	9.7054
Ina	_VHQLETQNR_.2	#N/A	9.2520	9.1912	9.4090	9.1961	9.0413	9.1048	8.8515	9.3238	9.0096	8.9973	9.0561	9.4194	8.8475	9.2600	9.7556	9.1146	9.4794	9.0391	9.3055	9.2043
Ina	_ALEAELAALR_.2	#N/A	9.0679	9.1334	9.0502	9.4106	9.0748	9.2011	9.0405	9.1672	8.7008	8.9214	9.0759	9.4773	9.2567	9.4057	9.0949	9.3143	9.1715	9.3848	9.3270	9.3289
Ina	_QILELEER_.2	#N/A	8.9361	8.8002	9.2162	9.2385	9.1709	8.9620	8.5814	8.8626	9.0655	9.1417	9.2292	9.2922	9.0029	9.1283	9.2090	9.3741	9.0330	8.6424	9.0453	9.4111
Ina	_AQLEEASSAR_.2	#N/A	8.9913	8.7013	9.1731	9.1212	8.8331	8.8830	8.7426	8.9532	9.0925	8.7489	8.9220	9.0041	9.0220	8.9066	9.4014	9.2217	8.7963	8.7604	9.0220	9.0155
Ina	_TIEIEGLR_.2	#N/A	8.1773	8.1747	8.4081	9.1592	8.4998	8.7250	8.5597	7.7866	8.7393	8.7857	8.8878	8.7137	8.7255	8.7549	8.9024	9.0955	8.1755	8.6267	8.6435	9.1616
Ina	_FANLNEQAAR_.2	#N/A	8.2039	8.4603	8.7380	8.7240	8.6678	8.5490	8.3918	8.6577	8.2752	8.3053	8.3059	8.5640	8.5556	8.9878	9.1021	8.5248	8.7190	8.2479	8.3668	8.6428
Ina	_NLQSAEEWYK_.2	#N/A	8.5695	8.1231	8.4456	8.5586	8.7317	8.3642	7.9231	8.7046	8.1047	8.3461	8.6380	8.7433	8.8023	8.7557	8.6811	8.7037	8.4479	8.3322	8.4349	8.6200
Ina	_KVESLLDELAFVR_.3	#N/A	6.8190	6.9881	7.1035	7.2349	7.0111	6.9081	6.8635	6.8135	7.2172	7.1250	6.9270	7.1380	7.1875	7.4213	7.3962	7.1903	7.2922	7.0830	7.2641	7.2849
Ina	_RLPASDGLDLSQAAAR_.3	#N/A	6.9485	7.0741	7.3030	7.6824	7.3736	7.3635	6.8924	7.0925	7.0852	7.5055	7.2565	7.4400	7.5934	7.5225	7.7871	7.4187	6.6259	7.2341	7.4840	7.5507
Ina	_DVDGATLAR_.2	#N/A	6.2786	6.6735	5.4013	7.6447	6.5460	7.3598	7.3811	5.5266	7.1085	7.3887	7.2140	7.4777	7.6019	7.2441	7.8599	7.3891	5.6505	7.4442	7.5460	7.7588
Ina	_LPASDGLDLSQAAAR_.2	#N/A	7.3955	6.8216	7.8233	7.2655	7.2545	7.1774	6.8839	7.0874	6.7462	7.1560	7.4557	7.0212	6.7979	7.6451	7.7361	7.2896	7.2635	6.8223	7.1155	6.7930
Ina	_AQALLER_.2	#N/A	5.2096	5.4976	4.5583	6.7038	6.0497	6.5880	6.7851	4.8176	6.7565	6.3493	6.3703	7.0688	6.8263	6.4747	7.0360	6.7787	5.3680	6.6564	6.7525	6.9130
Ina	_FAVFIEK_.2	#N/A	6.0154	6.0654	6.9117	6.6533	6.1915	5.8077	6.1814	5.3116	6.6393	6.2020	6.5406	6.2559	6.2592	6.6068	6.6247	6.6918	5.8306	6.4815	6.7686	7.1099
Ina	_AQYESLAAK_.2	#N/A	5.0956	6.1794	5.2360	6.5712	6.0781	6.2643	6.3349	4.4975	6.7983	6.4638	6.5287	6.7034	6.1873	6.3327	6.9625	6.3503	5.2408	5.9364	6.9248	6.5447
Ina	_VGELFQR_.2	#N/A	6.2209	5.6141	6.4178	6.4043	6.6818	6.3569	6.3773	6.3579	6.1980	5.8195	6.6654	6.7596	6.2352	6.5535	6.4791	5.9827	6.3294	6.5661	5.9695	6.2707
Ina	_KVFGDSSR_.2	#N/A	5.6736	5.5623	5.7833	4.8784	5.6886	5.4849	4.5845	5.2481	5.1414	4.8241	4.6620	5.3082	5.2343	5.3753	5.5553	4.8286	4.8592	5.1985	5.7050	5.5721
Ina	_LSGPGGSGSFR_.2	#N/A	7.2201	7.5483	7.9896	8.5449	8.0445	8.0958	7.8177	7.6074	7.5047	7.4173	8.1278	8.4012	8.0282	8.7816	8.2147	7.6269	8.2728	7.8984	8.1599	8.1662
Ina	_VESLLDELAFVR_.2	#N/A	5.0549	4.5534	5.5865	5.1745	4.2705	5.4489	3.9995	5.0505	4.6937	4.9060	5.5825	5.0688	3.3541	5.1852	5.1269	5.1811	3.7489	4.7282	4.4001	4.8092

Ina	_SNVASTAAC[Carbamidomethyl (C)]SSASSLGLGLAYR_.2	#N/A	5.5180	5.2141	4.4211	4.7938	5.7825	4.5093	4.5486	5.2199	5.6860	5.5010	5.6705	5.0478	5.9329	5.4753	4.7099	5.3210	6.0242	4.4824	5.1488	5.8065
Ina	_GANES[Phospho (STY)]LER_.2	P46660_S335_M1_Ina	3.7031	7.4188	3.2397	7.4196	3.9100	7.2901	6.4222	3.9288	6.6334	7.5934	6.7894	7.0362	3.2237	6.7791	7.3379	7.0129	5.9702	6.4985	6.7020	7.4820
Ina	_[Acetyl (Protein N-term)]SFGSEHYLC[Carbamidomethyl (C)]SASSYR_.2	#N/A	5.6976	5.4701	5.6029	5.1433	4.1762	4.7215	4.8398	4.8604	4.3207	5.5043	5.1237	4.5893	4.9142	6.0162	5.1300	5.0834	5.0132	4.9314	5.0637	4.6148
Ina	_[Acetyl (Protein N-term)]SFGSEHYLC[Carbamidomethyl (C)]SAS[Phospho (STY)]SYRK_.3	#N/A	3.9985	5.8519	3.9290	3.9084	3.6424	5.3205	5.4061	3.8696	3.8734	5.3687	3.6791	5.0832	6.2273	5.9511	5.3810	3.7480	3.9480	5.4040	3.7534	3.7407
Ina	_EEIHEYRR_.2	#N/A	5.2730	4.8972	4.4065	4.6295	5.1843	5.1803	5.6553	5.2820	5.5778	5.4389	5.7096	4.9841	3.4000	5.7124	5.3520	4.9504	5.5212	5.6873	5.5301	4.5726
Ina	_VSSAGLSLK_.2	#N/A	4.0187	3.4268	3.5553	3.5655	3.1874	4.2506	3.9790	4.0886	4.7013	4.1385	4.0950	3.7746	4.4089	4.4857	5.2032	4.6805	3.1254	2.7781	4.3109	4.0289
Ina	_VGEGFEETLGEAVISTK_.2	#N/A	8.3888	7.7709	8.2440	8.1020	7.8842	7.8837	7.1455	7.7655	7.4819	8.3346	8.1632	8.0643	8.4650	8.4948	7.2400	8.2747	8.3040	7.4024	7.8045	7.9627
Ina	_KEEEEEEEEASKEVSK_.3	#N/A	4.6980	5.2252	5.3778	4.2575	4.9896	4.9492	4.4651	5.1750	5.2826	5.1863	5.0024	4.9357	5.1256	5.7717	5.0522	5.1541	5.3922	4.5223	5.0959	4.9567
Ina	_SATEESTSS[Phospho (STY)]SQKM_.2	P46660_S497_M1_Ina	4.2159	4.9872	4.2931	5.0256	3.5537	3.7777	4.0349	3.9163	4.0940	3.9632	4.0059	3.7238	4.2020	5.8632	3.8691	4.1017	3.9949	3.5022	3.7047	3.7821
Ina	_HSAEVAGYQDSIGQLESDLR_.3	#N/A	7.0491	6.9368	6.9327	7.3571	7.1431	7.0199	7.1373	7.1818	7.0086	7.2632	7.2781	7.6818	7.3134	7.6647	7.3259	7.3244	6.9203	6.4095	6.9917	7.5533
Ina	_ILSSTASK_.2	#N/A	3.4519	2.4765	4.4893	3.8782	3.6588	3.6922	3.3893	3.6776	2.0244	3.2625	4.2890	3.5933	3.4828	4.5274	3.4677	4.3149	3.1748	3.9746	4.2737	4.2778
Adss2	_VVDLLAQDADIVC[Carbamidomethyl (C)]R_.2	#N/A	4.8067	4.9326	5.4767	4.6834	3.7460	5.0365	3.9690	5.1824	5.1252	5.6533	5.3171	5.1038	4.2645	4.8038	5.2104	5.0982	4.5927	4.7770	5.4785	5.7095
Adss2	_ELPVNAQNYVR_.2	#N/A	4.2085	6.4294	6.8993	6.3574	5.2975	6.0752	6.1405	6.1820	5.5196	4.0091	3.5424	5.8663	4.2292	5.8686	6.1844	6.0386	5.7363	6.1238	6.2727	5.1570
Nedd4	_RQIS[Phospho (STY)]EDVDGPDNR_.2	#N/A	8.2477	7.2265	8.3694	7.9929	7.5418	7.2682	6.8534	8.0284	7.4074	6.9596	7.5291	7.5370	8.0426	8.1266	6.6466	7.5453	7.9733	7.8261	7.6403	7.5832
Nedd4	_QIS[Phospho (STY)]EDVDGPDNR_.2	#N/A	8.8914	8.8266	9.0892	8.5363	8.1925	8.3257	8.3009	8.5676	8.3325	8.7185	8.2638	8.0996	8.6288	8.4185	7.3351	8.1468	8.3208	8.2291	8.1058	8.1894
Nedd4	_RPSPDDDLT[Phospho (STY)]DEDNDDM[Oxidation (M)]QLQAQR_.3	P46935_T287_M1_Nedd4	3.2549	4.0642	4.2923	4.3294	3.4618	3.1457	3.3355	3.4806	3.1777	3.7902	3.5521	3.7903	3.2857	4.3304	4.1966	4.1178	2.6806	4.1635	2.9699	4.0076
Yap1	_QAS[Phospho (STY)]TDAGTAGALTPQHVR_.2	#N/A	6.8917	6.5394	6.8004	6.8336	6.1156	6.7355	6.3816	3.4698	6.3642	6.9251	3.4375	6.5198	6.8776	7.0502	6.3174	6.5930	7.0006	6.2149	6.4782	6.5521
Yap1	_GDS[Phospho (STY)]ETDLEALFNAVM[Oxidation (M)]NPK_.2	P46938_S46_M1_Yap1	3.2399	6.4326	4.6105	4.4495	4.0784	3.9065	3.1749	4.4411	3.0951	3.4768	4.0746	6.0024	3.2606	6.5958	6.5157	4.9915	3.4779	4.3552	5.6611	3.7302
Gal	_EAGALDSLPGIPLATS[Phospho (STY)]SEDLEKS_.2	P47212_S117_M1_Gal	4.3775	3.6253	3.4130	3.3119	3.7152	4.2697	3.8258	4.0779	4.2328	4.5838	3.7114	3.5147	4.3982	3.4000	3.4030	8.5194	3.1147	8.9065	4.3670	3.3670
Rph3a	_LHLLPGASK_.2	#N/A	6.5565	6.9756	5.8609	6.8278	7.0266	6.3679	6.2729	7.0783	6.0880	6.5737	6.6876	7.0237	6.7775	7.1442	7.3300	6.7826	6.5218	6.9186	5.7848	6.7890
Rph3a	_KQEELTDEEKEIINR_.3	#N/A	5.8826	6.0295	6.4021	5.8751	6.2444	5.8907	5.4858	6.6632	5.2420	6.4291	6.0313	6.0822	4.3623	6.5974	5.6163	6.4365	6.6060	6.1570	6.1869	6.4137
Rph3a	_TKPQQPAGEPATQEQPTPESR_.3	#N/A	5.0937	6.1192	5.9706	4.0360	6.1522	5.2106	5.7428	5.8497	5.9683	6.0654	5.9265	5.6482	5.5126	6.3363	6.3779	5.9982	5.1321	5.8803	5.7415	6.4115
Rph3a	_TGPAGGFQAAPHTAAPYSQAAPAR_.3	#N/A	3.8952	3.4525	4.5586	4.1015	3.5880	4.2797	2.6034	4.2239	3.6766	3.0443	4.7398	4.6155	4.6164	4.5282	4.1217	3.8588	4.6237	3.7432	4.6486	3.7609
Rph3a	_DSEGWDHAHGGGTGDTSR_.3	#N/A	4.4817	4.2888	3.7027	3.7642	3.1892	4.6126	4.6556	3.6256	4.6996	4.2438	4.8498	4.2000	4.3200	3.6000	5.6505	3.8439	4.0778	4.7086	4.5205	4.5284
Rph3a	_WHQLQNENHVSS[Phospho (STY)]D_.2	#N/A	10.1420	10.5260	10.3744	10.3362	9.8294	9.7292	9.6154	10.3818	10.5560	10.0494	10.2105	10.7670	11.5459	11.3749	10.9064	11.1291	10.9192	10.9098	10.8304	10.9642
Rph3a	_RANS[Phospho (STY)]VQAAR_.2	#N/A	4.2073	4.1961	2.4060	3.8353	3.3945	4.0745	2.7991	3.4001	3.3191	4.7343	3.9848	3.6654	3.5470	4.0497	3.4867	2.1942	2.6500	3.6054	2.9870	4.4526
Rph3a	_DSEGWDHAHGGGTGDTSRS[Phospho (STY)]PAGLR_.4	P47708_S262_M1_Rph3a	3.4872	6.8596	7.2413	4.0971	6.4410	4.4143	3.3540	3.7128	3.4100	8.0747	3.7844	6.3129	7.4466	6.6927	3.3794	6.7283	3.2101	5.8664	6.3848	4.2399
Rph3a	_ANS[Phospho (STY)]VQAAR_.2	#N/A	3.3883	3.9308	4.4257	5.3250	3.5953	3.6286	3.4528	3.6140	3.3777	3.8923	4.3525	3.6569	3.5385	4.0411	3.4782	4.2513	3.1112	4.0773	3.7259	4.0081
Rph3a	_ISVC[Carbamidomethyl (C)]DEDKFGHNEFIGETR_.3	#N/A	3.9307	4.4110	3.9455	3.1753	3.2684	3.6082	4.2727	3.1338	4.3318	4.1370	3.2053	4.1371	4.4398	3.9836	3.8499	3.9265	4.1319	3.0176	3.3166	3.6609
Aldh2	_TIPIDGDFFSYTR_.2	#N/A	7.5116	7.7067	7.8257	7.7367	7.3712	7.4261	7.3597	7.4614	7.4747	7.2487	7.4806	6.9024	7.6357	7.5219	7.3392	7.6333	7.3810	7.2464	7.5654	7.3991
Aldh2	_AAFQLGSPWR_.2	#N/A	6.9668	7.3304	7.0834	7.4153	6.6531	7.0347	7.5045	7.4239	6.7731	7.3458	7.0448	7.3795	6.6291	7.3644	6.6567	7.0029	6.9413	7.0606	7.2134	7.3385
Aldh2	_VVGNPFDSR_.2	#N/A	5.9550	6.9944	5.0278	6.6346	7.2824	7.0598	6.4058	6.4953	6.6184	7.2047	6.5211	6.9006	7.3778	6.8194	7.1041	6.4975	6.3839	6.9192	6.7202	6.7156
Aldh2	_TFVQENVYDEFVER_.2	#N/A	6.5931	6.9290	6.6628	6.4665	6.3489	6.1315	6.4662	6.8656	6.2719	6.9617	6.7104	6.1776	7.6872	6.9041	5.7792	6.7814	6.7546	5.8119	5.7805	6.5083
Aldh2	_VAEQTPLTALYVANLIK_.2	#N/A	5.1143	5.7476	5.7730	5.3684	5.1915	4.8576	5.6519	5.6582	4.8395	5.6367	4.9715	5.1429	5.8537	5.3815	5.8220	5.0876	4.3398	5.5251	4.9601	5.6543
Aldh2	_TEQGPQVDETQFK_.2	#N/A	6.1602	6.0070	6.6261	6.2563	6.1014	6.0421	5.8091	6.5168	6.4076	6.7429	6.4238	6.1830	3.3720	5.6130	6.3675	5.9773	3.0641	5.5658	5.6965	6.0821
Aldh2	_TIEEVVGR_.2	#N/A	5.4437	5.5090	6.0466	5.8997	5.0624	6.5391	5.9648	5.0389	4.4566	6.2433	5.6139	6.1262	4.4577	3.8905	6.7658	6.2527	4.1497	5.9153	6.3664	6.1500
Aldh2	_LLC[Carbamidomethyl (C)]GGGAAADR_.2	#N/A	6.1302	6.2876	6.3196	6.6005	6.5945	6.8049	5.8424	4.0381	6.3691	3.8798	5.7579	6.5353	4.3584	6.6223	6.8777	6.2069	6.2970	6.5332	6.2685	6.5266
Aldh2	_ELGEYGLQAYTEVK_.2	#N/A	4.7798	5.0457	5.1047	5.4869	3.3221	5.1525	4.1519	5.1644	4.8871	4.6713	5.0040	4.6688	4.3861	3.9298	5.0582	4.3293	4.0781	3.0713	4.9423	4.8006
Aldh2	_LADLIER_.2	#N/A	3.8136	3.9939	4.3498	4.5590	3.1513	3.4054	4.1970	3.2509	4.6189	4.4606	4.8681	4.0787	4.5569	3.2748	3.4638	3.9640	4.2490	4.3450	4.9439	4.2359
Capza2	_EATDPRPYEAENAIESWR_.3	#N/A	8.1735	7.6933	7.8390	8.2609	8.0218	8.0854	7.8086	7.9625	8.1119	7.9566	7.9992	7.9326	8.5001	8.3879	7.5525	8.2792	8.0288	7.8070	8.0470	8.2394
Capza2	_LLLNNDNLLR_.2	#N/A	7.6864	7.9418	7.4475	7.7777	7.9209	7.8733	7.9274	7.8315	7.3791	7.2593	7.7371	7.6345	7.7252	7.6219	7.7189	7.7012	7.7155	7.9892	7.6682	7.3946
Capza2	_FTVTPSTTQVVGILK_.2	#N/A	6.2931	7.1757	7.5974	6.8618	7.0761	7.3789	6.9023	6.6880	7.1410	6.8306	7.1183	6.7360	7.4769	7.4043	7.0304	7.3590	7.3229	6.9321	7.4580	6.5948
Capza2	_EHYPNGVC[Carbamidomethyl (C)]TVYGK_.2	#N/A	6.1787	3.4755	3.3802	5.7732	6.4342	6.4501	5.8000	6.5318	6.0371	6.3177	6.0785	5.4953	6.4799	6.0502	6.1855	5.8624	3.5665	6.5858	3.8820	5.8547
Capza2	_TSVETALR_.2	#N/A	4.0441	5.1734	4.4954	3.6453	4.5935	5.0424	5.3655	2.5696	5.2807	5.9728	5.3908	5.1762	4.6605	4.7218	5.2287	5.4399	4.2822	5.8835	5.9186	5.4903
Capza2	_EGAAHAFAQYNLDQFTPVK_.3	#N/A	4.4400	3.8293	3.3431	3.6032	4.0636	3.9977	3.9994	4.3221	4.2795	4.1184	4.0274	3.2387	3.8374	4.5264	3.8688	4.5614	3.4857	3.6305	4.4936	3.5533
Capza2	_[Acetyl (Protein N-term)]ADLEEQLSDEEKVR_.2	#N/A	6.0283	6.3700	6.1800	6.0104	3.9223	6.2094	6.0871	5.8333	6.4470	6.1022	5.8641	5.7107	5.6758	6.7812	6.6179	5.7259	5.3625	6.3384	5.5162	5.7413
Capza2	_[Acetyl (Protein N-term)]ADLEEQLSDEEK_.2	#N/A	5.2834	5.8650	5.8426	5.0253	3.6551	5.9615	5.7970	3.8528	6.1870	5.3139	5.5013	5.0321	3.7828	6.4059	5.4869	4.7059	4.8951	6.0804	5.7981	5.6151
Capza2	_FIIHAPPGEFNEVFNDVR_.3	#N/A	6.3121	6.4203	6.1089	6.6475	6.3320	6.4793	6.2242	6.5440	6.2197	6.2694	6.4422	6.5843	6.4118	7.0947	6.7572	6.5432	6.6570	6.7018	6.7472	6.4427
Capza2	_DIQDSLTVSNEVQTAK_.2	#N/A	5.7549	5.5404	5.9234	3.3587	5.5631	4.5039	4.3079	4.7586	4.1483	5.7603	6.0308	5.6718	4.2564	5.9023	4.1419	5.3729	5.4518	3.2010	3.5001	5.4644
Capza2	_IVEAAENEYQTAISENYQTMSDTTFK_.3	#N/A	3.5548	4.4480	4.0911	4.1346	2.8925	3.1166	3.1477	3.2551	3.2068	3.7611	3.5812	4.3375	3.3149	2.6277	4.2258	3.8997	3.7928	4.1425	4.4416	4.0368
Capzb	_DYLLC[Carbamidomethyl (C)]DYNR_.2	#N/A	9.0369	8.8443	9.1532	9.2473	9.0016	8.9537	8.7561	9.1470	8.8700	9.1524	9.1510	8.8786	9.1067	9.1475	8.5551	8.7713	9.2020	8.7157	9.0642	9.0885
Capzb	_STLNEIYFGK_.2	#N/A	6.5738	6.8084	6.8571	6.8773	6.8349	6.7505	6.9525	7.0731	6.5267	6.8853	6.6915	6.3230	6.4949	6.6654	6.4563	6.6022	6.6850	6.7600	6.8972	6.6597
Capzb	_ELS[Phospho (STY)]QVLTQR_.2	P47757_S263_M1_Capzb	11.3842	11.2863	11.3779	11.0627	3.1055	10.7864	10.5415	10.9076	10.3340	11.7204	10.6586	10.4619	10.9971	10.8526	9.7272	10.7412	10.9551	10.4468	10.5332	10.6597
Capzb	_GC[Carbamidomethyl (C)]WDSIHVVEVQEK_.2	#N/A	4.6947	4.6052	4.9658	3.7427	3.7881	4.2732	4.0292	5.0142	3.6645	3.9012	4.6944	3.2031	4.5292	4.3663	3.4409	4.2087	4.3570	2.4723	4.1744	4.3860
Capzb	_KLEVEANNAFDQYR_.3	#N/A	6.2492	6.1414	6.6234	6.1752	6.0192	6.2189	6.2891	6.1436	6.0601	6.7788	6.2242	6.1175	6.6200	6.3716	6.0808	6.2210	6.0374	5.9849	6.3142	5.9447
Capzb	_LEVEANNAFDQYR_.2	#N/A	3.2637	5.7181	4.3011	4.3206	6.2134	4.7922	4.9598	6.4916	5.2424	4.5751	2.9763	3.7815	3.2945	6.1451	3.6029	4.1266	4.4874	5.5040	2.9611	5.1828
Capzb	_SGSGTM[Oxidation (M)]NLGGSLTR_.2	#N/A	6.9517	6.6978	6.2226	3.2115	6.6913	6.6287	6.7778	7.1937	6.6043	6.6712	6.0999	6.6402	6.9290	3.9474	6.1881	6.8350	6.5516	6.5926	6.4729	6.2665
Capzb	_[Acetyl (Protein N-term)]SDQQLDC[Carbamidomethyl (C)]ALDLMR_.2	#N/A	4.5280	3.1984	5.0016	4.3539	3.8657	4.6780	4.1043	4.2284	3.7311	3.4975	3.9726	3.3645	4.5487	4.3043	3.5218	3.3260	3.2652	3.0673	4.2168	3.0318
Gsr	_ALLTPVAIAAGR_.2	#N/A	5.9530	6.7614	3.5110	6.6938	6.8422	6.2897	6.3767	6.2313	6.1564	6.2437	6.0543	6.5349	6.9814	6.1631	6.3041	6.2166	6.4493	6.4063	6.2449	6.7244
Map2k4	_LC[Carbamidomethyl (C)]DFGISGQLVDSIAK_.2	#N/A	4.2471	4.1367	4.4816	5.3120	3.9566	4.7722	4.4853	4.8045	5.1597	4.6450	4.0488	4.9299	4.0440	4.2604	4.3105	4.8540	4.5764	3.5718	4.3267	4.4215
Map2k4	_LRTHS[Phospho (STY)]IESSGK_.3	#N/A	4.1472	3.6142	4.1739	4.8628	3.8317	3.9360	3.1603	3.7915	3.9464	5.1835	4.0600	4.0463	4.1119	4.3826	3.6820	4.6849	3.4633	3.7498	5.1393	3.7156
Map2k4	_YVYSVLDDVIPEEILGK_.2	#N/A	3.9063	5.0262	3.3099	3.7062	4.1132	4.7522	4.3692	4.6819	4.4283	3.0769	4.3280	4.5678	4.5214	1.2777	3.5918	3.9204	3.8724	4.2638	3.8193	3.4902
Map2k4	_FTLNPNTTGVQNPHIER_.3	#N/A	5.5887	4.5737	6.1802	4.2603	6.0969	5.6876	5.7750	3.1295	3.3325	6.2013	6.1748	5.6064	3.4405	6.3261	5.8565	6.4124	5.4971	5.4859	5.5516	5.9454
Map2k4	_THS[Phospho (STY)]IESSGK_.2	#N/A	5.2025	6.5876	5.0909	4.9647	4.3654	3.3860	4.9678	3.2403	3.9819	4.0305	5.0938	4.0306	4.5463	4.0901	3.9564	4.1691	4.2384	4.4119	3.2101	3.7674
Map2k4	_LKIS[Phospho (STY)]PEQHWDFTAEDLKDLGEIGR_.4	P47809_S88_M1_Map2k4	4.2691	4.2808	4.2583	3.9674	3.0597	3.2838	5.2402	3.4224	3.0396	3.9283	3.4140	4.1702	3.7427	4.2454	4.0585	4.0669	3.9601	3.8731	3.5217	4.3257
Map2k4	_ILDQM[Oxidation (M)]PATPSS[Phospho (STY)]PM[Oxidation (M)]YVD_.2	#N/A	12.5588	12.5123	11.3908	9.5694	3.7766	11.3454	11.8184	13.0932	11.4745	11.9345	3.7213	7.8222	12.6936	11.3120	5.7094	3.3439	12.1989	3.4603	7.3061	3.7932
Mapk14	_HTDDEM[Oxidation (M)]TGY[Phospho (STY)]VATR_.2	#N/A	3.9205	4.2996	3.8723	4.3484	3.7181	2.9651	3.2116	4.0839	3.4885	4.2398	3.6961	3.6983	3.7405	4.1408	3.8974	3.8132	4.2474	4.0133	4.5507	4.0479
Gfpt1	_VDS[Phospho (STY)]TTC[Carbamidomethyl (C)]LFPVEEK_.2	P47856_S259_M1_Gfpt1	3.9728	10.3383	10.0862	3.2552	4.1797	4.2130	4.3692	4.1345	4.2894	3.7834	3.7681	3.0724	4.4549	10.0362	2.8938	3.3349	3.1714	9.9818	4.3104	3.4237
Pfkm	_KRS[Phospho (STY)]GEAAV_.2	P47857_S775_M1_Pfkm	9.5820	9.5006	9.1704	9.4159	9.1112	9.3863	9.4091	9.1388	9.3370	9.2360	9.2888	9.0341	9.6200	9.1475	8.8877	9.3208	9.3908	9.3761	9.5121	9.5286
Pfkm	_ALVFQPVTELKDQTDFEHR_.3	#N/A	7.9866	8.0069	7.8201	7.8790	7.1233	7.5570	7.6466	8.1510	7.7559	7.7107	6.9044	7.3410	8.0679	7.4603	7.1763	7.1559	7.8262	7.4592	7.5909	7.3342
Pfkm	_RFDEAIK_.2	#N/A	7.6083	7.5443	7.6906	7.6839	7.2444	7.9860	7.8325	7.6660	7.5971	7.9268	7.6011	7.5645	7.3255	7.1763	7.3644	7.0983	7.4614	7.5246	7.5098	7.4352
Pfkm	_DLQVNVEHLVQK_.2	#N/A	7.1037	7.5425	7.1179	6.8706	6.9304	7.1459	6.9498	7.5962	6.9011	7.2276	6.8378	7.1523	7.3002	7.0026	6.2298	6.8428	7.0645	6.9467	7.0388	7.2549
Pfkm	_GQIEEAGWSYVGGWTGQGGSK_.2	#N/A	7.6575	7.4941	7.1665	7.2783	6.6808	6.6695	7.2900	7.9615	7.0617	7.0816	6.7395	6.7837	7.5797	7.6470	6.2645	7.2180	7.3560	6.4698	6.6344	6.7074
Pfkm	_ALVFQPVTELK_.2	#N/A	6.5694	6.4040	6.2871	6.5298	6.2888	6.6435	6.6737	6.2364	6.1390	6.3571	6.1687	5.5468	5.7430	6.2076	6.1641	6.1319	6.2898	6.4172	6.3859	5.9431
Pfkm	_NLEQISANITK_.2	#N/A	6.0299	6.5549	5.6445	6.3227	6.2253	5.8283	6.2722	6.1933	5.6552	5.3936	5.8586	6.3681	5.7948	5.8466	5.5479	5.6522	5.2736	6.4749	6.4552	5.8943
Pfkm	_IVEIVDAITTTAQSHQR_.2	#N/A	6.7220	6.4946	6.4601	6.2811	6.4545	5.8150	6.0664	6.4442	6.0941	6.3785	6.2147	5.8834	6.8073	5.8067	4.7414	5.5193	6.6419	5.8013	5.9970	6.2092
Pfkm	_SEWSDLLNDLQK_.2	#N/A	6.2419	5.9084	6.2749	6.2795	6.0298	6.1491	6.2349	6.3402	6.0384	5.6647	6.2327	5.3700	3.2507	5.9727	5.4062	5.5611	6.2397	6.0387	5.6411	5.8360
Pfkm	_GITNLC[Carbamidomethyl (C)]VIGGDGSLTGADTFR_.2	#N/A	5.0183	5.4437	4.6676	5.2214	4.8484	4.9778	4.9061	4.6553	3.9957	4.4977	4.9126	4.5022	5.7800	4.9706	4.1224	4.5067	4.0596	4.5026	4.9995	5.0020
Pfkm	_IFANTPDSGC[Carbamidomethyl (C)]VLGM[Oxidation (M)]R_.2	#N/A	6.5844	6.5258	6.2827	3.4824	6.0600	6.2241	6.3696	6.2295	5.7797	4.2481	5.5043	6.1228	5.9336	6.3880	5.6908	5.1628	5.7754	5.9117	5.6770	5.0152
Pfkm	_VLVVHDGFEGLAK_.2	#N/A	4.8611	4.4440	5.2893	4.5250	4.2359	4.5227	4.9814	5.2315	4.1227	4.9676	3.4088	4.6356	4.9318	4.7316	4.4671	3.7868	4.0065	4.6731	4.3151	4.3053
Pfkm	_AIAVLTSGGDAQGM[Oxidation (M)]NAAVR_.2	#N/A	5.8069	5.5657	5.8307	5.7534	5.3918	5.0474	5.2810	5.7806	4.9199	5.3871	4.8105	5.1276	5.4852	4.9591	4.6155	5.6285	5.4528	5.6751	5.2672	5.5221
Pfkm	_NVLGHM[Oxidation (M)]QQGGSPTPFDR_.3	#N/A	6.1492	6.1505	3.7615	5.9066	6.1157	5.5157	5.9965	6.3446	6.2107	6.0040	5.7376	6.0245	6.5137	5.6859	5.9770	5.6700	6.0691	6.0879	6.1007	6.2013
Pfkm	_LNIIIVAEGAIDK_.2	#N/A	4.4701	3.5327	3.5056	3.2193	3.2369	4.0319	3.7333	4.5322	3.7923	3.1756	4.1667	3.4222	3.9004	4.0649	3.3105	3.3142	3.2073	4.4047	4.2744	3.4596
Pfkm	_SSYLNIVGLVGSIDNDFC[Carbamidomethyl (C)]GTDM[Oxidation (M)]TIGTDSALHR_.3	#N/A	3.5396	3.7795	4.5770	4.0446	3.7465	3.7799	3.3016	3.0669	3.2218	3.3502	4.2012	3.5056	3.3872	3.8899	3.3269	4.4026	3.6045	4.2286	3.8772	3.8569
Pfkm	_VFFVHEGYQGLVDGGEHIR_.3	#N/A	6.6801	7.0161	4.5652	6.3037	6.3110	5.3871	6.8218	6.9313	5.7437	6.0079	6.6878	5.7790	4.3416	5.4291	3.7516	4.3738	6.1845	5.6237	3.2147	3.5626
Pfkm	_EATWESVSM[Oxidation (M)]M[Oxidation (M)]LQLGGTVIGSAR_.3	#N/A	4.1343	3.8685	3.1698	3.5550	3.4721	3.0120	4.0690	3.8347	3.9896	4.3407	3.4683	3.7579	4.1551	3.1569	3.6462	2.9784	4.3724	3.4608	3.1093	3.1239
Scg3	_ELS[Phospho (STY)]AERPLNEQIAEAEADK_.3	P47867_S40_M1_Scg3	3.4203	6.7348	5.2048	5.9905	3.6273	6.5550	3.2841	3.6460	4.8579	6.1651	5.3120	5.5667	3.4512	7.3051	5.0132	5.5819	4.8645	5.7345	5.8924	5.9835
Rpl6	_AVPQLQGYLR_.2	#N/A	6.5605	7.0791	5.5475	6.1647	6.5422	6.3599	7.0302	7.1240	6.6271	6.4917	6.4173	6.5385	6.2682	5.8195	6.9444	6.6588	6.8857	6.5629	6.5090	6.5648
Rpl6	_QLDSGLLLVTGPLVINR_.2	#N/A	4.4070	4.2452	4.7813	4.9774	4.8335	4.2639	4.1386	4.5696	4.8847	4.1483	3.9310	4.2393	5.0900	4.4768	4.7421	4.1097	3.2492	4.3509	4.3251	4.2496
Rpl6	_SSITPGTVLIILTGR_.2	#N/A	4.2304	5.2696	4.1946	5.2678	4.9350	5.3144	3.0209	5.5569	4.8265	4.7043	4.9498	3.8880	3.1880	4.7522	5.1396	5.2391	5.0924	4.8861	3.8065	5.4224
Rpl6	_KAGS[Phospho (STY)]DAAASRPR_.2	#N/A	5.0802	3.9094	3.7604	3.8226	3.7532	4.9868	2.7348	5.0100	4.0384	4.0344	4.3445	4.3222	5.2179	4.0722	4.1436	3.5859	4.5292	5.4358	4.2228	3.4757
Rpl6	_HQEGEIFDTEKEKYEITEQR_.4	#N/A	5.6014	3.9810	4.5581	4.4759	5.1558	3.1246	3.9568	3.7221	3.8770	5.7462	5.3103	4.1539	6.7616	4.5451	3.9822	5.5298	5.2487	3.5733	5.1843	5.9832
Rpl6	_AGS[Phospho (STY)]DAAASRPR_.2	#N/A	3.2742	3.2277	3.8105	4.4152	4.1128	4.3369	3.4283	4.4754	3.4874	3.4805	3.8618	3.1172	3.5954	3.7976	2.3890	3.6191	3.5123	4.3209	3.9695	4.3173
Fosl2	_S[Phospho (STY)]PPTSGLQSLR_.2	P47930_S200_M1_Fosl2	7.1266	6.7625	4.3440	4.2776	3.5135	7.0027	3.5346	3.5323	3.2294	5.7989	7.3926	3.7386	3.3375	7.9951	3.5599	7.2767	7.6579	4.1199	6.7858	7.1424
Crkl	_Y[Phospho (STY)]PSPPVGSVSAPNLPTAEENLEYVR_.3	P47941_Y105_M1_Crkl	7.9747	8.8720	8.1531	7.8333	7.0576	8.0220	8.3129	8.5568	7.4320	3.4364	7.2922	8.0995	8.5040	9.2107	7.9010	8.0574	8.2306	7.8465	8.1083	7.4197
Crkl	_IFDPQNPDDNE_.2	#N/A	8.4778	8.7496	8.7647	9.1381	8.8636	8.8259	8.4021	9.3456	8.7637	9.1808	8.7360	8.8064	9.3966	9.1467	8.7976	9.0073	9.1798	8.6012	8.8181	8.9846
Crkl	_DSS[Phospho (STY)]TC[Carbamidomethyl (C)]PGDYVLSVSENSR_.2	P47941_S42_M1_Crkl	3.4793	4.1234	6.7331	4.1675	4.1769	3.6570	5.7252	3.6820	5.2967	3.9451	3.5998	3.9220	3.6108	6.7055	3.7743	3.7172	3.9233	3.9170	4.0563	4.2465
Rplp1	_AAGVSVEPFWPGLFAK_.2	#N/A	7.3542	7.1144	8.0764	7.9681	7.3674	7.5655	7.1676	7.2063	7.5966	7.6154	7.5260	7.4898	7.6743	7.6318	7.8133	7.3736	7.1483	7.2849	7.5748	7.7262
Rplp1	_ALANVNIGSLIC[Carbamidomethyl (C)]NVGAGGPAPAAGAAPAGGAAPSTAAAPAEEK_.4	#N/A	3.9885	2.6772	3.5250	3.5958	0.4650	2.4416	4.3535	4.2141	2.0812	3.7991	2.0765	0.8870	4.4392	3.5631	2.6274	3.3506	3.7114	2.6800	3.7371	2.4717
Rplp1	_KEES[Phospho (STY)]EES[Phospho (STY)]EDDM[Oxidation (M)]GFGLFD_.2	#N/A	3.6287	9.0476	7.4250	5.8765	3.5658	11.7407	11.0044	3.5228	10.2531	7.3235	5.6247	5.0708	6.3137	11.6026	9.8685	5.9416	4.9715	12.0212	10.3051	7.4451
Rpl5	_EFNAEVHRK_.2	#N/A	3.2438	3.9253	3.5490	4.3404	3.4508	3.1567	3.1076	3.4695	3.1667	3.8013	4.1610	3.8014	3.2747	3.2417	3.6227	4.8824	3.9959	3.0778	2.9809	3.9966
Rpl5	_VGLTNYAAAYC[Carbamidomethyl (C)]TGLLLAR_.2	#N/A	4.5154	3.4948	3.3608	3.7600	3.6572	4.0772	3.6880	3.4399	3.2972	3.2209	4.1214	3.2210	3.8551	3.3989	3.2652	3.3594	3.5472	2.7147	3.9013	3.0762
Rpl5	_YLMEEDEDAYKK_.3	#N/A	4.1061	3.8967	3.1416	3.5833	3.4438	3.6679	2.9377	3.8065	4.1563	4.3124	3.0299	3.7861	4.2644	3.8082	3.6744	2.9502	4.3442	3.1930	3.4921	3.4854
Rpl13	_VDTWFNQPAR_.2	#N/A	6.9747	7.1908	7.0905	7.0288	7.2264	7.3257	7.3651	7.3485	7.3862	7.1448	7.1744	7.0841	7.5405	7.1351	7.2802	7.1806	7.5981	7.0755	7.3462	7.1826
Rpl13	_LATQLTGPVM[Oxidation (M)]PIR_.2	#N/A	6.8406	6.7801	5.8773	5.9272	6.2620	6.4509	6.6258	6.3807	6.4036	6.1449	6.1346	6.3980	7.0791	5.6280	6.4839	6.4739	6.2948	6.8908	6.5208	6.4578
Rpl13	_GFSLEELR_.2	#N/A	5.3447	6.0187	5.5881	5.1854	5.8519	5.4410	6.5703	4.6059	5.7257	6.3992	6.2588	6.5276	6.3455	6.4184	5.2214	6.3338	5.4241	6.3204	5.7994	6.3788
Rpl13	_KGDSS[Phospho (STY)]AEELK_.2	P47963_S140_M1_Rpl13	3.9983	5.6009	4.5346	3.6911	3.3360	5.9157	3.9334	3.6987	5.9457	4.2192	3.3322	3.8939	5.6311	4.5217	3.9588	5.7600	4.2364	3.5968	5.5558	2.9878
Rpl13	_TIGISVDPR_.2	#N/A	5.7256	6.1528	6.0118	5.8526	6.1257	6.0063	6.1735	5.6713	6.1523	5.7366	5.4481	5.8428	5.8908	5.8110	5.8824	5.9014	5.8180	6.2524	5.6467	5.6767
Rpl13	_STESLQANVQR_.2	#N/A	3.5761	5.3351	5.6350	5.3471	5.1658	5.6064	5.4613	5.3352	5.3872	5.5371	4.8720	5.6054	3.3507	5.8515	5.9286	2.9382	3.5681	5.8089	5.5583	3.8204
Rpl36	_EVC[Carbamidomethyl (C)]GFAPYER_.2	#N/A	7.0903	7.5610	7.6778	7.2788	7.4906	7.5956	7.4282	7.6609	7.6544	7.5582	7.4085	7.1528	7.8001	7.4632	7.4269	7.2675	7.6288	7.2467	7.2848	7.5791
Anxa5	_GTVTDFPGFDGR_.2	#N/A	9.1152	9.1425	9.1324	8.9330	8.8435	8.9676	8.7810	8.9603	8.6916	9.2092	8.8785	8.8389	9.2520	9.0435	8.8250	9.1342	9.0235	8.5861	8.6964	8.7501
Anxa5	_VLTEIIASR_.2	#N/A	8.1732	8.1124	8.1140	8.3065	8.3471	8.2796	8.1585	8.3698	8.1189	8.4844	8.0537	7.9129	8.1881	8.4556	7.7976	8.3500	8.1252	8.0800	8.3475	8.0987
Anxa5	_SEIDLFNIR_.2	#N/A	7.7451	7.9340	7.9517	7.8984	7.5605	7.7421	7.8662	7.7214	7.6171	7.7375	7.7698	7.7619	7.3264	7.7368	7.3677	7.6175	7.7296	7.4708	7.5328	7.7165
Anxa5	_GLGTDEDSILNLLTSR_.2	#N/A	7.1257	7.0731	7.6897	7.4549	7.5118	7.1956	6.8167	7.0955	7.5334	7.4689	7.2447	7.4237	7.2907	6.9580	7.2700	7.2997	7.3939	6.6544	6.9964	7.1175
Anxa5	_GAGTDDHTLIR_.2	#N/A	6.7496	7.5142	7.5017	6.8887	6.8717	7.1334	7.5913	7.3150	7.4410	7.3120	6.8873	6.8312	7.3515	6.8101	7.1751	6.2442	6.2184	7.1675	7.1728	6.7231
Anxa5	_QEIAQEFK_.2	#N/A	5.9394	5.7371	6.4270	6.1257	6.5411	6.5277	6.3288	6.5645	6.5788	6.2672	6.1778	6.3387	5.8878	5.9475	6.4949	6.2598	6.7162	6.3252	6.2048	5.9967
Anxa5	_ADAEVLRK_.2	#N/A	6.3153	6.3779	6.2134	6.2268	6.2972	6.1298	6.7978	6.5017	6.0130	6.3137	5.6693	6.1639	6.1911	6.0577	6.3743	6.0225	5.9274	6.0717	6.5070	6.2951
Anxa5	_DLVDDLKSELTGK_.2	#N/A	5.4017	4.8131	5.5356	5.1059	4.7730	5.7814	5.6443	5.1838	5.7368	5.5955	5.2578	5.1803	4.2875	5.5970	5.2570	5.4679	5.4593	4.8333	5.4522	4.9796
Anxa5	_ETSGNLEQLLLAVVK_.2	#N/A	4.3227	4.3241	4.8735	4.8519	4.2367	4.2913	4.3490	4.4128	4.9348	4.2942	5.3488	4.0927	4.2613	3.2512	4.6494	4.5676	3.8545	4.0461	4.5359	4.5717
Anxa5	_LYDAYELK_.2	#N/A	4.8777	4.8385	3.7709	5.7987	5.7541	5.2703	6.2051	5.0851	5.6048	6.2130	5.7276	5.4650	6.4027	5.4268	5.5484	5.6877	3.5249	6.2609	6.2190	5.6433
Anxa5	_TPEELSAIK_.2	#N/A	4.9966	5.2330	4.1585	5.8003	5.0259	4.9341	5.4208	5.3223	5.8934	5.5940	5.7942	5.3250	5.9879	4.2356	5.5436	5.1489	4.0231	5.5719	5.5837	5.7175
Epb41	_S[Phospho (STY)]LDGAAAAESTDR_.2	P48193_S543_M1_Epb41	9.0148	8.9674	8.9407	8.9156	8.7088	8.7081	8.5875	8.9265	8.6484	9.0907	8.6689	8.5249	8.8077	8.9214	8.0725	8.6160	8.8648	8.4600	8.7397	8.7066
Epb41	_LSTHS[Phospho (STY)]PFR_.2	P48193_S706_M1_Epb41	3.6137	4.3891	4.1500	4.0757	2.9514	3.5327	3.0888	3.3141	3.1479	3.8200	3.5223	4.2786	3.6344	4.1371	4.1669	5.0660	3.8517	3.9814	3.6300	4.1041
Epb41	_RAS[Phospho (STY)]RS[Phospho (STY)]LDGAAAAESTDR_.3	P48193_S541_M2_Epb41	3.8903	3.4269	4.1872	4.3963	1.6994	3.2127	3.7838	3.4136	3.1107	3.8572	3.4851	4.2414	3.2188	4.2634	4.1297	3.9958	4.4117	4.2386	2.2306	3.4266
Gad1	_GVPDS[Phospho (STY)]PERR_.2	P48318_S527_M1_Gad1	8.1934	7.7133	7.4070	7.6660	7.7046	7.4188	7.9510	7.8374	8.0941	7.6979	7.7014	7.8155	7.9754	7.5263	7.4149	7.7257	7.7854	8.2573	7.8449	7.5470
Gad1	_ANSVTWNPHK_.2	#N/A	6.7971	7.0538	6.9994	6.6383	6.8050	6.8907	7.3042	7.0057	7.0598	6.2652	6.8745	6.8342	7.0135	6.9754	7.4096	7.1002	6.7633	7.1279	6.6823	6.9112
Gad1	_GTVGFENQINK_.2	#N/A	6.0798	5.2273	6.3573	5.1532	6.4243	5.8428	5.7925	5.4872	6.1186	5.9550	5.8621	5.8094	6.0054	4.7668	5.0789	5.6437	6.2754	5.9148	5.5365	5.8525
Gad1	_NLLS[Phospho (STY)]C[Carbamidomethyl (C)]ENSDQGAR_.2	P48318_S77_M1_Gad1	8.4076	8.0977	8.6282	8.4450	7.8213	7.2741	7.8054	7.5474	7.5517	8.7723	7.9780	7.7024	8.3007	8.1083	4.9062	7.6776	7.7613	7.4737	7.7303	7.8981
Gad1	_TNS[Phospho (STY)]LEEKSR_.2	P48318_S55_M1_Gad1	5.9299	6.4864	5.2983	5.6375	5.1539	4.9704	5.3652	6.0693	4.6155	6.0650	4.8295	3.7056	6.5705	4.4151	4.2813	3.8441	5.1352	4.0869	5.0121	5.4157
Gad1	_ERQS[Phospho (STY)]SKNLLSC[Carbamidomethyl (C)]ENSDQGAR_.3	#N/A	3.3546	4.3546	4.1201	4.3119	3.5616	3.4725	3.4977	3.2976	3.2166	3.5798	3.9395	3.4559	3.3550	3.6606	3.5231	3.9548	3.6546	4.1851	3.4646	3.9477
Gad1	_QSS[Phospho (STY)]KNLLSC[Carbamidomethyl (C)]ENSDQGAR_.3	P48318_S72_M1_Gad1	4.0583	4.7502	3.3034	3.7865	3.7871	3.9143	4.2197	4.0404	3.5502	4.1281	4.1783	3.4570	3.6797	3.3894	3.3136	3.1199	3.5377	3.6288	4.5618	3.9274
Gad2	_M[Oxidation (M)]M[Oxidation (M)]GVPLQC[Carbamidomethyl (C)]SALLVR_.2	#N/A	7.1543	6.8036	4.5729	6.0675	7.1067	6.8396	6.9583	7.4787	6.9081	6.9813	6.4942	7.0234	7.3626	5.2232	6.0424	7.1630	6.7163	7.4900	6.7251	6.9323
Gad2	_HYDLSYDTGDK_.2	#N/A	6.2795	5.7129	6.5835	6.2657	5.9380	6.0390	6.1066	6.5418	6.2424	5.8825	5.4882	5.9364	5.3473	5.9043	4.7857	6.2336	6.1696	5.6352	5.8150	6.0141
Gad2	_M[Oxidation (M)]VISNPAATHQDIDFLIEEIER_.3	#N/A	5.1980	4.4227	3.8336	4.4934	4.3381	4.6039	3.8874	4.8116	5.1351	5.1803	4.4636	3.9053	3.5042	4.6935	4.1701	5.6083	5.1934	4.5856	4.4315	4.1837
Gad2	_[Acetyl (Protein N-term)]ASPGSGFWSFGS[Phospho (STY)]EDGSADPENPGTAR_.3	P48320_S13_M1_Gad2	5.5741	6.0944	6.6937	5.9867	3.2679	3.4920	5.4232	3.5131	6.5863	6.0737	3.2128	5.0696	5.8414	4.0936	3.8574	4.2751	4.1878	5.7495	3.3798	3.8936
Prox1	_DRAS[Phospho (STY)]PESLDLTR_.2	P48437_S511_M1_Prox1	3.9041	6.4231	4.4404	5.8128	3.2418	3.2423	3.8392	3.6045	3.7594	4.3134	3.2380	3.9882	3.9248	4.4275	3.8645	3.8650	4.1422	3.6910	3.3396	4.3945
Prox1	_GMSHS[Phospho (STY)]PSVALR_.2	P48437_S179_M1_Prox1	3.5870	4.0673	4.2892	4.3324	2.9247	3.1488	3.1155	3.4775	3.1746	3.7933	3.5490	3.7934	3.2827	4.3273	4.1936	3.9319	4.4755	4.1747	2.9730	4.0046
Ppp3cb	_GFS[Phospho (STY)]PPHR_.2	P48453_S472_M1_Ppp3cb	10.9299	10.7979	9.7384	10.6907	10.6848	10.5542	10.5250	10.7672	10.4860	10.7669	10.7047	10.5454	10.8988	10.7346	9.9849	10.3699	10.8733	10.5935	10.6131	10.2348
Ppp3cb	_LFEVGGSPANTR_.2	#N/A	9.1976	9.1616	8.5486	9.1418	8.9824	9.0117	9.0382	9.4140	8.8339	8.9400	9.1172	8.9684	9.3097	9.0853	8.7813	8.8723	9.3849	9.1493	9.0399	8.9825
Ppp3cb	_IC[Carbamidomethyl (C)]S[Phospho (STY)]FEEAK_.2	#N/A	8.2290	8.5753	8.5219	9.1084	7.8190	8.0208	8.5617	7.1143	7.7019	9.3817	8.7050	8.0548	8.1782	8.2853	7.0514	7.9680	7.1799	8.1607	8.4368	8.2765
Ppp3cb	_YLFLGDYVDR_.2	#N/A	9.2683	9.1380	9.1152	8.9558	8.8477	9.0449	9.1690	9.3284	8.8712	9.0603	9.1204	9.1930	9.1057	9.1070	8.0851	8.6736	9.3097	8.9322	8.9280	8.7724
Ppp3cb	_EESESVLTLK_.2	#N/A	8.3547	7.6779	8.8755	8.6560	8.1605	8.1599	8.2998	7.4727	8.4831	8.4816	8.5872	8.0571	8.1657	8.4669	8.0230	8.5905	7.8151	7.8964	8.4264	8.5633
Ppp3cb	_AHEAQDAGYR_.2	#N/A	7.8280	7.7426	7.8004	7.4673	7.7014	7.1325	8.0873	7.9028	7.2402	7.7027	7.7678	7.5529	7.7331	7.7488	8.2364	7.6581	7.4367	7.7539	7.7047	7.3589
Ppp3cb	_IINEGAAILR_.2	#N/A	6.6054	6.5823	6.9991	7.0013	6.9589	6.7720	7.3452	7.0627	6.4051	6.8219	6.8341	6.8277	6.9530	6.2436	6.3529	6.6094	7.1082	6.8880	6.7736	6.4254
Ppp3cb	_YENNVM[Oxidation (M)]NIR_.2	#N/A	7.7861	7.8878	7.8946	8.1719	7.7457	7.8261	8.1734	7.8301	7.8325	7.3529	8.0676	7.9997	7.7012	8.1599	8.3054	7.5936	7.8829	7.8845	7.9687	7.8682
Ppp3cb	_HLTEYFTFK_.2	#N/A	7.0154	6.6256	5.8932	6.8169	6.8957	6.6066	6.6625	6.3710	6.8448	6.7883	6.7013	6.5523	6.9427	6.6168	6.0450	6.2948	6.7381	6.4304	7.0487	6.4563
Ppp3cb	_IC[Carbamidomethyl (C)]S[Phospho (STY)]FEEAKGLDR_.2	#N/A	7.6327	8.0280	6.8921	7.9274	7.1067	6.5723	7.3286	7.2040	6.7834	7.9409	6.8585	7.3729	8.2336	7.1409	6.2751	7.2626	6.9641	7.1682	7.2767	7.3479
Ppp3cb	_VDEEIALR_.2	#N/A	3.3886	4.6142	3.9249	4.3008	4.2272	3.7578	3.3139	3.0890	3.2439	3.5949	4.2234	3.0028	3.4093	3.9120	2.8911	3.7335	3.6267	4.2065	2.8626	4.3228
Ppp3cc	_IRS[Phospho (STY)]FEEAR_.2	P48455_S462_M1_Ppp3cc	6.0549	6.1363	5.7125	6.5640	5.5515	5.3622	5.7869	5.2943	5.1932	6.4374	5.6280	4.6951	3.4762	5.5207	2.8863	5.4764	5.6808	5.9376	5.4770	5.3154

Kcnj9	_[Acetyl (Protein N-term)]AQENAAFS[Phospho (STY)]PGSEEPPR_.2	P48543_S9_M1_Kcnj9	8.7240	3.2158	3.6399	3.4809	3.5740	3.7981	3.9670	4.3290	4.0261	4.4426	2.8997	2.9419	4.1342	8.5540	3.5442	3.0804	3.8262	3.3232	3.6223	3.3552
Lmna	_KLES[Phospho (STY)]SESR_.2	P48678_S423_M1_Lmna	3.2899	9.2173	9.1444	9.3697	4.1285	9.1067	8.7845	3.7745	3.4717	9.4107	8.9142	8.9972	9.5216	4.6243	9.6753	9.3208	9.2048	3.8777	9.5152	9.1297
Lmna	_LADALQELR_.2	#N/A	6.0281	6.1387	6.3164	6.5930	6.5799	5.9986	6.2972	6.6148	5.9048	5.8957	5.6768	6.5406	6.7462	5.8929	6.1365	5.8376	5.9141	5.9172	6.0739	5.9998
Lmna	_LQEKEDLQELNDR_.3	#N/A	5.4338	6.0862	6.1666	6.5478	6.1937	6.0506	5.7260	5.7014	6.2570	6.1575	5.8010	5.9157	6.4174	5.9858	6.0058	5.8810	5.6597	5.9136	6.4042	6.3360
Lmna	_SGAQASSTPLS[Phospho (STY)]PTR_.2	P48678_S22_M1_Lmna	8.2119	8.4626	8.0957	8.2565	7.9543	7.9790	7.9468	8.4960	7.8648	8.2058	7.9152	8.1352	8.2161	8.5536	7.4994	7.7873	8.2387	4.6370	8.0844	8.0559
Lmna	_LSPS[Phospho (STY)]PTSQR_.2	P48678_S392_M1_Lmna	10.9559	11.2601	11.0968	11.4108	10.9046	10.9628	10.8733	11.1568	11.0013	11.2310	10.8002	11.0191	11.4749	11.4832	10.6650	11.0589	11.1697	10.8773	10.9646	11.0910
Lmna	_SS[Phospho (STY)]FSQHAR_.2	P48678_S429_M1_Lmna	4.5748	4.2451	4.1114	4.5103	3.1025	7.2767	4.4385	3.2996	6.3344	3.9711	3.3712	3.9712	4.6057	6.7986	7.7516	4.1097	4.2977	4.3526	3.1508	3.8267
Lmna	_GSHC[Carbamidomethyl (C)]S[Phospho (STY)]GSGDPAEYNLR_.2	P48678_S573_M1_Lmna	3.1030	3.5833	3.2724	3.8484	3.9416	4.2578	3.5995	3.9615	3.6586	3.3093	4.0330	3.3094	3.7667	3.3104	3.1767	3.4479	3.4587	3.6907	3.9898	2.9877
Lmna	_NSNLVGAAHEELQQSR_.2	#N/A	3.1117	4.2074	2.6125	2.5417	3.0307	2.1000	3.3574	3.3373	3.7832	4.4231	2.5396	3.5331	3.1425	4.1733	3.1930	3.4361	4.3354	4.2164	3.5843	2.7742
Lmna	_SVGGSGGGS[Phospho (STY)]FGDNLVTR_.2	P48678_S637_M1_Lmna	3.2383	6.3885	6.4460	4.3459	3.4453	6.8314	3.6028	3.4640	3.5231	4.5497	3.0017	3.8069	3.6885	4.3138	6.0841	4.1013	6.9149	6.5122	2.9864	4.1582
Lmna	_LRLS[Phospho (STY)]PS[Phospho (STY)]PTSQR_.2	P48678_S390_M2_Lmna	9.7131	9.7671	9.8859	9.8355	8.5475	9.5266	9.4615	9.5602	9.5855	9.8782	9.3653	9.5386	9.9240	10.1401	9.3134	9.6479	9.9447	9.5726	9.6508	9.6912
Lmna	_MQQQLDEYQELLDIK_.2	#N/A	3.7221	6.9878	6.9807	6.6375	3.9290	6.4132	3.1191	3.9478	3.0393	6.9456	7.0102	3.3231	3.2047	7.0664	3.1444	3.0842	7.4928	4.4111	4.0597	3.6744
Lmna	_NKS[Phospho (STY)]NEDQSM[Oxidation (M)]GNWQIR_.3	#N/A	4.2021	4.6522	4.6585	4.6364	3.6691	4.2239	4.3356	3.6660	4.4960	5.2340	4.5072	4.4757	5.0899	5.3478	4.4530	4.5772	4.0545	4.0273	4.8226	4.2359
Lmna	_ASS[Phospho (STY)]HSSQSQGGGSVTK_.2	P48678_S404_M1_Lmna	6.2718	6.3605	6.2814	6.1921	3.9829	6.0061	6.1066	6.2533	5.7397	5.9430	5.3223	5.7706	6.7076	6.4287	3.1693	5.3333	6.1188	5.6970	4.2876	5.8615
Lmna	_AQHEDQVEQYKK_.3	#N/A	4.5339	3.5134	3.3423	3.7785	3.8716	4.0957	3.7489	4.0314	3.7285	3.2394	3.8375	3.2395	3.8366	3.3804	5.0432	3.9371	3.5286	3.6208	3.9199	3.0577
Lmna	_LEAALGEAK_.2	#N/A	3.5164	3.8027	3.1919	4.0678	3.7233	3.7567	3.3248	3.7421	3.2450	3.3270	4.2245	3.5288	3.4104	4.5919	3.3502	4.3793	3.2393	4.2054	3.8540	3.8801
Hspa4l	_S[Phospho (STY)]FDDPIVQTER_.2	#N/A	13.5215	13.5537	13.5186	13.3697	12.9960	13.2867	13.0633	13.2405	13.2940	13.5019	13.3737	13.1778	13.3309	13.7670	12.7094	13.3323	13.8470	13.2640	13.4631	13.4523
Hspa4l	_LHGRS[Phospho (STY)]FDDPIVQTER_.3	#N/A	9.4437	10.0587	9.0187	9.4895	8.5480	9.1426	9.1346	9.7638	9.3395	9.4471	8.6098	10.0753	10.3945	9.7346	9.5034	9.8925	10.1519	9.7766	9.8861	9.6692
Hspa4l	_QLT[Phospho (STY)]QDLLNSYIENEGK_.2	P48722_T593_M1_Hspa4l	6.3612	6.2584	6.4013	6.5373	6.0427	6.3647	6.0633	6.3211	6.0126	6.4258	6.3123	6.1671	6.4155	6.3818	5.8316	6.4186	6.4635	6.2673	6.2985	6.4076
Hspa4l	_SGGIETIANEYSDR_.2	#N/A	7.2867	7.2581	7.6884	7.3469	7.2747	7.1638	6.9291	7.3743	7.3280	7.3879	7.2736	7.0122	7.0269	7.2252	6.5675	7.2111	7.4172	6.7684	6.8688	7.0537
Hspa4l	_VLATTFDPYLGGR_.2	#N/A	6.6129	6.8741	7.0620	6.7008	6.9035	6.4984	6.7542	6.9018	6.3029	6.7794	6.0435	6.4639	6.8216	6.8584	6.1609	6.3779	6.8840	6.8572	6.4877	6.5479
Hspa4l	_SIDLPIQSSLYR_.2	#N/A	6.8824	6.2984	7.0034	6.9475	6.5713	6.6927	5.9193	6.9370	6.6442	6.7331	6.3854	6.5022	6.9964	6.9954	6.7130	6.8031	6.7063	6.3910	6.8478	6.7153
Hspa4l	_SQIVTNVR_.2	#N/A	6.0377	6.3127	6.6788	6.5039	6.6853	6.4469	6.6765	6.5894	6.7174	6.2715	6.2939	6.2315	6.4494	6.5689	6.8734	6.3793	6.2378	6.3218	6.8344	6.3797
Hspa4l	_LSLTQDPVVK_.2	#N/A	5.8323	6.4940	5.5030	6.3697	5.9040	6.1042	6.4414	6.4686	6.1726	6.3245	5.7208	6.2001	6.0653	5.8181	5.7332	5.2649	5.9650	6.2641	6.0141	5.8848
Hspa4l	_DKLGTVYEK_.2	#N/A	3.5167	5.9538	6.2301	5.6743	4.9980	5.5225	5.9127	5.5042	5.3145	3.7231	5.9062	5.4912	6.2390	5.6383	5.7926	5.7850	5.6731	5.5919	5.1858	4.8217
Hspa4l	_QDLPSLDEKPR_.3	#N/A	4.8854	3.4106	5.5271	5.5061	3.7689	5.9996	5.3637	5.1769	5.5552	4.5403	5.3678	4.8802	3.9394	3.4831	5.3307	5.1578	4.9651	5.3366	5.1571	4.9011
Hspa4l	_AQFEQLC[Carbamidomethyl (C)]ASLLAR_.2	#N/A	4.2597	3.2391	4.3289	3.5043	3.5974	4.1878	3.9437	4.3423	4.1652	3.9174	3.2867	4.0417	4.1108	3.6546	3.3480	3.6616	4.1681	3.8852	4.3248	3.3319
Hspa4l	_C[Carbamidomethyl (C)]HAEHT[Phospho (STY)]PEEEIDHTGAK_.3	#N/A	5.2045	4.1884	6.1371	5.7533	5.7053	5.8449	5.8056	5.8393	5.9874	6.0825	5.8987	5.2311	6.0219	6.0641	5.6379	5.3490	6.2036	5.6629	5.9500	5.9716
Hspa4l	_GSSQHTDS[Phospho (STY)]GEMEVD_.2	P48722_S832_M1_Hspa4l	4.2039	3.5742	3.2820	6.5336	3.9146	4.1566	3.6091	3.9711	3.6682	3.3002	4.0426	3.3003	3.7763	7.2565	3.1863	3.4388	5.6658	3.6817	3.9807	3.8643
Hspa4l	_FITPEDMNK_.2	#N/A	3.3770	3.1249	5.2157	6.3088	4.2156	5.0627	5.2784	3.0774	3.7291	4.8068	5.5632	3.0144	3.3978	5.0076	2.9027	3.7219	3.6151	4.2181	3.8666	3.4642
Hspa4l	_TSFEEGTGEC[Carbamidomethyl (C)]EVFSK_.2	#N/A	3.8452	5.2598	4.4261	4.5627	4.0522	4.0855	2.9959	4.0709	4.5845	3.6558	3.8956	4.8554	3.8761	5.9289	3.0213	3.2074	3.5682	3.5813	3.8803	3.0972
Hspa4l	_SVM[Oxidation (M)]AAAQVAGLNC[Carbamidomethyl (C)]LR_.2	#N/A	3.6916	4.6351	4.3186	4.1726	3.5540	4.1489	4.5576	3.6942	3.1858	4.4027	3.5390	3.8687	5.1742	3.9074	3.3444	3.5981	3.6793	4.0301	3.8981	4.2235
Hspa4l	_NHPAPFSK_.2	#N/A	3.1361	4.1830	4.1735	3.9194	3.2412	3.3764	3.7051	3.4704	3.6253	4.4475	3.1039	2.5343	3.7907	4.2934	2.8897	3.9990	4.0081	3.8251	3.4737	4.2604
Hspa4l	_TGSEHNGPMDGQSGSET[Phospho (STY)]SPDPPK_.3	P48722_T818_M1_Hspa4l	3.9998	4.0030	4.5361	3.6896	3.3375	3.5616	4.2036	3.7002	4.2627	4.2061	3.1362	3.8925	4.0205	4.5232	3.7808	4.3447	4.2378	3.5953	3.2439	2.9893
Hspa4l	_[Acetyl (Protein N-term)]SVVGIDLGFLNC[Carbamidomethyl (C)]YIAVAR_.2	#N/A	4.0158	4.4961	3.8604	3.2604	3.3535	3.5776	4.1876	3.0487	4.2467	4.2221	3.1202	4.2222	4.3547	3.8985	3.7648	4.3607	4.0468	3.1027	3.4017	3.5758
Pcnt	_RNS[Phospho (STY)]EIDELK_.2	P48725_S1437_M1_Pcnt	5.0943	5.9256	5.5434	5.4670	4.4937	5.7345	5.4890	5.4226	4.3180	4.5858	5.3227	5.4940	5.1805	5.4050	4.1682	5.0297	4.9968	5.2975	5.3290	4.4830
Cbr1	_LQAEGLSPR_.2	#N/A	7.6721	7.7994	8.3139	8.1163	7.7342	7.8860	8.2199	8.1042	7.8086	7.8848	7.5731	7.8587	8.0568	7.9550	8.3017	7.8058	7.7175	8.0422	8.0120	7.9324
Cbr1	_ILLNAC[Carbamidomethyl (C)]C[Carbamidomethyl (C)]PGWVR_.2	#N/A	5.5419	6.3501	6.0296	6.7134	6.5196	6.6482	6.1973	5.8903	5.8037	6.3598	5.7552	6.1788	6.7456	6.6576	6.0877	5.9952	6.6023	6.5019	6.1426	6.0813
Cbr1	_FVEDTKK_.2	#N/A	4.2502	6.1895	6.0605	5.6213	5.6195	5.7571	5.7252	5.9037	5.7288	5.3456	5.5656	5.6029	5.3268	5.4320	6.3073	5.3424	5.1223	5.8000	5.5561	5.2603
Cbr1	_FHQLDIDNPQSIR_.3	#N/A	7.1330	7.2638	7.0904	7.1263	7.0204	7.0104	7.2974	7.3400	7.1763	7.0662	7.0290	7.0824	7.2765	7.3030	7.3143	7.3326	7.2074	7.0471	6.9998	7.1302
Cbr1	_GVHAEEGWPNSAYGVTK_.2	#N/A	3.9017	4.1011	4.4380	3.5839	6.5377	3.4635	5.4574	3.1628	4.3607	5.6594	5.7060	3.9905	4.4688	5.0702	4.5425	4.2466	4.1608	2.9886	3.2877	3.6898
Cbr1	_[Acetyl (Protein N-term)]SS[Phospho (STY)]SRPVALVTGANK_.2	P48758_S3_M1_Cbr1	3.4604	3.8586	4.4979	4.1238	3.6674	4.4410	3.3807	3.6861	3.3833	3.2710	3.7576	3.5848	3.4913	5.6765	3.4061	4.3234	3.1834	3.9661	4.2651	4.2132
Cbr1	_FSGDVVLAAR_.2	#N/A	4.3642	4.4557	3.9008	3.2200	3.3987	2.8884	3.9778	3.0891	3.8980	4.1748	3.3766	4.1818	4.0634	4.5868	4.0031	2.9623	3.5049	3.4702	3.3068	3.6162
Cox7a2	_GGASDALLYR_.2	#N/A	8.8006	9.1141	9.1749	9.0901	9.2122	8.9659	9.2536	9.1991	9.1115	9.0770	9.0880	9.1752	9.2576	9.2284	9.3567	8.9795	8.7258	9.1179	9.2653	8.9008
Cox7a2	_LFQEDNGM[Oxidation (M)]PVHLK_.2	#N/A	6.8043	6.4899	7.1375	6.7566	6.8143	6.6814	6.8103	6.9381	6.3554	6.9760	6.9902	6.8849	6.9363	7.1963	7.1916	6.7829	7.1448	6.9693	6.8112	6.8616
Gstm5	_IAAFLQSDR_.2	#N/A	7.8069	7.9059	7.9595	7.9859	7.7379	8.0408	8.2170	8.2179	7.6863	7.5306	7.6072	7.6378	7.1211	7.3914	6.5275	6.9921	7.4384	7.9451	7.2184	6.8321
Gstm5	_YIC[Carbamidomethyl (C)]GEAPDYDR_.2	#N/A	7.3334	7.4802	8.0769	7.8188	7.5500	7.7946	7.5837	7.5470	7.8498	7.9429	7.4154	7.1926	7.0484	6.5478	6.9090	7.1324	7.4824	7.1032	7.2692	7.1316
Gstm5	_SM[Oxidation (M)]VLGYWDIR_.2	#N/A	7.3092	6.9705	6.8197	6.9875	6.7969	7.1533	6.6506	6.9399	7.0705	6.7134	6.3345	6.4758	5.7156	6.3982	5.9842	6.4330	6.9451	6.9687	6.4761	6.4682
Gstm5	_SQWLDVK_.2	#N/A	3.2666	2.2873	7.9786	4.4228	4.1051	3.4620	4.0512	4.4678	4.4474	3.4729	2.3020	3.1248	3.2873	2.5170	3.7344	3.7371	3.5046	4.0009	3.8989	1.9265
Gstm5	_LTFVDFLTYDVLDQNR_.2	#N/A	3.7173	3.0540	4.2536	3.9720	3.0551	3.4290	2.9852	4.0220	3.5726	3.2140	3.0513	4.1749	3.7381	0.5121	4.0632	4.0622	3.9554	3.8778	3.5263	4.2077
Gstm5	_M[Oxidation (M)]LLEFTDTSYEEKR_.3	#N/A	3.3104	3.7907	4.5658	4.0558	4.1489	3.7492	4.0332	3.7541	3.4512	3.6123	3.8256	3.5168	3.5593	4.6039	2.9693	3.6553	3.2513	3.8981	4.1972	4.2812
Slc25a4	_QIFLGGVDR_.2	#N/A	11.3105	11.3460	11.1226	11.1273	11.0548	11.1073	11.3824	11.4411	11.0708	10.8690	11.0726	11.1794	11.2336	10.7180	10.4698	10.7162	11.3496	11.3128	11.0317	10.7748
Slc25a4	_EQGFLSFWR_.2	#N/A	10.9820	10.9484	10.8093	10.6536	10.6627	10.7482	10.9164	10.8902	10.6330	10.5320	10.6911	10.7856	10.9224	10.2707	9.9852	10.2858	10.8583	10.7836	10.5900	10.2972
Slc25a4	_EFNGLGDC[Carbamidomethyl (C)]LTK_.2	#N/A	10.3310	10.1182	10.2378	10.1538	9.9492	9.9561	10.3715	10.3133	10.2280	9.7383	10.1506	9.9560	10.0634	9.7363	9.6789	9.5827	10.1971	10.1562	9.9296	9.6395
Slc25a4	_[Acetyl (Protein N-term)]GDQALSFLK_.2	#N/A	8.4071	8.9124	8.6224	8.6837	8.6078	8.8067	9.0731	8.0208	8.6453	8.4259	8.6440	8.7445	8.5136	8.2063	8.3707	8.4158	8.4096	9.1177	8.4911	8.6465
Slc25a4	_DFLAGGIAAAVSK_.2	#N/A	7.9435	7.9988	8.3332	7.8961	7.8041	7.7266	7.9872	7.5034	8.0569	7.7869	8.2351	7.7886	7.3523	6.9135	7.2813	7.5386	7.9876	7.8746	7.6903	7.6898
Slc25a4	_AAYFGVYDTAK_.2	#N/A	7.5060	6.9807	7.3824	7.2634	6.1736	7.6789	7.4245	7.2261	7.6086	7.1062	6.9786	7.0636	6.6237	7.0874	6.2503	6.8146	6.9132	7.4155	7.3057	6.9025
Slc25a4	_LAADVGKGS[Phospho (STY)]SQR_.2	P48962_S149_M1_Slc25a4	7.7923	7.6549	4.8746	4.4352	7.3246	7.3865	7.6506	4.4485	5.2350	5.0808	5.3069	5.1006	5.2481	7.1205	6.3752	4.3781	7.8419	7.5347	4.8642	7.1111
Slc25a4	_IAKDEGANAFFK_.3	#N/A	4.8812	3.9790	5.2164	4.5398	4.8342	4.1771	5.4792	3.8917	4.7669	4.2337	4.9012	5.1081	4.5899	4.2264	3.4202	4.5179	3.3889	5.2260	4.1284	4.5028
Slc25a4	_GADIM[Oxidation (M)]YTGTLDC[Carbamidomethyl (C)]WR_.2	#N/A	6.7990	7.2087	7.3821	7.0787	5.6692	6.2178	7.1167	6.5938	6.5115	6.2241	6.1824	6.0080	6.4494	6.9513	5.8362	6.5998	6.5894	6.4619	6.7798	6.0288
Slc25a4	_DEGANAFFK_.2	#N/A	6.9261	4.2293	6.6999	6.0993	3.2967	6.8167	3.7514	3.3155	6.4962	4.4012	3.3870	3.9554	7.3375	5.8604	6.0274	3.9527	4.3135	4.3367	6.4051	6.3832
Slc25a4	_GSS[Phospho (STY)]QREFNGLGDC[Carbamidomethyl (C)]LTK_.3	P48962_S150_M1_Slc25a4	3.3681	3.9510	4.4055	4.2161	3.5750	3.0325	3.4731	3.5938	3.9324	3.6770	3.6653	3.6771	3.3990	4.4436	3.4984	4.2310	3.0910	4.0584	4.3575	4.1209
Cit	_RVS[Phospho (STY)]EVEAVLSQK_.2	#N/A	5.0107	6.2157	6.0617	4.7232	5.3852	5.8205	6.2396	5.2437	4.3495	5.9575	6.0613	6.0492	6.5214	7.0513	4.5554	6.0033	5.6454	6.4870	6.6033	5.5065
Cit	_MNS[Phospho (STY)]PGLQSK_.2	#N/A	3.3859	3.9332	5.7487	5.5122	3.5928	3.0147	4.4956	3.6116	3.3087	3.1965	5.1503	3.9087	3.4168	5.5380	3.6317	4.2488	4.5004	4.0407	4.3397	4.1386
Cit	_DKS[Phospho (STY)]PGRPLER_.2	#N/A	7.6013	7.5202	7.3633	7.2739	7.1586	7.1204	7.5109	6.9653	6.9478	7.4234	7.0866	6.9008	7.2369	7.4249	6.3801	6.6890	6.8235	7.1375	6.9889	6.9415
Cit	_DKMNS[Phospho (STY)]PGLQSK_.2	#N/A	4.1246	3.3340	3.5239	3.5991	3.9070	4.0224	4.1818	4.2241	4.1610	4.1713	4.1000	3.3756	4.3017	3.5601	3.2363	3.2180	3.5165	3.2566	5.3607	3.3836
Cit	_ERS[Phospho (STY)]PGRLFEDSSR_.3	P49025_S2021_M1_Cit	4.0791	3.9237	4.6154	3.6103	4.7915	3.0673	4.0142	3.7795	3.9344	4.1384	3.4130	5.0840	4.0998	4.6025	4.0396	3.6899	3.9692	3.5160	4.4780	3.0686
Cit	_S[Phospho (STY)]PEHQPSAMSLLAPPSSR_.3	P49025_S1348_M1_Cit	3.4004	4.4971	4.0420	4.1837	3.6073	3.6407	3.4408	3.2061	3.3610	3.2110	4.3404	4.3866	3.5264	6.8276	3.4661	4.2634	3.7437	4.0894	3.7380	3.9961
Camlg	_VVLGDS[Phospho (STY)]VDGGGADQLGGVAEVR_.2	P49070_S88_M1_Camlg	3.7046	5.4719	3.2412	3.8796	3.9115	1.6907	4.6905	3.9303	4.6297	3.5152	4.0363	3.3406	3.2222	3.2792	3.1619	3.0667	3.4275	5.4585	4.8918	3.6919
Camlg	_IMGFHRPGS[Phospho (STY)]GS[Phospho (STY)]EEENQTK_.3	P49070_S53_M2_Camlg	4.0828	3.2363	3.6194	3.5014	4.2898	4.3231	4.2592	4.3085	4.1794	3.8934	1.3765	2.9624	4.1137	3.6575	2.7837	3.4449	3.8057	3.3437	3.6428	3.3347
Hnrnpa1	_GFAFVTFDDHDSVDKIVIQK_.3	#N/A	8.0980	8.1083	7.7290	7.9015	7.0896	7.2238	7.6296	8.3348	7.5105	7.0924	6.6462	6.5449	8.7273	8.6397	7.4163	7.5291	6.9751	6.6799	7.5176	7.9465
Hnrnpa1	_SES[Phospho (STY)]PKEPEQLRK_.2	#N/A	11.5340	11.7121	11.4347	11.6018	11.2958	11.3328	11.3567	11.5887	11.4010	11.6326	11.2521	11.5581	12.1545	12.1427	11.5594	11.5154	11.5923	11.5829	11.5327	11.5817
Hnrnpa1	_LFIGGLSFETTDESLR_.2	#N/A	4.9836	5.0599	5.6964	5.2941	5.1681	4.0993	5.1335	5.1993	4.6626	5.6700	5.3469	4.7643	4.2644	5.0282	5.2934	5.0349	4.9078	4.4089	4.7442	5.4250
Hnrnpa1	_EDSQRPGAHLTVK_.3	#N/A	5.3178	5.5381	5.7091	6.0983	5.2405	5.6042	5.2406	5.2384	5.6603	5.9103	5.5386	5.6434	5.5033	5.6479	5.8411	6.0316	5.1670	5.4795	5.6955	5.6771
Hnrnpa1	_IEVIEIM[Oxidation (M)]TDR_.2	#N/A	6.1614	6.7677	7.0796	7.0266	6.2001	6.8690	6.5016	6.4360	6.4731	6.7834	6.2313	6.6086	6.7737	7.2087	6.5118	6.4078	6.5312	6.5496	6.7151	6.9586
Hnrnpa1	_[Acetyl (Protein N-term)]S[Phospho (STY)]KSES[Phospho (STY)]PKEPEQLR_.2	P49312_S2_M2_Hnrnpa1	9.3420	3.8715	10.2180	3.7840	5.9336	8.6607	3.9034	3.7839	3.8237	3.8321	3.8300	6.2345	6.5977	9.4245	3.7321	3.5541	9.0214	8.6399	3.6255	8.6869
Hnrnpa1	_SES[Phospho (STY)]PKEPEQLR_.2	#N/A	11.4746	11.6321	11.6675	11.5622	11.2435	11.3623	11.1676	11.6065	11.2768	11.5957	11.2431	11.4126	11.7922	12.0902	11.3604	11.3914	11.6919	11.4123	11.4223	11.4424
Hnrnpa1	_NQGGYGGSSSSSSYGSGR_.2	#N/A	4.2979	4.8757	4.5961	4.1528	4.9126	4.8193	5.2224	5.4055	4.5739	5.1631	4.4734	5.4768	4.5889	5.0353	5.5822	5.3935	5.3733	4.8823	5.1067	5.2082
Hnrnpa1	_GFAFVTFDDHDSVDK_.2	#N/A	3.4939	4.1896	4.2627	4.5929	3.5661	3.3049	3.0542	3.4391	3.3780	3.8568	3.6978	4.0060	3.5189	4.2758	2.7823	4.0799	4.2304	4.0425	3.7369	4.0795
Hnrnpa1	_GFGFVTYATVEEVDAAM[Oxidation (M)]NAR_.2	#N/A	4.5334	3.8001	4.5961	4.0653	4.1584	4.3825	3.3827	4.3200	3.4418	4.2999	3.8162	4.3502	3.5498	4.5945	2.9599	3.6647	4.8324	3.5400	4.2066	4.2717
Hnrnpa1	_SHFEQWGTLTDC[Carbamidomethyl (C)]VVM[Oxidation (M)]R_.3	#N/A	4.3709	3.7453	6.1129	4.8026	3.7086	3.9327	3.8325	4.1944	5.0270	5.0746	5.0916	3.6852	5.1756	3.5434	3.4904	3.2149	5.4665	3.4578	3.7568	5.6226
Hnrnpa1	_YHTVNGHNC[Carbamidomethyl (C)]EVR_.3	#N/A	3.1178	3.3842	3.6540	3.8632	3.9563	4.1804	4.8049	3.9467	3.5967	3.3241	4.0182	3.2737	3.7519	3.2957	3.1620	3.4626	3.4439	3.7055	4.0046	2.9730
Hnrnpa1	_DYFEQYGK_.2	#N/A	3.9225	4.4028	3.9537	3.1671	3.2602	3.4843	4.2808	3.1419	4.3399	4.1288	3.2135	4.1289	4.4480	3.9917	3.8580	4.2674	4.1400	3.0094	3.3085	3.6690
Inpp1	_TLADVLVQEVIK_.2	#N/A	4.8327	4.4466	5.0866	4.6153	4.5451	4.8563	4.7750	4.6194	3.7800	4.6330	2.8399	4.7390	4.2944	4.3888	4.6915	4.5051	4.8322	3.7995	4.5617	5.4002
Ppm1a	_NVIEAVYNR_.2	#N/A	6.6278	6.1232	5.4104	7.2234	6.1089	6.4734	6.7078	6.5415	5.8543	6.1295	5.8855	6.4910	6.6106	6.5406	6.5344	6.3951	6.5594	6.7383	6.4785	6.1034
Ppm1a	_ALGDFDYK_.2	#N/A	4.6654	5.0477	3.3140	5.5403	4.9212	5.2143	4.9593	5.2391	5.1722	4.8006	4.2715	4.9608	5.4778	4.9526	4.9713	5.4364	4.8890	5.2411	4.9404	5.1399
Ppm1a	_GPTEQLVSPEPEVHDIER_.3	#N/A	3.6201	4.8495	5.4776	4.6645	4.6393	5.2051	4.6735	4.8475	5.0546	5.1257	4.8663	4.9439	5.0237	5.3044	4.6530	5.3429	5.0449	4.8242	4.6455	4.7318
Ppm1a	_LNPYKNDDTDSAS[Phospho (STY)]TDDM[Oxidation (M)]W_.2	P49443_S377_M1_Ppm1a	3.6131	4.3981	4.1494	4.0847	3.8348	3.1749	4.5008	3.3134	3.1569	3.8194	3.5313	4.2875	3.5667	5.9445	3.5591	3.9580	3.8511	3.9904	5.4457	5.1478
Pcyt1a	_TS[Phospho (STY)]PSSSPASLSR_.2	P49586_S343_M1_Pcyt1a	8.8009	7.9865	8.1321	8.2488	7.8213	7.9613	8.4536	8.1700	7.5935	4.0745	7.7934	7.8958	8.3791	9.2662	7.5558	7.9116	8.9072	7.8681	7.9493	8.0176
Pcyt1a	_AVTC[Carbamidomethyl (C)]DIS[Phospho (STY)]EDEED_.2	P49586_S362_M1_Pcyt1a	9.4948	9.4106	9.5428	9.8179	8.6367	8.9980	8.8241	9.4354	9.2459	9.6864	9.0333	9.2644	9.3413	9.8592	9.1404	9.2534	9.6619	8.9332	9.3715	9.4611
Pcyt1a	_QS[Phospho (STY)]PSSS[Phospho (STY)]PTHER_.2	P49586_S323_M2_Pcyt1a	4.3784	4.3999	5.9572	4.2548	3.5001	3.9813	3.8205	3.6153	4.1568	3.9169	3.9780	4.9370	3.5979	4.5813	4.3401	4.2548	3.5157	4.8200	3.8979	4.3608
Pcyt1a	_SPS[Phospho (STY)]PSFR_.2	P49586_S331_M1_Pcyt1a	8.2048	8.5610	3.5692	5.1044	8.3162	8.4660	3.4930	4.2501	3.9725	9.7460	8.1746	3.7260	8.2739	8.7499	8.2189	8.4434	8.5269	3.3196	8.3525	8.7273
Pcyt1a	_MLQAIS[Phospho (STY)]PK_.2	P49586_S315_M1_Pcyt1a	4.5696	3.4332	3.6050	3.5972	3.9073	4.1314	3.6338	4.2700	3.6929	3.2751	4.0672	3.3227	3.8009	3.3447	3.2110	3.4136	3.3068	3.6565	3.9555	3.0220
Psma2	_HIGLVYSGM[Oxidation (M)]GPDYR_.3	#N/A	5.7975	4.6931	3.9855	5.3787	4.9386	5.2408	5.3360	3.1738	4.3717	5.6858	3.2453	5.8361	4.4798	6.2652	5.9986	5.7976	4.1718	6.0147	5.9995	6.0309
Psma2	_LAQQYYLVYQEPIPTAQLVQR_.3	#N/A	3.5109	3.6134	4.2013	3.5470	3.6242	3.4703	4.1574	4.0011	4.8553	4.6501	4.2300	3.5343	3.4159	4.1268	2.5927	3.5191	3.2338	3.3677	3.0193	4.5094
Psma2	_LVQIEYALAAVAGGAPSVGIK_.3	#N/A	3.7865	4.2163	4.3228	3.2185	3.1242	3.3483	4.4169	3.4868	0.9047	2.3310	0.9105	3.7368	3.7077	4.3098	3.9941	4.1314	4.0245	3.8086	3.4572	2.3098
Psma2	_YNEDLELEDAIHTAILTLK_.3	#N/A	4.4227	3.4021	3.4535	3.6673	3.7604	3.9845	3.6608	4.1426	3.8398	3.1282	4.2141	3.0670	3.9478	3.4916	3.1043	3.2667	3.6399	3.5096	3.8086	3.1810
Psen1	_AAVQELSGSILT[Phospho (STY)]SEDPEER_.2	P49769_T370_M1_Psen1	3.9103	5.9866	4.1119	3.7943	3.0813	5.2854	5.3251	3.8517	3.7939	5.3834	3.0452	4.2247	3.9352	6.3613	4.0278	3.8556	3.9568	5.8557	5.5600	5.1933
Tmod1	_NNDPELEEVNLNNIR_.2	#N/A	7.0631	6.9652	6.8568	7.3026	6.8855	7.0925	6.6452	7.1363	7.2961	7.2762	7.1719	7.1493	7.0308	7.7963	7.0595	7.3815	7.6242	7.0496	7.3454	7.3964
S100b	_AM[Oxidation (M)]VALIDVFHQYSGR_.2	#N/A	4.3796	4.5382	4.8080	4.6272	4.6246	4.3873	4.1408	4.3648	4.6767	4.9363	4.5508	4.6766	5.1285	4.6645	4.4391	4.5581	4.6039	4.5585	4.8426	4.8050
Bckdha	_S[Phospho (STY)]VDEVNYWDK_.2	#N/A	8.9367	8.7884	9.8219	9.3860	7.0928	8.9700	8.4618	7.2952	9.1207	9.1353	9.1590	8.1872	7.7800	9.3579	8.1735	8.6084	9.3325	8.1406	8.7396	8.9885
Bckdha	_IGHHS[Phospho (STY)]TSDDSSAYR_.2	#N/A	4.4317	3.9276	5.1559	5.8569	3.7104	5.9093	5.5293	5.1703	5.6103	4.7096	4.8880	4.5419	3.3501	5.8990	4.2486	3.7257	5.4611	5.7202	4.2798	5.5317
Bckdha	_S[Phospho (STY)]VDEVNYWDKQDHPISR_.3	#N/A	7.9532	8.6898	8.3871	8.7323	7.0816	8.4641	8.1450	7.3288	8.2924	8.4713	7.7193	8.2145	7.3611	8.9434	7.7064	8.3090	8.4050	8.0576	8.1816	8.4890
Bckdha	_IGHHS[Phospho (STY)]TSDDSSAY[Phospho (STY)]RSVDEVNYWDKQDHPISR_.5	P50136_S334_M2_Bckdha	3.5957	9.7386	4.1320	6.5877	2.9334	3.1575	3.1068	3.2961	3.1659	3.8020	3.5403	4.2965	3.2739	4.1191	4.1848	3.9406	3.8338	3.9994	3.6480	4.0861
Bckdha	_NNGYAISTPTSEQYR_.2	#N/A	3.0918	3.4102	3.6281	3.0968	3.9303	4.2242	3.6107	4.2930	3.6698	3.2981	4.2699	3.2996	3.7779	5.5330	3.1879	3.4367	4.8845	3.0025	4.1519	4.9249
Gng4	_EDPLIIPVPASENPFR_.2	#N/A	7.7320	7.5200	7.7651	7.7498	7.6468	8.0428	7.8439	7.7493	7.8982	7.8254	7.4474	7.3322	7.6465	7.0148	7.4080	7.4178	7.8741	7.6277	7.7367	7.4826
Gng4	_VSQAASDLLAYC[Carbamidomethyl (C)]EAHVR_.2	#N/A	4.2894	4.2944	3.9409	3.8491	4.0870	4.1675	4.7279	4.1485	3.8514	4.2509	3.7222	4.2960	3.8509	3.6026	3.9661	3.5931	4.4264	4.0980	4.2535	3.2966
Gng4	_EGMSNNSTT[Phospho (STY)]S[Phospho (STY)]ISQAR_.2	P50153_S12_M2_Gng4	4.3094	3.6934	6.3744	3.3800	3.6471	4.3379	5.2090	4.0097	4.1646	4.5157	3.6433	3.5829	4.3301	3.3319	3.4712	3.1534	4.5474	3.2857	4.4352	3.2989
Ahcy	_VAVVAGYGDVGK_.2	#N/A	5.7547	6.6691	6.3146	6.2645	5.7550	6.1909	5.9365	6.3703	5.9820	5.6277	5.8831	5.6745	6.5480	6.7610	5.7231	6.1125	6.1673	5.9575	5.7182	6.0123
Ahcy	_VADIGLAAWGR_.2	#N/A	5.4931	5.9190	4.7814	4.8238	5.7557	5.9116	6.0040	5.7571	5.5362	4.5335	3.4415	6.0018	5.3493	5.5050	5.4716	5.3151	5.2180	5.4100	5.8006	5.3265
Ahcy	_VPAINVNDSVTK_.2	#N/A	3.6201	6.0113	3.1567	6.4868	3.8271	3.8604	6.0976	3.8458	6.3742	3.4307	6.4965	5.7694	3.3067	3.1948	3.2464	2.9822	3.3430	6.2725	5.8907	6.0526
Ahcy	_KLDEAVAEAHLGK_.3	#N/A	4.3056	4.5144	3.8422	3.5880	3.0117	3.0450	4.0801	3.8017	3.9566	3.4896	2.6102	4.2405	4.1221	4.6247	4.0618	3.6677	4.3394	3.5642	4.0338	3.0909
Ahcy	_GISEETTTGVHNLYK_.2	#N/A	3.6268	3.0677	3.6742	4.5515	3.9765	2.9909	3.5646	4.3391	3.6237	3.3442	3.9981	3.2535	3.1586	3.5369	3.0278	3.4828	3.3760	4.4572	4.1058	3.6283
Gdi1	_KQNDVFGEADQ_.2	#N/A	10.8037	10.6646	10.9055	10.9770	10.6384	10.6748	10.4577	10.9220	10.7191	10.8002	10.4968	10.5736	11.0182	10.6500	10.3042	10.5352	10.9772	10.5191	10.5980	10.5621
Gdi1	_TDDYLDQPC[Carbamidomethyl (C)]LETINR_.2	#N/A	10.0391	9.7178	10.0254	9.9878	9.4649	9.7060	9.6740	9.9014	9.9165	9.7960	9.8248	9.5715	10.0194	9.6876	9.4870	9.6642	9.9186	9.7017	9.7574	9.5950
Gdi1	_QLIC[Carbamidomethyl (C)]DPSYIPDR_.2	#N/A	9.5664	9.2997	9.5053	9.2977	9.2029	9.2653	9.5130	9.4111	9.3028	9.4374	9.2641	9.2891	9.2744	9.2264	9.2030	9.1828	9.3983	9.0639	9.1617	9.0834
Gdi1	_IIC[Carbamidomethyl (C)]ILSHPIK_.2	#N/A	8.1066	8.2709	7.9277	8.1604	8.1909	8.2796	8.2970	8.6075	7.6691	8.0035	7.9176	8.2608	7.6584	7.9968	7.6666	7.4833	8.1851	8.0770	8.0226	7.7935
Gdi1	_FLVFVANFDENDPK_.2	#N/A	6.0915	5.8623	7.0550	6.3260	6.5560	6.2427	6.0678	5.7176	6.9147	6.3943	6.1024	5.8633	6.0139	5.5742	5.9517	6.3878	6.3840	5.8987	6.2499	6.5115
Gdi1	_VVEGSFVYK_.2	#N/A	5.0028	7.1909	6.0326	6.2540	5.7499	6.5335	6.8882	5.6776	6.5509	6.7196	6.3661	5.7680	6.7025	6.4331	6.0614	6.2780	5.1611	7.0045	6.8818	6.3679
Gdi1	_NPYYGGESSSITPLEELYKR_.3	#N/A	6.3896	6.5004	6.8928	6.6194	6.3581	6.2381	6.4415	6.5865	6.6864	6.4762	6.2640	6.3369	6.8634	6.4031	6.2697	6.3047	6.1236	6.3460	6.3296	6.2974
Gdi1	_NPYYGGESSSITPLEELYK_.2	#N/A	5.0921	5.4924	5.6254	5.5748	5.5621	6.1413	5.5862	5.5631	4.9411	5.6417	5.0355	5.5084	3.6521	5.4511	5.2165	5.1811	6.1975	5.3493	5.4522	5.0091
Gdi1	_M[Oxidation (M)]LLYTEVTR_.2	#N/A	8.7581	8.7101	8.5065	8.6976	8.7509	8.5843	8.6120	8.9451	8.5384	8.7111	8.2627	8.4271	8.8026	8.5889	7.8309	8.5841	8.4875	8.4533	8.4068	8.2675
Gdi1	_TFEGVDPQNTSM[Oxidation (M)]R_.2	#N/A	7.8900	7.8533	8.0054	8.1842	7.4584	7.8411	7.9181	7.7226	7.8935	7.8272	7.9160	7.7583	7.6455	8.0968	7.9189	7.1966	7.7100	7.5767	7.4713	7.3975
Gdi1	_QNDVFGEADQ_.2	#N/A	4.5217	4.2595	4.6105	4.9353	5.3371	3.7350	3.5197	3.2850	5.1555	4.9170	5.0768	4.5334	5.1561	4.6703	4.1451	5.0357	4.1956	4.6387	4.8613	4.6596
Gdi1	_YIAIASTTVETAEPEKEVEPALELLEPIDQK_.3	#N/A	3.1763	4.0229	3.5882	3.1377	4.0622	4.0956	4.2735	4.1448	3.8572	3.6659	3.8867	3.2538	4.2354	3.6205	2.6108	3.2174	3.2900	3.8125	4.1929	3.0306
Gdi1	_VPSTETEALASNLM[Oxidation (M)]GM[Oxidation (M)]FEK_.3	#N/A	4.3177	4.5022	3.8543	3.2665	3.0238	4.5210	4.5512	4.5434	3.9445	4.5562	3.4232	4.2284	4.1100	3.8923	3.0491	3.6798	4.0406	3.5059	3.1544	3.0788
Gdi1	_KSDIYVC[Carbamidomethyl (C)]M[Oxidation (M)]ISYAHNVAAQGK_.3	#N/A	4.4787	4.3412	4.0153	3.1055	2.8103	3.4227	3.8633	3.2035	3.9576	4.2893	3.2622	4.5104	4.5096	4.0533	3.8887	3.8408	4.2016	4.4487	3.2469	3.7306
Gdi1	_FDLGQDVIDFTGHALALYR_.3	#N/A	4.1312	4.0464	3.8501	3.7330	3.4689	3.6930	2.4834	3.9346	3.4886	3.5201	3.8630	3.9359	3.5967	3.1761	3.8242	3.6587	4.0361	3.9015	4.2006	3.6352
Atp6v1a	_LPANHPLLTGQR_.2	#N/A	7.8559	8.3738	8.1783	8.1694	8.0111	8.2433	8.2135	8.3063	7.7169	7.7365	7.9220	8.2884	7.8944	8.1046	8.5726	8.0313	7.6870	8.3004	8.1697	8.1114
Atp6v1a	_TVISQSLSK_.2	#N/A	8.5167	8.7633	8.7986	8.9480	8.7995	9.0070	8.9947	8.6907	8.9017	8.9018	8.6410	8.8593	8.9439	8.7141	9.1690	8.8666	8.4314	8.9551	8.9262	8.6854
Atp6v1a	_VGHSELVGEIIR_.2	#N/A	8.0766	7.6822	7.8665	8.2225	7.7700	7.7570	8.0517	8.0357	7.7288	7.9027	7.9471	7.9134	8.1758	7.9563	7.3334	7.6645	7.5650	7.7925	7.5720	7.7766
Atp6v1a	_LIKDDFLQQNGYTPYDR_.3	#N/A	8.4035	8.1432	8.4112	8.6273	8.3753	8.4982	8.1706	8.4710	8.3567	8.4751	8.0595	8.0064	8.7561	8.3167	8.6520	8.4501	8.0292	8.0543	8.4871	8.4548
Atp6v1a	_YSNSDVIIYVGC[Carbamidomethyl (C)]GER_.2	#N/A	8.5361	8.1999	8.6337	8.4777	7.4729	7.3898	7.7876	8.5549	7.9330	8.0441	7.7947	7.7705	8.5679	8.5776	7.9991	7.7424	8.0769	7.8967	8.1994	8.1148
Atp6v1a	_DIKWEFIPSK_.2	#N/A	7.9158	8.5660	8.0090	8.1460	7.8300	8.1756	8.2502	8.6781	8.1390	8.3328	7.6264	8.2387	8.5828	8.6098	7.9795	8.3762	8.3696	8.3044	8.0650	8.0713
Atp6v1a	_VGSHITGGDIYGIVNENSLIK_.3	#N/A	8.5298	8.5420	8.3938	8.3468	8.1762	8.2848	8.3400	8.3412	8.1201	8.2144	8.4344	8.4305	8.9441	8.4304	7.7964	8.1510	8.3369	7.9926	8.2477	8.5690
Atp6v1a	_GVNVSALSR_.2	#N/A	8.0369	8.3251	8.4725	8.2499	8.3116	8.1748	8.5964	8.0153	8.2587	8.3685	8.3184	8.2391	8.4521	8.3069	8.5273	8.2708	7.5530	8.4503	8.4413	8.4094
Atp6v1a	_AVETTAQSDNK_.2	#N/A	7.6560	7.8529	8.1760	8.1448	8.1575	8.2845	7.8225	8.2061	8.3132	7.9980	7.9730	8.1467	7.8081	8.1145	7.9803	7.6696	7.9451	8.1604	8.2511	7.9084
Atp6v1a	_ALDEYYDKHFTEFVPLR_.3	#N/A	8.3214	8.4091	7.8270	7.7904	7.9167	7.9971	7.9022	8.2056	7.9185	8.2723	7.9135	8.0830	8.2984	8.1284	7.7078	7.7396	8.0161	8.0071	8.0673	8.0739
Atp6v1a	_EILQEEEDLAEIVQLVGK_.2	#N/A	6.9347	6.5801	7.2059	6.9168	6.0082	5.9834	6.0681	6.9359	6.7031	7.0737	6.1666	6.2709	7.2836	7.8274	6.3899	7.0571	6.5104	6.5007	6.7153	6.8386
Atp6v1a	_HFTEFVPLR_.2	#N/A	7.0752	6.6569	7.0276	6.9178	7.2554	6.8247	7.2374	6.6622	6.6408	7.1830	7.1620	7.0522	6.8378	7.3225	6.8285	6.8473	6.7261	6.9198	6.8858	7.4719
Atp6v1a	_AKEILQEEEDLAEIVQLVGK_.3	#N/A	7.0501	7.2690	6.2311	7.2582	6.6990	6.3640	6.6301	7.4925	6.0032	6.9499	6.1423	7.0774	7.5939	7.7674	6.5215	6.1356	6.8104	6.6668	6.2348	6.8011
Atp6v1a	_IKADYAQLLEDM[Oxidation (M)]QNAFR_.3	#N/A	6.1516	6.2244	5.5041	6.1154	6.3386	5.3957	5.4699	7.0022	6.3549	6.4482	6.0497	6.6647	6.5880	6.0843	5.6264	6.1964	6.0295	6.5687	6.1244	6.3221
Atp6v1a	_QVRPVTEK_.2	#N/A	5.6147	5.4078	5.1997	4.7159	5.8040	5.4349	5.3896	5.3754	5.6753	5.2950	5.3501	5.4290	4.8115	5.4473	5.8456	5.4313	4.8800	4.1028	5.5128	4.8666
Atp6v1a	_LAEM[Oxidation (M)]PADSGYPAYLGAR_.2	#N/A	8.2514	8.8813	9.3130	9.4152	8.9985	8.9005	9.0726	8.9439	8.4780	8.4512	8.9542	8.8247	7.8228	9.4510	8.8473	8.5155	9.2143	8.5218	8.2737	8.7905
Atp6v1a	_TALVANTSNM[Oxidation (M)]PVAAR_.2	#N/A	7.4273	7.7109	7.8661	7.9250	7.6331	7.5096	7.7925	7.6274	7.6550	7.4408	7.4218	7.6128	7.5445	7.6940	7.4878	7.3760	7.2656	7.5735	7.4362	7.2367
Atp6v1a	_LASFYER_.2	#N/A	5.4486	6.1710	5.6868	7.0960	5.9571	6.3860	6.9465	5.5201	6.6954	7.1708	6.5192	6.8118	6.9842	6.2878	6.4189	6.9076	4.2274	6.6170	6.7527	6.2240
Atp6v1a	_ITWSIIR_.2	#N/A	5.5928	6.5589	6.6211	7.4652	6.2915	6.5844	6.7967	5.6254	6.8699	7.2804	6.5745	7.2170	5.8045	6.3559	6.1281	6.8555	6.0121	6.9267	6.9730	7.0009
Atp6v1a	_RAVETTAQSDNK_.2	#N/A	5.0389	5.0154	5.6596	5.4735	5.0686	5.4958	5.2027	4.5201	4.9352	5.3169	4.9197	4.1159	4.6892	5.2664	5.3474	5.6659	5.0846	4.4826	5.2559	5.2824
Atp6v1a	_GNEM[Oxidation (M)]SEVLR_.2	#N/A	7.6828	7.8557	8.4727	8.4088	7.9114	7.7054	8.1599	8.0355	7.9352	7.9552	7.9333	8.0798	7.6482	8.2902	8.1735	7.7614	7.7530	7.8943	7.6673	8.1498

Atp6v1a	_ALDEYYDK_.2	#N/A	4.7600	4.8587	5.0170	5.0867	4.1316	5.0368	5.3379	4.1166	5.1792	5.2926	4.9331	4.4389	3.8205	5.0362	4.5432	4.8549	3.4062	4.6619	5.4207	5.0851
Atp6v1a	_EHM[Oxidation (M)]GEILYK_.2	#N/A	6.9342	7.2355	7.4575	7.2441	6.8170	7.0921	7.2008	7.1357	6.7972	7.2999	7.2306	6.8508	7.0078	7.4708	7.2785	7.1310	6.6622	6.8447	6.8247	6.9924
Atp6v1a	_LEGDM[Oxidation (M)]ATIQVYEETSGVSVGDPVLR_.3	#N/A	8.4909	8.3862	8.7825	8.4795	7.6584	7.8854	7.5831	8.6900	8.0044	8.6959	7.8159	7.9276	8.6372	9.0220	7.8418	8.4514	8.2814	8.6048	7.9640	8.2077
Atp6v1a	_DFPELTM[Oxidation (M)]EVDGK_.2	#N/A	8.3385	7.6714	8.3623	7.9762	7.8407	8.1720	7.9235	7.9439	8.0606	8.2769	8.0242	7.8743	7.4692	8.0174	7.5448	7.9590	7.9808	7.8237	7.9430	8.0474
Atp6v1a	_FSMVQVWPVR_.2	#N/A	4.2428	5.4407	6.3259	6.7789	4.1596	4.4262	4.9699	4.4685	3.8658	4.5329	5.2552	4.4284	4.2737	6.7636	4.6454	3.6049	5.1273	3.1837	3.9353	3.9527
Atp6v1a	_FKDPVKDGEAK_.3	#N/A	4.2855	5.4846	3.3210	5.2146	3.6233	5.4332	6.4461	3.9859	5.9090	5.5069	6.5745	5.8988	6.2998	5.1250	6.5332	5.9640	3.8106	5.8136	5.4182	6.0772
Atp6v1a	_DDFLQQNGYTPYDR_.2	#N/A	7.5759	7.4096	7.9440	7.4930	7.4550	7.6461	7.5216	7.5869	7.4588	7.4112	7.4450	7.1784	4.4656	4.0094	7.1063	7.5115	7.6301	7.0896	7.3975	3.6867
Atp6v1a	_ADYAQLLEDM[Oxidation (M)]QNAFR_.2	#N/A	6.2386	6.2836	6.4468	5.9753	5.9896	5.9720	5.8555	6.2894	5.9466	6.0073	6.1292	5.9954	5.8994	6.2520	5.8768	5.8994	6.2019	6.0751	6.0443	5.9713
Atp6v1a	_ADYAQLLEDM[Oxidation (M)]QNAFRS[Phospho (STY)]LED_.2	P50516_S614_M1_Atp6v1a	4.0671	5.0286	3.6037	3.5171	4.2740	4.3074	4.2749	4.2928	4.1951	3.8777	3.6737	4.4703	5.1262	3.6417	2.2666	3.4292	3.5173	4.7368	3.6585	3.3190
Atp6v1a	_EASIYTGITLSEYFR_.2	#N/A	4.1990	3.4360	3.7356	3.4814	2.9051	4.4393	4.1430	3.9083	4.0632	4.0096	3.5419	4.3471	4.2287	1.0850	4.1684	4.1960	4.4460	3.3872	3.6593	3.1975
Atp6v1a	_TVGM[Oxidation (M)]LSNM[Oxidation (M)]ISFYDMAR_.2	#N/A	3.1169	4.2022	4.1543	4.4674	3.3238	3.2837	3.7243	3.3425	3.0397	3.9282	3.4141	3.9284	3.1477	4.1923	3.7497	3.9798	4.3406	4.3097	3.1079	3.8696
Atp6v1a	_ASLAETDKITLEVAK_.3	#N/A	4.1132	3.7090	3.3292	3.3956	4.3201	4.3534	4.2288	3.9941	4.1490	3.9238	3.6277	3.5985	4.3145	3.3163	4.2542	3.4753	4.5318	3.3013	2.9499	3.2833
Atp6v1a	_GSVTYIAPPGNYDASDVVLELEFEGVK_.3	#N/A	3.7828	3.5363	3.3194	3.8014	3.9898	4.0231	3.0583	4.0085	2.9785	3.5934	3.9580	3.2624	3.8137	3.3575	3.0837	3.1449	3.5057	3.2272	3.9428	3.0348
Atp6v1e1	_GALFGANANR_.2	#N/A	8.7126	9.0109	9.2680	9.1793	9.0462	8.9891	9.0109	9.2236	8.7081	9.0049	8.8967	9.0581	9.1496	9.5096	9.3656	8.7437	8.8091	8.9595	9.0675	8.9100
Atp6v1e1	_KQDFPLVK_.2	#N/A	7.9978	8.3952	7.9533	7.6746	8.3482	7.5644	7.9469	8.5224	7.7417	8.1967	7.9427	8.2976	7.8957	8.1835	8.5132	7.9515	7.8348	7.9709	8.2754	8.1276
Atp6v1e1	_[Acetyl (Protein N-term)]ALSDADVQK_.2	#N/A	5.8949	7.4340	7.2621	7.5463	6.3687	6.2813	6.8001	5.9416	6.8584	6.0478	5.6010	6.3043	7.4641	6.1311	7.1964	6.1077	5.6898	6.3623	6.2808	6.6697
Atp6v1e1	_VSNTLESR_.2	#N/A	6.3317	6.7711	7.0990	7.0400	6.6140	6.8680	7.3011	6.0634	7.4377	7.5518	7.1031	6.9209	6.9131	6.8990	7.8176	6.9807	6.2659	7.1580	7.2471	7.4228
Atp6v1e1	_AEEEFNIEK_.2	#N/A	6.2290	7.0196	6.7696	7.0761	6.2409	6.7492	6.7169	6.3098	7.1263	7.3111	7.1253	6.3863	7.0495	6.9714	6.9736	6.9928	6.0779	6.7971	6.7808	6.9627
Atp6v1e1	_DDLITDLLNEAK_.2	#N/A	5.7087	5.5869	5.3834	5.0665	5.5352	5.8730	5.5791	4.2835	5.1206	5.5385	5.8177	5.3567	5.7922	5.1202	5.4116	5.5526	5.5159	5.7060	5.2038	5.7538
Atp6v1e1	_LDLIAQQM[Oxidation (M)]M[Oxidation (M)]PEVR_.2	#N/A	8.1248	7.7594	7.1741	7.3033	8.0139	7.7025	7.6517	8.1095	7.8038	7.8213	7.3279	8.0627	8.3205	7.5972	7.6714	8.0843	7.8384	8.0514	7.9687	7.9128
Atp6v1e1	_ARDDLITDLLNEAK_.2	#N/A	6.6399	6.5725	6.3744	6.8175	6.5125	6.0456	6.2103	6.7950	6.6078	6.4270	6.5127	6.7790	6.6308	6.9804	6.7094	6.5266	6.6141	6.2505	6.2717	6.5979
Atp6v1e1	_IQM[Oxidation (M)]SNLM[Oxidation (M)]NQAR_.2	#N/A	5.6340	5.3699	3.9224	5.6283	5.6746	5.3665	5.2368	5.3346	5.4117	5.3684	6.8179	5.2959	5.8009	6.1042	5.4986	4.5556	3.7899	5.7337	5.7251	5.4119
Atp6v1e1	_IMEYYEK_.2	#N/A	4.0130	3.9898	4.2946	4.8120	3.3507	3.5748	4.6937	3.7134	4.2495	4.2193	3.9910	3.8793	4.3575	4.8498	4.5815	4.3579	4.2510	2.3917	3.3989	3.5786
Atp6v1e1	_IKVSNT[Phospho (STY)]LESR_.2	P50518_T195_M1_Atp6v1e1	3.3713	3.1306	3.9076	4.3181	4.2099	4.4340	3.3312	3.6931	3.3903	3.5777	3.7646	3.0201	4.4983	4.5429	2.9084	3.7162	3.1904	3.9591	4.2581	4.2202
Acadvl	_SGELAVQALDQFATVVEAK_.2	#N/A	4.4730	2.5023	4.2498	3.3101	3.3273	4.0040	3.8690	3.6343	3.7892	3.2848	3.2678	4.0730	3.9546	3.5787	3.8943	3.8351	3.9169	3.6612	3.6352	4.2243
Pa2g4	_[Acetyl (Protein N-term)]S[Phospho (STY)]GEDEQQEQTIAEDLVVTK_.2	P50580_S2_M1_Pa2g4	6.5876	8.3400	7.4095	6.7940	7.4490	8.3929	6.7915	7.2391	7.0461	3.6131	4.0011	7.6950	7.7961	6.2673	6.8328	6.6089	8.6197	8.4437	7.6426	7.9387
Cast	_EQKPFTPAS[Phospho (STY)]PVQSTPSKPSDK_.3	P51125_S219_M1_Cast	6.6202	6.6185	6.6495	6.4500	3.3322	6.1122	6.2932	6.2460	6.0050	6.7087	6.2432	5.8207	6.7554	7.5025	5.9193	6.2087	6.1077	6.7234	5.8961	6.7926
Cast	_SNDTSQT[Phospho (STY)]PPGETVPR_.2	P51125_T479_M1_Cast	3.7939	4.2089	4.3302	3.8955	7.9531	8.0142	4.4094	3.2705	8.1308	4.0002	7.9508	4.0983	9.0619	9.1577	3.9866	8.5239	8.8453	8.1791	3.1799	8.7141
Cast	_S[Phospho (STY)]NEQM[Oxidation (M)]VSEKPSESK_.2	P51125_S165_M1_Cast	4.4392	3.9361	3.7481	3.1450	3.1453	3.1786	3.6379	3.3681	3.8230	4.2498	3.3016	4.1068	4.6062	4.4911	3.9282	3.8013	4.2058	3.2209	3.2760	2.9573
Cast	_SLSES[Phospho (STY)]ELIGELSADFDR_.2	P51125_S446_M1_Cast	3.3148	3.7951	4.5614	4.0603	4.1534	4.3775	3.3877	3.7496	3.4468	3.5212	3.8212	3.5213	3.5548	4.5994	2.9649	3.6597	3.2469	3.9026	4.2016	4.2767
Rab7a	_NNIPYFETSAK_.2	#N/A	8.2261	8.0285	7.7966	8.1127	8.0915	7.7864	8.1250	8.0890	7.8971	8.1004	7.9322	7.9131	8.2598	7.5640	7.3796	7.8217	8.4545	7.7393	7.8793	7.6690
Rab7a	_VIILGDSGVGK_.2	#N/A	7.4165	7.6293	7.3553	7.3698	7.4915	7.7527	7.8438	7.5432	7.5353	7.0290	7.6327	7.6126	7.3225	7.2156	6.8386	7.2830	7.4429	7.5064	7.8003	7.2016
Rab7a	_FQSLGVAFYR_.2	#N/A	6.7914	6.4074	6.0893	6.2677	5.8645	6.0342	6.6237	6.4201	6.0750	5.7307	5.6902	5.9556	6.5597	6.5190	6.9403	5.8280	6.0242	5.8498	6.2499	6.2447
Rab7a	_EAINVEQAFQTIAR_.2	#N/A	6.3113	5.8130	5.7152	5.6333	5.5939	5.6086	5.9855	6.1357	5.8358	6.1319	5.4803	5.3856	6.6047	5.0158	5.0280	5.6998	5.8824	5.3563	5.8417	5.6140
Rab7a	_ATIGADFLTK_.2	#N/A	5.8329	6.3166	6.5441	6.3799	6.2995	6.4175	6.6008	6.2626	6.2955	6.3610	5.6596	6.0295	5.6931	6.2531	6.1027	6.2197	6.7124	6.4275	6.3718	6.0227
Rab7a	_TLDSWRDEFLIQASPR_.3	#N/A	5.8109	6.3410	5.8080	5.8447	3.3202	3.3535	6.0775	6.1289	5.6151	4.4247	3.1268	5.9689	6.3420	5.8987	4.9296	3.9762	4.0309	6.2715	5.0010	4.2496
Rab7a	_LVTM[Oxidation (M)]QIWDTAGQER_.2	#N/A	6.7907	6.5971	6.3639	6.3793	5.9386	6.1042	6.5019	6.6880	6.0848	6.0867	6.2438	5.8150	6.7378	5.9362	5.4965	5.8542	6.6022	6.4971	6.3715	6.0169
Acadl	_LPANALLGEENK_.2	#N/A	6.4800	6.3491	6.7113	6.3117	6.4478	6.3088	5.9677	6.2268	6.0680	5.9762	6.3678	5.3492	6.6011	6.0333	6.3901	5.5275	6.7043	5.6183	6.4013	5.5121
Acadl	_AQDTAELFFEDVR_.2	#N/A	3.2928	4.6269	6.1374	4.5213	4.4591	4.3555	3.4097	4.4941	3.4688	5.4034	5.3435	4.5834	5.0269	4.6214	4.7344	4.6388	5.3502	4.3598	4.2888	4.8866
Acadl	_THIC[Carbamidomethyl (C)]VTR_.2	#N/A	3.8008	3.5183	4.0165	3.7835	4.0077	5.8897	3.0404	5.1703	3.7236	3.1681	4.0979	5.6766	3.8316	3.3754	6.0569	5.0692	3.3527	5.7295	3.9248	3.0527
Acadl	_IFSS[Phospho (STY)]EHDIFR_.2	P51174_S55_M1_Acadl	3.9198	4.1077	3.8000	3.7943	3.2575	4.7984	3.3911	3.6201	3.7112	4.1261	3.2409	3.9971	3.7134	4.4231	3.8854	2.7238	4.1578	3.9584	4.7472	3.5324
Rpl9	_TLSNQTVDIPENVEITLK_.2	#N/A	3.5757	4.7933	5.0912	5.2982	4.7201	4.6853	5.3040	5.1353	5.1916	5.1317	4.6157	5.2528	5.0186	5.4466	4.5323	5.0270	5.2458	5.2678	4.6980	4.6665
Rpl9	_FLDGIYVSEK_.2	#N/A	3.2534	4.9030	5.2621	5.3013	3.4019	5.7149	5.5100	4.5041	3.1087	5.6105	5.0236	3.1380	5.4356	3.7768	4.9987	5.2057	5.1478	5.0491	3.9902	5.1462
Plcb3	_RNNS[Phospho (STY)]SEAK_.2	P51432_S1105_M1_Plcb3	3.5813	4.4215	4.1176	4.1081	2.9190	3.1431	3.1213	3.2816	3.1803	3.7876	3.5547	4.3110	3.2884	3.8267	4.1993	3.9262	3.8193	4.0138	3.6624	4.0716
Hsd17b4	_VDSEGIS[Phospho (STY)]PNR_.2	P51660_S308_M1_Hsd17b4	3.7094	6.0446	3.2459	6.8354	5.9541	3.9496	3.1318	4.3979	3.0520	6.2848	6.4510	6.0058	3.2174	3.7201	3.1572	3.0715	3.4348	4.3984	5.9886	3.6871
Hsd17b4	_VVLVTGAGGGLGR_.2	#N/A	4.4273	4.7470	4.6166	3.6091	3.0327	4.7056	5.0401	4.9673	4.9991	4.1372	3.4761	4.4107	4.1010	4.4031	4.4682	3.6888	4.3184	4.8352	4.8217	3.0698
Hsd17b4	_IDVVVNNAGILR_.2	#N/A	3.5208	3.7983	4.5582	4.0634	4.1851	4.1143	4.8059	4.3600	4.3419	3.3314	4.2201	3.5244	4.4096	4.5963	3.3457	4.3837	4.0819	3.9057	3.6744	4.2736
Hsd17b4	_ISDEDWDIIHR_.3	#N/A	3.7881	4.2147	4.3244	3.9013	3.1258	3.7232	3.4885	3.6434	4.4294	3.1220	4.1042	3.8088	4.3115	4.1330	3.7485	4.1330	4.0062	3.8070	3.4556	4.2785
Adcy9	_ASLGS[Phospho (STY)]DDSTQAK_.2	P51830_S1295_M1_Adcy9	3.7684	8.6522	8.7353	8.5707	8.4005	8.3484	3.9415	8.7977	8.5422	7.6131	8.2211	8.4032	8.7241	8.2449	7.5502	8.1300	8.6785	8.4396	8.0683	8.0614
Adcy9	_GQGTAS[Phospho (STY)]PGSVDLAQTVK_.2	P51830_S610_M1_Adcy9	9.3833	9.5408	9.0806	8.4729	8.0621	9.0981	9.4227	9.4459	9.0750	8.4777	8.3405	8.7771	9.4140	9.2940	7.1457	8.2242	9.2049	9.4242	9.1065	8.3370
Adcy9	_AIEKDS[Phospho (STY)]C[Carbamidomethyl (C)]EDIGVEEASELSK_.3	P51830_S1332_M1_Adcy9	4.5473	5.0310	4.6573	4.9148	3.8851	4.1092	5.0713	4.0180	3.7151	3.2528	4.0895	3.3449	3.8232	5.6559	3.2332	3.3914	3.5152	5.6697	3.3816	4.1876
Adcy9	_QGDEES[Phospho (STY)]ENSVKR_.2	P51830_S354_M1_Adcy9	4.2550	3.5557	3.4263	3.8881	3.9131	4.0093	4.2197	4.0340	3.7200	3.9154	4.1225	3.3928	3.7122	3.5795	3.2609	3.2401	3.2522	3.7887	4.0462	3.4590
Adcy9	_IIADDLMKQGDEES[Phospho (STY)]ENS[Phospho (STY)]VKR_.3	#N/A	3.7048	7.2454	3.0084	4.1217	4.1539	5.9854	6.7133	4.3327	3.8988	3.8972	3.7953	4.9727	3.7323	6.8604	3.8038	3.4727	4.3502	5.8627	4.9080	3.4951
Gss	_QIEINTISASFGGLASR_.2	#N/A	4.3146	3.6882	3.4146	3.3748	4.3520	4.3326	4.2497	4.0150	4.1699	3.9029	3.6485	3.5776	4.3353	4.1511	4.2750	3.4544	4.5527	3.2805	4.4299	3.3041
Hdgf	_AGDVLEDS[Phospho (STY)]PK_.2	#N/A	10.7638	10.7565	10.8597	10.9785	10.4407	11.0225	10.5001	10.3042	10.6770	11.1555	10.8949	10.4582	10.3060	11.1575	10.2517	10.3094	10.8953	10.3599	10.6324	10.8012
Hdgf	_RAGDVLEDS[Phospho (STY)]PK_.2	#N/A	8.2351	8.3788	8.6856	8.3629	8.2562	8.4023	7.7224	8.3740	8.3482	8.7988	8.4278	8.2650	7.9118	8.9324	8.0880	8.3138	8.5330	8.1964	8.3159	8.4744
Hdgf	_AGDVLEDS[Phospho (STY)]PKRPK_.2	#N/A	9.4174	9.6097	9.1475	9.5456	8.9968	9.4031	9.3690	9.5222	9.4768	9.6226	9.1105	9.5369	9.5899	9.9323	9.1686	9.2563	9.5754	9.3316	9.3858	9.4907
Hdgf	_GSAEGSS[Phospho (STY)]DEEGKLVIDEPAKEK_.3	#N/A	3.9568	6.7381	3.8787	6.9827	3.6890	7.8830	7.7490	6.0822	5.8238	7.6603	3.7491	7.6737	3.6798	8.6698	7.6614	7.8077	6.9054	6.9244	7.6959	7.5298
Hdgf	_GSAEGSS[Phospho (STY)]DEEGKLVIDEPAK_.3	#N/A	4.1373	8.4807	4.9794	4.9255	3.8267	8.8373	4.1306	3.8815	8.6241	4.2232	3.7844	4.0216	4.2697	9.2107	8.6676	8.6459	4.4192	8.1602	8.5108	8.9236
Hdgf	_GFSEGLWEIENNPTVK_.2	#N/A	6.2455	6.0426	3.1650	3.5598	5.1686	3.0168	5.4810	5.3929	4.1895	4.4955	4.9813	5.0783	5.3391	5.5887	5.6431	5.4703	4.3676	4.0223	4.6218	3.9972
Hdgf	_KGSAEGS[Phospho (STY)]S[Phospho (STY)]DEEGKLVIDEPAK_.3	#N/A	3.7449	3.5955	6.9226	4.1453	3.9438	6.7153	4.6667	3.6851	6.5060	5.1307	7.9780	3.3632	3.7249	7.3759	6.2058	4.7685	3.9638	4.7822	4.6030	6.5072
Hdgf	[Carbamidomethyl (C)]AAEPEVEPEAHEGDGKKGSAEGSS[Phospho (STY)]S[Phospho (STY)]DEEG	#N/A	3.6246	3.6945	3.1612	3.5996	3.8315	3.8649	3.2166	3.8503	3.1368	4.4352	4.1163	3.4206	3.3022	3.1992	3.2419	2.9867	3.3475	3.8019	4.1010	3.7719
Atp6v0d1	_LYPEGLAQLAR_.2	#N/A	9.8234	9.8701	9.3807	9.8960	9.8665	9.6725	9.9420	10.1608	9.4775	9.6569	9.7141	9.6865	9.6084	9.4273	9.4805	9.3901	9.6989	9.9155	9.8252	9.4623
Atp6v0d1	_NVADYYPEYK_.2	#N/A	9.2837	8.2186	8.9591	8.9607	9.2423	8.5656	8.4825	9.2977	8.8648	9.3151	9.2469	8.7297	9.2674	8.6768	8.6236	8.6777	8.9584	9.0067	8.7403	8.7280
Atp6v0d1	_FFEHVK_.2	#N/A	6.8539	7.1542	7.2804	6.7197	6.9556	7.0834	7.1738	6.7884	7.0639	6.9814	6.8214	7.2936	6.7273	6.9065	6.7927	6.3940	6.7462	7.1568	6.3438	6.8944
Atp6v0d1	_NIVWIAEC[Carbamidomethyl (C)]IAQR_.2	#N/A	7.2494	6.7322	7.2752	7.3056	7.0658	6.8968	6.8860	7.1804	6.5369	7.0327	7.0312	6.7310	7.0856	6.5648	5.9033	6.3993	6.8143	5.8868	6.7164	6.4187
Atp6v0d1	_ADDYEQVK_.2	#N/A	5.2169	6.1838	5.3301	5.6007	5.6870	6.4087	6.4015	5.2429	6.1184	6.1962	5.8314	5.8802	5.6107	5.9471	5.8448	5.3989	5.0917	6.0267	6.3934	5.3202
Atp6v0d1	_AYLESFYK_.2	#N/A	6.4114	5.5710	7.1782	7.4655	5.7821	5.9612	5.9604	5.0898	7.2123	6.7285	6.3929	6.1435	5.7678	4.9969	5.8644	6.5315	3.4602	5.6276	6.5483	7.0266
Atp6v0d1	_LKEQEC[Carbamidomethyl (C)]R_.2	#N/A	5.4879	5.9011	6.0467	5.9331	5.9553	5.8252	5.9246	6.4375	5.9963	6.5569	6.3649	6.6195	5.8357	5.6275	5.4714	3.7962	4.1570	6.2714	4.9442	5.6105
Atp6v0d1	_LLFEGAGSNPGDK_.2	#N/A	8.5558	8.1860	8.6657	8.5680	8.2934	8.4359	8.5874	8.5128	8.3631	8.7035	8.3046	8.3553	8.3075	8.4238	7.9540	8.2111	8.6886	8.3966	8.3973	8.2369
Atp6v0d1	_AFIITINSFGTELSKEDR_.2	#N/A	4.9932	5.6406	5.0848	5.4596	4.7074	4.5543	4.5632	5.7749	4.8292	5.0644	4.0774	5.3914	5.5905	6.0343	3.9717	5.5776	4.9322	5.2199	5.0516	5.5247
Atp6v0d1	_LHLQSTDYGNFLANEASPLTVSVIDDK_.3	#N/A	4.2719	3.5168	4.6308	3.4175	3.9938	2.8788	4.7972	4.4884	4.6832	4.8892	4.0458	3.6203	4.3333	4.6211	4.2324	3.4971	4.5100	3.8488	4.3131	3.7972
Atp6v0d1	_LLFEGAGSNPGDKTLEDR_.3	#N/A	6.3669	6.1199	4.2625	5.8403	5.4825	5.7008	5.9805	6.4517	5.4911	5.4077	5.7836	6.0833	6.2042	5.9259	5.1830	5.8915	6.0164	5.4673	6.2692	5.4996
Atp6v0d1	FC[Carbamidomethyl (C)]TLLGGTTADAM[Oxidation (M)]C[Carbamidomethyl (C)]PILEFEADRR	#N/A	5.0599	4.3828	5.2756	4.2106	3.2238	3.2166	3.0479	4.3390	4.7557	3.8611	3.4814	4.0432	3.2151	4.2378	4.1260	4.0419	4.8582	4.2426	4.2668	3.9370
Atp6v0d1	_AGVLSQADYLNLVQC[Carbamidomethyl (C)]ETLEDLK_.3	#N/A	4.1121	3.8908	3.1475	3.5773	3.4498	3.6739	4.0913	4.4532	4.1504	4.1184	3.0239	3.7802	4.2584	3.8022	3.6685	2.9561	4.3501	3.1990	3.4980	3.4795
Fabp7	_NTEINFQLGEEFEETSIDDR_.2	#N/A	3.1204	3.6683	3.9137	3.8658	3.9590	4.1830	3.5821	5.5737	4.8036	5.6675	4.0156	3.3268	6.0882	4.4196	3.3206	4.7078	3.4413	5.2837	5.2260	2.9703
Fabp7	_LTDSQNFDEYMK_.2	#N/A	3.8457	4.1571	4.3820	3.8437	3.1834	3.3007	4.3577	5.5461	3.7010	4.3718	3.1796	4.0466	3.8664	4.3691	3.9349	4.1906	4.0838	3.7494	3.3980	4.3361
Slc25a5	_GNLANVIR_.2	#N/A	11.2962	11.2765	11.1449	11.1971	11.2698	11.0415	11.5391	11.3544	11.1612	11.0433	11.1910	11.2424	11.4927	10.5601	11.1148	10.7224	11.0891	11.5424	11.3033	11.0726
Slc25a5	_TAVAPIER_.2	#N/A	10.3287	10.8047	10.2740	10.6817	10.3551	10.7371	11.3484	10.2257	10.8548	10.5915	10.8174	10.6108	10.7460	10.2159	10.4623	10.1799	11.1327	11.1791	10.8276	10.6696
Slc25a5	_YFPTQALNFAFK_.2	#N/A	10.7457	10.6982	10.3604	10.4867	10.4374	10.4337	10.7195	10.5742	10.3670	10.1826	10.3335	10.2416	10.7411	9.7835	9.7597	9.8290	10.5683	10.7048	10.3793	10.2823
Slc25a5	_EQGVLSFWR_.2	#N/A	10.5228	10.5039	10.2877	10.4019	10.2024	10.2229	10.4724	10.5451	10.3121	10.1501	10.3392	10.3428	10.2328	9.9681	9.5519	10.0103	10.4024	10.4439	10.1546	9.9699
Slc25a5	_LLLQVQHASK_.2	#N/A	9.5587	9.7327	8.8793	9.3399	9.8106	9.7097	9.6800	10.0036	9.1842	8.9471	9.6359	9.3496	9.7106	8.7578	8.9817	9.1985	9.8711	9.7184	9.3141	9.4378
Slc25a5	_QIFLGGVDKR_.2	#N/A	9.8106	9.9006	9.6416	9.4953	9.5581	9.6364	9.9164	10.0571	9.4721	9.3254	9.5538	9.7377	9.6576	9.2059	9.2668	9.2520	9.8376	9.6883	9.4612	9.3715
Slc25a5	_DFLAGGVAAAISK_.2	#N/A	8.9675	8.5314	9.0158	8.7028	8.5054	8.6543	8.7077	8.7488	8.7794	8.4024	8.7093	8.4297	8.8239	8.0059	8.0949	8.4308	9.0087	8.7581	8.7873	8.1361
Slc25a5	_GAWSNVLR_.2	#N/A	8.0178	8.2270	8.2679	8.9138	7.8952	8.3640	9.1072	8.6971	8.5947	8.5624	8.6606	8.3382	8.4448	7.7950	8.2861	8.4637	7.0587	9.1223	8.7968	8.6926
Slc25a5	_GLGDC[Carbamidomethyl (C)]LVK_.2	#N/A	6.8988	7.3760	7.3047	7.3701	7.6298	7.4736	7.9350	6.5446	7.7039	7.5240	7.7054	7.4432	7.7973	6.6835	7.2936	7.1800	6.6200	8.1134	7.8307	7.5112
Slc25a5	_AAYFGIYDTAK_.2	#N/A	6.7663	6.4456	6.9243	6.5240	5.5054	6.0065	6.9565	5.5215	6.6316	6.5564	6.5859	6.2105	6.6780	6.2715	5.2946	6.1759	5.5601	7.0354	6.5901	6.5824
Slc25a5	_QIFLGGVDK_.2	#N/A	6.3216	6.4098	6.8650	5.9428	6.8411	6.5250	6.8405	6.8731	6.4207	6.4436	6.1140	6.4216	6.1183	5.7848	6.3800	6.2258	6.3835	6.8107	6.7023	6.0314
Slc25a5	_[Acetyl (Protein N-term)]TDAAVSFAK_.2	#N/A	6.0404	5.4887	6.3412	6.4146	6.2733	6.1997	7.2357	4.4811	6.4961	6.0283	6.7778	6.0067	6.9207	3.1335	6.3899	6.1312	4.2185	7.2015	6.9956	6.6261
Slc25a5	_GM[Oxidation (M)]GGAFVLVLYDEIKK_.2	#N/A	4.3374	3.5858	4.1290	3.9571	3.6056	4.2682	3.6608	4.5031	3.6495	3.9084	3.9931	3.8122	3.9150	3.8396	2.8469	4.0216	3.6618	4.1938	4.0391	3.9431
Slc25a5	_MM[Oxidation (M)]MQSGR_.2	#N/A	3.8442	4.2320	3.7275	3.6507	3.1819	2.3979	5.1054	3.4355	3.8575	4.0505	4.2924	4.0649	4.1106	4.7523	3.9433	4.1891	4.1197	4.0309	3.3613	4.1555
Slc25a5	_GTDIMYTGTLDC[Carbamidomethyl (C)]WR_.2	#N/A	3.8269	5.5262	5.9426	3.7573	3.8504	4.0745	3.6906	4.0526	3.7497	3.2182	4.8844	3.2183	3.8578	3.4015	3.2678	3.3568	3.5498	3.5996	3.8987	3.0788
Slc25a5	_GM[Oxidation (M)]GGAFVLVLYDEIK_.2	#N/A	3.7646	4.2382	4.3009	3.9248	3.1023	3.3264	4.4387	3.4650	2.9970	3.9709	3.3714	4.1276	4.6059	4.2880	4.0159	4.1095	4.0027	4.3524	3.1506	3.8269
Nek1	_TC[Carbamidomethyl (C)]S[Phospho (STY)]LPDLSK_.2	P51954_S997_M1_Nek1	8.8289	8.8416	8.4552	8.5979	8.0350	8.4546	8.5083	8.4405	8.2461	8.4399	8.5716	8.3066	8.6333	8.7142	7.3178	8.8104	8.8580	8.4583	8.6025	8.4156
Nek1	_SSDVPLPLELLET[Phospho (STY)]GGSPSK_.2	P51954_T615_M1_Nek1	3.4091	3.9516	3.9454	5.5176	4.4433	5.2233	3.3333	3.9283	3.3924	3.6154	3.7667	3.8411	3.5004	3.8631	4.5586	5.3656	3.6471	5.7659	3.3619	4.2223
Nek1	_EQPGDEYS[Phospho (STY)]EEEESVLK_.2	P51954_S1071_M1_Nek1	3.7106	4.2922	4.2469	3.9788	3.0483	3.2724	2.9919	3.4109	3.0510	3.9169	3.4254	4.1817	3.1591	4.2339	4.0700	4.0555	3.9486	4.2983	3.0965	3.8810
Tst	_VLDASWYSPGTR_.2	#N/A	6.3299	3.4061	6.9365	6.8190	7.0499	6.8865	6.7543	6.7828	7.0510	6.8171	6.6630	6.3262	6.4268	6.5970	6.6406	6.3725	6.8237	6.5189	6.6435	6.3674
Usp10	_LLS[Phospho (STY)]PTHEK_.2	P52479_S541_M1_Usp10	7.2268	3.5099	3.5283	3.1965	7.2329	7.2188	7.1453	7.6667	7.1403	7.1983	7.4140	7.5668	6.9521	7.3154	6.3381	3.3169	7.6508	7.5368	7.0771	3.0987
Usp10	_C[Carbamidomethyl (C)]S[Phospho (STY)]PPVPSPLASEK_.2	P52479_S359_M1_Usp10	3.8343	4.1685	4.3706	3.8551	3.1720	7.4263	3.7693	3.5346	3.6895	7.5260	3.1682	4.0580	3.8550	4.3576	3.7947	4.1750	4.7338	3.7608	3.4094	3.1837
Usp10	_TC[Carbamidomethyl (C)]DS[Phospho (STY)]PQNPVDFISGPVPDSPFPR_.3	P52479_S208_M1_Usp10	4.4788	4.3411	4.0154	3.1054	3.1849	3.2182	3.8632	3.6285	3.7834	4.2894	3.2621	4.0673	3.9489	5.5831	3.8886	3.8409	4.1662	3.6669	3.3155	4.4185
Pkm	_LDIDSAPITAR_.2	#N/A	12.3882	12.3250	12.4807	12.3848	12.2731	12.2736	12.3322	12.5201	12.3060	12.2173	12.1478	12.3357	12.4573	12.2692	12.3343	12.2102	12.3397	12.1610	12.2486	12.0747
Pkm	_NTGIIC[Carbamidomethyl (C)]TIGPASR_.2	#N/A	11.6777	11.7164	11.9504	11.8900	11.6634	11.6306	11.6514	11.8111	11.6869	11.7503	11.5919	11.5975	11.8819	11.6893	11.5292	11.5752	11.7472	11.4297	11.5928	11.4571
Pkm	_GDYPLEAVR_.2	#N/A	11.1395	10.8858	11.2065	11.2050	11.0932	11.1161	11.2930	11.0871	11.2068	11.2549	11.2850	10.9565	11.3038	11.0773	11.0984	11.0080	10.5697	11.1205	11.0136	11.2014
Pkm	_APIIAVTR_.2	#N/A	10.7690	10.9746	11.0272	10.9646	11.0376	10.9212	11.0391	10.9619	10.8468	10.8376	10.8273	10.9209	10.9318	10.8008	10.7962	10.7072	10.4057	11.0626	10.9985	10.7034
Pkm	_DAVLNAWAEDVDLR_.2	#N/A	10.9530	10.5464	11.2355	10.8730	10.4836	10.6917	10.4353	10.6915	10.8400	10.9063	10.6907	10.4474	11.0699	10.5894	10.5121	10.7678	10.7160	10.3403	10.6429	10.7288
Pkm	_IENHEGVR_.2	#N/A	9.0152	9.3139	9.5012	8.9674	9.0834	9.3183	9.3235	9.2572	9.2351	9.2159	9.1133	9.0973	9.2248	9.1094	9.8315	9.2500	8.8978	9.2110	9.2275	9.1524
Pkm	_IYVDDGLISLQVK_.2	#N/A	9.0699	9.2180	9.3793	8.9990	8.9110	8.7577	8.7595	8.8778	9.3360	8.9268	8.8093	8.5461	9.0794	9.0444	8.6333	8.9328	8.9370	8.7137	8.9481	8.7389
Pkm	_C[Carbamidomethyl (C)]DENILWLDYK_.2	#N/A	8.1229	8.2644	8.8978	8.4013	7.8661	8.1799	8.0107	7.6207	8.5193	8.4258	8.2985	7.8180	8.3910	8.0368	7.9994	8.4311	8.3541	7.9737	8.2425	8.3516
Pkm	_GVNLPGAAVDLPAVSEK_.2	#N/A	8.4542	8.4047	8.9127	8.8976	8.2189	8.3509	8.3858	8.4372	8.6722	8.8072	8.5978	7.9300	8.1071	8.7869	8.1272	8.0781	8.2614	8.1998	8.4262	8.3789
Pkm	_KAADVHEVR_.2	#N/A	8.3449	8.0828	8.7462	8.0604	8.3110	8.4095	8.4437	8.7023	8.3755	8.3898	9.2470	7.9530	7.8898	8.0599	8.3186	8.5139	8.5989	7.8513	8.4328	8.4019
Pkm	_EKGADFLVTEVENGGSLGSK_.3	#N/A	7.5296	7.4729	7.5592	7.9150	7.1696	7.4597	7.0204	7.4530	7.5251	7.6733	7.5187	7.0598	7.2078	7.3710	7.4379	7.5723	7.6613	7.0381	7.4502	7.5504
Pkm	_AADVHEVRK_.2	#N/A	6.4359	6.7062	7.3561	6.5180	6.8695	6.9640	6.7199	7.1232	7.0418	7.0622	7.1622	6.4757	6.4267	7.0224	6.8303	7.3682	6.8557	6.3269	6.9020	6.6475
Pkm	_AADVHEVR_.2	#N/A	6.0675	6.8859	6.5512	6.3291	6.6132	7.0178	7.1846	6.6294	6.4628	6.5683	6.6095	6.2944	5.7526	6.4516	6.9856	6.4960	6.1261	6.3140	6.4296	6.4727
Pkm	_GDLGIEIPAEK_.2	#N/A	12.0285	11.8456	12.0315	11.9292	12.4185	11.8668	11.8409	12.0342	11.9631	11.9792	11.9571	11.8686	12.0697	11.9100	11.7195	11.9625	11.8883	11.7730	11.7379	11.8323
Pkm	_M[Oxidation (M)]QHIAR_.2	#N/A	8.9027	9.2908	9.2319	9.3263	9.0591	9.0971	9.2039	9.0302	9.0023	8.9824	8.5748	9.0440	8.9502	9.0776	9.3282	8.8339	8.7879	9.1738	9.0401	8.9563
Pkm	_ITLDNAYM[Oxidation (M)]EK_.2	#N/A	9.0585	8.6565	8.0885	8.0657	9.0698	8.9007	8.6602	9.1220	8.6495	8.8377	8.8351	8.7505	9.1645	8.5638	8.8636	8.9652	8.6993	8.8569	8.8833	8.9030
Pkm	_GVNLPGAAVDLPAVSEKDIQDLK_.3	#N/A	6.1134	6.9824	6.3413	6.3208	6.7557	6.3145	6.6251	6.4660	6.5390	6.3452	6.3559	6.4822	7.2888	6.9895	6.0762	6.1718	6.8482	6.4930	6.1604	6.5537
Pkm	_GADFLVTEVENGGSLGSK_.3	#N/A	7.0648	7.2218	3.3967	6.2044	6.9510	6.9158	6.8961	7.2587	6.3741	6.9734	6.6732	6.9919	7.2237	6.9711	6.1737	6.6826	7.0309	6.5097	6.0893	6.7581
Pkm	_VNLAM[Oxidation (M)]DVGK_.2	#N/A	8.9388	8.7134	8.0463	8.3510	8.9057	8.5220	8.6067	9.0067	8.4932	8.6730	8.2290	8.6785	8.6476	8.0508	8.5981	8.6545	8.4981	8.8238	8.6504	8.5110
Pkm	_AEGSDVANAVLDGADC[Carbamidomethyl (C)]IM[Oxidation (M)]LSGETAK_.3	#N/A	6.0686	6.6687	7.0823	7.1313	6.1286	6.8360	6.5852	5.9269	6.6321	6.3418	6.1676	5.9263	6.6605	7.1915	5.3015	5.5861	6.2332	6.0144	6.2339	6.5484
Pkm	_EATESFASDPILYR_.2	#N/A	4.8417	4.8741	5.2118	5.9314	5.3468	5.6914	4.9665	5.2836	4.6848	4.9474	5.4493	4.7992	4.8674	5.4478	5.4313	5.7588	5.6967	4.6336	4.7889	3.9239
Pkm	_AEGSDVANAVLDGADC[Carbamidomethyl (C)]IM[Oxidation (M)]LSGETAKGDYPLEAVR_.3	#N/A	3.3578	5.3071	3.9732	4.0508	3.8625	4.3154	4.4558	4.6728	2.5961	4.2262	2.8227	4.9207	5.6537	5.1748	4.0168	4.1697	4.3105	4.4810	4.0694	4.1443
Pkm	_FGVEQDVDM[Oxidation (M)]VFASFIR_.2	#N/A	6.7399	6.9143	6.9214	7.4242	6.7899	6.9171	7.1215	7.4739	7.3228	7.8149	6.4885	6.7790	7.1875	6.9542	6.7150	7.5003	7.4985	6.7795	7.4269	6.9311
Pkm	_EATESFASDPILYRPVAVALDTK_.3	#N/A	9.3439	9.3956	9.1672	9.2578	9.0208	9.3417	9.3067	9.3408	9.2947	9.2297	9.0971	9.8983	9.1605	9.4986	9.1466	9.0609	9.1422	9.2770	9.2107	9.0375
Pkm	_LAPITSDPTEAAAVGAVEASFK_.2	#N/A	6.0401	5.6113	5.4603	5.1728	5.0807	5.3514	5.2489	5.6872	5.1797	6.0345	5.1180	5.4636	5.8280	5.8224	4.8980	5.0598	5.2727	5.5795	5.1885	4.5993
Pkm	_GSGTAEVELKK_.2	#N/A	8.4837	8.7706	8.8547	8.4834	8.4562	7.7939	8.2595	8.6687	8.8903	8.4983	8.2554	8.1778	8.3183	8.5406	8.7684	8.5556	8.1383	8.5983	8.5174	8.6942
Pkm	_EATESFASDPILYRPVAVALDTKGPEIR_.4	#N/A	3.7402	5.0329	4.2765	3.9492	3.0779	3.4062	4.4632	5.3423	5.1947	5.2292	3.3958	4.7461	5.6678	4.3044	3.6306	5.1348	3.9782	5.3470	4.8015	4.6602
Pkm	_RFDEILEASDGIM[Oxidation (M)]VAR_.3	#N/A	7.6093	7.7533	7.9343	7.8909	7.3486	7.4623	7.3390	7.1242	7.3248	7.7290	7.3321	7.1839	7.7273	7.7433	7.1445	7.5083	7.4708	7.4083	7.5816	7.9754
Pkm	_GADFLVTEVENGGSLGSK_.2	#N/A	7.9809	8.0415	8.5502	8.1086	7.8754	7.8555	7.5891	8.0650	8.1212	8.1890	7.9641	7.6683	8.3095	8.0021	7.7736	7.8519	7.8835	7.5587	7.8505	7.9091
Pkm	_LNFSHGTHEYHAETIK_.3	#N/A	5.1279	5.0932	5.6224	5.5346	4.1103	4.6284	4.7938	4.4706	3.5564	4.6947	4.6194	4.4966	4.0252	4.8236	5.2426	3.3279	4.5050	4.5199	4.1200	3.9656
Pkm	_GSGTAEVELK_.2	#N/A	3.2532	6.0302	3.7895	6.7617	4.0917	6.2857	6.2628	4.4544	3.1085	6.2799	4.0879	3.1382	3.2739	6.2499	3.2136	3.5981	3.4912	4.3419	3.3905	3.7436
Pkm	_FDEILEASDGIMVAR_.2	#N/A	3.7597	4.2431	6.6077	3.9296	3.0975	3.3216	4.4436	3.4601	3.0019	3.9661	3.3762	4.1325	3.1099	4.2831	4.0208	4.1046	3.9978	3.8354	3.4839	4.2501
Ndufs6	_TGTC[Carbamidomethyl (C)]GYC[Carbamidomethyl (C)]GLQFK_.2	#N/A	4.3296	5.9862	3.8661	3.2547	5.3126	5.3949	5.0783	5.4630	3.9327	4.1401	5.4200	3.5594	4.0981	5.7014	4.0378	5.3617	4.9384	4.7721	5.1323	5.4561
Ndufs6	_EVNENFAIDLIAQQPVNEVEHR_.3	#N/A	4.0041	4.2313	3.1227	3.9981	3.7931	4.3524	3.4499	3.8118	4.4956	3.4590	3.8833	3.5627	3.6170	3.1608	4.2146	3.7560	4.6363	3.0856	4.1394	3.3822
Ndufs6	_ITHTGQVYDEK_.2	#N/A	5.7188	5.6926	6.1218	4.9234	5.6070	5.8138	5.3167	5.5507	5.6913	5.5470	5.8505	5.6484	5.0371	5.7206	6.3800	5.4707	5.5796	5.3740	5.2132	5.6443
Ndufs6	_VYINLDKETK_.2	#N/A	5.7689	6.6770	6.1323	6.4441	6.5607	6.2981	6.4786	6.2232	6.2106	6.2150	6.4165	6.3449	6.7965	6.7238	7.1267	6.8371	6.4569	6.5856	6.5747	6.5633
Ndufs6	_IIAC[Carbamidomethyl (C)]DGGGGALGHPK_.2	#N/A	5.2595	5.3407	5.3812	5.3712	5.3402	5.4575	5.4438	5.7929	5.6147	5.3464	5.3612	5.4358	5.2839	5.8487	6.4886	5.3743	5.2639	5.5782	5.5909	5.3260
Ndufs6	_QKEVNENFAIDLIAQQPVNEVEHR_.4	#N/A	3.3733	3.7695	3.9096	4.3161	4.2119	4.4360	3.3292	3.0737	3.5451	3.5796	3.7627	3.0181	3.4963	3.8967	4.7176	4.4667	3.6114	3.9611	4.2601	4.3485
Fgd1	_ALRFSY[Phospho (STY)]HLEGS[Phospho (STY)]QPR_.2	P52734_S98_M2_Fgd1	8.7715	8.6170	9.0567	8.5102	7.8291	8.8208	8.2804	9.0156	8.0670	7.9988	8.8079	8.7414	7.8797	9.1130	8.2800	8.1561	9.6518	8.0519	8.3849	8.8763
Fgd1	_SLS[Phospho (STY)]LDPGQSLEPHPEGPQR_.3	#N/A	5.7347	7.4212	3.9751	4.2510	4.2774	5.0955	5.1922	3.1392	3.2833	7.5232	3.9887	2.9531	6.3109	3.9622	5.9207	3.7837	5.6146	6.0547	3.8053	7.5283
Fgd1	_LLNSTNRDDEDT[Phospho (STY)]PPNSPNVDLGK_.3	P52734_T710_M1_Fgd1	4.2565	3.7463	4.9105	3.4329	3.5943	4.3907	5.1769	3.9569	4.1118	3.9610	3.5904	3.6357	4.2772	3.2791	3.5240	3.1006	4.4946	3.3386	5.3312	3.2461
Fgd1	_QES[Phospho (STY)]VELTVQQK_.2	P52734_S364_M1_Fgd1	4.5742	3.5536	3.3021	3.8188	3.9119	4.1360	3.6292	3.9912	3.6883	3.2796	4.0627	3.3181	3.7963	3.3401	3.2064	4.3182	3.4884	3.6611	3.9601	3.0174
Rida	_AAYQVAALPR_.2	#N/A	7.9140	8.5500	8.2089	8.0027	8.0726	8.2477	8.0993	8.3217	7.9941	7.9903	7.4322	7.9731	7.8490	7.6239	8.2739	7.8522	7.6817	8.2071	7.8792	7.9104
Rida	_APAAIGPYSQAVQVDR_.2	#N/A	7.5424	7.3690	7.2750	7.5990	7.2892	7.4355	7.6758	7.2624	7.8167	7.4486	6.9158	6.8174	7.2820	7.8517	7.6709	7.3711	7.5497	7.4660	7.1274	6.9533
Rida	_AAGC[Carbamidomethyl (C)]DFNNVVK_.2	#N/A	5.5825	5.7769	5.3297	5.6352	5.8575	5.8581	6.0124	6.0732	5.8042	5.7460	5.9206	5.7772	6.1404	5.6496	5.9706	5.1303	5.3967	5.9796	5.0867	5.2824
Rida	_TYFQSSLPAR_.2	#N/A	6.8028	7.9029	7.5701	7.1762	7.7461	7.6684	7.5975	7.7083	7.5607	7.7025	6.6104	7.4891	7.7253	6.8734	7.6096	7.4222	7.4496	7.2870	7.1797	6.7260
Rida	_TTVLLADM[Oxidation (M)]NDFGTVNEIYK_.2	#N/A	5.9198	5.4563	4.0311	5.0609	2.6022	5.1879	4.7622	5.3131	5.0564	5.0456	4.5825	5.1319	5.3762	5.3009	5.0893	5.3676	3.8239	4.9688	4.4305	5.0692
Rida	_TIHSGQVGLDPSSGQLVPGGVVEEAK_.3	#N/A	3.5787	4.4241	4.1150	4.1106	2.9165	3.1405	3.1238	3.2791	3.1829	3.7851	3.5572	4.3135	3.2909	4.3355	4.1214	3.9236	3.8168	4.1665	2.9647	4.0128
Hmga2	_KPAQEETEES[Phospho (STY)]AEED_.2	P52927_S104_M1_Hmga2	3.2327	4.0863	4.2702	4.0160	3.4397	3.2322	3.6084	3.3737	3.5286	4.5442	3.0073	3.8125	3.4692	4.1967	3.3050	4.0957	3.9114	3.9217	3.5703	4.1637

Dbt	_LSDIGEGIR_.2	#N/A	4.9915	4.9770	5.3198	5.5595	5.2441	5.0301	4.8415	5.4700	5.1086	5.1908	4.2826	5.2384	4.6599	4.8468	5.0470	3.9099	5.2827	5.0791	4.7426	5.1080
Cux1	_AAS[Phospho (STY)]REEPIEWEF_.2	P53564_S1506_M1_Cux1	5.7820	6.3837	7.0254	5.9505	5.4976	5.7997	5.9128	5.2916	5.8621	5.8139	6.1440	6.0309	6.3175	6.5114	6.2890	6.4974	6.3892	6.7157	6.6772	6.3495
Limk1	_SC[Carbamidomethyl (C)]S[Phospho (STY)]IDTSPGTSSLASPASQR_.2	P53668_S298_M1_Limk1	6.4989	6.4429	6.2127	3.5669	5.6964	6.2551	5.7826	5.8163	4.0121	6.1502	3.7936	3.6937	6.5044	6.1602	4.5439	5.6489	3.7452	3.4483	5.5810	5.2836
Limk1	_VQGVDPGC[Carbamidomethyl (C)]M[Oxidation (M)]S[Phospho (STY)]PDVK_.2	#N/A	7.1038	6.7024	7.7156	7.5264	6.8582	6.3263	6.5843	7.3417	6.6109	6.8247	6.9744	6.5477	6.4231	7.0728	5.7035	5.7594	6.8179	5.6933	5.8522	6.0724
Sox1	_SEPSGS[Phospho (STY)]PPAPAHSR_.2	P53783_S325_M1_Sox1	3.4085	9.5669	4.4459	8.0675	3.6154	8.8325	3.4327	3.6341	9.0350	9.0336	8.6372	9.3446	3.4393	9.9810	9.0757	7.9637	9.5146	8.9658	8.2933	4.1612
Pitpna	_LFTNFHR_.2	#N/A	6.6571	6.6751	6.0660	6.8784	7.1922	6.6066	6.9121	6.8710	6.6242	6.5651	6.6372	7.0023	6.3600	6.8172	6.8711	6.7257	6.6685	6.5760	6.5458	6.3977
Pitpna	_LEPEAWK_.2	#N/A	5.7670	5.6636	5.4403	5.7435	5.3520	5.6373	5.2773	5.4749	5.3085	4.9367	5.9297	3.2978	3.9474	5.6231	5.8749	5.3185	4.9102	5.8229	5.6078	5.5836
Pitpna	_VENFIHK_.2	#N/A	5.1023	5.2504	4.9694	4.6334	5.6882	5.6851	5.4979	5.2306	5.3148	5.3749	6.0433	5.3611	3.8049	5.1574	5.6123	5.3512	5.2145	5.7746	5.2905	5.1670
Pitpna	_VILPVSVDEYQVGQLYSVAEASK_.3	#N/A	5.6480	5.2508	5.4618	5.8379	4.8071	5.3811	2.9837	4.7599	5.4670	3.9251	3.4172	4.9398	6.1354	5.6549	4.0618	5.6188	5.0235	4.9220	4.9551	5.4672
Pitpna	_NETGGGEGVEVLVNEPYEKDDGEKGQYTHK_.4	#N/A	3.2991	4.9482	4.6059	5.1583	4.3747	4.4999	3.5283	4.5003	5.0169	5.0363	4.9813	5.2935	5.1750	5.1509	5.3046	4.7597	5.1266	5.2751	4.4407	4.9739
Pitpna	_HVEAIYIDIADR_.2	#N/A	3.8480	4.1548	4.3843	3.8413	3.1858	3.2983	4.5872	3.5484	4.8704	4.3695	3.1820	4.0442	3.8688	4.2914	3.9325	4.1929	4.0861	3.7471	4.5140	4.3384
Pitpna	_MLAPEGALNIHEK_.3	#N/A	3.2179	3.2840	3.7542	4.4715	4.0565	3.9285	3.4846	4.4191	3.0732	3.4242	4.0527	3.1735	3.2386	3.7413	3.0618	3.5628	3.4560	4.3772	4.0258	3.7083
Slc16a1	_SDANTDLIGGS[Phospho (STY)]PK_.2	#N/A	10.0776	9.8946	10.1492	9.3674	9.0714	9.5861	9.5685	9.8057	9.6595	9.3986	9.6453	9.4567	9.6548	9.5048	8.5570	9.4126	10.3054	9.6558	9.5646	9.4046
Slc16a1	_SKES[Phospho (STY)]LQEAGK_.2	#N/A	8.9020	9.4605	9.3589	9.0675	8.8499	9.1388	9.0285	9.0684	9.0438	8.7346	8.9447	8.4616	8.9026	8.9393	7.8832	8.7420	9.2462	9.2182	8.7737	8.5271
Slc16a1	_EGKEDEAS[Phospho (STY)]T[Phospho (STY)]DVDEKPK_.2	#N/A	6.1863	5.3447	6.0139	5.1777	5.3542	5.7074	6.4024	5.8729	5.5665	5.2494	6.1740	5.4328	5.2774	5.3262	4.5550	5.4419	5.9037	5.3316	5.7936	4.9689
Slc16a1	_LKS[Phospho (STY)]KES[Phospho (STY)]LQEAGK_.2	#N/A	9.1251	9.0190	9.0058	8.8933	8.6407	8.8310	8.7800	8.8485	8.8601	8.7996	8.6840	8.4608	8.8567	8.8426	7.6567	8.4647	9.1342	8.9289	8.5615	8.4567
Slc16a1	_AAQSPQQHSSGDPTEEES[Phospho (STY)]PV_.2	#N/A	8.7588	8.7515	8.4292	8.4252	8.3276	8.3922	8.5634	8.8950	8.3660	8.2766	8.2545	8.3114	8.7775	8.4917	7.0253	8.0088	8.9271	8.6064	8.2611	8.1520
Slc16a1	_SDANTDLIGGS[Phospho (STY)]PKGEK_.2	#N/A	6.9499	7.2415	6.6694	7.0650	6.8196	6.2274	6.7737	7.5794	6.2129	6.6182	6.0224	7.0755	7.8728	7.2541	5.3380	6.5074	7.0902	7.0826	6.6535	6.2720
Slc16a1	_S[Phospho (STY)]KESLQEAGKSDANTDLIGGSPK_.3	#N/A	3.7961	3.5314	3.3327	3.7966	4.0030	3.9609	3.4946	4.2847	3.1855	3.3279	4.1312	3.2576	3.6299	4.1341	3.2664	3.1582	3.5463	4.0085	3.9244	3.4618
Rab2a	_YIIIGDTGVGK_.2	#N/A	7.0965	6.9979	6.9584	6.7826	6.5587	7.0226	7.2839	7.3978	7.2749	6.9222	6.9163	6.5425	6.8742	6.2355	5.8248	6.5050	7.0307	6.9475	6.5639	6.2462
Rab2a	_TASNVEEAFINTAK_.2	#N/A	7.0233	6.8208	6.7007	6.5580	6.9170	6.6545	6.8720	7.0962	6.7791	6.6369	6.9319	6.3216	6.7528	6.5990	6.4180	6.3725	6.9916	6.8715	6.6016	6.4538
Rab2a	_IQEGVFDINNEANGIK_.2	#N/A	6.7313	6.7627	6.8368	6.7606	6.4477	6.6886	6.3547	6.8854	6.8714	6.7629	6.5274	6.4351	6.8645	6.5714	5.9663	6.0935	6.8601	6.8307	6.6974	5.9835
Rab2a	_LQIWDTAGQESFR_.2	#N/A	5.9128	5.9739	5.8030	6.4907	5.8826	5.7124	5.9555	5.7341	6.1550	6.1316	6.0105	5.8264	6.3193	5.6165	4.7252	5.9621	6.0927	6.1844	5.8465	5.7030
Rab2a	_DTFNHLTTWLEDAR_.3	#N/A	6.1312	6.4143	5.7094	6.0365	5.5231	6.0181	5.8727	6.3760	6.5053	5.9473	6.3702	5.9911	5.8859	6.2804	5.1363	6.0880	6.1726	6.4297	5.6417	5.1803
Anapc1	_SFDFEGSLS[Phospho (STY)]PVIAPK_.2	P53995_S688_M1_Anapc1	7.2982	7.1819	6.8812	6.8597	6.1147	6.8785	7.3276	7.1287	7.1746	6.8591	6.1855	6.6875	7.2038	3.6255	5.7046	6.4552	7.1148	7.1481	6.9690	6.1272
Idh2	_ATDFVVDR_.2	#N/A	6.6828	6.4328	6.8280	6.7083	6.4191	6.7890	6.6612	6.5542	7.1520	6.7872	6.4080	6.5307	6.7336	6.3961	6.5132	6.8959	5.9051	6.9540	6.1461	6.3518
Idh2	_GKLDGNQDLIR_.2	#N/A	6.4263	6.5112	5.4561	5.9033	6.5532	6.5593	5.9746	6.9724	6.2698	6.7301	6.4366	6.2187	6.3460	6.2470	6.2380	5.9152	6.6026	6.1705	6.5203	6.4503
Idh2	_LNEHFLNTTDFLDTIK_.3	#N/A	5.3759	5.4757	5.6515	5.9146	5.7862	6.0600	5.4134	6.0301	5.8920	5.9971	6.0178	6.1564	6.3096	6.3200	5.7664	5.0932	5.8578	5.7518	5.6015	6.1269
Idh2	_YFDLGLPNR_.2	#N/A	5.3299	6.1012	6.1846	6.6896	6.0432	6.3024	6.0912	5.4256	6.0916	5.7720	5.6608	6.2227	3.2377	6.0277	5.5098	5.8213	4.4306	5.7465	5.7629	4.9013
Idh2	_DQTNDQVTIDSALATQK_.2	#N/A	3.1472	4.1719	6.0790	5.4756	4.5622	5.6101	5.1573	3.4593	3.6142	4.4586	3.0928	3.8980	3.7796	6.1635	5.5866	5.0240	3.9970	3.8362	5.0901	5.9440
Idh2	_TIEAEAAHGTVTR_.2	#N/A	4.7121	4.5475	4.9982	4.2341	4.5721	5.1674	4.3664	4.0225	4.8749	4.6311	4.2900	4.8775	3.4760	4.8585	3.2948	3.7449	3.8016	3.9959	4.5122	4.0892
Idh2	_FAQTLEK_.2	#N/A	3.5787	3.7404	4.6161	4.0056	3.7856	4.3228	3.4424	3.8043	2.8703	2.3089	3.8759	3.4665	3.6095	3.1533	3.0196	2.9408	3.3016	3.8479	4.1469	4.3314
Stmn1	_DKHVEEVRK_.2	#N/A	8.0840	8.1457	10.0241	8.7144	8.4586	8.3973	7.9195	9.4249	8.6437	9.0556	9.4692	7.7074	7.5396	8.1014	7.5280	9.1616	9.7719	7.6205	8.4593	9.1752
Stmn1	_EHEKEVLQK_.2	#N/A	5.9951	6.3271	6.0847	6.5315	6.4235	6.1373	6.1088	7.0106	6.9165	6.5901	6.3501	6.9221	6.9461	6.3658	7.4357	7.1709	5.6053	6.2784	6.5277	6.9239
Stmn1	_SKESVPDFPLS[Phospho (STY)]PPKK_.3	#N/A	3.5315	8.0613	6.6141	6.9719	2.8692	6.5775	7.3303	7.4238	5.8826	6.4846	5.4701	7.8253	7.9931	9.0837	6.8824	6.2201	7.4050	7.7764	6.7694	6.5615
Stmn1	_SHEAEVLK_.2	#N/A	8.3937	8.6136	8.5693	8.4709	8.0429	7.2803	7.3203	7.9805	8.4510	8.8644	8.6415	8.0949	8.3079	8.8076	8.0535	8.5137	8.2514	7.1103	8.5733	8.7664
Stmn1	_ESVPDFPLS[Phospho (STY)]PPKKK_.3	#N/A	8.1710	8.5971	8.8241	9.0850	7.8233	8.7824	8.8658	9.7889	8.6891	9.5880	8.9863	9.7467	10.2478	10.4448	9.5253	9.3453	9.6190	9.6608	8.9745	9.1271
Stmn1	_ASGQAFELILS[Phospho (STY)]PR_.2	#N/A	11.5208	12.0930	11.4699	11.0244	10.0083	10.7762	11.5096	11.8956	10.9597	11.3457	10.6309	11.6153	12.1251	12.6777	11.3529	11.1369	11.3730	11.6347	11.6447	10.9526
Stmn1	_SKESVPDFPLS[Phospho (STY)]PPK_.2	#N/A	13.1910	13.4253	13.3485	13.2064	8.1108	12.8815	12.8462	8.3806	12.8810	13.2915	5.8179	8.1626	13.7271	13.9568	12.9690	13.1101	13.4681	13.1151	13.3203	13.2491
Stmn1	_RKS[Phospho (STY)]HEAEVLK_.2	#N/A	10.4675	10.1707	4.4149	10.1452	10.1250	10.0246	9.9968	9.8930	9.6308	10.6584	10.1311	10.0643	10.9225	11.1175	10.3222	10.7732	9.9709	10.3372	10.6321	10.7519
Stmn1	_KSHEAEVLK_.2	#N/A	3.1339	3.0361	5.4507	3.9128	3.6094	3.2667	2.9976	3.3596	4.5620	5.0384	5.1173	4.9734	3.1648	4.2094	5.1295	4.7350	5.4936	4.6317	4.9443	4.5512
Stmn1	_NKES[Phospho (STY)]KDPADETEAD_.2	P54227_S139_M1_Stmn1	5.9669	6.9487	5.3776	6.9602	6.8997	6.7821	6.4835	7.0195	6.9855	6.4353	5.8568	7.1428	7.5233	7.7033	7.3115	7.0869	6.1641	6.9544	6.3907	6.9282
Stmn1	_REHEKEVLQK_.3	#N/A	4.2792	4.5814	3.8158	5.4043	4.3222	3.0186	4.0628	5.6029	6.5033	7.1183	3.4616	4.2668	4.1484	3.1503	6.3289	7.3775	6.3193	3.4674	6.7304	5.4856
Stmn1	_[Acetyl (Protein N-term)]ASSDIQVKELEKR_.3	#N/A	3.6147	4.0805	4.1510	5.5607	2.9525	3.8720	3.0878	4.3226	3.1469	4.6318	4.2608	4.2775	3.9462	5.6056	4.8731	4.0863	4.7217	3.9804	3.6289	4.7709
Stmn1	_RAS[Phospho (STY)]GQAFELILS[Phospho (STY)]PR_.2	#N/A	5.3999	7.4312	7.1338	5.0155	3.6930	6.5997	7.0310	4.3768	6.8514	6.8248	5.3280	6.2425	6.5998	8.0128	6.9319	6.8146	5.6238	7.3601	6.8797	5.7419
Stmn1	_[Acetyl (Protein N-term)]ASSDIQVK_.2	#N/A	4.2416	4.5783	3.7782	3.3426	2.9477	3.2845	3.5645	4.4673	4.0206	4.0522	2.3673	4.3044	4.1860	3.1878	2.4534	3.8503	3.9646	3.2738	2.2470	3.5978
Stmn1	_ESVPDFPLS[Phospho (STY)]PPK_.2	#N/A	7.2739	4.0320	4.5071	3.7186	3.3085	3.5326	4.2326	3.0937	4.2917	4.1771	3.1652	2.9306	4.3997	3.9435	3.8098	4.3157	4.0918	3.0577	3.3567	3.6208
Atxn1	_EAS[Phospho (STY)]PSTLNDK_.2	P54254_S382_M1_Atxn1	4.2628	4.5571	8.4323	3.3214	8.6227	8.6346	8.3847	8.6010	8.1588	9.0264	8.3114	8.6808	4.1648	3.8375	4.1046	8.8990	3.9857	8.5324	8.5413	3.1336
Atxn1	_RWS[Phospho (STY)]APETR_.2	#N/A	8.2911	8.3560	4.4328	3.7929	7.7237	8.1820	7.7094	8.4223	7.6550	8.1932	8.0603	8.0632	8.5795	8.5228	7.7296	8.0134	8.3599	7.9932	7.6317	7.9533
Atxn1	_WS[Phospho (STY)]APETR_.2	#N/A	7.5650	7.5051	7.3092	7.7704	7.5895	7.7684	7.1740	7.9260	7.3531	7.8354	7.6034	7.8474	7.7171	7.7848	7.8313	7.7537	7.4011	7.5323	7.8182	7.7858
Atxn1	_ALS[Phospho (STY)]AGLDYSPPSAPR_.2	P54254_S81_M1_Atxn1	6.5563	7.2183	7.2764	4.1176	5.3160	5.8491	7.1166	5.9314	5.7033	6.8820	3.5079	6.9845	6.4028	7.0977	5.8798	6.7572	6.6717	7.2839	6.7949	7.3137
Atxn1	_HGS[Phospho (STY)]AGTSGEHGLQGM[Oxidation (M)]GLHK_.3	P54254_S62_M1_Atxn1	3.2185	4.1006	4.2560	4.0018	3.4255	3.4588	3.6226	4.8417	3.5428	4.5300	3.0215	3.8267	3.7083	4.4365	3.6480	4.0815	3.9256	3.9075	3.5561	4.1779
Atxn1	_RRWS[Phospho (STY)]APETR_.3	#N/A	3.4900	3.8290	4.5275	4.0942	3.6970	3.7303	1.5540	3.7157	3.2713	3.3006	4.2508	3.5552	3.4368	3.9394	3.3765	4.3530	3.2130	4.1790	3.8276	3.9064
Msh6	_SAAVSASS[Phospho (STY)]PEAK_.2	P54276_S63_M1_Msh6	3.4734	4.5294	4.0097	6.6585	4.3120	3.6729	3.2291	7.1775	6.5367	6.4195	4.3082	6.9400	3.4941	3.9968	2.8063	6.5054	3.7115	6.7830	3.7703	6.2524
Cacnb3	_VEEHS[Phospho (STY)]PLER_.2	P54285_S393_M1_Cacnb3	10.8709	10.6872	10.5046	10.7709	10.3191	10.4599	10.3028	10.6399	10.2351	10.6635	10.4626	10.5056	10.6314	11.0181	9.9784	10.4818	10.7628	10.4738	10.5174	10.4814
Cacnb3	_RS[Phospho (STY)]PPPSLAK_.2	P54285_S152_M1_Cacnb3	7.2094	6.9059	6.5447	5.8895	6.3136	6.7252	6.7659	6.1881	6.2802	6.2527	6.9006	3.4656	7.0265	6.9036	7.3916	7.0922	7.2769	6.0683	3.7778	6.6311
Cacnb3	_LLAQDS[Phospho (STY)]EHDHNDR_.3	P54285_S464_M1_Cacnb3	5.1098	5.8392	5.1504	4.4546	4.1627	5.3048	5.4439	4.1814	3.8786	5.9563	5.4211	3.0895	3.9866	4.4252	2.9108	4.2213	3.6786	4.6896	4.9694	3.8602
Cacnb3	_QAWTGSS[Phospho (STY)]QR_.2	P54285_S418_M1_Cacnb3	4.4428	3.5600	3.4783	3.2466	3.7805	4.0046	3.7605	4.1432	3.8196	6.1573	4.1940	3.4494	3.9277	3.4714	3.3377	3.2869	3.1800	3.5297	3.8288	3.1487
Cacnb3	_DSLMPS[Phospho (STY)]DEASESSR_.2	P54285_S403_M1_Cacnb3	3.4908	5.6536	6.5379	4.1254	3.6978	4.3683	4.9797	3.7165	3.4137	3.5863	6.7408	3.5864	3.5217	9.3451	6.4924	3.6844	7.3447	5.2857	3.5974	6.9150
Lipe	_SVS[Phospho (STY)]EAALAQPEGLLGTDTLK_.2	P54310_S559_M1_Lipe	6.5209	3.2849	3.5707	3.5501	4.2411	5.8582	6.6590	4.2598	3.9570	6.0219	5.3747	3.0111	4.0650	6.5479	2.8324	3.3963	3.7571	3.3924	6.1441	5.4185
Lipe	_TTS[Phospho (STY)]PTAESVRPTESMR_.3	P54310_S542_M1_Lipe	3.4966	3.8225	4.5340	4.0876	3.7035	3.7369	3.3446	3.7223	3.2648	3.3072	4.2443	3.5486	3.4302	4.5721	3.3700	4.3595	3.2195	4.1856	4.2290	3.8999
Rad23a	_NENLAANFLLSQNFDDE_.2	#N/A	4.2118	3.7910	4.7238	4.6074	3.5496	4.7207	4.9604	3.9122	3.9997	4.4182	2.9242	4.9563	4.1587	5.7145	4.1571	3.0559	4.0561	4.3771	4.9150	5.1507
Rad23b	_IDIDPEETVK_.2	#N/A	7.2617	7.0775	7.1078	7.0582	7.3116	6.8819	7.0974	7.2299	7.1380	7.1438	7.5073	7.1036	7.6470	7.6386	7.6634	7.2700	6.7565	6.9627	7.0687	7.0127
Rad23b	_ASFNNPDR_.2	#N/A	6.5974	6.0776	6.6721	6.3470	6.4722	6.1994	6.4324	6.1763	6.4268	6.7105	6.7933	6.4647	5.5881	6.3631	7.0063	6.2723	6.0004	6.0781	6.4639	6.6806
Rad23b	_TLQQTFK_.2	#N/A	4.5883	4.3723	5.2829	4.7758	4.9574	5.2212	4.6821	5.5659	3.4154	4.6039	4.7801	4.9876	4.6807	4.5681	5.5298	5.0230	4.6240	4.8234	5.3255	4.9874
Rad23b	_NENLAANFLLQQNFDED_.2	#N/A	4.6573	5.9601	3.1281	5.3333	3.7984	5.7776	5.8799	5.7730	5.6656	5.9099	5.8212	3.4537	3.3353	5.6032	6.0356	5.2458	3.5527	3.1505	3.9291	5.8897
Rad23b	_QIIQQNPSLLPALLQQIGR_.3	#N/A	3.5159	4.0654	4.4948	4.1524	4.0654	3.3378	3.2125	3.4286	3.6535	3.5331	4.0102	3.9053	3.3828	4.0928	3.7817	3.8291	3.5817	3.9528	3.5398	3.8715
Faf1	_RTS[Phospho (STY)]PVQTR_.2	P54731_S269_M1_Faf1	9.4224	9.6077	9.0916	9.4284	9.3188	9.2604	9.3993	9.6282	9.2549	9.3376	9.2024	9.3555	9.7685	9.3460	8.3872	9.0126	9.5579	9.4188	9.2609	9.2267
Faf1	_TPS[Phospho (STY)]GEFLER_.2	P54731_S581_M1_Faf1	4.1853	3.1337	3.7219	3.3989	2.8914	4.4256	4.1567	4.4110	4.0769	3.9959	3.5555	4.3607	4.2423	3.2441	4.1820	3.5475	3.9083	6.2137	3.0221	3.2111
Ephb3	_FLEDDPSDPTYTSS[Phospho (STY)]LGGK_.2	P54754_S790_M1_Ephb3	3.7147	3.3106	3.7277	4.4980	3.9216	3.9550	3.1265	4.3926	3.0467	3.5253	4.0262	3.3305	6.2003	3.7148	3.1518	3.0768	3.4295	4.4037	6.5645	3.6818
Ephb2	_FLEDDTS[Phospho (STY)]DPTYTSALGGK_.2	P54763_S776_M1_Ephb2	3.8173	4.1982	3.8239	3.8848	4.3012	5.2387	3.8233	3.8194	3.7920	3.7934	3.6606	2.9401	3.9370	4.0070	3.7119	3.6400	3.9907	3.7114	3.7982	3.7802
Psmc4	_ENAPAIIFIDEIDAIATK_.2	#N/A	3.9134	4.5846	3.9545	4.1636	3.4152	4.4808	2.7835	3.6463	3.9194	3.6245	3.7178	3.2752	3.4515	4.4961	3.9349	3.3384	3.1435	2.6744	4.3050	3.6258
Psmc4	_LQQELEFLEVQEEYIKDEQK_.3	#N/A	3.8644	3.4547	3.4010	3.7198	4.6493	4.0370	4.8612	5.7480	4.3978	3.6750	3.8764	3.1808	6.6112	6.2264	3.0021	3.2265	3.5873	5.0597	3.8612	5.3723
Slc12a2	_GEGTPAAGDGLGRPLGPTPS[Phospho (STY)]QSR_.3	#N/A	8.7852	4.1072	8.7472	8.6938	3.5283	8.2901	8.0710	8.8396	3.5772	3.7561	3.4175	8.4060	3.7239	4.3635	7.3132	3.8650	8.5684	3.8448	3.7344	7.8815
Slc12a2	_PLGPTPS[Phospho (STY)]QSR_.2	#N/A	3.8305	4.1723	4.3668	8.2391	3.1682	8.2722	3.7656	8.0585	3.6858	4.3870	3.1644	4.0617	3.8512	4.3539	3.7910	4.1754	4.0686	7.7906	3.4132	7.5592
Slc12a2	_KES[Phospho (STY)]KGPIVPLNVADQK_.3	P55012_S987_M1_Slc12a2	5.2727	5.4550	6.0733	5.7052	3.7188	5.3558	5.5512	6.0063	5.5692	5.4112	5.7134	5.1838	5.8696	5.6571	2.9527	4.3748	6.1145	6.1997	5.2958	5.2903
Slc12a2	_S[Phospho (STY)]PGTKDVVVNVDYSK_.2	P55012_S937_M1_Slc12a2	6.0763	7.4964	3.8173	6.5066	4.1195	3.8654	3.2160	4.4822	6.1342	5.8125	4.1157	3.1104	3.3017	3.8043	5.2377	6.2498	3.5190	6.5157	6.0540	6.1307
Ncan	_ELGGEVFYVGPAR_.2	#N/A	6.9428	7.3029	6.5758	7.1289	7.1296	7.2073	6.6777	6.9982	7.0723	7.2824	6.6621	6.9402	7.0342	7.2729	6.8909	7.3245	6.8241	6.8359	7.0524	6.7248
Ncan	_LSSATIAAPR_.2	#N/A	6.6501	6.9205	6.7347	6.6050	6.7996	6.9752	6.8828	6.8225	6.8464	7.0552	6.7031	7.1507	6.5835	7.0161	7.5634	6.5795	6.6297	6.6540	6.9473	6.6309
Ncan	_FDAYC[Carbamidomethyl (C)]FR_.2	#N/A	5.6680	6.2181	6.0042	6.7737	6.3305	6.7120	6.7972	6.2232	6.2005	6.6528	6.5413	6.1850	6.5247	6.1608	6.7310	6.0252	5.3130	6.2634	6.4829	6.7210
Ncan	_VSLPAYPR_.2	#N/A	7.3562	7.4467	6.7092	7.3976	7.5665	6.8929	7.6870	7.3137	7.1305	7.5682	7.4067	7.5442	7.7851	7.6105	7.8640	7.2552	7.1110	7.6640	7.4756	7.4072
Ncan	_YPIQTPR_.2	#N/A	6.4493	7.3994	7.4615	7.0736	7.6689	7.5239	7.2178	7.0189	6.8191	7.3363	6.7973	7.2861	7.1203	7.3287	7.3012	6.9750	6.8328	7.1131	7.0107	7.1139
Ncan	_AALAELVALPC[Carbamidomethyl (C)]FFTLQPR_.2	#N/A	3.7563	5.0150	3.8445	3.1519	3.5419	4.0930	4.2624	4.1022	3.8188	3.6081	3.5790	4.2775	3.0532	4.8168	3.7451	3.8137	4.0944	4.2662	3.9980	3.2168
Ncan	_YPITQSR_.2	#N/A	5.4148	5.9795	6.4896	5.7366	5.3534	5.6302	6.1398	4.4973	5.8471	5.7313	3.0680	5.7833	4.3025	4.9785	6.3834	5.7349	5.4677	5.5323	5.7375	5.5176
Ncan	_YQC[Carbamidomethyl (C)]DEGFSQHR_.2	#N/A	4.0070	3.3121	3.5435	3.5773	5.4570	4.7854	4.5485	5.0242	5.4103	5.3584	4.6751	4.6598	4.9993	5.3238	5.5429	5.7539	4.1422	4.6915	5.1636	5.8153
Ncan	_ASDSGLYR_.2	#N/A	4.5867	3.6657	3.3725	5.4538	4.3020	4.9284	5.4300	4.2282	5.7424	5.2289	5.4410	5.2191	4.0334	3.5772	5.7235	3.1811	3.0743	5.1987	5.5327	5.0511
Ncan	_YNVHATVR_.2	#N/A	4.0575	3.2616	3.5941	3.5267	5.3242	4.2978	5.5809	6.3431	4.2047	3.8681	3.6833	6.3376	5.3848	5.7151	6.1442	6.2135	5.0333	5.3396	5.4258	5.8188
Ncan	_DFQWTDNTGLQYENWR_.2	#N/A	5.7080	4.6034	5.4198	4.3122	3.4789	3.1286	3.1357	4.4544	4.6494	3.7731	3.5692	3.7732	5.1890	4.3475	3.9832	4.1350	4.4958	4.6152	4.2957	3.2778
Ncan	_C[Carbamidomethyl (C)]GGPAPGVR_.2	#N/A	4.8521	4.0857	4.2708	5.4310	3.4404	5.1947	5.4845	4.8418	3.5279	4.5449	3.0066	4.4244	3.6934	4.1960	5.4992	4.0964	3.9107	5.0722	5.2879	5.2508
Ncan	_HLQAAFEDGFDNC[Carbamidomethyl (C)]DAGWLSDR_.3	#N/A	4.8835	3.3800	3.4757	3.6451	4.9371	4.6875	5.3567	5.8503	5.5496	5.6947	4.4934	5.3015	4.4886	5.5317	4.9627	5.3688	5.8182	2.8175	4.7089	4.5483
Ncan	_FINSFGHENSWIGLNDR_.3	#N/A	4.0741	6.0586	5.4798	6.3245	6.2977	5.2192	5.6647	6.3248	5.4590	6.2214	6.1669	5.9594	6.5037	6.0230	6.2750	5.9556	6.1600	5.0076	6.0530	6.4221
Ncan	_QDLPILVAK_.2	#N/A	3.1294	3.6097	3.6657	3.8749	3.9680	4.1921	3.5731	3.9350	3.6322	3.3358	4.0066	3.2620	3.7402	3.2840	3.1503	3.4743	3.4323	3.7172	4.0162	2.9613
Ncan	_AQGM[Oxidation (M)]PTLTSTS[Phospho (STY)]SEGHPEPK_.3	P55066_S869_M1_Ncan	3.7714	4.2314	4.3077	3.9180	3.1091	3.3332	4.4320	3.4717	2.9902	3.9777	3.3646	4.1209	4.5991	4.2947	4.0092	4.1163	4.0094	3.8237	3.4723	4.2617
Aqp4	_GSYM[Oxidation (M)]EVEDNR_.2	#N/A	8.5620	9.4435	9.0178	8.1553	5.2543	8.4457	8.3815	9.3849	8.3445	8.0078	8.6147	8.6905	9.2380	8.0086	7.7629	8.3184	8.4213	8.2636	7.9246	8.7186
Tyro3	_AEQPTESGS[Phospho (STY)]PEVHC[Carbamidomethyl (C)]GER_.2	P55144_S808_M1_Tyro3	4.5791	3.4237	3.6145	3.1103	3.9168	4.1409	5.8683	4.2794	3.6834	6.0584	4.0578	3.3132	3.7914	3.6016	3.2015	3.4231	3.3163	3.0160	4.1655	3.5686
Sh3bp1	_ERTEADLPKPTS[Phospho (STY)]PK_.3	P55194_S535_M1_Sh3bp1	8.1466	4.4770	3.8795	8.9912	7.5894	3.0824	8.3184	7.6796	7.6335	9.0055	3.3979	7.3580	8.8548	8.8272	4.0244	3.7051	8.0847	3.5311	8.6971	8.6905
Sh3bp1	_DNHSQADHS[Phospho (STY)]PLTTAAPFSR_.3	P55194_S262_M1_Sh3bp1	7.7757	7.2434	6.9432	7.5165	6.7272	6.9012	6.8141	7.0407	6.5475	6.7950	6.8745	7.3071	7.8568	7.7637	6.3254	7.1471	7.3691	7.1242	6.9596	6.9491
Rab8a	_KLEGNS[Phospho (STY)]PQGS[Phospho (STY)]SHGVK_.2	#N/A	4.6889	6.4592	5.7427	6.3755	6.0345	5.7379	5.8634	5.4111	6.1874	5.2995	5.7542	5.3093	6.6035	5.2751	4.5452	5.7462	6.0447	5.9086	6.3673	5.9123
Rab8a	_LEGNS[Phospho (STY)]PQGS[Phospho (STY)]SHGVK_.2	#N/A	8.2233	8.3308	7.9028	8.1482	7.8712	7.1434	8.2626	8.1209	7.8421	6.8936	7.6784	7.9263	8.2434	7.8586	6.8976	7.8592	8.2359	8.0511	7.8252	7.8068
Adk	_VAQWLIQEPHK_.2	#N/A	4.3946	4.2889	4.8856	4.4755	3.5120	3.6063	4.4804	4.5917	4.1855	5.2994	3.6911	4.4087	4.0874	3.5644	3.7025	3.6477	4.3767	4.0631	4.3650	3.7519
Adk	_YAAENNR_.2	#N/A	4.4412	4.3787	3.9778	3.1430	3.1473	3.2344	4.3049	3.1660	4.3640	4.1047	3.2376	4.1048	4.4721	4.0158	3.9262	3.8033	4.1641	2.9853	3.2844	3.6931
Cdh11	_LADLYGS[Phospho (STY)]KDTFDDDS_.2	P55288_S788_M1_Cdh11	6.9700	8.4569	7.9750	3.3403	3.6868	7.7683	7.9832	5.2609	8.0367	7.7397	4.6007	7.9078	7.5090	8.4479	6.9260	7.5850	8.4178	8.2346	7.7865	7.7005
Cdh11	_PGLRPAPNS[Phospho (STY)]VDVDDFINTR_.3	P55288_S714_M1_Cdh11	4.0279	3.9749	4.5642	3.6615	3.3656	3.1185	4.1755	3.7282	3.8831	4.1897	3.3618	3.8644	4.0486	5.5435	3.7527	4.3728	4.2659	4.4312	3.2158	3.0174
Lrpap1	_IQEYNVLLDTLSR_.2	#N/A	4.1717	3.8311	5.1924	4.2697	4.5094	3.7533	3.7057	5.2993	5.0326	4.9597	4.5896	4.1481	4.1988	4.5641	4.8223	4.4388	4.4098	4.2220	4.4479	4.6143
Lrpap1	_RES[Phospho (STY)]GEEFR_.2	P55302_S53_M1_Lrpap1	3.8890	3.6117	3.8443	4.7473	4.1063	4.7139	4.7436	3.9657	4.3497	5.4404	4.6996	3.1562	4.0642	5.0555	5.0114	4.9863	4.4501	4.0779	4.6954	4.5553
Stmn2	_DLS[Phospho (STY)]LEEIQK_.2	#N/A	13.9097	14.1788	14.2698	14.1035	11.3712	13.8019	13.6704	13.7987	14.0038	14.1727	14.0876	13.9763	14.7653	14.4232	13.7861	14.2212	14.2867	13.8102	14.0970	14.1445
Stmn2	_DLS[Phospho (STY)]LEEIQKK_.2	#N/A	13.3170	13.5751	13.3400	13.3996	13.1337	13.0725	12.9321	13.3892	13.0843	13.4750	13.0628	13.2959	14.1675	13.8552	13.1571	13.4250	13.6132	13.1458	13.2895	13.3660
Stmn2	_KLEAAEER_.2	#N/A	8.1820	8.1779	8.5198	8.1897	8.1680	7.9090	8.0798	8.0654	8.1734	8.3765	8.2308	8.0236	8.4417	8.3677	9.1397	8.4750	8.1079	7.8169	8.5294	8.4294
Stmn2	_KDLS[Phospho (STY)]LEEIQK_.2	#N/A	6.4105	7.0437	6.1251	7.1861	6.4585	6.2620	5.8414	6.7855	6.1865	6.4808	6.3973	6.4473	6.8227	7.1636	7.1025	6.8117	6.9977	6.2644	6.6899	6.7883
Stmn2	_KKDLS[Phospho (STY)]LEEIQK_.2	#N/A	6.8482	7.1680	6.9676	6.9152	6.5564	7.0833	6.4898	7.2716	6.9224	7.0722	6.4670	7.0264	7.5151	7.4067	7.2474	7.2096	7.4401	6.9155	7.1378	6.9100
Stmn2	_RKS[Phospho (STY)]QEAQVLK_.2	P55821_S97_M1_Stmn2	5.2903	5.5790	5.1439	3.4275	4.7705	4.9763	5.3727	5.4536	4.6511	5.6708	4.9769	4.4937	5.8063	5.4547	3.7568	5.2778	5.7817	5.7251	5.3487	4.4583
Stmn2	_RAS[Phospho (STY)]GQAFELILKPPS[Phospho (STY)]PISEAPR_.3	#N/A	4.2615	5.6175	4.2388	3.9869	3.0402	3.2643	3.0000	3.3620	3.0591	3.9088	3.4335	4.1897	3.1672	5.2582	4.0780	4.0474	4.3600	5.1022	3.0885	3.8890
Stmn2	_AS[Phospho (STY)]GQAFELILKPPS[Phospho (STY)]PISEAPR_.3	#N/A	3.6785	4.2014	4.2203	4.4617	3.8854	3.4620	3.8784	4.2321	3.0893	3.4890	4.0687	3.3647	3.2547	4.6121	3.1944	3.0406	3.4720	4.6706	4.0160	3.7244
Stmn2	_NKELQVELS[Phospho (STY)]G_.2	P55821_S178_M1_Stmn2	3.2664	3.8515	3.8027	4.4230	4.1050	3.6508	3.4361	4.4676	3.4952	3.4727	3.8696	3.1250	3.6033	3.1470	3.0133	3.6113	3.5045	3.8541	4.1219	3.7013
Stmn2	_KDLS[Phospho (STY)]LEEIQKK_.3	#N/A	3.4846	3.8345	4.5220	4.0996	3.6915	4.4168	3.3483	3.7103	3.4074	1.9141	1.4107	1.8773	3.5155	4.5601	2.9255	2.7148	3.2075	3.9419	4.2410	1.4248
Golga3	_GFKEEGS[Phospho (STY)]PDR_.2	P55937_S60_M1_Golga3	9.9610	10.0293	10.0264	9.9134	9.8141	9.9842	9.8903	10.1474	9.8121	10.1109	9.8055	9.7897	10.0624	10.2755	9.5888	9.6763	10.0057	9.7226	9.8216	9.8295
Golga3	_LHNQNS[Phospho (STY)]VPR_.2	#N/A	7.0171	7.4345	7.6170	7.2197	6.6620	6.6903	6.6739	6.1101	7.1159	8.1000	7.4565	7.1858	4.4436	7.3825	6.3941	6.9797	6.2391	7.0633	7.1438	6.8086
Golga3	_LHNQNS[Phospho (STY)]VPRDGLGQ_.2	#N/A	6.3050	6.0207	5.7453	6.2962	5.4088	5.2812	5.4157	5.8357	5.2781	6.4293	5.5604	5.7684	6.0389	6.3531	3.2977	5.8008	5.6664	5.5932	5.8635	5.3075
Atp5mf	_DFTPSGIAGAFR_.2	#N/A	9.2931	9.4213	9.2649	9.2304	9.3042	9.2900	9.6291	9.4164	9.0085	9.0786	9.3302	9.3580	9.0991	8.9773	8.9921	9.0576	9.4812	9.5412	9.1748	9.0941
Rrp1	_EAGS[Phospho (STY)]EAESSSADPGPGR_.2	P56183_S434_M1_Rrp1	8.9062	8.8523	8.7439	8.7488	8.5732	8.9388	8.6932	8.7572	8.5942	8.8752	8.2594	8.4735	9.0392	8.6321	7.7948	8.3225	8.7365	8.7918	8.5415	8.2194
Arpp19	_VTS[Phospho (STY)]PEKAEEAK_.2	P56212_S23_M1_Arpp19	3.4827	7.8540	4.5201	7.5007	6.4124	7.8752	7.5004	3.1238	3.2787	7.8812	7.8067	7.1919	7.3550	7.7983	3.3838	7.4999	7.4482	7.4471	7.9667	3.9138
Arpp19	_YFDS[Phospho (STY)]GDYNM[Oxidation (M)]AK_.2	#N/A	10.9026	10.8263	10.9223	11.1433	10.5767	10.6479	10.6430	10.9520	10.9075	11.2773	10.8875	11.4349	11.7191	11.4847	11.0412	11.3332	11.0516	11.0905	11.2435	11.2569
Arpp19	_KPS[Phospho (STY)]LVASK_.2	P56212_S104_M1_Arpp19	4.2281	3.7747	5.6632	3.4612	6.3931	5.9203	4.1632	3.9285	4.0834	3.9894	3.5621	3.6641	4.2489	5.6384	4.1886	3.0722	4.4662	5.7544	3.0155	4.8651
Arpp19	_GQKYFDS[Phospho (STY)]GDYNM[Oxidation (M)]AK_.2	#N/A	3.8406	3.4785	3.3772	5.7341	4.0476	4.0809	3.0005	4.0663	4.4216	3.6512	3.9002	6.6143	7.3940	3.4153	3.0259	3.2027	3.5635	3.5859	5.8522	3.0926
Acyp2	_SVDYEVFGTVQGVC[Carbamidomethyl (C)]FR_.2	#N/A	5.1323	4.9231	5.9157	5.4641	4.1083	5.0210	4.5285	4.1271	5.5329	5.7260	4.5621	4.9763	6.1034	5.0522	5.2303	4.9255	3.6243	4.8027	5.7037	5.4235
Acyp2	_NTS[Phospho (STY)]KGTVTGQVQGPEEK_.2	P56375_S51_M1_Acyp2	4.5641	3.4387	3.5995	3.1253	3.9018	4.1259	3.6393	4.2645	3.6984	3.2696	4.0727	3.3282	3.8064	3.5866	3.2165	3.4081	3.3013	3.0310	4.1804	3.5536
Acyp1	_VQGVFFR_.2	#N/A	6.3527	6.7116	6.0028	5.8864	6.8490	6.0939	6.7679	6.4236	6.1284	5.5164	6.0600	6.7799	6.0130	6.5021	6.3907	6.3881	6.5288	6.1138	6.3841	6.2784
Acyp1	_[Acetyl (Protein N-term)]AEGDTLVSVDYEIFGK_.2	#N/A	5.7927	6.0595	6.8791	6.2595	5.4896	5.7321	5.5920	6.2332	6.1036	6.5402	5.1413	5.8997	7.1082	6.7341	6.4489	6.4927	6.3199	5.7367	5.6243	6.6382
Acyp1	_VIANLDYSDFQIVK_.2	#N/A	5.3690	5.5726	6.0552	5.7640	5.6196	5.6445	5.0560	5.3515	5.1945	6.1071	5.2168	5.1670	5.8815	4.4536	5.4311	5.6717	5.3216	4.6128	5.1577	6.1205
Acyp1	_FM[Oxidation (M)]QQWLETR_.2	#N/A	3.3348	6.3182	5.7423	4.3546	6.1502	6.3932	5.3178	6.3384	5.4011	6.2159	4.1696	6.0852	5.0598	3.8582	6.2881	6.4116	6.3110	6.2319	5.8812	6.0073
Acyp1	_LGLVGWVQNTDR_.2	#N/A	3.6115	4.3913	5.4112	5.8515	4.8732	3.5348	5.0959	4.9295	4.3685	6.2045	5.3170	4.7344	3.6323	5.3309	4.1690	4.9119	4.5029	5.1250	3.6321	5.0521
Acyp1	_GTVQGQLQGPVSK_.2	#N/A	3.6582	5.3183	4.2216	4.0312	2.9960	4.5620	3.5933	3.3586	3.5135	4.7624	2.9921	4.2340	3.6790	5.4698	5.3555	5.0601	3.8963	3.9369	5.1773	4.1486
Atp5mpl	_MFQTLIQK_.2	#N/A	3.9349	5.9481	6.3591	7.1714	5.1792	6.1103	6.3874	4.6957	5.2431	4.0953	6.0018	4.8923	4.4927	6.5394	4.6327	3.2970	5.1968	4.3307	4.1980	4.9575
Nudt2	_EETGIEASQLTIIEGFR_.2	#N/A	5.1558	4.8252	5.0913	5.0376	4.3153	5.0699	4.7552	4.4608	3.2246	5.1336	4.2040	3.0221	3.3900	5.1108	4.2969	4.7201	3.6073	4.2258	4.4054	3.8597
Nudt2	_ATLQEGHQFLC[Carbamidomethyl (C)]ST[Phospho (STY)]PA_.2	P56380_T145_M1_Nudt2	4.0080	4.5880	3.5446	3.6554	3.4827	3.9992	3.6809	4.2337	3.6011	3.8186	3.8120	3.8849	3.7665	3.5827	3.7063	3.3701	3.7309	3.6548	3.6979	3.8753
Atp5f1e	_QAGLSYIR_.2	#N/A	8.9220	8.8403	8.8332	8.8746	8.9157	8.7201	8.8341	8.9101	8.6540	8.8068	8.5672	8.8756	8.8358	8.5642	8.9434	8.6303	8.1283	8.8672	8.8206	8.7309
Atp5f1e	_DALKTEFK_.2	#N/A	8.0947	8.3254	8.6306	8.2693	8.2949	8.2346	8.3832	8.4905	8.4508	8.5477	8.2967	8.4512	8.8207	8.5487	8.6259	8.6581	8.5146	8.5277	8.5540	8.4584
Atp5f1e	_FSQIC[Carbamidomethyl (C)]AK_.2	#N/A	5.6943	5.9001	5.9692	5.5335	5.3277	6.3844	6.8977	5.1401	6.6346	5.9758	5.9228	6.2472	5.5138	6.0757	7.0349	6.0456	5.2122	6.0325	6.5994	6.0144
Cartpt	_ALDIYSAVDDAS[Phospho (STY)]HEK_.2	#N/A	5.6175	5.6471	6.8633	3.9060	3.8852	5.7198	3.1629	3.9039	6.1498	5.8535	3.9755	5.6198	4.8653	6.5302	5.3974	6.6604	6.3349	5.9874	5.8284	5.8879
Cartpt	_ALDIYSAVDDAS[Phospho (STY)]HEKELPR_.3	#N/A	3.6363	4.3665	4.1726	4.0531	2.9740	3.1981	5.3566	3.3367	5.5333	3.8426	3.4997	4.2560	3.2334	4.1597	4.1443	6.2511	6.3792	3.9588	6.4519	4.1267
Cox6b1	_SLC[Carbamidomethyl (C)]PVSWVSAWDDR_.2	#N/A	9.8234	9.7990	10.2413	9.9844	9.7860	9.6082	9.7856	9.8352	9.9133	9.8908	9.8791	10.0127	10.3449	10.2501	10.5363	10.0552	10.1837	9.7397	9.9082	9.9546
Cox6b1	_IAEGTFPGKI_.2	#N/A	9.1879	9.2309	8.8731	9.2899	9.3465	9.0030	9.2792	9.3647	9.3463	9.2362	9.3354	9.6121	9.6484	9.5215	10.2348	9.6166	9.9231	9.4891	9.4886	9.2578
Cox6b1	_NC[Carbamidomethyl (C)]WQNYLDFHR_.3	#N/A	7.8213	8.1269	8.0662	8.1080	7.9823	7.8784	8.0056	7.7937	7.8778	7.9582	8.0441	8.3350	8.3862	8.4066	8.6265	8.0953	8.0474	8.2174	8.0661	8.2782
Cox6b1	_IAEGTFPGK_.2	#N/A	7.2358	7.8244	7.8141	7.7497	7.5237	7.6148	8.0236	7.1459	7.9246	8.0116	7.8111	7.9400	7.6846	7.6073	8.8807	7.6793	7.4303	7.7816	8.0895	8.5041
Cox6b1	_[Acetyl (Protein N-term)]AEDIKTK_.2	#N/A	4.5940	5.5408	4.6941	4.5120	4.8006	4.0014	4.7456	5.4922	4.8108	4.9322	4.0583	5.4607	5.3049	4.0247	5.8150	5.1401	3.9857	4.5991	4.8942	4.8099
Cox6b1	_FPNQNQTK_.2	#N/A	8.7766	4.5947	7.8152	7.6206	8.4539	8.3941	8.7896	8.3573	9.3831	9.0586	9.1783	2.9833	3.4615	9.6344	10.5583	9.0852	3.6462	9.3117	7.4376	8.8964
Cox6b1	_GGDVSVC[Carbamidomethyl (C)]EWYR_.2	#N/A	7.7145	7.5150	8.2150	7.8616	7.4569	7.5374	7.3438	7.4439	8.1225	7.7346	7.8655	7.7052	8.0206	8.2347	8.4080	7.9674	7.8825	7.6948	7.7679	8.0322
Cox7b	_APSFHDK_.2	#N/A	5.8421	6.3741	4.9008	6.2718	5.9628	6.7785	6.5280	5.3007	5.3900	6.8727	5.9016	6.0808	4.8543	6.0689	6.0990	6.0340	6.1118	6.5137	6.7989	5.3777
Cyb5a	_FLEEHPGGEEVLR_.3	#N/A	6.0938	5.6384	5.6697	6.2404	6.0906	5.5715	5.7766	5.8667	6.4880	6.2171	6.5142	5.7273	6.5778	6.1802	6.6143	6.2584	6.0023	6.0803	5.9144	6.1582

Cyb5a	_YYTLEEIQK_.2	#N/A	5.9487	6.4733	6.3973	6.1261	6.3819	6.4039	6.2555	6.2431	5.4756	6.2443	5.9829	5.7867	5.9641	6.3752	6.4694	6.5516	5.7759	5.9598	6.2610	6.1804
Cyb5a	_EQAGGDATENFEDVGHSTDAR_.3	#N/A	6.8298	7.0039	6.0080	6.5320	5.8208	6.6553	6.3742	3.6918	6.7044	5.7615	5.9308	6.6482	5.7689	6.7179	6.1208	6.8916	5.9735	6.1632	6.6466	6.6472
Cyb5a	_[Acetyl (Protein N-term)]AGQS[Phospho (STY)]DKDVK_.2	P56395_S5_M1_Cyb5a	3.9349	3.5760	3.8813	4.1855	3.2726	4.4223	4.5591	4.5002	4.3275	4.8348	3.0618	4.8834	3.9633	4.5555	1.9835	4.2798	3.6548	3.0218	3.3209	4.4226
Cyb5a	_TYIIGELHPDDR_.2	#N/A	4.9258	5.3177	5.1155	5.3417	5.2500	5.2861	5.1374	5.7762	5.7795	3.4753	5.1897	4.8564	5.2928	5.2816	5.4354	5.6998	5.3046	5.5722	5.0111	5.3721
Usp5	_GTGLQPGEEELPDIAPPLVT[Phospho (STY)]PDEPK_.3	P56399_T623_M1_Usp5	10.7659	11.1684	10.6728	10.7566	9.6585	10.6049	11.0166	10.5702	10.5417	10.8547	10.3687	10.8225	11.0623	11.4353	10.2169	10.7873	10.7377	11.0929	10.7705	10.3840
Usp5	_ATNNS[Phospho (STY)]LER_.2	P56399_S751_M1_Usp5	3.5170	10.6419	4.0533	10.3103	10.2104	10.2242	10.2681	3.2174	10.2126	10.5083	10.6035	10.6258	11.1188	11.2358	10.4667	10.9129	10.8587	10.8579	10.8764	10.7573
Usp5	_DGLGGLPDIVR_.2	#N/A	7.3661	7.5919	7.2529	7.2347	7.5231	7.4684	7.8434	7.3681	7.5778	7.4369	7.5836	7.6397	6.7128	7.4483	7.0648	6.9436	7.6922	7.4593	7.4662	7.5853
Usp5	_IFQNAPTDPTQDFSTQVAK_.2	#N/A	7.1099	6.8717	6.9935	6.4723	6.8242	6.9847	6.8707	6.9199	7.1530	6.9252	6.7787	6.6777	6.7685	7.5579	6.9467	7.1519	6.8748	6.8848	6.5355	6.2262
Usp5	_IVILPDYLEIAR_.2	#N/A	5.5736	5.0487	5.7587	6.2119	6.5938	6.2738	5.5125	5.7187	5.7459	6.6235	6.2076	5.0389	5.6295	4.9834	6.3195	5.5703	5.6876	5.3881	5.7507	6.6521
Usp5	_WVIYNDQK_.2	#N/A	6.6207	6.2823	6.5836	6.2346	5.7491	6.1376	6.3791	6.6218	6.5277	6.3092	6.1530	6.2392	6.6389	6.3424	6.0738	6.0685	6.6042	6.3633	6.2660	5.6159
Usp5	_[Acetyl (Protein N-term)]AELSEEALLSVLPTIR_.2	#N/A	6.2278	5.3779	5.8466	6.1350	6.2483	6.0351	5.5370	5.9127	5.5690	5.5734	6.5156	5.8102	4.7116	4.7808	6.1738	5.9527	5.6972	5.2838	6.1543	6.1933
Usp5	_DLGYIYFYQR_.2	#N/A	5.7230	5.6562	6.1817	5.9373	5.7074	5.8765	5.2396	5.7259	5.8579	6.3773	6.1347	6.0957	4.9214	6.2082	5.9047	5.7792	6.3544	5.5006	5.5125	5.9673
Usp5	_ENLWLNLTDGSILC[Carbamidomethyl (C)]GR_.2	#N/A	5.1236	4.8846	6.1398	5.1781	5.4269	5.3095	4.8818	5.0178	5.8247	5.5954	5.7764	5.4059	6.1249	5.4380	5.2239	5.5142	5.7178	5.4138	4.8946	5.4921
Usp5	_SSENPNEVFR_.2	#N/A	7.4886	7.5845	7.1777	7.4156	7.3943	7.2680	7.6441	7.6262	7.5601	7.5084	7.5122	7.5016	3.3082	7.4923	7.5702	7.4035	7.4248	7.4560	7.4008	7.6790
Usp5	_ETGYPLAVK_.2	#N/A	5.4992	6.3010	5.3867	5.8856	6.3052	5.5230	6.1463	6.5536	6.5736	6.4958	6.0980	6.3486	3.5037	5.9368	6.3927	6.1901	5.2512	6.1143	6.1751	6.0172
Usp5	_AVDWIFSHIDDLDAEAAM[Oxidation (M)]DIS[Phospho (STY)]EGR_.3	P56399_S775_M1_Usp5	5.5662	5.7217	4.9080	4.0030	4.4302	4.4242	5.8855	3.0951	5.7697	5.3446	4.2294	5.6654	6.4199	6.4789	4.9798	5.3037	3.6327	5.7357	4.7497	3.5905
Usp5	_QAEEEKVPLPELVR_.3	#N/A	6.8987	6.4817	3.7841	7.1676	6.8979	7.1129	4.1113	6.6106	7.0936	7.1550	7.3520	6.9930	7.4704	6.8506	7.0752	6.9081	7.1377	6.6649	6.8709	6.9876
Usp5	_GHPEFSTNR_.2	#N/A	3.3096	6.5839	6.9369	6.7755	6.6399	6.7509	6.6758	6.8129	6.1599	6.7962	6.7436	6.6388	6.6664	7.0458	7.0114	6.6775	6.6320	6.6938	6.8989	6.6936
Usp5	_YFDGSGGNNHAVEHYR_.3	#N/A	5.9044	5.9941	6.7443	6.7745	6.0392	6.0291	5.8869	6.0558	6.4610	6.2349	6.0654	6.2564	6.6418	6.6383	6.5500	6.3915	6.2769	6.8382	6.1717	6.7043
Usp5	_FASFPDYLVIQIK_.2	#N/A	4.2666	4.5347	3.7610	4.7163	4.8372	4.4555	3.4178	4.3533	3.4769	4.6768	3.6932	4.1102	3.5850	4.0607	3.8348	3.4926	4.1555	3.6314	3.9238	3.6355
Usp5	_KQEVQAWDGEVR_.3	#N/A	5.6458	5.0855	5.6801	5.3907	5.5484	5.3938	5.4316	5.8307	5.4368	5.5691	5.7971	5.3327	5.3978	4.9032	5.5080	5.6530	5.4945	5.7839	5.8542	5.8194
Usp5	_SAAES[Phospho (STY)]ISESVPVGPK_.2	P56399_S783_M1_Usp5	10.5267	10.7769	11.0604	10.8739	10.1558	10.7016	10.2910	10.4346	10.6706	10.6299	10.3332	10.3377	10.5742	11.0338	9.9822	10.6457	10.7999	10.3395	10.6081	10.7187
Usp5	_VTSAVEALLSADSASR_.2	#N/A	5.8010	5.4305	5.7527	5.2069	5.4904	5.4018	4.9514	5.4244	5.9050	5.4658	5.4501	5.4790	5.8625	5.4450	5.3735	5.4918	5.7691	5.6528	5.6030	5.4290
Atp5f1b	_VLDSGAPIKIPVGPETLGR_.3	#N/A	10.4264	10.5059	10.2841	10.5871	10.5725	10.3218	10.6080	10.6586	10.2931	10.6116	10.4134	10.7440	10.9561	10.9598	10.7925	10.7300	10.5984	10.5078	10.5677	10.6562
Atp5f1b	_AIAELGIYPAVDPLDSTSR_.2	#N/A	9.6880	10.4446	10.2772	10.6544	9.7073	10.3767	10.3678	10.1122	10.5142	10.2376	10.2547	10.2519	10.5215	10.5086	10.7194	10.2878	10.4735	10.1193	10.3920	10.3022
Atp5f1b	_IPVGPETLGR_.2	#N/A	10.9278	10.8427	11.1563	10.8648	11.0567	10.9798	11.0005	11.0855	10.7727	10.8673	11.0421	11.1138	10.7340	11.0140	11.4265	10.7126	10.8922	11.0797	11.0353	10.9114
Atp5f1b	_LVPLKETIK_.2	#N/A	10.7019	11.0659	10.4812	11.0075	11.0714	10.6301	10.7910	11.3902	10.6245	10.6804	10.4594	11.2649	11.4654	10.9919	11.2633	10.8209	10.8063	11.0578	10.8990	10.9204
Atp5f1b	_VVDLLAPYAK_.2	#N/A	10.0839	10.8705	10.6681	10.8743	10.7357	10.4592	10.9357	10.3131	10.5526	10.5325	10.5872	11.3794	10.9061	10.7232	11.0433	10.6647	10.0527	11.1237	11.0659	10.8850
Atp5f1b	_VALTGLTVAEYFR_.2	#N/A	10.5325	10.4652	10.9315	10.7976	10.5062	10.4760	10.2481	10.4392	10.6755	10.5622	10.4765	10.4912	10.7769	10.4288	10.8316	10.6375	10.6496	10.3102	10.7480	10.7710
Atp5f1b	_IGLFGGAGVGK_.2	#N/A	9.2771	9.7944	9.7765	9.6331	9.3905	9.7188	9.7705	9.4199	9.3204	9.4728	9.0252	9.6929	9.6792	9.7834	9.5709	9.0781	9.3056	9.9324	9.5984	9.8738
Atp5f1b	_ADKLAEEHGS_.2	#N/A	8.3167	8.3924	8.4745	8.5596	8.5112	8.1573	8.3669	8.7697	8.4156	8.3612	8.1741	8.6304	9.0277	8.8082	9.1896	8.8233	8.4400	8.4403	8.5986	8.5596
Atp5f1b	_VLDSGAPIK_.2	#N/A	6.0682	6.9109	7.6439	7.4860	6.7568	7.2100	7.3827	6.1821	7.4860	7.5217	7.2990	7.2015	7.5943	7.5613	7.8334	7.3028	7.5843	7.3811	7.6425	7.4040
Atp5f1b	_IM[Oxidation (M)]NVIGEPIDER_.2	#N/A	11.7102	11.7021	11.8091	11.7539	11.8586	11.6062	11.6576	11.9130	11.6908	11.7713	11.7185	11.9168	11.8298	11.9431	11.9822	11.7668	11.8787	11.7220	11.7704	11.8699
Atp5f1b	_TIAM[Oxidation (M)]DGTEGLVR_.2	#N/A	9.9015	10.1229	10.3564	10.4652	10.1603	10.1049	10.0431	10.3375	10.0689	10.0688	9.9344	10.3298	10.4815	10.4248	10.5403	10.0332	10.2323	10.0195	10.1463	10.1041
Atp5f1b	_TVLIM[Oxidation (M)]ELINNVAK_.2	#N/A	8.7393	8.8595	9.2258	9.1532	9.0336	9.0052	8.5304	8.9710	8.8969	8.9761	8.9766	8.7354	8.9442	8.8927	8.9288	9.1061	9.0630	8.8642	9.1735	8.9556
Atp5f1b	_IM[Oxidation (M)]DPNIVGNEHYDVAR_.3	#N/A	7.9950	8.5696	8.8719	8.6029	8.2040	8.4387	8.4609	9.2223	8.6312	8.3728	8.2687	8.5028	8.5789	9.0095	8.9849	8.5512	8.4664	8.6014	8.4033	8.5331
Atp5f1b	_VALVYGQM[Oxidation (M)]NEPPGAR_.2	#N/A	10.7799	10.9313	10.5283	11.0452	10.9460	10.7432	10.8733	11.1361	10.6786	10.8735	10.7472	11.0394	11.0436	11.3052	11.0774	10.9877	10.7919	10.9562	10.9756	10.8092
Atp5f1b	_AHGGYSVFAGVGER_.3	#N/A	7.1426	5.7166	7.1327	6.8341	7.0724	6.5982	6.4096	6.3415	6.9716	6.7239	6.9459	6.7764	6.8283	5.7062	7.1051	7.2662	6.7534	5.7461	5.6697	6.6400
Atp5f1b	_FLSQPFQVAEVFTGHM[Oxidation (M)]GK_.3	#N/A	8.6861	8.7368	8.4382	8.5872	8.7351	8.6601	8.5860	8.9395	8.4113	8.9207	8.5685	8.8989	8.9508	8.8613	8.6562	8.9054	8.5461	8.9137	9.0237	8.8755
Atp5f1b	_KGSITSVQAIYVPADDLTDPAPATTFAHLDATTVLSR_.4	#N/A	3.7834	5.3634	5.1012	3.2677	4.2441	5.4985	4.6700	5.0341	5.3611	2.8795	4.2337	4.2295	5.0169	5.1826	3.5650	4.6177	6.0252	4.5426	4.0836	4.5010
Atp5f1b	_IPSAVGYQPTLATDM[Oxidation (M)]GTM[Oxidation (M)]QER_.3	#N/A	7.1470	6.9261	6.0102	6.5694	7.0210	6.9477	6.6393	7.3088	6.8556	7.0410	6.6117	7.1891	7.6129	6.4030	7.2273	7.4099	7.1989	7.1767	7.0461	6.8672
Atp5f1b	_FTQAGSEVSALLGR_.2	#N/A	6.1886	6.0103	4.4525	5.2475	3.2539	5.3110	5.7416	5.5086	5.0298	4.9490	4.9006	4.9608	6.2565	6.4564	4.4447	5.4160	6.0548	5.9475	5.4038	6.0667
Atp5f1b	_TREGNDLYHEM[Oxidation (M)]IESGVINLK_.4	#N/A	4.8850	5.5919	5.7916	5.9711	5.2184	5.0438	5.5793	4.9106	5.4997	6.1645	5.8740	4.7496	6.6455	5.8268	5.9655	6.0515	4.7455	5.4996	5.3739	6.0140
Atp5f1b	_EGNDLYHEM[Oxidation (M)]IESGVINLK_.3	#N/A	5.8938	5.7090	6.3300	6.2387	5.4762	5.5959	6.5326	5.1019	6.1825	5.6491	6.4684	5.3568	5.5807	6.6833	5.4710	5.2566	5.9187	5.8400	5.4088	5.5182
Atp5f1b	_DQEGQDVLLFIDNIFR_.2	#N/A	5.9911	6.6933	6.8988	6.1790	5.7767	6.2831	6.5518	6.3099	5.6681	6.0548	5.7832	6.3888	6.2489	7.0453	5.5361	5.8662	6.4761	6.5360	6.4927	6.1998
Atp5f1b	_IM[Oxidation (M)]NVIGEPIDERGPIK_.3	#N/A	4.3211	4.6184	3.9817	4.8178	4.2175	3.6146	4.7204	4.8186	4.1272	3.8012	3.8919	5.1778	5.5848	5.2708	5.2787	4.6380	4.5739	4.9606	4.2937	4.6705
Atp5f1b	_LVLEVAQHLGESTVR_.3	#N/A	7.7030	7.3267	8.0755	7.9538	8.1400	7.8144	7.4464	7.6460	8.2164	8.3519	8.0290	7.7710	8.0425	7.7659	8.0197	7.9042	7.7694	7.5878	8.0421	8.4677
Atp5f1b	_QFAPIHAEAPEFIEM[Oxidation (M)]SVEQEILVTGIK_.3	#N/A	4.0214	3.8088	4.9889	3.7242	3.9028	4.4651	3.7778	4.3069	4.8325	4.5069	4.1862	4.3832	4.3329	6.1346	6.1282	3.0399	5.1891	4.5595	3.6815	4.2768
Atp5f1b	_SLQDIIAILGM[Oxidation (M)]DELSEEDKLTVSR_.3	#N/A	3.5680	5.0064	4.6609	4.7551	4.0982	4.3189	4.3828	4.7904	4.2706	4.1719	2.9765	4.4085	5.0640	5.1942	3.9057	4.9398	5.0645	4.8789	4.2348	4.4533
Atp5f1b	_GFQQILAGEYDHLPEQAFYM[Oxidation (M)]VGPIEEAVAK_.4	#N/A	3.4088	3.6353	3.2783	3.7925	3.5630	3.4037	3.1993	3.9257	3.2017	4.0107	3.5241	3.8285	3.5971	3.7586	3.8019	4.1824	3.3132	2.7944	3.2067	3.9441
Atp5f1b	_IVAVIGAVVDVQFDEGLPPILNALEVQGR_.3	#N/A	3.2336	4.0855	4.2710	4.3506	3.4405	3.1670	3.6076	3.4593	3.1564	4.5450	3.5308	3.8116	3.2645	4.3091	1.4830	4.0966	4.4574	4.1929	2.9911	3.9864
Slc1a3	_TTTNVLGDSLGAGIVEHLSR_.2	#N/A	6.2225	5.8932	5.0411	5.1889	4.8509	5.4896	6.0680	5.5865	5.4710	5.5687	4.6296	4.4131	5.3324	5.4626	4.7756	5.0263	4.9044	6.2576	4.8679	5.1325
Slc1a3	_IVQVTAADAFLDLIR_.2	#N/A	4.7501	5.1105	4.4833	4.0882	4.3869	5.0306	5.3935	4.9517	4.5897	5.2917	5.0064	4.4859	4.5337	4.9089	3.8243	4.5604	5.3638	4.6931	4.1726	4.4118
Slc1a3	_DVEM[Oxidation (M)]GNS[Phospho (STY)]VIEENEMK_.2	#N/A	4.0725	7.7682	8.5637	8.1761	3.7252	7.2075	7.0676	3.9487	6.7260	5.9136	5.2110	3.6900	3.7887	6.8463	3.2660	3.5611	6.2065	4.0898	5.1480	3.6418
Slc1a3	_NRDVEMGNS[Phospho (STY)]VIEENEM[Oxidation (M)]K_.3	#N/A	3.6886	3.9016	4.2696	5.4726	4.1013	4.0955	3.7227	4.0341	3.6780	3.6210	3.8859	3.1761	3.8325	4.2495	3.0169	3.3983	3.4786	3.9468	4.0380	3.7309
S100a1	_DLLQTELSGFLDVQK_.2	#N/A	3.9442	4.6975	4.4752	4.0863	4.1512	4.2706	4.3978	4.1699	4.2345	4.4478	4.1469	3.1010	4.4834	4.1100	4.7287	3.1274	3.6672	3.1324	3.6969	3.9604
Wfs1	_LNATAS[Phospho (STY)]LEQDKIEPPR_.3	#N/A	3.6963	9.1262	4.4285	6.8805	3.4008	3.1468	3.8446	3.6031	3.5756	8.8416	8.6592	3.8404	7.0757	8.0549	7.1145	3.8808	4.3601	3.9272	8.8723	3.5645
Wfs1	_GITS[Phospho (STY)]ENEAEVK_.2	P56695_S158_M1_Wfs1	4.4777	4.3423	4.0142	7.8636	3.1838	3.2171	3.8643	8.5128	3.7845	7.5004	3.2632	4.0684	3.9500	4.4526	3.8897	7.5445	4.1673	3.4658	3.3144	4.4196
Wfs1	_LNATAS[Phospho (STY)]LEQDK_.2	#N/A	3.6491	3.6700	3.1857	4.4324	3.8561	3.8894	3.1920	4.4582	3.1122	3.4597	4.0917	3.3961	3.2777	3.7803	3.2174	3.0112	3.4950	4.3381	3.9867	3.7473
Wfs1	_[Acetyl (Protein N-term)]MNSGT[Phospho (STY)]PPPSPSGPPPPPAPQPQAR_.3	P56695_T5_M1_Wfs1	4.3673	3.3468	3.5089	3.6119	3.7050	3.9291	3.8360	4.1980	3.8951	4.5736	4.2695	3.0729	4.0032	5.6536	3.4132	3.2114	3.6952	3.4542	3.7533	3.2242
Pdcd5	_KVM[Oxidation (M)]DS[Phospho (STY)]DEDDADY_.2	#N/A	7.8015	8.6825	9.1263	9.3256	9.1102	7.9152	8.2082	7.5226	8.5408	7.8771	9.1846	9.0582	9.4699	9.8808	8.9342	9.2981	9.3123	8.7384	8.9536	9.4656
Pdcd5	_[Acetyl (Protein N-term)]ADEELEALRK_.2	#N/A	5.8868	6.2464	6.3344	5.2097	6.6401	6.2101	6.1872	6.3392	6.0341	5.8234	5.8917	6.6064	6.3054	6.1999	6.7074	6.4166	6.4766	6.3048	6.3194	5.8126
Pdcd5	_RKVM[Oxidation (M)]DS[Phospho (STY)]DEDDADY_.2	#N/A	7.3431	8.3496	6.9115	8.3319	7.3758	7.3891	7.5497	8.1116	7.2700	7.6987	7.0049	7.8053	8.8272	8.2530	7.9577	7.9404	7.4493	7.7102	7.6009	7.4967
Pdcd5	_VSEQGLIELK_.2	#N/A	4.3176	3.5154	4.8634	4.3611	4.8614	4.4643	4.0721	4.1257	4.6457	4.1434	4.3179	4.5653	3.9309	4.2575	5.0336	4.4813	3.7680	4.8219	4.1772	3.9582
Pdcd5	_VMDS[Phospho (STY)]DEDDADY_.2	#N/A	3.5840	6.8226	8.3148	7.9427	2.9217	6.2466	6.0630	3.2844	5.4470	5.2957	6.3404	6.3008	3.6047	8.6424	6.3947	3.9289	6.3907	4.2088	5.2356	5.9423
Fus	_AAIDWFDGK_.2	#N/A	6.0595	5.8887	6.4885	6.2832	6.6128	6.3706	5.9766	6.8297	6.3452	6.3905	6.6431	5.9437	5.9592	6.8479	6.5800	6.4292	6.4174	5.8188	6.0993	6.7517
Fus	_GEATVS[Phospho (STY)]FDDPPSAK_.2	P56959_S333_M1_Fus	3.9197	7.0646	3.5227	3.2022	4.1266	7.3951	6.5711	4.1876	7.0282	3.7303	3.8211	7.2429	7.5165	5.7722	6.9755	7.0445	7.6127	3.1079	7.4292	7.8243
Fus	_HDSEQDNSDNNTIFVQGLGENVTIESVADYFK_.3	#N/A	3.2887	3.2133	3.8250	4.0007	4.1273	4.3513	4.3138	3.7758	3.4729	3.4950	3.8473	3.1027	3.5810	4.6256	2.9910	3.6336	3.5268	3.8764	4.1755	4.3028
Nrg2	_SDS[Phospho (STY)]PPLC[Carbamidomethyl (C)]PAADSR_.2	P56974_S715_M1_Nrg2	9.5967	9.3906	9.3656	9.4390	2.9260	3.5581	9.1283	9.1905	3.1733	3.7946	3.5477	9.1281	9.5705	9.2474	4.1922	3.9332	3.8264	4.0068	3.6554	9.1442
Samsn1	_SSS[Phospho (STY)]FGNFDR_.2	P57725_S23_M1_Samsn1	3.3063	3.1957	3.8426	4.3831	4.1449	6.6921	3.2414	4.5075	3.1616	7.5205	4.1411	3.0851	3.3270	3.8297	2.9734	3.6512	3.5444	4.2888	3.9374	3.7967
Atp6v1d	_FTAGDFSTTVIQNVNK_.2	#N/A	8.2762	7.7413	8.7264	8.1732	7.8621	7.9489	7.7867	7.9284	7.9349	8.3477	8.2025	7.6379	8.7918	8.2944	7.3887	8.2455	8.1861	7.0722	7.9111	8.2580
Atp6v1d	_IEIFPSR_.2	#N/A	7.5935	7.5128	7.4568	7.2195	7.8878	7.3631	7.4924	6.9230	7.2826	7.7231	7.7487	7.8192	7.4486	7.1112	7.3085	7.5836	7.3561	7.5848	7.7940	7.4086
Atp6v1d	_VNAIEHVIIPR_.2	#N/A	5.4481	5.5492	5.4652	5.8584	5.6657	5.5802	5.5130	5.8017	5.4643	5.8887	5.1858	5.6526	6.1127	5.6775	5.4114	5.0934	5.8314	5.5081	5.3509	5.7659
Atp6v1d	_EAAFSLAEAK_.2	#N/A	6.5860	6.2760	6.9274	6.5919	6.0846	6.6333	7.0692	5.6656	6.3229	6.4611	6.6363	6.0548	6.8673	6.2552	5.8664	6.1518	5.3753	6.5758	6.4239	6.3200
Atp6v1d	_TLAYIITELDER_.2	#N/A	6.2203	6.3263	6.9420	6.4313	6.1076	6.5748	6.1292	6.0287	5.6589	5.6665	6.1412	5.7257	6.8171	6.1291	5.9765	5.6559	5.3365	6.1780	5.9132	6.1434
Npy	_SSPETLISDLLM[Oxidation (M)]KES[Phospho (STY)]TENAPR_.3	#N/A	4.2766	3.8907	3.8132	4.1558	3.7560	3.6908	3.7726	3.7747	3.7639	3.8882	3.7796	3.6168	3.8996	3.8513	3.5912	3.6387	6.4470	8.6686	6.7125	3.5285
Eef1d	_ITSLEVENQNLR_.2	#N/A	5.1678	6.0807	5.1500	6.0900	6.3872	6.3125	5.9159	5.8137	6.0957	6.2436	6.0128	5.8327	6.4929	6.4912	6.4897	6.0725	5.7440	5.7473	6.2019	6.3385
Eef1d	_[Acetyl (Protein N-term)]ATNFLAHEK_.2	#N/A	5.6229	5.5510	6.0410	5.5166	6.5853	5.9432	6.1742	5.5900	6.0149	5.7794	5.6993	6.4393	6.3134	6.2736	6.2567	5.7095	6.3147	6.0719	5.5966	6.4430
Eef1d	_ATAPQTQHVS[Phospho (STY)]PM[Oxidation (M)]R_.2	#N/A	11.4396	11.6343	11.6265	11.8028	11.3413	11.2518	11.1783	11.6324	11.2100	11.3535	11.1500	11.3141	11.7189	12.0441	11.2054	11.2535	11.5357	11.1976	11.1299	11.3718
Eef1d	_LSSLEKSS[Phospho (STY)]PTPR_.2	P57776_S119_M1_Eef1d	3.2198	5.9723	4.2573	4.3644	3.4268	3.1808	3.6213	3.4455	3.1427	3.8253	3.5170	5.6536	6.7033	6.0802	3.6467	5.8037	4.4436	4.2067	3.0049	5.1821
Eef1d	_GATPAEDDEDKDIDLFGSDEEEEDKEAAR_.4	#N/A	3.7568	9.1711	5.4202	5.4955	3.3879	8.4498	8.9392	4.1033	8.5667	4.6472	3.6391	4.5402	3.5847	10.4756	8.9575	4.7382	3.9238	9.8183	8.0714	5.0348
Eef1d	_DIDLFGS[Phospho (STY)]DEEEEDKEAAR_.3	#N/A	3.8897	3.8017	4.6406	3.7968	3.6393	3.8146	3.7966	3.8234	3.7723	3.8008	3.6878	3.6145	3.9145	4.5171	3.3503	3.3403	3.9433	3.6659	3.7214	3.8479
Actn4	_EAILAIHK_.2	#N/A	4.5928	5.1030	4.6612	5.3773	5.2216	4.9241	4.6477	4.8318	4.7044	4.2734	5.2238	4.5394	4.4532	4.7992	3.4001	0.9514	5.5341	4.4400	4.5805	4.2428
Actn4	_DGLAFNALIHR_.2	#N/A	4.3968	5.0432	4.9628	4.3223	4.1516	4.2191	4.4696	4.6667	3.7866	2.6776	4.1610	4.3214	4.5184	5.0173	3.7818	3.5750	5.0412	4.8511	4.9896	4.8280
Actn4	_M[Oxidation (M)]APYQGPDAAPGALDYK_.2	#N/A	6.9057	6.4760	5.9228	6.6933	6.9674	6.6789	7.0994	6.8329	6.4727	7.1563	6.6053	6.3958	6.7894	6.9414	6.6748	7.2398	6.9378	7.2600	6.4650	7.0403
Actn4	_AGTQIENIDEDFRDGLK_.3	#N/A	3.2468	4.5246	4.2843	5.5153	3.9559	4.6241	4.1900	3.4725	4.0615	4.5583	4.4379	3.7984	3.2777	4.3223	3.6197	4.4253	4.4706	4.1797	4.5230	5.0647
Actn4	_IC[Carbamidomethyl (C)]DQWDNLGSLTHSR_.3	#N/A	5.8869	4.3823	5.9996	6.1582	5.6735	6.5220	6.3165	5.9028	6.0249	6.3117	5.6100	6.0502	6.2331	6.4431	6.2708	6.2156	4.1605	6.4759	6.5960	6.1411
Actn4	_VLAVNQENEHLMEDYER_.3	#N/A	3.8898	4.3598	3.9864	3.1344	3.2275	3.4516	4.3135	3.1747	4.3726	4.0961	4.2587	4.0962	4.4807	4.9511	3.8907	4.2347	4.1727	2.9767	3.2758	3.7017
Snrpa1	_LTAELIEQAAQYTNAVR_.2	#N/A	4.2704	3.8663	4.1539	3.5529	2.9765	3.8594	4.0716	3.8369	3.5149	4.0810	3.4704	3.5787	4.1572	3.1590	4.0969	4.1524	4.3746	3.4586	4.1483	4.0765
Nadk	_S[Phospho (STY)]LSASPALGSTK_.2	P58058_S46_M1_Nadk	3.4901	4.5127	4.0264	4.2356	4.3287	3.0520	3.2124	3.5743	7.5116	7.7820	3.6458	4.4021	8.8195	4.4241	2.7896	3.8350	3.0715	8.0671	4.3769	4.3014
Eef2	_VFS[Phospho (STY)]GVVS[Phospho (STY)]T[Phospho (STY)]GLK_.2	P58252_S418_M3_Eef2	11.1230	10.8973	10.5378	11.3478	10.0229	9.5072	10.0815	9.5251	9.3059	11.6906	10.3654	10.4525	10.6259	11.1832	8.4433	10.5771	9.9660	10.2084	10.3214	10.6063
Eef2	_GEGQLSAAER_.2	#N/A	7.8872	7.8498	8.3513	7.8518	7.6730	7.8894	8.2202	8.0917	7.7570	7.5760	7.5643	7.8486	7.5655	7.8946	7.8664	7.6202	7.6158	7.8328	7.6514	7.7597
Eef2	_VNFTVDQIR_.2	#N/A	7.8116	8.0002	7.7691	7.8928	7.8952	7.8946	8.0136	8.0685	7.8296	7.8041	7.8539	7.6538	8.0519	7.8701	7.6310	7.4084	7.2841	7.7988	7.7337	7.4840
Eef2	_FSVSPVVR_.2	#N/A	7.8577	7.4932	7.3486	7.6627	7.7834	7.4964	7.6127	7.8341	7.1597	7.2304	7.2487	7.7178	7.0065	7.0535	7.6192	7.2476	7.3542	7.5438	7.6586	7.4159
Eef2	_YEWDVAEAR_.2	#N/A	7.5008	7.2644	7.8123	7.2455	7.1550	7.7032	7.3133	7.6518	7.4476	6.9289	7.4776	7.0568	6.9691	7.1932	6.5526	7.3436	7.3195	7.2211	6.8983	7.3330
Eef2	_STLTDSLVC[Carbamidomethyl (C)]K_.2	#N/A	7.4495	6.9331	5.7702	6.9852	6.8219	7.1869	7.3506	7.5451	6.9835	6.7120	6.9529	6.7198	7.4411	6.8073	7.2481	6.9762	7.1608	6.7083	6.7967	6.6289
Eef2	_DLEEDHAC[Carbamidomethyl (C)]IPIKK_.3	#N/A	6.7970	6.7937	7.0624	7.0341	6.9197	6.7844	7.3060	6.9194	7.2650	6.7998	7.1181	6.7469	6.7645	6.9697	7.0515	7.1835	6.4149	6.7399	7.1410	6.9564
Eef2	_SDPVVSYR_.2	#N/A	6.4547	6.7515	6.0050	6.4550	6.1166	6.1982	7.0051	5.1393	6.8129	6.4017	6.3634	6.4841	6.6436	6.7963	6.5260	6.5743	5.4379	6.5334	6.6256	6.5151
Eef2	_TFC[Carbamidomethyl (C)]QLILDPIFK_.2	#N/A	6.3696	6.3109	6.8908	6.4401	5.9713	6.5879	6.1471	5.7901	6.0739	6.1645	4.7757	5.9256	5.9682	5.3966	5.8618	6.8166	6.3276	5.8307	5.7906	5.6699
Eef2	_ETVSEESNVLC[Carbamidomethyl (C)]LSK_.2	#N/A	5.5084	6.3566	6.3030	5.7098	5.4355	5.6164	5.3124	5.3403	6.2498	6.2009	5.7706	5.2477	5.1301	6.4516	5.0595	5.1634	5.8658	5.3118	5.5950	6.0646
Eef2	_AYLPVNESFGFTADLR_.2	#N/A	6.3624	6.0779	6.4860	6.1744	6.2903	5.8998	6.1487	6.2600	6.4129	6.2722	5.7384	5.6714	6.0103	5.5647	2.9592	3.2695	3.6303	6.2236	6.3965	3.1593
Eef2	_C[Carbamidomethyl (C)]ELLYEGPPDDEAAM[Oxidation (M)]GIK_.2	#N/A	6.5700	5.4177	3.8494	4.3762	6.0047	5.8346	5.5934	5.7579	6.1287	5.7917	5.6281	5.3687	5.8588	5.2592	4.8720	5.9832	3.5512	5.6727	5.0823	5.4731
Eef2	_EGIPALDNFLDKL_.2	#N/A	5.1934	5.7673	5.0579	4.8450	5.5794	5.5629	5.5072	4.2542	3.7086	5.1897	4.0830	5.4493	3.8167	3.5763	4.3900	3.3979	3.2910	5.6607	4.6407	5.3433
Eef2	_FT[Phospho (STY)]DTRKDEQER_.2	#N/A	3.4678	4.5350	4.0041	6.5016	5.2879	5.5861	5.3537	3.5967	5.8118	3.6741	3.6682	4.4245	3.4019	4.4465	4.5577	3.8127	2.5256	4.0555	4.3546	4.1238
Eef2	_VFDAIMNFR_.2	#N/A	3.8065	5.2868	5.6557	3.7778	4.0134	4.9179	4.5972	4.0321	3.7293	3.2386	4.1037	3.7611	3.8373	5.2450	4.6016	3.1686	3.5294	3.6201	4.6825	3.4723
Eef2	_AGETRFT[Phospho (STY)]DTR_.2	#N/A	3.3082	4.0108	4.3457	4.2760	6.3301	6.1767	3.5329	3.5339	3.4531	6.2694	2.9318	5.8713	3.6186	4.3837	3.5583	4.1712	4.5320	6.0096	3.6458	4.0882
Eef2	_ALLELQLEPEELYQTFQR_.2	#N/A	4.4026	4.4319	4.8290	4.0557	4.5922	3.9861	4.4888	4.5112	3.9647	4.3273	4.3010	4.3558	4.0320	5.0832	4.3304	4.1453	4.5188	4.4438	4.2442	3.7954
Eef2	_YFDPANGK_.2	#N/A	4.1196	3.8832	3.1550	3.5698	3.4573	4.4936	5.4643	3.9557	3.9749	5.7238	5.8677	4.0363	4.1403	3.1421	3.6610	4.7074	4.3576	5.3511	5.1893	5.1963
Eef2	_EDLYLKPIQR_.3	#N/A	4.2135	4.6065	3.7500	3.3708	2.9196	4.4537	4.1285	3.8938	4.0487	4.0241	3.5274	4.3326	4.2142	3.2160	4.1539	3.5756	4.4315	3.4016	3.0502	3.1830
Eef2	_STAISLFYELSENDLNFIK_.2	#N/A	3.9949	3.3242	3.5315	3.5893	4.2018	3.9065	4.3471	4.2206	3.9177	3.8055	4.2921	3.0503	4.0258	3.5696	2.8716	3.3570	3.7178	3.4316	3.7307	3.2468
Opa1	_VVVVGDQSAGK_.2	#N/A	6.8747	6.8251	7.2849	6.7999	6.8871	6.9833	6.6234	7.4084	6.9992	6.9107	6.6744	6.7976	6.7590	6.3293	6.8595	6.8048	6.1934	6.6320	6.5931	6.5896
Opa1	_EVLEDFAEDGEK_.2	#N/A	6.8837	6.3497	6.4654	6.6864	6.8501	6.5213	6.3414	6.6545	6.7974	6.3749	6.4976	6.4506	6.6682	6.5017	6.5213	6.4510	6.7455	6.0379	6.2442	6.5048
Opa1	_ESVEQQADSFK_.2	#N/A	6.4051	6.4688	5.6271	6.2879	6.2306	5.9917	6.1405	6.9302	6.2875	6.1019	6.3665	6.4264	6.9119	5.8789	6.2228	6.2274	6.5722	6.6287	6.6688	6.2942
Opa1	_AVEVAWETLQEEFSR_.2	#N/A	5.8125	5.5090	6.1836	5.9678	5.4564	5.5247	5.2735	6.3851	5.1319	6.1933	6.1768	4.8953	6.2780	5.1512	5.3162	5.9385	5.9787	4.9126	4.9064	5.9535
Opa1	_EFDLTKEEDLAALR_.3	#N/A	5.4862	4.5188	5.3911	5.3952	5.2535	5.2252	5.8017	5.4592	5.2609	5.7985	5.8596	5.0144	5.3470	5.0537	5.5163	5.7774	5.3711	5.3873	4.9475	5.6507
Opa1	_FNLETEWK_.2	#N/A	3.0062	5.2331	4.3420	5.4982	3.9529	4.4077	4.2603	3.7128	4.7376	4.7253	4.2707	4.6609	4.6292	4.8565	4.5185	5.0356	4.2231	5.5882	4.7974	4.7179
Opa1	_TSVLEM[Oxidation (M)]IAQAR_.2	#N/A	6.2200	5.4773	6.0399	5.4990	5.8930	5.4299	5.0305	5.8175	5.1266	5.9579	5.6984	5.8029	6.0989	5.7825	5.3234	5.8598	5.5536	5.7168	6.3570	6.0227
Opa1	_DFFTAGSPGETAFR_.2	#N/A	6.4353	6.2008	6.3507	6.3371	5.9883	5.9074	6.3706	6.2601	6.1681	6.3711	5.4878	6.8357	5.2404	4.2850	5.9627	5.8765	6.2479	6.2170	6.4651	6.1784
Opa1	_VNDEHPAYLASDEITTVR_.3	#N/A	5.6246	5.3055	5.5658	5.2189	5.6494	5.1267	5.3898	4.8687	5.1064	5.1934	5.4434	5.1752	5.3245	5.5695	4.9370	5.0819	5.4900	5.4571	4.9271	5.1520
Opa1	_IQQIIEGK_.2	#N/A	3.5603	3.7588	4.5977	4.0239	3.9590	3.2599	3.7735	3.7860	3.7270	3.4848	4.6163	2.3271	3.5912	3.1349	3.0012	2.9610	3.2832	3.8898	3.1737	4.0726
Opa1	_IDQLQEELLHTQLK_.3	#N/A	3.1731	3.3289	3.7094	1.8433	3.2025	3.9733	2.4605	4.3743	2.6234	3.7090	4.0078	3.5735	3.1938	3.6965	2.4855	3.5180	3.4111	3.1221	3.9008	3.6635
Opa1	_LDAFIEALHQEK_.2	#N/A	5.7025	5.4893	4.7937	5.2673	4.5560	5.3907	5.1192	5.3746	4.9159	4.9555	5.2260	4.5403	5.6125	4.4283	5.4070	5.0881	5.4772	5.0435	5.3222	4.8174
Opa1	_SIVTDLVSQM[Oxidation (M)]DPHGR_.3	#N/A	4.3916	4.4284	3.9282	3.1927	3.0977	3.5099	3.9504	3.1164	4.3144	4.2022	3.1879	4.1545	4.4224	3.9662	3.3534	3.7537	4.1145	3.0350	3.3340	3.5816
Ptpa	_LVALLDTLDR_.2	#N/A	6.8727	7.4910	6.9594	7.0192	6.8960	6.9607	7.0292	7.1440	6.9400	7.0400	6.8292	7.1365	6.2722	6.9250	6.5400	6.3410	6.2300	6.9865	7.1709	6.8334
Ptpa	_FGSLLPIHPVTSG_.2	#N/A	5.5397	5.5764	5.8900	6.2165	4.3507	6.1102	6.3184	6.3061	5.9913	6.1589	5.3865	6.2155	6.2938	5.8925	4.6611	5.6694	6.2969	6.2038	5.3668	5.8543
Ptpa	_LDQEAENLVATVVPTHLAAAVPEVAVYLK_.3	#N/A	3.1879	4.4021	4.2853	4.5015	4.0265	4.0626	3.5146	2.3759	3.5737	3.3942	3.9481	3.2035	3.2086	3.7113	3.0918	3.5328	3.4260	4.4072	4.0558	3.6783
Strn4	_S[Phospho (STY)]LENGAGEPVEGAPR_.2	P58404_S206_M1_Strn4	11.3957	11.8123	11.7072	11.5388	10.5875	11.2520	11.2013	10.5836	11.0342	11.5267	11.1427	11.1450	11.2719	11.9480	10.5332	11.4174	11.4349	11.2591	11.5403	11.5978
Strn4	_AS[Phospho (STY)]PGPGLSGGESLLVK_.2	P58404_S223_M1_Strn4	9.6520	9.9311	9.8222	3.5019	8.9477	9.4576	9.2785	9.3874	9.4330	9.8296	2.9485	9.2128	9.7954	10.3982	9.0040	9.4772	9.7138	9.6182	3.0562	9.5589
Hrh3	_GSKPSASSAS[Phospho (STY)]LEKR_.3	P58406_S332_M1_Hrh3	3.8879	3.4311	3.4245	3.6963	4.0949	4.1282	3.8073	4.1136	4.3743	3.4581	4.4976	3.1573	4.5397	3.4626	2.9786	3.2501	3.6109	4.9760	3.8376	4.0962
Hrh3	_VQPHGS[Phospho (STY)]LEQC[Carbamidomethyl (C)]WK_.2	#N/A	8.4457	8.3855	8.3736	8.3898	8.0091	8.0911	7.8097	8.1313	7.9403	7.9134	8.2630	7.9640	8.4287	8.2142	7.0281	8.0921	8.8216	8.4177	8.2704	8.2082
Foxp2	_RDS[Phospho (STY)]SSHEETGASHTLYGHGVC[Carbamidomethyl (C)]K_.4	P58463_S329_M1_Foxp2	4.3921	4.4278	3.9287	3.1921	3.0982	3.1315	3.9499	3.1169	3.8701	4.2027	3.3488	4.1540	4.4230	3.9667	3.9753	3.7542	4.1150	3.0345	3.3335	3.6440
Fam207a	_AVEPALPQS[Phospho (STY)]PAGGAGAK_.2	P58468_S38_M1_Fam207a	9.1284	9.1961	8.6915	8.8185	8.3245	8.6910	8.7526	8.9216	8.7268	8.7696	8.8463	8.6946	9.4799	9.4209	8.5537	9.0111	9.1639	8.7184	8.6625	8.7198
	LEFQQGLGEAPGDAS[Phospho (STY)]P.2	P58686_S82_M1_	3.7041	8.8562	9.4141	3.8801	3.9111	8.5051	8.2450	5.2855	8.9513	7.5239	4.0013	7.9037	8.2808	9.5413	9.0717	8.4423	7.5511	8.8028	9.0635	8.5184
Aaas	_FS[Phospho (STY)]PVLGR_.2	P58742_S495_M1_Aaas	9.9416	4.1213	9.2975	9.6006	5.1027	9.7301	9.4342	9.6279	9.3263	9.8182	9.6375	9.3349	9.9919	9.5395	8.6168	9.3939	9.8894	9.5840	9.3221	9.4002
Tpm1	_KLVIIESDLERAEER_.3	#N/A	5.4900	5.9888	5.8733	6.8241	6.2408	5.4730	5.8012	7.2164	6.0995	6.6583	6.3927	6.5165	7.7054	6.9950	6.9233	6.2424	6.6257	6.6341	6.1146	6.4748
Tpm1	_KLVIIESDLER_.3	#N/A	3.4865	4.5163	4.9253	4.2320	2.4768	3.0484	3.9167	3.5779	3.9996	3.1645	4.5312	4.3660	3.3831	4.4277	4.5360	3.8314	3.0751	3.3518	4.3733	4.1050
Tpm1	_TVTNNLKS[Phospho (STY)]LEAQAEK_.2	P58771_S206_M1_Tpm1	3.5549	3.7642	4.5924	4.0293	3.7619	4.3465	3.2145	3.7806	3.4778	4.5015	3.8521	3.5784	3.5858	4.6598	3.7276	4.4179	3.2778	3.8716	4.1706	5.0525
Tpm1	_C[Carbamidomethyl (C)]AELEEELKTVTNNLK_.3	#N/A	3.4522	3.9325	4.4240	4.1976	4.2907	3.0140	3.2447	3.6123	3.3094	3.6585	3.6838	4.0184	3.4175	4.4621	2.9791	3.3301	3.1095	3.6564	4.3390	4.1394
Tpm1	_AQKDEEKMEIQEIQLK_.3	#N/A	3.9785	4.7643	4.1585	5.4639	4.7513	4.2736	3.5941	5.1460	4.9256	4.2291	5.2263	5.3708	4.4431	6.6352	4.7473	4.8827	4.8078	2.8604	5.0175	3.4119
Tpm1	_LVIIESDLERAEER_.3	#N/A	3.1300	4.1890	4.1675	4.4542	4.7520	3.3703	3.7111	3.4764	3.6313	4.2247	3.1100	4.5698	3.7968	4.2994	3.7365	5.1086	4.0141	3.8191	4.1880	4.2664
Tbc1d10a	_EPAAGGS[Phospho (STY)]LSGTR_.2	P58802_S18_M1_Tbc1d10a	4.1115	7.8528	3.6481	3.3940	4.3184	4.3518	4.2305	3.9958	4.1507	7.8591	3.6294	2.9337	4.3162	3.3180	4.2559	3.4736	4.5335	3.2997	2.9483	3.2850
Tnks1bp1	_ENYEDQEPLVGHES[Phospho (STY)]PITLAAR_.3	P58871_S602_M1_Tnks1bp1	8.7444	4.4991	8.3611	8.6644	7.5337	8.3024	8.2772	8.0354	8.3829	8.7459	8.6711	8.4006	3.5244	9.5226	8.1115	8.7442	9.0020	8.9062	8.8843	8.5476
Tnks1bp1	_LANISVPASES[Phospho (STY)]PR_.2	P58871_S300_M1_Tnks1bp1	3.6454	9.4277	8.4897	4.0634	3.8384	6.6489	8.0155	3.3458	3.9909	9.3309	3.5100	4.2663	8.4619	8.0456	3.5090	3.9903	7.8542	3.9691	3.6177	8.0587
Tnks1bp1	_VSGAGLS[Phospho (STY)]PSRK_.2	#N/A	7.5865	7.4072	7.4186	3.7758	6.7512	6.4767	7.1487	7.3091	3.5998	7.7730	6.8530	7.1723	3.8389	7.4339	3.8669	7.0763	7.0271	6.9339	7.0282	7.3262
Tnks1bp1	_DS[Phospho (STY)]LGSFSTR_.2	#N/A	4.5631	4.2568	8.1205	7.4731	3.2692	3.3026	3.7789	7.5529	6.9306	4.3737	7.6749	6.9905	7.7187	8.0123	6.5820	7.7070	7.4258	4.3643	7.4764	7.3912
Tnks1bp1	_VPSS[Phospho (STY)]DEEVVEEPQSR_.2	P58871_S1612_M1_Tnks1bp1	8.6681	10.2789	10.2696	10.4801	3.8373	10.1151	9.6678	7.6051	9.8583	10.3347	3.8404	10.0508	7.7706	10.6010	9.8916	10.1421	10.6966	10.0741	10.2573	10.4375
Tnks1bp1	_VSGAGLSPS[Phospho (STY)]R_.2	#N/A	5.5258	6.0317	6.0035	6.4334	10.1838	10.0418	3.0948	10.2502	10.0705	6.3981	3.9945	3.2317	5.5846	5.5936	3.1202	5.6350	3.3978	5.3498	5.7199	6.0881
Tnks1bp1	_SLSSGFS[Phospho (STY)]PEEAQQQDEEFEK_.2	P58871_S980_M1_Tnks1bp1	4.1155	5.2678	4.8446	3.7121	3.4571	3.5925	3.6417	3.6274	5.1732	4.6006	4.9823	4.8841	3.9580	3.9478	4.8017	3.6987	4.8663	4.7884	3.6629	3.6356
Tnks1bp1	_WLDDLLAS[Phospho (STY)]PPPNSGSAR_.2	P58871_S692_M1_Tnks1bp1	8.6967	9.0127	3.4239	3.3010	3.7262	7.5690	8.6965	4.0888	4.2437	3.8291	3.7223	3.5038	4.4091	9.8764	3.3921	3.2325	3.1256	8.7043	8.4070	7.2683

Tnks1bp1	_M[Oxidation (M)]QAESQS[Phospho (STY)]PTNVDLEDKER_.2	#N/A	3.9681	4.0386	4.6809	6.4606	5.0119	4.9924	5.1822	6.2847	6.3397	5.6569	5.8737	6.4374	5.9425	6.4634	4.9395	6.5285	5.9240	6.4275	6.2113	5.4280
Tnks1bp1	_SSGSLS[Phospho (STY)]PGLETEDPLEAR_.2	P58871_S1375_M1_Tnks1bp1	7.6532	8.7538	7.8602	7.7236	3.3738	7.5699	7.8300	7.4364	7.4782	8.0140	7.5272	7.5761	7.7465	9.7447	7.6214	7.7808	7.9868	9.0254	7.8284	7.8025
Tnks1bp1	_GEGVSQVGPGT[Phospho (STY)]PPAPESPR_.2	P58871_T533_M1_Tnks1bp1	7.7203	7.8356	8.0187	7.7423	7.3102	8.0758	7.4418	7.9691	7.7946	6.3321	7.1816	7.5426	8.2582	8.5853	7.7255	3.5683	8.6607	7.8253	7.9129	8.0753
Tnks1bp1	_RFS[Phospho (STY)]EGVLQPPSQDQEK_.2	#N/A	8.2663	7.8913	7.0789	5.4804	7.0949	3.8741	7.6460	3.6017	7.6906	3.7999	3.6732	7.4852	8.1059	8.5471	6.9631	7.7776	7.9934	8.0670	7.8781	8.1801
Tnks1bp1	_NM[Oxidation (M)]APGAGC[Carbamidomethyl (C)]S[Phospho (STY)]PGEPR_.2	#N/A	7.3400	7.6543	6.5386	7.2724	3.6613	6.8241	6.9783	6.1577	5.7243	7.6496	7.1769	6.6290	6.8596	7.7289	5.4625	7.0240	6.9158	6.6611	7.0909	7.1677
Tnks1bp1	_DVGHLEEGASGGLLS[Phospho (STY)]PSTPHSR_.3	P58871_S1022_M1_Tnks1bp1	6.3943	6.5994	5.8572	6.1495	5.3529	6.1479	6.2540	5.8717	3.7399	5.9121	5.9217	5.8825	6.4273	7.2138	3.2581	5.9675	6.0749	6.5978	6.1348	6.2057
Tnks1bp1	_GYSS[Phospho (STY)]QDAEEQDREFEKR_.3	P58871_S912_M1_Tnks1bp1	3.9556	3.7934	4.0009	6.4524	6.1135	6.2952	3.6604	6.3923	3.6609	6.9313	6.6754	6.4914	3.7928	3.8177	5.8345	6.6732	3.7287	6.8555	6.7980	7.0071
Tnks1bp1	_ASRVPS[Phospho (STY)]S[Phospho (STY)]DEEVVEEPQSR_.2	#N/A	3.8255	6.1850	4.0334	5.7468	3.2029	3.1048	6.2606	4.0512	6.0065	3.9635	3.4878	4.8579	3.7728	6.0815	5.1181	5.6136	6.0061	5.9637	4.9702	3.6003
Tnks1bp1	_RDS[Phospho (STY)]LGSFSTR_.2	#N/A	4.0430	3.9598	4.5793	3.6464	3.3807	3.1034	3.9781	5.7131	3.8983	5.9259	5.8932	3.8493	4.0637	5.9160	4.0034	3.7261	4.2811	3.3521	3.2007	5.1138
Tnks1bp1	_LDS[Phospho (STY)]PPPSPITEASEAAEAAEADSWAVSGR_.3	P58871_S496_M1_Tnks1bp1	4.2351	6.7315	4.0974	6.1438	5.4073	6.0763	6.4730	6.2458	5.9584	3.7841	5.6059	6.5853	6.6312	7.1687	5.5735	5.2432	6.3258	6.5723	3.5680	5.9115
Tnks1bp1	_HS[Phospho (STY)]LGQEVIGIGGSQDESEVPVR_.3	P58871_S796_M1_Tnks1bp1	6.3512	7.1189	6.9503	6.8322	4.5238	5.6672	6.4459	3.7809	4.8321	7.4263	6.5661	6.2368	3.9783	7.3288	4.7677	5.7116	4.0519	4.7812	6.5999	4.6261
Tnks1bp1	_NRS[Phospho (STY)]AEEGEVTESK_.2	P58871_S1657_M1_Tnks1bp1	3.1500	4.1691	4.1874	4.0591	3.3569	3.3902	3.7254	3.3756	3.6114	4.4614	3.0900	4.0777	4.2144	4.4240	3.7166	4.4830	4.3737	3.3112	4.0469	3.9027
Tnks1bp1	_S[Phospho (STY)]PPSGSQSLLEGIM[Oxidation (M)]TASSSK_.2	#N/A	5.9235	5.8959	3.9534	3.6992	3.1229	4.0774	5.7328	6.3454	3.8508	4.2274	3.3294	5.5412	5.3871	7.2978	4.3937	6.4231	4.6091	5.2661	4.5394	4.5982
Tnks1bp1	_DRQS[Phospho (STY)]PSTC[Carbamidomethyl (C)]SEGLLGWAQK_.3	P58871_S713_M1_Tnks1bp1	3.8887	4.3690	3.9875	3.1333	3.2264	3.4505	4.3147	3.1758	4.3738	4.0950	3.2473	4.0951	4.4818	4.0256	3.8919	4.2336	4.1739	2.9756	3.2746	3.7029
Bcl2l13	_S[Phospho (STY)]HTGEAAAVR_.2	#N/A	6.7575	6.6891	3.8689	4.5702	3.6327	6.9883	6.2479	6.5056	6.3208	6.9399	6.4087	5.8336	4.0138	3.8854	3.5040	3.5717	3.8677	3.8597	6.4646	3.6912
Bcl2l13	_ALQTILSQPVTYEAYR_.2	#N/A	4.7887	3.1625	4.4398	4.1815	4.8425	4.6880	4.6076	4.3575	4.1590	5.2745	4.9787	4.4337	3.4978	4.7642	4.4078	4.6475	4.7892	4.9167	3.7666	5.2522
Bcl2l13	_KKSHT[Phospho (STY)]GEAAAVR_.3	#N/A	7.2572	4.0311	3.7520	3.1846	6.7051	3.6755	6.7796	6.8928	3.8111	3.7958	3.7907	6.3809	4.4787	6.3333	3.6625	3.5702	3.7489	3.0590	3.8546	3.5751
Pcif1	_[Acetyl (Protein N-term)]ANENHGS[Phospho (STY)]PR_.2	P59114_S8_M1_Pcif1	8.0273	7.9000	8.3430	8.1953	7.3979	8.0789	7.7494	8.1704	8.0777	7.9721	7.8055	7.5758	7.7059	7.9876	6.8644	6.5389	8.2611	7.8216	7.3369	7.6353
Pcif1	_QLS[Phospho (STY)]EEQPSGNGVK_.2	P59114_S116_M1_Pcif1	3.6568	3.6623	3.1934	3.9274	3.8638	3.8971	3.1843	3.8825	3.1046	3.4674	4.0840	3.3884	3.6877	3.2315	3.2097	3.0189	3.3797	3.7697	4.0688	4.4096
Arhgap39	_AFS[Phospho (STY)]EDEALAQQDSK_.2	P59281_S597_M1_Arhgap39	11.6612	11.5222	11.8279	11.7446	10.3583	10.8990	11.1143	11.0734	10.9775	11.7927	11.0103	11.0048	11.3935	11.1583	9.7485	10.9808	11.4673	10.9743	11.0872	10.9706
Arhgap39	_KPSS[Phospho (STY)]ETDIENWASK_.2	P59281_S683_M1_Arhgap39	3.5953	4.1515	6.1042	5.8501	4.1219	5.3496	3.4333	3.6071	5.2506	6.1434	3.8667	3.1418	6.0394	3.6759	3.0105	5.5397	3.5738	5.5243	4.1758	3.6761
Arhgap39	_DGST[Phospho (STY)]GSSLEPELEER_.2	P59281_T133_M1_Arhgap39	4.3172	4.5028	3.8537	3.2671	3.0233	3.0566	4.0248	3.7901	3.9450	4.1278	3.4237	4.2289	4.1105	4.6131	4.0502	3.6793	4.3278	3.5053	3.1539	3.0793
Arhgap39	_QLVYVEQAGS[Phospho (STY)]SPK_.2	P59281_S402_M1_Arhgap39	4.3426	6.1969	3.3780	3.3468	4.2713	5.3977	4.2777	5.7630	4.1979	3.8749	3.6765	3.5497	4.3633	3.3651	2.8022	3.4265	3.0798	3.2525	5.3403	5.5763
Arhgap39	_EDTSSC[Carbamidomethyl (C)]S[Phospho (STY)]PPGVLLEK_.2	P59281_S169_M1_Arhgap39	3.8393	6.5882	4.3756	3.8501	3.1770	3.3071	4.3641	3.5397	3.6946	4.3782	3.1732	4.0530	3.8600	4.3627	3.9413	4.1842	4.0773	3.7558	3.4044	4.3297
Eif5	_SVS[Phospho (STY)]DQFYR_.2	P59325_S10_M1_Eif5	7.6716	3.3847	7.5417	8.3137	6.0138	4.0291	7.4634	7.1617	7.3850	7.9694	7.2684	7.6678	7.2653	7.6406	7.2554	7.7286	7.2604	7.3176	7.3238	7.7082
Eif5	_AM[Oxidation (M)]GPLVLTEVLFDEK_.2	#N/A	3.8478	3.9571	3.7793	3.9884	4.0815	4.3056	4.3596	4.1176	3.5186	2.8697	3.7595	4.2215	3.6267	3.1705	3.8454	3.8652	3.3187	2.5975	4.1617	4.3486
Eif5	_GLTLS[Phospho (STY)]DDLER_.2	P59325_S227_M1_Eif5	4.2876	3.7152	3.3231	3.5322	3.6253	2.7305	3.9158	4.2777	3.9748	4.4939	3.4392	3.6047	4.0829	3.6267	3.4930	3.1317	3.7749	3.3745	3.6736	3.3040
Eif5	_VETVKS[Phospho (STY)]DNKDDDIDIDAI_.2	P59325_S417_M1_Eif5	3.9219	3.7785	4.0658	3.7826	3.3841	4.1403	4.7572	3.5552	3.7101	3.7106	3.9117	3.5888	3.8756	3.6003	3.9489	4.0401	4.0929	3.8662	3.5148	3.5673
Eif5	_EEGIDSSDKEVAEAER_.3	#N/A	3.6371	4.3657	4.1734	4.0523	2.9748	2.8441	3.5722	3.3375	3.0905	4.5804	2.9710	4.2551	3.6578	4.1605	3.5975	3.9820	3.8752	3.9580	3.6066	4.1732
Inpp5j	_AAGPATS[Phospho (STY)]PPR_.2	P59644_S266_M1_Inpp5j	3.1311	3.3708	3.6674	4.5583	3.9697	4.0152	3.5714	4.3324	3.6305	3.3375	4.0048	3.2603	3.5159	7.9103	5.7837	3.4760	3.3692	4.4640	4.1126	8.0678
Inpp5j	_QLS[Phospho (STY)]PTSVGPK_.2	P59644_S171_M1_Inpp5j	6.7470	4.3004	4.0561	4.5656	5.9613	3.2589	3.8225	3.2443	5.6616	6.6911	3.2214	4.0266	4.6562	6.0804	3.8479	3.8816	4.2424	4.4079	3.2061	3.7714
Inpp5j	_SRS[Phospho (STY)]PGLAR_.2	P59644_S885_M1_Inpp5j	7.5697	3.3628	7.0158	4.4396	3.8632	6.8476	6.9766	4.4510	6.4043	6.8941	4.0845	6.5313	3.2705	3.7732	3.2102	6.2559	6.3347	4.3453	3.9939	3.7402
Inpp5j	_VKAPSGGPS[Phospho (STY)]PSGR_.2	P59644_S691_M1_Inpp5j	4.0483	3.9545	4.5846	3.6411	3.3860	3.0981	3.9834	3.7487	3.9036	6.5051	3.3822	3.8439	4.0690	4.5717	4.0088	4.3932	4.2864	3.5468	6.3242	3.0378
Inpp5j	_HRS[Phospho (STY)]RS[Phospho (STY)]PGLAR_.3	P59644_S883_M2_Inpp5j	3.5165	4.7709	4.0528	4.1729	2.8542	2.9112	3.4516	3.2169	3.3718	3.2002	4.3512	4.7868	3.5372	4.2684	3.4769	3.8614	3.7545	4.0786	6.6989	4.1922
Inpp5j	_GGSRS[Phospho (STY)]PS[Phospho (STY)]PQSR_.2	P59644_S909_M2_Inpp5j	4.4951	4.3248	4.0317	4.5899	3.2012	3.4063	4.3589	3.2200	4.4180	4.0508	3.2915	4.6567	4.5260	4.0698	3.9361	3.8573	4.2181	4.4322	3.2304	3.7471
Fxyd7	_S[Phospho (STY)]C[Carbamidomethyl (C)]KSELPSSAPGGGGV_.2	P59648_S65_M1_Fxyd7	3.9962	10.7738	11.4341	11.1264	10.4193	10.2712	10.3025	10.5600	10.5013	11.1104	10.5573	10.0790	10.2712	10.5126	3.8117	10.1426	10.6340	10.1140	10.2243	3.8254
Fxyd7	_SELPSS[Phospho (STY)]APGGGGV_.2	P59648_S73_M1_Fxyd7	6.5438	3.5478	6.9627	6.8555	5.1971	6.5254	4.9450	6.1129	5.6648	4.8216	6.7070	5.2933	6.3965	5.5088	5.0760	5.1109	4.9279	5.6217	5.3897	4.6365
Fxyd7	_ADSRS[Phospho (STY)]ES[Phospho (STY)]PTC[Carbamidomethyl (C)]K_.2	#N/A	5.5197	7.8918	3.7503	4.1502	4.5715	6.5276	6.4239	7.1623	6.3586	7.1758	5.0157	6.4418	6.4947	6.5236	3.2915	3.5758	5.1753	7.2501	4.0895	5.7861
Fxyd7	SESPTC[Carbamidomethyl (C)]KSC[Carbamidomethyl (C)]KS[Phospho (STY)]ELPSSAPGGGGV_.	#N/A	8.4291	8.7040	8.2461	3.4617	4.3295	7.3758	8.0077	4.3482	4.0454	3.9331	7.3537	7.2973	4.1534	7.5139	4.2448	3.4847	3.8455	3.3040	3.6030	7.2623
Fxyd7	_KADS[Phospho (STY)]RSES[Phospho (STY)]PTC[Carbamidomethyl (C)]K_.2	#N/A	3.5542	4.2443	4.2391	8.3063	5.2892	6.4295	5.7338	8.0742	7.9916	4.1397	3.3159	7.7648	3.8434	4.1911	3.9556	6.5132	4.2125	7.9125	3.2890	8.1932
Anks1a	_SES[Phospho (STY)]LSNC[Carbamidomethyl (C)]SIGK_.2	#N/A	8.1236	8.5155	8.3213	8.6219	7.9107	8.0696	8.0178	8.2615	8.0448	8.4151	7.7187	7.8255	8.5096	8.3818	7.5644	7.9208	8.3811	3.9478	4.2469	7.8876
Anks1a	_SES[Phospho (STY)]LSNC[Carbamidomethyl (C)]SIGKK_.2	#N/A	3.6627	3.6564	6.6157	3.9215	5.6456	5.3125	3.1785	3.8884	3.0987	3.4733	6.1216	5.9624	3.2641	6.4954	5.8357	6.2742	3.3856	6.3682	6.0751	5.9909
Anks1a	_GTESTQDAC[Carbamidomethyl (C)]AK_.2	#N/A	3.4319	3.8872	1.2898	4.1523	3.6389	4.4695	3.4092	3.6576	3.5347	3.6132	3.7291	3.6133	3.4628	4.5074	3.4346	4.2949	3.1548	3.9946	4.2937	4.1847
Dock4	_S[Phospho (STY)]DPNLSAPEK_.2	P59764_S1759_M1_Dock4	4.5046	4.1005	7.6670	7.1051	3.2107	7.4766	7.6702	3.6027	3.7576	7.6978	7.2023	4.0414	3.9230	4.4257	6.2028	7.2120	8.0136	3.6928	6.7385	7.4116
Dock4	_NSAPASM[Oxidation (M)]S[Phospho (STY)]PDGTR_.2	#N/A	3.9669	7.6395	8.6217	8.1494	8.3844	8.1888	7.6111	8.7875	8.3728	8.6558	8.6662	9.0123	8.9475	8.1993	7.2711	8.3499	8.3895	5.9650	8.2690	8.3448
Dock4	_AVNPTPSSWS[Phospho (STY)]LDSGK_.2	P59764_S1778_M1_Dock4	4.1675	3.5311	6.9554	3.4700	3.2261	5.7921	7.5607	3.9154	4.0703	4.0034	5.5845	4.0910	4.2357	5.7272	4.1754	3.2917	4.1728	3.3810	3.0296	5.7791
Dock4	_AS[Phospho (STY)]PLLSDK_.2	P59764_S1709_M1_Dock4	4.0079	3.9950	4.5441	3.2525	3.3456	3.5697	4.1955	3.0566	4.2546	4.2142	3.1281	3.8844	4.3626	3.9064	3.7727	4.3527	4.0547	3.0948	3.3938	3.5837
Sash1	_S[Phospho (STY)]LPVSIC[Carbamidomethyl (C)]R_.2	P59808_S805_M1_Sash1	8.7164	8.9792	8.9388	8.9116	8.4609	8.7056	8.6135	7.8988	8.7226	8.9728	8.7718	8.5980	9.3247	9.2903	8.0346	8.8080	8.5363	8.7848	8.6434	8.6802
Sash1	_EQS[Phospho (STY)]DDETEDSVK_.2	P59808_S241_M1_Sash1	7.7594	8.6838	8.2801	7.2911	2.8677	9.2258	8.8407	8.5924	8.0299	3.9722	7.3883	8.9537	9.2133	8.5259	7.9187	7.3346	9.2235	3.3497	8.4878	8.7707
Sash1	_AGGS[Phospho (STY)]VESLR_.2	#N/A	6.2871	3.5979	5.6982	4.5044	6.0674	3.9614	6.6168	5.6701	6.9523	7.0384	6.8372	3.3241	7.2113	6.9748	3.1454	6.8580	5.9077	4.4101	4.0587	7.0787
Sash1	_S[Phospho (STY)]C[Carbamidomethyl (C)]ETLEQPEPVESWPR_.2	P59808_S813_M1_Sash1	7.2398	8.6837	8.1466	7.0093	3.6259	8.1850	8.1385	3.1875	3.3424	3.2296	5.9971	7.0300	3.5078	8.9696	7.6302	7.1169	3.7252	8.1170	8.1495	7.7339
Sash1	_DSGC[Carbamidomethyl (C)]YES[Phospho (STY)]SENLENAK_.2	P59808_S723_M1_Sash1	3.4262	5.2537	4.0161	4.2095	3.6332	3.6665	3.4149	4.3856	3.3351	5.0658	3.8000	4.2401	5.4643	4.0032	3.4403	4.2892	5.4106	4.1153	3.7638	3.9702
Sash1	_LKAGGS[Phospho (STY)]VESLR_.3	#N/A	3.5587	4.5640	3.9751	4.2506	4.2774	4.6239	3.3739	3.1392	3.2941	3.2779	4.2736	2.9526	3.4595	3.9622	2.8409	3.7837	3.6769	4.7577	3.8049	5.0561
Sash1	_SHS[Phospho (STY)]LDDLQGDADVGK_.2	P59808_S831_M1_Sash1	4.3833	8.2228	7.1394	7.5979	3.0894	7.6310	7.8024	3.7240	3.8789	7.7989	6.3304	7.6673	7.3523	8.4206	7.0408	7.6666	8.0760	7.8027	3.2201	7.8574
Sash1	_AVLLTAVELLQEYDS[Phospho (STY)]NSDQSGSQEK_.3	P59808_S692_M1_Sash1	3.2729	4.0970	3.7227	4.0376	3.8270	3.9783	3.5001	3.5830	3.4887	3.2930	3.4878	3.9249	3.6255	3.7316	3.3272	3.9024	3.6066	3.9127	3.9085	4.2255
Arpc4	_ELLLQPVTISR_.2	#N/A	9.0382	8.7008	8.8375	8.9414	8.6451	8.8155	9.1712	9.1188	8.6313	8.7069	8.9248	8.6544	8.6894	8.7982	8.0594	8.1647	8.9130	8.9566	8.4948	8.4895
Arpc4	_VLIEGSINSVR_.2	#N/A	6.9072	7.0659	7.1356	6.8472	6.5040	6.7784	7.1777	6.8074	7.1696	6.9657	6.5082	6.5613	6.6603	6.4151	5.0898	5.9388	6.7141	6.8580	6.5099	6.1907
Arpc4	_AENFFILR_.2	#N/A	5.7851	6.2161	5.7797	6.7166	5.7030	5.9130	6.5373	4.5201	5.8555	5.8381	5.2571	6.2365	5.8299	5.0366	6.5407	5.0273	5.5738	6.4705	5.5518	6.3435
Arpc4	_IVAEEFLK_.2	#N/A	4.2968	4.4498	3.9259	5.2844	4.2089	4.5866	4.0062	3.2466	4.4064	4.5044	3.1131	3.3652	4.1264	3.8096	4.3923	4.0362	3.3328	4.1204	4.9099	4.1574
Arpc4	_ATLQAALC[Carbamidomethyl (C)]LENFSSQVVER_.2	#N/A	4.2013	4.6884	4.8329	3.2133	3.4477	4.0001	4.0139	5.0988	4.5309	3.7414	3.8100	3.7562	4.7308	4.8956	3.3253	3.2930	4.4730	3.1190	3.8258	4.8051
Rims4	_QASQLS[Phospho (STY)]LESTVGPC[Carbamidomethyl (C)]GERS_.2	#N/A	5.5648	5.8725	3.9533	3.7726	3.8104	4.4812	5.1053	3.9483	5.1238	5.8665	4.3176	4.8710	3.8604	5.5039	4.1842	5.0053	3.8720	3.8555	3.3551	3.6759
Rims4	_QASQLS[Phospho (STY)]LESTVGPC[Carbamidomethyl (C)]GER_.2	#N/A	3.4785	5.3789	6.0522	4.1480	3.9171	5.1967	3.3683	3.8303	3.7569	5.0867	3.7159	3.6089	3.2610	5.3022	3.2661	3.6835	3.7252	4.8825	4.8998	3.9112
Plp1	_TSASIGSLC[Carbamidomethyl (C)]ADAR_.2	#N/A	12.7840	13.1506	13.2042	12.8829	12.3383	13.0551	12.9461	12.9225	13.0411	12.2595	12.4387	12.7882	12.8341	12.5876	12.5713	12.3270	13.0402	12.9068	12.7216	12.4494
Plp1	_M[Oxidation (M)]YGVLPWNAFPGK_.2	#N/A	12.0002	11.9374	11.6557	11.7927	11.6377	12.0065	11.8115	11.8341	11.5585	11.3614	11.3385	11.7034	11.6869	10.9424	10.8772	11.0001	11.6395	11.7232	11.5867	11.3478
Plp1	_LIETYFSK_.2	#N/A	10.8306	10.8646	10.9869	11.2289	10.4525	11.1747	11.2536	10.1978	11.1265	10.8177	10.8616	10.7933	11.1955	10.3744	10.2825	10.5379	10.1927	11.0669	11.0489	10.6907
Plp1	_VC[Carbamidomethyl (C)]GSNLLSIC[Carbamidomethyl (C)]K_.2	#N/A	10.4345	10.7973	10.7967	10.7491	10.2637	10.8395	10.8111	10.4012	10.5226	10.3247	10.3697	10.3550	10.1086	10.3154	9.9758	9.9552	10.6411	10.5986	10.3276	10.0979
Plp1	_GLLEC[Carbamidomethyl (C)]C[Carbamidomethyl (C)]AR_.2	#N/A	10.2692	10.6400	10.2334	10.3931	10.0555	10.4236	10.4427	10.2049	10.2303	9.9810	10.0202	10.2897	10.2410	9.8083	9.7675	9.4493	10.3261	10.2146	9.9847	9.9029
Plp1	_QIFGDYK_.2	#N/A	9.9091	10.0349	9.9240	9.9622	9.8221	10.0351	10.3692	9.8023	10.1308	9.9149	9.9659	9.8955	10.2998	9.2812	9.5337	9.5872	9.2004	10.1743	9.8976	9.7997
Plp1	_VC[Carbamidomethyl (C)]HC[Carbamidomethyl (C)]LGK_.2	#N/A	8.1111	8.7128	8.5652	8.3595	8.1254	8.6965	8.6774	8.3645	8.5336	7.8672	8.1201	8.4156	8.2160	7.6743	8.4842	7.5953	8.2492	8.3786	8.3387	7.9165
Plp1	_WLGHPDK_.2	#N/A	7.5154	7.1290	7.8039	7.4573	7.1757	7.5894	8.0409	8.1074	7.6729	7.3485	7.5518	7.8211	7.3307	7.1167	7.2179	7.2019	7.5747	7.9242	7.7396	7.1421
Plp1	_GQHQAHSLER_.2	#N/A	10.6010	10.4366	11.4503	10.3350	10.1738	10.7722	10.3406	11.1583	10.4965	10.4639	11.0131	10.1493	10.2649	9.8074	9.7159	10.3681	11.0384	10.1378	10.3864	10.2003
Plp1	_GLSATVTGGQK_.2	#N/A	10.6785	10.7764	10.6734	10.6298	10.2009	10.7484	10.7962	10.5227	10.5978	10.2993	10.2094	10.5019	10.2507	10.0870	10.1338	10.0542	10.5433	10.5736	10.4198	10.1276
Pcbp1	_LVVPATQC[Carbamidomethyl (C)]GSLIGK_.2	#N/A	7.5067	7.9500	7.7924	7.9601	7.5676	7.7755	7.8469	7.7291	7.7830	7.5490	7.3902	7.9435	8.2126	7.9467	8.1322	7.6651	7.6299	7.7080	7.8091	7.7006
Pcbp1	_AITIAGVPQSVTEC[Carbamidomethyl (C)]VK_.2	#N/A	7.3480	7.1331	7.5367	7.3212	7.3186	7.4499	7.4069	7.1831	7.2389	7.3378	7.3084	6.9906	7.3490	6.9976	7.0018	7.3620	7.6893	7.0602	6.9930	7.3758
Pcbp1	_IITLTGPTNAIFK_.2	#N/A	6.7002	6.6641	6.7037	6.9350	6.8702	6.9457	6.9350	6.9970	6.9962	6.5772	6.5672	6.8537	6.9367	6.3611	6.6871	6.7486	6.4674	6.9253	7.0566	6.5202
Pcbp1	_IANPVEGSSGR_.2	#N/A	7.0291	7.2637	6.8051	6.7157	6.6089	6.8282	7.3202	6.8934	7.0215	7.2233	6.8875	6.8810	7.2986	6.6789	7.2314	6.9475	6.7618	6.9040	7.0157	6.6909
Pcbp1	_QGANINEIR_.2	#N/A	5.0926	5.3811	3.6235	5.0299	3.9258	5.0767	5.0561	4.2884	5.6644	5.6592	5.0506	4.7798	4.9750	5.4277	5.0130	4.9214	4.9166	4.9638	4.9627	5.0666
Pcbp1	_QVTITGSAASISLAQYLINAR_.2	#N/A	3.7145	4.2840	4.1653	3.7239	3.0564	2.7959	3.4437	2.4947	3.6198	4.1565	2.8450	4.0960	3.4447	4.1771	3.9538	3.9821	3.5480	4.1511	3.3708	3.7148
Pcbp1	_QIC[Carbamidomethyl (C)]LVM[Oxidation (M)]LETLSQS[Phospho (STY)]PQGR_.2	#N/A	7.1711	7.8820	5.2828	3.6095	3.5235	3.5857	7.6534	8.7179	4.3631	4.7247	3.6753	6.9075	7.0478	9.1232	3.7465	3.8469	4.1402	6.7854	6.1141	3.1236
Ppfia3	_GEGPAVPGDT[Phospho (STY)]PPPTPR_.2	P60469_T714_M1_Ppfia3	13.5405	13.3601	13.4539	13.3630	13.0485	13.0651	12.8888	13.6855	13.0557	13.4698	13.3072	13.2515	13.8712	13.5811	12.5439	13.2951	13.7672	13.1686	13.2385	13.2137
Ppfia3	_LAPPS[Phospho (STY)]PAR_.2	P60469_S683_M1_Ppfia3	10.6452	10.6182	10.1979	10.7356	9.9533	9.9358	10.1794	9.9852	9.8770	11.1019	10.3321	10.1063	10.1798	10.3394	9.0164	10.2625	9.9990	10.2132	10.2544	10.1654
Ppfia3	_QAQS[Phospho (STY)]PGGVSSEVEVLK_.2	#N/A	9.7884	9.6762	9.9002	9.8292	9.1286	9.3152	9.1916	9.5442	9.3843	9.7692	9.3813	9.4288	10.0155	9.9343	8.9692	9.5177	9.7845	9.3748	9.4792	9.3942
Ppfia3	_RGS[Phospho (STY)]ALGPDEAGGELER_.3	#N/A	7.7924	8.0957	7.8031	8.4941	7.3304	7.9794	8.3414	7.1501	7.4715	8.8393	8.0918	7.7441	7.9384	8.3468	6.5491	7.8851	7.3704	8.0873	7.9971	8.1629
Ppfia3	_YSQAPTLPSGAPLDPYGAGS[Phospho (STY)]GR_.2	P60469_S537_M1_Ppfia3	8.0750	3.7439	3.2944	7.3393	6.0936	3.8207	3.9444	6.9235	5.9953	4.4652	6.5932	3.6333	7.1812	8.0223	3.5216	3.1030	4.4970	3.3458	3.6449	3.3326
Ppfia3	_VSSSGLDS[Phospho (STY)]LGR_.2	P60469_S645_M1_Ppfia3	8.5948	8.0399	8.1808	7.9899	7.7284	7.2918	7.8984	7.8994	7.6298	8.4749	7.8975	7.8548	8.3897	7.9093	8.6406	7.8004	7.9572	7.7493	7.5602	7.5052
Ppfia3	_ESVSLAGTPSDETLATDPLGLAK_.2	#N/A	3.6588	5.3565	4.0740	3.6074	4.0120	4.6221	3.8720	4.7157	4.9456	4.2251	4.3268	4.5784	4.8076	4.5996	4.3283	3.7640	4.8596	4.6575	5.1262	4.4132
Ppfia3	_GS[Phospho (STY)]ALGPDEAGGELER_.2	#N/A	6.0220	4.3183	6.9670	6.0844	6.0212	6.0668	5.7270	3.3849	5.8044	6.9853	5.9801	5.4187	6.6492	4.2079	4.0961	6.4660	3.9225	5.8852	4.9122	6.4050
Ppfia3	_TAELEEALER_.2	#N/A	5.8792	5.5522	4.1347	4.4869	4.5153	5.0870	5.6213	6.2759	5.6474	5.4117	5.0514	5.6113	3.1282	4.9304	5.3012	5.5186	4.3210	5.6472	5.4767	5.8693
Ppfia3	_S[Phospho (STY)]LPGSALELR_.2	P60469_S508_M1_Ppfia3	12.1577	11.8910	11.6355	12.2728	3.2742	11.4938	11.5710	11.1451	11.3335	12.1833	3.2097	11.3940	11.9953	11.9212	10.4539	11.9456	11.7270	11.4925	11.6883	11.6751
Ppfia3	_M[Oxidation (M)]AQALALQAGS[Phospho (STY)]PEDGAPPR_.2	#N/A	9.7247	10.0747	9.8133	9.2794	7.7737	9.3380	9.6635	9.1755	9.2751	9.5583	8.4787	9.3448	9.0813	10.4771	8.3411	9.2179	9.6338	9.6866	9.4655	9.2987
Ppfia3	_RQAQS[Phospho (STY)]PGGVSSEVEVLK_.3	#N/A	3.2583	3.2437	3.7946	4.4311	4.0969	4.5600	3.4442	4.4595	3.5033	3.4646	3.8777	3.1331	3.6114	3.7817	3.2483	3.6032	3.4963	3.8460	3.9854	5.1473
Ppfia3	_QM[Oxidation (M)]S[Phospho (STY)]PLEQLQEEELQTAHR_.3	#N/A	6.0379	5.2729	3.4855	3.3949	5.1926	3.9356	3.9998	4.1601	5.7011	5.5634	5.5408	4.6980	4.1089	5.6523	5.0260	6.0180	4.6072	5.7663	6.1066	6.0400
Ppfia3	_WSGAKDESS[Phospho (STY)]KDWDR_.3	P60469_S554_M1_Ppfia3	4.0537	4.6581	3.5903	3.5305	4.2607	4.2940	4.2883	4.2794	4.2085	3.5067	3.6871	2.9915	4.3739	3.6284	2.8128	3.4159	3.7766	3.3728	3.6718	3.3057
Pdxp	_IVPGAPELLQR_.2	#N/A	8.3745	8.4596	8.2782	8.3380	8.4825	8.3661	8.4711	8.3284	7.9075	8.2039	8.1483	8.6040	8.4022	7.9440	8.5533	8.1555	8.1202	8.3143	7.9712	8.0277
Pdxp	_NTLFVSNNSR_.2	#N/A	7.6692	7.5023	8.1755	8.1856	8.1436	7.5246	7.6545	8.1491	7.6365	7.9881	7.8403	7.5856	7.9282	8.1355	7.6980	7.7207	7.7590	7.6356	7.7612	7.7498
Pdxp	_LAGDPGEDPR_.2	#N/A	6.9947	7.2052	7.0389	7.0299	6.6559	7.1557	7.2913	6.8152	6.9416	7.0568	6.6678	6.7140	6.9919	6.8051	7.2804	6.8661	6.6818	7.0726	6.6949	6.2424
Pdxp	_AEQLFSSALC[Carbamidomethyl (C)]AAR_.2	#N/A	6.5151	6.1315	6.1070	6.7718	6.2410	6.3145	6.2835	6.3037	6.7700	6.6280	5.9364	6.0116	6.2067	6.2029	6.5705	6.5985	5.8531	6.3766	6.5897	6.1353
Pdxp	_LSGPPDASGAVFVLGGEGLR_.2	#N/A	5.5185	5.6546	5.0806	5.7795	5.5017	5.1053	4.9881	4.9528	5.4705	5.7259	5.1993	5.6381	6.0177	5.2665	4.7899	5.4320	5.6090	4.4988	5.9134	5.8440
Pdxp	_TPGTGSLAAAVETASGR_.2	#N/A	6.3797	6.3269	6.9630	6.1343	6.4650	6.2949	6.2723	6.8781	5.5755	6.6370	6.1744	6.2985	6.1861	6.2711	6.7027	6.4106	6.5072	6.3661	5.8282	6.3575
Pdxp	_C[Carbamidomethyl (C)]GM[Oxidation (M)]TVLTLTGVSSLEEAQAYLTAGQR_.3	#N/A	3.3522	3.9669	4.3896	3.7552	3.5591	3.0484	3.2159	3.5779	3.6093	3.6929	1.6575	3.6930	3.3831	3.9800	3.5143	4.2152	3.6723	4.0743	4.3734	4.1050
Pdxp	_AVLVGYDEQFSFSR_.2	#N/A	3.8427	3.4763	3.3793	3.7415	4.4509	4.0830	2.9984	4.0684	4.4195	3.6533	3.8981	3.2025	4.5849	3.5867	3.0238	3.2049	3.5657	3.0309	4.1803	3.5537
Gabarapl2	_EDHS[Phospho (STY)]LEHR_.2	#N/A	5.2858	6.1823	5.9682	6.3439	5.7857	6.3678	5.5754	6.2924	5.4201	6.0692	6.0507	5.8379	5.4602	6.3324	6.0736	6.3058	6.1405	5.9416	6.1898	6.1088
Gabarapl2	_WM[Oxidation (M)]FKEDHS[Phospho (STY)]LEHR_.3	#N/A	4.4629	4.3570	3.9995	3.1213	3.1690	4.4385	4.3266	3.1877	4.3857	4.0830	3.2593	4.0832	4.4938	4.0375	3.9038	3.8250	4.1858	4.4645	3.2627	3.7148
Actb	_VAPEEHPVLLTEAPLNPK_.3	#N/A	13.2996	13.1930	13.3410	13.3051	13.0652	13.0567	13.1104	13.3516	13.2151	13.2920	13.1250	13.0598	13.5636	13.3605	13.1999	13.2840	13.2088	13.0046	13.1791	13.0418
Actb	_AGFAGDDAPR_.2	#N/A	13.6479	13.4999	13.6920	13.5462	13.4424	13.4677	13.8112	13.7393	13.6560	13.5437	13.4706	13.5793	13.4314	13.5474	13.4536	13.3256	13.3666	13.5366	13.2492	13.4539
Actb	_DSYVGDEAQSKR_.2	#N/A	12.1567	12.1517	12.1076	12.2352	12.0879	12.1149	12.2820	12.4609	12.3729	12.1659	11.8883	12.2861	12.4790	12.2355	12.2643	12.2692	12.1400	12.2214	12.1916	12.0961
Actb	_IIAPPER_.2	#N/A	12.2731	12.5915	12.4217	12.2654	12.3155	12.2584	12.7475	12.6168	12.0548	12.2485	11.6736	12.5049	12.3502	12.1754	12.6823	12.0592	12.0134	12.4405	12.2803	12.0797
Actb	_AVFPSIVGRPR_.2	#N/A	11.0672	11.0934	11.0738	11.2836	11.5108	11.1171	11.0627	11.2446	10.8229	11.2697	11.1358	11.1993	11.4019	10.7458	11.1082	11.1503	11.1177	11.0217	11.1143	11.1660
Actb	_DSYVGDEAQSK_.2	#N/A	11.9634	12.0003	12.2017	12.1306	11.8522	12.0464	12.0292	12.0894	12.2121	12.0608	11.9535	11.9548	12.0398	12.2185	12.1532	12.0379	12.0251	12.0372	12.1622	12.0701
Actb	_AVFPSIVGR_.2	#N/A	11.1614	11.0243	11.5049	11.4275	11.2497	11.2139	11.4362	10.7163	10.8567	11.2782	11.3852	11.2781	11.2389	11.0270	11.2501	11.0605	11.0316	11.2822	11.3772	11.3290
Actb	_C[Carbamidomethyl (C)]DVDIRK_.2	#N/A	10.5754	10.6675	10.8885	10.7218	10.6950	10.5916	10.9195	10.8096	10.9880	10.9358	10.7105	10.7166	10.9718	10.5456	11.3420	10.9110	10.3464	10.6393	10.7905	10.6689
Actb	_IKIIAPPER_.2	#N/A	9.1884	9.3486	8.4415	9.1621	9.1887	9.1518	9.3124	9.7236	8.9032	9.2331	8.6120	9.3189	9.9309	8.9987	8.6239	9.1724	9.2658	9.0120	9.1538	8.8439
Actb	_GYSFTTTAER_.2	#N/A	12.3213	12.5266	12.8725	12.5894	12.3045	12.3839	12.6347	12.5000	12.6391	12.3807	12.2070	12.2179	12.4274	12.6040	12.4380	12.4615	12.1398	12.4225	12.2500	12.3229
Actb	_TTGIVM[Oxidation (M)]DSGDGVTHTVPIYEGYALPHAILR_.3	#N/A	9.1062	8.0056	7.7356	7.9874	8.3458	7.6133	7.7174	9.3925	7.9060	8.8310	7.6425	8.6579	9.0760	8.2305	8.3425	8.7543	7.9657	8.8081	7.8736	8.5966
Actb	_KDLYANTVLSGGTTM[Oxidation (M)]YPGIADR_.3	#N/A	7.4267	7.8181	8.2757	8.3242	7.2280	7.6709	7.6774	8.7636	7.5430	7.7177	7.7970	7.4324	7.6310	8.3097	7.6198	8.6814	7.4781	7.2843	7.4798	7.3898
Actb	_[Acetyl (Protein N-term)]DDDIAALVVDNGSGMC[Carbamidomethyl (C)]K_.2	#N/A	7.3169	11.6770	12.0649	10.3424	9.4976	9.9833	11.7367	10.9161	9.9733	8.5532	10.5113	10.6242	11.0179	11.8073	8.7865	9.4033	11.2382	11.4897	9.2926	10.8011
Actb	tyl (Protein N-term)]DDDIAALVVDNGSGM[Oxidation (M)]C[Carbamidomethyl (C)]KAGFAGDDr	#N/A	8.4629	8.4348	8.9894	8.7044	7.7610	8.1974	8.4292	8.4197	8.3252	8.1547	8.1378	8.2950	8.6400	8.7659	7.6981	7.8678	8.8866	7.8759	8.0210	7.8535
Actb	_LC[Carbamidomethyl (C)]YVALDFEQEM[Oxidation (M)]ATAASSSSLEK_.3	#N/A	9.4656	9.1955	9.7993	9.4770	8.7944	8.8916	8.6753	9.2113	8.7212	9.4956	8.6230	8.8961	9.5120	9.8445	8.6474	9.2215	9.2502	8.5656	8.9573	9.2325
Actb	_EKLC[Carbamidomethyl (C)]YVALDFEQEM[Oxidation (M)]ATAASSSSLEK_.3	#N/A	7.6916	7.2068	7.2047	7.3205	6.4594	6.7172	6.6844	7.8919	6.2946	7.6023	6.1453	6.4233	8.2186	7.9029	6.0439	6.5253	7.2990	6.5118	7.0209	7.2501
Actb	_EITALAPSTM[Oxidation (M)]K_.2	#N/A	12.4584	12.5301	12.6627	12.4302	12.5915	12.3609	12.6063	12.8153	12.4821	12.3797	12.3587	12.5682	12.7835	12.4544	12.7472	12.3299	12.4237	12.5731	12.4839	12.3085
Actb	midomethyl (C)]PEALFQPSFLGM[Oxidation (M)]ESC[Carbamidomethyl (C)]GIHETTFNSIM[Oxida	#N/A	3.7743	6.9865	3.3108	6.7757	4.7382	5.3230	4.5491	6.1450	4.8659	5.8129	6.4751	5.7256	7.5630	6.6020	4.4997	5.5638	6.0625	5.8647	5.6290	6.2079
Actb	_HQGVM[Oxidation (M)]VGM[Oxidation (M)]GQK_.2	#N/A	8.5559	8.6410	8.3992	8.2212	8.6077	8.5087	8.6680	8.5769	8.5789	8.4334	8.2731	8.4724	8.2948	8.4859	8.8786	8.4014	8.1902	8.3531	8.4941	8.4753
Actb	_DLYANTVLSGGTTM[Oxidation (M)]YPGIADR_.2	#N/A	10.7765	10.9388	10.8367	10.7074	10.4835	10.6649	10.8390	10.8525	10.7936	10.9033	10.5195	10.6561	11.3569	10.7965	10.6987	10.8223	10.8419	10.6590	10.7311	10.6593
Actb	_IWHHTFYNELR_.3	#N/A	10.5379	9.3668	4.0738	9.8427	10.5378	9.3905	8.8790	10.7028	9.1665	10.1812	9.9643	9.8949	9.5152	6.7291	9.1249	10.3810	10.3775	9.2251	10.0105	10.0931
Actb	_QEYDESGPSIVHR_.2	#N/A	10.7502	10.6071	10.9781	10.7847	10.5781	10.5106	10.6968	10.8099	10.8540	10.8126	10.6803	10.6907	10.9754	10.8314	10.8936	10.8350	10.5940	10.5825	10.7425	10.7073
Actb	_QEYDESGPSIVHRK_.3	#N/A	4.8265	4.9869	4.1079	5.1491	5.4173	4.7182	5.6058	5.6669	4.9499	5.7163	4.6696	5.5290	6.1530	5.3563	5.2866	5.5043	4.4591	5.0409	5.3532	5.1276
Actb	_DLTDYLM[Oxidation (M)]K_.2	#N/A	10.7958	10.6125	10.2542	10.4868	10.8765	10.5295	10.7947	10.6472	10.7131	11.0911	10.6519	10.7977	11.3648	10.2515	10.2720	10.9831	10.4522	10.9992	10.9202	10.9838
Actb	idomethyl (C)]PEALFQPSFLGM[Oxidation (M)]ESC[Carbamidomethyl (C)]GIHETTFNSIM[Oxidati	#N/A	6.1366	6.2813	5.2585	5.4313	4.7963	5.7932	5.4984	6.1202	5.0984	6.2272	5.2131	5.5780	6.4752	6.4552	5.2578	5.5402	5.7219	5.9794	6.0711	5.9413
Actb	_DLTDYLM[Oxidation (M)]KILTER_.3	#N/A	3.3738	3.5983	3.9101	2.9987	4.2124	4.4365	4.4199	2.8976	4.1127	4.1080	3.7621	3.7096	3.3946	3.7519	2.9059	3.0140	4.1663	4.2213	3.1131	3.2660
Actb	_S[Phospho (STY)]YELPDGQVITIGNER_.2	P60710_S239_M1_Actb	8.7954	8.6664	9.1541	8.8086	6.8474	8.8454	8.5043	8.1912	8.6367	8.8813	8.7082	8.2140	8.3442	12.3103	8.1401	8.5256	8.9677	11.9406	8.5894	8.4684
Nrgn	_KGPGPGGPGGAGGAR_.2	#N/A	8.1571	8.7049	8.4299	8.4964	8.5808	8.0403	8.0627	8.3992	8.0304	7.7943	7.9926	8.4679	8.2625	8.7163	9.1300	8.5968	8.3427	8.1782	8.1966	7.9923
Nrgn	xidation (M)]DC[Carbamidomethyl (C)]C[Carbamidomethyl (C)]TESAC[Carbamidomethyl (C)]S	#N/A	4.9414	5.1010	5.0288	4.1769	4.6867	4.1670	4.8475	5.0056	4.7596	5.7157	4.8632	5.4531	4.0634	4.8814	5.6073	5.1256	5.4150	4.6614	5.1035	5.1346
Nrgn	_GPGPGGPGGAGGAR_.2	#N/A	3.7156	3.6034	3.2522	3.8686	3.9226	3.9559	3.1255	3.9413	3.0457	3.5262	4.0252	3.3296	3.2112	3.7138	3.1509	3.0778	3.4386	4.4047	4.0532	3.6808
Nrgn	_IKS[Phospho (STY)]GEC[Carbamidomethyl (C)]GR_.2	P60761_S48_M1_Nrgn	3.7231	4.2797	4.2594	3.9663	3.0608	3.4233	3.6581	3.4235	3.5784	4.4945	3.0570	4.1692	3.7438	4.2465	3.6835	4.0460	3.9611	3.8720	3.5206	4.2135
Cdc42	_QKPITPETAEK_.2	#N/A	7.7567	7.6507	7.8071	7.6155	7.9326	7.9639	7.9954	7.9325	7.7419	7.7773	7.7464	7.9084	7.9061	7.4936	7.5865	7.6133	7.7021	7.5936	7.5755	7.6120
Cdc42	_WVPEITHHC[Carbamidomethyl (C)]PK_.3	#N/A	5.0033	6.2546	6.3224	6.8233	6.8217	5.3211	6.9138	6.4830	6.6016	6.3582	7.0452	4.9202	6.5779	6.0689	7.1115	6.6157	6.0379	6.3011	6.0901	6.3430
Cdc42	_TPFLLVGTQIDLR_.2	#N/A	5.6000	6.1483	6.5763	5.1041	6.2967	5.6061	5.6104	5.6304	5.9803	6.1988	6.3234	4.9200	5.8948	5.6436	5.4175	5.0609	6.0042	5.3268	5.7605	5.3354
Cdc42	_TC[Carbamidomethyl (C)]LLISYTTNK_.2	#N/A	5.2710	5.2409	5.7086	5.4773	3.7322	4.1510	4.7499	5.3475	5.5713	5.1145	5.2382	4.8295	5.3869	5.6170	5.4648	5.2268	5.6915	5.5766	5.5677	5.2083
Cdc42	_TPFLLVGTQIDLRDDPSTIEK_.3	#N/A	4.5657	4.5457	3.3105	3.8103	3.4507	4.1453	3.6377	3.9996	3.8376	3.2712	4.0711	3.3266	3.8048	3.3486	3.2149	4.7623	3.2262	4.3722	4.8057	4.4143
Cdc42	_YVEC[Carbamidomethyl (C)]SALTQK_.2	#N/A	3.4428	6.3224	3.9791	5.5181	4.2814	5.3264	3.2597	3.1432	3.3188	7.0367	3.6932	5.8736	3.4269	3.9662	5.5374	3.7877	3.6808	4.0305	3.8009	4.1488
Cdc42	_LRPLSYPQTDVFLVC[Carbamidomethyl (C)]FSVVSPSSFENVK_.3	#N/A	3.7530	4.2498	4.2893	3.9364	3.0907	3.3934	4.4504	3.4534	3.6083	3.9593	3.0869	4.1393	3.7737	4.2764	4.0276	4.0979	3.9910	3.8421	3.4907	4.2434
Cirbp	_S[Phospho (STY)]QGGSYGYR_.2	P60824_S146_M1_Cirbp	4.3655	4.4544	6.3290	6.4744	3.0716	5.4610	6.1974	4.7363	3.8967	6.4982	3.3754	4.1806	4.0622	5.3625	4.0019	3.7276	4.0884	5.0806	3.2022	6.2800
Ensa	_KSS[Phospho (STY)]LVTSK_.2	P60840_S109_M1_Ensa	5.2436	3.5632	6.8123	4.1442	6.6057	6.7478	7.0867	5.1578	7.1336	7.2873	7.2496	6.3910	7.3127	6.7433	7.0078	6.9767	6.8077	6.8562	7.4974	6.9966
Ensa	_[Acetyl (Protein N-term)]S[Phospho (STY)]QKQEEENPAEETGEEK_.2	P60840_S2_M1_Ensa	4.1311	3.1880	3.6677	3.4136	4.3380	4.3714	4.2109	3.9762	4.1311	2.5832	3.6097	4.4149	3.2276	3.2984	4.2363	3.4932	4.5139	3.3193	2.9679	3.2654
Eif4a1	_ATQALVLAPTR_.2	#N/A	6.6670	6.6788	5.9153	6.5221	5.5856	6.3110	6.4637	6.3961	6.1297	6.3080	5.6910	5.9592	5.5490	6.5626	5.8182	5.8004	6.4496	6.3228	5.8292	5.1371
Eif4a1	_GYDVIAQAQSGTGK_.2	#N/A	4.2501	6.8698	7.4782	7.0502	6.4791	6.8341	6.7017	4.3152	7.0355	6.6317	4.3867	6.2981	6.4028	6.6521	6.3633	6.2535	6.6091	6.6190	6.6380	6.5981
Eif4a1	_GIDVQQVSLVINYDLPTNR_.2	#N/A	3.1129	4.2300	4.1503	4.4713	3.3198	4.6793	3.7283	3.3386	3.0357	4.4243	3.4101	3.6580	3.1438	4.1884	3.7537	3.9758	4.3366	3.5891	3.2079	1.5459
Lzts1	_FGFSQDS[Phospho (STY)]GRGK_.2	P60853_S50_M1_Lzts1	7.3417	7.2001	7.3992	7.3773	6.7975	3.4047	6.9528	6.6154	6.0042	7.4945	6.3818	4.0494	4.5276	7.0290	5.0217	4.1878	7.0850	4.4306	7.3364	6.6002
Lzts1	_ALSFS[Phospho (STY)]DGGSK_.2	P60853_S233_M1_Lzts1	3.6212	4.3816	4.1575	4.0682	2.9589	3.5252	3.5562	3.3215	3.4764	5.9288	2.9551	4.2711	3.6419	4.1445	4.1594	3.9661	3.8592	3.9739	3.6225	4.1115

Lzts1	_S[Phospho (STY)]FDEKEFASGQTFEERPR_.3	P60853_S283_M1_Lzts1	4.1581	4.7338	3.1936	3.5313	3.4958	3.7199	3.8883	3.8585	4.1043	4.3644	2.9779	4.9301	4.2124	3.1806	3.6224	3.0022	4.3962	4.4370	3.0856	3.1476
Rps20	_DTGKTPVEPEVAIHR_.3	#N/A	6.2679	6.4321	6.1665	6.3701	6.3990	6.5247	6.0919	6.6009	6.2554	6.0599	6.3437	6.2897	6.4331	6.7097	6.2264	6.1824	6.3376	5.9303	5.9160	5.7440
Rps20	_LIDLHSPSEIVK_.2	#N/A	5.4707	5.9197	3.7261	4.6728	5.3998	5.3865	6.0021	6.0511	5.2280	5.0901	5.4283	5.9151	4.5843	6.0681	6.0201	4.9570	5.9212	5.7928	5.3579	5.2092
Rps20	_VC[Carbamidomethyl (C)]ADLIR_.2	#N/A	4.4417	4.2209	3.1227	4.4515	3.7931	3.6923	3.2550	3.2113	3.8900	3.2979	4.1547	3.4591	3.3407	3.8014	4.7667	3.5289	3.5580	4.5913	4.0959	3.8104
Rps20	_TPVEPEVAIHR_.3	#N/A	4.3767	4.4432	3.9133	3.2140	3.2261	2.4735	1.8551	3.1016	2.9590	2.3982	2.8314	4.1693	4.4076	3.9514	4.6092	3.2144	4.0997	1.8110	3.3488	4.1751
Snap25	_RADQLADESLESTRR_.3	#N/A	5.8034	6.0640	5.3820	5.4966	5.7098	5.8175	6.0515	5.5258	5.6795	6.3353	5.6807	5.8985	6.3958	5.9869	5.8859	5.8879	5.6579	5.6837	6.0265	5.7688
Snap25	bamidomethyl (C)]GLC[Carbamidomethyl (C)]VC[Carbamidomethyl (C)]PC[Carbamidomethyl (	#N/A	6.9791	6.8697	7.1953	6.3888	6.6513	6.4623	7.0436	6.6616	6.3646	6.1757	6.4861	6.4872	5.9133	6.5612	6.9426	6.0058	6.6411	6.6609	6.1597	6.3968
Snap25	_ADQLADESLESTR_.2	#N/A	6.0982	5.6429	7.3895	5.6993	6.0461	6.7366	5.9016	5.8292	6.0261	6.4695	6.1976	5.8446	4.0873	5.5014	5.9110	5.3892	6.5408	6.1425	5.7318	5.9517
Snap25	_IEEGM[Oxidation (M)]DQINK_.2	#N/A	7.2492	7.3691	8.1903	7.9344	7.2927	7.5638	7.4122	6.9743	7.6700	7.5053	7.5443	7.3456	6.9311	7.7596	7.3324	6.9468	7.3597	7.4048	7.0364	7.5277
Snap25	_TLVM[Oxidation (M)]LDEQGEQLER_.2	#N/A	7.8787	7.9404	7.8697	7.8684	7.1945	7.6758	7.5637	7.6441	7.6159	7.7563	7.5916	7.3609	7.5546	8.3072	7.3093	7.3811	7.5184	7.4562	7.2488	7.5303
Snap25	_IDEANQR_.2	#N/A	5.3375	5.4597	6.1500	6.0220	5.8420	6.1425	6.4859	6.5358	3.3361	6.0318	5.2399	6.0324	6.2879	5.7661	6.6144	4.2882	5.9220	6.2561	5.8814	6.3672
Snap25	_EQM[Oxidation (M)]AISGGFIR_.2	#N/A	9.9599	9.4892	9.7522	9.5395	9.1771	9.2272	9.2534	9.3358	9.3178	9.6591	9.2210	9.1582	9.3054	8.9117	8.5099	9.1645	9.3933	9.3517	9.1063	9.1757
Snap25	_MLQLVEESK_.2	#N/A	3.4731	5.9805	7.4539	7.5356	5.7961	6.0851	6.4310	3.1334	5.0464	4.9707	6.9235	2.9584	3.4537	6.7823	5.9365	4.3361	5.3551	4.1621	5.3264	6.2555
Snap25	_AWGNNQDGVVASQPAR_.2	#N/A	8.8907	9.1553	9.2296	9.0429	8.7761	8.9808	9.1238	9.3108	9.0415	8.8377	8.7045	8.9754	9.2589	9.1035	8.8134	8.7267	9.2908	8.9876	8.9683	8.8848
Snap25	_RADQLADESLESTR_.3	#N/A	7.8210	4.0319	5.4711	3.7184	4.9602	5.6280	5.2492	4.9014	5.1143	5.5246	5.5287	4.6766	3.9917	5.5598	5.3595	5.2045	4.2090	3.6242	5.6071	5.7142
Snap25	_ENEM[Oxidation (M)]DENLEQVSGIIGNLR_.2	#N/A	8.1640	7.9677	7.9194	7.8160	7.5244	7.9343	7.7882	7.9599	7.5683	7.7480	7.6048	7.6892	7.7770	8.1591	7.3120	7.5601	7.8842	7.7215	7.7265	7.8857
Snap25	_NELEEMQR_.2	#N/A	3.5916	8.3882	4.3602	4.0978	2.9293	8.3437	3.1109	3.2919	3.1700	7.2109	4.2148	4.3007	3.2781	8.5868	4.1890	3.9365	7.8151	6.8418	2.9775	1.2251
Snap25	_ADQLADESLESTRR_.2	#N/A	4.3988	4.1624	4.7298	4.3544	5.0864	4.1248	4.8414	4.4360	5.3508	4.1407	4.7689	4.7256	5.3765	4.8643	4.9085	4.8073	4.5774	3.9989	4.7809	4.4763
Snap25	_HM[Oxidation (M)]ALDM[Oxidation (M)]GNEIDTQNR_.3	#N/A	6.1222	6.1693	6.2497	5.1742	6.0466	6.6449	6.4756	5.7947	6.8360	6.8836	6.3619	6.6827	7.2455	5.5551	6.8583	6.7357	5.9071	6.8151	6.7629	6.8418
Snap25	_M[Oxidation (M)]LQLVEESKDAGIR_.2	#N/A	6.0009	5.7887	5.0806	4.6401	5.3823	5.8711	5.3081	6.3227	5.5167	6.0537	5.2238	5.8368	6.7343	4.7594	5.7470	5.8449	5.5567	5.8453	5.2804	5.6829
Snap25	_[Acetyl (Protein N-term)]AEDADM[Oxidation (M)]RNELEEMQR_.2	#N/A	3.9144	4.0254	3.9118	4.5198	3.7475	3.9716	4.0354	4.0113	4.0171	4.2599	4.3201	3.9426	4.0377	4.9712	3.4477	3.2538	3.8932	3.4967	3.7957	3.7737
Snap25	_VVDEREQM[Oxidation (M)]AISGGFIR_.3	#N/A	3.7985	3.5206	3.3351	3.7857	4.0055	4.0388	3.0426	4.0242	4.4637	3.6091	3.9423	3.2467	7.0229	3.3732	3.0680	3.1606	3.5214	3.6280	3.9271	3.0504
Dnajc5	_EINNAHAILTDATKR_.3	#N/A	8.1768	7.7417	7.7497	7.9657	7.8101	7.8802	7.9592	7.9029	8.1328	7.5194	7.9118	7.7370	7.6006	7.4335	7.6861	7.6675	7.5883	8.1931	7.7287	7.4343
Dnajc5	_SLS[Phospho (STY)]TSGESLYHVLGLDKNATSDDIKK_.4	#N/A	4.2269	8.9752	3.4846	7.8578	3.9777	7.2725	4.1188	4.1063	4.0931	7.8401	6.5836	8.0604	4.2358	7.6706	3.1352	3.3015	7.7662	8.4070	8.1025	7.2438
Dnajc5	_NATSDDIKK_.2	#N/A	6.2904	5.5447	5.6358	5.3479	6.1079	5.7349	5.9117	6.7920	6.5970	6.3314	5.5047	5.1885	5.9624	4.9077	5.7707	5.8434	5.4573	5.7356	5.3217	6.1431
Dnajc5	_SLS[Phospho (STY)]TSGESLYHVLGLDK_.2	#N/A	12.4575	12.1869	12.0102	11.9338	11.3854	7.6523	12.1378	12.4985	11.7240	12.0348	11.8230	12.0435	12.6195	8.3275	10.9506	11.7679	12.2277	11.8230	11.8217	11.6020
Dnajc5	_EINNAHAILTDATK_.2	#N/A	3.4186	3.5858	4.4560	4.7513	3.7654	4.5728	3.7534	3.6443	3.9270	3.2292	4.1080	3.7947	3.5082	3.5205	3.6764	2.5817	4.3129	4.6062	4.3070	4.2860
Dnajc5	_APEGEETEFYVS[Phospho (STY)]PEDLEAQLQSDER_.3	P60904_S151_M1_Dnajc5	6.0210	6.6740	5.8143	5.7300	4.0475	6.0642	6.4550	4.4316	6.4038	5.9426	5.2211	5.5712	5.6773	5.8814	5.2940	3.6754	5.6432	6.3116	5.8991	5.0804
Dnajc5	_QRS[Phospho (STY)]LS[Phospho (STY)]TSGESLYHVLGLDK_.3	P60904_S10_M2_Dnajc5	4.2881	3.7147	3.3235	3.4013	3.6258	4.3592	4.2231	3.9884	4.1433	4.5450	3.6220	3.6042	4.3088	3.3106	4.2485	3.1321	4.5261	3.3070	2.9556	3.2776
Dnajc5	_YHPDKNPDNPEAADKFK_.4	#N/A	3.2921	3.2098	3.8284	3.9515	3.8209	3.8542	3.2272	4.4933	3.1474	3.4246	4.1269	3.0993	3.3128	3.8155	3.2526	2.9761	3.5302	4.3030	5.1575	3.7825
Rab5b	_GVDLHEQSQQNK_.2	#N/A	4.6990	4.4384	4.6153	4.6326	4.0129	4.4322	4.4228	5.4708	5.2102	4.9075	4.0921	4.4276	5.0686	3.3943	3.5517	4.7242	5.2282	4.3952	4.4236	3.6970
Rab5b	_GAQAAIVVYDITNQETFAR_.2	#N/A	7.1121	6.9952	6.5113	7.0103	6.5655	6.4802	6.7297	6.8967	6.3693	6.7611	6.2322	6.2067	7.6528	7.0709	5.3126	6.5796	7.0170	6.6835	5.9199	6.3786
Chp1	_DELLQVLR_.2	#N/A	3.9971	4.2306	3.0190	4.7128	4.4430	4.2374	3.0401	4.2228	4.0836	3.8077	4.1316	4.1615	4.4306	3.6278	3.5424	3.2322	3.7200	4.0971	4.1198	4.2236
Chp1	_IINAFFSEGEDQVNFR_.2	#N/A	3.5993	4.0796	4.2769	5.3391	4.8606	3.1611	3.8517	3.4652	3.1623	4.7286	3.5367	3.8453	3.2704	4.3150	4.1813	3.9442	4.4633	4.1870	2.6169	5.1286
Chp1	_ETGFSHSQITR_.3	#N/A	3.9951	3.3240	3.5317	3.5891	4.2021	4.2354	4.3469	4.2208	4.2671	3.8057	3.7457	3.0501	4.4325	3.5698	2.8714	3.3572	3.7180	3.1833	3.7305	3.4013
Rab10	_AFLTLAEDILR_.2	#N/A	7.3881	7.7171	7.1331	7.2335	7.1520	7.3696	7.3340	7.5551	7.5213	7.1448	7.3548	7.0928	7.0660	6.7916	7.0484	6.6390	7.1145	7.3523	7.6752	6.6093
Rab10	_GKGEQIAR_.2	#N/A	5.5961	6.3824	6.4293	6.4902	5.9256	5.3143	6.3880	6.1421	6.2778	5.8677	6.6454	5.3330	5.4690	5.5357	6.5502	6.0182	5.4556	6.4653	6.4172	5.7152
Rab10	_EPNSENVDISS[Phospho (STY)]GGGVTGWK_.2	#N/A	3.8646	6.8938	3.4012	3.7214	4.3299	6.6323	3.8914	4.0903	6.8859	6.4299	3.9757	5.9677	3.8955	3.4393	5.3643	3.2267	7.0563	6.9052	6.5978	3.1165
Rab10	_KTPVKEPNSENVDISS[Phospho (STY)]GGGVTGWK_.3	#N/A	3.9375	4.0653	4.4738	3.7519	3.2752	3.2089	5.6718	3.6378	3.7927	4.2801	3.2714	3.9548	3.9582	4.4608	3.8979	4.2824	4.1755	5.4729	3.3062	4.4278
Rab10	_ANINIEK_.2	#N/A	3.2093	3.1388	3.3878	4.3749	3.4163	4.4322	3.8919	3.9304	4.1661	3.2103	3.2895	3.3868	3.2402	3.5623	2.3124	3.7896	4.4331	3.8642	3.8221	3.5792
Rab10	_NIDEHANEDVER_.2	#N/A	5.2319	4.5375	5.0399	5.5643	4.9978	4.9371	4.0532	5.1866	3.2051	4.7746	5.5327	3.0415	3.3706	3.8732	5.1116	3.6948	3.5879	4.9692	5.0152	3.8402
Rab10	_TPVKEPNSENVDISS[Phospho (STY)]GGGVTGWK_.3	#N/A	4.4713	3.6387	4.0674	3.9038	3.2370	3.9194	5.1111	3.7301	4.7828	3.8829	3.5702	4.0777	3.8490	3.9140	4.2060	3.8930	4.4858	4.7863	3.8916	3.9381
Rab8b	_SSTNVEEAFFTLAR_.2	#N/A	4.5449	3.5129	4.7206	4.9529	4.5289	4.5530	4.5878	4.0754	3.2055	4.4193	4.1469	4.1844	3.8806	3.4243	3.2906	3.3340	3.9977	3.5768	3.8759	3.6311
Ube2d3	_SQWSPALTISK_.2	#N/A	7.7510	7.9340	8.0114	7.8760	7.8115	7.5593	7.7471	7.2871	7.7837	7.6854	7.7411	7.8287	7.9616	7.7614	7.8589	7.5342	7.7050	7.6837	7.3785	7.5751
Ube2d3	_ELS[Phospho (STY)]DLAR_.2	P61079_S11_M1_Ube2d3	3.9161	3.4030	3.4526	3.6682	4.1230	4.1563	4.4259	4.1417	3.2085	3.7267	3.8248	3.1291	3.9469	3.4907	2.9505	3.2782	3.6390	5.6756	3.8095	3.1680
Ube2d1	_ELS[Phospho (STY)]DLQR_.2	P61080_S11_M1_Ube2d1	5.7640	5.7301	5.7958	5.8211	5.2937	5.8396	4.5927	6.1379	5.2323	6.3049	5.6994	5.3621	3.6416	6.0706	5.2093	5.9265	5.8270	5.8712	5.6741	5.7999
Ube2m	_EAAEVLQNNR_.2	#N/A	9.4910	9.8831	9.6817	9.9001	9.6267	9.9344	9.5441	9.9896	9.7546	9.9836	9.7399	9.4938	9.7495	10.0903	9.5615	9.8265	9.9663	9.7457	9.8725	9.8714
Ube2m	_GGYIGSTYFER_.2	#N/A	4.7813	5.5361	4.0405	4.3564	5.1372	5.4988	5.1712	3.2287	5.0419	4.0421	5.7021	5.2086	4.5348	5.4628	5.6548	4.6933	5.6196	5.1118	5.2404	5.4932
Ube2m	_DINELNLPK_.2	#N/A	4.0193	4.1756	4.6745	4.5131	3.3570	4.1525	4.5844	3.7197	4.4080	4.1982	4.7180	3.8730	4.9544	4.5427	4.9084	3.7003	4.2573	3.7886	3.0410	3.0088
Ube2m	_TC[Carbamidomethyl (C)]DISFSDPDDLLNFK_.2	#N/A	3.9183	3.4008	3.4549	3.6659	3.4486	4.1203	4.4237	4.0821	4.3439	3.7289	3.8226	3.1269	4.5094	3.5112	4.1917	3.2804	3.6412	3.1065	4.2559	3.4782
Ube2n	_LLAEPVPGIK_.2	#N/A	9.3735	9.3704	9.3860	9.6122	9.5024	9.2204	9.4064	9.5621	9.2414	9.4917	9.2599	9.6904	9.3424	9.2054	9.8301	9.3857	9.4973	9.4792	9.4481	9.3552
Ube2n	_TNEAQAIETAR_.2	#N/A	9.0930	9.0608	9.4742	9.1986	8.9674	8.9014	9.0632	9.3311	9.0971	9.2470	8.8212	9.1319	9.2095	9.2209	9.4951	9.1394	8.9872	9.0895	8.9534	9.1290
Ube2n	_IYHPNVDK_.2	#N/A	7.8466	7.7354	8.0508	7.7673	7.9436	7.6147	7.9190	8.2507	7.8936	7.4817	8.0271	7.9362	7.8246	8.2701	8.6858	8.1521	7.8101	8.0665	7.9749	8.1960
Ube2n	_WSPALQIR_.2	#N/A	7.7710	7.6843	8.1076	8.0636	7.9024	7.6762	7.7172	7.8482	7.5165	8.0268	7.4259	8.0293	7.9185	7.8903	7.9559	7.8630	7.7288	7.7375	7.7415	7.8128
Ube2n	_YFHVVIAGPQDSPFEGGTFK_.3	#N/A	5.2770	6.1987	6.0361	5.7492	5.6106	5.4214	5.7638	5.6099	5.3768	5.6855	5.7789	5.6589	5.7747	6.2340	5.9617	5.6544	5.9498	5.7691	5.5206	6.1129
Ube2n	_LLAEPVPGIKAEPDESNAR_.3	#N/A	4.8631	5.3736	3.6375	4.8237	5.5599	5.3423	5.8646	6.0305	5.2191	5.3273	5.1749	5.6663	7.0440	6.0829	4.9751	5.6500	5.3005	5.1580	5.2347	5.0048
Ube2n	_LELFLPEEYPM[Oxidation (M)]AAPK_.2	#N/A	8.2070	8.0674	7.9070	8.0210	8.1412	8.0319	7.6183	8.0870	7.7953	7.9949	7.7651	8.0153	7.9694	7.6050	8.0294	8.0783	7.9412	8.0311	7.9332	8.0291
Ube2n	_AEPDESNAR_.2	#N/A	3.2123	3.6926	6.2314	3.9577	4.9820	6.5935	5.2357	5.6339	5.6093	3.4186	6.2978	3.1791	3.6574	3.2011	4.6674	6.3504	3.3494	3.8001	5.5631	5.8969
Ube2n	_TVLLSIQALLSAPNPDDPLANDVAEQWK_.3	#N/A	4.2915	3.7113	3.3270	3.3979	3.6292	3.8533	3.9118	3.9919	3.9709	4.4978	4.3453	3.6007	4.0790	3.3141	3.4890	1.5972	4.5296	3.3036	2.9522	3.2810
Actr2	_ILLTEPPM[Oxidation (M)]NPTK_.2	#N/A	8.6194	8.3404	8.0098	7.8734	8.5531	8.4568	8.3010	8.8131	8.2323	8.2514	8.0183	8.2612	8.6457	7.6646	7.9802	8.1313	8.1891	8.6592	8.3501	7.9821
Actr2	_HLWDYTFGPEK_.2	#N/A	5.7086	5.9940	5.9476	3.8357	5.4530	5.6615	6.1201	3.5541	6.0501	5.6438	5.7150	5.9294	6.3560	5.9399	4.3625	6.0232	5.0145	6.2910	5.3621	5.6000
Actr2	_DLM[Oxidation (M)]VGDEASELR_.2	#N/A	7.5958	7.1083	7.3114	7.3382	7.1819	7.3175	7.1363	7.2986	7.0131	7.2107	7.4452	6.9173	7.2978	7.0578	6.6821	7.1172	6.9737	7.2647	6.9512	7.0074
Actr2	_LC[Carbamidomethyl (C)]YVGYNIEQEQK_.2	#N/A	3.7150	6.6151	3.2516	6.1861	3.9219	6.2666	6.4181	3.9407	6.5079	3.5256	4.0259	5.4556	3.7459	3.2896	5.1655	5.7316	6.1181	6.4099	4.0106	6.2059
Actr2	_GYAFNHSADFETVR_.3	#N/A	3.9630	4.0398	4.4993	3.7264	3.3007	3.1834	3.8981	3.6634	3.8050	4.2545	4.5191	3.9292	3.9837	4.4864	4.5395	3.8060	4.2011	3.9899	3.2807	2.9526
Actr2	_HIVLSGGSTM[Oxidation (M)]YPGLPSR_.2	#N/A	3.3897	3.9293	4.4272	2.6160	3.0087	4.7238	3.5720	3.6154	4.4164	3.2003	3.8214	3.6555	3.4206	2.8468	3.4768	3.5262	4.5172	4.0368	4.3358	4.2466
Actr2	_LALETTVLVESYTLPDGR_.2	#N/A	3.5944	3.7247	3.1310	3.9898	2.8062	4.3070	3.9833	3.8201	3.5172	3.4507	3.8916	3.4508	3.6253	3.1690	3.0353	2.9565	3.3173	3.6897	4.1312	4.3472
Actr1a	_AQYYLPDGSTIEIGPSR_.2	#N/A	7.6614	7.9965	7.6977	7.5670	7.2280	7.5979	7.2733	7.6308	7.2500	7.5795	7.4257	7.1222	7.8110	7.7658	7.2272	7.4205	7.2545	7.2907	7.3674	7.0834
Actr1a	_DQLQTFSEEHPVLLTEAPLNPR_.3	#N/A	5.7983	5.5369	5.7855	5.9014	6.0863	5.7270	5.6512	5.8542	5.8165	5.8795	5.0415	5.5457	5.7529	6.1332	5.2203	5.9612	5.1310	5.4356	5.3574	5.4547
Actr1a	_AC[Carbamidomethyl (C)]YLSINPQKDETLETEK_.3	#N/A	4.3054	3.9013	5.7429	6.1742	3.0115	4.5430	4.8255	3.8019	5.5338	6.2829	3.4354	4.3508	4.1222	4.6249	5.7560	5.3375	4.3396	5.2448	4.2806	4.9744
Actr1a	_KEYEEDGAR_.2	#N/A	3.8181	3.5009	3.3547	3.7661	4.0251	4.0833	3.0230	4.0438	3.7410	3.2270	4.1153	3.2271	3.8490	3.3928	3.0484	3.1803	3.5410	3.6084	3.9074	3.0701
Arf3;Arf1	_NISFTVWDVGGQDK_.2	#N/A	8.0149	7.9307	8.3782	7.8960	7.6491	7.7798	8.1095	8.1014	8.2140	7.7938	7.9012	7.6664	8.3726	7.7020	7.1537	7.4766	8.1604	8.0259	7.8332	7.8232
Arf3;Arf1	_DAVLLVFANK_.2	#N/A	7.5361	7.5575	8.5335	7.5837	7.0086	7.9261	7.6763	7.2186	7.7236	7.4971	7.7313	6.9418	6.6039	7.6704	6.6676	7.2285	7.3159	7.6256	7.5264	7.4065
Arf3;Arf1	_LGEIVTTIPTIGFNVETVEYK_.2	#N/A	6.6623	7.0487	7.0810	7.0392	5.6895	6.7154	7.0992	7.0676	6.8970	6.6999	6.2543	6.2873	6.6078	7.1726	5.2462	5.9024	6.6958	6.5169	6.4928	6.3596
Arf3;Arf1	_M[Oxidation (M)]LAEDELRDAVLLVFANK_.3	#N/A	6.0994	6.5152	6.3917	6.3781	6.6104	6.6564	6.5199	6.5293	5.8803	6.4696	6.1655	5.9137	6.1556	5.1358	5.7566	6.1494	6.0803	6.4241	6.5068	5.8808
Arf3;Arf1	_M[Oxidation (M)]LAEDELR_.2	#N/A	6.4872	7.2781	7.6496	7.5188	6.6169	7.4781	7.5196	6.5194	6.8429	6.8702	7.5834	6.5126	6.2768	7.3812	6.7201	6.2185	6.4802	6.8110	6.8706	6.8805
Arf3;Arf1	_QDLPNAM[Oxidation (M)]NAAEITDK_.2	#N/A	7.8580	8.2606	8.7401	8.6116	7.7649	8.3012	8.2581	7.9384	8.2806	7.6848	8.4453	7.7340	7.9688	8.2914	7.6920	7.3902	8.2275	7.5933	7.6119	7.8880
Arf3;Arf1	_ILM[Oxidation (M)]VGLDAAGK_.2	#N/A	7.4118	7.9756	7.6399	7.9616	7.2469	7.6338	8.0516	7.5145	7.5911	7.2275	7.1470	7.5810	7.1992	7.4476	6.9742	7.0244	7.4915	7.9069	7.2279	7.4011
Arf3;Arf1	_HYFQNTQGLIFVVDSNDRER_.3	#N/A	7.0948	6.2721	5.5441	6.0382	7.0075	6.0376	5.6249	6.4862	6.5173	6.5960	6.9334	5.2141	7.0542	5.7211	4.9035	6.0666	6.2851	6.0412	6.0064	6.4196
Arf3;Arf1	_HYFQNTQGLIFVVDSNDR_.3	#N/A	5.2446	5.8885	5.8880	4.7735	3.8652	4.3650	3.1829	5.8953	4.6301	3.4688	4.0826	3.3870	3.2686	3.7712	4.8046	3.0204	5.2980	5.0769	3.9958	3.7382
Rap2b	_YDPTIEDFYR_.2	#N/A	7.6230	7.6195	7.8608	7.5815	7.5988	7.5881	7.3315	7.2822	7.5642	7.5045	8.0512	7.4891	7.4784	7.6475	7.5560	7.2492	7.4675	7.5578	7.2881	7.6458
Rap2b	_VVVLGSGGVGK_.2	#N/A	5.9248	6.6009	5.9161	6.8374	6.5743	6.4132	6.5974	6.3288	6.5214	6.4246	6.2736	6.6191	6.4455	6.3182	6.8219	6.8098	6.2278	6.9902	6.6795	6.6510
Rap2b	_ALAEEWSC[Carbamidomethyl (C)]PFM[Oxidation (M)]ETSAK_.2	#N/A	5.4770	5.1207	5.4316	5.6548	5.7514	5.0436	4.7739	5.8624	5.0059	5.3515	5.3694	4.5658	5.5491	4.9040	4.9549	5.6882	5.9258	5.8122	6.0441	5.0654
Rap2b	_ASVDELFAEIVR_.2	#N/A	3.7275	5.4712	4.4063	4.9666	4.5007	4.2687	4.8228	4.9092	4.4488	4.8609	4.4307	3.7252	4.9304	5.5153	4.7834	4.9621	5.3110	4.7148	4.9992	5.2226
Rap2b	_SALTVQFVTGSFIEK_.2	#N/A	4.0785	4.2975	3.7977	3.9027	4.2270	3.3532	4.9192	5.0302	5.1688	4.8926	4.4590	3.8137	4.2920	3.8357	4.7030	4.4311	4.3930	3.1654	5.0866	4.0115
Rap2b	_EIEVDSSPSVLEILDTAGTEQFASM[Oxidation (M)]R_.3	#N/A	3.6561	4.1067	4.6724	4.4015	2.9938	4.4338	3.4378	3.4084	3.8784	2.9745	3.2507	2.8713	4.5736	4.5060	3.1311	3.3085	4.4064	4.2438	3.1499	2.2949
Rpl26	_FNPFVTSDR_.2	#N/A	6.8350	7.1787	6.9703	6.9478	6.7854	7.0498	7.0883	6.8790	7.1577	6.5805	6.6757	6.9461	7.2278	6.8516	6.8218	7.0088	6.9812	7.0468	6.7963	6.7057
Rpl26	_YVIYIER_.2	#N/A	4.5580	4.2619	3.6161	4.5270	3.2641	5.0036	5.3872	3.2829	5.4703	4.2782	5.3217	3.8159	5.1677	4.8034	5.2473	5.7114	4.2809	5.3353	5.2188	5.1907
Stx1b	_TKQELEDLTADIKK_.3	#N/A	9.5470	9.1501	9.5256	9.0337	8.8427	9.1723	9.0959	9.1737	9.2454	8.7317	8.9216	9.1350	9.5974	8.6367	8.5354	8.8450	9.3480	9.2098	8.7911	8.9251
Stx1b	_LAIFTDDIK_.2	#N/A	7.7217	8.2011	7.8965	7.7462	7.7769	8.0242	8.1924	7.5927	8.0723	7.8245	8.1854	7.8243	8.1188	7.6001	7.2778	7.6379	7.7935	8.3316	8.0437	7.5619
Stx1b	_QLEITGR_.2	#N/A	7.0851	7.6682	6.9244	7.6196	7.4591	7.7762	8.1263	6.0840	8.1948	7.9720	7.8002	7.7111	7.6813	6.8795	7.3615	7.4617	7.1337	7.9882	7.9532	7.4597
Stx1b	_LSEDVEQVKK_.2	#N/A	8.1432	7.9457	8.1708	7.3778	7.8971	7.7208	8.2038	7.7310	7.6132	7.6216	7.4233	7.3876	7.8749	7.2795	7.4532	7.6108	7.7592	7.9515	7.4248	7.6206
Stx1b	_QALNEIETR_.2	#N/A	8.3341	7.5045	7.8814	7.2222	7.8703	8.0028	7.9045	7.8404	7.8978	7.3350	7.8769	7.2431	7.4446	7.3513	7.5322	7.1651	7.8410	7.5954	7.4737	7.3963
Stx1b	_LSEDVEQVK_.2	#N/A	6.1435	5.6556	5.3767	5.6940	4.3381	5.9095	5.7900	5.5711	5.4982	5.3123	5.6383	5.3162	5.2322	5.2713	5.3779	4.4980	5.4450	6.1018	5.1758	3.8119
Stx1b	_TQHSTLSR_.2	#N/A	7.1104	7.0168	7.2939	6.5443	6.9207	6.7246	7.0013	6.8656	6.1856	6.2396	6.8621	3.3739	6.3561	5.9054	7.1564	6.7227	5.6653	6.7133	6.9797	6.7554
Stx1b	_DHFM[Oxidation (M)]DEFFEQVEEIR_.3	#N/A	7.0355	6.4604	6.9804	6.5074	5.3499	6.4029	5.9258	6.3839	6.2610	6.6872	5.9283	5.9257	6.7229	6.5088	5.1425	6.1628	6.2357	6.1204	6.1133	6.0602
Stx1b	_TTTNEELEDM[Oxidation (M)]LESGK_.2	#N/A	5.8577	6.4727	6.2172	6.3108	5.6955	5.7032	6.1758	6.5232	6.3284	6.2336	5.8518	5.9829	5.5610	5.8146	5.2227	5.3247	6.5982	5.8779	5.4766	5.7958
Stx1b	_IEYNVEHSVDYVER_.3	#N/A	5.8100	5.7343	5.8802	5.1982	5.3482	5.5913	5.3844	4.8938	5.6571	5.4225	6.0974	5.0892	5.4997	5.1466	4.2840	5.2876	3.9267	5.2187	5.3936	5.1225
Stx1b	_SAKDS[Phospho (STY)]DDEEEVVHVDR_.3	#N/A	14.2248	14.1222	14.2484	14.1082	13.4853	13.9946	13.9815	13.6987	13.9239	13.9995	13.9647	13.7587	14.2319	13.9901	12.7479	13.5658	14.1970	13.9666	13.9442	13.7301
Stx1b	_AIEQSIEQEEGLNRS[Phospho (STY)]SADLR_.3	P61264_S108_M1_Stx1b	3.8264	3.5577	3.4945	3.2443	3.7968	4.8996	3.5246	4.1594	4.2221	4.1380	4.1411	4.1928	3.6634	4.9348	3.2770	3.3031	4.0644	3.2126	3.9411	3.4404
Stx1b	_KQHS[Phospho (STY)]AILAAPNPDEK_.3	#N/A	8.2652	8.1976	7.8909	7.8682	7.4279	7.9843	7.9793	7.6246	7.8564	7.4618	7.8725	7.6054	8.6414	7.7102	6.4673	7.6051	8.2545	8.1535	7.8149	7.5727
Stx1b	_AIEQSIEQEEGLNR_.2	#N/A	9.5863	9.6785	9.8766	9.5497	9.0165	9.5890	9.4720	9.6048	9.6668	9.2376	9.1320	9.0305	9.8456	9.3426	8.5534	8.9670	9.7112	9.5905	9.3819	9.2067
Stx1b	_LKAIEQSIEQEEGLNR_.3	#N/A	5.1684	5.7203	4.4644	5.3315	5.8588	4.7840	5.6441	6.0677	4.6753	5.5617	5.8018	5.7813	4.4424	4.7865	4.8462	5.2737	5.7911	5.8446	5.5101	4.3427
Stx1b	_QHSAILAAPNPDEK_.2	#N/A	5.2240	6.0766	6.4176	6.2975	6.0471	6.3570	6.3169	5.9445	6.3491	5.8317	6.2254	6.0982	5.2594	5.6985	6.1270	6.0347	5.7319	5.9547	6.0959	5.7409
Stx1b	_DS[Phospho (STY)]DDEEEVVHVDR_.2	#N/A	9.2338	8.6477	9.2897	8.7954	3.8770	8.9941	8.6972	7.7673	8.6631	8.6877	7.5065	8.2630	4.1932	8.7100	6.9216	8.1155	9.1215	8.3493	8.5972	8.3507
Stx1b	_TQELRS[Phospho (STY)]AKDS[Phospho (STY)]DDEEEVVHVDR_.4	#N/A	3.9244	3.7672	4.0614	3.7132	3.7472	3.6620	4.3538	3.9026	3.8127	3.8061	3.8035	3.5936	3.9500	4.0650	3.4573	3.8755	3.9361	3.5877	3.5897	6.0452
Stx1b	_FVEVM[Oxidation (M)]TEYNATQSK_.2	#N/A	3.4886	5.5269	4.2779	4.2773	4.3225	5.1556	3.2987	5.0510	4.7890	4.8438	4.0125	4.0180	3.4206	4.1935	3.8803	4.0958	4.1606	5.2579	4.2711	4.0097
Stx1b	_KFVEVM[Oxidation (M)]TEYNATQSK_.3	#N/A	3.7229	3.3188	4.7769	4.5062	4.8674	3.9632	5.4992	4.3844	3.0384	3.5335	4.0179	3.2083	3.2039	3.7065	3.1436	3.0851	3.4212	6.5002	4.0605	3.6735
Fgf12	_EPS[Phospho (STY)]LHEIGEK_.2	P61329_S212_M1_Fgf12	14.3403	14.1595	14.3561	14.3576	14.0981	14.1263	13.7559	14.2785	14.1087	14.1385	14.3055	13.9989	14.6553	14.5998	13.8489	14.2915	14.5711	14.0902	14.2653	14.1993
Fgf12	_VVAIQGVK_.2	#N/A	4.1177	3.8075	3.6543	4.4195	4.2425	2.7221	4.7292	3.4581	4.1598	4.1767	3.7330	4.7600	4.0038	3.6923	4.3503	4.2197	3.8406	4.8475	4.6327	4.1969
Fgf12	_KS[Phospho (STY)]SGTPTMNGGK_.2	P61329_S226_M1_Fgf12	4.2300	4.1234	4.3127	4.2358	3.0970	4.3117	5.0774	3.4915	3.6174	4.1352	3.3707	3.9012	3.7691	6.2843	4.0040	3.8668	4.8800	4.1259	3.1395	4.3031
Fgf12	_SRKS[Phospho (STY)]S[Phospho (STY)]GTPTMNGGK_.2	#N/A	3.6824	4.5738	5.2872	5.3671	3.2635	4.4586	4.3262	3.5736	3.7285	3.9567	4.2892	3.8413	3.8939	4.0799	4.3468	3.8355	4.4330	3.7634	3.4120	3.9990
Rpl27	_YSVDIPLDK_.2	#N/A	6.2565	6.3152	6.7337	6.3007	6.1823	6.5140	5.7726	6.0413	6.6173	6.7376	6.4140	5.4255	6.2330	6.5869	6.8131	6.4452	6.3084	5.9074	6.3581	6.3418
Rpl27	_NIDDGTSDRPYSHALVAGIDR_.3	#N/A	4.2691	3.3081	3.6415	4.1331	4.2545	4.2854	4.4142	4.1513	4.4556	3.8748	3.7458	3.6575	3.9565	4.0227	3.5218	4.2830	4.4623	4.0043	3.8556	3.3050
Smg6	_NWS[Phospho (STY)]GC[Carbamidomethyl (C)]GEGEQK_.2	P61406_S331_M1_Smg6	7.3114	6.6176	7.0550	7.0447	6.5954	6.4580	6.2990	6.3679	4.0856	3.9872	6.2115	6.2724	4.2510	3.7513	4.1908	5.7257	6.1225	5.6192	6.1977	6.4746
Snn	_LM[Oxidation (M)]TANS[Phospho (STY)]PEVHG_.2	#N/A	8.0812	8.0513	7.9767	8.0315	7.3300	7.1754	7.6429	7.8601	7.0949	7.2924	7.6043	7.0184	7.9454	6.4810	6.1820	7.0605	7.4554	7.3457	7.2578	6.9473
Abat	_GNYLVDVDGNR_.2	#N/A	12.1027	12.1670	12.1177	12.3040	12.0627	12.0469	11.9170	12.4504	12.1857	12.2584	12.0318	11.9108	12.4792	12.2621	11.4903	12.2322	12.3422	12.0395	12.1253	12.1066
Abat	_GTFC[Carbamidomethyl (C)]SFDTPDEAIR_.2	#N/A	8.5158	8.7010	8.2677	8.5577	8.0765	8.6414	8.3880	8.7797	8.6808	8.6113	8.4567	8.5808	8.5754	8.4352	8.5278	8.6293	8.7973	8.5122	8.6422	8.1407
Abat	_GVVLGGC[Carbamidomethyl (C)]GDK_.2	#N/A	7.6616	8.0196	8.0375	8.1729	8.0163	8.1604	8.5052	8.1758	8.3726	8.2608	8.2342	8.1637	8.0073	8.1423	8.3283	8.0916	7.9720	8.2715	8.2766	8.0603
Abat	_TVAGIIVEPIQSEGGDNHASDDFFR_.3	#N/A	8.2392	8.1084	8.0794	8.2284	7.7259	7.8672	8.0668	7.9565	8.0630	8.1315	7.9082	7.9857	8.2760	8.3762	7.5888	7.8507	7.7709	8.1029	8.1682	8.0075
Abat	_EDLLNNVAR_.2	#N/A	7.8362	7.8509	8.1485	7.8339	8.2630	7.7663	8.2416	8.1303	7.9116	7.7458	8.1017	7.8283	7.7229	8.0060	7.7492	7.8812	7.7356	8.0383	8.1306	7.9006
Abat	_IFNTWLGDPSK_.2	#N/A	7.4345	7.9721	7.9122	7.7980	7.8461	7.7695	8.0967	8.1478	7.8191	7.9287	7.7746	7.9101	7.4520	7.9437	7.5722	7.6949	7.6268	7.9183	7.7479	7.6084
Abat	_IDIPSFDWPIAPFPR_.2	#N/A	7.4950	7.9543	7.5270	7.6873	7.8257	7.5248	7.8059	7.5603	7.6772	7.5616	7.9673	7.9174	7.8853	7.6764	7.4200	7.6574	7.6219	7.6508	7.7949	7.4462
Abat	_LKYPLEEFTTDNQQEEAR_.3	#N/A	6.9929	7.7343	7.5850	7.3246	7.6102	7.6479	7.9030	7.6300	8.0882	6.9794	7.7661	7.5934	6.6500	7.2600	7.7784	8.0931	8.2261	7.7086	7.7817	7.4710
Abat	_M[Oxidation (M)]LDLYSQISSVPIGYNHPALAK_.3	#N/A	6.4025	6.3127	6.6102	6.4072	5.8885	5.5076	5.7363	6.3656	5.9853	6.0847	6.1782	5.9671	6.2671	5.3709	5.7182	6.5997	5.7611	5.8337	5.7726	5.8904
Abat	_NLLLAEVINIIK_.2	#N/A	5.9779	5.6039	6.1404	5.5363	5.7655	5.9478	6.2759	6.1774	5.3806	5.2278	5.7939	5.6419	3.4435	5.9273	5.2369	5.6994	6.0392	6.2405	5.9572	5.5526
Abat	_C[Carbamidomethyl (C)]LEEVEDLIVK_.2	#N/A	5.0695	4.7803	5.6208	4.7759	5.0937	5.4493	4.7785	4.9556	5.6092	5.3533	4.4977	5.0506	5.0151	4.9912	5.1090	5.3939	5.3154	5.0619	4.6882	5.0474
Abat	_TLLTGLLDLQAQYPQFISR_.2	#N/A	5.0608	5.7606	5.0320	4.9603	5.1954	5.5778	5.4678	5.9240	5.4983	5.9561	3.1907	5.9027	5.4274	5.9750	5.4221	5.4667	5.3201	5.9970	5.6160	5.0404
Abat	_EEFRPSAPYR_.3	#N/A	4.2449	5.0229	4.7376	5.9284	5.1725	5.4258	5.4232	3.0850	5.5325	6.1314	5.9673	4.7613	4.3910	5.5324	5.3504	5.8347	4.0831	5.5759	6.0260	5.9918
Abat	_REDLLNNVAR_.2	#N/A	6.4070	5.8901	4.6032	5.4864	6.0235	5.5197	5.5598	6.4030	4.1156	5.3145	6.0277	5.4729	4.1812	6.3088	4.6037	5.4358	5.6423	5.3196	5.0316	4.3842
Abat	_QLNTIQNAEAVHFFC[Carbamidomethyl (C)]NYEESR_.3	#N/A	4.0866	5.2432	3.7896	3.3312	4.9732	3.6484	4.1168	4.4787	5.5449	4.2929	5.2642	5.3747	5.2697	5.2034	4.6760	4.4315	5.2994	4.0378	4.8075	5.4357
Abat	_TM[Oxidation (M)]GC[Carbamidomethyl (C)]LATTHSK_.2	#N/A	5.6198	5.9458	6.3189	6.0910	5.8657	5.6233	6.1164	5.9814	5.8634	5.5318	5.3990	5.8840	5.3467	6.0929	6.0857	5.4582	5.2918	5.5136	5.5723	5.3205
Abat	_VDIEFDYDGPLM[Oxidation (M)]K_.2	#N/A	5.4674	5.6098	5.8824	5.9451	5.5121	5.9116	5.9548	5.5289	6.3183	6.0544	6.0248	5.2663	5.8451	5.7731	5.6021	5.9096	5.7752	5.9539	5.8005	5.5947
Abat	_YPLEEFTTDNQQEEAR_.2	#N/A	3.1859	3.3161	3.7222	4.5035	4.0244	5.5311	3.5166	4.3871	3.5757	3.3922	4.4241	3.2055	3.6838	3.7092	4.2181	3.5308	5.6702	4.4092	4.0578	4.6171
Abat	_GM[Oxidation (M)]SQLITMAC[Carbamidomethyl (C)]GSC[Carbamidomethyl (C)]SNENAFK_.2	#N/A	3.2320	3.2700	3.7683	4.4574	4.0706	4.2946	3.4705	4.4332	3.5296	3.4383	3.9040	3.1594	3.6377	3.7554	3.0477	3.5769	3.4701	4.3631	4.0117	3.7224
Ufm1	_VLSVPESTPFTAVLK_.2	#N/A	5.5696	5.6220	6.1995	6.1647	5.7393	6.0282	5.5498	5.4517	5.8532	5.6759	5.3437	5.6800	5.8496	5.1038	6.2112	5.9769	5.9188	6.0025	5.4784	5.5561
Ufm1	_ITLTSDPR_.2	#N/A	4.6582	5.0517	4.5130	5.5264	4.5895	5.2323	4.9608	4.7816	5.0523	5.4490	5.0827	5.0142	3.9975	5.7638	5.7528	4.9227	4.8876	4.7320	5.0231	5.1605
Dcaf7	_LEC[Carbamidomethyl (C)]LLNNNK_.2	#N/A	5.3453	5.7029	5.5717	6.3898	5.5128	6.0795	5.8571	6.5250	5.7379	5.7408	6.5419	5.8018	6.3392	5.9983	5.3646	5.6402	5.5808	5.6692	6.0795	5.9544
Dcaf7	_LALGSFVEEYNNK_.2	#N/A	5.8168	6.5492	3.6394	5.9496	4.8019	5.5864	5.7728	6.6054	5.4022	5.8161	5.8513	6.1368	6.6971	7.3648	5.7489	6.2426	6.5024	6.2089	5.6837	5.9686
Ap1s1	_AIEQADLLQEEDES[Phospho (STY)]PR_.2	P61967_S147_M1_Ap1s1	8.7020	9.5971	10.1470	9.4545	4.6293	9.7165	9.3167	6.0692	9.5827	9.3294	8.2103	8.8993	8.1793	9.5531	8.0996	8.6993	9.6897	9.3144	9.3431	9.1932
Hnrnpk	_TDYNASVSVPDSSGPER_.2	#N/A	8.6582	8.8438	9.0530	9.0574	8.3853	8.9841	8.6632	8.4713	8.9014	8.9032	8.3756	8.8346	8.6838	9.0641	8.8790	8.7570	8.6373	8.6045	8.6888	8.8688
Hnrnpk	_IILDLISESPIK_.2	#N/A	7.6401	7.3147	7.9596	8.0137	7.6671	7.6179	7.6415	7.7737	7.4230	8.0492	7.4891	7.8662	7.8393	7.7718	7.7447	7.8617	7.6904	7.4980	7.7724	7.4683
Hnrnpk	_GSDFDC[Carbamidomethyl (C)]ELR_.2	#N/A	6.4798	6.2973	6.5191	6.2483	6.8921	6.1041	6.5722	6.5133	6.4234	6.7258	6.5748	6.0157	6.7748	6.8866	6.5761	6.5707	6.2020	6.1648	6.3428	6.4788
Hnrnpk	_IITITGTQDQIQNAQYLLQNSVK_.3	#N/A	5.2823	5.7751	5.4705	5.6174	5.2483	5.8106	5.5230	5.6623	5.6945	6.4412	5.9015	5.4042	5.7969	6.7848	5.1141	6.1882	4.2216	5.1882	5.4603	6.7451
Hnrnpk	_DYDDM[Oxidation (M)]S[Phospho (STY)]PR_.2	#N/A	12.0184	12.0252	11.5734	12.0840	11.8136	11.6717	11.6702	11.8773	11.7143	11.9564	11.7898	11.8953	12.3156	12.2544	11.5694	11.9685	11.8151	11.8180	11.8358	11.9605
Hnrnpk	_LFQEC[Carbamidomethyl (C)]C[Carbamidomethyl (C)]PHSTDR_.3	#N/A	5.2196	6.6846	6.1206	6.2972	5.9981	6.2831	6.8499	6.0942	6.1212	6.4832	6.1222	6.3230	5.6172	6.8642	6.9264	6.2697	6.2022	5.8688	6.3345	6.4473
Hnrnpk	_DLAGSIIGK_.2	#N/A	4.8035	4.5311	5.0383	5.3916	5.0925	5.4554	6.2239	3.4490	6.2899	6.0060	6.0331	5.8925	5.8062	4.9330	5.5878	5.5753	4.4016	6.1337	5.9128	6.2442
Hnrnpk	_RDYDDM[Oxidation (M)]S[Phospho (STY)]PR_.2	#N/A	8.6127	9.6184	10.3618	10.7017	8.3835	8.4712	7.9560	8.6729	7.2675	8.9610	9.6921	8.3441	8.9291	10.6054	9.1030	8.6384	9.2802	8.6486	8.3587	9.1604
Hnrnpk	_RPAEDM[Oxidation (M)]EEEQAFKR_.3	#N/A	5.9498	6.1965	6.6785	5.7398	6.4321	6.1008	5.7063	6.2335	5.9854	5.6392	6.0151	6.2216	5.4164	5.6805	7.0511	6.1340	6.7827	5.8848	5.9148	6.2123
Hnrnpk	_GSYGDLGGPIITTQVTIPK_.2	#N/A	5.8484	5.8048	6.3269	5.7945	6.0408	6.0738	5.8096	6.1538	6.1481	6.2642	6.0671	5.8039	5.7920	6.1395	5.9031	5.9925	5.9579	5.6028	5.9993	6.1229

Hnrnpk	_LLIHQSLAGGIIGVK_.3	#N/A	4.6086	5.2291	5.3140	4.8539	5.6240	4.8024	4.0185	4.8998	5.3799	5.1765	4.7904	5.0987	4.8055	4.5638	5.0346	4.3023	4.8474	4.3958	5.1993	5.2129
Hnrnpk	_IIPTLEEGLQLPS[Phospho (STY)]PTATSQLPLESDAVEC[Carbamidomethyl (C)]LNYQHYK_.4	P61979_S116_M1_Hnrnpk	8.8136	9.4426	9.0659	8.7595	3.8637	8.8152	3.1844	4.4505	8.8996	9.5421	8.5182	9.5817	9.7381	10.5760	9.2153	9.1556	9.0194	9.1841	9.5081	9.0874
Hnrnpk	_NTDEMVELR_.2	#N/A	3.1854	6.6494	6.6372	7.0700	3.3923	5.4548	5.9208	3.4111	3.5760	4.4968	6.3869	3.8598	3.7414	6.9661	3.6811	4.0483	4.4091	3.8744	3.5230	5.8709
Hnrnpk	_GGDLM[Oxidation (M)]AYDRR_.2	#N/A	4.5265	3.5154	3.5254	3.2020	4.3835	4.7924	4.1987	4.8211	5.4313	5.5730	3.9333	3.4049	4.1683	4.1442	4.9625	4.5255	4.2874	5.5000	4.2571	3.4795
Hnrnpk	_ILSISADIETIGEILKK_.2	#N/A	3.9378	4.0650	4.4741	3.7515	3.2756	3.4997	4.2655	3.6382	4.3246	4.1442	3.1981	3.9544	4.4327	4.4612	3.8427	4.2827	4.1759	0.6385	3.3058	4.4282
Hnrnpk	_ILSISADIETIGEILK_.2	#N/A	4.5520	4.2679	4.0886	4.5330	3.2581	3.3494	3.7900	3.2769	2.3300	4.3626	3.3484	3.9940	4.5829	4.1267	3.8153	3.9142	4.2750	4.3753	3.1735	3.8040
Ywhag	_[Acetyl (Protein N-term)]VDREQLVQK_.2	#N/A	10.4701	10.7657	10.4325	10.4256	10.5781	10.4658	10.6205	10.8578	10.5266	10.4621	10.0845	10.7804	10.9347	10.5450	10.6785	10.5502	10.4832	10.5552	10.5089	10.3272
Ywhag	_MKGDYYR_.2	#N/A	7.5360	8.9096	9.5330	9.0029	7.9596	8.6143	9.0001	7.5676	8.4127	8.3862	8.8336	8.2348	7.4023	9.2189	9.2303	7.6402	8.0269	8.1325	8.1613	8.2665
Ywhag	_AYSEAHEISK_.2	#N/A	9.5999	9.4745	9.7815	9.4828	9.5983	9.4695	9.6871	9.6471	9.7941	9.7781	9.3938	9.5479	9.1886	9.3995	9.8904	9.5070	9.1513	9.4646	9.4497	9.3714
Ywhag	_ATVVESSEKAYSEAHEISK_.3	#N/A	8.6776	8.3635	7.2107	8.0526	7.8598	7.6471	7.3976	8.6860	7.7052	7.5888	8.0357	8.0895	9.1665	7.4170	7.2765	7.6656	7.5591	7.1457	7.8147	7.3770
Ywhag	_RATVVESSEK_.2	#N/A	5.6337	5.1261	5.8624	5.3774	5.3854	4.8214	5.5161	5.5141	5.4719	4.8065	4.7068	5.1091	5.2065	4.0033	5.4570	5.6749	5.0521	4.5965	4.5493	5.3120
Ywhag	_NC[Carbamidomethyl (C)]SETQYESK_.2	#N/A	6.9550	6.9928	7.4259	7.0662	6.8780	6.7846	7.0550	7.0761	7.1354	7.1129	6.9919	7.1897	6.9000	7.4167	7.3024	6.9744	6.7042	6.9631	6.7628	6.6094
Ywhag	_EHM[Oxidation (M)]QPTHPIR_.2	#N/A	8.7065	8.5055	8.8623	8.5096	8.4325	8.4549	8.7208	8.6307	8.6943	8.6387	8.2485	8.4840	8.5775	8.4794	9.1361	8.7923	8.4356	8.5711	8.8531	8.4882
Ywhag	_NVTELNEPLSNEER_.2	#N/A	11.0652	10.8792	11.1063	10.8842	10.7907	10.7995	10.8088	11.2064	10.9310	10.9461	10.6958	10.8380	11.2511	10.8950	10.7882	10.8828	10.9238	10.6978	10.8431	10.7450
Ywhag	_YLAEVATGEK_.2	#N/A	9.0169	9.1578	9.4571	9.2562	8.9776	9.2699	9.1440	9.1234	9.1289	9.2943	9.0358	8.8838	9.0113	9.2757	9.0483	8.9912	8.8287	8.9876	9.0576	9.0097
Ywhag	_NC[Carbamidomethyl (C)]SETQYES[Phospho (STY)]KVFYLK_.2	P61982_S119_M1_Ywhag	3.8576	4.1452	4.3939	3.8318	3.1953	3.4194	4.3457	3.5580	4.4048	4.0639	3.2784	4.0346	4.5129	4.3810	3.9229	4.2025	4.0957	3.7375	3.3861	4.3480
Ywhag	_DNLTLWT[Phospho (STY)]SDQQDDDGGEGNN_.2	P61982_T234_M1_Ywhag	3.8341	12.8846	14.4362	14.2215	14.1074	14.0898	14.0641	14.3756	14.0028	14.6348	14.3699	14.3320	4.1470	9.3722	13.7712	14.5334	14.4045	14.4504	14.3700	13.8463
Ywhag	_TAFDDAIAELDTLNEDSYK_.2	#N/A	7.9169	7.4427	7.9752	7.6658	7.6145	7.6924	7.4888	7.6360	7.8663	8.0418	8.0283	7.6777	7.5185	8.0730	7.4323	7.7667	7.7887	7.4974	7.6698	7.7830
Ywhag	_YLAEVATGEKR_.2	#N/A	8.3516	8.6637	8.4245	8.5373	8.3999	8.3760	8.5792	8.5247	8.5863	8.5575	8.0670	8.5720	8.6675	8.6304	8.5793	8.5904	8.1822	8.3679	8.4544	8.2371
Ywhag	_ELEAVC[Carbamidomethyl (C)]QDVLSLLDNYLIK_.2	#N/A	3.0687	4.3020	4.0236	3.9962	3.7853	3.9318	4.1117	4.3777	3.3820	2.9587	4.3615	3.0121	3.5474	5.2352	3.0294	3.6665	3.3763	4.3846	3.4440	4.0171
Ywhag	_VISSIEQKTS[Phospho (STY)]ADGNEKK_.2	#N/A	4.0511	3.2680	3.5877	3.5331	4.2580	4.2914	4.2909	4.2768	4.2111	3.8617	3.2170	2.9941	4.3766	3.6257	2.8155	3.4132	3.7740	3.3754	3.6745	3.3030
Ywhag	_VISSIEQKTS[Phospho (STY)]ADGNEK_.2	#N/A	7.9327	4.3977	7.1711	7.6432	7.8723	7.5306	7.5498	3.1470	7.5556	7.2248	7.2634	7.6284	7.7646	7.9546	7.6206	7.9606	7.8901	7.7766	7.2267	7.5211
Rras2	_FQEQEC[Carbamidomethyl (C)]PPS[Phospho (STY)]PEPTR_.2	#N/A	4.2249	10.6517	3.2604	11.0220	10.4336	10.6375	10.2628	10.9279	10.6203	10.7778	10.6290	10.4616	10.8615	10.9141	10.1446	10.4423	11.0326	10.5141	10.6675	10.6094
Rras2	_KFQEQEC[Carbamidomethyl (C)]PPS[Phospho (STY)]PEPTRK_.3	#N/A	8.5604	8.8086	8.5030	8.8524	8.6001	8.7327	8.5653	8.7014	8.7010	8.7148	8.5848	8.3631	9.2368	8.9734	8.2988	8.5133	8.8660	8.9430	8.7470	8.6074
Rras2	_FQEQEC[Carbamidomethyl (C)]PPS[Phospho (STY)]PEPTRK_.2	#N/A	6.6168	8.6088	6.6927	8.5538	8.2195	8.4239	8.2879	6.6146	7.4646	8.4335	8.2496	8.1547	7.5503	8.7989	6.9407	7.1868	8.0062	8.6088	8.3724	8.2795
Rras2	_KFQEQEC[Carbamidomethyl (C)]PPS[Phospho (STY)]PEPTR_.2	#N/A	7.1773	7.4431	7.0594	7.7441	7.0950	7.5263	7.3349	7.3672	4.2850	7.0145	7.1466	4.7890	5.9819	7.2470	6.9370	7.1320	4.3671	7.1502	7.1860	6.9797
Rras2	_[Acetyl (Protein N-term)]AAAGWRDGS[Phospho (STY)]GQEK_.2	P62071_S10_M1_Rras2	3.6602	4.3426	3.7694	4.9709	2.9979	3.4862	3.5953	3.3606	4.7574	4.5573	2.9941	4.6950	4.4369	5.9439	3.6207	4.8030	4.2138	4.1816	3.5835	5.2972
Timm10	_YLDIHER_.2	#N/A	6.5344	6.5943	6.7745	5.3304	6.5734	6.6829	6.7869	7.1127	6.5981	6.6277	6.2846	7.0565	6.7728	6.7298	7.2280	6.8962	6.6635	6.7444	6.4161	7.1289
Timm10	_GESVC[Carbamidomethyl (C)]LDR_.2	#N/A	3.5293	6.0592	5.5008	6.0532	5.2806	5.7500	5.7968	5.6835	3.2321	6.2836	6.4085	5.2239	3.3975	6.5531	7.1321	6.2233	5.8604	5.8926	6.4348	6.3515
Timm10	_EAELSKGESVC[Carbamidomethyl (C)]LDR_.2	#N/A	3.7644	3.7515	4.3733	3.8140	4.0174	2.7367	3.5237	4.3801	3.4375	3.3852	2.9957	3.7663	3.1996	4.5727	4.2557	3.3804	3.6440	3.7786	3.3311	3.7950
Timm13	_VQIAVANAQELLQR_.2	#N/A	5.8415	5.5581	5.4128	5.1387	5.5745	5.1536	5.3827	5.2961	5.3915	5.8910	5.2891	5.3079	5.9447	5.1296	5.9524	6.2043	6.0957	5.3539	5.4329	5.6056
Timm13	_YM[Oxidation (M)]DAWNTVSR_.2	#N/A	6.6401	6.4179	6.2818	6.2558	6.7465	6.4466	6.4864	7.1047	6.4433	7.0715	6.6616	6.4718	6.7945	4.9164	6.9328	7.1537	6.6486	6.4135	6.7663	6.6069
Timm13	_LDPGAIMEQVK_.2	#N/A	3.7608	3.5583	3.2973	3.8235	3.9677	4.8296	5.0429	3.9864	3.0006	3.5714	3.9801	3.2844	3.1660	4.7837	5.3920	3.1229	3.4837	3.6658	3.9648	3.0127
Timm8b	_FIDTTLAITGR_.2	#N/A	3.1209	3.3810	3.7320	3.8664	2.7718	4.1836	4.6581	3.9435	3.6407	3.3273	2.8892	3.2705	3.7487	3.2925	5.1028	4.4089	3.4407	4.0781	4.0077	2.9698
Rps7	_HVVFIAQR_.2	#N/A	6.0292	6.4470	6.1541	6.2821	6.5011	6.2092	6.4221	6.8065	6.1860	3.0907	5.8245	6.7024	6.4639	6.7866	6.2771	6.6309	5.9150	6.4499	6.5598	6.4073
Rps7	_AIIIFVPVPQLK_.2	#N/A	6.0807	6.1095	5.7422	5.8750	5.9744	6.1052	5.7694	6.0975	5.5723	6.0777	6.0719	5.4158	6.1581	5.5154	5.9780	5.8838	6.1613	5.7882	5.4398	5.5543
Ppp1ca	_LLEVQGS[Phospho (STY)]RPGK_.2	P62137_S22_M1_Ppp1ca	5.3309	5.3936	4.4166	4.5635	5.4262	5.0614	4.5222	4.5423	4.0129	4.2003	5.1349	4.5914	5.0753	5.1916	4.7977	5.3851	4.9434	4.7451	5.1230	5.5285
Ppp1ca	_[Acetyl (Protein N-term)]SDSEKLNLDSIIGR_.2	#N/A	4.2404	3.5019	4.2726	4.2165	3.8843	3.9434	5.1586	4.3889	3.3599	3.5190	4.2451	3.5162	3.4664	3.7471	5.3536	4.1195	3.4185	4.3256	4.2036	4.0804
Ppp1cb	_[Acetyl (Protein N-term)]ADGELNVDSLITR_.2	#N/A	5.4854	5.3644	5.5055	5.8875	5.8786	5.0269	5.8278	4.8018	6.2151	5.6973	5.6113	5.7723	5.5234	5.3595	6.0827	6.1252	5.5541	5.3138	5.5836	4.7944
Psmc1	_VAEEHAPSIVFIDEIDAIGTK_.3	#N/A	3.4571	4.0269	5.5447	3.4712	3.4097	3.2446	3.6384	3.4037	4.0283	3.0686	3.0372	4.0318	3.7240	4.4947	3.8510	4.0657	3.9414	3.5197	3.5037	3.3678
Psmc1	_AVANQTSATFLR_.2	#N/A	3.7684	5.2789	5.0963	4.5994	5.1674	4.1242	4.9932	5.0467	4.9174	4.4492	3.1023	4.1239	5.0997	4.1943	5.1642	4.1133	4.0064	4.8890	4.4878	5.1446
Psmc1	_APQETYADIGGLDNQIQEIK_.2	#N/A	7.2286	6.7767	4.2538	4.3678	6.8227	5.8067	3.0801	3.4421	3.1392	7.0648	3.5136	6.5157	3.2473	4.2919	4.1582	3.9673	4.4402	4.2101	3.0083	3.9692
Psmc5	_NDSYTLHK_.2	#N/A	4.4546	4.4866	3.5766	3.1482	3.8789	4.1029	4.1169	4.2415	3.7213	3.6450	4.2773	3.3511	4.5619	3.5637	4.0574	3.3852	3.2784	4.9139	3.9228	3.5307
Rps8	_LTPEEEEILNK_.2	#N/A	8.5131	8.5513	8.7620	8.1098	8.3508	8.3763	8.3893	8.5260	8.6111	8.2020	8.1922	8.3440	8.4081	8.2633	8.3285	8.1133	8.4122	8.4455	8.0396	8.4154
Rps8	_IIDVVYNASNNELVR_.2	#N/A	6.5965	5.8399	6.5476	6.3297	6.5624	6.0972	6.2786	6.6922	6.1322	6.2756	6.3152	5.5101	7.0950	5.9993	5.3740	6.0509	6.5652	5.4678	5.0900	6.1510
Rps8	_NC[Carbamidomethyl (C)]IVLIDSTPYR_.2	#N/A	5.0872	5.3012	5.3996	5.4746	5.1659	5.5013	5.3758	5.2615	5.7691	4.8730	5.6628	5.6765	5.9771	6.0349	4.8947	5.2713	6.0296	5.0415	4.9239	4.9667
Rps8	_ISSLLEEQFQQGK_.2	#N/A	5.0446	5.2665	4.6877	4.9580	5.2390	5.1288	5.0087	3.9353	5.4110	5.1614	4.7787	5.0760	5.4530	5.4811	4.7348	5.0499	5.2769	5.7081	4.8631	5.5436
Rps8	_LDVGNFSWGSEC[Carbamidomethyl (C)]C[Carbamidomethyl (C)]TR_.2	#N/A	3.1537	4.1654	5.1942	5.2656	3.3607	4.6210	4.9941	4.4546	5.1650	4.1581	3.0863	4.6695	3.7731	4.2292	4.3060	4.1336	4.3775	4.2728	4.8183	4.6404
Rps15a	_IVVNLTGR_.2	#N/A	5.2644	5.0975	5.4094	5.6873	5.1046	5.6990	6.2788	4.9320	5.7566	6.3048	5.7292	5.9005	4.3499	5.9460	5.1073	5.6612	4.4048	6.3702	6.2736	5.9559
Rps15a	_M[Oxidation (M)]NVLADALK_.2	#N/A	6.1577	3.8879	3.3482	3.3453	5.5027	4.9030	5.9234	6.2305	5.2720	5.6074	5.6828	5.8652	5.4202	3.3666	5.6746	4.9058	4.9495	5.5697	5.0046	5.4196
Rps15a	_HGYIGEFEIIDDHR_.3	#N/A	4.3442	4.8771	5.0033	4.9188	5.3582	5.4725	4.2793	5.2275	5.0334	3.8733	5.4325	4.6919	4.3649	3.3157	2.8038	3.1883	5.2926	3.5299	3.9190	5.5551
Ywhae	_EALQDVEDENQ_.2	#N/A	11.7525	11.7121	12.2115	11.9151	11.6160	11.6881	11.2750	11.8661	11.5330	11.7666	11.7530	11.4763	11.9316	11.9567	11.2047	11.4122	11.9161	11.3688	11.6001	11.6730
Ywhae	_YLAEFATGNDR_.2	#N/A	10.0386	10.0991	10.2901	10.0664	9.7994	10.1081	9.7200	10.0638	10.0318	10.0010	9.8926	9.6518	10.0243	10.2635	9.5723	9.9322	10.1131	9.7774	9.9432	9.9911
Ywhae	_LIC[Carbamidomethyl (C)]C[Carbamidomethyl (C)]DILDVLDK_.2	#N/A	9.3906	9.6844	10.3940	10.0711	9.6201	9.9218	9.8858	9.6240	9.7504	10.0978	9.7015	9.5464	9.1385	9.8653	10.1302	9.4298	9.8131	9.4742	9.6725	9.6065
Ywhae	_VAGM[Oxidation (M)]DVELTVEER_.2	#N/A	9.9030	9.6749	7.9824	9.1867	9.6131	9.8558	9.2544	9.9305	9.7540	9.5545	9.1595	8.0686	10.0457	9.6844	9.5157	9.7286	9.5718	9.8407	9.8659	9.8784
Ywhae	_EAAENSLVAYK_.2	#N/A	9.5430	9.6688	9.9862	9.6104	9.6127	9.6280	9.6076	9.7220	9.7136	9.6625	9.5688	9.4670	9.9324	9.6522	9.7981	9.7949	9.4600	9.5360	9.5621	9.6052
Ywhae	_IISSIEQK_.2	#N/A	7.3976	8.0295	8.0372	7.9310	7.5641	8.0250	8.5940	7.2111	8.2901	8.2545	8.1420	7.9047	7.8502	7.8931	8.6162	7.8867	7.0752	8.0805	8.2813	7.9630
Ywhae	_LIC[Carbamidomethyl (C)]C[Carbamidomethyl (C)]DILDVLDKHLIPAANTGESK_.4	#N/A	5.4267	5.8130	4.9576	5.4454	5.4428	4.8309	5.7392	5.7652	5.1241	5.6986	5.4395	5.6481	5.3057	5.9226	5.3501	5.6157	5.1327	5.5307	5.8317	5.4029
Ywhae	_HLIPAANTGESK_.2	#N/A	9.2650	9.2865	9.6217	9.2872	9.2679	9.3928	9.3051	9.2979	9.2436	9.2836	9.2439	9.2122	8.8510	9.6020	9.5783	9.2674	9.0013	9.2344	9.3112	9.1802
Ywhae	_QM[Oxidation (M)]VETELK_.2	#N/A	6.8320	6.9126	7.1333	7.0883	7.0521	7.0319	7.2792	6.5094	6.8399	6.9477	7.0355	6.7846	6.2370	7.3764	7.3652	7.0653	6.3958	6.7866	6.7801	7.0858
Ywhae	_YLAEFATGNDRK_.2	#N/A	8.2021	8.4758	8.2983	8.3793	8.0377	8.3236	8.2941	8.6116	8.3368	7.9931	7.9693	8.3398	8.5738	8.6232	8.2702	8.2399	8.1905	8.1375	8.1707	8.0545
Ywhae	_AAFDDAIAELDTLSEESYK_.2	#N/A	9.2600	9.0355	9.4591	8.8212	8.6177	8.8925	8.5206	8.9079	8.8113	9.1902	8.9213	8.8429	9.0135	9.8531	8.4872	9.1686	9.1161	8.8951	8.7441	9.2792
Ywhae	_[Acetyl (Protein N-term)]M[Oxidation (M)]DDREDLVYQAK_.2	#N/A	7.6199	7.7604	8.0694	7.6450	7.4232	7.5700	7.6543	7.7245	7.6228	7.6406	7.4050	7.5286	7.8473	7.9035	7.6737	7.8867	7.3806	7.5092	7.3438	7.7555
Ywhae	_M[Oxidation (M)]KGDYHR_.2	#N/A	5.2707	3.8679	5.6983	3.7937	5.0399	5.4996	5.7119	4.9340	6.6040	5.5826	3.9504	3.2547	3.1363	3.3651	3.0761	3.1526	6.8188	5.2287	5.5314	5.6729
Ywhae	_KEAAENSLVAYK_.2	#N/A	3.1747	4.4552	3.7110	4.5147	4.4275	5.1111	4.9670	4.3759	4.7076	3.3810	4.7744	2.8273	5.2756	4.9532	3.1050	4.8860	3.4127	4.6326	4.0690	5.4648
Ywhae	_AASDIAM[Oxidation (M)]TELPPTHPIR_.2	#N/A	5.6808	7.7162	6.3363	7.5436	8.3517	7.0997	7.7177	8.5985	7.2256	7.8466	7.9475	7.2444	6.6652	7.4808	8.3077	7.0975	7.6982	7.2403	8.5225	7.4247
Ywhae	_YDEM[Oxidation (M)]VESM[Oxidation (M)]KK_.2	#N/A	4.8200	5.6434	5.4515	5.7074	4.8617	5.4305	5.5817	4.7431	5.2716	5.2894	5.4932	5.0048	4.8674	5.9191	5.8633	4.8798	5.1609	5.2210	4.8124	5.2798
Ywhae	_EENKGGEDKLK_.2	#N/A	4.7447	4.8046	5.1556	4.4699	3.9660	4.4841	4.7960	3.6368	5.1116	4.7349	4.7680	4.8588	4.2754	4.3815	5.3599	4.6258	4.6022	4.4731	4.4387	4.8697
Ywhae	_IISSIEQKEENK_.2	#N/A	6.4009	5.6941	5.8531	5.5046	5.7882	5.8978	5.6918	5.6866	5.9412	5.8755	5.8382	5.8332	5.6853	6.4661	5.9062	6.0767	6.0252	5.6221	6.0772	5.7659
Ywhae	_KVAGM[Oxidation (M)]DVELTVEER_.3	#N/A	5.2025	5.3026	3.9151	5.3847	5.7309	4.8128	4.6646	5.5587	5.6484	5.4519	5.1782	5.2408	6.0262	5.0130	5.3879	5.7392	4.7264	5.4258	5.4067	5.4199
Ywhae	_YDEM[Oxidation (M)]VESMK_.2	#N/A	4.0180	3.9848	5.0244	4.7898	3.3557	3.5798	4.1854	3.7184	4.2444	4.2243	3.1180	3.8743	4.3525	4.5414	3.7626	4.3629	4.2560	3.5771	3.2257	3.0075
Ywhae	_LAEQAERYDEM[Oxidation (M)]VESM[Oxidation (M)]KK_.3	#N/A	6.8019	6.8898	5.8899	6.4162	6.5937	6.3174	6.4146	7.1744	6.8481	6.7681	6.1158	7.0359	7.7702	6.7290	6.8937	7.2887	6.1870	6.6347	6.7422	6.5513
Ywhae	_IISSIEQKEENKGGEDK_.3	#N/A	6.6591	6.3551	6.5844	6.5411	6.3510	6.5383	6.2478	6.1835	7.0118	6.3321	6.2500	6.1775	6.5613	6.5856	6.7704	6.3462	6.1338	6.0364	6.5175	6.1441
Ywhae	_DNLTLWTSDMQGDGEEQNK_.2	#N/A	3.6196	13.2375	11.8756	11.7547	12.3807	11.8616	11.9362	3.8453	11.5233	12.5079	3.7783	12.3316	3.8736	12.2042	11.1901	3.7247	4.0855	11.6557	11.4825	11.8343
Ywhae	_AAFDDAIAELDTLSEESYKDSTLIMQLLR_.3	#N/A	3.9560	3.9161	3.9791	3.6027	3.8203	3.9183	4.3201	3.8304	4.3126	3.9649	3.5004	3.8055	4.1508	2.7831	3.4739	3.8601	3.6809	3.5084	3.9706	3.9332
Ywhae	_LAEQAERYDEMVESMK_.3	#N/A	3.7959	3.5527	4.1683	3.8178	4.0029	4.1350	3.9677	4.0216	3.7188	3.6438	4.0931	3.2788	3.8268	3.3706	2.9487	3.1581	3.5188	3.6601	3.9592	3.8836
Rps14	_IEDVTPIPSDSTR_.2	#N/A	6.8284	6.9726	6.8102	7.0657	6.8164	7.6359	6.9508	6.9163	6.9729	7.0202	6.8328	7.3466	7.2165	6.9511	7.0973	6.6905	6.8058	7.2281	6.5182	6.9118
Rps14	_TPGPGAQSALR_.2	#N/A	5.4369	5.8203	6.1989	3.3011	5.4418	5.6350	6.0932	5.9991	5.9351	5.3608	4.2486	6.0970	5.4023	5.6831	6.3156	5.5529	5.6888	6.0285	3.7742	6.4157
Rps23	_ANPFGGASHAK_.2	#N/A	2.3984	6.2453	5.9935	5.6962	6.1900	6.3051	6.4678	5.8397	6.6018	6.2102	6.1439	6.5739	5.3411	6.3450	6.7568	6.4069	5.2577	6.5339	5.8412	6.0114
Rps18	_VLNTNIDGR_.2	#N/A	8.0527	7.6380	8.0312	7.9804	7.5971	7.8665	7.7741	7.5336	7.7871	8.2376	7.9986	7.8423	7.7645	7.7927	7.9588	8.0238	7.7587	7.6158	8.0660	8.1836
Rps18	_IPDWFLNR_.2	#N/A	7.0170	6.8299	7.2910	7.0799	6.8272	6.9429	7.1664	6.4212	6.7550	6.9406	6.9096	6.9141	6.7983	6.7285	6.8161	6.6889	6.8379	7.0431	7.1851	6.8522
Rps18	_YSQVLANGLDNK_.2	#N/A	7.3328	6.9384	7.2255	6.9210	6.7946	6.7899	7.0014	7.0559	7.0194	6.5008	7.2564	6.6269	7.0525	7.3512	6.8437	6.6573	6.6622	6.7643	6.5353	6.5977
Rps18	_LREDLER_.2	#N/A	5.6004	6.3354	6.4183	5.6606	6.2435	5.7484	5.8613	3.5000	3.8745	5.9978	5.8378	6.3026	5.8806	5.8229	6.0737	6.2541	4.8535	6.4934	5.9349	6.1786
Rps29	_GHQQLYWSHPR_.3	#N/A	6.2571	5.7086	3.8981	6.6070	6.0895	6.3633	6.1441	5.8066	5.6537	6.7178	5.9971	6.1578	5.6308	6.6507	7.0638	6.8625	5.7179	6.4506	6.3578	6.1230
Rps29	_DIGFIKLD_.2	#N/A	3.5716	2.9946	3.8708	4.3549	3.7785	2.2877	2.9933	4.5357	3.2168	3.3822	2.2340	3.2329	3.3552	3.8579	3.4035	4.2816	3.5726	2.8648	2.8925	3.8249
Rps11	_DYLHYIR_.2	#N/A	7.0270	6.3585	7.0621	6.6606	6.4337	6.6350	6.9523	7.0342	6.2547	6.5711	6.3609	6.2644	6.9394	6.2693	5.6239	5.7200	6.7376	6.5280	6.3741	5.4933
Rps11	_EAIEGTYIDKK_.2	#N/A	6.0926	6.1498	5.5864	5.6563	5.3393	5.7108	5.7223	6.1317	6.0198	5.3547	6.1128	5.4276	6.3009	5.4773	5.3499	5.8118	5.3305	5.3419	5.7750	5.9877
Rps13	_DSHGVAQVR_.2	#N/A	6.5375	6.4797	6.3986	6.5238	6.6463	6.3282	6.6970	6.9582	6.5949	6.7269	7.0066	6.4523	6.6930	6.3602	6.9648	6.7393	6.2427	6.8767	6.2316	6.5068
Rps13	_LILIESR_.2	#N/A	4.1222	3.7908	4.5657	4.0559	3.4063	4.6398	4.8996	3.8178	5.0956	3.5168	4.9983	3.0255	3.5592	4.4442	3.9868	3.5801	3.2512	4.7489	4.7239	4.6739
Snrpd2	_NNTQVLINC[Carbamidomethyl (C)]R_.2	#N/A	3.7016	5.0377	3.2382	5.7259	3.9085	5.2542	4.8755	4.8425	5.1690	5.5863	5.4098	5.4995	3.7325	3.2762	5.2140	5.1383	5.8300	5.4098	5.2877	5.3776
Snrpd3	_VAQLEQVYIR_.2	#N/A	5.2360	6.2831	5.5782	6.2013	5.1650	5.5902	5.6512	6.2050	5.8445	5.5876	5.4406	5.5964	5.1878	5.5971	5.2255	5.7096	5.4227	5.6685	5.7810	5.9251
Snrpd3	_FLILPDM[Oxidation (M)]LK_.2	#N/A	5.3874	5.3957	4.8365	5.9414	5.5009	5.0102	5.6110	5.8309	5.4996	5.5192	3.1650	5.3939	4.3995	4.2202	5.6486	5.9068	5.5671	6.1777	5.6702	3.6205
Lsm5	_[Acetyl (Protein N-term)]AANATTNPSQLLPLELVDK_.2	#N/A	6.0420	5.9066	5.7318	6.2528	6.2057	5.6381	5.6700	5.2510	5.8362	6.3367	5.8528	5.9528	6.4987	5.7288	6.5430	5.8973	6.0057	5.1149	5.7203	5.9260
Arf6	_FNVWDVGGQDK_.2	#N/A	7.1822	7.3222	6.4880	6.6175	6.9301	6.8676	7.0612	7.2462	7.0272	6.6903	6.8855	6.5324	7.0757	6.9243	6.0824	6.3433	7.0073	6.9961	6.7444	6.3292
Psmc6	_EVIELPLTNPELFQR_.2	#N/A	4.4705	4.6556	5.3483	4.3087	4.3494	4.6459	4.5986	3.8169	4.5049	4.2549	4.0015	5.0420	4.4369	5.2067	4.1735	4.5367	4.1982	4.5006	3.4654	4.3935
Psmc6	_ALQSVGQIVGEVLK_.2	#N/A	4.8287	3.8506	4.5573	3.4821	3.5679	4.1480	4.6222	4.5777	4.0551	5.0676	4.3335	4.8490	4.1632	3.7070	4.5065	4.7840	3.8552	4.5064	3.7893	4.8139
Psmc6	_ADHDFVVQEDFM[Oxidation (M)]K_.3	#N/A	3.9169	4.0859	4.4532	3.7725	3.2546	3.7932	4.3295	3.1476	5.3166	4.1232	3.2191	3.9754	4.4536	3.9974	5.1843	4.2618	4.1457	5.5130	3.3028	3.6747
Kcnab2	_RSS[Phospho (STY)]LVITTK_.2	#N/A	8.8901	8.6914	4.4846	3.1929	3.2860	8.4288	7.8981	8.4511	4.3141	4.1546	8.5553	3.9439	8.8372	3.9659	7.3715	4.2932	4.1142	3.0352	8.6043	3.6432
Kcnab2	_QTGS[Phospho (STY)]PGM[Oxidation (M)]IYSTR_.2	#N/A	8.2305	8.2776	9.3790	9.2801	8.4845	8.3589	7.6723	7.8947	8.0158	7.3871	8.1562	8.1687	8.1573	8.6695	3.1053	8.1209	8.5536	7.5436	7.3802	8.2131
Kcnab2	_AEVVLGNIIK_.2	#N/A	5.0214	4.8394	4.7405	4.6224	4.3032	4.4738	5.3770	3.7982	5.2290	3.4726	4.4772	4.9095	3.6034	5.3810	3.3439	2.9346	3.7873	5.0302	4.2220	4.6677
Kcnab2	_SS[Phospho (STY)]LVITTK_.2	#N/A	4.4966	4.9714	3.8026	3.1409	3.5548	4.2013	5.0713	3.7931	4.1200	3.6964	3.7686	3.7615	6.0830	3.8196	3.6090	4.5581	3.8061	5.0529	4.7534	5.2057
Kcnab2	_YPESTTGS[Phospho (STY)]PAR_.2	#N/A	3.8283	6.1292	6.7357	4.5738	3.1661	3.3902	4.3750	3.2361	4.4341	4.0347	3.3076	4.0639	4.5422	4.0859	3.9522	4.1732	5.6293	4.4161	3.2143	3.7632
Kcnab2	_[Acetyl (Protein N-term)]M[Oxidation (M)]YPESTTGS[Phospho (STY)]PAR_.2	#N/A	10.7698	10.8444	11.8990	11.9833	10.4010	10.7532	11.0926	10.6485	10.4906	11.0119	11.2618	10.4805	11.1126	10.5752	9.4758	10.3659	10.7112	10.5819	10.5037	10.8130
Kcnab2	_[Acetyl (Protein N-term)]MYPESTTGS[Phospho (STY)]PARLS[Phospho (STY)]LR_.2	P62482_S14_M2_Kcnab2	4.1040	5.4851	3.7410	3.8551	3.7955	3.9644	5.1670	3.7536	4.1807	3.5347	4.0394	3.2095	4.0739	6.4882	3.1651	3.9669	3.4427	3.7608	4.0617	3.6951
Kcnab2	_NEGVSSVLLGASNAEQLM[Oxidation (M)]ENIGAIQVLPK_.3	#N/A	3.4654	3.0672	4.4108	4.2109	4.3040	3.0272	3.2371	3.5990	3.2962	3.6717	3.6706	3.6718	3.4042	4.4489	2.8143	3.8103	3.0963	4.0532	4.3522	4.1261
Kcnab2	_LQLEYVDVVFANRPDPNTPM[Oxidation (M)]EETVR_.3	#N/A	3.8923	3.4881	3.5501	3.1747	4.0992	4.1325	4.4498	4.2151	4.3700	3.7028	3.8486	3.3776	4.5354	3.5372	2.9743	3.2544	3.2519	3.0804	4.2298	3.5042
Abi2	_EIGILTTNK_.2	#N/A	6.0829	5.5038	5.5058	6.3225	5.9757	5.9580	6.1572	5.4516	5.7664	5.8357	6.3306	5.9360	5.8085	6.1016	5.9863	5.7303	5.8077	6.1157	6.3131	6.0405
Abi2	_ALFDSYTNLER_.2	#N/A	5.5268	6.3261	6.2082	5.8251	5.6348	5.3122	5.1043	5.7246	6.1908	6.1374	6.0977	5.9611	6.4337	6.0123	6.0887	5.5455	5.6680	5.7941	5.8843	3.6380
Abi2	_VADYC[Carbamidomethyl (C)]ENNYIQSPDK_.2	#N/A	6.4231	3.1194	3.7362	6.2323	6.3452	6.5572	6.4492	6.9660	4.1225	6.0719	6.7141	6.5172	4.2305	7.2220	6.6752	6.7637	6.8415	3.2269	6.9792	6.7167
Abi2	_VADYC[Carbamidomethyl (C)]ENNYIQS[Phospho (STY)]PDKQR_.3	P62484_S40_M1_Abi2	4.1409	5.0120	3.6775	5.0699	4.3478	4.1614	4.5571	4.3666	4.0637	4.4051	4.9028	4.4052	4.1718	3.4916	4.2265	3.5030	3.8638	3.2856	3.5847	3.3928
Abi2	_NMAPSQQS[Phospho (STY)]PVR_.2	#N/A	3.5351	8.7369	9.4033	9.5655	7.1363	7.7404	8.3853	6.2873	7.3640	7.4622	8.7479	7.7826	3.3133	9.7990	7.1320	5.3765	8.0234	6.7283	6.9400	8.0110
Abi2	_HTPPTIGGS[Phospho (STY)]LPYR_.2	P62484_S301_M1_Abi2	9.9405	11.2297	9.3756	10.2329	10.0502	3.7910	3.3482	10.3615	3.4073	9.5413	3.7817	10.2098	10.7809	10.4306	3.7808	10.1089	8.7172	10.3932	7.5483	9.1506
Abi2	_[Acetyl (Protein N-term)]AELQM[Oxidation (M)]LLEEEIPGGR_.2	#N/A	4.5389	4.6485	3.5743	4.9002	4.8594	4.3796	4.0771	4.1362	3.9035	3.2444	4.0979	3.3534	3.8316	3.5614	4.1328	3.3829	3.2761	3.0562	4.0729	5.0267
Abi2	_TTPPTQKPPS[Phospho (STY)]PPM[Oxidation (M)]SGK_.2	#N/A	11.0472	11.5322	10.7223	11.3251	10.6662	10.9519	11.0941	11.5919	10.2791	10.7069	10.9351	11.3274	9.6742	12.5969	10.7192	10.7785	11.4188	11.1982	10.7105	10.9010
Rab11a	_ENDM[Oxidation (M)]S[Phospho (STY)]PSNNVVPIHVPPTTENKPK_.3	#N/A	7.4456	9.6261	4.8401	4.1590	3.9384	6.3980	7.2996	8.9480	6.3294	6.4317	3.6285	6.3308	9.7099	7.1652	5.6450	6.7175	3.8168	7.9178	4.0204	3.8452
Tsc22d1	_NSQLEQENNLLK_.2	#N/A	6.5990	6.3582	6.5648	6.6389	6.7523	6.2542	5.8556	6.9150	6.5658	6.1248	7.0811	7.3416	7.2180	7.7284	7.5452	7.1631	7.6775	7.0189	6.9581	7.0765
Tsc22d1	_EEVEVLK_.2	#N/A	3.8248	4.1780	4.3611	3.8645	3.1626	3.3215	4.3785	3.5252	3.6801	4.0312	3.1588	0.4168	3.8456	4.3482	3.9557	4.1697	4.0629	3.7703	3.4188	4.3152
Dynlrb1	_[Acetyl (Protein N-term)]AEVEETLKR_.2	#N/A	7.3039	8.0145	7.9200	7.8282	7.9179	7.7885	7.8402	7.8902	7.9954	7.6080	7.4563	7.8815	7.8471	8.2325	8.4961	7.9088	7.5722	7.8331	7.9451	7.8036
Dynlrb1	_EIDPQNDLTFLR_.2	#N/A	7.5809	7.7600	7.8032	7.0931	7.5542	7.6725	7.4968	7.5921	7.5231	7.7391	7.5534	7.6461	7.9198	7.9615	8.4800	7.9442	7.3484	7.6553	7.8124	7.3198
Dynlrb1	_GVQGIIVVNTEGIPIK_.2	#N/A	4.8491	4.3691	5.1488	3.5658	5.3162	4.3552	4.4268	4.6012	5.2951	5.3666	4.5213	3.4875	4.1610	4.3468	4.8371	4.1692	4.4952	4.5805	3.8970	4.5470
Eef1a2	_RYDEIVK_.2	#N/A	9.1900	9.5563	9.5794	8.8963	9.2675	9.4376	9.2737	9.4707	9.3947	9.5594	9.3626	9.2090	9.4532	9.2731	9.4156	9.2639	9.0213	9.3199	9.2892	9.2055
Eef1a2	_KLEDNPK_.2	#N/A	4.7097	5.2972	5.3396	5.1470	5.1411	5.3653	5.0498	4.6382	5.3374	5.3067	5.1354	5.1688	4.0598	4.6669	5.4974	3.1698	5.3310	4.4345	5.2527	4.6864
Eef1a2	_VETGILRPGM[Oxidation (M)]VVTFAPVNITTEVK_.3	#N/A	6.9841	6.8231	6.8665	6.5660	6.6128	6.8973	6.6728	6.9422	6.7698	6.5264	6.5979	6.4343	6.8027	6.8880	6.7178	6.5021	6.8254	7.6096	6.6911	6.9545
Eef1a2	_YYITIIDAPGHR_.2	#N/A	8.2248	8.1948	8.3663	8.1028	8.0740	8.1463	7.9355	8.3063	8.0338	8.0285	7.9133	7.8538	8.3179	7.9973	7.6629	8.1031	8.2851	8.0828	8.1862	7.9132
Eef1a2	_EVSAYIKK_.2	#N/A	3.9734	5.1401	3.8745	5.0167	5.0412	4.6106	4.0594	4.3661	3.4739	3.8974	4.4926	4.2081	4.0897	3.5252	4.3164	5.3213	4.3391	4.4535	3.1747	4.7844
Eef1a2	SGDAAIVEM[Oxidation (M)]VPGKPM[Oxidation (M)]C[Carbamidomethyl (C)]VESFSQYPPLGR	#N/A	4.2523	3.7505	3.2877	3.4371	3.5900	3.8141	3.9511	3.9526	4.0102	4.4586	4.3845	3.6400	4.1182	3.2748	3.5283	3.0963	4.4903	3.3392	2.9914	3.3393
Rps4x	_LSNIFVIGK_.2	#N/A	4.4257	4.2554	3.9411	4.8351	4.1857	3.4745	4.5136	3.1294	3.7258	4.2899	4.2894	4.8003	4.4354	4.5328	3.9096	4.3106	4.1274	5.1197	5.0298	4.3521
Rps4x	_EC[Carbamidomethyl (C)]LPLIIFLR_.2	#N/A	4.2747	4.0935	3.6015	4.0075	4.1849	4.5913	4.3774	4.9808	2.4716	4.2238	4.3622	4.7860	4.0958	3.6101	4.6026	3.1187	3.7879	4.2771	3.6606	3.5091
Rps4x	_TDITYPAGFM[Oxidation (M)]DVISIDK_.2	#N/A	5.9241	4.8211	4.4739	3.9250	4.5140	5.0265	5.2357	5.3544	4.8949	5.2488	4.5004	4.8632	5.7966	4.8802	4.5369	4.5287	5.3581	4.9125	3.7288	4.4685
Rps4x	_FDTGNLC[Carbamidomethyl (C)]M[Oxidation (M)]VTGGANLGR_.2	#N/A	4.3235	4.6456	3.1845	3.7616	3.7857	4.6443	3.7615	4.5338	4.5883	4.2664	4.6012	3.9655	3.8471	3.5642	4.3098	4.4807	3.9853	3.9034	3.6488	3.5312
Ppp2cb	_YSFLQFDPAPR_.2	#N/A	8.7800	8.7795	8.6989	8.6022	8.5275	8.7992	8.7665	8.6341	8.1615	8.7148	8.7677	8.6904	8.6178	8.8186	8.2972	8.6531	8.7093	8.8691	8.5649	8.6035
Ppp2cb	_NVVTIFSAPNYC[Carbamidomethyl (C)]YR_.2	#N/A	6.5497	7.0709	7.3285	6.8378	6.8178	7.1624	7.1578	7.4702	7.0417	6.7569	6.3900	6.9744	7.3266	6.6149	6.4274	6.6863	6.9386	6.7547	7.1129	6.7965
Ppp2cb	_ELDQWVEQLNEC[Carbamidomethyl (C)]K_.2	#N/A	6.4788	6.3461	5.8554	5.9596	6.4337	6.2212	6.1804	6.1293	6.1142	5.7762	6.2057	5.7564	6.9578	6.3120	5.9232	5.9294	6.0417	5.9263	5.8868	6.2671
Ppp2cb	_GAGYTFGQDISETFNHANGLTLVSR_.3	#N/A	5.4481	5.3250	4.5490	4.8632	5.1323	4.9989	4.7486	5.7331	5.0823	4.3768	5.2783	4.9896	5.7293	5.2691	3.8991	4.8465	5.0352	4.6883	4.9680	5.5759
Ppp2cb	_QITQVYGFYDEC[Carbamidomethyl (C)]LR_.2	#N/A	3.4297	4.9864	5.8137	4.8824	4.6633	3.6700	5.2523	4.7066	5.4532	5.2922	4.9343	4.3600	5.4450	5.2884	5.1701	5.0590	5.4249	4.7729	4.8964	4.7906
Ppp2cb	_SPDTNYLFM[Oxidation (M)]GDYVDR_.2	#N/A	6.1739	6.3253	6.3941	6.5864	5.5504	5.6991	6.2252	5.9959	5.6332	5.8058	5.9642	5.7980	5.9287	6.2414	5.1243	5.8742	5.4712	5.6677	5.5683	6.1347
Ppp2cb	_LQEVPHEGPM[Oxidation (M)]C[Carbamidomethyl (C)]DLLWSDPDDR_.3	#N/A	3.2046	5.9927	5.1622	4.4883	4.0431	5.7002	4.9209	4.4058	5.7115	3.3912	4.0575	3.1903	3.5334	5.9734	5.5629	3.5495	3.4426	5.9368	5.5136	5.3025
Ppp2cb	_AHQLVM[Oxidation (M)]EGYNWC[Carbamidomethyl (C)]HDR_.3	#N/A	4.6930	5.6555	4.3152	5.2983	5.3292	5.1110	5.6048	4.5376	5.4515	4.7945	4.9732	5.1021	4.1873	4.5138	4.7193	3.0107	5.7726	5.5610	6.2310	4.7016
Rpl18a	_SSGEIVYC[Carbamidomethyl (C)]GQVFEKS[Phospho (STY)]PLR_.3	P62717_S71_M1_Rpl18a	3.8134	4.8756	3.8123	3.9259	4.2924	4.7290	4.2404	3.8623	3.7438	3.7865	3.3808	3.8713	3.8404	3.8226	3.7110	3.6285	4.9036	3.8090	3.7205	3.6634
Rpl18a	_DLTTAGAVTQC[Carbamidomethyl (C)]YR_.2	#N/A	3.3756	3.9435	4.4131	4.2086	3.5826	3.0250	3.4655	3.6013	3.2985	3.1862	3.6728	3.6696	3.4065	4.4511	3.4909	4.2386	3.0985	4.0509	4.3499	4.1284
Acta2;Actg2	_YPIEHGIITNWDDM[Oxidation (M)]EK_.3	#N/A	11.2073	11.1580	11.3471	11.3233	11.0513	11.1394	11.0191	11.1809	11.1555	11.3181	11.0749	10.9509	11.0843	11.1401	11.1010	11.1420	11.1669	11.0245	11.2226	11.1323
Acta2;Actg2	_VAPEEHPTLLTEAPLNPK_.2	#N/A	8.7864	8.7707	8.6873	8.2524	8.4986	8.7135	8.8087	12.4716	12.3264	8.5091	8.6061	8.8871	8.8245	9.0857	6.0453	8.6094	8.9338	8.8143	8.7465	8.4940
Acta2;Actg2	_DLYANNVLSGGTTM[Oxidation (M)]YPGIADR_.2	#N/A	3.8822	6.5133	6.2994	3.8072	6.1902	6.0714	3.8173	3.5826	6.2129	4.3353	3.2161	4.0100	3.9029	4.4056	6.0245	6.0547	4.1203	6.4162	5.8160	5.7370
Ap2s1	_FILIQNR_.2	#N/A	9.5312	9.2371	9.3585	9.4074	9.3470	9.2508	9.4460	9.6461	9.0798	9.2700	9.2679	9.1216	9.4976	8.9446	8.4120	8.8450	9.4105	9.3877	9.2546	8.9823
Ap2s1	_HTNFVEFR_.2	#N/A	6.5665	6.2580	6.6294	6.7522	6.5562	6.5615	6.6562	6.8325	6.5122	6.3150	5.9460	6.3183	5.6081	5.6243	5.7871	5.5030	5.9552	6.2328	5.8371	6.0485
Ap2s1	_VYTVVDEM[Oxidation (M)]FLAGEIR_.2	#N/A	6.4808	6.0518	5.2932	4.0534	5.6303	5.2096	5.5988	3.3363	5.4982	5.8925	4.6961	5.0318	6.0991	3.2125	4.3076	5.0113	5.9835	5.2628	5.5736	4.5447
Rhob	_LVVVGDGAC[Carbamidomethyl (C)]GK_.2	#N/A	7.5322	7.8110	7.5819	7.8382	7.5384	7.7885	7.8095	7.4359	7.1870	7.5627	7.6355	7.7266	7.6275	7.3904	7.6357	7.1348	7.7657	7.2164	7.6158	7.3437
Rhob	_EVFETATR_.2	#N/A	7.2043	7.3758	7.3764	7.3764	7.0575	7.7021	7.4894	7.3908	7.5729	7.4916	7.5391	7.0653	6.9940	7.2179	7.6104	7.0334	6.8172	7.4589	7.2154	7.2164
Rhob	_IQAYDYLEC[Carbamidomethyl (C)]SAK_.2	#N/A	5.5580	5.1075	5.4291	5.2278	5.2183	4.9151	5.4273	3.4450	4.5364	4.6984	4.3205	5.0575	5.4692	5.0708	5.1685	3.9644	4.4431	5.3245	4.6493	4.8433

Rhob	_DLRS[Phospho (STY)]DEHVR_.2	P62746_S123_M1_Rhob	5.8956	5.5485	5.6646	4.8671	5.6002	5.6605	3.3829	4.9307	4.1774	5.4071	5.5151	4.4860	4.8713	6.3666	4.3014	4.1758	5.6862	5.1221	4.8495	4.8802
Hpcal1	_EHTEFTDHELQEWYK_.3	#N/A	4.0266	3.2925	3.2712	3.5576	4.0946	4.2944	4.3154	4.2523	2.9402	3.8372	5.1318	4.0965	4.4010	3.4028	3.9618	4.5424	3.7495	3.2148	4.3642	3.3698
Rpl23a	_LAPDYDALDVANK_.2	#N/A	8.9206	8.7736	8.4217	8.4012	8.5510	8.4476	8.5320	8.7897	8.6203	8.6364	8.6874	8.6620	8.9657	8.9006	8.6285	8.8145	8.7839	8.6066	8.3979	8.6868
Rpl23a	_IRTS[Phospho (STY)]PTFR_.2	P62751_S43_M1_Rpl23a	9.0448	9.3691	4.3049	3.9208	3.3445	3.3778	8.7861	9.4601	8.8066	4.4490	8.7983	4.1237	3.7893	9.3955	8.6713	4.0005	9.2933	8.8883	8.7595	4.2589
Rpl23a	_KLYDIDVAK_.2	#N/A	5.3054	5.4585	5.6104	5.4264	4.9919	5.3079	5.5096	5.9172	5.6294	3.1197	5.3058	5.0515	5.7116	5.3108	5.7015	5.6198	5.6644	5.3543	4.9844	5.9511
Rps6	_DIPGLTDTTVPR_.2	#N/A	7.6415	7.7697	7.5595	7.4515	7.6608	7.6363	8.0159	7.8772	8.0353	7.7435	8.0074	7.7925	8.0974	7.9716	7.7353	7.7099	7.7012	7.9568	7.5569	7.5378
Rps6	_LIEVDDER_.2	#N/A	5.7823	6.3986	5.7398	6.4215	5.9476	6.6588	6.3962	5.6390	6.5322	5.9289	6.7407	6.2569	6.4672	6.6620	6.0116	6.4899	5.9361	6.5607	6.1215	6.7117
Vsnl1	_FFPYGDASK_.2	#N/A	8.7066	9.5200	8.1274	9.9204	9.9058	9.6074	9.6156	9.2187	9.0785	9.5915	9.2999	10.0007	9.4856	8.7911	8.2764	9.1716	8.8810	9.0673	8.5616	8.7488
Vsnl1	_FAQHAFR_.2	#N/A	8.7121	8.8922	8.7952	8.7459	8.6182	8.6293	9.0747	8.7061	8.5013	8.7001	8.3017	8.9841	8.5524	8.7362	9.1344	8.5119	8.5397	8.6723	8.7333	8.4174
Vsnl1	_STEFNEHELK_.2	#N/A	7.8765	7.8083	8.1620	7.7786	7.8994	7.9466	7.9036	8.3095	8.0702	7.9968	8.0456	7.7947	7.7289	7.9877	8.3214	7.8669	8.0588	7.9316	8.1362	8.0543
Vsnl1	_EFIC[Carbamidomethyl (C)]ALSITSR_.2	#N/A	7.6364	7.5068	8.0792	7.3627	7.1128	7.4405	7.4556	7.1632	7.0791	7.4954	7.0776	7.3066	7.2059	7.5012	7.3743	7.4704	7.4159	7.0082	7.2614	7.4030
Vsnl1	_NKDDQITLDEFKEAAK_.3	#N/A	7.1282	6.9226	6.6152	6.9718	6.8442	6.9281	6.4685	7.2280	7.0675	7.1600	6.8739	7.2526	6.9956	7.1971	6.9930	7.2090	7.3948	6.9816	6.9628	6.9946
Vsnl1	_LNLEEFQQLYVK_.2	#N/A	6.9853	6.3569	7.2425	6.6867	7.0304	6.9523	6.6304	6.0015	6.5345	6.9286	6.5516	6.4457	6.8761	6.1873	6.8302	6.4953	6.6195	6.1193	6.9021	6.8699
Vsnl1	_NGDGTIDFR_.2	#N/A	6.0105	5.9582	6.7340	6.3639	6.5473	6.5340	6.3811	6.5617	6.2800	6.4232	6.6310	6.1888	4.1842	6.5487	5.5391	5.9122	5.8623	6.0816	6.1010	5.9148
Vsnl1	_LAPEVM[Oxidation (M)]EDLVK_.2	#N/A	8.3289	8.7794	8.6842	8.9551	8.7412	8.5211	8.7422	8.7644	8.4803	8.4543	8.6959	8.7916	8.8258	8.7850	8.6159	8.5024	8.8262	8.7189	8.5530	8.5853
Vsnl1	_MNEDGLTPEQR_.2	#N/A	4.4442	8.2593	9.1579	8.9556	7.9183	8.2735	8.4246	7.3418	7.6988	7.8735	8.4498	7.5831	3.9834	8.9865	8.0691	3.8064	7.8077	7.0181	7.2426	7.7726
Vsnl1	_LNWAFNM[Oxidation (M)]YDLDGDGK_.2	#N/A	6.6817	6.8419	7.1974	6.3166	6.1465	6.5375	6.4921	6.2861	6.6026	6.5780	6.4980	6.5832	6.2755	6.8185	6.5773	6.3174	6.4867	6.5482	6.5274	6.4685
Vsnl1	_M[Oxidation (M)]DKNKDDQITLDEFK_.3	#N/A	6.0621	6.4902	6.6824	6.8022	6.3233	6.3314	6.2915	5.9225	6.0994	6.2113	6.4316	6.3244	5.9832	6.5769	6.1831	6.7283	7.2014	5.8405	6.3502	6.3349
Vsnl1	_VEM[Oxidation (M)]LEIIEAIYK_.2	#N/A	6.6663	6.7006	6.9558	6.7458	6.8291	6.8929	6.4896	7.0308	6.6677	6.0328	6.2743	6.5415	5.5514	6.0489	6.4722	5.8784	6.5504	6.4758	7.6293	6.5339
Vsnl1	_TFDKNGDGTIDFR_.3	#N/A	4.2424	4.8829	3.2778	5.3533	5.0725	4.2546	5.4183	3.9427	4.7706	4.4487	4.3050	3.8654	4.2631	5.1954	3.5382	5.3150	4.4804	4.5079	5.4023	4.5802
Vsnl1	_NKDDQITLDEFK_.2	#N/A	4.9882	5.2270	5.9761	5.3320	5.1381	5.6525	4.9313	5.6771	5.4961	5.4513	5.6336	5.2247	5.1767	5.8809	5.2882	5.3397	5.8490	5.2318	5.0380	5.2249
Vsnl1	_SDPSIVLLLQC[Carbamidomethyl (C)]DIQK_.2	#N/A	5.9433	6.0692	6.6888	6.7616	6.2264	6.1280	6.3004	6.3242	6.1621	6.3663	6.4370	6.1033	6.1382	6.1124	6.3208	6.1608	6.4340	5.8183	6.5969	6.4396
Mtpn	_GPDGLTALEATDNQAIK_.2	#N/A	8.4467	8.3401	8.5665	8.5153	8.3264	8.3010	8.1180	8.4327	8.6064	8.5512	8.2543	8.3856	8.8552	8.6332	8.7812	8.6749	8.3239	8.2985	8.4747	8.5260
Mtpn	_GEDVNRT[Phospho (STY)]LEGGR_.2	#N/A	6.5548	6.9169	6.4918	7.0614	6.5786	6.7988	6.5564	6.3489	6.6690	7.2588	6.9694	6.8868	6.9278	7.3128	7.4065	7.2197	6.9273	7.1921	6.7483	7.1586
Mtpn	_NGDLDEVKDYVAK_.2	#N/A	5.0060	4.8259	5.2618	4.3965	4.4625	4.6116	5.0029	5.3579	4.5369	5.1701	4.8085	4.9920	4.5670	5.1440	4.9557	5.0979	5.1973	4.5001	4.3142	4.8261
H4c1	_VFLENVIR_.2	#N/A	10.8614	11.1499	11.0531	10.9269	10.7280	10.8227	11.3218	10.8405	10.5656	10.9688	10.5543	10.9248	10.9561	10.8441	10.4536	10.6151	10.4205	11.0411	10.7838	10.6220
H4c1	_DAVTYTEHAK_.2	#N/A	10.4890	10.5467	11.0074	10.3211	10.2430	10.5410	11.0302	10.8308	10.6824	10.5571	10.2956	10.2951	10.6932	10.5419	10.4598	10.3201	10.2602	10.4784	10.4841	10.2024
H4c1	_ISGLIYEETR_.2	#N/A	10.0035	10.4573	10.6839	10.1063	9.5395	10.0607	10.4307	10.1266	10.1862	10.0448	9.5035	9.6483	10.0437	10.3831	9.5544	9.9300	9.8994	10.1630	10.0451	9.8189
H4c1	_TVTAM[Oxidation (M)]DVVYALK_.2	#N/A	6.9071	6.9177	7.4775	6.9532	6.2762	6.9039	7.1838	6.7248	6.3681	6.6148	6.6258	6.5448	6.4180	6.5193	6.5743	6.5074	6.4292	6.6901	6.5652	6.3314
H4c1	_DNIQGITKPAIR_.2	#N/A	10.7327	10.9295	10.7115	10.3033	10.2928	10.6199	10.8062	10.8821	10.7356	10.4310	10.1047	10.4026	10.8957	10.5318	10.4611	10.1771	10.6208	10.6445	10.3430	10.0726
H4c1	_TVTAM[Oxidation (M)]DVVYALKR_.2	#N/A	5.7607	5.9589	5.6195	5.4613	5.9934	5.6280	6.3019	5.8488	5.8794	6.0719	5.5094	5.6300	6.6814	5.4691	5.4194	5.9332	6.0457	6.1582	6.0948	6.0927
H4c1	_RIS[Phospho (STY)]GLIYEETR_.2	P62806_S48_M1_H4c1	4.5560	5.0863	5.5687	5.3259	3.2621	5.7719	4.8338	3.2808	4.9549	4.3666	3.1848	3.9900	4.5869	5.3203	4.4347	3.9181	4.2789	4.3714	3.1696	3.8079
H4c1	_DAVTYTEHAKR_.3	#N/A	3.2430	3.7947	4.1994	4.0263	3.4499	3.6372	4.2610	3.3635	3.5184	4.2428	2.9970	4.2291	3.6838	4.1865	3.6236	4.0063	3.9012	3.9320	4.5179	4.9842
H4c1	_DNIQGITK_.2	#N/A	3.7106	3.6085	3.2471	3.8737	3.9175	4.3741	3.9199	3.9362	4.3684	3.5212	4.0303	3.3346	3.2163	3.2852	3.1560	3.0727	3.4335	4.2048	4.3174	3.6859
Gabra1	_NNTYAPTATSY[Phospho (STY)]TPNLAR_.2	P62812_Y374_M1_Gabra1	3.9136	7.8528	7.8846	9.0473	6.9474	6.6174	7.0467	6.9890	6.6355	8.3136	3.3919	6.5136	7.3684	7.1262	4.7073	7.1727	6.8793	6.8829	6.7379	6.7328
Atp6v1b2	_NFITQGPYENR_.2	#N/A	10.6043	10.8423	10.6488	10.7748	10.6117	10.6639	10.6253	11.1129	10.5617	10.8049	10.6376	10.7008	10.9644	10.7385	10.2378	10.5525	10.7904	10.5877	10.6265	10.6431
Atp6v1b2	_NYLSQPR_.2	#N/A	7.3019	8.6460	8.4329	9.1090	7.8123	8.7246	7.6096	7.8992	7.7648	7.4058	7.1527	7.1126	7.2018	7.3709	8.6042	8.9087	8.5161	8.5008	7.6133	8.7584
Atp6v1b2	_QIYPPINVLPSLSR_.2	#N/A	8.8139	8.7017	9.0909	8.7373	8.9784	8.9618	8.9324	8.9444	8.7161	9.1897	8.7821	8.7759	9.0168	8.8812	9.0252	8.9791	8.7712	8.6633	9.0113	8.8879
Atp6v1b2	_EQALAVSR_.2	#N/A	8.9551	8.2860	8.8775	9.0820	8.7329	8.7675	8.0978	8.7101	9.2960	8.0123	9.0935	9.0099	9.1922	9.0620	9.3848	9.1570	8.2354	9.0687	9.1992	9.0913
Atp6v1b2	_IPQSTLSEFYPR_.2	#N/A	9.0867	8.8080	9.1509	9.2875	8.9753	9.0651	8.9929	9.1158	8.8865	9.0453	8.8498	9.0316	8.8861	8.9607	9.0833	8.8683	8.7759	8.9103	8.8858	8.6612
Atp6v1b2	_AVVQVFEGTSGIDAK_.2	#N/A	8.7404	8.6515	9.2517	9.0150	8.7670	8.7377	8.5436	8.6952	8.9506	8.8945	8.7232	8.6319	8.8705	9.1499	8.6571	8.5908	8.9767	8.7196	8.7328	8.7645
Atp6v1b2	_GFPGYM[Oxidation (M)]YTDLATIYER_.2	#N/A	8.8449	8.5390	8.5601	8.1961	8.5407	8.6487	8.3997	9.0020	8.4210	8.8800	8.5489	8.9580	9.1420	8.0349	8.2954	8.8841	9.1509	8.6194	8.9861	8.3385
Atp6v1b2	_SGQVLEVSGSK_.2	#N/A	8.3447	8.4985	8.8658	8.4790	8.2448	8.5746	8.6924	8.4366	8.7652	8.6321	8.3932	8.3710	8.7286	8.5612	8.1967	8.3094	8.1702	8.3525	8.4347	8.4672
Atp6v1b2	_AVVGEEALTSDDLLYLEFLQK_.2	#N/A	6.0375	6.3896	6.5246	6.2936	5.9878	5.8601	6.3311	6.7824	6.3150	6.3953	5.7788	6.0624	6.7396	7.1515	5.8157	6.2658	6.4695	5.9044	6.0175	6.3374
Atp6v1b2	_EEVPGRR_.2	#N/A	6.5695	6.0947	6.5498	6.3415	6.8333	6.7499	6.8169	6.6503	6.7033	6.8528	6.7013	6.5353	7.1836	6.1782	7.1616	7.1022	6.2778	6.4201	6.6077	6.5562
Atp6v1b2	_IPIFSAAGLPHNEIAAQIC[Carbamidomethyl (C)]R_.3	#N/A	6.2324	6.2775	6.3752	6.7663	5.9403	6.6123	6.4952	6.2562	5.7517	6.2911	6.2071	6.1472	6.3443	6.7740	6.1437	6.0712	6.4225	6.2850	6.5814	6.4798
Atp6v1b2	_GPVVLAEDFLDIM[Oxidation (M)]GQPINPQC[Carbamidomethyl (C)]R_.3	#N/A	6.2449	6.2760	6.8314	6.2553	6.4880	6.3777	6.1613	6.0913	6.2647	6.6348	6.4879	5.6042	5.9296	6.0493	5.9545	6.3094	6.1380	6.2718	6.5079	5.9314
Atp6v1b2	_TSC[Carbamidomethyl (C)]EFTGDILR_.2	#N/A	6.0164	5.9947	6.3838	6.2905	5.8360	6.5749	6.2440	5.8085	6.0705	6.0689	5.7209	6.0728	3.8162	6.1283	6.1219	5.6476	6.1383	6.1671	6.0302	6.0953
Atp6v1b2	_TPVSEDMLGR_.2	#N/A	3.8464	8.1973	8.7100	8.8666	7.2803	7.7043	8.4486	5.4556	7.2108	6.8711	7.9612	6.8974	4.5813	8.8392	7.6563	3.2085	6.6903	6.3449	6.3867	6.5018
Atp6v1b2	_KTSC[Carbamidomethyl (C)]EFTGDILR_.3	#N/A	6.7369	6.9719	7.2090	7.4230	6.6716	6.9849	7.0849	6.8164	7.2720	6.9142	6.5724	6.6873	7.0918	7.0465	7.3141	7.0645	6.6942	6.5209	6.9191	6.9341
Atp6v1b2	_IYPEEM[Oxidation (M)]IQTGISAIDGM[Oxidation (M)]NSIAR_.3	#N/A	6.0874	5.8953	6.2176	5.9973	5.8058	7.0713	5.7996	5.7732	6.8116	6.0424	5.3479	6.4483	5.8646	5.2279	5.5474	6.5232	6.0426	5.4842	6.5699	5.6735
Atp6v1b2	_SAIGEGMTR_.2	#N/A	3.5432	7.4812	4.0795	8.3467	6.2669	7.3674	7.9055	3.2436	6.5490	6.2015	7.6290	6.4390	3.5640	8.5951	7.4778	3.8881	5.9789	4.0519	5.7757	5.9963
Atp6v1b2	_YAEIVHLTLPDGTKR_.3	#N/A	7.7590	7.1210	7.3535	7.3282	7.3132	7.3509	7.3759	7.7978	7.5235	7.6284	7.1641	7.3993	7.9182	7.4088	7.4037	7.4473	7.2535	7.3609	7.5554	7.3913
Atp6v1b2	_LALTTAEFLAYQC[Carbamidomethyl (C)]EK_.2	#N/A	6.5702	6.4020	7.0105	6.5660	6.4401	6.0998	6.6562	6.0558	6.4956	6.7204	6.4849	6.3074	6.9593	6.2191	5.9959	6.2950	6.4202	5.6860	6.1608	6.3045
Atp6v1b2	_YAEIVHLTLPDGTK_.2	#N/A	4.8501	4.9981	5.9173	5.2557	5.1654	5.3701	4.9423	5.4116	4.7172	4.7473	5.1004	4.8449	5.1194	5.5796	4.6831	5.0741	4.8741	5.1343	4.7316	5.1385
Atp6v1b2	_TVSGVNGPLVILDHVK_.3	#N/A	4.1985	3.7944	4.6818	5.3604	3.8280	3.4216	2.6247	3.9088	4.0637	5.0025	3.0280	3.6838	4.2291	3.2310	3.7095	3.5606	4.4465	3.3262	3.0353	3.5543
Atp6v1b2	_TVYETLDIGWQLLR_.2	#N/A	3.9890	5.0754	4.5253	4.7477	4.9198	3.5508	4.2143	3.6894	4.2734	4.1953	3.1470	3.9032	4.3815	4.6462	3.7915	4.3339	4.2271	4.8974	3.3750	3.6025
Atp6v1b2	_HVLVILTDM[Oxidation (M)]SSYAEALR_.3	#N/A	5.5858	5.4470	5.6820	5.5132	5.2222	4.2990	4.1434	5.7702	5.0210	5.6748	5.1951	3.8414	5.7753	5.1269	4.3475	5.4998	4.6622	4.8716	4.9000	4.9753
Atp6v1b2	_SDFEENGSM[Oxidation (M)]DNVC[Carbamidomethyl (C)]LFLNLANDPTIER_.3	#N/A	3.0914	3.4105	3.6277	3.0971	3.9300	4.0550	3.6111	4.2926	4.4475	3.2977	3.9262	3.3000	3.1121	3.6148	3.1883	3.4363	3.3295	3.0028	4.1523	3.5818
Rab1A	_QWLQEIDR_.2	#N/A	8.5542	8.3944	8.4634	8.4887	8.3549	8.3195	8.6765	8.8204	8.7612	8.1916	8.5791	8.3693	8.7199	7.9510	7.4646	7.7933	8.6727	8.5077	8.0139	8.1539
Rab1A	_EFADSLGIPFLETSAK_.2	#N/A	8.2815	7.7841	8.4991	8.2249	8.1420	8.2243	8.1317	7.8408	8.4448	8.0918	8.2695	8.0001	8.1285	7.2876	7.3880	7.8547	8.0370	7.7472	7.8923	7.8486
Rab1A	_IQST[Phospho (STY)]PVK_.2	P62821_T195_M1_Rab1A	7.6502	7.7953	7.7343	8.0080	7.6390	7.8450	4.2548	7.3120	4.3139	4.1548	3.1875	7.4471	8.5694	7.1330	3.8320	7.4408	4.1140	3.0354	7.9810	7.4471
Rab1A	_KVVDYTTAK_.2	#N/A	6.2949	6.7563	6.6398	6.3732	6.5462	6.3309	6.9034	6.9045	6.5137	6.3550	6.2446	6.5261	5.6028	5.9086	5.9813	6.0477	6.3826	6.6100	6.0202	6.0073
Rab1A	_TITS[Phospho (STY)]SYYR_.2	P62821_S78_M1_Rab1A	4.9760	6.2672	4.5379	3.9252	4.2647	4.9957	5.5673	3.7860	5.2146	3.7484	3.7005	4.6678	5.0791	4.2790	4.4604	4.4175	3.7203	6.2607	5.0017	4.2631
Rab1A	_GAHGIIVVYDVTDQESFNNVK_.3	#N/A	6.8811	6.7239	3.9931	6.0585	5.7383	5.3168	5.9511	6.8619	5.6682	5.3863	5.9942	6.0208	6.6095	6.8826	3.4173	4.7278	6.2686	6.0513	5.3355	5.2774
Rab1A	_YASENVNK_.2	#N/A	7.3819	7.6774	7.3217	7.4313	7.3157	7.3945	7.6201	7.6800	7.7798	7.2253	7.3422	7.0898	7.2517	7.2884	6.9525	6.9478	7.4994	7.5775	7.3330	7.2371
Rab1A	_FADDTYTESYISTIGVDFK_.2	#N/A	6.8888	5.7656	6.2311	5.7182	5.1911	5.2991	5.6644	6.5154	5.5910	5.7533	5.2631	5.7703	6.6193	6.1613	3.9674	5.4553	6.0760	5.6601	5.3229	5.2523
Rab1A	_QWLQEIDRYAS[Phospho (STY)]ENVNK_.3	P62821_S114_M1_Rab1A	3.2849	3.2171	3.8212	4.4045	4.1234	4.3475	3.4176	4.4861	3.4767	3.4912	3.8511	3.1065	3.5848	4.6294	2.9948	3.6298	3.5229	4.8373	4.1717	4.3067
Rab1A	_MGPGATAGGAEK_.2	#N/A	3.9142	3.4049	5.3505	3.6701	4.1211	3.9873	5.2469	4.1398	3.8370	3.7248	4.2114	3.1310	3.9450	3.4888	2.9524	3.2763	3.6371	3.5124	3.8114	3.1661
Rab3c	_TITTAYYR_.2	#N/A	8.2303	8.3555	7.8375	8.1804	7.7960	8.2077	8.6133	7.5274	8.4999	8.3601	8.3268	8.1903	8.5301	7.8701	7.6780	7.9895	7.2633	8.3949	8.0808	8.1018
Rab3c	_TYSWDNAQVILAGNK_.2	#N/A	7.1061	6.9202	6.9351	7.0019	6.5130	6.4482	6.7400	6.3611	6.4553	6.5373	6.5847	6.0405	6.8348	6.4334	5.4723	6.1793	6.6245	6.5457	6.1014	5.8303
Rab3c	_LVDIIC[Carbamidomethyl (C)]DK_.2	#N/A	6.6952	6.1220	7.9050	7.9958	7.9142	6.9083	6.7805	6.1649	6.4007	5.6422	6.8193	6.4999	4.5105	4.3834	6.3772	6.6275	7.3588	6.7923	6.8165	8.0580
Rab3c	_MSESLETDPAITAAK_.2	#N/A	6.8428	6.1469	4.9597	5.9256	5.4348	6.2587	5.3712	5.9132	6.2266	6.3961	5.2706	6.4459	6.5333	6.3264	5.6514	6.3412	6.6161	6.5601	6.1682	5.9266
Rab3c	_LGEQLGFEFFETSAK_.2	#N/A	5.3268	4.9108	4.2049	4.6235	5.0308	3.2331	4.8067	4.9475	5.0467	3.8776	5.4260	4.8903	4.5546	4.2430	4.1092	4.8090	4.9303	5.2210	4.6012	5.2120
Rab3c	_DNINVKQTFER_.3	#N/A	3.7008	4.3020	4.2371	4.4462	3.0385	3.2626	3.0017	3.3637	3.0608	3.9071	3.4352	4.1915	3.1689	4.2135	4.0798	4.0457	4.3617	4.2885	3.0868	3.8908
Ran	_VC[Carbamidomethyl (C)]ENIPIVLC[Carbamidomethyl (C)]GNK_.2	#N/A	8.5706	8.5129	9.1396	8.7560	8.4040	8.4075	8.4139	8.1971	8.5940	8.6985	8.6973	8.2455	8.0808	8.5781	8.7586	8.3854	8.7808	8.2679	8.4474	8.1498
Ran	_LVLVGDGGTGK_.2	#N/A	8.1672	8.6265	8.2638	8.3157	8.4442	8.4066	8.4739	8.2636	8.4329	8.4661	8.5845	8.0628	8.8679	8.3921	8.4749	8.4536	7.9173	8.3222	8.5421	8.2567
Ran	_[Acetyl (Protein N-term)]AAQGEPQVQFK_.2	#N/A	8.5675	8.1732	8.4683	8.4119	8.4719	8.6776	8.4692	8.5750	8.5382	8.3113	8.3603	8.4473	8.6788	8.3377	8.3215	8.3868	8.2797	8.4027	8.2030	8.5075
Ran	_NLQYYDISAK_.2	#N/A	8.3881	8.2005	8.6907	8.3937	8.2299	7.9847	8.0477	7.6607	8.2146	8.1397	8.0441	7.7997	7.7449	7.8492	8.1833	8.2278	7.5814	7.9143	7.8228	8.2630
Ran	_FNVWDTAGQEK_.2	#N/A	7.9727	8.2276	8.1411	8.3133	7.8843	8.1848	8.1765	8.4120	8.1091	8.1114	8.1651	8.1565	8.3646	8.2736	8.2421	8.0056	8.3654	8.2244	8.1210	8.1091
Ran	_HLTGEFEK_.2	#N/A	6.2944	5.8075	6.8057	6.2406	5.9911	6.1408	5.9284	6.1538	6.1196	6.1698	5.9772	5.6983	5.6422	5.4210	6.4954	5.3826	5.9677	5.0173	6.0859	6.0921
Ran	_YVATLGVEVHPLVFHTNR_.3	#N/A	3.8744	5.3892	6.5227	5.5574	4.0814	5.8333	5.3619	5.6705	4.3878	5.8523	4.7848	3.3597	4.5532	5.4296	2.9921	4.8109	5.7729	5.4259	4.8216	5.5052
Rpl23	_ISLGLPVGAVINC[Carbamidomethyl (C)]ADNTGAK_.2	#N/A	6.7798	6.5109	5.5814	6.0294	5.4867	6.2098	6.2699	6.3469	5.6427	5.7780	6.2827	6.3972	6.7079	5.7045	6.1217	6.4112	6.6428	6.5247	5.9629	6.3357
Rpl23	_GSAITGPVAK_.2	#N/A	5.2996	5.7924	5.9472	6.2028	4.2136	5.5836	6.4801	5.7833	6.2272	5.9189	5.8757	5.2837	5.7830	6.2208	6.3415	5.6152	5.8921	6.3172	5.7953	6.0288
Rap1a	_YDPTIEDSYR_.2	#N/A	8.8340	8.4442	8.2189	8.3793	8.3772	8.2422	8.2282	8.5686	8.3859	8.5015	8.2526	8.1545	8.6133	8.2273	7.9159	8.3073	8.3557	8.1723	8.1562	8.2439
Rap1a	_YDPTIEDSYRK_.2	#N/A	7.9786	7.8951	7.9491	7.8616	7.7872	7.8169	8.0319	7.8482	8.0050	7.2841	7.6806	7.5359	8.1540	7.8093	7.9283	7.8844	7.8059	7.9244	7.5961	7.9107
Rap1a	_LVVLGSGGVGK_.2	#N/A	7.4383	7.6131	7.0752	7.1939	6.9920	7.3538	7.7085	7.6606	6.8954	7.1097	6.9153	7.5584	7.1628	7.3147	6.8203	6.7462	6.5687	7.7133	7.0217	6.6713
Rap1a	_EQGQNLAR_.2	#N/A	6.6287	6.8819	7.0582	6.7756	5.9758	7.0950	6.7122	6.4872	7.1821	6.9297	6.9441	6.9195	7.0086	6.8281	7.3578	6.7583	6.9857	7.0533	7.1940	6.7868
Rap1a	_INVNEIFYDLVR_.2	#N/A	3.9492	4.9510	5.5109	5.4689	5.0681	4.8337	5.1555	5.0486	5.5206	4.5722	4.5857	4.6058	5.1493	4.5383	5.1763	5.2069	5.3265	5.1905	5.0765	4.7493
Rap1a	Carbamidomethyl (C)]QQC[Carbamidomethyl (C)]M[Oxidation (M)]LEILDTAGTEQFTAM[Oxidati	#N/A	4.6322	4.4467	4.0025	4.0329	3.1035	4.0299	3.4769	3.9918	4.2269	4.7023	3.8872	3.7862	3.4870	4.3018	4.0804	4.5028	4.3825	3.6370	3.8133	4.2518
Rap1a	_QWC[Carbamidomethyl (C)]NC[Carbamidomethyl (C)]AFLESSAK_.2	#N/A	4.3253	3.6775	3.3608	3.3640	3.6631	3.8871	3.8780	4.0257	3.9371	4.5317	4.3115	3.5669	4.0452	3.3479	4.0302	3.1694	3.0626	3.2698	3.7534	3.3149
Rps24	_TTPDVIFVFGFR_.2	#N/A	3.0831	4.2360	4.7686	4.2343	3.2900	2.9526	2.6376	3.2779	3.6783	4.3945	3.1569	3.9621	3.8437	4.3464	3.7835	3.7440	4.3068	3.4312	4.0659	4.3134
Rps25	_LITPAVVSER_.2	#N/A	7.9083	8.3202	7.7944	7.9116	8.1726	7.8683	8.2290	7.9861	7.8941	7.6427	7.8566	8.0918	7.7868	7.7696	8.4363	7.9950	7.8897	8.0543	7.7095	7.9875
Rps25	_AALQELLSK_.2	#N/A	6.0702	6.3405	6.2998	6.3902	6.4876	6.4532	6.6453	6.1314	5.9907	6.5965	5.9778	6.5302	6.5134	6.3913	6.8546	6.6222	6.2551	6.5062	6.1488	5.9942
Rps25	_AQVIYTR_.2	#N/A	5.1034	4.9427	5.0151	4.9440	4.5631	4.9216	5.1303	4.5290	4.7851	5.5397	5.4605	5.7269	5.7712	4.7428	5.8853	5.6432	4.4746	5.6583	5.2661	5.5503
Rps26	_DISEASVFDAYVLPK_.2	#N/A	3.8231	3.5034	5.0681	5.0108	3.1608	3.6529	4.0236	3.2413	5.1134	4.4615	3.9849	4.6738	4.5474	4.0911	4.8334	4.4722	3.3538	4.4109	3.2091	3.7793
Rps28	_EGDVLTLLESER_.2	#N/A	7.8401	7.3726	7.5369	7.6730	7.3990	7.5625	7.4784	7.8500	7.7256	7.5454	7.9636	7.3365	7.5001	7.7361	7.1144	7.4439	7.5686	7.3735	7.3341	7.3960
Rps28	_TGS[Phospho (STY)]QGQC[Carbamidomethyl (C)]TQVR_.2	P62858_S23_M1_Rps28	7.4991	3.2688	8.0116	7.7132	6.9889	7.0900	7.4904	7.3953	7.0024	7.9509	7.1184	7.3444	7.4716	7.7370	6.9402	6.9945	7.1762	7.0355	7.2878	7.1694
Elob	_ADDTFEALR_.2	#N/A	5.4741	6.1376	6.5089	6.2335	5.9854	6.2294	6.3857	5.8743	6.5358	6.2002	5.8154	5.6117	6.6187	6.7131	6.7227	6.0785	6.0225	5.9968	6.4418	6.4780
Elob	_ESSTVFELK_.2	#N/A	3.7572	4.4136	4.8469	5.1141	3.2710	4.9942	4.2112	4.1940	5.1280	5.0646	4.3781	4.1397	4.4372	4.4997	4.6640	4.6764	4.4891	4.8479	4.8727	4.3985
Elob	_TTIFTDAK_.2	#N/A	3.1236	4.1954	4.1611	4.4606	3.3306	3.4266	3.7175	3.3493	3.8810	3.4953	3.1164	3.4482	3.8032	4.1991	4.4141	3.3986	4.3474	3.5374	3.1011	3.8764
Gnb1	_VHAIPLR_.2	#N/A	11.0371	11.1878	11.3137	11.1742	11.1281	11.1453	11.4413	11.3398	10.8272	11.0427	10.6861	11.3262	11.1806	11.0832	11.4299	10.8495	10.8438	11.3143	11.2483	10.9367
Gnb1	_LLVSASQDGK_.2	#N/A	10.6323	10.6136	11.1127	10.9531	10.7821	10.8544	10.8728	10.7121	11.0966	10.9775	10.5948	10.6798	10.7089	10.7305	10.7459	10.8746	10.5621	10.5589	10.8302	10.7156
Gnb1	_AGVLAGHDNR_.2	#N/A	10.7528	10.6434	10.7284	10.6126	10.5206	10.6083	10.7629	10.9656	10.7173	10.4887	10.6310	10.6326	10.4169	10.4574	10.2687	10.1898	10.5178	10.5626	10.3301	10.4239
Gnb1	_LIIWDSYTTNK_.2	#N/A	10.2498	10.4437	10.7222	10.5625	10.1278	10.2738	10.1665	10.4290	10.3697	10.4783	10.1717	10.0878	10.5682	10.6170	9.9967	10.2751	10.3059	10.2746	10.3121	10.1982
Gnb1	_ELAGHTGYLSC[Carbamidomethyl (C)]C[Carbamidomethyl (C)]R_.3	#N/A	7.9176	8.0140	8.6737	8.4397	8.0577	8.1338	8.2918	7.9507	8.1657	8.1452	7.7712	8.0461	7.8947	7.9770	8.4361	7.9055	7.8874	7.7961	8.1933	7.8781
Gnb1	_LFVSGAC[Carbamidomethyl (C)]DASAK_.2	#N/A	8.3171	9.3063	8.9605	9.1929	8.4748	9.0082	9.4130	8.4895	8.6649	8.8056	8.2913	8.6747	8.5274	8.9844	8.8307	8.5561	8.5587	9.0723	8.8577	8.4912
Gnb1	_KAC[Carbamidomethyl (C)]ADATLSQITNNIDPVGR_.3	#N/A	8.6837	8.6676	8.5104	8.6921	8.3686	8.4930	8.5550	8.7969	8.5335	8.5570	8.5061	8.2646	8.6532	8.3870	8.3875	8.4969	8.8306	8.2957	8.4192	8.3372
Gnb1	_VSC[Carbamidomethyl (C)]LGVTDDGM[Oxidation (M)]AVATGSWDSFLK_.3	#N/A	7.5870	7.2788	7.1034	7.4083	7.0979	7.1559	6.9629	7.2733	7.2912	7.5969	6.9479	7.1714	8.1006	7.0927	6.6802	7.5142	7.9126	6.7929	7.1711	7.3156
Gnb1	_LLLAGYDDFNC[Carbamidomethyl (C)]NVWDALK_.2	#N/A	6.0420	7.2988	6.3482	7.0425	6.3857	6.5467	6.6583	6.8914	6.3410	6.6228	6.4332	6.5840	7.0593	7.2854	6.2720	6.7795	6.9571	6.7949	6.7426	6.2168
Gnb1	_ADQELM[Oxidation (M)]TYSHDNIIC[Carbamidomethyl (C)]GITSVSFSK_.3	#N/A	8.3937	8.6832	8.9380	8.5775	7.1105	8.0301	7.8210	9.9564	7.8204	9.7222	7.5435	7.9916	8.7919	9.4364	6.9347	8.2031	8.4544	9.4565	7.9637	9.4128
Gnb1	_[Acetyl (Protein N-term)]SELDQLRQEAEQLK_.2	#N/A	6.2811	6.7470	6.6563	6.7605	6.2985	6.7155	6.7035	6.8637	6.6752	6.7661	6.5515	6.8939	6.9065	6.9777	6.6943	6.6907	6.9309	6.7858	6.7876	6.6139
Gnb1	_[Acetyl (Protein N-term)]SELDQLRQEAEQLKNQIR_.3	#N/A	4.7489	6.2239	4.4878	4.7126	5.0770	4.6140	5.3880	5.4519	4.4285	5.5703	4.3126	5.1221	5.6814	5.6333	4.6462	4.7263	5.0555	4.6788	3.9673	5.1653
Gnb1	_IYAM[Oxidation (M)]HWGTDSR_.2	#N/A	7.9449	8.3636	8.3075	8.4292	8.0780	8.3302	8.2908	8.2659	8.2704	8.2105	7.7480	8.2491	8.4041	8.4272	8.3674	8.4181	8.4189	8.3713	8.3417	8.2184
Gnb1	_AC[Carbamidomethyl (C)]ADATLSQITNNIDPVGR_.2	#N/A	8.2638	8.2815	8.7705	8.0944	8.2162	8.4300	8.4595	8.1231	8.2364	8.3725	8.3053	8.0059	7.8553	8.5890	8.4456	8.1932	8.4822	8.0154	8.2074	8.2182
Gnb1	_[Acetyl (Protein N-term)]SELDQLR_.2	#N/A	4.3267	3.6761	3.3621	4.6376	3.6644	3.8885	3.3439	4.2386	3.9358	3.6903	4.6008	3.5656	4.0438	3.5876	3.4539	3.1707	3.7359	3.4136	3.2589	4.0099
Gnb1	on (M)]TC[Carbamidomethyl (C)]AYAPSGNYVAC[Carbamidomethyl (C)]GGLDNIC[Carbamidome	#N/A	3.1107	3.3913	3.6470	4.5787	3.9492	4.1733	3.5918	4.3119	3.6509	3.3170	4.0253	3.2807	3.7590	3.6340	3.1690	3.4556	3.3487	2.9836	4.1330	3.6010
Gnb1	_QTFTGHESDINAIC[Carbamidomethyl (C)]FFPNGNAFATGSDDATC[Carbamidomethyl (C)]R_.3	#N/A	4.3725	3.3519	3.5037	3.6171	3.7102	3.9343	3.8309	4.1928	3.8900	4.5788	4.2643	3.0781	3.9980	3.5418	3.4081	3.2165	3.6900	3.4594	3.7584	3.2191
Rbx1	_QVC[Carbamidomethyl (C)]PLDNR_.2	#N/A	3.4323	5.7155	6.3383	6.6553	4.2709	6.6950	6.6521	6.5253	6.4491	6.6584	3.7036	6.7803	6.4549	6.6230	7.0542	3.7772	3.1294	6.4458	4.3191	4.1592
Rbx1	_[Acetyl (Protein N-term)]AAAMDVDTPSGTNSGAGK_.2	#N/A	3.2445	3.2575	6.0391	5.9413	4.0830	4.3071	4.4256	4.4457	3.5171	2.0055	5.4929	3.1469	3.6252	3.7678	4.9917	3.5894	3.4825	4.3506	3.9992	3.7348
Gnb2	_TFVSGAC[Carbamidomethyl (C)]DASIK_.2	#N/A	8.1137	8.7546	8.8451	8.6824	7.4162	8.3284	8.2697	7.9870	8.4844	8.3039	7.8742	8.0991	8.2889	8.4871	8.6788	8.2731	8.1155	8.5571	8.3942	8.2159
Gnb2	_KAC[Carbamidomethyl (C)]GDSTLTQITAGLDPVGR_.3	#N/A	8.4850	8.4431	8.7422	8.6446	8.3155	8.3298	8.3324	8.5710	8.1080	8.5188	8.0533	8.4461	8.6873	8.7948	8.3032	8.4523	8.4148	8.3939	8.2196	8.2705
Gnb2	_AC[Carbamidomethyl (C)]GDSTLTQITAGLDPVGR_.2	#N/A	6.5291	6.4980	7.0656	6.6230	6.2294	6.8156	6.5764	6.3933	6.8225	6.5746	7.2341	6.0332	6.1134	6.8815	6.4865	6.4567	6.6223	6.2468	6.4334	6.5864
Gnb2	_[Acetyl (Protein N-term)]S[Phospho (STY)]ELEQLRQEAEQLR_.2	#N/A	5.6821	6.3624	5.4033	6.3127	5.3984	5.6580	5.9612	6.0912	5.3091	6.0349	5.4278	5.6813	6.1929	5.9034	5.5535	5.5714	6.0438	5.9164	5.9271	5.5605
Gnb2	_LLLAGYDDFNC[Carbamidomethyl (C)]NIWDAM[Oxidation (M)]K_.2	#N/A	6.8545	7.1069	7.2239	6.8288	6.9679	6.4384	6.6978	7.2208	7.0253	7.0802	6.7876	6.8559	7.1447	6.9932	6.7557	6.3747	7.1076	6.5899	6.9951	6.9020
Gnb2	_ELPGHTGYLSC[Carbamidomethyl (C)]C[Carbamidomethyl (C)]R_.3	#N/A	8.1615	8.3815	8.6329	8.4872	8.2968	8.2782	8.7062	8.4713	8.4057	8.2753	8.3417	8.1726	7.4080	8.4687	8.7815	8.1476	8.2269	8.3065	8.4235	8.1262
Gnb2	_[Acetyl (Protein N-term)]SELEQLR_.2	#N/A	3.4648	4.6188	4.3517	4.9354	4.7005	4.9628	4.4141	3.8796	4.6649	4.7778	5.2610	3.5624	3.8072	4.2420	4.8627	4.1721	3.9853	4.7590	4.6777	4.5416
Gnb2	_ADQELLM[Oxidation (M)]YSHDNIIC[Carbamidomethyl (C)]GITSVAFSR_.3	#N/A	6.4041	3.9123	4.5924	3.5989	2.8586	3.6570	4.1129	3.7955	3.8131	4.3015	3.0455	3.8018	4.2800	4.4138	4.1613	6.4571	4.4988	3.1821	3.1532	3.5011
Gnb2	_QTFIGHESDINAVAFFPNGYAFTTGSDDATC[Carbamidomethyl (C)]R_.3	#N/A	4.5423	3.4605	3.5778	3.1471	3.8801	4.1049	1.3573	4.2427	4.3976	3.6752	3.8762	3.3499	4.5630	3.5649	3.2382	3.3864	3.2795	3.0528	4.2022	3.5319
Gnb5	_SEAES[Phospho (STY)]LKGK_.2	#N/A	8.2661	4.5539	8.4291	8.1512	7.7931	8.0401	7.9727	7.9179	7.7691	8.3563	8.0930	7.9074	3.4208	8.5595	7.7357	7.7507	8.0258	7.9056	7.9662	8.0205
Gnb5	_VIVWDSFTTNK_.2	#N/A	6.1655	6.1487	6.0703	5.7513	5.7554	5.8410	5.4226	6.0127	6.2112	6.2706	6.1129	5.7733	5.7630	6.1240	5.2648	5.6399	6.0995	5.8149	6.1251	5.9623
Gnb5	_SEAES[Phospho (STY)]LKGKLEEER_.2	#N/A	3.2084	5.0844	4.2847	5.5872	3.4154	4.2609	3.6341	3.4610	3.5543	5.1915	3.0330	4.3561	3.7198	4.2976	4.7817	4.0714	4.2499	4.0219	3.2604	4.4468
Gnb5	_SGQC[Carbamidomethyl (C)]VQAFETHESDVNSVR_.3	#N/A	4.2756	4.7781	4.9990	4.4882	3.8300	4.2482	4.5834	5.2165	4.5136	4.2404	4.2698	4.0176	5.0025	5.2772	3.5870	4.4225	3.8749	4.7387	4.5436	4.7575
Gnb5	_LHDVELHQVAER_.3	#N/A	4.4036	4.5251	3.9402	4.8598	3.1097	4.2799	3.9384	3.7037	3.8586	4.2142	3.3372	4.1424	4.0240	4.1400	3.9637	4.8869	4.2413	3.5918	3.2404	4.6397
Gnb5	_VSPDGTAFC[Carbamidomethyl (C)]SGSWDHTLR_.2	#N/A	3.8172	4.1856	4.3535	3.8722	3.1549	5.4032	5.2006	4.6683	4.4452	4.0235	3.3188	4.0750	4.5533	4.3406	3.9633	4.1621	4.0553	4.4050	4.4703	3.7743
Rpl30	_VC[Carbamidomethyl (C)]TLAIIDPGDSDIIR_.2	#N/A	7.3562	7.2236	7.0060	7.3831	6.8100	6.9828	7.1554	6.8902	7.3288	7.0099	6.7960	6.7671	7.3593	7.3580	6.7925	7.0123	6.9328	6.8862	6.8531	6.6300
Rpl30	_LVILANNC[Carbamidomethyl (C)]PALR_.2	#N/A	5.9671	6.2037	6.0873	5.9595	6.1628	5.9993	6.2696	6.1251	5.6910	6.0902	6.2695	6.3127	5.6580	6.1057	5.7883	6.1985	6.0368	5.9405	6.2041	5.9999
Cycs	_TGPNLHGLFGR_.2	#N/A	9.7141	9.3599	9.5208	9.1735	9.3821	9.1567	9.2234	9.9063	9.0070	9.1287	9.0918	9.5941	9.9071	9.9460	9.5459	9.2829	9.6558	9.4748	9.3645	9.4170
Cycs	_TGQAAGFSYTDANK_.2	#N/A	6.7432	6.6493	7.4023	6.8194	6.1390	6.6814	6.2283	6.0684	7.1068	6.0166	6.3754	6.3025	6.5575	7.4592	6.2446	6.4209	6.9521	6.5946	6.6922	6.8055
Cycs	_GITWGEDTLM[Oxidation (M)]EYLENPKK_.3	#N/A	6.7401	6.6710	6.6540	6.5611	6.5070	6.4026	6.5235	6.5112	6.8665	6.6384	6.2081	6.7655	7.2530	6.8358	6.5219	6.5203	6.9381	6.9783	6.8992	6.7699
Cycs	_ADLIAYLK_.2	#N/A	3.8605	4.5219	5.6240	5.6451	5.0103	4.8907	5.0857	4.2079	4.9064	5.7296	4.8633	6.2573	3.9941	5.0681	5.9307	5.1693	3.2676	5.4689	5.8839	4.4254
Cycs	_KTGQAAGFSYTDANK_.3	#N/A	4.8341	5.0159	5.4829	4.9180	4.8637	5.3905	5.2856	3.8552	5.7225	5.2609	5.7243	5.2185	4.9897	5.5381	5.0715	5.9674	4.8546	5.1537	5.4653	5.4426
Rpl31	_SAINEVVTR_.2	#N/A	7.0885	7.1548	7.5140	7.2918	7.3065	7.0192	7.2315	7.3743	7.2798	7.0140	6.9464	6.7681	7.4443	6.6977	7.5687	7.2675	7.1712	7.2775	6.6273	6.8040
Rpl31	_EYTINIHK_.2	#N/A	5.9934	5.3880	5.6910	2.8930	5.5462	6.0769	5.5296	4.8406	5.6721	5.9452	5.3709	4.6658	5.1920	5.8838	5.7866	5.7692	5.4829	5.3639	5.7184	5.6585
Rpl31	_LYTLVTYVPVTTFK_.2	#N/A	5.0976	4.2251	4.2101	4.6016	4.3279	5.1003	4.8367	4.2042	4.6039	4.0148	3.5413	4.4151	5.1011	4.6988	4.3196	4.1183	4.6073	3.3028	3.8628	4.7039
Rpl31	_NLQTVNVDEN_.2	#N/A	3.7685	5.2671	5.5238	6.0453	4.5095	4.0088	4.6138	3.9942	4.6802	3.5791	4.0687	3.2767	5.7810	3.3432	4.2784	4.5054	4.8734	3.6580	4.1499	5.7102
Rps3	_DEILPTT[Phospho (STY)]PISEQK_.2	P62908_T221_M1_Rps3	10.7059	10.7158	11.0955	4.3057	10.0845	10.6158	3.6636	10.4271	4.0519	10.7690	3.6377	10.1239	10.6952	10.7363	9.6644	3.7309	11.0513	4.1480	10.6196	10.5563
Rps3	_AELNEFLTR_.2	#N/A	6.7685	6.9737	6.9107	6.9211	6.7246	6.6195	6.6486	7.1028	6.3175	6.6538	6.7524	7.0297	7.2668	6.4705	6.5129	6.4537	6.6484	6.8391	6.7271	6.4709
Rps3	_GLC[Carbamidomethyl (C)]AIAQAESLR_.2	#N/A	5.8623	6.9945	6.4920	6.1382	5.7478	6.2103	6.5534	6.3113	6.3426	6.1861	6.1786	6.3390	4.3497	6.1207	5.7142	6.0825	6.6731	6.0749	6.4132	6.0177

Rpl32	_SYC[Carbamidomethyl (C)]AEIAHNVSSK_.2	#N/A	3.5996	6.1486	5.4858	5.6872	5.0033	5.3715	5.3947	4.9009	4.9827	4.7945	4.7492	5.2944	3.2700	6.0241	5.8689	4.7578	5.6756	5.3716	4.9764	5.0869
Rpl8	_DIIHDPGR_.2	#N/A	6.8567	6.5551	6.1933	6.1000	5.8042	6.8360	6.9795	6.0663	6.4288	6.1322	6.6352	5.6518	6.5687	6.2408	6.4364	6.1945	6.1511	6.5729	6.0162	6.5530
Rpl8	_AVVGVVAGGGR_.2	#N/A	3.7882	5.4201	4.5837	5.7632	4.5834	5.5961	5.8797	3.4886	6.1207	5.3535	5.1332	5.4199	4.5823	4.9748	5.2976	4.5785	5.2501	4.8460	5.1965	5.4370
Cacng7	_YPDHLHISTS[Phospho (STY)]PC[Carbamidomethyl (C)]_.2	P62956_S273_M1_Cacng7	8.7291	9.1585	9.2592	8.9589	7.7898	8.8225	8.6677	8.2024	8.6486	8.4887	7.9895	8.3161	4.4188	8.5472	7.3800	8.5312	9.2367	8.5561	8.7394	8.3410
Cacng7	_LS[Phospho (STY)]DC[Carbamidomethyl (C)]SDYSGQFLQPEAWR_.2	P62956_S222_M1_Cacng7	3.3810	3.9380	4.4185	4.2032	3.5880	3.0196	8.6051	3.6067	3.3039	3.1916	3.6782	3.6642	3.4119	4.4565	3.4855	4.2440	3.1039	4.0455	4.3445	4.1338
Ybx1	_AADPPAENSS[Phospho (STY)]APEAEQGGAE_.2	P62960_S312_M1_Ybx1	3.7200	3.9626	4.4909	6.7669	3.4923	3.6099	3.8919	3.4557	3.9046	3.7711	6.4007	6.6748	4.0315	4.3055	3.6397	4.3082	3.7495	3.5480	3.8470	6.8294
Pfn1	_DSPSVWAAVPGK_.2	#N/A	9.6375	9.5279	9.8120	9.6199	9.5297	9.4636	9.1953	9.5480	9.4001	9.6689	9.2764	9.3607	9.4976	9.5482	9.4953	9.4577	9.5275	9.2369	9.2761	9.3195
Pfn1	_TFVSITPAEVGVLVGK_.2	#N/A	8.1385	7.9969	8.2028	8.1958	8.2298	8.1571	7.9204	8.2577	8.1169	8.1785	8.2030	8.1099	8.2071	7.8022	8.1137	8.0225	8.0479	7.8934	8.2431	8.0766
Pfn1	_STGGAPTFNVTVTM[Oxidation (M)]TAK_.2	#N/A	7.0080	3.1581	5.8285	6.3274	6.3349	5.9364	6.0269	5.7644	6.3591	6.3687	5.9014	6.0920	6.6020	5.1439	5.8724	6.6455	6.3119	5.9689	5.9415	6.6243
Pfn1	_DSLLQDGEFTM[Oxidation (M)]DLR_.2	#N/A	7.9745	7.4101	8.0695	7.9589	7.3616	7.6355	7.3015	8.0270	7.7878	7.9276	7.4585	7.5479	8.2238	7.4653	7.5211	7.6452	7.8630	7.2650	7.7341	7.4943
Pfn1	cetyl (Protein N-term)]AGWNAYIDSLM[Oxidation (M)]ADGTC[Carbamidomethyl (C)]QDAAIVG	#N/A	3.9994	3.5953	4.6508	4.8272	4.8525	4.8141	4.3912	1.9386	5.2884	5.2488	4.6619	3.8973	4.8859	3.7264	4.7275	4.8780	4.6023	4.5357	4.3370	5.2925
Pfn1	_C[Carbamidomethyl (C)]YEM[Oxidation (M)]ASHLR_.2	#N/A	3.9773	4.1826	5.5125	5.4767	4.1493	4.4458	4.0659	4.4406	4.3926	4.3358	3.6526	4.5173	4.2872	3.9267	5.8826	3.9219	4.5187	4.5350	3.9128	4.0683
Tra2b	_[Acetyl (Protein N-term)]S[Phospho (STY)]DSGEQNYGER_.2	P62996_S2_M1_Tra2b	11.1019	10.8134	10.9695	10.9021	10.4812	10.7539	10.6752	10.9106	10.7585	10.8199	10.4215	10.5976	10.9292	10.9134	9.9048	10.4932	10.9596	10.8395	10.6621	10.3659
Tra2b	_[Acetyl (Protein N-term)]SDSGEQNYGERES[Phospho (STY)]R_.2	P62996_S14_M1_Tra2b	6.4023	7.0034	7.2347	6.9610	5.5673	7.1348	6.5267	6.6911	6.0900	6.7921	6.5705	5.6492	3.8948	6.2200	3.3048	4.9897	6.4913	6.6913	6.4678	5.9532
Tra2b	_S[Phospho (STY)]RS[Phospho (STY)]YS[Phospho (STY)]RDYR_.2	P62996_S87_M3_Tra2b	3.3287	7.3309	3.8650	4.3609	6.3142	7.2238	7.2033	8.0124	6.9920	6.8485	3.9788	6.5078	6.9864	6.8932	2.9512	6.7070	3.5668	7.9860	6.5957	4.0405
Tra2b	_RPHT[Phospho (STY)]PTPGIYMGR_.3	P62996_T201_M1_Tra2b	4.5365	4.2834	5.2368	4.5486	3.2426	3.3649	3.8055	3.2613	4.4593	4.0094	3.9905	4.0095	4.5674	5.9140	3.8309	3.8986	4.2594	4.3909	3.1891	3.7884
Tra2b	_RHS[Phospho (STY)]HS[Phospho (STY)]HS[Phospho (STY)]PMSTR_.3	#N/A	3.9555	4.1902	5.3890	6.4437	3.5557	5.2527	5.8540	3.6383	4.1477	3.6666	5.7280	3.7450	3.9587	5.3754	3.8961	3.9816	3.6485	4.1883	4.8088	4.1592
Tra2b	_RRHS[Phospho (STY)]HS[Phospho (STY)]HS[Phospho (STY)]PM[Oxidation (M)]STR_.3	#N/A	4.0936	3.9407	3.7764	3.9939	3.5102	3.6463	4.9081	3.4757	5.1549	3.9371	3.5500	3.8888	4.1618	3.9227	3.6779	5.5800	4.3077	4.0877	3.7515	3.6895
Tra2b	_HSHS[Phospho (STY)]HS[Phospho (STY)]PMSTR_.2	#N/A	3.3401	3.1619	3.8764	4.3493	4.1786	4.4027	4.9778	4.5413	3.4215	3.5464	3.7959	3.0514	3.5296	4.5742	2.9397	3.6850	3.5781	3.9278	4.2269	4.2515
Rac1	_C[Carbamidomethyl (C)]VVVGDGAVGK_.2	#N/A	8.7274	8.4678	8.8144	8.6043	8.4453	8.4607	8.6308	8.5634	8.6924	8.3171	8.4192	8.4028	8.2844	8.1803	8.7452	8.1323	8.4017	8.4819	8.4359	8.4307
Rac1	_YLEC[Carbamidomethyl (C)]SALTQR_.2	#N/A	8.9027	8.8789	8.2555	8.5692	8.4205	8.7692	8.8570	8.9324	8.4688	8.3114	8.5295	8.6655	8.6955	8.3983	8.5033	8.2954	8.7370	8.7614	8.3570	8.3663
Rac1	_AVLC[Carbamidomethyl (C)]PPPVK_.2	#N/A	8.4776	8.5975	6.2266	8.2132	8.6318	8.5032	8.9781	8.4773	8.1531	8.0258	8.3931	8.6391	7.8243	7.8681	8.3279	6.7036	8.5749	9.1282	8.1485	8.4762
Rac1	_HHC[Carbamidomethyl (C)]PNTPIILVGTK_.3	#N/A	8.5046	8.3255	8.1415	8.1944	8.3336	8.2048	8.3540	8.8354	8.4253	7.9513	8.3106	8.2731	8.8022	8.0645	7.6967	7.7312	8.2426	8.2761	8.3282	7.8236
Rac1	_TVFDEAIR_.2	#N/A	7.9043	7.5483	8.0564	8.4724	7.9564	7.8946	8.5221	7.2951	8.1989	7.8736	8.4074	7.8383	8.4466	7.4839	8.0627	7.8188	6.6983	8.4870	8.1031	8.0477
Rac1	_LDLRDDKDTIEK_.3	#N/A	6.9836	7.0975	6.9048	7.2264	6.6800	7.0542	7.1921	6.6970	7.6810	7.2584	7.1032	7.1348	7.7508	6.8172	7.3530	7.1135	7.1467	7.0440	7.2870	7.2020
Rac1	_LTPITYPQGLAM[Oxidation (M)]AK_.2	#N/A	8.2129	7.7531	7.5089	7.5363	7.7990	7.9807	7.7570	8.0088	7.5416	7.6645	7.6497	7.6759	8.1645	7.0417	7.2547	7.2077	8.2080	8.0062	7.7762	7.4246
Rac1	_DDKDTIEK_.2	#N/A	4.1874	3.7872	5.3175	3.2105	4.6318	4.6312	4.9197	4.8995	5.5121	5.2653	4.4058	3.6925	4.2402	3.8000	4.9253	3.5495	3.8730	3.3756	3.9562	4.8294
Rac1	_LRPLSYPQTDVFLIC[Carbamidomethyl (C)]FSLVSPASFENVR_.3	#N/A	4.0458	3.9570	4.5821	3.6436	3.3835	3.1006	0.9602	3.7462	3.9011	4.1717	3.3797	3.8464	4.0665	4.5692	3.7347	4.3907	4.2839	3.5493	3.1979	3.0354
Pafah1b1	_TAPYVVTGSVDQTVK_.2	#N/A	6.4783	6.7101	7.0647	7.1200	6.3932	7.1041	7.0388	6.4604	6.7726	6.8744	6.0404	6.5345	6.2450	6.9259	6.7409	6.5896	6.2610	6.9799	6.6066	6.3291
Pafah1b1	_VWDYETGDFER_.2	#N/A	6.9613	7.1599	7.1077	7.2647	6.8748	6.5913	6.7781	6.5040	6.9629	7.0716	6.9527	6.8091	7.0373	6.5975	6.4457	6.6463	6.7674	6.5314	6.6452	6.4755
Pafah1b1	_KWTS[Phospho (STY)]VIR_.2	P63005_S57_M1_Pafah1b1	3.1049	6.9924	4.1423	6.3652	3.3118	3.2957	6.6815	7.2829	3.0277	4.4163	3.4021	6.7735	3.1357	7.0306	3.7617	6.0456	6.9664	4.3217	3.1199	5.7236
Pafah1b1	_FILSC[Carbamidomethyl (C)]ADDK_.2	#N/A	6.5620	6.3427	6.7449	6.6425	6.1951	6.9129	6.8788	6.7572	7.0597	7.0545	6.9466	6.4019	3.6228	6.6446	6.5608	6.3179	4.5278	6.8127	6.9241	6.7570
Pafah1b1	_YALSGHR_.2	#N/A	5.6928	6.6845	6.1905	5.9580	6.8263	6.2215	6.8917	5.9982	6.2433	6.2346	6.3578	5.8590	4.1815	6.4975	6.8463	6.2325	6.2105	6.8574	6.7859	6.4189
Pafah1b1	_LWDFQGFEC[Carbamidomethyl (C)]IR_.2	#N/A	5.9771	5.9886	6.1357	5.8390	3.9874	5.3653	5.6160	6.0787	6.0375	6.4412	4.0777	5.6523	6.0255	3.3551	4.8626	5.7829	3.5034	5.4422	5.7565	5.8384
Pafah1b1	_QRDELNR_.2	#N/A	4.2633	4.9126	4.5139	4.0351	3.1707	4.1006	4.1310	4.1359	4.5322	4.4696	4.5830	4.4901	4.1072	5.0076	5.0919	3.0169	4.5014	4.0835	4.8130	3.2528
Pafah1b1	_GHTDSVQDISFDHSGK_.3	#N/A	5.3837	6.2676	3.1750	3.9458	5.5990	5.5149	5.6594	3.8641	6.1736	5.9877	5.5630	5.2731	5.8266	5.9825	5.7292	5.1431	5.4090	3.7881	6.1456	6.1362
Rab3a	_M[Oxidation (M)]S[Phospho (STY)]ESLDTADPAVTGAK_.2	#N/A	10.9007	10.4138	10.4068	9.8146	10.0413	10.3316	10.2456	10.6796	10.4361	10.3553	7.9017	10.1884	10.8051	10.2061	9.4885	10.1806	10.6341	10.4024	10.3901	10.1829
Rab3a	_LVDVIC[Carbamidomethyl (C)]EK_.2	#N/A	8.3061	8.4297	8.3539	8.7087	8.3036	8.4413	9.0234	7.9214	8.6743	8.6288	8.7398	8.4308	8.7937	8.0453	8.4194	8.3813	7.8019	8.9474	8.5793	8.4608
Rab3a	_YADDSFTPAFVSTVGIDFK_.2	#N/A	6.8435	6.9540	6.1141	6.3154	6.3601	6.9244	7.1536	6.8146	6.7798	5.8087	6.4123	6.6590	6.6654	6.2585	5.6162	6.0960	6.8987	7.0722	6.6480	6.4105
Rab3a	_QLADHLGFEFFEASAK_.3	#N/A	6.2717	6.4133	6.8217	6.7537	6.2716	6.4236	6.6094	6.2869	6.3532	6.5746	6.5744	6.2479	6.8349	6.4603	5.1813	6.1313	6.3050	6.6407	6.2886	6.3569
Rab3a	_ESSDQNFDYMFK_.2	#N/A	3.7498	5.1876	6.0981	5.9248	4.6592	5.1570	5.2148	3.9755	4.8022	3.8104	5.6460	4.3847	3.9334	5.9543	4.3717	3.8598	4.6816	3.7758	4.2803	3.7435
Rab3a	_LVDVIC[Carbamidomethyl (C)]EKMS[Phospho (STY)]ESLDTADPAVTGAK_.3	#N/A	3.7354	4.2674	4.2717	3.9540	3.0731	3.2972	4.4679	3.4358	3.0262	3.9417	3.4006	4.1568	3.1342	5.4357	4.0451	4.0803	3.9735	3.8597	3.5083	4.2258
Hspa8	_VEIIANDQGNR_.2	#N/A	14.6588	14.8795	15.1351	15.1692	15.0288	14.8787	14.5214	15.1539	14.8152	15.0401	14.7636	14.7623	15.3673	15.3383	14.8090	15.0220	15.1412	14.6623	14.9028	15.1144
Hspa8	_TTPSYVAFTDTER_.2	#N/A	12.1905	12.0549	12.3931	12.3825	12.2534	12.1259	11.9365	12.3365	12.3289	12.3147	12.1222	12.1811	12.5160	12.3824	12.1029	12.3732	12.3449	11.8808	12.0315	12.3299
Hspa8	_TVTNAVVTVPAYFNDSQR_.2	#N/A	10.1298	10.3382	10.5243	10.4394	10.2111	10.1981	10.1293	10.3480	10.3963	10.3535	10.1488	10.2377	10.3089	10.6318	10.2696	10.3581	10.3756	10.1737	10.2121	10.2807
Hspa8	_LSKEDIER_.2	#N/A	10.9415	11.0566	10.9892	10.9318	11.0276	10.9507	10.9266	11.1553	11.1053	11.0206	10.7805	11.0460	11.1617	11.2220	11.3279	11.0951	10.9736	10.7925	10.8661	11.1062
Hspa8	_DAGTIAGLNVLR_.2	#N/A	10.7559	11.0939	11.0495	11.1399	10.9187	10.8821	10.9700	10.9522	10.7771	10.9728	10.7168	10.9402	11.0178	11.2063	10.9742	10.8966	10.8719	10.9795	10.8753	10.8947
Hspa8	_IINEPTAAAIAYGLDKK_.2	#N/A	8.8040	8.8402	9.0461	9.1186	9.1241	9.0105	8.9114	8.9510	8.9734	9.0176	8.9230	8.9222	9.0990	8.9115	9.3086	9.1199	9.0421	8.9674	9.1572	9.2477
Hspa8	_GTLDPVEK_.2	#N/A	9.4916	9.8278	9.5945	9.9587	9.8371	9.7700	10.0611	9.4066	10.0159	10.0246	9.8691	10.0867	10.1484	10.0377	10.5030	9.9794	9.2382	9.9999	9.9852	10.2220
Hspa8	_ITITNDKGR_.2	#N/A	9.3189	9.7139	9.3210	9.6049	9.5253	9.1821	9.5374	9.9540	9.5321	9.3785	8.9474	9.7544	10.0637	9.8338	9.9617	9.6183	9.2957	9.4872	9.5489	9.4540
Hspa8	_SQIHDIVLVGGSTR_.3	#N/A	7.7002	8.1738	8.7300	8.1272	7.7387	7.8209	7.9571	7.7068	8.0196	8.3223	8.0254	7.8125	8.0013	8.6327	8.3106	8.1140	7.3195	8.1030	7.9154	8.2743
Hspa8	_LLQDFFNGK_.2	#N/A	9.5599	9.4132	9.5115	9.7673	9.6078	9.5431	9.4405	9.4904	9.3439	9.3864	9.2751	9.6314	9.5362	9.4837	9.4243	9.3242	9.5124	9.2966	9.2116	9.5536
Hspa8	_VC[Carbamidomethyl (C)]NPIITK_.2	#N/A	8.4793	8.9584	9.2724	9.0080	9.1913	8.9412	9.2233	8.9013	8.8080	8.5592	8.7399	9.2164	8.7891	8.9928	9.6015	8.8019	8.8595	9.0719	9.0208	8.9298
Hspa8	_FEELNADLFR_.2	#N/A	8.9047	8.9646	9.1097	9.0904	8.9522	8.9649	8.7605	8.6031	8.6028	9.1259	9.0252	8.9862	8.8703	9.2180	9.0460	8.7363	8.8703	8.8802	8.8149	8.7950
Hspa8	_VQVEYKGETK_.2	#N/A	6.9571	7.4466	7.3567	7.6273	7.3306	7.3888	7.4395	7.4766	7.5733	7.5186	7.0743	7.3816	7.8184	7.5347	7.7205	7.7224	7.2982	7.2762	7.5367	7.5526
Hspa8	_EIAEAYLGK_.2	#N/A	7.7964	7.7197	8.1714	8.0136	8.2785	7.8352	7.7554	7.8123	7.9224	8.0310	8.1956	7.7637	7.6192	8.3020	7.9827	7.8612	7.6150	7.8469	7.8663	8.1719
Hspa8	_C[Carbamidomethyl (C)]NEIISWLDK_.2	#N/A	7.4631	7.2081	8.0301	7.3955	7.2565	7.5291	7.0527	6.8635	7.3766	7.8101	7.5794	7.2064	6.9282	7.6911	7.6930	7.2357	7.0928	7.1556	7.3334	7.4208
Hspa8	_YKAEDEKQR_.2	#N/A	6.8016	7.0230	6.8102	6.3560	7.0458	6.8081	6.7873	7.1387	7.2259	7.2255	7.0929	7.2246	7.2279	6.8716	7.7945	7.0823	7.2376	6.9099	6.4887	6.2754
Hspa8	_SFYPEEVSSM[Oxidation (M)]VLTK_.2	#N/A	9.8742	10.9387	11.1632	11.1058	10.0398	10.8109	10.7404	10.9753	10.8855	10.0094	10.0220	10.3319	11.1132	10.5634	10.9306	10.8628	10.9950	10.6439	10.4901	10.8731
Hspa8	_DIS[Phospho (STY)]ENKR_.2	P63017_S254_M1_Hspa8	8.9786	8.8683	8.0059	8.9572	9.0184	8.7555	9.0695	8.8865	8.8157	9.0405	8.9280	8.5453	9.3050	9.1273	8.7815	8.5646	9.1349	8.9069	8.7366	9.0516
Hspa8	_ARFEELNADLFR_.2	#N/A	7.1388	7.5052	7.5410	7.8200	7.3961	7.4521	7.0683	7.0907	6.9289	7.6672	7.3713	7.5417	7.5278	7.8355	7.3904	7.4971	7.4119	7.4079	7.3755	7.6435
Hspa8	_NQTAEKEEFEHQQK_.3	#N/A	6.0699	5.5547	7.3389	6.0619	6.7462	6.6701	5.8953	6.4026	6.6784	6.7620	6.5402	6.1105	5.5510	6.5315	6.8229	6.5534	6.7870	6.7371	6.5322	6.6932
Hspa8	_ITITNDK_.2	#N/A	6.4698	7.4071	7.3485	6.8021	5.8495	7.7883	7.6696	3.4209	7.8564	7.8531	7.8886	7.2067	7.5765	7.6599	8.1733	7.7509	6.5024	7.5881	8.0046	7.9754
Hspa8	_NSLESYAFNM[Oxidation (M)]K_.2	#N/A	8.8927	9.0598	9.0444	9.2119	9.1660	8.9659	8.8621	9.1587	8.9521	9.2073	8.8949	9.1327	9.1755	9.2057	9.1645	8.9576	9.0939	8.9463	9.0333	9.1316
Hspa8	_GPAVGIDLGTTYSC[Carbamidomethyl (C)]VGVFQHGK_.3	#N/A	5.6422	5.9180	5.6730	5.1658	5.6468	5.3392	5.7202	5.3904	5.2043	5.8554	4.6671	4.7169	6.0524	6.2253	5.1829	4.9192	6.3959	5.9095	5.4264	5.7851
Hspa8	_NQTAEKEEFEHQQKELEK_.4	#N/A	6.9079	6.4904	4.5501	5.7629	6.9728	6.6257	6.2240	6.6651	6.7755	6.9942	6.8441	6.0411	6.8750	6.1835	6.5345	6.9291	6.4352	7.0941	5.7333	6.6094
Hspa8	_HWPFM[Oxidation (M)]VVNDAGRPK_.3	#N/A	10.3144	9.8566	9.6740	9.7207	10.2847	9.8326	9.7432	10.3938	9.8523	10.2779	9.8675	10.1755	10.5030	10.0273	9.8356	9.9569	10.1073	9.8892	10.0576	10.1810
Hspa8	_NQVAM[Oxidation (M)]NPTNTVFDAK_.2	#N/A	9.8016	9.7934	10.0485	10.0584	9.7247	9.7167	9.8318	9.9808	9.9043	9.8308	9.7861	9.8124	9.9719	10.2515	10.0363	9.6911	9.9532	9.4833	9.6688	9.7227
Hspa8	_SINPDEAVAYGAAVQAAILSGDK_.2	#N/A	5.7568	5.9739	6.2602	5.6224	5.2932	5.4508	5.5941	5.8117	5.8691	5.5085	5.8899	5.7160	5.1513	6.3288	5.6047	5.4412	5.9960	5.5927	5.6203	5.7147
Hspa8	_FDDAVVQSDM[Oxidation (M)]K_.2	#N/A	7.8159	7.3844	8.1476	8.0783	8.1055	7.9396	7.9549	7.7732	8.0299	8.0767	7.8851	7.9152	7.3596	8.2283	8.1744	7.7808	7.5361	7.7143	7.5028	7.8889
Hspa8	_QTQTFTTYSDNQPGVLIQVYEGER_.3	#N/A	3.9869	5.7201	5.4273	3.7025	4.4401	4.6306	5.2150	5.5647	3.8422	4.2306	4.3615	3.9054	6.0341	5.7742	4.6653	3.7822	4.2249	4.9075	5.4520	5.0396
Hspa8	_NQVAM[Oxidation (M)]NPTNTVFDAKR_.3	#N/A	6.5471	7.0095	5.9608	6.6056	6.8959	6.3536	6.7952	7.2843	6.5844	6.6667	5.9361	6.9228	7.5097	7.0183	7.0133	6.8891	6.4428	6.7604	6.6000	6.1278
Hspa8	_GRLS[Phospho (STY)]KEDIER_.3	P63017_S511_M1_Hspa8	4.5134	4.7042	4.2352	4.4593	3.8512	3.7530	3.6899	4.0519	4.0886	4.1637	4.1871	3.3788	3.8571	4.5854	4.8880	3.3575	3.5491	4.0759	3.5624	5.1290
Hspa8	_STAGDTHLGGEDFDNR_.3	#N/A	11.0355	11.2999	11.4773	11.6128	11.1462	11.3249	11.2188	11.2129	11.4368	11.6003	11.3100	11.3711	11.8379	11.7405	11.4794	11.4901	11.2559	11.1761	11.4186	11.5478
Hspa8	_RFDDAVVQSDM[Oxidation (M)]K_.2	#N/A	6.7736	7.0092	7.2892	7.1412	7.1057	6.9948	6.8828	7.1071	6.7611	6.7255	6.9867	6.8759	6.9322	7.2190	7.2225	6.9506	7.0268	6.7879	6.7861	7.1165
Hspa8	_M[Oxidation (M)]KEIAEAYLGK_.2	#N/A	6.9833	7.6023	7.3713	7.3624	6.7830	7.3066	7.3833	7.4558	7.5122	7.0270	6.5745	7.2210	7.6698	7.8318	7.7951	7.6806	7.4084	7.3587	7.3818	8.2310
Hspa8	_MVNHFIAEFK_.2	#N/A	5.2921	4.9363	5.5604	5.3930	5.5563	6.2454	5.4572	4.2527	5.7131	5.2300	5.1397	5.0259	3.5651	5.6744	6.0860	4.0965	5.3837	5.4535	6.0573	4.8495
Hspa8	_IINEPTAAAIAYGLDK_.2	#N/A	8.0262	8.2017	8.4298	8.1912	8.0896	8.1072	8.1148	8.2435	8.0519	8.2169	8.1222	8.0580	8.1405	8.3014	8.1038	7.9322	8.0890	7.9177	7.9224	8.1764
Hspa8	_MVQEAEKYK_.2	#N/A	3.1814	3.3206	5.5699	4.5080	4.0199	3.9650	5.5677	4.3826	4.5059	3.5353	4.5577	3.2100	3.2021	5.7856	4.2684	3.5263	3.4194	4.4137	4.0623	3.8575
Hspa8	_LDKSQIHDIVLVGGSTR_.3	#N/A	6.3108	6.2574	5.7553	6.4862	6.3948	5.6009	5.8264	6.8639	6.1736	6.7075	6.0185	6.4678	7.6333	6.2179	6.0949	6.6939	6.1042	5.5129	6.2777	6.1747
Hspa8	_LYQSAGGM[Oxidation (M)]PGGM[Oxidation (M)]PGGFPGGGAPPSGGASSGPTIEEVD_.3	#N/A	4.0336	3.9692	4.5699	3.2782	3.3713	3.5954	4.1698	4.5317	4.2288	4.2399	3.1024	3.8587	4.3369	3.8807	3.7470	4.3785	4.0289	3.1205	3.4196	3.5580
Hspa8	_YKAEDEK_.2	#N/A	4.2153	4.6046	3.7519	3.3689	2.9214	4.4556	4.1267	3.8920	4.0469	4.0259	3.5256	4.3308	4.2124	3.2142	4.1521	3.5774	4.4297	3.4035	3.0520	3.1812
Tpt1	_DLISHDELFSDIYK_.2	#N/A	4.4961	4.6507	5.0310	5.3490	4.9310	4.1907	4.7613	4.4449	5.1950	4.9772	5.0458	5.0790	4.9639	5.0421	5.2849	5.3730	4.9993	4.8014	5.1159	5.7036
Tpt1	_LEEQKPER_.2	#N/A	4.7696	4.8095	5.1744	4.1960	4.5491	5.0840	4.3612	5.2015	4.4983	5.0918	5.0763	5.1401	3.9942	5.3134	5.9847	5.5887	3.6450	4.0733	4.8032	4.9695
Tpt1	_GKLEEQKPER_.3	#N/A	3.3930	4.3066	6.3673	5.6167	4.2316	5.5825	5.2601	3.0934	5.6368	5.8229	6.4429	4.1470	5.3695	4.5692	6.5636	6.1352	3.6311	5.1513	5.3790	6.1718
Tpt1	_EIADGLC[Carbamidomethyl (C)]LEVEGK_.2	#N/A	3.6509	3.2468	3.1875	5.1195	5.0968	3.8912	4.4634	5.0752	3.1105	4.1698	5.1080	3.3943	3.2759	3.7786	5.0335	5.3769	3.4932	4.3399	3.9885	5.0761
Mpc1	_KAADYVR_.2	#N/A	6.0226	5.6280	6.1681	5.3748	6.3862	6.0965	6.5579	5.9151	6.2218	6.2323	6.1872	5.8404	6.1207	5.5841	6.2768	5.1256	5.7521	6.3385	6.0947	6.0780
Dnaja1	_TIVITSHPGQIVK_.2	#N/A	6.2408	6.2844	5.9413	5.8133	5.8246	5.8876	5.9128	6.3628	5.4820	6.0269	5.3569	6.0198	6.2521	6.4607	6.4374	6.1159	5.7396	5.9270	5.5350	5.4643
Dnaja1	_QISQAYEVLADSK_.2	#N/A	3.1878	4.9141	5.3690	4.5015	3.7959	5.1176	4.1692	4.6764	4.4538	3.9905	4.2809	5.0521	3.2086	5.0602	4.9708	3.4043	4.4392	4.3593	4.0782	4.5998
Dnaja1	_DHAVFTR_.2	#N/A	3.8375	4.1654	4.5648	3.8519	4.2300	4.8098	3.9601	3.5378	4.7234	4.9379	3.7435	5.4215	3.8582	4.3608	5.1730	4.9287	3.8673	3.7576	4.9044	3.8253
Dnaja1	_ITFHGEGDQEPGLEPGDIIIVLDQK_.3	#N/A	3.6966	4.3062	4.2329	3.9927	3.8297	4.1437	5.2887	3.3970	4.4447	4.5209	3.6342	4.1085	3.7174	5.1197	4.4093	4.0415	3.9347	4.1450	4.5086	4.1870
Dnaja1	_NVVHQLS[Phospho (STY)]VTLEDLYNGATR_.3	P63037_S112_M1_Dnaja1	4.0625	4.2692	4.5988	3.6269	3.4002	3.0839	3.9975	3.7628	3.9177	4.1551	3.3964	3.8298	4.0832	5.1064	4.0229	3.7066	4.3005	3.5326	3.1812	3.0520
Hspd1	_VTDALNATR_.2	#N/A	8.2411	8.8334	8.4489	8.6527	8.6448	8.7232	8.8075	8.5804	8.5400	8.5399	8.5128	8.9055	8.7943	8.6584	9.3664	8.4726	8.3829	8.8186	8.8205	8.6949
Hspd1	_APGFGDNRK_.2	#N/A	7.8840	8.3026	8.3223	7.8225	8.2341	8.1800	8.5117	8.4905	8.1020	8.0278	8.0717	8.2976	8.2407	8.3322	8.8498	8.4790	8.1310	8.4666	8.2638	8.3078
Hspd1	_APGFGDNR_.2	#N/A	7.9125	8.3832	8.3086	8.2467	8.2339	8.3441	8.1386	8.1812	8.1793	8.3656	8.2983	8.4030	8.4816	8.4944	8.6543	8.3699	8.3078	8.3300	8.2604	8.5337
Hspd1	_AAVEEGIVLGGGC[Carbamidomethyl (C)]ALLR_.2	#N/A	8.2688	7.9737	8.7057	8.4904	8.2279	8.0264	7.9157	7.7357	8.1900	8.3365	8.2344	7.7988	8.1498	8.2239	7.7608	8.2891	8.1246	7.6940	8.0017	8.3652
Hspd1	_GANPVEIR_.2	#N/A	7.7120	7.9493	8.0738	8.2034	7.8990	7.8760	8.2949	7.5990	8.0948	8.2136	7.8337	8.0625	8.1010	8.1163	8.7550	8.2211	7.6193	8.0476	8.0511	8.3524
Hspd1	_LVQDVANNTNEEAGDGTTTATVLAR_.3	#N/A	7.8062	7.9764	8.3346	8.1103	7.9584	7.5831	7.8155	7.9202	7.9617	8.1986	7.9897	7.6406	8.3584	8.6240	8.0508	7.9188	8.1019	7.5721	7.8596	8.0992
Hspd1	_GYISPYFINTSK_.2	#N/A	8.0763	7.6763	8.0419	8.1614	8.0531	7.7927	7.8262	8.1583	7.9484	8.0317	7.9132	7.9669	8.1863	7.8873	8.3062	8.1118	8.2499	7.9766	7.9864	7.9700
Hspd1	_ISSVQSIVPALEIANAHR_.3	#N/A	6.6322	6.9367	6.9534	7.0691	7.4959	6.9957	6.6563	6.9102	6.8422	7.3321	7.1448	6.8728	6.7991	7.2754	7.2347	7.1340	6.6705	6.9437	7.1856	7.5834
Hspd1	_LSDGVAVLK_.2	#N/A	7.5094	7.3506	7.2344	7.6916	7.6519	7.3462	7.5258	7.9068	7.3620	7.5233	7.7811	7.8921	7.7561	7.8470	7.8236	7.5456	7.5090	8.0123	7.7680	7.9113
Hspd1	_VGLQVVAVK_.2	#N/A	7.1544	7.0520	7.5521	7.7146	7.2564	7.4291	7.2269	7.2752	7.4843	7.4111	7.2163	7.3486	6.8805	7.6470	7.1968	7.4806	7.1406	7.3935	6.9427	7.3862
Hspd1	_IQEITEQLDITTSEYEK_.2	#N/A	7.8345	7.1371	7.4105	7.0911	6.3724	6.6954	6.5406	7.5618	6.8186	7.6206	6.3410	6.9134	7.8518	8.4808	7.1609	7.2006	7.2434	7.2790	5.7531	7.1573
Hspd1	_C[Carbamidomethyl (C)]EFQDAYVLLSEK_.2	#N/A	6.2912	5.9945	6.2630	5.8536	5.8753	6.3463	4.3358	5.5911	6.2276	6.0602	6.1397	6.2967	6.0358	5.5113	6.0902	5.8893	5.2011	5.7320	5.5587	6.0322
Hspd1	_TVIIEQSWGSPK_.2	#N/A	7.1437	7.6222	7.4734	6.8863	6.5987	7.4514	7.2496	7.2376	7.6010	7.1741	6.8511	7.1674	7.1231	8.0392	7.5728	7.4089	7.2344	7.7927	7.6671	7.5914
Hspd1	_NAGVEGSLIVEK_.2	#N/A	6.4344	6.1411	6.0712	6.3432	6.7959	6.5981	6.6928	6.6166	6.9086	6.7272	6.9976	6.3871	3.5085	7.3411	7.0671	6.3131	6.6556	6.4707	7.0216	6.5819
Hspd1	_KPLVIIAEDVDGEALSTLVLNR_.3	#N/A	5.8835	6.7790	6.6253	6.6990	6.6962	6.8187	6.0321	6.5416	5.4985	6.2998	6.5787	6.7213	6.7207	8.2616	5.7791	6.0901	7.0496	7.0393	6.6327	6.0649
Hspd1	_SIDLKDK_.2	#N/A	5.4175	6.0466	5.0505	5.5219	5.8033	5.6838	6.3182	5.7270	5.7116	5.4623	5.9435	5.9021	6.6122	6.3291	6.1016	6.1928	5.9772	5.8252	5.5331	6.3435
Hspd1	_VGEVIVTK_.2	#N/A	4.7889	4.2261	3.9500	4.7046	3.9191	3.9508	4.7319	4.3972	5.3845	5.0351	3.7697	4.6560	3.2446	4.5196	4.7159	4.7137	4.2261	4.7469	4.5156	4.7832
Hspd1	_IQEITEQLDITTSEYEKEK_.3	#N/A	7.4971	7.4673	7.3880	7.6550	6.9214	6.5670	6.5035	7.5956	7.1416	7.4461	6.7197	7.4363	8.4926	8.2959	7.0669	8.0138	7.1808	6.6662	7.1573	7.7205
Hspd1	_C[Carbamidomethyl (C)]IPALDSLKPANEDQK_.2	#N/A	3.5783	4.4245	6.0470	3.5095	4.9321	5.3556	3.1243	3.2786	5.8284	3.7846	3.5577	3.3503	3.2914	6.0850	5.4828	3.9232	3.8163	4.0168	5.9440	5.7186
Hspd1	_TLNDELEIIEGM[Oxidation (M)]K_.2	#N/A	6.5270	6.4167	7.3470	6.9204	6.4081	6.7975	6.4550	6.3495	6.7451	6.8699	6.5943	6.5021	6.6410	7.0629	6.6640	6.9612	6.8422	6.4999	6.6861	6.8137
Hspd1	_VGGTSDVEVNEKK_.2	#N/A	4.2301	3.5621	3.9578	3.7164	4.4124	3.8827	3.6411	4.2618	4.4978	3.4040	4.0754	3.3308	3.1493	3.5005	3.2419	4.5103	4.3218	2.8151	3.6116	3.8936
Hspd1	_ALMLQGVDLLADAVAVTM[Oxidation (M)]GPK_.3	#N/A	3.7859	3.9070	4.5423	3.9038	3.5329	3.6560	3.8170	2.9342	3.8260	3.8479	3.8070	3.7193	3.8072	4.5542	3.5773	4.3591	3.8409	3.7727	3.8300	3.7390
Hspd1	TEIPKEEKDPGM[Oxidation (M)]GAM[Oxidation (M)]GGM[Oxidation (M)]GGGM[Oxidation (M)	#N/A	4.0304	3.5376	3.8458	3.2750	3.3681	3.5922	1.0541	3.1484	4.2321	4.2367	3.1056	4.2368	4.3401	3.0802	3.7502	4.3753	4.0322	3.1173	3.4163	3.5612
Hspd1	_ILQSSSEVGYDAM[Oxidation (M)]LGDFVNM[Oxidation (M)]VEK_.3	#N/A	4.3570	3.6458	3.3925	3.3323	3.6948	4.2902	4.2921	4.0574	4.2123	3.8605	3.6910	3.5352	4.3778	3.3796	4.2413	3.2011	3.0943	3.2381	4.3875	3.3466
Hspd1	_DM[Oxidation (M)]AIATGGAVFGEEGLNLNLEDVQAHDLGK_.3	#N/A	3.3735	4.4702	4.0689	4.1568	3.5804	3.6138	3.4677	3.2330	3.3879	3.1841	4.3674	4.3597	3.5533	4.0560	3.4930	4.2365	3.7707	2.2205	3.7111	4.0230
Cplx1	_KAIPPGC[Carbamidomethyl (C)]GDEPEEEDESILDTVIK_.3	#N/A	7.0954	7.2670	7.3666	7.1426	3.5381	7.2362	6.8503	6.8760	7.4773	6.9499	6.7692	7.3712	6.7542	7.6288	7.7087	7.6215	7.2616	7.4025	7.5995	7.4153
Cplx1	_EAEAQAAM[Oxidation (M)]EANSEGS[Phospho (STY)]LTRPK_.2	#N/A	6.1559	6.9084	5.7281	6.0551	5.8244	5.4842	5.5235	5.8334	3.9662	3.8749	5.4041	6.9687	6.4941	3.7084	6.9931	7.0996	7.0512	6.7346	6.1252	6.0821
Stmn4	_RRDPS[Phospho (STY)]LEEIQK_.3	#N/A	7.6854	8.0826	7.2524	7.6359	7.2680	7.8827	7.9454	7.4140	7.6597	8.3551	7.9054	7.8909	8.3380	8.0381	7.6022	7.9592	8.0652	8.0083	8.2304	8.0148
Vamp2	_DQKLS[Phospho (STY)]ELDDR_.2	P63044_S61_M1_Vamp2	8.6167	8.4092	7.6998	7.9398	7.3439	7.4047	7.7850	7.5995	7.0337	8.2921	7.7009	7.3651	7.7720	7.8538	5.3456	7.6395	7.9153	7.7337	7.4451	6.9057
Vamp2	_LSELDDR_.2	#N/A	4.9900	5.0872	4.4154	4.7145	4.7921	5.6428	5.5381	2.3372	5.3499	5.1359	5.6245	5.3234	5.3494	4.2051	4.2584	4.8594	4.1716	5.6371	5.2104	4.4158
Vamp2	_ADALQAGASQFETSAAK_.2	#N/A	9.4918	9.1203	9.4434	9.1049	8.0634	8.9726	8.8881	9.1410	9.0957	9.0035	8.8879	8.6501	9.1683	8.7428	8.1147	8.6791	9.2775	8.7913	8.8527	8.7361
Vamp2	_[Acetyl (Protein N-term)]SATAATVPPAAPAGEGGPPAPPPNLTSNR_.3	#N/A	9.3635	8.9430	11.0083	9.0280	3.7251	9.8303	7.5122	8.8880	10.1432	3.8301	3.7213	9.3581	4.4081	3.4100	10.4896	9.5383	3.1246	8.7792	8.4864	3.3769
Vamp2	_LQQTQAQVDEVVDIM[Oxidation (M)]R_.2	#N/A	7.2394	6.8358	7.1863	6.6198	6.6227	6.8833	6.7870	6.8551	6.9264	6.6940	6.7276	6.4441	7.0626	6.5729	5.4426	6.3122	6.6472	6.4572	6.6269	6.5849
Vamp2	_LSELDDRADALQAGASQFETSAAK_.3	#N/A	7.3260	7.5338	6.5349	6.9033	7.0190	7.1543	7.3177	7.2082	6.9326	6.6737	6.3072	7.3299	7.4324	6.9681	6.4165	6.7367	7.3850	7.0048	7.0033	6.8303
Sult4a1	_[Acetyl (Protein N-term)]AESEAETPGT[Phospho (STY)]PGEFESK_.2	P63046_T11_M1_Sult4a1	9.7630	10.2945	10.2116	10.1155	7.5129	9.3175	9.8555	5.8585	9.4034	10.6224	9.1312	9.6081	9.4188	9.7610	7.9104	9.5261	9.5541	9.5022	9.3795	9.5667
Pcp4	_AAVAIQSQFR_.2	#N/A	9.2086	10.1052	10.1223	9.9929	9.3828	9.6379	9.7532	9.7581	9.9015	9.5924	9.4538	9.8883	10.1057	10.3115	10.9749	10.3796	9.7712	10.2252	10.3512	10.2250
Pcp4	_VQEEFDIDM[Oxidation (M)]DAPETER_.2	#N/A	8.3267	8.1947	8.9256	8.4270	7.6855	7.8857	8.0843	7.9608	8.5337	8.3612	8.0891	8.5380	8.9204	9.0199	9.2152	9.0629	8.4751	8.4750	8.6194	8.7413
Pcp4	_KVQEEFDIDM[Oxidation (M)]DAPETER_.3	#N/A	6.7906	7.3981	7.6376	7.3458	7.0164	7.1342	6.7795	7.1949	7.3975	7.4349	7.4461	7.1319	8.1979	7.9258	8.4680	7.8145	7.0691	7.9039	7.3428	7.5294
Thra	_VEC[Carbamidomethyl (C)]GS[Phospho (STY)]DPEENSAR_.2	P63058_S12_M1_Thra	6.1005	6.2758	6.2402	3.6346	4.1566	6.5123	6.4048	4.1753	4.3126	6.1409	6.1963	3.0956	4.4780	6.6715	2.9169	3.3118	6.3668	5.7941	5.7628	3.4468
Eif4e	_DAVTHIGR_.2	#N/A	4.4913	5.0551	6.3020	5.7401	5.2203	5.9244	5.3683	5.9478	5.7207	5.8621	5.9295	5.4304	5.2204	5.6852	6.1215	5.4749	6.0375	5.5607	5.5453	5.5671
Eif4e	_IAIWTTEC[Carbamidomethyl (C)]ENR_.2	#N/A	3.9226	4.0803	4.4589	4.7688	3.2603	3.3976	4.7542	3.6229	4.2452	4.2950	3.2565	3.9697	3.9433	4.4459	4.1402	4.1792	4.1606	3.6725	3.3211	5.1465
Gabrb3	_NSAIS[Phospho (STY)]FDNSGIQYR_.2	P63080_S394_M1_Gabrb3	6.6005	6.5941	6.4589	5.6461	4.0789	3.9060	5.7857	4.4416	5.4797	3.4467	3.8956	3.1511	3.2611	6.0174	3.0394	5.1742	4.0987	6.1348	5.7973	4.6613
Gabrb3	_KQS[Phospho (STY)]MPKEGHGR_.3	P63080_S406_M1_Gabrb3	4.3738	3.6290	3.4093	3.3156	3.7115	4.2734	4.2341	4.0742	4.2291	3.8437	3.7077	3.5184	4.3945	3.3963	3.4067	3.2179	3.1110	3.2213	4.3707	3.3633
Mapk1	_[Acetyl (Protein N-term)]AAAAAAGPEM[Oxidation (M)]VR_.2	#N/A	9.1434	8.6329	8.4253	8.0968	8.9124	8.9432	8.9639	9.5119	9.0041	8.7763	8.2397	8.9348	9.1368	7.7776	8.5834	8.8633	8.6784	9.0293	8.8080	8.6208
Mapk1	_GQVFDVGPR_.2	#N/A	8.5156	8.3038	8.7113	8.5020	8.6651	8.2965	8.6306	8.8559	8.2781	8.6496	8.4223	8.3253	8.5971	8.0828	8.2781	8.1714	8.5294	8.5031	8.5002	7.9972
Mapk1	_NYLLSLPHK_.2	#N/A	8.2235	7.9815	8.3193	8.1475	8.4388	8.1546	8.3331	8.5196	7.9275	8.0276	7.6869	8.1165	7.9531	7.8152	7.8317	7.6219	8.0039	8.1476	8.2708	8.1139
Mapk1	_ELIFEETAR_.2	#N/A	5.0884	5.1765	5.7187	5.6877	5.2812	5.6366	6.0298	4.1232	6.3788	3.7081	5.7141	5.2714	5.7414	5.3189	5.1214	5.6625	3.6204	5.6798	6.0987	5.6230
Mapk1	_LKELIFEETAR_.3	#N/A	3.7829	2.9155	4.8433	3.8013	4.5498	4.5724	4.3860	4.0086	4.5417	3.6103	4.6583	3.2623	3.1439	3.3576	4.1938	3.7546	3.5058	3.8785	5.1072	4.9540
Mapk1	_DVYIVQDLM[Oxidation (M)]ETDLYK_.2	#N/A	6.8526	5.7172	6.5654	6.1019	6.5855	6.5577	6.0185	6.5104	6.3966	6.1872	5.7149	5.8737	6.9113	6.5059	6.0187	6.0746	6.2749	5.8736	6.0682	6.2315
Mapk1	_VADPDHDHTGFLTEYVATR_.3	#N/A	7.1910	8.2146	7.1592	7.5881	5.2464	7.4831	7.7785	5.5897	7.3458	8.2112	6.1501	7.2324	7.3255	8.3620	5.8292	7.1797	7.1531	7.8959	7.5232	7.6294
Mapk1	_ISPFEHQTYC[Carbamidomethyl (C)]QR_.3	#N/A	5.2837	5.3296	5.6671	5.6771	5.2637	5.5219	5.7928	5.9996	5.3284	5.4141	5.5736	4.9418	5.2496	5.0214	5.0560	4.6017	4.9043	5.1813	4.7907	5.2624
Mapk1	_YTNLSYIGEGAYGM[Oxidation (M)]VC[Carbamidomethyl (C)]SAYDNLNK_.3	#N/A	3.7360	3.8185	3.1602	3.7324	3.8306	4.2955	3.2175	4.4837	4.1165	5.0645	4.1172	4.5713	3.3032	3.8058	3.2429	2.9857	3.3678	3.7407	3.9612	3.7685
Mapk1	_APEIMLNSK_.2	#N/A	4.1559	4.0118	3.8771	5.5152	2.8620	3.9713	5.3063	4.3816	4.1063	3.9665	3.5850	4.3902	4.2718	5.2879	4.5341	3.5180	3.8788	3.6256	3.5697	3.4078
Mapk1	_YIHSANVLHR_.3	#N/A	3.7886	4.2142	4.3249	4.5340	3.1263	3.3504	3.4890	3.2759	2.9730	3.9949	3.2511	4.1037	4.5819	4.1257	3.9920	4.1335	1.7566	4.3763	3.9853	3.8030
Ppp1cc	_GNHEC[Carbamidomethyl (C)]AS[Phospho (STY)]INR_.2	P63087_S129_M1_Ppp1cc	7.2272	6.9819	7.0514	7.4129	7.1329	6.8393	7.0532	7.1942	7.0719	7.1587	6.9840	6.9407	7.1904	7.2681	7.2829	7.0031	7.2108	6.8563	6.7077	6.8500
Ppp1cc	_HDLDLIC[Carbamidomethyl (C)]R_.2	#N/A	7.0626	7.4253	7.9647	7.2962	7.5581	7.3277	7.2658	7.6019	7.3380	7.5019	7.6957	7.4170	7.2503	7.5155	7.7220	7.2809	7.2531	6.5347	7.4099	7.2623
Ppp1cc	_NVQLQENEIR_.2	#N/A	7.4790	7.0532	7.9933	7.7502	8.0077	6.9633	7.0614	7.5983	7.7204	7.6985	7.2881	7.1838	7.4851	7.2001	7.5452	7.8973	7.5625	6.9669	7.6193	7.6854

Ppp1cc	_IYGFYDEC[Carbamidomethyl (C)]K_.2	#N/A	7.4259	7.2797	7.1794	7.3892	7.2841	7.1239	7.1932	7.4086	7.2671	7.0860	7.3614	7.1010	7.4201	7.5192	7.3783	7.1899	7.5225	6.9886	6.7052	6.7383
Ppp1cc	_TFTDC[Carbamidomethyl (C)]FNC[Carbamidomethyl (C)]LPIAAIVDEK_.2	#N/A	7.2966	7.1367	6.6099	7.1654	6.7778	7.0380	7.2330	6.5981	6.8656	7.1288	7.0109	7.1516	7.1392	7.6966	7.3616	7.0049	7.1431	6.9964	6.9328	6.9020
Ppp1cc	_EIFLSQPILLELEAPLK_.2	#N/A	5.9046	5.4464	5.4647	5.1406	5.4155	5.5519	5.7272	6.1311	5.3257	5.4339	5.4946	5.7237	6.2285	5.5677	5.7164	5.5080	5.6131	5.9766	5.5969	5.7457
Ppp1cc	_YPENFFLLR_.2	#N/A	5.2723	4.2826	5.0484	4.9302	4.5133	4.5337	4.2009	4.7124	3.6023	5.2825	5.5300	5.2986	4.0431	5.4464	3.7750	3.7467	4.6031	4.1697	4.3806	5.2500
Ppp1cc	_[Acetyl (Protein N-term)]ADIDKLNIDSIIQR_.2	#N/A	5.3973	5.0295	3.3326	5.3016	5.0668	5.2222	4.6254	4.0217	6.0546	3.6066	5.3827	4.9896	5.8110	5.3469	5.7015	5.6170	5.7624	5.4875	5.4890	5.3924
Ppp1cc	_IFC[Carbamidomethyl (C)]C[Carbamidomethyl (C)]HGGLSPDLQSM[Oxidation (M)]EQIR_.3	#N/A	5.1477	4.7320	5.2075	5.0248	4.9837	5.1765	4.5876	5.3594	5.1263	5.8079	4.9707	5.1902	5.3059	5.3529	5.4496	5.0682	5.0543	4.8309	5.2914	5.4098
Ppp1cc	_AHQVVEDGYEFFAK_.2	#N/A	6.3760	5.7091	6.0339	6.1255	6.2026	5.4456	5.9358	6.0516	5.6689	6.4174	6.4217	5.8049	6.2273	5.8709	5.7413	6.0801	6.2809	5.2099	5.9491	6.1284
Ppp1cc	_QSLETIC[Carbamidomethyl (C)]LLLAYK_.2	#N/A	3.1677	4.1514	3.0913	4.4166	3.7151	2.0103	3.6735	3.3934	3.5937	3.3442	3.0723	3.0114	3.7591	4.2432	3.6989	4.0306	4.3914	3.8567	3.5053	3.6325
Ppp1cc	_IC[Carbamidomethyl (C)]GDIHGQYYDLLR_.3	#N/A	3.8249	4.1833	4.3612	4.1719	3.1626	3.6095	4.3838	3.4768	3.8370	4.0312	3.4494	4.0727	3.6093	4.3120	3.6419	4.1698	4.2407	4.0387	3.2312	3.4377
Ywhaz	_EKIETELR_.2	#N/A	10.7308	10.6962	11.2989	11.0073	10.7857	10.9541	10.7903	10.8820	11.0043	10.9743	11.1018	10.7407	10.8378	11.0418	11.2191	11.0294	10.8354	10.8060	11.0086	11.0466
Ywhaz	_GIVDQSQQAYQEAFEISKK_.3	#N/A	9.5427	9.8594	9.5732	9.6099	9.4162	9.3946	9.2096	10.0468	9.1894	9.6447	9.3580	9.8653	10.3760	10.1721	9.2875	9.5993	9.7457	9.4191	9.6099	9.7210
Ywhaz	_VVSSIEQK_.2	#N/A	6.8581	7.5506	7.6978	8.2920	7.8516	7.9978	8.2768	6.2295	8.0487	7.9279	8.4056	8.4159	8.5894	8.4202	8.7549	8.5281	7.9329	7.8388	8.0404	8.6527
Ywhaz	_YLAEVAAGDDK_.2	#N/A	7.1709	7.2096	7.3905	7.4764	6.8608	7.3950	7.2027	6.5113	7.3621	6.9029	7.5799	6.8866	6.9951	7.0966	7.0366	7.3388	6.8721	7.2407	7.3686	7.3105
Ywhaz	_FLIPNASQPESK_.2	#N/A	11.4514	11.4721	11.4600	11.4372	11.4408	11.3009	11.4665	11.5853	11.3287	11.3240	11.2674	11.5145	11.6738	11.5527	11.7299	11.4314	11.4806	11.3905	11.4481	11.3819
Ywhaz	_[Acetyl (Protein N-term)]M[Oxidation (M)]DKNELVQK_.2	#N/A	9.0579	8.1144	9.3406	9.2651	9.2080	8.7004	8.7646	9.3705	9.0903	8.1717	8.7508	9.3729	9.0806	9.4078	9.2506	9.2357	9.0869	9.2224	8.6408	9.3049
Ywhaz	_SVTEQGAELSNEER_.2	#N/A	10.4280	10.4423	10.6926	10.5692	10.3888	10.3192	10.3799	10.7064	10.6919	10.5284	10.3018	10.4991	10.6741	10.7970	10.7449	10.6387	10.4509	10.4306	10.3957	10.5885
Ywhaz	_KGIVDQSQQAYQEAFEISK_.3	#N/A	5.6302	5.2243	5.9485	5.7857	5.7804	5.1464	4.2450	5.4261	5.0363	5.2242	5.7431	4.9050	5.7703	6.0146	4.6539	4.2892	5.7181	3.9125	4.7911	5.1223
Ywhaz	_YDDM[Oxidation (M)]AAC[Carbamidomethyl (C)]M[Oxidation (M)]K_.2	#N/A	5.4579	5.5087	6.9276	6.7599	5.7190	6.0030	6.2628	5.5957	5.7011	5.1188	5.7754	5.4424	5.4786	6.4200	6.3145	5.4406	4.6743	5.7034	5.3454	5.4945
Ywhaz	_DIC[Carbamidomethyl (C)]NDVLSLLEK_.2	#N/A	9.5192	9.4148	9.8787	9.7852	9.4270	9.4542	9.5928	9.4436	9.5740	9.4896	9.4832	9.4416	8.9788	9.5170	9.6494	9.5046	9.6004	9.2208	9.5472	9.4036
Ywhaz	_TAFDEAIAELDTLSEESYK_.2	#N/A	10.0015	9.7056	10.0871	9.7972	9.0599	9.4587	9.4567	9.3855	9.6115	9.7409	9.3378	9.3145	9.7847	10.3463	9.6027	9.5060	9.8555	9.5014	9.5000	9.9479
Ywhaz	_YLAEVAAGDDKK_.2	#N/A	10.1188	10.1951	10.5716	10.4017	10.0399	10.2683	10.2131	10.2693	10.4133	10.3766	10.0122	10.2067	10.2159	10.4516	10.5294	10.4387	10.0265	10.2154	10.2615	10.3748
Ywhaz	_VVSS[Phospho (STY)]IEQKTEGAEKK_.3	#N/A	3.7486	3.3445	3.6938	4.5319	3.9556	3.9889	3.0925	4.3587	3.0128	3.5592	3.9922	3.2339	3.1782	3.6809	6.5318	3.1107	3.3955	4.4376	4.0862	3.6478
Ywhaz	_GIVDQSQQAYQEAFEISK_.2	#N/A	3.2346	5.9505	4.2720	4.3496	3.4415	3.5799	3.6066	3.4603	3.5268	4.5460	3.0054	3.8106	3.6922	10.8006	3.6319	4.0975	4.4583	9.8875	2.9902	4.1619
Ywhaz	_VVSS[Phospho (STY)]IEQKTEGAEK_.2	#N/A	4.5798	4.2402	4.1163	4.5053	3.2859	3.3192	3.7622	3.3046	3.6824	4.3904	3.4202	3.9663	3.8479	4.1544	3.7876	3.9419	4.3027	3.7679	3.3166	4.3175
Ywhaz	_TAFDEAIAELDTLSEESYKDSTLIMQLLR_.3	#N/A	4.1784	3.7742	3.2640	3.4608	2.8845	4.4186	4.1636	3.9290	4.0839	3.9889	3.5625	3.6637	4.2493	3.2511	4.1890	3.5405	4.4666	3.3665	3.0151	3.2181
Kcna2	_IPS[Phospho (STY)]S[Phospho (STY)]PDLKK_.2	#N/A	11.8834	11.5488	11.4151	11.7034	11.3836	11.5487	11.3058	11.4402	11.4139	11.3583	11.6577	11.2583	11.0150	11.1148	10.4284	10.8071	11.9738	11.4493	11.4607	11.3414
Kcna2	_ETEGEEQAQYLQVTS[Phospho (STY)]C[Carbamidomethyl (C)]PK_.2	#N/A	8.1486	8.4759	8.8597	8.6206	7.2552	8.3601	8.1951	7.4778	8.3913	8.2655	7.5741	7.7442	8.1982	8.1133	6.9794	3.9835	8.5696	8.3186	8.3830	8.0498
Kcna2	_IPS[Phospho (STY)]S[Phospho (STY)]PDLKKS[Phospho (STY)]R_.2	#N/A	10.9895	11.0653	10.5288	10.8565	10.4448	10.4763	10.7312	11.1093	10.7207	10.4282	10.3834	10.8559	10.6402	10.9627	9.9429	10.6559	11.2858	10.9790	10.7596	10.6503
Kcna2	_SDYMEIQEGVNNS[Phospho (STY)]NEDFREENLK_.3	#N/A	3.9965	4.7619	5.3850	4.8498	3.7016	4.6244	3.8624	3.9290	4.0170	3.7989	4.4041	3.6468	3.9910	3.8915	3.5351	4.2199	4.7162	3.4627	3.9946	3.7068
Kcna2	_IPS[Phospho (STY)]S[Phospho (STY)]PDLK_.2	#N/A	4.2689	3.7339	3.3044	3.4205	3.6066	3.8307	3.9344	3.9693	3.9935	4.4752	4.3679	3.6233	4.1016	3.6453	3.5116	3.1130	4.5070	3.3558	3.6549	3.3226
Ube2b	_VSAIVEQSWNDS_.2	#N/A	4.7481	4.6076	5.2564	4.2942	4.2338	5.2210	3.3073	3.0956	4.4932	3.6015	3.7408	2.9962	3.4744	3.9186	5.7718	3.7401	3.6333	4.1999	3.8485	3.8856
Hmgb1	_GKFEDMAK_.2	#N/A	4.1789	3.8239	3.2143	4.6680	3.5166	3.7407	4.0245	4.3864	4.0836	4.3852	2.9571	3.7134	4.1916	3.6983	3.8385	3.0229	3.8837	3.2658	3.5648	3.4127
Hmgb1	_M[Oxidation (M)]SSYAFFVQTC[Carbamidomethyl (C)]R_.2	#N/A	3.3690	3.9501	4.4064	4.2152	3.5759	3.6093	3.4722	3.2375	4.9571	6.2850	4.3718	3.6762	3.5578	4.0605	3.4975	4.2320	3.7751	4.0580	5.4484	4.0275
Sumo1	_[Acetyl (Protein N-term)]S[Phospho (STY)]DQEAKPSTEDLGDK_.2	#N/A	4.5382	5.5843	6.5653	3.1512	3.8759	4.1090	6.1971	4.2386	6.2293	5.8563	3.8721	3.3540	6.6041	6.6313	3.2423	6.4425	3.2754	6.5561	6.3813	3.5277
Sumo1	_[Acetyl (Protein N-term)]S[Phospho (STY)]DQEAKPSTEDLGDKK_.2	#N/A	8.6642	8.8889	8.9698	9.0289	8.5085	8.6920	8.5915	8.7605	8.6242	8.8320	8.4488	8.5118	9.0531	9.1711	8.5264	8.7550	8.9014	8.5350	8.6722	8.6449
Dynll1	_NADM[Oxidation (M)]SEEM[Oxidation (M)]QQDSVEC[Carbamidomethyl (C)]ATQALEK_.3	#N/A	6.9498	6.1824	5.2277	5.5292	5.6831	6.0271	5.6194	6.0985	5.6029	5.8860	5.7174	5.9793	6.6474	5.0652	4.6733	5.5956	6.3454	6.1947	5.8152	6.1239
Gng2	_[Acetyl (Protein N-term)]ASNNTAS[Phospho (STY)]IAQAR_.2	P63213_S8_M1_Gng2	9.3032	9.1246	9.0731	9.4159	9.0087	8.9850	9.0682	9.4283	9.0268	9.0171	8.8118	8.6937	9.4473	9.0253	8.3364	8.5941	8.8972	8.8459	8.9611	8.3276
Gng2	_EDPLLTPVPASENPFR_.2	#N/A	7.5364	7.7866	8.0588	7.9778	7.8842	7.9398	7.9035	7.9375	7.9385	7.6588	7.8191	7.6825	7.7619	7.7440	7.6017	7.4580	8.1979	7.7245	7.9041	7.5832
Gng2	_KLVEQLK_.2	#N/A	7.6438	7.6481	7.6611	7.4965	7.5746	7.6835	8.1350	7.6589	7.6489	7.3298	7.5170	7.5942	7.4161	7.5310	8.0030	7.5619	7.8712	7.8208	7.9671	7.5397
Gng3	_IEASLC[Carbamidomethyl (C)]R_.2	#N/A	4.7332	5.3027	5.5743	5.7098	5.4942	5.0834	5.7742	5.2082	5.7800	5.4562	5.0477	5.2827	5.0620	4.3726	5.2320	4.7557	5.1907	5.9722	5.5730	5.6528
Gng3	_GETPVNSTM[Oxidation (M)]S[Phospho (STY)]IGQAR_.2	#N/A	10.3794	9.9275	10.2100	10.1565	9.6767	9.9838	9.8454	10.1396	9.8643	9.9617	9.9307	9.6416	10.2659	9.9963	9.3062	9.7965	10.5080	9.9460	9.9351	9.7449
Gng3	_MKGET[Phospho (STY)]PVNSTM[Oxidation (M)]SIGQAR_.3	#N/A	4.2463	5.3850	3.8393	4.0861	3.6751	4.1642	5.0054	3.8362	4.1418	3.7760	4.1581	3.6829	4.2730	6.2570	3.4636	3.4177	3.6415	3.3097	3.8995	3.3962
Eif5a	_VHLVGIDIFTGK_.3	#N/A	5.7413	6.1761	5.8249	5.9062	5.6469	5.6280	5.5412	5.0755	5.7097	5.9302	5.6749	5.6757	5.6917	6.4002	6.0675	5.9562	4.6494	5.9470	5.8052	6.4422
Eif5a	_EDLRLPEGDLGK_.3	#N/A	1.9315	4.6404	2.8877	5.0453	4.5474	4.6304	3.6708	2.3637	4.4086	5.1615	3.3760	2.7209	4.3957	3.9592	4.7160	4.7319	4.0878	4.2437	3.4501	3.9986
Eif5a	_EDLRLPEGDLGKEIEQK_.3	#N/A	5.9868	6.1800	5.9600	6.2412	6.2540	6.1588	5.5595	5.8685	6.0651	6.0653	5.6211	5.9724	6.0699	5.6783	5.7969	6.4011	6.4361	5.4996	5.5235	6.5840
Eif5a	_RNDFQLIGIQDGYLSLLQDSGEVR_.3	#N/A	4.3640	3.6389	3.3994	3.3254	3.7017	3.9258	3.8394	4.0643	4.2203	4.5703	2.3261	3.5283	4.3847	3.7975	3.4166	3.2080	3.1012	3.2312	4.3806	2.3767
Eif5a	_IVEMSTSK_.2	#N/A	3.2484	3.2536	3.7847	4.4410	4.0870	3.8980	3.4541	4.4496	3.1037	3.4683	4.0832	3.1430	3.2691	3.7718	3.0313	3.5933	3.4865	4.3467	3.9953	3.7388
Pkia	_[Acetyl (Protein N-term)]TDVETTYADFIASGR_.2	#N/A	4.4463	3.6991	5.4708	3.6777	4.4076	4.3489	3.9038	4.3795	4.9563	4.7143	4.4249	4.6794	4.9328	4.5083	5.6581	5.0132	5.2525	4.0587	4.0651	4.7682
Pkia	_SSTEQSGEAQGEAAK_.2	#N/A	3.8296	3.9578	3.9375	3.6444	3.4864	4.0869	3.5093	3.7884	3.9387	4.4631	4.1448	3.7132	3.9498	3.0313	4.6955	3.8744	4.3261	3.6031	3.9430	3.6382
Pkia	_SSTEQSGEAQGEAAKS[Phospho (STY)]ES_.2	P63248_S74_M1_Pkia	3.7817	3.7583	3.6123	4.0234	2.7819	3.5354	3.2220	3.9454	3.6425	4.4456	3.4946	4.1800	3.7506	3.9899	3.8479	3.6502	4.1382	3.8657	3.3595	3.6672
Kcnj3	_ERHNS[Phospho (STY)]VEC[Carbamidomethyl (C)]LDGLDDISTK_.3	#N/A	5.0494	6.6924	6.9191	6.1159	3.7784	4.8452	5.5120	4.1246	5.2219	6.0570	4.1961	5.7862	3.9298	5.6327	3.7879	5.2988	5.7759	5.7389	6.4422	5.9217
Kcnj3	_HNS[Phospho (STY)]VEC[Carbamidomethyl (C)]LDGLDDISTK_.2	#N/A	4.3121	4.9397	4.5616	3.4696	3.3786	3.5338	4.7265	4.3542	4.2179	5.2105	3.9322	4.2194	4.0898	3.7032	3.8028	3.4297	3.8514	4.5470	3.6109	3.1836
Actg1	_[Acetyl (Protein N-term)]EEEIAALVIDNGSGM[Oxidation (M)]C[Carbamidomethyl (C)]K_.2	#N/A	11.9298	11.7197	12.2004	11.8386	11.5248	11.5308	11.7564	12.1925	11.5081	11.8598	11.7492	11.9004	12.5158	12.0530	11.5173	11.5506	11.9746	11.6206	12.2148	11.5658
Actg1	etyl (Protein N-term)]EEEIAALVIDNGSGM[Oxidation (M)]C[Carbamidomethyl (C)]KAGFAGDDA	#N/A	8.9449	9.0514	8.9932	8.3452	7.7559	8.6362	8.9737	9.2161	8.3921	8.7950	8.5415	9.1764	8.8418	9.2416	8.4534	8.1317	9.1769	8.7913	8.6069	8.4265
Prkcg	_AAPALT[Phospho (STY)]PPDR_.2	P63318_T655_M1_Prkcg	17.0819	17.0820	16.9209	17.0092	16.7147	16.9856	16.7534	17.2860	16.7267	16.9584	16.7896	16.6543	17.3737	16.9527	16.0607	16.6525	17.0796	16.7850	16.7606	16.6768
Prkcg	_ENVFPGSTTR_.2	#N/A	8.5723	8.4240	8.6631	8.5209	8.3537	8.3762	8.5556	8.7433	8.4174	8.4321	8.0781	8.4624	8.6175	8.2997	8.4582	7.8822	7.9760	8.4182	8.1760	8.1153
Prkcg	_DVIVQDDDVDC[Carbamidomethyl (C)]TLVEK_.2	#N/A	8.7841	8.6503	8.6691	8.4187	7.4825	8.0962	7.9776	8.2181	8.3867	8.3588	7.7100	7.9583	8.6718	9.1743	6.9339	7.8561	8.4384	7.8733	7.5435	8.0072
Prkcg	_RGS[Phospho (STY)]DELYAIK_.2	P63318_S373_M1_Prkcg	8.1808	8.2975	7.7947	8.0333	7.5448	8.4465	8.0399	4.1234	8.4687	8.0831	7.9296	8.1672	4.4437	8.2898	7.3609	7.7879	8.8677	8.3972	8.1636	7.9165
Prkcg	_FEAC[Carbamidomethyl (C)]NYPLELYER_.2	#N/A	7.5815	7.7981	7.8113	7.5611	7.0125	7.6006	7.4104	7.4172	7.4038	7.4461	7.4706	7.4895	7.4064	7.5485	6.9501	7.6291	7.4265	7.3466	7.5793	7.0967
Prkcg	_C[Carbamidomethyl (C)]FFGAS[Phospho (STY)]PGR_.2	P63318_S342_M1_Prkcg	7.5078	7.6395	7.5720	7.5304	6.2806	7.0382	6.9743	6.7125	7.0435	8.3338	7.1239	6.9269	6.4801	7.0021	6.2939	6.5150	7.0802	6.7725	6.7887	6.7807
Prkcg	_APVDGWYK_.2	#N/A	6.6079	6.5997	5.4897	6.1765	6.6281	6.2666	6.2589	6.0664	6.4672	5.9323	6.3744	5.9297	6.3828	5.7797	6.0807	6.1725	5.5495	6.4835	6.1341	5.6208
Prkcg	_LSVEVWDWDR_.2	#N/A	5.6629	5.2525	6.2452	6.1251	6.0726	6.1922	5.8846	6.2505	6.2765	6.0347	5.8412	5.2636	3.8679	5.4904	5.0784	5.2112	6.0708	5.5155	5.8562	6.0068
Prkcg	_S[Phospho (STY)]GENFDKFFTR_.2	P63318_S639_M1_Prkcg	7.1086	7.8278	7.0044	7.6738	3.4819	7.3495	7.5251	7.2463	7.4796	7.1239	2.9651	7.0359	7.2211	7.5740	6.1398	7.1794	7.4133	7.4513	6.8413	6.6012
Prkcg	_DVIVQDDDVDC[Carbamidomethyl (C)]TLVEKR_.3	#N/A	5.9169	6.5615	5.7373	6.0261	5.0718	5.1825	5.7443	5.8865	5.8695	6.2701	4.9875	5.7021	7.0081	6.5450	4.4056	5.6706	5.2767	6.2215	5.2724	5.8213
Prkcg	_KDVIVQDDDVDC[Carbamidomethyl (C)]TLVEK_.3	#N/A	4.9489	4.9944	4.9332	5.2980	3.9355	4.8311	4.9633	3.9676	3.7884	3.4323	4.6755	4.1905	3.7728	4.8875	3.1828	3.5992	4.1994	3.8859	3.8760	4.6404
Prkcg	_S[Phospho (STY)]PTSPVPVPVM_.2	P63318_S687_M1_Prkcg	5.4039	8.7136	9.8248	10.0457	7.2730	8.4437	8.7486	5.2505	5.4390	5.5509	8.8692	7.1282	4.8304	9.6815	4.8926	4.9792	7.8322	5.1122	6.9436	5.4277
Prkcg	_SVPSLC[Carbamidomethyl (C)]GVDHTER_.2	#N/A	7.5213	7.1470	6.4903	7.2917	6.2380	7.6367	6.9983	7.2713	7.2085	7.4031	6.1576	6.6166	5.9317	6.3396	6.7296	6.3068	7.0718	6.7472	6.7266	6.7648
Prkcg	_M[Oxidation (M)]GPSSSPIPSPS[Phospho (STY)]PS[Phospho (STY)]PTDSKR_.3	#N/A	6.9550	7.9335	8.2146	7.0329	3.8171	8.2192	7.6311	7.0483	8.0749	9.1715	5.8130	7.9780	3.9378	6.7233	7.2182	7.9022	8.2412	8.1868	8.1624	7.9680
Prkcg	_NDFM[Oxidation (M)]GAM[Oxidation (M)]SFGVSELLK_.2	#N/A	5.6367	4.2203	4.3979	3.4293	4.1707	5.0584	5.1065	5.1084	4.7078	5.3794	5.1775	4.8037	4.3235	4.3616	4.2950	5.4985	3.8025	4.7315	5.1605	3.3315
Prkcg	_APTS[Phospho (STY)]DEIHITVGEAR_.2	#N/A	4.2679	4.3048	4.8161	3.3790	3.1802	4.6901	4.0419	3.8114	4.7548	4.4569	3.2919	4.1782	4.1693	5.1383	3.9175	4.2071	4.1543	4.5359	4.6171	3.5952
Prkcg	_DLKLDNVMLDAEGHIK_.3	#N/A	3.7779	5.2481	5.5651	6.4705	3.9848	5.0669	5.7703	4.0036	2.9835	3.5885	5.6102	3.2673	3.1489	6.1398	3.0886	3.1400	3.5008	3.6486	3.9477	3.2131
Prkcg	_S[Phospho (STY)]VPSLC[Carbamidomethyl (C)]GVDHTERR_.3	P63318_S145_M1_Prkcg	4.0913	3.9115	3.1268	3.9154	3.4290	3.6531	4.1120	3.7917	4.1711	4.2976	3.0447	3.8009	4.2792	4.6147	3.6892	3.4164	4.3294	3.5038	3.1524	3.0808
Prkcg	_LLNQEEGEYYNVPVADADNC[Carbamidomethyl (C)]SLLQK_.3	#N/A	4.4521	5.0224	4.5227	3.9945	3.5282	5.0894	4.5937	5.3488	4.4863	4.2923	4.3293	3.6350	3.5193	4.7366	3.6120	3.1523	3.7542	4.8762	4.1423	3.7762
Prkcg	_GSDELYAIK_.2	#N/A	3.3338	3.1681	3.8701	4.3556	4.1724	3.8235	3.2767	4.5350	3.4278	2.6518	3.8021	3.0576	3.1848	3.8572	2.9459	2.9562	3.5719	4.2613	3.9099	4.2577
Prkcg	_LGSGPDGEPTIR_.2	#N/A	5.3990	3.2677	4.7036	5.4591	4.0728	4.2969	5.3094	5.6436	3.5273	3.4406	5.2823	5.9419	3.6354	3.1792	3.0454	3.5792	3.3274	3.8220	4.1211	4.3573
Prkcg	_MGPSSSPIPSPSPS[Phospho (STY)]PTDSK_.2	#N/A	3.9574	4.2572	3.8084	3.6514	3.3114	3.4164	3.9270	3.4420	3.9017	4.3353	3.1657	4.0854	4.0442	4.9470	3.7978	4.1040	4.1128	3.5415	3.3770	3.6833
Rala	_VIM[Oxidation (M)]VGSGGVGK_.2	#N/A	7.4329	7.5694	6.9676	7.0805	7.3618	7.7563	7.4760	7.9175	7.4886	7.8946	7.4713	7.6879	7.7346	6.8505	7.5552	7.6062	7.1312	7.7479	7.6629	7.5257
Rala	_ADQWNVNYVETSAK_.2	#N/A	6.3739	6.8378	6.8606	6.2839	5.7577	6.5082	6.1538	5.6703	6.4893	6.4540	5.6054	6.1848	6.4390	6.7485	5.9820	6.1982	6.2386	5.7932	5.7824	6.7533
Rala	_SDLEDKR_.2	#N/A	6.1670	6.1314	5.7146	5.2647	5.7011	6.0752	5.9600	6.3585	6.0705	5.8968	5.7582	5.8469	5.6642	5.7750	6.7588	6.3470	5.4338	6.4022	5.7279	4.9219
Rala	_VKEDENVPFLLVGNK_.3	#N/A	5.1934	5.9571	5.5259	5.6556	5.6796	5.9740	5.7258	4.6104	5.3763	5.2892	5.8113	5.8872	4.7741	5.3869	5.6038	5.8443	4.8872	5.2424	5.5989	6.0758
Rala	_GQNSLALHK_.2	#N/A	4.8331	5.0859	5.1943	4.7927	4.5133	4.8597	4.5638	4.8396	3.5561	4.3541	3.9730	4.7414	4.7317	4.6767	5.0149	4.6095	4.9557	5.3051	4.5632	5.0869
Rala	_VVLDGEEVQIDILDTAGQEDYAAIR_.3	#N/A	4.3975	4.2873	4.6348	5.7071	3.7352	3.9593	3.8059	4.9125	3.8515	5.2379	4.2393	4.9735	5.0415	5.0715	3.3831	3.2415	3.6650	3.4844	3.7834	3.1941
Rps12	_TALIHDGLAR_.2	#N/A	7.1689	7.8836	7.6634	7.0808	7.6226	7.4099	7.5003	7.6834	7.3739	7.2653	7.2607	7.4092	6.7620	7.5915	7.8383	7.5310	7.0896	7.7199	7.5666	7.3053
Rps12	_[Acetyl (Protein N-term)]AEEGIAAGGVM[Oxidation (M)]DVNTALQEVLK_.2	#N/A	5.2680	5.3528	5.6177	5.8909	4.8932	5.2564	5.0516	4.9319	4.5571	5.8349	5.7566	4.7774	4.9176	6.2537	5.2848	5.7695	4.8614	4.4401	3.6144	6.2159
Rps12	_DVIEEYFK_.2	#N/A	4.3837	4.4363	3.9203	2.7781	3.2937	3.5178	3.2677	3.1085	3.2969	2.2494	3.1800	3.1223	4.4145	3.9583	2.5978	3.8312	4.1066	3.2809	3.3419	3.8216
Rps10	_AEAGAGS[Phospho (STY)]ATEFQFR_.2	P63325_S146_M1_Rps10	7.1105	3.4069	3.4488	7.3232	3.7652	6.8978	7.0756	6.9078	6.5790	3.1329	4.2094	3.1330	3.9430	3.4868	6.0748	6.9829	3.6351	6.3669	3.8134	6.9082
Rps10	_IAIYELLFK_.2	#N/A	4.9071	3.8666	4.7393	4.8091	3.6931	4.3368	5.1309	3.7118	4.0661	5.0280	4.5801	4.9324	3.8344	4.3356	4.2291	4.3324	4.0847	5.0296	4.4595	5.2035
Ppp3ca	_IITEGASILR_.2	#N/A	9.9071	10.0485	9.8568	9.9196	9.7270	9.6861	10.0542	9.9750	9.7460	9.5764	9.6598	9.8234	9.9212	9.7050	9.6004	9.7092	9.8806	10.1205	9.8344	9.6502
Ppp3ca	_EVFDNDGKPR_.2	#N/A	8.7658	8.4937	8.7244	8.6591	8.1804	8.5162	8.8858	8.7635	8.3742	8.6489	8.6515	8.3316	8.4619	8.5254	8.9287	8.6805	8.2448	8.6105	8.6491	8.6981
Ppp3ca	_QTLQSATVEAIEADEAIK_.2	#N/A	6.9465	6.5237	6.6800	6.8710	6.8254	6.4276	6.2690	6.7654	6.4476	6.9605	7.1413	6.6125	6.9385	6.6681	6.3832	6.9722	6.9473	6.4773	6.6793	7.4096
Ppp3ca	_GFS[Phospho (STY)]PQHK_.2	#N/A	9.2127	8.7836	9.3559	8.9896	8.6671	8.3573	8.6175	8.6302	8.3939	9.0720	8.8208	8.6905	8.6038	8.7095	8.0011	8.6764	8.3831	8.3954	8.4998	8.3575
Ppp3ca	_ITSFEEAK_.2	#N/A	5.5973	6.9478	6.5824	6.9462	6.1226	6.3661	7.4834	5.5092	7.1123	6.7319	7.5875	6.8240	7.5276	6.7602	7.0751	7.5339	5.8137	7.3873	7.7659	6.9116
Ppp3ca	_TQEHFTHNTVR_.2	#N/A	5.8594	6.9871	6.8126	6.0862	7.1740	7.0018	7.4628	7.7685	6.6499	7.3786	7.0278	7.1593	7.0563	7.1334	7.4352	7.2702	7.2654	7.2336	6.9193	6.8701
Ppp3ca	_GLTPTGMLPSGVLSGGK_.2	#N/A	4.2085	7.9097	8.7243	8.7415	6.5702	7.2195	8.2580	5.0592	7.0550	6.5852	7.8276	6.8124	5.3560	8.9810	7.2010	3.5706	6.9611	6.2214	6.2709	6.5712
Ppp3ca	_DAM[Oxidation (M)]PSDANLNSINK_.2	#N/A	8.5866	8.9088	9.5469	9.3610	8.8707	8.7810	8.9063	5.1845	8.8804	8.9420	9.2694	8.8726	3.3487	9.3501	7.1271	8.6162	9.2747	8.7324	8.7958	9.0271
Ppp3ca	_ALASETNGTDSNGSNSSNIQ_.2	#N/A	5.1648	4.8525	4.3758	5.2100	5.3634	4.5941	5.0265	5.4832	7.2617	5.0543	4.6359	4.7705	7.0676	5.6851	4.4398	5.6324	8.0160	5.3229	4.9998	5.6886
Ppp3ca	_LEESVALR_.2	#N/A	4.3984	5.4961	5.5823	5.8581	5.1890	5.8278	6.7065	4.5209	6.4204	5.9833	5.8374	5.7714	5.3828	5.8810	5.9566	6.0814	3.7679	6.0869	6.2039	6.0847
Ppp3ca	_ITS[Phospho (STY)]FEEAKGLDR_.2	#N/A	9.8722	9.9480	8.4865	9.7618	4.2585	8.3007	9.0694	9.1749	3.2752	3.2968	4.2547	9.7362	10.7223	9.4852	2.8598	9.6139	8.8981	9.6091	9.5982	9.1640
Ppp3ca	_VTEMLVNVLNIC[Carbamidomethyl (C)]SDDELGSEEDGFDGATAAAR_.3	#N/A	3.8447	4.3250	4.0315	4.5902	3.1825	3.4065	4.3586	3.2197	4.4177	4.0511	3.2912	4.0512	4.5258	4.0695	3.9358	4.1896	4.2178	4.4325	3.2307	3.7468
Ppp3ca	_QTLQSATVEAIEADEAIKGFS[Phospho (STY)]PQHK_.3	#N/A	3.6187	3.7340	4.1550	4.2102	3.6677	3.8918	3.9346	4.0303	3.9937	3.8250	3.6568	3.6235	3.9028	4.1421	3.5118	3.9636	3.8567	7.3847	3.7645	2.9924
Ppp2ca	_ELDQWIEQLNEC[Carbamidomethyl (C)]K_.2	#N/A	7.1289	6.7873	7.5111	7.5092	6.9605	6.9674	7.0673	7.2597	7.0740	7.2568	6.9794	6.9332	7.0933	7.3476	6.7919	6.8282	6.9764	7.1255	6.9554	6.9033
Phb	_VLPSITTEILK_.2	#N/A	8.1160	8.1573	8.0240	8.1812	8.2996	8.1599	8.0188	7.9969	7.6174	8.2058	8.0581	8.2168	8.2153	7.9394	7.9689	8.0428	8.0170	8.3114	8.4222	8.0497
Phb	_DLQNVNITLR_.2	#N/A	6.8924	7.3300	7.6060	7.4057	6.8792	7.2961	7.0563	7.4735	7.3264	6.8767	6.6919	7.3752	7.3751	7.6345	7.7949	7.3514	7.2253	7.4016	7.5006	7.4581
Phb	_FDAGELITQR_.2	#N/A	7.0162	7.3778	7.3552	7.4806	6.9259	7.3339	7.3245	7.4656	7.3372	7.6686	6.8930	7.3286	7.4839	7.4868	7.5241	7.5636	7.3348	7.2693	7.3924	6.7717
Phb	_IYTSIGEDYDER_.2	#N/A	5.7110	6.6008	7.1663	6.4417	5.7488	6.2456	5.5885	6.2195	6.5180	6.4366	5.6002	5.9137	5.5719	6.4961	6.2985	6.5041	6.2033	6.0437	5.8258	6.0889
Phb	_NVPVITGSK_.2	#N/A	5.9819	5.7740	5.8715	6.3759	6.1863	5.3837	6.4503	5.8266	6.6906	6.1123	6.3537	6.5617	5.8937	6.0635	6.7955	6.3576	4.9774	6.1610	6.8644	6.7404
Phb	_NITYLPAGQSVLLQLPQ_.2	#N/A	5.9932	5.8226	5.7132	6.2636	6.0944	6.0676	5.8582	5.7139	6.1257	5.7857	6.1892	5.6233	6.9426	5.7135	6.0587	6.1726	6.3053	6.0234	6.0045	6.9883
Phb	_QVSDDLTER_.2	#N/A	7.2418	7.0966	7.5181	6.9894	7.2234	7.0959	7.6474	7.5776	7.2648	7.4374	4.1377	7.4276	7.3994	7.4790	7.4710	7.2731	6.9155	7.2614	7.2392	7.3039
Phb	_AAIISAEGDSK_.2	#N/A	3.4044	7.2462	8.0431	7.9004	3.6113	2.9962	7.7227	7.7037	8.2184	7.9110	4.3365	3.6409	8.0209	7.7591	8.2042	7.9360	7.4939	7.8216	7.6292	7.6472
Phb	_EFTEAVEAK_.2	#N/A	3.6958	5.5454	6.1673	6.1675	3.9028	5.7937	6.2107	4.7292	5.9909	3.5064	4.0450	5.6486	3.2310	5.7762	6.1350	6.1448	5.6170	5.7252	6.1603	5.8896
Phb	_AAELIANSLATAGDGLIELR_.3	#N/A	3.5650	3.3183	4.1268	4.0461	3.9691	3.1967	3.0754	3.7075	3.6728	4.2943	3.2388	4.0138	3.6950	4.4016	3.5521	4.2301	3.9203	3.8982	4.0795	3.9468
Csnk2b	_YQQGDFGYC[Carbamidomethyl (C)]PR_.2	#N/A	6.1400	6.8151	6.6129	6.7004	6.3844	6.5696	6.5598	6.5847	6.4386	6.2185	6.3933	6.2529	6.7874	6.5308	6.6460	6.2839	6.7333	6.6298	6.4464	6.4953
Csnk2b	_GNEFFC[Carbamidomethyl (C)]EVDEDYIQDK_.2	#N/A	5.4349	4.4375	4.8422	5.4787	4.2587	5.2941	4.0713	4.6815	5.0258	4.3129	4.8135	4.4878	5.6260	5.4894	5.0588	3.9102	5.1286	4.4770	5.0392	5.5722
Rpl22	_ITVTSEVPFSK_.2	#N/A	7.1629	6.5095	6.8938	6.2325	6.7489	6.8532	6.8236	6.7028	6.5153	6.3686	6.7070	6.1965	6.5111	6.6823	6.8071	6.6617	6.3127	6.1888	6.3992	6.7015
Rpl22	_AGNLGGGVVTIER_.2	#N/A	6.2951	5.7723	6.5389	6.2274	5.9675	5.5564	5.9067	6.2149	5.7209	6.1014	5.7563	5.4094	5.4750	5.7992	5.7716	5.7115	5.9925	5.5422	4.8146	5.3724
Rpl22	_YFQINQDEEEEEDED_.2	#N/A	5.3358	7.0503	6.8037	6.1723	3.7330	6.5697	6.0037	3.7517	6.8686	5.5916	4.2148	3.5192	3.4008	7.3412	5.5879	6.0669	3.2490	7.2592	6.6301	5.9683
Ube2l3	_TDQVIQSLIALVNDPQPEHPLR_.3	#N/A	4.8243	4.3537	3.9439	3.1180	3.0430	3.9429	3.7046	3.1911	3.7959	4.2769	3.2746	4.2397	3.9614	4.2291	4.3240	3.8284	4.1179	3.6544	3.4469	4.1053
Ube2l3	_NIQVDEANLLTWQGLIVPDNPPYDK_.3	#N/A	3.6058	4.3970	4.1421	4.0836	2.9435	3.1676	3.0967	3.3062	3.1558	3.8121	3.5302	4.2864	3.2638	4.3084	4.1747	3.9507	3.8439	4.1936	2.9918	3.9857
Rack1	_FSPNSSNPIIVSC[Carbamidomethyl (C)]GWDK_.2	#N/A	7.0674	6.9375	7.3225	7.4870	6.8517	7.0475	7.4358	7.5671	6.8314	7.2469	7.0986	7.2109	7.1789	7.4794	7.0286	7.2103	6.7866	7.1079	7.0419	6.4187
Rack1	_IIVDELKQEVISTSSK_.3	#N/A	4.6034	5.0645	5.5737	5.7950	4.9454	5.4595	5.3143	5.5158	5.0930	6.0360	5.2592	5.7243	5.6305	5.6621	4.6926	5.3150	4.8981	5.2857	5.1446	5.8066
Rack1	_DVLSVAFSSDNR_.2	#N/A	6.7510	6.6214	7.2846	6.7348	3.1026	6.0486	6.2333	6.8632	6.2949	6.8251	6.4599	6.4724	6.8217	6.8606	5.9593	6.5761	6.6395	6.4678	6.6690	6.2507
Rack1	_VWNLANC[Carbamidomethyl (C)]K_.2	#N/A	5.5260	5.7434	5.8099	5.1358	5.5753	5.2061	5.5396	5.8253	4.9118	4.9487	4.1674	5.6492	5.6486	5.4915	5.9602	5.1414	4.8710	5.3615	5.5983	5.4188
Rack1	_YWLC[Carbamidomethyl (C)]AATGPSIK_.2	#N/A	5.7143	6.0993	4.4433	6.1944	5.7642	6.1995	6.0107	3.1574	5.5520	4.1134	5.8933	6.1410	6.0618	5.3135	6.2906	5.7919	6.1616	5.9870	6.1386	5.8734
Rack1	_AEPPQC[Carbamidomethyl (C)]TSLAWSADGQTLFAGYTDNLVR_.3	#N/A	3.9846	4.0182	4.5209	3.7048	3.3223	3.1618	4.2188	3.6849	3.8398	4.2330	3.3185	2.2194	4.0053	4.5079	3.7960	2.1328	4.2226	3.6105	3.2591	2.9741
Rack1	_YTVQDESHSEWVSC[Carbamidomethyl (C)]VR_.3	#N/A	3.8063	3.4022	3.6361	4.5896	4.0133	4.0466	3.0348	4.3010	4.4559	4.7653	3.5649	3.2389	3.1205	3.6232	3.0602	3.1684	3.3378	2.9945	4.1439	3.5901
Prkacb	_AKEDFLR_.2	#N/A	6.9630	6.7763	6.8198	6.2498	7.1813	7.0559	7.3690	7.0908	6.7609	7.0250	7.2015	6.7500	7.4652	6.7473	7.0335	6.5527	6.9100	7.3018	7.1403	6.6351
Prkacb	_TLGTGSFGR_.2	#N/A	5.5692	6.0213	5.1267	6.4376	6.1629	6.0719	6.3699	4.3248	6.4124	6.5396	6.1962	6.2035	5.9531	5.2287	5.8092	5.4498	4.7671	6.4828	6.4344	5.3453
Prkacb	_ILQAVEFPFLVR_.2	#N/A	5.3585	5.4471	4.9847	5.4443	5.5089	5.2729	5.4363	5.9010	4.8499	5.7172	4.8737	4.7183	4.2272	3.8017	4.1570	4.8546	5.5668	5.6782	5.3924	4.5084
Prkacb	_TWTLC[Carbamidomethyl (C)]GTPEYLAPEIILSK_.2	#N/A	4.9676	5.8330	5.0148	5.2517	5.0984	5.2946	5.9422	5.7987	4.8683	4.9196	5.0119	5.2784	5.3479	5.5945	4.6434	4.8984	4.8840	5.3362	4.9772	4.9113
Prkacb	_FRGS[Phospho (STY)]GDTSNFDDYEEEIR_.3	#N/A	3.7741	5.8866	4.3104	3.9153	3.1118	6.1980	5.8765	3.4744	2.9875	3.9804	3.3619	4.1182	4.5964	6.8093	4.0065	4.1190	5.7303	6.3873	3.1600	6.2154
Prkacb	_GS[Phospho (STY)]GDTSNFDDYEEEIR_.2	#N/A	4.4414	3.5614	3.4768	3.2480	3.7791	4.0032	3.7620	4.1417	3.8211	3.1469	4.1954	3.4509	3.9291	3.4639	3.3392	3.2854	3.1786	3.5283	4.3032	3.1502
Prkacb	_ATEQYYAMK_.2	#N/A	3.8768	3.4727	3.5656	3.1592	4.0837	4.1171	4.4652	4.2305	4.3854	3.6874	3.8641	3.3621	4.5509	6.9977	2.9897	3.2389	3.2674	3.0650	4.2144	3.5197
Ywhaq	_AVTEQGAELSNEER_.2	#N/A	10.0034	9.6905	10.2957	10.1634	10.0780	9.7710	9.8032	10.2278	10.1182	10.0762	9.7597	9.8715	9.9741	9.8770	9.9411	9.9051	9.7693	9.7835	9.9270	9.9941
Ywhaq	_EKVESELR_.2	#N/A	9.3977	9.4059	9.4949	9.5360	9.3697	9.5539	9.6765	9.6716	9.5585	9.5973	9.3581	9.4302	9.0481	9.4742	9.6171	9.5097	9.4070	9.4022	9.4360	9.5280
Ywhaq	_YLAEVAC[Carbamidomethyl (C)]GDDR_.2	#N/A	9.5132	9.3130	9.6732	9.4893	9.4431	9.1680	9.1886	9.6801	9.4294	9.4814	9.3465	9.2680	9.4353	9.4079	9.1637	9.3502	9.4045	8.9767	9.4173	9.3791
Ywhaq	_YLIANATNPESK_.2	#N/A	8.8352	8.7270	8.9524	8.8060	8.6688	8.8029	8.6403	8.9788	8.8801	8.6348	8.8445	8.5724	8.5234	8.5798	8.7524	8.3320	8.5785	8.6332	8.5937	8.3790
Ywhaq	_SIC[Carbamidomethyl (C)]TTVLELLDK_.2	#N/A	7.3846	7.3955	7.8627	7.3807	7.4363	7.7365	7.6010	7.0705	6.9101	7.2050	7.3228	7.1971	6.4554	7.2976	7.0442	6.9144	7.3391	7.0931	7.3892	7.1836
Ywhaq	_YLAEVAC[Carbamidomethyl (C)]GDDRK_.2	#N/A	6.8128	6.9045	6.9404	6.6117	6.7728	7.0156	6.7824	6.9430	6.4387	6.8875	6.6319	6.5539	5.9401	7.0537	7.3640	6.7057	6.3774	6.8385	6.4886	6.8657
Ywhaq	_KQTIENSQGAYQEAFDISK_.3	#N/A	5.4660	5.2899	5.5209	4.6672	5.6539	4.6259	4.6253	5.6080	4.2718	5.5628	5.6428	4.8921	5.1744	5.2835	3.7287	4.8285	5.6454	4.4179	4.8646	4.8990
Ywhaq	_TAFDEAIAELDTLNEDSYK_.2	#N/A	7.1746	6.5442	6.5410	6.5489	6.3248	6.4459	6.1753	6.8937	6.6122	6.4518	6.6687	5.8550	6.2149	6.8701	6.0946	6.2706	6.6214	6.3343	6.1114	6.4034
Ywhaq	_KQTIENSQGAYQEAFDISKK_.3	#N/A	5.0349	4.5562	5.0174	5.3481	5.0233	4.2734	4.6081	5.0429	4.4118	4.8759	4.2494	4.5897	5.5469	4.0438	4.3860	4.2968	3.7585	4.4043	5.3420	4.7093
Ywhaq	_YDDMATC[Carbamidomethyl (C)]MK_.2	#N/A	4.3639	2.4905	4.5435	5.1420	3.0700	3.5375	4.2513	3.0887	4.2867	4.1820	3.1603	0.7649	4.3948	4.9165	3.8048	4.3206	4.0868	3.0626	3.3617	1.1514
Ywhaq	_QTIENSQGAYQEAFDISK_.2	#N/A	3.6001	4.4028	4.1364	4.0893	2.9378	3.5463	3.1025	3.5440	3.4553	3.1166	2.5262	4.2922	3.6208	4.1234	4.1805	3.9450	3.8381	3.9951	3.6436	4.0904
Tuba4a	_DVNAAIAAIK_.2	#N/A	10.0150	10.2070	10.4823	10.7219	10.4218	10.3332	10.4698	10.0126	10.4216	10.7124	9.8570	9.9124	9.6765	9.7843	9.8536	9.9724	9.2727	9.8778	9.9393	9.8703
Tuba4a	_EIIDPVLDR_.2	#N/A	13.9646	13.7105	13.9180	13.9470	3.0176	13.8278	13.7558	13.9746	13.7136	14.0384	13.3168	13.3417	13.3024	13.0258	13.2256	13.2324	13.2961	13.1871	13.1479	13.3342
Tuba4a	_AVFVDLEPTVIDEIR_.2	#N/A	11.3764	11.4397	11.5380	11.6137	11.2322	11.4053	11.3341	11.5883	11.5279	11.6594	10.7816	10.8127	11.0247	10.9791	10.8403	10.8568	10.9370	10.6128	10.8016	10.7422
Tuba4a	_TIGGGDDSFTTFFC[Carbamidomethyl (C)]ETGAGK_.2	#N/A	4.0209	7.8837	7.9131	7.8315	3.9944	7.6465	7.5207	7.7349	7.5261	7.9114	3.9953	7.5976	7.8773	7.9140	7.2967	7.3894	7.5479	7.0296	7.3741	7.5569
Tubb4b	_FPGQLNADLR_.2	#N/A	14.3769	14.6286	14.6434	14.5314	14.4590	14.6019	14.5920	14.4465	14.3026	14.5794	13.7754	13.9702	13.8036	13.9095	14.0604	13.7479	13.9380	13.9868	13.9018	13.8255
Tubb4b	_EVDEQM[Oxidation (M)]LNVQNK_.2	#N/A	12.4482	13.3605	12.9465	13.3934	12.2975	13.3243	12.4238	13.6322	13.6016	13.6313	11.7876	11.9646	13.2834	12.8051	11.9783	12.0063	12.0646	12.7195	11.9677	12.0212

Tubb4b	_AVLVDLEPGTM[Oxidation (M)]DSVR_.2	#N/A	12.9609	13.0128	12.9786	12.9036	12.8137	12.9170	12.8254	13.0789	12.8518	13.0963	12.1250	12.2718	12.6606	12.3764	12.2062	12.4738	12.4379	12.1658	12.2862	12.3213
Tubb4b	_LAVNM[Oxidation (M)]VPFPR_.2	#N/A	12.6488	13.0497	12.9557	13.0603	12.7730	12.8207	13.0247	12.8403	12.3795	12.8855	12.1193	12.2742	12.3330	12.3854	11.9825	11.8462	12.0763	12.1914	12.0727	12.1648
Tubb4b	_LHFFM[Oxidation (M)]PGFAPLTSR_.3	#N/A	12.0882	12.3381	11.9896	12.0775	12.0489	12.1578	12.0717	12.1277	11.7693	12.4056	11.4821	11.6544	11.6634	11.3732	11.2465	11.5639	11.4452	11.6753	11.5689	11.4885
Tubb4b	_TAVC[Carbamidomethyl (C)]DIPPR_.2	#N/A	13.2909	13.5421	13.1676	13.2424	13.6301	14.1067	14.1285	14.1278	14.0115	13.8024	12.2207	12.6864	11.1601	12.8916	13.6221	12.9618	12.4852	11.7534	13.2163	12.2432
Tubb4b	_FPGQLNADLRK_.2	#N/A	11.7856	12.1880	11.7345	11.9937	12.0649	11.9097	12.0236	12.2580	11.6880	12.0863	10.8692	11.4532	11.1949	11.3762	11.6512	11.2643	10.8943	11.4751	11.2718	11.2601
Tubb4b	_IREEYPDR_.2	#N/A	12.7398	12.8713	12.9755	12.8297	12.6036	12.8571	12.8676	12.8754	12.9212	13.0134	12.1021	12.1881	12.3770	12.3424	12.4200	12.2493	12.1615	12.0880	12.1075	12.1265
Tubb4b	_YLTVAAVFR_.2	#N/A	12.1010	12.3367	12.3434	12.2818	12.0600	12.0280	12.1985	11.8663	12.0648	12.4742	11.4278	11.4198	11.4607	11.4283	11.3405	11.4686	11.3357	11.5675	11.6172	11.5795
Tubb4b	_GHYTEGAELVDSVLDVVR_.2	#N/A	10.6626	10.9741	10.3407	10.6058	10.1254	10.5946	10.4598	10.4525	10.2844	10.9427	9.5473	9.8872	10.1860	10.7695	9.4162	9.9783	9.7972	10.0285	9.6771	10.2013
Tubb4b	_INVYYNEATGGK_.2	#N/A	10.4112	10.9570	10.9350	10.8895	10.3303	10.7718	10.8044	10.4852	10.7659	10.6144	9.7497	9.9573	10.1858	10.5438	10.2646	10.1892	9.8733	10.1535	10.0302	10.2231
Tubb4b	_IM[Oxidation (M)]NTFSVVPSPK_.2	#N/A	12.4688	12.5504	12.4301	12.4444	12.3918	12.4107	12.4575	12.5472	12.3335	12.7252	11.4818	11.8640	12.0215	11.7665	11.7890	11.8248	11.6766	11.8003	11.7712	11.7408
Tubb4b	_ALTVPELTQQM[Oxidation (M)]FDAK_.2	#N/A	12.3680	12.4127	12.4029	12.3722	12.3665	12.2979	12.2210	12.4267	12.0954	12.4712	11.5448	11.8091	11.9976	11.6522	11.5261	11.6618	11.7503	11.7568	11.6047	11.7672
Tubb4b	_M[Oxidation (M)]SATFIGNSTAIQELFKR_.3	#N/A	7.4558	7.5285	6.3683	7.2013	7.1657	6.8629	7.2632	7.4950	7.1472	7.8390	6.1611	7.3531	8.4427	7.1601	6.7705	7.2224	6.5424	6.9136	7.1184	7.3741
Tubb4b	_KLAVNM[Oxidation (M)]VPFPR_.3	#N/A	11.5343	11.3918	10.7073	11.0780	11.4697	11.2687	11.2836	11.5718	11.0579	11.7194	10.4544	10.9199	11.3250	10.4662	10.4132	10.7515	10.6770	10.8064	10.6460	10.8325
Tubb4b	_NM[Oxidation (M)]M[Oxidation (M)]AAC[Carbamidomethyl (C)]DPR_.2	#N/A	8.4544	9.9369	10.0268	9.9657	8.8726	9.5465	9.3231	8.7585	8.8209	8.9044	8.7071	7.9513	7.8921	9.2203	9.3212	8.0083	7.3656	8.0533	8.3449	8.5866
Tubb4b	_SGPFGQIFRPDNFVFGQSGAGNNWAK_.3	#N/A	8.7066	9.3312	7.5608	8.7967	6.9046	8.3011	8.7735	9.0938	8.1684	8.6670	7.3719	7.8903	8.7712	9.2780	7.1553	6.9244	8.0332	7.9503	7.5814	7.9322
Tubb4b	_NSSYFVEWIPNNVK_.2	#N/A	12.2724	12.3380	12.5859	12.3845	11.9986	12.1642	12.2174	12.3752	12.2090	12.4262	11.4965	11.6429	12.0483	12.0269	11.4514	11.6454	11.8214	11.4982	11.6168	11.6903
Tubb4b	_GHYTEGAELVDSVLDVVRK_.3	#N/A	10.3220	10.9129	9.8080	10.4413	9.6338	10.0623	10.0874	10.4226	10.4123	10.7146	9.1662	9.7390	10.7023	10.3817	9.6242	9.9791	9.6022	9.8781	9.8925	10.1238
Tubb4b	_LTTPTYGDLNHLVSATMSGVTTC[Carbamidomethyl (C)]LR_.3	#N/A	5.3142	7.4555	7.7727	7.9011	5.7604	6.7083	7.1357	4.8449	6.3988	6.7695	6.1170	5.1548	4.8890	7.8625	5.9318	5.0041	5.9508	4.4948	5.4880	6.0559
Tubb4b	SC[Carbamidomethyl (C)]DC[Carbamidomethyl (C)]LQGFQLTHSLGGGTGSGM[Oxidation (M)]GTI	#N/A	5.9526	5.8822	6.9285	6.1150	5.8317	6.0464	6.0493	5.5797	5.4491	6.5646	5.6728	5.6011	5.6465	6.1512	4.6260	4.7661	4.8273	4.7577	5.2710	5.3797
Tubb4b	:C[Carbamidomethyl (C)]DC[Carbamidomethyl (C)]LQGFQLTHSLGGGTGSGM[Oxidation (M)]GTL	#N/A	4.0398	3.8485	3.3822	4.7887	4.6136	4.2835	3.5658	3.7166	4.1102	4.4530	4.2791	3.3386	3.5018	3.4715	3.3277	3.7586	3.6232	3.2169	3.7174	4.6121
Tubb4b	_FWEVISDEHGIDPTGTYHGDSDLQLER_.4	#N/A	6.1766	6.6606	7.6117	6.8518	5.9204	6.1971	6.0596	6.0797	7.3289	7.1146	5.4043	5.6830	6.5826	6.5698	5.9549	5.6148	5.5322	5.6576	6.2329	6.1686
Tubb4b	_ISEQFTAM[Oxidation (M)]FR_.2	#N/A	11.2956	11.1575	10.8168	10.5725	11.1723	11.1077	10.9111	11.3978	11.2017	11.4822	10.1722	10.5174	10.7767	10.0711	10.2420	10.4764	10.4430	10.4719	10.5532	10.4711
Tubb4b	_TLKLTTPTYGDLNHLVSATM[Oxidation (M)]SGVTTC[Carbamidomethyl (C)]LR_.3	#N/A	3.7425	3.7117	3.9940	3.9769	3.7756	4.3587	4.2181	5.1080	5.3960	5.5240	3.3476	4.1528	3.7734	4.0321	4.2104	4.1340	4.1804	4.6711	5.0953	3.5122
Tubb4b	_MSATFIGNSTAIQELFK_.2	#N/A	4.4926	4.3273	3.9682	3.2706	3.1987	3.4088	2.0361	3.2174	4.4154	3.1344	3.2889	4.0535	4.5235	3.5846	2.1460	3.8547	4.2155	2.0529	3.2330	3.7445
Prkcb	_[Acetyl (Protein N-term)]ADPAAGPPPS[Phospho (STY)]EGEESTVR_.2	P68404_S11_M1_Prkcb	10.7175	10.8879	10.0853	10.7151	9.8021	10.8595	10.7020	7.8681	10.2255	10.8997	9.9614	10.4840	10.1448	10.9969	10.1219	7.8876	11.5678	10.9173	10.4793	10.6428
Prkcb	_LIPDPKS[Phospho (STY)]ESK_.2	P68404_S206_M1_Prkcb	9.1554	9.3253	8.9726	8.9267	3.7444	8.9979	9.1244	9.1260	8.8661	9.0526	9.1699	9.0808	9.4870	9.3926	8.5615	8.7576	9.4278	9.3137	8.9990	9.0311
Prkcb	_ENIWDGVTTK_.2	#N/A	8.3203	8.0170	7.9853	8.3975	8.0532	8.0994	7.9892	8.4810	8.4056	8.2838	8.4159	8.1242	8.1600	8.0684	8.1719	8.0411	8.5141	8.1872	8.0993	8.2003
Prkcb	_QPVELT[Phospho (STY)]PTDK_.2	P68404_T642_M1_Prkcb	3.5047	3.9850	4.3715	4.2502	4.3433	3.0665	3.1978	8.8400	8.6066	8.5938	3.6313	3.7111	3.3649	4.4095	7.8127	8.4858	4.5578	4.0925	4.3915	8.5027
Prkcb	_KGTDELYAVK_.2	#N/A	5.4584	5.0439	5.0102	5.7614	4.9138	5.1129	5.9850	4.9691	4.8875	5.2170	4.9368	5.4269	4.3674	5.5418	5.0369	3.8320	5.5330	5.3219	3.5384	5.1398
Prkcb	_C[Carbamidomethyl (C)]SLNPEWNETFR_.2	#N/A	3.5865	4.9868	5.4919	5.8394	5.5423	5.0307	5.1248	4.7326	5.3215	5.5517	6.2144	5.5544	3.2831	5.8438	5.6939	3.9438	5.5687	4.3468	5.4473	5.4025
Prkcb	_LSVEIWDWDLTSR_.2	#N/A	5.6908	4.4473	6.0997	5.5195	5.0193	5.5685	5.0703	5.0761	5.6427	5.7921	5.6751	4.0705	6.2295	5.4757	5.0851	5.8922	6.1147	5.1565	6.0453	5.8550
Prkcb	_DLKLDNVM[Oxidation (M)]LDSEGHIK_.3	#N/A	6.2907	6.5548	6.4226	6.1256	6.3822	5.9530	5.9268	6.0357	6.1820	6.5205	6.4212	5.7675	6.1547	6.3875	5.7399	5.7629	5.8034	6.2634	6.2484	6.1331
Prkcb	_AGVDGWFK_.2	#N/A	4.3071	4.3903	4.4465	5.7394	3.9672	3.9648	5.1082	4.3527	4.3118	4.2361	4.7397	3.8515	4.1579	4.1511	3.8346	2.9022	2.9110	5.0000	4.4406	4.6290
Prkcb	_NLVPM[Oxidation (M)]DPNGLSDPY[Phospho (STY)]VK_.2	#N/A	7.4528	7.2724	7.1979	6.8953	6.9604	7.1561	7.0629	7.3815	7.2109	7.3353	7.0944	7.3352	7.6376	7.2913	6.8286	7.3789	7.8050	7.7623	7.4226	7.3658
Prkcb	_C[Carbamidomethyl (C)]VM[Oxidation (M)]NVPSLC[Carbamidomethyl (C)]GTDHTER_.3	#N/A	3.6824	4.3204	4.2187	4.0070	3.0201	6.2335	3.0201	3.3828	3.0792	6.0209	6.1150	4.2098	3.7031	4.2058	4.0981	4.0273	3.9205	3.9127	4.7892	4.1728
Prkcb	_FQLKES[Phospho (STY)]DKDR_.3	P68404_S234_M1_Prkcb	3.8107	3.5084	3.3473	3.7735	4.0177	4.0907	3.0304	4.0364	3.7335	3.6213	4.1079	3.2345	3.8416	3.3854	3.0558	3.1728	3.5336	3.4728	3.9149	3.0626
Ywhah	_AVTELNEPLSNEDR_.2	#N/A	10.7539	10.5570	10.9122	10.6095	10.5522	10.6643	10.4922	10.7412	10.6920	10.6884	10.7202	10.4487	10.8365	10.8099	10.5814	10.5634	10.6551	10.5409	10.7156	10.6183
Ywhah	_NSVVEASEAAYK_.2	#N/A	8.9151	8.8130	9.4273	8.8223	8.6945	8.5468	8.5094	8.7026	8.8636	8.7779	8.5369	8.4725	8.9346	8.8505	8.7913	8.7811	8.8185	8.4725	8.6973	8.8182
Ywhah	_YLAEVASGEK_.2	#N/A	8.4542	8.4210	8.5877	8.6178	8.2963	8.5060	8.5160	8.2886	8.7180	8.7141	8.4113	8.4236	8.4500	8.5314	8.7150	8.6798	8.0766	8.2547	8.5874	8.6042
Ywhah	_YLAEVASGEKK_.2	#N/A	8.1675	8.3207	8.3826	8.2195	8.0850	8.1978	8.2880	8.0389	8.4671	8.2850	7.9829	8.2423	8.1918	8.2656	8.4311	8.3358	7.9308	7.9955	8.1997	8.1764
Ywhah	_NC[Carbamidomethyl (C)]NDFQYESK_.2	#N/A	7.6757	7.8588	8.2388	8.1607	7.9773	7.4894	7.9258	7.8805	8.1742	7.8901	7.8131	7.8707	7.7809	8.2576	8.0309	7.7559	7.7555	7.9283	7.9597	7.9133
Ywhah	_[Acetyl (Protein N-term)]GDREQLLQR_.2	#N/A	7.2538	7.7246	7.6665	7.4155	7.2435	7.5539	7.5033	7.7703	7.3945	7.2600	7.2809	7.7246	7.6039	7.6973	7.6866	7.3955	7.5978	7.2372	7.7192	7.1339
Ywhah	_ELETVC[Carbamidomethyl (C)]NDVLALLDK_.2	#N/A	6.6233	6.4615	7.0236	6.4695	6.5570	6.9693	6.6685	6.8818	6.6912	7.1718	6.9775	6.7832	5.7849	7.0368	6.9014	6.6256	6.8374	6.7166	6.7667	6.9479
Ywhah	_QAFDDAIAELDTLNEDSYK_.2	#N/A	6.9662	6.3948	7.0697	6.3654	6.6165	6.5851	6.5108	6.4371	6.6164	6.8644	6.7572	6.5727	6.2739	6.8970	6.7580	6.6095	7.2453	6.2052	6.6126	6.5409
Ywhah	_AVTELNEPLSNEDRNLLSVAYK_.3	#N/A	6.5024	7.0120	6.3730	6.9380	6.2831	6.4152	6.3243	7.0710	6.1821	5.9290	6.3260	7.1636	7.1699	6.7380	6.7756	6.8050	6.8963	6.8242	6.2969	6.4130
Ywhah	_NSVVEASEAAYKEAFEISK_.3	#N/A	5.6131	6.9967	5.4936	6.6109	6.4217	6.1096	6.1344	6.8208	5.9116	7.1377	6.1254	7.2679	8.4484	7.2307	5.9690	5.8859	5.9172	6.5597	6.2416	7.0132
Ywhah	_EAFEISK_.2	#N/A	4.7489	4.7047	3.1197	4.7816	3.1844	4.0790	3.8682	3.5579	4.8491	5.2382	4.6245	4.4065	5.1664	4.6502	5.3029	4.2012	3.3060	5.0699	4.7590	5.3689
Ywhah	_DNLTLWTSDQQDEEAGEGN_.2	#N/A	11.9981	12.4122	12.7765	11.8788	11.0933	11.9090	10.8418	11.0620	12.2334	13.0192	11.0439	10.5131	3.7163	12.6721	11.7243	12.7746	12.3456	12.1274	12.6083	12.1135
Ywhah	_YDDMASAMK_.2	#N/A	4.0144	3.9884	4.4960	3.6750	3.3522	3.5762	4.1889	3.7148	4.2480	4.2208	3.1215	3.8778	4.3561	5.4412	3.7661	4.3593	4.2525	3.5807	3.2293	3.0040
Kpnb1	_AAVENLPTFLVELSR_.2	#N/A	6.5323	6.0382	6.4134	6.2219	6.3913	6.5256	6.6900	6.3932	6.1710	6.5165	6.3458	6.4837	6.3086	5.9469	5.8798	6.3010	5.9108	6.3171	6.4159	5.7741
Kpnb1	_LAATNALLNSLEFTK_.2	#N/A	6.6618	5.3914	5.7963	6.1915	6.0850	5.8086	5.6351	5.9590	5.4190	5.6870	6.1322	5.7445	5.8802	5.3969	5.3759	5.5895	6.3767	5.9890	5.4849	5.4529
Kpnb1	_VLANPGNSQVAR_.2	#N/A	6.5957	6.5876	5.8380	6.3431	6.4312	6.5533	6.9988	6.7288	6.4200	6.3128	6.3907	6.2527	6.6930	5.7720	6.4604	6.1316	3.5538	6.4767	6.9141	6.1470
Kpnb1	_WLAIDANAR_.2	#N/A	4.7418	5.1713	4.8882	4.9949	4.1761	5.2275	5.5609	5.3263	3.4523	5.3869	2.9310	3.7362	3.6178	4.1204	4.7142	4.7341	4.0046	5.2629	4.7910	4.2406
Kpnb1	_SNEILTAIIQGM[Oxidation (M)]R_.2	#N/A	3.5898	5.4443	4.8058	4.9718	4.8937	5.6339	4.6864	4.7399	4.7469	3.4004	5.2374	4.4430	3.3370	3.8397	3.9276	2.9519	4.7188	5.0013	4.1803	4.7205
Kpnb1	_[Acetyl (Protein N-term)]M[Oxidation (M)]ELITILEK_.2	#N/A	3.0507	3.2390	3.6167	3.5041	3.5973	3.8214	3.9438	4.3058	4.0029	4.4659	4.3773	2.9651	4.1110	3.6547	3.5210	3.1036	3.8030	2.4442	3.0627	3.3320
Doc2b	_WHTLTNELPGAVLS[Phospho (STY)]D_.2	P70169_S411_M1_Doc2b	4.2987	7.3761	3.5775	3.5433	3.6364	3.8605	7.4418	4.2666	3.6607	4.5050	4.3382	3.0042	4.0718	8.8269	6.7856	3.1427	3.7639	8.0515	6.3632	3.2929
Hrh1	_SRS[Phospho (STY)]NS[Phospho (STY)]GLDYIK_.2	P70174_S381_M2_Hrh1	4.0104	3.3087	3.5470	3.5739	4.2173	4.2506	4.3316	4.0969	4.2518	3.8210	3.7305	3.0348	4.4173	3.4191	2.8562	3.3725	3.1338	3.1985	4.3480	3.3861
Hrh1	_SNS[Phospho (STY)]GLDYIK_.2	P70174_S383_M1_Hrh1	4.9848	4.1011	4.2554	4.3662	3.4249	4.6351	5.5119	3.4437	3.1408	3.8271	3.5152	3.8272	3.2489	4.2935	3.6485	4.0810	4.4418	4.2085	3.0067	3.9708
Hrh1	_RIS[Phospho (STY)]ETSEDQTLVDR_.2	P70174_S344_M1_Hrh1	3.4145	3.9046	4.4519	4.1698	3.6214	3.6547	3.4267	3.1920	3.3469	3.2251	4.3264	4.6116	3.5123	4.0150	3.4521	4.2774	3.8520	4.1035	3.6787	3.9820
Dlg3	_GVTSNTS[Phospho (STY)]DSESSSK_.2	P70175_S632_M1_Dlg3	3.9740	7.3836	7.7058	7.6326	7.0587	7.2987	7.2544	7.6679	3.6542	3.7846	7.1702	7.2950	7.5689	7.7691	7.0726	3.3361	7.5527	4.0014	7.4530	7.1902
Dlg3	_LLAVNNTNLQDVR_.2	#N/A	4.5903	4.9377	4.0585	4.9063	4.5296	4.9663	5.1485	5.0215	4.8049	4.4156	3.6837	4.6758	4.5528	4.0965	4.3542	4.8891	4.2448	5.2118	4.4125	3.6440
Dlg3	_EQMMNSSMS[Phospho (STY)]SGS[Phospho (STY)]GSLR_.2	P70175_S508_M2_Dlg3	5.2754	4.0311	5.5834	5.4816	3.3324	3.5025	4.0842	3.6697	4.1043	4.2751	6.7046	3.8580	5.0227	4.0454	3.8063	3.7604	4.3378	3.8498	3.1483	3.9176
Dlg3	_FIEAGQFNDNLYGTSIQSVR_.2	#N/A	4.0232	3.5143	4.5595	3.2678	3.1678	3.2399	3.6246	3.8992	3.6043	4.2296	3.9521	4.1536	4.3473	4.1778	3.6607	3.8297	4.0393	3.1102	2.5446	3.5683
Dlg3	_VNDDLISEFPHK_.3	#N/A	3.2022	3.6825	3.1732	3.9476	4.0407	4.2648	2.9753	3.8623	3.5594	3.4085	3.9338	3.0136	3.6675	3.2113	3.0775	2.2924	3.3595	1.6236	4.0890	1.7473
Dlg3	_ALFDYDR_.2	#N/A	3.7744	3.3702	3.6680	4.5577	3.9813	4.0146	3.0668	4.3329	2.9870	3.5850	3.9665	3.2597	3.1524	3.6551	3.0922	3.1365	3.3698	4.4634	4.1120	3.6221
Dlg3	_NATHEQAAAALKR_.3	#N/A	4.5583	4.2616	4.0949	4.5268	3.2644	3.2977	3.7837	3.2831	3.7039	4.3689	3.1826	3.9878	3.8694	4.1329	3.8091	3.9204	4.2812	3.7464	3.3950	4.3390
Dlg3	_VNEVDVSEVVHSR_.3	#N/A	4.2092	3.7936	3.2447	3.4801	3.5470	3.7711	3.9941	4.3561	4.0532	4.4156	2.9267	3.6830	4.1612	3.7050	3.5713	3.0533	3.8533	3.2962	3.5952	3.3823
Pip5k1b	_IQALKASPS[Phospho (STY)]K_.2	P70181_S407_M1_Pip5k1b	3.6451	4.3577	4.1814	4.0443	2.9828	3.5013	3.0574	3.3455	3.5004	7.6056	6.3636	4.2472	3.6658	4.1685	4.1355	3.9900	3.8832	3.9500	3.5986	4.1355
Pip5k1a	_IPLKPS[Phospho (STY)]PTK_.2	P70182_S443_M1_Pip5k1a	4.3228	3.6800	5.9710	3.3666	3.6605	4.3244	4.2579	4.0232	4.8547	5.7745	3.6567	3.5695	6.0806	3.3453	4.9667	5.6458	6.7705	3.2723	4.4217	3.3123
Pip5k1a	_S[Phospho (STY)]GPSGNSC[Carbamidomethyl (C)]TSQLMASGEHR_.3	P70182_S458_M1_Pip5k1a	3.7546	4.2482	4.2909	6.6730	3.0923	3.3164	4.4488	3.4550	3.0070	3.9609	2.5188	4.1377	3.1151	4.2780	4.0260	4.0995	3.9926	3.8405	3.4891	4.2450
Kifap3	_RDS[Phospho (STY)]LPGK_.2	#N/A	8.9480	9.1728	8.8534	8.3950	8.1525	8.9813	8.5757	8.8607	8.4908	8.7153	8.8453	8.4122	8.7702	8.8316	7.4289	8.2433	8.7872	8.9153	8.8788	8.3584
Kifap3	_RDS[Phospho (STY)]LPGKEK_.2	#N/A	6.7278	7.2026	6.6329	6.7028	5.9876	6.5629	6.2299	7.1298	6.2020	5.9858	6.0500	6.8814	6.9447	6.9169	5.5872	6.2812	6.6727	6.6805	6.7390	6.2859
Psmb7	_DGIVLGADTR_.2	#N/A	6.1407	6.2206	6.8556	6.3368	6.2904	6.2330	6.4152	6.1647	5.8269	6.2260	6.6364	5.7879	4.4324	5.9911	5.7379	5.9161	5.7267	4.9774	5.5389	5.6089
Psmb7	_VTPLEIEVLEETVQTM[Oxidation (M)]DTS_.2	#N/A	5.6820	5.9069	5.3850	5.3326	5.1987	4.8601	4.8777	6.2899	5.0531	5.5830	4.8033	5.3076	6.0052	5.5737	5.9101	5.6302	5.3211	5.2529	5.6162	5.2153
Psmb7	_ATEGMVVADKNC[Carbamidomethyl (C)]SK_.2	#N/A	4.2918	3.5188	3.8284	3.2924	2.9979	3.6096	2.0418	4.5175	4.2147	4.2541	3.0882	4.2542	4.3227	3.8665	2.6789	3.6540	4.0148	3.1347	4.4337	3.5438
Lxn	_LFLVQTVQQASK_.2	#N/A	5.4729	6.1475	6.3404	5.6824	5.9517	6.1637	6.0276	5.5922	4.6798	6.2326	5.5692	5.5558	5.3777	6.1436	5.9198	6.3225	5.7997	5.9709	6.0243	5.6527
Lxn	_FSVEEIIQK_.2	#N/A	4.4202	4.1004	4.7171	4.3841	4.3000	3.7233	4.3002	4.2536	3.9854	4.8933	3.9197	4.0609	3.6906	4.7166	3.4985	3.5023	3.5440	3.8801	2.6540	3.6756
Lxn	_AASVAENC[Carbamidomethyl (C)]INYQQGTPHK_.2	#N/A	3.8002	4.1672	4.2801	3.9761	3.5711	3.0600	3.2250	3.4684	3.8234	2.9339	3.5893	3.5954	3.9503	3.5736	3.0936	4.3876	3.7218	3.5300	3.8290	3.8394
Plxna1	_TASS[Phospho (STY)]PDSLR_.2	P70206_S1630_M1_Plxna1	4.0498	7.8711	7.0971	3.5344	6.6741	4.2901	7.7886	4.0575	7.5013	7.3071	7.5652	7.5017	4.3779	6.9222	2.8167	3.4119	7.5000	3.2380	7.7027	7.5905
Plxna1	_QTSAYNISNS[Phospho (STY)]STFTK_.2	P70206_S1611_M1_Plxna1	4.3479	4.4721	3.8844	3.2364	3.0540	3.0873	3.9941	4.4732	3.9144	4.1584	3.3930	4.1982	4.0798	3.9225	4.0195	3.7100	4.0708	3.0787	3.3777	3.5998
Plxna1	_LGKDS[Phospho (STY)]PSNK_.2	P70206_S1793_M1_Plxna1	4.0974	3.9054	3.1329	3.5920	3.4351	3.6592	3.1805	3.7978	4.1650	4.3037	3.0386	3.7948	4.2731	4.6208	3.6831	2.9415	4.3355	3.1843	3.4834	3.4941
Plxna2;Plxna4	_YTGS[Phospho (STY)]PDSLR_.2	P70207_S1630_M1_Plxna2;Plxna4	8.6091	8.7434	4.1318	4.4898	7.8428	8.1203	8.9125	8.1951	8.4851	8.5756	8.4185	8.0793	8.6250	7.8441	3.7721	3.9574	4.3182	8.4614	8.3458	3.8472
Plxna2;Plxna4	_YTGS[Phospho (STY)]PDS[Phospho (STY)]LRSR_.2	P70207_S1630_M2_Plxna2;Plxna4	6.7083	5.9422	3.9275	5.8153	3.0970	3.1303	3.9511	3.7164	3.8713	6.0372	5.1543	5.6858	4.0368	4.5394	3.9765	3.7530	5.4903	3.5790	3.2276	4.8009
Fv1	_S[Phospho (STY)]LSDLTSQK_.2	P70213_S244_M1_Fv1	6.5978	7.0094	6.4436	5.8235	3.8629	6.8313	3.1852	3.8817	3.1054	7.0018	6.8283	6.9585	3.2708	7.1707	3.2105	5.8436	3.3789	6.6019	6.0104	3.7405
Chl1	_DETFGEYS[Phospho (STY)]DSDEKPLKGS[Phospho (STY)]LR_.3	P70232_S1148_M2_Chl1	3.7882	4.2792	4.3245	3.9658	3.9113	3.0482	3.7643	3.4886	3.6464	3.9270	3.5486	3.4855	3.8089	4.3116	3.3415	4.1331	4.0262	4.1949	3.5201	3.5631
Nfic	_SPFNSPS[Phospho (STY)]PQDSPR_.2	P70255_S339_M1_Nfic	6.8701	4.6314	6.9610	3.9880	4.0812	6.8800	3.4599	6.9616	3.5190	7.1493	3.8934	3.4490	3.6271	3.1708	3.0371	3.5875	6.9256	3.8303	4.1294	4.3490
Nfic	_NWTEDIEGGISS[Phospho (STY)]PVKK_.2	#N/A	3.4617	3.4779	3.5085	4.8167	4.0802	4.7276	3.3347	5.0732	4.2345	5.2501	4.6050	3.2773	4.3994	3.2139	3.5514	3.3240	5.3285	4.4662	4.4411	4.2959
Nfix	_S[Phospho (STY)]IDDSEMESPVDDVFYPGTGR_.2	P70257_S280_M1_Nfix	4.1408	3.9672	3.9182	3.6537	3.4785	3.7761	3.6781	3.8412	3.6716	4.0027	3.7008	3.8566	3.8064	3.9053	3.4896	3.7268	3.6200	3.5595	3.9500	3.7471
Nfix	_S[Phospho (STY)]PAAGSSQSSGWPNDVDAGPASLKK_.3	#N/A	5.2920	3.8183	4.5382	4.2841	3.7077	3.7410	3.3404	4.7613	3.2606	3.3114	4.2401	3.7012	3.4261	4.4818	3.3658	3.8785	4.4692	4.1898	3.8384	3.8957
Nfix	_S[Phospho (STY)]PAAGSSQSSGWPNDVDAGPASLK_.2	#N/A	4.5116	4.3084	4.0481	4.5735	3.2177	3.3899	4.3753	3.2364	4.4344	4.0344	3.3079	4.0345	4.5424	4.0862	3.9525	3.8737	4.2345	4.4158	3.2140	3.7635
Pfkfb2	_NS[Phospho (STY)]FTPLSSSNTIR_.2	#N/A	6.7537	7.0490	3.6777	3.8869	3.9800	4.2041	3.5611	3.9231	3.6202	7.2205	3.9946	6.3265	6.5539	6.9101	3.1383	3.4863	3.4203	5.9491	4.0282	6.0000
Pfkfb2	_RNS[Phospho (STY)]FTPLSSSNTIR_.3	#N/A	3.2426	4.0765	4.2800	4.3416	3.4496	3.1580	2.5374	3.4683	3.1654	3.8025	3.5398	3.8026	3.2735	4.3181	3.6239	4.1056	4.4664	4.1839	2.9821	3.9954
Pkn1	_TDVSNFDEEFTGEAPTLS[Phospho (STY)]PPR_.2	P70268_S920_M1_Pkn1	3.1591	4.1600	4.1965	3.9424	3.3661	3.3994	3.6820	3.4473	3.6022	4.4706	3.0809	3.8861	3.7677	4.2703	3.7074	4.0221	3.9850	3.8481	3.4967	4.2373
Pdlim4	_RSS[Phospho (STY)]VSGISLEDNR_.2	#N/A	7.5006	7.3022	3.8825	7.5539	7.6342	7.3768	6.5199	3.7614	3.9163	7.4083	6.5972	6.5058	7.2335	7.4456	4.0214	6.9785	7.4735	6.7804	6.0294	7.0373
Pdlim4	_S[Phospho (STY)]SVSGISLEDNR_.2	P70271_S118_M1_Pdlim4	5.9292	3.8154	3.9034	4.0805	6.3360	3.5670	5.9884	3.9224	3.6195	6.8034	5.9727	4.2117	4.3977	3.9415	3.8078	3.8396	4.0898	5.6159	5.7722	3.6188
Pdlim4	_IHIDPESQDC[Carbamidomethyl (C)]S[Phospho (STY)]PATSR_.2	#N/A	4.3371	4.2028	4.3604	3.8894	3.1618	3.3859	3.7483	3.5245	3.8074	6.3039	4.1194	4.7274	5.2186	4.4102	4.4211	4.1690	4.0622	3.7404	3.1329	5.1767
Hdac2	_IAC[Carbamidomethyl (C)]DEEFS[Phospho (STY)]DS[Phospho (STY)]EDEGEGGRR_.2	#N/A	6.3862	6.1339	6.6808	6.4757	3.6740	6.0505	6.3037	5.7877	6.1916	6.6064	5.6795	6.2488	3.7991	6.9542	5.5859	6.5615	6.4936	6.1290	6.2795	6.0869
Hdac2	M[Oxidation (M)]LPHAPGVQM[Oxidation (M)]QAIPEDAVHEDS[Phospho (STY)]GDEDGEDPDKR	#N/A	3.9407	10.3448	9.1627	7.0363	3.5031	10.0966	9.9009	4.1077	9.7498	8.6887	2.9635	8.5633	7.2080	10.2241	9.2038	9.1910	7.4513	10.2215	10.1628	9.7849
Pebp1	_LYTLVLTDPDAPSR_.2	#N/A	10.9250	11.0317	11.3280	11.2186	10.7311	11.0244	10.8366	10.9141	11.0004	11.0125	10.8205	10.8957	11.1029	11.4299	11.1924	11.0432	11.0492	10.7541	10.9495	10.9892
Pebp1	_VDYAGVTVDELGK_.2	#N/A	10.8026	10.8641	11.1479	10.9847	10.6829	10.6963	10.6305	10.7918	11.0421	10.9491	10.6323	10.8463	11.1888	11.1875	11.1189	11.1371	10.7946	10.7048	10.6637	11.0440
Pebp1	_FKVETFR_.2	#N/A	10.2711	10.6228	10.4664	10.3120	10.5283	10.3498	10.4313	10.6378	10.2271	10.4202	10.2664	10.5310	10.8320	10.7320	10.9154	10.4764	10.4606	10.4146	10.3321	10.4262
Pebp1	_VLTPTQVM[Oxidation (M)]NRPSSISWDGLDPGK_.3	#N/A	10.0463	10.0507	9.4994	9.5817	9.7730	9.8170	9.7542	10.0792	9.9832	10.0394	9.6322	10.1328	10.2286	10.0491	10.3155	10.1662	10.1469	10.1086	10.1045	10.0126
Pebp1	_LYEQLSGK_.2	#N/A	6.4033	7.1018	7.0190	6.9907	7.2036	7.3570	7.5352	6.3365	7.7719	7.6831	7.4383	7.1353	6.8527	7.7523	8.1088	7.6899	6.7163	7.5505	6.9135	7.9037
Pebp1	_[Acetyl (Protein N-term)]AADISQWAGPLC[Carbamidomethyl (C)]LQEVDEPPQHALR_.3	#N/A	7.7544	8.0791	8.0051	8.3804	7.9638	8.3103	8.1937	8.5541	8.2250	8.5677	7.6841	7.9148	8.0679	8.5506	8.5362	8.1972	7.9969	8.0028	8.1847	7.7697
Pebp1	_GNDISSGTVLSDYVGSGPPSGTGLHR_.3	#N/A	7.4917	7.7897	7.3296	7.8735	8.3371	7.9072	7.8338	8.3812	7.5089	7.8343	8.2733	8.0612	7.8591	7.6538	8.0741	8.1642	8.1006	7.1017	7.1781	7.8656
Pebp1	_YVWLVYEQEQPLSC[Carbamidomethyl (C)]DEPILSNK_.3	#N/A	5.7865	4.9882	5.8486	5.8133	5.1229	5.2573	5.5511	5.0531	5.6601	5.8319	5.1612	5.1786	4.9590	6.3047	6.2196	5.4331	5.2769	5.0731	5.3940	5.5841
Pebp1	_YNLGAPVAGTC[Carbamidomethyl (C)]YQAEWDDYVPK_.3	#N/A	3.8286	4.1742	4.3649	3.8608	3.1663	3.3178	3.7637	3.5290	3.6839	4.3889	3.1625	4.0636	3.8493	4.3520	3.7890	4.1735	4.0667	3.7665	3.4151	4.3190
Pebp1	_EWHHFLVVNM[Oxidation (M)]K_.2	#N/A	2.8099	3.6399	3.3984	3.3264	3.7007	3.9247	3.8404	5.4140	3.8995	6.0510	4.6663	3.5293	5.5663	3.3855	3.4176	3.2070	3.1002	3.2322	4.3816	3.3525
Stam	_AS[Phospho (STY)]PALVAK_.2	P70297_S156_M1_Stam	3.5713	7.0346	6.1818	7.0213	6.2947	6.6654	6.7402	3.7970	3.1901	7.4004	6.8940	5.8346	6.6497	6.7699	3.2952	6.5266	3.2942	4.2603	6.6892	6.7776
Stim1	_NTGASSGATS[Phospho (STY)]EESTEAEFC[Carbamidomethyl (C)]R_.2	P70302_S40_M1_Stim1	3.5251	3.8753	3.8279	4.1404	3.7321	3.7654	3.2782	3.7508	4.0838	4.1020	3.5624	3.6014	4.2492	3.9131	3.3203	3.6535	4.0143	3.7183	3.7026	3.7849
Stim1	_DLTHS[Phospho (STY)]DSESSLHMSDR_.3	P70302_S519_M1_Stim1	4.0706	4.0336	5.8017	3.4941	3.4083	3.6324	3.4789	3.8409	3.5380	3.5217	3.9124	3.9230	3.6461	3.9450	3.8113	3.6603	4.0933	3.9031	3.4565	3.6223
Hnrnph2	_IQNGTSGVR_.2	#N/A	4.4043	4.0481	4.3084	4.3133	3.4779	4.6818	3.1347	3.4967	3.1938	5.5192	5.0707	4.1803	3.3018	4.7603	5.5115	4.1339	4.4947	4.1556	2.9538	4.3367
Rock2	_NDQWNWDNIR_.2	#N/A	7.1779	6.7074	5.9481	6.5468	6.7386	6.7582	6.4707	7.1643	6.5838	6.8212	6.7815	6.3043	7.0090	7.0479	6.0398	6.5214	7.2756	6.8022	6.7005	6.9334
Rock2	_TLTSDVANLANEKEELNNK_.3	#N/A	8.6460	8.7435	7.6910	8.0619	7.5443	7.8875	8.2139	8.6130	7.6399	8.2190	8.0117	8.1780	9.0980	9.1320	6.8577	7.8891	8.1659	8.2808	8.0828	7.8939
Rock2	_QIQQLESNNR_.2	#N/A	8.0164	7.0364	7.2096	7.6580	7.5353	6.5373	7.4758	8.1209	7.0669	7.5471	4.3523	5.8010	7.7281	8.0063	6.3677	7.3546	7.3967	6.0911	7.2807	5.5574
Rock2	_ENLLLSDS[Phospho (STY)]PPC[Carbamidomethyl (C)]RENDAIQTR_.3	P70336_S425_M1_Rock2	4.5234	8.1455	3.5589	3.1660	3.8611	7.5697	3.6800	4.2238	6.8783	3.2289	4.1134	3.3689	3.8471	8.0599	6.9778	7.0394	7.6155	3.6103	7.1319	7.2113
Rock2	_NLLLANSTEEQQK_.2	#N/A	3.3687	3.1333	5.2955	4.9499	4.5224	5.0605	3.4757	3.9113	4.6877	3.5750	4.2034	3.0202	3.3894	3.8975	4.0668	4.9152	4.3366	4.3639	4.4733	4.9631
Rock2	_KS[Phospho (STY)]EESQEIQKK_.2	P70336_S439_M1_Rock2	4.0067	3.9962	4.5430	3.6827	3.3444	3.5685	4.1967	3.7070	4.2558	4.2130	2.3037	3.8856	4.3638	3.9076	3.7739	4.3516	4.2447	3.0936	3.3926	3.5849
Rock2	_DSDIEQLR_.2	#N/A	3.6666	3.6525	3.2032	3.9176	3.8735	4.2348	3.5303	3.8923	3.5894	6.0514	3.9638	3.3786	3.6975	3.2413	3.1075	3.5171	3.3895	3.7599	6.2137	4.4194
Frat1	_AAC[Carbamidomethyl (C)]S[Phospho (STY)]DPGAFMGR_.2	P70339_S252_M1_Frat1	4.1379	7.1501	7.6103	7.4009	3.4756	6.1710	5.8152	4.4274	4.1245	4.3442	2.9981	4.3443	4.2326	7.0420	3.6426	2.9820	6.0787	3.2248	3.5239	5.5484
Hint1	_AQVAQPGGDTIFGK_.2	#N/A	9.3750	9.2800	9.3288	9.2842	9.3896	9.2584	9.2653	9.5292	9.2814	9.3897	9.2533	9.4971	9.6287	9.5845	9.9070	9.3687	9.2244	8.9598	9.4139	9.2056
Hint1	_IIFEDDR_.2	#N/A	7.4891	7.8788	8.0712	8.1956	7.9522	8.0649	8.2121	6.9759	8.4233	8.3746	8.1819	7.4621	8.4758	7.9738	8.2703	8.0246	7.3341	8.4915	8.3429	8.4436
Hint1	_M[Oxidation (M)]VVNEGADGGQSVYHIHLHVLGGR_.4	#N/A	3.9634	4.0239	3.9646	3.7104	3.1341	3.1674	5.0507	4.0248	3.8342	4.2386	4.4326	4.1181	3.9997	4.5023	3.9394	3.7901	4.2170	3.7212	5.5134	6.2071
Hint1	_C[Carbamidomethyl (C)]LAFHDIS[Phospho (STY)]PQAPTHFLVIPK_.3	P70349_S45_M1_Hint1	4.0304	3.6007	3.8356	3.7418	3.6995	3.8402	3.6057	3.7971	3.3747	3.1790	4.2730	3.5767	4.1175	3.8227	3.4070	3.8406	3.5373	3.6475	3.9994	3.7897
Hint1	_C[Carbamidomethyl (C)]AADLGLK_.2	#N/A	4.1221	3.8807	3.1575	3.3667	3.4598	3.6839	4.0813	4.4432	4.1404	4.3284	3.0139	3.7702	4.2484	3.7922	3.6585	2.9661	3.9405	3.2090	3.5080	3.4695
Ufd1	_FIAFSGEGQS[Phospho (STY)]LR_.2	P70362_S299_M1_Ufd1	8.2811	9.0435	8.6074	8.1735	4.3167	8.1067	7.7812	7.7433	7.4588	8.7178	4.3129	7.2667	7.5438	8.6658	7.2953	7.5293	7.7453	8.1899	7.7729	8.0027
Ncoa1	_LLQEGS[Phospho (STY)]PSDITTLSVEPEKK_.3	P70365_S702_M1_Ncoa1	3.9902	4.0126	4.5265	3.6992	3.3279	3.1562	4.2132	3.6906	4.2723	4.1965	3.1458	3.9021	4.0109	4.5136	3.7904	4.3351	4.2282	3.6049	3.2535	2.9797
Ncoa1	_KGS[Phospho (STY)]PC[Carbamidomethyl (C)]DTLASSTEKR_.3	#N/A	3.2966	3.7769	4.5796	4.0421	4.1352	4.3593	5.7329	3.7678	4.7475	3.5029	3.8394	3.5030	3.5730	4.6176	3.7081	3.6415	3.2651	4.3571	4.1834	4.2949
Ncoa1	_EHS[Phospho (STY)]GLSPQDDSNSGMSIPR_.3	P70365_S369_M1_Ncoa1	3.6563	4.0666	4.3930	6.4311	3.3688	3.4105	3.9496	3.2374	3.7852	4.0550	3.3089	3.8723	3.8933	5.5020	3.7277	4.2091	4.2355	3.7863	3.2347	3.7645
Ncoa1	_QMSSQNS[Phospho (STY)]PSR_.2	P70365_S570_M1_Ncoa1	3.6777	3.6414	3.2143	3.9065	3.8846	3.9180	3.1635	4.4296	3.0837	3.4883	4.0631	8.0100	3.2491	3.7518	3.1888	3.0398	3.4664	4.3667	4.0153	3.7188
Ncoa1	_KGS[Phospho (STY)]PC[Carbamidomethyl (C)]DTLASSTEK_.2	#N/A	4.2261	3.7767	3.2615	3.4633	3.5638	3.7879	3.9773	3.9264	4.3129	4.4324	2.9099	3.6662	4.1444	3.2486	3.5545	3.0701	4.4641	3.3690	3.0176	3.2156
Elavl1	_VLVDQTTGLSR_.2	#N/A	4.5569	5.3533	5.1149	5.3291	5.2356	5.1316	5.9625	4.3369	4.7179	5.2342	5.5007	5.5866	5.4946	5.6944	5.5864	5.3936	5.2352	4.9915	4.9406	5.3168
Elavl1	_FAANPNQNK_.2	#N/A	5.3035	3.7605	5.8492	4.0256	5.9984	4.3428	5.1136	3.7843	3.4814	3.4865	3.8558	5.2472	3.5895	6.0414	5.4526	4.4215	5.2639	5.1396	6.1770	4.3113
Elavl1	_TNLIVNYLPQNM[Oxidation (M)]TQEELR_.2	#N/A	4.0575	3.6538	4.1198	3.3404	3.8488	3.9623	4.9355	5.2656	4.3039	4.1002	3.4600	4.9404	4.3612	3.3096	4.6377	5.0476	5.3986	4.3770	4.3598	4.2366
Rasgrf2	_LGS[Phospho (STY)]LSLKK_.2	P70392_S484_M1_Rasgrf2	7.3836	7.7260	4.0209	6.9012	4.3232	3.6618	7.2508	3.1850	3.3399	3.6909	7.3040	6.3101	7.1907	6.4705	2.7951	5.6695	6.4460	7.2471	5.8615	5.7446
Rasgrf2	_KFS[Phospho (STY)]S[Phospho (STY)]PPPLAVSR_.2	P70392_S725_M2_Rasgrf2	3.7174	3.6017	8.9545	3.8668	3.9243	3.9577	8.1238	3.9431	3.0440	3.5280	4.0235	6.3836	3.7483	3.2920	3.1492	3.0795	3.4403	8.8929	8.2923	2.9693
Rasgrf2	_TLNS[Phospho (STY)]C[Carbamidomethyl (C)]KVPQIR_.2	P70392_S633_M1_Rasgrf2	4.0683	4.5486	3.8080	3.3129	3.4060	3.6301	4.1351	4.4971	4.1942	4.2746	3.0677	4.2747	4.3022	3.8460	3.7123	4.4131	3.9943	3.1552	6.9813	3.5233
Rasgrf2	_EHLVDGKS[Phospho (STY)]PR_.2	#N/A	6.4894	3.4334	5.9557	6.0091	5.8157	4.0674	5.5467	5.4905	4.4355	3.6377	5.5322	5.5234	4.0258	3.6028	3.0399	3.1892	3.3175	5.5782	5.1397	3.5698
Rasgrf2	_KLS[Phospho (STY)]LTSSLNSR_.2	P70392_S745_M1_Rasgrf2	3.8281	3.4240	3.3647	3.1105	4.0350	4.0684	6.1445	4.2792	4.4341	3.6387	3.9128	5.3745	4.5996	3.6014	3.0384	3.1902	3.3161	5.7994	4.1657	3.5684
Dlx5	_VPS[Phospho (STY)]AT[Phospho (STY)]S[Phospho (STY)]QPEK_.2	P70396_S113_M3_Dlx5	9.6469	9.1940	9.8033	9.6229	8.7491	9.3079	9.3544	9.3534	9.2743	9.4746	9.2530	8.4807	8.5452	9.0781	7.4475	8.3575	9.3891	9.1616	8.8769	8.6438
Usp9x	_AQENYEGGEEVS[Phospho (STY)]PPQTK_.2	P70398_S2547_M1_Usp9x	3.8196	8.1644	8.9950	8.2695	3.1573	8.7442	8.5805	8.5933	8.3939	8.4872	8.5902	8.3834	3.8403	8.4000	7.3417	4.1645	8.6360	8.6835	8.4041	8.2697
Tp53bp1	_STPFIVPS[Phospho (STY)]SPTEQGGR_.2	P70399_S381_M1_Tp53bp1	4.0862	10.8912	3.6007	10.2547	9.4414	4.0404	10.2507	10.1040	10.2503	4.1000	3.3738	10.2460	10.3962	3.6183	9.6719	4.0021	10.7619	10.3402	3.2122	3.4176
Tp53bp1	_SEDRPSS[Phospho (STY)]PQVSVAAVETK_.3	#N/A	6.1860	6.3603	7.9618	7.9896	7.4841	7.7657	7.8440	6.5946	7.9773	8.2612	7.9268	7.1236	8.5038	8.6639	7.5043	8.3498	7.7279	8.2788	8.3642	6.0364
Tp53bp1	_MESLGS[Phospho (STY)]PR_.2	#N/A	8.1306	9.3638	9.2816	9.6086	8.6604	9.1945	9.2878	8.5071	8.9476	8.9140	9.3203	8.5980	8.7250	9.8610	8.4936	8.5749	8.9661	8.7274	8.8943	9.0079
Tp53bp1	_AETTEKDAVTEDS[Phospho (STY)]PQPPLPSVR_.3	#N/A	3.4893	8.5846	7.5810	4.0949	3.6962	8.1196	3.3530	6.0356	3.4121	8.1723	6.9379	7.1850	7.6673	9.5819	3.3773	7.6222	7.2402	3.9373	4.2363	7.6530
Tp53bp1	_DAVTEDS[Phospho (STY)]PQPPLPSVR_.2	#N/A	8.7109	8.6831	9.1827	8.6712	3.7671	8.8222	8.3847	3.7309	8.5662	8.6077	8.0117	8.1723	7.5142	9.1032	7.7650	8.2724	8.9119	8.5131	8.5957	8.4040
Tp53bp1	_GGPGKLS[Phospho (STY)]PR_.2	P70399_S1359_M1_Tp53bp1	7.4161	7.6983	7.1929	7.2372	6.5127	7.3291	7.1521	7.2028	7.4659	7.0232	7.0886	7.2763	7.3029	7.6328	6.9167	6.6321	7.3666	7.1550	7.0856	7.4687
Tp53bp1	_ENTQIDDTEPLS[Phospho (STY)]PVSNSK_.2	#N/A	5.4091	4.7949	5.8533	5.9087	3.9963	5.7031	3.6357	5.2100	4.9737	5.2406	5.3300	5.1846	3.1375	3.6401	4.0528	5.4189	4.9734	4.9090	5.5284	5.3914
Tp53bp1	_KLPTSEEERS[Phospho (STY)]PAK_.3	P70399_S1675_M1_Tp53bp1	3.9979	3.3212	3.5345	3.5863	4.2048	5.9451	4.3441	4.2236	4.2643	3.8085	5.1573	3.0473	4.4297	3.5726	5.2711	3.3600	3.7208	3.1861	3.7277	5.2299

Tp53bp1	_ASQEPFS[Phospho (STY)]PAEDVM[Oxidation (M)]ETDLLEGLAANQDRPSK_.3	#N/A	5.6678	6.1446	5.1270	3.4744	3.7689	3.2863	5.8600	5.5809	4.1480	3.3978	3.3715	5.7206	3.5400	6.0670	3.6411	3.8826	3.5382	6.0009	5.2302	4.8533
Tp53bp1	_EAPKEEC[Carbamidomethyl (C)]PEAM[Oxidation (M)]EVETSVISIDS[Phospho (STY)]PQK_.3	P70399_S719_M1_Tp53bp1	4.4667	8.2117	8.1816	7.2217	3.8044	7.8212	6.3271	8.1779	8.5538	7.9836	6.6110	8.5089	8.3212	8.3822	7.2250	7.3719	8.8801	7.6604	7.1897	7.5073
Tp53bp1	_EHHPEEGFS[Phospho (STY)]GSEVEEVPETPC[Carbamidomethyl (C)]GSHR_.4	P70399_S652_M1_Tp53bp1	4.1716	4.9655	3.7851	3.9550	3.5093	4.9668	5.5052	3.6904	4.2287	5.0376	3.6089	3.8663	3.7158	6.5263	5.2693	3.7118	3.6794	5.9098	5.4998	4.9181
Tp53bp1	_S[Phospho (STY)]NISSPVTPTAASSSSTTPTR_.2	P70399_S1628_M1_Tp53bp1	3.4577	3.8614	4.4951	4.1266	3.6646	4.4438	3.3214	5.1694	3.3805	3.5875	3.7548	5.0527	3.4885	4.5331	2.8986	4.3206	3.1806	3.9689	4.2679	4.2104
Tp53bp1	GEKPASAPVDDTES[Phospho (STY)]LHS[Phospho (STY)]QGEEEFEM[Oxidation (M)]PQPPHGHVLH	#N/A	3.6998	5.9632	3.9719	4.1292	3.9822	3.6280	3.6515	4.1722	3.5548	3.9066	3.5999	3.8285	3.6762	7.4835	5.3157	3.4885	4.0400	7.9854	4.0414	3.7571
Tp53bp1	_TEEDRENTQIDDTEPLS[Phospho (STY)]PVSNSK_.3	#N/A	3.1321	3.3699	3.6684	4.5573	3.9707	4.0143	3.0672	4.3333	2.9874	3.5846	3.9669	3.2593	3.1528	4.3272	0.5428	3.4770	3.3702	4.4630	4.1116	3.6225
Tp53bp1	_SEALS[Phospho (STY)]S[Phospho (STY)]VLDQEEAM[Oxidation (M)]DTK_.2	#N/A	3.2782	3.7709	3.9652	4.0360	3.8056	3.9492	3.5016	3.6095	3.4962	3.2815	4.1762	3.4970	3.6305	3.6971	3.3099	3.9292	3.5895	4.8011	3.8906	3.5624
Idh3g	_ENTEGEYSSLEHESVAGVVESLK_.3	#N/A	10.2212	10.0620	8.9555	9.5316	9.1589	9.0354	9.2642	9.8218	8.8268	9.3815	9.2198	9.6885	10.2864	10.5666	8.3468	9.3986	9.8109	9.6273	9.2354	9.6174
Idh3g	_LHSYATSIR_.2	#N/A	7.0554	7.5074	7.2986	7.4914	7.3155	7.2085	7.5059	7.8392	7.0628	7.1381	7.0230	7.4702	7.0834	7.3978	7.3251	7.5965	3.7102	7.0703	7.4737	7.1413
Idh3g	_LGDGLFLQC[Carbamidomethyl (C)]C[Carbamidomethyl (C)]R_.2	#N/A	6.8602	6.9935	7.0645	6.9216	6.6358	7.1727	7.3899	7.0186	6.8438	6.7942	6.8315	6.9924	6.5387	7.0826	6.1138	6.4354	6.7599	6.9647	6.6766	6.7408
Idh3g	_HKDIDILIVR_.3	#N/A	5.2820	6.4428	4.5530	6.7793	6.6895	6.2287	5.9664	6.1677	6.3946	6.6412	6.3563	5.0290	7.0975	6.3140	6.2365	6.8559	6.2850	5.9035	2.9512	6.2527
Idh3g	_HAC[Carbamidomethyl (C)]VPVDFEEVHVSSNADEEDIR_.4	#N/A	6.1774	6.7034	6.4605	6.1638	5.6904	5.6368	6.0914	3.7531	6.4353	6.2048	6.1948	5.3241	6.2585	6.4295	4.9228	6.3038	5.9120	5.4113	6.2312	6.2753
Idh3g	_TSLDLYANVIHC[Carbamidomethyl (C)]K_.2	#N/A	5.9530	5.3551	6.3440	5.8756	5.7655	5.0853	5.8743	5.2693	5.5203	5.8003	5.2237	5.4439	5.3490	4.9369	5.8147	5.9043	4.6351	5.4448	6.1397	5.9653
Idh3g	_DIDILIVR_.2	#N/A	4.2623	3.7405	3.2978	4.0472	3.6000	3.8241	3.9410	3.9627	2.8301	4.3746	4.3745	3.6299	4.1082	3.2849	3.5182	3.1064	4.5004	3.3328	3.1212	3.2519
Idh3g	_AVLASM[Oxidation (M)]DNENM[Oxidation (M)]HTPDIGGQGTTSQAIQDIIR_.3	#N/A	3.8666	3.4969	4.2327	4.5252	2.5533	3.3965	2.9730	4.1369	3.5713	2.7554	2.0634	3.9862	3.7623	3.4500	3.1206	2.9573	2.8867	3.4864	3.2699	3.5007
Cdh10	_LAEMYGGGES[Phospho (STY)]DKDA_.2	#N/A	4.1160	8.6536	9.1372	9.2885	5.4987	8.0215	8.5597	5.8999	7.3601	7.3412	8.4708	7.4828	3.6514	9.3678	6.8801	3.9232	8.1384	6.8457	6.9441	7.5310
Evl	_TPSVAKS[Phospho (STY)]PEAK_.2	P70429_S347_M1_Evl	5.7624	5.1479	5.3656	5.2824	5.3849	5.6580	4.6884	4.4882	4.5182	6.0998	4.6933	5.0209	5.0098	4.7497	5.0303	5.2426	3.0950	5.4768	5.0667	4.6106
Evl	_SNS[Phospho (STY)]VEKPVSSLLSR_.2	#N/A	8.8607	9.2024	8.2392	8.2366	7.4414	7.8237	8.6661	9.2602	7.7109	8.9378	7.6595	8.4597	8.7794	9.5311	7.0116	8.4136	8.3559	8.6310	8.3544	8.0726
Evl	_QVQNGPS[Phospho (STY)]PEEM[Oxidation (M)]DIQR_.2	#N/A	5.6668	6.0502	5.2153	5.8734	5.2442	5.2855	5.6665	5.6515	5.3110	5.3997	4.7538	5.7717	5.9348	5.8622	5.2605	5.9677	5.8250	5.5783	5.2448	3.8977
Evl	_KEDESQTEDPSTS[Phospho (STY)]PSPGTR_.2	P70429_S302_M1_Evl	4.5793	3.4235	3.6148	3.1101	3.9170	5.0133	3.0135	4.2797	4.4346	3.6382	3.9132	3.3130	4.6000	3.6018	3.0389	3.4234	3.3165	3.0158	4.2995	3.5688
Evl	_VQRPEDAS[Phospho (STY)]GGSSPSGTSK_.2	P70429_S240_M1_Evl	4.3296	4.4903	3.8662	3.2546	3.0357	3.0691	4.0124	3.7777	3.9326	4.1402	3.4112	4.2164	4.0980	4.6007	4.0377	3.6917	4.3154	3.5178	3.1664	3.0668
Evl	_VKPAGS[Phospho (STY)]VNDVGLDALDLDR_.2	#N/A	4.1088	3.5992	3.3529	3.6093	3.8358	4.0813	3.8824	4.0290	3.8026	3.6516	3.9137	3.4077	3.9681	3.3636	3.2642	3.1693	3.2973	3.4768	4.1798	3.5693
Slc9a3r1	_SAS[Phospho (STY)]SDTSEELNSQDSPK_.2	#N/A	10.3683	10.5385	10.7727	10.6517	10.2023	10.0074	9.9397	10.1351	9.8452	10.6424	9.8759	10.0938	10.3437	10.8236	10.0375	10.3305	10.4935	9.9446	10.0453	10.3585
Slc9a3r1	_[Acetyl (Protein N-term)]S[Phospho (STY)]ADAAAGEPLPR_.2	P70441_S2_M1_Slc9a3r1	9.9937	10.1264	9.9634	10.0646	10.0992	9.8522	9.7257	10.1524	9.9633	10.2472	10.1117	10.0339	10.8107	10.6255	10.0995	10.1665	10.4394	9.9695	10.0627	10.2972
Slc9a3r1	_LLVVDPETDER_.2	#N/A	8.2091	8.0919	8.1996	8.6163	8.5447	8.2962	8.2729	8.4379	8.3523	8.5537	8.3594	8.1668	8.2645	8.6675	8.2846	8.3668	8.3497	8.1164	8.1680	8.4787
Slc9a3r1	_AVDPDSPAEASGLR_.2	#N/A	7.7885	7.9016	8.2236	7.9319	8.2474	7.7089	7.5713	7.9237	7.8739	7.8248	7.5195	7.7289	8.1827	7.6198	8.1272	8.0883	8.1670	7.9250	7.6259	7.6525
Slc9a3r1	_EALVEPASES[Phospho (STY)]PRPALAR_.3	#N/A	12.3889	12.3818	12.2178	12.4643	12.1168	12.0121	11.9071	12.3046	11.9270	12.4533	12.0482	12.0475	13.0200	12.4626	11.7192	12.1329	12.2652	12.0622	11.9907	12.1641
Slc9a3r1	_SEQAEPPAAADTHEAGDQNEAEK_.3	#N/A	4.2949	6.0806	3.3304	6.5682	6.0306	6.7269	6.1756	3.9953	6.5351	6.5947	5.9449	5.8893	6.2030	6.4860	6.3153	6.0737	5.6234	5.5430	6.0360	6.4108
Slc9a3r1	_LVEVNGENVEKETHQQVVSR_.3	#N/A	5.2435	6.0040	4.9861	5.9059	5.3684	5.3014	5.2916	6.1572	4.3951	5.1661	5.7765	5.9802	5.1711	3.3885	5.5056	3.1760	5.4829	5.2109	5.5777	5.1465
Slc9a3r1	_SASSDTSEELNSQDS[Phospho (STY)]PKR_.2	#N/A	7.9928	8.1411	7.3981	8.6131	7.7092	8.2822	7.7183	8.1653	8.1585	8.5393	7.6167	8.3474	8.5421	8.5902	8.3999	8.0971	8.4277	7.7601	8.0131	8.3187
Stx4	_QGDNIS[Phospho (STY)]DDEDEVR_.2	P70452_S15_M1_Stx4	9.7628	9.6635	9.4848	9.1642	8.9865	9.5022	9.3182	9.4484	9.2912	9.3422	9.0196	8.9927	9.5958	9.1438	7.6167	8.4767	9.6554	9.1383	8.8967	8.9251
Pde7a	_RGS[Phospho (STY)]HPYIDFR_.3	#N/A	3.6622	5.6988	6.4482	6.1285	5.3486	6.0818	5.0462	5.8659	5.4493	6.2844	3.8986	3.1119	6.0605	3.5872	3.0243	3.4647	4.6609	5.7764	4.1965	3.5542
Vasp	_KVS[Phospho (STY)]KQEEASGGPLAPK_.3	P70460_S235_M1_Vasp	4.0678	4.7333	3.6044	4.9063	4.8939	4.3081	4.2742	4.2935	4.1944	3.8784	4.9302	2.9774	5.3994	5.6853	2.7987	3.4299	3.7907	3.2560	4.4054	3.3286
Sparcl1	SSPNAEPSDEGNS[Phospho (STY)]REHSAGSC[Carbamidomethyl (C)]TNFQC[Carbamidomethyl (C	#N/A	3.8098	4.4245	4.1132	3.8627	4.6284	3.8389	3.5448	4.0194	4.6290	3.9090	3.6711	3.8464	4.3854	4.0527	4.5089	3.9248	3.9125	4.3409	4.8725	3.7350
Sparcl1	_HGAGDDY[Phospho (STY)]FIPS[Phospho (STY)]QEFLEAER_.2	P70663_S353_M2_Sparcl1	4.3772	4.0037	3.9438	3.2590	3.8087	3.8752	3.5864	4.1916	3.7127	3.1925	3.8787	3.5136	4.2488	6.0101	3.4733	3.4461	3.4104	5.4340	3.8268	3.1364
Sparcl1	_AAGS[Phospho (STY)]KEHIPHTEQQDQEGK_.3	#N/A	6.1801	6.5672	6.8016	6.3974	6.0008	6.4812	5.7517	6.0910	5.9158	6.2330	5.9939	5.6344	5.8243	6.8176	6.3947	5.9977	6.5062	5.7551	5.7409	6.2232
Sparcl1	_KAESSPNAEPSDEGNS[Phospho (STY)]R_.2	#N/A	4.2159	3.9544	4.8904	4.5361	4.3288	3.9109	4.2273	3.5524	3.9632	4.8130	3.8076	3.6805	3.6563	4.6927	4.1220	4.3620	4.1777	4.2432	4.1285	4.4180
Sparcl1	_HQSEQGNQGQESDSEAEGEDKAAGSKEHIPHT[Phospho (STY)]EQQDQEGK_.5	P70663_T290_M1_Sparcl1	4.0287	7.9169	3.8475	3.2733	3.3664	3.5905	6.9419	4.5366	4.2337	4.2350	7.3202	3.8636	4.3418	3.8856	3.7519	4.3736	4.0338	3.1156	3.4147	3.5629
Sparcl1	_AEKPSALNS[Phospho (STY)]EEETHEQSTEQDK_.3	P70663_S70_M1_Sparcl1	4.4463	3.4257	3.4299	3.6909	3.7840	4.0081	3.7571	4.1190	3.8162	3.1518	4.8668	3.7364	3.6965	6.4758	5.0476	3.2903	4.3299	3.5332	3.8322	3.1453
Naca	_SPASDTYIVFGEAK_.2	#N/A	5.7454	5.6527	6.0569	5.6722	5.9060	5.7814	5.2795	5.9622	5.5440	5.8598	5.5569	6.1620	6.2596	6.6529	5.7163	5.8961	5.8399	5.2075	4.8677	4.8117
Naca	_NNSNDIVNAIM[Oxidation (M)]ELTM[Oxidation (M)]_.2	#N/A	3.7630	4.6916	5.9918	5.0950	3.1007	5.3824	5.2735	5.5913	5.5023	3.9693	3.3730	4.1293	3.1067	4.2864	5.8252	5.6405	4.0010	4.6594	5.9253	3.8286
Naca	_DIELVMSQANVSR_.2	#N/A	3.4431	4.5597	3.9794	4.2463	4.2817	3.0049	3.2594	3.1435	3.3185	3.6494	3.6929	2.9483	3.4266	8.2389	2.8366	3.7880	3.6812	4.0309	3.8006	4.1484
Irf3	_SSLPHS[Phospho (STY)]QENLPK_.2	P70671_S135_M1_Irf3	8.6926	3.2172	8.4038	8.6618	8.6792	8.4124	8.1953	8.4027	8.1870	8.8852	7.9013	8.5121	8.8932	8.8428	8.0264	8.3719	8.8076	8.3716	8.2426	3.3538
Casp3	_S[Phospho (STY)]VDSGIYLDSSYK_.2	P70677_S26_M1_Casp3	4.4104	7.8072	8.6193	7.2511	3.7482	4.2368	4.3455	4.1108	4.2657	7.2075	3.7443	6.5788	4.4312	8.1246	6.8690	6.9154	3.1477	7.7970	4.3341	7.5615
Ctps1	_SGSSS[Phospho (STY)]PDSEITELKFPSISQD_.2	#N/A	3.2414	4.0776	4.2789	4.3428	2.9351	7.4633	7.1016	3.4671	3.1643	3.8037	3.5386	3.8038	3.2723	4.3169	4.1832	6.4940	4.4652	4.1851	2.9833	3.9942
Ctps1	_SGSSS[Phospho (STY)]PDSEITELK_.2	#N/A	3.5160	5.7606	6.6702	4.0683	3.7229	3.7562	3.3252	3.7416	3.2454	3.3265	4.2249	3.5293	3.4109	4.5914	3.3506	4.3789	3.2389	3.9106	4.2096	4.2687
Ctps1	_GC[Carbamidomethyl (C)]RLS[Phospho (STY)]PR_.2	P70698_S562_M1_Ctps1	9.2063	9.2741	8.8937	8.8584	9.0747	9.0340	9.0991	9.1565	8.7331	9.0280	9.1210	9.0609	9.6283	9.2557	8.5874	8.7479	9.1799	9.1327	8.9421	8.9972
Atp8a1	_RTVS[Phospho (STY)]EIR_.2	P70704_S9_M1_Atp8a1	9.8581	9.6605	9.3339	9.6205	8.9754	9.3091	9.6098	9.4193	9.1083	9.6855	4.1875	3.4919	9.4404	8.6849	7.1331	8.6638	3.2762	3.8732	8.8298	8.5301
Atp8a1	_IIYQAASPDEGALVR_.2	#N/A	6.7567	6.5528	6.5566	6.6148	6.7246	6.4799	6.6711	6.7728	6.5070	6.1457	6.6035	5.8647	6.1786	5.2716	5.7211	5.8076	6.7186	5.6748	6.2149	5.4439
Atp8a1	_LDGHGTVPLGADQILLR_.3	#N/A	5.7522	6.3969	5.1346	6.0558	5.3745	6.3313	6.1582	5.5892	5.8349	5.8531	5.4185	5.9771	6.0920	5.8634	5.0488	5.7156	6.1622	6.5503	5.6863	4.6668
Atp8a1	_T[Phospho (STY)]SLADQEEVR_.2	#N/A	3.8250	3.4209	3.3616	3.1075	4.0319	4.0653	3.0162	4.2823	4.4372	3.6356	3.9158	3.2202	4.6026	3.6045	3.0415	3.1871	3.3192	3.0132	4.1626	3.5715
Atp8a1	_TDDVS[Phospho (STY)]EKT[Phospho (STY)]S[Phospho (STY)]LADQEEVR_.2	#N/A	9.1278	9.0771	9.1024	8.9930	3.6636	8.6329	8.6199	6.5509	8.9377	8.7346	4.5014	8.4747	3.8404	8.8416	7.3598	8.2030	9.0497	8.7465	8.5749	7.9024
Atp8a1	_SESLQQNLLHGYAFSQDENGIVSQSEVIR_.3	#N/A	3.9521	3.3669	3.4887	3.2346	4.1591	4.1924	4.3899	4.1552	4.3101	3.7627	3.7887	3.0931	4.4755	3.4773	2.9144	3.3143	3.1920	3.1403	4.2897	3.4443
Cct7	_TC[Carbamidomethyl (C)]TIILR_.2	#N/A	8.8932	8.4339	8.6565	8.1361	5.5700	4.5643	5.2853	9.0636	8.3872	9.0916	8.4207	9.0181	8.4472	8.3949	8.9715	9.2788	8.8434	5.4778	8.8477	9.2712
Cct7	_LLDVVHPAAK_.2	#N/A	6.4037	6.2346	5.7202	5.7122	6.2166	5.9711	5.9841	6.5723	5.4176	5.3048	5.8246	6.1497	5.4807	5.9907	6.0650	5.3517	6.6028	5.9752	5.8274	5.9267
Cct7	_ATISNDGATILK_.2	#N/A	5.8094	5.8391	5.7441	5.7071	5.6076	5.2473	4.7130	5.0638	5.3891	4.0423	4.8626	5.1276	4.4066	5.2635	5.6877	5.2906	5.8788	4.9875	5.0053	5.5158
Cct7	_LPIGDVATQYFADR_.2	#N/A	5.8784	3.5695	6.4017	6.0828	5.2411	6.0071	6.2253	5.7500	5.1642	5.4522	5.5308	5.4850	3.7804	5.7549	5.2745	5.4429	6.1428	5.5498	5.2841	5.3583
Cct7	_SLHDAIM[Oxidation (M)]IVR_.2	#N/A	5.2137	5.2812	4.2923	4.9063	5.2106	5.3746	5.0430	6.3342	5.3923	4.8483	4.6937	5.6272	5.0984	3.4893	4.6813	4.8268	5.1469	3.5118	5.0875	4.6814
Cct7	_SQDAEVGDGTTSVTLLAAEFLK_.2	#N/A	5.0833	4.9874	4.7851	5.0386	4.7985	4.7052	4.6256	5.3037	4.7728	5.5359	4.7821	4.9913	5.6733	5.6157	4.0315	4.4392	5.0920	5.3484	4.3881	4.9137
Cct7	_QLC[Carbamidomethyl (C)]DNAGFDATNILNK_.2	#N/A	3.8951	3.1898	4.4314	3.7943	3.2328	3.4569	4.3082	3.5955	4.3673	4.1014	4.2425	3.5407	4.4754	4.0192	3.8854	3.5175	4.1332	2.9820	2.3127	3.6964
Cct7	_TATQLAVNK_.2	#N/A	3.9517	3.3674	3.4883	3.6325	2.4790	4.1920	3.7512	4.1556	2.7725	3.7623	1.9234	3.6327	4.4759	3.4778	3.1797	3.3138	3.1924	2.5473	4.2893	3.4448
Cct7	_ALEIIPR_.2	#N/A	4.3451	3.6577	3.3806	3.3443	3.6828	3.3461	3.5849	4.0455	4.8828	4.5514	4.2917	3.5620	4.0254	3.3676	4.1368	3.1892	3.0823	5.0267	3.9136	3.1362
Cct7	_INALTAASEAAC[Carbamidomethyl (C)]LIVSVDETIKNPR_.3	#N/A	3.2717	4.3243	4.3091	4.3126	3.4786	0.3781	3.5695	3.3348	3.4897	4.5831	2.9683	3.7735	3.6551	4.1578	3.5949	4.1346	3.8725	3.9607	3.6093	4.1248
Cct7	_INALTAASEAAC[Carbamidomethyl (C)]LIVSVDETIK_.3	#N/A	3.3888	4.6140	3.9251	4.3006	4.2274	4.4515	3.3137	3.0892	3.3728	3.5951	3.7472	3.0026	3.4808	3.9122	2.8909	3.7337	3.6269	4.2063	3.8549	3.8792
Cct2	_GATQQILDEAER_.2	#N/A	8.5938	8.1077	8.7421	8.4521	8.5475	8.3992	8.2641	8.8591	8.4197	8.6269	8.3864	8.3162	8.5423	8.3208	8.1302	8.7331	8.3664	8.3091	8.2435	8.4634
Cct2	_[Acetyl (Protein N-term)]ASLSLAPVNIFK_.2	#N/A	5.9421	6.6082	6.3938	7.0379	6.8551	6.8712	6.5158	5.8089	6.6470	6.6482	6.2708	6.9775	6.6779	6.2861	6.7484	6.6836	6.6854	6.2670	6.0529	6.8678
Cct2	_LSSFIGAIAIGDLVK_.2	#N/A	5.8539	4.5562	5.3086	5.8032	5.2070	4.8633	4.6861	4.3852	4.8065	5.4399	5.1296	4.4632	5.3077	4.7501	4.8714	4.4557	5.2186	5.1871	4.7364	5.4785
Cct2	_EALLSSAVDHGSDEAR_.2	#N/A	5.6505	5.4744	6.0270	5.6920	5.8655	5.8347	5.6166	5.6597	6.0807	6.0140	6.3298	5.9194	6.0535	5.8434	6.0615	5.5905	5.4530	5.4985	5.5652	5.9538
Cct2	_VQDDEVGDGTTSVTVLAAELLR_.2	#N/A	5.5926	5.4922	4.6009	4.8581	4.6972	4.5918	5.0954	5.6916	5.0547	5.6159	4.8943	4.9330	6.2707	6.3104	4.3596	5.1307	5.4896	5.3267	4.9996	5.2444
Cct2	_LAVEAVLR_.2	#N/A	4.4837	4.3875	4.0550	4.1707	5.6498	5.3591	4.7806	4.4542	4.8983	4.5796	6.6760	4.7806	5.1089	5.2188	4.5484	4.2504	3.7567	5.7254	4.8073	5.2303
Cct2	_FWQDLM[Oxidation (M)]NIAGTTLSSK_.2	#N/A	5.2232	4.9714	5.2966	4.5240	3.5200	6.2138	5.5614	5.1569	4.9030	5.5917	4.9488	3.7100	5.8209	5.4225	4.8621	4.9012	5.0810	5.1862	3.0614	3.1718
Cct2	_NIGVDNPAAK_.2	#N/A	4.1592	5.1897	3.7170	5.0192	3.4969	4.8083	4.4159	4.4061	4.6557	4.3655	2.9768	4.7501	4.2113	3.7551	5.1447	3.0033	4.0330	5.3439	3.5452	4.7296
Cct2	_M[Oxidation (M)]LPTIIADNAGYDSADLVAQLR_.2	#N/A	4.3267	4.2229	3.7922	4.3202	3.5831	4.1083	4.3482	4.1149	3.9371	4.2987	3.6265	4.1051	4.5680	4.2382	3.7686	3.9246	4.2436	4.4263	3.8395	4.0656
Cct2	_LALVTGGEIASTFDHPELVK_.3	#N/A	3.9997	4.0031	4.5360	3.6897	3.3374	3.5615	4.2037	3.7000	2.8495	4.2060	3.1363	3.8926	4.0204	4.5230	3.7809	4.3446	4.2377	3.5954	3.2440	2.9892
Cct2	_VAEIEHAEKEK_.3	#N/A	3.8588	4.1440	4.3951	3.8306	3.1965	3.2876	4.3446	3.5592	3.7141	4.3587	3.1927	4.0335	3.8795	4.3822	3.9218	4.2037	4.0969	3.7363	3.3849	4.3492
Cct4	_IDDVVNTR_.2	#N/A	6.1960	6.5152	6.0596	6.6601	6.0098	5.9690	6.6158	6.4446	6.4757	6.4089	6.2568	6.0186	5.6973	5.3544	6.7237	6.5559	5.7028	6.3568	6.3264	6.3337
Cct4	_VIDPATATSVDLR_.2	#N/A	4.3592	6.5348	5.9517	5.5820	5.6925	6.2171	6.1426	6.2442	5.3137	6.0551	6.3063	6.3801	5.9669	5.9177	5.8713	5.8605	5.8357	5.4687	5.9213	5.5648
Cct4	_LVIEEAER_.2	#N/A	5.7398	5.6460	4.9876	6.3140	5.6733	5.8433	5.5601	5.2259	6.3673	6.0360	5.9602	5.9383	6.3474	5.1549	5.5370	5.4711	5.0470	6.3912	6.3533	5.8643
Cct4	_DALSDLALHFLNK_.2	#N/A	4.2005	5.1461	4.3982	4.8788	4.7057	4.8736	3.9732	4.6267	4.2431	4.4245	4.2032	4.7733	3.1752	3.8038	3.8186	3.8042	4.2713	4.5687	4.0892	4.1222
Cct4	_ALIAGGGAPEIELALR_.2	#N/A	3.5893	4.6149	5.0698	4.4311	4.5585	4.9875	4.0296	3.8150	4.2956	3.3999	2.1659	3.7197	3.6202	3.9050	4.2464	3.6977	3.3122	4.3242	4.4830	4.3421
Cct4	_AQDIEAGDGTTSVVIIAGSLLDSC[Carbamidomethyl (C)]TK_.3	#N/A	3.1548	3.2840	3.9898	4.1989	2.9389	3.2889	2.7509	3.6141	3.3081	3.6598	3.7388	2.7304	3.8607	4.0272	2.8462	3.7410	3.2532	4.0412	1.7353	4.1381
Cct4	_ITGC[Carbamidomethyl (C)]TSPGK_.2	#N/A	3.7681	3.9213	3.7446	4.5135	3.8328	3.9404	5.3430	3.2964	5.1856	4.0224	4.8057	3.9745	4.6024	4.1462	4.9343	3.0258	4.2945	4.1136	4.6686	3.8235
Cct4	_VANSGITR_.2	#N/A	3.5166	3.9969	4.3596	4.0939	4.1222	4.3784	4.7892	3.5479	4.2156	4.2863	3.6194	3.0044	3.3531	4.3977	5.1042	3.8615	4.5460	4.1043	4.0221	4.3136
Cct4	_AFADAM[Oxidation (M)]EVIPSTLAENAGLNPISTVTELR_.3	#N/A	3.7554	3.5636	3.2920	3.8288	3.9624	3.9957	3.0857	3.9811	3.0059	3.5660	3.9854	3.2898	3.1714	3.6740	3.1111	3.1176	3.4784	4.4444	4.0930	3.6410
Cct4	_DVEREDIEFIC[Carbamidomethyl (C)]K_.3	#N/A	4.2204	4.5995	3.7570	3.3638	2.9265	3.6810	4.1216	4.4461	4.1433	4.3255	3.0168	4.3256	4.2513	3.7951	4.1469	3.5826	3.9434	1.2534	3.5051	3.4724
Cct5	_VLVDINNPEPLIQTAK_.2	#N/A	6.7385	6.3786	6.9587	6.4453	6.8434	6.6123	6.7080	6.7286	6.5881	6.7430	6.8510	6.6880	6.6671	7.0389	6.5816	6.8518	6.4328	6.6259	6.4340	6.7840
Cct5	_LGFAGVVQEISFGTTK_.2	#N/A	5.8411	5.2692	5.7453	5.4072	4.7334	5.0335	4.9398	5.8384	4.3861	5.5989	5.4757	4.8155	5.8376	5.1991	4.0181	3.8457	4.5490	4.9983	5.4702	5.5769
Cct5	_[Acetyl (Protein N-term)]ASVGTLAFDEYGRPFLIIK_.2	#N/A	3.9576	3.7512	1.3287	3.3714	4.1285	2.8390	2.3398	3.4804	3.3361	3.8170	2.9445	2.0264	2.5624	2.9734	4.0876	3.6397	1.5144	3.8115	2.7524	2.9336
Cct5	_TSLGPNGLDK_.2	#N/A	6.3388	4.3961	6.0299	5.8610	2.9445	6.0691	5.8833	5.9202	3.1549	3.8131	5.8123	6.1464	3.2629	6.3227	6.2467	5.7076	3.8448	5.7612	6.4905	5.7031
Cct5	_WVGGPEIELIAIATGGR_.2	#N/A	5.0420	4.6841	3.3066	5.1239	5.2810	4.3137	4.5353	4.6870	5.0885	5.0768	3.3151	3.6211	4.2919	4.5334	4.9352	4.7800	4.5092	4.4640	4.9857	4.9198
Cct5	_ESNPALGIDC[Carbamidomethyl (C)]LHK_.2	#N/A	5.2345	5.5335	5.5177	5.8970	5.8585	5.6635	5.2160	5.9620	5.7605	5.7003	5.7708	5.7815	3.6625	5.6743	5.6148	4.9503	5.6246	5.7513	4.7678	5.1699
Cct5	_DVDFELIK_.2	#N/A	3.8450	3.4741	3.3816	3.7392	4.0520	4.0564	3.7088	4.0707	3.7678	3.2001	0.9759	3.2002	3.8759	3.4197	3.2860	3.3387	3.5679	3.5815	3.8806	0.7316
Cct5	_DVDFELIKVEGK_.3	#N/A	4.4095	3.3890	3.4667	3.6541	3.7472	3.9713	3.7938	4.1558	3.8529	3.1150	4.2273	3.1151	3.9610	3.5047	3.3710	3.2536	3.6530	3.4964	3.7955	3.1820
Cct6a	_GLVLDHGAR_.2	#N/A	6.6158	6.6450	6.6090	6.1243	6.6019	6.5931	6.4770	6.3820	6.0290	6.0901	6.2102	6.6146	6.2353	6.7792	6.7924	6.4931	6.0524	6.7983	6.5112	6.4324
Cct6a	_GIDPFSLDALAK_.2	#N/A	5.4655	5.9275	5.8510	5.9350	6.0593	6.0783	5.8666	5.0552	5.4082	5.7603	5.5796	6.3612	6.3769	5.8419	5.7997	5.4994	5.5264	6.1511	5.8139	5.4065
Cct6a	_IITEGFEAAK_.2	#N/A	5.8107	6.1629	6.8647	6.0988	6.4557	6.0355	6.6437	6.5960	5.9365	6.5478	6.1865	6.3194	5.9986	6.4036	5.9587	5.9595	6.2845	6.1857	5.4967	5.8848
Cct6a	_VLAQNSGFDLQETLVK_.2	#N/A	4.4837	4.5595	5.2330	4.7845	3.9083	4.0207	2.4251	3.5161	3.5526	4.3995	3.8883	3.7823	4.1680	4.0294	3.7964	4.1085	4.2416	3.7162	3.9781	4.4869
Cct6a	_GLQDVLR_.2	#N/A	3.3606	3.1488	3.8895	4.3362	4.1918	4.3268	4.5836	3.0536	3.6904	3.1982	3.8832	3.2879	6.1605	4.0358	4.6463	4.1889	3.5913	4.3998	3.6148	4.6657
Cct6a	_VATAQDDITGDGTTSNVLIIGELLK_.3	#N/A	3.5656	3.4168	4.7793	4.4070	2.9034	2.8526	3.1369	3.9074	3.8636	4.7103	3.7629	4.3266	4.8044	4.6999	3.0569	3.1995	3.8037	2.4852	3.9241	3.6506
Cct6a	_EMDRETLIDVAR_.3	#N/A	3.8098	4.2901	4.0664	4.5553	3.1476	3.3716	4.1148	3.2546	4.4526	4.0162	3.3262	4.0163	4.5607	3.9241	3.9707	4.1547	4.2527	4.3976	3.1958	3.7817
Cct6a	_QADLYISEGLHPR_.2	#N/A	3.8221	3.3366	3.5789	3.8883	3.9229	3.9657	3.7295	4.2439	3.7886	4.0284	3.4415	3.4184	3.8227	3.5660	3.0788	3.2649	4.0601	3.9007	4.0339	3.5330
Cct3	_ISTPVDVNNR_.2	#N/A	7.6669	7.4322	7.9641	7.7778	7.2351	7.7545	7.6130	8.5544	7.2286	8.2188	8.0550	8.4803	9.0688	8.7001	7.6955	7.6835	8.3791	8.0363	7.9929	8.2269
Cct3	_AVAQALEVIPR_.2	#N/A	7.3661	7.1815	7.2307	7.6684	7.4869	7.3434	7.3960	7.8220	7.1347	7.1717	6.9209	7.5283	7.6446	7.3241	6.9706	7.3195	7.0110	7.5055	7.2100	7.1021
Cct3	_IPGGIIEDSC[Carbamidomethyl (C)]VLR_.2	#N/A	6.0736	6.8759	7.1469	6.0442	6.2842	6.9698	6.6857	6.2445	6.2762	5.9277	6.5003	6.3694	5.9030	6.8130	6.5667	6.4727	6.6641	6.7162	6.9283	6.3321
Cct3	_ELGIWEPLAVK_.2	#N/A	6.5753	6.6426	6.0842	6.6728	6.3608	5.9727	6.4147	6.3736	6.2121	6.1123	6.3819	6.8613	6.6324	6.1539	6.3153	6.2293	6.6071	6.8222	6.3272	6.2893
Cct3	_EIQVQHPAAK_.2	#N/A	6.4409	6.3225	5.6023	5.6532	5.7914	5.9352	6.3983	6.3772	6.2098	5.8984	5.9830	5.9460	5.6760	5.5215	6.0483	5.8672	5.6673	5.9725	5.7664	5.6835
Cct3	_EILSEVER_.2	#N/A	6.1181	6.6757	6.6628	6.8100	7.0685	6.8256	7.0286	3.1141	7.3879	7.3861	6.9365	6.6048	7.4026	6.4847	6.7520	7.1759	5.9409	6.8215	6.8824	7.0262
Cct3	_TAVETAVLLLR_.2	#N/A	4.3645	4.7711	5.7802	5.3724	5.1669	5.6525	4.6565	3.5169	5.1974	4.8188	4.5545	3.4698	4.0647	3.4230	5.2732	4.0863	4.5209	3.9896	5.6032	5.9762
Cct3	_IVLLDSSLEYK_.2	#N/A	4.7673	5.6019	5.6701	5.3582	5.0761	5.4711	5.0913	5.1104	5.8370	5.4302	5.4663	5.3793	5.0150	5.0242	4.7367	5.1066	5.5749	5.3400	5.5148	4.9747
Cct3	_NVLLDPQLVPGGGASEM[Oxidation (M)]AVAHALTEK_.3	#N/A	5.7019	5.0201	4.9506	3.1157	4.8989	5.3059	4.7656	5.9983	5.3266	5.7116	4.9188	5.3090	5.1927	5.8458	5.1505	4.7713	5.1573	5.3219	5.4492	5.1953
Cct3	_TLIQNC[Carbamidomethyl (C)]GASTIR_.2	#N/A	3.3552	5.6012	5.8394	4.3341	4.8574	5.3632	5.3756	5.6680	4.9726	3.5616	5.6760	4.7767	5.4885	5.8523	5.5805	3.7001	5.5273	5.5275	5.1172	3.8456
Cct3	_WSSLAC[Carbamidomethyl (C)]NIALDAVK_.2	#N/A	3.1827	3.7690	3.2693	3.6516	3.5716	4.2643	4.2571	3.9342	1.3801	4.3873	3.5678	4.0547	4.2546	3.2564	4.1943	3.2757	3.0288	3.8928	3.0098	2.6998
Cct3	_NLQDAM[Oxidation (M)]QVC[Carbamidomethyl (C)]R_.2	#N/A	3.7978	4.2050	4.3341	3.8916	3.1355	5.8308	4.4056	6.3246	5.7479	4.0041	3.3382	5.5857	5.5674	4.7251	5.8933	4.9820	4.0359	5.1818	3.4459	5.6627
Cct3	_QTGAPDAGQE_.1	#N/A	3.4653	3.8538	4.5027	4.1189	3.6722	4.4361	3.3290	3.6910	3.3881	3.5798	3.7625	3.5799	3.4962	4.5408	2.9062	4.3283	3.1882	3.9612	4.2603	4.2181
Cct3	_KGESQTDIEITREEDFTR_.4	#N/A	3.5023	3.5632	3.4098	3.5959	3.8780	4.2537	3.4308	4.0849	4.0632	3.5325	3.9203	3.3652	3.4054	3.4184	3.2190	3.2255	3.3349	3.4610	4.1305	3.9540
Ptprn2	_ADSVAGAIQSDPAEGS[Phospho (STY)]QESHGR_.3	#N/A	9.1845	9.3738	9.4949	9.5704	8.9539	9.3109	9.0494	9.1303	9.2541	9.5038	9.9445	9.8387	10.4535	10.2557	10.0903	10.2424	10.0302	9.6943	9.9366	10.0498
Ptprn2	_SEQPEEVLSS[Phospho (STY)]EEETAGVEHVR_.3	#N/A	7.8669	8.3482	8.3997	3.9307	7.2237	8.0675	8.0266	3.5873	3.4281	8.0094	4.0053	8.6269	9.2702	9.0103	8.5749	9.0224	8.8019	8.7556	9.0398	8.9138
Ptprn2	_INSVSSQFS[Phospho (STY)]DGPM[Oxidation (M)]PS[Phospho (STY)]PSAR_.2	#N/A	3.9086	4.2584	5.5366	3.8375	3.3449	3.3782	3.7805	3.9769	3.7007	3.7192	3.9500	5.5397	3.8661	3.9193	3.8058	5.9398	3.8554	3.7214	3.6248	5.8107
Ptprn2	_SLQQNADNEKWFS[Phospho (STY)]LER_.3	P80560_S139_M1_Ptprn2	4.3872	3.3667	3.4890	3.6318	3.7249	3.9490	3.8161	4.1781	3.8752	3.0927	4.2496	3.0928	3.9833	3.5271	3.3933	3.2313	3.6753	3.4741	3.7732	3.2043
Ptprn2	_KSEQPEEVLS[Phospho (STY)]S[Phospho (STY)]EEETAGVEHVR_.3	#N/A	4.0452	3.7518	3.2049	3.7256	3.5470	3.7140	3.8339	3.8825	3.8146	3.7685	3.7630	3.5707	3.9546	4.1128	3.6861	4.8735	3.9652	4.3061	3.6296	3.0392
Nucb2	_SGRLS[Phospho (STY)]QELDLVSHK_.3	P81117_S89_M1_Nucb2	4.4968	3.5060	3.5322	3.7414	3.8345	4.0586	3.7066	4.0685	3.7657	3.2022	4.1401	3.3955	3.8737	3.4175	3.2838	3.3408	3.5658	4.9173	3.8827	3.0948
Irs2	_VAS[Phospho (STY)]PTSGLKR_.2	P81122_S1089_M1_Irs2	3.5090	9.1947	4.5464	9.1534	8.6568	3.7492	8.4691	3.7346	8.3704	3.3196	4.2319	8.7934	3.4179	3.9205	7.8172	8.7726	8.7611	8.7693	8.8013	3.8875
Irs2	_RVS[Phospho (STY)]GDGAQDLDR_.2	P81122_S556_M1_Irs2	7.4801	7.7905	7.1815	7.5708	7.0263	7.3777	7.0596	7.2690	7.1548	7.7511	7.1350	7.4617	7.7576	8.0249	6.4514	7.3068	7.4591	7.3356	7.5519	7.3374
Irs2	_HNSAS[Phospho (STY)]VENVSLRK_.3	P81122_S1165_M1_Irs2	5.7066	4.2666	4.2725	3.9532	3.9114	3.2980	5.7887	3.3283	3.0254	5.9057	3.3998	4.1561	3.1335	4.1781	4.0444	5.8911	4.3264	4.3239	3.1221	3.8554
Irs2	_TYS[Phospho (STY)]LTTPAR_.2	P81122_S573_M1_Irs2	7.8006	8.2564	3.3143	7.8546	3.6166	8.0649	7.6329	8.2122	7.4502	7.6673	4.3579	7.4191	4.2996	3.3014	6.7638	7.6901	7.8137	8.0598	2.9648	7.7354
Irs2	_SNT[Phospho (STY)]PESIAETPPAR_.2	P81122_T517_M1_Irs2	6.8046	6.1710	6.6586	6.7841	6.4376	6.6511	6.2214	3.8303	6.7955	6.9682	6.3808	6.5514	6.8159	7.4320	5.5647	6.4437	6.8274	6.4033	6.9148	7.3726
Irs2	_ASSPAESS[Phospho (STY)]PEDSGYMR_.2	P81122_S728_M1_Irs2	3.9721	3.5159	3.3792	3.7810	3.7057	4.0982	3.7064	4.0683	3.7654	4.2762	5.6617	3.2420	3.8735	4.8780	3.3970	3.0365	3.5655	3.6233	3.9224	3.9140
Irs2	_QVPQPSSAS[Phospho (STY)]LDEYTLMR_.2	P81122_S590_M1_Irs2	3.1415	4.1775	6.7169	3.9248	3.3485	3.3818	3.6996	3.4649	3.6198	4.4530	3.0985	3.9037	3.7853	4.2879	3.7250	4.0045	4.0026	3.8306	3.4791	4.2549
Irs2	_GPGTGGDEASAAGGS[Phospho (STY)]PPQPPR_.2	P81122_S66_M1_Irs2	4.0766	3.2425	3.6132	3.5076	4.2836	4.3169	4.2654	4.0307	4.1856	9.1721	3.6642	2.9686	4.3510	3.3528	2.7899	3.4388	3.0675	3.2648	4.4142	3.3198
Stim2	_SIVPSS[Phospho (STY)]PQSQR_.2	P83093_S523_M1_Stim2	10.2589	3.2700	3.5857	10.3294	9.0983	4.2894	9.4904	9.6380	9.1801	3.8597	3.6918	2.9961	4.3786	3.6237	7.9291	3.4112	3.7720	3.2372	3.6765	3.3474
Stim2	_GS[Phospho (STY)]PEC[Carbamidomethyl (C)]VGLTETK_.2	#N/A	8.3614	8.1239	8.4005	8.5201	7.7600	7.9748	7.9343	8.1471	7.3541	4.2003	7.8061	7.3749	7.3319	7.8375	6.4458	7.3199	8.1608	3.0809	7.5785	7.2041
Stim2	_GESPVTADVSRGS[Phospho (STY)]PEC[Carbamidomethyl (C)]VGLTETK_.3	#N/A	4.3766	8.0642	7.6755	7.7470	3.0827	7.8988	7.8979	3.7307	7.7932	7.3738	3.3643	7.1327	4.0511	8.1711	6.6544	3.7387	7.5862	7.8395	7.5182	7.3547
Stim2	_DELS[Phospho (STY)]LEDSSR_.2	P83093_S631_M1_Stim2	6.7980	6.9665	7.4859	7.0107	3.9256	4.0593	6.7818	4.2883	3.6745	7.5626	4.0489	6.3749	3.1078	6.7730	3.1926	3.4320	5.8537	6.6588	6.4831	6.8561
Stim2	_DELSLEDSSRGES[Phospho (STY)]PVTADVSR_.3	P83093_S640_M1_Stim2	4.5119	3.4909	3.5473	3.7565	3.8496	3.3225	3.6915	4.0534	3.7506	3.2174	4.1249	3.3804	3.8586	3.4024	3.2687	3.3559	3.5507	3.5988	3.8978	3.0797
Stim2	_RAS[Phospho (STY)]GSAGAAASPSAAAAGER_.2	P83093_S28_M1_Stim2	3.1133	4.2058	4.1507	4.4709	3.3202	3.3536	3.7279	3.3390	3.6481	4.4247	3.1267	3.9319	3.8135	4.3162	3.7533	3.9762	4.3370	3.8023	3.4509	4.2832
Stim2	_S[Phospho (STY)]MIFSPASR_.2	P83093_S661_M1_Stim2	4.0519	3.9509	4.5882	3.6375	3.3896	4.8985	4.8162	4.5134	4.2106	4.2582	3.0841	3.8404	4.3186	3.8624	3.7287	4.3968	4.2899	3.1388	3.4378	3.5397
Tnik	_QNS[Phospho (STY)]DPTSENPPLPTR_.2	P83510_S611_M1_Tnik	12.7372	12.2441	12.5706	12.1588	11.9573	12.0006	11.8714	12.4265	12.1500	12.3157	12.2532	12.0527	12.8571	12.2162	11.1939	12.0408	12.7318	12.2084	12.2549	11.9496
Tnik	_TTSIS[Phospho (STY)]PALAR_.2	P83510_S651_M1_Tnik	3.9680	6.9730	7.7794	3.7694	3.3057	3.5298	3.5895	3.6698	3.6486	9.4615	7.7346	7.6575	8.2845	3.8177	3.8605	6.1159	4.4144	3.8939	5.7065	5.8890
Tnik	_ANS[Phospho (STY)]KSEGS[Phospho (STY)]PVLPHEPSK_.2	#N/A	8.7298	3.8505	7.0404	3.8666	7.2510	3.7686	3.5722	6.5564	6.3726	3.8134	3.9267	7.7954	3.6865	8.6152	5.1043	3.9532	6.6123	6.7067	6.9123	3.9649
Tnik	_VRANS[Phospho (STY)]KSEGS[Phospho (STY)]PVLPHEPSK_.3	#N/A	9.0734	9.2595	8.5088	9.0690	8.0411	3.6660	8.7129	3.5785	8.3002	10.1976	8.4104	9.3558	3.9441	3.9891	8.1312	8.3136	8.6148	8.7499	8.5765	8.1059
Tnik	_SES[Phospho (STY)]FSISGVQPAR_.2	P83510_S541_M1_Tnik	4.0882	6.2164	6.8641	5.9353	5.3196	3.2045	5.7592	3.6554	3.8103	4.2756	3.2889	3.9503	3.9757	5.7610	4.0087	3.9385	4.1931	3.6532	5.9433	5.2695
Tnik	_KNS[Phospho (STY)]PGNGSALGPR_.2	#N/A	8.1526	7.7127	7.4808	7.4147	7.3755	6.9569	7.3415	7.6513	6.9495	7.7687	7.0729	7.2285	7.5730	7.5062	5.7535	7.1495	7.6000	7.3093	7.0946	7.1257
Tnik	_SEGS[Phospho (STY)]PVLPHEPSK_.2	P83510_S740_M1_Tnik	13.4525	13.2933	13.0327	12.9984	12.7911	13.0422	12.9699	13.5187	12.9880	12.7365	12.9432	12.9559	13.7559	13.2201	12.0491	12.7837	13.6639	13.2442	13.0028	12.7930
Tnik	_VYQTSPT[Phospho (STY)]DEDEEDDESSAAALFTSELLR_.3	P83510_T950_M1_Tnik	3.6112	9.4447	4.1475	4.3567	2.9490	3.1730	3.0913	3.4532	3.1504	3.8176	3.5247	4.2810	3.2584	4.3030	4.1693	3.9561	4.4513	4.1990	2.9972	3.9803
Tnik	_AS[Phospho (STY)]NPDLR_.2	#N/A	3.5719	4.0522	4.3043	3.5236	2.9096	4.3679	3.1306	3.4925	1.7888	3.7782	3.5641	3.7783	3.2977	4.3424	4.2086	5.4712	3.2641	4.1597	2.9579	4.0196
Tnik	_LGSQPIRAS[Phospho (STY)]NPDLR_.3	#N/A	3.4676	3.8515	4.5050	4.1166	3.6746	3.7079	3.3735	3.6933	3.2938	3.2782	4.2732	3.5776	3.4985	4.5431	3.3989	4.3306	3.1905	3.9589	4.2580	4.2204
Wnk1	_GTEDGSGSPHS[Phospho (STY)]PPHLC[Carbamidomethyl (C)]SK_.3	P83741_S2027_M1_Wnk1	4.0747	5.9589	3.9569	5.2646	3.9761	5.4187	5.1896	4.1141	3.8545	5.1532	5.2449	4.9010	3.9862	6.1177	5.5728	4.2585	5.4634	5.7079	6.0174	5.5198
Rpl36a	_LEC[Carbamidomethyl (C)]VEPNC[Carbamidomethyl (C)]R_.2	#N/A	7.0275	7.1300	6.9563	6.7890	7.1992	6.8477	7.0338	7.2502	7.1086	6.7148	6.8809	6.8350	7.1102	6.9681	7.3124	6.3488	7.3645	7.0944	6.7697	7.2193
Cbx1	_KKVEEVLEEEEEEYVVEK_.3	#N/A	5.3731	5.5427	4.9152	5.5986	4.6518	3.5025	3.6873	5.5866	3.6075	4.4653	4.8237	5.5225	6.5164	6.3457	4.7183	5.2497	5.9656	3.8428	4.4351	5.8590

Cbx1	_VEEVLEEEEEYVVEK_.2	#N/A	6.4429	4.2818	4.2573	3.9684	3.0587	3.4254	3.6560	6.0332	4.2411	5.9581	3.0549	4.1745	6.1544	4.6585	3.6814	5.6611	3.9590	3.8741	3.5227	4.2114
Cbx1	_ADS[Phospho (STY)]DSEDKGEESKPK_.2	P83917_S89_M1_Cbx1	3.2253	3.2767	3.7616	4.4641	4.0638	4.2879	3.4772	4.4265	2.0268	3.4316	3.9107	3.1661	3.6444	1.9641	3.0544	3.5702	3.4633	4.3698	4.0184	4.3663
Eloc	_EIPSHVLSK_.2	#N/A	6.3480	5.7401	6.5237	6.1871	6.4542	6.3380	6.8566	6.5534	6.4156	6.4135	6.4095	6.6583	6.5242	6.4516	7.1974	6.0404	6.2214	6.4980	6.9160	6.5032
Eloc	_LISSDGHEFIVK_.2	#N/A	4.9744	5.0699	5.1854	4.9144	4.1194	4.8347	4.3662	5.0990	4.5727	4.2661	4.1777	4.6086	4.0805	4.8069	5.0626	3.8384	4.7051	4.4473	4.6622	4.6957
Eloc	_EHALTSGTIK_.2	#N/A	6.2559	3.7205	3.3177	3.5269	5.4267	2.6419	5.2159	5.9894	6.0924	3.1282	5.6629	6.0483	5.7259	6.1460	6.3046	6.2668	5.7563	6.0028	5.8473	5.9874
Hpca	_GAKS[Phospho (STY)]DPSIVR_.2	P84075_S175_M1_Hpca	10.5345	10.7289	10.3926	10.4917	10.2876	10.2905	10.1939	10.6532	10.2807	10.3717	9.9714	10.2499	10.7078	10.5556	9.9565	10.2211	10.5124	10.2717	10.3078	10.1618
Hpca	_LSLEEFIR_.2	#N/A	8.7321	8.6879	8.6886	8.9220	8.7798	8.8288	8.6657	8.5264	8.6273	8.6708	8.7457	8.9836	8.5000	8.7268	8.5961	8.6735	8.5271	9.0749	8.5974	8.5048
Hpca	_FAEHVFR_.2	#N/A	8.6447	8.7389	8.7544	8.6664	8.8765	8.7758	8.6972	8.7748	8.4643	8.7782	8.5793	8.9092	8.8182	8.6007	8.9894	8.4703	8.9058	8.8148	8.6764	8.6760
Hpca	_IYANFFPYGDASK_.2	#N/A	8.3556	8.4628	8.7072	8.5054	8.3588	8.2978	8.4254	8.2764	8.0670	8.2889	8.4124	8.3462	8.6748	8.5437	8.3460	8.5184	8.5440	8.3380	8.4712	8.4285
Hpca	_ENTEFSELELQEWYK_.2	#N/A	6.9994	6.8787	7.4291	7.4711	6.5538	6.7422	6.1729	7.0744	6.8663	6.9910	7.3230	6.4959	7.5176	7.6479	7.1468	7.2904	7.3884	6.4675	6.3303	7.1658
Hpca	_LLQC[Carbamidomethyl (C)]DPSSASQF_.2	#N/A	6.1204	6.4993	6.9674	6.0751	6.2610	6.3728	6.4626	6.3583	6.5169	6.0754	6.6715	6.3849	6.6732	6.4302	6.5211	6.3111	6.6877	6.3177	6.5515	6.1618
Hpca	_TFDTNSDGTIDFR_.2	#N/A	4.5248	3.4781	3.5602	5.4322	3.8625	5.7427	6.1041	4.2251	5.8355	5.5117	6.0767	6.2216	4.5455	6.2263	6.3170	3.3688	5.7185	6.9008	5.6880	5.6514
Hpca	_EEM[Oxidation (M)]LEIVQAIYK_.2	#N/A	5.3013	4.1297	5.6992	5.0916	4.0995	4.7296	4.2038	4.8056	4.9464	4.9341	5.1628	3.2468	4.5972	5.0202	4.6945	4.5847	5.2708	4.4250	4.4184	4.8622
Hpca	_LRPEMLQDLR_.2	#N/A	4.0837	6.2866	5.1221	6.4200	4.1462	5.7986	5.9746	4.1433	3.5282	3.8943	5.3490	5.0739	3.6937	6.1024	4.7833	3.4458	4.7544	3.9140	5.4002	3.6245
Hpca	_DC[Carbamidomethyl (C)]PTGILNVDEFKK_.3	#N/A	3.6061	3.7130	3.1427	3.0665	3.8130	3.8464	2.1003	3.8318	1.7449	3.4167	4.1347	3.4391	3.3207	3.8234	0.1816	2.9682	3.3290	4.2951	3.9437	2.8415
Hpca	_MPEDESTPEKR_.2	#N/A	4.3782	4.9222	5.5766	4.9167	3.0843	4.9993	5.2165	3.8432	4.7512	4.1888	4.9429	4.3689	4.2860	5.3052	5.0885	3.7403	5.0155	3.8684	3.1650	3.3608
Arf5	_VQESADELQK_.2	#N/A	5.9500	5.8529	5.7771	5.4278	5.6578	5.9078	5.8121	5.0143	6.0021	5.5462	5.5588	6.1543	6.3110	6.2854	5.3320	5.3735	6.1903	5.7678	5.2660	5.4628
Cplx2	_AALEQPC[Carbamidomethyl (C)]EGS[Phospho (STY)]LTRPK_.2	#N/A	9.4189	9.4756	9.4411	9.4501	8.9725	8.8766	8.8960	9.2774	8.8187	9.3936	9.3011	9.5841	9.7925	9.9111	9.4773	9.5369	9.6471	9.2002	7.3058	9.4041
Cplx2	_KAIPAGC[Carbamidomethyl (C)]GDEEEEEEESILDTVLK_.3	#N/A	8.4580	8.8136	8.6433	8.6584	7.9926	8.3634	8.0749	8.1611	8.4922	8.3768	8.3603	8.7845	9.5555	9.6982	9.1962	9.1149	8.9441	8.8247	8.4762	8.9584
Cplx2	_AIPAGC[Carbamidomethyl (C)]GDEEEEEEESILDTVLK_.3	#N/A	6.1811	6.4454	6.7774	6.4604	5.6964	5.6489	5.6449	5.5879	6.1893	6.7374	6.5575	6.5388	7.1750	7.3151	7.1279	7.0149	6.5045	6.4271	6.0445	7.0335
Cplx2	_YLPGPLQDMFK_.2	#N/A	3.4622	7.4665	8.4895	8.5981	6.2210	7.3094	7.8303	3.6879	6.1788	5.8238	7.9700	6.7620	3.4646	8.7055	7.6943	4.3252	6.7531	6.0109	6.0355	6.8734
Erh	_[Acetyl (Protein N-term)]SHTILLVQPTK_.2	#N/A	6.2912	6.5295	6.8937	6.8478	6.8492	6.9544	6.1319	6.6953	6.8788	6.5975	6.4609	6.7663	6.9732	7.2107	7.1849	7.1280	6.5925	6.4126	6.8223	6.5692
Erh	_TYADYESVNEC[Carbamidomethyl (C)]M[Oxidation (M)]EGVC[Carbamidomethyl (C)]K_.2	#N/A	6.0153	5.7893	4.4087	5.4220	5.0393	5.3099	4.8644	5.4710	4.8036	5.0676	4.8280	5.0165	6.0939	6.0474	4.7015	5.4104	5.6121	4.8544	5.2479	5.5186
Erh	_MYEEHLK_.2	#N/A	5.1463	4.8290	3.3710	3.7498	3.8430	6.0310	3.5754	4.0601	3.7572	5.4857	4.1316	3.2108	3.8653	2.0425	3.0114	3.3493	3.5573	1.6224	3.8912	1.9985
Erh	_ADTQTYQPYNK_.2	#N/A	3.6165	4.3863	4.1528	4.0729	2.9542	3.5299	3.5515	3.3168	3.4717	3.1002	2.9504	4.2758	3.6372	4.1398	3.5769	4.1526	3.8545	3.9786	3.6272	4.1068
Ap2m1	_TFITQQGIK_.2	#N/A	8.7502	8.5748	8.7306	8.7042	8.8471	8.5942	8.7666	9.0520	8.4532	8.4386	8.3957	8.4699	8.5220	7.7689	8.3483	8.0730	8.4923	8.6689	8.6898	8.1261
Ap2m1	_ESQISAEIELLPTNDK_.2	#N/A	7.8381	7.3834	8.3216	7.5365	7.3540	7.7286	7.6275	7.2679	8.1268	7.4552	7.7178	7.4537	7.4065	7.1528	6.6008	7.3259	7.5074	7.3214	7.6022	7.8565
Ap2m1	_VNVIHAR_.2	#N/A	7.5634	7.9025	7.4327	7.3049	7.2161	7.6025	8.0713	7.4345	7.5129	6.9772	7.1071	7.5914	7.3323	6.7240	7.3875	6.9341	7.4890	7.4923	7.5030	7.1320
Ap2m1	_DIILPFR_.2	#N/A	6.1679	6.9081	6.4604	6.8914	6.0818	6.5612	7.0052	5.7905	6.9662	6.6280	6.5005	6.7344	6.2128	5.9004	6.1416	6.5475	5.4111	7.0718	6.8024	6.8375
Ap2m1	_VYRDDIGR_.2	#N/A	6.5493	6.3690	6.1770	5.9181	5.6923	6.1870	6.4692	6.6932	6.2403	5.8085	5.6183	6.3812	6.3459	5.4532	5.7783	5.3488	5.4338	5.9408	5.8208	6.0037
Ap2m1	_TSFFHVK_.2	#N/A	6.5227	5.5499	5.1796	5.6197	6.2383	6.0937	6.2740	6.7193	5.5688	6.0157	5.6224	5.9091	6.1572	5.5644	5.5746	5.1047	5.8405	6.2493	5.9202	5.3393
Ap2m1	_M[Oxidation (M)]IGGLFIYNHK_.2	#N/A	6.5719	6.1188	4.8110	5.0430	5.7855	6.2672	6.0756	6.7488	5.9411	6.3027	5.6185	5.9645	6.5914	5.3240	5.1332	5.5145	5.4923	6.6554	5.8657	3.4376
Ap2m1	_ASENAIVWK_.2	#N/A	5.6719	5.5649	5.8784	5.3532	5.7482	5.5414	6.4692	4.8046	5.9878	5.5809	6.2875	5.4010	6.0287	5.6853	4.6190	5.8773	4.9458	5.9702	6.0785	5.2546
Ap2m1	_SNIWLAAVTK_.2	#N/A	6.0510	4.5985	6.4027	5.4456	5.7891	6.0614	6.4574	5.7577	5.7011	6.2343	3.0177	5.7448	5.7346	5.7495	4.4207	4.8873	5.4904	6.3404	5.7748	5.7965
Ap2m1	_S[Phospho (STY)]PVTNIAR_.2	P84091_S45_M1_Ap2m1	6.7636	7.4471	6.6962	5.5432	6.6387	7.3288	7.6270	6.6226	7.3462	5.3623	7.4240	6.9340	5.1986	6.9981	5.6812	6.8861	7.2890	7.3278	7.3097	7.2044
Ap2m1	_SGIYETR_.2	#N/A	4.2897	5.0917	3.1276	4.0827	4.1413	5.3742	5.5545	3.7617	5.3769	4.8473	5.2114	4.2784	3.5669	4.6115	4.8004	3.6477	3.2589	5.5140	4.3538	4.7501
Ap2m1	_QSIAIDDC[Carbamidomethyl (C)]TFHQC[Carbamidomethyl (C)]VR_.3	#N/A	5.4590	5.6182	6.4126	5.7087	5.4301	5.6438	5.8845	6.0584	5.8420	5.7429	5.5293	5.0378	5.7178	5.3734	5.2398	4.9191	5.5106	5.4140	5.0896	4.9947
Ap2m1	_LNYSDHDVIK_.2	#N/A	6.8324	6.6780	6.2903	6.5011	6.6694	6.1874	6.5163	6.7509	6.6123	6.2839	6.4330	6.0905	6.3572	6.4001	6.0641	5.9602	6.7421	6.3498	6.2511	6.2328
Ap2m1	_LS[Phospho (STY)]KFDSER_.2	P84091_S255_M1_Ap2m1	3.3422	3.1598	3.8785	4.3472	4.1807	5.7430	3.3603	4.5434	3.4194	3.5485	3.7938	3.0492	3.5275	3.8656	2.9375	3.6871	3.5802	3.9299	4.2290	4.2494
Ap2m1	_EEQSQITSQVTGQIGWR_.2	#N/A	3.8600	4.1909	4.1656	4.4561	3.3351	3.3684	3.7130	3.3538	1.9313	4.4396	3.1119	3.9171	3.7987	4.2036	3.7384	3.9911	4.3519	3.8172	3.4657	4.2683
Rhog	_EYIPTVFDNYSAQSAVDGR_.2	#N/A	5.6758	4.9566	4.7842	4.1136	5.0989	5.4590	5.0626	5.2475	5.2916	3.1589	4.9575	4.1735	3.9170	3.4608	3.3271	3.2975	3.6091	5.3063	5.2333	4.7352
Rhog	_YLEC[Carbamidomethyl (C)]SALQQDGVK_.2	#N/A	3.6933	5.4114	5.1728	4.4766	4.4308	3.9336	5.0782	7.4305	3.0680	3.5039	4.0475	4.6589	3.2335	3.7361	3.1732	3.0555	3.4508	4.4239	4.0309	3.7031
Rpl19	_VWLDPNETNEIANANSR_.2	#N/A	8.4671	8.5536	8.3557	7.9781	7.9813	8.1478	8.3953	8.6591	8.2498	8.3505	8.3392	8.4149	9.0553	8.9169	7.8401	8.2685	8.7383	8.3946	8.3049	8.1892
Rpl19	_LLADQAEAR_.2	#N/A	6.9409	6.9785	7.0208	6.3485	6.8930	6.9658	6.8365	7.0550	7.2680	6.3704	6.7388	6.7088	6.9042	6.6363	7.1688	6.7047	6.8136	7.3431	7.1431	6.8875
Rpl19	_TLS[Phospho (STY)]KEEETKK_.2	P84099_S189_M1_Rpl19	5.8341	4.9581	4.8000	3.5091	4.3305	5.0942	5.2815	4.2771	3.9742	5.0352	4.5862	5.2723	4.8916	5.9064	3.4923	4.4765	5.0120	5.8815	3.6742	3.3033
Srsf3	_S[Phospho (STY)]RS[Phospho (STY)]LS[Phospho (STY)]RDR_.2	P84104_S126_M3_Srsf3	7.3928	6.6839	6.4807	7.2652	5.6336	6.5699	6.9007	6.8559	6.9326	7.5654	6.6095	6.4426	6.2585	6.8681	6.4553	6.4511	6.8447	6.5000	7.2367	5.7566
Srsf3	_RRS[Phospho (STY)]PPPR_.2	P84104_S108_M1_Srsf3	9.7979	8.3416	7.3815	7.7808	8.2685	8.6083	8.7902	8.4785	8.3605	8.1612	8.4872	4.3034	8.2216	7.9564	5.9688	5.2343	8.5541	8.2836	6.4240	6.5731
Srsf3	_NPPGFAFVEFEDPRDAADAVR_.3	#N/A	5.3389	4.9762	3.6429	3.4779	4.3132	4.8154	5.7172	5.0688	4.0896	4.0209	4.8448	5.3047	3.7387	3.6809	4.3254	3.4684	3.8292	5.3257	4.7624	3.3981
Srsf3	_NPPGFAFVEFEDPR_.2	#N/A	5.2806	5.1112	6.2526	5.8789	5.7264	5.9073	4.4292	5.6775	2.9874	5.5119	5.4558	4.1181	4.5963	4.1401	3.6520	4.1191	5.8538	4.6027	6.0424	3.8174
Srsf3	_ERS[Phospho (STY)]LS[Phospho (STY)]RER_.2	#N/A	8.9674	8.8404	9.1532	9.0722	8.4941	8.7655	8.9731	8.7596	8.8279	8.9478	8.6393	8.5292	9.0176	8.6267	8.8406	8.6730	8.9069	8.6433	8.8904	8.2540
Srsf3	_VYVGNLGNNGNK_.2	#N/A	3.1678	3.3341	3.7041	6.8020	4.0064	7.3302	6.6031	7.1702	6.6867	7.0402	6.6399	6.9559	3.7018	7.5844	6.7096	3.5127	5.1517	7.5668	6.8850	4.4237
Srsf3	_NHKPS[Phospho (STY)]RS[Phospho (STY)]FSR_.3	P84104_S148_M2_Srsf3	3.8478	4.1550	4.3841	3.8416	3.1855	3.4096	4.3556	3.2167	4.4146	4.0541	3.2882	4.0445	4.5227	4.0665	3.9328	4.1927	4.2147	2.5614	3.2338	3.7437
Srsf3	tyl (Protein N-term)]MHRDS[Phospho (STY)]C[Carbamidomethyl (C)]PLDC[Carbamidomethyl (	P84104_S5_M1_Srsf3	3.5889	4.4140	4.1251	4.1005	2.9266	2.2330	3.1137	3.2892	3.1727	3.7952	3.5471	4.3034	3.2808	4.1122	4.1917	3.9337	3.8269	4.0063	3.6548	4.0792
Adcy5	_SVS[Phospho (STY)]PPGYAAQTAASPAPR_.2	P84309_S8_M1_Adcy5	10.8821	10.5859	9.5993	3.2323	7.9453	10.3633	10.4505	11.1363	9.7273	10.1916	10.3167	3.4352	11.1911	10.6721	8.9865	10.0591	11.0561	11.0475	10.4680	10.0916
Adcy5	_SGGAVT[Phospho (STY)]PQQQQR_.2	P84309_T62_M1_Adcy5	7.1405	6.5495	6.9042	6.5270	6.3200	6.1198	6.9146	6.1855	6.3623	6.9272	5.9291	6.3853	4.3901	5.9118	3.8002	3.7214	6.5707	6.5263	5.9019	5.3011
Adcy5	_S[Phospho (STY)]VELGLEER_.2	P84309_S156_M1_Adcy5	7.1498	8.8019	8.4622	8.5498	4.2709	8.6417	8.8477	3.1327	8.3381	8.1832	3.7036	7.7428	3.4373	8.5989	6.8043	8.1171	7.6985	8.8422	8.3518	8.7103
Adcy5	_DVAAHFLAR_.2	#N/A	4.1810	3.8218	3.2164	3.5084	3.5187	4.4662	4.8226	3.8814	4.0362	4.0366	4.3943	4.3416	4.2017	4.3093	4.1414	3.6288	4.4190	4.6229	4.2500	3.7240
Adcy5	_ILGDC[Carbamidomethyl (C)]YYC[Carbamidomethyl (C)]VSGLPEAR_.2	#N/A	3.0872	5.5915	3.6235	3.1014	3.9257	4.9834	3.0222	4.2884	5.1160	3.6295	3.9219	4.3111	3.1079	3.6105	3.1926	3.4321	3.3252	5.3684	4.1565	3.5775
Isoc2a	_DASHPQFK_.2	#N/A	4.1280	4.4402	4.1410	4.3093	4.2286	4.9190	4.4682	4.3011	4.6978	5.3247	4.6951	3.9416	3.1345	4.1791	4.0454	4.7723	4.2145	4.5674	4.1208	4.3711
Isoc2a	_QSGAFLATSESLILQLVR_.2	#N/A	4.1973	3.1768	3.6789	3.4419	3.5350	3.7591	4.0060	4.3680	4.0651	4.4036	2.9387	3.6949	4.1732	3.7170	3.5832	3.0414	3.8652	3.2842	3.5833	3.3942
Rpl10l;Rpl10	_LIPDGC[Carbamidomethyl (C)]GVK_.2	#N/A	3.8678	3.4513	5.4758	3.7164	4.0747	4.1081	6.0193	4.2395	4.3944	3.6784	4.6779	3.1774	4.5599	5.5602	6.1062	3.2299	3.2764	5.7681	4.2054	3.5287
Cdh8	_LGELYSVGES[Phospho (STY)]DKET_.2	P97291_S795_M1_Cdh8	4.3449	3.9408	4.5983	3.6274	3.0510	7.1295	3.9971	3.7624	6.6607	4.1555	3.3960	6.2685	4.0828	7.4580	4.0225	3.7070	6.7562	3.5331	6.7954	6.7564
Hrh2	_LNNS[Phospho (STY)]LLSR_.2	P97292_S321_M1_Hrh2	4.4061	5.5390	3.4416	3.2832	3.7439	3.9679	4.8625	4.1065	3.8563	5.6126	4.2307	3.4861	3.9644	5.0603	3.3744	3.2502	5.9956	3.1890	4.6233	3.3957
Nptn	_QNPSITWIR_.2	#N/A	8.1342	7.9664	7.8820	7.9167	7.9631	7.7319	8.1105	8.1332	7.8038	8.2076	8.0061	8.3274	7.6724	8.2007	7.4190	8.0289	7.9671	8.2880	7.8873	8.0059
Nptn	_IVTSEEVIR_.2	#N/A	7.8238	6.2354	8.0260	7.8234	7.6097	7.4731	7.5381	7.4393	7.6829	7.7182	7.2292	7.1180	7.2979	6.7271	7.8772	7.8127	7.6734	6.6220	7.3303	7.4593
Nptn	_AAPDITGHK_.2	#N/A	7.0889	7.5803	7.9422	6.7882	7.3770	7.3365	7.2291	7.5608	7.2893	7.1155	7.4241	6.9081	7.1484	7.3790	7.7712	6.9102	7.0313	7.4658	7.0886	7.5505
Nptn	_SVGYPHPEWIWR_.2	#N/A	7.4300	6.3053	7.2930	7.3630	7.8539	7.0234	7.1161	7.9312	6.7863	7.3948	7.1288	7.2729	7.1809	7.7948	6.5821	7.2534	7.7539	7.5469	7.1452	6.9447
Nptn	_VTVNTAYGSNGVSVLR_.2	#N/A	6.5678	5.7358	6.4161	6.3013	5.8697	6.2828	5.6271	5.9630	5.8624	6.4442	6.1041	5.4777	5.7552	5.2231	5.6996	5.5670	6.2011	5.4698	6.1036	6.0344
Nptn	_QLWDGAR_.2	#N/A	5.8786	5.8610	5.0836	5.3845	6.3600	6.1093	6.0782	6.6354	5.9956	5.4781	5.9895	6.2305	5.6691	5.5189	5.7986	5.7713	6.4085	6.3360	5.8302	5.7468
Nptn	_LTLEDSGTYEC[Carbamidomethyl (C)]R_.2	#N/A	4.4866	5.4893	5.7346	5.7281	4.7379	5.9980	5.7502	3.2114	5.7727	5.7165	5.5007	5.2130	4.5175	6.0093	5.1462	3.8487	4.2095	5.6383	4.9110	5.0240
Nptn	_NGVELTATR_.2	#N/A	3.6522	7.9656	6.4033	6.7687	5.8268	5.5176	8.0267	5.5749	3.1092	5.7193	6.1016	7.8213	3.6830	3.2268	3.2144	7.8823	3.3751	6.1232	8.0437	6.2547
Nptn	_AEDSGEYHC[Carbamidomethyl (C)]VYHFVSAPK_.3	#N/A	6.3764	5.8241	5.7938	5.7974	5.7869	5.1387	5.7043	6.0823	5.9315	5.9135	5.9644	5.4345	6.0454	6.0513	5.0301	5.6688	5.8621	5.1214	4.9355	5.6842
Nptn	_SENKNEGQDAMMYC[Carbamidomethyl (C)]K_.3	#N/A	3.7856	3.8985	3.9415	3.5709	3.5915	3.8881	3.8525	3.5852	2.9231	3.8218	3.6567	3.7218	4.1473	3.6911	3.5842	3.7552	3.8393	3.4132	3.7123	4.1123
Csrp1	_C[Carbamidomethyl (C)]SQAVYAAEK_.2	#N/A	5.7455	5.4914	5.7331	5.9097	6.2181	5.9666	6.0034	5.4435	6.6048	5.5230	5.3390	5.7785	5.7647	6.2446	6.3787	6.1567	5.3999	5.4657	6.3882	5.7649
Csrp1	_GFGFGQGAGALVHSE_.2	#N/A	7.5265	8.5861	8.3892	8.0684	5.5652	7.8796	7.9003	7.7110	8.0657	7.8852	5.6531	7.6343	8.3799	8.9900	8.1916	7.8389	8.0093	8.2256	7.9159	7.6454
Csrp1	_GYGYGQGAGTLSTDKGESLGIK_.3	#N/A	5.9330	5.7384	3.8799	5.7702	6.0175	5.6546	6.0426	5.7102	6.1254	6.4370	6.2955	5.7805	4.3742	6.6688	6.7377	5.7939	5.4191	5.4924	5.3531	5.9255
Csrp1	_TVYFAEEVQC[Carbamidomethyl (C)]EGNSFHK_.2	#N/A	4.9813	5.5032	5.6887	5.6471	4.8754	4.7570	4.4624	4.7469	4.8254	5.4127	4.5844	5.0899	6.1948	6.0023	4.4915	5.5529	4.4546	3.3794	4.8128	5.0179
Csrp1	_HEEAPGHRPTTNPNASK_.4	#N/A	3.8948	3.6853	4.0207	3.9504	4.6392	3.8985	4.4926	3.9964	4.7142	5.4731	3.5713	4.1249	3.8651	4.0588	9.4329	3.7358	3.9525	3.8147	3.7728	3.6763
Cdh6	_LADMYGGM[Oxidation (M)]DS[Phospho (STY)]DKDS_.2	#N/A	3.5444	4.0247	6.6378	6.0842	3.9168	4.1409	4.9059	3.5719	3.8144	3.7507	3.6434	3.9002	3.9225	7.4131	3.3325	3.8893	4.7431	3.6660	3.9651	4.3980
Rps3a	_LITEDVQGK_.2	#N/A	7.1535	6.9481	6.8751	6.5670	6.7252	6.9837	6.8218	6.7206	7.4051	6.5738	6.8254	6.5966	7.2708	6.5292	7.0940	6.8560	6.3066	6.8414	6.8056	6.9517
Rps3a	_TTDGYLLR_.2	#N/A	6.8774	6.4013	6.5350	6.1654	6.5341	6.5290	6.7987	6.1658	6.6982	6.7143	6.8193	6.6185	6.5966	6.4348	6.8753	6.3128	6.1297	6.9562	6.6270	6.2218
Rps3a	_ADGYEPPVQES[Phospho (STY)]V_.2	P97351_S263_M1_Rps3a	8.9015	9.1613	9.3901	9.2233	3.2618	8.8602	8.9691	3.5516	9.2103	9.2982	8.8876	8.9429	9.3235	9.2188	8.4831	9.0681	9.3791	9.0281	9.0557	9.0162
Rps3a	_VFEVSLADLQNDEVAFR_.2	#N/A	4.6258	4.7834	3.4024	3.9787	5.2443	4.6686	3.8365	4.3229	4.5718	4.7944	4.9033	4.2748	5.7075	3.5474	4.0055	5.4586	5.6467	4.3409	4.3298	4.5990
Rps3a	_KDWYDVK_.2	#N/A	3.5809	3.7382	3.5367	4.0033	3.7878	4.1273	3.4964	3.8066	3.1805	4.3039	4.0606	3.4643	3.3459	3.1556	4.1550	4.6459	2.3100	4.2699	4.1447	4.1983
Rps3a	_EVQTNDLKEVVNK_.3	#N/A	3.3481	3.9710	4.3855	4.2361	3.5551	3.0525	3.4930	3.5738	3.2709	3.1587	3.6453	3.6971	3.3790	4.4236	3.5184	4.2111	3.0710	4.0784	4.3775	4.1009
Rps3a	_LIPDSIGKDIEK_.3	#N/A	3.6038	3.7153	3.1404	3.9805	3.8107	4.2977	3.2374	3.8295	3.1576	3.4144	4.1371	3.4414	3.6346	3.1784	3.2628	2.9659	3.3267	3.8228	4.1218	4.3565
S100a13	_KGS[Phospho (STY)]LNINEFK_.2	P97352_S32_M1_S100a13	3.9479	4.3664	5.3814	5.1084	3.2856	5.5326	4.7148	3.1165	4.9273	5.2661	4.9357	4.5363	4.3864	4.8352	4.5552	4.2928	5.1479	5.4125	5.1614	5.2923
Evi5	_SDS[Phospho (STY)]NQYIR_.2	P97366_S689_M1_Evi5	3.7811	3.5380	3.3176	3.8032	3.9880	4.0213	5.4168	4.0067	2.9803	3.5917	3.9598	3.2641	3.1457	5.1558	3.0855	3.1432	3.5040	2.9692	4.1187	3.6154
Atp1b3	_LFIYNPSSGEFLGR_.2	#N/A	6.4861	6.5130	6.0893	6.6980	6.2921	6.2189	6.3550	6.1523	6.5858	6.2977	6.4226	6.1588	6.2511	5.9860	6.4983	5.6751	6.7331	6.1479	6.0010	5.9058
Atp1b3	_FDSGLNKK_.2	#N/A	4.3218	4.4981	4.8765	4.8750	3.0279	3.5796	5.2772	5.7552	4.4916	4.2241	5.3913	4.6363	5.2652	3.8965	4.9268	4.3627	4.0447	3.1047	3.4038	5.1097
G3bp2	_SAT[Phospho (STY)]PPPAEPASLPQEPPK_.2	P97379_T227_M1_G3bp2	14.9597	14.9803	15.1981	15.0579	14.7483	14.8093	14.3813	15.1150	14.7671	15.1331	14.8380	14.8317	15.4274	15.3906	14.5841	14.8766	15.3707	14.6729	14.8250	15.0148
G3bp2	_LPNFGFVVFDDSEPVQR_.2	#N/A	3.3763	3.7122	3.8582	4.2008	3.7380	4.0095	3.3114	4.4767	4.1601	3.6120	3.0562	3.6431	4.4085	4.2551	5.0201	4.3201	4.4056	3.5883	3.4484	3.9453
G3bp2	_VDAKPEVQSQPPR_.3	#N/A	4.5031	4.7701	4.4401	3.7856	3.2092	3.7247	5.0840	4.1271	4.9490	5.2435	4.7601	4.1426	3.9245	3.7827	5.3516	3.8652	4.1419	3.6913	3.9325	5.3604
G3bp2	_HLEELEEKS[Phospho (STY)]AT[Phospho (STY)]PPPAEPASLPQEPPK_.3	P97379_S225_M2_G3bp2	3.2015	3.3005	3.7378	3.9469	4.0401	4.2642	3.5010	3.8630	3.5601	3.4078	3.9345	3.1899	3.6682	6.8077	3.0782	3.5464	3.3602	4.7874	4.0883	4.3900
G3bp2	_GDMEQNDSDNRR_.2	#N/A	3.9427	4.0601	4.4790	3.7467	3.2804	3.5045	4.2607	3.6430	4.3198	4.1490	3.1933	3.9496	4.4278	4.4660	3.8379	4.2876	4.1807	3.0296	3.3010	3.6489
Anxa11	_DVQELYAAGENR_.2	#N/A	7.0712	7.8100	7.8687	7.6480	7.6328	7.4953	7.3764	7.8781	7.8542	7.9223	7.5435	7.4900	8.3730	7.7436	7.2092	7.2014	8.2198	7.7658	7.3206	7.8304
Anxa11	_SDTSGHFQR_.2	#N/A	4.1692	3.1499	3.7058	3.4516	2.8753	4.4094	4.1728	3.9381	4.6762	3.9798	5.1052	4.3769	4.2585	3.2603	4.1982	3.5313	4.4758	3.3573	4.5835	3.2273
Arhgap5	_GSEEDPLLS[Phospho (STY)]PVETWK_.2	#N/A	4.4290	7.7311	7.0155	6.9139	3.7667	7.2271	3.7743	6.6290	3.8334	6.7812	4.2078	3.1346	3.9415	7.0316	3.3515	6.1910	7.3110	7.1896	6.6769	3.1625
Arhgap5	_GGIDNPAITS[Phospho (STY)]DQEVDDKK_.2	#N/A	3.9761	4.1920	3.5127	3.6777	3.4616	3.4950	3.7141	3.8484	3.6343	3.7867	3.8343	3.9181	3.7997	3.5230	4.9239	3.3382	3.9575	3.9437	3.5923	3.4900
Arhgap5	_HRGSEEDPLLS[Phospho (STY)]PVETWK_.3	#N/A	4.5634	5.5439	4.1157	4.3875	3.3602	4.3202	4.7286	3.3936	4.3837	4.6543	3.9653	4.0904	3.5705	6.3653	4.4724	4.4180	4.5646	6.6674	4.3105	4.5064
Lyst	_QPEPFS[Phospho (STY)]PR_.2	P97412_S375_M1_Lyst	7.0644	7.4165	7.0729	7.2420	6.2519	7.2367	6.8838	7.3406	7.4038	6.9827	7.2544	7.0248	6.9011	6.1864	4.7317	6.5257	7.8254	7.8448	7.2057	6.8299
Lyst	_RM[Oxidation (M)]S[Phospho (STY)]QEHPSQASEAELAQR_.3	P97412_S2614_M1_Lyst	5.7062	5.1450	3.6325	3.7191	3.9347	3.9197	3.6063	5.1398	4.4523	4.4071	3.9309	3.2952	4.6709	3.6196	3.1835	3.4411	4.3173	4.2625	4.6356	3.0485
Lyst	_STANHDSES[Phospho (STY)]PVHS[Phospho (STY)]PSAHR_.3	P97412_S2560_M2_Lyst	6.4591	6.0634	6.4073	3.7117	3.3155	3.1686	5.7682	3.6781	5.5985	5.6229	5.0107	4.4077	5.6413	4.5011	3.9382	4.5698	5.1304	5.5657	3.2660	4.6776
Lyst	_MFLAYQDQS[Phospho (STY)]FDIPDR_.2	P97412_S3251_M1_Lyst	3.2870	4.0321	4.3244	4.2973	3.4939	3.5272	3.5542	3.5126	3.4744	3.0975	2.9531	3.7583	3.6399	4.3624	3.5796	4.1499	4.5107	4.1396	4.4386	4.1095
Crmp1	_KS[Phospho (STY)]IPHITSDR_.2	#N/A	10.0561	10.2586	9.8868	10.1493	9.8115	10.0213	9.6906	10.0605	9.7793	10.1321	8.6119	8.8417	9.3180	9.4027	8.5119	8.9697	9.0498	8.7448	8.6242	9.0249
Crmp1	_DNFTLIPEGVNGIEER_.2	#N/A	10.4358	10.6129	10.7675	10.6483	10.2213	10.4854	10.3908	10.4402	10.6098	10.7112	10.0419	9.9002	10.1663	10.3808	10.0443	9.9851	10.1105	9.7609	9.8883	9.8815
Crmp1	_VFGLHSVSR_.2	#N/A	8.7699	9.0513	8.5345	8.5032	8.3867	8.6509	8.8135	9.3288	8.3149	8.7354	7.8248	8.3976	8.2212	8.9511	7.7667	7.7258	8.4327	8.2196	7.9487	7.8657
Crmp1	_SAADIIALAR_.2	#N/A	9.7510	9.6660	9.4095	9.6904	9.7258	9.6660	9.6786	9.9045	9.4165	9.8133	8.9830	9.3385	9.3868	9.0428	8.9738	9.1572	9.1324	9.1599	9.0680	9.2636
Crmp1	_IFNLYPR_.2	#N/A	9.7505	9.4083	9.3730	9.1353	9.3399	9.5635	9.4116	9.8278	9.0377	9.8436	8.9095	8.5427	8.9871	8.6553	8.9967	8.9311	8.7333	8.6026	8.7038	8.9358
Crmp1	_INC[Carbamidomethyl (C)]PVYITK_.2	#N/A	9.0797	9.2028	9.0809	8.7569	9.0730	8.9603	9.1423	9.0000	8.8850	9.1362	8.2983	8.2963	8.2704	8.5713	8.8304	8.1394	8.4496	8.2884	8.3890	8.3330
Crmp1	_IVFEDGNISVSK_.2	#N/A	8.0266	8.1432	8.4497	8.1979	7.9705	8.1429	8.1630	8.3185	8.3183	8.4221	7.4210	7.3470	7.6143	7.9310	7.7468	7.5616	7.5495	7.4022	7.6793	7.7790
Crmp1	_KPFPEHLYQR_.3	#N/A	7.5215	8.3804	8.2390	8.0955	8.1260	8.3120	8.4151	7.6709	8.0610	8.2814	7.5081	7.7294	7.4081	7.6836	8.0915	7.5148	6.5489	7.3398	6.9225	7.5119
Crmp1	_AALAGGTTM[Oxidation (M)]IIDHVVPEPGSSLLTSFEK_.3	#N/A	7.2145	6.6827	6.8680	6.8960	6.7753	7.2442	6.2719	6.7497	6.5342	6.9785	6.6830	6.1168	6.6548	6.5302	6.3306	6.2829	7.0555	6.0698	6.1295	6.7197
Crmp1	_WHEAADTK_.2	#N/A	6.1790	6.5435	6.4736	6.2303	6.5207	6.2047	6.5525	6.8095	6.6608	6.8291	5.8814	6.0330	5.9332	6.1030	6.6748	5.7928	6.1668	6.1883	5.0385	5.6245
Crmp1	_DLYQM[Oxidation (M)]SDSQLYEAFTFLK_.2	#N/A	6.5765	6.7117	6.7316	6.5859	5.1932	6.0131	5.9685	7.2751	6.6390	7.1073	5.1643	5.5835	7.0603	7.4175	6.5258	6.4438	6.3787	5.4434	5.7549	6.0614
Crmp1	_IINDDQSFYADVYLEDGLIK_.2	#N/A	5.8385	6.7375	6.5432	6.0310	5.7208	5.9672	6.2046	6.4690	6.3176	6.5350	5.3262	5.5569	6.6141	6.3273	5.4317	5.8325	5.9894	5.7590	5.2264	5.9808
Crmp1	_STVEYNIFEGM[Oxidation (M)]EC[Carbamidomethyl (C)]HGSPLVVISQGK_.3	#N/A	6.3411	5.8476	5.4578	6.5415	5.4321	5.9171	5.9238	6.1982	5.8489	6.3362	5.3831	5.4664	6.3984	5.8020	5.4519	5.0151	5.9022	4.9585	5.3064	4.9213
Crmp1	_HAAPAPS[Phospho (STY)]AK_.2	#N/A	10.8247	10.9994	11.0408	11.1070	10.7528	10.7842	10.7943	10.6876	10.6791	10.9656	10.0159	9.7346	10.0818	10.4110	8.2766	9.4259	9.9768	9.8784	9.5853	10.0219
Crmp1	_S[Phospho (STY)]IPHITSDR_.2	#N/A	15.1623	15.1835	15.4146	15.3365	14.6691	14.8702	14.8321	15.1494	14.9418	15.2700	14.2992	14.3009	15.0906	14.8861	14.0473	14.3946	14.7031	14.1203	14.3331	14.5331
Crmp1	_GM[Oxidation (M)]YDGPVYEVPAT[Phospho (STY)]PK_.2	#N/A	13.5875	13.6309	13.8102	13.7207	13.3199	13.4067	13.1819	13.6682	13.4513	13.8627	12.7114	12.6104	13.3981	12.9585	12.2048	12.7247	13.0658	12.4666	12.7455	12.8066
Crmp1	_HAAPAPS[Phospho (STY)]AKSS[Phospho (STY)]PSK_.2	#N/A	9.5554	9.8709	9.2377	10.3860	9.8682	9.9492	9.8804	9.9027	9.4265	10.2841	6.7999	9.0051	9.2933	9.4507	8.1788	8.7376	9.2542	8.9372	9.0651	8.8554
Crmp1	_HAAPAPSAKS[Phospho (STY)]S[Phospho (STY)]PSKHQPPPIR_.4	#N/A	12.8301	13.1027	13.1888	13.2261	12.5049	12.5375	12.8345	12.8708	12.9251	13.1852	11.5768	11.8135	12.6491	12.2871	11.9154	12.0077	11.7981	11.9232	12.0297	11.9117
Crmp1	_M[Oxidation (M)]TVVWDK_.2	#N/A	4.2850	6.2255	5.1299	7.7423	6.8009	6.6367	5.1773	4.0951	6.3308	6.5163	4.3034	5.2446	4.5332	4.7928	6.2301	4.1106	4.9244	4.2098	5.3015	6.2221
Crmp1	_IVAPPGGRS[Phospho (STY)]NITS[Phospho (STY)]LG_.2	P97427_S570_M2_Crmp1	3.9116	10.4069	3.8864	3.9428	3.5740	3.7162	3.7320	3.7861	9.5560	3.9695	3.7689	3.6857	3.8614	8.7685	3.5370	3.7058	3.5913	3.5888	4.0057	8.1265
Crmp1	_SS[Phospho (STY)]PSKHQPPPIR_.2	#N/A	12.9479	13.2936	12.6734	3.7685	12.6578	12.3888	3.7124	4.0744	12.4061	12.9340	9.6916	12.4700	13.1203	3.4233	12.0779	12.4437	12.8469	12.3251	3.9098	12.2596
Crmp1	_EELEVLVQDK_.2	#N/A	3.9113	4.0915	6.6634	5.2360	5.7318	5.9632	3.8464	4.6537	6.4726	5.6209	3.2452	3.9809	3.9320	4.4347	4.8784	4.3850	4.1494	3.6838	5.0467	5.5175
Crmp1	_GLGAVILVHAENGDLIAQEQK_.3	#N/A	5.0945	5.6776	4.8599	5.5282	5.4374	5.2044	4.9628	5.6542	5.1326	5.3079	4.2261	4.3242	4.2527	3.7965	3.7126	4.5617	3.9448	4.4404	4.1834	4.7355
Crmp1	_NLHQSNFSLS[Phospho (STY)]GAQIDDNNPR_.3	#N/A	8.8126	8.8288	8.5939	8.1489	6.7473	7.8780	8.3771	7.9944	7.4421	8.8125	7.8955	7.4499	8.0016	8.9044	6.7004	7.3928	7.4868	7.8614	7.3966	7.6027
Crmp1	_NLHQSNFSLSGAQIDDNNPRR_.4	#N/A	5.3261	4.6044	5.5704	5.7660	6.0476	5.4235	5.6901	5.0292	5.3993	6.4490	5.2969	5.1494	6.2132	5.7342	5.7286	5.3832	4.6841	4.9164	4.9713	5.2460
Crmp1	_HQPPPIR_.2	#N/A	3.2685	7.4792	3.8048	1.9891	6.8207	7.7060	6.9675	4.4697	5.8347	7.4150	4.1033	6.7338	3.2892	6.4093	3.2290	3.8359	3.5066	6.0422	3.5469	3.7589
Crmp1	_IAVGSDADVVIWDPDKM[Oxidation (M)]K_.2	#N/A	4.9423	5.1547	4.8775	5.4281	5.1151	4.7725	5.3154	4.8357	5.1938	5.4793	4.8236	4.7917	4.3819	4.7862	4.5268	4.2653	4.7379	4.9943	5.0102	5.0734
Crmp1	_ILEM[Oxidation (M)]GITGPEGHALSRPEELEAEAVFR_.4	#N/A	5.0779	5.0579	5.0186	4.6155	4.8242	4.8722	4.3462	5.0755	4.3151	5.0793	3.8950	4.0766	3.9659	3.6925	4.6036	4.7018	4.1033	3.9538	4.4719	4.8149
Crmp1	_[Acetyl (Protein N-term)]S[Phospho (STY)]HQGKKS[Phospho (STY)]IPHITSDR_.3	P97427_S2_M2_Crmp1	4.5893	5.3678	3.9870	5.1835	3.1565	4.8377	4.7942	4.8628	3.6145	3.9868	3.4622	4.6786	5.2336	3.7836	4.0976	3.8126	4.3773	4.0752	3.0880	4.0522
Mprip	_SKS[Phospho (STY)]NPDFLK_.2	#N/A	3.1020	6.5483	6.7976	4.5874	3.9406	5.3436	5.8737	4.3033	3.6596	3.3084	4.0339	3.2894	3.7676	5.8282	3.1777	5.1418	6.1987	3.6898	5.9672	2.9887
Mprip	_SKS[Phospho (STY)]NPDFLKK_.2	#N/A	6.8193	6.7586	4.2221	6.8085	6.2223	6.1254	6.3320	6.2430	6.0335	7.2580	4.1593	6.2023	4.1343	6.7005	5.3500	6.2338	6.4138	6.2716	5.9675	6.5007
Mprip	_AEEQLPPLLS[Phospho (STY)]PPS[Phospho (STY)]PSTPHSR_.3	P97434_S289_M2_Mprip	3.2955	8.7382	9.1920	4.2887	3.5024	8.4865	9.5047	3.5212	8.6357	3.1061	2.9445	3.7497	3.6313	4.3710	3.5710	4.1585	4.5193	9.7467	4.4301	7.5129
Mprip	_DFASEAPTAPLSDAC[Carbamidomethyl (C)]PLS[Phospho (STY)]PHRR_.3	P97434_S364_M1_Mprip	3.1231	3.3788	3.6594	4.5663	3.9617	3.0470	3.8463	4.3243	3.6385	3.3294	4.0129	3.2683	3.7465	3.2903	3.1566	3.4680	3.3612	3.7109	4.0099	2.9676
Mprip	_AATEALGEKS[Phospho (STY)]PEGTTVSGYDIM[Oxidation (M)]K_.3	P97434_S976_M1_Mprip	8.2916	3.4772	3.5611	8.5305	3.8633	4.0874	3.6777	7.8710	3.7368	3.2311	4.1112	7.5057	3.8449	9.5727	3.2549	3.3697	3.2628	7.6257	3.9116	7.3121
Mprip	_DFASEAPTAPLS[Phospho (STY)]DAC[Carbamidomethyl (C)]PLSPHR_.3	P97434_S358_M1_Mprip	3.6229	4.3799	4.1592	4.0665	2.9606	3.5235	3.5579	3.3232	3.4781	3.0938	2.9568	4.2694	3.6436	4.1462	3.5833	3.9678	3.8609	1.6651	3.6208	4.1132
Kcnk2	_RLS[Phospho (STY)]VEIYDK_.2	P97438_S348_M1_Kcnk2	5.7481	3.4320	5.4918	5.4300	3.9085	5.5403	3.6325	4.2712	3.6916	3.2763	4.9377	4.3577	3.7997	3.5934	3.2097	3.4149	3.3080	3.6577	4.7482	5.7761
Slc30a3	_DPVPQSGLS[Phospho (STY)]PER_.2	P97441_S66_M1_Slc30a3	3.2913	4.0278	4.3287	9.4781	3.4982	3.1093	8.8878	9.6128	9.0544	9.0495	3.5885	3.7539	9.5428	4.3667	4.2330	4.1542	9.7644	9.1180	9.1675	4.0440
Slc30a3	_DRSS[Phospho (STY)]AGGGLR_.2	P97441_S23_M1_Slc30a3	4.3943	4.4256	3.9309	5.4340	3.1004	3.5071	3.9477	3.1192	3.8679	4.2049	3.3465	4.1517	4.4252	3.9690	3.9730	3.7564	4.1172	3.0322	3.3313	3.6463
Slc30a3	_SLFTEPS[Phospho (STY)]EPLPEEPK_.2	P97441_S38_M1_Slc30a3	3.4444	3.8746	4.4819	4.1398	3.6514	4.4570	3.3082	3.6701	3.3673	3.6007	3.7416	3.6008	3.4753	4.5199	2.8854	4.3074	3.1674	3.9821	4.2811	4.1972
Cacna1a	_ESPS[Phospho (STY)]WVTQR_.2	P97445_S1981_M1_Cacna1a	10.6588	10.1499	10.0674	3.6671	9.3708	3.1240	3.9574	3.7227	9.2047	10.2064	10.0669	9.4588	4.0430	4.5457	3.9828	3.7467	9.8752	3.5728	3.2214	9.3994
Cacna1a	_RAWPGS[Phospho (STY)]PER_.2	#N/A	9.2245	8.9549	8.3140	8.7656	8.2747	8.8392	8.6975	8.6197	8.4434	8.4558	8.7785	8.5363	8.6225	8.5133	7.0769	8.0431	8.9789	8.9157	8.4165	8.2761
Cacna1a	_WSRS[Phospho (STY)]PSEGR_.2	P97445_S2200_M1_Cacna1a	3.8639	4.1389	4.4002	7.5491	3.2016	3.2825	3.7989	3.5643	7.5421	4.3536	3.1978	7.0795	7.7441	7.2428	3.8243	3.9052	8.0017	3.7312	3.3798	4.3543
Cacna1a	_M[Oxidation (M)]AGPAAPPGGS[Phospho (STY)]PR_.2	#N/A	10.8862	10.6598	10.7997	10.8251	10.2977	10.2841	10.5922	10.7613	10.3269	10.1374	10.7088	10.6643	11.1302	10.7574	9.5057	10.3418	11.1567	10.9243	10.5058	10.5067
Cacna1a	_KQNLLAS[Phospho (STY)]R_.2	#N/A	11.5435	11.4316	10.9670	11.1373	10.8762	11.2476	3.7583	11.2442	11.0588	10.9641	11.1843	10.6949	11.2456	11.0268	9.6346	10.6361	11.4443	11.2676	11.1005	10.7528
Cacna1a	_PLVSYS[Phospho (STY)]PAPR_.2	P97445_S2252_M1_Cacna1a	4.1362	3.1829	3.6728	3.4480	4.3432	5.8062	4.0000	6.4245	4.0591	4.4097	6.8808	4.4098	4.1671	3.7109	4.2311	6.1428	3.8591	3.2903	3.5893	3.3882

Cacna1a	_AWPGS[Phospho (STY)]PER_.2	#N/A	3.1419	5.6651	4.8835	5.8562	5.3502	5.5644	5.4692	3.4645	4.4967	5.1650	6.9782	6.1033	3.7849	4.2875	3.7246	5.8340	6.8376	6.9538	6.8256	7.1097
Cacna1a	_KQNLLAS[Phospho (STY)]REALYGDAAER_.3	#N/A	3.9655	5.3224	3.5021	4.1806	4.1725	4.2058	4.3765	4.4756	3.8033	3.7761	3.7753	3.3583	5.4904	3.4639	2.9010	3.3276	3.6884	3.8674	4.3105	3.4309
Cacna1a	_GADYTEPDS[Phospho (STY)]PREPPGGAHDPAPR_.3	P97445_S2303_M1_Cacna1a	3.2581	4.0610	4.2955	4.3261	3.4650	3.1425	3.1218	3.4838	3.1809	3.7870	3.5553	3.7871	6.2297	4.3336	4.1999	3.9256	4.4819	4.1684	2.9666	4.0109
Cacna1a	_GADYTEPDSPREPPGGAHDPAPRS[Phospho (STY)]PR_.4	P97445_S2318_M1_Cacna1a	3.1795	6.7115	4.2169	6.3238	5.7302	3.4198	6.2308	3.4269	3.5818	4.4910	5.7259	6.0129	6.7287	4.2499	3.6870	4.0425	6.1385	5.7887	3.5171	4.2169
Cacna1a	_EEQNRT[Phospho (STY)]PLM[Oxidation (M)]FQR_.3	#N/A	4.6675	4.0364	3.5744	5.1579	3.3102	3.9957	4.6075	4.8427	4.6716	4.6551	4.9846	3.5668	4.1870	3.9007	3.8887	3.8625	4.0828	4.4862	3.7242	3.7287
Cacna1a	_GNDLSTISDTS[Phospho (STY)]PM[Oxidation (M)]KR_.2	#N/A	4.1291	6.6931	4.1228	3.6182	3.1041	3.4429	4.0199	3.2980	6.0771	4.3161	3.1500	4.1390	4.1562	6.1362	3.7943	5.6168	4.0662	3.5017	3.4099	5.7664
Cacna1a	_QGSSSVSGS[Phospho (STY)]PAPSTSGTSTPR_.2	P97445_S2220_M1_Cacna1a	4.3163	3.6542	3.3918	3.3407	3.4042	4.2986	3.9805	4.0567	4.2116	3.6224	3.6902	3.5436	4.3770	3.5781	3.8321	3.4159	3.9775	3.2464	3.7813	3.3458
Cacna1a	_AS[Phospho (STY)]IKS[Phospho (STY)]AKLENSTFFHK_.3	P97445_S459_M2_Cacna1a	3.9723	4.5372	4.5086	3.7170	3.3101	2.9807	3.6552	3.6727	3.8276	3.5234	3.3063	3.9199	3.9931	3.8381	3.8082	4.3172	4.2104	2.5221	3.2713	2.9619
Cacna1a	_EVAEVS[Phospho (STY)]PLS[Phospho (STY)]AANM[Oxidation (M)]SIAVK_.2	#N/A	3.8619	4.1556	4.3982	3.8422	3.1997	3.3747	4.0615	3.5623	4.1720	4.0683	3.2675	4.2497	3.7561	4.4007	3.9334	6.3792	4.1000	7.0506	3.2221	3.8666
Cacna1a	_RMAGPAAPPGGS[Phospho (STY)]PR_.2	#N/A	3.8889	3.4301	3.4255	4.5322	4.0959	4.1292	4.0964	4.1146	4.3733	3.6995	3.8519	3.1563	4.5387	3.4636	2.9776	4.9634	3.5971	3.5376	3.8366	3.1409
Cacna1a	_AKEVAEVS[Phospho (STY)]PLS[Phospho (STY)]AANM[Oxidation (M)]SIAVK_.3	#N/A	3.9090	3.8660	3.9168	3.9097	3.5854	3.8285	3.5348	3.7992	3.7862	4.2211	3.6259	3.6647	3.9330	3.9374	3.8698	3.6019	3.9530	4.3668	3.8467	3.7194
Cacna1a	_WSRS[Phospho (STY)]PS[Phospho (STY)]EGREHTTHR_.4	P97445_S2200_M2_Cacna1a	3.6629	4.3399	4.1992	4.0265	3.0006	3.4835	3.0396	3.3633	3.5182	4.5546	2.9968	4.2294	3.6836	4.1863	4.1177	4.0078	3.9010	3.9322	3.5808	4.1533
Cacna1a	_EM[Oxidation (M)]GTDGYSDSEHY[Phospho (STY)]LPMEGQTR_.3	P97445_Y2033_M1_Cacna1a	4.3716	3.6312	3.4071	3.3178	3.7093	4.2756	4.3067	4.0720	4.2269	3.8459	3.7055	3.5207	4.3923	3.3941	2.8312	3.2157	3.1088	3.2235	4.3729	3.3611
Atp5pf	_FEVIDKPQS_.2	#N/A	6.1404	6.0133	6.7702	5.8057	6.4026	6.5858	5.3198	6.2356	6.6173	6.2949	6.1936	6.0072	6.6729	6.7163	7.7624	6.1921	6.3930	6.1423	6.4912	6.2403
Atp5pf	_QASGGPVDIGPEYQQDLDRELYK_.3	#N/A	6.7517	6.3630	6.7939	6.9591	6.3893	6.3576	6.1017	6.8312	6.8730	6.6942	6.0705	6.8553	7.2927	7.4237	7.9512	6.8592	6.5075	6.3926	6.9844	6.5748
Atp5pf	_QASGGPVDIGPEYQQDLDR_.2	#N/A	4.7881	4.0834	5.6166	4.9164	4.9744	4.4959	4.3970	4.9346	5.5845	5.5495	5.0203	4.9888	4.5141	5.7958	6.0435	4.6408	4.8999	5.0015	4.6560	5.3224
Atp5pf	_GEM[Oxidation (M)]DTFPTFK_.2	#N/A	7.8302	7.5891	7.6638	7.8589	7.7269	7.4861	7.8466	7.8690	7.9908	7.7818	7.4315	7.9780	8.1582	8.2560	8.7849	8.1845	7.9675	8.0191	8.1709	7.9371
Atp5pf	_FDDPKFEVIDKPQS_.2	#N/A	8.1159	7.6478	8.0300	7.7693	7.6831	7.5699	7.4092	7.4092	8.2116	8.0325	7.9029	7.9254	8.4286	8.0642	8.6228	8.4740	8.2561	7.7654	8.2228	8.0866
Rps5	_VNQAIWLLC[Carbamidomethyl (C)]TGAR_.2	#N/A	5.8220	5.7836	5.4395	5.7018	4.9030	5.5748	5.1335	5.0661	5.2530	5.3455	5.3864	4.7983	4.9606	5.4712	4.5706	4.7963	5.9503	5.1994	5.4712	4.7201
Rps5	_GSSNSYAIK_.2	#N/A	4.8640	4.0519	4.6454	4.8793	3.5912	4.7927	4.6269	4.6923	4.8769	3.2228	4.8613	4.2097	3.9882	4.0117	5.0618	3.6467	4.2477	4.3784	4.4943	4.2234
Rps5	_WSTDDVQINDISLQDYIAVK_.2	#N/A	3.2129	2.0552	2.6108	3.8291	2.9176	4.0506	2.4627	4.0970	3.4868	2.8647	0.1554	2.5670	3.0085	3.0074	3.2917	3.8322	3.1128	2.2188	3.3841	2.4721
Rgs14	_PLREPGSSHLGS[Phospho (STY)]PDTAR_.3	#N/A	9.0165	9.1932	8.8585	9.0219	8.3849	8.7462	8.6211	8.4450	8.4101	8.9045	8.8084	8.8254	8.8120	9.4253	8.0331	8.7291	8.8924	8.8669	8.7772	8.7301
Rgs14	_EPGSSHLGS[Phospho (STY)]PDTAR_.2	#N/A	9.1461	8.7776	3.9152	9.1689	7.5203	3.1180	8.4499	7.8363	8.6279	9.0373	9.0755	8.4235	4.0491	9.3059	7.9754	8.8346	8.9990	8.8139	8.8783	8.6755
Rgs14	_S[Phospho (STY)]LGSGESESESRPGK_.2	#N/A	9.1187	9.0227	9.0693	9.1548	8.3996	8.6756	8.5131	8.9779	8.3777	9.0518	8.5244	8.8587	8.8862	9.2925	8.4331	8.9270	8.9733	8.8250	8.7418	8.9705
Rgs14	_S[Phospho (STY)]LPLGVEELGQLPLAEGPC[Carbamidomethyl (C)]GR_.2	#N/A	5.5417	6.1968	5.2187	5.0530	4.0383	3.7736	6.1058	5.7247	4.4445	5.4701	3.9897	5.8513	5.3905	7.2271	3.7852	3.9197	4.6194	5.6942	5.3317	4.4768
Rgs14	_SPLYQEC[Carbamidomethyl (C)]LLAEAEGRPLREPGSSHLGS[Phospho (STY)]PDTAR_.4	#N/A	3.9834	3.3357	3.5200	5.4795	4.1903	4.2237	4.3586	4.2091	4.2788	3.7940	3.7574	3.0618	4.4442	3.5581	2.8831	3.3455	3.7063	3.1716	3.7422	3.4131
Rgs14	_GLS[Phospho (STY)]LPDIK_.2	P97492_S339_M1_Rgs14	4.2481	3.7547	3.7461	3.8422	3.5858	3.8099	3.9552	3.9485	3.3220	4.4544	3.5717	3.0149	4.1224	3.2707	2.3612	1.8027	4.4862	3.3470	3.2678	3.7109
Rgs14	_S[Phospho (STY)]LGSGESESESR_.2	#N/A	5.9536	5.8701	6.3971	6.2931	4.5173	5.4526	5.7398	5.6681	5.6533	6.4946	5.9181	5.4897	5.5576	6.8144	5.7326	5.8235	5.7888	5.3794	5.9152	5.7664
Gclc	_GLLSQGS[Phospho (STY)]PLSWEETQR_.2	P97494_S8_M1_Gclc	6.5319	6.3764	4.4899	4.1317	5.7037	3.6928	6.5030	6.0467	4.9578	5.7549	6.0143	6.0788	6.4694	6.4479	5.0487	4.3155	3.1754	6.1569	6.0392	3.9439
Smarcc1	_RKPS[Phospho (STY)]PS[Phospho (STY)]PPPPTATESR_.3	#N/A	7.3266	7.4942	7.4605	7.6389	7.4305	7.2898	7.1050	6.9675	7.2508	7.6098	7.4468	7.2225	7.4477	7.8685	7.3010	7.3467	7.5328	7.4219	7.2419	7.7388
Smarcc1	_NEEPVRS[Phospho (STY)]PERR_.2	#N/A	3.4894	3.8460	3.8883	3.6525	3.4737	3.0715	3.6282	3.1961	4.0524	3.0042	4.1469	3.5952	4.1745	3.4771	3.3231	3.7138	3.9347	3.7726	4.2718	4.0232
Myt1l	_LEEQLS[Phospho (STY)]PR_.2	P97500_S391_M1_Myt1l	8.2023	8.5346	7.9963	8.0395	8.1433	8.2322	7.9672	8.3761	7.9794	8.2676	8.1676	8.2189	8.4195	8.5354	7.7626	8.0218	8.6754	8.1994	8.4718	8.1315
Myt1l	_KLS[Phospho (STY)]ETNPQDR_.2	P97500_S343_M1_Myt1l	3.2091	4.1100	4.2465	4.3751	3.4160	3.1915	3.0728	3.4348	3.1319	3.8360	3.5063	3.8361	3.2400	4.2846	4.1508	4.0720	4.9809	4.2175	3.0157	3.9618
Myt1l	Oxidation (M)]VEES[Phospho (STY)]DEEVC[Carbamidomethyl (C)]LSSLEC[Carbamidomethyl (C)]	P97500_S319_M1_Myt1l	4.3534	4.4665	3.8900	3.6358	3.0595	3.0928	3.9886	3.7539	3.9088	4.1640	3.3875	4.1926	4.0743	4.5769	4.0140	3.7155	4.2916	3.5416	3.1902	3.0431
Grm1	_SAFTTS[Phospho (STY)]DVVR_.2	P97772_S853_M1_Grm1	7.6120	8.0679	8.6818	8.2319	3.8243	4.0484	7.2776	4.0787	7.7225	8.1023	7.0892	7.4162	7.4082	3.4277	5.9818	7.0120	7.1058	6.9809	7.5574	7.0912
Mllt11	_APIASIHS[Phospho (STY)]VDLDLL_.2	P97783_S84_M1_Mllt11	3.5133	5.7600	3.9164	3.8591	3.6101	3.4778	3.4143	3.7563	4.0954	3.7197	3.7107	3.7389	5.9601	3.9199	3.6471	3.8582	3.7907	3.7335	3.7361	3.7988
Cxadr	_APQS[Phospho (STY)]PTLAPAK_.2	#N/A	10.2289	10.0270	10.1200	9.9272	3.6922	9.7442	9.9468	9.9056	3.9348	10.0361	7.1780	9.7065	9.9951	9.9510	8.8089	9.5039	10.1260	10.0116	7.9240	9.7069
Cxadr	_TQYNQVPSEDFERAPQS[Phospho (STY)]PTLAPAK_.3	#N/A	4.0338	7.0112	3.5843	5.9783	3.8325	5.3492	6.2820	4.2734	5.8372	4.0732	5.5837	3.8796	7.5412	7.0155	3.3325	5.8935	6.2758	6.4245	6.0885	5.0612
Sirpa	_HDQQPAITR_.2	#N/A	7.7231	7.5556	7.7715	7.3950	7.6973	7.7622	7.8402	7.5848	7.6873	7.8550	8.1015	7.4811	7.2810	7.4365	7.9494	7.7932	7.4261	7.8926	7.6302	7.8059
Sirpa	_LLIYSFAGEYVPR_.2	#N/A	5.8775	5.6398	5.8381	5.8445	5.9566	5.5949	5.8956	5.3108	5.7582	5.8795	6.0071	5.5216	5.8974	5.6295	5.2460	5.6036	5.5288	4.9704	5.3420	6.0228
Sirpa	_PSPPEVSGPADR_.2	#N/A	9.1833	9.1277	9.1736	9.0861	9.0805	9.1349	8.9965	8.9907	9.2369	8.8352	9.2522	8.9691	8.5954	9.1867	8.9974	8.9692	9.2208	9.0630	8.9284	9.2284
Sirpa	_NNM[Oxidation (M)]DFSIR_.2	#N/A	7.0709	7.1750	6.1279	6.8495	6.9042	6.9730	6.7085	7.5197	6.7743	6.9732	6.8115	6.8394	7.0675	6.8074	6.8961	6.7928	7.0848	7.1949	6.7954	6.9644
Sirpa	_GSTSS[Phospho (STY)]TRLHEPEK_.3	P97797_S409_M1_Sirpa	3.5395	5.8688	4.3400	4.0980	3.3626	4.0466	3.4374	3.5179	6.5720	3.3575	3.4189	5.6500	3.5652	4.3564	6.8473	4.1583	3.5511	3.9725	3.9743	5.9004
Sirpa	_DGQELHPLETTVNPSGK_.3	#N/A	4.5250	3.7374	3.9466	4.0470	3.7606	3.9330	4.2831	4.4163	4.3691	4.3118	4.7331	3.9020	3.8234	3.7523	3.2749	4.3789	3.8897	4.0748	3.7022	3.8178
Sirpa	_VIC[Carbamidomethyl (C)]EVAHITLDR_.2	#N/A	4.3688	4.0811	3.6910	4.0088	4.7463	4.5168	4.6214	5.3282	3.8986	4.5360	5.0645	4.2052	4.9213	3.8428	4.2122	4.0493	4.7226	3.1851	4.0283	4.4571
Sirpa	_VPRPEDTLTYADLDMVHLSR_.4	#N/A	4.2886	3.7142	3.3240	4.7094	3.6263	3.2737	3.9148	3.9889	3.9739	4.4949	4.0741	3.6037	4.0819	3.3111	3.4920	3.1326	4.5266	3.3755	3.6745	3.3030
Neo1	_NS[Phospho (STY)]QDITPVDNSM[Oxidation (M)]DSNIHQR_.3	#N/A	6.4221	6.6823	5.3409	6.6249	5.6352	6.3229	6.3835	5.4106	5.8984	6.7397	6.0072	5.4902	5.8131	6.4263	4.7617	5.8751	6.3179	6.1155	5.9197	6.0773
Smn1	_GTGQSDDS[Phospho (STY)]DIWDDTALIK_.2	P97801_S28_M1_Smn1	4.0839	4.1293	4.3729	3.6934	3.2913	4.5304	4.9779	3.7925	4.6773	3.9361	3.5834	3.9046	3.9647	4.2627	4.8894	3.9473	4.4497	4.8359	4.7884	3.6091
Smn1	_[Acetyl (Protein N-term)]AM[Oxidation (M)]GSGGAGS[Phospho (STY)]EQEDTVLFR_.2	#N/A	9.0201	8.9231	9.2399	8.9861	3.9348	8.8740	8.7092	8.3550	9.0261	9.1085	8.6201	8.4599	8.8152	9.2013	8.2569	9.0246	9.0799	8.6392	8.7939	8.7907
Smn1	_[Acetyl (Protein N-term)]M[Oxidation (M)]AM[Oxidation (M)]GSGGAGSEQEDTVLFR_.2	#N/A	4.2059	5.7519	3.7424	3.3784	2.9120	3.2706	4.1361	4.4315	5.9567	4.0164	3.5350	4.3402	4.6135	6.4252	4.1615	3.5680	3.9288	3.2207	3.5197	3.7686
Smn1	_RGT[Phospho (STY)]GQSDDSDIWDDTALIK_.3	P97801_T22_M1_Smn1	3.5347	4.2567	4.1984	4.2802	3.9695	3.0965	3.1798	3.5417	3.2389	3.7411	3.6132	3.5653	3.3469	4.3915	3.3744	3.8796	3.6563	4.1225	4.0177	4.0688
Fh	_SGLGELILPENEPGSSIM[Oxidation (M)]PGK_.2	#N/A	7.4428	7.3029	6.9733	7.0938	7.5182	7.4526	7.7578	8.2123	7.4233	7.5808	7.5246	7.8377	7.5359	7.6439	7.3229	7.6008	7.6184	7.6870	7.4948	7.5593
Fh	_IYELAAGGTAVGTGLNTR_.2	#N/A	7.3740	6.7615	7.8228	7.5448	7.3852	6.6659	6.6449	7.2638	7.3329	7.5589	7.4954	6.9945	7.5807	6.9216	7.5524	7.5294	7.5915	7.0835	7.2831	7.5165
Fh	_LHDALSAK_.2	#N/A	7.4951	7.1596	7.8876	6.3618	7.5887	6.7870	7.2317	7.4701	6.5288	7.4181	7.0449	7.2802	7.2866	7.5215	7.7973	6.3853	6.9547	7.1098	7.2054	7.6254
Fh	_AAAEVNQEYGLDPK_.2	#N/A	5.9721	3.1648	6.3227	6.4303	6.3032	6.1602	5.9091	6.3989	6.5349	6.0664	6.3550	6.3052	3.5325	7.1387	6.6331	6.0624	6.4208	6.0829	6.0468	6.2273
Fh	_YYGAQTVR_.2	#N/A	6.4780	7.3876	3.7272	6.9637	4.0294	7.4633	7.6517	4.3921	7.6721	7.3041	7.4167	7.3382	7.1114	7.4121	7.9841	7.1631	7.1929	7.4278	7.3185	7.7751
Fh	_VEFDTFGELK_.2	#N/A	4.6715	3.8994	5.7083	4.1645	4.2576	5.0595	4.8259	3.6454	5.1235	5.6734	5.3052	4.8659	3.4506	5.0037	5.4862	4.3663	3.1426	4.5907	4.7538	4.5987
Fh	_AIEM[Oxidation (M)]LGGELGSK_.2	#N/A	6.3107	7.0845	7.2142	7.1445	6.6595	6.7070	6.8887	6.7269	6.9592	6.3557	6.4659	6.9106	3.8423	7.1905	7.1539	6.8757	6.5707	6.8493	4.3793	6.8483
Fh	_MPIPVIQAFGILK_.2	#N/A	4.5543	5.6324	4.9266	6.1694	3.8866	4.7622	5.4872	4.2547	4.7202	3.2598	4.8573	4.4109	4.5750	5.0138	4.4817	5.9573	3.2915	3.6950	4.3960	5.5424
Fh	_ETAIELGYLTAEQFDEWVKPK_.3	#N/A	3.2088	3.4924	3.5258	3.5174	3.4157	3.4491	2.3811	3.3977	3.5526	4.5202	3.0312	2.4186	3.7180	3.6087	3.2698	4.0717	3.9354	3.8978	3.2822	2.1511
Fh	_LNDHFPLVVWQTGSGTQTNM[Oxidation (M)]NVNEVISNR_.3	#N/A	3.5787	4.4402	4.1150	4.1268	3.7792	4.0466	3.4836	3.2791	3.4659	4.1238	3.4252	3.6916	3.6053	4.1021	3.3306	3.9236	3.8168	4.0325	3.6811	4.1938
Fh	_SQSSNDTFPTAM[Oxidation (M)]HIAAAVEVHK_.3	#N/A	3.4704	4.5324	4.0067	4.2159	5.7890	3.0322	4.6606	5.7490	3.2912	3.6767	3.6656	4.4218	3.3992	4.4438	5.9757	5.8743	3.0913	4.0582	4.3572	6.7144
Fh	_THTQDAVPLTLGQEFSGYVQQVQYAMVR_.3	#N/A	3.0437	3.9932	3.8653	3.8994	3.4231	3.4564	3.7214	3.4867	3.6416	3.7773	3.8708	3.7664	3.8071	3.5594	3.7468	4.0791	4.0244	3.9051	3.5537	3.5264
Map4k4	_AASS[Phospho (STY)]PNLSNGETESVK_.2	P97820_S795_M1_Map4k4	7.9965	7.2122	6.9010	7.0831	6.7504	4.4024	6.9849	7.1438	4.1001	3.9728	6.9326	3.6475	7.3923	7.5279	4.2052	3.5243	7.5500	7.2747	6.6348	6.7289
Map4k4	_TTSRS[Phospho (STY)]PVLSR_.2	P97820_S621_M1_Map4k4	7.1821	7.4405	7.0900	7.4165	7.6861	7.1162	5.9835	7.8522	6.9764	6.7927	7.0017	7.0515	3.9053	7.7221	5.8892	7.5043	7.8711	7.5144	3.5086	7.4698
Map4k4	_RDS[Phospho (STY)]PLQGGGQQNSQAGQR_.3	#N/A	5.1138	4.3626	4.4441	4.9810	4.3550	4.2992	4.5345	4.1724	3.5815	4.6344	4.8566	3.8070	3.8837	4.2758	3.1146	4.2630	3.3727	4.3785	3.9779	4.5366
Map4k4	_SSS[Phospho (STY)]KSEGSPSPR_.2	P97820_S701_M1_Map4k4	5.8810	6.1644	5.5298	4.0561	3.8410	5.5592	5.4390	4.2036	3.9010	3.6720	5.2455	4.4592	5.2002	5.3531	3.3013	3.3473	4.0865	5.5677	4.2653	4.9516
Map4k4	_DSPLQGGGQQNSQAGQR_.2	#N/A	4.1790	3.1401	3.7156	3.4052	2.8851	4.8525	4.0427	4.4047	4.1018	4.3670	4.3139	4.3671	4.2099	3.7536	5.3303	3.5411	3.9019	3.2475	3.5466	3.4309
Map4k4	_S[Phospho (STY)]EGSPSPR_.2	P97820_S703_M1_Map4k4	3.5675	4.4353	4.1038	4.3129	2.9052	3.6094	3.1350	3.4970	3.1941	3.7738	3.5685	4.3247	3.3022	4.3468	4.2130	3.9124	4.4950	4.1552	2.9535	4.0240
Jpt1	_SSGGREDSES[Phospho (STY)]PGTQR_.2	#N/A	6.7848	6.9914	7.1410	6.7577	6.5150	6.6494	6.6547	6.8736	6.7100	7.1651	6.1660	6.4979	7.0123	6.9047	7.2945	7.2768	6.4408	6.3148	6.8234	6.4435
Jpt1	_SNS[Phospho (STY)]SEASSGDFLDLK_.2	P97825_S87_M1_Jpt1	3.5865	9.4300	10.2636	4.0910	3.3787	3.5480	4.0390	3.3097	3.1907	3.6288	3.9299	4.2939	3.6300	9.8627	3.7772	3.6518	4.3648	8.9723	3.6453	4.0997
G3bp1	_STS[Phospho (STY)]PAPADVAPAQEDLR_.2	P97855_S231_M1_G3bp1	12.8407	12.8278	12.9938	12.9316	12.4717	12.4574	12.2333	12.8106	12.4558	12.9338	12.4235	12.5946	13.3793	13.0242	12.2925	12.5984	12.9161	12.3012	12.5081	12.6240
G3bp1	_YQDEVFGGFVTEPQEES[Phospho (STY)]EEEVEEPEER_.3	P97855_S149_M1_G3bp1	4.5622	4.2577	4.0988	4.5228	3.2683	3.3392	3.7798	3.2871	2.9842	4.3728	3.3586	3.9838	4.5931	4.1369	3.8051	3.9243	4.2851	4.3651	3.1634	3.8142
Nfib	_KPEKPLFSSTS[Phospho (STY)]PQDSSPR_.3	P97863_S328_M1_Nfib	7.8987	7.9117	7.7303	7.8259	6.7872	7.6362	7.0722	7.5449	7.2081	3.5341	3.9285	6.9950	7.7705	7.7351	6.1883	3.3697	7.2388	7.1794	3.9179	6.7004
Rbbp6	_VEGTEIVKPS[Phospho (STY)]PK_.2	P97868_S1179_M1_Rbbp6	7.4899	7.4979	6.7752	7.3753	6.8139	7.3085	7.1960	7.5019	7.0352	6.9410	7.0292	6.5314	7.5077	6.9802	6.1031	6.9036	7.8442	7.5018	6.6700	7.0433
Rbbp6	_SRS[Phospho (STY)]PQAFR_.2	P97868_S773_M1_Rbbp6	6.2650	6.3882	6.4095	6.3066	5.3513	6.3350	6.5633	7.1897	3.3688	3.4081	6.1973	6.0464	6.6553	6.2300	3.1866	4.0497	6.4043	6.2736	3.9083	6.0355
Rbbp6	_YHS[Phospho (STY)]RS[Phospho (STY)]RS[Phospho (STY)]PQAFR_.3	P97868_S769_M3_Rbbp6	3.9227	4.0802	5.2644	5.4707	5.0985	3.2237	5.0574	3.6230	3.7779	5.8801	3.2566	3.9696	3.9434	4.4460	3.8831	5.3529	5.1649	5.2524	5.5611	5.4945
Rbbp6	_WDKDDFES[Phospho (STY)]EEEDVK_.2	P97868_S1329_M1_Rbbp6	3.7503	5.2474	5.6303	3.9391	3.0880	4.6883	4.4673	3.4507	4.1941	4.4672	3.0842	4.1420	3.7710	5.1249	3.7107	4.0188	3.9883	4.5098	4.8377	4.9685
Rbbp6	_PSANREDFS[Phospho (STY)]PER_.2	#N/A	7.2142	3.8641	3.9570	3.7731	6.3479	6.8708	6.9607	7.1343	6.8047	6.9211	3.5701	6.7322	7.1384	7.3482	3.5681	6.5540	7.0318	3.5719	7.0281	6.5228
Rbbp6	_VDGDREKS[Phospho (STY)]PR_.2	P97868_S1053_M1_Rbbp6	5.0973	4.4485	3.9080	3.2128	3.0775	3.4606	3.9389	4.9658	4.2942	4.1746	3.4186	3.8243	4.1375	3.9460	3.9960	3.7335	4.0943	3.0552	3.3542	4.1372
Rab33a	_S[Phospho (STY)]MVEHYYR_.2	P97950_S101_M1_Rab33a	3.4827	4.5201	5.6265	5.3699	4.3213	3.0445	5.2879	3.1831	3.2789	3.6890	3.6533	4.4095	3.3869	4.0061	2.7970	3.8276	3.7208	4.1124	3.7610	3.9731
Apba2	_SAS[Phospho (STY)]QDC[Carbamidomethyl (C)]IETTPGAQEGK_.2	#N/A	9.9815	9.8696	10.5121	10.3492	9.2688	8.8782	9.6329	9.3968	9.0738	10.4096	9.5167	9.5472	9.4885	9.8774	8.1166	9.3707	9.5862	9.2349	9.2704	9.3821
Apba2	_SAS[Phospho (STY)]QDC[Carbamidomethyl (C)]IETTPGAQEGKK_.2	#N/A	3.7686	6.7550	3.7481	7.2512	3.8145	5.1368	6.1948	6.2104	3.8444	7.0338	3.5106	6.3973	6.2577	6.6923	3.3625	6.1924	6.2652	5.7453	3.7917	6.1663
C1qa	_DSLSFSNTNNK_.2	#N/A	8.9756	8.8982	9.2856	9.4576	8.9995	9.1350	8.8529	9.0472	9.0001	9.5113	8.6357	8.4892	9.0069	9.2683	8.4830	8.9588	9.0021	8.5977	8.9382	8.8198
Cds1	_GDS[Phospho (STY)]DVPEVPPSSDR_.2	P98191_S56_M1_Cds1	9.9112	8.7426	4.1609	9.5707	7.6053	9.1201	8.2585	9.2874	8.4382	3.0921	8.6781	9.5186	8.3466	4.1479	3.5850	8.8797	10.4525	9.6137	8.7186	7.8140
Atp8a2	_ATS[Phospho (STY)]VGDQLEAPAR_.2	P98200_S6_M1_Atp8a2	11.0570	11.1883	10.9504	10.7399	9.8928	10.8416	10.8746	10.8383	10.7583	10.6654	10.5174	10.4309	10.9711	10.4853	9.1287	10.1901	11.1434	10.8128	10.5175	10.3054
Atp8a2	_EQS[Phospho (STY)]SDDFC[Carbamidomethyl (C)]R_.2	P98200_S421_M1_Atp8a2	3.9518	7.3027	4.0763	3.9591	3.2459	3.2792	3.8040	6.3021	3.7242	4.3504	6.4698	6.7852	7.1006	4.1144	3.8294	3.9019	4.2627	7.2753	6.9231	3.7917
Atp8a2	_[Acetyl (Protein N-term)]S[Phospho (STY)]RATS[Phospho (STY)]VGDQLEAPAR_.2	P98200_S2_M2_Atp8a2	3.1129	3.5932	3.2625	3.8584	3.9515	4.1756	3.5896	3.9516	3.6487	3.3192	4.0231	3.3193	3.7567	3.3005	3.1668	3.4578	3.4488	3.7007	3.9997	2.9778
Atp8a2	Oxidation (M)]TSC[Carbamidomethyl (C)]TNDS[Phospho (STY)]C[Carbamidomethyl (C)]DFNDF	#N/A	5.0005	4.6217	4.5187	5.0337	4.7396	4.3382	4.6298	4.9226	4.6270	4.9957	4.1543	4.8288	4.9990	4.2025	4.2487	4.5258	5.2468	4.3683	4.2225	3.8443
Arvcf	_DIPSYGS[Phospho (STY)]LSR_.2	P98203_S205_M1_Arvcf	4.5295	8.4966	8.3576	8.4378	3.2356	7.9005	7.9712	8.0645	3.7327	4.3401	7.6061	4.0166	3.8982	8.2541	3.8379	3.8916	4.2524	4.3979	7.9740	3.7814
Arvcf	_GSLGS[Phospho (STY)]LDR_.2	P98203_S337_M1_Arvcf	4.0923	3.2267	5.7223	3.4919	6.2859	4.3326	4.2497	4.3180	4.1699	3.9029	3.6485	2.9529	4.1232	7.0425	4.2750	3.4545	3.8153	3.3342	3.6332	3.3443
Arvcf	_S[Phospho (STY)]LAADDEGGPDLEPDYSTATR_.2	P98203_S269_M1_Arvcf	6.4029	8.0795	8.6036	7.5843	3.0347	7.9718	7.3069	3.0535	7.6224	7.6899	5.4248	6.6556	6.1137	7.9445	6.1949	7.2185	5.8252	7.3540	7.8710	7.4516
Arvcf	_RSPS[Phospho (STY)]VDSTR_.2	P98203_S347_M1_Arvcf	5.0989	4.6902	5.1944	3.5053	3.9371	4.6144	5.1324	4.2610	4.1494	5.3551	5.1150	4.5577	5.4176	5.0992	3.2254	3.2423	3.7583	3.8242	3.6911	4.9539
Arvcf	_S[Phospho (STY)]LPEHFQAEPYGLEDDTR_.3	P98203_S251_M1_Arvcf	3.2183	6.1455	4.2557	4.3659	3.4252	3.1823	5.4291	3.4440	3.1411	3.8268	3.5155	3.8269	3.2492	5.7060	3.6482	4.0813	4.4421	5.8457	5.5109	5.0569
Arvcf	_S[Phospho (STY)]PSVDSTR_.2	#N/A	3.2327	6.7284	3.7690	4.4567	4.0713	6.2486	6.4955	4.4339	5.3937	6.7991	6.6869	3.1587	3.6369	6.5412	3.0470	3.5776	3.4708	6.3818	4.1195	4.3588
Arvcf	_NFDT[Phospho (STY)]LDLPK_.2	P98203_T643_M1_Arvcf	3.3857	3.9334	4.4231	4.1985	3.5926	3.0149	3.2494	3.6114	3.3085	3.6594	3.6829	3.6595	3.4166	4.4612	2.8266	4.2486	3.1086	4.0409	4.3399	4.1384
Tubb5	_ISVYYNEATGGK_.2	#N/A	10.3406	10.9257	10.6920	9.6897	10.1045	9.8292	8.9523	10.3315	10.2456	10.5914	9.5551	9.3770	9.8107	10.1733	9.9002	9.5235	9.4391	9.7369	9.6906	9.5921
Tubb5	_M[Oxidation (M)]AVTFIGNSTAIQELFKR_.3	#N/A	5.6658	5.8425	5.6942	6.6399	5.3985	6.0907	5.8671	6.0328	5.9676	5.6821	5.4867	5.3839	6.9177	4.4447	5.3898	5.8879	5.3080	5.8324	5.3394	6.2020
Tubb5	_MAVTFIGNSTAIQELFK_.2	#N/A	4.6951	6.8137	7.3055	7.3108	5.3445	6.4792	5.7289	4.9347	5.4893	6.2815	5.7559	4.4177	5.4829	7.2169	4.9561	4.5029	5.9369	4.6553	4.9312	5.2128
Tubb5	_ALTVPELTQQVFDAK_.2	#N/A	11.1185	11.0231	11.2676	11.2578	11.1080	11.0938	11.0247	11.0721	11.1039	11.3728	10.4185	10.3929	10.5895	10.2355	10.5165	10.4295	10.3429	10.1868	10.4192	10.4912
Tubb5	_FWEVISDEHGIDPTGTYHGDSDLQLDR_.4	#N/A	7.0324	7.2235	8.8769	8.0018	7.2636	7.3311	7.1285	7.0758	7.1295	7.8240	6.7946	6.3186	7.2634	6.8342	7.1272	7.2562	6.6904	6.8041	6.9955	7.3272
Psmb4	_QPVLSQTEAR_.2	#N/A	6.2456	6.1491	6.9437	6.3862	6.6355	6.3367	6.7408	7.0294	6.7790	6.6258	6.5068	6.6585	6.8826	6.9002	7.0777	6.7465	6.2374	6.5063	6.3296	7.0167
Psmb4	_FQIATVTEK_.2	#N/A	5.9838	6.5236	6.3612	6.2814	6.0179	6.3708	6.5851	6.6812	6.0890	6.6062	6.2629	6.2088	6.6535	6.6262	6.6920	6.3060	6.0155	6.2671	6.5275	6.2890
Psmb4	_AIHSWLTR_.2	#N/A	6.6471	6.6875	4.3575	3.7538	7.0040	6.7835	6.6938	6.8010	5.9301	6.8699	6.3168	6.9148	6.2337	6.6605	6.6575	6.0568	5.0999	6.4519	6.4707	6.2863
Psmb4	_TQNPM[Oxidation (M)]VTGTSVLGVK_.2	#N/A	3.2099	3.2921	3.7462	4.4795	5.2915	5.2555	5.2786	5.2457	3.0652	5.0890	4.0446	5.6686	3.2306	5.2332	3.1703	3.5548	3.4479	5.2316	5.2064	3.7003
Psmb4	_FDGGVVIAADM[Oxidation (M)]LGSYGSLAR_.2	#N/A	4.5050	4.6930	4.1971	4.0568	3.8292	4.0032	4.4556	4.2919	4.2976	4.7592	3.9217	4.1421	4.2470	4.1355	4.2412	4.5884	4.4985	3.8251	4.1572	4.2312
Psmb4	_VNDSTM[Oxidation (M)]LGASGDYADFQYLK_.2	#N/A	3.9965	3.5972	3.5347	3.8322	3.9100	4.1091	3.5426	4.0354	4.0974	3.6420	4.0190	4.0611	4.4835	3.9100	4.1589	3.9537	3.8351	3.7146	3.4963	3.7544
Rplp2	_LASVPAGGAVAVSAAPGSAAPAAGSAPAAAEEK_.3	#N/A	5.8712	5.8530	6.9135	6.6456	5.5965	5.8443	5.3162	5.7717	6.4235	6.0361	5.1228	6.3443	5.9197	6.1934	7.0270	6.0929	5.6767	6.1422	5.0415	5.9578
Rplp2	_NIEDVIAQGVGK_.2	#N/A	9.9181	10.1400	10.1655	10.1761	3.0606	9.9879	9.7678	10.4583	9.7624	10.1726	9.9863	10.3459	10.7043	10.6090	10.4095	10.2129	10.3763	9.9878	10.0860	10.2879
Rplp2	_YVASYLLAALGGNSSPSAK_.2	#N/A	6.1682	6.1026	6.2738	6.5000	5.9395	5.9033	5.7351	6.1362	6.1202	6.4325	5.8909	5.8822	6.4032	6.2933	6.4846	6.1563	5.9385	5.5185	6.0828	6.2869
Rplp2	_ILDSVGIEADDDRLNK_.2	#N/A	4.6071	3.8878	4.2822	3.4351	3.9923	3.6529	3.6671	4.2531	4.2367	3.9201	4.1581	4.5299	4.4844	4.7140	4.9449	4.8374	4.7743	4.3542	4.1819	4.8336
Rplp2	_KEESEES[Phospho (STY)]DDDM[Oxidation (M)]GFGLFD_.2	#N/A	3.8839	8.2553	8.2195	3.4460	3.8883	9.1188	9.4548	4.1470	10.7328	3.4662	3.9573	3.3499	4.0619	10.2997	10.4059	5.8717	4.7264	12.8251	8.2840	5.8698
Rplp2	_LASVPAGGAVAVSAAPGSAAPAAGSAPAAAEEKKDEK_.4	#N/A	3.8410	5.1563	3.3775	3.7433	4.0479	4.0605	5.2930	4.0666	3.7265	3.6516	4.1382	5.0929	3.8718	5.4406	5.0599	3.2031	3.5639	3.5856	3.8846	4.4682
Uqcrh	_LELC[Carbamidomethyl (C)]DNR_.2	#N/A	8.3519	8.7185	8.9007	8.6970	8.8409	8.6445	8.6287	8.8532	8.7610	8.7554	8.7003	9.0680	9.0760	9.0827	9.3545	9.0836	8.8361	8.7907	9.0083	9.0092
Uqcrh	_EHC[Carbamidomethyl (C)]EQLEK_.2	#N/A	6.8292	7.4075	7.9100	7.2903	7.3585	7.4270	7.2591	7.4360	7.8048	7.6610	7.2624	7.7999	7.7381	7.7556	8.7885	7.7196	7.2659	7.6478	7.8508	7.6488
Uqcrh	_ERLELC[Carbamidomethyl (C)]DNR_.2	#N/A	7.2001	7.5181	7.5663	7.7786	7.2311	7.5007	7.4183	7.4986	7.4243	7.6819	7.5113	7.3718	7.9646	7.7848	7.9998	7.6947	7.5752	7.4918	7.8459	7.5486
Uqcrh	_SQTEEDC[Carbamidomethyl (C)]TEELFDFLHAR_.3	#N/A	6.5782	6.8382	7.3182	7.0662	6.8167	6.9844	6.7155	6.6777	7.1307	7.1053	6.8680	6.7539	7.3584	7.1556	7.5418	7.2995	6.9988	6.4801	6.9456	7.4931
Prdx5	_LLADPTGAFGK_.2	#N/A	10.3129	10.3777	10.4088	10.5139	10.6847	10.2841	10.5095	10.7050	10.1731	10.3085	10.2801	10.6605	10.6516	10.4399	10.6142	10.4715	10.3240	10.5290	10.4899	10.5048
Prdx5	_THLPGFVEQAGALK_.2	#N/A	8.6509	8.7575	8.8766	8.4833	8.7439	8.6305	8.5205	8.8937	8.5554	8.7637	8.5370	8.9945	8.8644	8.8772	9.1546	8.8675	8.7360	8.6955	8.6645	8.8489
Prdx5	_VGDAIPSVEVFEGEPGK_.2	#N/A	9.0230	9.0751	9.2270	9.1155	9.0522	8.9304	8.8254	8.9577	9.0588	9.0130	9.0406	8.8618	9.3021	9.2127	8.8166	8.9964	9.1001	8.7749	8.6502	9.1008
Prdx5	_VGDAIPSVEVFEGEPGKK_.2	#N/A	7.0411	7.6245	7.1088	7.0168	6.8906	7.2730	7.2826	7.3100	7.2362	7.0588	7.1080	7.2319	7.2014	7.8717	7.3297	7.4177	7.6120	7.2552	6.8556	7.4056
Prdx5	_KVNLAELFK_.2	#N/A	6.8680	7.0612	6.8382	6.9441	7.2975	6.9708	7.0863	7.3777	6.7256	7.0937	6.9209	6.9388	7.0329	7.4981	7.4055	7.0227	6.9912	6.8459	7.0926	7.1973
Prdx5	_ALNVEPDGTGLTC[Carbamidomethyl (C)]SLAPNILSQL_.2	#N/A	6.1791	6.5414	6.5828	6.1868	5.6057	6.0371	6.6480	6.4273	6.4271	6.3634	5.6476	6.4102	6.2498	7.0308	6.6998	5.9261	6.4371	6.5049	6.5805	5.9932
Prdx5	_VNLAELFK_.2	#N/A	5.6033	6.4700	5.8876	6.7668	6.2833	6.3107	6.1522	5.7484	6.0705	6.1383	5.8512	6.9354	5.7583	6.1349	6.6388	6.5312	4.9084	6.4646	6.8501	6.6574
Prdx5	_ATDLLLDDSLVSLFGNR_.2	#N/A	6.5785	6.1267	6.9020	6.2383	6.3430	6.7503	6.3400	6.4586	6.4798	6.2208	6.8936	6.6367	5.9116	5.6399	6.6880	6.3393	6.8629	5.8745	6.2539	6.5130
Prdx5	_ATDLLLDDSLVSLFGNRR_.3	#N/A	5.5833	6.2628	5.1332	5.9772	5.1000	5.3773	5.8621	5.6916	6.2024	5.8187	5.5163	6.3098	6.2182	6.4802	6.1068	6.1209	5.9405	5.8622	6.2987	6.0405
Prdx5	_FSM[Oxidation (M)]VIDNGIVK_.2	#N/A	7.3969	7.4909	7.6019	7.6908	7.5160	7.4265	7.2258	7.3998	7.3592	7.1297	7.3828	7.3657	7.3616	7.5439	7.3940	7.2954	7.2357	7.1928	7.2508	7.0296
Cpe	_SNAQGIDLNR_.2	#N/A	6.8836	7.1061	7.2048	7.6495	7.5333	7.3422	7.2602	7.5311	7.1825	7.4767	6.9672	6.9097	6.8785	7.6078	7.1286	7.1505	7.2516	6.8077	7.1522	7.0494
Cpe	_LTASAPGYLAITK_.2	#N/A	5.6993	5.4085	5.5508	5.3585	5.7899	5.7355	5.4208	6.0654	5.1659	5.5833	5.3285	5.8672	5.5908	5.7147	5.4143	5.2952	5.6016	4.7066	5.0851	5.4725
Cpe	_IVYVNEK_.2	#N/A	3.2489	4.5493	4.4202	4.7207	4.0875	4.6966	4.3042	3.8155	3.5127	4.1328	3.9346	3.1425	4.1044	4.0250	4.4894	3.5938	3.3128	4.4787	4.1357	4.6535
Cpe	_YIGNM[Oxidation (M)]HGNEAVGR_.3	#N/A	4.3145	3.9104	3.1279	3.5970	3.0206	3.9635	4.6357	5.0410	4.5430	4.1251	3.9466	4.5479	4.1131	4.6158	5.1617	3.6767	4.3305	4.6550	5.1577	3.0819
Cpe	_EALVSVWLQC[Carbamidomethyl (C)]TAISR_.2	#N/A	4.5031	4.3168	4.0397	3.7855	3.2092	3.2425	3.8389	3.6042	3.7591	4.3137	3.2378	4.0430	3.9246	4.4272	3.8643	3.8652	4.1419	2.9765	3.3398	4.3942
Il6st	_SHIAQWS[Phospho (STY)]PHT[Phospho (STY)]PPR_.3	Q00560_S665_M2_Il6st	5.9442	5.9791	4.1100	5.6557	2.9114	5.2080	4.8769	3.2741	4.4330	5.0359	2.9076	4.3185	3.5944	5.5432	3.5341	5.1002	4.1314	5.7688	5.3397	5.4801
G6pdx	_DVAGDIFHQQC[Carbamidomethyl (C)]K_.2	#N/A	3.0265	5.8170	3.7416	3.3792	2.9112	5.0122	5.2700	4.4154	3.8090	4.0157	4.2874	4.1646	5.9164	8.7039	4.1623	3.5672	3.9280	3.2215	4.5432	3.7159
Hnrnpul2	_S[Phospho (STY)]GDETPGSEAPGDK_.2	#N/A	12.5188	12.0895	12.8596	12.5331	12.0777	12.2796	11.9478	12.2513	12.2545	12.2924	12.0838	11.9423	11.6011	12.7379	11.2570	11.9741	12.4759	11.8978	11.9103	12.2417
Hnrnpul2	_DLLVQQASQC[Carbamidomethyl (C)]LSK_.2	#N/A	5.7807	6.2114	5.8774	5.9863	4.9488	5.6206	4.8469	4.9199	5.3480	5.8225	5.3126	5.2907	4.7213	5.1930	5.6594	5.3076	5.4832	5.0964	5.2535	5.3858
Hnrnpul2	_YNVLGAETVLTQM[Oxidation (M)]R_.2	#N/A	6.5615	6.1768	4.2115	6.3424	5.4230	5.6940	6.0238	6.0687	6.2426	6.0432	5.1853	6.0190	6.7128	5.1480	6.0570	5.8799	6.2191	5.9203	6.0907	6.1489
Hnrnpul2	_NFILDQC[Carbamidomethyl (C)]NVYNSGQR_.2	#N/A	3.6982	4.1785	6.3151	6.1668	3.0359	4.7918	5.4743	5.8400	5.6407	5.7255	3.4378	5.3945	3.1714	5.3865	5.1345	6.0398	4.3643	4.4958	5.4321	5.4488
Hnrnpul2	_AVEEQGDDQDS[Phospho (STY)]EK_.2	Q00PI9_S183_M1_Hnrnpul2	3.8289	5.4942	6.3071	5.9216	4.9504	6.1312	4.8582	5.1081	5.0790	5.5786	5.5117	4.6666	5.5753	6.9363	5.1861	4.4124	5.8510	6.0677	4.8363	6.0485
Hnrnpul2	_S[Phospho (STY)]KSPPPPEEEAKDEEEDQTLVNLDTYTSDLHFQISK_.5	Q00PI9_S224_M1_Hnrnpul2	3.6080	3.2039	3.8344	4.3913	3.8149	3.8483	3.2332	4.4993	3.1534	3.4186	6.2030	3.0933	3.3188	3.8215	3.2586	2.9701	3.5362	4.2970	3.9456	3.7885
Hnrnpul2	_S[Phospho (STY)]GDETPGSEAPGDKAVEEQGDDQDSEK_.3	#N/A	4.1876	3.1315	3.7242	3.3966	3.8002	3.7139	3.4852	4.4133	3.8027	4.3584	2.9839	4.3585	4.2185	3.7622	1.6685	3.5497	3.9105	3.2390	3.5669	3.4395
Hnrnpul2	_AVEEQGDDQDS[Phospho (STY)]EKS[Phospho (STY)]KPAGS[Phospho (STY)]DGER_.3	Q00PI9_S183_M3_Hnrnpul2	4.4331	4.3868	3.9697	3.1511	3.1392	3.1725	3.9089	3.1579	3.8291	4.2437	3.3078	4.1130	3.9946	4.0077	3.9343	3.7952	4.1560	3.6213	3.2698	2.9634
Pde4d	_QHEVEIPSPT[Phospho (STY)]QKEK_.3	Q01063_T289_M1_Pde4d	4.2783	3.2578	3.5979	3.5229	3.6161	3.8401	3.9250	4.2870	3.9841	5.3430	4.3585	2.9839	4.0922	3.6359	3.5022	3.1224	3.7842	3.3652	3.6643	3.3132
Pde4d	_NNFAALTNLQDR_.2	#N/A	5.1452	4.2328	3.8745	4.9908	3.0440	4.5812	5.1792	4.8223	3.9243	5.1605	5.2599	4.2081	4.0897	4.8978	4.5845	5.0625	4.8642	4.5483	4.0573	5.1331
Pde4d	_SDS[Phospho (STY)]DYDLSPK_.2	Q01063_S136_M1_Pde4d	6.5168	6.7306	6.7092	6.8773	3.5863	6.3163	6.7362	3.9358	4.9387	7.4558	3.9792	6.5202	7.0177	3.2669	5.4921	6.8823	5.8501	6.6381	6.8672	7.0605
Pde4d	_HSVSEMASNK_.2	#N/A	4.0515	3.2676	5.0973	5.4275	4.2585	4.9050	4.3892	4.2772	3.9744	5.6009	5.8323	5.1340	5.8380	5.6641	3.4925	5.4952	3.7744	5.8885	5.6297	3.3035
Pde4d	_QHEVEIPS[Phospho (STY)]PTQK_.2	Q01063_S287_M1_Pde4d	4.3879	4.4320	9.3431	3.1963	8.5632	3.1273	3.9541	3.1127	3.8743	4.1985	3.3530	4.1582	4.0398	4.5424	7.9362	3.7500	4.1108	3.5761	3.2246	3.0086
Pde1b	_TALQQLER_.2	#N/A	6.2492	6.3019	6.1616	6.2428	6.4700	6.1885	6.7701	6.6991	6.1110	5.7926	6.8886	6.7807	6.1269	5.4939	6.6191	6.3361	5.8466	6.5994	6.0973	6.8616
Pde1b	_TIVFELLTR_.2	#N/A	6.1071	4.6141	6.1202	5.6231	5.7205	5.6870	5.6712	5.6659	5.8513	4.5202	5.3655	5.6849	5.0161	4.4760	5.3719	5.0606	5.7856	5.6935	5.5973	5.8025

Pde1b	_QILDTEDELR_.2	#N/A	6.0419	5.8537	3.3935	5.8344	5.7644	6.1645	5.6619	5.7402	5.9108	5.0796	6.2007	6.1755	3.8878	5.4483	5.0060	5.4488	5.6719	6.3312	5.8647	5.3283
Pde1b	_DWLASTFTQQTR_.2	#N/A	4.2109	4.7067	4.9580	5.0734	2.9170	4.4512	4.7394	5.5385	5.1466	5.7224	4.9496	4.6507	5.4528	3.2186	4.7037	4.8884	4.4341	5.2932	4.9743	5.2387
Pde1b	_S[Phospho (STY)]PPEM[Oxidation (M)]LESDC[Carbamidomethyl (C)]PSPLELK_.2	#N/A	8.2563	8.5857	8.5102	8.2429	7.5089	8.6154	8.2731	8.1455	8.2266	7.8792	8.6961	8.2139	8.1440	9.0304	7.6324	7.9958	8.9382	8.7054	8.5674	8.4131
Pde1b	_QILDTEDELRELR_.3	#N/A	4.7060	4.3834	4.1557	4.0700	3.9000	2.7578	3.0831	3.4451	3.2571	3.8257	3.5166	4.2729	3.2503	4.2949	4.1612	5.0018	3.6659	3.7302	4.5304	4.0594
Pde1b	_NLEYTASLLEAVYIDETR_.2	#N/A	4.1907	4.4197	3.7272	3.3936	2.8968	4.4309	4.1513	4.4163	4.0715	4.0013	3.5502	4.3554	4.2370	3.7653	3.6351	3.2013	3.9136	3.3788	3.5349	3.2058
Grin2b	_S[Phospho (STY)]FTHSDC[Carbamidomethyl (C)]K_.2	Q01097_S940_M1_Grin2b	8.4171	7.9903	7.6737	7.8559	7.1676	7.7169	8.0441	7.7704	7.4387	8.1448	7.9132	7.4204	7.8984	7.4700	6.5781	7.3882	7.8818	7.7243	7.5967	7.3775
Grin2b	_YPQS[Phospho (STY)]PTNSK_.2	Q01097_S1284_M1_Grin2b	9.1567	9.0783	9.2300	3.6281	8.5387	8.8584	8.8374	8.7853	8.3922	9.0271	8.6652	8.3718	8.8407	8.2923	7.3098	8.2580	8.7926	8.8073	8.3918	8.3157
Grin2b	_HSQLSDLY[Phospho (STY)]GK_.2	Q01097_Y1039_M1_Grin2b	8.7292	8.3947	6.9808	8.5185	7.8268	8.0510	7.7214	7.8867	7.2976	8.4907	8.3019	7.4958	8.3832	8.0068	5.2446	7.5307	8.3678	8.0251	7.7666	7.8802
Grin2b	_SDRYS[Phospho (STY)]GHDDLIR_.3	Q01097_S1050_M1_Grin2b	3.2543	4.0648	4.2917	4.3300	3.4612	3.4945	3.5869	3.4799	3.5071	5.5265	2.9858	3.7909	3.6726	4.1752	3.6123	4.1172	4.4780	5.9935	5.8184	4.1422
Grin2b	_SVS[Phospho (STY)]LKDK_.2	Q01097_S1323_M1_Grin2b	8.3289	8.0806	8.4529	8.2786	7.2955	7.2774	7.7479	3.8729	4.0278	8.6312	7.3284	4.3116	7.8520	7.2403	5.4394	7.3947	6.9590	7.5832	7.1715	7.1923
Grin2b	_RQHS[Phospho (STY)]YDTFVDLQKEEAALAPR_.4	#N/A	4.4937	8.7416	3.5292	3.1957	3.8314	4.1535	7.8839	4.1941	7.7269	3.7238	3.8276	3.3985	7.9154	7.9770	3.2868	3.3378	3.2309	8.2144	4.2508	6.9920
Grin2b	_ENS[Phospho (STY)]PHWEHVDLTDIYK_.3	Q01097_S1143_M1_Grin2b	3.6440	5.7625	5.6141	3.9403	3.8509	5.1146	4.5214	3.8696	4.4652	3.4545	4.0969	3.4013	3.2829	5.4667	3.2226	3.0061	3.3669	5.5785	4.9901	4.4774
Grin2b	_RES[Phospho (STY)]S[Phospho (STY)]VYDISEHRR_.3	Q01097_S929_M2_Grin2b	4.7123	3.7675	3.1916	3.9292	3.8620	4.2464	5.4279	3.8807	5.1966	5.9039	3.9523	3.3902	5.6373	3.2297	3.2115	3.0172	4.9866	5.5324	4.0705	4.8190
Grin2b	_QHSY[Phospho (STY)]DTFVDLQKEEAALAPR_.3	#N/A	3.2761	3.7564	3.8124	4.0216	4.1147	4.3388	3.4264	3.7883	3.4855	3.4825	2.9343	3.1153	3.5935	3.1373	3.0036	3.6210	3.2856	3.8639	4.1629	4.3154
Grin2b	_RQHS[Phospho (STY)]YDTFVDLQK_.3	#N/A	6.8045	6.5376	7.3081	6.8833	3.1650	3.3891	6.5455	3.5276	6.3957	5.8903	3.3087	4.0650	4.5432	6.4406	4.1520	6.0309	6.0276	6.5072	5.8847	6.1702
Grin2b	_QHSY[Phospho (STY)]DTFVDLQK_.2	#N/A	3.7657	4.2707	4.3020	3.9572	3.1034	3.3275	3.7993	3.4660	3.8584	3.9720	3.4038	4.1601	3.9665	4.2890	4.0484	4.1106	4.0037	3.8630	3.5115	3.5841
Grin2b	_S[Phospho (STY)]DVSDISTHTVTYGNIEGNAAK_.3	Q01097_S1058_M1_Grin2b	3.7062	4.0299	3.7728	4.0568	3.4080	3.4526	3.9044	3.6113	3.7342	4.3976	3.2775	3.8336	3.7304	4.4460	3.6881	3.5929	4.3669	3.9225	3.0997	3.5688
Grin2b	_NM[Oxidation (M)]ANLSGVNGS[Phospho (STY)]PQSALDFIR_.2	Q01097_S917_M1_Grin2b	4.5590	5.0277	4.0956	4.5260	3.2651	3.3424	4.4228	3.2839	2.9810	3.9869	3.3554	3.9870	4.5899	5.8185	3.8083	3.9212	4.2820	4.3683	3.1665	3.8110
Grin2b	_AGNLYDIS[Phospho (STY)]EDNSLQELDQPAAPVAVSSNASTTK_.3	Q01097_S1255_M1_Grin2b	3.9503	3.3688	3.4868	3.6340	4.1572	4.1905	4.3917	4.1759	4.3119	3.7609	3.7906	3.0949	3.9811	3.5249	2.9163	3.3124	3.6732	3.7976	5.0224	3.2022
Grin2b	_ESSVY[Phospho (STY)]DISEHRR_.3	Q01097_Y932_M1_Grin2b	3.6662	3.6529	3.2027	3.9181	3.8731	3.9064	3.1750	3.8918	3.0952	3.4768	4.0747	3.3790	3.6970	3.2408	3.2004	3.0283	3.3891	3.7604	2.9968	4.4189
Creb1	_DLYC[Carbamidomethyl (C)]HKS[Phospho (STY)]D_.2	Q01147_S326_M1_Creb1	3.4154	5.3088	4.4528	4.1688	5.3735	5.0589	3.4257	4.4123	3.3459	3.2260	4.1564	4.6886	3.5114	5.9201	4.7452	4.2784	3.1383	4.0111	4.3102	4.5167
Creb1	_ILNDLS[Phospho (STY)]SDAPGVPR_.2	Q01147_S128_M1_Creb1	4.2842	4.5357	7.3569	6.9818	2.9903	3.0236	4.0578	4.5099	3.9780	4.0948	6.9198	6.7717	6.9073	7.4068	4.0832	6.8382	7.0193	7.2311	7.0961	7.4921
Adra2c	_TEVEPDES[Phospho (STY)]SAAER_.2	Q01337_S286_M1_Adra2c	3.3109	3.1911	5.7508	4.3785	4.1495	4.3736	3.3916	4.5121	3.4507	3.5172	3.8251	3.0805	3.5587	3.8343	2.9688	3.6558	3.5490	4.2842	3.9328	3.8013
Adra2a	_RGPGAAGPGASGS[Phospho (STY)]GHGEER_.3	Q01338_S362_M1_Adra2a	3.3736	3.1284	7.0759	4.3158	4.2121	6.9583	3.3289	3.0740	3.3880	3.5799	3.7624	3.0178	6.6890	3.8970	2.9061	3.7185	6.7786	7.1644	3.8701	3.8640
Adra2a	_TRAS[Phospho (STY)]QVKPGDS[Phospho (STY)]LPR_.3	Q01338_S339_M2_Adra2a	4.4370	4.3829	3.9736	3.7194	3.1431	3.5952	3.9050	3.6703	3.8252	4.2476	4.5636	4.1091	3.9907	4.4933	3.9304	3.7991	4.2080	3.6251	3.2737	2.9595
Adra2a	_ASQVKPGDS[Phospho (STY)]LPR_.2	#N/A	8.1770	8.0889	8.0196	7.7272	7.8436	7.9947	8.0833	7.7270	7.6990	7.9401	7.9478	7.6794	8.1103	7.6683	6.4701	7.4164	8.0494	8.1543	7.6276	7.4804
Adcy6	_NAEPPS[Phospho (STY)]PTPAAHTR_.2	#N/A	9.5286	9.5122	3.5294	8.8123	3.9276	9.2041	9.3172	9.3998	8.6863	9.0109	8.6994	8.5551	9.1689	3.5413	3.2221	3.1784	9.2523	8.8622	8.6648	8.4467
Nme2	_GLVGEIIKR_.2	#N/A	9.7341	9.7874	9.7565	9.7555	9.9373	9.9378	9.7659	10.1232	9.7133	10.0593	9.6460	9.9576	9.8347	9.9205	9.9932	9.8107	9.8305	9.6974	9.6724	9.8717
Nme2	_GDFC[Carbamidomethyl (C)]IQVGR_.2	#N/A	8.4020	9.0179	9.1433	9.0439	8.5318	8.9160	8.8065	8.2189	8.8613	8.7446	8.5405	8.6341	8.9856	9.0259	9.2062	8.8238	8.5064	8.9063	9.0990	8.8078
Nme2	_DRPFFPGLVK_.2	#N/A	8.1623	8.0716	7.7088	8.0261	8.0187	7.9174	8.0811	7.9764	7.6254	8.1364	7.9820	7.9459	8.1735	8.0016	8.2527	7.8423	7.6895	7.9704	7.9156	7.8994
Nme2	_SC[Carbamidomethyl (C)]AHDWVYE_.2	#N/A	7.1323	7.1781	7.8578	7.4771	7.4996	7.4010	7.3528	7.6816	7.6723	7.2025	7.5413	7.0844	7.8674	7.6476	7.7507	7.0294	6.9491	7.2224	7.2387	7.0567
Nme2	_GLVGEIIK_.2	#N/A	7.0517	6.1637	7.6167	7.6395	7.5275	7.1051	6.5450	5.5529	7.2586	7.4896	7.3919	6.8567	7.1792	6.1409	7.5890	7.7433	6.8469	6.9515	7.7327	7.6374
Nme2	_TFIAIKPDGVQR_.2	#N/A	9.5459	9.5524	9.4340	9.4995	9.5943	9.4287	9.4033	9.8022	9.4764	9.5965	9.3151	9.5373	9.6809	9.5942	9.5307	9.6015	9.5981	9.2162	9.4251	9.4788
Nme2	_NIIHGSDSVESAEK_.2	#N/A	5.3127	5.3225	6.0558	4.7392	4.9695	5.7024	4.9136	5.2720	5.2704	5.8025	5.3204	5.3142	3.6334	5.4618	5.7839	5.7288	5.0271	5.7937	5.2375	4.6688
Nme2	_VM[Oxidation (M)]LGETNPADSKPGTIR_.3	#N/A	7.7356	8.2617	7.6492	7.5607	8.0995	7.8778	8.1022	8.0838	8.3224	8.4549	8.0410	8.1698	8.6278	7.8730	8.5471	8.2857	7.6652	8.3469	8.4424	8.1510
Cacna1c	_SITADGES[Phospho (STY)]PPTTK_.2	Q01815_S815_M1_Cacna1c	3.4012	10.7163	4.4386	10.0339	3.6081	3.6414	10.5130	11.1510	10.4606	10.0635	4.3397	3.6440	3.5256	10.2038	9.4141	9.7203	10.8324	10.6672	10.6561	9.7153
Cacna1c	_EAVSAAS[Phospho (STY)]EDDIFR_.2	#N/A	3.8285	6.4120	4.3648	3.8609	3.1662	3.3179	4.3749	3.5288	3.6837	4.0348	3.1624	4.0638	3.8492	4.3518	3.9521	4.1734	4.0665	3.7666	3.4152	4.3188
Cacna1c	_TGNNQADTES[Phospho (STY)]PSHEK_.2	Q01815_S1742_M1_Cacna1c	4.8786	5.2405	4.2355	3.4948	3.1874	3.3882	5.6099	5.2124	5.3928	4.2269	4.7016	4.0761	5.3779	4.2421	3.9270	4.4217	5.0826	5.1035	4.8288	3.3013
Cacna1c	_EAVSAAS[Phospho (STY)]EDDIFRR_.2	#N/A	3.7668	3.8478	7.1363	4.0010	3.4662	3.0828	3.8716	3.6705	3.6457	4.7530	3.5092	3.7757	3.7910	3.8497	3.8969	3.6480	4.4248	3.6671	3.0414	3.6245
Vcp	_EVDIGIPDATGR_.2	#N/A	9.1578	8.8954	9.1904	9.0093	9.1977	9.2922	8.9315	9.2084	9.3378	9.0652	9.1115	9.3257	9.0562	9.4402	8.9660	9.2058	9.2178	8.7745	9.0541	9.3412
Vcp	_SVS[Phospho (STY)]DNDIR_.2	#N/A	9.1251	9.4217	8.8106	9.3772	8.9849	8.9583	9.0259	9.1193	8.9995	9.2009	8.9767	9.1806	9.4835	9.6804	8.8651	9.0576	9.2746	9.0931	8.8581	9.2556
Vcp	_LDQLIYIPLPDEK_.2	#N/A	8.5186	8.6967	8.9370	8.8492	8.7479	8.7156	8.5600	8.4634	8.8505	8.9248	8.3967	8.7031	8.9787	8.8854	8.9872	8.7040	8.7354	8.3534	8.4990	8.8101
Vcp	_GVLFYGPPGC[Carbamidomethyl (C)]GK_.2	#N/A	8.1908	8.3863	8.9420	8.7438	8.7506	8.6743	8.5991	8.5349	8.3992	8.8172	8.6260	8.5840	8.5288	8.7750	8.6354	8.5312	8.5690	8.3458	8.7097	8.7319
Vcp	_RSVS[Phospho (STY)]DNDIR_.2	#N/A	7.5409	7.6529	7.3627	7.6183	7.4631	3.4900	7.3685	7.7422	7.3491	7.5577	7.4241	7.5421	7.9265	8.2800	7.4199	7.5494	7.7205	7.6887	7.2852	7.6706
Vcp	_GGNIGDGGGAADR_.2	#N/A	8.1225	8.0825	8.0636	8.3759	8.0988	8.3499	8.0717	8.0486	8.2926	8.1994	8.1983	8.2473	8.0352	8.5466	8.3912	8.1273	8.2162	7.9244	8.0876	8.1320
Vcp	_WALSQSNPSALR_.2	#N/A	7.7920	7.9691	8.1876	7.5731	8.1081	7.6839	7.8134	8.0050	7.8014	7.9947	7.3956	7.6099	7.7160	8.1675	8.0059	7.8034	7.7750	7.7693	7.7798	7.8947
Vcp	_M[Oxidation (M)]DELQLFR_.2	#N/A	7.8798	7.3267	6.9074	6.6454	7.4283	7.3253	6.6158	7.6389	7.5644	7.4833	7.1634	7.4775	7.2890	7.1822	7.4354	7.6053	7.3589	7.4719	7.6292	7.5029
Vcp	_ETVVEVPQVTWEDIGGLEDVKR_.3	#N/A	7.3108	7.2659	7.5818	7.4809	7.0776	7.1003	6.7982	7.5101	7.4613	6.7660	6.8735	7.0249	7.7371	7.4751	7.5425	7.0562	7.4231	7.0897	7.2438	7.1795
Vcp	_LGDVISIQPC[Carbamidomethyl (C)]PDVK_.2	#N/A	6.8406	6.7930	6.6878	7.3015	7.0004	6.7919	6.5858	6.8205	6.8190	7.1007	6.6412	6.7798	5.9149	7.5428	6.5752	6.3055	6.9594	7.1187	6.5148	6.5454
Vcp	_LEILQIHTK_.2	#N/A	6.2902	7.1960	7.4032	6.9178	6.5971	6.8540	6.8125	6.9226	6.3924	6.7760	6.2449	6.6156	6.3150	6.8473	6.8612	6.8649	7.1162	6.4197	7.0720	6.9117
Vcp	_ESIESEIRR_.2	#N/A	5.5229	5.7399	5.9480	5.7278	5.9779	6.3669	5.7722	5.7114	6.3698	6.2999	6.2735	6.4160	6.0953	6.2356	6.4036	6.4060	5.8956	5.9128	6.3768	5.3863
Vcp	_AIANEC[Carbamidomethyl (C)]QANFISIK_.2	#N/A	5.6579	5.5913	5.8442	6.0889	5.5610	5.5343	5.0771	4.5317	5.2864	4.6962	5.3335	5.3419	4.9530	5.6177	5.3958	4.4238	4.9053	5.2471	5.2205	5.4525
Vcp	_SVS[Phospho (STY)]DNDIRK_.2	#N/A	3.5164	3.8027	4.5538	4.0678	5.7437	5.9575	3.3248	6.2214	3.2450	3.3270	4.2244	5.5547	6.1337	3.9131	3.3501	5.7857	3.2393	5.6738	3.8540	5.9590
Vcp	_LAGESESNLR_.2	#N/A	7.7017	7.3352	8.0913	7.7473	7.9080	7.6389	7.5756	3.3588	8.0808	7.9465	7.5122	7.6781	7.8758	8.0779	8.0483	7.5787	7.7553	7.5736	7.5978	7.8799
Vcp	_QAAPC[Carbamidomethyl (C)]VLFFDELDSIAK_.2	#N/A	4.5808	5.3096	4.9699	4.9921	5.2283	5.0656	4.5873	5.3687	4.2623	4.4292	3.3062	5.6598	4.7711	4.8419	4.9291	4.3596	4.3629	5.0927	4.0317	4.7913
Vcp	_LIVDEAINEDNSVVSLSQPK_.2	#N/A	7.7318	7.8984	7.7245	7.7978	7.2509	7.4177	7.2498	7.7849	7.4454	7.7402	7.1720	7.5488	8.0565	8.3653	7.2672	7.8137	7.9538	7.2384	7.2010	7.8418
Vcp	_ELQELVQYPVEHPDK_.2	#N/A	6.2861	6.2341	6.4616	6.6198	6.3072	6.3895	6.3431	5.6651	6.5100	6.4167	6.6744	6.1537	6.4438	6.5494	6.3698	6.5198	6.2577	6.0602	6.1142	6.6620
Vcp	_NAPAIIFIDELDAIAPK_.2	#N/A	6.0665	6.3426	6.1254	5.7942	5.9040	6.2202	6.2494	6.6973	6.2615	5.9844	6.1565	5.9124	3.2558	5.5564	6.4257	5.3372	4.4487	5.3964	5.7377	6.5472
Vcp	_EM[Oxidation (M)]VELPLR_.2	#N/A	7.3599	6.9059	7.5142	7.4081	7.6981	7.2666	7.4635	7.3887	7.3292	7.5839	7.5189	7.4446	6.8041	7.5643	7.6076	7.3773	7.4063	7.3360	7.4446	7.5439
Vcp	_DHFEEAM[Oxidation (M)]R_.2	#N/A	5.6492	5.9351	5.6021	5.9775	5.2463	5.3276	5.8271	5.4210	5.6338	5.1644	5.9971	5.2427	4.0239	6.6709	5.8329	5.2190	4.8548	4.9952	5.1920	5.0917
Vcp	_IVSQLLTLM[Oxidation (M)]DGLK_.2	#N/A	5.0062	4.6528	5.1846	5.0772	5.0440	4.8598	3.7200	5.2224	4.6195	5.0301	4.9749	4.6107	4.3722	4.1766	4.6750	4.9059	4.6519	4.9510	4.6766	5.0958
Vcp	_ESIESEIR_.2	#N/A	4.8248	5.5454	4.2988	6.0635	5.6826	5.1728	5.3936	5.2114	5.0050	5.7187	6.0636	5.6452	3.2923	5.8537	6.5001	5.2921	4.7489	4.1651	5.8997	6.0065
Vcp	_EAVC[Carbamidomethyl (C)]IVLSDDTC[Carbamidomethyl (C)]SDEK_.2	#N/A	6.4615	5.0888	6.1746	6.1571	3.7579	4.2270	3.7832	4.1206	5.7923	6.4221	5.4176	3.4721	4.8314	6.7519	3.3604	5.8133	5.9610	2.7590	3.6168	5.4474
Vcp	_[Acetyl (Protein N-term)]ASGADSKGDDLSTAILK_.2	#N/A	4.6912	4.6629	4.6660	5.0328	4.3807	4.5412	4.2375	4.8965	4.4404	4.7469	4.6105	4.8739	4.3889	5.2489	4.9109	4.7193	5.2237	4.8660	4.6860	4.8226
Vcp	_M[Oxidation (M)]TNGFSGADLTEIC[Carbamidomethyl (C)]QR_.2	#N/A	3.8835	4.8902	4.0332	4.6420	4.7674	4.6479	4.6261	5.0967	5.3434	5.2445	3.1663	4.0567	4.1567	6.0881	5.8302	5.1747	4.7865	3.4577	5.0962	5.1211
Vcp	_VINQILTEM[Oxidation (M)]DGM[Oxidation (M)]STK_.2	#N/A	7.6405	7.2675	5.9055	6.1749	7.5767	7.3070	6.4529	7.9275	7.1697	7.6094	6.8444	7.5213	7.6540	6.9094	7.2653	7.8063	7.6490	7.4109	7.3174	7.4169
Vcp	_EDEEES[Phospho (STY)]LNEVGYDDIGGC[Carbamidomethyl (C)]R_.2	Q01853_S197_M1_Vcp	3.6128	3.9073	4.6207	3.8222	3.8197	4.1968	4.0753	4.0031	4.1642	4.1759	4.0464	3.6979	3.8546	4.7462	3.3481	3.7275	3.8118	3.7002	3.6663	4.1922
Vcp	_LADDVDLEQVANETHGHVGADLAALC[Carbamidomethyl (C)]SEAALQAIR_.4	#N/A	3.5253	3.2761	4.0616	2.3839	2.8630	1.6103	3.4603	3.2256	3.3805	3.1914	4.3600	3.3316	3.5460	3.7226	3.1331	3.8702	3.7633	4.0698	3.1538	3.3246
Vcp	_DVDLEFLAK_.2	#N/A	3.7762	3.5429	4.2670	3.8080	3.9831	4.0165	4.4281	4.0019	2.9852	3.5868	3.9646	4.6498	3.1506	2.3159	3.5406	4.3222	3.4991	4.6550	4.1138	3.7761
Vcp	_EAVC[Carbamidomethyl (C)]IVLSDDTC[Carbamidomethyl (C)]SDEKIR_.3	#N/A	6.3075	3.1992	3.8391	4.3866	4.6982	4.3654	3.3997	5.9091	3.4588	3.5091	3.8332	3.0886	3.5669	3.8262	2.9769	3.6477	3.5408	4.2923	3.9409	4.2582
Vcp	_LAGESESNLRK_.3	#N/A	3.0948	4.2243	4.1322	4.4894	3.3018	3.3058	3.7463	3.3205	3.0176	3.9503	3.3920	3.9504	3.1257	4.1703	3.7717	3.9578	4.3186	4.3317	3.1299	3.8476
Uba1	_AENYDIS[Phospho (STY)]PADR_.2	Q02053_S876_M1_Uba1	9.3315	9.0933	9.3248	9.3205	9.1005	8.9918	8.9720	9.3092	9.0974	9.3242	8.9664	8.9787	9.2771	9.3059	8.5789	8.9814	9.2539	8.9191	8.8480	9.0453
Uba1	_DNPGVVTC[Carbamidomethyl (C)]LDEAR_.2	#N/A	9.0726	8.7237	8.8346	8.7262	8.5503	8.8426	8.9128	9.0442	9.1048	8.8927	8.5409	8.7182	8.8277	8.6491	8.6454	8.7793	8.9258	8.5645	8.7011	8.4771
Uba1	_RVS[Phospho (STY)]GPDPK_.2	Q02053_S13_M1_Uba1	8.0794	8.4756	8.4427	7.9435	7.7520	8.5521	8.1269	7.9900	8.2565	8.6684	8.2902	7.6818	7.9366	8.4846	7.3805	7.9374	8.6299	8.0749	8.3484	8.3356
Uba1	_YDGQVAVFGSDFQEK_.2	#N/A	8.5189	8.3900	8.3426	7.8998	7.5535	7.9695	7.9777	8.3386	8.4066	8.2417	8.0504	7.6124	8.2932	8.4035	7.7679	7.9683	8.3121	7.9234	7.7614	7.7630
Uba1	_[Acetyl (Protein N-term)]SSS[Phospho (STY)]PLSKK_.2	Q02053_S4_M1_Uba1	7.0982	7.3533	6.5318	7.4709	6.9473	7.0016	7.3665	7.1105	7.3040	7.3143	7.0183	6.9793	7.9675	6.9671	6.7635	7.1747	7.1529	3.5229	7.2292	6.9427
Uba1	_LKSDTAAAAVR_.2	#N/A	6.9247	7.9110	6.9433	7.3885	6.6491	7.7597	7.5186	7.1217	7.5378	7.2402	7.8884	6.7891	7.9361	7.0800	8.1653	6.8947	7.0553	7.2730	8.2405	6.9574
Uba1	_QNSLDEDLIR_.2	#N/A	7.1674	6.9852	7.5318	7.2842	6.9135	7.5509	6.8682	7.4690	7.2686	7.6136	7.3265	6.6924	7.3750	7.1966	7.0716	6.8210	7.2043	7.0317	6.9958	7.3390
Uba1	_QPAENVNQYLTDSK_.2	#N/A	7.7205	7.3837	7.4367	7.2113	6.6288	7.2012	7.3495	7.5554	7.4277	7.3367	7.2324	7.5041	7.1979	7.3251	7.0648	7.2262	7.6322	7.0703	7.1322	6.9552
Uba1	_SLPASLVEPDFVM[Oxidation (M)]TDFAK_.2	#N/A	7.3698	6.8250	6.6957	6.9747	7.3258	6.8482	7.2422	7.7442	7.3925	7.6169	6.8074	7.1144	6.7757	6.6279	6.8320	6.5746	7.2965	7.4284	7.1206	7.0962
Uba1	_AAVASLLQSVQVPEFTPK_.2	#N/A	5.8631	6.5745	6.6575	6.6638	6.1465	6.4318	6.0841	6.6030	6.9021	6.7381	6.4351	6.5197	6.7896	5.9885	6.3167	6.3914	6.4493	6.2143	6.5440	6.3254
Uba1	_SIPIC[Carbamidomethyl (C)]TLK_.2	#N/A	6.2289	5.2413	6.0531	6.4158	6.1564	6.7353	6.3120	5.8295	6.2447	6.2095	5.7185	6.4666	6.0396	5.8365	6.7942	5.7950	5.3063	6.4103	6.1392	5.5612
Uba1	_YFLVGAGAIGC[Carbamidomethyl (C)]ELLK_.2	#N/A	5.9873	4.9971	5.6427	5.5112	4.9706	5.7310	5.0805	5.8598	5.4642	5.4390	5.5323	5.0736	6.0961	4.5146	5.1131	5.5692	5.4870	4.6312	5.6112	5.1750
Uba1	_NEEDATELVGLAQAVNAR_.2	#N/A	7.7699	7.3720	7.2305	7.0875	7.0870	7.0907	7.4562	7.5784	7.1652	7.3108	7.1460	7.2829	7.5764	7.1752	7.0616	7.0354	7.2336	7.1504	7.4085	6.8137
Uba1	_ATLPS[Phospho (STY)]PDKLPGFK_.2	Q02053_S835_M1_Uba1	9.0726	9.9607	3.5430	3.5778	4.2134	4.2467	9.5605	4.2321	8.0008	3.8170	8.0163	9.3453	4.4212	10.7791	2.8601	8.1716	3.7293	3.4201	3.7192	3.2583
Uba1	_VGEFC[Carbamidomethyl (C)]HSR_.2	#N/A	6.9978	6.2829	6.4123	6.5396	6.9633	6.2588	6.5644	6.9839	7.3011	6.0806	6.1461	6.9198	6.5102	6.8303	6.1567	6.9533	7.1523	6.5185	6.4155	6.6245
Uba1	_LAGTQPLEVLEAVQR_.2	#N/A	7.2919	6.7906	7.0901	7.0399	7.1420	7.1181	7.0211	7.2895	6.8482	7.1364	7.3064	6.7135	6.6369	6.8855	6.6957	6.8302	6.8469	6.9138	7.1253	6.8022
Uba1	_NGS[Phospho (STY)]EADIDESLYSR_.2	Q02053_S46_M1_Uba1	4.4436	6.0339	3.9802	6.3765	3.1497	6.2795	4.3073	3.1684	6.7592	6.2942	3.2399	6.4212	4.4744	6.9338	5.2121	6.2922	6.7941	6.2055	5.9966	6.1372
Uba1	_VVQGHQQLDSYK_.2	#N/A	4.5267	4.5516	4.3724	4.4350	3.9120	4.5410	4.6887	4.0373	4.7231	4.2725	4.6078	4.4503	3.9874	3.6531	4.6020	3.9688	3.2058	3.7928	4.7213	3.8139
Uba1	_QFLDYFK_.2	#N/A	4.4155	3.9474	4.7455	4.8373	3.5803	4.6609	4.8849	2.7120	4.5029	3.8796	3.4627	4.3387	3.1963	3.5618	3.4180	4.2857	4.3922	4.5314	3.9717	4.0889
Uba1	_LDQPM[Oxidation (M)]TEIVSR_.2	#N/A	7.5708	7.3003	7.3134	7.2922	7.2232	7.4372	7.6149	7.5818	7.1972	7.3251	7.2134	7.1926	7.3148	7.1859	7.4443	7.2903	7.0855	7.3320	7.0917	7.1800
Uba1	_M[Oxidation (M)]LQTSSVLVSGLR_.2	#N/A	5.8093	5.0617	5.3239	5.0759	5.7487	5.6255	5.8449	5.6931	6.0188	5.3205	5.2968	5.3059	5.7188	4.8996	5.0336	5.9602	4.9976	5.9710	5.3324	5.3353
Uba1	_NFPNAIEHTLQWAR_.2	#N/A	5.6322	6.3391	5.2509	5.5775	5.7630	5.5153	5.6014	5.6150	5.4045	5.8370	5.6238	5.4138	4.5350	5.3404	3.4224	5.2803	5.4895	5.7011	5.3393	5.4697
Uba1	_LAYVAAGDLAPINAFIGGLAAQEVM[Oxidation (M)]K_.3	#N/A	3.1655	1.7014	1.9478	3.9109	2.9246	2.7565	2.1565	1.8077	3.5961	2.4694	3.9705	3.2259	2.9658	2.7908	3.1142	2.2088	2.8989	1.8077	2.7559	2.5143
Uba1	_NFAM[Oxidation (M)]IGLGC[Carbamidomethyl (C)]GEGGEVVVTDM[Oxidation (M)]DTIEK_.3	#N/A	6.5927	5.8969	5.7370	4.4827	5.8478	5.2880	3.4958	6.5166	5.3464	3.4130	3.9293	5.2163	5.3868	3.7301	3.0730	6.4352	3.4448	3.7944	4.9387	5.3017
Uba1	_DEFEGLFK_.2	#N/A	4.3148	4.2501	3.8514	5.1084	3.0209	4.0392	5.2834	4.5405	5.4481	2.7579	4.0079	5.0989	4.1128	4.6155	4.3562	4.7863	3.5448	3.9395	4.5045	5.2104
Uba1	_QM[Oxidation (M)]NPYIQVTSHQNR_.3	#N/A	4.8312	3.7153	4.2292	3.6329	4.7550	4.9853	4.9396	4.0194	4.7376	4.6999	3.4411	4.2621	4.1387	4.1093	4.7737	4.8919	4.0010	4.8311	4.0931	4.9376
Uba1	_IHVS[Phospho (STY)]DQELQSANASVDDSRLEELK_.3	Q02053_S810_M1_Uba1	4.5306	3.9503	3.3719	3.6467	4.3881	3.7782	3.7991	4.3253	3.7012	4.1922	4.1740	4.4490	3.9848	3.3757	3.3763	3.2644	4.0620	3.3911	3.7888	3.1108
Uba1	_IYDDDFFQNLDGVANALDNIDAR_.3	#N/A	4.1810	2.8435	3.7176	3.4032	2.8871	4.4213	4.1610	4.4067	4.0812	3.9916	3.5598	4.3650	4.2466	3.7557	4.1864	3.5431	3.9039	3.2455	3.5446	3.4329
Uba1	_VLGPYTFSIC[Carbamidomethyl (C)]DTSNFSDYIR_.2	#N/A	3.4344	3.8847	4.4718	4.1498	3.6413	4.4670	3.4068	3.6601	3.3572	3.6107	3.7316	3.6108	3.4653	4.5099	3.4322	4.2973	3.1573	3.9921	4.2912	4.1871
Uba1	_QNSLDEDLIRK_.3	#N/A	3.8204	3.4987	3.3570	3.7638	4.0274	4.0607	3.0207	4.0461	4.4418	3.6310	0.6489	3.2248	3.1064	3.6090	3.0461	3.1825	3.5433	3.0086	4.1580	3.5760
Ctnnb1	_TS[Phospho (STY)]M[Oxidation (M)]GGTQQQFVEGVR_.2	#N/A	9.4229	9.0553	9.1809	9.2691	8.7475	8.9119	8.8836	9.3698	8.8665	9.1440	6.7459	8.5494	9.5750	7.9746	4.9003	8.7201	9.2745	9.0505	9.0062	8.5999
Ctnnb1	_HAVVNLINYQDDAELATR_.3	#N/A	4.9593	4.8222	5.0976	5.0416	4.8275	4.3520	5.0756	5.0544	4.1514	5.3060	5.4638	5.0850	4.2022	4.6734	3.4451	3.7653	4.3971	3.0492	4.8173	4.9626
Ctnnb1	_RTS[Phospho (STY)]M[Oxidation (M)]GGTQQQFVEGVR_.3	#N/A	5.9176	7.4568	6.1039	3.4188	4.0095	4.8917	3.7758	4.1447	4.1737	5.0209	3.9274	3.2415	3.8871	5.3559	4.9306	3.3916	5.0608	5.7534	4.1496	3.3580
Ctnnb1	_TMQNTNDVETAR_.2	#N/A	4.5355	4.2844	4.8207	3.7625	3.2416	3.3659	4.3992	3.2603	4.4583	4.0105	5.2022	4.0106	4.5664	4.2422	3.9764	3.8976	4.2584	4.3919	3.1901	3.7874
Ank1	_GPPETGS[Phospho (STY)]PK_.2	Q02357_S1759_M1_Ank1	8.1941	3.7274	3.9784	8.5172	8.3371	3.8958	8.7260	3.8966	3.9560	8.7074	8.6057	8.3846	4.1215	8.4529	3.2748	3.8040	4.0269	3.6300	4.3378	3.8048
Ank1	_LDQVVES[Phospho (STY)]PAIPR_.2	Q02357_S852_M1_Ank1	8.1220	7.6033	7.6578	7.4927	6.7595	6.2173	6.5960	6.7235	5.6415	8.3585	6.5965	6.7898	7.1906	7.0328	4.2719	6.9370	7.0051	6.5699	6.1564	6.7243
Ank1	_AEDS[Phospho (STY)]DAIPEWK_.2	Q02357_S1589_M1_Ank1	4.1809	8.4263	8.1563	3.4634	2.8870	8.3110	7.9923	5.0790	7.9969	6.5474	3.5599	3.6662	4.2467	8.7298	6.9910	3.5430	5.8122	7.9034	7.9791	8.2451
Nfia	_SVEDEM[Oxidation (M)]DS[Phospho (STY)]PGEEPFYTGQGR_.2	#N/A	3.3228	7.4432	6.7411	5.4067	3.7814	6.9563	6.9130	3.3871	6.7528	3.9543	3.5491	6.1239	3.5767	7.1037	6.0295	4.0263	6.4118	6.7863	6.9339	6.5302
Nfia	_LKS[Phospho (STY)]VEDEM[Oxidation (M)]DS[Phospho (STY)]PGEEPFYTGQGR_.3	Q02780_S303_M2_Nfia	4.4310	4.3889	3.9676	3.1532	3.1371	3.1705	3.9110	3.1559	3.8312	4.2416	3.3098	4.1150	3.9966	4.0057	3.9364	3.7931	4.1539	3.6192	3.2678	2.9654
Nfia	_S[Phospho (STY)]PGSGSQSSGWHEVEPGLPSPSTLK_.3	Q02780_S323_M1_Nfia	3.9577	3.3613	3.4943	3.6265	4.1647	4.1980	4.3843	4.1496	4.3045	3.7683	3.7831	3.0875	4.4699	6.2797	2.9088	3.3199	3.6807	3.1459	4.2953	3.4387
Nucb1	_KVPEQPPELPQLDS[Phospho (STY)]QHL_.3	Q02819_S456_M1_Nucb1	5.8699	6.8931	5.2555	5.6652	5.3323	6.5675	6.0071	5.9703	5.7437	6.1557	6.0885	6.7847	5.5866	7.4687	6.1246	6.3461	5.7620	6.9386	6.7315	6.3451
Nucb1	_SGKLS[Phospho (STY)]QELDFVSHNVR_.3	Q02819_S85_M1_Nucb1	3.5782	3.7409	4.6233	4.0060	3.7852	4.3232	5.1649	6.1049	3.1831	4.9136	3.9878	5.9196	3.6091	6.9006	3.2883	4.5048	5.0055	4.8734	5.4057	4.3310
Nucb1	_AQRLS[Phospho (STY)]QETEALGR_.2	#N/A	4.3950	3.3497	3.5374	3.6148	3.7735	4.2925	3.9118	4.2265	3.8909	4.5815	3.8296	4.9820	4.0316	4.2320	3.9046	4.2912	3.7238	3.4571	3.7562	4.3794
Nucb1	_LSQELDFVSHNVR_.3	#N/A	3.1068	3.5871	3.2686	3.8522	3.9453	2.1296	3.5957	3.9577	3.6548	3.3131	4.0292	3.3132	3.7629	3.3067	3.1729	3.4517	3.4549	3.6945	3.9936	2.9839
Epha4	_NPNS[Phospho (STY)]LKR_.2	Q03137_S887_M1_Epha4	7.8379	7.7732	7.6888	7.7720	7.4278	7.4476	7.2827	7.4303	7.2507	7.5288	7.7005	7.4896	7.4194	7.8799	6.1935	6.8888	7.3910	7.7942	7.2036	7.3893
Epha4	_IDTIAADESFTQVDIGDR_.2	#N/A	5.9092	5.6773	3.1728	5.0605	3.8431	5.1867	5.2827	5.6159	3.5590	5.5828	5.1667	4.8160	5.2330	6.1061	3.0771	5.5390	5.4348	4.5964	5.4602	5.0279
Epha4	_TYVDPFTYEDPNQAVR_.2	#N/A	5.5319	4.7220	3.6600	3.6961	4.2608	4.9733	3.9284	4.7893	3.8486	4.2242	4.6438	4.7568	4.0141	4.5167	3.3968	4.5912	5.7879	4.7230	4.7007	4.6455
Mark3	_RYS[Phospho (STY)]DHAGPAIPSVVAYPK_.3	Q03141_S419_M1_Mark3	8.9651	9.1210	8.5895	4.3329	4.1951	8.0523	8.3253	8.5505	7.9867	8.5333	3.7795	8.5118	8.6329	4.5578	7.1192	8.0552	3.5946	8.5706	8.4450	7.9629
Mark3	_SRGS[Phospho (STY)]TNLFSK_.2	Q03141_S601_M1_Mark3	3.6482	4.3546	4.1845	4.0412	2.9859	5.8844	3.0543	3.3486	3.1134	3.8545	3.4878	4.2441	3.2215	4.1716	4.1324	3.9931	3.8863	3.9469	5.9588	4.1386
Mark3	_SAELDASDSSS[Phospho (STY)]SSNLSLAK_.2	Q03141_S378_M1_Mark3	3.8407	4.8188	4.2190	4.1133	3.7860	3.7779	4.5693	3.6838	4.2111	3.8093	3.3174	3.5831	4.0042	5.2176	3.9003	4.1227	4.7942	5.0613	4.2197	3.5215
Mark3	_IDIMVGM[Oxidation (M)]GYS[Phospho (STY)]QEEIQESLSK_.2	Q03141_S341_M1_Mark3	3.2538	4.0653	4.2912	4.3304	3.4608	3.4941	3.5873	3.4795	3.5076	4.5653	2.9862	3.7914	3.2847	4.3293	3.6127	4.1168	4.4776	4.1727	2.9709	4.0066
Mark3	_VRPNSDLSNSTGQS[Phospho (STY)]PHHK_.3	#N/A	6.1176	6.2021	5.0827	4.9927	5.1389	5.2557	5.2900	4.9335	4.9847	5.7327	5.0502	5.1656	5.4305	5.5513	4.4907	5.2363	5.3137	5.5528	5.1924	5.1372
Mark3	_TPVAS[Phospho (STY)]THS[Phospho (STY)]ISSATTPDR_.2	Q03141_S540_M2_Mark3	4.3041	4.5158	3.8407	3.2801	3.0102	3.0436	4.0379	4.5298	3.9581	4.1147	3.4367	4.2419	4.1235	3.8788	4.0632	3.6663	4.0271	3.1224	3.4214	3.5561
Cdk7	_SFGS[Phospho (STY)]PNR_.2	Q03147_S164_M1_Cdk7	3.3516	8.2919	6.9154	4.3377	3.7614	3.7947	3.2867	3.0520	3.2069	3.3650	4.1864	3.0398	3.3724	3.8750	3.3121	4.4174	3.5897	4.2435	7.9543	7.5921
Enah	_PSS[Phospho (STY)]PVNTPSSQPPAAK_.2	#N/A	3.4181	7.9319	8.3110	7.9641	4.2567	7.5259	7.5224	3.1185	7.2956	7.8807	3.7179	2.9733	3.4388	3.9415	2.8616	7.5239	3.6562	4.1770	7.5515	3.9085
Enah	_S[Phospho (STY)]PVISRPK_.2	Q03173_S740_M1_Enah	3.6486	6.1026	4.1849	4.0408	7.2857	3.2104	3.0539	3.3490	3.1130	7.5456	7.3249	4.2437	3.6693	4.1720	5.7009	3.9935	3.8866	3.9465	7.2541	4.1390
Enah	_NSRPSS[Phospho (STY)]PVNTPSSQPPAAK_.3	#N/A	9.0602	9.2641	8.8107	7.4340	3.5544	8.3387	8.5965	8.6053	8.4497	8.7918	8.5630	8.3268	8.7777	9.5808	8.3267	8.8605	8.7616	8.1985	8.5157	8.4586
Atp5f1a	_AVDSLVPIGR_.2	#N/A	12.2659	12.0484	12.0977	12.2214	12.2086	12.0499	12.2433	12.4645	11.7925	12.0677	12.2132	12.4356	12.5501	12.2423	12.1868	12.1060	12.1276	12.2752	12.0167	12.0453
Atp5f1a	_VVDALGNAIDGK_.2	#N/A	11.9856	11.8403	12.2161	11.9116	12.0024	11.7322	11.8722	12.1800	11.9401	11.9295	11.9426	11.8715	12.0462	12.1298	12.1483	11.9032	12.0000	11.8507	11.9087	11.8819
Atp5f1a	_VLSIGDGIAR_.2	#N/A	11.8952	11.8907	12.0126	12.0256	11.9872	11.8180	11.8663	12.0370	11.7659	11.9214	11.8300	11.9892	11.9680	11.8968	11.9254	11.9104	11.8450	11.8274	11.9233	11.8124
Atp5f1a	_TGAIVDVPVGEELLGR_.2	#N/A	11.4278	11.1844	11.5857	11.5892	11.1814	11.2898	11.2359	11.3719	11.4197	11.4721	11.4067	11.2124	11.6040	11.3943	10.9705	11.2597	11.3264	11.1543	11.1576	11.2472
Atp5f1a	_ELIIGDR_.2	#N/A	11.1654	10.9603	11.2836	11.2467	11.2574	11.0161	11.0809	11.2756	11.2492	11.3827	11.4260	11.3846	11.6489	11.3416	11.3410	11.3793	10.9119	11.2624	11.3340	11.3740
Atp5f1a	_LELAQYR_.2	#N/A	10.8051	10.7886	10.8688	10.9439	11.0469	10.8116	10.9650	11.0954	10.6852	10.8257	10.9552	11.1023	11.1957	10.9355	10.9915	10.8474	10.6250	11.0234	10.8551	11.0109
Atp5f1a	_TSIAIDTIINQK_.2	#N/A	9.5600	9.7866	10.0120	9.7795	9.5677	9.6168	9.4820	9.6797	9.9940	9.8095	9.6111	9.4416	9.9003	9.9588	9.6888	9.9091	9.8446	9.6587	9.8714	9.5907
Atp5f1a	_GYLDKLEPSK_.2	#N/A	10.0795	9.7538	9.7533	9.9400	10.1026	9.7148	9.6193	10.3341	9.6407	9.9886	9.8907	9.8348	10.4225	10.1214	9.7672	9.8702	10.0058	9.9416	9.5748	9.7142
Atp5f1a	_HALIIYDDLSK_.2	#N/A	9.4928	9.6335	9.7196	9.4705	9.3614	9.2744	9.3020	9.8023	9.4588	9.3033	9.3064	9.4738	9.9594	9.9713	9.5122	9.5719	9.4551	9.5526	9.5277	9.5012
Atp5f1a	_ILGADTSVDLEETGR_.2	#N/A	9.1398	9.0907	8.9486	9.3249	9.1291	9.2555	9.0768	9.4011	9.1991	9.0535	9.2664	8.8508	9.3581	9.1805	9.0576	9.1383	9.2830	9.3626	9.0568	9.1487
Atp5f1a	_VVDALGNAIDGKGPIGSK_.2	#N/A	6.6089	6.9267	6.4816	7.3135	7.2989	6.8777	6.9051	7.3156	7.0064	7.2675	6.8478	7.4811	7.7221	7.0543	7.1393	7.4776	6.5620	6.9026	7.0839	7.2458
Atp5f1a	_STVAQLVK_.2	#N/A	7.4967	7.6982	7.8706	8.1492	7.9030	7.9125	8.0823	7.7469	7.7590	8.0771	8.1359	8.4599	7.6541	8.1557	8.2814	7.5285	6.7325	8.1902	8.1606	8.0018
Atp5f1a	_RTGAIVDVPVGEELLGR_.3	#N/A	7.6015	7.6043	7.8981	7.6229	7.5400	7.5342	7.6109	7.7100	7.7341	8.1548	7.6446	7.6841	7.7685	7.7556	7.8440	8.0195	7.8612	7.7039	7.9003	8.0163
Atp5f1a	_EGDVVKR_.2	#N/A	7.9423	7.5302	7.9596	7.5741	7.5177	7.6417	7.8991	7.8514	7.7358	7.5596	7.9022	7.3884	7.6539	7.5302	7.9938	7.3335	7.2374	7.5553	6.8360	7.6309
Atp5f1a	_LKEIVTNFLAGFEP_.2	#N/A	6.7093	6.3644	7.1046	6.9926	6.7895	6.6847	6.5002	6.8530	6.9164	6.7475	6.6250	6.8547	7.4887	7.0489	6.6967	7.0128	6.7109	6.4712	6.9071	6.8559
Atp5f1a	_RFNDGTDEK_.2	#N/A	6.6445	6.4390	7.0689	6.4730	6.3689	6.5057	6.7002	7.0060	7.2038	7.1741	6.8900	6.9589	6.9359	7.1606	7.6730	7.1258	6.9743	6.5289	7.1064	6.4497
Atp5f1a	_EIVTNFLAGFEP_.2	#N/A	6.6290	6.6498	6.6733	7.1539	6.7371	6.5670	6.5962	5.9844	7.0294	7.1801	7.0797	6.8273	6.9101	6.8494	6.6114	6.6483	6.4956	6.3334	6.5876	6.8896
Atp5f1a	_EAYPGDVFYLHSR_.2	#N/A	10.8230	10.9351	10.7536	10.7068	10.7811	10.6097	10.6721	10.9991	10.5086	10.7342	10.6677	10.8258	11.1264	10.9876	10.5026	10.7908	10.9083	10.7716	10.6942	10.7557
Atp5f1a	_FNDGTDEK_.2	#N/A	3.0879	7.0380	6.2154	7.3023	5.6722	7.0056	7.1995	5.6319	7.1394	7.5137	7.8408	6.6348	7.0987	6.3813	7.6591	6.9406	5.7739	7.3089	7.4547	7.4452
Atp5f1a	_ISEQSDAK_.2	#N/A	6.6954	6.6085	6.5540	6.4244	7.0507	6.7109	6.9359	6.4031	7.4449	6.9494	7.2138	6.8689	7.4090	7.0762	8.2836	4.5010	6.6228	6.6081	6.7782	6.8906
Atp5f1a	_QM[Oxidation (M)]SLLLR_.2	#N/A	9.6051	9.3967	8.9834	9.5489	9.5634	9.4146	9.5412	9.9056	9.2234	9.4972	9.3033	9.7044	9.8705	9.3776	9.5143	9.6191	9.3946	9.6748	9.6713	9.6794

Atp5f1a	_RLTDADAM[Oxidation (M)]K_.2	#N/A	7.8155	7.9796	8.5290	8.2422	7.9253	7.9069	8.3478	7.8742	7.9062	7.8819	8.1385	8.1254	7.8822	8.4432	8.4226	7.8029	7.7306	7.8101	7.8004	8.0439
Atp5f1a	_FNDGTDEKK_.2	#N/A	4.4148	4.4052	5.1467	5.1590	4.7077	5.8337	3.9272	5.3937	3.8475	5.8863	5.7315	5.4129	4.9874	4.5156	5.9191	5.7286	5.4694	4.3813	5.7391	5.3292
Atp5f1a	_LYC[Carbamidomethyl (C)]IYVAIGQK_.2	#N/A	5.2380	4.8723	5.5117	4.4724	5.3759	5.5432	4.6161	5.6805	5.4906	3.9756	4.9357	5.5519	3.7900	4.2926	4.6177	5.7461	5.8199	4.9171	5.9848	5.5767
Atp5f1a	_GM[Oxidation (M)]SLNLEPDNVGVVVFGNDK_.2	#N/A	8.7478	8.7023	8.9470	8.6483	8.4226	8.5058	8.7223	8.8592	8.8305	8.8526	8.6327	8.7630	8.7008	9.0292	8.7144	8.8857	8.9380	8.7998	8.7703	8.8171
Atp5f1a	_QGQYSPM[Oxidation (M)]AIEEQVAVIYAGVR_.2	#N/A	4.9737	5.0555	5.3750	4.8530	5.3035	4.7327	5.3094	5.7817	4.4922	5.5122	5.6400	4.6949	5.3187	5.1078	4.6580	4.8829	5.2946	5.6481	5.2487	5.4280
Atp5f1a	_EPMQTGIK_.2	#N/A	3.3314	6.7545	7.7913	8.1185	4.1700	6.3857	6.6336	4.5326	6.0241	3.3853	7.3461	5.3974	3.3521	8.0774	7.5048	3.6763	3.5695	5.4654	5.5822	6.2674
Atp5f1a	_TGTAEM[Oxidation (M)]SSILEER_.2	#N/A	8.9961	9.2008	9.6036	9.3586	8.9306	9.0660	9.1439	9.1156	9.1312	9.0397	9.0216	8.9907	8.9815	9.4511	8.6094	8.8350	8.9160	8.9025	8.7903	9.0001
Atp5f1a	_FENAFLSHVISQHQSLLGNIR_.3	#N/A	5.5976	6.0417	6.0305	5.7749	5.9738	5.3997	5.7397	6.4726	5.6543	5.7296	5.8199	5.7904	6.2527	6.3575	5.5503	5.8227	6.1672	6.0335	6.2824	6.0941
Atp5f1a	_EVAAFAQFGSDLDAATQQLLSR_.3	#N/A	7.0675	7.4067	7.1845	7.1151	6.8600	7.1857	6.7928	6.9004	6.9477	7.3485	7.1677	6.9079	7.2985	7.6900	6.5622	7.1277	7.2645	6.9297	7.2605	7.3663
Atp5f1a	_LTDADAMK_.2	#N/A	3.8409	4.0888	4.0353	4.8201	3.1786	3.8924	5.1857	3.2235	2.9662	3.7607	4.8830	4.0473	4.5296	4.6056	4.5798	4.1858	4.2216	4.4287	3.2269	3.4640
Atp5f1a	_YTIVVSATASDAAPLQYLAPYSGC[Carbamidomethyl (C)]SM[Oxidation (M)]GEYFR_.3	#N/A	4.3346	4.4854	3.8711	3.2497	3.0407	3.5669	4.0074	3.0594	4.2574	4.1451	3.1309	4.2115	4.3654	3.9092	4.0328	3.6967	4.0575	3.0920	3.3910	3.5865
Atp5f1a	_NVQAEEMVEFSSGLK_.2	#N/A	4.5110	3.4918	3.5464	3.1784	3.8487	4.1362	3.6924	4.2113	4.3662	3.7066	3.8449	3.3813	4.5317	3.5335	3.2696	3.3550	3.2482	3.0841	4.2336	2.8260
Grin2d	_S[Phospho (STY)]LEDLSSC[Carbamidomethyl (C)]PR_.2	Q03391_S1258_M1_Grin2d	7.8766	7.9139	7.3793	7.7681	7.4893	7.4832	7.9293	7.2250	7.4996	7.6811	7.3874	6.7330	7.2294	7.2126	5.8083	7.0685	8.1013	7.6260	7.1204	7.3769
Grin2d	_LAFEDES[Phospho (STY)]PPAPSR_.2	Q03391_S1049_M1_Grin2d	3.7119	3.6072	3.2485	3.8723	3.9188	8.8809	3.1293	3.9376	3.0495	8.5471	4.0289	3.3333	3.2149	3.7176	3.1546	7.9227	8.9229	8.6949	7.9941	3.6846
Grin2d	_RGS[Phospho (STY)]AHFSSLESEV_.2	Q03391_S1313_M1_Grin2d	3.8148	3.5043	3.3514	3.7695	4.0217	4.0867	3.0264	4.0405	6.4004	3.2303	4.1120	3.2304	3.8456	3.3894	3.0518	3.1769	5.1463	3.6118	3.9108	3.0667
Scg2	_VAWIPDVESR_.2	#N/A	6.8669	7.5744	7.3414	7.3723	7.6851	7.1220	7.3884	7.3807	7.1071	7.3810	7.4627	7.9853	7.8067	7.6796	8.0069	7.6035	7.5163	7.3831	7.2695	7.0701
Scg2	_IETQTQEEVRDS[Phospho (STY)]KENTEK_.3	#N/A	5.7767	5.1872	4.0823	4.3561	3.0784	5.5550	3.5134	3.2584	5.9014	3.8740	5.5691	4.1828	5.1784	4.0949	6.2574	6.2018	5.9042	5.8333	6.1867	4.2106
Scg2	_IETQTQEEVRDS[Phospho (STY)]K_.2	#N/A	5.5141	5.0095	5.3530	6.0718	5.4154	5.7329	5.3916	4.4507	5.7282	5.8663	5.6146	5.3430	4.2559	4.8608	5.2023	4.6814	5.1599	3.2015	5.7163	5.7771
Scg2	_QAPYENLNDQELGEYLAR_.2	#N/A	4.7301	4.6575	4.6325	4.2016	5.1223	4.7704	5.0593	5.0878	5.3110	4.1776	4.2374	5.3389	4.1599	3.8433	4.9822	4.9491	5.0737	3.0728	4.5014	4.2546
Scg2	_EHLGPGSS[Phospho (STY)]QEM[Oxidation (M)]ER_.2	#N/A	9.5140	9.3406	8.9040	8.7161	3.1896	3.9005	9.6914	3.7653	9.8336	9.6098	3.8685	4.1435	4.0421	10.6828	7.9215	9.9556	9.7305	3.6171	9.8810	9.9093
Scg2	_VPSPVS[Phospho (STY)]SEDDLQEEEQLEQAIK_.3	Q03517_S532_M1_Scg2	3.6386	3.6804	3.1752	3.9456	3.8456	4.2628	3.2025	3.8643	7.2837	3.4065	3.9358	3.4066	3.6695	3.2133	3.2279	3.0008	3.3616	3.7879	4.0869	4.3914
Scg2	_SPYFPNQYSQDK_.2	#N/A	6.2618	4.1903	5.9961	5.8644	6.4853	6.1313	6.2575	6.4753	6.5106	6.4761	6.4723	6.4789	4.2039	6.3182	6.6587	6.6098	4.2057	5.9858	6.1525	6.1931
Scg2	_S[Phospho (STY)]GQLGLPDEENRR_.2	#N/A	6.2412	6.0017	5.9645	5.9338	6.0199	5.7801	5.9533	6.4140	6.0383	6.3254	5.8167	6.5895	6.6747	6.4807	5.9868	6.2130	6.1176	5.7503	6.1721	6.0629
Scg2	_ESKDQLSEDASK_.2	#N/A	4.3863	3.3657	3.4899	5.1618	3.7240	4.2631	4.4937	4.1790	3.3507	3.0918	3.7363	3.0919	3.9842	3.5280	4.9881	3.2303	3.6763	3.4732	4.5310	3.2053
Scg2	_VPSPVSSEDDLQEEEQLEQAIKEHLGPGS[Phospho (STY)]SQEM[Oxidation (M)]ER_.4	#N/A	4.6383	3.9994	4.3571	4.2645	3.5266	3.5600	3.5215	3.5454	3.4417	3.1303	2.9203	3.7255	3.6071	4.3952	3.5469	4.1826	4.5434	4.1068	4.4059	4.0768
Scg2	_FPLMYEENS[Phospho (STY)]RENPFKR_.3	Q03517_S176_M1_Scg2	4.4077	3.5951	3.4432	3.2817	3.7454	3.9695	3.7957	4.1576	3.8547	3.1132	4.2291	3.4846	3.9628	5.2067	3.3729	3.2518	3.6548	3.4946	3.7937	3.1839
Kcnb1	_NHFESSPLPTS[Phospho (STY)]PK_.2	#N/A	9.4664	9.1390	9.2422	9.1873	8.2728	3.9766	8.6466	8.3441	8.1907	9.1816	8.5381	8.5657	3.1905	8.4670	6.6946	8.5004	8.7090	8.6064	8.4023	3.6602
Kcnb1	_SLHGSTS[Phospho (STY)]PK_.2	Q03717_S782_M1_Kcnb1	7.3464	4.2396	4.1169	4.5047	5.9734	3.3198	7.0486	6.0977	3.6819	4.3909	6.7389	6.4648	3.8473	4.1550	3.7870	3.9425	4.3033	6.8911	3.1452	3.8323
Kcnb1	_VQDNHLS[Phospho (STY)]PNK_.2	#N/A	5.7192	5.8359	5.0617	5.6692	5.5066	5.2255	5.9949	4.9047	5.4493	5.3135	5.5011	5.1786	3.9809	5.8681	3.6885	5.3326	5.9305	5.5835	5.3478	5.3677
Kcnb1	_T[Phospho (STY)]PPRSPEK_.2	Q03717_T732_M1_Kcnb1	8.2128	8.2119	7.5973	7.8611	5.2517	6.7684	6.9629	5.8643	6.5279	7.1847	8.0268	7.4827	5.8092	6.8264	3.2335	5.9050	7.2628	8.0885	6.4356	6.9287
Kcnb1	_TQS[Phospho (STY)]QPILNTK_.2	Q03717_S541_M1_Kcnb1	8.7128	8.7657	8.6395	8.6890	7.3560	4.0325	7.8335	6.9800	7.0070	8.9656	8.2174	8.0401	7.6427	7.9628	5.1536	7.4600	7.7386	7.7603	7.6939	7.4059
Kcnb1	_TEKNHFESSPLPTS[Phospho (STY)]PK_.3	#N/A	3.7429	4.2599	6.5361	4.4883	3.0806	3.3047	4.4605	3.3216	3.0187	3.9492	6.7455	6.1766	8.2306	6.6678	5.0882	5.5877	4.3196	6.3508	3.1289	3.8487
Kcnb1	_VQDNHLS[Phospho (STY)]PNKWK_.3	#N/A	6.2658	6.7017	3.8905	6.5687	5.2686	5.8679	5.8982	5.9350	5.2837	6.0436	6.2728	5.7734	6.2181	6.0080	4.8356	5.9645	6.0666	6.5089	6.2723	6.0297
Kcnb1	_DKVQDNHLS[Phospho (STY)]PNKWK_.3	#N/A	5.6335	5.4056	5.5048	5.2556	5.4165	5.7568	5.6755	6.1752	4.9369	5.0856	5.6575	4.7803	4.9619	5.1865	4.4519	5.0438	5.5524	5.4286	5.3274	4.7063
Kcnb1	_SSSS[Phospho (STY)]PQHLNVQQLQDMYSK_.3	Q03717_S520_M1_Kcnb1	3.9202	4.3864	4.1232	5.7437	3.0297	4.4774	5.7085	3.7888	4.0024	5.0568	3.2887	4.2152	4.1104	4.0254	4.0799	3.9362	4.0293	3.9399	3.4477	3.8158
Kcnb1	_S[Phospho (STY)]IEM[Oxidation (M)]M[Oxidation (M)]DIVVEK_.2	#N/A	7.7791	7.0357	6.5792	6.4527	6.3066	6.2050	6.6689	6.3761	6.4873	7.2774	6.2766	6.6867	6.9398	5.7943	5.1549	6.7077	7.2288	7.1404	6.8733	6.6464
Kcnb1	_STSS[Phospho (STY)]LPPEPMEIVR_.2	Q03717_S15_M1_Kcnb1	3.8694	3.8253	4.0309	3.8241	3.6135	3.4314	3.7330	3.7998	3.6707	3.7482	3.8959	3.6371	3.8942	4.0550	3.4949	3.8518	4.9243	3.6928	3.7522	3.8167
Kcnb1	_NGS[Phospho (STY)]IVSMNMK_.2	Q03717_S444_M1_Kcnb1	4.5061	3.4967	3.5416	3.1833	3.8439	4.0679	3.6972	4.2065	3.7563	3.2116	4.1307	3.3861	3.8644	3.5287	3.2744	3.3502	3.2434	3.0890	4.2384	3.4957
Kcnb1	_EQGS[Phospho (STY)]PEKAR_.2	Q03717_S511_M1_Kcnb1	3.9708	4.0320	4.5071	3.7186	3.3085	3.5326	4.2326	3.6712	4.2917	4.1771	3.1652	3.9215	4.3997	4.4942	3.8098	4.3157	4.2088	3.6243	3.2729	2.9603
Kcnd1	_QNGGLEDSGS[Phospho (STY)]GDGQM[Oxidation (M)]LC[Carbamidomethyl (C)]VR_.2	#N/A	3.9218	4.1449	5.3633	3.8983	3.7661	3.8577	3.9011	3.6222	4.2837	4.0721	3.7904	4.0400	4.1889	4.9189	3.4774	3.7637	6.3411	4.2237	3.8933	3.7220
Pfdn6	_ETLAQLQQEFQR_.2	#N/A	4.1281	4.9013	5.3080	5.8579	5.1493	5.1170	5.3146	5.8810	5.3547	4.3345	4.6562	5.8813	5.3835	6.0264	6.0011	5.6827	5.4704	3.2150	5.2144	5.4013
Ckb	_VLTPELYAELR_.2	#N/A	11.4771	11.9227	11.8319	11.8643	11.5955	11.5845	11.6963	11.6961	11.6119	11.6453	11.4805	11.8402	11.8972	11.9634	11.9004	11.7278	11.8682	11.8364	11.8437	11.8972
Ckb	_FSEVLKR_.2	#N/A	10.5542	10.8696	10.9154	10.7610	10.8733	10.6370	10.9061	10.8656	10.6906	10.7716	10.5668	11.0721	11.0046	11.1090	11.4880	10.8506	10.8112	10.8626	10.9214	10.9064
Ckb	_DLFDPIIEER_.2	#N/A	11.5547	11.3997	11.8737	11.4588	11.4445	11.5142	11.3313	11.2697	11.5621	11.5368	11.9541	11.4902	11.1824	11.9567	11.6264	11.5539	11.6878	11.3571	11.3899	11.7500
Ckb	_GIWHNDNK_.2	#N/A	10.8708	10.8053	11.2952	11.0176	11.0460	10.9387	10.7467	11.1588	10.9680	11.0303	10.8977	11.1667	11.0568	11.3761	11.3423	11.0743	11.0906	10.9541	11.0102	11.1697
Ckb	_LLIEMEQR_.2	#N/A	8.9577	10.0247	10.5461	10.5211	9.4470	9.7892	9.9871	9.3728	9.4387	9.4310	9.9368	9.6867	9.6060	10.8469	9.7429	9.1482	9.7065	9.4419	9.3415	9.6861
Ckb	_HGGYQPSDEHKTDLNPDNLQGGDDLDPNYVLSSR_.5	#N/A	9.3841	9.0127	8.6702	9.6756	8.8663	8.7214	9.0442	9.3375	8.9225	9.5689	9.3445	9.5790	9.7266	9.8154	8.6933	9.3942	9.2326	8.9356	9.1113	9.4291
Ckb	_FC[Carbamidomethyl (C)]TGLTQIETLFK_.2	#N/A	10.0043	9.8870	10.0399	10.0487	9.8759	9.7606	9.7948	9.7346	9.9110	10.0161	9.8089	10.0619	10.1628	10.0858	10.2365	9.9397	9.9826	9.9264	9.7395	10.1791
Ckb	_TFLVWINEEDHLR_.2	#N/A	6.9931	6.7508	7.5857	7.7002	7.0253	6.4633	6.5512	6.0756	7.4088	7.1702	6.6997	7.1050	7.0863	7.9578	6.5784	6.8612	7.2148	6.8143	7.7136	7.4730
Ckb	_HEKFS[Phospho (STY)]EVLKR_.3	#N/A	7.6110	6.8455	7.6749	7.9056	7.7302	7.6346	7.1812	7.3582	7.2809	7.6002	7.5654	6.9128	7.5594	7.8817	7.4501	7.6282	7.3762	7.3660	7.1988	7.7431
Ckb	_NYEFM[Oxidation (M)]WNPHLGYILTC[Carbamidomethyl (C)]PSNLGTGLR_.3	#N/A	5.0596	5.9659	5.9971	5.7481	4.5273	5.0178	4.7946	5.3401	4.4502	5.6644	5.0313	5.0236	5.0441	6.0724	5.5223	5.0615	5.3960	5.5607	4.7069	4.2449
Ckb	_LEQGQAIDDLM[Oxidation (M)]PAQK_.2	#N/A	10.7491	11.8517	11.5208	11.4695	10.8410	10.8779	11.7412	11.9692	11.1419	10.9510	11.0802	11.1629	12.2098	11.7237	12.1703	11.8152	12.0291	11.6385	10.9009	11.0340
Ckb	_LAVEALSSLDGDLSGR_.2	#N/A	9.7051	9.4486	10.1775	10.0457	9.4826	9.3325	9.2266	9.4751	9.8340	9.8253	9.6183	9.6573	10.1303	9.9567	9.8711	9.9158	9.8288	9.3473	9.7472	9.8612
Ckb	_HEKFS[Phospho (STY)]EVLK_.2	#N/A	6.1171	5.8758	5.1187	4.9994	5.1114	5.7235	5.4409	5.8353	5.6065	5.1136	6.0189	5.7420	5.6438	5.3863	4.9277	5.1027	5.8353	5.6905	5.7710	6.0695
Ckb	_TDLNPDNLQGGDDLDPNYVLSSR_.3	#N/A	4.2408	5.2779	5.0750	5.1945	2.8075	3.9780	4.4138	4.4665	4.8031	4.6327	4.8361	4.5748	4.2716	5.4698	5.2078	4.8368	4.9268	4.3200	5.3553	4.6754
Ckb	_FPAEDEFPDLSSHNNHM[Oxidation (M)]AK_.3	#N/A	7.6200	7.9761	8.5186	8.3106	7.8741	7.9603	7.9656	7.4033	7.9560	8.2386	8.4342	7.8941	7.8587	8.5944	8.5300	8.1333	7.7545	7.6819	7.9701	8.1218
Ckb	_LGFSEVELVQM[Oxidation (M)]VVDGVK_.2	#N/A	7.7486	7.6729	8.0789	7.7412	7.5069	7.6285	7.4010	8.0133	7.2910	8.1354	7.7760	7.9893	8.1750	8.0439	7.1596	7.7800	7.9283	7.6498	7.6732	8.0794
Ckb	_PFSNSHNTQK_.2	#N/A	7.2378	7.5301	7.5191	7.3085	7.4260	7.3611	7.4061	7.5112	7.4900	7.5550	7.2311	7.6366	7.5014	7.8234	8.6937	7.5977	7.5197	7.2627	7.6713	7.5753
Ckb	_LRFPAEDEFPDLSSHNNHM[Oxidation (M)]AK_.4	#N/A	10.8035	10.2059	10.3105	10.3532	10.1974	10.0326	10.1136	10.1683	10.2686	10.3940	10.0960	10.4997	11.4321	10.7827	10.6741	10.5104	10.4763	10.0607	10.2791	10.5136
Ckb	_SMTEAEQQQLIDDHFLFDKPVSPLLLASGM[Oxidation (M)]AR_.4	#N/A	3.3925	5.0653	5.0196	5.4773	4.1038	4.7379	4.8062	4.0864	4.2177	4.0491	5.2798	4.7723	3.2942	5.8770	4.8346	4.2554	4.0581	4.7022	4.3213	4.6454
Ckb	_GTGGVDTAAVGGVFDVSNADR_.2	#N/A	8.7991	8.7113	8.8517	8.7867	7.3799	8.3231	9.0037	9.0693	8.6748	8.7344	8.5785	8.6700	9.0188	9.2068	8.8879	8.6877	8.8393	8.4602	8.7259	8.5639
Ckb	_HGGYQPSDEHK_.3	#N/A	3.8799	3.4392	3.4165	3.7043	4.0868	4.0215	4.4621	4.1056	3.8027	3.1652	5.0524	3.1653	3.9108	3.4545	3.2320	3.2420	3.6028	4.1565	4.3282	3.1318
Ckb	_RGTGGVDTAAVGGVFDVSNADR_.3	#N/A	3.8158	4.4542	3.3524	3.0983	4.6538	4.0561	3.0253	4.2915	5.1839	3.6264	4.7372	4.7433	3.1110	3.6136	4.1810	3.1779	3.3283	3.0040	4.9526	3.5806
Npy1r	_QAS[Phospho (STY)]PVAFKK_.2	#N/A	7.9439	8.2377	7.6552	7.7893	7.6997	7.6759	8.0803	7.9043	7.6306	7.8760	7.1028	7.4295	8.3507	7.6828	6.2575	7.2571	7.7036	7.8769	7.5337	7.4993
Npy1r	_QAS[Phospho (STY)]PVAFK_.2	#N/A	6.0658	6.3999	6.4077	5.9255	6.3223	6.1911	6.4163	6.0230	5.5499	6.2599	6.8587	5.6705	6.8482	6.1050	4.1869	6.0114	6.0232	6.5937	6.3204	6.4874
Npy1r	_TS[Phospho (STY)]LKQAS[Phospho (STY)]PVAFK_.2	#N/A	3.5468	5.2997	4.0831	4.1425	2.8846	3.5995	3.1557	3.2472	3.4021	3.7532	4.3816	4.3454	3.5676	4.0702	4.2337	3.8917	3.7849	5.0168	5.2878	4.0372
Npy1r	_TS[Phospho (STY)]LKQAS[Phospho (STY)]PVAFKK_.3	#N/A	3.0978	3.4042	3.6341	3.8432	3.9363	4.1604	3.6048	3.9667	3.6638	3.3041	4.0382	3.2937	3.7719	3.3157	3.1820	3.4427	3.4639	3.6855	3.9846	2.9930
Nf1	_S[Phospho (STY)]FDHLISDTK_.2	#N/A	11.4211	11.2493	10.9318	10.9510	10.2082	10.7988	10.8370	10.7882	10.8087	11.0380	10.8070	10.6461	11.0387	10.7769	9.1806	10.4884	11.2204	10.9203	10.6994	10.4343
Nf1	_KVS[Phospho (STY)]VSESNVLLDEEVLTDPK_.3	Q04690_S2599_M1_Nf1	6.5859	3.9638	4.3927	6.3057	5.5461	3.0453	6.3656	3.5809	3.2781	3.6898	6.2325	5.5627	3.3861	4.4307	3.5113	4.2182	6.4361	6.0190	6.0484	5.3450
Nf1	_SFS[Phospho (STY)]PGSYER_.2	Q04690_S2190_M1_Nf1	4.4816	4.3384	6.2976	6.2933	6.3265	7.1088	4.3453	6.6884	6.5863	6.6056	7.6122	7.1065	4.5124	6.4973	3.8858	6.5531	7.1144	7.4838	3.2440	6.7302
Nf1	_SSSGLATYS[Phospho (STY)]PPM[Oxidation (M)]GAVSER_.2	#N/A	4.1365	6.9525	7.5322	8.4198	3.8164	7.1240	6.8615	3.9113	6.0075	7.9299	7.2185	7.6554	8.7156	7.7788	3.8791	7.5979	7.5622	3.3944	8.0530	7.8087
Nf1	_KS[Phospho (STY)]FDHLISDTK_.2	#N/A	7.7149	7.4998	6.8557	7.1952	6.3569	6.8277	6.8407	7.2115	6.9423	7.0457	6.9414	6.7616	7.1266	6.6580	4.9079	6.6168	7.1613	7.2579	7.0224	6.4406
Nf1	_SM[Oxidation (M)]S[Phospho (STY)]LDM[Oxidation (M)]GQPSQANTK_.2	#N/A	3.3565	8.8228	6.9845	6.6599	7.3315	8.1557	8.2487	8.0041	3.9844	7.9657	6.8706	7.4451	8.1585	3.8265	6.0539	7.8482	5.8526	8.4031	7.8809	7.7816
Nf1	_SAGS[Phospho (STY)]FKR_.2	Q04690_S2831_M1_Nf1	7.8378	7.5654	4.1943	3.8908	7.0141	7.1663	7.5783	7.4385	7.0868	7.3831	6.9539	6.9512	7.3733	6.9170	6.0008	4.0137	7.3331	7.3842	6.9894	7.0716
Nf1	_GSEGYLAATYPAVGQTS[Phospho (STY)]PR_.2	Q04690_S2517_M1_Nf1	9.8095	9.9859	9.7730	9.6960	8.5155	9.2447	9.4006	3.2961	9.2488	9.5589	2.9296	9.0755	3.6164	9.1674	7.2934	9.0955	3.8338	3.9994	9.0430	9.0672
Nf1	_KS[Phospho (STY)]M[Oxidation (M)]SLDMGQPSQANTK_.2	#N/A	3.8579	3.7543	4.1314	4.0251	3.6692	3.7784	3.7861	3.8551	3.8042	3.8917	3.9901	3.6437	3.9289	3.8249	3.5152	3.8217	3.7956	3.9134	3.7690	3.6387
Nf1	_SMS[Phospho (STY)]LDMGQPSQANTKK_.2	#N/A	3.7223	4.2805	4.2586	3.9671	3.0600	3.2841	2.9802	3.3421	3.0393	3.9286	3.4137	4.1699	3.1473	4.3070	4.0582	4.0672	4.3402	4.3101	3.1083	3.8692
Cdk16	_KIS[Phospho (STY)]TEDINK_.2	#N/A	3.4899	6.2093	4.0262	4.1995	5.8786	3.6564	3.4250	5.5466	3.3452	3.2268	5.5170	5.6740	6.1480	4.0133	2.7898	5.5418	3.7280	5.6149	5.5986	3.9803
Cdk16	_KIS[Phospho (STY)]TEDINKR_.2	#N/A	4.2528	5.0704	4.7642	4.0139	3.3602	3.5269	4.7929	3.6231	3.6001	4.3074	3.7160	4.3876	3.8499	4.0602	3.7793	3.7865	4.2983	4.5280	3.8553	4.4623
Cdk16	_QLS[Phospho (STY)]MTLR_.2	Q04735_S12_M1_Cdk16	4.1007	4.5810	3.7755	6.8246	3.4384	3.6625	4.1027	4.4646	4.1618	4.3070	3.0353	4.3071	4.2698	6.3240	3.6799	2.9447	3.9619	3.1876	3.4866	3.4909
Sox10	_AS[Phospho (STY)]PGPGELGK_.2	Q04888_S45_M1_Sox10	6.9045	7.5367	7.2403	6.9219	6.8169	7.7926	6.8569	7.0918	6.8420	7.2811	7.2811	7.0091	6.6943	7.9680	6.9625	7.0291	7.4213	6.6949	6.9823	7.5476
Sox10	_[Acetyl (Protein N-term)]AEEQDLS[Phospho (STY)]EVELSPVGSEEPR_.2	Q04888_S8_M1_Sox10	3.7550	5.5090	3.2916	3.8292	3.9619	3.9953	3.0862	3.9807	3.0064	3.5656	3.9859	3.2902	3.1718	5.4598	4.8329	3.1171	3.4779	4.4440	4.0926	5.0568
Cdk18	_RAS[Phospho (STY)]LSDIGFGK_.2	#N/A	3.9004	9.1197	8.4256	8.5579	4.1074	8.4322	8.4898	8.2400	7.9450	8.5911	4.1976	8.0710	8.4264	8.9667	7.1542	7.9803	8.1411	8.3593	7.9424	8.1481
Cdk18	_RFS[Phospho (STY)]M[Oxidation (M)]EDLNKR_.3	#N/A	6.4020	6.8955	6.8270	7.0593	6.2505	6.4103	6.6313	5.7340	6.3940	7.0591	6.8970	6.3064	6.5121	6.5992	5.6325	6.2964	5.9677	6.1117	6.1999	6.6115
Cdk18	_RFS[Phospho (STY)]MEDLNK_.2	#N/A	4.2803	7.3559	7.7816	7.8811	5.5935	6.1280	6.6331	4.2850	4.3789	5.7273	6.0414	5.0154	4.0902	7.7243	5.4383	3.1244	5.8780	4.4343	4.3609	5.4628
Cdk18	_AS[Phospho (STY)]LSDIGFGK_.2	#N/A	3.1600	4.1632	4.1974	4.4283	3.3669	5.5572	3.6853	3.3856	3.6055	5.3462	3.0841	3.8893	3.7709	4.1759	3.7106	4.0229	4.3837	3.8490	4.2675	4.2406
Cdk18	_FS[Phospho (STY)]MEDLNK_.2	#N/A	3.3977	4.6051	3.9340	4.2917	4.2362	4.4603	3.3048	3.0980	3.3639	3.6040	3.7383	2.9937	3.4720	3.9210	2.8820	3.7426	3.6357	4.1974	3.8460	3.8880
Mark2	_SS[Phospho (STY)]DQAVPAIPTSNSYSK_.2	#N/A	8.0097	8.8265	8.1102	8.0395	3.6139	8.1012	8.1216	7.8376	8.1662	8.3239	3.4385	8.5173	4.2969	8.1548	6.7081	3.2850	8.1434	3.3226	5.9136	8.1681
Mark2	_SRNS[Phospho (STY)]PLLDR_.2	#N/A	9.8244	9.7391	9.7302	9.8547	8.7621	8.2660	8.9954	9.1037	8.3695	9.9741	8.6870	8.9580	8.9257	8.9640	3.7229	8.7159	8.6890	8.8001	8.5344	8.7125
Mark2	_RSS[Phospho (STY)]DQAVPAIPTSNSYSK_.3	#N/A	7.9132	6.9521	3.7802	3.7644	5.1436	7.4556	7.9644	5.9847	5.7045	7.7782	7.5249	7.2834	3.9158	3.7982	5.3634	5.7199	7.7002	6.5206	3.6720	3.6404
Mark2	_S[Phospho (STY)]SELEGDTITLKPR_.2	Q05512_S365_M1_Mark2	3.9845	6.9349	7.4718	6.6618	3.3222	3.5463	4.2189	5.1250	4.2780	4.1908	6.7131	4.1909	4.3860	3.9298	3.7961	6.5355	7.1455	3.0714	6.6522	3.6071
Mark2	_DQQNLPYGVTPAS[Phospho (STY)]PSGHSQGR_.3	Q05512_S616_M1_Mark2	4.2464	3.7564	8.1033	8.0711	3.5842	6.4943	3.9569	4.3189	4.0160	8.9842	4.3904	3.6458	4.1241	3.6678	3.5341	6.4782	6.9958	7.3065	3.6324	3.3451
Mark2	_NS[Phospho (STY)]PLLDR_.2	#N/A	3.5345	4.7570	3.6441	3.1596	3.1308	4.7671	4.2745	3.1495	3.5922	3.6521	3.7616	5.2768	4.0030	4.5056	3.9427	2.6166	4.1476	4.8204	3.2614	3.5751
Mark2	_PSADLTNSSAPS[Phospho (STY)]PSHK_.2	#N/A	5.7717	4.0304	5.4756	5.3499	3.4782	3.6331	4.8664	3.6897	3.8508	5.8095	5.0183	3.8539	3.9887	5.2974	3.7007	5.1494	4.8004	4.7507	4.7098	3.0829
Mark2	_GRNS[Phospho (STY)]ATSADEQPHIGNYR_.3	Q05512_S40_M1_Mark2	3.1990	3.3030	3.7353	3.9444	4.0376	4.2616	3.5035	3.8655	3.5626	3.4053	3.9370	3.1924	3.6707	3.2144	3.0807	3.5439	3.3627	3.7867	4.0858	4.3926
Mark2	_VPVAS[Phospho (STY)]PSAHNISSSSGAPDR_.3	Q05512_S566_M1_Mark2	4.0462	5.0818	3.5828	3.5380	4.2531	3.8552	3.9099	4.2719	3.9690	4.1906	4.1186	2.9990	4.0771	3.6209	3.4871	3.4083	3.7691	3.3803	3.6794	4.8943
Mark2	_NSATS[Phospho (STY)]ADEQPHIGNYR_.2	Q05512_S43_M1_Mark2	4.3043	3.6985	3.3398	3.3851	3.6420	5.1010	3.8990	4.0047	3.9581	4.5106	4.3325	3.5879	4.0662	3.3269	3.4762	3.1484	4.5424	3.2908	4.4402	3.2939
Fabp5	_ELGVGLALR_.2	#N/A	7.2878	7.4645	7.5453	7.5181	7.0890	7.0670	7.3572	7.0144	7.6759	7.2018	7.3008	7.2561	7.1061	7.0007	7.7334	7.1993	6.7754	7.6065	7.4675	7.2765
Fabp5	_[Acetyl (Protein N-term)]ASLKDLEGK_.2	#N/A	6.0516	6.6439	6.3861	6.1340	5.4653	6.4772	6.3288	5.5424	6.9659	6.9612	5.8774	6.7821	6.4875	6.9498	6.7938	6.8070	6.5179	6.7395	6.5658	6.7511
Fabp5	dation (M)]AAM[Oxidation (M)]AKPDC[Carbamidomethyl (C)]IITC[Carbamidomethyl (C)]DGNI	#N/A	4.5119	3.9728	4.3837	4.2379	4.6526	4.9122	4.1811	4.6534	4.2949	5.7198	5.3056	5.0582	5.9062	3.6716	5.3869	5.6002	3.0692	4.0218	4.9059	5.2173
Fabp5	(Oxidation (M)]IVEC[Carbamidomethyl (C)]VM[Oxidation (M)]NNATC[Carbamidomethyl (C)]T	#N/A	5.4811	5.3536	6.2283	5.0567	4.7867	4.8607	4.5305	5.7561	5.3188	4.9472	5.2693	5.3582	5.5075	6.1684	5.6849	5.4451	5.3149	5.7783	5.4824	5.1426
Fabp5	_TTVFSC[Carbamidomethyl (C)]NLGEKFDETTADGR_.3	#N/A	4.3032	4.5168	3.8397	4.4172	4.8042	3.5983	4.0388	4.5288	4.2260	4.1137	5.0355	4.2429	5.1238	3.8778	4.0642	3.6653	4.0261	3.1234	3.4224	3.5551
Fabp5	_TETVC[Carbamidomethyl (C)]TFQDGALVQHQQWDGK_.3	#N/A	4.4695	4.3504	4.0061	3.1147	3.1756	3.4319	4.3333	6.5324	4.3924	4.0764	3.2659	4.0765	4.5004	4.0442	3.8978	3.8317	4.1925	4.4578	3.2560	3.7215
Fabp5	_KTETVC[Carbamidomethyl (C)]TFQDGALVQHQQWDGK_.3	#N/A	3.1079	3.3941	3.6442	4.5815	3.9465	4.0385	3.5946	4.3091	4.4640	3.6088	3.9427	3.2835	3.1286	3.6313	3.1718	3.4528	3.3460	2.9864	4.1358	5.3498
Fabp5	_LMESHGFEEYMK_.2	#N/A	3.8010	3.5180	3.3376	3.7832	4.0080	4.0413	3.0401	4.0267	4.4612	3.6116	3.9398	3.2442	3.1258	3.3757	3.0655	3.1632	3.5240	2.9892	3.9245	3.5954
Gch1	_HRS[Phospho (STY)]EEENQVNLPK_.3	#N/A	4.3381	5.1841	3.3736	5.2236	4.2221	4.7777	4.7961	4.0385	4.3550	5.2757	4.3048	4.1631	4.4820	5.8719	4.2448	5.2474	3.9670	5.3378	5.1540	4.7943
Pc	_ALAVSDLNR_.2	#N/A	6.8281	7.1036	6.2886	6.7828	7.2461	6.6429	6.9849	7.0422	6.9860	7.1492	7.0499	7.0773	6.9938	6.9846	7.0414	7.0580	6.6747	6.7394	6.8410	7.0040
Pc	_DFTATFGPLDSLNTR_.2	#N/A	6.7456	6.5103	6.6665	6.7726	6.1456	6.4105	6.5437	6.7402	6.5278	6.2243	6.6266	6.2208	6.0687	6.5863	6.0869	6.5860	6.1982	6.4482	6.3784	6.1944
Pc	_GANAVGYTNYPDNVVFK_.2	#N/A	6.1574	5.6024	5.9961	6.0385	5.6147	5.5086	5.4761	5.0266	5.9484	5.8036	5.8670	5.6025	3.2320	5.1777	5.8175	5.3229	5.6555	5.3242	5.0340	5.6697
Pc	_FIGPSPEVVR_.2	#N/A	3.4965	6.9857	6.9794	7.3689	7.0680	7.0592	7.3569	6.9613	6.7930	6.7580	7.2618	6.5425	3.4303	7.0318	7.0829	6.6338	6.5842	6.9852	6.6353	6.9030
Pc	_AC[Carbamidomethyl (C)]TELGIR_.2	#N/A	3.7356	5.7577	4.7871	4.6023	4.8362	5.2537	6.1319	4.9643	5.0959	4.9246	5.3882	5.2250	5.1945	4.2753	5.2711	4.6520	4.3269	5.0454	5.1277	5.2331
Pc	_DM[Oxidation (M)]TLEGDDLILEIE_.2	#N/A	5.7250	3.5638	5.6346	3.9815	4.5351	4.6732	3.0670	3.7258	5.5306	4.1921	4.4821	4.8457	5.9903	5.2625	4.8401	5.6909	5.2800	3.6122	5.2787	4.8901
Pc	_VVEIAPATHLDPQLR_.2	#N/A	5.5412	5.8769	6.1424	4.8974	5.6207	5.8673	5.7225	6.0775	5.3353	5.1431	5.6870	5.7994	4.9683	5.8006	5.8065	4.3840	4.9782	5.7798	5.7820	5.6606
Pc	_DAHQSLLATR_.2	#N/A	3.7991	6.2837	6.4164	6.7662	6.4340	5.8619	6.3204	6.1608	5.6049	5.7940	5.4089	5.4165	6.1319	6.2281	5.9633	6.5212	6.0341	6.3282	6.0646	5.7660
Pc	_VFDYSEYWEGAR_.2	#N/A	5.6097	5.9628	6.3019	3.6738	4.9734	4.9319	5.4104	4.8396	5.3941	4.7012	5.3043	5.0074	3.9413	5.7910	4.8288	4.7954	5.7342	4.6392	5.2482	4.6670
Pc	_AEAEAQAEELSFPR_.2	#N/A	4.3905	5.2254	5.6707	5.4520	5.1094	4.9627	5.0260	3.7168	4.4504	4.4550	4.5622	3.5652	5.4866	4.7214	4.3957	4.4268	5.2544	4.4826	3.2272	5.0789
Pc	_EGPEGFAR_.2	#N/A	3.8017	3.5174	4.7950	5.8853	5.0244	4.9085	5.1402	4.0273	5.1877	3.2435	5.5888	5.3660	3.8325	3.3763	5.9192	5.5789	3.5246	5.4494	4.9844	5.6426
Pc	_VVHSYEELEENYTR_.3	#N/A	5.9616	5.1496	4.6605	5.4072	5.6261	5.2177	5.1111	4.6570	5.3631	5.2960	4.5301	4.6091	5.7247	5.3957	4.8135	5.2530	4.1149	4.9865	5.3651	5.5881
Pc	_ADFAQAC[Carbamidomethyl (C)]QDAGVR_.2	#N/A	4.5284	4.4448	3.3478	3.7531	3.8661	4.0902	5.1806	4.0369	3.7341	4.7089	5.7160	3.2340	3.8421	3.3859	5.1565	3.3724	5.2366	3.6153	5.2899	4.9601
Dnal1	_LSLS[Phospho (STY)]TNC[Carbamidomethyl (C)]IEK_.2	Q05A62_S56_M1_Dnal1	10.0579	10.0300	9.8191	10.2927	9.7417	9.9120	9.5499	10.0165	9.8040	9.9294	9.9125	9.8390	10.4716	10.2072	9.7251	9.9823	10.2523	9.8359	10.0985	9.9540
Drp2	_GQTT[Phospho (STY)]PDTEVADDVGSK_.2	#N/A	6.4641	6.1014	6.3291	6.0552	5.7630	5.4214	5.6748	6.4417	3.4864	6.0509	5.9408	5.7686	3.5944	6.0706	5.2637	5.4158	6.3744	5.8865	4.1620	5.8590
Drp2	_EKGQTT[Phospho (STY)]PDTEVADDVGSK_.3	#N/A	3.2363	4.3330	4.2061	4.0196	3.4432	3.4765	3.6049	3.3702	3.5251	4.5477	3.0038	4.2224	3.6906	4.1932	3.6303	4.0992	3.9079	3.9253	3.5739	6.0207
Eml1	_TSSS[Phospho (STY)]ERVS[Phospho (STY)]PGGR_.2	Q05BC3_S135_M2_Eml1	5.0427	4.5460	4.3985	4.0197	4.7650	3.2317	4.0272	3.5693	4.4703	4.2357	4.2966	4.6584	4.9485	4.3999	5.1336	5.7864	5.8181	5.8300	3.2235	4.2374
Larp7	_TAS[Phospho (STY)]EGS[Phospho (STY)]EAETPEAPK_.2	#N/A	8.3799	8.4599	9.0093	8.5207	8.4202	8.4833	8.1551	8.2738	8.4766	8.3476	8.6320	8.3263	8.4548	9.1448	8.2361	8.4669	8.9059	8.3638	8.2824	8.5703
Larp7	_TAS[Phospho (STY)]EGS[Phospho (STY)]EAETPEAPKQPAK_.2	#N/A	6.8112	7.1218	6.6914	6.8731	6.2525	6.4285	6.4871	6.7690	6.7013	6.6954	6.2059	6.7221	7.2912	7.2254	6.2757	6.7866	7.0645	6.8997	6.7080	6.5834
Larp7	_DLEFC[Carbamidomethyl (C)]S[Phospho (STY)]T[Phospho (STY)]EEEKETDR_.2	Q05CL8_S334_M2_Larp7	3.9615	3.1577	4.4978	3.7279	3.2992	4.0751	4.2419	3.6619	4.3682	4.1678	3.1745	3.9308	4.4090	3.9528	3.8191	2.9097	4.1995	3.0484	3.3474	3.6301
Eif5b	_IEPIPGES[Phospho (STY)]PK_.2	Q05D44_S1164_M1_Eif5b	8.4481	8.5796	8.2210	8.1505	7.9355	8.2544	7.8606	7.7349	7.9394	8.1860	7.9922	7.8696	8.0567	8.2388	7.2840	7.8979	8.6830	8.2692	8.1482	8.2188
Eif5b	_SVPTVDS[Phospho (STY)]GNEDDDSSFK_.2	Q05D44_S215_M1_Eif5b	13.3009	13.5147	13.6018	13.3155	3.8094	13.1925	13.1318	12.3999	13.1985	13.2313	12.4144	13.0963	13.1769	13.3900	12.3704	12.9807	13.6457	13.1686	13.0299	12.8720
Eif5b	_TARPNSEAPLS[Phospho (STY)]GSEDADDSNK_.3	#N/A	5.9070	6.2784	6.2766	6.2103	5.4790	6.3149	4.0768	5.5090	4.0427	6.6479	6.1800	4.0924	4.3049	6.3398	5.6131	5.8296	5.7040	3.9993	6.2999	3.4456
Eif5b	_KTARPNSEAPLS[Phospho (STY)]GSEDADDSNK_.3	#N/A	4.3906	4.4293	3.9272	3.1936	3.0967	5.8825	3.9514	3.1154	4.3134	4.1554	3.7608	3.8524	4.4832	3.9652	3.9768	3.7527	4.8071	3.0360	3.3350	4.4333
Eif5b	_PNSEAPLS[Phospho (STY)]GSEDADDSNK_.2	#N/A	4.4161	3.3956	3.4601	3.6607	3.7539	3.9779	3.7872	4.1492	3.8463	3.1216	4.1923	3.1217	3.9544	4.9995	3.3644	4.3369	3.6464	3.1435	4.3719	3.1754
Eif5b	_TSFDENDS[Phospho (STY)]EELEDKDSK_.2	#N/A	5.1495	4.2205	3.4944	3.4262	3.8336	4.0836	4.3944	3.8024	3.8790	3.6309	4.2534	4.0125	4.2606	4.3383	3.2068	3.9645	3.4764	3.6475	3.6234	3.3428
Eif5b	_KTS[Phospho (STY)]FDENDS[Phospho (STY)]EELEDKDSK_.3	#N/A	4.0376	3.8109	3.7451	3.6390	3.7339	3.4306	3.8206	3.8712	3.8181	4.1207	3.9048	3.7042	4.0786	3.7515	3.5793	3.5589	3.6953	3.6390	4.0681	3.5834
Cab39	_DVAQIFNNILR_.2	#N/A	4.6853	5.6246	5.4159	5.0716	5.0309	4.8010	5.5219	5.8539	5.3702	5.8058	5.1310	4.7539	5.7639	4.6472	4.9769	3.3106	5.0914	4.9760	5.5779	5.3406
Atp5me	_VPPVQVSPLIK_.2	#N/A	9.9491	10.0928	10.2047	10.1608	9.9889	9.6950	10.1786	10.0403	9.9012	10.2377	9.7018	10.2336	9.9693	9.7999	10.3992	10.1337	9.9087	10.2169	10.4068	9.9688
Atp5me	_ELAEAQDDSILK_.2	#N/A	9.6110	9.4337	9.8725	9.6669	9.5908	9.5118	9.3322	9.6050	9.6980	9.4908	9.4811	9.2476	9.6833	9.5452	9.8495	9.5818	9.6206	9.4769	9.6677	9.5838
Atp5me	_YSYLKPR_.2	#N/A	8.6820	9.1382	9.2684	8.8915	9.1643	8.9515	9.0343	8.9426	8.7970	9.0802	8.6752	9.1281	8.9754	9.1715	9.5329	8.8990	8.7882	9.1505	9.0927	8.8736
Atp5me	_YSALIIGM[Oxidation (M)]AYGAK_.2	#N/A	7.0768	6.9389	7.2017	6.6694	6.7527	6.7700	6.1786	6.6343	6.8479	6.9720	6.7319	6.7984	7.1301	6.3772	7.0743	6.8826	6.7250	6.9936	6.9480	6.5722
Sh3bp2	_S[Phospho (STY)]PPDGQSFR_.2	Q06649_S414_M1_Sh3bp2	6.5096	6.8742	4.4813	7.2796	6.5595	4.4576	7.1003	6.3903	7.1501	6.7185	6.5085	6.7192	6.5568	6.8397	3.4227	4.3068	3.1668	6.5191	6.9253	6.6318
Clu	_ASGIIDTLFQDR_.2	#N/A	5.9194	6.0169	5.7767	5.6067	5.8921	5.9280	5.9567	5.7068	6.2955	6.2920	5.7151	5.7031	5.1059	5.8265	6.1003	5.7044	3.4759	5.8549	5.8179	5.5723
Clu	_VSTVTTHSSDSEVPSR_.3	#N/A	4.4752	4.5779	4.5963	3.7198	3.8129	4.6112	4.9991	4.0901	5.2390	4.6466	4.1616	3.1808	3.8953	3.4390	5.7924	3.3193	3.5873	3.5621	3.8612	3.1163
Clu	_QQSQVLDAMQDSFAR_.2	#N/A	8.7136	5.8895	3.9196	8.6204	4.2219	6.1376	6.4223	3.6811	3.3783	3.5897	3.7527	3.0081	3.4863	6.3368	2.8964	3.7282	3.1784	5.4533	4.2701	7.7413
Anxa7	_SDTSGHFER_.2	#N/A	4.1169	4.2362	4.5326	4.7207	5.7247	5.4199	5.3349	4.4484	5.2747	4.4914	5.5092	5.3078	4.6990	5.1838	5.5285	5.4413	4.8278	3.8440	5.2527	4.8728
Anxa7	_GFGTDEQAIVDVVSNR_.2	#N/A	6.7505	6.2783	6.6670	6.4057	6.0824	5.9445	6.1445	6.3762	6.0580	6.2318	5.8867	5.7718	6.4193	6.3590	5.4868	6.0883	6.4551	5.6864	6.1576	5.7592
Anxa7	_LYQAGEGR_.2	#N/A	4.4375	4.3824	2.7791	3.1467	3.1436	3.6543	4.5702	3.1623	3.9771	4.1084	3.2339	3.7592	4.4684	4.0121	3.7993	3.1527	4.1604	3.6827	3.2881	3.6894
Igf2r	_AEALSSLHGDDQDS[Phospho (STY)]EDEVLTVPEVK_.3	Q07113_S2401_M1_Igf2r	4.2292	7.6134	3.2132	3.5116	2.9353	6.5926	4.1128	3.8781	4.0330	4.0398	3.5117	4.3169	4.1985	3.2003	5.8707	3.5913	4.4158	7.8821	3.0659	3.1673
Igf2r	_LVSFHDDS[Phospho (STY)]DEDLLHI_.2	#N/A	4.1133	3.5713	3.4037	3.5278	3.9206	3.8331	4.4944	4.0820	3.9937	4.1489	3.7757	3.3948	4.1017	3.6455	3.4259	3.2216	4.1342	3.3582	3.6572	3.3228
Mtf1	_[Acetyl (Protein N-term)]GEHS[Phospho (STY)]PDDNIIFFK_.2	Q07243_S5_M1_Mtf1	3.6710	4.3318	4.2073	4.0184	3.0087	3.8448	3.0315	3.3935	3.0906	3.8773	3.4650	4.2212	3.1987	4.9481	4.1846	4.0159	4.3915	4.6720	4.4208	3.9205
Tle3	_ESSTNNSVS[Phospho (STY)]PSESLR_.2	Q08122_S203_M1_Tle3	9.6811	9.6867	9.4426	9.6376	9.5572	9.1366	3.3915	9.7383	9.2628	3.5173	9.1069	9.3890	9.8453	10.0930	8.9924	8.7556	9.6005	9.0933	3.9327	9.4617

Tle3	_DAPTS[Phospho (STY)]PASVASSSSTPSSK_.2	Q08122_S286_M1_Tle3	3.7860	9.7488	9.5059	9.8078	9.3696	9.2773	4.4174	3.2785	9.1248	9.4419	3.3500	4.1063	4.5845	10.0412	3.9946	4.1309	9.6485	4.3737	9.4848	9.3422
Tle3	_VS[Phospho (STY)]PAHS[Phospho (STY)]PPENGLDK_.2	Q08122_S263_M2_Tle3	3.7515	4.2513	4.2878	3.9379	3.0892	8.0141	7.7401	3.4519	3.0101	3.9578	8.0241	4.1408	3.1182	8.9794	4.0291	4.0964	3.9895	3.8436	3.4922	4.2419
Dmwd	_SVVEGISSQPGSS[Phospho (STY)]PSGTVV_.2	Q08274_S659_M1_Dmwd	10.5131	10.1791	10.3488	10.5857	9.6224	9.8103	9.7284	9.8035	9.8292	10.3559	3.4891	9.7387	10.2878	10.0535	9.0696	10.0137	10.3262	3.9482	9.9466	4.1372
Dmwd	_YHS[Phospho (STY)]LGNISR_.2	#N/A	9.7347	9.7885	9.1882	9.7506	9.1884	9.3187	9.4314	9.5622	9.0900	9.7581	9.1518	9.2014	9.7016	9.6173	8.1439	9.3072	9.1647	9.1368	9.1604	9.1245
Dmwd	_TLPGT[Phospho (STY)]PGAT[Phospho (STY)]PPASGSSR_.2	Q08274_T457_M2_Dmwd	10.2314	10.0333	10.3167	4.0890	3.7021	4.4062	3.3589	3.7209	8.4113	10.3565	8.5604	8.7160	9.6861	4.5707	6.8252	9.6334	3.2181	9.0507	8.3131	6.2001
Dmwd	_RYHS[Phospho (STY)]LGNISR_.3	#N/A	5.1383	5.7734	3.8924	5.3743	4.0886	4.7616	4.6828	4.9892	4.7161	4.7637	5.2089	4.1902	5.4524	5.4364	3.9190	4.6904	4.0993	4.8056	4.8668	4.5521
Dmwd	_AFTDEETEAQAGQASWPRS[Phospho (STY)]PSK_.3	Q08274_S643_M1_Dmwd	4.0559	3.2632	3.5925	6.1622	4.2629	4.5457	4.2861	4.2816	4.2063	3.8665	3.6849	2.9893	4.3717	3.6306	3.7731	3.4180	3.7788	3.3706	3.6697	3.3078
Dmwd	_LGDPDGAGEPPST[Phospho (STY)]PSGLGAGGDR_.2	Q08274_T112_M1_Dmwd	4.1428	3.8600	3.1782	3.5466	3.4805	7.1856	4.0606	3.8431	3.9980	4.3491	3.4767	3.7495	4.1635	3.1653	3.6378	2.9868	4.3808	3.4523	3.1009	3.1323
Dmwd	_GGSGGNSS[Phospho (STY)]NDKLSGPAPR_.2	#N/A	3.5839	4.1765	4.2796	4.1626	3.2727	4.2198	4.0844	3.4361	3.2965	4.2764	4.3248	3.9833	4.2223	4.2591	3.8418	4.0965	3.9738	3.7664	3.5090	4.0134
Dmwd	_SNS[Phospho (STY)]LPHPAGGGK_.2	Q08274_S487_M1_Dmwd	11.7223	11.5863	11.7739	11.8878	11.2989	11.3170	10.9575	11.5797	11.2849	11.3537	11.1841	11.1895	11.6740	11.7550	10.7107	11.4364	11.4951	11.3446	11.3197	11.2932
Dmwd	_S[Phospho (STY)]IDLNKPIDKR_.3	Q08274_S145_M1_Dmwd	3.3870	4.3000	3.9233	4.3024	4.2256	4.6150	4.8637	3.0874	4.5292	4.5759	5.3338	4.3431	3.4077	4.0655	3.8982	3.7319	3.6251	4.5588	4.6983	4.9322
Calb2	_LLPVQENFLLK_.2	#N/A	9.0059	8.9009	9.0018	8.8893	9.1204	9.0920	8.5620	8.6449	8.7722	9.1414	8.7618	9.1383	9.2134	8.8098	9.4703	9.0865	8.9577	9.1113	9.3626	9.0805
Calb2	_SGYIEANELK_.2	#N/A	7.1326	7.1396	7.2893	7.3098	7.1343	7.2800	7.1632	7.7860	7.5254	7.6566	6.9670	7.4951	7.2491	7.5437	7.3687	7.7073	7.2680	7.1479	7.4687	7.5482
Calb2	_EM[Oxidation (M)]NIQQLTTYR_.2	#N/A	7.2891	6.7287	6.7141	6.6154	7.0820	7.4819	6.8372	7.4155	7.2678	7.5116	6.7537	7.2245	7.6823	6.5764	7.4102	7.3928	6.8266	6.9960	7.3846	6.9299
Calb2	_ELENFFQELEK_.2	#N/A	6.6894	6.4299	7.0111	6.8320	6.6730	6.7557	6.2622	6.7340	7.4823	6.6064	6.4745	6.5132	6.6558	6.6681	7.0112	7.2696	6.5111	6.3000	7.2818	7.0155
Calb2	_SVMSLAEAGK_.2	#N/A	4.4266	4.3933	3.9632	3.1576	3.1327	3.1660	4.4695	3.1514	3.8356	4.2372	3.3143	4.1194	4.0011	4.5037	3.9408	3.7887	4.1495	3.1062	3.2634	2.9699
Kcnma1	_GGYST[Phospho (STY)]PQTLANR_.2	Q08460_T1061_M1_Kcnma1	11.0903	10.8641	10.5056	10.6480	10.2458	10.6008	10.4419	10.8670	10.5777	10.3805	10.5820	10.4690	11.0408	10.7734	9.1077	10.0098	11.2735	10.9288	10.4314	10.3726
Kcnma1	_LEDEQPPTLS[Phospho (STY)]PK_.2	#N/A	4.1479	7.8905	7.7476	7.6124	8.0861	7.6892	9.0076	8.9054	9.1597	7.7485	2.9832	9.1077	9.1926	7.6777	6.1862	8.9280	8.3030	9.5277	7.7022	8.9992
Kcnma1	_RLEDEQPPTLS[Phospho (STY)]PK_.2	#N/A	9.8190	10.1201	9.6874	9.7403	8.4640	8.5173	9.7045	4.8296	8.3622	9.5599	8.7778	9.5442	9.9390	9.5869	8.1120	9.3202	10.2829	10.0158	9.6767	9.4583
Kcnma1	_SS[Phospho (STY)]PDNSPVHGMLR_.2	Q08460_S924_M1_Kcnma1	3.9426	7.2285	7.4785	7.2492	3.8069	6.3337	6.7950	3.5355	3.6663	3.9100	3.5548	5.7162	3.8101	3.9245	3.5745	3.7433	5.9679	3.8054	3.8791	3.6920
Eps8	_DSVSS[Phospho (STY)]VSDVSQYR_.2	Q08509_S58_M1_Eps8	4.0872	4.0046	7.1844	7.3104	3.4249	3.6490	3.4624	3.7872	3.5215	7.8601	6.6192	3.8940	3.6295	6.8242	5.4672	3.6893	6.6216	6.0306	6.1914	3.5933
Eps8	_QNSSSS[Phospho (STY)]DSGGSIVR_.2	Q08509_S661_M1_Eps8	3.3004	4.0186	4.3379	3.9342	3.5074	3.5407	3.5407	3.5261	3.4609	3.1110	2.9396	3.7448	3.3313	4.3759	3.5661	4.1634	4.5242	4.1261	4.4251	3.6816
Eps8	_RKS[Phospho (STY)]QMEEVQDELFQR_.3	Q08509_S684_M1_Eps8	4.1147	2.9392	2.6350	3.5746	3.4525	3.0316	2.9121	3.8151	4.1477	4.3211	3.0212	3.7775	4.1355	2.7669	3.6658	2.9588	4.3528	3.4804	3.1289	3.1043
Ssrp1	_EGINPGYDDY[Phospho (STY)]ADSDEDQHDAYLER_.3	Q08943_Y441_M1_Ssrp1	4.5095	7.3628	5.1484	3.7694	3.4297	6.3683	6.4000	3.6527	5.3279	3.8765	3.6283	3.8631	4.3079	8.0228	4.8113	3.7974	3.9596	7.5065	4.8251	3.9274
Agbl4	_SQTAPEAGNDTGNEDAIGGNVNK_.2	#N/A	3.1732	3.8031	4.2106	4.4110	3.5581	2.7618	2.7542	3.8348	3.5882	4.4846	2.8192	2.6106	3.7536	3.6743	3.6934	4.0240	4.3567	3.0384	3.5108	3.1083
Chd8	_TAS[Phospho (STY)]PSPLRPDAPVEK_.2	Q09XV5_S1997_M1_Chd8	6.0964	3.8382	3.9358	3.8956	3.6325	3.7762	5.8957	3.8547	3.6694	5.6995	3.7040	3.6328	3.8038	3.8856	4.7276	3.7551	6.1878	3.5792	3.9678	5.7354
Cnnm1	_GT[Phospho (STY)]PQT[Phospho (STY)]PKDDPVLTLLSNR_.3	Q0GA42_T821_M2_Cnnm1	6.1020	8.2134	7.9625	7.0427	3.8059	7.6979	8.0312	4.1685	7.4712	7.7658	4.1687	6.2086	7.3124	8.1399	5.8318	6.7532	6.5688	8.3377	7.6794	6.5287
Cnnm1	_SDGLRS[Phospho (STY)]PGEVVYLR_.3	Q0GA42_S850_M1_Cnnm1	3.9444	5.3321	5.4557	3.8878	2.9204	3.3448	3.6938	3.5664	4.1122	3.7007	3.9855	4.5667	3.8867	3.6742	4.1785	3.7561	3.7312	3.7935	3.4421	3.6412
Inf2	_SFSEDAVTDSS[Phospho (STY)]GSGTLPR_.2	Q0GNC1_S1226_M1_Inf2	4.9170	3.8930	5.2587	5.1443	3.9260	6.0905	4.0464	3.9269	6.5340	3.8506	3.6320	5.6362	4.1826	6.2894	4.5278	4.5485	4.0991	4.5782	5.8789	4.7759
Inf2	_AVLLASDAQAC[Carbamidomethyl (C)]TLEEVVER_.2	#N/A	1.1426	3.2982	1.1099	3.2063	4.0423	1.2491	2.8200	4.4050	2.6049	3.4101	3.9322	2.6858	3.6659	3.8250	3.0759	3.5487	3.1400	4.3913	2.3328	3.6941
Unc79	_ALS[Phospho (STY)]LPETLTSK_.2	Q0KK59_S1242_M1_Unc79	8.1976	7.7912	7.4924	7.6995	7.0579	7.2815	7.7768	7.5039	4.4284	7.3913	7.5396	7.2634	7.6368	7.4515	3.0327	7.4868	7.9663	7.6294	7.1415	7.1624
Unc79	_VDS[Phospho (STY)]PGKPAPR_.2	Q0KK59_S1596_M1_Unc79	4.5537	6.2562	5.3639	5.3197	5.7179	4.6259	5.9954	3.2785	4.3707	5.4707	3.1872	5.0498	5.0794	5.3122	3.5573	4.3609	5.8474	5.8956	4.9249	5.3489
Ubxn2b	_FKAFS[Phospho (STY)]GEGQK_.2	#N/A	9.2161	9.2471	8.5721	9.2089	8.4284	7.8792	8.6062	8.9320	7.9955	9.6771	8.4805	9.0432	9.5375	9.1823	7.4300	8.9676	8.6036	8.6822	8.7630	8.9575
Ubxn2b	_STPATC[Carbamidomethyl (C)]RS[Phospho (STY)]PRT[Phospho (STY)]PPHR_.3	Q0KL01_S56_M2_Ubxn2b	7.6615	7.6722	7.5629	7.5461	7.0402	5.9941	6.6792	6.3761	3.5698	8.6478	6.6467	7.0823	7.3651	7.3146	5.7817	7.1396	3.9526	7.2018	6.6799	7.2217
Ubxn2b	_AFS[Phospho (STY)]GEGQK_.2	#N/A	3.8739	4.3542	4.0023	3.1185	3.2117	3.4357	4.3294	3.1905	4.3885	4.0803	3.2620	4.0804	4.4966	4.0403	3.9066	4.2188	4.1886	4.4617	3.2599	3.7176
Trio	_DSDDSAAT[Phospho (STY)]PQDETIEER_.2	Q0KL02_T1824_M1_Trio	7.9556	7.5348	7.6793	7.7519	6.9230	7.0178	7.1178	7.2641	6.9199	7.7453	6.6716	6.6261	4.2921	3.8359	5.0825	3.6234	3.9842	6.9285	6.4631	6.5446
Trio	_NFLNALTS[Phospho (STY)]PIEYQR_.2	Q0KL02_S2282_M1_Trio	4.2386	5.7040	3.7752	3.3456	2.9447	3.6628	6.5113	4.4643	4.1614	4.3073	3.0350	4.3074	4.2695	6.1528	3.6795	3.6007	3.9615	3.1879	3.4870	3.4905
Trio	_SADAGSQKDS[Phospho (STY)]DDSAATPQDETIEER_.3	Q0KL02_S1818_M1_Trio	3.6926	4.3102	4.2289	3.9968	3.0304	3.4537	3.6277	3.3930	3.5479	4.5249	3.0266	4.1996	3.7134	4.2160	3.6531	4.0375	3.9307	3.9025	3.5511	4.1830
Spata18	_SARS[Phospho (STY)]PS[Phospho (STY)]PSTGTR_.2	Q0P557_S244_M2_Spata18	4.4850	4.3349	4.0216	3.0992	3.1911	3.2244	3.8570	3.2098	3.7772	4.2956	3.2559	4.0611	3.9427	4.0596	3.8824	3.8471	4.2079	3.6731	3.2406	4.4123
Spata18	_SNS[Phospho (STY)]PC[Carbamidomethyl (C)]TTVAK_.2	Q0P557_S273_M1_Spata18	4.2663	3.7365	3.3018	3.4231	3.6040	5.1155	5.7068	3.9667	5.1575	4.4726	4.3705	3.6259	4.1042	3.6480	3.5142	3.1104	4.5044	3.3532	3.6523	3.3252
Sobp	_SADS[Phospho (STY)]PPGSSGQALSLAPAER_.2	Q0P5V2_S597_M1_Sobp	4.5505	3.5300	3.3257	3.7951	3.8882	4.1123	3.6528	4.0148	8.2815	3.2560	7.5813	3.3417	3.8200	3.3638	3.2300	3.3946	8.6597	7.8918	3.9365	3.0410
Slc45a4	_GS[Phospho (STY)]PPINSLSR_.2	Q0P5V9_S408_M1_Slc45a4	7.5637	7.3724	3.3059	3.4190	7.2504	7.5816	7.4957	3.9708	7.3620	4.4768	4.3664	3.6218	4.1001	7.1388	3.5101	3.1145	4.5085	7.2935	6.5961	7.3602
Slc45a4	_S[Phospho (STY)]M[Oxidation (M)]SDLYDLQQR_.2	#N/A	7.0008	3.5172	3.4420	3.9758	5.5558	4.0240	6.3545	4.3259	7.0441	3.5943	3.9594	6.4554	6.8971	5.5827	3.0851	6.7796	7.1306	6.7578	6.8656	6.9233
Zc3h18	_LGVSVS[Phospho (STY)]PSR_.2	Q0P678_S530_M1_Zc3h18	4.3433	9.3059	8.9592	9.3054	8.8904	9.1471	9.2941	9.1072	9.0420	4.1539	3.3976	8.8046	9.4466	3.9179	4.0241	3.7054	9.0225	3.0833	3.3823	3.5952
Zc3h18	_KRPLS[Phospho (STY)]PQSK_.2	Q0P678_S888_M1_Zc3h18	3.4964	4.5064	4.0327	4.1929	4.3350	7.8585	7.8643	3.1968	3.2652	7.8531	3.6395	7.8219	8.1136	4.4178	7.1819	3.8413	8.2724	4.0842	4.3832	4.0951
Zc3h18	_KANLS[Phospho (STY)]PDR_.2	#N/A	7.2439	6.7763	6.6573	6.2059	3.3053	6.6130	6.7234	6.8736	6.8898	6.6913	6.3585	6.6656	6.7699	3.7122	6.1888	5.8745	6.5526	6.9082	6.5621	6.7156
Zc3h18	_ANLS[Phospho (STY)]PDRGSR_.2	#N/A	3.4422	6.2971	4.4796	5.7441	4.8094	5.4319	5.6841	3.1643	5.3544	3.2528	4.5920	3.6030	6.3154	5.4733	5.2848	4.3051	3.7020	5.6077	3.7798	5.0254
Zc3h18	_M[Oxidation (M)]GS[Phospho (STY)]PKPER_.2	#N/A	6.1060	7.0080	6.7149	6.7832	6.4150	6.5089	7.1136	6.2973	6.4977	6.3950	6.2444	6.2916	6.0815	7.1713	5.9318	5.8344	6.4862	6.6178	5.9596	6.2724
Zc3h18	_ANLS[Phospho (STY)]PDR_.2	#N/A	3.0859	5.9123	4.9757	6.0348	5.9588	6.1572	5.5266	3.5206	5.7716	5.7795	5.9369	5.5221	5.8017	5.3818	4.9182	5.8256	5.6705	3.7749	5.4272	6.0264
Zc3h18	_S[Phospho (STY)]PQPPSR_.2	Q0P678_S483_M1_Zc3h18	3.6471	5.6168	5.9702	3.9371	3.8540	5.2216	6.2793	3.8728	3.5699	5.6401	5.6879	3.3981	3.6780	3.2218	3.2194	3.3644	3.3700	5.6116	5.3712	4.6528
Zc3h18	_SQDQDS[Phospho (STY)]EAHELSR_.2	Q0P678_S80_M1_Zc3h18	3.1883	4.2850	4.2541	3.9716	3.3953	4.8872	4.1962	3.4181	3.5730	4.4998	3.0517	4.1745	3.7385	4.2411	3.6782	4.0513	3.9558	3.8773	3.5259	4.2081
Zc3h18	_VQSQEETRS[Phospho (STY)]DEEDRASEPK_.3	Q0P678_S64_M1_Zc3h18	3.6759	4.3343	4.2337	4.0209	3.4445	3.5629	3.9340	3.1778	3.9462	2.8123	3.8768	4.2238	4.0732	4.0142	3.6836	4.2619	3.9638	3.6559	3.5403	3.8424
Dact3	_S[Phospho (STY)]LGDASPSAPESVGAR_.2	Q0PHV7_S165_M1_Dact3	11.3460	11.0347	11.2951	10.9638	9.4899	10.5959	10.5378	10.1241	10.4840	10.9588	10.3894	10.3238	10.2822	10.6068	9.3575	10.3495	10.9036	10.5384	10.5081	10.3940
Dact3	_GRS[Phospho (STY)]VEQS[Phospho (STY)]PPR_.2	#N/A	9.3237	8.5079	8.9418	8.4845	8.0572	8.6147	9.1086	8.4108	8.3918	8.7603	8.7079	7.7284	8.1784	8.5426	7.2224	8.0723	8.4693	8.5077	8.6538	8.1122
Dact3	_SQS[Phospho (STY)]ETSLLGR_.2	Q0PHV7_S409_M1_Dact3	8.8091	8.8656	8.6820	8.7925	7.9594	8.2584	8.4790	3.9991	8.0803	8.6516	8.1147	7.5765	8.6949	7.8205	3.4818	8.0998	8.1787	8.3452	7.9261	8.1150
Dact3	_AFSFPVS[Phospho (STY)]PER_.2	Q0PHV7_S10_M1_Dact3	8.4811	8.9907	8.9274	8.0964	3.7538	8.7785	8.4854	7.8551	8.5125	6.3011	4.2136	7.9382	8.1820	8.6090	7.7062	7.5707	8.2975	8.6372	8.5360	8.3734
Dact3	_PRPPEAS[Phospho (STY)]PPPGGAR_.2	#N/A	3.7317	5.8163	3.5083	3.6444	3.5017	7.7484	5.2373	4.1974	3.8945	6.4081	7.0277	3.9371	6.9432	5.4306	3.7768	5.3152	7.4599	7.3607	5.5362	6.7815
Dact3	_SFS[Phospho (STY)]APYPTAAAGAETC[Carbamidomethyl (C)]SSAER_.2	Q0PHV7_S188_M1_Dact3	3.6799	3.6786	3.4038	4.0962	3.7061	3.8646	3.8792	4.3886	4.3191	3.8862	3.5558	3.5681	3.8116	3.7251	3.4564	3.6840	3.6875	3.0907	3.8501	3.5445
Dact3	_GRS[Phospho (STY)]VEQS[Phospho (STY)]PPRER_.3	#N/A	4.3434	3.6594	2.3999	3.3460	3.6811	4.3039	4.2784	4.0437	4.1986	3.8742	3.6773	3.5489	4.3641	3.3659	3.4372	3.1874	3.0806	3.2517	4.4012	3.3329
Stum	_GASSSS[Phospho (STY)]GVVVQVR_.2	Q0VBF8_S29_M1_Stum	8.5445	8.6796	8.5048	9.3656	8.1176	9.3723	7.7846	8.5621	8.9058	8.8255	8.9744	8.6400	9.1030	8.5915	7.4820	8.6615	9.4244	8.8322	8.4233	8.8290
Stum	_DAETAAAAAAVAAADPR_.2	#N/A	3.8230	3.4961	3.3596	3.7612	4.0300	1.2842	3.0181	4.0487	3.7458	3.2221	4.1202	3.2222	3.8539	3.3977	3.0435	3.1851	3.5459	3.6035	3.9026	3.0750
Rbm15	_SLS[Phospho (STY)]PGGAALGYR_.2	Q0VBL3_S293_M1_Rbm15	12.2887	12.2064	12.1326	12.2607	11.9801	11.9858	11.7090	12.3136	12.0010	12.1741	12.1165	12.0289	12.6669	12.4752	11.4549	12.1223	12.5506	11.9493	12.0420	12.1915
Rbm15	_LHSYSS[Phospho (STY)]PSTK_.2	Q0VBL3_S127_M1_Rbm15	7.6167	4.0947	4.2618	4.3599	3.4313	3.4646	3.6168	3.4500	3.5370	4.5358	3.0156	3.8208	3.7024	4.2998	3.6422	4.0873	4.4481	7.0119	7.1891	3.9771
Rbm15	_HLDRS[Phospho (STY)]PESERPR_.3	Q0VBL3_S655_M1_Rbm15	6.8539	6.7471	5.7404	3.8944	6.6885	6.8885	3.5535	7.3284	3.6126	3.3553	3.9870	3.3554	3.7207	3.2644	3.1307	3.0519	3.4127	3.7367	4.0358	4.4426
Rbm15	_S[Phospho (STY)]RS[Phospho (STY)]PLDKDAYAPSSSVVGTSVGSHR_.3	#N/A	4.0833	9.0811	7.8771	4.3559	3.4312	8.5934	8.7051	3.2560	8.1243	3.8939	3.8925	4.1879	3.7203	9.8697	7.6623	7.6428	4.0462	8.9282	7.2456	8.4858
Rbm15	HC[Carbamidomethyl (C)]T[Phospho (STY)]PS[Phospho (STY)]PDRS[Phospho (STY)]PELSSNR_.	Q0VBL3_S670_M3_Rbm15	4.0637	4.4183	4.6000	4.7109	3.4014	4.2889	4.7903	4.5016	3.7247	5.3522	4.6659	3.8285	4.0844	3.9523	3.7168	3.7772	3.9988	3.4812	3.8760	3.4824
Rbm15	_DRT[Phospho (STY)]PPLLYR_.2	#N/A	9.4798	9.5850	3.3785	9.4795	3.8810	9.3486	9.0260	9.5303	9.3224	9.5737	9.2233	9.2923	9.8955	9.9774	8.8598	9.4162	9.8608	9.1410	9.2742	9.4819
Fam83b	_KSEPKVDSS[Phospho (STY)]PR_.2	Q0VBM2_S827_M1_Fam83b	6.7384	7.0743	7.0542	8.1084	7.4474	7.1864	6.6234	6.6909	6.6149	7.5987	3.5862	4.8070	3.7598	4.8442	3.6964	3.5766	3.9771	3.9657	3.6143	3.7220
Ppp4r2	_TS[Phospho (STY)]APEPR_.2	Q0VGB7_S63_M1_Ppp4r2	4.4682	3.5346	3.5036	3.2212	3.8059	4.1790	3.7352	4.1685	4.3234	3.1736	3.8021	3.4241	4.4889	3.4907	3.3124	3.3122	3.2054	3.1269	4.2764	3.4577
Vgf	_LLQQGLAQVEAGR_.2	#N/A	3.8666	4.4900	3.6715	3.4493	3.5424	4.1626	3.9987	4.3606	4.0578	4.4110	4.5624	2.8357	4.1658	3.7096	4.8212	3.0300	4.1769	4.0184	3.5906	3.3869
Vgf	_S[Phospho (STY)]QEEAPGHR_.2	#N/A	11.7059	11.8719	11.8804	11.6677	11.6517	11.4215	11.5414	11.7811	11.7453	11.7235	11.6846	11.9743	12.2667	12.0386	11.8061	11.8561	11.9268	11.8050	11.6998	11.9648
Vgf	_QNALLFAEEEDGEAGAEDKRS[Phospho (STY)]QEEAPGHR_.4	#N/A	3.9027	5.6378	5.9951	5.7513	3.7369	4.7389	5.3842	4.7712	5.6766	6.0869	5.2177	5.1714	4.2164	6.0928	5.6672	4.9353	3.9084	4.8786	5.3880	4.6254
Tanc1	_SPC[Carbamidomethyl (C)]ETISS[Phospho (STY)]PSSTLESK_.2	Q0VGY8_S211_M1_Tanc1	3.6170	6.7866	3.1536	3.9672	6.0723	4.2844	6.0874	3.8427	3.5399	3.4281	3.9142	3.4282	6.2512	3.1917	3.0580	3.5666	3.3400	5.9255	5.8278	4.3698
Tanc1	_QQGPPPAPANDS[Phospho (STY)]DNEEDAPASSLK_.3	Q0VGY8_S1436_M1_Tanc1	4.0625	3.9403	4.5988	3.6269	3.4002	3.0839	3.9976	3.7629	3.8334	4.1550	3.3964	3.8298	4.0832	4.5859	3.7181	4.4074	4.3005	3.5326	3.1812	3.0520
Npepps	_VLGATLSPELIQK_.2	#N/A	6.3127	6.5235	6.1510	6.3679	6.2777	6.2270	6.9859	6.5998	6.2593	6.2963	5.9444	5.9857	5.2458	5.4028	6.1412	6.0884	6.9481	6.4475	6.3808	5.7938
Npepps	_DNWEELHNR_.2	#N/A	6.2271	6.0378	6.6615	6.1975	5.5683	6.3859	6.0201	7.0508	6.0961	6.6862	5.9524	5.9824	6.2839	5.9530	5.8093	5.4257	6.5226	5.9645	5.8797	5.9926
Npepps	_LSVEGFAVDK_.2	#N/A	6.0296	5.9673	5.9318	6.4274	6.0504	6.0773	6.2653	6.5570	5.9915	6.8689	6.0706	6.3926	5.6034	6.0204	5.4048	5.7380	6.5837	6.2687	5.8722	5.9044
Npepps	_LGLQNDLFSLAR_.2	#N/A	3.3634	5.4022	4.9250	3.4458	4.8963	5.1826	5.9890	4.6355	4.2414	4.9484	5.0528	5.0613	4.7374	4.7953	3.1368	4.3258	4.1272	5.3309	5.1785	4.1889
Npepps	_AGIISTVEVLK_.2	#N/A	7.3062	4.9084	5.6374	8.0385	4.7140	7.7028	7.8884	7.6871	7.8506	4.9098	5.0187	7.5562	7.8237	7.9810	7.8220	8.0974	7.4640	5.5680	7.8273	7.6375
Npepps	_QILSADLR_.2	#N/A	6.3554	6.4209	6.5904	6.7331	6.1696	6.0699	6.3539	6.3409	6.3906	5.8947	6.2542	5.9840	6.5862	5.8097	5.7761	6.2676	4.4195	5.8554	5.8058	5.8475
Npepps	_IDFVGELNDK_.2	#N/A	5.8135	6.4006	5.6559	6.0614	5.1208	5.9690	6.1414	5.0643	6.0125	6.2240	5.6695	5.6700	4.3035	6.0501	5.7186	5.0014	5.8385	6.1476	5.8702	5.6075
Npepps	_YQGGFLISR_.2	#N/A	4.2977	6.3828	6.3478	6.5803	6.2978	6.4395	6.0792	5.7097	6.0349	5.9248	5.4985	6.0952	6.0751	3.6166	3.4829	5.8794	5.5885	5.9105	5.8250	5.9381
Npepps	_YAAVTQFEATDAR_.2	#N/A	3.1095	4.3699	4.7623	5.2108	3.9481	5.0112	5.3306	4.3107	5.2297	4.9635	3.7358	4.1383	3.1302	4.6512	4.2148	4.7398	4.6160	5.3106	4.1342	4.5246
Npepps	_DVFSPIGER_.2	#N/A	6.0422	5.9048	6.4006	5.8870	5.8302	5.7785	6.3294	5.6751	5.7794	5.8694	6.0336	5.4463	5.6190	6.3111	5.1537	5.6726	5.8566	5.8010	5.4667	5.6155
Npepps	_ETALLIDPK_.2	#N/A	4.7940	4.3475	4.3182	4.3035	3.6463	3.8073	3.9087	3.5064	4.2777	3.7643	3.4694	3.5556	3.3116	4.3563	3.3022	4.3788	4.5045	4.0123	3.5651	3.9090
Npepps	_DADSIHQYLLQR_.2	#N/A	4.8138	5.5719	4.3294	4.4579	4.2006	4.6812	4.8082	5.0013	4.7821	4.8760	5.3673	4.9646	4.2444	4.0775	4.7422	4.7016	4.8685	5.1940	4.2436	4.6557
Npepps	_AFFESHPAPSAER_.2	#N/A	3.7461	6.2330	3.2827	6.1111	6.3131	5.9140	6.0559	6.9824	3.0153	3.5567	5.7050	6.0879	5.9918	3.6834	5.8566	6.1742	3.4427	5.9284	5.6793	5.6417
Npepps	_S[Phospho (STY)]PVYLTVLK_.2	Q11011_S746_M1_Npepps	3.3004	4.0187	4.3378	4.2838	3.5073	3.1002	3.1641	3.5261	3.2232	3.7447	3.5976	3.7448	3.3313	4.3759	4.2422	4.1633	4.5241	4.0075	4.4252	4.0532
Npepps	_VALSNMNVIDR_.2	#N/A	3.6801	5.3847	3.5432	6.5049	6.8814	7.4843	6.6346	5.8021	3.9308	6.2789	3.8303	3.1081	4.0740	7.5419	3.5724	3.4690	5.4183	3.5261	6.7198	3.9768
Npepps	_QM[Oxidation (M)]GFPLIYVEAEQVEDDR_.2	#N/A	4.6123	3.8843	4.3587	3.4068	3.0559	4.3698	4.3217	3.8405	4.4659	5.0090	3.7735	3.5004	4.2618	3.6502	3.3477	3.4463	3.8695	4.4466	3.8691	3.4054
Npepps	_ATFDISLVVPK_.2	#N/A	3.9152	4.0876	4.4515	3.7742	3.2529	3.2312	3.8502	3.6155	3.7704	2.7291	3.2491	3.1802	3.9359	4.4385	2.7470	4.2601	4.1532	3.6799	3.4715	0.6714
Pepd	_EAS[Phospho (STY)]FEGISK_.2	Q11136_S162_M1_Pepd	4.4786	6.0297	3.3976	3.7232	3.8164	4.0404	3.7247	4.8964	3.7838	3.1841	4.1582	3.1842	3.8919	6.0528	4.7590	3.3227	5.6151	3.5656	3.8646	4.9542
Pepd	_YAVDDVQYTDEIASVLTSR_.2	#N/A	4.1700	3.6944	4.1411	4.5210	3.6325	3.3517	3.2773	5.1675	3.0627	3.7530	3.5312	3.1817	4.5471	4.0425	3.4132	4.3388	4.1309	3.4799	3.3428	4.4693
Relch	_NYKSS[Phospho (STY)]PEIQEPIKPLEK_.3	#N/A	10.2358	3.4323	9.6880	9.8382	9.3588	9.6517	9.6803	10.0196	4.3754	9.8049	9.5715	9.5292	4.5408	9.9824	8.7233	9.5807	9.9197	9.9119	9.3749	9.4577
Relch	_YS[Phospho (STY)]DDGNRETDER_.2	Q148V7_S193_M1_Relch	7.2654	3.1857	3.6700	3.4509	4.3403	6.1820	4.2086	4.3591	4.0562	6.5393	2.9297	4.4127	4.1642	6.1059	4.2340	3.4955	6.2908	6.0433	3.5922	3.3853
Relch	_SS[Phospho (STY)]PEIQEPIKPLEK_.2	#N/A	10.1362	9.9739	10.1364	9.8558	9.3971	9.7516	9.6265	9.8909	9.8206	9.8213	9.6645	4.1766	9.9019	9.9813	8.4816	9.5282	10.3662	9.6042	9.6984	9.4265
Relch	_AGS[Phospho (STY)]ISTLDSLDFAR_.2	Q148V7_S180_M1_Relch	8.5484	8.2503	8.1086	7.8491	6.8744	7.2308	7.9057	7.3906	7.4802	8.0359	7.5202	6.9743	7.2679	7.4695	5.8624	3.3160	7.4512	7.5275	7.4427	6.9805
Relch	_VVPALITLSSDPEISVR_.2	#N/A	3.7318	4.5304	3.2684	4.0633	3.9387	3.3633	4.3329	3.2479	3.4431	3.3133	2.5075	3.3134	3.7627	3.3065	1.9660	3.0939	3.0795	3.8391	2.4849	2.9837
Relch	_AGSISTLDSLDFARY[Phospho (STY)]SDDGNRETDER_.3	Q148V7_Y192_M1_Relch	4.2161	3.1956	3.6601	3.4607	3.5538	3.7779	4.9838	4.3492	4.0463	4.4224	2.9199	3.6761	4.1544	3.6981	3.5644	3.0602	3.8464	5.1784	3.6021	3.3754
Relch	_[Acetyl (Protein N-term)]AAMAPGGGGSGSGVNPFLS[Phospho (STY)]DSDEDDDEVAATEDRR_.3	Q148V7_S20_M1_Relch	3.3117	4.0073	4.3492	4.2725	3.5187	3.5520	3.5294	3.5374	3.4496	3.1223	2.9283	3.7335	3.3426	4.3872	3.5548	4.1747	4.5355	4.1148	4.4138	4.0645
Traf3ip1	_QES[Phospho (STY)]TETLVVDR_.2	#N/A	3.8952	3.4239	3.4318	3.6890	4.1021	4.0062	3.7589	4.1209	3.8180	3.1499	4.1924	3.1500	3.9261	3.4698	2.9713	3.2573	3.6181	3.5313	3.8304	3.1471
Traf3ip1	_RQES[Phospho (STY)]TETLVVDR_.2	#N/A	3.7314	4.2715	4.2676	5.2241	3.0691	3.4150	4.4720	3.4317	3.5866	3.9377	3.0653	4.1609	3.7521	4.2547	4.0492	4.0762	3.9694	3.8638	3.5123	4.2217
Cdk12	_NNS[Phospho (STY)]PAPPQPAPVK_.2	#N/A	7.2221	6.5449	7.6037	6.9484	6.5486	7.2127	6.8378	6.8612	6.9122	6.9088	7.0702	6.6680	6.4176	7.2298	6.2048	7.4323	7.3216	6.5956	6.7046	6.7244
Cdk12	_ETTSGTTAEPVKNNS[Phospho (STY)]PAPPQPAPVK_.3	#N/A	4.4657	5.7534	3.5012	4.6826	5.2634	4.9609	4.4008	5.4763	4.3210	4.7394	4.9464	4.7900	5.5447	3.4882	2.9253	4.7338	5.6593	5.5743	3.4873	3.4552
Cdk12	_RRS[Phospho (STY)]S[Phospho (STY)]SPFLSK_.2	Q14AX6_S332_M2_Cdk12	7.0008	7.9226	7.0047	6.5542	6.7887	6.6525	3.8011	6.7950	6.7048	3.9801	6.4393	6.4974	3.9682	7.0118	4.0285	6.2583	4.3350	6.6703	3.1473	6.5206
Cdk12	_RQS[Phospho (STY)]IS[Phospho (STY)]PPYKEPSAYQSSTR_.3	Q14AX6_S273_M2_Cdk12	3.4554	6.5954	4.0531	5.8939	3.6623	6.0391	5.8790	3.6811	3.3060	3.2660	4.2855	5.6021	3.4714	6.2346	3.4112	4.7258	3.1783	4.1444	5.6569	5.7085
Cdk12	_S[Phospho (STY)]RS[Phospho (STY)]PAYSR_.2	Q14AX6_S356_M2_Cdk12	4.4580	3.5448	5.2364	5.4812	3.7957	4.0198	3.7454	4.1583	4.4260	3.1635	4.9657	5.4454	3.9125	3.4805	3.3226	3.3020	3.1952	3.1371	5.3336	4.9518
Kcnc2	_SVLSGDDSTGSEPPLS[Phospho (STY)]PPER_.2	Q14B80_S557_M1_Kcnc2	9.4455	9.1740	7.9259	7.8366	3.6818	9.1194	8.0584	8.7997	8.7616	8.0828	8.5645	7.5706	4.3648	8.9680	7.0660	7.5307	8.8657	3.2511	7.8935	8.1224
Kcnc2	M[Oxidation (M)]AC[Carbamidomethyl (C)]NSTQS[Phospho (STY)]DTC[Carbamidomethyl (C)]	#N/A	4.1782	3.4522	3.6304	3.2306	3.9327	3.8341	3.8461	3.8545	3.7614	3.8706	3.7277	3.8587	3.8738	3.6175	3.2299	3.3555	3.9023	3.4099	3.8298	3.1127
Map6d1	_AGS[Phospho (STY)]RDAAQERPSGPGGR_.3	Q14BB9_S82_M1_Map6d1	6.8779	7.3773	6.3151	6.3663	6.5812	6.8238	6.9257	6.8606	6.5974	7.3986	6.5734	6.3563	7.5087	6.8021	6.5713	6.7059	6.7673	6.6685	7.0240	6.9113
Map6d1	_TVYVLPVGDADAAVVATTSYR_.2	#N/A	5.4035	5.7706	5.0052	3.4870	5.0336	5.4378	4.6623	5.3899	5.1135	5.5760	5.1746	5.5621	5.6717	5.8394	4.8353	5.0900	3.8201	4.9414	3.6284	5.0966
Map6d1	_VVTTHS[Phospho (STY)]SGWDPS[Phospho (STY)]PGASFQVPEVR_.3	Q14BB9_S154_M2_Map6d1	4.7010	5.6229	4.1929	4.8028	4.1354	4.4365	5.7825	4.5447	4.1427	4.8296	4.3391	5.0646	3.9229	6.9036	4.2607	4.7746	4.2729	5.2085	4.1206	4.5879
Map6d1	_SDVAVPLTLHGYS[Phospho (STY)]DPGSEESGADC[Carbamidomethyl (C)]SVSR_.3	Q14BB9_S34_M1_Map6d1	3.8825	4.2727	4.1662	4.3437	3.2304	5.4641	4.7294	3.3469	3.4910	4.7589	3.2783	4.0633	3.6407	6.3153	3.9124	3.9864	4.2178	4.9334	4.9949	4.8484
Fam171b	_STVEDFEANTS[Phospho (STY)]PTK_.2	#N/A	11.0064	9.8178	3.4544	3.2705	3.7566	9.8731	9.5899	10.6909	10.2577	3.1244	10.4626	10.1156	11.0932	10.2044	3.3616	10.3013	11.1084	10.2504	10.4889	3.1726
Fam171b	_RDS[Phospho (STY)]KTNIWK_.2	#N/A	4.3096	6.1919	5.9869	4.2921	5.5725	5.4190	5.6903	3.4069	6.1266	6.0674	5.4042	5.7200	4.8306	5.7238	3.6519	5.9572	6.1392	5.9275	6.0909	5.6760
Fam171b	_DQTTS[Phospho (STY)]TTHINHISSVK_.3	Q14CH0_S405_M1_Fam171b	3.6509	5.9334	3.1875	3.9333	3.8579	4.2505	3.1902	3.8766	3.1104	3.4615	5.0236	3.3943	3.6818	4.9911	3.2156	3.0130	3.3738	3.7756	4.0747	4.4037
Fam171b	_HILEGGSGVIIEHPGEES[Phospho (STY)]PGRK_.3	#N/A	3.6016	4.4173	4.1379	4.1039	3.8021	3.5204	3.9798	3.3020	3.1626	3.8079	3.8996	3.6687	3.3024	4.5848	3.5570	3.9465	3.8397	5.8543	3.5061	3.8889
Fam171b	_KSTVEDFEANTS[Phospho (STY)]PTK_.2	#N/A	7.5697	7.3249	4.5017	4.1199	4.2130	7.0970	7.0465	7.4708	3.3871	7.0830	3.7615	6.8760	3.4952	4.5398	6.1772	6.4636	7.5097	7.1808	7.0225	6.5428
Fam171b	_STVEDFEANTSPT[Phospho (STY)]KK_.2	Q14CH0_T794_M1_Fam171b	3.5498	4.1575	4.2952	3.9299	3.2776	5.0857	4.0525	3.2806	3.8743	4.2745	3.3521	3.9662	3.9824	5.3450	3.8227	4.1116	5.7970	5.4433	3.1747	3.8077
Nsun2	_FQQPPQPEGEEDAS[Phospho (STY)]DGGR_.2	Q1HFZ0_S23_M1_Nsun2	8.7840	8.5995	8.6516	8.4937	8.2728	8.2739	8.3284	8.7631	8.3382	8.8690	8.1631	8.4948	8.2625	9.1640	7.7304	8.4657	9.0392	8.5412	7.9044	7.8399
Nsun2	_EGVILTNENAAS[Phospho (STY)]PEQPGDEDAK_.2	#N/A	3.4750	3.8441	4.5124	4.1092	3.6819	7.8475	3.3662	3.7007	3.2864	3.2856	4.2659	3.5702	3.4518	4.5505	3.3916	4.3379	3.1979	7.4408	7.2954	3.9215
Nsun2	_KKEGVILTNENAAS[Phospho (STY)]PEQPGDEDAK_.3	#N/A	3.3644	3.9547	3.9992	4.2198	3.5713	4.3059	3.4768	3.5901	4.9748	3.6807	3.6616	3.6808	3.3953	4.4399	4.3766	4.2274	3.0873	4.0621	4.3612	4.1172
Specc1l	_RSS[Phospho (STY)]TSSEPTPTVK_.2	Q2KN98_S833_M1_Specc1l	4.4692	7.9657	3.5046	3.2202	3.8069	6.9564	7.2791	4.1695	4.3244	7.7281	7.1403	3.4231	7.5527	7.6025	2.9288	7.4138	3.2064	7.2384	7.3756	3.4587
Specc1l	_SKS[Phospho (STY)]DNQISDK_.2	Q2KN98_S171_M1_Specc1l	5.6551	6.7050	6.2657	6.2710	6.2554	6.5759	6.6713	6.2082	5.8234	6.3449	6.1769	3.4018	6.7796	6.7704	5.7886	5.4132	6.8243	6.6550	5.0472	4.1873
Specc1l	_KGS[Phospho (STY)]SGNASEVSVAC[Carbamidomethyl (C)]LTER_.3	Q2KN98_S385_M1_Specc1l	3.5686	4.2049	5.8507	4.2763	3.2111	3.3580	3.8097	3.4074	3.2113	3.6728	3.3024	4.0712	3.3417	4.2489	3.9425	4.0808	3.8371	4.3241	4.8945	4.0215
Phactr1	_S[Phospho (STY)]KFANLGR_.2	Q2M3X8_S111_M1_Phactr1	7.8419	8.2068	7.8681	8.0002	8.2864	8.3035	8.1277	8.3940	8.2504	7.7681	8.2380	7.5074	8.6688	8.7021	8.1124	8.1791	8.3009	8.4942	8.6043	8.2518
Phactr1	_KLS[Phospho (STY)]QRPTVEELR_.3	Q2M3X8_S505_M1_Phactr1	7.2601	7.8333	3.4349	3.6859	7.2150	7.8292	7.0807	4.1240	7.0679	7.1584	7.0742	7.1035	7.7316	8.0380	7.3050	3.2604	7.2975	7.1476	7.6740	7.1652
Phactr1	_SDSLVPGTHT[Phospho (STY)]PPIR_.3	Q2M3X8_T104_M1_Phactr1	3.8744	3.4702	6.6102	3.1568	4.0813	5.1109	4.4676	4.2329	4.3878	7.8373	3.8665	4.8308	4.5533	3.5551	2.9922	3.2365	3.2698	4.6838	4.2120	3.5221
Phactr1	_RLS[Phospho (STY)]QRPTAEELEQR_.3	Q2M3X8_S467_M1_Phactr1	3.4957	3.8234	4.5331	4.0885	3.7026	3.3399	3.3455	3.7214	3.2657	3.3063	4.2451	3.5495	3.4311	4.5712	2.1034	4.3587	3.7940	4.1847	2.2280	3.4177
Phactr1	_SKS[Phospho (STY)]DTPYLAEAR_.2	Q2M3X8_S67_M1_Phactr1	10.4326	10.6428	10.5608	10.5444	10.0004	9.9062	10.1165	10.2825	9.7720	10.3019	10.0403	9.9271	10.4319	10.5723	9.2255	10.1379	10.1699	10.0663	9.9421	10.0786
Phactr1	_SDT[Phospho (STY)]PYLAEAR_.2	Q2M3X8_T69_M1_Phactr1	3.1313	3.3706	3.6676	4.5581	3.9699	4.1940	3.5712	3.9331	5.4839	3.3376	4.0047	3.2601	3.7383	3.2821	3.1484	3.4762	3.4304	3.7191	4.0181	2.9594
Phactr1	_NEQEEQEEKR_.2	#N/A	3.8004	3.5187	3.3370	3.7838	4.0074	4.0407	3.0407	4.0261	4.4618	3.6110	3.9404	3.2448	3.1264	3.6290	3.0661	3.1625	3.5233	2.9886	4.8813	3.5960
Gsk3a	_GEPNVSY[Phospho (STY)]IC[Carbamidomethyl (C)]SR_.2	Q2NL51_Y279_M1_Gsk3a	16.4673	15.9435	16.2231	13.5427	16.0200	15.7173	15.6826	16.5238	15.9933	16.0983	16.0182	15.7447	16.5782	15.9302	14.9507	15.6144	16.3860	15.8097	15.9043	15.6517
Gsk3a	_[Acetyl (Protein N-term)]S[Phospho (STY)]GGGPSGGGPGGSGR_.2	Q2NL51_S2_M1_Gsk3a	11.1340	11.1253	11.2072	11.1004	10.6240	10.7099	10.8216	11.1365	10.8887	10.8446	10.3761	10.4359	10.8638	10.5993	9.3998	10.4516	10.7437	10.5531	10.4896	10.3384
Gsk3a	_T[Phospho (STY)]SSFAEPGGGGGGGGGPGGSASGPGGTGGGK_.3	Q2NL51_T19_M1_Gsk3a	4.2692	4.5508	3.8057	3.3151	2.9753	3.0086	4.0728	4.4948	3.9930	4.0798	3.4717	5.9668	4.1585	3.1603	4.0982	3.6313	3.9921	3.4573	3.1059	3.1273
Psd3	_KSHS[Phospho (STY)]SPSLNPDASPVTAK_.3	#N/A	10.8901	11.4175	11.3655	11.2610	3.8833	11.2894	9.2798	10.9050	11.2846	11.6739	10.7962	11.0887	11.3879	11.4221	10.9311	11.3582	10.9269	11.1094	11.3842	10.7002
Psd3	_VLLFQTQSPEEM[Oxidation (M)]QGWINK_.2	#N/A	5.9782	5.9596	5.3683	6.1242	5.5598	5.1495	5.5096	5.8352	4.7023	5.2875	5.3792	5.5705	6.0092	5.2951	4.8530	5.6237	5.5915	5.7407	5.5208	5.4834
Psd3	_IGS[Phospho (STY)]TTNPFLDIPHDPNAAVYK_.3	#N/A	8.8182	9.1173	8.2892	8.0232	6.6675	6.8611	8.5428	8.2917	6.7652	8.5296	7.1882	8.3631	8.0091	9.0687	6.0478	7.2782	6.9551	8.2454	7.6994	7.1791
Psd3	_DHYLEFEK_.2	#N/A	5.1548	5.9718	5.5490	5.3802	5.6385	5.6715	4.9574	6.0127	5.1665	5.3819	5.2895	5.1083	5.3908	5.3597	5.2395	5.9620	4.3196	5.8112	5.2323	5.5491
Psd3	_SHS[Phospho (STY)]SPSLNPDASPVTAK_.2	#N/A	12.7129	12.8181	11.4255	12.9296	12.1172	12.2811	12.3527	12.6490	12.3463	12.8758	12.2211	12.4634	13.1157	12.8452	11.1578	12.4323	12.6777	12.2318	11.8346	12.4586
Psd3	_ALYNS[Phospho (STY)]IKNEK_.2	Q2PFD7_S719_M1_Psd3	7.0917	3.4958	7.0022	3.1824	3.8447	5.0801	3.6964	4.2073	3.7555	8.7009	4.1298	3.3853	4.5277	6.0372	3.2736	3.3510	3.2442	3.0881	4.7422	5.3801
Psd3	_INC[Carbamidomethyl (C)]VAAVFSAPPFPAAIGSQK_.2	#N/A	4.6922	4.4390	5.0681	4.3962	3.1085	3.8399	3.4084	4.1331	4.1511	4.5044	4.0100	5.1443	3.9243	4.8266	3.6284	4.6091	3.9399	4.0812	4.6516	4.1784
Psd3	_KDHRPET[Phospho (STY)]PGIK_.3	#N/A	7.4334	7.2383	7.8822	6.5346	6.5879	6.4186	6.8488	6.9417	5.7361	6.1481	6.7462	6.8973	3.5346	7.1721	5.6109	6.3262	6.6699	6.6367	5.9447	5.6171
Psd3	_LEWAVDDEEKKK_.3	#N/A	4.0971	4.5774	3.7791	3.3417	3.4349	3.6589	4.1062	2.3399	2.6137	2.9609	3.0388	4.3036	4.2734	3.8171	2.6604	2.9376	3.9654	3.1840	3.4831	3.4944
Psd3	_DEYKPEKS[Phospho (STY)]LSDEDLK_.2	#N/A	3.6934	4.1490	3.8052	3.9460	3.5385	3.7527	3.0793	3.6853	3.2996	4.1311	3.5973	3.8453	3.4651	3.9677	3.7107	3.6196	3.6917	4.1905	3.8391	3.9347
Pi4k2a	_VAAAAGSGPS[Phospho (STY)]PPC[Carbamidomethyl (C)]S[Phospho (STY)]PGHDRER_.3	#N/A	10.0841	10.2858	9.9849	10.2752	9.8139	9.7023	9.9114	9.9229	9.7059	10.1197	9.6445	10.1301	4.1726	3.7164	9.3430	3.0419	10.3045	9.9107	9.7040	9.8858
Pi4k2a	_SAS[Phospho (STY)]ESYTQSFQSR_.2	Q2TBE6_S462_M1_Pi4k2a	9.1709	9.2164	9.0172	9.1823	8.1576	8.7780	8.8221	9.1009	8.8076	8.7875	8.3405	8.6574	8.6183	9.2077	4.0361	8.6102	8.9967	8.7957	8.7730	8.4824
Pi4k2a	_[Acetyl (Protein N-term)]M[Oxidation (M)]DETS[Phospho (STY)]PLVS[Phospho (STY)]PER_.2	Q2TBE6_S5_M2_Pi4k2a	3.6787	10.1304	10.3498	9.9957	4.1632	9.7174	9.6702	4.3319	9.8916	10.6100	6.7372	9.7629	3.9970	9.7077	8.9171	9.9873	10.4841	9.8070	9.8218	9.8119
Pi4k2a	_VAAAAGSGPS[Phospho (STY)]PPC[Carbamidomethyl (C)]S[Phospho (STY)]PGHDR_.2	#N/A	7.6203	7.7400	4.0499	7.3972	7.0129	7.5857	7.4864	6.9056	6.5310	7.2539	6.8304	6.7258	7.2228	7.8204	6.3846	6.6897	7.0619	7.1310	7.3449	3.8409

Msto1	_M[Oxidation (M)]AEDEES[Phospho (STY)]PGELC[Carbamidomethyl (C)]PDVLYR_.2	Q2YDW2_S41_M1_Msto1	3.4259	6.3318	4.4633	4.1583	3.6328	4.4755	5.5823	3.6516	5.5120	3.2365	4.3149	3.6193	3.4568	6.9698	3.4406	4.2889	3.1488	6.7784	4.2997	4.1787
Acot10;Acot9	_DSYIEVLLPLGTDPELR_.2	#N/A	4.6616	4.3067	5.0079	4.1355	5.2483	4.4740	4.8196	4.7032	5.2249	5.2414	4.5577	4.2804	3.3550	4.8476	4.4245	5.2891	4.0715	4.7885	4.5181	4.4105
Cnnm3	_IPVYEEER_.2	#N/A	3.2663	2.9053	3.8026	4.0553	3.2254	3.8801	4.9137	4.4675	5.3926	4.9441	3.3624	3.7363	5.6065	3.6272	3.6672	4.4028	3.5043	5.0214	4.7019	3.8928
Osbp	_SLS[Phospho (STY)]ELESLKLPAESNEK_.2	#N/A	7.0318	7.7714	6.7982	6.9962	5.8683	6.8037	7.2449	6.8874	7.0359	6.7092	6.6557	6.5899	7.3185	7.3517	6.1749	3.5708	7.2468	7.4802	6.9247	6.6796
Osbp	_M[Oxidation (M)]LAES[Phospho (STY)]DDS[Phospho (STY)]GDEESVSQTDK_.2	#N/A	6.7727	6.7651	7.1117	6.3417	5.7246	6.5555	6.3981	6.4980	6.1462	6.2412	5.5806	6.4336	6.6953	6.9380	5.7016	6.1969	7.0750	6.6494	6.3525	5.8697
Osbp	_TGS[Phospho (STY)]NIS[Phospho (STY)]GASSDVSLDEQYK_.2	Q3B7Z2_S377_M2_Osbp	3.9448	3.7908	4.9755	3.8432	3.6470	4.3701	3.6656	3.6679	3.6677	4.0254	4.3143	3.5945	3.7989	3.8128	3.4490	3.8325	4.0223	3.8039	3.7365	3.6630
Osbp	_SLS[Phospho (STY)]ELESLK_.2	#N/A	4.4748	3.4543	3.4014	3.7194	3.8125	4.0366	3.7285	4.0905	3.7876	3.1803	4.1620	3.1804	3.8957	3.4395	3.3057	3.3189	3.5877	3.5617	3.8608	3.1167
Osbp	NPPGS[Phospho (STY)]AGS[Phospho (STY)]GKDQC[Carbamidomethyl (C)]C[Carbamidomethyl	Q3B7Z2_S333_M2_Osbp	3.7688	4.2340	4.3051	4.5143	3.1066	3.3306	4.4345	3.2956	2.9928	3.9752	3.3671	4.1234	4.6017	4.1454	4.0117	4.1137	4.2937	4.3566	4.7664	3.8227
Nhej1	_DSGETQASSSTS[Phospho (STY)]PR_.2	Q3KNJ2_S245_M1_Nhej1	3.7891	6.1417	3.7666	6.0438	5.9230	4.1419	5.7187	6.0196	3.7131	6.0410	3.8913	5.8610	3.8786	3.7781	3.8183	3.2638	3.7180	3.8700	6.0944	6.1006
Hddc2	_ELYELWEEYETQSSEEAK_.2	#N/A	6.5226	5.9918	6.0482	5.3952	4.8590	5.1218	5.4335	6.0728	5.4479	6.2582	4.9860	6.0492	7.1732	7.2077	6.0734	6.6434	6.1236	5.6382	5.0877	5.1570
Arhgap45	_YDFEPYVSNNSWS[Phospho (STY)]PIM[Oxidation (M)]R_.2	Q3TBD2_S568_M1_Arhgap45	3.8981	6.1707	3.4347	3.6861	4.1051	4.8689	5.3176	5.4794	4.3641	5.3544	3.8427	3.1471	3.9290	5.6557	2.9684	3.2603	3.6211	3.5284	5.1429	3.1501
Ppp1r21	_T[Phospho (STY)]PDSEAPDVESR_.2	#N/A	7.5623	7.7177	8.2886	7.4097	7.0101	7.5621	7.2092	6.9779	7.5633	7.8379	7.6072	7.2670	7.5007	8.0003	6.2033	7.6328	7.4937	7.0429	7.3300	7.9061
Ppp1r21	_SGESSSQLS[Phospho (STY)]QEQK_.2	Q3TDD9_S101_M1_Ppp1r21	6.8892	6.4200	6.5439	7.1021	6.1135	5.7635	6.1432	6.0158	5.6496	7.0722	6.2891	5.9177	5.7889	6.2135	4.2173	6.0777	5.5548	6.2139	5.8308	5.6903
Ppp1r21	_T[Phospho (STY)]PDSEAPDVESREDLIK_.2	#N/A	4.3568	4.4632	3.8934	3.6392	3.0629	3.0962	3.9852	3.7505	3.9054	4.1674	3.3841	4.1893	4.0709	5.2808	4.0106	3.7189	4.2882	3.5449	3.1935	3.0397
Rogdi	_LTTPATLTLPEIAASGLTR_.2	#N/A	4.3606	4.8879	3.7136	4.4810	4.4043	4.3379	5.0988	4.4572	3.5843	3.9550	4.2369	3.9144	4.7010	4.0460	4.1249	3.6590	3.4154	5.0906	5.0889	4.0049
Stt3b	_ENPPVEDS[Phospho (STY)]S[Phospho (STY)]DEDDKR_.2	#N/A	7.3862	7.1711	7.9413	7.7066	7.0625	7.3365	7.3603	6.5208	7.1177	6.8764	7.3442	6.6921	7.3887	6.4013	5.6210	6.5262	7.2897	7.2934	7.0235	6.7056
Stt3b	_ENPPVEDS[Phospho (STY)]S[Phospho (STY)]DEDDKRNPGNLYDK_.3	#N/A	3.8718	7.5505	6.9626	7.1132	3.7819	6.8582	7.2053	3.9678	6.9873	3.8583	3.5905	6.7480	3.8916	7.1564	4.2258	3.7205	7.2213	7.3678	6.7331	6.3218
Hp1bp3	_EASYSLIR_.2	#N/A	4.2407	3.7621	3.2762	1.5133	2.2311	3.8025	2.1357	3.9411	3.0252	3.0866	3.1895	2.5708	4.1298	3.2533	3.5398	1.0025	4.4788	2.7126	2.7767	3.3182
Rnf157	_TLS[Phospho (STY)]PLDR_.2	Q3TEL6_S414_M1_Rnf157	5.3341	3.6860	3.1697	3.9511	3.8401	3.8734	3.2080	3.8588	3.1282	3.4437	4.1077	3.4121	3.2937	3.7963	3.2334	2.9952	3.3560	4.3221	3.9707	3.7633
Iqsec3	_AQAPELQQEEERPGAVGS[Phospho (STY)]PR_.3	Q3TES0_S256_M1_Iqsec3	11.3159	11.3196	11.0222	11.1526	10.7302	10.8208	11.0364	11.2447	10.9848	11.1056	10.9905	10.9654	11.7505	10.9934	10.0054	10.9590	11.2487	11.0316	10.7726	10.7301
Iqsec3	_LQTSQHS[Phospho (STY)]PK_.2	Q3TES0_S1055_M1_Iqsec3	7.8981	8.3412	8.0491	7.8826	7.7613	7.9062	7.9327	8.2911	8.0927	8.0255	7.8091	7.8570	8.1096	8.1424	3.7974	7.5488	8.0464	8.1841	7.7274	7.7330
Iqsec3	_VQTPQS[Phospho (STY)]PHQHPVAPGAIADKEK_.4	#N/A	7.7738	6.3944	6.2058	5.9037	3.5679	3.7222	5.8958	3.6709	5.0951	7.1175	3.7435	3.7183	6.6087	7.0411	3.6065	3.6248	3.8978	5.8559	6.7814	3.4268
Iqsec3	_VQTPQS[Phospho (STY)]PHQHPVAPGAIADK_.3	#N/A	4.0659	3.9369	4.6022	3.6235	3.4036	4.0679	4.1375	3.7663	4.1965	4.2722	3.0701	5.5114	4.3046	3.8484	3.7147	4.4108	4.3040	3.1528	4.9862	3.5257
Iqsec3	_S[Phospho (STY)]PTLSTDTLR_.2	Q3TES0_S646_M1_Iqsec3	7.3669	3.9784	3.9257	4.3287	7.5464	6.4860	3.3419	3.9959	3.1065	7.4732	6.7235	3.8679	3.2419	4.0500	3.7562	6.5624	6.6523	4.3012	3.6227	3.8475
Eepd1	_GNSAQHS[Phospho (STY)]PSSLR_.2	#N/A	3.4634	3.8951	3.9997	4.2702	3.6135	5.3627	3.2833	3.9761	4.6935	5.6363	4.6004	3.7845	3.6986	5.3606	4.8678	5.1223	5.4358	4.1759	3.8245	4.2984
Eepd1	_GNSAQHS[Phospho (STY)]PSSLRR_.3	#N/A	3.9869	3.3322	3.5234	3.5974	4.1938	4.2271	6.0124	4.0424	6.2001	5.8085	3.7540	5.2674	6.5560	5.8261	5.5052	5.8200	5.8100	6.0233	4.3245	3.4096
Eepd1	_EHGPFRS[Phospho (STY)]VEDLVR_.3	#N/A	4.5230	5.9021	4.2863	3.1664	3.8607	4.1242	4.5895	4.2234	4.3783	3.6945	3.8569	3.3693	4.5437	7.2290	3.2575	3.3671	3.2602	6.7445	5.7834	3.5125
Eepd1	_S[Phospho (STY)]VEDLVR_.2	#N/A	5.7679	5.0106	4.2044	7.0175	3.3739	3.4072	3.6742	3.4395	3.5944	3.4281	3.0731	3.8783	3.7599	5.8329	2.0795	5.6519	3.9772	6.5656	3.7974	6.5273
Myl12b	_LNGTDPEDVIR_.2	#N/A	7.5353	6.8891	7.5552	7.3411	7.4498	7.3709	7.2444	7.3294	7.4866	7.2603	7.5665	7.4641	7.2719	7.6601	7.7658	7.3735	7.5667	7.2295	7.4246	7.6333
Myl12b	_GNFNYIEFTR_.2	#N/A	7.4718	7.6330	7.2507	7.4585	7.4203	7.2146	6.7078	7.2376	6.7893	7.6433	7.0632	7.3183	7.5684	7.8482	7.1588	7.5838	7.5224	7.2412	7.1754	7.0665
Myl12b	_FTDEEVDELYR_.2	#N/A	7.3568	6.9755	7.5218	6.9793	6.6005	7.1469	6.9499	7.2578	7.3067	6.8540	7.0764	6.9471	7.1623	7.4406	7.3501	7.4062	7.1488	7.0450	6.9374	7.3043
Myl12b	_ATSNVFAM[Oxidation (M)]FDQSQIQEFK_.2	#N/A	6.3625	6.4562	4.7814	4.6955	4.4249	4.7157	5.0290	6.8961	5.0783	5.7667	4.7122	5.0618	5.2377	6.0967	4.7615	5.2218	4.9145	4.7296	4.6926	5.0142
Myl12b	_EAFNMIDQNR_.2	#N/A	3.8772	7.0733	8.1497	7.8696	3.2149	6.4020	6.8416	3.1873	4.3852	4.0835	7.5861	5.8448	4.4933	7.7854	6.7631	4.2221	6.3934	5.5124	5.9866	6.3613
Aarsd1	_RAEAQALLQDYVSTQS[Phospho (STY)]AEE_.2	#N/A	3.6163	3.7027	3.1529	8.6273	3.8233	3.8566	3.2248	4.4910	3.1450	3.4269	4.1245	3.4289	3.3105	3.8131	3.2502	2.9785	8.0028	4.3053	3.9539	3.7801
Aarsd1	_ELS[Phospho (STY)]LDDPEVEQVR_.2	Q3THG9_S174_M1_Aarsd1	3.0843	9.0840	8.5973	7.5904	3.2912	8.8043	8.5983	3.3099	8.5159	7.8881	3.3815	8.8003	3.1151	9.6187	8.8692	8.6066	8.4239	9.0948	9.1741	8.9357
Aarsd1	_GAQADHFTESPLS[Phospho (STY)]PGSQVQVR_.3	#N/A	7.8915	8.0915	6.5863	6.9358	6.2576	7.4342	7.7253	8.0358	5.7870	6.5543	6.4578	7.7367	7.9934	8.6177	5.7940	6.8844	7.4750	7.6448	6.2574	6.2015
Aarsd1	_AEAQALLQDYVSTQS[Phospho (STY)]AEE_.2	#N/A	3.2059	4.1132	4.2433	4.3783	3.4129	3.4462	3.6352	3.4316	3.5554	4.5174	3.0341	3.8393	3.7209	4.2814	3.6606	4.0689	4.4297	3.8949	4.6480	4.1905
Gmps	_TLNM[Oxidation (M)]TTS[Phospho (STY)]PEEK_.2	#N/A	10.4023	10.8815	10.6626	10.6221	10.3939	10.6058	10.4715	10.6337	10.4112	10.7081	10.4966	10.2560	10.8088	10.9924	9.9565	10.2358	10.6247	10.2484	10.4293	10.4780
Gmps	_TLNMTTS[Phospho (STY)]PEEKR_.2	#N/A	3.8882	5.5440	4.4245	5.6201	3.2259	3.2582	5.6126	3.5885	4.3743	4.0113	3.2478	4.0041	3.9089	6.0788	3.8924	4.2331	4.1262	3.7069	3.8864	4.3785
Gmps	_NFLYDIAGC[Carbamidomethyl (C)]SGNFTVQNR_.2	#N/A	4.7358	4.0284	3.4758	3.9128	4.6069	2.7707	3.7951	4.2942	4.1822	5.1096	4.2285	0.8756	3.9622	3.7930	3.3723	3.2523	4.2960	3.6385	3.7942	3.1833
Gmps	_VIC[Carbamidomethyl (C)]AEEPYIC[Carbamidomethyl (C)]K_.2	#N/A	3.7026	4.3002	4.2389	3.9868	3.4535	3.4438	3.6377	3.4030	4.5839	4.5149	3.0365	4.1896	3.7233	4.2260	4.1019	4.0475	3.9407	3.8925	4.7239	5.1295
Gmps	_QADFEAHNILR_.3	#N/A	4.3575	3.6453	3.3929	3.3319	3.6952	4.2897	4.2925	4.0579	4.2127	3.8601	3.6914	3.5348	4.3782	3.3800	2.8171	3.2015	3.0947	3.2376	4.3871	3.3470
Mat2a	_FVIGGPQGDAGLTGR_.2	#N/A	3.7818	5.3579	3.8945	4.9686	4.7266	5.3981	5.4828	3.2826	4.4243	4.7978	4.7889	5.7315	5.6791	5.3465	5.0477	4.7755	3.5755	5.6654	5.2102	4.8536
Mat2a	_TQVTVQYMQDR_.2	#N/A	3.6015	3.7176	3.1381	3.9827	3.8084	3.8418	3.7318	3.8272	3.1599	3.4121	4.1393	3.4437	3.3253	3.8280	3.2650	2.9636	3.3244	4.2905	3.9391	3.7950
Usp39	_EADEDS[Phospho (STY)]EPEREVR_.2	#N/A	5.7268	6.5888	6.1497	5.4756	5.5212	5.8057	5.9232	6.3403	6.1062	5.9094	5.1579	5.1261	6.4588	6.0135	5.3048	5.3068	4.5197	5.5548	5.5629	5.3672
Usp39	_EREADEDS[Phospho (STY)]EPEREVR_.3	#N/A	5.1105	4.6590	4.1297	4.5044	3.1982	4.9075	4.8908	4.9071	4.6584	4.8103	4.0462	4.2492	4.6793	4.7770	4.0738	4.3842	4.0577	4.7459	4.4105	4.1011
Usp39	_EADEDS[Phospho (STY)]EPER_.2	#N/A	3.7467	6.9560	3.2833	3.8375	3.9536	6.2727	3.0945	3.9724	6.2907	5.3666	3.9941	3.2985	3.1801	4.9555	3.1198	3.5413	3.4696	5.6249	6.4794	2.9986
Usp39	_EREADEDS[Phospho (STY)]EPER_.2	#N/A	4.8548	5.4589	4.2327	4.3890	3.4022	3.4355	3.6459	4.2022	3.5661	4.5067	3.4364	3.8499	3.7316	4.2342	3.6713	4.0582	4.5672	3.8843	3.5329	4.2012
Smarca4	_DSEAGSST[Phospho (STY)]PTTSTR_.2	Q3TKT4_T1390_M1_Smarca4	4.3661	3.6367	3.4016	3.3233	3.7038	4.2811	4.3012	4.0665	4.2214	3.8514	3.7000	3.5261	4.3868	3.3886	2.8257	3.2102	3.1033	3.2290	4.3784	3.3556
Cwc27	_NFSLLSFGEEAEEEEEEVNR_.2	#N/A	3.2435	3.2410	3.7972	4.4285	4.0995	4.3236	3.4416	3.8035	3.2896	3.4672	3.8751	2.7118	3.6087	3.1525	3.1605	5.1093	3.3008	3.8487	3.9136	4.3306
Prrc2c	_S[Phospho (STY)]LEDVKPR_.2	Q3TLH4_S853_M1_Prrc2c	9.0604	9.3778	8.9095	9.0254	8.5751	8.8805	9.0212	8.8963	8.7446	9.2053	8.8325	9.0343	9.5221	9.7234	8.3572	8.8528	8.9691	9.0664	8.8880	8.9790
Prrc2c	_SFS[Phospho (STY)]SQRPGVDR_.2	Q3TLH4_S1516_M1_Prrc2c	3.5467	7.4231	4.0830	4.1427	2.8845	3.1085	3.1558	3.2471	3.2149	3.7531	3.5892	4.3455	6.8832	4.3675	5.7139	3.8916	3.7848	4.1345	4.4335	4.0448
Prrc2c	_SESS[Phospho (STY)]DFEVVPK_.2	Q3TLH4_S1222_M1_Prrc2c	4.3426	3.6602	3.3781	3.3467	3.6804	3.9044	3.8607	4.0430	3.9198	4.5490	4.2942	3.5496	4.0279	5.3315	3.4379	3.1867	3.0799	3.2525	4.4019	3.2489
Prrc2c	_S[Phospho (STY)]VSHGSNHAQNAEEQR_.3	#N/A	3.0738	3.7209	3.8751	4.1212	3.7578	3.9345	3.4452	4.0665	3.5042	4.0684	3.8786	3.1106	3.6123	3.9769	3.4976	3.6837	3.7758	4.0446	4.0533	3.6811
Trappc10	_SPS[Phospho (STY)]DNSLNTTGIIC[Carbamidomethyl (C)]R_.2	Q3TLI0_S684_M1_Trappc10	9.3165	9.3935	8.8980	8.8418	3.6284	8.8562	9.0834	3.9911	8.5902	8.7744	4.3461	8.4688	8.8980	8.9828	6.9542	8.2002	9.1946	9.0006	8.3298	3.3009
Tmem38a	_AKEELGEGS[Phospho (STY)]R_.2	#N/A	6.8566	6.4222	7.1322	6.5191	6.9506	6.7257	6.3225	7.1997	7.0934	6.1337	5.9854	6.2870	6.9707	5.8120	6.4844	6.1348	6.9838	6.4858	6.3338	6.6918
Tmem38a	_AKEELGEGS[Phospho (STY)]RK_.3	#N/A	3.4990	4.5957	3.9434	4.2823	3.7059	3.7393	3.3422	3.1075	3.2624	3.3096	4.2419	2.9843	3.4278	3.9305	3.3676	4.3619	3.6452	4.1880	1.6048	3.8975
Exoc5	_VC[Carbamidomethyl (C)]HLGDQLEGVNT[Phospho (STY)]PR_.3	Q3TPX4_T122_M1_Exoc5	3.7294	5.2602	4.2657	3.9600	3.0671	4.1284	4.6121	4.8542	3.0322	5.0667	5.1555	4.1159	3.1402	4.1848	4.0511	4.0743	4.1833	5.0387	4.6659	3.8621
	ADS[Phospho (STY)]ES[Phospho (STY)]EEDEQESEEVR.2	#N/A	6.2025	6.5945	5.6574	6.3915	6.0654	6.2163	5.6410	6.6672	6.0657	6.3675	5.8618	6.6432	6.8768	6.9083	6.2108	5.6644	6.6967	6.2697	6.5315	6.0110
	VGDTEKPEPERS[Phospho (STY)]PPNR.3	#N/A	8.1639	8.6941	8.1835	8.4605	7.9413	8.2290	8.1696	8.3300	8.1661	8.4933	8.2541	8.1327	8.8392	8.9045	7.9755	7.9798	8.3974	8.0504	8.0906	8.1190
	RRADS[Phospho (STY)]ES[Phospho (STY)]EEDEQESEEVR.2	#N/A	3.7712	4.3287	4.2909	4.4305	3.7442	3.2393	3.9939	4.4795	3.3431	3.8990	3.8452	3.5268	4.0270	3.9557	4.0890	3.7982	4.4614	4.2669	4.2952	3.4065
	RADS[Phospho (STY)]ES[Phospho (STY)]EEDEQESEEVR.2	#N/A	3.2965	4.6882	4.2562	3.9695	3.0576	3.6386	3.3536	3.4203	3.8362	4.3527	4.0727	3.8693	3.8290	4.2150	3.0076	3.4656	3.9580	3.8752	3.6333	3.2982
Fndc4	_GKGPEQS[Phospho (STY)]PQGRPVGTTR_.3	Q3TR08_S209_M1_Fndc4	5.0095	5.6692	4.8336	5.3099	3.8506	3.3302	5.0941	4.7873	4.9406	5.5011	3.3676	5.0695	5.2128	5.2923	5.4685	5.1348	4.1204	5.4631	5.1210	3.8231
Pnpla6	_DEGGS[Phospho (STY)]PEGASPSTASEVEEEK_.2	Q3TRM4_S1319_M1_Pnpla6	4.3630	4.4570	3.8995	3.2213	3.0691	3.1024	3.9790	3.7443	3.8992	4.1736	3.3779	4.1831	4.0647	4.5673	4.0044	3.7251	4.2820	3.5511	3.1997	3.0335
Map9	_ESPGGC[Carbamidomethyl (C)]TS[Phospho (STY)]PGSQEK_.2	Q3TRR0_S257_M1_Map9	3.2047	9.3976	9.7160	4.3795	9.1263	9.4590	3.6364	9.4989	9.3355	4.5162	9.3640	9.4421	9.5542	10.2377	9.4084	9.4307	9.5652	9.4690	9.5084	9.5795
Map9	_S[Phospho (STY)]PSAATSSHYLGSLK_.2	Q3TRR0_S389_M1_Map9	6.4643	6.0155	4.2468	3.9789	5.7509	5.2688	2.9920	3.4109	5.3424	6.0711	6.1992	4.1818	6.7205	4.2339	5.6432	6.4508	5.7363	3.8846	3.5332	4.2009
Map9	_KQS[Phospho (STY)]LEPDKADHIR_.3	#N/A	7.7370	7.8958	7.8671	8.2022	7.3957	7.3998	7.5351	6.7831	7.0697	8.4100	8.1362	7.9024	8.1647	8.1781	7.1998	8.0733	7.3917	7.7884	8.1869	8.2366
Map9	_STS[Phospho (STY)]SGETSSGLDADGHFKPSPQPR_.3	Q3TRR0_S160_M1_Map9	4.5405	3.4623	3.5760	6.1547	3.8782	4.1023	3.6628	4.2409	5.7311	4.6409	4.0963	3.3517	4.5612	3.5630	5.7744	3.3846	3.2777	3.0546	4.2040	3.5300
Map9	_RTS[Phospho (STY)]FQDELIR_.2	Q3TRR0_S23_M1_Map9	5.4326	5.9863	5.1149	4.8424	3.9999	4.0333	4.6790	4.0180	4.0418	6.2974	3.9479	3.2522	3.1338	6.3470	4.6446	5.4831	3.5159	5.2039	4.1306	5.0314
Map9	_M[Oxidation (M)]NDFHIS[Phospho (STY)]DDEEKNSPR_.3	#N/A	6.2671	5.9549	4.0439	5.4131	3.5286	5.5017	5.6995	3.7329	5.7108	3.9190	5.4837	3.8681	4.0592	7.0921	4.6045	6.3960	6.0120	6.4136	6.1681	6.3399
Cblb	_QNSPVTS[Phospho (STY)]PGSS[Phospho (STY)]PLAQR_.2	Q3TTA7_S480_M2_Cblb	3.6786	3.8155	3.8984	5.1355	3.8855	3.5831	4.0208	3.9786	3.8599	4.1724	3.9053	3.5416	3.9935	4.0485	4.0462	3.7240	3.9460	3.5673	3.5277	3.9106
Ccdc136	_GLS[Phospho (STY)]LTETELEELR_.2	Q3TVA9_S50_M1_Ccdc136	11.4156	11.2131	11.6106	11.5509	10.5638	10.9332	10.9476	10.4469	11.0599	11.5717	11.0863	10.7565	11.2686	10.8705	10.1396	10.9367	11.0574	10.6499	10.9337	10.9211
Hexim2	_TQS[Phospho (STY)]PGGC[Carbamidomethyl (C)]SVEAVLAR_.2	Q3TVI4_S75_M1_Hexim2	4.3528	4.4671	3.8894	6.9515	3.0589	3.5487	5.9428	6.3453	4.2756	4.1932	3.1491	4.1933	4.3837	7.4366	5.8417	3.7149	6.2341	3.0738	6.0626	6.1176
Pbxip1	_GREPS[Phospho (STY)]SSQPVVPVDVEDQAK_.3	Q3TVI8_S164_M1_Pbxip1	4.1560	3.9830	3.6610	3.6702	6.1074	3.7178	3.6459	4.0165	5.6316	3.8786	3.6220	3.8724	4.1161	3.7738	5.1910	3.4696	3.6395	3.5578	3.8710	3.6558
Tmem63b	_LTSVSS[Phospho (STY)]S[Phospho (STY)]VDFDQR_.2	Q3TWI9_S115_M2_Tmem63b	3.2783	6.4680	4.0567	4.3741	3.8722	5.8634	5.9854	3.2336	6.1304	3.8079	3.5503	3.5358	3.2840	5.7344	3.3798	3.8742	4.0336	4.1893	5.4697	4.0059
Srsf6	_SHS[Phospho (STY)]PLPAPPSK_.2	Q3TWW8_S303_M1_Srsf6	9.7496	9.6789	3.5485	9.3841	9.2429	9.5589	9.4538	9.7524	9.1478	9.4427	9.3068	9.1173	9.7378	9.5531	8.1344	9.0443	9.5832	9.5966	9.2083	9.2430
Srsf6	_LIVENLSSR_.2	#N/A	7.5309	5.6943	6.1260	5.9255	5.8643	6.0218	6.9656	5.7737	7.1028	6.0938	6.1113	5.7885	6.1700	5.7596	5.8472	6.6598	5.9507	6.0987	6.9666	6.3354
Srsf6	_ARS[Phospho (STY)]MS[Phospho (STY)]PPPK_.2	#N/A	3.7521	6.9047	7.7514	3.9373	3.0898	3.3139	7.0261	3.4524	3.0095	3.9584	7.5132	4.1402	3.1176	8.3365	4.0285	4.0970	3.9901	5.4822	3.4916	3.8395
Srsf6	_S[Phospho (STY)]M[Oxidation (M)]S[Phospho (STY)]PPPK_.2	#N/A	7.3149	6.5598	3.5483	6.2470	5.2941	7.9467	6.5106	6.8727	7.8065	7.0999	5.6726	3.0335	5.8876	5.9686	2.8548	7.4264	5.5318	8.7116	7.5782	6.8238
Srsf6	_SRS[Phospho (STY)]QS[Phospho (STY)]RS[Phospho (STY)]HS[Phospho (STY)]PLPAPPSK_.3	Q3TWW8_S297_M3_Srsf6	3.7547	3.5644	3.2913	3.8295	3.9617	4.0158	3.6184	3.9804	3.6775	3.2904	4.0519	3.2905	3.7856	3.3294	3.1956	3.1168	3.4776	3.6718	3.9709	3.0066
Srsf6	_ARS[Phospho (STY)]MS[Phospho (STY)]PPPKR_.3	#N/A	4.0922	3.2269	3.6288	3.4920	4.2992	4.3325	4.2498	4.0151	4.1700	3.9028	3.6486	2.9530	4.3354	3.3373	4.2752	3.4543	4.5528	3.2804	4.4298	3.3043
Psmd1	_TVGT[Phospho (STY)]PIASVPGSTNTGTVPGSEK_.2	Q3TXS7_T273_M1_Psmd1	6.7954	3.3641	3.6742	5.9058	3.9765	4.2005	6.5460	4.3391	3.6237	3.3442	6.1164	5.8065	3.7318	6.6549	4.0328	3.4828	6.9206	6.2257	5.6822	5.9003
Psmd1	_TPDAS[Phospho (STY)]PEPK_.2	Q3TXS7_S315_M1_Psmd1	6.5503	6.5420	6.8209	4.5471	7.1900	7.4381	4.4017	7.3142	4.4608	4.0080	6.8380	4.0906	7.5994	4.1126	6.5456	4.1465	7.2709	7.3010	6.8911	6.7980
Psmd1	_TASAVAGKT[Phospho (STY)]PDAS[Phospho (STY)]PEPKDQTLK_.3	#N/A	6.8774	7.4224	6.4431	6.9531	6.3564	6.4410	7.2559	7.4505	6.9272	7.2653	6.3760	7.2222	8.1282	6.6679	6.1079	6.8730	7.2748	6.9665	7.0692	6.7865
Psmd1	_IEEEEQEPEPPEPFEYIDD_.2	#N/A	3.7501	5.8231	3.2867	3.8341	3.9571	5.6815	5.4568	3.9758	5.3739	3.5607	3.9907	5.0311	3.7810	5.1777	4.4651	4.7454	5.1980	5.3294	4.8705	5.4722
Psmd1	_TASAVAGKT[Phospho (STY)]PDAS[Phospho (STY)]PEPK_.2	#N/A	10.4569	10.3017	10.4336	10.3519	9.9103	10.0544	10.0999	10.4558	10.2083	10.1991	9.8227	9.8627	10.3172	10.1944	9.0345	9.7014	10.3842	10.0777	9.9415	9.8483
Psmd1	_AAVESLGFILFR_.2	#N/A	4.2284	3.6624	3.2639	3.4610	2.1606	4.4188	3.9749	3.1296	4.0837	3.6991	4.0660	3.3717	4.2492	3.2510	3.5521	3.3874	3.2824	3.3667	3.8916	3.2180
Psmd1	_TPEQC[Carbamidomethyl (C)]PSVVSLLSESYNPHVR_.3	#N/A	3.7834	4.6208	3.3200	3.8008	3.9903	4.6533	5.0208	4.0091	4.4181	3.5940	3.9574	3.2618	3.1434	3.6461	2.1823	3.1455	3.5063	2.9716	4.1210	3.6131
Slc17a7	_WAPPLER_.2	#N/A	8.8069	8.9481	8.4125	8.5452	8.7749	8.8229	9.1061	9.2707	8.6435	8.4867	8.5447	8.5856	8.9426	7.7886	8.3627	7.9131	8.6564	9.0681	8.7474	8.0184
Slc17a7	_DPPVVDC[Carbamidomethyl (C)]TC[Carbamidomethyl (C)]FGLPR_.2	#N/A	6.4816	6.7037	6.4095	6.9033	6.5093	6.6835	6.4160	6.6127	6.9477	6.5634	6.9434	6.4615	6.4313	5.8747	6.3155	6.3804	7.0258	6.1693	6.5252	6.2605
Slc17a7	_YIEDAIGESAK_.2	#N/A	7.4961	6.9432	7.1375	7.3370	7.1783	7.3339	7.4077	7.4372	7.1841	6.7269	7.2458	6.6231	7.4244	6.7003	6.9397	6.7426	7.0157	7.3360	6.7626	6.8393
Slc17a7	_GGHVVVQK_.2	#N/A	5.8378	5.9120	4.5311	5.0105	5.1423	6.1483	5.2768	5.7334	6.0291	5.4568	5.3014	4.5934	4.5857	4.7349	5.3600	5.1090	4.4232	5.4485	4.9266	5.2222
Slc17a7	_KYIEDAIGESAK_.2	#N/A	5.6839	5.3799	3.8146	5.3924	5.2545	5.3624	5.1027	6.3488	5.3978	4.8912	5.0020	5.0356	4.3157	5.1877	4.5168	4.7675	4.5004	5.0004	4.1391	5.0834
Slc17a7	_HIM[Oxidation (M)]STTNVR_.2	#N/A	5.3280	5.7957	6.0859	5.4337	5.4997	5.9526	6.3361	5.8047	5.6071	5.3455	5.8572	5.5208	5.4925	5.6475	5.4817	4.5026	5.6196	5.5076	5.6866	4.8857
Slc17a7	_QPWAEPEEM[Oxidation (M)]S[Phospho (STY)]EEK_.2	#N/A	7.0809	7.2291	7.0359	6.6086	5.7762	7.2308	7.1397	7.0424	6.6367	6.8709	6.7586	6.8641	6.8482	7.1688	5.9603	6.6602	7.4544	7.3284	6.6565	6.4280
Slc17a7	ethyl (C)]GFVGHDQLAGS[Phospho (STY)]DES[Phospho (STY)]EMEDEAEPPGAPPAPPPSYGATHST\	Q3TXX4_S519_M2_Slc17a7	4.2629	4.5571	3.7995	3.3214	2.9690	3.6386	4.1266	4.4886	4.1857	4.2831	3.0592	4.2832	4.2937	3.8375	3.7038	3.6250	3.9858	3.1637	3.4627	3.5148
Slc17a7	_RQEGAETLELS[Phospho (STY)]ADGRPVTTHTR_.4	Q3TXX4_S36_M1_Slc17a7	3.6478	3.2437	3.7946	4.4311	3.8547	3.8881	5.5210	4.4595	3.1136	3.4584	4.0931	3.3974	3.2790	3.7817	3.2187	3.0099	3.4964	4.3368	3.9854	3.7487
Fam131b	_DTDAYSDLS[Phospho (STY)]DGEK_.2	#N/A	9.4667	9.3922	9.7721	9.5394	9.2494	9.5646	9.1221	9.3421	9.3921	9.0741	9.5286	9.2145	9.4014	10.0802	9.2353	9.1531	9.8422	9.2321	9.4076	9.5818
Fam131b	_NS[Phospho (STY)]NAYGIGALAK_.2	#N/A	10.0147	3.6508	9.8246	3.9160	9.6202	9.4587	3.1729	9.3464	9.2306	3.4788	9.6526	3.3770	9.7672	9.7332	8.7128	9.6378	9.9787	3.7583	4.0573	9.6468
Fam131b	_RNS[Phospho (STY)]NAYGIGALAK_.2	#N/A	3.9933	3.3258	3.5299	3.5909	5.8726	4.2336	6.2001	6.3129	4.2689	6.8862	6.7197	3.0519	6.7592	6.8316	2.8732	3.3554	3.1508	5.9071	4.3309	3.4032
Fam131b	_VSDVTSSGVQSFDEEEGDANN_.2	#N/A	7.4067	9.1776	9.3045	9.4398	5.8867	8.9485	8.6261	6.2391	8.8558	9.1980	6.1366	8.8881	7.9500	9.5313	8.7190	9.1027	8.9001	8.8394	8.8678	9.0263
Fam131b	_DLESLS[Phospho (STY)]PR_.2	#N/A	3.4102	6.7366	7.8121	7.7677	3.6171	3.6505	3.4310	3.6359	6.7575	7.7876	4.3307	6.6424	3.5166	4.4857	3.4563	4.2731	6.9037	4.0992	4.3154	3.9863
Fam131b	_DTDAYSDLS[Phospho (STY)]DGEKEAR_.2	#N/A	8.9100	9.2290	8.8207	9.1044	5.9814	8.8649	8.7045	8.7656	8.9128	8.8848	7.8253	9.1041	9.0875	9.6840	9.0785	9.1424	9.5600	9.0983	9.0155	9.1285
Fam131b	_RDTDAYSDLS[Phospho (STY)]DGEKEAR_.3	#N/A	6.2938	6.5261	6.0130	6.8079	5.5458	6.3852	6.1106	6.0139	6.3354	6.5137	6.1367	6.1504	6.5541	7.0420	6.4823	6.2591	6.8925	6.7271	6.2720	6.6343
Fam131b	_KVSDVTSSGVQS[Phospho (STY)]FDEEEGDANN_.2	#N/A	9.5215	9.9225	10.0370	9.9846	8.1907	9.7242	9.4730	9.0825	9.6599	9.9156	8.7629	9.6185	9.9787	10.3131	9.5584	9.9155	10.0799	9.6614	9.6945	9.8385
Fam131b	_APTIQPQHS[Phospho (STY)]HEAVRR_.3	Q3TY60_S102_M1_Fam131b	3.0896	4.1863	4.3528	3.8728	3.6035	3.3298	3.7516	3.5169	3.6718	6.4058	3.1505	4.0757	3.8373	5.3002	3.7770	4.7709	4.0546	3.7786	3.4271	4.3069
Fam131b	_RDTDAYSDLS[Phospho (STY)]DGEK_.2	#N/A	3.5615	3.7576	4.5989	4.0228	3.7684	4.1508	3.6885	3.7872	3.4843	3.4836	4.1963	3.6086	3.5923	4.6654	3.4969	2.9526	3.2844	3.8651	4.1641	4.3142
Fam131b	_DLESLS[Phospho (STY)]PREDPEM[Oxidation (M)]STALSR_.3	#N/A	3.6504	5.8164	4.7579	6.0826	5.0526	5.8068	5.0750	3.6948	3.7483	3.7342	6.0536	5.6670	6.0528	6.5980	5.5651	6.0484	3.5838	6.1116	6.1571	6.3838
Ica1l	_VALS[Phospho (STY)]EEEEEERFER_.2	#N/A	4.2857	4.1611	3.6114	3.1825	3.3191	4.0703	3.8749	3.5446	3.5669	4.1215	3.3563	3.9974	4.3119	3.7607	4.0608	3.4753	3.8191	3.1845	3.3736	3.6111
Ica1l	_ALPRDS[Phospho (STY)]LEGDDFEK_.3	#N/A	4.1554	3.9549	3.9958	3.4211	3.1781	3.3205	4.1280	3.4849	4.1234	4.1563	3.2988	3.8443	4.2574	4.3180	3.9335	4.0397	4.2130	3.2993	3.2635	3.3687
Aifm3	_GKEELSASGKGS[Phospho (STY)]PR_.2	#N/A	6.3663	6.3138	5.8976	5.9803	5.8556	6.0576	5.9837	6.0888	6.0931	6.3593	5.3417	5.5458	5.9657	5.5381	4.7687	5.6641	5.8883	5.8188	5.6249	5.2981
Aifm3	LPTPQPYPS[Phospho (STY)]PQDC[Carbamidomethyl (C)]VEATVC[Carbamidomethyl (C)]HVK_.	Q3TY86_S63_M1_Aifm3	3.3146	3.1874	3.8509	4.0600	4.1531	4.3772	3.3880	3.7499	3.4470	3.5209	3.8214	3.0769	3.5551	6.9147	2.9652	3.6595	3.2471	3.9023	4.2014	4.2770
Aifm3	_EELSASGKGS[Phospho (STY)]PR_.2	#N/A	3.6771	4.1574	4.1991	4.4225	3.0148	3.2389	3.0254	2.5555	3.0845	3.8834	3.4589	3.8835	3.1926	2.6179	4.1035	4.0220	4.3854	4.2648	3.0631	3.9145
Mphosph8	_GTVAVGDS[Phospho (STY)]EEDGEDVFEVER_.2	Q3TYA6_S51_M1_Mphosph8	3.9297	3.3894	3.4663	3.6545	4.1367	4.1700	4.4123	4.1776	4.3325	3.7403	3.8111	3.1155	4.4979	3.4998	2.9368	3.2918	3.2144	3.1179	4.2673	3.4668
Lmtk2	_GQS[Phospho (STY)]PPESVVPEESSDVR_.2	Q3TYD6_S1104_M1_Lmtk2	10.7754	10.8108	10.4792	10.0303	3.4338	10.2047	10.1252	10.5149	10.2536	10.5335	10.3679	10.1886	11.0421	10.4371	9.7916	10.0934	11.0651	10.3816	10.4218	10.1953
Lmtk2	_YFS[Phospho (STY)]PPPPAR_.2	Q3TYD6_S1418_M1_Lmtk2	3.3092	3.1927	3.8455	9.3170	4.1478	3.8371	8.7406	4.5105	8.1007	3.4075	7.9949	8.6950	8.6328	3.8326	3.2697	3.6541	3.5473	4.2859	7.2780	3.7996
Lmtk2	_HSGTSPQASPALLTEEGS[Phospho (STY)]PTAPTDPILKPEETK_.4	Q3TYD6_S797_M1_Lmtk2	3.0850	4.2341	4.1224	4.4993	4.6259	4.9937	3.7562	3.3107	3.6764	4.3964	3.1550	3.9602	3.8418	4.1605	4.4367	3.9479	5.6348	6.1167	4.9408	5.3082
Lmtk2	_NAGFTSALLES[Phospho (STY)]PQR_.2	Q3TYD6_S742_M1_Lmtk2	7.8381	8.3368	7.4805	7.4985	3.2344	7.0364	7.4145	7.2700	6.9408	7.6935	6.9502	6.3406	6.6488	7.8663	5.4184	6.5242	6.7167	7.3558	7.0132	6.1956
Esyt2	_SSS[Phospho (STY)]SLLASPSHIAAK_.2	Q3TZZ7_S662_M1_Esyt2	3.9290	3.3901	3.4656	3.6552	4.1359	3.9724	4.4130	4.1547	3.8518	3.1161	4.2262	3.1162	3.9599	6.8973	2.9376	3.2911	3.6519	6.7654	3.7966	3.1809
Tbc1d2b	_DTSPDKGELVS[Phospho (STY)]DEEEDT_.2	Q3U0J8_S959_M1_Tbc1d2b	6.4911	5.9443	3.6058	3.5150	4.2762	6.1141	5.9305	4.2949	3.9921	3.8798	4.3665	6.1698	4.1001	3.6439	4.1634	5.3522	6.0474	5.5979	4.8751	3.3212
Trappc9	_FNFESVPES[Phospho (STY)]PGEK_.2	Q3U0M1_S953_M1_Trappc9	9.3679	10.3904	10.5419	3.4669	3.5602	10.4924	10.1427	3.9228	9.9925	9.8963	2.9135	9.1468	7.9127	9.9749	8.2457	3.0665	10.2295	10.1558	9.8196	9.7774
Trappc9	_VESRPTNPS[Phospho (STY)]EGS[Phospho (STY)]KTGDLGHVK_.3	Q3U0M1_S840_M2_Trappc9	6.5848	4.4213	6.7359	3.7308	3.0183	6.6135	3.5796	3.2037	3.5532	4.0202	6.8924	4.2336	4.3371	4.0398	4.0915	4.1115	6.7708	6.5711	6.8656	6.5329
Rasip1	_AAS[Phospho (STY)]GGAALASPGPGSGSGTPTGSGGK_.2	Q3U0S6_S286_M1_Rasip1	3.3699	3.9492	5.4322	5.3784	3.5769	3.6102	4.7404	3.5956	4.3683	5.0897	4.3709	3.6753	3.4008	5.6225	3.4966	3.8367	3.0928	4.0842	4.1640	4.2806
Rasip1	_SVS[Phospho (STY)]ELSLQGR_.2	Q3U0S6_S322_M1_Rasip1	7.1706	8.4599	8.1014	8.2359	6.8128	8.1727	8.0030	7.4823	8.1876	6.9664	7.2151	7.8948	7.5783	7.9739	6.8088	7.8641	8.4746	8.0589	8.0323	7.8186
Khsrp	_GS[Phospho (STY)]PQQIDHAK_.2	Q3U0V1_S481_M1_Khsrp	7.0152	6.4513	6.8318	6.7067	6.7193	6.6645	6.5561	6.2282	6.6925	6.8310	6.4909	6.4508	6.7928	6.9153	6.8895	7.1029	7.2503	6.6090	7.1081	6.5369
Khsrp	_IINDLLQSLR_.2	#N/A	5.7269	5.9208	5.4541	5.4807	5.8417	5.6638	5.7987	5.4383	5.4399	5.7609	5.4964	5.7531	5.6987	5.5227	6.0414	5.4373	6.1226	5.7985	5.6085	5.6138
Khsrp	_SVSLTGAPESVQK_.2	#N/A	5.8695	5.7649	6.7476	6.0554	6.7768	5.6261	6.0970	6.0444	6.2102	6.3289	5.9571	5.9899	6.5566	6.2879	6.3784	6.0295	6.3131	6.0025	6.1055	5.8004
Khsrp	_VQIS[Phospho (STY)]PDSGGLPER_.2	Q3U0V1_S182_M1_Khsrp	10.2039	10.5754	10.5057	10.5256	9.9177	10.2951	9.9718	10.4088	10.2851	10.5543	10.0876	10.3813	10.9241	10.9413	10.2907	10.5189	10.8383	10.3105	10.3684	10.6468
Khsrp	_IGGDAATTVNNNTPDFGFGGQK_.2	#N/A	3.3166	3.7969	4.5596	4.0620	4.1551	3.8528	3.2332	4.6645	3.2181	4.1012	3.2223	3.5230	5.0511	4.5977	3.2347	3.4912	3.2451	3.9043	4.2034	3.6007
Pag1	_SSSS[Phospho (STY)]C[Carbamidomethyl (C)]NDLYATVK_.2	Q3U1F9_S351_M1_Pag1	3.2237	9.4303	9.5667	9.4441	9.0152	4.2863	8.9758	4.4249	9.0363	9.5787	8.9687	9.0028	9.5223	9.3040	8.3707	3.5686	9.5127	8.7062	8.9079	9.1170
Pag1	_RPS[Phospho (STY)]EEPEPDYEAIQTLNR_.3	Q3U1F9_S379_M1_Pag1	4.6513	5.0377	4.4691	5.4006	3.4621	5.0287	4.9210	3.9271	6.3140	5.5796	4.7503	3.9384	3.6024	5.5813	3.8267	3.6551	4.0316	4.6375	5.2931	5.6299
Pag1	_HSANAESILGT[Phospho (STY)]C[Carbamidomethyl (C)]SDLDEESPPPVPVK_.3	Q3U1F9_T245_M1_Pag1	3.4850	5.9405	4.3066	4.1596	4.0255	4.0721	5.7027	3.4856	4.5504	3.3406	4.0617	3.2569	3.5111	6.4834	4.8707	4.1256	3.4748	6.0714	5.1751	4.1195
Tfpt	_TTATLDPTS[Phospho (STY)]PAPGEGPSGR_.2	Q3U1J1_S180_M1_Tfpt	7.5898	7.5591	7.1811	3.2418	6.8033	6.8605	4.2061	7.1881	6.4881	7.5883	7.0891	7.1267	7.5859	7.9274	6.7712	7.4316	7.5017	7.2430	7.1930	3.5943
Tfpt	_EGLS[Phospho (STY)]PSQR_.2	Q3U1J1_S167_M1_Tfpt	4.0422	3.2769	3.5788	3.5420	4.2491	4.2825	4.2998	4.2679	4.2200	3.8528	3.6987	3.0030	4.3855	3.6168	2.8244	3.4043	5.7886	3.2304	3.6834	5.6573
Tfpt	_LLPYPTLAS[Phospho (STY)]PPFD_.2	Q3U1J1_S255_M1_Tfpt	3.2311	3.2709	3.7674	4.4583	4.0696	4.2937	3.4715	4.4323	3.5305	3.4374	3.9049	3.1604	3.6386	3.7544	3.0487	3.5760	3.4691	3.8188	4.0126	4.3605
Dennd1b	_ASKIS[Phospho (STY)]S[Phospho (STY)]EDGEETR_.2	Q3U1T9_S516_M2_Dennd1b	3.2351	5.1106	4.2725	4.3491	4.6887	5.5526	3.6060	5.2824	6.0392	4.5466	3.0049	4.8829	5.8494	4.9664	4.9707	5.4639	6.0244	5.4588	5.5930	4.7876
Uevld	_DGVLS[Phospho (STY)]PSSQAQLSSR_.2	Q3U1V6_S365_M1_Uevld	9.2914	9.2045	9.0673	9.2805	8.7425	9.0429	8.7849	9.1349	9.0449	9.2825	8.7571	8.6508	9.2849	9.1770	7.5092	8.6655	9.2114	8.7001	8.6644	8.4897
Map3k9	_SS[Phospho (STY)]PPASPTIIPR_.2	Q3U1V8_S541_M1_Map3k9	4.0744	4.4231	4.0273	3.7795	3.8732	3.2470	3.5675	7.7803	3.9482	3.8915	3.4729	7.1526	7.5995	4.2512	3.4344	7.1082	3.7483	4.2729	3.9208	3.9285
Dennd4b	_S[Phospho (STY)]APSS[Phospho (STY)]PAPR_.2	Q3U1Y4_S732_M2_Dennd4b	8.7826	8.0569	8.5904	8.8316	7.7461	7.6371	4.0454	7.5805	4.0400	4.2386	7.9783	7.8212	7.4036	5.9236	3.8537	3.3854	7.4525	8.0877	3.1769	7.8900
Dennd4b	_SGS[Phospho (STY)]LGSAR_.2	Q3U1Y4_S961_M1_Dennd4b	3.3539	3.1480	3.8902	4.3355	4.1925	4.4166	3.3486	3.0543	3.4077	3.5602	3.7821	3.0375	3.5157	3.8773	2.9258	3.6988	3.5920	4.2412	3.8898	3.8443
Gpsm3	_SAPPS[Phospho (STY)]PPPPGTR_.2	Q3U1Z5_S38_M1_Gpsm3	9.0274	8.7238	3.4816	9.4423	9.3873	9.9612	9.0230	4.1707	9.1193	9.8929	9.0157	9.9798	3.9759	10.4644	6.6300	9.4252	9.7189	9.8293	7.9442	3.1969
Mast3	_RLS[Phospho (STY)]ADLR_.2	Q3U214_S769_M1_Mast3	8.2315	8.6939	8.2083	8.4708	8.2983	8.0969	8.5418	8.1716	8.5177	8.3122	8.6466	7.8799	8.8196	8.0918	7.0772	8.5361	8.3822	8.9517	8.6354	8.4421
Mast3	_RGQS[Phospho (STY)]ADKLGLGTSER_.3	#N/A	8.9200	8.7093	8.4114	8.2235	7.6482	7.8692	8.3499	7.8476	8.0001	8.5806	8.3314	8.0111	8.3934	7.9864	6.8521	7.8052	8.1728	8.1728	7.8509	7.9937
Mast3	_DPLEEIQPLNDLDESQPPAPGS[Phospho (STY)]PESR_.3	Q3U214_S370_M1_Mast3	7.1755	3.9195	6.6346	7.0007	3.1493	3.9570	4.1597	6.5467	3.9441	4.1245	6.3486	6.6385	7.1280	7.5232	6.0765	6.6071	4.3269	7.4107	6.9444	6.3559
Mast3	_KQEAVQEVS[Phospho (STY)]FDEEPGPPR_.2	#N/A	6.3554	7.4324	7.0527	6.5173	3.4747	6.7398	7.0518	4.5582	6.7314	7.0262	4.5035	6.2066	6.4933	6.9821	5.5561	6.5660	5.7922	7.1099	6.6157	6.9282
Mast3	_GQS[Phospho (STY)]ADKLGLGTSER_.2	#N/A	9.6710	9.5256	8.9055	8.9843	8.1595	8.5812	8.7481	8.8054	8.2026	9.0525	8.7607	8.5757	9.1027	8.9674	7.1368	8.5948	8.9145	8.9221	8.4739	8.6508
Mast3	_QEAVQEVS[Phospho (STY)]FDEEPGPPR_.2	#N/A	3.2395	8.0424	7.9797	6.8354	3.4464	7.5626	7.2457	3.4652	7.2013	7.1810	5.6620	6.3009	6.8732	7.8010	6.2544	6.2784	7.3313	7.6044	7.1589	6.9681
Mast3	_SFS[Phospho (STY)]EDREDGWGQSLGDR_.3	#N/A	3.9871	4.7528	4.9732	3.7976	3.5488	4.7686	4.8523	4.0256	4.4695	5.3082	3.2412	3.6939	4.1023	3.7925	3.7926	3.5272	3.9363	4.9153	5.0498	3.6714
Mast3	_S[Phospho (STY)]SESVVDEDGGR_.2	Q3U214_S156_M1_Mast3	3.1424	4.1767	4.1798	3.9257	3.3493	3.3826	3.6988	3.4641	3.6190	4.4538	3.0976	3.9028	3.7844	4.2871	3.7242	8.4832	4.0018	3.8314	8.6775	4.2541

Mast3	_HLGS[Phospho (STY)]EDDETNDEESSTEIPQFSSC[Carbamidomethyl (C)]SHR_.4	Q3U214_S702_M1_Mast3	3.7357	3.5834	3.2723	4.5190	3.9427	3.9760	3.1054	4.3716	3.0256	3.5463	4.0051	3.3095	3.1911	3.8083	3.1308	3.0978	3.4084	4.4247	4.0733	3.6607
Mast3	_VY[Phospho (STY)]SSSEFLAVQPTPTFAER_.2	Q3U214_Y730_M1_Mast3	4.1278	5.7162	3.1633	3.5616	3.4656	3.0185	4.9945	5.1847	3.9831	4.0897	3.4618	3.7644	4.1486	3.1504	4.0883	2.9719	4.3659	6.4083	3.1159	3.1174
Fam160a2	_QQS[Phospho (STY)]LGGSESPGPVPR_.2	Q3U2I3_S510_M1_Fam160a2	8.2187	7.8579	3.7489	7.9301	7.3529	7.1850	4.1296	3.8950	4.0499	7.8850	7.2231	4.3337	4.2153	3.2171	4.1550	6.6413	4.4326	3.4005	6.5970	3.1841
Gse1	_ERS[Phospho (STY)]PS[Phospho (STY)]PPAVQC[Carbamidomethyl (C)]K_.2	Q3U3C9_S826_M2_Gse1	8.2195	8.2912	8.6411	8.1892	7.2452	8.3503	8.0350	8.4208	8.2908	8.2949	8.1993	8.3247	9.3116	9.0993	8.3645	8.7890	9.0552	7.9673	8.4117	8.6737
Gse1	_YSPDEM[Oxidation (M)]NNS[Phospho (STY)]PNFEEK_.2	Q3U3C9_S857_M1_Gse1	3.3841	6.0889	3.9204	4.3053	5.6464	5.9943	5.7402	6.5423	3.2394	3.5905	4.2189	3.0073	6.1495	3.9075	2.8956	5.8652	3.6222	4.2110	6.3107	3.8745
Gse1	_RQT[Phospho (STY)]PS[Phospho (STY)]PR_.2	Q3U3C9_S840_M2_Gse1	4.6106	3.5408	3.4974	3.2274	3.7997	4.0238	3.8304	2.7487	3.8005	3.1674	4.1749	3.4303	3.8578	3.4523	3.3186	3.3060	3.1992	3.5489	3.8479	3.1296
Fam117b	_SSS[Phospho (STY)]WGSTEQLK_.2	#N/A	3.3880	8.0632	9.0737	8.4062	8.9064	7.7926	8.1348	8.5928	3.3733	9.3152	8.8917	8.4226	3.5388	8.8523	8.2082	8.6163	9.0773	4.0770	8.5596	8.6334
Fam117b	_TS[Phospho (STY)]PTVATQTGASVTSTR_.2	Q3U3E2_S102_M1_Fam117b	3.6918	7.1980	4.1844	4.4372	3.0295	6.3110	3.0107	3.3727	3.0698	3.8981	3.4442	6.7809	3.1779	4.2225	4.0888	4.0367	6.2020	4.2795	3.0777	6.3840
Fam117b	_SPS[Phospho (STY)]APVC[Carbamidomethyl (C)]K_.2	#N/A	4.1284	3.8744	3.1638	3.5610	3.4661	3.0180	4.0634	3.8287	7.4076	5.6366	3.4623	3.7639	4.1491	3.1509	7.3420	2.9724	4.3664	6.5021	7.6413	3.1179
Fam117b	_SSS[Phospho (STY)]WGSTEQLKEIAK_.2	#N/A	4.3344	3.6939	3.3699	3.3805	3.6721	3.8962	3.8944	3.9449	3.9535	3.8830	3.6702	3.5833	4.0616	3.2816	3.4716	3.1785	4.0155	5.1739	3.7204	3.2826
Fam117b	_RSPS[Phospho (STY)]APVC[Carbamidomethyl (C)]K_.2	#N/A	4.2834	3.8793	3.1590	3.5659	2.9895	3.0229	4.0586	3.8239	3.9788	4.0940	3.4574	4.2626	4.1442	3.1461	4.0840	3.6455	4.3616	3.4716	3.1202	3.1130
Fam117b	_S[Phospho (STY)]PEQGRPSAEKR_.3	Q3U3E2_S176_M1_Fam117b	3.2902	3.2118	3.8265	4.3992	4.1287	1.9149	3.4123	4.4914	3.4714	3.4965	3.8458	3.1012	3.5795	3.8135	2.9895	3.6351	3.5282	4.3049	3.9535	3.7805
Fam117b	_SIDTQTPGGADKGS[Phospho (STY)]NNSSR_.3	Q3U3E2_S399_M1_Fam117b	4.0789	4.5592	3.7973	3.3235	3.4166	3.6407	4.1245	4.4864	4.1835	4.2852	3.0571	4.2853	4.2916	3.8354	3.7017	4.4238	3.9836	3.1658	3.4649	3.5126
Tepsin	_QQLQELGAGS[Phospho (STY)]PGPVTNK_.2	Q3U3N6_S400_M1_Tepsin	3.8471	7.1120	6.7174	6.4243	3.1849	3.4090	6.4319	6.3105	4.4153	6.6364	6.3894	4.0451	7.1872	5.9539	3.9334	4.1920	6.8192	3.7480	6.0800	6.3944
Prickle1	_S[Phospho (STY)]AESLQSLNSGLC[Carbamidomethyl (C)]PEK_.2	Q3U5C7_S592_M1_Prickle1	6.6163	7.2656	4.0475	6.4933	5.5161	6.2005	6.5784	6.7826	6.4918	6.6635	6.0224	6.4341	6.5097	6.9691	4.0102	4.0002	6.9139	6.8090	4.3323	6.2609
Prickle1	_NLFQQQS[Phospho (STY)]SEVDPR_.2	Q3U5C7_S433_M1_Prickle1	7.1214	4.5339	3.8226	3.2982	2.9922	3.6154	4.1498	4.5117	4.2089	4.2599	3.0824	4.2600	4.3169	3.8607	3.7270	3.6482	4.0090	3.1405	3.4395	3.5380
Prickle1	_KS[Phospho (STY)]RSDNALNLVTER_.3	#N/A	5.7270	5.5781	3.9385	4.2872	4.2408	4.4648	3.3003	3.1026	3.3594	5.9114	3.7338	2.9892	3.4675	3.9256	2.8775	3.7471	3.6403	4.5186	3.8415	3.8926
Prickle1	_SKS[Phospho (STY)]QSRPQQVK_.2	#N/A	3.1848	3.6652	3.1905	4.5270	4.7630	4.2475	3.5177	3.8796	3.5768	4.8977	4.1077	3.5051	4.8229	3.2286	3.0949	3.5297	3.3768	3.8501	4.0717	4.0782
Prickle1	_S[Phospho (STY)]QSRPQQVK_.2	#N/A	4.0536	3.9492	4.5899	3.6358	3.3913	3.6154	4.1498	3.7539	4.2089	4.2599	3.0824	3.8387	4.3169	3.8607	3.7270	4.3985	4.2916	3.1405	3.4395	3.5380
Prickle1	_S[Phospho (STY)]RSDNALNLVTER_.2	#N/A	3.9824	3.9061	3.9203	3.8724	3.4860	3.6499	3.6326	4.0085	3.8839	3.8680	3.7766	3.6829	4.0101	3.9338	3.6580	4.1420	3.8914	3.7591	3.6324	3.7565
Foxk2	_EGS[Phospho (STY)]PAPLEPEPGASQPK_.2	Q3UCQ1_S389_M1_Foxk2	9.8922	9.9620	9.9450	9.7648	9.3443	9.9800	9.5279	9.7944	9.7170	9.6233	9.7138	9.7613	9.9801	10.3929	9.0209	9.6078	10.1781	9.6633	9.8537	9.7135
Ttll12	_[Acetyl (Protein N-term)]M[Oxidation (M)]EIQSGPQPGS[Phospho (STY)]PGR_.2	#N/A	12.6660	12.4503	12.7334	12.5972	12.3121	12.3883	12.0611	12.5515	12.3010	12.6533	12.2710	12.1933	12.5056	12.5856	11.6790	12.1017	12.7168	12.0373	12.1824	12.3305
Ttll12	_[Acetyl (Protein N-term)]M[Oxidation (M)]EIQSGPQPGS[Phospho (STY)]PGRAER_.2	#N/A	6.0092	6.4614	5.1560	6.1241	5.9579	5.6290	6.1235	6.8918	5.9580	6.2367	5.6737	6.3383	7.0287	6.5859	5.5061	5.9143	6.2923	6.1246	5.9576	5.7856
Wdr41	_LEENGDLYPES[Phospho (STY)]P_.2	Q3UDP0_S459_M1_Wdr41	3.7000	10.6330	10.6784	10.3604	3.0377	10.4655	3.0025	6.1938	3.0616	10.1071	3.4360	7.9114	8.8923	10.3815	9.5475	4.0449	8.9207	10.4906	10.4116	10.4916
Hgsnat	_LINSELGS[Phospho (STY)]PSR_.2	Q3UDW8_S238_M1_Hgsnat	8.2947	7.7416	3.9785	3.9941	3.8081	7.9655	7.5786	3.9616	7.6153	7.5577	7.6983	7.2579	7.6066	7.3205	4.1929	3.7566	8.0748	7.4743	7.3319	7.4765
	VTSFS[Phospho (STY)]TPPT[Phospho (STY)]PER.2	Q3UE31_S289_M2_	9.6728	9.4204	3.6446	9.5503	4.3150	3.7934	8.8381	4.3337	4.0308	9.7948	8.3606	9.2377	4.1389	3.6827	8.2319	9.0710	3.8309	8.7370	8.5847	8.8787
	SRS[Phospho (STY)]LS[Phospho (STY)]GTGR.2	Q3UE31_S351_M2_	6.2841	6.7204	6.5636	6.3402	6.3143	3.7591	6.3038	5.9598	3.8145	6.4648	3.6557	5.5299	6.7603	6.2477	3.5282	6.4747	6.2716	6.5884	6.5362	5.6762
	SLVGS[Phospho (STY)]WLK.2	Q3UE31_S362_M1_	4.3625	6.2622	3.8991	3.2217	3.0686	3.5389	5.9005	4.2528	6.2140	4.1834	6.1531	5.6457	4.3934	3.9372	4.0048	6.3310	4.0855	3.0640	6.1319	3.6145
	SHS[Phospho (STY)]ANDSEEFFREDDSGADLHNATNLR.4	Q3UE31_S324_M1_	3.9122	3.4069	3.4488	3.6720	4.1192	4.1525	4.4298	4.1379	4.3500	3.7228	3.8286	3.1330	4.5154	3.5172	2.9543	3.2743	3.6351	3.1004	4.2498	3.4842
Frmd8	_SS[Phospho (STY)]VSS[Phospho (STY)]VGAR_.2	#N/A	5.6817	3.2708	3.5891	3.5360	5.6813	5.8010	5.9274	4.2782	5.8384	6.2043	5.7498	3.8966	4.3148	3.5852	3.7179	3.4146	3.7754	3.2310	3.9972	3.4023
Frmd8	_SHRSS[Phospho (STY)]VSS[Phospho (STY)]VGAR_.2	#N/A	5.5404	5.6565	4.8686	3.2655	5.2092	5.5459	5.0322	4.1353	3.8386	4.0163	5.2767	5.3635	3.9466	5.7676	3.3567	3.2790	5.2548	3.2253	3.9604	3.4226
Garem1	_TDWNEDC[Carbamidomethyl (C)]KS[Phospho (STY)]PK_.2	Q3UFT3_S356_M1_Garem1	5.1428	5.8330	6.2654	3.4303	2.8601	4.7673	4.8727	6.2128	4.9454	5.4390	3.5869	4.8268	4.2737	5.3787	4.9211	3.5161	4.7818	4.4636	4.1334	5.3382
Garem1	_STC[Carbamidomethyl (C)]APSS[Phospho (STY)]PPTPATLGATIK_.2	Q3UFT3_S493_M1_Garem1	3.9321	3.3870	3.4687	3.6521	4.1390	3.9693	3.7958	6.6108	3.8549	3.1130	4.2293	3.1131	3.9630	7.2374	2.9344	3.2942	3.6550	3.4944	3.7935	3.1840
Eif3j1	cetyl (Protein N-term)]AAAAAAAAAAGDS[Phospho (STY)]DSWDADTFSM[Oxidation (M)]EDPVF	Q3UGC7_S14_M1_Eif3j1	3.7113	3.6078	3.2479	3.8729	3.9183	4.1901	3.5750	3.9370	7.0885	3.3338	4.0085	3.3339	6.6012	7.5805	7.7923	5.8949	3.4342	3.7152	6.9222	2.9632
Hdhd2	_TFFLEALR_.2	#N/A	4.3566	4.9994	4.8105	5.0568	3.8641	4.3737	4.3580	3.8045	4.7592	4.6221	5.5410	5.1727	5.4810	3.2494	4.9921	5.2954	4.3422	4.9757	4.8205	4.6920
Hdhd2	_FVTNTTK_.2	#N/A	3.5932	4.4096	4.1295	4.0962	2.9309	3.5532	3.1093	3.2936	3.4485	3.1235	2.9271	4.2991	3.6139	5.5252	4.1874	3.9381	3.8312	4.0019	3.6505	4.0836
Mcrip1	_S[Phospho (STY)]PTNSSEIFTPAHEENVR_.2	#N/A	8.0929	8.0201	5.5087	3.8575	7.4422	5.5393	7.6243	7.7383	5.7036	3.5565	4.0205	3.5387	3.8760	6.3014	7.8649	6.5077	8.2789	6.1816	8.2425	7.8345
Arfgef3	_EHLS[Phospho (STY)]EPQSLR_.2	Q3UGY8_S1966_M1_Arfgef3	7.6085	7.4553	4.1723	4.0534	2.9737	6.9736	3.0666	6.9757	6.9356	7.1623	6.9700	4.2563	3.2337	4.2783	4.1446	3.9809	7.3037	6.8962	6.7374	3.9556
Arfgef3	_VAAPSSS[Phospho (STY)]PSAEAEYWR_.2	Q3UGY8_S1646_M1_Arfgef3	11.0717	3.8474	11.2376	11.4037	10.0253	10.5684	10.6852	10.7938	3.7954	10.7839	10.3949	10.3077	10.9598	10.0931	3.5262	5.2596	11.2815	3.4932	3.7922	10.2213
Arfgef3	_SGS[Phospho (STY)]TGSSLSVSVR_.2	Q3UGY8_S2090_M1_Arfgef3	4.4283	3.5745	3.4638	3.2611	3.7660	4.2189	7.6059	4.1287	4.2836	3.7892	3.7622	3.4639	4.4490	3.4508	2.8879	3.2724	3.1655	8.2004	4.3162	3.4178
Arfgef3	_YSESNFS[Phospho (STY)]VDDQDLSR_.2	Q3UGY8_S593_M1_Arfgef3	3.7449	5.6777	5.6664	3.8393	3.9519	5.0090	4.8813	3.9706	3.9019	3.3002	4.0421	3.3003	3.7758	3.3196	3.1859	3.1071	3.4678	3.6816	3.9806	2.9969
Arfgef3	_DGAESSRDS[Phospho (STY)]M[Oxidation (M)]EINEADFR_.3	#N/A	3.9997	4.0689	5.6160	3.6239	3.7167	3.8774	5.2417	3.5361	3.6447	5.8294	3.5974	3.6688	3.7845	4.9462	3.5136	3.7323	5.5401	3.7645	3.7941	5.6106
Arfgef3	_SDVSDIGS[Phospho (STY)]DNC[Carbamidomethyl (C)]SLADEEQTPR_.2	Q3UGY8_S628_M1_Arfgef3	4.4891	5.4313	4.4541	3.7716	3.1952	3.2285	4.8415	3.6182	3.7731	4.2997	3.2518	3.9744	3.9386	4.4412	3.8783	3.8512	4.1559	4.2760	3.3259	4.4082
Arfgef3	_HSFS[Phospho (STY)]AGPELLR_.2	Q3UGY8_S2074_M1_Arfgef3	4.4355	4.0314	4.5077	3.7179	3.1416	3.1749	3.9065	3.6718	3.8267	4.2461	3.3054	3.9208	3.9922	4.4948	3.9319	3.7976	4.2095	3.6237	3.2722	2.9610
Dip2b	_LLSPYSPQT[Phospho (STY)]QETDSIGQK_.2	Q3UH60_T56_M1_Dip2b	6.1730	3.8945	4.1914	4.2023	3.6664	3.7148	3.2955	3.9212	5.4102	3.5497	3.7778	3.7338	3.4640	3.9666	3.6027	5.3395	5.2321	4.1635	5.4547	4.9869
Wnk2	_AGS[Phospho (STY)]LGPETPSR_.2	Q3UH66_S1797_M1_Wnk2	9.3245	9.4651	9.4106	9.5957	8.6474	8.5417	8.4893	8.5833	8.3961	9.7195	8.6569	7.8727	4.1455	8.6338	7.0203	8.1614	8.5047	8.2829	8.5344	8.6274
Wnk2	_QSS[Phospho (STY)]LPGTGGVASDFVK_.2	Q3UH66_S1770_M1_Wnk2	10.5086	10.2271	10.1625	10.2304	3.7338	9.1052	9.4341	9.3678	9.2277	10.1827	9.6696	9.2599	9.8590	9.7751	8.1352	9.5355	9.6488	9.1063	9.3921	9.5404
Wnk2	_YS[Phospho (STY)]APPDVYLDEIPSSPEVK_.2	Q3UH66_S1713_M1_Wnk2	4.0680	3.9358	4.0232	3.6224	3.4057	3.0793	4.0030	3.7683	3.9232	4.1505	3.4019	3.8252	4.0887	4.5913	3.7135	3.8318	4.3060	3.5281	3.1767	3.0575
Limch1	_TSVPESIASAGTGS[Phospho (STY)]PSK_.2	Q3UH68_S523_M1_Limch1	4.0837	9.8929	10.4196	10.2224	3.4214	8.9998	9.3369	4.4816	4.1788	10.4907	9.2403	3.8086	4.2868	9.5402	3.6969	4.4286	9.4046	8.9237	3.4696	3.5079
Limch1	_S[Phospho (STY)]PEPEATLTFPFLDK_.2	Q3UH68_S719_M1_Limch1	8.4124	8.1920	8.6793	8.0261	7.1691	8.1471	7.8480	7.3306	7.8621	8.1933	7.7371	7.4519	3.3611	8.7798	7.4704	7.8608	8.0674	7.6162	8.0245	7.3964
Limch1	_QTPS[Phospho (STY)]PDVVLR_.2	Q3UH68_S217_M1_Limch1	4.0737	3.9291	4.6100	3.6157	9.9788	9.9566	4.1296	9.7746	9.5757	4.2800	3.4076	3.8185	4.0944	4.5971	8.9640	10.0632	4.3118	3.5214	3.1700	3.0632
Limch1	_S[Phospho (STY)]ASQDLIKK_.2	#N/A	3.9890	3.5848	6.1658	3.2714	4.1959	4.2293	4.3530	4.1183	4.2732	3.7996	5.9374	3.4743	4.4387	3.4405	2.8776	5.9765	3.1552	5.5457	4.3266	3.4075
Limch1	_SRQT[Phospho (STY)]PS[Phospho (STY)]PDVVLR_.2	Q3UH68_S217_M2_Limch1	3.7274	8.8188	8.3478	8.8054	3.9344	8.4768	8.1180	3.9531	7.7855	9.1377	4.0134	3.3178	3.7583	8.8218	7.4439	3.0896	3.4504	8.0711	8.2152	8.5876
Limch1	_DDS[Phospho (STY)]FDSLDSFGSR_.2	#N/A	3.7848	7.1562	8.0010	3.9046	3.1225	6.9405	4.4185	3.4852	6.6067	6.6239	3.3512	4.1074	4.5857	7.2614	5.5378	4.1297	4.0229	6.6058	6.1279	7.3589
Limch1	_SAS[Phospho (STY)]QDLIKKEEER_.3	Q3UH68_S379_M1_Limch1	3.8092	5.1931	3.8770	4.0565	3.6913	3.7856	3.8684	3.8530	3.8297	3.8095	3.6451	3.7824	3.9778	3.9494	3.6504	3.6922	4.0976	3.8988	3.4444	4.3248
Limch1	_SDSLS[Phospho (STY)]PPR_.2	Q3UH68_S192_M1_Limch1	4.3542	6.8095	3.3896	3.3352	7.6043	6.4339	7.0893	4.0546	5.6189	7.2970	4.2826	3.5381	4.0163	8.0559	3.4264	7.4674	6.9298	3.4411	3.7401	7.2922
Limch1	_KETDDIDS[Phospho (STY)]PKR_.3	Q3UH68_S169_M1_Limch1	3.6600	5.0681	3.1965	3.9243	3.8669	3.9002	4.3154	3.8856	3.1014	5.1761	4.0809	3.3852	3.6199	4.8143	4.7710	3.0221	3.3829	4.3490	4.6947	3.7365
Limch1	_GSSDGRGS[Phospho (STY)]DSESDLPHR_.3	#N/A	3.7297	9.0562	5.9061	8.8497	6.5968	8.7887	8.6195	8.5804	6.6873	9.0899	8.4457	8.4439	9.1099	8.9241	8.4797	8.6454	8.5552	8.5094	8.9321	8.8092
Limch1	_TINHQM[Oxidation (M)]ES[Phospho (STY)]PGER_.2	#N/A	7.6423	8.3604	8.3132	8.4706	7.3252	7.5317	8.0438	7.4786	7.3845	7.7528	7.3380	7.4226	7.8819	8.5285	7.4154	7.1187	7.7447	7.0361	7.1878	7.6318
Limch1	_HGRDDS[Phospho (STY)]FDSLDSFGSR_.3	#N/A	3.4590	6.8330	4.4964	6.3009	3.6659	5.5758	6.1464	3.6846	5.9924	6.3916	4.2819	4.7741	3.4678	5.9180	4.2365	4.9065	3.1819	5.7840	4.2666	6.0843
Shisa6	_LHSQDPLLS[Phospho (STY)]PER_.2	#N/A	7.1570	6.6915	6.5063	6.6904	6.5258	7.0440	6.8858	6.6256	6.2354	7.3241	6.9643	4.9344	6.7282	6.7427	5.0104	6.0655	7.0665	6.6840	6.5799	6.6766
Shisa6	_ETISAIDTS[Phospho (STY)]PKENTPVR_.2	Q3UH99_S247_M1_Shisa6	4.8732	6.0163	6.3009	6.1727	3.6807	6.4332	6.0327	3.4345	5.7824	3.9075	4.4379	5.7437	3.5602	6.5729	4.8110	5.9271	5.8178	5.6757	4.7218	6.1220
Shisa6	_ILSDEQLLS[Phospho (STY)]TER_.2	Q3UH99_S422_M1_Shisa6	3.7414	5.7471	3.6971	5.5202	4.2301	4.0301	5.3503	4.3900	3.8279	6.0474	5.4171	3.2752	5.8300	6.7739	2.8659	5.2606	5.4733	5.7380	5.5304	5.5400
Ccdc177	_YVLTS[Phospho (STY)]PR_.2	Q3UHB8_S90_M1_Ccdc177	10.5547	11.0043	10.5453	11.0954	4.2732	10.6833	11.0492	3.6299	10.9240	10.9698	11.0815	2.9568	11.4894	10.5771	9.7018	10.8673	9.9050	10.9341	10.8198	10.8920
Ccdc177	_SHS[Phospho (STY)]LDSLSR_.2	#N/A	8.2104	8.2110	6.8294	8.2883	7.0901	7.6471	7.9699	7.8246	7.6099	7.3553	8.0070	7.1172	8.6838	8.1402	6.7458	7.8464	8.0543	7.9913	7.7753	7.8159
Ccdc177	_RTS[Phospho (STY)]PS[Phospho (STY)]PPAR_.2	Q3UHB8_S209_M2_Ccdc177	10.2723	5.4728	10.0374	10.0972	3.7686	10.1530	10.2847	9.9920	3.7851	3.6299	10.2426	9.9557	10.5677	3.8801	3.5796	5.0893	10.3982	10.3030	10.0609	10.0984
Ccdc177	_NSC[Carbamidomethyl (C)]PAGSASS[Phospho (STY)]APNPLGR_.2	Q3UHB8_S281_M1_Ccdc177	3.9083	4.9430	4.9154	3.6469	4.0013	4.1562	3.9494	4.0759	4.6249	3.9883	4.6691	3.0517	4.0809	3.9969	3.1490	3.3847	3.4297	4.5967	4.0813	3.7457
Ccdc177	_KSHS[Phospho (STY)]LDS[Phospho (STY)]LSR_.2	#N/A	8.1983	8.0564	7.9370	7.3121	7.5255	7.8222	8.1186	7.7202	7.6063	7.8150	8.0046	8.0970	8.6422	8.0097	6.9003	8.0197	8.0698	8.0646	8.0171	7.8539
Ccdc177	_SFSLGDLSHS[Phospho (STY)]PQTAQHVER_.3	Q3UHB8_S310_M1_Ccdc177	4.1757	8.2487	3.6359	7.0369	5.9495	7.1746	8.1085	7.3287	4.0925	4.7169	6.1781	7.1449	4.2198	7.9530	6.0022	6.2799	6.6472	7.7588	6.6570	6.7716
Ccdc177	_DGALSSESGASSSSY[Phospho (STY)]SGESLR_.2	Q3UHB8_Y254_M1_Ccdc177	3.0921	3.6926	4.1296	3.6313	3.0844	3.3085	3.8488	3.3178	2.5152	3.2080	3.3893	3.9531	3.1230	4.1676	4.0339	4.0915	3.7683	4.3344	3.1326	3.8449
Tnrc6c	_DSSEATGWEEPS[Phospho (STY)]PPSIR_.2	#N/A	8.0882	8.6764	7.3715	7.9312	7.6532	8.6386	4.1856	5.5693	8.2741	8.6600	7.7875	8.2882	7.0782	3.8965	7.8776	8.4926	8.6710	3.1046	8.2243	8.6131
Tnrc6c	_AET[Phospho (STY)]PPSHQAGTQLNR_.3	Q3UHC0_T776_M1_Tnrc6c	3.3260	3.7338	4.3634	4.1093	3.5330	3.5663	3.5151	3.2805	3.4353	3.1366	2.9140	3.7192	3.6008	4.1034	3.1079	4.1890	3.8181	4.0150	4.5845	4.0704
Tnrc6c	_QKDSSEATGWEEPS[Phospho (STY)]PPSIR_.3	#N/A	4.4999	3.5029	3.5354	3.1895	3.8376	4.9083	3.7034	4.2003	3.7625	3.2054	4.1369	3.3923	3.8706	3.5224	3.5391	3.3440	3.2371	3.0952	4.2446	3.4894
Dab2ip	_GDS[Phospho (STY)]PELKPR_.2	#N/A	9.3417	9.3897	9.0155	9.2467	8.7185	9.2174	8.8044	9.1761	8.7530	9.1616	9.2913	8.8571	9.1043	9.3665	8.4516	8.8027	9.4607	9.2411	9.1876	9.0331
Dab2ip	_LPS[Phospho (STY)]PTPENK_.2	Q3UHC7_S702_M1_Dab2ip	8.0752	4.5071	8.2814	8.1435	8.1315	7.8310	7.9325	7.3942	7.6095	8.6213	8.4252	7.6550	7.4328	4.4185	2.7840	8.0156	3.7338	7.7165	7.5912	4.0958
Dab2ip	_RQMS[Phospho (STY)]LTEK_.2	Q3UHC7_S895_M1_Dab2ip	4.1724	3.1467	6.7360	3.4119	2.8785	3.7291	5.7344	4.3980	4.0952	3.9830	2.9687	4.3737	4.2032	3.7470	4.1950	3.5345	3.8953	3.2542	3.5532	3.4243
Dab2ip	_QQSSSSKGDS[Phospho (STY)]PELKPR_.3	#N/A	5.6851	6.1816	5.4060	5.6370	5.4309	5.3970	5.8016	5.7885	5.7719	6.0225	5.7322	5.5695	6.7711	5.9421	5.5738	5.9129	5.8980	6.0888	6.3602	5.7007
Adgrb1	_LHLHGS[Phospho (STY)]PR_.2	Q3UHD1_S1289_M1_Adgrb1	7.1004	7.4852	7.1102	6.7265	6.6077	6.8017	6.6735	7.0382	6.2291	6.6587	6.4637	7.1062	7.2124	6.2308	4.6178	6.2360	7.0265	6.7754	6.1624	6.1285
Adgrb1	_NENVATLSVSS[Phospho (STY)]LERR_.2	#N/A	3.9443	5.5125	4.4806	5.1855	3.5384	4.3810	5.2969	4.8830	4.1027	5.4145	3.1917	4.6290	4.4262	5.3635	3.8363	4.0161	4.1183	5.2888	4.4981	4.7405
Adgrb1	_NENVATLSVSS[Phospho (STY)]LER_.2	#N/A	9.6832	9.9448	9.7691	9.6814	3.2491	9.1555	9.5771	8.4674	8.7881	9.3218	9.0905	8.7179	9.1597	4.0029	7.4204	9.2157	9.4439	9.5310	9.2334	9.2951
Adgrb1	_RPS[Phospho (STY)]LPEEEKMK_.3	Q3UHD1_S1257_M1_Adgrb1	3.1666	4.1525	4.2040	4.4176	3.3735	3.2340	3.0303	3.3923	3.0894	3.8785	3.4638	3.8786	3.1975	4.2421	4.1084	4.0295	4.3903	4.2599	3.0582	3.9194
Mtus2	_QLS[Phospho (STY)]EENANLQEYVEK_.2	Q3UHD3_S1286_M1_Mtus2	4.4656	5.5126	3.5010	3.2238	3.8033	4.0274	5.2971	4.1659	3.7969	3.1711	4.1712	3.4267	3.9049	6.0727	3.3150	5.3962	3.2028	5.6778	3.8515	3.1260
Mtus2	_QLS[Phospho (STY)]EEHAVKR_.3	Q3UHD3_S109_M1_Mtus2	4.4975	4.4108	3.6929	5.4275	4.4152	3.9513	4.2263	3.8038	4.4210	4.9878	4.5373	3.2348	3.8625	3.6800	3.1231	3.5015	3.3947	3.9722	3.7022	5.2441
Mtus2	_SM[Oxidation (M)]S[Phospho (STY)]PVER_.2	#N/A	6.8719	7.2618	3.4582	3.6730	7.1635	7.2312	7.0986	4.1473	4.2368	3.7322	7.1463	7.3878	7.7535	7.2703	7.4892	3.2837	7.5524	7.1588	7.3714	3.4547
Snx27	_SES[Phospho (STY)]GYGFNVR_.2	#N/A	11.5809	11.5968	11.5517	11.6703	11.2565	11.4932	11.3931	11.5425	11.4484	11.6604	11.4897	11.3338	11.9810	11.6342	10.7263	11.5151	11.6829	11.4287	11.5846	11.4839
Snx27	_IVKSES[Phospho (STY)]GYGFNVR_.2	#N/A	4.2327	6.6870	3.6435	3.4773	3.5704	3.7945	3.9707	4.3326	4.0297	4.4390	2.9033	2.9383	4.1378	3.6816	3.5479	6.0285	3.8298	3.3196	3.6187	3.3589
Agap2	_ALS[Phospho (STY)]TDC[Carbamidomethyl (C)]TPSGDLSPLSR_.2	Q3UHD9_S802_M1_Agap2	12.5661	12.6376	12.2942	11.9778	10.9960	11.6119	12.0092	11.8370	11.3238	11.6205	11.7929	11.2992	12.1821	12.1591	3.7249	11.6004	11.9241	11.6988	11.5382	11.4184
Agap2	_GSEPRDPGS[Phospho (STY)]PR_.2	Q3UHD9_S54_M1_Agap2	9.1209	9.4942	7.8968	9.4042	8.8204	8.2957	8.8684	9.6890	9.0634	8.9530	8.5108	9.4707	10.1635	8.9272	8.0311	9.1122	9.2159	9.0353	8.5820	8.8333
Agap2	_GAGSRLS[Phospho (STY)]WPESEGKPR_.3	#N/A	8.1532	3.8039	7.9455	8.0487	7.3534	7.4818	7.6281	7.2686	7.6433	7.6152	7.1607	7.4809	4.1019	7.8529	3.4878	3.8557	8.2191	7.8309	7.7279	7.4536
Agap2	_RSS[Phospho (STY)]AASLGR_.2	#N/A	4.1804	7.2692	7.1268	3.4250	3.5181	6.8492	7.1587	6.0007	6.5420	6.5298	6.4943	6.3966	5.9719	6.3723	5.9368	6.6315	6.8626	6.8666	6.3697	6.6865
Agap2	_ELLGAELRTS[Phospho (STY)]PK_.2	Q3UHD9_S382_M1_Agap2	7.1042	7.7929	7.4851	7.4777	3.8459	4.2625	7.5086	3.8646	6.9913	3.4495	6.0137	6.8731	3.6698	3.2136	3.2276	7.0044	6.6591	3.7876	7.0784	6.8851
Agap2	_LRTDS[Phospho (STY)]QSEAVAIQAIR_.3	#N/A	3.4825	4.2090	4.2827	4.1552	3.3051	4.1042	3.2363	3.4606	3.3708	3.4148	5.9295	7.3476	3.5081	7.8768	3.8991	4.1009	3.4943	7.3824	7.0829	6.4287
Agap2	_FLTGS[Phospho (STY)]YQVLEK_.2	Q3UHD9_S424_M1_Agap2	4.1120	6.1873	3.1475	6.0331	3.4497	3.0344	5.6465	3.8124	3.9673	4.1055	5.7742	3.7802	4.1327	3.1346	4.0725	5.8523	4.3501	6.4392	5.7488	3.1015
Agap2	_LKS[Phospho (STY)]FGS[Phospho (STY)]LR_.2	Q3UHD9_S856_M2_Agap2	8.9764	12.8312	13.0972	12.5057	4.1207	12.3994	12.4391	4.4834	12.3900	12.4122	10.5612	11.8293	9.6117	12.4284	11.1293	12.2319	12.3180	12.5288	12.5276	12.4001
Agap2	_SGNS[Phospho (STY)]LNKEWK_.2	Q3UHD9_S684_M1_Agap2	6.0167	3.2069	3.8313	4.3944	3.8180	6.4727	5.7098	5.7419	6.2805	6.2999	5.9351	5.5716	3.3158	3.8184	3.2555	5.6923	5.6879	5.7889	5.9338	5.8791
Agap2	_TDS[Phospho (STY)]QSEAVAIQAIR_.2	#N/A	9.0192	9.5940	10.1839	9.2311	7.0206	9.0915	9.0601	3.8444	8.9520	9.2675	8.5631	3.4265	8.5590	9.4512	7.6099	8.7499	8.6873	9.2904	9.1277	9.0256
Agap2	_RTS[Phospho (STY)]LFANR_.2	Q3UHD9_S642_M1_Agap2	3.1251	5.5402	5.3796	4.4591	3.3321	3.3654	3.7160	3.3508	3.6362	4.4366	3.1149	4.8366	3.8017	6.2630	3.7414	3.9881	4.3489	4.3014	3.0996	3.8779
Agap2	_RRS[Phospho (STY)]S[Phospho (STY)]AASLGR_.2	Q3UHD9_S1171_M2_Agap2	8.4712	8.3967	8.7421	8.4882	8.2829	8.2467	8.0845	8.2838	8.0682	8.3991	8.4877	7.8944	8.5991	8.2143	7.5318	8.4009	8.2954	8.3771	8.4648	8.4504
Agap2	_ELTLVLTAIGNDTANR_.2	#N/A	3.1663	4.1804	4.2038	4.4179	3.3733	4.9691	3.0301	4.7852	5.6037	5.3103	3.4635	3.8789	3.1972	4.2418	5.2009	4.0293	4.3901	4.2602	3.0584	5.3119
Agap2	_LS[Phospho (STY)]WPESEGKPR_.2	#N/A	3.5714	3.7477	4.6088	4.0128	3.7783	3.8117	3.2698	3.7971	3.1900	3.3820	4.1695	3.4738	3.3554	7.5940	3.2951	2.9335	3.2943	3.8551	4.1542	4.3242
Agap2	_SS[Phospho (STY)]AASLGR_.2	#N/A	4.2102	4.6097	3.7468	3.3740	2.9163	4.4505	4.1318	4.4359	5.2147	4.0208	3.5306	4.3358	4.2174	3.2193	4.1572	3.5723	3.9331	3.3984	3.0470	3.1863
Agap2	_AEAAAVAGLSTPGS[Phospho (STY)]LHR_.2	Q3UHD9_S632_M1_Agap2	3.9018	3.4549	3.6322	3.9473	3.9344	4.0993	3.8687	4.2971	3.7570	3.7392	3.9306	3.3443	3.9225	3.6193	3.7514	3.3918	4.1398	3.8530	4.1966	3.5863
Agap2	_AVVNSQEWTLSR_.2	#N/A	3.9670	3.3521	3.5036	3.6172	4.1739	3.9344	3.8307	4.1927	3.8898	4.5790	4.2642	3.0782	3.9979	3.5416	2.6929	3.3291	3.6899	3.4595	3.7586	3.2189
Pitpnm3	_AQS[Phospho (STY)]QPESDKDHERPLPALSWAR_.4	Q3UHE1_S946_M1_Pitpnm3	8.4254	9.1593	7.8221	7.8943	4.2020	7.8940	8.6746	7.7501	3.2188	3.5698	7.2261	7.9281	3.3842	9.1465	2.9162	7.6453	3.6015	8.8515	8.1884	7.9525
Pitpnm3	_NVLSDS[Phospho (STY)]VESSDDEFFDAR_.2	Q3UHE1_S27_M1_Pitpnm3	4.4965	4.3235	4.0330	4.5886	3.2026	3.2359	3.8455	3.2213	3.7657	4.3071	3.2444	4.0496	3.9312	4.0711	3.8709	3.8586	4.2194	4.4309	3.2291	3.7484
Pitpnm3	_GS[Phospho (STY)]PPLLDAPAS[Phospho (STY)]PPQAPR_.2	#N/A	9.8300	10.7911	11.0958	9.8298	7.7421	10.7776	10.4944	8.2198	10.7309	10.4982	8.9917	10.4878	9.6019	10.9515	9.6077	10.1916	11.1040	10.7884	10.6748	10.4766
Pitpnm3	_KGS[Phospho (STY)]ISSTQDTPVVVEEDC[Carbamidomethyl (C)]SLASSK_.3	Q3UHE1_S295_M1_Pitpnm3	3.3724	3.9467	4.4098	4.6527	3.5794	3.0282	4.8712	3.5981	3.2952	3.6727	3.6696	3.6728	3.4033	4.4479	2.8134	3.8113	3.0953	4.0541	4.3532	4.1780
Hivep2	_GSHS[Phospho (STY)]FDER_.2	Q3UHF7_S565_M1_Hivep2	3.2240	7.4855	7.5256	4.3603	3.4309	3.4642	7.2790	3.4496	6.5590	4.5354	3.0161	3.8212	3.7029	4.2994	3.6426	4.0869	4.4477	3.9130	3.5616	4.1725
Hivep2	_QASFSGS[Phospho (STY)]PEIAQGEAGVDPSVK_.2	Q3UHF7_S1081_M1_Hivep2	3.7032	4.9187	4.4591	4.8034	3.4493	3.3079	3.8132	3.3620	3.8722	3.9525	3.4336	3.8024	3.9803	4.0796	3.6604	4.2758	4.7555	3.6461	3.9452	3.8891
Hivep2	_ELSLS[Phospho (STY)]TEEGNEPSPEK_.2	Q3UHF7_S2415_M1_Hivep2	4.1273	7.7528	7.9037	6.2079	3.4650	7.2478	7.0289	5.4460	7.1799	6.0116	3.5501	5.7832	4.0113	7.1444	6.0903	6.2153	6.1853	6.8760	6.0074	6.3481
Hivep2	_SPS[Phospho (STY)]LGSEDLPLAADPDK_.2	Q3UHF7_S764_M1_Hivep2	3.1920	4.1271	4.2294	4.3923	3.3989	3.4322	3.6492	3.4176	3.5694	4.5034	3.0480	3.8532	3.7348	4.2375	3.6746	4.0549	4.4157	3.8810	3.5296	4.2045
Hivep2	_EM[Oxidation (M)]S[Phospho (STY)]QGDASPR_.2	Q3UHF7_S2085_M1_Hivep2	4.3888	6.4595	3.4795	3.6413	3.7344	5.4134	3.6977	4.1486	4.2477	5.1490	4.2402	4.3619	4.9403	3.5176	3.3839	3.7218	3.6659	2.8772	3.7826	3.5951
Hivep2	_LADLEHSSGESS[Phospho (STY)]FESTGTGLSR_.3	Q3UHF7_S947_M1_Hivep2	5.9036	4.5948	3.7617	3.3591	2.9312	5.2393	4.1169	4.4508	4.0371	4.0357	3.5158	4.3210	4.2026	3.7997	4.1423	3.5872	3.9480	3.2014	3.5005	3.4770
Hivep2	_SES[Phospho (STY)]TEMVAC[Carbamidomethyl (C)]PQDK_.2	Q3UHF7_S811_M1_Hivep2	3.1885	7.8605	4.2259	8.3895	3.3954	3.4288	3.6527	3.4142	3.5729	4.4999	3.0515	3.8567	3.7383	4.2410	3.6780	4.0515	4.4123	3.8775	3.5261	4.2080
Hivep2	_S[Phospho (STY)]IEQAEDAHKK_.2	#N/A	5.0004	5.1708	5.2122	5.3747	4.1961	4.6735	4.9381	4.0342	4.2107	5.2053	4.4740	4.5309	4.5623	4.7337	3.9752	4.8038	4.7242	3.7568	4.7994	5.0183
Hivep2	_MSTFSS[Phospho (STY)]QEGPR_.2	Q3UHF7_S41_M1_Hivep2	4.2821	4.5378	3.8187	3.5646	2.9882	3.0216	4.0599	3.8252	3.9801	4.0927	3.4587	4.2639	4.1455	3.1474	4.0853	3.6442	4.3629	3.4703	3.1189	3.1143
Hivep2	_QSTS[Phospho (STY)]EDSLNSTER_.2	Q3UHF7_S2295_M1_Hivep2	4.4732	4.6877	5.4919	3.2162	3.8109	4.0350	3.7301	4.1736	3.7892	3.1787	4.1636	3.4190	3.8973	3.4957	3.3073	3.3173	3.2104	3.1219	4.2713	3.4627
Zswim8	_GWGS[Phospho (STY)]PGRPK_.2	Q3UHH1_S1132_M1_Zswim8	6.7985	7.2582	7.0154	6.7727	5.9813	6.6438	6.8054	6.7625	5.8618	6.8523	6.6240	6.2405	3.3312	5.9965	5.5003	6.1126	6.1903	6.3130	5.7002	6.0873
Zswim8	_QSAGPNS[Phospho (STY)]PTGGGGGGGSGGTR_.2	#N/A	3.6981	3.2939	3.7443	4.4814	3.9050	5.5270	3.1431	4.4092	4.8414	3.5087	5.4552	3.1834	3.2287	4.9885	3.1685	3.0602	3.4461	6.0399	6.0609	3.6984
Zswim8	_RLS[Phospho (STY)]AEGGDK_.2	Q3UHH1_S564_M1_Zswim8	6.3403	4.4708	4.0683	4.1574	2.8697	5.8270	5.6576	5.5238	5.8457	5.4316	5.5120	4.3602	3.5527	5.5729	4.2485	3.8769	5.8990	5.3940	5.8983	5.4571
Zswim8	_KQS[Phospho (STY)]AGPNS[Phospho (STY)]PTGGGGGGGSGGTR_.2	#N/A	3.7176	4.0367	3.4913	3.3805	2.6561	4.2512	3.9365	4.1660	4.0503	3.9864	3.6447	3.8783	3.9763	3.2505	3.9957	2.9314	4.3653	3.1704	3.1546	3.3539
Ccser2	_SSS[Phospho (STY)]GESLAQSPDNAK_.2	Q3UHI0_S198_M1_Ccser2	6.8276	7.0211	6.9656	6.7007	5.6883	5.8081	6.5016	6.0652	5.9196	6.0758	6.2888	5.4854	7.0178	6.0580	6.1472	6.3554	6.1702	6.8110	5.6424	6.5781
Ccser2	_HTSGNNLIS[Phospho (STY)]PDTDYR_.2	#N/A	4.1354	3.5480	3.4037	3.5300	3.9046	5.7300	5.3170	4.1687	5.2378	6.7186	4.0881	4.8105	4.3043	5.2418	4.0541	6.0314	4.6936	5.9805	5.1610	3.1949
Tmed8	_RGS[Phospho (STY)]AGGVGDRR_.2	#N/A	5.4844	5.8503	5.2576	5.7672	5.6269	5.5135	5.2906	5.6253	5.5001	5.4408	4.8652	5.1988	6.1287	5.9326	4.4941	4.9011	5.4141	5.4429	4.9712	5.0479
Tmed8	_RGS[Phospho (STY)]AGGVGDR_.2	#N/A	6.3416	5.3880	5.7288	5.9839	6.0580	6.2571	6.1450	5.2684	5.5417	5.6308	6.1012	5.2909	5.6620	6.2736	4.2370	3.4925	5.7211	6.0112	5.7263	6.1879
Aak1	_VGSLT[Phospho (STY)]PPSS[Phospho (STY)]PK_.2	#N/A	14.0092	13.7602	14.1715	13.8967	12.9304	13.4297	13.5082	13.5909	13.4502	14.0732	13.5096	13.4706	13.5492	13.8520	12.2085	13.3263	13.7702	13.2786	13.2444	13.2904
Aak1	_VQTT[Phospho (STY)]PPPTIQGQK_.2	Q3UHJ0_T604_M1_Aak1	13.5033	13.5962	13.4553	13.5702	13.3867	13.4354	13.2928	13.7901	13.3481	13.5863	13.2894	13.4158	14.1121	13.7642	13.0356	13.3964	13.7612	13.3301	13.3745	13.4233
Aak1	_EQGSSGLGSGSS[Phospho (STY)]GGGGSSSGLGSGYIGR_.3	#N/A	4.1014	3.2177	3.6380	3.4828	7.5874	3.8000	4.2406	4.3271	7.6353	8.5648	2.8978	2.9438	4.1323	7.7815	6.4723	3.4636	3.8243	3.3251	3.6241	7.9693
Aak1	_LTDPIPTTETSIAPR_.2	#N/A	8.2140	7.9932	7.8874	7.7447	8.0843	8.3363	8.0324	8.1865	8.1612	8.2070	8.0978	7.8751	8.0854	7.8316	8.0381	7.9119	7.8480	7.9449	7.8795	7.4646
Aak1	_VAEDEFDPIPVLITK_.2	#N/A	7.3746	6.8360	7.3221	7.2356	6.8181	7.2755	6.9168	7.1716	7.3740	6.9310	6.8462	6.9445	6.7442	7.0377	6.9895	7.1434	6.9194	6.6496	7.1447	6.5593
Aak1	_AAEDSNLISGFGVAEGSEK_.2	#N/A	6.8321	7.1851	6.3551	6.3250	6.4590	6.5224	6.5586	5.7041	6.4799	6.3862	6.2300	6.0102	6.5812	7.0400	5.6445	5.7526	6.3071	6.4021	6.0629	6.6509
Aak1	_DLKVENILLHDR_.3	#N/A	6.4513	6.2244	6.0214	6.1199	6.5669	5.9909	5.8483	6.2924	5.6791	6.3035	5.8160	5.9404	5.5576	6.5535	6.0910	5.9346	6.6610	5.1081	6.0894	6.0782
Aak1	_FQNPQAEGVNAVEDEIKK_.3	#N/A	6.4126	5.9532	6.3600	5.7037	6.7486	6.3190	6.1181	5.8220	6.2044	6.7286	6.1323	5.0028	5.8014	6.3442	6.0287	6.0540	6.1110	6.3406	5.8196	6.3160
Aak1	_SKS[Phospho (STY)]ATTT[Phospho (STY)]PSGS[Phospho (STY)]PR_.2	Q3UHJ0_S676_M3_Aak1	5.5197	7.6147	3.8456	7.7941	5.1602	5.4666	6.9644	5.3023	4.8847	7.5781	4.9167	5.2431	7.6126	6.7594	4.5987	5.2044	6.5294	4.9615	3.8514	4.9096
Aak1	_LPSQTESVTSNR_.2	#N/A	6.5310	7.0080	7.3110	6.7875	6.7363	7.2660	6.8418	7.0746	6.8002	6.7270	6.5040	6.5247	6.5042	6.6208	6.5418	6.6540	6.4922	6.4197	6.7421	6.0833
Aak1	_NSS[Phospho (STY)]GSSESSLPNLAR_.2	Q3UHJ0_S936_M1_Aak1	10.0435	10.0664	10.1818	4.0173	9.5728	9.4846	9.4427	9.5292	9.4113	10.2550	9.7147	9.6981	10.1128	10.1130	9.0125	9.7381	9.9276	9.5145	6.3911	9.7758
Aak1	_AQATPSQPLQSSQPK_.2	#N/A	4.4149	4.8527	5.3098	5.0832	4.5280	4.1997	5.0922	3.6924	4.8871	4.6192	5.1695	4.4921	4.0127	4.5154	4.6516	4.1745	4.6006	4.9363	4.6361	4.1754
Aak1	_SATTTPSGS[Phospho (STY)]PR_.2	#N/A	12.1188	12.1977	12.3387	12.3915	11.5950	11.4644	11.8304	11.6516	11.6380	12.2104	11.5263	11.7036	12.0797	11.7516	10.8987	11.6362	11.5675	11.5841	11.5576	11.5076
Aak1	_VGSLT[Phospho (STY)]PPSS[Phospho (STY)]PKTQR_.2	#N/A	3.4886	13.0067	10.5699	11.8111	11.1919	3.6826	11.9138	4.0334	11.1627	11.6299	10.3491	11.6956	12.5980	11.4757	10.4946	3.5858	11.3791	4.1313	10.4781	9.2733
Aak1	_REQGSSGLGSGSS[Phospho (STY)]GGGGSSSGLGSGYIGR_.3	#N/A	3.2826	3.2194	3.8189	4.3854	4.1212	2.7172	3.4199	3.7818	3.4790	3.4889	3.8534	3.1088	3.5870	3.1308	2.9971	3.6275	3.2791	3.6338	4.1694	4.3089
Aak1	_ILSDVTHSAVFGVPASK_.2	#N/A	4.4366	5.7236	4.7198	3.9472	4.4385	4.2761	4.6402	4.9851	4.5429	4.6959	4.2124	4.0409	4.7618	6.0819	3.8016	3.2990	3.6924	4.3832	4.1270	3.3946
Aak1	_STQLLQAAAAEASLNK_.2	#N/A	6.1401	5.4637	3.8949	5.7288	5.8818	5.2512	5.7626	6.3737	5.0876	5.2821	5.8790	3.0328	5.9453	3.9435	5.7289	3.7035	5.5873	4.8894	5.4677	5.3178
Aak1	_YTTLSYR_.2	#N/A	4.3821	3.9779	3.9186	3.6645	3.0882	4.4561	3.3678	3.7253	4.7372	4.6522	3.3588	4.1640	4.0456	4.5483	3.9853	3.7442	4.2629	3.5702	4.0677	3.0144
Aak1	_GHYVLC[Carbamidomethyl (C)]DFGSATNK_.3	#N/A	3.3630	3.1389	3.8993	4.3263	4.2016	2.2584	3.3395	3.0634	3.3986	3.5694	3.7729	3.0284	3.5066	4.5512	2.9167	3.7079	3.6011	3.9508	4.2498	4.2285
Aak1	_LQTGFTENEVLQIFC[Carbamidomethyl (C)]DTC[Carbamidomethyl (C)]EAVAR_.3	#N/A	3.2681	3.2339	3.8044	4.4213	4.1066	4.3307	3.4344	4.4693	3.4935	3.4744	3.8679	3.1233	3.6016	3.7915	3.9377	3.6130	3.5061	3.8558	3.9756	4.3235
Slc2a13	_GSNYHLSDNDAS[Phospho (STY)]DVE_.2	#N/A	10.2688	11.0499	11.2703	10.9210	5.4629	10.7914	10.8592	9.5324	10.9084	10.5165	9.0544	10.5901	8.5149	10.4398	9.3040	10.3456	11.2127	10.7444	10.4996	10.3374
Slc2a13	_KAS[Phospho (STY)]EDVEYTLR_.2	Q3UHK1_S6_M1_Slc2a13	6.8757	3.8122	7.6715	7.6554	6.0151	4.4566	7.1694	3.8910	4.0459	6.6866	6.5670	3.7017	4.2113	3.2131	4.1510	6.7814	4.4287	6.9474	6.7667	6.7290
Slc2a13	_VKGSNYHLS[Phospho (STY)]DNDASDVE_.2	#N/A	7.2052	6.1674	4.0922	6.1336	3.3275	6.5886	6.0840	5.8070	6.8208	5.3818	4.6431	5.4043	4.5271	5.6639	3.7793	6.1804	6.0040	6.9248	5.1867	3.5032
Slc2a13	_SLSS[Phospho (STY)]LMGER_.2	Q3UHK1_S18_M1_Slc2a13	3.7570	6.6157	3.2936	3.8272	3.9639	4.1445	3.0842	3.9827	3.6798	3.2881	4.0542	3.2882	3.7879	6.1773	3.1096	3.1191	3.4799	3.6696	3.9686	3.0089

Slc2a13	_SLLAAESAAS[Phospho (STY)]LQGAELER_.2	Q3UHK1_S47_M1_Slc2a13	4.6754	4.3475	4.0089	4.8804	2.9817	3.4388	3.6514	4.0328	4.3103	3.9724	3.2895	4.0589	3.8573	4.0678	4.1143	3.8249	3.9411	3.7980	3.7656	3.7451
Camkv	_AAAT[Phospho (STY)]PEPAVAQPDSTALEGATGQAPPSSK_.3	Q3UHL1_T470_M1_Camkv	9.0815	8.6853	9.4324	8.9999	7.8001	7.9817	8.2748	8.3627	7.9151	9.1612	8.5095	8.3383	8.2933	8.4892	7.0446	8.2474	8.2042	8.2079	8.3135	8.1994
Camkv	_TEEFC[Carbamidomethyl (C)]EIFR_.2	#N/A	6.8673	7.0771	7.2748	7.0463	6.9788	7.0565	6.9936	6.8817	7.2473	6.8841	7.1498	6.6540	6.5676	7.0078	6.6185	6.4533	7.0365	7.0629	7.0706	6.7335
Camkv	_EPC[Carbamidomethyl (C)]GTPEYLAPEVVGR_.2	#N/A	6.9707	6.2294	6.5293	6.6228	6.2812	6.6060	6.5359	6.4948	7.1516	6.8620	6.7649	6.1189	5.9228	7.1263	6.6673	6.4998	7.0176	6.5100	6.4851	6.6953
Camkv	_LENLVYYNR_.2	#N/A	7.1348	7.2657	6.7759	6.7065	6.2214	6.7586	6.9034	7.3874	6.9179	6.6699	7.0536	6.6973	6.4716	6.8711	5.7617	6.0188	6.6388	7.0043	6.6265	5.9784
Camkv	_IVISDFHLAK_.2	#N/A	6.4269	6.3711	5.9019	6.5591	6.1695	6.6054	6.2656	6.6536	5.9413	6.4794	6.0927	6.2022	6.3429	6.6252	5.6491	6.0449	6.7172	6.8838	5.1731	5.9552
Camkv	_AT[Phospho (STY)]PATEESTVPATQSSALPAAK_.2	#N/A	8.4651	8.0034	8.5947	5.0472	7.5272	8.0985	7.7854	5.2570	5.2116	8.1507	7.9665	7.5847	7.7648	8.0905	4.7948	7.6460	8.3091	7.7601	7.7783	7.7516
Camkv	_EVFDWILDQGYYSER_.2	#N/A	5.9060	5.1421	5.2981	5.7742	5.2164	4.9656	5.4054	5.7214	5.9560	3.2281	4.8044	5.4728	6.3738	5.3967	4.9747	5.7470	5.8225	5.0001	4.6352	5.1779
Camkv	_SDDIAS[Phospho (STY)]ADR_.1	#N/A	6.5457	6.2568	6.8477	6.4784	6.5475	6.5822	6.0432	6.4288	6.0304	6.4089	6.7385	6.2269	5.9630	5.9498	4.9772	6.3232	6.6669	6.0856	7.0141	6.1450
Camkv	_ITAEEAISHEWISGNAASDK_.3	#N/A	5.7602	5.8407	6.2342	5.2957	5.6930	5.5113	5.6556	6.3471	5.3912	5.6734	5.6416	6.0633	6.5292	5.5597	5.2865	5.5363	5.9624	5.1853	5.2581	5.9363
Camkv	_LMEVEQDQR_.2	#N/A	4.1790	3.1401	5.8071	5.8785	2.8851	5.3507	5.9377	3.9283	4.7190	3.7072	5.4416	4.4082	4.2487	6.2846	4.8374	3.5411	4.4660	3.0583	4.2433	3.2175
Camkv	_GEEATGC[Carbamidomethyl (C)]AQESQR_.2	#N/A	7.5237	3.3778	3.1370	4.5652	6.8359	7.4503	3.5783	4.3254	6.1139	3.3305	6.9981	3.2672	3.1449	3.6476	3.1555	7.2950	3.3622	2.9701	6.9211	3.6146
Camkv	_SAT[Phospho (STY)]PATDGR_.2	Q3UHL1_T438_M1_Camkv	4.2634	3.7557	3.2989	3.4423	3.6011	3.9794	3.9563	3.9638	4.2134	6.4112	3.4316	3.6452	4.3514	4.1483	3.5335	3.1075	3.8630	3.1874	3.8590	3.4448
Camkv	_NYNQPSEVTDR_.2	#N/A	3.9307	4.0721	4.4670	3.7586	3.2685	3.2156	4.2726	3.6311	3.7860	5.8776	6.0285	3.9615	3.9515	4.4541	3.8498	4.2756	4.1688	3.6644	3.3129	4.4211
Dop1b	_S[Phospho (STY)]DLSEEDLPYYVDLPDR_.2	Q3UHQ6_S1080_M1_Dop1b	4.1428	3.1763	3.6793	3.4415	4.3497	4.3830	4.1992	4.3684	4.1194	3.9534	3.5981	4.4033	4.2849	3.7174	4.2246	3.5049	3.8657	3.3309	2.9795	3.2537
Dop1b	_AALLAAFQPES[Phospho (STY)]PR_.2	Q3UHQ6_S1167_M1_Dop1b	4.3434	5.6063	4.0270	3.0938	3.1965	3.4110	4.9736	3.1959	4.4132	4.0555	3.2868	3.8406	4.5213	0.8480	1.9197	3.8525	4.0942	5.0024	3.2352	3.7423
Dop1b	_SEDSGIGLSASS[Phospho (STY)]PELSEHLR_.3	Q3UHQ6_S597_M1_Dop1b	3.9743	3.3448	3.5109	3.6099	4.1813	3.9271	4.3677	4.2000	3.8971	4.5716	4.2715	3.0709	4.0052	3.5490	2.8922	3.3364	3.6972	3.4522	3.7513	3.2262
Mtcl1	_QELGPGQETGTSS[Phospho (STY)]R_.2	Q3UHU5_S1795_M1_Mtcl1	7.8580	3.1742	3.6814	7.9406	7.8121	7.3028	6.7037	7.9722	4.0677	7.6032	7.7155	7.5079	4.1757	7.7193	3.5858	3.5070	3.8678	3.2817	3.5807	7.5307
Mtcl1	_EDVTPPLS[Phospho (STY)]PDDLK_.2	Q3UHU5_S1407_M1_Mtcl1	3.2441	5.4949	4.0352	4.3970	3.7184	6.3040	4.6466	4.0708	4.7605	3.7372	3.6272	3.5401	3.2695	6.7123	3.6207	3.8758	4.0242	6.7774	6.0244	3.8625
Mtcl1	_GLPSAGSKEDVT[Phospho (STY)]PPLS[Phospho (STY)]PDDLK_.3	#N/A	4.1752	3.9725	3.9175	3.6590	3.5129	3.7097	3.0296	3.8755	3.9109	3.7543	3.7099	3.8619	4.0600	7.2201	3.6862	3.7261	3.6192	6.6304	3.9202	3.8716
Mtcl1	_GRS[Phospho (STY)]PS[Phospho (STY)]PLGVGSETFR_.3	Q3UHU5_S1799_M2_Mtcl1	3.2573	3.2447	3.7936	4.4321	4.0958	4.3199	3.9058	4.4585	3.5043	3.4636	3.8787	3.1342	3.6124	3.7806	3.0225	3.6022	3.4953	4.0069	3.9864	4.3343
Pdap1	_SLDS[Phospho (STY)]DES[Phospho (STY)]EDEDDYQQK_.2	#N/A	8.3148	8.4945	7.9699	8.4958	7.2820	8.0337	7.9721	8.6509	8.2141	8.0618	7.4890	8.2646	8.5892	9.1983	8.6420	8.4050	8.5390	8.4324	8.0732	7.8147
Pdap1	_GVEGLIDIENPNR_.2	#N/A	6.1302	6.7577	7.0368	6.7085	6.0460	6.7993	6.2068	6.2655	6.7502	6.8964	6.3965	6.7101	6.5719	6.6844	6.8901	6.5269	6.7031	6.4578	6.7218	5.6786
Pdap1	_M[Oxidation (M)]QSLS[Phospho (STY)]LNK_.2	#N/A	6.8907	6.8865	6.2902	7.0500	6.0581	5.5243	6.0582	5.6344	5.5405	7.2500	5.8547	6.1743	6.9329	6.0598	5.3860	6.4052	5.6725	6.0142	6.2605	6.1678
Pdap1	_QYTS[Phospho (STY)]PEEIDAQLQAEK_.2	Q3UHX2_S19_M1_Pdap1	4.3816	4.5588	3.6029	3.7070	4.1836	5.7030	4.0148	3.8792	3.5907	4.2881	3.5009	4.6522	4.1247	4.8883	4.5566	4.2671	3.9914	4.0769	4.4850	4.0607
Pdap1	_SLDS[Phospho (STY)]DESEDEDDDYQQKR_.2	#N/A	5.0742	5.2859	4.4226	4.7242	4.1318	4.4105	4.6699	4.6406	5.2971	4.6506	3.7358	4.6451	4.9609	5.7627	4.9014	4.6562	5.0876	5.0171	4.7545	4.3441
Pdap1	_KSLDS[Phospho (STY)]DES[Phospho (STY)]EDEDDDYQQK_.2	#N/A	6.9452	7.1343	6.9678	7.1250	6.5745	6.4748	6.6635	6.8446	6.7177	6.7867	5.9276	6.9292	7.3263	7.6474	7.2215	6.8563	7.2780	6.9184	4.7612	6.7034
Pdap1	_KSLDS[Phospho (STY)]DESEDEDDDYQQKR_.2	#N/A	3.9215	4.3800	3.8020	3.9448	3.3743	3.3435	3.4801	4.0139	3.5589	3.8558	3.5813	3.8787	3.8191	3.8401	3.5533	3.6362	3.9884	3.7871	3.5665	4.1755
Arhgap17	_LGEQGPEPGPTPPQTPT[Phospho (STY)]PPST[Phospho (STY)]PPLAK_.3	#N/A	4.0111	6.5908	3.8431	6.4185	3.6519	6.2153	5.3145	3.8804	3.8499	6.0092	3.5759	3.6297	3.9907	3.5119	3.7291	3.6556	6.6601	3.4072	3.5213	3.4677
Plekha7	_SVDISLGGS[Phospho (STY)]PR_.2	Q3UIL6_S609_M1_Plekha7	6.7418	7.2829	7.4991	7.3757	4.2364	7.4043	7.4561	6.7126	7.3615	6.6839	5.9921	7.1006	6.8684	7.4108	6.4234	7.0098	7.2606	6.8802	6.9853	7.3235
Plekha7	_KVMS[Phospho (STY)]PLQS[Phospho (STY)]PTK_.2	Q3UIL6_S900_M2_Plekha7	3.7403	5.7169	3.7851	6.3391	3.7324	4.3847	5.4628	3.6785	4.0646	4.0591	3.5433	5.1046	4.2301	6.0383	3.4719	3.6106	3.9714	3.4366	3.8547	4.7165
Plekha7	_HLSGSSS[Phospho (STY)]PPPR_.2	Q3UIL6_S482_M1_Plekha7	3.2853	10.9538	4.5909	10.2396	4.1239	4.3479	11.1335	8.7609	3.4763	3.4916	10.8422	3.4917	3.5844	4.6290	2.9944	3.6302	3.2764	9.7572	4.1721	4.3063
Plekha7	_IEDVM[Oxidation (M)]AGLS[Phospho (STY)]ANKENY[Phospho (STY)]R_.3	Q3UIL6_S820_M2_Plekha7	4.2533	3.7354	3.2888	3.4361	5.4949	5.9360	4.1884	3.9537	4.5089	3.9642	3.5872	3.6389	4.2740	3.2759	4.2138	3.0974	4.4914	3.3418	2.9904	3.2429
Edc4	_DSQDTSAEQS[Phospho (STY)]DHDDEVASLASASGGFGSK_.3	Q3UJB9_S884_M1_Edc4	4.2504	7.8481	6.7442	3.5328	2.9565	6.3776	7.3692	3.8569	6.7203	6.4368	3.4905	4.2957	4.1773	8.2499	4.1170	3.6125	4.3946	7.8051	3.0871	3.1461
Edc4	_[Acetyl (Protein N-term)]ASC[Carbamidomethyl (C)]AS[Phospho (STY)]IDIEDATQHLR_.2	Q3UJB9_S6_M1_Edc4	5.7791	4.0474	3.1491	5.2796	3.4787	3.1289	3.7617	3.4974	3.1945	5.4789	3.5689	3.7700	3.3026	4.3472	3.5948	4.1347	4.4955	4.1548	2.9530	4.0245
Usp19	_RNS[Phospho (STY)]PVER_.2	Q3UJD6_S1258_M1_Usp19	8.5765	8.3556	8.2404	8.3261	4.2863	8.1028	4.2627	8.6138	8.0348	4.4666	4.3765	2.9659	4.1102	3.6540	2.7872	3.4415	3.8023	8.8899	3.6462	3.9004
Rmdn3	_SHS[Phospho (STY)]LPNSLDYAQASER_.2	#N/A	7.7177	8.2389	6.7129	8.4082	7.8011	7.8048	7.9509	8.1729	8.2257	8.3573	7.4598	7.5762	8.9615	8.0883	6.6664	7.7526	8.3798	7.7296	7.6490	8.1937
Rmdn3	_SLQGLAGEIVGEVR_.2	#N/A	4.2483	4.2649	4.0426	5.1238	4.4730	4.3361	4.4676	4.3282	4.7700	3.5898	3.9617	3.8301	3.1477	3.2755	4.4228	4.3718	4.3475	4.0070	4.9599	4.2693
Rmdn3	_AIPGEAGDAAILPS[Phospho (STY)]LSQEGQEK_.2	Q3UJU9_S81_M1_Rmdn3	3.7331	3.9596	4.4897	3.9561	3.4805	3.6035	3.8698	3.3716	3.8789	3.7950	3.8591	3.7720	3.9743	8.0315	3.6300	6.9324	3.7880	3.6323	3.9313	3.8987
Rmdn3	_DSDKES[Phospho (STY)]GDAEDEVSC[Carbamidomethyl (C)]ETVR_.2	Q3UJU9_S193_M1_Rmdn3	4.5061	4.3138	4.0427	4.5789	3.2122	3.6035	3.8359	3.2691	3.7561	4.3167	3.2347	4.0399	3.9215	4.0808	3.8613	3.8682	4.2290	4.4212	3.2195	3.7580
Rmdn3	_KDS[Phospho (STY)]LDLDVEAASSPAAAALEEDDSSGR_.3	Q3UJU9_S212_M1_Rmdn3	4.4512	3.5516	3.4867	3.2382	3.7889	4.0130	3.7521	4.1516	3.8112	3.1567	4.1856	3.4410	3.9193	3.4738	3.3293	3.2953	3.1884	3.5381	4.2933	3.1403
Tax1bp1	_KLEGQS[Phospho (STY)]PQQVSR_.2	Q3UKC1_S632_M1_Tax1bp1	4.0470	7.9959	4.5833	3.6423	7.8417	3.6089	4.1563	3.7474	4.2154	7.4475	7.2332	7.4419	8.1658	4.5704	3.7335	7.9670	4.2851	3.5481	7.5346	7.7208
Tax1bp1	_VPS[Phospho (STY)]WEDNVVC[Carbamidomethyl (C)]SQPAR_.2	Q3UKC1_S693_M1_Tax1bp1	7.9042	4.0336	4.3229	4.2988	3.4924	5.4389	7.8731	3.5111	7.7731	8.3936	7.6374	8.0061	3.6414	8.6178	5.9434	4.1484	4.5092	7.9790	7.7995	7.7386
Tax1bp1	_AAS[Phospho (STY)]PVEELLTM[Oxidation (M)]EDEGNSDM[Oxidation (M)]LVVTTK_.3	Q3UKC1_S124_M1_Tax1bp1	3.8875	6.0824	4.3135	4.6579	4.0944	4.9567	5.4144	5.2822	5.0986	5.1228	4.1847	3.1577	6.7255	5.1120	5.2859	4.8262	5.9832	6.3104	5.3189	5.6515
Ell2	_LSNAS[Phospho (STY)]PNPNEGVK_.2	Q3UKU1_S501_M1_Ell2	4.1618	3.7577	3.2805	3.4443	2.8679	4.4021	4.1802	3.9455	4.1004	7.1134	3.5790	3.6472	6.3360	3.2676	6.5783	3.5240	4.4831	3.3500	2.9986	3.2346
Arglu1	_ASS[Phospho (STY)]PPDRIDIFGR_.2	#N/A	9.3120	9.5529	8.8365	8.8483	3.7675	9.0022	9.2463	4.0005	8.7141	9.0141	3.5886	3.5285	9.4634	9.4821	7.8338	8.6005	9.3352	9.0957	8.8232	3.8499
Arglu1	_ERASS[Phospho (STY)]PPDR_.2	#N/A	3.1672	3.6475	3.2081	3.9127	4.0058	4.2299	3.5353	3.8972	3.5944	3.3736	3.9687	3.3737	3.7024	3.2462	3.1125	3.5121	3.3945	3.7550	4.0540	4.4243
Gpd1l	_ELLQTPNFR_.2	#N/A	7.1887	7.5229	6.8119	7.6468	7.6654	7.2786	7.0518	7.1387	7.2281	7.0898	7.0645	7.2284	7.2464	6.9653	7.0552	7.2331	7.6517	7.3530	7.2073	6.7336
Gpd1l	_IC[Carbamidomethyl (C)]DEITGR_.2	#N/A	6.5481	5.6978	6.0461	5.8068	5.6469	6.5415	6.8416	3.6286	6.2109	6.3425	6.3211	5.8610	3.5239	6.0791	6.2243	5.8698	5.6874	6.1746	6.0322	6.2631
Gpd1l	_GIDEGPDGLK_.2	#N/A	6.7135	6.5825	6.4802	6.8305	6.4807	6.8851	6.8728	6.7221	4.2785	6.9578	6.7917	6.6840	4.4440	6.4615	6.5158	6.8908	6.6917	6.5205	6.6378	6.5056
Gpd1l	_KLTDIINNDHENVK_.3	#N/A	4.6418	4.4623	4.7732	3.3523	4.7143	3.9838	3.4308	4.2282	4.4644	3.7695	4.7826	4.4084	4.7851	3.5772	4.6744	4.9603	3.7079	3.9932	3.7758	3.9722
Gpd1l	_FC[Carbamidomethyl (C)]ETTIGSK_.2	#N/A	5.0836	4.9584	4.7124	5.7233	5.1955	5.0136	5.7432	4.2638	4.4187	5.5561	5.2208	5.4447	5.7322	5.1286	5.6259	4.5882	3.3007	5.0355	2.9950	4.6977
Gpd1l	_LISDIIR_.2	#N/A	4.0374	4.3427	3.6422	5.4857	4.0573	4.7777	5.7124	3.1294	5.1892	3.2877	3.8691	4.3430	5.1434	4.4669	4.4898	4.3401	3.6670	5.4233	3.8147	4.6381
Gpd1l	_LQGPQTSAEVYR_.2	#N/A	3.8381	4.1647	5.7820	3.8512	3.1759	3.3999	4.8509	5.4472	5.5397	5.7816	5.3654	4.8076	4.5324	4.3615	3.9424	5.4621	4.0762	4.6773	4.7155	5.3845
Gpd1l	_VC[Carbamidomethyl (C)]IVGSGNWGSAVAK_.2	#N/A	5.1183	4.6144	3.9247	5.1084	4.2270	4.4511	3.3141	3.0888	3.3732	3.5948	3.7475	3.0030	3.4812	3.9118	2.8913	3.7333	3.6265	4.2067	3.8552	4.2031
Gpd1l	_TIEELEKELLNGQK_.3	#N/A	3.5642	4.0445	4.3120	4.3096	2.9019	3.1260	3.1383	3.5003	3.1974	3.7705	3.5718	4.3281	3.3055	4.3501	4.2164	3.9091	4.4984	4.1519	2.9501	4.0274
Ppp1r7	_ELYLSNNGIEVIEGLENNNK_.2	#N/A	9.9313	10.0329	8.7066	8.9636	8.4830	8.7646	9.5214	10.1761	8.6258	9.1019	8.8836	9.7589	9.9841	10.8481	8.9333	9.0134	9.3708	9.7547	9.1165	9.0596
Ppp1r7	_ELDLYDNQIK_.2	#N/A	7.2810	6.7807	7.5570	7.1844	6.8743	7.1647	6.9181	7.5605	7.4036	7.7217	7.3719	7.2786	7.4488	7.7111	7.3639	7.2908	7.2523	7.1806	7.2363	7.4351
Ppp1r7	_IEGLQSLVNLR_.2	#N/A	6.8927	7.4058	7.0129	7.4530	6.7896	7.0321	7.0153	7.2758	6.6849	7.1407	6.8771	7.4769	6.9031	7.4860	7.0681	7.2839	7.0286	7.2763	7.0083	7.2339
Ppp1r7	_QIDATYVR_.2	#N/A	5.9797	5.9465	6.4778	5.9102	6.0847	6.1510	6.1563	5.7526	6.5458	6.3156	6.1373	6.3963	5.7602	6.2167	6.7433	6.6123	6.0089	6.1838	6.4219	6.3815
Ppp1r7	_SLETVYLER_.2	#N/A	5.6219	8.0968	7.8090	7.6448	6.1326	7.9718	7.6980	7.8155	7.6338	8.1840	7.8546	8.0851	8.4823	8.4949	7.4911	8.2428	8.3254	8.1398	7.9630	8.3568
Ppp1r7	_C[Carbamidomethyl (C)]IENLEELQSLR_.2	#N/A	4.5874	5.1388	4.9852	5.1738	4.3139	5.4001	5.2124	4.5859	5.4535	5.4387	5.1121	5.5058	4.9616	5.5855	5.7865	5.3861	3.7682	5.1952	5.1404	5.2680
Ppp1r7	_HGGGGIVANLS[Phospho (STY)]EQSLKDGVDR_.3	#N/A	3.4777	6.9966	6.6205	4.3342	3.8826	6.2738	6.3855	4.7621	6.4426	5.6315	4.0816	3.7756	3.4603	7.7346	6.0440	4.9828	4.5224	7.0923	5.9003	6.2172
Ppp1r7	_LQNLDALTNLTVLSVQSNR_.2	#N/A	5.5982	5.9240	5.6021	5.6692	5.4263	5.9651	5.5503	5.5115	5.8808	6.2148	5.4517	5.9366	6.1248	6.4244	5.7764	5.3260	6.4064	5.6572	5.8725	6.2350
Ppp1r7	_RVES[Phospho (STY)]EES[Phospho (STY)]GDEEGKK_.2	#N/A	7.1561	7.7150	7.7365	7.8044	7.3101	7.6186	7.5477	7.6497	7.7558	7.7832	7.6075	7.5832	7.8754	8.1538	8.8195	8.3142	7.7485	7.5616	8.1892	7.7690
Ppp1r7	_LTMLDIASNR_.2	#N/A	3.5644	5.1723	6.3645	5.9228	3.7713	4.3527	5.6315	3.7901	3.9810	3.4807	5.7483	5.0967	3.5953	6.4731	4.1822	4.4274	3.2873	3.8621	4.1612	4.3818
Ppp1r7	_IENISNLHQLQM[Oxidation (M)]LELGSNR_.3	#N/A	4.5738	5.1338	5.0140	5.0970	5.0797	4.7281	3.9131	4.9641	4.3431	5.3003	4.7980	4.9277	5.0736	5.4450	4.5514	5.2054	4.8867	4.6764	4.6609	5.1735
Ppp1r7	_KHGGGGIVANLS[Phospho (STY)]EQS[Phospho (STY)]LKDGVDR_.3	#N/A	4.4381	3.8636	4.8116	4.1310	3.1442	4.5580	4.4326	3.5668	4.3921	4.2487	3.3140	3.8914	4.0008	5.4221	3.5445	3.9405	4.3118	4.6816	4.5718	4.1923
Ppp1r7	_VES[Phospho (STY)]EES[Phospho (STY)]GDEEGK_.2	#N/A	5.6238	6.7252	7.0973	6.2845	6.8791	7.3260	6.1430	6.5531	6.8820	6.8153	6.2810	7.5626	7.1624	7.8273	7.5253	6.6279	7.5373	6.3409	6.9872	7.7621
Ppp1r7	_VESEES[Phospho (STY)]GDEEGKKHGGGGIVANLSEQSLK_.4	#N/A	3.3033	7.1053	4.5729	4.0487	4.1418	4.9296	5.7258	3.7612	6.0302	3.5096	3.8327	5.5872	3.5664	7.4250	5.7107	3.6482	3.2584	7.1916	5.3472	4.2883
Ppp1r7	_HGGGGIVANLS[Phospho (STY)]EQSLK_.2	#N/A	3.2530	3.2490	5.9922	4.4364	4.0915	3.8934	3.4495	4.4542	3.1082	3.4637	4.0877	3.1384	3.2737	3.7763	3.0267	3.5979	3.4910	4.3421	3.9907	3.7433
Ppp1r7	_RVES[Phospho (STY)]EES[Phospho (STY)]GDEEGK_.2	#N/A	5.1137	5.0406	5.2915	5.2820	5.3117	5.4503	4.7157	4.9022	4.7535	5.1740	5.2817	5.2117	5.0384	5.7819	5.2457	3.9060	5.4217	4.9705	4.9119	5.3241
Ppp1r7	_GAGQQQS[Phospho (STY)]QEMM[Oxidation (M)]EVDR_.2	#N/A	4.2266	5.1699	6.2870	6.6090	3.5643	4.7023	5.2350	3.9270	4.4732	4.5629	6.1384	4.7520	4.1376	7.3913	3.5806	3.5757	3.4688	3.6714	3.8612	4.2685
Ppp1r7	_GAGQQQS[Phospho (STY)]QEM[Oxidation (M)]M[Oxidation (M)]EVDRR_.3	#N/A	3.8900	4.2468	4.1926	4.1647	3.0597	3.5213	3.7518	3.2547	3.8250	4.1299	3.6707	3.7659	3.9716	4.0692	3.6743	4.0210	3.9536	3.7347	3.2994	4.1626
Ppp1r7	_DAEDVDLTHYR_.3	#N/A	3.6128	5.2198	4.1491	5.9340	2.9506	3.5335	5.7850	3.3132	3.4681	3.1039	2.9467	4.2794	3.6336	5.8523	3.5733	3.9577	3.8509	4.7598	3.6309	4.1032
Ppp1r7	_ELDLYDNQIKK_.3	#N/A	3.7640	4.2388	4.3003	3.9254	3.1017	3.3824	4.4394	3.4643	2.9976	3.9703	3.3720	4.1283	3.7847	4.2873	4.0166	4.1089	4.0020	0.8120	3.4797	4.2543
Ppp1r7	_GAEDPEEEHELAVDMETINLDR_.3	#N/A	3.8197	3.4994	3.3562	3.6486	4.0266	4.0818	3.6834	4.0453	3.7425	3.2255	4.1168	3.2256	3.8505	3.4075	3.2606	3.1818	3.5426	3.6069	3.9059	3.0716
Smcr8	_VAS[Phospho (STY)]PANVGTLHTLSR_.2	#N/A	6.8716	6.8085	5.3213	4.1495	4.0335	5.3073	7.0730	8.4595	3.1762	5.5290	4.7185	7.0275	6.5263	8.1913	4.4623	4.2837	6.2374	6.9778	5.8277	3.8113
Smcr8	_SS[Phospho (STY)]VESVLIK_.2	Q3UMB5_S420_M1_Smcr8	3.1151	4.2040	4.1525	4.4691	3.3221	3.2855	3.7260	3.3408	3.6463	4.4266	3.1249	3.9301	3.1460	4.1906	3.7514	3.9781	4.3389	4.3114	3.1096	4.5493
Smcr8	_SDS[Phospho (STY)]QASLTVPLSPHVVR_.2	Q3UMB5_S488_M1_Smcr8	4.1584	3.5958	5.7030	3.6311	3.8475	5.3153	7.1472	6.6712	3.4600	5.7940	4.1160	6.1885	3.5921	7.3867	3.2504	3.1967	3.3038	6.1321	5.7838	5.1768
Washc4	_[Acetyl (Protein N-term)]AVDTLS[Phospho (STY)]PDWDFDRVDDGSQK_.3	#N/A	4.2885	4.6192	4.0807	3.4961	3.7860	3.8856	4.1535	3.9202	3.8432	3.7798	3.7960	3.6416	4.0497	3.8812	3.4827	3.4794	3.8320	5.2184	3.8991	3.3610
Cobll1	_RQSS[Phospho (STY)]LTFQSSDPEHVR_.3	#N/A	3.7050	3.6141	3.2415	3.8793	3.9119	4.1965	7.2369	7.3303	3.6278	3.5156	4.0022	3.3402	3.7358	3.2796	7.3568	3.0671	3.4279	3.7216	4.0206	2.9569
Cobll1	_TLS[Phospho (STY)]SPTGTETNPPK_.2	Q3UMF0_S896_M1_Cobll1	7.1599	6.7713	3.9641	3.1567	6.5281	6.6590	3.9145	3.1523	3.8347	7.3269	7.4962	4.1186	4.0002	8.1085	3.9399	3.7896	7.2517	3.6157	7.1848	2.9690
Cobll1	_QDS[Phospho (STY)]NPKPKPSNEITR_.3	Q3UMF0_S794_M1_Cobll1	3.1708	4.1483	4.2082	4.4134	3.3778	3.4111	3.6703	3.3965	3.5906	4.3521	3.0692	3.8744	3.2017	4.2463	3.6957	4.0338	2.7856	2.7543	3.0539	3.9236
Cobll1	_ITVEKDPDSALGIS[Phospho (STY)]DGETSPSSK_.3	Q3UMF0_S546_M1_Cobll1	3.3095	3.9498	4.3469	4.2748	3.5164	3.0911	3.1732	3.5351	3.2323	3.7356	3.6067	3.7357	3.3403	4.3849	3.5571	4.1724	4.5332	4.1171	4.4161	4.0622
Cobll1	_QSS[Phospho (STY)]LTFQSSDPEHVR_.2	#N/A	4.1734	3.1457	3.7100	3.4108	2.8795	4.4137	4.1686	4.3991	4.0888	3.9840	3.5675	4.3727	4.2543	3.2561	4.1940	3.5355	3.8963	3.3616	3.0101	6.6570
Faxc	_TETFEDEGAENS[Phospho (STY)]FSR_.2	Q3UMF9_S378_M1_Faxc	3.4054	4.8473	3.9417	4.2840	4.2440	4.4681	4.8883	3.1058	3.3562	3.6117	4.2091	4.9263	3.4261	3.9288	2.8743	3.7503	3.6435	4.1897	4.6893	4.6089
Ankrd27	_PSEVPAQS[Phospho (STY)]PTR_.2	Q3UMR0_S634_M1_Ankrd27	7.0401	3.4650	6.6578	6.9871	3.8755	6.8764	6.7283	7.0286	4.1052	7.2133	6.8643	6.2864	7.4041	7.1377	5.9692	3.3819	6.8202	6.7127	6.7227	6.8660
Mcu	_VAIYSPDGVR_.2	#N/A	5.9677	6.7366	6.3460	6.4262	6.1265	6.5455	6.2840	6.6605	6.3007	5.4311	6.3790	6.0846	5.9854	5.3385	5.9384	6.0929	6.5435	6.3794	6.4595	5.7231
Mcu	_DLLSHEDAATLNDVK_.3	#N/A	4.4755	3.4549	3.4007	3.7201	3.8132	3.1138	3.7279	4.0898	3.7870	3.1810	4.1613	3.1811	3.8950	2.7697	3.4647	3.3195	3.5871	4.2316	3.1938	3.1161
Ppp1r12c	_TGS[Phospho (STY)]TGALGPSER_.2	Q3UMT1_S431_M1_Ppp1r12c	10.0344	10.1795	10.1838	10.1482	9.6179	9.7112	9.7950	10.1595	9.7980	10.0908	9.4802	9.8510	10.0910	10.2086	8.9019	9.6035	9.9764	9.7917	9.3807	9.7603
Ppp1r12c	_ALNGVSS[Phospho (STY)]PVSSNPK_.2	Q3UMT1_S403_M1_Ppp1r12c	4.1474	3.1717	3.6839	3.4369	2.8535	4.3876	5.9590	7.1928	4.1149	3.9579	3.5935	7.2066	7.5031	7.5304	4.2200	7.1310	6.7733	3.2792	6.6090	6.8288
Hdgfl2	_GGSSEELHDS[Phospho (STY)]PR_.2	Q3UMU9_S635_M1_Hdgfl2	6.3995	5.8728	6.7693	6.3905	5.9747	6.5605	6.2857	5.8629	6.3933	6.6418	6.4517	5.6873	5.8433	6.0749	5.1308	5.6717	6.6674	6.3981	5.9903	5.5288
Hdgfl2	_GGS[Phospho (STY)]S[Phospho (STY)]GEELEDEEPVKK_.2	#N/A	10.3744	10.4129	10.2912	9.9955	7.2962	10.2825	10.1365	9.5702	10.3296	9.8977	9.3204	9.7805	4.4407	10.3644	8.4620	9.3866	10.3909	9.9096	9.8560	9.2891
Hdgfl2	_TRLAS[Phospho (STY)]ESANDDNEDS_.2	#N/A	5.3245	6.4042	3.1625	6.0529	4.4016	4.8632	5.7669	3.8274	5.6946	5.8838	5.2062	5.5521	6.8013	6.0746	5.5528	5.7145	6.2122	6.1259	5.2537	5.1693
Hdgfl2	_AERGGS[Phospho (STY)]S[Phospho (STY)]GEELEDEEPVK_.2	#N/A	4.7941	5.5199	4.8101	5.2647	3.0216	5.2210	5.4133	3.9279	5.9025	5.8607	3.1119	5.7660	4.3464	5.6107	4.7945	5.9350	5.7636	5.6142	5.4017	5.4702
Hdgfl2	_AQEDGQDS[Phospho (STY)]EDGPR_.2	Q3UMU9_S620_M1_Hdgfl2	4.5565	5.1177	3.5920	3.1329	3.8942	5.5812	5.6485	6.2587	6.2058	5.0540	4.0803	5.9191	6.0121	4.4160	4.4029	3.4006	3.2937	5.0824	5.3862	5.8039
Hdgfl2	_LAS[Phospho (STY)]ESANDDNEDS_.2	#N/A	6.6743	7.4539	7.0059	6.5310	6.6220	6.9937	6.7622	6.5903	7.0406	7.2339	6.9354	6.8755	7.0697	7.1450	6.3067	6.1167	7.1016	7.0609	7.0889	6.5713
Hdgfl2	_GGS[Phospho (STY)]S[Phospho (STY)]GEELEDEEPVKKR_.3	#N/A	4.7124	5.9640	4.1521	5.5851	3.4709	5.2924	5.7261	5.1076	6.1650	5.7223	4.8084	5.4082	4.9458	5.7810	5.1214	5.7486	5.4402	6.0439	5.8933	4.9717
Hdgfl2	_GGS[Phospho (STY)]SGEELEDEEPVK_.2	Q3UMU9_S366_M1_Hdgfl2	7.9577	9.7980	10.3558	9.9827	3.2127	9.8072	9.6579	3.7218	9.8754	9.8660	3.8338	9.5277	3.7833	9.8983	8.8926	9.5303	10.2318	9.4851	9.6686	9.6875
Hdgfl2	_KRS[Phospho (STY)]EGLSLER_.3	Q3UMU9_S450_M1_Hdgfl2	4.2864	5.0018	3.3219	3.4030	3.6242	4.3608	3.9169	3.9868	4.1417	3.9311	3.6204	3.6058	4.3072	3.3090	3.4941	3.1305	4.5245	3.3087	2.9573	3.2760
Hdgfl2	_AERGGS[Phospho (STY)]S[Phospho (STY)]GEELEDEEPVKK_.3	#N/A	3.9194	3.6734	4.0335	3.4103	3.7625	3.4219	4.3420	3.9101	3.8873	4.0978	4.2561	4.3753	4.2440	3.3010	3.5210	4.2266	3.5370	4.1789	4.4330	3.0640
Gpr162	_RLS[Phospho (STY)]HDETNIFSTPR_.3	Q3UN16_S413_M1_Gpr162	8.3163	8.2797	7.7621	7.6986	5.7646	7.7174	7.8079	7.3470	7.1992	7.2518	4.1679	7.2286	7.1505	7.1613	5.4455	6.6779	7.6632	7.8140	7.4550	7.0605
Gpr162	_RSS[Phospho (STY)]LDGSESAK_.2	Q3UN16_S256_M1_Gpr162	7.6603	3.7057	6.5753	3.3923	3.6348	6.8342	4.2322	3.9975	7.0313	7.4001	6.6403	3.5952	4.3178	3.3196	3.4835	3.1412	4.5351	7.0059	7.0134	6.3919
Gpr162	_C[Carbamidomethyl (C)]SLTGS[Phospho (STY)]EGSSR_.2	Q3UN16_S565_M1_Gpr162	4.1502	3.8526	3.1857	3.5391	6.1335	2.9961	4.0853	3.8506	4.0055	4.0673	3.4842	3.7420	4.1710	3.1728	4.1107	3.6188	4.3883	3.4449	3.0934	3.1398
Gpr162	_RLS[Phospho (STY)]LGSPDSR_.2	Q3UN16_S539_M1_Gpr162	8.4516	8.1988	8.2155	8.3966	6.1561	7.2262	5.9853	7.1297	7.2555	8.4483	7.3064	7.3296	6.1720	6.5322	4.4190	7.3057	7.7428	7.3066	6.6454	7.7008
Gpr162	_RC[Carbamidomethyl (C)]S[Phospho (STY)]LT[Phospho (STY)]GSEGSSR_.2	Q3UN16_T563_M2_Gpr162	7.1991	4.0455	4.8489	4.4950	3.3890	5.0463	4.9454	4.3911	3.6579	4.7880	4.3178	4.2300	3.7254	4.2280	3.7778	3.8107	3.9427	4.2181	3.6011	3.7311
Gprin1	_DLAAAAAQKS[Phospho (STY)]PSAEAAAPPPGPR_.3	#N/A	12.8525	7.8847	12.3205	11.9501	12.1611	6.7385	12.0186	12.8908	3.8132	12.4232	12.1018	12.4526	13.3002	3.8923	11.2382	12.0911	12.6402	3.7339	12.2481	12.2487
Gprin1	_KAS[Phospho (STY)]LPASEK_.2	#N/A	7.8167	7.9279	7.4089	7.7185	6.9650	7.3663	6.9857	6.4629	6.3687	7.9731	7.0992	7.0796	6.9203	7.7632	6.1430	7.2883	3.6324	6.8800	7.1055	7.0829
Gprin1	_SLGTWGS[Phospho (STY)]LSAAK_.2	Q3UNH4_S446_M1_Gprin1	7.9758	3.8757	7.9067	8.2255	3.6503	4.4580	6.8847	5.8940	6.6201	8.6264	3.7406	6.7792	3.4743	7.3145	3.4232	6.9953	3.1663	6.6992	4.2822	4.1961
Gprin1	_RERPGS[Phospho (STY)]TGEGDLVSLR_.3	#N/A	7.3597	7.0659	6.9188	7.1001	6.3645	7.2122	3.6320	6.7597	7.0746	4.5206	7.2781	6.8905	3.7177	7.7030	3.6574	6.9735	3.9350	3.8982	6.7035	7.0276
Gprin1	_VITQIPGATSPGKVETPS[Phospho (STY)]LQK_.3	Q3UNH4_S584_M1_Gprin1	3.4042	4.5986	3.9405	4.2852	6.5778	6.2933	3.2983	3.1046	3.2595	3.6106	4.2390	2.9872	3.4249	3.9276	2.8755	3.7491	3.6423	4.1909	3.8395	3.8946
Gprin1	_VDIVS[Phospho (STY)]PGGDNAGSLR_.2	#N/A	10.1454	10.1673	10.3963	10.3460	8.9866	9.0449	9.2165	8.8737	9.1088	10.7103	9.2331	9.5302	9.6821	9.9976	8.3973	9.6576	9.2392	9.1443	9.2497	9.5146
Gprin1	_ERPGS[Phospho (STY)]TGEGDLVSLR_.2	#N/A	8.4666	7.7562	7.7189	8.5925	6.7592	7.5972	7.0486	3.5079	7.3582	8.2503	8.4217	8.1756	7.4304	7.9534	8.0772	8.6970	7.7866	8.2544	7.2198	7.5720
Gprin1	_AETVS[Phospho (STY)]PGEVDAM[Oxidation (M)]TLGK_.2	#N/A	9.5872	9.8275	9.6778	9.6450	9.0133	9.6808	9.2201	9.5096	9.4735	9.7071	8.9478	9.3879	10.1862	9.9411	9.1565	9.5457	9.8471	9.3651	9.4753	9.6226
Gprin1	_KAS[Phospho (STY)]LPASEKTDPLASSK_.3	#N/A	5.9052	6.1242	3.2938	5.8498	5.1471	5.2158	5.4083	4.6047	5.2391	5.8777	5.3092	5.3954	6.3222	5.9705	3.6382	5.2236	4.8233	4.3034	5.2960	3.0092
Gprin1	_VDIVSPGGDNAGS[Phospho (STY)]LRK_.2	#N/A	6.7506	6.7302	6.0922	6.8657	5.0290	5.0043	5.2226	5.2935	5.3044	7.1810	5.2301	5.6976	5.1662	6.4126	4.1649	6.3240	4.9255	5.3898	4.9892	6.1562
Gprin1	_NSELVS[Phospho (STY)]PVKPER_.2	#N/A	11.0067	10.8631	10.6856	11.0145	10.1130	9.8071	10.1266	10.1074	9.6789	11.0413	10.1420	10.3556	10.6580	10.9225	9.2188	10.4144	9.9690	10.0579	9.9516	10.1554
Gprin1	_KTDPEFSGKLT[Phospho (STY)]PGSSGK_.3	Q3UNH4_T256_M1_Gprin1	3.9473	6.6873	7.0750	7.3133	6.3939	5.8684	6.2767	3.1172	6.0444	4.1536	6.7247	6.8086	4.4232	7.1610	3.8333	6.6933	4.1153	6.4490	3.3332	3.6443
Gprin1	_ADSASPS[Phospho (STY)]PR_.2	Q3UNH4_S622_M1_Gprin1	8.1330	8.4157	8.5149	8.4987	7.7578	7.5052	7.9175	7.7258	7.6603	8.9195	7.8700	8.2138	8.2386	8.0516	3.3821	7.5373	7.5484	7.7660	7.4118	8.3535
Gprin1	_APS[Phospho (STY)]PAPRPPSR_.2	#N/A	10.6339	10.4782	10.9128	10.6994	9.8378	9.4579	9.8666	9.7686	9.5675	11.1244	9.8757	10.1264	10.0021	10.5221	9.0412	10.0314	9.7436	9.7804	9.6796	10.0152
Gprin1	_SVATGPM[Oxidation (M)]T[Phospho (STY)]PQAAAPPAVPPVFPEVR_.3	#N/A	8.0651	8.7272	7.0707	3.9751	6.2068	6.3318	9.5433	10.0014	6.4710	7.4281	6.3202	8.8961	8.5066	9.9905	3.3708	3.6073	3.8158	9.7078	3.7235	6.6414
Gprin1	_APS[Phospho (STY)]WDAGAPPPR_.2	Q3UNH4_S714_M1_Gprin1	14.8551	14.6156	14.8462	14.5884	14.3971	14.5935	14.1659	14.6846	14.2585	15.0643	14.8927	14.4293	14.8168	15.2894	14.1196	14.6028	15.0044	14.5153	14.5850	14.6830
Gprin1	_S[Phospho (STY)]PSAEAAAPPPGPR_.2	#N/A	12.0945	11.9945	3.8851	12.2600	12.4439	3.7763	11.4070	11.4794	11.6616	12.3908	3.8021	3.9232	3.5358	12.4215	11.2858	11.7130	11.8365	11.4217	4.2356	11.8426
Gprin1	_MDGNS[Phospho (STY)]LKQADSTSTR_.2	Q3UNH4_S110_M1_Gprin1	4.1107	3.8921	3.1461	4.9072	3.4484	3.6725	4.0927	3.8110	4.1518	4.3170	3.0253	3.7816	4.2598	3.1332	3.6699	2.9547	4.3487	3.1976	3.1330	3.4809
Arhgef40	_IQQQLGEEAS[Phospho (STY)]PR_.2	Q3UPH7_S959_M1_Arhgef40	7.3718	7.1862	6.7668	7.0011	6.7524	6.3637	6.5538	6.8661	6.4937	7.6294	6.4420	6.3949	6.9939	6.2476	5.6901	5.9870	6.6963	6.4245	5.8720	6.3164
Sec31a	_LVTFESVAVPLQQGAEQQR_.2	#N/A	4.7971	4.2011	4.0388	4.7380	4.3546	5.2542	4.5396	4.8525	4.6551	4.3725	4.6421	4.5362	5.2175	4.5617	3.9361	4.5068	4.6493	4.5917	4.5155	3.9413
Sec31a	_DSDQVAQS[Phospho (STY)]DGEESPAAEEQLLGER_.3	#N/A	3.8539	8.2101	7.9307	3.9748	3.7927	8.4945	8.0208	4.1506	7.6408	6.3977	3.9519	5.9325	3.8534	7.8322	7.2232	6.0932	3.8963	8.2894	7.8590	7.1285
Sec31a	_AQDGSS[Phospho (STY)]PLSLQDLIEK_.2	Q3UPL0_S712_M1_Sec31a	3.4575	3.8616	4.4950	4.1267	3.6645	4.4439	3.3213	3.6832	3.3803	3.5876	3.7547	3.5877	3.4884	4.5330	2.8985	4.3205	3.1804	3.9690	4.2681	4.2103
Sec31a	_VDGPDVALKDSDQVAQS[Phospho (STY)]DGEESPAAEEQLLGER_.4	#N/A	4.5722	4.2477	4.1088	4.5128	3.2783	3.3292	4.4360	3.2971	2.9942	3.9737	3.3686	3.9738	4.6031	4.1469	4.0132	3.9343	4.2951	4.3551	4.8603	3.8242
Ag2	_S[Phospho (STY)]TQSLSLQR_.2	Q3UPL5_S217_M1_Ag2	7.6399	3.9846	3.8553	3.9240	3.8054	4.0295	5.5207	3.4441	6.7570	7.5250	6.7921	3.7598	4.0120	4.2939	3.4221	4.0498	3.7041	3.5546	3.8536	7.0475
Ag2	_TQPVTFDEIQEVEEEGVS[Phospho (STY)]PM[Oxidation (M)]EEEK_.3	#N/A	3.4674	4.6368	4.3622	4.1486	3.6744	4.2947	3.4055	3.6931	4.6873	4.1099	3.6362	3.6095	3.4912	3.8739	4.6815	3.6614	4.0222	3.9909	4.5159	4.2202
Fcho2	_NS[Phospho (STY)]SNEELTK_.2	Q3UQN2_S402_M1_Fcho2	4.4097	4.0056	4.5335	6.5279	3.1158	3.1492	3.9323	3.6976	3.8525	4.2203	3.3311	3.8950	4.0179	4.5206	3.9576	3.7718	4.2353	3.5979	3.2465	2.9867
Fcho2	_HS[Phospho (STY)]PVQMNR_.2	Q3UQN2_S394_M1_Fcho2	3.3474	3.9717	5.3431	4.4350	3.4655	3.0532	3.4938	3.5731	3.2702	3.6977	3.6446	3.6978	3.3783	4.4714	3.5192	4.2103	3.0703	4.0792	4.3782	4.1001
Rbm20	_SGS[Phospho (STY)]PGPLHSVSGYK_.2	Q3UQS8_S728_M1_Rbm20	3.6697	3.6494	3.2063	4.4530	3.8766	3.9100	3.1715	4.4376	3.0917	3.4803	4.0712	3.3755	3.2571	5.9196	3.1969	3.0318	3.4745	4.3587	4.0073	3.7268
Brd9	_EKEHC[Carbamidomethyl (C)]DS[Phospho (STY)]EGEADAFDPGKK_.3	#N/A	3.4956	6.0662	6.1300	5.6712	4.3342	5.9365	5.7186	5.0557	3.2660	6.4444	4.8149	5.2583	3.3740	6.4459	2.7841	5.3855	5.8149	6.0787	5.6900	5.3728
Brd9	_EKEHC[Carbamidomethyl (C)]DS[Phospho (STY)]EGEADAFDPGK_.3	#N/A	4.3823	4.5943	3.9724	3.9378	3.5307	3.3935	4.6473	3.7835	3.7814	3.9149	3.1909	3.7114	3.9129	4.5725	3.4407	3.7108	4.4891	4.4494	3.3548	3.6126
Tgfbrap1	_HTAPS[Phospho (STY)]SPSPGTR_.2	#N/A	4.1320	4.0972	6.5009	6.0806	3.6806	3.9062	4.2358	6.1254	5.5182	3.9426	3.5048	5.4474	4.4248	3.7081	3.6029	5.6330	6.0548	5.6596	3.9000	3.5183
Tgfbrap1	_HTAPSS[Phospho (STY)]PSPGTRT_.2	#N/A	6.1342	6.8649	4.0087	6.4769	3.9298	3.6788	5.4606	3.1728	3.3277	6.0646	4.3072	5.8498	3.4932	3.9958	3.2373	3.9931	3.7105	5.7486	5.5279	5.4526
Slmap	_ESDLSDTLS[Phospho (STY)]PSKEK_.2	Q3URD3_S469_M1_Slmap	3.8384	4.3553	4.0807	4.4172	4.6308	5.1064	4.1415	3.2618	3.5629	4.1986	3.3645	3.6847	3.5501	4.1037	4.3384	3.9012	4.1380	4.0792	4.7540	4.1714
Slmap	_SDVIHAPLPS[Phospho (STY)]PVDK_.2	#N/A	6.5173	6.7944	6.8732	6.7600	6.3935	6.8212	6.1252	3.8711	6.1428	6.7449	6.6397	5.9933	4.0958	6.6924	5.0319	6.3338	6.7527	6.3830	6.0282	6.3982
Slmap	_AKESDLSDTLSPS[Phospho (STY)]KEK_.2	Q3URD3_S471_M1_Slmap	4.3751	3.6277	3.4105	3.6197	3.7128	3.9369	3.8283	4.1902	3.8874	4.5814	4.2617	3.5172	3.9954	3.5392	3.4055	3.2191	3.6875	3.4620	3.7610	3.2165
Lingo2	_HKNS[Phospho (STY)]IDLEYVPR_.3	Q3URE9_S576_M1_Lingo2	5.2423	5.9910	5.5135	4.3351	3.4561	5.8654	6.0073	3.4748	3.5122	5.1366	5.6188	5.4816	5.5740	6.8594	3.6174	5.6721	4.4729	6.3595	2.9756	4.0019
Plcl1	_RSS[Phospho (STY)]IIKDPSNQK_.2	#N/A	8.3652	8.3695	8.0143	8.2578	7.9436	6.1243	8.2064	8.1184	8.0233	8.3879	7.8738	7.3797	3.6291	8.1127	6.9581	7.9851	7.8934	7.8137	3.6005	7.5879
Plcl1	_TVS[Phospho (STY)]FSSM[Oxidation (M)]PSEK_.2	#N/A	8.3887	8.8721	9.2573	9.1925	8.1297	8.6938	7.6669	8.2441	8.1711	8.0495	8.5578	3.8066	8.3646	7.3902	7.4276	7.7063	8.5680	7.8081	7.8287	7.7635
Plcl1	_S[Phospho (STY)]GVALPGAAGVPADSEAGLLEAAR_.2	Q3USB7_S48_M1_Plcl1	7.0265	4.1356	4.7733	3.8221	5.1751	5.0574	6.4358	3.5676	4.3952	4.0736	3.2687	4.0250	6.0068	4.3906	3.9133	4.2122	4.1053	5.3080	3.3764	4.3576
Plcl1	_RM[Oxidation (M)]S[Phospho (STY)]GDYNGEQK_.2	#N/A	4.0820	4.8903	3.6492	5.3378	4.3167	4.3827	3.6426	4.9769	4.4830	5.4269	3.7093	5.2699	5.4933	5.3109	3.2198	4.2247	4.1510	3.8345	5.2712	3.6033
Plcl1	_KKT[Phospho (STY)]VS[Phospho (STY)]FSSMPSEK_.2	#N/A	3.4740	5.2310	4.8350	5.6437	4.0956	4.6580	4.5376	3.9439	3.5007	4.7331	3.8751	3.1542	3.6087	5.7799	3.0176	3.4948	4.8023	3.6059	3.7443	3.7281
Plcl1	_KLPSESDLLEGEVT[Phospho (STY)]DEDEEAEM[Oxidation (M)]SR_.3	Q3USB7_T557_M1_Plcl1	3.6400	4.3628	4.1763	4.0494	2.9777	3.5064	3.0625	3.3404	3.4952	4.5776	2.9739	4.2523	3.6607	4.2127	4.1406	3.9849	3.8780	3.9551	3.6037	4.1303

Insyn2a	_STGVQTS[Phospho (STY)]PDLRK_.2	#N/A	7.6415	8.1162	8.0424	8.1429	8.0025	7.8495	7.9180	3.8922	8.0108	8.1017	2.9442	8.1199	8.6022	3.7224	7.6724	8.0122	8.3468	7.9028	3.5778	8.2687
Insyn2a	_STGVQTS[Phospho (STY)]PDLR_.2	#N/A	7.1636	7.3722	3.8950	7.5977	7.1350	7.2924	7.1659	3.0832	6.9799	7.7275	7.1054	7.1042	4.3893	3.9331	6.8231	7.4808	7.5391	6.5876	3.3672	7.4952
Sfswap	_GVS[Phospho (STY)]QEKDGQSSAIVSSVQSK_.3	Q3USH5_S903_M1_Sfswap	4.1013	3.2177	3.6379	4.1194	4.3083	4.3416	4.2407	4.0060	4.1609	3.9119	3.6395	2.9439	4.3263	6.3428	4.2660	3.4635	4.5437	5.5386	4.4389	3.2951
Sfswap	_VKLDDDS[Phospho (STY)]EEDEESR_.2	Q3USH5_S601_M1_Sfswap	3.8005	4.2023	4.3368	3.8889	3.1382	3.3459	3.7355	3.5008	3.6557	4.4171	3.1344	4.0918	3.8212	4.3238	3.9801	4.1454	4.0385	3.7946	3.4432	4.2908
Sorbs2	_RKS[Phospho (STY)]EPAVGPLR_.3	#N/A	10.3831	10.8136	10.7501	10.5856	10.3299	10.4591	10.3686	10.0497	10.2019	10.9022	10.7775	10.3249	10.9976	10.9658	10.1643	10.3512	10.3978	10.5822	10.7339	10.7462
Sorbs2	_RGS[Phospho (STY)]LPDNSILHR_.3	#N/A	9.4577	9.7374	8.5953	9.1127	8.3098	8.8975	9.4420	9.5600	8.4656	9.6287	9.2493	9.1998	9.8366	10.2424	7.6944	8.8536	8.7545	9.7097	9.1144	9.0423
Sorbs2	_ESQS[Phospho (STY)]PDSAWR_.2	Q3UTJ2_S40_M1_Sorbs2	9.9562	9.9557	10.0101	10.0290	9.7203	9.6241	9.4680	10.0139	9.5330	10.1818	9.6674	9.6957	10.2672	10.0009	9.2990	9.6951	10.0862	9.5394	9.7793	9.8624
Sorbs2	_S[Phospho (STY)]EPAVGPLR_.2	#N/A	9.9012	9.4351	10.3595	9.6506	9.6061	9.3758	9.0823	9.2639	9.1167	10.1060	10.0477	8.9181	9.3811	10.0971	8.9328	9.2468	9.6945	9.2088	9.4343	9.7142
Sorbs2	_GAEDYPDPPLPHSYS[Phospho (STY)]SDR_.2	#N/A	4.0581	7.2700	4.0650	3.8751	3.4718	3.5825	3.8921	3.6723	3.2952	3.9860	3.5768	7.9416	3.6467	9.5260	3.7138	3.7561	8.2645	3.7602	7.8318	4.1164
Sorbs2	_VQS[Phospho (STY)]SPNLLAAGR_.2	Q3UTJ2_S27_M1_Sorbs2	3.4502	3.8688	4.4877	4.1340	6.9019	4.4512	3.3140	3.6759	3.3731	3.5949	3.7474	3.5950	3.4811	4.5257	2.8912	3.7334	3.1732	3.9763	4.2753	4.2030
Sorbs2	_SFISSSPSS[Phospho (STY)]PSR_.2	Q3UTJ2_S382_M1_Sorbs2	10.0206	9.6616	10.3776	3.6201	6.4204	6.4176	9.1390	9.9400	9.6143	9.9849	10.0050	9.6742	10.1211	10.3969	9.7658	9.4422	10.2729	5.5499	9.9340	9.9337
Sorbs2	_TS[Phospho (STY)]PGRADLPGSSSTFTK_.3	#N/A	3.9332	3.7985	5.7045	3.6972	3.7394	5.0645	5.0965	6.1721	3.9141	6.2950	3.3307	3.6375	6.9649	6.4890	5.8040	3.2457	5.6454	3.4885	5.1014	5.2735
Sorbs2	_APHYPGIGPVDESGIPTAIR_.3	#N/A	5.7956	5.4288	5.4589	5.6087	5.2818	5.4569	5.2909	6.1254	4.9847	5.5041	5.8394	6.1398	4.5052	5.8112	5.7749	5.7093	5.8119	5.6960	5.5615	5.8748
Sorbs2	_SC[Carbamidomethyl (C)]DDLNDDC[Carbamidomethyl (C)]DS[Phospho (STY)]FPDPK_.2	Q3UTJ2_S474_M1_Sorbs2	3.8033	7.9883	7.1896	4.0744	3.5489	7.3378	7.2378	4.1031	6.3579	4.3479	7.3367	3.8626	7.3416	8.7196	6.4837	4.4907	3.9589	7.7494	6.3387	5.4847
Sorbs2	_GLLHGWSQSS[Phospho (STY)]TEEVPR_.2	#N/A	6.9318	7.0579	4.1168	3.9683	7.1742	6.7062	6.8746	3.8596	3.6576	6.7867	3.7608	6.6971	6.8497	7.6001	6.1036	3.8024	8.1407	3.8377	6.7962	3.9054
Sorbs2	_QNAEIWSSTEETVS[Phospho (STY)]PK_.2	Q3UTJ2_S456_M1_Sorbs2	3.6163	6.8633	7.4329	3.6375	4.0972	6.1557	6.1909	4.0219	5.7692	7.4885	3.8653	3.4199	4.1372	7.3397	3.0003	3.4343	3.2802	6.3683	4.1882	3.9887
Sorbs2	_SRS[Phospho (STY)]C[Carbamidomethyl (C)]DDLNDDC[Carbamidomethyl (C)]DSFPDPK_.3	#N/A	4.1303	5.4079	3.4392	3.5077	3.9692	4.2171	4.8499	4.4030	4.1002	4.3697	2.9737	4.1240	4.2082	5.9472	4.3764	3.2578	3.9003	3.2502	3.5493	4.6635
Sorbs2	_GS[Phospho (STY)]LPDNSILHR_.2	#N/A	7.7225	7.6305	7.4628	3.3549	7.2599	7.3479	6.6984	7.3107	6.8864	7.7500	7.6656	6.9916	7.5977	7.8576	6.5721	3.1775	7.5665	3.2244	7.4881	7.6275
Sorbs2	_RFS[Phospho (STY)]IESLLEEETQVR_.3	Q3UTJ2_S630_M1_Sorbs2	4.4356	4.3843	3.9722	3.1486	3.1417	3.5328	4.2993	3.1604	4.3584	4.1103	4.7152	4.1104	4.4665	4.0102	3.8765	3.7977	4.1585	2.9909	3.2900	3.6875
Sorbs2	_RRGS[Phospho (STY)]LPDNSILHR_.4	#N/A	4.4548	4.3652	3.9913	3.1295	3.1609	3.1942	3.8872	5.3262	3.8075	4.2653	3.2861	4.0913	3.9729	4.0294	3.9126	3.8169	4.1777	4.5107	3.2708	4.4426
Cdk5	_LLDRS[Phospho (STY)]PSR_.2	Q3UTQ8_S306_M1_Cdk5	4.1836	9.6410	9.7273	9.7149	8.5182	8.8072	9.2603	9.1533	8.7324	9.8884	8.8786	8.9763	4.2043	9.0050	4.1440	9.0439	8.9072	9.0721	3.0601	8.9438
Cdk5	_SNS[Phospho (STY)]KDIQNLSVGLPR_.3	#N/A	3.9312	3.4318	5.0235	3.6969	3.4501	3.8208	5.8819	6.3168	3.6759	3.9977	4.4627	5.6252	3.8035	7.0704	3.7921	3.2933	3.6541	6.3639	3.8383	3.9959
Cdk5	_NLSEGNNANYTEY[Phospho (STY)]VATR_.2	Q3UTQ8_Y171_M1_Cdk5	3.7368	4.3947	4.1258	5.4916	3.5413	3.6519	4.0738	3.3648	4.4267	5.0825	3.9019	3.6547	4.0422	5.4809	3.6323	3.7815	4.1644	3.5683	3.6404	4.3405
Cdk5	_VSS[Phospho (STY)]LPSDSSSGTNHSK_.2	Q3UTQ8_S738_M1_Cdk5	3.4565	6.0397	6.2896	4.1277	3.6634	4.4449	5.4726	3.6822	3.3793	5.6708	3.7537	3.5887	3.4874	4.5320	2.8974	4.3194	3.1794	4.2437	4.2691	4.2093
Cdk5	_DLTNNNIPHLLS[Phospho (STY)]PK_.2	Q3UTQ8_S407_M1_Cdk5	4.0535	3.9493	4.5898	3.6359	3.3912	3.6153	4.1499	3.7539	4.2090	4.2598	3.0825	3.8388	4.3170	8.8595	3.7271	4.3984	4.2915	7.3995	3.1902	3.0430
Trappc14	_KSS[Phospho (STY)]PSSPAVR_.2	Q3UTZ3_S495_M1_Trappc14	8.6057	7.6482	7.6831	7.4314	7.5418	3.6726	7.8040	8.3580	7.9289	8.4119	7.9192	6.7758	4.0363	6.5397	7.7284	3.6140	8.0489	8.0933	7.3803	7.7938
Trappc14	_SQS[Phospho (STY)]FSHQQPSR_.2	Q3UTZ3_S517_M1_Trappc14	6.4008	6.8615	6.7460	6.5463	6.3227	6.3982	6.4190	6.2626	6.5001	6.7063	5.9882	4.0128	5.9086	6.4913	3.4316	6.0945	5.9295	6.1121	5.7967	5.9351
Ankrd34b	_VAS[Phospho (STY)]LQEELQDTPEEEIAYK_.2	Q3UUF8_S260_M1_Ankrd34b	6.0458	6.1768	6.4470	6.1557	5.3408	5.7330	5.8084	4.5763	5.6911	6.3534	5.6305	5.6921	5.9038	6.4743	5.1194	5.8050	5.9009	5.7288	5.7550	5.7784
Ankrd34b	_NSGANHY[Phospho (STY)]SSDSQLAEGVTPPTVEDGK_.3	Q3UUF8_Y365_M1_Ankrd34b	2.8686	4.4655	4.0736	4.1521	2.8750	3.6091	3.4724	3.2377	3.3926	3.1794	4.3721	4.3549	3.5580	4.0607	4.2432	3.8822	3.7754	4.0578	3.7064	4.0277
Ankrd34b	_HQS[Phospho (STY)]IDVK_.2	Q3UUF8_S293_M1_Ankrd34b	3.7688	4.7745	3.3054	4.8853	3.9758	4.1326	4.6720	3.9945	5.3829	6.0863	5.1398	3.2764	3.7997	3.3435	4.8393	3.1310	3.4918	3.6577	3.9337	3.0208
Ankrd34b	_RGS[Phospho (STY)]GAFPLDHSLAQSR_.3	Q3UUF8_S426_M1_Ankrd34b	3.4654	3.4901	3.3656	3.7552	4.0360	4.0693	3.0121	4.0547	4.4332	3.6396	3.9118	3.2162	4.5986	3.4037	3.0375	3.1911	3.5519	0.7807	3.8966	3.5674
Tbc1d24	_SVS[Phospho (STY)]LSKR_.2	Q3UUG6_S324_M1_Tbc1d24	9.2208	9.1034	8.6837	8.8714	8.3245	8.5499	8.9692	9.2833	8.6543	8.8625	8.5322	8.8680	9.0266	8.6304	3.5020	3.1226	8.8656	8.9600	8.4997	8.4017
Tbc1d24	_QKS[Phospho (STY)]VSLSK_.2	Q3UUG6_S322_M1_Tbc1d24	6.4346	6.2203	3.8301	6.7369	5.8731	3.8525	6.8677	6.0522	3.1491	3.4229	4.1286	3.0976	5.9124	6.1832	3.2543	3.6387	5.5708	5.6839	6.0949	5.1802
Ksr2	_S[Phospho (STY)]KSHEFQLGNR_.3	Q3UVC0_S312_M1_Ksr2	3.8223	4.1805	4.3586	5.7102	3.1600	3.3841	4.3810	3.5227	4.4401	4.0286	3.3137	4.0699	4.5482	4.0919	3.9582	4.1672	4.0604	4.4101	3.2083	3.7692
Ksr2	_TES[Phospho (STY)]VPC[Carbamidomethyl (C)]DINNPVR_.2	Q3UVC0_S484_M1_Ksr2	3.1226	3.6029	3.6589	3.8680	3.9611	4.1852	3.5799	3.9419	3.6390	3.3289	4.0134	3.2688	3.7471	3.2908	3.1571	7.8420	3.4391	3.7103	7.4975	2.9681
Ksr2	_TPNVTTVT[Phospho (STY)]PPGT[Phospho (STY)]PPMR_.2	Q3UVC0_T273_M2_Ksr2	3.1638	5.7463	3.7001	4.5256	4.0024	4.2264	3.5387	3.9007	3.5978	3.3701	3.9722	3.2276	3.7059	3.2496	3.1159	3.5087	3.4019	3.7515	4.0506	4.4277
Fam91a1	_KLS[Phospho (STY)]EASDER_.2	Q3UVG3_S671_M1_Fam91a1	6.6034	6.1843	5.8910	3.7226	5.8935	6.5792	6.0172	5.6913	5.9014	5.9335	6.8032	5.8236	6.0287	6.6946	6.1018	6.1950	5.9689	6.2792	5.8702	6.0688
Vps51	_[Acetyl (Protein N-term)]AAAAAVGPGLGSGPGDS[Phospho (STY)]PEGPEADAPER_.3	#N/A	4.4207	11.7786	11.5241	9.9068	3.3699	11.5892	11.6342	4.0614	11.5132	10.2360	3.6876	10.4333	3.9988	11.7072	10.1852	10.2266	7.6293	11.8482	11.3608	10.7872
Vps51	_[Acetyl (Protein N-term)]AAAAAVGPGLGSGPGDS[Phospho (STY)]PEGPEADAPERR_.3	#N/A	9.9782	11.7890	11.5148	10.7761	3.4067	11.4910	11.5957	8.1897	11.3591	10.5992	3.0573	10.8637	10.4908	11.5648	3.6924	10.4887	11.0374	11.7043	11.3392	10.9004
Vps51	_IDVYS[Phospho (STY)]PVEFNK_.2	Q3UVL4_S690_M1_Vps51	3.7443	3.5748	3.2809	3.8399	3.9513	4.6196	4.4740	3.9700	3.6671	3.3008	4.0415	3.3009	3.7752	3.3190	3.7874	3.1064	3.4672	5.4439	4.7512	2.9963
Ttbk2	_SIHS[Phospho (STY)]FELEKR_.3	Q3UVR3_S448_M1_Ttbk2	4.0460	4.1942	3.6689	4.7303	3.3838	3.7185	4.1756	3.8945	4.2347	4.2524	4.1326	3.8996	4.3428	3.8865	3.7528	3.7479	4.0348	3.9907	4.3420	4.6228
Grm5	_RGS[Phospho (STY)]SGETLR_.2	#N/A	3.1041	5.8339	4.1415	5.4095	3.3110	3.3443	6.1194	6.2318	5.8445	4.4155	5.7885	3.9411	6.1024	5.3744	3.7625	3.9670	4.3278	5.8882	5.3096	3.8568
Grm5	_TDDDAPS[Phospho (STY)]LHS[Phospho (STY)]ETAAR_.2	Q3UVX5_S1037_M2_Grm5	7.2960	4.5746	3.9645	7.2274	5.6516	6.1373	3.2743	3.1286	6.0130	7.0040	5.7784	2.9632	3.4415	6.3624	2.8515	3.7731	3.6663	6.0372	5.7499	3.8291
Grm5	_SVTWAQNEKST[Phospho (STY)]R_.2	Q3UVX5_T923_M1_Grm5	4.4400	4.3800	3.9765	3.1443	3.1461	3.1794	3.9020	3.1648	3.8222	4.2506	3.3009	4.1061	3.9877	4.4903	3.9274	3.8021	4.1629	3.6281	3.2767	2.9565
Grm5	_DSVDSGTTPNS[Phospho (STY)]PVSESALC[Carbamidomethyl (C)]IPSSPK_.3	Q3UVX5_S1173_M1_Grm5	6.4799	3.6167	3.2389	3.8819	3.9093	4.7604	5.4206	3.9280	3.0590	3.5129	4.0385	3.3429	3.7332	3.2770	3.1642	3.0645	6.1444	6.0256	4.0232	2.9543
Grm5	_SSS[Phospho (STY)]LVNLWK_.2	Q3UVX5_S860_M1_Grm5	3.6461	7.8498	7.1984	7.5735	2.9838	3.2079	7.4200	3.3464	3.3156	3.8524	3.4899	4.2462	3.2236	7.0710	4.1345	3.9909	3.8841	7.5692	3.0320	3.9455
Grm5	_S[Phospho (STY)]PS[Phospho (STY)]PISTLSHLAGSAGR_.2	Q3UVX5_S1014_M2_Grm5	4.0424	7.3635	4.5787	3.6470	3.3801	3.1040	4.1609	3.7428	3.8977	4.2487	3.3763	3.8498	4.0631	6.1389	3.7381	4.3873	4.2805	7.0521	3.2013	3.0319
Grm5	_GS[Phospho (STY)]SGETLR_.2	#N/A	3.9884	3.5843	3.4540	3.2709	4.1954	4.2287	4.3536	4.1189	4.2738	3.7990	3.7524	3.4737	4.4392	3.4410	2.8781	3.3505	3.1557	3.1766	4.3260	3.4080
Tubal3	_SFGGGTGSGFTSLLM[Oxidation (M)]ER_.2	#N/A	6.5040	6.4641	6.5380	6.9050	6.2348	6.7761	6.3582	6.6765	6.3359	7.0225	6.1145	5.7005	5.4650	5.7242	5.2470	5.8339	5.9958	5.3771	5.8467	5.7554
Tubal3	_AVC[Carbamidomethyl (C)]M[Oxidation (M)]LSNTTAIVEAWAR_.2	#N/A	5.1444	6.0646	6.4342	5.9199	5.6132	5.4643	2.9966	8.2277	3.0557	3.9122	3.4301	7.6047	3.7266	4.2292	4.0747	4.0508	3.9439	5.1232	6.6160	5.6692
Fam81a	_IKDDIVSS[Phospho (STY)]LQK_.2	Q3UX26_S58_M1_Fam81a	3.9295	5.9788	5.0634	3.8551	4.0189	5.9351	5.7448	3.6299	5.4579	4.1358	3.6078	4.0580	5.3269	3.7037	3.9463	5.6429	6.5323	3.7609	5.0596	3.7233
Ppm1h	_GGVGAPGS[Phospho (STY)]PSTPPTR_.2	Q3UYC0_S220_M1_Ppm1h	10.9326	10.6072	10.6993	4.2215	3.8423	9.3164	10.1195	10.0310	9.5705	10.9507	10.0139	9.9918	10.0328	4.4109	3.3396	9.7154	3.9666	9.7559	9.6007	9.7806
Ppm1h	_SS[Phospho (STY)]LPNGEGLQLK_.2	#N/A	3.4566	4.2411	9.0884	8.7786	8.3324	8.4871	3.3178	3.4407	3.3050	3.4807	8.7843	4.0692	8.0920	8.6414	7.1208	4.0810	3.4626	8.0909	8.2110	4.1575
Ppm1h	_AGTITST[Phospho (STY)]PNR_.2	#N/A	3.1187	8.9809	8.2722	8.8321	7.7763	3.3590	8.3348	3.3444	3.6427	8.6152	8.0109	8.0463	3.1495	8.6704	3.7479	7.9018	8.1354	8.1653	3.1061	8.0827
Ppm1h	_KAGTITST[Phospho (STY)]PNR_.2	#N/A	7.3496	3.6041	3.4342	7.0354	4.2151	4.2485	4.3338	4.0991	4.2540	3.8188	3.7326	3.0370	4.4194	3.4213	2.8583	3.3703	3.1359	3.1964	4.3458	3.3883
Ppm1h	_AGTITSTPNRNS[Phospho (STY)]K_.2	Q3UYC0_S118_M1_Ppm1h	6.3531	6.5266	6.2149	6.4169	4.3190	3.0422	5.8684	3.1808	5.5252	6.4043	5.6674	4.4119	3.3893	4.4339	2.7993	5.0023	3.7185	4.0681	4.3672	4.1111
Ppm1h	_RSS[Phospho (STY)]LPNGEGLQLK_.2	#N/A	8.7532	8.2331	7.9611	3.6456	4.1456	7.7650	7.6676	8.1483	7.2200	8.1180	3.8022	7.4943	8.2730	8.2313	6.7871	7.6115	3.6616	7.6091	7.7084	7.8114
Macrod2	_GLS[Phospho (STY)]PPHKK_.2	#N/A	10.6906	10.8299	10.2279	10.5275	10.3411	10.5106	10.2468	10.5988	10.2815	10.5670	10.4256	10.5087	10.9900	10.8943	10.5342	10.7241	10.7757	10.6873	10.5617	10.5953
Macrod2	_GLS[Phospho (STY)]PPHK_.2	#N/A	8.8528	8.6647	8.2890	8.7210	8.6169	8.8154	8.6420	8.5243	8.5674	8.7600	8.8950	8.2984	8.2718	7.4790	8.4859	8.5486	8.0007	8.5022	8.5654	8.9292
Rgl3	_VIEPPAASC[Carbamidomethyl (C)]PSS[Phospho (STY)]PR_.2	Q3UYI5_S510_M1_Rgl3	3.7573	4.2817	4.2936	3.9683	3.9196	3.3191	9.3998	3.4577	3.8651	3.9636	3.4149	4.1711	3.9731	4.2807	4.0594	4.1022	7.2993	3.6688	3.9426	3.8704
Lrrfip1	_RGS[Phospho (STY)]GDTSISM[Oxidation (M)]DTEASIR_.3	Q3UZ39_S88_M1_Lrrfip1	3.7254	3.5937	3.2620	3.8588	3.9323	3.9657	3.2686	3.9511	3.0360	3.5360	4.0154	3.3198	3.2014	3.3001	3.1411	4.4024	3.4483	3.7011	4.0002	3.6711
Rcsd1	_S[Phospho (STY)]PDANMPEEEGVVR_.2	#N/A	3.8559	4.1469	6.3720	6.6992	3.1936	4.9078	4.3475	3.5562	3.7111	5.2888	5.8303	4.0364	3.8766	6.7784	4.6849	4.2008	4.0939	7.3392	3.3878	4.9110
Rcsd1	_LGQKS[Phospho (STY)]PDANMPEEEGVVR_.3	#N/A	3.5273	4.7310	5.1899	5.4847	3.2975	3.6979	4.6661	3.3256	3.2689	3.5793	3.1954	4.2160	3.4106	5.0617	3.8323	4.1143	3.9165	4.1587	3.3935	3.8978
Rcsd1	_S[Phospho (STY)]KDPGSPQLNQEAMADGVEGTPWSAEKPR_.4	Q3UZA1_S211_M1_Rcsd1	3.8957	3.9319	3.8409	3.6310	3.5457	3.8735	3.7637	3.8233	3.9782	3.9159	3.4568	3.7254	4.1436	3.9310	3.5756	3.6601	3.6456	3.5367	3.9708	3.8980
Garnl3	_SFSDVLPES[Phospho (STY)]PK_.2	Q3V0G7_S455_M1_Garnl3	8.3804	8.8906	8.8467	8.4493	6.9929	8.6361	8.0103	7.5264	8.1557	8.1728	7.5755	8.0628	7.7559	8.8206	7.6908	8.1543	8.7278	8.4897	8.3357	8.1703
Garnl3	_IC[Carbamidomethyl (C)]LPYS[Phospho (STY)]PTK_.2	Q3V0G7_S179_M1_Garnl3	4.1049	3.2142	6.3624	3.4794	4.3118	4.3451	4.2371	4.3305	4.1573	3.9155	3.6360	2.9403	4.3228	3.6795	4.2625	3.4670	3.8278	5.4934	5.5453	3.2916
Garnl3	_GASAHTS[Phospho (STY)]PQT[Phospho (STY)]PPAR_.2	Q3V0G7_S848_M2_Garnl3	3.8391	7.1502	7.4255	4.5846	7.0008	7.2774	4.3642	7.5193	7.5911	4.0454	3.2969	7.2894	4.5314	8.0019	3.9414	4.1840	7.9038	7.5773	3.2251	7.5524
Prr7	_ALELEPLELEGSLAGS[Phospho (STY)]PPGLAPPPPPHR_.3	Q3V0I2_S68_M1_Prr7	3.6846	3.6646	4.2209	4.0890	3.7855	4.0095	3.8652	3.7333	3.9243	3.8910	3.5356	4.5978	3.8119	5.8092	3.4424	4.0295	3.8288	3.7478	3.7883	3.7862
Nt5c2	_NRTS[Phospho (STY)]VDFK_.2	Q3V1L4_S511_M1_Nt5c2	3.8458	4.7607	3.5291	4.0693	3.8314	4.0554	4.9089	4.1940	5.5387	4.0522	5.1052	3.4191	3.8974	3.5162	3.3074	3.3377	4.0839	3.9750	4.2714	3.9715
Pacs2	_STS[Phospho (STY)]LKER_.2	Q3V3Q7_S408_M1_Pacs2	3.6561	9.4167	9.1765	9.1908	9.0167	9.2983	9.2455	3.4084	3.1055	9.2974	3.4799	9.1506	9.6263	9.2895	4.1244	8.9732	9.1801	9.2846	9.1665	9.3954
Pacs2	_TES[Phospho (STY)]LVIPSTR_.2	Q3V3Q7_S387_M1_Pacs2	7.0302	6.4262	4.1282	6.6723	6.4777	7.1866	6.4261	6.4490	3.0136	7.3336	6.3206	3.9544	3.1217	4.1663	4.9192	6.2931	6.1756	4.3357	3.1339	6.1871
Pacs2	_ANS[Phospho (STY)]LDNER_.2	#N/A	3.2866	7.5103	6.9574	6.3469	5.7376	7.3562	7.2873	6.9533	6.6002	7.5132	6.4901	6.1805	3.3073	7.4827	6.2033	5.9191	7.1987	6.8114	7.0206	7.0636
Pacs2	_ANS[Phospho (STY)]LDNERC[Carbamidomethyl (C)]PDTR_.2	#N/A	5.1268	5.4627	3.9756	4.7889	4.7346	3.8219	5.2931	5.1092	5.1753	3.3922	4.4839	4.7177	4.9790	5.4377	4.1857	3.8012	5.8679	5.0637	4.9836	3.8333
Pacs2	_EPSGQPEDS[Phospho (STY)]PEAETSTLDVFTEK_.3	Q3V3Q7_S361_M1_Pacs2	4.1315	6.4992	3.6681	3.4527	4.3384	3.7699	3.9952	4.3572	4.0543	4.4144	2.9279	4.4145	4.1624	3.7062	4.2359	3.4936	3.8544	6.3644	3.5941	3.3834
Carmil2	_LQRS[Phospho (STY)]PVLK_.2	Q3V3V9_S1242_M1_Carmil2	5.1492	4.8249	4.2029	4.5431	5.3886	4.9390	4.5749	5.1565	5.0690	5.0095	5.2760	6.1945	4.9570	6.0607	4.9603	5.4657	5.3411	5.2804	5.2302	5.3702
Carmil2	_GPRPDLETS[Phospho (STY)]PGAAAR_.2	#N/A	7.0700	6.8817	7.1301	3.6578	3.8666	4.0486	5.0284	4.0032	4.2257	4.0000	3.0092	3.5799	7.2951	3.8775	4.0946	4.6751	3.9764	3.9952	6.3160	3.5548
Carmil2	_AGS[Phospho (STY)]DGDIM[Oxidation (M)]DSSTETPPISK_.2	#N/A	8.7717	9.5144	9.4710	8.5863	5.8552	9.1220	8.9794	7.1983	9.1894	9.0925	6.4029	8.9258	8.4574	9.8231	8.7244	9.1031	9.1635	9.1715	8.8995	9.2217
Carmil2	_KAGS[Phospho (STY)]DGDIM[Oxidation (M)]DSSTETPPISK_.2	#N/A	6.0372	7.0767	5.2499	6.8581	3.9463	6.6343	7.1889	5.3480	6.4191	6.3513	6.1932	6.8538	6.4720	7.5554	6.5374	7.3158	5.9379	6.8508	6.9573	7.1222
Carmil2	_PLPPYPTEPSSPERSPPS[Phospho (STY)]PAT[Phospho (STY)]DQR_.3	Q3V3V9_S1284_M2_Carmil2	3.2751	4.0440	4.3125	4.3091	3.4821	3.1255	3.1389	3.5008	3.1979	3.7700	3.5723	3.7701	3.3060	4.3506	4.2169	4.1381	4.4989	4.1514	2.9496	4.0279
Vezt	_TEYVC[Carbamidomethyl (C)]DS[Phospho (STY)]PTEGPSK_.2	Q3ZK22_S667_M1_Vezt	3.6897	5.7223	5.7857	5.9650	3.8967	6.0393	3.4671	4.2900	3.4678	5.3327	5.7812	5.3881	3.5996	3.6233	3.8095	4.8923	6.1737	3.9912	3.9304	3.4764
Bahd1	_DLS[Phospho (STY)]PELAPDEGAR_.2	Q497V6_S182_M1_Bahd1	4.0648	6.8402	3.6014	6.3873	4.2718	6.3951	6.5232	4.2905	6.5079	6.7454	3.6760	6.3319	4.3628	7.4732	6.3652	6.4062	3.7877	7.1260	6.7531	3.3316
Shank3	_AHS[Phospho (STY)]PQGEGEIPLHR_.3	#N/A	10.6531	10.6313	10.3400	10.6345	10.2811	10.4250	10.1039	10.5619	10.2561	10.5841	10.5556	10.3958	11.1095	11.0313	10.0593	10.5172	10.8409	10.4824	10.4761	10.5845
Shank3	_S[Phospho (STY)]LGEEPVGGLGSLLDPAK_.2	#N/A	10.3665	10.5215	10.6544	10.1422	9.4817	9.5780	10.0774	9.6169	9.7773	10.4591	10.0030	9.9509	10.5444	10.4347	9.0975	9.8738	9.6595	10.0443	9.8699	9.7120
Shank3	_S[Phospho (STY)]KLWGDPVESR_.2	Q4ACU6_S1471_M1_Shank3	9.6451	10.0454	10.1632	10.1560	10.0311	9.9180	9.5321	10.0250	9.7148	9.7582	10.0562	9.2367	10.8000	10.3405	9.5090	10.1738	10.0778	10.1193	10.3136	10.0741
Shank3	_STS[Phospho (STY)]M[Oxidation (M)]QDTVR_.2	#N/A	3.6663	8.0746	9.4682	10.1430	6.7082	7.1690	7.7225	9.0597	6.6648	7.0893	7.7749	7.2808	8.3514	7.8592	3.0817	3.4453	8.7633	3.8475	3.9096	9.2886
Shank3	_S[Phospho (STY)]M[Oxidation (M)]TAELEELASIR_.2	#N/A	5.8235	5.1702	7.0343	6.7947	4.0561	5.3454	5.9537	3.7437	5.9801	5.9575	5.7539	5.1774	5.6524	5.6318	5.1782	5.6776	4.2814	3.6085	5.9287	5.9033
Shank3	_SAS[Phospho (STY)]DINLKGDQPAASPGPTLR_.3	#N/A	9.0158	9.5022	9.1678	4.4453	3.1857	8.3336	8.9354	8.6054	8.3359	8.8523	3.2060	8.2898	8.5433	9.5587	7.8234	8.4175	8.0956	9.0141	8.8333	8.6655
Shank3	_RAPSPVKPAS[Phospho (STY)]LER_.3	Q4ACU6_S1583_M1_Shank3	4.1419	6.8120	3.6785	3.4423	4.3489	6.2801	6.4041	4.3676	4.1203	3.9525	3.5989	4.4041	4.2857	3.2875	4.2255	3.5040	3.8648	6.5928	2.9787	3.2545
Shank3	_S[Phospho (STY)]KSM[Oxidation (M)]TAELEELASIR_.3	#N/A	3.9533	3.8657	5.5762	3.7674	3.5532	3.9810	3.8768	4.7925	3.9359	4.1287	3.8421	5.6510	4.0439	5.7185	4.7634	5.8280	4.0830	6.0488	3.6898	3.7725
Shank3	_TKS[Phospho (STY)]VGEDKLASLLEGR_.3	#N/A	10.2859	10.3639	10.3829	10.3565	3.0892	9.3572	9.6214	9.5160	9.5139	10.3738	3.2281	9.5667	9.8983	10.4129	8.8689	9.7114	9.6341	9.7305	9.7397	9.8519
Shank3	_FM[Oxidation (M)]DY[Phospho (STY)]VQLHSTDK_.2	#N/A	6.0302	6.2946	6.4338	6.6102	5.8955	5.8340	5.9083	5.6929	5.4066	6.2794	6.0520	5.9455	6.6283	7.1096	5.4873	6.3527	6.7058	6.5246	6.2264	6.5312
Shank3	_LFSSLGELSTS[Phospho (STY)]AQR_.2	#N/A	5.0545	4.7838	4.6157	5.1421	4.5634	5.1751	5.0797	3.2082	3.3631	3.9025	5.0026	4.9442	3.5285	4.9818	5.2753	5.5154	3.7459	3.8816	5.4199	4.4767
Shank3	_S[Phospho (STY)]LGEEPVGGLGSLLDPAKK_.2	#N/A	3.6617	7.2935	6.5112	3.9226	3.8686	3.9019	3.1795	3.8874	3.0997	6.4664	4.0792	3.3835	3.2651	3.7678	3.2049	3.0238	3.3846	4.3507	5.8224	3.7348
Shank3	_APS[Phospho (STY)]TTLTLR_.2	Q4ACU6_S685_M1_Shank3	4.5172	4.3028	4.0537	4.5679	3.1602	3.3843	4.3809	5.2663	4.4400	4.0288	3.3135	4.0289	4.5480	4.0918	3.9581	4.1674	4.2401	4.4102	3.2084	3.7691
Shank3	_HYTVGSY[Phospho (STY)]DSLTSHSDYVIDDK_.3	Q4ACU6_Y555_M1_Shank3	4.0922	3.6880	3.3502	3.7746	4.2991	4.3324	4.2499	4.0152	4.1701	3.4663	3.6487	3.5775	4.3355	2.0186	4.2752	3.4543	4.5528	1.5535	4.4297	3.3043
Shank3	_SAS[Phospho (STY)]DINLK_.2	#N/A	3.3774	3.1246	3.9137	4.3120	4.2159	3.7690	4.4541	3.0777	3.2326	3.3393	4.2121	3.0140	3.3981	3.9007	3.3378	3.7223	3.6154	4.2177	3.8663	3.8677
Shank3	_LDEILAVAAEPTLRPDIADADSR_.3	#N/A	3.8620	4.1408	4.3983	3.8274	3.1997	3.5360	4.3414	3.5623	4.4005	4.0683	3.2740	4.0303	4.5085	4.3853	4.2934	4.2069	4.1000	3.8117	3.3817	4.3523
Shank3	_GPRPGSLDY[Phospho (STY)]SSGEGLGLTFGGPSPGPVK_.3	Q4ACU6_Y998_M1_Shank3	4.2576	6.0157	3.2931	3.4318	3.5953	3.8194	3.9458	3.9580	4.0048	4.4639	4.3792	3.6347	4.2783	5.4524	3.5230	3.1017	4.4957	3.3375	2.9861	3.2471
Shank3	_SPTPVHS[Phospho (STY)]PDADRPGPLFVDVQTR_.3	Q4ACU6_S1134_M1_Shank3	4.3641	4.1261	3.3996	3.3253	3.7018	4.2831	3.8392	4.0645	4.2194	3.8534	3.6980	4.6055	4.3848	5.3615	3.4164	3.2082	3.1013	3.2310	4.3804	3.3537
Prpf38a	_VPS[Phospho (STY)]PDHR_.2	Q4FK66_S209_M1_Prpf38a	8.9703	8.8827	8.6443	8.8900	8.5316	8.8932	8.6952	8.7194	8.5418	8.6558	8.6500	8.3218	8.8631	8.2993	7.0321	8.2445	8.9060	8.6168	8.5580	8.3187
Fam189a2	_SKS[Phospho (STY)]DPVLHHSEER_.3	Q4FZH1_S114_M1_Fam189a2	4.1855	5.9631	5.4090	3.3987	3.8100	4.4258	4.1565	5.0898	4.0767	3.9961	5.2277	5.2259	4.2422	3.7601	4.1819	3.5476	5.5974	5.5967	3.5401	3.2110
Abi2	_DKS[Phospho (STY)]PSSLLEDAK_.2	Q4JIM5_S632_M1_Abi2	4.2633	8.4335	3.7998	8.6223	2.9694	3.0027	7.5800	3.8440	7.6227	4.0739	3.4776	4.2828	4.1644	3.1662	4.1041	3.6254	4.3817	7.6211	7.1118	7.4001
Abi2	_STQASSGS[Phospho (STY)]PALPR_.2	Q4JIM5_S621_M1_Abi2	8.8041	8.3388	8.6626	8.8423	8.4319	8.0596	7.9170	8.2188	7.8950	8.7145	8.0352	8.0425	4.0160	7.7277	3.9557	7.9052	8.3244	8.0015	7.9774	8.2283
Abi2	_S[Phospho (STY)]NSTSSM[Oxidation (M)]SSGLPEQDR_.2	Q4JIM5_S783_M1_Abi2	4.0714	6.6742	7.6357	3.5938	4.5866	3.8267	4.1820	3.9870	4.0632	4.1544	6.1709	4.0579	4.2070	4.3146	3.9195	4.1284	4.1414	3.3213	3.3422	3.3652
Abi2	_TVS[Phospho (STY)]TSSQPEENVDR_.2	Q4JIM5_S819_M1_Abi2	8.4429	4.4919	8.5359	4.1785	7.5374	3.0727	3.1916	7.5903	7.2491	3.7172	3.6251	4.3813	3.3587	7.2977	4.2696	3.8558	3.7490	7.0056	3.7328	6.9183
Abracl	_[Acetyl (Protein N-term)]M[Oxidation (M)]NVEHEVNLLVEEIHR_.3	#N/A	4.9694	5.6297	6.2776	5.6722	5.9328	5.2304	5.0172	5.3249	5.5230	6.3781	5.5603	5.8121	5.4480	4.9608	5.1858	5.7517	5.5625	5.2909	5.7318	5.4372
Oxr1	_SQS[Phospho (STY)]VDITAPGFNPLGGAGK_.2	Q4KMM3_S15_M1_Oxr1	7.9121	8.7150	7.9743	7.1514	4.0705	6.6961	8.2351	7.9776	4.9252	7.4981	6.6849	7.2509	7.9524	9.0732	6.1889	6.8813	6.7808	7.8657	7.4311	6.2377
Oxr1	_ESLPIELDQLS[Phospho (STY)]GR_.2	#N/A	7.9405	8.3610	8.7621	8.3601	7.2444	8.2102	8.0576	6.9448	8.3547	8.1284	7.8398	7.4994	7.8522	8.3053	7.4954	7.6873	8.2323	7.9233	8.1777	7.7969
Oxr1	_DTIQQVSQR_.2	#N/A	6.8593	7.0736	7.3622	7.2671	7.3126	7.2535	6.6736	7.1245	7.3582	6.2995	7.2174	7.4314	7.5132	7.7898	7.5399	6.7930	7.0709	7.0598	6.9757	7.2660
Oxr1	_GTIEYTVES[Phospho (STY)]R_.2	Q4KMM3_S104_M1_Oxr1	8.3484	8.1678	8.4046	8.2276	7.6880	8.1933	7.9815	8.1638	7.9977	8.2748	8.2756	7.9500	8.2047	8.1800	7.5134	8.0181	8.6743	8.0882	8.3307	8.1830
Oxr1	_TSSNPGALSHETGLSGLETATK_.3	#N/A	5.7776	5.5602	5.4472	5.3966	5.4775	5.6148	4.9264	5.6403	5.2945	5.6032	5.5740	5.4807	6.0315	5.6363	5.5070	5.3751	5.7714	5.1505	5.0034	4.9190
Oxr1	_DSETEVEELRK_.2	#N/A	5.2623	5.5022	5.8273	3.4523	5.8855	5.2661	5.7444	5.7064	6.1429	5.9682	5.3811	5.5702	5.4216	6.2332	5.6783	5.8721	6.2970	5.4821	5.6801	5.8757
Oxr1	_DQGNDS[Phospho (STY)]ASTAPR_.2	#N/A	4.1196	4.2051	5.5311	4.4544	3.7924	6.3208	4.4096	4.4651	4.5775	4.6570	5.3476	5.5680	5.5286	4.6764	4.7851	4.1944	4.8523	4.0221	5.4314	4.2578
Oxr1	_DQQGQELKR_.2	#N/A	4.3619	4.8210	3.4414	5.2492	4.5569	5.2179	3.9424	4.4505	4.7632	4.7857	5.1319	4.5815	4.8908	4.6099	5.4647	4.5420	4.4464	4.0213	5.2444	5.1165
Oxr1	_DQQGQELKRDS[Phospho (STY)]ETEVEELR_.3	#N/A	3.4889	4.5139	4.0252	4.2005	4.3275	3.6575	3.2136	3.1893	3.3442	3.2278	4.3237	5.7827	5.9098	4.0123	2.7908	3.8338	5.7508	4.1062	3.7548	3.9793
Oxr1	_RM[Oxidation (M)]S[Phospho (STY)]FQKPK_.2	#N/A	6.2411	7.4001	7.4231	7.5302	6.5710	7.0362	7.0533	6.5738	6.4376	6.6937	7.0293	6.0433	6.1498	7.6260	5.9085	6.5151	6.6628	6.2027	6.2306	6.3305
Oxr1	_VVSSTS[Phospho (STY)]EEEAFTEK_.2	Q4KMM3_S204_M1_Oxr1	11.6669	12.2252	12.4346	12.2762	9.1370	11.9020	11.8289	10.9596	11.8206	12.2592	10.7136	12.0031	12.1623	12.3612	11.6456	12.0267	12.3487	11.8945	12.1303	12.1091
Oxr1	_DSDGQVFGALASEPFK_.2	#N/A	7.1176	6.1744	6.5588	6.2325	6.8202	5.9520	6.1938	6.6646	3.3596	6.5978	6.7101	6.2709	7.1124	6.6148	5.7738	4.2647	6.7195	6.5433	6.0523	6.2555
Oxr1	_TPIEEEQNNSANTQK_.2	#N/A	5.8155	5.7155	6.5253	6.0820	6.1365	6.1338	6.3492	6.8006	6.1735	6.3356	6.1284	5.8809	6.1689	6.8627	6.2759	6.2866	6.5817	6.3059	5.9344	5.9830
Oxr1	_FDTTPNELVQLNK_.2	#N/A	6.1600	6.1960	5.6230	6.1958	6.3112	5.8595	6.3163	6.3772	5.8061	5.9851	6.1966	6.3305	6.2055	6.6653	5.8906	6.2403	6.4547	5.8739	6.1185	6.3148
Oxr1	_KATGVS[Phospho (STY)]AEDADPR_.2	Q4KMM3_S308_M1_Oxr1	4.5159	4.3040	4.0525	4.5691	3.2220	3.2254	3.8261	3.5914	3.7463	6.6554	6.1316	4.0301	3.9117	4.4144	3.8515	3.8780	4.1291	3.7041	3.3527	3.4814
Oxr1	_RDS[Phospho (STY)]ETEVEELRK_.3	#N/A	3.9611	6.5593	3.8395	6.3664	3.2988	6.4655	6.2648	3.8389	6.0300	4.1674	6.5781	3.8417	3.8332	6.6388	3.9218	3.5498	6.4142	3.7683	3.7015	3.6987
Oxr1	_ADLEPESFRPNLSDPSELLPDQIEK_.3	#N/A	5.0638	4.9019	3.6829	4.5428	3.9664	4.3948	4.4678	3.4378	4.6863	4.3772	4.5091	4.9503	3.1673	4.3802	4.8262	4.4478	4.7874	4.5878	4.3683	3.6370
Oxr1	_M[Oxidation (M)]S[Phospho (STY)]FQKPK_.2	#N/A	3.4688	4.3454	3.8890	4.0726	3.6863	3.0104	3.2101	4.0924	3.4809	4.3459	3.3586	3.3492	3.5098	3.8511	3.4719	4.0709	3.3425	3.9346	3.7402	3.7346
Oxr1	_IKESLPIELDQLSGR_.3	#N/A	5.1267	5.4980	5.1843	5.8685	5.2468	5.1309	5.1515	5.0765	4.8918	5.5042	5.3346	5.2290	5.2849	5.8391	5.1712	5.4981	5.0849	5.6741	5.0508	5.0002
Oxr1	_DSLNSIALK_.2	#N/A	3.8305	3.4886	3.6893	3.7538	3.2430	3.3387	4.8149	4.0561	3.7533	3.8649	3.8794	4.0389	3.8613	4.5663	4.1411	4.5998	3.5534	3.5961	3.8951	3.4373
Oxr1	_KS[Phospho (STY)]QSVDITAPGFNPLGGAGK_.3	#N/A	4.5131	4.3069	4.0496	4.5720	3.2192	3.2525	3.8289	3.5942	3.7491	4.3237	3.2278	4.0330	3.9146	4.4172	3.8543	3.8752	4.1319	3.7012	3.3498	3.3842
Oxr1	_TFVSQASATM[Oxidation (M)]QDYAQR_.2	#N/A	4.8639	4.3553	4.8280	4.7404	3.9818	4.3864	4.2452	4.1439	4.1937	4.3100	4.0400	3.3309	4.3308	3.4810	4.2082	3.7424	4.1984	3.7909	4.6328	4.3594
Oxr1	_SSDASSESVQTVSQMEVQSLTATSEAANVPDR_.3	#N/A	4.0085	5.5876	6.6812	5.6190	3.1046	5.0318	4.9955	3.7089	3.8638	4.2090	5.1172	4.7569	4.0292	7.8640	3.4026	3.7606	5.6138	3.5866	3.8261	2.9980
Oxr1	_ARDQGNDS[Phospho (STY)]ASTAPR_.2	#N/A	3.1327	3.3693	3.6690	3.8781	3.9712	4.1953	3.5699	3.9318	3.6289	3.3390	4.0033	3.2588	3.7370	3.2808	3.1471	3.4776	3.4290	3.7204	4.0195	2.9581
Oxr1	_HEYWFAVPQER_.2	#N/A	4.3981	4.4218	3.9347	3.1861	3.1042	4.2299	3.9439	3.1230	3.8641	4.2087	3.3427	4.1479	4.0295	3.9728	3.9692	3.7603	4.6059	4.5421	3.2349	2.9983
Unc13a	_ALS[Phospho (STY)]PTGSSR_.2	Q4KUS2_S255_M1_Unc13a	12.3698	12.3665	12.1586	12.2707	12.0447	12.3018	12.3201	12.4005	12.3150	11.8674	12.0203	12.0856	12.6045	12.6026	11.1935	11.8851	12.4109	12.4179	12.0271	12.0419
Unc13a	_ESYSDSM[Oxidation (M)]HS[Phospho (STY)]YEEFSEPR_.3	#N/A	3.9100	3.7835	3.8199	3.6336	3.9098	4.4390	4.3917	3.9243	3.9634	3.8492	3.6433	3.5217	4.1190	3.7675	3.3781	3.6378	3.4822	3.4422	4.1021	3.7345
Trp53i11	_HS[Phospho (STY)]QTDLVSR_.2	#N/A	7.5205	7.6578	7.1970	6.9353	6.4724	7.4612	7.5083	7.2144	7.1334	7.2301	7.4012	7.2934	7.4947	6.8717	6.2445	6.9454	7.4193	7.2438	6.8452	6.7120
Trp53i11	_KHS[Phospho (STY)]QTDLVSR_.2	#N/A	10.4339	10.3574	9.7949	10.0152	9.7319	9.8807	9.9670	10.2727	9.8523	9.8102	9.8596	9.9393	10.5125	10.0724	8.7415	9.6720	10.2746	10.4827	9.8147	9.7512
Pds5b	_APS[Phospho (STY)]PSQPPKK_.2	Q4VA53_S1381_M1_Pds5b	9.8429	9.0292	9.3689	9.7158	9.4753	9.8695	9.7425	9.6739	9.5622	9.7035	9.4400	9.2408	9.8056	9.4969	7.8597	9.7955	9.7737	9.6720	9.2866	9.3121
Pds5b	_TAS[Phospho (STY)]DSDEQQWPEEK_.2	Q4VA53_S1255_M1_Pds5b	9.9741	10.0644	9.9265	9.5651	9.4030	9.7628	9.6412	9.9171	9.0997	9.6169	9.5419	9.3674	9.2652	9.7678	9.0352	9.5894	10.1808	9.7392	9.3228	9.7348
Pds5b	_M[Oxidation (M)]ETVSNASSSSNPSS[Phospho (STY)]PGR_.2	#N/A	6.7732	6.4644	6.2564	6.1277	6.0260	6.4849	6.6894	7.2247	7.7604	6.5634	5.6967	6.4494	6.1479	5.7780	5.3193	6.1637	6.7499	7.1164	6.3696	6.0230
Pds5b	_AES[Phospho (STY)]PETSAVESTQSTPQK_.2	Q4VA53_S1356_M1_Pds5b	10.5611	10.5039	10.2671	10.0978	10.0035	10.2889	10.1427	10.7103	10.1716	9.9918	9.7836	9.9595	10.4775	10.1521	8.8256	9.4988	10.5007	10.2976	10.0426	9.6175
Pds5b	_HKEELLENEDEQNS[Phospho (STY)]PPKK_.4	Q4VA53_S1281_M1_Pds5b	3.9311	4.0745	3.4676	3.6532	3.7599	3.7477	4.7143	4.1567	4.4068	3.7417	5.4368	3.1141	5.7415	3.5057	2.9355	5.0030	3.6540	4.0746	3.7945	5.2631
Phactr4	_SSS[Phospho (STY)]PVLVEEEPER_.2	Q501J7_S118_M1_Phactr4	10.0676	10.1528	10.0562	10.2220	9.4402	9.9863	9.5726	10.1751	9.8909	10.2692	9.6341	9.9905	10.5622	10.4245	9.7709	10.0051	10.4029	9.7163	9.9462	9.9642
Lrrc47	_QHRES[Phospho (STY)]GEGEEVADSAR_.3	#N/A	5.2401	6.1416	4.4537	3.9206	5.6140	4.9624	5.2777	5.4108	5.0198	3.2410	5.7545	5.0724	5.6845	3.3787	4.7026	5.2755	5.2824	4.4737	5.3620	5.2846
Lrrc47	_KRQS[Phospho (STY)]VSGLHR_.3	#N/A	3.9307	4.4110	3.9455	3.1753	3.2685	3.4925	4.2726	3.1337	4.3317	4.1371	3.2052	4.7297	5.2177	3.9835	3.8498	4.2756	4.1318	3.0176	3.3167	3.6608
Lrrc47	_RQS[Phospho (STY)]VSGLHR_.2	#N/A	3.7050	4.2978	4.2894	3.9844	4.4130	3.2668	2.9975	3.9581	3.0566	5.4083	4.3410	4.1873	3.1647	4.2093	3.6429	4.0499	4.3576	4.2347	3.7626	3.8866
Lrrc47	_KQHRES[Phospho (STY)]GEGEEVADSAR_.3	#N/A	4.2829	3.5630	3.3931	3.5795	3.8719	4.2985	3.4499	4.0689	3.7806	5.4681	3.8320	4.0564	3.8005	3.3443	3.5175	3.8302	3.3321	3.6604	4.2712	3.9248
Kctd8	_IVS[Phospho (STY)]PKQEHEDR_.2	#N/A	6.4048	5.7663	3.1439	5.1875	5.3148	5.3291	5.5532	5.6845	5.3699	5.7431	5.1231	5.8660	6.1942	5.7096	5.4992	5.8103	5.5255	5.5578	5.2468	5.7750
Kctd8	_RNS[Phospho (STY)]ELFQSLISK_.2	Q5DIH3_S413_M1_Kctd8	4.2100	3.7928	3.2455	3.4794	3.5477	4.4372	5.1754	3.9104	4.0653	4.0075	5.3439	3.6823	4.2307	6.0850	4.1704	3.0541	4.4481	4.8585	3.0337	3.1995
C2cd3	_SDVGTSELPS[Phospho (STY)]PAPEEDGVEPSR_.2	Q52KB6_S453_M1_C2cd3	3.4951	3.2910	3.7473	4.4784	3.9020	4.4994	4.4523	4.4122	5.6420	3.5057	4.0457	3.3501	3.2317	3.7344	4.4186	3.0572	3.4490	5.5480	4.4186	4.6928
Ccnl1	_EVKAEEKS[Phospho (STY)]PVSINVK_.3	Q52KE7_S358_M1_Ccnl1	3.7571	3.5620	3.2937	3.8271	3.9641	4.1443	3.0840	3.9828	3.6799	3.5677	4.0543	4.3749	5.8780	3.3318	2.9536	3.1192	5.5694	3.6694	4.0680	4.1343

Ccnl1	_QQAS[Phospho (STY)]KSPYNGVR_.3	Q52KE7_S378_M1_Ccnl1	4.3139	3.6889	3.3493	3.3755	3.6516	3.8757	3.8895	4.0142	5.9824	4.5202	4.3230	3.5784	4.3346	3.3364	3.4667	3.1579	4.5519	3.2812	4.4307	3.3034
Spire1	_LRPVS[Phospho (STY)]PEEIRR_.3	Q52KF3_S229_M1_Spire1	8.0252	8.2098	7.6355	7.4342	7.0377	6.6636	7.4994	7.8332	6.8178	7.6437	7.4014	7.6752	7.6734	7.8533	5.3721	7.3060	7.4920	7.4432	7.4146	6.9114
Spire1	_S[Phospho (STY)]VDKSDEELQFPK_.2	#N/A	7.1308	7.7496	7.3144	7.0331	5.5633	7.0441	6.8564	3.5213	6.8680	6.8684	6.4050	6.2584	3.9976	7.0972	5.4915	6.4499	6.6211	6.6091	6.7164	6.8707
Spire1	_RHS[Phospho (STY)]IEKETPTNVR_.3	#N/A	6.7295	7.1115	3.5119	6.1971	5.5301	6.0023	6.7045	6.2569	5.7450	6.3678	4.6385	5.9833	6.8341	6.3730	5.3158	3.3315	5.8240	6.1389	6.0732	6.2156
Spire1	_SRS[Phospho (STY)]VDKSDEELQFPK_.3	#N/A	6.3798	6.4084	4.1339	3.7404	5.3504	5.8797	6.1030	5.5323	5.9139	6.2749	5.4943	5.7867	5.9028	4.0390	4.2664	5.6548	4.1872	6.2973	5.6703	5.8706
Spire1	_APTLAELDSS[Phospho (STY)]DS[Phospho (STY)]EEEKSLHK_.3	Q52KF3_S306_M2_Spire1	3.9362	5.1055	3.9615	3.9106	3.7111	4.7250	5.8951	4.0968	3.6197	3.6988	3.9152	3.5703	3.7628	6.9615	3.4818	3.5741	3.6632	3.8869	3.9454	3.8700
Spire1	_HS[Phospho (STY)]IEKETPTNVR_.3	#N/A	3.2460	4.0731	4.2834	4.3382	3.4530	3.1546	3.1098	3.4717	3.1688	3.7991	3.5432	3.7992	3.2769	4.3215	3.6205	4.1090	4.4698	4.1805	2.9787	3.9988
Srrm1	_RLS[Phospho (STY)]PS[Phospho (STY)]AS[Phospho (STY)]PPR_.2	Q52KI8_S387_M3_Srrm1	12.1877	12.0729	12.0153	12.1575	11.6242	11.8855	11.9461	12.0979	11.9603	11.9105	11.5479	11.7288	12.1060	11.9636	11.0720	11.5976	12.2089	11.8911	11.8214	11.7003
Srrm1	_TAS[Phospho (STY)]PPPPPKR_.2	#N/A	11.6532	11.6968	11.4985	11.5721	11.1554	11.4659	11.5116	11.7123	11.3968	11.0581	11.1228	11.2746	11.7292	11.3754	10.6880	11.0708	11.6065	11.4242	11.2898	11.0807
Srrm1	_RRT[Phospho (STY)]PS[Phospho (STY)]PPPR_.2	#N/A	12.0266	11.9087	11.8414	11.9146	11.5743	11.7833	11.8187	11.8416	11.7619	11.7140	11.5099	11.6193	11.9279	11.8216	11.1270	11.5000	11.9027	11.6645	11.5784	11.4097
Srrm1	_APQTSS[Phospho (STY)]PPPVR_.2	#N/A	11.7215	4.1708	4.3039	11.7010	11.2125	11.6331	11.5359	11.5620	11.4609	3.5495	11.5510	11.6499	12.2535	11.9964	10.9974	11.5804	12.0426	11.8448	11.5129	11.6253
Srrm1	_SRVS[Phospho (STY)]VS[Phospho (STY)]PGR_.2	#N/A	11.5413	11.6318	11.6412	11.5646	11.0804	11.4530	11.4579	11.4103	11.4408	11.4503	11.1763	11.1682	11.5559	11.3475	10.5991	11.1070	11.5607	11.2858	11.2589	11.2309
Srrm1	_AAS[Phospho (STY)]PSPQSVR_.2	Q52KI8_S779_M1_Srrm1	11.3934	11.4995	11.4925	11.3563	10.9603	11.3422	11.3188	11.4027	11.3477	11.2837	10.9804	11.0529	11.5786	11.3015	10.5373	10.9501	11.5331	11.3405	11.0601	11.1054
Srrm1	_RVS[Phospho (STY)]HS[Phospho (STY)]PPPK_.2	#N/A	11.6753	11.7525	11.9053	11.7653	11.4039	11.8358	11.5850	11.5047	11.6336	11.6584	11.5227	11.2524	11.1718	11.6487	10.3444	11.1659	11.8167	11.5498	11.3414	11.4609
Srrm1	_RRS[Phospho (STY)]PT[Phospho (STY)]PPPR_.2	#N/A	10.6816	10.7312	10.8408	10.5308	10.3351	10.6044	10.4726	10.6186	10.5363	10.6695	10.1800	10.3727	10.6356	10.6713	10.5847	10.3062	10.7028	10.1435	10.2953	11.0872
Srrm1	_S[Phospho (STY)]PS[Phospho (STY)]PPPAR_.2	#N/A	11.4888	11.1430	9.8118	11.4154	10.2670	11.1450	11.3404	10.0601	11.3419	11.2469	11.1430	10.5641	10.7587	8.7962	9.5400	10.6420	11.0148	11.0212	10.8844	10.8838
Srrm1	_RRS[Phospho (STY)]PS[Phospho (STY)]PPPAR_.2	#N/A	10.1256	10.0399	10.0851	10.0116	9.6730	9.9654	9.9068	10.0252	9.9207	9.9928	9.6687	9.7074	10.2225	9.9385	9.1388	9.6144	10.1801	9.8619	9.7511	9.6383
Srrm1	_RYS[Phospho (STY)]PPIQR_.2	Q52KI8_S616_M1_Srrm1	3.7621	11.4217	3.2986	10.9244	3.9690	11.3437	11.0783	11.3676	10.8642	3.5727	10.8878	10.7303	11.3664	11.2660	3.1045	10.5307	11.1282	11.1652	10.8327	10.6826
Srrm1	_WQS[Phospho (STY)]PVTK_.2	#N/A	10.4662	10.5384	10.1904	10.5164	10.1125	10.2946	10.2968	10.4801	10.3146	10.3433	10.0516	10.0019	10.4497	10.2874	9.4753	10.1738	10.3876	10.2161	10.3287	10.0371
Srrm1	_S[Phospho (STY)]PT[Phospho (STY)]PPPR_.2	#N/A	10.6662	9.0165	9.5498	9.7236	9.6703	9.6228	9.6850	8.8045	10.5501	10.5997	10.5500	10.0405	8.6993	10.3662	7.2418	10.1887	8.7138	9.1918	7.8455	8.7784
Srrm1	_RRS[Phospho (STY)]PS[Phospho (STY)]LSSK_.2	Q52KI8_S674_M2_Srrm1	8.3707	7.9354	7.3406	7.6306	7.3740	7.4178	7.3724	7.7506	7.8295	7.8130	7.2179	7.3116	7.8020	7.6581	7.2160	5.1778	8.0065	7.6425	7.6563	7.4670
Srrm1	_RAS[Phospho (STY)]PS[Phospho (STY)]PPPK_.2	#N/A	9.1219	8.9379	8.3838	9.2185	9.0387	9.2715	8.7448	8.7361	9.0936	9.1389	8.9301	8.9171	8.6028	8.1515	6.5675	8.7828	9.2497	8.9937	8.6050	8.6389
Srrm1	_S[Phospho (STY)]PELPEPSVR_.2	#N/A	9.2271	9.2152	8.9772	9.1135	8.8024	9.3062	8.6135	9.1761	9.0500	4.2298	9.2831	8.6569	4.3470	8.0661	3.7570	8.8910	9.2816	8.7042	8.8737	8.4438
Srrm1	_S[Phospho (STY)]RS[Phospho (STY)]YS[Phospho (STY)]PR_.2	Q52KI8_S304_M3_Srrm1	7.0175	7.7749	7.8754	8.2851	3.8973	3.9991	7.7524	7.3430	7.7184	4.0690	8.2710	3.4884	6.5039	6.9234	6.0144	3.9766	6.8999	7.3808	7.6752	3.1696
Srrm1	_AAS[Phospho (STY)]PS[Phospho (STY)]PQSVRR_.2	#N/A	8.6608	9.0930	3.7600	8.6796	8.3491	3.9227	8.8988	4.4249	8.7121	8.5728	4.0584	8.2692	8.9995	8.7619	3.0560	8.5973	9.0482	8.8504	8.7167	8.3450
Srrm1	_KAQVS[Phospho (STY)]PQS_.2	Q52KI8_S943_M1_Srrm1	7.9700	8.0965	7.6381	7.7159	7.3071	7.4637	7.9260	8.0343	3.5701	3.3978	7.5633	7.8277	7.9604	7.7504	6.1479	3.5364	8.0792	7.8990	7.4991	7.6820
Srrm1	_AKS[Phospho (STY)]PT[Phospho (STY)]PSLS[Phospho (STY)]PAR_.2	Q52KI8_S832_M3_Srrm1	3.8543	7.7678	7.1077	6.8555	6.6076	4.0471	3.7180	6.6552	7.1428	7.4102	7.0801	6.8784	3.8852	7.6368	6.3847	7.0933	7.4969	7.6420	7.3999	6.3648
Srrm1	_RWQS[Phospho (STY)]PVTK_.2	#N/A	3.2411	3.2609	3.7774	4.4483	4.0797	7.0447	6.4635	4.4423	6.6088	6.6320	6.3429	3.1503	3.2618	3.7645	3.0386	3.5860	3.4792	4.3540	4.0026	3.7315
Srrm1	_QRS[Phospho (STY)]PTVTK_.2	Q52KI8_S664_M1_Srrm1	3.2026	6.0971	4.2400	5.5605	3.4095	3.1980	3.0663	3.4282	6.1737	5.8388	5.7601	3.8427	6.0393	5.8799	5.2959	5.4788	6.1371	6.0456	3.0222	3.9553
Srrm1	_S[Phospho (STY)]PS[Phospho (STY)]PPPARR_.2	#N/A	7.1965	8.7082	6.2742	7.3052	8.8444	7.6476	4.6877	7.4954	6.9540	8.5170	8.0246	7.4428	7.8115	8.7597	6.6206	8.0010	8.9137	8.2755	8.5008	7.7433
Srrm1	_GAS[Phospho (STY)]AS[Phospho (STY)]PQGRQS[Phospho (STY)]PSPSTRPIR_.3	#N/A	5.7969	6.6937	5.6716	5.3243	5.2335	6.2810	6.2302	5.3961	5.5783	6.6138	6.0037	5.6533	5.6572	5.5087	4.9808	5.9262	6.4731	5.8142	5.2120	5.8537
Srrm1	_RS[Phospho (STY)]PT[Phospho (STY)]PPPR_.2	#N/A	3.7395	9.4599	8.3984	8.3250	9.0755	9.2624	9.5472	9.1767	9.2530	9.0279	8.5925	8.6750	3.1302	8.6116	8.2261	9.0995	8.2805	9.3914	7.5849	8.5823
Srrm1	_RS[Phospho (STY)]PS[Phospho (STY)]PPPAR_.2	#N/A	8.1957	7.3660	6.7125	7.9272	7.8089	7.2675	7.8246	7.3546	7.7451	9.5650	7.1301	7.0823	7.6714	7.1150	6.5068	7.4231	7.6585	6.6774	7.3366	6.3731
Srrm1	_APKPEPVPEPKEPS[Phospho (STY)]PEK_.3	#N/A	7.9372	8.0108	7.6328	7.4378	7.4563	7.6766	7.9230	8.0669	7.7982	7.9512	7.6754	7.5376	8.0785	7.5947	7.0758	7.4756	8.4454	7.5713	7.4968	7.3212
Srrm1	_RVS[Phospho (STY)]HS[Phospho (STY)]PPPKQR_.3	#N/A	7.2865	6.5526	6.0129	7.7998	6.4694	6.1200	7.3958	3.5559	7.5075	7.4832	6.2656	7.3533	7.1987	7.6672	4.2720	7.0516	5.6005	6.6741	7.7386	7.1811
Srrm1	_SVS[Phospho (STY)]GS[Phospho (STY)]PEPAAK_.2	Q52KI8_S795_M2_Srrm1	3.9269	3.4430	5.2627	5.5175	3.4360	3.8010	3.7682	4.1525	5.5062	3.7375	3.6503	3.9722	3.8205	3.5015	4.1712	5.1453	3.6498	5.0742	3.7749	3.6058
Srrm1	_TAS[Phospho (STY)]PPPPPK_.2	#N/A	10.1894	10.1688	9.8703	10.2422	9.7454	10.0463	9.9272	10.1945	10.0809	9.9286	9.7550	9.7652	10.2960	10.0111	9.0240	9.7765	10.2400	9.9715	9.6434	9.8206
Srrm1	_AS[Phospho (STY)]PS[Phospho (STY)]PPPKR_.2	#N/A	4.0163	8.3346	7.9635	7.9856	4.2232	8.0474	7.4028	8.1866	8.3864	7.8498	7.3162	8.0193	4.4113	6.5753	2.8502	6.8939	8.9894	8.3829	7.7067	8.1685
Srrm1	_RRS[Phospho (STY)]PS[Phospho (STY)]PAPPPPPPPPPPR_.3	#N/A	11.2245	10.7122	9.0618	10.6890	6.6367	9.7032	11.1492	10.9933	10.5315	10.6327	9.5320	10.7255	8.5926	11.5983	8.1131	9.7212	11.1158	11.0482	9.5679	8.7250
Srrm1	_VSHS[Phospho (STY)]PPPK_.2	Q52KI8_S657_M1_Srrm1	6.5561	6.5984	4.5251	4.0965	5.9537	3.7280	3.3535	3.1188	3.2737	3.2983	6.5454	3.5575	3.4391	6.5244	3.3789	6.3986	6.7579	4.1767	6.1769	6.4971
Srrm1	_KETES[Phospho (STY)]EAEDDNLDDLER_.2	#N/A	3.7056	7.3531	7.9664	7.4586	3.9462	7.5411	7.4052	3.7338	8.0975	5.7554	3.9117	7.0347	3.6734	7.7667	6.3198	6.8284	6.3406	7.5390	7.3115	7.3667
Srrm1	_S[Phospho (STY)]PSAPPPPPPPPPPR_.2	Q52KI8_S572_M1_Srrm1	3.9617	10.7357	9.4469	9.8700	3.6384	11.2032	10.6681	3.9956	10.5812	9.0616	4.0671	9.7942	3.8008	10.6982	8.3022	10.3972	10.6111	10.8437	10.4895	10.2984
Srrm1	_RES[Phospho (STY)]PS[Phospho (STY)]PAPKPR_.2	#N/A	8.7469	8.7841	8.6682	8.5549	8.2617	8.6325	8.6891	7.7165	7.8515	8.6129	8.4983	8.4929	8.7192	7.8715	7.8237	8.2259	8.8741	8.7735	8.6067	8.3192
Srrm1	_EKS[Phospho (STY)]PELPEPSVR_.2	#N/A	11.3566	11.2732	10.8975	11.0835	3.7085	11.0089	10.8838	11.2592	10.9041	10.9578	10.5625	10.8615	11.4347	11.0854	10.0632	10.7037	11.4758	10.8973	10.7825	10.6777
Srrm1	_RHS[Phospho (STY)]PS[Phospho (STY)]PRPR_.2	#N/A	7.7409	7.4643	7.1624	7.2157	6.5699	7.0640	7.7293	7.2273	7.3772	6.8205	7.0328	6.7662	7.1572	6.8239	5.1843	6.0337	7.3652	7.2656	6.5191	6.6132
Srrm1	_QS[Phospho (STY)]PSPSTRPIR_.2	Q52KI8_S731_M1_Srrm1	7.2782	3.8740	7.5232	3.8528	6.4240	7.4184	6.5614	6.4894	4.3270	3.7473	3.6183	6.4949	6.7512	7.2955	5.9337	3.6431	6.8052	6.9782	7.0559	6.8788
Srrm1	_RYS[Phospho (STY)]PS[Phospho (STY)]PPPK_.2	#N/A	13.0702	13.2562	13.2469	13.3166	12.5870	13.1111	13.0956	13.1057	12.9676	13.0697	12.7636	12.8433	13.1373	13.1192	12.2190	12.7988	13.3578	12.9629	12.7889	12.7569
Srrm1	_Y[Phospho (STY)]SPPIQR_.2	Q52KI8_Y615_M1_Srrm1	3.4980	3.8211	4.5354	4.0863	3.7049	3.7382	4.4326	3.7237	3.2634	3.3086	4.2429	3.5472	3.5288	4.5735	3.3686	4.3609	3.2209	3.9286	4.2276	4.2507
Srrm1	_S[Phospho (STY)]RS[Phospho (STY)]PS[Phospho (STY)]PAPEKK_.2	Q52KI8_S207_M3_Srrm1	4.5417	5.6244	4.9596	5.4164	3.8794	5.3412	5.0645	4.8379	4.8221	3.6758	5.6822	3.3506	4.9034	5.6439	3.0013	3.3857	5.1245	5.5150	4.2028	5.5403
Srrm1	_RVS[Phospho (STY)]RT[Phospho (STY)]PEPK_.2	#N/A	9.3451	9.3645	9.3999	9.3932	8.9987	9.2664	9.3187	9.3633	9.3049	9.3442	9.0382	9.0662	9.5513	9.3695	8.2355	9.1807	9.4429	9.2302	9.2615	9.0620
Srrm1	_TRHS[Phospho (STY)]PTPQQSNR_.3	#N/A	7.3281	7.2095	6.8635	5.9874	6.4175	6.7167	7.1314	7.0620	6.8200	6.3559	6.4567	6.8163	6.5789	7.2695	3.3374	5.7545	7.0795	7.1236	3.8417	6.3453
Srrm1	_RES[Phospho (STY)]PSPAPK_.2	#N/A	4.0641	3.9387	4.6004	3.6253	3.0490	3.0823	3.9991	3.7644	3.9193	4.1535	3.3980	3.8282	4.0848	4.5874	4.0245	3.7050	4.3021	3.5310	3.1796	3.0536
Srrm1	_RAS[Phospho (STY)]PS[Phospho (STY)]PPPKR_.2	#N/A	12.0850	11.9505	12.0025	12.1212	11.5784	11.7460	11.7586	12.0016	11.7147	11.6196	11.3125	11.7348	11.7675	11.8512	10.2390	11.6207	12.0054	11.7618	11.6368	11.0846
Srrm1	_KSRVS[Phospho (STY)]VS[Phospho (STY)]PGR_.2	#N/A	8.6971	8.7707	8.5101	8.7539	8.3319	8.4442	8.5854	8.6919	8.6802	8.4129	8.2097	8.3462	4.1114	8.3889	7.8261	8.3193	8.7106	8.4476	3.7259	8.1971
Srrm1	_RRT[Phospho (STY)]AS[Phospho (STY)]PPPPPK_.2	#N/A	8.2085	7.8107	8.2638	7.0440	5.1362	7.8588	6.8049	8.2529	7.9640	8.0234	7.7376	7.7144	8.2267	7.9324	7.4761	7.3525	8.3203	5.7527	7.3444	7.6397
Srrm1	_RYS[Phospho (STY)]PS[Phospho (STY)]PPPKR_.2	#N/A	3.4745	3.8446	9.6557	4.1098	8.3964	3.7147	3.3667	9.4881	9.4271	3.2851	8.3828	3.5707	9.2698	9.6292	9.0738	4.3374	3.6697	4.1635	9.3297	8.7689
Srrm1	_S[Phospho (STY)]PS[Phospho (STY)]PAPPPPPPPPPPR_.3	#N/A	3.1684	6.5350	3.2069	3.9139	4.0070	5.9072	5.8269	3.8960	3.5932	3.3747	3.9676	6.6212	3.7012	6.7995	3.1113	3.5133	6.9876	6.3337	5.0976	6.3147
Srrm1	_RRAS[Phospho (STY)]PS[Phospho (STY)]PPPK_.2	#N/A	7.8260	7.7148	7.0607	6.5762	5.6950	7.2145	7.5418	7.3420	6.8469	3.8621	5.3480	6.9189	7.0583	6.8859	5.1652	5.1670	6.6475	7.1765	6.6897	6.5857
Srrm1	_KVELS[Phospho (STY)]ES[Phospho (STY)]EEDKGSK_.2	Q52KI8_S461_M2_Srrm1	6.6967	6.7982	6.4692	6.4238	6.4544	6.5842	6.6391	6.8760	5.9600	6.3794	6.0405	6.1436	6.1943	6.4537	4.2774	6.3543	6.7379	6.5634	6.1678	5.9622
Srrm1	_HRPSS[Phospho (STY)]PAT[Phospho (STY)]PPPK_.2	Q52KI8_S401_M2_Srrm1	10.7765	10.9692	9.4655	10.8301	10.5884	3.4461	10.5595	10.2794	9.3216	10.6354	10.0487	4.1494	11.4164	4.1454	4.0104	9.5487	10.6569	4.1714	10.1043	10.6536
Srrm1	_KPPAPPS[Phospho (STY)]PVQSQS[Phospho (STY)]PSTNWSPAVPAK_.3	Q52KI8_S810_M2_Srrm1	3.4797	3.8394	4.5171	4.1046	3.6866	3.7200	8.2427	3.7054	3.2817	3.2903	4.2612	3.5655	3.4471	4.5552	3.3869	4.3426	3.2026	3.9469	4.7566	4.2324
Srrm1	_KPPAPPS[Phospho (STY)]PVQS[Phospho (STY)]QSPSTNWS[Phospho (STY)]PAVPAKK_.4	Q52KI8_S814_M3_Srrm1	3.7963	3.9479	3.8493	3.9787	3.4921	3.6267	3.8176	3.6979	5.7760	4.4802	2.9690	3.7504	3.9503	3.8709	3.6044	3.6693	4.4502	3.8440	3.0154	3.7275
Srrm1	_APQTSS[Phospho (STY)]PPPVRR_.2	#N/A	3.2689	4.0502	4.3063	4.3154	10.6413	10.7991	3.1326	11.1923	3.1917	10.8808	3.5661	3.7763	3.2997	4.3443	3.5977	4.1318	4.4926	4.1577	2.9559	10.6923
Srrm1	_RS[Phospho (STY)]PS[Phospho (STY)]PAPPPPPPPPPPR_.3	#N/A	3.4541	3.8650	4.4915	4.1301	3.6610	7.3270	5.9558	3.6798	6.2303	3.5910	3.7513	3.5911	3.4850	5.7203	4.6950	5.3442	6.1617	3.9724	5.7348	6.0916
Srrm1	_LS[Phospho (STY)]PSAS[Phospho (STY)]PPR_.2	#N/A	3.4452	4.5576	3.9815	4.2442	4.2837	3.0070	3.2573	3.1456	3.3164	3.6515	3.6908	2.9462	3.4245	3.9686	4.8539	3.7901	3.6832	4.1499	3.7985	5.8005
Srrm1	_VS[Phospho (STY)]VS[Phospho (STY)]PGR_.2	#N/A	4.5790	5.5031	4.3642	3.8614	3.2851	3.3184	3.7630	3.5283	3.6832	5.9247	3.1619	3.5994	3.8487	4.3513	3.7884	3.9411	4.0660	3.7672	5.3500	4.3183
Srrm1	_RGAS[Phospho (STY)]AS[Phospho (STY)]PQGR_.2	Q52KI8_S723_M2_Srrm1	3.2390	3.2629	3.7753	4.4504	4.0776	4.7920	3.4635	4.4403	3.5226	3.4454	3.8969	4.4916	3.6306	4.4850	3.0407	3.5839	3.4771	4.3561	4.0047	4.3525
Srrm1	_EARS[Phospho (STY)]PQPNK_.2	Q52KI8_S694_M1_Srrm1	4.4312	5.7989	3.4667	3.6758	3.7689	5.8820	5.5055	5.3889	3.8312	3.1367	4.2056	5.3046	5.9242	3.4830	3.3493	3.2753	5.6147	3.5181	3.8172	3.1603
Srrm1	_HS[Phospho (STY)]PS[Phospho (STY)]PRPR_.2	#N/A	3.8690	4.0016	4.4053	3.8203	3.2068	3.2773	5.1082	3.5694	3.7243	4.3485	3.7455	4.0232	3.8898	4.3924	3.8295	4.2139	4.5152	3.7261	3.3746	4.3594
Srrm1	_RRVS[Phospho (STY)]HS[Phospho (STY)]PPPK_.3	#N/A	4.5626	4.2573	4.0992	4.5225	3.2687	4.4893	3.7794	3.2875	3.6996	4.3732	3.1782	3.9834	3.8650	4.3677	3.5868	3.9247	4.2855	3.7508	3.3994	4.3347
Srrm1	_RRS[Phospho (STY)]PS[Phospho (STY)]PR_.2	Q52KI8_S609_M2_Srrm1	3.2661	4.0529	4.3036	4.3181	3.4731	3.5064	4.0793	3.4918	3.4952	4.5776	2.9739	3.7791	3.6607	4.3416	3.6004	4.1291	4.4899	4.1604	2.9586	4.1303
Prune2	_SSGQESESIPEYTAEEEREDNR_.3	#N/A	7.0557	8.1376	8.2595	8.1447	6.9149	8.0614	7.9287	7.1847	7.8921	7.9959	7.6984	7.6907	8.0704	6.9162	5.7985	7.8676	8.1650	8.0018	7.0317	6.7958
Prune2	_SSGQESES[Phospho (STY)]IPEYTAEEER_.2	#N/A	6.9426	5.7053	3.9623	3.8694	3.8277	5.9458	6.0951	5.2405	5.9142	6.0624	3.4677	5.4245	4.1545	5.4478	4.5058	3.7576	3.9874	3.5836	5.3373	3.8494
Prune2	_NLSLTC[Carbamidomethyl (C)]FAGEEPSS[Phospho (STY)]PER_.2	Q52KR3_S594_M1_Prune2	3.6695	3.6496	3.2061	4.4528	3.8764	3.9098	3.1717	4.4378	3.0919	3.4801	4.0714	3.3757	3.2573	3.7600	3.1971	3.0316	3.4747	4.3585	4.0071	3.7270
Prune2	_DTSLTS[Phospho (STY)]GSPTSDLGQTWNNSK_.2	Q52KR3_S876_M1_Prune2	3.7907	4.2149	4.3270	3.9015	3.1284	3.2909	4.4154	3.4911	3.4104	3.9970	3.3024	4.1043	3.8114	4.3141	3.9926	4.1356	4.0288	3.8072	3.4558	4.2811
Thrap3	_ASVSDLS[Phospho (STY)]PR_.2	Q569Z6_S243_M1_Thrap3	12.5513	12.6478	12.3272	12.4090	12.0272	12.3647	12.3786	12.4998	12.3203	12.2686	12.0729	12.1199	12.7605	12.2545	11.4287	11.9147	12.4661	12.2878	12.1136	11.9965
Thrap3	_ERS[Phospho (STY)]PALK_.2	Q569Z6_S248_M1_Thrap3	10.7405	10.5739	10.3160	10.3694	10.0540	10.4760	10.5496	10.5004	10.2115	10.4083	10.1884	10.1604	10.6043	10.3451	9.1750	9.7866	10.5869	10.4223	10.3518	10.0395
Thrap3	_S[Phospho (STY)]REEEWDPEYTPK_.2	Q569Z6_S860_M1_Thrap3	9.6860	9.7699	4.0876	9.6023	2.8890	9.0212	9.5985	3.2517	9.0473	9.6062	9.0006	9.2613	9.6000	9.8604	8.0200	9.2085	9.5813	9.5316	9.3635	9.0018
Thrap3	_NKKS[Phospho (STY)]PEIHR_.2	Q569Z6_S669_M1_Thrap3	9.5474	9.5361	9.2948	9.2937	9.0616	9.2619	9.3199	9.4525	8.9764	9.0731	8.9326	9.1329	9.8024	9.3894	7.0828	8.6577	9.6540	9.4189	8.8556	8.9790
Thrap3	_IDIS[Phospho (STY)]PSTFR_.2	#N/A	11.6802	11.5935	11.2993	11.1600	3.8452	11.3586	11.1787	11.2542	11.1710	11.1522	11.1902	10.8797	11.4517	11.3036	10.1150	3.0004	11.6336	11.1950	11.0612	11.0302
Thrap3	_ERS[Phospho (STY)]PALKS[Phospho (STY)]PLQSVVVR_.3	Q569Z6_S248_M2_Thrap3	7.1091	9.2004	8.2929	7.4348	3.3731	8.2862	8.4303	5.9019	7.8049	7.6876	5.6680	7.9816	6.4077	9.3912	7.3076	7.3350	7.7574	8.4973	8.4018	7.2797
Thrap3	_S[Phospho (STY)]YS[Phospho (STY)]PAHNR_.2	Q569Z6_S55_M2_Thrap3	4.0318	3.9760	3.9638	3.6625	3.3695	6.2153	5.7959	3.7321	6.1255	4.2963	3.1565	3.8654	4.1188	7.1725	3.7912	6.3353	7.5609	7.1156	6.2925	4.0348
Thrap3	_M[Oxidation (M)]DS[Phospho (STY)]FDEDLAR_.2	#N/A	7.2734	7.8794	8.6925	7.7797	4.3821	8.0333	7.9812	5.6913	7.7194	7.6982	5.4259	7.4003	6.8230	7.9484	6.3356	7.2915	8.0136	7.6919	7.5387	7.4647
Thrap3	_FSGEEGEIEDDESGT[Phospho (STY)]ENREEK_.3	Q569Z6_T937_M1_Thrap3	3.7029	4.4540	3.7376	4.5556	3.3909	3.4979	4.1484	3.5999	3.9272	5.1154	3.2269	4.0207	3.8010	4.5489	3.7074	3.5575	4.3478	3.8634	3.5805	3.6825
Thrap3	_IDIS[Phospho (STY)]PSTFRK_.2	#N/A	7.2126	3.7248	3.3135	3.4114	3.6157	3.8398	7.2480	3.9784	3.9844	4.4843	4.3588	3.6142	4.0925	3.3006	3.5025	6.5184	4.5161	7.2281	6.4136	3.3135
Thrap3	_S[Phospho (STY)]PRPSPVPK_.2	Q569Z6_S264_M1_Thrap3	3.7463	3.5730	4.8324	4.2085	3.9533	5.0818	3.3917	4.5757	3.3952	5.0486	4.0356	3.2991	3.5258	3.5298	3.1204	4.0136	3.4503	4.0902	4.0162	3.3771
Thrap3	_M[Oxidation (M)]DS[Phospho (STY)]FDEDLARPSGLLAQER_.3	#N/A	4.5338	6.0172	3.8860	3.9849	3.6371	5.4413	5.8649	3.8955	5.5439	3.8262	3.6849	4.9376	5.3798	5.3094	4.2933	5.1823	3.7851	6.7107	5.5571	4.7497
Thrap3	_S[Phospho (STY)]RS[Phospho (STY)]YS[Phospho (STY)]PAHNR_.2	Q569Z6_S51_M3_Thrap3	3.9688	3.4216	3.6269	3.6342	3.9292	4.2269	4.0172	4.0193	5.7968	5.6149	3.8651	5.2649	4.1317	3.3534	3.0002	3.4336	4.7038	3.5007	5.6954	5.4802
Thrap3	_WAHDKFS[Phospho (STY)]GEEGEIEDDESGTENR_.3	#N/A	3.7684	4.2344	4.3047	3.9209	3.1062	3.3779	3.7035	3.4688	3.6237	4.4491	3.1024	4.1238	3.7892	4.2918	3.7289	4.1133	4.0065	3.8267	3.4752	4.2588
Thrap3	_SS[Phospho (STY)]FSITR_.2	Q569Z6_S557_M1_Thrap3	3.6400	4.3628	4.1763	4.0494	2.9777	3.2018	3.0625	3.3404	3.1216	3.8463	3.4960	4.2522	3.2296	4.2742	4.1405	4.1642	3.8781	4.2278	3.0260	3.9515
Thrap3	_WAHDKFSGEEGEIEDDES[Phospho (STY)]GTENREEK_.4	#N/A	3.8618	4.1689	4.9307	3.8555	3.1995	3.5176	4.0669	3.5621	3.6176	4.3176	3.1244	4.0584	4.2151	3.9553	3.9467	3.7769	4.0998	3.7612	3.4098	3.8283
Thrap3	_FSGEEGEIEDDES[Phospho (STY)]GTENR_.2	#N/A	3.7854	3.3813	3.6570	4.5687	3.9923	4.0257	3.0558	4.3219	2.9760	3.5960	3.9554	3.2707	3.1414	3.6441	3.0811	3.1475	3.3587	2.9736	4.1230	3.6111
Scn2b	_EQKLS[Phospho (STY)]TDDLKTEEEGK_.3	#N/A	5.0072	4.1931	5.6173	5.4757	5.2229	5.9902	5.5446	5.4186	3.3481	4.9328	5.4725	5.4581	3.4561	5.6836	4.4546	5.6444	4.9406	5.6870	5.5828	5.5858
Scn2b	_EQKLS[Phospho (STY)]TDDLK_.2	#N/A	6.9934	3.4860	7.1922	6.5380	6.8019	6.9905	6.2107	6.9202	6.4519	6.8802	6.9846	6.8493	6.8571	6.9133	2.9764	6.7340	7.4380	7.1673	4.2277	6.3643
Scn2b	_LS[Phospho (STY)]TDDLKTEEEGK_.2	#N/A	3.6214	4.2912	4.0812	3.9188	3.3443	4.7074	3.9900	3.2695	3.8299	4.0172	4.4346	4.2304	3.9380	4.1193	3.9856	4.1803	4.2676	4.4416	3.1968	3.7966
Scn2b	_YDVSVTLK_.2	#N/A	4.2102	3.7926	3.2456	3.4792	3.5479	4.4370	3.9932	3.9106	3.1445	4.4165	3.5441	3.6821	4.2309	3.2327	3.5704	3.0542	4.4482	3.3849	3.0335	1.8892
Gpkow	_KHS[Phospho (STY)]PDRQDGPVPK_.3	#N/A	6.4402	6.6917	6.5258	6.0279	5.9171	5.8354	6.3803	6.5960	6.0116	6.2146	5.9352	6.3395	6.7356	6.6624	5.9524	6.3197	6.1945	6.4281	6.1681	5.9752
Znf608	_DRHS[Phospho (STY)]PFSQR_.3	Q56A10_S1452_M1_Znf608	3.8833	3.4358	3.4199	3.7009	4.0903	4.1236	4.4587	4.1090	4.3789	5.1456	3.8575	3.1619	4.5443	3.4580	2.9832	3.2454	3.6062	3.5432	4.0786	3.5131
Znf608	_APGS[Phospho (STY)]PGAGNPPGTPK_.2	Q56A10_S626_M1_Znf608	4.5567	4.2632	4.0933	4.5283	3.2628	3.2962	3.7853	3.2816	3.7055	4.3673	3.1841	3.9893	3.8709	4.3736	3.8107	3.9188	4.2796	3.7449	3.3935	4.3406
Deptor	_STSFMSVS[Phospho (STY)]PSK_.2	Q570Y9_S265_M1_Deptor	3.8342	3.4849	3.3708	3.7500	4.0412	4.0672	3.0069	4.0599	3.7571	3.2109	4.1314	3.2110	3.8651	3.4089	3.0323	3.1964	3.5571	3.5923	3.8913	3.0862
Prag1	_ANS[Phospho (STY)]LPAGTR_.2	Q571I4_S40_M1_Prag1	8.6922	8.3839	8.2421	8.3630	8.0094	4.0122	7.9220	7.8557	7.5836	8.3924	8.0993	7.9154	8.4144	8.5805	6.5658	8.0820	7.9744	7.9419	8.0165	8.1899
Tab3	_TLVHSSS[Phospho (STY)]DGHIDPQHTAGK_.3	#N/A	3.8865	4.8873	3.8227	4.5870	5.6575	4.0133	3.9610	4.0421	3.6979	3.7465	4.3393	3.5549	3.8349	3.6598	5.5146	3.6897	3.7455	3.8547	4.2710	4.7738
Tab3	_YLYM[Oxidation (M)]EYHS[Phospho (STY)]PEDNR_.2	#N/A	3.3002	4.8539	5.1302	4.1450	3.5071	4.8484	3.4071	3.4625	3.3894	5.7718	5.0378	3.9985	3.5288	4.3000	3.5676	5.9342	5.7816	4.0171	4.0425	4.1171
Tab3	_SSSSGSDDY[Phospho (STY)]AYTQALLLHQR_.2	Q571K4_Y516_M1_Tab3	3.6993	4.1796	4.1769	4.4447	3.0370	3.2611	3.4632	3.3652	3.0623	3.9056	3.4367	3.9057	3.1704	4.2150	4.0813	4.0442	4.3633	4.2870	3.0852	3.8923
Spag9	_ETDYPAGEELSES[Phospho (STY)]GQVDK_.2	Q58A65_S815_M1_Spag9	4.3067	5.5225	4.0237	4.8808	3.3395	5.1599	5.0623	4.0726	5.4067	5.0862	3.1488	4.9698	4.0986	4.1744	3.7832	4.6142	5.2327	4.8335	4.6396	5.0374
Spag9	_SASQSS[Phospho (STY)]LDKLDQELKEQQK_.3	#N/A	7.5136	7.9167	8.0512	7.4581	7.0782	7.8350	7.5827	7.6159	7.8300	7.2463	6.9773	7.0405	4.3576	8.1201	6.6631	7.4924	7.6720	6.3855	7.8448	7.5130
Spag9	_SASQSS[Phospho (STY)]LDKLDQELK_.2	#N/A	5.9512	7.3957	6.3581	4.2130	5.1149	7.1138	7.0908	5.6013	7.2621	7.1636	3.3351	5.2793	3.7124	7.4736	3.9689	5.6914	6.0983	5.6576	3.8259	6.8167
Pex1	_SQS[Phospho (STY)]GEDESLNQPGPIK_.2	Q5BL07_S1212_M1_Pex1	8.7576	8.5631	8.6022	8.3184	7.8482	8.4752	8.3953	8.6002	8.2710	8.4302	8.3281	8.3156	8.4822	8.8089	7.4709	8.3799	8.5837	8.3251	8.3594	8.5464
Pex1	_TPS[Phospho (STY)]QEGC[Carbamidomethyl (C)]QDLTQEQR_.2	Q5BL07_S1182_M1_Pex1	3.1244	6.5917	4.1618	4.4598	3.3314	6.2479	3.7167	3.3501	3.6369	6.5700	5.6350	5.6149	6.4042	4.1999	3.7421	6.0889	6.3143	4.3021	5.7751	3.8772
Tut7	_NTTEEVGS[Phospho (STY)]PAKEK_.2	Q5BLK4_S705_M1_Tut7	6.5963	6.6680	6.4915	5.9760	5.2533	5.9704	6.5661	6.4498	6.1255	5.5217	5.7917	6.0235	6.6792	5.6661	3.8049	5.3585	6.0292	6.4090	5.3337	5.8038
Stxbp5l	_SSS[Phospho (STY)]ISS[Phospho (STY)]IDKDSK_.2	Q5DQR4_S819_M2_Stxbp5l	10.8897	10.7216	8.8079	10.7410	10.3451	10.3970	10.4369	10.7193	10.5576	10.4301	10.3510	10.3017	10.7223	10.3848	9.2631	10.0662	10.5692	10.5140	10.2645	10.1879
Stxbp5l	_RLS[Phospho (STY)]SADVSK_.2	Q5DQR4_S770_M1_Stxbp5l	8.1243	3.8261	8.3770	8.1582	7.8837	7.9600	8.2609	8.0161	8.1175	4.0408	7.5525	3.7155	8.1461	7.1115	4.1372	7.4237	8.2057	8.0061	7.9484	3.1663
Stxbp5l	_SLSGSTNTVSS[Phospho (STY)]EGVTK_.2	Q5DQR4_S598_M1_Stxbp5l	8.6800	7.8173	8.0388	7.6063	7.1732	7.8711	7.7265	6.9709	7.7165	7.3916	7.9334	7.3801	7.6599	7.7954	6.5125	7.7665	8.0884	7.3990	7.9165	7.1558
Stxbp5l	_TSYQS[Phospho (STY)]LTELNDSPVPLELER_.2	Q5DQR4_S727_M1_Stxbp5l	4.5024	3.5004	3.5379	3.1870	3.8402	4.1448	3.7009	4.2028	3.7600	3.2079	4.1344	3.3898	4.5232	3.5250	3.2781	3.3465	3.2397	3.0927	4.2421	3.4920
Stxbp5l	_EEIQESLTSDYFQIC[Carbamidomethyl (C)]K_.2	#N/A	4.9499	5.2590	5.1482	4.3009	4.4009	3.4876	3.3106	4.4031	3.8452	4.6121	3.3238	4.2913	4.3991	4.9407	3.9503	3.0031	4.4692	3.6052	3.2538	3.8721
Stxbp5l	_SRSS[Phospho (STY)]S[Phospho (STY)]ISS[Phospho (STY)]IDKDSK_.2	#N/A	6.1772	6.6272	6.1165	6.5102	6.3948	6.3110	3.4961	6.0620	3.4784	6.1173	3.4375	6.2169	6.2876	4.3893	3.3432	3.8987	6.5053	6.4976	6.0794	4.2063
Slc4a10	_FPSKSSPS[Phospho (STY)]_.2	Q5DTL9_S1118_M1_Slc4a10	11.3554	10.6266	3.6896	3.6697	3.3696	3.5106	3.9357	11.0808	10.3958	4.3497	3.1028	10.2068	10.7233	4.1188	9.1975	10.7804	4.0172	10.6388	11.5192	3.9804
Slc4a10	_S[Phospho (STY)]FADIGKK_.2	#N/A	7.0449	6.8276	6.6235	6.9595	6.8139	7.1995	6.9362	7.1202	6.5898	6.7945	6.6739	6.3766	7.5392	6.2770	5.1360	5.9955	6.6757	6.5669	6.7436	6.3306
Slc4a10	_ENS[Phospho (STY)]TVDFSK_.2	Q5DTL9_S280_M1_Slc4a10	6.7534	7.0156	6.5616	6.7513	6.5575	3.8589	6.6327	6.5337	3.7552	7.2477	3.5806	6.0803	3.9027	3.8411	3.8096	3.6626	3.5558	3.6242	6.7935	6.5528
Slc4a10	_NAGQVVS[Phospho (STY)]PQSAPAC[Carbamidomethyl (C)]AENK_.2	Q5DTL9_S261_M1_Slc4a10	4.3868	4.4331	3.9234	3.1974	3.0929	3.1262	6.2466	3.1116	3.8754	4.1974	6.7871	4.1592	4.0408	6.0066	3.9806	3.7489	4.1097	3.5750	3.2236	3.0097
Slc4a10	_GHTS[Phospho (STY)]PC[Carbamidomethyl (C)]GMK_.2	Q5DTL9_S297_M1_Slc4a10	4.3192	4.5007	3.8558	3.2650	4.8988	3.0587	7.1251	3.0441	3.9430	4.1298	3.4216	5.8148	4.1084	3.8939	4.0482	3.6813	4.0421	3.1073	3.4064	3.5711
Slc4a10	_ERDSGLEDGRES[Phospho (STY)]PSFDTPSQR_.4	#N/A	5.6950	6.4877	3.6401	4.2552	3.7592	3.8348	5.0248	4.9444	5.5500	6.0925	5.7158	5.3374	6.6183	4.7594	5.0521	5.5658	5.9885	6.2183	5.7959	5.5030
Slc4a10	_DSGLEDGRES[Phospho (STY)]PSFDTPSQR_.3	#N/A	11.5329	11.6237	10.9492	11.4892	10.2289	11.2108	11.4649	11.0498	11.1893	11.4690	11.4462	10.9416	12.0516	11.2888	10.0259	11.0450	11.3791	11.2232	11.4708	11.2168
Slc4a10	_DDPSVINIS[Phospho (STY)]DEM[Oxidation (M)]SK_.2	#N/A	4.0673	7.8431	8.1963	6.8227	4.0687	7.7021	7.8632	3.7677	7.3036	3.6660	3.7829	7.0222	4.0911	7.8429	5.8087	7.0473	7.5752	7.4126	7.6268	7.1736
Slc4a10	_ESRFPSKS[Phospho (STY)]SPS_.2	Q5DTL9_S1115_M1_Slc4a10	3.3274	3.1746	3.8637	4.3620	4.1660	3.8190	3.3751	4.5286	3.4342	3.5337	3.8086	3.0640	3.3481	3.8508	2.9523	3.6723	3.5655	4.2677	3.9163	3.8178
Slc4a10	_KQS[Phospho (STY)]EPNSMDK_.2	Q5DTL9_S247_M1_Slc4a10	3.1775	5.7446	6.3004	5.9802	4.0161	5.1581	5.8824	4.3787	3.0328	3.5392	5.5994	3.2139	3.1982	3.7009	3.1022	3.5224	4.9894	4.4176	4.4189	3.6679
Slc4a10	_NDVS[Phospho (STY)]RENSTVDFSK_.2	Q5DTL9_S276_M1_Slc4a10	5.6581	4.2282	4.0457	4.3111	3.4491	4.4556	4.0963	3.8118	4.1554	5.1218	3.0290	3.7852	4.1321	4.0328	3.6735	3.8543	4.6006	3.4881	3.1367	3.1009
Slc4a10	_S[Phospho (STY)]FADIGK_.2	#N/A	4.4108	3.5920	3.4463	3.2786	3.7485	4.2364	3.7925	4.1112	4.2661	2.6649	3.7447	3.4814	4.4315	3.4333	3.3697	3.2549	3.1480	3.1843	4.3337	2.5768
Rnf20	_ALVVPEPEPDSDS[Phospho (STY)]NQER_.2	#N/A	3.5243	9.7249	9.7532	4.0654	3.7312	4.0150	3.3224	3.7499	9.1215	3.3423	4.1216	3.5264	3.5694	9.9583	3.3477	8.9617	3.2472	9.1044	4.2068	4.1771
Rnf20	_KALVVPEPEPDSDS[Phospho (STY)]NQER_.2	#N/A	4.1045	5.7821	3.3521	3.7671	3.8376	4.0843	5.4512	4.0507	3.7479	5.7678	4.1222	3.4079	3.8559	3.3997	3.2660	3.1686	3.5480	3.6094	3.9085	3.0770
Jakmip3	_S[Phospho (STY)]PAINFHHTPFVDGK_.3	Q5DTN8_S622_M1_Jakmip3	3.4736	5.3430	4.0099	4.2158	4.3121	4.2450	4.0825	5.1122	3.2880	3.6799	3.6624	4.4187	3.3961	5.9599	2.8061	3.8185	3.0881	4.4999	4.3604	4.1180
Jakmip3	_RPS[Phospho (STY)]SLNDLDQSQDER_.2	#N/A	4.0042	4.7595	3.9892	3.8941	4.4690	4.5982	3.8432	4.8276	3.6831	4.5974	3.7962	4.2090	3.8119	3.9199	3.4204	4.4249	3.7440	3.9436	3.8592	4.2606
Psd	_S[Phospho (STY)]LSELADPNPK_.2	Q5DTT2_S720_M1_Psd	12.2678	11.9640	11.9496	12.1935	2.9781	10.8319	11.3728	11.0145	10.7011	12.6570	2.9743	11.1832	11.5974	4.1638	4.1402	11.2981	11.1261	11.2309	11.1935	4.1308
Psd	_FC[Carbamidomethyl (C)]SEGDC[Carbamidomethyl (C)]AIS[Phospho (STY)]PPR_.2	Q5DTT2_S17_M1_Psd	9.8616	10.1497	10.5738	10.2115	9.8215	9.9129	9.7884	9.6634	9.9196	10.1712	10.1030	8.8098	9.5759	10.0844	8.9900	9.5159	10.1548	9.3205	9.4647	9.6606
Psd	_LEASTS[Phospho (STY)]DPLPAGGGSVLPGSR_.2	Q5DTT2_S156_M1_Psd	7.3007	4.2699	4.2692	3.9565	3.0706	7.3215	7.9130	3.3316	7.0747	7.0996	3.4031	4.1594	7.1786	4.1814	4.0477	4.0778	4.3296	8.0213	3.1189	3.8586
Psd	_LALGS[Phospho (STY)]TDTLSNGQK_.2	Q5DTT2_S543_M1_Psd	3.9383	6.5795	4.1361	6.7048	3.6200	5.1379	5.9121	4.0320	5.0215	7.2347	3.6655	5.6956	5.0608	4.1232	3.5820	6.0172	3.8379	5.4305	3.7599	4.0902
Psd	_AQRPGS[Phospho (STY)]ETR_.2	Q5DTT2_S1011_M1_Psd	3.5981	4.4047	5.2421	4.0913	2.9358	3.1599	3.1044	3.4664	3.9555	3.8044	3.5379	4.2942	3.2716	4.3162	4.1825	3.9430	4.4645	4.1858	2.9840	3.9935
Zc3h12c	_RQS[Phospho (STY)]DPSIR_.2	Q5DTV4_S492_M1_Zc3h12c	4.1763	8.5563	3.2118	8.0215	3.5140	4.4709	4.1114	3.8767	4.0316	4.0412	8.1954	3.7159	4.1970	8.6988	4.1367	3.5927	8.2976	3.4188	3.0674	3.1658
Jcad	_DFS[Phospho (STY)]PRPVSR_.2	Q5DTX6_S954_M1_Jcad	8.1148	4.2254	7.6306	7.8965	7.6609	7.3369	3.7475	7.7974	7.3379	7.8131	7.4254	7.9815	8.3549	8.1907	3.7728	3.9566	7.9937	7.5421	7.4715	7.6049
Jcad	_AAPLYC[Carbamidomethyl (C)]LSEPRGS[Phospho (STY)]QELTK_.3	Q5DTX6_S980_M1_Jcad	4.4378	4.3821	3.9744	3.7202	3.1439	3.1772	3.9042	3.6695	3.8244	4.2484	3.3031	4.1083	3.9899	4.4925	3.9296	3.7999	4.2072	3.6259	3.2745	2.9587
Jcad	_SADS[Phospho (STY)]VEEPDPLK_.2	Q5DTX6_S1248_M1_Jcad	4.2221	8.7085	9.0476	8.7060	3.5599	8.0225	8.0266	3.9225	8.0978	9.1262	3.8082	8.5594	3.9496	8.5897	3.6002	3.8821	3.7752	8.0865	8.2383	8.7532
Jcad	_S[Phospho (STY)]PPQHIPNPYLEDPVPR_.3	Q5DTX6_S351_M1_Jcad	3.8085	6.3788	5.4508	6.1255	3.1462	3.3379	5.8615	5.7629	3.6638	6.4311	6.5851	6.2271	3.8292	4.3319	3.9720	6.0524	4.0466	6.3318	5.8690	6.2955
Jcad	_GPT[Phospho (STY)]PS[Phospho (STY)]PVNEQSSAFVHSSPR_.3	#N/A	4.0917	5.9703	4.3162	6.5870	3.4857	6.3209	5.6312	3.5045	6.1331	5.6045	3.5488	3.8103	3.7436	5.2400	5.3819	5.2749	3.8146	6.2592	5.8025	6.1942
Jcad	_DQQTQTS[Phospho (STY)]FHEDSK_.2	Q5DTX6_S693_M1_Jcad	5.7580	4.4166	5.5228	5.1995	5.2544	5.3638	5.1622	3.2866	3.1754	6.3805	5.8011	5.8646	6.1168	5.9284	6.0516	5.7889	6.0646	4.0089	3.6575	5.4108
Jcad	_TAQQPAGLALENVAS[Phospho (STY)]PDRR_.3	Q5DTX6_S888_M1_Jcad	4.1268	5.9149	3.3156	3.4093	4.3338	4.3671	6.3501	5.4896	4.1688	3.9374	3.6140	6.0989	4.3008	6.8370	4.2405	3.4889	5.5791	5.5535	2.9636	5.2007
Jcad	_SESLVLATDTNNNDFK_.2	#N/A	3.6710	3.6481	4.9356	5.0013	3.8779	3.9113	3.1702	3.8967	3.0904	3.4816	4.0699	3.3742	3.2558	5.3969	3.8748	4.2970	3.3939	4.9555	4.5827	5.1433
Jcad	_AEGHASS[Phospho (STY)]PQPQSEGTC[Carbamidomethyl (C)]K_.2	Q5DTX6_S543_M1_Jcad	4.5557	3.2467	3.7916	4.8500	4.0939	3.8911	3.4472	4.4565	4.4055	4.5845	4.0901	3.1361	4.0935	3.7787	4.9633	3.6002	3.4934	4.1602	3.9884	3.7457
Jcad	_APQASIC[Carbamidomethyl (C)]SDVDGFPTSQAT[Phospho (STY)]SPEPGKKDEEAK_.4	Q5DTX6_T1164_M1_Jcad	4.2425	4.5774	3.7791	3.3417	2.9486	3.6589	4.0995	4.4682	4.0197	4.0531	3.4983	4.3035	4.2734	3.8172	4.1248	3.6046	3.9654	3.1840	3.4831	3.4945
Kctd16	_RKS[Phospho (STY)]DLLR_.2	Q5DTY9_S362_M1_Kctd16	9.7980	9.7831	9.1302	9.6787	9.3118	9.2585	9.5028	9.5007	9.2243	9.6986	9.4319	9.3353	10.1831	9.7356	8.5888	9.4109	9.7660	9.6391	9.4964	9.3497

Kctd16	_TLTSGS[Phospho (STY)]RESNISSKK_.3	#N/A	4.1122	3.8906	3.1477	3.5772	3.4499	3.0342	4.0473	3.8126	6.1318	4.1053	3.4461	3.7800	4.1329	3.1347	4.0727	3.6568	6.0413	3.4829	3.1315	3.1017
Kctd16	(STY)]HC[Carbamidomethyl (C)]DC[Carbamidomethyl (C)]C[Carbamidomethyl (C)]C[Carbamid	Q5DTY9_S286_M1_Kctd16	3.3726	3.1294	3.9089	4.3168	4.2112	4.4352	3.3299	3.6919	3.3890	3.5789	4.5145	3.0188	3.4971	3.7799	2.9071	3.7175	3.1891	5.1201	4.2594	4.2189
Kctd16	_TLTSGS[Phospho (STY)]RESNISSK_.2	#N/A	8.4953	8.2596	7.8271	7.9992	7.7956	7.8283	7.9889	8.1174	7.7374	8.2953	8.0527	7.8474	8.5643	8.1976	6.9921	7.9143	8.1178	8.1319	8.1248	7.7539
Kctd16	_QSPDEFC[Carbamidomethyl (C)]HS[Phospho (STY)]DFEDASQGSDTR_.3	Q5DTY9_S137_M1_Kctd16	3.9523	4.9461	4.1532	5.7140	3.0604	4.4317	5.3997	3.6005	4.8933	4.5072	3.2341	4.8822	3.9209	5.7971	3.9693	4.9655	4.6333	5.3212	5.7413	4.1574
Kctd16	_EKLS[Phospho (STY)]IEEELEK_.2	Q5DTY9_S389_M1_Kctd16	3.8535	3.4655	3.3901	3.7307	4.0605	4.7585	2.9876	4.2538	4.4087	3.6641	3.8873	4.5425	4.5741	3.5759	3.9978	3.2157	3.2906	4.1052	3.6303	4.8495
Kctd16	_QSNIQT[Phospho (STY)]LDRPIKK_.3	#N/A	4.3688	3.9646	4.5745	3.6512	3.0749	4.0477	3.9732	3.7386	3.8935	4.1794	3.3721	4.1773	4.0589	4.5616	3.9986	3.7309	4.2762	3.5569	3.2055	3.0277
Kctd16	_QSNIQT[Phospho (STY)]LDRPIK_.2	#N/A	4.2273	4.5926	3.7639	3.3569	2.9334	4.4676	4.1147	4.4530	4.0349	4.0379	3.5135	4.3187	4.2003	3.8020	4.1400	2.6707	3.9502	3.4155	3.4982	3.1691
Kctd16	_LLAPEDVKQS[Phospho (STY)]PDEFC[Carbamidomethyl (C)]HSDFEDASQGSDTR_.4	Q5DTY9_S130_M1_Kctd16	3.9171	3.6428	4.0598	3.9080	4.0011	3.4789	3.6324	3.9943	3.6915	4.1234	4.0658	3.3689	3.7995	4.0979	3.2096	3.5074	3.4916	3.7503	4.0493	3.7752
Usp22	_S[Phospho (STY)]PHIPYK_.2	Q5DU02_S237_M1_Usp22	7.0599	3.8869	6.3903	6.9777	7.0271	6.9289	6.8546	7.0116	7.0466	7.1884	6.6110	3.6130	6.6601	7.0949	5.3280	6.5266	7.1844	6.4622	6.6591	6.6002
Iqsec2	_DPLGARDS[Phospho (STY)]PGR_.2	#N/A	9.3088	9.2062	9.3790	9.0297	8.8691	9.1938	9.0393	8.5411	8.7524	8.9166	9.0814	8.3293	8.3833	9.0279	7.8276	8.2261	9.2213	8.8604	8.7059	8.5642
Iqsec2	_HSWDS[Phospho (STY)]PAFNNDVVQR_.2	#N/A	4.2073	6.2329	5.0472	6.1972	5.4596	5.6385	6.2959	3.7924	3.7840	3.9059	5.8635	5.3358	7.2776	6.4028	3.3916	6.0676	3.5373	7.4862	7.2626	6.3545
Iqsec2	_SSS[Phospho (STY)]PGAGGGHSTSTSTSPATTLQR_.3	#N/A	5.0901	8.5907	7.5115	7.7923	6.1085	3.6189	8.1573	5.9524	4.7077	7.8537	4.0325	3.6313	3.8602	6.1020	7.3860	7.7211	7.8627	8.1939	8.1794	7.5422
Iqsec2	_SS[Phospho (STY)]LEDTYGAGDGLK_.2	#N/A	3.4177	8.0293	4.0247	4.2010	3.6247	3.6580	3.4234	3.1887	3.3436	7.5529	4.3231	7.2681	3.5091	4.0117	3.4488	4.2807	3.7264	4.1067	3.7553	3.9787
Iqsec2	_LIEAFSQR_.2	#N/A	6.2599	6.7615	5.4091	5.4211	6.4909	6.4983	6.8029	7.1789	5.5391	6.1347	5.8729	6.5789	4.0862	6.4028	5.5569	5.0873	6.4208	6.5353	6.3270	5.8256
Iqsec2	_M[Oxidation (M)]QFS[Phospho (STY)]FEEYEK_.2	#N/A	9.4152	9.5058	9.4505	9.2395	7.4962	8.6603	8.8070	8.0836	8.8767	9.3147	8.2398	8.3508	8.6912	8.2337	7.2457	8.6701	8.4818	9.0669	8.6881	8.7480
Iqsec2	_GALSSS[Phospho (STY)]LR_.2	#N/A	4.5678	3.4350	3.6032	3.1216	3.9055	4.1296	3.6356	4.2682	6.0342	3.2733	6.4069	3.3245	3.8027	3.5903	3.2128	3.4118	3.3050	4.6099	4.1767	3.0238
Iqsec2	_QLVYEADGC[Carbamidomethyl (C)]S[Phospho (STY)]PHGTLK_.2	#N/A	4.0567	6.4076	7.0303	6.5554	4.0883	3.6185	5.6711	3.8987	6.4053	3.4560	5.3235	5.6548	4.5447	5.5845	3.0780	5.3992	5.8986	3.6809	5.2777	3.8134
Iqsec2	_ESQY[Phospho (STY)]QNLR_.2	Q5DU25_Y89_M1_Iqsec2	8.3253	7.7256	8.1641	8.3552	7.5794	7.3938	7.6694	7.8396	7.7590	7.7897	7.4362	7.6286	7.6349	7.5645	6.4815	7.0809	7.6191	7.6509	7.5986	7.5055
Iqsec2	_DPLGARDS[Phospho (STY)]PGRESQYQNLR_.3	#N/A	3.7838	4.2190	7.0797	6.5285	3.1215	3.3456	4.4196	3.4841	6.3452	3.9901	3.3522	4.1085	4.5867	4.1305	3.9968	4.1287	4.0218	6.7773	3.1697	3.8078
Iqsec2	_ETRHS[Phospho (STY)]WDSPAFNNDVVQR_.3	#N/A	3.7938	7.0016	3.8802	4.1031	3.6880	5.7519	6.6033	5.4582	5.6676	3.7433	3.8598	3.5122	3.6951	7.2141	3.4786	5.1005	3.7787	6.8944	5.5404	4.2539
Iqsec2	_[Acetyl (Protein N-term)]M[Oxidation (M)]EAGSGPPGGPGSES[Phospho (STY)]PNR_.2	#N/A	9.3230	9.5097	10.1094	9.9372	8.6692	9.3959	9.4116	9.0090	9.1177	9.3286	9.4602	8.9586	8.5010	10.0551	8.5150	8.7472	9.1420	9.0512	8.8764	9.1021
Iqsec2	_GELHRDPLGARDS[Phospho (STY)]PGR_.3	#N/A	7.9555	7.8138	7.4141	7.6290	7.3774	7.4492	7.4895	7.3853	7.5196	7.6494	7.4851	7.1577	7.8372	7.4519	6.7209	6.9742	7.9544	7.6171	7.4788	7.4074
Iqsec2	_SS[Phospho (STY)]LEDTYGAGDGLKR_.2	#N/A	7.4812	7.7744	7.3093	7.3535	5.7946	7.2468	7.5411	3.5217	7.1481	7.3946	3.3940	7.0134	7.3438	7.5685	5.8351	7.0455	7.4832	7.3092	7.3905	3.8496
Iqsec2	_AAHLPLLTIEPPSDSS[Phospho (STY)]VDLSDR_.3	Q5DU25_S593_M1_Iqsec2	4.1951	3.1240	3.7317	3.3891	2.9012	5.1603	4.1469	4.4208	4.0671	4.0057	3.5457	4.3509	4.2325	3.7698	4.1723	3.5572	3.9180	3.2314	3.5305	3.4470
Iqsec2	_DS[Phospho (STY)]PGRESQYQNLR_.2	#N/A	4.3245	3.6791	3.3600	3.3656	3.6622	4.3235	6.6468	4.0249	4.1798	3.8938	3.6584	3.5685	4.3452	3.3471	5.7789	6.5900	3.9830	3.2714	6.7061	3.3141
Iqsec2	_LRSS[Phospho (STY)]ASESR_.2	Q5DU25_S363_M1_Iqsec2	3.9748	3.6627	3.8500	3.7795	3.8252	3.7826	4.2484	3.9699	4.2516	3.9552	4.4131	3.4391	3.8288	3.9418	3.0682	3.6710	4.4037	3.4502	4.2357	3.7773
Iqsec2	_ALSDSYELS[Phospho (STY)]TDLQDK_.2	Q5DU25_S316_M1_Iqsec2	3.6621	3.6570	3.1986	3.9222	3.8690	4.4574	3.1791	3.8877	3.0993	3.4727	4.0788	5.8398	3.2648	3.7674	3.2045	3.0242	3.3850	4.3511	3.9997	3.7344
Iqsec2	_EAS[Phospho (STY)]YQLQDTTGYTAR_.2	Q5DU25_S138_M1_Iqsec2	3.2087	4.1104	6.1876	4.3756	3.4156	5.0589	3.6325	3.4343	3.5527	4.5201	3.0314	3.8365	3.7182	4.2208	3.6579	5.0874	5.5444	3.8977	4.8747	4.1878
Iqsec2	_AQNPAYFEGKPAS[Phospho (STY)]LDEGAM[Oxidation (M)]AGAR_.3	#N/A	8.6328	8.7055	7.8148	7.7817	6.6638	7.6409	7.7999	7.3768	7.4515	8.4376	7.1128	7.4128	7.9635	7.7679	6.2316	7.6399	7.4776	8.4268	7.7583	7.5970
Iqsec2	_GALSSS[Phospho (STY)]LRDLSDAGK_.2	#N/A	3.9410	4.0618	4.4773	4.7962	3.2787	3.2054	3.8761	3.6414	3.7963	5.1867	3.2749	3.9512	3.9325	4.4644	3.8395	4.2859	4.1791	3.6541	3.3027	4.4314
Iqsec2	_RNS[Phospho (STY)]VGS[Phospho (STY)]LDSTIEGSVISSPRPHQR_.4	Q5DU25_S1148_M2_Iqsec2	3.6354	4.3674	4.1717	4.0540	2.9731	3.5110	3.5705	3.3358	3.4907	4.5821	2.9693	4.2569	3.6561	4.1588	3.5958	3.9803	3.8735	7.5829	3.6083	4.1258
Zfyve26	_GPLGLRS[Phospho (STY)]PSES[Phospho (STY)]PQHIAATER_.3	Q5DU37_S605_M2_Zfyve26	4.0098	3.3093	3.5463	3.5745	4.2167	4.2500	4.3322	4.2354	4.2525	3.8203	3.7311	3.0355	4.4179	3.5844	2.8568	3.3719	3.7327	3.4168	3.7158	3.2617
Lrrc8b	_S[Phospho (STY)]KTLLSTSGGSADIDASK_.2	Q5DU41_S186_M1_Lrrc8b	4.1237	3.8791	3.1591	3.5657	3.4614	3.6855	5.9508	3.8241	4.1388	4.3300	3.0123	3.7686	5.7037	3.1462	3.6569	2.9677	4.3617	3.2106	3.1200	3.4679
Rassf5	_S[Phospho (STY)]PESTLTPTLNQNVC[Carbamidomethyl (C)]K_.2	Q5EBH1_S177_M1_Rassf5	6.8278	6.8380	6.5603	6.3389	6.4865	6.4857	3.8313	6.4227	3.7999	3.7860	3.6677	6.4454	3.9450	7.1578	6.0003	3.9211	3.5195	6.3016	6.1637	3.7868
Ermn	_HREGS[Phospho (STY)]PLKEESLAR_.3	#N/A	9.2198	9.5397	9.0422	9.2817	8.9829	9.1966	8.8464	9.2464	8.9443	9.1934	8.9676	9.3050	9.5921	9.8305	9.2577	9.1855	9.5024	9.0099	9.1335	9.2586
Ermn	_ILNENPEENLFVVHQAIK_.3	#N/A	4.5357	5.0471	4.9316	5.1478	4.1500	4.8402	4.1636	3.2606	4.2221	4.5594	3.7861	5.0986	4.5666	4.1104	4.9552	4.9310	4.3518	3.8601	4.0623	4.9607
Ermn	_YNTISYR_.2	#N/A	3.7906	4.0800	3.3271	3.7937	3.9975	4.0308	4.5840	4.0162	4.4218	4.2463	3.9503	3.6889	3.1362	4.5248	4.8150	3.1527	3.5135	3.8562	4.2718	3.6059
Ermn	_EDSPLGS[Phospho (STY)]PGSQPGTPDEQPVFGKK_.3	#N/A	4.2925	4.5576	3.5310	3.4383	2.9986	3.3184	3.8886	4.2109	4.2110	4.1031	3.4483	4.0333	4.1352	3.5497	4.2499	3.6546	4.1957	3.8609	3.2872	3.3429
Ermn	_EGS[Phospho (STY)]PLKEESLAR_.2	#N/A	10.6744	10.6425	10.5739	10.7507	10.2082	10.4748	10.0605	10.5703	10.3187	10.4917	10.2853	10.3277	10.7367	10.9803	10.0428	10.2172	10.7302	10.0647	10.2240	10.4278
Ermn	_EDSPLGS[Phospho (STY)]PGSQPGTPDEQPVFGK_.3	#N/A	4.1767	3.1561	3.6996	3.4213	3.5144	3.7385	4.9286	4.3887	4.0859	4.3830	2.9594	4.0998	4.1939	3.7377	3.6040	3.0207	3.8860	3.2636	3.5626	3.4150
Ermn	_SRPVDILHS[Phospho (STY)]K_.3	Q5EBJ4_S166_M1_Ermn	3.9051	3.4140	3.4417	3.1876	4.1120	4.1454	4.4369	4.2022	4.3571	3.7157	3.8358	3.1401	4.5226	3.5244	2.9614	3.2672	3.2391	3.0933	4.2427	3.4914
Prkaa1	_SGS[Phospho (STY)]ISNYR_.2	Q5EG47_S496_M1_Prkaa1	8.6009	8.9405	8.2467	8.8166	8.2309	8.5427	8.7886	8.0364	8.6416	9.2282	8.2495	8.3108	8.7694	8.5223	3.1358	8.0497	7.5571	8.2137	8.3633	8.4132
Prkaa1	_SIDDEITEAKS[Phospho (STY)]GTATPQR_.2	#N/A	3.9604	3.9712	5.7852	6.0498	3.6997	4.2165	4.6042	3.9075	3.7609	3.5904	3.8584	3.6973	5.1219	4.6838	4.3928	3.8547	4.4907	5.6903	3.7844	5.2440
Nufip2	_GADNDGS[Phospho (STY)]GSESGYTTPK_.2	Q5F2E7_S213_M1_Nufip2	7.6751	7.4068	7.6125	7.6052	7.6951	7.5216	7.4724	7.7025	7.7422	7.5871	7.4446	7.7644	8.0070	8.3473	7.6837	7.5576	7.9823	7.6842	7.6174	7.8319
Nufip2	_SLS[Phospho (STY)]SDEATNPISR_.2	Q5F2E7_S113_M1_Nufip2	7.2943	7.1275	3.7623	3.9983	7.1844	6.8769	3.7642	4.2275	6.8328	7.4138	6.8954	6.9513	3.8499	7.2273	6.4988	7.3637	7.5925	7.0006	7.0543	7.3865
Nufip2	_KSS[Phospho (STY)]DIKPGLSSK_.2	Q5F2E7_S304_M1_Nufip2	3.6606	3.6585	3.1972	3.9236	3.8675	4.2408	3.5243	3.8863	3.5834	3.3845	3.9578	3.3846	3.6915	3.2352	3.9809	3.0227	3.3835	3.7659	4.0650	4.4134
Nufip2	_DYEIENQNPLAS[Phospho (STY)]PTNTLLGSAK_.3	Q5F2E7_S626_M1_Nufip2	4.0177	3.9869	3.8585	3.2623	3.3554	3.5795	4.1857	3.0468	4.2448	4.2240	3.1183	4.2241	4.3528	3.8966	3.7629	4.3626	4.0449	3.1046	3.4036	3.5739
Nufip2	_NDS[Phospho (STY)]WGSFDLR_.2	Q5F2E7_S649_M1_Nufip2	4.2349	4.5850	3.7715	3.3493	2.9410	4.4752	4.1071	3.8724	4.0273	4.0455	3.5060	4.3112	4.1928	3.1946	4.1325	3.5970	4.4101	3.4231	3.0716	3.1616
Taok1	_NS[Phospho (STY)]PQALR_.2	#N/A	9.5769	9.1395	9.6605	9.5938	8.7833	8.9501	9.1299	8.5292	8.9200	9.9961	9.0948	8.9067	8.8563	9.3229	8.1029	8.9761	8.7482	8.9103	8.9216	9.0498
Taok1	_NS[Phospho (STY)]PQALRR_.2	#N/A	8.3085	7.7154	8.0159	8.3114	7.5317	7.2605	7.6748	7.7987	7.4207	8.5800	7.4500	7.6380	7.6796	7.6111	6.8637	7.6010	7.6232	7.4763	7.1220	7.6654
Taok1	_ASDPQS[Phospho (STY)]PPQVSR_.2	Q5F2E8_S421_M1_Taok1	12.1555	11.9109	12.1601	12.1511	12.0214	11.7073	11.6373	12.2682	11.7810	12.0433	11.9068	12.1471	12.4646	12.2708	11.7583	12.2188	12.3924	12.0410	11.9890	12.1449
Taok1	_AGS[Phospho (STY)]LKDPEIAELFFKEDPEK_.3	Q5F2E8_S9_M1_Taok1	3.3476	4.2446	4.3850	4.2367	3.5545	2.8976	3.3169	3.2589	4.8211	4.7926	4.3933	4.3356	3.5793	5.7783	3.2142	3.1747	3.7966	4.3074	3.4195	3.5463
Mlip	_AEYVFIVDS[Phospho (STY)]DGEDEATC[Carbamidomethyl (C)]R_.2	Q5FW52_S85_M1_Mlip	3.9757	3.7435	4.0750	3.6674	3.8316	3.9314	4.2985	3.9743	4.2896	3.9887	3.4835	3.5759	4.4253	7.9981	3.4420	3.8896	3.9674	7.6758	3.5033	3.5290
Arhgap1	_SSS[Phospho (STY)]PEPVTHLK_.2	#N/A	12.9749	12.9190	13.0998	12.9832	12.6380	12.8622	12.4985	13.0804	12.7429	12.9364	12.6177	12.6074	3.1662	13.0378	12.4272	12.5741	12.9796	12.7858	12.7272	12.6380
Arhgap1	_STQKS[Phospho (STY)]PATAPK_.2	Q5FWK3_S223_M1_Arhgap1	7.8661	7.8864	7.7068	7.7714	7.0480	7.6108	7.6551	7.7565	7.6773	7.7762	7.3500	7.4280	7.6739	7.5075	6.5022	6.7883	7.6950	7.4309	7.0107	7.1054
Arhgap1	_FLLDHQGELFPSTDAQGV_.2	#N/A	6.1441	6.5132	6.4479	6.4217	6.1160	5.8409	6.4476	6.0827	5.8912	6.6129	6.0787	6.2584	6.1151	5.9173	6.0799	6.6427	6.2390	6.1983	6.3915	5.9793
Arhgap1	_SDDSKSS[Phospho (STY)]S[Phospho (STY)]PEPVTHLK_.2	#N/A	3.7599	7.6262	7.9149	3.7812	7.1632	7.7458	7.1014	7.7880	7.5357	7.4542	6.7104	5.6033	7.3399	8.2844	3.4229	4.1498	7.8705	3.6235	7.4487	7.0048
Arhgap1	_LAS[Phospho (STY)]IDEK_.2	Q5FWK3_S27_M1_Arhgap1	3.1946	3.3074	3.7309	4.4948	4.0332	4.2572	4.8148	4.3958	3.5670	5.0199	4.8638	3.1968	3.6751	3.7180	3.0851	3.5395	3.4327	4.6254	5.1458	4.1888
Xkr7	_TKEGS[Phospho (STY)]AELVPR_.2	#N/A	3.4233	3.8957	4.4608	4.1609	6.8910	7.2078	3.4178	3.6490	3.3380	3.2339	4.3175	3.6219	3.4542	4.4988	3.4432	4.2863	3.1463	4.0032	4.3022	4.1761
Xkr7	_EGS[Phospho (STY)]AELVPR_.2	#N/A	7.4950	7.6442	6.9927	7.1078	2.9313	7.2004	7.3151	6.8443	6.8509	7.4730	7.0687	7.0539	4.2024	6.7788	4.1421	3.5873	6.9692	6.9545	6.5354	6.9434
Xkr4	_GS[Phospho (STY)]GAGSAALC[Carbamidomethyl (C)]LR_.2	Q5GH67_S87_M1_Xkr4	3.5126	3.8065	7.1168	4.0716	5.9432	3.7529	3.3286	6.4166	3.2488	3.3232	4.2283	6.4594	3.4142	4.5881	3.3539	4.3755	6.7790	3.9139	6.6019	3.8839
Xkr4	_TVVSSGSAAGEGEVRPST[Phospho (STY)]PQR_.3	Q5GH67_T208_M1_Xkr4	3.4057	3.9134	4.4431	6.4100	3.6127	2.9949	3.2694	3.6314	3.3285	3.6394	3.7029	3.6395	3.4366	4.4812	2.8466	3.7780	3.1286	6.4027	5.6152	4.1585
Vps13a	_EYTEAS[Phospho (STY)]PLEDKVVELDNIPVR_.3	Q5H8C4_S2581_M1_Vps13a	3.7887	4.2141	4.3250	3.9007	3.1264	3.3505	4.4147	3.2758	2.9729	3.9950	3.3473	4.1036	4.5818	4.1256	3.9919	4.1336	4.2739	4.3764	3.1746	3.8029
C2cd4c	_HGS[Phospho (STY)]LSADDSTPDTSPGVR_.2	#N/A	5.7594	5.9200	6.0810	3.6362	5.5526	5.5733	5.3693	4.0583	5.1809	5.6998	5.8916	5.5342	5.8541	6.2815	5.7766	5.7104	5.6959	5.8173	6.0235	5.2500
C2cd4c	_AQTSYGFATLAES[Phospho (STY)]PHTR_.2	#N/A	4.0960	4.1925	5.3590	5.0929	3.7804	4.5052	4.3964	4.0979	5.1741	4.1839	4.0749	4.6697	5.0086	5.4453	4.2965	3.6858	4.9996	5.0708	3.5595	5.0487
C2cd4c	_ESLFHSEHGALAQVGS[Phospho (STY)]PGAGR_.3	Q5HZI2_S175_M1_C2cd4c	5.2778	4.2937	5.1898	3.9803	3.0468	3.6086	5.1174	5.2853	3.0525	5.4328	4.5270	5.0805	5.7789	4.2325	4.0714	4.0540	3.9472	5.0092	3.5346	4.1995
C2cd4c	_RAT[Phospho (STY)]PEPGPESGQAPR_.3	Q5HZI2_T282_M1_C2cd4c	3.4890	4.5138	4.0253	4.2344	4.3276	3.0508	3.0761	3.5755	3.2726	3.6953	3.6470	4.4033	3.3807	4.4253	2.7907	3.8339	3.0727	4.0767	4.3758	4.1026
Cnrip1	_IQPNDGPVFFK_.2	#N/A	8.7584	8.8312	8.7143	8.6759	8.6997	8.7256	8.7430	8.9395	8.7242	8.9917	8.7134	8.5796	9.0738	9.0567	8.8698	8.8636	8.6696	8.8432	8.7917	8.8825
Cnrip1	_VVYTGIYDTEGVAPTK_.2	#N/A	7.7471	7.8159	8.3958	7.8999	7.5268	7.8279	7.4895	7.8339	7.6821	7.9033	7.5636	7.7378	7.7234	7.9703	7.9566	7.7994	7.7123	7.5129	7.4267	7.8175
Cnrip1	_IKPTTLQVENISIGGVLVPLELK_.3	#N/A	5.2748	5.0864	5.3428	4.9906	4.8368	5.2610	4.2635	5.0048	4.4942	4.8075	4.3551	4.6112	3.3348	5.2846	4.8112	5.4473	5.3230	5.0377	5.3096	4.9838
Cnrip1	_[Acetyl (Protein N-term)]GDLPGLVR_.2	#N/A	4.6512	5.1129	5.3240	4.6281	5.4720	5.4088	4.5785	4.6813	5.9815	5.3098	4.8712	5.3412	4.0044	3.3886	5.9382	5.3180	5.1226	5.3289	5.2940	5.5605
Cnrip1	_DHC[Carbamidomethyl (C)]QWGSPFSVIEYEC[Carbamidomethyl (C)]KPNETR_.4	#N/A	3.3768	4.1159	4.3803	4.4740	4.3070	3.4430	4.5928	3.2297	4.1045	3.4153	4.0499	4.3807	3.5500	4.5909	4.6415	4.6584	3.7673	4.5346	4.6474	4.2199
Cnrip1	_LLTGSSYK_.2	#N/A	2.8600	3.8961	1.8755	5.1780	4.0719	4.6324	4.7150	4.3992	4.4105	5.1625	4.4439	4.8703	4.6805	4.0331	4.9279	4.6779	3.8964	4.9994	4.4638	4.3236
Kctd1	_S[Phospho (STY)]PAS[Phospho (STY)]PLNNQGIPTPAQLTK_.2	Q5M956_S12_M2_Kctd1	4.3886	3.3681	3.4876	3.6332	3.7263	3.9504	3.8147	4.1767	3.8738	3.0941	4.2482	3.0942	3.9819	6.9761	3.3919	3.2327	3.6739	5.6997	3.7746	3.2029
Cobl	_TIS[Phospho (STY)]PPSAVVETDTPPIGK_.2	Q5NBX1_S826_M1_Cobl	9.2976	4.5662	3.7903	8.7726	8.5793	8.3553	8.1810	4.4794	8.1480	9.2069	8.7094	4.2923	4.2846	3.8284	8.1112	8.7840	9.0246	3.1728	8.5973	8.8626
Cobl	_AEHLGLSGADS[Phospho (STY)]DEDPAK_.2	Q5NBX1_S272_M1_Cobl	3.5444	4.4584	4.0807	4.1450	2.8821	3.6020	3.4795	3.2448	3.3997	7.3569	4.3792	4.3478	3.5651	4.0678	3.5049	7.3272	3.7825	4.0507	3.6993	4.0348
Cobl	_KEPDPS[Phospho (STY)]PPSQDNR_.2	Q5NBX1_S676_M1_Cobl	3.6863	3.2821	3.7561	7.6862	3.8932	3.9265	3.1549	4.4210	3.0751	3.4969	4.0546	7.3556	3.2405	3.7432	3.1803	3.0484	3.4579	4.3753	4.0239	3.7102
Cobl	_S[Phospho (STY)]SLGNDETDKEK_.2	#N/A	4.5635	4.2564	4.1001	4.5216	3.2696	3.3029	3.7785	3.5438	3.6987	4.3741	3.1773	3.9825	3.8641	4.3668	3.8039	3.9256	4.0815	3.7517	3.4003	3.7773
Cobl	_KSS[Phospho (STY)]LGNDETDKEK_.2	Q5NBX1_S235_M1_Cobl	4.2520	4.6043	4.8163	3.4674	3.9040	4.0430	4.5882	3.9993	4.4621	5.1061	4.4161	4.0304	5.0935	4.5862	4.0102	4.0780	4.3009	4.3913	4.3503	4.2617
Cobl	_S[Phospho (STY)]PPTRKDDAAPNLHSEAR_.4	Q5NBX1_S982_M1_Cobl	4.3463	3.6565	3.3817	3.3431	3.6840	3.9081	3.8571	4.0467	3.9162	4.5526	4.2905	3.5460	4.3670	3.3688	3.4343	3.1903	3.0835	3.2488	4.3982	3.3358
Dph1	ospho (STY)]PAPAC[Carbamidomethyl (C)]ESC[Carbamidomethyl (C)]NC[Carbamidomethyl (C)	Q5NCQ5_S418_M1_Dph1	3.9110	3.4080	4.0251	3.6798	4.1180	3.9904	4.4310	4.1367	3.8339	3.1341	4.2468	3.1342	3.9419	2.9362	2.9555	3.2732	3.6340	3.0082	3.8145	3.1630
Neurl4	_ALS[Phospho (STY)]PEGALRR_.2	#N/A	6.7110	5.9651	6.4442	5.2089	5.1939	5.1684	5.3565	6.0417	5.2234	5.4521	5.0271	3.4738	4.4392	5.6051	3.3621	5.5844	5.9033	5.6055	5.5271	3.4080
Neurl4	_ALS[Phospho (STY)]PEGALR_.2	#N/A	5.1892	3.8262	3.2121	5.5687	3.5143	4.8304	5.1019	3.8770	4.1553	4.3829	2.9594	4.8724	4.1939	4.8711	4.7283	4.5297	4.4147	4.8773	3.5626	4.7791
Neurl4	_S[Phospho (STY)]FPLHS[Phospho (STY)]PVAGVAHR_.3	Q5NCX5_S904_M2_Neurl4	4.4688	3.4483	3.4074	3.7134	3.8066	5.5271	4.6228	4.0965	5.1924	3.1743	4.1680	3.1744	3.9017	3.4454	3.3117	3.3129	3.5937	3.5557	3.8548	3.1227
Crad	_ESSDNQPPS[Phospho (STY)]PSLPEELK_.2	#N/A	3.9962	8.4908	9.0793	8.6566	7.9935	8.4451	7.9651	3.3879	8.4006	8.8593	8.6821	8.1555	4.0074	9.1658	3.4174	8.4520	9.0435	8.1219	8.2785	8.5990
Crad	_ETAQTDS[Phospho (STY)]PQPQER_.2	Q5PR69_S487_M1_Crad	7.5452	3.8067	7.7527	7.7158	7.3574	7.4479	7.2762	7.6760	3.2490	3.3230	4.2285	7.6037	3.4144	8.4132	7.1552	7.7609	8.1386	7.6453	7.7456	7.7794
Crad	_VNLS[Phospho (STY)]PVTPAK_.2	Q5PR69_S552_M1_Crad	3.9208	3.3983	3.4574	3.2032	4.1277	4.1611	4.6117	4.1865	4.3414	5.6625	3.8201	3.1244	4.5069	5.5390	2.9458	3.2829	3.2234	3.1089	4.2584	3.4757
Crad	_ESSDNQPPS[Phospho (STY)]PSLPEELKGQK_.3	#N/A	7.9661	8.1430	7.9387	4.0793	7.5431	7.8011	7.9153	4.4074	3.9456	8.2534	3.6221	8.1385	8.3559	4.4414	3.4637	8.1689	5.1187	3.4546	7.8583	8.2075
Crad	_DLPSFLVPGLPS[Phospho (STY)]PQK_.2	Q5PR69_S783_M1_Crad	6.7663	7.3243	5.8516	4.1419	2.8852	3.1093	7.2715	7.4031	3.2141	3.7538	3.5885	7.1148	6.6188	8.2075	4.2330	3.8924	3.7856	6.6725	5.9333	4.0379
Crad	_PASQT[Phospho (STY)]PPSS[Phospho (STY)]PLSK_.2	Q5PR69_S953_M2_Crad	3.1163	4.2130	4.3261	3.8996	3.3232	3.3566	3.7249	3.4902	3.6451	4.4277	3.1237	4.1024	3.8105	4.3132	3.7502	3.9792	4.0279	3.8053	3.4539	4.2802
Diras2	_C[Carbamidomethyl (C)]DES[Phospho (STY)]PNREVQSSEAEALAR_.3	Q5PR73_S126_M1_Diras2	3.1789	6.5997	6.7947	7.1555	5.3485	6.3527	6.7385	3.8856	3.5827	6.7152	3.9571	6.3227	6.7925	7.1105	6.1235	6.9050	6.7215	6.6835	7.1267	6.6097
Diras2	_VAVFGAGGVGK_.2	#N/A	5.6507	5.8781	3.9226	3.1982	3.0921	5.8028	5.7740	5.3009	5.5523	5.3539	5.6790	5.8928	5.5445	5.4591	3.9814	5.2482	4.5912	6.2874	5.2472	5.5819
Diras2	_ELFQELLNLEK_.2	#N/A	5.9661	6.0599	5.5888	5.9522	5.7663	5.5688	5.7814	6.1625	5.8429	3.2204	5.7853	5.7267	6.2412	5.8362	5.5464	5.4607	5.4839	5.9111	5.0604	5.5227
Diras2	_PEQSNDYR_.2	#N/A	3.8357	3.4834	3.3723	3.7486	3.7572	4.2815	4.6642	4.0614	4.1029	3.2094	4.1648	3.2095	4.7311	4.4573	4.9338	3.1978	3.6092	5.1333	4.1702	2.8022
Diras2	_GTFRES[Phospho (STY)]YIPTVEDTYR_.3	Q5PR73_S35_M1_Diras2	3.6541	3.6650	3.1907	3.9301	3.8610	4.2474	3.5178	3.8798	3.5769	3.3910	3.9513	3.3911	3.6850	3.2287	3.0950	3.5296	3.3770	3.7725	4.0715	4.4068
Mdc1	_VLLAADS[Phospho (STY)]EEEGDFPSGR_.2	Q5PSV9_S168_M1_Mdc1	4.3248	9.2777	3.3602	3.3646	3.6625	8.7344	8.6880	4.0252	8.6509	4.5311	4.3120	3.5675	4.0457	9.7866	8.3407	3.1688	3.0620	9.0841	8.4494	7.8613
Osbp2	_IVHSS[Phospho (STY)]PSSDGR_.2	Q5QNQ6_S760_M1_Osbp2	8.6230	8.7141	8.5343	8.4020	8.2569	3.2507	8.5733	8.8285	8.1901	8.4834	3.4471	8.4011	8.8202	8.8683	4.0917	8.5138	8.8037	8.8453	3.0748	8.2571
Brsk1	_STPLPGPPGS[Phospho (STY)]PR_.2	Q5RJI5_S508_M1_Brsk1	11.2830	10.6865	10.9839	10.6947	10.4609	9.5992	9.9978	10.5758	10.2814	10.8965	9.9647	10.4525	10.9116	10.3668	8.7943	9.9311	10.3338	10.1711	9.7358	10.3380
Brsk1	_SRLNS[Phospho (STY)]IR_.2	Q5RJI5_S555_M1_Brsk1	3.9299	6.3651	6.5724	3.1745	6.9595	6.3001	6.9211	6.1828	6.6259	4.1362	5.4573	6.2392	6.4951	5.7658	5.9079	6.8275	5.8932	5.8551	6.4552	5.8018
Brsk1	_NSFLGS[Phospho (STY)]PR_.2	Q5RJI5_S563_M1_Brsk1	13.5052	13.4095	13.3584	13.3869	4.1909	12.2344	12.7986	12.4624	12.3039	9.9765	3.7569	12.1974	13.0899	12.8229	2.8826	12.6537	12.4607	12.5594	3.7416	3.4125
Brsk1	_FQVDISSSEGPEPS[Phospho (STY)]PR_.2	Q5RJI5_S676_M1_Brsk1	4.1832	6.0599	3.2186	3.5062	3.5209	3.7450	4.0202	3.8835	4.7769	5.9810	2.9528	3.7091	4.1873	3.2057	3.5974	3.0272	4.4212	3.4119	3.0605	3.4084
Brsk1	_SSGGT[Phospho (STY)]PLHS[Phospho (STY)]PLHTPR_.2	#N/A	6.0342	5.9965	5.7904	5.1402	3.6648	4.5448	4.7666	4.6153	4.1644	6.3458	4.7969	5.0328	3.9236	5.6131	3.4645	4.5578	4.2923	4.7318	4.9914	4.5727
Brsk1	_S[Phospho (STY)]PVFSFSPEPGAGDEAR_.2	Q5RJI5_S450_M1_Brsk1	4.5670	3.5465	9.8528	3.8116	3.9047	4.1288	3.6363	3.9983	3.6954	9.5635	4.0698	3.3252	3.8035	3.3473	3.2135	3.4111	3.4955	3.6539	3.9530	3.0245
Brsk1	_SVSGASTGLSSS[Phospho (STY)]PLSS[Phospho (STY)]PR_.2	Q5RJI5_S443_M2_Brsk1	3.4572	4.8337	4.3139	4.1939	3.3750	3.4602	5.0815	4.9038	3.1507	5.1085	3.7830	4.1986	3.4840	5.1194	3.8898	4.8506	3.9165	4.6242	3.9083	4.1103
Brsk1	_S[Phospho (STY)]M[Oxidation (M)]EVLSITDAGSGGSPVPTR_.2	Q5RJI5_S399_M1_Brsk1	7.6567	8.0436	6.9974	4.1043	2.9229	3.1469	7.0777	7.2626	5.9321	6.6765	3.5508	6.3779	3.2845	4.3291	4.9817	5.9772	5.7897	4.1729	6.7054	5.6132
Brsk1	_VDS[Phospho (STY)]PMLSR_.2	Q5RJI5_S384_M1_Brsk1	3.3905	4.4872	4.0519	5.7695	3.5974	3.6308	3.4507	3.2160	3.3709	3.0737	4.3504	3.6547	3.5363	4.1262	3.4760	4.2535	3.7537	4.0795	3.7281	4.0060
Brsk1	_SLPS[Phospho (STY)]NGELDPDVLESM[Oxidation (M)]ASLGC[Carbamidomethyl (C)]FR_.3	Q5RJI5_S311_M1_Brsk1	4.7373	5.3840	3.9375	4.0565	3.1070	3.9926	3.9620	4.0617	3.8822	4.2115	3.3608	4.1660	4.0476	4.4058	3.9874	3.7630	4.0120	3.5891	3.4559	3.8688
Islr2	_EESLAGC[Carbamidomethyl (C)]S[Phospho (STY)]LVESQSK_.2	Q5RKR3_S700_M1_Islr2	3.7994	5.8787	6.6759	3.9096	3.9195	3.9166	3.5684	3.3647	4.3200	5.6632	4.9895	4.9257	3.8830	5.7772	3.3554	3.7273	6.7770	5.5763	5.2537	4.8107
Islr2	_ASYLES[Phospho (STY)]EKS[Phospho (STY)]YPAR_.2	Q5RKR3_S654_M2_Islr2	4.2193	4.6006	3.7559	3.3649	2.9254	4.4596	5.3236	4.4450	4.0429	4.0299	3.5215	4.3267	4.2502	3.7940	4.1480	3.5815	5.8550	5.1166	3.5062	3.4713
Lrrk2	_SNS[Phospho (STY)]ISVGEVYR_.2	Q5S006_S910_M1_Lrrk2	6.4171	3.5972	3.2585	3.8623	3.9288	6.3819	3.1193	3.9476	3.0395	3.5325	5.8813	6.1783	3.2049	5.7021	3.1446	3.0840	3.4448	4.4109	4.0595	6.4867
Ccdc88a	_AGS[Phospho (STY)]PGSEVVTLQQFLEESNK_.2	Q5SNZ0_S1677_M1_Ccdc88a	4.9026	4.6647	3.3843	4.0795	4.5310	4.5200	5.0764	4.8337	4.4063	4.9868	3.5535	4.0377	4.2403	3.6358	3.2951	3.5578	3.6219	4.3142	4.0060	4.1277
Ccdc88a	_FYDPS[Phospho (STY)]PPR_.2	Q5SNZ0_S1387_M1_Ccdc88a	3.6048	3.2007	3.8376	8.1705	8.8431	3.8451	3.2363	4.5025	8.5947	3.4154	4.1360	3.0902	3.3220	3.8246	3.2617	2.9670	3.5393	8.5242	3.9424	3.7916
Ccdc88a	_RT[Phospho (STY)]SIHDFLSK_.3	Q5SNZ0_T1821_M1_Ccdc88a	3.6730	3.6461	3.2096	3.9112	3.8799	3.9133	6.7601	4.4343	6.1974	3.4836	4.0679	3.3722	6.6598	3.7565	3.1935	3.0351	3.4712	4.3620	4.0106	3.7235
Ccdc88a	_QKS[Phospho (STY)]LTLTPTR_.2	Q5SNZ0_S1417_M1_Ccdc88a	7.3129	7.5136	3.3175	7.0865	6.8659	6.7001	3.9214	7.1475	3.9804	4.4883	6.1917	3.6103	6.6029	6.8840	3.4986	6.1325	3.7805	6.5524	6.3899	5.8052
Ccdc88a	_DT[Phospho (STY)]TSFEDISPQGISDDSSTGSR_.2	Q5SNZ0_T1560_M1_Ccdc88a	3.8524	6.3269	5.8625	3.7682	4.0593	4.6612	5.6840	4.0780	3.7752	4.0452	4.1496	5.6815	3.8832	6.5681	3.3883	3.2145	3.5753	3.6105	3.9096	4.1926
Ccdc88a	_SVS[Phospho (STY)]GKTPGDFYDRR_.3	Q5SNZ0_S1738_M1_Ccdc88a	4.4805	4.4196	3.5160	3.2089	3.8183	4.1667	4.4156	4.1809	3.0147	3.7370	3.8144	3.4117	4.5012	3.5031	3.1063	3.3246	3.2177	3.1146	4.2640	3.4701
Ccdc88a	_VHASRPAS[Phospho (STY)]LDSGR_.3	#N/A	4.0110	5.1841	3.5476	4.0614	3.5361	5.3278	3.6889	4.4074	3.6926	3.8216	3.5874	3.8929	5.1944	3.7435	3.7142	4.1686	3.1744	3.9480	4.7386	3.5665
Ccdc88a	_SSS[Phospho (STY)]QENLLDEVMK_.2	Q5SNZ0_S1704_M1_Ccdc88a	3.1956	4.1235	4.2330	4.3887	3.4025	3.4358	3.6456	3.4212	3.5658	4.5070	3.0445	3.8496	3.7313	4.2339	3.6710	4.0585	4.4193	3.8846	3.5332	4.2009
Ccdc88a	_S[Phospho (STY)]SPMLQHK_.2	Q5SNZ0_S1653_M1_Ccdc88a	3.6949	4.3079	4.2312	4.4404	3.0327	3.2568	3.0076	3.3695	3.0667	3.9013	3.4410	4.1973	3.1747	4.2193	4.0856	4.0398	4.3676	4.2827	3.0809	3.8966
Ccdc85a	_STS[Phospho (STY)]PEHLHKPR_.2	#N/A	9.4342	9.3802	9.1642	9.4021	9.0957	8.8809	3.4161	9.5702	3.7632	3.4237	9.2471	9.2505	9.7791	3.4150	8.9184	9.1772	9.3501	9.1824	8.7799	9.1583
Ccdc85a	_GTS[Phospho (STY)]PEHLK_.2	#N/A	4.3018	7.4668	7.3755	3.5843	7.1124	6.9148	4.0402	3.8055	3.9604	4.1124	6.7559	7.0463	4.1259	7.2013	6.8914	6.8736	4.3432	3.4900	7.2397	6.6205
Ccdc85a	_HALGGS[Phospho (STY)]LEHLPR_.3	#N/A	6.7731	6.1748	4.9194	6.3955	5.3640	5.3153	5.4585	5.1909	5.1641	7.0988	6.1664	5.6828	6.3499	5.9913	3.6878	5.7350	5.4432	5.8364	5.3577	6.0627
Ccdc85a	_ARGTS[Phospho (STY)]PEHLK_.2	#N/A	4.1930	3.8098	3.2284	3.4964	3.5307	3.7548	4.0104	3.8933	4.0695	4.3993	2.9430	3.6993	4.1775	3.7213	3.5876	4.3443	4.4310	3.2799	3.5789	3.3986
Ccdc85a	_HRPGGS[Phospho (STY)]PEHTR_.3	Q5SP85_S320_M1_Ccdc85a	3.9736	6.0352	3.5102	4.9192	4.1806	5.7026	3.8374	4.1993	6.0193	4.5723	4.2708	3.0716	4.0045	3.5483	2.8929	3.3358	3.6966	3.4529	3.7519	5.7896
Ccdc85a	_AAGGSAPAAESC[Carbamidomethyl (C)]PSAPAGAST[Phospho (STY)]PTGVDDLSK_.3	Q5SP85_T24_M1_Ccdc85a	5.8153	4.2675	4.0890	4.6782	3.2585	3.2919	3.7896	3.2773	3.7098	4.3630	3.1884	3.9936	3.8752	4.3779	3.8150	3.9145	4.2753	3.7406	3.3892	4.3449
Ccdc85a	_GS[Phospho (STY)]PEQQRHPHPGSS[Phospho (STY)]PEVLPK_.3	Q5SP85_S284_M2_Ccdc85a	4.5598	4.9309	3.8700	3.9834	3.6127	5.2337	4.7211	4.8544	3.7027	3.9266	3.7022	3.6929	5.5485	3.8116	3.5410	3.6887	4.7061	4.7605	4.5912	4.9329
Ccdc85a	_HASGSGGSGGGS[Phospho (STY)]REGTLR_.3	Q5SP85_S380_M1_Ccdc85a	3.1371	3.3649	3.6734	4.5523	3.9757	4.1997	3.5654	4.3383	3.6245	3.3434	3.9989	3.2543	3.7326	3.6605	3.1426	3.4820	3.3751	4.4580	4.1066	3.6275
Ccdc85a	_HVLSGS[Phospho (STY)]PEHFQK_.2	#N/A	3.6406	6.2837	3.9981	3.9821	6.1619	3.6558	5.5733	3.8662	3.9582	3.6640	5.9228	3.4431	6.4060	6.4852	2.9205	6.3725	5.9210	3.8244	6.1110	5.8933
Ccdc85a	_HHASGGS[Phospho (STY)]PEHLQKPR_.3	#N/A	4.2884	4.1555	3.6183	3.3660	4.5219	3.0761	3.9916	3.5387	3.9299	4.2924	3.4085	3.9500	4.0953	3.9963	3.8006	3.4376	4.2685	4.4136	4.7425	3.0641
Ccdc85a	_HSGGS[Phospho (STY)]PEHLQK_.2	Q5SP85_S330_M1_Ccdc85a	7.8742	7.9248	7.9644	8.2006	7.5830	7.8281	7.7290	8.1308	7.7186	8.2608	7.8031	8.0369	8.5718	8.2450	8.1513	8.0331	8.0498	7.9771	7.9749	7.8666
Ccdc85a	_HPHPGS[Phospho (STY)]SPEVLPK_.3	Q5SP85_S295_M1_Ccdc85a	3.6305	4.1108	4.2457	4.3760	2.9682	3.1923	3.0720	3.4339	3.1311	3.8368	3.5055	3.8369	3.2391	4.2837	4.1500	3.9754	4.4320	4.2183	3.0165	3.9610
Ccdc85a	_GPS[Phospho (STY)]PEHHKPLC[Carbamidomethyl (C)]K_.3	Q5SP85_S273_M1_Ccdc85a	3.7346	3.3305	3.7078	4.5179	3.9416	3.9749	3.1065	4.3727	3.0267	3.5452	4.0062	3.2200	3.1922	3.6948	3.1319	3.0968	3.4095	4.4236	4.0722	3.6618
Cyfip2	_SYLQDPIWR_.2	#N/A	6.5825	6.3003	5.8198	6.5647	6.6451	6.2115	6.6240	6.4547	5.9498	6.4301	6.3989	6.3896	6.1993	5.2630	5.3569	5.9291	6.2503	6.2582	5.9193	6.3506
Cyfip2	_YAPLHLVPLIER_.2	#N/A	6.4565	5.9597	4.5201	5.1841	5.1914	5.9172	6.3987	6.6933	4.5393	5.0541	5.9310	4.5618	5.9470	5.2382	3.4283	4.8397	6.1342	6.1412	5.7414	5.3723
Cyfip2	_FISELAR_.2	#N/A	5.4330	5.3164	5.4208	3.9362	5.6297	5.4129	5.5417	5.4629	5.5690	4.3093	5.7658	5.9182	3.5077	4.9568	4.8883	3.8608	5.5979	5.8781	5.6434	4.9274
Cyfip2	_QLQVVPLFGDM[Oxidation (M)]QIELAR_.2	#N/A	5.9986	5.2964	5.1704	4.7923	4.9213	5.7049	5.0604	5.9345	5.4073	4.6935	5.2591	5.6109	5.1011	3.2390	3.9829	3.8108	5.2877	5.4620	5.2603	4.5515
Cyfip2	_SLDQAISR_.2	#N/A	5.0011	5.1176	5.5869	5.7194	6.1376	5.1979	5.9297	6.2444	5.1527	4.5140	5.3316	4.4584	5.8590	6.8785	4.5701	3.8067	4.6705	5.0486	4.6240	3.4383
Cyfip2	_SVETDSSTVEHVR_.2	#N/A	3.6206	5.0562	4.8258	5.3806	3.8275	3.8608	4.7556	4.7875	5.4262	5.0835	5.0695	4.8616	4.7328	3.1952	4.5489	4.0014	4.7053	4.6220	4.6844	4.1032
Cyfip2	_LGT[Phospho (STY)]PQQIAIAR_.2	Q5SQX6_T1067_M1_Cyfip2	6.2299	5.6002	3.2219	3.4889	5.2535	4.8354	4.4009	5.3068	4.9651	4.3927	5.2462	3.7059	4.4617	4.9152	3.5942	5.1854	5.0689	5.4344	5.0109	3.9572
Cyfip2	_NAFVTGIAR_.2	#N/A	4.9710	4.7742	3.4378	5.1551	4.4569	4.2869	4.8184	4.4509	4.6819	3.9898	3.9143	3.7290	3.5270	4.5716	3.6968	4.3591	3.2191	4.6624	4.7074	4.1329
Cyfip2	_YSNSEVVTGSGLDSQK_.2	#N/A	6.7893	3.5899	6.5805	5.9765	3.9482	6.2257	6.2502	6.1524	5.9692	3.3159	4.0264	3.3160	3.7601	3.3038	5.6415	4.8639	5.8771	5.9010	5.4075	2.9811
Cyfip2	_M[Oxidation (M)]ESVFNQAIR_.2	#N/A	3.1246	6.3751	5.6253	4.9448	6.5308	5.8381	5.4617	6.6551	5.5435	6.5445	6.0258	5.5409	3.1453	5.5359	3.0850	5.5932	6.4927	6.3236	4.1191	5.6204
Cyfip2	_EANHNVSAPYGR_.2	#N/A	3.5820	3.7370	3.1186	4.0022	3.7890	3.8223	3.2591	3.8077	3.1793	3.3926	4.1588	3.4632	3.3448	3.1567	3.2845	2.9442	3.3049	3.8445	4.1435	3.8144
Tom1l2	_NNPPTIVQDK_.2	#N/A	7.8636	7.9608	8.2206	7.8762	7.8181	7.9614	7.9134	8.6231	8.2003	8.1901	8.2053	8.2608	8.4023	8.4083	8.2196	8.0347	8.2369	7.9614	7.7890	8.2372
Tom1l2	_FHLLVANR_.2	#N/A	6.5693	6.4353	6.3950	6.6613	6.6927	6.4256	6.6339	6.4398	6.1593	6.2977	6.5360	6.4872	6.5262	6.4917	6.3092	6.4373	6.2658	6.3267	6.5966	6.8300
Tom1l2	_TVTYEDPQAVGGLASALDNR_.2	#N/A	4.1603	4.1052	5.2395	3.4705	3.1574	4.3216	4.2737	4.4057	3.9215	4.7106	2.4870	4.7293	5.3907	5.4347	4.1096	4.1800	4.9044	4.7388	4.8560	4.0385
Tom1l2	_GNS[Phospho (STY)]LAEQR_.2	Q5SRX1_S394_M1_Tom1l2	8.3441	8.1670	8.3288	8.4906	7.4556	7.1918	7.7198	7.2328	7.0793	9.0897	7.5292	7.1961	7.3957	7.8868	6.5523	7.3246	6.7166	7.3194	7.4892	7.4422
Tom1l2	_GIEFPM[Oxidation (M)]ADLDALS[Phospho (STY)]PIHTPQR_.3	#N/A	7.8513	9.2522	7.6919	7.1247	5.2900	5.6866	8.8508	8.7492	7.2346	8.2978	6.3442	8.6054	8.3960	9.4677	7.0844	6.9950	6.8870	8.7177	7.8260	5.4705
Tom1l2	_IVELISR_.2	#N/A	3.9781	4.0247	2.6321	5.1213	3.3159	4.7530	5.0221	4.2032	5.0928	5.2708	4.7858	3.9141	3.9988	4.7510	4.5060	4.8510	4.2162	5.2449	3.2656	4.7087

Tom1l2	_AAETVPDLPS[Phospho (STY)]PPTEAPAPASNTSTR_.3	Q5SRX1_S479_M1_Tom1l2	12.9031	12.8816	11.6135	11.8705	10.8967	4.4156	12.5034	13.1327	11.4665	3.7850	11.4489	12.6859	13.1368	13.5384	11.9744	11.8843	12.1702	12.4447	12.1161	11.3226
Tom1l2	_DFIDSVLVK_.2	#N/A	3.1102	3.3918	3.6465	4.6859	4.0454	4.2323	3.0148	4.3114	4.9204	4.4587	3.9449	3.7437	4.0953	3.5704	2.9210	4.3311	3.3482	2.9841	4.1882	3.6006
Tom1l2	_EVM[Oxidation (M)]LALTVLETC[Carbamidomethyl (C)]VK_.2	#N/A	3.7494	3.3649	3.8328	4.0420	2.1802	3.2899	3.4060	2.7000	3.4651	3.5028	3.8395	3.0949	3.5732	4.4349	3.8739	3.9231	4.0891	3.8843	4.1833	4.2950
Tom1l2	_VSNEEVTEELLHVNDDLNNVFLR_.3	#N/A	3.1807	4.1992	4.2182	4.4035	3.3877	3.3890	3.6604	3.4064	3.3592	4.4922	3.0593	2.8477	3.7461	4.2461	3.6858	3.4558	4.4045	2.7105	3.5183	3.3711
Zzef1	_GDQEEELDRPVSS[Phospho (STY)]PGEAEQK_.3	Q5SSH7_S2407_M1_Zzef1	7.1476	4.1462	3.6307	6.8918	3.3154	3.4571	3.9903	7.1848	3.9862	7.1990	3.1594	6.7167	4.1199	3.6506	3.7941	6.0898	7.2412	3.2467	3.2010	6.2462
Zzef1	_S[Phospho (STY)]M[Oxidation (M)]EETRPVPTVK_.2	#N/A	7.5100	7.7112	6.6539	7.3683	6.8298	6.5381	7.1970	6.8711	6.3458	7.0996	7.1102	3.7858	3.4751	5.7723	3.6741	6.5125	7.0015	6.9576	3.8602	6.4000
Zzef1	_S[Phospho (STY)]PEMDKLK_.2	Q5SSH7_S240_M1_Zzef1	3.5244	6.4060	6.4175	6.8752	3.7313	5.6266	5.9023	3.7501	3.2370	3.3350	6.0805	4.2092	3.5553	6.4031	3.3422	4.3873	5.6242	3.9021	4.2012	4.2772
Zzef1	_LLPSSGPC[Carbamidomethyl (C)]VAEVSTAEEPS[Phospho (STY)]PPST[Phospho (STY)]PTR_.3	Q5SSH7_S1515_M2_Zzef1	5.8786	7.7486	3.9899	4.0371	3.1594	3.3982	6.5231	3.3639	4.0479	4.2527	3.1968	5.3744	4.1794	7.0715	3.8322	3.8155	4.1120	6.4259	3.3531	4.0499
Zzef1	_TSSVVEEHFQGSAS[Phospho (STY)]PTEAATPAAGDR_.3	Q5SSH7_S1475_M1_Zzef1	4.2295	4.5904	3.7661	3.3547	2.9356	3.6719	4.1125	4.4552	4.1523	4.0401	3.0258	4.3166	4.2604	3.8041	4.1379	4.7531	3.9524	3.1970	3.4961	3.4814
Utp18	_GQLHGS[Phospho (STY)]S[Phospho (STY)]DES[Phospho (STY)]EVENEAK_.2	Q5SSI6_S114_M3_Utp18	4.4172	4.4364	4.2199	4.4018	3.3894	4.3304	4.1644	3.8923	3.4221	5.0437	3.0576	4.5827	3.7444	5.5570	1.5128	3.1350	4.4062	4.2455	4.9467	4.2140
Abr	_SQGGGDSVS[Phospho (STY)]PTPPEGLAPGVEAGK_.2	#N/A	7.5804	7.9646	7.6329	7.8586	6.8602	7.1579	3.7968	3.8072	3.7683	8.2941	3.6555	7.3841	7.8765	7.6088	5.8148	7.3147	7.6991	3.8159	7.8321	7.1829
Abr	_GIEEVGIYR_.2	#N/A	3.2598	3.3795	3.4761	3.6447	4.1465	3.7232	4.3504	4.1652	4.3227	5.6126	4.6817	2.8176	3.9704	3.3092	2.9270	3.3017	3.6625	4.3502	4.1829	4.5108
Abr	_ISQNFLSSINEDIDPR_.2	#N/A	4.5650	3.4379	3.6004	4.9484	3.9027	4.1268	3.6384	4.2653	3.6975	3.2704	4.0719	3.3273	3.8055	3.3493	3.2156	3.4090	3.3022	3.5048	3.9509	3.0266
Abr	_GPPEGSETMPYIDES[Phospho (STY)]PTMSPQLSAR_.3	Q5SSL4_S53_M1_Abr	3.3213	3.9978	6.5630	4.2629	3.5282	3.0793	4.7998	3.5470	3.2441	3.7238	3.6185	3.7239	3.3522	7.1671	4.2630	4.1842	4.5450	4.1052	4.4043	4.0740
Abr	_C[Carbamidomethyl (C)]SQSNNQFQK_.2	#N/A	4.0502	4.5305	3.8260	3.2948	3.3879	3.6120	4.1531	4.5151	4.2122	4.2565	3.0858	4.2566	4.3203	3.8640	3.7303	4.3951	4.0123	3.1371	3.4362	3.5413
Arhgap44	_HSATDKRDS[Phospho (STY)]EEESESTAL_.3	#N/A	5.5720	7.1235	4.0231	7.1114	6.4980	4.9218	4.9486	5.4735	6.2548	5.9616	6.9840	6.6902	7.0872	5.6037	5.9209	7.0384	5.5239	7.0625	7.2917	5.1383
Arhgap44	_SKELS[Phospho (STY)]PGSGQK_.2	Q5SSM3_S598_M1_Arhgap44	4.4014	7.9048	7.6561	8.1979	7.5537	6.5517	7.0284	4.1018	3.8611	7.9085	7.2125	7.1766	7.7743	7.5495	6.7638	7.4382	7.2832	7.2872	4.3431	6.7662
Tns3	_RES[Phospho (STY)]PPSAER_.2	Q5SSZ5_S934_M1_Tns3	8.4101	4.1159	4.4232	3.8025	3.2261	3.2595	3.8220	3.5873	9.1913	4.3306	9.1914	4.0054	3.9076	4.4103	3.8473	3.8822	4.1249	3.7082	3.3568	9.2652
Coil	_VTGS[Phospho (STY)]PAEEDEEETK_.2	#N/A	3.2243	7.6950	3.7606	8.7178	5.6876	7.1684	7.5047	8.4808	7.6858	3.4306	7.8580	7.9757	7.2410	8.4866	7.1805	8.0030	3.4623	4.3708	7.3196	7.9359
Coil	_VTGS[Phospho (STY)]PAEEDEEETKK_.3	#N/A	3.9068	5.6937	4.4431	3.7826	3.2445	3.2396	5.2977	5.4021	4.3148	4.1131	4.2533	3.9854	6.3824	4.7695	4.1219	3.9546	5.5277	5.3056	4.7789	2.1225
Coil	_RQEDSGQNEKAS[Phospho (STY)]DLETK_.3	#N/A	3.7325	3.5307	3.8855	4.3740	4.1560	3.7873	3.8191	4.3111	4.3035	3.4085	4.0492	2.4653	4.2956	3.6329	2.3775	3.6623	3.8506	4.4196	4.2766	3.8078
Coil	_QEDSGQNEKAS[Phospho (STY)]DLETK_.2	#N/A	3.7550	3.8677	3.2916	3.8292	3.9619	3.9953	3.0862	3.9807	4.3551	3.5656	3.9859	3.2902	3.3589	3.3296	2.9546	3.1171	3.4779	3.6715	3.9706	3.0069
Luc7l3	_NEVNGTSEDIKSEGDTQS[Phospho (STY)]N_.2	Q5SUF2_S431_M1_Luc7l3	4.6888	4.3323	3.2747	3.6723	3.8296	3.9672	3.7345	3.9513	4.0756	4.0453	3.7600	3.4857	4.0453	3.8839	3.3117	4.2532	4.0770	3.9397	3.6001	3.9688
Synrg	_SLS[Phospho (STY)]LGDKEISR_.2	Q5SV85_S1067_M1_Synrg	9.8225	9.5922	9.7266	9.8002	9.1212	8.7951	8.8611	9.2029	8.8739	10.0022	9.0548	9.2101	9.5404	9.6665	3.2299	9.2285	9.2156	9.0988	9.1719	9.3272
Synrg	_S[Phospho (STY)]GSIDDSFTDFQEM[Oxidation (M)]PASSK_.2	#N/A	5.4144	3.7054	3.9022	4.9947	3.4254	6.5612	3.6337	4.1476	3.6279	5.7315	3.6612	6.1136	5.7402	7.0466	3.8804	3.7158	3.7641	3.9217	3.7319	6.9069
Synrg	_TADSVS[Phospho (STY)]PLEPPTK_.2	Q5SV85_S576_M1_Synrg	9.1238	8.4394	3.4622	3.2081	4.1326	7.6083	4.4164	4.1817	4.3366	9.8874	3.8152	3.1196	4.5020	7.1013	2.9409	8.5037	8.7135	3.1138	7.9559	3.4708
Synrg	_QLS[Phospho (STY)]LEGAGLAMEEFKENTSSTK_.3	#N/A	4.4545	3.5483	3.4899	3.2349	3.7922	4.1927	4.3896	4.1549	4.3098	3.7630	3.7884	3.4378	4.4752	5.1919	2.9141	3.2985	3.1917	3.1406	4.2900	3.4440
Synrg	_QLS[Phospho (STY)]LEGAGLAMEEFK_.2	#N/A	4.3583	3.3378	3.5179	3.6029	3.6961	3.9201	3.8450	4.2070	3.9041	4.5647	4.2785	3.0639	4.0122	3.5559	3.4222	3.2024	3.7042	3.4452	3.7443	3.2332
Rap1gap2	_SETSS[Phospho (STY)]NPSS[Phospho (STY)]PEIC[Carbamidomethyl (C)]PNK_.2	Q5SVL6_S590_M2_Rap1gap2	11.4842	11.5402	11.8482	11.7516	11.1621	11.1916	11.2478	11.4693	11.1496	11.6826	11.2431	10.9582	11.2073	11.9068	10.1499	11.2380	10.9041	10.4312	11.2844	11.3172
Rap1gap2	_NQSRS[Phospho (STY)]PIK_.2	#N/A	7.9609	7.7991	7.8470	7.6429	6.4892	6.4093	7.2620	6.3974	3.4240	8.2869	6.6567	7.1384	6.6170	6.7171	4.7828	6.3542	6.6851	6.6682	5.9947	6.7424
Rap1gap2	_TTFS[Phospho (STY)]PPVAAATAK_.2	Q5SVL6_S525_M1_Rap1gap2	6.2148	4.3857	3.9708	8.5838	3.1403	3.1737	3.9078	3.1591	3.8280	9.0169	3.3066	6.4015	3.9934	4.4961	3.9332	3.7963	4.1571	7.6043	3.2710	7.1441
Rap1gap2	_NQSRS[Phospho (STY)]PIKR_.2	#N/A	6.5900	3.7303	5.6555	4.3721	5.6479	3.8291	3.2524	5.6865	5.0749	6.8210	4.1520	3.4564	3.3380	3.8407	3.2777	2.9509	3.5554	5.8997	3.9264	5.7830
Rap1gap2	_TPDGGHSS[Phospho (STY)]QEIK_.2	Q5SVL6_S581_M1_Rap1gap2	5.3959	4.2401	4.2990	6.0024	3.1004	3.3837	4.0259	3.4630	2.9989	3.9690	3.3733	4.1296	6.1269	4.2860	4.6268	5.3952	4.0007	5.8364	3.4810	4.2530
Rap1gap2	_SHS[Phospho (STY)]M[Oxidation (M)]ETM[Oxidation (M)]VGSQR_.2	#N/A	4.8965	4.9959	4.8325	5.6044	4.2126	3.5596	4.5643	3.7557	4.6567	5.0023	3.9411	4.3823	4.3970	5.2158	3.8071	3.9152	4.0271	4.6339	4.0699	4.5101
Rap1gap2	_LHS[Phospho (STY)]GSEGQGDSR_.2	Q5SVL6_S553_M1_Rap1gap2	3.5221	4.1609	4.2921	4.1419	3.2928	3.4714	3.6120	3.4686	3.5322	3.9540	3.0108	4.3633	3.6976	4.3054	3.6373	4.1094	4.2610	4.0125	3.2575	4.1295
Kat7	_LSQSSQDSS[Phospho (STY)]PVR_.2	Q5SVQ0_S59_M1_Kat7	8.1813	8.1048	7.9880	8.0010	7.8109	7.8267	7.7932	7.9541	7.8735	8.2408	7.5433	7.6477	8.3152	7.8842	7.2640	7.7350	7.8273	7.8122	7.6663	7.5132
Kat7	_S[Phospho (STY)]SGSETEQVVDFSDR_.2	Q5SVQ0_S101_M1_Kat7	3.5134	3.8057	4.5508	4.0708	3.7203	3.7537	2.6218	3.7391	3.2480	3.3240	4.2275	3.5318	3.4134	4.5889	3.3532	4.3763	3.2363	3.9131	4.2122	4.2661
Tbc1d9b	_GTS[Phospho (STY)]PPDYR_.2	Q5SVR0_S997_M1_Tbc1d9b	9.5087	4.0252	8.9521	9.0716	9.4237	9.5268	9.1824	9.5488	8.9779	9.5886	9.4976	9.2882	9.3221	4.3694	8.4307	9.3393	9.7512	9.7249	9.3058	9.3887
Tbc1d9b	_VFQKNS[Phospho (STY)]PM[Oxidation (M)]EDLGAK_.2	#N/A	6.0495	6.1911	5.5774	3.9490	5.0945	5.2458	4.5955	5.3206	4.8331	5.0154	3.0743	5.7085	3.7611	4.2638	4.0401	4.6747	5.6327	5.7237	5.8543	4.7654
Tbc1d9b	_NS[Phospho (STY)]PMEDLGAK_.2	#N/A	3.3161	4.0030	4.3535	4.2681	3.5230	3.5564	3.5251	3.5418	3.4453	3.1267	4.6121	3.7291	3.6107	4.1134	3.5504	4.1791	4.5399	4.0051	3.6537	4.0804
Cluh	_AVEDM[Oxidation (M)]GS[Phospho (STY)]PQTAK_.2	#N/A	4.2589	6.6950	4.0383	7.5702	3.2433	3.3741	6.4836	3.2265	6.8762	4.2359	7.0691	3.9979	4.2093	4.0763	3.8532	4.0837	4.2246	3.4087	6.7202	4.0588
Ssh2	_IPHSSS[Phospho (STY)]SENIR_.2	Q5SW75_S1217_M1_Ssh2	8.6924	8.2961	8.3903	3.8737	4.1676	4.3000	3.9661	3.9713	7.7315	3.6576	7.9536	7.9341	4.0791	8.1790	2.9283	7.6706	3.4685	7.6578	4.0150	3.8239
Ssh2	_EM[Oxidation (M)]TTS[Phospho (STY)]ADQIAEVK_.2	Q5SW75_S487_M1_Ssh2	9.3112	8.6915	3.3110	8.6102	3.9029	8.8668	8.5434	9.1777	8.6995	9.2106	8.6014	8.7781	9.4961	8.4872	7.9915	9.0480	9.2056	8.8920	8.8940	8.8342
Nacad	_DEDS[Phospho (STY)]LEEDAQR_.2	#N/A	7.2279	6.4238	7.5583	7.0347	6.6097	6.9246	6.5400	6.6431	6.8463	7.3001	6.9901	6.0594	6.4540	6.9503	5.7102	6.3412	6.9506	6.3692	6.1378	6.8121
Nacad	_VSLS[Phospho (STY)]PHSTLNPK_.2	Q5SWP3_S1216_M1_Nacad	3.6478	4.1281	4.2284	4.3932	2.9855	3.2096	3.0548	6.3313	5.5418	3.8541	5.5920	6.3319	3.2219	4.2665	4.1328	5.6304	6.7031	5.9079	5.9658	3.9438
Nacad	_EFPALEQKDEDS[Phospho (STY)]LEEDAQR_.3	#N/A	3.9817	7.3651	6.5766	5.8320	3.8044	7.4554	7.4242	3.9598	6.9035	6.1328	3.6026	3.5361	4.2894	8.3980	6.6851	5.9023	3.9935	7.8053	7.2925	6.9097
Acaca	_SSM[Oxidation (M)]S[Phospho (STY)]GLHLVK_.2	#N/A	5.9972	5.8823	4.7215	3.5933	4.9600	4.4289	5.3704	4.0823	5.2183	5.9260	4.5959	4.4150	5.7305	3.7027	3.0790	3.0957	5.3962	3.8109	4.9420	5.5310
Acaca	_FIIGSVSEDNS[Phospho (STY)]EDEISNLVK_.2	Q5SWU9_S29_M1_Acaca	3.5237	5.8980	5.7580	4.8919	3.7307	5.7910	6.2600	5.4451	5.8206	3.8933	3.3101	5.3418	6.3969	3.9734	5.2992	3.8877	3.8770	6.2423	5.7164	3.9562
Wwc1	_LNRSDS[Phospho (STY)]DSSTLSK_.2	Q5SXA9_S939_M1_Wwc1	4.0305	4.2270	4.3121	3.9136	3.1136	3.3376	4.4275	3.2886	2.9858	3.9822	3.3602	4.1164	4.5947	4.1384	4.0047	2.9422	4.2867	4.3636	3.1618	3.8157
Specc1	_LGSS[Phospho (STY)]PTSSC[Carbamidomethyl (C)]NPTPTK_.2	#N/A	3.2317	6.2552	7.0919	4.4577	6.2766	4.2943	6.3269	6.0857	3.5299	6.2209	6.2843	5.6055	3.6380	3.7550	3.0481	5.3652	6.3600	6.0645	5.3178	5.5527
Specc1	_ASS[Phospho (STY)]EDTLNKPGSASSGVAR_.2	#N/A	6.7592	6.7561	6.8486	7.0572	6.1472	6.2280	6.4002	6.2336	5.8547	7.2951	6.4019	6.2760	4.7719	6.9005	5.8235	6.2248	3.8471	7.0122	6.5684	6.4048
Specc1	_SSKGS[Phospho (STY)]PTGSSPNNASELSLASLTEK_.3	Q5SXY1_S355_M1_Specc1	6.2259	6.6578	5.3422	3.7145	3.6559	4.0317	3.8393	6.2618	3.8984	3.6661	5.6910	3.6340	7.1061	7.6615	3.4165	6.1971	3.6985	6.4268	3.8558	3.7643
Specc1	_S[Phospho (STY)]STSLAFESR_.2	Q5SXY1_S37_M1_Specc1	3.9763	3.3428	5.8245	3.6079	4.1833	3.9251	4.3657	4.2020	3.8991	3.7869	3.7645	5.3938	4.0072	3.5510	2.8902	3.3384	3.6992	3.4502	6.1888	3.2283
Specc1	_T[Phospho (STY)]SPAPSDSATTVK_.2	Q5SXY1_T809_M1_Specc1	6.6499	4.2175	4.1390	6.0928	3.3085	3.2990	3.7396	5.5054	3.0244	3.9435	3.3988	3.9437	3.1324	4.1770	3.7650	3.9645	4.3253	4.3250	3.1232	3.8543
Specc1	_KLGSS[Phospho (STY)]PTSSC[Carbamidomethyl (C)]NPTPTK_.2	#N/A	5.3492	4.0550	3.6491	5.5792	3.5939	3.8621	5.2294	3.6126	4.0353	3.8812	4.5849	3.7812	4.1434	3.6872	5.4623	5.0683	5.5252	4.9126	5.1376	4.8649
Specc1	_S[Phospho (STY)]PLSGIPVR_.2	Q5SXY1_S847_M1_Specc1	4.0170	3.9858	4.5533	3.6724	3.3547	3.5788	4.1863	3.7174	4.2454	4.2233	3.1190	3.8752	4.3535	4.9769	3.7635	4.3619	4.2551	4.0787	3.2267	3.0065
Specc1	_GSPT[Phospho (STY)]GSS[Phospho (STY)]PNNASELSLASLTEK_.2	Q5SXY1_T357_M2_Specc1	4.3566	4.4633	3.8932	3.2276	3.0627	3.0961	3.9854	3.0815	3.9056	4.1672	3.3842	4.1894	4.0710	4.9826	4.0108	3.7187	4.0795	3.5448	3.1934	3.0398
Nkapl;Nkap	_IGELGAPEVWGLS[Phospho (STY)]PK_.2	Q5SZT7_S141_M1_Nkapl;Nkap	6.8121	6.9177	3.1370	5.9004	3.8074	5.9216	6.8000	7.0338	6.2596	6.8960	4.1404	6.3053	3.3264	7.0102	3.2661	5.9852	3.3234	6.8459	6.1043	3.7960
Rabl6	_ESS[Phospho (STY)]DEDRDSKLPSK_.2	#N/A	3.5136	6.9564	4.8355	5.0711	6.5176	7.7749	7.2506	4.1499	6.1832	6.2416	7.0418	6.7347	4.0372	7.3332	7.5452	6.5305	7.0345	7.4942	6.5306	3.5764
Rabl6	_VAPQQC[Carbamidomethyl (C)]S[Phospho (STY)]EPETK_.2	Q5U3K5_S502_M1_Rabl6	9.5768	9.5849	3.3476	3.7732	9.2303	9.3270	9.6470	4.0367	3.7338	3.6216	9.3058	3.2342	3.8419	9.8538	3.0555	3.1731	3.5339	3.6155	9.3224	3.0629
Rabl6	_EDLS[Phospho (STY)]DVTDEDTGPAQPPPPSK_.2	#N/A	3.7775	6.7318	7.2622	6.8231	3.9221	7.1016	4.0676	4.1826	6.7134	3.8514	3.6236	3.4893	4.2003	3.7705	3.2825	3.8350	7.1677	7.4693	4.0024	3.8214
Rabl6	_NISLSS[Phospho (STY)]EEEAEGLAGHPR_.3	#N/A	4.3369	7.5414	7.7229	4.3177	3.7382	7.7432	7.4371	3.9553	7.8644	3.8164	7.5067	7.5538	8.1520	8.0930	7.8057	7.6089	7.9639	9.1108	7.6808	7.7040
Rabl6	_FPVREDLS[Phospho (STY)]DVTDEDTGPAQPPPPSK_.3	#N/A	3.9277	5.7695	3.5389	3.3315	3.9792	4.2180	3.9437	4.2123	4.9329	4.7577	3.7709	3.2933	4.4577	8.6118	4.8504	3.3534	3.4270	7.6884	5.5121	3.4265
Tmprss13	_NKPGVYTK_.2	#N/A	4.4096	7.1935	7.2739	6.9802	6.8291	6.9310	7.0208	7.1635	6.4817	6.8119	6.7617	6.7391	6.9677	6.6102	7.6288	6.8689	7.0722	6.8620	6.6558	6.7697
Dnajc11	_[Acetyl (Protein N-term)]ATALS[Phospho (STY)]EEELDNEDYYSLLNVR_.3	Q5U458_S6_M1_Dnajc11	3.9635	3.3556	3.5001	3.6207	4.1704	3.9379	4.3785	4.1892	3.8863	3.7741	4.2607	3.0817	3.9944	5.5580	2.9030	3.3256	3.6864	3.4630	3.7621	4.4884
Dnajc11	_RTPAEIR_.2	#N/A	3.6913	0.9907	4.2276	4.4368	3.0291	3.2531	2.7622	3.3731	3.0703	1.4027	3.4446	4.2009	3.1783	4.2229	4.0892	4.0362	4.3712	1.4941	3.0773	1.2361
Gprasp1	_VESTS[Phospho (STY)]GSEC[Carbamidomethyl (C)]ENPTK_.2	Q5U4C1_S295_M1_Gprasp1	4.7764	4.4755	4.9944	4.1686	4.7583	4.8942	5.5645	3.5355	3.4591	3.8790	3.8356	3.9299	3.4930	5.4263	4.9077	3.9117	3.9350	4.0331	4.9699	4.5584
Scaf1	_APS[Phospho (STY)]PAPAVS[Phospho (STY)]PK_.2	#N/A	11.0614	10.9793	10.8031	10.4287	10.5963	10.8193	10.7666	10.7996	10.7532	10.7509	10.4458	10.5317	10.5351	10.9320	9.8994	10.4846	11.0837	10.6537	10.7076	10.4728
Scaf1	_S[Phospho (STY)]AS[Phospho (STY)]PPPAASSSSSR_.2	#N/A	10.6398	10.3690	10.6109	10.3338	10.0437	10.3523	10.1684	10.6891	10.3327	10.1499	9.9420	10.1799	10.2727	10.5046	9.1063	9.8718	10.6060	10.3107	10.0097	9.6340
Scaf1	_S[Phospho (STY)]AS[Phospho (STY)]PGPPPAR_.2	#N/A	9.9200	8.8805	9.3198	9.6303	8.6852	9.5945	9.4386	9.2590	9.3954	9.6635	9.3967	9.0101	8.9648	7.8854	7.0947	9.0249	9.4738	9.4810	8.8593	9.4586
Scaf1	_S[Phospho (STY)]PFLKPDER_.2	Q5U4C3_S821_M1_Scaf1	9.0155	9.1830	8.8184	8.8781	8.3356	8.8959	8.6164	8.7404	8.4061	8.9163	8.7208	8.0823	8.5706	8.6217	7.1892	7.8256	8.9973	8.4519	8.4745	8.1761
Scaf1	_FDIYDPFHPTDEAYS[Phospho (STY)]PPPAPEQK_.3	Q5U4C3_S240_M1_Scaf1	4.5370	7.1008	3.5725	6.7340	3.8748	6.7681	7.2597	6.7529	4.3923	6.9359	3.8710	3.3552	4.5578	7.1941	5.5356	6.4773	6.8452	7.0369	6.8486	5.1758
Scaf1	_GTEETSWS[Phospho (STY)]GEER_.2	Q5U4C3_S912_M1_Scaf1	7.6929	7.8773	7.1905	7.3196	7.1279	7.5830	7.6829	7.8845	7.0997	7.5583	6.9387	7.3423	7.6772	7.5331	6.3760	7.5138	3.2263	7.4240	7.3091	6.9026
Scaf1	_APS[Phospho (STY)]PAPAVS[Phospho (STY)]PKR_.2	#N/A	7.5955	6.5483	7.2406	7.1690	7.3348	8.1468	7.3628	7.2129	7.0916	4.2361	7.4063	5.9632	6.9992	7.8798	6.7546	7.5594	7.9385	7.5456	7.6572	7.6728
Scaf1	_SGDPAPPDS[Phospho (STY)]PSWEAK_.2	Q5U4C3_S518_M1_Scaf1	4.1865	3.4996	3.4794	7.9774	3.9981	4.1072	4.0970	4.3098	7.4423	4.2201	4.0499	6.5065	7.3764	7.0616	3.9392	6.7261	7.1537	6.8546	4.0979	8.0606
Scaf1	_QRS[Phospho (STY)]AS[Phospho (STY)]PGPPPAR_.2	#N/A	3.6485	7.6535	4.1848	7.1172	2.9862	7.4066	7.5249	3.3489	7.2250	4.5690	5.6255	7.4511	3.6692	7.4898	4.0200	7.2061	3.8866	7.4043	3.5952	4.1389
Scaf1	_QRS[Phospho (STY)]GDPAPPDS[Phospho (STY)]PSWEAK_.2	#N/A	3.3517	3.9674	6.4876	4.2325	3.5586	6.0287	6.3350	3.5774	6.3014	3.6934	3.6489	6.3515	3.3826	6.6215	2.7926	4.2147	6.7283	5.7318	4.3739	5.7972
Scaf1	_RRS[Phospho (STY)]AS[Phospho (STY)]PPPAASSSSSR_.3	#N/A	3.6710	4.5588	4.2073	4.0184	3.0088	3.2328	5.5276	3.3714	3.0906	3.8774	3.4649	4.2212	3.1986	4.1944	4.1095	4.0159	3.9091	4.6361	4.2613	3.9205
Scaf1	_REVLY[Phospho (STY)]DSEGLS[Phospho (STY)]ADER_.2	Q5U4C3_S695_M2_Scaf1	4.2497	4.5702	3.7863	3.3345	2.9558	2.9891	4.6771	4.4754	4.0125	4.0603	3.4912	4.2964	4.1780	3.1798	4.1177	3.6118	3.9726	3.4379	3.0864	3.1468
Dyrk2;Dyrk4	_VYTY[Phospho (STY)]IQSR_.2	Q5U4C9_Y380_M1_Dyrk2;Dyrk4	8.5545	8.7418	8.4384	8.4193	7.9851	8.3673	8.4409	8.3870	8.0910	8.2141	8.0317	7.9056	8.4171	8.0253	6.4790	7.7609	8.2795	8.4010	3.0954	7.6839
Dync2i2	_VGS[Phospho (STY)]AGAAALAAGGAGGAER_.2	Q5U4F6_S15_M1_Dync2i2	6.6577	6.5436	6.2805	6.6462	5.0595	5.9143	6.3556	6.7786	6.2306	6.8191	5.4413	6.0660	3.3712	4.4158	5.1432	5.7565	6.0585	6.2011	6.2058	6.3189
Hykk	_YMGALS[Phospho (STY)]QSR_.2	Q5U5V2_S195_M1_Hykk	4.0534	5.5302	3.5899	3.5309	4.2603	3.8481	4.2886	4.2790	3.9762	3.8640	4.3506	2.9918	4.0842	3.6280	2.8132	3.4155	3.7763	3.3732	3.6722	3.3053
Fam169a	_TS[Phospho (STY)]PTASVNK_.2	Q5XG69_S359_M1_Fam169a	3.8118	5.6993	3.8701	4.9742	5.2028	5.6970	5.4601	3.8315	3.9284	3.4910	5.3479	3.7940	4.0939	3.8316	5.3343	3.6854	3.7821	3.6334	5.8672	3.7986
Fam169a	_HFQDSEFSSS[Phospho (STY)]QGEDENVAK_.3	Q5XG69_S348_M1_Fam169a	3.6921	4.3107	4.2284	3.9973	3.0298	3.2539	3.0104	3.3724	3.0695	3.8984	5.6367	5.4581	3.1776	4.2222	4.0885	4.0370	3.9301	6.1823	3.0780	3.8995
Fam169a	_TLLGS[Phospho (STY)]SDNVATVSNIEK_.2	Q5XG69_S523_M1_Fam169a	3.6634	4.3395	4.1997	7.1548	6.5017	6.8295	6.5269	3.4011	7.0087	3.8697	7.1694	4.2289	3.2063	4.2509	6.6284	4.0083	4.3992	4.2511	3.0493	3.9282
Fam169a	_TSPDTDSEM[Oxidation (M)]LIDQSPSDDKGHTEENLS[Phospho (STY)]PVSK_.4	#N/A	6.7354	3.5869	3.3785	6.0686	5.4485	3.9962	3.9726	5.5807	3.8928	3.5665	3.9958	6.3246	7.3139	3.3878	5.9917	7.0064	5.9395	7.0773	4.1578	3.6514
Fam169a	_AVDS[Phospho (STY)]S[Phospho (STY)]SEEIEVEVPVVDRR_.2	#N/A	4.4178	4.4032	3.9544	3.4192	3.1239	3.2911	3.9252	3.4806	3.8455	4.2284	3.3241	4.1293	4.2844	4.3145	3.9506	3.7799	4.2233	3.2993	3.2428	3.4037
Fam169a	_SQSEEQSEASS[Phospho (STY)]EHLEQFTQSAEK_.3	Q5XG69_S614_M1_Fam169a	3.8024	4.2418	3.4953	3.9353	3.9147	4.0756	3.5428	4.1673	3.4808	3.8920	3.6401	3.3741	3.6985	3.8300	3.2418	3.3089	3.8928	3.9919	3.6550	3.7544
Leo1	_KYVIS[Phospho (STY)]DEEEEEDD_.2	#N/A	3.2822	7.2717	6.7867	7.0245	3.4891	6.8967	7.1774	4.8278	7.0532	8.0211	2.9578	7.5806	3.6446	7.8327	6.5502	7.3308	8.1653	7.5986	3.6198	7.2024
Leo1	_YVIS[Phospho (STY)]DEEEEEDD_.2	#N/A	4.4833	3.5195	3.5188	3.2060	3.8211	4.0452	3.7200	4.1837	3.7791	3.1888	4.1535	3.4089	3.8871	6.5852	3.2972	3.3274	3.2206	3.5703	3.8693	3.1082
Leo1	_LNS[Phospho (STY)]DEEGESSGKR_.2	#N/A	4.3320	3.6709	3.3674	3.3574	3.6697	3.8938	3.8714	4.1174	3.9305	4.5383	4.3049	3.5603	4.0385	4.2388	3.4486	3.1760	3.0692	3.2632	4.4126	3.3215
Leo1	_KLNS[Phospho (STY)]DEEGESSGKR_.3	#N/A	3.1246	3.3814	3.6706	4.0598	4.1441	4.5110	3.6589	4.1880	3.8663	3.6339	3.8016	3.0972	4.0317	3.6800	3.8963	3.6692	3.6026	3.6743	3.6746	3.5260
Leo1	_[Acetyl (Protein N-term)]ADM[Oxidation (M)]EDLFGS[Phospho (STY)]EAESEAER_.2	#N/A	3.4234	4.2817	4.2347	4.2152	3.9769	4.7162	3.2995	3.6615	4.2975	3.6093	3.7330	3.3282	3.4667	4.5113	2.8767	3.7479	3.1587	3.9907	4.2898	4.1886
Leo1	_KLNS[Phospho (STY)]DEEGESSGK_.2	#N/A	3.1509	4.2494	4.1884	4.4333	3.3579	4.8605	3.6902	3.3766	3.0738	3.8942	3.4481	3.8943	3.1818	4.3993	3.7847	4.0139	4.3747	3.8720	4.1088	3.9037
Leo1	_MQNT[Phospho (STY)]DDEDR_.2	Q5XJE5_T189_M1_Leo1	3.6136	3.7054	3.1502	3.9706	3.8206	3.8539	3.2275	3.8393	3.1477	3.4242	4.1272	4.3316	3.3132	3.1883	3.2529	2.9758	3.3365	3.8129	4.1119	4.3664
Lmtk3	_S[Phospho (STY)]PSVSSEYYIR_.2	Q5XJV6_S531_M1_Lmtk3	4.4588	8.5247	8.0436	8.0563	5.9742	7.8161	8.0299	4.1065	7.6587	8.1659	7.4358	3.4334	3.9117	7.8478	6.6555	6.3586	3.6037	8.0468	7.7035	7.9086
Lmtk3	_ALVNGEPM[Oxidation (M)]S[Phospho (STY)]PEAGEK_.2	#N/A	8.8778	9.1186	8.5875	8.6035	8.4350	8.8335	8.7203	8.8622	8.4528	8.7987	8.6652	8.5965	8.9207	8.8083	7.6266	8.5277	8.9980	8.9626	8.2661	8.4840
Lmtk3	_AQRPPEGM[Oxidation (M)]S[Phospho (STY)]PELPPR_.3	#N/A	6.6463	6.2426	6.4958	6.8892	4.8691	7.2007	7.1205	7.3051	6.1956	7.4947	7.2027	6.6939	7.5077	6.3764	5.3374	5.8513	7.4684	7.7784	5.6323	5.3363
Lmtk3	_VAENGLES[Phospho (STY)]PEKEER_.2	Q5XJV6_S947_M1_Lmtk3	6.9131	7.2696	6.6599	6.8617	6.2289	6.8005	7.0956	7.3238	6.7449	6.5744	6.4959	6.5200	6.8477	6.7759	5.8670	3.1169	6.8819	7.1226	6.4318	6.6475
Zdhhc8	_QGLPS[Phospho (STY)]PPGTPR_.2	Q5Y5T5_S672_M1_Zdhhc8	3.7948	3.5243	3.3314	3.7894	4.0018	4.1066	3.0463	7.9158	3.7176	3.2503	4.0920	3.2504	3.8257	3.3695	3.0717	3.1569	8.4356	3.6317	3.9308	3.0467
Zdhhc8	_TPRPGS[Phospho (STY)]AESALSVQR_.2	#N/A	3.6988	5.1205	3.9431	4.0292	3.9058	3.6259	3.9938	3.9245	4.5103	5.1197	3.8719	3.4902	3.9492	3.9812	4.0192	3.7686	4.1294	3.8715	3.3774	3.6585
Zdhhc8	_TS[Phospho (STY)]PPTPAMYK_.2	Q5Y5T5_S346_M1_Zdhhc8	3.8930	4.1098	4.4293	3.7964	3.2307	3.4548	4.3104	3.5934	4.3694	4.0993	3.2430	3.9993	4.4775	4.4164	3.8876	4.2379	4.1311	3.7021	3.3507	3.6986
Kif1b	_MADTGS[Phospho (STY)]PGM[Oxidation (M)]QR_.2	#N/A	3.5948	8.3238	8.3770	4.1069	3.8049	4.8657	6.9647	3.2520	3.1814	7.0606	3.9175	3.6813	3.3211	4.0894	7.8051	3.9397	3.8328	4.2273	3.4880	3.9081
Kif1b	_IVEGQGQSSEVIS[Phospho (STY)]PPEEVNR_.2	Q60575_S1075_M1_Kif1b	3.5236	4.4792	4.0599	4.1658	2.8613	3.6228	3.1789	3.2240	3.3789	3.7299	4.3584	4.3686	3.5443	4.0470	4.2569	3.8685	3.7617	4.0715	3.7201	4.0140
Kif1b	_SGLS[Phospho (STY)]LEELR_.2	Q60575_S1057_M1_Kif1b	8.1396	8.0438	3.5561	3.1688	3.8583	7.7124	8.0502	7.0783	7.5655	8.2622	3.8545	7.7407	4.5413	7.6850	5.6017	7.5652	7.7435	7.9797	7.6726	7.6717
Kif1b	_EFSQVHGSIS[Phospho (STY)]DC[Carbamidomethyl (C)]K_.2	Q60575_S1605_M1_Kif1b	3.2592	3.2427	3.7955	4.4302	3.2640	4.3219	3.5040	4.4605	3.5024	3.4656	3.8767	3.1322	3.6104	3.7826	3.0205	3.6041	3.4973	4.3359	3.7038	3.7496
Kif1b	_GDS[Phospho (STY)]LILEHQWELEK_.2	Q60575_S1487_M1_Kif1b	3.6326	3.6865	3.1692	3.9516	3.8396	4.2688	3.5619	3.8583	3.1288	3.4432	4.1082	3.4126	3.6635	3.2073	3.2339	2.9947	3.3555	3.7939	4.0930	4.3854
Mast2	_KLS[Phospho (STY)]NPDIFAPTGK_.2	Q60592_S14_M1_Mast2	5.6956	5.9355	5.4342	3.7883	5.8679	5.6249	5.2801	4.0216	4.4663	5.8837	3.9449	3.2493	3.8268	5.9971	3.0706	3.1580	3.5188	5.3271	3.9297	4.7556
Mast2	_SLS[Phospho (STY)]EEKEDHSDGLAGLK_.3	Q60592_S817_M1_Mast2	5.3297	5.6188	4.4620	4.1597	3.6315	5.2931	3.4166	3.6502	4.2113	5.0186	4.3163	4.5597	3.5023	5.1587	3.4420	4.2875	3.1474	4.9604	3.8695	3.9719
Xrcc1	_TQAAGPS[Phospho (STY)]SPPRPPT[Phospho (STY)]PK_.2	Q60596_S445_M2_Xrcc1	7.4887	6.6387	3.8199	7.3630	5.6373	3.6187	6.9549	4.5090	6.9452	4.2106	7.0328	7.0089	4.3142	6.5286	6.3547	4.1021	6.2908	6.9689	7.0411	6.9240
Ogdh	_SSPYPTDVAR_.2	#N/A	8.2704	8.1496	8.2899	8.4006	8.2056	8.3392	8.2426	8.3170	8.3814	8.4719	8.0887	8.2431	8.3112	8.3535	8.6140	8.2160	8.1226	8.4364	8.2097	8.3060
Ogdh	_LNVLANVIR_.2	#N/A	7.9168	8.2948	8.0982	8.2913	8.0563	8.0660	8.3541	8.2794	7.4740	8.1776	8.1998	8.2540	8.1369	8.0892	7.7912	7.9932	7.9532	8.2668	7.9351	8.2085
Ogdh	_FEEFLQR_.2	#N/A	7.6823	7.7544	7.8262	7.9447	7.8813	7.5487	7.9858	7.5327	7.7422	7.8296	7.6374	7.6037	7.2099	7.5514	7.6338	7.4660	7.4933	7.6574	7.7099	7.3667
Ogdh	_DVVVDLVC[Carbamidomethyl (C)]YR_.2	#N/A	6.9363	7.0847	7.7454	6.9611	6.9089	7.1018	6.7439	7.1232	6.9731	7.0339	6.6867	6.9673	6.3726	7.3336	6.4344	6.9194	7.2164	6.6947	7.4040	7.1290
Ogdh	_QILLPFR_.2	#N/A	6.9373	6.8506	7.0929	6.8871	7.2509	6.7976	7.1436	7.1773	6.4646	6.8346	6.5438	7.2858	6.9611	6.3109	6.7912	6.8476	6.7606	7.4820	6.8736	6.6414
Ogdh	_IC[Carbamidomethyl (C)]EEAFTR_.2	#N/A	6.8507	6.4889	7.0590	6.9251	6.5273	6.7313	6.6267	7.1172	6.9515	6.7438	6.6059	6.4369	6.6960	6.7729	6.6686	5.9241	7.0722	6.9232	6.0842	6.4855
Ogdh	_THLTELQR_.2	#N/A	5.5085	6.0563	6.4527	5.8328	5.7044	5.5816	6.0718	5.2106	5.9107	5.8947	6.0360	5.7610	6.5596	5.7035	6.1250	4.1141	5.4271	6.1929	6.0512	5.6905
Ogdh	_ELEQIFC[Carbamidomethyl (C)]QFDSK_.2	#N/A	4.7991	4.9984	5.5752	5.1729	4.9563	4.1344	3.3034	4.2610	4.3760	4.9571	4.9027	4.4622	3.4706	5.4560	4.1650	5.0702	5.5679	4.5364	4.2859	4.8943
Ogdh	_TSFDEM[Oxidation (M)]LPGTHFQR_.3	#N/A	7.0783	5.7294	3.9859	4.4764	6.5423	6.1543	5.8054	6.5816	6.3273	7.1596	6.3993	6.6522	6.6545	5.1927	6.0075	5.7416	6.3302	6.5466	6.4945	6.6090
Ogdh	_VASSVPVENFTIHGGLSR_.3	#N/A	4.1367	4.2494	5.1785	5.6385	5.1694	6.0454	5.8973	5.2403	5.0286	6.5752	5.6653	3.7482	4.0213	5.5606	5.4132	5.8562	6.1197	6.6590	5.5290	6.3066
Ogdh	_SWDIFFR_.2	#N/A	3.9596	5.0169	3.4962	5.2372	2.9130	4.3083	4.4478	4.7053	4.8780	3.7702	4.7177	3.0856	4.7407	3.5280	4.7602	4.9889	3.6825	4.5563	5.6800	5.5222
Ogdh	_LVEDHLAVQSLIR_.3	#N/A	3.8667	4.2646	4.9143	4.3004	3.7669	3.9333	3.8323	3.9429	4.4379	4.5374	4.2150	3.9625	4.8124	3.5251	4.6373	3.9988	3.5123	4.2060	4.6624	4.5650
Ogdh	_VVNAPIFHVNSDDPEAVMYVC[Carbamidomethyl (C)]K_.3	#N/A	3.3590	3.8393	3.1299	4.1044	4.1976	4.4216	4.3822	3.7055	3.4026	3.5653	3.7770	3.5654	3.5107	4.0533	2.9207	3.7039	3.2027	3.9467	4.2458	4.2325
Ogdh	_TKAEQFYC[Carbamidomethyl (C)]GDTEGKK_.3	#N/A	4.3482	3.6546	3.3836	3.3412	3.6859	3.9100	3.8552	4.2171	3.9143	4.5545	5.1153	4.5433	4.0223	4.8468	5.5245	3.1922	3.7144	3.4351	4.4729	3.2434
Ogdh	_VFHLPTTTFIGGQEPALPLR_.3	#N/A	3.5853	3.7337	3.1219	3.9989	3.7923	3.8256	3.2558	4.5220	3.6592	3.3959	4.1555	3.4599	3.3415	3.8441	3.2812	2.9475	3.5588	4.2744	3.9229	3.8111
Ogdh	_HHVLHDQNVDKR_.3	#N/A	3.6103	4.3925	1.9639	4.0791	2.9480	3.5361	3.0922	3.3107	3.4656	3.1064	2.9442	4.2820	3.6310	4.1337	4.1703	3.9552	3.8483	3.9848	3.6334	4.1007
Cttn	_TVPIEAVTSK_.2	#N/A	6.4940	6.9865	6.3692	6.6357	6.9720	6.8886	6.9375	6.4433	6.8099	6.7424	6.5764	7.0676	7.0463	6.9137	7.6870	6.9768	6.3879	6.6445	6.8354	6.7320
Cttn	_NAST[Phospho (STY)]FEEVVQVPSAYQK_.2	Q60598_T323_M1_Cttn	4.3906	3.3701	7.7503	3.6352	3.7283	3.9524	6.2716	4.1747	3.8718	3.0961	4.2462	6.3546	3.9799	3.5236	3.3899	3.2347	3.6719	6.9585	3.7766	3.2009
Cttn	_SAVGFDYQGK_.2	#N/A	5.5535	5.9721	6.3212	3.4813	6.2414	5.8942	5.6802	5.8369	6.2646	6.0213	5.7250	5.2453	4.1338	6.7248	6.4254	6.0648	3.8258	5.6862	5.6591	6.3640
Cttn	_HC[Carbamidomethyl (C)]S[Phospho (STY)]QVDSVR_.2	Q60598_S113_M1_Cttn	3.2816	3.8325	4.3192	5.0585	4.2739	4.7669	4.9286	4.6020	5.1996	5.0070	4.9354	4.6055	3.6228	4.3598	5.0432	4.4185	4.4905	4.7161	4.7219	4.7097
Cttn	_YGVQADRVDK_.2	#N/A	4.4776	4.3558	4.5511	5.0788	3.1702	4.3145	4.9191	3.1890	5.4292	4.2601	4.9980	5.0780	4.7321	5.4174	4.4618	4.8339	4.5964	4.5814	4.2713	4.6612
Cttn	_SAVGFEYQGK_.2	#N/A	5.3189	4.1965	5.7132	5.6961	5.1375	5.1208	5.6297	5.6001	5.8206	5.8586	5.7956	5.5403	4.7840	4.1981	5.8639	5.6737	5.2918	5.3788	5.2681	3.8754
Cttn	_TVQGSGHQEHINIHK_.3	#N/A	3.8157	4.1871	6.1079	3.8737	5.3723	5.8184	4.3877	5.8742	4.4468	5.6164	5.3453	5.6328	5.8957	5.4342	7.0579	5.8325	4.0537	5.3383	5.5917	5.7696
Cttn	_KQT[Phospho (STY)]PPASPSPQPIEDRPPSSPIYEDAAPFK_.4	Q60598_T401_M1_Cttn	7.2798	7.9917	7.8378	8.3106	6.5503	7.9377	7.4778	7.9486	7.2290	7.2018	7.1716	7.9006	7.8711	9.1246	7.8917	8.3608	7.6336	8.3757	7.8594	7.6199
Cttn	_VDQSAVGFEYQGK_.2	#N/A	4.1179	3.2011	3.6545	5.9160	4.3249	4.3582	5.3476	4.3436	5.9069	3.9285	3.6229	2.9273	4.3097	7.0479	5.9699	3.4801	3.8408	3.3061	5.7731	3.2785
Cttn	_SAVGHEYQSK_.2	#N/A	3.7574	4.3600	4.6059	3.1243	3.1660	4.8182	3.8821	5.2152	5.3842	4.2705	3.2810	4.0862	4.1558	4.4704	5.6485	3.8220	4.1828	4.9752	4.0392	5.3247
Cttn	_YGIDKDKVDK_.2	#N/A	4.4221	4.3834	4.3241	4.0700	3.4936	3.5269	3.5545	3.3198	3.4747	3.0973	3.0566	4.3884	4.7590	4.1428	3.5799	5.5650	4.6940	4.9322	3.8894	5.0593
Cttn	_DYSSGFGGK_.2	#N/A	4.1042	3.8986	3.1396	3.5852	3.4419	3.6660	4.0992	3.8045	4.1583	4.3105	3.0318	3.7881	4.4075	4.7189	3.6764	2.9482	4.3422	3.4909	2.9700	3.5278
Kcnh1	_S[Phospho (STY)]PQDRS[Phospho (STY)]PILAEVK_.2	Q60603_S899_M2_Kcnh1	4.1717	5.1190	3.7083	3.4125	2.8778	5.0276	4.0354	4.3974	4.0945	4.3742	2.9681	4.3743	4.2026	3.7464	3.6126	3.0120	3.8946	3.2548	3.5539	3.4236
Myl6	_ALGQNPTNAEVLK_.2	#N/A	7.7185	7.5040	7.5311	7.5748	7.8427	7.2675	7.6691	7.6384	7.6912	7.4035	7.3967	7.7071	8.0644	7.3451	8.3347	7.7745	7.6953	7.4324	7.5556	7.5381
Myl6	_NKDQGTYEDYVEGLR_.3	#N/A	7.4865	7.3997	7.6231	7.4944	7.3878	7.1058	7.1531	7.4880	7.8219	7.3205	7.4583	7.6355	7.4255	7.5054	7.9735	7.8866	7.7663	7.2328	7.6514	7.6850
Myl6	_EAFQLFDR_.2	#N/A	7.3794	7.3741	7.8559	7.6153	7.2971	7.6525	7.3708	7.3197	7.2013	7.7112	7.6500	7.5357	7.4728	7.3302	7.6342	7.7525	7.1410	7.2987	7.5037	7.5191
Myl6	_HVLVTLGEK_.2	#N/A	3.8383	4.9897	4.5421	4.5078	4.9702	4.6477	4.2396	4.9622	3.5646	4.6522	4.7683	4.9836	4.5322	5.6318	5.0478	4.9884	5.4942	4.7926	4.8785	5.2148
Myl6	_ILYSQC[Carbamidomethyl (C)]GDVMR_.2	#N/A	4.4918	3.5110	3.5272	3.1976	3.8295	4.0536	3.7116	4.1922	3.7707	3.1973	4.1450	3.4005	3.8787	5.0714	4.5728	3.3358	3.2290	3.5787	3.8777	3.0998
Tiam1	_ANS[Phospho (STY)]LGDLYAQK_.2	Q60610_S231_M1_Tiam1	9.9949	10.4258	10.1510	10.2714	8.3910	9.8866	9.7680	8.9992	9.4879	10.2011	9.1370	9.4593	9.7962	9.6787	8.3679	9.4892	9.6117	9.6980	9.4989	9.5557
Tiam1	_TES[Phospho (STY)]LPSAQQYVPFGGK_.2	Q60610_S1407_M1_Tiam1	7.1965	7.6727	7.9189	7.4021	6.4068	7.1438	7.0168	6.8323	6.7466	7.1710	6.7244	6.1276	4.3059	3.6946	3.5950	6.7734	3.6724	6.9013	6.9475	6.3455
Tiam1	_SNATNSSYS[Phospho (STY)]PPTGR_.2	#N/A	9.4496	8.9677	8.3212	9.0772	3.5611	9.4555	3.8627	9.4062	3.8787	8.9578	8.8208	7.3376	9.1154	3.9347	3.5993	8.8951	9.3198	9.3554	8.5300	3.7358
Tiam1	_SKS[Phospho (STY)]ADIWR_.2	Q60610_S172_M1_Tiam1	4.1031	3.2160	3.6397	3.4811	4.3101	3.7983	4.2389	4.3288	4.0259	4.4428	2.8995	2.9421	4.1340	3.6778	4.2642	5.7575	3.8260	3.3234	3.6225	6.5924
Tiam1	_RSNATNSSYS[Phospho (STY)]PPTGR_.2	#N/A	4.0309	3.2882	3.5675	3.5533	4.2378	8.0407	3.8946	8.9589	8.5783	4.5150	4.3281	3.0143	4.0618	3.6055	2.8356	3.3930	8.4973	3.3956	3.6947	3.2828

Tiam1	_S[Phospho (STY)]LEGIFDDTVPDGK_.2	#N/A	4.5319	4.2880	4.0685	4.5532	3.2380	3.2713	3.8101	3.2567	3.7303	4.3425	3.2090	4.0142	3.8958	4.1065	3.8355	3.8940	4.2548	4.3955	3.1937	3.7838
Tiam1	_FTIDSDAISASS[Phospho (STY)]PEKEPQQPAGGGDTDR_.3	Q60610_S1466_M1_Tiam1	3.9858	3.5291	4.5221	3.7036	3.3236	3.1605	2.5139	3.6862	3.8411	4.2317	3.3197	3.9064	4.0065	4.5092	3.7947	4.3307	4.2239	3.6093	3.2579	2.9754
Tiam1	_S[Phospho (STY)]LEGIFDDTVPDGKR_.2	#N/A	4.4711	3.5169	3.4315	3.5135	3.8088	4.0329	3.7327	4.1081	3.7918	3.1765	4.1662	3.3293	3.8999	6.6111	3.3099	3.3151	3.3874	3.5580	3.8570	3.1209
Satb1	_LEQNGS[Phospho (STY)]PLGR_.2	Q60611_S38_M1_Satb1	6.8427	6.4286	6.0089	6.8736	6.9130	6.3468	6.0396	6.1048	5.7660	6.9257	6.1601	6.3276	3.1106	7.0752	6.1110	6.1122	6.6950	5.8429	5.7820	6.0457
Icam5	_LDDLDC[Carbamidomethyl (C)]PR_.2	#N/A	5.5864	5.3508	5.4151	5.6569	5.6051	6.2964	6.0779	5.5018	5.7013	5.8873	5.8724	5.9357	5.9323	6.1191	6.2632	5.5859	5.4748	6.3152	5.5647	5.8073
Icam5	_SFVGEPPR_.2	#N/A	3.5681	5.6456	4.6055	4.0162	6.3880	6.4122	6.4105	6.3710	6.3743	6.0840	5.4990	6.5346	6.7578	5.5745	6.6784	5.7073	6.1895	6.5950	6.4893	6.1661
Icam5	_ASLTLTLLR_.2	#N/A	3.4520	5.1465	4.4130	5.1032	4.5395	4.9658	4.6819	3.7884	5.0266	4.8241	4.3490	4.8700	3.3641	5.5551	5.5144	3.8987	3.2857	4.6084	5.0631	4.8986
Icam5	_QLM[Oxidation (M)]C[Carbamidomethyl (C)]VVTLGGESR_.2	#N/A	3.7563	4.2465	4.2926	3.9331	3.0941	3.3900	3.6914	3.4567	3.6116	4.4612	3.0902	4.1359	3.7770	5.7911	4.4877	4.1012	3.9944	5.6443	3.6196	4.2467
Icam5	_C[Carbamidomethyl (C)]TAVNGQGQAVK_.2	#N/A	4.1690	4.3255	4.2136	3.4098	3.0151	4.4088	3.0252	3.3777	3.8433	3.8837	3.4586	4.2149	3.1923	2.6097	4.1032	2.9716	3.9154	4.2651	3.0633	3.9142
Icam5	_SGKEEVMEGPLR_.3	#N/A	3.7289	4.2739	4.2652	3.9605	3.0666	3.4175	3.6639	3.4292	3.5841	4.4887	3.0628	4.1634	3.7496	4.2522	3.6893	4.0738	3.9669	3.8662	3.5148	4.2192
Grb2	_NYVTPVNR_.2	#N/A	6.8472	7.2591	7.3129	7.3867	7.0791	7.3255	7.0542	7.5418	7.0725	7.0953	7.0802	7.2272	7.2750	7.0673	7.6381	6.8317	6.6761	7.2288	6.8741	7.3859
Grb2	_NQQIFLR_.2	#N/A	6.4545	6.2503	5.9857	6.3347	6.8984	6.5529	6.5213	6.8080	6.3790	6.1331	6.4256	6.7768	6.7306	6.2753	7.0665	6.2329	6.8087	6.4911	6.6982	6.6529
Grb2	_FNSLNELVDYHR_.2	#N/A	4.8466	5.4676	5.2482	5.4528	5.2501	4.9145	4.7693	5.6047	4.8365	5.6126	4.6130	5.3709	3.3455	5.1551	5.2336	5.3878	5.4590	5.2639	5.2817	4.9367
Grb2	_ATADDELSFK_.2	#N/A	3.1397	3.4177	4.7472	4.4446	3.3466	3.3799	3.7015	5.2870	3.6217	3.2217	3.1004	5.5227	3.1705	4.2151	3.7269	3.3864	4.3634	4.2869	3.0851	4.3227
Flot2	_NVVLQTLEGHLR_.2	#N/A	4.8208	4.8788	4.5442	3.5012	4.3693	4.7471	4.6953	4.9154	4.3034	4.2310	3.2546	4.1325	3.9829	4.9560	3.9470	2.7071	3.9552	4.5607	3.9237	3.8288
Hnrnpd	_IFVGGLSPDTPEEK_.2	#N/A	8.1306	8.5341	8.0949	8.0526	7.3531	8.1938	8.0075	8.0334	8.0797	8.2342	7.9210	8.3929	8.5931	8.5768	8.3006	8.2429	8.5237	8.1454	8.1431	8.2229
Hnrnpd	_YHNVGLSK_.2	#N/A	4.5436	5.1412	5.5429	5.1490	5.9350	5.5128	5.6191	4.2816	5.5271	5.5322	5.4506	5.3873	5.6158	6.0595	6.0116	5.4297	4.7531	5.2595	5.3054	5.2243
Hnrnpd	_IDASKNEEDEGHSNSS[Phospho (STY)]PR_.3	#N/A	7.8556	9.1883	8.8167	8.9007	8.3295	8.6863	8.8600	8.9470	8.6547	9.0121	8.5907	8.8317	9.5776	8.8895	8.8467	9.0206	8.9274	8.8476	9.0632	8.9730
Hnrnpd	_IREYFGGFGEVESIELPM[Oxidation (M)]DNK_.3	#N/A	5.8008	5.8538	5.1995	3.1804	5.4548	5.2130	4.4441	6.2513	5.4175	3.7085	5.2397	5.8852	6.6752	6.3098	4.8930	6.1089	5.8117	5.7285	5.8896	6.0804
Hnrnpd	_EYFGGFGEVESIELPM[Oxidation (M)]DNK_.2	#N/A	6.2994	6.1776	6.6566	6.1334	5.7211	6.0342	6.5006	6.0512	6.1664	6.3446	5.8444	6.4019	6.7165	6.6443	6.3759	6.5266	6.6033	6.1474	6.2220	6.3776
Hnrnpd	_NEEDEGHSNS[Phospho (STY)]SPR_.2	Q60668_S82_M1_Hnrnpd	4.9910	6.7964	6.3907	5.9245	3.9878	5.6454	6.8230	4.3278	5.0181	3.5915	6.5009	7.1161	7.3713	7.9984	3.0871	3.1430	3.3647	4.1018	4.1185	5.9664
Hnrnpd	_IDASKNEEDEGHSNS[Phospho (STY)]S[Phospho (STY)]PRHTEAAAAQR_.4	#N/A	4.5046	4.1005	4.0412	3.7870	3.2107	3.2440	3.8374	3.6027	3.7576	4.3152	3.2363	4.0415	3.9231	4.4257	3.8628	3.8667	4.1404	3.6928	3.3413	4.3927
Ptprn	_AEDS[Phospho (STY)]SEGHEEEVLGGRGEK_.3	#N/A	3.6928	3.2887	3.7496	4.4761	3.8997	3.9331	3.1484	4.4145	7.9042	7.5339	4.0481	3.3524	8.9884	3.7367	3.1737	8.4245	3.4514	4.3818	4.0304	3.7037
Ptprn	_AEDS[Phospho (STY)]SEGHEEEVLGGR_.3	#N/A	6.9279	6.3559	7.4390	6.8612	3.3441	4.3526	6.3781	3.8555	4.5296	4.5175	7.5815	6.9285	7.0094	7.0229	5.8949	3.8238	7.3578	6.2741	4.3877	3.9104
Ppp5c	_SVVDSLDIESM[Oxidation (M)]TIEDEYSGPK_.2	#N/A	7.8776	6.3051	6.8132	7.0679	6.6407	6.6582	6.2546	7.7765	6.1185	6.3786	5.8678	6.8193	6.9077	7.7125	6.4438	7.4483	6.4701	6.8066	6.5930	5.7661
Ppp5c	_AEGYEVAHGGR_.2	#N/A	5.2424	5.5746	5.1371	4.5782	5.4193	4.9556	5.7900	5.2042	5.4053	3.7647	5.3322	4.7751	4.4735	4.8714	5.5217	5.1394	4.8142	5.1095	5.3548	5.2141
Ppp5c	_AFLEENQLDYIIR_.2	#N/A	5.1711	4.0955	5.2390	5.3818	5.0954	4.6087	5.0436	4.2594	4.9853	4.7905	4.5148	5.0440	5.7576	4.6829	3.3560	4.7128	4.9798	3.8624	4.8267	4.3486
Ppp5c	_FYSQAIELNPGNAIYYGNR_.2	#N/A	5.5870	5.5077	3.3741	5.3754	4.0445	5.3245	5.1715	5.5136	5.3834	3.6481	5.5839	5.4714	5.0171	5.2960	5.1456	5.4166	5.9153	5.7769	5.3915	4.6347
Ppp5c	_TQANDYFK_.2	#N/A	4.5719	4.8329	3.5580	4.5865	3.9946	4.1112	5.1343	3.6836	4.3098	4.2114	4.2422	3.3562	4.5567	4.6791	4.6738	4.1561	3.2732	4.3547	4.2085	3.5749
Ppp5c	_TEC[Carbamidomethyl (C)]YGYALGDATR_.2	#N/A	4.4160	3.3955	5.9936	6.0355	6.1457	6.4388	6.1571	4.1493	6.5172	3.1215	6.0796	3.1216	3.9545	5.6843	5.0392	6.0901	3.6465	3.5029	6.0567	3.1755
Ppp5c	_GNHETDNMNQIYGFEGEVK_.3	#N/A	3.3881	3.9310	4.4255	4.1961	3.5950	3.6284	3.4531	3.2184	3.3733	3.1987	4.3528	3.6571	3.5387	4.0414	3.4784	4.2511	3.7561	4.0771	3.7257	4.0084
Psmb6	_LAAIQESGVER_.2	#N/A	6.3506	6.9273	7.1181	6.6581	6.5560	6.9119	6.6925	6.8335	7.1296	6.9330	6.0465	6.4824	6.6894	7.0830	7.0469	6.8272	6.7963	6.9405	7.0408	6.9126
Psmb6	_QVLLGDQIPK_.2	#N/A	6.0083	6.3787	6.3402	6.1687	6.1607	6.3972	6.5856	6.6122	6.0142	6.6281	6.4742	6.7046	6.3975	5.6554	6.8447	5.9022	5.3855	6.4371	6.1109	6.2551
Psmb6	_TTTGSYIANR_.2	#N/A	6.7175	5.8786	7.9894	9.0998	7.3940	5.4125	5.8791	7.7960	5.8033	5.7378	5.3977	6.2661	5.5299	5.7852	5.9921	5.7476	5.7142	5.8554	5.7078	5.1937
Tcf4	_SSSGSWGTGGHPS[Phospho (STY)]PSR_.2	Q60722_S66_M1_Tcf4	4.0701	3.9327	4.6064	3.3147	3.4078	3.6319	4.1333	4.4952	4.6937	4.0033	3.0659	4.9301	4.3004	5.4897	3.7105	4.4150	5.8180	3.1570	5.1190	3.5215
Tcf4	_NGGQASSS[Phospho (STY)]PNYEGPLHSLQSR_.3	Q60722_S371_M1_Tcf4	4.0699	3.5929	3.3726	3.5571	3.6544	5.5649	3.9922	4.0506	3.9751	3.6174	3.9382	3.8639	4.1142	3.3874	3.6853	3.4320	4.1165	3.4296	3.4518	3.2030
Tcf4	_DLGSHDNLS[Phospho (STY)]PPFVNSR_.3	Q60722_S92_M1_Tcf4	3.6254	5.9253	3.1620	4.4087	3.8324	3.8657	4.6595	4.4819	3.1359	4.4215	4.1154	3.4198	3.3014	3.8040	3.2411	2.9875	3.5187	4.3144	3.9630	3.7710
Csnk2a1	_QLYQTLTDYDIR_.2	#N/A	4.2946	5.1183	3.8312	5.9216	5.0655	4.8130	4.6486	5.5965	4.5934	4.8049	3.0909	5.0215	4.6161	5.2901	4.4291	4.5974	4.4791	4.8785	4.7869	4.5961
Csnk2a1	_FNDILGR_.2	#N/A	4.4045	4.1853	4.1712	4.8685	3.3407	4.4008	4.6049	2.9638	4.5892	4.3827	5.0512	4.3238	4.7165	4.9025	5.0869	5.0346	4.0104	5.1921	4.4821	4.4290
Csnk2a1	_FVHSENQHLVSPEALDFLDK_.4	#N/A	4.3879	4.3547	3.4957	4.5089	4.5935	4.5288	4.8054	4.3818	4.2684	4.2361	4.0154	4.0845	3.2013	4.1948	4.8869	3.9327	3.4187	4.6750	4.0632	5.0684
Csnk2a1	_YSEVFEAINITNNEK_.2	#N/A	3.6800	4.3228	12.6810	12.7629	3.0177	3.4664	3.6150	3.3804	3.5352	4.5376	12.6436	5.0414	3.7007	12.8938	4.1006	7.3929	12.5239	12.4878	3.5637	12.5390
Csnk2a1	_VLGTEDLYDYIDKYNIELDPR_.3	#N/A	4.2879	4.5321	3.5659	3.8403	2.9940	2.7662	4.0541	3.8194	3.9743	4.0985	3.4530	4.2582	3.7417	3.7447	3.4202	3.7321	4.0108	1.1825	3.1246	3.1086
Csnk2a1	_TPALVFEHVNNTDFK_.2	#N/A	3.6501	4.4325	4.1318	4.1191	3.4485	3.8638	3.5693	3.2959	3.6933	3.6896	3.8580	4.1730	4.1226	4.0320	3.9192	4.2540	3.8336	4.2848	3.7040	3.4387
Csnk2a1	_EYWDYESHVVEWGNQDDYQLVR_.3	#N/A	4.5557	1.8205	3.5911	3.1337	3.8934	4.0916	3.6477	4.2560	4.4109	3.2611	3.8896	3.3366	4.5764	3.5102	1.5307	3.3997	3.2929	3.0395	4.1889	3.5452
Khdrbs1	_KDDEENYLDLFSHK_.3	#N/A	7.2543	7.1181	7.4758	7.2262	7.0467	6.8565	6.6371	7.3724	7.2167	7.0802	7.2104	7.0501	7.4491	7.3198	7.3791	6.8745	7.2934	6.6000	6.9678	6.9850
Khdrbs1	_DPSGAHPSVR_.2	#N/A	4.0821	6.0917	5.8680	6.2009	6.5803	5.5273	6.2905	6.3209	6.5121	5.9760	5.3680	6.4052	5.2787	6.3308	6.5116	6.0613	6.0334	6.0287	5.5636	5.9683
Khdrbs1	_S[Phospho (STY)]C[Carbamidomethyl (C)]SKDPSGAHPSVR_.2	#N/A	3.8918	5.0049	3.7560	3.8629	4.4709	3.3603	3.8602	3.9300	3.8077	4.0910	3.5200	4.0318	3.9186	3.7827	4.0252	4.8076	4.2408	3.7223	3.2602	3.5285
Cldn11	_MGHEPGVAK_.2	#N/A	4.1565	6.2728	6.4963	6.3356	5.2609	6.2271	6.5220	3.8476	5.4769	3.5812	5.5923	5.5085	4.2140	6.1185	5.1383	3.0006	5.6575	5.1932	3.9529	4.9482
Cldn11	_KM[Oxidation (M)]DELGSK_.2	#N/A	6.1916	6.9155	7.0336	6.5000	6.0612	6.9068	6.8578	6.2035	6.4180	5.8926	6.6263	5.9580	6.0246	6.3385	6.6200	6.2327	6.2149	6.1507	6.2899	6.0309
Cldn11	_FYYSSGSSS[Phospho (STY)]PTHAK_.2	#N/A	13.7027	13.4671	13.4965	13.6654	10.5806	13.0811	10.5170	13.5473	13.3918	12.9861	12.8530	13.1154	13.7596	13.3250	12.1826	12.8083	13.6202	12.8903	13.0708	12.9757
Cldn11	_FYYSSGSSS[Phospho (STY)]PTHAKSAHV_.3	#N/A	6.7762	7.2130	5.5674	6.5076	6.7432	7.6301	7.0814	5.5215	5.9029	6.8469	7.6243	3.5980	5.5142	3.8077	5.0932	6.3948	5.3141	6.1662	6.0566	5.5991
Gas7	_KSTGDSQNLGSSS[Phospho (STY)]PGRK_.2	#N/A	4.0431	6.4004	4.7696	4.7775	5.3167	4.8232	4.3180	4.3355	5.2374	6.7663	5.5908	5.0005	6.2271	6.1363	6.4850	6.4487	3.6354	5.8208	5.5068	5.7649
Gas7	_HETDM[Oxidation (M)]FNQSTVEPVDQLLR_.3	#N/A	4.6968	5.3837	5.2381	4.3642	5.0270	4.8714	4.8515	3.2240	4.8701	4.9355	4.0354	5.4793	5.3038	4.0470	5.2836	5.5436	5.1963	5.1899	4.9983	5.6355
Gas7	_STGDSQNLGSSS[Phospho (STY)]PGR_.2	#N/A	8.8089	9.1514	9.4252	8.8857	7.8717	7.7318	8.4598	8.6401	8.3123	8.7349	7.9570	8.5363	8.6963	9.2830	8.3829	8.2343	8.0730	8.6078	8.3999	7.9929
Gas7	_STGDSQNLGSSS[Phospho (STY)]PGRK_.2	#N/A	8.2252	8.4741	8.3819	8.5338	7.9065	7.8158	7.8272	8.0095	8.0003	8.4743	7.8753	8.1228	8.7887	8.6561	7.9797	8.2118	8.3835	4.3435	8.1843	8.1174
Gas7	_KSTGDSQNLGS[Phospho (STY)]SSPGR_.2	Q60780_S106_M1_Gas7	3.9531	4.0497	4.4894	3.7363	3.2908	3.1933	6.5274	3.6535	3.8084	4.2644	3.2870	3.9391	3.9738	4.4765	3.9136	4.2980	4.1912	3.6420	3.2906	4.4435
Gas7	_C[Carbamidomethyl (C)]VDLYNQAQSK_.2	#N/A	4.5478	4.2721	4.0844	4.5372	3.2539	3.2873	3.7942	3.5595	3.7144	4.7312	3.1930	3.9982	3.8798	4.3825	3.8195	3.9100	4.0971	3.7360	3.3846	4.9211
Rasa3	_YGS[Phospho (STY)]QEHPIGDK_.2	#N/A	4.5053	9.9856	9.3369	9.2406	9.0244	9.6723	9.6352	9.4560	9.3878	9.8886	9.3518	9.1124	9.9195	9.6643	8.4720	9.0731	9.6065	9.6937	9.5244	9.3622
Rasa3	_T[Phospho (STY)]RYGSQEHPIGDK_.3	Q60790_T805_M1_Rasa3	4.4975	4.7806	3.9758	3.9702	3.7276	4.6039	3.9311	4.4135	4.5759	5.0193	3.9422	4.1351	4.0168	4.3845	3.8416	3.8013	4.8966	4.7096	4.6240	4.9892
Ppp1r1b	_DGNSEDQVEGR_.2	#N/A	6.3478	7.1177	6.8739	7.1101	6.7872	7.2963	6.9146	6.8511	7.2117	7.1098	7.6875	7.4702	7.5170	8.0395	7.8730	7.4167	7.3327	7.6606	7.4825	7.6782
Ppp1r1b	_ATLSEPGEEPQHPS[Phospho (STY)]PP_.2	Q60829_S192_M1_Ppp1r1b	15.0977	15.0471	15.0144	15.1438	15.0230	14.9126	14.5491	15.4765	14.8931	15.1552	15.1542	15.2688	16.0313	15.6211	15.3712	15.3473	15.5584	15.1207	15.0955	15.5092
Ppp1r1b	_GLEGPWERPPPLDEPQR_.3	#N/A	6.6436	7.3179	6.8899	7.2809	7.0086	7.1621	7.0671	7.0135	7.0529	7.1215	7.8558	7.7440	7.6441	7.8667	8.4262	8.2291	7.5616	6.4375	7.6334	8.2345
Ppp1r1b	_GTVGQKPTC[Carbamidomethyl (C)]GR_.2	#N/A	3.1696	6.1799	6.0325	6.7062	6.3170	5.9328	6.1555	6.4049	6.4335	4.4810	6.8445	7.0899	6.6280	6.8522	8.1215	6.9729	6.3639	6.6212	6.8306	7.2354
Ppp1r1b	_VSEHSS[Phospho (STY)]PEEEASPHQR_.3	#N/A	11.0696	11.5736	11.1484	11.3561	10.9184	11.2142	11.1432	11.1668	11.3071	11.5872	11.2630	11.0158	11.9411	11.6028	11.8027	11.4781	11.2150	11.3611	11.4298	11.6765
Ppp1r1b	_IQFSVPAPPSQLDPR_.2	#N/A	3.5465	4.7759	6.5164	4.1429	2.8842	5.3812	5.5880	5.8263	3.2151	4.5747	5.5500	6.3051	7.1942	4.0227	6.7085	6.2790	5.7181	6.2181	5.6628	6.4588
Ppp1r1b	_GLEGPWERPPPLDEPQRDGNSEDQVEGR_.4	#N/A	5.6206	5.6838	4.5799	5.8061	5.0538	4.5251	5.5053	6.4488	5.6037	4.4229	6.4035	6.6212	6.7681	7.1792	6.6943	6.6689	6.3656	6.4006	5.7342	5.4644
Ppp1r1b	_ELGYPQEDDEEDEDEEEDEEEDSQAEVLK_.3	#N/A	4.0419	3.2772	3.5785	3.5423	3.6354	3.8595	3.9057	4.2676	3.9647	4.5040	4.3391	3.0033	4.0728	3.6166	3.4829	3.1418	3.7648	3.3846	3.6837	3.2938
Serpinb6	_FYEAELEELDFQGATEESR_.2	#N/A	7.8751	7.2374	7.3521	7.4939	6.7725	6.8130	6.8364	7.5630	6.4894	7.7067	6.7463	7.1019	7.9328	7.7241	6.8201	7.6966	6.9511	6.7750	6.8179	6.8093
Stip1	_LAYINPDLALEEK_.2	#N/A	7.8854	8.0227	8.2934	7.9010	8.1009	7.7917	8.1284	7.8547	8.1698	8.0628	7.6502	8.1119	8.2359	8.3042	8.0204	7.9864	8.1549	8.2220	7.6295	8.2064
Stip1	_LDPQNHVLYSNR_.3	#N/A	6.9037	6.7620	7.3839	7.0543	7.0345	7.0059	6.7670	6.5726	6.8860	7.1167	7.1387	6.8088	6.5372	6.9941	7.4445	7.0086	6.5974	6.2633	6.9682	7.2030
Stip1	_HDS[Phospho (STY)]PEDVKR_.2	#N/A	7.0343	7.2012	6.1339	7.1077	7.2772	6.6873	6.5874	7.1457	6.4876	6.9797	7.0737	7.2374	7.4857	7.8199	7.4025	7.5056	7.3438	7.1616	7.0694	7.3955
Stip1	_ALDLDSSC[Carbamidomethyl (C)]K_.2	#N/A	6.0357	6.1154	6.5399	6.2831	6.2427	6.2914	6.6801	5.8654	6.3796	6.7234	6.7327	6.5006	6.3724	6.9672	7.1749	6.7201	5.8964	6.2486	6.3690	6.2843
Stip1	_DPQALSEHLK_.2	#N/A	5.7909	6.3376	6.7733	6.3474	6.3496	6.1780	5.8518	5.9263	6.0467	5.4815	5.9872	5.9377	4.7128	6.0543	6.5299	5.4639	5.9998	5.9675	5.9241	6.1014
Stip1	_ALSAGNIDDALQC[Carbamidomethyl (C)]YSEAIK_.2	#N/A	6.1497	6.2334	5.9978	6.2986	5.8502	5.9786	6.0727	6.1224	5.9411	5.8292	5.7949	5.9728	6.2515	6.8998	6.0128	5.9446	6.4986	6.0314	5.0856	6.1211
Stip1	_DPQALSEHLKNPVIAQK_.3	#N/A	4.9686	6.6483	6.3430	6.1049	6.3144	6.2615	6.0498	6.0647	5.8286	6.5086	5.4594	6.0788	6.5657	6.1070	6.9748	5.1596	6.1151	6.2146	6.0746	6.0093
Stip1	_TPDVLKK_.2	#N/A	5.1646	5.6806	5.2795	5.9565	6.2373	5.4289	5.8587	6.1963	5.1251	4.0472	5.6153	5.9656	5.6184	6.0695	5.8898	5.0878	5.1743	5.7631	5.5500	5.5816
Stip1	_HDS[Phospho (STY)]PEDVK_.2	#N/A	7.2589	8.1007	7.0470	7.7846	7.6347	7.6519	7.7384	7.7601	7.7206	7.7442	7.7099	7.7168	8.0944	8.3852	7.7821	7.6564	7.9736	7.7090	7.7951	8.1389
Stip1	_ELIEQLQNKPSDLGTK_.3	#N/A	5.7771	5.6554	5.7559	6.3082	6.0846	6.1826	5.7550	5.7750	5.8290	5.8085	6.2566	6.1316	5.9340	6.6161	6.2102	6.4550	5.9850	5.9426	6.1542	6.2949
Stip1	_LM[Oxidation (M)]DVGLIAIR_.2	#N/A	6.6622	7.0086	6.9375	6.9002	7.0808	6.9146	6.7144	6.9509	6.6751	5.8836	7.0026	6.9447	6.4151	6.8644	6.8536	6.8453	6.8374	6.7712	6.6304	6.9581
Stip1	_HYTEAIKR_.2	#N/A	6.0789	6.2030	6.6851	5.8834	6.4643	6.1744	6.2036	6.5003	5.2683	6.5403	6.9518	6.3570	3.1879	6.3046	6.0995	6.7401	6.7524	6.2451	6.5598	6.5643
Stip1	_HDS[Phospho (STY)]PEDVKRR_.3	#N/A	3.9922	5.8770	3.7017	4.9945	4.9231	4.7892	5.2886	5.5566	5.2155	5.6631	4.5127	5.4008	7.2359	5.7020	5.8939	5.9443	5.5934	5.6857	5.8288	5.4074
Stip1	_HYTEAIK_.2	#N/A	3.3822	1.9410	1.7282	1.2197	3.0891	3.1192	3.0925	2.9155	1.6706	2.1889	2.8334	3.2999	3.4029	3.9056	3.1111	3.2980	3.6203	2.5222	3.3751	3.7327
Stip1	_S[Phospho (STY)]LLSDPTYR_.2	Q60864_S145_M1_Stip1	5.6108	3.6007	3.4376	3.2873	4.2118	5.5200	5.1226	4.1025	4.2574	6.3673	5.0859	3.4901	4.4228	3.4246	2.8617	6.0529	5.8499	5.7127	4.3424	5.8634
Stip1	_LILEQMQK_.2	#N/A	4.2846	4.8352	3.1578	5.7340	2.9907	3.9727	4.9235	3.8227	3.9776	3.4070	4.3709	4.0151	4.1431	5.5475	4.3186	3.6467	4.3604	3.4727	3.1213	3.1119
Stip1	_DAIHFYNK_.2	#N/A	5.0837	3.6925	4.3643	4.9478	5.2288	4.3369	4.8951	4.4207	4.1656	4.6411	4.5328	3.3923	4.3310	4.5419	4.3660	3.1543	4.1786	4.3317	4.4343	3.2998
Stip1	_TYEEGLKHEANNLQLK_.3	#N/A	6.6537	6.7215	6.5347	6.8326	6.2772	6.3200	6.3518	6.8538	6.7988	6.4392	6.0531	6.6451	6.9684	6.9353	6.8339	6.3969	6.5462	6.1412	6.5091	6.6472
Stip1	_IGNSYFKEEK_.2	#N/A	4.4411	4.3783	5.1823	3.6159	4.3264	4.3462	4.7696	4.8548	4.7814	4.9736	4.6780	4.9421	4.4516	5.4263	5.2321	4.9768	4.0321	4.5403	4.5984	4.9160
Stip1	_NKGNEC[Carbamidomethyl (C)]FQK_.2	#N/A	3.3317	4.4284	4.7328	4.1150	3.5387	4.3534	4.5712	3.2747	3.4296	4.4811	4.3572	3.9717	3.5951	4.2019	4.9017	4.0729	3.8124	3.5359	3.6693	4.0647
Stip1	_AMDVYQK_.2	#N/A	4.1554	3.8474	3.1909	3.1869	3.4932	3.7173	4.0479	1.6668	4.1070	4.3618	2.9805	3.7368	4.2150	3.7588	3.6251	2.9995	4.3935	3.2424	3.5414	3.4361
Stip1	_AMADPEVQQIMSDPAMR_.2	#N/A	4.0077	3.9951	4.5440	3.6817	3.3454	3.1387	4.1957	3.7081	4.2547	4.2140	3.1283	3.8846	4.0284	4.5311	3.7728	4.3526	4.2458	3.5874	3.2360	2.9972
Caprin1	_YQEVTNNLEFAK_.2	#N/A	3.1948	4.2915	4.2476	3.9781	3.4017	3.4351	3.6464	3.4117	4.6621	4.5062	3.0452	3.7442	3.7320	5.6569	4.6675	4.0577	3.9494	3.8838	3.5324	4.2017
Reep5	_HESQVDSVVK_.2	#N/A	5.9792	5.9354	6.4690	5.9800	6.0150	6.0705	6.4606	5.8458	6.4495	5.7370	5.6799	5.6008	5.9266	5.6389	5.5606	5.9781	5.6960	6.0681	6.1568	5.7077
Arhgef2	_EAQELGS[Phospho (STY)]PEDR_.2	#N/A	13.3652	12.6760	13.2639	13.1436	12.7098	12.1114	12.3461	12.9844	12.7128	12.4882	12.5955	12.5479	12.8530	13.0606	11.6068	12.5406	13.0021	12.7721	12.5686	12.6464
Arhgef2	_LS[Phospho (STY)]PPHSPR_.2	#N/A	10.1011	9.9692	10.1280	9.7428	9.4885	10.1315	9.7232	9.8921	9.6087	9.7198	9.6914	9.3384	8.9778	9.8315	8.2836	9.0113	3.3645	9.0287	9.4501	9.3814
Arhgef2	_LQDSS[Phospho (STY)]DPDTGSEEEVSSR_.2	Q60875_S940_M1_Arhgef2	6.0486	4.2301	6.2153	3.4595	5.3047	6.1499	4.0800	5.3834	6.1702	4.2062	3.2451	4.0279	4.2104	5.4658	5.2209	5.7752	6.1266	5.4542	5.5186	3.3248
Arhgef2	_LES[Phospho (STY)]FESLR_.2	#N/A	7.6371	7.5638	3.2325	7.6020	3.9028	7.7923	7.4013	6.2384	7.2071	3.5065	7.5977	6.8374	3.2309	7.8377	3.1707	7.1172	7.1525	7.5566	7.4242	3.7006
Arhgef2	_SLHRPFDDREAQELGS[Phospho (STY)]PEDR_.3	#N/A	6.2249	10.1095	8.6635	9.2532	3.7568	9.3067	9.3174	3.4929	8.8358	8.4472	3.5644	8.2299	4.0646	9.4178	7.9648	9.1422	7.5307	9.3247	9.0016	8.8273
Arhgef2	_LQEIYNR_.2	#N/A	4.6634	4.7954	4.2328	4.6316	4.3654	3.3050	5.5282	4.7274	4.6254	4.3761	4.0844	4.5839	3.8621	2.9569	4.8103	3.5476	4.7721	4.6538	4.5235	4.9805
Arhgef2	_TFNGS[Phospho (STY)]IELC[Carbamidomethyl (C)]R_.2	Q60875_S710_M1_Arhgef2	4.0022	4.0006	4.5385	3.6871	3.3400	3.1441	3.9373	3.7026	3.8575	6.6215	3.3362	3.8900	4.0230	4.5256	3.7783	4.3471	4.2403	3.5929	3.2414	2.9918
Arhgef2	_QILSQS[Phospho (STY)]TDSLNMR_.2	Q60875_S174_M1_Arhgef2	3.3414	5.6795	6.0801	6.2276	3.5242	4.4494	5.7205	3.9989	3.1258	3.5477	5.4415	3.9197	3.2604	4.0769	3.8080	3.6863	5.2473	4.2899	4.2611	3.8699
Arhgef2	_S[Phospho (STY)]LPAGDALYLSFNPPQPSR_.2	Q60875_S885_M1_Arhgef2	4.0396	8.9166	3.5762	3.3221	4.2466	4.2799	9.2211	9.1832	6.4461	3.8502	3.7012	7.9159	8.2499	3.3898	5.9384	3.4018	3.1045	3.2278	7.5616	3.3568
Arhgef2	_LES[Phospho (STY)]FESLRGER_.2	#N/A	7.5205	7.5385	7.7278	4.5615	3.1537	3.3778	4.3873	3.2484	4.4464	4.0223	3.3200	4.0762	4.5545	4.0982	3.9645	7.7577	7.8583	4.4038	7.5057	3.7755
Arhgef2	_SVSTTNIAGHFNDES[Phospho (STY)]PLGLR_.3	Q60875_S163_M1_Arhgef2	3.7676	6.0469	3.9800	4.5509	3.9745	4.0078	3.0736	4.3397	2.9938	3.5782	3.9733	3.2776	3.1592	6.1652	3.0990	3.1297	3.3766	5.7240	4.1052	3.6289
Arhgef2	_LQDSSDPDTGSEEEVSSRLS[Phospho (STY)]PPHSPR_.3	#N/A	5.8828	7.0110	7.1942	5.6634	5.1222	6.4197	6.4078	4.0303	3.8522	5.7510	5.2182	5.7143	3.9991	6.2567	4.1603	5.8279	3.3765	3.5446	4.1091	5.6255
Elavl2	_VSYARPSS[Phospho (STY)]ASIR_.2	Q60899_S119_M1_Elavl2	3.5250	7.3024	4.0613	4.1643	6.6517	3.0869	6.8115	6.8808	6.8232	3.7314	6.6328	7.0013	3.3446	4.0484	4.2555	6.6417	3.7631	6.9104	3.7186	4.0154
Elavl2	_ELEQLFSQYGR_.2	#N/A	6.1130	5.6707	6.0520	5.7466	6.0416	5.9134	6.1761	6.1369	5.5188	6.1674	3.7327	6.0778	5.4668	6.3204	5.7338	5.8323	6.1004	5.9453	5.8594	5.7473
Elavl2	_IEAEEAIK_.2	#N/A	3.8365	5.9167	4.5371	4.4152	3.7066	3.7400	3.3415	3.7254	3.2617	5.2709	4.2411	3.5455	3.4271	6.0944	3.3668	4.8007	3.2226	4.1887	4.8869	3.8968
Elavl2	_TNQAILSQLYQS[Phospho (STY)]PNR_.2	Q60899_S221_M1_Elavl2	6.4609	8.0991	4.3721	5.9400	3.3377	5.0743	6.8707	7.5144	5.3261	6.2861	3.1254	6.8641	6.8552	7.9693	3.7614	4.1731	6.5654	3.7679	3.4165	5.3574
Elavl2	_FANNPSQK_.2	#N/A	3.4958	3.8233	4.5332	4.2791	3.7027	3.7361	3.3454	3.1107	3.2656	3.3064	3.7403	3.5494	3.4310	3.9337	3.3707	4.3588	3.6483	4.1848	3.8334	3.1580
Eps15l1	_LQQEETQLEQSIQAGR_.2	#N/A	6.6408	5.2483	6.1591	5.0559	5.3142	5.2259	5.2847	5.2305	5.2951	5.4496	4.8645	5.5710	6.6976	3.1563	5.3150	4.7855	5.6322	5.1729	5.2325	4.5385
Eps15l1	_STPSHGSVSSLNSTGS[Phospho (STY)]LS[Phospho (STY)]PK_.2	#N/A	3.8973	3.5283	5.0433	3.7660	3.6698	4.6326	3.8838	4.6584	5.3088	3.7079	3.8506	4.2575	4.1131	4.8957	4.8208	4.4268	5.1051	4.2997	5.2224	4.7896
Eps15l1	_ESDPFHS[Phospho (STY)]SSSDDFFKK_.3	Q60902_S669_M1_Eps15l1	3.3227	3.6550	4.1254	4.3146	3.8871	3.9087	3.4532	3.3034	3.3116	3.6629	3.6860	3.4406	3.4196	4.4642	3.2876	3.9438	4.1250	4.0443	4.3433	4.1415
Eps15l1	_GIDPPQVLS[Phospho (STY)]PDM[Oxidation (M)]VPPSER_.2	Q60902_S353_M1_Eps15l1	7.0009	7.0638	7.1402	6.9712	8.4402	7.8367	8.2853	7.9126	9.2205	9.1081	7.8652	9.5640	8.7797	10.0000	8.5524	9.2394	8.3496	10.2919	10.1134	7.0663
Eps15l1	_TVFAGAVPVLPAS[Phospho (STY)]PPPK_.2	#N/A	4.5276	5.9338	4.0642	4.5575	3.2337	3.2671	6.1758	7.1461	3.7346	4.3382	3.2132	4.0184	3.9000	8.0991	3.8398	3.8897	4.2505	4.3998	3.1980	3.7795
Eps15l1	_TVFAGAVPVLPAS[Phospho (STY)]PPPKDSLR_.3	#N/A	3.6377	7.6282	4.0362	3.7927	3.8446	3.8780	6.2435	3.9415	3.9789	3.4483	4.1503	3.4547	4.1444	7.9775	3.2760	3.8079	3.1751	5.9379	4.2955	3.8423
Mef2a	_SEPIS[Phospho (STY)]PPRDR_.2	Q60929_S406_M1_Mef2a	3.4168	9.2817	9.2836	8.4785	4.2553	7.6378	3.2858	3.6477	7.0458	8.2313	3.7192	7.0796	3.4529	9.1450	2.8630	3.7616	8.7229	8.7542	4.3036	4.1748
Vdac2	_YQLDPTASISAK_.2	#N/A	9.3541	9.5704	9.6775	9.4885	9.4201	9.4706	9.5833	9.5857	9.7017	9.4501	9.2116	9.2762	9.4141	9.6534	9.6328	9.4722	9.1766	9.3116	9.5245	9.2292
Vdac2	(Protein N-term)]AEC[Carbamidomethyl (C)]C[Carbamidomethyl (C)]VPVC[Carbamidomethyl	#N/A	8.5077	8.3734	9.4153	9.1912	8.8890	8.8100	8.8489	8.9012	9.0103	9.0798	8.9381	8.9636	8.4680	9.1515	9.1610	8.5226	8.8214	8.8456	9.1192	8.6905
Vdac2	_SNFAVGYR_.2	#N/A	7.4186	7.9411	7.8465	8.2423	7.9236	8.2729	8.6098	7.6439	8.5356	8.7759	8.2774	8.3879	8.5617	8.2979	8.4292	8.3642	6.2996	8.4118	8.5754	8.3985
Vdac2	_LTLSALVDGK_.2	#N/A	7.2303	7.8260	8.1897	7.9174	7.7207	7.5397	7.7127	7.2675	7.6482	7.8870	7.2845	8.1905	7.6198	7.5611	7.7651	7.8398	6.3376	8.0041	7.7769	7.6298
Vdac2	_WC[Carbamidomethyl (C)]EYGLTFTEK_.2	#N/A	7.3268	6.9992	7.8848	7.6067	6.6820	7.0910	6.8776	7.3305	7.2177	7.4740	6.7572	6.7990	6.7193	7.2320	6.9266	6.9807	6.9679	6.8806	7.2861	6.7404
Vdac2	_GFGFGLVK_.2	#N/A	5.7505	6.1353	6.4940	6.6987	6.3039	6.1209	6.1672	4.7670	6.4203	6.9631	6.0705	6.8523	6.3271	6.2545	6.4739	6.3229	5.4875	6.2384	6.7477	6.6843
Vdac2	_LTFDTTFSPNTGK_.2	#N/A	6.4800	6.9592	7.5955	6.8629	6.5339	6.9165	6.5603	6.7809	7.0544	6.9737	6.9239	6.4451	6.2913	6.7222	7.0369	6.7354	6.7430	6.8153	6.8384	6.4244
Vdac2	_SFNAGGHK_.2	#N/A	7.4543	7.3778	7.5614	7.1101	7.0408	7.4798	7.5949	6.5683	7.2974	7.4763	7.8151	3.9780	7.3683	7.5528	8.3709	7.1711	7.0794	7.5867	7.6599	7.2502
Vdac2	_VNNSSLIGVGYTQTLRPGVK_.2	#N/A	6.0194	5.5902	5.8370	5.7384	5.9544	5.4256	5.5154	5.7848	5.5643	5.8432	6.0490	5.3700	5.3727	5.8340	5.7127	5.5540	5.0138	5.5400	6.1822	5.8534
Vdac2	_LTFDTTFSPNTGKK_.2	#N/A	6.3586	6.5470	6.7241	6.9118	6.5608	6.4550	6.6215	6.6088	6.4515	6.6300	4.3493	6.6272	6.6285	7.1494	6.7814	6.7225	6.6981	6.5935	6.5882	6.4154
Vdac2	_TGDFQLHTNVNNGTEFGGSIYQK_.3	#N/A	6.3109	4.5103	4.0288	6.3663	6.3643	3.0543	3.2100	6.8098	5.2660	3.4851	5.8012	4.3997	3.3772	4.4218	2.7872	4.9817	5.5421	4.0803	4.3793	4.7363
Vdac2	_WNTDNTLGTEIAIEDQIC[Carbamidomethyl (C)]QGLK_.3	#N/A	3.4100	4.0496	3.9463	3.8029	4.1107	4.4727	4.2426	3.6544	3.3516	3.6164	3.7259	2.9814	4.7008	4.5042	2.8697	3.7549	3.1516	3.9978	3.6378	4.1815
Vdac2	_SC[Carbamidomethyl (C)]SGVEFSTSGSSNTDTGK_.2	#N/A	3.6634	4.3394	4.1997	4.4089	3.0012	3.2252	3.0391	3.4010	3.0982	10.2434	3.4725	4.2288	3.2062	4.2508	4.1171	4.0083	4.3991	4.2512	3.0494	3.9281
Vdac3	tyl (Protein N-term)]C[Carbamidomethyl (C)]NT[Phospho (STY)]PTYC[Carbamidomethyl (C)]D	Q60931_T4_M1_Vdac3	8.5814	8.7724	8.5738	8.4768	8.4123	8.4806	8.4342	8.7097	8.4293	8.7846	8.7291	8.4486	8.7894	8.8843	8.2383	8.5238	8.9984	8.5034	8.5582	8.5824
Vdac3	_LTLDTIFVPNTGK_.2	#N/A	8.5835	8.5592	9.0396	8.5820	8.3790	8.7089	8.5944	8.5373	8.7128	8.5664	8.6357	8.2708	8.6869	8.6409	8.4551	8.6609	8.8790	8.6396	8.7719	8.4738
Vdac3	_LSQNNFALGYK_.2	#N/A	7.7372	7.9758	8.3940	8.0651	8.2081	7.9288	8.1775	8.1139	8.1783	7.8405	7.7529	7.6757	7.4900	8.2839	7.7517	7.7115	7.6085	7.9078	7.8998	8.0324
Vdac3	_LTLDTIFVPNTGKK_.2	#N/A	8.1532	7.9545	7.8631	7.8292	7.7282	7.7674	7.6904	7.7935	8.0830	8.0156	7.8621	7.7494	8.5724	7.9690	7.4144	7.9639	7.8271	7.8219	8.0167	7.9006
Vdac3	_VC[Carbamidomethyl (C)]NYGLTFTQK_.2	#N/A	7.1318	7.2152	7.0643	7.3337	6.6445	7.3974	6.9547	6.8736	7.0462	7.4986	7.4887	6.7715	7.0120	7.5776	7.0437	6.8240	7.0832	6.9033	6.8875	6.9405
Vdac3	_NFNAGGHK_.2	#N/A	7.2523	7.2778	7.6543	7.1337	7.1818	7.1977	7.4248	6.4221	7.1509	7.3965	7.6386	7.2028	7.0433	7.2970	8.3005	6.9968	7.0551	7.2850	7.4158	7.1572
Vdac3	_VNNASLIGLGYTQTLRPGVK_.2	#N/A	5.6113	5.7849	6.1371	5.7300	5.8441	5.6481	5.8861	5.4730	5.6096	5.5842	5.6510	5.5933	6.2887	6.0884	5.2599	5.6276	5.8129	5.7617	5.9143	5.4450
Vdac3	_IETSINLAWTAGSNNTR_.2	#N/A	6.7460	5.9901	6.3791	7.1091	3.2463	5.4470	6.3118	6.4821	4.8927	4.3508	6.5272	4.0058	6.7812	6.1154	5.3486	4.9579	6.9866	5.0951	6.2179	6.4789
Vdac3	_WNTDNTLGTEISWENK_.2	#N/A	3.1877	4.1314	4.2251	4.3966	4.8331	5.4515	5.5717	5.2688	5.7284	5.4928	5.5766	5.4944	3.7391	4.2418	3.6789	4.0506	6.4064	5.4509	5.3122	5.9016
Vdac1	_YQVDPDAC[Carbamidomethyl (C)]FSAK_.2	#N/A	9.6642	9.9764	10.2214	10.0797	9.3347	9.8291	9.8910	9.8453	10.1328	9.6373	9.5666	9.8272	9.8456	10.1466	10.1394	9.8216	9.6449	9.9129	9.7122	9.8735
Vdac1	_LTFDSSFS[Phospho (STY)]PNTGK_.2	#N/A	8.7512	9.1390	9.2172	9.1670	8.4101	9.0328	8.5672	7.0287	8.9618	9.1081	8.8883	8.6988	8.6388	9.4261	8.6812	8.9757	9.0300	8.8985	8.9828	8.9962
Vdac1	_VTQSNFAVGYK_.2	#N/A	9.4385	9.4276	9.7677	9.5163	9.5668	9.3924	9.4179	9.5954	9.5764	9.5587	9.3590	9.3955	9.5179	9.7515	9.7142	9.5224	9.2466	9.3417	9.4779	9.5863
Vdac1	_WTEYGLTFTEK_.2	#N/A	9.3865	9.3283	9.6885	9.6514	9.1957	9.1664	9.0944	9.4669	9.4820	9.5000	9.3803	9.2282	9.4017	9.4947	9.4377	9.4763	9.2758	9.2121	9.4958	9.3817
Vdac1	_TDEFQLHTNVNDGTEFGGSIYQK_.3	#N/A	9.1064	8.7368	8.5675	9.1898	8.7329	8.8143	8.3756	9.1898	8.8095	8.7474	8.9739	9.1015	9.1514	9.5435	8.5706	8.7088	9.2950	8.5819	8.9993	8.8594
Vdac1	_GYGFGLIK_.2	#N/A	7.8547	7.5546	7.7010	8.3284	8.2638	7.8321	8.3342	7.7103	7.8238	8.4154	8.3536	8.4579	8.4890	8.2468	8.1460	8.4714	8.0939	8.3601	8.7156	8.8457
Vdac1	_SENGLEFTSSGSANTETTK_.2	#N/A	6.3040	7.1145	6.1390	6.0836	5.4291	6.1158	6.4161	6.4952	6.2255	4.3636	5.9116	6.1043	6.2453	8.3190	5.6391	6.2007	3.9053	6.6950	5.9915	2.7708
Vdac1	_TKSENGLEFTSSGSANTETTK_.3	#N/A	5.9948	6.7374	4.1246	6.0177	3.9547	4.5633	5.2467	5.4313	5.2210	4.4830	5.1596	5.2951	3.1180	5.9444	5.7488	5.7941	4.5723	5.4006	5.1297	5.1705
Vdac1	_LTFDSSFS[Phospho (STY)]PNTGKK_.2	#N/A	9.9151	10.1902	9.8178	9.9842	9.3258	9.5532	9.6379	9.9954	9.5877	9.7738	9.4088	9.7284	10.1787	10.5591	9.5432	9.7752	10.0143	9.8550	9.8426	9.7956
Vdac1	_LETAVNLAWTAGNSNTR_.2	#N/A	6.8972	3.6256	6.8450	6.5902	6.3241	5.9106	5.7318	5.7762	6.4774	6.4505	6.0155	6.0027	6.0312	3.2682	5.4530	5.7422	7.0625	3.7330	5.6113	6.3930
Vdac1	_VNNSSLIGLGYTQTLKPGIK_.2	#N/A	8.1274	8.0209	8.3715	8.1151	8.1086	8.1112	8.0030	8.2199	8.1386	8.2479	8.0422	8.2286	8.4989	8.3743	8.1562	8.2153	8.2475	7.9683	8.0827	8.3671

Vdac1	_EHINLGC[Carbamidomethyl (C)]DVDFDIAGPSIR_.3	#N/A	7.6610	7.4557	7.7628	7.8249	7.8468	7.8759	7.4417	7.0119	7.9962	8.1571	8.0778	7.4897	6.9542	8.0475	7.8155	8.0813	7.9843	7.5080	7.6498	8.3842
Vdac1	_WNTDNTLGTEITVEDQLAR_.2	#N/A	6.3135	6.0990	5.8167	6.3241	5.8471	6.1179	6.1387	6.3779	6.3192	6.3789	6.3734	5.8232	6.5745	6.8508	6.2032	6.1878	6.3541	6.1347	6.4977	6.3554
Vdac1	_KLETAVNLAWTAGNSNTR_.3	#N/A	5.7908	3.6129	4.7972	6.0244	5.8101	4.4180	3.8998	5.7155	4.5214	6.0125	5.5373	5.4222	6.2765	3.4826	4.1110	5.9676	5.7510	3.4458	5.4328	5.8397
Tbc1d1	_KSFS[Phospho (STY)]QPGLR_.2	#N/A	6.8917	4.5843	3.9548	6.5597	4.2571	4.4812	3.2840	3.6459	3.3431	3.6248	3.7175	2.9729	3.4511	4.4957	2.8612	3.7634	3.1432	4.0063	4.3053	4.1730
Tbc1d1	_QSARPST[Phospho (STY)]PEPDC[Carbamidomethyl (C)]TQLEPTGD_.2	Q60949_T1242_M1_Tbc1d1	6.8487	6.7536	3.7932	3.3276	2.9627	6.3471	4.0854	4.4823	6.2557	4.0672	5.5666	4.2894	4.1710	3.8313	4.7993	3.6188	7.1087	3.1699	5.3978	5.7670
Tbc1d1	_LNPSAS[Phospho (STY)]SPNFFK_.2	Q60949_S660_M1_Tbc1d1	7.9329	7.7816	4.5986	4.0230	3.7681	3.8015	3.2800	8.1337	3.2002	3.3718	4.1797	3.4840	3.3656	3.8683	3.3054	4.4241	7.1140	4.2502	3.8988	3.8353
Tbc1d1	_SFS[Phospho (STY)]QPGLR_.2	#N/A	3.1132	3.3887	3.6495	4.5762	5.4071	6.4909	6.5286	4.3144	3.6484	3.3195	6.0209	3.2782	7.0487	3.6366	3.1665	3.4581	3.3513	3.7010	6.6843	6.5107
Pml	_VPAPPGS[Phospho (STY)]PC[Carbamidomethyl (C)]R_.2	Q60953_S17_M1_Pml	9.3964	9.8625	9.8499	9.6495	9.4705	9.7236	9.4686	9.6739	9.1649	9.7062	9.5918	9.1560	9.6216	10.0256	9.0354	9.2554	9.8019	9.5533	9.5656	9.4400
Pml	_LATSS[Phospho (STY)]PEQSWPSTFK_.2	Q60953_S515_M1_Pml	6.5258	3.5506	4.0709	4.2769	3.8400	4.1594	6.0583	4.1247	5.8962	3.4437	6.1142	3.4160	3.4157	3.4575	3.2373	5.8844	3.3609	4.1563	5.8772	4.0327
Rbbp4	_[Acetyl (Protein N-term)]ADKEAAFDDAVEER_.2	#N/A	6.0202	5.7216	4.3325	5.6388	5.4734	5.2366	4.9775	5.8798	5.6960	6.1595	5.8848	5.3277	5.3994	5.9955	5.8426	5.1667	5.3400	5.4884	5.4164	5.5689
Ncor1	_AQLS[Phospho (STY)]PGLYDDSSAR_.2	Q60974_S1481_M1_Ncor1	7.1011	7.4742	6.4810	7.7078	6.3986	7.2816	6.8657	7.3203	6.7379	7.2608	6.2071	6.5674	6.9707	7.1257	6.1205	6.8526	7.1839	6.6851	6.6640	7.0094
Ncor1	_VS[Phospho (STY)]PENLVDK_.2	Q60974_S2134_M1_Ncor1	6.7720	6.7430	6.6611	6.8453	5.9461	6.2908	5.8789	6.5417	5.9901	6.6977	6.5270	5.9179	6.1557	6.4565	6.0775	6.0194	6.5068	6.5158	6.1251	6.6188
Ncor1	_SHIPSEPYEPIS[Phospho (STY)]PPQGPAVHEK_.3	Q60974_S2165_M1_Ncor1	3.4424	4.5605	3.9786	4.2470	4.2809	5.2606	3.2602	3.1427	3.3193	3.6487	3.6936	2.9491	3.4273	6.2077	2.8374	3.7872	3.6804	5.9633	4.3292	5.8506
Ncor1	_EPAPLLSAQYETLS[Phospho (STY)]DSDD_.2	Q60974_S2449_M1_Ncor1	4.3499	3.6529	3.3854	3.5945	3.6876	3.9117	3.8535	4.2154	3.9125	4.5562	4.2869	3.5424	4.0206	3.5644	3.4307	3.1940	3.7126	5.8253	3.7359	3.2417
Ncor1	_YS[Phospho (STY)]PESQSQTVLHPR_.2	Q60974_S2116_M1_Ncor1	3.3801	3.9390	4.4175	4.2041	3.5871	3.0205	3.2439	3.6058	3.3029	3.6650	3.6773	3.6651	3.4110	4.4556	3.4864	4.2431	3.1030	4.0464	4.3455	4.1329
Klf3	_GS[Phospho (STY)]PPAAGGSPSSLK_.2	#N/A	3.9119	4.0909	4.4482	3.7775	8.7813	3.2344	3.8470	9.7732	9.2148	4.3056	3.2458	3.9803	3.9326	4.4353	3.8686	4.2568	4.1500	3.6832	3.3318	4.4023
Ppp2r5c	_RKS[Phospho (STY)]ELPQDPHTEK_.3	#N/A	5.2177	6.4691	5.2456	5.1026	5.4390	4.9481	5.4274	5.5126	5.6479	5.8658	5.0487	3.6653	4.9800	4.9392	3.4005	4.4792	3.9670	5.3420	4.9029	4.1638
Gng7	_[Acetyl (Protein N-term)]S[Phospho (STY)]GTNNVAQAR_.2	Q61016_S2_M1_Gng7	11.0791	11.1248	10.9730	11.5129	11.0730	10.9940	10.7535	11.4948	11.0796	11.1607	11.6329	11.7795	12.1988	12.1572	11.6155	11.8504	11.9691	11.8553	11.6812	11.9094
Gng7	_KLVEQLR_.2	#N/A	8.0072	8.2299	8.0930	7.8968	8.1922	8.0834	8.3129	8.3194	7.7561	7.8915	8.2799	8.3485	8.1880	8.4299	9.0852	8.3641	8.3587	8.7711	8.7233	8.4217
Gng7	_NDPLLVGVPASENPFK_.2	#N/A	7.2936	7.1376	7.2863	7.4371	7.2235	7.1345	7.4425	7.4041	7.2160	7.4025	7.6572	7.5058	7.4764	7.4899	7.7451	7.3940	7.7000	7.6945	7.4406	7.3979
Gng7	_NDPLLVGVPASENPFKDK_.3	#N/A	3.2878	4.5528	3.8241	4.4016	4.1263	3.7153	4.1952	4.4890	3.8387	3.4941	3.8482	3.5337	3.3085	3.8111	4.4255	4.8670	3.5258	4.3397	3.9559	5.0591
Gng7	_ASSDLMGYC[Carbamidomethyl (C)]EQHAR_.2	#N/A	3.4525	4.2600	4.2554	4.7476	3.6594	3.9477	3.8334	3.6782	3.2901	3.8339	4.2902	3.7736	3.5172	4.5389	4.0358	3.9889	3.8848	4.0181	3.8891	4.1040
Ncoa2	_LLQDSSS[Phospho (STY)]PVDLAK_.2	Q61026_S699_M1_Ncoa2	4.4485	8.1974	8.1859	3.7310	7.1115	7.9200	7.5645	3.6588	3.8137	4.2591	3.2923	3.9339	3.9791	4.4818	7.4026	3.8107	8.1251	3.6367	3.2853	4.4488
Tmpo	_SST[Phospho (STY)]PLPTVSSSAENTR_.2	Q61029_T159_M1_Tmpo	10.7390	10.7715	10.6044	10.7687	10.2226	10.4103	10.4250	10.7420	10.3423	10.5030	10.1180	10.1096	11.0504	10.4760	9.3202	10.1333	10.7281	10.2295	10.1678	10.0938
Tmpo	_EQGTESRSS[Phospho (STY)]T[Phospho (STY)]PLPTVSSSAENTR_.3	#N/A	7.7241	8.3167	3.6573	7.1723	3.9595	4.1836	3.5816	3.9435	7.5330	7.3318	4.0150	3.2705	3.7487	7.8695	7.1875	3.4659	7.6260	7.8465	7.4129	7.2257
Tmpo	_QNGSNDSDRYS[Phospho (STY)]DNDEDSKIELK_.3	Q61029_S183_M1_Tmpo	3.6286	4.3742	4.1649	4.0608	2.9663	0.7129	3.1557	3.3290	3.4839	4.5889	2.9625	4.2637	3.6493	4.1186	3.5890	4.1405	3.8666	4.4361	4.3012	4.1190
Hars1	_DQGGELLSLR_.2	#N/A	5.9629	5.2157	5.5715	5.9296	5.6089	4.9722	5.6221	5.6884	5.4217	5.1533	6.3048	5.7485	5.8616	6.1663	5.7172	6.0889	5.7376	5.6795	5.6445	5.5647
Hars1	_IFSIVEQR_.2	#N/A	5.2529	4.6296	5.2652	3.5269	5.5305	4.9188	4.7662	4.2830	4.9201	4.7185	4.7932	5.2974	6.3365	4.9444	4.8612	5.2153	5.3555	5.2748	5.2579	4.7416
Hars1	_AALEELVR_.2	#N/A	3.0983	4.3269	4.6676	4.7455	4.4257	4.5701	5.0795	4.3074	5.2399	4.2803	4.4693	4.9169	4.9058	4.9117	5.2789	4.5453	4.0856	4.8839	5.4824	5.7725
Hars1	_YDLTVPFAR_.2	#N/A	5.4280	5.5468	3.6167	5.9129	5.0579	5.5265	5.5700	5.4183	4.8702	5.8994	5.5545	5.4810	3.7892	5.1270	5.5541	3.4253	4.9400	5.5953	5.9336	5.2234
Hars1	_ASAEQIEEEVTK_.2	#N/A	6.6674	6.0857	3.4868	6.2829	6.5879	5.9792	5.8357	4.1517	4.3066	3.7662	3.7852	6.0033	4.4720	6.2245	6.1998	6.2184	6.4937	6.3260	5.9795	6.7825
Hars1	_TIC[Carbamidomethyl (C)]SSVDKLDK_.3	#N/A	4.3972	3.6056	3.4327	3.2922	2.3087	3.6633	2.7094	4.0976	3.8652	3.1027	4.2396	1.1777	3.9733	3.4197	2.5293	3.1416	3.1344	3.4841	4.3473	3.1943
Pak3	_[Acetyl (Protein N-term)]SDS[Phospho (STY)]LDNEEKPPAPPLR_.2	Q61036_S4_M1_Pak3	9.3819	9.3196	9.2293	9.2279	5.3717	8.6130	8.7092	5.3465	5.3537	9.5162	5.1211	8.8836	9.0677	10.0174	7.8319	9.0154	5.2636	8.8293	8.8527	8.9637
Tsc2	_STS[Phospho (STY)]LNERPK_.2	Q61037_S939_M1_Tsc2	9.4777	9.3526	9.4084	9.3389	8.6431	8.5646	9.0753	8.6957	8.6228	9.4013	8.6770	8.5373	9.0412	4.4924	7.3422	8.3717	8.7244	8.7402	8.6582	8.4572
Tsc2	_IDIGRLS[Phospho (STY)]PEAK_.2	Q61037_S1412_M1_Tsc2	7.7890	7.3366	7.4171	7.6898	6.6979	7.6075	7.2594	7.4135	7.0938	6.6804	7.5981	6.5011	6.9914	6.5187	5.5184	6.3302	7.8927	7.6273	6.8532	7.1065
Tsc2	_S[Phospho (STY)]PSGLRPR_.2	Q61037_S1455_M1_Tsc2	8.3784	8.1222	7.7805	4.5591	7.4604	7.2613	7.8555	4.3315	2.9856	8.1754	7.7093	7.6459	7.7006	3.6537	3.0908	7.3753	7.5476	7.7012	7.4654	7.4034
Tsc2	_SSS[Phospho (STY)]SPELQTLQDILGDLGDK_.2	Q61037_S1388_M1_Tsc2	4.3468	3.9427	4.5964	3.6293	3.0529	3.0862	3.9952	3.7605	3.9154	4.1574	3.3940	4.6592	4.0808	4.5835	4.0206	3.7089	4.2982	3.5350	3.1836	3.0497
Tsc2	_SQS[Phospho (STY)]GILDGEAATWSATGEESR_.2	Q61037_S1421_M1_Tsc2	4.2105	5.3142	3.7470	3.3738	2.9166	4.4507	5.3339	4.4361	5.0695	4.0211	5.3304	4.3356	4.2172	5.3475	4.1569	5.5726	3.9334	4.5913	3.8593	3.1478
Tsc2	_SIS[Phospho (STY)]VSEHVVR_.2	Q61037_S981_M1_Tsc2	6.3922	4.0866	4.4525	6.6448	3.1969	5.8672	3.8512	3.6166	5.9059	4.3013	3.2501	3.9761	3.9369	4.4396	3.8766	3.8529	4.1542	3.6789	3.3275	5.7911
Tsc2	_SM[Oxidation (M)]S[Phospho (STY)]GGHGLR_.2	#N/A	4.0277	4.0462	3.6915	3.4433	4.6528	4.2378	4.2480	4.3806	4.5141	4.4050	4.7260	4.7288	4.8604	3.7296	3.3606	3.9705	3.8778	4.8405	3.5847	4.3156
Tsc2	_LISS[Phospho (STY)]VDDFTEFV_.2	Q61037_S1806_M1_Tsc2	3.1597	4.1594	4.1971	4.4246	3.3666	3.2409	3.0234	3.3853	3.0825	3.8855	3.4569	3.8856	3.1905	4.2351	4.1014	4.0226	4.3834	4.2669	3.0651	3.9124
Wbp4	_S[Phospho (STY)]LDKAKEEEK_.2	Q61048_S51_M1_Wbp4	3.5160	4.1224	1.0346	4.1734	2.3024	3.6304	1.4667	2.8414	2.6627	2.7594	3.6200	3.2441	3.0057	2.8616	2.9363	2.7048	2.3100	1.8586	1.2587	4.0064
Usf1;Usf2	_THPYS[Phospho (STY)]PK_.2	Q61069_S186_M1_Usf1;Usf2	3.8367	6.7916	7.1467	6.3961	3.1744	6.7778	4.3666	6.8380	4.4257	4.0430	7.0000	7.3385	7.2421	7.1241	3.9438	4.1816	4.0748	6.9970	7.0225	4.3271
Ei24	_RPQS[Phospho (STY)]VER_.2	Q61070_S56_M1_Ei24	6.3231	6.4670	5.5969	6.2395	5.4447	5.8937	6.1031	5.0423	5.4335	6.3667	5.5252	5.9563	6.6831	5.1857	4.2137	4.9486	5.4986	6.0404	5.4858	5.5476
Ei24	_FPS[Phospho (STY)]PHPS[Phospho (STY)]PAK_.2	Q61070_S326_M2_Ei24	9.2347	8.8840	8.9116	8.4912	8.0268	8.4605	8.3284	8.2306	8.4921	9.0411	8.3986	7.9933	3.8526	7.7460	3.2626	3.3620	7.6184	8.4778	6.3986	7.3460
Ppm1g	_KLEEALST[Phospho (STY)]EGAEDTGNSDK_.2	Q61074_T525_M1_Ppm1g	3.9436	4.2658	4.6504	3.9131	3.5776	3.5054	3.8758	3.6877	3.9348	4.1499	3.9376	4.0044	4.3341	4.0752	4.0259	3.8263	4.3849	4.0545	3.1248	3.9076
Map3k2	_AQS[Phospho (STY)]YPDNHQEFTDYDNPIFEK_.3	Q61083_S239_M1_Map3k2	5.5590	6.4587	4.5637	4.0579	4.1510	4.4888	3.3900	3.7520	3.4491	3.5188	3.8235	4.4946	3.5572	6.0890	2.9672	3.6574	3.2492	3.9002	4.7036	4.4088
Map3k3	_AIDILDRS[Phospho (STY)]S[Phospho (STY)]SM[Oxidation (M)]K_.2	Q61084_S111_M2_Map3k3	4.0648	4.4257	8.7106	3.7969	3.8768	3.1394	8.2759	3.3831	3.5503	4.0226	3.4616	4.2396	3.6927	4.2329	4.0983	3.8409	4.3812	4.2921	3.9382	4.2678
Ksr1	_TES[Phospho (STY)]VPSDINNPVDR_.2	Q61097_S392_M1_Ksr1	3.3054	8.7199	3.8417	9.1393	4.1440	4.3681	3.3971	3.7590	8.1219	8.9531	7.6976	3.0860	3.5642	4.6088	2.9743	3.6503	3.5435	7.2928	4.1922	7.7059
Ksr1	_SKS[Phospho (STY)]HESQLGNR_.2	Q61097_S297_M1_Ksr1	8.4612	8.6936	8.1497	8.3896	7.7562	8.1478	8.1721	8.2808	7.4071	8.5455	7.6517	7.5633	4.4077	8.2764	7.4555	7.9048	3.7428	7.7538	8.0358	7.6765
Dpf2	_VDDDS[Phospho (STY)]LGEFPVSNSR_.2	Q61103_S142_M1_Dpf2	4.3449	8.7212	9.1091	7.8663	3.6826	8.9349	9.5766	4.0452	8.9566	8.2727	4.2920	7.7589	4.0256	9.2569	8.3500	3.1889	3.0821	9.2141	8.9441	8.4457
Shc3	_QGS[Phospho (STY)]LDIYSTAEGK_.2	Q61120_S282_M1_Shc3	8.6153	4.2773	8.9271	8.1646	3.2487	3.3588	4.4063	3.2674	4.4654	8.3145	3.3389	4.0035	7.7144	4.1172	3.9835	7.5099	4.2655	4.3848	7.7393	7.5102
Shc3	_AAS[Phospho (STY)]VEC[Carbamidomethyl (C)]ISPVTPR_.2	Q61120_S354_M1_Shc3	9.8793	9.7813	10.1404	9.9360	8.7855	8.8064	9.2160	8.9222	8.6174	10.0701	9.1946	8.8548	8.9308	9.1438	7.5079	8.8857	9.1124	8.9025	8.9720	9.0397
Nab2	_SFS[Phospho (STY)]PKS[Phospho (STY)]PLELGEK_.2	Q61127_S159_M2_Nab2	3.1818	3.3202	3.7181	4.5076	4.0204	7.1541	6.7974	4.3830	3.5798	3.3881	3.9542	3.2096	3.6878	8.0824	7.1118	3.5267	3.4199	7.9427	6.0772	4.4097
Prpf4b	_KS[Phospho (STY)]KS[Phospho (STY)]PPLR_.2	#N/A	11.0100	10.9856	10.8998	10.8202	10.5931	10.7796	10.8199	10.9061	10.7928	10.6906	10.6666	10.4827	10.7976	10.4867	9.4930	10.3857	10.9343	10.7068	10.6963	10.4513
Prpf4b	_KKS[Phospho (STY)]PIVNER_.2	Q61136_S278_M1_Prpf4b	9.5470	9.6083	9.1716	9.2332	8.9093	9.1418	9.0697	9.5228	9.1499	9.1109	9.0123	8.9064	9.5949	9.3248	8.2782	8.9918	9.5005	9.3585	9.0565	8.9404
Prpf4b	_SKDAS[Phospho (STY)]PINRWS[Phospho (STY)]PTR_.2	#N/A	8.2776	8.6127	8.0695	8.2068	7.2993	8.2040	8.4781	8.1706	8.3295	8.5207	8.1686	8.0137	8.8933	8.2670	7.0872	8.1682	8.1248	8.3649	8.1723	6.9304
Prpf4b	_S[Phospho (STY)]RS[Phospho (STY)]PLLNDR_.2	#N/A	9.3341	9.2952	9.2957	9.0602	8.7132	9.0759	8.9719	9.1675	8.9987	8.9850	8.8713	8.6479	9.2689	9.0596	8.1549	8.7342	9.2911	8.8802	8.8406	8.6526
Prpf4b	_RRS[Phospho (STY)]LS[Phospho (STY)]PK_.2	Q61136_S354_M2_Prpf4b	8.1801	8.4942	8.1361	8.7898	8.1563	8.4802	8.3405	8.4839	8.7176	8.5060	8.2017	7.9996	8.9621	8.1659	7.4204	8.3193	8.4315	8.5913	8.4908	7.9154
Prpf4b	_S[Phospho (STY)]RS[Phospho (STY)]PLRR_.2	Q61136_S451_M2_Prpf4b	8.2927	8.2559	7.8557	8.0545	7.5926	8.0512	8.2641	7.9024	7.8450	7.9354	7.5403	7.8403	7.6543	7.8950	7.1464	7.9085	8.4135	8.0061	8.1875	7.7163
Prpf4b	_AKS[Phospho (STY)]RS[Phospho (STY)]LER_.2	Q61136_S394_M2_Prpf4b	7.2680	7.2923	7.7030	7.2788	7.0093	7.2544	7.1778	7.5246	7.6097	7.3061	6.6350	6.6427	6.6738	7.4019	5.8888	7.2666	7.2356	7.3081	7.4484	6.6940
Prpf4b	_TLS[Phospho (STY)]PGRR_.2	Q61136_S387_M1_Prpf4b	7.5786	7.7722	4.0928	7.3412	7.0898	7.2362	7.1358	7.5967	3.2051	7.3617	7.3490	7.0386	7.7770	4.0798	5.9472	6.8491	7.1749	4.0386	6.9966	6.8152
Prpf4b	_SKDAS[Phospho (STY)]PINR_.2	Q61136_S431_M1_Prpf4b	7.6823	7.7720	3.2747	3.4501	3.5770	7.4997	7.0083	7.2864	4.0945	3.9783	7.2530	6.6311	6.9497	7.2473	3.5413	6.4479	7.1370	7.2601	3.0044	7.0970
Prpf4b	_S[Phospho (STY)]KS[Phospho (STY)]PVDLR_.2	#N/A	10.4750	10.2730	10.7377	10.3534	9.5792	10.5395	10.0660	9.7778	10.2433	10.3373	10.2244	9.6411	9.3528	10.2060	8.6764	9.7126	10.4044	9.7777	10.0399	9.9838
Prpf4b	_S[Phospho (STY)]RS[Phospho (STY)]PLLNDRR_.2	#N/A	7.7673	7.2041	7.5042	7.4781	7.3119	6.7408	7.7543	7.4866	7.5795	7.9383	7.5720	7.3158	7.8904	7.5096	6.7196	7.5629	7.8839	7.6722	7.7682	7.4415
Prpf4b	_DAS[Phospho (STY)]PINRWS[Phospho (STY)]PTR_.2	#N/A	8.0210	8.6075	4.2110	4.4106	3.3806	8.4588	8.3871	3.3993	8.0163	8.5689	6.4624	3.8716	3.7532	8.3316	3.6929	8.0372	8.7722	8.0084	7.9524	4.2228
Prpf4b	_EVIEASDKEGLS[Phospho (STY)]PAKR_.3	#N/A	7.0470	7.7711	7.3084	7.5208	5.2002	7.5804	7.2656	6.5686	7.4008	7.4101	6.8406	6.8349	7.2861	7.6188	6.5149	7.1637	7.3057	7.4690	7.6247	7.4451
Prpf4b	_LC[Carbamidomethyl (C)]DFGSASHVADNDITPY[Phospho (STY)]LVSR_.3	Q61136_Y849_M1_Prpf4b	8.2004	9.0941	7.7183	6.6735	2.8542	6.5931	8.8819	8.4100	6.8722	7.1158	4.3512	8.4123	8.1729	9.7993	6.1640	6.1786	7.1161	8.6591	7.2832	5.7443
Prpf4b	_S[Phospho (STY)]KS[Phospho (STY)]PVDLRDK_.2	#N/A	7.5198	7.2705	6.6698	7.5656	6.5753	7.0419	7.9642	7.6310	6.7627	8.3945	8.0296	7.6441	8.1491	8.0365	7.3476	7.6104	8.3900	7.9522	8.0674	8.0751
Prpf4b	_RS[Phospho (STY)]RS[Phospho (STY)]PLLNDR_.3	#N/A	3.6005	1.9871	4.1368	4.0889	2.9382	3.5459	3.1020	3.3009	3.1513	3.8068	3.9570	4.2918	3.6212	4.1239	1.1101	3.9454	3.8386	2.5143	3.6432	4.0909
Prpf4b	_S[Phospho (STY)]KS[Phospho (STY)]PPLR_.2	#N/A	3.2997	4.0194	4.3371	4.2846	3.5066	3.5400	3.5415	3.5254	3.4617	3.1103	2.9403	3.7455	3.6271	3.2198	3.5669	4.1626	4.5234	3.9887	4.4259	4.0968
Astn1	_VGS[Phospho (STY)]PRS[Phospho (STY)]PVNK_.2	Q61137_S376_M2_Astn1	7.6441	7.6405	7.3192	7.2729	6.5741	6.7772	7.4516	7.1832	6.8726	7.3011	6.7547	6.7752	7.2185	7.1028	6.1500	6.5132	7.2037	7.0253	6.6977	6.6414
Astn1	_SAS[Phospho (STY)]AEAANEIHYIPSVLIGGHGR_.3	Q61137_S194_M1_Astn1	6.8300	7.3337	4.5077	7.2614	4.0699	6.7517	3.2497	7.4294	3.2399	6.7206	6.4795	7.1134	7.3352	7.8941	3.0717	5.7317	6.6668	6.9673	6.8186	5.6283
Astn1	_GNSPSDES[Phospho (STY)]EERERDPK_.3	#N/A	5.0169	5.4878	4.8186	4.4433	4.8426	5.3382	5.4536	4.7662	5.5471	5.5319	5.3424	3.6335	5.7207	4.1878	3.4819	3.9898	5.3193	3.7816	5.3848	3.4703
Astn1	_VQGHNS[Phospho (STY)]SGTLSIR_.2	Q61137_S227_M1_Astn1	3.4507	6.2415	6.8270	7.0560	4.0104	3.3884	6.3230	3.4353	6.7054	3.4744	6.3735	6.2700	3.4482	6.6442	3.1547	4.0755	7.0163	4.0895	4.0912	6.6113
Astn1	_GNSPSDES[Phospho (STY)]EERER_.2	#N/A	3.1862	4.9909	3.7225	4.5032	4.0248	4.2489	3.5163	4.3874	3.5754	3.3925	3.9498	3.2052	3.6834	3.7096	3.0935	3.5311	3.4243	4.4089	4.0575	4.4053
Bcar1	_GS[Phospho (STY)]PGFTPEDLDR_.2	Q61140_S572_M1_Bcar1	4.1948	3.1242	3.7314	3.3894	2.9009	3.7066	4.1472	4.4205	4.1177	4.3511	2.9912	4.3512	4.2257	3.7695	4.1725	7.2545	7.6104	7.0393	3.5307	3.4468
Psen2	_TQES[Phospho (STY)]EEDC[Carbamidomethyl (C)]EEDPDR_.2	Q61144_S52_M1_Psen2	3.4014	3.8817	4.4748	4.7974	4.2399	4.4640	3.3011	3.6631	3.3602	3.6077	3.7346	5.0766	3.4683	4.5129	2.8783	3.7463	5.3985	3.9891	4.2882	4.1902
Psen2	_TSLMSAES[Phospho (STY)]PTSR_.2	Q61144_S25_M1_Psen2	4.2071	4.6128	3.7437	3.4896	2.9132	4.4474	4.1349	3.9002	4.0551	4.0177	3.5337	4.3389	4.2206	3.2224	4.1603	3.5692	4.4379	3.3953	3.0439	3.1894
Ocln	_YSS[Phospho (STY)]NGNLETPSKR_.2	#N/A	6.8771	3.9316	4.6075	3.6181	3.4090	6.3297	6.3658	4.4941	4.1912	4.2776	3.0647	3.8210	6.0498	3.8430	3.7093	4.4161	3.9913	6.2683	3.4572	3.5203
Ocln	_YSS[Phospho (STY)]NGNLETPSK_.2	#N/A	3.8445	3.4746	3.3811	3.7397	4.0514	4.0848	2.9967	4.0702	4.4177	3.6551	3.8963	3.2007	4.5831	3.4192	3.0220	3.2066	3.5674	7.3317	4.1821	3.5520
Ocln	_NVS[Phospho (STY)]AGTQDM[Oxidation (M)]PPPPSDYAER_.2	#N/A	5.8780	5.5624	3.8558	5.2563	5.5900	5.5232	5.9694	6.4391	5.7750	3.5583	3.9035	5.1983	5.9903	5.2738	4.2852	5.1325	6.1798	5.4580	5.7365	5.2711
Ppp2r5e	_SQS[Phospho (STY)]SSQFR_.2	Q61151_S32_M1_Ppp2r5e	3.3775	3.5618	9.3961	4.2956	6.6282	6.8751	7.0803	9.6109	6.6678	7.4257	4.0891	6.4017	7.0335	3.4382	3.2125	6.4717	9.4839	4.1754	4.1217	4.0231
Map4k2	_TPS[Phospho (STY)]EIQFHQVK_.2	Q61161_S328_M1_Map4k2	8.6757	8.4783	8.4426	3.7829	8.1682	8.5161	3.0398	8.8626	8.2119	8.6442	3.9395	8.4996	3.8322	8.8869	3.0652	8.3332	8.7409	8.5784	8.4792	8.5003
Map4k2	_ASDPHLGTLS[Phospho (STY)]PEDSELETHDMFPDTIHSR_.4	Q61161_S298_M1_Map4k2	4.3353	3.6676	3.3707	3.3541	3.6730	3.8971	3.8681	4.0356	3.9272	4.5416	4.3016	3.5570	4.0352	3.3578	3.4453	3.1793	3.0725	3.2598	4.4093	3.2563
Ctcf	_[Acetyl (Protein N-term)]M[Oxidation (M)]EGEAVEAIVEESETFIK_.2	#N/A	5.9917	5.5773	6.1561	5.3236	4.8836	4.8585	4.7852	6.1743	5.1594	6.1959	4.3998	5.4364	7.0969	6.6367	5.6665	6.3300	6.1862	5.3108	4.4528	5.0038
Slc9a1	_LDS[Phospho (STY)]PTLSR_.2	#N/A	11.0734	11.2141	11.1008	11.1240	10.5819	11.2398	11.1257	10.5364	10.8370	10.9808	11.1806	10.5555	11.0499	10.7271	9.6133	10.4577	11.0956	11.1424	10.8114	10.7640
Slc9a1	_IGS[Phospho (STY)]DPLAYEPK_.2	Q61165_S707_M1_Slc9a1	10.3952	10.3043	9.9761	10.1914	9.2263	9.6131	9.9559	9.4664	9.6938	10.0393	9.6808	9.4822	10.0092	9.5035	7.6739	9.4188	10.3293	9.8716	9.6264	9.4547
Slc9a1	_ADLPVITIDPAS[Phospho (STY)]PQSPESVDLVNEELK_.3	Q61165_S727_M1_Slc9a1	5.6166	4.5501	3.9890	4.1982	4.2913	4.4210	6.2509	3.6118	3.3089	3.6590	3.6833	2.9387	3.4170	5.4138	2.8270	3.7976	3.1090	4.0405	4.3395	4.1388
Slc9a1	_ITNYLTVPAHKLDS[Phospho (STY)]PTLSR_.3	#N/A	3.7421	3.5770	5.4210	3.8421	3.9491	3.9824	7.5270	8.3094	3.0192	3.5527	3.9987	3.3031	3.1847	7.9624	4.0075	3.1043	3.4020	4.4311	5.3609	3.6543
Slc9a1	_C[Carbamidomethyl (C)]LS[Phospho (STY)]DPGPHPEPGEGEPFIPK_.3	Q61165_S801_M1_Slc9a1	3.5574	3.9665	5.1652	4.3028	2.8951	3.7341	4.4905	3.5071	3.2042	3.7637	3.5786	3.7638	3.3123	4.3569	4.2231	3.9023	4.5051	4.1452	2.9434	4.0341
Mapre1	_[Acetyl (Protein N-term)]AVNVYSTSVTSDNLSR_.2	#N/A	6.2479	6.5713	7.2082	5.8568	6.1532	6.4529	5.9159	6.6043	6.2895	6.9358	6.0653	5.5266	6.5320	6.1348	6.1817	5.9094	6.1587	5.9125	6.0243	5.8749
Mapre1	_NIELIC[Carbamidomethyl (C)]QENEGENDPVLQR_.2	#N/A	5.6078	5.4223	5.3408	5.1379	4.8841	5.4971	4.4944	5.4537	5.2365	5.1947	4.5096	4.9450	4.8400	5.8460	5.2962	4.9312	4.7548	4.5125	5.1618	4.6839
Mapre1	_LEHEYIQNFK_.3	#N/A	5.1243	4.6665	3.7500	5.3319	4.2583	4.5518	4.9527	3.7502	4.5244	4.5034	4.9597	4.6730	4.5629	4.1807	5.0280	4.7545	3.0665	4.8869	5.4158	5.1182
Mapre1	_LTVEDLEKER_.2	#N/A	5.9436	5.6307	5.4626	5.7064	5.6455	6.0179	4.8238	5.6440	5.4885	5.5949	5.3421	5.3561	5.8668	5.6696	5.3519	5.6283	5.2555	5.2308	5.2549	5.0132
Prdx2	_QITVNDLPVGR_.2	#N/A	10.0334	9.9710	10.3721	10.0960	10.0878	9.9829	9.8932	10.2639	10.1766	10.2066	9.9227	10.1659	10.2612	10.1820	10.3603	10.3232	10.0735	9.9032	10.1884	9.9703
Prdx2	_SLSQNYGVLK_.2	#N/A	8.9792	9.2668	9.3559	9.1558	9.1747	8.9450	9.0693	9.3014	8.9813	9.1854	8.8083	9.1528	9.3886	9.1226	9.5672	9.0664	9.1148	9.2102	9.0576	8.9891
Prdx2	_NDEGIAYR_.2	#N/A	8.2203	8.3488	8.3796	8.8307	8.4481	8.1069	8.0734	8.1673	8.8876	8.6805	8.5886	8.7936	8.9797	8.8688	9.3988	8.9197	7.9359	8.7847	8.7991	8.9114
Prdx2	_EGGLGPLNIPLLADVTK_.2	#N/A	6.7695	6.8993	6.1179	7.2131	6.7987	6.5958	6.6026	6.3512	6.8965	6.7711	6.9947	6.7953	7.1152	6.6238	6.5908	6.8127	6.7702	6.3293	6.4106	6.9889
Prdx2	_GLFIIDAK_.2	#N/A	4.8455	5.5190	6.2706	6.8817	5.7887	6.2230	5.6950	4.8131	6.2925	6.4802	6.1072	7.0420	5.2645	6.0569	6.6519	6.7714	4.6754	6.4151	6.9974	6.8599
Prdx2	_KEGGLGPLNIPLLADVTK_.3	#N/A	8.2375	8.3106	8.0777	8.3293	8.2151	8.1127	8.3000	7.8996	7.9606	8.3070	8.1979	8.2370	8.3610	8.3566	8.5478	8.1759	8.0782	8.1800	7.8940	8.1127
Prdx2	_SAPDFTATAVVDGAFK_.2	#N/A	6.9543	6.8888	7.2852	6.7058	6.7358	6.9542	6.5825	6.6437	6.9804	7.0969	6.3680	6.3975	6.8374	6.6886	7.0317	6.9230	6.6069	6.6694	7.0312	6.8120
Prdx2	_LGC[Carbamidomethyl (C)]EVLGVSVDSQFTHLAWINTPR_.3	#N/A	4.3983	3.9941	4.5450	3.6807	3.1043	3.1377	3.9438	3.7091	3.8640	4.2088	3.3426	3.8836	4.0294	4.5321	3.9691	3.7604	4.2467	3.5864	3.2350	2.9982
Rgl2	_VSC[Carbamidomethyl (C)]EVEPPGTSDS[Phospho (STY)]PAAR_.2	Q61193_S518_M1_Rgl2	3.9225	3.4026	3.4591	6.5286	4.1294	4.1628	3.8168	4.0732	4.3458	3.7331	3.8244	3.1288	4.5112	3.4087	4.4782	3.2846	3.3552	3.4145	4.1425	3.2389
Rgl2	_SASC[Carbamidomethyl (C)]GS[Phospho (STY)]PLSGNTGEGTSR_.2	Q61193_S622_M1_Rgl2	3.2030	3.2990	3.7393	4.4864	4.0416	3.9434	3.4995	4.4042	3.0583	3.5137	4.6432	3.1884	3.2237	3.7264	3.0767	3.5479	3.4410	4.3921	4.0407	3.6934
Pafah1b3	_LLAQDQGQGIPLPETAS_.2	#N/A	3.7048	5.6137	6.1885	4.4503	6.5026	5.9655	6.0620	6.8962	6.2160	6.3613	6.1024	6.2318	6.8773	5.9407	6.1355	6.7138	6.4972	5.8661	6.1475	6.2707
Pafah1b2	_IIVLGLLPR_.2	#N/A	8.2903	8.5506	8.3794	8.6717	8.7567	8.6729	8.5238	8.4637	8.2729	8.6820	8.3965	8.7181	8.7203	8.5576	8.7375	8.5736	8.3178	8.5391	8.5107	8.4671
Pafah1b2	_GEKPNPLR_.2	#N/A	7.8151	8.1389	7.9497	7.8321	8.3113	8.3519	8.4942	8.1240	8.5212	8.1123	8.0430	8.4818	8.3767	8.5172	9.0409	8.2455	8.1203	8.1271	8.3363	8.2585
Pafah1b2	_[Acetyl (Protein N-term)]SQGDSNPAAIPHAAEDIQGDDR_.3	#N/A	4.0408	4.6851	4.5493	3.6796	4.4011	4.9917	4.6892	3.6539	4.4608	4.8396	4.8462	4.2897	4.0908	5.2423	4.4419	4.4068	4.7725	4.8810	4.8251	5.1890
Pafah1b2	_ELFSPLHALNFGIGGDTTR_.3	#N/A	5.6359	6.1479	5.8855	5.6829	5.8488	6.0356	5.7224	5.8476	5.7321	5.8626	5.6606	5.5850	5.4293	6.3234	6.2075	5.9130	5.7002	5.7176	5.5845	6.0422
Pafah1b2	_[Acetyl (Protein N-term)]SQGDSNPAAIPHAAEDIQGDDRWM[Oxidation (M)]SQHNR_.4	#N/A	6.5871	6.2514	5.9103	5.2804	5.9161	5.8485	5.9264	6.9784	6.3639	6.5540	5.9252	6.5981	7.6157	5.9583	6.5476	6.3638	6.3901	6.3172	6.1065	6.2454
Psap	_GC[Carbamidomethyl (C)]SFLPDPYQK_.2	#N/A	9.6652	10.0352	10.1006	10.0074	9.8868	9.6980	9.7974	9.7340	10.0945	10.0623	9.6001	10.0329	10.1293	10.0810	10.4847	10.1073	10.0147	9.8131	9.9792	9.8605
Psap	_SLPC[Carbamidomethyl (C)]DIC[Carbamidomethyl (C)]K_.2	#N/A	7.3591	8.6320	8.0040	8.3101	8.4440	8.4730	8.4174	7.9317	8.3500	8.1180	7.8054	8.2714	8.5531	8.1939	8.8647	7.8352	7.8145	8.0352	8.2437	8.2483
Psap	_TVVTEAGNLLK_.2	#N/A	6.9828	6.8362	7.0231	7.1699	6.5505	6.6479	6.4287	6.3118	6.5000	6.5508	6.5672	7.0318	6.3363	6.8557	7.3210	6.2133	6.4094	6.5318	6.7249	6.3032
Psap	_LVSDVQTAVK_.2	#N/A	6.0024	6.0595	6.0540	5.5269	6.2682	5.8074	6.1159	6.0544	6.2690	5.3863	5.7504	5.9868	5.5705	5.2401	6.5194	5.7760	5.5949	5.9021	5.4207	5.7644
Psap	_LVLYLEHNLEK_.2	#N/A	5.1399	5.6202	5.4217	5.4472	4.8621	5.1616	4.7604	4.5042	5.1948	5.5563	5.0812	3.9156	5.1287	5.7821	5.4773	5.1660	4.4505	4.9828	5.2530	4.6406
Psap	_LGPGVSDIC[Carbamidomethyl (C)]K_.2	#N/A	5.0457	4.4911	3.7235	5.4370	5.1388	5.5243	5.3014	5.1891	4.8589	5.6444	5.2508	4.8662	5.3882	5.8895	5.2705	4.3714	4.7735	5.3447	5.1271	5.6427
Psap	_EVVDSYLPVILDM[Oxidation (M)]IK_.2	#N/A	6.1653	6.0236	6.1608	6.2066	6.2939	6.0326	5.8872	5.7845	6.0638	6.1467	5.7889	6.1905	5.8427	5.8766	6.4557	6.3858	6.0943	5.9207	6.0070	5.9130
Psap	_TC[Carbamidomethyl (C)]EWIHDSSLSASC[Carbamidomethyl (C)]K_.3	#N/A	3.9358	4.5604	5.4271	5.0564	3.7728	4.1333	4.2912	3.9227	4.1122	4.5158	3.8078	3.5169	4.2203	4.1900	4.9913	4.7861	3.9390	4.8717	3.9152	4.0224
Dyrk1a	_IYQY[Phospho (STY)]IQSR_.2	Q61214_Y321_M1_Dyrk1a	12.3143	12.3055	12.1279	12.2828	11.9282	12.0662	12.1277	12.2974	12.0662	12.0004	12.0795	11.9337	12.5613	12.0456	11.1462	11.7923	12.3213	12.0309	12.0545	11.8292
Lcp1	_FSLVGIAGQDLNEGNR_.2	#N/A	3.2633	5.1489	5.5082	5.2630	2.9132	5.7771	5.2911	5.5656	4.7018	4.7265	5.5084	4.8540	5.0603	5.2384	4.2771	5.1923	5.6129	5.2723	4.7649	5.2224
Lcp1	_GS[Phospho (STY)]VSDEEMMELR_.2	Q61233_S5_M1_Lcp1	3.4627	4.5594	3.9797	4.2460	3.6696	3.7029	3.3785	3.1438	3.2987	3.2733	4.2782	2.9480	3.4641	3.9668	3.4039	4.3256	3.6815	4.1517	3.8003	3.9338
Lcp1	_EGES[Phospho (STY)]LEDLMK_.2	Q61233_S257_M1_Lcp1	3.9434	4.4878	3.4800	4.2604	4.1503	4.1837	4.3986	4.1691	4.3188	3.7540	3.7974	3.1018	4.4842	3.5180	2.9231	3.3055	3.6663	3.1316	4.2810	3.4531
Snta1	_QPSS[Phospho (STY)]PGPQPR_.2	Q61234_S195_M1_Snta1	3.4469	6.2443	3.6807	9.0154	8.7242	5.9088	8.5516	6.7683	3.9095	9.0447	3.9298	3.2529	8.8339	8.7966	5.0227	3.4360	5.8268	6.0404	8.5030	8.7433
Snta1	_NSAGGTSVGWDS[Phospho (STY)]PPASPLQR_.2	Q61234_S183_M1_Snta1	6.6021	8.3960	7.3703	5.7555	4.5699	7.3540	8.1048	7.5772	6.8820	6.4603	3.9738	4.8658	6.5095	9.1230	6.0092	6.1030	6.0311	8.0506	6.5859	7.0147
Snta1	_YLEIC[Carbamidomethyl (C)]AADGQDAVFLR_.2	#N/A	4.2263	3.7765	3.2617	3.4709	3.5640	3.7881	3.9771	4.3390	4.0362	4.4326	2.9097	3.6660	4.1442	3.6880	3.5543	3.0703	3.8363	3.1332	3.6122	3.3653
Sntb2	_GPAGEASAS[Phospho (STY)]PPVR_.2	#N/A	3.4660	3.8530	4.5035	4.1182	3.6730	9.0016	3.3751	3.6917	3.2953	3.2766	8.7078	3.5792	3.4608	3.9634	3.4005	4.3290	3.1889	4.1550	7.4858	3.9304
Sntb2	_GLGPPS[Phospho (STY)]PPAPPR_.2	Q61235_S75_M1_Sntb2	9.9343	9.9944	7.6522	9.5176	9.2981	9.2235	8.4226	10.2472	8.9354	9.6190	9.4542	9.3607	3.6855	9.7418	7.3927	8.5945	9.4965	9.1910	9.2332	9.2372
Sntb2	_GPAGEASAS[Phospho (STY)]PPVRR_.3	#N/A	3.0978	3.4042	3.6341	3.8432	3.9364	4.1604	3.6047	3.9667	3.6638	3.3041	4.0382	3.2936	3.7719	3.3156	3.1819	3.4427	3.4639	3.6855	3.9846	2.9929
Igbp1	_[Acetyl (Protein N-term)]AAS[Phospho (STY)]EDELLLPR_.2	Q61249_S4_M1_Igbp1	6.1683	7.1096	6.4233	6.7166	3.7472	6.8781	6.6085	4.1099	6.9918	7.1784	5.5581	6.5970	6.8573	7.1047	6.2481	7.2744	6.9299	6.2975	7.1574	6.5536
Abcd2	_AEAYS[Phospho (STY)]PAENR_.2	Q61285_S58_M1_Abcd2	7.8977	3.4976	3.3580	7.2591	4.0284	4.0617	3.0197	4.0471	4.4407	3.6321	6.9097	3.2237	7.3574	7.1061	3.0451	3.1836	3.5444	3.0096	4.1591	3.5750
Tcf12	_TSS[Phospho (STY)]TNEDEDLNPEQK_.2	Q61286_S583_M1_Tcf12	3.1492	3.3528	3.6855	4.5402	3.9877	3.9972	3.5533	4.3504	3.0045	3.5675	3.9839	3.2422	3.1699	3.6725	3.1305	3.4941	3.3872	4.4459	4.0945	3.6395
Cacna1e	_GTQADWES[Phospho (STY)]PER_.2	#N/A	9.6130	9.0296	8.8006	9.1710	8.4226	8.3640	8.7616	8.6401	8.0877	9.4401	8.4652	8.1154	8.8105	8.5065	6.4225	7.9318	8.4155	8.3973	8.1519	8.0368
Cacna1e	_QGT[Phospho (STY)]PVPASGPAAAYK_.2	Q61290_T29_M1_Cacna1e	8.8674	3.9989	4.3577	8.1912	8.0172	7.9521	8.1233	8.3056	7.9413	8.4437	8.1405	7.5500	3.6066	7.6331	3.5463	7.2443	8.2524	8.1363	7.9489	7.7890
Cacna1e	_GTQADWES[Phospho (STY)]PERR_.2	#N/A	8.1530	7.9642	7.6339	7.3452	7.0014	7.4647	7.8235	7.9689	7.1546	7.7977	7.4538	7.2938	7.8795	7.4830	6.1130	6.9673	7.5499	7.6481	7.0504	7.0317
Cacna1e	_S[Phospho (STY)]YHSSLR_.2	Q61290_S2013_M1_Cacna1e	8.3266	7.9560	7.4876	7.2613	7.1902	7.2972	8.0257	7.0879	6.9336	8.2195	7.6708	6.6483	4.1640	7.0752	4.8337	7.1105	7.1273	7.4979	6.9535	6.9714
Cacna1e	_HLLS[Phospho (STY)]PDVSR_.2	#N/A	8.4889	8.0201	7.5541	8.1872	6.8773	6.6765	7.4573	7.3504	6.7384	8.1053	3.5970	7.1684	7.5151	6.9681	4.2415	7.0751	7.5954	7.5038	7.2963	6.8269
Cacna1e	_KHLLS[Phospho (STY)]PDVSR_.2	#N/A	7.4636	7.3139	6.7092	3.6240	3.8439	3.8772	6.8689	4.0209	5.7770	6.8750	6.9276	3.9009	4.1602	4.0250	4.0999	6.0219	4.0104	7.1212	6.2654	6.4115
Cacna1e	_AS[Phospho (STY)]IKS[Phospho (STY)]TKVDGASYFR_.3	Q61290_S447_M2_Cacna1e	4.4308	5.3554	3.9674	4.6334	3.1369	5.3712	5.0668	4.1290	4.7565	4.2971	3.1517	4.8396	4.1295	5.0511	3.7870	4.9194	4.0667	3.4574	5.0002	4.8744
Cacna1e	_FGEAVVGRPGS[Phospho (STY)]GDGDSDQSR_.3	#N/A	5.6803	8.1025	7.1749	7.4607	3.5170	7.7859	8.1076	3.8267	7.3149	7.1259	3.3908	6.8812	4.0226	7.9260	5.8601	7.1044	7.3661	8.4009	7.0700	7.3367
Cacna1e	_DSSDEHC[Carbamidomethyl (C)]VDISSVGT[Phospho (STY)]PLAR_.3	Q61290_T441_M1_Cacna1e	5.3074	6.3377	4.3051	5.8432	3.2322	6.1724	5.3013	4.4609	4.8265	5.5675	4.9460	4.8151	4.7004	4.2895	4.2358	4.8383	5.4074	5.4869	4.9121	4.9478
Cacna1e	[illegible]SQS[Phospho (STY)]EPDLSC[Carbamidomethyl (C)]M[Oxidation (M)]TANM[Oxidation (M)]DK	#N/A	7.8665	7.4231	6.9133	6.8307	6.0015	6.7170	6.3721	7.0288	6.4572	6.9840	6.6495	6.5871	7.0661	7.1798	3.8470	6.0651	7.0182	7.2069	6.3260	3.9733
Cacna1e	_SAS[Phospho (STY)]QERS[Phospho (STY)]LDEGVSVEGEKEHEPHSSHR_.4	Q61290_S948_M2_Cacna1e	5.6103	3.7379	3.8619	5.6756	3.6985	3.7509	3.3922	4.1793	3.3124	3.8531	4.7338	5.0663	3.4779	6.0073	3.4176	4.8627	5.0309	6.0620	5.7666	5.2962
Cacna1e	_TDGEAS[Phospho (STY)]PLKEAETKEEEEEVEK_.3	Q61290_S1099_M1_Cacna1e	3.9937	4.0091	4.8657	3.6957	3.3314	3.5555	4.2097	3.6941	4.2687	4.2000	3.9248	3.8986	4.3768	3.9206	3.7868	1.5585	3.4367	3.0806	2.2059	3.5978
Cacna1e	_S[Phospho (STY)]LDEGVSVEGEKEHEPHSSHR_.4	#N/A	3.9095	6.3612	5.9528	3.8095	4.2642	5.3761	6.2532	3.8293	3.7367	5.5442	5.0752	3.6074	3.9022	4.8427	4.1598	3.8526	3.9756	5.5775	4.5402	4.7559
Cacna1e	_EVS[Phospho (STY)]PM[Oxidation (M)]SAPNM[Oxidation (M)]PSIER_.2	#N/A	8.1905	5.9925	6.4185	6.6283	7.6432	7.0357	7.1027	7.8894	6.7781	6.6979	7.5416	7.0704	7.8448	7.2912	5.6245	6.8177	7.6015	7.5553	7.2005	6.9980
Cacna1e	_GGS[Phospho (STY)]LKGDIGGLTSALDNQR_.3	#N/A	4.6639	5.3178	3.9160	4.0903	3.4255	3.6496	4.7256	4.2387	3.5253	4.4703	3.8996	4.7191	3.9084	4.8867	3.7515	3.7246	3.6177	4.7092	4.0073	3.8366
Cacna1e	_HMQMSS[Phospho (STY)]QEALNK_.2	Q61290_S794_M1_Cacna1e	4.3470	3.6558	3.3825	3.3424	3.6847	4.3002	4.4246	4.0474	4.2023	3.8705	3.6809	3.5452	4.3677	3.3695	2.8066	3.1911	3.0842	3.2481	4.3975	3.3365
Cacna1e	_AKEVS[Phospho (STY)]PMSAPNMPSIER_.3	#N/A	3.7781	4.2584	4.0981	4.5236	3.1158	3.3399	4.4252	3.2863	2.9835	3.9844	3.3579	3.9845	4.5924	4.1361	4.0024	4.1230	4.2844	4.3659	3.1641	3.8134

Ctnna2	_VYGTAAVNS[Phospho (STY)]PVVSWK_.2	Q61301_S901_M1_Ctnna2	8.3354	8.4683	7.5968	7.3701	6.5731	6.7008	8.1375	8.2340	7.1459	7.7043	6.8745	7.7520	8.2618	4.4681	6.0144	7.2172	3.1155	7.9270	7.3879	7.0174
Ctnna2	_NATNEQDLANR_.2	#N/A	5.9147	5.7965	6.0442	5.9398	6.2757	6.2287	5.9830	5.9945	5.9969	6.3403	5.9762	5.5182	5.9511	5.7012	4.5485	5.6934	6.4276	5.9122	5.5882	5.9165
Ctnna2	_GHTGIGELAAALNEFDNK_.3	#N/A	5.6225	6.3613	5.2667	5.8845	5.7425	5.9748	5.8337	6.9195	5.7247	6.0533	5.9149	6.0547	6.5305	6.5729	5.2569	4.8658	5.9585	6.3297	6.2129	5.1784
Ctnna2	_QVQEAIAGISSAAQATS[Phospho (STY)]PTDEAK_.2	#N/A	9.8514	10.2365	9.5361	9.2107	3.7236	9.3953	10.0779	10.0172	9.1880	9.1860	8.8411	9.6148	10.3198	10.6828	8.6906	9.0694	9.5169	10.2901	9.6566	8.9787
Ctnna2	_[Acetyl (Protein N-term)]TSATS[Phospho (STY)]PIILK_.2	Q61301_S6_M1_Ctnna2	10.3046	10.1754	10.0716	10.2640	2.9632	9.6627	9.7403	10.0723	9.7396	9.9663	2.9594	9.6799	10.2527	9.8712	8.9524	9.9279	10.2945	9.7225	9.9350	9.5991
Ctnna2	_LVYDGVR_.2	#N/A	3.8059	4.1871	3.2380	5.0383	4.6769	5.2256	5.6317	4.7581	4.9534	4.6858	4.6792	4.9346	5.4153	4.8025	3.6614	5.1052	4.4414	5.3360	5.2371	3.5030
Ctnna2	_IVEEALEAVK_.2	#N/A	4.3161	4.6802	4.6170	4.1213	4.8308	4.8400	4.8297	5.4037	4.4612	4.8688	4.4750	4.5118	3.4938	4.1626	3.9068	4.7347	4.1130	3.8140	5.1146	4.2243
Ctnna2	_LLEPLVTQVTTLVNTSNK_.2	#N/A	5.2484	4.2583	4.3259	4.2921	4.4521	4.8216	4.8490	4.0430	4.1829	4.4630	4.6807	4.7862	3.6384	4.5321	3.7293	4.1514	4.8262	4.4004	4.3154	4.1080
Ctnna2	_TPEELEDDSDFEQEDYDVR_.2	#N/A	3.7221	6.3959	4.3866	4.4324	3.3255	7.5458	6.2749	3.5325	5.5185	3.8418	3.5991	4.0448	3.8519	7.6038	5.2235	4.1004	4.3820	4.0094	4.3154	4.0832
Ctnna2	_SKLDAEVAK_.2	#N/A	4.5962	4.5216	3.2819	3.4429	3.5842	4.8357	5.6167	5.5055	5.4038	4.4528	5.0960	4.7619	4.1240	4.4362	4.2820	3.0905	4.8003	3.3334	3.1663	5.0827
Ctnna2	_NATM[Oxidation (M)]LYTASQAFLR_.2	#N/A	4.1173	3.2017	5.3684	3.4669	4.6302	4.3245	3.9811	4.3430	4.0402	4.4286	4.4508	4.1095	4.1482	3.6920	3.5583	4.0569	3.8402	4.0294	3.6082	4.1228
Ctnna2	_LVEVANLAC[Carbamidomethyl (C)]SSNNEEGVK_.2	#N/A	3.2933	3.2087	4.4002	4.3961	2.7947	3.8531	3.2284	4.4945	2.7437	3.4234	2.7448	3.0981	2.4030	3.8167	2.9864	3.1301	1.6962	4.3018	3.4492	3.7837
Ctnna2	_VLTEAVDDITSVDDFLSVSENHILEDVNK_.3	#N/A	4.3669	3.0556	3.9034	3.2174	3.0730	3.1063	3.9751	3.7404	3.8953	4.1775	3.3740	4.1792	4.0608	3.8749	4.0005	3.7290	4.2781	3.5550	3.2036	3.0296
Ctnna2	_HIS[Phospho (STY)]PVQALSEFK_.2	Q61301_S939_M1_Ctnna2	4.4361	3.5667	3.4716	3.2533	3.7738	4.2111	3.7673	4.1365	3.8263	3.1416	4.2007	3.4562	4.4568	6.3775	3.3445	3.2802	3.1733	3.1590	4.3084	3.4256
Apc	_QSVGSGS[Phospho (STY)]PVQTVGLETR_.2	Q61315_S2713_M1_Apc	10.2643	10.1894	10.0709	9.9151	9.6014	9.8708	9.6548	10.1824	9.8297	9.6116	9.9144	9.8417	10.3707	9.9757	9.1281	9.8464	10.4313	9.9390	9.7706	9.7007
Apc	_SELS[Phospho (STY)]PITR_.2	Q61315_S2283_M1_Apc	3.7555	7.4265	7.2680	3.9339	7.2409	3.3909	6.5650	7.0841	3.6107	7.3076	6.9586	6.5270	7.2265	7.6746	6.0753	7.0790	7.6049	6.7177	6.8684	4.2458
Apc	_SGRS[Phospho (STY)]PTGNTPPVIDSVSEK_.3	#N/A	3.8054	3.5245	4.1796	6.2243	3.6631	5.4630	5.8892	4.0942	3.7913	3.7716	3.4394	5.7014	3.8994	4.2176	3.7103	3.6030	3.5914	3.6319	6.2148	3.8949
Apc	_AVEFSSGAKS[Phospho (STY)]PSK_.2	Q61315_S1359_M1_Apc	6.3448	5.2051	6.1200	3.1587	4.0832	4.4795	5.2137	4.2310	4.6998	6.1972	4.9888	3.1689	4.5514	5.4494	4.0885	4.9465	3.2679	5.4807	5.8814	5.3792
Apc	_HSS[Phospho (STY)]PSGTVAAR_.2	Q61315_S2774_M1_Apc	4.0932	3.6891	3.3492	3.3757	4.3002	5.0607	4.2488	4.0141	4.1690	3.9038	3.6476	3.5785	4.3344	5.4287	4.2742	3.4553	4.5518	3.2814	4.4308	3.3032
Apc	_SPT[Phospho (STY)]GNTPPVIDSVSEK_.2	Q61315_T2676_M1_Apc	3.3407	5.8873	6.2739	6.2592	3.5477	5.4203	5.6263	3.2824	5.4422	5.7539	6.2360	5.2166	3.6028	6.1766	4.5938	5.9065	3.8201	6.3689	3.6783	5.7553
Apc	_YSDEQLNSGRQS[Phospho (STY)]PSQNER_.3	#N/A	4.4870	4.3478	3.9084	4.8345	3.6416	5.3948	5.9626	3.8683	4.1464	4.2015	4.8096	4.3492	3.5734	5.2523	3.5132	3.5630	5.1190	5.5003	5.2508	5.0511
Apc	_ALEAELDAQHLSETFDNIDNLS[Phospho (STY)]PK_.3	Q61315_S778_M1_Apc	6.0901	6.5344	5.7227	3.8580	3.9511	5.2363	6.3352	3.9519	3.6491	3.3188	4.0235	5.3990	6.4781	3.3009	5.1591	3.4574	3.4492	6.2177	5.6663	2.9782
Apc	_T[Phospho (STY)]GSSSSILSASSESSEK_.2	Q61315_T2567_M1_Apc	3.4180	3.9011	4.4554	4.1663	3.6249	2.9826	3.4232	3.6436	3.3408	3.2286	3.7152	3.6272	3.4488	4.4934	3.3486	4.2809	3.1409	4.0086	4.3076	4.1707
Apc	_QAS[Phospho (STY)]SDSDSILSLK_.2	Q61315_S2129_M1_Apc	3.4542	7.4002	3.9905	4.2352	4.2928	3.0160	7.0396	3.1546	3.3074	3.6605	3.6818	2.9372	3.4155	4.4601	2.8255	3.7991	3.6922	4.0419	6.2100	4.1374
Apc	_LKSESEKQS[Phospho (STY)]PR_.2	#N/A	5.3561	4.7288	3.9375	5.0386	3.8993	4.6454	4.4704	4.8679	4.3835	4.9367	4.0639	3.9995	4.7535	4.3984	3.2295	3.6407	4.8181	4.8226	4.1233	3.6394
Apc	_SHS[Phospho (STY)]ES[Phospho (STY)]PSRLPINR_.2	#N/A	3.6708	4.0496	3.8692	4.9457	3.3855	4.1895	4.4914	4.7505	3.9385	5.0872	3.0755	3.8558	4.0689	4.0066	3.7113	4.2249	4.2448	4.2276	4.1804	4.1016
Apc	_C[Carbamidomethyl (C)]TSVSS[Phospho (STY)]LDSFESR_.2	Q61315_S1391_M1_Apc	3.6917	3.2782	3.6712	4.0795	4.1586	4.2838	3.9455	4.0775	3.9178	3.6573	3.8106	3.0888	4.0613	3.4265	4.3664	4.9427	3.5747	3.6060	5.2235	3.9364
Apc	_S[Phospho (STY)]PPEHYVQETPLVFSR_.2	Q61315_S1385_M1_Apc	3.8088	4.8629	3.7962	3.9488	3.6797	4.4074	3.8286	3.8631	3.7998	3.7775	3.6855	4.7886	3.8824	3.8071	3.7211	3.7646	3.7427	4.9775	4.8595	3.8736
Apc	_NDS[Phospho (STY)]LSSLDFDDDDVDLSR_.2	Q61315_S1859_M1_Apc	3.9569	3.3622	3.4935	3.6273	4.1639	4.1972	5.8076	4.1826	4.3053	3.7675	3.7839	3.0883	3.9878	3.5316	2.9096	3.3190	3.6798	6.6895	3.7687	3.2088
Apc	_SKT[Phospho (STY)]PPPPPQTVQAK_.2	Q61315_T1437_M1_Apc	9.4508	9.1468	9.4156	8.7974	8.8413	8.6594	8.5353	9.2288	8.9160	4.5175	8.6184	9.1204	9.3036	9.0473	8.0078	9.2423	9.4252	9.3168	8.9096	8.8573
Apc	_TPASKS[Phospho (STY)]PSEGPGATTSPR_.2	#N/A	3.6272	4.2813	4.4246	4.0111	4.1436	3.6393	4.2142	3.6024	3.3830	4.0624	4.1820	3.8872	4.1530	4.6455	3.7505	4.2428	4.8666	3.6345	3.7366	4.0181
Apc	_KPTS[Phospho (STY)]PVKPMPQNTEYR_.3	Q61315_S1772_M1_Apc	3.8523	3.4667	4.6791	3.7319	4.0593	4.0926	2.9888	4.0780	4.4099	3.6629	3.8885	3.1929	4.5753	3.5771	3.0142	3.2145	3.5753	3.0405	4.1899	3.5441
Apc	_SFHVEDTPVC[Carbamidomethyl (C)]FS[Phospho (STY)]R_.3	Q61315_S2021_M1_Apc	4.2242	4.5957	3.7608	3.3600	2.9303	4.4645	4.1178	4.4499	4.0380	4.0348	3.5167	4.3219	4.2035	3.7988	4.1432	3.5863	3.9471	3.2024	3.5014	3.4761
Hspa4	_GC[Carbamidomethyl (C)]ALQC[Carbamidomethyl (C)]AILSPAFK_.2	#N/A	7.0268	7.2824	7.6269	7.2882	7.2006	7.2345	7.3177	7.2543	7.1604	7.2061	6.6878	7.0409	7.3506	7.2148	7.4169	6.8068	6.9727	7.2124	7.0120	7.0799
Hspa4	_EFSITDVVPYPISLR_.2	#N/A	7.1427	7.0655	6.9384	7.1039	6.8635	7.2415	6.9635	6.9685	6.9137	6.8685	6.9150	7.0795	7.0268	6.8909	6.9637	7.2472	7.1862	6.8758	6.9744	6.9413
Hspa4	_AGGIETIANEYSDR_.2	#N/A	7.7331	6.9003	7.6346	7.3724	7.1390	7.1127	7.1272	7.5709	7.2030	7.8309	7.3417	7.1037	7.1484	7.4110	6.5859	6.9001	7.4188	7.0419	6.7651	7.0128
Hspa4	_VLATAFDTTLGGR_.2	#N/A	6.6037	6.8187	7.1300	6.7024	6.9538	6.6697	6.6435	6.7742	6.6972	6.4412	6.4682	6.8966	5.8463	6.9244	6.6608	6.7556	6.9941	6.8333	6.9455	6.6800
Hspa4	_QSLTVDPVVK_.2	#N/A	6.6204	6.9603	6.7251	6.5634	7.3692	6.3886	6.8047	6.8092	7.0042	6.1705	6.2981	6.6276	6.3600	6.6415	7.2927	6.9036	6.7820	6.7286	6.6819	6.8878
Hspa4	_NHAAPFS[Phospho (STY)]K_.2	Q61316_S429_M1_Hspa4	5.8447	5.8788	4.4686	5.6401	5.4531	5.9102	5.8493	5.5909	5.8020	5.7566	5.5642	5.7433	5.8640	6.1041	6.0472	5.9416	5.7672	6.1901	5.7842	6.1143
Hspa4	_SNLAYDIVQLPTGLTGIK_.2	#N/A	6.6290	5.7448	5.8778	5.6950	5.9949	6.4022	6.1581	6.4320	5.4850	6.1562	6.1277	5.8510	6.2263	6.3407	5.6829	6.4216	6.8229	6.4372	6.2139	6.4684
Hspa4	_LEDTENWLYEDGEDQPK_.2	#N/A	5.8098	6.1952	6.1881	6.0993	5.7395	6.0651	6.2060	5.9264	5.9697	5.4124	6.4100	6.2725	6.3513	6.4663	5.3608	6.5146	6.0740	5.9937	5.9093	5.5151
Hspa4	_KPVVDC[Carbamidomethyl (C)]VVSVPSFYTDAER_.3	#N/A	5.7283	5.3064	5.2466	5.6059	5.5239	5.9257	4.9565	4.9600	5.0001	5.8360	5.6363	5.2330	4.8223	6.0936	5.8662	5.4742	5.6880	4.7793	5.6962	5.7214
Hspa4	_LFEELGK_.2	#N/A	4.6108	5.1144	4.8061	5.8496	5.9797	4.9675	5.4048	4.1480	4.8184	4.8806	5.4239	5.4814	5.3287	5.6907	5.2263	5.3999	4.2560	5.1840	5.7629	4.9921
Hspa4	_DKLSGEYEK_.2	#N/A	6.5761	6.5661	6.7209	6.7576	6.6787	6.7562	7.0942	6.9749	6.8460	6.9472	6.5985	6.3934	6.7683	5.7717	7.1963	6.7187	6.4644	3.0962	6.5530	6.8101
Hspa4	_FQESEERPK_.2	#N/A	6.1839	5.3719	6.3898	5.3923	5.7337	5.5141	5.8359	5.9718	5.8375	5.1524	5.4888	5.9604	5.9745	5.5054	6.2434	5.7670	4.6126	5.7386	5.7094	6.0213
Hspa4	_LM[Oxidation (M)]NETTAVALAYGIYK_.2	#N/A	3.8502	5.3927	5.4731	4.7136	5.2814	5.0392	5.2125	5.6318	5.1633	5.3148	4.6283	5.0886	6.0819	4.3736	4.9595	4.9167	5.3010	5.3302	5.7129	5.6490
Hspa4	_LKETAESVLK_.2	#N/A	5.6285	5.6208	5.4595	5.3462	4.3380	5.1776	5.7921	6.0113	3.5304	4.5425	4.8644	3.8142	5.5004	4.1984	6.0042	4.0940	5.4045	5.2153	5.7649	4.6868
Hspa4	_QDLPALEEKPR_.3	#N/A	7.3785	3.2060	7.0240	8.4670	8.1978	8.5131	8.8414	4.4972	3.1513	7.0157	8.3847	7.1967	7.5599	8.0492	8.8227	7.9583	7.5458	8.2564	8.5352	8.5632
Hspa4	_SVM[Oxidation (M)]DATQIAGLNC[Carbamidomethyl (C)]LR_.2	#N/A	5.9612	5.9556	6.6656	5.9857	5.9211	5.6792	5.5500	5.8957	5.8800	5.7545	5.6733	5.6639	6.0646	6.0058	5.8705	5.7261	5.9434	5.9009	5.8768	5.6663
Hspa4	_AFSDPFVEAEK_.2	#N/A	7.2916	6.6573	6.7156	6.9252	6.8587	6.7512	6.6224	7.0175	6.8285	6.9400	7.0492	6.7491	6.9860	7.8609	6.5953	6.6471	6.9259	6.2524	6.3427	6.9963
Hspa4	_NKEDQYEHLDAADVTK_.3	#N/A	4.3155	4.8005	4.5816	4.3056	4.7927	4.3637	4.8445	4.6504	4.7487	4.5516	4.8109	4.6753	4.8461	4.6946	4.9755	4.5740	4.4084	4.9376	5.2647	5.0315
Hspa4	_FLEM[Oxidation (M)]C[Carbamidomethyl (C)]DDLLAR_.2	#N/A	4.9764	4.7854	3.8790	4.1177	3.0753	3.9000	3.9197	4.3041	4.0375	4.0629	3.8297	4.1676	4.7002	4.5844	4.5360	3.9230	4.0332	4.5306	4.3934	4.1039
Hspa4	_NAVEEYVYEMR_.2	#N/A	3.1855	4.1336	5.2166	6.2053	3.3924	4.0650	4.8176	3.4111	3.1083	3.8596	4.9025	3.8597	3.2163	6.2338	3.8012	4.0484	4.4092	4.2411	3.0393	3.9382
Hspa4	_WNSPAEEGLS[Phospho (STY)]DC[Carbamidomethyl (C)]EVFPK_.2	Q61316_S415_M1_Hspa4	3.7755	3.5436	3.3120	3.8088	3.9824	4.0157	3.0657	4.0011	2.9859	3.5861	3.9654	3.2697	3.1514	3.3501	3.0911	3.1376	3.4984	3.6511	3.9501	3.0274
Hspa4	_SQVISNAK_.2	#N/A	4.5535	3.5329	3.3227	3.7981	3.8912	4.1153	3.3063	4.0118	4.4828	3.2590	4.0833	3.2591	3.8170	3.3608	3.2271	3.3975	3.5091	3.7238	4.9879	3.0381
Hspa4	_ELSTTLNADEAVTR_.2	#N/A	3.2649	4.0542	4.3023	4.3193	3.4718	5.4365	4.9614	3.4906	3.1877	3.7802	3.5621	3.7803	3.2958	4.3404	3.6016	4.1278	4.4886	5.6663	2.9599	4.0177
Cntn2	_TTGPGDGIPAEVHIVR_.3	#N/A	3.1612	3.3408	3.6975	3.4673	3.9997	4.5880	5.1387	4.6136	4.8640	4.3838	5.0250	4.8792	3.1819	5.1162	4.6643	4.9776	3.3992	3.1793	4.0825	2.9711
Cntn2	_FAQLNLAAEDPR_.2	#N/A	4.3722	3.6306	3.4077	3.6168	3.7100	3.9340	3.8311	4.0663	4.0615	4.5786	4.2646	5.3200	3.9983	3.5420	8.2421	3.2163	3.6903	3.4591	3.7582	3.2193
Bad	_S[Phospho (STY)]APPNLWAAQR_.2	Q61337_S136_M1_Bad	10.3417	9.3408	10.4137	10.2798	8.5573	10.0575	10.0672	9.8050	10.7630	10.5003	10.2728	10.2787	10.5044	11.0185	10.6133	10.3088	9.5824	9.4981	10.8879	10.7329
Bad	_S[Phospho (STY)]AGTATQMR_.2	Q61337_S170_M1_Bad	3.5342	8.8732	9.5409	9.7005	7.2252	8.1902	8.5209	3.2345	3.2274	6.9429	8.8933	7.4743	3.3355	9.9257	8.0823	3.8791	7.7244	4.1219	6.9450	7.7636
Bad	_RM[Oxidation (M)]S[Phospho (STY)]DEFEGSFK_.2	#N/A	6.5450	5.9154	6.9481	6.8092	6.2216	6.3692	6.1607	6.7357	6.5923	6.6748	6.1981	6.9031	6.9800	6.4474	6.5826	7.0638	6.8190	6.2997	6.6140	6.8020
Bcan	_ELETPS[Phospho (STY)]EEK_.2	#N/A	10.2278	9.6941	4.0341	4.5875	9.8031	10.2666	9.6486	9.8747	9.7762	9.9124	10.3106	10.0262	10.2216	10.8468	9.9614	10.1665	10.4673	10.1754	10.1685	10.5283
Bcan	_EYQWGLNDR_.2	#N/A	9.6173	9.7192	9.9807	9.8850	9.4837	9.5585	9.3764	9.7412	9.6690	9.9226	9.6256	9.5834	9.6886	9.8522	9.4347	9.7839	9.8264	9.4226	9.5920	9.7144
Bcan	_EVGGETGS[Phospho (STY)]PELSGVPR_.2	Q61361_S546_M1_Bcan	9.2454	9.4294	9.3442	9.1217	8.5261	9.3029	8.7662	9.1516	8.7224	9.5066	8.7251	8.9537	9.2971	9.4618	8.3260	8.9778	9.4149	8.9861	8.9681	9.0611
Bcan	_SWEEAESQC[Carbamidomethyl (C)]R_.2	#N/A	7.7298	7.9112	8.3389	8.1751	7.9040	7.8554	7.9275	8.0308	7.9824	7.9327	7.5646	7.7725	8.0496	8.2377	8.1572	8.0926	8.1312	7.7681	8.1352	8.0289
Bcan	_YPIITPSQR_.2	#N/A	7.7471	8.2157	7.4583	7.9399	8.4186	8.0506	8.1086	7.9976	7.6940	7.9098	7.5036	7.9517	7.6078	7.9933	8.1728	7.8371	7.6610	7.8941	8.0954	7.7793
Bcan	_FNVYC[Carbamidomethyl (C)]FR_.2	#N/A	5.8600	6.9311	6.6507	7.0618	6.7534	6.6054	6.6236	6.1027	6.1936	6.3503	6.5541	6.2743	6.2779	6.7269	6.5433	6.0502	4.9181	6.6209	6.4595	6.6311
Bcan	_C[Carbamidomethyl (C)]QENGLWEAPQSC[Carbamidomethyl (C)]VPR_.2	#N/A	5.5931	6.1811	5.5781	6.8576	4.8144	5.7710	6.7333	5.4200	5.6902	5.7894	4.5265	6.1303	3.6239	7.0777	5.8898	5.6766	5.6048	5.9836	5.7171	5.1809
Bcan	_LTWEEAR_.2	#N/A	4.8158	5.4088	3.3282	6.0265	4.2658	5.6762	6.2442	4.6105	5.7084	5.5997	5.3387	5.3674	5.5802	5.1154	5.8223	5.3246	3.3525	5.5418	5.4887	5.8198
Bcan	_GAIYSIPS[Phospho (STY)]EDGGGGSSTPEDPAEAPR_.3	Q61361_S406_M1_Bcan	4.4188	3.5840	3.4542	3.2706	3.7565	4.2284	3.7846	4.1191	4.2740	3.1243	3.7527	3.4735	4.4395	6.1037	3.3618	3.2628	5.1157	3.1763	4.3258	3.4083
Bcan	_ALGAHLTSIC[Carbamidomethyl (C)]TPEEQDFVNDR_.3	#N/A	8.0569	8.4153	8.4136	7.9607	7.7125	8.0570	8.1053	8.5871	7.7047	8.3221	8.1041	8.1549	8.3416	9.0316	7.7962	7.5532	8.1242	7.8137	7.7165	7.9267
Bcan	_C[Carbamidomethyl (C)]SPGWLADGSVR_.2	#N/A	3.2936	4.0255	7.0349	6.8149	6.7141	6.6046	6.6573	3.5192	6.9507	6.4602	6.0047	5.5454	3.3244	6.0739	6.5811	6.1520	4.5173	6.1370	6.0644	6.2751
Bcan	_ELETPS[Phospho (STY)]EEKSGR_.2	#N/A	10.6593	10.9519	10.1609	10.5239	10.4878	10.7271	10.3858	10.9813	10.3911	10.6671	10.5534	10.9261	11.5819	11.5654	10.8216	10.9801	11.1505	10.9975	10.8185	11.0075
Bcan	_DSAHPSAS[Phospho (STY)]SEASSPASDGLEAIVTVTEK_.3	Q61361_S361_M1_Bcan	4.0974	3.2217	3.6339	3.4869	4.3043	4.3376	4.2446	4.3230	4.1649	3.9080	3.6435	2.9479	4.3303	3.6720	4.2700	4.3595	3.8203	3.3292	3.6282	3.3493
Bcan	_ALGAHLTSIC[Carbamidomethyl (C)]TPEEQDFVNDRYR_.4	#N/A	3.1855	6.2598	3.7218	4.5038	4.0241	4.8723	5.3357	5.3694	5.0913	5.8201	5.4867	6.3316	5.5001	5.5438	5.5605	5.6021	4.3236	5.4223	5.2959	5.2656
Bcan	_EVEVLVAR_.2	#N/A	4.3919	4.4280	3.9332	3.1923	3.0980	4.2439	4.7227	3.1168	4.4087	4.6781	3.3489	4.1541	4.0357	4.2122	3.3702	5.2506	4.1148	2.1360	4.5419	5.1634
Bcan	_C[Carbamidomethyl (C)]EVQHGIDDSSDAVEVK_.2	#N/A	3.7934	3.6512	3.9337	3.6618	3.7336	3.3726	2.5585	3.3431	3.3656	4.0995	3.8299	2.0381	3.6299	3.3661	2.4248	4.0582	3.5944	3.9172	3.8925	3.9600
Bcan	_EAC[Carbamidomethyl (C)]S[Phospho (STY)]GDM[Oxidation (M)]DGYPGVR_.2	#N/A	3.7039	4.4273	5.3179	4.0309	3.4741	4.1886	3.6312	3.3965	3.5514	3.8805	3.8524	3.5552	3.7169	6.6166	3.6566	4.1105	3.9342	3.9366	3.5852	4.1865
Cox7a2l	_NKVPELQK_.2	#N/A	5.5027	6.4941	5.6681	5.7168	5.5968	6.2789	6.8595	6.0735	5.6719	5.8104	6.3114	5.2587	6.5252	5.9158	6.2878	5.7322	5.7644	6.0573	6.1327	6.3858
Cox7a2l	_LTSSVTAYDYSGK_.2	#N/A	3.5114	3.8077	4.5488	4.0729	5.8523	6.9183	6.6165	3.7370	3.2500	3.3220	4.2295	6.3497	3.4154	4.5868	5.6030	4.3743	3.2343	3.9152	4.2142	4.2641
Prkg2	_LQDVHVQGGS[Phospho (STY)]PLQAS[Phospho (STY)]PDKVPLDVHR_.4	Q61410_S92_M2_Prkg2	4.4126	3.3513	3.4636	3.6572	3.7503	3.4279	2.7334	4.1527	3.8499	3.1180	4.2243	3.4797	3.9579	3.5017	1.8496	3.2566	3.6500	4.1288	3.7985	3.1790
Prkg2	_ALS[Phospho (STY)]LEMIQLK_.2	Q61410_S431_M1_Prkg2	3.1381	3.3639	3.4428	4.5513	3.9767	4.0083	3.6021	4.3393	2.9934	3.3444	3.9728	3.2533	3.1588	4.7020	3.1416	4.4830	3.3761	4.4570	4.1056	3.6285
Hras	_SFEDIHQYR_.2	#N/A	6.8651	6.4388	6.6578	6.4392	6.3247	6.3563	6.9036	6.6517	6.6879	6.9734	6.8009	6.7518	7.0484	6.8916	7.0725	6.9019	7.0286	6.6304	6.1775	6.8784
Hras	_QGVEDAFYTLVR_.2	#N/A	4.8078	5.3297	4.7451	5.2707	3.8406	4.4050	4.6793	5.1656	4.8236	4.7594	4.3415	4.1954	5.0123	4.8650	4.2939	5.1529	4.6331	4.6249	5.0593	4.7360
Kcna4	_GAYSSHDPQGS[Phospho (STY)]RGSR_.3	Q61423_S79_M1_Kcna4	3.3825	3.9476	4.6171	4.2128	3.5895	3.9746	3.4697	3.6082	3.5998	4.5303	3.7443	3.6737	3.5553	4.4580	3.4951	4.2455	3.9812	4.0551	3.7291	4.1353
Kcna4	_QSSFPHC[Carbamidomethyl (C)]SDLMPS[Phospho (STY)]GSEEK_.2	Q61423_S111_M1_Kcna4	3.2803	3.2216	3.8166	4.4091	4.1189	3.2965	3.4222	4.4815	3.4813	3.4867	3.8556	3.1111	3.3010	3.8037	2.9994	3.6252	3.5184	3.5732	3.9634	3.7707
Hadh	_DTPGFIVNR_.2	#N/A	6.4952	6.3735	4.2488	6.7596	3.4183	6.4224	6.6117	6.6282	6.3454	6.4280	6.0522	5.7966	6.4688	6.6433	6.5762	5.8796	6.3427	6.1725	6.0532	5.9772
Hadh	_TFESLVDFC[Carbamidomethyl (C)]K_.2	#N/A	4.2430	4.5769	3.7796	4.8445	2.9902	4.6097	5.0707	4.4687	4.6987	4.0536	4.1472	3.8591	4.2739	3.8177	4.1603	4.6852	3.9659	4.1994	3.4510	4.5654
Hadh	_AGDIEFVEK_.2	#N/A	4.1966	3.8062	3.2321	3.4928	3.5343	4.4506	4.0068	3.8970	2.5322	4.4029	3.5305	3.6957	4.2173	3.2191	2.6523	3.0407	4.4347	2.8063	3.0471	3.1861
Hadh	_FAAEHTIFASNTSSLQITNIANATTR_.3	#N/A	3.5099	4.6910	4.5473	3.4213	3.7168	3.7502	3.2125	4.7617	3.2515	3.3205	3.2465	3.5232	3.5408	4.5854	3.3566	4.3729	3.2328	3.9166	2.9881	4.2627
Znf638	_SYRS[Phospho (STY)]AS[Phospho (STY)]PER_.2	Q61464_S560_M2_Znf638	8.7411	8.8234	8.5043	8.6669	8.2860	8.2140	8.7503	9.0239	8.3016	8.4705	8.1231	8.6960	9.5428	8.7717	8.0320	8.6108	8.7202	8.6449	8.4489	8.3250
Znf638	_GLS[Phospho (STY)]PAQKPK_.2	#N/A	8.2577	8.2093	8.4954	8.1212	8.0292	8.2147	7.9000	7.9106	7.8051	8.0289	8.2295	7.8075	7.6901	8.6407	7.5386	8.1583	8.3340	8.0056	8.1758	7.9791
Znf638	_QSSVTQVTEQS[Phospho (STY)]PK_.2	Q61464_S128_M1_Znf638	7.1854	6.6864	7.3259	6.9875	7.0104	6.6842	6.1369	7.0594	6.7339	7.4618	7.3295	7.1589	7.5920	7.5608	6.7636	7.2005	7.6482	7.1284	7.0123	6.9822
Znf638	_HTETVDKGLS[Phospho (STY)]PAQKPK_.3	#N/A	3.6968	3.6223	3.2333	3.8875	3.9037	3.9370	3.1444	3.9224	3.0646	4.6644	4.0441	5.0161	3.5069	3.7327	3.1698	3.0589	4.1341	4.3858	4.2927	3.6997
Znf638	_GSTFS[Phospho (STY)]PDLK_.2	Q61464_S1099_M1_Znf638	3.4967	3.8224	4.5341	4.0876	4.1807	4.4048	3.3604	3.7223	4.5131	3.5484	3.7939	3.5485	3.5275	4.5722	2.9376	3.6870	3.2196	3.9299	4.2289	4.2494
Mcl1	(Protein N-term)]M[Oxidation (M)]ETDAPQPGLASPhospho (STY)]PDSPHDPC[Carbamidometh	#N/A	4.3910	4.2544	4.2938	3.8926	3.6684	3.0658	3.7296	3.7978	3.6203	3.9417	3.9497	3.9569	4.0181	3.7467	3.4432	4.1543	4.1890	3.8665	3.7650	3.9295
Pde1a	_NNLVDIIQQNK_.2	#N/A	5.0035	5.9744	5.3601	5.0338	4.6579	5.2333	4.9456	6.2407	5.2110	5.0209	5.8339	5.5459	6.5696	5.8728	4.7493	4.2819	5.9525	5.3749	4.8724	5.5766
Pde1a	_NSLQQPEGIDR_.2	#N/A	7.1232	7.5457	4.2807	7.7165	7.2008	7.5999	7.3559	7.6590	7.4672	7.4034	7.4316	7.2598	7.8862	7.6126	6.8375	7.4116	7.8002	7.3155	7.4390	7.2934
Pde1a	_[Acetyl (Protein N-term)]GS[Phospho (STY)]TDTDIEELENATYK_.2	Q61481_S3_M1_Pde1a	3.6428	4.1148	8.4351	3.8590	7.3196	3.2193	3.7206	3.3949	8.0863	8.4595	3.4988	7.3168	8.0924	8.0978	3.8722	7.7393	8.1943	8.1487	3.6537	3.9544
Alcam	_APFLETDQLK_.2	#N/A	5.6835	6.2028	5.0410	5.5036	5.9319	6.0136	6.1491	6.0891	6.3146	5.6887	6.5553	6.0598	6.5842	6.2771	5.8279	5.9584	6.0916	6.2509	6.3594	6.3038
Alcam	_SSNTYTLTDVR_.2	#N/A	5.3992	5.2376	5.2192	4.8974	5.6048	5.3654	5.2856	5.2195	5.5107	5.5683	5.6728	5.6997	6.1104	5.5302	5.5710	5.4154	5.8267	5.1753	5.9313	5.2129
Alcam	_EIDPGTQLYTVTSSLEYK_.2	#N/A	4.8768	5.8216	5.2391	5.2258	5.5357	5.4683	5.2414	5.7772	5.4369	5.4480	5.4368	5.4592	6.0505	6.2857	5.0980	6.0828	5.6052	5.4646	5.6334	5.7320
Alcam	_VLQPVEGEVAILFK_.2	#N/A	4.0098	3.7075	3.1214	4.0569	4.1929	4.7457	4.4226	4.3520	4.7384	4.2326	4.0385	3.8755	4.2846	4.5019	2.6807	4.1876	4.3240	4.7766	4.4688	5.0193
Alcam	_NM[Oxidation (M)]AASTTITVHYLDLSLNPSGEVTK_.3	#N/A	4.3429	4.2879	3.9188	4.1653	3.6177	4.2546	4.1162	3.2103	3.6443	4.0605	1.5790	4.0606	4.5163	5.4930	4.3177	3.9770	5.0774	4.2281	4.9690	4.8940
Alcam	_KTDPSGLSK_.2	#N/A	5.3428	4.2206	3.3438	3.3810	3.8629	4.4388	4.9114	3.3479	4.1636	3.7065	5.1826	3.5839	4.3291	4.3011	4.4416	3.9257	4.5464	3.2867	5.4092	4.9376
Alcam	_SVQYDDVPEYKDR_.3	#N/A	3.5658	4.4370	4.1021	4.1236	2.9035	3.1276	3.1367	3.2662	3.1958	3.7721	3.5702	4.3264	3.3038	4.3484	0.9690	3.9107	3.8039	4.1536	1.2947	4.0257
Snap91	_DALEIYKR_.2	#N/A	9.5264	9.2536	9.4330	9.2900	9.6038	9.2844	9.2811	9.6197	9.5675	9.2550	9.0739	9.0089	9.1435	9.2044	8.9655	9.1937	9.3534	9.0657	9.2037	9.2184
Snap91	_NTLFNLSNFLDK_.2	#N/A	7.8635	7.5306	7.3244	7.9968	7.6141	7.3991	7.4180	7.5802	7.8100	7.7283	7.5038	7.2892	7.9772	6.9451	6.6222	7.3299	7.4909	7.5263	7.5527	7.2207
Snap91	_GLGSDLDSSLASLVGNLGISGTTSK_.2	#N/A	5.9081	6.0865	5.0762	5.2371	5.6266	5.5895	5.8184	6.1508	5.3159	6.2673	5.5916	5.6913	6.0089	5.8475	4.3860	5.8709	5.4075	5.4112	5.2565	5.6528
Snap91	_DPLADLNIKDFL_.2	#N/A	5.8980	5.4652	4.8552	5.5727	5.4084	5.7593	5.6545	5.0366	4.5821	5.6927	5.1909	5.2883	4.8367	5.2195	5.1745	5.5898	5.2360	5.5115	4.5977	5.2356
Snap91	_SSPATTVTS[Phospho (STY)]PNSTPAK_.2	Q61548_S313_M1_Snap91	12.4411	12.6695	12.6463	12.3930	11.9723	11.0383	12.3514	12.6230	12.0282	12.6561	12.2077	11.7981	12.5540	12.5915	11.4832	12.0474	12.5491	12.2725	12.3610	11.7571
Snap91	_QM[Oxidation (M)]AFDFAR_.2	#N/A	7.5132	7.3600	7.1281	7.3085	7.4122	7.3692	7.4778	7.7486	7.2210	7.2900	7.2066	7.4397	7.0084	6.8584	6.5781	6.9783	7.0901	7.4081	7.2084	6.8114
Snap91	_IAAAQYSVTGSAVAR_.2	#N/A	6.2400	6.6304	3.7661	4.4596	6.5296	6.8901	6.8970	6.2433	6.6861	3.4362	6.1666	6.4870	6.3398	6.5064	6.3875	6.5243	6.2427	6.2416	6.6752	6.8593
Snap91	_ATTHEVM[Oxidation (M)]GPK_.2	#N/A	6.1853	6.2835	6.3368	5.9643	6.0284	6.0662	6.3869	6.2327	5.8096	5.9316	6.3881	6.0613	5.7090	5.9696	6.0544	5.5343	5.4801	5.9076	5.8057	5.0020
Snap91	_LTGGANWQPK_.2	#N/A	6.1216	6.2747	4.0412	6.1260	6.2032	5.6028	6.4976	5.7635	5.6581	3.7112	5.4930	4.3874	5.1290	5.3231	5.9605	4.6050	5.7292	5.6840	5.5445	5.6784
Snap91	_KPGNNEGSGAPS[Phospho (STY)]PLSK_.2	#N/A	12.0552	12.1205	11.9389	11.8616	11.3939	11.7183	11.8050	12.0345	11.6592	11.9958	11.5005	11.6584	12.2066	12.1950	10.8901	11.6083	11.9955	11.8554	11.6799	11.5986
Snap91	_GASPVPESSLTADLLSVDAFAAPSPASTASPAK_.3	#N/A	6.2797	6.4454	5.8857	5.7933	5.0520	5.6072	6.7395	6.3933	5.8440	6.5065	5.6588	6.1126	6.6547	6.6537	5.3751	3.9686	5.5796	5.7665	5.3038	5.2752
Snap91	_KGDLQWNAGEK_.2	#N/A	5.5123	4.1761	5.6629	4.6276	4.3054	4.9682	4.7356	4.6353	5.8977	5.0128	4.3956	2.9468	4.0972	3.6731	6.0542	3.4606	5.9416	4.8313	5.1832	5.5281
Snap91	_TIDTS[Phospho (STY)]PPVDIFATASAAAPVSSAK_.2	#N/A	3.6640	7.8641	4.1216	5.7451	3.6190	5.9627	8.1706	8.2505	6.1546	3.6275	3.9243	7.3902	3.2854	8.7120	3.5639	5.7526	3.5740	7.5992	6.8357	3.9334
Snap91	_PDAAPSIDLFGTDAFSS[Phospho (STY)]PPR_.2	Q61548_S594_M1_Snap91	5.8678	4.3517	4.1874	4.0383	2.9888	3.2129	6.7655	6.4250	3.1105	3.8574	3.4849	4.2412	3.2186	7.2128	5.0595	3.9960	3.8891	4.2388	3.0371	3.9405
Snap91	_SGSHGYDM[Oxidation (M)]STFIR_.3	#N/A	5.4175	5.0703	3.7675	3.9012	5.1915	5.6110	7.6585	4.8323	5.4044	5.5611	4.6133	5.2276	4.9256	4.8332	5.0217	4.8860	3.8379	5.5636	5.2611	4.8915
Snap91	_DALEIYK_.2	#N/A	4.1223	4.6026	3.7539	3.3669	3.4601	3.6841	4.0810	4.4430	4.1401	3.5958	3.0136	3.7699	4.2482	3.7919	3.6582	2.9664	3.9402	3.2092	1.8147	3.4692
Fscn1	_VNASASS[Phospho (STY)]LKK_.2	#N/A	9.0295	9.0597	9.1257	9.0669	8.7726	8.7669	8.8199	9.0175	8.9237	8.7896	8.9452	8.9588	9.2716	9.2664	8.8429	8.9860	9.2014	9.0878	8.9223	9.0733
Fscn1	_FLVVAHDDGR_.2	#N/A	8.1843	8.6964	8.7802	8.4007	8.6179	8.4903	8.4786	8.5914	8.4008	8.3916	8.5111	8.6274	8.4531	8.6988	8.4320	8.4385	8.6064	8.2906	8.5509	8.5323
Fscn1	_YLAPSGPSGTLK_.2	#N/A	8.5155	8.5050	8.4366	8.2888	8.4133	8.1583	8.5202	8.5664	8.2628	8.2407	8.3565	8.5155	8.9244	8.4750	8.7971	8.2430	8.2928	8.5884	8.2640	8.4604
Fscn1	_GEHGFIGC[Carbamidomethyl (C)]R_.2	#N/A	7.3998	7.6760	8.0300	7.8099	7.4134	7.6901	7.8769	8.1134	7.6087	7.6107	7.8050	7.4895	7.2618	7.9343	8.1920	7.5228	7.4400	7.8492	7.5358	7.7365
Fscn1	_LSC[Carbamidomethyl (C)]FAQSVSPAEK_.2	#N/A	7.6910	7.3682	7.9479	7.4464	7.5521	7.2535	7.5202	8.1313	7.8831	7.5454	7.3705	7.7310	7.7686	7.8205	8.1051	7.3212	7.3824	7.4608	7.6650	7.4384
Fscn1	_KVTGTLDANR_.2	#N/A	6.5246	7.0219	6.8550	6.9467	6.4805	6.5504	6.7706	6.5442	6.8843	6.0698	6.5261	6.4629	5.9459	6.6183	7.2338	6.7895	6.9385	6.9654	6.6208	6.7493
Fscn1	_VNASASS[Phospho (STY)]LKK_.2	#N/A	6.2603	6.8739	3.7589	4.4668	6.1433	5.9189	6.3647	7.0995	6.1694	6.5635	6.4178	6.6714	7.2847	6.5388	6.5872	6.6981	6.9575	4.3725	6.6986	3.7130
Fscn1	_YSVQTSDHR_.2	#N/A	7.2802	7.0383	7.6258	6.8060	7.0294	7.1414	7.5023	6.9879	7.1224	6.9515	7.2920	6.9345	6.9822	7.0475	7.7046	7.0572	7.2271	7.0878	7.1507	7.4025
Fscn1	_LVARPEPATGFTLEFR_.3	#N/A	7.4003	6.9988	7.0484	7.4501	7.3221	7.0171	7.0385	7.3427	6.9985	7.5806	7.3446	7.1241	7.2174	7.4241	7.0968	6.9898	7.2121	7.3818	7.5709	7.1701
Fscn1	_WSLQSEAHR_.2	#N/A	6.7230	6.9531	6.8990	7.1120	7.3176	6.9717	7.0040	7.2035	6.9289	7.0953	6.5721	6.9203	7.1071	7.0141	7.2255	7.0144	7.2532	7.3535	7.2929	7.0560
Fscn1	_YFGGTEDR_.2	#N/A	6.6150	6.6426	6.8585	6.6377	6.5419	6.5258	6.9761	6.9201	6.3810	6.9437	6.6538	6.7606	6.6761	6.4203	6.8945	6.8484	6.3823	6.7107	6.9737	6.9747
Fscn1	_YLTAEAFGFK_.2	#N/A	6.7985	6.6350	6.0241	6.7300	6.5106	6.2787	6.1308	6.0548	6.6289	6.6662	6.4757	6.6576	6.7909	6.2110	6.2830	6.1631	6.3956	6.6744	6.7179	6.6935
Fscn1	_AC[Carbamidomethyl (C)]AETIDPASLWEY_.2	#N/A	5.9420	5.5957	6.7709	5.3726	6.1701	5.7812	5.7339	6.0571	6.4244	5.7158	5.7827	6.5159	5.5112	5.8163	6.4833	6.1008	5.9912	5.7242	6.0471	5.9246
Fscn1	_SSYDVFQLEFNDGAYNIK_.2	#N/A	6.4476	6.2679	6.4422	6.2228	6.3132	6.0979	5.1399	6.3835	6.1319	6.6908	6.0504	6.5168	6.5095	6.7246	6.0743	6.1951	6.2913	6.1129	5.6800	6.2601
Fscn1	_VGKDELFALEQSC[Carbamidomethyl (C)]AQVVLQAANER_.3	#N/A	6.2638	5.8000	5.2522	6.2593	5.6860	5.3975	5.5745	6.6562	5.8186	5.8350	6.1924	6.0543	6.5324	6.4279	4.6970	5.6528	6.6162	5.6228	6.2110	6.0508
Fscn1	_VTGTLDANR_.2	#N/A	5.0099	5.2458	5.6256	5.9730	4.8225	4.8635	5.5390	5.8376	5.9087	5.4073	4.9524	5.1936	4.9156	4.9956	5.6100	5.7703	5.7145	5.7594	5.6704	5.0419
Fscn1	_YLAADKDGNVTC[Carbamidomethyl (C)]ER_.2	#N/A	6.0246	6.2615	6.0924	6.1974	5.9779	6.2395	5.7467	5.9349	6.2361	6.5535	6.4609	5.8688	5.8292	6.4989	6.5342	6.2713	6.0623	6.0411	6.2192	6.1385
Fscn1	_YAHLSAR_.2	#N/A	4.7724	5.6355	5.8583	5.1745	5.0352	5.9702	6.4383	3.9814	6.1069	6.0797	6.1693	5.7451	5.2450	5.1625	6.5079	5.8274	5.2211	6.0558	6.3589	5.3582
Fscn1	_QIWTLEQPPDEAGSAAVC[Carbamidomethyl (C)]LR_.2	#N/A	4.3681	3.7210	4.8162	5.2978	4.8019	5.1977	4.2168	4.4690	4.7523	5.0320	3.6157	4.6902	4.3025	3.3043	5.0830	4.5845	5.8488	3.3133	2.9619	4.9006
Fscn1	_DVPWGVDSLITLAFQDQR_.2	#N/A	3.7826	3.5365	4.6981	3.8016	3.9895	4.1286	3.4748	4.9967	3.7054	4.6150	4.0798	3.2626	3.8135	4.7070	3.4182	3.8776	3.5055	3.9043	4.1720	3.5986
Fscn1	_VNASASS[Phospho (STY)]LK_.2	#N/A	6.1700	6.2515	6.5137	6.6679	6.4632	6.3658	6.1505	6.0040	6.2557	6.6398	6.7817	6.3774	6.1946	6.7005	6.1688	6.7047	6.7528	6.5221	6.4974	6.7086
Fscn1	_QGM[Oxidation (M)]DLSANQDEETDQETFQLEIDR_.3	#N/A	5.0211	5.1972	4.5319	4.0898	3.4277	4.4172	3.3467	5.2215	4.8199	4.2150	3.7917	3.1612	3.5253	4.5699	3.3721	4.4644	3.4853	2.7770	4.2311	4.2472
Ktn1	_EIQNGTLRES[Phospho (STY)]DSEHVPR_.3	Q61595_S75_M1_Ktn1	6.3893	6.2591	5.6044	3.6422	3.9009	3.5368	5.9606	4.0248	6.1953	6.1327	5.4985	6.1092	5.8417	4.0951	4.5364	5.8973	3.9926	6.1851	3.4813	3.9573
Ktn1	_DLLT[Phospho (STY)]ELQK_.2	Q61595_T1211_M1_Ktn1	4.7781	6.2784	5.7504	5.7899	3.9106	5.8172	5.2405	4.2733	5.4638	5.9140	6.0220	5.3465	6.6958	6.6836	6.8240	6.4198	5.3453	5.6803	5.8978	5.8932
Gdi2	_NTNDANSC[Carbamidomethyl (C)]QIIIPQNQVNR_.2	#N/A	10.0625	10.0993	10.8046	10.1110	9.8142	10.0020	10.0794	10.4718	9.9955	10.4411	9.9247	10.0197	10.2258	10.6958	9.5932	9.6723	10.1975	9.7156	9.9222	9.9855
Gdi2	_LYSESLAR_.2	#N/A	8.7270	8.8872	8.8535	8.8273	8.7617	9.0428	9.3272	8.5132	8.9103	8.9552	8.8535	8.9115	9.0244	8.7489	9.1576	8.7458	7.9570	9.2462	8.9174	9.0536
Gdi2	_TDDYLDQPC[Carbamidomethyl (C)]C[Carbamidomethyl (C)]ETINR_.2	#N/A	7.4648	7.7199	7.4995	8.0645	7.5541	7.5894	7.7770	7.8308	7.9611	7.4791	7.5589	7.6741	7.7318	7.7266	7.1323	6.9814	7.8340	7.1363	7.5614	7.4917

Gdi2	_VIC[Carbamidomethyl (C)]ILSHPIK_.2	#N/A	6.9351	7.2196	7.7162	6.6460	6.6601	6.9969	6.8452	7.3304	6.8794	6.8746	6.2963	6.8746	6.9478	6.7217	6.5518	6.3480	6.9314	6.7630	7.1009	6.5739
Gdi2	_FVSISDLFVPK_.2	#N/A	6.6834	6.5223	6.7226	6.8009	6.0782	6.7697	6.1472	6.6614	6.4571	6.4293	6.2869	6.4161	6.8502	6.4435	6.2050	5.8754	6.6705	6.5922	6.5067	6.1515
Gdi2	_DLGTDSQIFISR_.2	#N/A	6.3793	5.7488	6.4206	6.5272	5.9892	6.2018	6.1680	5.6470	6.5068	6.3843	5.1663	5.7104	5.6649	6.2449	6.1333	6.2651	5.2326	6.2910	6.1674	5.7627
Gdi2	_M[Oxidation (M)]LLFTEVTR_.2	#N/A	7.1869	7.3106	6.9450	7.0278	7.0119	7.1351	7.1624	7.3211	6.8087	7.4027	6.4823	7.0478	6.1557	7.0071	5.4406	6.8189	6.3184	6.8284	5.7637	6.8628
Gdi2	_FLM[Oxidation (M)]ANGQLVK_.2	#N/A	5.8747	6.6165	6.8154	6.7372	6.9452	6.2490	6.7509	6.1245	5.6747	5.9212	6.7069	6.2852	6.3921	6.2858	6.3736	6.2460	6.2160	6.5434	6.3329	6.3344
Gdi2	_SPYLYPLYGLGELPQGFAR_.2	#N/A	7.2318	7.1444	6.8300	7.0423	6.6802	6.6450	7.2707	7.2996	6.9530	7.0113	6.5987	6.9720	7.2532	7.5707	6.5572	6.8588	6.9909	7.2022	6.9394	6.4475
Gdi2	_AYDATTHFETTC[Carbamidomethyl (C)]DDIKDIYKR_.4	#N/A	5.7644	5.1492	4.6641	5.0656	4.1529	4.1131	4.5544	5.6860	5.0221	5.1339	4.7270	5.4645	5.3445	4.7458	2.8797	5.0429	4.1457	2.9710	4.1204	4.6157
Gdi2	_VLHM[Oxidation (M)]DQNPYYGGESASITPLEDLYK_.3	#N/A	4.7623	3.9845	3.1368	3.7070	3.6510	2.6261	3.3220	4.4640	3.6648	4.0965	3.6762	3.4989	3.2349	3.5655	2.9217	3.3751	3.9612	3.1882	3.8807	3.6342
Gdi2	_FLVYVANFDEKDPR_.2	#N/A	4.1327	4.5829	4.8299	4.1627	3.6136	3.9539	4.3386	4.2689	3.7450	4.4253	4.2945	4.4371	4.1841	4.4482	3.8410	4.1668	4.5997	3.9632	3.9952	4.0474
Gdi2	_VIEGSFVYK_.2	#N/A	3.3266	3.9925	3.4267	6.9708	3.5336	5.9100	4.7508	3.3027	4.3234	6.3832	6.1325	3.7186	3.6002	4.4021	4.0327	2.8127	4.5504	4.4991	4.3658	3.3905
Gdi2	_FDLGQDVIDFTGHSLALYR_.3	#N/A	4.4791	3.8764	3.8040	4.0317	3.5134	3.4967	3.2305	3.7394	3.5107	3.7586	3.8850	4.0314	3.8903	3.8229	3.5961	3.6231	3.8245	3.7866	3.4969	3.6969
Gdi2	_MTGSEFDFEEMKR_.3	#N/A	4.3718	4.4481	4.3940	4.8073	3.0779	3.1112	3.9702	3.0966	3.8904	4.1824	3.3691	4.1743	4.4027	3.9464	3.9956	3.7339	4.0947	3.0547	3.3538	3.6237
Slc20a1	_EVKSS[Phospho (STY)]PSES[Phospho (STY)]PLMEKK_.2	#N/A	3.9441	3.8570	4.1650	3.9416	3.4964	3.6649	3.9024	3.8177	3.9232	4.4412	3.5933	3.6433	4.0467	4.1805	3.6222	3.9577	4.1546	3.8014	3.3680	4.0179
Slc20a1	_EVKSS[Phospho (STY)]PSES[Phospho (STY)]PLM[Oxidation (M)]EK_.2	#N/A	5.7184	3.9519	3.6043	7.0976	3.7831	6.6894	4.0280	3.8358	3.9877	3.7848	4.7254	7.1950	5.2428	3.6123	6.0524	6.4120	7.5553	3.1549	5.9253	3.4664
Slc20a1	_SS[Phospho (STY)]PSES[Phospho (STY)]PLMEKK_.2	#N/A	4.2794	7.3939	3.1630	3.5618	2.9855	3.0188	4.0626	3.8279	3.9828	4.0900	3.4615	4.2667	4.1483	3.1501	4.0880	3.6415	4.3656	3.4675	3.1161	3.1171
Slc20a1	_SS[Phospho (STY)]PSESPLMEK_.2	#N/A	3.8805	6.4295	7.0447	7.5414	3.8099	3.8947	3.9929	4.0031	3.9648	4.2901	3.2401	4.1672	4.1085	3.8104	3.8719	3.5942	5.5885	3.5191	3.3696	3.6865
Drd1	_IQPVTHSGQHS[Phospho (STY)]T_.2	Q61616_S445_M1_Drd1	11.3144	11.2494	11.0549	3.4197	10.8021	10.9963	10.8851	11.5193	11.0663	3.9478	11.4874	11.4734	11.9073	11.4289	10.5176	11.3453	11.7705	11.7111	2.9740	3.2592
Znf148	_GGLLTSEEDS[Phospho (STY)]GFSTSPKDNSLPK_.3	Q61624_S301_M1_Znf148	3.5323	4.4705	4.0686	4.1571	2.8700	3.6141	3.4673	3.2326	3.3875	3.1844	4.3670	4.3600	3.5530	4.0556	4.2483	3.8772	3.7703	4.0628	3.7114	4.0226
Znf148	_GGLLTSEEDSGFSTS[Phospho (STY)]PK_.2	Q61624_S306_M1_Znf148	3.2879	3.7682	4.5883	4.0334	4.1265	4.2865	3.4146	3.7765	3.4737	3.4942	3.8481	3.4943	3.5817	4.6263	3.1037	3.6328	3.2738	3.8757	4.1747	4.3036
Znf148	_AGMTSS[Phospho (STY)]PDATTGQTFG_.2	Q61624_S784_M1_Znf148	3.8968	4.3638	4.3491	4.2991	3.5186	3.8599	3.5561	3.5374	3.5096	3.7837	3.8501	3.7601	3.6576	4.3872	3.5814	4.1746	3.6197	4.1414	3.8132	4.0644
Grid2	_VPS[Phospho (STY)]KEDDKEIDLEHLHR_.4	Q61625_S865_M1_Grid2	5.5764	6.1623	3.7978	5.0994	2.8986	5.9454	6.2550	3.5036	6.4656	5.4965	6.2719	6.2637	3.3088	6.5912	4.2196	3.9058	5.0168	6.4256	5.6310	5.6773
Pacsin1	_TEQSVT[Phospho (STY)]PEQQK_.2	#N/A	10.9920	10.9843	11.0568	11.1504	10.9054	10.7733	10.7578	11.1136	10.8910	11.0407	10.7862	11.0244	11.3080	11.4890	10.9834	11.0587	11.1481	10.9322	10.9985	11.1269
Pacsin1	_GPQYGSLER_.2	#N/A	9.6659	9.8033	9.9183	10.1170	9.6323	8.5707	9.5857	9.6817	9.6344	9.8164	9.7944	9.6888	10.2163	9.9531	10.0165	9.8099	9.7643	9.4118	9.5615	9.8050
Pacsin1	_GRLDS[Phospho (STY)]GQLGLYPANYVEAI_.2	#N/A	8.5230	8.9993	8.3601	8.5203	8.2695	8.4994	8.5304	8.5402	8.7069	8.7951	8.6645	8.7675	9.3026	9.3683	8.8908	8.8477	8.9312	8.7721	9.0343	8.8952
Pacsin1	_EVLLDIKR_.2	#N/A	9.2550	9.7779	9.4941	9.6021	9.4158	9.2975	9.3609	9.6874	9.4374	9.3753	9.1597	9.7489	10.0075	9.8683	9.7902	9.6879	9.5667	9.4270	9.5121	9.7189
Pacsin1	_NSLLNEDLEK_.2	#N/A	9.2433	9.2000	9.6409	9.7459	9.4452	9.5633	9.2133	9.8622	9.5574	9.6186	9.7376	9.4854	9.6309	9.8168	9.8252	9.6919	9.8596	9.4722	9.5739	9.8821
Pacsin1	_AYAQQLTDWAK_.2	#N/A	8.5122	8.5837	8.8524	8.7052	8.4119	8.3193	8.6358	8.9012	8.6013	8.3320	8.4482	8.8935	8.6540	9.1020	9.1200	8.5563	8.7091	8.5465	8.6645	8.7558
Pacsin1	_LGEEDEQGWC[Carbamidomethyl (C)]R_.2	#N/A	8.1521	7.8910	8.5173	8.4347	8.1471	8.0180	8.1388	8.5774	8.3744	8.4402	8.4370	8.2794	8.3579	8.3202	8.2828	8.1038	8.4014	7.9110	8.1034	8.3244
Pacsin1	_GADAQEDLR_.2	#N/A	7.6935	7.9944	7.8997	8.3655	8.0810	8.2480	8.2196	7.8990	8.0833	8.4984	8.0703	8.3330	8.4633	7.9974	8.8516	8.2785	7.8324	8.1146	8.0914	8.2322
Pacsin1	_ALYDYDGQEQDELSFK_.2	#N/A	8.1711	7.6368	8.0748	8.2181	7.7999	7.8802	7.2112	8.0149	7.9540	8.4429	8.0722	7.9493	8.6843	8.3690	8.0939	8.2862	8.1419	7.6564	7.5318	8.1756
Pacsin1	_EAEDGFRK_.2	#N/A	7.4277	7.5820	7.5538	7.4601	7.6672	7.6039	7.6044	7.5231	7.8011	7.3679	7.8184	7.6758	7.4149	7.6801	7.9360	7.6350	7.3492	7.5201	7.5329	7.9659
Pacsin1	_LDS[Phospho (STY)]GQLGLYPANYVEAI_.2	#N/A	5.4441	6.1962	6.9894	6.9716	6.3293	6.4762	6.2114	5.7271	6.5834	6.0211	7.0006	6.3867	5.4353	5.7123	6.8521	7.2504	6.7781	5.7012	6.4759	6.6722
Pacsin1	_NSLLNEDLEKVK_.2	#N/A	5.4757	6.1157	3.8286	6.1685	6.0216	5.3737	4.8490	6.5477	5.8892	4.8262	5.6329	6.5350	6.3592	6.0237	5.4210	5.7898	6.2155	5.3740	5.3494	5.3003
Pacsin1	_KAEGATLSNATGAVESTSQAGDR_.3	#N/A	7.2254	7.4759	7.4805	7.0813	6.9792	7.3229	7.4384	7.4324	7.1727	7.4154	7.2842	7.6659	7.8080	8.0535	7.4526	7.1171	7.4911	7.2511	7.3659	7.5611
Pacsin1	_AYHLAC[Carbamidomethyl (C)]K_.2	#N/A	4.6418	4.9884	5.4654	5.5960	5.0814	4.7378	5.8211	5.3993	4.3300	5.5011	4.2045	5.0920	3.3905	5.7493	6.1811	5.3514	4.9997	5.3490	5.6530	5.7184
Pacsin1	_LC[Carbamidomethyl (C)]NDLM[Oxidation (M)]SC[Carbamidomethyl (C)]VQER_.2	#N/A	4.6011	6.5335	7.0134	7.1107	5.4731	6.2112	6.5505	5.5641	6.1448	5.6741	6.1576	5.9141	5.3873	7.4261	6.8762	5.5676	5.3176	5.6225	6.0794	6.3960
Pacsin1	_AYHLAC[Carbamidomethyl (C)]KEER_.2	#N/A	6.0063	6.1210	5.5012	6.0210	6.2289	5.7769	5.6140	6.6810	5.7579	6.1234	6.2000	6.0753	6.1756	6.3224	6.2107	6.7877	6.3066	6.3190	5.9899	6.1504
Pacsin1	_GSVSS[Phospho (STY)]YDR_.2	Q61644_S346_M1_Pacsin1	7.6796	7.9612	7.2558	8.8587	3.8044	8.2536	8.5918	4.2645	8.3399	9.1266	8.7688	8.4342	8.8115	3.6028	8.3475	8.6829	3.5957	4.1296	8.9472	9.0786
Pacsin1	_TEQSVT[Phospho (STY)]PEQQKK_.2	#N/A	8.2498	8.4607	8.2693	8.5100	8.1419	8.2480	8.0363	8.5715	8.0593	8.4958	8.0311	8.4339	8.9372	9.0333	8.5849	8.5022	8.6339	8.5379	8.2703	8.5203
Pacsin1	_HLNLAENS[Phospho (STY)]SYM[Oxidation (M)]HVYR_.3	#N/A	3.8287	6.5065	5.7667	6.7314	6.5133	6.5617	3.6612	6.8709	6.1444	7.0473	6.2254	6.6031	7.3247	3.5045	6.7765	6.8280	7.0681	6.8670	7.0120	6.8898
Pacsin1	_[Acetyl (Protein N-term)]SGSYDEASEEITDSFWEVGNYKR_.3	#N/A	6.4713	5.6883	6.5359	6.9104	5.4898	4.5773	4.7507	6.1194	6.1495	6.5916	5.5765	6.6174	7.7541	7.6908	6.4905	6.7722	5.9482	6.0065	5.5472	6.3162
Pacsin1	_AWGAM[Oxidation (M)]M[Oxidation (M)]TEADKVSELHQEVK_.3	#N/A	6.6571	6.5284	6.4200	6.1754	6.8977	6.6493	5.8699	7.2177	6.4232	6.5371	5.9602	6.7680	7.8326	6.9628	6.6798	6.9100	6.9887	6.9550	6.8647	7.1842
Pacsin1	_KAEGATLSNATGAVESTS[Phospho (STY)]QAGDRGSVSSYDR_.4	#N/A	6.8237	4.3125	4.1921	3.8824	3.4856	3.9077	6.6744	3.9210	5.9767	3.7126	6.5839	6.8047	3.8476	3.7175	3.4944	3.5186	3.7006	8.6487	3.8123	6.5878
Pacsin1	_AEGATLSNATGAVESTSQAGDR_.2	#N/A	4.4508	5.9591	3.4863	3.2385	3.7886	4.0126	4.2719	4.1512	4.6845	3.7423	4.1860	3.4414	4.4716	4.4074	3.3297	3.2949	3.1881	3.1443	4.2937	3.4404
Pacsin1	_TTPQYM[Oxidation (M)]EGM[Oxidation (M)]EQVFEQC[Carbamidomethyl (C)]QQFEEK_.3	#N/A	3.7372	3.8491	4.0585	3.2194	3.5160	3.5909	4.0497	3.4915	4.1088	4.3846	3.8270	3.4958	4.1990	4.4454	3.6269	3.8671	3.7603	3.4414	3.9347	3.1679
Pacsin1	_STSGPGM[Oxidation (M)]PM[Oxidation (M)]NWPQFEEWNPDLPHTTAK_.3	#N/A	3.7407	3.3366	3.7017	4.5240	3.9477	3.9810	3.1004	4.3666	3.0206	3.5513	4.0001	3.2260	3.1861	3.6887	3.1258	3.1028	3.4034	4.4297	4.0783	3.6557
Ddx5	_APILIATDVASR_.2	#N/A	7.8286	8.0111	7.8164	8.1041	7.6320	7.8564	7.7376	8.0616	7.8322	7.6724	7.6338	7.9531	7.8631	7.9431	7.6305	7.8688	7.6249	7.8793	7.9377	7.7637
Ddx5	_QVSDLISVLR_.2	#N/A	4.3425	4.7076	6.1871	4.6123	5.5021	5.8601	5.0859	4.0281	4.8831	5.6549	5.6152	5.9184	5.6359	4.9049	5.1536	5.5742	4.6543	5.0116	5.6991	5.7459
Ddx5	_LIDFLEC[Carbamidomethyl (C)]GK_.2	#N/A	4.8162	4.5286	4.4848	4.6514	4.8820	5.0465	3.7563	4.6831	4.7783	5.0929	4.1617	4.5891	4.8563	4.8089	3.9980	4.0243	3.8553	3.9743	4.2728	5.1487
Ddx5	_GVEIC[Carbamidomethyl (C)]IATPGR_.2	#N/A	5.3836	5.4283	6.2675	5.6687	5.7839	5.9817	4.8347	6.0707	5.8989	5.2255	5.0261	5.6768	5.6375	5.4041	5.2020	4.3718	6.2904	5.4206	5.2983	3.8876
Ddx5	_ELAQQVQQVAAEYC[Carbamidomethyl (C)]R_.2	#N/A	6.8227	5.8648	6.4761	6.7391	3.3562	6.0596	6.2128	5.6827	6.0346	3.8958	6.2402	3.8959	6.5771	6.3006	5.8961	6.3446	6.5172	5.7166	6.5456	6.0610
Ddx5	_LLQLVEDR_.2	#N/A	1.7202	4.7238	3.3399	3.7810	5.4638	5.1734	5.0777	4.5140	6.0550	5.7010	5.5169	4.8710	5.7143	5.7677	5.6026	5.4182	3.5262	5.2459	6.1381	5.3527
Ddx5	_STC[Carbamidomethyl (C)]IYGGAPK_.2	#N/A	5.4106	5.4181	4.0551	4.1706	4.8521	5.6623	5.8615	3.2192	5.9584	5.6189	4.6882	5.4918	3.3508	5.9318	5.3121	5.2385	3.7569	5.4831	5.4887	5.5082
Ddx5	_NFYQEHPDLAR_.2	#N/A	5.7712	5.5148	5.0421	5.4928	5.6015	5.6995	5.6614	5.7647	5.4532	5.3060	5.6640	5.4820	5.4900	6.0255	5.6480	5.4635	5.4425	5.4745	5.0248	5.2824
Ddx5	_FVINYDYPNSSEDYIHR_.3	#N/A	3.6963	3.6228	3.2329	5.6088	5.4103	5.5423	3.5600	3.9220	3.6191	3.3488	3.9935	3.3489	4.9619	3.2709	4.7793	5.1729	4.9173	3.7302	6.2574	4.9741
Ddx5	_DWVLNEFK_.2	#N/A	3.8347	4.1681	3.7428	3.8547	3.1725	3.3965	4.6922	3.5351	4.4277	5.0293	3.3012	4.4904	4.5358	4.3581	4.6192	4.1796	4.0728	4.0211	3.4090	4.3251
Cbx5	_KSS[Phospho (STY)]FSNSADDIK_.2	#N/A	8.5305	8.3402	8.8202	8.4867	4.0359	7.8856	7.7857	8.6826	8.4093	8.4250	8.1579	8.3793	8.8830	8.7952	8.2655	3.5422	8.9535	8.3635	8.2738	8.3904
Cbx5	_SS[Phospho (STY)]FSNSADDIK_.2	#N/A	6.8874	6.7057	7.1105	6.7039	6.3395	6.6580	6.3591	6.5127	6.3339	4.0389	6.5984	6.4956	3.5881	7.2968	6.3373	6.6637	7.3718	4.0374	6.6389	4.0577
Cbx5	_TADSSSS[Phospho (STY)]EDEEEYVVEK_.2	Q61686_S14_M1_Cbx5	3.2080	3.2982	3.7443	4.4856	4.0465	4.2706	3.5758	4.2365	4.1826	4.5780	3.9322	3.9319	3.6659	3.2096	4.0134	5.1995	4.5725	3.7957	4.0948	4.4592
Atrx	_EENVNS[Phospho (STY)]PEDKR_.2	Q61687_S912_M1_Atrx	5.8287	5.9864	5.1990	5.4988	5.1918	5.9290	5.4166	4.7877	5.3832	6.3046	4.8992	5.5143	5.8803	5.9171	4.9015	5.0594	6.0516	5.6827	4.9303	4.9212
Atrx	_LTLS[Phospho (STY)]DGES[Phospho (STY)]GEEKPTKPK_.2	#N/A	6.1632	5.9896	6.4774	6.3286	4.3050	6.1007	5.9240	5.8083	6.1163	6.1644	5.9671	5.8085	5.5582	5.9531	3.7500	5.4218	6.5105	5.6729	5.4026	4.2362
Atrx	_VC[Carbamidomethyl (C)]DQTSKFS[Phospho (STY)]PK_.2	Q61687_S315_M1_Atrx	6.2968	3.5354	3.3202	6.9319	6.3887	7.4395	7.3603	6.7239	6.9291	6.8913	6.8295	6.9842	6.9372	7.3383	6.4396	7.0521	7.7587	3.6429	7.1826	6.7942
Atrx	_QNYSESSNYDS[Phospho (STY)]ELER_.2	Q61687_S801_M1_Atrx	5.7247	5.7142	5.7919	5.2247	2.9988	5.6006	3.0414	3.3615	3.1005	4.5637	3.4749	4.2311	3.2085	4.2531	4.1194	4.0060	3.8992	4.2489	3.0471	4.6110
Atrx	_YVES[Phospho (STY)]DDEKPTDENVNEK_.2	#N/A	5.0845	6.2105	4.3777	5.1343	4.4525	5.3920	5.7082	5.4147	4.7364	5.6603	5.0145	5.2804	6.2255	6.0209	5.0962	5.7346	6.0232	6.1166	5.2173	5.2027
Atrx	_TTSTSNPS[Phospho (STY)]S[Phospho (STY)]PAPDWYK_.2	Q61687_S1978_M2_Atrx	3.2817	3.2203	3.8180	4.4077	4.1203	4.3444	3.4208	3.7828	3.4799	3.4880	3.8543	3.1097	3.5879	3.1317	2.9980	3.6266	3.2800	3.8695	4.1685	4.3098
Atrx	_EEIS[Phospho (STY)]DHENNVTILLEDSDLR_.3	Q61687_S626_M1_Atrx	3.8165	3.5026	3.3531	3.7678	4.0234	4.0777	3.0247	4.0422	4.4457	3.6271	3.9244	3.2287	3.1103	3.3911	3.0501	3.1786	3.5394	3.6101	3.9091	3.0684
Atrx	_TTQEGS[Phospho (STY)]S[Phospho (STY)]ADDTGDTEGR_.2	Q61687_S854_M2_Atrx	3.1239	3.8461	3.9309	4.3905	3.6828	3.8108	4.6727	3.6665	4.0519	3.6644	3.7380	3.6331	3.4717	3.8064	3.4806	3.7494	3.9547	4.0458	3.6349	3.4837
Atrx	_AKQPVIGDQNS[Phospho (STY)]DS[Phospho (STY)]DEM[Oxidation (M)]LAVLK_.3	#N/A	4.4737	5.1738	3.3840	3.5612	3.8114	4.4951	5.1241	4.0606	4.0109	3.5621	3.9238	3.4222	4.1532	7.4691	3.0596	3.3177	3.3397	7.8011	4.1528	3.2279
Atrx	_IGKQS[Phospho (STY)]EES[Phospho (STY)]PADDGELRR_.3	Q61687_S1287_M2_Atrx	4.2852	4.5347	3.8218	3.2990	2.9913	3.6162	3.3990	4.5109	4.2080	4.2608	3.0815	4.2609	4.3161	3.8598	4.0822	3.6473	4.0081	3.1413	3.4080	3.5371
Hsph1	_IES[Phospho (STY)]PKLER_.2	#N/A	13.6481	13.8025	13.5219	13.4685	13.4626	13.3700	13.3059	13.8648	13.3153	13.5342	13.2827	13.5092	14.0838	13.9112	13.0778	13.4159	13.8047	13.5770	13.4057	13.4612
Hsph1	_PKIES[Phospho (STY)]PKLER_.2	#N/A	7.0065	6.8765	6.5952	6.6383	6.9987	6.8951	6.3953	7.1872	6.1302	6.6849	6.7419	6.5979	6.9244	6.9847	5.4452	6.8098	7.1211	6.6826	7.0104	6.4798
Hsph1	_SVLDAAQIVGLNC[Carbamidomethyl (C)]LR_.2	#N/A	6.7806	6.7021	7.3515	7.0996	7.2907	6.7882	6.1883	7.1428	6.6271	7.0421	6.7878	6.9761	6.4503	6.4997	6.8537	6.6092	7.2419	6.7815	6.7915	6.8386
Hsph1	_FVVQNVSAQK_.2	#N/A	6.9568	6.4636	6.1507	6.6667	6.6867	6.1601	6.7806	6.6187	6.5922	6.6627	6.9231	6.5164	6.1840	6.6092	6.7555	6.1198	6.5317	7.0189	6.5428	6.1401
Hsph1	_AGGIETIANEFSDR_.2	#N/A	7.2441	6.4586	6.4117	6.4623	6.7569	6.8053	6.5298	6.6478	6.5215	6.4691	6.7098	6.8277	6.8713	6.8748	5.8113	6.4925	6.9796	6.5533	6.5533	6.4436
Hsph1	_[Acetyl (Protein N-term)]SVVGLDVGSQSC[Carbamidomethyl (C)]YIAVAR_.2	#N/A	6.6030	6.0777	7.2145	6.8819	6.6096	5.9453	5.4597	6.7826	5.8756	6.9203	6.7937	6.0086	7.1243	7.0157	5.2054	6.2593	6.5708	5.9833	6.2336	6.8940
Hsph1	_FIC[Carbamidomethyl (C)]EQEHEK_.2	#N/A	5.7964	4.8613	5.6871	6.1017	5.6435	5.3385	6.0198	6.1617	5.4882	6.0106	6.0231	5.9257	3.7269	5.6703	5.7728	5.9945	5.4407	6.0011	5.9635	6.1731
Hsph1	_LLTETEDWLYEEGEDQAK_.2	#N/A	6.4565	6.7212	6.4796	6.3876	5.9988	6.3036	5.9516	6.4059	5.8825	6.4588	6.4112	6.1887	6.8853	7.3824	5.4344	6.3804	6.6063	6.4203	5.7123	6.3125
Hsph1	_NAVEEC[Carbamidomethyl (C)]VYEFR_.2	#N/A	3.3817	6.0673	6.2650	6.0468	5.8938	6.2212	5.8161	5.7255	3.3796	6.0464	5.7257	5.7475	6.2118	6.4623	5.1644	5.9387	5.4844	6.0563	5.4813	5.2098
Hsph1	_SQFEELC[Carbamidomethyl (C)]AELLQK_.2	#N/A	4.5035	3.7603	4.2633	4.0123	4.2838	4.0468	2.9918	4.4197	4.5812	4.0424	4.5761	4.7461	5.2817	5.2585	3.6516	4.3346	4.6668	3.9943	4.2539	4.1039
Hsph1	_S[Phospho (STY)]LDQDPVVR_.2	Q61699_S774_M1_Hsph1	6.7736	6.6118	3.8026	6.0814	3.4114	6.1496	5.7421	6.8117	4.1888	5.9253	3.0623	6.2868	4.2969	6.8262	5.9435	6.6378	6.4206	6.3397	5.8037	6.3043
Hsph1	_VVNVELPVEANLVWQLGR_.2	#N/A	4.3332	4.5091	4.8947	3.4491	4.1228	4.1876	3.9630	4.6333	4.0221	4.1286	4.0633	3.6249	3.8576	4.8108	6.4460	4.8557	5.0959	4.3245	4.4433	3.9627
Hsph1	_VLEELGQR_.2	#N/A	6.8825	3.5424	6.7053	3.2290	6.0290	6.4508	6.4486	4.1608	6.3008	6.3113	6.3110	3.4319	4.4811	6.4463	6.9383	5.8327	3.1976	6.5598	6.2088	6.4885
Hsph1	_QDLPNAEEKPR_.3	#N/A	3.6169	2.6878	5.0501	4.4352	4.2437	2.7583	4.3801	4.4554	4.9208	4.2581	4.0433	3.6264	3.2749	3.7776	3.7292	3.1928	3.4923	3.0448	3.8795	3.7446
Hsph1	_NQQITHANNTVSSFK_.2	#N/A	3.9180	3.4011	3.4546	3.6662	3.0215	2.3136	3.0213	4.5118	3.8408	3.1271	4.2152	4.0216	3.9489	3.4927	4.4786	4.6105	3.6409	3.8214	3.8076	3.1699
Hsph1	_T[Phospho (STY)]PNGPNIDKKEDLEGK_.3	Q61699_T816_M1_Hsph1	3.1568	5.6447	4.1943	5.5265	3.3638	3.2438	4.8034	3.3825	3.0797	3.8883	4.8475	3.8884	3.1877	4.2323	3.7097	4.0198	5.3958	7.3582	3.0679	3.9096
Elavl4	_SSQALLSQLYQS[Phospho (STY)]PNR_.2	Q61701_S233_M1_Elavl4	4.3213	7.4864	3.8579	3.2629	3.0274	3.0608	7.3529	3.0462	3.9409	4.1319	3.4195	7.1037	4.1063	8.7188	4.0460	3.6834	6.8451	6.8124	3.1581	3.0751
Elavl4	_ILVDQVTGVSR_.2	#N/A	5.0071	3.3192	4.3436	4.5066	4.0213	5.2453	4.9249	4.3840	4.5738	5.0814	3.9532	5.2067	3.6869	5.1002	5.1031	3.5277	4.8752	5.3708	4.7998	4.4088
Elavl4	_NC[Carbamidomethyl (C)]PS[Phospho (STY)]PMQTGAATDDSK_.2	#N/A	3.5046	10.0122	11.0445	11.1398	8.5987	9.2345	9.8957	3.4758	8.4482	8.5884	9.9453	8.4144	3.3698	11.2130	8.2291	3.8495	8.9762	4.0093	7.6682	8.4358
Cd47	_ISVSDLINGIASLK_.2	#N/A	6.7224	6.2778	5.7921	6.4111	5.4616	5.8511	5.8790	5.8570	6.1063	6.0035	5.6788	5.4401	5.3488	5.6374	5.4630	5.4419	5.8581	6.0066	5.4193	5.9575
Cd47	_FVASNQR_.2	#N/A	4.5330	3.4698	3.5684	3.1564	3.8707	4.0948	5.5124	4.7453	3.7295	4.8375	4.1038	4.9491	3.8375	3.5555	5.2922	4.7242	3.2702	5.4185	4.7250	3.0586
Cd47	_NVEAQSTEEMFVK_.2	#N/A	4.4518	8.4708	3.4873	7.9432	3.7895	7.4217	3.7515	4.1522	3.8106	3.1573	4.1850	3.0390	3.9187	8.8850	6.7725	3.2959	8.1065	8.0056	7.5491	3.1397
Phgdh	_GTIQVVTQGTSLK_.2	#N/A	6.9934	7.2104	7.3472	7.4378	6.6471	7.4262	7.0432	7.1462	6.8104	6.7714	5.7190	6.8021	7.2838	6.7168	6.6320	6.8547	6.7592	6.9812	6.6235	6.8473
Phgdh	_GGIVDEGALLR_.2	#N/A	7.0427	7.0047	7.2416	7.5052	6.9884	7.1660	6.8884	7.2352	7.2661	7.4636	7.0492	6.8394	6.6846	6.6411	6.9938	6.5170	6.9616	6.8487	6.8573	6.9252
Phgdh	_VTADVINAAEK_.2	#N/A	7.1260	6.9312	6.8944	6.7317	6.9256	7.3071	7.0591	7.3068	6.9432	7.1741	6.6920	6.6545	6.7713	6.5300	6.7409	6.8362	6.3619	6.6351	6.4274	6.5457
Phgdh	_QADVNLVNAK_.2	#N/A	5.5352	6.0075	6.1754	5.8536	5.6775	5.5693	5.1502	5.5995	5.5216	5.8469	4.9421	4.6150	4.9532	5.8776	5.2875	5.8585	5.2688	5.5234	5.0061	5.6564
Phgdh	_NAGTC[Carbamidomethyl (C)]LSPAVIVGLLR_.2	#N/A	4.4092	4.7453	5.6371	5.8646	5.1987	5.0974	3.7778	4.6452	5.3703	5.0518	4.9811	5.4079	3.8936	5.2920	5.5274	4.7712	3.9511	4.8883	4.9960	5.2491
Phgdh	_VLISDSLDPC[Carbamidomethyl (C)]C[Carbamidomethyl (C)]R_.2	#N/A	5.8833	6.4542	5.9524	6.3666	6.1111	6.3516	6.0572	6.1758	6.6297	3.9279	2.9137	5.8618	5.9666	6.4318	6.3752	5.3189	5.6476	5.5764	6.0558	5.4130
Phgdh	_ILQDGGLQVVEK_.2	#N/A	4.2672	4.5502	4.0985	3.3395	4.2158	4.5573	4.4388	4.1799	4.5474	4.6297	3.6838	3.6705	4.3706	3.8885	4.2379	4.1910	3.9753	4.3820	3.0628	3.3394
Phgdh	_AGTGVDNVDLEAATR_.2	#N/A	5.6976	5.4575	5.5320	5.5243	4.8408	5.1109	5.2517	4.6835	5.2226	5.7134	4.3452	4.9519	3.6541	4.9801	4.5468	5.2932	5.0342	5.3505	3.6103	5.3492
Phgdh	_ALVDHENVISC[Carbamidomethyl (C)]PHLGASTK_.3	#N/A	4.2980	4.7127	4.8162	4.8082	4.4379	5.0350	4.3905	4.5276	3.3051	3.3939	3.6532	4.4622	4.1704	4.9422	4.0681	4.0817	4.2385	3.7301	4.0321	3.4732
Kcna5	_RES[Phospho (STY)]GLDTGGQR_.2	Q61762_S535_M1_Kcna5	4.4753	6.5249	5.9009	3.7199	3.8130	6.0125	3.7280	4.0900	6.2785	3.1808	6.0257	3.4169	6.6453	3.4390	3.3052	3.3194	3.5872	6.1902	6.4700	5.8983
Kif3b	_DYEGPAIS[Phospho (STY)]PK_.2	Q61771_S681_M1_Kif3b	10.0305	10.1087	9.8857	10.0993	9.5590	9.9123	9.6949	9.9438	9.5946	10.1845	9.8322	9.9455	10.1193	10.3046	9.3131	9.9175	10.1981	9.8265	9.8186	9.8727
Kif3b	_SQNQQYLVR_.2	#N/A	4.0741	4.4915	3.6107	3.5101	3.5783	4.3144	4.2679	4.2998	4.1881	3.8847	3.6667	3.6248	4.3535	3.6488	3.8176	3.4362	3.7970	3.2623	3.6514	3.8510
Lasp1	_GFSVVADTPELQR_.2	#N/A	7.3476	7.8608	7.4538	7.2648	7.5606	7.8422	7.6493	7.2344	7.2965	7.5269	7.5750	7.6133	8.0325	8.1257	8.2475	7.9603	7.7068	7.5547	7.6129	7.9710
Lasp1	_YHEEFEK_.2	#N/A	7.2062	7.2607	7.1336	6.7541	6.7246	7.4104	7.0324	6.8698	6.7734	7.5151	7.1339	7.2752	7.2989	7.5924	7.9026	7.3944	6.9189	7.4206	7.5029	7.3034
Lasp1	_YKEEFEK_.2	#N/A	6.9389	7.2817	7.1593	7.0824	6.8096	7.0407	7.1602	6.5454	6.8257	7.4727	7.1549	7.2549	7.3533	7.2445	7.7168	7.2828	6.8207	7.2457	7.3890	7.4117
Lasp1	_QSFTM[Oxidation (M)]VADTPENLR_.2	#N/A	7.1507	7.0656	6.3348	6.8526	6.8307	6.9996	6.6411	7.1008	6.8941	7.0246	6.6347	6.9054	7.5777	6.7318	7.0353	7.4378	6.8818	6.4766	6.9469	7.4013
Lasp1	_TQDQISNIK_.2	#N/A	6.4194	6.1441	6.2115	7.0659	6.4233	6.3633	6.0950	6.5492	6.8842	6.4103	5.8442	6.4774	5.7553	6.9727	6.8343	6.3061	5.9937	6.2594	6.0911	6.7369
Lasp1	_IVYPTEK_.2	#N/A	3.4928	5.3281	4.5302	5.7648	4.8423	5.5373	4.4219	4.2609	5.1457	4.8845	5.1634	5.3348	3.4340	4.5683	5.8744	5.3112	3.2157	5.6989	5.0655	5.3058
Lasp1	_EAQDSSSYR_.2	#N/A	4.4339	3.9729	2.8969	3.6785	3.7716	4.2768	4.7352	4.1314	3.9590	3.1393	4.3113	4.4152	3.6215	3.8308	7.0645	3.2779	3.6287	4.5803	4.4792	4.4096
Lasp1	_TGDTGMLPANYVEAI_.2	#N/A	4.5552	4.3802	4.5199	4.6675	3.2613	3.7345	4.3658	3.5521	3.7070	4.3658	4.5037	3.9908	3.8724	4.6175	2.9037	3.9173	4.0898	3.7434	3.3920	4.3421
Lasp1	_MGPSGGEGVEPERR_.3	#N/A	3.8468	3.4723	3.3834	3.7374	4.0537	4.0871	3.8922	4.0725	4.4154	3.6574	3.8941	3.1984	4.5809	4.4180	3.0198	3.2089	3.5697	3.0349	4.1844	3.5497
Lasp1	_LKQQSELQSQVR_.3	#N/A	4.0824	3.2367	3.6190	3.3649	4.2894	4.3227	4.2596	4.0249	4.1798	3.8930	3.6584	2.9628	4.3452	3.3470	2.7841	3.4445	3.0617	4.8098	4.4200	3.3140
Rai1	_S[Phospho (STY)]PALPER_.2	Q61818_S1332_M1_Rai1	3.6993	4.3035	4.2356	4.9056	3.0370	4.3508	3.0033	3.3996	3.0623	5.2960	3.4367	4.4189	3.1704	4.2226	4.2393	4.0442	3.9373	3.8958	5.1597	4.1896
Pdcd4	_SGVAVPTS[Phospho (STY)]PK_.2	#N/A	10.5338	10.5307	10.3135	10.8957	10.0928	10.4280	10.1864	10.4044	10.1827	10.5781	10.2895	10.1033	10.4217	10.4048	9.0820	9.8081	10.2783	10.1860	3.2521	10.1118
Pdcd4	_SGVAVPTS[Phospho (STY)]PKGR_.2	#N/A	7.6503	3.9733	6.5424	3.6599	3.3673	7.2184	7.5022	8.2900	7.2885	7.4136	3.1064	7.9022	8.7545	3.8847	6.6356	7.4193	7.3936	7.6113	7.1567	6.9646
Myh10	_TSDVNDTQPPQS[Phospho (STY)]E_.2	Q61879_S1975_M1_Myh10	11.8352	11.6274	11.4545	11.6135	11.0512	11.3226	11.2571	11.5593	11.3318	11.2722	11.2476	11.1903	11.3849	11.1662	10.2552	10.9987	11.6696	11.3063	10.9750	11.0893
Myh10	_SDLLLEGFNNYR_.2	#N/A	5.9350	6.5996	6.2498	5.9097	5.2883	5.9884	6.2880	7.1123	5.8197	6.0309	5.7529	5.8777	6.6692	5.6979	4.6077	4.9482	5.9288	6.4287	6.2389	4.9049
Myh10	_IVFQEFR_.2	#N/A	5.5197	5.9099	5.7656	6.2587	5.9918	5.8931	6.0498	6.0190	5.3652	6.0995	5.5540	5.8038	5.6554	5.5641	4.5567	5.8834	5.1980	5.8986	5.4889	5.7405
Myh10	_TQLEELEDELQATEDAK_.2	#N/A	4.8162	5.1207	4.8936	5.1575	4.7695	5.3723	4.4508	4.8007	5.6508	4.9904	5.2466	4.8390	5.0376	4.3995	4.2258	4.6417	5.6446	5.4513	5.2034	4.5263
Myh10	_GGPISFS[Phospho (STY)]SSR_.2	Q61879_S1937_M1_Myh10	3.9737	4.2115	4.1735	3.8133	3.3431	3.3764	3.7336	3.3618	3.6538	3.7843	3.1324	7.9100	3.8192	4.4567	3.7589	3.9991	4.3599	3.8756	3.9390	3.6299
Myh10	_EQEVAELKK_.2	#N/A	5.8642	5.3313	5.5829	5.5442	5.5461	5.7363	5.3813	6.1507	6.0329	6.2128	5.3423	5.0807	5.8345	5.1256	5.3163	5.8036	6.1603	5.3823	4.8803	4.6910
Myh10	_LQQELDDLTVDLDHQR_.3	#N/A	3.3465	5.0183	4.4022	5.1640	3.5534	4.8634	4.9572	3.2600	4.7262	4.9529	5.6210	5.3743	5.1883	4.6898	4.1530	4.3772	4.8149	5.0489	4.8020	4.8245
Myh10	_LDPHLVLDQLR_.2	#N/A	6.4298	5.7727	5.6546	6.0293	6.3811	6.4063	6.5180	6.5543	6.0601	5.9763	6.1489	6.1442	3.7726	5.7465	5.4850	5.2898	6.3058	6.6151	6.0944	6.2589
Myh10	_DAAGLESQLQDTQELLQEETR_.2	#N/A	5.5847	5.0125	5.0936	4.9965	4.6618	5.4972	5.1137	4.8360	5.2455	4.9084	5.1191	4.5075	4.9867	4.9759	4.0594	4.3225	5.3555	5.4025	4.6660	4.2885
Myh10	_QLLQANPILESFGNAK_.2	#N/A	3.5583	4.2682	3.7716	4.0847	3.8167	4.2980	4.2934	4.4365	3.2367	4.0344	4.2126	3.1561	4.2298	2.1799	3.3902	4.1458	3.3156	3.2947	3.6676	3.4779
Myh10	_LVQEQGSHSK_.2	#N/A	3.2896	3.1631	3.6538	3.0886	1.8904	3.6865	3.5010	3.1973	2.1732	3.8162	3.0417	4.0736	4.3503	4.0471	4.2672	3.8346	3.6753	2.4137	2.3846	2.8690
Myh10	_DLEAQIEAANK_.2	#N/A	3.5663	3.7528	4.6037	4.0179	4.6986	5.9281	5.3564	3.7920	5.4436	3.3769	4.7801	3.4789	3.3605	3.1409	5.0175	4.4293	5.0091	4.9531	5.8001	5.4690
Myh10	_QLHIEGAS[Phospho (STY)]LELSDDDTESK_.3	#N/A	4.0510	6.8085	6.9188	3.9000	3.5948	7.0454	6.9570	3.9293	6.9368	4.4553	3.4557	3.9090	4.1018	7.1681	5.9386	3.7492	4.0928	7.3194	6.5715	4.5676
Myh10	_ELQAQIAELQEDFESEK_.2	#N/A	5.9550	6.1864	5.4385	5.6097	5.4329	5.6840	5.7373	5.6041	5.7979	5.7383	5.4742	5.5973	5.6608	6.1596	4.6430	4.8521	5.7356	5.6981	5.2016	5.2760
Myh10	_IAQLEEELEEEQSNM[Oxidation (M)]ELLNDR_.3	#N/A	6.9622	6.6470	6.3725	6.0564	5.8552	6.1779	6.3336	6.4295	6.4534	6.3836	5.9635	5.9928	7.3207	6.0266	5.2071	6.1887	6.9419	6.4559	5.8699	6.2863
Myh10	_SLEAEILQLQEELASSER_.2	#N/A	4.0856	1.2912	3.4634	3.6574	4.1338	3.2479	4.4151	4.1525	3.1694	4.0411	3.8140	3.1183	4.5008	4.4991	2.9397	3.5506	3.6498	2.1139	3.7987	3.1788
Myh10	_RQLHIEGASLELS[Phospho (STY)]DDDTESK_.3	#N/A	4.1033	5.4157	3.9041	3.7067	3.9091	5.1240	5.2637	4.0425	3.7464	3.8575	4.1208	3.5784	4.0509	6.3805	3.2645	3.4154	3.3936	6.2545	5.4410	3.6816
Myh10	_VIQYLAHVASSHK_.3	#N/A	5.2414	4.5659	3.7679	4.5061	3.2680	3.4920	3.2820	3.6306	4.3322	4.1366	5.1379	3.9620	4.4403	3.5174	3.8503	4.2751	4.3849	4.6896	3.3135	3.6613
Myh10	_IAEC[Carbamidomethyl (C)]SSQLAEEEEKAK_.2	#N/A	3.8924	4.3727	2.7515	3.1370	3.2301	3.4542	4.3110	2.2542	1.3763	4.0987	3.2436	4.0988	4.4781	4.0219	3.8882	4.2373	3.1123	2.9793	3.2784	3.6992
Myh10	_AVIYNPATQADWTAK_.2	#N/A	4.5429	5.8992	3.5783	3.1465	3.8806	6.4402	4.4779	4.2433	4.3982	5.0076	3.8768	3.3494	4.5636	3.5654	3.0025	3.3869	3.2801	3.0522	4.2016	3.5324
Myh10	_NKQEVMISDLEER_.2	#N/A	3.5030	2.8455	4.5404	4.0813	3.7099	1.6055	3.3382	3.7286	3.4258	3.5421	3.8002	3.5422	3.5338	4.5785	3.3636	4.3659	3.2259	3.9236	4.2226	4.2557
Myh10	_LQQLFNHTM[Oxidation (M)]FILEQEEYQR_.3	#N/A	3.2844	3.2176	3.8207	4.0298	4.1229	4.3470	1.1602	3.7801	3.4772	3.4907	3.8516	1.9136	3.5853	3.1290	2.9953	3.6293	3.2773	3.8721	4.1712	4.3072
Mog	_VIGPGYPIR_.2	#N/A	9.7756	9.6004	9.2134	9.3810	9.2721	9.6531	9.5519	9.8011	9.0408	8.8979	9.0913	9.3137	9.2574	8.5548	8.8566	8.5824	9.3321	9.4755	9.0451	8.7594
Mog	_ALVGDEAELPC[Carbamidomethyl (C)]R_.2	#N/A	9.3344	9.0482	8.9926	8.8920	8.8601	9.3444	9.0422	9.2235	9.1334	9.0368	8.6991	8.7341	8.8427	8.3232	8.3868	8.5865	8.9910	8.7109	8.5698	8.3899
Mog	_AEVENLHR_.2	#N/A	8.4809	8.1463	8.4299	7.8647	7.8819	8.2965	8.3793	8.2625	8.1399	7.8512	7.9496	8.0545	7.6859	7.8697	7.8266	7.7586	8.0572	7.8282	7.7908	7.7397
Mog	_TFDPHFLR_.2	#N/A	7.9442	7.7565	7.2550	7.9092	7.9239	7.7835	7.9606	8.0915	7.4190	6.7324	7.4906	7.6198	7.5605	7.0716	6.9156	6.5186	7.9991	7.6441	7.3333	7.1653
Mog	_FSDEGGYTC[Carbamidomethyl (C)]FFR_.2	#N/A	6.9497	7.0889	7.2386	6.4884	6.6835	6.8914	6.5804	6.3913	6.9290	6.2655	6.7488	6.1792	6.2918	6.2285	5.7511	5.6778	6.6901	6.4788	5.2664	6.1904
Mog	_LAGQFLEELR_.2	#N/A	6.4344	6.4620	6.5902	6.4813	6.6022	7.0725	6.6394	6.3468	6.4056	5.9746	6.2469	6.4759	6.1620	5.9797	5.7258	5.7782	6.6492	6.5143	6.3676	5.9288
Mog	_LAGQFLEELRNPF_.2	#N/A	5.0195	5.5927	4.5749	5.8897	5.1551	5.7057	5.2746	4.8262	5.6078	5.5711	5.2954	4.5769	3.5684	4.7276	4.6330	4.6822	5.5571	5.9376	4.9383	4.3565
Mog	_DQDAEQAPEYR_.2	#N/A	6.9165	6.9647	4.3908	7.1129	7.1657	7.6038	7.0099	5.9762	6.4978	6.4290	7.4064	6.7920	6.2226	7.2634	6.7298	6.5731	7.2627	7.0533	6.8000	6.8246
Mog	_LRAEVENLHR_.3	#N/A	4.9883	5.4542	4.0039	5.0534	5.2401	5.0385	5.3952	5.8339	5.9382	4.6026	4.8123	5.2261	5.0787	4.6769	4.8011	3.8294	4.5929	5.1068	5.4922	4.4850
Mog	_DHSYQEEAAM[Oxidation (M)]ELK_.2	#N/A	5.1582	4.4551	4.8068	3.6026	4.4961	5.0158	4.7111	5.2571	4.5234	4.7286	4.7930	4.0300	4.4387	3.6647	3.7349	3.6764	4.9454	4.3601	4.6507	4.1387
Mog	_NGKDQDAEQAPEYR_.2	#N/A	3.7694	3.5497	6.3476	6.6113	3.9763	5.2526	6.5479	3.9951	5.9486	3.5800	4.0666	3.2758	3.8003	3.3440	3.0971	3.1315	3.4923	5.4184	3.9562	3.0213

Runx1t1	_STT[Phospho (STY)]PGTPSTIETTPR_.2	Q61909_T565_M1_Runx1t1	7.4738	4.2287	4.1278	7.9090	7.6256	3.3102	4.4549	7.9789	7.2303	3.9547	3.3876	7.5350	7.9318	4.1659	7.3081	7.1409	7.6439	7.4467	7.5674	7.4165
Runx1t1	_QQS[Phospho (STY)]PVNPDPVALDAHR_.3	#N/A	5.5567	5.4503	5.8321	6.0203	5.6824	5.8572	5.0947	5.8664	5.6855	6.2154	6.0682	5.5534	6.2120	6.0917	5.4062	5.9451	4.7701	5.7168	5.5408	5.7281
Kcna6	_S[Phospho (STY)]LTLAAPGEVR_.2	#N/A	8.7062	4.3141	8.1696	4.4341	3.0264	3.2505	3.0139	3.3758	7.4982	3.8950	8.1138	4.2036	3.1810	4.2256	6.0973	4.0336	8.4345	4.2764	3.0746	7.3492
Kcna6	_SEKS[Phospho (STY)]LTLAAPGEVR_.2	#N/A	7.4919	4.5206	6.7838	4.2072	6.4136	3.0440	3.2203	3.5823	6.5098	3.6885	6.3596	4.4101	7.0390	6.1963	2.7975	3.8271	6.5843	4.0699	4.3690	4.1094
Kcna6	_MRS[Phospho (STY)]EKS[Phospho (STY)]LTLAAPGEVR_.3	Q61923_S3_M2_Kcna6	3.7786	3.5405	3.3151	3.8057	3.9855	4.0188	3.0626	4.0042	2.9828	3.5892	3.9623	3.2666	3.1482	3.3532	4.1251	3.1407	3.5015	3.6480	3.9470	3.0305
Npm1	_M[Oxidation (M)]TDQEAIQDLWQWR_.2	#N/A	6.5821	6.0845	5.9169	5.5267	6.4282	6.3023	5.7831	6.8462	5.8815	6.7389	6.3709	6.2909	6.3958	5.9874	6.2740	6.5390	5.6258	6.3009	6.5166	6.4874
Npm1	_GPSSVEDIK_.2	#N/A	3.2542	5.8781	5.8117	6.0546	5.5428	5.5558	5.7152	4.4194	5.2889	4.5656	5.9308	5.4282	6.3935	5.4737	6.3093	5.8428	5.3697	5.4197	5.5625	6.2022
Npm1	_VDNDENEHQLSLR_.2	#N/A	4.5025	4.7938	5.2072	4.6718	4.4173	4.5066	4.1658	4.5268	5.1746	4.9121	4.2930	4.7596	4.2454	5.1888	5.5016	4.7908	5.1032	5.2017	4.3670	4.4386
Npm1	_DELHIVEAEAM[Oxidation (M)]NYEGSPIK_.3	#N/A	5.5480	4.0607	4.2388	4.3828	4.5204	5.2603	3.0651	3.3771	3.1242	5.0424	3.4986	3.6190	3.2323	3.8319	5.3319	4.6549	5.3175	4.0960	4.1337	3.9541
Npm1	_MSVQPTVSLGGFEITPPVVLR_.2	#N/A	3.9935	3.3256	4.6850	3.5907	4.2004	4.4822	4.1270	4.2192	4.2687	3.8041	3.7473	3.0517	4.4341	5.6891	4.1062	3.3556	3.7164	3.4330	3.7321	3.4030
Npm1	bamidomethyl (C)]GSGPVHISGQHLVAVEEDAES[Phospho (STY)]EDEDEEDVKLLGM[Oxidation (M]	Q61937_S125_M1_Npm1	3.5004	4.5971	3.9420	4.2837	3.7073	3.7407	3.3407	3.1061	3.2610	3.3110	4.2404	2.9857	3.4264	3.9291	3.3661	4.3634	3.6437	4.1894	3.8380	3.8961
Pcbp2	_LVVPASQC[Carbamidomethyl (C)]GSLIGK_.2	#N/A	8.0955	8.2344	8.4953	8.3004	8.0997	8.0257	8.2530	8.0956	8.0614	7.8618	7.8150	8.2289	8.0973	8.2224	8.1780	7.9546	8.0660	8.2518	8.0971	7.9324
Pcbp2	_INISEGNC[Carbamidomethyl (C)]PER_.2	#N/A	8.1628	7.6536	8.3446	8.0033	8.1523	8.0325	7.8473	8.0961	8.1873	8.0847	7.9238	8.0680	7.8765	7.9351	7.9745	7.8247	7.7188	7.8690	7.5712	7.7259
Pcbp2	_AITIAGIPQSIIEC[Carbamidomethyl (C)]VK_.2	#N/A	7.9839	7.7040	7.9032	7.9622	7.6679	7.7540	7.9279	7.5765	7.7138	7.9220	7.0762	6.8244	7.4406	7.2926	7.5298	7.6027	7.1486	6.8406	7.0011	7.3966
Pcbp2	_IITLAGPTNAIFK_.2	#N/A	6.8181	7.3102	6.7738	7.1122	7.1850	6.8616	7.1399	6.9539	6.7282	6.9580	6.8520	6.8915	6.8351	7.0835	7.0297	7.2711	6.9335	7.2088	7.5821	7.0215
Pcbp2	_PSSS[Phospho (STY)]PVIFAGGQDR_.2	Q61990_S185_M1_Pcbp2	3.5473	3.8063	4.0836	5.9184	3.6786	3.8915	3.6153	5.5690	3.6001	4.0025	3.5754	3.6957	3.7385	3.8696	3.5840	3.8922	3.7337	3.8013	3.8147	4.3316
Pcbp2	_ESTGAQVQVAGDM[Oxidation (M)]LPNSTER_.2	#N/A	4.7564	5.2742	5.2106	4.3676	4.6210	4.8185	4.9704	4.6026	4.5211	5.0535	4.2778	5.1878	3.8525	4.8052	5.0497	4.8658	4.6650	5.4330	4.7688	4.9356
Pcbp2	_QVTITGSAASISLAQYLINVR_.2	#N/A	4.2387	4.5812	3.7753	3.3455	2.9448	0.1553	4.1024	4.4644	4.1615	4.3072	3.0351	4.3074	4.2696	3.8133	3.6796	3.6008	3.9616	3.1878	3.4869	3.4906
Ctr9	_KGGEFDEFVNDDT[Phospho (STY)]DDDLPVSK_.3	#N/A	4.3383	4.4817	3.8748	3.2460	3.0444	3.0777	4.0037	3.0631	3.9240	4.1489	3.4026	4.2078	4.0894	3.9129	4.0291	3.7004	4.0612	7.2378	3.3873	3.5902
Ctr9	_GGEFDEFVNDDT[Phospho (STY)]DDDLPVSK_.2	#N/A	3.9833	4.0195	4.5196	3.7061	3.3210	3.1631	3.9184	3.6837	3.8386	4.2342	3.3172	3.9089	4.0040	4.5067	3.7972	4.3282	4.2214	3.6118	3.2604	2.9728
Ctr9	_NSNS[Phospho (STY)]DS[Phospho (STY)]DDDERPNR_.2	Q62018_S1039_M2_Ctr9	4.2515	4.5685	3.7881	3.3328	2.9576	2.9909	4.0905	4.4772	4.0107	4.0621	3.4894	4.2946	4.1762	3.8261	4.1159	3.6136	3.9744	3.1751	3.4741	3.1450
Ctr9	_KGS[Phospho (STY)]GS[Phospho (STY)]EQEGEEEEGGER_.2	#N/A	4.4606	4.0564	4.4827	3.7430	3.1667	3.2000	3.8814	3.6467	3.8016	4.2712	3.2803	3.9459	3.9671	4.4697	3.9068	3.8227	4.1844	3.6487	3.2973	4.4367
Ctr9	_RKGS[Phospho (STY)]GS[Phospho (STY)]EQEGEEEEGGER_.2	#N/A	3.2548	3.2472	3.7911	4.4346	4.0933	3.8916	3.1898	4.4560	3.1100	3.4619	4.0895	3.1366	3.2755	3.7781	3.2152	3.5997	1.4723	4.3403	3.9889	3.7451
Cep131	_SNS[Phospho (STY)]TTQVNQSWTGSPR_.2	Q62036_S78_M1_Cep131	3.9916	4.2868	4.1056	5.2975	2.9651	4.5304	3.6461	3.2939	3.6168	3.9618	3.4988	4.0129	3.7610	4.1437	4.1349	3.7995	4.2919	3.8646	3.1925	4.6685
Pea15	_QPS[Phospho (STY)]EEEIIK_.2	#N/A	14.0782	14.1610	14.4067	14.3037	14.0277	14.1979	13.8517	13.7493	14.0536	14.6012	14.1747	13.9692	14.5227	14.4419	14.0192	14.2173	13.8661	13.7808	14.1842	14.2517
Pea15	_SAC[Carbamidomethyl (C)]KEDIPSEK_.2	#N/A	6.0811	6.9720	7.0520	6.6103	6.5498	6.7621	6.8682	6.8489	6.8954	6.6350	6.3086	6.6406	6.5305	6.6404	7.0919	6.3156	6.2556	6.7596	6.3885	6.6555
Pea15	_YKDIIRQPS[Phospho (STY)]EEEIIK_.3	#N/A	11.3129	11.9107	10.7798	11.7142	10.9520	11.2990	11.4025	11.5003	11.5124	11.6017	10.7542	11.6445	12.6696	11.8826	11.4818	11.8847	11.3931	11.5746	11.6001	11.6474
Pea15	_LTRIPS[Phospho (STY)]AK_.2	Q62048_S104_M1_Pea15	5.8418	7.3100	4.4889	6.2973	5.8133	4.7680	5.6192	6.5061	5.6679	6.5235	4.9543	6.8857	8.4950	6.2055	6.6779	6.9612	3.1744	6.2277	6.1427	5.8284
Pea15	_DIIRQPS[Phospho (STY)]EEEIIK_.2	#N/A	11.5765	11.7961	11.5295	11.8300	11.1290	11.2363	11.2842	11.6063	11.4178	11.8425	10.8686	11.5295	12.4484	11.8238	11.1628	11.7935	11.6646	11.3767	11.5176	11.5756
Pea15	_RPDLLTM[Oxidation (M)]VVDYR_.2	#N/A	4.1738	5.0934	5.4605	5.0581	4.5936	5.1325	4.7807	5.2033	5.1995	5.7438	4.7044	5.2255	5.0402	4.7631	5.3575	5.2916	4.9391	5.0577	5.5783	5.4571
Pea15	_[Acetyl (Protein N-term)]AEYGTLLQDLTNNITLEDLEQLK_.3	#N/A	3.7596	3.7031	4.2959	3.9298	3.0973	5.2126	3.5563	5.0716	3.7976	4.5491	3.3764	4.6085	3.1100	4.6986	4.4878	4.1751	4.3029	3.8587	4.2273	4.3860
Pea15	_QPS[Phospho (STY)]EEEIIKLAPPPK_.2	#N/A	7.5240	7.7656	6.6366	7.3512	7.7767	8.2876	6.2260	7.8397	7.7203	8.1245	7.6523	6.4834	7.0364	7.8656	6.9613	8.0757	7.8972	7.5179	7.2723	7.7766
Pea15	_SEEITTGSAWFSFLESHNK_.2	#N/A	4.2322	4.1208	4.9088	3.3412	3.5688	3.7427	3.8017	4.5567	4.0166	4.6059	3.1479	4.0422	4.3468	4.7508	3.1228	4.4940	4.6291	3.7613	3.7037	4.3245
Pea15	_DNLSYIEHIFEISR_.2	#N/A	4.3492	4.1012	3.6129	3.2985	3.9490	3.8106	4.0639	3.6159	3.9841	3.4593	3.4704	3.4368	4.1496	3.5711	3.5828	3.3100	4.3381	3.3405	2.7119	2.7437
Vcan	_YEINSLIR_.2	#N/A	6.5308	6.6442	6.0971	6.8504	6.8387	6.6866	6.4026	6.9464	5.7965	6.7714	6.5756	6.7570	6.7938	6.8968	6.8265	6.8418	6.5189	6.6972	6.7397	6.6750
Vcan	_AQC[Carbamidomethyl (C)]GGGLLGVR_.2	#N/A	6.4843	6.3669	6.8285	6.9899	6.7616	6.3658	6.7001	7.1718	6.9946	6.5014	6.1535	6.7330	6.0099	6.7933	6.9494	6.2756	6.0815	6.2291	6.8967	6.2296
Vcan	_GTVAC[Carbamidomethyl (C)]GQPPVVENAK_.2	#N/A	6.3102	6.0385	6.2676	6.3354	6.3762	5.7957	6.0483	5.9023	6.4019	6.5043	5.8597	5.7276	6.5387	5.7885	6.2785	5.8356	6.2759	5.6179	6.0122	6.3391
Vcan	_LATVGELQAAWR_.2	#N/A	4.4673	6.0608	5.2105	5.1732	5.7409	5.5683	5.4133	5.7621	5.3451	5.6108	5.5855	5.5174	5.8825	5.2989	5.9165	5.9620	5.6606	4.9740	5.4935	5.9616
Vcan	_FTFEEAEAEC[Carbamidomethyl (C)]TSR_.2	#N/A	3.8857	4.1171	5.9108	6.2980	5.4515	3.4476	5.6322	5.5228	4.4747	6.0685	3.2502	5.4398	4.4847	6.0944	5.2137	4.2306	4.1238	2.9727	3.3579	5.4532
Vcan	_NGFDQC[Carbamidomethyl (C)]DYGWLSDASVR_.2	#N/A	6.0186	4.1499	6.8191	4.4151	3.3761	3.4094	3.6720	3.3948	6.2986	6.5726	3.0709	3.8761	3.7577	4.2446	6.2260	4.0321	6.6344	3.8581	3.0556	6.5477
Vcan	_VGHDYQWIGLNDK_.3	#N/A	3.8833	4.1195	4.4196	5.2352	3.2210	3.2631	4.3201	3.5837	4.5238	4.0896	3.2172	4.0090	3.9040	4.4067	4.5765	4.2282	4.1213	3.7118	4.7250	4.3737
Map3k7	_S[Phospho (STY)]IQDLTVTGTEPGQVSSR_.2	Q62073_S412_M1_Map3k7	7.5015	9.7395	10.0067	9.4909	3.5434	9.3733	9.2400	3.9060	9.3370	9.1109	3.5396	8.8263	7.3152	9.4786	8.3132	3.0497	9.0676	9.0633	9.3347	9.3259
Plcg1	_AREGS[Phospho (STY)]FEAR_.2	Q62077_S1248_M1_Plcg1	6.8178	7.1030	6.7627	6.1655	6.4790	6.3890	6.0424	6.4691	6.3275	6.7792	6.1189	5.9559	6.9216	4.2208	5.3578	6.3392	6.1358	6.4574	6.4503	6.4703
Srsf2	_S[Phospho (STY)]RS[Phospho (STY)]PPPVSK_.2	Q62093_S189_M2_Srsf2	12.5144	12.3116	12.6486	12.3379	11.7672	12.3516	12.3050	11.9887	12.1241	12.2234	12.1422	11.6264	11.5450	12.0655	10.8701	11.4806	12.2676	11.9805	12.0101	11.6910
Srsf2	_TS[Phospho (STY)]PDTLRR_.2	#N/A	8.6099	8.5959	8.3976	3.9893	3.8887	8.3895	8.5039	8.5098	8.3111	8.0983	7.9865	7.9613	8.4623	8.1158	7.3866	7.9999	8.3852	8.4412	8.0861	7.6265
Srsf2	_VGDVYIPR_.2	#N/A	6.2337	6.3567	4.9086	5.4350	6.5045	5.8858	6.7709	6.6412	5.8925	6.1386	5.8620	4.8773	5.9145	5.0081	5.8635	5.6364	5.7394	5.9487	5.3478	5.8310
Srsf2	_S[Phospho (STY)]KS[Phospho (STY)]PPKS[Phospho (STY)]PEEEGAVSS_.2	Q62093_S206_M3_Srsf2	8.4161	7.7221	8.5542	8.3406	7.8594	8.1329	7.9233	8.0464	7.7573	7.6696	8.2320	7.4584	7.1776	8.1554	6.7378	7.5586	8.4169	7.9070	7.7922	7.7395
Srsf2	_VDNLTYRTS[Phospho (STY)]PDTLR_.2	#N/A	7.9359	4.2493	7.5657	7.7405	3.2031	7.4316	7.6029	8.1710	7.3357	7.6783	7.3782	7.3251	8.4506	3.9468	3.9001	7.4577	8.1379	3.0544	7.7218	7.3944
Srsf2	_TS[Phospho (STY)]PDTLR_.2	#N/A	3.5478	4.4550	4.0841	4.1416	2.8856	3.5985	3.1547	3.2482	3.4031	3.1689	6.2048	4.3444	3.5686	4.0712	4.2327	3.8927	3.7859	4.0473	3.6959	4.0382
Srsf2	_S[Phospho (STY)]RS[Phospho (STY)]RS[Phospho (STY)]PPPVSK_.2	#N/A	9.8066	9.5653	9.9675	9.7806	9.0381	9.6931	9.6998	9.4778	9.5745	9.7381	9.6452	8.9329	8.8798	9.3468	8.0742	8.9124	9.4844	9.2979	8.8252	9.0368
Srsf2	_VDNLTYR_.2	#N/A	4.1143	3.2048	3.6508	3.4700	4.3212	4.3545	4.2277	4.3399	4.1480	4.0699	3.6266	2.9310	4.3134	3.6889	4.2531	3.4764	3.8372	3.3123	3.6113	3.3662
Ddx3y	_YLVLDEADR_.2	#N/A	6.3499	6.7552	6.3162	6.0569	6.2010	6.3969	6.3189	6.5855	6.5193	6.2797	6.5044	6.3978	7.1012	6.4019	6.0557	6.2500	6.7523	6.1223	6.1410	5.9049
Ddx3y	_GKPNYFSDRGS[Phospho (STY)]GSR_.3	Q62095_S90_M1_Ddx3y	3.5169	3.4228	3.5616	4.1642	5.9688	3.9199	3.2810	4.2364	6.0310	3.4902	4.0627	3.2226	3.2487	3.5696	3.0754	3.3771	3.4801	4.0446	6.2800	3.4234
Ddx3y	_FS[Phospho (STY)]GGFGAR_.2	Q62095_S592_M1_Ddx3y	5.4213	5.9703	6.1896	5.7494	4.8647	5.7775	5.4339	3.9648	5.3340	5.8892	4.9593	5.0676	5.3590	5.6068	4.4976	5.0703	5.4151	6.0296	4.8865	4.9854
Ddx3y	_HVINFDLPSDIEEYVHR_.3	#N/A	5.0810	5.6972	5.5706	5.4180	5.1547	4.9712	5.3453	5.5037	4.9777	6.0000	3.8419	4.8700	3.5755	6.1348	5.2033	5.3184	5.3115	5.6018	4.1809	5.6645
Ddx3y	_DLLDLLVEAK_.2	#N/A	4.6910	4.1891	4.2781	4.8108	4.6955	4.4385	4.6699	4.1926	4.3204	4.2983	4.7035	3.6240	4.1022	4.4587	4.4039	4.0794	4.5063	4.7718	4.8198	4.9742
Ddx3y	_VGNLGLATSFFNER_.2	#N/A	3.6896	3.6295	4.6329	3.8947	3.8965	4.1927	4.2427	4.7939	4.0572	3.5189	3.9868	4.0203	3.7204	3.2642	3.1305	3.9098	3.4125	3.6808	3.4237	4.4423
Dlg4	_RYS[Phospho (STY)]PVAK_.2	Q62108_S295_M1_Dlg4	12.9536	12.8224	12.6113	12.7458	12.3489	12.5135	12.6436	12.8485	12.5548	12.6200	12.5815	12.6108	13.0954	12.5583	11.8629	12.3780	12.8331	12.7883	12.5946	12.4733
Dlg4	_IIPGGAAAQDGR_.2	#N/A	8.0370	8.3620	7.7004	8.0415	7.9874	8.1540	8.7206	8.4821	8.3922	7.9383	8.1690	8.3275	8.0628	8.2062	8.5818	7.9097	8.2800	8.3959	8.3041	8.0662
Dlg4	_DYHFVSSR_.2	#N/A	6.4941	6.5287	6.3039	6.6190	6.5260	6.8300	6.8355	6.5785	6.7687	6.5154	6.7404	6.3829	6.4862	6.6874	6.4564	6.6812	6.7122	6.8029	6.5886	6.1886
Dlg4	_VIEDLSGPYIWVPAR_.2	#N/A	7.7127	7.1960	7.3252	7.5223	7.2370	7.5411	7.5464	7.4905	7.1297	7.7940	7.5488	7.5357	7.3752	7.0906	7.5387	7.4550	7.6932	7.5344	7.4207	7.4065
Dlg4	_PIIILGPTK_.2	#N/A	7.5122	6.9309	7.3546	7.3672	7.4738	7.1536	7.1979	7.1698	6.9219	7.0992	7.2483	6.8659	7.1928	7.1890	7.0297	7.2576	7.3994	7.4634	7.2005	7.2516
Dlg4	_EVTHSAAVEALK_.2	#N/A	5.0579	5.2326	4.2459	5.3478	5.0112	4.6408	5.2894	5.2453	4.7849	4.6328	4.4663	4.5644	5.0177	4.6923	5.3764	4.8637	4.7284	5.3316	5.0232	5.2508
Dlg4	_VHSDS[Phospho (STY)]ETDDIGFIPSKR_.3	#N/A	3.4702	4.5669	3.9722	4.2535	3.6772	3.7105	8.7869	3.1362	3.2911	3.2808	4.2706	8.8997	3.4566	3.9592	8.1121	4.3332	3.6739	4.1592	3.8078	3.9262
Dlg4	_REYEIDGR_.2	#N/A	5.0867	5.3873	3.5083	3.5584	5.4084	5.2294	5.5358	5.3903	5.5409	5.4383	5.6880	4.9710	4.2395	5.2577	5.7137	4.9798	4.3690	5.0066	5.5098	5.0957
Dlg4	_VNDSILFVNEVDVR_.2	#N/A	6.5488	4.1747	6.7165	6.3500	5.9779	5.9351	3.0082	5.9711	5.6854	6.8340	6.6796	6.1142	3.1753	5.7947	5.4174	5.7574	6.5008	6.0053	6.4126	5.8098
Dlg4	_NTYDVVY[Phospho (STY)]LK_.2	Q62108_Y240_M1_Dlg4	8.2323	8.1093	7.7719	8.4792	3.9713	8.1885	8.1432	7.8191	8.0897	8.4223	8.6224	8.2587	8.8284	8.7111	7.5560	8.5249	8.7678	8.6999	8.6125	8.5615
Dlg4	_AKDWGS[Phospho (STY)]SSGSQGR_.2	Q62108_S509_M1_Dlg4	4.1711	3.5603	4.8160	3.5037	3.7806	4.1805	4.1743	4.1143	4.4980	3.9817	3.2299	3.3874	4.1593	3.4520	4.1997	3.5332	4.1430	3.3785	3.3775	3.2572
Dlg4	_EQLM[Oxidation (M)]NSSLGS[Phospho (STY)]GTASLR_.2	#N/A	9.2202	6.0049	6.0318	6.0514	5.8281	5.6483	8.6897	5.8894	8.6297	8.8026	8.4980	8.7056	9.3999	8.5218	4.3857	5.3990	7.8360	5.6074	8.7856	6.9045
Dlg4	_RVHSDS[Phospho (STY)]ETDDIGFIPSK_.3	#N/A	4.2296	3.9987	3.7662	3.3575	2.9357	5.3727	4.1153	4.4553	4.1579	4.1208	3.3534	4.3193	4.2605	4.9780	4.1406	3.5917	3.9525	5.0867	6.6223	6.3831
Dlg4	_VHSDSETDDIGFIPSK_.3	#N/A	4.6833	6.0544	6.3435	5.1504	4.6646	6.5006	5.7688	3.9615	4.7348	5.1216	5.0982	4.5489	4.3407	7.1955	5.5807	4.3821	6.2613	6.7087	5.0463	6.2600
Dlg4	_HC[Carbamidomethyl (C)]ILDVSANAVR_.2	#N/A	3.5412	4.2476	4.5786	4.3245	4.5599	4.4475	4.1627	3.0653	4.0499	3.3518	4.1997	4.0442	3.3856	3.8883	3.7056	4.4041	4.0795	3.8064	4.1869	3.8553
Dlg4	_RVHS[Phospho (STY)]DS[Phospho (STY)]ETDDIGFIPSKR_.3	#N/A	3.7816	6.2507	3.9292	3.8000	3.9885	6.3081	6.1846	3.6724	5.7349	4.8930	3.3059	3.6699	3.9927	3.7594	5.4847	4.9607	4.2100	6.2115	6.2842	6.3447
Dlg4	_EVTHSAAVEALKEAGS[Phospho (STY)]IVR_.3	Q62108_S142_M1_Dlg4	3.6170	6.8038	4.1533	4.0724	2.9547	3.1788	6.1359	3.3174	3.1446	4.9531	3.5190	5.5048	5.8318	7.1965	4.1635	5.4698	3.8551	6.4970	5.3291	4.3397
Dlg4	_DWGSSSGS[Phospho (STY)]QGR_.2	#N/A	3.8442	4.1925	5.0159	3.8791	6.3353	5.3539	5.3310	6.8271	5.0126	3.8201	4.6910	4.0820	3.9101	3.9812	5.1287	3.6663	4.7174	4.7771	6.7872	4.9416
Dlg4	_GNS[Phospho (STY)]GLGFSIAGGTDNPHIGDDPSIFITK_.3	Q62108_S73_M1_Dlg4	1.7206	4.1470	4.4816	4.1401	4.2332	4.4573	4.2038	3.7316	3.3670	3.6009	3.7414	2.8512	3.4750	5.0497	2.8851	3.7395	3.1671	4.0345	4.2814	4.1969
Dlg4	_FIEAGQYNSHLYGTSVQSVR_.3	#N/A	6.0478	4.7631	4.5841	4.4019	5.1837	4.2128	5.1555	6.4067	3.1427	5.1236	5.5927	5.7773	5.9888	3.8108	3.2479	2.9808	3.5255	4.3077	5.1902	5.6894
Dlg4	_DLLGEEDIPREPR_.3	#N/A	4.5052	3.4846	3.5406	3.7498	3.8429	4.0670	3.1569	4.0601	3.7338	3.2107	2.8302	3.3871	3.8653	3.4091	0.9142	3.2034	3.5573	3.5921	3.8911	3.0864
Dlg4	_EQLMNSSLGSGT[Phospho (STY)]ASLRS[Phospho (STY)]NPKR_.3	Q62108_S425_M2_Dlg4	3.4749	4.4962	4.0432	4.1828	3.2960	3.4030	3.3032	3.2073	3.2886	3.5042	4.0080	4.3857	3.4267	4.4781	3.9053	4.0924	3.7450	4.0885	3.7371	3.9973
Dlg4	_S[Phospho (STY)]LENVLEINKR_.2	Q62108_S654_M1_Dlg4	3.8438	3.4753	3.3804	3.7404	4.0507	4.0841	2.9974	4.0695	4.4184	3.6544	3.8971	3.2014	3.8747	3.2029	3.0227	3.2059	3.5667	3.5827	3.8818	3.0957
Dlg4	_ILAVNSVGLEDVMHEDAVAALK_.3	#N/A	3.7962	3.7478	3.1566	3.8932	3.1339	3.3580	4.4072	3.4965	4.4663	4.0025	3.3398	4.0961	4.5743	4.1181	3.9844	4.1411	4.0342	4.3839	3.1821	3.7954
Dlg4	_IIEGGAAHKDGR_.3	#N/A	3.1685	4.1506	4.2059	4.4158	3.3754	3.4087	3.6727	3.4380	3.5929	4.4799	3.0715	3.8767	3.7583	4.2610	3.6981	4.0314	3.9757	3.8575	3.5061	4.2280
Jak2	_TNGISDVQIS[Phospho (STY)]PTLQR_.2	Q62120_S523_M1_Jak2	8.1805	8.0242	7.4914	7.7646	6.9205	7.5157	7.7232	7.8791	3.1016	7.8776	7.3474	3.1451	3.2670	3.7697	5.8166	7.1291	3.4843	7.6709	7.1133	6.8914
Ptprr	_NQEIHLS[Phospho (STY)]PIAR_.2	#N/A	7.1312	6.9184	6.5618	6.5323	6.4924	6.4696	6.6503	6.9024	6.2292	6.1567	6.6293	6.2459	7.1637	6.3074	4.9452	6.1652	7.2895	6.6854	6.2636	5.8537
Ralbp1	_TPS[Phospho (STY)]SEEISPTK_.2	Q62172_S29_M1_Ralbp1	6.9258	3.5968	6.9908	6.8825	4.2495	7.0038	6.7965	6.7527	6.9812	7.8921	3.7122	6.8201	3.9892	7.2001	6.0067	3.5885	7.2987	3.6601	4.0033	3.8314
Ralbp1	_[Acetyl (Protein N-term)]TEC[Carbamidomethyl (C)]FLPPSS[Phospho (STY)]SPSEHR_.2	Q62172_S10_M1_Ralbp1	3.8186	7.1276	3.7692	7.4337	3.4653	7.4497	3.5169	7.5416	7.5195	7.6593	7.4197	3.7894	3.6623	7.5328	3.7373	6.9356	4.2009	6.9146	3.5492	6.4700
Ralbp1	_[Acetyl (Protein N-term)]TEC[Carbamidomethyl (C)]FLPPS[Phospho (STY)]SSPSEHRR_.3	#N/A	3.8814	6.6120	4.0370	5.8149	3.6876	3.8649	6.2208	3.6127	6.4941	3.9175	3.6842	3.6261	4.1261	3.6699	3.4886	7.1763	3.8181	3.3900	3.6891	3.3472
Ralbp1	_TEGYAAFQEDS[Phospho (STY)]S[Phospho (STY)]GDEAESPSK_.2	#N/A	4.4197	7.9567	6.9757	7.0867	3.7574	7.9660	8.0373	4.1201	7.4773	3.1252	4.2171	3.4726	4.4404	8.1118	3.3609	7.0178	3.1569	7.9426	7.3424	7.6936
Ralbp1	_TPS[Phospho (STY)]SEEIS[Phospho (STY)]PTKFPGLYR_.2	#N/A	3.8716	3.8993	3.6461	3.9344	4.0069	3.6916	3.4363	3.9273	3.4590	3.9991	4.0118	3.7099	3.5818	7.5176	3.7038	3.4126	3.9812	4.7925	6.4881	3.7141
Ralbp1	_TGEPS[Phospho (STY)]PPHDVLHEPPDT[Phospho (STY)]VSDDDKDHGK_.4	Q62172_T60_M2_Ralbp1	4.0377	7.9170	4.2327	3.7564	3.8521	3.1306	9.5153	3.4210	3.5549	4.0190	3.4774	3.8758	3.6966	4.4460	8.1425	3.8300	3.7606	9.7225	3.9270	4.2748
Ralbp1	_RTEGYAAFQEDS[Phospho (STY)]S[Phospho (STY)]GDEAESPSK_.3	#N/A	4.5267	4.2932	4.0633	4.5584	3.2328	3.3747	4.3904	3.2515	4.4495	4.0193	3.3230	4.0194	4.5576	4.9512	3.9676	3.8888	4.2496	4.4007	3.1989	3.7786
Sema4a	_TSAS[Phospho (STY)]DVDADNNHLGAEVA_.2	Q62178_S746_M1_Sema4a	4.2955	3.8970	3.8321	3.3094	3.8543	3.9684	4.2765	3.8730	5.8723	4.0077	3.4536	3.6231	4.4048	3.7189	4.0881	3.6576	4.0184	3.1439	3.1170	3.5128
Dpysl3	_DNFTAIPEGTNGVEER_.2	#N/A	9.0500	9.2452	9.1017	9.1080	8.8689	9.2357	9.1281	9.0466	9.3298	9.4873	8.2543	8.2079	8.0352	8.7366	8.1284	8.1261	8.5836	7.9455	8.1433	8.2922
Dpysl3	_GAPLVVIC[Carbamidomethyl (C)]QGK_.2	#N/A	8.0202	8.0985	8.1295	8.2648	7.8179	8.3276	7.9823	8.5530	8.1328	8.2624	6.6204	7.4467	6.9167	6.8352	6.7744	7.0361	7.5249	6.4909	6.7013	6.9403
Dpysl3	_QIGDNLIVPGGVK_.2	#N/A	7.9506	8.1319	7.8624	8.2559	7.9661	8.3792	8.0697	7.8793	7.9504	8.0760	7.1330	6.9004	7.1965	7.0056	7.4214	6.9689	7.3034	7.2859	7.0155	6.9405
Dpysl3	_IAVGSDSDLVIWDPDALK_.2	#N/A	7.9533	8.1677	8.2646	8.4323	7.3626	7.8778	7.9453	7.8644	8.4604	8.4958	6.8860	6.9905	7.1850	7.1808	7.4274	7.3022	6.9967	7.0214	6.8900	7.2085
Dpysl3	_AITVASQTNC[Carbamidomethyl (C)]PLYVTK_.2	#N/A	7.8454	7.7588	7.9103	7.3601	7.4025	7.7885	7.5535	7.5186	7.6536	7.8205	6.2553	6.6427	7.0827	6.9623	7.0500	6.8473	7.0368	6.4038	6.4635	6.2815
Dpysl3	_GM[Oxidation (M)]YDGPVFDLTTT[Phospho (STY)]PK_.2	#N/A	12.6215	12.7739	12.9654	12.8268	12.1492	12.6526	12.3093	12.3964	12.5566	12.8844	11.6396	11.4363	11.8095	11.9699	11.3646	11.6248	11.9940	11.4129	11.6111	11.7008
Dpysl3	_SAADLISQAR_.2	#N/A	8.2731	8.9220	8.8646	8.4341	8.5347	8.9089	8.6597	8.9364	8.6963	8.9142	7.4214	7.6235	7.7645	7.9763	8.3066	7.5783	7.9733	7.6634	7.6478	7.4915
Dpysl3	_GS[Phospho (STY)]PTRPNPPVR_.2	#N/A	13.4794	13.8054	13.6502	13.4165	13.0373	13.6086	13.3673	13.3573	13.4862	13.8033	12.8340	12.8016	13.3659	13.4075	12.8555	12.9033	13.1070	12.8008	12.9100	13.0261
Dpysl3	_GGT[Phospho (STY)]PAGSTRGS[Phospho (STY)]PTRPNPPVR_.3	#N/A	12.7177	13.0792	12.9104	13.0517	12.3539	12.8596	12.7431	12.3905	12.8516	13.3168	10.9459	11.4690	11.8659	12.1303	10.7776	11.6245	11.4141	11.5084	11.7924	11.7947
Dpysl3	_IVAPPGGRS[Phospho (STY)]NITS[Phospho (STY)]LS_.2	Q62188_S564_M2_Dpysl3	4.4400	13.3471	12.6516	3.1443	3.1461	12.8891	12.8138	3.1648	12.5994	10.3536	3.3009	4.1061	3.9877	11.8891	10.4173	10.0304	4.1629	11.1791	11.1899	3.6919
Dpysl3	_NLHQSGFS[Phospho (STY)]LSGTQVDEGVR_.3	#N/A	8.2170	9.2322	8.6247	8.8191	8.4161	8.5471	8.6724	7.9820	8.2873	9.1415	7.5110	6.7680	7.8036	7.9799	5.2770	7.0710	7.2637	7.4354	7.3364	7.3710
Dpysl3	_GM[Oxidation (M)]TTVDDFFQGTK_.2	#N/A	7.0586	7.0492	6.9501	6.9115	6.9876	7.1822	6.5760	6.9253	7.2512	7.3774	5.6841	6.0354	6.8774	6.1051	6.4244	6.4907	6.0688	6.1044	6.1383	5.9567
Dpysl3	_NHQSVAEYNIFEGM[Oxidation (M)]ELR_.3	#N/A	6.7183	6.4443	7.1750	7.0070	6.4778	6.5271	6.0314	6.5997	6.8872	6.8238	5.4733	5.2136	6.1802	5.7035	5.5513	5.8973	5.8716	5.3141	6.0370	6.3340
Dpysl3	_GM[Oxidation (M)]YDGPVFDLTTT[Phospho (STY)]PKGGT[Phospho (STY)]PAGSTR_.3	#N/A	9.5432	10.3398	9.6491	9.8022	6.0710	9.6126	9.9135	8.9573	9.6940	9.8979	5.1857	8.3189	8.7428	9.2375	8.2260	3.6901	5.2053	8.6976	9.4271	8.2133
Dpysl3	_AALAGGTTM[Oxidation (M)]IIDHVVPEPESSLTEAYEK_.3	#N/A	5.9400	6.2421	5.8886	5.7164	5.5320	6.1787	5.9859	6.1076	5.5253	6.3135	4.7341	4.1923	5.4873	5.7636	5.1325	5.3972	5.5945	5.5551	4.9694	5.3454
Dpysl3	_MADLHAVPR_.2	#N/A	3.3760	6.8632	6.9470	7.0881	5.3063	6.3999	7.2747	3.2305	5.6131	5.5265	5.2160	3.6692	3.5508	6.7709	5.8559	4.2389	5.3850	4.0650	4.4619	4.0205
Dpysl3	_IM[Oxidation (M)]LEDGNLHVTQGAGR_.3	#N/A	5.4525	6.2705	5.6195	6.2382	5.2946	6.5720	5.5806	5.7559	6.2914	6.0660	4.2968	5.5892	5.3033	5.1802	6.1646	5.9073	4.8278	4.7716	5.1509	4.8850
Dpysl3	_SC[Carbamidomethyl (C)]C[Carbamidomethyl (C)]DYALHVDITHWNDSVK_.3	#N/A	5.4196	3.6338	5.2681	5.1399	4.8963	5.3719	5.0139	5.0749	4.4843	4.9159	3.6947	3.9324	4.7871	4.9000	3.3569	3.1711	4.0774	3.8718	4.2018	3.9166
Dpysl3	_NLHQSGFS[Phospho (STY)]LSGTQVDEGVRS[Phospho (STY)]ASKR_.4	Q62188_S551_M2_Dpysl3	3.2389	4.0802	4.2763	4.3453	2.9376	3.1617	3.1027	3.4646	3.1617	3.8062	3.5361	3.8063	3.2698	4.3144	4.1807	3.9448	4.4627	4.1876	2.9858	3.9917
Scn9a	_S[Phospho (STY)]GS[Phospho (STY)]EES[Phospho (STY)]IRKK_.2	Q62205_S502_M3_Scn9a	4.6623	4.9946	4.1220	5.0752	4.4656	4.7011	4.8326	4.8137	4.7861	4.8674	4.4557	4.3899	4.0325	4.2875	4.0682	4.3812	4.3724	5.0483	4.6455	4.4092
Tgfb1i1	_EGC[Carbamidomethyl (C)]PS[Phospho (STY)]PPGQTSK_.2	Q62219_S194_M1_Tgfb1i1	9.8287	9.8095	9.7861	3.7483	9.4738	9.3273	3.6997	8.8595	9.0484	9.4600	9.6197	9.3191	9.8427	10.2912	9.2073	8.7628	9.9508	9.4730	9.2575	9.7884
Sos1	_IPESETESTASAPNS[Phospho (STY)]PR_.2	Q62245_S1082_M1_Sos1	7.7342	7.3709	4.3748	7.1888	3.5443	3.0633	7.1210	7.6470	6.4957	3.7078	6.8843	3.7079	6.5674	6.8140	3.5292	5.8841	7.2094	6.8992	4.3882	6.3665
Sos1	_RLS[Phospho (STY)]ESAC[Carbamidomethyl (C)]R_.2	Q62245_S401_M1_Sos1	3.7705	3.5486	3.3071	4.2620	3.9774	4.1309	3.7526	3.9962	3.6933	3.2746	4.0677	3.2747	3.8014	3.3451	3.2114	3.1326	3.5002	3.6560	3.9551	3.0224
Sos1	_SAS[Phospho (STY)]VSSISLSK_.2	Q62245_S1120_M1_Sos1	6.6108	3.3482	7.0091	5.8743	3.9593	3.9926	6.0337	4.3550	3.0090	3.5629	3.9885	3.2376	5.8353	3.6771	3.1142	3.1144	3.3918	4.4413	4.0899	3.6441
Sptbn1	_ETWLSENQR_.2	#N/A	9.6661	9.2252	9.3605	9.2113	9.3210	9.4038	9.4471	9.7512	9.4208	9.2453	9.3729	9.2655	9.6875	9.2812	8.7944	9.1605	9.4208	9.4011	9.1993	9.1726
Sptbn1	_DVEDEILWVGER_.2	#N/A	9.0700	8.8221	9.3213	8.8139	8.6052	9.0669	8.8186	8.8735	9.2031	8.8593	9.0876	8.5999	8.9730	8.7609	8.1180	8.7669	9.1831	8.7525	8.8615	8.8312
Sptbn1	_DQNTVETLQR_.2	#N/A	8.7822	8.4747	8.9425	8.7591	8.6677	8.6930	8.7588	8.8589	8.7948	8.6096	8.7160	8.4756	8.4681	8.9045	8.3412	8.5828	8.7005	8.7211	8.5624	8.7441
Sptbn1	_SALPAQSAATLPAR_.2	#N/A	8.4791	8.4480	8.6799	8.5045	8.4130	8.6443	8.7565	8.9454	8.6079	8.1781	8.5224	8.5569	8.6017	8.2232	8.4172	8.2534	8.3458	8.5283	8.5041	8.3260
Sptbn1	_EIGQSVDEVEK_.2	#N/A	7.7458	7.5412	7.5780	7.5412	7.3248	7.5865	7.6163	7.7778	7.3504	7.5151	7.7455	7.3706	7.7329	7.4379	7.1343	7.2662	7.6822	7.4845	7.2782	7.3980
Sptbn1	_VAVVNQIAR_.2	#N/A	8.3508	8.2666	8.2476	7.9283	8.2624	8.1881	8.4821	8.4409	8.3368	7.7634	8.2981	7.9642	8.4827	8.1005	7.9129	7.7398	8.1276	8.1199	8.1020	7.8633
Sptbn1	_FM[Oxidation (M)]ELLEPLSER_.2	#N/A	8.7072	8.2064	7.8316	7.8804	8.0936	8.2876	8.4689	8.6926	8.1814	8.2328	7.9335	8.3530	8.2344	7.9306	7.4856	8.0096	8.4251	8.6707	8.4298	8.0626
Sptbn1	_LLEVLSGER_.2	#N/A	8.5651	8.3362	8.2037	8.1867	8.0580	8.2943	8.6528	8.4219	8.1559	8.0977	8.5206	8.2567	8.1130	8.1795	7.3266	7.9492	8.3225	8.6167	8.1071	8.2336
Sptbn1	_IHC[Carbamidomethyl (C)]LENVDK_.2	#N/A	8.1437	8.2608	8.1001	7.8859	8.0466	8.1892	8.2691	8.1740	8.0894	7.9994	8.0205	7.8351	8.0032	7.9093	7.8648	7.7274	8.0032	8.0408	7.9103	8.1748
Sptbn1	_VLLLSQDYGK_.2	#N/A	8.3042	8.1466	8.0962	8.0693	8.3687	8.0121	8.1517	8.2377	7.8393	8.1370	8.1280	8.0511	8.2421	7.8626	7.3230	7.7821	8.3570	8.2045	7.6664	7.5601
Sptbn1	_EVDDLEQWIAER_.2	#N/A	8.0635	7.8049	8.2337	7.4731	7.4510	7.8780	7.5268	7.7011	8.1565	7.6725	7.8839	7.5845	8.2032	7.4441	7.4671	7.6075	7.8804	7.6253	7.5684	8.0273
Sptbn1	_EIEELQSQAQALSQEGK_.2	#N/A	8.0491	7.4585	8.1586	7.5470	7.9318	7.6533	7.3712	7.7670	7.6390	7.9271	7.9874	7.1906	7.2521	7.5854	7.0537	7.6148	8.1433	7.3988	7.4079	7.5190
Sptbn1	_ENEVLEAWK_.2	#N/A	8.1830	7.6421	7.8335	7.8703	7.4888	7.5894	7.6017	7.4719	8.2022	7.9378	7.9013	7.4212	8.3062	7.0032	7.4778	7.8886	7.5460	7.8426	8.0777	7.9242
Sptbn1	_SQNIITDSSSLNAEAIR_.2	#N/A	7.7689	7.3719	7.9261	7.7139	7.5760	7.5092	7.5987	7.8048	7.6190	7.8011	7.5994	7.3663	7.4442	7.4248	7.3136	7.5474	7.5552	7.4058	7.6457	7.3950
Sptbn1	_LVSDGNINSDR_.2	#N/A	7.9457	7.6073	7.8438	7.9233	7.6134	7.8424	7.8275	7.8754	7.8761	7.7052	7.8180	7.6220	7.3680	7.5879	7.4792	7.3956	7.8939	7.6664	7.6610	7.6013
Sptbn1	_KLPEELGR_.2	#N/A	7.4438	7.7132	7.5234	7.1621	7.5306	7.5246	7.8058	7.8711	7.6925	7.3267	7.7405	7.6776	7.1277	7.6059	7.3450	6.4456	7.3900	7.7192	7.7377	7.6163
Sptbn1	_LVDTGDKFR_.2	#N/A	7.5429	7.8266	7.4270	7.0541	7.5533	7.6649	7.6094	7.9245	7.4158	7.2138	7.1975	7.7348	6.9858	7.2588	7.4510	6.9898	7.7974	7.6221	7.4218	7.1481
Sptbn1	_LTTLELLEVR_.2	#N/A	7.5952	7.5820	7.5437	7.4810	6.9717	7.4805	7.6590	7.5282	7.3570	7.3635	7.3486	7.5560	7.3368	7.2962	6.9848	7.3809	7.3758	7.4275	7.2969	7.2903
Sptbn1	_ITDLYTDLR_.2	#N/A	7.7940	7.3082	7.1597	7.5778	7.4766	7.5247	7.5283	7.4138	7.1966	7.4319	7.4658	7.2193	7.6438	6.9722	6.5738	7.1430	7.3952	7.6851	7.6242	7.0060
Sptbn1	_VLDNAIETEK_.2	#N/A	7.4071	6.9964	7.6575	7.4382	6.9457	7.1717	7.3194	7.2921	7.5656	7.1269	6.9994	7.3371	7.3711	7.0634	6.9686	6.6605	7.6911	7.3029	7.1825	6.9073
Sptbn1	_SLLDAC[Carbamidomethyl (C)]EGR_.2	#N/A	6.9845	6.9998	7.3128	7.0078	6.9084	6.9948	7.3132	7.1927	7.1110	6.6312	7.4558	6.5642	6.7230	7.1126	6.8816	6.6336	6.5692	6.9137	7.0086	6.7477
Sptbn1	_VQAVVAVAR_.2	#N/A	6.9557	7.1548	6.7807	6.8111	6.3855	6.7758	7.3732	6.8759	7.0219	7.3737	6.8290	7.1773	7.3028	6.5565	6.6760	6.3660	6.4621	7.0965	6.8459	6.1278
Sptbn1	_IDDIFER_.2	#N/A	6.6938	6.7314	6.5265	7.1794	6.1376	6.9168	7.6803	6.4033	7.3818	6.9280	7.0738	7.0992	6.6567	6.4349	6.5199	6.6558	5.5540	7.4376	7.6123	7.5344
Sptbn1	_LVSQDNFGFDLPAVEAATK_.2	#N/A	7.0882	7.4967	6.9907	6.9361	6.4739	7.1125	7.0214	7.3938	7.0489	7.2852	6.9948	7.1815	7.0811	7.0872	6.4466	6.8724	6.9432	7.0825	7.0875	6.5913
Sptbn1	_LQALDTGWNELHK_.2	#N/A	6.3002	6.2421	6.1093	5.6433	6.2662	6.2004	6.0982	6.3197	5.4580	5.7329	6.4382	6.0570	6.1088	5.8652	6.0752	6.0335	5.9771	6.2272	5.7227	5.7193
Sptbn1	_TQTAIASEDM[Oxidation (M)]PNTLTEAEK_.2	#N/A	7.5729	6.5450	7.3700	6.3379	6.8694	7.1269	6.7147	7.2616	7.1625	7.3349	6.9157	6.9610	7.3832	5.9893	6.7279	6.9049	7.1866	6.9631	6.9410	6.9477
Sptbn1	_LQAAYAGDKADDIQKR_.3	#N/A	7.0727	7.1650	6.7127	6.2036	6.3800	6.7835	7.1319	6.8693	6.9252	6.8220	6.3079	6.9140	6.9904	6.1405	6.6565	6.9892	6.6149	7.0393	6.7351	6.7962
Sptbn1	_TAGYPNVNIHNFTTSWR_.3	#N/A	6.2418	7.1884	6.4510	7.0485	6.5161	6.8492	6.9832	7.0291	6.3007	6.2792	6.8305	6.5947	6.2011	6.7486	6.2285	6.4661	6.0958	6.9941	6.7541	6.5004
Sptbn1	_HLLGVEDLLQK_.2	#N/A	7.1973	6.9966	6.1575	7.0054	6.8158	6.9009	6.9297	7.2184	6.5988	6.7371	7.0803	6.6710	6.8626	6.9258	6.1518	6.3299	6.8645	7.2716	6.7823	6.5819
Sptbn1	_LSDGNEYLFQAK_.2	#N/A	6.6090	6.6125	6.9304	6.4001	6.1427	6.8658	6.6397	6.4739	6.8172	6.5471	6.5483	6.3827	6.4791	6.5322	6.3661	6.4373	6.8131	6.7761	6.1704	6.4391
Sptbn1	_EQWANLEQLSAIR_.2	#N/A	6.9108	6.6859	6.4079	6.7247	5.7084	6.9752	6.7511	6.7897	6.8691	6.8876	6.6958	6.4613	6.5006	6.1059	6.2929	6.5668	7.1704	6.3391	6.5485	6.3773
Sptbn1	_IVSSNDVGHDEYSTQSLVK_.3	#N/A	5.9555	5.6705	6.3704	5.7874	5.5394	5.5127	5.6749	5.6894	6.2756	5.6535	5.6743	4.9520	6.4077	5.5146	5.7228	6.1950	6.5115	6.0110	5.9190	6.4109
Sptbn1	_EAEKLESEHPDQAQAILSR_.3	#N/A	5.8444	5.3978	6.3415	4.7951	5.8141	5.2882	4.8706	6.1326	5.4800	5.8181	5.4436	5.4374	6.1119	5.9078	5.4935	5.6326	5.9709	5.8405	5.1265	5.3952

Sptbn1	_LQAAYAGDK_.2	#N/A	5.1155	4.8000	5.7225	4.0188	5.3162	5.2238	6.1645	4.2118	5.9626	5.6196	5.3659	5.1765	4.9761	4.6929	5.4811	4.5331	5.6579	5.1976	5.6710	5.6227
Sptbn1	_QLWGLLIEETEKR_.2	#N/A	4.0337	4.7686	4.6119	3.6037	4.1113	3.8183	4.3676	4.2683	4.1741	4.5732	4.9931	4.0955	4.7271	4.6222	3.9376	4.3325	5.2074	4.6370	4.3055	4.0110
Sptbn1	_RPPS[Phospho (STY)]PDPNTK_.2	#N/A	14.8426	14.7215	14.5686	14.6182	14.2768	14.5440	14.5481	14.8918	14.4121	14.3580	14.4084	14.4992	14.9359	14.7733	13.6966	14.2947	14.7729	14.7172	14.5026	14.3601
Sptbn1	_TSS[Phospho (STY)]KESS[Phospho (STY)]PVPS[Phospho (STY)]PTLDRK_.3	#N/A	10.2844	11.5460	11.5066	11.6245	3.7720	11.2816	11.4345	10.5461	11.3585	11.7255	11.2599	10.5649	10.0185	10.4196	9.1991	10.6373	11.4468	11.6735	11.7505	9.8402
Sptbn1	_[Acetyl (Protein N-term)]TTTVATDYDNIEIQQQYSDVNNR_.3	#N/A	12.1745	11.9132	12.1085	12.0593	11.2316	11.8002	11.5367	11.7343	11.7425	11.8577	11.8186	11.1059	12.1593	11.9128	10.6244	11.7251	12.1779	11.7774	11.7705	11.5273
Sptbn1	_ESS[Phospho (STY)]PVPSPTLDR_.2	#N/A	11.0845	11.0542	11.6790	3.8618	3.8401	11.3416	10.9666	9.6125	9.1840	10.6819	9.1755	10.9619	4.1015	11.3778	10.7673	8.6721	12.0006	10.7636	9.1255	10.5634
Sptbn1	_FQIQDISVETEDNK_.2	#N/A	8.1597	8.0824	8.0993	8.1627	3.9695	7.6979	7.4970	8.2502	7.8210	7.7028	7.9472	7.6560	8.2774	7.8512	6.4940	7.1731	8.3478	7.9684	7.7077	7.5128
Sptbn1	_EIQGHQPR_.2	#N/A	3.5688	5.5007	6.0622	6.1181	6.1354	5.5063	5.9102	6.6125	6.3310	6.2536	6.1938	6.2260	5.7143	5.7065	6.4599	6.1598	5.9670	5.8088	6.1252	6.0294
Sptbn1	_QIEAQEKPR_.2	#N/A	5.2540	3.9309	3.2866	4.3608	4.1287	4.8010	4.6350	5.1976	5.4003	4.6234	5.2254	4.0232	4.3821	3.9545	5.4814	5.0837	4.6502	5.2869	5.2273	4.7868
Sptbn1	_QALQDTLALYK_.2	#N/A	4.4029	4.7818	3.9943	5.0256	4.9476	4.7331	4.6705	4.0389	4.6380	4.4652	4.4844	4.6995	4.0487	4.6806	3.7048	5.3704	5.4715	5.2905	3.4640	4.3382
Sptbn1	_DLDDFQSWLSR_.2	#N/A	5.7077	6.6458	6.7363	6.6881	3.4183	6.8246	6.4486	6.3314	7.0848	5.9888	3.0286	6.1485	5.9906	6.6596	6.6120	6.2476	6.9062	6.8093	6.8690	6.4912
Sptbn1	_HQILEQAVEDYAETVHQLSK_.3	#N/A	6.9631	5.6234	3.3590	5.6690	6.6316	6.2216	5.3203	6.4257	6.1947	6.4784	6.3564	5.5255	4.3442	5.0623	6.1374	5.2562	6.6476	6.1537	6.0303	6.5900
Sptbn1	_SAATWDER_.2	#N/A	5.2911	5.5855	4.9660	5.5842	5.2135	6.0471	5.6829	4.7631	4.0262	5.6231	5.7650	5.8132	5.8455	5.0552	6.0395	5.8447	4.4090	5.8747	5.5694	5.6153
Sptbn1	_LPEELGR_.2	#N/A	5.9497	5.8087	5.1429	5.3986	5.0474	5.8645	5.9985	6.1693	5.5484	4.4629	5.0894	5.5240	3.3409	4.3855	5.5867	5.4141	5.5551	6.0197	5.6360	5.4281
Sptbn1	_AQTLPTSVVTITSESS[Phospho (STY)]PGKR_.3	#N/A	8.3558	9.4835	6.3695	6.3487	6.3890	5.7557	8.9967	5.6262	7.3045	6.0447	6.7847	8.2394	8.4631	9.8375	5.6952	6.9296	7.7013	9.1010	7.7562	5.7700
Sptbn1	_FATDGEGYKPC[Carbamidomethyl (C)]DPQVIR_.3	#N/A	6.1269	5.9291	4.2430	6.7667	6.6524	6.0533	6.3306	6.4920	5.9731	6.4240	7.0903	5.8649	3.2364	6.7587	6.1160	5.8510	5.9480	6.8333	6.2260	5.3972
Sptbn1	_EAIC[Carbamidomethyl (C)]EVALDYK_.2	#N/A	6.4005	6.0885	6.4509	4.5088	4.0191	5.7783	6.0602	5.6158	6.1513	6.2238	6.6688	5.9633	3.6891	6.0100	5.3338	5.3639	5.7740	5.9849	5.3278	6.2923
Sptbn1	_ILSSDDYGK_.2	#N/A	3.5856	4.7122	3.9380	5.2224	3.7691	5.0525	4.4510	4.0948	4.0659	4.3035	3.7658	3.3082	3.3412	3.4387	4.4297	3.7734	3.3086	3.8784	4.3663	3.9755
Sptbn1	_HRPDLIDFDK_.3	#N/A	6.7217	6.3327	4.5238	6.1502	6.7700	6.2054	5.9552	6.5777	6.4480	6.8164	7.2424	5.9657	6.8934	6.1219	6.0299	6.1279	6.0199	6.5805	6.6141	6.2125
Sptbn1	_SAASGIPYHSEVPVSLK_.3	#N/A	6.5978	6.1655	6.6884	6.2374	6.2010	6.4933	6.6208	5.9902	6.3624	6.1136	6.9378	6.5455	5.9765	6.2560	5.7904	6.8528	6.3043	3.6357	6.1717	6.1091
Sptbn1	_FANSLVGVQQQLQAFNTYR_.2	#N/A	4.5113	4.8807	4.5428	5.0709	4.4242	5.0277	4.8434	4.4471	4.6983	4.2284	4.5540	4.2708	5.3447	5.2293	3.9004	4.5345	4.6832	5.3023	4.6088	4.6948
Sptbn1	_VIESTQDLGNDLAGVM[Oxidation (M)]ALQR_.3	#N/A	6.0915	6.2760	6.2810	5.9453	5.9776	6.1334	5.8652	6.4579	6.3336	6.1999	5.9022	5.7088	6.5314	5.9992	5.7978	6.0367	6.2534	6.3565	6.2440	6.0978
Sptbn1	_LQAAYAGDKADDIQK_.2	#N/A	4.9938	4.4513	4.3474	4.1379	2.8892	4.9282	3.7673	3.2518	4.2988	4.2307	4.4451	4.3408	3.5722	4.2254	4.0768	3.9093	4.3941	3.1823	3.7337	2.5517
Sptbn1	_ESS[Phospho (STY)]PVPS[Phospho (STY)]PTLDRK_.2	#N/A	10.1015	10.0487	10.2284	9.9479	6.8013	10.1191	9.6414	10.2043	10.0954	9.8615	9.8312	9.9767	9.8782	10.2907	9.4249	9.6540	10.5970	9.9706	10.0266	9.7754
Sptbn1	_FESLEPEM[Oxidation (M)]NNQASR_.2	#N/A	7.9475	8.0714	8.0627	7.9316	7.3102	7.8578	7.8666	7.9666	7.9580	7.4448	7.7726	7.5198	7.9227	7.8537	7.2873	7.3551	7.9722	7.7671	7.4717	7.3861
Sptbn1	_DDEEMNTWIQAISSAISSDKHDTSASTQS[Phospho (STY)]TPASSR_.4	#N/A	3.6094	4.0897	4.2668	4.3549	2.9472	3.1712	3.0931	3.4550	3.1522	3.8158	3.5265	3.8159	3.2602	7.1579	4.1711	3.9543	6.7184	4.1972	2.9954	3.9821
Sptbn1	_INAVVETGR_.2	#N/A	3.3153	4.7080	5.5190	4.6179	3.5223	4.6962	5.3346	3.5410	5.2811	5.1921	5.6580	5.0575	3.3462	4.3908	5.4412	5.2270	5.2422	4.9388	4.7567	4.0681
Sptbn1	_AQTLPTSVVTITSESSPGK_.2	#N/A	4.2903	4.7455	6.0878	4.2504	3.6280	4.0193	3.9576	4.2481	4.7169	4.3497	4.0887	3.5371	4.3078	3.5851	4.0445	3.2817	4.6047	4.3698	3.9210	3.9376
Sptbn1	_STDEVDSKR_.2	#N/A	4.5172	3.9561	4.2220	4.0172	4.3761	4.4476	5.1905	4.2175	4.9028	3.4503	3.4197	4.3000	4.5379	4.0789	2.9768	3.3612	4.2484	3.9987	2.9791	4.1287
Sptbn1	_TSS[Phospho (STY)]KESS[Phospho (STY)]PVPSPTLDR_.2	#N/A	8.0942	4.0548	8.9687	6.6924	3.5208	8.4825	7.9524	3.7919	8.3768	7.9156	3.8833	8.0161	8.1616	8.9074	3.7083	8.4523	8.8177	8.1275	8.3018	3.9330
Sptbn1	_GDQVSQNGLPAEQGS[Phospho (STY)]PR_.2	#N/A	11.4215	10.9910	7.9674	11.4076	10.8935	11.0158	10.6146	11.6626	10.8700	10.8169	11.4437	8.3785	10.7183	11.4467	10.0880	11.1547	11.4804	10.7239	11.3368	11.3035
Sptbn1	_EGM[Oxidation (M)]QLISEKPETEAVVK_.3	#N/A	6.1697	4.8398	6.0120	5.6216	5.4943	6.0682	5.9723	6.1595	6.3022	5.9855	6.1397	5.7387	6.5892	6.0401	5.0842	6.2933	5.9650	5.9875	6.0244	6.1772
Sptbn1	_DLVAIEAK_.2	#N/A	3.7632	3.1723	3.8140	3.2149	3.8122	3.8506	4.5872	4.0908	4.8407	5.3337	4.3384	3.5466	3.8960	4.0603	3.4289	4.8365	3.5880	4.2641	4.3318	4.5593
Sptbn1	_ELEAENYHDIKR_.3	#N/A	5.4247	5.4609	5.4599	5.5985	5.4079	5.7915	5.3707	5.5187	5.8886	5.4130	5.5808	5.2620	5.0270	5.2346	5.2748	5.6136	5.2381	5.5323	5.5769	5.5792
Sptbn1	_LILEVHQFSR_.2	#N/A	5.7833	5.4989	4.9684	4.8712	5.2702	4.8916	5.3008	6.1514	5.0340	5.4498	5.3335	5.1784	5.5256	4.3013	4.7841	4.6754	5.4808	5.5206	5.1074	5.0631
Sptbn1	_LISDINK_.2	#N/A	3.3603	4.1565	4.3144	4.2239	4.4628	4.2372	5.5063	3.5860	4.2489	4.5791	4.6096	3.4658	3.5665	3.4809	4.5751	3.2503	2.5214	5.1390	5.0815	4.0976
Sptbn1	_HDTSASTQS[Phospho (STY)]T[Phospho (STY)]PASSR_.2	Q62261_T2319_M2_Sptbn1	5.9021	6.0526	6.2856	5.9755	5.5087	5.6688	5.0839	5.2518	5.6353	5.4666	5.8687	5.3675	5.0789	5.7718	3.8525	5.5076	5.5476	5.0140	5.2486	5.4057
Sptbn1	_QNLLSQSHAYQQFLR_.3	#N/A	4.7983	4.4265	4.6640	5.3802	4.9514	5.3309	4.9024	4.9288	4.7015	4.8812	5.0432	5.0013	5.3888	3.2626	4.5961	4.0587	4.7520	4.8516	4.8535	5.2314
Sptbn1	_DVSSVELLM[Oxidation (M)]NNHQGIK_.2	#N/A	3.8603	4.2661	3.3924	3.5669	4.1877	4.6234	4.1111	4.2452	4.3100	4.3350	4.3611	3.8731	4.1530	4.2887	3.4174	4.5342	4.3349	4.8090	4.5987	4.2436
Sptbn1	_VDTVNNM[Oxidation (M)]ADELINSGHSDAATIAEWK_.3	#N/A	4.6691	5.0506	4.6222	4.4191	4.4451	4.1077	4.5843	4.8984	4.2821	4.1446	4.0284	4.7050	4.7478	5.7093	3.8562	4.4126	5.1142	4.9148	4.5245	4.3427
Sptbn1	_EAASELLMR_.2	#N/A	3.1567	4.9440	5.0450	4.9100	3.3636	2.7256	5.0627	3.3824	2.6988	2.6342	5.2220	3.8885	3.1876	5.4419	3.6434	4.0196	4.3804	4.2698	3.0681	2.2279
Sptbn1	_DASVAEAWLLGQEPYLSSR_.2	#N/A	5.9094	5.7385	5.9577	5.8401	5.2121	5.7259	5.3237	5.9360	5.2829	5.6354	5.6966	5.5986	3.3474	5.5664	5.1368	5.3585	6.0974	5.6903	5.1516	5.3343
Sptbn1	_MWEVLESTTQTK_.2	#N/A	4.0769	5.0204	6.1510	5.2327	4.2838	5.1219	4.9745	4.3026	3.9997	4.4691	5.2522	2.9683	4.1078	5.6721	4.9969	3.4390	4.9325	3.3496	3.6487	3.3288
Sptbn1	_DALLSALSIQNYHLEC[Carbamidomethyl (C)]NETK_.3	#N/A	3.2400	3.6248	4.3434	3.8001	3.5718	3.4150	3.2486	3.6266	3.8459	3.6299	3.7579	2.7132	3.4916	2.8384	3.6705	3.7685	3.6617	3.6989	4.0405	3.9843
Sptbn1	_LAEISDVWEEMK_.2	#N/A	3.4683	5.1975	5.3705	4.8550	3.6753	2.6968	4.6754	3.1381	3.2930	3.2789	4.4972	3.5769	3.4585	5.2890	3.3982	4.3313	4.4606	4.1573	3.8059	3.9281
Sptbn1	_WDVDDWDNENS[Phospho (STY)]SAR_.2	Q62261_S35_M1_Sptbn1	3.4279	4.5749	3.9642	4.1734	4.2665	5.6818	3.2746	3.6365	3.3337	3.6343	3.7080	2.9635	3.4417	4.4863	2.8518	3.7728	3.1338	4.0157	4.3147	4.1636
Sptbn1	_GNLEVLLFTIQSK_.2	#N/A	4.0639	3.1475	4.1850	3.3873	3.4643	3.4977	3.6267	4.4503	3.5040	3.9642	3.4465	4.2436	3.6694	4.1721	3.6091	4.3006	3.8868	2.9962	3.7594	4.0342
Sptbn1	_RRPPS[Phospho (STY)]PDPNTK_.2	#N/A	3.1799	3.3221	3.7162	4.5095	4.0184	8.7486	3.5226	9.1121	8.5650	8.6424	3.9561	3.2115	9.1050	8.8642	3.0998	3.5248	3.3818	3.7676	8.3960	4.4117
Sptbn1	_VAHM[Oxidation (M)]EFC[Carbamidomethyl (C)]YQELC[Carbamidomethyl (C)]QLAAER_.3	#N/A	5.9890	5.7678	5.6439	5.2711	5.5783	5.8111	5.5164	6.2495	5.7269	6.4738	5.8594	5.7519	6.1121	5.7511	5.0020	5.5416	5.9356	5.4947	5.4717	6.2685
Sptbn1	_TQILAASYELHK_.2	#N/A	5.9525	6.5092	6.4552	6.4106	6.0440	6.4825	6.3812	6.6033	6.3928	6.2058	6.2443	5.0381	6.7136	6.0223	5.7450	5.7246	6.0184	6.7895	6.5350	5.5745
Sptbn1	_FQIQDISVETEDNKEK_.3	#N/A	4.4287	4.1883	3.7828	4.4562	4.2320	4.5399	4.6460	4.2665	4.2443	4.6666	4.8225	3.7486	3.8475	4.5890	3.5915	4.1376	3.8285	4.6960	4.6630	4.2709
Sptbn1	_RPPS[Phospho (STY)]PDPNTKVSEEAESQQWDTSK_.3	#N/A	5.7002	5.5671	5.1559	5.5956	4.0676	3.3135	4.4517	5.9753	5.3110	4.8942	3.6188	4.3532	6.2133	5.7540	4.6473	4.8262	5.1897	6.0680	3.9771	4.5533
Sptbn1	_TLETPAAQM[Oxidation (M)]EGFLNR_.2	#N/A	8.3338	8.0748	6.9376	7.2320	6.9024	7.0637	7.8814	8.4712	7.4241	7.6684	7.3107	7.8470	8.0413	8.0327	6.7533	7.1354	7.7799	8.0462	7.4462	7.4008
Sptbn1	_LLDPEDISVDHPDEK_.2	#N/A	7.2675	6.5528	6.5692	6.3962	6.9085	6.7236	6.7453	6.9744	6.5455	6.1324	6.9471	6.4442	6.6893	6.3440	5.7976	6.3852	6.8095	6.9205	6.3582	6.3288
Sptbn1	_EVVAGSHELGQDYEHVTM[Oxidation (M)]LQER_.3	#N/A	5.7178	5.8783	4.2544	5.2675	5.1524	4.9397	4.8143	5.9298	5.1809	5.0464	4.8468	4.8308	5.0676	5.7812	3.6840	5.1070	5.5507	5.3051	4.5751	4.8800
Sptbn1	_KHEAIETDIAAYEER_.3	#N/A	7.2198	6.5156	8.1680	7.2355	7.1002	7.2935	6.4424	7.6382	7.0716	7.0365	7.0873	6.5096	7.0121	5.5539	5.8083	6.9623	7.7665	5.8156	7.5154	6.9407
Sptbn1	_ITDLYTDLRDGR_.3	#N/A	3.2585	3.8667	3.7948	4.4308	4.0971	4.3212	4.1084	4.4598	3.9806	3.4649	3.8209	3.1329	3.6111	3.7819	4.1420	3.6034	3.4966	4.3366	3.8411	3.5355
Sptbn1	_LEMNLGLQK_.2	#N/A	4.1110	3.8918	3.1465	3.5784	3.4488	3.0353	3.3078	3.8114	4.1514	4.3174	2.5696	3.7812	4.1318	3.1336	3.6695	2.9551	4.3491	3.4841	3.1327	3.1006
Sptbn1	_M[Oxidation (M)]HTTFEHDIQALGTQVR_.3	#N/A	6.0226	5.2592	4.8109	5.2330	5.4347	5.0866	4.9468	5.8918	5.0834	5.0686	4.7164	5.2260	5.6731	4.4671	4.8839	5.2325	5.5806	4.9106	5.4863	4.9839
Sptbn1	_VSEEAESQQWDTSKGDQVSQNGLPAEQGS[Phospho (STY)]PR_.4	#N/A	7.9472	8.5319	8.3482	7.5219	3.7088	4.2761	4.3062	4.0715	8.1139	7.2638	3.7050	6.4433	4.3918	7.1213	6.5929	7.6520	3.1083	8.6834	7.8910	7.0325
Sptbn1	_IQEKVDSIDDR_.3	#N/A	4.0661	3.2530	3.6027	3.5181	4.2730	4.3301	4.2759	4.2918	4.5429	3.8767	3.6748	2.9791	4.3616	3.6407	2.8004	4.5289	3.7890	3.2543	3.6595	3.3304
Sptbn1	_IFQEM[Oxidation (M)]LYIM[Oxidation (M)]DWM[Oxidation (M)]DEM[Oxidation (M)]K_.2	#N/A	4.3605	4.4594	3.8971	3.2237	3.0666	3.1000	3.9815	3.0854	3.9017	4.1711	3.3803	4.1855	4.3914	3.9352	4.0068	3.7227	4.0835	3.0660	3.3650	3.6125
Sptbn1	_QAEAFLNNQEYVLAHTEM[Oxidation (M)]PTTLEGAEAAIK_.3	#N/A	2.7398	4.2184	3.2711	4.1353	4.0483	4.0402	3.3881	3.3948	3.4472	3.5264	3.8216	3.2259	3.5396	3.6063	2.9653	3.4152	3.4987	4.0130	3.9815	4.1502
Sptbn1	_HYASEEIKEK_.3	#N/A	4.2418	3.8377	3.2006	3.5242	2.9479	2.9812	4.1002	3.8655	4.0204	4.0524	2.2309	4.3043	4.1859	3.1877	4.1256	3.6039	4.4032	3.4300	3.0785	3.1547
Sptbn1	_SNAHYNLQNAFNLAEQHLGLTK_.3	#N/A	4.0154	4.4958	4.5517	3.2601	3.3532	3.5773	4.1879	3.0490	4.2470	4.2218	3.1205	3.8768	4.3550	3.2808	1.9365	4.3603	4.0471	3.1024	3.4014	3.5761
Sptbn1	_MLTAQDMSYDEAR_.2	#N/A	4.2768	4.5432	5.2883	3.3075	2.9829	3.0162	4.0652	4.5024	3.9854	4.0874	3.4641	4.2693	4.1509	5.0964	4.0906	3.6389	3.9997	3.1498	3.4488	3.5287
Sptbn1	_FFWEM[Oxidation (M)]AEEEGWIR_.2	#N/A	4.4783	4.3417	5.5048	3.1060	3.1844	3.3995	4.3420	3.2031	4.4011	4.0677	3.2746	4.0678	4.5091	4.0529	3.9192	3.8404	4.2012	4.4491	3.2473	3.7302
Sptbn1	_EAIC[Carbamidomethyl (C)]EVALDYKK_.3	#N/A	3.5312	4.4716	4.0675	4.1582	3.5818	3.6152	3.4663	3.2316	3.3865	3.1855	4.3660	4.3611	3.5519	4.0546	3.4916	4.2379	3.7693	4.0639	3.7125	4.0216
Sptbn1	_AQQYYFDAAEAEAWM[Oxidation (M)]SEQELYM[Oxidation (M)]M[Oxidation (M)]SEEK_.3	#N/A	3.8221	3.4969	3.3587	3.1046	4.0291	4.0624	3.0190	4.2852	4.4401	3.6327	3.9187	3.2231	4.6055	3.6073	3.0444	3.1843	3.3220	3.0103	4.1597	3.5743
Syp	_M[Oxidation (M)]ATDPENIIK_.2	#N/A	8.7883	9.1154	9.3106	9.3860	8.9131	9.0798	9.3694	9.1027	9.0202	8.6951	9.1621	8.7704	8.5019	8.7845	8.5618	8.1096	8.9263	8.9846	8.6051	8.6526
Syp	_LHQVY[Phospho (STY)]FDAPSC[Carbamidomethyl (C)]VK_.2	#N/A	9.5787	9.5201	9.2598	9.1284	8.7948	8.9479	9.4144	9.6907	8.8682	8.9241	8.9709	9.1958	9.5647	9.3080	8.2766	8.6581	9.4006	9.3469	9.0029	8.7362
Syp	_ETGWAAPFM[Oxidation (M)]R_.2	#N/A	8.4382	8.2352	8.1196	8.1029	7.9896	8.0487	8.4559	8.4216	7.9357	7.9681	8.0035	8.1861	8.2608	7.7387	7.5094	7.7103	8.2173	8.2800	7.8159	7.7394
Syp	_M[Oxidation (M)]LLLADM[Oxidation (M)]DVVNQLVAGGQFR_.2	#N/A	4.2962	4.5237	3.8328	3.5786	3.3041	3.0356	3.8025	3.8111	3.9660	4.1068	4.2975	4.2499	4.1315	3.1333	4.0712	3.6583	4.3488	4.0790	3.1329	3.1003
Tspan7	_AVDHVQR_.2	#N/A	6.6675	6.5111	6.6890	5.7897	6.1340	6.0414	6.5304	6.2619	6.2635	6.3540	6.8380	6.2115	6.2099	5.1182	6.4758	5.6257	6.1435	6.1463	6.1810	5.6686
Tspan7	_TYTDAM[Oxidation (M)]QNYNGNDER_.2	#N/A	6.1913	6.7105	6.3057	6.6868	5.6418	6.4467	6.5400	6.6724	6.2819	5.7431	6.5698	5.7563	5.9851	6.7535	5.0858	5.7265	7.3439	6.1456	6.1692	5.9975
Tgoln1;Tgoln2	_DADS[Phospho (STY)]GDSQNPPNQPSK_.2	Q62313_S28_M1_Tgoln1;Tgoln2	3.9614	6.7667	3.9148	6.0750	6.4409	7.0161	6.3440	6.6462	5.7420	6.4277	6.4968	5.9089	4.4091	6.6532	5.8856	4.3063	4.1011	3.0483	3.3474	3.6302
Tgoln1;Tgoln2	_TES[Phospho (STY)]GETLAGDSDFSLKPEKGDK_.3	#N/A	3.6543	4.3485	4.1906	4.0351	2.9920	5.1122	3.5893	3.3546	3.9145	4.5633	2.9882	4.2380	3.6750	4.1776	3.6147	4.3275	3.8923	3.9408	3.5894	4.1446
Tgoln1;Tgoln2	_TES[Phospho (STY)]GETLAGDSDFSLKPEK_.2	#N/A	3.7325	3.9847	3.8340	3.9099	3.5865	3.7792	3.3555	3.7299	3.2757	4.1492	3.5541	3.8168	3.4412	3.8392	3.6827	3.6485	3.7253	3.7867	3.7648	3.7085
Trim28	_S[Phospho (STY)]GEGEVSGLLR_.2	#N/A	4.5015	10.1296	10.5120	10.2211	3.8393	9.9067	9.7720	9.3797	10.0990	10.3752	10.0464	9.8151	9.9809	10.2262	9.4596	9.8422	10.2364	9.6859	10.2295	10.1625
Trim28	_SRS[Phospho (STY)]GEGEVSGLLR_.2	#N/A	7.6334	7.4210	7.1838	6.8630	6.3594	8.0679	6.7968	6.9092	7.3651	7.3791	7.7513	7.6032	7.4118	7.3628	6.5316	7.5651	7.9627	8.2880	6.9208	6.8389
Trim28	_[Acetyl (Protein N-term)]AASAAATAAASAATAASAASGS[Phospho (STY)]PGSGEGSAGGEK_.3	Q62318_S23_M1_Trim28	8.4373	10.0294	8.4443	3.8922	3.5017	8.2173	9.6344	9.9836	7.7999	8.7515	4.1234	9.3904	3.8448	10.8346	7.7137	7.9379	8.8784	9.5210	8.9959	7.6002
Trim28	_LSPPYSSPQEFAQDVGR_.2	#N/A	3.8714	4.1314	4.4077	3.8180	7.9561	3.2750	7.7187	7.9837	3.7267	4.0777	7.7806	4.0209	7.4336	8.1343	8.0585	4.2163	4.1094	6.8885	7.5373	5.8613
Trim28	_VS[Phospho (STY)]LERLDLDLTSDSQPPVFK_.3	Q62318_S489_M1_Trim28	4.1140	3.8888	3.1495	3.5754	3.4517	4.0074	4.0893	3.8144	4.1484	4.3203	3.0220	4.9572	4.1347	3.1366	3.6665	2.9581	4.3521	3.4811	3.1297	3.4046
Trim28	AAAASAAASS[Phospho (STY)]PAGGGGEAQELLEHC[Carbamidomethyl (C)]GVC[Carbamidometh	Q62318_S51_M1_Trim28	4.3852	3.6176	3.4206	3.3042	3.7229	4.2620	3.8182	4.0856	4.2405	3.0907	3.7191	3.5071	4.4059	8.8069	3.3954	3.2292	3.1224	3.2099	4.3593	3.3747
Trim28	_[Acetyl (Protein N-term)]AASAAATAAASAATAASAASGSPGSGEGS[Phospho (STY)]AGGEKR_.3	Q62318_S30_M1_Trim28	4.5686	5.8037	4.1052	4.5165	3.2747	3.3329	4.4323	3.2934	2.9906	3.9774	3.3649	3.9775	4.5994	4.1432	4.0095	3.9307	4.2915	5.1443	3.1570	3.8205
Snrnp70	_YDERPGPS[Phospho (STY)]PLPHR_.3	#N/A	8.0448	9.0181	8.7432	8.9957	8.4828	8.8562	8.9028	7.8810	8.7313	8.8315	8.7868	8.5930	8.9923	9.0652	7.6487	8.0467	8.6069	8.6430	8.1214	8.2938
Snrnp70	_GGGGS[Phospho (STY)]GQDNGLEGLGSDGR_.2	Q62376_S408_M1_Snrnp70	7.1120	7.4068	3.7951	7.5363	2.9646	7.2204	7.2155	7.2818	6.8839	7.2484	7.1004	6.9411	6.8526	7.8609	6.2718	6.9011	7.4871	7.0660	6.7071	7.0403
Tpd52	_NSPTFKS[Phospho (STY)]FEEK_.2	#N/A	11.2239	10.9256	10.9716	10.9484	10.2556	10.4223	10.1495	10.5866	10.1357	11.1511	10.6938	10.5309	10.8455	11.2339	9.9226	10.5996	10.7113	10.3405	10.5315	10.8055
Tpd52	_S[Phospho (STY)]FEEKVENLK_.2	#N/A	10.9573	10.7942	11.0187	10.9511	9.9719	10.2004	9.9861	9.9941	9.9653	10.9689	10.4681	10.2933	10.0901	11.1408	9.8169	10.4240	10.6565	10.0952	10.5109	10.5691
Tpd52	_NSPTFKS[Phospho (STY)]FEEKVENLK_.3	#N/A	9.0610	9.3891	8.5273	8.5974	7.7046	7.8363	8.6350	8.8827	7.9325	8.9347	7.9451	8.6808	9.3434	10.0157	7.8728	8.4338	8.5656	8.8058	8.5436	8.2811
Tpd52	_ASAAFSSVGSVITK_.2	#N/A	3.2199	3.2821	3.7562	4.2568	4.0584	3.0622	3.7463	4.4211	3.5417	3.4262	3.9161	3.1715	3.6498	3.7432	3.6803	3.1253	3.4579	3.3958	4.1067	4.3717
Tpd52	_PAGGDFGEVLNSTANATSTM[Oxidation (M)]TTEPPPEQMTES[Phospho (STY)]P_.4	Q62393_S223_M1_Tpd52	4.0025	4.4828	3.8737	3.2471	3.3402	3.5643	4.2008	3.0620	4.2599	4.2088	3.1335	4.2089	4.3680	6.6330	3.7780	4.3474	4.0600	3.0894	3.3885	3.5890
Speg	_SSS[Phospho (STY)]FSQGEAEPR_.2	Q62407_S2135_M1_Speg	10.1744	10.1281	10.3561	4.5181	9.4836	9.6413	9.6893	9.8019	9.6173	9.9063	9.4708	3.2201	10.0222	9.8595	8.4909	9.4438	9.6003	9.5686	9.4695	9.5127
Speg	_AAS[Phospho (STY)]VELPQR_.2	#N/A	9.7487	9.6432	9.6163	9.3236	8.7834	9.1741	9.1311	9.0981	8.7844	9.2290	9.3002	8.7638	9.0089	8.9345	7.6042	8.8002	9.1289	9.0800	8.8727	9.1338
Speg	_ARS[Phospho (STY)]LEQPK_.2	Q62407_S439_M1_Speg	8.7942	8.8819	8.6283	8.9544	8.2823	8.6614	8.6002	9.0627	8.6064	8.7938	8.6063	8.6943	9.2653	8.9673	8.3590	8.5935	8.9102	8.8396	8.5238	8.5470
Speg	_AAS[Phospho (STY)]VELPQRR_.2	#N/A	7.2261	7.4116	6.9039	7.4489	7.2073	7.0625	6.8999	6.6057	6.5702	6.8387	7.2490	6.9202	7.3235	7.1904	5.6102	6.9180	7.2882	6.9482	6.9625	6.7276
Speg	_AS[Phospho (STY)]PVLAVR_.2	Q62407_S2451_M1_Speg	6.6460	6.5051	6.1676	6.2398	4.0200	5.1174	4.8687	5.2172	5.5554	6.0684	5.3263	5.7744	4.2993	5.0461	4.4699	5.2664	5.3786	4.8103	4.4551	4.7189
Speg	_SRS[Phospho (STY)]VQDLR_.2	Q62407_S2396_M1_Speg	7.0742	4.5616	7.4626	4.2482	6.6044	3.0030	3.2613	3.1416	3.3204	3.6475	3.6948	6.5525	3.4284	3.9646	5.3799	6.3792	3.6793	7.1075	6.7544	6.7862
Speg	_SATS[Phospho (STY)]DLELR_.2	Q62407_S493_M1_Speg	3.2462	8.0447	7.3957	8.1286	3.4531	7.7503	3.5950	6.6497	3.5152	7.7625	2.9938	3.7990	7.2538	4.3217	3.6203	4.1091	4.4699	7.7573	2.9786	7.5725
Speg	_LSPS[Phospho (STY)]QDHDSSDSSSK_.2	Q62407_S860_M1_Speg	5.8826	6.7037	7.0353	4.4525	5.5216	3.9095	5.8615	5.4054	5.1024	6.2278	4.0716	5.2079	3.2576	5.0686	4.3257	5.5338	5.2051	5.6704	5.7870	3.7272
Speg	_ATS[Phospho (STY)]EGESLRR_.2	Q62407_S2499_M1_Speg	4.2470	6.4332	3.7836	3.3372	2.9531	2.9864	5.6304	4.4727	4.0152	4.0576	3.4938	4.2990	6.1134	3.8216	4.1204	3.6091	5.1946	5.7212	3.4786	3.1495
Speg	_SEGGAAWGT[Phospho (STY)]PEASQEELR_.2	Q62407_T453_M1_Speg	5.7721	6.4998	6.7219	6.2782	5.4443	6.7751	6.2003	6.0398	6.6800	5.7080	5.8539	5.3029	5.9184	6.6174	5.7789	5.9345	6.2749	6.3849	6.5330	6.6543
Speg	_SEGGAAWGT[Phospho (STY)]PEAS[Phospho (STY)]QEELRSPR_.3	#N/A	4.0487	3.9541	4.5850	6.1191	3.3865	3.6105	4.1546	3.7491	4.2137	4.2551	3.0872	3.8435	4.3218	4.5721	3.7318	4.3936	4.2868	3.5464	3.1950	3.0383
Speg	_S[Phospho (STY)]TS[Phospho (STY)]REELVR_.2	Q62407_S509_M2_Speg	3.5607	3.3843	3.5817	3.8741	5.3373	5.5275	3.2587	5.9087	4.0519	4.2893	4.1668	5.4786	5.8452	3.4064	4.5419	3.3976	3.3222	3.4747	4.1431	4.0160
Speg	_AAS[Phospho (STY)]SEAAPHHQPPPESR_.3	Q62407_S2114_M1_Speg	4.0979	5.7744	3.3710	3.5602	4.6755	5.1531	5.6575	4.0485	4.2635	3.8192	3.7421	3.4019	4.1230	3.3847	3.2622	3.1884	4.1359	3.4313	3.4668	5.0732
Speg	_RGSS[Phospho (STY)]AESALPR_.2	Q62407_S2020_M1_Speg	4.2995	6.9078	3.3349	3.3899	3.6372	3.8613	3.9039	3.9998	3.9630	4.5058	4.3373	3.5928	4.3202	3.3220	3.4811	3.1435	4.5375	7.1458	2.9442	3.2890
Speg	_RLS[Phospho (STY)]STLER_.2	Q62407_S2461_M1_Speg	3.7987	3.3946	4.6250	4.5820	4.0056	4.0390	3.0425	4.3086	4.4635	3.6093	3.9422	3.2840	3.1281	3.6308	3.0678	3.1608	3.3455	2.9869	4.1363	3.5978
Speg	_MPS[Phospho (STY)]IPEEPEHGDLER_.3	#N/A	3.9001	3.9110	3.8635	4.0779	3.5577	3.7011	3.7493	3.8389	3.7440	3.6089	3.7805	3.7060	3.8782	6.1765	3.9692	3.6829	4.0765	3.8017	3.6428	3.8084
Speg	_QRS[Phospho (STY)]ATS[Phospho (STY)]DLELR_.2	Q62407_S490_M2_Speg	4.3295	3.6734	3.3649	3.3599	3.6672	3.8913	3.8739	4.2358	3.9330	4.5358	4.3074	3.5628	4.0410	3.5848	3.4511	3.1735	3.7331	3.4164	3.7154	3.2621
Ppp1r13b	_SS[Phospho (STY)]ITEPEGPGGPNIQK_.2	Q62415_S708_M1_Ppp1r13b	4.3708	4.4491	3.9074	3.2134	3.0769	3.5306	4.2345	3.0956	4.2936	4.1751	3.1672	4.1752	4.4017	3.9455	3.8117	3.7329	4.0937	3.0557	3.3548	3.6227
Ppp1r13b	_VNGTSSPQS[Phospho (STY)]PLSTSGR_.2	Q62415_S335_M1_Ppp1r13b	3.2943	3.2076	3.8306	4.3950	5.6967	3.8520	3.4082	4.4956	3.4673	3.5007	3.8416	3.0971	3.3151	3.8177	2.9854	3.6392	3.5324	4.3008	3.9493	3.7847
Sorbs1	_AGEQDPVPTPAELTS[Phospho (STY)]PGR_.2	#N/A	3.7095	3.6096	3.2461	3.8747	3.9164	3.9498	3.1317	3.9352	3.0519	3.5201	9.2256	3.3357	3.2173	3.7200	3.1571	3.0716	9.5694	4.3985	9.0515	9.3618
Sorbs1	_SATVS[Phospho (STY)]PQQPQAQQR_.2	Q62417_S1209_M1_Sorbs1	3.0941	9.4167	9.5379	9.7329	9.3519	9.0236	8.9030	9.0828	9.0925	9.4082	8.6448	3.9511	9.5676	9.3798	8.5406	8.9783	9.1003	3.7831	8.7065	8.9504
Sorbs1	_NNNPQSELAAAHGDSES[Phospho (STY)]PR_.3	#N/A	7.4685	7.2225	8.4836	7.3666	7.1076	7.0618	6.9768	6.9190	7.1396	7.4533	7.0495	7.1758	6.8014	7.7777	6.9119	6.9750	7.4544	3.8636	7.0954	7.2159
Sorbs1	_S[Phospho (STY)]AESLLESTK_.2	Q62417_S722_M1_Sorbs1	7.5889	7.6870	7.5068	8.3839	3.3737	7.6159	7.7297	7.2122	7.5368	8.9179	7.8805	7.4948	8.2039	8.3973	7.1299	7.9900	7.7095	7.8850	7.9924	8.3442
Sorbs1	_EGS[Phospho (STY)]GGSVHGDFPK_.2	Q62417_S791_M1_Sorbs1	6.3533	6.0156	5.9592	3.4306	7.3337	5.5531	5.4638	4.1768	5.9233	6.2195	7.6629	5.6500	5.8656	8.3116	2.8195	7.7345	6.4165	4.9778	7.5341	5.7792
Sorbs1	_RAGEQDPVPTPAELTS[Phospho (STY)]PGR_.3	#N/A	4.4319	3.4113	3.4444	9.9327	3.7696	3.9937	3.7715	4.1335	3.8306	10.0871	9.1020	9.0170	3.9386	3.4824	3.3487	3.2759	3.6307	9.1045	3.8178	3.1597
Sorbs1	_SAT[Phospho (STY)]LPLPAR_.2	Q62417_T286_M1_Sorbs1	4.0199	7.9636	8.2217	3.3024	4.2269	4.2602	7.0950	4.0874	7.8522	3.8305	7.6610	3.5053	4.4077	3.4095	2.8466	7.5767	3.1242	7.1128	7.6667	7.7032
Sorbs1	_S[Phospho (STY)]M[Oxidation (M)]PSLDFSGR_.2	#N/A	8.1205	8.4111	7.8948	8.6737	7.9613	8.0138	7.9269	7.6320	7.4386	8.4397	8.2526	7.4041	8.1588	8.5703	6.7766	7.7491	8.1521	7.7961	7.8530	7.9152
Sorbs1	_GTPSSSPVSPQES[Phospho (STY)]PKHESK_.3	#N/A	10.4567	10.6774	10.5091	10.7519	10.1405	10.2231	10.3130	10.4267	10.3779	10.8731	10.3026	10.4630	11.0736	10.8922	10.3257	10.6240	10.6139	10.3845	10.4279	10.5568
Sorbs1	_SKS[Phospho (STY)]EM[Oxidation (M)]NYIEGEK_.2	#N/A	7.2430	5.9045	3.7705	6.4532	4.0728	6.9128	7.2716	7.1088	7.0743	7.2343	6.1661	3.1706	3.6488	4.0621	6.8963	6.9429	6.8939	3.8220	4.1211	4.6962
Sorbs1	_GTPSSSPVS[Phospho (STY)]PQESPK_.2	Q62417_S58_M1_Sorbs1	7.5079	3.8847	4.0634	8.3494	7.7828	7.5950	8.0079	7.2886	4.1240	8.4698	8.2950	7.5842	4.2594	8.3869	3.9484	7.7323	7.8352	7.7614	7.7560	8.0500
Sorbs1	_DIS[Phospho (STY)]PEEIDLK_.2	#N/A	4.4074	8.3259	8.7261	7.0439	3.7452	8.2515	8.0090	4.1078	7.8384	7.8995	4.2294	6.9751	4.4282	9.0645	3.3731	7.3458	7.7927	8.0755	7.8533	8.0539
Sorbs1	_SVLEGGDIPLQGLSGLK_.2	#N/A	4.4946	3.7463	4.8041	4.0114	5.2140	3.1259	3.2684	3.7985	4.5561	3.3834	4.1680	4.0886	3.3540	3.8567	3.2937	4.0840	3.2957	4.2618	3.9104	4.6318
Sorbs1	_S[Phospho (STY)]PTPVLSR_.2	Q62417_S707_M1_Sorbs1	5.2913	3.8084	5.2049	3.7336	3.7626	3.6965	4.1161	3.9061	4.9511	5.4385	4.8925	3.6404	4.2833	4.0156	3.6933	3.8249	3.9016	5.0182	3.5886	3.8849
Sorbs1	_SSS[Phospho (STY)]LKSSPER_.2	Q62417_S295_M1_Sorbs1	3.7906	5.1368	4.3269	3.8987	3.1284	3.3557	4.4127	3.4910	4.4278	4.4269	3.1246	4.1016	3.8114	4.9268	3.9899	4.1355	4.0287	4.7820	3.4530	3.2288
Sorbs1	_RES[Phospho (STY)]DGTPGGLASLENER_.2	Q62417_S432_M1_Sorbs1	3.8741	4.1287	4.4104	3.8153	3.2118	2.9719	4.3293	3.5744	3.7293	4.0804	3.2080	4.0182	3.8948	4.3974	3.9065	4.2190	4.1121	3.7210	3.3696	4.3644
Sorbs1	_YS[Phospho (STY)]FSDDTKSPLSVPR_.3	#N/A	4.2380	3.7648	3.2734	3.4514	3.5757	3.7998	3.9654	4.3273	4.0245	4.4443	2.8980	3.6543	4.1325	3.6763	3.5426	3.0820	3.8245	3.3249	3.6239	3.3536
Sorbs1	_AQS[Phospho (STY)]C[Carbamidomethyl (C)]ENLC[Carbamidomethyl (C)]VSLNDSK_.2	#N/A	4.0601	3.9427	4.5964	3.6293	3.3979	3.6219	4.1432	4.5052	4.2023	5.7384	3.0758	3.8321	4.3104	3.8541	3.7204	4.4050	4.0024	3.1470	3.4461	3.5314
Sorbs1	_SAQDLSDVST[Phospho (STY)]DEVGIPLR_.2	Q62417_T213_M1_Sorbs1	3.8955	3.7863	3.1681	3.7633	3.6722	4.7697	3.7188	3.8456	3.5740	3.9448	3.7760	3.6042	3.7394	4.2421	3.4645	3.8958	3.9341	4.0017	3.6503	3.4317
Sorbs1	_DDDSDVHS[Phospho (STY)]PR_.2	Q62417_S250_M1_Sorbs1	3.2355	3.2665	3.2901	4.4539	4.0741	3.9109	3.1706	4.4367	3.0908	3.4812	4.0702	3.1559	3.2562	3.7589	3.1959	3.0327	3.4736	4.3596	4.0082	3.7259
Sorbs1	_YS[Phospho (STY)]FSDDTK_.2	#N/A	4.3049	4.5150	3.8415	3.2793	3.0110	3.0444	4.0371	4.5306	3.9573	3.3724	3.9348	4.2411	4.1227	3.8796	4.0625	3.6670	4.0278	3.1216	3.4207	2.3477
Sorbs1	_AQS[Phospho (STY)]C[Carbamidomethyl (C)]ENLC[Carbamidomethyl (C)]VSLNDSKR_.3	#N/A	3.8537	5.8283	4.0225	3.0983	3.1914	3.4155	4.3497	3.2108	4.4088	4.0600	3.2823	4.0601	4.5168	5.8515	3.9269	4.1986	4.2088	4.4414	3.2396	3.7379
Sorbs1	_TPVDYIDLPYSSS[Phospho (STY)]PSR_.2	Q62417_S1201_M1_Sorbs1	3.3271	3.9919	4.3646	4.2571	3.5341	3.5674	3.5140	3.5528	3.4342	3.1377	2.9129	3.7181	3.3580	9.1917	3.5394	4.1901	4.5509	8.2644	4.3984	4.0799
Sorbs1	_DDDSDVHSPRYS[Phospho (STY)]FSDDTK_.3	#N/A	4.4923	3.5105	4.8078	3.1971	3.8300	4.1549	4.4274	4.1927	4.3476	3.7252	3.8262	3.3999	4.5130	3.5149	2.9519	3.3364	3.2295	3.1028	4.2522	3.4818
Sorbs1	_VGGS[Phospho (STY)]IENLLMR_.2	Q62417_S576_M1_Sorbs1	3.9539	4.2679	4.3118	3.7355	3.2916	3.1925	4.2495	3.6543	4.3086	4.1602	3.1821	3.9384	3.9746	5.9154	3.8267	4.2988	4.1919	3.6412	3.2898	4.4443
Sorbs1	_DIS[Phospho (STY)]PEEIDLKNEPWYK_.3	#N/A	3.4168	4.2697	4.2111	4.2226	3.9915	3.6114	3.4710	3.3887	3.3912	3.1818	3.7887	4.0942	3.5567	4.2269	3.4964	4.0292	4.2030	4.0963	4.1061	4.0369
Sorbs1	_GELGAS[Phospho (STY)]QEGSEHIPK_.2	Q62417_S666_M1_Sorbs1	3.9889	4.0139	4.5252	3.7005	3.3266	3.5507	4.2145	3.6893	4.2736	4.9429	3.1471	3.9034	4.3816	4.5122	3.7917	4.3338	4.2269	3.0758	3.2548	3.6027
Dbnl	_NGPALQEAYVR_.2	#N/A	6.5919	6.3242	6.4422	6.4268	6.2554	6.2453	6.0765	6.7369	6.4399	5.9826	6.3881	6.3271	6.2984	6.8899	6.5870	6.1096	6.0699	6.2995	5.9198	6.7186
Dbnl	_VAGTGEGGLEELVEELNSGK_.2	#N/A	4.2162	4.8131	5.0509	5.0227	4.2030	5.2280	5.2929	5.4596	4.7508	5.2057	5.1828	4.8704	4.7198	5.4649	4.4733	5.8239	5.6272	5.3416	5.4176	5.0440
Dbnl	_ESTSFQDVGPQAPVGSVYQK_.2	#N/A	4.5634	4.7580	4.8665	4.6736	4.7616	4.8845	5.0941	5.0927	5.3570	5.0106	4.3217	5.1011	5.6422	5.0999	5.2470	4.9605	5.3322	4.6765	4.6541	4.5794
Dbnl	_SPTDWALFTYEGNSNDIR_.2	#N/A	4.0630	5.1418	4.4116	5.1421	3.5912	4.9826	3.3789	4.5185	4.4424	4.7980	5.0440	5.0449	4.4480	4.7983	4.6460	5.1465	5.6534	4.1245	4.7825	4.6891
Dbnl	_TRQEWESAGQQAPHPR_.3	#N/A	3.7175	7.1704	4.2538	3.9718	6.1976	6.8364	7.0963	6.9008	6.5802	6.5368	5.7907	7.1473	7.1494	7.5055	6.8934	6.9162	3.9556	7.2865	6.3762	6.3073
Dbnl	_VVTEKS[Phospho (STY)]PTDWALFTYEGNSNDIR_.3	Q62418_S24_M1_Dbnl	3.3625	4.4642	4.0285	4.3354	3.8236	3.3714	3.2061	4.1960	3.2159	4.1294	3.8281	3.3612	4.9583	5.9948	4.7232	4.0641	3.9267	5.4441	4.6814	3.9818
Dbnl	_VKDPNSGLPK_.2	#N/A	4.2144	6.4834	3.2498	3.4750	6.2628	4.4328	3.9890	5.2298	5.9921	4.0031	3.5483	5.9764	4.2351	3.2369	6.3872	6.2170	5.7013	6.3630	5.6668	5.9349
Dbnl	_FVLINWTGEGVNDVR_.2	#N/A	4.6739	3.4022	3.6360	4.5896	4.6290	5.6341	3.6028	4.8488	3.6619	3.3061	4.7613	4.9069	3.1205	5.0004	4.5608	4.6914	3.3378	4.6440	4.6952	4.5675
Dbnl	_AMS[Phospho (STY)]TTSVTSSQPGK_.2	Q62418_S277_M1_Dbnl	3.7256	5.4439	6.7707	6.6395	4.6447	3.4200	4.8190	3.7153	3.8526	3.7683	5.1539	3.7261	3.7724	5.9232	4.2515	3.7755	4.3942	3.9960	3.8175	3.7868
Dbnl	_QLT[Phospho (STY)]QPETSYGR_.2	Q62418_T299_M1_Dbnl	3.4085	4.5052	7.4168	4.1918	3.6154	3.6487	4.3427	3.1980	3.3529	3.2191	4.3324	4.3946	3.5184	4.0210	3.4581	4.2714	3.7357	4.0975	5.4622	3.9880
Dbnl	_AEEDVEPEC[Carbamidomethyl (C)]IMEK_.2	#N/A	3.9596	4.6016	5.2267	3.6247	4.1665	3.9419	3.8233	4.1852	3.8824	4.5864	4.2567	3.0857	3.9904	5.5533	2.9070	3.3217	3.6825	3.4670	3.7660	3.2115

Dbnl	_ASGANYSFHK_.2	#N/A	3.9509	4.0519	4.4872	3.7385	3.2886	4.8102	4.2525	3.6513	4.4815	4.5172	3.2848	3.9414	3.9716	4.8069	3.8297	5.0619	4.1889	3.6442	3.2928	4.4413
Sh3gl1	_ITAS[Phospho (STY)]SSFR_.2	Q62419_S286_M1_Sh3gl1	4.2186	4.6013	3.7552	3.3656	2.9247	4.4589	4.1234	4.4443	4.0436	4.0292	3.9343	4.3274	4.2090	3.7933	4.1488	3.5807	3.9415	3.2079	3.5070	3.1779
Sh3gl2	_TIEYLQPNPASR_.2	#N/A	10.6806	10.6325	10.8893	10.7504	10.6714	10.5676	10.5669	10.8688	10.7891	10.5626	10.4737	10.6679	10.9584	10.9522	11.0029	10.7295	10.7498	10.4722	10.7483	10.6239
Sh3gl2	_IPDEELR_.2	#N/A	9.5425	9.7202	9.7564	9.8071	9.7751	9.8711	10.0537	9.6278	9.8757	9.8630	9.9162	9.9709	9.9522	9.7854	10.3668	10.0616	9.3841	10.0474	10.0057	10.0833
Sh3gl2	_ALYDFEPENEGELGFK_.2	#N/A	9.8907	9.7756	10.0477	9.8219	9.4312	9.6117	9.5246	9.6692	9.8398	9.9495	9.7759	9.6178	10.0118	10.0786	9.8643	9.8757	9.8681	9.4708	9.6368	9.6665
Sh3gl2	_RLDFDYK_.2	#N/A	9.1528	8.6623	9.1618	8.9114	8.9683	9.0488	8.7930	9.2505	8.8901	8.9844	9.1232	9.0166	8.9927	9.2799	9.0359	9.0969	9.0133	8.8848	9.0321	9.2138
Sh3gl2	_KVDVTSR_.2	#N/A	8.4009	8.5113	8.6648	8.4297	8.7705	8.3861	8.6504	8.4392	8.5724	8.6547	8.7330	8.6793	8.6591	8.6116	9.2099	8.6827	8.4303	8.5962	8.7052	8.6082
Sh3gl2	_[Acetyl (Protein N-term)]S[Phospho (STY)]VAGLKK_.2	Q62420_S2_M1_Sh3gl2	8.7873	8.8040	8.6404	8.3526	7.4322	7.3802	7.6422	6.7749	7.1224	9.0206	8.2883	7.6527	8.2697	7.9403	6.6855	8.0437	7.5404	7.9361	8.3012	8.1962
Sh3gl2	_RLDFDYKK_.2	#N/A	8.0784	7.7494	7.4785	7.4881	7.8439	7.7507	7.6328	8.2947	7.5587	7.8376	7.8798	7.8533	7.6276	8.2742	8.0544	7.9697	8.3054	7.7163	7.8685	7.6710
Sh3gl2	_QAVQILQQVTVR_.2	#N/A	7.0348	7.3212	7.6305	7.4825	6.9407	7.3139	7.0600	7.0789	7.4851	7.6764	7.3300	7.0772	7.6050	7.0625	7.8222	7.0992	7.3926	6.9770	7.5462	7.4196
Sh3gl2	_EIQHHLK_.2	#N/A	7.0352	6.8108	8.0562	6.2900	7.2011	7.0625	6.9389	7.6983	7.3242	7.2613	6.0790	6.7249	6.2002	7.1274	6.9660	7.4135	7.1624	6.5218	7.6276	7.2537
Sh3gl2	_EYQPKPR_.2	#N/A	6.7836	6.8418	6.5226	6.6327	6.3026	6.8223	6.3732	6.5900	6.8332	6.8279	6.4097	6.3805	6.6399	6.7992	7.0996	6.8060	6.1525	6.6956	6.3476	6.6156
Sh3gl2	_QNFIDPLQNLHDKDLR_.3	#N/A	7.9360	8.0096	7.8548	8.2085	7.9078	7.9177	8.0151	8.1402	8.0313	8.2744	7.9651	8.1748	7.9347	8.5319	8.2539	8.4430	8.2881	7.9400	8.0419	7.7664
Sh3gl2	_ELGDDC[Carbamidomethyl (C)]NFGPALGEVGEAM[Oxidation (M)]R_.2	#N/A	6.5026	6.3151	5.9364	6.6795	5.9921	6.4510	6.7598	6.7261	6.4548	6.6325	6.2382	6.5080	6.6190	7.3445	6.7871	6.8947	6.5486	6.3287	6.5318	6.2907
Sh3gl2	_GPGYPQAEALLAEAM[Oxidation (M)]LK_.2	#N/A	5.4664	5.6622	6.2492	6.7538	5.8806	5.6896	5.6075	5.8331	5.2119	5.4162	5.0266	5.4556	5.7227	5.9993	5.7254	5.7115	5.6124	5.3767	5.6078	5.6442
Sh3gl2	_VGGAEGTKLDDDFKEM[Oxidation (M)]ER_.3	#N/A	7.0192	7.2041	7.0509	7.4412	6.8153	7.0038	6.8596	6.1774	7.0468	7.0469	7.3252	6.7489	6.6927	7.6245	6.9764	6.6596	6.8781	6.5538	6.4795	6.8803
Sh3gl2	_ELSEVKDSLDM[Oxidation (M)]EVK_.2	#N/A	6.6158	6.4668	6.6757	6.4446	6.1942	7.0604	5.9876	6.9000	6.1175	6.8738	6.0599	6.5278	7.6873	7.4691	7.3199	6.9811	6.9513	7.3545	6.8549	6.2324
Sh3gl2	_AVMEIMTK_.2	#N/A	4.4031	4.4168	5.0376	5.4557	3.1092	3.1425	3.8314	3.1279	3.8591	4.2137	3.8705	3.1209	4.4340	5.7272	3.9643	3.7652	4.1260	3.0234	1.6610	3.6550
Sh3gl2	_LDDDFKEMER_.3	#N/A	4.3326	4.4873	3.8692	3.2516	3.0387	1.1890	4.0094	3.0575	3.9296	4.1432	2.6101	4.2134	4.3635	3.9073	4.0347	3.6947	4.0555	3.0939	3.3930	3.5846
Ostf1	_TLSNAEDYLDDEDS[Phospho (STY)]D_.2	Q62422_S214_M1_Ostf1	3.3611	11.1378	4.5197	4.2231	3.5681	11.0772	10.5378	3.5868	9.4881	3.1717	4.3797	3.6841	3.5657	11.7429	9.4166	4.2241	4.0175	10.9489	8.0850	4.0353
Ndufa4	_LALFNPDVSWDR_.2	#N/A	9.8560	9.9342	10.1199	10.1155	9.8220	9.9121	9.9032	10.0152	9.7173	9.9403	9.8466	9.7742	9.9576	10.0753	9.9817	9.8182	9.8158	9.7513	10.0375	9.6866
Ndufa4	_FYSVNVDYSK_.2	#N/A	9.3853	9.3722	9.6300	9.6489	9.3939	9.4192	9.3140	9.4705	9.4545	9.3891	9.4274	9.1497	9.4010	9.5446	9.3308	9.6022	9.4089	9.3338	9.3593	9.4210
Ndufa4	_KNNPEPWNK_.2	#N/A	8.1506	8.4303	8.0001	8.2372	8.0046	8.3692	8.5063	8.2778	8.0299	8.1628	8.4474	8.1156	8.0923	8.0360	8.4840	8.4120	8.1543	8.3113	8.4570	8.0501
Ndufa4	_LGPNEQYK_.2	#N/A	7.0081	7.1220	6.8120	6.6390	6.3614	6.8082	7.2257	6.5680	6.8506	6.3572	7.0540	6.7407	6.6022	6.8007	7.0677	6.7648	6.8198	6.8611	6.3807	6.9129
Ndufa4	_LALFNPDVSWDRK_.3	#N/A	5.0153	5.4237	5.1387	5.6700	5.6575	5.1064	5.3963	4.6523	4.7304	5.6037	4.5673	5.2603	5.8009	5.8329	5.2290	5.6887	4.9173	5.3418	5.4325	5.4793
Ndufa4	_NNPEPWNK_.2	#N/A	4.4648	7.2733	6.6964	7.0422	7.1423	7.3291	7.5041	7.0024	6.9782	7.6717	7.2794	6.8538	7.3203	7.5243	7.5000	7.2133	7.5376	7.0151	7.4834	7.7275
Ndrg1	_TAS[Phospho (STY)]GSSVTSLEGTR_.2	Q62433_S330_M1_Ndrg1	9.8793	9.5023	10.5098	10.2708	9.1436	9.3275	9.2006	9.7117	9.9352	9.5473	9.2796	9.1991	9.6303	9.4707	9.7445	9.2416	9.6899	8.8387	9.4703	9.6939
Ndrg1	_LNITPNSGATGNNAGPK_.2	#N/A	6.2218	7.3744	6.7637	7.0532	6.8244	6.7909	6.7597	7.3352	6.8828	7.0266	7.0294	7.1441	6.8466	7.4925	7.1688	6.9322	7.2559	7.0693	6.7434	7.2224
Ndrg1	_[Acetyl (Protein N-term)]S[Phospho (STY)]RELHDVDLAEVKPLVEK_.3	Q62433_S2_M1_Ndrg1	9.3230	10.0010	9.3152	9.3358	8.3125	9.2248	9.3847	9.3963	9.2536	9.1329	8.8626	9.3868	10.1264	10.2563	9.1200	9.3285	9.7611	9.6738	9.3650	9.2643
Ndrg1	_SRT[Phospho (STY)]AS[Phospho (STY)]GSSVTSLEGTR_.2	#N/A	3.5029	4.1854	4.2642	4.1764	3.0312	4.4567	5.1725	3.9609	5.1536	4.8850	3.4448	3.9994	3.1785	4.2766	4.0893	4.5367	4.6648	4.0459	3.2763	4.9888
Tle1	_DASGS[Phospho (STY)]PASTASSGSSSLK_.2	Q62440_S284_M1_Tle1	3.8033	4.1995	5.2449	4.8500	3.1410	3.3651	4.4001	5.4751	4.4592	4.0096	3.3327	4.0890	5.8362	4.3266	3.9773	4.1482	5.1644	3.7918	3.4404	5.2727
Tle4	_DAPIS[Phospho (STY)]PASVASSSSTPSSK_.2	Q62441_S292_M1_Tle4	8.9505	9.6770	9.5418	9.6105	9.1068	9.0061	9.1300	9.8652	9.3325	9.0916	9.3798	9.6001	10.1585	10.0835	8.6106	9.4587	9.9824	9.5579	9.2625	9.2818
Tle4	_SSVS[Phospho (STY)]PSASFR_.2	Q62441_S208_M1_Tle4	8.5372	9.5618	9.6920	9.5336	8.2749	9.0974	9.0291	8.6500	9.2413	9.4843	9.0887	9.2980	9.0183	9.6505	8.6714	9.3710	9.7015	8.2625	9.2748	9.2537
Nptx1	C[Carbamidomethyl (C)]TSVPVDADMC[Oxidation (M)]C[Carbamidomethyl (C)]AASVAAGGAEELI	#N/A	4.8581	5.3656	4.1638	4.4286	4.0993	3.4955	4.0501	4.9445	3.5504	3.4671	4.4270	4.4980	4.3880	4.9795	4.5539	4.5140	3.3009	4.9073	4.6716	5.0633
Fkbp3	_FLQDHGSDS[Phospho (STY)]FLAEHK_.3	Q62446_S36_M1_Fkbp3	4.8955	5.6285	4.0368	4.0732	4.5131	5.1893	3.5544	5.1571	3.4427	4.7702	4.9927	3.2495	5.0614	5.7596	5.1082	5.2271	4.3498	5.3381	5.1417	3.6167
Eif4g2	_TQT[Phospho (STY)]PPLGQTPQLGLK_.2	Q62448_T507_M1_Eif4g2	9.0634	9.1033	8.7224	3.4893	4.3019	7.4188	8.8980	9.3436	6.5615	8.3892	7.3706	2.9503	8.0057	9.4231	5.3359	3.4571	8.2584	8.4409	3.6306	6.4941
Eif4g2	_LTEAVVTDYLNSGNANDAVSGVR_.2	#N/A	3.2382	4.2786	4.2756	4.3460	3.4452	3.4785	4.5836	4.9142	4.0054	4.5497	2.4410	4.1525	3.4247	4.7897	3.3180	3.3384	4.3754	4.5909	3.5922	3.1034
Eif4g2	_FS[Phospho (STY)]PTMGR_.2	Q62448_S394_M1_Eif4g2	3.7716	4.7303	6.1968	3.9178	3.1093	3.3748	4.6517	3.4720	3.6269	4.4459	3.1055	4.1206	3.7923	4.2950	3.7320	3.9974	4.0097	3.8235	3.4721	4.2620
Vat1	_VLLVPGPEKET_.2	#N/A	6.7745	6.9274	8.6010	8.4216	6.7829	8.7055	8.9047	6.9718	8.7746	8.5566	6.8509	8.6850	6.7208	6.5626	6.4321	6.5593	8.8019	8.4742	8.4330	8.4828
Spen	_SQS[Phospho (STY)]PVHLR_.2	Q62504_S749_M1_Spen	8.0627	8.0718	7.6912	6.5455	6.7596	7.7589	7.6761	8.1180	7.6171	7.7176	6.3346	7.5510	8.0851	7.7982	6.8222	7.3463	8.0858	7.7554	7.4605	7.5607
Spen	_DLEPGEVPS[Phospho (STY)]DS[Phospho (STY)]DEDAEHR_.2	Q62504_S1395_M2_Spen	4.3208	7.0742	3.3562	6.4355	3.6585	6.5484	3.6630	4.2445	5.1556	5.7128	4.3161	5.7337	4.0497	6.2856	3.4598	5.4667	6.5359	6.7599	6.2696	5.2572
Spen	_S[Phospho (STY)]RSPNRFDAHDPR_.4	Q62504_S188_M1_Spen	4.5442	3.8597	4.0263	3.5463	3.5960	3.7713	4.1579	3.9232	4.0781	4.0744	4.3579	3.7491	4.2435	4.0134	3.4504	3.6259	3.7280	3.4520	4.0146	3.9804
Spen	_EAGPAAAS[Phospho (STY)]PQESESPQK_.2	Q62504_S2128_M1_Spen	3.6917	3.6274	3.2282	3.8926	3.8986	3.9319	3.1495	3.9173	3.0697	3.5023	4.0492	3.3535	3.7225	3.2663	3.1749	3.0538	4.1446	1.5080	4.0339	4.4444
Spen	_RPQS[Phospho (STY)]PGVS[Phospho (STY)]PAHSER_.3	Q62504_S758_M2_Spen	3.3405	3.8208	4.5357	4.0859	4.1791	4.4031	3.3620	3.7240	3.4211	3.0202	3.7955	3.0509	3.5292	4.5738	2.9392	3.6854	3.2212	3.9282	4.2273	4.2510
Zyx	_S[Phospho (STY)]PGGPGPLTLK_.2	Q62523_S336_M1_Zyx	10.5315	10.3433	10.3872	10.7040	3.2002	9.6564	9.8571	9.7136	9.6787	11.0461	10.1984	10.0622	10.4204	10.6358	9.5657	10.2131	10.2406	9.6870	9.7983	10.2042
Ppp3r1	_DTQLQQIVDK_.2	#N/A	6.2553	6.5475	8.0825	7.1920	6.8432	6.5553	6.0086	6.3060	7.3807	6.7288	7.6802	7.0655	7.0012	7.2821	8.2856	7.5063	7.6580	7.0660	7.3484	7.5218
Ppp3r1	_VIDIFDTDGNGEVDFK_.2	#N/A	6.4327	5.9852	7.0257	6.1274	5.8157	6.2330	5.7079	5.2070	6.1244	6.1710	6.8441	5.4641	5.5000	6.4405	6.6619	6.4328	5.9347	5.7368	5.7665	6.7429
Ppp3r1	_IYDM[Oxidation (M)]DKDGYISNGELFQVLK_.3	#N/A	5.3422	4.9204	5.4371	4.7637	4.8468	4.8251	4.7327	5.3530	5.1412	5.5931	5.6190	4.6747	4.8561	5.6743	6.2900	5.6771	5.4377	4.7241	5.4087	5.7071
Ppp3r1	_EFIEGVSQFSVK_.2	#N/A	4.2688	5.2951	7.3079	6.3310	6.3712	6.2257	5.6598	6.1109	5.6721	6.0136	6.3350	5.7275	6.0907	6.2806	6.7562	6.1079	5.9697	5.6053	6.5162	6.3508
Ppp3r1	_ISFEEFC[Carbamidomethyl (C)]AVVGGLDIHK_.3	#N/A	3.6709	4.4641	3.8924	3.2284	3.0619	3.0952	3.9862	3.0806	3.9064	4.1664	3.3850	3.4125	4.0718	4.3136	4.0116	3.7179	4.0787	3.5440	3.1926	4.4670
Ppp3r1	_KLDLDNSGSLSVEEFM[Oxidation (M)]SLPELQQNPLVQR_.3	#N/A	4.0236	3.2955	3.5602	3.5606	4.2305	4.2639	4.3184	4.2493	4.2386	3.8342	3.7173	3.0216	4.4041	3.5982	2.8430	3.3857	3.7465	3.4030	3.7020	3.3729
Ppp3r1	_LDLDNSGSLSVEEFM[Oxidation (M)]SLPELQQNPLVQR_.3	#N/A	4.2416	4.5784	3.7781	3.3427	2.9477	3.6599	4.1053	4.4672	4.1644	4.3044	3.0379	4.3045	4.2724	3.8162	3.6825	3.6037	3.9645	3.1850	3.4840	3.4935
Ppp3r1	_TIINADKDGDGR_.3	#N/A	3.6925	3.2884	3.7499	4.4758	3.8995	3.9328	3.1486	4.4148	3.0688	3.5031	4.0483	3.1778	3.2343	3.7369	3.1740	3.0546	3.4516	4.3815	4.0301	3.7039
Mapk3	_[Acetyl (Protein N-term)]AAAAAAPGGGGGEPR_.2	#N/A	8.0478	8.1543	8.1981	8.1547	8.4237	8.5371	8.5447	8.6124	8.2629	8.3167	7.6240	7.8302	7.9691	7.6210	7.8490	7.6609	8.1251	8.2543	7.8627	7.7811
Mapk3	_IADPEHDHTGFLTEY[Phospho (STY)]VATR_.3	Q63844_Y205_M1_Mapk3	7.0018	7.3638	6.3997	6.5860	5.2417	7.0257	6.4167	6.3914	5.8280	6.8211	5.5799	6.0934	6.7456	7.4543	4.7897	6.3320	6.3278	6.8345	7.0536	6.4254
Nup62	_ELEDLLS[Phospho (STY)]PLEESVK_.2	Q63850_S412_M1_Nup62	4.2093	3.1887	3.6669	3.4539	3.5470	6.1383	3.9941	4.3560	4.0532	5.8365	2.9267	3.6830	4.1612	3.7050	3.5713	3.0533	3.8533	3.2962	3.5952	3.3823
Omg	_TLDISNNR_.2	#N/A	9.6825	9.8941	10.0364	10.0459	9.7602	10.1745	9.7255	9.9039	9.9219	9.9314	10.0746	9.9419	9.9947	10.4042	9.6756	9.8951	10.1595	10.0402	9.9224	10.1670
Omg	_LESLPAQLPR_.2	#N/A	8.4958	8.6043	8.6973	8.7316	8.6959	8.8071	8.6183	8.6626	8.7902	8.5124	8.7676	8.8246	8.6157	8.7124	8.8226	8.7176	8.9621	8.7471	8.6144	8.7460
Omg	_LWTVPTNM[Oxidation (M)]PSK_.2	#N/A	8.2752	7.8933	7.3216	7.1303	8.0803	7.9151	7.8929	8.2738	7.9675	8.0292	7.7304	8.2043	8.2715	7.7731	7.7894	8.0443	7.9701	8.1083	7.9936	8.1167
Omg	_AHVIGTPC[Carbamidomethyl (C)]SK_.2	#N/A	6.4637	6.5463	6.4698	6.6541	6.6808	5.9993	6.7128	6.0119	6.1951	5.7763	6.6416	6.3056	6.6303	6.7331	7.3186	6.3938	6.1729	7.0801	6.5143	6.4556
Omg	_SDTAYQWNLK_.2	#N/A	7.1835	6.6071	6.9836	6.6887	6.5655	7.0376	6.5122	3.4373	6.9889	6.3119	6.6361	4.1554	6.2471	6.8972	6.7230	6.7707	3.9749	6.8513	6.5664	7.3037
Omg	_FTFIPEQSFDQLLQLQDTLHNNR_.3	#N/A	4.9083	5.1285	4.1516	3.7762	0.9781	5.2602	4.3036	4.6850	4.1696	4.2992	3.0431	4.9589	4.4088	5.4254	4.1372	4.3971	5.0491	4.9721	5.2027	4.8713
Cavin2	_S[Phospho (STY)]SPFKVS[Phospho (STY)]PLSFGR_.2	Q63918_S287_M2_Cavin2	4.2058	3.7974	3.2412	3.4840	3.5435	4.1195	4.8062	3.9061	4.0829	4.4121	3.2909	3.6869	4.2200	5.0889	3.9287	3.0498	4.4438	3.3178	4.1308	3.3178
Map2k2	_LKQPST[Phospho (STY)]PTR_.2	Q63932_T395_M1_Map2k2	12.1943	4.4284	12.1098	4.1150	12.0789	3.5720	11.9907	12.3391	3.4297	12.2501	11.9508	12.0490	12.4670	12.3516	4.2061	12.0802	12.1554	12.0457	12.0760	12.0929
Mef2d	_SEPVS[Phospho (STY)]PSRER_.2	Q63943_S437_M1_Mef2d	4.3294	9.2285	3.3649	8.9974	8.7634	8.5267	8.8002	8.7513	8.7345	4.5358	7.9768	8.8236	4.0411	3.3520	8.5650	8.6330	3.0667	8.9309	8.4133	8.5812
Kcnc3	_LAPLATPPGS[Phospho (STY)]PR_.2	Q63959_S754_M1_Kcnc3	7.7441	10.1450	10.1014	9.4892	3.2108	8.2358	8.9912	3.5671	8.4867	8.8922	3.2837	8.6275	3.8698	8.6965	3.9282	7.5654	8.1497	8.1321	7.8600	8.7253
Rab34	_INS[Phospho (STY)]DDKNLYLTASK_.2	Q64008_S241_M1_Rab34	3.4937	5.4892	4.2488	5.6986	4.3323	4.3399	4.0518	3.5708	3.2679	4.2605	3.6423	4.3985	3.3760	4.4206	2.7860	3.8386	4.9786	4.0815	4.2258	4.0978
Crk	_QEAVALLQGQR_.2	#N/A	5.8661	6.6565	6.8090	6.6663	6.8076	6.2269	6.0850	5.9214	6.3888	6.6539	6.5782	6.1886	6.1357	6.8549	6.8954	5.9155	6.3096	5.5547	6.3547	6.6701
Crk	_QGS[Phospho (STY)]GVILR_.2	Q64010_S125_M1_Crk	5.9354	5.5878	5.5683	6.0021	3.7310	5.8005	5.0969	5.1837	6.2886	5.8580	5.2403	4.8758	3.4028	5.6973	3.3425	5.2388	5.1002	4.2131	3.8616	5.8496
Crk	_ALFDFNGNDEEDLPFK_.2	#N/A	4.4642	5.1370	4.0008	3.1201	5.2011	5.4514	4.3279	5.4656	5.4341	6.1188	5.1394	5.2303	4.4950	4.0388	5.4582	4.8718	5.1166	4.7368	5.6906	5.4422
Crk	_DSS[Phospho (STY)]TSPGDYVLSVSENSR_.2	Q64010_S41_M1_Crk	10.7985	11.0174	11.4308	10.9516	4.9641	10.7384	10.4197	4.4609	10.9478	10.9639	5.2909	11.3834	10.5084	11.4307	11.0825	10.7645	11.0746	10.7528	10.9242	10.7523
Crk	_LLDQQNPDEDFS_.2	#N/A	4.5597	3.4431	3.5952	3.1297	4.8655	4.5559	5.3098	4.2601	5.3667	3.2652	3.8936	5.0694	5.7882	3.5822	5.1910	5.1453	3.2969	3.0354	4.9117	4.8902
Crk	_IHYLDTTTLIEPVAR_.3	#N/A	5.1498	5.8004	5.9210	5.7544	5.7676	6.1467	5.8456	5.0229	5.9716	5.4683	5.2456	5.3443	5.0546	5.8542	5.5709	6.0363	5.5620	6.1498	6.0042	5.7396
Raly	_GRLS[Phospho (STY)]PVPVPR_.2	#N/A	9.2026	9.4559	8.3058	7.9583	7.3595	8.0832	9.1741	10.1544	7.7227	8.6909	7.6651	9.3770	9.5175	10.2027	7.5916	7.9244	8.9560	9.1401	8.4105	8.0336
Raly	_LPAPQEDTAS[Phospho (STY)]EAGTPQGEVQTR_.3	#N/A	8.7820	9.0895	9.0839	9.2217	8.4949	8.9075	8.4593	8.9025	8.5189	8.8841	8.6385	8.9455	9.1372	9.3405	8.3175	8.9428	9.0870	8.8085	6.8182	8.8014
Raly	_LS[Phospho (STY)]PVPVPR_.2	#N/A	4.2839	3.7190	3.3193	3.4055	3.5080	2.1480	3.4580	3.9842	4.1391	4.4902	4.3530	3.6084	4.3046	4.8960	4.0875	3.1279	4.5219	3.3113	4.0113	3.2734
Tfe3	_RS[Phospho (STY)]SFSMEEES_.2	Q64092_S564_M1_Tfe3	3.3959	4.9624	5.4194	4.1886	3.6028	4.1270	3.4456	3.6216	3.3658	3.2065	4.3453	3.6496	3.5312	6.1251	3.4710	4.2589	3.1188	4.0309	4.3300	4.0980
Mef2l	_TSS[Phospho (STY)]TGEEEESLAIIR_.2	Q64096_S621_M1_Mef2l	4.4393	7.3019	4.4768	4.1449	3.6463	3.6796	3.4018	3.6650	3.3220	3.2499	4.3015	3.6059	3.4875	7.8946	3.4272	4.3023	3.1622	7.0253	3.7769	3.9571
Mef2l	_QLC[Carbamidomethyl (C)]PEPAEILS[Phospho (STY)]PEPV_.2	Q64096_S1145_M1_Mef2l	4.1252	3.1939	3.6617	3.4591	4.3321	5.6432	4.2168	4.3508	5.3359	3.9358	3.6157	4.4209	4.3025	5.5634	4.6588	3.4873	3.8481	5.4824	2.9619	3.2713
Mef2l	_SSS[Phospho (STY)]AQC[Carbamidomethyl (C)]LSSGK_.2	Q64096_S1120_M1_Mef2l	3.7957	7.6630	4.3320	3.8937	3.1334	3.3506	3.7308	3.4961	7.2477	4.4218	3.1296	4.0965	3.8164	7.7026	3.9848	4.1406	4.0338	3.7994	7.0300	4.2861
Arl13b	_EALSQLDS[Phospho (STY)]EDEQDQR_.2	Q640N2_S328_M1_Arl13b	3.7498	7.5999	7.6010	7.8893	3.0875	7.9200	7.6075	3.4501	7.6990	7.8022	3.3862	7.2326	3.1199	7.6131	6.4589	7.5707	7.8171	7.4448	7.5864	3.8418
Hepacam	_THTS[Phospho (STY)]PPR_.2	#N/A	4.3380	3.6648	3.3735	9.5513	3.6758	10.0499	9.6502	9.3125	3.9244	9.6524	9.6779	3.5542	9.4864	9.3627	7.8457	3.1821	9.6651	9.5909	3.7240	9.3613
Hepacam	_QNS[Phospho (STY)]LEYM[Oxidation (M)]DQNDDR_.2	#N/A	9.6910	9.6452	10.2051	9.7618	9.0442	9.7255	9.5489	9.3799	9.4681	9.0448	9.3281	9.0881	9.4370	8.6300	8.1921	8.8252	9.7135	9.2426	9.1961	9.1258
Hepacam	_STTEPGPPQYSVS[Phospho (STY)]PPVPGR_.2	Q640R3_S345_M1_Hepacam	8.9819	8.4876	6.7840	5.6304	3.4546	8.0412	8.0243	8.3307	5.6336	8.6927	6.0735	7.4841	7.7532	9.0599	3.6637	7.5202	6.8958	8.0675	7.5589	3.3052
Hepacam	_SPARS[Phospho (STY)]PATGR_.2	Q640R3_S370_M1_Hepacam	8.6603	8.0402	5.8807	6.9801	7.0167	6.5070	7.6411	6.7753	6.0082	8.3588	6.3508	3.4106	6.3506	4.0867	3.3134	3.9447	6.5866	3.7919	4.0909	6.2255
Hepacam	_THTS[Phospho (STY)]PPRAPSS[Phospho (STY)]PGR_.2	#N/A	12.5246	12.6175	12.1794	12.4785	11.9900	12.3269	12.4052	12.1674	10.0322	12.3688	11.6233	11.8106	4.1643	11.9622	11.2433	11.8541	12.3517	12.1825	11.9815	11.9307
Hepacam	_QNS[Phospho (STY)]LEYM[Oxidation (M)]DQNDDRLK_.3	#N/A	9.8257	9.5534	7.0845	6.4180	7.4389	9.4010	7.7140	9.4632	9.4602	9.4912	8.8299	9.2727	10.0302	7.6163	8.0586	7.5004	9.6505	9.4152	9.3390	7.4571
Hepacam	_DKDSS[Phospho (STY)]EPDENPATEPR_.2	Q640R3_S321_M1_Hepacam	7.2779	4.3655	6.7699	6.7465	6.7553	6.8587	7.1392	3.2460	6.6741	6.9750	3.4987	6.6957	7.2215	6.8033	6.1294	6.5160	4.0382	7.1015	3.4797	3.6689
Hepacam	_SEADTLPRS[Phospho (STY)]GEQER_.2	#N/A	5.4417	5.0302	5.0396	5.1663	4.9542	3.6417	4.2522	3.6058	3.7607	4.0526	3.3772	4.2267	5.7494	4.5779	4.1600	4.4870	5.1432	3.7082	3.3568	3.9180
Hepacam	_S[Phospho (STY)]PGLPIR_.2	Q640R3_S352_M1_Hepacam	12.8075	4.0478	11.4354	4.3129	2.9052	3.1293	3.1350	3.4970	3.1941	3.7738	3.5685	3.7739	11.4545	4.3468	4.2131	3.9124	4.4951	4.1552	2.9534	4.0241
Spr	_ETSKDPELR_.2	#N/A	5.8596	5.9749	5.9543	5.9997	5.8719	6.3116	5.9648	6.2977	6.3698	6.0725	5.9631	5.5013	5.3247	5.9989	5.9884	6.4067	5.5898	5.6169	5.3810	5.6729
Spr	_ALAPQLAR_.2	#N/A	4.9059	5.6939	4.4899	6.0113	5.7271	5.2658	5.5665	5.1044	5.6277	6.0900	5.4261	5.1198	5.2969	4.0712	6.5136	3.8936	4.6262	5.4531	5.8886	5.4554
Spr	_MEADGLGC[Carbamidomethyl (C)]AVC[Carbamidomethyl (C)]VLTGASR_.2	#N/A	5.8746	5.6038	4.6343	4.7330	5.0557	5.1824	5.5971	6.1757	5.4246	5.7406	5.0665	3.9626	5.6621	5.5031	4.9539	5.7399	4.8318	5.5934	5.4325	6.1333
Spr	_LLLINNAATLGDVSK_.2	#N/A	5.4734	5.3674	5.2538	5.3234	4.8577	4.6666	4.8786	4.7804	5.5923	3.7720	3.6045	4.3534	4.7944	4.7711	4.9152	4.8885	5.5918	3.2482	4.6402	4.3211
Spr	_DM[Oxidation (M)]LYQVLAAEEPSVR_.2	#N/A	6.9826	6.2855	6.0584	3.8888	6.1568	6.4123	6.5649	6.9151	6.6747	4.4169	6.2432	6.5265	6.6195	6.2241	6.3020	5.9998	6.9438	6.4782	6.2882	6.5339
Spr	_LKS[Phospho (STY)]DGALVDC[Carbamidomethyl (C)]GTSAQK_.2	Q64105_S228_M1_Spr	3.8322	6.1150	3.3688	3.1147	4.8424	5.5763	5.8016	5.5824	4.4300	6.1503	5.3309	5.6308	4.5954	5.3972	4.9274	5.3023	5.8103	5.0277	5.1341	5.3642
Trim24	_SEWSDASQKS[Phospho (STY)]PVHVGETR_.3	Q64127_S812_M1_Trim24	4.7931	5.1152	5.3484	4.2617	4.4376	3.8167	4.5542	5.0598	3.1849	3.3870	4.1644	4.2303	3.3504	3.8530	3.2901	2.9385	3.5677	5.1350	3.9140	4.7513
Trim24	_HEAEDC[Carbamidomethyl (C)]KFS[Phospho (STY)]DDS[Phospho (STY)]DDDFVQPR_.3	Q64127_S1026_M2_Trim24	4.1877	3.1314	3.7243	3.3965	3.4896	3.7137	1.7262	4.4134	4.1105	4.3582	2.9841	4.3583	4.2186	3.7624	3.6286	2.9960	3.9106	3.2388	3.5379	3.4396
Maoa	_EIPVDAPWQAR_.2	#N/A	6.4465	6.6898	6.4428	6.2770	6.7779	6.7266	6.6225	6.1664	6.2877	6.2710	6.6101	6.4977	5.8664	6.4966	6.1553	6.3048	6.5609	6.7425	5.6684	6.5547
Maoa	_DVPALEITHTFLER_.2	#N/A	6.6843	5.7197	3.7873	5.5400	5.8364	6.0250	5.5676	5.8226	5.1259	5.8708	5.8303	4.9201	5.5166	5.5648	4.2004	4.6129	5.6487	5.8493	5.5326	4.8627
Maoa	_VLGSQEALS[Phospho (STY)]PVHYEEK_.2	#N/A	4.0430	4.6251	3.8196	4.2048	3.2014	4.0679	4.2667	4.8950	4.1560	4.4162	4.4340	3.8467	4.0638	3.9603	3.2426	4.1433	4.2811	3.8551	3.2060	4.0359
Maoa	_IYFAGTETATQWSGYM[Oxidation (M)]EGAVEAGER_.3	#N/A	4.4422	4.0381	4.5010	3.7247	3.1483	3.1816	3.8998	3.6651	3.8200	4.2528	3.2986	3.9275	3.9854	4.4881	3.9252	3.8043	4.2028	3.6304	3.2790	2.9543
Agtpbp1	_VTS[Phospho (STY)]PTTVVLDEDEPR_.2	Q641K1_S1160_M1_Agtpbp1	8.1605	5.3904	6.8243	3.8555	3.9487	4.1728	5.5924	3.9544	3.6515	3.3164	4.0259	3.3165	3.7596	6.7945	3.3696	3.4550	3.4516	3.6979	6.7870	2.9806
Nuak1	_ENDFPQSGQDSVPES[Phospho (STY)]PSK_.2	Q641K5_S389_M1_Nuak1	3.6263	7.3080	4.1626	7.8684	2.9641	3.1881	3.0762	3.4381	3.1153	7.6276	7.3476	7.0057	3.2433	7.9705	4.1542	3.9712	7.7662	7.4903	7.5635	7.7511
Nuak1	_SSVC[Carbamidomethyl (C)]DC[Carbamidomethyl (C)]DALPDSES[Phospho (STY)]PLLAR_.2	Q641K5_S326_M1_Nuak1	3.7035	5.7188	5.3643	6.1248	3.9105	3.9438	3.1376	4.4038	5.1965	5.6014	4.0373	4.1612	3.2233	5.5372	4.9396	3.0657	5.4235	5.1005	5.8901	5.6000
Nuak1	_TAIPLPSS[Phospho (STY)]PEADM[Oxidation (M)]SGK_.2	#N/A	4.0990	3.5880	3.3932	3.5402	7.3018	3.8117	7.7123	4.0717	4.0162	4.4562	3.7919	3.4128	4.1243	5.9734	3.5343	3.2113	4.1161	3.3368	3.4228	3.3453
Actr3b	_LSQELSGGR_.2	#N/A	4.5096	4.2972	4.0462	4.5755	4.5308	3.2490	3.8324	3.6136	3.7526	4.3202	3.6330	4.0364	3.9181	4.0842	3.8372	3.8717	4.2325	4.9130	3.2160	3.5875
Actr3b	_TLTGIVIDSGDGVTHVIPVAEGYVIGSC[Carbamidomethyl (C)]IK_.3	#N/A	1.4272	2.4038	3.2695	3.4554	3.5718	1.4696	3.9693	4.3313	4.0284	4.4404	2.9019	3.6582	4.1365	3.6802	3.5465	3.0781	4.4721	3.3209	3.6200	1.9083
Sf1	_TGDLGIPPNPEDRSPSPEPIY[Phospho (STY)]NS[Phospho (STY)]EGKR_.4	#N/A	3.9254	7.7823	3.7419	3.8681	3.4546	6.5740	7.5716	3.8807	6.1315	4.6566	7.6354	3.8623	4.0899	7.9435	6.3555	3.6239	4.1533	8.8843	7.0416	5.5613
Sf1	_TGDLGIPPNPEDRSPSPEPIY[Phospho (STY)]NS[Phospho (STY)]EGK_.3	#N/A	3.8170	4.2973	4.0592	4.5624	3.1547	3.3788	4.3864	3.2475	4.4455	4.0233	3.3190	4.0234	4.5535	4.0973	3.9636	4.1619	4.2456	5.9608	3.2029	3.7746
Zeb1	Phospho (STY)]C[Carbamidomethyl (C)]TSS[Phospho (STY)]PSPLNLC[Carbamidomethyl (C)]SAI	Q64318_S682_M2_Zeb1	3.5679	4.1497	4.2181	4.3293	3.3322	3.4515	4.0400	3.2882	4.5051	3.6225	3.1550	3.8758	4.1789	4.1245	3.7918	4.1513	4.0719	4.1987	4.9333	4.6638
Npdc1	_GPTS[Phospho (STY)]PSTPR_.2	Q64322_S236_M1_Npdc1	10.3697	10.4238	10.0978	10.2519	10.1389	10.3790	10.3794	10.2358	10.3563	10.0963	9.9832	10.0073	10.3528	9.8826	9.3897	9.8595	10.3525	10.3452	9.9799	9.9928
Npdc1	_IS[Phospho (STY)]PGDQR_.2	Q64322_S243_M1_Npdc1	8.4036	8.5891	8.0901	8.3680	8.2143	8.7355	8.6302	8.1184	8.6249	8.1988	8.3397	8.3017	7.9204	8.2482	7.1744	7.9751	8.0316	8.1143	8.2071	8.0295
Npdc1	_GPTS[Phospho (STY)]PST[Phospho (STY)]PRIS[Phospho (STY)]PGDQR_.2	Q64322_S236_M3_Npdc1	3.8907	3.4527	7.5877	4.0740	4.0976	4.1310	7.4367	4.3554	3.7404	7.6513	5.8648	3.1788	3.9058	3.6904	3.0002	6.7221	3.6308	3.9585	3.8558	3.5247
Syn2	_VLLVVDEPHTDWAK_.2	#N/A	8.2360	8.2824	8.4490	8.3298	8.1974	7.9812	8.2720	8.3584	8.2272	8.1946	8.1415	7.8928	8.4620	8.1143	8.0803	8.3326	8.2920	8.0276	8.0697	8.2470
Syn2	_FPLIEQTYYPNHR_.3	#N/A	7.8363	7.5823	8.4362	8.1155	8.0255	8.0848	7.9981	7.4485	7.8654	8.3249	8.3970	7.7362	7.5120	8.0648	7.8876	8.1298	7.5829	7.6724	8.0924	8.4481
Syn2	_TLGGEKFPLIEQTYYPNHR_.3	#N/A	8.6085	8.8230	8.7777	8.5904	8.8365	8.7600	8.8506	9.3442	8.8107	8.8498	8.6688	8.9161	8.9986	9.0699	8.8004	9.0924	8.9473	8.8714	8.8257	8.9463
Syn2	_QLITDLVISK_.2	#N/A	7.7194	7.9256	8.6362	8.3888	7.6024	7.9181	8.0604	7.9605	8.0782	8.3322	7.9680	7.8877	8.0304	8.2904	8.0543	8.4468	7.7178	8.1873	8.2254	7.9978
Syn2	_ILGDYDIK_.2	#N/A	7.8092	7.5923	7.8465	7.9122	7.7140	7.9846	7.8872	7.3465	7.7669	8.0201	8.1743	7.7191	7.8364	7.6644	7.8866	7.3843	7.4559	8.1267	7.8129	8.1832
Syn2	_SFRPDFVLIR_.2	#N/A	7.4992	7.4824	7.3956	7.7458	7.4687	7.3406	7.3893	7.7582	7.0629	7.7350	7.3528	7.6116	7.3303	7.6834	7.2147	7.6333	7.3006	7.5427	7.5821	7.3385
Syn2	_EM[Oxidation (M)]LTLPTFPVVVK_.2	#N/A	9.1727	8.8622	9.1757	9.2902	9.2736	9.1867	9.1547	9.2377	9.3062	9.4259	9.0817	9.2283	9.4185	9.8818	9.1717	9.2518	9.1952	9.1960	9.1314	9.1916
Syn2	_M[Oxidation (M)]NQLLSR_.2	#N/A	7.0932	7.5146	7.0695	7.4794	7.5598	7.5942	7.8341	7.3463	7.3091	7.2382	6.9533	7.6676	7.3897	7.5903	8.1120	7.5706	7.0474	7.8059	7.7007	7.4024
Syn2	_QTAASAGLVDAPAPSAASR_.2	#N/A	7.7104	7.9354	8.0550	7.8470	7.7083	7.6790	7.7624	7.9774	7.9878	7.7532	7.4950	7.8026	7.9231	7.7571	7.7462	8.0518	7.8846	7.5399	7.9202	7.6192
Syn2	_KSFASLFSD_.2	#N/A	5.1356	6.2154	5.8293	5.6006	5.1405	5.8845	5.8738	5.1049	5.9215	5.9409	5.1464	4.9367	5.2934	6.4133	5.3920	5.5749	4.9967	6.3813	6.2488	6.3512
Syn2	_TNTGSAM[Oxidation (M)]LEQIAM[Oxidation (M)]SDR_.2	#N/A	8.0010	7.8232	7.8435	8.4806	7.7570	7.9250	8.3528	10.0805	9.4111	8.1322	7.6798	8.8251	8.2379	8.4595	7.8170	7.9355	7.9841	7.8883	8.1100	7.8352
Syn2	_T[Phospho (STY)]PALS[Phospho (STY)]PQRPLTTQQPQSGTLK_.3	#N/A	5.1126	6.1649	6.6851	6.3200	3.7039	6.1294	6.2219	6.1265	5.3826	6.0144	5.3196	6.3320	3.7224	7.2655	4.8786	5.5310	5.5772	5.8690	6.0824	5.2174
Syn2	_VEQAEFSELNLVAHADGTYAVDM[Oxidation (M)]QVLR_.3	#N/A	5.8083	4.2768	5.4547	5.8745	5.1049	5.2730	4.8072	5.8912	3.8178	4.9683	4.3203	5.0638	5.3676	6.0446	4.4633	4.4567	3.2358	5.1948	4.9227	5.4620
Syn2	_TPALS[Phospho (STY)]PQRPLTTQQPQSGTLKEPDSSK_.4	#N/A	6.9011	7.0900	5.8579	6.7307	5.5039	6.2284	7.3638	7.4851	5.6833	7.1144	6.5798	7.1911	5.6803	8.3824	6.2154	6.4149	6.4878	7.1850	6.7501	6.5189
Syn2	_SS[Phospho (STY)]ANEDEAKAETIR_.2	#N/A	6.8850	6.3949	7.1624	7.0306	6.0792	4.9354	6.1667	5.9698	6.1244	7.2779	6.1296	6.1299	6.0575	6.5843	5.6008	6.3682	5.9525	6.0955	5.1771	6.2361
Syn2	_LWVDAC[Carbamidomethyl (C)]SEM[Oxidation (M)]FGGLDIC[Carbamidomethyl (C)]AVK_.2	#N/A	4.6517	5.9965	4.5434	3.9489	3.9715	5.0619	4.6439	5.4818	3.0782	5.4188	4.8575	4.0439	3.1863	6.0114	4.3735	4.4302	4.5146	4.5237	4.4145	3.9082
Syn2	_QHAFGM[Oxidation (M)]AENEDFR_.3	#N/A	5.4560	5.3135	5.8373	5.6968	5.2946	5.6003	5.5840	4.9861	5.4389	5.6687	5.6121	5.2965	5.3533	5.8052	5.6346	5.8946	5.2960	5.2655	5.5105	5.7136
Syn2	_TSISGNWK_.2	#N/A	6.6236	7.0650	7.6231	7.4503	7.2362	7.5941	7.5142	7.2934	7.5286	7.4506	7.2979	7.6253	7.4564	7.2663	8.0339	7.7354	6.9909	7.5246	7.6898	7.4124
Syn2	_DGKDYIFEVM[Oxidation (M)]DC[Carbamidomethyl (C)]SM[Oxidation (M)]PLIGEHQVEDR_.4	#N/A	6.7641	7.1639	5.7914	5.9023	5.8499	6.2288	6.0367	6.9270	6.7778	6.0209	5.8924	6.5610	4.0478	6.5311	5.2272	6.1940	6.1141	6.3269	6.5241	6.3489
Syn2	_SQS[Phospho (STY)]LTNAFSFSESSFFR_.2	Q64332_S546_M1_Syn2	4.0394	3.7107	4.1108	3.6188	3.9108	3.5904	6.2597	4.0525	4.2470	3.9767	4.3569	4.1984	4.3551	6.5677	3.7651	3.9259	6.4641	3.1155	3.4146	4.2573
Syn2	_VENHYDFQDIASVVALTQTYATAEPFIDAK_.3	#N/A	3.9135	2.8532	4.4498	3.7759	3.2512	3.2329	2.1871	3.6138	3.7687	4.1198	3.2474	0.5506	3.9342	4.4368	3.8671	4.2584	4.1515	3.6816	3.3302	4.4038
Tbr1	_S[Phospho (STY)]PPQYC[Carbamidomethyl (C)]GAK_.2	Q64336_S549_M1_Tbr1	8.9281	9.0610	9.0863	9.3038	8.5831	8.9611	8.8430	8.5349	8.6285	4.0835	8.4329	7.7083	7.8684	8.9659	8.1898	8.3275	8.6698	7.9684	8.3111	8.3758
Tbr1	_LTPS[Phospho (STY)]PNDSPR_.2	Q64336_S410_M1_Tbr1	3.3949	8.8407	4.4323	4.1782	3.6018	8.8745	8.4260	3.2116	3.3665	9.0916	4.3460	8.3769	9.0072	9.3112	3.4717	8.9331	3.7493	8.4353	3.7325	4.0016
Tbr1	_SIDS[Phospho (STY)]SDSGIYEQAK_.2	Q64336_S625_M1_Tbr1	4.2551	4.5648	5.8595	3.5376	2.9612	2.9945	4.0869	3.8522	4.0071	4.0657	3.4857	4.2909	4.1725	3.1744	4.1123	3.6172	4.3899	3.4433	3.0919	3.1414
Tbr1	_RIS[Phospho (STY)]PADTPVSESSSPLK_.3	Q64336_S640_M1_Tbr1	3.4371	4.5657	3.9734	4.1826	4.2757	2.9989	3.2654	3.6274	3.3245	3.6434	3.6989	4.0647	4.3325	4.4772	2.8426	3.7820	3.1246	4.0249	4.3239	4.1544
Tbr1	_SKLS[Phospho (STY)]PVLDGVS[Phospho (STY)]ELRHSFDGSAADR_.4	Q64336_S84_M2_Tbr1	4.1507	3.1684	3.6873	3.4332	2.8568	4.3910	4.1913	3.9566	4.1115	3.9613	3.5901	4.3953	4.2769	3.2787	4.2167	3.5128	4.4943	3.3389	2.9875	3.2457
Pde1c	_RSS[Phospho (STY)]LNSINSSDAKR_.3	Q64338_S469_M1_Pde1c	5.6617	6.6284	4.2439	6.1417	3.0826	6.2451	6.2209	3.7315	5.1672	6.2221	6.3617	6.7377	6.8638	6.3477	6.2025	3.7386	6.0347	3.5647	6.7094	3.0207
Pde1c	_[Acetyl (Protein N-term)]MES[Phospho (STY)]PTKEIEEFESNSLK_.3	Q64338_S3_M1_Pde1c	3.8795	3.4396	3.4161	3.7047	4.0864	4.1198	4.4625	4.1052	4.3827	3.6901	3.8614	3.1657	4.5482	3.4541	2.9870	3.2416	3.6024	3.5470	3.8461	3.1314
Aktip	_TLAGDVKTS[Phospho (STY)]PPR_.2	Q64362_S30_M1_Aktip	9.2353	9.2763	8.2341	8.5280	8.7137	7.5806	8.7541	8.1142	8.4614	8.7042	8.3951	8.5306	9.2064	8.7570	8.3493	8.7411	9.3033	8.4001	8.4983	8.3358
Atp7a	_VSIASEVESTASS[Phospho (STY)]PSSSLQK_.2	Q64430_S357_M1_Atp7a	3.3533	5.0794	3.8896	4.3360	4.1919	4.4160	3.3492	3.0537	3.4083	3.5597	3.7826	3.0381	3.5163	4.6242	2.9264	3.6982	3.5914	4.2418	3.8903	3.8437
Hspe1	_VLLPEYGGTK_.2	#N/A	10.4346	10.3481	10.4691	10.4106	10.6369	10.3286	10.4624	10.6547	10.3307	10.3799	10.4269	10.7317	10.9818	10.7316	11.1102	10.7024	10.3876	10.6159	10.5292	10.5953
Hspe1	_FLPLFDR_.2	#N/A	8.8349	9.1683	8.8948	9.2802	9.2596	9.1490	9.0554	8.8557	8.9380	9.2601	9.0383	9.4296	9.1633	8.9615	9.2967	9.4533	9.0912	9.1945	9.4015	9.0530
Hspe1	_KFLPLFDR_.2	#N/A	8.9006	9.2867	8.9109	9.2258	9.2034	8.8945	9.2433	9.1252	8.5891	9.9802	9.0016	9.4890	9.2807	9.3669	9.2543	9.2897	9.0780	9.1712	9.2308	9.0702
Hspe1	_SGEIEPVSVK_.2	#N/A	8.6134	8.8842	8.9361	8.6539	8.8436	8.5757	8.9448	8.9330	9.0777	8.7718	8.8755	8.8349	9.0744	9.1564	9.4512	9.0487	8.5551	8.8547	8.7041	8.9682
Hspe1	_VVLDDKDYFLFR_.2	#N/A	7.1114	7.3726	7.4900	7.2601	7.3576	6.9139	7.1955	7.1694	7.3601	7.5493	7.2533	7.5291	7.3310	7.4493	7.7673	7.4005	6.9447	7.2463	7.1381	7.5181

Hspe1	_VLQATVVAVGSGGK_.2	#N/A	7.4909	7.6542	8.1829	7.7021	7.6022	7.7491	7.4283	7.4058	7.8858	7.3933	7.4930	7.8199	7.9067	8.2162	8.0144	7.9478	7.4599	7.6895	8.0229	7.8881
Hspe1	_GKSGEIEPVSVK_.2	#N/A	4.9051	4.1524	4.6895	5.1980	5.5466	5.2298	4.4367	4.6074	4.5998	4.8173	5.1606	5.2038	4.3668	4.5959	5.5307	5.3263	5.1066	4.3565	5.4233	5.7477
Hspe1	_GGIM[Oxidation (M)]LPEK_.2	#N/A	6.6043	6.7202	6.2699	6.6807	6.8160	6.7752	7.1520	6.7009	6.9113	7.3574	6.8592	7.3895	7.4662	7.0056	7.7132	7.0361	6.3752	7.3976	6.9675	7.0484
Ptprd	_KS[Phospho (STY)]LPNSK_.2	Q64487_S1303_M1_Ptprd	8.9035	8.8604	8.5748	8.9417	8.4898	8.7142	4.0360	8.8396	8.7723	8.4216	8.5501	4.2401	4.1217	8.5987	4.0614	8.2790	8.8931	9.0151	8.6676	8.4405
Ptprd	_DFLPVDTSNNNGR_.2	#N/A	7.6647	7.5317	7.2667	7.6388	7.2493	7.4856	7.4653	7.7542	7.0356	7.7158	7.6269	7.3286	7.7879	8.6267	7.0156	7.5023	7.6885	7.4893	7.2038	7.3048
Ptprd	_YANVIAYDHSR_.2	#N/A	4.9182	5.5905	4.4632	5.5638	5.3166	5.2383	5.2358	5.2801	5.4564	3.9028	5.2464	4.9091	4.6959	4.9744	4.4951	4.7079	5.3598	5.1701	5.1661	4.4068
Ptprd	_GALQIEQSEESDQGKYEC[Carbamidomethyl (C)]VATNSAGTR_.3	#N/A	3.1403	3.8857	4.4306	2.4554	3.2473	3.1268	2.3887	4.2754	3.3682	3.2038	4.3612	3.2172	3.4240	2.5088	3.4734	4.1006	4.2561	4.0334	4.3324	4.1459
Top2b	_ASGS[Phospho (STY)]ENEGDYNPGR_.2	Q64511_S1539_M1_Top2b	7.2248	7.2600	7.0786	8.2796	6.3173	6.6591	6.9659	7.0196	6.7243	3.9310	6.2054	3.4614	4.0735	3.3554	6.5393	7.5539	7.3558	3.4657	3.4775	6.5874
Top2b	_KAS[Phospho (STY)]GS[Phospho (STY)]ENEGDYNPGR_.2	Q64511_S1537_M2_Top2b	6.4542	5.9843	6.2961	6.5232	6.1749	6.7666	6.7228	6.4800	6.4837	6.3224	6.2319	6.5863	6.7047	6.3026	5.3992	4.9062	6.6241	6.5445	6.3907	6.2773
Top2b	_YFAES[Phospho (STY)]DEEEDVDFAM[Oxidation (M)]FN_.2	Q64511_S1600_M1_Top2b	3.4339	3.8852	6.9375	4.1503	3.6409	3.6742	3.4072	3.6596	7.8767	3.2445	4.3069	3.6113	3.4929	4.5094	7.2890	4.2969	3.1568	3.9926	6.8002	3.9625
Top2b	_IVETINS[Phospho (STY)]DS[Phospho (STY)]DSEFGIPKK_.2	#N/A	3.7277	3.9142	4.0800	4.0376	3.5613	3.5683	3.6628	3.7125	3.5486	4.2163	3.7039	3.6684	3.6744	4.1079	3.5306	3.9021	3.7033	6.0655	4.0616	3.8366
Top2b	_IVETINSDS[Phospho (STY)]DSEFGIPK_.2	#N/A	3.8041	3.8200	4.1367	4.0852	3.6296	3.6903	2.9824	3.7883	3.4296	3.4785	4.0100	3.5461	3.5548	6.5255	3.6533	3.9552	3.8360	7.2134	3.7708	3.6973
Top2b	_KIVETINS[Phospho (STY)]DS[Phospho (STY)]DSEFGIPKK_.3	#N/A	3.6166	4.3862	4.1529	4.0728	2.9543	3.5298	3.0860	3.3169	3.4718	3.8229	2.9505	4.2757	3.6373	4.1399	4.1640	3.9615	3.8546	3.9785	3.6271	4.1069
Top2b	_KTSFDQDS[Phospho (STY)]DVDIFPSDFTSEPPALPR_.3	Q64511_S1568_M1_Top2b	4.0919	3.9109	3.1274	3.5975	3.4296	3.6537	4.1114	4.4734	4.1705	4.2982	3.0441	3.8003	4.2786	3.8223	4.6886	4.3300	2.9360	3.1788	3.4779	3.4996
Top2b	_KIVETINS[Phospho (STY)]DS[Phospho (STY)]DSEFGIPK_.2	#N/A	4.0130	4.2247	3.8185	3.7630	3.5823	3.7046	3.9211	4.4994	3.9222	4.0674	3.5205	3.9787	4.0418	3.8394	3.6841	3.9321	3.8575	3.6380	3.6345	3.6196
Top2b	_AS[Phospho (STY)]PITNDGEDEFVPSDGLDKDEYAFSSGK_.3	#N/A	5.4838	5.9526	4.9420	4.3253	3.7490	5.0215	5.0576	3.0645	4.7952	3.3526	4.1988	4.6419	5.8140	5.7531	4.3076	4.4114	5.6412	5.1119	4.7157	3.8545
Top2b	_VKAS[Phospho (STY)]PITNDGEDEFVPS[Phospho (STY)]DGLDKDEYAFSSGK_.4	Q64511_S1387_M2_Top2b	3.6500	3.2148	4.1863	3.7003	3.7934	4.0175	3.9071	3.4634	3.9662	3.8563	3.5349	3.5960	4.0743	4.3132	3.4843	3.9949	3.7663	3.5426	3.8417	3.9904
Sdc3	_KKDEGS[Phospho (STY)]YTLEEPK_.3	Q64519_S418_M1_Sdc3	3.2147	3.2872	3.7510	4.4746	4.0533	3.9316	3.4878	4.4160	3.0700	3.5019	4.0495	3.1767	3.2355	3.7381	3.0650	3.5596	3.4528	4.3804	4.0289	3.7051
Sdc3	_QAS[Phospho (STY)]VTYQKPDKQEEFYA_.3	Q64519_S428_M1_Sdc3	3.5898	4.4130	4.1261	4.0995	2.9276	3.5565	3.1127	3.2902	3.4451	3.1268	2.9238	4.3024	3.6106	4.1132	4.1907	3.9347	3.8279	4.0053	3.6538	4.0802
Guk1	_IC[Carbamidomethyl (C)]VLDVDLQGVR_.2	#N/A	4.7354	4.9144	4.6614	4.6432	4.6478	4.9305	4.3425	4.5264	4.0608	4.9169	4.3118	3.9039	4.3316	4.9916	4.7859	4.3451	5.2665	4.3522	4.5128	4.4054
Gpd2	_NYLSSDVEVR_.2	#N/A	6.5474	6.8246	6.3399	6.5086	6.5193	6.4858	6.4393	6.4674	6.6690	6.4591	6.0447	6.1747	6.3587	6.5601	6.1773	5.9816	5.9894	6.4785	6.4405	6.0783
Gpd2	_ELNWSELR_.2	#N/A	6.5314	6.5469	6.1155	6.5875	6.3545	6.4315	6.7493	6.0991	6.2042	6.6014	6.4818	6.6007	6.6573	6.2382	5.9953	6.3250	6.6847	6.4454	6.3125	6.9086
Gpd2	_TVGLFLQGGK_.2	#N/A	5.9495	6.5528	6.5099	5.3619	5.8290	6.4025	6.3293	5.7435	5.7683	5.8591	6.0014	6.2317	4.4571	6.0219	5.7918	5.7426	5.3580	6.3107	6.2057	6.2941
Gpd2	_LAFLNVQAAEEALPR_.2	#N/A	5.9234	5.7320	5.9599	6.5707	6.2089	5.7325	6.1327	5.8902	5.8065	6.0988	5.8327	5.7485	5.4207	5.1818	5.6888	5.9077	5.0439	5.7612	5.8510	5.8842
Gpd2	_KQEELETATR_.2	#N/A	5.3964	5.5617	5.3526	5.3441	5.2285	5.4519	5.6333	5.2229	5.3282	5.7427	5.3373	4.3467	5.1704	5.5322	5.7890	5.5709	5.4802	5.7928	5.1563	5.0888
Gpd2	_FHNLNAGPSR_.2	#N/A	7.1825	7.2664	3.3310	6.6523	6.6703	6.8439	7.5073	6.8622	6.7562	6.7095	6.3795	6.9878	6.6777	6.8093	7.3225	7.0145	6.6932	6.8549	7.0977	6.8993
Gpd2	_DWSPTLYIR_.2	#N/A	5.0153	5.7025	5.0920	5.2578	5.6665	5.6653	5.7140	4.1501	5.5238	5.2642	5.7757	5.5715	5.9317	5.8558	5.2043	5.5578	5.3518	6.5330	4.9497	5.8674
Gpd2	_TEQLTDSTEISLLPSDIDR_.2	#N/A	6.8155	3.6992	6.8264	6.7805	6.5731	7.0403	6.9254	6.5939	7.0444	7.2194	6.4589	6.7451	6.1794	7.5364	6.4099	6.5107	6.5350	6.7949	6.9317	6.4861
Gpd2	_LVQDYGLESEVAQHLAK_.2	#N/A	4.9821	5.1385	5.2768	4.6388	4.7564	4.5904	5.1211	4.9698	4.5287	5.1116	4.4284	4.8021	5.2430	5.3174	4.9405	5.0851	4.7155	5.2461	4.3002	5.0027
Gpd2	_YGAATANYM[Oxidation (M)]EVVSLLK_.2	#N/A	4.2824	4.1996	2.5876	4.0526	4.4829	3.9497	3.4606	2.8761	2.9108	3.7367	2.5811	3.4217	3.5462	3.7071	4.2550	3.6173	3.7636	4.1096	3.7182	3.6702
Gpd2	_C[Carbamidomethyl (C)]VINASGPFTDSVR_.2	#N/A	3.1177	5.2054	4.1551	4.4665	5.3383	3.2829	3.3938	4.8443	4.9180	3.9274	3.4149	3.9275	3.1486	4.0066	4.9104	5.1638	5.9493	5.2096	5.0498	4.7754
Gpd2	_M[Oxidation (M)]NLAIALTAAR_.2	#N/A	5.2919	3.7945	4.5620	4.0118	4.5785	4.8721	3.9564	3.0818	3.2367	3.3352	4.2162	4.7082	3.4022	3.9048	4.5212	4.5954	4.4796	6.0222	5.4415	5.2410
Gpd2	_NTPEFDILVIGGGATGC[Carbamidomethyl (C)]GC[Carbamidomethyl (C)]ALDAVTR_.3	#N/A	3.7160	5.1085	3.2526	5.4038	4.1874	4.2253	3.1252	3.9417	4.1102	3.5266	4.2466	3.3292	3.2108	4.5220	3.1505	3.0781	4.7402	4.2576	4.3616	4.7179
Gpd2	_AIM[Oxidation (M)]NLDVEQYR_.2	#N/A	3.7438	6.9732	7.7234	6.9064	6.6150	6.9419	6.6965	7.2620	7.0834	6.4962	4.0168	6.7676	4.0090	6.7977	6.6573	6.8777	7.7836	6.9525	6.6979	6.4969
Gpd2	_TALVERDDFSSGTSSR_.3	#N/A	3.9587	4.5131	3.4064	3.6256	4.1656	4.1989	4.5160	4.1843	4.3103	3.7693	3.7822	3.0865	4.4690	3.5333	3.6604	3.3208	3.6816	3.4679	3.7669	3.2106
Gpd2	_LVGAIVYYDGQHNDAR_.2	#N/A	4.2149	3.8372	3.9888	3.7797	3.6862	3.7233	3.4737	3.8390	3.7463	4.0255	3.7425	3.6646	3.8833	2.5096	3.4991	3.5771	3.8662	3.6546	3.6526	3.8697
Gpd2	_EYAC[Carbamidomethyl (C)]TAVDMSR_.2	#N/A	4.2475	3.7553	5.4842	3.4921	3.5852	3.8093	3.9559	4.3178	4.0150	4.4538	4.3893	3.6448	4.1230	3.6668	3.5331	4.7806	3.8151	3.3344	3.6334	3.3441
Gpd2	_M[Oxidation (M)]TIAGTTDTPTDVTHHPIPSEEDINFILNEVR_.4	#N/A	3.8039	4.1989	4.3402	3.8855	3.1417	3.3657	4.3994	3.5043	4.4585	4.0103	3.3320	4.0883	4.5666	4.3273	3.9766	4.1488	4.0420	4.3917	3.1899	3.7876
H2ac20;Hist2h2aa1	_AGLQFPVGR_.2	#N/A	11.4939	11.4510	11.2923	11.4204	11.3805	11.3327	11.5718	11.5350	11.2562	11.3057	11.1373	11.3524	11.4263	11.0541	10.9542	10.8909	11.2323	11.3304	11.0286	11.1674
H2ac20;Hist2h2aa1	_HLQLAIR_.2	#N/A	10.6890	11.4244	11.0389	10.9673	10.4521	11.1054	11.1276	10.8256	10.9633	10.5265	10.3306	10.8746	10.8654	11.1579	11.0416	10.8968	10.8260	11.1619	10.8602	10.9636
H2ac20;Hist2h2aa1	_VTIAQGGVLPNIQAVLLPK_.2	#N/A	8.9925	9.1908	8.9017	9.1658	8.6884	9.1900	8.9892	8.9806	9.0970	8.8494	8.7202	8.9513	9.3441	9.1517	9.0645	8.8255	8.9280	9.0231	8.8153	9.1526
H2ac20;Hist2h2aa1	_NDEELNKLLGK_.2	#N/A	7.5326	7.6986	6.9109	7.4741	7.3768	7.3011	7.3378	8.1510	7.6752	7.6872	7.2454	7.7295	8.3904	7.5485	7.6057	7.7203	7.6305	7.5986	7.6014	7.5228
H2ac20;Hist2h2aa1	_NDEELNK_.2	#N/A	2.3365	4.4030	4.1361	4.0895	2.9376	3.1617	3.1027	3.3002	3.1618	3.8062	3.1246	4.2924	3.2698	4.1232	4.1807	3.9447	3.8379	3.9953	3.6438	4.0902
H2ac20;Hist2h2aa1	_KGNYAER_.2	#N/A	3.1582	4.1609	4.1957	4.4260	3.3652	3.6422	3.6829	3.3839	3.2927	4.4697	3.0818	3.8870	3.1891	4.2337	3.7083	4.0212	4.3820	2.9016	3.0665	3.9110
Srm	_YQDILVFR_.2	#N/A	5.1795	5.4784	4.8765	4.2835	4.7048	5.4374	5.2541	5.4216	4.9485	5.4378	4.6393	5.5496	4.4015	6.1382	5.0384	5.1980	5.6932	5.1665	5.2194	5.1110
Srm	_AAFVLPEFTR_.2	#N/A	6.5079	7.2389	7.0625	4.0917	6.6470	7.0770	6.4816	6.5819	7.4595	7.8040	6.7922	7.2235	6.8847	7.2493	6.5972	7.1622	7.1189	7.2735	6.6834	6.7484
Srm	_VLIIGGGDGGVLR_.2	#N/A	5.1409	5.9142	5.0668	5.7862	3.9166	5.6219	4.7147	4.2792	5.2249	4.7319	5.5577	3.1334	4.5996	6.0098	5.2717	3.7502	5.6448	5.0810	5.1607	5.3058
Rbl2	_AQPAGS[Phospho (STY)]PSHQIQQR_.2	Q64700_S37_M1_Rbl2	4.2411	4.5788	3.7777	3.3431	2.9472	3.6603	4.1048	4.4668	4.1639	4.3048	3.0375	4.3049	4.2720	3.8158	4.1263	3.6032	3.9640	3.1854	3.4845	3.4930
Znrf1	_GREEDSS[Phospho (STY)]PGPQSQSHR_.3	#N/A	3.6349	3.7141	4.1712	4.1443	3.7298	4.5043	3.6953	4.0925	3.6767	3.9092	3.6300	3.6036	3.6556	4.1583	4.1427	3.9798	3.8730	4.0500	4.6263	4.4106
Znrf1	_ALEAPPEEDDDVS[Phospho (STY)]ANEELAER_.2	Q64707_S50_M1_Znrf1	3.1215	7.9065	7.7271	7.0154	3.3284	7.5537	7.2330	3.3472	7.7156	6.6881	3.1185	3.9237	3.8053	7.2821	7.2152	3.9844	4.3452	6.7473	7.3146	6.2393
Znrf1	_GREEDSS[Phospho (STY)]PGPQSQSHR_.3	#N/A	3.4674	3.8517	4.5048	4.1168	3.6743	4.4340	3.3738	3.6931	3.3902	3.2780	3.7646	3.5778	3.4983	4.5429	3.3991	4.3303	3.1903	3.9591	4.2582	4.2202
Vcl	_DPNAS[Phospho (STY)]PGDAGEQAIR_.2	#N/A	8.9849	9.0003	9.4937	9.2519	8.8280	8.8913	8.6704	9.0045	8.8061	8.9292	8.9898	8.6028	8.6837	9.4344	8.3327	8.6141	9.2306	8.7535	8.7927	8.9511
Vcl	_GWLRDPNAS[Phospho (STY)]PGDAGEQAIR_.3	#N/A	3.8021	7.0061	6.8974	4.9572	4.0091	6.8570	6.8067	4.1645	6.9480	4.9463	3.9388	6.0902	3.1247	8.0010	6.3524	5.8329	6.0491	7.4794	6.6392	6.7765
Vcl	_GQGAS[Phospho (STY)]PVAMQK_.2	Q64727_S346_M1_Vcl	3.9949	3.5908	6.8117	6.6679	4.2019	5.0219	5.4134	4.1124	5.3227	3.8055	5.6887	3.4803	4.4327	7.2534	4.8829	3.3571	3.1492	3.1831	4.3325	3.4015
Sp9	_KGS[Phospho (STY)]DS[Phospho (STY)]DTDASNLETPR_.2	Q64HY3_S424_M2_Sp9	4.5065	3.4859	3.3697	3.7511	4.4920	4.0683	3.6969	4.0588	4.4158	4.5572	4.2788	3.8468	3.8640	5.9763	4.6047	3.3505	3.5561	4.6886	4.9273	4.9414
Sp9	_SES[Phospho (STY)]PDLILHDSGVSAAR_.3	Q64HY3_S440_M1_Sp9	3.6464	3.6727	3.1830	3.9378	3.8533	3.8867	3.1948	3.8721	3.1150	3.4570	4.0944	3.3988	3.2804	3.7831	4.7874	3.0085	3.3693	4.3354	3.9840	4.4902
Ctnna3	_TPEELEDVS[Phospho (STY)]DLEDDHEVR_.2	#N/A	4.1207	3.3132	3.7441	4.0770	3.8108	4.3614	3.9594	3.6283	3.4971	3.2964	4.1524	3.2027	3.6291	4.3043	3.2869	3.9803	3.5666	3.9572	3.8991	3.5110
Wapl	_TES[Phospho (STY)]PSESC[Carbamidomethyl (C)]PVK_.2	#N/A	7.7857	7.6006	7.6114	4.4933	3.9169	7.4597	7.6255	4.3973	7.4988	7.4817	7.3313	7.2435	7.9570	7.5176	3.1566	6.7810	7.7910	7.5011	7.4470	7.2400
Wapl	_RTES[Phospho (STY)]PSESC[Carbamidomethyl (C)]PVK_.2	#N/A	4.0975	3.9772	3.6341	3.4867	4.3045	4.3378	4.2445	4.3232	4.1647	3.6752	3.6433	2.9477	4.3301	3.3319	4.2698	3.4597	3.8205	3.2857	4.4351	3.2989
Eif3j2	tyl (Protein N-term)]AAAAAAAAAAAAGDS[Phospho (STY)]DSWDADTFSM[Oxidation (M)]EDPV	Q66J56_S16_M1_Eif3j2	3.8628	7.0481	3.9212	4.0426	3.7527	4.5969	6.7041	3.7737	5.0409	6.2513	3.5923	3.7429	6.5058	7.0799	3.6114	3.7365	3.8921	3.8285	3.8593	4.1105
Rbm12b2;Rbm12b1	_RVS[Phospho (STY)]VEDLR_.2	Q6JIV4_S681_M1_Rbm12b2;Rbm12b1	7.3626	7.6709	6.8387	6.8589	6.7261	6.9822	6.6961	7.4376	6.7303	6.9931	6.7754	6.6828	7.7683	7.5402	5.9505	6.9751	7.5101	7.1802	7.0144	6.8595
Rbm12b2;Rbm12b1	_S[Phospho (STY)]RS[Phospho (STY)]PLGFYVHLK_.3	Q6JIV4_S277_M2_Rbm12b2;Rbm12b1	3.1449	4.1742	4.1823	3.9282	3.3518	3.3852	3.6963	3.4616	3.6165	4.4563	3.0951	3.9003	3.7819	4.9856	3.7217	4.0078	3.9993	4.7972	3.4825	4.2516
Rbm12b2;Rbm12b1	_MRS[Phospho (STY)]EEHS[Phospho (STY)]PSR_.2	Q6JIV4_S250_M2_Rbm12b2;Rbm12b1	4.5102	3.4927	3.5456	3.1792	3.8479	4.0720	3.6932	4.2105	3.7523	3.2156	4.1267	3.3821	3.8603	3.4041	3.2704	3.3542	3.2474	3.5971	3.8961	3.0814
Map3k10	_GSDGAS[Phospho (STY)]PPASPSIIPR_.2	#N/A	8.5423	8.0771	7.9743	7.9495	7.9024	8.1462	7.8543	8.2851	7.9533	8.3292	8.3877	7.7940	8.7497	8.4331	6.9852	8.1496	8.6284	8.2843	8.1033	7.9122
Map3k10	_QWSSS[Phospho (STY)]APNLGK_.2	Q66L42_S583_M1_Map3k10	4.3640	4.4559	3.9006	7.5907	3.0701	6.9453	3.9780	3.7433	3.8982	4.1746	3.3768	4.1820	6.6200	4.5663	4.0034	3.7261	4.2810	3.5522	3.2008	3.0325
Map3k10	_KGSDGAS[Phospho (STY)]PPASPSIIPR_.3	#N/A	6.6791	6.8805	6.6101	5.9598	3.8419	4.1690	6.4920	4.1664	6.1645	3.4790	3.9703	7.1767	6.7083	3.4970	3.2829	3.3482	6.0813	6.7215	5.9659	6.3282
Cbarp	_HAS[Phospho (STY)]LDGASPYFK_.2	#N/A	9.9243	10.0636	9.2735	9.9067	8.8864	9.5064	9.4267	9.3103	9.1610	9.5042	9.0882	9.0376	9.4908	9.5829	7.7289	9.1641	9.3785	9.4934	9.0557	9.3225
Cbarp	_ASS[Phospho (STY)]LDTRGS[Phospho (STY)]PK_.2	#N/A	7.8622	7.5898	7.8295	7.7033	7.3878	7.1021	7.3177	6.8208	7.1765	7.5439	7.3698	6.7729	7.1708	2.8536	5.7579	7.0923	7.1814	4.2564	7.2249	7.2981
Cbarp	_RDYS[Phospho (STY)]IDEK_.2	Q66L44_S537_M1_Cbarp	8.3173	3.9572	8.3252	4.2223	3.5688	3.6022	3.4793	7.2203	3.3995	3.1725	7.6308	3.6833	7.2798	7.3757	3.5047	6.8946	3.0848	4.0509	3.6995	7.6199
Cbarp	_GAGDEVSELPAPARS[Phospho (STY)]PPR_.3	#N/A	8.8008	8.6542	8.6874	8.6189	8.8453	8.9041	8.3523	8.7583	8.7748	8.2173	8.6697	7.9091	8.8297	8.2319	7.4370	8.0274	8.5155	8.4991	8.1589	8.4118
Cbarp	_LAEPVASSQVLVAAAAPTS[Phospho (STY)]PDHSPA_.3	Q66L44_S692_M1_Cbarp	6.6087	7.5201	6.0260	3.9494	3.6324	3.7823	6.9987	3.8655	5.5424	3.9594	3.6853	6.7770	4.2360	7.4152	3.5127	3.7172	3.9094	3.8101	3.7526	3.9526
Cbarp	_RHAS[Phospho (STY)]LDGASPYFK_.3	#N/A	5.7395	5.9472	3.4419	5.7069	4.1123	4.7798	5.7631	4.1310	5.7978	5.2015	5.3624	4.9160	6.3943	5.4067	3.2853	3.2675	4.6960	5.8480	5.3662	5.1029
Cbarp	_GAGDEVSELPAPARS[Phospho (STY)]PPRS[Phospho (STY)]PR_.3	Q66L44_S520_M2_Cbarp	8.2531	8.8197	9.0253	9.6232	8.2983	8.0628	8.6329	9.4509	8.2879	9.4856	7.0584	8.2774	8.4391	7.9145	6.5719	8.1483	8.0977	7.8916	7.8552	8.3988
Cbarp	_ASS[Phospho (STY)]LDTRGS[Phospho (STY)]PKR_.2	#N/A	7.4272	7.0919	7.2432	7.5850	7.8590	6.8441	6.8543	6.9645	6.6266	7.4822	6.6669	6.6994	7.0930	3.2267	5.9883	6.6618	6.6259	6.8190	6.5120	6.4604
Cbarp	_LLQMDSGYAS[Phospho (STY)]IEGR_.2	#N/A	3.9663	3.2390	5.9554	5.3292	3.5010	4.2066	3.7371	4.3876	3.6573	3.7769	3.8083	3.9412	3.8228	5.3258	3.7625	3.3284	3.6743	3.9505	3.8330	3.5505
Cbarp	_RGDS[Phospho (STY)]VDC[Carbamidomethyl (C)]PPEGR_.2	Q66L44_S610_M1_Cbarp	3.3472	3.8275	4.5290	4.0926	4.1857	7.4605	3.3553	3.7173	3.4144	6.9945	6.9825	3.5536	3.5225	4.5671	2.9325	3.6921	3.2145	3.9349	4.2340	4.2444
Cbarp	AS[Phospho (STY)]PET[Phospho (STY)]PPEHGIS[Phospho (STY)]LGPEHAQQQDPQQEQDAEHAQC[Carbamidomet	Q66L44_S389_M2_Cbarp	3.7152	4.2876	4.2515	3.9741	3.0530	3.2770	2.9873	3.4156	3.0464	3.9216	3.4207	4.1770	3.1544	4.2386	4.0653	4.0601	3.9533	3.8799	3.5284	4.2056
Cbarp	_RVS[Phospho (STY)]FNEAALFEQSR_.3	Q66L44_S129_M1_Cbarp	4.2291	4.5908	3.7657	3.3551	2.9352	4.4694	4.1129	4.4548	4.0331	4.0397	3.5517	3.2448	4.1985	3.8038	4.1382	3.5913	3.9521	3.4173	3.0659	3.1673
Tusc1	_GHGREEEPGS[Phospho (STY)]PR_.3	#N/A	3.6258	4.1721	3.5406	3.9240	3.7483	3.5974	4.0782	4.2168	4.3444	3.6661	4.3417	3.8111	4.9290	4.8816	6.8758	4.0020	5.6823	5.6519	4.5959	6.3192
Bcl9l	_EAPGS[Phospho (STY)]PPLSPR_.2	Q67FY2_S21_M1_Bcl9l	8.2490	7.7787	6.4437	8.8822	3.8708	8.6789	8.1916	8.5655	7.3879	9.1817	6.9137	7.5840	3.5986	9.2524	8.5012	7.7908	8.9706	7.6385	7.6526	8.7535
Bcl9l	_TAMPS[Phospho (STY)]PGVSQNK_.2	Q67FY2_S1014_M1_Bcl9l	4.3315	5.8447	3.8681	3.2527	3.0376	3.5699	4.0105	3.0564	4.2543	4.1421	3.1279	4.2145	4.3624	3.9062	4.0359	4.6936	4.0544	3.0950	3.3941	3.5834
Grm7	_TELC[Carbamidomethyl (C)]ENVDPNS[Phospho (STY)]PAAK_.2	Q68ED2_S900_M1_Grm7	10.8715	10.5576	10.7357	10.5898	9.8937	10.2976	10.3967	10.6614	10.3198	10.2580	10.2690	10.2338	10.9839	10.4093	9.2215	9.9836	10.8433	10.5087	10.3606	10.0342
Crtc1	_RPLS[Phospho (STY)]VDKHGR_.3	#N/A	4.4488	6.3077	3.4274	5.6679	4.2234	4.9096	5.5823	4.8129	4.8356	5.8268	5.8570	4.8847	5.3194	5.3090	3.3318	6.5468	4.3793	2.9910	6.5066	3.1428
Crtc1	_RPLS[Phospho (STY)]VDK_.2	#N/A	8.1253	5.4492	5.8831	8.1547	4.9183	4.6973	4.4121	7.9366	7.6624	5.1530	4.6696	7.9942	4.2962	5.8560	4.9561	5.2231	4.1090	5.0441	5.5494	5.8753
Rab3ip	_TLVLSSSPTS[Phospho (STY)]PTQEPLAAAK_.2	Q68EF0_S218_M1_Rab3ip	6.7586	7.0300	7.5576	6.1575	3.0669	3.2910	6.3713	7.4672	6.5171	6.5362	6.0143	6.5854	7.2230	7.4931	5.3145	4.0741	6.6737	6.2422	7.2003	6.0274
Begain	_AS[Phospho (STY)]PLYATYK_.2	Q68EF6_S473_M1_Begain	8.8188	8.8105	8.8573	9.0621	8.4375	8.7067	8.9001	8.8589	8.5526	8.8350	8.9186	8.5418	9.0046	8.9652	8.1282	8.6569	8.9836	8.8812	8.6331	8.6094
Begain	_AGGDLSLS[Phospho (STY)]PSR_.2	Q68EF6_S510_M1_Begain	4.0275	3.6234	9.0243	9.0768	4.2345	8.5559	4.3145	8.6370	8.4842	8.7032	8.6976	8.5843	4.4001	3.4019	7.6625	8.2549	3.1166	8.6507	4.3651	8.5725
Begain	_VLEKPDPGS[Phospho (STY)]LSSR_.2	Q68EF6_S194_M1_Begain	7.9737	4.4373	3.9192	3.6651	3.0887	3.1221	3.5994	3.7247	3.8796	4.1932	3.3582	4.1634	4.0450	4.5477	7.0889	3.7447	4.2624	3.5708	3.2194	3.0139
Begain	_GDIYC[Carbamidomethyl (C)]S[Phospho (STY)]DPALYC[Carbamidomethyl (C)]PDER_.2	Q68EF6_S229_M1_Begain	3.9774	10.1172	10.4722	10.0021	3.4227	10.1366	9.9543	6.4112	9.9719	9.5261	8.7842	9.6423	8.5420	10.6116	9.2764	9.4606	9.9484	10.0032	9.9572	10.0622
Begain	_YYGGGGGGGAAGGGS[Phospho (STY)]PGDKAEGR_.3	#N/A	5.2928	4.2683	4.2030	3.5246	3.1862	4.8746	3.3520	3.6293	5.7520	5.4158	4.0709	3.9496	4.4523	4.1047	4.8214	4.4163	4.3281	4.9538	5.2015	5.6854
Begain	_KPSATYGS[Phospho (STY)]GPR_.2	Q68EF6_S344_M1_Begain	4.0290	3.2901	3.5656	3.5552	4.2359	5.4479	4.3130	4.2547	5.8943	3.8396	5.4158	3.0162	4.3987	5.3298	2.8375	3.3911	3.7519	6.3034	3.6966	3.3675
Begain	_AQNST[Phospho (STY)]DSAAEEEEEAEAAAFPEAYR_.3	Q68EF6_T263_M1_Begain	4.2677	5.5793	3.4820	3.3884	3.5870	6.0566	3.9589	4.1566	5.6756	4.4556	4.0122	5.6842	5.3888	6.5146	5.5237	6.0053	5.7889	6.3294	5.3937	5.4160
Begain	_SADALAGYAAS[Phospho (STY)]DGDGDRLR_.2	#N/A	3.8202	3.7831	4.5340	4.1858	3.8660	4.0901	6.5642	3.8424	3.7928	4.0265	3.4591	3.4227	3.9009	4.2374	3.8669	3.0834	3.9745	5.1365	4.7007	5.2659
Begain	_LS[Phospho (STY)]PQQAFPR_.2	Q68EF6_S570_M1_Begain	3.9581	4.0447	4.4944	6.8620	5.5212	6.4800	5.3178	3.6585	6.1879	5.9702	8.5759	2.7088	4.4124	2.6237	3.8224	6.0145	4.1962	3.0450	5.2927	5.9228
Begain	_VQLC[Carbamidomethyl (C)]GAGS[Phospho (STY)]S[Phospho (STY)]PEPEHGSR_.2	Q68EF6_S590_M2_Begain	5.0040	4.8751	5.9350	5.4618	5.1029	4.5657	4.6614	5.4164	5.0865	4.9132	4.5939	5.7253	5.7506	5.7752	5.4607	5.4422	5.8268	5.5263	5.3761	5.3401
Begain	_ADS[Phospho (STY)]FSEGDDLSQGHLAEPC[Carbamidomethyl (C)]FLR_.3	Q68EF6_S483_M1_Begain	3.7626	4.2402	7.2905	6.9665	3.1004	3.3837	4.4407	3.4630	6.8300	3.9690	3.0966	4.1296	3.7834	4.2860	4.0179	4.1075	4.0007	7.0691	3.4810	6.2085
Begain	_YYGGGGGGGAAGGGS[Phospho (STY)]PGDK_.2	#N/A	3.1655	3.3365	3.7018	4.5239	4.0041	3.9809	3.5841	4.3667	3.5961	3.3718	3.9705	3.2259	3.1862	3.6889	3.1142	3.5815	3.4036	4.4296	4.0782	3.6559
Begain	_GDIY[Phospho (STY)]C[Carbamidomethyl (C)]SDPALYC[Carbamidomethyl (C)]PDERHAR_.4	#N/A	3.6304	3.6886	3.1670	3.9538	3.8374	3.9312	3.7182	3.8561	3.5533	3.4147	3.9276	3.4148	3.6613	3.2051	3.0714	2.9926	3.3533	3.7961	4.0951	4.7937
Begain	_AASTLGS[Phospho (STY)]PLEAQVAPGFAR_.2	Q68EF6_S357_M1_Begain	3.0800	4.2391	4.1175	4.5042	3.2870	3.3206	4.4446	3.3057	3.0029	3.9651	3.3772	3.9652	3.1109	4.1555	4.0218	3.9430	4.3038	4.3465	3.1447	3.8328
Begain	_RPS[Phospho (STY)]VDT[Phospho (STY)]PVTDVGFLR_.2	Q68EF6_S246_M2_Begain	3.8863	4.1165	4.4226	3.1309	3.2240	3.4481	4.3171	3.1782	4.3761	4.0926	3.2497	4.0060	4.4842	4.0280	3.8942	4.2312	4.1762	3.8183	3.2723	3.7052
Begain	_ESLEPS[Phospho (STY)]SMEAS[Phospho (STY)]PEMHPPTR_.2	Q68EF6_S555_M2_Begain	3.5037	3.8153	4.5412	4.0805	3.7107	4.3977	3.3675	3.7294	3.4266	5.5414	3.8009	3.5415	3.5346	4.5792	2.9447	4.3667	3.2267	3.9228	4.2218	4.2565
Cltc	_IYIDSNNNPER_.2	#N/A	13.3673	13.3030	13.0118	13.1858	12.9166	13.1932	13.1554	13.6181	13.0869	12.9517	12.9315	12.8998	13.3825	13.1201	11.8375	12.7022	13.3149	13.1909	13.0106	12.7659
Cltc	_LLLPWLEAR_.2	#N/A	10.4155	10.2417	10.0542	10.1251	10.0254	10.2807	10.4019	10.2558	9.9710	9.9348	9.9946	10.1043	10.0008	9.2684	9.0837	9.6215	10.0507	10.1595	9.7942	9.5528
Cltc	_NNLAGAEELFAR_.2	#N/A	9.7349	9.8293	9.6804	9.7242	9.4116	9.7036	9.7480	9.9949	9.4444	9.5308	9.5829	9.3987	9.6941	9.2215	8.8878	9.1374	9.5726	9.6005	9.3084	9.3511
Cltc	_DPHLAC[Carbamidomethyl (C)]VAYER_.2	#N/A	8.4940	8.2259	8.4689	8.0765	8.3384	8.2927	8.5317	8.5211	8.0134	8.0734	8.3778	7.9336	8.1324	7.8081	7.4860	8.2130	8.2135	8.1793	8.0400	8.1262
Cltc	_LLYNNVSNFGR_.2	#N/A	9.4788	9.3609	9.1279	9.2867	9.2438	9.2505	9.4301	9.7017	9.0218	9.1291	9.8727	9.0636	9.1514	8.4479	9.5280	8.6941	9.3604	9.2330	8.9676	8.6412
Cltc	_ESNC[Carbamidomethyl (C)]YDPER_.2	#N/A	9.0998	8.9476	9.0376	8.8255	8.6677	8.9729	9.2171	8.9749	9.1712	8.8600	8.8112	8.6648	8.5482	8.3488	8.5430	8.2394	8.7173	8.9179	8.5931	8.6598
Cltc	_LHIIEVGTPPTGNQPFPK_.3	#N/A	8.0178	8.0248	7.7900	7.9199	7.7033	7.9284	8.1262	8.2890	7.7028	7.9722	7.8337	7.5715	7.9812	7.4579	6.7146	7.4957	7.7365	7.7614	7.4355	7.5195
Cltc	_EVC[Carbamidomethyl (C)]FAC[Carbamidomethyl (C)]VDGK_.2	#N/A	9.1433	8.9206	9.1821	9.0584	8.7935	8.8741	9.0844	9.4039	9.0128	8.8360	8.8902	8.6994	8.6083	8.4971	7.9767	8.2878	8.5808	8.5795	8.3751	8.5695
Cltc	_LAELEEFINGPNNAHIQQVGDR_.3	#N/A	9.0469	9.0872	8.9920	8.9789	8.2002	8.7086	9.1455	9.1024	8.5837	8.6305	8.8470	8.6118	8.7823	8.8071	7.8033	8.0072	9.0735	9.0656	8.5729	8.3136
Cltc	_AHIAQLC[Carbamidomethyl (C)]EK_.2	#N/A	8.6671	8.3276	8.8787	8.6457	8.3797	8.6925	8.9583	8.7803	8.4131	7.9668	8.3525	8.3915	8.2718	8.2068	8.2850	7.9092	8.5094	8.5438	8.2693	8.2688
Cltc	_GQFSTDELVAEVEKR_.2	#N/A	7.4910	7.2881	7.4459	6.9872	7.0756	7.1653	7.2228	7.4812	7.1520	7.0067	7.0770	7.1332	7.3729	7.2251	5.9545	6.5637	7.1189	7.1123	7.0498	6.6536
Cltc	_ENPYYDSR_.2	#N/A	8.2582	8.2838	8.0343	8.0121	8.2189	7.9509	8.5630	7.8014	8.2618	8.0064	7.6521	7.5261	7.9288	7.1857	7.3877	7.6211	7.2756	8.1289	7.7951	7.8548
Cltc	_LTDQLPLIIVC[Carbamidomethyl (C)]DR_.2	#N/A	8.1694	7.7789	8.4512	7.7332	7.6515	7.9643	8.1026	7.8607	8.0210	8.0353	7.8847	7.5461	7.3474	7.3412	6.9076	7.1161	8.0658	7.7046	7.3360	7.4555
Cltc	_C[Carbamidomethyl (C)]NEPAVWSQLAK_.2	#N/A	8.1997	7.7807	7.4973	7.6498	7.6404	7.8420	8.2822	8.1979	7.7865	7.7113	7.4691	7.8752	7.7094	7.3155	7.5972	7.3269	7.8739	7.9782	7.5797	7.0205
Cltc	_SVDPTLALSVYLR_.2	#N/A	8.0040	8.1588	8.1482	7.5418	7.8035	7.5533	7.8173	7.9394	8.1291	7.7273	7.5979	7.6871	7.5109	7.1337	7.0694	7.0854	7.7366	7.9904	7.8533	7.5721
Cltc	_NLQNLLILTAIK_.2	#N/A	8.0861	8.1030	7.7853	7.9339	7.5229	7.7189	8.3496	8.2378	7.6188	7.7500	7.8547	7.8641	8.0416	6.8742	6.9559	6.8318	7.9972	8.0151	7.8644	7.3834
Cltc	_EHLELFWSR_.2	#N/A	8.0079	7.5983	7.9701	7.9432	7.7287	7.8879	7.8565	8.0518	7.6324	7.7696	7.9849	7.3950	7.8030	7.2497	6.6889	7.2745	7.7642	7.8588	7.5305	7.3079
Cltc	_HELIEFR_.2	#N/A	8.1840	7.8045	7.7543	7.5161	7.3231	8.1274	8.1942	8.3726	7.7891	7.1233	7.9841	7.7946	7.6912	7.3703	6.7033	7.1343	7.7863	7.5160	7.1127	7.4269
Cltc	_WLLLTGISAQQNR_.2	#N/A	8.2255	7.7541	8.1077	7.7749	6.9529	7.6411	7.5711	7.5191	7.8451	7.5683	7.5295	6.9221	8.0395	7.1309	6.5997	6.8794	7.6526	7.3983	7.2340	7.5631
Cltc	_HDVVFLITK_.2	#N/A	7.7842	7.5875	7.9325	7.5433	7.1193	7.5868	8.1274	7.7033	7.5089	7.0148	7.3890	6.9517	7.2249	7.2187	5.2328	6.6510	7.3787	7.7508	6.5188	6.9185
Cltc	_LASTLVHLGEYQAAVDGAR_.3	#N/A	6.4274	6.4054	6.4020	6.4746	6.6775	6.5079	6.7619	6.9756	6.3995	6.7574	6.4219	6.2639	6.0819	6.1435	5.1320	5.9042	6.3659	6.1653	5.9751	6.2737
Cltc	_AFM[Oxidation (M)]TADLPNELIELLEK_.2	#N/A	7.3250	7.3712	6.6669	6.7804	6.9224	7.2197	7.6537	7.5185	6.8762	6.9057	6.8484	7.0396	6.7064	6.7683	6.3048	6.4529	7.0067	7.1869	6.9861	6.6996
Cltc	_VIQC[Carbamidomethyl (C)]FAETGQVQK_.2	#N/A	6.7731	7.3133	7.3346	6.9680	6.2531	7.2916	7.4191	6.6872	7.1969	6.7339	6.1256	6.0191	6.0981	6.4546	6.4053	5.3467	6.8897	6.8146	6.3872	6.2235
Cltc	_LEKHELIEFR_.3	#N/A	6.6516	6.6960	6.3271	6.5394	6.7176	7.0014	7.3646	7.5017	6.9205	7.1535	6.7217	7.1978	7.2922	6.7982	5.6417	6.7038	6.8311	7.5745	7.1034	6.5450
Cltc	_IAAYLFK_.2	#N/A	7.6291	7.2962	7.0205	7.3966	7.1095	7.1693	7.5241	7.4178	6.2997	7.0809	7.1453	7.0127	7.6248	5.7947	6.5580	5.8988	6.9741	7.1344	6.8945	6.9291
Cltc	_YIEIYVQK_.2	#N/A	6.7848	7.0531	7.5503	6.8413	6.4480	6.6992	7.1743	6.6480	6.8590	6.2042	6.4343	6.4492	6.9627	6.0133	5.6654	6.3228	5.6833	7.2215	6.8480	6.8171
Cltc	_SVNESLNNLFIEEDYQALR_.2	#N/A	6.3262	6.1991	5.7364	5.8344	5.4721	6.0315	5.9624	6.7658	5.5860	6.1718	5.5369	5.6600	6.4980	6.1109	4.4897	5.2562	6.1040	6.0156	5.8937	5.8110
Cltc	_ISGETIFVTAPHEATAGIIGVNR_.3	#N/A	7.1846	7.4743	6.3562	6.4936	6.1421	6.2906	7.3330	6.9522	5.8127	6.5867	6.4790	6.8407	7.2874	6.5331	5.4069	5.7368	6.3672	6.9953	5.6057	5.5097
Cltc	_YESLELC[Carbamidomethyl (C)]RPVLQQGR_.3	#N/A	5.9931	6.4023	6.7324	5.9197	5.9831	6.3406	6.7107	5.8481	5.5968	6.5688	6.3448	5.0688	6.1378	6.1078	4.7565	5.5992	6.3611	6.1944	6.3487	5.6840
Cltc	_QSVELC[Carbamidomethyl (C)]KK_.2	#N/A	6.3873	5.3885	6.6032	6.0902	5.5922	5.7624	5.6515	6.0009	5.8249	5.5198	5.2636	6.2672	4.5774	6.5121	5.0702	6.7658	6.5688	6.3990	5.4263	5.9929
Cltc	_RPLIDQVVQTALSETQDPEEVSVTVK_.3	#N/A	6.0923	6.3607	6.5519	5.3396	5.9281	6.0628	5.9419	6.5333	6.5422	5.6790	5.5282	5.2795	6.2585	5.9358	4.6384	5.5154	6.8782	6.5570	6.1719	5.8591
Cltc	_EAIDSYIK_.2	#N/A	4.7371	4.8268	5.0717	5.2553	5.4825	5.1939	6.1473	4.3071	5.8181	5.8901	6.1330	4.8435	5.5939	4.6139	5.1312	6.0905	4.5190	5.9641	5.8291	6.1741
Cltc	_GQFSTDELVAEVEK_.2	#N/A	5.9825	5.6317	5.6287	5.7976	5.5323	5.7041	5.8655	5.9746	6.0135	5.5983	5.7118	5.3066	5.8921	5.5161	4.2174	5.1257	5.9968	4.9275	4.8683	5.4087
Cltc	_IVLDNSVFSEHR_.2	#N/A	8.1206	8.1275	7.9023	8.0008	7.6140	7.9892	8.1567	8.4039	7.9718	7.8613	7.5487	7.3737	7.8984	7.4930	6.8763	7.4263	8.1014	7.8823	7.5230	7.3729
Cltc	_KFNALFAQGNYSEAAK_.3	#N/A	6.9661	6.0082	7.0208	6.7842	6.4563	6.6897	6.8147	6.5441	6.5022	6.6321	6.5014	6.1521	6.4736	5.7166	5.9275	6.5670	6.2085	6.4810	6.4324	5.9956
Cltc	_AYEFAER_.2	#N/A	5.6523	6.1638	5.6996	6.8081	5.7643	6.4853	6.8920	4.4164	7.1229	7.0076	6.4495	6.8098	6.7967	5.3475	6.4084	6.0134	3.0132	6.9285	6.9249	5.9971
Cltc	_RDPHLAC[Carbamidomethyl (C)]VAYER_.3	#N/A	7.0355	7.3088	6.5491	7.5145	6.9236	7.2918	7.5879	4.3037	7.0914	6.5115	7.0710	6.8887	7.1724	3.6259	6.7509	6.4103	6.6943	7.0887	6.8218	6.8379
Cltc	_VGYTPDWIFLLR_.2	#N/A	5.2468	5.8553	5.8441	5.1921	4.7827	5.4723	5.2848	5.2303	5.3234	4.3881	4.7044	4.9655	4.1965	4.5230	3.5780	3.5651	5.5096	5.9868	4.9180	4.8963
Cltc	_QSVELC[Carbamidomethyl (C)]K_.2	#N/A	5.0077	5.5318	4.9033	5.2203	3.4408	4.2385	5.2848	4.2137	4.5042	4.5960	4.7611	5.1555	4.2675	3.8112	5.5141	2.9471	4.5436	5.0114	5.1403	4.6205
Cltc	_VM[Oxidation (M)]EYINR_.2	#N/A	6.3833	6.4051	5.8201	6.6014	5.9962	6.4600	6.9877	6.5985	6.3639	5.6967	7.1013	5.7497	6.0130	6.4127	5.5058	5.3926	5.9636	6.2660	5.6956	5.6683
Cltc	_VANVELYYK_.2	#N/A	4.3401	4.9827	4.0087	3.0879	3.7551	2.7761	4.6076	4.8576	3.7740	4.4154	3.9441	3.8838	2.9339	4.4236	3.7113	4.4111	3.9017	4.9250	4.5611	4.3009
Cltc	_VVGAM[Oxidation (M)]QLYSVDR_.2	#N/A	6.3471	6.2794	6.1775	5.9634	5.2564	5.9325	6.4028	5.6334	5.5974	5.2863	5.9579	5.5911	5.6968	5.7432	4.4533	4.9768	5.8734	5.7173	5.2878	5.0578
Cltc	_M[Oxidation (M)]EGNAEESTLFC[Carbamidomethyl (C)]FAVR_.2	#N/A	7.4979	6.7607	6.4106	6.3801	6.2420	6.6362	6.6856	7.2156	6.3745	6.6878	6.0476	6.9150	7.3706	6.6425	3.3414	6.2756	6.6327	6.4894	6.4796	5.8465
Cltc	_LLEM[Oxidation (M)]NLM[Oxidation (M)]HAPQVADAILGNQM[Oxidation (M)]FTHYDR_.4	#N/A	3.8170	5.8908	4.8197	3.7672	5.5653	5.6283	5.6544	5.6895	5.6475	5.7766	5.2269	5.5930	3.8479	3.3916	4.6553	5.0616	5.5944	5.8695	5.3921	5.6509
Cltc	_FNALFAQGNYSEAAK_.2	#N/A	5.1104	5.8404	5.0380	4.5920	5.5370	5.4949	5.2524	4.4181	5.3084	5.5129	4.5978	5.4821	3.2376	5.1178	4.0128	4.1485	5.1056	4.4803	5.4083	3.7073
Cltc	_TLQIFNIEMK_.2	#N/A	4.0368	4.4555	5.9272	6.0981	4.3136	4.4789	5.8193	3.7372	3.4909	2.7582	5.1315	3.8555	4.3337	5.8003	2.4290	4.3817	4.6741	2.4817	3.2069	4.0391
Cltc	_VDKLDASESLR_.2	#N/A	6.9886	7.1771	7.1963	7.0301	6.6036	7.0132	7.3348	6.8472	7.1259	6.3554	6.5160	6.3228	6.4585	6.2367	6.7245	6.7358	6.7754	6.6893	6.8757	6.5083
Cltc	_VSQPIEGHAASFAQFK_.3	#N/A	7.0436	6.5774	6.5293	6.0534	5.7076	5.7566	6.6890	6.9798	6.7087	6.0894	6.1968	6.3910	6.8936	5.8206	5.8639	6.1457	6.2027	6.1121	5.2362	5.4680

Cltc	_GQC[Carbamidomethyl (C)]DLELINVC[Carbamidomethyl (C)]NENSLFK_.2	#N/A	6.4534	6.6974	6.4517	6.3408	5.6000	6.2369	6.6751	6.1750	6.2868	6.2750	6.2285	5.9727	6.3491	6.2509	5.5443	5.6953	6.4600	6.2026	6.0870	6.0307
Cltc	_AM[Oxidation (M)]LSANIR_.2	#N/A	5.5278	6.1070	5.8327	5.7275	5.2130	6.1770	6.3317	5.5756	5.9196	5.3718	5.4910	5.5027	5.3987	5.5837	5.2614	4.8061	5.6294	5.9595	6.4805	5.3549
Cltc	_ALEHFTDLYDIK_.2	#N/A	6.1970	5.5791	6.2520	5.6925	5.2813	5.5561	6.3438	5.6493	5.7051	5.6841	6.2064	5.4210	5.3899	5.4256	5.0343	5.3436	5.8806	6.1290	5.8532	5.7498
Cltc	_ESYVETELIFALAK_.2	#N/A	4.4191	4.0149	5.1618	3.7015	4.6694	3.4486	4.4857	5.7157	4.9919	3.9992	4.7516	3.7480	4.4911	4.2335	3.1430	3.7812	4.2259	5.3936	5.1532	4.3164
Cltc	_HSSLAGC[Carbamidomethyl (C)]QIINYR_.3	#N/A	5.2692	4.8411	6.2466	5.9208	5.1206	5.2811	5.6723	5.4822	5.7587	5.9793	5.6606	4.7344	5.2828	4.8880	4.7979	4.5501	5.3182	5.6768	5.6716	5.2009
Cltc	_DAMQYASESK_.2	#N/A	4.0977	6.1516	7.2351	7.2241	4.3046	5.8644	6.2890	4.0096	5.7574	5.9083	6.2232	4.6802	4.3299	6.6900	5.4471	4.4598	4.5473	3.2859	4.4353	3.2988
Cltc	_IHEGC[Carbamidomethyl (C)]EEPATHNALAK_.3	#N/A	6.2418	5.9393	6.0537	5.2122	6.0442	6.1641	6.6306	6.0325	6.2722	6.0179	6.1609	5.7712	5.9856	5.5095	5.8042	5.5995	5.9721	6.1633	5.9419	5.6949
Cltc	_ALEHFTDLYDIKR_.3	#N/A	8.1433	7.6864	7.2629	7.8098	7.6197	7.5406	7.6567	8.1796	7.7192	7.8539	7.4717	7.5061	8.0305	6.8710	6.7907	7.3605	7.7496	7.4455	7.4508	7.2813
Cltc	_LPVVIGGLLDVDC[Carbamidomethyl (C)]SEDVIK_.2	#N/A	7.0927	6.8394	6.1775	6.7497	6.0965	6.5121	6.8578	6.7364	6.3094	6.4274	6.2391	6.2057	6.7774	6.4900	5.4616	6.0689	6.7443	6.6989	5.9770	6.2954
Cltc	_ADDPSSYM[Oxidation (M)]EVVQAANASGNWEELVK_.3	#N/A	6.2891	5.3037	5.0409	5.2725	4.8619	4.9473	4.8203	6.1435	5.0494	5.2260	5.1245	4.8576	5.7706	4.8613	4.1204	5.1480	5.2885	4.7500	4.9126	4.8036
Cltc	_FQSVPAQPGQTSPLLQYFGILLDQGQLNK_.3	#N/A	3.3421	4.4530	3.4612	3.2636	3.7635	4.4653	4.7348	4.6483	4.2810	3.1622	3.7009	4.0548	3.2803	5.5818	3.3548	2.7630	4.9599	4.3519	3.5986	3.4153
Cltc	_GILRT[Phospho (STY)]PDTIR_.2	Q68FD5_T394_M1_Cltc	3.2616	3.2403	3.7979	4.0071	4.1002	4.3243	3.4409	3.8028	3.5000	3.4679	3.8744	3.1298	3.6080	7.8555	3.0181	3.6065	3.3001	8.1212	4.1484	4.3299
Cltc	_KFDVNTSAVQVLIEHIGNLDR_.3	#N/A	5.7164	5.0997	5.5153	4.7970	3.6381	5.6752	4.5366	4.4823	4.6946	4.9510	4.6593	3.5918	4.7115	5.0766	3.4801	3.1445	3.7621	4.7964	4.7246	3.2911
Cltc	_TSIDAYDNFDNISLAQR_.2	#N/A	7.4612	7.3024	7.3869	7.1365	6.8090	7.0619	7.3346	7.1605	7.1071	7.2232	6.9964	6.8694	7.2434	6.6310	5.9018	6.7278	7.3010	7.0030	6.6166	6.6943
Cltc	_AVNYFSK_.2	#N/A	3.6639	3.6552	3.2004	3.9204	3.8708	3.9041	4.2656	3.8895	3.0975	3.4745	3.1291	2.3264	3.0859	3.0473	3.2719	3.5512	3.3868	2.9314	3.0486	3.1606
Cltc	_YIQAAC[Carbamidomethyl (C)]K_.2	#N/A	4.1015	3.8997	3.1370	3.5879	4.4816	4.7766	5.1473	3.8019	5.1119	4.3078	4.9495	3.7907	5.0394	3.8127	4.8451	3.7359	4.3396	5.0657	4.8698	4.0877
Cltc	_NNRPSEGPLQTR_.2	#N/A	4.8990	5.2968	4.7505	5.4586	4.0406	5.3671	6.3786	5.6728	5.7204	5.3614	5.9134	4.6738	5.5148	5.2790	5.0929	4.6555	5.3825	5.6073	5.5548	5.4162
Cltc	_VGEQAQVVIIDM[Oxidation (M)]NDPSNPIR_.2	#N/A	4.2822	4.4063	5.0162	4.5701	4.2837	4.5600	4.4684	4.2544	4.4726	4.3192	4.2009	3.9699	4.4831	4.0705	3.0974	4.4922	4.1588	4.2540	4.1896	4.5271
Cltc	_AHM[Oxidation (M)]GM[Oxidation (M)]FTELAILYSK_.2	#N/A	3.4956	5.0353	4.5330	4.0100	3.7025	3.3922	3.3456	3.7213	5.0211	3.3062	4.0775	3.5496	4.7599	4.5711	3.3709	4.3586	4.2630	4.3017	4.1227	4.2484
Cltc	_AHTM[Oxidation (M)]TDDVTFWK_.2	#N/A	4.2394	3.7634	3.2748	3.4500	5.8664	5.0787	5.9141	5.9383	4.8977	5.4131	5.5733	4.8154	4.2601	3.2619	5.3412	3.0834	5.1114	5.7326	4.3352	4.7451
Cltc	_RPIS[Phospho (STY)]ADSAIMNPASK_.2	Q68FD5_S67_M1_Cltc	3.7073	4.2955	4.2436	3.9821	3.0450	4.9731	2.9952	3.4077	3.0543	3.9136	3.4287	4.1849	3.1623	4.2307	4.0732	4.0522	3.9454	4.2951	5.3364	3.8842
Cltc	_FDVNTSAVQVLIEHIGNLDR_.3	#N/A	3.5727	2.8860	4.6101	4.3560	3.7796	3.8130	3.2685	4.5346	3.1887	3.3833	4.1681	3.4725	3.3541	3.8568	3.2938	2.9348	3.5714	4.2617	3.9103	3.8238
Cltc	_YGYIHLYDLETGTC[Carbamidomethyl (C)]YM[Oxidation (M)]NR_.3	#N/A	3.8628	4.1400	4.3991	3.8266	3.2005	3.2836	4.0028	3.5632	3.7181	4.0691	3.1967	4.0295	3.8835	4.3862	3.9178	4.2077	4.1008	3.7323	3.3809	4.3531
Cltc	_EDKLEC[Carbamidomethyl (C)]SEELGDLVK_.3	#N/A	3.7289	3.5902	3.2655	4.5122	3.9358	3.9692	3.1123	4.3784	3.0325	3.5395	4.0120	3.3163	3.1979	3.7006	3.1376	3.0910	3.4153	4.4179	4.0665	3.6676
Fnip1	_NESSDSALGDS[Phospho (STY)]ESEDTGPDIR_.2	#N/A	3.6276	5.6647	4.1639	4.0618	2.9653	3.5188	4.9128	3.3280	5.5936	3.8339	2.9615	4.2646	3.6483	6.0411	4.4276	5.0126	6.2310	5.6389	5.2316	5.3552
Fnip1	_NESSDSALGDS[Phospho (STY)]ESEDTGPDIRR_.3	#N/A	3.7220	3.5970	3.2586	3.8622	3.9290	3.9623	3.1191	3.9477	3.0393	6.0590	4.0188	3.2332	3.7529	3.2967	3.1445	3.0842	3.4450	3.7045	4.0035	2.9740
Kiaa1549	_DHTTPSENGDVPS[Phospho (STY)]PK_.2	Q68FD9_S1384_M1_Kiaa1549	8.2450	7.7711	7.5451	7.3852	7.5742	7.7537	7.5322	8.0631	7.8445	7.3331	7.6894	7.6750	7.9167	8.1094	7.0185	7.2430	8.3595	7.9028	7.7138	7.1547
Kiaa1549	_LITTDS[Phospho (STY)]DGTYK_.2	Q68FD9_S1613_M1_Kiaa1549	8.7812	8.5414	9.1151	8.7457	6.1113	8.7828	8.3393	4.1277	8.7975	9.0106	6.7148	7.8607	4.4931	8.3917	7.3895	8.2359	9.4045	8.4371	8.4026	8.6256
Kiaa1549	_GHYEFPVVDDLS[Phospho (STY)]SGDTKER_.3	#N/A	4.1647	6.6217	6.5035	4.8841	3.9646	5.9712	6.0102	3.8651	3.8572	3.5627	3.8996	4.9514	3.9910	8.4221	5.3516	5.5043	4.5544	8.2429	6.0607	3.6248
Kiaa1549	_GLQGPGLGYPTSS[Phospho (STY)]TEDLQPGHSSASLVK_.3	Q68FD9_S1904_M1_Kiaa1549	5.8081	3.8657	4.0218	3.5523	3.5427	3.8376	4.0663	6.0083	4.3734	3.6919	3.5743	3.7552	4.5098	4.0089	3.6435	3.8304	3.7236	3.4580	3.8881	3.6106
Kiaa1549	_GHYEFPVVDDLS[Phospho (STY)]SGDTK_.2	#N/A	3.3152	6.0062	4.0357	4.3574	3.9755	6.0356	3.4528	3.5889	3.3833	3.6105	3.7576	3.8753	3.4913	6.5170	3.7435	3.8512	3.9554	3.9919	5.6674	3.7828
Kiaa1549	_RGHYEFPVVDDLS[Phospho (STY)]S[Phospho (STY)]GDTKER_.4	#N/A	3.7833	3.5358	3.3199	3.8009	3.9903	4.0236	3.0578	4.0090	2.9780	3.5939	3.9575	3.2619	3.1435	3.3580	3.0832	3.1454	3.5062	3.6432	3.9423	3.6131
Atg9a	_LEASYSDS[Phospho (STY)]PPGEEDLLVHVAEGSK_.3	Q68FE2_S18_M1_Atg9a	7.4683	8.2115	7.3591	6.8689	4.0614	7.4587	7.9076	6.5051	7.6019	6.8579	7.0576	7.2661	7.6905	7.4828	6.7701	5.6817	7.2492	7.9224	7.1204	6.7785
Ripor1	_FSTYS[Phospho (STY)]PPDTPSLR_.2	Q68FE6_S347_M1_Ripor1	11.1402	3.7721	10.6194	3.4587	10.1638	10.4765	10.6751	11.0698	10.2939	10.8236	10.3837	10.5126	11.1264	11.0628	9.0886	10.4562	10.9272	10.6617	10.3930	10.2702
Ripor1	_PLTSPAPDS[Phospho (STY)]PEQIPK_.2	Q68FE6_S748_M1_Ripor1	3.8960	8.3173	8.4122	7.6838	3.2337	8.3553	7.9681	8.2048	8.3156	4.3215	7.7804	7.7255	3.9167	8.5287	7.5235	7.8900	8.7644	7.9764	8.0997	7.9152
Ripor1	_HTSS[Phospho (STY)]PEVVAEDR_.2	Q68FE6_S875_M1_Ripor1	6.8717	3.9047	6.6208	7.0943	3.5343	3.6682	3.7766	3.6522	5.4475	3.9177	6.8044	6.4212	3.9096	3.7587	6.1148	6.5486	4.1899	3.6881	3.6234	3.7789
Tmem151b	_SGS[Phospho (STY)]VNEASC[Carbamidomethyl (C)]PTEQTR_.2	Q68FE7_S488_M1_Tmem151b	3.9129	4.6933	4.5850	4.6715	3.8070	4.0932	3.6808	4.2493	3.7399	3.2369	4.1143	3.9057	3.8480	3.5840	3.2580	3.3755	3.5196	4.6579	3.9599	3.9249
Znf280d	_SVNS[Phospho (STY)]DAS[Phospho (STY)]DPGSVR_.2	Q68FE8_S904_M2_Znf280d	4.0038	5.9593	3.5404	3.2862	4.2107	4.2441	4.3382	4.1035	4.2584	5.5755	3.7371	5.0829	5.6362	3.4257	2.8628	3.3659	6.6283	3.1920	4.3414	3.3927
Git1	_LQAENLQLR_.2	#N/A	5.6760	5.9301	6.1060	6.1549	5.7519	5.5792	5.9355	5.8025	5.4033	5.5338	5.5359	5.4910	5.5601	6.0670	5.7143	5.2093	5.6276	5.7396	5.9351	5.7090
Git1	_SAVPFLPVNPEYSATR_.2	#N/A	5.6182	5.6594	6.1459	5.8813	6.1141	5.1294	5.7269	5.7789	6.1471	6.2696	5.7513	5.7218	5.9983	5.6116	5.6042	5.3888	5.5397	5.5333	5.2134	5.4099
Git1	_S[Phospho (STY)]M[Oxidation (M)]DSSDLSDGAVTLQEYLELKK_.3	#N/A	6.6664	6.1976	5.7787	3.3699	2.9204	5.5469	4.1277	4.4400	5.3949	6.6165	5.3266	4.3318	4.2134	5.4491	5.2495	3.3609	3.9372	3.2122	6.4517	5.5233
Git1	_SLSS[Phospho (STY)]PTDNLELSAR_.2	Q68FF6_S371_M1_Git1	11.6885	11.9722	11.9125	11.8198	10.9392	11.4902	11.5187	11.3405	11.6200	11.5501	11.2398	11.5678	11.9037	12.2228	11.0943	11.8161	12.0058	11.6084	11.6214	11.7121
Git1	_ARS[Phospho (STY)]M[Oxidation (M)]DSSDLSDGAVTLQEYLELKK_.3	#N/A	3.9046	5.7613	3.7329	4.0026	3.9387	4.1628	3.6522	3.7752	3.7113	5.9781	4.0856	3.3411	3.8193	3.7430	3.2294	3.4450	3.6712	3.6879	5.0778	3.8449
Git1	_S[Phospho (STY)]M[Oxidation (M)]DSSDLSDGAVTLQEYLELK_.2	#N/A	3.3448	3.9742	4.3823	4.2394	3.5518	3.5851	3.4963	3.5705	3.4165	3.1554	4.3960	3.7004	3.5820	4.4203	3.5217	4.2078	3.0678	4.0339	4.3807	4.0516
Git1	_HQS[Phospho (STY)]GADSDYENTQSGDPLLGLEGK_.3	#N/A	4.1853	3.9729	7.3029	3.5155	3.6080	3.9888	6.9329	4.2191	3.8881	3.6970	3.8018	6.4015	3.9962	7.2048	3.4442	4.5287	3.5827	7.2339	3.8130	6.7770
Git1	_HQS[Phospho (STY)]GADSDYENTQSGDPLLGLEGKR_.4	#N/A	3.9334	3.8934	3.9546	3.7746	2.9256	3.9394	3.7123	3.9715	3.6793	3.9152	3.5349	4.3160	3.8250	3.8659	4.1704	3.7705	3.8845	3.8477	3.0150	3.6430
Git1	_AEVC[Carbamidomethyl (C)]ADC[Carbamidomethyl (C)]SAPDPGWASISR_.2	#N/A	3.3443	3.9748	1.8593	4.3242	3.5512	3.3248	3.2191	3.5700	3.2671	3.7008	2.5278	1.9968	3.3751	4.4198	2.7852	3.7517	3.0672	4.0823	4.3813	4.0970
Git1	_SQSELDDQHDDSVASDEDT[Phospho (STY)]DQEPLPSAGATR_.4	Q68FF6_T401_M1_Git1	3.9837	6.4689	6.6892	4.1753	3.3214	3.2369	4.2067	3.5416	6.4317	4.0831	3.3660	3.9201	4.3381	3.6721	4.0011	3.7022	4.2808	6.8899	6.0318	3.4555
Git1	_ENDAVWLATQNHSTLVTER_.3	#N/A	4.4825	3.5203	3.5180	3.2069	3.8203	4.1647	2.9371	3.4855	4.3378	3.1880	3.8164	4.2240	4.5032	3.5051	1.7923	3.3266	3.2197	3.1126	2.8203	3.4721
Slain1	_S[Phospho (STY)]MPNLAR_.2	Q68FF7_S401_M1_Slain1	4.4901	5.7166	6.1154	6.5146	3.8279	5.7423	5.4514	4.0752	4.9013	3.1956	6.1696	4.6155	3.8804	7.1971	3.2904	3.3342	4.7330	3.9361	3.8761	4.4061
Slain1	_SSQS[Phospho (STY)]FDSSLHGAGSGVSR_.2	#N/A	3.5322	3.7090	4.0426	3.9741	4.0470	4.1869	3.2581	3.8427	3.2554	3.5276	3.9233	3.4351	3.3886	4.2178	3.2101	3.4030	3.3399	4.1782	4.1336	4.0042
Pkp4	_VGS[Phospho (STY)]PLTLTDAQTR_.2	Q68FH0_S313_M1_Pkp4	12.9168	12.7793	12.4067	12.5856	12.2516	12.3812	12.2827	12.7192	12.3344	12.6247	12.4915	12.3010	13.0477	12.6133	11.2275	12.2182	12.8504	12.3703	12.2686	12.2714
Pkp4	_AQS[Phospho (STY)]PSVYTSTGVSPSR_.2	Q68FH0_S220_M1_Pkp4	10.7191	10.6621	10.6951	10.7754	9.7915	9.8273	10.2604	10.3080	10.4104	9.9881	9.8204	9.7507	10.7138	10.1530	9.7693	10.3034	10.7467	10.2315	9.8512	9.7099
Pkp4	_LYLDS[Phospho (STY)]PR_.2	Q68FH0_S1133_M1_Pkp4	11.0349	11.1366	4.3007	10.9669	10.7594	10.8541	10.8279	10.9504	10.6061	11.0557	10.9707	10.8434	11.3163	4.2878	9.7507	10.7581	11.0574	10.7913	10.7786	10.6860
Pkp4	_TGS[Phospho (STY)]VGISNLQR_.2	Q68FH0_S446_M1_Pkp4	10.3785	10.3239	10.1914	10.1948	9.5773	9.6129	9.4567	9.9765	9.5978	10.4023	9.6826	9.6635	9.9337	9.8239	8.1100	9.1910	[illegible]	9.4712	9.4037	9.4022
Pkp4	_VAS[Phospho (STY)]PSQGQVGSSSPK_.2	Q68FH0_S326_M1_Pkp4	8.2404	8.4433	3.9904	7.8475	8.2547	3.6212	7.4699	7.8893	7.3617	8.3609	8.9391	7.7863	8.5642	9.2176	7.9076	3.8057	8.5877	4.0101	8.9250	3.7603
Pkp4	_PSPIYSSYSS[Phospho (STY)]PAR_.2	#N/A	3.6678	8.7030	9.4630	4.0216	8.6254	8.8357	3.6029	3.3682	8.5284	9.0406	8.8253	4.2244	3.6886	4.1912	4.1127	8.5099	6.8788	8.2200	8.6461	8.3761
Pkp4	_TYYS[Phospho (STY)]PVYR_.2	Q68FH0_S421_M1_Pkp4	7.7208	7.8964	7.1537	7.5811	7.0326	7.7283	7.4209	7.3536	7.1969	7.5505	7.3871	7.3776	7.9140	7.5183	4.0137	7.3158	7.3120	3.5419	7.1905	7.4813
Pkp4	_S[Phospho (STY)]YEDPYC[Carbamidomethyl (C)]DDR_.2	Q68FH0_S1136_M1_Pkp4	6.5266	6.5303	6.7685	6.3286	6.1424	6.3817	7.2814	7.1215	5.6905	6.0355	6.0746	5.4375	6.1986	5.9444	5.3157	7.7660	6.4432	4.1740	6.3038	5.6370
Pkp4	_PAS[Phospho (STY)]PTAVR_.2	#N/A	3.6163	4.3865	4.1526	4.0731	2.9541	3.1781	3.0862	3.3167	3.1453	7.2180	3.5196	4.2759	3.2533	4.1397	4.1642	6.6603	3.8544	4.2041	3.0023	7.1227
Pkp4	_AAS[Phospho (STY)]PYSQRPAS[Phospho (STY)]PTAVRR_.3	#N/A	4.5321	3.4707	3.5675	3.1573	3.8698	4.0939	3.6713	4.2325	3.7304	3.2376	6.5778	6.3089	3.8384	3.3822	3.2485	3.3761	3.2693	3.6190	3.9181	3.0595
Pkp4	_TSS[Phospho (STY)]QRS[Phospho (STY)]TLTYQR_.2	Q68FH0_S457_M2_Pkp4	4.0709	3.9319	4.6072	3.6185	3.4086	3.6327	4.1324	3.7713	4.1915	4.2772	3.0651	3.8213	4.2996	4.5943	3.7096	4.4158	4.3090	3.1578	4.8667	3.5206
Pkp4	_AASPYSQRPAS[Phospho (STY)]PTAVR_.3	#N/A	7.6964	7.0630	4.3764	6.7152	8.2825	7.9781	7.8663	6.8580	3.6949	6.6913	8.0014	3.5096	8.2909	7.1869	7.0070	7.5885	8.5291	7.7610	7.8070	7.5255
Pkp4	_GLYPGSSKSPSY[Phospho (STY)]SSYS[Phospho (STY)]SPAR_.3	Q68FH0_S1098_M2_Pkp4	4.0507	3.5603	3.4901	3.8642	3.7923	4.0164	6.0368	5.5666	5.2844	4.2570	4.1943	3.4498	3.9280	3.4771	3.3381	3.2987	3.1918	6.1670	4.3020	3.3775
Pkp4	_VASPSQGQVGSSS[Phospho (STY)]PKR_.2	#N/A	8.3216	9.5796	9.3876	8.7139	8.3783	9.3483	9.3416	8.9853	9.2904	9.4471	8.3562	9.2451	9.6340	9.6293	8.6966	8.0169	9.5512	9.4411	9.2853	4.8812
Pkp4	_LQHTGPADDGATRS[Phospho (STY)]PS[Phospho (STY)]DSIQKDPR_.4	#N/A	6.7875	7.2347	7.1215	7.0952	3.8991	7.4445	7.2705	3.8289	7.3158	3.3355	4.0875	7.3194	3.6637	6.5738	7.0552	7.3508	6.9577	3.8331	7.5029	7.5750
Pkp4	_SAVS[Phospho (STY)]PDLHITPIYEGR_.2	#N/A	7.6293	7.8892	7.4672	7.6839	3.7112	7.5907	7.4772	7.5581	7.4976	7.6877	3.8436	7.5327	7.8869	8.1941	6.7896	7.6495	7.7589	8.2740	7.5430	7.4308
Pkp4	_LQHTGPADDGATRS[Phospho (STY)]PS[Phospho (STY)]DSIQK_.3	#N/A	5.4809	4.5178	5.2341	4.5158	3.5366	5.8867	5.5681	4.1244	5.3349	5.2331	3.7580	5.2512	3.7114	5.7814	5.3301	5.0830	6.3227	5.5870	5.0810	5.6004
Pkp4	_S[Phospho (STY)]PNHGTVELQGSQTALYR_.3	Q68FH0_S426_M1_Pkp4	3.8394	3.8935	3.9348	3.9053	3.9411	3.4012	3.7105	3.7356	3.7696	4.0457	3.3800	3.7015	3.8776	4.9207	3.2877	3.4474	3.8843	3.7726	3.8457	3.7252
Pkp4	_AEQYPGS[Phospho (STY)]PDSWV_.2	Q68FH0_S1185_M1_Pkp4	3.5726	4.4302	4.1089	4.1168	2.9103	3.1344	3.1300	3.2729	3.1890	3.7789	3.5634	4.3197	3.2971	4.3417	4.2080	3.9175	3.8106	4.1603	2.9585	4.0190
Pkp4	_SSYAS[Phospho (STY)]QHS[Phospho (STY)]QLGQELR_.2	Q68FH0_S391_M2_Pkp4	3.3125	4.0065	4.3500	4.2717	3.5195	3.5528	3.5286	3.2939	3.4488	4.7900	2.9275	3.7327	3.6143	4.1169	3.5540	4.1755	4.5363	4.0016	3.6501	4.0839
Ahcyl2	_DGGEALVS[Phospho (STY)]PDGTVTEAPR_.2	#N/A	10.0032	10.2394	10.5534	9.9699	8.4934	10.0937	10.0130	9.3903	10.1044	10.1217	9.5646	9.6312	9.8411	10.2578	8.4875	9.6320	10.4310	9.7192	9.9679	9.8650
Ahcyl2	_IVLLAEGR_.2	#N/A	7.4523	7.5151	8.2115	5.6370	8.0854	5.1791	8.0580	7.1468	7.7016	8.2875	8.1949	8.0441	7.4771	7.6857	8.3309	7.9274	6.9674	8.1142	8.2656	8.0716
Ahcyl2	_GESEDDFWWC[Carbamidomethyl (C)]IDR_.2	#N/A	7.1756	7.4159	7.5467	7.4667	6.5613	6.7800	6.9434	6.4750	7.4504	7.2801	7.1810	6.8514	7.6186	7.1337	6.1846	6.8256	6.8225	6.7647	7.2104	7.0029
Ahcyl2	_AQGEKPLAGAK_.2	#N/A	6.7408	6.8756	6.0556	6.3129	5.7308	6.7997	6.6975	6.7763	6.4112	6.0326	6.3802	6.5379	5.4089	6.0858	6.4002	6.3495	6.0884	6.3319	6.1888	6.2641
Ahcyl2	_TPELTWER_.2	#N/A	6.4803	5.7670	6.7250	7.4223	7.0323	6.5200	7.2965	6.5781	7.0831	7.0392	7.0586	6.7736	7.0329	6.6608	6.7079	7.0511	5.6001	6.4585	6.5500	7.0227
Ahcyl2	_HRDGGEALVS[Phospho (STY)]PDGTVTEAPR_.3	#N/A	8.2880	8.2983	8.2426	8.2532	7.7200	8.2445	8.1276	8.1869	7.9220	8.3714	7.8275	8.0631	8.5851	8.3607	7.2869	8.1952	8.4804	8.1088	8.1650	8.0284
Ahcyl2	_LC[Carbamidomethyl (C)]VPAM[Oxidation (M)]NVNDSVTK_.2	#N/A	6.8901	6.8166	7.0693	6.6251	6.8721	7.1053	7.2647	7.2261	6.6892	7.0231	6.4493	6.8422	7.0103	6.2574	6.7910	6.8834	6.9556	7.0182	6.8871	6.6113
Ahcyl2	_GIVEESVTGVHR_.2	#N/A	6.7576	7.0757	6.7181	6.0018	6.3825	7.1679	6.9153	6.4509	6.6484	6.5903	6.4027	6.0892	6.2723	[illegible]	6.4269	6.2906	6.5551	6.5077	6.7007	6.2729
Ahcyl2	_FDNLYC[Carbamidomethyl (C)]C[Carbamidomethyl (C)]R_.2	#N/A	3.2810	2.9346	3.4198	2.2376	5.3035	2.2635	3.1447	4.5029	3.2038	3.7641	4.6213	3.7643	5.0326	4.3564	0.6979	4.1439	4.5047	4.1456	5.1106	4.0037
Ahcyl2	_QVVVC[Carbamidomethyl (C)]GYGEVGK_.2	#N/A	4.0350	3.2841	3.5715	3.5493	4.2419	4.2752	4.3070	4.2606	4.2272	3.8456	3.7059	3.0102	4.3927	3.6096	3.6182	3.3971	3.7579	4.4606	3.6906	3.2869
Mars1	_NNSELLNNLGNFINR_.2	#N/A	5.5637	5.1703	4.1382	4.0875	4.7602	5.3638	5.4566	5.6946	5.4916	5.9260	5.5569	5.6859	5.8928	5.6349	4.9069	5.9468	5.5528	5.4818	5.6578	5.6618
Elfn2	_DSTYSQLS[Phospho (STY)]PR_.2	Q68FM6_S750_M1_Elfn2	10.7163	10.9332	11.0326	10.6952	10.1393	10.4296	10.6904	10.8344	10.5102	10.4444	10.2826	10.3530	11.0585	10.4236	9.4978	10.1052	10.8042	10.4501	10.0942	10.2450
Elfn2	_QLS[Phospho (STY)]ADAAVSRK_.2	#N/A	6.0686	6.6414	5.9846	5.9881	5.3469	5.3902	5.6720	6.0978	5.4821	4.0980	5.7367	5.9535	6.3779	5.6697	4.3780	5.8120	5.3192	5.7547	5.1557	5.1460
Elfn2	_QLS[Phospho (STY)]ADAAVSR_.2	#N/A	8.9971	8.9619	9.0764	8.9785	8.1523	8.4370	8.5853	8.2379	8.3340	8.8760	8.3127	7.6578	8.4507	8.3138	8.2740	8.5131	8.3154	8.3173	7.8642	8.4482
Elfn2	_YIEKGS[Phospho (STY)]PLNSPLDR_.2	#N/A	7.2504	6.8395	7.0293	6.5761	6.3136	6.7476	6.8206	7.0601	6.6100	3.2499	6.7697	4.4566	3.8341	3.3778	3.2441	5.8520	7.0124	4.8806	6.0957	5.0761
Elfn2	_GS[Phospho (STY)]PLNSPLDR_.2	#N/A	6.6402	6.3527	3.3534	3.5625	3.6556	6.4942	3.8855	6.0861	3.9445	4.5242	4.3189	3.5743	4.0526	3.5964	3.4626	3.1620	6.3567	3.4048	5.2713	3.2736
Elfn2	_HYYSGYSS[Phospho (STY)]S[Phospho (STY)]PEYSSESTHK_.3	Q68FM6_S760_M2_Elfn2	3.7488	5.5423	4.2851	3.9559	3.0865	4.9499	5.6454	3.4492	4.9673	3.9551	4.6515	4.1587	3.6282	2.7722	4.0470	5.1745	3.9868	3.9624	3.5102	5.3008
Ttc41	_SISELVS[Phospho (STY)]EK_.2	Q69ZV3_S1279_M1_Ttc41	3.9570	3.7893	3.9192	3.2016	3.2947	3.5188	6.4674	3.1075	4.8060	4.1633	3.1790	4.1634	4.4135	3.9573	3.8236	4.3019	4.1056	3.0439	4.3548	3.6346
Peak1	_ANTLS[Phospho (STY)]PVR_.2	Q69Z38_S282_M1_Peak1	3.8949	6.3979	3.9813	3.1395	7.2215	3.4567	6.1018	6.7103	4.3675	4.1013	7.2637	4.1014	4.4756	4.0193	6.0327	4.2398	4.1676	2.9818	7.2912	3.6966
Peak1	_DPSTKPVT[Phospho (STY)]SPPSK_.2	Q69Z38_T851_M1_Peak1	3.5697	3.7641	3.9725	4.0293	3.7766	6.1396	3.2862	3.7953	3.2064	3.3802	4.1859	3.4902	6.2018	4.0106	3.3116	3.7980	6.4313	6.8490	4.1833	6.7521
Peak1	_SYSSTHSS[Phospho (STY)]PAK_.2	Q69Z38_S727_M1_Peak1	4.4772	4.3428	4.0137	3.1071	3.1833	3.2166	3.9859	3.2020	3.7850	3.3990	3.4931	4.0689	3.6144	2.7787	3.8902	3.8393	3.6645	4.4502	3.2484	4.4201
Peak1	_SLFTSQSSGEGEAHQTTES[Phospho (STY)]PT[Phospho (STY)]AK_.3	Q69Z38_S836_M2_Peak1	3.8693	2.9536	3.4058	3.1517	4.0762	4.1095	4.4727	4.2381	4.3930	3.6799	3.8716	3.1759	4.5584	3.5602	2.9973	3.2314	3.2749	3.0574	4.2069	3.5272
Radil	_GTPALTSEAAQSS[Phospho (STY)]PPTR_.2	Q69Z89_S235_M1_Radil	9.0697	8.9530	8.8006	8.7794	8.1650	8.5166	8.7014	9.0996	8.5848	8.3889	8.4418	8.4982	9.1448	8.6314	7.5168	8.3702	8.9934	8.6992	8.5455	8.1620
Radil	_S[Phospho (STY)]LLLEFEPDVEDTLLQR_.2	Q69Z89_S432_M1_Radil	3.6145	3.4425	4.2617	4.3599	2.9522	3.1763	3.4623	3.4500	4.3100	3.8208	3.5215	3.8209	3.2552	4.2998	4.1661	3.9594	4.4481	4.2570	2.1203	3.9771
Brsk2	_TRLNS[Phospho (STY)]IK_.2	Q69Z98_S482_M1_Brsk2	7.6122	8.3401	8.0501	7.6920	7.5185	7.7461	7.5447	8.0330	7.2269	8.0963	7.5005	6.8646	7.4104	7.1944	6.9139	6.9200	7.5626	7.7486	7.3718	7.1250
Brsk2	_VTPHPS[Phospho (STY)]PR_.2	Q69Z98_S436_M1_Brsk2	8.0117	7.5695	8.1420	7.9344	7.4229	2.9863	4.0433	6.9539	4.1024	8.3391	7.1615	7.3722	6.7657	3.1826	6.0837	7.1103	7.1683	7.4818	7.1885	7.2496
Brsk2	_S[Phospho (STY)]M[Oxidation (M)]EVLSVTDGGSPVPAR_.2	#N/A	8.5223	9.3167	8.8080	8.0707	6.8904	8.4216	8.7943	7.7465	8.2371	9.1159	7.3397	8.1079	7.7474	8.6843	6.5800	8.2751	7.8910	8.7906	8.5088	8.4693
Brsk2	_VDS[Phospho (STY)]PMLNR_.2	#N/A	4.3343	6.5521	7.2338	7.5713	4.3621	5.7128	6.7265	3.0591	4.2153	5.6279	5.7644	4.6849	4.3651	7.4988	4.0331	3.6964	4.5736	3.0923	3.6559	4.5827
Brsk2	_S[Phospho (STY)]SGASSGLSTS[Phospho (STY)]PLSSPR_.2	Q69Z98_S413_M2_Brsk2	3.7725	4.1693	3.9135	4.0354	3.4218	6.0890	6.7316	4.1703	6.2128	3.8025	3.6534	3.9914	3.7318	6.8943	3.8534	3.7274	3.7863	6.5414	6.7054	4.0500
Brsk2	_VTPHPS[Phospho (STY)]PRGS[Phospho (STY)]PLPTPK_.3	Q69Z98_S436_M2_Brsk2	3.8715	4.1313	4.4078	5.1270	3.2093	4.4333	4.3318	3.5719	4.3909	4.0779	3.2644	4.0207	4.4990	4.3949	3.9090	4.2164	4.1096	3.7236	3.3721	4.3619
Brsk2	_GS[Phospho (STY)]PLPTPK_.2	Q69Z98_S440_M1_Brsk2	4.2266	4.5934	3.7632	3.3577	2.9327	3.6749	4.0903	4.4523	4.1494	4.3194	3.0229	4.3195	4.2574	3.5769	4.1408	3.5887	3.9495	3.2000	4.4990	3.4785
Brsk2	_KLQVPTPEEM[Oxidation (M)]SNLT[Phospho (STY)]PES[Phospho (STY)]PELAK_.3	#N/A	3.5362	8.2590	7.3209	4.1717	3.7314	4.0213	7.8739	4.2365	6.7527	7.1321	3.4462	5.3084	3.6121	7.4569	3.6195	3.8811	3.7742	4.0774	3.7608	6.3884
Brsk2	_LQVPTPEEM[Oxidation (M)]SNLT[Phospho (STY)]PES[Phospho (STY)]PELAK_.3	#N/A	3.8118	4.0797	3.7635	4.0129	3.6080	4.2186	3.8305	4.4406	3.6366	3.7668	3.8142	3.8544	3.8020	3.5029	3.6968	3.5806	3.7277	3.9683	4.9805	3.4699
Brsk2	_LQVPTPEEMSNLT[Phospho (STY)]PES[Phospho (STY)]PELAKK_.3	#N/A	3.7484	4.2544	4.2847	3.9410	3.0861	3.3980	4.4550	3.4487	3.6036	4.4692	3.0823	4.1439	3.7691	4.2717	4.0322	4.0933	3.9864	3.8647	3.4953	4.2387
Brsk2	_RVDS[Phospho (STY)]PMLNR_.2	#N/A	3.9965	4.0063	5.7437	6.1425	3.3342	3.5583	4.7747	3.6969	4.2660	5.1333	3.1395	3.8958	4.3740	4.5199	3.7861	4.3414	4.2345	3.0796	3.2472	3.5951
Cdk13	_GGDVS[Phospho (STY)]PSPYSSSSWR_.2	Q69ZA1_S384_M1_Cdk13	3.5704	7.9554	4.6078	7.8861	7.1891	7.8234	7.7100	7.7153	7.2508	7.7900	7.2864	3.4748	8.0184	7.8225	6.4582	7.2236	7.9859	7.2232	7.4955	7.2913
Cdk13	_QTDPST[Phospho (STY)]PQQESSK_.2	Q69ZA1_T1147_M1_Cdk13	6.0776	6.0878	4.5032	3.7261	5.2568	3.4366	5.8920	3.6672	5.6347	4.2847	5.1802	5.5301	6.5804	6.0798	3.7993	5.7982	6.2121	3.4420	3.8675	4.0275
Cdk13	_TEHAPS[Phospho (STY)]PSSGGTVK_.2	Q69ZA1_S526_M1_Cdk13	3.4113	7.0615	3.9476	7.6326	4.2499	6.4740	6.8028	3.6031	6.6538	7.2591	6.7606	2.9801	7.8593	4.5029	6.3796	7.7562	3.1504	3.9991	4.2981	4.1802
Cdk13	_HSS[Phospho (STY)]TS[Phospho (STY)]PSTLTLK_.2	Q69ZA1_S438_M2_Cdk13	3.2482	7.4858	4.2856	4.3360	3.4551	4.4885	7.2118	3.3583	7.5914	4.5596	2.9918	3.7970	3.6786	4.1813	3.6183	4.1112	3.8960	7.5824	3.5858	7.1834
Cdk13	_S[Phospho (STY)]LS[Phospho (STY)]PLGGRDES[Phospho (STY)]PVSHR_.3	#N/A	3.2964	4.4175	4.0075	4.3783	3.4824	4.4109	5.0998	3.7454	5.1370	4.0015	3.5869	4.4423	3.2397	5.4858	4.5203	4.9427	3.9175	5.4328	5.1650	5.4280
Cdk13	_S[Phospho (STY)]RS[Phospho (STY)]PY[Phospho (STY)]SPVLR_.2	Q69ZA1_Y400_M3_Cdk13	3.8524	3.8698	3.9487	3.7919	3.7108	3.7058	4.1067	3.8526	5.8252	3.9272	3.7707	3.7026	3.8785	6.8435	4.6280	4.7634	3.8425	6.2803	4.6437	7.2514
Cdk13	_S[Phospho (STY)]RS[Phospho (STY)]PYS[Phospho (STY)]PVLRR_.3	#N/A	3.2164	3.6967	3.1589	3.8693	4.0550	4.2791	3.4861	3.8480	3.5452	3.4228	3.9195	3.4229	3.6532	3.1970	3.0633	3.5613	3.3452	3.8042	4.1032	4.3751
Tspyl5	_AAAEDAWHDEKPPQS[Phospho (STY)]PR_.3	Q69ZB3_S41_M1_Tspyl5	5.4321	4.4685	3.8880	4.4931	3.0575	3.0908	3.9906	3.0762	3.9108	5.7921	5.5585	6.0651	5.4784	3.9260	4.0160	5.1427	5.3758	3.9448	5.3670	5.4006
Cnnm4	_NSLLHRAS[Phospho (STY)]EEETI_.2	Q69ZF7_S766_M1_Cnnm4	6.5427	6.7220	3.4529	6.4660	3.7551	3.9792	3.7859	4.1178	3.8450	6.5880	5.8804	5.6631	3.9531	6.0866	3.3631	5.8323	3.1546	6.7670	5.9760	5.7342
Arhgap23	_SYS[Phospho (STY)]PSFQR_.2	Q69ZH9_S421_M1_Arhgap23	8.1563	8.5839	8.4915	8.6524	8.0220	8.4879	8.1706	8.5605	8.4968	9.0703	3.1074	3.8636	8.9261	4.5520	8.1172	8.4265	8.4162	8.4255	8.3912	8.3326
Arhgap23	_RHS[Phospho (STY)]TSDLSDATFSDIR_.3	#N/A	7.5571	4.4258	4.1172	7.9262	6.6094	3.1427	7.2001	3.2813	7.5959	7.9555	3.5590	7.3712	3.3780	7.7356	6.5126	3.9258	7.3872	4.0181	7.4661	7.7872
Arhgap23	_S[Phospho (STY)]AEALGPGALVSPR_.2	Q69ZH9_S361_M1_Arhgap23	11.4417	12.0137	11.7209	11.3112	8.0551	11.4253	11.5328	11.1368	11.3855	11.6562	11.0978	11.1243	11.5066	12.2204	10.5985	11.2429	11.4044	11.6182	11.5462	11.4538
Arhgap23	_SKS[Phospho (STY)]C[Carbamidomethyl (C)]DDGLNTFRDEGR_.3	#N/A	8.4100	8.8424	8.4456	8.8927	7.8235	8.4037	8.3931	8.2308	8.1770	8.8434	8.4103	8.1774	8.9364	8.9513	7.6033	8.2984	8.2073	8.5410	8.4163	8.2930
Arhgap23	_HYS[Phospho (STY)]QDC[Carbamidomethyl (C)]SSIK_.2	Q69ZH9_S594_M1_Arhgap23	6.5367	6.8447	6.6236	6.5642	6.5340	6.3752	6.3968	6.2983	5.9731	6.6272	5.8751	6.4853	6.1037	6.5574	5.6314	6.2177	6.1436	6.2418	6.4003	6.3960
Arhgap23	_HS[Phospho (STY)]TSDLSDATFSDIRR_.3	#N/A	4.0251	6.7526	3.5616	6.9140	4.2320	6.2154	6.3685	4.2507	6.4580	7.1494	3.7158	6.3603	4.4026	6.8570	5.4127	6.6336	3.7480	6.5378	6.2775	6.6923
Arhgap23	_QMNLGFGDES[Phospho (STY)]PEPEAR_.2	Q69ZH9_S515_M1_Arhgap23	4.4703	5.6786	5.8852	5.3306	3.1764	5.2161	5.0883	3.1951	5.4429	4.2809	3.2706	4.0758	3.9574	5.8992	3.8971	3.8324	4.1932	3.6660	3.3070	4.4270
Arhgap23	_ARS[Phospho (STY)]DDYLSR_.2	Q69ZH9_S351_M1_Arhgap23	8.9067	8.8932	8.7904	8.4514	8.2945	8.1499	8.2356	7.7153	8.1995	8.4941	8.4264	8.6025	9.0865	8.9797	7.9194	8.4779	8.9325	8.3828	8.3442	8.5182
Arhgap23	_SKS[Phospho (STY)]C[Carbamidomethyl (C)]DDGLNTFR_.2	#N/A	8.2100	7.9493	8.2971	7.9052	7.4454	7.6664	7.4323	7.5042	7.5079	8.0468	8.1981	7.4190	7.4315	8.1084	6.8954	7.6784	8.0176	7.3521	7.5065	7.8217
Arhgap23	_HS[Phospho (STY)]TSDLSDATFSDIR_.2	#N/A	3.1546	4.1645	4.1920	4.4297	3.3615	3.3948	3.6866	3.3802	3.6068	4.4660	3.0854	3.8906	3.1854	6.1010	3.7120	3.5172	4.3783	4.2720	3.0702	5.4778
Arhgap23	_RS[Phospho (STY)]S[Phospho (STY)]YLLAITTER_.2	Q69ZH9_S606_M2_Arhgap23	4.2850	4.5349	3.8216	3.2992	2.9911	3.6164	4.0570	4.5107	4.2079	4.0956	3.0814	4.2610	4.3159	3.8597	4.0823	3.6472	4.0079	3.1415	3.4405	3.5370
Sh3rf1	_TSS[Phospho (STY)]LDSAVPIAPPPR_.2	Q69ZI1_S805_M1_Sh3rf1	4.3319	9.3736	3.8684	3.2524	3.0380	3.5696	8.7661	3.0567	8.4605	9.2987	3.1282	4.2142	4.3627	3.9065	3.7728	3.6940	4.0548	8.9290	8.6198	3.5838
Prex1	_DSVLS[Phospho (STY)]YTSVR_.2	Q69ZK0_S1173_M1_Prex1	3.9261	4.0768	4.4623	3.7633	3.2638	3.2203	3.8611	3.6294	5.4462	4.2915	3.2600	3.9662	3.9468	4.4494	3.8865	4.2710	5.1790	3.6691	3.3176	4.4164
Jmjd1c	_LVSRT[Phospho (STY)]PT[Phospho (STY)]PK_.2	Q69ZK6_T322_M2_Jmjd1c	4.9637	6.1134	4.4078	4.2138	3.5774	3.0302	3.4707	3.5961	3.3910	3.1810	5.1464	3.6748	3.4013	5.2943	5.2004	4.2334	3.0933	5.5804	4.3552	5.7433
Nlgn2	_RLS[Phospho (STY)]PPGGSGSGVPGGPLLPTAGR_.3	Q69ZK9_S714_M1_Nlgn2	3.9175	8.9606	6.9987	6.9217	4.1244	4.1577	4.4245	4.1431	6.7008	3.7281	3.8234	8.9131	8.7999	10.3924	6.7564	3.2796	3.6404	8.6358	3.8081	3.1694
Fgd6	_AAS[Phospho (STY)]EELVEK_.2	Q69ZL1_S494_M1_Fgd6	4.5297	5.5491	5.2644	5.1366	4.2156	3.9290	5.3062	5.1363	3.6195	5.4751	5.6415	3.0143	5.6189	4.7295	3.3819	4.3926	5.5016	4.1608	4.2226	3.6453
Fgd6	_S[Phospho (STY)]LDEDSERKEEVSPLGAK_.3	Q69ZL1_S1167_M1_Fgd6	3.9096	3.4095	3.4462	3.6746	4.1165	4.0126	4.4324	4.1353	3.4526	3.7202	3.8313	3.1356	4.5181	3.4842	2.9570	3.2717	3.6325	3.0977	3.4476	3.4869
Hectd1	_SSS[Phospho (STY)]DNNTNTLGR_.2	Q69ZR2_S1576_M1_Hectd1	8.7399	8.4782	8.5363	3.4186	2.8717	7.7202	8.0395	8.2892	4.0966	8.2897	7.8809	8.0953	8.3180	7.7025	4.2017	7.7457	3.8885	7.9257	3.5599	7.8074
Hectd1	_RLDS[Phospho (STY)]SGER_.2	Q69ZR2_S357_M1_Hectd1	4.0368	6.4182	3.5733	3.5475	5.4457	6.0330	4.3052	4.2624	4.2254	3.8474	3.7041	4.9734	6.1811	5.7709	2.8298	6.3795	6.1065	3.2249	4.3744	3.3597
Hectd1	_AS[Phospho (STY)]LALIR_.2	Q69ZR2_S545_M1_Hectd1	4.4834	4.3365	4.0200	3.1008	3.1895	3.4180	4.3471	3.2083	4.4062	4.0625	3.2798	4.0626	4.5143	4.0581	3.8840	3.8455	4.2063	4.4440	3.2422	3.7353
Tasor	_NNS[Phospho (STY)]RDETTEPEQQK_.2	Q69ZR9_S653_M1_Tasor	4.4732	3.5296	3.5087	3.2162	3.8110	4.0350	3.7301	4.1736	3.7892	3.3519	4.1636	3.4190	3.8973	3.4410	3.3073	3.3173	3.2104	3.5601	3.8592	3.5559
Hbs1l	_SSQS[Phospho (STY)]ESEIVPK_.2	Q69ZS7_S150_M1_Hbs1l	7.0459	4.2683	4.0882	4.5334	6.3442	3.2911	3.7904	3.2765	3.7106	4.3622	6.3166	3.9944	6.4098	4.3787	3.8157	6.5288	4.2745	3.7398	3.3884	4.3457
Kazn	_SSTPSDINS[Phospho (STY)]PR_.2	Q69ZS8_S343_M1_Kazn	9.8472	10.0058	10.1592	10.2795	9.2942	9.1097	9.2714	9.4716	9.4233	10.0757	9.3978	9.6433	9.6395	9.8142	8.9127	9.4770	9.3655	9.2383	9.1062	9.8287
Kazn	_VS[Phospho (STY)]PC[Carbamidomethyl (C)]HSR_.2	#N/A	4.5737	5.5565	5.9325	6.4665	5.9475	5.2097	5.4618	5.2472	5.4268	5.6049	5.7723	5.8585	5.3285	5.8144	3.2068	3.4178	5.5609	5.0110	4.6205	5.2570
Kazn	_THSLC[Carbamidomethyl (C)]NGDS[Phospho (STY)]PGPVQK_.2	Q69ZS8_S356_M1_Kazn	6.4068	7.0989	6.1795	7.6866	5.9981	6.1264	6.0493	6.0060	6.3085	6.3671	6.2813	6.6354	5.4702	7.0850	6.2072	6.8639	3.2006	6.5420	6.0957	6.2257
Kazn	_QVRVS[Phospho (STY)]PC[Carbamidomethyl (C)]HSR_.3	#N/A	3.9294	4.0734	4.4657	4.1757	3.2671	3.4912	4.2740	3.1351	4.3331	4.1357	3.2066	3.9629	4.4411	4.1238	3.8512	4.2743	4.1332	3.0163	4.4200	3.3622
Kazn	_S[Phospho (STY)]LDPGLFDDSDSQC[Carbamidomethyl (C)]SPTR_.2	Q69ZS8_S404_M1_Kazn	4.0445	5.9007	4.5808	3.6449	3.3823	3.6063	4.1588	3.7449	4.2179	4.2509	3.0914	3.8477	4.4260	4.5679	3.7360	4.3894	4.2826	3.5506	3.1992	3.0341
Ehbp1	_S[Phospho (STY)]IQEDTKR_.2	#N/A	10.3747	10.2500	10.2731	10.1837	9.5216	9.4965	9.5588	9.6668	9.3843	10.2111	9.5121	9.5186	9.7639	9.7557	9.2236	9.7204	9.3761	9.4194	9.5061	9.7247
Ehbp1	_QRS[Phospho (STY)]IQEDTKR_.3	#N/A	5.1035	5.5491	5.6199	5.6102	4.6022	4.4546	4.9386	4.4881	4.6720	5.4499	4.3867	5.0811	4.9513	4.4712	5.0838	4.4398	4.8997	4.3915	5.1626	4.8978
Ehbp1	_SRS[Phospho (STY)]LEC[Carbamidomethyl (C)]R_.2	Q69ZW3_S747_M1_Ehbp1	3.6088	5.1057	4.1451	5.0100	3.6743	4.4417	3.9419	4.3614	4.1658	3.8633	3.6974	4.7085	3.8703	4.8129	3.5191	4.6322	4.4938	4.9836	5.0351	4.9075
Ehbp1	_T[Phospho (STY)]SGSDDPGLSSSTDSAQALASLGK_.2	Q69ZW3_T673_M1_Ehbp1	3.4272	3.9075	4.4643	4.1726	3.6298	3.8539	3.2906	3.6525	4.8577	3.6335	3.7240	3.6336	3.4577	3.8664	3.7327	3.7721	4.0147	4.0149	3.6781	4.1796
Ehbp1	_QRS[Phospho (STY)]IQEDTK_.2	#N/A	3.8657	4.9416	4.4023	4.5986	4.2961	4.1060	4.4763	4.9928	4.3965	5.0409	3.8752	4.2094	4.5620	3.4403	3.0009	4.4960	4.3203	3.5608	3.8599	3.1176
Ehbp1	_LDGELAITKPNVSS[Phospho (STY)]PSK_.2	Q69ZW3_S765_M1_Ehbp1	3.9229	4.0799	4.4592	3.7665	3.2606	3.2235	3.8580	3.6233	3.7782	4.2946	3.2568	3.9693	3.9436	4.4463	4.3615	4.2678	4.1610	3.6722	5.7589	4.4133
Ehbp1	_VGNYETDTNSS[Phospho (STY)]VDQEK_.2	Q69ZW3_S588_M1_Ehbp1	3.1830	4.2798	4.2593	3.9663	3.3900	3.4233	3.6581	3.4234	3.5783	4.4945	3.0570	4.1692	3.7438	4.2464	3.6835	4.0460	3.9611	3.8721	3.5206	4.2134

Ehbp1	_ITEIVNQLNALSS[Phospho (STY)]LDEDQDDC[Carbamidomethyl (C)]IK_.3	Q69ZW3_S222_M1_Ehbp1	3.2072	3.2948	3.7435	4.4822	4.0457	3.9392	3.4953	4.4084	3.0624	3.4135	4.0419	3.1842	3.2279	3.7305	3.0725	3.5521	3.4452	4.3879	4.0365	3.6975
Morc2a	_S[Phospho (STY)]PPLPAVIK_.2	Q69ZX6_S614_M1_Morc2a	8.6833	8.5557	8.4068	8.2532	4.3202	8.5662	8.0368	8.3810	8.5334	8.8150	3.6276	8.0014	8.5448	8.5789	7.5874	8.2223	8.4017	8.2092	8.5501	8.4476
Ablim3	_ASS[Phospho (STY)]PGYIDSPTYSR_.2	Q69ZX8_S373_M1_Ablim3	7.2191	6.7332	3.8356	7.8027	3.0051	3.0384	4.0430	6.1336	6.7276	4.1096	3.4419	6.9108	6.6216	3.1305	4.0684	3.6611	4.3460	6.6555	3.1357	3.0975
Ablim3	_SS[Phospho (STY)]PYHSQLDVR_.2	#N/A	3.5351	3.7839	7.1957	4.0491	3.7421	3.7754	3.3060	3.7608	3.2262	3.3457	4.2057	6.7171	5.6867	4.6106	6.0783	6.6771	3.2581	3.8914	4.1904	3.8613
Ablim3	_SGPESGRSS[Phospho (STY)]PYHSQLDVR_.3	#N/A	3.6197	4.3831	4.1560	5.4967	2.9575	3.1815	3.0828	3.3201	3.1419	3.8261	3.5162	4.2725	5.9269	4.1431	4.1608	3.9646	3.8578	4.2075	3.6240	3.9718
Ablim3	_RFS[Phospho (STY)]SGGEEEDFDR_.2	Q69ZX8_S502_M1_Ablim3	3.5146	4.8310	6.0820	6.3951	3.7067	5.8534	5.6974	5.4596	4.4496	6.7464	5.2585	4.6628	5.9921	5.7267	5.5063	5.1133	5.7947	4.9447	6.2229	5.4044
Ablim3	_YTS[Phospho (STY)]DEMLER_.2	Q69ZX8_S337_M1_Ablim3	3.9485	3.3706	7.8310	3.6357	4.1554	3.9530	4.3935	4.1742	3.8713	3.7591	4.2457	3.0967	3.9794	3.5231	2.9181	3.3106	3.6714	3.4781	3.7771	3.2004
Ablim3	_TSETSIS[Phospho (STY)]PPGSSIGSPNR_.2	Q69ZX8_S282_M1_Ablim3	8.5029	8.6077	8.2751	8.5601	8.2858	8.3222	8.4199	8.8626	8.6182	8.4825	8.3898	8.2963	9.0505	8.9657	8.1896	8.5956	8.9133	8.3486	8.5582	8.5217
Ablim3	_SAS[Phospho (STY)]LPAYR_.2	Q69ZX8_S584_M1_Ablim3	3.8478	4.1550	6.2268	3.8415	3.1856	3.2985	4.3555	3.5482	3.7031	4.0542	3.1818	4.0444	3.8686	5.9667	3.9327	6.3806	4.0859	3.7473	5.4328	4.3382
Ablim3	_SHYLADS[Phospho (STY)]DPLISK_.2	#N/A	3.7439	6.3735	6.3004	6.1388	3.0816	6.0082	6.2088	3.4442	3.5991	6.4382	3.9197	4.1745	3.7646	6.1499	5.3320	6.1428	5.6888	5.7849	6.2690	5.9984
Ablim3	_RAS[Phospho (STY)]SPGYIDSPTYSR_.2	Q69ZX8_S372_M1_Ablim3	3.3947	5.1320	3.9310	4.2947	5.0716	3.7517	3.3078	5.7413	3.3669	3.6010	5.4472	2.9967	3.4154	3.9181	2.8850	3.7396	3.6327	5.5950	4.8178	3.8851
Ablim3	_RTS[Phospho (STY)]ETSIS[Phospho (STY)]PPGSSIGSPNR_.2	Q69ZX8_S277_M2_Ablim3	3.0885	4.2306	4.1259	4.4957	3.2954	3.3288	3.7527	3.3142	3.6729	4.3999	3.1515	3.9567	3.8383	4.3410	3.7780	3.9515	4.3123	3.7775	3.4261	4.3080
Tmcc1	_NKFGS[Phospho (STY)]ADNIPNLK_.2	#N/A	6.4695	5.7125	5.6562	5.7467	4.6906	6.0311	5.7928	5.6421	5.6448	5.9968	5.6774	5.6401	4.2521	5.8919	4.2912	5.2739	6.0577	5.7139	4.5252	5.3256
Tmcc1	_FGS[Phospho (STY)]ADNIPNLK_.2	#N/A	6.4564	6.3863	5.2151	4.2990	4.2290	6.3753	6.2920	3.0908	5.4027	4.5567	5.5592	5.5959	3.4792	5.7988	2.8893	3.7353	6.3032	5.7265	4.0582	4.2011
Kiaa0754	_GS[Phospho (STY)]PPDC[Carbamidomethyl (C)]TC[Carbamidomethyl (C)]VPAGDTVISEVK_.2	Q69ZZ9_S276_M1_Kiaa0754	3.7244	4.2784	4.2607	3.9650	3.0621	3.4220	4.4790	3.4248	3.5797	4.4931	3.0583	4.1679	3.7451	4.2478	4.0562	4.0693	3.9625	3.8707	3.5193	4.2148
N4bp1	_KQFS[Phospho (STY)]LENVPEGELLPDGK_.2	#N/A	4.2885	3.6628	3.3797	3.3494	4.0844	4.1968	4.0057	4.0446	3.9871	3.5985	3.9399	3.5523	4.1269	3.3937	3.8558	3.3900	3.9809	3.4221	3.4516	3.2127
Cep170	_ALLHSGS[Phospho (STY)]SSSK_.2	#N/A	3.9151	8.4224	8.8153	8.6061	7.5572	4.1553	7.7878	4.1407	4.3471	8.8209	3.8258	7.7464	7.4913	7.7523	5.9049	3.2772	7.1439	7.2648	7.1173	7.6886
Cep170	_SKTS[Phospho (STY)]PVASGSTSK_.2	Q6A065_S1069_M1_Cep170	7.0888	7.1702	3.3937	7.0188	6.9728	6.9160	7.1197	4.0828	6.8153	6.7844	6.5707	7.1682	7.5489	7.3763	6.3668	3.2192	7.2182	7.1230	7.1829	7.2323
Cep170	_SRS[Phospho (STY)]ADIGAR_.2	Q6A065_S985_M1_Cep170	4.2083	4.6117	3.7448	6.3216	5.8606	5.9253	5.5390	4.4339	4.1311	6.6776	5.6264	4.3378	6.4917	3.7829	4.1591	3.5704	5.2761	5.8925	5.8300	3.4602
Cep170	_LGEAS[Phospho (STY)]DSELADADK_.2	Q6A065_S1102_M1_Cep170	7.6379	4.5024	3.8541	7.1731	3.0236	6.3721	4.0245	3.7898	3.9447	4.1281	3.4233	6.7641	4.1102	7.2006	4.0499	3.6796	4.3275	3.5057	3.1543	3.0790
Cep170	_LRTS[Phospho (STY)]PALK_.2	Q6A065_S1250_M1_Cep170	3.9448	3.3743	3.4814	3.6394	6.2796	4.1851	4.3972	4.1625	6.0278	3.7554	3.7961	3.1004	4.4829	3.4847	2.9217	3.3069	6.3365	3.1330	6.4253	3.4517
Cep170	_SIKS[Phospho (STY)]DVPVYLKR_.3	#N/A	3.7682	6.4404	4.8821	4.0377	4.5320	4.3436	5.9600	4.2755	5.3458	5.3431	3.8583	6.2377	6.3852	7.2650	5.5081	5.9965	6.0695	6.4048	6.8685	4.6924
Cep170	_S[Phospho (STY)]VDSRPQPAEHPDHLTITR_.4	Q6A065_S1384_M1_Cep170	7.0218	3.6181	3.2376	3.8832	3.9079	3.9413	3.1402	3.9267	3.0604	3.5116	4.0399	5.7103	3.7319	3.2756	3.1655	3.0631	3.4239	3.7255	4.0246	2.9529
Cep170	_SIKS[Phospho (STY)]DVPVYLK_.2	#N/A	6.1469	7.1519	6.8898	6.8796	3.5563	7.4882	7.1339	5.9008	7.2180	6.5348	6.1310	6.7594	6.5306	8.4387	6.8824	6.9979	7.2666	6.8989	6.4966	6.7408
Cep170	_TEEDSKS[Phospho (STY)]IKS[Phospho (STY)]DVPVYLK_.2	#N/A	3.6183	5.5340	8.3516	3.9219	3.8252	7.4100	7.4552	3.8388	7.8415	3.8034	3.6522	6.4458	3.9872	8.2885	7.3672	3.7311	3.9345	7.5069	7.9056	7.1495
Cep170	_QGS[Phospho (STY)]FTIDKPSSNIPIELIPHINK_.3	Q6A065_S829_M1_Cep170	4.5357	4.2842	4.0723	4.5493	3.2418	3.2752	3.8063	7.7243	3.7265	4.3463	3.2051	4.0103	3.8919	4.1104	3.8316	3.8979	4.2587	4.3916	3.1898	3.7877
Cep170	_C[Carbamidomethyl (C)]STSS[Phospho (STY)]PSKDVTK_.2	Q6A065_S963_M1_Cep170	3.9747	3.3480	3.5113	3.6131	4.1816	3.9304	4.0552	4.2435	3.6756	3.9766	4.2719	3.0741	4.0056	3.5493	3.1049	3.3368	3.6976	3.4555	3.7545	3.2266
Cep170	_ALLHSGS[Phospho (STY)]SSSKEK_.2	#N/A	3.4641	3.8550	4.5015	4.1202	3.6710	3.7043	3.3771	3.6897	3.2973	3.2747	2.7154	3.5811	3.4627	4.5396	3.4025	4.3270	3.1870	3.4737	4.2615	4.2168
Cep170	_SDS[Phospho (STY)]EATISR_.2	Q6A065_S1157_M1_Cep170	3.7867	7.1660	4.3230	3.9398	3.1244	3.3485	3.7760	3.4871	3.8351	7.1273	3.3864	4.1426	6.4010	4.3101	4.0309	4.1316	4.0248	3.8455	3.4941	3.5994
Cep170	_SDS[Phospho (STY)]LDTDSSM[Oxidation (M)]DTTLILK_.2	#N/A	5.5791	7.1576	7.1374	7.1991	3.7226	6.3836	3.7973	3.4108	5.9629	7.1495	6.2208	5.4746	3.8523	4.3033	3.5171	6.1657	6.3542	6.1502	3.6323	6.0410
Cep170	_GNKHDDGTQS[Phospho (STY)]DSENAGAHR_.3	#N/A	4.5252	4.3741	4.0893	4.3412	4.0873	4.0062	3.4890	3.2664	4.2003	4.1869	3.5783	3.7787	3.3309	4.7041	3.3398	4.4710	4.1364	4.2135	3.4817	3.9201
Cep170	_HDDGTQS[Phospho (STY)]DSENAGAHR_.3	#N/A	4.1209	4.3265	4.1378	3.5927	3.1104	3.4372	3.8362	3.3124	4.0174	4.3003	3.1541	4.1308	4.1477	4.1472	3.8616	3.8770	4.0698	3.4747	3.3977	3.9932
Cep170	_RFPTDYAS[Phospho (STY)]TS[Phospho (STY)]EDEFGSNR_.2	Q6A065_S1229_M2_Cep170	3.8502	4.1638	4.3865	3.8504	3.1880	3.4825	3.9706	3.5506	3.9756	4.3484	3.1085	4.0533	4.1056	6.5311	3.7445	4.1906	4.0883	3.7561	3.4047	3.7151
Cep170	_NQATSASSEKDNDDDQSDKGTY[Phospho (STY)]TIELENPNSEEVEAR_.4	Q6A065_Y499_M1_Cep170	3.6248	4.3780	4.1611	4.0646	2.9625	3.1866	3.0777	3.3252	3.1368	3.8311	1.9302	4.2675	3.2449	4.1482	4.1558	3.9697	3.8629	4.2125	3.6189	3.9668
Cep170	_GNKHDDGTQS[Phospho (STY)]DSENAGAHRR_.4	#N/A	4.5302	4.2897	4.0668	4.5548	3.2363	3.2697	3.8118	3.2551	3.7320	4.3408	3.2106	4.0158	3.8974	4.1049	3.8372	3.8923	4.2531	4.3971	3.1954	3.7821
Larp4b	_SPS[Phospho (STY)]PVHLPEDPKVAEK_.3	#N/A	8.6048	3.3673	7.9118	3.6324	8.7193	8.1712	8.1345	8.6494	3.8746	3.0933	8.3194	3.0934	9.4864	8.7284	8.3298	8.7970	3.6747	8.3746	3.7738	3.2037
Larp4b	_EQNTPPKS[Phospho (STY)]PQ_.2	Q6A0A2_S739_M1_Larp4b	6.7889	8.2107	7.7006	8.7688	3.5445	7.2744	7.0777	8.2133	7.3208	7.9102	8.0117	8.4900	7.5751	9.1504	7.3308	7.2717	7.2269	8.5065	7.4536	7.7077
Larp4b	_RAS[Phospho (STY)]PPAAGKR_.2	#N/A	8.1370	8.1386	7.4832	7.9361	7.8599	7.5953	7.5552	8.0061	7.4461	7.8129	7.4066	8.1136	8.1851	8.5314	7.2534	8.4534	8.1217	6.5181	7.8046	7.9050
Larp4b	_KNS[Phospho (STY)]FGYR_.2	Q6A0A2_S500_M1_Larp4b	8.1938	8.3329	8.3871	8.3514	7.9321	7.6153	7.9943	7.6183	3.1456	8.5409	7.7204	8.0271	3.3111	8.1826	7.2753	7.6321	7.8565	4.3047	7.7928	8.0695
Larp4b	_RAS[Phospho (STY)]PPAAGK_.2	#N/A	7.2413	7.0933	6.1637	6.9196	6.8735	7.0980	6.3383	6.9960	6.6560	6.6438	6.7186	4.3057	7.0036	7.2017	3.3759	5.7325	6.1120	6.6598	6.7619	7.0410
Larp4b	_SPS[Phospho (STY)]PVHLPEDPK_.2	#N/A	9.9686	9.9053	10.3378	9.9896	9.7006	9.5787	9.3736	9.6985	4.1688	10.3156	9.9357	9.6438	4.3343	10.4221	8.8440	3.4555	3.8163	3.3331	9.4638	10.0168
Rftn1	_DSVALRHS[Phospho (STY)]NPR_.2	#N/A	4.7421	5.4934	4.1876	4.7603	3.1612	5.3234	4.0117	4.6731	4.8497	4.5895	4.3961	4.2563	4.2880	4.0740	3.9639	4.5530	4.5753	4.0780	4.6124	4.6733
Cgnl1	_SYGSQPNS[Phospho (STY)]PTSEDLAK_.2	Q6AW69_S203_M1_Cgnl1	4.1048	6.4665	4.2807	4.3576	3.4502	3.4835	3.6146	3.5719	3.5348	3.9154	5.1407	3.8186	3.7002	4.4088	4.6628	4.1062	3.6658	3.9088	3.9482	6.4759
Cgnl1	_RQDS[Phospho (STY)]AGPILDGAR_.2	Q6AW69_S284_M1_Cgnl1	4.3142	6.3119	6.6245	5.5016	5.4471	3.0537	4.0278	5.7362	5.1085	5.5043	5.8960	4.2318	4.3451	6.3131	5.2257	5.3999	5.7544	5.3396	5.9021	5.7993
Nlrp14	_KVM[Oxidation (M)]LPESYLLVATR_.2	#N/A	11.9524	11.1837	12.5350	12.3605	11.7487	11.9707	11.0574	11.3654	11.8655	12.3621	12.1281	11.1308	12.3829	11.2980	11.8729	12.2051	12.0210	10.9877	11.6867	12.0445
Arhgap21	_DQGEPVS[Phospho (STY)]PSEDEPFSWPGPK_.2	Q6DFV3_S41_M1_Arhgap21	10.7787	11.0812	10.7622	10.5475	9.9424	10.8560	10.6140	3.9910	10.7098	4.4969	10.5554	10.3617	10.5822	11.3319	9.7243	10.5016	11.0523	10.9951	3.6766	10.4197
Arhgap21	_NRS[Phospho (STY)]PTLSC[Carbamidomethyl (C)]R_.2	Q6DFV3_S1501_M1_Arhgap21	7.9878	8.0695	7.6523	7.6713	7.6388	7.7304	7.6723	7.8014	7.5020	7.6793	7.2076	7.5803	7.7768	7.8513	7.4579	7.1081	7.7886	7.5485	7.4883	7.2951
Arhgap21	_QLS[Phospho (STY)]HDQESVGPPSLDGQHSSK_.3	#N/A	8.8386	4.1811	8.4565	4.4463	3.3449	8.1965	8.3586	8.5765	3.6234	8.5787	8.4188	8.0115	8.7203	8.6478	6.8301	8.3930	4.3617	4.2886	3.0868	8.4609
Arhgap21	_VGGS[Phospho (STY)]IRPWK_.2	Q6DFV3_S947_M1_Arhgap21	7.9756	8.4077	7.6522	7.8902	6.9252	7.1919	7.8963	7.4010	6.5808	7.9877	7.5759	7.6512	7.5653	8.0459	4.0747	7.0708	7.1795	7.5874	7.2362	7.4299
Arhgap21	_LLSKDDTS[Phospho (STY)]PPKDK_.3	Q6DFV3_S1108_M1_Arhgap21	6.1885	3.5980	3.4402	3.2846	3.7425	4.2424	4.3399	4.1052	4.2601	3.8127	3.7387	3.4875	4.4255	3.4273	2.8644	3.2488	3.1420	6.4058	4.3397	3.3943
Arhgap21	_KGS[Phospho (STY)]SSEVFSDAAR_.2	Q6DFV3_S917_M1_Arhgap21	3.6849	5.5128	6.2166	5.5103	5.9101	6.9813	6.5677	6.1555	6.7889	5.6693	3.5379	6.0619	6.0762	7.0841	5.9801	6.5055	7.1338	6.7193	6.9137	6.0451
Arhgap21	_SKS[Phospho (STY)]YDEGLDDYREDAK_.3	#N/A	8.3051	8.2700	8.0402	8.3090	3.8406	7.7899	3.9834	7.6047	7.6224	8.3351	7.5429	7.9801	7.8507	8.5291	6.6383	7.9642	7.7327	8.2172	7.9224	7.9263
Arhgap21	_VRTS[Phospho (STY)]ASDLSR_.2	Q6DFV3_S1847_M1_Arhgap21	3.3911	3.9280	4.4286	4.1931	3.5981	3.6314	3.4500	3.6168	3.3702	3.2017	4.3497	3.6541	3.5357	4.4666	3.4754	4.2541	3.1140	4.0354	4.1214	4.1439
Arhgap21	_EKPQS[Phospho (STY)]GRQTPQPLR_.3	Q6DFV3_S736_M1_Arhgap21	4.1198	6.3771	3.6563	3.4645	5.9364	3.7817	4.2223	4.3454	4.0426	4.4262	2.9161	4.4263	6.2160	3.6944	4.2476	3.4819	3.8427	6.1881	3.6058	5.6437
Arhgap21	_T[Phospho (STY)]SQGFGFTLR_.2	Q6DFV3_T61_M1_Arhgap21	3.3600	3.9591	4.3974	4.2242	3.5669	3.0406	3.4812	3.5857	3.2828	3.6851	3.6572	3.6852	3.3909	4.4355	3.5065	4.2229	3.0829	6.0252	4.3656	4.1128
Arhgap21	_S[Phospho (STY)]TS[Phospho (STY)]HDRVPQSVQIR_.2	#N/A	3.7672	3.9566	4.1068	3.8634	3.7324	4.4816	4.1104	3.7318	4.0646	3.8598	3.5665	3.7935	4.1940	4.1149	3.5096	3.9712	3.4759	3.8582	4.1313	3.7812
Arhgap21	_RQLS[Phospho (STY)]HDQESVGPPSLDGQHSSK_.4	#N/A	7.7569	7.8637	7.2605	7.2671	7.0460	7.2852	7.3125	7.5258	7.0447	7.3498	7.5039	7.6510	7.8677	7.6324	6.0690	5.1228	7.3274	7.5555	7.2128	7.5407
Arhgap21	_TQSPHS[Phospho (STY)]PKEESER_.3	Q6DFV3_S1092_M1_Arhgap21	4.6307	4.7992	4.4382	3.2433	4.1282	3.5605	4.9852	3.0659	4.5002	4.8838	4.1790	4.2134	5.0216	3.9157	3.9378	4.5481	4.0640	4.4626	3.3847	3.5930
Arhgap21	_GIPS[Phospho (STY)]IVR_.2	Q6DFV3_S1122_M1_Arhgap21	5.0287	4.2237	5.3778	3.1965	3.8306	4.0547	5.7459	4.1932	5.5222	6.6134	5.5471	4.5624	3.8776	5.0135	3.2877	5.2178	3.2301	5.7364	3.8788	5.6991
Arhgap21	_SVS[Phospho (STY)]QERLEDSVLMK_.2	Q6DFV3_S453_M1_Arhgap21	3.5233	3.7958	5.7301	4.0609	3.7302	4.3781	4.3240	3.7490	3.4461	3.5218	3.8205	3.5219	3.5542	4.5988	3.3432	4.3863	3.2462	3.9032	4.2023	4.2761
Ncoa7	_EVLDSLGSS[Phospho (STY)]LDK_.2	Q6DFV7_S612_M1_Ncoa7	7.1222	3.6871	3.1686	3.9522	3.8390	4.2694	5.7898	3.8577	3.5548	3.4131	3.9292	3.4132	3.6629	3.2067	3.2345	5.9540	3.3549	3.7945	4.0936	4.3848
Ncoa7	_HSGS[Phospho (STY)]PENLGESTLNIHEDLDKIK_.4	Q6DFV7_S500_M1_Ncoa7	3.6286	4.3742	4.1649	4.0608	2.9664	3.1904	3.0739	3.3290	3.1330	3.8350	3.5073	4.2636	3.2410	4.2856	4.1519	3.9735	3.8667	5.5866	3.0146	3.9629
Ncoa7	_VLS[Phospho (STY)]STSEEDEPGVVK_.2	Q6DFV7_S211_M1_Ncoa7	5.5459	5.3417	6.3253	5.7643	3.3636	3.6241	3.7520	3.7904	5.0790	5.6674	3.5740	3.9561	5.0466	6.8582	3.7774	6.3753	5.3688	3.8098	5.5939	3.8844
Ncoa7	_YYS[Phospho (STY)]VDDNQNK_.2	Q6DFV7_S92_M1_Ncoa7	7.1471	6.8407	7.4865	6.9988	3.7648	3.0067	4.1284	3.5166	6.0091	3.8828	6.5623	4.1661	6.3720	7.0691	6.1996	6.4794	6.7318	4.0075	4.1110	3.2076
Ncoa7	_SVRS[Phospho (STY)]PEESTER_.2	Q6DFV7_S353_M1_Ncoa7	7.2724	4.0157	4.5235	3.7022	3.3249	6.2095	7.2439	6.9763	6.7224	6.7634	7.0504	3.9051	7.5852	3.9271	6.4082	6.8626	4.0754	3.0741	6.5296	3.6044
	[Acetyl (Protein N-term)]STIC[Carbamidomethyl (C)]PPPS[Phospho (STY)]PAVAK.2	Q6DFW0_S9_M1_	4.2248	4.5951	3.7614	7.0480	3.4525	7.4859	4.0886	4.4505	4.1476	7.0693	7.2764	4.3212	4.2557	3.7995	3.6658	2.9589	7.9059	3.2017	3.5008	7.4177
Nop58	_EEPLS[Phospho (STY)]EEEPC[Carbamidomethyl (C)]TSTAVPS[Phospho (STY)]PEK_.2	#N/A	5.0978	4.2412	7.2935	5.1490	4.0169	3.8988	6.9744	3.4470	5.4202	3.6255	3.7179	3.2948	3.4516	5.7837	3.1425	5.8421	5.0237	6.8691	4.3060	4.1735
Nop58	_HIKEEPLS[Phospho (STY)]EEEPC[Carbamidomethyl (C)]TSTAVPS[Phospho (STY)]PEK_.3	#N/A	3.9698	4.0580	3.7259	4.1006	3.3075	4.7632	5.6794	3.6051	3.5912	4.0161	3.4327	4.7837	3.7378	3.7466	3.8375	3.6547	4.3477	3.9646	3.1184	3.5280
Nol4l	_NTPES[Phospho (STY)]PPYSSGSYDSIK_.2	Q6DIB4_S189_M1_Nol4l	3.7729	3.5462	8.0593	3.8113	3.9798	8.2260	3.0683	7.6374	2.9885	8.4178	3.9680	7.3205	3.1539	3.3475	7.0521	3.1350	3.4958	3.6536	3.9527	7.7320
Nol4l	_LEIYQSS[Phospho (STY)]QEEPIALDK_.2	Q6DIB4_S354_M1_Nol4l	7.7624	7.9332	8.3380	8.4223	3.8255	4.1595	7.4429	4.1881	4.3430	8.3976	3.8216	3.4045	4.5085	8.4236	3.2928	3.3318	3.2250	7.6061	4.2568	7.7305
Scaf8	_ASEPVKEPVQTAQS[Phospho (STY)]PAPVEK_.3	#N/A	7.7956	7.7839	7.6901	7.6829	7.2774	7.6998	7.6199	7.5283	7.5905	7.7891	7.6104	7.5627	7.7882	7.9052	7.0306	7.6848	8.0270	7.6405	7.5188	7.4592
Lemd2	_WTKPSSFS[Phospho (STY)]DSER_.2	Q6DVA0_S507_M1_Lemd2	3.8967	3.8126	4.0172	7.5221	3.6223	7.5649	7.2541	7.5848	3.7361	3.6831	3.9548	3.6209	3.9212	6.9857	3.4772	6.9865	7.5451	3.6911	7.1487	3.8073
Lemd2	_AS[Phospho (STY)]VRGS[Phospho (STY)]SEDDEDTRTPDR_.3	Q6DVA0_S145_M2_Lemd2	3.9779	3.7418	4.0753	3.7597	3.2759	4.0624	3.6271	3.8911	3.7917	4.0531	5.9576	3.5481	3.9572	4.0980	3.4037	3.8957	3.1532	3.6264	4.3179	3.8679
Utp14b	_S[Phospho (STY)]KAIMAKY[Phospho (STY)]DPEAR_.2	Q6EJB6_S307_M2_Utp14b	7.9617	9.1634	8.4423	8.3686	4.0868	7.2432	8.4820	6.9813	8.3561	8.6357	8.2784	7.1486	7.2752	7.0796	6.8944	8.3068	8.8271	8.7137	7.9900	7.5733
Tmem151a	_SLS[Phospho (STY)]GGPLGR_.2	Q6GQT5_S423_M1_Tmem151a	3.5968	4.9865	5.2130	5.2749	3.8037	4.9275	4.9634	3.8225	3.7540	3.9232	7.2019	4.4872	3.8886	3.9058	3.5709	4.5722	4.0540	4.7601	5.0757	5.0991
Ralgapa1	_SQT[Phospho (STY)]PS[Phospho (STY)]PSTLSR_.2	Q6GYP7_S1003_M2_Ralgapa1	9.4018	8.8142	9.2905	9.2944	9.0265	4.1679	8.6823	8.8664	8.5157	9.1833	8.9419	8.3975	8.9535	8.2148	6.6832	8.3853	8.8842	8.5492	8.6924	8.7787
Ralgapa1	_SSS[Phospho (STY)]TSDILEPFTVER_.2	Q6GYP7_S796_M1_Ralgapa1	8.1534	4.2038	4.2261	3.7432	6.8749	4.0604	8.0177	7.0002	3.8866	3.9298	7.6329	3.7447	3.9946	4.2641	3.4047	3.6967	3.6867	3.5855	7.1271	3.9414
Ralgapa1	_SATTTGS[Phospho (STY)]PGTEK_.2	Q6GYP7_S739_M1_Ralgapa1	10.0633	9.8566	9.5888	9.5140	9.2512	9.0436	9.7537	9.6330	9.2230	9.5392	9.0241	9.2865	9.6061	9.0056	3.8212	3.9083	9.4862	9.5740	8.9688	8.9306
Ralgapa1	_RGS[Phospho (STY)]SPGSLEIPK_.2	Q6GYP7_S859_M1_Ralgapa1	4.1325	8.6350	7.8399	8.6841	7.2399	7.3734	8.2545	7.5845	7.3278	7.4327	7.3240	7.0105	3.8686	3.3939	3.2347	3.1992	8.4761	8.0081	6.9531	6.9204
Ralgapa1	_GVGHEFQKVS[Phospho (STY)]VDK_.3	Q6GYP7_S710_M1_Ralgapa1	3.6530	3.6661	3.1895	3.9313	3.8599	3.8932	3.2255	4.4544	3.1084	3.4635	3.3693	2.7293	3.2739	3.7765	3.2136	3.5167	3.4912	2.7007	3.9905	3.6492
Ralgapa1	_GWS[Phospho (STY)]RDQPGQAPMR_.2	#N/A	3.8750	4.2910	4.1581	3.8624	3.3276	3.6879	3.7742	3.9342	3.7813	3.8952	4.0918	4.0171	3.9104	3.8886	3.5410	3.9836	3.8210	3.7458	3.1502	3.7282
Hecw2	_ANS[Phospho (STY)]DTDLVTSESR_.2	Q6I6G8_S48_M1_Hecw2	12.0687	11.8203	11.9815	11.8640	11.1863	11.1222	11.1853	11.6188	11.1020	11.7674	11.2731	11.2694	11.7684	11.3061	10.2137	11.0661	11.6424	11.2232	11.1645	11.2526
Hecw2	_SHS[Phospho (STY)]AGEVGEDSR_.2	#N/A	6.2364	6.9050	5.3878	6.2466	5.9022	5.5515	6.4580	6.4239	5.6389	3.5463	5.7825	6.6442	6.8669	6.5201	5.3676	5.1343	6.4022	6.1409	5.6416	6.0644
Hecw2	_TSSTLEIDTEDLISTSSRNS[Phospho (STY)]PPR_.3	Q6I6G8_S406_M1_Hecw2	3.5692	3.7499	7.4763	4.0151	3.7761	4.3323	3.2720	3.7949	3.4920	3.3798	7.4030	3.4760	3.6000	3.1438	3.2974	4.4321	3.2921	3.8574	4.1564	4.3219
Hecw2	_SNS[Phospho (STY)]IQQMEQLNR_.2	#N/A	4.0728	7.0117	8.1431	7.8829	5.6867	6.1881	6.6573	3.4239	5.4642	6.0177	6.9129	5.3724	4.0743	6.8377	4.7789	3.8358	6.1288	4.2527	3.8962	5.5844
Hecw2	_QRSHS[Phospho (STY)]AGEVGEDSR_.3	#N/A	3.9814	4.3798	4.1242	4.2777	3.4540	3.5321	3.7877	3.1794	4.1247	3.8451	3.6731	3.7780	4.2433	3.6972	3.6687	3.9382	4.0489	4.3589	3.4693	3.7938
Tmem132e	_DQTEDPASS[Phospho (STY)]PTSK_.2	#N/A	3.3826	3.9365	4.4200	7.4883	3.5895	3.0180	6.9788	3.6082	3.3788	6.7800	4.3583	3.6627	3.4134	6.9172	3.4840	4.2455	3.1055	7.5924	4.3430	4.1353
Tmem132e	_DQTEDPASS[Phospho (STY)]PTSKR_.2	#N/A	5.9941	3.9499	4.4082	5.8829	3.5777	3.3973	5.7175	3.5964	3.2949	5.4767	4.0135	5.5280	6.0996	4.4462	3.9130	4.2337	4.0079	4.0574	4.3564	4.1235
Arhgap20	_KSS[Phospho (STY)]C[Carbamidomethyl (C)]DAVLSR_.2	Q6IFT4_S730_M1_Arhgap20	6.3965	3.5810	6.8018	6.1549	5.2404	3.9809	3.4822	6.3218	6.0301	5.9503	5.9783	3.3912	6.2743	6.2579	5.1973	5.9246	6.8064	6.2811	5.9810	6.0729
Arhgap20	_S[Phospho (STY)]APSLILDK_.2	Q6IFT4_S46_M1_Arhgap20	4.1566	3.8462	3.1920	6.6227	3.4943	2.9898	5.9953	3.8569	6.8065	4.3629	2.9794	3.7357	4.1773	3.1791	3.6240	5.3410	4.3946	6.4916	6.9165	3.1461
Arhgap20	_SS[Phospho (STY)]EPSIDYLDTK_.2	#N/A	4.1555	3.8473	3.1910	3.5338	3.4933	3.7174	4.0478	3.8559	4.1069	4.3619	2.9804	3.7367	4.2150	3.1781	3.6250	2.9996	4.3936	4.9386	3.0881	3.4360
Arhgap20	_RC[Carbamidomethyl (C)]S[Phospho (STY)]EPSIDDQNYK_.2	Q6IFT4_S847_M1_Arhgap20	3.1814	4.2781	4.2610	3.9647	3.3883	3.4217	5.5324	3.4251	3.5800	4.4928	3.0586	3.8638	3.7454	4.2481	3.6851	4.0444	3.9627	3.8704	3.5190	4.2151
Arhgap20	_RSS[Phospho (STY)]EPSIDYLDTK_.2	#N/A	3.7826	3.5365	3.3191	3.8017	3.9895	4.0228	3.0586	4.0082	2.9788	3.5932	3.9583	3.2627	3.1443	3.3572	3.0840	3.1447	3.5055	3.6440	3.9430	3.6139
Gpsm1	_AAQSSVTAS[Phospho (STY)]PQTEEFFDLIASSQSR_.3	Q6IR34_S543_M1_Gpsm1	3.2463	8.4825	3.7826	3.9917	4.0848	4.3089	3.4562	3.8182	3.5153	3.4526	3.8897	3.1451	3.6234	3.1671	3.0334	3.5912	3.3154	3.8340	4.1331	4.3453
Gpsm1	_APS[Phospho (STY)]SDEEC[Carbamidomethyl (C)]FFDLLSK_.2	Q6IR34_S490_M1_Gpsm1	7.3802	7.6410	7.9176	3.5550	3.4721	7.3316	7.4967	3.8347	7.7085	7.9039	3.4683	7.3460	4.1551	8.2168	7.2942	7.6832	7.4798	7.6558	7.8077	7.3954
Gpsm1	_AS[Phospho (STY)]VGSLPGLR_.2	Q6IR34_S567_M1_Gpsm1	7.6308	7.4637	4.2824	3.9433	3.0838	6.4423	4.4573	3.4464	3.0155	3.9524	3.3899	4.1462	3.1236	7.4708	4.0345	4.0910	3.9841	6.7155	7.9007	5.9495
Gpsm1	_EGSHS[Phospho (STY)]PLDSADVR_.2	Q6IR34_S469_M1_Gpsm1	6.5789	4.5517	3.8048	3.3160	2.9743	3.0077	4.0738	4.4939	3.9940	4.0788	3.4726	4.2778	4.1594	6.1456	4.0992	3.6303	3.9911	3.1583	3.4574	3.5201
Gpsm1	_VDLAGS[Phospho (STY)]PEQEASGLPDPQQQC[Carbamidomethyl (C)]PPGAS_.3	Q6IR34_S653_M1_Gpsm1	3.3634	3.9557	4.4008	6.0798	3.5703	3.6037	6.4115	3.5891	3.3980	3.1740	6.1872	3.6818	3.5634	4.4389	3.5032	4.2263	3.0863	4.0632	4.3622	4.0331
Cltb	_DLEEWNQR_.2	#N/A	9.1811	9.3969	9.4083	9.5416	9.4838	9.3470	9.1506	9.3167	9.3204	9.4629	9.4538	9.4271	9.9674	9.6759	9.7464	9.1854	9.0816	9.0697	9.0279	9.2485
Cltb	_LTQEPESIRK_.2	#N/A	8.5926	8.5227	8.7729	8.4320	8.7033	8.3768	8.8658	8.8071	8.8770	8.6845	8.4649	8.7525	8.7249	8.7246	9.3195	8.9269	8.4276	8.6755	8.6102	8.7008
Cltb	_VAQLC[Carbamidomethyl (C)]DFNPK_.2	#N/A	8.2496	8.4801	8.6055	8.6485	8.4718	8.4009	8.5171	8.4493	8.4152	8.3797	8.4616	8.7472	8.8364	8.6323	9.1006	8.3311	8.4904	8.4034	8.0372	8.7024
Cltb	_LTQEPESIR_.2	#N/A	8.3222	8.4439	9.1182	8.7533	8.5245	8.4934	8.4459	8.5532	8.5892	8.3130	8.4342	8.5612	8.5249	8.4301	9.1116	8.5794	7.9782	8.3621	8.1796	8.4318
Cltb	_LQELDAASK_.2	#N/A	8.1526	8.0975	8.5733	8.2927	8.3325	8.0703	8.0283	8.0070	8.3449	8.1594	8.0679	8.1340	8.5373	8.3895	8.7484	8.4057	8.0757	7.8533	8.1832	8.4778
Cltb	_VTEQEWR_.2	#N/A	7.3320	7.9255	7.8115	6.7926	7.1693	7.6462	6.6563	7.2638	7.3772	7.4399	7.7708	7.1318	7.3392	7.7590	7.4195	7.5721	7.3194	6.8456	7.4711	7.3496
Cltb	_KDLEEWNQR_.2	#N/A	4.3325	3.6450	4.7332	4.4574	5.9184	4.5362	4.3265	5.2075	6.0775	4.5632	4.0667	3.3711	3.2527	3.7553	5.5882	4.5393	3.4700	4.0000	4.1367	4.5648
Cltb	_ESKEETPGTEWEK_.2	#N/A	5.6512	5.2403	5.8960	5.6790	5.9565	5.9060	5.6009	5.7738	5.6023	5.8459	5.3394	5.5058	5.8681	5.7328	6.6591	6.0683	5.6095	5.4809	5.8622	5.9380
Cltb	_AFYQQPDADTIGYVASEEAFVK_.3	#N/A	5.3089	4.5063	3.2720	5.3186	3.9423	4.1660	3.1058	3.9611	3.6582	3.5460	4.0326	4.1836	3.7663	3.3100	4.4848	5.3689	3.4583	3.6911	3.9902	4.2028
Nipbl	_AITSLLGGGS[Phospho (STY)]PK_.2	Q6KCD5_S2652_M1_Nipbl	7.5208	8.2463	6.9315	5.9315	3.8680	7.0945	7.9631	8.2421	6.2094	6.6561	4.0798	7.2575	7.5302	7.8869	5.4885	6.5250	7.6048	7.7726	3.9986	3.7354
Dgkb	_FPHSS[Phospho (STY)]PNVK_.2	Q6NS52_S95_M1_Dgkb	9.5002	3.6302	9.5480	3.3168	3.7103	8.5525	8.5106	4.0730	8.7212	9.7005	4.2642	8.7015	8.9554	8.9993	7.7302	3.2166	8.9443	3.4595	4.3720	3.2190
Dgkb	_VLAC[Carbamidomethyl (C)]GGDGTVGWILDC[Carbamidomethyl (C)]IEK_.2	#N/A	3.8095	4.2898	4.0667	4.5550	3.1473	3.3713	4.3938	3.2549	2.8830	4.0159	3.3264	4.0827	4.8973	4.1047	3.9710	4.1544	4.2530	4.3973	3.0656	3.7618
Dgkb	_QDILNQTIDFEGFK_.2	#N/A	3.8184	4.1844	4.3547	4.5638	3.1561	3.3802	4.3850	3.2461	4.4441	5.6953	3.3176	4.0739	4.5521	4.0959	3.1476	3.5546	4.2441	4.4061	3.2043	3.7732
Fbxo41	_GGGGS[Phospho (STY)]ASGPGVR_.2	Q6NS60_S363_M1_Fbxo41	3.1674	3.3346	3.7037	6.5361	4.0059	4.2300	3.5351	4.3686	3.5942	3.3737	6.7766	3.2240	3.7023	3.2461	4.8853	6.7334	7.0685	3.7551	4.0542	4.4242
Fbxo41	_EHHAGSAVPSTYAVS[Phospho (STY)]R_.3	Q6NS60_S391_M1_Fbxo41	5.4212	4.2156	5.7030	5.0238	4.2201	4.1973	5.1569	4.2461	3.9957	5.4653	4.4623	4.0004	4.2758	3.5837	3.8470	4.5237	4.6661	3.2437	4.0395	4.6505
Fbxo41	_RHS[Phospho (STY)]TEGEEGDVSDVGSR_.3	#N/A	8.2826	8.7250	4.8845	5.6204	7.5289	8.5732	8.6305	8.2144	8.6394	8.3518	8.4912	8.1952	9.2624	5.6912	7.7734	8.1153	8.5844	8.6780	8.6273	8.3031
Fbxo41	_LSARPEGGS[Phospho (STY)]GR_.2	Q6NS60_S526_M1_Fbxo41	3.7807	11.9118	4.3170	3.9087	3.1184	3.3657	11.5580	3.4811	3.6360	4.4368	3.1146	4.1116	3.8014	4.3041	8.9363	4.1256	4.0187	3.8144	8.8755	4.2711
Fbxo41	_[Acetyl (Protein N-term)]AS[Phospho (STY)]LDLPYR_.2	Q6NS60_S3_M1_Fbxo41	3.3414	4.4381	4.1010	4.1247	3.5483	6.5975	6.3596	3.2651	6.5863	3.1520	2.8986	6.3505	3.5854	4.0881	3.5252	4.2043	3.8028	6.8504	3.6790	5.9642
Fbxo41	_HGSS[Phospho (STY)]PSTGASSR_.2	Q6NS60_S396_M1_Fbxo41	4.5333	3.4695	3.5687	3.1561	3.8710	4.1139	3.8374	4.2336	4.3885	3.2388	3.8672	3.3590	4.5540	3.5558	3.2473	3.3773	3.2705	3.0618	4.2113	3.5228
Amer3	_SSQTS[Phospho (STY)]PC[Carbamidomethyl (C)]SQGYGR_.2	Q6NS69_S52_M1_Amer3	3.8565	4.3368	4.0197	3.1011	3.1942	3.4183	4.3469	3.2080	4.4060	4.0628	3.2795	4.0358	4.5140	4.0578	3.9241	4.2014	4.2061	4.4442	3.2424	3.7351
Amer3	_DGPES[Phospho (STY)]PAHS[Phospho (STY)]PQALDC[Carbamidomethyl (C)]K_.2	Q6NS69_S263_M2_Amer3	3.6241	3.5207	2.8727	4.0081	3.9759	4.3132	3.4489	3.8108	3.1508	3.9275	3.8823	3.4691	3.6160	5.2128	3.1039	3.6076	3.3081	3.8504	5.0425	4.3379
Retreg2	_SAPPAGDEPLAETES[Phospho (STY)]ESEAELAGFSPVVDVK_.3	Q6NS82_S276_M1_Retreg2	3.3408	4.3404	4.1214	4.3092	3.9322	3.4571	3.5551	3.2983	3.2265	3.5857	3.9611	4.1605	3.3661	4.1357	3.3120	3.9390	4.1003	4.1811	3.4439	3.9537
Sphkap	_ASSC[Carbamidomethyl (C)]ES[Phospho (STY)]IPEEDSEAR_.2	Q6NSW3_S1070_M1_Sphkap	7.2409	8.1470	8.2833	6.8173	6.8221	3.6922	7.9077	7.9442	6.3171	7.2093	6.9654	7.8334	7.4239	7.2086	5.8360	3.5913	8.1556	7.9945	7.0173	6.8943
Sphkap	_SNS[Phospho (STY)]LLESTDYWLQNQR_.2	Q6NSW3_S50_M1_Sphkap	4.4881	7.0197	5.8680	5.7399	3.8258	6.3092	6.6130	5.8072	5.7989	6.2729	5.5393	3.4042	6.0797	6.9958	5.3135	6.1186	3.2253	6.7962	4.2564	3.4776
Sphkap	_ANS[Phospho (STY)]LDGFAQNC[Carbamidomethyl (C)]PQDSVNVQPVSR_.3	Q6NSW3_S1244_M1_Sphkap	6.5050	6.3848	6.4560	5.2438	3.3399	4.8272	6.3510	5.6722	3.8853	4.9120	3.1338	5.6964	5.5038	7.4562	3.7784	4.3471	4.2183	6.2300	4.9013	4.3578
Sphkap	_QSS[Phospho (STY)]C[Carbamidomethyl (C)]ESITDEFSR_.2	Q6NSW3_S1106_M1_Sphkap	4.0152	7.2176	7.6708	8.1488	5.4947	3.8479	5.4925	3.7288	7.1046	3.9492	3.6201	6.1054	3.7880	6.0291	4.9229	6.2087	6.6630	3.7530	7.3128	7.0393
Sphkap	_RAS[Phospho (STY)]TDLGK_.2	Q6NSW3_S841_M1_Sphkap	4.1367	3.8661	3.1721	3.3813	3.4744	3.6985	4.0667	4.4286	4.1258	4.3430	2.9993	3.7556	4.2338	3.7776	3.6439	2.9807	3.9259	3.2236	3.5226	5.8323
Sphkap	_LKAS[Phospho (STY)]S[Phospho (STY)]C[Carbamidomethyl (C)]ESIPEEDSEAR_.2	Q6NSW3_S1066_M2_Sphkap	5.4652	5.2671	4.2692	4.8674	2.7159	4.0663	4.5805	3.4461	4.5330	3.5329	3.2362	4.7579	3.4313	3.4789	4.1919	4.0868	5.2820	4.8674	4.8215	4.0158
Sphkap	_QSS[Phospho (STY)]TESITEEFYR_.2	Q6NSW3_S1171_M1_Sphkap	3.9460	7.3017	3.4826	3.6382	4.1529	6.5242	4.3960	4.1717	6.5054	7.0632	3.7949	3.0992	4.4817	3.5206	5.6991	3.3081	3.6689	6.9567	3.7796	3.4505
Sphkap	_EILKEQS[Phospho (STY)]ES[Phospho (STY)]IKEHASGLPGR_.3	Q6NSW3_S1574_M2_Sphkap	4.5311	4.2888	4.0677	4.5540	3.2372	3.3703	3.5021	3.2559	4.4539	4.3417	3.2098	4.0150	4.5620	4.1057	3.8363	3.8932	4.2540	4.3963	3.1945	3.7830
Sphkap	_ASSSGLC[Carbamidomethyl (C)]KS[Phospho (STY)]DSC[Carbamidomethyl (C)]LYR_.3	Q6NSW3_S1273_M1_Sphkap	3.5822	3.7369	3.1188	4.0020	3.7892	3.8225	3.2589	4.5251	3.1792	3.3928	4.1586	3.4630	3.3446	3.8473	3.2843	2.9443	3.5619	4.2712	3.9198	3.8143
U2surp	_RVKS[Phospho (STY)]PS[Phospho (STY)]PK_.2	#N/A	3.7730	4.4912	3.3096	5.1699	3.9800	4.0133	5.4798	5.0354	5.0101	5.2854	3.9678	3.2722	3.1538	3.3477	3.0935	3.1351	5.1154	5.6270	4.1106	3.6234
U2surp	_RHS[Phospho (STY)]TS[Phospho (STY)]PS[Phospho (STY)]PSR_.2	Q6NV83_S930_M3_U2surp	3.2669	3.2351	3.8032	4.4225	4.1055	3.8795	3.2020	4.4681	3.1222	3.4498	4.1016	3.1245	3.2876	3.7903	3.2273	3.6118	3.5049	4.3282	3.9768	3.7573
U2surp	_VKS[Phospho (STY)]PS[Phospho (STY)]PK_.2	#N/A	3.9352	4.0676	4.4715	3.7542	3.2729	3.4970	4.2682	3.6356	4.3273	4.1415	3.2008	3.9571	4.4353	3.9791	3.8454	4.2801	2.8123	3.1811	3.3211	3.6564
Wdr44	_EAENTANQAGNES[Phospho (STY)]PVQELR_.2	#N/A	10.7257	10.6256	10.6308	10.7484	10.2431	10.2869	10.2037	10.5544	10.2585	10.7823	10.2374	10.3212	11.0434	10.7375	9.8036	10.5305	10.7309	10.1270	10.3075	10.3146
Wdr44	_S[Phospho (STY)]NSGRELTDEEILASVMIK_.3	Q6NVE8_S344_M1_Wdr44	3.8895	3.4296	3.4261	3.6947	4.0965	4.1298	4.4525	4.1152	4.3727	3.7001	3.8513	3.1557	4.5381	3.5399	2.9770	3.2516	3.6124	3.0777	4.2271	3.5069
Wdr44	_EYVSNDATQS[Phospho (STY)]DDEEK_.2	#N/A	4.0131	3.3060	3.5497	3.5711	4.2200	3.8883	3.3911	4.2388	3.3171	3.8237	4.3103	3.0321	4.0440	5.1559	2.8534	4.5746	3.7360	3.8140	3.5740	3.2650
Wdr44	_TKEYVSNDATQS[Phospho (STY)]DDEEK_.2	#N/A	3.4341	3.4663	3.3894	3.1353	4.0598	3.3625	2.9883	4.2545	4.4094	3.6634	3.8880	3.1924	4.5748	3.5766	3.0137	3.2149	3.2913	3.0410	4.1904	3.5436
Wdr44	_TKEYVSNDATQS[Phospho (STY)]DDEEKLQSQQTDTDGGR_.4	#N/A	3.7364	4.2664	4.2727	3.9530	3.0741	3.2982	4.4670	3.4367	3.0252	3.9427	3.3996	4.1559	3.1333	4.2597	4.0442	4.0813	3.9744	4.3241	3.5073	3.8552
Cpsf6	_S[Phospho (STY)]RDRHDDYYR_.3	Q6NVF9_S513_M1_Cpsf6	3.7761	5.2218	4.8953	4.4722	3.1138	4.8191	4.4272	3.2884	6.0290	5.4859	5.4673	4.1161	4.5944	4.9930	5.2007	4.1210	5.2095	5.2867	6.2523	4.7711
Rprd2	_VS[Phospho (STY)]SSC[Carbamidomethyl (C)]LDLPDSTEEK_.2	Q6NXI6_S1086_M1_Rprd2	4.4886	4.3313	4.0252	3.7711	3.1947	3.2281	3.8534	3.6187	3.7736	4.2992	6.8181	4.0574	3.9390	4.4417	3.8787	3.8508	6.6417	3.6768	3.3254	4.4087
Rprd2	_SFNYS[Phospho (STY)]PSSSTSEVSSTSASK_.2	Q6NXI6_S612_M1_Rprd2	3.7318	3.6299	4.0831	3.8950	3.9387	5.7921	3.9667	3.9575	3.9160	3.5424	3.7707	3.3560	4.0618	6.5372	3.1773	6.2883	3.4547	5.6286	4.2801	3.8779
Rprd2	_DVEDM[Oxidation (M)]ELS[Phospho (STY)]DVEDDGSK_.2	#N/A	3.8743	8.5578	8.1989	7.3798	3.5588	8.5172	7.9985	3.9313	7.9228	7.5934	3.8103	7.6228	3.8792	8.8543	7.2398	7.8187	7.0402	7.9790	8.1193	8.0273
Rprd2	_ASVGQS[Phospho (STY)]PVLPSTTFK_.2	Q6NXI6_S633_M1_Rprd2	4.2436	6.1848	3.2791	3.4458	3.5813	3.8054	5.7653	3.9440	4.0188	4.4499	4.3932	5.1536	4.2643	6.7653	3.5369	3.0877	5.3936	3.3515	4.8089	3.2331
Rprd2	_GPTSTSVDSIDGT[Phospho (STY)]PVRDER_.3	Q6NXI6_T742_M1_Rprd2	4.0870	4.4342	3.9292	3.1985	3.0955	3.2337	4.2101	4.0066	4.2018	4.0790	3.3704	4.1528	4.3413	3.2706	4.0055	3.9494	4.1155	3.4132	3.4807	3.5425
Wwc2	_SDS[Phospho (STY)]DSSTLAKK_.2	#N/A	6.0440	5.8759	5.1857	4.9495	6.0412	5.8974	5.3087	5.8906	5.6940	5.8027	5.1010	5.8629	3.4798	5.3414	3.4176	4.3118	3.1718	3.9776	4.2767	4.2017
Wwc2	_LNRSDS[Phospho (STY)]DSSTLAK_.2	#N/A	3.6222	4.3806	4.1585	4.3676	4.2025	3.1840	3.0804	3.4423	3.1394	4.1387	3.5138	2.5054	3.2475	4.2921	4.1584	3.0522	4.4404	4.2099	3.0081	3.9694
Znf532	_AADKS[Phospho (STY)]PESQNLIDGTKK_.3	Q6NXK2_S313_M1_Znf532	3.6307	6.3910	4.1670	4.0587	2.9684	5.8730	3.0718	3.4338	3.1309	3.8370	3.5053	4.2616	3.2390	4.2836	4.1499	6.3599	4.4318	5.5486	5.6184	6.2413
Znf532	_KAASDSC[Carbamidomethyl (C)]KEPVANS[Phospho (STY)]R_.3	Q6NXK2_S293_M1_Znf532	3.7632	4.2396	4.2995	3.9262	3.1010	3.1594	4.4401	3.4636	3.6185	4.4543	3.0971	4.1290	3.7839	4.2866	4.0173	4.1081	4.0013	3.8319	3.4805	4.2536
Dpp10	_LYASAFSER_.2	#N/A	6.6385	6.8701	6.5994	6.4977	6.7114	6.6013	6.7499	6.6043	6.4433	6.4493	6.6918	6.3693	6.8118	6.2727	5.9734	6.3912	6.4793	7.0104	6.6248	6.5049
Dpp10	_ATQSQPADQELGS[Phospho (STY)]NSPPQR_.2	#N/A	9.3147	9.0876	9.0340	8.8421	8.8224	9.0727	8.8449	9.2694	8.8982	8.8055	8.9889	9.0464	8.9703	9.1788	3.5461	8.8338	9.7806	9.4393	9.2338	8.9199

Dpp10	_QNEEPVFSR_.2	#N/A	6.2040	4.8812	3.5118	3.2577	5.2877	5.6527	5.2566	5.9286	5.7644	5.1811	5.7336	3.0700	4.4524	5.4699	5.4805	5.1373	5.9379	5.1982	5.7521	5.6739
Szrd1	_ILGSAS[Phospho (STY)]PEEEQEKPILDRPTR_.3	#N/A	6.5912	7.4026	6.0374	6.9858	5.4711	3.7289	6.5242	6.1079	5.9921	7.1442	5.7917	6.6657	7.1543	7.2025	6.3342	6.7140	6.7853	6.3313	6.2016	6.4716
Sec23ip	_KLS[Phospho (STY)]VGAYVSSVR_.2	Q6NZC7_S748_M1_Sec23ip	4.8728	6.1363	4.9483	4.6648	3.3554	3.3887	3.6927	7.0441	3.6129	5.7295	3.0915	5.5490	5.6051	7.6701	4.4046	4.0114	5.1643	6.4248	3.0763	4.2480
Sec23ip	_EM[Oxidation (M)]AS[Phospho (STY)]PSSESNESKR_.2	Q6NZC7_S735_M1_Sec23ip	4.2164	4.6035	3.7530	3.3678	2.9225	3.6850	4.0802	4.4421	3.6714	4.3295	3.0128	4.3296	4.2473	3.7911	4.1510	3.5785	2.5083	2.3709	3.5092	3.4683
Zc3h11a	_KLS[Phospho (STY)]VGGDSDPPLKR_.3	Q6NZF1_S289_M1_Zc3h11a	3.4851	4.5177	4.0214	4.2043	5.6932	5.8294	3.2175	3.1854	3.2765	7.1049	3.6509	6.2602	6.7454	7.0177	2.7947	6.6883	3.7231	6.3654	6.6765	6.6565
Eif4g1	_KAAS[Phospho (STY)]LTEDR_.2	#N/A	9.8320	9.8561	9.4982	9.2335	9.0083	9.1518	9.2527	9.2683	9.0542	9.4798	8.9611	8.8498	9.1588	8.9779	7.4697	8.8987	3.2665	9.0867	8.7129	8.7022
Eif4g1	_SFS[Phospho (STY)]KEVEER_.2	Q6NZJ6_S1189_M1_Eif4g1	9.5197	9.4078	8.9899	9.2568	8.4089	8.3925	8.9962	8.4854	8.1443	9.2493	8.3900	8.6846	9.1355	8.6842	5.4525	8.3124	8.2118	8.8138	8.4764	8.5197
Eif4g1	_EATLPPVS[Phospho (STY)]PPK_.2	Q6NZJ6_S1231_M1_Eif4g1	10.2935	8.8489	3.1503	3.5746	7.5241	10.1105	9.3888	10.1385	4.1476	9.0381	8.9385	3.7774	4.2557	8.4197	3.6657	2.9589	9.6705	10.0618	7.2594	10.1056
Eif4g1	_AAS[Phospho (STY)]LTEDR_.2	#N/A	3.4976	4.5053	4.5343	4.1918	4.3361	3.0594	3.2050	3.5669	3.2640	3.7039	6.7039	4.3947	3.3721	4.4167	2.7822	3.8424	4.9268	5.9461	6.0256	4.0940
Eif4g1	_SS[Phospho (STY)]LS[Phospho (STY)]RER_.2	Q6NZJ6_S1147_M2_Eif4g1	4.1814	3.1609	3.6948	3.4260	3.5191	3.7432	4.0220	4.3839	4.0810	6.3675	2.9546	4.3878	4.1891	3.7329	3.5991	3.0255	3.8811	3.2683	3.5674	3.4101
Ptpdc1	_QLSYS[Phospho (STY)]DSDLKR_.2	Q6NZK8_S394_M1_Ptpdc1	3.8361	7.4637	4.3724	3.8533	3.2769	7.1937	3.7712	7.5713	7.4229	4.3814	3.1700	6.8829	8.0436	7.3327	5.9749	6.9670	4.0742	6.9860	3.4076	4.3265
Soga3	_EQSES[Phospho (STY)]LSELR_.2	Q6NZL0_S639_M1_Soga3	10.1853	9.9391	9.9940	4.0787	9.5817	9.7158	9.4951	4.0091	9.5659	9.5285	8.8530	9.4466	9.0837	9.6803	8.5243	8.4874	10.0944	9.4854	8.0508	9.4555
Soga3	_RGS[Phospho (STY)]KDLPK_.2	Q6NZL0_S501_M1_Soga3	9.5933	9.6990	9.0507	9.2835	8.9042	9.2668	9.3497	9.4005	9.0409	9.3397	8.9105	8.8496	9.2200	9.1947	7.6498	8.4034	9.2928	9.2512	8.6838	8.6763
Soga3	_EGPIGGES[Phospho (STY)]DSEEVR_.2	#N/A	9.5733	9.0367	8.7311	9.2265	3.5496	8.7977	8.8137	9.1292	8.9161	9.2742	8.4747	8.2142	3.5841	8.4937	7.0694	8.1558	8.9968	8.4095	8.4267	8.3919
Soga3	_MKELS[Phospho (STY)]LK_.2	Q6NZL0_S495_M1_Soga3	4.6074	8.3841	8.5757	8.8938	6.7151	7.1534	7.9861	6.3394	6.6401	6.7208	8.1351	6.7808	4.0907	8.4731	5.3085	3.4266	7.1530	6.4376	6.1079	6.5487
Soga3	_AQS[Phospho (STY)]PARPR_.2	Q6NZL0_S41_M1_Soga3	3.2336	3.9503	4.2996	2.5450	3.5518	3.7759	3.3382	4.3512	2.5701	4.4204	2.0958	4.4205	3.1574	3.8803	3.5665	3.0581	3.3102	3.3010	3.6000	3.3775
Soga3	_KREGPIGGES[Phospho (STY)]DSEEVR_.3	#N/A	3.2269	4.0921	4.2644	4.3573	3.4339	3.1736	3.6142	3.4526	3.1498	4.8448	3.5241	3.8183	5.5618	4.3024	3.6396	4.0899	4.4507	4.1996	2.9978	3.9797
Soga3	_LIADTSTIITEAR_.2	#N/A	3.3651	3.4509	3.4048	3.1506	4.0751	4.1853	2.6609	3.6561	4.3940	3.7024	3.8727	3.1770	4.5595	3.5613	2.9984	3.2303	3.6374	3.0563	4.2058	3.5283
Hdac4	_QEPIES[Phospho (STY)]EEEEAEATR_.2	Q6NZM9_S562_M1_Hdac4	7.2133	7.6956	3.8983	7.7810	4.2006	7.9504	7.0714	3.0624	7.3659	7.9760	4.1968	7.4755	3.3827	7.9493	6.6072	6.7117	3.6001	7.5320	7.6248	7.3103
Rbm26	_RLNHS[Phospho (STY)]PPQSSSR_.3	#N/A	6.7386	4.1623	6.3537	6.5944	6.3925	6.7442	8.1189	7.1215	7.0043	7.1638	3.4892	6.9662	7.2369	8.4525	6.9925	3.8111	7.1078	7.2461	6.9165	6.7534
Rbm26	_LNHS[Phospho (STY)]PPQSSSR_.2	#N/A	3.7985	8.0704	8.2471	3.8909	9.2875	9.1350	4.4048	3.4989	8.6119	7.9550	8.8887	8.6501	3.8192	8.8266	8.4865	8.7914	8.6019	8.7771	8.6331	8.7763
Znf740	_AGS[Phospho (STY)]PDVLR_.2	Q6NZQ6_S19_M1_Znf740	3.9311	4.0717	4.4674	3.7583	3.2688	3.2153	3.3990	3.6315	3.7864	4.2864	3.2650	3.9612	3.9518	3.5902	3.8495	4.2760	4.1692	3.6640	3.3126	4.4215
Marchf1	_LSVC[Carbamidomethyl (C)]PS[Phospho (STY)]TQDIC[Carbamidomethyl (C)]R_.2	Q6NZQ8_S75_M1_Marchf1	6.8333	6.5742	4.2061	4.4152	3.0075	3.2316	3.0327	3.3947	3.0918	3.8761	6.6634	4.2225	3.1999	4.2445	4.1108	4.0147	7.1555	4.2575	3.0558	3.9218
Marchf1	_SSNISKASSPT[Phospho (STY)]T[Phospho (STY)]GTAPR_.2	Q6NZQ8_T59_M2_Marchf1	3.4547	4.3263	4.2130	4.0129	3.2252	3.3431	3.3639	3.3770	3.3413	4.2335	3.2679	4.2158	3.4827	4.2000	3.9049	4.0418	4.5716	3.9186	3.5672	4.0621
Sri	_QHFISFDSDR_.3	#N/A	4.4207	4.0177	3.5252	3.8666	4.2645	2.1620	3.9368	3.1456	4.1142	4.2313	3.3201	4.1253	4.0069	3.9916	3.3444	3.7828	4.1436	2.3771	4.2950	2.9757
Sri	_ITFDDYIAC[Carbamidomethyl (C)]C[Carbamidomethyl (C)]VK_.2	#N/A	4.0387	4.8498	3.4037	3.3212	4.2457	4.2790	4.3033	4.0686	4.2235	3.8493	3.7021	3.5240	4.3889	3.3908	2.8278	3.4008	3.1054	3.2269	4.3763	3.3578
Sri	_ALTDSFR_.2	#N/A	3.2597	3.2423	3.7960	4.4297	4.0982	3.8867	3.4429	4.4609	3.1149	3.4660	4.0944	3.1318	3.2804	3.7830	3.0200	3.6046	3.4977	4.3354	3.9840	3.7500
Ppp2r2a	_LFEEPEDPSNR_.2	#N/A	7.9709	8.1649	7.8683	7.5393	8.1664	8.0588	7.9424	8.2830	7.9660	8.1573	8.0510	8.3060	8.5207	7.6985	8.0398	8.0444	8.2166	7.7127	7.6931	8.1144
Ppp2r2a	_VVIFQQEQENK_.2	#N/A	7.5223	7.3835	7.8867	7.9600	7.4986	7.6153	7.2055	7.8631	7.7751	7.4511	7.9234	7.3788	7.4821	8.0385	7.7933	7.7204	8.0258	7.4433	7.6316	7.7572
Ppp2r2a	_NAAQFLLSTNDK_.2	#N/A	6.1302	7.1338	6.2726	6.5806	6.3445	6.7532	6.5901	7.0500	6.5265	6.5676	6.1792	6.9141	6.0123	7.1909	6.6246	6.5886	6.3927	7.1883	6.6514	6.9638
Ppp2r2a	_ENIIAVATTNNLYIFQDK_.2	#N/A	5.4631	5.5279	5.7140	4.5133	4.9485	4.7368	5.1462	5.5868	5.1497	5.3654	4.8527	5.3270	6.2889	5.9940	5.1049	5.8313	5.3251	4.8458	3.8816	5.3312
Ppp2r2a	_SFFSEIISSISDVK_.2	#N/A	5.0587	4.3736	5.3169	5.4387	5.3355	5.5742	5.1358	5.9954	5.4908	5.3963	4.8073	5.5504	5.7573	5.7544	5.2561	5.6017	5.4944	5.3987	4.9561	5.1909
Ppp2r2a	_INLWHLEITDR_.3	#N/A	4.5679	5.3068	4.4694	4.6598	5.5401	4.8168	4.6487	4.9862	5.0082	5.2027	5.0685	4.7444	3.4239	4.9421	4.5411	4.3978	5.2936	4.8634	4.6882	5.1235
Ppp2r2a	_[Acetyl (Protein N-term)]AGAGGGNDIQWC[Carbamidomethyl (C)]FSQVK_.2	#N/A	4.3224	3.6799	5.0386	3.2618	4.5091	4.9925	4.1907	4.8763	5.2051	4.5481	4.3900	4.4069	4.1053	3.8970	4.6749	4.2436	5.1738	4.1642	4.4203	3.8692
Ppp2r2a	_GEYNVYSTFQSHEPEFDYLK_.3	#N/A	4.5625	4.2574	4.6943	5.6360	4.5906	4.4075	4.5686	5.2900	2.9845	4.3731	4.3898	3.9835	4.5934	6.0800	4.9079	3.9246	4.2854	5.4600	3.1631	4.6465
Ppp2r2a	_ENIIAVATTNNLYIFQDKVN_.2	#N/A	3.9607	4.0862	3.4817	3.6958	2.9340	3.0420	4.2224	5.1584	4.3015	3.7822	3.7801	3.8560	4.3239	5.3595	3.0252	4.5740	3.1834	4.7855	4.7288	4.1429
Ppp2r2a	_GAVDDDVAEADIISTVEFNHSGELLATGDK_.3	#N/A	4.5508	4.2691	4.0874	4.5343	3.2569	3.2902	3.7912	3.2756	3.7114	4.3614	3.1900	3.9952	3.8768	4.1254	3.8166	3.9129	4.2737	4.3766	3.1748	3.8027
Psd2	_KTHS[Phospho (STY)]S[Phospho (STY)]PALSLGHGPVTGSK_.3	Q6P1I6_S745_M2_Psd2	3.9130	5.7349	3.4496	3.6712	4.1200	4.1533	4.4290	4.1387	4.3492	3.7236	3.8278	3.1322	4.5146	3.4877	2.9535	3.8060	3.6360	4.2604	3.8125	3.1650
Inpp4b	_KPPS[Phospho (STY)]PNVSTEEK_.2	Q6P1Y8_S470_M1_Inpp4b	6.9336	7.3311	3.1430	6.8624	3.4453	3.0388	4.0958	6.5683	3.9628	4.1100	6.2552	3.7847	6.3108	3.1301	3.6730	2.9516	4.3456	3.4875	3.1361	3.0971
Ppp4r3a	_TNLSGRQS[Phospho (STY)]PSFK_.2	Q6P2K6_S728_M1_Ppp4r3a	6.3629	6.3767	5.7629	6.4501	5.6167	5.7321	3.9555	3.1113	6.1190	4.1971	5.5995	4.1596	6.3544	3.9611	3.9809	3.7486	6.0232	3.0401	3.3391	3.6384
Nsd3	_SEKPAQSASS[Phospho (STY)]PEATSGSAGPVEK_.3	Q6P2L6_S561_M1_Nsd3	4.5711	4.2488	4.1077	4.5139	3.2772	4.1934	4.1617	3.2960	2.9931	4.3817	3.3675	4.0875	4.6020	4.1458	3.7963	4.4139	4.2940	4.5515	3.1545	3.8230
Golm2	_FFDENES[Phospho (STY)]PVDPQHGSK_.2	#N/A	7.2451	3.8256	7.1421	7.1116	6.2956	6.9898	6.8718	7.0055	7.0719	7.6023	6.4221	7.0540	5.3010	7.2847	6.6010	5.5948	7.3657	6.9038	6.9679	7.0152
Sik3	_TWC[Carbamidomethyl (C)]GS[Phospho (STY)]PPYAAPELFEGK_.2	Q6P4S6_S167_M1_Sik3	3.5937	7.4761	3.1302	3.9906	6.2180	3.8339	3.2475	6.7340	3.1677	3.4043	6.7581	3.4515	3.3332	3.8358	3.2729	2.9558	7.0296	4.2827	7.0237	6.8124
Sik3	_QLS[Phospho (STY)]ADSAEAHSLNMNR_.2	#N/A	3.8768	5.6887	6.3043	3.7912	3.5857	3.6190	4.4181	3.9675	3.4793	3.6874	3.9478	3.7631	3.7564	5.3536	3.5845	3.7942	3.7207	3.9885	4.0062	3.7312
Sik3	_RAS[Phospho (STY)]DGGANIQLHAQQLLK_.3	Q6P4S6_S493_M1_Sik3	4.2239	4.5960	3.7605	3.3603	2.9300	3.6775	2.9368	4.4496	4.1467	4.3220	3.0203	4.3221	4.2548	3.7985	4.1435	3.5860	3.9468	3.2026	3.5017	3.4758
Snrnp200	_EEAS[Phospho (STY)]DDDM[Oxidation (M)]EGDEAVVR_.2	#N/A	9.8785	9.7782	9.8578	9.6671	8.7991	9.6748	9.4452	9.4350	9.3542	9.3097	8.9529	8.9873	8.9776	9.6941	7.7154	8.4942	9.4994	9.3562	8.9908	8.7691
Abcf1	_EAEQGS[Phospho (STY)]GEEKEEKEGDLK_.2	#N/A	7.7597	7.3532	7.5692	7.5670	7.3946	7.2960	7.4570	7.7245	7.5428	7.7719	7.4184	7.4995	7.7324	7.8039	7.2050	7.4617	7.5100	7.4543	7.3398	7.4476
Abcf1	_SKPAAADS[Phospho (STY)]EGEEEEEDTAKEK_.3	#N/A	3.4588	4.5440	3.9951	4.2306	4.2973	3.0206	3.2437	3.1592	3.3028	6.5892	3.6772	5.4924	3.4109	3.9822	5.7410	3.8037	5.8926	6.1398	5.8427	3.9492
Abcf1	_QLSVPAS[Phospho (STY)]DEEDEVPAPIPR_.2	#N/A	4.9052	10.5164	10.0735	4.7232	4.3212	4.7847	10.0314	4.3035	9.9600	4.6372	4.6947	4.0866	4.1306	11.1502	4.3567	4.7108	4.9711	10.5004	4.5530	4.5231
Abcf1	_LKQLSVPAS[Phospho (STY)]DEEDEVPAPIPR_.3	#N/A	3.8009	6.4944	4.1526	3.8257	4.0078	4.1429	3.6293	4.0265	3.7237	4.0472	4.0981	3.2867	3.8317	8.3635	3.1080	3.9781	3.5238	6.8954	3.9671	3.8679
Abcf1	_SKPAAADS[Phospho (STY)]EGEEEEEDTAK_.2	#N/A	5.0448	5.0338	5.4741	5.5392	4.6585	5.5846	5.1480	4.9468	5.5339	5.2503	5.5261	2.9636	4.3461	4.6199	4.4557	4.8936	5.3824	4.9486	4.9122	4.5182
Abcf1	_GGNVFEALIQDDS[Phospho (STY)]EEEEEEEENR_.3	#N/A	3.2801	3.2218	3.8164	4.4093	4.1187	4.3428	3.4224	4.4813	3.4815	3.4865	3.8558	3.1113	3.3008	3.8035	2.9996	3.6250	3.5182	4.3150	3.9636	3.7705
Abcf1	_AKGGNVFEALIQDDS[Phospho (STY)]EEEEEEEENR_.3	#N/A	4.0011	3.3180	3.5377	3.5832	4.2080	3.9004	3.8648	4.2268	3.9239	4.5449	4.2983	3.0441	4.0319	3.5757	3.4420	3.3632	3.7240	3.4255	3.7245	3.2530
Rrp12	_GDSIEEILADS[Phospho (STY)]EDEDEEERGR_.3	#N/A	3.5567	3.1525	3.8857	4.3400	3.7636	3.7969	3.2845	3.0498	3.2047	3.3673	4.1842	3.0420	3.3701	3.8728	3.3099	4.4196	3.5875	6.7467	3.8943	3.8398
Rrp12	_GDSIEEILADS[Phospho (STY)]EDEDEEER_.2	#N/A	3.0876	3.4143	3.6239	3.1009	3.9262	4.1503	3.6149	4.2888	3.6740	3.2939	4.0484	3.3038	3.7820	3.6110	3.1921	3.4325	3.3257	3.0066	4.1561	3.5780
Dgkq	_TWPGSGS[Phospho (STY)]PR_.2	Q6P5E8_S17_M1_Dgkq	8.3440	7.4748	8.6247	3.6380	7.8941	8.0698	4.3958	7.1007	4.3160	9.7710	7.7018	3.0990	8.9246	3.5209	6.6767	8.9220	7.1206	3.4803	9.0405	8.7707
Dgkq	_LGS[Phospho (STY)]PAGS[Phospho (STY)]PVLGISGR_.2	Q6P5E8_S22_M2_Dgkq	4.1425	9.4703	10.0410	6.7424	3.4802	8.8817	9.0407	3.8428	8.9889	9.7306	2.9935	8.2084	4.2280	9.0696	7.3009	7.1851	7.6561	8.7832	9.0655	9.1443
Ttyh3	_YLATSQPRPDS[Phospho (STY)]SGS[Phospho (STY)]GH_.2	Q6P5F7_S519_M2_Ttyh3	3.2920	4.0469	4.3295	4.3583	3.4990	3.5323	4.7112	3.2493	3.4892	3.9570	3.8222	3.7731	3.6547	4.0866	3.5944	4.1550	4.0499	4.8161	3.4902	3.9055
Ttyh3	_C[Carbamidomethyl (C)]ENTPLIGRES[Phospho (STY)]PPPSYTSSMR_.3	#N/A	4.1483	5.5663	5.4734	6.2523	3.9215	4.0389	5.6047	4.1814	4.1458	4.1991	4.0752	3.8760	3.9866	6.4513	3.2114	3.2362	3.6786	4.0414	3.7708	3.2077
Mapk4	_GYLS[Phospho (STY)]EGLVTK_.2	Q6P5G0_S186_M1_Mapk4	3.1814	3.3206	3.7177	6.6914	5.7287	4.2440	5.9257	4.3826	3.5802	3.3877	5.9764	3.2100	6.6814	3.7048	3.0983	6.4994	6.4591	5.8149	5.8780	3.6718
Mapk4	_VGS[Phospho (STY)]PSYLDK_.2	Q6P5G0_S430_M1_Mapk4	3.1457	6.9128	4.1832	6.5692	5.6247	3.2549	6.0242	6.3679	3.0686	4.4572	6.7852	6.4276	3.1766	6.8143	3.7208	6.2694	6.5505	6.3547	5.7997	3.8985
Mapk4	_AGST[Phospho (STY)]PLAEDVQVDPR_.2	Q6P5G0_T383_M1_Mapk4	4.0593	4.7385	3.8053	3.8147	3.1937	5.0906	5.5504	3.9935	5.0799	5.6100	3.3803	5.5264	3.9856	4.5571	4.1367	3.4257	5.2218	4.0880	5.5551	3.5796
Ubxn7	_SESLIDASEDS[Phospho (STY)]QLEAAIR_.2	Q6P5G6_S266_M1_Ubxn7	3.8509	3.5653	3.9953	4.0440	3.8532	4.2321	3.5339	4.1202	3.6029	3.2964	3.9773	3.3923	3.7110	3.9648	3.2562	3.8121	3.2889	3.7572	4.0934	3.5555
Frmd5	_S[Phospho (STY)]C[Carbamidomethyl (C)]PSITHGPR_.2	Q6P5H6_S351_M1_Frmd5	3.2018	3.3002	3.7381	4.4876	6.2349	3.9446	7.1050	7.0873	3.0571	3.5149	7.3215	3.1896	3.2225	6.7051	3.0779	3.5467	3.4399	4.3933	4.0419	7.1571
Frmd5	_SSS[Phospho (STY)]HGDTFLPHVR_.3	Q6P5H6_S396_M1_Frmd5	4.0391	7.8848	4.0754	3.7510	4.0432	4.3214	3.5281	3.9464	3.9301	3.6723	3.7765	3.5115	4.0765	4.0964	3.1053	3.5496	3.1908	3.6153	4.2746	7.4588
Nwd2	_VS[Phospho (STY)]VDC[Carbamidomethyl (C)]LWR_.2	Q6P5U7_S1685_M1_Nwd2	4.3861	3.2898	3.4216	3.3033	3.7238	3.9479	3.8172	4.0865	3.8763	3.0916	4.2507	3.5061	3.9844	3.5281	3.3944	3.2302	3.1233	3.4730	3.7721	3.2054
Adat2	_VESTTT[Phospho (STY)]PDGPC[Carbamidomethyl (C)]VVSVQETEK_.2	Q6P6J0_T10_M1_Adat2	4.3469	4.4730	3.8835	5.0059	3.0530	3.0864	3.9951	3.0718	3.9153	4.6222	3.3939	4.1991	4.3778	5.8543	4.0204	5.1715	5.4994	3.0796	3.3787	3.5989
Sepsecs	_LLLEQGK_.2	#N/A	6.3174	7.0082	6.6429	6.3648	6.5242	6.9304	6.8052	6.2451	6.7293	6.9881	6.9763	6.6559	6.4650	6.6061	6.3825	6.7793	6.3401	6.9462	6.9295	6.6263
Pcnp	_T[Phospho (STY)]LSVAAAFNEDEDSEPEEM[Oxidation (M)]PPEAK_.3	Q6P8I4_T106_M1_Pcnp	3.4835	4.0835	4.3391	4.1593	3.3994	3.3681	3.8097	3.5196	5.6137	3.6584	3.2295	3.8726	3.5787	4.2557	6.2485	4.1591	3.9822	4.0006	3.9201	4.1240
Snx6	_SLVDYENANK_.2	#N/A	6.5609	6.5185	6.1140	6.3261	6.3867	6.5269	6.3963	7.0407	6.6834	6.1110	6.6277	6.9693	6.8327	6.4403	6.4068	5.6962	6.7287	5.9297	6.5690	4.0960
Snx6	_[Acetyl (Protein N-term)]M[Oxidation (M)]EGLDDGPDFLS[Phospho (STY)]EEDR_.2	Q6P8X1_S13_M1_Snx6	3.9742	3.3449	3.5107	3.6101	4.1811	4.2144	4.3678	4.1998	4.2880	3.7848	3.7667	3.0710	4.4535	3.5488	2.8924	3.3363	3.6971	3.1623	4.3118	3.4223
Caskin1	_AQPGS[Phospho (STY)]PQALGGPHGPATAK_.2	#N/A	11.4166	11.5758	11.2850	11.4665	10.9512	10.8413	11.1315	11.1267	10.8275	11.6081	11.0543	11.1853	11.6937	11.5845	10.3528	11.1070	11.2285	11.0451	10.9817	11.0204
Caskin1	_KVPLPGPGS[Phospho (STY)]PEVKR_.3	#N/A	9.7568	10.0240	8.6622	8.7079	7.9255	8.4789	9.8829	10.5641	8.1561	9.2849	8.2223	9.8476	10.2978	10.7805	8.2035	8.7607	9.1178	9.7664	9.1026	8.3822
Caskin1	_VPLPGPGS[Phospho (STY)]PEVK_.2	#N/A	10.3025	9.7975	9.7520	9.9002	9.5574	9.7183	9.4071	9.7704	9.3850	9.9287	9.9400	9.4221	9.3113	9.8493	8.3386	9.5170	9.8761	9.2670	9.3352	9.5308
Caskin1	_SGS[Phospho (STY)]LSNVAGGR_.2	Q6P9K8_S390_M1_Caskin1	8.7819	8.3364	8.9306	8.7765	8.3744	7.8391	7.9806	3.8946	7.9199	9.1134	8.0877	7.9038	8.2557	8.5480	7.0721	7.9351	8.0310	8.2298	8.1511	8.0715
Caskin1	_SLPQS[Phospho (STY)]PTHR_.2	Q6P9K8_S828_M1_Caskin1	10.4850	10.0717	10.4724	10.2303	3.6672	4.4412	3.3809	9.6431	3.3830	3.2708	3.7574	3.5850	3.4911	10.0283	3.4063	9.7437	3.1831	3.9663	9.4968	4.2130
Caskin1	_PAS[Phospho (STY)]PEPGR_.2	#N/A	8.2897	8.2283	8.1106	8.4925	7.3979	7.5019	7.7575	6.8530	7.3221	8.7323	7.9562	7.6517	7.5541	8.1393	5.5925	7.3719	7.4116	7.5592	7.2285	7.9781
Caskin1	_VPLPGPGS[Phospho (STY)]PEVKR_.2	#N/A	7.6172	7.5642	7.3429	7.6374	7.4873	7.0374	7.3422	7.9065	6.5695	7.5068	7.2827	7.2300	7.2298	7.6254	6.3220	7.2255	7.3970	7.3516	6.9159	7.0426
Caskin1	_IQGS[Phospho (STY)]PTPASK_.2	#N/A	9.0675	8.7280	9.0405	9.0571	8.6846	8.0516	8.5790	8.4171	8.1340	9.0506	8.3928	8.5582	8.6276	8.5294	7.4901	8.5744	8.4407	8.4319	8.1719	8.3797
Caskin1	_RRAS[Phospho (STY)]DLAGSVDTGSAGSVK_.3	#N/A	4.2611	3.3076	5.1275	3.5727	3.6658	5.1724	5.6282	5.1524	5.1134	4.5344	4.7305	5.9361	4.9692	6.4966	4.7040	5.3328	3.7344	3.4150	3.7141	3.2635
Caskin1	_KVPLPGPGS[Phospho (STY)]PEVK_.2	#N/A	10.9615	10.7939	10.4445	10.4999	10.2474	10.3207	10.2899	10.7933	10.2375	10.4402	10.5658	10.5106	10.9178	10.9923	9.6526	10.3273	10.9013	10.4583	10.3310	10.4119
Caskin1	_AAASVVSGPPVASDC[Carbamidomethyl (C)]AS[Phospho (STY)]PGDSAR_.2	#N/A	8.4181	8.8137	8.9520	9.1077	4.7046	7.8780	8.5693	7.4820	8.0970	8.9267	3.9191	8.5333	8.1736	9.1223	7.1435	8.3065	8.5087	8.1203	8.1614	8.0973
Caskin1	_TPLDLAC[Carbamidomethyl (C)]EFGR_.2	#N/A	1.5803	4.6100	4.6601	4.6572	5.0630	4.5038	5.4310	3.4726	3.7742	4.7645	4.4836	4.4310	3.8823	5.2305	5.2759	4.4739	4.6025	4.5078	3.2510	4.3960
Caskin1	_TGSEPS[Phospho (STY)]PPQGGGSLGPSAPPEEIWVLR_.3	Q6P9K8_S358_M1_Caskin1	3.5804	7.9966	3.1170	5.6197	3.7873	3.8207	7.7351	4.5269	3.1810	3.3910	4.1604	7.3947	3.3464	8.5681	3.2861	2.9425	3.5637	7.3822	3.9180	3.8161
Caskin1	_IQGS[Phospho (STY)]PTPASKK_.2	#N/A	4.0523	3.9505	4.5886	3.6371	3.3900	3.0940	3.9874	3.7527	4.6623	5.6472	3.3862	3.8399	4.0730	4.5757	4.0128	4.3972	5.2905	3.5428	3.1914	3.0419
Caskin1	_RPPEGHPTPRPAS[Phospho (STY)]PEPGR_.4	#N/A	4.5120	4.3079	4.0486	7.6892	3.2181	3.3894	3.8300	3.2368	6.6990	8.4323	3.3083	4.0341	4.5429	4.0866	3.8554	7.4538	4.2349	4.4154	3.2136	3.7639
Caskin1	_S[Phospho (STY)]QEYLLDEGM[Oxidation (M)]APGTPPK_.2	#N/A	8.7632	3.9463	6.9178	8.8367	5.8259	7.0300	7.5988	7.3062	7.7636	3.1894	7.5602	7.7386	7.7275	7.1720	6.3336	7.9666	7.9732	7.6526	7.5825	7.8252
Caskin1	_RAS[Phospho (STY)]DLAGSVDTGSAGSVK_.2	#N/A	5.1081	5.9014	5.9050	5.9281	5.1614	5.6767	5.8153	5.6762	4.0132	6.4519	5.2113	3.0325	5.5617	5.5438	3.7487	5.0637	5.5205	6.0096	5.1275	5.1043
Caskin1	_SVSESS[Phospho (STY)]PGDSPVKPPEGSSGAAR_.3	Q6P9K8_S428_M1_Caskin1	5.0621	3.8128	3.7701	5.8108	3.7441	3.9613	3.7687	3.9073	5.1126	4.0264	3.6043	3.6614	4.2911	3.7705	3.2447	3.5779	3.6129	3.5582	3.9458	3.7746
Caskin1	_RGS[Phospho (STY)]GEPAEQSHFMEDGTAR_.3	#N/A	3.5182	3.9218	3.6880	5.2546	4.2457	4.3015	5.4107	3.8751	4.0905	4.5898	4.4103	3.0997	4.1986	6.0169	3.2634	4.4641	4.0452	4.6414	4.7454	4.5075
Caskin1	EM[Oxidation (M)]M[Oxidation (M)]AIESPPPSEPAAAEC[Carbamidomethyl (C)]QS[Phospho (ST	#N/A	8.0122	7.9648	3.8730	3.7113	6.3652	7.4641	7.5507	7.6369	6.6246	7.8128	6.5442	7.9115	4.0985	7.8419	3.7529	7.3859	8.1342	8.1839	7.6803	7.2248
Caskin1	_Y[Phospho (STY)]AASDSEPERDELLVPAAAGPYATVQR_.3	Q6P9K8_Y888_M1_Caskin1	4.0058	3.9970	4.5421	6.1728	3.3435	3.5676	4.1976	3.7062	4.2567	4.2121	3.1302	3.8865	4.3647	6.5392	3.7748	4.3507	4.2438	5.8194	3.3917	3.5858
Caskin1	_HISSS[Phospho (STY)]QELLGDGPPGPGSPMSR_.3	Q6P9K8_S710_M1_Caskin1	3.6780	5.3614	4.1982	4.4234	3.0157	3.2398	4.4366	3.3865	3.0836	3.8843	3.4580	4.2142	3.1917	5.3787	4.1025	4.0229	4.3845	4.2657	3.0640	3.9135
Caskin1	_AGDIITVLEQHPDGR_.3	#N/A	3.5470	4.4558	4.0833	4.2924	2.8847	3.1088	3.1555	3.5175	3.2146	3.7533	3.5890	4.3453	3.3227	4.3673	4.2336	3.8919	4.5155	4.1347	4.4338	4.0446
Fam126a	_S[Phospho (STY)]FEQVSGAPVPR_.2	Q6P9N1_S453_M1_Fam126a	9.5810	9.1543	9.3490	9.0994	8.2052	8.5505	8.5230	8.2853	8.3684	9.1962	8.5966	8.0997	8.1576	8.4887	7.0857	8.2661	8.8132	8.2222	8.3166	8.1997
Fhod1	_S[Phospho (STY)]LEGGGC[Carbamidomethyl (C)]PVR_.2	Q6P9Q4_S370_M1_Fhod1	7.2962	7.1862	6.7814	7.0567	7.1118	7.0307	6.6006	7.0455	6.5820	7.0809	6.7707	6.4716	6.8927	6.3635	5.5926	3.6595	6.8536	6.9784	6.6908	6.4313
Fkbp15	_S[Phospho (STY)]NSLSEQLTVNSNPDTVK_.2	Q6P9Q6_S342_M1_Fkbp15	3.9847	3.3623	4.4568	4.5654	3.5294	4.7599	3.7230	4.2104	3.6462	5.2841	3.5553	3.9270	4.0156	5.0803	3.7483	3.3468	3.7076	4.5450	3.7688	3.2366
Fkbp15	_VTS[Phospho (STY)]LEEELTDLR_.2	Q6P9Q6_S722_M1_Fkbp15	4.1495	3.1695	3.6861	3.4347	2.8556	3.7519	4.0133	4.3752	4.0724	4.3964	2.9459	4.3965	4.1804	3.7242	4.2178	3.5117	3.8725	3.2770	3.5760	3.4015
Fkbp15	_[Acetyl (Protein N-term)]MFGAGDEDDTDFLS[Phospho (STY)]PSGGAK_.2	Q6P9Q6_S14_M1_Fkbp15	3.2425	3.2595	3.7788	4.4469	4.0810	4.3051	3.4600	4.4437	3.5191	3.4488	3.8935	3.1489	3.6272	3.1709	3.0372	3.5874	3.4805	3.8302	4.1293	4.3491
Fkbp15	_HNQDSQHC[Carbamidomethyl (C)]SLS[Phospho (STY)]GDEEDELFK_.3	Q6P9Q6_S1159_M1_Fkbp15	4.5531	4.2669	4.0896	4.5320	3.2592	4.5757	5.3978	3.2779	4.4191	4.3636	3.3494	3.9930	4.5839	5.7038	3.8143	5.0305	4.2760	4.3743	3.1725	3.5220
Mtss2	_TPTVPDSPGY[Phospho (STY)]VGPTR_.2	Q6P9S0_Y567_M1_Mtss2	8.1966	8.2191	8.2042	8.1937	7.0700	8.1002	7.5822	7.3663	7.3538	9.5965	7.6312	7.7673	7.8001	3.7298	5.8488	7.6945	3.6443	7.4796	3.8209	3.5810
Mtss2	_GLS[Phospho (STY)]LEHQK_.2	Q6P9S0_S419_M1_Mtss2	8.8079	8.8829	8.8395	8.6681	8.2196	7.8104	8.4936	8.1461	7.7444	8.8879	7.9879	7.7989	8.3270	8.2073	6.8517	7.9896	7.8937	8.0798	7.8768	8.2965
Mtss2	_GSDYSWSYQTPPS[Phospho (STY)]SPSSSNSR_.2	Q6P9S0_S260_M1_Mtss2	3.2188	4.1003	4.2562	6.0759	3.4257	3.4591	3.6224	3.3877	6.3011	4.5302	3.0212	3.8264	3.7080	4.2107	3.6478	4.0817	3.9254	3.9078	3.5564	4.1777
Mtss2	_AGS[Phospho (STY)]EEC[Carbamidomethyl (C)]VFYTDEVASPLAPDLAK_.3	Q6P9S0_S575_M1_Mtss2	4.0379	4.5182	3.8383	5.7354	3.3756	3.5997	4.1655	4.5274	4.2245	4.2442	3.0981	4.2443	4.3326	3.8764	3.7427	4.3828	4.0246	3.1248	3.4239	3.5537
Bcr	_ASAPRPPPAPADGADPAPVEESEARPDGEGS[Phospho (STY)]PSK_.4	Q6PAJ1_S122_M1_Bcr	3.5780	8.3452	4.2537	8.5001	7.5314	3.4820	8.0884	3.4259	8.1288	3.7958	8.5165	3.6364	3.8064	4.2581	7.5285	8.1314	8.6572	8.5633	8.2270	8.3756
Bcr	_VS[Phospho (STY)]PSPTTYR_.2	Q6PAJ1_S356_M1_Bcr	8.6816	8.4304	4.1431	8.4525	7.6799	7.9405	8.2759	7.8484	7.5713	3.1099	2.9407	7.6901	7.9339	8.0789	3.5672	3.9517	7.8658	8.0646	7.6361	7.9663
Bcr	_RHQDGLPYIDDS[Phospho (STY)]PSSSPHLSSK_.4	#N/A	3.8227	4.1801	4.3590	5.6945	3.1604	3.3237	7.7866	3.5231	7.8220	7.7171	3.1566	4.0695	7.9459	4.3461	3.9578	7.8005	4.0608	3.7724	3.4210	4.3131
Bcr	_STLVLHDLLK_.2	#N/A	5.4891	5.2807	4.5499	3.6900	5.0059	5.5199	4.8807	5.4363	5.2760	5.6602	4.9519	4.4570	4.3711	4.9353	4.5435	5.3692	4.5035	5.1142	4.7736	5.3843
Bcr	_HQDGLPYIDDS[Phospho (STY)]PSSSPHLSSK_.3	#N/A	3.8997	8.3099	7.7386	8.3668	3.7488	8.4499	8.1619	7.8949	8.2391	7.9458	8.1081	7.9733	8.4148	8.5553	6.8793	8.5542	8.5619	8.5334	8.3639	3.7224
Ahdc1	_ANS[Phospho (STY)]EASSSEGQSSLSSLEK_.2	Q6PAL7_S1180_M1_Ahdc1	6.3420	5.8973	6.4867	3.3084	5.5943	4.9601	5.6800	5.7379	4.1987	6.2007	3.0722	4.2702	5.5329	6.4172	3.7168	5.9239	6.3390	3.1507	5.4423	5.5629
Ahdc1	_AYS[Phospho (STY)]PTC[Carbamidomethyl (C)]S[Phospho (STY)]PTLGFK_.2	Q6PAL7_S1392_M2_Ahdc1	4.3585	4.4614	3.8951	3.2257	3.0646	3.5429	4.2222	3.0833	4.2813	4.1875	3.1548	4.1876	4.3894	3.9331	4.0089	3.7206	4.0814	7.5159	3.3671	3.6104
Ahdc1	_ASTVS[Phospho (STY)]PGGYM[Oxidation (M)]VPK_.2	Q6PAL7_S1060_M1_Ahdc1	7.5884	4.4471	3.9094	3.2114	3.0789	3.1122	3.9692	3.0977	3.8894	4.1834	3.3680	4.1732	7.9109	4.5575	3.9946	3.7349	4.0957	7.1395	3.2096	3.0237
Dennd5a	_FNS[Phospho (STY)]YDISR_.2	Q6PAL8_S193_M1_Dennd5a	7.2086	7.4255	7.1154	3.6205	3.4066	7.2310	7.4611	7.1011	7.1539	7.3079	7.2153	6.9787	7.3878	6.7529	5.6389	6.6307	4.3070	7.5518	6.9660	6.9541
Prkab2	_S[Phospho (STY)]HNDFVAILDLPEGEHQYK_.3	Q6PAM0_S107_M1_Prkab2	7.9494	8.3274	8.1365	7.9429	6.6309	7.7559	7.6442	6.9059	7.7485	7.8869	7.4728	7.4002	7.7111	8.2991	7.0271	7.3566	7.5465	7.1658	7.8170	7.4488
Prkab2	_IM[Oxidation (M)]VGS[Phospho (STY)]TDDPSVFSLPDSK_.2	#N/A	3.9773	7.0222	6.7041	7.4127	3.7792	4.0033	6.5748	3.7182	3.9101	3.6587	3.7897	7.7206	4.0181	8.3367	5.2187	3.7973	7.4682	8.0850	7.7127	6.1862
Prkab2	_DLSSS[Phospho (STY)]PPGPYGQEM[Oxidation (M)]YVFR_.2	#N/A	10.5611	10.9570	10.4847	10.7046	9.8526	10.6531	10.3766	3.6496	10.2054	10.5518	8.7098	10.4047	10.7702	11.2171	10.1123	10.5384	3.7952	10.5416	10.4292	10.2418
Txlna	_EQGVESPGAQPASS[Phospho (STY)]PR_.2	Q6PAM1_S523_M1_Txlna	3.8550	3.7821	7.3983	6.7416	6.8224	5.4897	5.4377	3.8925	5.4176	7.5111	5.7016	3.5687	7.7590	6.4544	7.0397	7.5394	6.2912	7.4113	7.1948	3.5207
Gapvd1	_SRS[Phospho (STY)]SDIVSSVR_.2	#N/A	9.1105	8.7928	8.9992	8.6553	8.1420	4.0181	8.3698	8.6370	8.0985	8.7448	8.1230	7.9426	8.5091	8.0853	6.6940	7.6912	8.2848	8.1035	8.0383	8.0479
Gapvd1	_S[Phospho (STY)]SDIVSSVR_.2	#N/A	8.1928	7.9951	8.1054	8.0555	6.7425	7.1310	7.5405	7.0515	7.0865	8.1294	7.5986	6.8651	7.3351	6.9654	3.6163	6.7208	6.7790	3.6917	3.9908	7.3240
Gapvd1	_LQELESC[Carbamidomethyl (C)]SGLGS[Phospho (STY)]TSDDTDVR_.2	Q6PAR5_S746_M1_Gapvd1	4.3110	4.5089	3.8476	3.2732	3.0171	3.0504	4.0310	4.5367	3.9512	4.1216	3.4298	4.2350	4.1166	3.8856	4.0564	3.6731	4.0339	3.1155	3.4146	3.0855
Gapvd1	_FSLC[Carbamidomethyl (C)]S[Phospho (STY)]DNLEGISEGPSNR_.2	Q6PAR5_S569_M1_Gapvd1	3.2745	3.7548	4.6017	4.0199	4.1130	4.3371	3.4280	3.7900	3.4871	3.4808	3.8615	3.4809	3.5952	3.1389	3.0052	3.6194	3.2872	3.8622	4.1613	4.3171
Ptpn23	_REEGEAVEAGDT[Phospho (STY)]PEELR_.3	#N/A	4.7750	5.6674	3.2819	5.8581	3.7910	5.2417	5.7092	4.2437	4.6394	5.7359	4.8854	4.9471	4.2666	5.6762	4.2933	5.5882	5.1402	5.4024	5.6648	5.5718
Ptpn23	_ELQEAQEHDAR_.2	#N/A	3.6127	3.7064	2.3618	3.9715	3.8196	4.2887	3.5678	3.8384	3.5355	3.4324	3.9099	0.1464	3.6436	3.1873	3.0536	3.5710	1.0164	3.8138	4.1129	4.3655
Ano8	_GPS[Phospho (STY)]PPRPGK_.2	Q6PB70_S1009_M1_Ano8	7.9719	8.2385	8.0366	7.3535	7.9314	8.3875	7.8322	7.4022	5.3063	7.9172	7.6695	7.2623	6.8107	6.5355	3.9529	7.2158	7.5737	8.0390	7.1201	7.6672
Ano8	_VESIGQWQK_.2	#N/A	7.9457	7.9632	8.4619	3.8706	3.9205	7.9636	7.0036	3.9393	7.4233	7.5606	4.0273	7.7247	8.0478	8.6617	7.8966	3.0757	7.4033	7.1497	7.6524	7.6285
Ttbk1	_S[Phospho (STY)]PGLPASTAR_.2	Q6PCN3_S1222_M1_Ttbk1	9.0845	9.0970	9.1612	9.1866	8.2937	7.9427	8.5393	8.3250	8.0293	9.3324	8.7208	8.8057	8.6112	8.9951	7.2922	8.6846	8.5085	8.5125	8.5997	8.5869
Ttbk1	_APPDS[Phospho (STY)]PTTPVR_.2	Q6PCN3_S441_M1_Ttbk1	9.6824	4.4289	9.5791	8.7787	3.1016	3.1349	3.9510	10.1198	9.2159	4.2061	3.3498	4.1550	4.0366	4.5393	3.9764	3.7576	4.2657	3.5836	3.2322	3.9040
Ttbk1	_RVNS[Phospho (STY)]PESER_.2	#N/A	4.4589	4.3610	3.9955	3.1253	3.1650	7.7438	7.8143	7.6167	7.5683	8.0547	7.5551	7.8172	8.3221	7.8986	3.9085	7.8752	7.9062	7.7332	3.2667	3.7108
Ttbk1	_GVGVPSS[Phospho (STY)]PVR_.2	Q6PCN3_S433_M1_Ttbk1	4.1600	3.1591	3.6966	8.3289	2.8661	3.7414	4.1820	4.3857	4.0828	3.9706	2.9564	4.3860	4.1909	3.7347	4.2073	7.9920	8.1098	3.2665	3.5656	3.4120
Ttbk1	_KGTTISPS[Phospho (STY)]R_.2	Q6PCN3_S1036_M1_Ttbk1	3.6378	6.0288	3.1744	3.9464	3.8448	3.8781	3.2033	3.8635	3.1235	3.4484	4.1030	3.4074	3.2890	3.7916	3.2287	2.9999	3.3607	4.3268	3.9754	3.7586
Ttbk1	_S[Phospho (STY)]QEGAPSTLLADDQK_.2	Q6PCN3_S794_M1_Ttbk1	4.0436	7.8300	8.3003	3.2882	3.3813	7.8807	7.3951	4.5217	8.0094	7.4550	7.4262	7.3843	7.4215	8.2421	3.7369	7.4624	4.0189	7.7156	7.3982	7.5233
Ttbk1	_AGTDALLSTSTST[Phospho (STY)]PPQQNTR_.2	Q6PCN3_T325_M1_Ttbk1	4.2371	3.7657	6.5565	3.4523	3.5748	4.4101	3.9663	6.5438	4.0924	6.5016	4.9334	3.6552	4.2578	3.2596	3.5435	3.0812	7.0256	6.3739	3.0066	5.8939
Ttbk1	_SETSQPPTPGS[Phospho (STY)]PSHSPLHSGPR_.3	Q6PCN3_S638_M1_Ttbk1	4.0710	3.9318	4.6073	3.6183	3.4088	3.6329	4.1323	3.7714	4.1914	4.2774	3.0649	3.8212	4.0918	7.3036	3.7095	6.1202	4.3091	3.5241	3.1726	3.0606
Ttbk1	_VNS[Phospho (STY)]PESER_.2	#N/A	4.3431	6.0106	6.1540	3.3463	3.6808	6.0768	4.2782	4.0435	4.1984	3.8744	6.1910	3.5491	4.3638	3.3656	5.6006	5.6721	3.0803	6.1534	6.1683	6.2547
Ttbk1	_MDLPGS[Phospho (STY)]PSR_.2	#N/A	3.4987	7.7708	4.5361	8.8698	3.7057	7.4595	7.4904	3.7244	3.2627	7.3408	8.0466	5.9603	3.4281	8.2103	3.3678	4.3617	7.6202	4.1877	3.8363	3.8978
Ttbk1	_RVNS[Phospho (STY)]PES[Phospho (STY)]ERLSTAADGR_.3	Q6PCN3_S456_M2_Ttbk1	6.9147	3.5480	3.4902	3.2346	3.7925	6.1054	5.9335	4.1552	6.5057	7.4095	6.3421	3.4375	3.9157	6.8050	5.2640	3.2988	3.1920	5.8269	3.8407	6.3807
Ttbk1	_SETSQPPTPGSPSHS[Phospho (STY)]PLHS[Phospho (STY)]GPRPR_.3	Q6PCN3_S646_M2_Ttbk1	4.1899	4.2510	4.2310	3.4950	3.1947	3.4953	4.2699	3.4050	4.3290	4.1398	3.2025	5.8649	4.4371	3.9808	3.9045	5.5805	5.0962	3.0204	3.3194	3.6581
Ttbk1	_GVGVPSS[Phospho (STY)]PVRAPPDS[Phospho (STY)]PTTPVR_.3	Q6PCN3_S433_M2_Ttbk1	4.4086	4.0324	3.7452	4.0829	3.4277	7.4138	4.2225	3.6456	3.6054	4.2233	3.1551	3.8291	4.3896	3.7652	3.7997	3.5647	4.3816	7.4342	3.0878	3.5506
Ttbk1	_SRMDLPGS[Phospho (STY)]PSR_.2	#N/A	4.5201	4.1159	6.9888	3.8025	3.2262	3.2595	3.8219	3.5873	3.7422	4.3307	3.2208	4.0054	3.9076	4.4103	3.8473	3.8822	4.1249	3.7082	3.3568	4.3772
Ttbk1	_RRES[Phospho (STY)]DPTGPQR_.2	Q6PCN3_S655_M1_Ttbk1	4.4701	4.0660	4.4731	5.9761	3.1762	3.2096	3.8719	3.6372	3.7921	4.2807	3.2707	3.9555	3.9575	4.4602	3.8972	3.8323	4.1748	3.6583	3.3069	4.4272
Ttbk1	_GRAS[Phospho (STY)]MADGDLEPEEGSK_.2	Q6PCN3_S815_M1_Ttbk1	3.2358	4.0833	4.2732	4.5365	3.4427	3.4760	3.6054	3.3707	3.5256	4.5472	3.0042	3.8094	3.6910	4.1937	3.6308	4.0987	4.4595	3.9248	3.5734	4.1607
Mff	_NDSIVTPS[Phospho (STY)]PPQAR_.2	Q6PCP5_S151_M1_Mff	12.2941	12.9305	12.5961	12.8831	12.1150	12.8135	12.6913	12.7916	12.7592	12.5037	12.2349	12.7429	12.9291	13.0180	12.0573	12.7444	13.3322	12.8406	12.7821	12.7332
Mff	_ERS[Phospho (STY)]M[Oxidation (M)]SENAVR_.2	#N/A	4.4584	5.5700	3.8651	6.2891	4.0658	4.2094	4.4467	4.2604	3.6663	6.0099	3.4008	3.5410	4.7126	4.0098	4.7387	4.2984	4.6113	4.3038	4.1697	4.2379
Mff	_SM[Oxidation (M)]S[Phospho (STY)]ENAVR_.2	Q6PCP5_S131_M1_Mff	5.7044	3.7210	3.8441	4.1330	5.4867	3.9476	8.3912	4.0529	8.7573	4.2573	3.8071	3.5384	6.1453	4.9568	8.7498	5.5197	8.1955	6.9883	3.6462	3.6605
Ankrd13d	_GPES[Phospho (STY)]PQKT[Phospho (STY)]PPSPAPPSFEEQLR_.3	Q6PD24_S552_M2_Ankrd13d	9.6303	9.9597	9.6078	9.8431	6.3890	9.1562	9.5907	8.6361	9.3983	9.5891	9.0302	9.6321	9.8233	10.6511	8.7148	9.6475	9.7527	9.5441	9.7662	9.3150
Ppp2r5b	_SHS[Phospho (STY)]SSQFR_.2	Q6PD28_S46_M1_Ppp2r5b	4.2408	4.5791	5.6474	3.3434	2.9469	5.3429	4.1012	4.4665	4.1637	4.0514	3.0372	5.5701	4.2717	5.4484	4.1265	3.6029	3.9637	3.1857	3.4848	4.9028
Rundc3b	_SNDC[Carbamidomethyl (C)]LASPTTELTS[Phospho (STY)]PGLTPS_.2	Q6PDC0_S402_M1_Rundc3b	5.9965	6.2778	6.3792	6.2027	3.1859	3.2231	6.9368	3.6248	5.3250	6.3915	3.2583	3.9690	5.4472	6.0127	3.9105	5.1713	4.1625	5.5037	3.3204	3.8321
Rundc3b	_FEQSSDSISS[Phospho (STY)]DEEELR_.2	Q6PDC0_S217_M1_Rundc3b	4.0523	5.1573	4.5886	3.6371	3.3900	3.0941	4.7444	3.7527	3.9076	4.2586	3.3862	4.5921	4.0730	4.5757	3.7283	4.3972	4.2904	3.5428	3.1914	3.0418
Smarcc2	_S[Phospho (STY)]PS[Phospho (STY)]PSPTPEAK_.2	#N/A	9.4077	9.9303	9.5616	10.0515	9.4786	9.7264	9.3088	9.4145	8.7790	9.9196	9.7529	8.9863	8.8589	9.9192	8.0625	9.7601	9.9299	9.5144	9.6672	9.6383

Smarcc2	_TLTDEVNS[Phospho (STY)]PDSDRR_.2	#N/A	6.5405	6.6338	6.6102	6.5712	6.4953	7.0521	6.6350	6.7137	6.7004	7.0231	6.8235	7.2109	6.9692	7.9270	6.8125	6.7074	7.1904	7.1224	7.4186	7.1305
Smarcc2	_RS[Phospho (STY)]PS[Phospho (STY)]PSPTPEAK_.2	#N/A	4.0280	7.4629	3.5646	7.1537	4.2350	7.1600	7.5069	4.2537	7.3811	7.4675	7.1887	7.4880	7.6351	7.6390	6.7999	7.6292	7.5832	3.3985	7.3962	6.6081
Smarcc2	_KRS[Phospho (STY)]PS[Phospho (STY)]PSPTPEAK_.2	#N/A	10.1766	10.2623	10.3617	10.3033	10.0344	9.9904	9.9103	10.2747	10.0731	10.2156	9.7954	10.1019	10.4849	10.4326	9.8704	10.1090	10.2199	9.9644	10.0313	10.0796
Smarcc2	_DM[Oxidation (M)]DEPS[Phospho (STY)]PVPNVEEVTLPK_.2	#N/A	10.0222	10.2008	9.9225	9.2874	3.5391	10.0863	9.7692	9.6058	9.8262	10.2048	9.5798	9.6045	9.9402	10.4248	9.3182	9.8285	10.1998	10.2469	9.9976	9.9290
Smarcc2	_TLTDEVNS[Phospho (STY)]PDS[Phospho (STY)]DRRDK_.3	#N/A	3.7617	3.9187	3.9694	3.6053	3.6138	4.2683	4.1958	4.2189	4.2980	4.1335	3.5517	3.7851	4.3562	4.7143	4.4166	4.1593	4.3737	4.0395	3.8757	4.2896
Smarcc2	_YYEAADTVTQFDNVR_.2	#N/A	4.5165	4.3034	4.0531	4.5686	4.9816	3.2559	3.8255	5.5975	3.7457	4.3271	3.2244	5.4325	3.9112	4.0911	4.6196	4.6843	4.2394	4.4109	3.2091	3.7684
Mon1a	_S[Phospho (STY)]YEDLTELEDR_.2	#N/A	9.7620	10.8967	10.9491	10.3632	3.5005	10.6447	10.4954	6.9479	10.6184	10.3794	8.8193	9.9781	4.2077	10.5553	9.0542	9.9110	10.5976	10.3661	10.3312	10.1027
Mon1a	_S[Phospho (STY)]YEDLTELEDREASGDSPK_.3	#N/A	3.5995	4.0798	4.2767	4.3450	2.9372	5.2867	5.7679	3.4649	3.1621	3.8058	3.5365	3.8059	3.2701	4.3148	4.1810	5.2263	4.4630	6.3984	6.0939	3.9920
Phldb1	_TLQPPES[Phospho (STY)]PR_.2	Q6PDH0_S445_M1_Phldb1	10.2201	10.4638	10.3538	10.1915	9.7938	10.4670	9.7010	10.5462	10.2931	10.3002	9.7946	10.1486	10.2594	10.3998	9.6335	10.1108	10.1064	9.7007	9.7895	10.1158
Phldb1	_KLS[Phospho (STY)]SGDLR_.2	Q6PDH0_S565_M1_Phldb1	3.9558	8.9541	8.2123	3.6284	8.0258	8.5885	4.3862	8.3230	4.3064	8.9654	7.9782	7.8964	8.5798	8.5424	7.2734	8.2698	8.3532	8.4690	8.0757	8.2636
Phldb1	_SS[Phospho (STY)]PPPLPAK_.2	Q6PDH0_S951_M1_Phldb1	4.1764	3.8571	4.0456	3.6791	3.5141	3.6116	4.0576	3.9474	3.8486	4.3827	3.8563	3.7465	3.9848	4.0818	7.2975	3.8542	3.9608	8.2835	3.5443	3.9556
Phldb1	_SQPASIPGS[Phospho (STY)]PK_.2	Q6PDH0_S382_M1_Phldb1	6.5011	7.3632	6.4157	7.1456	6.1834	6.6057	6.6111	6.4419	6.0979	6.8515	6.4584	6.8341	3.6775	6.4782	5.7919	4.1123	6.0479	4.1772	6.1695	6.3216
Phldb1	_SPS[Phospho (STY)]PTLGESLAPR_.2	Q6PDH0_S522_M1_Phldb1	7.8419	6.3333	7.3355	3.6452	3.7895	8.5993	6.1547	3.9705	8.2335	4.0895	7.8823	7.3719	7.5777	7.3062	3.3286	3.1106	8.3740	8.2640	6.8777	7.5544
Phldb1	_T[Phospho (STY)]RSPS[Phospho (STY)]PTLGESLAPR_.2	#N/A	3.4495	7.1434	7.0425	4.1155	3.3782	7.3650	6.3876	3.4218	3.3301	4.7144	3.7256	5.8972	3.4795	4.2548	5.8129	6.0347	5.7032	4.1108	6.1388	6.4365
Phldb1	_KNS[Phospho (STY)]ITEISDNEDELLEYHRR_.4	Q6PDH0_S580_M1_Phldb1	4.2781	4.6055	4.1110	3.4663	3.6158	3.8399	4.4138	3.7742	3.6914	3.7811	3.7104	3.6692	4.1474	3.7641	3.4533	3.9196	3.8128	3.3650	3.8455	3.3685
Phldb1	_KNS[Phospho (STY)]ITEIS[Phospho (STY)]DNEDELLEYHR_.3	Q6PDH0_S580_M2_Phldb1	3.9266	4.4069	3.9496	3.1712	3.2644	3.4884	4.2767	3.1378	4.3358	4.1330	3.2093	4.1331	4.4439	3.9876	3.8539	4.2715	4.1359	3.0135	3.3126	3.6649
Phldb1	_NSITEIS[Phospho (STY)]DNEDELLEYHR_.3	#N/A	3.9539	4.0489	4.4902	3.7355	3.2916	3.1925	4.2494	3.6543	3.5253	4.1602	3.2878	3.9383	3.9746	4.4773	3.8266	4.2988	4.1920	3.6412	3.2898	4.4443
Mindy2	_VTAS[Phospho (STY)]PELAEAAAGR_.2	Q6PDI6_S99_M1_Mindy2	3.8045	8.9158	3.3410	3.7798	4.0114	4.0970	8.3651	4.0301	3.7273	3.2406	4.1017	3.2407	9.4580	3.3791	3.2454	3.3792	9.3698	8.5708	3.9211	8.8000
Mindy2	_VS[Phospho (STY)]PPEGR_.2	Q6PDI6_S21_M1_Mindy2	6.6889	6.5505	7.3367	4.4167	6.1048	7.8081	7.5112	3.3932	7.0466	7.7868	8.1512	6.8100	7.7123	7.8666	5.6394	6.5316	4.1988	7.4179	7.4345	7.2224
Fbxo42	_RGS[Phospho (STY)]LPDQK_.2	Q6PDJ6_S488_M1_Fbxo42	9.5393	8.9837	8.8369	8.1450	8.6875	8.5193	8.7266	8.7783	7.7576	9.0305	8.4038	8.4841	8.3705	8.5817	7.1190	7.8540	8.5517	8.3504	8.2537	8.3549
Kmt2d	_ASQVEPQS[Phospho (STY)]PGLGLR_.2	Q6PDK2_S2299_M1_Kmt2d	3.3637	8.0533	8.0851	7.5802	7.3618	7.7784	7.4849	7.8739	7.5401	8.0072	7.5765	4.3499	3.5631	8.2254	6.8084	7.6471	7.9950	7.5238	7.6850	7.6240
Kmt2d	_LC[Carbamidomethyl (C)]PQPEEPYLS[Phospho (STY)]PQPEEPR_.2	Q6PDK2_S727_M1_Kmt2d	4.5749	3.4279	3.6103	3.1145	3.9126	6.4540	6.1657	4.2752	6.5328	3.2804	4.0619	6.4514	7.1876	7.0832	3.2057	6.3437	7.1249	6.0988	4.1697	3.5644
Dync1li2	_GGPASVPSAS[Phospho (STY)]PGTSVK_.2	Q6PDL0_S407_M1_Dync1li2	4.2088	9.8005	10.2955	3.3755	9.6446	8.8612	9.2478	4.4345	4.0534	4.0194	3.5321	9.5811	9.6556	9.1907	4.1586	9.2834	3.9317	3.2178	3.5168	9.1106
Dync1li2	_TGSPGS[Phospho (STY)]PSAGGVQSTAK_.2	#N/A	4.3087	8.0640	3.8453	8.0958	7.4662	7.0719	7.8437	4.5344	7.4531	8.4994	7.2681	7.4253	7.2831	7.5862	4.0586	7.0386	7.2324	7.0506	6.7994	7.0912
Dync1li2	_EFQDYIEPEEGC[Carbamidomethyl (C)]QGS[Phospho (STY)]PQRR_.3	#N/A	8.6263	8.6363	8.7292	8.7466	6.9179	8.3128	8.3384	8.2206	8.2444	8.8565	8.0533	8.0685	8.5044	9.6400	8.0602	8.2360	8.4767	8.3141	8.4837	7.7172
Dync1li2	_EFQDYIEPEEGC[Carbamidomethyl (C)]QGS[Phospho (STY)]PQR_.2	#N/A	9.5179	9.9131	10.2444	10.0572	8.4288	9.6794	9.4247	8.9584	9.6594	9.9186	9.2445	9.4121	9.4110	9.9637	9.2429	9.5480	10.0662	9.4421	9.8251	9.6411
Dync1li2	_FAVSAESLR_.2	#N/A	4.6179	4.9440	4.4209	6.1013	5.6149	5.0314	5.6831	4.8376	5.5631	4.8676	5.4361	5.7187	3.9053	5.4192	3.8960	4.2295	4.6942	5.1615	4.1652	4.7238
Dync1li2	_KPDSM[Oxidation (M)]VTNSS[Phospho (STY)]TENEA_.2	#N/A	7.0110	7.5805	3.5293	3.5016	6.9648	6.7986	3.9378	7.1398	3.9968	4.0781	3.9415	3.7471	4.1049	3.6487	5.1336	7.3617	4.0893	3.3864	6.5419	3.3259
Dync1li2	_KTGSPGS[Phospho (STY)]PSAGGVQSTAK_.2	#N/A	4.4506	6.1602	6.3760	3.2388	6.0574	4.1966	3.7528	4.1509	5.6314	6.8063	5.4390	5.3317	5.7820	5.0734	4.4032	3.2946	5.7242	3.1445	5.1864	4.7529
Dync1li2	_TSES[Phospho (STY)]PARGPSGSPR_.2	Q6PDL0_S383_M1_Dync1li2	3.7912	4.2116	4.3275	3.8982	3.1290	3.3551	4.4121	3.4916	3.6465	3.9976	3.1251	4.1010	3.8119	5.3692	3.9893	4.1361	4.0293	3.8039	3.4525	4.2816
Srsf1	_VKVDGPRS[Phospho (STY)]PS[Phospho (STY)]YGR_.3	#N/A	10.5741	11.1075	10.7936	10.7943	10.3962	11.0260	10.9828	10.6449	10.7433	11.1036	10.6448	10.2122	10.9816	10.5691	9.9203	10.5473	10.5258	10.6550	10.9049	9.4515
Srsf1	_YS[Phospho (STY)]PRHS[Phospho (STY)]R_.2	Q6PDM2_S242_M2_Srsf1	8.8422	3.8759	9.2493	9.0102	8.2522	8.8886	8.6033	8.6259	8.7810	9.1276	8.7490	3.6020	3.4837	8.5535	7.2027	4.3061	3.1661	8.4523	4.2824	8.4097
Srsf1	_DGTGVVEFVR_.2	#N/A	6.7439	6.5094	5.8242	6.4887	5.8423	5.9362	5.9513	6.4195	6.0747	6.1845	6.2320	5.7728	6.3403	6.1214	5.3644	5.4115	5.7784	5.8564	5.6228	5.8115
Srsf1	_VDGPRS[Phospho (STY)]PSYGRS[Phospho (STY)]R_.2	#N/A	3.5419	6.7207	4.2235	4.1710	3.2477	6.2035	3.2542	3.3981	3.2708	6.3709	4.2502	3.7324	7.3383	4.1396	5.9821	6.6046	3.5681	4.1842	3.8328	5.9128
Srsf1	_VDGPRS[Phospho (STY)]PSYGR_.2	#N/A	11.0949	10.3302	11.1771	10.1722	10.5336	10.9791	10.9042	10.3972	10.8414	10.9223	10.7944	8.9796	10.4574	10.2758	3.5640	10.4876	10.2586	10.7382	9.3593	10.5015
Srsf1	_GS[Phospho (STY)]PRYS[Phospho (STY)]PR_.2	#N/A	4.3555	11.4064	11.6585	3.6002	10.8586	3.9174	11.4355	11.2397	3.9069	11.3988	11.1683	10.8102	11.2396	3.5587	10.4376	3.1996	11.2717	11.0539	3.7415	10.8846
Srsf1	_SRGS[Phospho (STY)]PRYS[Phospho (STY)]PR_.2	#N/A	4.5588	10.0571	4.0954	4.5263	9.6255	9.8904	3.7832	3.2836	2.9808	9.7306	9.5696	9.4280	4.5896	9.7442	3.8086	3.9209	4.2817	9.7664	3.1668	9.5233
Mylk	_KSS[Phospho (STY)]TGSPTSPINAEK_.2	Q6PDN3_S1795_M1_Mylk	5.6573	4.2846	5.9847	3.9712	3.3949	3.4282	3.6532	3.4185	3.5734	4.4994	3.0521	4.1741	3.7389	4.2415	4.7782	4.0509	3.9562	3.8769	3.5255	5.7349
Chd4	_MSQPGS[Phospho (STY)]PSPK_.2	Q6PDQ2_S1528_M1_Chd4	4.5676	3.4352	3.6031	7.6219	3.9054	4.1294	7.6572	3.9977	3.6948	3.2731	7.3765	3.3246	3.8029	3.3466	3.2129	3.4117	6.6820	3.6545	3.9536	3.0239
Chd4	_KMS[Phospho (STY)]QPGS[Phospho (STY)]PSPK_.2	Q6PDQ2_S1528_M2_Chd4	3.8532	4.2361	3.6494	3.9226	3.9517	8.2554	3.7357	3.5535	3.7070	3.8703	3.5927	3.3854	3.8509	6.9255	3.6168	3.4580	4.0912	3.8284	3.4769	3.6035
Sarm1	_EM[Oxidation (M)]LHS[Phospho (STY)]PLPC[Carbamidomethyl (C)]TGGK_.2	#N/A	6.6651	6.5182	7.0066	5.8324	6.2863	6.5488	6.3161	6.3666	6.6463	6.2628	6.2096	6.2957	6.7268	6.1098	5.3875	6.1802	6.6986	6.5448	6.0266	5.8379
Prrt3	_S[Phospho (STY)]LTEVC[Carbamidomethyl (C)]LR_.2	#N/A	8.5016	8.4372	8.6214	8.9020	3.7456	8.4682	8.5040	4.3581	8.4589	8.8045	8.9580	8.0178	3.9975	8.3697	6.2593	8.2688	8.3280	8.4492	8.7933	8.4631
Prrt3	_SSESHSAS[Phospho (STY)]SDTIEL_.2	Q6PE13_S965_M1_Prrt3	3.9803	4.1879	4.2381	3.7318	3.4076	3.4409	3.7099	3.4263	9.1872	8.7010	3.8884	3.9140	3.7956	4.2761	3.7353	4.0636	3.7032	3.7266	6.9244	3.7653
Prrt3	_RGS[Phospho (STY)]QPDAELDGAGTSLLR_.3	#N/A	4.7942	8.8755	9.2383	9.7220	3.6544	6.5055	7.4869	3.8858	9.3959	7.2645	3.6899	3.6363	4.0442	6.8213	3.4852	6.7529	6.7732	9.6836	8.4156	3.7411
Prrt3	_C[Carbamidomethyl (C)]RS[Phospho (STY)]LTEVC[Carbamidomethyl (C)]LR_.2	#N/A	6.5889	4.4933	4.0458	4.1799	4.3481	3.0713	3.1930	3.2099	3.2521	3.7158	3.6265	4.3827	3.5302	4.0329	4.2710	3.8544	3.7476	4.0856	3.7342	3.9999
Prrt3	_GS[Phospho (STY)]QPDAELDGAGTSLLR_.2	#N/A	3.9968	3.5927	3.4456	3.2793	4.2037	4.2371	4.3452	4.1105	4.2654	3.8074	3.7441	3.4821	4.4309	3.4327	2.8697	3.3589	3.1474	3.1850	4.3344	3.3997
Prrt3	_GLASS[Phospho (STY)]PTGGALRPR_.2	Q6PE13_S833_M1_Prrt3	3.3289	8.6183	3.8652	4.0743	4.1674	4.3915	8.7914	9.0569	3.4327	6.9639	3.8071	8.0842	3.5408	4.5854	2.9509	3.6738	3.2328	8.2004	4.2157	4.2627
Prrt3	_TSLPQHVM[Oxidation (M)]EPPVGAAAAGTSGS[Phospho (STY)]SLDSFSK_.3	Q6PE13_S894_M1_Prrt3	3.6760	4.3268	4.2123	4.0134	3.0137	3.2378	3.0265	8.3171	3.0856	3.8823	3.4600	4.2162	3.1936	4.2382	4.1045	4.0209	3.9141	4.2638	3.0620	3.9155
Prrt3	_GSVGPAPSLSELDLRPPS[Phospho (STY)]PINLSR_.3	Q6PE13_S799_M1_Prrt3	5.5927	3.8442	4.5123	4.1093	3.6818	4.4265	3.3386	6.5947	3.3977	3.5702	3.7721	3.5703	3.5058	7.0009	3.3917	4.3378	3.1978	5.0321	4.2507	4.2276
Prrt3	_S[Phospho (STY)]IDAALFR_.2	Q6PE13_S806_M1_Prrt3	4.1228	3.8800	3.1583	3.5666	3.4605	3.6846	4.0805	4.4425	4.1396	4.3291	3.0132	3.7694	4.2477	7.6488	3.6577	2.9669	3.9397	3.2097	3.5088	3.4687
Gpatch2l	_EVAPLTNFSDS[Phospho (STY)]DDTVVAK_.2	Q6PE65_S88_M1_Gpatch2l	3.7963	5.2703	3.3328	3.6626	4.0032	5.5784	3.9157	3.9917	4.9462	3.6069	3.9678	3.2722	4.0013	3.6564	3.0935	3.1584	3.2522	3.8320	4.1339	3.6234
Mob4	_DNLIVPILEEEVQNSVSGESEA_.2	#N/A	8.4924	8.0871	7.5876	7.5948	7.5386	7.3547	7.9156	8.4292	7.6163	7.9100	7.8355	8.0599	8.6316	8.8152	7.0749	7.6453	7.9776	7.9948	7.2963	7.7862
Mob4	_HTLDGAAC[Carbamidomethyl (C)]LLNSNK_.3	#N/A	4.4824	4.3376	4.0189	3.1019	3.1885	3.2218	3.5140	3.6249	3.7798	4.2930	3.2585	4.0637	3.9453	4.4479	3.8850	3.8445	4.1626	3.6705	3.3191	4.4149
Ctif	_S[Phospho (STY)]QEIEELER_.2	Q6PEE2_S18_M1_Ctif	9.9719	9.6697	10.2653	9.7938	8.4740	9.7424	9.1714	9.2794	9.6024	9.7631	9.7802	9.3889	9.3271	9.6519	9.1154	9.4493	9.9995	9.1039	9.6063	9.6345
Slc46a1	_VNPHPEFQQFPQS[Phospho (STY)]P_.2	Q6PEM8_S458_M1_Slc46a1	9.0936	9.2088	8.6067	8.4256	7.9332	8.5525	8.8274	9.0392	8.6119	8.3262	8.2913	8.4742	8.7758	8.7637	6.9349	8.2640	9.1563	8.9869	8.5912	8.0999
Mapre3	_LSNVAPPC[Carbamidomethyl (C)]ILR_.2	#N/A	8.5309	8.7784	8.6434	8.2973	8.5706	8.4302	8.7608	8.8430	8.3569	8.6621	8.0959	8.2293	8.0284	8.3657	8.4392	7.9515	8.0115	8.2000	8.0907	8.1360
Mapre3	_LEHEYIHNFK_.2	#N/A	6.1032	6.2754	6.3660	5.8331	6.1259	6.1089	6.1724	6.2438	5.9449	5.8147	6.0004	5.9546	5.7559	6.3405	5.6314	5.6174	5.7366	5.5921	5.9432	5.2298
Mapre3	_FFDANYDGKDYNPLLAR_.3	#N/A	7.2798	7.0108	7.0807	7.4459	7.0205	6.7400	6.7240	7.0683	7.3477	7.1077	6.3812	6.8558	6.7206	6.9151	6.7480	6.8359	6.6567	6.6002	6.7175	6.2326
Mapre3	_QGQDVAPPPNPGDQIFNK_.2	#N/A	7.2305	6.8989	7.5730	7.2217	6.4721	6.7019	6.9984	7.3179	6.9170	6.9500	5.9373	6.4871	6.4427	6.1990	5.8632	5.9592	6.9058	6.8873	6.2612	6.3392
Mapre3	_FFDANYDGK_.2	#N/A	6.5191	6.6479	6.9895	6.3615	7.0577	6.9793	6.8054	6.4055	6.8151	7.1856	5.9239	5.9021	5.9416	6.3746	6.4153	6.1845	6.2446	6.2739	6.3958	5.9443
Mapre3	_KLIGTAVPQRT[Phospho (STY)]SPTGPK_.3	#N/A	3.6451	3.6740	3.1817	3.9391	3.8521	5.8026	3.1960	3.8708	3.1162	3.4557	5.5442	3.4001	3.2817	3.2198	3.2214	3.0072	3.3680	3.7814	4.0805	3.7513
Mapre3	_LTVDGLEK_.2	#N/A	6.1014	6.3867	6.1478	6.6275	6.7886	6.4966	6.3146	6.1854	6.6249	6.5418	6.5566	6.1089	4.0260	6.1299	6.2182	6.7569	4.8424	6.2645	6.6062	6.3359
Mapre3	_DYNPLLAR_.2	#N/A	5.3677	5.6636	6.4723	5.8034	5.8879	6.3802	6.7805	3.7675	5.8464	6.3715	5.5322	5.2587	5.4982	5.4083	6.1539	5.6918	4.3052	6.2601	6.3236	6.1792
Mapre3	_[Acetyl (Protein N-term)]AVNVYSTSVTSENLSR_.2	#N/A	7.7525	7.8400	7.9966	7.8193	7.6833	7.0259	7.5269	7.8832	7.3336	7.6469	7.2657	6.6416	7.4594	7.2914	6.7615	7.0491	7.2088	6.6161	6.6596	7.0978
Mapre3	_LIGTAVPQRTS[Phospho (STY)]PTGPK_.2	Q6PER3_S162_M1_Mapre3	6.2447	7.1669	4.0419	3.6927	3.7910	3.8243	3.8816	8.1405	4.9831	4.9065	3.7820	3.7610	3.9672	3.7873	3.4319	3.9242	3.6865	6.2230	5.3349	3.6483
Wipf2	_HSSS[Phospho (STY)]APPPPPGR_.2	Q6PEV3_S175_M1_Wipf2	4.0770	9.3820	9.3788	3.7935	9.3221	3.8939	8.0638	3.7842	3.5483	3.2375	8.2014	3.1693	9.4342	9.6038	3.3464	9.2702	9.4905	3.8914	4.0216	3.5805
Pik3c3	_DGDESS[Phospho (STY)]PILTSFELVK_.2	Q6PF93_S244_M1_Pik3c3	6.3966	6.9849	4.3930	6.2149	3.2563	6.4855	6.2688	3.5571	6.5418	6.1273	3.1906	6.1962	6.1244	4.3801	5.2285	3.9123	4.0948	3.7384	5.9606	6.2531
Dlgap3	_SHS[Phospho (STY)]LEAPGKR_.2	#N/A	10.7885	10.9802	10.2854	11.0321	10.3030	9.8640	10.3994	10.4093	10.0466	11.0144	10.2182	10.3986	10.8452	10.5365	9.3879	10.2765	10.0770	10.3803	10.1421	10.4277
Dlgap3	_S[Phospho (STY)]LDSVDR_.2	#N/A	8.0040	3.8783	8.1980	7.8751	6.5121	3.6810	7.6966	7.8266	7.9712	7.5267	9.2205	8.7529	9.0537	9.0989	7.9422	8.9209	8.7542	8.8443	8.8060	9.1825
Dlgap3	_SHS[Phospho (STY)]LEAPGK_.2	#N/A	3.2409	7.4700	3.7772	4.4485	6.9385	4.3035	3.4616	6.1133	3.5207	7.5780	6.8302	3.1505	7.2433	7.4442	3.0388	6.7536	3.4789	6.5167	6.7741	7.2447
Dlgap3	_S[Phospho (STY)]LPDS[Phospho (STY)]GRTSPC[Carbamidomethyl (C)]PR_.2	Q6PFD5_S783_M2_Dlgap3	6.8618	7.2654	7.5108	7.8784	6.7716	6.3023	3.6594	6.8684	6.9017	7.5493	6.7972	6.9992	6.8972	7.2392	6.3559	6.8584	6.8279	6.4684	6.4863	7.1546
Dlgap3	_LFAKS[Phospho (STY)]HS[Phospho (STY)]LEAPGKR_.3	Q6PFD5_S183_M2_Dlgap3	5.3556	5.7388	4.3360	5.8369	3.5055	4.5005	5.4668	5.1951	4.6519	3.1092	5.2971	5.5564	3.6282	5.9065	3.5679	5.6968	5.2262	5.5665	6.2207	5.2798
Dlgap3	_RDS[Phospho (STY)]WMER_.2	Q6PFD5_S771_M1_Dlgap3	4.2936	6.3502	6.5645	6.5084	2.9997	5.3907	5.6030	3.8138	3.9687	5.3383	6.0930	4.2723	4.1341	6.6599	4.4169	3.6557	4.5737	4.7807	4.3236	4.3460
Dlgap3	_ERS[Phospho (STY)]LDSVDR_.2	#N/A	8.9632	8.9292	8.9528	8.4442	8.2832	8.0584	8.2931	8.7023	8.1416	8.8351	8.4819	8.4834	8.9900	8.9207	7.9383	8.6900	8.2224	8.6051	8.5850	8.4826
Dlgap3	_HAS[Phospho (STY)]EPQPGPR_.2	Q6PFD5_S712_M1_Dlgap3	6.5865	6.4750	8.6994	7.1930	6.7206	6.2826	6.4529	6.3666	7.3738	8.6574	6.2043	5.8435	6.4608	7.5502	8.1794	6.4378	6.2310	6.4712	6.4559	6.4453
Dlgap3	_WRPSIGVQVETIS[Phospho (STY)]DSDTENR_.3	Q6PFD5_S641_M1_Dlgap3	4.1537	3.1654	3.6903	3.4305	2.8598	4.3940	4.1883	4.3794	5.1702	3.9643	3.5871	4.3923	4.2739	5.5278	4.1281	3.5158	3.8766	5.3939	3.5719	3.2428
Dlgap3	_S[Phospho (STY)]NSVTAGVQADLELEGLAGLATVATEDK_.3	Q6PFD5_S672_M1_Dlgap3	3.9186	4.3989	3.9576	5.3595	3.2563	3.4804	4.2848	3.1459	4.3439	4.1249	3.2174	3.9737	4.4519	3.9957	3.8620	4.2635	4.1440	3.0055	3.3045	3.6730
Dlgap3	_HSSATES[Phospho (STY)]ADSIEIYIPEAQTR_.3	#N/A	6.0666	7.5970	7.1341	4.1819	3.6243	6.2754	6.9124	3.9589	6.5448	7.4670	6.1595	5.9121	6.6415	7.8691	6.3909	5.5569	6.0015	6.7693	6.7671	6.5856
Dlgap3	_AMGDEESGDS[Phospho (STY)]DGS[Phospho (STY)]PKTS[Phospho (STY)]PK_.2	Q6PFD5_S407_M3_Dlgap3	3.5764	4.0932	5.5344	5.1716	4.2013	3.3666	3.1374	3.9220	3.7870	3.5974	3.9720	3.3378	4.0570	4.7693	3.6650	3.6141	3.2417	4.0920	4.2006	3.8840
Dlgap3	IQAGC[Carbamidomethyl (C)]S[Phospho (STY)]QDDDC[Carbamidomethyl (C)]LPLLAAPASVSGR	Q6PFD5_S510_M1_Dlgap3	4.1091	7.6542	3.1446	3.5803	3.4468	3.0373	7.5408	3.8095	3.9644	4.1084	3.4430	3.7832	4.1298	3.1316	3.6714	2.9532	4.3472	3.4860	6.3622	3.0986
Dlgap3	_RSSS[Phospho (STY)]VDTAR_.2	Q6PFD5_S429_M1_Dlgap3	3.6613	5.9726	5.8813	5.8683	3.8682	3.9015	4.7520	5.4221	3.1005	3.4719	4.0800	3.2450	3.2660	3.7686	3.2057	3.0234	3.4833	4.3503	3.9989	3.7356
Dlgap3	_S[Phospho (STY)]LPDSGR_.2	Q6PFD5_S779_M1_Dlgap3	4.4798	4.0526	4.3039	4.3177	3.4734	3.5068	5.9830	3.4922	3.4949	4.5779	2.9735	5.2579	3.6603	4.3420	3.6000	4.1294	4.1714	4.1600	6.0443	4.0193
Dlgap3	_AMGDEESGDSDGS[Phospho (STY)]PK_.2	Q6PFD5_S410_M1_Dlgap3	3.8530	4.2343	3.8905	4.4309	3.7455	4.5191	3.6051	3.5534	3.7083	4.2968	4.8241	4.1237	3.8737	5.6812	4.1002	3.6991	4.0911	3.8266	3.4752	3.8445
Dlgap3	_EAEDYELPEEILEK_.2	#N/A	3.1806	4.1384	5.9105	4.4036	3.3876	3.4209	3.6605	3.4258	3.5807	4.4921	3.0594	3.8646	3.7462	4.2488	3.6859	4.0436	3.9635	6.7838	3.5182	4.2158
Dlgap3	_EGYPLPYEPPATDGS[Phospho (STY)]PGPTPVPAPGPGSGR_.3	Q6PFD5_S753_M1_Dlgap3	3.5276	4.0079	4.3486	4.2730	2.8653	3.0894	3.1750	3.5369	3.2340	3.7339	3.6084	3.7340	3.3421	5.6304	4.2530	3.8725	4.5350	4.8991	4.4144	4.0640
Dlgap3	_C[Carbamidomethyl (C)]S[Phospho (STY)]SADGLDGPTMGAR_.2	Q6PFD5_S578_M1_Dlgap3	3.4559	3.8749	3.8663	4.1401	3.6629	3.4979	3.2224	3.6816	3.2118	3.5681	3.9139	3.6011	3.3483	3.9044	3.8545	3.6918	4.0526	4.1906	4.2814	3.9497
Dlgap3	_DSWMERGS[Phospho (STY)]RS[Phospho (STY)]LPDSGR_.3	Q6PFD5_S777_M2_Dlgap3	3.2488	4.0703	4.2862	4.3354	3.4557	3.4891	3.5924	3.4745	3.5126	4.5602	2.9912	3.7964	3.6780	4.3243	3.6178	4.1117	4.4725	3.9378	2.9760	4.1477
Nup98	_YGLQDS[Phospho (STY)]DEEEEEHPPK_.2	#N/A	3.5091	3.4305	3.5546	4.1713	4.0175	4.0403	5.3406	4.2293	5.2466	3.5005	4.0585	4.9426	3.5356	3.5625	3.0830	3.3700	5.7220	4.0516	3.9929	3.4166
Nup98	_GETVQEIC[Carbamidomethyl (C)]S[Phospho (STY)]PR_.2	Q6PFD9_S1027_M1_Nup98	3.1534	4.1657	4.1908	3.9367	3.3603	5.2714	3.6878	3.4531	3.6080	4.4648	3.0866	3.8918	3.7734	4.2761	3.7132	4.0163	3.9908	3.8424	3.4910	4.2431
Tra2a	_[Acetyl (Protein N-term)]S[Phospho (STY)]DVEENNFEGR_.2	Q6PFR5_S2_M1_Tra2a	9.9296	9.7403	10.1475	9.8817	5.4984	9.8361	9.6441	9.1665	9.5722	9.7615	8.7694	9.3547	9.0792	9.7698	8.7545	9.2905	10.0415	9.5257	9.6420	9.5775
Tra2a	_S[Phospho (STY)]RS[Phospho (STY)]YT[Phospho (STY)]PEYR_.2	Q6PFR5_S82_M3_Tra2a	3.7188	4.2840	4.2551	3.9705	3.0566	3.2806	2.9837	3.4192	3.0428	3.9252	8.8185	4.1734	3.1508	4.1954	4.0617	4.0637	3.9569	4.3066	3.1048	3.8727
Tra2a	_RRS[Phospho (STY)]PS[Phospho (STY)]PYYSR_.2	#N/A	10.9624	10.9130	11.1213	11.0927	10.5068	10.8657	10.0074	10.6554	10.9308	11.0896	10.7591	10.4959	11.0211	10.6836	10.2385	10.5957	10.8778	10.6379	10.9403	10.5902
Tra2a	_S[Phospho (STY)]PSPYYSR_.2	#N/A	8.6970	8.9665	8.4347	9.0890	8.3523	9.0157	9.0634	8.1111	8.9865	8.9722	8.7851	8.7120	8.9328	8.8328	8.1231	8.4766	8.6243	8.7723	8.6623	8.9468
Tra2a	_RS[Phospho (STY)]PS[Phospho (STY)]PYYSR_.2	#N/A	9.7245	9.8904	9.8526	9.7643	9.3587	9.6327	9.7088	9.6975	9.7312	9.5852	9.3038	9.2186	9.7943	9.5421	8.8203	9.2376	9.6570	9.5053	9.4742	9.1675
Tra2a	_[Acetyl (Protein N-term)]S[Phospho (STY)]DVEENNFEGRES[Phospho (STY)]R_.2	Q6PFR5_S14_M2_Tra2a	6.0579	5.7014	6.7219	5.6772	3.4331	6.1218	5.2494	3.6319	5.1287	5.9905	3.4777	5.1758	3.7443	5.9201	4.0556	5.5066	5.9606	5.3379	5.2416	5.2252
Tra2a	_S[Phospho (STY)]RS[Phospho (STY)]HS[Phospho (STY)]PMSNR_.2	Q6PFR5_S94_M3_Tra2a	4.5807	7.0688	7.9035	7.7746	4.7349	6.3111	7.1886	3.5267	6.4535	4.3912	3.1602	5.1596	3.8470	7.6381	5.4865	3.9428	5.7432	3.7688	3.4174	5.4875
Tra2a	_S[Phospho (STY)]HS[Phospho (STY)]PMSNR_.2	Q6PFR5_S96_M2_Tra2a	4.2775	4.5424	4.4413	3.3067	2.9836	3.8822	4.0645	4.5032	4.2003	4.0881	3.0739	4.2685	4.3084	3.8521	4.0899	3.6396	4.0004	3.1490	3.4481	3.5294
Tra2a	_AHT[Phospho (STY)]PTPGIYMGR_.2	#N/A	3.5192	3.4204	3.5769	4.1498	4.0354	4.2968	5.0471	4.2514	3.5327	3.4405	3.9071	3.2180	3.6408	5.8560	3.0509	3.5791	3.4892	3.8219	4.1210	4.3627
Nyap1	_RPPAKPR_.2	#N/A	3.5520	4.2196	4.5894	4.3352	3.7589	4.8880	4.0867	3.0545	3.2094	3.3626	4.3924	3.6152	3.3749	4.3643	4.1692	3.9889	3.7385	4.2410	3.8895	4.3206
Slc6a7	_KYGGITSFENT[Phospho (STY)]AIEVDR_.3	Q6PGE7_T620_M1_Slc6a7	5.6865	5.8977	5.1954	3.6329	3.5101	3.9501	3.8452	4.2315	3.8240	3.7537	3.7606	3.8921	4.0367	5.2050	3.8398	3.3679	3.7287	3.4752	3.7742	4.0559
Morn4	_NEGLFENNK_.2	#N/A	3.1492	5.3969	4.9659	4.2180	5.7488	5.2602	5.3315	5.7459	5.2099	5.0401	5.4470	5.6548	3.1800	6.2591	6.1197	4.0121	4.3729	5.6538	5.9055	5.2880
Gmip	_DLS[Phospho (STY)]PEGPAPLTEEELDLR_.2	Q6PGG2_S75_M1_Gmip	3.6518	4.2911	4.1881	4.0376	2.9895	5.7828	3.0507	3.3522	3.5071	4.5657	2.9857	4.2404	3.6725	6.3456	5.4575	3.9967	3.8899	3.9433	5.9738	4.1422
Gmip	_S[Phospho (STY)]QGSPEDPPSQASPGSNK_.2	Q6PGG2_S235_M1_Gmip	3.9913	4.8524	3.5279	3.5951	4.1983	4.1354	4.0685	4.2170	5.1059	3.5313	3.9881	3.0560	4.1885	3.4477	4.0400	3.3308	3.7142	3.3688	4.1175	3.2708
Washc2	_VS[Phospho (STY)]PEVGSADVASIAQK_.2	#N/A	9.1589	8.5966	9.0917	8.9655	8.1024	8.7510	8.3367	8.4353	8.6070	8.9463	8.5475	8.2981	8.4716	9.0463	7.7844	8.4311	9.0953	8.0456	8.2036	8.4687
Washc2	_VPLLFS[Phospho (STY)]DEEDSEVPSGVKPEDLK_.3	Q6PGL7_S723_M1_Washc2	4.2228	3.7800	3.2583	3.4666	3.5606	7.4825	3.9805	3.9232	4.0396	4.4292	2.9131	3.6694	4.2436	3.2454	7.5515	3.0669	4.4609	8.1770	3.0209	7.4972
Washc2	_VDNARVS[Phospho (STY)]PEVGSADVASIAQK_.3	#N/A	7.3301	7.3838	6.8006	7.2356	6.1266	5.8679	6.4944	7.0752	6.4890	6.8888	6.4392	7.0071	7.3371	7.7088	6.2222	6.6381	7.1173	6.3255	6.6918	6.1658
Washc2	_LAAQESS[Phospho (STY)]EAEDVTVDR_.2	Q6PGL7_S1049_M1_Washc2	4.1969	3.8710	3.5894	3.7967	3.5883	3.6941	3.6752	4.0671	3.6483	4.1702	3.9558	4.3750	3.7915	6.2650	3.5506	3.3843	4.8846	4.4991	3.6895	4.7826
Washc2	_EGLLPASDQEAGGPSDIFSSSS[Phospho (STY)]PLDK_.3	Q6PGL7_S783_M1_Washc2	3.3471	4.4438	4.0953	4.1304	3.5541	3.5874	3.4940	3.2594	3.4142	3.1577	4.3937	4.6322	3.5797	4.0823	3.5194	4.2101	3.7970	4.0361	3.6847	4.0493
Washc2	_GLFS[Phospho (STY)]DEEDSEDLFSSQSSSKPK_.3	#N/A	4.4868	8.2822	8.6641	3.7693	3.1929	3.2263	7.9529	3.6205	3.7754	4.2974	3.2540	3.9721	3.9408	8.3619	3.8806	3.8489	4.1582	8.1132	3.3236	4.4105
Washc2	_TSALLFSS[Phospho (STY)]DEEDQWNIADSHTK_.3	Q6PGL7_S614_M1_Washc2	3.5630	6.5520	4.6004	4.0212	3.7699	4.3384	3.4267	3.7887	5.4850	3.4821	3.8602	5.2643	3.5939	3.1377	5.6586	3.6207	3.2859	6.4756	4.1626	5.4854
Washc2	_VASIFDDPLNAFGS[Phospho (STY)]Q_.2	Q6PGL7_S1333_M1_Washc2	4.3331	4.7591	3.8697	3.2511	3.0392	3.5684	5.4496	3.0579	4.2559	4.2129	3.1294	4.2130	4.3640	4.6057	3.9129	3.6952	4.0560	4.2165	3.3925	3.5850
Washc2	_GLFS[Phospho (STY)]DEEDSEDLFSSQSSSK_.2	#N/A	4.5749	4.2450	4.1115	4.5102	3.2810	3.3143	3.7671	3.2997	3.6873	4.3855	3.1660	3.9712	4.6058	4.1495	3.7925	3.9370	4.2978	4.3525	3.1507	3.8268
Washc2	_AGNS[Phospho (STY)]DS[Phospho (STY)]EEDDANER_.2	#N/A	3.8370	3.4821	3.3735	3.1194	4.0439	4.0772	3.0042	4.2704	4.4252	3.6476	3.9039	3.2082	4.5907	3.5925	3.0296	3.1991	3.3072	3.0251	4.1746	3.5595
Washc2	_AGNS[Phospho (STY)]DS[Phospho (STY)]EEDDANERVDLILEPK_.3	#N/A	4.5699	4.2500	4.1065	3.8524	3.2760	3.3093	3.7721	3.5374	3.6923	4.3805	3.1709	3.9761	3.8577	4.3604	3.7975	3.9320	4.0751	3.7581	3.4067	4.3274
Dclk2	_SSSSS[Phospho (STY)]PTSPGSFR_.2	Q6PGN3_S340_M1_Dclk2	9.4163	9.3041	8.1430	9.1315	7.5628	7.9300	8.7654	9.0517	7.8381	9.1395	7.6265	8.4621	9.2618	8.1007	7.2134	8.5466	8.1273	3.8064	7.8011	7.3525
Dclk2	_C[Carbamidomethyl (C)]LS[Phospho (STY)]PEGVNGNR_.2	Q6PGN3_S374_M1_Dclk2	9.4684	9.3836	9.3154	9.1625	9.1312	9.2575	9.2723	9.3732	9.2399	9.2262	9.0979	8.9609	9.3015	9.5858	8.6259	9.2496	9.3677	9.0645	9.0451	8.9934
Dclk2	_SSS[Phospho (STY)]NVNGGPELDR_.2	Q6PGN3_S361_M1_Dclk2	3.8913	3.4278	3.4279	7.9674	4.0983	4.1316	4.4507	4.1170	4.3709	8.3441	7.4068	3.1539	3.9222	3.4660	2.9752	3.2534	3.6142	3.5352	3.8343	7.5763
Dclk2	_S[Phospho (STY)]IELEHFEER_.2	Q6PGN3_S6_M1_Dclk2	7.9121	7.7017	7.3326	6.9148	3.9968	5.9847	6.9782	4.3594	6.3262	7.8623	5.8684	6.1465	6.3575	7.2883	4.3670	6.3785	5.7684	6.4847	6.8547	6.7037
Dclk2	_YSGSRS[Phospho (STY)]PGFSR_.2	Q6PGN3_S307_M1_Dclk2	4.5195	3.4833	3.5550	3.1699	3.8572	4.1277	3.6838	4.2199	4.3748	7.4045	3.8534	3.3727	8.4208	3.5420	3.2610	3.3636	3.2567	3.0756	4.2250	3.5090
Erc2	_TISNLEGS[Phospho (STY)]PSR_.2	Q6PH08_S14_M1_Erc2	10.1827	10.2365	10.5013	10.3247	10.0532	9.9418	4.2446	10.2359	10.1015	10.4396	10.0262	3.9335	10.6090	10.6908	9.9799	10.1695	4.1968	9.9951	3.2849	10.2905
Erc2	_S[Phospho (STY)]QLQPEPAK_.2	Q6PH08_S364_M1_Erc2	6.6362	6.8670	6.8570	7.3727	6.1178	5.9546	6.4434	6.5561	6.3604	6.7825	6.4556	6.4763	6.5350	6.9951	6.3297	6.7769	6.9811	6.8132	6.5395	7.0292
Erc2	_AAILQTEVDALR_.2	#N/A	5.4118	6.1263	5.9060	6.1305	5.5969	5.0177	5.7008	5.5381	5.3961	5.8392	4.8522	4.9035	6.0873	5.8514	5.9779	4.7155	5.8338	5.6294	5.6530	5.6940
Erc2	_DLNHLLQQESGNR_.3	#N/A	7.0973	7.2936	6.9444	7.0198	6.6179	6.5357	6.8214	7.2026	6.4486	7.3106	7.3371	7.1914	7.5740	7.9148	6.6864	7.1519	7.3838	7.0491	6.9823	7.1339
Erc2	_ESELLALQTK_.2	#N/A	5.6091	5.8959	5.8235	3.7851	5.0574	5.7404	5.8251	4.8861	5.7668	5.7660	5.9642	5.6558	5.6940	6.0105	5.2812	5.4712	5.4654	5.8995	5.3936	3.5935
Erc2	_SQHSNHRPS[Phospho (STY)]PDQDDEEGIWA_.3	#N/A	9.7593	10.5355	9.6384	9.9406	3.5708	9.9285	9.9553	9.0367	9.5751	10.1725	3.7757	10.2267	3.9090	11.2752	9.8666	10.2674	10.4811	10.3737	10.1008	9.9910
Erc2	_IAELESLTLR_.2	#N/A	3.1523	4.0585	3.6886	4.5371	3.9908	4.1498	4.1806	4.6374	3.9042	3.3586	4.5671	4.2499	3.7174	3.2612	4.5346	3.4972	3.5047	3.2588	3.9332	3.9742
Erc2	_TISNLEGS[Phospho (STY)]PSRS[Phospho (STY)]PR_.2	Q6PH08_S14_M2_Erc2	3.9341	4.1955	4.0484	3.7947	3.2718	3.2942	3.8548	3.9897	3.8365	4.0716	3.3764	3.9679	3.9656	4.3111	4.3273	3.3283	4.2952	3.5380	4.1795	3.7847
Rab35	_DYDHLFK_.2	#N/A	4.9513	4.1305	3.3992	4.4943	4.6160	4.0459	5.2981	5.0240	4.6404	4.1317	4.5239	4.1558	4.4111	3.8533	3.4114	4.0002	4.5673	4.8050	3.5477	4.4052
Rab35	_QQQQQQNDVVK_.2	#N/A	2.5522	2.6720	1.4652	3.8056	3.7112	4.0404	4.7496	3.3138	3.6514	3.8907	3.4069	4.1416	1.8623	2.4205	3.3487	2.9523	4.6112	3.3720	1.9342	3.0305
Rab35	_FAGQM[Oxidation (M)]GIQLFETSAK_.2	#N/A	4.8592	4.1356	4.2209	4.4007	5.0093	4.8333	4.8539	4.8515	4.6630	4.4777	4.7379	3.8617	4.4621	4.4147	4.1824	4.0465	5.4301	4.1240	4.2512	4.4466
Rab35	_WLHEINQNC[Carbamidomethyl (C)]DDVC[Carbamidomethyl (C)]R_.3	#N/A	3.3767	4.3105	4.1720	4.2623	3.9501	3.9627	4.7527	3.2321	3.3870	3.1892	4.2942	3.3993	3.5524	4.8831	3.2454	3.9901	3.7698	4.0768	3.7162	4.0221
Sort1	_SGYHDDS[Phospho (STY)]DEDLLE_.2	Q6PHU5_S819_M1_Sort1	6.1711	10.8998	10.9036	10.5592	3.7534	10.3657	10.5193	3.5977	10.5453	10.3924	4.2079	9.9085	5.3699	3.8965	8.7668	9.9060	9.9943	10.4133	10.1820	10.2964
Camk2d	_FTDEYQLFEELGK_.2	#N/A	9.4768	7.3262	7.5107	6.9812	6.7486	7.1380	7.0579	7.0603	7.3892	6.9686	7.4285	6.9346	7.0634	8.9254	6.7583	7.2718	7.3736	6.7632	9.2360	7.1211
Camk2d	_FYFENALSK_.2	#N/A	5.8402	6.3731	5.8996	5.6918	5.8688	6.5692	6.1205	5.7036	6.3215	5.6773	5.5805	6.1627	5.7847	5.1182	5.9606	5.7828	5.3273	6.2532	6.2561	5.8642
Camk2d	_IPTGQEYAAK_.2	#N/A	6.4355	6.4585	6.9713	6.5929	6.8802	6.9920	7.0012	6.9876	7.0276	6.4955	6.2071	6.5688	6.9458	6.7226	6.9809	6.7850	3.1212	7.0966	6.5565	6.3266
Camk2d	_ESTESSNTT[Phospho (STY)]IEDEDVK_.2	Q6PHZ2_T337_M1_Camk2d	3.8883	4.1145	4.2246	3.8011	3.2260	5.8517	4.3151	3.5886	4.3742	4.0946	3.2477	4.0040	4.4822	4.4116	3.8923	4.2332	4.1263	3.7068	3.3554	4.3786
Camk2d	_HPWIC[Carbamidomethyl (C)]QR_.2	#N/A	3.5095	5.4699	4.5469	5.2773	4.7715	4.6365	4.9692	3.7351	4.8336	3.3200	4.4483	3.5358	3.4174	4.5849	4.9421	4.3724	4.4842	5.2562	4.9880	5.1422
Camk2d	_IC[Carbamidomethyl (C)]DPGLTAFEPEALGNLVEGM[Oxidation (M)]DFHR_.3	#N/A	3.4810	6.3098	6.9030	4.1032	5.9456	6.3291	5.8444	3.7067	5.7711	3.5641	6.1359	4.4210	5.9497	6.8388	5.7394	4.3439	3.2039	4.7779	4.2446	4.2338
Camk2d	_VTEQLIEAINNGDFEAYTK_.2	#N/A	5.1652	5.5388	5.3326	5.0218	5.0784	5.3932	5.2594	5.8828	4.3660	5.2844	5.2830	5.5187	5.2396	5.6863	4.8034	5.1475	5.6613	5.1901	4.5831	5.5267
Camk2d	_ITASEALK_.2	#N/A	4.0934	3.6893	3.3490	3.3759	4.3003	3.3294	4.2486	1.7978	2.1530	3.9040	2.9547	2.9518	3.5896	3.3361	3.4441	3.4555	1.7905	3.2816	3.4111	3.3031
Rbm15b	_ASGDPGAGGAS[Phospho (STY)]PR_.2	Q6PHZ5_S107_M1_Rbm15b	8.5342	8.7333	8.2024	8.6334	8.4764	8.5909	8.4543	8.6633	8.5656	8.3167	8.2358	8.2704	8.6013	8.8347	7.8091	8.1882	8.3303	8.4902	8.3095	7.9266
Rbm15b	_TTHS[Phospho (STY)]PYEER_.2	Q6PHZ5_S606_M1_Rbm15b	4.3693	3.3487	3.5069	5.8809	6.2268	5.9565	3.8341	6.4040	5.8946	4.5756	5.8294	6.0471	6.2281	3.5450	3.4113	3.2133	3.6933	3.4562	5.5797	3.2223
Rbm15b	_QRS[Phospho (STY)]LS[Phospho (STY)]PVAAPPLREPR_.3	Q6PHZ5_S261_M2_Rbm15b	3.7497	4.3961	6.3417	3.9397	3.0874	5.5926	5.4887	3.3148	3.0119	3.9560	3.3863	4.1119	3.1200	5.5215	3.4664	5.4174	4.3128	4.7928	5.1417	4.4805
Rbm15b	_RNS[Phospho (STY)]LEGYSR_.2	Q6PHZ5_S559_M1_Rbm15b	3.1900	3.3120	3.7263	4.4994	4.0286	4.1533	3.1251	4.3912	4.5040	4.9199	4.0248	3.2014	3.2107	3.7134	3.0897	3.5349	3.4281	4.8559	4.2289	4.7062
Kcnip4	_TSS[Phospho (STY)]PAIQNS[Phospho (STY)]VEDELEM[Oxidation (M)]ATVR_.2	Q6PHZ8_S50_M2_Kcnip4	4.3404	10.3434	3.9933	3.5820	3.3033	4.4980	5.1569	10.6213	3.9289	4.4658	3.7505	4.0386	4.0757	4.0065	4.9216	4.8531	10.4487	4.9299	4.4540	3.2193
Atp1a3	_YQLSIHETEDPNDNR_.3	#N/A	11.0866	11.2139	11.7425	11.0841	10.3751	11.1543	10.9154	10.8343	11.3228	10.9361	10.7506	10.5463	10.8012	11.0104	10.1456	10.7723	11.2712	11.2023	11.1533	10.9748
Atp1a3	_C[Carbamidomethyl (C)]IELSSGS[Phospho (STY)]VK_.2	Q6PIC6_S456_M1_Atp1a3	8.5539	8.5602	8.6336	8.5234	8.1333	8.4998	8.7940	8.5658	8.7381	8.5230	8.7294	8.3100	9.0044	8.2621	8.2120	8.2621	8.5735	8.6921	8.7962	8.4588
Atp1a3	_TDKLVNER_.2	#N/A	10.3432	10.2587	10.1194	10.0962	9.9718	10.1512	10.5737	10.3570	10.3937	9.8891	10.1443	10.1157	10.2794	9.4425	9.8563	9.8523	10.0910	10.4318	9.8955	9.7471
Atp1a3	_GGQDNIPVLK_.2	#N/A	10.2092	10.0999	10.1897	9.9930	9.9675	10.0715	10.3270	10.3066	10.1199	9.7408	10.1563	9.8578	9.9187	9.6139	9.5498	9.3915	10.2311	10.1572	9.7581	9.6498

Atp1a3	_IATLASGLEVGK_.2	#N/A	9.3561	9.7969	9.5902	9.6686	9.0861	9.6030	9.8231	9.6638	9.6300	9.2033	9.2181	9.3596	9.3051	9.3912	9.1058	9.0077	9.3946	9.9213	9.3221	9.0338
Atp1a3	_GGQDNIPVLKR_.2	#N/A	9.5576	9.4987	9.0559	9.2739	9.3174	9.1231	9.4315	9.8166	9.6109	9.0718	8.7217	9.1485	9.6314	8.8366	9.0069	9.0473	9.4951	9.4366	9.2295	8.9607
Atp1a3	_IISAHGC[Carbamidomethyl (C)]K_.2	#N/A	8.3142	8.3090	8.2569	8.0624	8.0744	8.1508	8.4585	8.2808	7.9902	7.9270	7.6449	8.0254	8.0505	7.5551	8.0548	7.3890	7.9813	8.3162	7.9752	7.7862
Atp1a3	_VLGFC[Carbamidomethyl (C)]HYYLPEEQFPK_.3	#N/A	8.4521	8.6901	7.2474	7.9796	8.0264	8.0929	8.6723	8.5075	7.8972	7.7500	7.9300	8.3266	8.3421	8.3725	7.0314	7.4356	8.3435	8.6985	7.4334	7.4507
Atp1a3	_GVVVATGDR_.2	#N/A	8.6199	7.7833	8.6307	8.7807	8.3729	7.7454	7.9129	8.0651	8.9665	7.5697	9.0720	8.9021	9.3890	8.3449	8.6419	8.8166	8.1097	9.1841	8.8766	9.0966
Atp1a3	_AC[Carbamidomethyl (C)]VIHGTDLK_.2	#N/A	8.7704	8.6372	8.9158	8.4112	8.1104	8.8713	9.1563	8.7618	8.8561	8.3121	8.8111	8.2857	8.0379	8.2455	8.7419	8.1836	8.6431	8.7190	8.5853	8.1589
Atp1a3	_C[Carbamidomethyl (C)]ATILLQGK_.2	#N/A	8.0674	8.2090	7.9697	8.4654	8.3170	7.8873	8.4523	8.2294	8.3562	8.0174	7.8941	7.9567	7.7918	7.5083	7.4727	7.8468	8.2096	8.1136	8.1763	7.9515
Atp1a3	_RDLDDLKK_.2	#N/A	8.5280	8.1072	9.0227	8.1511	8.0108	8.3333	8.3151	8.8997	8.2847	8.1454	8.4826	8.1596	8.0592	7.8462	7.2393	8.1720	8.7571	8.3183	8.0766	7.9447
Atp1a3	_RNPGGWVEK_.2	#N/A	8.3477	8.3086	8.4755	8.1483	7.9768	8.4390	8.3869	8.5787	8.2521	7.8788	7.9477	7.7793	7.9806	7.8150	8.1501	7.7548	8.2705	8.3722	8.0274	8.0317
Atp1a3	_EAFQNAYLELGGLGER_.2	#N/A	7.3225	7.2682	7.6445	6.6053	6.6901	7.0826	7.2277	7.1329	7.0114	7.0143	7.1630	6.4267	6.8461	6.6810	6.5333	6.6485	7.5486	6.8360	6.6913	6.7184
Atp1a3	_AQEILAR_.2	#N/A	4.8017	6.3014	5.7562	7.3394	5.5753	7.1365	7.5038	5.0694	7.2988	7.2023	7.2065	6.7381	7.4210	6.3157	6.8341	6.6526	4.4643	7.3225	7.4561	7.5299
Atp1a3	_NITFFSTNC[Carbamidomethyl (C)]VEGTAR_.2	#N/A	7.3005	6.5986	7.7005	6.6894	6.6890	6.4268	6.1944	6.5675	7.1351	6.8302	6.7509	6.0014	7.3202	6.1446	6.0105	5.4441	6.3552	6.2060	6.8462	6.4228
Atp1a3	_NPGGWVEKETYY_.2	#N/A	6.8424	6.3732	5.9502	6.0507	6.4595	6.1241	6.4346	6.6042	6.5358	6.3502	6.1300	6.1883	7.5524	5.4939	5.3125	6.2130	6.6622	6.5363	6.2986	5.9662
Atp1a3	_EQPLDEEM[Oxidation (M)]KEAFQNAYLELGGLGER_.3	#N/A	6.8518	7.3909	5.6762	5.7130	5.1646	6.1724	7.0376	7.5764	6.1707	6.2813	5.9635	6.9913	7.0346	6.9403	5.4952	5.8362	7.0595	7.6112	6.7982	6.0283
Atp1a3	_NPGGWVEK_.2	#N/A	5.4310	5.3172	5.7799	5.8449	5.5773	6.2853	6.2447	5.0693	5.9955	5.2721	5.8263	5.6284	6.1000	4.7703	5.4422	5.5528	5.1412	6.2279	6.2917	6.0826
Atp1a3	_M[Oxidation (M)]QVNAEEVVVGDLVEIK_.2	#N/A	10.4417	9.9494	9.8462	9.6972	9.1996	9.4325	9.4233	10.2798	9.5196	9.8111	9.3678	9.5713	10.5123	9.7583	8.6695	9.8458	9.9021	9.7685	9.3527	9.5326
Atp1a3	_KYNTDC[Carbamidomethyl (C)]VQGLTHSK_.3	#N/A	8.1882	8.3992	8.8516	8.4859	8.0347	8.3729	8.5062	8.2614	8.4995	8.5106	8.3748	8.3953	8.6165	7.8690	8.3415	8.0218	8.1602	8.2435	8.1848	7.8914
Atp1a3	_DFTSEQIDEILQNHTEIVFAR_.3	#N/A	6.0684	6.0082	5.2368	5.3564	5.1975	5.4394	5.1840	6.0964	5.0006	5.1532	3.0963	5.6220	5.5329	6.0165	3.9967	4.6588	6.1530	5.6762	5.5800	4.1357
Atp1a3	_S[Phospho (STY)]PDC[Carbamidomethyl (C)]THDNPLETR_.2	#N/A	10.1533	9.9725	10.1858	10.1158	9.7237	9.8424	10.0662	10.0701	10.0826	9.7015	9.9490	9.7696	10.0848	10.0211	9.2195	9.5479	10.1998	10.0481	9.8105	9.6293
Atp1a3	_MSVEEVC[Carbamidomethyl (C)]R_.2	#N/A	7.8032	8.3980	8.6284	8.5824	7.8866	8.2470	8.6273	7.8611	8.3992	7.7854	8.3424	7.9273	7.8759	8.2150	7.7735	7.2433	7.9078	7.9291	7.8192	7.8278
Atp1a3	_EVAMTEHK_.2	#N/A	3.6699	6.5593	7.4371	7.2961	5.9778	6.1314	7.2259	3.3703	6.0337	5.5246	7.1882	4.3525	3.1998	6.8212	6.0508	4.0148	5.7880	5.0608	4.7361	5.5160
Atp1a3	rbamidomethyl (C)]DDVNFTTDNLC[Carbamidomethyl (C)]FVGLM[Oxidation (M)]SM[Oxidation	#N/A	3.4079	6.0389	4.6931	5.6498	4.2982	5.1330	5.9030	6.5063	4.2775	5.5061	4.9364	5.5915	4.6220	6.5014	3.6961	5.1308	6.5018	5.6055	5.3326	4.9828
Atp1a3	_SSHTWVALSHIAGLC[Carbamidomethyl (C)]NR_.3	#N/A	8.6184	8.4809	8.2570	8.5466	8.0081	7.9605	8.1764	8.4244	8.0694	8.2727	8.2125	8.1207	8.7178	8.2382	7.4001	7.7149	8.3221	8.2340	7.8662	8.3069
Atp1a3	_DVAGDASESALLK_.2	#N/A	7.9858	8.9544	8.7740	8.5822	7.4935	8.1167	8.9030	8.1199	8.9158	7.6078	7.7677	7.5780	7.3231	7.9228	7.6736	7.7059	7.7069	8.8353	7.8923	7.8529
Atp1a3	_ADIGVAM[Oxidation (M)]GIAGSDVSK_.2	#N/A	7.3340	7.7528	7.1092	7.3274	6.6489	7.4404	7.5869	7.4478	7.6910	7.1654	6.8026	7.2910	6.8693	6.8763	6.6535	7.1526	7.2831	7.8291	7.1765	7.2156
Atp1a3	_KADIGVAM[Oxidation (M)]GIAGSDVSK_.2	#N/A	5.0204	5.2629	5.3361	5.6420	4.4601	6.4175	6.4560	5.3057	6.0230	5.7428	5.3299	5.6182	5.5174	5.3106	5.6118	5.7939	4.2938	6.3254	6.2388	6.0896
Atp1a3	_MSVEEVC[Carbamidomethyl (C)]RK_.2	#N/A	3.6314	4.3714	5.9541	6.4676	2.9691	3.5150	6.2552	3.3318	5.3536	4.5861	3.3973	4.2609	3.6521	6.2655	5.7411	3.9763	3.8694	3.9637	4.6713	4.1218
Atp1a3	_TVNDLEDSYGQQWTYEQR_.2	#N/A	9.8476	9.0203	8.7488	8.9053	8.4175	8.5393	8.4444	9.3903	8.6148	8.9524	8.8455	8.7158	9.6365	8.8273	7.3120	8.4891	9.3634	8.5429	8.1946	8.4472
Atp1a3	_YNTDC[Carbamidomethyl (C)]VQGLTHSK_.2	#N/A	7.0361	6.8966	6.8968	6.9871	6.7567	6.9555	6.9438	7.2261	7.0317	6.7648	6.8173	6.7611	6.9296	6.8814	6.6091	6.2532	7.2973	7.0246	6.5585	6.6398
Atp1a3	_M[Oxidation (M)]TVAHM[Oxidation (M)]WFDNQIHEADTTEDQSGTSFDK_.4	#N/A	8.5252	7.6359	4.4072	6.6928	7.3733	7.3325	7.5945	7.6925	7.0632	8.2818	7.1059	7.3829	8.0592	6.5857	5.9850	7.4517	8.0297	7.5589	7.3557	7.8738
Atp1a3	_VDNSSLTGESEPQTRS[Phospho (STY)]PDC[Carbamidomethyl (C)]THDNPLETR_.4	#N/A	3.2841	4.0350	4.3215	4.3002	3.4910	3.5243	3.5571	3.5097	3.4773	3.0947	2.9559	3.7611	3.6427	4.3595	3.5825	4.1470	4.5078	3.9731	3.6217	4.1124
Atp1a3	_AC[Carbamidomethyl (C)]VIHGTDLKDFTSEQIDEILQNHTEIVFAR_.4	#N/A	3.2907	3.2112	3.8270	4.3987	4.1293	4.3534	2.2702	4.4919	3.4709	3.4971	3.8452	3.1007	4.6773	4.6235	2.9890	3.6356	4.0282	3.8785	4.1775	4.3008
Atp1a2	_VDNSS[Phospho (STY)]LTGESEPQTR_.2	Q6PIE5_S215_M1_Atp1a2	12.4558	12.3240	12.2966	12.1892	11.9272	12.1488	12.2457	12.4984	12.1891	11.9487	11.9973	11.9928	12.4327	12.1112	11.1717	11.7378	12.4403	12.2973	11.9927	11.8021
Atp1a2	_NM[Oxidation (M)]VPQQALVIR_.2	#N/A	11.2887	11.3208	11.1188	11.0904	11.1756	11.1283	11.3916	11.4766	10.9916	10.8837	10.9961	11.0807	11.3123	10.5836	10.3842	10.6034	11.1387	11.2370	10.9687	10.6785
Atp1a2	_LIIVEGC[Carbamidomethyl (C)]QR_.2	#N/A	11.9326	11.7857	12.1118	11.9250	11.7076	11.7521	11.9362	12.0609	11.8658	11.5925	11.6136	11.5523	11.6514	11.3417	10.9595	11.2730	11.8922	11.6963	11.6420	11.3449
Atp1a2	_LIFDNLKK_.2	#N/A	11.0769	10.8836	10.6041	10.5620	10.8172	10.6646	10.9269	11.2184	10.3973	10.4445	10.4896	10.7576	10.9417	10.2241	10.0961	10.1002	10.9183	10.8310	10.4921	10.3898
Atp1a2	_AAVPDAVGK_.2	#N/A	9.5471	9.8937	9.4628	9.7365	9.3040	9.6316	10.2546	9.4021	9.9300	9.5483	9.6177	9.7403	9.7711	9.1606	9.6270	9.2576	9.1022	10.0711	9.5148	9.5669
Atp1a2	_C[Carbamidomethyl (C)]IELSC[Carbamidomethyl (C)]GS[Phospho (STY)]VR_.2	#N/A	8.8375	8.6704	8.6080	8.5900	8.2616	8.5593	8.5707	8.7091	8.7121	8.4241	8.4835	8.1505	8.4745	8.1642	7.2711	7.9790	8.9324	8.3864	8.2758	8.1951
Atp1a2	_TGTLTQNR_.2	#N/A	9.4758	9.5260	9.2873	9.1606	9.1718	9.4088	9.7825	9.7190	9.4927	9.0682	9.2190	9.2240	9.4145	8.8740	9.1551	8.5449	9.1515	9.5595	9.1567	9.0484
Atp1a2	_IAGLC[Carbamidomethyl (C)]NR_.2	#N/A	8.7572	8.8999	8.7170	8.7094	8.5912	8.6159	9.2204	8.9350	8.7258	8.1932	8.2451	8.7149	8.6375	8.4213	8.6342	8.0199	8.5935	8.9082	8.8031	8.2058
Atp1a2	_DHTEIVFAR_.2	#N/A	8.6072	8.7505	8.8263	8.5838	8.4080	8.7996	8.7430	8.7270	8.9785	8.2899	8.2837	8.1244	7.3200	8.1428	7.5752	7.8274	8.6925	8.4468	8.2894	7.9053
Atp1a2	_LSLDELGR_.2	#N/A	8.4147	8.5917	8.8061	8.5101	8.2527	8.0774	8.8738	8.4900	8.6754	8.2473	8.1945	8.1265	8.6259	8.0130	8.1377	7.8225	8.3140	8.6139	8.4508	7.8350
Atp1a2	_FDTDELNFPTEK_.2	#N/A	8.5021	7.6800	8.3874	7.6962	7.9104	8.2717	8.1708	7.9219	7.7135	8.0539	8.0749	7.3486	7.7969	7.3670	7.2467	7.3991	8.0977	7.7726	7.5665	7.5617
Atp1a2	_VLGFC[Carbamidomethyl (C)]QLNLPSGK_.2	#N/A	8.0823	8.1141	8.2948	7.6275	7.7374	7.8964	8.0824	8.1488	7.9533	7.6347	7.5821	7.6136	7.8253	7.2418	7.5888	6.9407	7.8886	7.9301	7.5242	7.4116
Atp1a2	_KYQVDLSK_.2	#N/A	7.2981	7.1145	7.0871	7.0294	6.7656	7.4047	7.1404	6.9199	7.0137	6.6840	6.9672	6.6621	6.5281	6.7198	6.0470	6.3188	7.3972	7.0590	6.6551	6.7469
Atp1a2	_YQLSIHER_.2	#N/A	7.6647	7.2200	7.7276	6.5737	7.0610	7.4153	7.5026	7.7635	7.5032	6.4264	7.0584	7.1343	7.2079	7.0960	6.8835	7.0503	7.8938	7.7405	7.4611	7.2307
Atp1a2	_TTNDLEDSYGQEWTYEQR_.2	#N/A	7.8901	7.2135	7.5588	7.0974	5.8877	6.4792	6.5019	6.8517	6.6939	5.8747	6.1365	6.1383	6.8230	6.6561	5.6666	5.6641	7.1975	6.5450	5.6971	5.6734
Atp1a2	_NLEAVETLGSTSTIC[Carbamidomethyl (C)]SDKTGTLTQNR_.3	#N/A	7.4943	7.1753	5.3921	6.8296	6.6036	5.9418	6.5395	7.8253	6.6047	6.5199	6.3351	7.1655	7.9670	6.5288	5.9131	6.7103	7.5441	6.6962	6.7603	6.6447
Atp1a2	_LIFDNLK_.2	#N/A	6.1984	6.2749	6.9919	6.8537	6.4407	6.8250	7.6459	5.2352	6.7030	6.7591	6.3390	6.5247	5.8010	6.0289	6.1139	6.6453	5.5633	7.2876	7.0988	6.6587
Atp1a2	_YPGGWVEK_.2	#N/A	6.7696	7.0298	6.5920	6.8476	6.4725	6.5301	7.0521	6.8799	6.5223	6.1336	6.8227	6.5291	7.2901	5.7409	5.7080	5.6851	5.9379	6.6694	5.9253	6.4040
Atp1a2	_ELDELKK_.2	#N/A	6.2404	6.4571	6.2550	6.0976	6.1231	6.3786	6.9755	6.5625	6.5851	6.1832	6.2639	6.0190	5.8876	5.5284	6.0793	5.4908	5.9942	6.4055	5.9724	5.0809
Atp1a2	_C[Carbamidomethyl (C)]STILVQGK_.2	#N/A	5.4873	6.4408	6.2255	5.3430	5.7362	5.9302	6.0054	5.5443	5.8299	5.4018	6.0847	5.5101	5.4301	5.0340	5.7542	5.8380	5.7097	6.1075	5.5690	5.7636
Atp1a2	_VIM[Oxidation (M)]VTGDHPITAK_.2	#N/A	8.9628	9.0758	9.1015	8.5707	8.8805	8.8539	9.3140	9.0798	9.0403	8.7506	8.6768	8.7631	9.1502	8.2903	8.3430	8.6152	9.0862	8.5937	8.7011	8.3170
Atp1a2	_NLEAVETLGSTSTIC[Carbamidomethyl (C)]SDK_.2	#N/A	9.2214	8.9183	9.1642	8.7952	8.1133	8.4468	8.4553	8.9632	8.5750	8.6273	8.6876	8.4585	8.9651	8.6585	7.7629	8.3189	8.8543	8.3506	8.3540	8.0474
Atp1a2	_AC[Carbamidomethyl (C)]VVHGSDLK_.2	#N/A	7.6694	7.5813	7.8133	7.4900	7.1624	7.5330	7.5871	7.4169	7.4325	7.4198	7.3725	7.1233	7.1922	6.9802	7.4126	6.8556	7.3217	7.5612	7.4735	7.0251
Atp1a2	_DTAGDASESALLK_.2	#N/A	7.9244	7.5483	7.6648	7.5306	7.1976	7.5658	7.2980	7.8493	7.6806	7.6144	7.3402	7.4004	7.7991	6.7520	6.6349	7.2570	7.3791	7.1253	7.5024	7.0984
Atp1a2	_LC[Carbamidomethyl (C)]FVGLM[Oxidation (M)]SM[Oxidation (M)]IDPPR_.2	#N/A	9.0656	8.4337	8.2126	7.0470	7.8798	7.8810	8.2084	8.6369	8.4574	8.4973	7.7438	8.0544	7.5703	4.0427	7.1154	8.0825	8.0075	8.7296	8.2814	7.7190
Atp1a2	_AGQENISVSK_.2	#N/A	7.5242	6.9011	7.2094	7.0631	6.5934	6.9197	7.2881	7.4350	7.3065	6.9342	6.5387	6.3673	4.4123	6.1499	6.3414	6.2937	6.9510	6.5588	6.2599	6.0494
Atp1a2	_NSVFQQGM[Oxidation (M)]K_.2	#N/A	7.8896	8.1597	8.2312	8.0493	7.7790	7.9143	8.4713	8.0451	7.5689	7.4987	7.9239	7.9501	7.4184	7.6689	7.7333	7.1745	7.7991	8.1959	7.6887	7.3905
Atp1a2	_GFKFDTDELNFPTEK_.3	#N/A	5.2259	6.4788	6.1523	6.2624	5.5126	5.3283	5.9569	4.3034	6.4601	5.9790	5.7174	6.1115	5.6811	5.4233	5.7090	5.9785	4.8179	6.3998	6.1198	6.0742
Atp1a2	_QGAIVAVTGDGVNDSPALK_.2	#N/A	8.0831	8.5737	8.1673	8.2677	7.4168	8.3949	8.5476	8.0716	8.5533	7.5414	7.7118	8.0117	7.8426	8.0125	6.8675	7.8519	8.4321	8.3749	8.0024	7.9755
Atp1a2	_DM[Oxidation (M)]TSEQLDEILR_.2	#N/A	7.2948	7.0005	7.5804	6.9935	6.5889	7.1976	6.9592	6.7413	7.2117	6.7393	6.9995	6.4309	6.3151	6.4450	6.2840	6.3194	7.0579	6.7619	6.5730	6.6089
Atp1a2	_QAADM[Oxidation (M)]ILLDDNFASIVTGVEEGR_.3	#N/A	6.7554	6.2995	7.0733	7.0438	5.8775	6.8798	6.1063	6.3325	6.3826	7.0617	6.9112	6.0609	7.1456	6.5169	6.1497	7.0506	6.6608	6.3613	6.4775	6.7895
Atp1a2	_DGPNALTPPPTTPEWVK_.2	#N/A	10.2846	10.1441	10.2113	9.9845	9.9066	9.8926	10.0697	10.1794	10.0880	9.8707	10.0832	9.6794	10.0046	9.7658	9.4615	9.5464	10.2598	9.8965	9.7116	9.5950
Atp1a2	_AIAKGVGIISEGNETVEDIAAR_.3	#N/A	6.0393	6.2783	4.4658	5.4587	4.1089	5.2680	5.4226	5.5817	5.1673	5.4768	4.9489	4.7389	4.5691	5.7931	3.6170	4.1439	5.6884	5.0861	4.5497	5.3611
Atp1a2	_QGAIVAVTGDGVNDSPALKK_.2	#N/A	5.4132	5.9907	5.8508	5.6539	4.7916	5.0771	5.5333	4.8641	5.7007	4.7987	5.1739	4.7452	3.7794	5.7862	4.3139	4.8480	5.2716	5.6452	5.2653	4.9448
Atp1a2	_NSQTDKLVNER_.2	#N/A	5.3574	5.7125	3.4291	4.2057	4.8221	5.2526	5.8985	5.4142	5.6406	5.4388	4.9839	3.9467	5.1296	5.2113	5.2418	4.4524	5.2988	5.4228	5.1875	4.6488
Atp1a2	_IISSHGC[Carbamidomethyl (C)]K_.2	#N/A	5.8215	5.9797	5.7435	5.4626	4.9308	5.6917	6.1910	5.5512	5.7395	5.5026	4.7738	5.5550	5.5089	4.9689	5.9952	4.6983	5.6000	5.9489	5.5138	4.7449
Atp1a2	_EIPLDKEM[Oxidation (M)]QDAFQNAYM[Oxidation (M)]ELGGLGER_.3	#N/A	5.0862	7.0651	5.5266	4.2045	3.9901	4.8051	4.7712	5.5028	4.5648	6.2703	4.3196	6.3690	6.4619	6.6447	5.1462	3.9819	6.3488	7.0375	6.6875	3.9348
Atp1a2	_YQVDLSK_.2	#N/A	3.3494	3.7482	4.4109	4.3645	3.2123	3.2583	5.0942	3.5750	3.4298	4.2679	3.5648	4.4771	3.8953	4.3980	4.5900	4.0636	3.1493	4.2489	4.8326	4.2972
Atp1a2	_M[Oxidation (M)]QINAEEVVVGDLVEVK_.2	#N/A	8.1153	7.7317	7.5250	7.4399	6.8279	7.1216	7.1283	7.8113	7.2100	7.3362	6.8907	7.0871	8.1535	7.2300	6.0082	7.3912	7.4428	7.4237	6.9495	7.0347
Atp1a2	_LNIPVSQVNPR_.2	#N/A	11.4284	11.4181	11.1670	11.1385	11.2092	11.2726	11.5662	11.6830	11.1919	10.9482	11.1508	11.2090	11.4628	10.8952	10.7820	10.8300	11.3503	11.3609	11.1445	10.8204
Atp1a2	_ADIGIAM[Oxidation (M)]GISGSDVSK_.2	#N/A	6.1195	6.3549	5.9602	3.1538	5.5843	5.7428	5.9089	6.0686	6.6239	4.2410	5.6360	5.2040	6.0532	5.1417	3.9369	3.7926	6.0897	6.0347	5.8237	3.6824
Atp1a2	_VAEIPFNSTNK_.2	#N/A	11.1657	11.0200	11.0933	10.7869	10.9209	10.8879	11.0860	11.3284	11.1279	10.8954	10.8092	10.7899	11.2237	10.5520	10.5400	10.4416	11.0564	10.9302	10.6823	10.7273
Atp1a2	_SPEFTHENPLETR_.2	#N/A	6.8815	6.4522	6.4706	6.1935	6.5246	6.7758	6.8869	6.8547	6.7295	6.4432	6.8579	6.2698	6.5648	5.8549	6.0312	6.2265	6.5931	6.8643	6.2774	6.0844
Atp1a2	_AGQENIS[Phospho (STY)]VSKR_.2	Q6PIE5_S439_M1_Atp1a2	7.9406	7.7785	7.6648	7.8402	7.3723	7.6943	7.6028	5.1915	7.6544	7.3465	5.4479	6.5551	7.4254	7.0228	4.9381	4.9762	5.1608	7.4388	7.2768	4.9037
Atp1a2	_GVGIISEGNETVEDIAAR_.2	#N/A	10.1217	9.8817	9.7582	9.7326	5.4433	9.3079	9.5076	9.9266	9.4388	9.4085	9.4061	9.4861	10.1440	9.7309	8.2887	9.3221	9.9707	9.4972	9.3170	9.2811
Atp1a2	_SPTWTALSR_.2	#N/A	7.8399	7.8902	7.8453	7.8172	7.6813	7.7886	8.1072	7.8590	7.5313	7.5211	7.5529	7.5820	8.1062	7.2193	7.0740	7.2060	7.3356	7.9081	7.4594	7.1711
Atp1a2	_C[Carbamidomethyl (C)]IELSC[Carbamidomethyl (C)]GS[Phospho (STY)]VRK_.2	#N/A	8.1060	8.1733	7.8704	7.8185	7.9309	8.1425	7.9942	8.0906	7.8528	7.6177	7.5042	7.4027	8.2218	7.8318	6.6351	7.5227	8.5177	7.8437	7.5488	7.4728
Atp1a2	_EDSPQSHVLVM[Oxidation (M)]K_.2	#N/A	6.5690	6.5192	7.2474	6.3071	6.4124	6.6061	6.8282	6.5259	6.4655	6.0674	6.5265	6.2789	5.3478	6.0097	5.7698	5.9544	6.6733	6.4166	5.9754	5.8949
Atp1a2	_NIC[Carbamidomethyl (C)]FFSTNC[Carbamidomethyl (C)]VEGTAR_.2	#N/A	6.2644	3.5063	3.3493	6.3462	5.3889	4.0887	5.7446	4.0384	2.4725	3.2323	5.5087	5.5302	3.8436	3.3874	6.0626	4.5038	3.5357	3.7729	3.9128	6.0382
Atp1a2	_YQVDLS[Phospho (STY)]KGLTNQR_.3	#N/A	4.3873	3.3668	3.4889	3.6319	3.7250	3.9491	3.8161	4.1780	3.8751	3.0928	4.2495	3.0929	3.9832	3.5270	3.3933	3.2314	3.6752	3.4742	3.1302	3.2043
Atp1a2	_M[Oxidation (M)]TVAHM[Oxidation (M)]WFDNQIHEADTTEDQSGATFDKR_.4	#N/A	4.3398	3.6630	3.3753	3.5844	3.6776	3.9016	3.8635	4.2255	3.9226	4.5462	4.2970	3.5524	4.0307	3.5744	3.4407	3.1839	3.7227	3.4267	3.7258	3.2517
Nfrkb	_SPS[Phospho (STY)]PAVPLR_.2	Q6PIJ4_S228_M1_Nfrkb	4.1493	3.1697	3.6859	3.4349	2.8554	4.3896	4.1927	4.3750	4.1129	7.8220	3.5915	4.3967	4.2783	3.7240	4.2180	3.5115	3.8722	3.2772	3.5762	3.4013
Rhbdf1	_QTS[Phospho (STY)]ITQTIR_.2	Q6PIX5_S76_M1_Rhbdf1	8.4252	8.0322	8.3020	4.1336	7.1816	7.3000	7.0749	7.3406	7.4281	7.5179	7.3760	3.5946	3.4815	4.5261	5.6804	6.8299	3.1735	3.9759	4.2750	6.7359
Dmxl1	_DSS[Phospho (STY)]LKLDVR_.2	Q6PNC0_S1904_M1_Dmxl1	7.1169	7.9157	7.1197	6.9922	3.7578	7.7912	7.2752	3.7766	7.5696	6.2685	6.3901	7.0593	7.3302	7.4236	6.0373	6.8269	7.5603	7.8029	4.1747	7.2516
Dmxl1	_TGS[Phospho (STY)]ESS[Phospho (STY)]LKK_.2	Q6PNC0_S1426_M2_Dmxl1	3.6172	4.9045	4.0194	3.9820	3.8241	4.5765	3.2106	3.8429	3.2077	3.5748	3.8765	3.4430	3.6481	4.0575	3.2643	3.8450	3.3401	4.7886	4.1233	3.7348
Dmxl1	_WDS[Phospho (STY)]DNDEENEDPPISMK_.2	Q6PNC0_S1965_M1_Dmxl1	3.5867	8.0457	8.4562	3.9975	3.7937	7.3961	7.8415	3.8124	6.6572	3.3973	4.1541	3.4585	3.3401	8.5392	4.9584	2.9489	3.3097	5.1933	2.0177	3.8097
Dmxl1	_ISEASWLPEEHY[Phospho (STY)]SSSPEK_.2	#N/A	3.5445	6.8292	4.2606	4.1886	5.9091	4.3247	3.8099	6.4839	3.4767	3.6357	3.8503	6.2570	6.7295	3.9890	3.5098	4.0760	3.7630	4.2641	4.0297	3.8553
Dmxl1	_GSSFLTSKDS[Phospho (STY)]S[Phospho (STY)]LKLDVR_.3	#N/A	3.7135	5.1926	4.6086	3.8182	3.8793	4.4984	4.1631	4.0924	4.3426	3.7853	3.6702	3.4840	4.2665	4.2667	3.3917	3.7662	3.7294	4.8193	4.3782	3.6944
Atp2b4	_TQDGVALEIQPLNS[Phospho (STY)]QEGLDSEDKEK_.3	#N/A	7.4466	8.6444	7.6412	7.3128	3.1116	7.9368	8.1715	7.2219	8.0592	7.5502	7.2262	6.5101	7.4118	8.2699	6.9593	7.0227	7.6971	8.4984	7.4949	7.1245
Atp2b4	_GIIDSTAGEQR_.2	#N/A	6.2486	5.9295	5.5703	5.5717	5.7527	6.5027	6.8495	5.9535	6.1917	5.7830	5.9680	5.7510	5.0629	5.4145	5.7272	6.2786	5.8453	6.4392	5.7331	5.8558
Atp2b4	_EAGHGS[Phospho (STY)]DKEDISR_.3	Q6Q477_S1065_M1_Atp2b4	3.7683	4.2345	4.3046	3.9211	3.1060	3.3781	4.4350	3.4687	5.6027	4.4492	5.6939	4.4217	3.7890	4.2917	4.0122	4.1132	4.0064	5.7289	3.4754	4.2587
Atp2b4	_TQDGVALEIQPLNS[Phospho (STY)]QEGLDSEDKEKK_.3	#N/A	6.0105	6.9854	6.5958	6.1242	4.0703	6.3058	6.4637	5.8276	6.2562	5.9188	5.5899	5.3700	6.2836	6.4566	5.3640	5.4809	5.9183	6.5995	6.6253	5.7799
Atp2b4	_DDFLC[Carbamidomethyl (C)]LEGK_.2	#N/A	3.9579	4.5955	3.4052	3.3196	4.2441	4.2774	4.3048	4.0702	4.2250	4.5945	3.7037	3.5225	4.3905	3.3923	2.8294	3.3993	3.1070	1.2950	4.3748	3.3593
Atp2b4	_NEKGEVEQEKLDK_.3	#N/A	4.1269	4.6072	3.7493	3.3715	3.4646	3.6887	4.0765	4.4384	4.1356	3.7280	3.0091	4.3333	4.2436	3.7874	3.6537	2.9709	3.9357	3.2138	3.3836	3.4647
Rictor	_NDS[Phospho (STY)]GEENVPLDLTR_.2	#N/A	3.8501	9.2287	9.3757	8.2345	3.1878	9.1357	8.8526	3.5505	8.7314	8.6622	3.1840	8.0712	3.8708	8.7353	7.0653	8.0340	8.9300	8.9239	8.7801	8.3968
Rictor	_GRNDS[Phospho (STY)]GEENVPLDLTR_.3	#N/A	3.5408	5.3130	3.9015	4.3241	3.7478	5.3890	5.0266	3.0656	3.2205	3.3514	4.2000	3.0262	3.3860	3.8886	3.3257	5.0006	3.6033	4.2299	3.8784	3.8556
Camk2n1	_[Acetyl (Protein N-term)]S[Phospho (STY)]EVLPYGDEK_.2	Q6QWF9_S2_M1_Camk2n1	4.5781	3.4247	3.6135	7.0235	3.9158	6.7136	3.0123	4.2785	7.0489	3.6394	3.9120	3.3142	4.5988	7.8435	3.2025	7.0409	3.3153	7.3526	7.5634	7.9595
Camk2n1	_LSPYGDGGDVGQIFSC[Carbamidomethyl (C)]R_.2	#N/A	3.5218	3.7973	4.5916	4.0625	3.7287	3.7620	3.3194	3.7474	4.8221	3.3324	4.5902	3.8694	3.4050	4.5972	3.9037	4.3847	3.2447	4.2108	4.2165	4.2044
Camk2n1	_LQDTNNFFGAGQSK_.2	#N/A	4.2359	3.7669	5.2520	4.4535	3.5736	4.4113	3.9675	3.9362	5.3166	3.9817	5.8760	5.8235	4.2566	3.2584	5.7112	3.0799	4.4739	3.3592	5.8653	3.2254
Gnas	_ISTAS[Phospho (STY)]GDGR_.2	Q6R0H7_S1091_M1_Gnas	3.8950	3.4708	3.5991	3.9895	3.9014	4.1255	3.6713	4.2640	8.6649	4.1013	4.1048	3.3602	3.8385	3.3822	3.2485	3.4077	3.3009	3.6506	3.9496	3.8917
Gnas	_ALWEDEGVR_.2	#N/A	7.0338	6.5484	6.3990	6.4350	6.4777	6.0550	6.4235	6.6061	6.4568	6.1693	6.3240	6.4468	6.5018	3.9737	5.9768	6.3875	6.0892	6.1028	6.3837	6.1206
Gnas	_VLTSGIFETK_.2	#N/A	4.4176	5.4751	3.1343	4.0697	3.4365	5.1819	5.0383	4.8433	5.1735	5.3533	4.9255	5.3837	4.2717	5.2584	3.6817	4.8586	5.1884	5.1883	4.4870	4.7778
Gnas	_EAAEM[Oxidation (M)]EGS[Phospho (STY)]PTTATAVEGK_.2	Q6R0H7_S367_M1_Gnas	4.4625	4.0583	4.4808	3.7449	3.1686	3.2019	3.8795	3.6449	3.7998	4.2730	3.2784	3.9478	3.9652	4.4679	3.9049	3.8246	4.1825	3.6506	3.2992	4.4349
Ppp1r9b	_AQLEQSVEENKER_.2	#N/A	3.0869	4.6906	3.8256	3.9277	4.1478	4.5367	2.7226	3.5857	2.1566	3.7979	3.7624	4.0369	3.2776	3.8915	4.7488	3.3029	3.8967	3.6891	4.2989	4.1544
Ppp1r9b	_ERPGEQSEVAQLIQQTLEQER_.3	#N/A	5.2515	5.0128	4.1326	4.4601	3.2867	4.8710	3.1439	5.4697	4.5258	4.8934	3.5773	4.3336	5.6218	5.7085	3.8133	5.1020	5.4450	5.1671	4.9771	4.9137
Ppp1r9b	_ASS[Phospho (STY)]LNENVDHSALLK_.2	#N/A	9.6501	9.5816	9.9905	9.8656	8.7012	8.3766	8.7845	8.3506	8.2630	10.1026	8.7814	8.8479	9.0956	9.5940	7.3687	9.1113	8.5000	8.7498	8.9569	9.1407
Ppp1r9b	_TVTEGGAAHR_.2	#N/A	4.4959	1.3836	2.9362	3.1934	2.3985	2.9188	2.9346	4.1963	2.8468	3.7426	3.9469	3.3963	3.3518	3.2315	4.5391	3.2731	3.2332	2.7596	4.2486	2.0497
Ppp1r9b	_ETQAQYQALER_.2	#N/A	3.3411	3.1608	3.8774	5.9087	6.1743	6.5882	6.3649	4.5423	3.4205	6.2301	3.7949	3.0503	6.6842	6.8134	6.7868	3.6860	6.4337	4.2540	3.9026	6.4050
Magi1	_KDS[Phospho (STY)]QNSSQHSVSSHR_.3	Q6RHR9_S733_M1_Magi1	6.1298	6.3246	4.2732	6.5563	4.9046	5.8906	6.1007	5.1945	5.9538	6.9430	6.0036	3.8095	6.0361	6.3959	3.6308	5.3116	5.5534	5.8144	5.1149	5.7476
Magi1	_SLHTASPSHGIQVLPEY[Phospho (STY)]LPADAPAPDQTDSSGQK_.4	Q6RHR9_Y762_M1_Magi1	3.6833	2.3514	3.7591	4.4666	3.8902	3.9235	3.1579	4.4240	3.0781	3.4939	4.0576	3.1686	3.2435	3.7462	3.1833	3.0454	3.4609	4.3723	4.0209	3.7132
Sdk2	_S[Phospho (STY)]PPRPSPGSLHYS[Phospho (STY)]DEDVTK_.3	#N/A	3.3709	3.7446	4.2101	4.3108	3.7843	5.0668	3.3680	4.0874	3.4470	3.8730	3.7581	3.3375	3.3990	3.7893	3.3554	3.4404	3.8135	4.1686	3.7865	3.5627
Ranbp10	_SQDSYPGSPSLS[Phospho (STY)]PR_.2	Q6VN19_S369_M1_Ranbp10	8.0392	7.0916	7.3446	7.1879	3.1286	7.2990	6.9087	7.4549	3.9821	6.8888	7.0629	7.1729	4.1246	7.5738	3.9613	3.7846	7.7804	8.0955	7.1973	7.2769
Wdfy3	_LKISS[Phospho (STY)]PVR_.2	Q6VNB8_S3483_M1_Wdfy3	4.3704	3.6324	3.4059	3.3190	3.7081	4.2768	7.2519	4.0708	4.2257	7.0220	6.7508	7.2423	4.3911	3.3929	6.1253	6.5408	3.1076	7.3408	6.9870	3.3599
Wdfy3	_SQS[Phospho (STY)]EYC[Carbamidomethyl (C)]NVGTK_.2	Q6VNB8_S1942_M1_Wdfy3	5.7624	5.8380	5.9156	5.4715	3.7848	5.9270	3.7563	4.1474	5.6923	3.1525	4.8622	3.4452	4.4678	4.4454	3.3335	3.2911	5.2577	5.2935	5.3269	4.6589
Ntrk3	_VFLAEC[Carbamidomethyl (C)]YNLS[Phospho (STY)]PTKDK_.2	#N/A	6.2270	6.2158	5.8431	5.9035	3.0451	6.2632	3.8933	6.2677	3.9524	3.9137	3.4346	4.1491	6.4001	4.1257	5.3083	4.0523	4.0721	6.2720	3.4254	5.8258
Ntrk3	_VFLAEC[Carbamidomethyl (C)]YNLS[Phospho (STY)]PTK_.2	#N/A	4.4590	4.3609	3.9956	3.1252	3.1651	3.1985	3.8830	3.1839	3.8032	4.2696	3.2818	4.0870	3.9686	4.0337	3.9084	3.8211	4.1819	3.6472	3.2958	4.4383
Npr2	_GSS[Phospho (STY)]YGSLM[Oxidation (M)]TAHGK_.2	Q6VVW5_S523_M1_Npr2	3.4614	4.5414	3.9977	4.2279	4.3000	3.6849	5.4827	7.0512	6.8318	6.0671	4.2962	2.9300	6.2102	3.9848	3.4219	6.2733	3.6995	4.1337	3.7822	3.9518
Pcp4l1	_TPPAANQAS[Phospho (STY)]DPEEK_.2	#N/A	9.1284	9.4205	9.5145	9.3634	9.3459	9.0115	8.7783	9.3676	9.0582	9.4568	9.2047	9.1515	9.5803	9.7054	9.7470	9.6956	9.2828	9.2028	9.5076	9.5595
Pcp4l1	_[Acetyl (Protein N-term)]SELNTKT[Phospho (STY)]PPAANQASDPEEK_.2	#N/A	3.1448	3.6251	4.8352	3.8903	3.9834	6.0869	3.5577	6.9269	3.6168	3.3511	5.7363	6.4248	3.7248	7.0305	6.5895	6.6001	3.4169	3.7326	4.0316	5.8303
Pcp4l1	_TPPAANQAS[Phospho (STY)]DPEEKGKPGSIK_.3	#N/A	3.5466	6.8741	4.0829	4.1428	2.8843	5.5325	3.1559	3.2470	5.3279	3.1701	4.3814	6.6650	3.5673	6.3851	7.2276	6.5854	3.7847	6.3345	6.6453	6.2157
Pcp4l1	_KAEEEEEIDIDLTAPETEK_.3	#N/A	4.5278	3.5073	3.3484	3.7724	3.8655	4.0896	3.6755	4.0375	3.7346	3.2333	4.1090	3.2334	3.8427	3.3864	3.2527	3.3719	3.5347	3.6147	4.8624	3.0637
Dagla	_TSTPTGHGAS[Phospho (STY)]PTKQDDLVISAR_.3	Q6WQJ1_S1032_M1_Dagla	6.8564	4.2426	3.5296	5.5265	5.6190	3.0051	4.0764	5.9070	3.9966	4.0763	3.4752	4.0334	4.1620	3.1638	3.8825	5.5269	4.7871	3.4538	3.1024	3.1308
Dagla	_GS[Phospho (STY)]PSLHAVLER_.2	#N/A	2.5365	3.8804	7.0947	3.9543	3.6247	3.7461	3.3658	3.8573	3.4249	3.6239	3.7993	3.6701	3.5330	3.8434	3.7097	3.7113	3.9041	4.0053	3.5702	3.5207
Dagla	_HSQDTQPLEAALGSGGVTPERPPS[Phospho (STY)]AT[Phospho (STY)]IEEEEAAGGSEGGGVAPR_.4	Q6WQJ1_S874_M2_Dagla	4.1837	3.1354	3.7203	4.2555	2.8898	5.0927	5.9213	4.4094	4.1065	4.3623	2.9800	4.3624	4.2145	3.7583	3.4525	3.5458	3.9066	3.2429	3.7976	3.4356
Kctd12	_DLQLVLPDYFPER_.2	#N/A	6.0907	6.1994	6.9393	6.6054	5.4012	6.4527	6.2841	5.9088	5.9145	6.4110	6.0197	6.3070	6.6612	6.6357	6.4863	6.5891	6.3361	6.3913	6.2813	6.1772
Kctd12	_EAEYFELPELVR_.2	#N/A	6.0998	6.0674	6.4317	6.3068	5.9403	5.9226	6.1556	5.6739	6.4497	6.1622	5.9946	5.6752	5.9361	5.4573	6.0562	6.4971	5.4930	5.8739	6.0249	6.0592
Kctd12	_YILDYLR_.2	#N/A	3.5607	5.7228	4.9417	5.3879	5.0267	5.7025	5.3330	4.7826	5.5354	5.8486	5.3483	5.1338	3.3395	4.4877	4.5474	5.1251	4.3274	5.3830	5.5349	5.3251
Kctd12	_S[Phospho (STY)]PSGGAAGPLLTPSQSLDGSR_.2	#N/A	8.6524	8.7685	4.0432	7.8430	6.8957	7.6145	5.2916	8.2678	6.8715	3.7766	3.8098	8.1512	9.0219	9.0124	6.7985	7.7522	7.6855	3.5473	7.9292	7.2573
Kctd12	_SPSGGAAGPLLT[Phospho (STY)]PS[Phospho (STY)]QSLDGSRR_.3	#N/A	4.0879	6.1486	6.1662	2.8914	3.2310	3.2643	3.8508	3.7898	5.7064	6.3037	3.2496	4.0548	3.9364	6.6356	5.0888	6.7516	5.7083	6.1287	6.7568	6.2710
Kctd12	_MFTQQQPQELAR_.2	#N/A	4.1257	3.1934	3.6623	3.4585	4.3327	4.3660	4.2163	4.3514	4.1365	3.9363	3.6151	4.4203	4.3019	3.7004	4.2416	3.4878	3.8486	3.3008	3.5999	3.3777
Tacc1	_EADEQLVAEVIEKC[Carbamidomethyl (C)]S[Phospho (STY)]PDTC[Carbamidomethyl (C)]SR_.3	Q6Y685_S111_M1_Tacc1	4.1284	6.8287	3.6650	3.4558	4.3353	5.4727	6.3147	4.3541	4.0512	4.4176	5.4558	4.4177	6.7040	7.0799	6.1029	3.4905	3.8513	6.2538	3.5972	5.5057
Tacc1	_KYEAQPLDLDAC[Carbamidomethyl (C)]S[Phospho (STY)]QDEGAVISK_.3	Q6Y685_S480_M1_Tacc1	3.4218	5.5579	4.4544	4.1672	4.2603	5.1968	4.8718	3.6427	5.0268	3.6281	3.7142	3.6282	3.4479	6.1193	4.1290	4.8244	3.1399	5.0427	4.3850	4.1698
Tacc1	_ESC[Carbamidomethyl (C)]DSS[Phospho (STY)]PGLAEPEAKPQESR_.3	Q6Y685_S82_M1_Tacc1	4.1169	4.5972	3.1523	3.3615	3.4546	3.6787	4.0865	4.4484	4.1456	4.3232	3.0191	3.7754	4.2536	3.7974	3.6637	2.9609	3.9457	3.2038	3.5028	3.4747
Gigyf2	_SQS[Phospho (STY)]WEER_.2	#N/A	8.9568	9.1542	8.8267	3.9246	8.6421	8.7379	3.6998	8.8295	3.6200	9.1725	8.7042	8.7391	3.7855	9.3388	3.7252	4.0043	8.9119	8.7209	8.6421	4.2551
Gigyf2	_WRPHS[Phospho (STY)]PDGPR_.3	#N/A	8.6157	8.7336	8.6808	8.6336	8.0917	8.2480	8.2692	8.3309	8.1658	8.7813	8.2965	8.2280	7.6749	7.7267	6.9891	8.3240	8.1356	8.2616	7.5007	8.2434
Gigyf2	_S[Phospho (STY)]FDEVEGVFGR_.2	Q6Y7W8_S140_M1_Gigyf2	3.6493	6.0124	5.8869	4.5578	2.9870	5.5226	5.5348	3.3497	4.2084	3.8556	3.4867	4.2430	3.6700	6.5486	4.6795	3.9942	3.8873	5.8681	5.0786	4.1397
Gigyf2	_SQS[Phospho (STY)]WEERGDR_.2	#N/A	3.1240	7.4809	4.1614	4.4602	3.3310	3.2766	2.9877	3.3497	3.0468	3.9211	6.9585	3.9212	3.1549	4.1995	3.7425	3.9870	4.3478	6.1026	3.1007	6.9525
Gigyf2	_ALSSGGSITS[Phospho (STY)]PPLSPALPK_.2	Q6Y7W8_S26_M1_Gigyf2	4.1509	3.1682	3.6874	3.4334	3.5265	3.7506	4.0146	4.3765	4.0737	4.3951	2.9472	4.3952	4.1817	8.8410	3.5918	3.0328	3.8738	6.6488	3.5747	3.4028
Gigyf2	_QQQQQQQQQQHQQSNR_.3	#N/A	3.2634	3.7437	4.6128	4.0088	4.1020	4.3261	3.4391	3.8011	3.4982	3.4697	3.8726	3.4698	3.6063	3.1500	3.0163	3.6083	3.2983	3.8512	4.1502	4.3281
Sbf1	_GS[Phospho (STY)]GEGQGQILQR_.2	Q6ZPE2_S20_M1_Sbf1	8.9076	8.4709	8.7810	8.7081	8.3996	8.3030	8.2771	8.8805	8.6180	8.3936	8.2806	8.4399	9.0630	8.3373	7.3613	7.9801	8.8742	8.5121	8.3562	8.2357
Sbf1	_DKGPS[Phospho (STY)]FR_.2	Q6ZPE2_S1047_M1_Sbf1	8.9042	8.6337	8.2753	8.5816	7.8597	7.5178	8.3423	7.7876	7.1556	9.2150	7.7917	7.7162	8.3170	7.7015	5.4627	7.2059	7.6409	8.1575	7.4810	7.3215
Sbf1	_ALYLEPSDGVS[Phospho (STY)]PTQETGEAQSQDDER_.3	Q6ZPE2_S706_M1_Sbf1	3.7824	3.5367	3.3190	3.8018	3.9894	4.1190	3.6461	4.0081	3.7052	3.2627	4.0796	3.2628	3.8133	6.5826	3.2233	3.1445	3.5053	3.6441	3.9432	3.0343
Sbf1	_C[Carbamidomethyl (C)]SGPHINS[Phospho (STY)]AR_.2	Q6ZPE2_S559_M1_Sbf1	3.2592	3.2428	2.8380	4.4302	4.0977	3.8872	3.4434	4.4604	3.1144	3.4655	4.0939	3.8267	3.2799	3.7825	3.1529	3.9302	3.4972	5.1483	3.9845	4.6232
Tiam2	_TSSS[Phospho (STY)]SEWPSEPSK_.2	Q6ZPF3_S1524_M1_Tiam2	8.8303	8.4941	9.1846	3.8463	3.2862	3.7040	3.7417	3.8810	7.7480	3.9495	3.8239	4.0256	3.8750	3.8891	3.5252	3.5582	3.8050	3.7275	3.6603	3.7394
Tiam2	_QGS[Phospho (STY)]PTKDIEIQFQR_.2	#N/A	8.5929	3.8998	8.5157	7.1356	3.5353	8.2252	7.9965	3.7335	8.5759	3.6695	3.5801	3.7060	8.2161	8.5633	7.1806	3.7371	3.8929	8.0866	8.0305	8.6837
Tiam2	_HLS[Phospho (STY)]DADR_.2	Q6ZPF3_S1116_M1_Tiam2	4.0860	3.7831	3.0409	3.7234	4.0545	4.1702	4.5619	3.0581	3.8424	4.3660	4.5725	4.5467	4.6547	4.8202	3.4164	4.2807	4.4614	4.9467	4.1450	4.7951
Tiam2	_ESDILSDEDEDFHHPLK_.3	#N/A	4.9966	10.2134	9.5355	5.2494	4.5880	10.0251	9.8655	3.8014	9.5282	4.7894	5.3016	4.5996	4.1740	10.4834	8.4710	4.9645	4.9905	10.0266	9.7026	5.1708

Tiam2	_HQS[Phospho (STY)]LDSHPETASIDLNLVLER_.3	Q6ZPF3_S1672_M1_Tiam2	4.5253	3.3887	3.3509	3.7699	3.8630	4.0871	3.6781	4.0400	3.7372	3.2307	4.1116	3.2309	3.8452	3.3890	3.2553	3.3693	3.5373	3.6122	3.9112	3.0663
Tex2	_TAPSS[Phospho (STY)]PLT[Phospho (STY)]SPSDTR_.2	Q6ZPJ0_T268_M2_Tex2	7.6776	8.2028	8.8274	8.6667	6.4568	6.4627	6.6404	7.5874	7.2907	8.5766	7.8369	3.7983	7.4702	6.4080	5.9256	6.4822	7.2293	4.1554	7.5856	3.8244
Tex2	_SLS[Phospho (STY)]TEVEPK_.2	Q6ZPJ0_S195_M1_Tex2	4.4333	3.5695	3.4688	3.2561	3.7710	3.9951	3.7701	4.1337	3.8291	5.8318	4.2035	3.4590	3.9372	3.4810	3.3472	3.5786	3.1705	3.5202	4.9747	3.1582
Ube2o	_KEEKPEVQS[Phospho (STY)]PVK_.2	Q6ZPJ3_S893_M1_Ube2o	6.5653	6.0852	7.0024	5.3956	6.0664	5.9595	6.1783	6.5246	5.8605	6.2355	6.4361	6.1005	5.3025	6.7873	4.9042	5.8706	7.0502	6.7737	6.2552	6.3935
Ube2o	_LIHGEDS[Phospho (STY)]DS[Phospho (STY)]EGDDDGR_.2	#N/A	6.3381	6.4160	5.8498	6.2789	5.8010	6.0854	5.9588	6.4133	5.8252	6.0664	6.4438	5.6410	3.6873	5.8529	4.8162	5.7061	6.3715	5.8083	5.6933	5.7067
Ube2o	_DSSSLEPMAAAELS[Phospho (STY)]DSGR_.2	Q6ZPJ3_S1175_M1_Ube2o	4.0739	4.6682	5.6812	3.5104	4.2808	3.8276	4.2681	4.2995	3.9967	4.4721	4.3711	2.9713	4.1047	6.1502	2.7927	3.4360	3.7968	3.3527	3.6517	3.3258
Ube2o	_DHS[Phospho (STY)]M[Oxidation (M)]EDPDKKGEAR_.2	Q6ZPJ3_S406_M1_Ube2o	3.4541	5.0700	4.9310	4.7912	4.2927	4.4197	4.6848	3.1545	3.3075	4.1601	4.4078	2.9373	3.4749	5.1086	2.8256	3.7990	3.6922	4.1410	3.7896	3.6612
Ube2o	_S[Phospho (STY)]IPLSIK_.2	Q6ZPJ3_S510_M1_Ube2o	4.0086	3.3105	3.5452	3.5756	4.2156	3.8928	4.3334	4.2343	4.2536	3.8192	3.7322	3.0366	4.0395	3.5833	2.8579	3.3707	3.7315	3.4179	3.7170	3.2605
Ube2o	_NM[Oxidation (M)]TVEQLLTGSPTS[Phospho (STY)]PTVEPEKPTR_.3	Q6ZPJ3_S836_M1_Ube2o	3.9410	5.1766	4.2335	3.8708	3.2660	2.9647	3.9657	5.3486	3.8121	4.2939	3.3061	4.8625	4.9071	5.4859	3.8274	4.0523	4.2439	3.7325	3.2211	3.5631
Ube2o	_NC[Carbamidomethyl (C)]AQGEGS[Phospho (STY)]M[Oxidation (M)]AKK_.2	Q6ZPJ3_S376_M1_Ube2o	3.4069	3.9122	4.4443	3.1687	3.6138	3.6472	3.4343	3.6326	3.3545	3.2175	4.3339	3.6383	3.4378	4.4824	3.4596	4.2699	3.1298	4.0196	4.3187	4.1597
Zfyve28	_LKSGS[Phospho (STY)]DEEER_.2	Q6ZPK7_S394_M1_Zfyve28	3.5295	3.4153	3.5508	4.1784	4.0226	3.9883	3.2896	4.2261	3.9297	3.5586	3.9943	3.6833	3.1803	3.6251	3.0734	3.3667	3.4807	3.8511	4.0856	3.6499
Ddx55	_TVQLTDLGVS[Phospho (STY)]DLEEDS_.2	Q6ZPL9_S594_M1_Ddx55	3.5135	3.8056	4.5509	4.0707	3.7204	4.3879	3.3772	3.7392	3.4363	3.5316	3.8107	3.5317	3.5444	4.5890	2.9544	4.3765	3.2364	3.9130	4.2121	4.2663
Pitpnm2	_KQWS[Phospho (STY)]TSSK_.2	Q6ZPQ6_S302_M1_Pitpnm2	8.4423	8.4482	8.1741	4.1005	7.6376	8.0601	3.3575	8.2637	7.7959	3.2943	7.9696	7.8114	8.1346	4.5592	3.3828	7.8316	8.0818	3.9428	4.2419	7.4205
Pitpnm2	_GAS[Phospho (STY)]PSRHS[Phospho (STY)]ISEWR_.3	#N/A	8.3417	7.5682	8.6136	8.7048	6.4821	7.6204	7.5801	6.6984	7.2629	7.9912	7.6548	6.5764	8.1065	7.8975	5.9640	7.3694	7.6388	7.4268	7.5413	7.2671
Pitpnm2	_HS[Phospho (STY)]ISEWR_.2	Q6ZPQ6_S321_M1_Pitpnm2	4.1063	4.7059	3.3361	4.7883	3.6219	5.5761	4.2357	4.1606	4.2126	3.9168	3.6346	3.9304	4.3214	4.4231	4.2611	3.4684	4.5387	3.2944	4.4265	3.9349
Pitpnm2	_TDTGENNNVFNLS[Phospho (STY)]PVEK_.2	Q6ZPQ6_S129_M1_Pitpnm2	7.2033	7.2534	6.5280	6.9429	3.6840	7.0983	6.8796	7.1210	6.8749	6.2479	6.7236	6.3886	3.5079	7.4443	6.2446	6.7830	7.4276	6.7768	6.6870	6.7985
Pitpnm2	_WNS[Phospho (STY)]NDLMDK_.2	Q6ZPQ6_S365_M1_Pitpnm2	4.4794	6.6072	7.1056	6.9607	3.8171	4.9709	5.6158	4.1797	3.7831	3.1849	6.4324	3.4129	3.8911	6.3548	3.3012	3.3234	3.2166	3.5663	3.7183	3.1122
Pitpnm2	_RGAS[Phospho (STY)]PSRHS[Phospho (STY)]ISEWR_.3	#N/A	3.6809	3.2768	3.7614	8.4233	3.8879	7.5888	3.1602	7.3886	7.0327	3.4915	4.0599	3.1663	3.2459	7.3618	3.1856	7.3662	3.4632	7.6130	7.3852	7.3550
Pitpnm2	_S[Phospho (STY)]NIDIPR_.2	Q6ZPQ6_S630_M1_Pitpnm2	3.7060	3.9924	4.3641	4.6038	3.1575	4.3578	5.1382	3.2798	4.0969	3.1373	2.9133	3.2339	3.6001	4.1028	3.5399	4.1896	3.8175	4.2762	4.6689	4.5621
Pitpnm2	_QWSTSSKSS[Phospho (STY)]R_.2	Q6ZPQ6_S308_M1_Pitpnm2	4.1084	3.2107	3.6450	3.4758	4.3154	4.3487	4.2336	4.3341	4.1538	3.9190	3.6324	2.9368	4.3192	3.3210	4.2590	3.4705	3.8313	3.2966	2.9452	3.2880
Kcnt1	_GGGSVGS[Phospho (STY)]DVGQR_.2	Q6ZPR4_S47_M1_Kcnt1	4.3736	7.7685	7.2774	3.2111	3.0797	3.1130	7.3069	3.0984	3.8891	7.2059	6.9864	4.1729	4.0545	6.5892	5.3507	3.7357	6.9930	3.5618	3.3082	6.6017
Ankib1	_GVAPADS[Phospho (STY)]PDAPR_.2	#N/A	9.9671	10.0510	9.9891	10.0121	9.8457	10.0146	9.6269	10.1197	9.7489	10.0230	9.6948	9.7106	10.2954	10.1617	9.4462	9.9352	9.9066	9.7427	9.7524	9.9633
Ankib1	_GVAPADS[Phospho (STY)]PDAPRR_.2	#N/A	8.9574	8.8801	8.6655	8.8713	8.6296	8.6276	8.6547	9.0686	8.6926	8.9197	8.5955	8.6453	9.1432	8.9996	8.1706	8.7523	8.8193	8.7625	8.7100	8.7993
Klhl9	_AC[Carbamidomethyl (C)]TLTVFPPEENPGS[Phospho (STY)]PSR_.2	Q6ZPT1_S603_M1_Klhl9	7.9807	6.7737	7.5864	7.8988	4.0643	8.6561	7.3995	4.2499	7.2310	3.6680	8.3455	8.0826	4.5703	8.4781	6.9030	7.9033	8.1610	3.0455	7.6185	7.6083
Zc3h4	_TGTGS[Phospho (STY)]PFAGNS[Phospho (STY)]PAR_.2	Q6ZPZ3_S1270_M2_Zc3h4	8.1544	7.9943	7.9772	8.1334	7.2286	7.8756	7.7612	7.9436	7.6743	8.1210	7.8657	7.7417	7.3989	8.2898	4.9465	7.2274	8.1763	7.5946	7.5334	7.7940
Efr3b	_S[Phospho (STY)]PSPLQAPEK_.2	Q6ZQ18_S214_M1_Efr3b	3.6398	3.7191	3.9954	3.9842	7.8727	3.6195	8.3360	3.8655	3.5626	8.2406	8.3972	3.4452	3.6707	4.0334	3.2665	3.8209	3.3627	3.8265	4.1255	3.7107
Cand1	_SVILEAFSSPSEEVK_.2	#N/A	6.5375	6.7887	7.4085	7.1469	6.3900	6.8940	6.6859	6.4702	6.2476	7.8023	6.5157	6.1742	6.5000	6.3690	5.4834	5.7165	5.8968	6.4548	5.8453	5.9825
Cand1	_LTLIDPETLLPR_.2	#N/A	6.6717	6.3385	5.8945	6.4583	6.4076	6.4957	6.4027	6.5269	6.5661	6.1582	6.3047	5.8139	6.5043	5.4881	4.7475	5.0782	6.2829	6.3554	5.7404	5.8817
Cand1	_AVAALLTIPEAEK_.2	#N/A	6.7577	6.5038	6.6690	6.1870	5.9059	6.2031	6.2574	6.0862	6.1130	5.7835	5.6156	5.6907	5.3734	5.0796	5.2646	5.7557	6.4189	6.0986	5.7859	5.7358
Cand1	_EGPAVVGQFIQDVK_.2	#N/A	6.2209	6.1960	6.0146	6.3045	5.9252	5.8645	6.1695	6.0042	5.8957	6.1363	6.1971	5.6048	5.6256	5.7911	4.5222	4.9083	6.3438	5.9200	5.6085	5.8829
Cand1	_ITSEALLVTQQLVK_.2	#N/A	5.9729	5.6087	5.7760	5.3889	5.2898	5.2376	5.2616	5.3461	5.3092	4.4160	4.9666	5.4534	5.7501	3.3372	3.3514	5.1199	5.3167	5.4061	4.2520	4.7561
Cand1	_[Acetyl (Protein N-term)]ASASYHISNLLEK_.2	#N/A	5.0249	4.2355	3.8751	4.7772	3.9221	3.9622	4.8218	5.5353	4.3963	3.1769	3.9719	3.9444	5.3790	4.0533	3.8895	2.9311	5.1575	4.6563	4.3145	3.9213
Cand1	_ISGSILNELIGLVR_.2	#N/A	4.5433	4.2782	3.9296	3.5217	3.9808	3.7927	3.9167	3.2669	3.5078	3.4238	3.6324	2.9926	4.1884	1.3991	4.1281	1.7796	4.0716	4.6375	3.8984	3.2155
Cand1	_DS[Phospho (STY)]SSTNLESMDTS_.2	Q6ZQ38_S1219_M1_Cand1	4.1408	3.8620	3.1763	7.6389	3.4786	3.7027	6.4573	3.8412	4.1216	5.1822	2.9951	3.7514	4.2297	3.1634	3.6397	2.9849	4.3789	3.4543	3.1028	3.1304
Cand1	_NVVAEC[Carbamidomethyl (C)]LGK_.2	#N/A	4.8304	5.2124	4.0734	4.1523	3.9250	4.8092	4.6837	4.8297	4.5919	4.9272	4.3718	4.3552	3.5578	4.0604	4.3064	3.7744	3.7751	4.8111	5.5702	4.8350
Cand1	_QEFEKQDELKR_.3	#N/A	3.6693	4.4915	3.2059	3.9149	3.8763	3.3882	3.1793	4.2308	4.2561	3.4799	3.5740	3.3759	3.7002	3.2440	4.1048	4.4672	3.3922	3.9000	4.2294	2.8789
Cand1	_HTVDDGLDIRK_.3	#N/A	3.2872	3.8765	4.4800	4.1417	3.2744	2.8136	3.7327	3.6682	3.3654	3.6026	3.7397	3.6027	3.4734	4.5180	3.4240	4.3055	3.1655	3.9840	3.3856	2.5019
Cand1	_DLLDSVLPHLYNETK_.3	#N/A	3.8230	3.4961	3.3596	3.7612	4.0299	1.8278	2.7231	4.0487	1.5613	3.6336	3.9178	3.2222	3.8539	1.8525	3.0435	3.1851	3.5459	3.6035	2.2313	3.0749
Cand1	_QSYYSIAK_.2	#N/A	3.1562	4.2529	4.2862	3.9395	3.3631	3.6060	4.0223	3.4503	4.2257	4.4676	3.0838	3.8890	3.7706	4.2733	3.7104	4.0191	3.9880	3.8616	3.4938	4.2403
Cand1	_HC[Carbamidomethyl (C)]EC[Carbamidomethyl (C)]AEEGTR_.2	#N/A	4.4098	0.8981	3.9464	3.1744	3.1159	3.4916	3.9322	3.1346	4.3326	4.1361	3.2062	4.1362	4.4407	3.9845	3.9576	3.7719	4.1327	3.0167	3.3158	3.6617
Larp1	_GLSAS[Phospho (STY)]LPDLDSESWIEVK_.2	Q6ZQ58_S525_M1_Larp1	7.3314	7.6477	6.9022	7.1011	5.9012	6.7421	7.5721	7.2312	7.1452	7.0599	6.3319	3.8285	7.6394	3.7971	3.6712	6.8641	6.8742	7.5304	6.8101	6.9871
Larp1	_S[Phospho (STY)]LPTTVPESPNYR_.2	Q6ZQ58_S743_M1_Larp1	11.6598	11.8994	11.3364	11.4093	10.7355	11.1799	11.3408	10.8660	11.2070	11.4450	10.9815	10.9631	11.4197	11.2397	9.6485	11.0943	11.1226	10.8140	10.8526	10.8896
Larp1	_ETESAPGS[Phospho (STY)]PR_.2	Q6ZQ58_S498_M1_Larp1	9.6479	9.7698	9.8386	9.8384	9.4723	9.4886	9.3696	9.6186	9.5222	9.6087	9.4110	9.4240	9.8219	9.7912	8.9370	9.6201	9.4991	8.9753	9.2758	9.4150
Larp1	_ESPRPPAAAEAPAGS[Phospho (STY)]DGEDGGRR_.3	#N/A	7.1597	7.1821	7.0310	7.0974	6.8859	7.2288	6.3233	7.0206	6.8008	6.8376	6.8314	6.8686	6.9104	7.2453	6.5894	6.8528	7.0876	6.9587	6.7076	6.8122
Larp1	_NTFTAWSEEDS[Phospho (STY)]DYEIDDRDVNK_.3	Q6ZQ58_S608_M1_Larp1	3.7568	4.2523	4.2931	3.9389	3.0945	3.4608	3.2424	3.3253	3.7720	4.1896	3.2579	4.1418	3.8967	4.1658	4.0301	4.1017	4.1781	3.5007	3.3069	3.9502
Larp1	_TASISSS[Phospho (STY)]PSEGTPAVGSYGC[Carbamidomethyl (C)]TPQSLPK_.3	Q6ZQ58_S828_M1_Larp1	3.3467	3.1553	3.8830	4.3427	4.1853	4.4093	5.7432	3.0471	3.4149	3.5530	3.7893	3.0447	3.5230	3.8701	2.9330	3.6916	3.5848	3.9344	4.5570	4.2448
Larp1	_QHYQKET[Phospho (STY)]ESAPGS[Phospho (STY)]PR_.2	Q6ZQ58_T492_M2_Larp1	4.5557	3.4471	3.5912	3.1337	3.8935	4.1175	3.6476	4.2561	3.7067	3.2612	4.0811	3.3365	4.5764	3.5783	3.2248	3.3998	3.2929	3.0394	4.1888	3.5453
Larp1	_KNTFTAWS[Phospho (STY)]EEDSDY[Phospho (STY)]EIDDRDVNK_.3	#N/A	4.0643	4.3829	3.9855	4.0207	3.0611	3.1958	4.2484	4.0472	3.5508	4.0490	3.4001	4.1090	4.3811	4.0236	4.0355	3.9086	4.1718	3.7234	3.2886	3.7008
Arhgap26	_DSQSEGTAQLDSIGFSIIR_.2	#N/A	3.8170	3.6308	4.1349	3.8227	3.1548	3.7667	4.3863	4.2192	4.4454	3.2005	3.3189	2.0043	4.2941	4.3205	3.9635	2.2462	4.2455	3.0118	3.2030	3.8367
Kdm1a	_NGY[Phospho (STY)]S[Phospho (STY)]C[Carbamidomethyl (C)]VPVALAEGLDIK_.3	Q6ZQ88_S573_M2_Kdm1a	5.7838	3.4753	5.7233	6.3719	3.8336	4.9500	5.1754	4.0694	3.7666	5.3839	5.6330	3.2015	3.8746	6.3082	3.2847	3.3399	3.5667	5.4900	6.3389	3.0957
Kdm1a	_QATPGVPAQQS[Phospho (STY)]PSM_.2	Q6ZQ88_S850_M1_Kdm1a	3.9052	3.4139	3.4418	3.6790	4.1121	3.9962	4.4368	4.1309	3.8280	3.7158	4.2024	3.1400	3.9361	3.4798	2.9613	3.2673	3.6281	3.5213	3.8204	3.1571
Usp34	_DSSIIDPGTEQDLPS[Phospho (STY)]PENSSVK_.2	Q6ZQ93_S3443_M1_Usp34	6.3226	6.2092	6.4742	5.7564	4.2349	4.4590	3.3062	3.6682	5.8653	3.6026	3.7397	5.4821	5.8879	6.1627	2.8834	5.6146	6.1274	3.9841	4.2831	4.1952
Usp34	_RVS[Phospho (STY)]S[Phospho (STY)]DEEHTVDSC[Carbamidomethyl (C)]IGDIK_.3	Q6ZQ93_S3395_M2_Usp34	4.4442	3.5586	3.4797	3.2452	3.7819	4.2030	4.3793	4.1446	4.2995	3.7733	3.7781	3.4480	4.4649	3.4667	2.9038	3.3248	3.1814	3.1509	4.3003	3.4337
Acap2	_FVDKYSALLS[Phospho (STY)]PSEQEKR_.3	#N/A	6.9558	7.0983	5.7041	3.6094	5.5092	6.1558	7.0826	7.9605	5.8838	6.4729	5.8444	6.7462	7.6391	7.2452	5.5616	6.2040	6.5917	6.9638	6.1759	6.3502
Acap2	_KS[Phospho (STY)]SPSTGSLDSGNESK_.2	Q6ZQK5_S378_M1_Acap2	5.3737	8.9483	4.0694	3.9253	8.3648	8.2957	3.9168	7.8328	3.9250	3.9988	8.5560	8.6910	4.0537	4.0763	4.7234	8.9690	5.3545	3.6247	3.1271	8.8094
Acap2	_YSALLS[Phospho (STY)]PSEQEK_.2	#N/A	7.8832	7.5987	7.8565	7.6188	3.5467	7.4527	7.0631	7.6525	6.8531	3.1503	7.6751	7.1795	3.5871	4.0897	3.5268	7.2377	7.9629	7.4295	7.3192	7.7186
Acap2	_SSPSTGS[Phospho (STY)]LDSGNESK_.2	#N/A	9.0361	9.4358	9.0280	4.5115	9.0064	8.9548	4.2504	8.9696	8.7011	9.2424	9.0821	8.8750	9.0024	7.8751	8.5667	8.5537	9.5193	9.3851	8.7035	8.6690
Acap2	_SSPSTGS[Phospho (STY)]LDSGNESKEK_.2	#N/A	4.2552	3.7959	4.0971	3.4825	3.5930	5.5967	3.9965	3.9556	4.0556	3.7671	3.7355	3.6854	4.1636	4.0842	3.5737	3.9057	5.7545	3.0762	3.8432	5.5823
Acap2	_FVDKYSALLS[Phospho (STY)]PSEQEK_.3	#N/A	3.5334	4.4694	4.0697	4.1559	2.8712	3.6129	5.3896	3.2338	3.3887	3.1832	4.3682	4.7192	3.5542	4.0568	3.4939	3.8783	3.7715	4.5959	3.7102	4.5693
Acap2	_ESLPSTVS[Phospho (STY)]ANSLYEPEGER_.2	Q6ZQK5_S573_M1_Acap2	3.9886	4.3343	4.2672	4.0209	3.7817	3.9485	3.6542	4.4312	3.6305	3.9430	5.3205	3.5110	3.7724	4.4477	3.5200	3.7559	3.9689	3.8524	3.5752	3.7135
Acap2	_YSALLS[Phospho (STY)]PSEQEKR_.2	#N/A	7.8404	7.6219	7.6731	7.6752	7.3331	7.5022	7.2522	7.7969	7.1844	7.5177	7.6249	7.2723	7.8303	7.7293	3.9998	7.0618	7.9864	7.5162	7.5038	3.4654
Rps9	_LIGEYGLR_.2	#N/A	6.8933	6.5795	6.2565	6.5440	6.3249	6.0347	7.0315	7.0667	6.5288	5.8393	6.3449	6.3981	5.0945	6.2663	5.4976	6.3824	6.9143	6.6734	6.4899	5.9234
Rps9	_LFEGNALLR_.2	#N/A	6.4191	6.5803	6.4100	5.7265	5.8970	6.5230	6.2643	6.2420	5.8988	5.5457	6.3980	5.4910	6.4816	5.6360	5.4399	5.3686	6.0664	6.2850	5.7701	5.7198
Rps9	_IEDFLER_.2	#N/A	4.3772	5.2523	3.2032	3.5217	3.5054	4.8009	5.8436	3.8681	5.1564	4.3740	5.3192	4.2933	4.2028	3.9232	4.7510	3.9612	4.4058	4.9764	4.0975	4.6339
Rps9	_ELLTLDEKDPR_.3	#N/A	3.8905	3.4286	3.4271	3.6937	4.0974	4.0109	4.4515	4.1162	3.8133	3.7011	4.1877	3.1547	3.9214	3.4651	1.4653	3.2526	3.6134	3.5360	2.4750	3.1424
Syne2	_SISEGHPWHVPDS[Phospho (STY)]PSHSK_.3	Q6ZWQ0_S6448_M1_Syne2	6.9500	6.8653	6.3391	6.4327	3.2670	3.3405	6.2851	3.2858	5.9716	3.9850	3.3573	3.9851	6.9567	6.0423	4.8434	6.0565	6.4593	4.3664	6.1373	5.3875
Syne2	_LTSHTPGLDDEKEAS[Phospho (STY)]ENETDIEDPR_.3	Q6ZWQ0_S6348_M1_Syne2	4.0716	3.2475	3.6081	3.5127	4.2785	4.3118	4.2704	4.2972	4.1907	3.8821	3.6693	2.9737	4.3561	3.6462	2.7950	5.6328	3.7945	3.3550	3.6540	3.3235
Syne1	_NPET[Phospho (STY)]PTELQFIEADLR_.2	Q6ZWR6_T2268_M1_Syne1	3.0857	3.5660	3.2896	3.8312	3.9243	4.4837	3.6168	3.9787	3.6759	3.2136	4.0503	3.2921	3.7839	3.3277	4.7666	3.4306	3.4760	3.6735	3.9725	3.0050
Syne1	_YLFQTGSS[Phospho (STY)]HER_.2	Q6ZWR6_S1360_M1_Syne1	8.3837	7.9917	7.4271	8.3302	4.1555	6.7669	7.1906	7.0228	6.0952	3.3998	7.5705	7.3532	7.3646	6.9841	5.3661	6.9974	7.4711	7.3194	7.0763	6.9336
Syne1	_TLPS[Phospho (STY)]EDEEGEEDKEFYLR_.3	Q6ZWR6_S8308_M1_Syne1	3.9571	3.5530	3.4937	3.2396	4.1641	4.1974	7.5820	4.1502	4.3051	3.7677	3.7837	3.0881	4.4705	7.2430	2.9094	3.3192	3.1870	3.1453	4.2947	3.4393
Rps27	_LTEGC[Carbamidomethyl (C)]SFR_.2	#N/A	5.5106	5.9995	6.6850	6.0158	6.2841	5.8829	6.8142	5.9857	5.7469	5.9406	6.0737	5.7903	4.0482	5.6332	6.0095	5.9401	5.3764	6.4352	5.9938	5.8741
Rps27	_DLLHPS[Phospho (STY)]PEEEKRK_.3	Q6ZWU9_S11_M1_Rps27	6.4409	6.8417	5.8619	6.0541	6.0214	6.1449	6.5164	6.0355	6.2098	5.8641	6.5758	6.2600	6.4999	6.8359	6.1409	6.3572	6.8957	6.5687	6.4554	6.3221
Rpl35	_VLTVINQTQK_.2	#N/A	5.8563	5.8486	6.1334	5.2413	5.5496	5.9414	5.7350	6.3002	5.4267	5.5772	5.5850	4.7916	4.8375	6.0080	5.9618	5.9592	6.0703	6.2623	6.0593	5.3682
Rpl35	_QLDDLKVELSQLR_.3	#N/A	3.9092	3.2847	3.5709	3.3168	2.9762	3.8534	3.8248	4.0730	2.9681	2.6735	4.1155	3.0109	4.3933	4.0828	2.8322	3.3965	3.1098	4.0325	3.4529	4.4036
Eif2s1	_ADIEVAC[Carbamidomethyl (C)]YGYEGIDAVK_.2	#N/A	5.8362	4.0900	4.9091	4.6941	4.8367	4.5143	4.0239	3.4547	3.1519	4.5405	3.5263	3.8161	5.6271	4.3045	3.6375	4.9347	4.4528	4.1975	2.9957	4.2050
Tmsb10	_ETIEQEKR_.2	#N/A	7.3198	7.6307	8.2885	7.6233	7.9997	7.3264	7.9325	8.1359	7.7991	7.8720	7.9915	7.8298	7.9615	7.7749	9.4378	8.3248	7.6570	7.6083	8.2910	7.8079
Tmsb10	_TET[Phospho (STY)]QEKNTLPTK_.2	Q6ZWY8_T23_M1_Tmsb10	6.4438	6.1649	6.3359	3.8895	5.5163	5.5249	5.4144	3.5003	5.6190	8.0985	6.5719	6.1823	6.3966	4.3233	3.7603	3.9691	6.2627	3.7952	6.5630	5.8971
Tmsb10	_NTLPTKETIEQEKR_.3	#N/A	3.9408	6.2543	5.2050	5.2901	5.9198	5.2399	5.3703	5.8388	5.9740	5.4198	4.7637	5.5954	7.2240	5.3263	6.8113	5.9706	4.1218	5.5045	6.0209	5.7601
Tmsb10	_[Acetyl (Protein N-term)]ADKPDM[Oxidation (M)]GEIASFDK_.2	#N/A	6.0952	6.4072	6.6787	6.7161	6.2036	6.1888	6.4260	5.5634	6.4050	5.6010	6.3333	6.4193	6.1478	7.0011	7.2317	6.8243	5.8028	5.8982	6.4286	6.1883
Tmsb10	_[Acetyl (Protein N-term)]ADKPDMGEIAS[Phospho (STY)]FDKAK_.3	Q6ZWY8_S12_M1_Tmsb10	3.9073	4.0819	4.4436	3.7820	3.2451	3.2390	3.8424	3.6077	3.7626	4.3102	3.2413	3.9849	3.9281	4.4307	3.8678	3.8617	4.1454	3.6878	3.3363	4.3977
Synm	_IDFSTPFQVEEVDDVSPS[Phospho (STY)]PK_.2	Q70IV5_S780_M1_Synm	3.5445	4.1897	3.6293	4.4047	4.0780	4.0085	3.2825	3.5506	5.5967	3.3416	4.1153	3.9851	3.5706	3.6354	4.6664	3.4442	3.5286	3.9832	4.7899	4.1817
Synm	_QFTQS[Phospho (STY)]PGAEEEATSFPDTK_.2	Q70IV5_S655_M1_Synm	3.0909	3.4110	3.6272	3.0976	3.9295	4.0554	3.0260	4.2921	4.4470	3.6258	3.9257	3.3005	3.1116	3.6143	3.0514	3.1773	3.3290	3.0033	4.1528	3.5813
Znrf2	_S[Phospho (STY)]LGGAVGSASGGR_.2	Q71FD5_S75_M1_Znrf2	8.4946	8.6409	8.7629	8.9345	8.3712	8.1628	8.1617	8.1900	8.4373	8.8820	8.4855	8.2033	8.6368	8.9963	8.1573	8.6095	8.5585	7.8050	8.3893	8.5519
Znrf2	_AYS[Phospho (STY)]GSDLPSGTGSGGGGADGAR_.2	Q71FD5_S18_M1_Znrf2	8.7579	3.4666	8.9577	3.1531	8.3829	4.0980	3.6671	4.2366	3.7262	3.2417	4.1006	8.6373	9.3021	3.3780	8.5993	8.8026	3.2735	3.6231	3.9222	3.0553
Tln2	_TSS[Phospho (STY)]PEESIR_.2	Q71LX4_S2083_M1_Tln2	3.9743	4.0286	4.5105	3.7151	3.3120	3.5361	4.2291	3.0902	4.2882	4.1806	3.1617	5.3335	4.3962	3.9400	3.8063	4.3191	4.0883	5.5972	3.3602	6.2658
Tln2	_DRFGLEGDEESTM[Oxidation (M)]LEESVS[Phospho (STY)]PK_.3	Q71LX4_S428_M1_Tln2	3.6183	3.7007	3.1549	3.9659	3.8253	5.8241	5.2942	3.8440	3.1430	6.3458	4.9702	3.5207	3.3085	3.8111	3.2482	5.8799	3.3412	5.6507	6.0411	4.8787
Cspg5	_VSS[Phospho (STY)]PHAR_.2	Q71M36_S498_M1_Cspg5	7.2582	3.2007	3.8376	4.3881	4.1398	4.3639	3.4012	4.5025	3.4603	3.5076	6.7825	3.0901	6.9446	3.8246	2.9784	3.6462	3.5393	4.2938	3.9424	6.3039
Cspg5	_GDQDDLGVNC[Carbamidomethyl (C)]LQNNLT_.2	#N/A	3.9594	5.6281	6.0848	6.1787	3.2972	5.8498	5.4066	3.6598	3.8147	5.8607	5.9657	5.2982	3.9802	5.3860	4.8122	4.7838	6.0851	5.1148	5.9199	5.3343
Cspg5	_LEGGKGDQDDLGVNC[Carbamidomethyl (C)]LQNNLT_.2	#N/A	5.7237	5.9097	5.7821	5.2988	4.4207	5.2040	5.7646	5.6215	5.6943	5.3495	5.3442	5.7987	6.2386	6.5587	4.4821	5.2303	5.4042	5.7131	5.2619	5.1872
Cspg5	_KFC[Carbamidomethyl (C)]DTPRVS[Phospho (STY)]S[Phospho (STY)]PHAR_.3	Q71M36_S497_M2_Cspg5	3.4706	3.8485	4.5080	6.2250	3.6775	4.4308	3.3343	3.6963	3.3934	3.5745	3.7678	3.5746	6.0012	4.5461	3.3960	5.9500	3.1935	3.9559	5.7766	4.2233
Cspg5	_LKEEESFNIQNSMS[Phospho (STY)]PK_.2	#N/A	3.8808	3.6856	4.4799	3.9508	3.7959	3.8835	3.7674	3.9882	3.7337	3.8647	3.5846	4.1319	3.8797	4.0573	4.2200	3.7232	4.2056	3.8026	2.9654	3.6885
Ssh1	_SSS[Phospho (STY)]SDSIHSVR_.2	Q76I79_S929_M1_Ssh1	8.3726	4.4938	8.1535	7.9914	8.2897	8.2031	7.9832	8.5449	8.0241	8.2928	7.6357	7.9563	8.3569	8.0424	7.3269	7.9236	8.2658	8.1298	8.0801	7.7547
Ssh1	_SDS[Phospho (STY)]LPQVEELEK_.2	Q76I79_S585_M1_Ssh1	7.4534	7.6507	6.8541	7.6778	4.1758	4.3999	6.9813	3.7272	3.4243	3.5436	3.7987	3.5437	3.5324	4.5770	2.9424	6.7890	6.7917	3.9250	4.2241	4.2543
Mindy1	_TRAPS[Phospho (STY)]PQGR_.2	Q76LS9_S440_M1_Mindy1	10.1493	10.1851	9.9236	10.2335	9.9871	9.9211	9.9512	10.2034	9.8044	10.1918	9.6845	9.9551	10.3267	10.3665	9.7248	10.1205	10.0966	9.8630	10.0821	9.9676
Mindy1	_VETAEVC[Carbamidomethyl (C)]SRPQELPQS[Phospho (STY)]PR_.3	#N/A	7.3168	7.8494	7.5082	7.6807	6.7806	7.2359	7.3937	3.9264	6.4587	7.7390	7.0267	7.1688	7.3468	7.9797	6.6504	6.9055	7.3332	7.4525	7.1398	6.9416
Mindy1	_PQELPQS[Phospho (STY)]PR_.2	#N/A	6.2404	5.8493	6.5959	6.6850	5.9954	6.3044	6.0937	5.8269	6.0258	6.5266	5.9826	5.7375	5.4457	6.0701	5.1236	5.7173	6.5860	6.0231	5.7302	5.8046
Ppp2r1a	_LAGGDWFTSR_.2	#N/A	8.1930	8.1263	7.9870	8.3884	7.9275	8.1609	8.0821	8.3860	7.9405	7.8431	8.2365	7.9551	8.2465	7.8831	7.9090	7.9779	8.1868	7.9925	7.8907	7.8024
Ppp2r1a	_DKAVESLR_.2	#N/A	7.8288	7.8449	8.0353	7.5796	7.6691	7.9340	7.8937	8.3317	7.7938	7.8209	7.8396	8.0018	7.6754	7.8959	7.9021	7.9284	7.7685	7.6284	7.8925	7.9720
Ppp2r1a	_IGPILDNSTLQSEVKPILEK_.3	#N/A	7.7591	7.7586	7.9010	7.4914	7.7257	7.9504	7.8610	8.1488	7.3798	7.9673	7.7051	7.2371	7.8994	7.4720	7.3237	7.4967	7.6696	7.8377	7.2511	7.9011
Ppp2r1a	_VLELDNVK_.2	#N/A	7.3253	7.6361	7.8673	7.8326	7.8516	7.7335	7.9819	7.2722	7.8001	7.8614	7.9302	7.8009	8.0498	7.6070	7.5364	7.6817	7.3845	8.0648	7.6109	8.0086
Ppp2r1a	_NEDVQLR_.2	#N/A	6.8789	6.2647	7.0154	7.2560	6.7492	6.8094	7.6761	6.8489	7.2880	7.3363	7.4980	7.0978	6.5076	6.8637	7.2478	6.8846	6.5496	7.0391	7.2177	7.2619
Ppp2r1a	_AVGPEITK_.2	#N/A	6.6434	6.5351	6.2623	6.1169	6.5538	6.8808	6.5364	6.0821	7.0248	6.6647	7.0618	6.6431	6.8015	5.5054	7.1015	6.7079	6.7208	7.3136	6.5825	6.9576
Ppp2r1a	_LSTIALALGVER_.2	#N/A	6.9451	6.5496	6.1321	6.2604	6.6125	6.3775	6.8101	6.9384	6.7624	6.6433	6.6832	6.6489	6.7582	6.3370	6.8392	6.2550	6.6978	6.7751	6.9940	6.7412
Ppp2r1a	_EWAHATIIPK_.2	#N/A	6.1705	7.0393	6.5228	6.7899	6.6897	6.3148	6.8530	6.9066	6.4887	6.5412	6.3821	6.4491	7.2940	6.6785	6.0872	6.6574	6.3115	6.5997	6.8159	6.3179
Ppp2r1a	_SALASVIM[Oxidation (M)]GLSPILGK_.2	#N/A	6.7099	6.3672	6.1157	6.2859	6.7936	6.2163	6.0310	6.2984	5.0345	6.1758	6.0636	6.4881	5.6584	5.6386	6.4838	6.3772	6.1745	6.3347	6.1888	5.5425
Ppp2r1a	_LTQDQDVDVK_.2	#N/A	8.1327	7.2785	7.9861	7.5236	6.9610	7.0445	7.5845	7.6716	3.1154	7.5789	7.3104	7.2468	7.0804	7.8844	7.4523	7.4062	7.2764	7.3200	7.2682	7.2978
Ppp2r1a	_TSAC[Carbamidomethyl (C)]GLFSVC[Carbamidomethyl (C)]YPR_.2	#N/A	5.9464	3.9500	6.8712	7.0010	6.1205	6.2658	6.8233	6.7602	6.6887	6.8712	6.7549	6.6602	6.6102	6.1638	6.7042	6.0732	6.5069	6.1285	6.2026	6.4671
Ppp2r1a	_AISHEHSPSDLEAHFVPLVK_.3	#N/A	6.0090	5.5792	5.9833	6.2684	5.7732	5.9041	6.2306	6.1214	5.5592	5.0106	5.3139	4.9732	3.7669	3.6261	5.2913	6.7277	6.6253	5.8427	5.5200	6.6257
Ppp2r1a	_QLSQSLLPAIVELAEDAK_.2	#N/A	4.4688	5.2770	4.9165	4.8733	4.1123	5.2327	5.3096	4.7806	5.2927	5.4248	5.3375	5.2117	4.7936	5.2003	4.5570	5.0819	5.0458	5.4568	4.6645	4.8178
Ppp2r1a	_SEIIPM[Oxidation (M)]FSNLASDEQDSVR_.2	#N/A	7.8518	7.9590	6.6088	6.8123	6.4609	6.8728	6.6357	7.1559	6.6212	7.5691	6.8268	7.9210	6.7920	7.2288	7.4386	6.5757	7.8535	6.7505	6.5419	6.2930
Ppp2r1a	_NLC[Carbamidomethyl (C)]SDDTPM[Oxidation (M)]VR_.2	#N/A	6.5211	6.5166	7.3922	6.0095	6.6831	6.4337	6.7186	6.5762	6.7264	6.6559	7.0543	6.5419	6.5725	6.0488	6.6400	6.4134	6.5837	6.5980	6.5478	6.6105
Ppp2r1a	_MAGDPVANVR_.2	#N/A	3.2343	7.5616	8.0017	8.1080	6.5596	7.0767	7.8150	3.4599	6.9988	3.5092	7.3469	6.9184	3.2651	8.0742	7.0520	7.7467	7.1059	6.6035	6.4383	6.2223
Ppp2r1a	_YFAQEALTVLSLA_.2	#N/A	3.6455	5.1189	3.3879	4.9011	4.4620	3.9142	4.5100	4.5182	4.8649	4.2178	4.5157	4.9603	4.0181	5.1624	4.3280	4.6995	4.8365	4.0668	4.2253	3.3419
Ppp2r1a	_LNIISNLDC[Carbamidomethyl (C)]VNEVIGIR_.2	#N/A	3.7826	5.7430	5.2129	5.7464	4.5614	5.0245	5.0212	4.7258	5.2057	5.3176	5.2919	4.9431	5.8604	5.9007	5.1290	5.5219	5.4960	5.3550	5.3826	5.2183
Ppp2r1a	_HM[Oxidation (M)]LPTVLR_.2	#N/A	6.9681	7.3889	6.5161	6.9265	7.3410	7.0859	7.3714	7.6757	6.4872	6.7557	6.9048	7.2460	7.4826	6.4240	6.9307	6.9291	6.8665	7.3950	6.9414	7.2638
Ppp2r1a	_ENVIM[Oxidation (M)]TQILPC[Carbamidomethyl (C)]IK_.2	#N/A	6.3439	6.1823	6.5953	6.3556	6.5872	6.1074	6.2131	6.6164	6.4477	6.4616	6.2460	6.2583	6.4827	5.9309	6.0305	6.1706	6.2873	5.7837	6.7358	6.2813
Ppp2r1a	_VLAM[Oxidation (M)]SGDPNYLHR_.2	#N/A	6.2431	5.7224	6.5124	6.0827	6.2339	6.1933	6.4283	6.7725	5.9745	6.2278	4.9543	6.2448	6.0350	5.9431	6.2073	6.1808	6.2146	6.1303	6.1054	6.0320
Ppp2r1a	_ELVSDANQHVK_.2	#N/A	7.5409	6.9204	7.4131	6.6585	6.7909	7.3992	7.2992	7.5799	7.8316	7.1659	7.0321	7.5387	7.4270	7.7841	7.7326	7.8787	7.4206	7.7614	7.0578	7.4784
Ppp2r1a	_LNSLC[Carbamidomethyl (C)]M[Oxidation (M)]AWLVDHVYAIR_.3	#N/A	4.4797	4.4881	4.0163	3.1045	3.1858	3.3423	3.8623	3.2045	4.4025	4.2903	3.2761	4.0663	4.5106	4.0543	3.8877	3.8418	4.2026	3.7027	3.2459	3.7316
Ppp2r1a	_VKEFC[Carbamidomethyl (C)]ENLSADC[Carbamidomethyl (C)]R_.2	#N/A	4.3646	3.3441	3.5116	3.6092	3.7023	3.9264	3.8387	4.2007	3.8978	4.5709	4.2722	3.0702	4.0059	3.5497	3.4159	3.2087	3.6979	3.4515	3.7506	3.2269
Stambpl1	_SDGSNFANYS[Phospho (STY)]PPVNR_.2	Q76N33_S242_M1_Stambpl1	10.9895	10.8502	10.8651	10.9371	10.2516	10.2932	10.2695	10.4339	10.2433	4.5375	10.4695	10.4617	10.9097	11.0399	9.3566	10.6688	10.7113	10.3935	10.3658	10.5291
Mvb12a	_GLS[Phospho (STY)]LDPPKEPSK_.2	Q78HU3_S168_M1_Mvb12a	10.0298	9.9174	10.2145	10.0472	8.3129	9.1431	3.9169	8.4216	8.6262	10.1001	9.1564	8.3274	8.9789	9.6279	8.1306	9.2751	8.9300	8.8261	8.9575	9.0967
Mvb12a	_TLS[Phospho (STY)]QDMR_.2	Q78HU3_S161_M1_Mvb12a	3.7198	3.5993	3.2564	3.8644	3.9268	3.9601	3.1213	3.9455	3.0416	3.5304	4.0210	3.3254	3.2070	3.7097	3.1467	3.0819	3.4427	4.4088	4.0574	3.6766
Atp5md	_YFNSYTLTGR_.2	#N/A	7.0760	7.2067	7.3938	7.2064	7.2815	7.3381	7.4142	7.2992	7.3564	7.1078	7.3485	7.3182	6.9361	7.0972	6.9485	7.1072	7.2466	7.4329	7.1578	7.2193
Atp5md	_AGAESDGQFQFTGIK_.2	#N/A	3.3435	3.1585	3.8798	4.3459	4.1820	4.4061	4.0745	3.0439	3.4181	3.5498	3.7925	3.7060	3.5262	3.8669	2.9362	3.6884	4.2639	3.9312	3.9002	4.2481
Ccdc25	_VENM[Oxidation (M)]SSNQDGNDS[Phospho (STY)]DEFM[Oxidation (M)]_.2	#N/A	8.3740	8.6759	8.5092	8.5080	7.9757	8.2048	8.1780	8.4231	8.1669	8.5174	7.1723	8.3777	8.5750	8.8201	8.0456	8.3797	8.7546	8.2881	8.2270	8.3002
Fam102a	_SSS[Phospho (STY)]LSDLTHR_.2	Q78T81_S252_M1_Fam102a	7.4516	7.2037	7.0806	7.4785	3.0907	6.4662	3.9574	3.1094	6.5232	7.4722	6.7159	4.1615	4.4154	3.9592	3.9828	3.7467	4.1075	6.5499	3.3410	6.4662
Nap1l4	_FYEEVHDLER_.2	#N/A	7.2849	6.6215	7.5382	6.8599	6.7850	6.7817	6.7526	6.9844	6.8277	7.0069	6.6850	6.9897	6.7210	7.0097	7.1172	7.0665	6.7657	6.8174	7.3929	7.1607
Nap1l4	_LTDQVM[Oxidation (M)]QNPQVLAALQER_.2	#N/A	4.7581	4.7815	4.3260	4.7252	4.5827	4.0779	3.9733	4.6556	4.8908	4.4778	4.1283	4.9487	4.9592	5.5776	4.9803	5.1645	4.6771	5.0146	4.7077	4.5537
Nap1l4	_[Acetyl (Protein N-term)]AENSLSDGGPADSVEAAK_.2	#N/A	7.3392	8.2883	8.1884	8.4828	5.8209	8.4933	8.1180	6.1398	8.2237	8.6202	5.7998	8.2121	5.8448	8.9622	8.1138	8.5169	8.2491	8.2156	8.1931	8.5128
Nap1l4	_FY[Phospho (STY)]EEVHDLERK_.3	Q78ZA7_Y85_M1_Nap1l4	8.1663	8.3632	8.0726	3.7659	7.3914	7.4796	7.9135	7.2350	7.4760	3.9538	7.3758	7.2955	4.5152	8.0363	6.7747	7.6736	7.3880	3.5661	7.4257	7.2068
Nap1l4	_EFITGDVEPTDAES[Phospho (STY)]AWHSENEEEDK_.3	#N/A	3.9835	4.1594	4.1636	4.0479	3.3331	3.3664	3.7335	3.5815	3.6537	3.7941	3.1324	3.9376	3.6800	4.8311	3.7589	3.9891	4.3560	3.9161	3.1280	4.2527
Nap1l4	_EFITGDVEPTDAESAWHS[Phospho (STY)]ENEEEDKLAGDM[Oxidation (M)]K_.4	#N/A	5.6523	6.6020	3.7661	6.3462	4.8226	6.2594	5.9336	4.9772	3.6531	3.9925	5.6343	5.7400	6.5503	3.7894	7.1595	3.5440	3.8270	6.4993	3.8082	6.4870
Nap1l4	_LDNVSHTPSSYIETLPK_.3	#N/A	4.4589	4.1782	4.7526	4.4361	4.4084	4.2706	4.0988	4.3543	4.0618	4.5214	4.2610	5.0296	4.9794	4.5800	4.6326	4.5245	4.3681	4.2418	4.8928	4.1464
Nap1l4	_REFITGDVEPTDAESAWHS[Phospho (STY)]ENEEEDKLAGDM[Oxidation (M)]K_.4	#N/A	3.5146	3.9949	4.3616	4.2601	2.8523	3.0764	3.1879	3.5498	3.2470	3.7209	3.6214	3.7210	3.3550	4.3996	4.2659	3.8595	4.5479	4.1024	4.4014	4.0769
Mtch1	_RVS[Phospho (STY)]SGSC[Carbamidomethyl (C)]FALE_.2	Q791T5_S381_M1_Mtch1	3.9874	4.4677	8.3344	8.3514	3.3251	8.2952	8.0872	7.3951	8.5310	7.3988	3.1486	7.5926	7.4263	8.1548	6.3306	7.8023	8.4018	8.1581	3.3733	8.0709
Mtch2	_VLQYYQESEKPEELGSVTVQK_.3	#N/A	5.4472	5.8108	6.7506	5.9020	4.8847	5.2497	5.7902	5.8658	5.6718	4.9661	5.1626	5.9109	5.2809	5.2078	4.1098	4.5965	5.6992	5.5859	5.3246	5.0174
Mtch2	_TYC[Carbamidomethyl (C)]YDLR_.2	#N/A	5.6111	3.2925	4.5323	4.4799	4.0480	4.3778	5.6854	3.8550	5.4492	5.2161	4.9708	3.1820	3.6602	3.1298	5.3459	4.1426	3.3577	4.9349	6.2305	5.2906
Mtch2	_EYSSSFDR_.2	#N/A	3.1105	5.2251	3.2649	3.8559	3.9490	4.1731	5.6685	3.9540	5.5342	5.7510	4.0255	3.3169	3.7592	3.3030	5.1138	3.4554	3.4512	5.7761	3.9973	5.7561
Picalm	_ATTLSNAVSSLASTGLSLTK_.2	#N/A	4.2153	3.9138	4.5666	3.0217	3.8967	4.3289	3.6769	3.9878	3.8459	4.5581	3.6738	3.5725	3.5085	5.1268	3.6643	2.4972	4.7400	3.9052	2.5179	4.0861
Scn4b	_EC[Carbamidomethyl (C)]LVSSSGNDNTENGLPGSK_.2	#N/A	3.4240	4.5732	4.4615	4.5891	3.6310	3.6691	3.4171	3.1824	3.8449	3.0598	4.0227	4.7035	5.1841	3.8944	3.3924	3.5312	5.5880	5.3883	4.8573	3.9724
Opalin	_IS[Phospho (STY)]ENPRRSPTHEM[Oxidation (M)]NPR_.4	#N/A	8.4188	8.4005	7.6161	7.7834	5.6562	8.0012	8.5434	8.3185	9.4663	8.4502	7.7246	8.4277	9.3891	8.1258	8.7293	8.4436	8.3069	8.3500	8.4827	8.2794
Opalin	_RS[Phospho (STY)]PTHEM[Oxidation (M)]NPR_.3	#N/A	4.0409	6.3827	6.6389	6.6325	4.5918	4.7466	6.0138	3.9506	5.6242	6.0302	6.8176	4.8469	5.0621	8.0006	6.6878	4.9688	4.3189	5.0365	5.4543	4.6503
Opalin	_GS[Phospho (STY)]QEGHIYVK_.2	Q7M750_S100_M1_Opalin	8.0795	8.6590	8.1102	8.4615	8.2456	8.4376	7.8178	8.3130	8.2639	8.5161	8.1974	8.4963	8.3849	8.8042	8.4924	8.6112	8.6622	8.1313	8.2431	8.4983
Opalin	_TVSGSEEPLPDTYRPPEELER_.3	#N/A	5.6318	5.9867	5.6394	6.4484	5.6843	5.9093	5.2825	6.6712	5.5592	5.5817	5.7958	5.9911	5.8550	6.2775	6.0017	6.4426	6.2878	5.4431	6.1014	5.2199
Opalin	_S[Phospho (STY)]PTHEMNPR_.2	#N/A	3.7615	6.9642	7.8022	8.0258	3.8003	6.9261	6.7674	4.4941	5.9403	5.9225	7.3709	5.6383	3.7422	8.1668	6.5120	3.7672	5.8956	5.1384	3.8745	6.5369
Asap2	_LLHEDLDES[Phospho (STY)]DDDVDEK_.2	#N/A	3.9020	9.1517	9.1565	9.1054	3.7983	8.8827	8.7328	3.9504	8.6321	8.9675	3.5812	8.6850	3.8513	9.2910	7.9462	8.7806	9.0457	8.7876	8.9124	8.9228
Asap2	_LLHEDLDES[Phospho (STY)]DDDVDEKLQPSPNRR_.4	#N/A	3.6142	7.8357	3.1508	3.9700	3.8212	7.2363	7.2715	3.8399	3.1471	3.4248	4.1266	3.4310	3.3126	8.8635	6.9161	2.9764	3.3371	8.3632	4.1113	4.3670
Asap2	_LLHEDLDES[Phospho (STY)]DDDVDEKLQPSPNR_.4	#N/A	3.9165	6.0116	4.4527	3.7729	3.2542	3.2299	5.3583	3.6168	3.7717	4.3011	3.2504	3.9758	3.9372	7.4587	3.8641	4.2613	4.1545	6.8927	3.3272	4.4068

Cyfip1	_SGDGEST[Phospho (STY)]PVEHVR_.2	Q7TMB8_T1234_M1_Cyfip1	7.1675	7.2931	4.4434	7.1826	6.3019	6.9921	3.2697	7.0181	3.3288	6.6508	6.8098	6.8753	3.4368	6.4025	5.5512	6.6610	3.1289	4.0206	6.5857	6.6803
Plppr4	_PGSSQLVHIPEETQENISTS[Phospho (STY)]PK_.3	Q7TME0_S549_M1_Plppr4	10.9484	11.0460	4.4726	10.2802	8.9079	3.6754	10.6341	10.5599	3.3262	10.2877	9.8447	10.0900	10.5275	10.8188	8.2333	4.2981	3.1580	10.6810	4.2904	3.9613
Plppr4	_DASS[Phospho (STY)]LTNLKR_.2	Q7TME0_S386_M1_Plppr4	9.5166	9.4373	9.2577	8.9801	8.3522	8.3405	8.7725	8.5971	8.4085	9.4940	8.4179	8.2952	8.8447	8.5965	6.4279	8.2357	8.3875	8.7444	8.2234	7.8292
Plppr4	_QQDVLQSS[Phospho (STY)]PK_.2	Q7TME0_S594_M1_Plppr4	9.2418	3.5346	3.3211	3.7997	3.9914	4.1169	3.6482	8.9792	7.9789	9.1918	9.2092	9.4917	9.6156	9.4147	8.6493	9.1517	3.5074	9.4638	9.4496	9.1363
Plppr4	_DALRS[Phospho (STY)]LTDLNQDPSR_.2	#N/A	6.4543	6.9313	6.0635	5.6123	4.5300	6.2903	6.4719	6.0362	6.4781	6.5671	5.5616	5.7363	6.4991	7.0323	5.6171	5.9980	4.5829	7.0155	6.4404	5.7222
Plppr4	_M[Oxidation (M)]SLQVM[Oxidation (M)]DTEPEGQS[Phospho (STY)]PPR_.2	#N/A	8.3551	7.5547	7.7606	7.8024	6.8284	7.1331	6.9648	7.1247	6.8897	7.8296	6.5720	6.5974	7.3972	6.9447	4.9253	7.3577	7.3674	7.1012	6.9906	7.0597
Plppr4	_TVHTS[Phospho (STY)]PGGGLGAR_.2	#N/A	3.9053	3.7906	3.7148	3.9234	3.8024	7.3751	3.8791	3.8829	3.7994	3.9386	3.7326	3.5620	4.0724	3.7676	3.3355	3.4901	3.7708	3.4208	4.1016	4.1215
Plppr4	_S[Phospho (STY)]LTDLNQDPSR_.2	#N/A	7.8623	7.8909	7.6360	7.1599	6.7575	7.5730	7.2451	7.5475	7.4617	7.9040	7.5890	6.7823	7.2167	8.1667	6.0974	7.4844	7.7236	7.4365	7.2903	3.2770
Plppr4	_NASHAS[Phospho (STY)]M[Oxidation (M)]DSAR_.2	Q7TME0_S439_M1_Plppr4	3.2151	4.1040	4.2525	4.3691	3.4220	3.4554	3.6261	5.5671	3.5463	5.5140	3.0249	5.0816	3.7117	4.2144	3.6514	4.0780	4.4388	3.9041	3.5527	4.1814
Plppr4	_KM[Oxidation (M)]SLQVMDTEPEGQS[Phospho (STY)]PPR_.3	#N/A	3.9502	4.9751	5.6776	6.7216	4.1572	4.1905	5.1665	4.1680	4.3120	3.7608	3.7906	3.0950	4.4774	3.5037	2.9163	3.3124	3.4548	3.3158	4.0528	3.3308
Plppr4	_DSESC[Carbamidomethyl (C)]SLKDSFGS[Phospho (STY)]GDR_.2	#N/A	3.8137	3.5897	4.2447	3.8804	4.0207	4.0540	3.8747	3.6400	3.7949	3.6243	4.0115	4.0788	3.9604	3.7001	3.9001	3.8899	4.1777	3.7649	4.4135	3.6671
Plppr4	_SNS[Phospho (STY)]PENTR_.2	Q7TME0_S746_M1_Plppr4	4.4090	3.3885	3.4672	3.6536	3.7467	3.9708	3.7943	4.1563	3.8534	3.1145	4.2278	3.1146	3.9615	3.5053	3.3715	3.2531	3.6535	3.4959	3.7950	3.1825
Ndufa12	_FNVSATPEQYVPYSTTR_.2	#N/A	8.0454	8.1130	7.7016	7.9318	7.8827	7.6448	8.1518	8.0255	7.9943	7.8122	8.1751	8.1353	7.9745	8.3746	8.0366	8.2553	8.3341	7.9521	8.2974	7.7046
Ndufa12	_IGTLVGEDK_.2	#N/A	5.5285	5.7545	5.8885	6.0094	5.0326	5.7726	5.4271	5.4074	5.5995	5.7024	5.2504	5.9265	4.2365	6.1965	5.7015	5.6936	5.4276	6.2111	5.2047	6.1751
Ndufa12	_[Acetyl (Protein N-term)]M[Oxidation (M)]ELVEVLKR_.2	#N/A	7.6664	7.7747	7.4736	7.7379	7.8656	7.5758	7.8288	7.8563	7.3200	7.8057	7.6460	7.8793	7.5414	7.8327	7.5148	7.6007	7.5618	7.7462	7.5847	7.7418
Ndufa12	_GVQQVTGHGGLR_.2	#N/A	7.1750	7.1321	7.2213	7.3697	7.1225	6.6698	6.9186	7.6794	7.3600	6.8220	7.0567	7.3709	7.3642	7.4891	8.0294	7.1818	6.7375	7.0840	7.7444	7.4850
Ndufa12	_WLHC[Carbamidomethyl (C)]M[Oxidation (M)]TDDPPTTNPPTAR_.3	#N/A	3.9936	4.0092	4.5299	3.6958	3.3313	3.1528	4.1689	6.3775	3.8488	4.2240	3.3275	3.8987	4.0143	4.5169	5.6468	4.3385	4.2316	6.4068	6.5196	5.8268
Ndufa12	_[Acetyl (Protein N-term)]M[Oxidation (M)]ELVEVLK_.2	#N/A	3.6471	3.6720	3.1836	3.9372	3.8540	4.0939	2.9471	3.8727	2.7671	3.3980	3.9443	3.3981	3.6779	3.2217	2.7670	3.0092	3.3700	3.7795	3.5813	4.3998
Uhrf2	_RPAS[Phospho (STY)]DDEC[Carbamidomethyl (C)]PGDSK_.2	Q7TMI3_S668_M1_Uhrf2	3.4475	3.8716	4.2624	3.7122	3.6544	3.6878	3.3937	3.6732	3.3139	3.2581	4.2933	3.5977	3.4793	3.9820	3.4190	3.8757	3.1704	4.1365	3.7851	3.9490
Syncrip	_NLANTVTEEILEK_.2	#N/A	5.2099	4.5000	4.9840	5.3150	4.8588	4.7094	4.7549	4.3792	4.7857	4.5404	4.3470	3.7477	4.4581	4.2507	4.6387	4.3941	5.0067	5.0152	4.7666	4.5657
Syncrip	_DLFEDELVPLFEK_.2	#N/A	5.9659	5.5845	5.4482	5.5856	4.7521	5.9027	4.9198	4.9695	5.0405	5.2752	4.7604	5.5612	5.3416	5.0503	5.2037	5.7726	5.0726	5.0520	5.0163	5.9367
Syncrip	_DSDLSHVQNK_.2	#N/A	4.0300	4.5800	4.7700	4.8625	1.6889	4.6907	4.3924	4.3911	4.5248	4.3592	4.3066	4.1708	4.8076	5.3090	4.6495	4.9547	3.7529	4.0188	3.9700	5.1872
Syncrip	_LYNNHEIR_.2	#N/A	3.3620	5.7007	4.8007	4.1075	4.2006	5.6565	3.3405	5.1726	3.6574	3.5683	3.7740	5.1758	3.5076	4.5522	4.3761	5.2896	3.1997	3.9498	4.2488	5.2924
Syncrip	_TGYTLDVTTGQR_.2	#N/A	3.9238	3.5197	3.5186	3.2063	4.1307	5.8565	5.7450	5.7341	5.9800	3.7344	5.2168	3.1214	4.5038	6.0246	5.0158	5.7868	3.2203	5.8832	4.2614	3.4727
Syncrip	_GFC[Carbamidomethyl (C)]FLEYEDHK_.3	#N/A	4.5181	3.4847	3.5536	3.1712	3.8559	4.1291	3.6852	4.2185	4.3734	3.2236	3.8521	3.3741	4.5389	5.3407	3.2624	3.3622	3.2554	3.0770	4.2264	3.5077
Syncrip	_VTEGLTDVILYHQPDDK_.3	#N/A	3.4680	3.8511	4.5054	4.1163	3.6749	3.7082	3.3732	3.6936	3.2934	3.2786	4.2729	3.5772	3.4588	4.5434	3.3986	4.3309	3.1909	4.1570	3.8056	3.9285
Tubb2a	_AILVDLEPGTM[Oxidation (M)]DSVR_.2	#N/A	13.5912	13.6657	13.9163	13.8262	13.5335	13.6697	13.7472	13.6819	13.8050	13.8392	12.8603	12.8284	13.2180	12.9376	12.9932	13.1711	13.0129	12.7440	13.0274	13.0271
Tubb2a	_ALTVPELTQQM[Oxidation (M)]FDSK_.2	#N/A	12.1733	12.3450	12.2786	12.3150	12.2106	12.2618	12.1620	12.3411	12.1270	12.4052	11.5334	11.7243	11.8047	11.6333	11.5706	11.5277	11.6731	11.6109	11.5197	11.6646
Tubb2a	_YLTVAAIFR_.2	#N/A	11.8353	12.1870	12.0849	12.1576	11.8325	12.0865	12.0953	11.8162	11.9317	12.2700	11.3809	11.3253	11.3973	11.3958	11.2455	11.3753	11.1873	11.4581	11.5686	11.4396
Tubb2a	_INVYYNEAAGNK_.2	#N/A	11.7467	12.7464	12.8027	12.5297	11.8522	12.7571	12.4216	12.2852	12.4303	12.4095	11.4335	11.5329	12.1236	12.2234	11.6793	11.8998	11.7464	12.0333	11.8159	12.0386
Tubb2a	IC[Carbamidomethyl (C)]DC[Carbamidomethyl (C)]LQGFQLTHSLGGGTGSGM[Oxidation (M)]GTI	#N/A	5.6739	6.3374	6.9807	6.9635	6.0281	6.6082	6.4719	5.3057	6.0124	6.5501	6.2317	5.6659	5.5815	6.4858	4.9746	5.2729	5.5211	5.1334	4.8602	5.9987
Tubb2a	_IM[Oxidation (M)]NTFSVM[Oxidation (M)]PSPK_.2	#N/A	9.6948	10.1009	9.9784	9.5460	9.9338	10.0372	10.0288	9.8448	10.0739	10.0810	8.9408	9.2310	9.2102	9.5541	9.4818	9.2127	9.0979	9.3558	9.2675	9.2999
Tubb2a	_FWEVISDEHGIDPTGSYHGDSDLQLER_.4	#N/A	6.8722	7.1160	8.8416	7.8176	7.2562	7.1364	6.9669	6.9817	7.5722	7.6568	6.6771	6.4782	6.9223	6.6945	7.0422	6.9681	5.9817	6.5929	6.6775	7.2613
Tubb2a	_INVYYNEAAGNKYVPR_.3	#N/A	5.1783	5.0695	4.0737	5.0912	4.8638	4.8901	5.1376	5.1267	4.8130	5.1214	4.5337	4.7772	5.2499	4.1676	4.7330	4.5926	3.7755	4.1518	4.2351	4.4497
Tubb2a	C[Carbamidomethyl (C)]DC[Carbamidomethyl (C)]LQGFQLTHSLGGGTGSGM[Oxidation (M)]GTL	#N/A	4.1811	4.1831	3.0866	3.3524	4.3557	3.5253	3.6956	4.4825	4.1444	4.3243	3.0180	3.7742	3.9590	4.6668	3.6625	2.9621	3.9445	2.8305	3.5040	3.4735
Huwe1	_AES[Phospho (STY)]PEEVAC[Carbamidomethyl (C)]R_.2	#N/A	11.0583	11.0145	11.0399	10.9990	10.6363	10.8309	10.8414	11.1191	10.6804	10.6457	10.7333	10.4261	10.5895	10.5997	9.3468	10.0663	11.0495	10.6764	10.5124	10.4971
Huwe1	_RAQC[Carbamidomethyl (C)]ETLS[Phospho (STY)]PDGLPEEQPQTTK_.3	#N/A	7.6468	7.8546	4.6001	4.0215	4.1147	7.3577	7.5062	7.3652	3.4855	7.0215	6.6635	7.0141	7.7750	7.0222	3.0036	6.4781	7.5878	7.4717	4.1629	6.3836
Huwe1	_ESNQPPEDSS[Phospho (STY)]PPASSESSSTR_.2	Q7TMY8_S2918_M1_Huwe1	8.9071	6.3830	4.1526	4.0731	3.4967	3.5301	3.5514	3.3167	3.4716	7.6564	7.9882	4.2759	8.5706	4.1397	3.5767	4.1527	7.8789	3.9788	3.6274	4.3067
Huwe1	_GSGTAS[Phospho (STY)]DDEFENLR_.2	Q7TMY8_S1907_M1_Huwe1	9.6667	10.3252	9.8513	9.9730	3.0413	9.8638	9.6930	3.0600	10.0168	9.6659	3.4056	9.4641	9.6999	9.7146	8.2557	9.4099	10.2119	9.9352	9.6821	9.5168
Huwe1	_AQC[Carbamidomethyl (C)]ETLS[Phospho (STY)]PDGLPEEQPQTTK_.2	#N/A	7.2381	7.8635	7.7072	7.4003	5.6391	5.9320	7.2042	6.8184	7.1482	6.7522	7.3117	6.8441	7.0545	7.4063	5.9779	5.2766	7.9043	7.5528	6.9412	7.2332
Huwe1	_DGGS[Phospho (STY)]GNSTIVSR_.2	Q7TMY8_S2362_M1_Huwe1	3.3412	4.4379	6.7232	6.2076	3.5481	6.0749	6.4186	3.2653	3.4202	3.1518	2.8988	4.3273	3.5856	4.0883	3.5253	4.2041	3.8030	4.0302	3.6788	6.6478
Huwe1	_AES[Phospho (STY)]PEEVAC[Carbamidomethyl (C)]RK_.2	#N/A	7.9116	7.8417	7.6298	7.0835	3.8389	7.9767	7.9069	7.6015	7.3702	7.2835	7.7378	6.8457	7.3604	7.3237	5.8359	6.3177	7.8640	7.5040	7.1996	7.2936
Huwe1	_DLS[Phospho (STY)]MSEEDQMMR_.2	Q7TMY8_S1368_M1_Huwe1	3.6112	3.7079	3.1478	3.9730	3.8182	4.2902	3.2299	3.8369	3.5340	3.4218	3.9084	3.4340	3.6421	3.1859	3.2553	2.9733	3.3341	3.8153	4.1144	3.4640
Smap2	_DLDLLASVPS[Phospho (STY)]PSSVSR_.2	Q7TN29_S219_M1_Smap2	7.1815	3.9015	8.3224	4.1666	3.4505	3.6746	8.5963	3.8073	4.1961	4.3191	3.8788	7.9832	8.1493	9.2452	6.9262	6.9581	3.9961	8.0191	7.4315	3.5252
Smap2	_S[Phospho (STY)]LDINVLR_.2	Q7TN29_S127_M1_Smap2	3.7917	4.2111	4.3280	3.8530	3.1294	2.9946	3.3113	3.4920	3.4669	4.4259	3.1256	3.1683	3.8124	4.3150	3.9889	4.1366	4.0297	3.8034	3.4520	4.2820
Smap2	_KAVGSMPTAGSAGS[Phospho (STY)]VPENLNLFPEPGSK_.3	Q7TN29_S239_M1_Smap2	3.7880	4.2148	4.3243	3.9014	3.1257	3.3584	2.7819	3.4884	3.6433	4.4295	3.1219	4.1042	3.8087	9.4540	3.9925	4.1329	4.0261	3.8071	4.4557	4.2784
Cpeb4	_QQLSPS[Phospho (STY)]PGQEAGILPETEK_.2	Q7TN98_S99_M1_Cpeb4	6.1756	7.3734	7.4447	4.0166	6.5134	5.8205	5.6754	5.6507	5.8790	5.9446	6.9640	7.1499	6.1796	7.6769	6.2916	7.2676	6.1956	3.8589	3.6982	3.9212
Cpeb4	_S[Phospho (STY)]PASPHPPPFTHR_.3	Q7TN98_S252_M1_Cpeb4	3.6117	3.7074	3.1482	3.9726	3.8186	4.2898	3.2295	3.8373	3.5345	3.4223	3.9089	3.4335	3.6425	3.1863	3.2549	2.9738	3.3346	3.8149	4.1139	3.7545
Cpeb3	_SPAS[Phospho (STY)]PSQAPYAQR_.2	Q7TN99_S197_M1_Cpeb3	8.8859	8.8112	9.0712	9.0332	7.9146	8.3920	8.4567	8.7566	8.4823	9.0550	8.3549	8.4903	8.6539	9.0432	8.0691	8.5052	8.7120	8.3039	8.2911	7.9896
Cpeb3	_RS[Phospho (STY)]PAS[Phospho (STY)]PSQAPYAQR_.2	#N/A	4.1380	4.6183	6.1599	3.3826	3.4757	3.9151	4.0653	4.4273	4.1244	4.3443	3.8525	4.3444	4.2325	3.7762	3.6425	2.9821	3.9245	3.2249	3.5240	3.4535
Spag7	_EFAPS[Phospho (STY)]DEELDSYR_.2	Q7TNE3_S114_M1_Spag7	4.3517	4.4682	3.8883	3.6341	3.0578	6.4357	3.9903	3.7556	5.3777	4.1623	3.3892	4.1944	4.0760	7.1516	5.4203	3.7138	4.2933	6.1682	3.1884	6.1140
Tspoap1	_GGGS[Phospho (STY)]PEKLPNR_.2	Q7TNF8_S1402_M1_Tspoap1	3.1509	5.2606	4.8378	5.7586	4.8607	5.7373	5.1401	3.3766	5.2271	5.5823	4.7129	4.4503	6.0087	5.4171	3.4941	4.0139	5.2266	5.0687	5.3069	5.2357
Trim46	_LQESFGAPDVIS[Phospho (STY)]PR_.2	Q7TNM2_S627_M1_Trim46	9.8536	10.3902	10.0203	9.7860	8.9639	9.9598	9.8578	9.8604	9.9234	9.8938	9.6392	9.3700	10.1076	10.3919	9.0930	9.5393	10.0457	8.8738	9.7211	9.6684
Plppr3	_SVS[Phospho (STY)]TDELGPPGR_.2	Q7TP80_S320_M1_Plppr3	10.3933	10.1827	3.4864	10.0297	9.4643	9.6421	9.8963	10.0198	9.7320	10.0963	9.8319	9.7234	10.3264	9.6546	7.9002	9.5242	10.1602	9.7984	9.7154	9.5135
Plppr3	_GLGLPDEAS[Phospho (STY)]PVHLR_.2	#N/A	7.6940	7.2326	6.8712	7.0281	5.8234	7.2126	6.6424	6.9944	6.3921	7.2586	6.7794	6.6304	6.4431	6.8084	5.0232	6.6459	6.7475	7.2311	7.1044	6.6784
Plppr3	_QKS[Phospho (STY)]LEGR_.2	Q7TP80_S413_M1_Plppr3	4.0216	4.6357	3.5885	3.9427	3.8908	4.1149	5.3098	4.8198	3.9100	3.2586	4.0837	4.5201	3.8174	3.8222	4.3997	3.3971	4.4002	3.4014	4.6831	4.2970
Plppr3	_EKTS[Phospho (STY)]LGS[Phospho (STY)]LKR_.2	Q7TP80_S343_M2_Plppr3	7.0816	6.8674	4.2370	7.2464	3.4065	3.4398	6.5100	3.4069	6.6815	4.5110	6.4889	6.6413	3.7273	6.6179	3.6670	6.6479	7.0864	3.8886	3.5372	6.1790
Plppr3	_RAS[Phospho (STY)]VDVDLLAPR_.2	Q7TP80_S351_M1_Plppr3	3.2284	3.4148	4.7569	4.3558	3.4353	3.1722	4.9446	3.4541	4.4042	4.5398	3.0116	4.4689	3.2593	3.9214	2.9044	4.0913	4.2714	4.1981	4.1070	4.7566
Plppr3	_QPGM[Oxidation (M)]GPGS[Phospho (STY)]PVSDVDQEEPR_.2	#N/A	5.3860	5.3760	4.9693	4.0832	3.3493	3.6871	4.7532	5.3627	5.2634	5.1390	4.6435	5.0462	3.5943	4.2866	3.8642	5.0093	4.0155	4.4841	4.6788	4.9930
Slc7a6os	_EFDYDSPHGLDS[Phospho (STY)]D_.2	Q7TPE5_S305_M1_Slc7a6os	4.0353	7.6812	8.0281	3.6541	3.3730	7.9064	7.0959	3.7357	7.4462	4.2416	3.1007	3.8570	4.3352	7.5984	7.1735	4.3802	4.2734	6.9509	6.8770	7.2303
Mycbp2	_SKS[Phospho (STY)]DSYTLDPDTLR_.2	Q7TPH6_S2943_M1_Mycbp2	3.8657	8.9879	3.4022	3.7186	4.0726	4.1059	8.5114	7.4890	8.5247	3.6763	3.8752	8.0029	4.5620	7.9533	3.0009	7.8986	3.5886	8.4543	8.1419	3.5308
Mycbp2	_LISGDAEPT[Phospho (STY)]PEGEEK_.2	Q7TPH6_T3992_M1_Mycbp2	4.4192	8.5410	3.4546	3.2702	3.7569	8.2206	3.7842	8.6970	7.9208	7.9864	4.2176	3.4731	8.6426	8.1723	3.3614	3.2632	8.4208	8.5272	8.0791	7.5335
Mycbp2	_ALLSS[Phospho (STY)]PEGEEK_.2	Q7TPH6_S4003_M1_Mycbp2	3.7217	7.7463	7.4869	7.1386	3.8599	7.0651	6.7429	3.4221	7.0301	5.6687	3.5261	5.5261	3.7470	6.3223	5.4289	6.3185	6.2448	6.7692	7.0683	6.6151
Mycbp2	_SSS[Phospho (STY)]PQDKNLPQK_.2	Q7TPH6_S2916_M1_Mycbp2	3.9387	8.6209	3.4753	3.6455	8.0127	8.0741	8.3437	8.3197	8.2068	3.7493	7.6922	8.0441	8.8725	7.6452	2.9278	7.8902	8.3752	8.3014	7.8844	7.4503
Mycbp2	_STS[Phospho (STY)]PKPKFVPK_.2	Q7TPH6_S2970_M1_Mycbp2	5.2747	3.1769	6.1712	5.4108	5.1391	4.3825	4.8970	4.3679	4.1200	4.5996	4.5142	4.4039	4.1731	4.8857	4.2252	3.5043	3.8651	3.2844	4.1651	3.3941
Mycbp2	_DPKDSPGS[Phospho (STY)]ENR_.2	Q7TPH6_S2986_M1_Mycbp2	3.7607	3.3565	5.4298	4.5439	3.9676	4.0009	3.0805	4.3467	3.0007	3.5712	3.9802	5.5883	3.1662	3.6688	3.1059	3.1228	3.3835	4.4497	4.0982	3.6358
Mycbp2	_SLS[Phospho (STY)]PNHNTLQTLK_.2	#N/A	5.9574	3.8288	3.9914	3.7604	3.7233	4.1999	4.8625	3.8704	4.1481	4.7509	3.8148	3.6591	4.2562	4.1538	3.5244	3.8058	3.8776	4.4513	3.5587	3.4772
Mycbp2	_QVS[Phospho (STY)]TENDSTLVHR_.2	Q7TPH6_S1621_M1_Mycbp2	7.8461	7.5814	7.5595	6.8563	6.9034	7.2821	7.1954	7.3006	6.6988	7.1779	6.7190	7.1918	7.5699	6.6000	5.5571	6.7299	7.2449	7.2655	6.9738	6.7759
Prrc2b	_ESGS[Phospho (STY)]PAQEFSK_.2	Q7TPM1_S621_M1_Prrc2b	7.9535	7.7228	8.4644	3.7799	7.9431	7.9765	7.6335	3.6099	7.6720	8.1353	8.1560	7.5002	7.2002	8.0163	6.8734	7.5078	8.1546	7.5615	7.5985	7.8585
Prrc2b	_ALS[Phospho (STY)]LSSADSTDAKR_.2	Q7TPM1_S415_M1_Prrc2b	3.8822	4.1206	4.4185	3.8072	5.5480	3.4440	4.3211	5.8001	4.3802	4.0886	3.2537	4.0100	4.4883	6.1324	3.8983	4.2271	4.1203	5.2543	3.3615	6.3510
Prrc2b	_LKFS[Phospho (STY)]DDEDEEDVVKDGR_.3	#N/A	4.3295	6.7549	6.4453	6.2703	3.0356	6.4261	6.5430	4.0778	6.0631	6.2493	3.4246	5.5501	4.1115	7.2371	5.8715	6.1157	6.1540	6.6051	5.8477	5.7949
Prrc2b	_LKFS[Phospho (STY)]DDEDEEDVVK_.2	#N/A	4.2878	3.6032	3.8244	3.2964	2.9939	4.6517	4.0542	4.5135	3.9744	4.0984	3.4531	4.2583	4.1399	4.6037	4.0796	3.6499	4.2351	3.8354	4.1116	4.1773
Prrc2b	_S[Phospho (STY)]PDEALPGGLGSHSPYALER_.3	Q7TPM1_S793_M1_Prrc2b	3.8224	3.4967	3.3590	3.7618	3.8549	4.0790	3.6861	4.0481	3.7452	3.2227	4.1196	3.2228	3.8533	3.3970	3.2633	3.3613	3.5453	3.6041	3.9032	3.0743
Prrc2b	_QQQQQQQQQQQQQQQGEQLYK_.3	#N/A	3.8056	3.5135	3.3422	3.7786	4.0125	4.0958	3.0356	4.0313	3.7284	3.2395	4.1028	3.2396	3.8365	3.3803	3.0609	3.1677	3.5285	3.6209	3.9200	3.0575
Fsd1	_TAS[Phospho (STY)]PVNSPAR_.2	Q7TPM6_S313_M1_Fsd1	9.7857	9.7362	10.2189	10.0204	9.1889	8.5195	9.3577	9.2415	8.9419	10.0185	9.2061	9.2525	7.6701	9.3646	7.4693	9.2607	9.0332	8.9603	8.9099	9.1144
Fsd1	_RGS[Phospho (STY)]ATSSSNTSLT_.2	Q7TPM6_S486_M1_Fsd1	3.8840	10.0418	9.5921	9.8810	9.3186	9.2805	3.6751	4.2726	9.1916	9.8632	8.9467	8.7783	3.9913	9.1838	3.2523	8.5964	8.7713	8.6661	8.7721	8.7442
Fsd1	_LDGSTSHQNLR_.2	#N/A	2.2415	3.3055	3.5501	3.5707	3.6638	3.9873	4.5362	3.7819	4.0657	4.1223	4.3107	3.5669	4.0444	3.5882	4.3447	3.1701	3.7365	3.4130	3.7120	3.2655
Fsd1	_ALESSELLETANQTLQASDSEDFSQAAK_.3	#N/A	3.8629	4.1399	1.9203	3.8265	3.2006	3.4247	4.3405	3.5633	4.3995	4.0692	3.2731	4.0294	3.8836	4.3863	3.9177	4.2078	4.1010	3.7322	3.3808	4.3533
Mtmr10	_IWLS[Phospho (STY)]TETLANED_.2	Q7TPM9_S763_M1_Mtmr10	4.5550	3.4478	3.5905	3.1344	3.8928	4.0922	3.6483	4.2554	4.4103	3.6625	3.8890	3.3372	4.5758	3.5776	3.2255	3.3991	3.2923	3.0401	4.1895	3.5446
Mtmr10	_RNS[Phospho (STY)]LVLK_.2	Q7TPM9_S603_M1_Mtmr10	4.2014	3.8014	7.6076	3.4880	3.5391	4.4458	4.0019	3.9018	4.0567	4.4077	3.5353	3.6908	4.2221	3.2239	3.5791	3.0455	4.4395	3.3937	3.0423	3.1909
Actn1	_ATLPDADKER_.2	#N/A	8.2786	7.9765	8.4068	8.1141	7.9545	7.8606	8.3378	8.6507	8.2726	8.0723	8.2451	8.2048	8.3562	8.2720	8.0501	8.2341	8.1789	8.4366	8.0921	7.9174
Actn1	_TINEVENQILTR_.2	#N/A	7.9551	7.9919	7.6130	7.6574	7.8262	7.7405	7.9135	8.1934	7.4591	7.8590	8.0678	7.6343	8.1333	7.6574	7.8851	7.7404	8.0459	8.0057	7.8992	7.2063
Actn1	_LASDLLEWIR_.2	#N/A	7.4440	7.5782	7.7209	7.9044	7.1968	6.9881	7.6578	7.3343	7.3522	7.5720	7.6801	7.2008	7.3905	7.6732	6.9043	7.5382	7.9801	7.6788	7.6065	7.5670
Actn1	_ILAGDKNYITEDELR_.3	#N/A	7.8499	7.6109	7.0528	7.0044	6.8336	7.0063	7.2229	7.1879	7.6475	7.5890	7.4744	7.0885	7.4742	7.7982	6.9540	7.3798	7.5327	7.6492	7.2400	7.5707
Actn1	_EGLLLWC[Carbamidomethyl (C)]QR_.2	#N/A	7.4888	7.4740	7.4606	7.4432	7.3144	7.1153	7.4196	7.4452	6.9816	6.9113	7.6067	7.2357	7.3493	7.3178	6.7302	6.9068	7.4543	7.6546	6.7586	6.9655
Actn1	_GYEEWLLNEIR_.2	#N/A	6.9527	6.8695	7.0440	6.8869	6.8009	6.8416	7.3560	7.5343	6.6782	7.1334	7.2695	6.9367	7.0325	7.0421	6.7032	6.7380	7.1808	6.9648	7.0334	7.0316
Actn1	_VGWEQLLTTIAR_.2	#N/A	7.5018	7.1333	6.9095	7.1786	6.8090	7.4482	7.2271	7.2926	6.9937	7.3721	7.2101	7.4409	7.0591	7.3134	6.3694	7.1725	7.3998	7.3124	7.3016	7.0251
Actn1	_TIPWLENR_.2	#N/A	7.3069	6.8821	6.9963	7.1124	7.1123	6.8305	6.5591	6.6946	7.0492	6.5204	7.1028	7.4164	6.9054	6.6252	7.2290	7.0616	6.9948	7.3710	6.7753	7.1295
Actn1	_IC[Carbamidomethyl (C)]DQWDNLGALTQK_.2	#N/A	6.9658	6.8628	6.9277	6.6710	6.2710	6.5503	6.3521	6.7501	6.9782	6.3576	6.4728	6.8392	7.0886	7.1135	6.5878	6.7661	7.1743	6.5638	6.7526	6.4463
Actn1	_FAIQDISVEETSAK_.2	#N/A	7.3121	6.7292	6.8303	6.8768	6.5432	6.4099	6.7841	6.9388	6.5117	6.5418	6.5537	6.4386	7.0205	6.9039	5.6972	6.4118	6.6944	6.7627	6.3866	6.7483
Actn1	_LAILGHNEVSK_.2	#N/A	6.1340	6.5065	6.2788	6.3121	5.4505	5.7811	6.2448	6.5268	6.3150	5.7730	5.8002	6.4279	6.2719	6.5181	5.5435	6.1407	6.6898	6.5791	6.3167	6.3292
Actn1	_ASFNHFDR_.2	#N/A	5.7643	6.0028	5.4141	5.6524	5.4172	6.3106	5.5657	6.7265	5.6405	5.7989	6.0065	6.2087	6.1077	6.0892	6.2406	5.9932	5.8662	6.3016	6.0383	5.6833
Actn1	_DLLLDPAWEK_.2	#N/A	5.8716	6.0025	6.7187	5.9292	6.1007	5.9909	5.9496	6.1250	5.7028	5.9736	6.5570	6.0883	5.0800	5.9049	5.8202	5.8156	6.0131	6.0735	5.9810	5.9230
Actn1	_KDDPLTNLNTAFDVAER_.3	#N/A	4.7477	4.9834	4.7681	6.2654	5.2381	5.5823	5.5510	5.4120	5.6018	5.7997	5.6661	5.7622	6.1732	5.3798	6.0875	5.3729	5.5334	5.6536	5.8947	5.6534
Actn1	_LLETIDQLYLEYAK_.2	#N/A	5.5552	5.2651	5.6910	5.1952	4.9923	4.1378	4.9075	5.1941	4.8986	4.4207	5.2459	4.8616	6.1646	4.9836	5.2392	5.3069	5.1484	5.4041	5.0191	5.2147
Actn1	_RDQALTEEHAR_.3	#N/A	5.6382	5.7640	6.7735	5.7811	6.1619	6.0120	6.3695	6.2054	6.1839	6.6317	7.0102	6.0654	6.3818	6.1881	6.5580	6.6329	6.6148	6.0493	6.7581	6.3510
Actn1	_DDPLTNLNTAFDVAER_.2	#N/A	5.6893	5.5504	5.7899	6.3402	5.5261	4.8725	5.5356	5.7635	6.1056	5.4864	6.1835	5.9016	3.1949	5.9654	6.0304	5.7010	6.0930	6.3039	5.6634	6.1508
Actn1	_DGLGFC[Carbamidomethyl (C)]ALIHR_.2	#N/A	4.8381	4.9066	4.8039	5.5218	4.7698	4.3221	4.9465	4.9792	4.4058	4.3613	5.1506	3.8031	4.2819	4.4752	3.4534	3.5407	5.1955	5.3314	5.1759	4.9428
Actn1	_IDQLEC[Carbamidomethyl (C)]DHQLIQEALIFDNK_.3	#N/A	4.1545	5.0654	3.8214	4.3058	4.6962	4.5502	5.0363	5.5223	5.2142	5.0221	4.2668	5.5759	4.5875	5.5906	2.8445	4.6672	5.3582	5.0420	4.7950	4.4676
Actn1	_QFGAQANVIGPWIQTK_.2	#N/A	3.4284	5.1052	5.2136	4.8493	4.8984	5.0297	4.7242	3.6541	3.3330	4.9510	5.1622	4.9140	5.5537	5.1948	4.1172	5.1355	5.4306	5.3294	5.5238	5.1276
Actn1	_HRPELIDYGK_.3	#N/A	6.1629	4.9269	3.1744	5.6240	5.5842	3.8781	5.3159	6.2304	3.1235	5.1003	5.8304	4.6979	5.9571	5.5285	5.3317	5.7503	5.2822	6.2192	5.8620	4.9064
Actn1	_VLAVNQENEQLM[Oxidation (M)]EDYEK_.2	#N/A	4.8761	4.5841	4.3955	5.2897	4.4647	4.1455	4.5937	4.5399	4.2260	4.4166	4.3936	4.5642	5.7501	5.8046	3.9926	4.8225	5.3120	4.6076	4.3497	5.4238
Actn1	_GISQEQM[Oxidation (M)]NEFR_.2	#N/A	6.1389	6.1480	4.1623	4.0634	6.2112	6.1560	6.0169	6.2688	5.9753	6.3544	5.9448	6.4797	7.1940	5.6717	6.3711	6.0563	5.8791	6.8820	6.1719	6.2462
Actn1	_ALDFIASK_.2	#N/A	3.6723	3.3941	4.2748	5.2619	2.0561	4.3822	4.4844	4.2286	4.9013	5.2009	5.2220	3.6898	3.7031	5.7751	5.5852	5.0531	3.3952	4.9525	4.6004	5.1118
Actn1	_C[Carbamidomethyl (C)]QLEINFNTLQTK_.2	#N/A	3.2397	5.0452	4.2027	5.4597	4.8107	3.4799	3.6015	3.3668	5.3687	4.5511	5.7718	4.4744	3.6872	4.1898	5.6499	5.6410	3.9045	4.8337	5.4813	5.6223
Actn1	_IMSIVDPNR_.2	#N/A	3.5006	4.5022	5.9682	5.8216	4.3392	3.9200	5.1985	3.2010	3.3559	3.2161	5.6595	4.3916	3.5213	6.4303	5.2885	4.2684	3.7387	3.8472	2.6878	3.9910
Actn1	_NVNIQNFHISWK_.2	#N/A	3.3929	4.6099	4.4365	4.2965	4.2315	3.9938	4.2953	3.0933	3.3687	4.9935	5.1661	4.5064	3.4768	4.6091	2.8868	4.7610	3.6310	4.2022	3.5168	3.8833
Actn1	_HTNYNM[Oxidation (M)]EHIR_.3	#N/A	3.4735	4.2299	4.2837	4.2464	4.0284	3.3006	3.2996	5.1722	3.9735	6.5522	6.0064	3.2794	3.4249	3.9666	4.3880	7.6129	6.3143	4.5246	3.8007	4.0245
Actn1	_MVSDINNAWGC[Carbamidomethyl (C)]LEQAEK_.2	#N/A	3.8097	4.2900	5.8353	5.9469	3.1474	5.2050	5.2915	3.2547	4.4527	4.0160	5.7326	4.0161	4.5608	6.0307	4.9820	4.1546	4.2528	4.3975	3.1957	3.7818
Actn1	_AIM[Oxidation (M)]TYVSSFYHAFSGAQK_.2	#N/A	4.4246	4.1058	3.6608	3.9818	3.7933	3.3363	3.2636	3.9121	3.5092	3.4717	3.3980	3.5253	4.6051	4.3182	3.2958	3.5862	4.2822	4.4349	3.2607	4.3852
Actn1	_ASIHEAWTDGK_.2	#N/A	5.3034	3.5863	5.7149	4.9224	5.3775	5.4079	5.2161	5.3957	4.0064	3.2624	5.2778	5.1317	4.3681	5.8152	4.8767	5.1881	5.7408	5.0429	5.1945	5.3010
Actn1	_KHEAFESDLAAHQDR_.3	#N/A	4.0962	3.6539	6.3927	3.6874	4.4953	7.0837	3.5203	3.9955	3.0664	5.0368	5.2626	3.4556	3.6526	3.7281	4.6033	3.9598	3.2447	5.0037	3.8765	3.5321
Actn1	_VEQIAAIAQELNELDYYDSPSVNAR_.3	#N/A	3.1639	2.4382	3.7002	4.5255	4.0024	3.2505	3.0989	4.3651	3.0191	3.5528	3.9986	3.2276	3.1846	3.6872	3.9115	3.5088	3.3586	4.4312	4.0798	4.1887
Actn1	_QKDYETATLSEIK_.2	#N/A	3.6973	3.7723	3.7072	4.5267	3.6689	4.0878	3.4316	4.4097	4.3478	3.5079	3.8153	3.6460	3.9127	4.0030	3.4935	3.8904	3.9218	3.7080	4.1652	3.1380
Actn1	_ISIEM[Oxidation (M)]HGTLEDQLSHLR_.3	#N/A	5.5979	5.5484	4.5054	4.6847	5.3622	3.3553	5.6188	5.6458	4.4689	5.9082	5.7224	5.2594	6.0809	4.3169	4.9297	5.5695	6.1070	3.8016	3.4502	5.3366
Actn1	_MLDAEDIVGTARPDEK_.3	#N/A	4.2660	4.5539	3.8026	3.3182	2.9721	3.6354	4.1298	4.4917	4.1888	4.2799	3.0624	4.2800	4.2969	3.8407	3.7070	3.6281	3.9889	3.1605	3.4596	3.5180
C2cd5	_NQTYSFS[Phospho (STY)]PSK_.2	Q7TPS5_S295_M1_C2cd5	4.1835	3.1356	3.7201	3.4007	7.0541	3.7179	4.0473	4.4092	4.1064	4.3624	2.9799	4.3625	4.2144	3.7582	3.6245	3.5456	7.1625	3.2430	3.5421	6.7838
C2cd5	_SQS[Phospho (STY)]ESSDEVTELDLSHGKK_.3	#N/A	5.3414	7.0674	6.6127	7.0308	5.0485	5.6924	6.5550	3.4406	5.6037	6.5806	5.3065	6.4341	5.4580	6.1397	5.2124	5.4299	6.1510	6.6793	6.6303	6.5713
C2cd5	_SQSESS[Phospho (STY)]DEVTELDLSHGK_.3	#N/A	3.5183	3.6228	3.8835	4.1802	3.9346	3.8001	3.8807	4.0942	3.6469	3.7022	3.8313	3.3916	3.5463	3.9054	3.2315	3.7036	3.9316	4.7523	4.2367	3.6796
C2cd5	_LSSPAAFLPAC[Carbamidomethyl (C)]S[Phospho (STY)]SPSR_.2	Q7TPS5_S259_M1_C2cd5	3.9069	3.4180	3.4434	3.6832	4.1138	4.1641	3.8332	8.2760	4.0254	3.5476	3.9391	6.9275	4.1677	8.4615	2.9655	3.2690	3.3550	7.1919	4.1382	3.2426
Mybbp1a	_SPAPSNPTLS[Phospho (STY)]PSTPAK_.2	Q7TPV4_S1253_M1_Mybbp1a	3.8643	3.9412	3.8978	3.8288	5.6765	3.6875	5.9350	3.8965	5.9183	5.8640	2.9065	3.7830	3.8910	3.9064	4.2331	5.8747	3.8218	3.7099	3.6435	3.7570
Nexn	_TVS[Phospho (STY)]QESLTPGK_.2	Q7TPW1_S296_M1_Nexn	8.4162	8.5100	8.6142	8.6263	8.3738	8.1446	8.4058	8.0133	8.0584	8.5477	8.9741	8.6117	8.6411	9.5898	8.5263	8.7516	9.2300	8.6803	8.3826	8.8942
Nexn	_EM[Oxidation (M)]LAS[Phospho (STY)]DDEEESPK_.2	#N/A	8.8220	9.1762	8.8962	8.8719	8.7160	8.8272	8.3757	9.1442	8.5457	9.0939	9.2687	9.3941	9.5535	10.2839	9.1882	9.2977	9.5260	9.2299	8.7154	9.4276
Nexn	_EMLAS[Phospho (STY)]DDEEESSPKIEK_.2	#N/A	3.7861	4.2167	4.3224	3.9033	3.1238	3.3603	4.4172	3.4865	2.9755	3.9924	3.3499	4.1061	3.8068	4.3095	3.9944	4.1310	4.0242	3.8090	3.4576	4.2765
Lnpk	_ADS[Phospho (STY)]VPNLEPSEESLVTK_.2	Q7TQ95_S411_M1_Lnpk	12.6831	12.6532	12.5739	12.3387	3.5516	12.1786	12.1067	11.9521	12.0126	12.4578	12.0341	11.8667	12.4546	12.2777	10.8961	11.9866	12.5077	12.3345	12.1921	12.0414
Lnpk	_IVEYLVGDGPQNR_.2	#N/A	6.4538	3.6890	5.4481	3.9541	5.2282	5.9376	5.3288	5.6057	5.8397	6.0474	5.9565	5.9824	3.2967	3.7994	4.7518	5.9930	6.1142	5.6035	5.9414	4.6700
Tppp	_AANKT[Phospho (STY)]PPKS[Phospho (STY)]PGDPAR_.2	#N/A	13.0218	12.9463	12.9790	13.2879	12.4460	12.3365	12.4570	12.4051	12.3728	13.1710	12.5651	12.8802	13.1412	13.2234	12.4772	12.9235	12.6083	12.5778	12.6624	12.8200
Tppp	_AVSS[Phospho (STY)]PTVSR_.2	Q7TQD2_S159_M1_Tppp	10.3928	10.7935	10.9870	11.1355	10.0071	9.9550	10.3368	9.9409	10.4201	11.4486	10.2580	10.4380	10.6071	10.4622	10.7046	10.4527	10.1427	10.0021	10.2355	10.4919
Tppp	_VDLVDESGYVPGYK_.2	#N/A	10.5353	10.1005	10.5364	10.4091	10.3651	10.3669	10.0770	10.5233	10.5056	10.4180	10.7696	10.5808	10.9170	10.8628	10.9370	10.9406	10.7080	10.4320	10.5674	10.8134
Tppp	_APVISGVTK_.2	#N/A	8.2816	8.5045	8.5705	8.9811	8.6591	8.7602	8.8338	8.3516	8.7571	8.7174	8.8663	9.1820	9.3042	9.1025	9.5645	9.1160	8.3452	9.1765	9.1034	9.1098
Tppp	_FAVHGDTR_.2	#N/A	8.6850	8.5170	8.9284	8.7725	8.6363	8.7295	8.6092	9.0011	8.6326	8.5964	8.8133	8.9987	8.7476	9.0565	9.4332	8.9691	8.7507	8.7012	8.7638	8.8977
Tppp	_NVTVTDVDIVFSK_.2	#N/A	7.1876	7.2089	8.0332	7.8185	6.9724	6.6706	6.5528	6.9997	7.4458	7.3424	7.1862	7.1215	8.0798	7.6267	7.7596	7.6153	7.5047	6.9441	7.5546	7.6852
Tppp	_TITFEQFQEALEELAK_.2	#N/A	5.7155	5.5352	5.3421	5.6590	5.6242	5.0923	5.0965	5.0491	4.7182	5.8140	5.4474	5.7958	5.9872	6.4918	6.0332	5.7274	5.8505	5.1294	5.2317	5.7357
Tppp	_RLS[Phospho (STY)]LESEGANEGATAAPELSALEEAFR_.3	#N/A	10.8939	11.2017	10.2804	10.5083	9.1405	10.1613	10.9469	10.5615	10.4564	10.3786	9.9242	11.0271	11.3780	11.9927	10.4902	10.2789	10.7358	11.0896	10.8291	10.3019
Tppp	_LSLESEGANEGATAAPELSALEEAFR_.3	#N/A	4.5520	4.3745	4.7395	4.4414	3.9809	4.3670	4.2311	3.9379	5.0208	4.0537	4.9578	4.6585	4.5240	4.6712	5.2946	4.5429	5.2017	4.3153	4.5425	5.0072
Tppp	_DC[Carbamidomethyl (C)]HVIDGK_.2	#N/A	6.7528	3.3865	6.8531	7.0006	6.7270	6.5306	6.8653	7.0682	6.9047	6.8281	3.9502	6.8595	3.1362	3.6388	3.1642	3.4604	6.3507	6.3438	6.8836	7.0112
Tppp	_RLS[Phospho (STY)]LESEGANEGATAAPELSALEEAFRR_.4	#N/A	6.6028	8.4082	7.0951	6.8123	5.3870	6.6580	8.3198	7.0894	7.2909	7.5329	5.4787	8.0150	9.0347	8.6033	7.7902	7.2943	6.6793	7.4589	7.8418	7.4609
Tppp	_LSLESEGANEGATAAPELSALEEAFRR_.3	#N/A	4.7291	4.5701	3.7864	3.3344	5.2623	3.6516	4.6400	5.3277	4.7585	4.2962	3.0461	5.7965	5.3232	3.8244	5.1531	5.5270	3.9727	3.1767	5.2675	5.6822
Tppp	_TPPKS[Phospho (STY)]PGDPAR_.2	#N/A	11.2904	3.6379	3.2840	11.4318	3.6873	4.2433	10.6281	3.9652	10.5116	11.3642	10.8949	11.1083	3.9585	11.5671	10.6859	11.1535	10.8929	10.7528	10.7163	10.9095
Tppp	_DKSSEEAVR_.2	#N/A	3.5648	3.4993	3.5390	3.8845	3.8413	3.3316	4.4386	4.2039	4.3588	3.7140	4.0451	3.7177	4.5243	3.5261	3.2770	3.3476	5.4804	3.0916	4.0188	4.1409
Tppp	_[Acetyl (Protein N-term)]ADS[Phospho (STY)]KAKPAK_.2	Q7TQD2_S4_M1_Tppp	4.4489	3.1248	3.4844	3.2405	3.7866	4.1983	3.7544	4.1493	3.8135	3.1544	4.1879	3.4433	3.5618	3.4714	3.3316	3.2930	3.1861	3.1462	4.2956	3.5114
Maco1	_NASGVVNSS[Phospho (STY)]PR_.2	Q7TQE6_S332_M1_Maco1	8.5198	8.5397	8.2710	8.2328	4.0531	7.9656	8.0723	7.9935	7.9843	7.8641	7.6283	7.5910	8.1724	7.5911	6.7278	7.4318	8.2931	7.7808	7.6892	7.5035
Fbxo10	_EAEVGS[Phospho (STY)]DGER_.2	#N/A	6.4350	7.4207	5.7199	7.7499	6.5714	7.4756	6.8683	7.3593	7.0648	6.8117	6.1666	6.0270	7.3126	7.1427	5.6711	5.7938	7.4176	7.6065	6.7220	6.8673
Amph	_AALPAGEGGS[Phospho (STY)]PEGAK_.2	Q7TQF7_S500_M1_Amph	14.9208	14.6224	15.1356	15.0564	14.6147	14.4862	14.2726	14.9652	14.6747	14.7939	14.5006	14.5885	14.8998	15.2525	14.5372	14.6451	14.9724	14.5034	14.5898	14.7443
Amph	_GLFPENFTR_.2	#N/A	8.8550	9.0930	8.9445	8.9854	8.9239	8.7785	8.8175	9.0643	8.5435	9.1650	8.8153	8.9946	8.8825	8.9881	9.0514	9.0100	8.9956	8.9945	8.9548	8.9035
Amph	_KLVDYDSAR_.2	#N/A	8.8636	8.7390	9.0492	8.9059	8.8188	8.9593	8.8624	8.9975	8.9589	8.8780	8.6357	8.9724	8.9242	8.8755	9.1929	9.0481	8.7971	8.7852	8.7716	8.9466
Amph	_ESDWLQYR_.2	#N/A	8.5285	7.9172	8.8088	8.3374	8.3819	8.0761	7.8631	8.1636	8.1860	8.1785	8.3620	8.1451	8.3660	8.2614	8.3211	8.2873	8.3155	8.0472	8.0587	8.2652
Amph	_EIAVLC[Carbamidomethyl (C)]HK_.2	#N/A	6.9714	7.6068	7.5385	7.6797	7.2793	6.9692	7.4592	7.4121	6.9930	7.3999	7.2513	7.6119	7.0767	7.8193	7.8629	7.2867	7.6058	6.9377	7.6535	7.3960
Amph	_VGPYVNTFK_.2	#N/A	6.4702	6.6264	6.2702	6.5826	6.1857	6.4927	6.6017	6.2644	6.2397	6.0674	6.4162	6.8861	6.6782	6.3450	5.4754	6.2510	6.5633	6.4465	6.1389	6.6302

Amph	_LVDYDSAR_.2	#N/A	5.0204	5.4257	5.6035	6.4549	6.0528	6.6064	6.5420	5.0758	6.5029	6.2902	6.4050	6.5506	6.6225	6.5775	7.0249	6.6757	4.9065	6.4234	6.5065	7.0662
Amph	_ADETKDEQFEEYVQNFKR_.3	#N/A	5.6480	5.7191	5.2392	5.7179	5.5645	5.6751	5.3624	5.9345	5.8666	5.7423	5.4142	6.1149	6.3083	6.4084	5.7229	6.2802	5.4966	5.5688	5.6013	6.0740
Amph	_EAAEDVAAQGSAGEK_.2	#N/A	6.2214	5.6684	5.6235	5.4831	6.6201	6.0365	5.8739	4.3097	6.1966	5.4256	6.3565	5.3633	4.1149	6.0739	6.1118	6.2463	6.0551	5.6269	5.6533	6.1069
Amph	_GDVVLVVPSDSEADQDAGWLVGVK_.3	#N/A	5.3985	5.2151	5.4663	5.0919	4.8426	5.6323	5.0733	4.7064	5.9660	5.4151	5.0244	3.0448	5.4406	6.0233	5.6308	5.5722	3.2151	5.4104	5.1451	5.0883
Amph	_ADETKDEQFEEYVQNFK_.3	#N/A	6.7892	6.3187	6.5371	6.2621	6.3341	6.5093	5.9093	6.2872	6.6858	6.4940	6.2247	6.3766	6.4637	7.4431	6.1906	6.4726	6.5270	6.3428	6.0962	7.0843
Amph	_LVDGSLLTLDTYLGQFPDIK_.2	#N/A	5.6667	6.1289	5.2644	5.2211	5.0283	5.7653	5.8637	5.6406	5.2941	5.7093	5.5104	5.7357	6.0361	6.0803	5.5901	5.3874	5.7269	6.0355	5.0395	5.7754
Amph	_AFSIQGAPSDS[Phospho (STY)]GPLR_.2	Q7TQF7_S252_M1_Amph	7.4987	10.6448	10.0188	10.1267	7.7830	9.7740	9.9772	7.3504	9.7377	9.9534	9.0457	9.8295	7.3561	11.0647	9.3757	9.9094	10.0120	7.2216	9.9237	9.9486
Amph	_TPS[Phospho (STY)]PPEEPSPLPSPTASPNHTLAPASPAPVRPR_.4	Q7TQF7_S262_M1_Amph	3.8741	3.9797	4.0536	4.2448	3.4343	3.6040	9.9566	3.6877	3.5384	3.8806	3.6409	3.6996	3.6681	10.3433	3.8005	3.7721	4.0810	9.3402	4.0021	3.8926
Amph	_VETLHDFEAANSDELNLQR_.3	#N/A	6.1202	5.9055	6.1997	6.3097	5.9743	6.3134	6.4822	5.9982	6.3058	5.7573	5.8900	5.4750	6.5007	6.7519	6.5113	6.3901	5.7631	6.2150	5.9768	6.0992
Amph	_LTESLHEVYEPDWYGREDVK_.3	#N/A	4.4829	4.4308	3.8714	3.9928	4.3657	3.9030	4.2212	3.5776	4.6064	4.5513	3.3010	4.4719	3.9831	4.4006	4.5653	4.2656	4.1153	3.7601	4.1591	4.5764
Amph	_LYEVM[Oxidation (M)]TK_.2	#N/A	3.5591	4.4882	4.0584	4.3227	3.3767	3.4845	3.1647	3.2871	3.2231	3.2862	3.9272	0.9743	3.3437	3.7470	3.6170	4.0597	4.0988	4.1939	3.4770	3.9368
Amph	_C[Carbamidomethyl (C)]DVLWEDFHQK_.3	#N/A	4.4932	3.5096	3.5286	3.1962	3.8309	4.0550	3.7102	4.0721	3.7693	3.1987	4.1436	3.3991	3.8773	3.4211	3.2874	3.3372	3.5694	3.5801	3.8791	3.0984
Plekha6	_TKS[Phospho (STY)]PAEEELTPSAVVR_.2	#N/A	8.3995	8.5653	7.3897	8.4273	7.8211	7.1957	8.1998	8.4370	7.3157	7.6007	7.1217	8.3188	8.9306	8.7971	7.8026	8.2381	7.8660	8.2885	8.3136	7.1264
Plekha6	_DPSQTS[Phospho (STY)]PLGTPR_.2	#N/A	8.2083	8.2967	8.3384	8.2479	3.9572	4.1512	7.7582	8.3224	3.6731	8.3670	8.1169	7.9612	8.2310	8.6469	7.4916	8.1360	8.6292	7.8750	7.9856	8.4062
Plekha6	_SHSVPRS[Phospho (STY)]PSQGSYSR_.3	#N/A	4.2229	4.0843	4.4548	3.7709	3.2172	3.3511	5.2245	3.6189	5.5455	5.2176	3.2436	4.0248	6.3963	4.2445	4.9245	4.0631	4.1566	3.3711	4.9111	4.4786
Plekha6	_S[Phospho (STY)]PSQGSYSR_.2	#N/A	3.8098	4.0782	3.7428	4.0561	3.5661	3.7300	5.4010	4.4204	3.8197	3.7577	3.7552	3.8557	3.9605	3.7566	3.6992	3.5603	5.2563	3.9431	3.8134	3.5779
Plekha6	_ANGLPSGPETAS[Phospho (STY)]EPGSPYPDGPR_.2	Q7TQG1_S247_M1_Plekha6	3.7505	3.5686	3.2871	3.8337	3.9574	3.9908	3.0907	3.9762	3.0109	3.5611	3.9904	5.9378	3.1763	3.6790	3.1161	3.1126	3.4734	5.5109	4.0881	3.6460
Plekha6	_QPVFYDELDAASGS[Phospho (STY)]LRR_.3	Q7TQG1_S443_M1_Plekha6	4.2625	7.3010	6.8719	6.1512	2.9686	3.0019	6.8197	3.8449	6.2931	7.1459	3.4784	6.8480	4.1652	6.9314	4.1049	3.6246	4.3825	3.4506	6.1239	3.1340
Plekha6	_MVENSS[Phospho (STY)]PR_.2	Q7TQG1_S574_M1_Plekha6	3.7881	4.2147	4.3244	4.5335	3.1258	3.3499	4.4153	3.2764	2.9735	3.9944	5.7640	4.1042	4.5824	6.3483	4.3292	4.1330	4.2744	3.8978	3.1740	3.8035
Plekha6	_KDPSQTS[Phospho (STY)]PLGTPR_.2	#N/A	8.4978	8.5430	8.4612	8.4457	8.1555	8.1237	3.7892	9.4667	8.1793	8.4541	8.0956	8.3049	8.6268	8.8979	7.9816	3.6772	8.5447	8.1703	8.1922	8.1782
Plekha6	_SISS[Phospho (STY)]PKYDYLGDR_.2	Q7TQG1_S506_M1_Plekha6	3.9560	3.3631	3.4926	3.6282	4.1629	6.2307	4.3860	4.1817	4.3062	5.7043	3.7848	3.0892	4.4716	3.4735	2.9105	3.3181	6.5074	3.1442	4.2936	6.0707
Plekha6	_QDGTVWIPS[Phospho (STY)]PSR_.2	Q7TQG1_S426_M1_Plekha6	9.0259	8.9063	3.6680	8.9495	8.4170	3.6453	8.4011	8.5369	3.4783	8.8654	7.2194	3.7622	3.6438	4.1464	3.5835	8.5908	3.8119	4.0941	8.4548	8.4277
Plekha6	_IYS[Phospho (STY)]PVRS[Phospho (STY)]PSAR_.2	Q7TQG1_S472_M2_Plekha6	9.7595	9.4890	9.7474	9.7709	9.4914	9.0528	9.2726	3.3944	9.3400	9.6534	9.2685	9.3023	3.1757	9.6660	8.5563	9.4149	9.4761	8.9484	8.9397	9.2040
Plekha6	_AYVPLES[Phospho (STY)]PPTVPPLPNESR_.2	#N/A	8.4657	4.0643	4.3893	7.2796	3.4511	3.4844	8.6151	3.5658	3.5326	7.8394	3.8759	3.8164	8.8294	9.6709	3.6378	7.7529	3.6591	8.9335	7.4273	4.2261
Plekha6	_GVPPPEDLRS[Phospho (STY)]PSR_.2	Q7TQG1_S336_M1_Plekha6	3.9431	3.3760	3.4797	11.4436	4.1500	4.1834	4.3989	4.1688	10.4325	3.7537	3.7977	3.1021	3.9740	3.5177	2.9234	3.3052	3.6660	3.4834	3.7825	3.1950
Plekha6	_S[Phospho (STY)]LQLPASPAPEPSTRPAYK_.3	Q7TQG1_S967_M1_Plekha6	4.0721	5.5029	4.1580	3.9417	4.0348	4.2589	5.6379	3.8792	3.5764	3.4026	3.9508	3.4286	3.6844	6.5938	3.0945	3.5412	3.3765	3.7840	4.0831	3.7813
Atxn2l	_LQPSSSPET[Phospho (STY)]GLDPFPSR_.2	Q7TQH0_T565_M1_Atxn2l	3.9240	8.2638	3.7020	3.8699	4.0043	3.8645	7.6517	8.9782	3.7590	3.8274	3.6259	4.0727	3.9041	8.3754	3.6798	7.5605	7.7075	3.7472	3.8351	3.7442
Atxn2l	_EVDGLLTSDPM[Oxidation (M)]GS[Phospho (STY)]PVSSK_.2	#N/A	9.4708	8.9450	9.1882	8.7668	9.6382	8.5909	8.3707	3.2065	8.7311	9.3331	9.0221	8.8391	9.4142	9.3295	8.3463	8.9919	9.3174	8.7714	8.8752	8.9143
Atxn2l	_EKEVDGLLTSDPM[Oxidation (M)]GS[Phospho (STY)]PVSSK_.3	#N/A	4.9004	5.4246	5.1733	5.9160	3.5560	4.2277	4.8477	3.5515	3.6683	4.9455	3.4980	4.5377	5.4553	5.9589	4.3540	5.1671	4.4625	4.9226	5.0972	5.0221
Atxn2l	_GPPQS[Phospho (STY)]PVFEGVYNNSR_.2	#N/A	9.9246	10.0907	8.5453	9.6335	8.9706	9.3785	9.5479	8.9198	9.2555	9.8164	9.3574	9.7983	9.9484	10.6947	8.9706	9.8187	9.9102	9.7297	9.6197	9.5960
Atxn2l	_QGSGRES[Phospho (STY)]PSLVSR_.2	#N/A	3.6291	5.8324	5.9036	6.1166	3.8360	4.0840	4.9419	3.6574	5.1559	5.7972	3.9962	3.2875	3.3674	6.0817	3.2461	5.4260	3.4538	3.9518	5.2422	4.7574
Atxn2l	_ES[Phospho (STY)]PSLVSR_.2	#N/A	3.6882	4.3146	4.2245	4.0012	3.0259	3.4582	3.6233	3.3886	3.5435	4.5293	3.0221	4.2040	3.7089	4.2116	4.0923	4.0331	3.9263	3.9069	3.5555	4.1786
Otub1	_IQQEIAVQNPLVSER_.2	#N/A	8.0023	7.8196	8.3660	8.0469	7.9221	8.1455	7.7406	8.1263	8.2246	8.1716	7.9084	7.9532	8.0000	8.3725	8.1971	8.0918	7.8074	7.8808	8.2101	7.8959
Otub1	_LLTSGYLQR_.2	#N/A	7.9732	8.2335	7.6711	7.5499	8.0132	7.7585	8.1064	8.1697	7.4227	7.9691	7.6999	8.2321	8.0342	7.8826	7.8307	7.4345	7.7605	7.8979	7.8907	7.4422
Otub1	_EYAEDDNIYQQK_.2	#N/A	7.7094	7.2699	7.6291	7.4938	7.1109	7.6591	7.6469	7.8677	7.5401	7.8804	7.6329	7.7391	7.7112	7.7000	7.3770	7.5990	7.3511	7.3302	7.5428	7.2632
Otub1	_FFEHFIEGGR_.3	#N/A	7.1761	6.8661	6.7745	7.1856	6.9960	6.6551	6.9184	7.2470	6.7816	7.3286	6.8552	7.0158	7.4108	6.6945	6.5383	6.9462	7.0918	6.9496	6.8201	6.8516
Otub1	_LELSVLYK_.2	#N/A	4.1354	4.8499	3.7383	5.3787	4.6430	4.6273	5.4463	4.4274	4.6338	5.7171	5.1455	5.5401	4.7370	5.2986	5.1926	5.0931	3.9247	5.1758	5.3766	5.4801
Otub1	QEPLGSDS[Phospho (STY)]EGVNC[Carbamidomethyl (C)]LAYDEAIM[Oxidation (M)]AQQDR_.3	#N/A	3.4591	4.1666	3.9954	4.2398	3.9033	3.8441	5.9362	3.4163	3.2960	3.5608	3.8997	5.4346	3.4233	4.2505	3.5950	4.0142	3.6059	5.7834	3.9073	5.2727
Otub1	_EFC[Carbamidomethyl (C)]QQEVEPMC[Carbamidomethyl (C)]K_.2	#N/A	3.5211	6.0000	6.8015	7.0592	2.8588	3.0829	6.0802	3.2215	3.2405	3.7274	5.8826	4.3711	3.3485	6.8658	4.2594	3.8660	3.7592	4.0740	3.7226	4.7969
Otub1	_AFGFSHLEALLDDSKELQR_.3	#N/A	3.2646	3.3837	3.8009	3.8389	4.1031	4.3272	3.4381	3.2726	3.4970	3.4709	3.8714	3.1289	3.6051	3.7880	3.0151	3.6095	3.5026	3.9279	3.4204	3.9718
Otub1	_GEGGTTNPHVFPEGSEPK_.3	#N/A	3.5943	4.4085	8.1327	4.0951	7.4856	7.6319	8.1606	3.2947	3.4495	3.1224	2.9282	7.3782	3.6150	8.6753	3.5547	4.1748	8.1719	7.8514	7.9891	4.0846
Abtb2	_SPTVDS[Phospho (STY)]GDIR_.2	Q7TQI7_S444_M1_Abtb2	5.7858	3.1682	3.6875	3.4333	2.8570	3.7505	4.1911	4.3766	4.0737	4.3950	2.9473	4.3951	4.1818	3.7256	4.2165	3.5130	3.8738	6.0688	3.5747	3.4028
Ccdc93	_QSELSAEES[Phospho (STY)]PEKLGTSQLHQR_.3	#N/A	7.5670	7.9334	7.4748	7.5862	7.0027	7.6664	7.6018	7.4540	7.3262	7.5992	7.2679	7.1857	7.3056	8.1195	6.8553	7.8263	7.7679	7.7889	7.6084	7.4233
Ccdc93	_QSELSAEES[Phospho (STY)]PEK_.2	#N/A	5.7424	5.4182	6.3155	3.6116	5.8078	5.7414	4.3693	4.1983	4.2896	5.9030	6.0702	3.0726	4.4550	6.0957	2.8939	5.8815	3.6956	3.4539	5.3530	5.6191
Prrc2a	_KGTS[Phospho (STY)]PGSELPPPK_.2	#N/A	4.0003	3.3866	3.6638	3.9459	3.9661	3.9740	3.5872	4.3288	3.4789	7.0266	4.1184	3.2761	3.6095	3.6509	3.1644	3.4724	3.3656	8.3959	3.9337	3.4587
Prrc2a	_SDS[Phospho (STY)]GGSSSEPFER_.2	#N/A	9.4780	9.2350	9.0414	9.5024	9.0010	8.8054	9.0152	8.8859	8.7912	9.6191	3.8467	9.0752	9.4620	9.8279	8.3040	9.1877	9.2958	8.8835	8.7190	9.2355
Prrc2a	_GTS[Phospho (STY)]PGSELPPPK_.2	#N/A	3.6800	4.3228	4.2163	4.4255	3.0178	3.2418	3.0225	3.3844	3.0816	3.8864	3.4559	9.4251	3.1896	4.2342	4.1005	4.0249	4.3825	4.2678	3.0660	3.9115
Prrc2a	_EGPEPPEEVPPPTT[Phospho (STY)]PPAPK_.2	Q7TSC1_T609_M1_Prrc2a	3.2657	3.2362	3.8020	4.4236	4.1043	4.3284	3.4368	3.7987	3.4959	3.4721	3.8702	3.1257	3.6039	3.1477	3.0140	3.6106	3.5038	9.3075	4.1525	4.3258
Prrc2a	_S[Phospho (STY)]EGSEYEEIPKR_.2	#N/A	6.9000	4.0551	6.5713	4.3202	2.9125	3.1366	3.1277	7.2819	3.1868	3.7811	3.5612	3.7812	3.2949	4.3395	4.2057	3.9197	4.4877	6.9019	2.9608	4.0167
Prrc2a	_ETPPGGNLS[Phospho (STY)]PAPR_.2	Q7TSC1_S1002_M1_Prrc2a	12.0561	11.8318	12.3558	12.2507	11.3505	10.8501	11.2871	10.2254	10.9472	12.5080	11.2496	11.5404	11.7724	11.8111	9.9302	11.3936	10.9184	10.8339	10.7529	11.4718
Prrc2a	_KQS[Phospho (STY)]SSEISLAVER_.2	Q7TSC1_S454_M1_Prrc2a	3.9200	4.0828	6.9113	3.7694	3.2577	5.6237	4.2833	3.6204	5.2176	4.1263	3.2160	3.9722	4.4505	4.4434	3.8605	4.2649	4.1581	3.6751	3.3237	6.1809
Prrc2a	_TASETRS[Phospho (STY)]EGSEYEEIPKR_.3	#N/A	3.7015	4.4667	3.8373	4.1581	3.7312	3.9558	3.5361	6.4911	4.9888	4.3045	5.9386	3.5404	3.8358	3.8477	6.5365	5.1488	3.7790	4.0401	6.1369	3.6881
Prrc2a	_ERS[Phospho (STY)]DS[Phospho (STY)]GGSSSEPFER_.2	Q7TSC1_S759_M2_Prrc2a	4.7141	5.1556	3.8019	5.0860	7.3933	4.3786	3.6018	3.9595	3.6609	5.1427	4.6214	7.8260	5.3816	5.0613	6.5381	4.8679	5.0163	7.3247	4.4760	3.6809
Prrc2a	_TAS[Phospho (STY)]ETRS[Phospho (STY)]EGSEYEEIPK_.2	#N/A	4.2831	3.7579	3.2017	3.8891	3.7090	3.8090	3.5897	4.0370	4.0387	4.2471	3.8486	3.4906	4.1872	3.8934	3.4551	4.2010	3.7342	3.4361	3.8126	3.1081
Prrc2a	_FS[Phospho (STY)]REEFPTLQAAGDQDK_.3	Q7TSC1_S166_M1_Prrc2a	3.1755	4.1436	4.2129	4.4087	3.3824	3.4158	3.6657	3.4310	3.5859	4.4869	3.0645	3.8697	3.7513	4.2540	3.6911	4.0384	3.9687	4.7357	3.5131	4.2210
Prrc2a	_S[Phospho (STY)]EGSEYEEIPK_.2	#N/A	3.6792	4.3236	4.2155	5.5444	3.0170	5.7031	3.0233	3.3796	5.3655	3.8856	3.4567	4.2130	3.1904	4.2026	4.1013	4.0241	3.9173	4.2670	3.5645	5.0469
Prrc2a	_GSETGSETHES[Phospho (STY)]DLAPSDK_.2	Q7TSC1_S1113_M1_Prrc2a	3.1973	3.3046	3.7336	4.4921	4.0359	3.9490	3.5052	4.3985	3.0526	3.4036	4.0321	3.1941	3.2180	3.7207	3.6201	3.5422	3.4354	4.3978	4.0464	3.6877
Prrc2a	_LKFS[Phospho (STY)]DEEDGRDS[Phospho (STY)]DEEGAEGHK_.4	#N/A	3.9306	4.3656	4.0899	4.7640	3.2768	3.3101	3.7883	3.2650	4.6973	4.3813	3.1872	4.1729	3.8740	4.1002	3.8137	3.9328	4.0306	3.7588	3.4428	3.7035
Lrrc75a	_AS[Phospho (STY)]PGAAPGPR_.2	#N/A	9.8964	9.8670	10.1038	9.9478	9.3752	9.6082	9.5710	9.7279	9.5009	9.7345	9.3989	8.9517	9.3058	9.6444	8.6067	9.3641	9.4874	9.2656	9.4447	9.4766
Lrrc75a	_EGTDQQDPIGS[Phospho (STY)]PVTPAR_.2	Q7TSF4_S322_M1_Lrrc75a	8.5518	8.6230	8.3503	8.6485	7.4919	8.2916	8.3140	8.5062	8.0505	8.7512	8.1080	8.0652	8.0542	8.6608	7.0247	8.0669	8.5523	8.1477	8.3639	8.1807
Lrrc75a	_GSLAERAS[Phospho (STY)]PGAAPGPR_.3	#N/A	4.0809	3.9219	3.1163	3.6085	3.0321	3.0655	4.0160	3.7813	3.9362	4.1366	3.4148	3.8114	4.1016	4.6043	4.0413	4.7284	4.3189	3.5142	3.1628	3.0704
Ctdp1	_DNS[Phospho (STY)]PAVFPDR_.2	Q7TSG2_S730_M1_Ctdp1	7.0783	7.0901	7.0986	7.4982	3.3324	3.2751	6.9743	6.6608	6.8279	6.8358	6.9723	6.5483	6.2225	6.9268	3.7411	6.8726	6.8519	6.1088	6.6984	6.8026
Phkb	_[Acetyl (Protein N-term)]ANS[Phospho (STY)]PDAAFSSPALLR_.2	Q7TSH2_S4_M1_Phkb	7.4865	7.6911	6.9725	7.0699	4.0480	7.3381	7.4742	7.4047	6.9785	6.8897	6.7279	6.9048	3.6602	7.3063	3.0702	3.5544	7.6647	4.3856	7.1952	7.0876
Phkb	_QSS[Phospho (STY)]TADAPEAQHEPGITITEWK_.3	Q7TSH2_S693_M1_Phkb	3.6890	7.2700	3.2256	3.8952	3.8960	3.9293	3.1521	3.9147	6.8023	3.4996	4.0518	3.3562	3.2378	3.7405	3.1775	3.0511	3.4119	4.3780	4.0266	3.7075
Kcnf1	_LSHS[Phospho (STY)]DTFIPLLTEEK_.2	#N/A	4.0504	3.4606	3.4147	3.7257	3.9510	5.3351	3.8479	4.1038	5.5440	3.4910	3.9803	3.1867	4.0472	3.4528	3.1716	3.2157	3.6011	6.0450	3.8671	3.7534
Tmem94	_LPS[Phospho (STY)]AETLK_.2	Q7TSH8_S664_M1_Tmem94	4.5205	4.2994	4.0571	6.6048	3.2266	3.2599	3.8215	3.2453	3.7417	4.3311	6.6626	4.0256	3.9072	4.4098	3.8469	3.8826	4.2434	5.8916	3.3572	3.4232
Tmem94	_VEPPHSS[Phospho (STY)]HEDLTDDLSTR_.3	#N/A	3.6187	9.5074	7.8003	7.5338	3.8965	8.0021	7.8598	4.0456	7.5422	8.7886	8.3723	7.2322	9.0540	7.5950	6.1182	6.8259	6.8243	9.4128	7.5726	7.5094
Ppp6r1	_NTVDLVNTHHLHSSS[Phospho (STY)]DDEDDRLK_.4	Q7TSI3_S531_M1_Ppp6r1	4.0139	4.8815	3.6008	3.8591	3.4582	4.3720	3.8366	4.2747	4.6111	4.0971	3.4708	4.6160	3.9536	3.6069	3.5227	3.2092	3.9798	3.7499	3.5765	3.4714
Ppp6r1	_EQM[Oxidation (M)]IQGQDS[Phospho (STY)]PEPDQLLATLEK_.3	#N/A	4.0296	4.0510	3.8352	3.7376	3.3673	3.5914	3.5208	3.7300	3.5799	4.2359	3.9543	3.9404	3.6880	3.8223	3.7786	3.6438	3.5370	3.6433	3.6134	3.6601
Ppp6r1	_EADMSSIQIPSS[Phospho (STY)]PPAHGSPQLR_.3	Q7TSI3_S739_M1_Ppp6r1	3.6421	3.2380	3.8003	4.4254	3.8490	3.8824	3.1991	4.4652	3.1193	3.4527	4.0987	3.1274	3.2847	3.7874	3.2244	3.0042	3.5020	4.3311	3.9797	3.7544
Map6	_ATGPAPGPS[Phospho (STY)]VDRETVAAPGR_.3	#N/A	8.9058	9.0797	8.2775	8.8719	8.0250	8.3788	8.4762	8.4377	8.0985	8.7822	8.0125	8.0200	8.5544	8.5812	7.7293	7.9323	7.8742	8.0812	7.9588	8.0895
Map6	_SEYQPSDAPFER_.2	#N/A	9.3919	9.0632	8.9654	9.0481	9.4562	9.2466	8.8708	9.6218	9.0476	9.5050	9.0920	8.9131	9.6344	9.2570	9.0026	9.0443	9.0356	8.6593	8.7247	8.8855
Map6	_DQSFPTPAPR_.2	#N/A	8.7713	9.0888	9.2858	9.0453	9.0403	9.0848	9.2068	9.4776	9.0713	9.1342	9.0697	8.9721	9.2150	9.1135	9.3987	8.9267	9.0414	8.7139	8.9131	9.0969
Map6	_DQVPVVPGSLK_.2	#N/A	8.2091	8.6562	8.5924	8.7897	8.5567	8.6639	8.7846	8.5882	8.4116	8.8601	8.4675	8.4867	8.3771	8.7434	8.7297	8.5200	8.6230	8.1814	8.3203	8.6060
Map6	_KDPGPVIPEPEKDR_.3	#N/A	8.2335	8.1860	8.9472	8.2135	8.3458	8.4838	8.2768	7.9551	8.5570	8.6476	8.4153	8.3508	8.2336	8.4361	8.4417	8.4043	8.3116	7.9887	8.1891	8.5225
Map6	_AVADALNR_.2	#N/A	7.5695	7.9826	7.8823	7.9108	7.9593	7.9546	8.0075	7.8109	7.9680	7.7889	7.5597	7.9398	6.9517	7.8953	8.3619	7.6148	7.3423	7.8325	7.6053	7.7523
Map6	_ADIAVPLVFTK_.2	#N/A	7.9534	7.9444	8.1743	8.0886	7.9711	7.8548	8.0831	7.6708	7.6374	7.7907	7.4145	7.8685	7.6932	7.9106	7.8147	7.6858	7.5786	7.7254	7.6083	7.8701
Map6	_NQGPVIPEPVK_.2	#N/A	8.0143	7.7841	8.0213	7.9655	8.0167	7.7575	7.7549	7.8822	8.1269	8.0530	7.8135	7.9248	7.4157	7.8180	8.1573	7.5436	8.0198	7.5687	7.4244	7.9028
Map6	_DQGPTAPDPLK_.2	#N/A	7.5280	7.3747	8.1334	7.5386	7.7123	7.7724	7.5407	7.6623	7.7944	7.3704	7.6792	7.4458	7.4039	8.0501	7.7798	7.5715	7.5560	7.2357	7.3033	7.7535
Map6	_NQGLAGPELVK_.2	#N/A	7.3956	7.6375	7.8539	7.8474	7.5877	7.7354	7.6187	7.4471	7.6620	7.7282	7.5822	7.7160	6.9927	7.5798	7.9031	7.3899	7.4956	7.3460	7.3585	7.5501
Map6	_NKDSVPLAPAK_.2	#N/A	6.8891	7.0702	6.9016	6.9687	6.7566	7.0427	6.9448	6.6089	6.9305	6.6090	6.5892	7.1419	7.0270	7.1091	7.4855	7.1127	6.7324	6.9238	6.8598	6.7766
Map6	_DQGAVLLGPVK_.2	#N/A	6.2994	6.6302	7.5543	6.8836	6.9399	6.9542	6.8333	7.2318	6.8468	7.2993	6.9950	6.5387	6.4543	7.1783	7.2107	6.9257	6.7090	6.5235	6.6578	6.5859
Map6	_NQSPVVPASTK_.2	#N/A	5.9795	6.0974	7.2067	6.8356	6.4140	5.4805	6.0921	6.7592	6.5646	6.4647	6.9971	6.5021	6.2906	6.9055	6.7896	6.6609	6.6834	6.3025	6.3032	6.6871
Map6	_GHDSVFVAPVK_.2	#N/A	6.8817	6.6165	6.6135	6.8258	6.1188	6.6828	6.5577	6.9678	6.6418	6.3056	6.4538	6.5879	5.9874	6.8613	6.7977	6.2857	6.4413	6.2499	6.1099	6.5398
Map6	_DTGTDTTAPR_.2	#N/A	5.6856	6.2923	6.4392	5.9413	6.7181	6.2692	6.6550	6.4168	6.4770	6.9166	6.4583	6.1604	6.1112	6.6967	6.7890	6.3756	6.0853	6.2932	6.2370	6.3058
Map6	_DKQVVSGQAAK_.2	#N/A	6.9459	7.4207	6.1155	6.6476	6.2342	6.5229	6.9092	6.7312	6.6297	6.5160	7.0590	5.9413	6.8051	6.3399	6.3035	6.7550	7.1153	6.3558	6.2800	7.0786
Map6	_FWNQLDK_.2	#N/A	5.6552	6.0687	5.7635	6.4812	6.0532	5.3918	6.3439	5.7448	5.5965	5.9432	5.8965	5.9817	5.5584	5.5781	5.8403	6.0845	5.4052	6.1580	5.8303	6.3241
Map6	_DPEGAGGAGVLAAGK_.2	#N/A	3.0377	3.2277	4.9237	4.4273	3.7835	3.5630	3.9255	3.6180	4.4002	4.7228	3.3218	3.7667	4.4118	4.2441	3.6117	3.6165	3.2156	2.8341	3.7707	4.9631
Map6	_AVAIETQPAQGESDAVAR_.2	#N/A	8.6966	8.8736	9.1401	9.1189	8.2150	8.6910	8.6677	8.6947	8.7742	8.8962	8.1969	8.4308	8.8193	8.6151	8.4171	8.5380	8.6208	8.2284	8.3026	8.5789
Map6	_ATGPAPGPS[Phospho (STY)]VDR_.2	#N/A	8.3930	8.2167	8.7059	8.3911	8.0670	7.9970	7.8644	7.1154	8.0681	8.4088	7.8183	7.5103	7.5553	8.3540	7.8224	7.7534	7.6341	7.4321	7.6628	7.8213
Map6	_GQS[Phospho (STY)]PTAPGPTKDQGAVLLGPVK_.3	#N/A	9.2553	10.1264	4.1100	4.1157	2.9114	6.9809	9.3446	10.2936	3.4290	8.5762	2.9076	9.5169	3.5944	10.3673	3.5341	4.1954	3.8117	7.0493	3.6700	7.0851
Map6	_NQS[Phospho (STY)]PVVLGPIK_.2	Q7TSJ2_S832_M1_Map6	8.1429	7.5403	8.3892	7.5148	3.6440	7.0098	6.9660	6.7504	6.7791	8.3210	7.4620	6.9036	6.4039	7.7930	5.3254	7.0197	6.9213	6.7613	6.5859	7.2355
Map6	_VQDHIASELLK_.2	#N/A	5.5176	5.5712	5.8003	5.4215	5.4407	5.3235	5.3649	5.9248	5.1693	5.5716	5.3603	5.5175	5.8573	5.6604	5.6660	5.0659	5.1955	5.0087	5.4981	5.6392
Map6	_AGPAWM[Oxidation (M)]VTR_.2	#N/A	8.3027	8.6143	8.3702	8.6524	8.4019	8.3057	8.5173	8.4514	7.8986	8.1963	8.0314	8.2886	8.3181	8.3580	8.0664	7.6627	7.7683	8.0443	7.8075	8.0024
Map6	_AQS[Phospho (STY)]PLLPEPLKNQSPVVPASTK_.3	#N/A	7.8612	8.8339	7.0251	7.0886	3.2367	5.6459	8.1810	8.3150	5.5138	8.0448	4.6977	8.0236	7.2094	9.3418	5.6562	6.5517	6.5435	7.3712	6.5952	5.6851
Map6	_RPQS[Phospho (STY)]QERGPM[Oxidation (M)]QLSADAR_.3	#N/A	3.6587	6.4666	5.9908	6.3155	5.0029	5.8058	6.2182	3.6349	5.4633	6.0542	5.8560	3.9259	3.6843	3.8029	4.9045	5.5589	5.3460	5.6734	5.3067	4.2809
Map6	_GPM[Oxidation (M)]QLS[Phospho (STY)]ADAR_.2	#N/A	5.6731	6.7586	7.0659	7.0861	6.7442	6.9999	6.9463	6.5049	6.6099	6.7882	6.7032	6.6678	6.3649	6.7874	6.6214	6.8061	6.7305	6.6047	6.6570	6.8419
Map6	_S[Phospho (STY)]LYSEPFKEC[Carbamidomethyl (C)]PK_.2	Q7TSJ2_S491_M1_Map6	6.0844	4.0124	4.5267	3.6990	3.3281	4.9277	4.2129	3.0741	4.2720	4.1967	4.6190	3.9018	4.3801	3.9239	3.7901	4.3353	4.0721	3.0773	3.3764	3.6011
Map6	_DQVPVVPGSLKGQS[Phospho (STY)]PTAPGPTK_.3	#N/A	9.5251	10.1152	3.7356	4.2166	7.5951	7.7996	3.7366	10.4030	3.7957	3.7717	3.7056	9.7465	10.2279	10.3690	7.9383	8.4924	8.4885	9.1941	8.6431	3.4419
Map6	_EPHKDQGSVAPGLPK_.3	#N/A	3.2838	5.9407	5.8338	4.3004	4.9819	5.7736	5.4496	3.5095	5.3696	5.6940	5.7301	3.7614	3.6430	5.4604	5.5304	5.7175	4.5076	4.4565	5.7549	5.2852
Map6	_RSEGHEQT[Phospho (STY)]PAAHAQGTGPEGGK_.3	Q7TSJ2_T392_M1_Map6	4.5638	5.1224	3.9599	5.1472	4.7486	4.9009	5.2569	4.9293	5.1044	4.6288	4.5445	4.1982	4.8630	5.1725	4.0726	4.3350	4.0075	4.5981	4.9607	4.0635
Map6	_AKPQYKPPDDK_.3	#N/A	4.8060	4.0996	5.1673	4.9922	3.4264	4.6416	5.3936	3.4452	4.7151	6.3206	3.5167	3.8257	4.8489	3.9807	5.9862	5.4848	4.7986	4.4465	5.1747	4.9642
Map6	_EEVASTVSSSYR_.2	#N/A	6.1587	5.8294	6.8753	6.2374	3.6906	6.3527	5.8154	4.0939	6.3342	3.8963	6.3271	5.8121	4.1542	3.4309	6.2587	6.2647	5.8313	5.5982	5.6069	6.2904
Map6	_GQS[Phospho (STY)]PTAPGPTK_.2	#N/A	14.4570	14.3414	14.6600	14.6578	14.1821	13.9774	14.0662	14.2705	14.0668	14.4193	13.8412	14.0523	14.2605	14.4114	13.6436	14.0573	14.0636	13.8193	13.7839	14.0530
Map6	_TEGHEETPLPPAQSQTQEGGPAAGK_.3	#N/A	6.9467	6.7272	7.1139	5.6562	4.5440	5.8789	6.5544	6.4385	5.9070	6.4436	5.7572	6.3700	3.6893	6.2680	6.3252	3.0206	3.3814	6.0945	4.0671	5.8332
Map6	_TTEGPSATKPDDKEQSK_.3	#N/A	4.0675	3.3507	5.3171	4.2595	3.4333	4.0600	3.7161	3.6123	3.5528	4.1162	3.8753	3.8313	3.6939	2.9597	4.0653	4.4961	4.0479	3.7611	4.6892	4.2747
Map6	_YSEATEHPGAPPQPPAPLQPALAPPSR_.3	#N/A	4.5596	5.5503	5.7128	4.8697	3.2657	3.2990	4.8987	5.1537	5.4647	5.1652	3.1813	3.9865	3.8681	5.8913	5.4307	5.4348	5.1361	4.6424	4.0915	4.3377
Map6	_S[Phospho (STY)]EGHEQTPAAHAQGTGPEGGK_.3	#N/A	3.9301	4.1564	3.4667	3.6632	4.1370	3.9597	4.6859	4.4777	3.7949	4.1307	3.7749	2.8823	3.6708	3.1390	2.9455	3.3130	3.6530	3.2060	3.3518	3.4459
Map6	_GTQLPTVSPSPPVM[Oxidation (M)]IPTVPHTEYIEGS[Phospho (STY)]P_.3	#N/A	6.9735	8.4106	3.7874	3.3337	3.2324	3.3795	8.0756	8.9931	4.0732	4.2046	3.2639	8.3787	7.4040	10.2442	5.4198	3.3342	5.9984	8.5136	6.4932	3.5028
Map6	_GPM[Oxidation (M)]QLS[Phospho (STY)]ADARDPEGAGGAGVLAAGK_.3	#N/A	4.3832	4.2320	3.9198	4.0762	3.0893	4.1553	3.4102	4.1794	3.3851	4.0619	3.3767	4.1819	4.0635	4.8158	3.0694	3.3287	3.3457	3.4064	2.7151	3.9774
Map6	_M[Oxidation (M)]VHETSYSAQFK_.3	#N/A	3.6706	4.5471	3.9931	4.0091	3.9985	4.2363	4.1172	3.8152	3.2071	4.0873	3.2283	3.4701	3.3409	3.2405	3.9050	3.7833	3.3124	4.4950	4.2802	4.4739
Map6	_GEANKPSAADNK_.2	#N/A	5.7734	5.7104	4.5858	3.6399	3.3873	4.9536	5.2719	5.8603	5.6344	5.1853	5.1311	4.1014	5.4693	5.3905	4.0100	4.3944	5.4709	5.0744	3.1942	3.0391
Map6	_SEGHEEKPLPPAQSQTQEGGPAAGK_.4	#N/A	3.6380	3.6811	3.1745	3.9463	3.8449	3.8782	3.2032	3.8636	3.1234	3.4486	4.1029	3.4072	3.6688	3.2126	4.4365	3.0001	3.3609	5.0081	4.0876	4.3907
Map6	_SGLGLGAASASTSGSGPADSVM[Oxidation (M)]R_.2	#N/A	3.8983	4.5484	3.9495	3.4903	4.3120	3.4310	4.0850	3.6131	4.2694	4.1718	3.6846	4.2369	3.9292	4.3031	3.5746	4.2106	3.6213	3.8603	3.7299	3.8084
Map6	_RRPQS[Phospho (STY)]QER_.2	#N/A	3.5193	4.5170	3.9231	4.3026	3.7262	5.1765	3.3219	3.0872	3.2421	3.3299	4.2216	3.0046	3.4075	3.9102	3.3473	4.3822	3.6249	4.2083	3.8569	3.8772
Map6	_AQS[Phospho (STY)]PLLPEPLK_.2	#N/A	4.5097	4.3103	4.0462	4.5754	3.2158	3.3918	3.8323	3.2345	4.4325	4.0363	3.3060	4.0364	4.5405	12.5943	3.8577	3.8718	4.2326	4.4177	3.2159	4.7714
Map6	_NEGHEEKPLPPAQSQTQEGGPAAGK_.4	#N/A	3.4190	4.5838	3.9553	6.0192	4.2576	4.4817	3.2835	3.6454	3.3426	3.6254	3.7169	2.9724	3.4506	4.4952	2.8607	3.7639	3.1427	4.0068	4.3058	4.1725
Sez6	_S[Phospho (STY)]PLQLPR_.2	Q7TSK2_S952_M1_Sez6	7.8823	7.1515	7.6102	7.7200	7.5182	7.3135	6.6711	6.9665	7.3260	8.1732	7.9411	6.8378	6.9596	7.5195	6.9303	7.7193	8.0087	7.6554	7.4310	7.7117
Pcdh8	_EERPGAAGGGAS[Phospho (STY)]APGS[Phospho (STY)]PDETAR_.2	Q7TSK3_S798_M2_Pcdh8	4.7699	3.9828	3.7211	4.2479	3.6179	4.3028	3.5049	3.6367	3.9285	3.9952	3.6773	3.7089	4.0525	4.2954	3.5302	4.1796	3.9075	4.0902	3.6620	3.4365
Pcdh8	_DSGKGDSDFNDS[Phospho (STY)]DSDISGDALKK_.3	Q7TSK3_S926_M1_Pcdh8	4.3981	4.4218	3.9347	3.1861	3.1042	3.1375	3.9439	3.1229	3.8641	4.2087	3.3427	4.1479	4.0295	4.5322	3.9693	3.7602	4.1210	3.5863	3.2349	2.9984
Pgm2	_[Acetyl (Protein N-term)]AAAT[Phospho (STY)]PTETPAPEGSGLGMDAR_.2	Q7TSV4_T5_M1_Pgm2	4.0065	3.3126	3.5431	3.5777	4.2134	4.2468	4.3355	4.2322	4.2557	3.8171	3.7343	3.0387	4.4211	3.5812	2.8600	3.3686	3.7294	3.1947	4.3441	3.3900
Atf7ip	_NKQEDLNSEALS[Phospho (STY)]PSITC[Carbamidomethyl (C)]DLSSR_.3	Q7TT18_S112_M1_Atf7ip	3.7641	3.5549	5.8081	3.8201	3.9711	4.0044	3.0770	4.3432	4.9845	3.5747	3.9767	3.2811	6.3413	6.9144	3.1024	3.1263	3.3800	5.3494	5.8832	3.6323
Atf7ip	_SKS[Phospho (STY)]EDMDSVESKR_.2	#N/A	4.0756	3.2435	5.3622	4.9490	4.2825	3.8259	3.9393	4.3012	3.9984	4.4704	4.3728	2.9697	4.1064	3.6502	3.5165	4.3477	3.7985	3.3510	3.6500	3.3275
Atf7ip	_SKS[Phospho (STY)]EDMDSVESK_.2	#N/A	3.1603	4.8939	4.9325	4.5291	3.9988	4.2229	3.5422	4.3615	3.6013	3.3666	3.9757	3.2311	3.1810	3.6836	3.1194	3.5052	3.3983	4.4348	4.0834	3.6506
Atf7ip	_EAFLVLS[Phospho (STY)]DEEDLSC[Carbamidomethyl (C)]GKDESEAVAQSK_.3	Q7TT18_S533_M1_Atf7ip	3.7700	3.5491	3.3065	3.8143	3.9769	4.1315	3.6337	3.9956	3.6928	3.2751	4.0672	3.2752	3.8008	3.3446	3.0966	3.1321	3.4929	3.6566	3.9556	3.0219
Rexo1	_S[Phospho (STY)]LDEGAPQDTPK_.2	Q7TT28_S487_M1_Rexo1	8.1653	8.1912	7.6295	7.7995	3.6592	8.0204	8.0265	7.9921	7.4863	7.7755	7.7128	3.5708	7.9536	7.6171	6.8942	7.4578	8.0299	8.0294	7.8723	7.4707
Rexo1	_ALS[Phospho (STY)]HAELFGDES[Phospho (STY)]EEEDSSLGAGAPR_.3	Q7TT28_S505_M2_Rexo1	3.1046	3.3974	3.6409	4.5848	3.9432	4.1672	3.5979	4.3058	3.6570	3.3109	4.0314	3.2868	3.7651	3.6280	3.1751	3.4495	3.3427	2.9897	4.1391	3.5950
Elp1	_SSGNSANDLANVAVIDGNR_.2	#N/A	4.9153	5.6418	5.9928	5.5934	5.6895	4.7473	5.6534	5.8033	4.1148	5.2981	4.8548	5.9661	5.9962	5.5182	3.6329	5.5699	5.9199	5.6789	5.1721	5.5729
Cdc42bpb	_ALGNS[Phospho (STY)]NRDKEIK_.3	#N/A	6.1728	5.9678	5.3128	5.3664	4.0744	4.6212	5.8065	4.9729	4.5047	6.0847	5.3222	5.0472	5.3852	5.2335	4.0813	5.2034	4.9641	5.2774	5.4770	4.8447
Cdc42bpb	_HSTPSNSSNPSGPPS[Phospho (STY)]PNS[Phospho (STY)]PHR_.3	Q7TT50_S1692_M2_Cdc42bpb	7.3909	7.1287	7.0812	7.3023	6.5377	7.3328	7.1420	7.0248	6.8145	6.8985	6.8548	6.5669	6.7144	7.0350	6.1203	6.6457	7.3257	6.9959	6.9355	6.6316
Cdc42bpb	_SM[Oxidation (M)]S[Phospho (STY)]DPDQDFDKEPDSDSTK_.2	Q7TT50_S1661_M1_Cdc42bpb	3.6908	3.6283	3.2274	3.8934	3.8978	4.2106	3.1503	3.9165	3.0783	3.5014	3.9880	3.3544	3.7217	3.2655	3.1757	3.0529	4.4678	3.7357	4.0348	4.4436
Ahcyl1	_VVLLAEGR_.2	#N/A	5.9926	5.6866	6.4889	6.3792	6.4861	6.4347	7.0072	5.6501	6.7453	6.4628	6.3232	7.5366	6.5479	6.2097	5.9647	6.1883	5.2101	7.0008	6.5177	6.2037
Ahcyl1	Acetyl (Protein N-term)]S[Phospho (STY)]M[Oxidation (M)]PDAM[Oxidation (M)]PLPGVGEELK	#N/A	7.8138	7.9320	8.0183	7.2058	6.5859	7.3653	7.5546	7.4857	7.6426	7.3912	7.2602	7.6037	7.8725	7.5534	6.5238	7.3388	7.8960	7.5518	7.5181	7.2595
Ahcyl1	_EIEIAEQDM[Oxidation (M)]SALISLR_.2	#N/A	7.0228	6.3660	6.2372	6.3520	5.7376	6.1720	6.1099	6.6792	6.5945	6.8131	5.4123	5.6024	6.7721	6.1434	5.4036	6.5738	6.4820	6.1316	6.0666	6.2436
Ahcyl1	_QVDVVITC[Carbamidomethyl (C)]TGNK_.2	#N/A	4.0839	6.2341	3.1194	3.6055	3.4216	6.2411	4.1194	3.7843	3.9392	5.6903	3.4178	3.8083	4.1046	4.6073	3.6966	4.4288	4.3220	3.5112	3.1598	5.7823
Ahcyl1	_GSSNFC[Carbamidomethyl (C)]VK_.2	#N/A	3.7552	3.5639	3.2918	3.8290	2.0808	3.9955	3.0860	3.9809	3.0062	2.4310	3.9857	3.2900	3.7477	3.3298	2.4403	3.1173	3.4781	3.6713	3.9704	2.7541
Ahcyl1	_TTDVMFGGK_.2	#N/A	3.3660	3.1360	3.9023	4.1115	4.2046	4.4287	4.1816	3.6985	3.3956	3.5723	4.7183	3.0254	3.5036	3.8104	2.9137	3.7109	3.1957	3.9538	4.2528	4.2255
Ahcyl1	_NSC[Carbamidomethyl (C)]IVC[Carbamidomethyl (C)]NM[Oxidation (M)]GHSNTEIDVTSLR_.3	#N/A	4.2860	3.3401	3.5277	3.7012	3.6587	4.2116	3.7350	4.2168	4.1659	3.6068	3.9104	3.0662	4.1125	3.5658	3.7603	3.3533	3.7141	3.4475	3.7466	3.2431
Mib1	_VAPAGSAISNAS[Phospho (STY)]GER_.2	Q80SY4_S411_M1_Mib1	4.5160	3.4868	3.5515	3.1734	8.7157	4.0778	3.6873	4.2164	3.7464	3.2215	4.1208	3.3762	8.7406	3.3982	3.2645	8.5189	3.2532	3.6029	3.9020	3.0755
Sytl5	_SYS[Phospho (STY)]LDLDSQNLQSFK_.2	Q80T23_S211_M1_Sytl5	6.5241	3.5781	6.7229	3.8432	3.9363	5.3429	5.7707	3.9667	5.1909	3.3041	4.9807	4.1963	3.7719	6.0436	3.1819	3.4427	3.4639	5.7841	3.9846	5.7223
Sytl5	_NTSGTPSIAVSGTSLS[Phospho (STY)]SER_.2	Q80T23_S319_M1_Sytl5	3.6562	5.6202	3.7862	4.4395	3.8631	3.8965	3.1850	4.4511	3.1052	3.4668	5.2415	3.3890	3.2706	3.7733	3.2104	3.0183	3.4880	4.3452	3.9938	3.7403
Sytl5	_DLNS[Phospho (STY)]LEDTEDGVDLVSSR_.2	Q80T23_S358_M1_Sytl5	4.3770	3.6258	3.4124	3.3124	3.7147	3.9388	3.8264	4.1883	3.8855	4.5833	4.2598	3.5153	3.9935	3.5373	3.4036	3.2210	3.6855	3.4639	3.7629	3.2146
Gabbr2	_TSTS[Phospho (STY)]VTSVNQASTSR_.2	Q80T41_S775_M1_Gabbr2	7.2358	7.0352	3.7949	6.7227	6.4875	7.7639	6.4425	6.7220	3.6780	7.0523	6.7172	6.3781	4.3762	6.5677	5.3487	6.7593	7.1664	4.7838	6.9559	3.7964
Gabbr2	_KEDS[Phospho (STY)]KTSTSVTSVNQASTSR_.3	Q80T41_S770_M1_Gabbr2	3.1872	4.1319	4.2247	4.3970	3.7217	3.2134	4.1093	3.4129	3.1101	4.4987	3.4844	3.8580	3.2181	4.2627	3.6793	4.0502	4.4110	4.2393	3.0375	3.9400
Gabbr2	_ITELDKDLEEVTM[Oxidation (M)]QLQDT[Phospho (STY)]PEK_.3	#N/A	4.7278	5.6145	5.9361	3.3690	3.6596	4.3268	5.4816	4.4669	5.4126	4.7576	4.5011	4.4412	4.9161	4.8544	4.6641	3.1659	3.9742	6.0859	5.7100	3.3114
Gabbr2	_TC[Carbamidomethyl (C)]KDPIEDINS[Phospho (STY)]PEHIQR_.3	#N/A	9.0072	9.1242	8.4437	8.5872	7.6541	8.4797	8.7529	8.4218	7.9487	8.6629	8.5063	8.0671	8.8709	8.3912	6.8828	7.9899	8.9245	8.7247	8.1828	8.0706
Gabbr2	_FTQFQDSR_.2	#N/A	4.3220	4.6466	4.0374	5.2552	3.2069	3.2403	5.1483	3.2257	5.4923	4.3114	3.2400	3.8440	3.9268	4.0755	3.8665	3.8629	5.0686	5.3238	3.2248	4.3965
Gabbr2	_DPIEDINS[Phospho (STY)]PEHIQR_.3	#N/A	3.6419	4.3613	5.8961	4.2018	2.9797	3.3363	5.3762	3.4086	3.3463	3.8483	3.7946	4.2508	3.4871	4.1132	4.1391	5.2327	4.2011	4.0724	4.1208	5.2662
Gabbr2	_DLEEVTMQLQDT[Phospho (STY)]PEK_.2	#N/A	3.3145	3.7931	4.3519	4.2697	3.5215	3.0861	3.5266	3.5402	3.2373	3.1251	2.9255	3.7307	3.3454	4.3900	3.5520	4.1775	4.5383	4.1120	4.4111	4.0673

Mtmr12	_QLS[Phospho (STY)]LPLTQSK_.2	Q80TA6_S564_M1_Mtmr12	8.7322	8.2229	7.9639	8.0151	3.5318	7.5206	7.7072	6.6833	7.2401	8.5021	2.9151	7.3580	7.7355	7.5477	5.3220	6.1517	7.9547	7.5400	7.0270	7.2541
Vat1l	_ISAEGVDIVLDC[Carbamidomethyl (C)]LC[Carbamidomethyl (C)]GDNTGK_.2	#N/A	4.7284	4.8757	4.7932	5.2284	4.2433	5.4161	4.7086	5.5290	5.2304	4.1288	5.4226	4.9472	5.4934	5.4692	5.0699	4.3508	5.4374	5.1871	4.5870	3.9871
Vat1l	_DSVTHLFDR_.2	#N/A	4.5131	4.8204	5.9229	5.2347	5.8251	6.6371	6.0494	5.8013	6.0446	5.6813	5.7365	6.3336	3.9145	5.6686	5.4784	5.4714	5.2859	5.7036	5.5930	5.5763
Vat1l	_TPLVPGFEC[Carbamidomethyl (C)]SGIVEALGDSVK_.2	#N/A	4.8718	5.4221	5.4604	4.7941	5.0268	5.6542	5.6005	4.3205	5.2353	5.0369	5.0975	5.2178	4.5634	5.0936	4.4077	5.0520	4.1548	4.5460	4.5830	4.5605
Vat1l	_AC[Carbamidomethyl (C)]GLNFIDLM[Oxidation (M)]VR_.2	#N/A	5.0826	4.0915	5.5800	5.0665	3.4345	5.2249	5.0456	4.3526	5.2720	4.8878	3.0125	5.0364	3.6993	4.5402	4.5934	4.0905	5.3423	5.0108	4.9727	5.1192
Vat1l	_TPTPLM[Oxidation (M)]ANDS[Phospho (STY)]TETSEAGEEEEDHEGDSENKER_.4	#N/A	4.1245	7.1764	3.8263	3.9140	3.4745	7.2734	6.3434	4.0421	3.8634	4.0861	5.7319	7.2071	3.9876	3.8751	3.7040	7.6163	4.0126	7.3519	3.6479	3.6862
Vat1l	_LILDVEK_.2	#N/A	4.2274	4.5925	3.7640	3.2981	2.9335	3.4441	4.8274	4.4531	2.5543	4.0380	3.5134	4.3186	4.2002	4.3150	4.1400	3.5895	3.9503	4.7442	3.0642	1.9119
Vat1l	_ETSKEPAEGGDGSHR_.3	#N/A	3.2602	4.0589	4.2976	4.3240	3.4671	3.9517	3.5810	3.3463	3.5012	4.5716	2.9798	3.7850	3.6666	4.1693	3.6064	4.1231	4.4839	3.9492	3.5978	4.1363
Sipa1l2	_EYGS[Phospho (STY)]TSSIDR_.2	Q80TE4_S195_M1_Sipa1l2	3.5429	3.7762	4.5803	4.0413	3.7499	4.3585	3.2982	3.7686	3.4657	3.3535	3.8401	8.1982	8.4302	4.6184	3.3236	8.0711	3.2658	3.8836	4.1827	7.5903
Sipa1l2	_TLS[Phospho (STY)]DESVC[Carbamidomethyl (C)]SNR_.2	Q80TE4_S1488_M1_Sipa1l2	8.0339	8.1707	8.3579	8.3155	7.8343	7.9331	7.7914	8.1848	7.8277	8.0101	8.0064	7.4873	4.0652	7.9758	6.8773	7.8251	8.1103	7.8056	7.7026	7.8807
Sipa1l2	_YTIGDIFVHS[Phospho (STY)]PQR_.2	Q80TE4_S149_M1_Sipa1l2	5.6143	6.1466	5.7972	5.3717	4.7509	4.0147	5.1945	5.3896	4.2943	5.7588	5.6514	5.5260	3.7721	5.0327	3.7118	4.0177	5.6778	5.6238	4.6745	4.8272
Sipa1l2	_SEGS[Phospho (STY)]PPPEEPEVTEC[Carbamidomethyl (C)]PR_.2	Q80TE4_S1406_M1_Sipa1l2	7.1987	4.4926	4.1087	7.9604	6.3404	3.6897	3.5075	6.8073	7.1659	6.2392	5.7262	3.6668	6.9178	6.5833	3.5328	6.8440	5.7487	7.2053	7.1798	4.0628
Sipa1l2	_NEFWFS[Phospho (STY)]DGSLSDK_.2	Q80TE4_S1549_M1_Sipa1l2	4.3389	4.4810	6.5656	3.2453	3.0450	3.5625	4.0031	3.0638	5.9938	4.1495	3.1353	4.2071	4.3698	3.9136	6.2894	3.7010	4.0618	3.0876	3.3867	3.5909
Lrrc7	_TPS[Phospho (STY)]PFEDR_.2	Q80TE7_S850_M1_Lrrc7	9.7757	4.0015	4.5376	9.9077	3.3390	8.8819	4.2021	9.0061	8.6095	10.4521	9.1825	8.5670	9.6347	4.5247	3.7793	9.2011	9.3903	9.1076	3.3872	9.1428
Lrrc7	_GVIAISKS[Phospho (STY)]TER_.2	Q80TE7_S928_M1_Lrrc7	8.8869	9.0455	8.6814	8.5090	8.6716	8.3355	8.4900	8.8019	8.1009	8.3801	2.9695	8.3674	4.2040	3.1891	7.4495	3.0106	8.5903	8.9321	8.7179	8.5264
Lrrc7	_S[Phospho (STY)]M[Oxidation (M)]DGYPEQFC[Carbamidomethyl (C)]VR_.2	#N/A	13.6253	13.7118	13.5821	13.5874	12.8271	13.1142	13.1038	13.0006	13.1513	13.4397	13.1192	12.8609	13.2264	13.4588	12.4199	13.0914	13.3961	12.9430	13.1804	13.2621
Lrrc7	_LETTPTTS[Phospho (STY)]PLPER_.2	#N/A	3.7953	7.7794	7.9099	5.6918	3.8759	5.1968	7.0108	3.6506	3.6645	8.5697	6.7760	6.0281	3.8186	4.0025	3.0757	4.9071	6.7650	3.8721	4.2005	3.8402
Lrrc7	_VYNIPLENYASGSDHLGS[Phospho (STY)]HERPDK_.3	#N/A	3.6218	3.3498	3.5993	6.5059	4.0875	4.0664	4.0155	4.2757	3.7717	3.5866	4.1460	3.1607	3.8797	3.6107	3.0181	3.4159	3.5507	3.5915	3.9353	3.1008
Lrrc7	_TAFPSKLETT[Phospho (STY)]PTTS[Phospho (STY)]PLPERK_.3	#N/A	4.5059	4.3140	7.8641	8.0412	3.2120	3.3955	4.3696	3.2308	4.4287	4.0400	3.3023	4.0401	4.5368	4.0806	3.8615	3.8680	4.2288	7.2832	3.2197	3.7578
Lrrc7	_SQS[Phospho (STY)]IDEIDVGTYK_.2	#N/A	10.7845	11.2011	10.8907	10.5477	9.8478	10.6626	10.4598	10.0000	10.3251	10.6138	10.1253	9.8675	10.3704	11.0556	9.8205	10.2087	10.3272	10.3581	10.2190	10.3019
Lrrc7	_KSQS[Phospho (STY)]IDEIDVGTYK_.2	#N/A	3.7619	7.6284	7.3944	6.3215	5.8928	6.7413	7.0067	6.2992	7.1181	3.8825	5.7615	6.4628	3.7245	7.6497	6.1502	6.3730	6.6492	6.9329	6.6220	5.3346
Lrrc7	_S[Phospho (STY)]VPAHGR_.2	Q80TE7_S801_M1_Lrrc7	6.9169	6.4711	6.8513	6.6453	6.1918	5.8230	6.7322	4.9418	5.5283	6.7585	6.8191	3.1716	5.6902	6.0356	2.9929	5.5253	6.0273	6.7410	5.9663	6.2818
Lrrc7	_FKKS[Phospho (STY)]QS[Phospho (STY)]IDEIDVGTYK_.3	#N/A	3.8493	9.2353	8.9402	7.2865	4.0562	8.4109	8.5471	4.2647	8.3941	4.9599	3.8983	7.5747	3.9739	8.8177	8.1837	7.7466	5.7068	8.7215	8.7096	8.9609
Lrrc7	_SYS[Phospho (STY)]TESYGASQTR_.2	#N/A	8.5324	8.0756	3.6105	8.2549	3.9127	7.7505	7.7781	8.0120	7.5520	8.2067	7.9381	3.3172	7.8557	8.5819	7.2282	7.6898	7.9993	7.6372	7.4775	7.8484
Lrrc7	_STERLS[Phospho (STY)]PLM[Oxidation (M)]K_.2	Q80TE7_S933_M1_Lrrc7	3.9805	7.3330	7.5347	7.1101	3.6754	3.8102	6.5590	7.2348	6.5318	4.0641	6.6333	6.8820	7.4137	3.7888	3.4213	6.7690	3.7011	3.8260	6.7711	6.8017
Lrrc7	_LETT[Phospho (STY)]PTTS[Phospho (STY)]PLPERK_.2	#N/A	4.2062	6.6568	3.8927	6.6215	3.7541	4.0064	5.9518	4.0221	4.0848	7.2643	4.0188	3.4933	4.2288	6.6597	3.3570	3.7095	6.1642	3.4870	4.2840	4.6066
Lrrc7	_SYS[Phospho (STY)]TESYGASQTRPVSAR_.2	#N/A	6.0729	5.5596	5.9578	3.7133	3.8057	5.5158	4.3406	5.0674	4.3339	5.7610	5.7220	4.7521	4.4687	5.6071	3.4998	3.8359	6.3979	3.5276	5.0393	3.9110
Lrrc7	_TLAVDENFLPELPR_.2	#N/A	5.7278	3.6160	3.2397	6.4473	3.9100	4.1983	3.1381	5.4807	5.9413	3.5137	4.0003	5.0936	5.8357	5.5501	5.7179	3.0652	3.4260	5.5384	4.0225	5.3464
Lrrc7	_S[Phospho (STY)]REQQPYEGNINK_.2	#N/A	7.1876	6.8328	7.3074	7.0388	6.5031	6.1389	6.3300	6.3726	6.1943	6.6567	6.6323	6.7163	6.8014	7.0526	5.4073	6.4719	5.9380	6.5275	6.5316	6.5561
Lrrc7	_IPS[Phospho (STY)]DYNLGNYGDK_.2	Q80TE7_S1233_M1_Lrrc7	4.3301	8.6235	3.3656	3.3593	3.6678	3.8919	3.8733	4.2352	3.9323	4.5364	4.3067	3.5622	4.0404	8.8522	6.6867	8.1350	3.7324	8.5151	3.7161	8.4199
Lrrc7	_S[Phospho (STY)]QSVPMLDDEMLM[Oxidation (M)]YGSSK_.2	Q80TE7_S997_M1_Lrrc7	4.0985	3.7777	5.5350	3.4003	3.6334	3.9899	4.1994	4.4156	4.1065	4.0474	3.7770	3.9810	4.2336	3.7646	3.6838	3.8517	3.9129	3.2839	3.5416	3.3745
Lrrc7	_RVS[Phospho (STY)]EPHELPPGDR_.3	Q80TE7_S1132_M1_Lrrc7	3.6081	3.7110	3.1447	4.3914	3.8151	5.6530	6.3770	4.4992	3.1532	3.4187	4.1327	3.4371	3.3187	3.8213	3.2584	2.9702	3.5360	4.2971	3.9457	3.7883
Pcdh19	_DKES[Phospho (STY)]PSVKR_.2	Q80TF3_S1134_M1_Pcdh19	4.4455	4.3744	3.9821	3.1387	3.1516	3.1849	3.8965	5.4186	3.8167	4.2561	3.2953	4.1005	3.9821	4.0201	3.9219	3.8076	4.1684	3.6337	3.2801	4.4518
Pcdh19	_SKS[Phospho (STY)]PEHVR_.2	Q80TF3_S983_M1_Pcdh19	4.3536	5.1897	6.2602	3.2306	3.0597	3.5478	4.2174	3.0785	4.2765	4.1923	3.1500	4.1924	4.3845	3.9283	4.0137	3.7158	4.0766	5.9338	3.3719	3.6056
Pcdh19	_EAGNGC[Carbamidomethyl (C)]EAIS[Phospho (STY)]PVTS[Phospho (STY)]PLHLK_.2	Q80TF3_S1063_M2_Pcdh19	4.4906	3.4701	3.5261	3.7352	3.8283	4.0524	3.7128	4.0747	3.7718	3.1961	4.1462	3.4017	3.8799	3.4237	3.2900	3.3347	3.5719	3.5775	3.8766	3.1009
Erbin	_SES[Phospho (STY)]AENHSYAK_.2	Q80TH2_S1013_M1_Erbin	4.4206	6.4635	5.7237	3.7031	4.2673	5.3266	5.9493	3.6867	5.0234	6.4969	5.0572	4.7120	5.8189	5.3401	3.9467	3.7827	4.2244	5.7325	5.2193	5.4985
Erbin	_VRQEDENFNSLLQNGVNLNNS[Phospho (STY)]PEEK_.3	Q80TH2_S712_M1_Erbin	4.0114	3.3077	3.5480	3.5728	4.2184	4.2517	5.0104	4.0959	4.2508	3.8220	3.7294	3.0338	4.4162	3.4180	2.8551	3.3735	3.7343	3.1996	4.3490	4.2532
Erbin	_STEDLS[Phospho (STY)]PQR_.2	Q80TH2_S854_M1_Erbin	7.7517	7.6875	7.3500	7.7232	7.2625	7.2864	7.5009	7.6523	7.2787	7.6040	7.0525	7.3486	7.8347	7.8837	6.9723	7.3011	7.6177	7.5179	7.0489	7.6557
Gramd1b	_STPAC[Carbamidomethyl (C)]S[Phospho (STY)]PILR_.2	Q80TI0_S21_M1_Gramd1b	3.8774	9.4171	8.8014	8.9555	8.5073	8.5631	8.7406	8.9737	8.5919	8.6919	3.2113	8.5266	8.9636	8.4005	7.1473	4.2223	8.8884	8.6732	8.7174	8.3829
Gramd1b	_TESTYLAEIHRQS[Phospho (STY)]PK_.3	#N/A	6.0162	7.7239	6.7659	7.3566	6.3907	7.4290	7.4100	6.9327	7.5379	7.5497	7.3566	7.0823	7.4279	6.8730	5.8938	6.8130	6.7384	7.7024	7.4283	7.0965
Gramd1b	_SSIETKPDAS[Phospho (STY)]PQLPKK_.3	#N/A	6.2512	6.1222	3.2111	3.5137	5.2514	5.6138	4.0277	3.8761	5.7906	6.4072	5.6379	5.3213	4.1948	3.1982	3.7188	3.0197	4.2741	5.9132	3.0680	5.5464
Gramd1b	_S[Phospho (STY)]RSPTPQNQDGDTMVEK_.3	Q80TI0_S28_M1_Gramd1b	3.2882	5.5898	4.1618	5.8374	3.7593	3.1587	4.8679	3.3880	3.4104	3.9826	5.0477	3.5993	3.5411	5.2495	3.7209	3.8821	4.1122	3.9889	4.1243	4.1675
Gramd1b	_SSIETKPDAS[Phospho (STY)]PQLPK_.2	#N/A	9.7385	9.6412	9.4217	9.2446	3.5732	8.8235	9.0757	9.3029	8.7782	9.3200	8.7768	8.7469	9.5722	8.9737	7.3584	8.5122	9.2524	8.9468	8.6317	8.5659
Gramd1b	_S[Phospho (STY)]PTPQNQDGDTM[Oxidation (M)]VEK_.2	#N/A	3.1804	4.1387	5.4311	5.3326	5.6821	4.6772	5.0285	4.7453	5.7623	5.4370	5.5744	3.8648	3.2113	4.2559	4.1222	5.5051	4.4042	4.2461	5.2666	4.9753
Plekhh1	_SC[Carbamidomethyl (C)]DS[Phospho (STY)]DEDYEAGGTGR_.2	Q80TI1_S739_M1_Plekhh1	4.3085	3.6943	3.3440	3.3809	3.6462	3.8703	3.8948	4.0089	3.9359	5.0028	4.3283	3.5837	4.0620	4.6482	3.4720	3.1526	4.5466	4.1003	4.4360	3.2981
Cadps	_VILHPTPNS[Phospho (STY)]PK_.2	Q80TJ1_S486_M1_Cadps	8.6978	9.2851	3.3545	8.4838	8.1194	8.5686	8.7671	9.1601	8.2296	8.5073	8.5114	3.2273	8.7839	8.7051	3.0486	8.0754	9.1447	8.7721	8.4865	8.1239
Cadps	_DIVTPVPQEEVK_.2	#N/A	8.5838	7.8758	8.2168	7.6907	7.8940	7.7855	7.9830	8.4213	8.2774	7.8409	8.0941	7.5314	8.0464	7.2147	7.2906	7.5524	7.9666	7.8025	7.9085	7.6913
Cadps	_LDALQTFIR_.2	#N/A	6.3895	6.5599	5.6128	5.7100	6.1548	6.3831	6.8459	6.6550	5.9128	6.1044	6.2498	6.1700	6.0991	4.6085	5.5131	5.7206	6.1134	6.5117	6.3445	6.0835
Cadps	_EGDTVIFASDDEQDR_.2	#N/A	7.1546	6.2883	6.3298	6.3987	5.7251	5.8730	6.2237	6.2980	6.8504	5.8321	6.0541	6.0830	5.9310	6.4492	4.6040	5.5705	6.6528	6.0776	6.1395	5.8498
Cadps	_S[Phospho (STY)]LPEIDGLSK_.2	Q80TJ1_S219_M1_Cadps	9.3355	9.3880	9.1088	9.0617	3.5597	8.9983	8.8933	8.7595	9.0164	8.9032	9.1023	8.3980	8.5439	8.7564	7.3865	8.5894	9.4375	8.9525	8.9123	8.5460
Cadps	_SLTSNLPNVNLPNVNLPK_.2	#N/A	5.3154	5.9606	3.6751	5.2327	4.8684	5.8163	6.3267	5.8608	5.9335	5.4775	5.0841	5.5387	4.2891	5.4646	4.6917	5.1720	5.4440	6.0105	5.5234	4.9507
Cadps	_LQGVLDSTLNSK_.2	#N/A	5.8849	5.6573	5.7957	5.8284	3.9450	5.6642	5.9527	5.0213	5.1134	3.7429	5.0166	5.2623	4.4954	4.8219	4.6142	4.7641	4.8234	5.2078	4.9918	3.4642
Cadps	_HSQDVLR_.2	#N/A	6.1304	5.9025	3.4623	5.9660	5.4676	5.9403	6.3296	6.0453	5.8761	5.8736	5.7936	5.6681	5.7540	7.8824	5.8785	5.3960	6.4410	5.7214	5.6650	5.0934
Cadps	_ATLSLLER_.2	#N/A	4.3649	5.5604	4.2790	5.3318	4.2350	4.6859	5.8875	3.3521	5.1137	5.5046	5.2911	4.1766	4.5222	4.9502	3.5396	4.2410	3.1021	5.0934	5.2244	4.5687
Cadps	_LQKEEEER_.2	#N/A	3.5196	4.9808	4.7295	4.0647	3.7265	5.4855	4.8184	5.7033	5.2251	3.3302	4.7653	4.8685	5.0747	4.7356	4.9529	4.7076	3.2425	4.5882	4.4492	3.8769
Cadps	_EQLYEM[Oxidation (M)]FQNILGIK_.2	#N/A	4.3725	2.9144	4.1863	3.8139	2.9183	2.5012	3.6085	3.6562	3.4497	2.6062	3.6001	3.9429	3.1565	3.3450	3.0962	3.3623	3.5092	3.6562	3.5496	3.2936
Cadps	_VLLENQITHFR_.2	#N/A	5.6214	5.3118	4.5081	5.0751	5.1023	4.8005	4.8669	5.4239	4.5223	4.4099	4.6816	5.6262	5.5878	4.6216	4.1125	4.4801	5.2695	5.3803	4.6113	4.2829
Cadps	_LFTESTGVLALEDKELGR_.3	#N/A	4.8758	4.4855	4.0852	5.7276	2.8550	4.2647	5.1417	3.2176	3.2443	3.7236	3.6187	4.3750	3.3524	4.7088	4.2633	3.8622	5.8177	4.8783	3.7264	4.0076
Cadps	_YVDLM[Oxidation (M)]ESSIAQSIHR_.2	#N/A	4.1679	3.2998	3.3966	3.5992	4.1077	3.9854	3.2534	4.4360	3.9091	3.5557	4.0039	3.0682	4.0745	3.4045	3.1295	3.2124	3.3254	3.4291	4.0827	3.2850
Cadps	_M[Oxidation (M)]VQSGGC[Carbamidomethyl (C)]SANDSR_.2	#N/A	4.3718	4.4596	3.9083	4.1522	3.0779	3.1901	3.9817	3.5811	3.7307	4.1824	2.7742	4.1857	4.3677	3.6964	4.0071	3.7339	4.3158	4.1444	3.1546	3.4826
Phf8	_DAEYIYPSLES[Phospho (STY)]DDDDPALK_.2	Q80TJ7_S820_M1_Phf8	3.7764	4.2264	4.3127	3.9130	3.1141	3.3700	3.7114	3.4767	3.6316	4.4412	3.1103	4.1159	3.7971	4.2997	3.7368	4.1213	4.0144	3.8187	3.4673	4.2667
Phf8	_SSGSSSSGLGTVSSS[Phospho (STY)]PASQR_.2	Q80TJ7_S768_M1_Phf8	4.2194	4.6005	3.7560	3.3648	2.9255	3.6821	3.2467	4.4451	4.1422	4.3266	3.0157	4.3267	4.2503	3.7940	4.1480	3.5815	3.9423	3.2072	3.5062	3.4713
Kiaa1107	_S[Phospho (STY)]LDSC[Carbamidomethyl (C)]PSQGLPQEGQVK_.2	Q80TK0_S1051_M1_Kiaa1107	9.2846	4.1058	4.4333	9.5311	3.2347	3.2494	3.8321	3.5974	3.7523	4.3205	3.2309	7.7574	3.9177	4.4204	3.8575	4.2419	8.0390	3.6981	3.3467	4.3874
Kiaa1107	_SSS[Phospho (STY)]DTSTPEELK_.2	#N/A	4.1842	3.5116	3.4876	3.4531	6.8309	4.4192	6.7675	4.1666	4.0835	7.5630	7.1004	4.0776	4.2099	3.5044	5.8568	6.8160	7.0327	6.6304	3.3250	7.0075
Kiaa1107	_SAPPTLAQAQGSQGESPHS[Phospho (STY)]VK_.2	Q80TK0_S343_M1_Kiaa1107	3.3931	4.3521	4.1347	4.2305	5.1754	3.5350	3.5515	3.3074	3.4717	4.4538	3.6028	5.0580	3.6372	4.1398	3.5769	4.9062	4.0263	4.8719	3.6323	4.7523
Kiaa1107	_KQGGGDHQIHS[Phospho (STY)]AS[Phospho (STY)]DDEIPR_.3	#N/A	5.8021	5.7522	5.2050	5.6363	4.4118	5.1756	5.8197	3.6241	5.2504	6.2743	5.3871	5.6026	3.9571	5.9977	4.7564	5.8927	6.1904	6.0758	5.1448	5.6345
Kiaa1107	_SEDYDAGGS[Phospho (STY)]QDDEGSHDR_.2	#N/A	3.5911	3.9790	4.2823	3.9336	4.0801	4.4902	3.5527	4.1185	4.1026	3.5611	4.2425	4.1506	3.9226	4.7950	4.0930	4.2988	4.4153	4.0671	4.5580	3.7652
Kiaa1107	_QGGGDHQIHS[Phospho (STY)]AS[Phospho (STY)]DDEIPR_.2	#N/A	8.0784	7.8083	3.9527	7.7543	3.8471	3.9065	7.6277	5.2808	5.1644	8.2361	7.5907	7.3411	4.2916	5.0002	4.2145	4.8348	8.1705	7.5756	4.4062	4.1119
Kiaa1107	_SSS[Phospho (STY)]DTSTPEELKVHEGDLR_.3	#N/A	3.9859	7.5431	3.6126	3.5676	3.9149	6.4003	4.0782	4.0823	4.0487	7.5276	3.9297	3.3174	4.1930	7.3510	3.0649	3.4212	3.3448	3.4343	4.1266	7.0710
Kiaa1107	_SSVSSRQS[Phospho (STY)]DENVTR_.2	Q80TK0_S353_M1_Kiaa1107	4.1754	3.8274	3.2108	3.4200	3.5131	3.7372	4.0280	4.3899	4.0871	4.3817	2.9606	3.7169	4.1951	3.7389	3.6052	3.0194	3.8872	3.2623	3.5613	3.4162
Ppm1e	_YNC[Carbamidomethyl (C)]PSFLAAALAR_.2	#N/A	6.4644	5.5697	5.2799	5.2868	5.4364	5.7188	5.6446	5.5853	5.8369	5.8544	5.4356	5.5838	4.4774	5.4730	5.5390	5.0502	5.2161	5.4915	5.6894	5.1390
Ppm1e	_RPWPQHQC[Carbamidomethyl (C)]S[Phospho (STY)]APADLGYEGR_.3	#N/A	9.4617	9.5365	8.5883	8.3590	8.0911	8.3880	8.8893	9.2910	7.5410	8.6611	8.5628	8.6431	9.3985	10.2320	7.2853	7.7923	8.4656	8.8411	8.8642	7.6075
Ppm1e	_VDS[Phospho (STY)]FTDR_.2	Q80TL0_S545_M1_Ppm1e	4.0301	3.9727	4.5664	3.6593	3.0829	3.1163	3.9652	3.7305	3.8854	4.1874	3.3640	3.8622	4.0508	4.5535	3.9905	3.7390	4.2682	3.5650	3.2136	3.0196
Ppm1e	_AVNVS[Phospho (STY)]EESEWTENSFQGGQEDGGDDKETHGEC[Carbamidomethyl (C)]K_.4	Q80TL0_S495_M1_Ppm1e	3.6612	3.6579	3.1978	3.9230	3.8681	3.9015	3.1800	4.4461	3.1002	3.4718	4.0797	3.3840	3.2656	3.7683	3.2053	3.7258	3.4830	4.3502	3.9988	3.7353
Adcy2	_S[Phospho (STY)]PQHLFR_.2	#N/A	9.5341	9.1467	9.1727	8.9672	8.6360	3.7184	8.9885	8.7175	8.8647	9.1220	9.2955	8.4442	8.7447	8.8237	7.3033	7.8273	9.3826	9.0308	8.8002	8.5616
Adcy2	_S[Phospho (STY)]PQHLFRPR_.3	#N/A	6.5066	7.1956	4.7767	3.4801	4.2754	6.0564	7.5912	7.2226	4.9280	5.4472	5.0704	6.8958	6.2888	7.5431	3.2437	4.4179	6.6684	7.1097	5.8585	5.8345
Phf24	_VIPDC[Carbamidomethyl (C)]SLTLEDFVR_.2	#N/A	8.7183	8.7024	9.0907	8.7613	8.6632	8.4411	8.8004	8.6829	8.7271	8.8928	8.7839	8.5996	8.9350	8.5886	8.7849	8.6831	8.7817	8.4034	8.8400	8.6043
Phf24	_ALLPTEQESR_.2	#N/A	8.6528	8.0400	8.4041	8.2709	8.5378	8.3581	8.3747	8.3962	8.3556	8.4534	8.3974	8.3285	8.3578	8.3129	8.2309	8.3675	8.3951	8.3358	8.4217	8.3009
Phf24	_GVEPEEFDR_.2	#N/A	7.9650	7.8665	7.8804	8.0552	8.0854	7.9882	8.1462	8.1509	7.7382	7.8114	8.1886	8.0854	8.1065	8.0759	8.0613	7.7603	7.6127	8.0139	8.0485	8.1524
Phf24	_QFAALDPEQR_.2	#N/A	7.8910	7.1046	7.5009	7.8175	7.8573	7.7616	7.9143	8.0319	7.7574	7.5858	7.8252	7.8007	7.5655	7.8981	7.9773	7.6793	7.6209	7.7871	7.6594	7.7509
Phf24	_ALTDEQEEQAAR_.2	#N/A	7.7056	7.4944	7.2112	7.5555	7.3347	7.4457	7.4871	7.4917	7.4938	7.6693	7.3046	7.3548	7.5855	7.3118	7.5432	7.7478	7.4443	7.5108	7.2396	7.6359
Phf24	_YVDWPTFLR_.2	#N/A	6.6495	6.3608	6.4107	7.0670	6.9255	6.7019	7.0742	6.8176	6.8265	7.0285	6.7976	7.0480	6.4859	6.2104	6.5221	7.0582	6.4774	6.8472	6.4473	6.5030
Phf24	_ENVIYILAAR_.2	#N/A	5.2322	5.2193	6.9902	6.0151	6.3507	5.5360	4.7078	6.1803	5.7876	5.8377	6.2375	5.2963	4.3076	5.7925	5.7467	5.2278	5.8808	5.2749	5.7497	5.4746
Phf24	_KLDDDKPPDIC[Carbamidomethyl (C)]LEPR_.3	#N/A	6.6299	6.4577	6.4931	6.8788	5.8169	6.3094	5.8254	6.2977	7.0061	6.5702	6.8953	6.3973	6.8937	6.8985	6.7959	6.7878	6.2396	6.3452	6.5083	6.5910
Phf24	_VFHDGC[Carbamidomethyl (C)]LR_.2	#N/A	6.7931	6.9531	3.2991	6.5041	7.1040	6.6602	6.8608	6.7176	6.4873	6.4708	6.6458	6.9716	3.7934	6.6250	7.3744	6.0305	6.7728	6.8077	6.5223	6.8656
Phf24	_GSTISEAEC[Carbamidomethyl (C)]HHAR_.3	#N/A	6.0214	5.7364	6.8030	5.2260	5.9620	6.1915	6.6642	5.9499	6.7522	6.6017	6.8328	6.1957	6.2325	5.9027	7.2077	6.1380	5.9908	5.8430	6.6521	6.2985
Phf24	_EAEASAPVVQEESS[Phospho (STY)]INR_.2	Q80TL4_S73_M1_Phf24	6.8496	7.2782	7.4908	6.9486	4.7056	6.6733	5.9000	5.6168	5.8181	6.2300	6.6100	4.7534	3.7486	7.4984	5.3644	6.2332	6.9859	5.8858	5.9694	6.9441
Phf24	_LTEAPSC[Carbamidomethyl (C)]SVSISHVGPIADSS[Phospho (STY)]PAASSSK_.3	Q80TL4_S348_M1_Phf24	3.8858	3.5542	6.6693	3.9904	4.1236	5.2903	7.5296	3.8918	5.5253	6.5254	3.7927	3.3752	6.9042	9.0726	3.0147	5.7692	3.3334	3.7305	6.4670	4.0125
Phf24	_RGT[Phospho (STY)]VEGSVQEVQEEKEAEASAPVVQEESSINR_.4	#N/A	3.8857	3.4334	3.4223	3.6985	4.0926	4.0157	4.4563	4.6260	3.8085	7.6594	4.1829	3.1595	3.9166	3.4603	2.9808	3.2478	3.6086	3.5408	6.1093	6.1690
Phf24	_RGT[Phospho (STY)]VEGSVQEVQEEK_.2	#N/A	3.3856	3.8659	4.4906	4.1310	4.2242	4.4482	3.3169	3.6789	3.3760	5.1852	3.7504	3.5920	3.4841	4.5287	2.8941	3.7305	3.1761	3.4672	4.2724	4.2060
Mon2	_DEQAAS[Phospho (STY)]DPM[Oxidation (M)]DQETVSR_.2	#N/A	4.5775	4.3364	4.3097	4.3700	3.5255	3.6873	3.9613	3.3554	3.6917	4.1153	3.7597	4.0057	4.5089	4.2052	3.4312	4.1177	4.1627	4.2803	3.5553	3.4548
R3hdm2	_PWSS[Phospho (STY)]TDSDGSVR_.2	#N/A	4.0702	3.2489	3.6067	6.7714	6.2757	3.8313	3.9339	4.2958	3.9930	7.1581	6.0807	2.9750	4.1010	3.6448	2.7964	6.1015	3.7931	3.3564	6.6363	3.3221
R3hdm2	_DSS[Phospho (STY)]QEYTDSTGIDLHEFLVNTLK_.3	Q80TM6_S143_M1_R3hdm2	3.8569	4.1459	4.3932	3.8325	3.1946	3.4187	4.3465	3.2076	6.4494	4.0632	3.2791	4.0354	4.5136	4.0574	3.9237	6.6377	4.2057	4.4446	3.2428	3.7347
R3hdm2	_SKS[Phospho (STY)]IEEREEEYQR_.3	#N/A	7.6949	7.7625	7.5590	8.0088	6.6743	7.1332	4.3751	6.5380	6.9593	7.7372	6.7349	4.1929	7.2237	5.4405	6.6564	7.4465	6.5934	4.8742	6.8254	6.9015
R3hdm2	_ASS[Phospho (STY)]FSGISLTR_.2	Q80TM6_S381_M1_R3hdm2	8.3091	9.0398	7.5358	3.7723	4.0188	4.0522	3.0293	9.6958	4.4503	3.6225	3.9289	8.5993	3.8428	10.4778	3.0546	6.2094	7.6409	8.4028	7.3760	3.0638
R3hdm2	_SLEPRPWSS[Phospho (STY)]TDSDGSVR_.2	#N/A	3.6783	7.2124	7.5563	4.0185	4.8194	6.0460	6.6144	6.3497	5.9549	6.9106	3.9778	6.5355	3.6397	7.0788	5.7707	6.7826	7.1399	7.3949	3.6841	7.7809
R3hdm2	_S[Phospho (STY)]IEEREEEYQR_.2	#N/A	8.1874	4.2672	4.0893	4.5323	3.1246	3.3487	4.4165	3.2776	7.2474	3.9932	3.3491	7.0353	4.5836	8.1256	3.9937	7.5009	7.4148	7.1842	7.4334	3.8047
R3hdm2	_SAS[Phospho (STY)]TDLGTADVVLGR_.2	Q80TM6_S923_M1_R3hdm2	4.4397	3.5631	3.4751	3.2497	3.7774	4.2075	3.7637	4.1401	4.2949	3.7779	3.7736	3.4526	4.4604	11.3457	3.3409	3.2837	3.1769	3.1554	4.3049	3.4292
R3hdm2	_TPS[Phospho (STY)]KEDVEKEGEENGLR_.3	Q80TM6_S37_M1_R3hdm2	3.4690	3.8501	4.5064	4.1152	3.6759	4.4324	3.3327	3.6947	3.3918	3.5761	3.7662	3.5762	3.4999	4.5445	2.9099	3.7147	3.1919	3.9575	4.2566	4.2217
R3hdm2	_SKS[Phospho (STY)]IEER_.2	#N/A	3.3674	3.1345	3.9037	4.3219	4.2060	3.7789	3.3351	3.0678	3.2227	3.3492	4.2022	3.0240	3.3882	4.9497	2.9123	3.7123	3.6055	4.8740	3.8762	5.6602
R3hdm2	_LEQEILDFINDNNNQFK_.2	#N/A	4.4071	4.9657	4.1192	4.1065	2.9206	3.9166	4.0593	5.2494	3.1787	4.5395	4.3032	4.5635	3.6036	6.0407	4.1977	3.9278	5.0075	4.0122	4.5299	4.0732
Nisch	_TPS[Phospho (STY)]PEPVDKDFYSEFGDK_.3	#N/A	3.7776	5.1576	4.8742	3.8893	3.7880	5.0686	4.8606	3.9426	3.5156	5.2764	5.3639	3.6946	3.6384	3.9826	4.4397	3.4900	4.5960	5.0813	4.6985	4.6802
Nisch	_AC[Carbamidomethyl (C)]SDS[Phospho (STY)]LESIPAGQVASDDLR_.2	Q80TM9_S545_M1_Nisch	5.1986	6.2909	4.3486	4.0227	3.3011	6.2049	5.6836	3.5219	5.1500	5.6737	3.7186	5.0984	3.5356	6.4778	3.8806	4.1636	5.4043	6.2245	3.8093	5.5695
Nav3	_SQS[Phospho (STY)]FNSIDK_.2	Q80TN7_S275_M1_Nav3	4.4326	6.1857	6.4742	5.7252	5.4443	5.7060	5.8243	4.1327	4.9581	6.3811	4.2042	3.4597	3.9379	5.8282	3.3480	5.3977	3.6299	3.5195	3.8186	3.1589
Nav3	_S[Phospho (STY)]LPRPSK_.2	Q80TN7_S1140_M1_Nav3	8.9619	10.2270	3.6528	4.5728	8.1555	4.1792	3.5860	7.9705	3.6451	10.3121	4.0194	8.2659	9.2099	8.4896	8.4442	8.2718	8.0308	8.1619	9.5866	2.9742
Nav3	_KTS[Phospho (STY)]LDGSQNQDDVVLHVSSK_.3	Q80TN7_S1116_M1_Nav3	3.3503	5.4017	3.8866	4.3390	4.1889	4.4130	3.3522	3.0507	3.4113	3.5567	3.7856	3.0411	3.5193	3.8737	2.9294	3.6952	3.5884	3.9381	4.2371	4.2412
Ubr5	_WLDGAS[Phospho (STY)]FDNER_.2	Q80TP3_S321_M1_Ubr5	6.6127	7.8132	7.2788	7.3094	3.9242	6.8097	7.1415	3.9788	6.4926	7.3440	4.4425	6.2654	3.7840	6.3851	4.5306	6.0286	6.8640	6.9028	6.8100	6.8483
Cyld	_SELFYTLNGSS[Phospho (STY)]VDSQQSK_.2	Q80TQ2_S359_M1_Cyld	6.6263	6.4875	6.8644	6.5382	3.9844	3.9990	5.9230	5.4775	5.8828	3.3636	4.0929	5.0730	3.5886	6.4095	3.2280	5.8033	3.5079	6.4310	6.2962	5.4864
Cyld	_VTGSTS[Phospho (STY)]DPGSR_.2	Q80TQ2_S341_M1_Cyld	3.9542	3.7464	4.0763	4.4268	3.7903	5.6867	4.3203	3.7397	4.3081	3.9765	5.3020	3.5752	4.2144	5.8675	3.4044	5.8640	3.7353	3.2751	3.1789	5.8085
Adgrl1	_YYTGTQS[Phospho (STY)]R_.2	Q80TR1_S1141_M1_Adgrl1	8.2750	8.1209	7.9662	8.1289	7.6497	7.8367	8.0384	7.9188	7.6768	7.9518	7.5157	7.0815	7.7135	7.2710	5.8319	7.2788	7.6847	8.0242	7.4808	7.3851
Adgrl1	_SPPGGTHGS[Phospho (STY)]LK_.2	Q80TR1_S1123_M1_Adgrl1	8.6401	8.2362	7.8218	8.9308	8.1316	8.5798	8.0823	3.3966	8.0337	7.9125	8.0816	7.6463	7.4127	7.5240	6.2979	7.6927	8.5446	8.2882	7.9118	7.6225
Adgrl1	_PLSS[Phospho (STY)]PPGR_.2	Q80TR1_S1369_M1_Adgrl1	3.5424	3.7767	4.5798	4.0419	3.7493	7.5488	3.2988	3.7680	3.2190	3.3530	4.1985	3.5028	3.3844	4.6179	3.3242	4.4053	3.2653	3.8842	4.1832	4.2951
Adgrl1	_QTESSFM[Oxidation (M)]AGDINST[Phospho (STY)]PTLNR_.2	#N/A	3.4586	3.9088	3.9949	4.2600	3.6326	3.8567	3.2731	3.6077	3.3322	4.5213	3.7066	3.7983	3.4403	3.7588	3.6866	3.8035	3.6330	4.0464	3.8838	4.1621
Adgrl1	_RPS[Phospho (STY)]HEGYLAAPSLEGPGPDGDGQM[Oxidation (M)]QLVTSL_.3	Q80TR1_S1439_M1_Adgrl1	3.1675	4.1515	4.2050	4.4167	3.3745	3.4078	3.6736	3.3932	3.5938	4.4790	3.0725	3.8777	3.7593	4.2619	3.6990	4.0305	4.3913	3.8565	3.5051	4.2289
Adgrl1	_TDTLTEYASWEDYVAAR_.2	#N/A	3.9780	3.3411	3.5146	3.6062	4.1849	4.2183	4.3640	4.1293	4.2842	3.7886	3.7628	3.0672	4.4496	3.4515	2.8885	3.3401	3.1661	3.1662	4.3156	3.4185
Adgrl1	_SVYVDDDSEAAGNRVDYAFNTNANR_.3	#N/A	3.4409	3.8782	4.4783	4.1433	2.9772	4.4605	3.3046	3.6666	3.3637	3.6042	3.7381	3.6043	3.4718	4.5164	2.8818	4.3038	3.1638	3.9856	4.2847	4.1936
Adgrl3	_GPAHLVTS[Phospho (STY)]L_.2	Q80TS3_S1536_M1_Adgrl3	4.0230	4.2958	4.0865	3.9027	2.9996	3.3773	6.1658	3.2747	3.8145	3.9952	3.3462	4.2429	3.9226	4.1245	4.0991	5.4538	4.2728	3.7451	3.2015	3.8018
Adgrl3	_S[Phospho (STY)]MPNLGSR_.2	Q80TS3_S1485_M1_Adgrl3	3.8415	3.4776	5.8468	6.3262	4.0484	5.3233	5.8027	4.0672	3.8867	5.1282	6.5371	3.2037	3.8724	6.1620	3.2824	3.2036	4.1275	3.5850	3.8841	3.0934
Adgrl3	_GSSDGFIVPPNKDGAS[Phospho (STY)]PEGTSK_.3	#N/A	3.5802	4.1312	3.9849	4.0541	3.9079	5.6392	6.4088	5.9261	5.5520	5.5268	5.6635	3.4783	3.7211	3.9817	5.4132	4.4013	6.1702	6.5777	6.3369	5.8248
Adgrl3	_STESSIGS[Phospho (STY)]GK_.2	Q80TS3_S1222_M1_Adgrl3	4.4064	4.4135	3.9430	3.1778	3.1125	3.1459	3.9356	3.1313	3.8558	4.2170	3.3344	4.1396	4.0212	4.5239	3.9610	3.7685	4.1293	3.5946	3.2432	2.9900
Ddn	_S[Phospho (STY)]PLGQPRPEPR_.2	#N/A	7.9118	8.3356	7.6522	8.0978	7.4721	8.0027	7.8415	7.2652	7.9288	8.1255	8.0869	7.6348	8.3577	8.0980	7.2176	7.9097	8.4328	8.1864	7.7108	8.1959
Ddn	_LRPGS[Phospho (STY)]PEPPPR_.2	Q80TS7_S77_M1_Ddn	9.3594	9.3547	8.9282	8.9680	8.8770	9.1000	9.0810	9.3770	8.9583	9.1718	8.9992	9.0995	9.7290	9.0195	8.4424	9.1177	9.4404	9.4070	9.0739	9.0441
Ddn	_RS[Phospho (STY)]PLGQPRPEPR_.3	#N/A	3.6863	3.6212	4.2226	4.0031	3.0240	3.4601	2.6529	3.3866	3.5415	4.5313	3.0202	4.2060	3.7070	4.2096	4.0943	4.0312	3.0950	3.8990	3.2899	4.1766
Baiap3	_SGS[Phospho (STY)]PAPAEPVDPNR_.2	Q80TT2_S78_M1_Baiap3	11.9513	11.7446	11.9591	11.8580	11.7502	12.0558	12.3887	12.2698	12.4941	11.3121	11.9519	12.1811	12.4037	11.9665	10.1151	11.7077	12.4319	12.0936	11.4902	11.5234
Cul9	_VGLQS[Phospho (STY)]PSVETR_.2	Q80TT8_S1775_M1_Cul9	8.8183	8.6178	8.1546	8.8671	8.1092	8.4890	8.1993	8.6438	8.3062	8.4274	8.7653	8.2501	8.7412	3.5705	6.7212	7.8575	8.9101	8.5124	8.1668	8.3395
Cul9	_AVLETPSTQGQDGS[Phospho (STY)]PESLIR_.2	Q80TT8_S284_M1_Cul9	4.3600	5.8388	3.3955	3.3294	3.6977	3.9218	3.8434	4.0604	3.9024	4.5663	4.2768	3.5323	4.3807	3.3825	3.4205	3.2041	3.0972	3.2351	4.3845	5.4306
Clasp1	_NSSNTGVGS[Phospho (STY)]PSNTIGR_.2	Q80TV8_S1088_M1_Clasp1	11.4155	11.1506	10.9763	4.0104	10.5566	3.4674	10.9436	11.0620	3.5343	10.8295	10.5553	10.6244	10.9136	10.6848	4.1015	10.3897	11.0536	10.9594	10.5415	10.2620
Clasp1	_S[Phospho (STY)]QEDLNEPIKR_.2	#N/A	8.7647	8.3850	7.8038	8.3975	7.7525	7.9369	7.8367	7.3321	7.3933	8.5144	8.1806	7.5424	7.7449	7.6274	5.6370	7.5159	7.9108	7.5494	7.4234	7.7356
Clasp1	_FSFRS[Phospho (STY)]QEDLNEPIKR_.3	#N/A	4.2334	4.5865	3.7700	3.3508	2.9395	4.4737	7.1834	4.4591	4.0288	4.0440	3.5074	4.3126	7.9221	7.3179	4.1339	3.5955	3.9563	7.5167	3.4921	6.7545
Clasp1	_DGGAAS[Phospho (STY)]PATEGR_.2	Q80TV8_S1220_M1_Clasp1	9.0661	9.0170	8.5416	8.6193	8.2745	8.3852	8.7945	8.5596	8.2727	8.7642	8.4368	8.1901	8.6257	7.7537	3.2063	7.7960	8.4716	8.4007	7.7577	8.1157
Clasp1	_SRS[Phospho (STY)]ANPAGAGSR_.2	Q80TV8_S676_M1_Clasp1	3.7086	3.6127	5.2833	4.8927	4.3663	4.5603	5.3675	5.0188	4.7756	3.6884	4.3767	3.7313	5.4707	4.1371	2.8976	4.5785	3.4745	3.7202	4.0192	3.8144
Clasp1	_TS[Phospho (STY)]PLTS[Phospho (STY)]PTNC[Carbamidomethyl (C)]SHGGLSPSR_.2	Q80TV8_S1106_M2_Clasp1	5.9165	3.9215	3.9949	3.3176	3.4358	3.6598	4.1220	3.5947	4.1811	4.3044	3.9161	3.8109	4.2892	5.6418	4.6555	3.8035	4.3166	5.5800	3.1683	3.4614
Clasp1	_SRS[Phospho (STY)]DIDVNAAASAK_.2	Q80TV8_S600_M1_Clasp1	6.3050	6.5923	3.7685	6.4305	5.8873	5.9764	6.3602	6.1687	5.9450	6.0218	5.7907	5.9415	5.5066	5.4943	4.7387	6.1005	6.0182	6.1278	3.6191	6.1764
Clasp1	_RS[Phospho (STY)]PTGSTASR_.2	Q80TV8_S572_M1_Clasp1	4.2542	4.6478	3.7908	3.3301	2.9603	2.9936	4.0878	4.4799	4.0080	4.6568	3.4867	4.2919	4.1735	3.8288	4.1132	3.6163	3.9771	3.4423	3.4714	3.1423
Clasp1	_NFDDEDS[Phospho (STY)]VDGNRPSSASSSSSK_.3	Q80TV8_S246_M1_Clasp1	4.4614	3.5414	3.4969	3.2279	3.7992	4.0232	3.7419	4.1618	3.8010	3.1669	4.1754	3.4308	3.9091	3.4528	3.3191	3.3055	3.1987	3.5483	3.8474	3.1301
Fnbp1	_TVS[Phospho (STY)]DNSLSSSK_.2	Q80TY0_S296_M1_Fnbp1	11.0559	11.1001	10.9841	11.1876	10.9037	10.8580	10.6764	11.1251	10.8057	11.0796	10.6899	10.9956	11.3854	11.4846	10.5745	10.9350	11.1500	10.8613	10.7163	10.9797
Fnbp1	_TVS[Phospho (STY)]DNS[Phospho (STY)]LSSSKEGKPELR_.3	#N/A	4.5620	4.2579	4.0986	4.5231	3.2681	3.3014	4.6283	3.2868	3.7002	4.3726	3.1788	4.4590	3.8657	4.3683	3.8054	3.9241	4.2849	3.7502	3.3988	4.3353
Vps13b	ospho (STY)]EM[Oxidation (M)]PVPES[Phospho (STY)]APQM[Oxidation (M)]PS[Phospho (STY)]F	#N/A	3.8352	7.4982	6.2357	3.9380	3.6873	7.0735	6.9704	3.8937	5.1517	3.9396	3.7114	3.8387	3.8624	9.1962	4.8278	3.3876	3.7645	5.2339	4.5581	4.3965
Vps13b	_RKEDELS[Phospho (STY)]IGS[Phospho (STY)]APLGK_.3	Q80TY5_S1001_M2_Vps13b	3.8515	4.1513	4.3878	3.8378	3.1893	3.2948	3.7866	3.5519	3.7068	4.3660	3.1855	4.0407	3.8723	4.3749	3.8120	3.9175	4.0896	3.7436	3.3921	4.3419
Dnajc6	_RTATSASAS[Phospho (STY)]PTLR_.2	#N/A	7.8745	7.8559	7.4046	7.3942	6.9146	7.0879	7.4378	7.1705	4.9450	8.4806	7.0705	3.9104	7.1240	6.8797	5.9084	3.8689	6.6866	7.2076	6.7157	6.7809
Dnajc6	_SAATS[Phospho (STY)]PTGSSHGTPTHQSK_.3	Q80TZ3_S683_M1_Dnajc6	5.7034	6.1244	3.4388	3.6820	4.1092	4.1425	4.4397	4.1279	4.3691	6.0824	5.6097	5.1770	4.5254	5.6595	2.9643	5.5017	3.6252	5.5663	5.6490	5.0092
Dnajc6	_VSEC[Carbamidomethyl (C)]SWPIR_.2	#N/A	6.5576	6.6738	5.7792	5.2743	5.7958	6.2506	6.2660	3.9874	6.4406	6.3632	5.9076	6.2099	6.2293	5.1816	5.7626	5.7851	6.2092	6.0177	5.9309	6.0243
Dnajc6	_IYSTC[Carbamidomethyl (C)]TDFER_.2	#N/A	5.7285	5.6276	5.4896	5.8801	5.0351	5.1026	6.2386	5.4079	5.3628	5.7216	5.6356	5.1650	3.3107	5.0743	5.3672	5.3757	5.0397	5.3338	5.0350	5.3170
Dnajc6	_HLDHYTVYNLS[Phospho (STY)]PK_.3	Q80TZ3_S137_M1_Dnajc6	3.8031	4.1997	4.3394	3.8863	3.1409	3.3432	6.7121	6.0036	3.6584	7.1982	6.6052	6.4450	3.8238	6.2058	3.7636	4.1480	4.0412	7.1216	6.8178	6.5053
Dnajc6	_TATSASAS[Phospho (STY)]PTLR_.2	#N/A	10.4396	10.3610	10.4337	10.4098	3.8605	9.5889	9.8961	9.7794	9.5233	10.3644	9.6003	9.5915	9.7980	9.8559	8.3795	3.2528	9.7787	9.6364	9.4904	9.5087
Dnajc6	_GASS[Phospho (STY)]PDM[Oxidation (M)]EPSYGGGLFDMVK_.2	#N/A	4.1137	5.4125	6.4639	7.1486	3.5406	5.4682	5.6929	3.9909	4.9657	4.0794	5.2678	3.6501	3.9673	7.3881	4.6599	3.2996	3.7380	4.4227	3.6903	3.7107
Dnajc6	_DSENKGASS[Phospho (STY)]PDM[Oxidation (M)]EPSYGGGLFDM[Oxidation (M)]VK_.3	#N/A	3.4683	7.9766	6.6892	4.1057	3.6891	3.9132	7.0543	8.0395	7.5689	3.6747	3.6763	7.6446	3.4100	4.4546	6.5270	7.6840	3.9851	7.7416	7.4361	6.8475
Dnajc6	_AADFEDLLSSQGFNAHK_.2	#N/A	4.1537	4.7471	4.4276	4.7265	4.4718	4.9887	4.3331	4.9682	4.1901	4.7985	4.9193	3.8450	4.1699	5.0990	4.3961	4.0478	4.5268	4.3152	4.2901	4.1829
Dnajc6	_ATGQPYEQYAK_.2	#N/A	3.9597	6.2602	6.2340	3.7296	5.4014	6.2577	4.2436	3.6601	5.9303	4.1661	3.2937	6.2455	3.9805	4.4831	5.7014	4.3046	4.1978	6.1881	6.4388	6.3552

Dnajc6	bamidomethyl (C)]EEDHAALVNQES[Phospho (STY)]EQS[Phospho (STY)]DDELLTLSSPHGNAEGDK	#N/A	3.8733	8.7734	5.1540	3.8285	3.7942	7.4879	7.4512	4.1334	6.8945	4.1053	3.5784	4.6314	3.9974	8.8969	7.6593	3.6106	4.1233	9.0725	8.1658	3.6503
Dnajc6	amidomethyl (C)]EEDHAALVNQES[Phospho (STY)]EQS[Phospho (STY)]DDELLTLSSPHGNAEGDKP	#N/A	3.3927	3.9264	4.4301	4.1916	3.5996	3.6329	3.4485	3.6183	3.3687	3.2033	4.3482	3.6525	3.5341	4.4682	3.4739	4.2556	3.1156	4.0339	4.3329	4.1454
Rere	_EKVAS[Phospho (STY)]DTEDTDR_.2	#N/A	4.2478	4.7752	5.5679	5.2424	4.5230	4.8070	4.5473	4.5234	4.7841	4.6079	4.8607	4.3201	4.2328	4.9328	3.8486	2.9817	4.9526	4.3336	4.7767	5.0056
Rere	_PHVM[Oxidation (M)]S[Phospho (STY)]PTNR_.2	Q80TZ9_S1258_M1_Rere	3.9652	3.3539	3.5018	3.6190	4.1722	3.9362	3.8290	5.2297	3.8880	4.5807	4.2624	3.0800	3.9961	3.5399	3.4061	3.3273	3.6881	3.4613	3.7604	3.2171
Rere	_KQPTS[Phospho (STY)]PDGRAS[Phospho (STY)]PINEDIR_.3	#N/A	3.6392	4.3636	4.1755	4.0502	2.9769	5.0342	3.0633	3.3396	5.5780	3.8455	5.1061	4.2530	3.2304	4.8298	4.1413	3.9841	3.8773	4.2270	3.6045	5.1472
Rere	_AS[Phospho (STY)]PINEDIR_.2	Q80TZ9_S600_M1_Rere	6.3858	5.9035	5.8856	6.6545	5.8431	6.3118	6.4721	5.5588	5.7763	7.1003	6.3671	6.0435	4.0189	6.1234	3.7823	6.4373	6.5754	6.0560	6.0096	6.1774
Rere	_VAS[Phospho (STY)]DTEDTDR_.2	#N/A	3.1670	3.3350	3.7033	4.5224	4.0056	4.2296	3.9623	4.3682	3.5946	5.6344	3.9690	3.2244	4.8988	3.6904	3.1127	3.5119	3.4051	4.4281	4.0767	4.6134
Rere	_EKVAS[Phospho (STY)]DTEDTDRITSK_.3	#N/A	4.0769	3.9259	4.6132	3.6125	3.4147	3.6387	4.1264	3.7773	4.0072	4.2833	3.6738	3.8153	4.2936	4.6003	3.7036	4.4218	4.3150	3.5182	3.1668	3.0665
Rere	_QPTS[Phospho (STY)]PDGRAS[Phospho (STY)]PINEDIR_.2	#N/A	4.3858	3.6170	3.4212	3.3036	3.7235	4.2614	4.3209	4.0862	4.2411	3.8317	3.7197	3.5065	4.4065	3.4083	3.3948	3.2298	3.1230	3.2093	4.3587	3.3753
Pja2	_ANHHGS[Phospho (STY)]SPEQVVRPK_.4	Q80U04_S305_M1_Pja2	4.5360	4.2839	4.0726	4.5490	3.2421	3.2755	3.8060	3.2609	3.7262	4.3466	3.2048	4.0100	3.8916	4.1107	3.8313	3.8982	4.2590	4.3913	3.1895	4.3613
Sgsm2	_S[Phospho (STY)]IDVEELGVGR_.2	Q80U12_S402_M1_Sgsm2	7.4525	7.8990	8.5909	8.3746	3.0696	7.7068	7.6330	6.4129	7.6593	8.1902	6.3319	6.9331	3.1378	7.4232	6.0158	7.1479	7.4100	7.1913	7.5102	7.1500
Sgsm2	_FSSGSS[Phospho (STY)]IDSHVQR_.2	Q80U12_S637_M1_Sgsm2	6.2811	6.4351	3.5888	3.1360	3.8911	4.1152	3.6500	4.2537	3.7091	3.2588	4.0835	3.3389	3.8171	3.5759	3.2272	3.3974	3.2906	3.0417	4.1912	3.5429
Ripor2	_SQS[Phospho (STY)]FAGFSGLQER_.2	Q80U16_S46_M1_Ripor2	3.5765	4.0568	4.2997	4.3219	5.7591	6.4466	6.3074	6.2275	3.1851	6.7367	6.6186	4.3158	3.2932	6.1265	4.2041	3.9214	6.5343	5.9934	6.2163	5.8756
Daam2	_ELGS[Phospho (STY)]TEDIYITSR_.2	Q80U19_S704_M1_Daam2	3.3231	10.0800	9.8472	9.3515	5.6782	9.8486	9.6509	3.5651	3.2622	8.8727	3.6366	9.2933	8.7816	9.7813	8.0684	9.0696	9.7493	9.8226	9.4435	9.3223
Rusc2	_LSEGPAALAGPAS[Phospho (STY)]PPR_.2	Q80U22_S534_M1_Rusc2	7.6881	7.9211	6.8153	6.9146	7.6193	7.4631	6.4604	8.0502	7.0934	7.5992	6.8635	7.3309	7.8843	6.7346	5.7816	6.6392	7.9392	7.3712	7.1279	7.4964
Rusc2	_NAGPEPETSRPSPLGSYS[Phospho (STY)]PVR_.3	Q80U22_S776_M1_Rusc2	3.9528	3.3663	3.4894	3.6314	6.3272	4.1931	4.3892	4.1785	4.3094	3.7634	3.7880	3.0924	4.4748	3.5274	2.9137	3.3149	3.6757	3.4737	3.7728	3.2047
Rusc2	_ASVGDSSQEFS[Phospho (STY)]PIQEAQQDR_.2	Q80U22_S572_M1_Rusc2	7.1238	7.1787	6.4547	4.4317	4.0963	6.8947	6.3203	4.4589	6.2186	6.5673	4.0925	6.0096	3.2784	6.8100	5.0475	6.3289	3.4958	4.3374	6.4755	5.7908
Rusc2	_AAGSGS[Phospho (STY)]PPLR_.2	Q80U22_S557_M1_Rusc2	3.7983	6.8588	3.3348	3.7860	4.0052	6.7849	3.6620	4.0239	3.7211	3.2468	4.0955	3.2469	3.8291	3.3729	3.2392	7.2478	3.5212	3.6283	7.3458	7.1336
Snph	_RTS[Phospho (STY)]PPVSVR_.2	#N/A	11.1061	3.6733	10.7347	10.8253	10.7997	10.9186	10.7761	11.2008	10.7170	10.8133	3.6244	10.8511	11.3776	4.1991	10.4255	10.7744	11.1835	4.0104	10.7625	10.8628
Snph	_EEGTGESAGGS[Phospho (STY)]PAR_.2	Q80U23_S204_M1_Snph	9.2309	9.2754	9.4226	9.4329	8.8159	9.2119	8.8780	9.1647	8.9092	9.3220	9.0034	8.7564	8.9633	9.3512	8.5723	8.8446	9.0988	8.8584	9.0645	8.9213
Snph	_TS[Phospho (STY)]PPVSVR_.2	#N/A	3.3788	3.9908	3.9151	4.3371	3.5577	3.6124	3.3503	3.9203	6.9165	3.5851	3.8052	3.8803	3.3995	3.9022	3.7686	3.7237	3.6169	7.1969	3.8914	7.5814
Snph	_AIQTDFVQYQPDLNTILEK_.2	#N/A	4.4124	5.5028	3.5833	3.9198	4.3513	3.9827	4.7889	3.6568	3.9488	4.3333	4.7421	4.8983	3.5523	5.0915	4.2207	4.1009	3.5147	4.3146	3.5277	3.8451
Snph	_QPGDPSNTS[Phospho (STY)]AEDGADSGYVAADDTLSR_.3	Q80U23_S236_M1_Snph	3.4366	4.5333	4.0058	4.2199	3.6435	3.6768	6.1575	3.1699	3.3248	3.2472	4.3043	3.6086	3.4902	3.9929	3.4300	4.2995	3.7076	6.4052	3.7742	3.9599
Snph	_SSTYTKLS[Phospho (STY)]DPAVC[Carbamidomethyl (C)]GDR_.2	#N/A	4.0905	4.9179	4.0013	3.5450	3.2786	3.9200	5.2664	4.1795	3.9503	5.0193	4.8100	3.8511	5.3454	5.0800	3.8682	4.6802	6.1807	3.4123	3.4851	3.8306
Snph	_EEGTGESAGGS[Phospho (STY)]PARS[Phospho (STY)]LTR_.2	Q80U23_S208_M2_Snph	5.7744	4.5967	3.9424	6.2269	4.2447	4.4688	3.2964	6.0832	5.7381	3.6124	5.7707	2.9853	3.4635	3.9295	2.8736	5.7446	6.3731	5.8024	5.5098	5.3784
Madd	_EKPAGS[Phospho (STY)]PIR_.2	#N/A	11.9643	11.7801	11.3951	11.6124	11.3933	11.4474	11.6772	11.8242	11.4278	11.4722	11.3577	11.4395	11.9340	11.3819	10.6186	11.2053	11.6537	11.5677	11.3107	11.1928
Madd	_S[Phospho (STY)]PTENVNTPVGK_.2	#N/A	10.2270	9.9020	10.3760	10.1174	9.7749	9.9852	9.7645	10.0523	9.8994	9.9747	10.0347	9.5689	10.0774	10.2618	9.6498	9.8616	10.3426	9.8119	10.0569	9.8692
Madd	_KRS[Phospho (STY)]PTENVNTPVGK_.3	#N/A	9.5827	9.4644	9.5980	9.2945	8.9821	9.2335	9.2782	9.1810	9.0313	9.2405	9.0332	8.8661	9.5072	9.2723	8.5123	8.7341	9.4528	9.3197	9.2122	8.9740
Madd	_REPSS[Phospho (STY)]PQGR_.2	Q80U28_S929_M1_Madd	3.6076	4.0772	10.3084	4.0333	3.4154	9.3466	9.6782	3.5766	8.5965	4.0367	3.4701	3.9009	3.6330	4.4146	3.7635	8.9364	9.6367	3.9067	3.9200	4.2262
Madd	_LAS[Phospho (STY)]DSDAESDSR_.2	Q80U28_S817_M1_Madd	8.1730	7.2627	8.1189	7.8241	7.3940	7.8885	7.7755	7.8891	7.2294	7.8360	7.8404	7.6166	8.1301	7.7163	7.0731	7.6184	8.2800	7.7575	7.6600	7.5661
Madd	_S[Phospho (STY)]LKEENFVASVELWNK_.3	#N/A	6.6122	7.2364	6.7682	6.8286	5.6394	6.0467	7.0486	6.4780	6.3750	6.8490	6.5263	6.3674	7.2551	6.1622	5.6516	6.4286	6.3354	6.7685	6.8590	6.4471
Madd	_LLRPNS[Phospho (STY)]LK_.2	Q80U28_S812_M1_Madd	7.9384	7.8036	6.8723	7.0857	6.4318	6.4360	7.3876	7.6700	5.8061	7.5173	7.1677	7.1444	7.2729	7.4679	5.4540	6.3091	7.1132	7.4531	6.8468	6.5218
Madd	_S[Phospho (STY)]PTENVNTPVGKDPGLAGR_.2	#N/A	3.6666	4.0390	4.4131	6.5564	3.8401	6.3430	3.9012	3.5894	3.9603	3.8729	3.4989	3.5901	4.0684	4.2772	3.4784	5.5950	3.6882	6.5898	3.8883	3.9545
Madd	_SSVIKHS[Phospho (STY)]PTVK_.2	#N/A	3.6058	7.2311	3.8777	7.0401	6.8772	6.9062	6.8587	3.8140	6.7720	6.5222	6.4225	6.9456	7.1519	6.9406	5.8330	6.7043	6.9298	3.8218	6.4014	6.7298
Madd	_EKPAGS[Phospho (STY)]PIRSSEDVSQR_.3	#N/A	3.8321	3.2030	3.7467	5.9738	5.4968	3.8167	3.7223	4.4231	6.4048	4.0101	3.6478	6.2122	3.7924	3.7580	5.4835	5.4694	3.6961	3.8865	5.6192	3.5937
Madd	_RNGS[Phospho (STY)]ETQLNK_.2	Q80U28_S1499_M1_Madd	3.3071	4.4317	4.3445	4.2771	3.5140	3.5474	3.5341	3.5328	3.4543	4.6071	2.9329	3.7381	3.3380	4.3826	3.5595	4.1700	4.5308	3.4003	4.4185	4.0598
Madd	_AT[Phospho (STY)]LS[Phospho (STY)]DSEIETNSATSAIFGK_.2	Q80U28_S1237_M2_Madd	4.0086	4.0312	4.4181	4.0287	3.3649	3.3983	3.7049	3.5939	3.6251	3.8192	3.1037	3.9089	3.7905	4.4299	3.7302	4.0210	4.3732	3.9645	3.9557	4.2623
Clec16a	_RGS[Phospho (STY)]SDPTVQR_.2	Q80U30_S848_M1_Clec16a	4.4860	3.5169	6.3665	3.2034	3.8237	4.0478	3.7174	4.1863	3.7765	3.1914	4.1509	3.4063	3.8845	3.5085	3.2946	3.3300	5.8168	3.5729	3.8719	3.1056
Arhgef17	_LPSLDHRDS[Phospho (STY)]PR_.3	Q80U35_S2005_M1_Arhgef17	4.7960	5.5809	3.7343	5.9512	4.4927	5.7191	5.3193	4.9113	5.4212	4.6387	5.0828	4.8922	5.7270	4.6972	3.6059	3.6908	4.1306	3.7510	5.4503	5.4624
Arhgef17	_SLS[Phospho (STY)]DPIPQR_.2	Q80U35_S728_M1_Arhgef17	3.2665	3.2354	3.8028	4.4229	4.1051	3.8799	6.6846	4.4677	6.1345	6.6762	4.1013	3.1249	3.2872	3.7899	3.0132	3.6114	3.5046	4.3286	6.4141	3.7569
Rimbp2	_RGTS[Phospho (STY)]VDDFLK_.2	Q80U40_S737_M1_Rimbp2	3.7634	4.2394	3.8690	3.9260	3.1011	3.3252	4.4399	3.4638	2.9982	3.9697	3.3726	4.1288	3.1062	4.1509	4.0171	4.1083	4.0015	4.3512	3.1494	3.8281
Rimbp2	_DHLS[Phospho (STY)]PDFYDESETDPGAEELPAR_.3	Q80U40_S852_M1_Rimbp2	3.9268	4.9520	4.4631	3.7684	3.2645	3.4886	5.8422	3.6272	4.3414	4.1331	3.2150	4.9186	5.7906	7.5990	3.8596	4.2717	4.1649	3.6741	3.3227	3.8093
Cep170b	_GS[Phospho (STY)]LDWPEEER_.2	#N/A	9.7144	10.1023	10.6247	10.3005	7.3431	9.8933	9.6178	7.5404	9.4954	10.1826	8.7730	9.2971	9.0527	10.1609	8.5349	9.6938	9.6882	9.3768	9.5873	10.1186
Cep170b	_SQS[Phospho (STY)]FTHTPPADPK_.2	Q80U49_S421_M1_Cep170b	8.0971	4.5869	8.8586	8.9945	8.1616	4.4786	3.2866	7.5760	3.3457	3.6223	3.7200	8.0146	8.0143	3.9393	2.8638	3.7608	3.6540	7.9018	3.8278	8.1221
Cep170b	_HEDGTQS[Phospho (STY)]DSEDPLAK_.2	#N/A	3.3229	3.1791	3.8592	4.3665	4.1615	4.3856	3.3796	3.7416	3.4387	3.5292	8.4609	3.0685	3.5468	9.3320	2.9568	8.2390	3.2388	3.9107	8.2280	8.4474
Cep170b	_VVQTS[Phospho (STY)]PSAR_.2	Q80U49_S819_M1_Cep170b	4.4348	4.3852	9.5668	9.8786	3.1409	3.1742	3.9072	3.1596	9.1403	9.7785	9.2592	9.5722	3.9929	4.4955	3.9326	9.5465	4.1577	3.6229	3.2715	2.9617
Cep170b	_AGS[Phospho (STY)]FTGPSDSETAPAR_.2	Q80U49_S1166_M1_Cep170b	8.1800	8.4609	8.6387	8.4698	7.9948	7.9475	7.8114	7.8974	7.6469	8.0259	7.8323	7.7550	7.8170	8.4404	7.0116	7.8856	8.1930	8.0106	7.9373	7.9086
Cep170b	_ESPLS[Phospho (STY)]PPTVPDPGGATPGSAR_.2	Q80U49_S981_M1_Cep170b	8.0883	3.3091	6.4199	6.3946	6.6426	7.7466	8.0662	8.4121	7.3095	7.6338	4.3071	8.5495	8.6762	9.5991	3.4509	7.8931	8.5128	3.4166	3.7156	7.5489
Cep170b	_LGDAS[Phospho (STY)]DTEAVDGER_.2	Q80U49_S1122_M1_Cep170b	4.2344	3.2138	3.6418	8.1231	7.6062	3.7962	3.9690	4.3309	4.0281	7.9803	7.7392	7.8100	4.1361	3.6799	3.5462	7.4253	3.8282	7.6911	7.5050	7.5803
Cep170b	_GASPVTPSTT[Phospho (STY)]PPPPTDPQLTK_.2	Q80U49_T541_M1_Cep170b	5.3423	7.0775	4.0504	7.2001	6.5076	3.9802	6.8307	3.8393	3.3504	3.3713	6.9709	4.3287	7.2817	7.5806	3.2799	7.5291	3.9687	7.0963	6.8432	7.5704
Cep170b	_DGPGDDRHS[Phospho (STY)]TKS[Phospho (STY)]DLPVHTR_.3	#N/A	6.1332	6.3968	6.8714	6.7701	6.9202	7.3342	3.4707	6.6191	7.3049	7.6058	5.9393	7.2246	3.8750	3.9337	5.5234	6.7866	4.0923	5.9383	7.1437	7.3024
Cep170b	_QES[Phospho (STY)]FTKEPTSGPPAPGK_.3	#N/A	5.3244	3.7082	6.4232	6.4231	4.8120	3.9106	5.7614	3.8756	4.9508	6.2216	4.2191	5.2768	3.6708	3.8708	4.6002	3.8843	3.5855	3.6089	3.9305	3.6084
Cep170b	_EIHDVAGDGDSLGS[Phospho (STY)]PGPTR_.2	#N/A	7.9698	8.3445	8.2807	8.5710	6.6642	7.2771	7.7733	3.6545	7.4232	8.0982	7.5296	7.8121	7.8007	8.1158	7.2628	8.0003	7.6198	7.2770	7.5312	7.9578
Cep170b	_GHGSRGS[Phospho (STY)]LDWPEEER_.3	#N/A	3.7388	6.5754	6.8907	7.1248	4.1054	6.2774	6.6020	4.3875	5.8682	7.6568	5.5717	6.0957	3.9357	6.6404	4.2676	6.3405	5.9107	6.0067	6.6147	6.6637
Cep170b	_GHKHEDGTQS[Phospho (STY)]DSEDPLAK_.3	#N/A	7.2349	7.3550	7.0659	7.4721	6.2635	7.4227	7.4728	3.5194	7.1598	7.1410	7.3627	7.2487	7.9270	7.8198	7.1772	7.5243	7.5094	7.3487	7.4258	7.4607
Cep170b	_NGPS[Phospho (STY)]PTTPQTPGPQK_.2	Q80U49_S965_M1_Cep170b	9.9628	9.7665	9.8171	10.0319	9.4335	9.6297	9.5991	9.7991	9.6046	9.7501	9.5955	9.7205	9.8832	10.3603	9.0970	9.5235	10.0828	9.6610	9.5932	3.0324
Cep170b	_SNS[Phospho (STY)]LSTPRPTR_.2	#N/A	4.1895	7.9201	7.7859	6.2560	6.4771	3.7119	4.1525	4.4152	4.1123	8.2370	7.7175	4.3565	7.7051	8.1562	4.1778	7.4740	3.9124	7.3218	6.8435	7.9179
Cep170b	_RGHGS[Phospho (STY)]RGS[Phospho (STY)]LDWPEEER_.3	#N/A	5.8146	6.1544	6.5041	6.5303	3.6891	5.1817	5.5137	3.7584	5.1462	6.5091	3.8695	5.7722	4.0502	5.9937	4.3825	5.5837	5.6115	5.5790	5.5528	5.4007
Cep170b	_S[Phospho (STY)]PSLGNVPNTPASTISAR_.2	Q80U49_S1347_M1_Cep170b	4.1208	4.0820	3.6574	3.4763	3.6978	3.9621	5.7156	4.3465	4.1543	3.9314	3.6329	3.8082	4.3681	3.6954	3.6295	3.4829	3.8437	3.1709	3.6177	3.4612
Cep170b	_SNS[Phospho (STY)]LSTPR_.2	#N/A	3.5468	3.7723	4.5842	3.9068	3.7538	3.7871	3.2943	3.7725	8.7105	3.3574	4.1940	3.4984	3.3800	3.8826	3.3197	4.4098	3.2697	4.2358	3.8844	3.8496
Avl9	_DSLPSDS[Phospho (STY)]PPITVQPQANNR_.2	Q80U56_S368_M1_Avl9	9.4517	9.5302	9.1411	9.0619	5.0790	9.3516	9.3063	5.8209	9.1010	9.2503	8.5706	9.0562	9.2349	9.3896	7.9939	9.0691	9.6927	9.5069	9.1539	8.8517
Avl9	_TEKPYFQVEGNNNK_.2	#N/A	4.6234	4.8720	4.8923	4.6477	4.1491	5.0888	3.9202	4.9772	5.1291	4.8845	4.2256	4.2025	3.9906	5.2708	3.6924	4.4735	4.8915	4.9628	4.4640	3.9514
Rims3	_RLS[Phospho (STY)]QSS[Phospho (STY)]LESATSPSC[Carbamidomethyl (C)]S_.2	#N/A	9.3181	9.3067	11.6723	9.4740	9.3236	10.8527	11.0213	9.5379	11.0598	11.2895	10.2765	9.1597	9.6742	11.5405	10.5827	11.0404	11.6257	10.8882	11.1141	10.9933
Rims3	_LSQSS[Phospho (STY)]LESATSPSC[Carbamidomethyl (C)]S_.2	#N/A	3.5306	4.4722	4.0669	4.1588	2.8683	3.0924	3.1719	8.4698	3.2310	3.7369	3.6054	4.3617	3.5513	8.6159	4.2500	3.8755	3.7686	7.8785	3.7131	4.0209
Rims3	_SSS[Phospho (STY)]ISGEIC[Carbamidomethyl (C)]GSQQAGGGAGTTTAK_.2	Q80U57_S22_M1_Rims3	4.2864	4.5335	3.8230	3.2978	2.9925	3.0259	4.0556	3.8209	3.9758	4.0970	3.4544	4.2596	4.1412	3.1430	4.0810	3.6485	4.3586	3.4746	3.1232	3.1100
Rims3	_QGSRESTDGSTNS[Phospho (STY)]NSSEGTFIFPTR_.3	Q80U57_S114_M1_Rims3	3.8894	3.4297	3.4260	3.6948	4.0964	4.1297	4.4526	4.1151	4.3728	3.7000	3.8514	3.1558	4.5382	3.4641	2.9771	3.2515	3.6123	3.0776	4.2270	3.5070
Pum2	_QAS[Phospho (STY)]PTEVVER_.2	Q80U58_S181_M1_Pum2	9.6157	9.9255	3.9815	9.7821	9.4350	9.6559	9.3712	9.9318	9.5190	9.8467	9.4572	9.6475	10.0738	9.9860	2.8345	9.5471	9.8081	4.1499	3.7985	9.6026
Pum2	_GKAS[Phospho (STY)]PFEEDQNR_.2	Q80U58_S136_M1_Pum2	3.2559	8.5256	3.7922	8.5012	7.7014	8.0186	7.7796	8.5567	8.0260	8.1870	7.4216	7.8569	8.2160	8.7615	7.6666	7.8062	8.1944	7.9628	7.6293	7.8880
Kiaa0232	_SAQTS[Phospho (STY)]LDS[Phospho (STY)]QEEATGILPK_.2	Q80U59_S1194_M2_Kiaa0232	3.8491	4.0423	3.8002	3.9767	3.6441	3.5099	3.7934	4.1865	3.6237	3.8039	3.8200	3.8171	3.7892	5.8104	3.6595	3.6173	4.0065	3.9586	3.6072	3.4756
Rubcn	_KQES[Phospho (STY)]PLLVTK_.2	Q80U62_S198_M1_Rubcn	4.3251	3.6777	3.3605	3.5697	3.6628	3.8869	3.8783	4.2402	3.9374	4.5314	4.3118	3.5672	4.0454	3.5892	3.4555	3.1691	3.7375	3.4120	3.7110	3.5882
Suz12	_ASM[Oxidation (M)]SEFLES[Phospho (STY)]EDGEVEQQR_.2	Q80U70_S548_M1_Suz12	4.2815	3.3562	3.8181	3.3027	2.9876	3.6199	4.7359	4.5072	3.9903	5.6089	3.0778	3.4849	4.3124	3.8561	3.7224	4.5524	4.0044	3.1450	4.6988	3.5334
Scrib	_NS[Phospho (STY)]LESISSIDR_.2	Q80U72_S1206_M1_Scrib	8.6844	8.2005	8.9455	8.3945	3.7557	8.5813	3.2924	3.0577	8.3446	8.5565	8.6031	3.0341	7.7133	8.6121	7.0759	8.0048	8.7870	7.8752	8.1036	8.4053
Scrib	_M[Oxidation (M)]QS[Phospho (STY)]PELPAPER_.2	#N/A	9.3088	8.7771	8.0391	9.1338	8.9248	8.6784	8.5180	8.8669	8.4404	9.3416	8.9009	8.5815	9.1170	9.0767	7.4574	8.7956	9.0053	8.6033	8.6132	8.8083
Scrib	_VS[Phospho (STY)]PTGAAGR_.2	Q80U72_S1126_M1_Scrib	4.0488	3.2702	4.5633	3.3313	5.0492	4.2891	5.3710	4.0585	4.2134	3.8594	3.6920	2.9964	4.3788	3.3806	2.8177	3.4110	3.0953	3.2370	5.5692	3.3476
Scrib	_M[Oxidation (M)]KS[Phospho (STY)]LEQDALR_.2	#N/A	6.5500	6.1241	6.1622	6.2797	5.8038	5.8153	5.7079	5.4923	6.3607	5.5560	5.8820	5.4898	6.2861	6.1366	4.7911	5.8307	5.9690	7.6533	6.8020	5.9740
Scrib	_EDGES[Phospho (STY)]EEGQPEAAWPLPSGR_.2	Q80U72_S583_M1_Scrib	3.2079	6.2331	3.7442	4.4815	4.0464	6.5953	6.1980	4.4091	6.2893	3.4142	3.9281	3.1835	3.6618	7.3657	6.1838	3.5528	3.4459	6.4971	4.0358	4.3837
Pum1	_RDS[Phospho (STY)]LTGSSDLYKR_.3	#N/A	6.6232	6.7621	6.2311	6.4655	6.3555	6.2988	6.6310	6.0790	6.2076	7.0648	6.2933	5.9926	7.0095	6.4647	5.7880	6.2350	5.8650	6.4544	6.3470	6.3946
Pum1	_RDS[Phospho (STY)]LTGSSDLYK_.2	#N/A	3.1370	5.0786	5.6240	4.4472	3.3439	5.2754	3.7042	3.4695	4.9802	5.1094	6.1381	3.9082	3.7898	4.2925	3.7295	3.9999	4.0072	5.1907	3.4746	4.2595
Pum1	_S[Phospho (STY)]MDELNHDFQALALEGR_.3	#N/A	3.6122	4.2792	4.1775	4.2542	2.9499	3.1740	3.0909	3.3542	3.1500	3.8185	3.5244	4.0977	3.2581	4.2490	4.1690	3.9571	4.1514	4.1254	3.3793	4.0123
Usp8	_SYS[Phospho (STY)]SPDITQALQEEEK_.2	#N/A	8.7292	8.9161	8.8464	8.7498	8.2181	8.6864	8.6276	8.5943	8.7300	8.7477	8.4470	8.4903	8.9545	9.0133	7.7417	8.4169	8.9031	8.6184	8.6857	8.0427
Usp8	_SYS[Phospho (STY)]SPDITQALQEEEKR_.2	#N/A	6.8661	7.2704	6.2588	7.4078	5.3283	6.8934	5.8251	6.1711	6.1073	4.4719	3.8285	6.5782	6.0670	4.0214	5.1072	6.0083	5.1197	7.0863	7.2787	6.6029
Usp8	_RSY[Phospho (STY)]SSPDITQALQEEEKR_.3	Q80U87_Y679_M1_Usp8	3.8270	4.1909	4.3633	3.8775	3.1647	3.3888	5.2649	3.2413	3.8150	4.0333	3.3241	4.0804	3.9230	4.0764	3.9687	4.8487	4.0056	3.5532	3.4667	3.9182
Usp8	_S[Phospho (STY)]VENLLDSK_.2	Q80U87_S153_M1_Usp8	3.3980	3.8924	4.4354	4.1863	3.6049	3.0026	4.2994	3.6236	3.3208	3.6471	3.6952	3.6472	3.4288	4.4734	3.4686	4.2609	3.1209	3.6503	4.1356	3.6555
Septin9	_S[Phospho (STY)]FEVEEIEPPNSTPPR_.2	#N/A	11.8150	12.0803	12.2994	11.9911	10.0897	10.7915	11.2225	9.4394	10.6655	12.2813	10.7933	10.6416	10.6339	12.0496	9.4970	11.0247	10.5199	10.9784	11.2665	11.2129
Septin9	_S[Phospho (STY)]PKPSLR_.2	Q80UG5_S85_M1_Septin9	9.5364	9.4264	9.6282	3.4975	9.1077	9.0798	9.2128	8.6270	8.8054	9.7311	8.9257	8.4167	4.3409	9.1042	7.9335	8.8019	8.8080	8.7452	8.9159	3.3386
Septin9	_YLQEEVNINR_.2	#N/A	6.7052	7.7753	7.5049	7.8529	6.9288	7.2956	7.2172	6.7819	6.8032	6.5547	6.9352	6.9467	6.8180	8.2188	6.7135	6.7820	7.6699	7.2408	6.8789	7.3446
Septin9	_SVQPT[Phospho (STY)]SEERIPK_.2	Q80UG5_T329_M1_Septin9	3.1521	4.1670	7.3719	4.4321	3.3591	3.2485	3.6890	3.3778	3.0749	3.8930	3.4493	3.8931	3.1830	4.2276	3.7144	4.0151	4.3759	7.9749	3.0726	3.9049
Septin9	_KSVQPTS[Phospho (STY)]EERIPK_.3	Q80UG5_S330_M1_Septin9	4.3717	3.6311	3.4072	4.4787	3.7095	4.2755	3.8316	4.0721	3.8907	4.5781	4.2651	3.5205	5.5360	3.3943	3.4088	3.2158	3.1090	3.2234	4.3728	3.3613
Septin9	_S[Phospho (STY)]FEVEEIEPPNSTPPRR_.3	#N/A	4.0702	6.8471	7.1243	6.6768	3.4079	6.3629	6.6886	3.7705	3.9254	7.4066	6.1721	3.8221	4.0909	7.3770	4.0306	6.4289	4.3082	5.5381	6.2594	6.3987
Rab3gap1	_KTS[Phospho (STY)]LSDSTTSAYPGDAGK_.2	Q80UJ7_S536_M1_Rab3gap1	4.1553	3.1637	3.6919	3.4289	2.8614	6.1857	4.1867	4.3810	4.1069	6.4397	3.5855	4.3907	4.1862	3.7300	4.2120	5.8442	3.8782	6.1888	6.0267	3.4073
Rab3gap1	_FSVTHHYLVQES[Phospho (STY)]PDKER_.3	#N/A	3.8309	4.4573	4.0104	3.7184	3.2212	3.5676	3.8682	4.0256	3.8842	4.4284	3.0242	4.2607	4.0149	4.0195	3.8936	3.8772	3.9398	4.5757	3.5266	3.8671
Naa15	_ITVNGDS[Phospho (STY)]SAETEELANEL_.2	Q80UM3_S854_M1_Naa15	4.0882	4.4130	4.0380	4.3985	3.8805	3.5280	3.9759	3.2143	3.9889	3.6872	3.9069	4.2285	4.1157	4.0509	3.4204	3.8551	4.0031	4.2687	3.5071	3.8772
Dgkz	_RPQNTLKAS[Phospho (STY)]K_.2	#N/A	5.3836	5.5213	4.4924	3.9388	4.8063	3.5620	3.7942	4.3326	4.2202	4.8913	4.4997	3.7843	4.8331	4.5064	3.4723	4.7441	5.3375	4.8297	3.8425	4.8930
Slc20a2	_ESALSRAS[Phospho (STY)]DESLR_.2	#N/A	3.3049	8.8945	7.8678	4.0882	3.5118	9.4818	3.5363	3.3016	3.4565	3.1155	8.3656	4.2910	3.6219	4.1246	3.5617	8.5884	9.1535	8.5435	3.6425	4.0916
Slc20a2	_ESALSRAS[Phospho (STY)]DESLRK_.3	#N/A	4.3351	6.0929	4.5724	4.4343	5.5698	4.9586	5.5022	3.8271	4.9096	4.7734	4.8492	3.4502	5.0385	3.7922	5.1239	4.3909	3.8795	5.9277	4.9401	4.1896
Slc20a2	_AS[Phospho (STY)]DESLRK_.2	#N/A	4.5020	5.8468	3.5374	3.1874	3.8397	4.1452	3.7014	4.2024	5.5835	3.7156	5.3470	5.6018	5.8217	3.5245	4.6180	5.4024	3.2392	3.0931	4.2426	3.4915
Slc20a2	_ALS[Phospho (STY)]MTHGSAK_.2	Q80UP8_S316_M1_Slc20a2	3.2518	4.3485	4.1906	4.0351	3.4587	3.4921	3.5894	3.3547	3.5096	4.5632	2.9882	4.2380	3.6750	4.1777	3.6147	4.1148	3.8923	3.9408	3.5894	4.1447
Slc20a2	_AS[Phospho (STY)]DES[Phospho (STY)]LRKVQEAESPGFK_.3	#N/A	3.2636	3.2384	3.7999	4.4258	4.1021	3.5534	3.4389	3.8009	3.4980	3.4699	3.8724	3.1278	3.6061	3.1498	3.0161	3.6085	3.2981	3.8513	4.1504	4.3280
Pgrmc2	_GLC[Carbamidomethyl (C)]SGPGAGEES[Phospho (STY)]PAATLPR_.2	Q80UU9_S84_M1_Pgrmc2	7.5425	7.7201	7.4868	7.3815	6.5691	7.3653	7.2019	7.5502	7.5193	7.1257	7.3717	7.3684	7.6978	7.7085	6.6717	7.1666	8.2715	7.6347	7.2947	7.3886
Pgrmc2	_DFS[Phospho (STY)]LEQLR_.2	Q80UU9_S98_M1_Pgrmc2	4.2949	5.4333	3.8315	3.2893	3.0010	5.8025	4.6163	4.5206	5.4170	5.2362	3.4460	5.0978	4.1328	3.1346	4.3444	3.6570	4.0178	5.6475	5.2980	3.1016
Pgrmc2	_LLKPGEEPSEYT[Phospho (STY)]DEEDTKDHSK_.4	#N/A	4.5509	4.2690	6.0310	4.5341	3.2570	5.8025	3.7911	5.2428	6.0034	4.3615	3.1899	3.9951	3.8767	4.1256	3.8164	3.9130	4.2738	4.3764	3.1747	3.8029
Pgrmc2	_LLKPGEEPSEYT[Phospho (STY)]DEEDTK_.3	#N/A	3.3784	3.1236	3.9147	4.3110	4.2169	3.7680	3.3241	3.0788	3.2337	3.3383	4.2131	3.0130	3.3991	3.9018	2.9013	3.7233	3.6164	3.9883	3.8653	3.8687
Fbxo2	_VLAELPATELVQAC[Carbamidomethyl (C)]R_.2	#N/A	7.9385	8.0664	7.9443	8.1070	7.9001	7.8094	7.7975	7.9906	8.0092	7.9705	7.5971	7.4851	7.9638	7.9535	7.8190	7.7152	8.0435	7.5411	7.7695	7.8163
Fbxo2	_TDAGSLYELTVR_.2	#N/A	6.1520	6.1831	6.9487	6.7690	5.9152	6.2021	6.0407	6.5284	6.7877	3.6613	5.9604	6.4991	3.2378	6.9413	6.3559	6.2482	6.4418	6.3914	6.3569	6.0714
Fbxo2	_NPC[Carbamidomethyl (C)]GEEDLEGWS[Phospho (STY)]DVEHGGDGWR_.3	Q80UW2_S141_M1_Fbxo2	3.7873	4.7306	4.3236	3.9021	3.1250	4.7991	3.9810	3.4877	4.8538	3.9936	3.3487	4.1050	4.5832	5.3098	3.8382	4.1322	4.0253	4.4504	4.5010	3.8043
Fbxo2	_C[Carbamidomethyl (C)]QQEGLVPEGS[Phospho (STY)]ADEERDHWQQFYFLSK_.4	Q80UW2_S106_M1_Fbxo2	4.4310	3.5718	3.4665	3.2584	3.7687	3.9928	3.7723	4.1314	3.8314	3.1365	4.2058	3.4612	3.9395	3.4535	3.3495	3.2751	3.1682	3.1641	4.3135	3.4205
Fbxo2	_EAEEEEEAEAVEYLAELPEPLLLR_.3	#N/A	3.5498	1.6137	4.5872	4.0345	3.7567	4.3517	1.8175	3.7754	3.4726	2.7250	3.8470	3.4955	3.5806	3.9796	2.9907	4.4127	3.2727	3.8768	2.4286	2.4717
Fbxo2	_FEHGGQDSVYWK_.3	#N/A	4.0802	4.5605	4.6165	3.3248	3.4180	3.6420	2.5054	4.4851	4.1822	2.0547	2.4585	3.8120	4.2903	3.8340	3.7003	4.4251	3.9823	3.1671	3.4662	3.5113
Kcmf1	vl (Protein N-term)]S[Phospho (STY)]RHEGVSC[Carbamidomethyl (C)]DAC[Carbamidomethyl ((	Q80UY2_S2_M1_Kcmf1	4.0391	3.9637	4.5193	4.8496	4.7649	4.5181	4.1643	5.0592	4.2234	4.2454	4.2358	4.9930	4.3314	5.5252	3.7415	4.3840	5.3470	4.8276	3.4250	3.5525
Ap4e1	_IIEQPEC[Carbamidomethyl (C)]SS[Phospho (STY)]PVIETER_.2	Q80V94_S962_M1_Ap4e1	7.7864	3.7889	4.5676	7.6306	3.7371	7.6949	7.7888	7.1160	7.6640	6.9644	7.1978	7.1808	3.5610	7.8486	6.0122	7.3713	3.2531	7.9014	7.3772	7.1110
Camsap3	_DLPDGHAVS[Phospho (STY)]PR_.2	Q80VC9_S334_M1_Camsap3	9.3119	9.5285	9.3268	9.3107	8.7722	8.8015	9.1412	8.8064	8.7487	9.4396	8.9166	8.7480	9.6367	9.0294	7.6564	8.9521	9.3060	8.9881	8.7636	9.0358
Camsap3	_AVASS[Phospho (STY)]PAANNSEVK_.2	Q80VC9_S555_M1_Camsap3	8.6086	7.9198	8.2233	8.5771	7.8267	7.4837	7.6141	8.0745	7.3005	8.4599	7.2942	7.7082	8.0861	7.5076	6.0926	7.5767	7.8725	7.3497	7.4127	7.0880
Camsap3	_RAS[Phospho (STY)]LLER_.2	Q80VC9_S903_M1_Camsap3	5.7865	5.2542	5.2259	5.9077	5.2109	5.4466	5.1382	6.4470	4.9643	5.7279	5.6736	5.2762	5.2710	5.3161	4.4233	5.4693	5.6368	5.4453	5.6973	4.6449
Camsap3	_NTETVPS[Phospho (STY)]QNNSGSSSPVFNFR_.2	Q80VC9_S343_M1_Camsap3	3.3302	4.2324	4.2742	4.1011	3.8062	3.5411	5.6958	3.4472	3.4748	3.7229	4.0457	4.0791	3.6403	4.1429	3.5800	4.0209	3.6796	5.5132	3.6384	4.1099
Camsap3	_KFS[Phospho (STY)]PSQVPVQTR_.2	Q80VC9_S812_M1_Camsap3	5.4834	5.6787	4.3113	4.3166	3.3149	3.1330	5.7496	7.0459	3.1967	4.5245	4.2704	5.8773	6.1227	6.5145	3.8418	4.1226	5.5333	5.6913	4.8133	4.0266
Camsap3	_APSPSGLM[Oxidation (M)]S[Phospho (STY)]PSR_.2	Q80VC9_S1083_M1_Camsap3	8.4573	3.4644	3.3913	3.7295	4.0616	4.0950	2.9865	4.0804	4.4075	8.2175	3.8861	3.1905	4.5729	3.4294	3.0118	3.2168	7.6881	3.5718	3.8709	3.5418
Camsap3	_RS[Phospho (STY)]PGPGPSPTPR_.2	Q80VC9_S767_M1_Camsap3	3.3659	4.9882	3.9022	5.2580	4.2045	5.1745	3.3366	3.0663	4.4518	3.5722	3.7701	3.0255	3.5038	4.5484	2.9138	3.7108	5.2821	3.9536	4.2527	4.2257
Camsap3	_SVS[Phospho (STY)]SDSLGPPRPVSTSSR_.3	Q80VC9_S431_M1_Camsap3	3.9328	4.0700	4.4691	3.7566	3.2705	3.4946	4.2706	3.6332	4.3297	4.1391	3.2032	3.9595	4.4377	5.0962	3.8478	4.2777	4.1708	3.6623	3.3109	3.6588
Znf579	_[Acetyl (Protein N-term)]M[Oxidation (M)]DPQPPPPAQGS[Phospho (STY)]PPHR_.2	Q80VM4_S12_M1_Znf579	8.5348	8.3796	4.2999	4.3217	7.4926	7.6570	3.1262	8.5278	7.7217	8.1625	3.5597	8.1340	9.1891	4.3380	6.6589	8.0443	8.3314	8.3092	2.9622	7.3287
Tecpr1	_GSGTESAPSDTDAS[Phospho (STY)]LEVER_.2	Q80VP0_S418_M1_Tecpr1	5.2337	6.0951	4.1235	5.1116	3.5454	4.6174	5.5738	3.8037	5.0233	5.5543	3.7181	5.3519	3.9979	4.9027	3.6319	5.1774	5.2151	4.6773	5.0307	4.5543
Epn1	_TALPTSGS[Phospho (STY)]STGELELLAGEVPAR_.2	#N/A	9.3862	9.8073	9.3384	8.8410	5.2045	5.5039	9.2679	8.0380	7.7604	10.2040	7.3851	6.0609	9.9026	5.2432	5.4052	8.1238	8.4172	4.8341	8.6380	7.9140
Epn1	_[Acetyl (Protein N-term)]STSS[Phospho (STY)]LRR_.2	Q80VP1_S5_M1_Epn1	10.2339	9.8423	10.2639	6.8659	9.7365	9.2980	9.4794	9.6564	9.2831	10.3488	9.2532	9.5717	9.4241	9.7250	8.9755	9.5802	9.0653	9.2796	9.4518	9.5215
Epn1	_NIVHNYSEAEIK_.2	#N/A	4.0994	5.0123	4.9731	3.5963	3.6484	4.5518	4.5484	4.5412	5.1104	4.6062	4.1070	4.3013	3.9174	5.0854	4.5912	4.9241	3.2650	4.4425	4.2301	4.7134
Epn1	_T[Phospho (STY)]PESFLGPNAALVDLDSLVSR_.2	Q80VP1_T469_M1_Epn1	3.9113	4.0915	4.4476	3.7781	3.2490	3.4731	4.2921	3.1532	4.3512	4.1176	3.2247	3.9810	4.4592	4.0030	3.8693	4.2562	4.1493	2.9982	3.2972	3.6803
Epn1	_ENMYAVQTLK_.2	#N/A	4.1966	3.8750	3.7332	3.4790	2.9027	4.4369	4.0421	3.9107	4.0656	4.0072	3.5443	4.3495	4.2311	4.7554	4.1708	3.5587	4.4484	3.3848	3.0333	3.1999
Inka2	_HIETPATSS[Phospho (STY)]PK_.2	Q80VY2_S281_M1_Inka2	4.3940	3.9898	4.5493	3.6764	3.1001	3.1334	5.1500	3.7133	3.8682	4.2046	3.3469	3.8793	4.0337	5.4096	3.9734	3.7561	4.2510	4.5116	3.2307	4.9238
Tmcc2	_GS[Phospho (STY)]PHLLR_.2	Q80W04_S189_M1_Tmcc2	6.6795	7.0401	6.9896	6.5624	5.7333	5.9621	5.6513	6.0203	5.9501	6.4206	5.8958	5.6158	5.3252	3.8319	4.1404	5.4973	5.6908	5.4989	5.6947	5.9024
Tmcc2	_NKFGS[Phospho (STY)]ADNIAHLK_.3	#N/A	5.3126	5.8620	4.3170	5.5223	2.9379	5.2154	5.5746	4.6894	4.4287	4.6633	4.9237	4.5077	6.1149	5.4671	3.8794	4.0309	4.9568	5.6941	3.6435	4.6174
Tmcc2	_ALSGSATLVSS[Phospho (STY)]PK_.2	Q80W04_S467_M1_Tmcc2	6.4553	6.1525	3.6821	3.4387	4.7269	4.3858	5.2321	6.3569	4.1167	3.9561	3.5953	4.9350	4.2821	3.7202	4.2219	3.5076	3.8684	3.2810	5.7254	3.2510
Tmcc2	_NKFGS[Phospho (STY)]ADNIAHLKDPMEDGPPEEAAR_.4	#N/A	4.3743	3.6155	3.3393	3.5919	3.7120	4.2323	3.3079	4.0168	3.9510	3.6301	3.8953	3.4336	4.0913	3.8363	3.4158	3.2184	4.1042	3.4630	3.4985	3.1748
Tmcc2	bamidomethyl (C)]KS[Phospho (STY)]DELQQQQGEEDGAGMEDAAC[Carbamidomethyl (C)]LLPG/	Q80W04_S6_M1_Tmcc2	4.0541	3.2649	3.5907	3.5301	4.2611	4.2944	4.2879	4.2798	4.2081	3.8647	3.6867	2.9911	4.3735	3.6288	2.8124	3.4163	3.7771	3.3724	3.6714	3.3423
Gstm7	_IRVDILENQLM[Oxidation (M)]DNR_.3	#N/A	4.7130	4.9978	5.1659	5.3779	3.8902	5.3065	5.0301	4.9019	4.9431	4.9902	4.0843	5.2570	6.0038	5.3978	4.5411	5.2901	3.2897	4.6149	4.1920	5.6208
Wipi2	_GAYVPSS[Phospho (STY)]PTR_.2	Q80W47_S395_M1_Wipi2	12.8785	12.8718	12.7789	12.8734	12.5219	12.4821	12.5299	12.9705	12.5143	12.8232	12.4377	12.5630	13.0854	12.6998	11.8435	12.5406	7.0341	6.2351	12.6457	12.5156
Agfg2	_ELGGC[Carbamidomethyl (C)]SQAGNR_.2	#N/A	4.5486	4.2713	4.0809	4.4328	3.2547	3.6358	3.6607	3.9845	3.5954	4.3592	4.1273	3.9974	3.8790	4.1233	3.9204	3.4184	4.2201	4.3787	4.2709	4.3316
Mtdh	_SETNWES[Phospho (STY)]PK_.2	#N/A	10.0019	9.9629	9.6897	9.9238	9.4774	9.6241	9.7291	9.8722	9.6377	9.7237	9.6479	9.2687	9.8798	9.6965	8.8593	9.2143	10.0246	9.6801	9.5369	9.4905
Mtdh	_SQEPISNDQKVS[Phospho (STY)]DDDKEK_.3	Q80WJ7_S423_M1_Mtdh	6.1379	6.2131	6.2786	6.3705	5.3678	6.3769	3.6585	4.3283	6.0505	6.1964	6.3693	5.9283	6.1262	6.0872	5.4686	5.5644	5.9986	5.9527	5.9843	5.3903
Mtdh	_SETNWES[Phospho (STY)]PKQIK_.2	#N/A	8.2853	8.5084	6.6195	7.8772	7.5320	7.4760	7.7523	8.3541	7.6908	7.5581	7.0688	7.8713	8.6024	7.6205	3.3257	7.7290	7.7276	7.7483	7.1715	7.4195
Mtdh	_SETNWES[Phospho (STY)]PKQIKK_.2	#N/A	5.3560	5.2214	4.3967	3.8289	4.5024	3.2859	4.7773	6.2464	3.7157	4.0668	3.1944	4.0318	3.8812	4.3838	3.9201	4.2053	5.7327	3.7347	3.3832	4.3508
Trmt9b	_S[Phospho (STY)]LDESTLRK_.2	#N/A	7.2971	7.9873	7.2725	4.4149	7.1465	6.9503	6.9930	7.2660	7.0122	7.2452	6.4836	4.2227	7.3068	7.8136	6.8109	7.3756	7.4338	7.4101	3.0555	7.1826
Trmt9b	_S[Phospho (STY)]LDESTLR_.2	#N/A	3.6209	3.6982	3.1575	3.9633	3.8278	3.8612	3.2203	4.7562	3.1405	3.4315	4.1199	5.3724	3.3059	3.8086	3.2456	2.9830	3.5232	5.7944	3.9585	6.4945
Trmt9b	_S[Phospho (STY)]HSM[Oxidation (M)]DYGSAVAR_.2	Q80WQ4_S210_M1_Trmt9b	4.5540	4.2659	4.0906	4.5310	3.2601	3.2935	3.7880	3.2789	3.7082	4.3646	3.1868	3.9920	3.8736	4.1287	3.8133	3.9162	4.2769	3.7422	3.1715	4.3433
Aftph	_SFS[Phospho (STY)]PGDFR_.2	Q80WT5_S151_M1_Aftph	4.1428	7.5143	3.1782	7.2972	3.4805	7.1475	4.0606	3.8431	7.2645	4.3491	2.9932	6.4582	4.2277	3.7715	6.1624	2.9868	4.3808	3.2297	3.5287	3.4488
Aftph	_HLLETHSS[Phospho (STY)]PAK_.2	#N/A	5.9751	5.6948	5.4302	3.5784	5.5149	5.3989	5.1999	5.6877	5.4286	5.8220	5.3605	5.5572	6.1287	5.8366	5.4652	3.8482	5.5095	5.4147	5.4497	5.6221
Aftph	_S[Phospho (STY)]PDPDPTGQNALDDSAASMK_.2	Q80WT5_S395_M1_Aftph	4.3306	3.6722	3.4648	3.3588	3.6683	3.8924	3.8728	4.0309	3.9319	4.5369	4.3063	3.5617	4.0399	3.5837	3.4500	3.1746	3.0678	3.4175	3.7165	3.2610
Srrm3	_ARPAS[Phospho (STY)]TS[Phospho (STY)]PSPGTR_.2	Q80WV7_S464_M2_Srrm3	6.7199	3.8000	4.0859	4.9214	3.6117	3.7663	6.8598	5.9024	3.7165	3.8937	3.6995	3.6894	3.8543	3.9775	3.5657	3.6661	3.7877	5.4568	3.6917	3.8583
Srrm3	_RRDS[Phospho (STY)]PSFMEPR_.3	#N/A	3.3999	3.9639	3.9362	4.3239	3.5651	3.9210	3.5422	3.6366	3.5335	3.2438	3.5495	3.8533	3.6692	3.7572	3.7416	3.6869	3.6401	4.3722	4.7097	4.2741
Ubap2l	_RYPSSISSS[Phospho (STY)]PQKDLTQAK_.3	#N/A	8.3406	8.8650	8.2234	8.3120	7.9567	8.1242	8.4399	8.8979	7.9043	8.5443	8.0790	8.4557	9.2880	9.3109	7.9455	3.6424	8.2964	8.3613	8.1005	8.1353
Ubap2l	_RYPSSISSS[Phospho (STY)]PQK_.2	#N/A	8.4218	8.5351	8.7306	8.6799	8.3045	8.4292	8.0335	7.9946	8.2749	8.8637	8.3957	8.1971	8.4987	9.1427	4.1450	8.4211	8.6315	8.1669	8.4232	8.4537
Ubap2l	_YPSSISSS[Phospho (STY)]PQK_.2	#N/A	3.4222	4.5189	7.5265	4.2055	3.6291	6.8406	3.4190	3.1843	3.3392	7.0350	4.3187	4.4083	3.5046	7.4993	3.4444	7.2073	3.7220	4.1112	6.6367	7.2247

Ubap2l	_TATEEWGTEDWNEDLSETK_.2	#N/A	6.3981	5.8103	5.2971	6.1268	4.3345	5.1593	5.2567	5.5587	5.6005	5.4367	4.6292	5.1979	6.5599	6.7807	4.3956	5.7784	6.0868	5.4621	4.4141	5.6922
Ubap2l	_YPSSISSS[Phospho (STY)]PQKDLTQAK_.2	#N/A	5.4209	5.4384	5.6956	6.3830	5.2105	4.0027	4.2104	4.1578	4.1173	4.0466	5.3052	3.8461	4.2827	3.4389	4.5338	3.2962	6.0072	4.9640	5.3133	5.1025
Ubap2l	_NQDEC[Carbamidomethyl (C)]VIALHDC[Carbamidomethyl (C)]NGDVNR_.3	#N/A	5.1583	5.1200	5.5518	5.4033	5.0310	4.9034	4.3981	5.0909	5.0429	4.8735	5.1774	4.9889	4.0998	5.8811	3.7979	5.1661	5.3072	4.6416	4.4608	5.4081
Ubap2l	_STSAPQMS[Phospho (STY)]PGSSDNQSSSPQPAQQK_.3	#N/A	4.3192	4.9474	7.4493	7.9156	5.4656	3.9159	3.5558	4.6453	3.9639	5.8126	6.8260	6.1083	4.1147	9.1031	3.4391	3.4970	6.3562	5.2070	5.3653	4.1648
Btbd10	_[Acetyl (Protein N-term)]AGRPHPYDSNSS[Phospho (STY)]DPENWDR_.3	Q80X66_S13_M1_Btbd10	4.3064	4.5135	3.8430	3.2778	3.0125	3.5950	4.1702	4.5321	4.2293	4.2395	3.1028	4.2396	4.3373	3.8811	3.7474	4.3781	4.0294	3.1201	3.4191	3.5584
C2cd2l	_NLGT[Phospho (STY)]PTSSTPR_.2	#N/A	10.2488	10.0819	4.1980	10.1608	9.8365	10.0565	9.8568	10.0440	9.8541	9.8567	10.2341	9.8160	10.0310	10.3400	9.0334	9.8396	10.3888	10.1738	9.7692	10.0721
C2cd2l	_VDGKLDS[Phospho (STY)]PSR_.2	#N/A	10.1573	3.7326	3.3056	9.8056	9.9740	10.0751	10.1666	10.6539	10.1643	9.7796	4.3667	10.1111	10.6415	10.1255	9.6846	10.0302	10.5923	10.4831	10.1293	10.0698
C2cd2l	_LDS[Phospho (STY)]PSRS[Phospho (STY)]PSKVEVTEK_.3	#N/A	7.9992	8.4654	8.7649	8.0293	7.7800	3.7445	6.1755	8.9599	8.1563	3.6311	8.1664	8.9734	3.6882	8.5390	7.1666	6.2179	8.0053	6.6517	3.8511	8.2849
C2cd2l	_NLGT[Phospho (STY)]PTS[Phospho (STY)]STPRPSITPTKK_.3	Q80X80_S420_M2_C2cd2l	3.4299	3.9444	3.7637	7.4922	3.6368	3.8231	7.2353	3.6556	3.3527	3.9924	3.7271	3.6705	7.7616	3.8018	3.4918	3.5893	3.9501	4.0518	3.6473	4.1827
C2cd2l	_LDS[Phospho (STY)]PSRSPSK_.2	#N/A	8.1238	7.6526	7.6414	7.8029	7.4133	7.8533	7.8384	8.3053	7.9964	7.5971	7.2695	7.9654	8.1875	7.9849	6.7576	7.6972	8.2857	8.0365	7.6525	7.8044
C2cd2l	_KAGS[Phospho (STY)]FSR_.2	#N/A	3.3904	8.7773	3.9267	8.4946	4.2290	4.4530	8.2758	8.3211	3.3712	3.5967	8.0119	7.6157	8.1582	3.9138	2.8893	7.8297	3.6285	4.2047	3.8533	3.8808
C2cd2l	_EAGLSQS[Phospho (STY)]HDDLSNTTATPSVR_.3	#N/A	8.3199	8.2426	8.0559	8.4960	6.6910	4.0679	7.9498	6.3578	6.8162	9.1403	7.7050	6.9909	7.1961	7.7708	5.0992	7.6636	7.3408	7.4716	7.1918	7.6953
C2cd2l	_S[Phospho (STY)]PSKVEVTEK_.2	#N/A	6.0894	6.2340	3.5117	3.2131	3.8140	6.3502	5.1036	4.1766	4.3315	6.2390	6.1825	3.4160	4.4970	6.0132	2.9358	5.4704	6.1959	3.1189	4.2683	3.4658
C2cd2l	_TST[Phospho (STY)]PEEAGVR_.2	Q80X80_T79_M1_C2cd2l	4.2420	3.2214	3.2774	3.4866	6.4738	3.8038	6.5043	4.3233	4.0205	6.2109	4.3949	3.6503	4.1285	6.0896	3.5386	5.7242	3.8206	6.6492	5.9459	3.3496
C2cd2l	_KKAGS[Phospho (STY)]FSR_.2	#N/A	4.2503	5.9758	3.2858	3.4392	4.8929	4.1177	5.5862	3.9507	4.9776	4.2163	4.2927	3.6421	4.2138	4.8033	3.5304	3.0944	4.8806	3.3449	2.9935	3.2399
C2cd2l	_NLGT[Phospho (STY)]PTSSTPRPSIT[Phospho (STY)]PTK_.2	#N/A	3.3989	7.4612	4.1397	4.0886	3.1917	3.3048	4.6546	6.1451	3.3779	3.8828	3.3275	4.2915	3.5179	4.1268	3.9649	3.9994	6.7970	5.4591	3.6429	4.0938
C2cd2l	_ESHLS[Phospho (STY)]NGLDPVAETAIR_.3	#N/A	4.4516	6.0259	3.7728	5.6559	3.4994	3.7197	6.5044	3.7592	6.9327	3.6615	4.8047	5.4529	4.2671	3.8109	4.6569	6.1889	6.3289	6.0346	5.6688	3.4881
C2cd2l	_VDGKLDS[Phospho (STY)]PSRS[Phospho (STY)]PSK_.2	#N/A	3.9590	4.0494	4.4953	5.1743	3.2967	3.2740	4.2499	3.4977	5.0964	4.1653	3.3253	3.9388	4.3015	4.3316	3.8271	4.3039	4.1970	4.0719	3.2257	3.4207
C2cd2l	_SLSPAATVTAELHYEQGS[Phospho (STY)]PR_.3	#N/A	9.6316	9.9562	9.5767	9.2071	8.7329	9.4382	9.4755	9.3150	9.4164	9.5725	9.3901	9.4076	10.1438	9.6671	8.7611	9.0960	9.7772	9.9362	9.8120	9.3421
C2cd2l	_QKEAGLSQS[Phospho (STY)]HDDLSNTTATPSVR_.3	#N/A	3.5553	5.6374	4.5928	4.0289	3.7623	3.7956	4.7200	3.0511	3.2060	3.3659	4.1855	3.4395	3.3715	3.8741	3.3112	4.4183	3.5888	4.2443	3.8929	3.8411
C2cd2l	_SDISERPSVDDVES[Phospho (STY)]ETGSTGALETR_.3	Q80X80_S619_M1_C2cd2l	3.8123	5.0880	3.8975	3.9722	3.6505	4.4223	3.9781	4.0132	4.5111	4.0186	3.4188	3.6670	4.1452	5.4524	3.5553	3.4475	4.0503	5.2918	3.0756	3.7957
Sympk	_TSS[Phospho (STY)]PETREPESK_.2	Q80X82_S1276_M1_Sympk	3.7009	3.6182	6.3851	3.8834	3.9078	3.9412	5.9318	3.9266	6.1891	3.5115	5.8745	5.3837	6.2838	5.2458	3.1657	4.8693	3.4238	6.4350	5.8522	2.9528
Reps2	_APS[Phospho (STY)]QAAESSPTK_.2	Q80XA6_S405_M1_Reps2	8.3716	7.2142	7.1486	8.2984	6.3023	6.3665	7.7862	3.9732	6.8417	6.7986	6.4488	6.3219	7.5398	7.0145	3.7262	7.7399	6.2513	7.5385	6.3024	6.4270
Reps2	_ASS[Phospho (STY)]LDLNK_.2	Q80XA6_S354_M1_Reps2	3.1089	4.2101	4.1464	4.4753	3.3159	3.3492	5.6325	3.3346	3.6524	5.3508	4.7836	5.2536	3.8179	4.1844	3.7576	3.9719	4.3327	3.7980	3.1158	5.4544
Reps2	_LNS[Phospho (STY)]ELQQLK_.2	Q80XA6_S492_M1_Reps2	5.7579	5.9480	6.1657	5.5392	5.8921	5.6564	5.8720	5.6943	5.5641	6.2001	5.9812	5.5637	6.2447	6.1295	5.4389	5.5289	6.1059	5.7157	5.9679	5.9862
Reps2	_VS[Phospho (STY)]LSHGYSK_.2	Q80XA6_S67_M1_Reps2	5.1898	5.6212	5.6982	5.6212	2.9115	3.1356	3.1287	3.4906	3.1878	6.3503	3.5622	3.7802	3.2958	4.3404	4.7730	3.9187	4.4887	4.1616	4.3732	4.0177
Reps2	_SYS[Phospho (STY)]STSIEEAMK_.2	Q80XA6_S324_M1_Reps2	3.8513	3.4678	3.3879	3.7329	4.0583	4.0501	3.7151	4.0770	3.7741	3.1938	4.1485	3.1939	3.8822	3.4260	3.0152	3.2134	3.5742	3.5752	3.8743	3.1032
Reps2	_SFS[Phospho (STY)]VEREPQENNSNYPDEPWR_.3	Q80XA6_S119_M1_Reps2	3.6975	3.6216	3.2340	3.8868	3.9044	3.9377	3.1437	4.4099	3.0639	3.5081	4.0434	3.3477	3.2294	3.7320	3.1691	3.0596	3.4467	4.3865	4.0351	3.6990
Reps2	_APSQAAESSPT[Phospho (STY)]KMDAPHAQPPSKPIR_.4	Q80XA6_T413_M1_Reps2	4.4054	4.4145	3.9420	3.1788	3.1115	3.1448	3.9366	3.1303	3.8568	4.2160	3.3354	4.1406	4.0222	4.5249	3.9620	3.7675	4.1283	3.5936	3.2422	2.9911
Reps2	_RGEDPPT[Phospho (STY)]PPPRPQK_.3	Q80XA6_T340_M1_Reps2	3.4989	3.8202	4.5363	4.0854	3.7058	4.4026	3.3423	3.7245	3.4217	3.3095	3.7961	3.5463	3.5297	4.5744	3.3677	4.3618	3.2218	3.9277	4.2267	4.2516
Usp6nl	_SVGRPS[Phospho (STY)]PK_.2	Q80XC3_S394_M1_Usp6nl	6.7616	7.0391	6.5418	5.6766	6.2712	4.2395	6.9453	6.8093	6.9963	6.8595	6.3670	6.2897	6.4218	3.2366	5.3763	3.0241	6.7694	6.6979	6.7454	6.0877
Ric8b	_GDNWYS[Phospho (STY)]EDEDTDTEEYK_.2	Q80XE1_S468_M1_Ric8b	3.2503	5.0558	5.2863	4.8784	4.0888	4.9167	3.4525	4.0625	6.6621	3.4566	3.9583	4.1384	3.5153	4.8461	3.9849	4.3284	5.0857	4.0244	4.5914	4.1703
Lrrtm4	_AYEAVSIEQDDS[Phospho (STY)]PSLELGR_.2	Q80XG9_S560_M1_Lrrtm4	4.0735	3.6590	4.1464	3.6840	3.7781	3.9107	3.5356	3.9803	3.5381	5.9266	4.1831	3.4632	3.6692	3.3143	3.3179	3.9666	3.2366	3.9370	6.1090	5.1895
Eif4g3	_TSS[Phospho (STY)]PTSLPPLAR_.2	Q80XI3_S267_M1_Eif4g3	14.5736	14.3986	13.9652	14.0084	13.7685	13.8704	13.9035	14.4009	13.6747	14.1385	14.1174	13.8247	14.4837	14.4326	12.7302	13.7664	14.4430	13.9334	13.8167	13.7166
Eif4g3	_NPT[Phospho (STY)]PPIGR_.2	Q80XI3_T168_M1_Eif4g3	10.2030	10.3798	9.6833	10.0566	8.8209	9.8643	9.6424	9.9850	10.1292	10.2211	10.0991	8.7268	8.3774	10.6764	9.0362	8.9952	10.2659	10.0259	9.6492	9.7900
Eif4g3	_GSS[Phospho (STY)]KDLLDNQSEEQRR_.3	#N/A	7.6700	7.4560	3.1916	7.5129	3.8619	3.8953	7.1824	3.8807	6.5348	3.4656	7.2252	6.7841	7.2490	7.6792	3.2115	6.6861	3.3779	7.0656	3.9926	6.5625
Eif4g3	_GSS[Phospho (STY)]KDLLDNQSEEQR_.2	#N/A	3.7544	3.3263	4.2907	5.3171	3.0921	3.3162	3.7843	3.4548	3.0072	3.9607	3.3816	4.1379	3.1153	4.4412	4.0262	4.0993	3.9925	2.9424	3.1404	3.8372
Eif4g3	_T[Phospho (STY)]PDEVLEAEAEPK_.2	Q80XI3_T513_M1_Eif4g3	3.1678	6.3210	6.7956	4.5216	4.0064	6.5086	6.1016	3.8966	5.7256	3.3742	3.9681	3.2236	3.7018	6.6485	3.1119	3.5127	6.3923	6.0658	5.9378	4.4237
Eif4g3	_AESES[Phospho (STY)]DGQAEETADPQSLHSGR_.3	Q80XI3_S472_M1_Eif4g3	3.6226	3.3490	3.6001	3.7493	4.0883	4.0665	3.7127	4.0746	3.7718	4.0781	4.1462	3.1599	3.8798	3.4236	3.0173	3.4167	3.5719	3.5916	3.8906	3.1009
Pip4k2b	_FGIDDQDYQNSVTR_.2	#N/A	5.7862	5.6018	5.4135	4.5366	5.6214	5.3181	5.4233	5.3811	4.9104	5.2082	5.2735	5.5518	3.4243	6.0666	4.7789	5.2706	3.2254	5.6144	5.3787	4.9843
Atg2b	_DFATAPPTS[Phospho (STY)]PAK_.2	Q80XK6_S1571_M1_Atg2b	9.8454	9.3855	9.7150	9.5567	9.0470	8.9996	9.0412	9.3290	9.0118	9.5755	9.2577	8.9107	9.5259	9.0685	3.3961	8.9328	9.3213	9.0165	8.8615	3.8507
Atg2b	_LS[Phospho (STY)]PSWNPK_.2	Q80XK6_S255_M1_Atg2b	6.7602	6.2024	6.8541	6.7843	6.2656	5.5947	5.8078	6.1846	5.6054	7.1633	3.6154	6.2245	5.7600	6.7026	4.2419	3.4876	4.5195	3.3136	6.0704	6.2698
Atg2b	_S[Phospho (STY)]SPVC[Carbamidomethyl (C)]STAPVETEPK_.2	Q80XK6_S239_M1_Atg2b	3.5093	4.4936	4.0455	4.1801	4.3478	3.6371	3.1933	3.2096	3.3645	3.2074	4.3440	4.3830	7.5944	4.0326	4.2713	3.8541	3.7473	4.0859	3.7344	3.9996
Atg2b	_S[Phospho (STY)]PGADVTC[Carbamidomethyl (C)]SLPR_.2	Q80XK6_S1741_M1_Atg2b	6.7329	6.1515	3.8062	7.0517	5.7608	5.9435	6.0997	4.4953	5.5983	6.6403	6.5849	5.7238	4.3005	6.5317	4.0977	6.0555	6.7360	5.2750	5.8671	6.1037
Atg2b	_SAS[Phospho (STY)]VDESRPEFIC[Carbamidomethyl (C)]R_.2	#N/A	3.8466	5.4850	4.1366	3.8265	3.5172	4.8644	5.6459	3.8879	3.3999	6.2479	4.6295	4.6592	4.8310	4.2038	3.8112	4.2975	3.9185	5.1160	4.5754	4.6226
Atg2b	_AFTEVFLDDSHS[Phospho (STY)]PANR_.3	Q80XK6_S734_M1_Atg2b	4.2476	4.5723	3.7842	3.3366	2.9537	3.6538	4.0944	4.4733	4.1705	4.0582	3.0440	4.2984	4.2785	3.8223	4.1197	3.6098	3.9706	3.1789	3.4779	3.4996
Bdh1	_VSVVEPGNFIAATSLYSPER_.2	#N/A	5.8534	6.3037	6.2679	6.6845	6.1166	6.2858	6.3675	6.2644	6.3697	6.3818	6.6214	6.5741	6.5411	6.5764	6.4163	6.5472	6.1986	6.3270	6.4998	6.3573
Bdh1	_FGIEAFSDC[Carbamidomethyl (C)]LR_.2	#N/A	6.1552	5.8836	6.6289	6.3782	5.7967	5.9529	6.0935	5.8294	5.5709	6.1840	5.5345	6.3636	6.1921	5.7163	5.2452	5.7947	5.3844	5.9568	5.9128	6.2067
Bdh1	_AIILTGC[Carbamidomethyl (C)]DSGFGFSLAK_.2	#N/A	3.8098	4.1976	3.2541	3.0967	3.5796	3.7175	3.6833	4.1471	3.6629	4.4907	3.9613	3.3328	4.8851	3.5129	2.6823	4.3354	4.8926	3.0774	4.6974	3.6010
Bdh1	_VVNISSMLGR_.2	#N/A	3.1598	6.0751	6.7450	6.4791	4.7415	5.4051	6.4261	3.9047	4.7639	3.5198	6.1797	4.3938	3.7099	6.6487	5.4986	3.5047	5.3264	4.6759	4.1329	4.4317
Bdh1	_SPYC[Carbamidomethyl (C)]ITK_.2	#N/A	4.2634	3.7394	3.2989	3.4260	2.1585	3.8252	3.9399	3.9638	3.9990	4.4698	4.3734	4.1349	4.1071	3.2860	5.2112	3.1075	4.5015	4.3764	2.9803	3.2530
Fam76b	_VSSLS[Phospho (STY)]PEQEQGLWK_.2	Q80XP8_S193_M1_Fam76b	3.8001	8.2003	3.6423	8.0556	4.0070	4.0404	3.0411	4.3072	4.4621	3.6107	8.0015	7.9993	3.1267	8.5595	7.8034	3.1622	8.4398	7.8061	7.9490	8.1820
Wnk3	_EGETTVNTETSS[Phospho (STY)]PK_.2	Q80XP9_S1039_M1_Wnk3	8.2438	8.3110	4.4122	4.2094	8.0030	3.0258	8.3008	8.4792	8.1122	8.3846	3.6720	8.1668	8.1722	8.5865	2.8157	8.0796	3.0977	4.0517	8.0011	8.0575
Wnk3	_S[Phospho (STY)]VEM[Oxidation (M)]TEDDKVAESSR_.2	Q80XP9_S62_M1_Wnk3	3.3060	3.1960	3.8423	4.3834	4.1446	3.8404	3.2411	4.5072	3.1613	3.4107	4.1408	3.0854	3.3267	3.8294	2.9737	3.6509	3.5441	4.2891	3.9377	3.7964
Tbc1d5	_TISSSPS[Phospho (STY)]IESLPGGR_.2	Q80XQ2_S565_M1_Tbc1d5	8.8167	8.9431	8.5642	8.4601	7.9120	8.5138	8.5044	8.4653	8.4226	8.5603	8.4880	8.0955	3.8329	8.5855	5.3261	7.9476	8.7557	8.4504	8.2882	8.4852
Tbc1d5	_EFTGS[Phospho (STY)]PPPSATKK_.2	Q80XQ2_S578_M1_Tbc1d5	4.3300	7.9437	7.4112	3.2542	8.0053	3.0695	4.0120	3.7773	3.9322	4.1406	8.2748	4.2160	4.0976	4.6003	7.8097	3.6921	4.3150	3.5182	8.7202	3.0664
Tbc1d5	_SES[Phospho (STY)]MPVQLNK_.2	Q80XQ2_S546_M1_Tbc1d5	3.5779	5.8783	4.1142	7.0143	2.9156	3.5685	3.5129	3.2782	3.4331	3.1388	5.6762	4.3144	3.5986	4.1012	3.5383	3.9228	3.8159	4.0172	3.6658	4.0682
Fam168b	_[Acetyl (Protein N-term)]M[Oxidation (M)]NPVYS[Phospho (STY)]PGSSGVPYANAK_.2	#N/A	4.0158	7.6384	3.9317	3.8152	3.1385	3.5213	4.2558	3.7517	3.9092	4.0802	3.1915	4.1659	7.7487	6.9556	3.9821	4.0515	4.1180	3.6575	3.3454	7.1021
Nucks1	_ATVTPS[Phospho (STY)]PVKGK_.2	#N/A	10.9334	11.1069	10.7621	10.6371	10.3256	10.6242	10.8664	10.9126	10.4659	10.5849	10.3752	10.1287	10.9640	10.5823	9.4584	9.9517	10.7983	10.7114	10.3585	10.0784
Nucks1	_ATVTPS[Phospho (STY)]PVK_.2	#N/A	6.3704	7.2189	3.5614	3.1635	6.7142	4.0877	7.2870	4.2263	3.7365	3.2314	7.6462	6.1852	3.8446	6.4558	3.2546	3.3700	6.2066	6.9640	3.9119	6.8810
Nucks1	_LKATVTPS[Phospho (STY)]PVK_.2	#N/A	7.6873	3.3192	3.5365	3.5843	4.2068	3.9015	4.3421	7.0451	6.3931	3.8105	7.2412	6.3091	7.5718	3.5745	5.0536	3.3620	3.7228	3.4266	3.7257	3.2518
Nucks1	_TPSPKEEDEEAES[Phospho (STY)]PPEKK_.3	#N/A	7.4634	7.6831	7.2899	7.6244	6.9032	7.9145	7.0532	3.2772	7.6920	7.7347	7.6646	3.7160	7.2314	7.0844	3.5373	6.7998	7.7417	7.1733	7.5936	7.1492
Nucks1	_EEDEEAES[Phospho (STY)]PPEKK_.2	#N/A	7.0018	5.8864	3.2283	3.4966	3.5305	6.3278	5.7933	5.8875	5.0860	6.1235	7.1127	3.8535	4.2135	6.2437	3.5878	3.0369	6.0570	4.9471	5.5314	4.2985
Nucks1	_KVVDYSQFQES[Phospho (STY)]DDADEDYGRDSGPPAK_.4	#N/A	4.0339	3.2852	3.5704	3.5504	4.2408	4.2741	4.3082	4.0735	4.2284	3.8444	3.7070	3.0114	4.3938	3.3956	2.8327	3.3960	3.1103	3.2220	4.3714	3.3626
Nucks1	_KVVDYSQFQES[Phospho (STY)]DDADEDYGR_.3	#N/A	4.0195	8.6620	8.1372	7.6656	3.0090	8.2714	8.6554	4.0598	8.0883	6.9350	3.4513	6.5982	3.7374	8.8641	7.1554	6.8427	3.9684	8.7073	8.0465	7.4900
Nucks1	_VVDYSQFQESDDADEDY[Phospho (STY)]GRDSGPPAK_.3	Q80XU3_Y26_M1_Nucks1	3.8758	5.6307	3.5204	3.4307	4.0827	4.1160	5.3562	4.1968	4.3870	3.6864	3.8657	3.2396	4.5525	3.5318	2.9913	3.2379	3.4717	3.2990	4.4650	3.5213
Nucks1	_LKATVT[Phospho (STY)]PS[Phospho (STY)]PVKGK_.2	Q80XU3_S181_M2_Nucks1	3.6190	4.3838	4.1553	4.0704	2.9567	3.1808	3.0835	3.3193	3.1426	3.8253	3.5170	4.2733	3.2507	4.1423	4.1616	3.9639	6.7440	3.9761	3.6247	4.1093
Nucks1	_NSQEDS[Phospho (STY)]EDS[Phospho (STY)]EEKDVK_.2	#N/A	8.5024	8.3576	7.1657	8.2652	7.8029	8.3978	8.7014	8.3624	8.3737	8.1146	8.0959	7.8869	8.4013	8.0598	7.1641	7.1583	8.4679	8.3671	8.1638	7.5805
Nucks1	_VVDYSQFQES[Phospho (STY)]DDADEDYGR_.2	#N/A	3.8094	9.4264	9.5147	7.1746	3.8089	9.3289	9.1773	4.1274	8.9390	5.7893	3.8958	6.6036	3.8331	9.4238	7.8979	6.5999	3.3068	9.1664	8.5528	8.4081
Nucks1	_VVDYSQFQES[Phospho (STY)]DDADEDYGRDSGPPAKK_.4	#N/A	3.8717	4.0249	3.8377	4.7594	3.6980	3.4553	5.5665	3.9360	4.2512	3.7619	3.8724	3.7976	3.8229	4.9534	3.6392	3.6545	3.7948	3.9040	3.7001	3.5281
Nucks1	_SGKNS[Phospho (STY)]QEDS[Phospho (STY)]EDS[Phospho (STY)]EEKDVK_.2	Q80XU3_S54_M3_Nucks1	4.0096	3.8892	3.8912	4.4110	3.9780	3.9316	3.7772	4.3832	4.0555	4.3396	3.0966	3.2359	3.7383	4.0561	3.4932	3.7096	3.9703	3.4130	3.8022	3.2416
Nucks1	_DDSHS[Phospho (STY)]AEDS[Phospho (STY)]EDEKDDHKNVR_.4	Q80XU3_S75_M2_Nucks1	3.7005	3.7079	3.9074	4.0543	3.5890	3.5113	3.6722	3.4012	3.7901	3.6978	3.9811	3.5353	3.7216	4.2242	3.5122	3.9864	3.9389	3.9600	3.6086	3.7114
Nucks1	_EM[Oxidation (M)]LLEDVGS[Phospho (STY)]EEEPEEDDEAPFQEK_.3	Q80XU3_S113_M1_Nucks1	4.4801	6.1325	4.0167	3.1042	3.1862	3.2195	3.8619	3.2049	5.3957	4.2907	3.2608	4.0660	3.9476	6.1791	3.8873	3.8422	4.2030	6.4792	3.3168	1.9880
Nucks1	_KDDS[Phospho (STY)]HS[Phospho (STY)]AEDS[Phospho (STY)]EDEKDDHK_.4	#N/A	3.5197	4.4831	4.0560	4.1697	2.8575	3.0815	3.1828	3.2201	3.2419	3.7261	3.6162	4.3725	3.3499	4.3945	4.2608	3.8646	3.7578	4.1075	4.4065	4.0718
Lrfn4	_SC[Carbamidomethyl (C)]S[Phospho (STY)]LDLGDTGGC[Carbamidomethyl (C)]YGYAR_.2	Q80XU8_S585_M1_Lrfn4	8.3614	9.0191	8.9659	9.3523	2.9854	8.6617	8.6321	8.2079	8.5678	8.5089	3.4615	4.2667	8.9178	9.2738	7.1457	8.1672	8.8316	8.7994	8.7126	8.6450
Lrfn4	_GVGGS[Phospho (STY)]AERLEESVV_.2	Q80XU8_S627_M1_Lrfn4	10.3156	10.0626	10.0816	10.2796	4.2769	9.8259	9.7869	9.9339	9.8166	10.0965	3.6709	9.7798	4.1008	10.0244	8.8941	10.0009	10.5382	9.9209	9.9337	9.9726
Glrx5	_SALVDEKDQDS[Phospho (STY)]K_.2	Q80Y14_S151_M1_Glrx5	5.5939	5.8082	5.9702	5.5054	5.4260	5.4280	5.1629	5.8820	5.6404	5.6361	5.5119	5.4320	5.9045	6.2735	5.9604	5.8362	5.4428	5.7157	5.7783	5.7924
Llgl1	_S[Phospho (STY)]LRQS[Phospho (STY)]FRR_.2	#N/A	7.8924	6.2794	8.3066	7.8475	7.3262	5.6801	5.9683	7.8206	6.9134	6.3982	7.1245	6.7711	7.1045	7.3185	6.3132	7.1138	6.8931	6.4637	6.8898	7.0186
Llgl1	_S[Phospho (STY)]LRQS[Phospho (STY)]FR_.2	#N/A	6.6091	6.1538	6.5814	6.4399	5.7927	5.7925	5.8035	6.4085	5.9943	6.6440	6.1106	5.7482	5.6417	5.6928	4.5652	5.5728	5.2151	5.4374	5.8015	5.1460
Llgl1	_DIILAPES[Phospho (STY)]C[Carbamidomethyl (C)]EGS[Phospho (STY)]PSSAHSKR_.3	#N/A	4.2977	4.5222	3.8343	3.2865	3.0038	3.0371	4.0443	4.5234	3.9645	6.6747	3.4431	6.2766	4.1299	3.8723	4.0697	3.6598	7.0986	3.1288	3.4279	6.2990
Llgl1	_DIILAPESC[Carbamidomethyl (C)]EGS[Phospho (STY)]PSSAHSK_.2	#N/A	7.3089	7.9981	6.6221	7.6689	5.2141	8.5283	7.8207	7.7648	7.6984	8.2259	3.3713	7.7138	7.6232	7.3855	7.1836	3.8566	8.0218	7.0908	6.8790	6.4322
Prickle2	_S[Phospho (STY)]FQESLGQGSR_.2	#N/A	10.4407	10.2395	10.7711	10.4195	9.7366	9.7450	9.5677	9.8836	9.5427	10.2563	9.9879	9.3908	9.8880	9.8719	8.7407	9.6728	9.9009	9.3835	9.5698	9.8109
Prickle2	_YVTS[Phospho (STY)]DELLHK_.2	Q80Y24_S807_M1_Prickle2	9.8439	9.6191	8.9967	9.8378	8.7348	9.3108	9.2441	3.7661	8.9219	10.0793	9.1438	9.0245	3.5712	9.4518	8.1347	9.3501	9.6796	9.2999	9.2126	9.1821
Prickle2	_AC[Carbamidomethyl (C)]SAGEDPNGS[Phospho (STY)]DSSDSAFQNAR_.2	Q80Y24_S319_M1_Prickle2	7.0117	4.0774	3.8450	6.8298	6.8048	7.1321	3.1861	4.0450	6.5367	3.5973	3.8583	3.0953	3.3154	7.8809	2.9921	7.0598	8.0052	4.2404	6.8353	6.8048
Prickle2	_S[Phospho (STY)]DNALHLASER_.2	#N/A	9.2430	8.5988	8.4460	8.4941	8.2026	8.4167	8.1267	8.5278	8.1267	9.1973	8.5037	8.0937	8.4706	8.6566	7.3620	8.5307	8.9505	8.1800	8.1762	8.6780
Prickle2	_GHGGS[Phospho (STY)]FIQEC[Carbamidomethyl (C)]R_.2	Q80Y24_S463_M1_Prickle2	7.2224	7.5372	7.7265	7.2175	7.3913	7.3498	7.0651	7.0732	6.4672	7.2545	7.1174	6.5706	6.8121	7.1274	6.3955	6.7397	7.1552	6.6795	6.9234	6.7781
Prickle2	_S[Phospho (STY)]REEPFHYGNK_.3	#N/A	7.5941	7.9641	8.2292	7.3335	7.6933	7.6801	7.7114	7.4575	7.1646	7.5241	7.7573	6.9268	7.8888	7.7894	6.8786	7.3294	7.6602	7.9733	7.7422	7.7241
Prickle2	_SRS[Phospho (STY)]DNALHLASER_.3	#N/A	8.4145	5.7202	3.7365	8.6904	7.8538	8.1470	8.4070	7.0630	4.7628	4.1612	8.2096	8.0274	8.9577	8.7310	3.8732	8.1836	7.7692	8.2561	8.1726	8.4214
Prickle2	_QRS[Phospho (STY)]FQESLGQGSR_.2	#N/A	5.6066	5.6482	6.1981	5.6142	5.1834	5.0547	5.4162	5.5304	3.8252	5.6737	5.1058	3.9289	5.9154	5.2097	4.7653	3.9023	5.0481	3.9612	3.2804	5.2122
Prickle2	_GS[Phospho (STY)]M[Oxidation (M)]ESLALSNATGLSAEGGAK_.2	#N/A	3.5969	5.7579	3.8632	4.1057	3.8038	3.8371	4.0923	3.8226	4.0125	4.1372	3.4911	3.5666	4.1779	3.9012	3.3880	3.6887	4.0495	3.9480	3.4759	3.5785
Prickle2	_YTEDM[Oxidation (M)]T[Phospho (STY)]PTEQTPR_.2	#N/A	4.0635	3.9830	3.6587	3.6696	5.1062	4.9121	4.8107	3.7639	5.3538	5.9514	3.3803	3.8725	5.2164	3.6417	3.7608	4.7356	4.3015	3.7514	3.0504	3.4723
Prickle2	_TVS[Phospho (STY)]DLALQNAFGER_.2	Q80Y24_S754_M1_Prickle2	3.9880	4.0148	4.5243	3.7014	3.3257	3.1584	4.2154	3.6883	3.8432	4.2296	3.3219	3.9043	4.0087	4.5113	3.7926	4.3329	4.2260	7.9636	3.2557	2.9775
Prickle2	_M[Oxidation (M)]HQS[Phospho (STY)]FDFDGGIASSK_.2	Q80Y24_S643_M1_Prickle2	3.8210	4.1818	4.3573	3.8684	3.1587	3.3828	4.3824	3.5214	4.4415	4.0273	3.3150	4.0713	4.5495	4.0933	3.9596	4.1659	4.0590	4.4087	3.2069	3.9836
Prickle2	_LMS[Phospho (STY)]QESYSDMSSQSFNETR_.2	Q80Y24_S479_M1_Prickle2	3.5675	3.7516	4.6049	4.0167	3.7744	3.8078	3.2737	3.7932	3.1939	3.3781	4.1733	3.4777	3.3593	3.8620	3.2990	4.4305	3.2904	4.2565	3.9051	3.8290
Prickle2	_LPGQEGVHIQPMS[Phospho (STY)]ER_.3	Q80Y24_S667_M1_Prickle2	3.3678	3.8481	4.5084	4.1132	4.2063	4.4304	3.3347	3.6967	3.3938	3.5741	3.7682	3.0236	3.5019	4.5465	2.9119	3.7127	3.1939	3.9555	4.2546	4.2238
Bsdc1	_ADQSISEEPGWEEEEEELEGIVPS[Phospho (STY)]PK_.3	Q80Y55_S234_M1_Bsdc1	4.0684	6.3934	7.0550	5.6642	4.0884	5.7248	5.6227	3.7687	6.0312	6.4502	3.9262	5.8660	5.6408	5.9454	6.9582	5.9448	3.4879	6.1715	6.4706	6.2614
Rbsn	_DSLSTHTS[Phospho (STY)]PSQS[Phospho (STY)]PNSVHGSR_.3	#N/A	6.2435	6.5469	6.4002	7.2430	6.5770	7.0402	6.8862	6.4694	7.1738	7.2129	6.0356	6.5315	3.7900	7.3835	6.4512	7.3728	6.1154	7.0272	6.6983	6.8039
Rbsn	_[Acetyl (Protein N-term)]AS[Phospho (STY)]LDDPGEVR_.2	Q80Y56_S3_M1_Rbsn	3.4127	6.0469	5.7956	5.9429	3.6196	2.9879	3.4285	3.6383	5.8005	5.9258	3.7098	5.3755	3.4435	6.4332	3.4539	6.0431	3.1356	6.0475	5.1677	5.9378
Elac2	_TS[Phospho (STY)]PNRLS[Phospho (STY)]PK_.2	Q80Y81_S195_M2_Elac2	8.3284	8.0167	8.6523	8.1631	7.8272	7.9948	8.1453	7.8546	8.0476	7.9944	8.0562	7.7456	7.5892	8.1619	3.5829	7.8427	3.8605	3.9726	7.6859	8.0511
Elac2	_AHTDEPHS[Phospho (STY)]PQSK_.2	Q80Y81_S815_M1_Elac2	3.9636	3.3555	5.8880	6.4156	4.1705	4.2038	4.3784	6.8421	6.4988	3.7742	6.1172	6.1650	6.7192	3.5382	2.9030	3.3257	3.6865	6.5306	5.8597	3.2155
Elac2	_QQPS[Phospho (STY)]QSPRTS[Phospho (STY)]PNRLS[Phospho (STY)]PK_.3	Q80Y81_S189_M3_Elac2	3.5062	4.4966	4.0425	4.1832	4.3448	5.4301	3.1963	3.5582	3.2554	3.7126	3.6297	4.3860	3.3634	4.4080	4.2743	3.8511	4.5563	4.0940	4.3930	4.0853
Elac2	_QQPSQS[Phospho (STY)]PR_.2	Q80Y81_S191_M1_Elac2	3.5475	3.7716	4.5849	4.0367	3.7545	4.3539	3.4112	3.7732	3.4703	3.4976	3.8447	3.4977	3.5784	4.6230	2.9884	3.6362	3.2704	3.8790	4.1781	4.3003
Dixdc1	_TDEPDS[Phospho (STY)]PSSR_.2	Q80Y83_S287_M1_Dixdc1	3.7251	8.9092	4.2614	3.9643	3.0628	3.2869	4.4782	9.1463	3.0365	3.9314	3.4109	4.1671	9.2676	9.2996	8.9875	4.0700	9.1952	3.8700	8.8698	3.8664
Cnksr2	_GSES[Phospho (STY)]PNSFLDQEYR_.2	#N/A	11.9098	11.7921	11.8682	11.8254	3.3630	11.3670	11.3185	11.5226	11.3813	11.7044	11.2190	11.2467	11.8307	11.6352	10.6153	11.3451	11.9351	11.2166	11.3320	11.1836
Cnksr2	_RENS[Phospho (STY)]LLR_.2	Q80YA9_S467_M1_Cnksr2	6.5320	6.1013	6.4526	5.9196	5.9008	5.9525	5.0786	6.0304	5.5156	6.1290	5.6243	5.5484	5.9392	6.1837	4.8636	5.2178	6.0198	5.9808	5.9132	5.8419
Cnksr2	_LGDS[Phospho (STY)]LQDLYR_.2	#N/A	7.5071	11.5923	11.1386	10.3937	3.7838	11.4784	11.2283	6.9356	11.1098	10.1278	4.1907	10.8993	3.9244	11.8425	10.0476	10.6660	10.5101	11.4755	11.0130	11.1569
Cnksr2	_GSES[Phospho (STY)]PNSFLDQEYRK_.2	#N/A	6.5353	6.9400	4.2972	6.4602	6.0920	6.2541	3.6353	3.4735	3.5555	4.5196	6.0512	6.1523	3.7209	4.3101	3.6607	6.0634	6.7338	6.4387	6.1762	5.9918
Cnksr2	_STYDGLHVITGTTENS[Phospho (STY)]PADR_.3	Q80YA9_S248_M1_Cnksr2	4.4456	5.2905	5.5652	3.2438	3.7833	5.2155	5.2021	4.1460	5.5562	3.7720	3.7795	3.4467	4.4663	5.3146	5.5658	3.2896	3.1828	5.1496	4.2990	3.4351
Cnksr2	_QEVTGSSAVS[Phospho (STY)]PIRK_.2	#N/A	6.1492	6.1351	6.5985	6.2479	5.4040	5.8342	5.5962	3.5743	5.5672	6.6524	5.8599	5.1240	3.7446	6.2061	4.5340	5.5759	5.9318	5.4862	5.7392	5.5867
Cnksr2	_QEVTGSSAVS[Phospho (STY)]PIR_.2	#N/A	5.7697	5.9867	3.4731	5.5016	4.8442	4.9923	4.9583	4.1708	4.3257	6.1624	5.6953	3.1087	4.4911	5.5320	2.9300	3.2987	5.7615	4.6126	4.6607	3.4599
Cnksr2	_IKQEQDYWS[Phospho (STY)]ES[Phospho (STY)]DKEEADTPSTPK_.3	Q80YA9_S685_M2_Cnksr2	3.9932	4.5885	4.1010	3.7365	3.7131	4.4113	3.6105	3.9092	4.7524	4.0666	4.2496	3.5288	3.7455	3.7314	3.3847	3.9215	3.1737	4.1067	4.5120	4.4107
Cnksr2	_EEKLGDS[Phospho (STY)]LQDLYR_.2	#N/A	3.7647	5.2942	3.7109	4.0716	3.5493	4.3296	3.8797	4.3881	3.8565	3.7176	3.7797	3.9010	3.8034	3.7240	3.7437	3.5281	3.6778	3.9577	3.8363	3.4446
Cnksr2	_SPTSSVATPSSTIS[Phospho (STY)]T[Phospho (STY)]PTKR_.2	Q80YA9_S338_M2_Cnksr2	4.4300	4.3899	3.9666	3.7125	3.1361	3.1694	3.9120	3.6773	3.8322	4.2406	3.3108	4.1160	3.9976	4.5003	3.9374	3.7921	7.8058	3.6182	3.2668	5.3006
Aatk	_EQDLRS[Phospho (STY)]PPS[Phospho (STY)]PGHQSVQAFPR_.3	Q80YE4_S1032_M2_Aatk	3.5750	4.4278	4.1113	4.1144	2.9127	6.9516	3.1275	3.2754	5.6465	3.7813	3.5610	4.3173	3.2947	4.3393	4.2056	3.9199	7.0039	4.1627	2.9609	4.0166
Aatk	_AVDSGYDTENYES[Phospho (STY)]PEFVLK_.2	Q80YE4_S950_M1_Aatk	3.7053	4.2975	4.2416	3.9841	3.0431	3.4410	2.9972	3.4057	3.5606	4.5122	3.0392	4.1869	3.7260	4.2287	4.4264	4.0502	3.9434	5.0053	3.5384	4.1957
Aatk	_SPPS[Phospho (STY)]PGHQSVQAFPR_.3	Q80YE4_S1035_M1_Aatk	5.9032	4.0816	4.2750	5.4966	3.4445	3.1631	6.2201	5.8947	3.1604	3.8076	3.5347	3.8077	3.2684	4.3130	3.6290	4.1005	4.4613	5.8285	2.9872	3.9903
Aatk	_AS[Phospho (STY)]PEVGHLLSQEDPR_.2	#N/A	6.4801	6.0921	6.1592	4.1932	5.0898	5.2773	4.2755	5.2023	4.5959	6.1832	5.9781	5.0693	5.5181	5.7202	3.1267	4.9416	5.8047	5.8508	5.7819	5.5004
Arhgap33	_QQS[Phospho (STY)]DGSLVR_.2	Q80YF9_S995_M1_Arhgap33	7.1121	6.8044	3.7149	6.3032	4.0172	4.2413	3.5239	3.8858	3.5830	6.6876	6.1943	3.2128	6.3476	3.2348	3.1011	3.5235	3.3831	3.7664	4.0654	4.4129
Arhgap33	_TPAS[Phospho (STY)]PVER_.2	Q80YF9_S594_M1_Arhgap33	9.4590	9.2177	9.3058	9.1832	4.0501	9.0883	8.9366	9.3890	8.9799	9.1896	8.7390	9.0548	9.3806	8.7210	7.9877	8.7578	9.3177	9.2484	8.4584	8.7706
Arhgap33	_TSAWLDDGDDLDFS[Phospho (STY)]PPR_.2	Q80YF9_S749_M1_Arhgap33	7.6921	8.5463	7.7835	7.3724	5.2248	8.2287	8.5288	6.1319	8.2488	7.8438	3.3168	7.0937	7.8368	7.7967	7.6701	7.5147	7.9300	8.6196	7.9694	5.8125
Arhgap33	_S[Phospho (STY)]AKSEESLSSQASGAGLQR_.3	Q80YF9_S657_M1_Arhgap33	4.3846	3.7626	3.7513	3.2631	3.8541	3.6712	3.9515	4.0012	3.8304	5.5516	3.6115	3.5339	3.6217	3.6698	3.1354	3.6536	3.8081	3.9866	3.6505	3.2998
Arhgap33	_AQQVAEQQSQQEQGGTPPAPHS[Phospho (STY)]PFR_.3	Q80YF9_S949_M1_Arhgap33	3.6802	3.9557	4.4964	3.9385	3.8117	3.9395	3.6335	3.6728	3.6121	3.9649	3.5465	3.7720	3.7530	3.8290	3.4923	4.0251	3.7714	3.8089	3.8513	4.3345
Bcas1	_RQS[Phospho (STY)]LGGFLK_.2	#N/A	10.2709	10.3816	9.5757	9.8637	5.3996	9.5408	10.0715	10.5762	8.4682	10.2720	9.2444	9.6788	10.5442	11.1433	8.9068	9.3512	10.1035	9.8136	9.6139	9.4002
Bcas1	_LGLS[Phospho (STY)]FRK_.2	Q80YN3_S347_M1_Bcas1	9.1802	9.5541	8.9674	8.9015	8.3326	8.8406	8.7889	9.8243	8.7295	8.5571	8.5634	9.2289	9.4983	9.8996	8.3039	8.2219	9.4297	8.8632	8.4358	8.6028
Bcas1	_ANFTPQETR_.2	#N/A	7.5189	7.2533	7.5505	7.1935	7.2913	7.4093	7.2757	7.1522	7.5593	7.4220	7.3409	7.0619	7.7880	7.6580	8.3491	7.2055	7.1445	7.5935	7.0964	7.8429
Bcas1	_AGPTS[Phospho (STY)]LPLGK_.2	Q80YN3_S414_M1_Bcas1	4.5143	3.4937	8.0343	8.3805	3.8520	4.0761	3.6891	8.3545	7.8992	8.3321	4.1225	3.2199	8.3506	8.3971	7.6054	7.5833	8.4369	3.6012	3.9002	7.9630
Bcas1	_TLVSPNKTETK_.2	#N/A	5.6771	5.6537	5.7189	5.1580	5.0636	5.0949	5.0349	5.7699	4.6109	5.5057	5.5043	5.0859	5.5389	5.2970	5.7746	5.0727	5.7439	4.9087	5.1832	5.0763
Bcas1	_ADS[Phospho (STY)]VC[Carbamidomethyl (C)]DGHAAGQK_.2	Q80YN3_S316_M1_Bcas1	5.5917	5.2754	5.8090	5.6886	5.0171	5.4282	4.9353	5.0480	5.6462	5.1687	5.1013	5.1859	4.5123	5.7377	5.9800	5.1875	5.3524	4.8389	4.8038	5.3902
Bcas1	_M[Oxidation (M)]SDAQVQTDPVSIGPVGK_.2	#N/A	6.6103	6.2595	6.3842	5.8478	4.8411	6.1799	5.5218	6.0214	6.2934	6.2738	4.7031	5.6530	6.1971	5.8330	6.2524	6.1245	6.1294	5.5884	6.0631	5.8186
Bcas1	_TPS[Phospho (STY)]PPEPEPAGTAQK_.2	Q80YN3_S525_M1_Bcas1	11.8474	11.9890	12.2515	12.1014	11.7506	12.1086	11.3263	12.1112	11.8065	11.8364	11.8037	11.9332	12.0084	12.6630	12.1091	11.8196	12.2820	11.7096	11.7823	12.0377
Bcas1	_EPAPC[Carbamidomethyl (C)]VQPPTVEANAM[Oxidation (M)]QTGDKT[Phospho (STY)]PK_.3	#N/A	6.3177	7.0040	4.0466	3.9431	4.8649	3.7413	6.0162	3.8919	3.5519	5.8625	5.1456	5.9602	5.8032	7.2592	5.9595	6.3274	3.3892	3.8948	5.9079	3.5750
Bcas1	_SC[Carbamidomethyl (C)]S[Phospho (STY)]PPPPPPEPTSEGR_.2	Q80YN3_S390_M1_Bcas1	8.5276	8.8298	8.3354	8.9342	8.7470	8.0360	8.3116	8.7353	8.3671	8.3955	8.2364	8.7935	4.3257	4.3645	9.0809	8.6550	8.1396	8.4053	8.4251	9.0099
Bcas1	_RM[Oxidation (M)]S[Phospho (STY)]DAQVQTDPVSIGPVGK_.3	#N/A	5.6029	6.1186	5.3243	5.5199	3.9711	5.6824	5.3683	5.2937	5.0805	5.4614	4.7684	5.6496	5.7437	6.5717	5.2652	5.5811	4.9669	5.5759	5.8848	5.4873
Bcas1	_S[Phospho (STY)]VKEDTLSTGAEENAVC[Carbamidomethyl (C)]ESPVETVR_.3	#N/A	3.7898	3.5446	3.3264	3.8098	3.9968	4.0301	3.6084	4.0262	3.8515	3.6004	3.9664	3.2707	4.0170	3.3603	3.0921	3.1519	3.2863	3.9357	4.1833	3.6220
Bcas1	_KS[Phospho (STY)]VKEDTLSTGAEENAVC[Carbamidomethyl (C)]ESPVETVR_.3	#N/A	3.5206	4.4822	4.0569	4.1688	2.8584	3.0824	1.6064	3.2210	3.2410	3.7270	3.6153	4.3716	3.5414	4.0440	4.2599	3.8655	3.7587	4.0745	3.7231	4.0110
Bcas1	_EDTLSTGAEENAVC[Carbamidomethyl (C)]ES[Phospho (STY)]PVETVR_.3	Q80YN3_S443_M1_Bcas1	3.1046	4.2144	4.1421	3.8879	3.3116	3.3449	3.7365	3.5018	3.6567	4.4161	3.1354	3.9406	3.8222	4.3248	3.7619	3.9676	4.0395	3.7936	3.4422	4.2918
Bcas1	_QS[Phospho (STY)]LGGFLK_.2	#N/A	3.5711	4.4317	4.1074	4.1183	2.9088	3.5753	3.1314	3.2715	3.4264	3.7774	2.9050	4.3212	3.5918	6.0856	4.2095	3.9160	3.8091	4.0240	3.6726	4.0615
Safb2	_MEEEGS[Phospho (STY)]EDNGLEEDSR_.2	Q80YR5_S95_M1_Safb2	4.4158	3.5870	3.4513	3.2736	3.7535	4.2314	5.7178	4.1162	4.2711	3.8017	3.7497	3.4764	4.4365	3.4383	2.8754	3.2599	3.1530	3.1793	4.3287	3.4053
Afap1	_SGTSSPQS[Phospho (STY)]PVFR_.2	Q80YS6_S669_M1_Afap1	8.9458	8.8897	8.6648	8.6270	8.4619	8.4026	8.3042	8.6495	8.0896	8.7662	8.4357	8.5961	9.1055	8.8732	7.0213	7.9251	8.8062	8.4125	8.0511	8.1283
Zc3hc1	_LC[Carbamidomethyl (C)]SSSS[Phospho (STY)]SDTSPR_.2	Q80YV2_S409_M1_Zc3hc1	6.4494	6.2549	5.1361	6.8688	4.5313	6.3722	6.1518	6.1083	6.2374	6.7646	4.9108	6.1732	5.6088	6.6775	4.7067	5.6518	4.6848	6.5745	4.7446	6.1419
Zc3hc1	_SQDATVSPGSEQS[Phospho (STY)]EKSPGPIVSR_.3	Q80YV2_S340_M1_Zc3hc1	3.4176	6.3707	4.2919	4.2183	4.0749	4.6188	3.3330	3.4740	3.3442	4.8570	4.3434	3.4552	3.4500	6.9413	4.9309	3.9563	4.6927	4.0077	5.9966	4.1719
Zc3hc1	_SQDATVS[Phospho (STY)]PGSEQSEK_.2	Q80YV2_S334_M1_Zc3hc1	5.9708	3.6533	6.3500	6.6567	5.7491	5.9538	4.2846	6.2684	6.3364	3.8680	5.8201	5.9628	5.9781	3.3721	5.0396	5.6489	6.0530	3.2456	4.3950	3.3391
Zc3hc1	_DTAATFQSVDGS[Phospho (STY)]PQAEQSPLESTSK_.3	Q80YV2_S62_M1_Zc3hc1	6.3225	6.1227	6.5333	6.7237	3.7620	5.6958	5.8731	5.2996	5.4973	6.4068	5.7407	5.8208	5.4190	6.8465	5.4814	6.4318	6.3876	5.9029	5.9837	5.8981
Zc3hc1	_SWESSSPVDRPELEAAS[Phospho (STY)]PTTR_.3	Q80YV2_S369_M1_Zc3hc1	4.1374	3.8654	3.1729	3.5520	3.4752	6.3588	4.0725	3.8378	5.9329	4.0801	3.4713	3.7548	4.1581	3.1600	3.6431	5.9717	4.3755	6.2666	3.1063	3.1270
Pank4	_VRS[Phospho (STY)]FDHPGK_.2	Q80YV4_S63_M1_Pank4	7.0093	7.6623	3.1559	3.9649	7.5010	7.3021	7.3729	3.8450	6.7195	7.3016	7.2412	6.7010	7.2360	7.3404	6.5209	7.1895	7.4783	7.3295	7.1827	7.5261
Negr1	_VIVNFAPTIQEIK_.2	#N/A	7.1460	7.0787	6.7198	6.6989	6.8566	6.9403	7.1752	7.2232	6.7633	6.7906	7.1711	7.1356	7.5526	7.3183	7.2057	7.0293	6.9568	7.1962	7.0196	7.2403
Negr1	_DQAGEYEC[Carbamidomethyl (C)]SAENDVSFPDVK_.2	#N/A	7.1801	6.7038	7.1069	6.6320	6.4713	7.0041	6.5104	6.8164	6.7699	6.1315	6.9299	6.6103	6.6634	7.3041	5.4233	6.1132	7.2105	6.4769	6.1487	6.8435
Negr1	_LFNGQQGIIIQNFSTR_.2	#N/A	3.7030	4.3625	4.7773	3.9864	4.4361	4.7858	6.8936	7.6702	4.5715	5.2737	5.0656	4.8062	3.1667	4.2113	4.0776	3.9993	5.4220	4.2059	6.8750	5.0636
Topors	_LQQTVPADAS[Phospho (STY)]PDSK_.2	Q80Z37_S99_M1_Topors	4.4623	4.3576	3.9989	3.1219	3.1684	6.8686	3.8797	3.1871	4.3851	7.0827	7.1411	4.0838	4.4932	4.0369	3.9051	3.8244	4.1852	4.4651	3.2633	6.8540

Shank2	_APSPVVS[Phospho (STY)]PTELSK_.2	#N/A	3.7660	7.5676	4.3023	3.9234	3.1037	3.3804	7.3224	3.4664	3.6213	8.1495	7.7704	4.1263	7.5139	4.2894	5.9327	7.1255	7.7408	3.8291	7.1454	7.7304
Shank2	_NSPAFLS[Phospho (STY)]TDLGDEDVGLGPPAPR_.3	#N/A	3.7785	3.5405	3.3151	3.8057	3.9855	4.1229	3.6423	4.0042	3.7014	3.2666	4.0757	3.2667	3.8094	8.7518	3.2195	3.1407	3.5014	3.6480	3.9470	7.2599
Shank2	_RNSPAFLS[Phospho (STY)]TDLGDEDVGLGPPAPR_.3	#N/A	4.1116	4.4083	3.9482	3.1726	3.1177	3.4898	8.4798	3.1364	4.3344	4.1343	3.2080	4.1344	4.4425	9.3381	3.9558	3.7737	4.1345	3.0149	3.3140	3.6635
Shank2	_QSNVEDS[Phospho (STY)]PEK_.2	#N/A	5.8728	7.2858	7.5627	7.0475	7.4660	3.9905	6.4396	4.1291	3.8337	3.1342	4.2081	3.4635	6.8135	7.6856	3.3518	5.6492	3.1660	3.1664	7.4170	3.4183
Shank2	_TIVAAGS[Phospho (STY)]VEEAVIPFR_.2	Q80Z38_S1005_M1_Shank2	7.5968	7.7216	4.4956	4.1261	3.6651	4.4433	7.3992	3.6838	3.3810	3.2688	7.3554	7.7548	3.4890	4.5336	3.4084	4.3211	3.1811	3.9684	4.2674	4.2109
Shank2	_S[Phospho (STY)]KSM[Oxidation (M)]TAELEELGLSLVDK_.3	Q80Z38_S370_M1_Shank2	4.4655	3.5373	3.5010	3.2238	3.8033	4.0274	3.7378	4.1659	3.7969	5.4025	4.8101	3.4267	3.9049	3.4487	3.3150	3.3096	3.2028	3.5525	3.8515	3.1260
Shank2	_ELDRFS[Phospho (STY)]LDSEDVYSR_.2	#N/A	3.3025	6.6872	7.3702	6.3495	4.1411	5.6179	6.4628	3.8427	4.9038	5.8522	4.1372	3.9560	3.3232	6.7367	5.2098	6.5125	3.5406	6.4823	5.6242	3.7929
Shank2	_S[Phospho (STY)]NSDNNLNAGAPEWAVC[Carbamidomethyl (C)]SAATSHR_.3	Q80Z38_S41_M1_Shank2	3.9555	6.5917	3.2242	3.7093	3.7253	3.5476	3.6938	3.9014	3.6140	3.8127	3.7874	3.5443	3.9805	3.2374	4.2194	3.0414	3.9979	5.4201	4.9369	3.0617
Shank2	_ANVISELNSLQQM[Oxidation (M)]NR_.2	#N/A	4.1098	5.5756	3.1453	4.7674	4.9418	5.2428	4.0935	4.4555	5.1544	5.5395	5.0778	3.7824	4.2606	3.8044	3.6707	2.9539	5.5281	3.1968	4.8620	4.1888
Shank2	_QSNVEDSPEKTC[Carbamidomethyl (C)]S[Phospho (STY)]PIPTIVK_.3	Q80Z38_S654_M1_Shank2	4.1771	5.7751	3.4469	3.4971	3.5148	3.4341	5.7380	4.8422	4.0297	4.2514	3.2317	4.1170	4.1662	3.3640	4.4600	3.0211	4.1450	5.5963	4.8652	3.2665
Shank2	_DNEGFGFVLR_.2	#N/A	4.7271	3.7186	4.9772	3.9837	3.8075	3.8408	3.2406	3.8262	4.6122	4.8179	4.6722	3.4447	4.4597	5.2824	4.3004	4.1149	3.3234	4.4007	4.6016	5.2443
Shank2	_RAPSPVVS[Phospho (STY)]PTELSK_.2	#N/A	8.0224	8.3932	8.7446	8.6947	7.4057	6.6520	8.3272	8.3397	7.8512	9.8542	7.1283	7.7269	8.4434	9.2526	6.6631	8.3837	8.4196	8.2391	8.3777	7.9899
Shank2	_RELDRFS[Phospho (STY)]LDSEDVYSR_.3	#N/A	3.9215	4.0813	4.4578	3.1661	3.2592	3.4833	4.2819	3.1430	4.3410	4.1278	3.2145	3.9708	4.4490	3.9928	3.8591	4.2664	4.1411	3.0084	3.3074	3.6701
Shank2	_IFLSGITEEER_.2	#N/A	4.5792	3.3351	3.5205	5.1938	4.3197	4.4223	4.4344	4.1196	4.2783	4.6277	4.9010	3.9244	4.4437	4.8688	3.7932	4.2828	3.7069	3.1721	4.2080	5.2186
Shank2	_SM[Oxidation (M)]T[Phospho (STY)]AELEELGLSLVDK_.2	Q80Z38_T374_M1_Shank2	4.3715	4.0687	3.7150	4.0603	3.3816	3.5203	3.9237	3.9649	3.9219	4.3645	3.0852	3.8677	4.0547	3.9686	3.7202	3.5347	4.1169	3.9026	3.3556	4.2667
Amigo1	_RM[Oxidation (M)]S[Phospho (STY)]DPESVSSVFSDTPIVV_.2	#N/A	3.8454	5.1646	3.7843	4.0071	3.6143	4.5369	3.8022	4.4617	3.7801	3.7955	3.7102	3.8199	3.9213	3.7978	3.6631	3.6016	3.7549	3.5061	3.7676	4.0207
Adgrb3	_SETGSTISM[Oxidation (M)]S[Phospho (STY)]LER_.2	#N/A	9.7548	8.9618	9.2951	9.3839	8.9701	8.6735	8.6477	9.6837	8.8101	8.4618	8.6363	8.1416	9.0101	8.6992	7.4942	7.7486	9.0076	8.3618	8.4606	8.3437
Wdtc1	_QSPS[Phospho (STY)]AGVHTFC[Carbamidomethyl (C)]DR_.2	Q80ZK9_S229_M1_Wdtc1	3.7774	4.0560	3.8407	3.7426	3.1662	4.0177	3.9222	3.6875	3.8424	4.2707	3.3211	3.9454	4.0079	3.8278	3.9476	3.8222	4.2252	3.6483	4.8180	4.1267
Wdtc1	_KDS[Phospho (STY)]ISEDEM[Oxidation (M)]VLR_.2	Q80ZK9_S511_M1_Wdtc1	6.1705	6.3713	3.1190	4.0018	3.7894	5.7815	6.4677	6.1391	5.7185	6.3636	4.1584	6.3868	6.4428	5.3101	5.1432	6.3924	6.3149	5.5343	4.1431	5.5539
Wdtc1	_GC[Carbamidomethyl (C)]S[Phospho (STY)]PQVELPPYLER_.2	Q80ZK9_S352_M1_Wdtc1	4.3003	4.6025	3.9366	4.2891	4.2388	4.4629	3.3022	3.1006	3.7785	3.6066	3.7357	2.9911	3.4694	3.9236	2.8794	3.7452	3.6383	4.1948	3.8434	3.8906
Spag1	_ESGVDPSQVLLS[Phospho (STY)]PDSSEAAR_.2	Q80ZX8_S703_M1_Spag1	6.8233	7.0301	6.6157	7.3236	3.9981	6.5286	6.4013	6.6483	6.6688	3.3659	3.9764	6.9974	7.0391	7.2244	5.9955	7.1277	7.0008	6.6891	6.6609	6.6176
Ddx42	_YM[Oxidation (M)]AENPTAGVVQEEEEDNLEY[Phospho (STY)]DSDGNPIAPSKK_.4	Q810A7_Y183_M1_Ddx42	3.6306	4.3722	5.7676	4.0588	2.9683	5.0632	4.6071	3.3310	5.3461	3.8369	3.5054	4.2617	3.6513	4.1540	5.1282	3.9755	3.8687	5.8109	3.6131	4.1210
Ankfy1	_ESGAAEQVDNK_.2	#N/A	4.8210	4.3039	4.9187	3.7686	4.8780	4.0063	4.1102	3.4544	4.5100	3.3968	4.5051	4.0830	4.0300	4.8891	4.0606	3.6042	4.0915	4.6698	3.6336	3.8583
Slitrk1	_VYDC[Carbamidomethyl (C)]GSHSLS[Phospho (STY)]D_.2	Q810C1_S695_M1_Slitrk1	7.2892	7.1152	6.5734	7.1141	6.2575	6.7572	6.6894	7.1646	7.0194	6.8106	7.1377	6.9053	3.7558	4.2465	6.1621	4.0340	7.4208	6.8544	6.6723	6.9939
Nfasc	_NLILAPGEDGR_.2	#N/A	8.6068	8.1871	7.9646	8.2754	8.2200	8.4699	8.2904	8.6026	8.3924	8.1331	8.2487	8.1755	8.0536	7.8202	7.7180	8.0422	8.4131	8.1819	7.9555	7.9090
Nfasc	_DLELTDLAER_.2	#N/A	8.1188	7.7294	8.0247	7.5480	7.5921	8.0981	7.7905	8.0282	8.0803	7.6436	8.1262	7.6395	7.3622	7.3734	7.4075	7.6256	8.0855	7.8549	7.8262	7.8022
Nfasc	_DNILIEC[Carbamidomethyl (C)]EAK_.2	#N/A	7.3896	6.6842	6.8485	7.0300	6.8900	6.9687	6.7791	6.1822	7.4886	6.9565	6.9384	6.8088	6.6085	6.7938	6.0072	6.6890	6.7461	6.6205	6.9315	6.8049
Nfasc	_YVVGQTPVYVPYEIR_.2	#N/A	6.5971	6.6022	6.9592	6.8416	6.6646	6.7718	6.6432	6.8265	6.8344	6.3781	6.7654	6.8203	6.7065	6.3944	6.3617	6.3312	6.4605	6.7915	6.4904	6.0534
Nfasc	_EVAGDTIIFR_.2	#N/A	6.4880	7.0171	7.3753	6.8174	6.7859	7.0265	6.7557	6.2123	6.8948	6.4726	6.1525	6.4199	6.1816	6.8668	5.6806	6.8453	6.4083	6.6998	6.5111	6.3843
Nfasc	_EDDSLTIFGVAER_.2	#N/A	6.6102	6.2548	6.8467	6.5198	6.0786	6.5065	6.4017	5.7658	6.7953	6.4855	6.4958	5.6131	6.6205	6.3417	5.5701	6.1354	6.9400	6.3813	5.9750	5.8235
Nfasc	_AYLTVLADQATPTNR_.2	#N/A	5.7050	5.6234	5.7156	5.4707	5.2637	5.4263	5.6380	5.6533	5.7140	4.7563	5.6842	5.0383	4.2333	5.3692	5.2716	4.1671	5.5115	5.5315	5.4849	4.8692
Nfasc	_LDC[Carbamidomethyl (C)]PFFGSPIPTLR_.2	#N/A	5.5854	5.4531	6.1125	5.7951	5.2005	5.8366	5.8900	4.2130	5.0837	5.1517	5.5599	5.3003	3.3243	4.9805	4.7964	4.3457	5.5796	5.3053	5.1156	5.3360
Nfasc	_ITNVSEEDSGEYFC[Carbamidomethyl (C)]LASNK_.2	#N/A	7.0379	6.3284	5.7902	5.7745	5.4804	5.1548	5.7473	6.4755	5.6114	3.8727	5.3812	4.5485	6.6405	7.0088	4.3357	5.0202	6.2875	5.1747	4.7710	4.8989
Nfasc	_VIAVNEVGSSHPSLPSER_.2	#N/A	4.5376	4.9263	5.3357	5.5938	4.0818	5.6542	5.5542	4.2721	5.8604	3.9757	5.9678	5.7178	3.9834	3.7959	5.2343	3.2445	4.4558	4.3435	5.7347	5.5347
Nfasc	_GTTVQLEC[Carbamidomethyl (C)]IR_.2	#N/A	3.7863	5.0394	4.0899	4.5317	5.6239	5.4931	5.7568	3.2782	6.0675	5.2067	6.0262	4.5009	5.9419	5.8448	5.1838	4.1312	5.2643	4.3740	3.1722	5.3158
Nfasc	_SGTLVIDFR_.2	#N/A	3.9092	3.5051	5.4887	3.1917	4.1161	5.0514	4.0652	4.9946	4.4667	3.7198	3.8316	3.3945	4.5948	4.4269	3.2635	7.4358	4.3168	3.7555	5.0999	3.4873
Nfasc	_DQGSYTC[Carbamidomethyl (C)]MASTELDQLDAK_.2	#N/A	4.1227	3.5571	2.8338	2.1972	4.3296	2.3450	3.5979	4.3484	4.1395	3.9333	2.2488	4.4234	4.1536	4.6877	4.2447	3.4848	3.8456	3.3038	3.6029	3.3746
Nfasc	_DKEETEGNESSEATS[Phospho (STY)]PVNAIYSLA_.3	Q810U3_S1231_M1_Nfasc	4.5700	4.3428	3.6055	3.3194	3.9077	4.1318	3.6333	4.2704	3.6924	3.2755	4.0668	3.3222	4.5907	3.5926	3.2105	3.4141	3.3072	3.0251	2.1292	3.5596
Nrcam	_SVQLTWTPGDDNNSPITK_.2	#N/A	7.6291	8.0187	7.5551	7.4653	7.3234	7.5790	7.2780	8.0120	7.5757	7.6042	7.5764	7.5547	7.9571	8.3085	7.6958	7.7856	8.0940	7.3779	7.6907	7.8287
Nrcam	_GFHTPEGVYSAPSSLK_.3	#N/A	6.8800	7.1085	6.8492	6.8335	6.4964	6.5028	7.0615	7.5433	6.5903	7.0785	6.7881	7.2171	7.4205	7.3548	7.6183	7.1879	7.0436	7.3924	7.4631	6.8187
Nrcam	_DNGELPNDER_.2	#N/A	8.6562	8.7523	9.1074	9.1633	8.8998	9.0368	8.6230	8.9921	8.8537	9.0537	8.7110	8.9226	8.9247	9.5354	8.7673	8.9063	9.1498	8.6846	8.9022	9.2653
Nrcam	_ENIVQC[Carbamidomethyl (C)]EAK_.2	#N/A	5.3762	3.7428	6.8070	6.7423	7.1023	6.6629	6.3645	6.6644	6.2707	6.2480	6.6061	6.1354	5.8324	6.6016	6.8336	6.7796	7.0782	5.9678	6.8095	6.8283
Nrcam	_DSTGTYTC[Carbamidomethyl (C)]VAR_.2	#N/A	5.9841	4.3507	6.1848	6.3584	5.9588	5.6312	5.9357	5.9899	6.3397	3.8584	2.9860	5.6975	3.6728	5.5840	6.4587	5.9443	5.6477	5.7502	5.9818	6.0007
Nrcam	_EDDGTFGEYS[Phospho (STY)]DAEDHKPLK_.3	Q810U4_S1178_M1_Nrcam	3.1219	4.1972	4.1593	4.4624	3.3288	3.2787	2.9856	3.3476	3.0447	3.9232	3.4191	3.9233	3.1527	4.1974	4.0636	4.0618	4.3456	4.3047	5.7315	3.8746
Nrcam	_NEVHLEIKDPTR_.3	#N/A	3.6693	5.8678	6.0308	3.9149	5.1739	5.1461	4.6608	3.8950	3.5921	5.4822	5.2485	4.8545	5.2106	5.5447	5.3486	5.7652	5.1951	5.4280	4.7817	5.4090
Nrcam	_ISWLTNGVPIEIALDDPSR_.2	#N/A	4.2035	3.3426	3.8591	3.4739	2.5852	3.2185	3.0498	3.7060	3.6658	3.7570	2.9999	4.0896	4.2344	3.7094	2.9436	2.9802	4.4158	3.6990	3.5221	3.5919
Nrcam	_TLQITHVSEADSGNYQC[Carbamidomethyl (C)]IAK_.3	#N/A	3.6298	3.6893	3.1664	3.9544	3.8367	2.4949	2.3738	3.8555	3.1316	3.4404	4.1110	3.1316	3.2970	4.5546	3.2367	2.9919	3.3527	4.3188	3.9674	3.7667
Nrcam	_HQAEVSGTQTTAQLK_.3	#N/A	3.5428	4.5690	3.6495	3.8961	3.7497	3.6259	4.2082	3.9073	4.0672	4.1301	3.6708	3.0863	4.0655	3.9663	3.3957	4.2854	4.1579	3.7571	3.7120	4.8600
Nrcam	_EPAEGNESSEAPS[Phospho (STY)]PVNAMNSFV_.2	#N/A	4.0984	4.2286	3.1338	3.5910	3.4361	3.0480	4.1050	3.7987	3.9536	4.3047	3.4323	3.7939	4.1191	4.1100	3.6822	2.9424	4.3364	3.4967	3.1453	3.0879
Nrcam	_EKEPAEGNESSEAPS[Phospho (STY)]PVNAM[Oxidation (M)]NSFV_.3	#N/A	4.1373	3.8655	3.1728	3.5521	3.4750	3.6991	5.1953	3.8377	4.1251	3.8478	5.1910	5.3173	4.2232	3.7770	3.6432	2.9814	4.3754	5.9913	3.5233	3.4542
Nrcam	_AETYEGVYQC[Carbamidomethyl (C)]TAR_.2	#N/A	3.1094	3.5897	3.2659	3.8549	3.9480	4.1721	3.5931	3.9550	3.5622	3.3158	4.0265	3.3159	3.7602	3.3040	3.1703	3.4543	3.4523	3.6972	3.9962	2.9813
Atg4c	_RFST[Phospho (STY)]EEFVLL_.2	Q811C2_T452_M1_Atg4c	3.5226	4.4802	4.0589	4.1668	2.8604	3.0844	3.1799	3.2230	3.2390	3.7290	3.6133	4.3686	3.3470	4.0460	4.2579	4.9465	3.7607	4.5294	3.7211	4.0130
Atg4c	_DFDFTSTAAS[Phospho (STY)]EEDLFSEDERR_.3	Q811C2_S434_M1_Atg4c	3.3428	3.9763	4.3802	4.2414	3.5497	3.5831	3.4984	3.2637	3.4186	3.1534	2.8972	3.7024	3.5840	4.0867	3.5237	4.2057	3.8014	4.0318	3.6804	4.0537
Dlg1	_DYHFVTSR_.2	#N/A	6.8288	6.9455	6.8085	6.5674	6.7310	6.0914	5.9984	6.6125	6.4807	6.7712	6.7661	6.4719	7.3031	7.0799	6.9600	6.6168	6.8727	6.3713	6.8761	6.8966
Dlg1	_AVEALKEAGS[Phospho (STY)]IVR_.2	#N/A	6.1880	7.3031	5.1752	5.8081	5.1679	5.8336	6.4707	7.2264	5.4522	6.7607	4.9895	6.6408	7.3173	7.4100	6.2450	6.7012	6.7114	7.2363	6.4579	5.5960
Dlg1	_QVTPDGESDEVGVIPSKR_.3	#N/A	6.3740	5.8025	6.6088	5.9935	6.6301	5.6702	6.3912	6.5454	6.2349	6.0824	6.3094	5.9397	6.1751	5.6667	6.4286	6.3914	6.0341	6.1269	6.2334	6.7107
Dlg1	_QIIEEQSGPYIWVPAK_.2	#N/A	4.8456	5.2800	5.4203	5.3650	5.3640	5.1891	5.1522	5.1542	4.9206	4.6862	5.6081	5.3881	5.8088	5.9534	5.3324	5.4232	5.7688	5.4840	5.2788	5.3332
Dlg1	_VNDC[Carbamidomethyl (C)]ILR_.2	#N/A	4.9039	5.4165	5.6817	5.2426	5.2134	5.6557	5.6985	5.4376	4.5446	5.1407	5.9626	5.4136	4.5174	5.2811	5.5545	5.2702	4.9335	5.7214	5.2686	5.4577
Dlg1	_IISVNSVDLR_.2	#N/A	6.6084	6.1806	5.8933	5.9811	6.0072	6.5054	6.2601	6.4534	4.8904	6.4344	6.0329	6.4489	5.9342	6.7078	5.4835	5.9384	6.9115	6.4747	6.0298	6.4622
Dlg1	_IIEGGAAHK_.2	#N/A	4.4751	4.5856	5.2150	5.0953	4.3115	3.4814	4.8522	3.1854	4.2982	4.1592	5.0274	4.9606	3.3030	5.0007	4.2875	4.0895	3.2501	5.3117	4.4859	5.2485
Dlg1	_IITGGAAAQDGR_.2	#N/A	5.6919	3.9559	4.4006	6.3677	3.5701	6.1150	6.7560	3.5889	3.2860	5.7838	6.5545	5.7953	3.3941	4.4387	3.5033	6.1824	5.5735	5.5007	4.3624	4.1160
Dlg1	_EQM[Oxidation (M)]M[Oxidation (M)]NSSVSSGSGS[Phospho (STY)]LR_.2	#N/A	10.2021	9.8186	9.4105	9.5342	9.1364	9.2552	9.4382	10.0312	9.4213	10.4305	9.0787	9.2593	10.0690	9.1010	8.5393	9.8841	9.6280	9.5509	9.4659	9.6577
Dlg1	_ALFDYDK_.2	#N/A	3.6541	4.3487	4.1904	4.0353	2.9918	3.4923	3.1140	3.3545	1.8184	4.5635	2.5476	4.2382	3.6748	4.1775	2.6265	2.4424	3.8921	3.4935	3.5896	4.1444
Dlg1	_NLSEIENVHGFVSHSHIS[Phospho (STY)]PIK_.3	Q811D0_S158_M1_Dlg1	5.5685	3.9520	4.5871	3.6386	3.3885	3.6126	4.1525	3.7512	4.2116	4.2571	3.0852	3.8414	4.0715	4.5742	3.7297	4.3957	4.2889	3.5105	3.1929	3.0403
Dlg1	_T[Phospho (STY)]KDSGLPSQGLNFR_.3	Q811D0_T595_M1_Dlg1	3.7514	4.2514	4.2877	3.9380	3.0891	3.3132	4.4520	3.4518	3.0102	3.9577	3.3846	4.1409	3.7721	4.2747	4.0292	4.0963	3.9894	3.8437	3.4923	4.2417
Dlg1	_HC[Carbamidomethyl (C)]LDVSGNAIK_.2	#N/A	3.5166	4.4862	4.0529	4.2620	2.8543	3.0201	3.3536	3.5479	3.1471	3.7229	3.6194	4.3757	3.3531	4.3977	2.9372	3.5662	4.5882	4.1043	4.4034	4.0750
Dlg1	_HVTSNAS[Phospho (STY)]DSESSYR_.2	Q811D0_S688_M1_Dlg1	3.1110	3.3910	3.6473	4.5784	3.9495	4.1736	3.5915	4.3122	3.6506	3.3173	4.0250	3.2804	4.9733	3.6344	3.3187	3.4559	3.3490	2.9833	4.1327	3.6014
Dlg1	_AASHEQAAAALK_.2	#N/A	3.3141	3.1879	3.8504	4.3753	4.1526	4.3767	2.4657	4.5153	3.4475	3.5204	3.3315	3.0774	3.3348	3.8374	2.9657	3.6590	3.5521	4.2810	3.1409	3.8044
Dlg1	_TRGDKGEIPDDM[Oxidation (M)]GS[Phospho (STY)]K_.3	#N/A	4.4495	3.9628	4.2505	4.0259	3.0954	3.2134	3.7395	3.1743	3.5324	4.6512	3.3823	4.1055	4.3637	4.4046	4.0177	3.9260	4.1724	3.6076	4.0566	3.7014
Dlg1	_FIEAGQYNNHLYGTSVQSVR_.3	#N/A	3.8072	4.1956	4.3435	4.5527	5.0426	3.3691	4.3961	3.2572	4.4552	4.0136	5.8312	4.0850	4.5632	4.1070	3.9733	4.1521	4.2553	4.3950	4.1052	3.7843
Dlg1	_NAGQAVTIVAQYRPEEYSR_.3	#N/A	4.3796	4.4403	3.9162	3.2046	3.0857	3.5218	3.9624	3.1044	3.5373	4.1664	3.1759	4.1165	4.4105	3.9542	3.9878	3.7417	4.1025	3.0469	4.7684	3.6315
Dlg1	_EAGS[Phospho (STY)]IVR_.2	#N/A	3.8562	4.3365	4.0200	3.1008	3.1939	3.4180	4.3472	3.2083	4.4063	4.0625	3.2798	4.0626	4.5143	4.0581	3.9244	4.2011	4.2064	4.4439	3.2421	3.7354
Atpaf1	_RGS[Phospho (STY)]GLPPEGADGSGVSELEANPFYDR_.3	Q811I0_S52_M1_Atpaf1	3.7764	8.1715	3.9714	7.2271	6.8148	3.7113	7.7016	3.1355	3.2904	7.2559	7.3307	2.9563	3.7721	8.4197	6.2618	6.9328	3.6732	8.0049	6.8942	3.9255
Mast4	_TPSPTPQPTS[Phospho (STY)]PQR_.2	Q811L6_S1389_M1_Mast4	7.6131	7.2509	7.6196	4.4369	6.7542	7.1525	3.4500	4.4537	3.5091	3.4588	3.8835	6.9159	7.4154	3.1609	3.0272	6.8984	7.2907	6.9786	6.9841	4.3390
Mast4	_SDS[Phospho (STY)]LPSFR_.2	Q811L6_S2517_M1_Mast4	3.2023	4.2990	7.1738	7.3288	3.4092	3.4426	3.6389	3.4042	6.0364	4.5137	3.0377	6.4287	3.7245	4.2272	3.6643	4.0652	3.9419	3.8913	6.7874	4.1942
Mast4	_KHS[Phospho (STY)]LEVTQEEVQR_.3	Q811L6_S1465_M1_Mast4	4.9140	4.9449	3.4139	3.7069	3.8001	4.0241	5.0508	2.9740	3.8001	3.1678	4.1745	2.4349	3.9082	4.6969	3.3182	3.8596	3.6002	3.5492	3.8483	3.1292
Mast4	_SLSYSAS[Phospho (STY)]FPEAQPGVR_.2	Q811L6_S2461_M1_Mast4	4.5155	3.4950	3.3607	3.7601	3.8532	4.0773	3.6878	4.0498	3.7469	3.2210	4.1213	3.2211	3.8550	3.3987	3.2650	5.8474	3.5470	3.6024	3.9015	3.0760
Arhgap32	_AVS[Phospho (STY)]PEGDER_.2	Q811P8_S1823_M1_Arhgap32	8.7835	8.7754	8.2869	8.7868	8.2472	8.2911	8.5685	8.1728	8.0713	8.9034	8.3223	8.0920	8.7886	8.5940	7.8719	8.3772	8.2667	8.3405	8.4065	8.3301
Arhgap32	_DVINRS[Phospho (STY)]PTQLQLGK_.2	#N/A	8.3993	7.6945	7.7213	8.0749	6.9995	7.4685	7.3188	8.2030	6.2371	6.3184	6.4943	7.4146	7.7284	7.9193	6.3953	6.5056	4.7705	7.6941	7.6165	7.5962
Arhgap32	_S[Phospho (STY)]PTQLQLGK_.2	#N/A	6.7007	3.8240	6.5588	3.8498	3.6134	6.4023	3.6998	3.8160	5.7164	6.5064	6.5089	5.1913	3.8333	3.7205	3.4826	5.9579	3.9881	3.7713	5.9065	3.6955
Arhgap32	_TQAQVSS[Phospho (STY)]PIVTENK_.2	Q811P8_S613_M1_Arhgap32	6.6743	4.6056	5.9353	6.4879	5.7920	5.9167	5.4811	3.0976	3.3644	6.2937	6.6086	6.4414	3.4724	6.0727	2.8825	3.7421	6.1781	4.1979	5.7993	3.8876
Arhgap32	_NES[Phospho (STY)]EPSEM[Oxidation (M)]K_.2	#N/A	4.7795	7.1918	7.4675	7.9377	3.5282	6.3389	5.2728	3.7310	5.6969	7.0142	7.2349	6.1061	5.4012	7.6396	6.2612	5.4440	5.2912	5.5866	6.5358	6.5786
Arhgap32	_KLHDM[Oxidation (M)]GC[Carbamidomethyl (C)]S[Phospho (STY)]LPEHR_.3	Q811P8_S1876_M1_Arhgap32	6.4987	4.5328	6.0078	3.2971	5.8761	3.0265	6.1917	4.5128	5.8530	7.1466	5.6379	5.5579	6.8267	6.5441	5.2667	5.8356	6.2318	6.0270	6.6122	5.6860
Arhgap32	_LSPFFTLDLS[Phospho (STY)]PTDDK_.2	Q811P8_S871_M1_Arhgap32	3.8788	6.7305	6.1653	3.8106	3.2166	5.8967	4.3245	5.3017	4.3836	4.0852	3.2571	4.0134	4.4917	6.7630	3.9017	4.2237	4.1169	4.4666	3.2648	3.7127
Arhgap32	_DAEAGGSQSQTPGSTASSEPVS[Phospho (STY)]PVQEK_.3	Q811P8_S856_M1_Arhgap32	9.3968	9.5525	9.2939	9.2125	8.2986	8.7646	8.9807	9.2782	8.8715	9.1239	8.7613	8.8808	9.4606	9.4985	8.0551	8.7687	9.2431	9.0780	8.9475	8.7075
Arhgap32	_S[Phospho (STY)]SSDALSASFNGDVLGNR_.2	Q811P8_S730_M1_Arhgap32	4.1868	4.0475	3.7330	3.2061	3.7183	3.7516	4.1909	3.7360	5.8103	3.9974	3.5897	3.7195	4.4569	3.7495	3.5408	3.5489	3.7325	3.0808	3.8810	3.5548
Arhgap32	_SAKS[Phospho (STY)]EESLTSLHAVDGDSK_.3	#N/A	7.9339	8.1332	7.9793	8.0518	6.7403	7.4157	7.3199	7.7530	7.2005	8.0505	7.1998	7.4943	8.0925	7.5891	6.6436	7.2074	7.8848	7.5603	7.5548	7.1428
Arhgap32	_SLLVSS[Phospho (STY)]PSTK_.2	Q811P8_S592_M1_Arhgap32	3.9518	4.0510	4.4881	3.7376	3.2895	3.5136	4.2515	3.6522	4.3106	4.1581	3.1842	4.2479	4.4187	4.4752	3.8287	4.2967	4.1899	3.6433	3.2919	3.7382
Arhgap32	_S[Phospho (STY)]EESLTSLHAVDGDSK_.2	#N/A	3.2897	3.2123	7.2753	4.3997	4.1282	3.8567	3.2248	4.4909	6.7879	3.4270	4.1244	3.1017	3.3104	7.2219	2.9900	3.6346	3.5277	4.3054	3.9540	3.7801
Arhgap32	_LSS[Phospho (STY)]RDFAFYNPR_.2	Q811P8_S1689_M1_Arhgap32	3.5708	3.7483	4.6082	4.0134	3.7777	3.8111	3.2704	3.7965	3.1906	3.3814	4.1701	3.4744	3.3560	3.1454	3.2957	4.4337	3.2937	3.8557	4.1548	4.3236
Arhgap32	_S[Phospho (STY)]FFNLGK_.2	Q811P8_S662_M1_Arhgap32	3.8540	0.2923	3.3906	3.7302	4.0609	4.0475	2.9872	4.0797	4.4082	3.6646	3.8869	3.1912	3.8849	3.4286	3.0126	3.2161	3.5769	3.5726	3.8716	3.1059
Arhgap32	_AES[Phospho (STY)]FPGHSC[Carbamidomethyl (C)]GFAAPVPPTR_.3	Q811P8_S1405_M1_Arhgap32	4.1145	3.2046	3.6510	3.4698	4.3214	4.3547	4.5606	4.3401	4.1478	3.9250	3.6264	2.9308	4.3132	3.6891	4.2529	3.4766	3.8374	3.3026	2.9512	3.2820
Pcyt1b	_QS[Phospho (STY)]PVSSPTR_.2	Q811Q9_S319_M1_Pcyt1b	4.4168	7.0630	3.9534	3.1674	3.1229	6.6051	7.0197	6.0608	3.8454	6.6906	3.3240	4.1292	4.0108	3.9915	3.9505	3.7790	4.1398	6.7994	6.8403	3.6688
Pcyt1b	_M[Oxidation (M)]LQALS[Phospho (STY)]PK_.2	#N/A	5.5222	4.6007	4.6250	5.3788	5.6547	5.9521	4.9084	4.9892	4.4812	6.4205	4.9250	5.3212	4.9799	5.1959	3.7665	5.1771	4.7006	6.2926	5.0391	5.9384
Pcyt1b	_AASASSS[Phospho (STY)]MS[Phospho (STY)]EGDEDEK_.2	Q811Q9_S360_M2_Pcyt1b	3.8946	3.7886	3.1631	4.3528	3.6641	3.9879	3.7158	3.8404	3.8954	3.9238	3.5612	3.6052	4.0035	5.6539	3.4649	2.9803	3.9372	3.5130	3.8121	4.0168
Pcyt1b	_MLQALS[Phospho (STY)]PKQS[Phospho (STY)]PVSSPTR_.3	Q811Q9_S315_M2_Pcyt1b	3.8725	4.5287	5.5408	3.7118	4.0794	4.1127	4.4695	4.0981	4.3897	3.6831	3.8684	3.1727	4.5552	5.4929	2.9941	3.2346	4.4246	3.5541	3.8531	3.5240
Srgap3	_GRIES[Phospho (STY)]PEK_.2	#N/A	8.2137	7.9855	7.8217	7.7701	7.4510	7.8116	7.6582	8.1068	7.7421	7.6099	7.7506	7.6862	7.8992	7.8208	6.6332	7.3362	7.7156	7.8203	7.6453	7.6084
Srgap3	_GRIES[Phospho (STY)]PEKR_.3	#N/A	7.4809	7.6063	7.6269	6.9978	6.7465	7.1041	7.5050	7.1443	6.6671	7.3464	6.7310	6.9244	7.3361	7.2443	5.4789	6.7322	7.1266	7.5016	6.7435	6.6996
Srgap3	_SGGDTHS[Phospho (STY)]PPR_.2	#N/A	8.3312	8.3533	8.1317	8.2233	7.9354	8.5433	8.2556	8.2632	8.0210	8.0745	8.0025	8.0724	7.8133	7.9942	3.6581	6.9137	8.3282	8.1978	7.9189	7.8569
Srgap3	_ADSEASS[Phospho (STY)]GPLLDDK_.2	Q812A2_S821_M1_Srgap3	4.0046	3.6979	4.1239	3.6335	3.8431	3.8022	4.2695	3.9924	4.0665	4.0257	3.5451	3.5272	4.0304	4.1283	4.8021	3.9382	6.9802	3.5091	3.4991	3.1318
Srgap3	_SSS[Phospho (STY)]SSSSGVGSPAVTPTEK_.2	Q812A2_S1070_M1_Srgap3	9.5688	8.6091	4.3686	3.8346	3.5382	4.0240	8.1755	9.0888	9.9797	9.8666	3.7363	9.5985	3.8056	9.8844	3.6319	3.7680	3.5494	3.6769	8.4554	4.0840
Srgap3	_RRS[Phospho (STY)]GGDTHS[Phospho (STY)]PPR_.2	#N/A	5.2546	4.1850	3.9156	5.2294	3.7278	5.2742	3.7904	3.6631	4.7886	3.4573	3.9750	3.5549	5.3030	3.9164	3.5642	4.1526	3.7627	5.3517	5.4104	3.8101
Srgap3	_IES[Phospho (STY)]PEKR_.2	#N/A	4.5217	12.3692	12.3736	12.3444	5.9389	12.0593	12.1268	12.1760	11.9328	12.5888	11.9894	3.3706	12.2753	3.5442	3.2589	6.0433	12.2902	12.1918	12.3465	3.5112
Srgap3	_NDLQS[Phospho (STY)]PTEHSDYGFGGVM[Oxidation (M)]GR_.3	Q812A2_S837_M1_Srgap3	3.8332	6.4071	3.1783	5.9390	3.5934	7.3842	8.3197	3.8555	7.0050	5.8700	3.7289	7.5923	3.9360	8.7447	5.7989	6.9170	3.9547	8.4234	7.6790	3.9041
Srgap3	_SGGDTHS[Phospho (STY)]PPRGLPSIDTPPR_.3	#N/A	7.4058	4.3577	4.5548	4.0443	6.6994	3.2069	5.2231	7.9538	6.7437	3.8514	5.9743	4.2471	3.2245	6.8636	4.1354	6.2909	3.8832	3.9500	3.5986	4.1355
Srgap3	_MATFGS[Phospho (STY)]AGSINYPDKK_.2	#N/A	3.6518	4.9885	3.4828	3.9567	3.9786	3.5777	3.6355	3.7609	3.6342	3.7552	3.6947	3.3353	3.7668	3.7391	3.2236	3.4850	3.7098	3.7937	3.9458	3.9654
Srgap3	_LRS[Phospho (STY)]DGAAIPR_.2	Q812A2_S858_M1_Srgap3	7.4318	5.2820	6.5417	4.8687	3.6660	3.6994	5.9136	3.6848	5.2989	7.5916	4.2817	6.1633	3.4677	5.4796	3.4074	6.1329	5.4019	6.1183	3.7967	5.6800
Srgap3	_AAAC[Carbamidomethyl (C)]PSS[Phospho (STY)]PHKIPLSR_.3	Q812A2_S895_M1_Srgap3	3.1596	4.1595	5.2778	4.4246	3.3666	3.2410	3.6815	3.3853	3.0825	3.8855	3.4568	3.8856	3.1905	6.4408	3.7069	4.0226	4.3834	4.2669	3.0651	3.9124
Srgap3	_HSS[Phospho (STY)]LGDHKS[Phospho (STY)]LEAEALAEDEK_.3	Q812A2_S948_M2_Srgap3	4.7608	4.2011	4.3324	3.8939	3.1976	3.3509	3.7312	3.4965	3.6514	4.4220	3.1301	4.0968	3.8169	4.3195	3.7566	3.9736	4.0342	4.4576	3.4482	4.2865
Srgap3	_SLEAEALAEDEK_.2	#N/A	4.3578	3.8253	3.8944	4.0905	3.8967	3.5748	4.0154	3.9155	3.6126	4.3910	3.1542	4.2194	4.3887	3.9324	4.1344	3.7199	4.0807	3.9328	3.3990	3.6097
Prr5	_S[Phospho (STY)]SPTPHSGPC[Carbamidomethyl (C)]PSR_.2	Q812A5_S310_M1_Prr5	3.6002	3.1961	3.8422	4.3835	3.8071	3.8405	3.2410	4.5071	3.1612	3.4108	4.1406	3.0855	3.3266	3.8293	3.2663	2.9623	3.5439	4.2892	3.9378	3.7963
Tufm	_LLDAVDTYIPVPTR_.2	#N/A	7.9657	7.9322	8.4298	8.3278	8.0819	7.9322	7.8744	8.1511	7.9587	7.9829	7.8737	8.0681	8.5825	8.0540	8.0071	8.0828	8.2681	8.0073	7.9271	7.8760
Tufm	_GTVVTGTLER_.2	#N/A	7.7165	7.8581	8.0180	8.1536	7.7149	7.5073	7.9539	7.4634	7.7199	7.4089	7.4093	7.6474	7.7480	7.7729	7.8274	7.7673	7.4562	7.9438	7.5590	8.0412
Tufm	_AEAGDNLGALVR_.2	#N/A	7.8408	7.5501	7.9382	7.7761	7.6178	7.6594	7.6275	8.0307	8.0284	7.6178	7.8151	7.9736	8.0733	8.1248	7.7757	7.8173	7.4640	7.4875	7.7815	7.7592
Tufm	_TVVTGIEM[Oxidation (M)]FHK_.2	#N/A	6.1321	6.4637	5.4824	4.3820	5.8893	6.4059	6.2068	6.4862	6.2749	5.8377	4.4202	5.8890	5.9565	6.1090	6.1249	6.3876	6.4193	6.4063	6.3534	6.1665
Tufm	_TIGTGLVTDVPAM[Oxidation (M)]TEEDKNIK_.3	#N/A	5.5476	4.3838	4.8712	4.7846	5.0494	4.0738	4.4351	5.6558	5.4100	5.3261	4.5610	5.1384	5.5408	4.9378	5.3105	5.2904	5.4460	4.8846	4.5742	5.0967
Tufm	_KYEEIDNAPEER_.3	#N/A	6.2816	5.8469	6.7196	5.9777	6.2937	6.0998	6.4521	5.1926	6.7559	6.8279	6.5995	5.7055	3.9246	6.3375	6.8936	6.4728	6.4656	6.2297	6.1905	6.5772
Tufm	_KGDEC[Carbamidomethyl (C)]ELLGHNK_.3	#N/A	4.0445	5.2591	5.5634	5.6906	6.1051	5.1937	5.4628	5.4280	5.6622	6.0919	5.9847	4.9431	5.7174	5.3864	6.1696	6.3825	6.3012	5.0113	5.6995	6.4464
Tufm	_TTLTAAITK_.2	#N/A	4.6281	5.4144	5.0183	6.0083	5.3109	5.7666	5.5729	4.2763	5.0491	4.3905	5.5609	5.4060	5.5065	5.6017	5.6897	5.8481	4.5326	5.5702	5.3174	6.2009
Tufm	_YEEIDNAPEER_.2	#N/A	3.2268	7.2519	3.1486	7.3666	7.4992	7.2000	7.1589	3.8377	7.3825	7.0261	7.1115	7.1245	7.0873	3.1866	7.8141	7.3398	7.2800	7.2164	7.4999	7.4033
Tufm	_ILAEGGGAK_.2	#N/A	3.1353	5.0911	4.1728	4.4489	5.4911	5.1084	5.7170	3.3610	5.3709	4.7356	5.0823	5.1117	4.4709	5.0585	5.9728	5.2143	4.3591	5.6858	5.1522	5.1571
Tufm	_ELAM[Oxidation (M)]PGEDLK_.2	#N/A	7.1531	5.4306	7.6669	7.2232	7.4142	7.2767	7.1376	7.3264	7.3832	6.8815	6.3162	7.1218	7.3705	7.3955	7.5834	7.1001	7.2549	7.2184	7.1587	7.4876
Tufm	_ELLTEFGYKGEETPVIVGSALC[Carbamidomethyl (C)]ALEQR_.3	#N/A	3.6622	2.4873	2.7767	3.9221	2.3841	3.4402	2.8638	3.0907	1.5967	3.1957	4.0787	0.7906	3.2647	3.9537	3.2044	1.7668	2.1459	3.0097	3.4352	1.5694
Tufm	_GEETPVIVGSALC[Carbamidomethyl (C)]ALEQR_.2	#N/A	4.5319	4.5499	3.8066	4.5897	3.5931	3.8296	3.9926	3.8373	3.6450	4.0806	3.7334	4.2760	4.1577	4.5059	3.3113	4.5065	3.8016	3.4582	4.3633	3.8857
Tufm	_GDEC[Carbamidomethyl (C)]ELLGHNK_.2	#N/A	4.6962	5.1916	5.0657	3.3732	4.2976	6.2597	6.3231	4.0166	4.8017	6.7486	6.6486	2.9545	4.3369	7.1417	4.8667	3.4528	6.4185	5.2390	4.4283	3.4417
Tufm	NMEOxidation (M)]TGTAPLDGC[Carbamidomethyl (C)]ILVVAANDGPM[Oxidation (M)]PQTR .	#N/A	4.1308	4.3989	3.1662	3.5586	4.1906	5.0088	4.6828	3.8312	3.6769	5.0032	3.9145	3.7615	4.2397	3.1533	3.3594	5.2753	4.3688	4.3577	2.6605	4.8230
Tufm	_GITINAAHVEYSTAAR_.2	#N/A	3.2922	3.9994	4.6072	3.5104	3.9889	4.0260	3.6028	3.5557	3.8291	3.8510	4.5923	4.3928	3.8567	3.2142	3.7091	4.1358	4.1026	4.3515	4.6324	4.7423
Tufm	_ADAVQDSEM[Oxidation (M)]VELVELEIR_.2	#N/A	5.9481	6.3894	6.2159	6.0647	5.8816	5.6455	5.9040	6.5121	6.0694	6.3271	5.7809	6.1959	7.6468	7.1123	6.5357	6.5770	6.3946	5.8471	5.9197	6.0564
Tufm	_DKPHVNVGTIGHVDHGK_.4	#N/A	4.1877	3.1314	3.7242	3.3966	2.8938	4.4279	4.1543	4.4133	4.0745	3.9983	3.5532	4.3584	4.2400	4.1832	4.1797	4.1779	3.9106	4.0982	3.6487	3.4396
Tufm	_HYAHTDC[Carbamidomethyl (C)]PGHADYVK_.3	#N/A	4.4566	4.3633	5.1733	3.1276	3.1627	3.3448	4.3203	3.1815	4.3794	4.0893	3.2530	4.0894	4.4875	4.0313	4.6685	3.8187	4.1795	2.9699	3.2690	3.7085
Tufm	_QIGVEHVVVYVNK_.2	#N/A	3.9888	4.0140	4.5251	3.7006	3.3266	3.1575	4.2145	3.6892	3.8441	4.1952	3.3227	3.9034	4.0095	4.5122	3.7917	4.3337	4.2269	3.6063	3.2549	2.9784
Svop	_SEDDAAS[Phospho (STY)]GEHDIQEGVR_.3	#N/A	6.4363	4.3672	3.3753	6.7542	4.9322	6.6908	6.9754	4.0482	4.3322	6.7753	3.8284	3.4642	4.0909	6.7264	5.5099	6.0571	4.3287	6.9127	6.5873	6.6453
Svop	_RTGESARS[Phospho (STY)]EDDAAS[Phospho (STY)]GEHDIQEGVR_.4	#N/A	3.5450	4.1698	4.2860	4.1846	3.2650	3.3942	4.0587	3.4610	4.0636	4.2598	3.1969	3.9769	3.8675	4.2963	3.8329	4.1023	4.2264	4.0825	3.2468	4.3372
Rnf214	_SDESLPDGLS[Phospho (STY)]PK_.2	Q8BFU3_S36_M1_Rnf214	9.1979	9.7878	9.9411	9.9843	7.0514	9.5202	9.2732	7.5430	9.5379	10.0044	8.9807	9.5214	9.3253	9.8535	9.1725	9.7884	9.9950	9.4131	9.5981	9.8093
Rnf214	_VDQDDDQDS[Phospho (STY)]SSLK_.2	Q8BFU3_S194_M1_Rnf214	4.0563	3.2628	3.5929	3.3388	4.2633	4.2966	4.2857	4.0510	4.2059	3.8669	3.6845	2.9889	4.3713	6.0492	2.8102	3.4184	3.0878	3.2445	4.3939	3.3401
Rnf214	rotein N-term)]AASEVAGLGAGT[Phospho (STY)]PS[Phosoho (STY)]PSESSALC[Carbamidometh	Q8BFU3_S15_M2_Rnf214	4.4649	4.0608	4.4783	3.7474	3.1710	3.2043	3.8771	3.6424	3.7973	4.2755	3.2760	3.9502	3.9628	4.4654	3.4800	3.8270	4.1801	5.6902	3.3017	4.4324
Rnf214	_NLS[Phospho (STY)]PPSVSSQMRTK_.2	Q8BFU3_S48_M1_Rnf214	3.7108	4.2920	4.2471	3.9786	3.0485	3.2726	6.8660	3.4112	3.0508	3.9171	3.4252	4.1814	3.1588	4.2035	4.0697	4.0557	3.9489	4.2986	3.0968	3.8807
Pef1	_VC[Carbamidomethyl (C)]TQLDVLTEAFR_.2	#N/A	3.8902	4.1126	4.8097	4.7930	3.2279	4.8394	4.3132	3.5905	4.3723	4.0965	3.2458	4.0021	4.7769	4.4135	3.7042	4.2351	4.1306	3.9705	4.5751	4.2126
Actbl2	_VAPDEHPILLTEAPLNPK_.3	#N/A	13.9808	13.8613	14.0586	14.0264	13.5643	13.7388	13.7946	14.0110	13.9195	13.9981	13.8258	13.7291	14.2603	14.0375	13.8851	13.9751	13.8623	13.6629	13.8638	13.7403
Hnrnpa3	_IFVGGIKEDTEEYNLR_.3	#N/A	7.3078	7.1899	6.8493	7.5843	6.8372	7.1161	6.7217	7.2537	7.4222	7.6617	7.2980	7.2461	7.9207	7.9584	7.3751	7.3364	7.2857	6.5795	7.3767	7.6659
Hnrnpa3	_GFAFVTFDDHDTVDKIVVQK_.3	#N/A	8.5250	8.4436	7.8525	8.0594	7.3851	7.5170	7.6605	7.2497	7.7730	7.6591	7.1983	8.1817	9.2118	8.8999	7.6941	7.8567	8.1418	8.1294	7.8988	8.1875
Hnrnpa3	_GFAFVTFDDHDTVDK_.2	#N/A	6.1775	5.9664	6.2642	6.1956	5.3612	5.6921	5.8073	5.8987	6.3392	6.0062	5.7438	5.5697	6.1830	5.9688	5.7314	6.5158	6.0401	5.8271	5.9799	5.8267
Hnrnpa3	_EDTEEYNLR_.2	#N/A	6.6677	6.0294	6.7526	6.8083	6.6066	6.6476	6.4053	5.3418	6.0258	6.4890	7.0286	6.3544	4.0107	6.7477	7.2788	6.6659	6.6844	6.7205	6.4460	6.6399
Hnrnpa3	_IETIEVM[Oxidation (M)]EDR_.2	#N/A	7.7952	8.2923	8.7713	8.3370	7.8048	8.2293	7.8499	7.5430	8.3060	8.2827	7.9283	7.8524	7.6656	8.6470	8.0167	8.1021	7.9947	7.6870	7.8533	8.2412
Hnrnpa3	_EDSVKPGAHLTVK_.2	#N/A	6.2731	6.5657	6.4340	6.0706	6.1730	6.0871	6.3350	6.2412	6.0434	6.1850	6.6384	6.1633	6.2831	6.6141	6.8070	6.3766	6.2355	5.7809	6.2645	6.1584
Hnrnpa3	_SSGS[Phospho (STY)]PYGGGYGSGGGSGGYGSR_.2	Q8BG05_S359_M1_Hnrnpa3	4.0524	7.3773	3.6758	10.9623	7.8092	4.3045	7.2555	7.7169	7.0494	7.7577	7.7165	7.3819	7.7215	8.1849	8.6619	7.7976	7.5771	7.3581	10.4640	7.5237
Hnrnpa3	_LFIGGLSFETTDDSLREHFEK_.3	#N/A	6.3303	7.0323	6.2296	5.8756	5.9073	5.5699	5.7731	6.4675	5.9550	6.0309	5.5466	6.3553	7.8266	7.0062	5.8116	3.3880	6.1419	5.8136	6.3421	6.4572
Hnrnpa3	_LFIGGLSFETTDDSLR_.2	#N/A	4.1126	3.8199	3.6492	3.8969	4.1024	4.2898	4.1883	3.9825	4.1506	3.9232	4.4825	3.7814	3.6630	3.9356	3.9162	3.4747	3.9014	5.1114	5.3191	3.7479
Hnrnpa3	_GEEGHDPKEPEQLRK_.4	#N/A	4.3201	4.4999	3.8566	3.2642	3.0262	3.0595	3.0741	3.0449	3.9422	4.1306	3.4208	4.2260	4.1076	3.8947	4.0473	3.6822	4.0430	3.1065	3.1606	3.5720

Hnrnpa3	_GFGFVTYSC[Carbamidomethyl (C)]VEEVDAAM[Oxidation (M)]C[Carbamidomethyl (C)]AR_.2	#N/A	3.8806	3.8072	3.1417	3.7182	3.6373	4.0354	3.7977	4.0780	3.8568	3.9605	3.6513	3.6214	3.9648	3.4171	2.7420	2.9587	3.6569	3.5605	3.8596	3.5629
Hnrnpa3	_WGTLTDC[Carbamidomethyl (C)]VVMR_.2	#N/A	3.6924	4.3104	4.2287	3.9969	3.0302	3.4539	4.5200	3.3928	3.5477	3.8988	3.0264	4.1998	3.7132	4.2158	4.0881	4.0373	4.3042	3.9027	3.5512	4.1828
Hnrnpa3	_YHTINGHNC[Carbamidomethyl (C)]EVK_.3	#N/A	3.7899	3.5291	3.3265	3.7943	3.9969	4.1115	3.0512	4.0156	3.7128	3.6005	3.0028	3.2553	3.8208	3.3646	3.0766	3.1521	3.5129	3.6366	3.9356	3.0419
Hnrnpa3	_YGKIETIEVMEDR_.3	#N/A	3.1003	4.2188	4.1377	4.4839	3.3072	3.3003	4.4648	3.3260	3.0231	3.9448	3.3975	3.9449	3.1312	4.1758	3.7663	3.9632	4.3240	4.3263	3.1245	3.8530
Slc6a15	_QSGS[Phospho (STY)]PTLDTAPNGR_.2	#N/A	7.8581	8.3139	4.2613	4.3604	3.4308	7.9174	7.7671	8.0174	8.0679	7.2579	3.0162	7.6417	7.5990	4.2993	7.0422	7.2601	7.8623	4.2027	7.6535	7.2189
Slc6a15	_IPSEMSS[Phospho (STY)]PNFGK_.2	Q8BG16_S687_M1_Slc6a15	4.1426	3.8602	3.1781	3.5468	3.4804	3.7044	4.0607	4.4227	6.6849	4.3490	2.9933	3.7496	4.2279	3.7716	3.6379	2.9867	4.3807	3.2295	3.5286	6.7403
Slc6a15	_KQS[Phospho (STY)]GS[Phospho (STY)]PTLDTAPNGR_.2	Q8BG16_S699_M2_Slc6a15	7.3911	5.5396	3.5075	7.5364	3.4615	6.8124	6.6570	7.0168	6.6595	6.6814	6.4948	6.3048	6.6907	6.5781	3.7454	6.3479	6.6121	7.0068	6.4318	6.2639
Slc6a15	_DTDAEDGS[Phospho (STY)]EADDERPAWNSK_.2	Q8BG16_S55_M1_Slc6a15	3.9344	4.0684	4.4707	3.7550	3.2721	3.2120	4.2690	3.6348	3.0482	4.1407	3.2683	3.9579	3.9551	4.4578	3.8462	4.2793	4.1724	3.6607	3.3093	1.6065
Necab1	_ETSPSSNNS[Phospho (STY)]SEELSSTLQLSK_.2	Q8BG18_S14_M1_Necab1	4.0206	3.6791	3.8191	3.8028	3.8701	3.8447	3.9194	4.0185	3.7411	3.9447	3.9017	3.4533	3.8665	4.2241	3.2955	3.6398	3.6862	5.2124	3.8370	4.0097
Psmd11	_AQS[Phospho (STY)]LLSTDR_.2	Q8BG32_S14_M1_Psmd11	7.9740	8.1241	8.0370	8.1658	7.1207	7.1985	6.9811	6.7207	6.5986	8.5360	7.6164	6.8652	6.9951	7.5811	5.1268	7.3231	6.8943	7.4008	7.4060	7.3971
Psmd11	_LYDNLLEQNLIR_.2	#N/A	4.4924	4.9794	3.4500	3.2749	5.3181	5.5861	5.0905	5.4595	4.7397	4.9494	4.5474	5.3204	4.4353	4.8981	4.8090	3.2586	5.7511	5.4724	5.3415	4.5661
Psmd11	_YQEALHLGSQLLR_.2	#N/A	3.8049	4.2852	4.0713	4.5504	3.1426	3.2550	1.8300	3.2596	4.4575	4.0112	3.3311	4.0113	3.8620	4.1094	2.7416	4.1498	4.2576	4.3927	3.1909	3.7866
Psmd11	_TAYSYFYEAFEGYDSIDSPK_.2	#N/A	3.6044	4.0847	4.2718	4.3499	2.9422	3.1663	3.0981	3.3193	3.1572	3.8108	3.5315	3.8109	3.2652	3.8229	4.1761	3.9493	4.4581	4.1922	2.9904	3.9871
Sv2b	_DNYEGYAPSDGYYR_.2	#N/A	8.4398	7.6905	7.9626	7.7462	7.8181	8.0359	7.6999	7.8665	7.9591	7.9107	8.0385	7.7108	7.5794	7.4508	7.2290	7.4978	8.1266	7.9043	7.7271	7.5018
Sv2b	_YFQDEEYKS[Phospho (STY)]K_.2	Q8BG39_S425_M1_Sv2b	6.8261	6.9263	6.6097	6.3625	6.2871	6.8901	6.4655	6.1383	6.3449	6.6350	6.3104	6.1447	6.6820	6.3316	5.1486	6.2357	6.5720	6.9469	6.3183	6.6612
Sv2b	_YFQDEEYK_.2	#N/A	6.5120	6.5339	6.3957	6.5380	5.6922	6.9140	6.8159	5.8520	6.2474	6.6793	6.7836	6.2827	6.0307	5.8875	5.9857	5.7857	6.1786	6.6459	6.2064	6.0561
Sv2b	_HVLFEDTFFDK_.2	#N/A	5.9378	5.3664	4.5113	5.7085	4.3166	4.9441	4.4483	5.6808	4.8976	5.1129	4.9977	5.2680	5.0131	4.5129	4.4992	5.0228	4.5035	5.3191	5.3396	4.8786
Sv2b	_QVHDTNMR_.2	#N/A	4.3717	4.4988	5.9740	5.5100	3.7095	4.2547	5.0622	4.0721	3.8907	4.5781	4.8416	3.5205	3.9987	4.5782	4.9463	3.2158	3.1090	3.0535	3.7577	0.9390
Sv2b	_DMC[Carbamidomethyl (C)]LSS[Phospho (STY)]SKK_.2	Q8BG39_S144_M1_Sv2b	4.1795	5.1668	3.2149	3.5099	3.5172	3.7413	4.0239	4.3858	4.0830	4.3858	2.9565	3.7128	4.1910	3.7348	3.6011	3.0235	4.4175	3.2664	3.5654	3.4121
Sv2b	_ADGLGGQADLMAER_.2	#N/A	3.2160	4.1030	5.6659	6.0504	3.4230	3.1845	5.2778	3.4417	3.1389	3.8291	3.5132	3.8292	3.2469	4.2915	3.6505	4.0790	4.4398	4.2105	3.0087	3.9688
Sv2b	_SNEQNQEEDAQS[Phospho (STY)]DVT[Phospho (STY)]EGHDEEDEIYEGEYQGIPHPDDVK_.5	Q8BG39_S33_M2_Sv2b	3.9016	4.3819	3.9746	3.1462	3.2393	3.4634	4.3018	3.1629	4.3609	4.1079	3.2344	4.1080	4.4689	4.0127	3.8790	4.2465	4.1609	9.0191	3.2875	3.6900
Tmem169	yl (Protein N-term)]M[Oxidation (M)]EESAPVESQGQLPS[Phospho (STY)]PHHGS[Phospho (STY)	#N/A	7.2240	7.6598	5.8963	6.6655	5.3160	5.3629	6.9712	6.6219	5.7981	6.1643	5.9077	6.3469	6.2115	7.0898	4.4286	5.8992	6.2220	6.6286	5.8534	5.5160
Tmem169	_KDS[Phospho (STY)]RPESIIIYR_.3	Q8BG50_S47_M1_Tmem169	4.5506	4.1465	4.3926	3.8330	3.2567	3.2900	5.6846	3.5567	3.8529	5.7533	3.1903	4.8134	3.8771	4.3797	3.8168	5.1521	4.0944	5.7088	5.5729	4.3467
Tmem169	_[Acetyl (Protein N-term)]MEESAPVESQGQLPS[Phospho (STY)]PHHGS[Phospho (STY)]LR_.3	#N/A	4.1369	3.8659	3.1724	3.5525	3.4746	3.0095	4.4091	3.8373	1.5130	4.3432	2.9991	3.7554	4.1576	3.1594	3.6437	2.9810	4.3749	3.4582	3.1068	3.1264
Efr3a	_NLYFLT[Phospho (STY)]NK_.2	Q8BG67_T660_M1_Efr3a	13.3780	4.2603	7.4158	3.9469	6.9663	6.8731	6.7439	6.8577	6.4623	7.0857	7.4350	6.7605	7.6971	13.8158	6.7258	7.1356	12.9758	12.7633	7.3142	7.7169
Efr3a	_LGPPSS[Phospho (STY)]PSAADKEENPAVLAESC[Carbamidomethyl (C)]FR_.3	Q8BG67_S220_M1_Efr3a	4.3609	5.5474	3.5817	3.3487	3.6986	3.9227	3.8532	4.2593	3.9123	3.9432	3.6626	3.1940	4.0203	3.8173	3.4304	3.2049	3.5567	3.4478	3.7469	3.2414
Sh3bgrl2	_FLEANKIEFEEVDITM[Oxidation (M)]SEEQR_.3	#N/A	7.2717	6.9054	7.6216	7.6244	6.4708	6.7977	6.1480	7.4236	6.5356	6.9950	6.3064	6.4492	7.6480	7.6261	7.4307	7.3698	7.4686	6.9088	6.6213	7.1505
Znf365	_AIHEPAES[Phospho (STY)]PR_.2	Q8BG89_S370_M1_Znf365	8.7216	8.8112	8.8373	8.8441	8.2545	8.6699	8.4517	8.3316	8.3065	8.6532	8.4397	8.3018	8.0556	8.8574	7.7014	7.9937	8.6312	8.3734	8.3479	8.4140
Znf365	_ADSLDAPC[Carbamidomethyl (C)]AS[Phospho (STY)]PGLPTQDTK_.2	Q8BG89_S146_M1_Znf365	8.1009	9.2673	9.3015	8.2640	7.0759	8.7955	8.4616	8.3333	8.7308	8.4571	8.2440	8.3712	8.8048	9.3945	8.6477	8.2557	9.1816	8.8043	8.9782	8.9521
Znf365	_MVEAVDRT[Phospho (STY)]IEKR_.3	#N/A	3.6890	5.3311	6.2711	6.6535	2.7706	4.7775	5.0219	4.4183	4.2031	3.4996	5.5726	3.3562	3.2378	5.8094	4.2576	3.0511	3.4552	4.3780	4.9636	3.7075
Znf365	_MVEAVDRT[Phospho (STY)]IEK_.2	#N/A	3.3027	4.1135	2.6807	3.7935	4.1412	2.5498	4.1384	4.5039	3.4751	3.0394	3.2887	3.0887	3.3234	4.0907	2.9770	3.6476	3.5407	4.2314	3.9410	3.7930
Clvs2	_DVDKELS[Phospho (STY)]PK_.2	Q8BG92_S293_M1_Clvs2	8.7509	8.5381	8.5280	7.8513	8.0230	8.6693	8.5133	8.3072	8.2223	8.6551	8.3687	8.0890	8.2831	8.5266	7.2992	7.9881	8.8757	8.4488	8.2236	7.9381
Clvs2	_SQS[Phospho (STY)]VVDPTALK_.2	#N/A	8.5646	8.2786	3.6479	8.3050	8.0477	7.7642	7.5531	4.3128	4.4677	8.8034	7.7361	3.2798	7.2588	7.7347	3.1681	6.9674	3.3497	7.5858	4.1321	7.7478
Clvs2	_SQS[Phospho (STY)]VVDPTALKR_.2	#N/A	9.1592	9.0398	8.9601	3.1497	8.2215	8.5125	8.2499	8.3634	3.7228	9.2630	8.1516	7.9213	8.2860	8.3301	6.1085	7.7918	7.6188	8.0565	8.4148	7.8762
Limd2	_SKS[Phospho (STY)]FSLR_.2	Q8BGB5_S30_M1_Limd2	11.4701	11.6100	11.6284	11.5826	11.1292	11.0854	10.8497	11.1401	10.8605	11.4392	10.8703	10.5077	11.4587	11.3209	11.0141	11.2228	10.9142	10.8202	10.9990	11.0671
Limd2	_[Acetyl (Protein N-term)]MFQAAGAAQATPSHEAK_.2	#N/A	3.1063	3.3957	3.6426	4.5831	3.9448	4.1689	3.5962	4.3075	3.6553	3.3126	4.0297	3.2851	3.7634	3.4935	3.1734	3.4512	3.3443	3.6940	3.9931	2.9844
Htatsf1	_VLDEEGS[Phospho (STY)]ER_.2	Q8BGC0_S613_M1_Htatsf1	9.4552	9.2270	9.5896	9.4573	8.8759	9.6248	9.2783	9.1546	9.2268	9.3485	9.3610	8.4512	8.6111	9.1779	7.9100	8.4284	9.0481	8.9164	9.2063	8.8742
Htatsf1	_[Acetyl (Protein N-term)]SGNNLSGNDEFDEQLR_.2	#N/A	6.3476	7.0120	5.8334	4.4928	5.9664	7.0144	6.7813	6.6442	6.3194	6.1715	5.7415	6.0543	6.8876	7.2654	3.7751	6.3599	4.0527	6.7676	6.4552	5.6955
Htatsf1	_LFDDS[Phospho (STY)]DER_.2	#N/A	7.5457	7.4349	7.2481	7.6454	4.2525	7.8104	7.5107	6.8907	7.6828	7.9730	7.7346	6.8377	7.1738	7.1975	6.3502	6.9414	7.2753	7.4324	4.3007	7.4937
Htatsf1	_AEEGGES[Phospho (STY)]EGDAS[Phospho (STY)]EKDAK_.2	Q8BGC0_S441_M2_Htatsf1	5.0781	4.8369	5.5803	4.7047	3.5792	5.5500	4.6318	4.8708	4.4416	4.4961	4.2672	4.3067	4.9737	5.1059	4.2822	3.7507	5.0385	4.4419	5.1298	4.7945
Htatsf1	_LFDDS[Phospho (STY)]DERGTVGNVK_.2	#N/A	3.5278	3.8580	4.2086	4.2473	3.5456	4.3315	3.9539	3.8974	4.0391	3.9020	3.5572	3.6813	3.6583	5.1680	3.6835	4.0406	4.0862	4.6366	4.0486	3.8241
Htatsf1	_VFDDDS[Phospho (STY)]DDIEEEEEADEK_.2	Q8BGC0_S645_M1_Htatsf1	4.2980	4.5219	3.8346	3.2862	3.0041	6.3487	4.0440	4.5237	3.9642	4.1086	3.4429	4.2481	4.1297	3.1315	4.0694	3.6601	4.0209	3.4862	3.1347	3.0985
Htatsf1	_QITAQAWDGTTDYQVEETSR_.2	#N/A	3.3323	3.8126	4.5439	4.0778	4.1709	4.3950	3.3702	3.7321	3.4293	3.5386	3.8037	3.5387	3.5373	4.5819	2.9474	3.6772	3.2294	3.9201	4.2191	4.2592
Creg2	_RES[Phospho (STY)]GS[Phospho (STY)]SEASPGPR_.2	Q8BGC9_S109_M2_Creg2	3.4475	4.5553	3.9838	4.2419	4.2860	3.0093	3.5475	3.1479	3.3141	3.6538	3.6885	2.9439	3.4682	3.9709	2.8322	3.7924	3.6855	3.2814	3.5274	3.9379
Coa6	_FEAGGFQSSQSTENS_.2	#N/A	6.3569	7.1014	7.3353	7.3983	6.3933	6.6134	6.2344	6.5847	6.8589	6.7608	6.7217	6.5770	7.0892	7.5222	7.0953	6.9799	7.2700	7.0898	7.0078	6.9710
Coa6	_EKFEAGGFQSSQSTENS_.2	#N/A	4.1537	3.8491	5.8211	5.9818	5.8453	3.7155	4.0497	3.8541	4.1088	4.3600	7.0230	4.1431	4.2168	5.8853	5.4349	2.9977	5.9816	3.4414	3.0900	3.1432
Eif4b	_SQSS[Phospho (STY)]DTEQPS[Phospho (STY)]PTSGGGK_.2	#N/A	12.2844	11.6025	12.4994	12.5785	12.2326	12.2108	11.3502	12.3209	12.2544	12.3870	12.0517	12.3982	12.6545	12.8502	12.3877	12.4698	12.4593	12.1410	12.1588	12.4519
Eif4b	_AFGS[Phospho (STY)]GYRR_.2	#N/A	7.3736	6.8837	7.0773	7.2206	7.1213	4.3733	6.8975	7.3924	6.1536	7.7350	3.8254	3.5170	7.5649	7.4589	2.9691	7.0907	7.0906	6.8234	6.7426	4.2809
Eif4b	_SWSS[Phospho (STY)]RDDYSR_.2	Q8BGD9_S312_M1_Eif4b	4.3967	3.6061	6.3257	3.2927	3.7345	3.9585	3.8066	4.0971	3.8657	3.1022	4.2401	3.4955	3.9738	3.4193	3.3838	3.2408	3.1339	3.1984	4.3478	6.1863
Eif4b	_TGS[Phospho (STY)]ESSQTGASATSGR_.2	#N/A	7.9914	7.8451	8.1473	7.8511	7.3683	7.2030	7.6111	7.3806	7.2524	8.3925	7.6999	7.4099	7.6092	8.2739	6.9605	7.5257	7.5375	7.4857	7.3789	7.7030
Eif4b	_ERHPS[Phospho (STY)]WR_.2	#N/A	6.8446	7.2956	6.5587	6.6140	6.4978	6.8211	6.3944	6.3992	4.3787	7.2487	6.5289	6.0365	6.7184	7.0036	3.9115	6.9329	6.6740	6.3718	6.2082	3.7078
Eif4b	_RAFGS[Phospho (STY)]GYR_.2	#N/A	4.3438	3.3232	3.3792	3.5884	4.5110	3.9056	3.8596	4.2215	6.3021	5.1498	4.2931	3.5485	4.0267	3.5705	3.4368	3.1878	3.7188	3.4307	3.7297	3.2478
Eif4b	_YAALS[Phospho (STY)]VDGEDEDEGDDC[Carbamidomethyl (C)]TE_.2	Q8BGD9_S597_M1_Eif4b	3.9547	9.0433	9.2932	8.4647	3.6833	8.3992	8.4084	3.9605	7.9323	8.4165	3.5372	4.2076	3.9534	10.0324	8.4061	8.8845	5.9941	8.8916	8.4949	8.9727
Eif4b	_ARPTT[Phospho (STY)]DSFDDYPPR_.2	Q8BGD9_T205_M1_Eif4b	3.8526	7.5137	8.6705	8.5804	3.2107	3.1429	3.5617	3.3341	3.4437	8.8919	7.6790	6.9658	3.7814	4.2271	3.9173	8.6275	4.1959	7.9291	8.0414	7.4984
Eif4b	_SRTGS[Phospho (STY)]ESSQTGASATSGR_.2	#N/A	3.9003	3.4188	3.4369	3.1827	4.1072	4.1405	4.4417	4.2070	2.9425	3.7109	3.8406	3.1449	4.5274	3.5292	2.9663	1.8426	3.2439	3.0884	4.2379	3.4962
Eif4b	_RGDDSFGDKYR_.3	#N/A	4.1413	3.6552	5.1309	4.8804	3.7237	3.9064	3.8695	4.0450	3.9179	3.9332	4.6130	4.6694	4.0259	4.9001	5.0547	3.9149	3.0818	3.4315	3.7306	4.6374
Eif4b	_ERHPS[Phospho (STY)]WRS[Phospho (STY)]EETQER_.3	Q8BGD9_S406_M2_Eif4b	3.6182	3.9563	4.4341	4.2214	3.5606	4.0960	4.0874	3.6224	3.2610	3.6175	4.2821	3.5533	3.9992	4.0712	3.5949	4.3103	3.6912	4.0637	3.9202	4.1495
Eif4b	_SAPKEDDASASTSQSSR_.2	#N/A	4.8003	3.7155	3.1402	3.9807	3.9458	3.6524	3.2376	3.8293	3.5204	3.5250	2.4725	4.8191	4.7778	4.3273	3.2630	2.9657	3.3265	3.8230	4.1220	4.3563
Atg4b	_FFDS[Phospho (STY)]EDEDFEILSL_.2	Q8BGE6_S383_M1_Atg4b	3.5870	5.3453	6.8689	5.6508	3.7940	6.6651	7.4709	3.8127	7.2942	5.9836	4.1538	3.4582	6.1271	4.9494	6.9025	6.6315	4.9733	6.5163	6.8491	6.1950
Pan2	_VPEPESQTS[Phospho (STY)]PK_.2	Q8BGF7_S1188_M1_Pan2	7.4528	4.1257	7.1338	4.0831	3.3643	3.5921	3.2277	3.2618	7.0502	7.1704	6.8410	3.9490	7.3061	6.9629	3.8115	4.1668	7.3325	7.0944	3.6894	4.0518
Slc35d3	_DYLM[Oxidation (M)]ENEALPS[Phospho (STY)]P_.2	#N/A	9.0129	10.1053	9.5938	8.1320	6.7873	9.9528	9.6223	7.5855	10.2410	9.1614	8.2955	8.8739	9.3995	8.5398	8.3871	9.3257	9.1133	9.9716	9.4872	9.4978
Slc35d3	_KDYLMENEALPS[Phospho (STY)]P_.2	#N/A	3.3973	3.8776	9.2619	8.3951	4.2358	8.0037	8.6732	3.6672	3.3643	3.6036	3.7387	3.6037	3.4724	8.9618	6.4949	3.7422	3.1644	6.1021	4.2841	6.2608
Samm50	_FYLGGPTSVR_.2	#N/A	6.9182	6.4108	6.3583	6.9003	7.0006	6.4713	7.1804	6.7533	6.5302	6.7001	7.0508	6.9798	6.9714	6.7584	6.7519	6.6365	6.9432	7.1401	6.4378	6.8763
Samm50	_LELNYC[Carbamidomethyl (C)]IPM[Oxidation (M)]GVQGGDR_.2	#N/A	3.4825	3.8366	4.5199	4.1018	5.3940	5.0867	5.7874	5.7235	5.6201	5.7892	5.3484	5.2050	3.4443	4.5579	4.8931	4.3454	3.2054	3.9441	5.7122	5.2386
Samm50	_THFFLNAGNLC[Carbamidomethyl (C)]NLNYGEGPK_.3	#N/A	4.4966	4.3233	4.0332	4.5885	3.2027	5.2590	3.8454	5.6963	4.4194	4.0494	3.2929	4.0495	4.5275	4.0712	3.8708	3.8587	4.2195	4.6945	4.9896	3.8370
Samm50	_DVVVQHVHFDGLGR_.3	#N/A	3.4677	3.8514	4.5051	4.1165	3.6746	3.0591	3.3735	3.6934	3.2937	3.2783	4.2732	3.5775	3.4591	4.5432	3.3988	4.3306	3.1906	3.9588	4.2579	4.2205
Samm50	_SSLSHAM[Oxidation (M)]VIDSR_.2	#N/A	3.4823	3.8368	4.5197	4.2656	3.6892	3.7226	3.3589	3.1242	3.2791	3.2929	5.2592	3.5629	3.4445	3.9472	3.3842	4.3453	3.6619	4.1713	5.2192	3.9142
Reep1	_SFS[Phospho (STY)]MQDLTTIR_.2	Q8BGH4_S152_M1_Reep1	4.2618	5.0591	6.0887	3.4276	3.5995	3.8236	3.9415	3.9622	4.0006	4.4681	4.3750	3.6304	4.1087	5.2415	3.5187	3.1059	4.4999	3.3487	2.9819	3.3297
Slc7a6	_[Acetyl (Protein N-term)]M[Oxidation (M)]EAQELGS[Phospho (STY)]PTPTYHLLPK_.2	#N/A	4.3375	4.9307	3.2235	3.7061	3.6752	3.8993	3.9042	3.9014	3.9633	3.8641	3.6579	3.5931	4.0714	4.1134	3.4814	4.0026	3.9708	3.5784	3.6027	3.0547
Slc7a6	_EDAGS[Phospho (STY)]PSQGSPETMQLK_.2	Q8BGK6_S30_M1_Slc7a6	4.3476	3.6552	3.3831	5.4479	3.6853	5.2819	3.8557	4.0480	3.9148	4.5539	4.2892	3.5446	4.0229	3.5667	3.4329	3.1917	3.0848	3.4345	3.7336	3.2439
Enpp6	_VDYLTPDFPSLS[Phospho (STY)]YPNYYTLM[Oxidation (M)]TGR_.3	Q8BGN3_S71_M1_Enpp6	3.8610	3.4581	3.3975	3.7233	4.0679	4.0405	3.7247	4.0866	3.7838	3.1841	4.1582	3.1842	3.8918	3.4356	3.0056	3.2231	3.5839	3.5656	3.8646	3.1129
Vipas39	_AFT[Phospho (STY)]FDDEDDELSQLK_.2	Q8BGQ1_T21_M1_Vipas39	4.3146	3.6882	5.7143	3.3747	3.6524	4.3326	4.4709	4.0150	4.1699	3.9029	3.6486	3.5776	4.3354	3.3372	4.2751	3.1587	4.5527	3.2805	4.4299	3.3042
Vipas39	_ETAGS[Phospho (STY)]SGSTPEGR_.2	Q8BGQ1_S77_M1_Vipas39	3.5102	4.4926	4.0465	4.1792	4.3487	3.0720	3.1923	3.2106	3.2514	3.7165	3.6258	4.3821	3.3595	4.0336	4.2704	3.8551	3.7482	4.0849	3.7335	4.0006
Vipas39	_TRPGS[Phospho (STY)]FQSLSDALSDTPAK_.3	Q8BGQ1_S119_M1_Vipas39	4.5325	3.8303	4.0563	3.5169	3.5900	3.7426	3.7422	3.9472	3.7299	3.8916	3.7230	3.7198	3.8670	3.9796	3.5724	3.6576	3.7580	3.6707	3.6710	3.8576
Aars1	_IVAVTGAEAQK_.2	#N/A	4.0761	4.9218	5.7343	5.8925	4.9966	5.2944	6.0052	4.0623	5.0989	5.4707	3.4101	5.0106	5.3577	4.3618	5.3220	4.3562	4.3142	5.7279	5.3351	5.1406
Aars1	_ITC[Carbamidomethyl (C)]LC[Carbamidomethyl (C)]QVPQNAANR_.2	#N/A	4.4694	3.5334	7.1571	7.2014	3.8071	6.8342	6.6027	7.0500	6.6066	6.6002	6.4138	3.4228	4.4901	3.4919	6.9432	6.3113	6.7520	6.5205	4.2751	6.8199
Aars1	_QIWQNLGLDEAR_.2	#N/A	6.0823	5.7945	5.5706	5.5751	5.6457	6.2066	3.5853	3.4816	3.1787	3.7892	5.9978	5.8824	5.9123	6.2591	5.1952	5.6980	4.4796	5.7613	5.4895	6.2831
Aars1	_M[Oxidation (M)]ALELLTQEFGIPVER_.2	#N/A	6.2159	3.6535	3.3848	3.3401	3.6870	6.1115	5.4272	5.8121	6.0190	5.8468	4.2875	4.5052	4.0212	3.3718	3.4312	6.0791	3.0865	5.9788	6.1043	3.2422
Aars1	_LYVTYFGGDEAAGLEPDLEC[Carbamidomethyl (C)]R_.2	#N/A	4.1330	4.1473	3.6696	3.4512	4.3399	4.0530	4.2090	4.3587	4.1756	3.9436	3.6079	2.7236	4.2947	3.2965	3.4730	4.3471	3.8559	3.3212	2.9697	3.3110
Aars1	_MFVDEVVTGQEC[Carbamidomethyl (C)]GVVLDK_.2	#N/A	3.1435	3.3584	4.4151	4.9780	3.9821	4.0028	3.0786	4.3447	1.6003	3.5732	3.9783	3.2479	3.1642	4.3414	3.1040	3.4884	3.3816	4.4516	4.1002	3.6339
Fam53b	_SSS[Phospho (STY)]FSLPAR_.2	Q8BGR5_S168_M1_Fam53b	8.5509	3.6077	3.4306	3.6398	3.7329	3.9570	7.7704	4.1702	3.8673	8.7201	8.2077	3.4971	3.9753	8.6106	7.8026	8.4128	3.6674	7.8082	3.7811	3.1964
Fam53b	_S[Phospho (STY)]RSQPC[Carbamidomethyl (C)]VLNDKK_.3	Q8BGR5_S267_M1_Fam53b	4.4839	3.5189	3.5194	3.2055	3.8217	5.5420	5.5324	4.1843	3.7785	7.2264	5.4761	6.2778	3.8866	6.3830	3.2966	3.3280	3.2212	5.4139	4.2606	3.4735
Zkscan1	_ETTGLS[Phospho (STY)]PQAAQEK_.2	Q8BGS3_S13_M1_Zkscan1	3.6807	4.3221	4.2170	4.0087	3.0184	7.4862	3.0218	3.3810	3.0809	3.8870	3.4553	4.2116	3.1890	4.2336	4.0999	4.0256	3.9187	4.2684	3.0666	3.9109
Micall1	_KPS[Phospho (STY)]PSASPTVR_.2	Q8BGT6_S647_M1_Micall1	7.1091	7.6642	3.9631	7.7341	6.2555	5.9121	5.8682	3.9123	4.0672	6.3469	6.1450	7.6868	3.4489	3.9515	6.8795	7.8852	5.5878	6.3821	6.7133	7.7602
Micall1	_SLHPWYGITPT[Phospho (STY)]SSPK_.2	Q8BGT6_T479_M1_Micall1	3.8754	3.5823	3.3670	3.5853	4.0823	4.1156	3.8532	4.0434	3.7734	3.6860	3.8763	3.3929	4.1415	3.3784	3.0019	3.2375	5.0567	3.4536	4.1671	3.2135
Micall1	_APS[Phospho (STY)]AS[Phospho (STY)]PLAIHASR_.2	Q8BGT6_S494_M2_Micall1	4.4324	6.9341	3.4679	3.6770	3.7702	3.9942	3.7709	4.1329	3.8300	8.0982	4.2044	3.4598	3.9381	3.4818	3.3481	3.2765	3.6301	3.5193	3.8184	3.1591
Micall1	_DLS[Phospho (STY)]SS[Phospho (STY)]FR_.2	Q8BGT6_S28_M2_Micall1	3.7739	4.2289	4.3102	3.9155	3.1116	3.3357	4.4295	3.4743	2.9877	3.9802	3.3621	4.1184	4.5966	4.2973	4.0067	4.1188	4.0120	3.8212	3.4698	4.2643
Phyhipl	_VHLTQLLEK_.2	#N/A	6.4803	6.5445	5.9228	7.0169	6.0886	6.3261	6.2424	6.3488	2.2421	6.1862	5.9929	6.9255	5.4198	6.5214	5.3179	6.6331	5.8390	7.0969	6.3566	6.7853
Phyhipl	_TC[Carbamidomethyl (C)]NIS[Phospho (STY)]VGR_.2	Q8BGT8_S372_M1_Phyhipl	5.9790	5.9601	5.9929	6.4973	6.6133	6.9688	6.5430	6.3565	7.0844	6.2631	6.8928	6.8975	6.9866	5.9677	7.1442	6.7309	6.1263	7.0187	6.2101	6.9997
Phyhipl	_SQDSGIAEM[Oxidation (M)]EELPVPHNIK_.3	#N/A	6.0938	6.1063	5.3309	6.4031	6.5833	6.2880	6.0097	6.6693	5.6715	6.2747	5.8795	6.2958	6.0982	4.9383	6.1935	6.4605	6.5169	5.9280	6.0154	6.0427
Phyhipl	_NLSLEAIQLC[Carbamidomethyl (C)]DR_.2	#N/A	4.5372	7.6122	8.0873	7.5463	4.1802	7.2792	7.3568	3.8876	7.3710	7.4319	4.3031	4.3422	5.9984	7.7301	6.3819	4.1864	7.2787	7.1954	7.2509	6.9801
Phyhipl	_LDHALSSPSS[Phospho (STY)]PC[Carbamidomethyl (C)]EEIK_.2	#N/A	4.1530	8.1147	7.8514	3.3281	3.4861	7.4347	7.5806	4.1781	7.3122	8.2019	7.3540	7.2894	4.3104	7.8062	4.5256	7.1224	7.6835	7.1927	3.7210	7.4436
Phyhipl	_GHWFLSPR_.2	#N/A	7.1533	8.0763	7.3732	7.1641	7.3351	7.0761	7.2094	7.3619	6.7879	6.9464	6.7117	6.9893	7.0405	8.2978	6.5914	6.6623	7.1208	7.1390	6.9862	7.0085
Phyhipl	_TEYTVAVQTASK_.2	#N/A	3.7649	4.2379	5.1524	4.8267	3.1026	3.3815	4.4385	3.4653	4.1081	4.4527	3.0988	4.1274	3.7856	4.2883	4.0157	5.5316	4.0029	5.0598	3.6327	4.2552
Phyhipl	_ISWDMDSK_.2	#N/A	3.4654	3.9457	4.5723	3.8557	4.3040	3.0272	3.2913	3.5990	3.2962	3.6717	3.6706	3.6718	3.4042	2.9777	2.1214	3.8103	3.0963	4.0532	4.3522	4.1261
Phyhipl	_DNSGSHGSPISGK_.2	#N/A	4.3739	3.6289	3.4094	3.3155	3.7116	4.2733	3.8294	4.0743	4.2292	4.6610	3.7078	3.5183	4.3946	3.3964	3.8964	3.2180	3.1111	3.2212	4.3706	3.3634
Phyhipl	_FEIAAEK_.2	#N/A	3.9062	4.0966	4.4425	3.7832	3.2439	3.4680	4.2972	3.6065	4.3563	4.1125	3.2298	3.9861	3.9269	4.4295	3.8744	4.2511	4.1442	3.6889	4.0563	4.1859
Phyhipl	_NLS[Phospho (STY)]LEAIQLC[Carbamidomethyl (C)]DRDGNK_.3	#N/A	3.2755	3.2265	3.8118	4.4139	4.1141	4.3382	3.4270	4.4767	3.4861	3.4818	3.8605	3.1159	3.5941	3.7989	3.0042	3.6204	3.5136	4.3196	3.9682	3.7659
Ccny	_SAS[Phospho (STY)]ADNLILPR_.2	#N/A	15.2776	15.2204	14.8944	14.8943	11.5305	14.9418	14.8060	14.9278	14.8800	14.8640	14.8977	14.6204	15.4578	14.8661	13.5612	14.5353	15.3134	14.9370	14.7735	14.6165
Ccny	_ASTIFLSKSQT[Phospho (STY)]DVR_.2	Q8BGU5_T75_M1_Ccny	4.2531	4.5669	3.7897	3.3312	3.4784	2.8000	4.1168	4.4788	4.1759	4.2929	3.0494	4.2930	4.2839	3.8277	3.6940	3.6152	3.9760	3.1735	3.4725	3.5050
Ccny	_LESYRPDTDLS[Phospho (STY)]R_.2	#N/A	5.7932	5.2964	3.7814	5.5419	3.2971	5.8971	3.5933	3.6598	5.5520	5.3329	5.8902	3.9965	3.7604	3.9332	3.8848	4.9805	6.0059	5.0475	5.4500	5.3166
Ccny	_RSAS[Phospho (STY)]ADNLILPR_.2	#N/A	8.0543	8.4051	7.7013	7.5411	3.9893	7.7404	3.0588	7.7340	2.9790	3.5929	7.6103	3.2629	8.6533	3.6471	3.0842	3.1445	8.4244	8.0938	7.6688	7.3468
Ccny	_LESYRPDTDLS[Phospho (STY)]REDTGC[Carbamidomethyl (C)]NLQHISDR_.4	#N/A	3.5901	4.4127	4.8598	4.0993	2.9278	3.1519	3.1124	3.4744	3.1715	3.7964	3.5459	4.3022	3.2796	4.3242	4.1905	3.9350	4.4724	4.1778	2.9761	4.0014
Mief1	_AISAPTS[Phospho (STY)]PTR_.2	Q8BGV8_S59_M1_Mief1	11.3300	11.2803	10.9268	10.9924	8.3215	8.4754	8.4012	11.2304	8.4468	8.9427	10.9654	11.0333	11.4885	3.9511	10.2596	8.3232	8.5924	8.2891	11.0929	10.9660
Wbp1l	_GGVS[Phospho (STY)]PDSEDKTPGR_.2	Q8BGW2_S238_M1_Wbp1l	4.9200	4.1378	4.2187	3.9645	3.3882	3.4215	3.6599	3.4252	3.5801	4.4927	3.0588	3.8640	3.7456	4.2482	3.6853	4.0442	3.9629	3.8703	3.5188	4.2152
Wbp1l	_SSTRPPSVADPQS[Phospho (STY)]PEVPTDR_.3	Q8BGW2_S183_M1_Wbp1l	5.5262	5.7450	4.5690	3.2773	3.3704	5.2758	4.1707	4.5326	4.2298	4.2390	4.6754	3.8596	4.3378	5.3166	4.5470	4.3776	4.0299	5.0098	3.4186	3.5589
Klhl26	_[Acetyl (Protein N-term)]AESGGSSGSSQS[Phospho (STY)]PERPSSLADR_.3	Q8BGY4_S13_M1_Klhl26	4.0455	6.1366	3.9090	3.9187	5.6996	6.4176	5.9832	5.8959	5.4990	3.8211	6.2938	5.5536	6.7873	6.4582	5.4180	5.9311	6.4794	6.4254	5.9227	5.5734
Slc5a7	_QS[Phospho (STY)]LTLSSTFTNK_.2	Q8BGY9_S550_M1_Slc5a7	6.9306	3.4046	3.6337	7.0539	3.9360	6.8471	7.0148	6.9096	6.8220	6.3879	4.0386	3.2940	3.7723	7.7457	5.4089	3.4423	6.9008	7.6069	6.6757	6.3403
Hpcal4	_EFIC[Carbamidomethyl (C)]ALSVTSR_.2	#N/A	6.6672	6.3486	7.3917	6.5559	6.1274	6.7126	6.2004	6.4579	6.4060	6.7583	6.2570	5.9809	6.3533	6.5689	6.2783	6.0579	6.3495	5.2651	6.5895	5.5464
Hpcal4	_SDPSIVLLLQC[Carbamidomethyl (C)]DM[Oxidation (M)]QK_.2	#N/A	5.5666	6.1931	5.8191	5.9875	5.7672	6.0381	5.9981	5.7203	5.9122	5.8319	5.3253	5.8165	6.3188	5.6701	5.3858	6.1876	5.4955	6.0083	6.0088	5.4856
Hpcal4	_LNWAFEM[Oxidation (M)]YDLDGDGR_.2	#N/A	5.6554	5.9899	5.6488	4.0822	5.2776	5.9367	6.4987	5.1682	6.3401	6.1145	5.9615	6.2264	5.8824	5.3318	5.2614	5.4866	5.7162	6.0868	6.0505	5.5291
Hpcal4	_M[Oxidation (M)]NQDGLTPQQR_.2	#N/A	7.6400	7.4283	7.7045	7.8961	7.6339	7.8665	7.7238	7.7995	7.7213	7.9529	7.4780	7.5249	7.3961	7.4014	7.6598	7.3004	7.3912	7.4470	7.2824	7.4789
Hpcal4	_LAPEVLEDLVQNTEFSEQELK_.3	#N/A	4.0282	4.8667	4.6915	4.3942	4.2351	4.2718	4.4709	4.1928	3.7969	4.3692	3.7127	3.4963	4.4757	3.6028	4.1679	4.9196	3.7063	3.6317	3.6974	4.5453
Hpcal4	_LEM[Oxidation (M)]LEIIEAIYK_.2	#N/A	4.1688	4.3250	4.5631	4.9166	3.3645	5.7950	4.6782	5.0327	3.8820	4.6527	4.8174	3.8655	4.0475	4.7078	4.7734	4.3717	4.2648	3.5683	6.8845	3.0163
Hpcal4	_MDQDKDDQITLEEFK_.3	#N/A	4.0800	3.2391	3.6166	3.3625	4.2870	4.3203	4.2620	4.0273	4.1822	3.8906	3.6608	2.9652	4.3476	3.3494	2.7865	3.4421	3.0641	3.2682	4.4176	3.3164
Hpcal4	_M[Oxidation (M)]DQDKDDQITLEEFKEAAK_.4	#N/A	4.4199	4.0157	4.5234	3.7023	3.1260	3.1593	3.9221	3.6874	3.8423	4.2305	3.3210	3.9052	4.0078	4.5104	3.9475	3.7820	4.2251	3.6080	3.2566	2.9766
Fam168a	_[Acetyl (Protein N-term)]M[Oxidation (M)]NPVYS[Phospho (STY)]PVQPGAPYGNPK_.2	#N/A	3.9563	4.7213	4.0950	3.9908	3.6079	3.6413	6.1620	6.9794	3.9589	4.2301	4.4054	6.6249	3.5982	5.6470	6.3572	3.7816	4.0984	6.5727	3.4697	3.9148
Pck2	_YVAAAFPS[Phospho (STY)]AC[Carbamidomethyl (C)]GK_.2	Q8BH04_S304_M1_Pck2	8.9531	9.1614	9.2580	9.5344	8.6534	9.1378	8.7610	8.9642	8.9505	9.1576	9.1080	8.8663	9.4001	9.3442	8.4848	9.0852	9.4213	8.7432	9.1149	9.0823
Mfsd8	_[Acetyl (Protein N-term)]ANLGSEAEREPLLGPGSPGS[Phospho (STY)]R_.3	Q8BH31_S21_M1_Mfsd8	3.6358	4.3670	4.1721	4.0536	2.9735	3.1976	3.0667	3.3362	3.1258	8.9185	3.5002	4.2565	3.2339	4.1592	4.1448	3.9807	3.8738	3.9593	3.6079	8.6641
Ubap1	_VLS[Phospho (STY)]PPHTK_.2	Q8BH48_S146_M1_Ubap1	7.7085	8.3127	4.1471	7.7874	2.9485	7.7656	7.2932	3.3112	7.7292	3.8171	7.9471	8.0175	7.7168	7.8803	7.7832	7.5607	3.8489	8.2388	7.7866	4.1012
	RDS[Phospho (STY)]SESQLASTESDKPTTGR.3	Q8BH50_S66_M1_	4.0636	4.0042	4.0196	3.6928	3.1891	4.6068	3.9504	4.5425	3.8706	4.1017	4.0639	4.0902	4.0360	3.8326	3.8224	4.1941	4.0958	3.6546	3.4998	3.9399
	DSSESQLASTES[Phospho (STY)]DKPTTGR.2	Q8BH50_S76_M1_	4.5559	4.2648	3.5914	3.1335	3.8937	4.1177	3.6474	4.2563	3.7065	3.2614	4.0809	3.3363	3.8146	3.3583	3.2246	3.4000	3.2931	3.6428	3.9419	3.0356
	S[Phospho (STY)]RSESETSTMAAK.2	Q8BH50_S143_M1_	3.5484	3.6863	4.1303	4.2877	3.9723	4.0839	4.2482	3.8544	3.2253	3.4876	4.0076	3.2445	3.3343	4.2349	3.1384	3.8370	3.4514	4.9508	4.0311	3.9797
Wdr48	_DTNNNVAYWDVLK_.2	#N/A	5.8844	5.8580	6.5779	6.0750	5.1267	6.4027	6.3118	6.1764	6.3152	6.1655	6.0109	6.0770	6.2164	6.5897	5.6356	6.1307	6.1808	5.8679	6.2407	5.7464
Tiprl	_IDPNPVDSQST[Phospho (STY)]PSE_.2	Q8BH58_T268_M1_Tiprl	8.3864	7.6360	8.3290	8.2712	5.7778	7.9213	7.3728	3.7442	8.0574	8.4060	7.9955	7.6086	3.8070	7.8328	3.5293	8.0084	8.2596	7.3824	7.8016	8.0187
Slc25a12	_LTVNDFVR_.2	#N/A	8.6126	8.2962	8.1850	8.4656	8.5242	8.4979	8.5074	8.3859	7.9812	8.2884	8.3470	8.4431	8.3813	8.1096	7.8756	7.9687	8.2013	8.4871	8.3703	8.4207
Slc25a12	_LLLADENGR_.2	#N/A	8.4438	8.5065	8.1930	7.9946	8.2541	8.0442	8.5600	8.3307	8.2203	8.2593	8.4113	8.0469	8.3195	7.7360	7.7740	7.9045	7.8086	8.2628	8.2682	8.0726
Slc25a12	_YLGLYNDPNSNPK_.2	#N/A	8.0874	8.0851	7.3958	7.7720	7.7888	7.9907	8.0753	8.2397	7.6054	7.5207	7.9080	7.5991	7.8543	7.2641	7.3543	7.7013	7.6584	7.9100	7.8099	7.5160
Slc25a12	_LTLADIER_.2	#N/A	7.2838	7.4879	7.6112	7.8635	7.7602	7.6349	8.0623	7.5823	7.8697	8.0934	8.1025	7.8658	7.6803	7.1300	6.9849	7.8626	7.4672	7.9314	8.1366	7.7301
Slc25a12	_IAPLAEGALPYNLAELQR_.2	#N/A	6.6465	6.8807	5.9224	6.5613	6.4766	6.4902	6.9690	6.9880	6.0727	6.2798	6.5528	6.4525	6.3630	6.3105	6.0750	6.1808	6.5836	6.7955	6.7121	6.3808
Slc25a12	_IVQLLAGVADQTK_.2	#N/A	7.3221	7.4649	6.9054	7.2690	6.8884	7.1464	7.4047	7.2334	7.3882	6.9819	7.2406	6.9888	7.1230	7.1103	6.4357	7.1959	7.2943	7.5014	7.0596	7.2727
Slc25a12	_LATATFAGIENK_.2	#N/A	7.1677	7.4267	7.5821	7.1006	6.4861	7.3142	7.5237	6.8901	7.0451	6.6638	6.9193	6.8372	6.6598	7.4072	6.6983	6.7971	7.0524	7.1281	6.7351	6.6570
Slc25a12	_YEGFFGLYR_.2	#N/A	7.0651	7.7157	6.4060	7.0123	6.8860	7.0605	7.2411	7.3562	7.0707	7.0195	7.1195	7.2340	6.6008	6.8660	6.1547	6.7788	7.0614	7.0229	6.9848	7.0222
Slc25a12	_SGNGEVTFENVK_.2	#N/A	6.8030	6.9328	7.1673	6.7158	6.9981	6.3376	6.7970	7.3201	6.7627	7.0299	7.2451	6.5879	7.4753	7.1730	6.4624	6.5615	6.5933	7.0771	6.4368	6.4329
Slc25a12	_IYSTLAGTR_.2	#N/A	5.8603	5.9817	6.1658	4.8556	5.3750	5.9547	6.0897	5.5869	6.0343	4.5974	5.7926	5.4272	5.5767	5.6698	6.0732	5.0349	5.3201	5.9502	5.9287	5.3963
Slc25a12	_VGGINLLTAGALAGVPAASLVTPADVIK_.3	#N/A	4.3705	4.3488	3.3681	5.0333	4.6947	3.6382	3.9852	2.9428	3.9442	4.8129	4.7002	4.4447	3.8125	3.5682	3.7044	3.3595	3.0124	4.2739	5.0378	4.2618
Slc25a12	_DIEVTKEEFAQSAIR_.3	#N/A	5.1395	5.2153	6.3204	6.1637	5.0135	4.8450	5.1507	5.2083	5.4302	5.7542	5.2725	5.0572	6.0425	5.5745	5.2969	5.2518	4.8898	5.1357	5.5634	5.9845
Slc25a12	_LQVAGEITTGPR_.2	#N/A	5.6795	6.3248	5.6121	5.8363	5.6497	5.8225	6.1607	5.9721	6.0540	5.5488	6.5805	5.3744	4.5992	5.8182	5.4491	4.2358	6.1740	5.9906	5.9378	5.6394
Slc25a12	_EEGPSAFWK_.2	#N/A	5.9430	5.5140	5.8165	5.6750	5.2703	6.0954	5.8712	5.2295	5.4385	4.9948	5.2892	5.6677	5.8662	6.1382	5.8444	5.2551	5.4480	3.9389	5.8258	5.9529
Slc25a12	_AGQTTYSGVVDC[Carbamidomethyl (C)]FR_.2	#N/A	5.4455	5.9284	5.0266	5.3903	5.1539	5.5615	5.6558	5.6796	5.7005	5.6072	6.0728	5.5674	3.4747	6.0670	5.3540	5.3404	5.8140	5.4344	5.3733	5.5209
Slc25a12	_FTLGSVAGAVGATAVYPIDLVK_.2	#N/A	4.7544	4.9614	4.2701	3.9675	3.8737	4.0741	4.2322	4.1060	4.2764	4.3300	3.8502	4.4805	4.4955	4.3140	3.6065	4.1064	3.7949	4.0681	3.7156	3.6136
Slc25a12	_SSPQFGVTLVTYELLQR_.2	#N/A	4.5587	4.1059	3.5942	3.1307	3.9402	4.1971	3.7068	4.0117	3.4383	3.9890	4.0781	3.3336	3.8968	3.5812	2.9094	3.4028	3.2959	3.9793	4.4320	3.0329
Slc25a12	_NSFDC[Carbamidomethyl (C)]FK_.2	#N/A	3.3156	4.0035	4.3530	4.2687	3.5225	3.6180	3.5256	3.5412	2.9967	3.1262	3.5288	3.7297	3.6113	4.3910	3.5510	2.4692	4.5393	4.1110	4.4100	3.4230
Slc25a12	_NIFLQYASTEVDGEHYM[Oxidation (M)]TPEDFVQR_.3	#N/A	3.2289	3.2731	3.7652	3.9743	4.0674	4.2915	3.4736	3.8356	3.5327	3.4352	3.9071	3.1625	3.6408	3.1846	3.0508	3.5738	3.3328	3.8166	4.1157	4.3627
Atl1	_ILIPWLLSPER_.2	#N/A	5.8619	5.9715	5.5592	5.5750	5.3876	5.8908	5.9255	5.9023	5.3151	5.6887	5.2139	5.5450	3.4645	4.9276	4.8062	5.3384	5.2223	5.3604	5.0792	5.0248
Atl1	_SSDWSS[Phospho (STY)]EEEEPVR_.2	#N/A	7.7314	8.9467	8.8129	9.1941	5.7229	9.2387	9.1317	3.8079	8.9937	7.8957	6.6326	8.6673	3.7367	8.7311	7.4267	8.7905	9.1304	9.1077	8.7556	8.5477
Atl1	_SSDWSS[Phospho (STY)]EEEEPVRK_.2	#N/A	8.2822	7.8502	6.6997	7.7741	7.7376	7.6193	7.8476	8.1577	7.8127	7.4183	7.4617	7.5059	8.2757	7.7126	6.7637	7.3081	7.4194	7.9956	7.6544	7.2741
Atl1	_DDHSFELDEAALNR_.3	#N/A	3.8154	3.5036	3.3520	3.7688	4.0224	3.6276	1.4530	4.0411	4.4468	3.6260	3.9254	3.2298	3.1114	3.3901	3.0511	3.1776	3.5383	2.7248	1.8144	3.5810
Atl1	_DRNS[Phospho (STY)]WGGFSEK_.2	#N/A	8.0375	3.9992	3.8059	7.6195	3.6104	6.9423	7.4404	7.7683	7.3499	7.4483	3.6271	3.7791	8.0940	3.8205	3.5064	6.9361	3.7875	3.9040	6.9152	3.6368

Atl1	_VAVLLM[Oxidation (M)]DTQGTFDSQSTLR_.2	#N/A	2.3865	4.5280	4.0111	4.2146	4.3134	1.7291	3.2277	2.9262	3.3301	3.6811	2.4747	4.4174	3.4955	3.9982	2.8049	3.8197	2.3009	4.1203	3.7689	3.9652
Fbxl4	_TS[Phospho (STY)]PLNAEVVQYAK_.2	Q8BH70_S45_M1_Fbxl4	3.6995	3.9896	4.7162	3.9388	4.3131	3.6507	4.5794	3.6748	3.3923	3.7300	3.6875	3.8152	3.5578	4.0604	3.6785	3.6557	3.7140	4.0995	4.9256	4.0274
Nup107	_HPDISYILGTEGRS[Phospho (STY)]PR_.3	Q8BH74_S87_M1_Nup107	4.0059	6.8572	4.3360	3.2884	4.2128	4.2462	4.3361	5.7078	5.2437	3.8165	3.7349	3.0393	4.4218	6.0248	3.6718	3.3680	5.1010	6.3390	5.0107	3.3906
Napepld	_S[Phospho (STY)]FKLDYR_.2	Q8BH82_S43_M1_Napepld	4.8327	5.8638	3.9406	6.4804	3.2984	5.0427	4.2426	6.1915	5.7621	5.5187	5.0971	5.3229	4.4098	5.6367	5.7697	4.3056	5.6319	5.8379	6.1129	5.0455
Napepld	_QNS[Phospho (STY)]VQNSGGSVSSR_.2	Q8BH82_S27_M1_Napepld	3.6274	4.3754	4.1637	4.0620	2.9652	3.1892	3.0751	3.3278	3.1342	3.9698	3.5085	4.2648	3.2422	4.1508	4.1531	3.4745	3.8655	4.2152	3.6163	3.9641
Echs1	_AQFGQPEILLGTIPGAGGTQR_.2	#N/A	5.9736	6.1734	6.8871	6.6322	6.5816	6.4007	6.3930	6.5518	6.7148	6.8526	6.6549	6.5491	6.0748	6.8536	6.0047	6.8205	6.2605	6.4118	6.6203	6.6647
Echs1	_LVEEAIQC[Carbamidomethyl (C)]AEK_.2	#N/A	6.4662	6.4571	6.4882	6.7520	6.3135	6.5402	6.1380	3.6381	6.4661	5.9326	6.7955	3.6327	3.4433	4.4879	6.5232	6.1759	6.6787	6.2003	4.3131	6.6424
Echs1	_FLSHWDHITR_.3	#N/A	6.2779	3.8475	3.1908	3.5341	6.2842	3.7171	6.2344	6.0391	4.1071	4.3616	2.9807	3.7369	5.6796	5.8763	4.9390	5.9933	5.3812	6.0516	3.0884	4.9974
Echs1	_AFAAGADIK_.2	#N/A	4.1872	3.1318	3.7238	3.3970	2.8933	3.8301	4.0510	4.4129	3.7053	4.3587	2.9836	3.9141	4.2181	3.7619	4.2236	3.5494	3.9102	4.2762	4.0210	4.4608
Echs1	_LFYSTFATDDRR_.2	#N/A	3.9233	4.2721	3.8066	4.2646	3.1707	3.2619	3.9264	3.1478	3.0612	3.9064	3.4365	4.0857	3.4421	3.4312	4.0811	4.1623	4.2677	3.6408	3.3622	4.0194
Echs1	_EGMTAFVEK_.2	#N/A	4.2732	3.7296	3.3087	3.9095	3.6109	3.8350	3.9301	4.2921	3.9892	4.4795	4.3636	3.6190	4.0973	3.6410	3.5073	3.1173	4.8638	3.3601	3.6592	3.3183
Rab39b	_SGFVPNVVHSS[Phospho (STY)]EEVIK_.2	#N/A	7.3721	7.1120	3.8467	6.8333	3.6704	4.9139	6.7597	6.6956	6.4682	6.1446	6.6192	6.3322	7.0787	7.1584	3.4267	3.6723	7.1735	6.9983	6.9728	3.8955
Gemin8	_S[Phospho (STY)]LEPPSEKPWER_.2	Q8BHE1_S181_M1_Gemin8	3.1856	3.6659	3.1898	3.9310	4.0241	4.2482	9.1319	3.8789	3.5760	3.3919	3.9504	3.3920	3.6841	3.2279	3.0941	3.5305	3.3761	3.7733	4.0724	4.4060
Atcay	_LGGAVEDSSS[Phospho (STY)]PPSTLNLSGAHR_.3	Q8BHE3_S46_M1_Atcay	10.4722	10.9452	9.7707	9.7627	8.8623	9.8703	10.7485	10.4880	3.1204	9.7248	9.4537	9.7064	3.2858	11.0655	7.6868	9.1033	9.7537	4.3300	9.6194	9.4170
Atcay	_IQYVHSLEELER_.3	#N/A	4.0269	3.9759	4.5632	3.6624	3.8028	3.5887	4.1764	3.7273	4.2576	4.2333	3.6312	3.5143	4.3436	3.8873	3.3759	4.3718	4.2650	3.1138	3.4129	3.7694
Atcay	_AAEATEDQETS[Phospho (STY)]MS_.2	Q8BHE3_S370_M1_Atcay	4.0217	3.2974	3.5583	3.5626	4.2286	3.8689	3.8854	4.2474	3.9445	4.5243	4.3189	3.0235	4.0525	3.5963	2.8449	3.3838	3.7446	3.4049	3.7039	3.2736
Tmem108	_EIQSLETSEDQLS[Phospho (STY)]EPR_.2	Q8BHE4_S538_M1_Tmem108	3.1022	7.6815	7.6183	6.9112	3.9408	7.3286	7.2380	3.9622	6.9716	6.9484	4.0338	6.9414	3.7674	7.7574	6.2692	7.0044	7.7202	7.7456	7.1924	7.2389
Nrdc	_LGADES[Phospho (STY)]EEEGR_.2	Q8BHG1_S85_M1_Nrdc	7.7597	8.0684	7.1382	7.7500	6.9667	7.6566	7.8297	8.0954	7.5915	7.7997	7.6493	7.7174	7.6866	7.8576	6.1260	7.3275	7.8503	7.7329	7.5843	7.2305
Serinc5	_SSS[Phospho (STY)]DALQSR_.2	Q8BHJ6_S339_M1_Serinc5	7.9221	7.8540	4.2643	7.8925	7.2078	3.4672	3.6143	3.3796	7.5564	7.8948	3.0131	7.1836	7.3983	7.5573	6.3529	4.0899	3.9173	3.9159	7.4794	6.9733
Slu7	_LVEQANS[Phospho (STY)]PK_.2	Q8BHJ9_S215_M1_Slu7	8.6803	8.8882	8.8860	8.7423	8.6892	8.8149	8.4301	8.9549	8.6869	8.5079	8.5279	8.5825	8.6607	9.1497	8.6262	8.5675	8.8319	8.4830	8.6236	8.6430
Scn3b	_AEEAAQENASDY[Phospho (STY)]LAIPSENK_.2	Q8BHK2_Y198_M1_Scn3b	4.0698	3.9330	4.6061	3.3144	3.4075	6.3789	6.3209	4.4955	6.4947	4.2761	3.0662	3.8225	2.5325	3.8445	3.7108	4.4147	3.9927	6.2413	6.1599	5.3823
Tbc1d10b	_ASPVPGPGT[Phospho (STY)]PTR_.2	Q8BHL3_T136_M1_Tbc1d10b	9.9048	9.9830	10.0689	9.9046	8.7526	8.8210	4.0006	9.2795	8.9073	10.0967	9.0728	9.3057	4.1677	3.7115	3.5778	8.8296	9.3065	9.1670	3.5888	3.3888
Tbc1d10b	_HGAPAAPS[Phospho (STY)]PPPR_.2	#N/A	7.8336	7.0074	7.1862	8.0807	7.6178	8.6005	6.3293	8.5249	7.6581	7.8960	7.5501	7.7245	7.7413	6.5223	5.2957	7.2655	8.5839	8.3897	6.2493	6.9092
Tbc1d10b	_RHGAPAAPS[Phospho (STY)]PPPR_.3	#N/A	7.8899	8.7739	7.0967	5.7446	5.6712	6.7117	8.6572	7.7497	6.1469	7.6401	6.4117	6.8504	7.4090	7.8436	3.3024	4.4271	7.6878	7.6852	6.7605	5.5137
Tbc1d10b	_ELLEQNPGKFEELER_.3	#N/A	3.3726	4.0149	3.9089	4.3168	3.7343	3.5168	3.3233	3.0488	3.8839	3.3441	4.2074	3.3247	3.3933	2.4314	2.7910	4.2513	3.8116	3.4900	3.8711	3.8630
Txlng	_KHS[Phospho (STY)]LEGDEGSDFITK_.3	Q8BHN1_S79_M1_Txlng	3.1706	4.1506	4.2718	5.7544	3.3775	2.5073	3.9580	3.4359	3.7763	5.1113	3.0694	3.5830	3.7562	4.9135	3.7573	4.0336	3.9736	4.3607	4.1944	4.2259
Ganab	_NPEPELLVR_.2	#N/A	6.5101	5.9215	5.7673	6.1406	6.3040	6.3191	6.1731	5.6923	5.8231	5.9300	6.0059	6.0877	5.7523	5.6664	6.2171	5.7463	6.0731	5.5218	5.4801	5.5933
Ganab	_LDLLEDR_.2	#N/A	5.5738	6.1034	5.5726	5.7341	5.4830	5.7483	5.4478	5.9903	4.8313	5.7649	6.2077	5.7760	3.2373	5.8512	5.9018	5.9212	5.2190	5.7459	6.1295	6.1003
Ganab	_IILTAQPFR_.2	#N/A	5.7616	5.9870	8.7941	6.2397	5.8213	5.8284	5.8874	6.5219	8.2926	4.0070	5.7101	5.9241	8.1140	5.6847	5.9191	5.6664	5.8802	5.7817	6.0931	5.7479
Ganab	_M[Oxidation (M)]LDYLQGSGETPQTDIR_.2	#N/A	3.9384	4.5758	3.4577	5.0914	4.6390	4.7250	4.7433	3.9354	4.9036	4.4812	4.2464	4.5281	2.9475	3.3297	4.4449	5.0226	4.3518	4.4993	3.2666	5.0287
Ganab	_DAVHYGGWEHR_.3	#N/A	3.4362	4.5667	3.9724	4.2532	4.2747	3.7102	3.2664	3.1365	3.3255	3.6425	3.6998	2.9553	3.4569	3.9595	1.8440	2.5290	3.6742	4.1590	3.8075	3.9265
	KLDAES[Phospho (STY)]DGSTEETDESET.2	Q8BHR8_S319_M1_	5.6983	4.6147	3.9631	4.0846	4.4866	4.6855	4.3442	4.5689	4.7132	5.1131	4.0123	4.8172	4.7058	5.7923	2.6714	4.4883	5.4599	4.7454	3.6379	4.3134
Armcx3	_YNDWS[Phospho (STY)]DDDDDSNESK_.2	Q8BHS6_S61_M1_Armcx3	9.5105	9.3482	9.1056	9.2384	8.8286	9.1575	9.0508	9.3773	9.0217	9.0468	8.9219	9.1582	9.5270	9.3744	8.3577	9.0113	9.5266	9.3647	8.8301	9.0167
Armcx3	_ASPNSDDTVLS[Phospho (STY)]PQELQK_.2	Q8BHS6_S119_M1_Armcx3	3.4802	5.9787	6.8084	6.9926	4.3188	7.0376	6.8673	3.5842	6.5377	3.6865	6.6196	4.4120	6.6868	6.9724	5.7448	6.9602	6.8205	6.7717	7.1640	6.4134
Armcx3	_RAS[Phospho (STY)]PNSDDTVLSPQELQK_.3	Q8BHS6_S110_M1_Armcx3	3.5386	3.7805	4.5760	4.3219	3.7455	3.7789	3.3026	3.0679	3.2228	3.3492	4.2023	3.5066	3.3882	3.8909	3.3279	6.0858	3.6056	4.2276	3.8762	3.8579
Sybu	_TPSDTGHSPIGFC[Carbamidomethyl (C)]PGS[Phospho (STY)]DEDFTR_.3	Q8BHS8_S111_M1_Sybu	3.4999	6.1629	5.6541	4.1895	4.3384	3.0617	3.2026	3.2003	3.2617	3.7062	3.6361	4.3924	3.3698	6.8816	4.5580	3.8448	3.7379	6.4264	3.7438	4.3109
Sybu	_SGSS[Phospho (STY)]PPS[Phospho (STY)]PR_.2	Q8BHS8_S199_M2_Sybu	4.1416	3.8612	3.1771	3.3862	3.4793	3.7034	4.0617	4.4237	4.1208	4.3479	2.9944	3.7506	4.2289	3.7727	3.6389	2.9857	3.9209	3.2285	3.5276	3.4499
Cyria	_DAIQNPNDIQLQEK_.2	#N/A	6.7457	6.5798	7.1510	6.2343	6.6242	6.7082	6.3325	7.0407	6.7960	6.1564	6.4771	6.9418	7.1166	6.3175	6.0162	6.3116	6.5779	6.6813	6.5902	6.2901
Cyria	_EQAPDSVEGLLNALR_.2	#N/A	5.3994	4.9750	5.0548	5.8418	5.2469	4.9418	4.7263	4.6070	5.1333	5.0990	5.1770	4.8110	3.5435	2.6012	4.0834	4.8853	4.9479	4.7759	5.4608	4.9678
Cyria	_EIENYPHFFLDFENAQPTEGER_.3	#N/A	5.7232	5.0399	3.1590	3.5658	3.4613	4.2431	5.5270	4.4889	4.3571	4.4518	3.8284	5.0599	4.7346	3.1461	3.8649	2.9676	5.1636	4.9504	3.1201	3.1131
Mal2	_VTLPAGPDILR_.2	#N/A	8.5509	8.5505	8.4513	8.3442	8.3327	8.2703	8.5787	8.2498	8.2635	8.2300	8.0511	8.0019	8.1824	7.8864	7.8393	7.4945	8.3592	8.5680	8.0522	7.7827
Mal2	_[Acetyl (Protein N-term)]SAGGAVPPPPNPAVSFPAPR_.2	#N/A	8.4357	8.6819	8.3080	8.2147	8.0192	8.1872	8.3373	8.4666	8.1804	7.9241	8.0823	8.0149	8.1706	9.2082	7.7329	7.9208	8.3752	8.0948	7.9742	7.6748
Cdkn2aip	_SAAQQENSS[Phospho (STY)]PSR_.2	Q8BI72_S169_M1_Cdkn2aip	7.8429	7.7769	3.2292	7.1966	3.9847	7.0890	7.8646	8.0039	7.2877	7.4462	3.9898	6.9025	7.3035	7.4774	3.1335	3.4911	7.0762	7.5230	4.0330	6.3292
Mia3	_EEAKPAS[Phospho (STY)]PSSVQDR_.2	#N/A	3.4829	4.5200	4.0191	4.2065	4.3214	3.6635	3.4179	3.1832	3.3381	3.2338	4.3176	4.4094	3.5036	4.0062	3.4433	3.8277	3.7209	4.1123	3.7608	3.9732
Mia3	_DSLPSGPREEAKPAS[Phospho (STY)]PSSVQDR_.3	#N/A	6.9702	6.8705	4.4123	6.3610	5.9450	3.0257	6.2181	6.8340	6.0457	6.3266	6.1329	6.2967	7.6713	4.4504	2.8158	6.1680	6.6295	6.2725	4.3507	6.1653
Mia3	_DETAAQTPGS[Phospho (STY)]GEAVLSK_.2	Q8BI84_S820_M1_Mia3	6.6345	6.1038	6.3754	6.2972	5.1499	5.7892	5.3404	6.2238	3.9890	5.9762	6.1902	3.6188	4.0971	3.2960	3.5071	6.2387	6.4978	6.8628	6.2594	5.9961
Mia3	_SEFGS[Phospho (STY)]LDR_.2	Q8BI84_S1733_M1_Mia3	3.9840	5.7429	3.5154	3.6054	4.1857	3.9226	3.8425	4.2045	3.9016	4.5671	4.2760	3.0664	4.0034	3.5535	3.4197	3.3409	3.7017	5.0497	3.7468	3.2307
Mia3	_HSAS[Phospho (STY)]DPGPAPVVNSSS[Phospho (STY)]R_.2	Q8BI84_S1754_M2_Mia3	3.3518	3.9673	4.3892	4.2324	3.5588	3.0488	5.6021	3.5775	3.2746	3.1624	3.6490	3.6934	3.3827	4.4273	3.5147	4.2148	3.0747	4.0747	4.3738	4.1046
Frmd4a	_LLGSENDTGS[Phospho (STY)]PDFYTPR_.2	Q8BIE6_S712_M1_Frmd4a	3.5547	4.4481	6.7683	4.1346	2.8925	3.5916	3.4898	3.2551	3.4100	7.0375	4.3895	4.3375	3.5755	4.0781	4.2258	3.8996	3.7928	6.1350	3.6889	4.0451
Frmd4a	_MWSESS[Phospho (STY)]LDEPYEK_.2	Q8BIE6_S601_M1_Frmd4a	3.7062	6.6765	6.8917	6.2461	3.9132	5.7872	3.9838	3.9319	3.9040	3.5168	4.1055	3.4098	4.0694	6.0268	3.2311	3.8463	4.4094	3.6724	4.2599	3.7611
Frmd4a	_SSS[Phospho (STY)]LESQGK_.2	Q8BIE6_S696_M1_Frmd4a	4.1033	3.2158	3.6399	3.4809	4.3102	4.3436	4.2387	4.3290	4.1589	3.9139	7.1217	2.9419	4.3243	3.3262	4.2641	3.4654	3.8262	3.2915	4.4409	3.2932
Frmd4a	_TSNS[Phospho (STY)]YTAGGLFK_.2	Q8BIE6_S854_M1_Frmd4a	3.6162	3.7029	3.1528	3.9680	4.5957	3.8565	3.2249	3.8419	3.1451	3.4268	4.1246	3.4290	3.3106	3.1909	3.2503	2.9783	3.3391	3.8103	4.1093	3.7802
Frmd4a	_SVDIS[Phospho (STY)]PTR_.2	Q8BIE6_S679_M1_Frmd4a	3.9248	7.2870	3.4613	3.6595	4.1317	4.1650	4.4173	4.1826	4.3375	3.7353	3.8161	3.1205	4.5029	3.5047	2.9418	3.2869	3.2194	7.2262	7.3931	3.4717
Frmd4a	_IISGSSGSLLS[Phospho (STY)]SGSQESDSSQSAK_.2	Q8BIE6_S356_M1_Frmd4a	3.9031	4.3834	3.9731	3.1477	3.2408	3.4649	4.3003	3.1614	4.3594	4.1094	3.2329	4.1095	4.4674	4.0112	3.8775	4.2480	4.1594	2.9900	3.2890	3.6885
Cd99l2	_QETQS[Phospho (STY)]AEPPPPEPPRI_.2	Q8BIF0_S226_M1_Cd99l2	4.1939	3.1252	3.7304	3.3904	2.9000	4.4341	4.1481	4.4195	4.0684	4.0045	3.5470	7.5806	4.2338	3.2356	4.1735	3.5560	3.9168	3.3820	3.0306	3.2026
Cd99l2	_GENLEAVVC[Carbamidomethyl (C)]EEPQVTYSK_.2	#N/A	4.3770	4.4429	5.1948	4.7981	3.9066	3.6344	4.5308	3.7303	4.1771	4.1876	4.4021	4.1690	4.0506	4.5533	3.9903	4.7881	4.2680	3.5652	5.6300	3.0194
Rbfox3;Rbfox2;Rbfox1	_KIEVNNATAR_.2	#N/A	5.0204	5.0427	4.3321	3.5297	4.5567	5.1231	5.0408	3.8601	5.0269	4.8393	4.9472	4.7973	4.2230	5.3876	5.3419	5.0064	5.2314	5.1525	4.4437	5.3452
Iars2	_VLSELGVDAQSLSAM[Oxidation (M)]EIR_.2	#N/A	3.9323	4.9742	4.4865	4.0326	3.8277	3.2141	4.1615	5.4413	4.8188	4.3607	4.3565	5.2701	5.1670	4.4557	3.5159	5.0350	5.1135	4.1269	4.6660	4.6521
Rufy1	_SQLPS[Phospho (STY)]PGELR_.2	Q8BIJ7_S52_M1_Rufy1	9.1045	8.4445	8.2185	4.1848	3.6064	8.7292	8.5820	8.0415	3.3222	9.2866	3.6966	8.0810	3.4303	8.2345	3.4671	7.1594	3.1223	4.0271	4.3262	4.1522
Dock9	_GSLISTDS[Phospho (STY)]GNSLPDRNPEK_.3	Q8BIK4_S1270_M1_Dock9	3.2940	3.5411	4.0144	4.3628	3.9543	4.0610	3.4591	3.7860	3.3753	3.6054	3.7497	3.3555	3.4834	4.0225	3.2143	3.8300	3.9340	4.2484	4.6192	4.0935
Dock9	_LDKLPVHVYEVDEEADKDEDAAS[Phospho (STY)]LGSQK_.4	Q8BIK4_S178_M1_Dock9	4.1085	3.7043	3.3339	3.3909	4.3154	3.4487	4.2335	3.9989	4.1538	3.9190	3.6324	3.5938	4.3192	3.3210	4.2589	3.4706	4.5365	3.5831	2.9452	3.2880
Cstf2	_SLGTGAPVIESPYGESIS[Phospho (STY)]PEDAPESISK_.3	Q8BIQ5_S120_M1_Cstf2	3.4641	4.5221	4.0004	4.2253	4.3026	3.0259	3.2384	3.1644	5.1249	3.6704	3.6719	2.9273	3.4056	3.9874	2.8156	3.8090	3.7021	4.1310	3.7796	3.9544
Anks1b	_NVSKS[Phospho (STY)]DSDLIAYPSKDK_.3	#N/A	3.8086	8.3526	4.3448	3.8808	3.1463	3.3704	4.3948	3.5089	8.1720	4.0149	3.3274	8.3213	3.8293	8.7295	7.2625	8.4745	8.8849	8.7100	3.4351	4.2989
Anks1b	_TLANLPWIVEPGQEAK_.2	#N/A	7.0507	6.9831	6.8849	7.1185	6.9218	6.9750	6.8306	6.9742	6.9549	6.6564	7.4265	6.6992	6.9911	6.7056	6.9873	6.7871	7.2431	6.8320	7.0228	6.6345
Anks1b	_NIIAEHEIR_.2	#N/A	6.9621	6.8272	6.5763	6.9280	6.4505	6.6928	6.8499	7.2294	6.4344	6.8440	6.6770	6.7028	6.4073	6.8808	5.9612	6.5421	6.6283	6.8250	6.6169	6.2468
Anks1b	_LIFQSC[Carbamidomethyl (C)]DYK_.2	#N/A	5.4981	5.9389	5.5902	5.5057	5.6105	6.0602	5.8053	4.2133	5.8288	3.7047	5.7800	5.9802	4.5336	5.5131	5.2905	5.7292	5.5775	6.0046	5.9210	4.6051
Anks1b	_NVSKS[Phospho (STY)]DSDLIAYPSK_.2	#N/A	3.4302	7.4512	7.6719	4.2487	3.8800	3.9134	7.0907	4.4353	3.0893	3.4837	4.0688	3.3087	3.2548	7.5749	3.1945	3.0352	7.4904	7.0844	4.0107	3.7244
Anks1b	_NSSTGPTPDC[Carbamidomethyl (C)]S[Phospho (STY)]PPS[Phospho (STY)]PDTALK_.2	Q8BIZ1_S507_M2_Anks1b	3.5169	5.4595	7.1969	4.8972	3.6093	4.7683	6.9710	4.6149	4.4426	3.8443	3.6539	4.4136	3.9943	4.0402	6.0739	3.7613	4.7817	7.2076	7.1760	6.6272
Snip1	_LS[Phospho (STY)]PEPVAHR_.2	#N/A	7.2983	7.3068	7.2402	7.2761	7.1027	7.3012	7.2399	6.9569	7.2113	7.1365	7.2316	7.1504	7.4616	7.8143	7.0744	7.3284	7.6302	7.0652	7.1132	7.3704
Snip1	_QERLS[Phospho (STY)]PEPVAHR_.3	#N/A	6.2232	7.4209	7.3390	6.9188	6.8111	6.6797	6.6760	6.2815	6.7283	7.0591	6.5548	7.0019	7.0038	7.0730	6.8590	7.1134	7.3226	6.9299	6.9722	6.8002
Snip1	_S[Phospho (STY)]GDALTTVVVK_.2	#N/A	7.2414	7.8184	7.3883	7.2459	3.1742	7.4197	7.3474	3.1929	6.9556	6.7329	3.2645	7.0100	6.8668	4.0428	5.8067	3.8302	4.1910	7.0240	6.6441	6.5940
Snip1	_RPDAPAAS[Phospho (STY)]LS[Phospho (STY)]PPAAEPGHSGHR_.3	Q8BIZ6_S48_M2_Snip1	3.4252	8.2306	4.4626	8.8271	3.6321	8.0289	3.4160	3.6509	7.2835	7.1280	3.7224	3.6200	3.4561	4.5007	8.2761	8.1723	3.1481	8.0942	8.0223	4.1780
Snip1	_HRS[Phospho (STY)]GDALTTVVVK_.3	#N/A	4.5483	4.2716	4.0849	4.5368	3.2544	3.3531	3.7937	3.2731	1.1698	3.9977	3.3446	3.9978	4.5792	4.1229	3.4469	3.9104	4.2712	4.3791	3.1773	5.0425
Zc3h14	_DLVQPDKPAS[Phospho (STY)]PK_.2	Q8BJ05_S515_M1_Zc3h14	9.1552	9.4187	8.9099	9.2204	9.0048	9.1092	8.7684	9.4029	9.0360	9.3185	8.9865	9.2614	9.6985	9.8082	8.9975	9.3060	9.3454	9.1934	9.2173	9.2485
Zc3h14	_WIRPQS[Phospho (STY)]SE_.2	Q8BJ05_S733_M1_Zc3h14	3.8510	6.1631	6.0757	6.1815	3.1887	6.0428	5.8050	3.5513	4.4115	6.4587	6.3778	6.4606	6.9364	4.0633	3.9296	4.1959	4.0890	6.2736	3.2369	3.7406
Zc3h14	_IS[Phospho (STY)]PPVKEEEAKGDNTGK_.3	Q8BJ05_S409_M1_Zc3h14	4.8850	5.3586	4.3721	3.8536	4.1230	3.3106	5.3483	4.7500	3.6911	4.3817	3.1697	4.0564	5.7158	6.3418	3.7963	3.9332	4.0739	4.3333	4.4153	5.3027
Zc3h14	_RFS[Phospho (STY)]HDGEEEEEDEDYGTR_.3	Q8BJ05_S309_M1_Zc3h14	3.1270	3.8776	3.6633	4.5624	3.9656	4.0194	3.5755	4.3282	2.9133	4.3045	2.6607	2.9885	3.1477	3.6504	3.1390	3.4719	3.3651	4.4681	3.6248	2.5863
Dlgap2	_EKS[Phospho (STY)]LDLPDR_.2	#N/A	14.1022	14.0693	13.9378	13.9005	12.1724	13.3025	13.4429	13.6062	13.2716	13.9779	13.3018	13.3957	13.6670	13.9915	12.7315	13.5671	13.6185	13.4270	13.4594	13.5467
Dlgap2	_SDVETATDS[Phospho (STY)]DTESR_.2	Q8BJ42_S745_M1_Dlgap2	11.8128	11.6760	11.7676	11.8649	11.4829	11.4219	11.3843	11.8193	11.4628	11.7856	11.5359	11.5721	12.0838	11.9247	11.2271	11.7338	11.8672	11.5360	11.5572	11.7038
Dlgap2	_IIES[Phospho (STY)]PERKEER_.3	#N/A	9.0722	9.5523	8.9010	9.3998	8.8793	9.0314	9.4009	9.3216	9.2835	9.5968	9.2802	9.5170	10.2200	9.6317	9.5077	9.5231	9.4461	9.5524	9.6814	9.4325
Dlgap2	_IIES[Phospho (STY)]PERK_.2	#N/A	10.4637	10.2783	10.3642	10.4490	10.0324	10.2474	10.1866	10.3448	10.1022	10.3851	10.4428	10.2706	10.4571	10.6585	9.9019	10.2013	10.6279	10.3934	10.3559	10.4760
Dlgap2	_S[Phospho (STY)]IDKAVLASK_.2	Q8BJ42_S700_M1_Dlgap2	9.3147	9.2734	9.3172	9.2251	8.5041	8.1972	8.6514	8.4172	8.0183	9.3671	8.9642	8.7945	9.4082	9.2014	7.4761	8.8864	8.2367	9.1342	9.1300	8.7713
Dlgap2	_EKS[Phospho (STY)]LDLPDRQR_.3	#N/A	6.5091	7.7848	5.2910	7.6580	6.9236	5.8622	7.1754	6.9536	6.3614	7.3919	5.8616	7.2991	8.8745	6.9349	6.9996	7.7555	6.1280	7.1997	6.8649	7.1473
Dlgap2	_GLQFGSS[Phospho (STY)]FQR_.2	Q8BJ42_S806_M1_Dlgap2	4.2371	3.7657	7.3844	3.4523	3.5748	6.4246	3.9662	3.9375	5.5164	6.5563	2.8989	3.6551	6.0020	3.2597	3.5434	3.0812	4.4752	3.3240	3.0066	3.3544
Dlgap2	_SHS[Phospho (STY)]LEGSSK_.2	Q8BJ42_S248_M1_Dlgap2	7.6557	7.1808	6.6485	7.4026	6.8372	6.4988	7.1346	6.6426	6.4607	7.3771	6.6963	7.0005	7.2738	6.9580	2.9034	3.7212	3.1854	6.8768	4.2631	6.8513
Dlgap2	_C[Carbamidomethyl (C)]SS[Phospho (STY)]IGVQDSEFPDHQPYPR_.3	Q8BJ42_S720_M1_Dlgap2	3.7547	4.2481	6.2689	3.9347	3.0924	5.0219	5.2120	3.4551	3.0069	3.9610	3.3813	4.1375	3.1149	4.1595	4.0258	4.0996	3.9928	5.5872	5.7398	5.5780
Dlgap2	_GGLYNS[Phospho (STY)]M[Oxidation (M)]DSLDSNK_.2	#N/A	8.9082	8.3238	7.5150	8.1330	7.2618	7.4435	7.8350	7.6882	7.3773	8.9191	7.6925	7.8610	7.9521	7.9675	6.3316	8.4266	8.0994	7.6687	8.4156	8.4401
Dlgap2	_T[Phospho (STY)]PPPVPPR_.2	Q8BJ42_T616_M1_Dlgap2	9.2463	9.4235	9.0939	9.3023	8.6123	8.2983	9.3072	7.9905	9.3919	9.9097	9.2910	8.5509	4.5968	9.0773	9.3120	9.3337	8.6844	8.1985	8.6704	9.5369
Dlgap2	_ADS[Phospho (STY)]IEIYIPEAQTRL_.2	#N/A	3.9381	6.1786	5.4295	3.2205	4.1450	4.1783	4.4039	4.1693	4.3241	3.7487	3.8028	3.1071	4.4896	3.4914	2.9285	3.3002	3.2061	3.1262	5.6201	3.4584
Dlgap2	_EAEENDLLEDILGK_.2	#N/A	4.5955	4.2364	4.8626	1.1212	4.0351	3.8639	4.4012	3.1497	3.4671	4.8187	4.7939	4.3375	4.5995	5.4434	4.1513	5.4269	3.3160	3.0163	4.1537	3.7607
Dlgap2	_TTSKPLISVTAQS[Phospho (STY)]STESTQDAYQDSR_.3	Q8BJ42_S636_M1_Dlgap2	4.0644	6.2386	3.9910	3.6268	3.9724	3.8378	3.8186	4.0954	3.7596	4.2114	3.9031	3.8694	3.9391	6.7774	3.7085	3.8128	3.4568	3.4864	4.0752	3.7631
Dlgap2	_IIES[Phospho (STY)]PER_.2	#N/A	3.3969	5.4576	4.4343	4.1873	3.6038	4.9028	5.2543	3.6226	3.3645	3.2075	6.5897	6.1814	6.3315	6.7732	6.1907	5.8350	6.3358	6.6018	6.0752	6.6292
Dlgap2	_QNSATERADS[Phospho (STY)]IEIYIPEAQTR_.3	#N/A	3.6018	7.1423	6.4192	6.4258	4.0074	6.0378	6.4346	4.1639	6.1455	7.0433	3.4773	3.9954	3.7821	7.4551	4.2047	3.7451	3.7942	7.5161	3.7006	6.0700
Dlgap2	_HS[Phospho (STY)]EPSTPTQYGALR_.2	Q8BJ42_S811_M1_Dlgap2	4.2387	7.6036	7.6202	7.0864	3.5764	6.8392	3.9647	3.9390	4.0238	7.6930	2.8973	3.6536	7.1019	8.0695	3.5419	7.3484	7.4143	3.3564	7.4491	6.9152
Dlgap2	_SGM[Oxidation (M)]SSWWS[Phospho (STY)]SDDNLDSDSTYR_.2	#N/A	4.0765	3.7400	4.5295	3.6511	3.6659	4.3861	3.7428	3.9160	3.7691	4.2007	3.9215	3.5757	3.8904	3.5257	3.8764	3.6893	3.7385	3.6897	4.2242	3.5406
Dlgap2	_MRS[Phospho (STY)]GSYIK_.2	Q8BJ42_S438_M1_Dlgap2	4.5581	4.2618	4.0947	4.5270	3.2642	3.2975	3.7839	3.5492	3.7041	4.3687	3.1828	3.9880	3.8696	4.3722	3.8093	3.9202	4.2810	3.7463	3.3948	4.3392
Dlgap2	_AMGDEESGESDSS[Phospho (STY)]PK_.2	Q8BJ42_S456_M1_Dlgap2	4.0738	4.0589	3.4336	4.2712	3.5164	4.2076	4.1285	4.1151	3.8773	4.0451	4.5787	3.8443	4.1007	4.4146	3.6485	3.2538	4.0215	3.4600	3.9917	3.7955
Dlgap2	_ADSIEIYIPEAQTR_.2	#N/A	6.4536	6.1424	6.6536	6.5864	5.7087	6.4866	9.5395	7.1897	6.0594	8.2608	6.1076	3.6440	9.7198	3.6573	9.5545	6.5693	6.4123	5.3445	6.0534	9.4699
Dlgap2	_S[Phospho (STY)]LDLPDR_.2	#N/A	3.6200	4.3828	4.1563	4.0694	2.9577	3.1818	3.0825	3.3204	3.9250	3.8263	3.5160	4.2723	3.2497	4.2943	4.1606	3.9649	3.8580	4.2077	4.9843	4.7134
Dlgap2	_DGS[Phospho (STY)]WFLK_.2	Q8BJ42_S873_M1_Dlgap2	4.2918	3.7110	3.3272	3.3976	3.6295	3.8536	3.9116	4.2735	3.9707	4.4981	4.3450	3.6005	4.0787	3.6225	3.4888	3.1358	3.7708	3.3787	3.6777	3.2998
Chmp2b	_ATIS[Phospho (STY)]DEEIER_.2	Q8BJF9_S199_M1_Chmp2b	9.9216	10.0718	4.1217	9.8764	9.2879	9.6597	9.6343	9.9273	9.4601	9.9933	9.3886	9.6417	10.1669	9.9768	9.3296	9.8503	9.8796	9.7558	9.7788	9.6860
Zc2hc1a	_NTT[Phospho (STY)]PPSLAR_.2	Q8BJH1_T243_M1_Zc2hc1a	12.3476	12.3346	11.9804	12.2832	12.0371	4.1961	11.8634	11.8590	11.7928	12.3262	11.9759	11.8193	12.4821	12.4913	11.6689	12.1227	11.9510	11.7842	4.2934	11.9064
Slc6a17	_YGSGYLLAST[Phospho (STY)]PESEL_.2	Q8BJI1_T722_M1_Slc6a17	9.2784	8.7313	9.2144	9.3008	8.0174	8.0121	8.6561	8.1959	8.6889	9.0757	8.6656	7.9808	8.7956	8.1252	6.4982	8.0725	8.5914	8.2500	7.9335	8.0573
Slc6a17	_DIS[Phospho (STY)]NLEENDETR_.2	Q8BJI1_S665_M1_Slc6a17	10.8513	10.6606	10.7667	11.0457	9.5274	9.9684	10.1102	9.5895	9.7399	11.0409	10.1968	9.3955	9.9270	10.0220	7.9335	9.8495	9.9346	9.7863	9.7766	9.6580
Slc6a17	_EHS[Phospho (STY)]NEHVTESVADLLALEEPVDYK_.3	#N/A	9.7528	10.2823	8.8668	9.5256	3.6761	9.1852	9.8964	9.2046	9.3610	9.5026	8.9182	9.2718	9.8126	9.7547	8.2568	8.9862	9.4886	10.0079	9.6782	8.9828
Slc6a17	_SYLGPGSTS[Phospho (STY)]PLDNSNNPNGR_.2	#N/A	9.4145	9.1559	9.7989	9.3557	8.1085	8.6222	8.6632	8.5455	8.4353	9.1233	8.2845	8.4493	8.7286	8.5863	3.2923	3.2265	8.8834	8.6336	8.3247	8.1666
Slc6a17	_ILGYLNSNVLSR_.2	#N/A	4.0404	3.4963	4.4869	4.2256	4.5004	4.2928	4.2528	4.7961	2.6818	4.1569	3.7501	4.5039	4.4199	3.9065	3.8300	3.7062	3.9783	4.4740	0.9771	3.0796
Slc6a17	_VPS[Phospho (STY)]EAPSPM[Oxidation (M)]PTHR_.2	Q8BJI1_S682_M1_Slc6a17	6.0625	6.1910	8.2610	8.3524	5.8604	5.2312	5.1781	7.2206	6.4375	7.5735	5.8194	3.6294	5.7980	6.4090	3.4803	5.4229	5.8964	5.9703	5.5017	5.0339
Sgta	_ALELDPDNDTYK_.2	#N/A	6.4173	6.5261	6.3170	6.5872	6.3347	6.7067	5.9542	6.8469	6.6960	6.3350	6.1666	6.8792	3.3525	6.9618	6.9173	6.9404	6.6464	6.2210	6.4097	6.6029
Sgta	_TPS[Phospho (STY)]ASHEEQQE_.2	#N/A	6.1360	6.6659	6.4466	5.9728	5.9797	6.0262	5.8141	5.9181	6.8067	6.8550	6.3160	5.7894	6.0914	6.7913	5.7984	6.3848	5.7888	5.9031	6.3558	3.1820
Sgta	_AIELNPANAVYFC[Carbamidomethyl (C)]NR_.2	#N/A	4.4276	4.3923	3.3799	5.0531	3.4817	4.5466	4.8996	4.6768	4.3278	4.1184	4.4583	4.2985	4.3201	5.5143	4.6983	4.7358	4.3976	4.6055	4.7128	4.7337
Sgta	_SRTPS[Phospho (STY)]ASHEEQQE_.2	#N/A	5.2649	3.5117	4.7898	6.1100	4.9442	5.3394	5.7555	5.6073	5.7697	6.5100	4.8556	5.9745	5.4417	6.3209	6.1733	6.2005	6.2604	5.7022	4.1120	5.5838
Sgta	_APDRT[Phospho (STY)]PPSEEDSAEAER_.3	#N/A	7.8583	8.2035	7.8042	8.0196	7.5804	6.6498	6.6024	5.6815	6.7095	8.1268	6.6854	8.0252	7.0783	6.8967	8.1819	8.0263	8.1190	6.6953	8.0106	7.1470
Sgta	_HAEAVAYYK_.2	#N/A	4.3750	3.4104	3.9115	3.2093	3.0811	3.1144	1.3602	3.0998	3.8872	4.1856	3.3659	4.1711	4.0527	4.5553	3.9924	3.7371	4.0979	3.5631	3.2117	3.0215
Psmd5	_VFTAIADQPWAQR_.2	#N/A	3.3430	6.0002	4.3804	5.8418	4.5243	3.0576	5.6828	3.5687	4.8149	3.1536	3.6402	3.7022	3.3739	6.8613	3.5235	4.1215	3.0659	5.6606	4.3826	4.0958
Psmd5	_IVENSEAVTEILNNAELLK_.2	#N/A	3.4358	4.0588	4.4803	3.7454	2.4817	2.3980	3.7015	3.6444	2.1532	4.2735	3.2779	3.7426	4.1369	3.6756	2.5563	4.2889	4.1820	3.6511	3.4577	3.3189
Fbxo9	_S[Phospho (STY)]PDGDVGSGYIEENEDASK_.2	Q8BK06_S126_M1_Fbxo9	4.1270	3.8758	3.1625	5.7340	3.4647	3.0194	4.1498	3.8274	4.3182	4.0905	3.4609	3.7652	4.1477	3.1496	3.6535	4.4677	4.3651	3.4681	3.1167	3.1165
Ndufv3	_TQSVLKEPEPTDTTTYK_.3	#N/A	6.3316	6.3245	6.2123	6.7119	6.5636	6.5671	6.1337	6.6720	6.7147	7.0652	6.9488	6.9602	7.4886	7.2752	7.6722	7.0577	5.8980	6.8369	7.0268	7.3031
Ndufv3	_LPQPSSGRES[Phospho (STY)]PRH_.3	#N/A	4.1742	3.1449	3.7108	3.4100	2.8803	4.4145	4.1678	4.3999	7.8229	7.6590	7.6647	4.3718	8.1318	3.7489	7.6279	7.7352	7.3914	3.2523	3.5514	3.2222
Ndufv3	_LPQPSSGR_.2	#N/A	5.5563	6.2296	3.6370	6.1502	6.6411	5.7767	6.5438	5.5182	6.6913	6.4794	6.2440	6.2499	5.7721	6.7760	7.5987	6.7218	6.0303	6.4617	6.5560	6.1640
Ndufv3	_LPQPSSGRES[Phospho (STY)]PR_.2	#N/A	4.0504	3.9524	4.5867	3.2950	3.3882	3.6122	4.1529	4.5149	4.2120	4.2568	3.0855	3.8418	4.3201	4.7590	3.7301	4.3953	4.0121	3.1373	3.4364	3.5411
Nop53	_EM[Oxidation (M)]C[Carbamidomethyl (C)]EGLLEES[Phospho (STY)]DGEDEHEAGR_.3	#N/A	4.0179	4.5737	3.4594	4.1685	4.1445	3.6946	3.8553	4.1485	4.2018	4.7401	3.5625	4.6609	3.9537	4.8159	4.0436	3.9448	4.3995	4.8297	4.3872	3.5265
Csnk1a1	_AAQQAASSSGQGQQAQT[Phospho (STY)]PTGK_.2	Q8BK63_T321_M1_Csnk1a1	4.2499	5.9016	3.7864	5.8377	4.9440	4.2658	6.2568	4.9207	5.3931	4.0604	5.1126	4.5066	4.1778	5.5901	4.1175	4.2611	4.3951	4.6432	4.6319	4.3340
Ahsa1	_ETFLTSPEELYR_.2	#N/A	6.7840	6.6228	6.7562	6.6925	6.7566	6.7183	6.6382	6.5590	6.9554	6.7518	6.2874	6.8599	6.2919	6.7009	6.7760	6.6262	6.5764	6.3475	6.8319	6.6036
Ahsa1	_VFTTQELVQAFTHAPAALEADR_.3	#N/A	3.6228	3.7146	4.1591	3.0702	3.2282	3.1846	3.0797	3.5578	3.3214	3.8291	3.5132	3.3100	3.2468	4.2914	2.1574	3.4766	4.4397	4.2106	3.0088	3.4374
Ahsa1	_QTFGYGAR_.2	#N/A	3.6303	4.2065	3.9519	4.4136	4.0753	4.1230	3.2109	4.7211	3.1311	3.4409	4.1106	3.0592	3.2965	3.7992	3.0839	2.9924	3.5139	4.4855	3.9679	3.9415
Ahsa1	_ADATNVNNWHWTER_.2	#N/A	3.6653	4.3375	4.2016	4.0241	5.2927	3.4810	3.6004	3.3657	3.5206	4.5522	5.8848	4.2269	3.6861	4.1887	3.6258	4.0102	3.9034	3.9298	3.5784	4.1557
Srrm4	_DPT[Phospho (STY)]PPPSSRGK_.2	Q8BKA3_T101_M1_Srrm4	3.6488	4.3540	8.6933	4.0406	2.9865	3.2106	3.0537	8.7783	3.1128	3.8551	8.3359	4.2435	3.2209	9.1875	8.7438	3.9937	8.8116	4.2365	3.0347	3.9428
Srrm4	_SAS[Phospho (STY)]PLAATR_.2	Q8BKA3_S271_M1_Srrm4	6.9157	6.8904	3.4318	6.0535	4.1021	4.1355	6.8827	6.7292	6.7049	6.3065	6.2718	3.1500	4.5325	3.5343	2.9713	6.4976	3.2490	3.0834	6.3980	6.3480
Srrm4	_GQEKGS[Phospho (STY)]PGGDLSK_.2	#N/A	3.3285	3.9906	4.3659	4.2557	3.5354	4.8066	3.5127	3.5542	3.4329	3.1391	4.8562	3.7167	3.5983	4.1010	3.5380	4.1915	4.5523	4.0175	3.6661	4.0680
Srrm4	_SRGQEKGS[Phospho (STY)]PGGDLSK_.3	#N/A	3.4298	3.9102	4.4464	4.1753	4.2684	2.9917	3.2727	3.6346	3.3318	3.6362	3.7061	3.6363	3.4398	4.4844	2.8499	3.7747	3.1318	4.0176	4.3166	4.1617
Ipo5	_NLIDEDGNNQWPEGLK_.2	#N/A	8.8797	8.6762	8.6231	8.6231	8.4384	8.6446	8.3684	8.8113	8.3499	8.5745	8.7116	7.8874	8.8597	8.5410	7.2760	8.4024	8.9278	8.4273	8.3045	8.2034
Ipo5	_LVLEQVVTSIASVADTAEEK_.3	#N/A	3.8046	5.2522	5.4783	4.8622	3.4072	4.9971	4.2360	5.0582	3.9234	5.0342	4.7346	4.4744	4.1246	5.4667	3.4518	4.4248	4.7315	4.8974	4.8172	4.3493
Ipo5	_HIVENAVQK_.2	#N/A	3.4489	4.1237	3.9852	4.2405	4.2874	4.0602	3.2536	3.1493	3.3127	3.6552	3.6871	2.9425	3.4208	3.9723	2.8308	4.0325	3.6869	4.1462	3.7948	4.1427
Pi4kb	_S[Phospho (STY)]VENLPEC[Carbamidomethyl (C)]GITHEQR_.2	#N/A	7.8697	7.8290	7.4503	7.6542	6.5868	6.9324	7.4968	6.9786	6.8908	7.5805	7.0975	6.9935	7.1024	7.0683	3.6055	7.1073	7.2903	7.2385	7.0979	6.9264
Mapkap1	_RTS[Phospho (STY)]FSFQK_.2	#N/A	6.7710	6.9435	6.7808	3.3192	3.6120	6.5112	6.4039	4.2908	6.3618	6.8735	6.4629	6.0047	6.8431	6.3206	3.5215	3.1183	6.2701	3.1926	3.9750	3.4394
Mapkap1	_YSS[Phospho (STY)]PGLTSK_.2	Q8BKH7_S270_M1_Mapkap1	5.6996	4.6135	3.7430	3.3778	2.9125	5.9207	4.1356	4.4321	4.0558	5.7380	5.0988	4.3396	4.2212	3.7811	4.3928	3.5685	3.9293	3.2201	3.5192	3.1900
Mapkap1	_TS[Phospho (STY)]FSFQK_.2	#N/A	3.8688	3.4503	3.4054	3.7154	4.0758	4.0326	4.4732	4.0945	3.7916	3.6794	4.1660	3.1764	3.8997	3.4435	2.9977	3.2309	3.5917	3.5577	3.8568	3.1207
Tnrc6b	_DDEPSGWEEPS[Phospho (STY)]PQSISR_.2	Q8BKI2_S913_M1_Tnrc6b	7.5553	3.4068	7.2869	7.5840	3.8721	6.7923	6.4347	4.2479	4.1961	7.0688	6.4583	3.1329	6.4160	7.3781	3.2489	3.1918	6.9759	6.3034	6.6541	6.7421
Tnrc6b	_M[Oxidation (M)]GS[Phospho (STY)]PAPLLPGDLLGGGSDSI_.2	Q8BKI2_S1793_M1_Tnrc6b	6.1020	4.8730	5.3149	4.7416	3.7236	3.0483	5.1036	5.5463	4.1550	5.9733	3.4320	5.4869	5.2631	5.6340	4.0585	4.3453	4.9913	4.6658	3.8539	4.4518
Tnrc6b	_SSSS[Phospho (STY)]AGSEVGGQSTGSNHK_.2	Q8BKI2_S601_M1_Tnrc6b	4.0091	3.3100	3.5456	3.5752	4.2160	4.2493	4.3329	4.2347	4.2532	3.8197	3.7318	3.0362	4.4186	3.4204	2.8575	3.3712	3.7320	3.1972	4.3466	3.3874
Baiap2	_EGDLITLLVPEAR_.2	#N/A	6.2311	5.8437	5.9663	6.4836	5.7090	6.3654	5.6508	5.9817	5.9272	6.1609	5.9695	6.1159	5.7196	5.2309	5.7508	6.4122	5.6753	5.5950	5.8201	5.9676
Baiap2	_QIQNQLEETLK_.2	#N/A	5.0962	5.3083	5.4531	4.5699	5.2776	4.3897	5.0924	5.0172	4.6982	5.6585	5.7156	4.9944	5.3675	5.6468	5.5941	5.1949	5.3861	5.3448	5.8852	4.6514
Baiap2	_YSDKELQYIDAISNK_.2	#N/A	4.9069	5.6084	5.9555	6.1065	5.5044	5.3592	5.0824	5.5521	5.9116	5.9736	6.0925	6.1307	5.8447	6.4669	5.9622	6.3663	5.7284	5.7323	6.2773	6.2446
Baiap2	_LSDSYSNTLPVR_.2	#N/A	5.7709	5.2368	5.2784	5.1341	5.5950	5.8449	5.3434	5.1841	4.8157	5.7193	5.5325	5.0156	4.8201	5.6364	5.8665	5.2835	5.3634	5.7426	5.2626	5.7574
Baiap2	_SLS[Phospho (STY)]PPQSQSK_.2	Q8BKX1_S326_M1_Baiap2	14.3398	13.7127	14.2403	14.4350	13.6457	13.5612	13.3742	14.4627	13.5266	13.7595	14.5249	14.7686	15.2324	15.2850	14.5829	14.8396	14.9759	14.6794	14.6261	14.8595
Baiap2	_LPLWQQAC[Carbamidomethyl (C)]ADPNKIPDR_.3	#N/A	3.7368	5.9371	4.2731	4.0608	5.2690	5.4841	5.6413	5.4162	3.0248	5.3701	5.6366	5.5326	5.9806	6.2640	5.6226	5.4694	5.6790	5.2595	5.7380	5.7847
Baiap2	_SFHNELLTQLEQK_.3	#N/A	4.3382	4.1705	3.8748	3.6207	4.4799	5.4142	4.4677	3.7691	4.5177	5.1551	4.8614	4.0863	4.8542	5.1103	5.1828	5.5839	4.5151	5.2919	5.6845	5.8320
Baiap2	_SSS[Phospho (STY)]M[Oxidation (M)]AAGLER_.2	#N/A	3.8714	5.7703	6.2084	6.7799	5.7472	4.7539	5.4232	3.8868	4.2818	6.3644	3.8625	4.9923	5.7169	4.2561	3.7637	5.9425	3.8472	5.4665	6.0694	3.7650

Baiap2	_SNLVISDPIPGAKPLPVPPELAPFVGR_3	#N/A	3.4570	2.8816	4.4945	4.5487	3.6640	4.0489	3.9305	4.9773	3.9529	3.2676	4.4083	4.1362	3.4698	3.9724	3.3960	4.3200	3.6871	3.2035	3.7946	3.9314
Baiap2	_TIM[Oxidation (M)]EQFNPSLR_2	#N/A	5.6803	5.2128	5.0693	5.7728	5.6809	6.0753	4.1170	5.5180	5.9279	5.5382	4.4214	5.8690	6.0654	5.8025	5.8993	5.8582	5.9516	5.4035	5.6814	6.5659
Baiap2	_AIFSHAAGDNSTLLSFK_2	#N/A	4.4339	4.6315	3.9705	3.1503	3.1400	3.8229	2.5681	3.1588	2.5464	3.4330	4.4430	3.7480	3.9937	4.0086	3.8119	4.4572	4.9125	2.9926	4.2640	4.2791
Baiap2	_LTENVYK_2	#N/A	3.8274	4.1754	4.3265	3.8620	3.1651	3.3892	4.3759	3.5278	4.4350	4.0337	3.3086	4.0648	4.5431	4.0869	3.9531	4.1723	4.0655	4.4152	3.2134	3.7641
Baiap2	_ELGDVLFQM[Oxidation (M)]AEVHR_3	#N/A	3.8898	3.7983	3.1505	3.7892	3.8120	4.0361	3.7933	4.1553	3.8524	3.7094	3.5118	3.6123	3.9605	3.5042	3.3705	3.3183	3.6525	3.5612	3.8602	3.9674
Pdhx	_VVDDELATR_2	#N/A	6.9213	7.2122	7.3588	7.1699	7.2193	7.2908	7.2228	7.1767	7.2003	7.2373	7.2759	7.5528	7.6715	7.5559	7.9716	7.1240	6.8832	7.3742	7.2288	7.3061
Pdhx	_VSVNDFIIR_2	#N/A	6.4947	6.6291	6.6124	6.6227	6.9069	6.9198	6.9098	6.9354	6.3440	6.3862	6.6626	7.0116	7.4076	6.9708	6.7660	6.5135	6.7861	6.8227	6.6075	6.9009
Pdhx	_DLVKDDIK_2	#N/A	5.0306	5.0422	3.1933	5.1934	5.2126	4.9240	4.4023	5.4536	5.4447	5.0453	4.8681	5.2695	4.8636	5.1018	5.6273	5.4820	4.7986	5.0604	5.2041	5.2550
Pdhx	_GIQEIADSVK_2	#N/A	5.1082	3.8844	5.8890	5.6903	5.4342	5.3970	5.4691	5.2790	5.1491	5.9275	5.9419	5.3532	4.1391	5.4107	6.0042	5.6420	4.3564	5.3387	5.6153	4.8754
Pdhx	_HSLDASQGTATGPR_2	#N/A	3.2823	4.0367	4.3198	4.3019	3.4853	3.5226	3.5588	3.5080	3.4790	3.0929	2.9577	3.7629	3.6645	4.1471	3.5842	4.1453	4.5061	3.9713	3.6199	3.0420
Pdhx	_LTEDEGNPQLQQHQLITVTM[Oxidation (M)]SSDSR_3	#N/A	4.0996	4.5799	3.1351	3.3442	3.4373	3.6614	4.6068	4.4657	4.1628	4.3059	3.0364	3.7927	4.2709	3.8147	3.6810	2.9437	3.9629	3.1865	3.4856	3.4920
Usp54	_NSSS[Phospho (STY)]PVSLDAAPPDSVNVYR_2	Q8BL06_S613_M1_Usp54	4.1801	3.8227	3.2156	8.3387	3.5179	3.7420	4.0232	4.3852	4.0823	4.3865	2.9558	3.7121	4.1904	8.2478	3.6004	3.0242	8.4922	8.1579	3.5661	7.8524
Ablim2	_S[Phospho (STY)]PQHYSR_2	#N/A	10.3707	10.1739	10.7041	10.6444	10.0717	9.9824	10.1523	9.8050	9.9990	10.6166	10.3798	9.9148	9.7184	10.4352	9.1914	10.1837	10.1469	9.9104	9.9836	10.3924
Ablim2	_IYPYDSLIVTNR_2	#N/A	5.9776	5.6540	6.1519	6.1317	5.5847	5.7229	5.8935	6.3574	6.0322	6.4794	5.4546	6.4193	5.9019	7.0186	6.5761	6.2131	5.4433	6.2134	6.1177	6.3428
Ablim2	_TSSESIVSVPASSTSGS[Phospho (STY)]PSR_2	#N/A	12.4807	12.3932	12.4797	3.6201	12.1317	12.2560	11.8525	12.7222	12.3580	12.3680	12.5509	12.5976	13.3100	13.1014	12.5345	12.6849	13.1282	12.4265	12.5670	12.7314
Ablim2	_TSS[Phospho (STY)]PSSAGSVSLGHYTPTSR_3	#N/A	4.0652	3.6001	3.9077	3.7038	3.4543	5.9181	6.9180	4.0649	6.5853	4.3903	3.6397	4.9349	7.2114	8.4099	7.2791	3.7291	4.0748	6.6784	7.5626	6.6524
Ablim2	_S[Phospho (STY)]PQHYSRPGSESGR_3	#N/A	5.1599	3.6156	4.9923	5.4873	5.3598	6.4506	5.7108	3.9830	6.1429	6.7748	6.3129	6.3823	3.7882	3.3320	5.9824	5.6703	6.2583	5.4608	6.2042	6.3453
Ablim2	_RLDVEDSS[Phospho (STY)]FOQDSR_2	Q8BL65_S453_M1_Ablim2	3.2802	6.2181	4.4395	4.1891	4.1588	4.0495	4.1673	6.2278	3.6652	4.5111	4.0880	3.5258	3.6192	4.4083	4.3450	4.1374	3.9944	4.1336	3.9553	4.6008
Ablim2	_T[Phospho (STY)]NSPOLDSQSLSLSSGTDQEPLQR_3	Q8BL65_T475_M1_Ablim2	3.9860	4.0434	3.7996	3.9769	3.4756	3.7659	3.7049	4.4767	3.7487	6.2050	3.6702	3.9089	3.8756	8.1523	5.2388	3.3481	3.7639	7.1534	3.7446	3.6392
Eea1	_SLGS[Phospho (STY)]ADELFK_2	Q8BL66_S52_M1_Eea1	4.3038	4.5162	3.8403	3.2805	3.0099	3.0432	4.0382	4.5294	3.9584	4.1144	3.4371	4.2423	4.1239	5.4007	4.0636	3.6659	4.0267	5.2349	4.8468	3.0927
Eea1	_LQQQSSQAAQELAAEK_2	#N/A	3.1718	4.0974	4.4417	3.7840	3.2432	1.7005	3.4480	3.6058	4.5379	4.1118	2.0685	3.0936	3.9261	4.4288	3.7069	3.0858	3.3790	2.6441	3.3383	2.9000
Gtf3c2	_VSS[Phospho (STY)]PTKPK_2	Q8BL74_S219_M1_Gtf3c2	3.8956	5.7101	4.4319	3.7938	3.2333	5.4679	4.3078	3.5960	4.3669	5.4052	4.6538	3.9967	5.9579	5.8441	3.8850	4.2405	4.1336	6.3844	3.3481	4.3860
Arhgap22	_LEEELDQER_2	#N/A	11.6319	11.5413	11.9680	11.8220	11.7109	11.6567	11.6472	11.8782	11.9926	11.7999	11.6381	11.8383	11.8598	12.0373	12.2965	12.0412	11.5818	11.5582	11.5998	11.9147
Srsf7	_S[Phospho (STY)]RS[Phospho (STY)]AS[Phospho (STY)]LRR_2	Q8BL97_S200_M3_Srsf7	7.0709	6.9102	7.1719	7.1002	6.4565	7.0088	7.2528	6.9248	7.1399	7.1082	6.9125	6.9709	5.1482	6.8623	6.9484	6.7978	7.0810	6.8938	7.1812	6.6990
Srsf7	_S[Phospho (STY)]RS[Phospho (STY)]VSLR_2	Q8BL97_S192_M2_Srsf7	5.3273	4.4460	4.6529	4.0630	3.3437	5.3291	3.7044	4.1846	4.6111	4.4482	3.1033	3.5643	5.1733	4.2122	3.7298	3.9997	5.6411	3.8649	3.4743	4.2597
Srsf7	_S[Phospho (STY)]RS[Phospho (STY)]ASLR_2	#N/A	3.6965	5.6205	4.9930	3.9929	3.0342	3.2583	3.0060	4.8198	3.0651	5.4409	5.4544	4.1958	5.3980	5.9654	4.0841	4.0414	5.8917	3.8986	3.5472	5.2809
Srsf7	_S[Phospho (STY)]RS[Phospho (STY)]GS[Phospho (STY)]IIGSR_2	Q8BL97_S208_M3_Srsf7	9.3724	9.2984	9.5366	9.3801	8.9275	8.9969	9.2672	9.4254	9.2511	9.3414	8.9042	8.9908	9.3930	9.4189	8.3414	8.8881	9.4505	9.1139	9.1375	8.8931
Srsf7	_SGS[Phospho (STY)]IGSR_2	Q8BL97_S212_M1_Srsf7	4.1404	3.9742	3.5310	3.5247	8.2752	4.2110	5.2528	7.6006	4.0414	5.2823	9.0161	4.3409	9.4468	3.5561	3.7561	4.2069	8.3510	3.3892	5.3103	4.4097
Ankrd34c	_VLNEPGS[Phospho (STY)]PTR_2	Q8BL88_S231_M1_Ankrd34c	3.3876	3.9315	7.7133	7.4374	4.2898	3.0130	3.2513	3.6133	3.3104	7.8724	3.6848	3.6576	3.4185	4.4631	6.8822	3.7961	3.1105	4.0389	4.3380	4.1403
Ankrd34c	_T[Phospho (STY)]NSIDSKDPTLPFTVQEQVLK_3	Q8BL88_T299_M1_Ankrd34c	4.1544	3.4925	3.5505	3.1791	4.1035	4.1369	3.8469	5.9034	4.3703	3.7072	4.5960	3.3819	3.9326	3.5375	2.9746	3.2587	3.2522	5.4258	4.2342	3.5045
Ankrd34c	_RGS[Phospho (STY)]GTLLLDR_2	Q8BL88_S446_M1_Ankrd34c	4.5384	5.4164	4.4048	5.0221	3.2445	3.2778	3.8036	3.5689	4.1165	4.3490	3.2024	4.5196	3.8893	4.3919	3.8217	3.9005	4.1066	3.7266	3.3752	4.3858
Hirip3	_AVES[Phospho (STY)]TDEDHQTDLDAK_2	Q8BLH7_S134_M1_Hirip3	3.7427	5.1272	4.4980	3.9494	3.4878	3.6114	4.9733	5.6154	3.8686	3.8056	5.8689	4.3132	5.8521	4.5098	3.6212	4.3148	3.7988	4.5275	3.8768	3.5843
Plcxd3	_LNYVFNEGEANT_2	#N/A	6.5757	7.0120	7.1677	6.8976	6.5954	6.9116	6.9010	6.9060	7.1292	7.1388	3.4371	6.5701	7.4068	7.1112	6.2584	7.0904	7.1899	6.4552	6.2872	7.2213
Lsamp	_AANEVSSADVK_2	#N/A	7.4852	7.7335	7.5782	7.3179	7.5639	7.7433	8.0246	7.8124	7.8267	7.5723	7.6399	7.7317	7.5930	7.9398	7.5917	7.4673	7.2180	7.9468	7.5726	7.8338
Lsamp	_HALEYSLR_2	#N/A	7.0774	7.2634	6.8115	6.7414	7.2109	6.8127	7.4288	7.2737	6.9718	6.8362	7.3714	7.2416	7.3034	7.6403	7.0212	7.1414	6.8377	7.3282	6.7433	7.3123
Lsamp	_QGDTAILR_2	#N/A	6.2448	6.2067	5.2381	5.6507	6.2407	5.8202	5.7514	5.6527	6.2665	5.7665	6.1129	5.2259	6.2780	5.5476	5.7186	5.4524	4.7701	6.0703	5.2776	5.5122
Lsamp	_C[Carbamidomethyl (C)]EASAVPAPDFEWYR_2	#N/A	3.2911	4.4214	3.8274	5.3204	4.8978	5.3336	5.0605	5.3089	4.8357	5.3336	5.2880	4.8737	3.5786	4.4303	5.2890	4.3360	4.5294	4.5083	4.6054	4.3809
Lsamp	_EFEGEEEYLEILGITR_2	#N/A	6.8956	6.9453	6.6379	6.8914	5.8025	6.4020	6.2049	6.8164	6.4149	6.6597	6.0498	6.9306	7.4062	7.7460	6.3689	6.7662	6.7337	6.5344	6.3664	6.5686
Lsamp	_C[Carbamidomethyl (C)]EASAVPAPDFEWYRDDTR_3	#N/A	3.4521	5.2148	4.4895	4.1322	4.6206	4.9015	3.3891	3.6777	3.3093	3.2627	4.2888	3.5931	3.4747	5.6730	3.5586	5.4304	3.1750	5.4163	5.2003	5.6179
Lsamp	_SGIFAGHDK_2	#N/A	3.9799	3.3392	6.2697	3.6043	4.1868	3.9215	3.8436	4.2056	3.9027	4.5660	4.2771	3.0653	6.2807	3.3545	3.4208	3.2038	3.7028	6.1082	5.8088	5.9197
Lsamp	_VDVYDEGSYTC[Carbamidomethyl (C)]SVQTQHEPK_3	#N/A	3.5967	3.7224	3.1333	3.9875	3.8036	4.3067	3.2445	3.8224	3.5195	3.4484	4.2812	1.1371	3.8850	4.8843	3.2699	2.9588	3.3196	3.8298	4.1289	3.4494
Lsamp	_IQKVDVYDEGSYTC[Carbamidomethyl (C)]SVQTQHEPK_3	#N/A	4.3243	3.9202	3.1181	3.6068	3.0304	3.0637	4.0177	3.7830	3.9379	4.1349	3.4165	5.0843	4.1034	4.4520	4.0431	3.6864	4.3207	3.5125	3.7206	3.0722
Rps6kc1	_VC[Carbamidomethyl (C)]LQQPSAS[Phospho (STY)]PQGGSSFESR_2	Q8BLK9_S448_M1_Rps6kc1	7.1917	3.6335	7.7329	3.3200	6.5395	7.0304	7.5252	4.0857	7.0112	4.5257	6.9931	7.0328	4.3616	7.5699	5.7118	6.6580	3.9857	6.6515	6.5397	4.8177
Rps6kc1	_SS[Phospho (STY)]PDQFLFSSLR_2	Q8BLK9_S779_M1_Rps6kc1	4.1748	6.9020	3.7114	3.4811	3.6580	5.6916	3.5150	6.6721	5.6166	5.9679	5.2623	3.7191	3.6007	4.0280	5.4071	5.4821	3.8977	6.4830	3.6361	3.9081
Rps6kc1	_S[Phospho (STY)]SQESLDIK_2	Q8BLK9_S422_M1_Rps6kc1	3.8914	4.1114	4.4277	3.7980	3.2291	3.2550	4.8189	3.5918	3.7467	4.0977	3.2253	4.0008	3.9121	4.4148	3.8891	4.2363	4.1295	3.7037	3.3523	4.3818
Rps6kc1	_VFSGGEDLEAVSS[Phospho (STY)]PPTSESLSR_2	Q8BLK9_S577_M1_Rps6kc1	3.9865	3.9626	4.4191	3.7905	3.4151	4.8449	3.9693	4.6428	3.7593	4.3645	3.6479	3.9476	3.9031	4.1179	3.7126	3.8599	3.9463	5.1434	5.5362	4.0221
Rps6kc1	_GVDLLLEGVQGESS[Phospho (STY)]PTR_2	Q8BLK9_S281_M1_Rps6kc1	4.2622	3.6769	4.1421	3.6040	3.8239	3.8572	4.0991	4.0328	4.0193	4.0728	3.4980	3.5084	4.1848	4.1472	4.4793	5.0793	4.0319	3.4806	3.4610	3.1683
Rps6kc1	_FLNRSS[Phospho (STY)]QES[Phospho (STY)]LDIK_2	Q8BLK9_S423_M2_Rps6kc1	4.0513	4.7689	4.2345	4.0330	3.8286	4.6602	3.6333	3.3986	3.5535	3.8762	3.8481	3.6141	3.7189	5.4258	3.6586	3.8113	3.9362	3.9387	5.5300	4.1886
Rps6kc1	_ESEAQDSVSRGS[Phospho (STY)]DDSVPVISFK_3	Q8BLK9_S658_M1_Rps6kc1	3.8999	6.8713	4.1165	3.7271	3.2860	6.0210	6.3742	3.1856	5.9550	3.7105	3.1995	4.0047	4.0379	7.1467	5.6276	3.9420	3.9510	6.6230	3.5222	3.8619
Rps6kc1	_ALFTLEDGDS[Phospho (STY)]PSQSLDPGESK_2	#N/A	4.1356	3.1835	3.6721	3.4487	4.3425	4.3758	4.2064	4.3612	4.1266	3.9462	3.6053	4.4105	4.2921	3.2939	4.2318	3.4977	3.8585	3.3237	2.9723	3.2609
Rps6kc1	_ALFTLEDGDS[Phospho (STY)]PSQSLDPGESKR_3	#N/A	3.6769	3.6422	3.2135	3.9073	3.8838	3.9172	3.1643	3.9026	3.0845	3.4875	4.0640	3.3683	3.2499	3.7526	3.1897	3.0390	3.3998	3.4659	4.0145	3.7196
Unc80	_GRS[Phospho (STY)]PTVGNKR_2	Q8BLN6_S1261_M1_Unc80	3.8116	3.5075	3.3482	3.7403	3.6625	4.0519	3.0296	4.0373	4.4506	3.6222	3.9293	3.2336	3.1152	3.6179	3.0549	3.1737	3.5345	2.9988	4.1492	3.5849
Unc80	_VAS[Phospho (STY)]QS[Phospho (STY)]EPGQQNVLLQQPLGR_3	Q8BLN6_S3045_M2_Unc80	3.4011	4.6017	3.9374	4.2883	4.2396	4.4637	3.3014	3.1015	3.3605	3.6074	3.7349	2.9903	3.4686	3.9245	2.8786	3.7460	3.6391	3.9888	3.8426	4.1905
Slc7a4	_ASPPSS[Phospho (STY)]PC[Carbamidomethyl (C)]LAS[Phospho (STY)]PGPTAK_2	Q8BLQ7_S427_M2_Slc7a4	4.1137	8.0232	5.7860	3.8761	3.7745	8.2206	3.7555	7.7910	7.5902	7.7850	7.5058	7.5491	3.8868	7.6249	6.2596	3.5642	7.8357	3.7688	6.7028	3.4384
Slc7a4	KAS[Phospho (STY)]PPSS[Phospho (STY)]PC[Carbamidomethyl (C)]LAS[Phospho (STY)]PGPTA	#N/A	4.0604	4.4623	3.9902	3.7948	3.4534	3.5776	4.2176	3.1666	3.9472	3.7304	3.8531	3.6194	4.0737	4.0034	3.6780	4.1094	3.9582	4.3148	3.5583	3.8280
Cadm2	_DGAELPDPDR_2	#N/A	10.0749	9.8246	9.8844	10.0065	9.7102	9.8822	9.8836	10.0991	9.9770	9.8668	10.1076	9.8129	9.8579	10.0688	9.4151	9.5952	10.2733	9.7817	9.7170	9.9584
Cadm2	_GAEDAPDADTAINAEGS[Phospho (STY)]QVNAEEKK_3	#N/A	8.9240	8.6328	8.7248	8.5426	7.7736	7.9356	8.2069	7.8674	6.5439	7.0363	6.4262	7.9861	8.0064	8.9815	5.8188	7.8563	8.1180	8.0298	5.8670	8.0458
Cadm2	_SDDGVAVIC[Carbamidomethyl (C)]R_2	#N/A	8.6193	8.6607	8.8634	9.0578	8.7189	8.6869	8.7621	8.9140	8.7856	8.4246	8.8199	8.4036	8.8049	8.6332	8.6284	8.4527	8.3910	8.6128	8.4304	8.6295
Cadm2	_TSGSKPAADIR_2	#N/A	8.0711	7.7012	7.9832	8.0977	7.7924	7.6528	7.7578	8.1551	7.8986	7.9533	7.6831	7.8008	7.8600	7.9317	8.1942	7.5696	7.4242	7.8054	7.3803	7.5447
Cadm2	_TFTVSSTLDFR_2	#N/A	6.5930	6.4763	6.3076	6.6738	6.2001	5.9719	5.4835	6.4391	6.5531	6.1365	6.1010	4.2206	4.3563	6.3308	6.4062	6.0485	6.0359	5.7186	6.3300	6.3990
Cadm2	_IIPSTPPFQEGQALTLTC[Carbamidomethyl (C)]ESK_2	#N/A	5.5755	5.8747	5.4107	5.8220	5.6147	5.6650	5.8169	6.1362	6.0407	5.5469	5.8391	5.8660	5.6001	6.1657	5.4963	5.0020	6.0608	5.7010	5.5072	5.3194
Cadm2	_YLKEEDANRK_3	#N/A	3.9280	2.5507	3.4646	3.6562	4.1349	2.9318	4.0318	4.1537	2.8728	3.7542	3.3729	2.6419	3.9589	3.5026	4.1712	2.3399	2.6280	3.4985	4.1550	3.1799
Cadm2	_GAEDAPDADTAINAEGS[Phospho (STY)]QVNAEEK_3	#N/A	4.5509	4.2690	4.0875	4.5341	3.2570	3.3505	3.7911	3.2758	2.9729	3.9950	3.3473	3.9951	4.5818	4.1256	3.8164	3.9131	4.2739	3.4764	3.1746	4.3665
Cadm2	_GAEDAPDADTAINAEGS[Phospho (STY)]QVNAEEKKEYFI_3	#N/A	3.5076	4.7167	4.3562	4.1597	3.4119	3.8077	3.1679	3.5667	3.5716	4.5303	3.3750	3.4236	4.3626	4.6791	3.5279	4.1760	3.8064	3.8046	4.1154	4.2666
Sowaha	_QRS[Phospho (STY)]EEPSPR_2	Q8BLS7_S242_M1_Sowaha	3.8255	3.8536	4.5029	4.1188	3.6724	3.1468	2.8835	3.1410	2.0626	4.1119	3.5572	3.5798	3.4614	3.7565	3.4011	4.3284	3.1698	3.8550	3.6946	3.9310
Slc9a7	_TKS[Phospho (STY)]SSEEVLER_2	Q8BLV3_S694_M1_Slc9a7	4.2101	4.6099	3.7466	3.3742	2.9162	3.6914	4.1319	4.4357	4.1329	4.0207	4.8164	4.3360	4.2409	3.7847	4.1573	3.5722	4.9802	3.2165	3.5155	3.4620
Slc9a7	_VGVEELS[Phospho (STY)]EEDQNENR_2	Q8BLV3_S546_M1_Slc9a7	6.1138	5.7569	4.6086	4.5625	3.9862	5.0276	5.4697	4.3281	2.9821	5.4318	3.9616	3.2660	3.1476	3.6502	3.0873	3.1414	3.3649	5.0184	4.1168	3.6172
Plekhm3	_S[Phospho (STY)]RSDVTHVDWR_3	Q8BM47_S150_M1_Plekhm3	4.8606	4.2202	4.0157	4.4853	3.3058	3.3017	3.7423	3.3246	3.0217	3.9462	3.3961	3.9463	3.1298	4.1744	3.7677	3.9618	4.3226	4.3276	3.1259	3.8516
Nyap2	_LSTS[Phospho (STY)]SETVNSTASK_2	Q8BM65_S154_M1_Nyap2	8.2160	7.8609	7.7884	8.3767	7.5936	6.9986	7.2313	7.1587	7.3177	8.6240	7.7711	7.6411	7.7222	8.1604	6.2865	7.8430	7.7678	7.3387	7.4908	7.7966
Nyap2	_RNPNTQLSTS[Phospho (STY)]FDETYIKK_3	Q8BM65_S238_M1_Nyap2	4.3720	3.6308	3.4074	3.3174	3.7097	4.2753	5.7139	4.0723	4.2272	3.8456	3.7059	3.5203	4.3927	3.3945	2.8316	3.2160	3.1092	3.2231	4.3726	3.3615
Nyap2	_FTVPASHS[Phospho (STY)]PPR_2	Q8BM65_S184_M1_Nyap2	8.8284	8.7860	7.8727	4.1995	8.7770	3.6564	8.4250	8.8752	3.3452	3.2268	8.5672	8.4692	9.0789	9.4078	3.4504	3.8348	9.3246	4.1052	8.2526	3.9803
Nyap2	_KEEDDQSEAVY[Phospho (STY)]EEM[Oxidation (M)]KYPIFDDLGHDSK_4	#N/A	3.8552	3.2735	3.7853	4.0658	3.5179	4.0422	4.6973	3.8675	3.7836	4.4965	3.8253	3.9751	3.9175	3.9376	3.5928	3.3725	4.0859	4.1566	4.1386	3.6149
Nyap2	_DQTGALGPAPGASLSSS[Phospho (STY)]PPPPSTLYR_3	Q8BM65_S442_M1_Nyap2	3.8144	3.4103	3.6280	3.0969	4.0214	4.0547	3.0267	4.2929	4.4478	3.6250	3.9264	3.2298	4.0669	3.6150	3.0521	3.1766	3.3297	3.0026	4.1520	3.5820
Emsy	_TNS[Phospho (STY)]SSSPVVLK_2	Q8BMB0_S173_M1_Emsy	6.8889	4.0111	4.3454	7.2387	3.5149	3.5483	3.5332	3.2985	3.4534	7.0347	2.9320	3.7372	3.6188	6.9534	6.3940	6.9236	6.9460	6.5557	3.6456	6.9673
Me3	_VRGEEYDDLLDEFM[Oxidation (M)]QAVTDK_3	#N/A	4.5702	5.2530	4.6787	4.1818	3.9862	4.7159	4.9015	5.2517	3.9790	5.1454	3.7768	4.4010	4.4742	4.5425	4.6918	5.6485	4.6929	5.1246	5.6170	4.5950
Me3	_TFTPGQGNNAYVFPGVALGVIAGGIR_3	#N/A	4.3099	3.2842	2.9019	3.7028	2.9286	4.1652	2.8402	3.7343	3.7100	3.3294	3.7084	2.1025	3.4182	3.9266	3.4577	4.3775	3.2315	4.3391	4.2377	4.6229
Me3	_EMFAQDHPEVNSLEEVVR_3	#N/A	4.5190	4.3009	4.0556	4.7002	3.2251	3.2585	3.4104	3.2439	3.7432	4.3296	3.6153	4.0270	3.9086	4.0937	3.8483	3.8812	4.2420	4.4083	3.2065	4.3783
Dlat	_ILVPEGTR_2	#N/A	9.8563	10.0556	9.9448	10.1745	10.1348	9.9427	10.2776	10.2349	10.0361	9.9219	9.9112	10.1968	10.1866	10.1804	10.5591	10.0302	9.8564	10.2096	9.9361	10.0552
Dlat	_DVPLGAPLC[Carbamidomethyl (C)]IIVEK_2	#N/A	7.7932	7.8410	8.2510	8.3808	8.2273	8.0744	7.8773	7.8784	8.1459	8.3023	7.8826	7.8288	7.6386	8.1364	8.0695	8.1255	8.1792	7.2099	7.6197	8.0568
Dlat	_GLETIASDVVSLASK_2	#N/A	7.7267	7.4859	7.8554	7.8113	7.8024	7.6469	7.4393	7.6738	7.6153	8.0125	7.5633	7.6955	7.7645	8.0821	7.5971	7.7426	7.3094	7.5402	7.6873	8.1370
Dlat	_VPLPSLSPTM[Oxidation (M)]QAGTIAR_2	#N/A	7.9761	7.7801	7.4095	7.3668	7.8751	7.7237	7.5090	8.1149	7.5969	7.7455	7.3505	7.7830	8.4546	7.6868	7.7719	7.8958	7.9369	8.0610	7.8565	7.8646
Dlat	_DIDSFVPSK_2	#N/A	6.5317	7.2230	7.4868	8.0529	7.0940	7.2131	7.4092	6.7962	7.7500	6.9899	7.4514	7.5156	7.7765	7.2445	8.0595	7.7782	6.5721	7.5703	7.6227	7.9062
Dlat	_VFVSPLAK_2	#N/A	6.7146	7.0548	7.2739	7.5811	7.3339	6.8983	7.5132	6.9966	6.9855	7.3926	7.4724	7.2403	7.5725	7.2326	7.6967	6.9423	5.7034	7.5212	7.5142	7.8127
Dlat	_YLEKPITM[Oxidation (M)]LL_2	#N/A	5.8018	5.5946	4.1353	5.4422	5.3998	5.3296	5.4059	5.6426	4.0605	5.9081	5.1523	6.2078	5.7512	5.4858	5.2517	5.2878	5.5951	5.7442	5.3013	5.9149
Dlat	_ISVNDFIIK_2	#N/A	4.9919	5.7524	4.6679	5.1468	5.2406	5.2728	5.2789	6.2011	3.7829	5.7078	4.7717	4.9929	5.9171	6.0811	4.7582	5.5313	4.8725	5.6634	4.6139	4.4585
Dlat	_VAPAPAGVFTDIPISNIR_2	#N/A	7.9947	8.0554	8.3676	8.3225	8.2837	8.1851	8.3727	8.2185	8.4476	8.1605	8.1703	8.3389	8.4805	8.5741	8.4992	8.2670	7.9601	8.4009	8.2162	8.3568
Dlat	_DVPVGSIIC[Carbamidomethyl (C)]ITVEKPQDIEAFK_3	#N/A	6.5844	7.2586	7.3307	6.8209	6.4655	6.9051	6.4276	6.6358	6.5099	6.2806	6.5357	6.8174	6.3241	7.3455	6.9181	6.5503	6.7590	6.6127	7.1073	6.8060
Dlat	_LQPHEFQGGTFTISNLGM[Oxidation (M)]FGIK_3	#N/A	5.4254	5.5835	5.3159	3.9655	5.0596	5.3310	4.7947	5.6862	4.6802	4.7041	5.1364	4.8399	5.3627	5.5675	4.4042	5.4497	5.7128	4.7333	5.4962	6.0182
Dlat	_VPEANSSWM[Oxidation (M)]DTVIR_2	#N/A	6.5067	7.1656	7.4397	7.0640	6.0865	6.5400	6.8205	5.0141	6.4934	6.2876	6.7240	6.5762	7.1269	7.0470	6.8871	6.6260	6.3459	6.2903	6.4488	6.4275
Dlat	_AAPAAAAAM[Oxidation (M)]APPGPR_2	#N/A	6.0075	4.8178	5.0635	6.0806	6.2850	6.1351	5.4974	6.0604	5.7172	5.9943	5.6031	6.2468	5.9233	5.7806	5.6002	5.8989	5.7226	6.2240	5.6789	6.3465
Dlat	_ASALAC[Carbamidomethyl (C)]LK_2	#N/A	4.1554	3.3092	3.6920	3.4288	2.8615	3.7460	3.6547	4.3811	4.0782	3.9639	2.9518	3.8732	4.1863	3.7301	4.5648	3.5175	3.8783	4.1338	3.4321	3.4074
Dlat	_VVDGAVGAQWLAEFK_2	#N/A	3.6765	3.2724	3.7659	4.4598	3.8834	3.9168	3.1647	4.4308	3.0849	3.4871	4.0643	3.1618	3.2503	3.7530	3.1900	3.0386	3.4676	4.3655	4.0141	3.7200
Rab3gap2	_GGFS[Phospho (STY)]PFGNTQGPSR_2	Q8BMG7_S448_M1_Rab3gap2	10.9073	10.6079	10.0219	10.3729	9.5397	9.7206	10.2666	10.4152	9.4354	10.3777	9.9258	9.8537	10.2790	10.2852	7.2600	9.7082	9.9650	9.9439	9.5551	9.6021
Rab3gap2	_AKS[Phospho (STY)]PRPDSFETEIK_3	Q8BMG7_S566_M1_Rab3gap2	3.4524	2.9034	3.9887	4.2370	4.2909	3.0142	3.2501	3.1527	4.5607	3.6730	4.3972	2.9390	3.4173	3.9757	2.8273	3.7973	3.6904	4.1427	3.7913	4.1392
Rab3gap2	_TSS[Phospho (STY)]WEDDSWGAWEETEPR_2	Q8BMG7_S42_M1_Rab3gap2	4.4755	4.3444	4.0121	3.1087	3.1816	3.4259	4.3393	3.2004	4.3984	4.0704	3.2719	4.0705	4.5064	4.0502	3.8918	3.8377	4.1985	4.4518	3.2500	3.7275
Gga3	_LAS[Phospho (STY)]ETEDNDNSLGDILQASDNLSR_3	Q8BMI3_S267_M1_Gga3	3.2183	3.2836	3.7546	4.4711	4.0569	3.9280	3.1534	4.4195	3.0736	3.4984	4.0531	3.1731	3.2390	3.7417	3.1788	3.0499	3.4564	4.3768	4.0254	3.7087
Ckap4	_VGAHGS[Phospho (STY)]EEGAVFK_2	Q8BMK4_S367_M1_Ckap4	5.7577	3.5165	3.5218	3.2031	5.6068	5.7053	5.1634	4.1867	3.7761	3.1918	5.6669	5.5640	4.5070	6.7034	4.4274	3.5515	5.8148	6.3020	5.5140	5.9051
Hadha	_FVDLYGAQK_2	#N/A	5.7285	6.2494	7.7354	3.9616	5.8218	5.8418	5.8678	6.1277	6.4597	5.9477	5.8190	6.3157	5.9364	5.3339	6.2464	5.6326	6.1039	5.6556	5.5697	5.5011
Hadha	_DGPGFYTTR_2	#N/A	6.2955	6.3180	5.9711	6.3744	6.4900	6.4405	6.5434	6.8661	6.6782	6.1947	6.3774	6.1084	6.1262	5.9409	5.6862	6.1388	5.9327	6.5778	6.0120	6.0596
Hadha	_AGLEQGSDAGYLAESQK_2	#N/A	4.5186	4.2765	3.1875	4.6867	4.1058	3.7650	3.2413	5.0023	4.1054	3.9172	3.1975	3.0292	3.6818	3.6487	3.2156	4.4568	4.5275	3.8092	4.1707	4.8144
Hadha	_SLNSEM[Oxidation (M)]DNILANLR_2	#N/A	3.4948	3.8243	4.5322	5.1569	5.6236	5.2857	3.3585	6.0751	5.7622	6.0061	5.1380	5.1364	3.5257	4.5703	5.4037	5.7295	5.8388	5.6909	4.2308	5.8255
Hadha	_DTTASAVAVGLR_2	#N/A	4.2194	4.5229	5.4616	5.1220	2.9255	4.5263	4.2010	4.4451	5.0568	4.0300	3.5215	4.1457	4.2083	4.7894	4.5673	3.5815	5.0743	3.8335	4.3396	4.2385
Hadha	_TVLGVPEVLLGILPGAGGTQR_2	#N/A	3.7294	3.5740	4.0435	3.7463	3.9364	3.9569	4.2813	3.6380	4.1645	3.5716	3.6576	3.5910	4.0490	3.5436	3.2486	3.4495	3.8184	3.7981	3.2734	3.1500
Hadha	_LPAKPEVSSDEDVQYR_3	#N/A	3.2483	3.7286	3.1271	3.9937	4.0868	4.3109	3.4542	3.8162	3.5133	3.4546	3.6666	3.4547	3.6214	4.2036	4.4330	3.5932	3.3134	3.8446	4.1351	4.3433
Larp6	_SPGAS[Phospho (STY)]PLLSR_2	Q8BN59_S451_M1_Larp6	3.5418	6.1460	5.6738	4.1476	6.1138	6.5250	3.1607	3.2421	6.1469	5.9730	3.5942	4.3505	3.3279	6.7005	4.2388	3.8867	6.9870	6.3878	5.6890	4.0498
Larp6	_YLSPGWGSAS[Phospho (STY)]EEPSR_2	Q8BN59_S58_M1_Larp6	3.5523	3.4066	3.5463	4.1946	4.0283	4.0681	3.9278	4.2223	7.0999	3.6384	3.9149	3.2147	4.0135	3.5569	3.0406	7.0159	3.4895	5.9116	3.9864	3.5705
Spata2l	_SGDLAPPESPSS[Phospho (STY)]PGQASPR_2	Q8BNN1_S329_M1_Spata2l	3.9044	8.5540	3.6774	3.8207	3.5417	8.1758	3.9172	3.1693	8.2593	8.7843	3.6378	8.4198	4.0387	8.0060	7.7853	7.8248	9.1656	3.7133	7.7390	7.8527
Kbtbd11	_ASAAEGSEAS[Phospho (STY)]PPSLR_2	Q8BNW9_S70_M1_Kbtbd11	12.3650	12.4174	12.4952	12.4810	11.9358	12.3114	12.1773	12.4761	12.4972	12.1563	12.0569	11.9958	12.6454	12.4283	11.9645	12.3556	12.5928	12.2492	12.3980	12.5149
Kbtbd11	_LLADAYSGR_2	#N/A	5.8142	5.3246	4.1583	5.0514	3.3278	5.5441	5.1513	3.3465	3.0437	5.4420	4.8744	5.6180	4.7237	4.1964	5.0548	5.6968	4.6772	5.6248	4.5750	5.4397
Kbtbd11	_AC[Carbamidomethyl (C)]LLAAALGTTGER_2	#N/A	4.1090	3.8938	5.3607	5.2491	3.4467	5.4083	4.9885	5.2319	4.8040	4.1085	4.8179	3.7833	4.1297	5.5259	4.9032	5.5824	5.2229	4.8815	5.1017	5.2042
Kbtbd11	_SGS[Phospho (STY)]RPQS[Phospho (STY)]PSGDAESR_2	Q8BNW9_S316_M2_Kbtbd11	8.7203	7.6570	8.7420	8.7511	8.2731	8.5950	8.4813	8.5205	8.5180	8.6761	8.5576	8.4276	8.3539	7.7295	8.7700	7.2414	8.7415	7.6081	8.3967	8.1207
Kbtbd11	_GDAAVYC[Carbamidomethyl (C)]YQAEAGEWR_2	#N/A	4.9979	3.6975	5.2936	3.3841	3.6430	3.8671	3.8980	4.5971	3.9571	4.5116	4.4315	4.6657	5.2765	5.6262	3.4752	3.1494	4.8585	3.3922	3.5953	3.2862
Kbtbd11	_PQS[Phospho (STY)]PSGDAESR_2	#N/A	3.2893	4.0298	4.3267	4.2949	3.4963	3.1113	3.5518	3.5150	3.2121	3.7558	3.5865	3.7559	3.3202	4.2985	3.5772	4.1523	4.7083	4.1372	4.4363	4.0421
Fam92a	_ANSKS[Phospho (STY)]PLQR_2	Q8BP22_S312_M1_Fam92a	4.5070	4.3542	3.5425	5.4278	3.8447	4.5960	3.6964	4.0583	4.0958	4.9994	4.1298	3.3853	3.8635	3.4073	3.2736	4.5319	3.5555	3.5939	4.9628	3.0845
Sfr1	_S[Phospho (STY)]SSHSPC[Carbamidomethyl (C)]SVVK_2	Q8BP27_S139_M1_Sfr1	3.8709	3.7402	6.9981	3.8371	3.7595	3.8262	4.0284	3.8898	3.7552	6.0199	4.0667	3.5229	6.4159	6.5778	3.3686	6.3358	6.8614	6.3563	3.9992	3.5587
Sfr1	_ENPPS[Phospho (STY)]PHSNSSGKQPLSGTPK_3	#N/A	4.2519	3.7509	3.2874	3.4375	3.5896	7.1140	4.1870	9.2740	4.1072	8.8092	3.5858	3.6404	4.2726	3.2744	3.5287	3.0960	9.1866	3.3432	2.9918	9.0991
Sfr1	_ENPPS[Phospho (STY)]PPTS[Phospho (STY)]PAAPQPR_2	#N/A	12.4038	14.4552	14.6885	14.5962	13.7818	12.1641	13.9535	14.5607	14.3088	12.3813	14.1096	14.4073	14.9534	12.7420	14.3735	14.5017	14.7469	14.1102	14.4050	14.3854
Sfr1	_ENPPS[Phospho (STY)]PHSNSSGK_2	#N/A	10.4848	10.6456	10.6446	10.5817	10.3714	10.5535	10.3532	10.6717	10.3449	10.5703	10.3959	10.4798	10.8983	11.3429	10.6391	10.7208	10.5992	10.6588	10.6671	10.6474
NARS1	_ISALEGYR_2	#N/A	6.2186	5.7009	5.3113	5.9532	5.8921	5.9792	5.8219	6.2608	5.2348	4.8776	5.5462	5.7625	5.6640	5.7854	4.5445	3.7705	5.8968	6.0301	5.2550	5.1555
NARS1	_DVC[Carbamidomethyl (C)]LYPR_2	#N/A	5.2439	4.3929	3.1685	5.5255	4.1702	4.2900	5.0698	4.1889	3.9170	6.0471	4.8206	5.2427	5.6133	5.1382	5.6213	4.4860	5.0052	5.5781	3.7623	5.2087
NARS1	_SWDSEEILEGYKR_3	#N/A	3.5996	4.0799	4.2766	4.3450	2.9373	3.1614	3.1029	3.4649	2.3652	3.8059	3.5364	3.8060	3.2701	4.3147	4.1810	3.9445	4.4630	4.1873	2.9855	3.9920
Fra10ac1	_TTPEC[Carbamidomethyl (C)]DES[Phospho (STY)]PRKK_2	Q8BP78_S243_M1_Fra10ac1	5.6062	5.7964	3.3496	3.3753	5.3273	5.4595	5.7968	4.0145	5.5686	5.0576	4.3227	6.2835	6.2910	3.6001	6.1727	3.3590	5.6966	5.5826	5.9016	3.2774
Fra10ac1	_S[Phospho (STY)]RSPPSEEASK_2	Q8BP78_S248_M1_Fra10ac1	3.7256	6.2970	5.0933	4.4885	5.3730	3.6382	3.9081	4.0035	4.6237	3.7638	4.4237	3.6451	4.0501	3.9564	3.6419	4.9956	3.9222	5.0494	4.8628	3.7736
Fra10ac1	_NAEEEDSAS[Phospho (STY)]DSELWK_2	Q8BP78_S285_M1_Fra10ac1	4.2716	4.1875	3.5857	3.3805	3.3197	3.6977	4.0019	4.4365	4.1336	3.4422	3.0072	4.6805	4.2417	3.7854	3.8254	4.3044	3.9337	3.2228	3.5219	3.4627
Rcn2	_EALLGVQEDVDEYVK_2	#N/A	5.6314	5.5767	5.8279	5.0645	5.1417	4.7616	5.2877	4.6113	4.7646	5.3777	4.9670	5.2407	5.4626	5.9502	5.1753	4.5430	5.3400	5.2010	4.7937	5.4487
Rcn2	_RDPTANEDPEWILVEK_3	#N/A	3.6460	4.5241	5.0461	5.1592	3.8688	4.2872	3.7603	3.8399	4.6901	4.6658	4.0719	4.1806	3.7988	4.3547	4.4088	4.5832	3.1227	3.8123	4.6669	4.1791
Rcn2	_VIDFDENTALDDTEEGSFR_2	#N/A	4.6234	4.2399	3.1642	3.5607	3.4664	4.7523	4.0638	4.3229	3.1677	4.8798	4.4746	3.9178	4.5038	5.0884	4.7942	4.1449	4.3668	3.4664	3.1150	4.4857
Rcn2	_LSEEELENQDLFLTSEATDYGR_3	#N/A	4.5340	4.2859	4.0706	4.5510	3.2401	3.2735	3.8080	3.2589	4.1933	4.3446	3.2068	4.0120	3.8936	4.1087	3.8333	3.8962	4.2570	4.3933	3.1915	4.3633
Rcn2	_HYAMQEAK_2	#N/A	3.4413	4.5380	4.0011	4.2246	3.6482	3.6816	3.3999	3.1652	3.3201	3.2519	4.2996	2.9266	3.4855	3.9882	3.4252	4.3043	3.7029	4.1303	3.7789	3.9552
	_SSS[Phospho (STY)]ESPSDNQVFQATR_2	Q8BP99_S63_M1_	3.8454	3.4737	3.3820	6.3204	5.7143	4.0857	2.9957	4.0711	4.9618	3.6560	3.8954	3.1998	5.5546	3.5840	3.0211	5.3827	3.5683	3.0336	4.1830	3.5510
Daam1	_ESS[Phospho (STY)]EESGEFDDLVSALR_2	Q8BPM0_S1026_M1_Daam1	3.9764	3.3427	3.5130	3.6078	4.1834	4.2167	4.3656	4.2021	4.2858	3.7870	3.7644	3.0688	4.4512	3.5511	2.8901	3.3385	3.6993	3.4501	3.7492	3.2283
Dmxl2	_LDHELS[Phospho (STY)]LDR_2	#N/A	10.2452	9.7739	9.9531	9.4858	8.6871	9.7476	9.5912	9.4022	9.0926	9.4434	9.4673	8.9137	8.6380	9.4928	7.6624	8.7036	9.5417	9.3112	9.1154	8.6845
Dmxl2	_KQS[Phospho (STY)]EVEADLGYPGGK_2	#N/A	9.1032	8.8628	3.4543	8.5748	8.1843	8.6480	8.4491	9.0106	8.6552	8.6263	8.4022	8.1441	9.0234	8.3464	3.3617	7.9523	9.1672	8.6833	8.4842	8.0822
Dmxl2	_NLAS[Phospho (STY)]PEGTLATLGLK_2	Q8BPN8_S1856_M1_Dmxl2	8.1561	8.5511	6.7220	6.5019	4.1386	3.8464	8.6904	9.3674	6.1526	6.9712	5.9653	8.2764	8.4497	8.6408	4.8864	3.6449	7.2182	8.4921	7.4207	5.5624
Dmxl2	_ESKSS[Phospho (STY)]DDIDYR_2	#N/A	7.7067	7.6857	6.9744	7.2108	6.8440	7.2803	7.3667	7.4451	4.1759	7.3517	7.1182	4.2929	4.2840	6.9933	3.6940	6.4049	7.5713	7.3898	3.4725	6.6673
Dmxl2	_YLDEYNPGIFR_2	#N/A	5.8212	5.8663	5.2587	4.6513	5.7814	6.0024	5.6974	5.4635	5.6144	5.6788	5.6715	5.4583	6.2454	5.3102	4.8730	5.1446	5.7510	5.5769	4.8814	5.5184
Dmxl2	_LVYSQPLDLPEAVEVIR_2	#N/A	5.0125	5.1732	3.5726	4.9138	5.1856	5.0386	4.8387	4.7330	4.6079	5.0282	5.1008	4.9852	5.1163	3.9253	4.0153	5.1183	4.8804	4.6512	4.8945	4.7875
Dmxl2	_ALLT[Phospho (STY)]PQDEEC[Carbamidomethyl (C)]ADGDTEVDVIAEQLK_3	Q8BPN8_T2017_M1_Dmxl2	3.4276	3.8915	4.4650	4.2109	3.6345	3.6679	3.4136	3.1789	3.3338	3.2382	4.3133	7.0512	3.4992	4.0019	3.4389	4.2905	3.7166	7.4173	3.7652	3.9689
Dmxl2	_FGNVDADS[Phospho (STY)]PVEETIQDHSALK_3	#N/A	7.8834	10.2911	8.8925	7.8501	3.2842	9.2946	9.6239	3.5142	9.2386	7.6720	6.8234	7.7966	8.6107	11.0971	8.0135	7.4046	7.2223	11.1398	8.8245	8.0014
Dmxl2	_M[Oxidation (M)]KLDHELS[Phospho (STY)]LDR_3	#N/A	7.5896	7.7262	8.6648	8.7937	6.2488	7.9486	7.9134	5.7971	8.0144	7.9324	7.2678	7.1549	5.4834	8.3089	6.5792	7.4174	6.9236	7.5587	7.9672	7.8193
Dmxl2	_VDPIGPLSYTGGVSELAR_2	#N/A	6.9541	5.7967	3.9267	6.1035	4.2290	6.4714	6.5890	6.2188	6.0319	5.3967	6.1399	6.4799	5.5542	4.5239	5.4513	6.1736	6.4812	5.8235	5.9823	6.0192
Dmxl2	_RQS[Phospho (STY)]ESIAAPPVASEDM[Oxidation (M)]DKHR_4	#N/A	7.4838	7.4443	7.9733	8.1734	6.9103	7.2043	5.1821	7.0257	7.4626	7.2674	7.1342	6.6595	8.1488	4.0581	6.5229	7.0229	7.3501	7.1630	7.1379	6.8934
Dmxl2	_QLDHES[Phospho (STY)]DDADREDEER_3	#N/A	4.7321	5.2776	4.0266	3.6135	4.0987	5.0354	5.2384	4.7383	4.7092	4.9831	5.1761	4.2892	5.4905	4.7846	4.2058	4.3291	4.6949	5.2492	4.8659	3.9736
Dmxl2	_LYQAVVDAR_2	#N/A	4.4951	5.1086	4.5362	4.0854	5.0463	4.6575	5.2257	5.6839	4.6826	5.1983	4.2629	5.2341	4.5760	4.9211	2.9397	3.6849	3.2217	5.3337	4.2268	4.2516
Dmxl2	_VS[Phospho (STY)]VDSNLFVYSK_2	Q8BPN8_S1141_M1_Dmxl2	7.8088	7.7698	7.7028	3.9412	6.4544	7.4625	7.3401	6.9382	7.8299	7.6061	3.9677	6.5647	7.3304	6.7844	6.3633	6.6744	7.7980	7.6559	7.3930	6.8685
Dmxl2	_RQS[Phospho (STY)]ESIAAPPVASEDMDK_2	#N/A	4.2450	3.9916	3.5947	4.8038	3.8757	3.9353	4.1199	4.2758	4.1390	3.8095	3.7923	3.7185	4.2703	4.2414	3.2800	3.2495	3.6382	3.2706	3.8678	3.3933
Dmxl2	_RKQS[Phospho (STY)]EVEADLGYPGGK_3	#N/A	3.9487	3.3704	4.7536	4.4408	4.1557	5.7200	4.3933	5.5429	4.6315	3.0864	5.7856	4.7639	3.9796	3.5234	2.9178	3.3108	3.6716	5.5281	3.7769	3.2006
Dmxl2	_SS[Phospho (STY)]DDIDYR_2	#N/A	4.0381	3.6928	4.1260	3.6102	3.9036	4.0014	4.2384	4.0426	4.2311	5.4537	3.4180	4.2024	4.3662	3.8502	4.0523	3.9408	4.0238	3.3925	5.5445	3.4728
Dmxl2	_LDHELSLDRES[Phospho (STY)]EAGTGSSEHEDGERGS[Phospho (STY)]PR_4	Q8BPN8_S456_M2_Dmxl2	3.3427	3.9764	4.3801	4.2416	3.5496	3.0579	3.4985	3.5683	3.2655	3.1533	3.6399	3.7025	3.3735	4.4181	3.5239	4.2056	3.0656	4.1161	4.3829	4.0954

Dmxl2	_ESEAGTGSSEHEDGEREGS[Phospho (STY)]PR_.3	Q8BPN8_S473_M1_Dmxl2	4.7193	4.6825	4.1244	3.8166	4.0009	4.8232	5.1271	4.7457	4.8274	3.9496	4.1577	4.1900	4.6203	4.4542	4.5438	4.1302	4.4574	4.5782	4.3986	3.8565
Dmxl2	_NIGAGVMQIAISQDNR_.2	#N/A	4.3815	4.4384	6.3001	3.2027	3.0876	4.7637	4.7840	3.1064	4.1702	4.1921	5.3240	4.1645	4.4124	3.9562	3.9858	3.7437	4.1044	3.0450	3.3440	3.6335
Dmxl2	_VGSVLDSRVS[Phospho (STY)]VDS[Phospho (STY)]NLFVYSK_.3	#N/A	3.8149	4.1880	4.3512	3.8745	3.1526	3.3315	3.7499	3.5152	5.7636	4.4027	3.1488	4.0774	3.8356	4.3382	3.7753	4.1598	4.0529	3.7802	3.4288	4.3052
Dmxl2	_QLDHES[Phospho (STY)]DDADREDEERSQDER_.4	#N/A	3.4830	4.1329	4.0193	4.1081	3.4470	3.7735	3.3719	3.5397	3.3809	3.4115	3.5008	4.0223	3.5093	4.3820	3.6881	3.8279	3.9688	4.0079	4.3987	4.1457
Sgsm1	_C[Carbamidomethyl (C)]SSGAS[Phospho (STY)]LDSHLHR_.3	#N/A	4.4978	3.5356	4.3781	4.6829	3.6903	4.4047	4.5908	4.1106	3.9260	4.8630	3.5791	3.9055	4.0815	3.4520	3.5877	3.7401	4.2559	4.4169	5.1268	3.9902
Sgsm1	_QDS[Phospho (STY)]PTKRPALC[Carbamidomethyl (C)]IQK_.3	Q8BPQ7_S215_M1_Sgsm1	5.1017	4.4830	4.0561	4.1696	3.5933	3.6266	3.4548	3.2201	3.3750	4.7584	4.3545	4.3725	3.5405	4.0431	3.4802	4.2493	3.7578	4.0753	3.7239	2.5142
Sgsm1	_RHS[Phospho (STY)]S[Phospho (STY)]GSM[Oxidation (M)]DDRPSISAR_.3	#N/A	5.8939	5.1131	5.2947	4.0031	5.3663	5.2261	5.2206	5.5530	5.4778	6.1107	5.5974	5.2490	3.8584	4.6655	3.5544	5.5161	3.5635	5.4638	5.8268	5.2838
Sgsm1	_HSSGS[Phospho (STY)]M[Oxidation (M)]DDRPSISAR_.2	#N/A	3.8822	3.9187	4.1693	3.7735	3.5559	3.6472	3.7847	3.7708	4.1988	4.0680	3.4871	3.7688	3.9085	4.1662	3.6204	3.8557	3.9758	3.6584	3.5511	4.0813
Sgsm1	_SPQGSSESTSS[Phospho (STY)]DKEDDEATDYVFR_.3	Q8BPQ7_S407_M1_Sgsm1	4.4842	2.8586	3.3920	3.7288	3.8219	4.0460	3.7191	4.0811	3.7782	3.1897	4.1526	3.1898	3.8863	3.4300	3.2963	2.7357	3.5783	3.5711	3.8702	3.1073
Elmo1	_IAFDAESEPNNSSGS[Phospho (STY)]M[Oxidation (M)]EK_.2	#N/A	7.9046	3.8400	4.2310	3.7575	3.7160	7.3078	3.9552	7.8455	7.2793	7.5463	5.3888	3.6740	4.0408	6.8225	3.5149	3.8377	3.6206	3.6638	7.2194	7.2172
Ppp1r18	_SC[Carbamidomethyl (C)]LVKGS[Phospho (STY)]PER_.2	Q8BQ30_S510_M1_Ppp1r18	7.1421	7.6691	7.1670	7.2554	6.8918	6.9667	7.3173	7.6967	6.8175	7.3772	6.9619	7.0372	7.8154	7.8131	6.8647	7.2752	7.3361	6.7204	7.0694	6.8939
Fam102b	_SSS[Phospho (STY)]FSEFC[Carbamidomethyl (C)]HR_.2	Q8BQS4_S197_M1_Fam102b	8.5355	3.9041	3.1341	8.1239	8.0023	3.6605	7.9304	8.2801	7.6795	4.3050	3.0373	7.6112	7.4598	8.5597	7.1686	8.2263	8.3540	7.7989	8.2058	7.9940
Fam102b	_C[Carbamidomethyl (C)]PVKQDS[Phospho (STY)]VESQLKR_.3	Q8BQS4_S267_M1_Fam102b	3.3426	3.9765	4.3800	4.2416	3.5495	5.8198	5.8010	3.5683	3.2654	6.1105	5.4354	5.6774	6.1932	4.4181	5.4318	3.8411	3.0655	5.8687	5.9480	4.0954
Fam102b	_SAS[Phospho (STY)]VPDELGAWGHSR_.2	Q8BQS4_S162_M1_Fam102b	4.5320	3.4708	3.5675	3.1573	3.8698	4.0938	3.6713	4.2324	3.7304	7.3797	4.1048	3.3602	3.8385	3.5546	3.2485	3.3761	3.2693	3.0631	4.2125	3.5216
Ralgapb	_SDS[Phospho (STY)]APPTPVNR_.2	Q8BQZ4_S359_M1_Ralgapb	11.9216	11.6733	11.5599	11.5819	9.2847	11.3778	11.4719	11.7360	11.4557	11.2720	11.0912	8.8404	11.6293	10.8789	10.0103	10.9531	11.6527	11.3862	11.1300	10.9643
Bicd1	_SGS[Phospho (STY)]LKGPDDPR_.2	Q8BR07_S548_M1_Bicd1	3.2486	8.4391	3.7849	4.4408	7.6463	8.1167	8.0649	4.4498	7.6217	8.6394	3.8874	7.6577	8.2546	3.7720	7.0997	3.5935	3.4867	4.3465	3.9951	7.7708
Fam177a1	_RVIHFVSGET[Phospho (STY)]M[Oxidation (M)]EEY[Phospho (STY)]STDEDEVDGLDKK_.4	Q8BR63_T60_M2_Fam177a1	3.8980	3.6221	3.6367	3.7984	3.8264	3.5474	3.8257	3.8335	3.7970	4.0262	4.1021	3.8322	3.9410	3.6406	3.7063	3.6600	3.9852	3.6870	3.0763	3.5175
Palm2	_EES[Phospho (STY)]LATDPAPGTQK_.2	Q8BR92_S355_M1_Palm2	8.4950	8.0424	8.7767	8.6308	8.2499	8.4543	7.6300	8.3051	8.0991	8.3219	8.4692	7.8006	7.6756	8.5867	7.4749	8.2226	8.3182	7.8172	7.8050	8.2385
Palm2	_LEQEIQALESEESQISAK_.2	#N/A	7.3225	6.4267	3.6451	5.5878	5.9633	5.3766	5.8004	7.0614	4.7136	6.0941	6.6866	6.0814	6.8255	6.9885	5.0574	5.8944	6.7764	5.8664	5.8416	6.1593
Palm2	_AELVLIDEDDEKS[Phospho (STY)]LREK_.3	#N/A	8.5205	9.2947	9.8740	9.3813	3.5087	9.1148	9.0118	6.8666	9.3027	9.4157	8.2654	8.8293	8.6592	9.9200	8.8148	9.2288	9.0177	9.2848	9.3234	9.1790
Palm2	_AELVLIDEDDEKS[Phospho (STY)]LR_.2	#N/A	6.6357	9.3315	9.7790	8.4087	3.8822	9.1949	8.8842	4.6955	9.0503	8.6971	7.0557	7.7722	7.1456	9.8950	8.1530	8.3392	8.1276	9.0559	8.6530	8.6600
Scfd1	_VNLEESTGVENS[Phospho (STY)]PAGARPK_.2	#N/A	4.4300	4.3283	4.1580	3.5678	3.1361	5.2410	5.2969	4.1381	3.8404	4.2406	3.3190	4.5599	4.0058	5.5648	3.9455	3.7921	4.0760	5.8638	3.3897	4.6510
Rell2	_SWGS[Phospho (STY)]GGGQEPGGSQAAGGGQPR_.2	Q8BRJ3_S195_M1_Rell2	3.1029	3.3990	3.6392	4.5865	3.9415	4.0434	3.0380	4.3041	4.4590	3.6138	3.9377	3.2885	3.1236	3.6263	3.1768	3.4478	3.3410	5.8767	4.1408	3.5933
Rell2	_YGLHEHRDGS[Phospho (STY)]PTDR_.3	Q8BRJ3_S187_M1_Rell2	4.7478	6.7804	3.3561	3.3688	3.6584	4.8174	4.2557	6.4423	4.1759	3.8969	3.6546	6.1362	4.3414	3.3432	4.2811	3.1647	4.5587	6.2158	4.4239	4.7917
Rell2	_GS[Phospho (STY)]EPDDAQLQPPEDDVNEDTVER_.3	Q8BRJ3_S52_M1_Rell2	4.0438	3.2753	3.5804	3.5404	4.2508	3.8576	4.2982	4.2695	3.9666	3.8544	4.3410	3.0014	4.0747	6.1440	2.8227	3.4059	3.7667	3.3827	3.6818	3.2958
Prkaa2	_SC[Carbamidomethyl (C)]S[Phospho (STY)]AAGLHR_.2	Q8BRK8_S491_M1_Prkaa2	7.1314	3.4104	7.3588	3.6756	6.5355	6.2983	6.7696	6.5100	3.8315	3.1364	4.2059	3.1365	3.9395	3.4833	3.3496	5.9919	6.4632	6.2164	6.4422	3.1606
Prkaa2	_TSC[Carbamidomethyl (C)]GS[Phospho (STY)]PNYAAPEVISGR_.2	Q8BRK8_S176_M1_Prkaa2	3.9425	3.3766	3.4791	3.6417	4.1495	3.9589	5.8053	4.1682	3.8653	3.1026	4.2397	3.1027	3.9734	3.5172	3.3835	3.3046	3.6654	3.4840	3.7831	3.1945
Prkaa2	_MPPLIADS[Phospho (STY)]PK_.2	Q8BRK8_S377_M1_Prkaa2	3.2761	3.2259	5.2019	4.0215	4.1146	4.3387	3.4264	3.7884	3.4855	3.4824	3.8599	3.1153	3.5936	3.1373	3.0036	3.6210	3.2856	3.8638	4.1629	4.3155
Clasp2	_ESSRDTS[Phospho (STY)]PVR_.2	Q8BRT1_S547_M1_Clasp2	8.9115	9.1971	8.6026	9.0871	8.4985	8.8417	8.9580	9.0743	8.8889	8.6985	8.2842	8.5901	9.4874	8.6479	7.4317	8.4228	8.9546	9.2841	8.8320	8.3856
Clasp2	_AQT[Phospho (STY)]GSAGADPTADVSGQS_.2	#N/A	3.4493	3.8698	4.4868	4.1349	4.2280	4.4521	3.3131	3.6750	3.3721	6.9809	3.7465	3.5959	3.4802	4.5248	2.8903	3.7343	3.1722	3.9772	4.2763	4.2021
Clasp2	_RAQTGS[Phospho (STY)]AGADPTADVSGQS_.2	Q8BRT1_S1273_M1_Clasp2	6.6463	6.3549	3.4753	6.7885	6.2444	6.4315	6.2598	6.7988	6.1608	6.0019	5.9379	6.0036	4.2233	6.2998	4.9779	5.7612	6.7116	6.5206	6.1096	6.0597
Clasp2	_VLTTTALSTVSSGAQR_.2	#N/A	4.0294	4.8538	4.1997	4.3690	4.6588	4.6584	4.9033	4.1889	4.6189	3.6458	4.1969	4.5426	4.5831	3.5849	2.8965	4.1090	3.9616	4.2636	4.6757	4.4141
Clasp2	_MVSQSQPGS[Phospho (STY)]R_.2	Q8BRT1_S465_M1_Clasp2	3.6723	8.3322	9.2126	9.0284	7.2340	7.6737	8.0588	3.8980	7.5521	7.3105	7.9320	6.8269	3.2545	8.3877	6.3529	3.0344	7.5167	6.6059	6.5104	6.9381
Clasp2	_SSSSS[Phospho (STY)]QESLNRPFSSK_.2	#N/A	6.8859	6.5429	4.0878	6.6221	5.8247	3.9104	3.5361	3.8298	5.1769	6.0888	4.2047	3.4869	3.6350	6.5437	3.3386	5.7243	3.3271	6.3155	5.9620	5.7951
Clasp2	_SRS[Phospho (STY)]DIDVNAAAGAK_.2	Q8BRT1_S376_M1_Clasp2	9.2141	9.1407	9.3361	9.0339	8.5399	8.6493	8.6524	8.8530	8.5810	8.7879	8.5006	8.3990	3.9213	8.4496	7.4884	8.5564	8.7903	8.6024	8.4984	8.3558
Clasp2	_IPRPSVS[Phospho (STY)]QGC[Carbamidomethyl (C)]S[Phospho (STY)]R_.2	Q8BRT1_S531_M2_Clasp2	3.8815	4.2866	4.1397	3.8856	3.3093	6.2627	6.2700	3.5740	5.9726	6.8442	3.9867	6.1077	3.8943	3.6753	3.8341	3.9653	4.1117	6.3961	6.2100	3.6423
Clasp2	_S[Phospho (STY)]QEDMSEPVR_.2	Q8BRT1_S947_M1_Clasp2	4.0318	3.6276	7.0588	7.3563	4.2387	4.2720	4.3103	4.0756	4.2305	3.8423	6.4384	3.5171	4.3959	6.8454	2.8348	3.3939	3.1124	3.2199	4.3693	3.3647
Clasp2	_EILSDDKHDWDQR_.3	#N/A	4.2949	5.0434	3.3303	3.3945	3.6326	4.8282	5.4632	3.9952	4.1501	4.9463	4.4428	3.5974	4.3156	3.3174	4.7657	3.1389	4.5329	4.9814	2.9488	3.6627
Clasp2	_IC[Carbamidomethyl (C)]DYKS[Phospho (STY)]FDDEESVDGNRPSSAASAFK_.3	Q8BRT1_S14_M1_Clasp2	4.0649	3.2542	3.6015	3.5193	4.2719	4.3052	4.2771	4.2906	4.1973	3.8755	3.6759	2.9803	4.0958	3.6396	2.8016	3.4270	3.7878	3.3616	3.6607	3.3168
Kiaa1671	_RDES[Phospho (STY)]DEEPPRVER_.2	#N/A	9.1634	9.0784	8.6666	9.2298	9.0363	9.3176	9.3808	8.6189	8.9249	9.5798	9.2274	9.2548	9.8587	9.3740	9.0305	8.7532	9.0074	9.6233	9.4418	9.2310
She	_KNS[Phospho (STY)]AAELGASK_.2	Q8BSD5_S80_M1_She	4.0802	5.1188	4.6165	3.6092	3.4179	4.6043	5.0899	3.7806	4.5666	4.1373	3.4141	4.5059	4.1009	4.6036	4.0406	4.3734	4.3183	4.1058	3.1635	3.9707
Ppfia2	_C[Carbamidomethyl (C)]ETS[Phospho (STY)]PPPTPR_.2	Q8BSS9_S774_M1_Ppfia2	4.0997	11.1799	11.2279	11.0938	10.8350	4.3400	11.0117	11.1250	10.7753	10.6041	10.2874	11.0583	11.5864	11.2690	10.6751	11.1274	11.3096	11.0047	11.0026	3.3516
Ppfia2	_QAQS[Phospho (STY)]PSGVSSEVEVLK_.2	#N/A	9.0811	8.8960	8.9844	8.9867	8.4475	8.4974	8.6615	8.8742	8.6078	9.1719	8.4093	8.6489	9.4671	9.0562	8.0319	8.8996	8.8912	8.5889	8.5420	8.7182
Ppfia2	_S[Phospho (STY)]LGDHEWNR_.2	#N/A	5.8880	6.2839	6.6459	6.4372	5.4470	5.1577	5.6879	5.2032	5.6401	5.7714	5.1883	5.3895	4.2299	5.9821	4.2196	5.3686	5.5254	5.9485	5.4517	5.7980
Ppfia2	_VKS[Phospho (STY)]LGDHEWNR_.2	#N/A	5.1791	5.1269	5.0486	4.6420	4.9297	4.2606	4.4963	3.3072	4.1224	5.2985	4.1156	4.8046	3.5636	5.2940	4.0370	4.5421	5.0077	4.4235	4.8271	4.1123
Ppfia2	_RQAQS[Phospho (STY)]PSGVSSEVEVLK_.3	#N/A	3.7234	4.2794	4.2597	3.9660	3.0611	3.2957	3.6585	3.4238	3.5787	4.4941	3.0573	4.1689	3.7441	4.2468	3.6838	4.0683	3.9614	3.8717	3.5203	4.2138
Ppfia2	_VAS[Phospho (STY)]VS[Phospho (STY)]LEGLNLAR_.2	Q8BSS9_S687_M2_Ppfia2	3.5022	3.8886	4.0385	4.1972	3.7188	3.5044	6.7522	3.2739	3.1887	3.7085	3.9062	3.7781	3.3267	7.8149	3.6664	3.8471	4.0888	6.8527	3.4988	3.9216
Ppfia2	_KMVSSEGS[Phospho (STY)]TES[Phospho (STY)]EHLEGMEAGQK_.3	Q8BSS9_S236_M2_Ppfia2	4.2429	4.5770	3.7795	3.3413	2.9490	2.9824	4.0991	4.4686	4.0193	4.7591	3.4979	4.3031	4.2738	3.8176	4.1244	3.6051	3.9659	3.1836	3.4826	3.4949
Tcp11l1	_VGRPHS[Phospho (STY)]SPAR_.2	Q8BTG3_S55_M1_Tcp11l1	6.4631	3.9013	4.4552	4.1664	5.6021	5.2977	5.6790	3.6435	3.3406	3.6273	5.0044	3.6274	5.0993	4.4933	2.8587	5.3391	3.1407	4.0087	4.9394	4.1705
Ndrg4	_LDPTTTTFLK_.2	#N/A	6.7963	5.7602	6.2730	5.3329	6.2502	5.7003	6.1597	6.6702	4.1424	5.7568	5.7238	6.1240	5.7607	5.6457	5.5709	5.2240	5.8397	6.2641	5.5694	5.0313
Ndrg4	_TAS[Phospho (STY)]LTSASSVDGSR_.2	Q8BTG7_S317_M1_Ndrg4	6.6846	4.3725	6.7669	7.0929	2.9680	6.6116	6.4751	3.4342	6.5208	3.8366	6.8738	4.2619	6.5573	4.2840	6.3066	3.9752	6.5488	4.2181	5.9546	3.9612
Ndrg4	_LSGGAVPSAS[Phospho (STY)]MTR_.2	Q8BTG7_S306_M1_Ndrg4	4.0268	7.1621	6.8922	7.9777	5.2085	5.9042	6.9007	3.9562	4.0925	4.1393	7.3527	5.1748	4.2744	7.0322	3.9646	4.0614	5.8544	3.8506	3.3394	3.9480
Ndrg4	_RLS[Phospho (STY)]GGAVPS[Phospho (STY)]ASMTR_.2	Q8BTG7_S304_M2_Ndrg4	4.1568	5.1907	6.9358	4.6690	3.7621	4.1792	5.9554	4.0055	5.9002	3.5009	3.9808	3.8888	4.0257	7.5859	4.9026	3.1469	5.5882	4.4604	4.8997	4.8312
Ndrg4	_DLDINRPGTVPNAK_.2	#N/A	3.8088	4.1609	4.4063	3.8952	4.8559	4.6397	4.6058	5.2656	3.8209	4.0151	3.3534	5.3668	3.9289	4.1317	3.9979	4.1537	4.2799	4.4926	4.7157	4.9379
Srrm2	_SSS[Phospho (STY)]PVTELTAR_.2	Q8BTI8_S1068_M1_Srrm2	13.1153	12.9777	13.0801	12.9194	12.7127	12.9239	12.6472	13.0440	12.7629	13.0094	12.8648	12.5957	13.1995	12.9170	11.9343	12.5555	13.2582	12.6699	12.7211	12.6910
Srrm2	_S[Phospho (STY)]RT[Phospho (STY)]PPVTR_.2	Q8BTI8_S1878_M2_Srrm2	13.4514	13.3604	13.5009	13.4343	13.0994	13.3848	13.3081	13.2829	13.3350	13.4049	13.1800	12.9571	13.1488	13.3089	12.5656	12.9557	13.4992	13.1561	13.1836	13.0988
Srrm2	_S[Phospho (STY)]RTS[Phospho (STY)]PVTR_.2	Q8BTI8_S1924_M2_Srrm2	10.2976	10.7419	10.5999	10.7927	10.4120	10.6397	10.6082	10.6286	10.5965	10.7160	10.4966	10.3040	10.6490	10.3025	9.6265	10.4541	10.4983	10.4423	10.2080	10.4643
Srrm2	_S[Phospho (STY)]RT[Phospho (STY)]PPAIR_.2	Q8BTI8_S1972_M2_Srrm2	12.9448	12.9140	12.9861	12.9275	12.5608	12.8700	12.6594	12.7566	12.8386	12.8547	12.6972	12.5046	12.6911	13.0358	12.0808	12.4537	13.0475	12.6338	12.7466	12.6584
Srrm2	_SST[Phospho (STY)]PPRQS[Phospho (STY)]PSR_.2	Q8BTI8_S882_M2_Srrm2	11.7733	11.8265	11.9243	11.8035	11.4339	11.6566	11.7076	11.7454	11.6314	11.6938	11.3928	11.5830	11.8284	11.9068	10.7805	11.5215	11.8964	11.7363	11.7014	11.5463
Srrm2	_VSS[Phospho (STY)]PVLETVQQR_.2	Q8BTI8_S1360_M1_Srrm2	13.1359	13.0103	12.7275	12.5367	12.2743	12.6824	12.6342	13.0648	12.4108	12.6195	12.5711	12.5613	12.9756	13.1416	11.5247	12.1475	13.0840	12.6705	12.4301	12.3443
Srrm2	_S[Phospho (STY)]RS[Phospho (STY)]PLAIR_.2	Q8BTI8_S1996_M2_Srrm2	12.6137	12.5469	12.6027	12.4967	7.5315	12.2824	12.2400	12.3637	12.3893	12.4296	12.2181	12.1585	12.4747	12.5964	11.6449	12.1632	12.7656	12.1454	12.2815	12.2110
Srrm2	_S[Phospho (STY)]RTS[Phospho (STY)]PAPWK_.2	#N/A	9.5143	7.1037	9.7550	9.4954	9.1636	7.0018	9.2629	9.3891	9.4004	9.4671	9.3648	8.9756	9.2212	9.6376	5.9007	9.0073	9.7174	8.9004	9.1676	8.9195
Srrm2	_AAETPAVASC[Carbamidomethyl (C)]WSGPQVS[Phospho (STY)]PEHK_.3	#N/A	10.4602	10.4896	9.1369	10.1123	9.5368	9.9656	10.0440	10.4157	9.7767	9.8982	10.0245	9.9787	10.4587	10.6644	9.0179	8.5601	10.4776	10.1425	9.9217	9.7428
Srrm2	_SSS[Phospho (STY)]PVTELTARS[Phospho (STY)]PVKQDK_.3	#N/A	6.7253	9.5639	9.1442	9.2844	3.6877	9.0669	9.4237	9.1519	9.2958	9.2996	3.9595	9.2593	10.1891	9.6054	8.6020	9.3531	3.5899	9.4410	10.3514	9.4118
Srrm2	_SRHS[Phospho (STY)]PRLS[Phospho (STY)]R_.3	#N/A	9.0507	9.3423	9.2684	9.2269	8.6200	9.1276	9.2363	8.5758	9.2481	9.3804	8.9986	8.7898	9.3962	9.1367	8.8782	8.8833	9.2452	9.0371	9.2617	7.5803
Srrm2	_S[Phospho (STY)]PVPSAFSDQSR_.2	Q8BTI8_S2404_M1_Srrm2	10.8290	10.2607	10.9052	10.4631	10.0529	10.2926	9.9716	10.0590	10.1228	10.5233	10.4125	9.7362	9.9511	10.2935	9.2197	9.8826	10.5309	9.7680	10.0136	9.9794
Srrm2	_S[Phospho (STY)]RTS[Phospho (STY)]PVSR_.2	Q8BTI8_S1849_M2_Srrm2	7.6821	7.9546	4.1493	8.0943	7.8032	6.0898	6.1494	7.9191	8.0027	7.4240	5.7901	7.3454	7.4494	6.3352	6.6054	6.0643	4.1020	7.9748	7.1402	5.6862
Srrm2	_S[Phospho (STY)]RS[Phospho (STY)]PQRPGWSR_.3	#N/A	8.8455	8.7477	8.9880	9.0180	8.5532	8.8027	8.9765	8.8437	8.9694	9.3086	8.7793	8.6924	9.1433	9.1182	8.5380	8.8782	9.0570	8.9527	9.0349	8.7895
Srrm2	_S[Phospho (STY)]RS[Phospho (STY)]IS[Phospho (STY)]PC[Carbamidomethyl (C)]PK_.2	Q8BTI8_S924_M3_Srrm2	9.0816	9.2471	9.2052	9.2433	8.7728	8.5119	8.9954	9.2895	8.8902	9.0622	8.6677	8.5388	8.8575	9.0082	7.2894	8.6245	9.1645	8.6186	8.5811	8.6686
Srrm2	_NSGPVSEVNTGFS[Phospho (STY)]PEVK_.2	Q8BTI8_S1305_M1_Srrm2	9.2165	8.9910	9.2468	8.9586	8.1800	8.9474	8.5133	8.6925	8.8149	8.8177	8.7092	8.6212	8.8233	9.2019	7.8339	8.5249	9.2720	8.6324	8.5251	8.6867
Srrm2	_ERAPS[Phospho (STY)]PASR_.2	Q8BTI8_S2381_M1_Srrm2	9.9786	9.9109	9.7524	9.4238	9.5287	9.6422	9.6945	9.9244	9.5055	9.7030	9.3632	9.5758	10.1528	9.7615	9.0749	9.5078	9.9215	9.8652	9.5051	9.4783
Srrm2	_ALPQT[Phospho (STY)]PR_.2	Q8BTI8_T1448_M1_Srrm2	9.3913	8.8849	9.1562	9.4577	8.6475	8.6424	8.5442	9.5889	8.9998	8.7217	9.3458	9.6609	10.2272	9.7606	8.7607	9.2283	9.9612	9.6511	9.3605	9.4026
Srrm2	_DKFS[Phospho (STY)]PTQDRPESSTVLK_.3	Q8BTI8_S1151_M1_Srrm2	9.6296	9.2119	9.0972	9.0477	4.2544	9.0817	8.8979	8.9998	9.0819	9.3744	9.2387	9.0302	9.8891	9.5826	8.3779	9.1145	9.3745	9.0806	9.0931	9.0140
Srrm2	_S[Phospho (STY)]RT[Phospho (STY)]PLISR_.2	Q8BTI8_T1836_M2_Srrm2	9.5279	9.4378	9.1516	3.8321	8.8276	9.1713	9.2163	9.3821	9.2670	9.4005	9.1547	9.0741	9.2835	9.5745	8.5854	8.9222	9.5532	9.1526	9.1343	8.9571
Srrm2	_RVPS[Phospho (STY)]PTPVPK_.2	#N/A	4.1735	9.1331	3.4818	3.4644	8.6921	4.4480	8.7258	4.1610	4.0551	8.7589	8.7917	8.5421	9.4090	3.4991	3.9293	3.3004	4.1761	3.3400	3.3305	3.3005
Srrm2	_REIS[Phospho (STY)]S[Phospho (STY)]SPTSK_.2	Q8BTI8_S453_M2_Srrm2	9.1906	3.6063	8.4008	7.8978	8.2089	4.0150	7.9929	7.9445	8.5313	8.4480	3.3958	7.9370	8.4666	7.9808	7.0674	3.2032	8.5921	8.4748	7.8289	3.3773
Srrm2	_SGAGS[Phospho (STY)]PPGKR_.2	Q8BTI8_S1179_M1_Srrm2	9.5978	8.9173	3.2609	7.6045	8.8122	8.5439	3.9779	9.0492	8.6943	8.7985	8.8207	3.6668	4.2461	3.2479	3.5551	7.6351	8.9881	8.8010	3.0183	8.7927
Srrm2	_T[Phospho (STY)]PPVALSSSR_.2	Q8BTI8_T2056_M1_Srrm2	8.6995	8.5693	8.4105	8.6193	7.9979	8.8209	8.3805	8.2388	8.3832	8.6869	8.5145	3.6743	3.4017	8.6051	7.4488	8.1112	8.7763	8.1491	8.0026	8.1040
Srrm2	_S[Phospho (STY)]RSRT[Phospho (STY)]S[Phospho (STY)]PAPWK_.2	Q8BTI8_S1808_M3_Srrm2	8.2590	3.9485	3.9402	3.6351	7.5119	8.2566	4.1491	3.8170	8.4537	7.9856	7.0071	3.8380	3.8974	7.3857	7.2477	6.8828	3.6420	3.6518	3.6243	3.8369
Srrm2	_VPS[Phospho (STY)]PTPVPK_.2	#N/A	9.0575	7.9398	8.5218	8.8684	8.9777	9.2220	3.4743	9.1672	3.3945	3.1774	9.1321	7.7796	8.1290	8.0511	3.4997	8.4293	3.0898	7.7314	8.2997	4.0296
Srrm2	_GHS[Phospho (STY)]RS[Phospho (STY)]RS[Phospho (STY)]PQWR_.3	Q8BTI8_S503_M3_Srrm2	7.1604	7.8879	6.7920	7.7685	7.0918	7.7414	7.7609	6.5495	7.7909	8.1834	7.8045	7.3997	8.1020	7.4337	6.6395	7.5646	7.6583	7.1860	7.9452	7.6622
Srrm2	_S[Phospho (STY)]RS[Phospho (STY)]RTS[Phospho (STY)]PVSR_.2	Q8BTI8_S1844_M3_Srrm2	6.7831	4.4501	4.0890	4.1367	6.3301	3.5937	7.0774	3.2530	7.1259	7.3824	6.9912	6.3083	3.5734	7.1857	4.2279	3.8976	6.8834	7.1520	3.6910	4.0430
Srrm2	_S[Phospho (STY)]RS[Phospho (STY)]RS[Phospho (STY)]LVR_.2	Q8BTI8_S700_M3_Srrm2	7.7548	7.4012	7.9229	7.7979	7.0180	7.5650	7.5538	7.2941	7.5757	7.3247	7.3624	7.1391	7.5503	7.3581	6.5799	7.0633	7.5931	7.3232	6.9691	7.1883
Srrm2	_SSS[Phospho (STY)]PVTELTARS[Phospho (STY)]PVK_.2	#N/A	3.8362	4.0544	3.7846	7.3109	3.6254	3.7818	6.8623	4.4617	7.8328	3.9394	3.6519	6.8136	3.8638	7.1904	6.0230	6.5790	3.7505	6.9579	6.9262	3.6234
Srrm2	_SRS[Phospho (STY)]T[Phospho (STY)]T[Phospho (STY)]PAPK_.2	Q8BTI8_S250_M3_Srrm2	6.5458	6.4482	3.8144	7.2734	6.5351	3.8774	3.9724	6.7094	4.0315	3.7838	6.5083	3.7615	6.8868	6.6212	3.5496	3.9223	6.1795	5.8350	6.0750	3.3606
Srrm2	_TS[Phospho (STY)]PAPWKR_.2	#N/A	6.5525	7.1300	3.9985	6.2304	2.9835	6.6518	6.2345	3.5202	6.2946	3.9821	6.2160	6.6884	3.8405	6.8483	4.1193	3.8150	6.9637	3.8166	6.3791	6.3940
Srrm2	_ARS[Phospho (STY)]RT[Phospho (STY)]PPSAPSQSR_.3	#N/A	8.9554	7.9287	8.3414	8.3486	8.5951	7.7022	8.5253	7.4154	8.2104	8.8418	8.2346	8.1380	8.8445	8.3319	7.4430	8.2838	8.6398	8.3232	8.4139	8.0321
Srrm2	_RKET[Phospho (STY)]PS[Phospho (STY)]PR_.2	#N/A	9.1684	8.6518	8.2968	8.7613	7.9111	8.3991	8.8584	8.3559	8.2581	8.5568	8.3735	8.1319	8.6152	7.7065	3.7393	6.7002	8.8193	8.4610	7.6107	7.8851
Srrm2	_HSGST[Phospho (STY)]SPYLK_.2	Q8BTI8_T963_M1_Srrm2	3.8101	3.5090	3.3467	3.7741	4.0170	4.0504	3.0311	4.0358	4.4521	3.6207	3.9307	3.2351	7.9264	3.3847	3.0564	3.1722	3.5330	3.6164	3.9155	3.0620
Srrm2	_GRS[Phospho (STY)]PS[Phospho (STY)]PKPR_.2	Q8BTI8_S1718_M2_Srrm2	8.4432	7.7331	7.5284	7.8642	7.2574	7.6165	7.9904	7.6104	7.6820	7.9042	7.4316	7.3296	7.8219	7.2573	3.0355	5.6098	8.0821	7.8030	6.3360	7.2834
Srrm2	_NNKS[Phospho (STY)]VT[Phospho (STY)]PQR_.2	#N/A	7.7181	7.2950	7.7039	7.2460	6.2407	7.4987	7.3945	7.1156	6.9601	7.4547	7.2657	6.9734	6.4532	7.6035	4.1460	5.7700	7.3932	7.4788	6.4889	6.3666
Srrm2	_VSS[Phospho (STY)]PVLETVQQRT[Phospho (STY)]PSR_.3	Q8BTI8_S1360_M2_Srrm2	3.4270	4.5758	3.9633	4.2624	4.2655	3.7194	3.2755	3.1273	3.2822	3.2897	4.2617	2.9644	3.4477	3.9503	2.8527	3.7719	3.6650	4.1681	3.8167	3.9173
Srrm2	_TS[Phospho (STY)]PLM[Oxidation (M)]LDR_.2	#N/A	10.0966	9.9334	8.2581	9.5586	8.7130	9.4294	9.6025	9.0941	9.8195	8.9157	8.8372	7.2829	4.0503	10.2890	6.9341	8.5148	10.4181	3.8509	4.1106	7.2418
Srrm2	_SLSYS[Phospho (STY)]PVER_.2	Q8BTI8_S2648_M1_Srrm2	8.2747	9.6192	9.2355	9.0670	7.8317	9.5445	8.6969	8.3657	9.3465	9.3828	7.8069	9.2233	8.3649	9.5295	6.9605	9.2180	9.8529	9.3947	9.3776	9.4114
Srrm2	_S[Phospho (STY)]RTS[Phospho (STY)]PAPWKR_.2	#N/A	3.9068	8.3593	3.7987	3.7463	7.8520	8.0786	7.9319	4.0477	8.2160	8.3297	7.6887	7.8931	3.9660	8.2910	7.5031	8.0286	8.3264	7.9147	8.0836	7.9391
Srrm2	_GRS[Phospho (STY)]RT[Phospho (STY)]PLTSR_.2	#N/A	9.2051	9.2834	9.3863	9.4779	8.9748	9.2288	9.1957	9.2983	9.2420	9.2556	9.0653	8.9895	9.4853	9.2205	8.5553	9.0260	9.3236	8.9980	9.2436	9.0655
Srrm2	_RSS[Phospho (STY)]SELSPEVVEK_.2	Q8BTI8_S1339_M1_Srrm2	7.1464	3.9837	7.0594	6.8298	3.4183	6.7635	6.3342	6.8280	6.5622	6.9342	6.4203	6.5554	6.7953	6.6846	5.9927	6.4292	7.1358	6.5400	6.2763	6.5012
Srrm2	_HAS[Phospho (STY)]S[Phospho (STY)]SPESLKPTPAPGSR_.3	Q8BTI8_S434_M2_Srrm2	6.3475	6.4722	3.9966	6.2095	4.6295	6.1413	6.1686	6.3646	6.0337	6.5746	5.9243	6.2489	3.9677	6.6294	5.4637	6.0663	6.5923	6.3024	6.2047	6.2143
Srrm2	_LKSGM[Oxidation (M)]S[Phospho (STY)]PEQSK_.2	#N/A	6.8652	6.7386	6.7422	7.2019	6.4861	7.1335	6.5890	4.1419	4.1137	6.9763	3.9840	3.2815	3.4773	7.2111	6.9540	3.4548	7.3473	6.9122	7.1533	6.3495
Srrm2	_GRS[Phospho (STY)]EC[Carbamidomethyl (C)]DS[Phospho (STY)]S[Phospho (STY)]PEPK_.2	Q8BTI8_S1434_M3_Srrm2	4.0459	6.1891	5.1042	4.8177	4.2670	5.5534	4.8951	5.5616	4.9412	4.9513	4.9263	4.6746	6.1169	5.1492	5.3555	5.0487	5.6116	5.9963	4.6050	5.5188
Srrm2	_SSSELS[Phospho (STY)]PEVVEK_.2	Q8BTI8_S1343_M1_Srrm2	4.2901	4.1772	5.9786	3.3479	3.3453	5.9962	3.9841	4.2777	5.4713	5.8159	3.9686	3.9648	6.3852	3.6158	6.3960	3.4171	5.9055	3.2235	6.5362	3.1911
Srrm2	_SM[Oxidation (M)]LQT[Phospho (STY)]PPDQNLSGSK_.2	#N/A	8.1364	8.6518	8.0087	8.2308	6.8711	8.3862	8.5035	8.4576	7.9598	8.1328	8.8779	8.2096	8.7177	8.4401	7.5840	6.7368	6.8229	8.1428	8.2474	8.6111
Srrm2	_PSM[Oxidation (M)]S[Phospho (STY)]PTPLDR_.2	#N/A	7.6930	8.2732	3.9149	6.5977	7.1962	7.2382	6.5393	7.7177	7.1539	7.5621	3.7977	7.0824	3.5314	6.9381	6.4887	7.1833	7.5899	7.3313	3.5827	7.3387
Srrm2	_S[Phospho (STY)]PVKQDKSEISTDPK_.2	#N/A	5.9140	6.1408	4.0756	5.4837	4.6092	5.4970	5.5180	5.7328	5.3430	5.7946	3.8359	5.3442	5.7134	5.2967	5.1963	5.5690	5.8142	5.6597	5.7584	5.4112
Srrm2	_VSSPVLETVQQRT[Phospho (STY)]PS[Phospho (STY)]RER_.3	Q8BTI8_S1372_M2_Srrm2	4.1502	3.1689	6.0427	3.4340	2.8563	6.6345	6.3509	4.3759	6.3680	6.2315	2.9466	6.7006	4.1811	6.8631	4.2172	3.5123	6.8238	7.2359	6.1012	6.1930
Srrm2	_S[Phospho (STY)]RT[Phospho (STY)]PLLPR_.2	Q8BTI8_S1984_M2_Srrm2	11.7755	11.6512	11.9908	12.0281	4.2250	11.8667	11.5240	7.2739	11.1755	3.3303	8.2256	5.7709	10.5726	3.9098	11.0950	11.6787	12.2104	11.5652	3.8573	4.9663
Srrm2	_S[Phospho (STY)]RAS[Phospho (STY)]PVSR_.2	#N/A	8.2202	7.8451	7.1375	7.0201	7.7458	7.7040	7.7514	7.9906	7.6876	7.0406	7.7421	7.5508	7.8979	7.9233	5.9507	6.6277	8.0152	7.6637	6.5479	7.5237
Srrm2	_QSHSESS[Phospho (STY)]PDGEVK_.2	Q8BTI8_S851_M1_Srrm2	5.8192	4.1813	5.7728	3.7189	3.9392	6.1624	6.6461	4.0040	6.3219	3.9451	3.6580	3.9492	7.0178	6.7641	3.7987	3.6402	3.8249	6.9343	3.5047	6.4218
Srrm2	_AQS[Phospho (STY)]GTDS[Phospho (STY)]S[Phospho (STY)]PEHKIPAPR_.2	#N/A	8.8654	8.1901	8.0018	7.3410	3.7908	7.1752	8.0437	8.0500	7.0889	8.2689	7.3666	8.0181	3.7635	3.9765	6.7161	7.1529	8.9372	7.2577	7.8768	3.2888
Srrm2	_NHSGS[Phospho (STY)]RT[Phospho (STY)]PPVALSSSR_.3	Q8BTI8_S2054_M2_Srrm2	7.0415	7.9635	6.8930	7.5605	6.9427	7.8508	6.3961	4.2779	7.8565	7.2163	7.5911	6.3950	3.8204	7.9306	6.5960	7.7120	5.4694	7.8110	6.1714	3.9107
Srrm2	_SLS[Phospho (STY)]GSS[Phospho (STY)]PC[Carbamidomethyl (C)]PK_.2	#N/A	3.7779	4.8072	6.1193	6.1441	3.6422	3.6851	4.0293	4.7884	3.5487	6.7829	3.5794	3.7182	6.3644	5.7642	6.1827	6.3642	6.3348	4.0766	3.4425	7.0540
Srrm2	_SQTS[Phospho (STY)]PKGS[Phospho (STY)]LSR_.2	Q8BTI8_S1058_M2_Srrm2	3.4584	6.7508	4.2521	5.9898	3.2699	3.6075	3.3243	4.3209	3.3942	5.2643	4.3737	6.4842	3.5596	6.4339	5.7994	4.4721	6.3947	6.4548	3.7048	4.0293
Srrm2	_EISSS[Phospho (STY)]PTSK_.2	Q8BTI8_S454_M1_Srrm2	3.0809	3.4211	3.6172	3.1077	3.9194	4.1435	5.6306	4.2821	3.6807	3.2872	4.0551	3.3105	3.7888	3.6042	3.1988	3.4258	3.3189	3.0134	4.1628	3.5712
Srrm2	_SRSGS[Phospho (STY)]S[Phospho (STY)]PGLR_.2	Q8BTI8_S1399_M2_Srrm2	4.3591	4.0860	3.6871	3.2929	3.3893	3.9341	3.9194	3.6132	3.9132	6.7620	3.0959	5.2968	4.1125	3.7063	5.8037	3.5063	4.3412	6.1842	6.2109	3.9451
Srrm2	_S[Phospho (STY)]VT[Phospho (STY)]PQRER_.2	#N/A	5.5748	5.3642	6.1181	5.9846	4.1797	5.9722	5.8474	3.0568	5.4441	3.3602	5.3632	4.2982	3.3772	6.0109	2.9233	3.7013	5.3223	5.2378	5.2581	5.7210
Srrm2	_SAVRPS[Phospho (STY)]PS[Phospho (STY)]PER_.2	Q8BTI8_S349_M2_Srrm2	11.0971	11.1091	11.1840	11.1498	10.8195	10.8966	10.8478	11.1430	10.8861	10.9809	10.6730	10.7897	11.1915	11.0778	10.2878	10.7900	11.1439	10.8906	10.7783	10.7556
Srrm2	_RQPS[Phospho (STY)]PQPS[Phospho (STY)]PR_.2	Q8BTI8_S2656_M2_Srrm2	9.5289	9.2048	9.3957	9.4085	9.7243	10.0134	9.1322	9.4264	9.2233	9.8467	9.6175	9.1638	9.2495	9.4783	8.2996	9.8044	9.5159	9.1573	9.8876	9.0879
Srrm2	_S[Phospho (STY)]PQRPGWSR_.2	Q8BTI8_S533_M1_Srrm2	5.6538	5.3897	6.5194	4.4893	5.2946	3.9463	5.8293	6.1452	6.0795	3.4064	4.0349	5.8118	3.2208	5.9244	3.0796	3.5450	3.4382	4.3950	4.0436	5.8849
Srrm2	_GGS[Phospho (STY)]GYHS[Phospho (STY)]RS[Phospho (STY)]PTRQESSR_.3	#N/A	4.0109	3.7644	4.0637	3.6624	3.0231	3.5398	4.3211	3.9598	4.1696	4.3614	3.0750	4.2278	4.4132	3.9570	4.0801	3.8790	4.1053	3.9175	3.0311	3.4899
Srrm2	_S[Phospho (STY)]RS[Phospho (STY)]RT[Phospho (STY)]PLLPR_.2	#N/A	9.0862	9.5196	9.5466	9.2654	3.9771	9.3744	9.1327	3.9958	9.4476	9.4539	4.3604	9.2279	3.8476	9.4760	8.7002	9.3466	9.9206	9.4009	9.3888	9.3237
Srrm2	_SS[Phospho (STY)]RSS[Phospho (STY)]PELTR_.2	#N/A	6.1818	8.7291	8.6508	3.4074	8.2594	5.6557	8.4960	8.5560	8.5059	8.5151	8.1333	8.2596	4.1437	8.5027	3.7636	8.1368	8.7276	8.2313	8.2648	8.2410
Srrm2	_S[Phospho (STY)]LTRS[Phospho (STY)]PPAIR_.2	Q8BTI8_S2019_M2_Srrm2	11.1840	11.6332	11.8006	11.2011	3.1551	11.3236	11.3269	7.2150	11.5087	11.6230	7.5247	10.8962	3.5246	11.6591	10.7929	11.3127	11.5346	11.3143	11.1210	11.4263
Srrm2	_S[Phospho (STY)]RS[Phospho (STY)]RT[Phospho (STY)]PLISR_.2	#N/A	9.8834	9.8741	9.9830	10.0404	9.5334	9.7508	9.7876	10.0052	9.8203	9.8996	9.5734	9.6040	10.0836	10.0023	9.1055	9.6303	10.1276	9.7316	9.6841	9.6240
Srrm2	_S[Phospho (STY)]RS[Phospho (STY)]RS[Phospho (STY)]PLAIR_.2	#N/A	8.0904	7.9725	8.0644	7.8826	4.2558	7.8019	7.5802	7.3909	7.9007	8.0191	7.3207	7.7856	3.3883	7.8028	7.1986	7.7974	8.1072	7.6475	7.9379	7.9446
Srrm2	_KET[Phospho (STY)]PS[Phospho (STY)]PR_.2	#N/A	3.7340	5.5117	3.2706	6.3771	5.5697	6.7461	5.7958	6.0620	5.9826	3.5446	4.0312	3.3112	3.7649	6.6399	3.1325	5.6479	6.8880	3.6925	5.6498	2.9860
Srrm2	_SRT[Phospho (STY)]PPSAPSQSR_.2	Q8BTI8_T2362_M1_Srrm2	9.4165	9.4811	9.3734	9.2548	9.0126	9.2953	9.2688	9.1731	9.3814	9.3513	9.0771	9.1618	7.6558	9.6256	8.7033	9.0606	9.1775	9.3234	9.2272	9.0519
Srrm2	_RGS[Phospho (STY)]RSS[Phospho (STY)]IEPK_.2	Q8BTI8_S1631_M2_Srrm2	8.9375	9.0210	8.8786	3.3294	3.8026	4.0142	8.7356	8.9609	9.0345	8.8752	8.6285	8.6576	4.3343	3.6339	7.8535	3.5722	9.0112	3.2049	8.6336	8.7602
Srrm2	_SSRRS[Phospho (STY)]S[Phospho (STY)]SELS[Phospho (STY)]PEVVEK_.2	#N/A	3.9329	5.9236	4.1534	3.9140	3.7787	4.0009	3.9027	3.7968	3.6329	3.6218	3.7622	5.3402	3.7606	6.0830	3.4799	3.7604	3.6571	4.4016	3.8875	3.9636
Srrm2	_S[Phospho (STY)]RS[Phospho (STY)]RAS[Phospho (STY)]PVSR_.2	Q8BTI8_S1864_M3_Srrm2	6.5116	6.8623	7.2515	6.9008	6.7066	3.2074	6.4955	6.7991	7.1991	7.1056	6.5153	6.6607	6.8293	6.9905	6.5116	7.0716	7.0129	6.7470	6.9364	6.8035
Srrm2	_SEQPLSQVLPS[Phospho (STY)]LSPEHK_.2	#N/A	3.9050	7.2545	3.9934	3.8218	3.2427	3.4668	3.9347	3.5678	5.5769	4.1113	3.2684	4.0246	3.5677	7.9274	3.9129	3.8020	7.4405	6.9459	3.2546	3.7239
Srrm2	_S[Phospho (STY)]RT[Phospho (STY)]PLTSR_.2	#N/A	10.4935	10.3698	10.4639	10.4215	10.0999	10.2351	10.1081	10.3173	10.2940	10.1726	9.8508	9.9352	10.5021	10.2868	9.5900	10.0488	10.4665	10.2203	9.9671	10.0531
Srrm2	_RRS[Phospho (STY)]PS[Phospho (STY)]VS[Phospho (STY)]S[Phospho (STY)]PEPTEK_.2	Q8BTI8_S1683_M3_Srrm2	3.1293	7.2748	4.1667	4.4549	3.3362	6.5194	6.9639	3.3550	3.0521	5.9590	3.4265	3.9159	3.1602	4.2048	3.7373	5.4274	7.3757	7.2209	6.0583	4.8502
Srrm2	_S[Phospho (STY)]SRSS[Phospho (STY)]PELTRK_.2	#N/A	4.1366	3.4796	3.5660	4.0536	3.8772	4.0018	3.4655	4.2388	3.4736	5.5924	4.0936	3.3690	3.6023	3.5735	3.2291	3.2052	3.5091	4.0662	3.9704	3.4110
Srrm2	_DTPQT[Phospho (STY)]PSR_.2	Q8BTI8_T1428_M1_Srrm2	3.9039	3.4152	3.4405	3.6803	4.1108	4.1442	4.4381	4.1296	4.3583	3.7145	3.8370	3.1413	3.9348	3.4785	2.9626	3.2660	3.6268	3.5226	3.8217	3.1558
Srrm2	_S[Phospho (STY)]RS[Phospho (STY)]RT[Phospho (STY)]PPVTR_.2	#N/A	10.6375	10.6729	10.7686	10.7306	10.2644	10.4371	10.5289	10.5321	10.5795	10.5310	10.2624	10.1546	10.6726	10.3941	9.3772	10.2069	3.6178	10.4598	10.2054	10.1891
Srrm2	_SRS[Phospho (STY)]PQWR_.2	Q8BTI8_S507_M1_Srrm2	3.5808	8.5051	8.8645	4.3025	3.7062	3.6283	3.5159	8.2042	3.4361	3.9863	3.7024	3.6610	7.6761	8.2922	6.8890	3.9257	3.8189	4.0770	3.7256	4.0712
Srrm2	_S[Phospho (STY)]RS[Phospho (STY)]RT[Phospho (STY)]PPAIR_.2	Q8BTI8_S1970_M3_Srrm2	10.9695	10.9887	11.0492	10.9656	10.4368	10.7243	10.6915	10.8407	10.8202	10.8658	10.6367	10.4487	11.0386	11.1198	10.1081	10.7518	11.0886	10.8100	10.7638	10.7517
Srrm2	_S[Phospho (STY)]RS[Phospho (STY)]RTS[Phospho (STY)]PVTR_.2	Q8BTI8_S1927_M3_Srrm2	8.4497	7.9930	8.6445	7.8911	8.1050	3.5024	7.7282	4.4716	3.6346	8.0812	8.0828	7.9532	9.2811	8.5821	3.7397	8.3614	7.9879	7.6807	7.7942	3.6349
Srrm2	_ELSHS[Phospho (STY)]PPRENSFESSLEFK_.3	Q8BTI8_S1278_M1_Srrm2	5.6895	5.6889	5.4838	3.8039	3.9699	5.1919	5.3829	3.9028	5.2507	3.9562	4.0414	4.8569	5.5162	3.8528	3.1796	3.3648	3.8531	5.1586	3.8164	5.1798
Srrm2	_NNKS[Phospho (STY)]VT[Phospho (STY)]PQRER_.2	#N/A	6.5873	6.6396	7.2414	6.7262	6.3010	6.8050	6.8920	6.6836	7.0990	6.5505	6.5469	6.5361	6.8839	6.9598	5.6164	6.7439	7.1222	6.9873	6.5750	6.4422
Srrm2	_QSSS[Phospho (STY)]PYEDKDKK_.2	Q8BTI8_S333_M1_Srrm2	3.6446	3.6745	5.7661	3.9396	5.7476	5.2486	3.1966	3.8703	5.9568	6.1052	3.9418	3.4006	3.6755	3.2192	3.2219	3.0067	5.8869	3.7819	5.7827	4.3974
Srrm2	_ARS[Phospho (STY)]HS[Phospho (STY)]PS[Phospho (STY)]SPER_.2	Q8BTI8_S1453_M3_Srrm2	6.6540	3.6823	3.3560	7.2046	6.7162	7.1497	3.8828	7.0363	6.8866	4.5269	6.8493	3.5717	6.9297	7.0048	5.5486	5.0447	6.9784	3.4075	6.4083	6.8142
Srrm2	_WGKS[Phospho (STY)]RS[Phospho (STY)]PQR_.2	Q8BTI8_S522_M2_Srrm2	5.8053	5.5944	5.7022	5.6571	5.0845	5.0987	5.8033	5.5641	5.2592	5.6879	4.5487	5.4507	5.3677	5.1493	4.3474	4.8036	4.3532	5.6920	5.2459	5.1208
Srrm2	_SEC[Carbamidomethyl (C)]DS[Phospho (STY)]S[Phospho (STY)]PEPK_.2	#N/A	3.6280	4.5641	4.1643	4.0624	2.9657	3.9107	3.0755	3.3461	3.2816	3.5463	4.3483	4.2652	3.4221	3.9347	4.1535	3.9729	4.1409	4.1340	3.8527	4.0057
Srrm2	_SSS[Phospho (STY)]AS[Phospho (STY)]PELKDGLPR_.2	Q8BTI8_S1378_M2_Srrm2	4.5452	8.0662	3.5806	8.1458	3.8829	8.0720	7.8772	4.2456	7.9994	7.6194	3.8791	7.8776	4.5659	8.3133	3.2354	7.9736	8.2971	7.8795	7.8970	7.7882
Srrm2	[Oxidation (M)]LQT[Phospho (STY)]PPDQNLS[Phospho (STY)]GSKSPC[Carbamidomethyl (C)]PC	Q8BTI8_S980_M2_Srrm2	4.0708	3.2483	3.6074	3.5134	4.2777	3.8306	3.9345	4.2965	3.9936	4.4751	4.3680	2.9744	4.1017	3.6454	3.5117	3.1129	3.7937	5.3068	3.6548	3.3227
Srrm2	_SGS[Phospho (STY)]S[Phospho (STY)]PEM[Oxidation (M)]KDKPR_.2	#N/A	4.7421	5.0296	4.0660	4.4643	5.1351	4.8099	4.9565	4.4104	3.6657	4.9844	5.2283	4.8925	5.1949	5.8712	4.1735	4.6297	5.3462	5.0324	5.3343	4.8567
Srrm2	_RSSRS[Phospho (STY)]S[Phospho (STY)]PELTR_.2	#N/A	3.9997	3.6185	3.9335	3.6906	3.4190	4.2351	3.8730	4.2033	3.8778	4.0307	3.4653	5.7792	6.2291	4.0742	3.6473	3.4578	3.7006	6.2004	3.8186	5.7662
Srrm2	_GGSGYHS[Phospho (STY)]RS[Phospho (STY)]PTR_.2	#N/A	5.1804	5.9214	6.2089	5.2931	5.3029	5.3108	5.2762	5.1996	5.5549	5.4893	4.7169	3.8201	5.7647	5.8924	5.3953	5.4992	5.6006	5.6126	5.7003	5.4456

Srrm2	_SGMS[Phospho (STY)]PEQSK_.2	#N/A	3.3883	3.9308	4.4257	7.4703	3.5952	5.7730	3.4529	3.6139	4.9063	3.9981	3.6855	3.6569	3.4191	4.4637	3.4783	4.2512	3.1112	4.0383	4.3373	4.1410
Srrm2	_SGT[Phospho (STY)]PPRPGSVTNMQADEC[Carbamidomethyl (C)]TAT[Phospho (STY)]PQR_.3	Q8BTI8_T823_M2_Srrm2	3.9216	4.0812	4.4579	3.7678	3.2593	3.4834	4.2817	3.1428	4.3408	4.1279	3.2144	3.9706	4.4489	3.9927	3.8589	4.2665	4.1409	3.0085	3.3076	3.6699
Srrm2	_SGSSQELDGKPSAS[Phospho (STY)]PQER_.2	Q8BTI8_S1508_M1_Srrm2	4.0202	3.2989	3.5568	3.5640	4.2272	9.7627	10.0524	9.7209	9.2098	11.0955	3.7206	3.0250	4.0511	3.5949	2.8463	3.3823	3.7431	9.6778	3.7054	3.2722
Srrm2	_MELGT[Phospho (STY)]PLR_.2	Q8BTI8_T955_M1_Srrm2	3.2858	4.0333	4.3232	5.7683	3.4927	3.5261	5.4794	3.3207	3.4756	3.0964	2.9542	3.7594	3.6410	4.1437	3.5807	4.1488	3.8583	3.9748	3.6234	4.1107
Srrm2	_S[Phospho (STY)]RS[Phospho (STY)]RAS[Phospho (STY)]PATHR_.2	Q8BTI8_S1820_M3_Srrm2	4.3426	5.3630	5.4321	5.2443	3.3529	4.7341	5.1525	4.7029	4.9658	4.3440	4.9212	4.0731	4.0958	4.1046	3.7431	4.1956	4.0229	5.1869	3.4428	4.9027
Srrm2	_SS[Phospho (STY)]PELTR_.2	#N/A	4.2195	3.7833	3.2550	3.4699	3.5572	3.7813	3.9838	4.3458	4.0429	4.4258	2.9165	3.6727	4.1510	3.6947	3.5610	3.0636	3.8430	3.3064	3.6055	3.3720
Srrm2	_RGGSGYHS[Phospho (STY)]RS[Phospho (STY)]PTR_.3	#N/A	3.9065	4.0963	4.4428	3.7829	3.2442	3.4683	4.2968	3.6069	4.3559	4.1128	3.2295	3.9857	4.4640	4.0077	3.8740	4.2514	4.1446	2.9934	3.2925	3.6850
Srrm2	_C[Carbamidomethyl (C)]RS[Phospho (STY)]PGMLEPLGSAR_.3	Q8BTI8_S2084_M1_Srrm2	4.0842	3.6801	3.3582	3.3667	4.2911	4.3245	4.2578	4.0231	4.1780	3.8948	3.6566	3.5695	4.3434	6.8812	2.7823	3.4463	4.5608	3.2724	4.4218	3.3123
Srrm2	_SGS[Phospho (STY)]SPGLR_.2	Q8BTI8_S1399_M1_Srrm2	3.3510	3.9681	4.3884	4.2332	3.5580	3.0496	4.4781	3.5767	3.2738	5.4858	3.6482	3.6942	3.3819	4.4265	2.7919	4.2140	3.0739	4.0755	4.3746	4.1038
Srrm2	MS[Phospho (STY)]C[Carbamidomethyl (C)]FS[Phospho (STY)]RPSM[Oxidation (M)]SPTPLDR .	Q8BTI8_S2067_M2_Srrm2	4.2554	4.5645	3.7920	3.3288	2.9615	2.9949	3.4227	4.4811	4.0068	4.0660	3.4854	4.2906	4.1722	3.8301	4.1120	3.6175	3.9783	3.1711	3.7837	3.5073
Srrm2	_TPAAAAAMNLAS[Phospho (STY)]PR_.2	Q8BTI8_S2224_M1_Srrm2	3.4720	3.8471	4.5094	4.1122	3.6790	4.4294	3.3691	3.6977	3.3948	3.5731	3.7692	3.5732	3.5029	3.8859	3.3945	4.3350	3.1949	3.9545	4.2536	4.2248
Srrm2	_S[Phospho (STY)]RS[Phospho (STY)]RT[Phospho (STY)]PAR_.2	Q8BTI8_S570_M3_Srrm2	4.2799	3.7229	3.3154	3.4094	3.6177	4.3673	3.9234	3.9803	4.1352	3.9376	3.6139	3.4154	4.3007	3.3025	3.5006	3.1240	4.5180	3.3152	2.9637	3.2695
Cpsf7	_SSS[Phospho (STY)]TEPPPPVR_.2	Q8BTV2_S48_M1_Cpsf7	4.4570	11.0551	10.8597	3.1272	10.5537	3.1965	3.8850	3.1819	3.8052	11.0196	10.8165	10.5288	3.9706	4.4733	10.0717	3.8191	10.9815	3.6452	10.8867	4.4403
Cpsf7	_DSSDSADGRAT[Phospho (STY)]PSENLVPSSAR_.3	Q8BTV2_T203_M1_Cpsf7	7.0516	7.1677	6.9423	3.9091	6.4778	7.0970	6.9168	3.9602	7.0916	7.3291	7.2760	6.4571	7.5389	7.3947	6.7064	3.6733	3.8154	7.0573	7.2799	7.2120
Pak4	_RES[Phospho (STY)]PPPPAR_.2	Q8BTW9_S104_M1_Pak4	8.2704	6.3795	7.5202	8.3366	8.1346	8.4004	7.8165	8.2244	7.5924	7.9867	7.9137	6.7678	7.3377	8.1406	3.4645	8.1348	7.8656	5.9976	6.7685	8.0208
Gmppb	_LYSGPGIVGNVLVDPSAR_.2	#N/A	3.9772	3.3418	4.4711	3.6070	4.0660	4.2175	4.2501	4.1831	3.9350	3.7878	3.7636	3.9675	3.2027	3.5450	4.0390	3.3394	4.5626	4.4642	4.3148	3.7773
Tox4	_LSTTPS[Phospho (STY)]PT[Phospho (STY)]NSLHEDGVDDFRR_.3	Q8BU11_S178_M2_Tox4	3.9502	3.5461	3.4921	3.2327	4.1572	4.1905	4.3918	4.1571	4.3120	3.7608	3.7906	3.4356	4.4774	3.4792	2.9163	3.3124	3.1939	3.1384	4.2878	3.4462
Sec62	_EDSKKEETPGT[Phospho (STY)]PK_.2	#N/A	3.5539	4.3358	4.0902	4.2007	3.6762	3.9003	4.0115	4.1440	4.0575	3.8107	3.6473	4.3116	4.0308	3.5411	3.5756	3.8988	3.8946	4.6294	3.6797	3.7332
Sec62	_SQHS[Phospho (STY)]SGNGNDFEM[Oxidation (M)]ITK_.2	Q8BU14_S356_M1_Sec62	3.8660	4.3716	3.4026	3.7182	4.0729	5.5362	4.4760	4.0917	4.3962	3.6766	3.8749	3.1792	4.5617	3.4406	3.0005	3.2281	5.3482	3.5605	5.5944	3.1179
Sec62	_EELEQQT[Phospho (STY)]DGDC[Carbamidomethyl (C)]DEEDDDKDGEVPK_.3	Q8BU14_T375_M1_Sec62	4.3079	3.2874	3.5683	3.5525	3.6456	3.8697	3.8955	4.2574	3.9545	4.5142	4.3289	3.0135	4.0626	3.6064	3.4726	3.1520	3.7546	3.3948	3.6939	3.2836
Hook3	_LNQSDSIEDPNS[Phospho (STY)]PAGR_.2	#N/A	9.2822	9.0005	9.6354	9.3587	9.0179	9.1279	8.9518	9.2911	8.8173	9.3451	9.1222	8.9290	9.3935	9.6676	8.5985	8.7503	9.2672	8.7019	8.9455	9.0015
Hook3	_LNQSDSIEDPNS[Phospho (STY)]PAGRR_.2	#N/A	7.1913	7.3624	6.9969	7.0352	6.9743	6.9993	6.8234	7.1754	7.0183	7.5201	6.9048	6.9661	7.6694	7.4457	6.5940	7.1583	7.1289	7.1627	7.1371	7.1420
Plekha1	_RLS[Phospho (STY)]NPC[Carbamidomethyl (C)]IQR_.2	Q8BUL6_S308_M1_Plekha1	7.6219	7.8266	7.6654	7.5418	7.3793	7.1962	7.4778	7.4101	7.0005	7.7419	6.4420	7.3298	7.1522	7.6514	6.0819	6.8462	7.2236	7.2907	6.9173	7.1533
Ptpn7	_AQS[Phospho (STY)]QEDSDYINANYIR_.2	Q8BUM3_S143_M1_Ptpn7	3.1637	4.1553	4.2012	4.4205	3.3707	3.9254	3.6774	3.3894	3.5976	4.4752	3.0763	3.8815	3.7631	5.5268	3.7028	4.0267	4.3875	4.9829	3.0610	3.9165
Foxj3	_VTLYNADQDGSDS[Phospho (STY)]PR_.2	Q8BUR3_S223_M1_Foxj3	7.8143	7.7218	7.9520	7.9348	3.3406	7.5840	7.3120	6.6702	7.4556	8.0359	6.4631	7.2961	7.2638	7.4957	7.3311	7.6826	8.1139	7.0027	7.3149	7.5170
Gphn	_ASHS[Phospho (STY)]AVDITK_.2	Q8BUV3_S338_M1_Gphn	8.0063	8.3354	8.0365	8.2355	7.9030	7.4953	7.7301	7.9058	7.3374	8.4818	7.4011	7.7684	8.0867	8.0462	6.8713	7.6903	7.6126	7.7935	7.8347	8.2182
Gphn	_IPDSIISR_.2	#N/A	5.6427	5.4184	4.4503	5.7478	5.8621	6.2924	6.2337	4.8594	5.5087	5.7458	5.9372	5.8250	5.9876	5.5183	6.2451	6.2897	5.7489	6.2481	5.5605	6.1062
Gphn	_VLAQDVYAK_.2	#N/A	5.3578	5.2369	5.8088	5.8398	5.6626	5.8903	5.4953	5.5511	5.1134	5.3384	5.5495	5.8735	4.4574	4.9155	6.2607	5.5392	5.4888	4.9144	5.8319	5.6214
Gphn	_DTASLSTTPSES[Phospho (STY)]PR_.2	#N/A	11.5403	11.9720	12.1454	12.3091	11.8069	11.4519	11.6702	12.0002	11.4545	12.0019	11.3523	11.3889	11.8052	12.3778	11.1247	11.9767	11.7493	11.3243	11.6496	12.0117
Gphn	_DLVQDPSLLGGTISAYK_.2	#N/A	5.7857	5.2143	4.5912	5.6943	5.3570	5.1758	5.6472	5.3244	5.5790	4.9349	4.7278	5.1799	5.8096	5.7308	5.5846	5.6939	5.1188	4.9560	5.1060	6.0151
Gphn	_VGVLTVSDSC[Carbamidomethyl (C)]FR_.2	#N/A	3.4640	5.4873	4.5015	5.8075	4.8251	5.7551	4.8402	5.1569	5.2504	3.5811	3.7612	5.1001	3.4949	5.5035	4.9489	5.5119	5.6613	5.2890	4.9238	5.1493
Gphn	_DTASLSTT[Phospho (STY)]PSES[Phospho (STY)]PRAQATSR_.3	#N/A	3.9798	3.9486	3.6485	5.8005	4.0699	3.9685	3.9166	3.8725	5.1251	3.4102	4.1124	3.2016	4.0278	3.6420	3.2447	3.4621	3.5115	3.5786	4.1628	3.6090
Gphn	_LSTAS[Phospho (STY)]C[Carbamidomethyl (C)]PT[Phospho (STY)]PK_.2	Q8BUV3_T286_M2_Gphn	7.5865	6.3304	8.1513	8.2873	7.8321	4.9015	6.0943	8.1414	5.5250	7.5621	7.9669	8.0400	8.2687	8.1144	6.4908	8.1324	7.7517	8.0260	7.2666	7.8326
Gphn	_C[Carbamidomethyl (C)]SS[Phospho (STY)]KENILR_.2	Q8BUV3_S328_M1_Gphn	8.6118	8.7469	8.6216	8.6438	8.2962	4.7847	8.3744	8.3977	8.2603	8.9446	3.3619	8.4895	8.8103	8.7494	8.0860	8.7551	4.1828	5.8861	8.4907	8.6453
Gphn	AQATSRLS[Phospho (STY)]TAS[Phospho (STY)]C[Carbamidomethyl (C)]PT[Phospho (STY)]PK .	Q8BUV3_T286_M3_Gphn	6.5992	5.9830	6.3636	6.7618	6.9185	6.5514	6.8104	5.8548	6.2051	6.6655	6.7148	5.9174	6.2434	7.5522	6.7689	6.4940	5.6033	6.0384	6.9400	6.3261
Gphn	_EVHDELEDLPS[Phospho (STY)]PPPPLS[Phospho (STY)]PPPTTSPHK_.4	#N/A	4.0924	8.0004	6.2186	6.0608	3.6987	7.4482	7.6065	4.2030	7.3635	6.0981	3.8820	6.2075	3.8878	8.7835	7.1214	5.4815	5.8705	8.1157	7.0519	5.4824
Gphn	VKEVHDELEDLPS[Phospho (STY)]PPPPLS[Phospho (STY)]PPPT[Phospho (STY)]TSPHKQTEDK .5	Q8BUV3_T198_M3_Gphn	3.9892	6.8917	3.9157	3.8922	3.7315	3.9778	6.3829	3.9874	5.7439	3.9347	3.5713	3.6769	4.0974	3.9299	3.5209	3.7333	3.8926	3.5029	3.6458	4.0269
Gphn	_VKEVHDELEDLPS[Phospho (STY)]PPPPLS[Phospho (STY)]PPPTTSPHK_.4	#N/A	4.0746	3.2445	3.6112	3.5096	4.2815	4.3149	4.2674	4.3003	4.1876	3.8852	3.6663	2.9706	4.3531	6.4798	2.7919	3.4367	3.7975	3.2628	3.6510	3.3219
Lsm11	_DSS[Phospho (STY)]LTLTR_.2	Q8BUV6_S220_M1_Lsm11	4.1281	3.1909	3.6647	3.4561	4.3351	4.3684	4.2139	4.3538	4.1341	3.9387	3.6127	4.4179	4.2995	3.7028	4.2392	3.4903	3.8511	3.3163	2.9649	3.2683
Lsm11	_S[Phospho (STY)]VPSSLQASAR_.2	Q8BUV6_S281_M1_Lsm11	3.3670	3.9521	4.4044	4.2173	3.5739	3.6072	6.2839	3.5926	3.3944	3.1776	4.3739	3.6782	3.3978	4.4424	3.4996	4.2299	3.0899	5.2346	4.3586	4.1197
Gpr107	_KVS[Phospho (STY)]NGAVEPQGSWEGTA_.2	Q8BUV8_S537_M1_Gpr107	7.9022	8.0659	7.8115	7.4840	6.5271	7.7477	7.4954	7.7317	6.9976	7.6602	7.1679	7.4430	7.9714	7.6142	6.2186	6.8326	7.8152	7.6952	7.5696	6.9707
Gprasp2	_QLQIQIDNQNDPEEGQ_.2	#N/A	3.5644	5.2772	5.8706	5.4240	4.7205	4.8030	4.8105	5.3441	5.6300	5.0552	3.6976	4.5364	3.5851	5.4451	4.2467	5.6734	4.9360	4.8458	4.5965	3.9926
Atp6v1h	_QLQSEQPQTAAARS[Phospho (STY)]_.2	Q8BVE3_S483_M1_Atp6v1h	9.2954	9.2906	9.1391	9.1339	8.8159	9.0614	9.1903	9.2636	9.1374	9.0915	8.7480	8.8987	9.4099	8.6701	7.9230	8.6969	9.0647	9.1200	9.0660	8.7093
Atp6v1h	_GAVDAAVPTNIIAAK_.2	#N/A	9.0285	8.7585	8.6844	8.5534	8.3285	8.5451	8.8727	8.7088	8.8259	8.4929	8.4569	8.3721	9.1918	8.3557	8.2717	8.2389	8.8561	8.5375	8.4839	7.9613
Atp6v1h	_LEWSPVHK_.2	#N/A	6.4520	6.4458	5.7632	5.1601	6.4293	6.2708	6.4891	6.8622	6.0936	6.5686	5.8335	6.1255	6.7576	6.3727	5.3782	5.7481	6.1367	6.8694	6.1999	6.0547
Atp6v1h	_LLEVSDDPQVLAVAAHDVGEYVR_.3	#N/A	6.4031	5.8449	5.9554	6.0394	5.5982	5.5457	5.8587	6.0527	5.8476	6.2902	6.1997	5.6823	5.7700	5.7166	4.6836	5.5501	5.4762	6.2044	5.6203	4.9932
Atp6v1h	_LGESVQDLSSFDEYSSELK_.2	#N/A	6.4086	6.6306	5.4749	5.9724	5.0375	5.2564	6.4356	6.0093	5.4902	5.6075	5.7954	5.3472	6.0625	6.2122	4.1653	5.6081	6.5997	5.7234	5.3522	5.8017
Atp6v1h	_QLQSEQPQTAAAR_.2	#N/A	6.9377	6.9559	7.3409	6.9216	6.7186	6.7271	7.6394	7.3636	7.6607	7.1380	6.7653	6.5139	3.4289	6.7769	6.7372	6.9382	6.7789	6.9211	7.1091	6.8789
Atp6v1h	_YNIIPVLSDILQESVK_.2	#N/A	6.2695	6.0047	5.5329	4.7549	5.6719	5.8260	4.6557	6.3008	5.0081	5.9676	5.5284	5.1949	4.1009	5.9063	4.2928	3.1136	4.9565	6.1789	5.2365	5.0992
Atp6v1h	_QLENLEQQKYDDEDISEDIK_.3	#N/A	3.6758	5.2621	4.5536	4.3264	3.6054	2.2341	5.1684	6.0421	4.6348	3.8836	3.4518	4.9105	4.3546	5.2941	4.1708	3.3852	5.1932	5.1129	4.4555	2.7283
Atp6v1h	_RSS[Phospho (STY)]EDKQEM[Oxidation (M)]LQTEGSQC[Carbamidomethyl (C)]AK_.3	#N/A	3.5386	4.1747	5.5551	5.2899	3.7096	3.3709	5.0973	3.4554	3.8375	4.3164	3.2562	3.7566	3.9684	4.2907	3.3961	4.0966	4.5348	4.9354	3.2524	3.6817
Atp6v1h	_VIEQLGGK_.2	#N/A	3.7565	4.1549	4.2928	4.8312	3.0942	4.6010	5.1989	3.3148	4.3788	3.8772	4.1235	4.2035	3.1132	4.1578	4.4117	4.4405	3.3172	3.7428	3.9375	4.7248
Nsd2	_SSS[Phospho (STY)]AENQEGDPGTDK_.2	Q8BVE8_S408_M1_Nsd2	3.4105	3.9086	4.4479	6.8523	3.6174	2.9901	7.7493	3.6361	3.3333	3.2211	7.5618	3.6347	7.6557	4.4859	3.4561	8.0604	7.8972	7.8149	4.3151	7.6629
Dpp9	Protein N-term)]C[Carbamidomethyl (C)]SGVS[Phospho (STY)]PVEQVAAGDM[Oxidation (M)]I	#N/A	3.9464	6.8551	6.2712	5.3629	3.8044	6.2809	5.9936	3.9463	4.0086	6.3938	3.4873	3.6081	3.9724	6.6959	5.0403	5.9743	6.0685	6.3016	6.0090	6.1097
Qdpr	_EGGLLTLAGAK_.2	#N/A	7.4605	7.1905	7.3394	7.2302	7.3921	7.5446	7.7490	7.3354	7.2008	7.4007	7.2478	7.7399	7.2035	6.8828	6.9173	7.0579	6.9869	7.2191	7.4526	7.3393
Qdpr	_VDAILC[Carbamidomethyl (C)]VAGGWAGGNAK_.2	#N/A	6.9246	6.2454	7.0667	7.0414	6.4370	5.9343	3.6635	4.2402	6.3151	6.8489	4.0970	3.3524	3.8307	6.3913	3.2407	5.6901	6.6589	3.0553	6.2852	6.1567
Qdpr	_AALDGTPGMIGYGM[Oxidation (M)]AK_.2	#N/A	4.3349	3.6679	3.3703	3.3545	3.6726	3.8967	3.8685	4.2304	3.9276	4.5412	4.3019	3.5574	4.0356	5.8793	3.4457	3.1789	3.7276	3.4218	3.7208	3.2567
Qdpr	_MTDSFTEQADQVTADVGK_.2	#N/A	4.0173	5.7759	6.9929	6.7188	5.0984	5.6355	5.7935	4.3272	5.1666	5.4019	5.7142	4.5570	4.3532	7.2762	5.0165	4.3622	5.2496	3.1042	4.7671	4.8453
Qdpr	_GAVHQLC[Carbamidomethyl (C)]QSLAGK_.3	#N/A	3.6622	3.6569	3.1988	3.9220	3.2026	3.9025	3.1789	3.8879	4.5398	4.1147	3.2376	4.9203	3.2646	3.2369	3.7089	3.0243	3.3851	3.7643	4.6088	4.4717
Qdpr	_QSM[Oxidation (M)]WTSTISSHLATK_.2	#N/A	3.6912	3.6699	4.0434	3.9350	3.4924	3.8331	2.9771	3.9168	3.6104	3.1822	3.8605	3.4208	3.7425	3.7715	2.6756	3.8878	3.4141	3.8424	3.7852	3.6261
Stx16	_S[Phospho (STY)]PPKWVDGVDEIQYDVGR_.3	#N/A	5.8465	6.5897	5.6085	6.4196	5.5255	5.7903	6.0842	5.6764	5.7997	5.7108	6.1655	6.2844	6.5983	6.6510	5.4423	5.7740	5.7544	6.5042	6.1217	5.8664
Stx16	_QLLAEQVSS[Phospho (STY)]HTTSSPLHSR_.3	Q8BVI5_S30_M1_Stx16	4.4532	3.5496	3.4887	3.6978	3.7910	4.0150	3.7501	5.4456	3.8092	3.1587	4.1836	3.4390	3.9173	3.4610	3.3273	3.2973	3.6093	3.5401	3.8392	3.1383
Stx16	_SIAAELDELADDR_.2	#N/A	4.3914	5.6116	3.9136	4.0720	3.4058	3.5545	4.2408	3.9366	3.6337	4.1990	3.1734	4.1991	4.4079	5.3129	3.6989	3.5512	4.1000	5.4212	3.3787	3.6290
Snx17	_VTSSVPLPS[Phospho (STY)]GGTSSPSR_.2	Q8BVL3_S331_M1_Snx17	3.9190	3.6548	6.7149	3.7033	5.5912	6.1762	3.9932	3.9796	6.0159	3.8810	3.7881	8.5842	4.1503	3.9047	3.3040	3.7098	7.0418	3.5668	3.6769	5.7381
Snx17	_SPPLLES[Phospho (STY)]PDASR_.2	Q8BVL3_S421_M1_Snx17	7.6618	8.0599	8.4771	8.3503	5.9814	8.1809	8.0381	7.3144	7.7772	7.8189	6.8966	7.3402	7.3935	6.0421	6.6804	7.7455	7.6967	4.0038	7.5764	8.0181
Snx17	_VLVTVLTSDQTEDVLEAVAAK_.2	#N/A	4.1120	2.3416	3.2508	3.2847	2.8354	3.3354	4.2300	3.3270	4.1502	3.8869	3.6289	2.9332	4.3157	2.4488	4.2554	3.4741	3.8746	1.9072	2.9487	3.7405
Snx17	_SDSQQAVKS[Phospho (STY)]PPLLES[Phospho (STY)]PDASR_.3	#N/A	3.7272	6.1404	7.2527	4.4727	3.0650	5.1091	6.7896	3.3372	5.9544	4.9768	3.4087	4.1650	3.1424	4.1870	5.2437	6.4896	4.3353	6.4999	6.1746	6.4434
Jakmip1	_HTS[Phospho (STY)]LNDLSLTR_.2	Q8BVL9_S382_M1_Jakmip1	7.9192	7.7070	7.8362	7.6246	7.2282	7.7155	7.4750	6.6238	7.3187	7.7497	7.3998	3.7444	6.8840	8.2689	6.3766	7.0099	4.3859	7.3602	7.2882	7.3531
Ppme1	_QC[Carbamidomethyl (C)]EGITS[Phospho (STY)]PEGSK_.2	Q8BVQ5_S243_M1_Ppme1	12.1338	11.4746	11.9362	12.0866	11.7521	11.4360	11.4768	11.9762	11.4988	11.5400	12.2225	12.4848	12.8523	3.3557	11.7631	12.3664	12.6956	12.3879	12.3255	12.3600
Ppme1	_SIVEGIIEEEEEDEEGSESVNKR_.3	#N/A	9.1178	8.7156	8.8776	8.9090	7.2924	8.1379	8.1128	8.4512	8.4941	8.5921	8.1435	8.0433	9.0790	9.3544	7.3485	8.5559	8.8997	7.8113	8.0669	8.3191
Ppme1	_SLENAIEWSVK_.2	#N/A	4.1475	4.5274	3.1830	4.9569	4.6662	3.7093	4.4389	3.8479	4.7191	4.3538	2.9885	4.3328	4.2230	3.1701	3.9802	4.3784	4.3856	4.3430	4.4021	4.4239
Ppme1	_FAEPIGGFQC[Carbamidomethyl (C)]VFPGC[Carbamidomethyl (C)]_.2	#N/A	3.7941	4.9033	5.3346	4.5439	4.0010	4.0343	5.7519	4.9050	4.4682	3.6046	5.0377	5.0237	4.0597	3.3687	3.0725	3.1562	5.8621	5.8019	5.3803	3.0460
Maob	_YVDLGGSYVGPTQNR_.2	#N/A	6.5493	6.8676	6.2768	6.1488	3.5963	5.9962	6.5777	7.2602	3.3720	6.1959	5.8482	6.1947	6.5624	4.4649	5.8597	4.2524	6.0529	6.6387	6.1824	5.9045
Maob	_FIGGSGQVSER_.2	#N/A	4.5160	3.4955	4.9699	3.7606	3.8537	4.0778	3.6873	4.0493	3.7464	3.2215	4.1208	3.2216	3.8545	3.3982	3.2645	3.3601	3.5465	3.6029	3.9020	3.0755
Camk1d	_DLKPENLLYYSQDEESK_.3	#N/A	3.6952	4.2313	4.6478	5.2577	4.3596	4.8891	5.0398	4.2375	4.9110	3.9015	4.6599	4.4573	3.1745	3.9068	3.3889	4.0401	4.2702	3.9671	3.5485	4.1714
Aldh5a1	_AAYDAFNSWK_.2	#N/A	6.7632	6.9887	7.5874	7.7800	7.2370	7.2983	7.4549	7.3741	7.1567	7.4696	6.9768	7.1955	7.2570	7.0647	7.4569	7.4917	7.2218	7.2680	7.4958	7.2069
Aldh5a1	_EVGEVLC[Carbamidomethyl (C)]TDPLVSK_.2	#N/A	6.7661	7.0120	6.9773	7.0071	6.6325	6.9638	6.3898	6.6062	7.0741	6.3357	6.9794	6.8301	6.8405	7.1151	7.2169	6.9112	7.2794	6.7583	7.0959	6.6510
Aldh5a1	_GIHDSFVTK_.2	#N/A	6.5203	6.2492	6.7464	6.4032	6.2354	6.0663	6.5502	6.5671	7.0566	6.5623	6.0785	6.9662	7.1301	6.4730	7.3091	6.4430	6.2062	6.6995	6.1529	6.6932
Aldh5a1	_NAGQTC[Carbamidomethyl (C)]VC[Carbamidomethyl (C)]SNR_.2	#N/A	5.9479	6.2834	7.0611	6.8134	6.8000	6.5886	6.3465	7.0460	6.4333	6.6586	6.6753	6.8077	6.7921	7.3113	6.5336	6.3816	6.6264	6.5718	6.9134	6.2027
Aldh5a1	_ISFTGSTATGK_.2	#N/A	6.5754	6.4879	6.9178	6.9413	6.3430	6.2296	6.7066	6.1885	5.8509	6.3257	6.2878	6.5225	6.1741	6.5743	6.5925	6.3432	5.8467	6.6524	6.1197	6.6661
Aldh5a1	_LGTVADC[Carbamidomethyl (C)]GVPEAR_.2	#N/A	6.3393	6.6171	6.4333	5.9182	6.6434	6.4469	6.8563	6.1962	6.3885	6.1333	6.2880	6.6869	6.2178	6.7279	6.2721	5.7785	6.3427	6.5241	6.1661	6.0306
Aldh5a1	_WLPAPATFPVYDPASGAK_.2	#N/A	4.4929	8.1070	8.3981	8.3329	7.9772	8.0091	7.9189	7.8923	7.9484	8.2020	7.8539	8.1890	8.1411	7.9450	8.0837	8.2058	7.8831	7.9903	7.9694	8.0340
Aldh5a1	_DM[Oxidation (M)]LC[Carbamidomethyl (C)]ITEETFGPLAPVIK_.2	#N/A	6.0100	5.5668	6.1068	5.6745	5.2200	5.5349	5.0776	5.1323	5.1079	5.6417	5.7169	5.4173	4.7880	5.1344	5.3410	5.4331	6.0606	5.8152	4.7239	5.4456
Aldh5a1	_VAEQLEVGM[Oxidation (M)]VGVNEGLISSVEC[Carbamidomethyl (C)]PFGGVK_.3	#N/A	5.6069	4.6213	4.6695	4.8712	4.0199	4.6592	4.5578	5.3100	4.4111	4.7705	4.5044	4.9289	5.3778	5.5843	3.2980	5.2186	5.1087	4.9214	4.8263	4.3792
Aldh5a1	_YGIDEYLEVK_.2	#N/A	4.8050	4.7202	4.1959	4.8066	5.3745	5.1418	4.0816	4.8213	4.4557	5.2678	4.5457	4.5657	4.8391	4.0608	4.6966	4.7880	3.7754	5.0896	4.4894	4.3185
Aldh5a1	_QPVGVAAIITPWNFPSAM[Oxidation (M)]ITR_.2	#N/A	3.6678	2.1950	3.7921	3.7260	4.0930	2.8988	3.5455	3.1556	4.1877	3.2395	3.6576	4.2848	4.2957	3.8395	3.4492	4.0181	2.0238	3.6067	3.4305	3.6448
Aldh5a1	_HQSGGNFFEPTLLSNVTR_.2	#N/A	4.1979	2.5353	3.2733	3.4516	3.5755	0.5595	1.6091	2.0232	4.0246	3.0546	2.8982	2.8386	4.2585	3.0189	3.5427	1.9437	3.4335	3.3573	1.9024	3.2274
Arrb1	_DDKDEEDDGTGS[Phospho (STY)]PHLNNR_.2	#N/A	3.6868	4.9445	4.6873	4.0379	4.1721	4.6538	3.9117	4.3712	3.5839	5.0175	4.2166	4.6337	4.5917	5.2901	3.6275	4.2385	4.7842	4.4338	4.4419	3.5491
Arrb1	_GM[Oxidation (M)]KDDKDEEDDGTGS[Phospho (STY)]PHLNNR_.4	#N/A	5.0999	5.6932	5.1040	5.8318	5.1338	5.4751	5.9089	5.4409	6.5430	6.7005	5.6580	5.4680	6.6562	5.4362	6.0964	5.7543	4.2110	5.9121	6.7073	5.8427
Phka2	_S[Phospho (STY)]LNLVDSPQPLLK_.2	Q8BWJ3_S729_M1_Phka2	4.0063	3.3128	6.1607	3.5779	4.2132	3.8951	5.3299	4.2320	3.9291	5.6833	4.3035	4.8878	5.2221	4.6193	2.8602	3.3684	3.7292	5.8718	5.5010	3.2582
Gprin3	_S[Phospho (STY)]VSTS[Phospho (STY)]PSILPAYLK_.2	Q8BWS5_S327_M2_Gprin3	10.6593	10.6342	10.5944	10.6391	9.8112	10.2626	10.3554	10.2765	10.5463	10.6878	10.7613	10.6034	11.7628	11.4855	10.7300	10.7849	10.9904	10.7578	10.6768	10.5416
Acaa2	_LEDTLWAGLTDQHVK_.2	#N/A	5.5798	5.2517	3.9750	4.9079	5.0122	5.2848	4.9676	5.8691	4.9174	5.2929	4.0601	4.2846	5.2199	5.4796	4.7021	5.3537	5.0878	4.4978	3.8474	4.6885
Acaa2	_VGVPTETGALTLNR_.2	#N/A	3.5134	6.0466	6.3425	5.8806	5.9928	6.0545	6.3777	6.3785	5.8209	3.3240	5.6164	5.6474	6.1703	5.0479	6.1178	5.8827	5.4702	6.2621	5.9136	5.8761
Acaa2	_TNVSGGAIALGHPLGGSGSR_.2	#N/A	3.9313	3.3878	4.1180	4.5406	3.1918	3.5848	4.0117	5.0862	3.4906	3.7419	3.3764	3.8324	4.4964	4.2666	4.1990	4.0324	4.0484	3.8599	4.2357	4.1235
Acaa2	_DFSATDLTEFAAR_.2	#N/A	4.9301	5.3446	3.8805	5.3795	3.0500	3.0833	5.2663	5.3303	3.9183	5.4641	5.2426	5.1759	4.0837	4.8627	5.4418	5.3458	5.0963	5.0208	5.3050	5.4885
Acaa2	_YALQSQQR_.2	#N/A	4.5770	5.9162	5.9682	4.5081	3.2831	5.8092	5.4242	3.3018	6.2337	5.8186	3.1639	3.9691	3.1070	4.1516	5.9659	3.9391	4.2999	5.6789	3.1486	5.5987
Acaa2	_VPPETIDSVIVGNVM[Oxidation (M)]QSSSDAAYLAR_.3	#N/A	3.5408	3.7947	3.9999	3.7974	3.5053	3.9358	4.0247	4.2820	3.2515	4.2432	3.6259	2.7990	3.7314	4.7281	3.4220	3.5239	3.9879	3.6257	3.5839	3.4476
Gemin5	_APSQPPS[Phospho (STY)]PTEER_.2	Q8BX17_S1414_M1_Gemin5	3.4227	3.8964	4.4601	4.1615	3.6297	4.4787	3.4184	8.9437	8.8181	3.6224	3.7199	3.6225	3.4536	4.4982	7.6853	4.2857	9.0404	8.7583	8.6266	4.1755
Vps13c	_EVST[Phospho (STY)]PQDVHTTQGVPAAR_.3	Q8BX70_T1397_M1_Vps13c	3.8800	6.4298	3.2457	6.4087	4.0869	6.3301	6.2276	5.8475	6.1534	3.6949	3.7787	5.6037	4.0220	3.2568	3.7803	3.2421	5.9814	3.5776	3.6437	3.9255
Vps13c	_LS[Phospho (STY)]FHNDSFR_.2	Q8BX70_S1956_M1_Vps13c	7.0087	6.8657	7.2099	3.3066	6.2358	4.2644	6.5306	6.0455	4.2381	3.8347	6.8779	3.5095	4.4035	3.4053	2.8424	3.2268	3.1200	6.4226	6.0858	3.3723
Vps13c	_ILLENLGEASSQPS[Phospho (STY)]PTQYAQEAAR_.3	Q8BX70_S1891_M1_Vps13c	4.4028	7.5769	3.4382	3.3020	3.7405	6.6314	7.2207	7.5827	6.1890	7.1030	6.4409	6.9196	7.4914	3.6444	5.5528	6.3424	6.7113	7.2982	7.0896	3.4684
Vps13c	_SST[Phospho (STY)]PS[Phospho (STY)]PERQVASIPVLSGGTK_.3	Q8BX70_S841_M2_Vps13c	3.8842	4.1186	4.4205	3.8052	3.2219	3.4460	6.7568	3.5846	4.3782	4.0905	3.2518	4.0080	4.4863	6.7824	3.8963	4.2291	4.1223	2.9711	3.2702	3.7073
Vps13c	_TANAS[Phospho (STY)]LEEIIDK_.2	Q8BX70_S736_M1_Vps13c	4.2321	4.5879	3.7686	3.3522	2.9382	4.4723	4.1099	4.4577	4.1503	4.0427	3.5088	4.3140	4.1956	7.0031	4.1353	3.5942	3.9550	3.4202	3.0688	3.1644
Slco2b1	_KVS[Phospho (STY)]VEPQDSHQDAQPR_.3	#N/A	4.8335	4.4648	3.6512	4.9083	4.3607	5.3818	4.4496	4.6256	4.0065	3.6275	4.4430	3.9429	4.8958	4.5339	2.9669	4.0054	5.2145	4.4771	4.8181	3.5026
Agap1	_VFQDVAQK_.2	#N/A	4.9744	4.8268	4.4821	4.3645	4.9898	4.1515	4.4254	4.3367	4.2486	3.1259	4.2900	4.5586	5.4106	4.8299	4.1453	4.3631	3.6031	4.4578	4.6419	4.0312
Agap1	_ATSAC[Carbamidomethyl (C)]APISS[Phospho (STY)]PK_.2	Q8BXK8_S422_M1_Agap1	4.1989	8.1771	8.2183	8.0736	7.6133	3.7190	7.6507	3.7856	4.1217	4.3635	7.3035	7.1508	7.3021	7.8035	3.7751	7.2847	7.4760	7.2437	7.3238	7.1458
Agap1	_LDPPPS[Phospho (STY)]PHANR_.2	Q8BXK8_S521_M1_Agap1	11.5425	11.3682	11.6000	11.3602	10.7724	10.7395	10.6956	11.2281	10.8566	11.0277	10.4944	10.9928	11.0711	11.3417	9.9769	10.8334	11.1018	10.8485	10.5667	10.5857
Agap1	_YLTGTYVQEES[Phospho (STY)]PEGGR_.2	Q8BXK8_S101_M1_Agap1	4.4831	4.3368	4.3853	4.2724	3.1892	3.3170	5.3014	3.2080	4.1331	4.1761	3.2754	4.0630	4.2640	4.0578	3.9111	3.9833	4.2060	5.4225	3.2425	4.1210
Fam53c	_GNAGGGGPQVPQQS[Phospho (STY)]PPKR_.2	#N/A	4.1994	5.8407	6.3393	6.5846	6.9263	7.0480	6.6218	5.8396	6.7824	6.2212	7.1358	6.9455	7.6512	8.1053	6.9277	7.0535	6.9264	7.0994	7.0272	7.1254
Fam53c	_SRS[Phospho (STY)]QPC[Carbamidomethyl (C)]DLDAR_.2	Q8BXQ8_S273_M1_Fam53c	3.6634	7.3110	4.1997	4.4088	6.7426	6.9505	3.0391	7.1284	6.3744	3.8697	6.4668	4.2289	3.2063	4.2509	4.1172	4.0083	6.9986	6.5001	3.0493	3.9282
Fam53c	_S[Phospho (STY)]LSVPVDLSR_.2	Q8BXQ8_S120_M1_Fam53c	3.6692	3.6499	3.2058	3.9150	3.8761	3.9095	3.1720	3.8949	3.0922	3.4798	4.0716	3.3760	3.2576	3.2438	3.1973	3.0313	3.3921	3.7573	4.0564	3.7273
Slc7a14	_QNSEALIANDELDC[Carbamidomethyl (C)]S[Phospho (STY)]PE_.2	#N/A	3.5179	10.5169	10.5032	8.7228	4.5760	10.6381	10.6040	6.0991	10.4086	9.0455	7.5726	9.5612	7.3446	10.6956	9.2188	8.4331	9.4594	10.6012	10.4320	9.9168
Slc7a14	_HKQNSEALIANDELDC[Carbamidomethyl (C)]S[Phospho (STY)]PE_.2	#N/A	4.0162	9.0461	8.4814	7.8914	4.2231	8.5636	8.8191	4.2133	8.7053	7.8392	4.0935	7.5526	5.7650	8.6766	7.3858	7.9577	7.2803	8.8640	8.4421	8.2969
Slc7a14	spho (STY)]C[Carbamidomethyl (C)]SPVS[Phospho (STY)]EGEEFSSPATNTC[Carbamidomethyl (C	Q8BXR1_S468_M2_Slc7a14	4.0830	3.2361	3.6196	3.5012	4.2899	4.3233	4.2590	4.3087	4.1792	3.8936	3.6579	2.9622	4.1139	3.6576	2.7835	3.4451	3.8059	3.3435	3.6426	3.3349
Slc7a14	_NLPS[Phospho (STY)]LGDNEM[Oxidation (M)]LIGK_.2	#N/A	4.0414	7.1341	6.1271	6.4831	6.4376	6.6917	6.9915	6.6689	6.6741	6.5307	3.5681	6.6413	7.6003	7.2708	5.2790	6.6000	6.7005	7.1282	6.4838	6.6247
Nalcn	_GKS[Phospho (STY)]LETLTQDHSNTVR_.3	Q8BXR5_S781_M1_Nalcn	6.4369	6.9130	3.8614	4.3643	4.1636	5.3525	5.9237	6.4189	5.1028	6.5642	4.1598	5.5626	3.3458	5.9670	3.2855	2.9431	3.5631	6.9223	5.7623	3.8154
Osbpl6	_TAS[Phospho (STY)]SSTEPSVSR_.2	Q8BXR9_S44_M1_Osbpl6	11.2368	11.0996	11.3433	11.1421	10.3495	10.2959	10.5254	10.6167	10.3773	10.9754	10.2558	10.1174	10.5964	4.4985	9.2801	10.2564	3.1459	10.3306	10.2863	4.1757
Osbpl6	_[Acetyl (Protein N-term)]S[Phospho (STY)]SDEKGIS[Phospho (STY)]PAHK_.2	Q8BXR9_S9_M2_Osbpl6	3.7768	4.8225	4.3131	3.9176	3.1145	3.3386	3.8270	4.0807	3.8861	5.3378	4.5621	4.7785	3.9941	4.3002	4.0087	4.1217	4.0149	5.3043	4.7204	4.7082
Rgs8	_RLS[Phospho (STY)]TEEATR_.2	Q8BXT1_S46_M1_Rgs8	4.5490	4.2914	4.9114	4.0683	3.4919	3.3111	3.5562	3.9895	4.3124	5.1022	4.0732	5.0662	5.7255	5.4541	3.4709	4.8326	4.3378	5.3363	4.5322	4.3605
Rgs8	_SDS[Phospho (STY)]C[Carbamidomethyl (C)]SDFTAILPDKPNR_.2	Q8BXT1_S26_M1_Rgs8	3.2018	4.1173	4.2392	4.3824	3.4087	3.4421	3.6394	3.4275	3.5596	4.5132	3.0382	3.8434	3.7250	4.2277	3.6647	4.0648	4.4255	6.1146	3.5394	4.1947
Fgd2	_TPEGPDEEDVS[Phospho (STY)]D_.2	Q8BY35_S654_M1_Fgd2	7.6582	7.0946	7.0697	7.3550	7.3308	7.3393	6.8593	7.4658	7.3551	7.1629	7.0120	7.4881	7.8868	7.7376	7.3455	7.3413	7.7603	7.4909	7.6902	7.5099
Usp47	_STETSDFENIES[Phospho (STY)]PLNER_.2	Q8BY87_S911_M1_Usp47	6.7538	7.9767	7.2956	6.9079	3.4400	7.7992	7.3394	5.6712	7.3351	7.1755	3.5303	6.6722	6.6369	8.2407	7.0860	7.1314	7.2412	7.8462	7.5482	7.5042
Usp47	_LFVLLPEQS[Phospho (STY)]PGSYSK_.2	Q8BY87_S832_M1_Usp47	3.8542	6.4574	4.3904	3.8352	3.1919	4.9400	6.1793	6.6234	4.4083	4.0605	3.2818	4.0381	6.2492	6.9078	3.9264	4.1990	4.4761	6.4933	5.3168	4.3445
Usp47	_ELEQHIQTS[Phospho (STY)]DPENFQSEER_.3	Q8BY87_S934_M1_Usp47	4.4795	4.3405	4.0160	3.1048	3.1856	3.4220	3.8625	3.2043	4.4023	4.2901	3.2758	4.0666	4.5103	4.0541	3.8879	3.8416	4.2024	4.9482	3.2461	3.7314
Sowahd	_PC[Carbamidomethyl (C)]HY[Phospho (STY)]LRPDAS[Phospho (STY)]LNLR_.2	Q8BY98_S236_M2_Sowahd	9.2911	4.3084	4.0983	3.8766	3.2678	8.7572	7.5991	8.1515	8.4644	5.4656	3.2293	7.9880	3.8957	4.1363	8.2064	7.8525	9.1244	8.0847	8.8440	8.2784
Sowahd	_PDAS[Phospho (STY)]LNLR_.2	Q8BY98_S236_M1_Sowahd	3.8253	3.4937	3.3619	3.7589	4.0323	2.6137	5.1227	4.0510	3.3754	3.2198	4.2788	3.2199	3.8562	4.6146	3.0412	3.1875	3.5483	3.6012	3.9002	3.0773
Tbc1d17	_YLLLASS[Phospho (STY)]PQDSR_.2	Q8BYH7_S176_M1_Tbc1d17	5.8529	4.1046	4.2519	4.3697	3.4215	3.4548	3.6266	3.4402	4.5341	4.5260	3.8819	3.8307	3.7123	4.2900	3.6520	4.0775	4.4383	3.9035	3.0102	4.1819
Tnr	_LILNYSPR_.2	#N/A	8.4896	8.3892	7.8329	8.3303	8.6556	8.2680	8.4999	8.4929	8.0604	8.8804	8.4880	8.6022	8.6312	8.1618	8.1365	7.8845	8.7602	8.4728	8.2928	8.5743
Tnr	_LYPATEYEISLNSVR_.2	#N/A	8.3148	8.3858	8.6507	8.4914	8.4098	8.3777	8.2626	8.3742	8.3671	8.5533	8.0595	8.2835	8.7056	8.4577	8.2988	8.3616	8.4925	8.0288	8.4458	8.4225
Tnr	_ASGPIDHYR_.2	#N/A	7.7749	7.3343	7.9507	7.8441	7.9051	8.0221	7.9839	8.2541	7.8843	6.0501	7.8942	7.9760	8.0359	8.3074	8.4143	7.9407	7.8044	7.8353	7.7565	7.8647
Tnr	_YEVSISAVR_.2	#N/A	7.1804	7.4562	7.7545	8.0066	7.4267	7.6235	7.6674	7.2999	7.7077	7.6433	7.6626	7.3749	7.7673	7.5170	7.7962	7.7849	7.0198	7.3620	7.7138	7.7768
Tnr	_YGLVGGEGGK_.2	#N/A	7.6974	7.3391	7.7786	7.4703	7.6968	7.4501	7.6113	7.9216	7.7100	7.2233	7.4480	7.8244	7.7326	7.6446	7.5861	7.0829	7.1738	7.9071	7.2620	7.8539
Tnr	_SSLTSTVFTTGGR_.2	#N/A	6.9621	7.3210	7.0431	7.7372	7.1279	6.9543	7.1009	6.9934	7.0121	7.0068	7.0762	7.4358	7.0960	7.4628	7.1901	7.3026	6.5636	6.9932	7.3661	7.2770
Tnr	_AAIENYVLTYK_.2	#N/A	6.9912	7.3044	7.3272	7.3216	7.2835	7.0751	7.2202	7.1456	6.7129	7.2063	7.1039	7.1043	7.3290	7.2821	7.0056	7.3872	7.3251	7.0616	7.1189	6.9291
Tnr	_QSALISWQPPR_.2	#N/A	6.3333	7.6020	7.1261	6.6711	6.9471	7.3942	6.6725	6.7111	4.7583	7.3463	6.9782	6.8487	5.3895	6.9857	6.9395	7.5297	6.5362	7.0999	7.0452	7.0788
Tnr	_ELIVDAEDTWIR_.2	#N/A	7.0644	6.6842	7.6091	6.8292	6.9648	6.7372	6.5728	7.2344	7.6701	6.6074	6.9812	6.8737	7.2650	7.3040	6.8985	7.0892	7.2793	6.4751	6.8496	7.2546
Tnr	_LDSSVVPNTVTEFAITR_.2	#N/A	6.6925	6.5744	6.7438	6.2498	6.5487	6.9195	6.6796	6.0150	6.0585	6.7701	6.1712	6.4401	6.5977	6.9451	6.6070	6.9154	6.1743	6.6128	6.4027	6.4853
Tnr	_TATSLDLEWDNSEAEAQEYK_.2	#N/A	7.2664	7.3012	6.0610	6.8065	6.6727	6.0809	6.1777	7.0655	6.1973	6.4248	6.3837	6.6665	7.5361	7.7164	6.1943	6.6469	7.0471	6.4430	6.1387	6.5553
Tnr	_DGQEAVFAYYDK_.2	#N/A	6.6880	6.6338	6.8901	6.7293	6.1993	6.4436	6.6292	5.9675	6.9386	6.5980	6.3930	6.3145	6.6529	6.7015	5.3832	5.8207	6.8506	6.4050	6.4030	6.7998
Tnr	_VATHLSTPQGLQFK_.3	#N/A	6.2196	6.4716	6.4303	6.3128	6.6461	6.4595	6.2516	6.2413	6.5259	6.1637	6.2236	6.4365	6.2824	6.5736	6.6256	6.3288	6.1762	6.4512	6.2117	6.7141
Tnr	_DKEEDM[Oxidation (M)]LEVLLDATKR_.3	#N/A	7.6981	7.6359	7.3412	7.8213	7.4307	7.4727	7.4005	7.6749	7.5862	8.0093	7.6488	7.6130	8.0315	7.9847	7.4017	7.6643	7.8229	7.2533	7.6872	7.5472
Tnr	_SPPTSASVSTVIDGPTQILVR_.2	#N/A	4.7295	4.8395	5.4081	5.3374	4.6700	5.2071	5.0066	4.9532	5.1717	5.0549	5.0702	4.8271	4.9897	5.7833	5.0839	5.3641	5.2462	5.2434	5.4136	5.4610
Tnr	_GHEFSIPFVEM[Oxidation (M)]K_.2	#N/A	6.7482	3.9127	6.6176	4.1779	7.1023	6.7669	6.3057	7.1436	6.5072	6.9411	6.3335	6.9436	6.8455	6.9479	6.9415	7.0153	7.0513	6.8944	6.6292	6.6353
Tnr	_LEGLSENTDYTVLLQAAQEATR_.3	#N/A	4.9424	5.6550	3.3453	6.7687	4.0157	6.2685	5.0686	6.1132	3.7316	5.9593	5.3458	6.0470	6.5064	6.4644	5.4962	5.9551	6.0412	5.4342	5.3953	6.6821
Tnr	_QSIPATM[Oxidation (M)]NAR_.2	#N/A	5.9157	5.8490	6.5457	6.3818	6.2713	6.2239	6.2966	6.5142	6.1506	6.0314	6.2639	6.2737	6.3523	6.3776	6.6448	6.2730	6.0566	6.1082	6.4338	5.8947
Tnr	_KELIVDAEDTWIR_.3	#N/A	4.9204	4.1283	4.7002	6.1043	3.2123	4.8176	4.2865	3.5749	3.0556	4.0809	4.4161	4.0177	4.4960	4.5579	4.8712	4.2194	4.1126	4.6149	4.3939	5.0608
Tnr	_VVYSTLAGEQYHEVLVPK_.2	#N/A	4.1149	3.5478	4.5546	4.4273	4.1521	4.7558	4.3753	5.0212	4.5247	4.1711	3.8905	4.1640	5.1597	3.9437	4.1468	3.8090	4.4737	4.0461	4.3630	4.5323
Tnr	_VGFGNLEDEFWLGLDNIHR_.3	#N/A	4.0476	4.0060	3.5842	3.5366	4.2546	4.1080	4.1844	3.4770	3.9704	4.4983	4.0236	4.2603	4.0785	5.0536	2.5532	4.4696	3.7705	4.6663	3.6780	3.2996
Tnr	_DVSDTVAFVEWTPPR_.2	#N/A	7.5332	7.5436	7.7954	7.9126	7.1080	7.4851	7.4606	7.6985	7.5717	7.3453	7.1094	7.1089	7.7451	7.1652	7.4365	7.4622	7.6291	7.2700	7.4318	7.3038

Tnr	_ITFTPSSGISSEVTVPR_.2	#N/A	7.2692	6.6084	7.1732	7.3824	6.7023	6.9065	6.8128	6.9081	7.1228	7.3233	6.8726	6.9288	7.1861	10.0462	7.1647	6.9785	7.0476	8.5537	7.0334	7.0922
Tnr	_C[Carbamidomethyl (C)]LNAC[Carbamidomethyl (C)]SGR_.2	#N/A	4.5280	3.6476	4.0646	4.5177	3.2341	3.3734	3.8140	3.2529	4.4509	4.3386	3.3244	4.8064	4.5589	4.1027	4.6297	3.8902	4.2509	4.3993	3.1975	3.7800
Tnr	_DSLTVSWSSPVAPFDYYR_.2	#N/A	3.9601	3.3590	3.4967	4.3713	4.1670	3.9413	3.8238	4.9691	3.8829	4.5858	4.2573	3.0851	3.9910	3.5347	4.0697	4.3642	3.6830	4.8189	4.9581	4.5576
Tnr	_LQVYC[Carbamidomethyl (C)]DM[Oxidation (M)]TTDGGGWIVFQR_.2	#N/A	4.6798	4.1888	3.5247	4.0891	4.0488	4.3788	4.5319	4.7900	3.7142	3.4184	4.4540	3.2643	4.5630	5.2146	3.1168	5.1375	3.4402	3.9708	4.0989	3.8273
Tnr	_DGQEAVFAYYDKFAVEDSR_.3	#N/A	3.8849	3.4342	3.4215	3.3728	4.0918	4.1252	4.4571	4.2224	4.3773	3.6955	3.8560	3.1603	4.5428	3.5446	2.9817	3.2470	3.2593	3.0730	4.2225	3.5116
Nlgn3	_RQEPLRQPS[Phospho (STY)]PQR_.3	#N/A	8.6397	8.8216	8.4612	8.8147	8.5336	8.6579	8.6775	8.7223	8.6492	8.9819	8.5817	8.3315	9.0897	8.8322	8.0112	8.5692	8.7237	8.6359	8.7101	8.4904
Nlgn3	_QEPLRQPS[Phospho (STY)]PQR_.2	#N/A	9.1498	10.1963	9.2480	9.1913	8.5869	9.7799	9.9316	9.3556	9.5369	10.0727	9.7978	9.8299	10.3255	10.1320	9.4928	9.9908	10.1856	9.9638	9.9669	9.8730
Nlgn3	_LTALPDYTLTLR_.2	#N/A	3.3696	3.9495	4.8629	4.3535	4.9192	4.9416	5.1663	3.2369	4.6014	3.1802	4.5603	5.1123	3.5572	4.7499	4.8211	5.3212	5.2452	4.4935	3.7072	4.4484
Fsd1l	_SENGMTGSTSS[Phospho (STY)]LNNVTQ_.2	Q8BYN5_S501_M1_Fsd1l	4.0087	3.9941	4.5450	3.6807	3.3464	4.9062	5.2945	3.7091	3.8640	4.2150	3.3426	3.8835	4.0294	4.5321	3.7718	4.3536	4.2468	3.5864	3.2350	2.9983
Cadps2	_PVSPSPSVLS[Phospho (STY)]EGR_.2	Q8BYR5_S68_M1_Cadps2	7.9402	8.0691	7.0627	8.0512	6.6144	6.8465	7.1638	6.5059	6.6598	8.2044	7.4602	7.0428	7.1219	6.9603	5.0122	7.1950	7.4619	7.2203	6.9396	7.0574
Arhgap25	_SKDAPIS[Phospho (STY)]PPAQK_.2	#N/A	9.7133	9.7339	9.1094	9.6006	9.5960	9.3916	9.0790	9.5826	3.1468	9.7312	9.4456	9.2651	9.2573	9.4972	8.8590	9.3276	9.5802	9.2065	8.8707	9.3837
Arhgap25	_S[Phospho (STY)]SVGWDATEDPPLSR_.2	Q8BYW1_S379_M1_Arhgap25	8.8291	9.4138	3.3079	3.8129	3.9783	4.1301	8.0941	3.9970	3.6942	3.2738	4.0685	3.2739	3.8022	3.3460	3.2123	3.1334	3.4942	3.6552	3.9542	3.0233
Arhgap25	_DAPIS[Phospho (STY)]PPAQK_.2	#N/A	6.3074	6.6802	8.5323	7.0872	8.3561	7.1561	8.3740	8.7040	3.6790	8.2130	7.8399	7.0446	3.8445	8.7298	7.5683	8.8835	7.8840	8.5497	8.3082	8.1612
Znf609	_TNS[Phospho (STY)]M[Oxidation (M)]GSATGPLPGTK_.2	Q8BZ47_S467_M1_Znf609	3.2530	3.7333	3.1224	3.9984	4.0916	4.3156	3.4495	3.8115	3.5086	7.0390	3.8830	6.9238	7.3728	7.2231	3.0267	7.2622	7.7100	3.8407	4.1398	4.3386
Znf609	_LVEPHS[Phospho (STY)]PSPSSK_.2	Q8BZ47_S575_M1_Znf609	4.4764	3.5264	3.5119	3.2129	3.8142	4.0382	3.7269	4.1768	3.7860	3.1819	4.1604	3.4158	4.4972	3.4990	6.2284	3.3205	3.2137	3.1187	4.2681	6.5589
Znf609	_AEADKIYSFTDNAPS[Phospho (STY)]PSIGGSSR_.3	Q8BZ47_S803_M1_Znf609	3.4159	4.5869	3.9522	4.2735	4.2545	3.7305	3.3510	3.1163	3.2712	3.3008	4.2507	2.9755	3.4366	3.9393	3.3764	3.7608	3.6540	4.1792	3.8278	3.9063
Znf609	_APS[Phospho (STY)]LTDLVK_.2	Q8BZ47_S1057_M1_Znf609	3.9089	4.0939	4.4452	3.7805	3.2466	3.4707	4.2945	3.1556	4.3535	4.1152	3.2271	3.9834	4.4616	4.0054	3.8717	4.2538	4.1536	5.5790	3.2949	3.6827
Znf609	_FC[Carbamidomethyl (C)]DS[Phospho (STY)]PTSDLEMR_.2	Q8BZ47_S358_M1_Znf609	3.6268	3.6922	3.1634	3.9574	3.8338	3.8671	3.2143	3.8525	3.1345	3.4374	4.1140	3.4184	3.3000	3.8026	3.2397	2.9890	3.3498	4.3158	3.9644	3.7696
Dnm3	_S[Phospho (STY)]PPPSPTTQR_.2	#N/A	13.0447	13.3824	13.1282	12.9614	12.8848	12.9527	13.1147	13.1237	12.9817	13.0611	12.9033	12.6768	13.0177	13.1369	12.2668	12.8017	13.1835	13.0030	12.9738	13.0517
Dnm3	_ENC[Carbamidomethyl (C)]LILAVTPANTDLANSDALK_.2	#N/A	5.1226	4.6427	5.0328	7.9976	4.6909	3.0179	5.0181	5.3038	5.2778	4.9870	4.1005	4.2249	4.8961	4.8179	4.4725	8.0857	4.2415	8.0447	4.7262	3.9337
Dnm3	_STIGNQVIR_.2	#N/A	5.9899	6.1197	5.7706	4.2857	5.7039	5.4893	6.1600	6.1033	6.1231	6.0172	6.1057	6.2255	5.1448	5.9259	5.9829	5.7420	5.1612	5.8131	5.6940	5.6832
Dnm3	_VPVGDQPPDIEYQIR_.2	#N/A	3.3056	6.9873	6.2776	6.8615	6.3035	7.2693	7.1272	6.5438	6.5145	7.1014	6.8299	6.7109	7.1984	7.3457	6.4460	6.8607	7.0769	6.7248	6.7571	4.2859
Dnm3	_FLELAC[Carbamidomethyl (C)]DSQEDVDSWK_.2	#N/A	3.3426	5.5131	6.3225	4.7079	4.8907	5.4225	5.9699	3.0429	5.4550	5.9792	5.0402	5.0063	6.0991	3.8659	4.8427	3.6875	6.2561	5.4028	4.8359	5.1334
Dnm3	_RS[Phospho (STY)]PPPSPTTQR_.2	#N/A	11.8723	11.8415	11.4898	11.6803	11.2758	11.5493	11.2988	11.8362	11.3807	11.9621	11.4610	11.4729	11.8651	11.7192	10.5944	11.1282	11.5144	11.5418	11.5701	11.1072
Dnm3	_APPSVPSR_.2	#N/A	3.4288	3.8903	4.4662	4.1554	3.6358	4.6557	5.5675	3.6545	3.3325	3.2394	4.3120	3.6164	3.4597	5.0051	5.9664	4.6225	3.1517	3.9977	5.3440	4.1816
Dnm3	_LQGQLLSIEHEVEAFK_.3	#N/A	3.3920	3.9270	4.4295	4.1922	3.5990	3.6323	2.2443	3.2144	3.3693	3.2026	2.4215	3.6532	3.5348	4.0374	3.4745	4.2550	3.7521	3.0977	2.9302	4.0044
Dnm3	_RS[Phospho (STY)]PPPS[Phospho (STY)]PTTQRR_.3	#N/A	3.2037	4.1154	4.2411	4.3805	3.4106	3.4440	3.6375	3.4294	3.5577	4.5151	3.0363	3.8415	3.7231	4.2792	3.6628	4.0666	4.4274	4.2228	3.0211	3.9565
Tigar	_IIQGQGVDAPLSETGFR_.2	#N/A	4.8353	4.4700	7.2196	4.1566	2.8705	4.8110	4.9927	3.2331	3.2288	3.7391	3.6032	4.3595	3.3369	4.0561	4.8477	5.0257	3.7708	4.7339	4.1693	4.0588
Pogz	_SLDAEPSVPSAAKPS[Phospho (STY)]SPEK_.2	Q8BZH4_S421_M1_Pogz	6.9520	6.5862	4.2842	6.8773	5.9161	6.6656	3.7347	3.4447	6.3903	6.7019	6.1762	5.9500	6.5853	7.0570	3.4666	4.0983	6.9727	3.9073	6.5452	6.7145
	SDLAVSNISPPS[Phospho (STY)]PDSK.2	Q8BZJ8_S111_M1_	3.2829	8.0380	3.6862	7.1366	6.5421	5.6853	6.7704	3.6051	6.5465	7.6014	7.0080	7.3816	3.6259	8.0090	8.0776	3.9342	3.5847	6.2169	7.6517	7.7195
Dock10	_DM[Oxidation (M)]SQS[Phospho (STY)]PTSSFVR_.2	#N/A	4.0834	7.0729	3.6200	3.3472	3.6844	7.5135	7.3677	7.5909	4.1841	3.8940	5.7240	3.8619	4.3496	3.3514	3.6832	3.4456	5.5004	3.2716	7.2513	3.3184
Dock10	_ASLASLDS[Phospho (STY)]NPSTTEK_.2	Q8BZN6_S1295_M1_Dock10	6.1588	7.5645	7.2091	7.0840	3.4579	6.8524	7.2282	5.7298	5.8017	6.4362	6.1825	3.9501	5.5156	6.8232	3.7714	6.5312	3.6719	7.1163	6.9475	5.7875
Dock10	_HATS[Phospho (STY)]VDTSFSK_.2	Q8BZN6_S1257_M1_Dock10	4.5023	4.3177	4.0388	4.5828	3.2083	3.3992	3.8398	3.2271	4.4251	4.0437	4.2715	4.0438	4.5331	4.0769	3.8651	3.8644	4.2252	4.2693	3.2233	3.7542
Dock10	_EKDM[Oxidation (M)]SQS[Phospho (STY)]PTSSFVR_.2	#N/A	3.4765	6.3425	4.2341	4.8798	3.2256	3.3482	4.1267	3.3501	3.3449	4.2282	3.2496	4.0279	3.4878	4.2263	3.8861	4.0509	4.3482	4.1055	3.3196	4.0610
Ncbp3	_AEAPAGPALGLPS[Phospho (STY)]PEVESGLER_.2	Q8BZR9_S25_M1_Ncbp3	3.9156	3.4035	3.4522	3.6686	4.0716	4.1559	4.4264	4.1917	4.3466	3.7262	3.8253	3.1296	4.5121	3.5139	2.9509	3.2777	3.2286	3.1038	4.2532	3.4809
Sowahb	_VAGPDPS[Phospho (STY)]PEDSPNQR_.2	Q8BZW2_S132_M1_Sowahb	3.4019	7.9804	4.4393	4.1852	3.6089	3.6422	7.9031	3.2046	3.3595	3.2125	7.6099	7.6158	7.9978	4.0276	3.4646	7.5660	7.9972	7.7902	3.7395	3.9945
Dzank1	_FTQGLEDQLS[Phospho (STY)]PPGNR_.2	Q8C008_S768_M1_Dzank1	10.8361	11.3344	9.9421	10.2918	8.6592	10.6315	10.8058	10.8739	10.6272	10.6209	10.2465	10.9162	11.0858	11.8081	10.2309	10.8763	11.0978	11.0545	10.8767	10.8977
Pak5	_S[Phospho (STY)]LVGTPYWM[Oxidation (M)]APEVISR_.2	#N/A	7.7844	8.4628	7.0336	4.0008	5.2476	6.1641	8.2762	8.8732	3.9354	7.0376	5.4731	7.7964	8.1298	9.0812	5.9042	5.9214	7.0797	7.9324	7.2610	3.8676
Pak5	_ESPPTPDQGAAS[Phospho (STY)]R_.2	Q8C015_S114_M1_Pak5	3.2120	3.2899	3.7483	4.4774	4.0506	3.9343	3.1471	4.4132	3.0673	8.8466	4.0468	8.9998	3.2327	3.7354	3.0677	3.5569	3.4501	8.6701	5.5747	3.7024
Pak5	_ASSSS[Phospho (STY)]PLDYSFQLTPSR_.2	Q8C015_S226_M1_Pak5	4.3302	4.1100	3.6726	3.6963	3.3416	3.1532	5.9678	3.7022	3.8571	4.2244	3.3357	3.9087	4.0225	3.9073	3.7611	3.4923	4.2399	3.6020	3.2506	3.8743
Pak5	_SNS[Phospho (STY)]LRKES[Phospho (STY)]PPT[Phospho (STY)]PDQGAASR_.3	Q8C015_S99_M3_Pak5	3.9614	5.9026	5.2988	3.7559	3.6679	4.2232	5.1937	4.9703	3.8408	5.9004	4.1336	4.6093	3.9857	4.4229	3.6064	3.4885	5.9235	3.6437	4.5814	4.6231
Pak5	_KES[Phospho (STY)]PPT[Phospho (STY)]PDQGAASR_.2	Q8C015_T107_M2_Pak5	9.6725	9.8260	9.4651	9.6714	9.4511	9.5658	9.4517	9.6451	9.3516	9.6171	9.4256	9.6191	9.8350	10.0385	9.1273	9.6210	9.8469	9.6132	9.7297	9.4412
Pak5	_SSYLHQTS[Phospho (STY)]PQPAMR_.2	#N/A	3.9483	5.7861	6.6518	6.4874	3.5379	4.6812	3.5458	3.7880	3.8194	3.1962	5.2086	3.8048	3.9713	6.2296	3.4255	3.9222	3.4595	3.7427	4.0741	3.9639
Pak5	_AQMVFS[Phospho (STY)]PPLSGSDTYPR_.2	Q8C015_S341_M1_Pak5	3.5612	3.7579	4.5987	4.0230	3.7682	4.3402	3.2799	3.7869	3.4841	3.4839	3.8584	3.4840	3.5921	3.1359	3.3053	4.4242	3.2841	3.8653	4.1644	4.3140
Lrrc4c	_M[Oxidation (M)]NS[Phospho (STY)]KDNVQETQI_.2	#N/A	10.4745	10.1838	10.0983	10.1831	9.7946	9.7608	9.7831	10.3189	9.7008	10.1566	9.9388	9.8087	10.5188	10.1335	8.9954	9.8918	10.2310	9.9860	9.9720	9.8746
Arhgef10	_NDLSSS[Phospho (STY)]SGS[Phospho (STY)]LNLSHGSSSLEHR_.3	Q8C033_S1259_M2_Arhgef10	3.5058	3.8132	6.5098	4.0784	4.1715	4.3956	3.3696	3.7315	6.1175	3.5393	3.8030	3.5394	3.5367	4.5813	2.9468	3.6778	3.2288	3.9207	4.2197	4.2586
Arhgef10	_T[Phospho (STY)]RSLVSQDHR_.3	Q8C033_T353_M1_Arhgef10	3.8446	3.4405	3.5978	3.1270	4.0515	4.0849	2.9966	4.2627	4.4176	5.1416	4.8496	3.3299	4.5831	3.5849	3.0220	3.2067	3.2996	3.0328	4.1822	3.5519
Rps6ka5	_TLQPSNPTEGSNPDTLFQFS[Phospho (STY)]D_.2	Q8C050_S862_M1_Rps6ka5	3.8665	7.5668	3.4030	3.7178	4.0734	4.1067	7.1628	4.2409	7.0787	3.6770	3.8744	3.1788	4.5612	5.7995	3.0001	3.2286	3.5894	7.4821	6.9809	3.5300
Map1s	_RPVVTTHDLEAPS[Phospho (STY)]RANS[Phospho (STY)]QDSLASR_.4	Q8C052_S458_M2_Map1s	3.9051	5.8864	3.4416	6.5367	4.1120	4.1453	5.3349	4.2022	6.0902	4.9535	3.8358	5.5900	4.5226	3.5244	4.4925	5.4978	5.5454	5.8786	5.6558	3.4914
Map1s	_ANS[Phospho (STY)]QDSLASR_.2	Q8C052_S462_M1_Map1s	11.6438	11.5627	11.5729	11.5203	11.1855	11.5010	11.4498	11.6717	11.4276	11.4042	11.2486	11.2829	11.3112	11.2767	10.9782	11.2027	11.6174	11.3893	11.3132	11.2408
Camkk2	_GGPC[Carbamidomethyl (C)]VESWGAPAPGS[Phospho (STY)]PPR_.2	Q8C078_S572_M1_Camkk2	11.4893	11.4029	11.0640	11.0329	10.6941	10.8230	10.9392	11.4172	10.5505	11.1307	11.0841	10.8867	11.2010	11.7626	9.9477	10.6352	11.6202	10.8867	10.8914	10.7559
Camkk2	_S[Phospho (STY)]FGNPFEGSR_.2	#N/A	11.6923	11.6712	11.7388	11.7402	3.9779	11.6341	11.2552	11.1336	11.5447	11.8175	11.7766	11.3368	11.6126	12.0202	10.9302	11.5422	11.8734	11.3874	11.7736	11.7175
Camkk2	_SLS[Phospho (STY)]APGNLLTK_.2	Q8C078_S511_M1_Camkk2	9.7831	9.7199	9.0036	9.0761	4.2604	8.6393	9.0264	9.4600	8.0560	9.6151	9.0695	2.9696	9.6264	9.8124	7.7523	8.9484	9.1434	9.1023	8.9658	8.8247
Camkk2	_DQPPEADGQELPLEAS[Phospho (STY)]DPESR_.2	#N/A	3.8087	4.3064	4.1656	5.3415	3.2191	3.7152	3.5537	3.8943	8.4848	8.2079	3.5671	7.8814	3.8156	6.6645	5.1675	6.0265	5.0646	3.7574	8.4044	8.2445
Camkk2	arbamidomethyl (C)]C[Carbamidomethyl (C)]PSLSY[Phospho (STY)]SPASS[Phospho (STY)]PQS	Q8C078_S133_M2_Camkk2	3.2709	4.0482	4.3083	4.3133	3.4778	3.5112	3.5703	3.4966	3.4905	4.5823	2.9691	3.7743	3.6559	6.1253	3.5956	4.1339	4.4947	4.1556	2.9538	4.0237
Camkk2	_KRS[Phospho (STY)]FGNPFEGSR_.3	#N/A	3.9438	6.3003	3.4804	3.6404	4.1507	4.1841	6.0919	6.4876	4.3184	4.6216	4.7012	6.5006	6.6003	7.4436	2.9227	3.3059	5.5480	6.5911	5.6647	4.7410
Camkk2	_KMS[Phospho (STY)]LQEPSQGGPASSSNSLDMNGR_.3	Q8C078_S99_M1_Camkk2	3.8270	3.4921	3.3635	3.7573	4.0339	4.0672	4.4325	4.2804	4.4353	3.6375	3.9139	3.2183	4.6007	3.6025	3.0396	3.1891	3.3172	3.0151	4.1645	3.5695
Strip1	_AAS[Phospho (STY)]PPASASDLIEQQQKR_.3	#N/A	3.8901	3.4290	3.4267	3.6941	4.0970	4.0113	4.4519	4.1158	3.8129	3.7007	4.1873	3.1551	3.9210	3.4647	2.9765	7.2958	3.6130	3.5364	3.8355	3.1420
Strip1	_AAS[Phospho (STY)]PPASASDLIEQQQK_.2	#N/A	12.4700	12.1267	12.1773	11.9840	11.5037	11.8178	11.8335	12.2905	11.7685	11.8079	11.9281	11.5780	12.3578	11.7613	10.3858	11.4549	12.4675	11.9116	11.7148	11.5427
Snx16	_SSS[Phospho (STY)]FGSVSTSSTSSK_.2	Q8C080_S29_M1_Snx16	10.5264	10.2925	10.6911	10.6228	9.9555	9.4887	9.6160	9.8078	9.5624	10.7607	10.0082	9.8936	10.3660	10.6500	9.1473	9.9790	9.9473	9.8153	9.7950	10.1058
Snx16	_EFLC[Carbamidomethyl (C)]LDDPPGPFDS[Phospho (STY)]LEESR_.2	Q8C080_S222_M1_Snx16	3.2320	3.2699	3.7683	4.4573	4.0706	4.2947	3.4705	4.4333	3.5296	3.4384	3.9039	3.1594	3.6376	3.1814	3.0477	3.5769	3.4701	3.8198	4.1188	4.3595
Ccser1	_GES[Phospho (STY)]PLAHAGEPALR_.2	#N/A	5.9824	5.4173	4.8099	5.9204	5.0570	4.6827	4.8134	4.1121	4.5570	5.6632	5.1126	4.7363	5.7185	5.5051	4.4225	4.8811	5.5536	5.0503	4.0314	4.6153
Farsa	_SLQALGEVIEAELR_.2	#N/A	3.8726	4.3529	4.0036	3.1172	3.2103	4.5676	4.3307	3.1918	4.3898	4.0789	3.7430	4.0790	4.4979	4.0417	3.5380	4.0517	4.1899	3.5955	4.0970	3.8088
Farsa	_LQLVQAGQAEK_.2	#N/A	3.9076	4.0323	5.0925	3.7818	3.2453	4.8581	4.2958	3.1569	4.3549	4.1139	3.2284	3.1206	4.4629	4.7172	3.9824	4.2525	4.6838	2.9945	4.7010	3.6840
Arhgap12	_ATT[Phospho (STY)]PPNQGRPDS[Phospho (STY)]PVYANLQELK_.3	Q8C0D4_S238_M2_Arhgap12	3.8749	4.1279	4.4112	3.8145	3.2126	3.4367	9.1780	3.1896	4.3876	4.0812	7.7659	4.0174	4.4956	10.0165	3.9057	8.1611	4.1877	9.2309	3.2608	3.7167
Arhgap12	_TSFSQEQSC[Carbamidomethyl (C)]DS[Phospho (STY)]AGEGSER_.2	Q8C0D4_S199_M1_Arhgap12	4.2250	4.3359	4.4150	4.8290	4.1029	3.4479	4.6086	4.6538	3.7844	5.0447	3.8823	4.1438	4.6475	5.1121	3.9677	4.5718	4.0608	4.5022	4.3040	4.7961
Arhgap12	_IQQDSES[Phospho (STY)]GDELSSSSTEQM[Oxidation (M)]R_.2	#N/A	3.8809	4.7899	3.6683	2.8918	4.3233	4.4052	3.5671	3.7625	3.6992	3.9544	3.9138	4.1168	4.2000	4.5589	3.9271	4.2447	3.3179	4.3731	4.4283	4.1900
Vps26b	_TPGQLSDNNSR_.2	#N/A	7.2497	7.1211	7.0424	7.1060	6.8106	7.0138	6.9666	7.2766	7.2288	6.8680	6.9330	6.9258	6.5162	6.7922	5.8381	6.3408	6.9501	7.1298	6.7244	6.7860
Vps26b	_SM[Oxidation (M)]S[Phospho (STY)]HQAAIASQR_.2	#N/A	9.9710	10.0979	9.9402	10.1377	9.5636	9.7194	9.8637	9.6467	9.7503	9.9884	9.7364	9.2921	9.7198	9.9589	8.4723	9.4703	9.7229	9.5221	9.4795	9.5100
Vps26b	_KSM[Oxidation (M)]S[Phospho (STY)]HQAAIASQR_.3	#N/A	5.3824	5.1477	4.8446	3.2921	4.8578	3.4238	5.7411	4.1443	5.3827	5.9156	5.2610	4.4363	5.7785	5.6101	3.8783	3.7282	4.0183	4.7285	3.4334	4.4985
Vps26b	_TPGQLSDNNSRQ_.2	#N/A	4.0183	7.5085	6.7167	6.9396	6.6217	6.6655	6.7719	6.9392	6.8868	7.1275	5.6833	6.6445	3.9172	6.5046	5.9263	6.3492	6.4039	6.8674	6.3032	6.0933
Vps26b	_ETTGTGPNVYHENDTIAK_.3	#N/A	3.4503	4.3444	4.4877	4.1339	3.6573	3.6906	2.9911	3.5466	3.3111	3.2609	4.2905	3.5949	3.4765	4.5258	3.4162	4.3133	3.1732	4.1393	4.2753	3.5869
Agps	_AASAAGAS[Phospho (STY)]PAATPAAPESGTIPK_.2	Q8C0I1_S57_M1_Agps	5.0396	5.0023	4.9802	5.0757	5.1154	4.9401	4.0135	5.4489	4.3124	5.1266	4.7754	4.5546	3.3020	5.0891	4.2932	4.7266	4.7156	4.6964	4.5306	4.7083
Sowahc	_FC[Carbamidomethyl (C)]TGDS[Phospho (STY)]PPLEAK_.2	Q8C0J6_S82_M1_Sowahc	9.6172	9.6252	9.5582	9.7114	9.1364	9.5145	9.2107	4.3447	9.2696	9.5455	3.9782	9.1786	9.9440	9.8114	3.1039	9.2561	9.6096	9.0806	4.1002	9.3726
Sowahc	_NLVGALDEDDGDS[Phospho (STY)]PAAR_.2	Q8C0J6_S391_M1_Sowahc	4.5758	6.3761	5.2290	4.5093	3.2819	5.7910	5.6647	3.3007	4.7635	3.9701	3.3722	3.9702	4.1134	7.1363	3.7916	3.9379	4.2987	5.3526	5.3791	6.1930
Tmx4	_EDGAHPADTQDVVEDALR_.3	#N/A	4.8439	5.4629	4.1217	5.0395	4.2155	4.4396	3.3256	5.1000	5.0593	3.5832	4.3690	4.5873	3.4927	4.8116	4.4222	3.7218	3.6150	5.0193	4.5258	4.2146
Gpcpd1	_VKLTLEGLEEDEDDDDKVS[Phospho (STY)]PTVLHK_.4	#N/A	3.7641	4.2387	4.3004	3.9253	3.1018	3.3259	4.4393	3.3004	2.9975	3.9704	3.3719	4.1282	4.6064	4.1502	4.0165	4.1090	4.2984	4.3518	3.1500	3.8275
Gpcpd1	_LTLEGLEEDEDDDDKVS[Phospho (STY)]PTVLHK_.3	#N/A	3.5154	3.8535	4.1201	4.1187	3.7223	3.7962	3.9281	3.7410	3.5277	3.7977	3.8632	3.5796	3.6718	5.2728	3.5782	3.9456	3.7923	3.9837	3.8034	3.9768
Wdr59	_AVS[Phospho (STY)]PTEPTPR_.2	Q8C0M0_S564_M1_Wdr59	8.6225	8.2459	4.3362	8.7215	8.4345	8.5766	8.1879	3.5244	8.5506	3.1093	2.9413	3.7465	3.6281	8.6743	3.5678	8.3517	8.8162	8.5393	8.3503	8.5780
Wdr59	_ETEHISSPWGESS[Phospho (STY)]PEELR_.3	Q8C0M0_S840_M1_Wdr59	3.1151	6.4157	4.1525	4.4692	3.3220	6.2763	6.7476	3.3407	6.5021	4.8741	3.1249	5.5516	3.1459	6.6530	3.7515	6.2554	4.3388	6.4114	6.5002	6.2895
Wdr59	_LYSGS[Phospho (STY)]PTR_.2	Q8C0M0_S603_M1_Wdr59	3.1453	4.1738	4.1827	4.4389	3.0312	3.2553	5.2468	3.3710	4.7321	3.8998	3.4425	3.8999	3.1762	4.2208	4.0871	4.0384	4.3691	4.2812	3.0794	3.8981
Asrgl1	_LALFHVEQGK_.2	#N/A	5.8108	6.5892	5.6346	6.7422	6.2823	6.4348	6.3844	6.6267	6.0266	6.5476	5.6163	6.4474	6.4943	6.3414	6.2831	6.0856	6.3896	6.2049	6.1502	6.3867
Asrgl1	_DLSAGAVSAVR_.2	#N/A	6.4464	6.3502	6.2601	6.3463	5.9608	5.9505	6.1054	6.2865	5.7026	5.5391	5.8712	5.8562	6.3240	5.9509	6.3857	3.8642	5.7675	5.6403	6.1839	5.6052
Asrgl1	_GNLAYATSTGGIVNK_.2	#N/A	5.5177	5.7662	6.1445	5.7580	5.6242	6.0091	5.5555	5.3007	5.8438	6.0415	5.1982	5.0415	4.3819	4.8512	6.2887	5.0820	5.2441	5.2351	5.9121	5.8394
Asrgl1	_FAEDMGIPQVPVEK_.2	#N/A	4.3178	6.5765	6.9997	6.6535	4.9307	5.7595	6.5842	4.5435	5.5849	4.1284	6.4907	4.2282	4.1098	7.4013	6.4389	3.6799	4.0407	4.9560	4.8288	3.5697
Asrgl1	_TVEEAAQLALDYMK_.2	#N/A	3.8171	3.5020	3.3537	3.7671	4.0241	4.0574	3.0240	4.2902	4.4451	3.6277	3.9237	3.2281	3.1097	3.6124	3.0494	3.1792	3.3270	3.0053	4.1547	3.5794
Rapgef5	_HTVDEYS[Phospho (STY)]PQKK_.2	Q8C0Q9_S445_M1_Rapgef5	4.7598	3.1877	3.6680	3.4528	4.3383	3.7700	3.9951	4.3571	4.0542	4.4145	2.9278	4.4146	4.1623	3.7061	4.2360	3.4935	4.2029	3.2951	3.5942	3.3833
Sipa1l1	_TLS[Phospho (STY)]DESIYSSQR_.2	Q8C0T5_S1564_M1_Sipa1l1	13.5534	13.5012	13.4331	13.4796	12.7378	13.0470	13.0761	13.2900	13.0061	13.5149	12.9537	13.1840	13.6210	13.4849	12.4422	13.1751	13.5885	13.1712	13.1566	13.1354
Sipa1l1	_LIDLES[Phospho (STY)]PTPESQK_.2	#N/A	11.2104	11.4049	11.0109	10.9531	10.1786	10.6400	10.9036	10.4460	10.5015	11.5926	10.5744	10.6705	11.2693	11.1995	9.2500	10.8608	11.1176	10.7483	10.7487	10.7540
Sipa1l1	_QDPVVHLS[Phospho (STY)]PNK_.2	Q8C0T5_S1249_M1_Sipa1l1	9.3217	9.2541	9.6658	9.0949	8.4162	8.2814	8.9852	8.6054	8.3977	9.4430	8.6702	8.6856	8.7836	8.8718	7.6660	8.6829	8.7503	8.6632	8.5852	8.4007
Sipa1l1	_DLQAS[Phospho (STY)]PKPTSK_.2	Q8C0T5_S1507_M1_Sipa1l1	7.2011	3.5347	6.9786	3.7999	5.5241	4.1171	5.7131	4.0100	6.3442	3.2607	4.0816	6.3541	5.3539	3.3590	5.1113	3.1465	3.5073	3.6422	3.9412	3.0363
Sipa1l1	_SIS[Phospho (STY)]SDGRPLER_.2	Q8C0T5_S1116_M1_Sipa1l1	3.5949	8.2601	4.1312	7.3787	2.9327	3.1567	3.1076	3.2953	3.1667	3.8013	3.5410	4.2973	3.2747	4.1183	4.1856	3.9398	3.8330	4.1827	7.3886	3.9966
Sipa1l1	_KLIDLES[Phospho (STY)]PTPESQK_.2	#N/A	4.2176	4.6024	3.7542	3.3667	2.9237	6.5816	4.1244	3.8897	4.0446	4.0282	3.5233	6.1047	7.1054	3.2119	4.1498	6.2482	4.4274	5.9911	5.8682	6.7935
Sipa1l1	_GSGFS[Phospho (STY)]LDVIDGPISQR_.2	Q8C0T5_S258_M1_Sipa1l1	6.4788	7.0764	6.5923	6.0721	3.7413	5.3837	6.1834	4.1145	5.5521	6.3100	5.9773	5.8385	3.9197	5.9167	4.6603	3.0659	5.1279	6.0143	6.1536	5.6548
Sipa1l1	_EYGS[Phospho (STY)]TSSIDK_.2	Q8C0T5_S208_M1_Sipa1l1	4.2287	4.5913	3.7652	3.3556	2.9348	3.6728	4.0924	4.4543	4.1515	8.1193	3.0250	4.3174	4.2595	3.8033	3.6696	2.9550	3.9516	3.1979	3.4969	3.4806
Sipa1l1	_RSPAS[Phospho (STY)]IDR_.2	Q8C0T5_S1184_M1_Sipa1l1	3.1268	6.8581	3.6631	4.5626	3.9654	4.0195	3.5757	4.3280	2.9821	3.5899	3.9616	3.2646	3.1475	3.6502	3.1529	3.4717	3.3649	4.4683	4.1169	3.6172
Sipa1l1	_HS[Phospho (STY)]AS[Phospho (STY)]PVVFSSAR_.2	Q8C0T5_S1410_M2_Sipa1l1	3.7754	5.7034	4.3117	6.4614	3.1131	5.6886	6.0664	3.2890	2.9862	7.1702	3.3606	6.2556	4.5951	6.8740	4.0051	6.7410	5.9527	6.0818	6.1645	6.5213
Sipa1l1	_SKS[Phospho (STY)]ETGDSSIFR_.2	#N/A	8.5166	8.7029	8.3001	3.8116	3.9796	4.1288	8.1887	7.4456	3.6955	8.2801	4.0699	7.3119	5.8727	7.7870	3.0939	3.1348	3.4956	3.6539	3.9529	3.0246
Sipa1l1	_SKS[Phospho (STY)]ETGDSSIFRK_.2	#N/A	5.1532	4.5209	3.4527	4.9106	3.9629	4.0108	4.1763	4.1241	4.1696	4.5968	3.8890	3.3771	5.1182	4.2837	3.1612	3.4693	3.9838	3.3570	4.5353	3.9952
Sipa1l1	_SYSSKDPS[Phospho (STY)]PTLASK_.2	Q8C0T5_S1712_M1_Sipa1l1	7.0905	3.6300	7.2770	7.0610	6.8008	3.9157	3.8610	7.4155	5.1349	6.1814	3.6679	3.4492	7.5387	7.7221	3.4382	6.9258	7.3553	3.4736	4.8402	3.2492
Sipa1l1	_QKS[Phospho (STY)]M[Oxidation (M)]PEGFGVSR_.2	#N/A	3.3862	3.9329	4.4236	6.7630	3.5931	3.6265	3.4550	3.6119	3.3752	3.1968	4.3546	3.6590	3.4171	4.4617	3.4803	6.3050	3.1091	6.4716	6.6660	4.1390
Sipa1l1	_SLHGEFSAS[Phospho (STY)]DSSLTDIQETR_.3	Q8C0T5_S1626_M1_Sipa1l1	4.0434	4.4025	4.0642	3.6650	3.0354	3.4447	3.6022	4.0817	3.5691	4.0251	3.4251	4.2061	3.8646	4.0483	4.0606	3.8801	3.9943	3.8307	3.2689	3.7256
Sipa1l1	_S[Phospho (STY)]MPEGFGVSR_.2	#N/A	4.1061	3.2130	3.6427	6.7216	4.3130	3.7554	3.9698	4.3318	4.0289	4.4398	2.9025	2.9391	4.1370	3.6807	3.5470	3.0776	3.8290	3.3204	3.6195	3.3580
Tlk1	_ISDYFEYQGGNGSS[Phospho (STY)]PVR_.2	Q8C0V0_S159_M1_Tlk1	4.0003	5.7790	3.5369	3.2828	4.2073	5.8739	5.7627	4.1070	4.2619	3.8109	3.7405	3.0449	4.4273	6.0133	2.8662	3.3624	3.1438	5.7567	4.3379	3.3962
Tlk1	_LSTS[Phospho (STY)]PTPGSAAAAR_.2	Q8C0V0_S22_M1_Tlk1	7.6840	3.4916	7.4238	3.7567	4.0344	4.0678	6.7044	4.0532	4.4347	3.6381	3.9134	3.2177	4.6002	3.4021	3.0390	3.1896	7.6162	3.5990	3.8981	3.0794
Frmd6	_HSLS[Phospho (STY)]LDDIR_.2	Q8C0V9_S544_M1_Frmd6	3.7605	7.3519	6.5706	3.8237	3.9674	4.0008	3.0807	3.9862	6.5183	3.5711	3.9804	3.2847	3.1663	3.6690	3.1061	3.1226	3.4834	7.1583	4.0981	6.7779
Frmd6	_LRGQS[Phospho (STY)]TDS[Phospho (STY)]LPQTIC[Carbamidomethyl (C)]R_.2	Q8C0V9_S522_M2_Frmd6	4.3166	3.6862	3.3520	3.3728	3.6543	4.3306	4.2516	4.0170	4.1719	3.9010	3.6505	3.5757	4.3373	3.3391	4.2770	3.1606	4.5546	3.2785	4.4280	3.3061
Ppp4r4	_SQS[Phospho (STY)]FNNQAFHAK_.2	Q8C0Y0_S777_M1_Ppp4r4	8.6041	8.5769	8.0615	8.2449	8.0664	8.0082	7.8115	8.0137	7.9810	8.2475	7.8393	7.1739	7.6731	7.7968	5.9719	7.3837	8.1751	7.7184	7.4751	7.6688
Ldlrap1	_RDS[Phospho (STY)]TPSLK_.2	Q8C142_S198_M1_Ldlrap1	4.1197	3.4965	4.3029	3.5022	4.1553	4.2630	3.6867	3.9883	3.9097	3.2222	3.8776	3.3063	3.8538	3.4647	4.6955	3.3560	5.9169	3.3702	4.0798	4.2584
Slc39a6	_YDSQLS[Phospho (STY)]SNEEK_.2	Q8C145_S488_M1_Slc39a6	3.7288	3.5903	5.4222	3.8554	3.9358	3.9691	3.1123	3.9545	3.0325	3.5394	4.0120	3.3164	3.1980	3.3035	3.1377	3.0909	3.4517	4.4178	4.6661	4.6273
Cpne1	_NC[Carbamidomethyl (C)]SS[Phospho (STY)]PEFSK_.2	Q8C166_S54_M1_Cpne1	7.4409	4.1669	7.1883	7.2947	6.9850	7.6063	7.2967	7.4528	3.0750	7.5938	7.2143	7.0604	7.8824	7.7015	6.8975	6.9234	7.6886	7.2824	3.0726	3.9049
Cpne1	_APPT[Phospho (STY)]PGKGPAQAPQA_.2	Q8C166_T525_M1_Cpne1	7.0742	6.6142	7.5106	7.5943	6.9595	7.1234	5.3607	3.6521	3.3492	3.6187	6.8286	6.4463	3.4573	7.2944	5.8505	6.8727	3.1493	6.3422	6.6046	6.2698
Cpne1	_FAAQAAQQR_.2	#N/A	3.4766	5.8103	6.5888	5.8307	4.3151	3.0384	3.2260	3.1769	3.2850	3.6829	3.6594	4.4157	3.3931	3.9999	6.5442	3.8215	3.7146	4.1185	6.2708	5.8077
Myo9a	_DWNES[Phospho (STY)]PVR_.2	Q8C170_S1950_M1_Myo9a	7.5343	8.0185	7.9117	8.2165	7.4419	7.8036	7.9411	7.1741	7.6697	7.3204	7.9876	7.3946	7.7268	7.4558	5.8067	7.4057	7.8209	7.7420	7.6532	7.3095
Myo9a	_GNYPSPSS[Phospho (STY)]PVIVR_.2	Q8C170_S2296_M1_Myo9a	8.7417	8.6121	8.1407	4.4516	7.4009	7.1978	3.1729	4.4390	3.0931	8.1024	7.5013	3.1536	7.7235	3.7612	3.1983	7.0687	8.0266	4.3573	7.6760	6.9160
Myo9a	_S[Phospho (STY)]LEDLHQK_.2	Q8C170_S1259_M1_Myo9a	5.3421	6.2962	5.9575	6.0390	2.8523	5.4227	6.1506	4.3719	4.9806	5.2642	5.7645	5.4078	4.1771	5.6104	4.2212	5.4894	5.3108	5.6252	5.2861	4.8333
Myo9a	_S[Phospho (STY)]LEILQR_.2	Q8C170_S755_M1_Myo9a	3.5417	3.7774	7.0246	4.0425	3.7486	5.2179	3.2995	3.7674	6.0061	6.5441	4.1992	3.5035	3.3851	4.6172	3.3248	4.4046	4.7591	3.8848	6.1097	4.2945
Myo9a	_RQS[Phospho (STY)]GTDLQEDVIVR_.2	Q8C170_S1243_M1_Myo9a	3.5165	3.8026	4.5539	4.0677	3.7234	3.7568	3.3247	3.7422	3.2449	3.3271	4.2243	3.5287	3.4103	3.9130	3.3500	4.3795	3.2394	4.2055	3.8541	3.8800
Myo9a	_AAS[Phospho (STY)]AC[Carbamidomethyl (C)]EAQGTEGPLGQAK_.2	Q8C170_S2458_M1_Myo9a	4.5105	5.6226	5.1936	4.5746	3.2166	3.2499	3.8315	3.5968	3.7517	4.3211	3.2303	4.0355	3.9172	4.3081	3.8569	3.8726	4.1345	3.6987	3.3473	4.3868
Thop1	_WDLSAQQIR_.2	#N/A	5.0696	5.4180	3.6785	5.5444	5.2519	5.4155	6.3109	5.7375	4.5100	5.6421	4.1334	5.3117	3.3194	4.2075	5.2103	5.6259	4.3696	5.2077	5.4168	3.9737
Thop1	_EELGGLPEDFLSSLEK_.2	#N/A	3.5898	4.2295	4.9585	4.8570	4.6980	3.4577	4.3572	4.4185	4.6858	3.7954	4.8162	4.6736	4.4428	5.0728	4.1212	4.4556	3.1593	3.9667	4.8875	4.7518
Thop1	_TSQTVATFLDELAQK_.2	#N/A	5.2740	4.7123	3.7945	5.1115	3.4195	4.4721	4.7388	5.4129	4.9427	4.8889	3.0542	4.9656	5.6551	4.9079	3.6988	5.0404	5.5380	4.8498	4.7569	5.3530
Thop1	_VDQVLHTQTDADPAEEYAR_.3	#N/A	5.4733	5.4202	5.8323	6.4262	5.8467	4.5465	5.5915	4.0309	5.6503	3.2399	5.1239	3.2400	3.8361	3.3798	5.6672	3.3785	3.5281	5.5565	3.9204	3.0571
Thop1	_NLNEDTTFLPFTR_.2	#N/A	4.1358	3.2344	3.8039	4.4218	4.1061	3.8788	4.3937	5.0721	3.1228	3.4491	4.1023	3.1239	3.2883	3.7909	3.0122	4.6883	4.9952	4.3275	5.0757	3.7579
Thop1	_LLEEAFNC[Carbamidomethyl (C)]R_.2	#N/A	4.5409	5.4429	4.0775	4.5441	3.2470	3.2804	3.8011	4.0590	3.7213	4.3515	3.1999	4.0051	3.8867	4.3894	4.2272	3.9031	4.1040	4.8478	3.3777	4.3564
Camsap2	_SVS[Phospho (STY)]NEGLTLNNSR_.2	Q8C1B1_S439_M1_Camsap2	12.4428	12.1383	12.1054	12.2637	11.3663	11.3187	11.6799	11.5858	11.4186	12.3306	11.4967	11.4706	11.8347	11.5382	9.8593	11.4588	11.7756	11.5016	11.4896	11.3759
Camsap2	_LNQSS[Phospho (STY)]PDNLTDTK_.2	Q8C1B1_S573_M1_Camsap2	10.3988	10.4004	10.3359	10.4321	9.9913	10.1497	10.0586	10.4230	10.0710	10.2829	10.0380	9.9404	10.6349	10.1811	8.9072	9.9096	10.5001	9.9208	10.0013	10.0391
Camsap2	_WLKS[Phospho (STY)]PTTPVDPER_.2	Q8C1B1_S836_M1_Camsap2	7.6126	4.1858	4.3533	3.8724	3.2960	3.3294	3.7521	6.4779	3.6723	4.4005	3.1509	6.7847	3.8377	4.3404	3.7775	3.9520	4.0551	6.6284	3.4267	4.3074
Camsap2	_EQQAWVIS[Phospho (STY)]PPQPSPQK_.2	Q8C1B1_S905_M1_Camsap2	7.8434	8.0964	3.7950	8.0357	6.9542	7.2759	7.3803	8.0150	3.5034	6.7772	3.8778	3.1332	7.7083	4.2575	3.0215	5.6187	8.0713	8.0312	7.2180	6.0575
Camsap2	_ADVSVEKLDGES[Phospho (STY)]DKEQFDDDQK_.3	#N/A	4.5222	4.1803	4.0548	4.5628	3.2283	3.3792	4.3860	3.2471	2.9028	3.9097	3.3186	4.0238	4.5531	4.0969	3.9632	3.8843	4.2451	2.5078	3.2033	3.7742
Camsap2	_SES[Phospho (STY)]VEGFLSPSR_.2	Q8C1B1_S1285_M1_Camsap2	9.9273	9.8294	9.9331	8.8877	3.7302	8.9203	8.1706	8.6440	8.8847	9.8573	7.8154	8.8994	7.9871	9.2457	6.0576	8.0263	3.6656	7.9981	7.8668	9.1354
Camsap2	_SKS[Phospho (STY)]LADIK_.2	Q8C1B1_S818_M1_Camsap2	4.2709	4.1344	4.6415	1.9657	4.4670	4.3763	3.6796	3.9713	4.1262	5.1877	3.7492	3.2198	4.2916	4.1826	3.5097	3.1149	4.5089	3.3242	2.9728	3.2604
Camsap2	_SSS[Phospho (STY)]MSYVDGFIGTWPK_.2	Q8C1B1_S391_M1_Camsap2	3.2889	4.0320	4.8039	4.7627	3.4959	3.3867	3.3150	3.5146	3.2967	3.5165	3.2517	3.7581	3.4363	4.2054	3.8892	4.1519	3.9245	4.1114	3.3733	4.0236
Camsap2	_DHIES[Phospho (STY)]PK_.2	Q8C1B1_S1248_M1_Camsap2	3.3525	4.3768	4.3899	4.2317	2.9747	4.3300	3.3466	3.7013	3.4089	3.1631	4.4156	3.7339	4.1364	4.4280	3.5140	3.0895	4.6400	4.0740	4.5501	3.1679
Camsap2	_LDGES[Phospho (STY)]DKEQFDDDQK_.2	#N/A	4.4026	4.5146	3.2454	4.4893	4.9520	4.9161	4.4662	4.5826	4.9696	4.9699	5.5704	4.1064	3.2179	3.2835	3.4353	2.8178	3.4318	4.5627	3.9533	4.1594
Septin11	_SYELQESNVR_.2	#N/A	7.8518	7.4102	8.2173	7.9927	7.9194	7.6399	7.4603	8.2423	8.0451	7.8227	7.8678	7.8103	8.0238	8.1084	8.0271	8.0387	8.0348	7.4968	7.6661	7.9570
Septin11	_ELEEEVSNFQK_.2	#N/A	7.4717	7.5253	8.0522	7.5985	7.5413	7.3826	7.3539	7.4223	7.6806	7.3594	7.7668	7.6747	7.4908	7.7730	7.6100	7.5090	7.6656	6.9478	7.1163	7.4380
Septin11	_STSQGFC[Carbamidomethyl (C)]FNILC[Carbamidomethyl (C)]VGETGIGK_.2	#N/A	6.3321	5.8069	5.2964	6.3475	5.7165	5.8580	4.7905	6.2894	5.2487	5.4959	5.2491	5.6512	6.1101	6.6920	5.6609	6.0282	5.8963	4.9687	5.4269	5.8186
Septin11	_[Acetyl (Protein N-term)]AVAVGRPS[Phospho (STY)]NEELR_.2	Q8C1B7_S9_M1_Septin11	6.5944	6.4220	6.3721	6.1281	6.5894	6.4098	5.9863	6.5886	6.1540	6.5048	6.4582	6.4916	6.4127	6.9678	6.2585	6.2715	6.6409	6.2463	6.4873	6.8823
Septin11	_FESDPATHNEPGVR_.3	#N/A	5.2445	6.1815	6.2392	5.1768	5.2521	6.2706	5.6547	5.3284	5.7850	6.1704	6.3661	6.3906	3.2900	6.4619	6.7654	6.6866	3.7397	6.4912	6.5661	6.2418
Septin11	_SLFNYHDTR_.2	#N/A	7.4677	4.5469	7.2106	6.8613	2.9792	3.6284	6.9620	7.4868	6.2981	4.2729	7.3185	7.5874	4.3039	3.8477	6.9098	3.6352	7.3297	7.4363	3.4525	3.5250
Septin11	_AAAQLLQSQAQQSGAQQTK_.2	#N/A	5.7048	6.2724	6.3635	5.5346	6.2287	6.2453	6.4387	6.9722	6.1421	6.5000	6.5254	6.6419	6.9877	7.1696	6.4383	6.5041	6.5019	6.1421	6.3455	5.8020
Septin11	_KKELEEEVSNFQK_.3	#N/A	4.9525	5.1859	5.4954	5.0118	5.4991	4.6705	5.2735	5.3368	3.9185	5.4989	6.2822	4.1215	4.0839	4.3280	5.1262	6.4250	5.5578	5.0147	5.6437	5.2786
Septin11	_NLSLSGHVGFDSLPDQLVNK_.3	#N/A	5.7913	6.7002	5.7405	5.8762	5.5490	5.1841	6.3914	5.8547	6.2274	6.1380	5.8765	5.4136	5.2484	5.8557	5.4917	5.5629	5.7877	5.2969	6.1359	5.8768
Septin11	_LTIVDTVGFGDQINK_.2	#N/A	3.8033	4.1995	5.4132	3.8860	3.2277	3.3652	4.4000	3.5037	3.9379	4.0097	3.3326	4.0889	4.5671	4.3267	3.9772	4.1482	4.0414	4.3911	4.8178	3.7882
Iws1	_EAEDS[Phospho (STY)]DS[Phospho (STY)]DDNIKR_.2	Q8C1D8_S458_M2_Iws1	6.3450	6.7516	7.0667	5.5668	6.2154	6.5876	6.9307	6.3125	6.2265	6.4874	6.6130	5.8808	6.2160	6.2712	4.1034	5.1422	6.1103	6.3471	6.2703	6.2239
Iws1	_VVC[Carbamidomethyl (C)]DADDS[Phospho (STY)]DSDVVSDK_.2	Q8C1D8_S366_M1_Iws1	4.3314	3.8691	5.9174	3.2671	3.0375	4.0097	6.2266	6.0193	4.2448	5.7305	3.4202	5.3932	4.3788	3.7000	5.2589	5.7227	6.3069	5.5739	3.1421	5.4433
Iws1	_IS[Phospho (STY)]DS[Phospho (STY)]ESEELPKPR_.2	Q8C1D8_S183_M2_Iws1	4.2498	6.2192	6.1301	5.6411	4.0317	6.1402	5.2802	4.1332	5.5747	7.5233	2.9409	5.5747	4.0756	7.5285	4.6002	5.0553	4.5017	5.8675	5.4034	6.6382
Iws1	_KAAVLS[Phospho (STY)]DS[Phospho (STY)]EDDAGNASAK_.2	#N/A	5.8102	5.7554	3.2083	3.9125	5.6478	5.5891	5.3141	3.8974	5.5531	5.7761	4.8517	5.4121	3.2551	3.2463	3.1948	5.3326	5.9032	5.6159	4.7161	5.2000

Iws1	_IDS[Phospho (STY)]DDDEEKEGDEEK_.2	#N/A	3.0952	3.7717	3.6315	4.5715	2.0853	3.2651	3.5555	4.3315	3.5907	2.9774	3.5836	3.5882	3.8373	4.4910	3.1846	3.4401	2.9839	3.2993	4.0477	2.9956
Iws1	_AAVLS[Phospho (STY)]DS[Phospho (STY)]EDDAGNASAK_.2	#N/A	8.0686	8.2280	7.7389	8.0174	6.4993	7.8540	7.6763	7.8675	8.0177	8.0594	7.5202	7.4783	6.2222	8.1436	6.9940	7.6141	8.2151	7.6813	7.6939	7.5710
Iws1	_ETTVAS[Phospho (STY)]DS[Phospho (STY)]EEEAGKEESSVK_.2	#N/A	4.0891	4.6764	4.1458	4.5141	4.4385	4.2693	4.3848	3.7762	3.7764	5.1541	4.0724	3.9551	3.8786	4.3688	3.2928	3.8585	3.6262	4.4866	3.6783	3.9216
Iws1	_GLHS[Phospho (STY)]S[Phospho (STY)]DS[Phospho (STY)]EEEEPKR_.2	#N/A	5.6735	5.6675	5.3381	5.7291	5.1639	5.7126	5.4314	5.8916	6.0782	5.7020	4.7321	4.9243	5.5568	4.9299	3.6013	5.1207	5.0888	5.0608	5.4016	4.8805
Iws1	_AAVLS[Phospho (STY)]DS[Phospho (STY)]EDDAGNASAKK_.2	#N/A	5.2566	5.0417	4.5705	4.0511	3.7400	5.2968	5.2801	4.9835	5.0767	4.9097	4.2077	3.5121	3.3937	4.7381	3.3334	4.3961	3.2560	4.9227	4.8256	3.8634
Iws1	_QKIDS[Phospho (STY)]DDDEEKEGDEEK_.2	#N/A	4.3123	3.8580	3.8488	4.1232	3.8100	3.6952	3.6895	3.8287	3.7728	3.2074	3.5752	3.1790	3.9217	2.8350	3.4055	2.7906	3.4308	3.8799	3.8542	3.6336
Iws1	_IDS[Phospho (STY)]DDDEEKEGDEEKVAK_.3	#N/A	3.7178	4.1981	4.1584	4.4632	2.0012	3.2796	2.9847	3.3467	3.0438	3.9241	3.4182	4.1745	4.9298	4.1965	4.0628	4.0627	4.3447	4.1347	3.6702	2.8653
Iws1	_KGLHS[Phospho (STY)]S[Phospho (STY)]DS[Phospho (STY)]EEEEPKR_.2	#N/A	3.6405	4.7220	4.1768	4.0489	3.7699	4.2450	4.3640	3.3409	3.1211	3.8468	3.4955	4.2517	3.2291	4.1639	4.1400	3.9854	3.8786	3.4069	3.6032	3.9510
Vash1	_GTGPPS[Phospho (STY)]PTKDR_.2	Q8C1W1_S323_M1_Vash1	4.3941	3.6087	7.7394	3.2953	7.2874	7.8535	3.8093	4.0945	3.8684	7.6750	7.4505	3.4982	3.9764	3.4166	3.3865	3.2381	3.1313	3.2010	4.3504	7.6403
Ccz1	_TFS[Phospho (STY)]PSKPAK_.2	Q8C1Y8_S74_M1_Ccz1	8.0944	8.3213	7.8347	7.9376	7.7815	8.0360	8.2241	8.0400	3.3039	8.0004	7.9880	7.4866	8.2805	3.9720	7.0155	7.7055	8.4103	8.0523	7.9334	7.6746
Ccz1	_HIEPELAGRDS[Phospho (STY)]PVR_.3	Q8C1Y8_S264_M1_Ccz1	7.9041	7.6628	7.0311	7.2129	6.5616	6.8128	7.0471	6.5469	6.5098	7.4506	7.1329	6.3588	6.8645	7.0503	4.9543	6.4658	7.0713	6.6265	6.7609	7.0323
Rbm14	_TRLS[Phospho (STY)]PPR_.2	Q8C2Q3_S582_M1_Rbm14	11.2438	11.4426	11.1705	11.2984	11.1296	11.2728	11.3013	11.4850	11.1144	11.1975	11.1324	11.1928	11.6337	11.6330	11.1639	11.1330	11.3318	11.1829	11.1589	10.6114
Rbm14	_DRS[Phospho (STY)]PLRR_.2	Q8C2Q3_S215_M1_Rbm14	8.8149	8.6056	8.7378	8.3587	8.3901	8.6102	8.3868	8.6192	8.3550	8.7006	8.4521	8.4080	8.8108	9.1275	8.0022	8.5307	8.9053	8.5432	8.5663	8.5248
Rbm14	_S[Phospho (STY)]PTKSSLDYR_.2	Q8C2Q3_S627_M1_Rbm14	7.0349	7.5577	3.4681	3.2567	6.9332	7.3303	7.0241	6.7437	6.8795	6.9878	6.6350	7.4617	7.9784	6.7365	7.2122	7.3070	7.0381	6.8477	7.1285	6.6310
Rbm14	_QPT[Phospho (STY)]PPFFGR_.2	Q8C2Q3_T206_M1_Rbm14	11.9262	12.3899	11.7086	11.8775	4.1035	11.6622	11.6832	11.7046	11.6760	12.0730	3.8711	11.6972	12.4226	12.6810	8.8656	11.9106	12.1299	11.9261	11.9201	11.8622
Rbm14	_LSESQLSFR_.2	#N/A	3.8504	4.6554	4.7954	3.8400	3.1881	3.8802	4.0346	3.2141	3.3771	4.6597	4.8430	4.0568	4.5201	4.6417	5.1880	4.4094	4.1284	3.9047	4.9929	4.4663
Rbm14	_RLS[Phospho (STY)]ESQLSFR_.2	Q8C2Q3_S618_M1_Rbm14	3.1751	4.1439	4.2126	3.9584	3.3821	3.4154	3.6660	3.4313	3.5862	4.4866	3.0649	5.3053	3.7517	6.0177	3.6914	4.0381	3.9690	3.8641	3.5127	5.9915
Rbm14	_IFVGNVSAAC[Carbamidomethyl (C)]TSQELR_.2	#N/A	5.1016	3.3360	3.2767	4.5234	3.9471	3.9804	3.1010	4.3672	3.0212	3.5507	2.8393	3.3051	3.1867	3.6893	3.0128	3.1023	3.4040	4.4291	4.0777	3.6563
Suco	_SKS[Phospho (STY)]LQFTGK_.2	Q8C341_S1077_M1_Suco	7.4261	7.4162	7.4157	7.0772	6.4517	6.7862	6.8359	7.1451	6.5920	6.8634	6.5738	6.4702	7.0196	6.2918	5.7835	3.9040	6.9170	6.9148	6.3838	6.3068
Layn	_KQS[Phospho (STY)]EADLAEPRPDLK_.3	Q8C351_S299_M1_Layn	6.5029	5.8887	4.4932	4.1285	5.5940	5.6016	6.1489	5.7828	4.9297	5.4460	5.7368	5.4736	5.7753	5.7503	4.9720	5.6600	5.6369	5.6920	5.1653	5.3294
Layn	_QENS[Phospho (STY)]PNLDVYNVIR_.2	Q8C351_S286_M1_Layn	4.2170	3.7858	5.2936	3.4724	3.5547	4.4302	4.1520	3.9173	4.5536	4.0006	3.5509	3.6753	4.2377	3.2395	4.1774	3.0610	4.4550	4.8033	5.5259	3.2065
Mettl3	_S[Phospho (STY)]DSPVPTAPTSSGPKPSTTSVAPELATDPELEKK_.4	Q8C3P7_S48_M1_Mettl3	3.3839	3.9495	4.4214	4.2146	3.3883	3.4947	6.2086	3.6096	3.1987	3.5918	3.9159	3.6756	3.3366	4.1223	3.8189	4.1835	3.9740	5.9302	3.4893	3.9317
Shisa7	_EHLLS[Phospho (STY)]PPR_.2	Q8C3Q5_S451_M1_Shisa7	11.2173	10.7874	10.5987	10.9310	10.5883	10.9017	9.3405	10.6463	9.1193	11.0144	11.1730	10.4181	10.6550	11.1517	9.8135	10.7562	11.2045	10.7049	10.5267	10.0949
Shisa7	_SSS[Phospho (STY)]LTPGLGGPDSM[Oxidation (M)]APR_.2	#N/A	11.3884	4.1874	10.7920	10.5785	10.6791	10.6150	10.4351	3.3578	6.2853	6.9499	10.7844	5.9248	11.2648	11.0273	9.2558	10.7055	6.6424	10.6621	3.0930	10.3831
Shisa7	_RVMS[Phospho (STY)]QEHLLGDGSR_.3	Q8C3Q5_S405_M1_Shisa7	4.3473	5.7906	6.9598	6.6752	3.0534	3.0867	4.7835	3.0721	3.9149	5.2727	5.8663	4.1988	4.0804	6.7806	4.0201	3.7094	4.0702	4.6583	4.5923	3.0492
Shisa7	_EHLLS[Phospho (STY)]PPRS[Phospho (STY)]PALPPDPTTR_.3	Q8C3Q5_S451_M2_Shisa7	5.1187	6.5632	4.4562	4.2020	3.6257	7.7523	6.7250	6.7049	6.0038	3.2294	4.3221	3.6265	3.5081	5.9168	3.4478	5.7945	3.7254	5.7003	6.8013	6.6998
Shisa7	_LVSQEHLLLSS[Phospho (STY)]PEALR_.2	#N/A	3.9401	5.6928	3.9850	3.8423	3.6325	3.7679	5.6763	6.0201	3.6765	4.0248	4.3302	3.6110	4.9902	6.3040	4.2609	3.8050	3.4381	6.0636	3.7210	3.6870
Tango6	_EAIS[Phospho (STY)]DDEDEALYQK_.2	Q8C3S2_S561_M1_Tango6	3.1231	3.3789	6.9341	7.5199	3.9617	4.1857	6.9739	3.9414	6.7860	3.3294	4.0129	3.2683	3.7466	7.2415	6.2999	3.4680	3.4386	7.1519	6.9339	6.8878
	SSS[Phospho (STY)]LDALGPAR.2	Q8C3W1_S176_M1_	11.1388	4.0353	10.9978	11.1996	4.2942	10.6786	10.3527	10.6218	10.5216	11.4472	10.6507	4.1067	11.0323	10.8682	10.2912	10.8324	11.0346	10.2325	10.5679	10.9174
Gpr158	_SAS[Phospho (STY)]AHNLSSEK_.2	Q8C419_S881_M1_Gpr158	10.7043	10.3346	10.3662	10.4861	9.1504	8.6274	9.9898	9.3757	8.8849	10.5227	9.5360	9.4716	9.5859	9.4371	7.1463	9.1739	9.1848	9.4337	9.1023	9.3123
Gpr158	_SLS[Phospho (STY)]VIASAK_.2	Q8C419_S904_M1_Gpr158	7.1642	7.0941	7.3334	7.6545	6.3855	6.7948	7.6187	4.2536	7.0708	8.1252	7.8208	3.5805	7.8660	6.8809	3.4688	7.6110	3.7508	7.8580	7.2726	7.4958
Gpr158	_LKEDS[Phospho (STY)]EAESTESVPLVC[Carbamidomethyl (C)]K_.2	#N/A	3.7896	7.2792	7.1809	6.7048	3.9054	4.1628	3.5486	4.7885	6.4602	7.5336	5.3689	3.3866	3.8265	6.5612	3.2534	3.3049	3.6456	6.6439	6.5595	4.0827
Gpr158	_EQTVQDDLQWYQALVR_.2	#N/A	3.7300	3.5701	3.6064	3.9077	3.9901	4.1328	4.2638	3.8963	3.6915	3.2765	3.7061	3.4000	3.7995	3.5935	3.8788	4.4199	4.5729	2.7932	4.1736	4.6049
Gpr158	_EES[Phospho (STY)]LKNR_.2	Q8C419_S806_M1_Gpr158	7.6984	7.3661	4.2502	4.4593	3.0516	6.5695	7.0600	3.3506	3.0477	8.0138	6.6022	6.2874	3.1558	6.1093	4.0666	4.0588	4.3486	4.3016	3.0999	3.8776
Gpr158	_VS[Phospho (STY)]LPTANPDVSSGITQIK_.2	Q8C419_S976_M1_Gpr158	3.2073	3.2946	3.7436	4.4821	4.0459	4.2700	3.4952	4.4086	3.5543	3.4137	3.9286	3.1841	3.6623	5.6012	3.0724	3.5522	3.4454	3.7951	4.0941	4.3842
Gpr158	_S[Phospho (STY)]IDKTEVC[Carbamidomethyl (C)]PWEIHSQSLLEDENR_.3	Q8C419_S1059_M1_Gpr158	4.0756	3.9922	3.8298	4.9308	3.4134	3.6375	3.1282	3.7745	3.5340	3.5100	3.9084	3.8290	3.7868	3.8366	3.8241	3.6486	3.7364	3.7781	3.7350	3.6972
Gpr158	_QC[Carbamidomethyl (C)]S[Phospho (STY)]KEDKEGTDHSAAK_.3	Q8C419_S770_M1_Gpr158	3.7599	4.2429	4.2962	3.9295	3.0976	3.3217	4.4434	3.3045	3.0017	3.9662	3.3761	4.1323	3.1097	4.1543	4.0206	4.1048	3.9980	4.3477	3.1459	3.8316
Pex5l	_KGS[Phospho (STY)]PGLTR_.2	#N/A	11.1518	11.2256	10.6910	11.0163	10.7239	10.6590	10.8372	11.0100	10.7254	10.9471	10.8019	10.9763	11.1408	11.2968	10.2146	10.6895	11.0814	10.9318	10.8444	11.0628
Pex5l	_S[Phospho (STY)]PVDSSVLEGVK_.2	#N/A	8.7133	8.8716	8.9416	9.1024	8.3882	8.8693	8.2918	3.9138	8.6664	9.1831	9.0082	8.4416	8.6040	3.2628	3.1291	8.6620	9.2478	8.3235	8.7489	8.8226
Pex5l	_TGS[Phospho (STY)]KELLWSAEHR_.2	#N/A	6.0026	6.2647	5.6767	5.9491	5.6468	6.1273	5.6461	6.0675	5.3092	6.3691	5.8196	5.7362	6.7706	6.4222	4.7362	5.3143	6.2961	5.8169	6.7140	5.8216
Pex5l	_SALNSES[Phospho (STY)]ASELELVAPAQAR_.2	Q8C437_S172_M1_Pex5l	8.3464	8.4517	3.4434	3.6526	3.7457	3.9698	7.7085	7.5459	3.8545	8.3135	4.2288	3.4843	7.5889	8.5542	6.6437	3.2520	3.6545	3.4949	7.5853	3.1836
Pex5l	_NKKGS[Phospho (STY)]PGLTR_.2	#N/A	3.7075	3.6116	3.2441	6.7200	6.7039	3.9478	3.1337	7.5265	3.0539	3.5181	5.6428	7.4489	8.1731	3.2821	3.1591	6.5449	6.5492	4.3965	6.7544	6.1680
Pex5l	_S[Phospho (STY)]QPELSTGK_.2	Q8C437_S157_M1_Pex5l	8.0869	3.6328	3.4055	8.9138	7.9824	7.9809	8.5707	7.2262	4.2253	9.0911	8.4988	8.1343	4.3907	8.2679	3.4105	7.2068	3.1072	3.2251	4.3745	8.6034
Pex5l	_GPETS[Phospho (STY)]SLDLDIQTQLEK_.2	Q8C437_S105_M1_Pex5l	3.2021	3.2999	3.7384	4.4873	4.0406	4.2647	3.5004	3.8624	3.5595	3.4084	3.9339	3.1893	3.6676	3.2113	3.0776	3.5470	3.3596	3.7898	4.0889	4.3895
Pex5l	_KGS[Phospho (STY)]PGLTRR_.2	#N/A	5.1317	4.1823	4.3568	3.8689	3.1582	3.3823	4.3829	3.5208	4.4420	4.0268	3.3155	5.6169	6.5358	4.3438	3.9601	4.1654	4.0585	6.0469	3.4232	5.1979
Pex5l	_NHS[Phospho (STY)]LEEEFER_.2	#N/A	8.8238	8.9581	6.8028	9.2618	8.5898	8.1804	8.3283	8.8401	8.6387	9.1813	8.2919	8.6469	9.7891	9.1098	7.7850	8.6310	8.7297	8.2994	8.4649	9.1075
Pex5l	_M[Oxidation (M)]SKS[Phospho (STY)]PVDSSVLEGVK_.2	#N/A	6.3208	7.4100	4.9747	4.1015	5.6612	5.8395	6.3845	6.5876	5.7801	3.7672	6.8026	6.0002	7.0469	7.7889	4.9830	3.6928	6.2484	6.1865	5.8725	3.5826
Pex5l	_RM[Oxidation (M)]S[Phospho (STY)]KS[Phospho (STY)]PVDSSVLEGVK_.3	#N/A	3.8710	5.0891	5.3678	5.8712	3.4721	5.2336	5.3377	3.6634	5.3408	5.4741	3.5681	5.4291	4.1887	5.5732	5.2391	5.7577	5.1972	5.4495	5.4138	5.9883
Tmem145	_VVTM[Oxidation (M)]AEPGAAS[Phospho (STY)]PPPPAR_.2	#N/A	9.1638	8.1270	8.3659	8.7371	8.0032	8.1468	8.7909	8.8916	8.0190	8.1078	7.9033	7.9716	8.7022	8.3362	6.6699	7.6827	8.5978	8.4328	8.0483	7.7675
Fbxl3	_GGRDS[Phospho (STY)]DQDS[Phospho (STY)]AEEGTAEKPK_.3	Q8C4V4_S12_M2_Fbxl3	4.5593	4.2607	4.0958	4.5258	3.2654	3.2987	3.7827	3.2841	3.7030	4.3699	3.1816	3.9868	3.8684	4.3711	3.8081	3.9214	4.2822	3.7474	3.3960	4.3380
Mb21d2	_GSTTSIPS[Phospho (STY)]PQSDGGDPNQPDDR_.2	Q8C525_S376_M1_Mb21d2	5.9186	5.5453	3.8920	4.3715	3.5154	3.7395	4.5948	3.7466	3.4437	3.5621	3.8181	3.8954	3.5518	3.9175	3.8506	3.7006	5.6378	3.9435	3.5636	5.0270
Cnot2	_TNSMSSSGLGS[Phospho (STY)]PNR_.2	Q8C5L3_S165_M1_Cnot2	3.6214	7.8052	8.8523	8.9141	6.8982	6.8599	7.2309	4.4859	5.5986	7.0783	7.6290	6.0323	3.3054	8.5076	5.6578	2.9835	6.3543	5.2956	5.2082	6.4147
Cwc22	_GGGQSS[Phospho (STY)]PQEEPTWK_.2	Q8C5N3_S106_M1_Cwc22	9.2529	9.0237	9.0332	9.1335	8.3979	8.8232	4.1635	8.9543	8.7672	8.7513	3.5624	8.5171	8.8564	8.8312	7.2550	3.5406	9.3858	8.8195	8.6103	8.2940
Clmn	_IKES[Phospho (STY)]PSEQGSR_.2	Q8C5W0_S317_M1_Clmn	7.7739	8.1629	4.1760	7.8502	7.0884	7.7683	7.8528	8.0760	7.8266	7.7675	7.5053	7.6068	8.3695	8.0288	7.2474	7.4009	7.9057	7.8920	7.8024	7.1505
Clmn	_NSHS[Phospho (STY)]DSSIYIR_.2	Q8C5W0_S925_M1_Clmn	4.1926	3.7884	8.1080	8.1051	7.8752	7.9394	7.6183	3.9148	7.6591	7.9343	7.9374	7.7095	8.4628	7.6487	6.4637	3.5547	8.2194	7.6682	7.4562	7.8972
Clmn	_SNS[Phospho (STY)]LPVKK_.2	Q8C5W0_S419_M1_Clmn	8.0435	3.8698	7.5437	7.4080	7.0562	7.6301	4.0703	7.2970	7.8084	7.8819	7.4529	7.1899	7.6671	3.1556	6.4618	7.6050	7.8443	3.4621	7.7848	7.0840
Clmn	VFVC[Carbamidomethyl (C)]DQLESPT[Phospho (STY)]GFC[Carbamidomethyl (C)]LDSAPSHK_.	Q8C5W0_T360_M1_Clmn	3.7454	4.5275	3.2820	3.8388	3.9524	3.9857	3.0957	4.7461	3.0159	3.5560	3.9954	3.2998	3.1814	3.3201	3.1211	3.1076	3.4684	3.6811	3.9801	2.9974
Tbcel	_YS[Phospho (STY)]PENFPYR_.2	Q8C5W3_S18_M1_Tbcel	7.7050	7.6748	7.2891	7.4554	7.0418	7.4056	6.9260	7.3359	7.3251	7.2457	7.4101	6.8929	7.4709	7.4467	6.7819	7.1400	7.8361	3.6696	3.6658	7.4541
Spata33	_AS[Phospho (STY)]NETLISYGIPDNDEQR_.2	Q8C624_S87_M1_Spata33	4.1610	6.2385	3.1965	3.5284	2.9520	2.9854	4.0961	3.8614	4.0163	4.0565	3.4949	3.7312	4.1817	3.1836	6.0747	3.6080	4.3991	3.4341	5.8430	3.1505
Rtkn	_RPSDS[Phospho (STY)]VQPAQHSPC[Carbamidomethyl (C)]R_.3	Q8C6B2_S108_M1_Rtkn	4.1075	4.3289	4.0926	4.8924	4.0806	4.4450	3.7375	4.0422	4.2355	4.1339	3.4704	3.7175	4.7026	4.0720	3.8916	4.2742	4.1764	3.9921	3.7476	3.6817
Rtkn	_TFS[Phospho (STY)]LDAAPADHSLGPSR_.2	#N/A	5.4334	4.2078	3.6854	5.2701	3.1807	4.5458	4.9328	3.5433	4.7854	5.4951	4.0873	4.2433	3.7623	3.6690	4.1372	4.1879	4.0810	4.9736	4.4437	4.1442
Wdr26	_RLS[Phospho (STY)]QSDEDVIR_.2	#N/A	6.3110	4.2318	4.3073	3.9184	3.1087	3.3753	3.7061	3.4714	5.8493	4.4465	3.1049	4.1212	7.2341	4.2944	6.1721	6.3869	4.0091	3.8241	3.4727	5.7173
Wdr26	_LSQS[Phospho (STY)]DEDVIR_.2	Q8C6G8_S103_M1_Wdr26	6.6163	4.5141	4.0250	6.7133	4.3273	3.6576	5.9702	3.1891	3.3440	3.2279	4.3235	4.4035	3.5095	4.0121	6.1129	6.7867	3.7268	6.3662	6.2911	3.9791
Wdr26	_KRLS[Phospho (STY)]QSDEDVIR_.3	#N/A	3.3348	3.1671	3.8711	4.3546	4.1734	4.3975	3.3677	4.5360	6.2995	3.5411	3.8012	3.0566	6.9347	3.8582	6.2068	6.2134	6.0676	3.9226	6.6289	4.2567
Usp20	_EAQPPS[Phospho (STY)]PR_.2	Q8C6M1_S369_M1_Usp20	10.0845	9.9964	9.8831	10.0693	9.5642	9.6949	9.8871	9.9664	9.8101	9.7326	9.3847	9.5252	9.7720	9.4563	8.5098	9.3337	9.7149	9.7853	9.6235	9.4019
Usp20	_LSEQDS[Phospho (STY)]PPPSHPLK_.2	Q8C6M1_S112_M1_Usp20	3.2873	8.7381	7.5406	8.5015	7.5456	3.8591	7.3592	9.0543	3.1426	8.2970	8.1258	8.3668	8.6450	3.8107	7.5599	3.6322	3.5253	8.4801	3.9564	3.7776
Usp20	_KFS[Phospho (STY)]WGQQR_.2	Q8C6M1_S334_M1_Usp20	7.6403	7.5060	7.5466	7.8376	6.6491	6.6096	6.8663	6.9054	7.1682	7.4389	6.9839	6.8359	6.7775	6.4563	2.8528	6.5405	6.9475	4.0146	6.5582	6.1888
Usp20	_AVPIAVADEGESES[Phospho (STY)]EDDDLKPR_.3	Q8C6M1_S134_M1_Usp20	4.0728	6.2432	3.8219	3.8472	3.7259	6.0422	3.8390	3.9294	3.8294	3.6070	3.8394	3.4514	3.9654	8.0189	3.5627	3.5617	3.8437	8.1079	3.8105	3.5659
Usp20	_STS[Phospho (STY)]PC[Carbamidomethyl (C)]QTPEPDNEAHIR_.3	#N/A	5.4908	3.7929	4.1051	5.7976	3.5854	5.8638	5.3859	3.9480	3.8250	3.7898	3.8124	3.6824	3.9626	6.2025	3.6714	4.8159	3.8069	3.5753	3.5798	3.0583
Usp20	_LSSSPPRAS[Phospho (STY)]PVR_.2	Q8C6M1_S415_M1_Usp20	4.2279	4.5920	3.7645	3.3563	2.9340	4.4682	4.1141	3.8794	4.0343	4.0385	3.5130	4.3182	4.1998	3.2016	4.1395	3.5900	3.9508	4.7732	3.0646	3.1686
Fam126b	_TAITTAS[Phospho (STY)]IRR_.2	Q8C729_S321_M1_Fam126b	4.1168	7.6821	8.3674	7.9077	7.3037	3.7847	4.2252	4.3424	6.9836	7.8855	2.9131	2.9284	4.1476	6.5512	4.2506	3.4789	3.8397	3.3098	3.6088	6.9582
Fam126b	_VEVT[Phospho (STY)]PTVPR_.2	Q8C729_T306_M1_Fam126b	3.7017	7.8395	3.2383	3.8825	7.2825	3.9420	3.1395	3.9274	3.0597	3.5123	4.0392	3.3435	6.9863	3.2763	3.1649	3.0638	3.4246	3.7249	4.0239	2.9536
Fam126b	_NSLPFDAPDSS[Phospho (STY)]QEGQK_.2	Q8C729_S294_M1_Fam126b	7.4028	8.0435	3.4806	7.9520	3.9665	7.3927	3.3712	7.0599	7.8345	7.8586	7.3905	8.1082	8.4742	3.5615	7.1461	7.4705	8.8884	8.2478	8.3300	8.2552
Fam126b	_TNS[Phospho (STY)]LSLIR_.2	Q8C729_S442_M1_Fam126b	3.2002	3.3018	6.8311	6.7032	4.0387	3.9462	6.8342	4.4014	3.0554	7.2770	6.9586	3.1912	3.2209	6.7950	3.0795	3.5451	6.5951	6.5260	6.5849	3.6905
Fam126b	_YSTIS[Phospho (STY)]LQEDR_.2	Q8C729_S491_M1_Fam126b	4.3999	4.4200	3.9365	6.7712	3.1060	3.5015	3.9421	3.1248	4.3227	4.2105	3.1963	4.1461	4.4308	3.9746	3.9675	3.7620	4.1228	3.0266	3.3257	3.6518
Fam126b	_S[Phospho (STY)]AKDKETALAIK_.3	Q8C729_S383_M1_Fam126b	3.2266	3.2753	3.7629	4.4628	4.0652	4.2893	2.6207	4.4278	3.5350	3.7824	3.9093	3.1648	3.6430	3.7500	3.0531	3.5715	3.4647	2.6500	3.4531	3.7170
Fam110b	_SKS[Phospho (STY)]DLSDR_.2	Q8C739_S257_M1_Fam110b	9.3259	9.3025	9.3271	9.1918	8.9399	8.9601	9.0636	8.8365	3.4536	9.5381	8.9607	8.9559	9.1577	4.1217	4.1822	9.0109	8.9107	8.7846	9.0549	9.1784
Fam110b	_ANS[Phospho (STY)]DIISLNFR_.2	Q8C739_S297_M1_Fam110b	3.1214	3.3806	3.6577	4.5680	3.9600	4.1840	3.5811	4.3226	3.6402	3.3277	4.0146	3.2700	3.7483	8.1739	6.4401	3.4663	3.3595	7.6298	7.1250	2.9693
Fam110b	_VYPTPGHGS[Phospho (STY)]PQESSSHVSR_.3	Q8C739_S172_M1_Fam110b	4.5449	4.2751	4.0815	7.6380	3.2510	3.2843	3.7971	3.2697	3.7173	4.3555	3.1960	4.0012	3.8828	4.3854	7.2527	3.9070	4.2678	6.5564	3.3816	4.3524
Fam110b	_VAAMKS[Phospho (STY)]PEADQVEPAC[Carbamidomethyl (C)]GVSR_.3	#N/A	4.0100	4.8238	4.8355	5.2418	3.0140	4.0310	4.4170	3.6796	3.8345	4.2698	4.2370	4.2317	3.9999	5.4661	4.0848	3.8654	4.2173	3.6473	3.2959	3.8012
Cstf2t	_QGGGQPSSFS[Phospho (STY)]PGQSQVTPQDQEK_.3	Q8C7E9_S579_M1_Cstf2t	4.4325	4.3875	3.9690	3.1518	3.1385	3.4690	4.2962	3.1573	4.3553	4.1135	4.8945	4.1136	4.4633	4.0071	3.9349	5.5616	4.1554	5.0676	5.5888	3.6844
Trim9	_NILVQTPESES[Phospho (STY)]PQSR_.2	Q8C7M3_S46_M1_Trim9	10.9903	10.6498	10.8240	11.0724	10.4064	10.3022	10.2935	8.7902	10.2837	11.1882	10.4476	10.4546	11.0843	10.7265	9.6714	10.6128	10.9202	10.3197	10.3206	10.4794
Trim9	_RAS[Phospho (STY)]GSVSDYDYLDLDK_.2	#N/A	3.6800	6.1470	3.7770	5.9612	3.7402	5.7926	5.8054	4.4545	3.7449	5.5138	3.6292	3.8223	3.8708	6.3723	5.0802	5.5816	5.9733	6.0614	5.8720	4.9159
Srsf12	_S[Phospho (STY)]KS[Phospho (STY)]LPKR_.2	Q8C8K3_S187_M2_Srsf12	9.3468	9.1987	9.5530	9.2945	8.7741	9.3986	9.2635	8.7919	9.3135	9.4869	9.1367	8.5076	8.6579	8.9866	8.2492	8.5905	9.1553	8.9374	9.1656	8.7827
Srsf12	IC[Carbamidomethyl (C)]DSIARS[Phospho (STY)]PC[Carbamidomethyl (C)]KS[Phospho (STY)]PI	#N/A	3.8033	4.6601	4.1388	4.3596	4.8255	4.5690	4.8542	4.2845	4.8358	5.1076	4.8020	4.6109	4.9595	4.9331	3.9935	4.1204	4.1607	4.3243	4.7322	4.2302
Srsf12	_QSTSLRQS[Phospho (STY)]RT[Phospho (STY)]PR_.3	Q8C8K3_T174_M2_Srsf12	3.6995	4.0525	3.7694	3.9599	3.5800	3.6746	3.0828	3.9412	3.4056	3.7006	3.7548	3.8892	4.0893	4.0337	3.7571	3.5845	4.0378	3.6809	4.3088	3.8241
Ank2	_VGT[Phospho (STY)]PTDIHSEK_.2	#N/A	11.3968	11.2196	11.5181	11.1980	10.8819	11.4604	11.1637	11.2661	11.0993	11.1930	11.3539	10.8355	10.8858	11.3144	10.0802	10.7192	11.5912	11.3067	11.2073	11.1497
Ank2	_S[Phospho (STY)]YIETETESR_.2	#N/A	12.1041	12.0598	11.8353	11.9392	11.5693	11.8204	11.6875	11.9590	11.6820	11.7262	11.9822	11.8472	12.2622	12.2469	11.2635	11.8583	12.2496	12.0196	11.8975	11.8469
Ank2	_SS[Phospho (STY)]PEDVVTNSSNR_.2	Q8C8R3_S2924_M1_Ank2	8.7333	8.5152	8.7386	8.4982	8.2895	8.4287	8.2905	8.5782	8.3422	5.6131	5.5144	5.5377	8.7690	8.7123	4.9964	8.4015	8.9701	8.3929	8.4529	8.5259
Ank2	_TEWQPS[Phospho (STY)]PTDIPLQK_.2	Q8C8R3_S3315_M1_Ank2	9.8325	10.4150	9.9982	9.7298	3.2750	9.6381	9.9250	3.5384	9.6886	4.3795	9.2181	4.0543	3.8587	10.4890	3.7984	9.6030	10.0335	10.1318	9.8573	9.6106
Ank2	_QPPIS[Phospho (STY)]PTSK_.2	Q8C8R3_S1936_M1_Ank2	11.1451	11.1073	11.2156	4.5497	3.9783	4.2023	3.5628	10.7553	3.6219	3.3460	3.9963	3.2517	3.7300	11.0682	3.1400	3.4846	3.3778	3.7274	4.0265	2.9510
Ank2	_DSLESS[Phospho (STY)]PVEPK_.2	Q8C8R3_S2431_M1_Ank2	8.7052	8.6238	9.4604	8.8378	8.5557	8.9302	4.4114	8.7840	8.7533	8.1801	3.3440	8.3383	8.7111	8.9018	7.9463	8.4383	9.2342	8.3767	3.1780	8.5871
Ank2	_NERHS[Phospho (STY)]PVSSLK_.2	#N/A	8.2937	8.3332	5.7906	7.7808	7.8601	8.1745	7.9284	4.3506	3.3714	6.5070	8.0193	7.9855	8.1572	3.6728	5.8742	7.8923	8.3828	8.3252	7.9703	7.9619
Ank2	_TQDQQEYYVTT[Phospho (STY)]PGAEVEDPQK_.3	Q8C8R3_T3718_M1_Ank2	7.4829	7.6299	7.5459	7.4313	6.4214	7.1770	7.4269	3.9344	6.4873	8.1913	6.9764	6.8220	7.2970	7.2874	3.5466	7.2428	6.0878	3.3611	7.1698	7.1435
Ank2	_LSPVS[Phospho (STY)]PSAK_.2	Q8C8R3_S1819_M1_Ank2	7.3169	3.5945	3.4043	7.4429	7.2043	7.3641	7.7766	6.8382	3.9899	7.6934	7.9227	3.4220	8.0717	7.5951	3.5800	7.5960	7.6728	7.9538	7.9395	8.0298
Ank2	_VVTEEVTTTTTTITEK_.2	#N/A	7.7273	8.4047	7.3934	7.2857	6.2802	6.9630	7.4213	8.6351	7.0139	6.9345	6.5156	7.5912	8.4341	8.9766	6.1948	7.0992	7.2211	8.2254	6.2994	5.9329
Ank2	_HTPVS[Phospho (STY)]PSSK_.2	#N/A	8.8349	4.2362	9.0614	3.9228	3.3464	3.3798	3.7017	3.4670	3.6219	4.4509	3.1005	4.1256	3.7873	4.2900	3.7270	4.0024	8.5901	8.8146	3.4771	8.3750
Ank2	_ATFSPIVTLEPR_.2	#N/A	6.6955	7.0850	6.5456	6.8858	6.5925	6.9637	7.1975	7.2078	7.0924	7.0341	6.7558	7.0194	7.4656	6.8153	6.6848	6.9039	6.7753	7.1094	7.0014	6.8051
Ank2	_AREEQTPLHIASR_.3	#N/A	7.1036	5.4728	6.7283	6.5617	6.7998	7.0190	7.1255	6.8397	6.9028	6.3135	6.5047	6.5750	5.5450	6.5792	6.9443	6.5706	7.4177	6.8952	6.8714	6.8148
Ank2	_FWLIDC[Carbamidomethyl (C)]R_.2	#N/A	6.2347	6.5537	6.8026	6.0317	6.2201	6.6385	6.6707	6.2774	5.7814	6.0630	6.4405	6.6324	6.6775	6.6659	5.7729	6.2150	6.2629	6.5418	6.3477	6.2375
Ank2	_Y[Phospho (STY)]SLEGGR_.2	Q8C8R3_Y889_M1_Ank2	3.8846	3.8626	3.9328	3.8948	5.6591	4.0151	3.7306	3.7850	3.8338	3.9687	3.3125	6.3878	6.3976	3.9543	3.5185	3.7528	4.5362	6.7618	3.8538	3.7302
Ank2	_HS[Phospho (STY)]PVSSLK_.2	#N/A	6.3323	5.9494	3.5543	6.2029	5.1417	5.9338	5.8136	4.0619	5.4770	5.4142	6.0447	5.0258	4.0486	6.2778	4.8522	3.3799	6.0103	6.0832	5.5762	5.9769
Ank2	_HTPVS[Phospho (STY)]PSSKNEK_.2	#N/A	6.0033	6.3618	6.0540	6.2004	5.3326	3.8631	6.0409	6.3675	3.9611	5.6176	5.2441	6.0482	4.0692	3.6130	5.0434	3.1454	6.0658	5.9217	5.9610	3.2902
Ank2	_ERAEGFES[Phospho (STY)]ESEDGATKPK_.3	#N/A	6.7945	3.3272	5.9208	3.5924	4.1988	4.2321	6.5533	4.1155	6.3029	3.8024	6.1613	3.0534	4.4358	3.4376	2.8747	6.0152	3.1523	6.5572	4.3294	3.4046
Ank2	_SVYSGQDDES[Phospho (STY)]PESS[Phospho (STY)]PEEQK_.2	Q8C8R3_S3226_M2_Ank2	3.8743	4.3577	5.7480	5.6361	4.0073	3.7103	4.8946	6.4131	4.8744	3.9546	5.6958	5.3568	5.8545	3.8335	4.7141	5.4962	5.8438	5.0140	5.3371	5.2379
Ank2	_GS[Phospho (STY)]PIVQEPEEASEPK_.2	#N/A	7.2205	6.3781	7.5329	6.7378	5.9060	7.1392	6.2580	6.7008	6.4732	6.7517	7.3802	5.7470	4.2655	7.1892	5.4569	6.4626	7.3212	6.6839	6.4871	6.3400
Ank2	TC[Carbamidomethyl (C)]DGHGC[Carbamidomethyl (C)]ETVSPSNSATPVSLGVQS[Phospho (STY	Q8C8R3_S2755_M1_Ank2	6.3627	6.1307	7.9700	6.2560	6.8108	7.6698	6.1343	5.8177	5.8965	7.5046	6.1125	5.6743	5.7848	6.5264	5.2538	5.8589	6.3200	4.1442	5.9789	5.8309
Ank2	_ATS[Phospho (STY)]PLIEETPIGSIK_.2	#N/A	10.2330	9.7651	10.0627	9.8555	3.0283	8.8756	9.3634	3.7851	8.8965	10.1296	3.4186	9.2048	9.3508	9.5781	4.0452	9.2346	9.2634	9.3254	9.1673	8.8496
Ank2	_ATAVPDSLC[Carbamidomethyl (C)]K_.2	#N/A	6.5578	6.2670	6.5066	6.5805	5.7781	6.6568	6.8768	6.3603	6.4501	6.9052	6.3016	6.0537	6.3979	6.6045	6.5959	5.6989	7.0840	6.7338	6.3277	6.1105
Ank2	_HLPVS[Phospho (STY)]PGK_.2	#N/A	6.4342	6.1827	5.8038	6.5903	5.3340	6.1870	6.0526	5.4595	5.5980	7.1378	6.9234	6.1116	5.8134	5.6577	4.8178	6.4040	6.3683	6.4408	6.2118	6.4012
Ank2	_ELEEEWVIVS[Phospho (STY)]DEEIQEAK_.2	Q8C8R3_S1562_M1_Ank2	9.5564	10.0754	9.6277	9.5415	7.4089	9.5465	9.7603	7.3645	9.6640	9.4568	8.6973	8.5944	9.8999	10.2406	8.7948	9.3261	9.8375	9.9698	9.5775	9.4389
Ank2	_GADSGLLTEPVIR_.2	#N/A	5.3662	5.3675	5.3971	3.5247	4.2665	4.7783	5.3791	4.8218	4.8913	3.9453	5.6093	4.2413	5.3609	4.2347	4.9067	5.2259	5.3687	4.5674	5.0660	5.0875
Ank2	_TLEQQENFSEVAR_.2	#N/A	3.8946	5.6066	3.5478	5.7765	4.8413	4.7574	4.5426	4.5655	4.9755	5.4004	4.6722	4.2433	4.5330	4.8606	4.6797	4.4670	5.1673	4.5414	4.2322	4.6324
Ank2	_KTS[Phospho (STY)]LVIVESTDDQSQVFER_.3	#N/A	6.8001	3.8183	3.7804	4.1432	5.7787	9.2478	6.0603	6.5236	3.6360	9.7462	9.2458	3.6694	3.7874	3.7706	8.1720	9.2067	6.5477	3.9632	9.2840	3.7376
Ank2	_TEKHPPVS[Phospho (STY)]PGKTEK_.2	#N/A	6.5673	6.5708	5.8783	6.0567	5.5662	6.1199	5.8765	5.2706	3.6988	7.0543	6.2811	7.1595	7.2699	5.1027	6.2883	6.3600	5.8270	6.8006	6.1465	6.6041
Ank2	_GAAVDFTAR_.2	#N/A	4.7415	5.0166	5.3554	4.5155	4.3164	5.0435	5.1482	1.5594	5.2805	5.1289	4.5709	4.2207	4.9381	4.2586	4.4043	4.4071	4.3003	4.9332	5.1741	5.0834
Ank2	_TDADADSWSEIREDDAAFEAR_.3	#N/A	5.5028	4.9724	4.9128	3.3867	3.4798	5.0578	4.6012	4.4232	4.8061	5.3974	5.3839	5.2357	6.3481	5.8646	3.7096	5.5723	5.2385	5.1534	4.3099	5.1233
Ank2	_EGHVGLVQELLGR_.3	#N/A	4.0365	4.2794	3.7166	4.7144	3.9945	4.3741	4.3366	3.1907	4.9072	4.2276	3.4962	4.6718	4.0583	3.5931	3.1163	3.5008	4.5500	4.2944	3.8742	4.8642
Ank2	_ARS[Phospho (STY)]YIETETESR_.2	#N/A	8.3967	3.7737	8.4697	8.0904	7.2509	3.7569	6.2516	8.5067	7.9010	3.9884	4.9184	8.0301	8.3888	8.4071	7.8626	8.0436	8.5077	8.0638	7.8406	7.7550
Ank2	_ESPC[Carbamidomethyl (C)]RDSLES[Phospho (STY)]S[Phospho (STY)]PVEPK_.2	Q8C8R3_S2431_M2_Ank2	3.5627	4.1843	3.6365	5.1526	4.0892	4.5335	3.4303	3.5745	5.3392	3.4915	4.2100	4.7456	3.5974	4.8740	3.2318	3.9324	3.5411	4.4206	4.5286	4.3193
Ank2	arbamidomethyl (C)]KTPEDIS[Phospho (STY)]T[Phospho (STY)]PPEGTKPC[Carbamidomethyl (C	Q8C8R3_S3744_M2_Ank2	3.6146	4.1401	6.2084	4.0463	4.1372	5.8609	6.9245	3.6296	3.5129	3.4791	3.8873	3.1222	3.6410	3.6892	3.0310	3.6177	3.5898	6.8883	3.8811	3.5494
Ank2	_GSPIVQEPEEASEPKEESS[Phospho (STY)]PR_.3	#N/A	12.0357	12.3647	12.1484	12.0872	7.6123	11.8789	6.7516	7.2005	11.7579	12.0088	12.7126	11.7990	7.5362	11.4639	7.2439	11.7268	8.2316	11.3267	12.0958	11.8269
Ank2	_TERHS[Phospho (STY)]PVFSGKPEK_.3	#N/A	7.1489	7.3677	4.0114	7.0403	6.1297	6.6589	6.8091	4.0159	2.8091	7.0933	6.7454	6.6311	7.0007	7.1317	5.7072	6.4343	7.1047	7.0490	6.7679	4.1500
Ank2	_GSPIVQEPEEASEPKEESS[Phospho (STY)]PRK_.3	#N/A	3.9444	3.9463	3.8929	3.8870	3.7338	4.0383	4.9264	7.0096	3.9163	3.9010	4.4525	3.7215	4.0244	5.9803	3.4345	3.7100	3.8587	3.5634	3.8625	3.9644
Ank2	_TELKKGS[Phospho (STY)]S[Phospho (STY)]EESVDEDR_.2	#N/A	3.6459	4.3569	4.1822	4.0435	2.7337	3.2905	2.4155	3.0296	3.7506	3.2940	4.0227	1.3122	3.2238	4.1422	2.4161	3.9908	2.3570	4.2336	3.0318	4.0911
Ank2	_NEKLS[Phospho (STY)]PVS[Phospho (STY)]PSAK_.2	#N/A	11.9065	11.7724	11.7918	11.8415	11.3161	11.2612	11.3932	11.5121	11.3475	11.6772	11.4806	11.5499	11.8397	11.9092	10.5065	11.5310	11.9043	11.6868	11.5556	11.5144
Ank2	_HLPGS[Phospho (STY)]PSIR_.2	Q8C8R3_S1900_M1_Ank2	7.5040	3.6943	3.3439	3.5531	3.6462	7.1753	3.8949	4.2568	3.9540	4.5148	4.3284	3.5838	4.0620	3.6058	3.4721	3.1525	3.7541	3.3954	3.6944	3.2831
Ank2	_NERHS[Phospho (STY)]PVSSLKTER_.4	#N/A	3.2052	3.6855	3.1702	3.9506	4.0438	4.2678	5.5542	3.8593	5.2819	3.4115	3.9308	3.4116	3.6645	3.2082	3.0745	3.5501	3.3565	3.7929	4.0920	4.3863
Ank2	_MDIVHLLETNTEPLQER_.3	#N/A	3.4331	4.5999	5.3333	5.8662	4.7100	4.9352	4.7784	3.1335	3.3285	3.6394	5.7947	3.8295	3.4538	5.8191	2.8466	3.7780	5.1161	3.7412	3.8106	3.9054
Ank2	_KSDSNAS[Phospho (STY)]FLR_.2	#N/A	6.9816	7.0315	6.6250	8.4655	7.8310	6.9065	6.7082	5.9518	8.3449	6.6806	6.7787	6.7643	8.7304	8.3726	7.2180	6.8062	7.4332	8.3210	6.9122	8.3271
Ank2	_S[Phospho (STY)]PLSPDTPSSEEVSYEVTPK_.2	Q8C8R3_S2493_M1_Ank2	3.8663	4.6806	3.7437	3.8681	3.7729	4.4996	5.1537	3.8964	3.8603	4.3774	3.7232	3.5610	3.8929	5.8516	3.7834	3.5653	4.2367	5.5245	3.2920	3.5179
Ank2	_ETTKVGT[Phospho (STY)]PTDIHSEK_.2	#N/A	5.2809	5.4933	3.5647	4.0070	3.6152	5.4114	4.7441	5.5034	4.7305	3.9204	3.7181	4.8383	3.8028	3.5518	3.5000	3.8655	4.0201	5.3297	4.4852	4.8502
Ank2	_IVDEQGDM[Oxidation (M)]DFQIS[Phospho (STY)]PDRK_.2	#N/A	7.5449	8.5189	8.1114	7.6696	5.3532	8.1118	8.1252	6.4534	8.1003	7.5508	6.5876	7.5902	8.0705	8.4700	7.1734	8.1595	7.6966	8.8995	8.1662	8.0569
Ank2	_HPPVS[Phospho (STY)]PGKTEK_.2	#N/A	9.0550	8.5861	8.8041	8.8334	8.3179	8.0927	8.1682	8.5659	4.0143	8.6144	4.0022	8.4710	8.6506	8.5009	3.6218	8.3372	4.1396	8.5056	8.1566	8.4570
Ank2	_THPAVS[Phospho (STY)]PSSK_.2	Q8C8R3_S1777_M1_Ank2	4.6240	9.0925	8.8672	3.8403	8.6666	8.5181	8.8530	3.9763	3.6734	8.8338	4.5975	8.8777	9.0852	4.0829	4.3965	8.7992	3.4735	4.7974	8.7107	8.6697
Ank2	_VSSSSS[Phospho (STY)]ESEPELTQLSK_.2	Q8C8R3_S2624_M1_Ank2	3.8458	5.6861	4.3544	5.4526	3.6810	5.1852	3.5876	3.4692	3.5502	3.7561	3.3757	3.6576	3.6978	4.3111	3.4944	3.9228	3.8515	4.0991	3.7230	4.0792
Ank2	_HLPVS[Phospho (STY)]PGKTEK_.2	#N/A	3.4146	8.5439	8.0642	8.3350	3.5412	7.4511	7.6059	3.9039	4.0588	4.0425	7.7931	3.8274	8.3923	8.1112	3.7157	3.7595	8.1389	3.5303	3.8908	7.5318
Ank2	_KGS[Phospho (STY)]S[Phospho (STY)]EESVDEDR_.2	#N/A	5.1383	5.9963	6.7699	6.1174	5.7755	6.8208	6.3387	5.6376	6.3480	6.0547	6.2887	5.6425	5.4054	6.0155	5.5293	3.8381	6.7448	6.4656	6.3521	6.1608
Ank2	_ATS[Phospho (STY)]PLIEETPIGSIKDK_.2	#N/A	3.5256	6.4378	4.0619	6.3094	3.6541	4.4517	5.5150	5.0076	3.0131	3.7774	4.7161	5.2394	5.8165	3.9367	3.6333	5.5107	3.9137	4.2712	3.6749	3.7461
Ank2	_AEGFES[Phospho (STY)]ESEDGATKPK_.2	#N/A	5.0908	5.3752	5.0514	4.1482	4.6073	4.9613	5.0963	5.0985	5.2557	5.3086	4.7665	3.0797	3.6046	3.5955	3.0830	4.4572	5.0315	5.1736	4.6064	4.4970
Ank2	_SDSNAS[Phospho (STY)]FLR_.2	#N/A	4.7142	9.6919	9.6823	10.1657	8.6832	8.3586	8.6014	8.9885	8.6351	9.7190	8.9517	7.9592	9.4280	8.4609	8.8315	8.6153	9.6451	8.6450	8.5992	9.7645
Ank2	_IVDEQGDM[Oxidation (M)]DFQIS[Phospho (STY)]PDR_.2	#N/A	7.1794	7.7917	7.8047	7.1143	3.7126	7.5694	7.5148	5.1826	7.3288	7.2474	5.9866	6.8979	6.7801	7.6028	6.0619	7.3780	7.5933	8.1023	7.2195	7.5114
Ank2	_SKS[Phospho (STY)]ESDASSLDAK_.2	Q8C8R3_S3343_M1_Ank2	11.3663	11.2660	11.0905	11.0639	10.8977	11.0112	11.1210	11.4969	10.9577	10.9134	11.0633	11.2528	11.7873	11.4628	10.6739	11.1486	11.5425	11.4301	11.2366	11.1323
Ank2	_STSSSGRPGTS[Phospho (STY)]PTR_.2	Q8C8R3_S3411_M1_Ank2	9.5456	9.5162	3.8868	9.4320	9.0855	9.0598	9.1489	9.4770	8.9042	9.0897	9.1929	9.2128	10.0167	9.6252	8.7698	9.3814	9.5916	9.6588	9.3098	9.2565
Ank2	_RFT[Phospho (STY)]PEEEMFK_.2	#N/A	4.1050	5.8297	6.4408	5.1419	3.4427	3.6668	4.0984	4.4603	4.1575	4.3113	3.0310	4.3114	4.2655	5.9814	3.6756	2.9490	3.9576	3.1919	3.4909	3.4866
Ank2	_SHLVNEVPVLAS[Phospho (STY)]PDLLSEVSEM[Oxidation (M)]K_.3	Q8C8R3_S1467_M1_Ank2	3.2064	5.9183	4.2438	4.3778	3.4134	4.5361	5.7002	5.7592	4.5545	3.8387	3.5036	5.3728	5.7349	6.0469	4.1482	4.0694	4.4302	5.8226	3.0183	3.9592
Ank2	_ELDFTEEQIHQIR_.3	#N/A	4.2606	3.8116	4.4573	3.5052	3.5983	3.8224	4.1671	4.3047	4.9523	5.0643	4.3763	3.6317	4.1099	3.6537	3.5200	3.1046	3.8020	4.7546	3.6465	3.1993
Ank2	_HSS[Phospho (STY)]LSSSAKPER_.2	Q8C8R3_S1793_M1_Ank2	6.2856	5.6758	6.0863	6.0394	5.0599	5.0963	5.2587	4.9330	3.8751	6.1659	5.0742	3.4288	4.8527	4.9708	3.9803	5.2819	4.1873	3.7896	3.8743	3.7801
Ank2	_KGS[Phospho (STY)]S[Phospho (STY)]EESVDEDRGLVPEPLPTAK_.3	#N/A	4.0317	8.6468	7.4630	3.7092	3.5423	8.4223	8.6904	3.8993	8.1729	3.7485	3.7783	3.5517	3.9732	9.1288	7.6612	3.3938	3.9856	8.6679	7.7751	3.0570

Ank2	_HEGLAETPETSPESLSFS[Phospho (STY)]PK_.3	Q8C8R3_S2213_M1_Ank2	4.2251	4.2362	3.5375	3.4142	3.2753	3.8979	4.1109	3.8763	4.0312	4.0420	3.5098	4.9143	4.1966	6.0608	4.1363	3.5935	4.4139	3.4195	3.0681	5.8801
Ank2	_VGT[Phospho (STY)]PTDIHSEKELPITNDITDSSQK_.3	#N/A	4.2381	5.5884	3.2735	3.4514	3.5758	4.1241	5.1600	3.9384	4.0716	4.2182	3.2863	5.2828	5.4507	6.3467	3.9198	3.0821	4.9390	5.7184	3.0057	3.2276
Ank2	_SGHDQVVELLLER_.2	#N/A	4.4120	3.9544	3.8357	2.9305	3.7679	4.1515	3.5318	4.4645	3.6078	3.5121	3.8653	3.8626	3.7571	4.6752	3.7086	3.9688	3.3495	3.9425	4.4559	3.6289
Ank2	_ESES[Phospho (STY)]DQEPEEEIGMTSEK_.2	Q8C8R3_S1428_M1_Ank2	3.5134	8.7243	9.9994	9.4517	3.9085	8.3228	8.6057	3.8562	7.5487	6.7969	5.7117	6.8270	3.8860	9.7722	3.5114	4.4116	7.9985	6.6471	6.6779	7.1536
Ank2	_TPEDIST[Phospho (STY)]PPEGTKPC[Carbamidomethyl (C)]LQTPVTSER_.3	Q8C8R3_T3745_M1_Ank2	4.2504	5.4303	3.7870	3.3338	2.9565	4.8342	4.0916	4.4761	4.1732	4.2955	3.0468	4.2956	5.3875	3.8250	4.1170	3.6125	3.9733	3.1761	3.9033	3.5023
Ank2	_ASSDVM[Oxidation (M)]LNGFGGDAPTLR_.2	#N/A	3.8424	3.4767	4.9334	3.7419	4.0493	4.0826	5.1898	3.9418	4.4198	3.6530	3.8985	4.2579	4.5853	3.4170	5.0603	3.2045	3.5653	3.5842	4.9416	3.0943
Ank2	_NS[Phospho (STY)]IEFFEEISDEASK_.2	Q8C8R3_S3427_M1_Ank2	3.9492	4.4108	5.4773	3.7402	3.2869	3.5110	4.2542	3.6496	4.3132	4.9120	3.1868	3.9431	4.4213	4.4726	3.8313	4.2941	4.1873	3.0361	3.2945	3.6423
Ank2	_IENPNSLQDQSHALLK_.3	#N/A	3.3602	3.1418	4.5037	4.3882	3.9887	3.7862	3.2952	3.0605	3.2154	3.3565	2.7718	4.2044	3.3809	3.8835	4.7393	3.7051	3.5982	4.6926	4.6092	4.7826
Ank2	_HSPVFSGKPEKHS[Phospho (STY)]PGS[Phospho (STY)]PSTK_.3	Q8C8R3_S1839_M2_Ank2	5.9550	4.4238	4.1153	4.1104	5.7873	3.5674	3.1236	6.3988	3.4342	5.9409	6.6251	4.3133	3.5997	5.3747	4.2016	5.7968	6.6150	5.9752	5.7940	4.0693
Ank2	_KRFT[Phospho (STY)]PEEEMFK_.3	#N/A	4.2424	3.7604	3.2779	4.8420	3.5802	3.9327	3.7266	4.3229	4.0200	4.4488	4.3944	3.6498	4.1281	5.6445	3.5381	3.0865	3.8201	3.3293	3.6284	3.3491
Ank2	_KS[Phospho (STY)]EEQIGEAK_.2	Q8C8R3_S2217_M1_Ank2	3.2664	3.0441	4.0589	4.2200	3.3987	2.8154	2.7954	3.1568	3.2922	3.7290	3.5219	3.7218	4.2001	3.6302	3.7263	3.4609	3.9315	4.1104	2.9450	4.1222
Ank2	_TEGDSPAAALS[Phospho (STY)]PQMHQEPVQQDFSGK_.3	#N/A	5.9776	6.2830	6.1488	6.4845	3.4612	5.3311	6.7136	5.6522	5.0446	5.8299	5.8338	5.9557	6.3733	7.7624	3.9423	4.9519	5.9051	5.5434	5.3235	4.4590
Ank2	_SAALLLQNDHNADVQSK_.3	#N/A	3.5437	3.6527	3.9722	3.8719	3.7788	4.0895	3.7869	3.7633	3.3391	3.7544	3.5964	3.6731	3.8725	4.5992	3.6062	3.9985	3.1233	3.7396	3.8085	3.6076
Ank2	_NEKLS[Phospho (STY)]PVS[Phospho (STY)]PSAKTER_.2	#N/A	4.0497	4.3955	4.0103	3.9850	4.1922	3.9862	3.9874	3.1863	3.9189	3.9167	3.4522	4.2529	5.0489	4.0226	4.1115	3.8272	3.9701	3.6422	3.5296	3.8522
Ank2	_DPDGS[Phospho (STY)]AEDDSLEQTSLMESSGK_.2	#N/A	3.9605	4.0423	4.5145	3.7289	3.2982	3.5223	4.2428	3.6609	4.3019	4.1668	3.1755	3.9317	4.4100	4.4839	3.8200	4.3054	4.1986	3.6346	3.2832	3.6310
Ank2	_TAEGTEPKPQGAIRS[Phospho (STY)]PQGLELPLPNR_.4	#N/A	3.2702	8.0167	4.3076	4.3140	3.4771	3.5105	7.5737	8.5128	3.4912	4.5816	2.9698	7.7663	7.6800	8.8626	3.5964	4.1331	3.8740	7.6752	3.6078	4.1263
Ank2	_EIASPSS[Phospho (STY)]PVK_.2	Q8C8R3_S2827_M1_Ank2	4.3068	3.6960	3.3422	3.3826	3.6445	3.8686	3.8966	4.2585	6.1136	4.5131	4.3300	3.5855	4.0637	3.6075	3.4738	3.1508	3.7558	3.3937	3.6927	3.2848
Ank2	_LLRDPDGS[Phospho (STY)]AEDDSLEQTSLMESSGK_.3	#N/A	3.8500	5.6170	4.1003	3.6243	3.8114	3.8898	4.0291	3.8086	3.8704	3.8100	3.7641	3.7857	4.1481	6.3869	3.4383	4.0784	3.7804	4.0300	3.6187	3.6475
Ank2	_HTPVSPSS[Phospho (STY)]KNEKLS[Phospho (STY)]PVS[Phospho (STY)]PSAK_.3	Q8C8R3_S1810_M3_Ank2	3.4602	4.2356	4.2645	4.2119	4.0225	3.6689	3.3149	3.1779	3.3328	3.4855	4.3122	4.0633	3.4982	5.1738	3.1479	4.0829	3.7155	4.1176	3.7662	3.9679
Ank2	_S[Phospho (STY)]PQGLELPLPNR_.2	#N/A	3.9593	11.9580	4.4956	3.7301	3.2970	3.1871	11.7198	3.6597	3.8146	4.2582	3.2932	3.9329	3.9800	12.3230	3.9198	3.8097	4.1974	11.6345	3.2844	4.4497
Ank2	_VVLKSDT[Phospho (STY)]QQS[Phospho (STY)]EDNNE_.2	Q8C8R3_S3893_M2_Ank2	4.1724	4.5834	4.2049	5.6809	4.1544	3.3072	4.6698	3.1564	4.1371	6.0507	2.9662	4.0718	4.2690	4.0062	3.3246	4.0206	4.1544	3.3199	4.0281	3.3894
Ank2	_FQVSAATESR_.2	#N/A	3.6058	4.3970	4.1421	4.0836	2.9435	5.6490	5.3948	3.3062	3.1558	3.8121	3.5302	4.2865	3.2639	4.1291	4.1748	3.9507	5.7840	5.0945	3.6379	4.0961
Ank2	_TPTEEGT[Phospho (STY)]PTSEQNPFLFQEGK_.3	Q8C8R3_T3050_M1_Ank2	3.5282	5.2320	4.5656	4.0560	3.7352	4.5312	3.3129	3.7539	3.2332	3.3388	4.2126	3.5170	3.3986	3.9013	3.0578	4.3912	3.2511	4.2172	3.8658	3.8682
Ank2	_ELGDDS[Phospho (STY)]LPSSQFLDGM[Oxidation (M)]NYLR_.2	Q8C8R3_S874_M1_Ank2	3.9586	3.7321	4.0997	3.7262	3.7095	4.0404	3.6451	3.8931	3.8236	4.0189	3.5162	3.5452	3.9834	3.2285	3.4035	3.0332	4.0114	3.5954	3.6500	3.9195
Ank2	_HPPVS[Phospho (STY)]PGK_.2	#N/A	3.4555	3.8636	4.4929	4.1288	3.6624	3.6957	3.3857	3.6811	3.3059	3.2660	4.2854	3.3456	3.4714	4.5309	3.4111	4.3184	3.1784	3.9711	3.6100	3.9410
Ank2	_DSEVLSPMADESLAVSHKDS[Phospho (STY)]LEAS[Phospho (STY)]PVLEDNSSHK_.4	Q8C8R3_S2395_M2_Ank2	4.2463	3.8421	3.1961	3.5287	2.9524	2.9857	4.0957	3.8610	4.0159	4.0569	3.4946	3.7316	4.1814	3.1832	4.1211	3.6084	4.3987	3.4344	3.0830	3.1502
Ank2	_PQGAIRS[Phospho (STY)]PQGLELPLPNR_.3	#N/A	4.4175	3.3969	3.4587	3.6621	3.7552	3.9793	3.7859	4.1478	3.8450	3.1230	4.2193	3.1231	3.9530	1.4981	3.3631	3.2615	3.6451	3.5044	3.8034	3.1741
Elfn1	_AS[Phospho (STY)]PSIWER_.2	Q8C8T7_S773_M1_Elfn1	9.4699	9.0928	8.9953	9.4094	8.5383	8.8460	8.9792	8.9049	8.8275	8.6876	8.9469	8.9221	9.1263	8.6899	7.5506	8.6763	8.9582	9.0715	8.5590	3.5877
Elfn1	_AVAAAAGS[Phospho (STY)]LKK_.2	Q8C8T7_S460_M1_Elfn1	8.3996	8.0662	8.3361	8.2821	7.6791	7.7030	7.8751	7.3975	7.4616	8.0473	8.0007	7.6062	7.8711	7.3763	6.4339	7.4472	7.9125	7.9799	7.3811	7.6679
Ppfibp1	_SSS[Phospho (STY)]LGNLKK_.2	Q8C8U0_S435_M1_Ppfibp1	3.3637	8.1863	7.8849	8.3472	7.9461	8.0213	7.7749	8.4066	8.1345	8.2229	7.5686	3.6815	3.5631	8.1784	7.0345	7.9299	7.9431	8.2136	3.7013	7.9312
Tmem163	_ISES[Phospho (STY)]GQFSDGLEDR_.2	Q8C996_S56_M1_Tmem163	3.9804	5.5195	7.1693	7.2023	3.6131	7.1295	6.0469	3.6308	6.9006	6.4459	5.6863	6.4373	3.7900	7.2489	5.3725	6.3821	6.8676	7.2331	6.6338	6.6186
Tmem163	_GHAPSTAAPAPSPAPM[Oxidation (M)]SSS[Phospho (STY)]VQSDEER_.3	#N/A	3.7013	5.3025	4.0448	4.5553	3.9082	3.8152	3.9948	5.6510	3.6289	4.9178	4.2835	3.3919	3.7608	5.2365	5.6728	3.8704	5.5504	5.5468	4.6482	3.6414
Tmem163	_GHAPSTAAPAPSPAPM[Oxidation (M)]SSSVQS[Phospho (STY)]DEERQPR_.4	#N/A	6.6175	4.2146	6.0998	3.8844	3.1452	5.8554	3.9092	3.4228	3.8294	4.2497	7.0808	6.8488	3.9948	4.2613	5.7133	3.8012	4.2433	3.4641	6.9601	3.6024
Tmem163	_S[Phospho (STY)]PPGPGVPRPPPR_.3	Q8C996_S11_M1_Tmem163	3.9492	4.6726	4.5143	4.1073	3.6838	2.4535	6.1878	5.3243	3.2845	3.2875	4.2640	5.0727	3.5078	6.2039	3.3896	4.3398	4.7547	4.8565	4.2487	4.2297
Dido1	_GLS[Phospho (STY)]PSYLGGPR_.2	Q8C9B9_S1840_M1_Dido1	4.3267	4.4932	6.0319	3.2575	3.0328	3.5747	4.1904	3.0515	4.2495	4.2193	3.1230	4.2194	4.3576	3.9013	4.0407	6.3657	4.0496	3.0998	3.3989	5.5103
Strip2	_AAS[Phospho (STY)]PPSYTLDLGESQLAPPPSK_.2	#N/A	9.3035	9.4879	4.2846	8.2345	7.8031	8.4871	9.0343	6.8381	8.2671	8.6540	8.7899	3.6497	4.2593	9.4680	3.9661	4.0969	9.2258	9.4665	8.9730	8.0600
Strip2	_QDS[Phospho (STY)]LDIYNER_.2	#N/A	9.5830	10.0689	9.8788	9.7895	3.0314	9.5395	9.5766	8.8655	9.2248	9.6362	9.4222	9.1567	9.5365	9.3741	7.8531	9.3614	9.6795	9.4568	9.2044	9.3579
Strip2	_QLLTKQDS[Phospho (STY)]LDIYNER_.3	#N/A	3.7060	3.3018	3.7364	4.4893	3.9129	3.9462	3.1352	4.4013	3.0554	3.5166	4.0349	3.3392	3.2208	3.7235	3.1606	3.0681	3.4382	4.3950	4.0436	3.6905
Shisa4	_DLTLLITER_.2	#N/A	3.2636	5.2929	4.6126	5.0909	4.5311	4.8985	4.2557	3.8009	5.2290	3.4699	4.4173	4.7572	5.0320	5.3937	4.1271	4.5551	4.5596	4.9023	5.0255	4.8860
Pde10a	_ST[Phospho (STY)]PEKLNVK_.2	Q8CA95_T780_M1_Pde10a	5.1323	3.2500	5.1601	5.8694	3.8611	3.8944	3.1870	4.4531	3.1072	5.3444	6.5860	5.4291	3.2726	5.4925	3.2124	5.1355	5.8576	6.4242	3.9918	5.9617
Pde10a	_AYLSLHPQVLDEFVSESVSAETVEK_.3	#N/A	3.1619	3.3400	5.0525	4.1339	4.0005	3.1631	3.0970	4.7776	3.0172	3.6735	4.2339	3.0320	5.3685	5.4254	3.1224	4.6771	3.4000	4.3589	3.9828	3.6523
Pde10a	_EVDLYTGYTTR_.2	#N/A	4.4517	4.3683	3.9883	3.1326	3.1578	3.1911	3.8903	3.1765	5.4993	4.2623	3.2892	4.0944	3.9760	4.0263	3.9157	3.8138	5.6161	5.9360	3.2739	3.7036
Pgm2l1	_IVLATDPDADRLAVAELQENGR_.3	#N/A	5.4976	5.9037	4.8696	5.4997	4.9243	5.1213	5.1990	5.4663	3.3049	5.6211	4.9283	5.0568	5.1649	5.6949	4.9559	4.6308	5.4961	5.5530	4.4627	4.1926
Pgm2l1	_LIDALIENFLEPSK_.2	#N/A	4.0091	4.3222	5.3566	4.1355	4.6597	4.3751	4.4148	4.8789	5.0761	4.8008	4.6369	4.7862	4.2970	4.6104	4.5973	5.3910	5.5816	4.5948	5.1800	4.4694
Pgm2l1	_DITTGYDSSQPNKK_.2	#N/A	3.2919	3.2901	3.5655	3.5553	4.2359	3.8725	4.3131	4.4964	4.7991	3.8395	4.5963	5.1984	4.0598	3.6036	4.3111	4.3234	4.3373	4.2252	3.5487	3.2809
Pgm2l1	_AVAGVMITAS[Phospho (STY)]HNR_.2	Q8CAA7_S175_M1_Pgm2l1	3.3483	3.1537	3.8846	4.3411	4.1869	4.4109	3.3542	3.0487	3.4133	3.5546	3.7877	3.0431	3.5214	6.6917	2.9314	3.6932	3.5864	4.2468	3.8954	3.8387
Podxl2	_RDPEDS[Phospho (STY)]DVFEEDTHL_.2	#N/A	3.8036	3.9861	3.7924	4.0353	3.4720	3.9602	3.8424	3.6890	3.9002	4.2124	3.3789	3.7833	4.0657	3.9135	3.6350	3.6119	4.4262	4.6715	3.2385	3.8535
Nhsl1	_SLS[Phospho (STY)]FSGPR_.2	Q8CAF4_S1482_M1_Nhsl1	3.5052	3.8138	5.2227	4.0790	3.7122	3.7455	4.7739	3.7309	3.2561	5.9476	4.2356	3.5400	3.4216	4.5807	3.3613	4.3682	3.2282	4.8402	5.4707	5.0768
Nhsl1	_KNS[Phospho (STY)]GAGAVLFSEPSAQEQR_.3	#N/A	5.3226	5.5550	5.5872	5.7197	3.7869	5.3080	5.6411	5.1202	4.9932	6.1483	5.2635	4.5325	5.1036	4.2994	4.1272	5.5304	4.0140	5.4747	3.5348	4.7970
Nhsl1	_NHS[Phospho (STY)]PS[Phospho (STY)]PPVTPTSAAPNLASPK_.3	Q8CAF4_S1373_M2_Nhsl1	3.8592	4.1436	4.3955	3.8302	3.1969	4.3087	4.3442	3.5596	4.4033	4.0655	3.2768	4.0331	4.5113	4.3826	3.9214	4.2041	4.0972	3.6883	3.3845	4.5285
Nhsl1	_SEPSADSPDSPSSC[Carbamidomethyl (C)]S[Phospho (STY)]PNK_.2	Q8CAF4_S1459_M1_Nhsl1	3.1389	3.3630	3.6752	4.5505	3.9775	4.2016	3.5636	3.9255	3.6227	7.7315	3.9971	3.2525	3.7307	3.2745	3.1408	3.4838	3.4228	3.7267	4.0257	2.9518
Shfl	_FHGKVS[Phospho (STY)]PK_.2	#N/A	9.2913	9.4926	9.0903	9.3693	9.4398	9.4252	9.1899	9.6625	9.1480	9.3452	9.3236	9.7953	10.0233	10.2202	9.9921	9.7375	9.7509	9.6373	9.6031	9.8297
Shfl	_EKFHGKVS[Phospho (STY)]PK_.2	#N/A	3.4684	5.1759	4.5392	4.4879	5.0728	5.4663	5.2004	5.5897	3.3912	5.5785	5.5977	4.8781	4.2107	5.9565	5.4781	5.4876	4.4279	5.2994	5.0528	4.9027
Immt	_SLEDALNR_.2	#N/A	8.0787	8.3652	8.2377	8.5565	8.5310	8.4829	8.5006	8.3431	8.3154	8.2135	8.3571	8.1598	8.8188	8.4646	8.1361	8.2981	7.9951	8.6567	8.5103	8.5973
Immt	_SEIQAEQDR_.2	#N/A	7.8489	7.6110	7.4665	7.9426	7.6013	7.7215	7.9558	7.7450	7.9141	7.7158	7.5347	7.6587	7.6105	7.5357	7.8268	7.5529	7.7433	7.6799	7.6708	7.4525
Immt	_ELDSITPDITPGWK_.2	#N/A	7.6791	7.2553	7.9802	7.4442	7.4973	7.3826	7.5805	7.4765	7.5569	7.7627	7.2636	7.4752	7.2793	7.5559	7.6292	7.6730	7.8460	7.3976	7.5256	7.0605
Immt	_LSEQELEFR_.2	#N/A	7.4230	7.0743	7.5206	7.5371	7.3657	7.0972	7.1372	7.4264	7.4288	7.4938	7.4460	7.5501	7.2373	7.4129	6.8194	7.4287	7.1684	7.0876	7.1887	7.2860
Immt	_AVDEAADALLK_.2	#N/A	6.5399	6.5469	6.7330	6.3510	6.3817	6.7828	6.8040	6.1511	6.5367	6.5206	6.3643	6.6662	6.5700	6.1322	6.7993	6.4318	6.3397	6.9320	6.5495	6.5726
Immt	_GVYSEETLR_.2	#N/A	5.4081	6.3393	6.5979	6.7596	5.8138	6.7010	7.3737	5.9024	6.7540	7.1084	6.3250	6.7497	6.0202	6.6467	6.6421	6.8181	5.0841	6.2212	6.6244	6.8889
Immt	_TSSAEM[Oxidation (M)]PTIPLGSAVEAIR_.2	#N/A	6.6209	6.3973	6.4860	6.4706	6.4349	6.6489	6.3185	6.3038	6.5069	6.5991	6.2407	6.3859	6.5231	6.5899	6.2495	6.4948	6.3728	6.3955	6.3812	6.1537
Immt	_EIAGATPHITAAEGR_.3	#N/A	5.2538	6.5520	6.3760	5.8240	5.7834	6.1562	6.1225	5.3796	6.1571	6.2694	6.3436	5.7790	5.8115	6.0465	6.4009	6.4303	5.5903	5.8616	6.0680	6.0514
Immt	_TSSVTLQTITAQNAAVQAVK_.2	#N/A	5.7527	5.1439	5.3611	4.9117	5.5657	4.2454	4.8157	5.5260	5.2566	5.3528	5.6809	5.3077	5.7529	4.4157	4.4377	4.7668	5.6757	4.8102	4.4134	4.0930
Immt	_QAAAHTDHLR_.2	#N/A	5.0033	4.9473	5.9950	4.9991	4.1727	5.4231	5.3120	5.0758	4.6271	5.0314	5.7387	4.6324	3.6050	5.2816	5.2190	4.9862	5.2610	4.3543	5.0976	5.3017
Immt	_STSETTEEAFSSSVR_.2	#N/A	5.6974	6.6537	5.9497	6.2416	3.7306	6.3183	5.5822	5.5576	3.2378	5.9962	4.2172	6.5183	6.4819	7.2337	5.8849	6.1129	6.6395	6.3938	6.0560	6.3344
Immt	_QHIELALEK_.2	#N/A	4.7129	3.7672	6.2224	5.5616	5.3838	4.8495	5.1024	5.9514	5.1504	5.1102	4.1889	4.8458	4.5826	5.5974	5.2300	5.1929	4.9999	5.2237	4.9523	5.4816
Immt	_TFDSAVAK_.2	#N/A	3.7024	4.5984	5.0582	4.7655	4.7842	4.7667	5.5590	3.9281	5.3515	4.9637	5.0438	4.5226	3.7333	4.9489	5.3931	5.1172	4.0961	4.1061	4.0232	5.0872
Immt	_VVSQYHELVVQAR_.2	#N/A	4.5437	4.8709	5.3771	4.3626	3.8244	4.5694	4.6517	5.0668	4.0124	4.5187	4.4001	4.4426	5.0724	5.0609	4.6895	4.1924	4.9513	3.9170	4.5487	3.9375
Immt	_GIEQAVQSHAVAEEEAR_.3	#N/A	5.8950	5.4609	4.9245	5.2949	4.7982	5.2766	5.7400	5.2666	4.7060	5.8228	5.9141	5.3465	6.4166	5.0602	5.2688	5.3888	5.2878	5.9329	5.3312	5.1282
Immt	_RIDQLNR_.2	#N/A	4.9789	5.5560	3.2937	4.7334	3.9640	3.9973	4.6431	3.4680	5.5311	4.3983	5.1656	3.2881	3.1697	3.6724	2.7538	3.1192	3.8982	3.6971	4.4206	4.3458
Immt	_LSTDDLNSLIAHAHR_.3	#N/A	6.4704	6.2620	5.7416	6.0983	6.0173	5.8052	6.1336	6.4750	5.5768	6.0456	5.9324	6.0373	6.1723	6.3171	5.8736	5.6888	6.0768	6.1213	6.0139	5.7598
Immt	_ISSVSEVMK_.2	#N/A	3.3593	3.1427	6.1351	5.9683	4.1978	4.4219	3.8304	3.7052	3.4023	3.5656	4.8138	3.0321	3.5104	5.4194	4.3885	3.7042	3.2024	3.9470	4.2461	4.2323
Immt	_SQEQM[Oxidation (M)]DSFTLDINTAYAR_.2	#N/A	4.9140	3.9466	4.5925	3.6331	3.3940	3.0901	3.6748	5.3123	4.1232	5.5707	3.3902	3.8360	4.0770	4.5796	4.0167	4.4011	4.2943	3.6140	3.1874	3.6579
Immt	_YSTSSSSGLTAGK_.2	#N/A	3.9330	4.0698	4.4693	3.7564	3.2707	3.2134	4.2704	3.6333	3.7882	4.2846	3.2669	3.9593	3.9537	4.4563	2.8739	4.2779	4.1710	3.6621	3.3982	4.4233
Immt	_VQEQELKYEFEQGLSEK_.3	#N/A	3.6237	3.6953	3.1603	3.9605	3.8307	3.8640	3.2174	3.8494	3.1376	3.4343	4.1171	3.5755	3.3031	3.1984	3.2428	2.9859	3.3466	4.3127	4.1018	3.7727
Acat2	_VAVLSQNR_.2	#N/A	5.3651	5.7019	5.0953	5.4830	5.0116	6.1452	6.3204	5.6465	5.6464	6.0283	5.1832	5.6903	4.3659	5.2631	6.1756	5.2977	5.1704	5.7117	5.7661	6.1254
Acat2	rbamidomethyl (C)]LAAQSIAM[Oxidation (M)]GDSTIVVAGGM[Oxidation (M)]ENM[Oxidation (	#N/A	4.4452	2.1091	3.3481	3.3933	2.9279	4.1619	4.1202	4.4591	2.5934	4.3584	4.4012	3.7260	4.7422	3.2076	3.7479	4.1514	3.9447	4.5050	3.1763	3.9856
Acat2	_VNIDGGAIALGHPLGASGC[Carbamidomethyl (C)]R_.3	#N/A	5.0037	4.7725	4.9631	4.2007	2.8774	4.0773	5.4623	4.1081	4.5018	3.9819	3.5696	4.6875	4.2564	5.2674	4.2347	3.5334	4.4737	4.8617	3.0080	4.1595
Eloa	_SYS[Phospho (STY)]PEHR_.2	Q8CB77_S125_M1_Eloa	8.0732	3.6520	7.7203	7.8747	7.8130	8.1329	8.2812	4.0512	8.1270	8.3099	4.2860	7.7534	8.3078	3.5635	8.2866	8.0948	8.3077	3.4377	7.9754	8.0550
Eloa	_S[Phospho (STY)]PEEDQEPIISHQKPGK_.3	Q8CB77_S195_M1_Eloa	5.2475	5.6277	6.2342	5.5685	4.6419	6.0345	5.6746	3.2436	5.6727	5.8187	5.7539	5.7495	5.4711	6.7475	5.3336	5.3920	6.0956	5.5705	5.4563	5.7613
Wdr37	_SHS[Phospho (STY)]LSIR_.2	#N/A	8.1148	8.0852	7.4357	8.1503	7.8995	7.3996	7.6104	7.3253	3.7047	9.0560	7.8356	7.6416	7.8327	8.1148	6.3666	7.6351	7.3937	7.8904	7.5064	7.6973
Wdr37	_STLLELFGQIER_.2	#N/A	5.2845	5.3825	4.7728	5.1548	4.9153	4.8127	4.8566	4.5789	5.1629	5.2204	4.9422	3.9947	3.9064	4.2048	4.8279	4.7177	4.9008	4.9792	4.6913	5.0173
Wdr37	_ASHSTSQLS[Phospho (STY)]QK_.2	Q8CBE3_S119_M1_Wdr37	7.6227	7.5034	7.8085	8.0033	6.7563	6.7508	6.7065	6.8757	6.1925	7.9547	7.0234	6.6032	6.9006	7.2445	6.0056	7.1556	6.9437	6.7527	6.8525	6.6271
Wdr37	_KSHS[Phospho (STY)]LSIR_.2	#N/A	3.4457	4.5571	6.7988	7.2698	6.3665	3.7007	6.6663	3.1461	3.3009	7.2827	6.6650	6.5476	3.4664	3.9690	2.8340	6.6467	6.5551	6.1661	6.7074	6.7273
Wdr37	_DPSIHSVNVFQGHTDTVTSAVFTVGDNVVSGSDDR_.4	#N/A	3.6867	3.2825	3.7557	4.4700	3.8936	3.9270	3.1545	4.4206	3.0747	3.4973	4.0542	3.1720	3.2401	3.7428	3.1799	3.0488	3.4575	4.3757	4.0243	3.7098
Ephb1	_YLQDDTS[Phospho (STY)]DPTYTSSLGGK_.2	Q8CBF3_S774_M1_Ephb1	4.1515	4.0314	5.7875	3.4641	3.4471	5.8802	5.3941	3.7088	4.0743	3.7574	3.7803	5.4051	4.1824	5.6894	3.7276	4.8700	5.5908	5.4768	3.6055	5.2806
Mfsd6	_VAILT[Phospho (STY)]DDEEEQKRK_.3	#N/A	7.7214	7.8010	7.4670	7.3917	6.2730	6.9700	7.3492	7.2785	6.3141	6.8609	7.4870	6.8187	7.2798	7.2807	5.5203	6.6572	7.4768	7.5463	6.6430	6.7001
Mfsd6	_[Acetyl (Protein N-term)]AADDKVAILT[Phospho (STY)]DDEEEQKR_.3	#N/A	4.1819	7.3500	7.4385	6.3207	3.5196	6.6034	6.5126	3.8823	6.6894	6.3429	3.5158	5.4958	4.2026	6.7793	5.8210	6.7145	4.4199	6.8002	6.9929	6.5763
Mfsd6	_[Acetyl (Protein N-term)]AADDKVAILT[Phospho (STY)]DDEEEQK_.2	#N/A	3.9521	7.0047	6.9694	3.7373	3.2899	6.6995	6.5449	3.6525	5.7114	4.2654	3.2860	3.9401	3.9728	6.2144	5.4641	4.2970	4.1902	6.6237	6.3942	5.8645
Mfsd6	_IPVPSSPVPIAT[Phospho (STY)]IDLVQQTEDVM[Oxidation (M)]PR_.3	Q8CBH5_T653_M1_Mfsd6	4.4786	3.5242	3.5141	3.2108	3.8163	4.0404	3.7247	4.1790	3.7838	3.1841	4.1582	3.4136	4.4993	3.5011	3.3019	3.3227	3.2158	3.1165	4.2659	3.4681
Mfsd6	_VAILT[Phospho (STY)]DDEEEQK_.2	#N/A	3.2028	4.1163	4.2402	4.3814	3.4097	4.9185	3.6384	3.4285	3.5586	4.5142	3.0372	3.8424	3.7240	4.2783	3.6637	4.8623	4.4265	4.7161	3.0219	4.1937
Abi1	_TNPPTQKPPS[Phospho (STY)]PPVSGR_.2	#N/A	10.5594	11.7560	8.4075	10.7794	9.3893	10.7566	11.4440	11.9574	10.3523	11.2130	10.5473	11.5341	11.8565	11.8196	10.5721	10.8313	11.2983	11.5204	11.0941	10.6673
Abi1	_NDDGWFEGVC[Carbamidomethyl (C)]NR_.2	#N/A	8.1775	7.8614	8.4230	8.5914	8.0416	8.2510	7.9232	8.2917	8.1250	8.5996	8.3696	8.2208	8.3104	8.4887	7.9719	8.0201	8.4374	7.9727	8.0527	8.3134
Abi1	_TAS[Phospho (STY)]LNQRPR_.2	Q8CBW3_S231_M1_Abi1	4.2756	7.4719	8.1195	7.6972	7.7304	7.0435	6.9982	7.6131	7.0939	7.9017	6.8588	4.2704	4.1520	6.9802	6.4028	7.2464	7.1070	7.5766	6.8776	6.9335
Abi1	_HNS[Phospho (STY)]TTSSTSSGGYR_.2	Q8CBW3_S296_M1_Abi1	5.1115	5.6587	4.9647	5.6303	5.1092	5.2105	5.3107	4.3320	4.8869	5.8030	4.9574	3.7743	4.1386	4.6773	3.6276	4.5736	4.4700	4.2331	4.9270	5.3018
Abi1	_ALIESYQNLTR_.2	#N/A	6.3719	6.2699	6.5280	5.8599	6.1914	6.1634	5.5433	3.7462	6.2226	5.7548	5.9891	6.0734	5.8107	6.6564	6.2816	6.2617	3.2435	5.8106	6.2292	5.8022
Abi1	_LGSQHS[Phospho (STY)]PGR_.2	Q8CBW3_S225_M1_Abi1	8.7444	8.7870	8.7980	8.7974	8.5067	8.3398	8.2535	8.3981	8.0561	9.1283	8.2028	8.3611	8.4087	8.5792	7.3294	8.0926	8.4777	8.1510	7.9848	8.4357
Abi1	_KNDDGWFEGVC[Carbamidomethyl (C)]NR_.3	#N/A	3.6895	5.7078	4.2258	5.5870	4.7620	5.4303	3.6246	4.7498	3.5448	5.2885	3.0234	5.4220	3.7102	5.3336	5.1547	5.4974	4.8625	5.4793	5.2945	5.1022
Abi1	_[Acetyl (Protein N-term)]AELQM[Oxidation (M)]LLEEEIPSGKR_.2	#N/A	3.4212	4.3647	3.1817	3.6710	4.7239	3.8134	4.2003	3.8708	4.6658	4.9142	4.0957	3.9574	3.2817	3.2198	5.3575	4.9926	3.3681	3.7814	4.8038	3.5123
Abi1	_DKDDELSFK_.2	#N/A	3.1231	4.1960	4.1605	4.4612	3.3300	4.9602	3.7181	3.3487	5.3660	4.4345	4.9470	4.0655	3.8037	4.7865	4.4220	4.2523	4.3468	4.3529	3.1017	4.6075
Chic1	_VVS[Phospho (STY)]EEHLR_.2	Q8CBW7_S80_M1_Chic1	5.0436	5.5757	5.3285	4.7625	5.1115	4.9211	5.5237	5.2524	5.2121	4.0061	5.3493	4.8183	5.5051	5.0799	3.9870	4.1715	5.3815	5.6802	4.4969	4.7253
Tmem63c	_S[Phospho (STY)]SPSEVSLEAER_.2	Q8CBX0_S88_M1_Tmem63c	4.0576	3.2615	3.5942	3.5266	4.2646	3.8438	4.2844	4.2833	3.9805	3.8682	4.3548	2.9876	4.0885	3.6323	2.8089	3.4197	3.7805	3.3689	3.6679	3.3096
Tmem63c	_LALLIHNDS[Phospho (STY)]LTSLIYGEQSEK_.3	Q8CBX0_S75_M1_Tmem63c	3.3505	3.9686	4.3879	4.2338	3.5574	3.0501	3.4907	3.5761	3.2733	3.1611	3.6477	3.6947	3.3813	4.4259	3.5161	4.2134	3.0734	4.0761	4.3751	4.1032
Samd4a	_AYSS[Phospho (STY)]PSTTPEVR_.2	Q8CBY1_S420_M1_Samd4a	3.9823	4.0205	4.5186	7.5095	3.3200	3.5441	7.2658	3.0822	4.2802	8.0493	7.4951	3.9100	4.3882	3.9320	3.7983	4.3272	4.0803	7.5254	7.3530	3.6093
Dctn4	_LLQPDLQPVSASQLYPR_.2	#N/A	5.2464	5.2944	5.1421	4.5581	4.9393	4.4127	4.9397	4.6396	4.8999	5.2703	4.8927	5.1083	4.5361	5.3613	5.3439	5.3034	5.5211	4.6141	4.9096	5.2984
Dctn4	_IQLVAVNYIPEVR_.2	#N/A	3.7620	4.5912	4.3414	3.8342	4.8200	3.3239	4.9635	3.3024	4.3242	4.7720	4.6974	5.2840	3.9140	4.7156	4.0577	4.4269	4.6463	4.3498	5.1764	3.8295
Dctn4	_LIEYYQQLAQK_.2	#N/A	4.1837	4.2974	4.3415	2.8944	4.4099	3.2097	2.9394	3.5298	3.4573	3.1147	3.0071	3.5707	3.6227	4.2268	3.5624	4.1670	4.5278	3.9150	3.1940	3.3859
Cacnb2	_SGGNSSS[Phospho (STY)]SLGDIVPSSR_.2	Q8CC27_S202_M1_Cacnb2	3.5895	4.1237	3.6955	4.0514	3.4398	7.9060	7.7669	3.5846	7.9803	3.6052	3.7726	7.8672	3.5063	8.4667	3.8207	7.6932	8.2651	3.9279	3.9089	4.2212
Cacnb2	_SAS[Phospho (STY)]QAEEEPC[Carbamidomethyl (C)]LEPVKK_.2	#N/A	7.6745	7.4312	6.8639	7.5065	4.1118	6.6201	4.4372	7.1582	4.3574	3.7154	6.5503	3.3902	4.5228	3.5246	2.9617	3.2670	3.2393	7.3543	4.2424	3.4916
Cacnb2	_SAS[Phospho (STY)]QAEEEPC[Carbamidomethyl (C)]LEPVK_.2	#N/A	4.3476	3.9435	3.8842	3.6301	3.0537	3.0871	5.1594	3.7597	3.9146	5.2783	3.3932	4.1984	4.0800	4.5827	4.0198	3.7097	4.2974	3.5358	3.1844	3.0488
Synpo	_VASLS[Phospho (STY)]PAR_.2	Q8CC35_S765_M1_Synpo	10.6780	10.9029	10.6553	11.2339	10.3213	10.3060	10.4893	10.1375	10.3651	11.2365	10.9356	10.8727	11.2789	11.2682	10.1255	11.0258	10.6747	10.8540	3.1162	11.2429
Synpo	_VRSPPSY[Phospho (STY)]STLYPSSDPKPSHLK_.4	Q8CC35_Y571_M1_Synpo	8.6720	8.3817	8.7391	7.1415	6.2950	6.5098	9.2219	10.2866	6.5443	7.8199	8.2604	8.4311	8.7953	9.8863	7.4964	8.9120	3.6752	8.1960	8.4092	8.3940
Synpo	_VTPNPDLLDLVQTADEK_.2	#N/A	7.6144	7.5483	7.6433	7.9954	7.4171	7.3765	7.6486	7.7789	7.3310	7.8600	8.0103	7.9779	8.1740	8.1615	8.4299	8.1408	7.9125	7.8154	7.9957	8.0234
Synpo	_ASPAAAEEAVPEWASC[Carbamidomethyl (C)]LK_.2	#N/A	6.5746	6.6690	6.8791	7.0712	6.6840	6.6150	6.3591	6.6874	6.6570	6.8935	7.3847	6.9213	6.9102	7.5487	7.2488	7.1620	7.2404	6.7077	6.8344	7.1682
Synpo	_YTTNAPGGFR_.2	#N/A	6.9695	6.5491	6.3768	6.9268	6.8697	6.9054	6.9546	6.5750	6.7844	7.2453	7.0260	7.2685	7.0113	7.5418	7.8237	7.2366	6.8410	7.1468	7.2828	7.3753
Synpo	_HPSPQS[Phospho (STY)]PSR_.2	Q8CC35_S230_M1_Synpo	5.7173	5.9054	6.5031	3.2814	3.2243	5.7233	4.0832	5.5831	4.0776	4.2008	3.2575	4.2433	6.1587	3.8777	3.8920	4.0884	4.0260	3.1237	5.6675	6.0115
Synpo	_VAS[Phospho (STY)]EEEVPLVVYLK_.2	#N/A	12.1652	12.0828	12.3401	12.1462	11.4225	11.7448	11.9923	11.5454	11.9851	12.5003	12.2372	12.1263	12.6079	12.7377	12.0646	12.3858	12.5174	12.1005	12.3080	12.2748
Synpo	_SSPGLYTAPVQDSLQPTAVS[Phospho (STY)]PTYSSDISPVSPSR_.3	Q8CC35_S882_M1_Synpo	4.4169	6.4854	4.1634	3.8608	3.6702	3.7482	5.8634	6.7320	4.2818	3.6730	4.0223	4.6863	6.5483	8.4293	4.0570	4.4743	4.5958	5.5801	6.0854	4.1362
Synpo	_LLGQRS[Phospho (STY)]PVLER_.2	#N/A	7.1182	7.9902	6.6611	6.8056	6.0522	6.1613	7.1410	7.5420	6.2270	7.0984	6.6901	7.4507	7.7581	8.4409	6.2162	7.0723	7.3615	7.3532	6.7488	6.7974
Synpo	_PSLYALS[Phospho (STY)]PVKEPAK_.2	#N/A	6.6943	6.3411	6.8806	6.3799	3.1940	5.2796	4.0541	4.1583	4.9299	6.6893	6.0135	6.0853	4.1398	6.6766	4.0795	6.0542	6.0295	5.8500	5.8477	5.7845
Synpo	_ASPAAAEEAVPEWASC[Carbamidomethyl (C)]LKS[Phospho (STY)]PR_.2	Q8CC35_S689_M1_Synpo	4.8894	5.9050	3.6485	3.4723	3.5654	3.7895	3.9757	4.3376	4.0347	4.4340	2.9083	2.9333	4.1428	6.5519	3.5529	3.0718	3.8348	4.6546	3.8383	3.3639
Synpo	_C[Carbamidomethyl (C)]PS[Phospho (STY)]PTM[Oxidation (M)]SLPSSWK_.2	#N/A	9.1292	9.1260	9.2962	8.0456	7.1779	7.6489	8.7680	9.9653	7.6790	8.6115	8.0187	9.3172	9.6105	10.9816	7.8752	8.3785	9.1802	9.2278	8.6938	8.1606
Synpo	_AAS[Phospho (STY)]PAKPSSLDLVPNLPR_.3	#N/A	3.8924	4.2738	4.1691	3.7989	3.3386	3.6754	8.4362	4.1181	3.7978	3.8776	3.7989	4.0000	3.9233	10.4705	3.8213	3.9946	3.6153	8.2393	3.9402	3.8845
Synpo	_PKPNQNLSEASGK_.3	#N/A	3.3795	3.1224	3.9158	4.3098	4.2181	3.7668	3.3146	3.0799	3.2348	3.3371	3.4180	3.0119	4.0364	3.7366	3.3400	3.7244	3.6176	4.2156	3.9761	3.3648
Synpo	_ENAALLTANGLHLSQSR_.2	#N/A	3.2679	4.3702	4.1689	4.0568	2.6386	3.5137	1.2133	3.3330	3.4879	2.9952	2.4529	4.2596	3.6533	4.1560	1.8373	3.9775	3.8707	3.9625	2.9875	4.1230
Synpo	_DRAS[Phospho (STY)]PAAAEEAVPEWASC[Carbamidomethyl (C)]LK_.3	#N/A	4.2542	4.0448	4.4949	3.7314	3.2571	3.3936	4.0499	3.6590	4.0496	4.2427	3.2061	3.9343	3.9793	4.4820	3.8413	4.1078	4.1967	9.2667	3.2857	3.8711
Synpo	_AWAPPASSMADRS[Phospho (STY)]PQPQR_.3	Q8CC35_S512_M1_Synpo	3.6163	3.7028	3.1528	6.8816	3.8232	3.8565	5.1065	3.8419	3.1451	3.4268	4.1246	3.4290	3.3106	7.2361	3.2503	2.9784	3.3392	3.8103	4.1093	4.3690
Rybp	_S[Phospho (STY)]ST[Phospho (STY)]PKGDMSAVNDESF_.2	Q8CCI5_T215_M2_Rybp	4.1976	4.2427	4.2460	3.4799	3.2070	3.3455	4.0984	3.4199	4.0967	4.1902	3.2595	4.0419	4.4157	4.9460	3.8945	4.0614	4.1627	3.0418	3.3408	3.6368
Amer2	_TVPLVDSEGGS[Phospho (STY)]GR_.2	Q8CCJ4_S340_M1_Amer2	4.0190	10.6404	10.9077	10.9580	4.2260	10.2641	10.0597	10.6132	3.9418	10.8394	10.5270	10.3860	11.0214	3.5937	10.1140	3.3811	10.8483	10.1659	10.4377	10.5224
Amer2	_IC[Carbamidomethyl (C)]LEAGS[Phospho (STY)]PTGSGDQSSR_.2	Q8CCJ4_S280_M1_Amer2	7.8197	7.9999	8.6343	7.9213	7.5963	7.6941	7.5915	7.9080	7.6639	7.7782	7.7249	7.7841	7.9321	8.7392	8.0056	7.8383	8.1917	8.0684	8.1763	8.2849
Amer2	_ASAVPDPSSVDPPS[Phospho (STY)]DPSADR_.2	Q8CCJ4_S356_M1_Amer2	4.2789	7.1802	7.4686	3.3054	4.7348	7.4472	6.9477	5.8453	5.0874	6.9985	5.3116	6.8948	6.3357	7.6482	6.7746	7.3047	7.4545	7.0704	7.0857	7.0523
Amer2	_DMSS[Phospho (STY)]VKR_.2	Q8CCJ4_S488_M1_Amer2	3.2004	3.3016	3.7367	4.4890	4.0390	4.2630	3.5021	4.4016	3.5612	3.4067	3.9356	3.1910	3.6693	3.7238	3.0793	3.5453	3.4384	4.3947	4.0433	3.6908
Amer2	_AAGPGNLVLPGS[Phospho (STY)]LTAS[Phospho (STY)]LEC[Carbamidomethyl (C)]VKEEPPR_.3	Q8CCJ4_S223_M2_Amer2	4.4424	4.3775	3.9790	3.1418	3.1485	3.4590	3.8996	3.1672	4.3652	5.6832	3.2388	4.1037	4.4733	4.0170	3.9250	3.8045	4.1653	2.9841	3.2832	3.6943
Amer2	_RPDS[Phospho (STY)]PGQDASR_.2	Q8CCJ4_S244_M1_Amer2	10.0206	10.5284	10.0810	10.2680	10.0532	10.1562	10.1844	10.3604	10.0746	10.3475	9.8472	10.2375	10.7440	10.7506	10.1006	10.5388	10.2180	10.2660	10.3453	10.2561
Amer2	_S[Phospho (STY)]GGTMPSIFGVK_.2	Q8CCJ4_S71_M1_Amer2	3.1464	3.3555	3.6827	4.5430	3.9850	4.2091	3.5561	4.3476	3.6152	3.3528	3.9896	3.2450	3.1671	7.3887	3.1333	3.4913	3.3845	4.4487	4.0973	3.6368
Amer2	_HAAGEPAGGEQAPAS[Phospho (STY)]AESAPER_.3	Q8CCJ4_S266_M1_Amer2	4.0658	3.9370	4.6021	3.6236	3.4035	3.0806	4.1375	3.7662	3.9211	4.2721	3.3997	3.8264	4.0865	4.5892	3.1542	4.4107	4.3039	3.5293	3.1779	3.0554
Bcas3	_VKS[Phospho (STY)]PPQISPSK_.2	Q8CCN5_S565_M1_Bcas3	4.5513	7.5801	8.2677	4.5337	8.1679	3.3501	3.7907	3.2762	2.9733	4.3619	3.3477	6.6483	4.5822	4.1260	3.8160	3.9135	4.2743	4.3760	3.1742	8.0159
Bcas3	_EGS[Phospho (STY)]IETLSNSSGSTSGSIPR_.2	Q8CCN5_S886_M1_Bcas3	6.4053	6.6545	6.3464	6.2264	3.5311	6.0168	6.2737	4.8351	5.5766	6.3127	5.9197	5.6432	4.1771	6.4198	3.5872	5.7973	5.4207	6.0531	5.4887	5.5582
Bcas3	_DVVGS[Phospho (STY)]GTELQR_.2	Q8CCN5_S877_M1_Bcas3	3.2504	6.9522	4.2878	4.3338	3.4573	3.4907	3.5908	7.0523	3.5110	7.4172	2.9896	3.7948	3.2813	4.3259	5.9882	4.1134	4.4741	4.1761	6.9985	4.0032
Bcas3	_LADAM[Oxidation (M)]AES[Phospho (STY)]PSR_.2	#N/A	6.8107	4.0082	3.8103	3.9320	6.1235	3.7825	3.0621	3.7100	3.2646	3.7242	3.5538	3.8406	3.4300	6.1515	3.7067	3.6250	3.7029	4.2312	3.8798	3.8997
Bcas3	_LTSQDS[Phospho (STY)]YNNFTNNNPGNPR_.2	Q8CCN5_S503_M1_Bcas3	6.1401	6.0782	4.1437	6.2168	3.5129	3.6743	5.3933	5.4279	5.1372	6.4350	5.3349	5.7613	5.0611	5.5176	4.0119	5.5525	3.5247	3.7722	4.9083	5.5848
Bcas3	_LADAM[Oxidation (M)]AES[Phospho (STY)]PSRDVVGSGTELQR_.3	#N/A	3.7877	3.3836	3.6547	4.5710	3.9947	4.0280	3.0534	4.3196	5.3539	3.5983	3.9531	3.2731	3.1391	3.6417	3.0788	3.1499	3.3564	2.9759	4.1253	6.6705
Bcas3	_HGSY[Phospho (STY)]DSLASDHSGQEDEEWLSQVEIVTHTGPHR_.5	Q8CCN5_Y707_M1_Bcas3	3.1777	3.3243	3.7140	6.3732	4.0163	4.2404	3.5248	4.3789	3.5839	3.3840	3.9583	5.4974	3.6919	3.2357	3.1020	5.5119	3.4158	5.7348	4.0645	4.4138
Bcas3	_C[Carbamidomethyl (C)]SPVPGLS[Phospho (STY)]SSPSGS[Phospho (STY)]PLHGK_.2	Q8CCN5_S486_M2_Bcas3	4.0266	3.9762	4.5629	3.6628	3.3643	3.1198	3.9616	3.7269	3.8818	4.1910	3.3605	3.8657	4.0473	4.5499	3.7540	4.3715	4.2646	3.5685	3.2171	3.0161
Pabpn1	_QMNMS[Phospho (STY)]PPPGNAGPVIMSLEEK_.3	Q8CCS6_S146_M1_Pabpn1	4.0885	3.6844	3.3539	3.3709	4.2954	4.3288	4.2535	4.0188	4.1737	3.8991	3.6524	3.5738	4.3392	3.3410	4.2789	3.4506	4.5565	3.2767	4.4261	3.3080
Tceal5	_RPAEDYVPR_.2	#N/A	7.0580	7.9581	7.4751	6.8333	6.9966	8.0705	7.5915	7.0653	6.9679	7.8232	7.7950	7.1150	7.8185	7.0134	7.5430	7.6657	7.1137	7.7112	7.7829	7.0677
Tceal5	_DAQDALVPR_.2	#N/A	6.0876	5.6010	6.0851	4.5982	6.2533	6.2245	6.2522	6.0589	6.2495	6.0461	6.1790	6.4913	5.7429	5.7313	7.2226	6.3218	5.7937	6.2550	6.2102	5.8526
Tceal5	_TDRGTDDS[Phospho (STY)]PKNSQEDLQDR_.3	#N/A	6.4246	7.0114	3.6335	6.4114	5.8413	6.5752	6.1286	6.3499	6.6060	6.7049	5.8178	6.6729	7.4968	6.9953	6.9808	6.6637	6.4481	6.4658	6.3661	6.3424
Tceal5	_GTDDS[Phospho (STY)]PKNSQEDLQDR_.2	#N/A	10.7251	10.8175	10.6900	10.9800	10.2730	10.4286	10.5466	10.8080	10.5836	10.8500	10.3275	10.5408	11.4532	11.1425	10.7469	10.6108	10.7105	10.5611	10.6555	10.5753
Tceal5	_M[Oxidation (M)]GGFHWVPR_.2	#N/A	6.4582	6.4641	5.7498	3.7012	5.8895	6.8092	6.5168	6.3074	5.7876	5.2597	5.3979	6.9541	7.1118	5.5072	6.4969	5.7107	4.2262	6.5777	3.5155	6.1705

Tceal5	_ADEDKS[Phospho (STY)]DAEGKPAR_.2	Q8CCT4_S35_M1_Tceal5	3.3089	3.7625	4.5673	4.2438	4.2470	4.4263	3.5201	2.4740	3.1541	3.5153	3.8941	3.3933	3.5607	4.6053	2.9708	3.6538	1.5047	3.2384	4.7281	2.9114
Inpp5f	_S[Phospho (STY)]PSADSIHTR_.2	Q8CDA1_S941_M1_Inpp5f	3.4183	3.9569	3.7510	7.5563	3.5332	7.5710	7.3722	3.6439	7.4970	3.7309	7.1521	3.6830	3.1948	7.5228	5.8026	7.0684	7.4516	7.4223	7.4605	7.2839
Inpp5f	_GLES[Phospho (STY)]PLKK_.2	Q8CDA1_S936_M1_Inpp5f	7.5157	7.4109	7.5264	7.3199	7.3910	7.0865	7.2238	7.2734	7.2170	7.6139	7.3093	6.6819	7.7791	7.3679	6.4193	6.9231	7.4590	7.0659	6.6910	6.8167
Inpp5f	_IIPS[Phospho (STY)]PDDSK_.2	Q8CDA1_S123_M1_Inpp5f	7.3350	7.8089	7.5513	3.0973	3.9298	7.5170	7.7639	4.2924	7.2532	7.6556	7.6682	3.3002	7.1266	3.6146	3.1885	7.2865	3.3293	7.3579	7.1542	3.5816
Inpp5f	_VQKS[Phospho (STY)]PAEPEAVNEIQQNELK_.3	Q8CDA1_S1103_M1_Inpp5f	6.3167	6.7199	5.8346	6.5256	6.3403	5.4806	5.7904	6.0320	4.4603	6.6699	5.2861	5.6471	6.1939	5.8170	4.4102	5.2790	6.2785	5.7486	5.3945	5.8174
Inpp5f	_SQS[Phospho (STY)]ASSIDVSTHAPSEAAAGPGSELGK_.3	Q8CDA1_S908_M1_Inpp5f	3.8052	5.4907	5.4878	4.5885	4.0173	5.5531	4.5693	4.3021	4.4570	5.5434	5.5868	3.2400	3.1216	5.9823	3.0613	3.1673	3.3389	2.9934	4.1428	5.0038
Inpp5f	_GLES[Phospho (STY)]PLKKS[Phospho (STY)]PSADSIHTR_.3	#N/A	6.4950	6.1306	6.2117	6.1319	5.6944	5.7319	5.7267	5.7724	5.4746	6.6015	5.8577	5.8017	6.0872	6.3027	5.1025	5.9921	6.1358	5.6279	5.2906	5.3127
Inpp5f	_KSPS[Phospho (STY)]ADSIHTR_.2	#N/A	6.3704	6.7072	5.6082	6.2944	3.5950	6.0656	5.5600	6.5024	5.8350	5.3676	5.9300	6.1238	7.0619	6.1709	5.6876	5.9014	6.4674	5.9621	5.6088	5.3569
Vcpip1	_ESS[Phospho (STY)]PSHGLLK_.2	#N/A	9.1564	9.4262	9.1868	9.2677	8.7029	8.4598	8.7914	3.4073	3.5622	9.5340	8.7381	4.1854	9.3388	9.2592	8.0673	8.8006	3.9449	3.8882	8.6139	8.8965
Vcpip1	_DGPSS[Phospho (STY)]APATPTK_.2	Q8CDG3_S756_M1_Vcpip1	10.4276	10.2280	10.6580	10.5453	9.6904	9.6282	9.9591	9.8926	9.8038	10.4006	9.9246	9.7675	9.9101	10.3574	8.6102	9.8049	9.8860	9.5411	9.6607	9.8050
Vcpip1	_TVS[Phospho (STY)]PSTIR_.2	Q8CDG3_S746_M1_Vcpip1	9.3687	9.4575	9.5327	9.6530	9.0789	9.1483	9.4005	9.0552	9.1541	9.8035	9.3893	3.0939	9.4515	9.4852	8.4490	9.1635	8.9390	9.1481	8.9458	9.2584
Vcpip1	_APYS[Phospho (STY)]PTTSK_.2	Q8CDG3_S767_M1_Vcpip1	8.4159	8.0774	3.4230	8.3727	7.8456	7.9096	8.0116	7.8320	7.8532	8.1498	3.7652	3.5472	3.9601	3.4100	7.1002	7.9107	7.7810	3.2501	8.0214	3.3770
Vcpip1	_S[Phospho (STY)]RESS[Phospho (STY)]PSHGLLK_.2	Q8CDG3_S993_M2_Vcpip1	3.9672	3.3977	3.6061	3.8533	3.7716	4.1462	3.9477	4.2795	3.9510	4.6547	3.5576	3.8120	5.0947	4.7311	3.3806	3.2780	4.0330	3.7433	4.0508	3.4813
Fam160b1	_TS[Phospho (STY)]PDHPKNDGKTEVHK_.3	Q8CDM8_S552_M1_Fam160b1	4.4711	3.5317	3.5066	3.2183	3.8088	4.0329	3.7322	4.1715	3.7913	3.1766	4.1657	3.4211	3.8994	3.4936	3.3094	3.3152	3.2083	3.1240	4.2734	3.1204
Txnl1	_QHLENDPGS[Phospho (STY)]NEDADIPK_.2	#N/A	5.5929	5.3882	5.8599	5.0115	4.8766	5.5474	5.5241	5.8415	5.2516	5.8491	5.8977	5.6385	5.9324	6.2271	4.6962	5.4702	5.8826	5.4683	5.6881	5.9522
Txnl1	_IKQHLENDPGS[Phospho (STY)]NEDADIPK_.3	#N/A	5.7211	6.2371	5.8991	6.2921	5.4829	6.4652	5.7812	6.1777	5.9519	6.1688	5.4699	6.0386	6.6233	6.7539	5.9938	6.5399	6.1197	6.0996	6.3088	6.4361
Txnl1	_FQNVNSVTLFVQSNQGEEETTR_.3	#N/A	3.6056	4.3972	4.3864	4.0838	2.9433	3.1674	3.0970	3.3059	3.1560	3.8119	3.5304	4.2867	3.6263	4.1289	4.1750	3.6022	3.8436	3.9895	3.6381	4.5422
Hectd2	_DLS[Phospho (STY)]PGAPPAVAAAAPEER_.2	Q8CDU6_S9_M1_Hectd2	3.4071	7.4377	7.7037	7.3739	4.2456	6.9171	6.8954	3.6574	6.8519	6.6720	6.4260	3.6135	7.3536	6.5078	6.0001	6.9230	3.1546	6.6346	5.9695	4.1845
Scyl2	_RGS[Phospho (STY)]LTLEEK_.2	#N/A	6.5024	6.1345	3.3924	6.0284	3.6947	5.9141	6.3202	6.4495	5.8786	6.2881	5.9949	3.5353	4.3776	6.2453	3.4236	3.2010	5.8920	6.0410	5.8679	3.3465
Scyl2	_GS[Phospho (STY)]LTLEEK_.2	#N/A	4.3902	3.6127	3.4256	3.2992	3.7279	3.9520	3.8132	4.0905	4.4724	3.0956	4.2467	3.5021	3.9803	3.4127	3.3904	3.2342	3.1274	3.2049	4.3544	3.3797
Nedd4l	_TS[Phospho (STY)]PQELSEEVSR_.2	Q8CFI0_S332_M1_Nedd4l	8.8349	8.5593	3.5257	3.2029	3.8279	4.1607	8.1119	7.8003	4.3455	3.7310	3.8241	3.4058	4.5109	3.5127	6.5498	3.3343	3.2274	3.1086	7.8938	3.4797
Nedd4l	_DT[Phospho (STY)]LSNPQSPQPSPYNSPKPQHK_.3	Q8CFI0_T502_M1_Nedd4l	4.1438	5.0472	3.8723	4.0302	4.2792	3.5479	4.6453	3.6076	3.6975	4.8903	4.8421	4.0804	3.8746	3.8727	3.4948	3.6853	4.6550	4.3182	3.9635	4.3642
Nedd4l	_SLS[Phospho (STY)]SPTVTLSAPLEGAKDS[Phospho (STY)]PIRR_.3	Q8CFI0_S477_M2_Nedd4l	3.1229	4.1962	4.1603	4.4613	3.3298	3.3632	3.7183	3.3486	3.6385	4.4343	3.1171	3.9223	3.8039	4.1984	3.7436	3.9858	4.3466	5.7648	3.1019	4.2736
Nedd4l	_DTLS[Phospho (STY)]NPQSPQPSPYNSPK_.2	#N/A	3.4239	4.5789	3.9602	5.3366	4.2625	2.9857	4.5787	3.6405	3.3377	3.6303	3.7121	2.9675	3.4457	4.4903	2.8558	3.7688	3.1378	4.0117	4.3107	4.1676
Mef2c	_NS[Phospho (STY)]PGLLVSPGNLNK_.2	Q8CFN5_S222_M1_Mef2c	9.4261	9.5307	9.0190	8.9704	3.8022	8.1792	9.1231	9.4259	8.2256	8.8327	8.0764	8.8347	8.7315	9.7605	7.8158	8.1010	8.7522	8.5966	8.4974	3.7858
Mef2c	_PSPDERES[Phospho (STY)]PSVKR_.3	Q8CFN5_S460_M1_Mef2c	4.2205	2.7853	3.2560	3.4651	3.5582	3.7823	3.9828	4.3448	4.0419	4.4268	2.9155	3.6717	4.1500	3.6937	3.5600	3.0646	3.8420	3.3074	3.1044	3.3710
Mef2c	_S[Phospho (STY)]PPPMNLGMNNR_.2	#N/A	3.3841	5.9895	8.2590	8.1712	4.2227	3.8357	5.2321	3.6870	3.3842	3.5904	7.6290	4.0240	3.4922	7.9401	2.9023	3.7290	3.1843	3.9719	4.2709	4.2141
Nrn1	Carbamidomethyl (C)]TYWEDFHSC[Carbamidomethyl (C)]TVTALTDC[Carbamidomethyl (C)]QE	#N/A	3.7487	3.8182	4.6886	4.7037	2.9551	4.3716	4.0187	5.0924	3.8812	4.3909	2.9514	3.8499	4.1859	5.5502	3.1878	3.0287	5.1700	3.8281	4.7999	4.1653
Akr7a2	_ALQTTYGTNAPR_.2	#N/A	5.2920	5.3728	5.7769	5.0163	5.6107	4.6184	5.5666	3.4692	4.9772	5.4557	3.5407	4.8838	3.2744	5.7291	6.2496	3.9401	5.6113	5.6040	5.4338	5.2517
Akr7a2	_VDLFYLHAPDHSTPVEETLR_.3	#N/A	4.6805	4.2377	3.4810	3.7612	3.9553	4.2573	3.9275	4.0139	3.7702	3.4472	3.9570	3.3504	4.1527	3.7871	3.5861	3.8908	3.4009	4.0071	4.2788	3.3198
Tp53bp2	_IPRPLS[Phospho (STY)]PTK_.2	Q8CG79_S697_M1_Tp53bp2	6.8003	6.8900	4.2513	7.6240	6.3945	3.1731	3.5376	6.6749	3.5138	4.0596	6.3929	6.6400	3.6557	4.4026	4.0690	6.3631	7.0263	3.9215	3.9750	6.4350
Tp53bp2	_KNQS[Phospho (STY)]SEDILR_.2	Q8CG79_S479_M1_Tp53bp2	4.5252	4.2948	4.0617	4.5599	3.2313	3.2646	3.8168	3.5821	3.7370	4.3358	3.2157	4.0209	3.9025	5.4931	3.8422	3.8873	4.1198	4.8242	3.3619	4.3721
Ppm1f	_IAEELVAVAR_.2	#N/A	3.3924	3.8727	4.0410	4.1378	4.2309	4.4550	3.4459	3.6721	3.3692	3.5987	3.7436	3.2496	3.4773	4.5219	2.8874	3.7373	3.0601	3.9801	3.4775	2.9789
Tns2	_GPLDGSPY[Phospho (STY)]AQVQR_.2	Q8CGB6_Y483_M1_Tns2	3.5540	6.4319	6.8657	4.1353	6.2232	3.1159	3.1485	6.3941	3.2076	3.7604	6.4727	4.3382	3.3156	5.8739	4.2265	3.8989	3.7921	6.1093	4.4408	4.0375
Tns2	_APHSC[Carbamidomethyl (C)]GS[Phospho (STY)]PSEGR_.2	Q8CGB6_S811_M1_Tns2	3.4308	3.8883	4.4682	4.2141	3.6378	3.6711	3.4103	3.1757	3.3306	3.2414	4.3100	3.6144	3.4960	3.9987	3.4357	4.2938	3.7133	4.1198	5.5284	3.9657
Tns2	_HLPGS[Phospho (STY)]GQQPSPPAR_.2	Q8CGB6_S1082_M1_Tns2	3.8171	3.9377	7.3979	3.8438	3.6993	3.6179	3.8604	3.8320	3.5525	4.2625	7.7035	7.5192	3.6606	3.8917	3.6427	3.6999	8.0197	3.7228	3.6786	3.7519
Tns2	_SFS[Phospho (STY)]LDPLMER_.2	Q8CGB6_S120_M1_Tns2	3.5398	3.7793	4.5772	4.0445	3.7467	3.7800	4.4741	3.7654	3.2216	3.3503	4.2011	3.5055	3.3871	5.3388	3.3268	4.4027	3.2627	3.8868	4.1858	4.2925
Tns2	_GYPSPGAHS[Phospho (STY)]PR_.2	Q8CGB6_S825_M1_Tns2	6.3772	3.1485	3.7072	3.4136	2.8767	3.7308	6.7927	4.3963	7.1502	6.0315	2.9669	4.3755	4.2015	3.7452	4.1968	3.5327	3.8935	3.2559	6.4807	3.4225
Tns2	_WDS[Phospho (STY)]YENFNQHHEDSVDGALAHTR_.4	Q8CGB6_S455_M1_Tns2	3.8499	3.4692	3.3864	3.7344	4.0568	4.0516	2.9913	4.0755	4.7589	3.6604	3.8910	3.1954	3.8807	3.4245	3.0167	3.2120	3.5728	3.5767	3.8757	3.1018
Rgs12	_TLPDSQQVPSS[Phospho (STY)]PASK_.2	Q8CGE9_S850_M1_Rgs12	12.1461	3.5955	12.1850	3.8607	11.5670	12.0258	3.5873	12.2039	11.8425	12.1836	11.7648	3.3217	3.7544	12.3756	3.1430	11.8478	12.3214	11.9155	11.9102	11.8655
Rgs12	_S[Phospho (STY)]LNEDVGEEDSEKK_.2	#N/A	6.7057	5.9722	6.7544	6.8429	6.1744	5.9069	6.6276	6.2223	6.0467	7.0395	6.2191	6.2413	6.3824	7.2897	5.8978	6.4087	6.8163	6.6749	6.5866	6.4157
Rgs12	_S[Phospho (STY)]LNEDVGEEDSEK_.2	#N/A	9.5470	9.0758	9.3106	9.4917	8.8703	8.7507	8.8250	8.6486	8.6046	9.3909	8.7220	8.4119	8.8249	9.0508	4.1748	8.7854	8.9221	8.4470	8.6316	8.6197
Rgs12	_QNSAVNSS[Phospho (STY)]PR_.2	Q8CGE9_S1130_M1_Rgs12	5.7502	5.8858	5.9749	3.5315	3.4705	3.8071	5.1697	5.2108	3.8453	5.9190	5.3938	4.8584	5.3109	3.6795	4.6086	4.3316	4.6018	5.0759	5.2789	4.0172
Rgs12	_ESQGSVSS[Phospho (STY)]AGSLDLSEAC[Carbamidomethyl (C)]R_.2	Q8CGE9_S940_M1_Rgs12	3.6135	3.7056	3.1501	3.9707	4.0638	6.8666	3.4773	3.8392	3.5363	3.4316	3.9107	3.4317	3.6444	3.1882	5.5915	3.5702	3.3364	3.8130	4.1121	4.3663
Rgs12	_LPAGSSELALSS[Phospho (STY)]PPPVK_.2	Q8CGE9_S1233_M1_Rgs12	4.0185	3.3006	3.5551	3.5657	4.2254	5.3854	3.8822	4.2442	3.9413	4.5275	4.3157	3.0267	4.0494	3.5931	3.4594	3.3806	3.7414	3.4081	3.7071	3.2704
Rgs12	_SKS[Phospho (STY)]GRS[Phospho (STY)]LNEDVGEEDSEK_.2	Q8CGE9_S876_M2_Rgs12	2.5929	4.5778	3.1330	3.3421	3.4352	3.6593	4.1058	4.4678	4.1649	4.3038	3.0385	2.6060	4.2730	3.8168	3.6830	2.9416	3.9650	3.1844	3.4835	3.4940
Rgs12	_HSISS[Phospho (STY)]DHSNVSTPK_.2	Q8CGE9_S859_M1_Rgs12	4.0795	3.9233	4.6158	3.6099	3.4173	3.0668	4.1238	3.7799	3.9348	4.2859	3.4135	3.8127	4.1003	4.6029	3.7010	4.4244	4.3176	3.5156	3.1642	3.0691
Wdr47	_RIS[Phospho (STY)]DLGNK_.2	Q8CGF6_S332_M1_Wdr47	6.3864	6.9003	6.3457	5.3886	6.0512	6.3140	6.0688	6.3568	5.9006	5.7649	5.5088	3.1973	6.6970	3.4226	5.3892	5.9018	5.5785	6.6720	3.8777	6.2402
Wdr47	_LSPYPSS[Phospho (STY)]PMR_.2	Q8CGF6_S297_M1_Wdr47	3.7407	8.2498	3.2773	9.2729	3.9476	3.9810	3.1005	3.9664	3.0207	3.5513	4.0002	3.3045	3.1861	3.3153	3.1258	3.1028	3.4636	4.4297	4.0783	3.6558
Wdr47	_RPQS[Phospho (STY)]ADAYM[Oxidation (M)]TR_.2	#N/A	5.3282	6.8250	3.4168	6.6171	5.0301	4.6380	6.2721	3.7815	4.7916	5.2307	4.6846	4.7959	5.4603	3.3865	3.4041	4.5502	4.0155	4.9392	3.4674	3.2091
Wdr47	_YQQHLEQK_.2	#N/A	3.4147	3.9044	4.4521	4.1695	3.6216	2.9859	3.2784	3.6404	3.3375	3.6304	3.0125	3.6305	3.0028	4.4902	2.8556	4.2777	3.1376	4.0118	4.3109	4.1675
Fam193a	_LILASS[Phospho (STY)]PQPK_.2	Q8CGI1_S1151_M1_Fam193a	3.8685	3.4644	3.5739	7.7421	4.0755	4.1088	4.4735	4.2388	6.5238	3.6791	6.4565	3.3539	7.1158	3.5609	2.9980	3.2307	3.2756	3.0567	4.2061	3.5279
Lonp1	_VLEFIAVSQLR_.2	#N/A	5.4229	4.7551	5.3153	5.4804	4.3584	4.8267	3.5092	4.8466	4.9906	3.5816	5.4747	3.8082	4.2183	4.6740	4.4314	5.2308	4.7506	4.0906	4.9554	4.2736
Lonp1	_LALLDNHSSEFNVTR_.3	#N/A	3.9510	4.0518	4.4873	3.7384	3.2887	3.5128	4.2523	3.1135	4.3114	4.1573	3.1850	3.9412	4.4195	3.9633	3.8295	2.9099	4.1115	2.2241	3.3370	3.6405
Gnal	_VLTSGIFETR_.2	#N/A	5.4973	5.9784	5.3004	5.8572	5.6366	4.2545	5.8158	5.8964	5.1262	4.4817	5.9344	5.9330	5.5605	5.5285	4.7687	5.5697	5.4381	6.0519	5.7532	3.2649
Gnal	_ESLDLFESIWNNR_.2	#N/A	4.1066	4.9989	5.8313	5.0899	5.1360	5.4544	5.1881	6.0226	5.6281	6.0296	6.1032	5.8657	6.1246	5.6835	4.0671	2.9507	6.0193	5.8205	5.1066	5.7641
Gnal	_IDSVSLVDYTPTDQDLLR_.2	#N/A	4.0145	3.7560	4.0742	4.7632	4.1943	3.6172	4.5915	5.1586	3.5935	3.1957	4.0038	4.5618	4.9312	4.7560	3.3612	4.3730	5.1289	4.5583	4.1458	3.1955
Gnal	_SNEYQLIDC[Carbamidomethyl (C)]AQYFLER_.2	#N/A	3.1335	4.1856	4.1709	4.4507	3.3404	3.3738	1.9906	3.3592	3.6279	2.6011	3.1065	2.4630	3.7933	4.2090	3.7330	3.9965	4.3573	3.8225	3.4711	3.6934
Adgrb2	_LS[Phospho (STY)]LDEDEEPK_.2	Q8CGM1_S1269_M1_Adgrb2	6.0463	6.2059	6.2201	6.4639	3.8451	6.4199	6.3558	4.2077	6.5960	6.6159	6.4898	5.2580	4.5281	5.6838	5.0722	5.8569	5.9989	6.5719	6.4551	6.4169
Bcor	_TDVLC[Carbamidomethyl (C)]TGEDEDC[Carbamidomethyl (C)]QAAS[Phospho (STY)]PLQK_.2	Q8CGN4_S1348_M1_Bcor	4.0135	4.0253	3.5501	2.4171	4.2204	4.0430	3.1870	4.2392	3.9363	4.1571	4.3107	2.8338	4.0444	4.6801	4.0890	3.7281	3.7364	4.0152	3.5520	4.0131
Bcor	_DGGS[Phospho (STY)]PPLLEK_.2	Q8CGN4_S423_M1_Bcor	3.6234	3.6957	3.1600	4.4067	3.8303	3.8637	3.2178	4.4839	3.1380	3.4340	4.1174	3.4218	3.3034	3.8061	3.2431	5.6598	6.2247	5.0898	3.9610	3.7731
H2bu1	_PDPS[Phospho (STY)]KSAPAPK_.2	Q8CGP0_S5_M1_H2bu1	3.3541	3.9650	4.3915	4.2301	3.5611	7.8345	3.4870	3.5798	3.4072	8.0721	4.3867	3.6911	3.3850	4.4296	3.5124	4.2171	3.0770	4.0724	4.3715	4.1069
H2bu1	_ESYSIYVYK_.2	#N/A	3.4451	4.5577	5.2919	4.2442	4.2837	4.5861	4.8707	3.1455	4.5949	4.7973	3.6908	2.9463	3.4245	3.9685	4.7501	5.6550	3.6832	4.9324	4.9435	4.8522
Calcoco1	_LPSYGLC[Carbamidomethyl (C)]ESGNTSSS[Phospho (STY)]PPGPR_.2	Q8CGU1_S563_M1_Calcoco1	6.2934	4.3182	7.0241	6.7766	3.8713	4.4954	3.6506	3.4159	3.5708	3.8654	3.8828	6.5538	3.7363	4.2389	3.4306	3.8454	6.7344	6.9254	6.4796	6.7873
Tph2	:S[Phospho (STY)]S[Phospho (STY)]EVEIFVDC[Carbamidomethyl (C)]EC[Carbamidomethyl (C)]C	Q8CGV2_S101_M2_Tph2	4.0624	5.1235	4.5987	3.6270	3.4002	3.6242	4.8196	3.7628	4.2000	4.2688	3.0736	3.8298	4.3081	5.4546	4.1982	4.4073	4.3005	5.5691	3.4484	3.5291
Tph2	_GLS[Phospho (STY)]LDSAVPEDHQLLGSLTQNK_.3	Q8CGV2_S19_M1_Tph2	3.9896	5.5484	4.4882	3.6998	3.3273	3.5514	5.2286	3.6899	4.2729	4.1959	3.1464	5.0456	4.3809	6.1895	3.7910	4.3345	4.2276	3.0765	4.9196	3.6020
Ogt	_DSGNIPEAIASYR_.2	#N/A	7.0227	6.7695	6.3705	6.2905	7.0560	6.5010	7.0265	7.1357	7.0039	4.4769	6.5613	6.7108	6.6302	6.8228	6.7186	6.5842	6.7432	6.5253	6.2938	6.6895
Ogt	_AIQINPAFADAHSNLASIHK_.3	#N/A	3.2280	5.6232	4.9570	5.6640	5.5924	4.9263	5.5985	5.9399	5.6672	6.4918	6.0753	5.6054	6.0290	5.8777	5.2517	5.5050	6.1534	5.6540	6.0643	4.9074
Ogt	_SDLGNLLK_.2	#N/A	4.0988	4.5791	3.7774	3.8229	3.2070	3.4037	4.1045	4.4665	4.4628	4.3100	4.0019	2.8954	4.2717	3.2087	3.4960	2.9429	2.8877	3.1857	4.4050	4.7694
Sugp1	_DIDASPS[Phospho (STY)]PLSVQDLK_.2	Q8CH02_S409_M1_Sugp1	3.3819	4.4786	4.0605	4.1652	3.5888	6.8533	3.4593	3.2246	3.3795	3.1925	4.3589	4.3681	3.5449	4.0476	3.4846	4.2449	3.7622	6.6363	6.6052	6.6940
Sugp1	_ALQQHQHGYDS[Phospho (STY)]DEEVDSELGTWEHQLR_.4	Q8CH02_S483_M1_Sugp1	3.9891	4.0625	3.8304	3.7491	2.9613	3.9512	3.6989	3.6895	3.6634	3.9464	3.5025	3.9520	4.0098	3.8175	4.1376	3.8129	4.2272	3.6548	3.3034	3.7845
Sugp2	_GRS[Phospho (STY)]LNIADQEGTLLGK_.3	#N/A	7.2045	7.5217	6.0743	7.1290	4.7642	5.8271	7.1596	6.6772	6.6091	7.3414	6.6271	6.6753	7.0797	7.4936	5.6188	6.6604	6.8794	7.0029	6.9996	6.8813
Sltm	_DVQDAIAQS[Phospho (STY)]PEK_.2	#N/A	11.0150	10.7808	11.3780	11.0901	10.6358	10.7955	10.4589	10.8035	10.6720	10.8965	10.7985	10.4744	10.8591	11.3264	10.4270	10.5723	11.1675	10.4505	10.6167	10.7569
Sltm	_S[Phospho (STY)]PGHM[Oxidation (M)]VILNQTK_.2	#N/A	6.7326	6.7160	6.7849	6.6472	6.2740	6.7372	6.7166	6.7669	6.1629	6.6550	6.7127	6.0647	6.3797	7.8992	5.8309	6.3758	6.8898	6.6266	6.2362	6.5581
Sltm	_DVQDAIAQS[Phospho (STY)]PEKEAK_.2	#N/A	10.5497	10.6109	10.4306	10.5318	10.1819	10.2029	10.0484	10.6778	10.1986	10.5213	10.1411	10.3247	11.0040	11.0554	9.9179	10.2816	10.7974	10.2928	10.1287	10.3108
Sltm	_ISS[Phospho (STY)]KS[Phospho (STY)]PGHM[Oxidation (M)]VILNQTK_.3	#N/A	3.7906	4.1018	3.6416	3.7984	3.6886	3.7797	3.6108	4.5561	4.0660	4.2415	3.3629	3.5864	4.2119	5.0914	3.4494	3.5265	3.7914	3.6251	3.6861	3.7468
Sltm	_AGAGMITQHSSTAS[Phospho (STY)]PVNR_.2	#N/A	3.7785	3.5544	3.3151	3.5760	3.9855	4.1662	3.9605	4.0816	4.0514	3.5308	3.9257	3.2729	4.1961	3.8286	3.0942	3.1406	3.3406	3.4423	4.1282	4.0504
Trim32	_EM[Oxidation (M)]DM[Oxidation (M)]S[Phospho (STY)]PEEVAPSPR_.2	#N/A	11.4143	11.0857	9.6661	10.9217	10.8325	10.9333	10.7135	11.5452	11.0574	11.1312	9.1971	11.0990	11.7625	10.9478	10.5967	11.2107	11.5065	11.0342	11.1154	11.0511
Trim32	_EMDMSPEEVAPS[Phospho (STY)]PRAS[Phospho (STY)]PAK_.2	#N/A	4.0223	4.1971	4.1647	4.2976	3.3342	3.3676	3.7192	3.3300	3.6394	4.4387	3.1180	3.9232	4.2040	4.1658	3.7445	3.9903	4.1057	7.7085	3.3801	4.0005
Nav1	_SIGS[Phospho (STY)]PESTPK_.2	Q8CH77_S762_M1_Nav1	7.5222	7.5798	7.2361	7.7354	4.1369	7.3158	7.4080	4.1556	7.0124	3.1152	7.4268	7.6616	3.9608	3.5046	6.8426	3.2920	7.3038	7.5907	7.7033	7.7460
Nav1	_S[Phospho (STY)]LTNLSFLTDSEKK_.2	#N/A	4.5664	6.5650	6.5453	4.5187	3.2725	5.8730	5.2759	5.7578	3.6958	4.3770	3.1744	4.8764	3.8612	6.4721	4.7362	3.9285	4.2893	5.9548	5.6326	3.8183
Nav1	_SS[Phospho (STY)]PLSWR_.2	Q8CH77_S315_M1_Nav1	3.3244	3.1775	3.8607	4.3650	4.1630	4.3871	5.3350	4.5256	3.4372	3.5307	5.6681	3.0670	3.5452	3.8478	2.9553	3.6693	3.5625	3.9122	3.9193	5.5638
Nav1	_LYHLPPPS[Phospho (STY)]VGPHSTASPPEDR_.3	Q8CH77_S1816_M1_Nav1	3.8183	4.1845	4.3546	7.1581	3.1560	3.3281	3.7534	3.5187	3.6736	6.9570	3.1522	4.0739	3.8390	4.3417	3.7788	4.1632	4.0564	3.7768	3.4254	4.3087
Nav1	_S[Phospho (STY)]LTNLSFLTDSEK_.2	#N/A	3.8188	3.9258	5.2954	5.1123	3.2304	5.4857	3.8743	3.7160	5.1671	3.6294	3.2732	3.7294	3.9600	5.3684	4.9964	4.7699	4.4711	5.1359	4.7961	3.7242
Nav1	_RSS[Phospho (STY)]TIVLR_.2	Q8CH77_S455_M1_Nav1	4.0231	3.6190	3.4193	4.6394	4.2300	4.2634	4.3189	6.4328	5.3755	3.8337	3.7177	3.5084	4.4045	5.5503	2.8434	5.4284	5.8497	3.2113	4.3607	3.3734
Nav1	_VNSNS[Phospho (STY)]LDLPSSSDTHASK_.3	#N/A	6.4995	6.1107	6.3595	5.5652	3.5674	5.9884	5.2649	3.7817	5.1471	6.1954	5.1676	4.6762	3.7778	5.8351	3.7017	4.8842	5.2534	4.9391	3.6823	3.4278
Nav1	_LELVES[Phospho (STY)]LDSDEVDLK_.2	Q8CH77_S380_M1_Nav1	4.0415	3.9613	4.5778	3.6478	3.3793	3.6034	4.1618	3.7419	4.2209	4.2479	3.0944	3.8507	4.3289	4.5649	3.7390	4.3864	4.2796	5.9919	3.2021	3.5500
Nav1	_SVQPEVELS[Phospho (STY)]GGSGSGGDEGADESR_.2	Q8CH77_S16_M1_Nav1	3.7885	4.3948	3.9926	3.9925	3.1621	4.7219	4.2332	4.0142	4.4965	4.2666	3.3158	4.1209	4.0026	4.0306	3.9423	3.8181	4.1789	3.5544	4.6607	4.7463
Nav1	_SLAESGLNWFS[Phospho (STY)]ESEEK_.2	Q8CH77_S477_M1_Nav1	4.3161	4.5039	3.8526	3.2682	3.0222	3.5854	4.1798	4.5417	4.2389	4.2299	3.1124	4.2300	4.3469	5.0980	3.7570	3.6782	4.0390	3.1105	3.4095	3.5680
Nav1	_APEAAVS[Phospho (STY)]DDGKS[Phospho (STY)]DDELLSSK_.2	#N/A	4.2411	3.8041	3.7777	3.5707	3.5104	3.4335	3.9625	3.6772	3.7880	4.0517	3.4634	3.8948	4.3170	3.7909	3.7162	3.6032	3.9806	3.2877	3.6951	3.3781
Nav1	_ERPES[Phospho (STY)]C[Carbamidomethyl (C)]DDASK_.2	Q8CH77_S510_M1_Nav1	3.2676	4.0515	4.3050	4.3166	3.4745	3.1330	4.7432	3.4933	3.1904	4.5790	3.5648	3.7776	3.2985	4.3431	3.5989	4.1306	4.4914	4.1589	2.9571	4.0204
Nav1	_SGYMS[Phospho (STY)]DSDLMGK_.2	Q8CH77_S394_M1_Nav1	3.9320	3.3871	3.4686	3.6522	4.1389	3.9694	3.7957	4.1577	3.8548	3.1131	4.2292	3.1132	3.9629	3.5066	2.9345	3.2941	3.6549	3.4945	3.7936	3.1839
Nav1	_SLAESGLNWFS[Phospho (STY)]ES[Phospho (STY)]EEKTPK_.3	Q8CH77_S477_M2_Nav1	3.3682	3.9509	4.4056	4.2161	3.5751	3.0324	3.4730	3.5938	3.2910	3.1788	3.6654	3.6770	3.3990	4.4437	3.4984	4.2311	3.0911	4.0584	4.3574	4.1209
Synj1	_EFGGVGAPPS[Phospho (STY)]PGVAR_.2	Q8CHC4_S1147_M1_Synj1	11.5158	11.0897	11.3963	11.1772	10.5288	10.2437	10.3356	10.6280	10.2873	11.5531	10.7606	10.5021	11.0495	10.1609	8.8541	10.2442	10.9162	10.3763	10.2582	10.4971
Synj1	_TNC[Carbamidomethyl (C)]LDC[Carbamidomethyl (C)]LDR_.2	#N/A	7.4095	6.9862	7.2009	7.1925	7.5123	7.2861	7.3514	7.5654	7.1651	7.1314	7.3609	6.8553	7.0584	6.6140	7.0591	6.9781	7.2133	7.1134	7.2145	6.8679
Synj1	_REIEAPKS[Phospho (STY)]PGTAR_.3	#N/A	3.7766	3.9539	6.6506	7.4305	6.1382	6.5240	7.2024	5.1012	4.7749	8.2864	5.1400	3.7965	7.3087	5.7987	3.9369	3.8859	4.8646	6.4111	3.6505	5.2083
Synj1	_IYAGTGALEGK_.2	#N/A	6.6490	6.6472	6.5875	6.4249	6.6297	6.8917	6.9591	7.1285	6.8519	6.0771	6.5480	6.1167	6.2383	5.7137	6.0208	6.6412	6.4916	6.7373	6.9568	6.4861
Synj1	_GFEANAPAFDR_.2	#N/A	7.2486	7.4430	7.0361	7.1352	7.2591	7.4779	7.4146	7.3452	6.6375	7.3860	7.4465	7.1996	7.0444	6.7137	6.3847	6.5323	7.2032	7.5199	6.8713	6.9742
Synj1	_AGVISAPQSQAR_.2	#N/A	6.8593	6.7923	6.5172	7.1879	6.9759	6.9125	7.3697	7.4748	6.6780	6.9141	6.3825	6.6359	4.2145	6.2344	6.4783	6.5470	7.0667	7.0229	6.1979	6.5017
Synj1	_IDLPNEEVK_.2	#N/A	7.3254	6.2904	6.0552	6.4468	6.8603	6.6858	6.4457	7.0131	6.5610	6.4192	6.5801	6.5879	7.1159	6.2770	5.9908	5.8566	5.9396	6.8780	6.3822	6.2648
Synj1	_QIVVNLLGSK_.2	#N/A	5.6030	5.7539	6.2053	4.8168	5.8749	6.0602	5.9237	6.1723	5.9543	5.5238	6.1090	5.6142	3.5129	5.9378	5.3882	5.2255	6.3775	6.1244	5.4278	5.2754
Synj1	_EVIAVQGPPDGTVLVSIK_.2	#N/A	5.8903	5.2778	5.3979	4.1028	5.0989	4.6922	5.0496	5.2714	4.8983	4.8386	4.4526	3.5638	4.2695	5.1708	4.7990	4.4391	5.1676	5.3823	4.8993	5.1029
Synj1	_QQNWDSLIAGDQLINQK_.2	#N/A	5.7804	5.2550	5.4533	4.6124	2.9745	5.4049	4.7433	5.4237	5.2000	5.5107	5.2259	4.2555	3.2329	4.2775	3.8469	5.0568	4.0914	4.6327	4.1215	4.6753
Synj1	_TPGPPSSQGS[Phospho (STY)]PVDTQPAAQK_.2	#N/A	11.2938	11.1821	11.3462	11.2863	10.7158	10.7976	10.9136	11.2708	10.9986	11.0564	10.7350	10.8153	11.3315	11.0477	9.9018	10.7258	11.2980	10.8193	10.8090	10.7382
Synj1	_EIEAPKS[Phospho (STY)]PGTAR_.2	#N/A	11.0768	10.4467	10.9360	11.0605	9.8249	9.4437	9.2660	10.0108	9.6995	10.4880	9.7665	10.1417	10.2361	10.1672	8.3216	9.8300	9.7748	9.9857	9.8402	9.8877
Synj1	_VSEQTLQSASSK_.2	#N/A	7.1851	3.5344	7.1680	7.1444	6.5101	4.0302	3.7349	7.2456	7.0856	3.1739	4.1684	6.6989	6.6652	6.5039	6.5592	6.4038	6.8498	6.6338	4.2761	6.6263
Synj1	_SAEDLDLLNASFQDESK_.2	#N/A	6.8457	6.8477	6.8795	5.9584	5.6644	6.4677	6.6417	5.9008	6.7595	6.1383	5.9756	6.0503	6.1517	5.5963	5.8331	5.6257	6.6829	6.6976	6.4171	6.1085
Synj1	_ERNEDFVEIAR_.3	#N/A	4.9066	4.9176	5.5613	5.0322	4.3597	5.5428	5.7283	3.4303	4.8883	5.1228	4.7352	4.8886	3.2355	4.4132	4.1318	5.0306	4.4284	5.3241	5.1129	3.9574
Synj1	_NEDFVEIAR_.2	#N/A	5.7755	3.7296	3.3087	6.0403	3.6110	5.1232	5.8641	4.2921	5.6343	5.5728	5.6976	3.6190	5.9319	5.2005	5.6901	5.1616	3.7893	5.7197	5.7955	3.3183
Synj1	_VTSTEFISLR_.2	#N/A	3.5222	4.0250	4.5596	4.4388	3.7292	4.4407	3.3189	3.7479	4.7867	3.3328	4.6135	3.5230	3.4046	4.5977	3.3443	3.6160	3.9426	3.9131	4.2667	3.8743
Synj1	_SSAQESTFFDDALIDELLR_.2	#N/A	2.5128	3.8528	2.7572	2.0157	2.3888	2.5477	3.5499	3.2189	2.7697	3.0096	3.3010	3.1314	3.7171	3.2608	3.1271	3.4975	2.2672	3.4896	4.0394	4.4390
Synj1	_NQPS[Phospho (STY)]PQAGLAGPGPAGYGAAR_.2	Q8CHC4_S1175_M1_Synj1	8.3313	8.0661	7.9981	3.9954	6.5026	3.8291	7.3758	4.5185	3.1726	8.0090	7.0410	7.3238	3.3380	7.0658	3.2777	6.6299	7.2575	6.9194	3.9264	6.6855
Synj1	_T[Phospho (STY)]SPC[Carbamidomethyl (C)]QS[Phospho (STY)]PTVPEYSAPSLPIRPSR_.3	Q8CHC4_S1053_M2_Synj1	3.5738	4.4290	4.1101	4.1156	2.9115	3.1356	3.1287	3.2742	3.1878	3.7801	3.5622	4.3185	3.2959	4.3405	4.2068	3.9187	3.8118	5.6766	2.9597	4.0178
Synj1	_QEAIDVLLLGNTLNSDLADK_.2	#N/A	4.3011	3.1461	3.3365	3.3883	3.6388	4.3461	2.8605	4.0015	3.4365	3.9164	3.5302	3.5912	4.3218	4.1559	4.2615	3.1451	3.2331	2.8961	3.8651	3.2906
Synj1	_TPAWTDR_.2	#N/A	3.6482	3.6709	3.1847	3.7410	3.8551	3.8884	4.5662	3.8738	3.5110	4.6177	4.0927	3.3970	3.2787	3.2228	3.2184	3.0103	3.3711	4.4776	4.0315	4.4009
Synj1	_HLEEMS[Phospho (STY)]LEK_.2	Q8CHC4_S982_M1_Synj1	3.8771	3.4729	3.5653	3.9897	4.0840	4.1173	4.4649	4.2302	4.3851	3.6877	3.8638	3.3624	4.5506	3.5524	2.9895	3.2392	3.2671	3.0652	4.2147	3.5194
Synj1	_IDLPNEEVKELIR_.3	#N/A	3.9589	1.8795	3.4955	3.6253	4.1658	3.9425	4.3831	4.1846	2.8989	3.7695	4.2561	3.0863	3.9898	3.5335	2.9077	3.3210	3.6818	3.4676	2.5497	3.2108
Synj1	_FLDYGFFYFDGSEVQR_.2	#N/A	4.4277	4.3922	3.9643	3.1565	3.1338	3.4737	4.2914	3.1525	4.3505	4.1182	3.2241	4.1183	4.4586	4.0023	3.8686	3.7898	4.1506	2.9988	3.2979	3.6796
Rapgef2	_RSS[Phospho (STY)]FLNAK_.2	Q8CHG7_S960_M1_Rapgef2	10.6296	10.6308	10.2441	10.4989	10.0080	10.2309	10.2398	10.4398	10.0690	10.3965	10.1166	10.0321	10.5899	10.4950	3.6297	9.9977	10.4790	10.3144	10.2163	10.0566
Rapgef2	_SETS[Phospho (STY)]PVAPR_.2	Q8CHG7_S1022_M1_Rapgef2	9.6393	9.3862	9.4982	9.6536	8.6703	8.7960	9.1755	8.8534	8.6911	9.6149	8.8598	8.6300	9.3393	8.7684	7.3508	8.5422	8.5527	8.5484	8.6563	8.6010
Rapgef2	_HIPAALPVSGTLSSS[Phospho (STY)]NPDLLQSHHR_.4	Q8CHG7_S585_M1_Rapgef2	8.6427	9.3955	4.0820	4.1437	2.8835	3.1075	9.0571	3.2461	3.2159	3.7521	3.5902	8.6364	8.4792	4.3685	4.2348	3.8906	3.7838	4.1335	4.4325	4.0458
Rapgef2	_ILS[Phospho (STY)]LSEEGSLER_.2	Q8CHG7_S1088_M1_Rapgef2	4.4876	4.0834	4.4557	3.7700	3.1937	5.7138	5.6473	3.6197	3.7746	4.2982	3.2533	3.9729	3.9401	4.4427	3.8798	3.8497	4.1574	5.4893	5.6232	6.0397
Rapgef2	_QAEDTISNASSQLSS[Phospho (STY)]PPTS[Phospho (STY)]PQSSPR_.3	#N/A	4.1198	4.0961	7.6078	7.7967	3.4907	3.5968	6.7052	4.1315	4.0229	7.0449	3.5016	6.8989	4.1884	7.0580	3.6956	6.7846	3.9980	3.5243	6.6771	3.6666
Rapgef2	_SSIVSNSS[Phospho (STY)]FDSVPVSLHDERR_.3	#N/A	3.7158	5.1294	4.1604	4.4612	3.0535	3.2776	2.9868	3.3487	3.0458	3.9221	3.4202	3.9222	3.1539	4.1985	4.0648	4.0607	4.3468	4.3035	3.1017	3.8758
Rapgef2	_S[Phospho (STY)]LGSLSQGSANATVLDVAQTGGHK_.3	Q8CHG7_S930_M1_Rapgef2	7.4864	7.4749	6.4991	3.9284	3.8628	3.8961	7.5605	8.3031	5.4969	3.4664	4.0850	7.0801	7.2813	8.1267	3.2107	3.0179	3.3787	6.9614	6.3909	4.8047
Rapgef2	_SSIVSNSS[Phospho (STY)]FDSVPVSLHDER_.3	#N/A	3.1988	4.1202	4.2363	4.3854	3.4058	3.2017	3.6423	3.4245	3.1217	4.5103	3.4960	3.8464	3.2297	4.2743	3.6677	4.0618	4.4226	4.2277	3.0259	3.9516
Rapgef2	_NNM[Oxidation (M)]ETETLC[Carbamidomethyl (C)]S[Phospho (STY)]DEDAQELLR_.2	Q8CHG7_S698_M1_Rapgef2	3.0997	3.4022	3.6360	4.5897	3.9383	4.0466	3.9369	4.3009	4.4558	3.6170	3.9345	3.2917	3.1204	4.3544	3.1800	3.4446	3.3378	4.5247	4.1440	3.5901
Septin8	_EFLSELQR_.2	#N/A	7.4956	7.5231	7.5887	7.3859	7.7406	7.6509	7.8767	7.3773	7.2432	7.6832	7.8132	7.6738	7.5072	7.1507	7.3174	7.2581	7.6176	7.7394	7.3185	7.2725
Septin8	_ELEEETNAFNC[Carbamidomethyl (C)]R_.2	#N/A	6.5083	6.5857	6.3914	6.3317	6.7313	6.6944	6.6654	6.4131	6.6497	6.4425	6.5738	6.1575	5.8648	6.5764	6.5082	6.6590	6.7700	6.1144	6.9841	7.0305
Septin8	_SLFDYHDTR_.2	#N/A	6.5861	6.6505	5.9992	6.9177	5.4184	6.4684	6.7894	6.6946	6.5019	6.6120	4.0653	6.3444	6.6830	6.7015	6.3269	6.4120	6.7820	6.5510	6.4688	6.7461
Septin8	_SVTQGFSFNILC[Carbamidomethyl (C)]VGETGIGK_.2	#N/A	4.5897	4.7903	4.3648	3.6926	3.7489	4.2222	4.0777	5.3729	3.5012	4.1534	4.1554	4.2860	4.7866	5.6678	4.4641	4.4341	5.0998	4.6821	4.5552	4.0383
Septin8	_LTIVDAVGFGDQINK_.2	#N/A	3.3909	4.8897	5.2709	4.2985	4.2295	5.1631	3.2117	3.6736	4.9559	4.6403	5.3076	4.2451	5.3040	4.4585	4.2958	3.7358	4.0984	3.3551	4.9209	5.3668
Septin8	_[Acetyl (Protein N-term)]AATDLERVS[Phospho (STY)]NAEPEPR_.2	#N/A	9.4860	9.7592	9.9047	9.9992	9.2523	9.7595	9.1948	9.4661	9.7211	9.7879	9.4485	9.3499	9.7729	10.0314	9.3714	9.6344	9.9402	9.4080	9.5914	9.8667
Septin8	_SLSLGGHVGFDSLPDQLVSK_.3	#N/A	5.8698	5.8106	5.5622	5.9313	5.8332	5.4716	5.4857	5.8335	6.0157	5.8722	5.7662	5.6923	5.7487	6.1032	5.4110	5.7135	6.0292	5.8718	5.8454	5.8489
Ep400	_S[Phospho (STY)]SPVNRPSSATNK_.2	Q8CHI8_S720_M1_Ep400	3.9604	4.0424	4.4967	3.7290	3.2981	3.1860	3.8955	3.6608	3.8157	4.2571	3.2943	3.9318	3.9811	4.4838	3.8201	4.3053	4.1985	3.6347	3.2833	4.4508
Phc3	_ISQRDPS[Phospho (STY)]PESK_.2	Q8CHP6_S261_M1_Phc3	8.7339	9.1836	8.8909	9.0241	8.6429	8.7484	8.6114	9.2745	8.7797	8.9066	8.2411	8.8658	9.4810	9.1773	8.8636	8.9904	8.9639	2.9705	8.7713	8.8639
Phc3	_KGGS[Phospho (STY)]PGLESR_.2	Q8CHP6_S269_M1_Phc3	7.6688	3.5272	7.7228	8.0507	7.4867	7.8599	7.8506	7.9213	7.6829	7.5845	7.5891	7.6799	7.8383	7.7777	7.2414	3.3197	7.7078	3.5625	3.8616	7.6280
Phc3	_M[Oxidation (M)]DRT[Phospho (STY)]PPPPTLS[Phospho (STY)]PAAVTVGR_.3	#N/A	3.7504	6.6281	5.7161	5.1021	3.8071	5.1301	5.7520	4.8516	5.4853	3.8485	4.1728	6.1410	3.5809	7.2461	5.2092	3.2053	6.0230	6.0769	3.7536	5.9628
Pgp	_FIFDC[Carbamidomethyl (C)]VSQEYGINPER_.2	#N/A	6.4760	6.5706	6.5499	6.9030	6.4871	6.3043	6.3488	6.4729	6.2995	6.4353	6.2038	6.2405	6.2944	6.2424	6.3546	6.0860	6.5086	5.8643	5.9037	5.6034
Pgp	_GETAVPGAPETLR_.2	#N/A	3.1543	6.9551	7.2355	7.2299	7.5626	7.4648	6.8900	7.5391	7.4387	6.9171	7.3475	7.3557	7.3090	7.2190	7.5105	7.2868	7.4724	7.0982	7.2657	7.5616
Pgp	_FIAGTGC[Carbamidomethyl (C)]LVR_.2	#N/A	3.5502	5.3548	5.7749	6.4266	5.7041	5.6016	5.7354	5.7511	5.2506	5.9246	6.0106	2.6486	5.1175	6.3363	5.8097	6.1463	5.4730	5.8261	5.6003	5.8045
Pgp	_TILTLTGVSSLEDVK_.2	#N/A	5.5499	4.2882	5.3737	5.0562	4.4997	4.3789	4.6972	4.3871	5.0153	5.2635	4.5925	3.7054	5.3569	3.5805	4.6390	5.1286	4.8686	4.1784	3.9779	4.9421
Pgp	_SNQESDC[Carbamidomethyl (C)]MFK_.2	#N/A	3.5212	4.4816	4.0575	4.2667	2.8589	3.0830	3.1813	3.5432	3.2404	3.7275	3.6148	4.3710	3.3484	4.3930	4.2593	3.8661	4.5413	4.1090	4.4080	4.0703
Fbxo4	_GAGS[Phospho (STY)]PPPASDWGR_.2	Q8CHQ0_S11_M1_Fbxo4	11.0461	10.7763	10.8722	10.9210	10.9382	10.9129	10.5175	11.2974	10.7257	10.9539	10.8836	10.7585	11.3294	11.0951	10.3870	10.8379	11.0877	10.7585	10.8250	9.8975
Ngef	_TKFVS[Phospho (STY)]FTSR_.2	#N/A	9.8703	9.7983	8.5099	9.4851	8.4467	3.5609	9.2721	9.8756	8.2916	9.3724	3.1369	9.1400	9.7885	9.3682	7.2385	8.7210	9.2095	9.4112	9.0776	8.7368
Ngef	_FVS[Phospho (STY)]FTSR_.2	#N/A	9.7302	9.4623	9.6154	9.9200	2.9148	9.0983	9.5795	3.8986	8.9575	10.2045	9.8935	9.1389	9.7820	9.3961	7.4978	9.3352	8.7022	9.7953	9.7147	9.6222
Ngef	_MEDPQRS[Phospho (STY)]QNKDR_.2	#N/A	4.1364	6.7998	7.3253	7.0766	4.8850	5.7867	6.6047	4.5131	5.3253	6.4147	6.5000	5.2293	5.1592	7.1118	5.1794	5.0550	4.9330	5.2744	5.1426	5.5941
Ngef	_VEELEDQGQTLANVFILR_.2	#N/A	3.6623	3.1469	4.1986	4.0271	3.0000	3.1603	3.0402	3.6437	3.0993	3.8686	2.4205	4.2300	3.9672	4.4950	3.2597	4.0072	3.9003	4.2500	3.7686	3.0318
Ngef	_MEDPQRS[Phospho (STY)]QNK_.2	#N/A	4.0790	6.7293	7.7501	7.0392	5.2894	5.8803	7.1011	4.4863	4.9484	6.0401	6.9147	4.9865	4.2915	7.0499	5.4885	4.4239	5.5896	5.1787	5.3510	5.6602

Epn2	_TTS[Phospho (STY)]PDLFESQSLTSASSK_.2	#N/A	9.2396	9.1396	4.1921	9.2518	8.3341	9.0018	8.4663	8.4644	8.5874	9.1213	8.7595	8.5718	8.7719	9.3596	8.2619	4.1133	8.9495	8.6020	8.8482	8.8188
Epn2	_TTS[Phospho (STY)]PDLFESQSLTSASSKPSSAR_.3	#N/A	3.6485	9.2978	4.1848	8.4909	6.5460	7.7335	3.0540	8.7554	3.1131	3.8548	3.4875	8.6692	9.1885	10.4321	7.2924	8.3121	3.8865	8.9949	3.5952	7.4408
Epn2	_AGGS[Phospho (STY)]PASYHGSTSPR_.2	Q8CHU3_S192_M1_Epn2	7.5644	7.1516	7.1047	7.3327	5.8133	7.0247	6.6886	7.3804	6.7830	7.3077	6.6996	7.2123	7.3723	7.4756	6.1951	6.8395	7.2730	6.7462	6.8068	6.7050
Epn2	_GSS[Phospho (STY)]QPNLSTSYSEQEYGK_.2	Q8CHU3_S173_M1_Epn2	3.9927	8.8948	9.2924	9.3400	8.5242	8.7406	8.5628	8.6987	8.6105	9.2154	8.5338	3.5573	8.6666	9.1302	8.1972	8.9905	8.7669	3.5267	8.5351	8.8274
Epn2	_NSDPWAASQQPASNAGK_.2	#N/A	4.0197	3.2994	5.4104	3.5646	5.4379	3.8818	3.8834	5.5789	3.9425	4.5263	5.3576	3.0256	4.0505	3.5943	4.6005	3.2439	3.7426	5.1956	4.7693	3.2716
Eif2b5	_AGS[Phospho (STY)]PQLDDIR_.2	Q8CHW4_S540_M1_Eif2b5	12.5240	12.3869	12.1233	12.3166	11.0203	12.2381	12.0558	12.0081	12.1562	12.1755	11.7225	12.0232	12.1408	12.0270	10.8728	11.7484	12.7037	12.1560	12.0421	11.9472
Rftn2	_NSS[Phospho (STY)]PSYANPK_.2	Q8CHX7_S228_M1_Rftn2	4.1664	3.8364	3.2019	3.5230	3.5042	6.0822	4.0369	9.2612	4.0960	5.6256	2.9695	3.7258	4.2041	3.7478	3.6141	3.0105	3.8961	3.2533	3.5524	3.4251
Rftn2	_S[Phospho (STY)]LGLDTNASQAAGGR_.2	Q8CHX7_S429_M1_Rftn2	6.1267	6.2282	4.1562	4.0694	2.9577	3.1818	4.9933	3.3203	3.1417	6.2140	3.5160	4.2723	3.2497	4.1433	4.1606	3.9648	3.8580	5.4333	3.6237	5.8605
Rftn2	_PVTWNSAAQT[Phospho (STY)]PERK_.3	#N/A	6.3776	5.9341	5.9185	3.5764	5.6039	6.3110	5.7242	6.4949	5.8628	6.1263	6.2779	5.2083	5.4509	6.5205	5.3724	5.2798	5.8465	6.3941	6.0747	5.8935
Rftn2	_PVTWNSAAQT[Phospho (STY)]PER_.2	#N/A	7.4945	7.3309	7.4382	7.5403	6.9036	7.0756	6.9203	7.0961	7.0042	7.3813	6.9947	6.1787	6.9613	7.0021	5.5849	6.4340	6.9313	6.6769	6.4723	6.4957
Rftn2	_EERPAQSDS[Phospho (STY)]FSGFSSSDSVLR_.3	Q8CHX7_S469_M1_Rftn2	3.8667	4.2383	4.7749	3.9249	3.9492	3.7406	4.3571	3.5671	3.7241	3.9540	3.7472	3.3717	3.8536	3.6340	3.4818	3.5357	4.1047	3.9303	3.4792	3.6010
Gatad2a	_RPPS[Phospho (STY)]PDVIVLS[Phospho (STY)]DSEQPSSPR_.3	Q8CHY6_S103_M2_Gatad2a	3.5532	4.4496	4.0895	4.1362	2.8909	3.5932	4.3448	3.2536	3.4085	3.1635	4.3880	4.3390	3.5739	4.0766	4.2273	3.8981	3.7913	4.0419	3.6905	4.0436
Flywch1	_SHC[Carbamidomethyl (C)]HS[Phospho (STY)]PDIEGLQAR_.3	Q8CI03_S294_M1_Flywch1	3.7834	3.5357	6.7827	3.8008	7.1056	4.1180	3.6472	4.0091	6.7652	7.3512	4.0806	6.6977	7.3910	3.3581	6.4205	3.1455	3.5063	3.6431	7.0998	6.7148
Slain2	_S[Phospho (STY)]LPNLSR_.2	Q8CI08_S414_M1_Slain2	8.3962	8.7287	7.8696	8.5785	8.2240	8.1185	7.9688	7.9981	8.0630	8.9115	8.5590	8.2853	8.9924	8.6576	7.8479	8.4047	8.1081	8.2385	8.1825	8.4482
Slain2	_RTS[Phospho (STY)]SEDLR_.2	Q8CI08_S87_M1_Slain2	3.1506	4.1684	4.1881	6.1556	3.3576	6.2124	3.6905	3.4558	3.6107	4.4621	3.0894	3.8946	3.7762	4.2788	3.7159	4.0136	3.9935	3.8396	3.4882	5.7684
Gnl3	_ELS[Phospho (STY)]PEQSTAGKPSDGSSALDR_.3	Q8CI11_S505_M1_Gnl3	3.6236	4.3792	4.1599	4.0658	5.7061	3.1854	5.0871	3.3240	3.1380	6.0866	5.9384	4.2686	3.2461	4.1470	4.1569	5.3898	3.8617	4.2114	3.6201	5.8799
Pdlim5	_RGS[Phospho (STY)]QGDIK_.2	Q8CI51_S228_M1_Pdlim5	8.6005	8.1281	8.4072	8.0193	8.0420	7.6304	8.3076	6.3774	7.5630	8.5318	8.1316	7.5109	7.9557	7.7370	3.8983	4.2271	7.8447	7.1784	6.3615	8.4740
Bag4	_S[Phospho (STY)]PGNSPTPMSR_.2	Q8CI61_S175_M1_Bag4	4.3020	4.5179	6.9681	7.1599	3.0081	3.0415	4.0400	4.5277	3.9602	4.1126	5.6255	4.2440	4.3329	7.5226	4.0653	3.6641	4.0249	3.1245	3.4236	3.5540
Vps50	_SVS[Phospho (STY)]RETLK_.2	Q8CI71_S585_M1_Vps50	9.0667	8.7119	7.7623	3.4626	8.0838	7.6852	8.6288	4.3473	7.6546	8.6788	8.1387	7.8395	8.4226	8.0013	6.1187	7.8958	8.3085	8.3151	7.8043	7.8707
Vps50	_KKS[Phospho (STY)]DYSLNK_.2	Q8CI71_S595_M1_Vps50	4.0815	3.9213	5.8230	3.6079	3.4192	5.5454	6.2874	5.7696	4.1810	6.3499	3.0545	4.9141	4.2890	5.2754	3.6991	4.4264	5.3931	5.0944	5.3190	3.0710
Vps50	_SAYQDYDS[Phospho (STY)]DSDVPEELKR_.2	#N/A	4.0418	7.1057	6.9901	5.3295	3.6602	8.5988	8.5886	3.9536	8.0205	4.1553	3.9009	6.7130	3.9459	8.5083	6.7297	3.6565	3.8251	6.9263	7.9138	8.2906
Vps50	_FLEQSRS[Phospho (STY)]PSVSPSK_.2	#N/A	8.2033	6.1529	5.3415	7.3670	5.1740	3.5745	7.3155	3.7728	7.0567	7.3137	6.9875	7.1388	8.3397	7.4933	3.6888	3.7227	8.8093	3.6666	6.1314	5.6142
Vps50	_SAYQDY[Phospho (STY)]DSDSDVPEELK_.2	Q8CI71_Y557_M1_Vps50	3.5823	3.7368	3.1189	4.0019	3.7892	3.8226	3.2589	3.8080	3.1791	3.3929	4.1585	3.4629	3.3445	3.1570	3.2842	2.9444	3.3052	3.8442	4.1433	4.3351
Vps50	_S[Phospho (STY)]PSVSPSK_.2	#N/A	3.1612	4.1579	4.1986	3.9445	3.3681	3.4015	3.6800	3.4453	3.6002	4.4726	3.0788	3.8840	3.7656	4.2683	3.7053	4.0241	3.9830	3.8502	3.4988	4.2353
Dis3l2	_RPGLEKAS[Phospho (STY)]DEEPED_.2	Q8CI75_S864_M1_Dis3l2	11.3807	11.0347	11.1893	11.0906	10.5610	10.5946	10.9058	11.4146	10.8369	10.7875	10.7993	11.0166	11.6031	11.1871	9.6831	10.6404	11.3207	11.2325	10.7234	10.7962
Pygb	_QKQIS[Phospho (STY)]VR_.2	Q8CI94_S15_M1_Pygb	12.3980	12.6347	11.4545	12.3691	11.8922	12.0349	12.3141	12.4173	12.1750	12.6590	11.3226	11.6965	12.4489	11.6333	11.0645	11.6072	11.5496	11.6944	12.0391	11.2446
Pygb	_[Acetyl (Protein N-term)]AKPLTDSER_.2	#N/A	8.5481	8.4389	8.5442	8.5242	8.4944	8.2890	8.6902	8.5844	8.4805	8.4873	7.8724	8.2759	7.9118	8.2486	8.1393	8.0642	7.9893	8.3391	7.9278	7.8661
Pygb	_GIAGLGDVAEVR_.2	#N/A	7.5413	7.9984	7.7653	7.7064	7.5621	7.7339	7.9322	7.6875	7.7835	7.5013	6.9251	7.3381	7.2699	7.2985	6.8133	7.0377	7.2406	7.3028	7.1392	7.0423
Pygb	_NLAENISR_.2	#N/A	7.1556	7.9541	7.4325	7.7582	7.4753	7.8125	8.1004	6.8893	8.0308	7.8095	7.0205	7.2213	7.3468	6.9460	7.3039	6.8859	6.7930	7.5975	7.2341	6.9738
Pygb	_VIFLENYR_.2	#N/A	7.4789	7.7197	8.0952	7.8224	7.5127	7.3932	7.8952	7.4186	7.7018	8.0120	7.5273	7.2843	7.5412	7.0918	6.2870	7.2242	6.6317	7.2621	7.2122	7.3421
Pygb	_VEDVEALDQK_.2	#N/A	6.9663	6.4746	7.0457	7.1595	6.8720	6.9731	7.3121	7.0049	6.9874	6.5142	6.5516	6.4026	7.0236	6.5304	6.5073	6.3829	6.5287	6.5946	6.6097	6.0957
Pygb	_DYFFALAHTVR_.2	#N/A	6.7885	6.7585	6.7661	6.8823	6.7479	6.5917	7.2303	6.6781	6.6414	7.0492	6.2677	6.8659	6.1013	5.9274	5.4096	5.8734	6.5340	6.7186	6.2146	5.9319
Pygb	_DFYELEPEKFQNK_.2	#N/A	6.6736	6.7137	6.7576	6.7666	6.9968	6.7754	6.4388	6.1918	6.9622	6.9222	6.5648	6.4187	5.9101	5.9226	5.1328	5.8485	6.7086	5.5549	6.0290	6.1983
Pygb	_LLSLVDDEAFIR_.2	#N/A	6.2697	6.5123	6.4004	6.5227	6.0331	6.8294	6.3293	6.5785	6.7600	6.4578	6.1506	5.4579	5.3432	6.5475	6.0147	5.5996	6.0206	6.1693	5.9932	5.9360
Pygb	_TC[Carbamidomethyl (C)]AYTNHTVLPEALER_.3	#N/A	6.1067	6.0081	6.0228	6.6332	5.9537	6.3168	6.2320	6.0516	6.3010	6.6078	5.8228	5.6475	6.0229	5.2198	5.4593	5.6718	5.2011	6.0756	5.9156	5.6564
Pygb	_YEFGIFNQK_.2	#N/A	6.6583	6.6314	5.6589	5.9890	6.2684	6.3848	6.2420	5.8658	5.9721	4.3895	6.1062	5.7080	5.7140	5.8193	4.6367	5.5760	5.4698	6.2750	5.4486	4.6284
Pygb	_QAVDQISSGFFSPK_.2	#N/A	5.2711	5.4710	6.4701	6.1253	5.4756	5.7589	5.8915	5.4368	5.7477	5.9741	5.6707	4.7657	5.7172	5.9650	5.3437	5.6690	4.8952	5.0488	4.9549	4.7109
Pygb	_LVTSIGDVVNHDPVVGDR_.3	#N/A	7.2790	7.3485	7.3220	7.0272	6.8417	6.9839	7.1283	7.4915	7.1052	7.3013	6.6138	6.7700	7.0722	7.0159	6.4882	6.9388	6.6255	6.7403	6.8690	6.6946
Pygb	_HLDHVAALFPGDVDR_.3	#N/A	7.9422	8.1863	8.1422	7.7427	8.1238	8.1428	8.0971	8.0490	7.9137	8.0383	7.8577	7.7601	7.6517	7.5031	6.7408	7.0954	7.6319	7.6962	7.4532	7.6451
Pygb	_IVNGWQVEEADDWLR_.2	#N/A	4.7226	4.3201	4.8244	4.3016	4.0984	4.0167	4.4241	4.8578	3.7948	3.6803	3.2734	4.4075	3.4387	4.0420	3.6716	3.7831	4.4778	4.9590	3.6446	3.7350
Pygb	_LLSLVDDEAFIRDVAK_.3	#N/A	3.4244	5.1138	4.0180	4.3282	4.2032	4.8436	4.9856	4.6516	4.6698	3.2350	4.3165	4.7557	4.4260	4.2819	3.5641	4.2873	3.7198	4.8587	5.3474	4.3421
Pygb	_DFNVGDYIEAVLDR_.2	#N/A	4.6869	3.1632	4.9520	3.4283	4.6666	3.7455	5.0792	4.4471	4.0787	4.3901	4.0687	4.1816	4.1868	4.0408	3.5968	3.5180	3.8788	4.7341	4.5714	2.3961
Pygb	_VIPAADLSQQISTAGTEASGTGNM[Oxidation (M)]K_.3	#N/A	5.8844	5.5702	5.3701	5.0733	3.3640	5.3264	5.2703	5.4329	5.6145	5.9101	5.0459	5.2210	4.6018	5.2529	5.2611	5.2688	4.3298	5.2382	4.9280	4.7438
Pygb	_VFADYEAYIQC[Carbamidomethyl (C)]QAQVDR_.2	#N/A	5.4881	4.0493	4.6128	5.6392	3.7017	4.1022	4.2498	3.8187	4.3127	4.1599	4.7859	3.4956	3.8720	4.3531	3.8270	4.2984	4.8048	3.9366	3.2901	3.3198
Pygb	_HLEIIYAINQR_.2	#N/A	4.8122	4.1855	4.1710	4.4506	4.2930	4.4392	4.1997	4.5273	4.4604	3.9115	4.3742	4.2697	3.1645	4.2091	2.5133	3.5130	4.3574	3.6532	3.4544	2.8525
Pygb	_NNTVNTMR_.2	#N/A	3.9531	3.3660	5.0389	3.2356	4.1601	3.3097	4.3889	4.1542	4.3091	3.7637	3.7877	3.0921	4.4745	3.4764	2.9134	3.3152	3.1910	3.1413	4.2907	3.4434
Pygb	_WLLLC[Carbamidomethyl (C)]NPGLAEIIVER_.2	#N/A	1.9763	3.5741	3.2816	3.8392	3.9519	3.9853	3.0962	3.9707	3.0164	3.5556	3.9958	3.3002	3.7759	3.3196	3.1215	3.1071	3.4679	3.6815	3.9806	2.9969
Osbpl11	_SFSLASSGNS[Phospho (STY)]PISQR_.2	Q8CI95_S186_M1_Osbpl11	7.4040	3.9987	7.0085	6.3960	3.0058	3.7572	7.0216	7.4441	3.6837	7.8187	6.4059	6.8828	7.2671	7.5228	5.4298	6.2729	3.9433	6.7267	6.7054	6.3083
Clip4	_SWS[Phospho (STY)]SSTTAGGLEGTVK_.2	Q8CI96_S608_M1_Clip4	4.5201	4.2998	4.0567	4.5650	3.1572	3.3813	4.3838	3.2449	4.4429	4.0258	3.3165	4.5388	4.5510	4.0947	3.9610	4.1644	4.2430	4.4073	3.2055	5.1124
Tmem230	_LAS[Phospho (STY)]TDDGYIDLQFK_.2	Q8CIB6_S24_M1_Tmem230	3.9711	3.3480	3.5077	3.6131	4.1780	4.2114	4.3709	4.1362	4.2911	3.7817	3.7698	3.0741	4.4566	3.4584	2.8955	3.3332	3.1731	6.6632	4.3087	3.4254
Copa	_NLS[Phospho (STY)]PGAVESDVR_.2	Q8CIE6_S173_M1_Copa	4.0985	3.9043	9.0711	9.1460	8.5630	9.2806	8.9549	4.4668	8.9697	8.8862	3.0375	8.7714	4.2720	3.8158	3.6821	2.9425	9.7768	9.0231	3.4844	8.8949
Pak2	_[Acetyl (Protein N-term)]S[Phospho (STY)]DNGELEDKPPAPPVR_.2	Q8CIN4_S2_M1_Pak2	8.7159	8.8356	9.0438	8.9202	3.9324	8.6204	8.2431	8.6830	8.5894	8.9498	3.9286	8.6870	3.1146	9.1147	8.4170	8.6920	8.9398	8.4846	8.5973	8.7057
Mark4	_RS[Phospho (STY)]PTSTGDTELKEER_.3	Q8CIP4_S438_M1_Mark4	8.1931	8.7379	3.2047	3.5202	7.5943	7.8033	8.4892	7.7361	8.0763	8.3500	8.1781	7.9678	8.8787	7.7298	7.3806	8.1204	7.8375	8.1101	8.4643	7.7517
Mark4	_KAS[Phospho (STY)]C[Carbamidomethyl (C)]SAVGSGSR_.2	Q8CIP4_S458_M1_Mark4	5.0096	5.2733	4.2577	5.2345	3.7741	3.9982	4.8126	4.6211	4.3473	3.1418	4.4686	4.6359	3.9341	3.4779	3.3442	3.2804	3.1736	5.0638	5.0202	4.3678
Dock3	_GS[Phospho (STY)]PSLPDKYR_.2	Q8CIQ7_S1763_M1_Dock3	9.3155	3.8801	9.2594	8.8556	8.6006	8.4547	8.8329	3.6647	8.2549	3.2496	8.5575	8.3469	3.4699	4.5145	3.4275	4.3020	3.1619	3.9875	8.0899	8.1696
Dock3	_GNVLASHSPMS[Phospho (STY)]PENIK_.2	#N/A	3.9081	6.0769	5.6495	6.5944	3.8175	4.0143	5.8471	3.6815	3.8364	3.5846	5.5744	3.9807	4.0019	6.2861	3.9416	3.8575	4.2192	3.6836	3.3321	3.7501
Mical3	_GTSQVSS[Phospho (STY)]PSQPPEK_.2	Q8CJ19_S1154_M1_Mical3	11.1212	11.1238	11.3481	11.4108	10.7993	10.7859	11.1631	11.1070	11.0465	3.7175	6.6197	10.6894	11.2595	11.1722	9.8207	10.7454	11.4076	11.1602	10.7482	10.7556
Mical3	_RKT[Phospho (STY)]SQSEEEEPPR_.3	Q8CJ19_T684_M1_Mical3	6.3268	3.5740	3.2818	3.8391	5.8548	6.4470	8.1624	6.3688	3.0162	7.0334	3.9957	6.0889	3.1817	3.5269	3.1214	3.1074	3.4474	6.7527	4.0191	3.6513
Mical3	_QVGVFS[Phospho (STY)]PAR_.2	Q8CJ19_S1167_M1_Mical3	8.3576	8.1418	8.0268	8.0000	7.7427	7.6559	8.0661	7.8310	7.1595	8.0911	8.0906	7.6179	8.2969	7.8014	6.1679	7.6199	8.1584	8.0602	7.7759	7.7328
Mical3	_GRS[Phospho (STY)]EEELEASK_.2	Q8CJ19_S977_M1_Mical3	8.3298	8.4725	8.0327	8.2300	7.4555	7.5839	7.8677	8.0534	7.4073	8.1723	7.7128	8.0108	8.0368	7.8005	6.5662	7.5781	7.8954	7.7244	7.7889	7.5318
Mical3	_SVFS[Phospho (STY)]GYKK_.2	Q8CJ19_S1736_M1_Mical3	8.0234	7.7753	7.3849	7.8252	7.1637	4.1372	7.1024	4.1226	6.0641	7.7519	3.8439	7.1671	4.5307	7.3109	2.9696	3.2591	3.6198	6.8421	3.8286	6.4432
Mical3	_KTS[Phospho (STY)]QSEEEEPPR_.2	#N/A	4.2076	7.7935	3.2431	6.7515	6.6140	3.7694	7.4520	3.9080	4.0548	6.9871	2.9284	7.3460	4.1629	3.2301	3.5729	3.0517	4.4457	7.7543	3.0361	3.1971
Mical3	_S[Phospho (STY)]PGTEEAKSPLATK_.2	Q8CJ19_S1171_M1_Mical3	7.8608	6.8848	7.6880	8.0634	6.1014	6.6483	7.3975	6.9378	6.0590	7.8935	7.4600	7.1344	6.7362	6.7313	4.8737	6.9389	6.0725	7.3130	7.2271	6.1085
Mical3	_AVLS[Phospho (STY)]PIR_.2	Q8CJ19_S1216_M1_Mical3	8.6270	7.9050	8.1641	8.2045	7.0747	7.3687	8.0102	4.5196	3.1737	8.6683	8.5155	7.6504	8.6976	7.8459	6.1377	8.1327	7.6936	8.3871	8.1422	8.0728
Mical3	_SAGDQPPLLTPKS[Phospho (STY)]PS[Phospho (STY)]DKELR_.3	Q8CJ19_S1404_M2_Mical3	4.1695	8.8167	7.9838	3.4148	2.8756	8.0245	4.0332	4.3952	7.7728	4.3765	2.9658	4.3766	4.2004	8.3517	6.2908	3.5316	3.8924	8.3113	6.9274	7.7358
Mical3	_QVGVFSPARS[Phospho (STY)]PGT[Phospho (STY)]EEAK_.3	Q8CJ19_T1174_M2_Mical3	3.6128	5.2822	4.0299	5.1356	3.5478	4.7317	4.1008	3.6608	4.9419	3.7410	3.6658	3.7759	3.9702	4.0471	3.8325	3.8485	4.8295	3.9131	4.9265	4.2254
Mical3	_VKS[Phospho (STY)]PEEPLFPTPLLLR_.3	Q8CJ19_S1187_M1_Mical3	5.7143	6.4123	3.6565	3.8657	3.9588	4.1829	6.6375	6.5475	3.6414	3.3265	4.0158	6.0056	3.7494	7.2304	3.1595	3.4651	3.4415	6.1111	4.0070	2.9705
Mical3	_RQLS[Phospho (STY)]FSEDSDLSSDDILER_.3	Q8CJ19_S1783_M1_Mical3	4.2130	3.7898	3.2485	3.4764	3.5508	3.7748	3.9903	3.9134	4.0494	4.4194	2.9229	3.6792	4.1575	3.2356	3.5675	3.0571	4.4511	3.3821	3.0307	3.2026
Crocc	_GFSGQRT[Phospho (STY)]PT[Phospho (STY)]PPRHS[Phospho (STY)]PGR_.3	Q8CJ40_T494_M3_Crocc	6.7935	7.2255	6.9170	6.9622	6.6057	7.0272	6.9733	3.1933	7.9918	5.9857	6.2239	7.1947	7.1034	7.5607	7.0605	4.1012	7.2111	7.4304	6.8534	8.3357
Crocc	_EAPAGGSGDGLS[Phospho (STY)]SPSPLEYSPR_.2	Q8CJ40_S1468_M1_Crocc	4.3169	5.7236	4.3542	4.1607	4.3718	3.2340	4.2864	3.5283	6.6242	4.0884	6.8566	6.8504	4.3435	4.3626	3.8636	4.1698	4.2763	4.0254	6.8457	3.4656
Crocc	_DLAQAALS[Phospho (STY)]DTESGVQLSSSER_.2	Q8CJ40_S464_M1_Crocc	4.1934	5.3207	3.4880	3.4456	4.0079	4.3679	4.2144	4.1665	4.1346	3.9383	3.6133	3.3188	4.2190	3.5039	4.2398	3.3061	4.2101	4.7427	3.3277	3.3132
Crocc	_TPTPPRHS[Phospho (STY)]PGR_.3	Q8CJ40_S501_M1_Crocc	3.7662	4.1196	4.3143	3.6545	3.5493	3.6646	3.4583	3.9957	3.8666	3.8126	3.7631	3.8457	3.9928	3.9183	4.0359	3.6768	3.8751	3.7721	3.6465	3.7416
Cmtm4	_TES[Phospho (STY)]RDVDSRPEIQR_.3	Q8CJ61_S194_M1_Cmtm4	6.8851	4.1210	3.6512	3.5534	3.3758	3.5241	3.9411	4.0338	3.9320	4.3465	3.9455	3.9117	4.0678	4.1586	3.7608	3.4701	4.0403	3.4735	3.4586	3.1712
Syn3	_SPGS[Phospho (STY)]PQLSR_.2	Q8JZP2_S483_M1_Syn3	12.0828	11.9656	11.8443	12.3647	11.5630	11.4289	11.6458	11.6601	11.3823	12.2160	11.1909	11.5216	11.8998	11.7040	10.6275	11.5593	11.4206	11.3148	11.2955	11.3550
Syn3	_LSPQGQQPVS[Phospho (STY)]PQSGSPQQQR_.3	#N/A	9.3465	9.3609	9.3830	9.5542	8.6565	8.5217	8.9880	9.2304	8.4355	9.1616	8.7356	9.0895	9.0853	9.4988	7.6818	8.8598	8.8572	8.9798	8.7098	8.6259
Syn3	_SQS[Phospho (STY)]LTNSLSTSDTSHR_.2	#N/A	8.8995	8.6020	9.2563	9.1845	8.3263	7.7046	8.4177	8.1237	8.0844	9.0057	8.3638	8.1436	8.4433	8.6926	7.3075	8.4930	8.3343	8.1922	8.5405	8.2261
Syn3	_STS[Phospho (STY)]QQGEEPQK_.2	Q8JZP2_S519_M1_Syn3	9.2805	9.3491	9.8115	4.2029	8.7618	3.8498	3.9013	8.3524	3.5904	9.8656	8.9813	8.8335	8.9405	4.0703	3.6273	9.0948	8.3219	8.8524	8.8957	9.0423
Syn3	_SAS[Phospho (STY)]PHPHLNK_.2	Q8JZP2_S530_M1_Syn3	3.1763	4.1427	4.2138	4.4079	3.3833	3.2242	3.6648	3.4020	3.0992	3.8688	3.4735	3.8689	3.2072	4.2518	3.6902	4.0393	4.4001	4.2502	3.0484	3.9291
Gas2l1	_TGTFS[Phospho (STY)]PQR_.2	Q8JZP9_S292_M1_Gas2l1	4.5120	7.9451	4.0486	4.5730	3.2181	3.2515	3.8300	3.2369	7.3170	6.7869	7.1415	4.0340	7.7330	7.1728	3.8553	3.8742	4.2350	7.5959	7.0653	7.0897
Gas2l1	_GSPTPSPRPGS[Phospho (STY)]PVPGSER_.3	Q8JZP9_S306_M1_Gas2l1	3.8947	3.9726	4.5928	4.0441	3.7575	3.8558	4.3809	3.7576	4.3732	3.9044	3.5621	5.1643	5.9865	5.4147	5.0649	3.8006	3.8135	3.9051	3.8618	3.6143
Afg3l2	_NLETLQQELGIEGENR_.2	#N/A	3.1972	4.1218	6.5074	4.3870	3.4042	5.8588	5.9370	3.4229	5.4459	3.8479	3.4944	3.8480	3.2281	4.2727	5.3939	4.0602	4.4210	5.6579	3.0275	3.9500
Eif3b	_GHPSAGAEEEGGS[Phospho (STY)]DGS[Phospho (STY)]AAEAEPR_.2	#N/A	7.2209	7.3052	7.3483	7.4585	6.8164	7.1687	7.2013	6.9959	7.2384	7.2678	6.8432	6.9160	7.2546	7.4001	6.8572	7.2337	7.3864	7.1508	7.4115	6.8077
Eif3b	_AKPAAQSEEETATS[Phospho (STY)]PAAS[Phospho (STY)]PTPQSAER_.3	Q8JZQ9_S75_M2_Eif3b	10.8635	10.7298	10.6158	10.8499	10.3336	10.2491	10.3611	11.0127	10.3225	10.4814	10.4281	10.7216	11.1658	11.3299	10.3347	10.7750	11.0234	10.8493	10.8745	10.6075
Eif3b	_SPS[Phospho (STY)]QEPSAPGK_.2	Q8JZQ9_S90_M1_Eif3b	3.7271	8.0164	4.1071	8.1973	4.1338	7.9589	4.0047	3.9528	3.6499	8.0272	7.9401	7.2836	3.7580	4.1452	6.9281	3.4777	8.0845	7.0864	4.0238	3.8224
Slc4a8	_KLLSS[Phospho (STY)]PGK_.2	Q8JZR6_S1046_M1_Slc4a8	4.3989	3.3784	3.4773	8.5985	3.7367	3.9607	8.9345	4.1664	8.7272	3.1044	8.3231	8.4665	9.0458	3.5153	3.3816	3.2430	8.9100	8.9127	3.7849	8.3334
Slc4a8	_ALS[Phospho (STY)]INSGNTK_.2	Q8JZR6_S1076_M1_Slc4a8	7.0593	6.8968	7.3518	7.5102	4.2825	5.2637	6.4836	4.9240	5.4912	6.7167	5.8954	6.0136	4.3521	3.3539	2.7910	5.5465	5.7559	5.7183	5.4392	5.1435
Slc4a8	_DGQTVS[Phospho (STY)]PQSATNLEVK_.2	Q8JZR6_S259_M1_Slc4a8	4.3631	6.8150	3.3985	3.3263	3.7008	6.7700	3.8403	4.0635	7.0953	3.8545	3.6970	3.5292	4.3838	3.3856	3.4175	5.8738	6.9830	3.2320	6.0098	3.3526
Slc4a8	_KQS[Phospho (STY)]DPHSMDR_.2	Q8JZR6_S246_M1_Slc4a8	3.9607	4.0421	4.4970	3.7287	3.2984	3.1857	4.2426	3.6611	3.8160	4.1670	3.2946	3.9315	3.9814	4.6944	3.8198	4.3056	4.1988	3.6344	3.2830	4.4511
Lin7a	_EQNS[Phospho (STY)]PIYISR_.2	Q8JZS0_S130_M1_Lin7a	9.5436	9.4019	9.5566	9.5498	9.2558	9.2486	9.0840	9.5393	9.2624	9.3649	9.3229	9.3484	9.8001	9.7410	9.2029	9.4825	9.5815	9.3023	9.3833	9.4589
Lin7a	_ATVAAFAASEGHSHPR_.3	#N/A	5.8793	5.7480	4.4367	4.1850	5.8979	5.4716	5.8424	3.6249	5.9819	6.0698	5.8908	5.3647	3.4301	6.3993	6.1986	5.3511	3.1222	5.7391	5.3551	4.1520
Lin7a	_VLQSEFC[Carbamidomethyl (C)]TAIR_.2	#N/A	3.5911	4.4117	5.4767	4.3365	2.9288	2.7187	5.4650	3.4734	5.4193	3.7974	3.5449	4.3012	3.2786	4.9811	5.1254	4.8705	4.4714	4.9209	2.9771	4.0005
Lin7a	_VLEEMEAR_.2	#N/A	3.2041	3.2978	5.4098	5.9735	4.0427	5.0384	4.8025	4.4054	3.0594	4.2393	4.0389	3.1873	3.2249	5.2278	3.0756	3.5490	3.4422	4.3910	4.0395	3.7672
Lin7a	_QQQQLLIQQQQQQQQQPQQNHM[Oxidation (M)]S_.3	#N/A	4.1485	3.6769	3.6850	3.4829	3.6628	3.2996	3.3269	3.6815	3.8821	3.5284	3.5714	3.6954	4.4925	4.0252	3.3486	3.5106	3.8714	3.0460	3.1434	3.5865
Lin7a	_LQESGEVPVHK_.3	#N/A	4.2452	4.5747	3.7818	3.5277	2.9513	2.9847	1.7126	3.8621	4.0170	4.0558	3.4956	4.3008	4.1824	3.1842	4.1221	3.6073	4.3998	4.3334	3.0820	3.1512
Cpne5	_DIVQFVPFR_.2	#N/A	7.2642	7.7046	7.6580	7.4201	7.7753	7.9383	7.9478	7.3323	7.4980	7.5866	7.6582	8.0949	7.7121	7.9705	7.6000	7.8328	7.4140	7.7995	7.8578	7.6597
Cpne5	vl (Protein N-term)]M[Oxidation (M)]EQPEDM[Oxidation (M)]AS[Phospho (STY)]LSEFDSLAGSIF	Q8JZW4_S9_M1_Cpne5	3.1690	4.1501	4.2064	4.4152	3.3760	3.4093	3.6721	3.3947	3.5923	4.4805	3.0710	3.8762	3.7578	4.2604	3.6975	4.0320	4.3928	3.8580	3.5066	4.2274
Cpne5	_FDLYDVDSKS[Phospho (STY)]PDLSK_.2	#N/A	3.3333	7.2308	6.6925	6.5066	3.5403	6.8948	6.3310	3.3887	6.7945	6.3516	3.8283	6.5676	3.5943	7.7802	6.7126	4.1963	7.5504	7.0739	7.0025	7.0759
Cpne5	PAAPAQS[Phospho (STY)]PPQS[Phospho (STY)]PAHS[Phospho (STY)]PPGSPVHT[Phospho (STY	Q8JZW4_T591_M3_Cpne5	3.7786	10.0688	10.4954	8.3047	3.4729	3.4892	9.4490	3.6793	9.2773	6.2684	3.2627	9.2572	3.7950	10.9226	9.8511	8.8162	5.9248	10.1732	9.8448	10.5777
Cpne5	RPPPAAPAQS[Phospho (STY)]PPQS[Phospho (STY)]PAHS[Phospho (STY)]PPGS[Phospho (STY)]I	#N/A	4.3864	5.5195	3.4263	3.5062	3.7242	3.9482	4.7658	4.1036	3.8766	4.0149	4.2510	3.3437	3.9847	7.3633	3.3947	3.2305	3.3963	5.0839	4.1248	3.2631
Sh2d5	_LGNPYC[Carbamidomethyl (C)]S[Phospho (STY)]PTLVR_.2	Q8JZW5_S237_M1_Sh2d5	3.9678	10.2145	3.5044	10.3064	4.1748	9.3173	9.8569	10.0817	4.2944	3.7784	10.0059	3.0774	4.4598	3.4616	2.8987	8.7663	3.1763	3.1560	4.3054	3.4286
Sh2d5	_RAS[Phospho (STY)]DC[Carbamidomethyl (C)]GPAPYRPR_.3	Q8JZW5_S12_M1_Sh2d5	4.1436	3.7394	5.3651	3.4260	5.0878	5.6947	4.1984	3.9637	5.8830	6.0892	4.8732	4.4025	5.6326	5.3075	4.5548	3.5057	4.9719	5.5366	2.9803	3.2529
Rbm17	_RPDPDS[Phospho (STY)]DEDEDYER_.2	#N/A	4.3481	3.6547	3.3835	7.9445	7.4215	7.8256	7.6369	4.0485	6.9990	3.8695	3.6820	3.5442	4.3688	3.3706	7.0169	3.1921	3.0853	3.2470	4.3965	7.4332
Rbm17	_RPDPDS[Phospho (STY)]DEDEDYERER_.3	#N/A	4.0783	6.7727	6.3894	3.6111	6.6014	3.0681	4.1251	6.1272	7.1571	4.2846	6.8035	6.7231	7.5786	6.8082	3.7023	4.4232	6.9587	3.5168	7.3676	6.9051
Adgrl2	_S[Phospho (STY)]ENEDIYYK_.2	Q8JZZ7_S1428_M1_Adgrl2	6.5065	6.0052	6.6262	3.9248	3.1024	5.4617	6.1983	5.9762	5.8902	5.9896	6.3876	5.7218	3.7854	4.2880	3.6935	5.2527	5.7994	5.1824	5.6180	5.7636
Adgrl2	_GNS[Phospho (STY)]DGYIIPINK_.2	Q8JZZ7_S1458_M1_Adgrl2	11.2462	11.3021	11.3155	11.2101	3.3417	10.6099	10.9164	3.0605	10.8392	11.0776	3.1320	10.3290	4.3665	10.7864	9.3955	10.4786	10.7519	10.6648	10.5889	10.4937
Adgrl2	_S[Phospho (STY)]MPNLGAGR_.2	Q8JZZ7_S1437_M1_Adgrl2	3.9744	4.0795	5.3961	5.7177	3.3121	3.1720	4.3531	3.6748	3.8296	4.2432	4.5716	3.9179	3.9951	4.7650	3.9348	4.3193	4.2124	3.6207	3.2693	2.9639
Spata2	_S[Phospho (STY)]VDAYDSYWESR_.2	Q8K004_S247_M1_Spata2	9.9635	10.4719	10.5054	10.0992	6.4868	9.8903	9.9168	8.1280	9.9233	10.0053	9.2220	9.6810	9.4896	10.0301	8.5730	9.7048	9.8731	10.0094	10.0053	9.8122
Fnbp1l	_TIS[Phospho (STY)]DGTISAAK_.2	Q8K012_S295_M1_Fnbp1l	10.1967	10.3305	10.2242	10.5334	9.7945	9.6558	9.7503	9.6656	9.6737	10.4257	10.1402	10.2420	10.7009	10.4181	9.3529	10.1852	10.0987	9.9709	9.8989	10.2384
Fnbp1l	_ES[Phospho (STY)]PEGSYTDDANQEVR_.2	#N/A	6.8878	6.6316	4.5231	4.0985	5.7245	6.8423	6.0875	6.6916	6.0640	6.9443	6.4320	6.2468	6.2150	7.5081	3.3808	6.6964	7.0275	6.3036	4.2399	6.9289
Fnbp1l	_HSSDINHLVTQGRES[Phospho (STY)]PEGSYTDDANQEVR_.4	#N/A	4.0303	3.6888	4.8620	4.8537	3.5929	3.8705	5.1430	4.0288	3.7228	4.5216	3.4827	5.0562	4.1695	5.9172	4.1092	3.9444	4.0221	3.4897	3.4665	3.1629
Bclaf1	_EVQS[Phospho (STY)]PEQVK_.2	#N/A	10.9082	10.9558	10.9926	10.9202	10.4213	10.9028	10.8235	10.7162	10.6551	10.7159	10.5564	10.5110	10.7652	10.8509	9.9758	10.3104	11.0057	10.7443	10.5798	10.5252
Bclaf1	_QKS[Phospho (STY)]PEIHR_.2	#N/A	10.4498	10.5858	10.3684	10.1740	10.1161	10.3461	10.4290	10.5946	10.0941	10.3180	9.9534	10.1117	10.7913	10.3531	10.0795	9.9960	10.3982	10.3124	10.3561	10.0326
Bclaf1	_KEVQS[Phospho (STY)]PEQVK_.2	#N/A	9.3360	9.0232	9.1917	8.9389	8.7800	9.1294	9.0019	9.1416	8.9650	9.1039	8.8659	8.7058	9.0729	9.0516	8.2465	8.5758	9.2278	9.0559	8.6568	8.8185
Bclaf1	_KAEGEPQEES[Phospho (STY)]PLKSK_.2	#N/A	8.3572	7.8374	6.4379	7.0348	7.6319	7.3783	8.3238	8.0270	8.0961	7.4991	6.9115	7.4998	9.0343	8.0134	7.6904	8.1538	7.7227	7.6893	7.1457	8.1260
Bclaf1	_KAEGEPQEES[Phospho (STY)]PLK_.2	#N/A	8.1014	8.6021	7.9370	7.4900	7.4784	8.7506	8.6494	8.0507	7.9198	8.7205	8.6282	8.0861	8.6870	8.6864	7.4083	7.9925	8.6743	8.5772	8.3584	8.3139
Bclaf1	_SATSGDIWPGLSAYDNS[Phospho (STY)]PR_.2	Q8K019_S221_M1_Bclaf1	9.1042	3.8760	3.1623	3.5626	7.9021	3.6886	4.0765	4.4385	4.1356	4.3331	3.0092	3.7654	4.2437	9.0253	3.6537	2.9709	9.0448	3.2137	3.5128	3.4647
Bclaf1	_AEGEPQEES[Phospho (STY)]PLKSK_.2	#N/A	3.5303	3.7888	7.4611	4.0540	6.7660	3.7705	3.3109	7.8678	7.2523	3.3409	4.2106	7.4554	7.6184	3.8992	3.3363	4.3932	3.2532	4.2193	3.8679	7.1869
Bclaf1	_TIT[Phospho (STY)]PQNAPR_.2	Q8K019_T305_M1_Bclaf1	8.3423	8.1878	3.8504	4.3753	4.1527	7.7147	3.3884	8.2279	8.0082	3.5204	7.4723	7.8534	7.9853	7.8120	6.9545	7.4426	8.1626	8.0408	7.4533	3.8045
Bclaf1	_KEVQS[Phospho (STY)]PEQVKSEK_.3	#N/A	4.0248	6.0242	3.8021	5.2420	3.3625	5.3583	6.0578	3.7252	4.4913	6.1780	4.7330	5.4116	6.3038	3.8531	5.8392	5.9375	3.5038	5.4452	5.8071	5.3785
Bclaf1	_RIDIS[Phospho (STY)]PSALR_.2	Q8K019_S656_M1_Bclaf1	4.1086	3.8942	3.1440	3.5808	3.4463	8.3525	4.0948	4.4567	4.1539	4.3149	3.0274	3.7837	4.2619	3.8057	3.6720	2.9526	3.9540	3.1955	3.4945	7.1739
Bclaf1	_ELFDYS[Phospho (STY)]PPLHK_.2	#N/A	4.0000	9.3605	3.5366	3.5842	4.2070	3.9014	9.1668	8.6664	8.5995	8.7862	4.2972	3.0452	4.0309	8.3548	7.6462	8.5485	3.7229	8.7565	8.8082	3.2520
Bclaf1	_AEGEPQEES[Phospho (STY)]PLK_.2	#N/A	7.6617	7.2849	7.8387	7.2490	7.5853	7.5179	7.0166	7.4563	7.6986	3.3076	7.7355	6.3842	3.4298	7.4180	5.7507	6.4785	7.3743	7.0024	7.1655	7.0910
Bclaf1	_QKS[Phospho (STY)]PEIHRR_.2	#N/A	5.6861	5.8847	5.8030	5.7032	5.4369	6.0243	6.0318	5.9474	5.6194	6.4887	5.1749	5.8484	6.6299	5.5418	3.2163	5.1522	7.2849	6.0499	5.7485	5.9324
Bclaf1	_S[Phospho (STY)]PEIHRR_.2	#N/A	6.3257	6.0925	7.3129	6.1741	6.3741	6.3928	5.4448	5.8948	6.1545	3.1232	6.1580	5.3470	4.4385	3.4403	3.3628	3.2618	6.4948	6.0347	4.3268	5.7914
Bclaf1	_FHDS[Phospho (STY)]EGDDTEETEDYR_.2	#N/A	5.9524	6.3620	5.9733	5.7360	5.7688	6.0118	6.5786	5.6978	6.0358	6.2105	6.3653	5.1472	6.0919	6.0821	5.5785	5.8948	5.4667	6.3794	6.5480	5.9793
Bclaf1	_YS[Phospho (STY)]PSQNS[Phospho (STY)]PIHHIPSR_.3	#N/A	8.1358	7.3684	4.2288	4.0114	6.6629	7.4726	7.4725	6.6698	3.8212	8.2244	8.3077	7.4255	6.9026	7.1704	6.1284	7.5857	6.9763	3.7419	7.2312	3.9600
Bclaf1	_NTPSQHSHSIQHS[Phospho (STY)]PER_.3	Q8K019_S267_M1_Bclaf1	7.9397	8.2322	7.7914	7.8236	7.3023	7.8710	7.9859	7.7309	7.9946	7.8647	7.7765	7.6554	8.2951	8.0150	7.2283	7.4697	7.9474	8.0476	7.8757	7.5295
Bclaf1	_LKELFDYS[Phospho (STY)]PPLHK_.3	#N/A	7.2434	7.9902	6.6137	6.1431	3.7152	6.8498	7.9381	7.8858	3.5792	6.3897	3.9536	7.3464	7.8426	8.2865	5.5987	6.3571	6.7542	7.6916	7.1297	3.7502
Bclaf1	_SIFREES[Phospho (STY)]PLR_.2	#N/A	9.5991	9.7634	9.5116	9.4243	9.1169	9.5227	9.2849	9.6680	9.3592	9.3665	9.0894	9.2567	10.0251	9.4536	8.6070	9.2080	9.8852	9.4907	9.3473	9.3392
Bclaf1	_QKFHDS[Phospho (STY)]EGDDTEETEDYR_.3	#N/A	5.7413	5.8592	5.1497	5.3358	4.7208	5.2928	5.6330	5.5043	5.3869	5.8157	5.7786	5.4246	6.2138	5.8124	5.3194	5.3680	5.2567	5.5437	5.5452	5.1083
Bclaf1	_EKSIFREES[Phospho (STY)]PLR_.3	#N/A	5.4923	5.8558	4.1597	4.1158	5.0927	3.6353	3.5719	5.1515	3.7945	4.9488	4.8336	5.8896	6.3309	3.8934	4.9838	5.8780	5.6966	5.6504	5.3875	5.7176
Bclaf1	_EVQS[Phospho (STY)]PEQVKSEK_.2	#N/A	9.4721	9.8624	8.8131	9.2817	9.2408	9.3307	9.4902	9.7369	9.2336	9.1838	8.7382	9.3555	10.1057	9.2846	9.0031	9.2049	9.5356	9.6341	9.0792	9.0397
Bclaf1	_YS[Phospho (STY)]PSQNS[Phospho (STY)]PIHHIPSRR_.3	#N/A	8.2175	8.3518	3.2454	8.6840	7.9673	8.3473	8.3458	3.9104	4.0525	4.4163	8.2214	8.1706	4.1605	8.4116	3.5706	7.9891	8.3665	3.3851	3.0337	7.8897
Bclaf1	_GRADGDWDDQEVLDYFS[Phospho (STY)]DKESAK_.4	#N/A	4.2163	4.2348	3.5372	3.4285	3.2557	3.4031	4.0585	4.2170	4.0502	4.2277	3.2417	4.0264	4.1857	3.1484	3.8758	3.3562	4.2059	3.3574	3.1179	3.2904
Bclaf1	_ADGDWDDQEVLDYFS[Phospho (STY)]DKESAK_.3	#N/A	3.8058	4.2862	4.0704	4.5513	3.1436	3.3677	4.3975	3.2586	4.4566	4.0122	3.3301	4.0123	4.5646	4.1084	3.9747	4.1507	4.2567	4.3936	3.1918	3.7857
Bclaf1	_SQEEPKDTFEHDPSES[Phospho (STY)]IDEFNK_.3	Q8K019_S198_M1_Bclaf1	4.3845	3.6183	3.4199	3.3049	3.7222	3.9463	3.8189	4.1808	3.8780	3.0900	4.2523	3.5078	3.9860	3.5298	3.3961	3.2285	3.6780	3.4714	3.7704	3.2071
Bclaf1	_LRC[Carbamidomethyl (C)]DS[Phospho (STY)]ADLR_.2	Q8K019_S688_M1_Bclaf1	4.0819	3.9209	3.1174	3.6075	3.4196	3.6437	4.1215	4.4834	4.1805	4.2882	3.0541	3.8104	4.2886	3.8324	3.6987	4.4268	3.9806	3.1688	3.4679	3.5096
Scamp1	_TVQTAAANAASTAATSAAQNAFK_.3	#N/A	5.4572	5.2688	4.9119	5.5066	5.0331	5.3543	5.3390	4.9931	5.4803	4.9780	3.5344	5.1302	5.1579	5.3302	4.4706	4.5176	5.4411	5.1939	5.2676	4.8692
Scamp1	_EMQNLS[Phospho (STY)]QHGR_.2	Q8K021_S112_M1_Scamp1	3.1787	5.9686	5.7411	6.1908	4.0173	5.5341	5.2805	4.9983	3.5829	3.3850	5.8455	3.2127	3.6909	6.2262	3.6465	3.5236	4.9798	2.7230	4.0655	4.4128
Stard8	_SLS[Phospho (STY)]IESLC[Carbamidomethyl (C)]PDEGR_.2	Q8K031_S234_M1_Stard8	7.6066	8.0057	4.1054	7.7509	3.6772	7.3881	7.3916	7.0966	7.3167	7.9232	7.7443	3.6702	8.1287	4.0925	3.5585	7.9475	3.8072	4.1035	8.0037	7.9143
	IQPQLPDEDGNHS[Phospho (STY)]DKEDEQPQVVVLKK.4	#N/A	4.5503	8.5552	7.6550	6.4510	3.2564	7.4266	8.0212	3.2752	7.5031	7.0256	3.1905	7.8297	4.5812	9.1917	8.1096	7.6125	6.9190	8.1140	7.7119	7.6318
	KIQPQLPDEDGNHS[Phospho (STY)]DKEDEQPQVVVLK.4	#N/A	3.7226	4.6864	5.5951	3.9668	3.0603	4.7746	3.8908	3.4229	5.2178	4.4950	3.0565	4.1697	3.7433	5.7643	5.0662	4.7784	3.9606	5.0348	5.0375	5.1668
	IQPQLPDEDGNHS[Phospho (STY)]DKEDEQPQVVVLK.4	#N/A	4.0072	6.1317	5.8762	4.0793	3.6581	6.1108	5.7830	3.7548	5.6004	5.6521	3.6740	3.6121	3.7793	6.8641	5.7512	5.3754	3.7419	5.5906	6.2515	5.6251
	NSSLLSFDS[Phospho (STY)]EDENE.2	Q8K039_S150_M1_	3.8131	3.8903	5.6924	3.8251	3.6527	7.2338	3.8685	3.7076	7.0891	3.6693	3.7791	3.7197	4.2539	7.7983	6.2585	3.7456	3.9459	7.2324	3.5899	3.4749
Samd14	_S[Phospho (STY)]LDEDEPPPSPLAR_.2	Q8K070_S108_M1_Samd14	12.2638	12.6561	12.5859	12.7663	9.4652	12.3309	12.2693	10.3891	12.3252	12.7796	11.5205	12.2128	11.9123	12.6082	11.2331	12.4245	12.4203	12.1622	12.2425	12.4186
Samd14	_VTDGC[Carbamidomethyl (C)]GS[Phospho (STY)]PLHR_.2	Q8K070_S77_M1_Samd14	7.2691	4.0687	6.8111	7.1589	7.2305	6.8732	3.8691	7.0242	6.5683	7.1865	6.9128	7.2906	6.6928	6.8886	6.2740	6.1396	8.1461	6.7527	6.4047	6.7911
Samd14	_HEGFS[Phospho (STY)]PK_.2	Q8K070_S267_M1_Samd14	5.1313	5.3700	4.3091	5.1485	5.1048	4.8118	5.0038	5.0420	5.3642	4.6822	4.8563	4.8909	5.8154	5.6730	4.4068	4.8503	4.3183	3.5218	5.5727	5.0169
Samd14	_AEPHSEDDSRDAS[Phospho (STY)]PPEPASPTIGLDKK_.4	#N/A	3.8079	5.8148	3.6864	6.0435	5.2482	6.0618	5.5072	3.7660	5.5923	6.2368	6.1104	5.2216	3.7729	3.7127	6.9224	6.7280	6.6556	6.9262	6.7139	3.9685
Samd14	_GSASSGSTTSPT[Phospho (STY)]C[Carbamidomethyl (C)]SPK_.2	Q8K070_T258_M1_Samd14	3.8248	3.8765	3.7483	3.7589	3.6486	3.8727	3.9668	4.8249	4.0259	4.8534	3.4221	4.0105	3.9585	3.8971	3.5440	3.7120	4.0390	3.5885	3.6919	4.1445

Samd14	_DAS[Phospho (STY)]PPEPASPTIGLDKK_.2	#N/A	4.0354	3.6858	6.5842	3.7357	3.4801	4.0747	3.8694	3.9919	7.4651	3.9332	3.5974	4.0407	3.9754	3.9057	3.7973	3.6519	3.8507	3.5990	3.7105	3.6799
Samd14	_DAS[Phospho (STY)]PPEPASPTIGLDK_.2	#N/A	4.0624	3.2567	3.5990	3.5218	4.2694	10.0892	3.9261	4.2881	3.9852	4.4835	4.3596	2.9828	4.0933	3.6371	8.7023	3.4245	9.5212	9.0501	3.6632	3.3143
Samd14	_DSASSAEDGEGS[Phospho (STY)]DGPGGK_.2	Q8K070_S64_M1_Samd14	3.5945	3.6445	4.0905	3.9097	3.5771	3.8968	3.2142	3.9160	3.6132	3.5805	3.8388	3.3707	3.7212	4.1285	3.0028	3.4421	4.3133	3.7520	4.2499	3.8058
Znf536	_GSGS[Phospho (STY)]DQESQSVSR_.2	Q8K083_S678_M1_Znf536	4.2391	3.7637	3.2746	3.4503	3.5768	3.8009	3.9643	3.9395	4.0233	4.4454	2.8969	3.6532	4.2598	3.2616	5.5665	3.0832	4.4771	3.3560	5.6167	3.2286
Cdk17	_RLS[Phospho (STY)]LPADIR_.2	Q8K0D0_S146_M1_Cdk17	8.5155	8.2906	7.3390	7.7290	6.0910	6.8180	7.1614	7.8058	6.9488	8.5785	7.2650	6.9532	7.3040	7.8832	5.5879	7.5281	7.7044	7.5618	7.3285	7.3776
Cdk17	_RAS[Phospho (STY)]LSEGFGK_.2	Q8K0D0_S180_M1_Cdk17	7.9117	7.4941	7.6442	3.2580	7.0939	3.5752	6.8687	7.7660	6.7760	7.6711	3.1226	3.8789	4.3571	7.5509	3.7672	6.7958	7.1511	6.9546	6.8880	7.0560
Cdk17	_RIS[Phospho (STY)]M[Oxidation (M)]EDLNKR_.3	#N/A	5.8375	5.5448	5.9696	5.7591	5.4679	4.8202	5.9432	5.0194	5.6766	6.0638	5.6939	4.9379	5.9987	5.3777	5.4554	5.6887	5.3252	5.3332	5.9144	5.4550
Cdk17	_RIS[Phospho (STY)]MEDLNK_.2	#N/A	4.4508	5.9303	6.8450	6.5625	3.7885	4.6950	5.7383	4.1511	3.8117	3.1562	5.6440	4.8730	3.9197	6.8489	3.3298	3.2948	3.1880	3.1443	3.5857	3.4403
Cdk17	_MGSDGESDQASGTSSDEVQS[Phospho (STY)]PTGVC[Carbamidomethyl (C)]LR_.3	Q8K0D0_S122_M1_Cdk17	3.8972	4.6244	6.0324	5.4794	3.2349	3.4590	3.9669	3.1672	4.3652	4.1035	3.2388	3.9950	4.4733	4.0171	3.8833	4.2421	4.1353	2.9841	3.2832	3.6943
Cdk17	_LQSS[Phospho (STY)]PPFDQPMSR_.2	Q8K0D0_S165_M1_Cdk17	3.0897	5.5697	3.6260	3.0989	3.9282	4.0567	3.6128	4.2909	4.4458	3.2960	3.9244	3.3017	3.1104	7.0709	3.1900	3.4346	3.3277	3.0046	4.1540	3.5801
Phyhip	_ISWAM[Oxidation (M)]EDSDLER_.2	#N/A	7.1769	6.8738	7.0259	6.0850	6.6645	6.9031	6.6579	7.0828	7.0507	7.0022	6.7427	6.6413	7.1769	6.4775	6.3017	7.2462	6.9963	7.0135	7.1000	6.7220
Phyhip	_EYFQHAR_.2	#N/A	6.5927	6.8591	7.0504	6.5619	6.5862	6.7937	6.7934	6.7963	6.5153	6.5477	6.7093	6.2515	6.0602	6.2530	6.4589	6.0965	6.1069	6.6181	6.3753	6.1651
Phyhip	_THC[Carbamidomethyl (C)]GNVLQPYLK_.3	#N/A	4.0996	3.2195	3.6362	4.7571	4.3066	4.7234	5.4681	4.3253	4.0224	5.6397	4.7997	4.3498	5.4714	3.6743	4.5758	4.5107	4.1664	4.8770	4.8321	5.1228
Phyhip	_FLTC[Carbamidomethyl (C)]SVEDGELIFR_.2	#N/A	5.5613	4.5691	6.0541	4.2557	4.2723	5.2844	5.5378	6.0116	3.3279	5.9274	5.8504	2.9577	3.4360	3.9571	5.1963	5.5511	3.6718	4.9090	5.3932	3.9241
Phyhip	_[Acetyl (Protein N-term)]M[Oxidation (M)]ELLSTPHSIEINNITC[Carbamidomethyl (C)]DSFR_.3	#N/A	3.5393	4.4635	4.0756	4.1501	2.8770	3.7592	3.1632	3.2397	4.6665	3.7456	3.5967	4.3529	3.5600	4.0627	4.2412	4.0847	3.7774	4.2351	4.8750	3.9790
Erich5	_LSSERDS[Phospho (STY)]PGAPQAGTM[Oxidation (M)]K_.2	#N/A	4.7906	4.8087	3.9895	4.2542	4.7668	3.4185	3.8139	4.9230	3.3124	4.4832	3.7596	4.8803	4.8749	3.9766	4.7812	4.6198	3.6913	4.2237	3.7945	4.1423
Rtn1	_HQAQVDQYLGLVR_.3	#N/A	6.6603	6.3485	6.2180	6.8212	6.5626	6.5842	6.9879	6.4347	6.5902	6.7038	6.4478	5.8299	6.5804	5.6984	5.6680	6.3691	6.3688	6.3397	6.3982	6.1486
Rtn1	_THINTVVAK_.2	#N/A	7.7606	7.6710	7.2726	6.8559	7.1432	7.6570	8.1052	7.5038	7.6662	7.4024	7.0660	7.2088	7.7404	6.7027	7.0831	7.2612	7.5786	7.4100	7.2705	7.0103
Rtn1	_TDEGHPFK_.2	#N/A	7.6174	7.1303	7.4765	7.0331	6.8748	7.2275	7.6353	7.3863	7.7696	7.0520	7.3093	7.2027	6.5800	6.7248	7.0874	6.9439	7.1960	7.3444	7.1048	7.0776
Rtn1	_VSLVTAPVK_.2	#N/A	4.9601	4.3746	5.1228	5.0916	5.0695	5.1138	5.5096	4.0042	4.7151	4.4830	4.4071	4.8690	5.5371	3.9358	4.9677	5.7049	4.0173	5.8007	5.2745	5.4883
Rtn1	_APNQPAPVEGK_.2	#N/A	7.5911	6.7840	4.4737	6.9745	7.1359	7.2883	7.3166	7.7001	7.3699	3.6088	7.1527	7.2637	7.0215	6.5064	7.3469	6.8361	6.9892	7.2123	6.7878	6.9312
Rtn1	_ELC[Carbamidomethyl (C)]SGPAR_.2	#N/A	4.2869	5.8369	4.4553	5.1973	2.9930	4.7360	5.5524	5.6085	5.5505	4.2591	5.2498	5.0010	4.3177	4.8380	5.8734	4.9273	4.0098	5.5781	5.0918	5.4699
Rtn1	_EQDS[Phospho (STY)]PPM[Oxidation (M)]KPGALDAIR_.2	#N/A	9.9443	10.0531	9.9251	9.7656	8.8835	9.8672	9.7405	9.7562	9.5282	9.4581	9.7028	9.2077	9.5474	10.1042	8.4539	9.0779	10.1146	9.7481	9.4663	9.3355
Rtn1	_[Acetyl (Protein N-term)]AAPPDLQDEPLSLGS[Phospho (STY)]PGSQWFGGR_.3	Q8K0T0_S16_M1_Rtn1	5.9491	6.9923	3.4971	3.2278	3.7993	4.1502	6.9122	6.9190	4.3169	3.7559	3.7955	6.1313	4.4823	7.4418	3.3190	3.3057	3.1988	6.4213	4.2829	3.4511
Rtn1	_REQDS[Phospho (STY)]PPM[Oxidation (M)]KPGALDAIR_.3	#N/A	6.3596	5.5270	3.5119	4.0758	4.5625	5.8426	6.8302	6.3660	5.6003	5.6294	5.5860	5.8939	6.0384	5.7390	5.4924	5.5147	6.2177	6.3938	6.0537	3.4659
Rtn1	_GDGEDEATAVMGAR_.2	#N/A	3.8925	4.1103	6.5040	3.7969	3.2205	6.5419	5.8092	3.5929	3.7478	4.3250	6.4010	3.9998	3.9132	6.8841	3.8529	3.8766	4.1305	3.7026	3.3512	3.3829
Rtn1	_GS[Phospho (STY)]VSEDELIAAIK_.2	Q8K0T0_S350_M1_Rtn1	4.0853	3.9361	3.7803	3.3766	3.7745	3.5926	3.8741	3.7932	3.8648	4.1141	3.8765	3.6623	4.0007	4.0934	3.4836	3.6059	3.9667	5.6181	3.5265	3.9660
Unc13c	_NAWLSPNDS[Phospho (STY)]DRELESDLSR_.3	Q8K0T7_S450_M1_Unc13c	4.0296	3.2895	3.5662	3.5546	4.2365	4.2699	5.6814	4.2553	4.2326	3.8402	3.7113	3.0156	4.3981	3.6042	2.8369	3.3917	3.7525	3.3969	3.6960	3.2815
Hspa12a	_ETAPTSTYSS[Phospho (STY)]PAR_.2	Q8K0U4_S24_M1_Hspa12a	11.9651	11.4850	11.8747	11.9618	11.4090	11.1003	11.0620	11.6498	11.1908	11.8665	11.5446	11.6042	12.0069	11.8870	10.3749	11.5725	11.6208	11.5956	11.5956	11.5030
Hspa12a	_LDLTGS[Phospho (STY)]GGTAVPAR_.2	Q8K0U4_S631_M1_Hspa12a	8.7336	8.8469	8.9525	8.9828	8.3559	8.7642	8.4406	8.4656	8.6543	8.7687	8.7377	8.3942	8.5597	9.1630	7.8754	8.6609	9.1064	8.8538	9.5440	8.8118
Hspa12a	_IIIPQDVGLTILK_.2	#N/A	7.5170	7.1809	7.1035	7.2337	7.3012	7.3453	7.4630	7.2871	7.3406	7.1004	7.3800	7.1744	6.9609	7.2703	7.2681	7.1628	7.1951	7.4059	7.3746	7.2539
Hspa12a	_TPTTILLTPER_.2	#N/A	7.2717	7.2858	7.3755	7.2308	7.4856	7.4470	7.4606	7.2619	7.6125	7.6116	7.4329	7.3375	7.3708	7.4829	7.3912	7.3253	7.1996	7.5098	7.6997	6.8443
Hspa12a	_[Acetyl (Protein N-term)]ADKEAGGGDAGPR_.2	#N/A	6.9717	6.6791	7.1827	7.1297	6.3550	7.0777	7.0577	7.1196	7.2673	6.5824	6.7908	7.1832	6.8871	7.3214	7.1426	7.1941	6.8750	6.9131	7.1099	7.1251
Hspa12a	_ELSDQAGSDFENSDVR_.2	#N/A	6.7963	6.2806	7.2844	6.7122	6.4899	6.6040	6.7500	7.0711	6.8952	6.7285	6.8157	6.2369	6.4967	6.5065	6.1765	6.2858	6.8565	6.4176	6.3687	6.8664
Hspa12a	_GAVLFGLDPAVIK_.2	#N/A	6.6899	7.0405	6.6115	6.3763	6.8872	6.8004	6.4972	6.6821	6.4910	6.5891	6.3922	6.8472	6.6578	5.8669	6.6466	6.7461	6.5496	6.9198	6.7560	7.1518
Hspa12a	_IFGEDFIEQFK_.2	#N/A	5.9842	5.5899	5.5803	5.9184	5.4982	5.9014	5.7581	5.4971	5.5084	5.5216	5.8889	5.9857	5.9045	5.3111	5.3391	5.4772	6.0658	5.6282	5.3503	5.9644
Hspa12a	_WEGGDPGVSNQK_.2	#N/A	6.9557	6.7004	7.2291	7.2876	6.6227	6.8054	6.9781	6.3823	6.7715	6.9582	6.7195	6.0841	6.7482	6.9793	6.7774	6.9258	6.8177	6.9163	6.6131	6.4994
Hspa12a	_ATAVDITTSK_.2	#N/A	3.9853	5.4786	4.1733	3.7041	3.3230	5.5741	5.7319	3.6856	5.1313	4.1916	5.3210	4.5736	4.3852	3.8804	5.5634	5.3708	4.5108	5.9008	5.6546	5.1700
Hspa12a	_FISADQSVALGELVK_.2	#N/A	3.0051	3.3036	4.9672	3.6744	3.1329	4.1486	3.9749	4.3996	4.0824	3.2849	3.9376	3.2727	3.6713	2.9958	3.5903	3.4711	3.4364	4.4023	3.1823	2.8708
Hspa12a	_ATGGPYGSLGVDYEFEK_.2	#N/A	5.7420	3.4718	6.3650	3.7370	3.8301	4.9006	5.4321	5.8075	6.1612	6.1636	4.1444	6.3029	3.8781	3.4219	5.9625	3.3364	3.5702	6.0183	3.8783	5.9305
Hspa12a	_DFYHDLDPSEAK_.3	#N/A	5.0385	4.4876	6.1518	5.5099	2.8530	5.8165	5.3135	3.2156	5.8148	5.9610	5.4500	4.8224	5.7256	4.0386	4.3009	5.4262	3.7533	5.8942	4.9201	5.1003
Hspa12a	_EIQTIMQFGDTEIK_.2	#N/A	4.3754	5.7703	5.8741	6.2913	3.0815	3.1148	4.8687	3.1002	3.8868	4.1860	3.3654	4.1706	4.0522	5.6709	3.9920	3.7375	5.1363	3.0511	3.3502	3.6273
Hspa12a	_WSS[Phospho (STY)]QGMLR_.2	Q8K0U4_S437_M1_Hspa12a	3.3672	3.9519	4.4046	4.2170	3.5741	3.6075	3.4740	3.2393	3.3942	3.1778	4.3736	3.6780	3.5596	6.8935	3.4993	4.2302	3.7769	4.0562	3.7048	4.0293
Hspa12a	_TNPLNITLPFSFIDYYK_.2	#N/A	3.9941	4.0087	4.5304	3.6953	3.3318	3.1523	3.9292	3.6945	3.8494	4.2234	3.3280	1.4437	4.0148	3.2242	3.7864	2.0981	4.2322	2.4969	3.2496	2.9836
Hspa12a	_M[Oxidation (M)]SPDAM[Oxidation (M)]NALFKPTIDSIIEHLR_.4	#N/A	3.9515	1.1592	3.4880	3.6328	4.1584	3.9500	3.8152	4.1771	3.8743	3.0936	4.2487	3.0938	3.9823	3.5261	3.3924	2.1894	3.6744	2.4343	3.7741	3.2034
Cnot3	_STDSEVSQS[Phospho (STY)]PAK_.2	Q8K0V4_S299_M1_Cnot3	9.3629	9.3938	9.5370	9.4162	8.8554	8.9643	9.1896	9.1911	9.0410	9.3576	8.9034	8.8440	9.5680	9.4279	8.4913	9.0360	9.3567	9.0517	9.2303	8.9575
Rnf208	_KGS[Phospho (STY)]SELGFPR_.2	Q8K0W3_S103_M1_Rnf208	7.8045	4.2633	4.2758	4.4849	6.5707	3.3013	4.4639	3.3250	6.7286	3.9458	7.3129	7.1673	7.1572	6.7136	4.0411	4.0844	4.3231	4.3272	3.1254	3.8521
Fez1	_GLS[Phospho (STY)]LQSNR_.2	Q8K0X8_S298_M1_Fez1	3.8545	3.4646	3.3911	3.7297	4.0615	6.5972	6.5556	4.0802	4.4077	7.4343	6.9387	6.3013	3.8854	3.4292	3.0120	6.4396	5.1103	3.5720	3.8711	3.1064
Fez1	_S[Phospho (STY)]M[Oxidation (M)]EDLVNEFDEK_.2	#N/A	8.3847	9.2889	9.2919	8.8508	8.6250	9.2611	8.6609	5.8463	9.1073	9.3098	3.7066	8.8386	7.6315	9.8066	8.5165	8.9649	9.0149	9.1780	9.1286	9.1218
Plekho2	_SSS[Phospho (STY)]LGDLLR_.2	Q8K124_S395_M1_Plekho2	4.0162	3.3029	3.5527	3.5681	4.2231	3.8853	3.8799	4.2418	3.9390	4.5298	4.3134	3.0290	4.0470	3.5908	2.8504	3.3783	3.7391	3.4104	3.7094	3.2681
Pdxk	_AEAGEGQKPSPAQLEIR_.3	#N/A	8.6017	8.7978	8.8644	8.7948	8.6945	8.4904	8.7027	9.0672	8.5602	8.7681	8.5655	8.8272	9.2389	8.8886	8.9595	8.6494	8.6347	8.5444	8.7107	8.5869
Pdxk	_VLSIQSHVVR_.2	#N/A	7.1293	8.0703	7.8626	7.8374	7.4606	7.8142	7.7572	7.2591	7.5973	7.3709	7.1027	7.3421	7.3769	7.9410	7.7368	7.5640	7.4971	7.5826	7.5905	7.3630
Pdxk	_DKSFLAM[Oxidation (M)]VVDIVR_.2	#N/A	4.0214	4.1252	3.3292	4.1714	4.8505	3.2967	3.6261	4.4214	4.5091	4.2169	4.7165	4.4520	3.5472	4.4762	4.0702	4.3556	4.8510	3.7180	3.9602	3.7407
Pdxk	_VVPVADITPNQFEAELLSGR_.2	#N/A	5.8845	6.1963	5.5049	6.1590	5.7367	6.0419	5.9547	6.0109	5.7751	5.5965	5.7563	5.5536	6.0718	6.4676	6.0021	5.5786	5.9860	5.9247	5.4320	5.6350
Pdxk	_VNDVNKYDYVLTGYTR_.3	#N/A	7.5895	7.3553	7.6895	8.0852	7.7484	7.2657	7.0706	7.8866	7.5908	7.8948	7.7125	7.6311	7.3544	7.7541	7.3858	7.8205	7.3580	7.1025	7.2998	7.5593
Pdxk	_SQELHELYEGLK_.2	#N/A	5.8024	5.4183	5.4193	6.1862	5.7527	5.5893	5.5069	6.1350	5.3090	6.1582	6.1110	6.0331	5.9829	6.1866	5.9973	5.9182	5.9439	5.5027	5.1454	5.9055
Pdxk	_DKVVPVADITPNQFEAELLSGR_.3	#N/A	3.7224	4.6630	5.1526	5.0114	4.9899	5.4927	4.5281	4.5809	5.8004	5.4176	4.9605	5.2066	5.3115	4.1918	5.6851	4.0312	5.6418	5.0625	5.2693	5.2933
Pdxk	_SFLAM[Oxidation (M)]VVDIVR_.2	#N/A	3.4277	4.0008	5.0264	4.2006	4.6862	4.1516	4.2994	4.0060	4.4526	4.3735	4.2375	4.1367	3.5095	3.4875	4.6422	4.3209	5.0821	4.4399	4.8028	4.2402
Pdxk	_KPDGSTVTQR_.2	#N/A	6.8582	6.5197	6.9409	6.8478	6.6846	6.7866	5.9146	6.2454	3.5381	4.5347	6.7873	6.5976	7.3913	7.1146	7.4916	7.6810	4.4470	6.9667	6.4153	5.7274
Pdxk	_TVSAMQHVLQR_.2	#N/A	4.4235	5.8687	6.7280	5.2507	3.7612	4.8145	6.0937	4.1418	3.1639	3.4201	5.0449	3.8092	3.9470	6.7077	4.7914	3.2676	3.9372	3.9062	3.7477	4.6240
Pdxk	_DIEDPEIVVQATVL_.2	#N/A	3.3275	3.9916	4.0861	4.2567	3.9997	3.5678	2.9515	3.2790	4.7655	3.1381	2.9125	3.7177	3.5993	4.3597	4.8985	4.1904	4.9264	4.0165	3.6651	3.8577
Cc2d1a	_LANHDEGS[Phospho (STY)]DDEEEETPKK_.3	#N/A	6.2903	6.7864	6.8878	6.1537	6.1905	6.6857	6.8104	7.0239	6.7611	7.0582	6.9822	6.0285	7.0464	6.7977	6.3051	6.1933	6.4458	6.9554	6.9934	6.5350
Cc2d1a	_LANHDEGS[Phospho (STY)]DDEEEETPKKQNTPAASTTQLK_.4	#N/A	4.2968	4.5231	3.8334	3.5793	3.0029	3.0363	4.0452	3.8105	3.9654	4.1074	3.4440	4.2492	6.5204	3.1126	4.0705	3.6589	4.3482	3.4850	3.1336	3.0096
Cc2d1a	_SSPS[Phospho (STY)]KAPPSGPAPAGK_.2	Q8K1A6_S461_M1_Cc2d1a	4.4790	4.0749	4.4642	8.5092	3.1851	3.2184	3.8630	3.6283	3.7832	4.2896	3.2619	4.0671	3.9487	4.4513	3.8884	3.8411	4.1660	3.6672	3.3157	4.4183
Cc2d1a	_LANHDEGS[Phospho (STY)]DDEEEETPK_.2	#N/A	5.1647	4.4075	4.7597	5.1615	4.8875	5.2878	4.5452	5.2455	4.9297	4.7193	3.4522	4.9777	5.2668	4.3497	4.4434	4.9225	3.8595	4.7347	4.7095	4.9750
Enho	_SADVDS[Phospho (STY)]LSESSPNSSPGPC[Carbamidomethyl (C)]PEK_.2	Q8K1D8_S43_M1_Enho	4.2121	3.7908	3.2475	3.4567	3.5498	3.7739	3.9913	4.3532	4.0504	4.4184	2.9239	3.6802	4.1584	3.7022	3.8264	4.7748	3.8505	4.6933	3.5980	3.3795
Tcp11l2	_QC[Carbamidomethyl (C)]VSEDQQS[Phospho (STY)]DSESSR_.2	Q8K1H7_S16_M1_Tcp11l2	4.0946	3.9082	3.1301	3.5948	3.4323	3.0518	4.0297	3.7950	3.5329	1.8337	3.4285	3.7976	4.1153	4.6180	4.0550	3.6744	3.3895	3.5005	2.3910	3.0841
Sde2	_EDGIDAVEVAADRPGS[Phospho (STY)]PR_.2	#N/A	5.0575	5.5667	5.6228	5.3986	4.1428	5.7993	5.9197	4.4502	5.2295	6.0168	5.1522	5.0227	3.6311	6.0478	5.1068	5.0863	5.5813	6.0079	5.6647	5.8491
Ppp1r11	_AFGESSTESDEEEEEGC[Carbamidomethyl (C)]S[Phospho (STY)]HK_.3	Q8K1L5_S91_M1_Ppp1r11	3.4500	3.4897	3.5095	4.2174	4.4867	3.8759	3.2056	4.1839	3.1259	3.4463	4.1053	3.2871	3.2913	3.5168	5.4016	3.3248	3.4209	4.0969	4.8725	4.5999
Dnm1l	_ELPSAGS[Phospho (STY)]RDK_.2	Q8K1M6_S535_M1_Dnm1l	10.4251	10.2038	9.6872	10.1359	9.3793	8.9563	9.4530	8.9021	2.9890	10.4607	9.3908	9.3252	9.2977	3.6571	3.0941	9.2546	8.8636	9.3048	9.1918	9.2720
Dnm1l	_SQLDINNKK_.2	#N/A	6.7337	6.7190	6.9502	6.6907	6.7860	6.7092	6.6165	6.5980	6.5106	6.5187	6.6041	6.5492	6.6159	7.1581	6.5686	6.4505	6.7432	6.9060	6.5732	6.9113
Dnm1l	_LYTDFDEIRQEIENETER_.3	#N/A	6.5590	6.2510	6.7212	5.8359	5.7263	6.5408	6.0794	6.3752	6.2983	6.4577	6.3966	6.1881	6.5368	6.5204	5.7576	5.9978	5.2959	6.2960	6.4925	6.5112
Dnm1l	_DTLQSELVGQLYK_.2	#N/A	5.9303	5.4305	6.2610	6.2301	5.9662	5.9487	6.0794	6.5512	6.3665	6.2481	6.3751	6.4619	6.1408	6.0410	6.2279	5.9279	5.8753	6.5273	6.0666	5.8222
Dnm1l	_VFSPNVVNLTLVDLPGM[Oxidation (M)]TK_.2	#N/A	5.9034	5.8843	5.1493	4.4095	6.0367	4.6352	4.9933	5.9869	5.7890	5.4193	6.2193	4.6728	5.0033	5.5915	5.8063	5.6511	5.5065	5.4256	5.6170	5.4270
Dnm1l	_ALQGASQIAEIR_.2	#N/A	4.8063	4.4158	5.4465	5.5666	4.7620	5.0668	5.1368	5.3429	5.7136	5.2964	5.2869	5.4379	4.7366	4.2470	5.1069	5.0617	5.1066	4.7703	4.8424	5.4667
Dnm1l	_SVTDSIRDEYAFLQK_.3	#N/A	6.5029	5.9820	6.9416	6.4484	6.1049	6.4098	6.2852	5.7727	6.7341	6.6436	6.4632	5.7835	5.3612	6.5762	6.3686	6.7731	6.3476	6.0135	6.3043	6.7291
Dnm1l	_SQLDINNK_.2	#N/A	6.6057	5.7450	6.9719	4.4866	6.3800	6.6916	6.6541	7.1140	6.6821	6.3273	6.8193	6.6127	7.1260	7.2095	6.5208	6.7335	6.8800	6.6694	6.9240	6.8036
Dnm1l	_GHAVNLLDVPVPVAR_.2	#N/A	5.5463	4.7854	4.8211	5.0274	5.0572	4.5449	4.1264	5.2733	5.2456	5.1879	4.6225	4.8397	4.2935	5.2023	4.3101	4.4480	4.7679	5.1568	4.5805	4.0009
Dnm1l	_RLEEPSLR_.2	#N/A	5.2872	4.9931	5.2166	3.2676	4.9176	4.7694	5.3349	4.9242	5.1980	4.7276	5.0691	4.4197	5.0736	4.7228	5.4561	4.6470	3.1590	4.3236	4.3227	4.4113
Dnm1l	_VPSALAPASQEPPPAASAEADGK_.2	#N/A	5.8611	4.6040	5.5255	5.3682	4.0047	5.5082	5.6138	5.4600	5.8245	5.2918	4.9300	4.8706	3.9064	3.4502	4.8354	3.2376	3.5984	4.8684	4.7994	4.0559
Dnm1l	_DC[Carbamidomethyl (C)]EVIER_.2	#N/A	3.7699	3.3141	3.5416	3.5792	4.2119	4.2453	5.1247	4.2307	3.6748	4.6054	5.2773	3.0402	4.4226	3.5797	5.6025	4.6700	3.7279	4.6703	4.8317	3.3915
Dnm1l	_SKPIPIM[Oxidation (M)]PAS[Phospho (STY)]PQK_.2	#N/A	8.6443	9.3689	8.9590	8.3580	6.8758	6.3150	8.6239	8.8190	6.1434	8.6695	7.3276	8.3097	7.0882	9.4935	3.8888	7.8362	7.2972	8.1472	7.3478	6.6381
Dnm1l	_TLESVDPLGGLNTIDILTAIR_.2	#N/A	4.2442	4.8204	3.9678	4.1364	4.2650	3.9476	4.9593	4.5504	4.8319	4.0003	3.3532	4.2350	4.5452	4.6710	4.2636	4.0886	4.4637	4.5123	4.2773	3.5180
Dnm1l	_SSLLDDLLTESEDM[Oxidation (M)]AQR_.2	#N/A	5.7555	5.7813	6.0854	5.8785	5.6720	5.5342	5.6069	5.5603	4.9703	6.2983	5.5230	5.6135	5.9378	5.4506	5.4979	5.7662	5.6561	5.4957	5.6823	5.5444
Dnm1l	_LQDVFNTVGADIIQLPQIVVVGTQSSGK_.3	#N/A	3.7961	4.2067	4.3324	3.8933	3.1338	2.9204	3.7312	3.4965	3.6514	4.4214	3.1300	0.4684	3.8168	4.3195	3.7566	4.1410	4.0342	2.5343	3.3536	4.2865
Dnm1l	_YIETSELC[Carbamidomethyl (C)]GGAR_.2	#N/A	3.7571	3.5620	3.2937	3.8271	3.9640	3.9974	3.0841	4.3502	3.0043	3.5677	3.9837	3.2881	3.1697	3.6724	3.1094	3.1192	3.3870	4.4461	4.0947	3.6394
Dnm1l	_IIQHC[Carbamidomethyl (C)]SNYSTQELLR_.3	#N/A	3.8951	3.4605	3.6294	3.9490	3.9317	3.8310	3.6269	4.4129	3.6332	4.0452	4.2615	3.3499	3.7627	3.7473	3.6221	3.9228	3.6767	3.8827	4.0268	3.6676
Dnm1l	_IC[Carbamidomethyl (C)]YIFHETFGR_.2	#N/A	4.3306	3.8145	4.0067	3.7232	3.8027	3.9147	4.0199	3.9469	3.9401	4.1853	4.1751	4.0639	4.1056	4.0133	4.3461	3.8218	3.9119	3.5629	3.2404	3.8750
Dnm1l	_FATEYC[Carbamidomethyl (C)]NTIEGTAK_.2	#N/A	4.2467	3.2262	3.6295	3.4913	3.5845	3.8085	3.9566	4.3186	4.0157	2.1550	4.3901	3.4635	4.1238	3.6675	3.5338	3.0908	3.8158	3.1216	3.6327	3.3448
Dnm1l	_FISNPNSIILAVTAANTDM[Oxidation (M)]ATSEALK_.3	#N/A	4.0653	3.2538	3.6019	3.3478	4.2723	4.3056	4.2767	4.0420	4.1969	3.8759	3.6755	2.9799	4.3623	3.3641	2.8012	3.4274	3.0788	3.2535	4.4029	2.2381
Phldb2	_RLS[Phospho (STY)]AGTTVADVQK_.2	Q8K1N2_S465_M1_Phldb2	3.6292	3.6899	3.1658	3.9550	3.8361	4.2722	3.2120	3.8549	3.5520	4.4398	3.9264	4.3160	3.6601	3.2038	3.2374	2.9913	3.3521	3.7973	4.0964	4.3819
Spire2	_DRS[Phospho (STY)]FSEHDLAQLR_.3	Q8K1S6_S443_M1_Spire2	3.4200	3.6043	5.0114	4.1592	4.0535	4.2943	3.5820	4.0660	3.4575	3.4908	3.8319	3.3728	5.0547	4.2458	4.0393	4.9254	3.3706	4.0517	4.1918	4.7761
Wdr11	_VYSSPHSS[Phospho (STY)]PAHNK_.2	#N/A	4.0015	5.6271	4.1432	4.7072	4.6990	5.1263	4.9663	5.1478	5.3831	4.6341	5.5882	4.9494	5.4295	4.6184	4.5893	3.9579	4.0341	4.7728	4.5562	3.6035
Coq9	_YTDQS[Phospho (STY)]GEEEEDYESEEQLQHR_.3	Q8K1Z0_S81_M1_Coq9	5.6440	6.3107	6.1134	6.4739	4.5087	6.2648	5.8100	5.3286	6.1691	6.0307	6.0412	5.9183	5.6918	6.2970	6.1076	6.2824	6.1082	5.3586	6.3039	6.0191
Coq9	_LVQLGQAEK_.2	#N/A	4.3944	4.5754	4.0099	4.7904	4.7558	5.0015	4.2132	4.0948	3.9603	4.5789	4.7773	4.6561	3.8946	4.8385	5.1205	5.0761	5.1213	4.0793	5.2049	4.9676
	GRDYC[Carbamidomethyl (C)]S[Phospho (STY)]EEEDT.2	Q8K207_S115_M1_	10.3851	10.5104	10.5644	10.5314	9.9249	9.9887	10.0792	10.4463	10.2102	10.4829	10.0520	10.3856	11.0047	10.6916	10.3065	10.3705	10.6055	10.1391	10.2052	10.4497
	ANM[Oxidation (M)]HS[Phospho (STY)]ESQEFFR.3	Q8K207_S93_M1_	4.1195	7.3243	3.6561	3.4648	4.3264	3.7820	3.9832	4.3452	4.0423	4.4265	2.9158	2.9257	4.1503	3.6841	4.2479	3.4816	3.8424	3.3071	3.6061	3.3714
Adgrg1	_LPISSGS[Phospho (STY)]TSSSR_.2	Q8K209_S681_M1_Adgrg1	3.9937	4.0091	4.5300	3.6956	3.3315	3.5556	4.2096	7.4810	4.2687	4.2001	3.1422	3.8985	4.3767	4.5171	3.7868	4.3386	4.2318	3.6014	3.2499	2.9833
Adgrg1	_NNSDS[Phospho (STY)]AKLPISSGSTSSSR_.3	#N/A	4.1402	3.5469	6.6420	3.2240	3.8984	4.7006	4.8111	4.8199	3.9596	3.7897	4.0844	3.3614	4.4914	3.4932	3.2202	3.2192	3.5054	3.1297	4.2792	3.4602
Pacs1	_QLS[Phospho (STY)]KPLSER_.2	Q8K212_S517_M1_Pacs1	10.9627	10.7413	11.1311	11.0007	10.2410	9.6631	10.3536	10.1814	9.8566	11.2795	10.2365	10.1294	10.2143	10.3999	8.3671	10.1321	9.9925	10.1914	10.2169	9.9893
Pacs1	_DVSVPVAEIK_.2	#N/A	6.3172	5.6532	6.2726	4.9033	5.4394	6.3834	6.3266	5.4404	5.7621	5.7784	6.3107	6.1728	5.7971	5.9699	5.5719	6.0106	6.2693	5.8686	4.8173	5.3959
Pacs1	_S[Phospho (STY)]TPLKER_.2	Q8K212_S508_M1_Pacs1	3.9471	3.5430	3.4952	3.2296	4.1541	4.1874	4.3949	4.1602	4.3151	3.7577	3.7937	3.0981	4.4805	3.4823	2.9194	3.3093	5.2942	3.1353	4.2847	3.4493
Pacs1	_ADLQGSAS[Phospho (STY)]PSKVEGTHTPR_.3	Q8K212_S493_M1_Pacs1	4.0694	3.2497	3.6060	3.5148	3.6079	6.5926	6.6832	4.2951	3.9922	4.4765	4.3666	5.5871	4.1003	3.6441	3.5103	3.1143	3.7923	3.7355	3.6562	3.3213
Pacs1	_VAAVGSQSYLSSILR_.2	#N/A	3.6327	1.1876	4.0306	2.8017	2.7372	3.3484	3.8515	1.8994	4.0346	3.2517	4.3662	3.6141	3.9013	4.4040	3.8410	4.2255	3.3672	3.9265	3.4674	0.9999
Pacs1	_DTTS[Phospho (STY)]PM[Oxidation (M)]ELAALEK_.2	#N/A	8.3323	4.2138	6.2991	4.4789	6.9557	3.2553	3.4678	3.3340	3.4411	8.4522	7.2343	3.9399	3.5842	4.1838	3.9768	3.9589	7.4945	6.9786	3.2250	7.3837
Pacs1	_YSSTFLDSAWR_.2	#N/A	4.9643	5.5539	4.3982	3.9792	5.4315	5.4780	5.0880	4.8307	4.8020	4.4400	5.3228	5.3549	4.7433	5.7680	4.6576	5.0283	5.3280	3.8215	4.1426	4.2773
Pacs1	_TNS[Phospho (STY)]SDSERSPDLGHSTQIPR_.3	Q8K212_S526_M1_Pacs1	8.0928	7.8445	7.4071	7.9868	7.0572	7.0533	7.2968	5.1953	7.1954	8.0789	7.0243	5.9866	8.0832	6.0143	5.8561	7.2136	7.5476	7.5919	7.3608	7.3571
Pacs1	_GSGVAQS[Phospho (STY)]PQQQPQQQPPQPQQPTPPK_.3	Q8K212_S28_M1_Pacs1	4.2789	3.3326	3.8603	3.5779	3.6166	3.8025	3.7555	4.0013	3.7878	2.8582	3.9315	4.5301	4.1504	5.6658	3.5530	3.6689	3.7100	3.4084	3.8506	3.7664
Pacs1	_GS[Phospho (STY)]LGKDTTSPM[Oxidation (M)]ELAALEK_.3	Q8K212_S421_M1_Pacs1	4.2644	4.5556	3.8010	3.3198	2.9705	3.0038	4.0776	4.4901	3.9978	3.9579	3.4765	4.2817	4.1633	3.8390	4.1030	3.5024	3.9873	4.4525	3.1011	3.6948
Arfip2	_HPSHSTS[Phospho (STY)]PSGPGDEVAR_.3	Q8K221_S76_M1_Arfip2	3.0363	3.8898	3.9293	3.6434	4.8255	5.3436	4.0710	3.8363	5.3749	4.1715	5.1793	4.7959	4.1566	3.9156	4.9855	5.0130	4.6303	5.0141	3.9548	5.4494
Fcho1	_GHS[Phospho (STY)]PSC[Carbamidomethyl (C)]LAR_.2	Q8K285_S628_M1_Fcho1	3.9973	4.0055	4.5336	3.6921	3.3351	5.4906	4.2060	3.6977	4.2651	4.2037	3.1386	3.8949	4.3732	4.5207	3.7832	3.4422	4.2354	3.5978	3.2464	3.5942
Mtmr3	_RSS[Phospho (STY)]DPSLNEK_.2	Q8K296_S633_M1_Mtmr3	9.5740	9.6233	4.3807	9.4051	9.1428	9.2923	3.2070	9.6312	9.3671	3.7018	8.9484	9.1441	9.6737	9.3483	8.5405	4.2062	9.4820	9.4058	9.1731	8.8555
Anln	_DSNLSAAS[Phospho (STY)]PKPGK_.2	Q8K298_S637_M1_Anln	10.5975	10.8119	10.6374	10.6574	10.4099	10.5724	10.1299	10.6476	10.2430	10.4184	10.0992	10.3316	10.8567	10.9002	10.0989	10.4042	10.6761	10.2001	10.2888	10.4201
Anln	_AES[Phospho (STY)]ADS[Phospho (STY)]LGSEDR_.2	#N/A	3.7799	3.5391	8.7300	3.8043	3.9869	8.8135	8.0922	4.0056	8.2350	8.4235	7.9042	7.6673	3.8108	8.2355	7.4718	8.0513	8.2049	8.0260	8.4116	7.9861
Anln	_AESADS[Phospho (STY)]LGSEDRDLLYSIDAYR_.3	Q8K298_S656_M1_Anln	4.0091	4.3433	5.2730	4.4323	3.3851	3.3951	4.0972	4.0664	4.0564	4.1694	3.1834	4.2303	4.0855	4.3471	4.6419	4.4928	4.0515	3.7270	3.7330	3.6546
Anln	_KDS[Phospho (STY)]LGPDKK_.2	Q8K298_S887_M1_Anln	4.0779	3.9637	4.1493	4.4723	3.3188	4.6440	3.8930	3.3376	3.9375	3.2905	3.6327	3.6323	4.9797	3.9277	3.7546	3.9749	4.6270	4.5420	3.4049	3.8032
Anln	_LNQVIVDIR_.2	#N/A	4.0498	3.2693	2.9647	3.5345	4.2567	4.2900	3.3812	4.2755	4.2124	3.8604	3.6911	2.9954	4.3779	3.6244	-0.0451	3.4119	3.7727	3.2379	3.6758	3.4723
Anln	_EVEMS[Phospho (STY)]VDEDINSSR_.2	Q8K298_S548_M1_Anln	3.8650	3.4541	4.4542	3.7193	4.0719	4.1052	4.4770	4.0906	4.3972	3.6756	3.8759	3.1802	4.5627	3.5645	3.0016	3.2271	3.5879	3.0531	4.2026	3.5315
Sdha	_GEGGILINSQGER_.2	#N/A	8.6422	8.7128	8.9477	8.6714	8.5358	8.5362	8.4459	8.6729	8.9041	8.8600	8.5282	8.4140	8.6076	8.6679	8.4423	8.6586	8.7347	8.3765	8.6602	8.6432
Sdha	_NTVIATGGYGR_.2	#N/A	7.6094	7.9688	8.0280	7.9561	7.7275	7.6684	7.7397	8.0305	7.5977	7.7708	7.5932	7.5996	7.5381	8.1981	8.1504	7.7617	7.7474	7.5867	7.8756	7.7319
Sdha	_ISQLYGDLK_.2	#N/A	6.8766	6.3134	6.2872	6.8273	6.9644	6.4694	6.9471	6.5631	6.8629	7.0141	7.1757	6.4940	6.5510	7.3501	6.7871	6.9448	6.3731	6.7436	7.3275	6.6014
Sdha	_AAFGLSEAGFNTAC[Carbamidomethyl (C)]LTK_.2	#N/A	5.9364	5.6824	6.4858	5.5887	6.1741	5.5413	4.9229	5.5704	5.8061	5.7645	6.2243	5.4498	6.3179	5.4533	5.6861	5.3140	5.6430	4.8287	5.0447	5.8140
Sdha	_VGSVLQEGC[Carbamidomethyl (C)]EK_.2	#N/A	7.9107	7.7228	7.9151	7.9125	7.8829	7.6902	7.8953	8.0034	8.1851	7.8556	7.7523	7.8545	7.6371	8.1368	7.7413	7.6574	7.8003	7.8627	7.7085	7.9897
Sdha	_VSDAISTQYPVVDHEFDAVVVGAGGAGLR_.3	#N/A	5.1150	6.1339	5.8027	5.6472	5.3402	5.7135	5.5166	6.0105	6.0044	5.8365	5.7705	5.6114	3.6944	5.6823	5.6091	5.7062	5.5606	5.1655	5.9085	5.6415
Sdha	_LGANSLLDLVVFGR_.2	#N/A	5.3931	5.1569	5.3487	4.7602	5.3208	5.4324	4.9161	6.0346	5.6436	5.5499	5.1926	5.7088	4.8018	5.2964	4.7035	5.0419	5.6878	5.7079	5.5622	4.7332
Sdha	_WHFYDTVK_.2	#N/A	3.8121	5.4386	4.3484	5.2152	5.4685	4.9947	5.5272	4.7575	5.5560	4.6682	5.5016	5.6089	5.0819	5.2187	5.5006	5.0374	5.4435	5.6721	5.5390	5.0545
Sdha	_AFGGQSLK_.2	#N/A	3.9546	4.6274	3.4912	5.2939	4.1615	5.0986	4.4886	3.4143	5.1583	4.3415	4.3005	5.3882	5.2884	4.3123	5.4386	4.1471	3.6775	6.1375	5.6722	5.3067
Sdha	_SM[Oxidation (M)]TLEIR_.2	#N/A	4.0639	3.8075	2.5562	3.1579	4.4005	4.1158	4.5798	4.2318	4.2074	5.3477	5.2737	4.5072	4.2722	3.5540	5.0135	4.4910	3.2352	5.5210	5.2677	6.2399
Sdha	_SM[Oxidation (M)]QNHAAVFR_.2	#N/A	4.4281	6.0676	3.9646	3.1562	3.1342	6.3867	3.9139	5.9151	6.1683	6.2227	5.6024	6.4630	6.3455	4.0027	6.6521	6.5445	6.0221	6.4233	6.4945	5.9200
Sdha	_ANAGEESVM[Oxidation (M)]NLDK_.2	#N/A	5.7861	5.2137	5.6034	6.1443	5.6859	6.4722	5.6185	5.6947	5.9427	5.8762	6.0166	5.5896	6.0931	6.1323	6.0136	6.2421	5.9709	5.7878	5.5597	5.9068
Sdha	_GVIALC[Carbamidomethyl (C)]IEDGSIHR_.2	#N/A	4.7225	4.5515	5.1805	4.8150	5.0276	4.4797	4.0539	4.7764	4.6383	4.4498	4.3170	5.1606	4.8096	4.9791	4.8716	4.3371	5.0517	4.7467	4.6652	4.9375
Sdha	_VTLEYRPVIDK_.2	#N/A	5.9349	6.1851	6.1700	6.1434	6.0368	6.0500	6.1552	6.3127	5.7263	6.0016	5.8507	6.1904	5.6410	6.3835	6.2113	6.2022	5.8528	6.2695	6.1889	6.4346
Sdha	_VDEYDYSKPIQGQQK_.3	#N/A	3.7052	4.2977	4.2414	4.6711	3.0429	3.2670	3.1490	3.4055	4.2652	3.6821	4.8291	4.1871	3.1645	3.5853	4.4523	3.9426	3.9432	3.9126	3.5385	3.8353
Sdha	_TYFSC[Carbamidomethyl (C)]TSAHTSTGDGTAM[Oxidation (M)]VTR_.3	#N/A	4.3821	4.4379	3.9186	3.2022	3.0882	5.9301	3.9599	3.1069	5.5051	6.2131	3.3588	4.1640	4.0456	5.6696	3.9853	3.7442	4.1050	3.5702	3.2188	3.0144
Sdha	_TGHSLLHTLYGR_.3	#N/A	4.7679	3.5170	3.3387	3.7821	5.2762	4.0424	4.9232	4.0278	4.4601	4.8801	5.5175	5.4814	3.1247	5.2843	3.0645	5.2380	3.5250	4.3595	5.2798	5.4674
Sdha	_SHTVAAQGGINAALGNMEEDNWR_.3	#N/A	3.5530	6.7445	4.0893	6.0452	2.8907	4.6807	6.1941	3.2534	3.2086	4.9902	4.9532	5.4531	3.3166	8.1196	4.2275	3.8979	3.7911	4.0421	3.6907	4.0434
Sdha	_HVNGQDQIVPGLYAC[Carbamidomethyl (C)]GEAAC[Carbamidomethyl (C)]ASVHGANR_.4	#N/A	4.0101	4.0042	3.9201	5.4215	4.5936	4.8685	3.9459	3.7105	4.9708	5.3802	5.2637	3.8937	4.0308	3.9072	5.0395	4.4865	4.2482	3.5965	5.4784	4.8620
Hacd3	_EGS[Phospho (STY)]PETLTNLKK_.2	#N/A	3.2236	4.0955	4.2610	4.3607	6.9107	6.9908	3.6176	3.3829	3.5378	6.7964	3.0164	3.8216	7.1879	4.2059	3.6430	6.2623	7.1589	3.9126	6.7173	6.2552
Hacd3	_EGS[Phospho (STY)]PETLTNLK_.2	#N/A	7.2673	4.5706	7.3920	4.2572	3.6809	3.7142	6.9170	3.1326	7.3664	7.0002	4.2669	6.8086	3.4529	3.9556	3.3926	4.3369	3.6702	4.1629	3.8115	3.9226
Hacd3	_WLDES[Phospho (STY)]DAEM[Oxidation (M)]ELR_.2	#N/A	8.7743	8.7405	9.4550	8.6518	3.4685	8.8833	8.7135	6.2865	8.4586	8.8119	7.9718	8.3547	7.4551	9.5699	7.1625	8.6040	9.1353	9.3248	9.6004	8.8897
Edc3	_SQDVAIS[Phospho (STY)]PQQQQC[Carbamidomethyl (C)]SK_.2	Q8K2D3_S131_M1_Edc3	9.4655	9.6782	9.6955	9.4254	9.1115	8.8584	9.1856	9.3443	9.0583	9.6343	8.8101	9.2247	9.3118	9.9481	8.3973	9.3057	9.3584	9.1126	8.9977	8.9599
Edc3	_HNS[Phospho (STY)]WSSSSR_.2	Q8K2D3_S161_M1_Edc3	4.0176	6.1538	6.3000	3.5666	5.8611	5.9372	6.2900	4.2433	5.7382	6.9159	6.5590	5.7205	6.3003	6.6452	3.4585	5.6511	5.8402	3.4089	6.2488	3.2695
Brd3	_SES[Phospho (STY)]PPPLSEPK_.2	Q8K2F0_S262_M1_Brd3	11.0921	10.9523	9.7857	10.7494	10.7046	10.8746	10.6746	11.2179	10.7020	3.2750	10.6078	10.5776	11.2232	10.7140	9.5412	10.5705	11.1901	10.7212	10.5411	10.5234
Lsm14a	_SS[Phospho (STY)]PQLDPLR_.2	#N/A	10.1987	3.7431	10.2288	10.1081	9.9535	10.3308	9.9121	10.2878	10.2582	10.1800	10.1872	10.0609	4.4669	10.5569	9.8652	3.9096	10.6776	9.9086	10.2787	10.3065
Lsm14a	_SFFDNIS[Phospho (STY)]C[Carbamidomethyl (C)]DDNRER_.2	#N/A	3.6781	4.0544	5.0217	3.9709	3.5450	3.8143	3.0979	3.6835	3.2211	3.6859	5.3282	3.8887	3.3866	3.7704	3.7556	3.5793	3.6630	4.6774	4.6515	3.6356
Lsm14a	_SS[Phospho (STY)]PQLDPLRK_.3	#N/A	10.5072	10.3432	10.4343	10.2864	10.0867	10.1546	9.7192	10.3330	10.0844	10.4330	10.2417	9.9559	4.1050	10.5593	9.6439	10.0925	10.4773	9.8708	9.9424	10.1658
Lsm14a	_S[Phospho (STY)]PVPARPLPPTSQK_.2	Q8K2F8_S216_M1_Lsm14a	3.8517	7.5852	7.7865	3.0963	3.1894	6.9855	4.3517	7.1767	4.4108	6.5737	3.2843	8.4363	4.5188	8.2099	3.9289	4.1966	4.2109	7.1356	3.2376	7.2577
Pphln1	_DAS[Phospho (STY)]PSSSSAVASSK_.2	Q8K2H1_S219_M1_Pphln1	11.1357	11.2957	11.2238	11.0781	10.9148	10.9575	10.7559	10.9519	10.8584	11.0423	10.7340	10.9931	11.3248	11.4131	10.9900	11.0423	10.9852	10.9063	10.7978	10.9727
Pphln1	_DAPFFRES[Phospho (STY)]PVGR_.2	#N/A	7.1952	7.3291	7.3694	7.2735	3.2789	6.9878	6.8102	6.8204	7.1256	7.1263	6.5522	7.1077	7.5402	7.7722	6.8234	7.3032	7.5253	7.1039	6.7958	7.2940

Pphln1	_DAPFFRES[Phospho (STY)]PVGRK_.3	#N/A	3.8981	4.1047	4.4344	3.1427	3.2358	3.4599	4.3052	3.1664	4.3643	4.1044	3.2379	3.7259	4.4724	4.0822	3.2853	4.2430	4.1644	3.4318	3.2841	3.6934
Otud6b	_[Acetyl (Protein N-term)]MEEVVAEELDDEEQLVR_.2	#N/A	4.2583	3.7445	4.8733	5.7407	3.5960	3.8201	3.9450	3.9587	3.2140	4.4646	3.9727	3.6339	4.2790	6.6822	3.4758	3.1024	4.4964	3.3368	2.9854	3.8952
Fntb	_PVPGFEEC[Carbamidomethyl (C)]EDEVTS[Phospho (STY)]DPATD_.2	Q8K2I1_S432_M1_Fntb	4.1571	7.0765	3.1926	3.5323	3.4948	3.7189	4.0462	3.8575	6.1864	4.3634	2.9789	3.7351	4.2134	3.1797	3.6234	3.0012	4.3952	6.8267	3.0866	3.4344
Agfg1	_GTPSQS[Phospho (STY)]PVVGR_.2	Q8K2K6_S181_M1_Agfg1	10.4892	10.6311	10.6721	10.6929	9.8817	9.4090	9.7581	9.6845	9.4729	11.2501	10.3213	10.5475	10.8752	11.0699	9.5894	10.6607	10.3950	10.2641	10.2333	10.5195
Agfg1	_SSS[Phospho (STY)]ADFGTFSTSQSHQTASTVSK_.3	Q8K2K6_S293_M1_Agfg1	3.8954	3.4237	3.4319	3.6889	4.1023	4.0061	6.5603	4.1210	3.8182	3.1497	4.1926	3.1498	3.9262	3.4700	2.9712	3.2575	3.6183	3.5312	3.8302	3.1473
Agfg1	_SISM[Oxidation (M)]TTFTQQEIEFLQK_.2	#N/A	4.2186	4.6014	3.7552	3.3657	2.9247	3.6829	4.0823	4.4443	4.1414	4.3274	3.0149	3.1884	3.9612	3.7932	4.1488	3.5807	3.9415	3.2080	3.5070	3.4705
Trappc12	_LSSLTAPVGEKS[Phospho (STY)]PDSTSPSYSTR_.3	Q8K2L8_S309_M1_Trappc12	7.7106	7.4147	7.2709	7.5836	6.5651	6.7912	7.3817	7.1947	6.5668	7.6714	7.1064	6.9889	7.5841	7.7162	4.8974	6.7862	7.0638	7.5082	7.3893	6.7999
Slc38a1	_S[Phospho (STY)]LTNSHLEK_.2	#N/A	7.2695	8.4858	7.3334	7.4474	6.6999	8.0585	8.2321	7.5486	7.8913	7.7501	7.8107	7.6068	8.2071	7.8601	6.2428	7.4352	8.0361	8.6938	7.7307	3.3923
Slc38a1	_RS[Phospho (STY)]LTNSHLEK_.2	#N/A	7.1497	7.5255	6.4805	6.4814	6.6370	6.9462	6.9026	7.2107	6.9398	6.4587	6.0984	6.2769	6.9060	6.3661	5.1702	6.3398	7.0662	7.0083	6.4855	6.2351
Slc38a1	_S[Phospho (STY)]LTNSHLEKR_.2	#N/A	7.0737	6.9426	4.5027	3.7274	6.3095	6.3409	6.6351	6.9453	6.5018	4.2920	6.0631	6.6426	6.7056	4.4718	4.9353	6.3199	6.4812	6.5413	5.5933	5.7789
Slc38a1	_RS[Phospho (STY)]LTNS[Phospho (STY)]HLEKR_.3	#N/A	3.8107	3.8730	4.5790	3.8559	3.5785	3.8572	3.6601	3.7554	3.6948	3.8822	3.6290	3.6893	3.8357	4.0078	3.5489	4.3962	3.8542	3.7264	3.7689	3.8045
Ipo11	_NFEDSS[Phospho (STY)]PETLEAHK_.2	Q8K2V6_S343_M1_Ipo11	7.2031	6.5697	3.9905	3.1303	6.1798	6.6462	4.3177	3.1788	4.3768	4.0920	6.0741	6.4921	4.4848	6.0255	3.8949	4.2306	7.0267	2.9726	6.4526	3.7059
Obi1	_LKAEVDNRS[Phospho (STY)]PQK_.2	#N/A	3.4750	4.8440	4.9314	5.4846	5.2156	3.8205	3.4809	3.4584	3.6275	3.9880	3.9331	4.0498	4.8937	4.4802	3.6315	3.7917	3.4817	3.8400	4.3316	4.1008
Obi1	_SVEVEGPSKS[Phospho (STY)]PQGR_.2	Q8K2Y0_S566_M1_Obi1	6.0097	6.0924	6.2464	7.0075	5.5718	5.8380	6.2162	5.8548	4.0473	4.0928	5.8202	6.0154	4.2127	6.5579	3.4338	6.3388	6.2363	3.9938	6.0757	6.0753
Ccm2	_TAQDPGIS[Phospho (STY)]PSQSLC[Carbamidomethyl (C)]AESSR_.2	Q8K2Y9_S164_M1_Ccm2	9.0596	9.0094	8.9037	9.4281	4.1697	8.5517	8.4617	8.7179	8.4020	9.1234	8.3506	8.6672	8.6157	9.0532	7.8966	8.7365	8.6831	8.6399	8.7196	8.5178
Ccm2	_GIITDS[Phospho (STY)]FGR_.2	Q8K2Y9_S393_M1_Ccm2	3.6448	3.2407	9.5714	9.8863	7.5814	3.8851	3.1963	4.4625	3.1165	3.4554	4.0960	9.2688	3.2820	3.7846	8.4089	9.3851	3.4993	9.5455	3.9824	9.4399
Matr3	_RDS[Phospho (STY)]FDDR_.2	#N/A	8.1940	8.6037	8.9827	8.3974	7.8093	8.8814	8.4044	8.1530	8.6368	8.7008	8.5994	8.3404	8.3418	8.8857	8.5859	8.1672	8.5925	8.4878	8.4389	8.5436
Matr3	_S[Phospho (STY)]ATREPPYR_.2	#N/A	9.0551	3.9217	8.7513	8.6880	8.4674	3.0032	8.9392	8.6138	8.3427	9.0065	8.8091	8.5079	7.9456	9.4399	8.1204	7.6866	8.7969	8.5916	4.3282	4.1501
Matr3	_ITPENLPQILLQLK_.2	#N/A	6.3461	6.6477	7.2656	7.2850	6.9601	6.9277	6.7229	6.4868	6.8567	7.2799	6.9782	7.0327	6.9295	6.7469	6.9480	6.6888	6.2169	6.5188	6.8493	7.1104
Matr3	_GDTDQASNILASFGLSAR_.2	#N/A	5.9166	5.5880	6.7931	6.4597	6.1279	6.1184	6.0127	5.4018	6.0935	6.6093	6.4496	5.8527	6.3514	5.5697	6.2170	5.9211	6.1631	5.2639	5.4819	6.1900
Matr3	_IGPYQPNVPVGIDYVIPK_.2	#N/A	6.6942	5.9996	5.6426	5.6044	5.8952	6.3021	5.9045	5.9988	6.2064	6.1652	5.9735	5.9049	6.1758	6.6023	5.4610	5.7659	6.1218	6.0712	5.6268	5.9297
Matr3	_KRS[Phospho (STY)]YS[Phospho (STY)]PDGK_.2	#N/A	6.2591	6.7879	4.1608	6.7728	3.3303	3.3636	3.7178	6.0212	3.6380	6.9049	3.1167	6.3811	3.8035	4.1988	3.7432	3.9863	4.3471	6.1253	6.7542	3.8761
Matr3	_S[Phospho (STY)]QESGYYDR_.2	Q8K310_S208_M1_Matr3	7.8840	7.4039	7.9172	8.0137	7.7486	7.7343	7.1727	7.9744	7.5162	7.8764	7.8482	7.3898	3.7762	8.0068	6.8983	4.0136	7.8330	7.4368	7.5791	7.5505
Matr3	_TES[Phospho (STY)]PAEGKEQEEK_.2	#N/A	6.9565	6.9657	7.1771	7.0322	6.8169	7.2184	6.8413	7.0635	6.9486	7.0328	7.0158	6.6551	7.2551	4.5881	4.7162	7.0401	7.3363	6.9729	7.2560	7.2607
Matr3	_SFQQSSLGR_.2	#N/A	5.9658	6.4578	6.5400	6.4910	6.4252	5.9172	6.4849	6.1993	6.1587	6.7770	6.1952	6.0307	6.1656	6.8522	6.3666	6.3414	5.5463	5.9284	6.6310	5.8972
Matr3	_TEEGPTLSYGR_.2	#N/A	7.1061	6.4939	6.9901	6.3820	7.2105	6.9562	6.5474	6.7896	6.7915	7.1258	6.5475	6.9059	3.6778	6.4313	7.0830	7.1592	6.6879	6.4027	6.5619	6.7536
Matr3	_TDAQKT[Phospho (STY)]ESPAEGK_.2	Q8K310_T617_M1_Matr3	3.9420	4.6448	4.0960	4.4630	4.3934	4.4983	3.7985	4.7449	4.5025	4.9182	3.5551	3.5663	5.3531	4.9502	3.4289	3.9097	4.2950	3.3744	3.6735	3.7710
Matr3	_RDS[Phospho (STY)]FDDRGPSLNPVLDYDHGSR_.4	#N/A	8.9555	10.7715	10.0199	9.4761	6.6812	10.0858	10.0253	6.7119	10.0610	9.6030	4.0609	9.3947	9.8949	11.2809	9.8834	9.2909	9.7102	10.7250	10.3188	9.8401
Matr3	_RSYS[Phospho (STY)]PDGK_.2	#N/A	4.0693	3.2498	3.6059	3.3517	4.2762	4.3095	4.2727	4.0380	4.1929	3.8799	3.6716	2.9759	4.3584	3.3602	2.7973	3.4314	3.0749	3.2574	4.4069	3.3272
Matr3	_SYS[Phospho (STY)]PDGKES[Phospho (STY)]PSDKK_.2	#N/A	10.8958	11.0052	10.7642	10.9119	10.6316	10.7801	10.8567	11.0293	10.9066	11.0607	10.6540	10.8039	11.3677	11.3438	10.9307	10.9060	10.9297	10.9281	11.0229	10.8998
Matr3	_TDAQKTES[Phospho (STY)]PAEGKEQEEK_.3	#N/A	3.3600	6.7208	4.3975	6.3699	3.5670	3.0405	3.4811	3.5857	6.5109	6.5895	6.0785	6.9214	3.3909	4.4355	6.6763	6.3947	6.6245	6.4256	6.6828	6.3910
Matr3	_DDWEEKR_.2	#N/A	4.0241	6.8538	6.1053	6.2306	6.2749	6.7917	6.2299	5.9444	6.4673	7.0360	6.9147	5.7747	6.4026	6.5770	6.7191	4.3690	6.1269	6.4038	6.7445	6.5804
Matr3	_LC[Carbamidomethyl (C)]SLFYTNEEVAK_.2	#N/A	3.9791	3.3400	3.5157	5.4009	5.2277	4.6139	5.0090	5.3183	4.2831	4.9020	4.4562	3.0661	4.4486	3.4504	4.7687	4.4819	3.1651	5.0701	5.3331	5.2570
Matr3	_SFQQSSLGRDS[Phospho (STY)]QGHGR_.3	Q8K310_S14_M1_Matr3	5.1856	4.2769	4.2622	5.1265	5.5635	5.3981	4.4775	4.5131	3.0357	3.9322	3.4101	5.2465	5.4240	5.2751	4.0547	4.7088	4.9294	4.3136	3.1118	3.8657
Matr3	_S[Phospho (STY)]ATREPPYRVPR_.3	#N/A	4.5184	3.4844	3.5538	3.1710	3.8561	4.0802	3.6850	4.2188	3.7441	3.2239	4.1184	3.3739	3.8521	3.5409	3.2622	3.3624	3.2556	3.0767	4.2262	3.0732
Matr3	_RRT[Phospho (STY)]EEGPTLSYGR_.3	Q8K310_T150_M1_Matr3	3.6279	4.3749	5.4204	4.0615	4.4525	5.2141	4.9483	3.3283	4.9886	3.0888	2.9618	4.6267	3.6486	5.8176	5.5770	3.9728	5.4580	5.2958	3.6158	5.3002
Matr3	_SYS[Phospho (STY)]PDGKESPSDK_.2	#N/A	8.1125	8.2490	7.7235	8.0246	7.6479	7.5254	7.6255	8.1012	7.6023	7.5759	7.4054	7.6307	8.0620	7.9828	7.4944	7.6669	7.6724	7.8032	7.6908	7.7541
Matr3	_DSFDDRGPSLNPVLDYDHGSR_.4	#N/A	4.4737	4.7555	3.6262	4.7546	4.2282	4.6609	4.2299	4.5568	4.8813	4.8360	4.2768	4.2231	3.9011	3.9424	4.7689	5.1065	3.3380	4.1469	4.1993	4.8880
Matr3	_VVHIMDFQR_.2	#N/A	4.0675	4.9792	5.5922	3.3121	3.4052	4.2864	4.7957	4.4978	4.1950	4.2738	4.8157	4.2739	4.3030	6.0430	3.7131	4.4124	3.9950	3.1544	3.4534	3.5241
Matr3	_VIHLSNLPHSGYSDSAVLK_.3	#N/A	6.0320	6.2820	3.9464	6.3868	6.3963	6.1547	6.5001	6.7476	5.8238	6.1725	6.1119	6.5596	6.8047	6.0356	5.8646	6.2866	5.6131	6.6184	6.3745	6.2995
Matr3	_GPSLNPVLDYDHGSR_.3	#N/A	3.5518	3.7673	4.2039	4.0324	3.7587	3.8021	3.4155	3.7775	3.2780	3.4933	3.8490	3.4277	3.5827	4.6273	3.2282	4.4147	3.2747	3.6711	4.4274	3.6094
Matr3	_DLSAAGIGLLAAATQSLSM[Oxidation (M)]PASLGR_.2	#N/A	3.1800	3.3220	3.7163	4.5094	4.0185	3.9664	3.1150	4.3812	3.0352	3.5367	4.0147	3.2115	3.2007	3.7033	3.1404	3.5249	3.4180	4.4151	4.0637	3.6703
Champ1	_AVLPAS[Phospho (STY)]PEPR_.2	Q8K327_S532_M1_Champ1	8.2212	8.3190	7.6872	7.7389	7.6990	7.3131	7.5800	7.8664	7.1887	8.0240	7.7742	7.4561	8.4638	8.3465	6.9471	7.9085	8.1537	7.4146	7.7883	7.7075
Champ1	_KPSSS[Phospho (STY)]PDLWK_.2	Q8K327_S434_M1_Champ1	3.4422	3.8769	4.4796	4.1420	3.6492	7.0793	3.3989	3.1642	3.3191	7.0759	4.2986	3.6030	6.8260	3.9872	3.4243	4.3052	3.7019	4.1312	3.7798	3.9542
Champ1	_TAS[Phospho (STY)]PEPR_.2	Q8K327_S265_M1_Champ1	3.6869	3.6322	5.5522	6.2584	6.1639	4.2146	6.0761	3.9125	6.6283	6.2363	5.8979	5.8105	3.7177	5.5710	3.1797	3.0490	3.4098	3.7397	4.0387	4.4396
Champ1	_DSQENS[Phospho (STY)]DAELSSSEYIR_.2	Q8K327_S617_M1_Champ1	4.3984	8.1348	7.9505	8.0601	3.1045	7.8012	7.4095	3.7089	7.9766	7.2041	3.3424	7.1758	4.0293	8.4457	7.5000	7.8703	7.5666	7.8371	7.9474	8.1096
Champ1	_SSC[Carbamidomethyl (C)]GS[Phospho (STY)]PPDLWK_.2	Q8K327_S464_M1_Champ1	4.0855	3.2335	8.3178	3.4987	4.2925	4.3258	4.2565	7.6682	4.1767	3.8961	3.6553	7.2057	4.3421	3.3439	2.7810	3.4477	3.8085	3.2737	4.4231	3.3109
Champ1	_RPGPPLS[Phospho (STY)]PEIR_.2	#N/A	5.5712	6.3589	5.1597	5.3303	4.8193	5.6629	5.2571	5.6533	4.6888	5.2202	3.1643	5.7765	4.3989	5.9743	4.1042	3.7301	4.0909	5.4980	4.8602	4.4130
Champ1	_EQPSKDTESGKS[Phospho (STY)]PSPPER_.3	Q8K327_S108_M1_Champ1	4.0427	4.0148	3.6944	4.2800	3.7248	3.8825	3.6347	3.5815	3.6184	3.9778	3.5863	3.7410	3.7572	3.7325	2.7112	3.7597	2.5722	4.1223	4.1729	4.3482
Champ1	_LM[Oxidation (M)]EALES[Phospho (STY)]PLEEQQI_.2	Q8K327_S795_M1_Champ1	3.0970	4.2221	4.1344	4.4872	3.3040	3.3373	3.7441	3.3227	3.6644	4.4084	3.1430	3.9482	3.8298	5.1008	3.7695	3.9600	4.3208	5.1534	3.1277	3.8498
Champ1	_GQES[Phospho (STY)]SS[Phospho (STY)]DQEQVDVESIDFSK_.2	Q8K327_S641_M2_Champ1	4.1951	3.1240	3.7317	3.3891	2.9012	4.4354	4.1469	4.4208	4.0671	4.0057	3.5458	4.3510	4.2326	3.2344	4.1723	3.5572	3.9180	3.3833	3.0318	3.2014
Champ1	_LKKDS[Phospho (STY)]QENS[Phospho (STY)]DAELSSSEYIR_.3	Q8K327_S613_M2_Champ1	4.1951	3.1240	3.7317	3.3892	2.9012	4.4354	4.1469	4.4208	4.0671	4.0057	3.5458	4.3510	4.2326	3.7697	4.1723	3.5572	3.9180	3.2315	3.5305	3.2014
Ssh3	_QAS[Phospho (STY)]VDDSREEDKA_.2	Q8K330_S639_M1_Ssh3	4.2243	6.5122	5.7307	5.0182	2.9304	5.5434	5.6445	5.9223	5.2554	5.7938	5.0204	4.3218	6.5674	5.9163	5.5985	5.7988	5.5723	5.4641	3.5013	4.9543
Ssh3	_S[Phospho (STY)]PPASGHSTPVGPTQDR_.2	#N/A	4.0798	3.9243	4.0315	3.5182	3.4175	3.4265	5.6788	5.6124	4.0202	4.2579	3.2019	3.8138	4.1547	4.2252	3.7021	3.8401	6.1931	3.3956	3.3692	3.2635
Atat1	_SSS[Phospho (STY)]LGNSPDR_.2	Q8K341_S272_M1_Atat1	4.0134	7.9545	8.2434	7.9988	7.3839	4.2537	4.3286	7.2830	4.2488	3.8240	3.7274	3.0318	6.7636	7.2315	2.8531	3.3756	3.7364	7.1247	6.9678	7.1785
Atat1	_LLLATDPGGS[Phospho (STY)]PAQR_.2	Q8K341_S315_M1_Atat1	9.8536	9.8806	9.8549	9.6192	4.0967	9.5851	9.4494	9.6958	9.5346	9.7295	9.5630	9.6415	10.1374	10.0967	9.0138	9.3493	10.0559	9.5281	9.3852	9.6024
Atat1	_RAT[Phospho (STY)]PPAHPPPR_.3	Q8K341_T261_M1_Atat1	3.6330	4.3698	4.5616	4.0564	2.9707	3.2370	3.5680	3.3333	3.4882	4.5846	2.9669	4.2593	3.6537	3.4879	3.5934	3.9779	3.8710	3.9621	3.6107	3.0583
Sash3	_SSS[Phospho (STY)]FKDFAK_.2	Q8K352_S27_M1_Sash3	3.9926	3.3265	3.5292	3.5916	3.6847	3.9088	3.8563	4.2183	3.9154	4.5533	5.4887	3.0526	4.0235	3.5672	4.3335	3.1911	3.7155	3.4339	6.0216	3.2445
Cbr3	_FHQLDIDDPQSIR_.3	#N/A	7.7488	7.7953	7.9318	7.7130	7.7789	7.7869	7.6858	7.6798	7.6335	7.9357	7.8134	7.4076	7.9059	6.6575	7.8109	7.5762	7.9380	6.4432	6.3182	7.7834
Cbr3	_EYGGLNVLVNNAGIAFR_.2	#N/A	3.2339	4.0852	4.2714	4.3503	3.4409	3.4742	3.6072	3.4596	3.5274	5.2193	3.0061	3.8113	3.6929	4.3094	3.0715	4.0969	4.4577	3.9229	2.9908	4.1625
Dennd1a	_S[Phospho (STY)]LEDLRAPK_.2	Q8K382_S592_M1_Dennd1a	10.8917	10.9144	9.9683	10.3556	9.4486	10.2973	10.5792	10.7766	10.0861	10.7010	10.1560	10.4682	11.0181	10.6272	9.1977	10.1935	10.6830	10.5904	10.2377	10.1792
Dennd1a	_RPKS[Phospho (STY)]NITVEGR_.3	Q8K382_S510_M1_Dennd1a	3.1749	4.1442	5.7013	4.4093	3.3819	3.4152	3.6662	3.4315	5.4936	4.4864	3.0651	3.8703	3.7519	4.2545	3.6916	5.6556	5.0343	3.8639	3.5125	4.2215
Dennd1a	_TM[Oxidation (M)]PS[Phospho (STY)]PQAETQDPR_.2	#N/A	9.4485	9.8300	10.1596	10.2589	8.8649	8.8348	9.9249	9.2162	8.7841	9.4071	9.4828	8.9056	9.3884	9.8360	8.0306	4.0225	9.4880	9.1640	8.2724	8.5442
Dennd1a	_ESDSAEGDETES[Phospho (STY)]PEQLVR_.2	Q8K382_S546_M1_Dennd1a	5.3486	5.5394	5.9100	5.4438	3.2347	4.3484	5.9447	3.4907	5.4089	5.7446	3.0755	4.9274	4.3155	5.3234	4.8181	3.6836	5.4923	5.4940	4.0651	5.3079
Plcl2	_RS[Phospho (STY)]LEAIPEK_.2	#N/A	10.0424	9.7588	9.7969	9.6214	9.2172	9.6620	9.4819	9.6053	9.3630	9.5986	9.7689	9.1515	9.4326	9.7440	8.0568	9.3009	9.8333	9.5595	9.5722	9.4117
Plcl2	_S[Phospho (STY)]LEAIPEKASDENGD_.2	#N/A	9.7336	9.9872	9.7031	9.9437	3.4447	9.5046	9.5743	3.4634	9.4681	9.8515	8.3852	9.4211	9.5651	9.9277	8.5058	9.7088	10.2765	9.7225	9.6637	9.5665
Plcl2	_LSSNC[Carbamidomethyl (C)]SGVEGDVT[Phospho (STY)]DEDEGAEM[Oxidation (M)]SQR_.3	#N/A	6.6965	7.2373	7.1592	7.0310	3.8784	6.6171	6.9656	6.0703	6.5472	6.6934	5.6907	6.4102	3.5685	7.2330	5.7014	6.5345	6.8445	6.8810	6.6910	6.5938
Plcl2	_S[Phospho (STY)]LEAIPEK_.2	#N/A	4.2938	5.3228	3.1486	6.3441	2.9999	6.1761	6.6757	3.8135	3.9684	6.3108	3.4470	3.7791	4.1338	5.5556	4.0735	5.4537	5.4073	6.0764	6.1507	5.6246
Plcl2	_TPS[Phospho (STY)]PAAGPVPR_.2	Q8K394_S87_M1_Plcl2	4.1973	3.1218	3.7339	3.4797	2.9034	4.4376	4.1447	3.9100	4.0649	4.0079	5.3277	4.3488	4.2304	3.2322	4.1701	3.5594	4.4477	3.3855	4.1241	3.1992
Plcl2	_RS[Phospho (STY)]LEAIPEKASDENGD_.2	#N/A	3.4059	6.0557	5.5344	4.0956	4.2187	5.4892	5.4290	6.2511	5.3021	4.3461	3.1148	5.5468	6.2435	5.8816	3.7509	5.8348	4.3117	5.7997	5.4946	5.4997
Mepce	_ALNAET[Phospho (STY)]PKSS[Phospho (STY)]PLPAK_.2	Q8K3A9_S192_M2_Mepce	8.6567	8.5568	8.7782	6.4046	7.3971	7.7982	7.6923	8.4457	7.9899	6.5249	3.4162	8.2180	8.3656	8.3463	5.7568	8.2468	8.5339	8.1877	7.8933	8.1459
Swi5	_ELYPDFDLNLND_.2	#N/A	4.1356	3.6998	3.9230	3.4725	4.3425	3.7658	2.8431	4.3613	3.9546	3.9767	3.9087	4.4104	4.9634	3.7102	4.2933	3.4977	2.4725	3.2909	3.9873	3.3875
Ahi1	_ERS[Phospho (STY)]PPLTPK_.2	Q8K3E5_S974_M1_Ahi1	9.1824	3.9406	4.5985	3.6272	9.2303	9.7453	9.4530	9.9140	9.7735	4.2685	9.4747	9.7942	9.9269	9.8714	3.7184	9.6459	9.4241	3.5329	9.3849	3.0517
Ahi1	_GKTPTADDS[Phospho (STY)]DDSR_.2	#N/A	3.2713	4.0478	4.3087	4.3129	3.4783	4.8981	5.0621	3.4970	3.4900	4.5828	2.9687	3.7739	3.6555	4.6241	3.5952	4.5375	4.4951	5.1927	2.9534	4.6795
Ahi1	_NLSM[Oxidation (M)]TS[Phospho (STY)]PPPGPAK_.2	#N/A	9.9124	9.5076	9.3899	8.7157	9.7264	10.0451	9.6267	10.3817	10.0342	3.8525	10.0790	10.0621	10.2733	9.4729	9.0136	10.0312	9.8359	9.7143	9.6353	9.5774
Ahi1	_AVSNDNEDTNGDGVHEITSRDS[Phospho (STY)]PVHPK_.4	Q8K3E5_S181_M1_Ahi1	6.2395	3.9382	6.9935	4.2033	6.2738	6.8455	3.2446	6.3767	6.3847	3.6642	6.6454	3.6643	3.4118	6.6338	5.8484	7.1390	6.6121	6.4478	6.5784	6.7129
Ahi1	_GKTPTADDS[Phospho (STY)]DDSREK_.2	#N/A	5.9107	5.6303	3.9887	5.4737	5.4343	5.9733	5.7892	6.1202	5.7891	5.6808	5.5348	6.2201	6.2642	6.2160	5.3483	6.1730	5.8840	3.8496	5.8097	5.5771
Ahi1	_TPTADDS[Phospho (STY)]DDSREK_.2	#N/A	5.7978	3.4373	6.0037	6.3632	5.9660	6.1737	6.3293	6.5800	6.4249	3.6924	5.9981	6.8344	4.5459	6.7048	2.9848	6.8298	6.6966	6.6582	6.5557	6.2533
Ahi1	_TPTADDS[Phospho (STY)]DDSR_.2	#N/A	4.2378	4.5821	3.7744	3.3464	2.9439	4.4781	4.1042	4.4635	4.0244	4.0484	3.5030	4.3082	4.1898	3.8124	4.1296	3.5999	3.9607	3.1887	3.4878	3.4897
Ahi1	_VKERS[Phospho (STY)]PPLT[Phospho (STY)]PK_.3	#N/A	3.5592	4.4436	1.9440	4.1302	2.8969	3.1210	3.1433	3.2596	1.1785	3.7655	3.5768	4.3331	3.3105	4.0826	4.2214	3.9041	3.7972	4.0359	3.6845	4.0496
Ahi1	_ERS[Phospho (STY)]PPLT[Phospho (STY)]PKEK_.3	#N/A	3.3976	3.9215	4.4351	4.1866	3.6046	3.6379	3.4435	3.6233	3.3637	3.2082	4.3432	3.6476	3.5292	4.0318	3.4689	4.2606	3.1205	4.0866	3.7352	3.9988
Kcnq3	_GSAFTYPSQQS[Phospho (STY)]PR_.2	Q8K3F6_S599_M1_Kcnq3	10.3136	10.1594	9.9097	10.0539	9.1875	9.8506	9.7267	9.8822	9.6611	9.9229	9.7306	9.3912	10.2616	9.2911	8.1372	9.2892	10.2933	9.8616	9.4281	9.4692
Kcnq3	_T[Phospho (STY)]PQGIGLLAK_.2	Q8K3F6_T82_M1_Kcnq3	3.1396	4.1795	4.1770	4.4447	3.3465	3.3798	3.7016	3.3652	5.4327	4.4510	3.1004	3.9056	3.7873	4.2150	3.7270	4.0025	4.3633	4.2870	3.0852	4.2569
Vrk3	_DLNSSFET[Phospho (STY)]SPK_.2	Q8K3G5_T58_M1_Vrk3	6.3514	6.2660	5.9999	4.1372	3.7930	3.8368	6.0639	5.9456	6.1313	3.8828	6.2079	5.8043	4.5299	6.4419	5.6538	3.6598	4.0206	6.0237	6.2541	3.6363
Appl2	_YVLLNDQADDTGGS[Phospho (STY)]PSENR_.2	Q8K3G9_S651_M1_Appl2	5.8977	4.2961	6.2673	5.8460	3.1493	3.4079	5.4068	3.3344	5.8343	6.3766	3.3264	5.4016	5.5514	5.9246	4.9769	4.1565	6.0295	5.5165	5.4430	4.0105
Appl1	_SESNLSSVC[Carbamidomethyl (C)]YIFESNNEGEK_.2	#N/A	4.1319	4.7774	4.1380	5.0529	3.5385	4.0147	5.0543	5.0850	4.2762	4.3798	3.0450	4.0071	4.2991	5.3360	3.8998	3.8485	5.6037	3.4361	5.3743	4.2015
Appl1	_VNQSALEAVTPS[Phospho (STY)]PSFQQR_.2	Q8K3H0_S401_M1_Appl1	6.9119	6.9094	5.6549	5.0908	4.4749	4.7224	6.4897	7.0482	4.4481	5.8587	4.5240	5.9867	5.6755	4.3262	4.3834	5.0166	5.2565	6.0666	5.4817	4.8035
Appl1	_EVFQIASNDHDAAINR_.3	#N/A	3.9069	4.0959	4.4432	3.7825	3.2446	3.2395	3.8420	3.6073	3.7622	4.3106	3.2408	3.9854	3.9276	4.4303	3.8673	4.2518	4.1449	3.6882	3.3368	4.3973
Myrip	_GLPS[Phospho (STY)]PGAHPR_.2	Q8K3I4_S540_M1_Myrip	8.0327	7.9899	7.9953	7.3969	7.3006	7.3752	7.5142	7.6937	7.3557	8.3306	7.5744	7.6160	7.9231	7.6534	6.4727	7.6412	7.6321	6.8769	7.0919	7.3881
Myrip	_GSLS[Phospho (STY)]SEENNQGVQEELK_.2	Q8K3I4_S618_M1_Myrip	6.7363	3.7396	3.2987	6.7690	3.6010	4.3840	3.9401	3.9636	4.1185	6.8553	3.5972	6.1665	4.2840	3.2858	3.5173	6.0214	4.5013	3.3319	2.9804	6.1853
Glcci1	_SAS[Phospho (STY)]WGSADQLK_.2	#N/A	10.6036	10.2275	10.8732	10.7333	10.1364	9.9498	9.7238	9.8082	9.9985	10.8029	10.3298	9.9884	10.0979	10.4206	9.2514	10.0895	10.4378	9.7174	10.1001	10.2198
Glcci1	_S[Phospho (STY)]PGS[Phospho (STY)]PVC[Carbamidomethyl (C)]R_.2	Q8K3I9_S136_M2_Glcci1	8.3222	8.2921	8.3279	8.3543	8.0699	7.9106	8.1282	7.5863	8.0202	8.3673	8.0374	8.1127	7.9125	8.4321	7.1780	8.0899	7.8907	7.7364	7.7453	8.0292
Glcci1	_VPC[Carbamidomethyl (C)]NVEGIS[Phospho (STY)]PELEK_.2	Q8K3I9_S293_M1_Glcci1	9.1059	9.0664	8.8381	8.8696	7.2775	8.6269	8.4479	8.2047	8.1609	9.2282	8.3361	8.5155	8.6214	8.5088	7.5900	8.1442	9.1146	8.4160	8.5424	8.3782
Glcci1	_SAS[Phospho (STY)]WGSADQLKEIAK_.2	#N/A	6.9192	7.9086	3.4505	7.8032	6.3489	3.9875	3.7776	4.1396	6.6019	3.1312	4.2111	3.1313	7.9743	7.2193	2.9527	7.0658	7.2950	6.9397	6.7711	7.1014
Glcci1	_S[Phospho (STY)]IDTQTPSVQER_.2	Q8K3I9_S335_M1_Glcci1	10.1525	10.0427	9.8795	10.1801	9.4864	9.3288	9.3490	9.2892	9.2233	10.3243	9.5505	9.5421	9.9239	10.2037	8.4877	9.6718	9.8647	9.5004	9.5204	9.7586
Glcci1	_STAGS[Phospho (STY)]PPAAAGSGTGPAGSC[Carbamidomethyl (C)]APAAGAGR_.2	#N/A	4.5377	4.2466	4.2127	5.9050	3.2438	4.0778	3.8052	3.3876	3.7254	4.3483	3.2041	4.0524	3.8909	7.7306	3.8306	3.8998	4.1860	3.7259	4.5113	4.3605
Glcci1	_TSS[Phospho (STY)]LDTITGPYLTGQWPR_.2	Q8K3I9_S163_M1_Glcci1	4.0010	3.3181	3.5376	3.5832	4.2079	4.2413	4.3410	4.2267	4.2612	3.8116	3.7399	3.0442	4.4267	7.1421	2.8656	3.3631	3.7239	3.4256	3.7246	3.2529
Ndufs8	_EPATINYPFEK_.2	#N/A	9.0713	8.8413	8.8761	8.8395	9.0109	8.8180	8.8248	9.0700	9.0233	9.1044	9.0531	9.1363	9.0648	9.2196	9.2809	9.1923	8.9763	8.8745	8.7949	8.9862
Ndufs8	_LC[Carbamidomethyl (C)]EAIC[Carbamidomethyl (C)]PAQAITIEAEPR_.2	#N/A	6.4617	6.2959	6.3974	6.7660	6.3791	6.5910	6.4022	6.2467	6.9192	6.1391	6.3232	6.8453	6.2248	7.0331	7.0979	7.1171	6.4875	6.4045	6.4547	6.9034
Ndufs8	_GLGM[Oxidation (M)]TLSYLFR_.2	#N/A	6.4761	6.4355	6.6321	5.9223	6.6073	6.3650	6.0686	6.6333	6.2838	5.7690	5.8854	6.2415	6.0792	5.8011	6.6656	6.3346	6.4635	6.3482	6.9138	6.3279
Ndufs8	_RYPSGEER_.2	#N/A	5.1707	4.9979	5.7221	5.0226	4.5997	4.4486	4.9846	4.8590	4.4891	5.4361	5.4403	4.9225	5.3420	5.3693	6.2016	5.6046	5.3570	4.4380	4.9686	5.3852
Ndufs8	_ILM[Oxidation (M)]WTELIR_.2	#N/A	6.2643	6.4423	6.1967	6.6928	6.1112	6.5380	6.7605	6.2384	6.7021	6.9593	7.1533	7.2944	6.0443	6.9590	7.4373	6.8049	6.1814	7.0639	7.1304	6.3710
Ndufs8	_YDIDMTK_.2	#N/A	3.1066	3.5869	3.2687	3.8521	3.9452	4.1693	3.5959	3.9578	3.6550	3.3130	4.0293	3.3131	3.7630	3.3068	3.7615	3.4515	3.4551	3.6944	3.9934	2.9841
Optn	_T[Phospho (STY)]SDSDQQTYLFQR_.2	Q8K3K8_T529_M1_Optn	4.0567	3.9461	4.5930	3.6327	3.3944	3.0897	4.1467	3.7571	3.9120	4.1608	3.3906	3.8355	4.0774	4.5801	3.7238	4.4016	4.2948	3.5384	3.1870	3.0462
Minar1	_SWS[Phospho (STY)]LNTEEVPDFER_.2	Q8K3V7_S370_M1_Minar1	3.0929	3.4091	3.6292	3.0957	3.9314	4.1555	6.1774	4.2941	6.6133	3.2992	4.0431	3.2986	3.7768	3.3206	5.4316	3.4378	6.1494	3.6806	6.3555	2.9979
Babam2	_[Acetyl (Protein N-term)]M[Oxidation (M)]S[Phospho (STY)]PEIALNR_.2	#N/A	6.5962	6.5620	5.8803	6.5880	5.6499	5.9286	6.2460	6.4429	6.2229	6.1034	5.9104	6.2553	6.7779	6.4494	5.0796	6.1224	6.3693	6.2464	6.1935	6.0289
Casc3	_FGSS[Phospho (STY)]PQRDPNWIGDR_.3	Q8K3W3_S263_M1_Casc3	6.0033	3.2434	3.6122	3.5086	4.2826	3.8258	6.3411	4.3013	3.9985	3.8863	6.6153	2.9696	4.1065	3.6503	2.7909	3.4378	3.7986	6.2446	5.9450	5.9805
Casc3	_GTVTGERQS[Phospho (STY)]GDGQESTEPVENK_.3	Q8K3W3_S145_M1_Casc3	5.6512	3.1245	4.1568	3.4771	4.7970	4.4349	4.6074	3.9127	4.3845	4.0052	4.6133	3.9300	4.9044	4.0855	4.7839	4.7860	4.5181	3.3828	3.0314	3.2019
Irf2bpl	_NSSS[Phospho (STY)]PVSPASVPGQR_.2	Q8K3X4_S638_M1_Irf2bpl	9.6809	9.4618	9.6825	9.7315	9.4227	9.1199	8.9352	9.4812	9.3531	9.7046	7.8908	9.3013	9.8763	9.9733	9.2411	9.5467	9.7713	9.2678	9.2642	9.5792
Irf2bpl	_S[Phospho (STY)]PGPPPPVGVK_.2	Q8K3X4_S69_M1_Irf2bpl	8.2843	7.6462	8.0615	7.6633	6.7854	8.0190	7.8585	8.1357	7.5173	8.9859	8.5456	7.5054	7.6133	9.1968	7.3612	7.1159	8.5857	7.0715	8.2785	8.0821
Irf2bpl	_RKAS[Phospho (STY)]PEPPDSAESALK_.3	#N/A	6.7303	7.0758	6.3640	6.8414	6.4274	6.5585	6.5513	6.7107	6.3893	6.9833	3.1922	7.1163	7.1407	6.9787	7.0983	6.6660	6.2846	4.3787	3.1770	6.5155
Irf2bpl	_RNS[Phospho (STY)]SS[Phospho (STY)]PVS[Phospho (STY)]PASVPGQR_.2	Q8K3X4_S638_M3_Irf2bpl	7.1656	6.3905	6.3630	4.3305	6.6216	7.6844	7.7718	6.5943	5.3366	7.7182	7.2313	7.7811	7.3514	8.3516	7.7500	6.5009	7.8746	7.4215	7.3934	7.8896
Irf2bpl	_KAS[Phospho (STY)]PEPPDSAESALK_.2	#N/A	8.4294	8.5915	8.2783	8.2368	8.1606	8.2381	7.9679	8.5486	8.1146	8.4066	8.1415	8.3306	8.9480	9.1230	7.9555	8.1932	8.7536	8.2561	8.3433	8.2621
Irf2bpl	_RPGS[Phospho (STY)]VSSTDQER_.2	Q8K3X4_S313_M1_Irf2bpl	4.8394	4.6189	4.1924	3.4112	3.1942	4.4205	3.9252	3.9785	3.9843	4.4845	4.3587	4.1223	4.8486	3.6361	4.5521	4.3359	3.9215	4.6676	3.5424	5.0183
Pom121	_SGPTSS[Phospho (STY)]PFSSPASSR_.2	Q8K3Z9_S409_M1_Pom121	4.0411	3.7235	4.0632	3.7632	6.5592	6.5980	3.9936	6.9709	5.8610	6.0517	6.8269	3.5234	7.0661	6.7809	5.6324	5.1615	7.2975	3.7037	6.5746	6.6441
Pom121	_SQT[Phospho (STY)]PERPAKK_.2	Q8K3Z9_T421_M1_Pom121	3.8156	5.5961	4.3519	3.8737	4.2805	4.9716	5.3663	3.5160	5.0117	4.6288	3.3203	4.0766	3.8364	4.3390	3.9649	4.1605	5.7516	3.7795	3.4280	4.3060
Stxbp5	_SSS[Phospho (STY)]VTS[Phospho (STY)]IDKESR_.2	Q8K400_S783_M2_Stxbp5	10.6579	10.4977	10.4753	10.5228	9.9603	8.2132	10.1703	10.5298	9.8636	10.1432	9.9091	9.9363	10.5196	10.1328	8.8172	7.8989	10.3611	10.1252	9.9325	9.6088
Stxbp5	_QPS[Phospho (STY)]GAGLC[Carbamidomethyl (C)]DITEGTVVPEDR_.2	Q8K400_S693_M1_Stxbp5	3.1607	8.5690	10.0916	4.5287	3.9993	3.9856	9.5131	4.3619	3.0160	3.5560	3.9955	3.2307	8.0633	3.6841	3.1212	8.6055	3.3988	4.4344	4.0830	3.6511
Stxbp5	_RPVSVS[Phospho (STY)]PSSSQEISENQYAVIC[Carbamidomethyl (C)]SEK_.3	Q8K400_S903_M1_Stxbp5	4.0752	4.0756	3.8221	3.7622	3.7457	3.8861	3.5594	3.8073	3.5563	3.2257	4.2259	3.9651	3.6896	3.5666	3.3599	3.8888	3.5239	3.6679	3.9962	3.6005
Mta1	_SAPVINNGS[Phospho (STY)]PTILGK_.2	Q8K4B0_S576_M1_Mta1	4.0311	3.2880	3.5676	3.5532	4.2380	4.2713	5.2268	6.3887	4.2311	3.8417	4.9934	3.0141	4.3966	3.6057	4.0958	3.3932	3.7540	3.3955	3.6945	5.1171
Mta1	_NNM[Oxidation (M)]S[Phospho (STY)]PHGIPAR_.2	#N/A	8.7071	9.0630	8.6964	8.9038	8.3827	8.5159	8.5580	8.7237	8.4388	8.6406	8.4345	8.3996	8.9044	9.1345	7.9829	8.1912	8.7086	8.4980	8.4519	8.3597
Mta1	_ATVVNGTGTPGQS[Phospho (STY)]PGAGR_.2	Q8K4B0_S386_M1_Mta1	3.1076	3.3944	3.6439	4.5818	3.9462	4.1703	3.5949	3.9569	3.6540	3.3139	4.0284	3.2838	3.7621	6.3195	3.1721	3.4525	3.4541	3.6954	3.9944	2.9831
Ablim1	_QSLGES[Phospho (STY)]PR_.2	Q8K4G5_S470_M1_Ablim1	9.3755	9.3051	9.5376	9.7172	9.4463	9.1818	9.0789	9.2706	9.0782	9.5491	9.0877	9.2952	9.4757	9.6700	8.9055	9.4417	9.2924	8.9854	9.0921	9.3186
Ablim1	_TS[Phospho (STY)]SESIYSR_.2	Q8K4G5_S396_M1_Ablim1	4.3633	4.1338	3.6561	7.3947	7.0681	7.5495	3.9600	7.0618	4.0191	3.8177	7.5501	3.9167	4.1272	7.7520	3.5372	3.4742	6.9574	6.9666	3.6545	3.3482
Ablim1	_STS[Phospho (STY)]QGSINSPVYSR_.2	Q8K4G5_S496_M1_Ablim1	11.6472	11.6969	11.9572	12.0103	11.4508	11.1148	11.0555	11.3676	11.1341	11.9239	11.3050	11.2857	11.8299	11.6874	10.8132	11.4519	11.4248	10.9323	11.1747	11.4367
Ablim1	_TSS[Phospho (STY)]LPGYGK_.2	Q8K4G5_S738_M1_Ablim1	4.3584	7.2849	6.7655	8.1479	6.4379	6.8561	7.4366	3.0832	4.2812	8.2670	7.6427	4.1877	4.3893	7.3978	3.7993	7.7816	5.7998	3.0682	7.4207	3.6103
Ablim1	methyl (C)]LC[Carbamidomethyl (C)]QLC[Carbamidomethyl (C)]AQPM[Oxidation (M)]SSS[Pho	#N/A	8.0743	7.9905	8.7627	7.8701	7.7690	8.1217	7.7208	7.7249	7.8785	8.2845	8.0287	5.1504	8.5684	8.8719	7.8624	8.0949	8.9156	7.9625	3.4863	8.0867
Ablim1	_RSS[Phospho (STY)]GREEDEEELLR_.3	#N/A	4.1045	3.6529	4.1370	3.6897	3.7915	3.9281	3.5154	4.0009	3.5151	3.2762	4.3911	3.4537	3.6473	4.6397	3.6694	3.9569	3.2475	3.5543	4.2177	3.9368
Ablim1	_TLS[Phospho (STY)]PTPSAEGYQDVR_.2	#N/A	11.4389	11.7646	11.9367	11.9534	11.3002	11.3383	11.1630	11.3936	11.3084	11.8278	11.2803	11.3656	11.8786	11.9528	11.1632	11.5815	11.8120	11.2675	11.4707	11.6239
Ablim1	_SS[Phospho (STY)]GREEDEEELLR_.3	#N/A	4.1669	4.5070	3.8496	3.2713	3.1920	3.5885	4.1147	3.9705	4.2359	4.2330	3.1094	4.2331	4.3439	3.8877	3.9219	4.0549	4.0359	4.1973	3.4126	3.5650
Ablim1	_YDS[Phospho (STY)]PLHSASHAPSSK_.3	#N/A	5.7361	7.1287	6.3915	6.6221	4.5607	4.2450	3.7006	4.2421	5.0540	6.0087	4.8622	3.9047	5.2805	4.5009	4.7656	5.2706	4.2533	3.4751	3.5098	3.1623
Ablim1	_QERQS[Phospho (STY)]LGES[Phospho (STY)]PR_.2	#N/A	3.1196	3.8886	3.9810	3.3980	2.8644	2.0645	4.1837	3.9490	4.1039	3.9689	3.5825	3.7665	4.2693	1.4336	3.6465	4.6205	2.3206	4.4250	2.9951	3.7097
Ablim1	_TLS[Phospho (STY)]PTPSAEGYQDVRDR_.3	#N/A	3.9096	4.0932	4.4459	3.7798	3.2473	3.4714	4.2937	3.6100	4.3528	4.1159	3.2264	3.9826	6.7819	4.4330	3.8709	4.2545	4.1477	3.6855	3.3341	3.6819
Ablim1	_HSYTPTTSR_.2	#N/A	3.8647	3.5657	5.8821	3.8309	3.9603	3.9936	3.0878	3.9790	3.0080	3.5640	3.9875	3.2918	3.1734	3.3280	3.1132	3.1155	3.4763	3.6732	3.9722	3.0053
Ablim1	_GVS[Phospho (STY)]MPNMLEPK_.2	Q8K4G5_S789_M1_Ablim1	3.9207	4.0821	4.4570	5.3455	3.2584	3.4825	4.2827	3.1438	4.3418	4.1270	3.2153	3.9716	4.4498	3.9936	3.8599	4.2656	4.1418	3.0076	3.3067	3.6709
Arhgef6	_KAS[Phospho (STY)]EEEYVIR_.2	#N/A	7.1031	4.3925	7.0710	4.0791	2.9480	3.1721	6.6273	6.7784	6.8634	7.2407	6.5828	6.7173	6.8570	6.8550	4.9480	6.6553	3.8483	6.7824	6.9077	6.7443
Arhgef6	_EIKPSERPLS[Phospho (STY)]PK_.2	#N/A	3.4037	6.7729	6.1494	6.3468	6.3373	6.3808	6.4311	3.3095	6.1384	6.3076	6.2411	6.1866	6.8668	4.1423	4.4951	6.1386	6.6451	6.6645	6.5110	3.9927
Arhgef6	_KAS[Phospho (STY)]EEEYVIRK_.2	#N/A	4.1380	4.3928	3.9890	3.7548	4.0030	4.1974	4.1207	3.8850	4.4911	4.2907	4.1957	4.7036	5.3162	3.9031	3.6979	4.0316	3.8804	4.7569	3.5240	3.6059
Arhgef6	_KDS[Phospho (STY)]VPQVLLPEEEKLIIEETR_.3	Q8K4I3_S679_M1_Arhgef6	3.5505	4.0600	4.5879	4.0337	3.7574	3.7908	3.2907	4.5784	3.2109	3.6681	4.1904	3.4947	3.3763	4.6260	3.3161	4.4134	3.2734	3.2977	3.7641	4.3032
Arhgef6	_MS[Phospho (STY)]GFMYQGK_.2	Q8K4I3_S487_M1_Arhgef6	3.4941	4.5305	3.9483	5.8414	3.7010	3.7344	3.3471	3.1124	3.2673	3.3047	4.9468	2.9794	3.4327	3.9354	3.3724	4.3570	3.6501	4.1831	3.8317	3.9024
Mrtfa	_FGSTGST[Phospho (STY)]PPVSPTPSER_.2	Q8K4J6_T488_M1_Mrtfa	7.1225	7.3137	6.9605	7.3365	6.8345	6.9054	7.1391	3.7746	6.9455	3.7715	6.9468	6.9564	7.6047	7.5816	3.6755	6.5783	7.6379	3.7644	6.8923	6.8636
Mrtfa	_AQQPAPASS[Phospho (STY)]PVKR_.2	Q8K4J6_S606_M1_Mrtfa	3.3682	7.0091	4.4057	6.9741	3.5752	3.0324	3.4729	3.5939	3.2911	3.1788	3.6654	3.6770	3.3991	4.4437	3.4983	4.2312	3.0911	4.0583	4.3573	4.1210
Ddx54	_ALPSFPTSEC[Carbamidomethyl (C)]VS[Phospho (STY)]DVEPDTR_.2	Q8K4L0_S74_M1_Ddx54	4.1169	3.8859	3.1524	3.5725	3.4546	3.0295	4.0864	3.8173	4.1455	4.3232	3.0191	3.7753	4.1376	3.1394	3.6636	2.9610	4.3550	3.4782	3.1268	3.1064
Ddx54	_IDDRDS[Phospho (STY)]EEEGPSNQR_.2	Q8K4L0_S774_M1_Ddx54	3.3921	4.6107	3.9284	4.2973	4.2307	4.4548	3.3104	3.0925	3.3695	3.5985	3.7438	2.9993	3.4775	4.5221	2.8876	3.7370	3.6302	3.9799	4.2789	4.1994
Wdr33	_[Acetyl (Protein N-term)]ATEIGS[Phospho (STY)]PPR_.2	Q8K4P0_S7_M1_Wdr33	4.3138	6.8192	3.3493	3.3756	3.6515	7.7663	7.7789	8.9192	4.1691	8.7509	5.9768	8.7049	4.3345	8.4874	4.2743	8.4294	4.5519	3.2813	4.4307	3.3034
Wdr33	_SSS[Phospho (STY)]LDGDHHDGYHR_.3	Q8K4P0_S1273_M1_Wdr33	4.5790	4.9721	4.1156	4.5060	3.2851	3.3185	3.7630	3.3039	3.6832	4.3896	3.1618	3.9670	3.8486	4.3513	3.7884	3.9411	4.3019	4.2873	3.4158	2.5433
Wdr33	_DAPRPDHPPHDGHS[Phospho (STY)]PASR_.4	Q8K4P0_S1204_M1_Wdr33	3.5631	3.7559	4.6006	4.0211	3.7701	3.8034	3.2780	3.7888	3.1982	3.3737	5.8558	3.4821	3.5940	3.1378	3.3034	4.4261	3.2861	3.8634	4.1624	4.3159
Hecw1	_STS[Phospho (STY)]DTDLVTSDSR_.2	Q8K4P8_S68_M1_Hecw1	11.6723	11.4081	11.3975	11.6038	10.6608	10.4832	10.9549	11.1657	10.5910	11.5362	10.8538	10.8197	11.2910	11.0872	9.0675	10.4978	10.8998	10.7838	10.5658	10.6193
Hecw1	_SYS[Phospho (STY)]AGEASEVSR_.2	Q8K4P8_S1073_M1_Hecw1	9.3544	9.0546	9.2766	9.5171	8.9652	8.2557	8.9299	8.8150	8.1519	9.2173	8.6964	8.5125	8.8383	8.5829	6.8054	8.6281	8.8035	8.6571	8.6711	8.6457

Hecw1	_EGSLS[Phospho (STY)]PVNSQK_.2	#N/A	9.1403	8.7569	8.5928	8.6390	8.1030	8.4593	8.5612	8.6343	8.1978	8.2611	8.4000	8.6150	8.5217	8.5293	7.0020	7.8077	8.8286	8.4505	7.9354	8.2585
Hecw1	_GST[Phospho (STY)]TEEEDGLEEESTLK_.2	Q8K4P8_T579_M1_Hecw1	6.1984	3.5053	3.3504	5.2113	4.0207	5.2097	5.0521	4.1778	4.1417	3.4355	4.0277	3.2314	3.5305	3.5123	3.9660	3.1759	4.9061	3.3337	4.0308	3.3530
Hecw1	_REGSLS[Phospho (STY)]PVNSQK_.2	#N/A	6.3584	4.1812	4.1840	4.0944	3.3536	3.3869	5.8645	4.9059	5.3753	4.4581	4.7512	3.9073	5.2576	4.3652	3.7286	4.6945	4.3077	5.4687	5.1534	4.1981
Hecw1	_C[Carbamidomethyl (C)]SPC[Carbamidomethyl (C)]SS[Phospho (STY)]PQNSPGLQR_.2	Q8K4P8_S1203_M1_Hecw1	3.5046	3.9849	4.3716	4.2501	4.3432	3.0664	3.1979	3.5599	3.2570	3.7109	3.6314	3.7110	3.3650	4.4097	4.2759	3.8495	4.5579	4.0924	4.3914	4.0869
Hecw1	_APS[Phospho (STY)]PYRR_.2	Q8K4P8_S1219_M1_Hecw1	4.0835	3.9193	3.1190	3.6059	3.4212	3.6453	4.1199	4.4818	4.1789	4.2898	3.0525	3.8088	4.2870	3.8308	3.6970	4.4284	3.9790	3.1704	3.4695	3.5080
Hecw1	_SGS[Phospho (STY)]VHQMEQLNR_.2	Q8K4P8_S871_M1_Hecw1	3.4935	4.7614	3.9489	4.2768	3.7004	3.7338	3.3477	3.1130	3.2679	3.3041	4.2474	3.5517	3.4333	3.9360	3.3730	4.3564	3.6507	4.1825	3.8311	3.9030
Rptor	_SVSS[Phospho (STY)]YGNIR_.2	Q8K4Q0_S722_M1_Rptor	9.7194	9.7336	9.4915	9.6915	9.1409	9.2144	9.4476	9.4776	9.2833	9.4558	9.0282	9.0524	9.3378	3.8785	7.6903	8.8550	9.5870	9.3563	3.4217	8.8993
Rptor	_ILDTSSLTQSAPAS[Phospho (STY)]PTNK_.2	Q8K4Q0_S863_M1_Rptor	9.5332	9.6495	9.1386	8.8767	7.1255	7.9529	9.6122	9.7587	8.1093	9.0128	7.6749	8.9664	9.1622	9.6890	6.1715	8.0469	8.9951	9.3111	8.4601	7.4368
Pitpnc1	_KKS[Phospho (STY)]APETLTLPDPEK_.3	#N/A	4.4470	7.9823	6.8209	8.2782	7.8639	7.7895	3.8950	7.6909	6.7043	4.2576	8.2424	7.3406	7.6787	7.7137	5.6490	7.7330	4.1980	3.6352	7.4527	4.4503
Pitpnc1	_S[Phospho (STY)]APSSAPSTPLSTDAPEFLSIPK_.2	Q8K4R4_S270_M1_Pitpnc1	5.6948	6.9000	4.1447	4.2401	3.6212	4.9931	6.7511	4.8798	3.2343	5.1297	3.5806	5.0001	3.3830	4.1465	3.6170	3.9597	4.1927	4.2484	3.9298	3.9059
Pitpnc1	_YEDNKGS[Phospho (STY)]NDSIFDSEAK_.3	#N/A	3.4661	6.2360	6.0198	5.4589	4.0641	5.3782	5.9531	4.0463	4.9578	5.0681	5.0988	4.8257	3.5985	5.3610	5.2384	5.5768	5.7584	5.7529	5.7898	6.2850
Pitpnc1	_S[Phospho (STY)]APETLTLPDPEKK_.2	#N/A	4.2134	3.7894	9.8663	3.4760	3.5511	3.7752	9.6813	3.9138	4.0490	10.0902	2.9226	3.6788	9.6318	3.7009	3.5671	9.7269	4.4515	9.5968	9.6630	3.3781
Naxe	_GNPSGIQPDLLISLTAPK_.2	#N/A	6.9796	6.9422	7.1317	6.9417	6.8118	6.2986	6.9650	6.6368	7.4876	7.0799	6.6873	7.0600	7.6933	6.9115	7.3396	7.3940	7.3248	7.1264	6.8917	7.2469
Naxe	_YHYLGGR_.2	#N/A	6.4006	5.7422	7.5860	7.2047	6.5728	6.3977	6.4452	6.6329	6.9208	6.2365	6.7542	7.1186	6.7094	6.8527	7.1164	6.8994	7.1160	6.7862	6.6407	6.9182
Naxe	_SPPTVLVIC[Carbamidomethyl (C)]GPGNNGGDGLVC[Carbamidomethyl (C)]AR_.2	#N/A	5.3819	5.4846	5.3048	5.7550	5.4063	5.7799	5.0726	5.2969	4.7991	5.7421	5.5566	5.8413	5.8452	6.5914	5.2733	5.1668	5.5902	5.6056	5.5252	5.8256
Naxe	_RGS[Phospho (STY)]ETMAGAAVK_.2	Q8K4Z3_S43_M1_Naxe	4.0628	8.2335	9.7142	9.7382	7.3405	4.3031	7.8439	4.0445	7.6846	3.8734	8.3520	7.7477	4.3648	9.8292	6.7419	3.4249	7.9704	6.3116	6.1347	7.2249
Slc8a2	_GIS[Phospho (STY)]ALLLNQGNGDK_.2	#N/A	9.0136	8.6101	8.1926	8.5204	7.8256	8.1996	8.2412	8.4722	8.0211	8.5450	8.1311	7.8967	8.2859	8.3719	6.2811	7.9151	8.4862	8.2747	8.1504	7.4918
Slc8a2	_S[Phospho (STY)]GIIIGAEGDPPK_.2	Q8K596_S266_M1_Slc8a2	7.2171	7.6492	7.9702	4.3542	4.1738	7.3002	3.3673	4.5364	7.4622	7.3765	6.4467	6.4715	3.3559	7.1698	2.9445	6.4570	7.1882	7.1561	7.1107	7.1508
Slc8a2	_C[Carbamidomethyl (C)]QPGVLLPVWEPEDPSLGDK_.2	#N/A	4.1597	3.8431	3.1951	3.9502	3.4974	2.9867	4.0947	3.8600	4.0149	4.0579	4.3936	4.2444	4.1804	3.1822	4.1454	3.1667	4.3977	4.6420	4.6776	4.5892
Slc8a2	_TNLALVIGTHSWR_.2	#N/A	4.5705	4.2494	4.3703	3.3198	3.0345	3.8672	3.7715	3.2953	2.2194	3.9754	3.4641	3.9755	4.6014	4.1451	3.8004	3.9326	2.3330	4.3569	2.0083	2.6650
Slc8a2	_GIS[Phospho (STY)]ALLLNQGNGDKK_.2	#N/A	3.3711	3.8514	3.9450	4.1166	4.2097	4.4338	3.3314	3.6933	3.3905	3.5775	3.7648	3.5776	3.4985	4.5431	2.9086	3.7160	3.1906	3.9589	4.2579	4.2204
Dcaf8	_GHGHS[Phospho (STY)]DEEDEEQPR_.2	#N/A	4.5090	5.1552	4.8221	5.2416	4.6416	5.3299	5.3218	5.0249	5.2499	5.0639	4.8289	5.1294	5.4137	5.1617	5.5037	5.2354	5.2384	5.2905	5.2970	4.9786
Flcn	_AHSPAEGASSESSS[Phospho (STY)]PGPK_.2	Q8QZS3_S73_M1_Flcn	4.1421	3.1770	3.6787	3.4421	4.3490	3.7593	4.1999	4.3678	4.9140	4.4038	2.9385	4.4039	4.1730	3.7167	4.2253	3.5042	3.8650	3.2844	3.5835	3.3940
Acat1	_FASEITPITISVK_.2	#N/A	8.0452	8.4929	8.5728	8.4745	8.4556	8.1217	8.4284	8.4794	8.6324	8.3916	8.4227	8.4322	8.3514	8.6976	8.6049	8.5940	8.6336	8.4608	8.5317	8.6946
Acat1	_QEQDTYALSSYTR_.2	#N/A	8.0293	8.1732	8.3891	8.3093	7.8329	7.9436	7.8303	7.8949	8.4403	8.1349	7.9710	7.8037	7.8790	8.3654	8.4262	8.4521	8.2355	7.7861	8.0114	8.1177
Acat1	_QATLGAGLPISTPC[Carbamidomethyl (C)]TTVNK_.2	#N/A	7.3634	7.3150	7.7984	7.6699	7.2917	7.3234	7.3838	7.1398	7.2977	7.7011	7.2540	7.0645	7.2526	7.5275	7.9368	7.7390	7.3163	7.2918	7.2588	7.2739
Acat1	_LGTAAIQGAIEK_.2	#N/A	6.9377	7.5794	7.0218	6.9142	7.2210	7.1821	7.2013	7.2855	7.4632	7.1982	6.6434	6.9888	6.7837	7.4691	7.2551	7.0892	7.3656	7.5592	6.9364	7.4368
Acat1	_TPIGSFLGSLASQPATK_.2	#N/A	6.5931	6.6727	6.9809	6.9260	6.9239	7.1480	6.6600	6.5341	6.6665	6.9332	6.8882	7.2222	7.3972	7.0245	7.0483	6.8652	6.5731	6.8582	6.3626	7.1222
Acat1	_SKEAWDAGK_.2	#N/A	6.2133	6.3763	6.1250	6.6974	6.2158	6.5024	6.2953	6.4110	7.1312	6.3443	6.7306	6.8665	5.8447	6.7486	7.0178	6.8138	6.3089	6.4055	6.3674	6.8393
Acat1	_M[Oxidation (M)]LEIDPQK_.2	#N/A	6.3255	3.1341	5.6417	5.4584	6.9398	6.0653	5.7308	6.5366	6.3452	6.6907	5.5701	6.3478	7.0839	5.9733	6.6341	6.6014	6.1854	6.5117	6.2254	6.5000
Acat1	_LEDLIVK_.2	#N/A	4.6762	3.9223	4.5886	4.6972	5.0314	4.8381	5.3297	3.7527	4.8194	5.1097	5.9857	5.0994	3.1935	5.8009	5.9156	5.5725	2.0563	5.2745	5.4557	5.9836
Acat1	_EVYM[Oxidation (M)]GNVIQGGEGQAPTR_.2	#N/A	6.2152	6.0668	6.1107	6.0917	5.6476	5.9820	5.8726	6.2095	6.0115	6.1972	5.8228	5.7743	6.0425	6.2405	6.1418	6.1646	5.9751	6.0649	6.0577	5.9357
Acat1	_GATPYGGVK_.2	#N/A	5.8983	4.3296	5.7015	5.6940	4.9172	3.2350	5.6396	4.7211	5.4729	3.8796	5.0395	5.2098	4.9742	5.3550	4.1073	5.5313	3.9113	5.2197	3.0592	5.1103
Acat1	_ENGTITAANASTLNDGAAALVLM[Oxidation (M)]TAEAAQR_.3	#N/A	4.7399	3.8584	4.2790	4.0886	3.9542	3.4819	4.1450	3.4827	4.8806	4.8883	3.7081	3.8578	3.2725	4.3171	4.6189	3.7564	4.4656	3.5468	4.5740	4.2208
Acat1	_DGLTDVYNK_.2	#N/A	4.2182	3.8646	4.3613	5.9372	3.5309	4.8715	5.5450	4.6211	5.0902	6.0660	5.7303	3.7213	3.6029	5.2626	5.1685	4.7541	3.8202	5.4158	3.6615	5.9108
Acat1	_EEVKEVYMGNVIQGGEGQAPTR_.3	#N/A	4.3095	5.0069	5.4093	3.2747	3.0156	3.0490	5.5700	4.5352	3.9527	4.1201	4.4588	4.2365	4.1181	6.1602	3.2231	3.6716	4.0324	3.4977	3.1463	3.0869
Acat1	_IAAFADAAVDPIDFPLAPAYAVPK_.3	#N/A	3.4938	3.9620	4.3945	4.2271	4.2792	3.5974	3.4841	3.5828	3.4043	3.1677	4.3837	3.6881	3.3880	4.4326	3.3414	3.6877	3.0800	4.0694	4.3685	4.1099
Ccsap	_AHS[Phospho (STY)]VDVEK_.2	#N/A	11.1186	11.2142	11.1137	11.1767	10.6542	10.9617	10.7102	10.7884	10.7437	11.1648	10.8889	10.7818	11.2863	11.4957	10.7908	11.0104	11.0488	10.9184	10.9857	11.1859
Ccsap	_ETDGAPS[Phospho (STY)]GPGPR_.2	Q8QZT2_S129_M1_Ccsap	3.1071	4.2038	7.8362	3.8904	7.8723	3.3474	8.2889	3.4993	3.6542	8.1660	8.1918	3.9381	8.9576	4.3223	8.6876	3.9701	8.5720	8.6000	8.5060	4.2893
Ccsap	_GALGEPS[Phospho (STY)]APS[Phospho (STY)]AREEEQPVGER_.3	Q8QZT2_S76_M2_Ccsap	4.2072	5.6170	5.1743	5.7516	2.9133	5.6353	5.0543	4.1626	5.3318	5.8110	3.5342	5.9090	4.2211	6.4509	5.9492	6.1079	5.9234	6.1285	6.0170	6.1410
Ccsap	_QRAHS[Phospho (STY)]VDVEK_.3	#N/A	3.6391	4.5702	4.3649	4.4725	4.1939	4.2741	4.7037	3.6375	3.8855	4.7466	4.4961	4.5115	5.4980	4.9749	4.4710	4.9828	4.6736	3.0423	5.0894	4.9602
Ccsap	_QMDMGS[Phospho (STY)]QK_.2	Q8QZT2_S180_M1_Ccsap	3.7560	4.2468	4.2923	3.9334	3.0937	3.3904	3.6911	2.8908	3.6113	4.4615	3.0899	3.2824	3.7767	4.2794	3.7164	4.1009	3.9941	3.8391	3.4877	4.2464
Stk32c	_GSSAAAPPSS[Phospho (STY)]PPPGR_.2	#N/A	14.4671	14.2228	14.5966	14.3941	13.9134	13.9504	14.0026	14.3892	13.9584	14.0213	14.3627	14.5192	15.1260	14.7553	13.9140	14.5123	14.7967	14.6921	14.5372	14.3886
Stk32c	_RGS[Phospho (STY)]SAAAPPSS[Phospho (STY)]PPPGR_.2	Q8QZV4_S18_M2_Stk32c	3.8789	9.2469	4.0870	3.8240	3.2188	3.5739	9.1423	3.8166	4.0398	3.9412	6.3017	4.2583	7.8197	3.9824	7.3110	3.7842	3.9289	3.9769	7.4014	3.7711
Stk32c	_M[Oxidation (M)]S[Phospho (STY)]MSSISSGSAR_.2	#N/A	3.4105	4.9671	6.4017	4.2269	3.9755	3.3424	3.3658	3.3906	3.3520	3.4375	5.1938	3.3538	3.4897	4.2291	3.2007	4.0309	3.4204	4.0484	4.1952	4.0853
Fam161a	_SS[Phospho (STY)]NPKPR_.2	Q8QZV6_S384_M1_Fam161a	4.4427	4.3772	3.9793	3.1415	3.1488	5.4007	3.8993	3.1675	3.8195	4.2533	3.2982	4.1033	3.9850	4.0173	3.9247	3.8048	4.1656	3.6309	3.2829	2.9538
Mapre2	_SDKDLETQVIQLNEQVHSLK_.3	#N/A	4.5318	5.3517	5.4715	5.0775	5.3105	4.8339	4.7235	5.7515	4.6176	5.1680	5.4141	5.2076	5.0484	5.4534	4.9675	5.4787	5.5251	5.1593	4.8569	5.7291
Mapre2	_LALEGVEK_.2	#N/A	3.7065	3.6126	6.0419	6.1471	3.9134	6.4819	6.8807	3.9322	6.2511	3.1615	6.5792	7.0157	3.2203	6.8514	3.5396	3.0686	3.4846	6.8822	6.8566	6.6712
Mapre2	_SSPASKPGSTPSRPSS[Phospho (STY)]AK_.3	Q8R001_S222_M1_Mapre2	3.4609	4.2534	4.2779	4.1804	4.6441	3.7430	5.0294	3.4566	3.2587	3.4761	4.2382	3.2847	3.4242	4.2959	3.1336	4.0967	4.3555	4.1917	4.0372	3.8938
Mapre2	_EIELLC[Carbamidomethyl (C)]QEHGQENDDLVQR_.3	#N/A	5.8291	4.9430	5.1344	5.3640	4.8973	4.5887	4.8004	4.8581	4.9444	5.2850	5.4587	4.4512	5.3947	4.6814	4.3918	4.7256	5.0454	3.9745	4.6356	4.6786
Mapre2	_SHHANS[Phospho (STY)]PTAGAAK_.2	Q8R001_S199_M1_Mapre2	7.8309	7.1918	6.9257	7.0464	6.7531	4.3642	7.1522	6.8946	6.5108	6.9116	6.6032	4.4221	4.3037	6.1466	4.2435	3.4860	4.5211	6.5320	6.4363	6.1809
Blmh	_NLVHSGATK_.2	#N/A	3.5230	4.0033	4.3532	4.2685	2.8607	4.5791	3.1795	3.5414	4.7840	3.7293	5.2454	3.7294	3.3466	4.3912	4.2575	3.8679	4.5395	4.1108	4.4098	4.0685
Blmh	_VENSWGEDHGHK_.3	#N/A	3.6021	4.4007	4.1384	4.0873	2.9398	3.1639	3.1004	3.3025	3.1595	3.8084	3.5339	4.2901	3.2676	4.1255	4.1784	2.3266	3.8402	3.9930	3.6416	4.0925
Gspt1	_ENLSAAFSR_.2	#N/A	4.7341	3.6528	4.8077	3.9179	4.5976	3.8337	4.1865	4.7120	3.5891	3.3788	4.2920	3.3789	3.6972	3.2409	4.4642	3.0284	4.9840	3.7602	4.5077	4.1169
Itpka	_DTLEISDFFR_.2	#N/A	4.4570	4.5740	4.8659	3.8330	3.8244	4.5917	5.0600	4.1871	3.7757	5.6797	4.1501	4.4408	4.9872	3.5092	4.6946	5.1169	3.2239	3.8421	4.8744	4.6744
Itpka	_TTPLPDGQILDHR_.2	#N/A	5.7134	5.1446	5.5876	4.9020	5.2429	5.2706	5.2319	5.8697	3.8001	5.4582	5.3851	5.4872	4.5421	5.5742	5.4740	5.3641	5.9774	5.3470	5.0994	4.5291
Hnrnpl	_SSSGLLEWDSK_.2	#N/A	7.1616	7.0633	7.7242	7.0185	6.8794	7.0044	6.6679	6.6289	7.2195	6.2734	6.8395	6.6031	7.1436	6.9806	6.9082	6.7464	6.4902	6.5903	6.6580	7.3170
Hnrnpl	_FSTPEQAAK_.2	#N/A	6.9408	6.7440	6.8021	6.9118	6.8470	6.9019	6.9396	6.7557	7.2286	6.6943	7.0815	6.9777	6.9858	7.0585	7.5885	6.8000	6.6138	7.1404	7.1092	7.4833
Hnrnpl	_IEYAKPTR_.2	#N/A	6.4082	6.9666	6.7861	6.9027	6.8142	6.5656	6.9172	6.5995	6.5784	6.7096	6.8093	7.2932	6.6200	6.7274	7.5228	6.5368	6.9367	6.8606	6.6691	6.4522
Hnrnpl	_VFNVFC[Carbamidomethyl (C)]LYGNVEK_.2	#N/A	5.3206	5.2270	5.8732	5.6864	5.1483	5.8002	5.5093	4.9280	5.3925	5.9389	4.9894	5.2296	5.0303	5.2877	5.8506	5.7758	5.3822	5.4178	4.7869	5.8888
Hnrnpl	_YYGGGNEGGR_.2	#N/A	6.0106	6.1172	6.2887	6.2927	5.6239	6.5276	6.2987	6.1528	6.2679	6.0726	6.0370	5.9296	6.3978	6.0218	6.4275	6.4114	6.1795	6.3639	5.9567	6.0034
Hnrnpl	_NDQDTWDYTNPNLSGQGDPGSNPNKR_.3	#N/A	3.8377	4.1651	4.3740	3.8517	3.1755	3.3995	4.3656	3.5381	4.4247	4.0441	3.2982	4.0545	4.5328	4.3611	3.9428	4.1826	4.0758	3.7574	1.0158	4.3281
Hnrnpl	_ISRPGDS[Phospho (STY)]DDSR_.2	Q8R081_S182_M1_Hnrnpl	6.3995	7.0582	4.2940	6.6037	6.2239	6.5992	6.5011	6.5058	6.3938	6.9011	6.4418	6.2421	6.9788	7.0787	6.0850	6.4734	6.6919	4.1114	6.6798	6.4579
Hnrnpl	_ASLNGADIYSGC[Carbamidomethyl (C)]C[Carbamidomethyl (C)]TLK_.2	#N/A	3.2308	6.7400	5.8503	4.3534	2.9457	6.4110	6.6724	6.5737	5.9216	3.8143	3.5280	6.0100	3.2617	6.9648	5.6817	3.9528	4.4546	4.1957	5.9176	5.9633
Hnrnpl	_LC[Carbamidomethyl (C)]FSTAQHAS_.2	#N/A	4.3734	4.4466	3.9099	3.2109	3.0795	3.1128	3.9686	3.0982	3.8889	4.1839	3.3675	4.1727	4.0543	3.9480	3.9940	3.7355	4.0963	3.0532	3.3522	4.8569
Hnrnpl	_SKPGAAMVEMADGYAVDR_.3	#N/A	3.7006	3.6185	3.5467	3.8837	3.9075	4.2009	3.1406	3.9262	2.9979	3.5112	3.5336	3.3446	3.7314	3.2752	3.1660	3.0627	3.4235	3.7260	4.0250	2.9525
Gtf2f2	_HYQTEEKS[Phospho (STY)]D_.2	Q8R0A0_S248_M1_Gtf2f2	4.2161	5.0962	3.1896	3.5352	3.4919	5.5008	4.8059	6.0632	5.9128	5.8163	5.3280	3.7381	6.8653	5.7391	5.5355	2.9982	6.0574	5.5479	3.0895	6.9197
Zbtb44	_DGSIS[Phospho (STY)]PVSSEC[Carbamidomethyl (C)]SAVER_.2	Q8R0A2_S161_M1_Zbtb44	5.9598	6.3882	5.6431	6.5597	3.5990	6.1245	5.4981	6.2729	5.9217	5.8875	5.7958	3.6532	5.2671	5.9114	5.6853	5.6423	6.5380	5.9107	5.6342	5.8802
Tceal3	_[Acetyl (Protein N-term)]MEEVRGENEGKLEK_.3	#N/A	4.0190	4.0809	5.7656	5.4091	3.3567	3.7413	4.7766	3.8294	4.3086	4.0082	4.4805	3.9703	3.8338	5.5350	5.0066	3.6129	3.7752	3.7449	3.6917	3.9505
Kiaa0513	_SANSWLAEK_.2	#N/A	4.9121	4.4391	5.5334	4.3865	5.4978	4.9974	2.9032	3.6742	4.4864	4.3067	3.8889	3.8804	5.3756	3.9555	4.2938	3.9667	4.9206	4.0863	4.2263	4.0188
Kiaa0513	_TVTM[Oxidation (M)]IS[Phospho (STY)]PEDEQK_.2	#N/A	9.5349	9.1153	9.4554	9.3035	8.8469	9.0552	9.1110	9.2948	8.9663	9.3445	8.7566	8.6940	9.2129	9.3473	8.1343	8.7825	9.3779	9.2103	8.9140	8.9168
Kiaa0513	_FWNAAFFDAVHC[Carbamidomethyl (C)]ER_.2	#N/A	3.9783	3.3755	4.5146	2.7356	3.6518	3.4668	4.2251	3.7492	3.2796	4.1846	4.3997	3.8335	3.9990	4.5017	1.7343	4.3232	4.0091	3.0191	2.9324	3.3315
Kiaa0513	_QAVIGNLDEEQYK_.2	#N/A	4.2115	4.5699	4.1873	4.5309	3.1563	4.7782	4.6382	3.1751	4.9609	4.5261	4.3041	4.4457	5.0202	4.0249	4.3014	3.8123	4.1497	3.9125	3.4284	4.5541
Kiaa0513	_TVTM[Oxidation (M)]IS[Phospho (STY)]PEDEQKGEK_.2	#N/A	10.7518	10.7773	10.5418	10.5261	10.1420	10.3413	10.4710	10.9011	10.4098	10.2886	10.2340	10.4035	11.0816	10.8059	9.5617	10.2226	10.8153	10.5915	10.2758	10.2495
Kiaa0513	_S[Phospho (STY)]SSNESFSSNQSADSAPDEETLALR_.3	Q8R0A7_S72_M1_Kiaa0513	3.9633	2.2975	4.4996	3.7261	3.3010	3.5251	4.2401	3.6637	4.2992	4.1696	3.1727	3.9290	4.4072	4.4867	3.8173	4.3082	4.2013	3.0502	3.2804	3.6283
Kiaa0513	_IFSGGEDLDQEEK_.2	#N/A	3.9989	4.0039	4.5352	3.6904	3.3367	3.5607	4.2044	3.0655	4.2635	4.2053	3.1370	3.8933	4.3716	3.9153	3.7816	4.3438	4.0636	3.0858	3.3849	3.5926
Fahd1	_IITLEEGDLILTGTPK_.2	#N/A	3.0505	3.6627	4.3975	3.6416	2.9967	3.0742	3.3761	3.8133	3.2926	3.9815	3.3894	3.7777	3.9215	3.1905	3.9342	4.0915	2.7493	4.3343	3.9067	3.7410
Tada3	_ESGADGASTS[Phospho (STY)]PR_.2	Q8R0L9_S298_M1_Tada3	4.4805	3.5223	3.5159	7.2687	6.7772	4.1667	6.3856	4.1809	6.8201	6.0950	3.8144	3.4118	4.5012	3.5030	2.9401	3.2886	3.2177	6.0675	4.2640	7.0146
Aamdc	_ETGTEHS[Phospho (STY)]PGVQPADVK_.2	#N/A	4.2690	3.7907	6.1050	3.1795	3.6068	6.3670	3.9912	3.7755	4.0503	6.3260	5.8545	5.9883	4.1584	3.7705	5.8774	6.0937	6.0080	3.3560	3.6550	3.3794
Atf7	_TDSVIIADQTPT[Phospho (STY)]PTR_.2	Q8R0S1_T53_M1_Atf7	7.1366	3.5746	4.1281	7.2670	6.9741	6.9782	6.8492	7.3438	7.4758	7.4039	7.0805	7.3305	7.4237	4.0200	6.7612	7.6372	7.6745	7.1443	7.0443	7.3559
Iqsec1	_SLGGQQGS[Phospho (STY)]PK_.2	Q8R0S2_S415_M1_Iqsec1	11.9142	12.1830	12.1088	11.9297	11.2822	11.5638	11.9451	11.9251	11.7628	11.6701	11.4670	11.6486	11.8664	11.4561	10.5966	11.2335	11.8357	11.6541	11.5252	11.2934
Iqsec1	_LQHSTS[Phospho (STY)]VLR_.2	#N/A	9.9043	9.7996	9.8538	9.5472	9.0351	9.2671	9.1672	9.4722	4.1970	9.2550	8.9068	8.4110	9.0847	8.9752	7.8509	8.7418	9.2127	8.9119	8.8968	8.8007
Iqsec1	_SALSSS[Phospho (STY)]LRDLSEAGKR_.3	#N/A	3.3817	8.5184	7.6112	4.2892	3.9277	7.3808	3.3389	4.0067	3.2591	3.3165	4.2386	3.8937	3.4245	7.8949	6.0096	6.7878	7.6546	4.1949	3.8435	6.9610
Iqsec1	_M[Oxidation (M)]QFS[Phospho (STY)]FEGPEK_.2	#N/A	11.9449	11.9147	11.8436	11.8675	10.3877	10.9689	11.3289	11.0313	10.9352	11.8675	11.0658	10.9261	11.4384	11.2636	9.7936	10.8744	11.4536	10.8944	10.9248	10.9940
Iqsec1	_SALSSS[Phospho (STY)]LR_.2	#N/A	3.8415	4.1613	4.3778	3.8479	3.1792	3.4033	7.3268	3.2230	4.4210	4.0478	3.2945	4.0508	4.5290	4.0728	3.9391	4.1864	4.2210	4.4292	7.0418	6.4050
Iqsec1	_SLAES[Phospho (STY)]IDDALNC[Carbamidomethyl (C)]R_.2	Q8R0S2_S251_M1_Iqsec1	3.3048	8.1641	8.3475	7.2134	4.1433	7.1072	7.6004	4.5060	3.4568	3.5111	3.8312	3.0866	3.5649	7.9706	6.5144	3.6497	3.5428	4.2903	6.8090	6.5118
Iqsec1	_ETLIGIYER_.2	#N/A	4.3090	3.6658	5.0993	5.3424	3.0151	2.2273	5.1726	4.5347	5.3770	2.7647	4.3608	3.1346	4.3399	2.8706	4.0584	3.3193	4.0319	5.4187	5.2557	4.7432
Iqsec1	_VLINFNAPNPQDR_.2	#N/A	3.6415	5.4164	4.1778	5.0631	2.9792	5.3114	5.7537	5.7954	5.6067	5.0097	5.1504	4.2562	3.2282	4.2728	4.5868	4.9639	5.6588	5.1140	4.8492	5.2824
Iqsec1	_ETRNS[Phospho (STY)]WDSPAFSNDVIR_.3	#N/A	5.3254	5.8896	4.1514	4.2656	3.6087	3.5780	3.8167	3.8873	3.6263	5.6298	3.6211	5.3839	4.8643	4.1552	4.6899	5.5531	4.8606	4.1468	5.7389	4.0320
Iqsec1	_LQHSTS[Phospho (STY)]VLRK_.2	#N/A	4.3217	4.4983	3.8582	3.2626	3.0278	3.0611	3.1483	3.0465	3.9405	4.1323	3.4192	4.2244	4.3525	3.8963	4.0457	3.6838	4.0446	3.1049	3.4039	3.5736
Iqsec1	_SALSSS[Phospho (STY)]LRDLSEAGK_.2	#N/A	5.8103	5.8302	5.8964	3.8934	3.5512	3.6021	3.5380	3.7150	5.4496	5.8109	3.7296	3.7553	3.6236	5.2071	3.8737	3.7246	5.6573	5.4684	5.4277	5.7228
Iqsec1	_GVDDGEDIPR_.2	#N/A	4.5684	3.4344	5.9045	3.1210	4.6269	4.9340	5.3113	4.2688	5.2505	3.6491	3.9023	4.2643	5.2427	3.5909	4.9955	5.3505	3.2077	4.9175	4.9492	4.7287
Iqsec1	_S[Phospho (STY)]LHSEEVPASDTAR_.2	Q8R0S2_S260_M1_Iqsec1	4.1006	7.5933	3.1361	3.5888	3.4384	7.1057	4.1027	3.8010	7.2233	7.4104	6.8373	6.7123	4.2699	4.6240	6.5842	6.5238	7.5788	3.4945	3.1431	7.1999
Iqsec1	_STPS[Phospho (STY)]LERPEPR_.2	Q8R0S2_S362_M1_Iqsec1	4.2299	3.2094	3.6463	3.4745	3.5677	3.7917	3.9734	4.3354	4.0325	4.4363	2.9060	3.6623	4.1406	3.6843	3.5506	3.0740	3.8326	7.4141	6.9407	3.3616
Iqsec1	_LRSS[Phospho (STY)]MSENR_.2	Q8R0S2_S159_M1_Iqsec1	3.4236	3.8955	4.4610	4.1606	3.6305	3.6639	3.4176	3.6493	3.3378	3.2342	4.3173	3.6216	3.5032	4.0059	3.2989	4.2866	3.1465	4.1126	3.7612	3.9729
Iqsec1	_SLS[Phospho (STY)]ESYELSSDLQDK_.2	Q8R0S2_S106_M1_Iqsec1	3.8204	3.4986	3.3570	3.7638	4.0274	4.0810	3.6842	4.0461	3.7433	3.2247	4.1176	3.2248	3.8513	3.3951	3.2614	3.1826	3.5434	3.6061	7.1198	3.0724
Iqsec1	_NSWDS[Phospho (STY)]PAFSNDVIR_.2	Q8R0S2_S513_M1_Iqsec1	3.6949	3.6553	4.2312	4.1155	3.7599	3.7835	3.7333	4.1225	3.7475	3.9316	3.6442	3.5447	3.8738	10.7558	3.4759	4.0398	3.9330	10.3670	3.7655	3.6226
Iqsec1	_SRS[Phospho (STY)]LS[Phospho (STY)]ESYELSSDLQDK_.2	#N/A	3.6642	4.9403	3.3068	3.8304	4.0538	4.2355	3.7596	3.9959	3.8562	3.5337	3.8941	3.2000	3.8010	5.6593	3.0435	3.3365	3.4931	3.6727	3.9718	4.1113
Cacnb4	_HSNHSTENS[Phospho (STY)]PIER_.2	#N/A	6.2990	6.2958	6.5196	6.5874	5.9534	6.3972	5.8001	5.7496	5.7182	7.0483	5.9826	5.5871	6.4589	6.2520	5.2351	5.9812	5.4299	5.4809	5.6763	5.7900
Cacnb4	_HSNHS[Phospho (STY)]TENS[Phospho (STY)]PIERR_.3	#N/A	4.0150	4.6334	3.8047	3.8112	3.6444	3.6188	4.7320	3.9374	4.0461	4.9984	4.2274	3.4811	3.9573	3.8298	3.6447	3.7939	3.8805	4.4104	4.2214	3.5857
Cacnb4	_ATHTSSS[Phospho (STY)]TPM[Oxidation (M)]T[Phospho (STY)]PLLGR_.2	#N/A	8.2298	7.6026	7.2773	8.0054	3.8189	3.9591	7.1398	4.0795	7.5305	8.0563	7.5075	3.4591	5.0339	3.4123	7.3173	8.3280	4.7254	7.7341	3.8129	5.0769
Cacnb4	_SNTRSS[Phospho (STY)]LAEVQSEIER_.3	Q8R0S4_S287_M1_Cacnb4	3.8392	3.4799	3.3758	3.1217	4.0461	4.3633	3.0020	4.2681	4.4230	3.6498	3.9016	3.2060	4.5884	3.5903	3.0273	3.2013	3.3049	3.0274	4.1768	5.2111
Cacnb4	_SDGS[Phospho (STY)]TTSTS[Phospho (STY)]FILR_.2	Q8R0S4_S38_M2_Cacnb4	3.4078	4.1834	6.5723	4.4486	3.1604	3.2823	5.0602	3.3624	3.4089	4.1640	3.3172	3.9095	3.1676	5.5478	3.9538	3.9843	4.3605	4.2909	5.0971	3.8895
Cacnb4	_NRGS[Phospho (STY)]PGGC[Carbamidomethyl (C)]SHDSR_.2	#N/A	5.3867	3.5131	3.5988	3.9580	3.9011	4.4151	3.7137	5.4868	4.9403	4.0436	4.7905	3.4026	3.8808	5.6165	3.2909	3.4074	5.2587	4.9729	3.9493	3.8766
Cacnb4	_NRLS[Phospho (STY)]SSSQHSR_.2	Q8R0S4_S474_M1_Cacnb4	3.7377	4.4123	3.8975	4.5917	3.5985	3.9582	2.8066	3.7405	3.6560	4.1194	3.3725	3.9846	2.8845	4.7792	3.9915	3.7713	2.4038	4.1422	3.3497	3.4844
Aldh1l1	_ILPNVPEVEDSTDFFK_.2	#N/A	6.1752	5.9769	6.5896	6.4577	6.1240	6.1781	6.0128	6.3254	5.8797	6.9640	5.8859	5.5130	5.8917	5.5615	5.2159	5.5591	6.0828	5.8767	5.8004	6.1041
Aldh1l1	_LIAEGTAPR_.2	#N/A	6.1887	6.9041	3.9795	6.4378	6.5280	6.5550	6.8187	6.4995	6.3976	6.2702	5.7981	6.3772	6.2276	5.2888	6.3527	5.5375	5.2740	6.7707	5.8495	5.9973
Aldh1l1	_KVS[Phospho (STY)]LELGGK_.2	Q8R0Y6_S671_M1_Aldh1l1	5.2117	6.2538	6.2012	5.6649	5.5327	5.5631	5.5818	5.1819	5.0734	5.6901	5.5054	5.5720	5.4654	6.0901	4.2318	5.4838	5.1171	5.5218	5.4768	5.6968
Aldh1l1	_KLVEYC[Carbamidomethyl (C)]QR_.2	#N/A	4.2992	7.0288	6.8013	7.6634	3.0053	7.3821	6.7241	6.2445	6.2361	7.0668	6.3554	5.8741	5.8798	5.9858	5.9067	6.3219	7.0379	6.4885	6.6472	6.2804
Aldh1l1	_EC[Carbamidomethyl (C)]DVLPDDTVSTLYNR_.2	#N/A	5.5724	5.1949	5.3653	5.8552	5.1318	5.1452	5.1332	4.1160	5.3338	4.7222	4.2493	3.8593	3.9742	3.5180	4.8708	4.2554	3.8100	4.3636	5.0043	5.0681
Aldh1l1	_AGLILFGNDDR_.2	#N/A	6.0322	6.1756	5.5264	6.1274	5.7833	6.2322	5.9400	6.3072	6.0501	6.2804	5.6326	5.2399	3.9202	6.1154	4.4919	5.6130	5.6910	5.7624	5.5373	3.1413
Aldh1l1	_DLGEAALNEYLR_.2	#N/A	5.1337	5.9492	5.3412	5.2761	5.7287	5.8485	5.2312	3.4316	5.4068	5.1894	4.9450	5.2937	3.7519	5.2906	5.0656	5.4551	4.8434	5.1952	4.4081	4.7913
Aldh1l1	_ANATEFGLASGVFTR_.2	#N/A	5.3747	4.5893	5.3030	5.5359	5.0850	5.1390	4.5288	4.7588	4.9725	3.8905	4.7003	2.6161	3.2851	3.7878	3.7720	4.6253	3.5024	4.3307	4.1045	3.7548
Aldh1l1	_GSASSALELTEEELATAEAVR_.2	#N/A	4.8400	5.3920	4.6416	5.2027	5.1130	5.0045	4.6550	4.3713	4.8421	4.9584	4.6053	4.2862	4.8813	5.2039	3.7933	3.4823	5.2265	4.5930	4.5579	4.7824
Aldh1l1	_LRGEDGESEC[Carbamidomethyl (C)]VINYVEK_.3	#N/A	5.1776	5.1766	4.2450	4.3766	3.4145	4.4347	3.0713	5.3547	3.2316	3.8375	4.9343	3.6898	3.2385	4.2831	3.5803	4.1581	4.0839	4.2189	3.0171	4.4092
Aldh1l1	_IAVIGQSLFGQEVYC[Carbamidomethyl (C)]QLR_.2	#N/A	3.8264	4.1764	4.3627	3.7866	3.1641	3.3882	4.3769	3.5268	4.4360	4.0327	3.3096	4.0658	2.6694	3.6170	3.1601	4.1713	4.0645	4.4142	3.2124	3.6878
Aldh1l1	_TYSTINPTDGSVIC[Carbamidomethyl (C)]QVSLAQVSDVDK_.3	#N/A	3.8962	4.1066	4.8335	3.7931	3.2340	3.4580	4.3071	3.5966	3.3365	4.1026	3.2397	3.9960	4.4743	3.9868	3.8843	4.2411	4.1343	3.6989	3.3474	3.6953
Aldh1l1	_DTNHGPQNHEAHLR_.4	#N/A	4.0373	3.6331	3.4051	3.3197	4.2442	4.2776	4.3047	4.0700	4.2249	3.8479	3.7036	3.5226	4.3904	3.3922	1.1132	3.3994	3.1069	3.2254	4.3749	3.3592
Vps37c	_LALES[Phospho (STY)]PEVQDLQLER_.2	Q8R105_S29_M1_Vps37c	3.9131	4.0897	4.4494	3.7762	3.2509	3.4750	4.2902	3.6135	4.3493	4.1195	3.2228	3.9791	4.4574	7.3122	3.8674	4.2580	4.1512	6.3360	3.3305	3.6784
Flad1	_C[Carbamidomethyl (C)]LS[Phospho (STY)]PGGHPVYR_.2	#N/A	3.7743	4.3072	6.1481	3.9938	3.8811	5.6359	3.7760	5.5919	3.6743	3.9807	3.5830	5.0621	3.8169	4.5579	3.3532	4.1192	3.8494	3.7713	3.8315	3.7374
Apeh	_ALDVSAS[Phospho (STY)]DEEM[Oxidation (M)]ARPK_.2	#N/A	4.9555	4.6377	4.0238	4.0943	4.9924	5.5436	4.9192	4.8061	5.3445	5.1487	4.6287	4.9730	4.2702	5.7293	3.4289	5.4311	5.1797	5.0406	5.0412	5.1766
Bud13	_VRHDT[Phospho (STY)]PDLS[Phospho (STY)]PPRR_.3	#N/A	6.2232	6.1827	6.4913	6.7923	5.1468	6.6192	6.2445	5.3970	6.0690	6.0869	6.4757	5.8318	6.4215	6.7471	5.4779	6.4392	5.4357	6.8973	6.4115	6.4566
Bud13	_HDT[Phospho (STY)]PDPS[Phospho (STY)]PPRR_.2	#N/A	6.2698	6.0425	3.9460	6.8468	3.7404	6.3547	6.1760	5.1982	6.3989	6.7429	5.8652	6.3337	6.7127	3.9841	5.1698	5.3783	5.4079	6.1979	5.9439	6.3213
Bud13	_AHHES[Phospho (STY)]PDLELHK_.3	Q8R149_S297_M1_Bud13	2.8224	3.9276	4.4289	4.1927	3.5984	3.0091	3.4497	3.6172	3.3699	3.2021	4.3493	3.6537	3.4224	4.4670	3.4750	4.2545	3.1144	4.0350	4.3341	4.1443
Bud13	_HDLDAS[Phospho (STY)]PPRK_.3	Q8R149_S238_M1_Bud13	4.3295	3.8302	3.8661	3.2547	3.0356	3.0689	3.1040	3.0543	3.9327	4.1401	3.4113	4.2165	4.0981	3.9041	4.0379	3.6916	1.3675	3.0970	3.3961	3.5814
Bud13	_HDT[Phospho (STY)]PDLS[Phospho (STY)]PPRR_.3	#N/A	3.9697	4.0331	4.5060	3.7197	3.3074	3.1767	3.9048	3.6701	3.8250	4.2478	3.3036	3.9225	3.9904	4.4931	3.8108	4.3146	4.2078	3.6254	3.2740	2.9592
Bud13	_VRHDT[Phospho (STY)]PDLS[Phospho (STY)]PPR_.3	#N/A	4.1113	3.2078	3.6478	3.4730	4.3182	4.3515	4.2307	4.3369	4.1509	3.9219	3.6296	2.9339	4.3164	3.3182	4.2561	3.4734	3.8342	4.0797	2.9480	3.2852
Bphl	_TDFAPQLQSLNK_.2	#N/A	6.5037	6.9167	6.6508	6.9068	6.8892	7.1988	6.8414	7.1145	6.9577	6.6511	7.0854	6.6692	7.0059	7.1085	7.3458	7.1060	7.3642	6.6405	6.6533	6.7531
Bphl	_FTLVAWDPR_.2	#N/A	5.4778	5.8160	5.9172	5.3344	5.6874	5.4177	5.5429	5.5911	5.1902	4.5074	5.3951	5.7104	4.9644	6.1163	5.6651	5.7867	3.7615	5.4431	3.6870	5.7145
Bphl	_TC[Carbamidomethyl (C)]EDWVDGISQFK_.2	#N/A	3.9442	4.4866	4.5419	3.1888	3.2819	4.4094	4.7604	3.1542	4.6813	5.3977	4.8888	4.9667	4.4263	3.9701	5.2380	4.8084	4.1184	4.1872	3.8906	5.4332
Bphl	_FADEFNR_.2	#N/A	4.5075	3.1963	3.5429	2.3963	3.8452	4.1398	3.2853	4.2078	2.2339	2.0431	2.6970	2.6394	3.1059	3.7725	2.9671	3.3515	3.2447	2.1225	2.3493	3.4970
Bphl	_QVSLLGWSDGGITALIAAAK_.2	#N/A	3.2032	4.1159	4.2406	4.3810	3.4101	1.5252	3.6380	3.4289	3.5582	1.3910	3.0368	2.8834	3.7236	4.2787	3.6633	4.0662	4.4270	4.2233	3.0215	1.4878
Syngr3	_AGAAFDPVSFAR_.2	#N/A	9.4090	9.0125	9.1028	8.9195	8.8641	8.8649	9.2033	9.2051	8.9965	8.6018	8.9692	8.7445	8.5751	8.6001	8.2217	8.3781	9.0909	8.9356	8.5435	8.4699
Syngr3	_FQQISSVR_.2	#N/A	5.9964	6.6309	6.1113	6.1338	6.0199	6.2622	6.6372	6.4243	6.7638	6.3356	5.9925	5.7091	5.5653	5.9058	6.1209	6.3155	6.0480	6.0131	5.9419	5.6638
Syngr3	_TTPGPGTAQAGDAAR_.2	#N/A	8.6133	8.4760	8.4684	8.4446	8.0083	8.3104	8.8486	8.5324	8.6052	8.3058	8.2127	8.0597	8.3376	8.1144	7.7665	7.8308	8.3613	8.3796	7.9617	7.8662
Syngr3	_[Acetyl (Protein N-term)]MEGASFGAGR_.2	#N/A	3.6379	5.0184	5.2513	5.2135	3.3619	5.0146	4.7402	4.0470	4.2019	3.8204	3.4616	3.6048	3.6586	4.9769	3.3161	3.9828	3.8760	3.1902	3.7484	3.3361
Dock7	_SLSNS[Phospho (STY)]NPDISGTPTSPDDEVR_.2	Q8R1A4_S900_M1_Dock7	4.4245	4.3955	3.9610	3.1598	3.1306	3.4770	3.9175	3.1493	4.3473	4.2351	3.2208	4.1216	4.4553	3.9991	3.9429	3.7866	4.1474	3.0021	7.9188	7.8681
Dock7	_SNS[Phospho (STY)]WVNTGPK_.2	Q8R1A4_S929_M1_Dock7	9.3728	9.3355	9.2780	9.2892	8.4975	8.7373	9.0005	9.1133	8.7399	9.1008	8.6322	8.4363	8.8825	8.3240	3.3209	8.1800	8.7126	8.7457	8.4080	8.1279
Dock7	_SPS[Phospho (STY)]GSAFGSQENLR_.2	Q8R1A4_S1422_M1_Dock7	8.6393	8.0687	8.2918	4.8596	7.6183	7.4748	7.6261	7.9178	7.2880	7.9811	7.6216	6.9301	7.6013	3.8857	3.5435	3.6866	7.5755	3.8395	3.7867	7.1924
Dock7	_KGS[Phospho (STY)]WSER_.2	Q8R1A4_S432_M1_Dock7	3.4354	4.5674	3.9717	5.2534	4.2740	2.9972	3.2671	3.1358	3.3262	3.6417	3.7006	2.9560	3.4343	3.9588	2.8443	3.7803	3.6734	4.1597	3.8083	3.9258
Dock7	_TTS[Phospho (STY)]GDDAC[Carbamidomethyl (C)]NLTSFR_.2	Q8R1A4_S452_M1_Dock7	6.0925	4.2088	4.2803	4.0865	3.2227	5.7595	5.7352	3.4530	3.3198	5.5366	3.0954	5.3551	4.0279	4.2374	3.7292	5.1765	4.1723	4.1510	5.6026	4.0121
Dock7	_RNS[Phospho (STY)]SLVGR_.2	Q8R1A4_S439_M1_Dock7	4.4785	3.5243	5.6981	3.2109	3.8162	4.0403	3.7249	4.1789	3.7840	3.1840	4.1583	4.3812	3.8920	3.5010	3.3021	4.7082	3.2157	3.5654	3.8644	3.1131
Dock7	_M[Oxidation (M)]NS[Phospho (STY)]LTFKK_.2	#N/A	6.1367	3.7951	4.1173	5.9949	5.1993	5.3079	5.2072	5.8120	5.8209	5.9253	3.7826	3.6846	6.1619	5.3593	3.5729	5.4327	6.0618	6.0572	5.4372	3.3839
Dock7	_SMS[Phospho (STY)]IDDTPR_.2	Q8R1A4_S182_M1_Dock7	3.8806	6.6235	4.4169	7.2032	3.2183	3.4424	4.3228	3.5810	4.3819	5.7524	3.2554	4.0117	4.4899	6.6313	3.9000	4.2255	5.6483	3.7145	3.3631	4.3709
Stac2	_SKS[Phospho (STY)]VENFFLR_.2	Q8R1B0_S48_M1_Stac2	4.3660	7.9041	3.4015	6.6659	3.7038	3.9278	3.8373	4.0664	6.4350	4.5724	4.2708	3.5262	4.3868	7.7624	3.4145	3.2101	3.1033	3.2291	4.3785	3.3556
Eif3c	_QPLLLS[Phospho (STY)]EDEEDTKR_.2	#N/A	8.2733	8.5550	9.2889	9.2564	6.2709	9.0220	9.1026	7.9642	9.1860	9.2647	8.5909	8.6137	9.0035	8.1811	6.9065	9.0394	8.8284	9.4376	9.2551	9.0663
Cplx3	_NETDESQIQLAGGDVELPR_.2	#N/A	5.0528	5.7032	4.6582	4.6663	3.4701	4.5195	3.8770	3.8328	4.8007	4.3387	4.2440	3.7599	4.2068	3.1549	4.4960	5.0549	4.9167	4.3958	3.6952	4.6861
Mettl22	_AAS[Phospho (STY)]DPNPAEPAR_.2	#N/A	8.8862	8.9626	9.0213	8.7009	8.2373	8.7392	8.6371	8.7906	8.7802	8.7844	8.5668	8.4171	8.7584	8.6248	8.0067	8.5607	8.8135	8.5896	8.5207	8.1916
Mettl22	_AAS[Phospho (STY)]DPNPAEPARDK_.2	#N/A	5.5601	6.3395	6.3503	6.2108	5.2641	5.4816	5.8119	6.2050	5.6792	6.0830	5.7731	5.9692	6.7271	5.6447	5.8352	5.7036	5.9920	5.7530	6.0854	5.4296

Hid1	_TGS[Phospho (STY)]QEGTSM[Oxidation (M)]EGSRPAAPAEPGTLK_.3	#N/A	3.9866	6.9434	5.4724	5.4099	3.5940	4.7081	7.1284	4.4535	6.5005	4.1042	4.5894	3.8171	6.9018	3.7930	3.5229	6.0627	6.6902	6.9739	3.6738	3.5786
Hid1	_TGS[Phospho (STY)]QEGTSMEGSR_.2	#N/A	4.1311	3.1880	3.6677	3.4531	4.3380	4.3714	5.0996	4.3568	4.1311	3.9417	3.6097	4.4149	4.2965	3.7057	4.2363	3.4932	3.8540	3.3193	3.5945	3.2654
Uqcr10	_AFDQGADAIYEHINEGK_.2	#N/A	5.6899	5.1915	5.7362	5.4976	5.3031	5.4770	5.0751	5.8817	5.5130	5.6401	5.5401	5.2205	5.3561	4.3848	4.8487	4.7494	5.0988	5.1311	5.2957	5.4546
Dync1li1	_NVLLLGEDGAGK_.2	#N/A	6.4537	6.9157	7.0046	6.7875	7.0048	6.9176	6.6115	6.9898	6.8781	7.0803	6.6702	6.9967	6.7851	7.1586	6.8553	6.4942	6.7229	6.5634	6.9714	6.7622
Dync1li1	_DAVFIPAGWDNDKK_.2	#N/A	6.5221	6.5166	6.5557	6.9883	6.6758	7.1751	6.6478	6.8747	6.5962	6.4991	6.6596	6.6753	6.6773	7.1554	6.5745	6.8795	6.4553	6.2968	6.5684	6.4116
Dync1li1	_FSLDALSLR_.2	#N/A	5.6427	6.5288	6.0132	6.2124	6.5299	6.5082	6.3215	6.4016	6.2751	6.3822	6.3539	6.2400	5.6434	6.0793	6.1103	6.0328	5.8600	6.4982	6.3254	6.4861
Dync1li1	_KPASVSPTTPTS[Phospho (STY)]PTEGEAS_.2	Q8R1Q8_S516_M1_Dync1li1	13.1358	13.2740	13.3118	12.0567	12.7249	13.1031	12.8936	13.5031	13.0476	13.2295	12.8225	13.0644	13.6079	13.1619	12.6410	12.7843	13.3896	12.9400	13.0527	13.0117
Dync1li1	_DFQEYVEPGEDFPAS[Phospho (STY)]PQRR_.3	#N/A	12.2042	12.7881	12.2862	12.3026	11.3823	12.2461	12.3114	11.7881	12.0085	12.3729	11.8942	12.0445	12.7251	12.9672	11.5858	12.2473	12.1710	12.4784	12.4144	12.2939
Dync1li1	_DFQEYVEPGEDFPAS[Phospho (STY)]PQR_.2	#N/A	12.5393	12.8860	13.0237	12.6292	11.7873	12.6970	12.4227	11.9513	12.4763	12.6999	12.3239	12.2075	12.3863	13.3124	12.1637	12.4631	12.6609	12.5560	12.5458	12.4907
Dync1li1	_IPAVVVEK_.2	#N/A	3.8943	4.3308	3.2795	4.4808	4.0262	4.2283	4.5600	3.5625	4.7977	3.8996	3.8227	4.2365	4.1264	4.7373	4.0063	3.4660	3.5154	4.3189	4.8754	4.8392
Dync1li1	_SVSS[Phospho (STY)]NVASVSPIPAGSK_.2	Q8R1Q8_S415_M1_Dync1li1	4.0744	3.2446	3.6110	3.3569	4.2814	4.3147	4.2676	4.0329	4.1878	3.8850	3.6664	2.9708	4.3532	3.3550	2.7921	3.4366	5.4448	3.2626	4.4120	3.3220
Dync1li1	_VPGGS[Phospho (STY)]PRT[Phospho (STY)]PNR_.2	Q8R1Q8_S405_M2_Dync1li1	4.2252	3.7776	3.2606	3.4642	3.5629	4.4221	3.9782	3.9255	4.9191	7.2956	3.5591	3.6671	4.2459	3.2477	3.5554	3.0692	4.4632	3.3699	3.0185	3.2147
Dync1li1	_GLEYLYLNVHDEDRDDQTR_.3	#N/A	3.6786	4.4610	3.2152	3.9056	3.8856	3.9189	3.1625	3.9043	3.0827	3.4892	4.0622	3.3666	3.2482	3.7508	3.9266	3.0408	4.2380	4.3676	4.0162	3.7178
Dync1li1	_NIDLVYK_.2	#N/A	4.0014	3.3177	3.5380	3.5828	4.2083	4.2417	4.3406	4.1059	4.2608	3.8120	3.7395	3.0438	4.4263	3.4281	2.8652	3.3635	3.1428	2.0818	4.3390	1.1081
Stard7	_ATAPS[Phospho (STY)]PERK_.2	Q8R1R3_S356_M1_Stard7	3.0889	6.6218	6.0087	6.6806	3.9274	4.0575	3.6136	6.7471	4.4450	3.2952	3.9236	6.5819	3.1096	3.6123	5.6617	3.4338	3.3269	5.9691	4.1548	6.7377
Chmp7	_VS[Phospho (STY)]PVNDVDVGVYQLM[Oxidation (M)]QSEQLLSR_.3	#N/A	3.4848	4.4192	3.8242	4.1313	3.6917	3.6155	4.3794	4.7595	3.0708	3.7084	3.7709	4.3589	4.8282	5.3481	4.1415	3.4088	4.0106	4.4767	4.5574	3.8093
Spart	_S[Phospho (STY)]PEQESVSTAPQR_.2	#N/A	8.5970	8.1726	8.4478	8.3515	7.8198	8.1795	7.8326	7.7832	7.8569	8.4242	7.9008	7.5960	7.3645	8.5728	7.2026	7.8299	8.0579	7.7521	7.6065	7.9515
Spart	_DAC[Carbamidomethyl (C)]KKS[Phospho (STY)]PEQESVSTAPQR_.3	#N/A	5.9114	6.1868	6.2547	5.7508	5.1877	5.8433	5.5232	5.7810	4.8969	3.1685	6.1071	5.6875	6.1079	6.3960	5.2148	4.2208	3.7863	5.5424	4.9380	5.6003
Spart	_KS[Phospho (STY)]PEQESVSTAPQR_.2	#N/A	4.3983	5.9966	5.5714	5.6882	4.8730	5.2981	5.3248	5.5508	3.8642	5.5328	4.2385	4.8194	5.6597	5.8142	4.6479	5.1155	3.6642	5.7823	4.8880	4.8954
Aagab	_VASAESC[Carbamidomethyl (C)]HSEQQEPS[Phospho (STY)]PTAER_.3	Q8R2R3_S211_M1_Aagab	6.4870	7.3869	3.7187	3.4021	6.3325	6.7103	6.9623	6.3847	4.1050	7.3624	6.2590	4.3639	4.2130	6.6673	6.1113	3.5443	6.5914	6.9683	6.9530	6.0785
Aagab	_AFWM[Oxidation (M)]AIGGDRDEIEGLSS[Phospho (STY)]DDEH_.3	Q8R2R3_S312_M1_Aagab	3.2798	4.0393	4.3172	4.3045	3.4867	3.1208	3.5614	3.5054	3.2026	3.0903	3.5770	3.7655	3.3106	4.3552	3.5868	4.1427	4.5035	4.1468	2.9450	4.0325
Actr8	_RIPVS[Phospho (STY)]PEQTR_.2	Q8R2S9_S132_M1_Actr8	3.9984	4.0044	4.5347	3.2430	7.5572	3.5602	4.2049	3.0660	4.2640	4.2048	3.1376	6.4879	4.3721	3.9158	3.7821	4.3433	4.0641	3.0853	3.3844	3.5931
Tmcc3	_PGS[Phospho (STY)]DTALTVDR_.2	Q8R310_S4_M1_Tmcc3	6.8226	3.7307	5.9774	3.5167	3.6098	6.3753	6.1987	6.3842	3.9904	4.4784	5.7329	5.9946	6.8633	3.6422	3.5085	3.1161	3.7904	6.2261	5.4366	5.8678
Ubqln1	_ALSNLESIPGGYNALR_.2	#N/A	6.6482	6.4715	6.6794	7.0147	6.8045	6.1081	6.4104	4.4900	6.2863	6.4665	6.3961	6.7038	6.5522	6.2597	6.8160	6.1542	6.4916	5.6577	6.6311	6.4446
Ubqln1	_EANLQALIATGGDINAAIER_.2	#N/A	3.9859	3.9218	4.5925	3.2033	4.2466	4.6522	3.5431	3.5008	3.6485	3.3651	4.4551	2.8263	4.1616	4.9397	2.9844	3.6370	3.7888	3.6955	4.3779	4.7757
Ubqln1	_FQQQLEQLSAM[Oxidation (M)]GFLNR_.2	#N/A	4.0480	3.2711	3.5846	3.5362	2.6202	4.2883	4.2940	4.2737	4.2142	3.8586	3.6928	4.5807	4.3796	3.6227	4.0477	3.4101	3.7680	3.2362	3.6776	3.3484
Ubqln1	_[Acetyl (Protein N-term)]AESAESGGPPGAQDSAADGGPAEPK_.2	#N/A	3.5975	3.7216	3.1341	3.9867	3.8044	4.3039	3.4612	3.8232	3.5203	3.4476	3.8947	3.4477	3.6284	4.1286	3.0384	3.5862	3.3204	3.8290	4.1281	4.3503
Pspc1	_LFVGNLPTDITEEDFK_.2	#N/A	4.2615	4.0408	3.2970	3.4279	3.5992	3.9941	4.3284	3.2784	4.1168	3.9560	3.5954	3.8630	4.2822	3.8426	3.5190	4.5741	2.9959	3.3336	3.9249	3.6482
Pspc1	_FAQPGTFEFEYASR_.2	#N/A	3.8974	5.8569	3.4339	6.1072	5.6394	5.4699	5.9861	4.1230	4.3649	5.9292	3.8435	5.5986	3.9282	3.4720	6.1618	5.6842	5.0455	6.0651	6.1697	5.7342
Ccdc12	_GQEDSLAS[Phospho (STY)]AVDATTGQEAC[Carbamidomethyl (C)]DSD_.2	Q8R344_S152_M1_Ccdc12	3.8341	4.0077	3.8471	3.8847	4.3220	4.0526	3.8763	3.6416	3.7965	3.6230	4.0108	3.8523	3.9619	4.5651	3.9016	3.6631	4.1793	4.2527	3.4143	3.6664
Rab11fip5	_TYS[Phospho (STY)]DEASQLR_.2	#N/A	10.0382	9.9828	10.1519	10.5915	9.5713	9.0795	9.6228	9.3310	9.4633	10.2922	9.5503	9.5300	10.0289	10.0093	8.4657	9.7528	9.6698	9.3329	9.4201	9.7308
Rab11fip5	_S[Phospho (STY)]PSHSSWLSTEGGR_.2	#N/A	8.6546	8.6898	8.4811	8.6488	7.2651	7.5787	8.1139	7.9548	4.0849	8.9239	7.8160	7.8669	4.2162	8.3067	3.3018	7.9593	7.9780	7.8398	7.6565	8.0404
Rab11fip5	_RTYS[Phospho (STY)]DEASQLR_.2	#N/A	5.3053	5.6619	3.9282	5.6034	4.2304	4.4545	3.3106	3.0923	3.3697	3.5982	4.8345	5.4432	4.5667	5.8201	4.4787	3.7368	3.6299	5.2248	5.6467	3.8822
Rab11fip5	_GSPSLGAS[Phospho (STY)]PHHSSTGEEK_.3	#N/A	6.6394	7.3694	7.4217	7.3434	6.5929	5.8601	6.9290	3.9229	4.6120	7.6885	4.5427	6.8306	3.9633	4.4478	3.6676	3.5622	5.9351	6.7272	3.8813	4.7739
Rab11fip5	_DKPRS[Phospho (STY)]PFSK_.2	Q8R361_S188_M1_Rab11fip5	3.7462	3.5729	3.2827	3.8381	3.9531	4.1553	3.0950	3.9718	3.6690	3.5568	4.0434	5.8640	3.7770	3.3208	3.1204	3.1083	3.4691	3.6804	3.9794	2.9981
Rab11fip5	_ATVALDEVFR_.2	#N/A	3.3646	4.1047	4.4020	4.4054	3.5716	3.6049	4.3073	3.4630	3.3968	3.1752	4.4427	3.3613	3.5622	4.0648	4.1443	4.2276	3.0875	4.0536	3.7022	4.0318
Rab11fip5	_RGS[Phospho (STY)]VGEKGS[Phospho (STY)]PSLGASPHHSSTGEEK_.4	Q8R361_S474_M2_Rab11fip5	3.9568	4.0460	4.4931	3.7326	3.2945	3.1896	3.8919	3.6572	3.8121	4.2607	3.2907	3.9355	3.9775	4.4802	3.9172	3.8123	4.1948	3.6383	3.2869	4.4472
Igsf8	_VLPDELQVSAAPPGPR_.2	#N/A	8.7414	8.2826	8.5955	8.4692	8.2009	8.5412	8.4784	8.8644	8.4268	8.0944	8.3990	8.1126	8.3275	8.0833	7.9804	7.8621	8.7360	8.3567	8.2765	8.1948
Igsf8	_AIPEAPVGR_.2	#N/A	6.8807	7.3985	6.7992	7.7064	7.4755	6.9709	7.5788	7.3869	7.2740	7.0045	7.0359	7.3483	7.5849	6.5484	7.2812	6.5910	7.0675	7.2672	7.0475	6.8328
Igsf8	_LVAQLDTEGIGSLGPGYEDR_.2	#N/A	6.0684	5.4123	5.7228	5.1695	5.5756	5.7469	6.0368	5.7332	6.0290	5.7820	5.4704	5.7615	5.5371	5.6262	4.9125	5.5978	5.8284	5.7899	5.2662	6.0220
Igsf8	_DSQFSYAVFGPR_.2	#N/A	6.2126	6.2552	6.5343	6.5376	5.9714	6.3171	5.9813	5.9466	6.5121	6.0776	5.5304	6.4055	6.2635	6.1493	5.4624	5.9183	6.4258	5.8553	5.3868	5.9773
Igsf8	_VASGDLQVQR_.2	#N/A	6.0247	6.6758	6.9841	6.3780	5.9222	6.6261	6.5398	5.9895	6.3350	3.9914	5.8713	6.0309	6.1269	6.0616	5.7536	6.0923	6.4455	6.2281	6.2704	6.0437
Igsf8	_ATLQEVVGLR_.2	#N/A	6.0515	5.5369	5.7889	5.5852	5.2913	5.5737	6.1535	4.7372	5.8898	4.8236	4.8801	5.6360	3.6822	4.5808	5.0664	4.3347	3.3743	5.2999	5.9989	5.6180
Igsf8	_HAAYSVGWEM[Oxidation (M)]APAGAPGPGR_.3	#N/A	4.2897	4.5302	3.8263	4.8492	2.9958	5.2626	5.9368	5.6742	5.4819	4.1003	5.2241	4.2563	4.1379	3.1397	4.8829	3.6518	6.1295	6.0533	5.5656	5.1952
Igsf8	_LEAARPADAGTYR_.3	#N/A	4.2481	3.8440	3.1943	3.5306	3.4708	4.2166	5.6651	3.8592	4.4670	4.0587	4.1106	3.7334	4.1795	3.1813	4.8664	3.7129	4.3969	3.4363	3.0849	4.2487
Igsf8	_LTVHEGQELALGC[Carbamidomethyl (C)]LAQTK_.3	#N/A	4.4789	5.5322	3.5144	3.2105	3.8166	4.1683	4.4140	4.1793	4.3342	5.1273	3.8128	3.4133	4.4996	3.5014	3.3016	4.6659	3.2161	4.6333	4.2656	3.4684
Igsf8	_AVLAHVDVQTLSSQLAVTVGPGER_.3	#N/A	4.2376	3.2170	3.6386	3.4822	3.5753	4.3915	3.9658	4.3277	4.0249	4.4439	2.8984	2.9432	4.1160	3.6767	3.5430	3.0816	3.8250	3.3245	3.6235	3.3540
Igsf8	_EEGVVLEAVAWLAGGTVYR_.2	#N/A	3.1928	3.3092	6.1501	4.4966	4.0313	3.9536	3.5097	4.3940	3.0481	3.5239	4.0275	3.1986	3.2135	3.7162	3.0869	3.5377	3.4308	4.4023	4.0509	3.6832
Clmp	_LVKPSSSSSGS[Phospho (STY)]R_.2	Q8R373_S293_M1_Clmp	3.1543	4.1647	4.1918	4.4299	3.3613	3.3946	3.6868	3.3800	3.6070	4.4658	3.0857	3.8909	3.7725	4.2298	3.7122	4.0173	4.3781	4.2722	3.0704	4.2421
Mcmbp	_VS[Phospho (STY)]PSTSYTPSR_.2	Q8R3C0_S154_M1_Mcmbp	8.1430	8.1511	8.3141	8.6567	7.8386	7.8589	7.8756	8.3027	7.9513	8.2114	7.8757	7.6328	8.1216	8.2034	7.6214	7.9720	8.1513	8.0772	7.5590	3.8007
Tbc1d13	_SGVTNMSS[Phospho (STY)]PHK_.2	Q8R3D1_S184_M1_Tbc1d13	4.2301	5.6967	3.7667	5.7303	2.9362	4.3682	4.1119	4.4558	4.0321	4.0407	4.8920	4.3159	4.1975	3.8048	4.1373	3.5922	3.9530	3.4183	3.0669	3.1663
Mcat	_DSS[Phospho (STY)]VAEEGAQEAVAR_.2	Q8R3F5_S41_M1_Mcat	5.9941	5.7109	3.4520	3.2729	4.1973	5.7799	5.6309	4.1169	4.2718	5.4236	5.9681	5.9247	4.4373	3.4391	2.8761	3.3525	3.1538	3.1786	4.3280	5.7982
Mboat2	_RTS[Phospho (STY)]TQENVHLSQAK_.3	Q8R3I2_S472_M1_Mboat2	4.3551	3.6477	3.3906	3.3343	3.6928	4.2921	3.8483	4.0555	4.2104	3.8624	3.6890	3.5372	4.3758	3.3776	4.7937	3.1992	3.0923	3.2400	4.3894	3.3446
Cluap1	_KPEPLDES[Phospho (STY)]DNDF_.2	#N/A	3.1029	7.4445	4.1403	4.4813	3.3099	7.5037	6.8442	3.3286	6.7098	4.4144	3.1371	3.9423	3.8239	6.6645	5.9069	5.0119	4.3267	6.9818	7.2724	6.6109
Cluap1	_IRKPEPLDES[Phospho (STY)]DNDF_.2	#N/A	3.5490	7.0913	6.0712	6.3451	4.0866	6.9467	6.8952	3.5518	6.4314	3.4043	4.2013	5.7867	3.3873	7.1465	5.4211	5.4208	3.5364	6.7028	6.3618	6.1854
Ppp6r2	_NVPGLAAPSS[Phospho (STY)]PTQK_.2	Q8R3Q2_S670_M1_Ppp6r2	12.6844	4.0907	12.2451	12.1967	11.6225	3.4838	11.8090	12.3189	4.3504	4.1283	12.1669	12.0225	12.6223	12.1271	10.5679	11.8701	12.6046	11.8359	12.0267	11.6334
Ppp6r2	_VGTEGLVDS[Phospho (STY)]FSQGLER_.2	Q8R3Q2_S289_M1_Ppp6r2	4.4106	3.5922	3.4460	3.6552	3.7483	3.9724	3.7928	4.1547	3.8519	3.1161	4.2262	3.4817	3.9599	3.5037	3.3700	3.2546	3.6520	3.4975	3.7965	3.1810
Exoc1	_LTGSTSS[Phospho (STY)]LNK_.2	Q8R3S6_S473_M1_Exoc1	7.5702	7.3184	6.9533	7.5053	6.5243	6.0228	6.5182	6.4914	6.5998	3.7479	6.5092	6.6330	6.7162	6.5813	4.4683	3.7666	6.5315	6.9838	5.0791	6.1532
Sh3glb2	_GKVPVTYLELLS[Phospho (STY)]_.2	#N/A	7.1652	8.2573	7.0517	6.8331	6.0221	6.8557	7.8298	7.7062	7.1292	7.7201	6.4288	7.7002	8.0354	8.8552	7.7846	7.1853	7.4903	7.8577	7.7322	7.0500
Sh3glb2	_QTEVLLQPNPSAR_.2	#N/A	7.0058	6.8231	7.2628	6.9411	7.2986	7.2288	7.2502	7.3802	7.2002	6.9795	6.9374	7.4138	7.5747	7.3157	7.6968	7.1469	7.2075	7.1186	7.4628	7.1513
Sh3glb2	_NFLEGDWK_.2	#N/A	5.8987	6.2645	5.9844	5.8752	6.1702	6.1277	6.0700	5.1659	6.1706	6.3360	6.4508	6.4058	6.5410	6.4187	6.1597	6.7290	6.1553	6.2074	6.2042	6.6467
Sh3glb2	_VAQTEFDR_.2	#N/A	5.6234	5.6895	5.9482	6.1668	6.1243	6.0688	6.0219	4.8886	6.0781	6.0626	6.1659	5.9429	5.9023	6.1000	6.6654	5.9653	4.0620	5.8271	6.2077	6.2872
Sh3glb2	_VEEFLYEK_.2	#N/A	3.8591	3.7631	3.7950	4.5805	4.2493	4.1567	4.6533	3.2366	4.9146	5.3504	5.5769	4.1522	4.7604	4.9619	5.2080	4.6474	4.2295	4.9098	5.1152	4.7440
Sh3glb2	_SQTTYYAQC[Carbamidomethyl (C)]YR_.2	#N/A	3.9317	5.4956	3.4682	3.6526	6.2888	5.6076	6.1370	6.1123	6.1901	6.4402	6.0478	5.8981	4.4960	3.5063	6.0397	6.1608	6.0320	5.1293	5.8432	5.7861
Sh3glb2	_LASDAGIFFTR_.2	#N/A	4.3077	4.5815	3.9395	3.3714	4.6411	4.4453	4.7913	4.0183	4.5183	3.8996	3.8961	4.2464	2.7073	4.1188	4.0834	3.4511	4.9856	4.4335	3.5456	3.9020
Sh3glb2	_DFIHTASLSFLTPLR_.2	#N/A	3.1682	3.1089	4.2396	3.9136	3.8837	3.8599	3.4858	3.8963	1.6230	3.9809	3.7600	3.6719	3.5410	3.7061	3.4067	3.9158	2.9085	4.2202	4.0857	3.8730
Sh3glb2	_TELDAHFENLLAR_.2	#N/A	4.3471	3.7839	3.9883	3.5801	4.2110	3.4464	3.7155	3.8992	3.2419	3.9350	4.7881	3.7478	4.3231	4.2882	3.9300	4.1742	4.2427	3.7014	4.0629	3.4546
Sh3glb2	_RLDLDAC[Carbamidomethyl (C)]K_.2	#N/A	3.6551	3.2510	3.7873	4.4384	6.6953	6.3263	6.4898	6.3597	6.5840	6.4689	6.6422	6.5718	6.2945	6.7384	7.1926	6.2655	6.4978	6.5137	6.8602	6.4568
Sh3glb2	_LLLEGISSTHVNHLR_.3	#N/A	6.4672	4.3252	5.9950	4.0118	6.6556	4.7613	5.7128	3.3780	5.6564	5.4243	3.4584	5.2463	3.1920	4.2366	6.3678	6.2055	3.9157	4.5617	3.0636	5.4230
Sh3glb2	_AVQFTEEK_.2	#N/A	4.3003	3.7025	3.3358	3.3891	3.6380	3.4500	3.5606	4.0007	3.7040	3.7583	4.3365	3.5919	4.3210	3.3229	3.9165	3.1444	4.5384	4.5379	3.4618	3.3771
Sh3glb2	_VPVTYLELLS[Phospho (STY)]_.2	#N/A	3.7554	4.2474	4.2917	4.5008	3.0931	3.3172	4.4480	3.3091	3.0062	3.9617	3.3806	4.1369	3.1143	4.7753	4.0252	4.1003	4.3072	4.3596	3.1413	3.8362
Irf2bp1	_NVAEALGHS[Phospho (STY)]PKDPGGGGGSVR_.3	#N/A	7.6140	7.9212	3.1428	3.9780	7.1371	7.5656	7.1457	7.4499	3.1551	7.4783	7.5955	7.1786	8.1664	8.2513	7.3282	7.6269	3.5379	7.4217	7.1528	7.3915
Irf2bp1	_KAS[Phospho (STY)]PEPEGETAGK_.2	#N/A	7.0722	6.9505	6.8619	7.3600	6.7180	7.0585	7.0240	7.2544	7.2599	7.1607	6.7347	6.9339	7.6174	7.4115	7.0590	7.3075	7.2132	6.9691	6.9315	6.8904
Irf2bp1	_NVAEALGHS[Phospho (STY)]PK_.2	#N/A	5.1749	5.3600	4.5542	4.5964	4.1763	4.8924	5.0634	3.3177	4.5553	5.1530	5.2600	4.4790	3.4403	5.5970	4.0442	5.0847	5.2193	4.3896	4.3405	5.4947
Irf2bp1	_AGGAS[Phospho (STY)]PAASSTTQPPAQHR_.2	#N/A	5.7966	6.1118	3.3673	5.9076	4.0377	5.9399	3.8973	6.0825	5.4989	6.0591	5.5676	5.6946	6.2320	3.6140	3.0469	5.8285	5.6376	5.5991	5.3564	5.6856
Irf2bp1	_RKAS[Phospho (STY)]PEPEGETAGK_.2	#N/A	3.9411	4.5300	3.9201	3.8309	3.7947	4.1653	3.6008	4.1032	3.2498	4.6265	3.9324	3.7330	4.8828	3.9315	3.5738	3.7368	3.8708	3.7152	3.6141	3.5881
Irf2bp1	_S[Phospho (STY)]PGPPALKHPTSK_.3	#N/A	3.2959	5.8054	4.3333	4.3050	3.5029	4.6304	5.1779	3.5745	3.5032	5.9999	3.4581	6.2555	3.6429	5.3498	3.5873	4.1589	3.6498	4.8827	4.5026	5.3155
Irf2bp1	_S[Phospho (STY)]PGPPALK_.2	#N/A	3.3526	3.1494	3.8889	4.3368	4.1911	4.4152	3.3500	3.0529	3.4090	3.5589	3.7834	3.0389	3.5171	3.8759	2.9271	3.6975	3.5906	4.2425	3.8911	4.2390
Cacnb1	_QDT[Phospho (STY)]FDADTPGSR_.2	Q8R3Z5_T499_M1_Cacnb1	7.4969	7.4993	8.0238	8.0568	7.5841	7.5795	7.1970	7.4343	7.3050	7.8560	7.5250	6.8742	4.0052	3.5489	6.9621	7.1736	7.0253	6.7208	6.8708	7.2904
Cacnb1	_SDGS[Phospho (STY)]TSSDTTSNSFVR_.2	Q8R3Z5_S44_M1_Cacnb1	6.8346	6.8343	7.5012	7.1951	7.1718	7.0468	6.9331	7.3838	6.3645	7.4815	6.1579	7.0080	7.1995	6.4314	6.4651	6.9805	7.2214	6.5636	6.4825	6.9294
Cacnb1	_LSSSKS[Phospho (STY)]GDNSSSSLGDVVTGTR_.3	#N/A	3.4312	3.5137	5.8908	4.2769	3.9242	4.1874	3.3881	4.1276	3.6370	6.0173	4.0114	6.2852	3.7451	3.4614	3.1719	5.7706	3.3822	6.4474	4.0844	4.0848
Cacnb1	_S[Phospho (STY)]GDNSSSSLGDVVTGTR_.2	#N/A	8.7418	8.8897	9.5768	9.1738	4.0225	8.3560	6.8070	3.6949	8.4930	9.1494	8.0604	8.1939	7.5870	8.7465	6.7967	8.3259	8.4189	8.2315	6.3269	8.4755
Cacnb1	_QGS[Phospho (STY)]WEDEEDYEEEM[Oxidation (M)]TDNR_.2	#N/A	3.5421	6.7674	6.5412	6.2052	3.2883	6.4145	6.3458	3.3430	6.2416	6.2569	3.1849	6.2334	5.3238	7.0802	5.3577	6.4210	6.0504	6.2976	7.0614	6.0875
Cacnb1	_ATHPPSS[Phospho (STY)]TPPNPLLNR_.3	Q8R3Z5_S417_M1_Cacnb1	3.4805	4.6285	4.6824	4.2092	4.3191	3.0424	7.0158	7.9895	3.2814	3.6869	3.6558	5.6694	3.3894	4.0039	3.7262	3.8254	3.7186	4.1149	5.1779	3.9709
Cacnb1	_ALS[Phospho (STY)]RQDT[Phospho (STY)]FDADTPGSR_.2	#N/A	6.2820	6.1419	3.2743	3.6568	3.7789	5.6944	5.4616	3.9518	5.3616	5.7029	3.8305	5.2253	4.0261	5.3619	4.4904	5.5060	5.7136	5.3607	5.4239	5.2485
Cacnb1	_GPYPPSQEIPM[Oxidation (M)]EVFDPS[Phospho (STY)]PQGK_.3	#N/A	5.1230	6.1297	3.7492	5.6482	3.7861	5.3616	5.6759	4.0949	5.2457	5.9271	3.7547	5.3899	3.4013	6.7576	4.3496	5.4332	3.6186	5.7799	5.5960	3.8709
Micos13	_DSWNSGIISVM[Oxidation (M)]SALSVAPSK_.2	#N/A	5.2302	5.0854	5.7089	5.7452	5.3610	4.8808	4.9363	5.7591	4.9852	6.1329	3.3549	5.3062	6.2993	5.1089	4.2820	5.1572	5.1628	5.0530	3.2227	5.7523
Micos13	_GSVAGGAVYLVYDQELLGPSDK_.2	#N/A	4.7721	4.6067	4.9197	4.2159	3.9960	5.1493	4.2539	4.7670	4.3454	4.8853	4.6902	4.3634	4.4535	4.5254	4.0779	4.9613	4.1455	4.1238	4.5201	3.6000
Cadm4	_VWSVASTVR_.2	#N/A	5.7380	5.4470	3.1225	5.8919	3.4248	5.4224	5.2880	2.9052	5.1062	5.1282	5.1810	4.9034	4.2834	4.8740	1.9335	5.0702	4.3251	4.8432	5.1461	5.5414
Cadm4	_GSYLTHEAS[Phospho (STY)]GLDEQGEAR_.3	#N/A	4.8516	5.5914	4.4130	6.2067	5.0250	5.2389	5.5460	4.0203	5.7442	5.3511	4.7544	4.1110	4.2219	4.6730	3.7979	4.1361	4.4282	5.5396	5.3023	4.5340
Cadm4	_FQLEEFSPR_.2	#N/A	5.5295	6.1372	3.3195	5.8537	3.6218	5.9925	6.0782	5.6035	5.5353	4.4904	4.3527	5.9629	5.8239	5.3174	5.0601	3.1281	6.4351	5.8311	2.9596	5.0518
Cadm4	_LHQYDGSIVVIQNPAR_.3	#N/A	3.3989	4.6039	3.9352	4.2905	4.2374	4.4615	4.4675	3.6656	3.3627	3.6052	4.0794	2.9925	3.4708	4.5154	2.8808	3.7438	3.1628	4.1923	4.2857	4.1927
Purg	_RAS[Phospho (STY)]GEEQEC[Carbamidomethyl (C)]LD_.2	Q8R4E6_S342_M1_Purg	10.8452	10.8784	10.9210	11.3212	10.3958	10.5854	10.7457	10.3292	10.9134	10.8700	10.6191	10.6233	11.0757	11.1806	10.4616	10.7438	10.9060	10.8681	10.8784	10.6827
Purg	_DALVQLIEDYGEGDIEER_.2	#N/A	6.2101	6.0027	5.9433	5.5292	5.5877	5.0215	5.4618	5.9165	5.4669	5.6087	5.1906	5.3483	6.7354	6.3507	5.3807	5.5575	5.7751	5.3691	5.2247	4.0191
Purg	_HSAPS[Phospho (STY)]PPVSVGS[Phospho (STY)]EEHPHSVLK_.3	#N/A	5.8158	4.2762	6.5869	6.8268	4.3812	6.7869	3.6976	3.3908	6.6331	6.9727	6.6261	6.5598	6.4805	6.6218	4.1501	7.1112	6.5382	6.5385	8.2608	3.9246
Purg	_QKHSAPS[Phospho (STY)]PPVS[Phospho (STY)]VGS[Phospho (STY)]EEHPHSVLK_.4	#N/A	3.4490	6.4510	3.9853	4.3464	3.6614	3.5246	6.4312	4.0930	3.1797	3.6025	3.8861	5.6231	3.3166	6.0098	5.6800	5.5978	4.0965	6.7392	6.5234	5.9069
Arhgef12	_SEGVQDAEPQSLVGS[Phospho (STY)]PSTR_.2	Q8R4H2_S341_M1_Arhgef12	3.9731	4.0297	8.9393	9.0055	3.3108	3.5349	8.5560	8.9144	4.2893	9.1701	8.7114	8.5573	8.8588	8.8585	3.8074	8.6679	8.9199	8.5820	8.6298	2.9626
Arhgef12	_TGTGDIATC[Carbamidomethyl (C)]DS[Phospho (STY)]PR_.2	Q8R4H2_S1327_M1_Arhgef12	7.4192	7.4661	7.0783	7.0492	6.9800	6.9984	7.4329	7.5454	6.8485	7.8148	7.3400	6.9098	7.4213	7.5177	5.7482	6.6028	7.5549	7.2418	6.6024	7.1006
Arhgef12	_ITS[Phospho (STY)]PVLVGEENNVVHNQK_.3	#N/A	6.2446	6.0075	4.4208	4.0101	5.2495	5.5439	5.5008	3.5981	3.5204	4.0755	3.4878	5.6299	3.6592	6.5914	3.3798	5.6261	3.6590	5.8651	3.9020	5.7228
Arhgef12	_HLSTPSSVS[Phospho (STY)]PEPQDPAK_.2	Q8R4H2_S637_M1_Arhgef12	3.5187	4.4841	4.0550	4.1706	2.8565	3.0806	3.1838	3.5457	3.2429	3.7251	3.6172	4.3735	3.3509	4.3955	4.2618	3.8636	4.5438	4.1065	4.4055	4.0728
Arhgef12	_TDWSSGDASRPSSDSADS[Phospho (STY)]PK_.3	Q8R4H2_S309_M1_Arhgef12	4.0412	3.9616	4.5775	3.6482	3.3789	5.5336	4.1622	3.7416	4.2213	4.2475	3.0948	3.8511	4.3293	3.8731	3.7394	4.3861	4.2792	3.1281	3.4271	3.5504
Arhgef12	_VEHHDLSVAGLQS[Phospho (STY)]PDR_.3	Q8R4H2_S1176_M1_Arhgef12	6.5569	6.2851	6.0439	6.2695	3.3386	5.2876	5.5251	3.3574	3.6297	6.3977	5.3229	4.8531	3.7951	4.2978	4.1566	3.9946	4.3554	5.6311	5.4173	5.4929
Arhgef12	_SSS[Phospho (STY)]HDFDPTDSSSK_.2	#N/A	3.1058	4.2133	4.1432	8.3070	7.3429	3.3461	3.7354	7.9936	7.2409	4.4172	3.1342	7.2569	3.8210	4.1813	3.7607	7.4493	4.3296	7.4593	3.1189	7.1639
Arhgef12	_SSS[Phospho (STY)]HDFDPTDSSSKK_.2	#N/A	3.8618	4.1505	4.3981	3.8371	5.2621	3.4727	5.3783	3.2758	4.3797	4.3367	4.5298	4.0399	4.1132	5.2409	3.7543	4.2067	4.0999	3.5121	4.3598	3.9561
Gtf2a1l	_LLPGNELR_.2	#N/A	6.3965	7.5508	6.8829	7.2731	7.2417	6.9557	7.5912	7.0052	7.0914	6.9766	6.8682	7.5117	7.4149	6.1396	7.8934	7.0771	6.9159	7.3002	7.6608	7.1991
Neto1	_GTGATADFADVAEDFENY[Phospho (STY)]HK_.3	Q8R4I7_Y417_M1_Neto1	3.4396	3.8795	4.4770	4.1447	3.6465	3.6798	2.6896	3.6652	3.3218	3.2502	4.3013	3.6056	3.4872	3.1412	3.4270	4.3025	3.1625	3.5868	3.7772	3.9569
Clybl	_VDC[Carbamidomethyl (C)]AVLDC[Carbamidomethyl (C)]EDGVAENK_.2	#N/A	4.4217	3.4012	3.4545	3.6663	3.7594	4.0197	3.7816	4.1436	3.8407	3.1272	4.2151	3.1273	3.9488	3.4926	3.3588	3.2658	4.4447	3.5086	3.8077	3.1698
Nup35	_TLGT[Phospho (STY)]PTQSGSTPR_.2	Q8R4R6_T272_M1_Nup35	4.4687	6.5990	6.4357	3.2206	3.8065	4.1785	6.0713	4.1691	4.3240	3.7488	6.4382	6.5920	7.0089	7.1156	6.9181	6.7817	3.2060	6.0617	6.3976	6.6664
Ppp1r14c	_KLS[Phospho (STY)]PPQKK_.2	Q8R4S0_S157_M1_Ppp1r14c	6.6917	6.8626	7.6146	7.0119	6.7911	7.7828	7.6187	7.9326	6.9140	7.5756	7.0337	6.5236	7.1100	7.9571	7.2363	7.1806	6.4809	7.4974	6.4174	7.1519
Cys1	_LAPAVS[Phospho (STY)]PEKK_.2	Q8R4T1_S95_M1_Cys1	6.9945	8.1641	7.4770	7.1742	6.8852	7.5093	7.7849	6.7362	7.4191	8.4160	7.0850	7.5046	8.0837	8.0425	7.0313	7.5478	7.6517	7.4174	7.7264	7.9723
Cys1	_GNPEDSC[Carbamidomethyl (C)]ASEAPGNS[Phospho (STY)]PK_.2	Q8R4T1_S116_M1_Cys1	6.5635	6.6965	7.2444	6.8444	6.7834	6.5171	6.3543	6.1910	6.1506	7.2912	6.3825	5.4950	4.5340	7.0189	5.6818	5.9014	4.0745	6.6105	5.8892	5.7490
Luzp1	_ELS[Phospho (STY)]PQHKR_.2	#N/A	8.7992	8.8436	8.4812	8.3518	8.7655	8.4049	8.3592	8.9937	8.3099	8.7830	8.2165	8.9151	9.1461	9.0959	7.6822	8.7469	8.6694	8.6227	8.5330	8.3750
Luzp1	_KGS[Phospho (STY)]LDYLK_.2	#N/A	6.5534	6.7986	3.7223	6.3534	5.7887	5.8443	5.9558	4.4114	5.5200	6.6544	5.9543	5.5445	4.2419	6.3399	4.1816	3.5479	5.8794	5.6455	5.7387	3.4377
Luzp1	_RKGS[Phospho (STY)]LDYLK_.2	#N/A	6.4010	6.9030	4.0186	3.1022	3.1953	5.5699	4.3458	6.5112	4.4049	6.3386	3.2784	6.7037	4.5129	6.6073	5.2060	5.6190	4.2049	5.8458	6.1555	6.5549
Luzp1	_ELS[Phospho (STY)]PQHK_.2	#N/A	5.6347	5.9824	5.9568	3.9467	5.7041	5.8487	5.6746	6.1528	5.4714	6.3524	6.1655	5.5875	5.4174	2.5607	3.2719	4.4390	4.9525	5.6789	4.9760	3.5397
Luzp1	_NQRPFS[Phospho (STY)]PR_.2	Q8R4U7_S746_M1_Luzp1	6.9103	6.3095	7.0630	4.3645	7.0337	5.6255	5.6982	4.5261	5.9673	6.9608	5.9686	5.6466	6.2737	7.5898	2.9549	6.6649	6.3989	6.5650	5.6087	6.3520
Luzp1	_S[Phospho (STY)]QENILQGFSLPNK_.2	Q8R4U7_S612_M1_Luzp1	3.9396	7.3154	6.5215	3.1842	3.2773	3.5014	6.9744	3.1249	4.3228	4.1459	3.1964	3.9527	4.4309	7.5272	3.8410	4.2845	4.1229	3.0265	5.7738	3.6520
Luzp1	_EFALS[Phospho (STY)]NEHYSLSSK_.2	#N/A	5.5049	5.6344	6.3074	5.8017	4.2179	4.8379	4.0197	3.6958	4.5978	5.5746	4.6469	4.1000	3.5122	5.3819	2.6743	4.5636	4.4386	4.7891	5.2026	4.9731
Luzp1	_EKPDS[Phospho (STY)]DDDLDIESFVTAK_.2	#N/A	3.6443	4.0541	3.7696	3.9931	3.4777	3.5821	3.1299	4.0215	3.1173	3.6679	3.8281	3.8824	3.2545	4.0009	3.7469	3.5838	3.7156	4.2832	3.7914	3.8523
Luzp1	_TFS[Phospho (STY)]DSTHVSVPNDIVGK_.2	Q8R4U7_S513_M1_Luzp1	3.6806	4.1609	4.1956	4.4260	3.0183	3.2424	3.0220	3.3839	3.0810	3.8869	3.4554	4.2117	3.1891	4.2337	4.1000	4.0255	4.3820	4.2683	3.0665	3.9110
Lrrc8c	_SQS[Phospho (STY)]LKS[Phospho (STY)]IPEK_.2	#N/A	10.1887	10.2277	10.4246	10.2804	9.8122	9.9536	10.1191	10.2617	9.9024	9.9351	9.9369	9.6742	10.2433	9.7730	8.6428	9.5815	10.3168	9.8764	9.8736	9.8585
Lrrc8c	_SGT[Phospho (STY)]IQSGPEGNLVR_.2	Q8R502_T198_M1_Lrrc8c	4.5403	3.9235	3.8510	4.0018	3.5928	3.7440	3.7334	3.8282	3.7222	5.7875	3.7224	5.2555	4.2651	3.8685	3.5600	3.6696	3.8587	3.8622	3.7890	5.8691
Lrrc8c	_S[Phospho (STY)]QSLKS[Phospho (STY)]IPEKFVVDK_.2	Q8R502_S210_M2_Lrrc8c	3.6374	3.3460	3.6024	4.1605	4.0953	4.1574	3.8155	4.2791	4.3538	3.7278	3.8324	3.1590	3.6648	3.2714	2.9581	3.2793	3.5600	3.4931	3.9358	3.4453
Mib2	_RVS[Phospho (STY)]ADGQPFQR_.2	#N/A	8.8228	8.9000	8.5069	8.7251	8.4004	8.2687	8.3170	8.6597	8.2578	8.7098	8.2955	8.2976	8.9863	9.0430	7.7085	8.5338	8.6989	8.4599	8.1947	8.4431
Mib2	_HGS[Phospho (STY)]PSTPDR_.2	Q8R516_S44_M1_Mib2	6.3537	6.5528	3.8319	6.2571	3.0014	6.2795	5.7194	4.5210	3.9669	5.8697	3.4455	6.0684	4.1323	6.4030	4.0720	3.6575	4.0183	6.6487	5.8385	6.5155
Mib2	_VS[Phospho (STY)]ADGQPFQR_.2	#N/A	3.2837	3.2182	3.8200	4.4056	4.1223	4.3464	3.4188	4.4850	3.4779	3.4901	3.8522	3.1077	3.3045	3.8071	2.9960	3.6286	3.5218	4.3114	3.9599	3.7741
Sh3kbp1	_S[Phospho (STY)]IEVENDFLPVEK_.2	Q8R550_S274_M1_Sh3kbp1	8.7933	9.2997	9.3677	9.2594	8.1627	8.4175	8.4887	7.0494	8.5240	9.8353	8.6621	8.5927	8.8367	9.6647	8.0798	9.0112	8.3932	9.0791	8.7891	9.1816
Sh3kbp1	_ELS[Phospho (STY)]GESDELGISQDEQLSK_.2	Q8R550_S156_M1_Sh3kbp1	6.0498	7.0005	6.6136	5.8785	3.9578	6.2086	5.9802	3.2300	6.1156	6.8385	5.6176	4.1536	3.7598	7.5813	6.5982	6.2297	4.0856	6.9214	7.4910	4.0918
Sh3kbp1	_ASS[Phospho (STY)]PSLFSTEGKPK_.2	Q8R550_S631_M1_Sh3kbp1	10.3143	10.2007	10.2003	10.2243	9.9257	9.6651	9.3078	10.0275	4.3649	10.3496	9.8228	10.1696	10.5495	10.3377	9.5305	9.8746	10.2854	3.5292	9.4766	9.8046
Otud7a	_AAGGAAS[Phospho (STY)]PGPGGGAR_.2	Q8R554_S749_M1_Otud7a	8.2801	8.2619	8.2617	8.4392	7.5881	7.5344	7.7790	7.9893	7.7168	8.1285	7.4803	7.8190	8.3962	8.3983	6.5317	7.6751	7.6010	7.8010	7.7954	7.9073
Otud7a	_AS[Phospho (STY)]PGPTAAQPTQLVLK_.2	Q8R554_S711_M1_Otud7a	3.5179	4.6146	3.9245	4.3011	3.7248	3.7581	3.3233	3.0886	3.2435	3.3284	4.2230	3.0032	3.4090	3.9116	3.3487	4.3808	3.6263	4.2069	3.8554	3.8786
Snap47	_ELLLSQPGTAANIPSHVTR_.3	#N/A	5.0792	5.4185	5.6411	5.1817	5.3869	4.7376	5.4843	5.4629	5.0693	5.5296	5.4268	5.0165	5.4856	5.2149	4.8982	5.0542	5.2532	4.7776	4.8970	5.1872
Snap47	_IELLEDALVLR_.2	#N/A	4.5993	4.3441	3.9446	4.3283	4.2481	4.7230	4.4601	4.0133	4.1467	4.3922	4.2981	4.3198	4.6570	4.3798	3.8069	2.5287	4.2215	4.2911	3.5174	4.5183
Snap47	_ELPTGGQEGEQLQLQK_.2	#N/A	3.3953	6.5669	6.9319	6.9715	4.2339	5.8116	6.3051	5.9483	6.9175	5.8357	5.7805	5.9897	6.2240	6.6637	5.5106	6.0354	3.6334	6.7385	6.5574	6.4625
Prpsap2	_LGIAVIHGEAQDAESDLVDGRHS[Phospho (STY)]PPM[Oxidation (M)]VR_.4	#N/A	3.5940	3.7251	3.1306	3.9902	3.8009	4.3074	8.6908	9.4498	3.5168	3.4511	3.8912	8.3079	3.6249	8.9483	3.0349	6.4298	7.4413	3.8325	7.6603	6.6807
Prpsap2	_HS[Phospho (STY)]PPMVR_.2	#N/A	4.1188	3.7146	3.3236	3.4012	4.3257	4.3591	4.2232	3.9885	4.1434	3.9294	3.6221	3.6041	4.3089	6.8718	4.2486	3.4809	4.5262	3.3069	2.9555	3.2777
Apbb1ip	_S[Phospho (STY)]SDTC[Carbamidomethyl (C)]GSPALPSK_.2	Q8R5A3_S531_M1_Apbb1ip	3.4935	3.9738	4.3827	4.2389	4.3320	8.0040	7.6393	3.5710	3.2681	3.6998	7.9402	3.6999	3.3762	4.4208	2.7862	3.8384	3.0682	4.0812	4.3803	4.0981
Apbb1ip	_RSSDT[Phospho (STY)]C[Carbamidomethyl (C)]GSPALPSK_.2	Q8R5A3_T534_M1_Apbb1ip	3.5331	3.7860	4.5705	4.0511	3.7401	4.3683	3.3080	3.7588	3.4559	7.0770	3.8303	3.5121	3.5640	4.6086	3.3334	4.3961	3.2560	3.8934	4.1925	4.2859
Apbb1ip	_QGLGNHS[Phospho (STY)]PGATR_.2	Q8R5A3_S507_M1_Apbb1ip	4.4943	4.3257	4.0308	4.9122	3.2004	3.2337	6.0784	3.6131	3.7680	4.3049	5.9658	4.0518	6.2110	6.1323	3.8731	3.8564	4.1507	5.7170	3.3310	4.4030
Tbc1d22a	_SVS[Phospho (STY)]ESHTPC[Carbamidomethyl (C)]PSESTGDTVPLQR_.3	Q8R5A6_S144_M1_Tbc1d22a	9.0256	9.0230	3.5667	8.7659	3.8689	8.1484	8.7598	4.2316	8.1830	3.2367	8.5595	8.2798	9.1593	3.5538	3.2493	3.3753	8.6036	3.0639	4.2133	3.5208
Actr1b	_AGFAGDQIPK_.2	#N/A	8.0715	7.8192	8.0816	7.7850	7.9297	7.6458	7.8097	8.1147	7.4469	7.3065	7.7291	7.8660	8.0100	7.5598	7.0371	7.4712	7.6101	7.6009	7.5561	7.4354
Actr1b	_VQYTLPDGSTLDVGPAR_.2	#N/A	6.6531	6.4963	7.1375	6.8309	6.6352	6.9012	6.6883	6.7338	6.6095	6.9439	6.8596	5.9542	6.8083	6.9351	6.1953	6.2698	6.5894	6.4748	6.1491	6.4954
Actr1b	_TLFSNIVLSGGSTLFK_.2	#N/A	6.2351	6.7098	6.2083	6.5923	6.1965	6.3192	6.2185	6.3368	6.2876	6.2084	6.0989	5.9825	6.0069	5.7938	5.5088	5.7098	6.2037	6.1420	6.3985	6.3570
Actr1b	_DQLQTFSEEHPVLLTEAPLNPSK_.3	#N/A	6.0331	6.1996	6.0366	6.0167	5.9264	5.7197	6.1177	6.0084	6.1557	6.0894	6.1888	5.2581	5.7773	6.2401	5.2592	5.7952	6.2515	5.7598	5.6156	5.3084
Actr1b	_TTGVVLDSGDGVTHAVPIYEGFAM[Oxidation (M)]PHSIM[Oxidation (M)]R_.3	#N/A	4.6794	4.7066	4.3096	4.5604	4.6695	4.5984	4.1190	5.4611	4.4159	4.7365	4.1036	4.6216	5.4992	4.5794	4.4113	4.5848	4.9463	4.7761	4.7545	4.6861
Actr1b	_AC[Carbamidomethyl (C)]YLSINPQKDEALETEK_.3	#N/A	3.2827	3.2192	3.8190	4.4066	4.1213	5.0615	4.5965	4.4840	4.8833	5.6612	5.1308	5.1939	3.3035	4.8109	2.9970	3.6276	4.6860	4.3677	3.9609	3.7731
Actr1b	_KEYEEDGSR_.2	#N/A	4.1505	4.2058	3.6871	3.4337	2.8566	3.7509	4.0142	4.3762	4.0733	4.3954	2.9469	4.3955	4.1814	3.7251	3.5914	3.5126	3.8734	3.2760	3.5751	3.4024
Usp15	_S[Phospho (STY)]PGASNFSTLPK_.2	Q8R5H1_S229_M1_Usp15	3.3171	7.2098	8.0289	8.0238	6.5294	4.3798	6.9180	3.7473	3.4445	8.3701	3.8188	6.9863	3.5525	7.1577	2.9626	3.6620	6.9187	3.9049	6.7160	6.9795
Usp15	_WYYFDDSSVSTASEDQIVSK_.2	#N/A	3.2917	4.0274	4.3291	3.5289	3.4986	3.5320	3.5495	5.2612	3.4697	3.1023	3.3221	3.7535	4.7306	6.4918	3.5748	4.4950	3.8524	3.9148	4.3126	4.1048

Wasf1	_IENDVATILSR_.2	#N/A	6.7840	7.0826	6.5441	6.7935	6.9319	7.3630	6.7212	7.0481	6.8631	6.9637	6.5632	7.2259	7.4649	7.1476	7.4609	7.1234	7.0496	7.2048	7.0980	7.1283
Wasf1	_LAQGPELAEDDADLLHK_.3	#N/A	5.3599	4.4177	5.3988	5.4793	5.4460	5.5433	4.6610	4.0601	5.2369	6.2227	6.0546	4.5361	5.5224	5.3261	5.4847	5.4856	5.5215	5.7270	5.1941	6.2707
Wasf1	_SSTIQDQQLFDRK_.2	#N/A	4.0998	4.4247	4.0320	4.3055	3.8450	3.3376	3.4844	3.7568	2.9062	3.9437	3.2919	3.2673	3.9390	4.4551	3.3352	3.8494	3.8122	3.8893	3.9304	3.7398
Arl6ip5	_AWDDFFPGSDR_.2	#N/A	7.0179	6.9750	6.6433	6.2822	6.6344	6.7805	7.2582	7.1201	6.6195	6.7937	6.7955	6.9983	7.2182	6.6159	6.2601	6.8158	7.2038	7.3030	6.3719	6.8380
Arl6ip5	_TPMGIILDALEQQEDNINK_.2	#N/A	4.1866	3.1325	3.7231	3.3977	2.8927	3.7149	4.1554	4.4122	4.1094	3.9972	2.9829	4.3595	4.2174	4.6942	4.1808	3.5487	3.9095	3.2400	3.5390	3.4385
Cadm1	_SDDSVIQLLNPNR_.2	#N/A	7.9369	7.2810	7.7740	7.6876	7.3102	7.4547	7.4670	7.5704	7.0507	7.2686	7.7644	7.3383	6.9409	8.0550	6.7986	7.1126	8.0627	7.2421	7.2264	7.7566
Cadm1	_C[Carbamidomethyl (C)]EASNIVGK_.2	#N/A	2.2506	3.9714	3.6378	2.9857	4.3874	2.6313	3.7453	2.4596	4.4973	3.9118	3.4839	3.0224	4.1321	3.8718	4.1358	3.5990	4.4940	1.6822	4.3890	3.7229
Cadm1	_GTYFTHEAK_.2	#N/A	5.7058	5.8827	4.5220	6.0352	5.7981	5.9287	3.3483	6.3098	5.8531	5.6555	5.7221	5.4376	3.5154	5.9098	5.7364	5.8829	5.1238	5.9378	4.2410	5.8035
Cadm1	_GADDAADADTAIINAEGGQNNSEEKKEYFI_.3	#N/A	3.6926	2.7928	4.2289	4.4380	3.0303	3.2544	2.2948	3.3719	3.0690	3.8989	3.4434	4.1997	3.1771	2.4085	4.0880	1.6075	4.3699	4.2803	3.0786	3.8990
Tmx1	_VEEEQEADEEDVS[Phospho (STY)]EEEAEDREGASK_.3	#N/A	4.2867	4.1704	4.9617	3.3542	3.1877	3.8892	4.5022	3.8158	4.6065	4.0629	3.4933	4.6094	4.1801	5.0957	4.0734	3.4212	4.5991	5.1149	4.4579	3.1489
Tmx1	_KVEEEQEADEEDVS[Phospho (STY)]EEEAEDR_.3	#N/A	4.3902	3.6126	3.4257	3.2991	3.7280	4.2570	3.8131	4.0906	4.2455	3.0957	3.7242	3.5020	4.4110	3.4128	3.3903	3.2343	3.1275	3.2049	3.4543	3.3798
Cops8	_SANSELGGIWSVGQR_.2	#N/A	3.3810	3.8613	5.5805	5.5065	5.2694	5.0752	4.8705	4.6146	5.3753	5.5519	5.1564	4.5702	6.1513	5.2152	5.3367	5.1603	5.4795	5.3391	5.1626	5.7025
Cops8	_GVLEQGWQADSTTR_.2	#N/A	4.4808	4.5929	5.1925	3.2086	3.8185	4.3328	4.5594	4.1811	3.7817	3.1862	4.1561	3.4115	3.8897	4.3261	4.6448	5.1561	3.2180	3.5677	4.9826	3.1108
Slc6a8	_GLTTLT[Phospho (STY)]PVS[Phospho (STY)]ESSK_.2	Q8VBW1_S628_M2_Slc6a8	3.1428	4.1763	4.1802	4.4415	3.3497	6.2786	5.7461	3.3684	3.0656	3.9023	3.4400	3.9024	3.1736	4.2183	3.7238	5.8134	4.3665	5.7039	5.6947	3.8955
Nae1	_LLQSVGQAPESISEK_.2	#N/A	4.3892	4.4189	3.4247	3.9272	4.8716	4.5416	4.9819	4.5096	3.8900	3.8283	4.2673	4.8269	4.0605	4.5316	4.7288	4.7906	3.1265	4.4625	4.6963	4.5267
Mpdz	_DNSQTPAVPAPDLEPIPSTS[Phospho (STY)]R_.2	Q8VBX6_S1590_M1_Mpdz	6.4217	3.5745	3.2812	3.8396	3.9515	3.9849	3.0966	3.9703	3.0168	3.5552	3.9962	3.3006	3.1822	3.3193	3.1219	3.1067	3.4675	3.6819	3.9810	2.9965
Camkk1	_KFS[Phospho (STY)]LQER_.2	Q8VBY2_S74_M1_Camkk1	5.4127	6.4719	6.8935	7.3894	5.9280	6.5836	5.8518	5.7210	6.5298	6.4188	6.3091	5.9466	6.5103	6.0053	5.1231	5.6857	5.9070	5.7129	6.0804	6.0158
Camkk1	_S[Phospho (STY)]FGNPFEPQAR_.2	#N/A	12.7804	12.8525	12.6615	12.7936	3.3953	12.6759	12.3387	12.1521	12.4222	12.8501	3.0784	12.1813	12.1550	13.0465	11.5980	12.5139	12.8794	12.2847	12.6095	12.5314
Camkk1	EGC[Carbamidomethyl (C)]GEGC[Carbamidomethyl (C)]KS[Phospho (STY)]PELPGVQEDEAAS .	#N/A	6.3077	7.5991	6.6085	7.4335	3.8375	6.8241	6.9466	4.0070	6.7775	7.0126	3.4732	6.9500	6.7033	7.6795	5.4460	6.3757	7.4027	6.9818	6.8530	6.9002
Camkk1	_SM[Oxidation (M)]S[Phospho (STY)]APGSLLMK_.2	#N/A	4.2564	4.8356	7.5338	6.6645	3.5941	5.7354	6.3161	3.6568	4.0772	3.7395	3.7283	3.7396	4.1853	7.8458	3.5953	3.8781	6.2230	3.3433	3.6424	5.1755
Camkk1	_KRS[Phospho (STY)]FGNPFEPQAR_.3	#N/A	3.4393	4.5635	3.9756	4.2501	4.2778	3.7071	6.2840	3.1396	3.2945	3.6456	4.2740	2.9521	3.4600	7.2395	2.8404	3.7842	3.6773	5.8884	3.8044	3.9296
Ccdc9	_S[Phospho (STY)]M[Oxidation (M)]EKENM[Oxidation (M)]AVEEK_.2	#N/A	5.1154	7.2431	4.7642	4.9531	4.3002	7.1236	4.4111	7.6701	4.4688	5.5812	4.3871	4.5230	5.1541	5.2367	4.1542	5.0154	4.6053	4.1080	4.5569	8.8389
Rapgef3	_LENGC[Carbamidomethyl (C)]GNVS[Phospho (STY)]PQTK_.2	Q8VCC8_S531_M1_Rapgef3	3.9654	4.0374	4.5017	3.7240	6.2311	3.5272	4.2379	3.6658	4.2970	4.1717	3.1706	3.9268	4.0051	4.4888	3.8151	3.3103	4.2035	3.6297	3.2783	2.9549
Reep2	_TETS[Phospho (STY)]EDDLGDKAPK_.2	#N/A	4.1005	6.7507	4.2029	6.4064	5.6658	3.4599	6.0226	6.7270	5.9786	4.3213	6.0679	4.2116	4.1265	6.4319	3.6690	6.1758	6.6057	3.9881	3.3970	3.2323
Reep2	_TETS[Phospho (STY)]EDDLGDKAPK_.3	#N/A	5.5991	4.2431	4.1134	4.5082	3.2829	3.3246	3.7652	3.3017	2.9988	3.9691	3.3732	4.7384	3.1069	4.1515	3.7905	3.9390	5.7973	5.3277	3.1487	3.8288
Reep2	_TETS[Phospho (STY)]EDDLGDK_.2	#N/A	3.5866	4.6092	4.1229	4.1028	2.9244	3.1484	3.1159	3.4778	3.1750	3.7930	3.5493	4.3056	3.2830	4.3276	4.1939	3.9315	4.4759	4.1744	2.9726	4.0049
Gpn1	_GTLDEEDEEADS[Phospho (STY)]DTDDIDHR_.3	#N/A	3.6493	5.5677	4.6754	5.6860	5.6232	5.5030	4.0760	3.5611	5.3488	6.1030	3.9680	5.2874	5.3079	6.0220	4.7307	6.2800	4.6918	6.0531	5.8375	5.4754
Gpn1	_GNAS[Phospho (STY)]PVLPSDULTR_.2	Q8VCE2_S314_M1_Gpn1	4.2136	5.4738	3.2490	3.4758	3.5513	3.7754	5.8069	3.9140	4.0489	4.4199	2.9224	5.2383	4.2343	5.8871	3.5670	3.0576	5.4010	3.5723	3.0301	3.2031
Rfxap	_[Acetyl (Protein N-term)]MEAQAVPEGSGPSTAS[Phospho (STY)]PR_.2	Q8VCG9_S16_M1_Rfxap	3.6757	3.6434	3.2123	3.9085	4.0016	4.2257	3.5395	3.9014	3.5985	3.3694	3.9729	3.3695	3.7066	3.2504	3.1166	3.5080	3.3986	3.7508	4.0499	4.4285
Pex19	_AKPS[Phospho (STY)]PEHAPTISAPDASGPQK_.3	#N/A	9.3683	9.3106	9.4364	9.5061	9.0840	9.0507	8.6611	9.3352	9.0705	9.5778	9.3228	9.3038	9.9073	10.2084	9.2495	9.4066	9.7705	9.0702	9.2751	9.4180
Pex19	_IC[Carbamidomethyl (C)]EQFEAET[Phospho (STY)]PTDSEATQR_.2	#N/A	3.2182	3.9516	3.9461	4.3181	3.7618	3.7751	3.3449	3.9311	3.7929	3.3454	3.5647	5.0200	3.4305	3.9332	3.3702	3.8563	3.6479	4.2238	3.8724	4.2753
Pex19	_AKPS[Phospho (STY)]PEHAPTISAPDASGPQKR_.4	#N/A	4.4046	4.4153	3.9412	3.6871	3.1107	3.1441	3.9374	3.7027	3.8576	4.2152	3.3362	4.1414	4.0230	4.2557	3.9627	3.7668	4.2403	3.5928	3.2414	2.9918
Plppr2	_LSPWEDLSQAPTM[Oxidation (M)]DS[Phospho (STY)]PLEK_.2	#N/A	5.6528	6.5356	4.9825	3.7149	3.4380	4.1538	6.1470	4.0356	3.8118	4.3051	3.6464	5.1487	3.9589	3.8542	3.8307	3.6566	3.8300	6.6166	5.5330	3.6645
Gripap1	_KQES[Phospho (STY)]FC[Carbamidomethyl (C)]R_.2	#N/A	6.2042	6.5376	6.1746	5.7539	5.9917	6.5906	6.3734	6.6877	6.1520	6.6918	6.5343	6.4689	6.5825	7.0777	6.3722	6.3499	6.6571	6.4213	6.6742	7.0654
Gripap1	_EC[Carbamidomethyl (C)]VGS[Phospho (STY)]PDPDLEPGEAN_.2	#N/A	10.5097	12.2526	12.4109	12.4782	3.2150	12.0775	11.8422	5.7351	11.8629	12.2884	8.8219	12.0789	11.2250	12.5603	11.5439	12.1041	12.5096	11.6307	12.0307	12.2695
Gripap1	_LSKEC[Carbamidomethyl (C)]VGS[Phospho (STY)]PDPDLEPGEAN_.2	#N/A	4.2287	3.7741	3.2641	3.4607	3.5664	3.7905	6.9569	3.9290	4.0338	7.0688	2.9073	7.7652	4.1418	3.2512	3.5519	4.7022	4.4667	6.9279	7.1442	3.2182
Gripap1	_TQTGDSSS[Phospho (STY)]VSSFSYR_.2	Q8VD04_S631_M1_Gripap1	3.6759	5.3992	3.6620	4.0839	4.1469	4.1003	3.9614	4.3382	5.6161	6.5834	3.9005	3.0981	3.9412	5.8853	2.9550	3.4785	3.6091	4.9521	4.1976	3.5562
Gripap1	_KKQES[Phospho (STY)]FC[Carbamidomethyl (C)]R_.2	#N/A	3.0829	4.2165	3.6192	3.8826	3.5274	4.2772	3.1745	4.1979	3.8302	4.0226	4.0353	3.3085	3.1782	5.1208	3.9713	3.8107	4.3728	4.2034	3.9007	4.3730
Gripap1	_TGLEELVLSEM[Oxidation (M)]NS[Phospho (STY)]PSR_.2	#N/A	3.9106	8.4100	4.2731	3.8404	6.2826	4.4535	7.6230	3.7238	4.2636	5.9067	3.8048	7.2862	3.9050	6.3865	4.1245	4.4082	4.8467	3.7192	6.5147	7.2816
Znf385a	_QPGS[Phospho (STY)]PSPPSVPESGQGVTK_.2	Q8VD12_S160_M1_Znf385a	3.4214	4.5814	3.9577	4.2680	4.2600	3.7250	3.3565	3.1218	3.2767	3.2953	4.2562	2.9700	3.4421	3.9448	3.3818	4.3476	3.6595	4.1737	3.8223	3.9118
Sgtb	_SFS[Phospho (STY)]SSADEHS_.2	#N/A	7.7243	7.6000	7.6189	5.9627	7.4426	7.3638	7.2975	7.0544	6.8097	7.9742	7.4891	7.5859	7.6719	7.8147	6.1520	7.5792	7.3079	6.9565	7.4923	7.8650
Sgtb	_SRS[Phospho (STY)]FSSSADEHS_.3	Q8VD33_S295_M1_Sgtb	4.2385	4.5817	3.7750	7.3572	6.2771	3.6632	4.1038	4.4641	4.1613	4.0490	3.0348	6.6456	4.2693	7.0921	6.6580	3.6006	3.9614	6.8356	6.6327	6.7998
Sgtb	_NDIRPLSNS[Phospho (STY)]PEDVGK_.3	Q8VD33_S77_M1_Sgtb	4.5205	3.4823	3.5559	3.1689	3.8582	4.1268	4.4555	4.2208	4.3757	3.6971	4.4189	3.3718	4.5412	3.5430	3.2601	3.3645	3.2577	3.0746	4.2241	3.5100
Sgip1	_TGS[Phospho (STY)]PLTVATGNDQAATEAK_.2	Q8VD37_S265_M1_Sgip1	11.8065	11.4925	11.7550	11.7026	11.2364	11.3318	11.1879	11.7202	11.2260	11.6961	11.3982	11.3203	12.0278	11.8716	10.5993	11.2523	11.8392	11.2103	11.2193	11.3289
Sgip1	_AT[Phospho (STY)]PPPPPPPTYR_.2	#N/A	12.7606	12.7007	12.8506	12.7282	12.3036	12.5585	12.1470	12.5229	12.3974	12.8756	12.5925	12.5338	12.4686	13.1454	11.6888	12.2672	12.9582	12.2537	12.4150	12.5243
Sgip1	_RST[Phospho (STY)]PT[Phospho (STY)]PELTSK_.2	#N/A	10.9851	11.2029	10.7441	10.8731	10.2858	10.7609	10.8582	10.5726	10.6074	11.4507	10.7479	10.9653	11.2715	11.4192	10.1156	10.8773	11.0515	10.7048	10.7238	10.8400
Sgip1	_TVPAT[Phospho (STY)]PPR_.2	Q8VD37_T259_M1_Sgip1	11.4740	4.0121	11.6624	11.6032	11.5798	11.7268	11.3739	11.7714	11.5635	11.5128	11.3871	11.4731	3.3379	4.3825	11.5864	11.4862	11.9308	4.1195	4.4186	11.7649
Sgip1	_TVPATPPRT[Phospho (STY)]GS[Phospho (STY)]PLTVATGNDQAATEAK_.3	#N/A	4.1410	3.6807	3.9138	3.4732	3.9943	9.2951	9.6327	3.8117	4.2500	6.8325	9.0072	9.6549	9.4690	8.3604	3.2398	3.7683	3.5196	9.5462	4.1085	9.1112
Sgip1	_RS[Phospho (STY)]PGAIKR_.2	#N/A	10.4500	10.5551	10.2762	10.6591	9.9201	9.5707	9.8777	10.1497	9.6273	10.3769	9.4965	10.1294	10.4833	10.4065	9.0612	10.1179	9.6797	9.8703	10.0258	9.8129
Sgip1	_STPT[Phospho (STY)]PELTSK_.2	#N/A	9.4312	9.6858	9.6425	9.8508	9.2960	9.0918	9.4137	9.3487	9.0196	10.1164	9.2590	9.3454	9.8100	9.8720	8.3170	9.1732	9.1649	9.3078	9.2853	9.3848
Sgip1	_NLS[Phospho (STY)]SEEVAR_.2	#N/A	9.4705	9.6483	9.4776	9.6603	9.0512	9.3628	9.2947	8.8329	9.0971	9.7188	9.3847	9.2046	9.8434	9.7438	8.3757	9.3133	9.1981	9.1866	9.3962	9.4410
Sgip1	_VAEQTFIKDDYLETLSS[Phospho (STY)]PK_.3	#N/A	7.7696	5.2252	5.3428	8.0082	6.6974	7.4841	7.6282	6.9935	7.2550	8.0079	7.7063	7.3371	7.2295	8.0195	6.6394	4.8749	7.6197	7.4645	3.8130	7.7580
Sgip1	_S[Phospho (STY)]PGAIKR_.2	#N/A	9.1696	9.2317	9.0574	9.3090	8.8705	8.8419	8.8366	8.9508	8.6519	9.5261	8.8832	8.8713	8.8454	9.5055	8.0597	9.0621	8.5025	8.9183	8.9377	9.0650
Sgip1	_HVPS[Phospho (STY)]PLNLEEVQKK_.3	#N/A	9.1854	9.0195	8.4146	8.7011	8.3406	7.8351	8.3434	8.8121	7.8957	9.1266	8.3372	8.7644	9.3351	9.0813	7.5262	8.5212	8.3764	8.2759	8.2456	8.3279
Sgip1	_RS[Phospho (STY)]PGAIK_.2	#N/A	8.5829	8.7448	8.7691	8.8968	8.2282	7.9208	8.2702	7.7428	7.5280	8.4393	8.4378	8.1721	8.3116	8.4248	6.6748	7.9724	8.1476	8.0692	7.9149	8.2551
Sgip1	_RNLS[Phospho (STY)]SEEVARPR_.3	#N/A	6.5883	4.4320	6.2353	6.7985	3.2895	3.5135	4.2516	3.1127	4.3107	4.1580	3.1843	4.1582	4.4188	6.6816	3.8288	6.2507	4.1108	6.4137	3.3377	3.6398
Sgip1	_VAEQTFIKDDYLETLSS[Phospho (STY)]PKEC[Carbamidomethyl (C)]GLGQR_.4	#N/A	3.6269	3.6922	3.1635	3.9573	3.8338	4.2745	3.4906	3.8526	3.5497	3.4182	3.9241	3.4183	3.6578	7.5169	3.0678	2.9890	3.3498	6.8921	4.0987	4.3796
Sgip1	_TVVSS[Phospho (STY)]PGPGSGSGTGTTSGASSPAR_.2	Q8VD37_S424_M1_Sgip1	3.3938	6.1698	6.5504	5.8710	4.7279	5.9356	5.2925	5.7348	5.0874	5.8357	5.5561	6.0387	6.0733	6.9256	3.3542	5.6049	6.5128	6.1233	3.8499	5.7185
Sgip1	_ITGEM[Oxidation (M)]VLSFPAGITR_.2	#N/A	5.9878	6.1609	4.3601	4.2616	6.2074	6.3346	6.2834	6.2335	6.0604	5.6250	3.6199	5.7694	6.5999	4.3982	6.0043	6.0548	6.2927	6.0170	6.0154	6.0890
Sgip1	_IEKLPS[Phospho (STY)]SDLDSIFGPVLS[Phospho (STY)]PK_.3	Q8VD37_S287_M2_Sgip1	5.4800	4.2070	4.2475	4.2199	3.2278	3.3563	8.2245	3.4226	4.1036	4.2233	3.2349	4.0147	8.3217	9.2307	3.8709	4.0638	6.2020	5.8616	7.2266	3.7892
Sgip1	_YNS[Phospho (STY)]PELDEEGYSIRPEEPGSTK_.3	Q8VD37_S78_M1_Sgip1	5.6150	3.8047	3.7939	5.9646	4.4476	5.9965	3.6771	4.0917	5.5882	6.2496	3.7521	3.6090	3.8257	6.5272	3.3379	5.6927	3.6846	3.8525	5.8911	7.0154
Sgip1	_KVAEQTFIKDDYLETLSS[Phospho (STY)]PK_.3	#N/A	4.4783	4.2424	4.2304	4.2012	3.5483	3.5816	3.5000	3.4077	3.4202	3.1519	3.8285	4.0653	3.5856	4.2456	3.5254	4.2043	4.2175	4.0304	4.0905	4.0553
Sgip1	_IPDISQK_.2	#N/A	4.6667	5.1202	4.2168	4.4259	3.0182	3.2423	5.4805	4.6297	5.2855	4.1656	4.5517	4.2118	3.1892	4.3677	5.4192	4.6027	4.2532	4.2682	4.9787	5.2238
Sgip1	_NLS[Phospho (STY)]SEEVARPR_.2	#N/A	11.4611	11.2796	11.4635	11.5433	10.9795	10.6726	10.8459	11.3606	10.9233	11.3407	10.7346	11.0864	11.7582	11.6617	10.5262	11.0607	11.0896	10.9056	10.9788	10.9537
Sgip1	_EC[Carbamidomethyl (C)]GLGQRAT[Phospho (STY)]PPPPPPPTYR_.3	#N/A	3.2664	3.2356	3.8027	4.4230	4.1050	4.8883	3.2015	4.4676	3.1217	3.4503	4.1011	3.1250	3.2871	3.7898	3.0133	3.6113	5.6661	6.3415	5.1587	3.7568
Sgip1	_DDYLETLSS[Phospho (STY)]PKEC[Carbamidomethyl (C)]GLGQR_.3	#N/A	3.3772	3.1247	3.9135	4.3122	4.2158	4.4399	3.3253	3.0776	3.3844	3.5836	3.7587	3.0142	3.4924	3.9006	2.9025	3.7221	3.6153	4.7428	3.8665	3.8676
Sgip1	_WVHFSDAS[Phospho (STY)]PEHVT[Phospho (STY)]PELTPR_.3	#N/A	10.5339	11.2909	10.9034	10.5091	8.8583	10.6652	10.9168	10.0628	10.6907	10.7131	10.1569	10.4778	10.5979	11.9882	10.4854	10.5538	10.5848	11.0737	10.8493	10.4950
Sgip1	_HVPS[Phospho (STY)]PLNLEEVQK_.2	#N/A	8.1210	7.7236	7.7177	7.8892	6.9768	7.0791	7.5620	7.2539	6.8426	7.9577	7.4240	7.3617	7.8084	8.0104	6.3014	7.3880	7.6762	7.4081	7.0471	7.2098
Sgip1	_ASIGNIALSPSPVR_.2	#N/A	6.5188	5.8484	6.0495	6.6804	6.4186	6.4380	6.6493	6.2685	6.4634	6.5815	6.3970	6.3026	6.5540	6.8893	6.7574	6.6137	6.6809	6.5686	6.3436	5.9116
Sgip1	_DDYLETLSS[Phospho (STY)]PK_.2	#N/A	3.3894	3.9297	4.4268	4.1949	3.5963	6.2528	3.4518	3.2171	3.3720	3.2000	4.3515	3.6658	3.5374	4.0401	3.4772	4.2523	3.7548	4.0784	3.7270	4.0071
Sgip1	_ASIGNIALS[Phospho (STY)]PS[Phospho (STY)]PVRK_.2	Q8VD37_S149_M2_Sgip1	4.1603	5.9036	3.1957	3.5291	3.4980	4.7355	4.0431	4.4050	4.1022	4.3666	2.9757	3.7320	4.2102	5.3865	3.6331	3.0043	3.9023	3.2472	3.5462	3.4313
Sgip1	_ASIGNIALS[Phospho (STY)]PS[Phospho (STY)]PVRKS[Phospho (STY)]PR_.3	Q8VD37_S149_M3_Sgip1	3.2718	4.0473	4.3092	4.3124	3.4787	3.2335	3.5694	3.4975	3.4896	4.5832	2.9682	3.7734	3.3027	5.6975	3.5947	4.1348	4.4956	4.1547	2.9529	4.0246
Sgip1	_YNTDAM[Oxidation (M)]STAVALNNVQFLVPIDGGVTK_.3	#N/A	3.6023	3.7168	3.1389	4.8238	3.8093	4.6778	3.2388	3.8280	4.2288	4.4129	4.1385	3.4429	3.6332	3.1770	3.2642	2.9644	3.3252	3.8242	4.1233	4.6792
Sgip1	_EQVVT[Phospho (STY)]PPAASDIPADSPAPAPPGPTGSAGPPGPPGPR_.4	Q8VD37_T335_M1_Sgip1	3.3975	4.4942	4.0449	4.1808	3.6044	3.6377	6.6999	6.9063	3.3639	3.2081	4.3434	6.5489	3.5294	8.2476	3.4691	5.0890	3.7467	6.6077	3.7351	3.9990
Sgip1	_GPS[Phospho (STY)]PLTM[Oxidation (M)]GAQDTLPVAAAFTETVNAYFK_.3	Q8VD37_S528_M1_Sgip1	3.6354	4.3674	4.1717	4.0540	2.9731	3.1972	3.0671	3.3358	3.3162	3.8417	3.5006	1.0735	3.2343	2.0073	4.1452	3.9803	3.8734	3.9597	3.6083	3.9562
Sgip1	_LPS[Phospho (STY)]SDLDSIFGPVLSPK_.2	#N/A	3.2165	3.2855	3.7528	4.4729	4.0551	3.9299	2.7719	4.4177	3.0718	3.5002	4.0513	3.1749	3.2372	1.9905	3.0632	3.5614	3.4546	4.3786	4.0272	3.7069
Sgip1	_GKHFY[Phospho (STY)]SS[Phospho (STY)]S[Phospho (STY)]ES[Phospho (STY)]EEEESHKK_.3	Q8VD37_S104_M3_Sgip1	4.3455	4.2873	3.5307	3.5901	3.6833	3.9073	3.8578	4.2198	3.9169	4.5519	4.2913	3.0511	4.0250	3.5687	4.3350	3.1896	3.7170	4.6324	3.7315	3.2460
Tspyl4	_GLEDAPS[Phospho (STY)]PSKGGDAPSAPVAADDSSK_.3	#N/A	6.8085	3.9242	6.9126	6.9991	7.3011	6.3078	6.4821	6.0581	3.7220	7.1294	6.5100	5.9258	3.4801	3.8357	6.1983	3.7791	6.0403	4.0219	6.5699	6.3455
Tspyl4	_NGC[Carbamidomethyl (C)]QLEGHS[Phospho (STY)]PAKPK_.2	#N/A	4.9166	4.4218	4.3740	4.3779	3.9080	3.7069	4.6524	3.5501	3.9408	4.3125	4.0453	4.2643	3.6992	5.0468	4.1720	4.5385	4.3365	4.6497	4.9995	4.6943
Tspyl4	_GLEDAPS[Phospho (STY)]PSK_.2	#N/A	4.4041	3.5987	3.4396	3.6487	3.7418	3.9659	3.7993	4.1612	3.8583	3.1096	4.2327	3.4882	3.9664	3.5102	3.3764	3.2482	3.6584	3.4910	3.7901	3.1874
Myh9	_VVFQEFR_.2	#N/A	4.6783	3.7602	3.4457	4.5516	4.6469	4.7917	4.3461	4.5366	4.5245	5.7529	3.4782	4.4717	5.5772	5.5559	4.3364	4.9141	4.7735	4.6265	3.6141	4.5295
Myh9	_KGTGDC[Carbamidomethyl (C)]S[Phospho (STY)]DEEVDGKADGADAK_.3	#N/A	6.4540	6.6464	6.3474	5.5767	6.0609	6.3178	6.3992	6.1106	6.2545	6.5084	6.0117	5.9253	6.6959	6.0560	6.9832	5.5243	6.5680	6.3814	6.1794	6.7496
Myh9	_GTGDC[Carbamidomethyl (C)]S[Phospho (STY)]DEEVDGK_.2	#N/A	4.2392	3.7636	3.2747	3.4502	3.5769	4.4080	4.1743	3.9396	4.0945	3.9783	3.5731	3.6531	4.2599	3.2617	4.1996	3.0833	4.4773	3.3559	3.0045	3.2287
Myh9	_GTGDC[Carbamidomethyl (C)]S[Phospho (STY)]DEEVDGKADGADAK_.2	#N/A	4.6871	5.8774	3.2296	4.4388	3.5319	3.7560	4.0092	5.7402	4.0683	4.4005	5.1732	4.7301	5.8272	5.7768	3.5864	3.0382	5.8223	3.2811	4.8171	4.4719
Myh9	_LQQELDDLLVDLDHQR_.3	#N/A	4.0892	3.2590	3.3532	3.3717	4.2962	3.8545	4.2528	4.0181	3.6515	3.8998	3.6516	3.5746	4.3384	3.3402	2.8043	3.4514	4.5558	4.0859	4.4268	4.1377
Myh9	_KGTGDC[Carbamidomethyl (C)]S[Phospho (STY)]DEEVDGK_.3	#N/A	3.8688	4.1489	3.5543	5.1031	4.3378	4.6536	5.3363	3.5087	3.9405	3.8165	4.7235	3.8751	4.0486	5.0488	3.6403	3.6187	3.1257	4.6606	4.4244	4.0358
Myh9	_KQELEEIC[Carbamidomethyl (C)]HDLEAR_.3	#N/A	4.4711	3.5317	3.5066	3.2183	4.1022	4.2250	3.7322	4.1715	3.7913	3.1766	4.1657	4.5548	4.4918	3.4936	4.4489	3.3152	3.5857	4.4173	4.2734	4.2945
Myh9	_DFSALESQLQDTQELLQEENR_.3	#N/A	4.5629	4.3334	4.0995	4.5221	3.2690	4.0342	5.1818	3.2878	3.6993	4.3735	3.1779	4.4453	4.5938	5.8036	3.8044	3.9251	5.2343	4.7533	3.1626	3.8149
Myh9	_ANLQIDQINTDLNLER_.2	#N/A	3.4560	4.5527	3.9864	4.2393	4.1255	4.9579	4.3169	3.1504	3.3053	3.2666	4.2848	2.9414	3.4708	3.9734	3.4105	4.3190	4.5765	4.1450	3.7936	3.9404
Myh9	_ELETQISELQEDLESER_.2	#N/A	3.4613	3.8578	4.4987	4.2446	3.6682	3.7015	3.3799	3.1452	3.3001	3.2719	4.2796	4.2700	3.4655	3.9682	3.4053	4.3242	3.6829	3.8016	3.7989	3.9352
Phip	_TAFYNEDDS[Phospho (STY)]EEEQR_.2	Q8VDD9_S1783_M1_Phip	8.2289	8.4919	8.2806	7.7668	3.5280	7.7395	7.8038	7.9018	7.9689	7.7828	6.9686	7.0430	7.6288	7.7831	6.1329	6.9760	8.0221	7.6710	7.3731	6.9440
Phip	_VLS[Phospho (STY)]DS[Phospho (STY)]EEEEKDADVPGTSTR_.2	#N/A	4.2637	6.6598	3.5971	4.5418	3.2871	6.2498	5.5457	3.7536	6.0898	4.2650	3.1905	5.6772	4.0740	5.5422	4.6326	3.4165	6.1658	6.3346	5.1842	3.0428
Phip	_KVLS[Phospho (STY)]DS[Phospho (STY)]EEEEKDADVPGTSTR_.3	#N/A	3.6067	3.6887	4.8857	6.3070	3.8136	5.0895	4.7754	3.8324	5.9431	3.4173	3.9039	3.4385	3.6376	6.1053	5.7764	2.9688	3.3296	4.5541	4.1189	4.3594
Parn	_TLS[Phospho (STY)]PDPR_.2	Q8VDG3_S556_M1_Parn	4.3355	3.4496	3.5887	3.1362	3.8909	5.8645	3.6501	4.3545	4.4085	6.0733	6.0667	5.5499	4.5739	3.5757	4.4963	3.3973	3.2904	3.0419	4.1913	5.1945
Hdlbp	_ETDPGS[Phospho (STY)]PR_.2	#N/A	8.9486	9.4735	8.3995	6.9519	8.4242	3.7677	3.9974	9.1683	4.0565	9.6406	2.9301	9.2253	8.3857	9.3352	3.5746	7.5690	8.3632	9.5488	8.1373	3.3856
Hdlbp	_EAKETDPGS[Phospho (STY)]PR_.2	#N/A	7.1854	7.7096	4.4286	7.6474	7.0635	7.1604	7.4598	7.7901	7.1472	7.3070	4.3497	7.7189	8.7671	7.4240	7.7595	7.8834	7.5078	7.6163	7.2066	7.2238
Hdlbp	_ETDPGS[Phospho (STY)]PRR_.2	#N/A	3.4551	4.5477	3.9914	7.2888	4.2936	6.8020	3.3901	3.1555	3.3104	3.2616	4.2898	7.0072	3.4758	7.4434	3.4155	7.0394	3.6931	4.1400	7.0403	7.1878
Hdlbp	_VATLNS[Phospho (STY)]EEENDPPTYK_.2	Q8VDJ3_S31_M1_Hdlbp	8.4789	8.3464	3.9737	8.3536	7.3690	8.1195	3.9633	3.8605	8.0636	8.5813	7.9717	7.9767	4.0735	8.8630	7.3715	8.2658	8.4404	8.1208	8.3378	8.4080
Zgpat	_GKS[Phospho (STY)]LDGC[Carbamidomethyl (C)]AEILQK_.2	Q8VDM1_S353_M1_Zgpat	5.4432	4.4063	4.8886	4.6085	3.1764	3.2733	4.0016	3.5390	3.3305	3.5738	3.7071	4.0773	3.8866	4.2647	3.2137	3.6484	3.1933	3.6133	4.3277	4.6783
Zgpat	_LLSTVDQES[Phospho (STY)]PAQEDAEYLAFQK_.3	Q8VDM1_S70_M1_Zgpat	3.1623	4.5429	3.9962	4.2295	4.2985	3.6865	3.2426	4.1548	3.2568	4.2946	2.9315	3.4806	3.9833	4.8765	4.6371	3.6979	4.1352	3.7838	[illegible]	2.1441
Psmd2	_TPVQSQPSATT[Phospho (STY)]PSGADEK_.2	#N/A	3.1371	8.5084	4.1745	4.4471	8.3236	8.3950	8.2468	8.8503	8.2067	8.1985	7.9466	3.9081	8.0259	8.3394	7.3928	7.8611	8.5638	8.2616	8.0634	8.0560
Psmd2	_DKTPVQSQPSATT[Phospho (STY)]PSGADEK_.3	#N/A	5.0722	6.9769	5.6298	6.3581	3.2697	6.1172	6.6913	3.3927	5.2897	5.1264	3.2150	5.3747	3.1979	5.8533	4.6519	4.0900	5.6757	5.7016	3.0608	5.3327
Psmd2	_TITGFQTHTTPVLLAHGER_.3	#N/A	4.7203	5.3982	4.3314	4.2903	4.4726	4.2893	4.8933	5.6136	3.9569	4.9630	4.5006	3.7513	5.7548	4.1355	3.7765	4.5846	4.8722	3.7442	4.6197	4.1906
Psmd2	_FGGS[Phospho (STY)]GSQVDSAR_.2	Q8VDM4_S361_M1_Psmd2	3.7110	4.2910	5.3504	4.7233	5.2682	3.6800	5.9527	3.7084	4.3236	5.9625	5.3170	3.9491	4.1813	6.3316	3.9489	6.0206	6.1892	4.2540	4.1038	4.0329
Psmd2	_LVGSQEELASWGHEYVR_.3	#N/A	3.5297	4.4731	4.0660	4.1597	2.8674	3.0915	3.1728	3.5348	3.2319	3.7360	3.6063	4.3626	3.3400	4.3846	4.2509	3.8746	4.5329	4.1174	4.4165	4.0619
Hnrnpul1	_GRS[Phospho (STY)]PQPPAEEDEDDFDDTLVAIDTYNC[Carbamidomethyl (C)]DLHFK_.4	#N/A	6.8509	8.0883	7.7459	4.0848	3.7063	7.5968	6.9792	6.2199	7.4046	7.6974	4.2415	7.0395	3.4274	7.4522	6.6795	7.1057	6.6294	7.2955	7.0789	7.1912
Hnrnpul1	_NYLDQTNVYGS[Phospho (STY)]AQR_.2	Q8VDM6_S513_M1_Hnrnpul1	7.1409	6.9215	7.1681	7.1441	6.1458	6.3889	6.3335	3.8998	6.6343	4.4057	6.5333	6.5819	7.2250	3.2220	5.5767	6.6517	7.2355	6.9301	6.9877	6.2576
Hnrnpul1	_S[Phospho (STY)]PQPPAEEDEDDFDDTLVAIDTYNC[Carbamidomethyl (C)]DLHFK_.3	#N/A	3.1843	6.2654	3.7206	5.3947	4.0229	4.2470	3.5182	3.8801	3.5773	3.3907	3.9516	4.8499	3.6853	6.0397	3.3054	3.5292	5.6075	5.4298	4.0711	4.4072
Atp1a1	_S[Phospho (STY)]PDFTNENPLETR_.2	Q8VDN2_S228_M1_Atp1a1	12.5665	12.1880	12.3196	12.1643	11.5772	12.1042	11.9156	12.1107	12.0503	12.0184	12.3075	11.8700	12.2529	12.1269	10.9480	11.8867	12.8598	12.1129	12.1681	12.0329
Atp1a1	_IVEIPFNSTNK_.2	#N/A	10.6445	10.3693	10.4343	10.3749	10.2826	10.3078	10.4212	10.6168	10.2006	10.2447	10.4074	10.2019	10.4846	10.0400	9.8862	10.0456	10.5228	10.3559	10.2174	10.0234
Atp1a1	_LNIPVNQVNPR_.2	#N/A	9.9920	9.8081	9.7300	9.7143	9.5902	9.5261	9.8883	10.0052	9.8488	9.4557	9.7792	9.5152	10.1790	9.4967	9.3434	9.2039	9.9411	9.7022	9.6675	9.2309
Atp1a1	_YHTEIVFAR_.2	#N/A	7.8834	7.8120	7.7012	7.3101	7.1571	7.7440	7.5974	7.8899	7.7384	7.3885	7.5855	7.0549	7.4329	7.4690	6.7225	7.2364	7.8981	7.7576	7.6905	7.5230
Atp1a1	_LSLDELHR_.2	#N/A	8.9144	9.0044	8.5872	8.6420	8.5849	8.6525	8.8870	9.1555	8.3654	8.4561	8.7871	8.6608	8.5822	8.3797	8.4313	8.1485	8.9497	8.8680	8.7486	8.3804
Atp1a1	_AVFQANQENLPILK_.2	#N/A	8.4149	8.1126	8.3966	8.5395	7.8584	8.0468	8.2888	8.5217	8.2033	8.7224	8.2076	8.7853	8.0901	8.1629	7.5468	8.1587	8.2835	8.1333	8.0391	7.9906
Atp1a1	_TSATWFALSR_.2	#N/A	7.8340	8.2776	8.0055	8.0244	7.4470	7.8184	8.0765	8.0251	7.8877	7.2897	7.5539	7.6855	7.8845	7.7922	7.2319	7.5382	8.1414	8.2582	7.9105	7.5629
Atp1a1	_GIVVYTGDR_.2	#N/A	7.7426	7.5928	7.4293	7.7451	7.4853	7.8948	8.1691	7.1512	8.0851	7.9385	8.1198	7.6072	8.2886	7.2284	7.2959	7.9799	6.7460	8.0456	8.2911	7.7135
Atp1a1	_YQLSIHK_.2	#N/A	7.2518	7.1383	7.3662	6.6891	6.9821	7.3243	7.2865	7.1136	7.2777	6.7877	7.5451	6.8285	7.1907	7.2212	6.9765	6.8750	7.6002	7.2942	7.0364	7.0668
Atp1a1	_KYGTDLSR_.2	#N/A	7.4188	7.5137	7.1693	7.2396	7.1761	7.1148	7.7030	7.2980	7.4593	7.1527	7.4835	6.9955	7.2154	6.3534	6.9062	6.9828	7.6175	7.3079	7.4622	7.0108
Atp1a1	_C[Carbamidomethyl (C)]SSILHGK_.2	#N/A	5.9732	6.2018	6.3645	5.9632	6.0489	5.9214	5.9166	6.3053	6.3184	5.9659	5.6911	6.2771	5.1448	5.5268	6.2787	5.5572	5.6128	6.1399	6.5338	5.5408
Atp1a1	_YGTDLSR_.2	#N/A	5.5901	5.7569	4.5214	5.5175	4.8201	5.3275	6.5757	5.2892	6.1264	5.9629	5.7655	5.5671	5.4918	3.8982	4.9117	4.8074	4.9348	5.9483	5.9692	5.5806
Atp1a1	_NIAFFSTNC[Carbamidomethyl (C)]VEGTAR_.2	#N/A	6.8981	6.1948	6.8089	5.9642	6.3750	6.2571	5.9204	6.2447	6.3698	6.8005	6.4619	4.3806	6.7387	4.7541	4.4168	5.7263	6.8626	5.3124	6.0517	6.1769
Atp1a1	_IISANGC[Carbamidomethyl (C)]K_.2	#N/A	6.1073	6.2046	5.7732	5.1449	5.8061	5.5798	6.3585	6.1045	5.9738	4.9708	6.0834	5.2782	3.8257	4.6604	6.3160	5.1248	5.6030	5.9305	6.0455	4.8865
Atp1a1	_AVAGDASESALLK_.2	#N/A	6.2682	7.3361	7.2623	6.4129	5.7799	6.2497	7.1929	6.3796	6.8849	5.6699	5.8406	6.6456	6.0608	6.3837	6.0730	6.0305	7.2753	7.4179	5.8888	6.3162
Atp1a1	_DM[Oxidation (M)]DELKK_.2	#N/A	4.8966	5.6127	6.0438	6.1558	4.9113	5.1916	5.8840	5.7975	5.6094	4.6295	6.0095	5.1940	5.1399	5.4838	5.6860	4.7816	4.8455	5.4617	5.0168	4.5154
Atp1a1	_EVS[Phospho (STY)]MDDHK_.2	#N/A	4.4408	6.9865	7.4615	7.4211	4.3291	6.2781	7.1611	3.6665	6.3471	4.2514	7.2301	4.7425	3.9869	7.5811	5.6727	3.8029	6.3177	5.0693	5.4325	5.2732
Atp1a1	_WVNDVEDSYGQQWTYEQR_.2	#N/A	6.3403	5.0544	4.5852	4.7758	4.2347	4.9632	4.7370	6.2136	4.8795	4.4756	5.0736	4.7848	6.1264	5.3552	3.0431	3.7679	5.1883	4.7461	4.7154	4.2434
Atp1a1	_M[Oxidation (M)]SINAEDVVVGDLVEVK_.2	#N/A	7.9272	7.6213	7.2670	7.3353	6.8579	7.0420	7.2279	7.7143	6.9806	7.3789	6.9617	7.2230	7.7668	7.3333	5.9544	7.4411	7.7850	7.2933	7.1966	7.3435
Atp1a1	_DM[Oxidation (M)]TSEELDDILR_.2	#N/A	6.8091	6.3363	7.2175	6.8652	6.0932	6.7579	6.8043	6.4392	6.8218	6.1114	6.8124	6.1597	6.4559	6.2618	6.1466	6.3908	7.0334	6.5804	6.2772	6.6575
Atp1a1	_ADIGVAM[Oxidation (M)]GIVGSDVSK_.2	#N/A	5.7094	6.0739	6.2130	5.3802	5.4010	5.6925	5.4485	5.5925	5.3095	5.5708	5.3845	5.3121	5.7602	5.2099	5.1823	5.6975	5.0965	5.7753	5.8579	5.3225
Atp1a1	_EQPLDEELKDAFQNAYLELGGLGER_.3	#N/A	3.4537	4.5798	4.4911	4.1305	2.5403	4.1620	4.3665	4.5196	3.3765	3.5914	3.7509	3.5915	3.4846	4.4780	2.8946	3.4602	4.3523	4.7843	4.6818	4.2065
Atp1a1	_DKYEPAAVSEHGDKK_.3	#N/A	6.1635	5.9325	6.3558	5.6945	5.1328	5.3256	6.0333	5.1014	5.4056	6.0773	5.9531	5.2950	5.2018	5.0694	4.9696	5.6489	5.5569	5.7357	5.7919	5.2350
Atp1a1	_EVS[Phospho (STY)]M[Oxidation (M)]DDHKLSLDELHR_.3	#N/A	5.8664	6.0001	4.8111	5.2637	3.5142	5.4142	5.8471	5.7369	5.1516	4.9485	4.7934	6.0072	4.0771	6.1899	3.5181	5.6369	6.4213	6.5161	4.3171	5.7040
Atp1a1	_AC[Carbamidomethyl (C)]VVHGSDLKDM[Oxidation (M)]TSEELDDILR_.4	#N/A	4.0524	4.5952	3.7085	3.6524	4.6953	4.6934	4.2084	4.4517	4.7530	4.4568	3.8679	4.4002	4.1608	4.6746	4.0730	4.4755	3.2823	4.8352	4.6737	4.7929
Atp1a1	_M[Oxidation (M)]TVAHM[Oxidation (M)]WFDNQIHEADTTENQSGVSFDK_.4	#N/A	7.4672	4.5263	4.0128	4.2128	5.5660	3.0384	5.8108	7.1550	5.9645	6.3305	5.7168	6.3639	7.4836	6.0974	2.8032	5.9415	6.0839	6.6486	6.2836	4.1150
Atp1a1	_EVS[Phospho (STY)]M[Oxidation (M)]DDHKLS[Phospho (STY)]LDELHRK_.3	#N/A	4.0070	5.1731	4.0497	3.6933	3.6694	3.8038	4.5215	3.9593	4.2601	4.2924	4.1293	4.2973	3.9006	4.1398	3.3993	4.2349	4.6287	4.5217	3.7806	4.2008
Ccdc92	_KSLS[Phospho (STY)]APLHPEFEEVYR_.3	#N/A	2.7171	4.2702	4.4948	4.1268	3.6643	3.6977	3.9808	3.6831	3.3040	2.2474	4.2835	3.3087	3.6685	4.7684	2.9757	4.3203	3.1803	3.7648	4.2682	4.2102
Ccdc92	_SATGQHS[Phospho (STY)]PAR_.2	Q8VDN4_S261_M1_Ccdc92	3.1743	3.3276	3.7106	4.5151	4.0129	4.2370	3.5282	3.8901	3.5873	3.3807	3.9616	3.2171	3.6953	3.2391	3.3054	3.5192	3.3874	3.7621	4.0611	4.4172
Ccdc92	_SLS[Phospho (STY)]APLHPEFEEVYR_.2	#N/A	3.4913	4.0318	3.8563	3.9643	3.7443	3.8930	3.5739	3.6652	3.5645	4.0812	3.5759	3.5878	3.7004	3.7934	3.3947	3.8359	3.6741	3.8403	3.8269	4.3040
Mical1	_VS[Phospho (STY)]PVPSPSQPAR_.2	Q8VDP3_S777_M1_Mical1	8.2482	8.1719	9.2002	9.0351	3.6530	8.1026	8.6222	8.7250	7.8651	8.7911	8.0445	7.3752	3.5543	7.1331	6.4917	7.4811	3.7717	4.1350	6.7591	7.6149
Ccar2	_EAAPDTGAEPS[Phospho (STY)]PEDSDPTYSSK_.2	Q8VDP4_S298_M1_Ccar2	6.7610	6.6235	3.4979	6.1091	5.2945	6.3284	5.8503	6.7484	4.2370	6.5953	6.2443	6.1951	6.6516	7.1597	5.6252	6.2834	7.0473	6.4212	6.4225	6.6084
Ccar2	_HDLS[Phospho (STY)]PYR_.2	Q8VDP4_S222_M1_Ccar2	7.6859	7.6614	6.8866	7.2990	7.1872	7.5153	7.3810	7.4977	7.3634	4.3094	7.6267	7.6582	7.9022	7.8843	7.1118	7.4590	7.6737	7.4346	3.3356	7.5607
Ccar2	_RPSS[Phospho (STY)]GGEEEEKAR_.2	#N/A	3.5421	3.4135	3.5412	4.1969	4.0213	4.0531	3.0297	4.2170	3.8657	3.6234	3.9294	3.2208	4.0312	3.5515	3.0768	3.3574	3.4819	4.0800	3.9916	3.3923
Ccar2	_S[Phospho (STY)]VAS[Phospho (STY)]NQSEM[Oxidation (M)]EYSSLQDM[Oxidation (M)]PK_.2	#N/A	4.1783	6.4233	5.8272	5.9832	4.1234	6.3843	6.1931	3.9917	6.4687	6.4973	4.6238	6.4690	4.2466	5.9494	5.5999	6.4365	6.8549	6.4043	6.6276	6.3266
Ptgr2	_VVGIC[Carbamidomethyl (C)]GTQEK_.2	#N/A	3.0957	4.6072	4.1331	4.4885	3.3027	4.9109	4.4603	3.3214	5.0945	3.9494	3.3929	4.4159	3.1266	4.1712	5.2578	3.9587	4.3195	4.3308	3.1290	4.8664
Sirt2	_LLDELTLEGVTR_.2	#N/A	7.9751	8.2901	8.3584	8.2858	7.7143	8.2230	7.8562	7.9687	8.0119	7.7294	7.5635	8.0904	7.8271	8.0489	7.8558	7.9245	8.1749	7.7016	8.0148	7.9527
Sirt2	_IFSEATPR_.2	#N/A	7.8554	7.8455	7.8980	8.1762	7.6661	7.9787	7.8424	8.0748	7.9722	7.9391	7.7149	7.9198	7.4864	7.7387	8.3837	7.9319	7.5808	7.9447	7.9446	8.1629
Sirt2	_NLFTQTLGLGSQK_.2	#N/A	7.6972	7.8271	7.8198	7.9062	7.6949	7.5439	7.5458	7.7764	7.6687	7.6945	7.4926	7.8030	7.7129	7.5390	7.6199	7.8307	7.4291	7.8582	7.4878	7.6267
Sirt2	_SPSTGLYANLEK_.2	#N/A	7.1616	7.2809	7.3642	7.3046	7.3111	7.2797	7.3239	7.0838	7.5484	6.9644	7.2089	7.1229	6.8193	7.2370	6.8743	7.2035	7.0184	6.8454	6.5838	6.9715
Sirt2	_ELEDLVR_.2	#N/A	4.9655	4.7949	5.1564	6.3374	5.0389	4.9322	5.8968	3.9750	5.7894	5.7077	5.6739	5.3784	5.5846	4.5603	5.8513	5.7983	3.5701	5.9428	6.3021	5.7792

Sirt2	_C[Carbamidomethyl (C)]EQC[Carbamidomethyl (C)]QSVVKPDIVFFGENLPSR_.3	#N/A	5.3215	4.8046	5.6793	5.2900	4.7779	5.4894	5.1152	5.3198	5.9761	5.0636	5.1600	4.8112	4.7778	5.8967	4.9628	4.9363	5.1980	4.5365	5.1954	4.7186
Sirt2	_VAGLEPQDLVEAHGTFYTSHC[Carbamidomethyl (C)]VNTSC[Carbamidomethyl (C)]R_.4	#N/A	6.3571	6.5677	7.2700	6.5181	6.0867	6.3905	5.9346	6.7268	6.0091	6.6183	6.3028	6.2453	6.4229	6.5287	5.7324	6.1234	6.0616	3.3054	6.4410	6.1653
Sirt2	_EHANIDAQSGSQAPNPSTTISPGK_.3	#N/A	5.3053	6.2723	5.9580	6.0778	5.0976	6.1253	5.8680	5.9670	6.0863	5.3147	5.5980	5.7394	5.7082	7.0004	5.8087	5.6761	5.3327	5.7337	5.8750	5.7532
Sirt2	_EHANIDAQSGSQAPNPSTTISPGKS[Phospho (STY)]PPPAK_.4	Q8VDQ8_S372_M1_Sirt2	7.4522	4.0663	7.2330	7.6393	3.5704	3.6038	4.0898	6.5175	4.0100	7.3697	3.4886	3.8869	4.1754	7.7068	3.6854	6.6133	5.9134	3.4662	3.7386	4.5902
Sirt2	_FFSC[Carbamidomethyl (C)]M[Oxidation (M)]QSDFSK_.2	#N/A	3.4113	4.8192	4.4488	4.1729	3.6183	2.9892	3.2751	3.6370	3.3342	3.6338	3.7085	3.6339	3.4422	4.4868	5.0247	3.7956	3.1343	4.0152	4.3142	4.1641
Kri1	_YVDEDNS[Phospho (STY)]DGETVDHR_.2	Q8VDQ9_S142_M1_Kri1	3.3388	6.1994	4.3763	4.1221	5.2355	3.5791	6.1845	4.8109	5.8013	3.1494	5.5985	5.5696	6.5076	5.7165	4.6886	5.7026	6.2832	5.6134	6.0323	5.1590
Caap1	_ILEGDNGLDS[Phospho (STY)]DMEEEADDGC[Carbamidomethyl (C)]K_.2	Q8VDY9_S183_M1_Caap1	3.2098	4.4776	3.7461	4.4796	4.0483	4.3559	3.7035	4.4110	3.0651	3.4161	4.0445	3.1816	3.2305	6.2419	2.7807	3.5547	3.4478	2.5215	4.0339	3.7001
Caap1	_SVNEILGLAESS[Phospho (STY)]PKEPK_.2	Q8VDY9_S307_M1_Caap1	5.0025	4.0041	5.0214	3.9479	3.5156	5.0713	4.8914	3.6697	5.0261	4.0677	3.6634	5.0812	5.7897	5.7528	4.9527	4.9613	3.6992	4.1251	3.7737	3.9988
Zdhhc5	_GLGS[Phospho (STY)]PEPGTTAPYLGR_.2	Q8VDZ4_S621_M1_Zdhhc5	9.4883	9.3485	8.9827	8.7301	8.1689	8.4334	8.7968	8.2267	8.1035	9.4261	8.5611	8.4905	9.1951	8.7792	2.9369	8.0787	8.8948	8.8346	8.1161	7.9909
Zdhhc5	_LSRGDS[Phospho (STY)]LKEPTSIADSSR_.3	#N/A	5.9546	6.3210	4.0662	5.2067	3.8362	5.2287	5.7520	5.8010	3.1362	4.6853	4.1157	5.0645	3.5664	5.9844	3.2413	5.3024	5.6055	5.7365	4.8195	4.7466
Zdhhc5	_SIGSASPGPGQPPLSS[Phospho (STY)]PTR_.2	Q8VDZ4_S694_M1_Zdhhc5	8.9344	3.4853	7.9851	3.7504	4.0407	4.0676	3.6975	4.0595	3.7566	3.2113	4.1310	3.2114	3.8647	9.1261	3.0327	3.1959	3.5567	8.5815	3.8918	7.2742
Zdhhc5	_VLC[Carbamidomethyl (C)]SS[Phospho (STY)]PAPR_.2	Q8VDZ4_S247_M1_Zdhhc5	4.2143	4.6056	3.7509	3.3699	2.9204	3.6872	4.1277	4.4400	4.1371	4.3317	3.0106	4.3318	4.2452	3.7889	4.1531	3.5764	5.4006	3.2123	3.5113	5.6625
Zdhhc5	_GDS[Phospho (STY)]LKEPTSIADSSR_.2	#N/A	9.1675	8.8726	8.8488	8.7959	8.2632	8.3033	8.3286	8.6780	8.3056	8.5819	8.4622	8.2241	8.7785	8.4490	6.7378	8.0422	8.5680	8.4239	8.2285	8.0770
Zdhhc5	_SEPS[Phospho (STY)]LEPESFRS[Phospho (STY)]PTFGK_.2	Q8VDZ4_S401_M2_Zdhhc5	3.6471	3.7438	3.9892	3.7946	3.8540	3.8874	4.0486	3.8140	3.9689	3.6508	4.1427	3.4471	4.1343	3.8314	3.2684	3.8148	3.5461	3.6408	3.9847	3.7984
Zdhhc5	_SFHFDPLSSGS[Phospho (STY)]R_.2	Q8VDZ4_S425_M1_Zdhhc5	8.2555	3.3114	8.2263	4.4988	7.0221	4.2532	3.5119	4.3918	7.5585	3.3969	3.9454	7.5727	7.1569	7.4632	3.0891	7.0667	3.4286	3.7783	4.0774	4.4010
Smg8	_LYEVAIDGKEEDLGS[Phospho (STY)]PTGELTSK_.3	Q8VE18_S469_M1_Smg8	4.7493	4.0044	4.5347	3.6910	3.1146	3.1480	5.0603	3.6988	3.8537	4.2191	3.3323	3.8938	4.0191	4.5218	3.4470	4.2838	5.0144	4.5167	3.2453	2.9879
Mios	_GFSQYGVSGSPT[Phospho (STY)]K_.2	Q8VE19_T768_M1_Mios	3.3515	9.4594	9.4184	4.3380	9.4765	9.0114	9.1291	10.1294	3.2068	9.0346	9.6392	9.5057	3.3723	9.7648	9.0430	9.3848	3.5896	9.4986	9.5092	8.8977
Mios	_QYTEDNDQKS[Phospho (STY)]PGNK_.2	Q8VE19_S444_M1_Mios	3.6862	4.8770	5.1402	4.6434	4.2010	4.6329	4.9695	4.0351	3.8562	3.8925	3.4498	5.1044	3.7609	4.7269	4.7347	4.9973	4.5654	4.3162	4.9847	2.7292
Gdap1l1	_DNIISDYDQIIDYVER_.2	#N/A	6.8594	6.5972	6.5573	6.2964	5.3307	6.0380	6.5280	6.8505	6.8242	6.4012	5.9237	6.0634	7.2995	6.7020	5.5219	6.2198	6.4822	6.2852	6.2635	6.4522
Gdap1l1	_LDHEEEPQLSEPYLSK_.3	#N/A	5.9697	5.7345	4.8436	4.7847	5.6486	6.2058	5.7799	6.3573	5.8539	6.3862	6.1956	5.3514	4.0759	5.0368	4.8953	4.6957	5.9064	6.1212	5.1314	5.4469
Gdap1l1	_LNLGEEVPVIIHR_.3	#N/A	3.5293	4.1955	4.2220	5.1281	2.7162	4.8126	4.5044	3.3475	4.8748	4.4462	4.6520	3.5598	3.9121	3.5702	4.0888	5.1078	4.5523	4.7143	5.0649	4.2864
Gdap1l1	_TFTGEHVVALM[Oxidation (M)]PEAGS[Phospho (STY)]PQHAR_.3	#N/A	4.8492	4.7988	3.4892	3.2378	3.7915	4.0156	4.5273	5.9114	3.8108	3.1592	4.1852	4.1858	3.9189	5.7689	3.3290	3.2978	3.1910	4.8580	4.5459	3.7088
Uba5	_KTEDSASEVTVEDSGESLEDLMAR_.3	#N/A	3.7587	4.2390	4.1175	5.0210	3.0964	3.3205	4.1705	3.3058	3.0029	3.9650	4.2028	3.9651	3.1110	5.7709	4.0218	4.1036	4.3038	4.3464	3.1447	3.8328
Ogfrl1	_IS[Phospho (STY)]PGASHVNSK_.2	#N/A	7.1958	7.1133	7.3737	7.0390	6.7629	7.4565	6.8225	6.8468	6.9014	7.3517	7.0617	6.9859	6.6638	7.0019	6.7728	6.8463	7.3502	7.2352	6.9230	6.6622
Ogfrl1	_EPGEEADKPS[Phospho (STY)]PEPGSGDPKPR_.3	Q8VE52_S382_M1_Ogfrl1	4.0161	5.4652	5.0195	5.8570	4.2231	5.2054	5.8560	5.7486	3.9389	4.5298	5.1386	4.8923	6.2988	5.9960	2.8504	3.3782	5.5308	5.5039	6.0525	3.5502
Ogfrl1	_AS[Phospho (STY)]PVPEDHAEAAGAEQGGESTEGNAKPK_.4	#N/A	4.0664	4.1099	3.7863	3.7996	4.0885	3.6283	3.4940	3.9183	3.5530	4.8456	3.9274	3.9993	4.4252	4.0478	3.3319	3.5949	3.1564	3.6632	4.3112	3.8314
Ogfrl1	_DSAADQSDS[Phospho (STY)]PPEK_.2	Q8VE52_S406_M1_Ogfrl1	4.1662	3.0943	3.2017	3.5232	3.5039	4.4895	4.0372	3.8666	4.0962	4.3725	2.9698	3.7261	4.1869	4.2435	3.6144	3.0103	4.4043	3.4289	3.0775	3.5844
Ogfrl1	_DSKIS[Phospho (STY)]PGASHVNSK_.3	#N/A	4.1130	3.2060	3.6496	3.3955	4.3200	4.3533	4.2290	3.9943	4.1492	3.9236	3.6278	2.9322	4.3146	3.3164	4.2543	3.4752	4.5320	3.3012	2.9498	3.2834
Taf12	_IAGTPGT[Phospho (STY)]GGRLSPENNQVLTK_.3	Q8VE65_T46_M1_Taf12	3.6691	5.8161	4.2054	4.6463	3.8163	3.5235	3.8914	6.1290	3.5243	4.5381	3.8125	3.5780	3.6898	4.1924	3.6357	4.0140	5.0844	3.9722	3.6208	4.1594
Pdcd10	_IPDEINDR_.2	#N/A	3.7773	5.5287	6.0302	4.8047	5.7745	5.9775	5.4693	5.1531	5.7949	5.5942	5.9064	5.0139	5.1527	5.6616	5.7520	5.1367	5.1561	5.3860	5.2129	5.7344
Fam114a2	_AETSLGIPS[Phospho (STY)]PTEISAEVK_.2	Q8VE88_S119_M1_Fam114a2	3.8827	9.5074	4.4190	9.1443	8.4281	3.2636	3.8178	3.5831	8.8428	9.3072	3.2166	9.0238	3.9035	4.4061	8.4382	4.2276	9.0652	9.0895	9.1044	8.9185
Ccdc115	_ADAQT[Phospho (STY)]PEEVGPSEASLR_.2	Q8VE99_T90_M1_Ccdc115	7.2833	7.3990	6.4157	6.6385	5.2778	6.2999	6.6922	6.8872	6.4783	7.3347	6.1346	6.1991	7.0386	7.8426	6.8500	6.4166	7.2848	6.5976	6.9480	6.9535
Grk5	_INSNHINSNSTGS[Phospho (STY)]S_.2	Q8VEB1_S589_M1_Grk5	3.7630	5.4959	3.2996	4.9271	4.6618	5.4655	5.5098	3.9887	3.6859	6.3682	5.7115	6.0309	6.0174	3.3377	5.6499	5.7745	5.5743	3.6635	5.8451	5.5777
MACIR	_S[Phospho (STY)]LDYLNLDK_.2	Q8VEB3_S167_M1_MACIR	3.7164	4.2864	5.3366	3.9730	3.0541	3.2782	2.9861	3.4168	3.0452	5.4811	3.4196	4.1758	3.1533	4.2398	4.0641	4.0613	3.9545	3.8787	4.8170	3.8751
Bloc1s4	_[Acetyl (Protein N-term)]MEEGPAVGTLS[Phospho (STY)]R_.2	Q8VED2_S11_M1_Bloc1s4	4.2609	3.2404	3.6153	3.5055	3.5986	3.8227	3.9424	4.3044	4.0015	4.4672	4.3759	2.9665	4.1096	3.6534	3.5196	3.1050	3.8016	3.3478	3.6469	3.3306
Lgalsl	_IQTLSAIDTIK_.2	#N/A	5.9322	6.0142	6.8399	6.3747	6.4208	6.2738	6.3287	6.4329	6.4666	6.4450	6.4040	6.8954	6.8800	6.7434	6.8021	6.5382	6.3693	6.1929	6.4654	6.4998
Lgalsl	_[Acetyl (Protein N-term)]AGS[Phospho (STY)]VADSDAVVK_.2	Q8VED9_S4_M1_Lgalsl	12.0369	11.9451	12.2434	12.4119	11.7425	11.9356	11.5695	11.8941	11.9659	12.2403	12.0494	12.2112	12.5393	12.5981	12.1853	12.4340	12.7462	11.9918	12.3608	12.4211
Lgalsl	_GEEQSAIPYFPFIPDQPFR_.2	#N/A	5.0002	4.8407	4.6563	4.1114	5.0535	4.5289	4.6411	4.9194	4.3979	5.1026	4.5878	4.5481	5.2422	5.7696	5.2722	5.2074	4.8821	5.3444	5.1049	5.3204
Lgalsl	_VFVDGHQLFDFYHR_.3	#N/A	3.8060	4.7372	3.3426	5.2873	4.8694	3.9771	4.6079	5.4732	4.4090	4.8506	4.1516	5.3729	4.0567	4.8419	4.5083	4.2579	4.6851	4.7237	4.5039	5.1194
Lgalsl	_LDDGHLNNS[Phospho (STY)]LGS[Phospho (STY)]PVQADVYFPR_.3	Q8VED9_S22_M2_Lgalsl	4.8501	4.4822	4.3956	3.6455	3.2851	4.8340	3.9497	3.4380	3.9279	4.4443	3.1770	4.4138	4.0690	9.4453	3.8701	4.8806	4.5282	8.5338	5.5880	3.6609
Lgalsl	_[Acetyl (Protein N-term)]AGSVADSDAVVKLDDGHLNNSLGS[Phospho (STY)]PVQADVYFPR_.4	#N/A	3.6656	4.2277	4.2019	4.0238	3.0033	3.4808	3.0369	3.3660	3.5209	4.5519	2.9995	4.2267	5.9202	4.1890	3.9649	4.0105	3.9037	3.9295	3.5781	4.1560
Rpe	l (C)]AEAGANM[Oxidation (M)]IVS[Phospho (STY)]GS[Phospho (STY)]AIM[Oxidation (M)]RS[Ph	Q8VEE0_S196_M3_Rpe	3.3054	4.6541	3.8417	4.3840	3.8076	3.8410	3.2405	4.5066	3.1607	3.4113	4.1401	3.0860	3.3261	3.8288	3.2658	2.9628	3.5434	4.2897	3.9383	3.7958
Gramd1a	_S[Phospho (STY)]SPSSSPSLR_.2	Q8VEF1_S11_M1_Gramd1a	4.3293	7.3246	3.6306	7.2426	3.3935	7.2262	7.3172	7.2897	7.3143	4.3564	3.9177	3.9358	4.0717	3.7160	3.7827	3.4489	7.5275	7.1688	3.6017	3.1664
Arl8a	_EIC[Carbamidomethyl (C)]C[Carbamidomethyl (C)]YSISC[Carbamidomethyl (C)]K_.2	#N/A	6.4120	5.3825	5.6648	5.6163	5.6384	5.3240	6.0271	5.5215	5.8428	6.1775	5.3051	4.3887	5.7251	5.4789	4.9640	4.9412	5.4934	5.2587	5.0323	5.3596
Arl8a	_M[Oxidation (M)]NLSAIQDR_.2	#N/A	6.3673	6.0893	6.6413	6.2576	6.1890	6.3277	6.5521	6.4582	6.0622	5.8334	5.6826	5.5608	5.9251	5.0496	5.6454	6.0328	5.7672	5.9168	6.2451	5.7687
Arl8a	_DLAGALDEK_.2	#N/A	3.7195	4.1998	4.1567	4.4649	3.0572	3.2813	4.1131	3.3450	3.0421	3.9258	3.4165	4.1728	3.1502	4.1948	4.0611	4.0644	4.3430	4.3072	3.1055	4.5314
Vps4a	_ENQSEGKGS[Phospho (STY)]DS[Phospho (STY)]DSEGDNPEK_.2	Q8VEJ9_S95_M2_Vps4a	4.0607	3.9421	4.5970	3.6287	3.2291	3.0857	4.1427	3.7611	3.9160	4.2670	3.3946	4.0838	4.0814	4.5841	3.7199	4.4056	3.8471	3.5344	3.1830	3.0502
Tmem30a	_EIEIDYTGTEPSS[Phospho (STY)]PC[Carbamidomethyl (C)]NK_.2	Q8VEK0_S89_M1_Tmem30a	3.8199	3.2266	5.0176	4.1636	4.3790	4.7003	3.5119	4.7523	4.5623	4.7192	4.0804	3.2294	3.6585	3.7601	4.1278	3.5712	3.6231	3.9613	3.8637	3.7949
Hnrnpu	_DLPEHAVLK_.2	#N/A	8.1482	8.3399	8.6842	8.5084	8.4861	8.3336	8.2174	8.2155	8.4632	8.1301	8.1057	8.2127	8.2220	8.2408	8.4900	8.5503	8.1734	8.1424	8.4547	8.5870
Hnrnpu	_SSGPTSLFAVTVAPPGAR_.2	#N/A	8.2339	8.3919	8.6984	8.2431	8.1041	8.1017	8.0030	8.1103	8.6327	8.2160	7.8704	8.0471	8.5363	8.3296	8.2915	8.4040	8.4989	8.2719	8.5407	8.1024
Hnrnpu	_DIDIHEVR_.2	#N/A	7.4284	7.8290	7.4043	7.5049	7.3906	7.3535	7.3174	7.3076	7.2429	7.5386	7.4499	7.4579	7.6910	7.7899	7.4176	7.5089	7.1250	7.3801	7.3643	7.3743
Hnrnpu	_LNTLLQR_.2	#N/A	6.8067	6.9861	7.4071	7.2174	7.3631	6.9372	6.6772	7.3516	6.6709	6.5262	7.1962	7.0841	7.3420	7.0173	7.5351	7.0132	7.2656	6.7250	7.3674	6.8710
Hnrnpu	_EKPYFPIPEDC[Carbamidomethyl (C)]TFIQNVPLEDR_.3	#N/A	6.9964	7.2207	6.4700	6.8848	6.6555	6.7306	6.9241	7.1475	6.6485	6.8514	6.7658	7.0384	7.0265	7.1577	6.9158	6.5128	7.0383	7.0398	6.5650	6.5780
Hnrnpu	_NFILDQTNVSAAAQR_.2	#N/A	6.5670	6.4310	6.6604	6.6790	6.1824	6.3810	6.1609	6.7178	6.0262	6.6811	6.5423	5.7128	6.7445	5.9011	6.3403	6.4011	6.4133	5.7105	6.0003	6.8256
Hnrnpu	_LLEQYKEESKK_.3	#N/A	5.4924	6.3249	5.9061	5.1787	5.8287	6.1606	6.2698	5.0161	5.5659	5.9100	5.8546	5.8551	5.9217	5.2344	6.6488	6.3759	5.1744	5.7192	6.3249	5.6881
Hnrnpu	Phospho (STY)]PQPPVEEEDEHFDDTVVC[Carbamidomethyl (C)]LDTYNC[Carbamidomethyl (C)]D	#N/A	9.4148	9.7139	9.6981	9.4536	8.7092	8.9200	9.3350	9.0455	9.1595	9.4608	8.9519	9.0360	9.7719	9.7270	8.5286	8.8343	9.4281	9.2904	9.2892	9.1643
Hnrnpu	hospho (STY)]PQPPVEEEDEHFDDTVVC[Carbamidomethyl (C)]LDTYNC[Carbamidomethyl (C)]DL	#N/A	3.8960	3.4231	6.5614	5.9586	4.1029	4.1363	4.4460	4.1217	5.9979	3.7066	3.8448	5.4294	4.5316	3.4707	2.9705	5.4962	3.6189	3.0842	6.2155	3.5005
Hnrnpu	_GYFEYIEENKYSR_.2	#N/A	5.5125	4.8308	4.3987	3.4406	4.6653	4.3984	3.9546	3.9492	4.7749	5.2533	5.1021	4.2511	4.2695	3.2713	4.5157	4.9369	4.9018	4.6623	4.3004	5.0732
Hnrnpu	_[Acetyl (Protein N-term)]SSS[Phospho (STY)]PVNVKK_.2	#N/A	12.8038	12.8573	12.8365	13.1210	12.5465	12.4730	12.4687	12.9146	12.6746	12.7641	12.3371	12.6979	13.3092	13.0154	12.5107	12.7955	12.8663	12.6119	12.6163	12.6314
Hnrnpu	_FIEIAAR_.2	#N/A	3.2309	3.2711	4.0273	4.4585	4.0695	4.2935	4.4057	3.1386	3.9088	3.9710	3.9051	3.8226	3.6388	3.7741	3.6296	5.3692	3.3308	5.5412	4.2972	5.2172
Hnrnpu	_[Acetyl (Protein N-term)]SSS[Phospho (STY)]PVNVK_.2	#N/A	3.9896	3.7537	3.9518	4.0189	3.3273	3.5514	3.5628	3.9248	3.6219	4.1959	3.9963	3.7461	3.7300	3.9899	3.8562	3.6183	4.1381	3.8612	3.3755	3.6672
Hnrnpu	_NGQDLGVAFK_.2	#N/A	4.3546	4.4653	3.8912	3.2296	3.0607	3.0941	3.9874	3.0795	3.9076	4.1652	3.3862	4.1914	4.0730	2.8217	4.1397	3.7168	4.0776	3.0719	3.3709	3.0418
Hnrnpu	_VSELKEELKK_.3	#N/A	4.3100	4.5099	3.8466	3.2742	3.0161	3.0494	2.7901	3.7973	3.9522	4.1206	3.4309	4.2360	4.1177	4.6203	1.8176	3.6721	4.3350	3.4982	3.1468	2.1340
Hnrnpu	_PAMEPGNGS[Phospho (STY)]LDLGGDAAGR_.2	#N/A	3.7231	4.2797	4.2594	3.9663	3.0608	3.2849	2.9794	3.4235	3.0385	3.9294	3.4129	4.1691	3.1465	6.4970	4.0574	4.0680	3.9612	4.3109	3.1091	3.8684
Hnrnpu	_LQAALDNEAGGRPAMEPGNGS[Phospho (STY)]LDLGGDAAGR_.3	#N/A	3.6911	4.3384	4.2274	4.0249	3.8698	3.2529	3.8791	3.3914	3.9382	3.8974	3.4715	4.2278	3.6636	4.2144	3.4563	4.0360	3.9291	3.9307	3.5792	4.2429
Hnrnpu	_LSASSLTM[Oxidation (M)]ESFAFLWAGGR_.2	#N/A	4.2276	3.7752	3.2631	3.4618	3.5654	4.4196	2.3599	3.9280	4.0829	3.9899	3.5616	3.6646	4.2484	3.2502	4.1881	3.5414	4.4657	3.3675	3.0161	3.2172
Rem2	_GHAGGQRPEPSS[Phospho (STY)]PDGPAPPTRR_.4	#N/A	4.1891	3.8157	3.2246	3.5213	3.5269	3.7509	4.0162	4.3782	4.0753	4.3955	3.8602	3.7051	4.1834	3.7271	3.5934	3.0332	4.1226	3.2760	3.5751	4.9883
Rem2	_QAS[Phospho (STY)]PLGTPTPEADTTLLK_.2	Q8VEL9_S27_M1_Rem2	8.2093	8.2906	8.2984	8.2105	6.8720	7.4840	7.7131	8.1293	7.7757	8.2355	8.0047	7.8043	8.3131	8.6017	6.9092	8.1018	7.9241	7.8283	7.3537	7.8824
Rem2	_GHAGGQRPEPSS[Phospho (STY)]PDGPAPPTR_.3	#N/A	8.2854	8.3425	3.6336	8.1328	7.4840	7.8942	7.8378	8.1097	4.0199	8.0638	8.0460	8.0371	8.1823	8.4633	3.5380	7.7635	7.9070	7.9303	7.4747	8.0089
Rem2	_RGS[Phospho (STY)]M[Oxidation (M)]PVPYK_.2	#N/A	4.0120	6.4594	6.0789	7.0095	2.9943	6.0161	3.8169	3.5822	4.5574	6.9241	5.8983	4.2470	3.9025	4.4052	4.7106	3.8337	6.1061	5.9653	6.1422	6.4265
Rem2	_SRS[Phospho (STY)]C[Carbamidomethyl (C)]HDLSVL_.2	Q8VEL9_S334_M1_Rem2	3.5750	4.1252	4.3253	4.1291	3.3113	3.3238	4.0269	3.5008	3.8898	4.3106	3.3834	3.9355	3.9978	4.3365	3.7927	4.1419	4.2750	3.9972	3.2086	3.8389
Rem2	_RRGS[Phospho (STY)]M[Oxidation (M)]PVPYK_.3	#N/A	4.1461	3.8567	3.1816	3.5433	3.4838	3.7079	4.0572	4.4192	4.1163	4.3524	2.9899	3.7461	4.2244	3.7681	3.6344	2.9902	3.9164	3.2330	3.5321	3.4454
Rem2	_GS[Phospho (STY)]MPVPYK_.2	#N/A	3.9448	3.3743	3.4814	3.6394	4.1517	4.1851	4.3972	4.1705	4.3174	3.7554	3.7961	3.1004	4.4829	3.5194	2.9218	3.3069	3.6677	3.4818	3.7808	3.1967
Slc25a3	_IQTQPGYANTLR_.2	#N/A	10.6381	10.2477	10.5513	10.1968	10.3335	10.3016	10.3493	10.6292	10.4494	10.0835	10.2186	10.1673	10.3658	9.6214	10.1682	9.8332	10.4800	10.2740	10.1061	9.9538
Slc25a3	_GSTASQVLQR_.2	#N/A	8.7597	8.6322	8.1714	8.3086	8.3206	8.4581	8.9258	9.0225	8.5501	8.2643	8.3511	8.3714	8.5609	8.2431	8.2173	8.2595	8.5572	8.7038	8.5331	8.3227
Slc25a3	_EEGLNAFYK_.2	#N/A	6.3973	5.1362	6.5676	6.0809	6.1716	6.5240	6.4487	3.6481	5.6470	6.2673	6.0881	5.6761	5.2457	5.4274	5.8863	5.1923	5.7454	6.2503	6.3567	6.6049
Slc25a3	_FGFYEVFK_.2	#N/A	5.4930	5.6727	5.5377	5.3200	5.0438	5.0153	5.5480	4.8149	5.2009	4.4975	5.2199	5.0501	5.2207	4.9144	3.9305	4.6519	5.0093	5.1355	5.0132	4.4525
Slc25a3	_GIFNGFSITLK_.2	#N/A	5.5699	5.2233	5.9291	5.0704	4.8822	5.3976	5.5048	3.9494	5.5558	4.3471	5.9652	4.0641	5.6144	4.7982	4.0736	4.9326	5.3366	3.4071	5.3392	5.1484
Slc25a3	_MYKEEGLNAFYK_.2	#N/A	3.6012	7.2203	7.5126	7.4418	5.5032	6.2185	7.1107	5.3882	5.3881	3.4118	6.4148	6.1132	3.3256	7.1149	5.5945	2.9633	6.1964	5.5403	5.4900	5.6623
Slc25a3	_YYALC[Carbamidomethyl (C)]GFGGVLSC[Carbamidomethyl (C)]GLTHTAVVPLDLVK_.3	#N/A	3.9438	3.3753	3.4804	3.6404	4.1507	4.1841	4.3982	4.1695	4.3184	3.7544	3.7970	3.1014	4.4838	3.5184	2.9227	3.3059	3.6667	3.4827	3.7818	3.4527
Rbm39	_YRS[Phospho (STY)]PYSGPK_.2	Q8VH51_S97_M1_Rbm39	8.7962	8.7133	8.3091	8.3151	8.1974	8.4669	8.5805	8.8036	8.3693	8.4673	7.9840	8.2193	8.7295	8.5048	7.4131	7.8766	8.7367	8.4867	8.3020	8.1238
Rbm39	_DKS[Phospho (STY)]PVREPIDNLTPEER_.3	#N/A	10.6359	10.3342	10.5451	10.2813	9.6341	10.3682	10.1001	10.1936	10.1221	10.4814	10.1055	9.9213	10.7141	10.6136	9.2823	9.9598	10.6203	10.0814	10.1743	9.7488
Rbm39	_S[Phospho (STY)]KS[Phospho (STY)]PFRK_.2	Q8VH51_S127_M2_Rbm39	9.7123	9.6892	10.0098	9.8631	9.2802	9.6979	9.6806	9.2795	9.6909	9.7632	9.5261	9.1227	9.4576	9.3554	8.5168	9.2163	9.8389	9.4150	9.6501	9.1069
Amot	_DTTVISHS[Phospho (STY)]PNTSYDTALEAR_.3	Q8VHG2_S693_M1_Amot	3.8578	3.7634	3.7696	6.4887	3.7746	5.7932	3.9758	5.4724	3.9366	3.9916	3.4779	3.5504	4.0357	3.6448	5.7728	6.3612	4.1284	5.8108	6.0779	6.3085
Agap3	_IEDLALQNQIR_.2	#N/A	6.3681	6.2774	5.8173	6.3402	6.0773	6.5633	6.4999	5.8519	5.8853	5.8741	5.7393	5.7120	5.7042	6.1486	6.2685	5.6562	6.4683	5.8511	5.9619	5.6712
Wasf3	_LSQSVHHGASSEGSLS[Phospho (STY)]PDTR_.3	Q8VHI6_S235_M1_Wasf3	3.7145	3.9148	4.1021	3.9944	3.6380	4.2102	4.2491	3.7553	4.1292	4.1426	4.0879	3.7028	4.0728	4.1181	3.5507	4.0396	4.0964	3.9673	3.4292	4.3559
Mark1	_SAAYNGPPAS[Phospho (STY)]PSHETGAFAHAR_.3	Q8VHJ5_S633_M1_Mark1	3.5167	9.1455	9.3543	8.8059	8.2342	8.0962	8.3020	3.5478	7.9369	3.7230	8.3622	8.5005	3.3530	4.3976	4.2639	8.0632	4.5459	8.6268	7.9413	7.9410
Mark1	_RGT[Phospho (STY)]STGIISK_.2	Q8VHJ5_T648_M1_Mark1	3.2529	3.2491	6.4338	4.4365	4.0914	3.8935	3.1879	4.4541	3.1082	6.7896	4.0876	3.1385	7.0692	3.7763	3.2133	3.5978	3.4909	4.3422	3.9908	3.7433
Mark1	_SRPSS[Phospho (STY)]DLNNSTLQSPAHLK_.3	Q8VHJ5_S394_M1_Mark1	5.8218	6.6493	7.3145	3.7039	3.6478	5.5293	7.1508	6.8613	4.0489	6.8967	3.8359	6.6367	6.5252	7.3595	3.4190	5.4190	6.2772	5.8281	3.8403	6.7185
Mark1	_RDPS[Phospho (STY)]EGEASGR_.2	Q8VHJ5_S666_M1_Mark1	6.2758	6.6169	6.4696	6.6750	6.5175	6.4024	6.4801	6.8868	5.2290	6.8917	3.7744	3.0298	7.3424	6.4596	2.9181	6.3842	3.2001	3.9493	6.9565	6.2167
Mark1	_KPPEFEGGESLS[Phospho (STY)]SGSLC[Carbamidomethyl (C)]QR_.3	#N/A	6.0364	6.9312	4.3071	6.7734	3.2779	6.4016	4.0340	3.4816	6.0025	6.4911	3.2611	3.9637	4.4956	4.0394	5.8217	4.1230	4.2367	3.8627	7.0224	6.2337
Mark1	_STAS[Phospho (STY)]PSNNVYSGGSMAR_.2	Q8VHJ5_S488_M1_Mark1	3.7395	5.4505	5.6732	3.8447	3.9465	3.9798	3.1016	3.9652	3.0218	3.5501	4.0013	3.3057	3.1873	3.3142	3.1270	3.1016	3.4624	4.4285	3.4147	3.6569
Mark1	_RPQANS[Phospho (STY)]VESEQKEEWGK_.3	Q8VHJ5_S444_M1_Mark1	4.0548	3.9480	4.5911	3.6346	3.3925	3.6166	4.1486	3.7551	4.2077	4.2611	3.0812	3.8375	4.3157	4.5781	3.7258	4.3997	4.2928	3.5403	3.1889	3.0443
Mark1	_SIS[Phospho (STY)]ANQK_.2	Q8VHJ5_S414_M1_Mark1	4.2209	4.5990	3.7575	3.3633	2.9270	3.6805	4.1211	4.4466	4.1437	4.0315	3.0172	4.3252	4.2518	3.7955	4.1465	3.5830	3.9438	3.2056	3.5047	3.4728
Caskin2	_SPS[Phospho (STY)]QES[Phospho (STY)]IGAR_.2	Q8VHK1_S696_M2_Caskin2	7.1498	7.6118	4.6034	3.6223	7.0986	3.0793	7.0245	3.7674	7.0233	7.7016	7.1565	3.8252	7.2209	4.5904	3.7135	7.1838	4.3051	7.1336	7.1636	7.5920
Caskin2	_T[Phospho (STY)]SPSLTPTR_.2	Q8VHK1_T840_M1_Caskin2	4.5799	4.1758	5.8423	3.8624	3.2860	3.3193	3.7621	3.5274	3.6823	4.3905	3.1609	4.0652	3.8477	4.3504	3.7875	3.9420	4.0651	3.7681	3.4167	4.3174
Setd7	_IAYVYPDQR_.2	#N/A	6.0724	6.8123	7.0955	6.8511	6.3464	6.0627	3.2179	6.1799	6.0158	5.9851	5.2811	6.1309	6.0851	6.2899	6.4866	6.1530	6.0602	5.7120	5.9516	6.1951
Setd7	_VYVADSLISSAGEGLFSK_.2	#N/A	4.7121	4.0298	4.3928	4.5482	4.4488	3.2942	4.4072	4.6677	4.6909	4.6914	3.4041	4.0563	5.2142	3.9143	3.6040	4.4129	5.0905	4.2377	4.0994	4.7235
Setd7	_SGPEAPEWYQVELK_.2	#N/A	5.6627	6.0282	6.0233	6.2444	6.3958	5.7237	4.0805	5.9970	5.7060	5.8186	5.6410	5.8670	4.2477	6.5707	5.3643	5.9705	3.9397	5.5628	4.8346	5.8276
Setd7	_AFQATQQK_.2	#N/A	4.2071	3.8979	4.2502	3.9755	3.0516	3.4325	2.9886	5.0241	4.3052	3.9202	3.9090	4.1784	3.7346	4.2373	4.1952	4.0588	3.9519	3.8812	5.3298	4.9637
Setd7	_AVEAEEELTVAYGYDHS[Phospho (STY)]PPGK_.3	Q8VHL1_S340_M1_Setd7	3.1662	3.6465	3.2092	3.9116	4.0048	4.2288	3.5363	3.8983	3.5954	3.3725	3.9698	3.3726	3.7035	3.2472	3.1135	3.5111	3.3955	3.7540	4.0530	3.5084
Ppp1r16b	_RAS[Phospho (STY)]LSDR_.2	Q8VHQ3_S350_M1_Ppp1r16b	4.1686	6.5624	3.2041	3.5208	3.5063	5.9142	5.2908	3.8690	4.0239	4.0489	3.5025	3.7236	5.2457	3.1911	4.1290	3.0127	4.4067	3.4265	3.0751	3.1581
Ppp1r16b	_EQS[Phospho (STY)]PQTLLELKR_.3	Q8VHQ3_S477_M1_Ppp1r16b	4.5786	3.4242	3.6141	3.1108	3.9163	4.1404	3.6247	4.2790	3.6838	5.7237	4.2663	3.3136	3.7919	4.8458	3.2019	3.4227	3.3158	4.4899	3.9646	3.0129
Rab40c	_[Acetyl (Protein N-term)]GTQGS[Phospho (STY)]PVKSYDYLLK_.2	Q8VHQ4_S6_M1_Rab40c	5.5521	5.6499	3.9622	4.2635	5.5863	3.7205	3.3610	5.1095	5.3586	3.2908	5.0987	2.9655	3.4466	3.9493	3.3863	3.7708	3.6640	5.1973	3.8178	5.2999
Gatad2b	_LQQQAALS[Phospho (STY)]PTTAPAVSSVSK_.2	Q8VHR5_S487_M1_Gatad2b	6.1675	6.9040	3.7849	3.3359	2.9545	4.0831	6.5366	7.2996	4.1712	5.4030	3.0447	6.4391	4.2792	7.7777	4.1190	3.6105	5.8154	3.1782	5.2249	3.5003
Gatad2b	_S[Phospho (STY)]LDPADERDDVLAKR_.3	#N/A	6.6032	7.2711	6.6201	6.7303	3.9787	6.6281	6.4247	4.3413	6.3900	7.2116	3.9959	6.7491	3.7295	7.4154	6.6566	6.9434	7.3099	6.6714	6.7288	7.2441
Gatad2b	_GRLT[Phospho (STY)]PSPDIIVLS[Phospho (STY)]DNEASSPR_.3	#N/A	3.7314	3.3650	4.1344	3.9569	3.8522	4.1887	3.6481	3.8667	3.7072	3.8539	3.5929	5.3925	3.8152	3.6388	3.3807	3.8293	4.8751	3.8267	3.8748	4.0484
Gatad2b	_VSS[Phospho (STY)]PLPS[Phospho (STY)]PSAM[Oxidation (M)]SDAANSQAAAK_.2	#N/A	5.7771	5.6125	4.5468	3.8949	5.5403	3.4465	4.7325	3.4649	3.6198	4.8848	3.8837	3.7163	3.7852	4.2879	3.7249	4.3637	4.9560	4.9022	3.5438	3.5394
Gatad2b	_LTPSPDIIVLS[Phospho (STY)]DNEASS[Phospho (STY)]PR_.2	#N/A	3.9748	4.1916	4.2494	3.7187	3.4189	3.4523	3.7137	3.4377	3.6339	3.7854	3.8753	3.9177	3.7993	4.6033	3.7390	4.0750	3.6977	3.7206	3.7740	3.7957
Gatad2b	_S[Phospho (STY)]LDPADERDDVLAK_.2	#N/A	3.5045	4.4983	4.0408	4.1849	4.3430	3.0663	3.1980	3.2048	3.2571	3.7108	3.6315	4.3878	7.9156	4.0278	4.2761	3.8494	3.7425	8.0502	3.7392	3.9948
Cacng8	_AGGGAGGS[Phospho (STY)]GGSGPSAILR_.2	Q8VHW2_S251_M1_Cacng8	7.3909	7.8416	7.3715	6.1255	5.7028	7.6188	6.7084	6.9788	7.1068	3.5136	6.8310	5.6669	7.0593	6.8286	4.9859	6.1835	7.3881	7.7794	7.4804	7.2908
Cacng8	_DAS[Phospho (STY)]PGGPGGPGFASTDISM[Oxidation (M)]YTLSR_.3	#N/A	5.0485	4.8334	4.0332	3.9732	3.8298	4.6013	4.8916	3.6090	3.7556	3.4335	4.1536	4.5120	4.9930	3.6660	3.2555	3.8587	3.5673	4.8055	5.0047	3.5256
Cacng8	_SSS[Phospho (STY)]RGS[Phospho (STY)]SEASPSR_.2	Q8VHW2_S277_M2_Cacng8	4.0230	4.3268	4.1083	3.6046	3.8907	3.7247	4.2541	3.8087	3.9636	4.0304	3.4422	4.2168	4.1290	3.9778	4.0669	3.9232	4.3463	3.5103	3.1589	3.9448
Satb2	_SGS[Phospho (STY)]PDVKGPPPVK_.2	Q8VI24_S20_M1_Satb2	8.0106	8.2693	4.2472	4.3744	3.4167	7.4721	3.0735	3.4355	3.1326	7.9899	7.5794	3.8354	3.2407	4.2853	7.2729	4.0728	4.4336	7.9595	3.0149	3.9626
Satb2	_ES[Phospho (STY)]SPPREEAPPPPPPTEDSC[Carbamidomethyl (C)]AK_.3	Q8VI24_S593_M1_Satb2	6.4607	4.2174	3.6026	4.1293	4.1330	5.1254	3.4212	3.5342	3.4170	3.3625	4.0916	3.2131	3.5508	3.6090	2.9921	3.6394	5.7471	4.0076	3.9702	4.1636
Satb2	_SES[Phospho (STY)]PC[Carbamidomethyl (C)]LRDS[Phospho (STY)]PDRR_.2	#N/A	3.6925	4.0358	3.8698	3.9317	3.8994	3.9327	4.0020	3.7695	3.9222	3.5030	4.1010	3.4054	4.0876	4.4895	3.2267	3.8552	4.8482	3.6813	4.0300	4.4970
Pxn	_YAHQQPPS[Phospho (STY)]PLPVYSSSAK_.3	Q8VI36_S83_M1_Pxn	5.9627	6.4006	3.6983	6.7191	6.1925	6.0177	5.9712	6.1696	5.8640	3.6888	6.1589	5.7046	6.5752	6.9048	5.9670	3.5150	6.1727	4.4768	6.0082	5.8309
Lrp4	_RGS[Phospho (STY)]LPDTGWK_.2	#N/A	4.4629	7.6022	3.9995	3.1214	3.1690	7.0440	3.8791	7.3844	4.3857	7.5091	3.2592	4.0832	6.6303	7.1369	3.9045	6.2644	7.5378	4.4645	6.6902	6.5497
Lrp4	_GS[Phospho (STY)]LPDTGWK_.2	#N/A	3.7826	4.2202	4.3189	3.9068	3.1204	3.3637	6.4221	3.4830	2.9790	3.9890	3.3533	4.1096	3.8034	4.3060	3.9979	4.1275	4.0207	6.2889	3.4611	5.4813
Pcnx3	_SQAPLDLSLSPDVSSEAS[Phospho (STY)]PAR_.2	Q8VI59_S1980_M1_Pcnx3	3.3175	4.0016	4.5215	4.2668	3.5244	3.0831	3.1812	3.5431	3.2403	3.7276	3.6147	3.7278	3.3483	4.3929	4.2592	3.8662	4.5412	4.1091	4.4081	4.0702
Nr3c2	_SSVSS[Phospho (STY)]PANLNNSR_.2	Q8VII8_S299_M1_Nr3c2	7.8219	3.4959	7.3417	6.9081	6.6808	7.5875	4.4420	7.6477	7.1194	7.2832	7.1276	7.1662	7.4210	7.6961	6.4836	6.8519	7.3593	7.4688	4.2376	6.7315
Nr3c2	_DAEYTYDQQNQQGS[Phospho (STY)]LSPAK_.2	Q8VII8_S127_M1_Nr3c2	4.9354	4.9010	4.7747	4.1311	3.1031	4.6279	4.2232	4.8344	4.2925	4.2076	3.9205	4.1491	5.2566	4.1700	3.9704	3.7592	4.8450	3.5852	3.4961	3.5164
Sfpq	_YGEPGEVFINK_.2	#N/A	7.0495	7.6393	7.6231	7.3783	7.7044	7.3897	7.4879	7.4068	7.0899	7.5021	7.2822	7.5623	7.6480	7.4046	7.2230	7.3073	7.8088	7.3497	7.3283	7.4907
Sfpq	_LFVGNLPADITEDEFKR_.3	#N/A	6.3896	6.4868	6.5895	6.5049	6.8663	6.8224	6.7387	6.1957	6.6479	7.0378	6.6037	6.9493	6.8806	7.1122	6.8561	6.9399	7.0903	6.8088	6.9120	7.0238
Sfpq	_FATHAAALSVR_.2	#N/A	5.6248	6.5508	6.0138	6.1623	6.5974	6.3468	5.8520	6.5508	5.1741	6.1247	5.9230	5.8156	5.8979	6.4787	6.5220	6.0816	6.1912	5.8475	6.0246	6.0605
Sfpq	_FGQGGAGPVGGQGPR_.2	#N/A	6.4628	7.0884	6.4941	6.3404	6.4118	6.6940	6.6510	6.6439	6.5502	6.7971	6.1636	6.7451	3.1547	6.7339	6.8656	6.1746	6.7059	6.7461	6.5930	6.9653
Sfpq	_PVIVEPLEQLDDEDGLPEK_.2	#N/A	3.6031	5.5591	5.3865	5.4499	4.7312	5.6817	5.7394	5.0128	5.0824	5.4555	5.9263	5.5752	5.1650	6.3647	5.5211	5.3991	5.7686	5.9466	3.9839	5.3575
Sfpq	_LFVGNLPADITEDEFK_.2	#N/A	3.8244	4.7977	4.3607	5.2088	4.8302	4.3632	4.4671	3.5247	3.6796	5.6255	4.6296	4.0679	3.8451	4.3477	5.2420	4.1693	4.0624	3.8910	4.6345	5.2158
Ciapin1	_EPVETAEVNNDK_.2	#N/A	5.3468	4.1565	5.6414	5.6302	5.6185	4.9736	4.8310	4.3555	5.1917	4.0526	6.4559	5.3206	4.7651	6.5069	5.3758	5.7570	5.5564	5.3602	6.0416	6.1359
Ciapin1	_EALS[Phospho (STY)]PEEVQSVQEHLGYHSDSLR_.3	Q8WTY4_S136_M1_Ciapin1	3.2929	5.8320	5.6527	4.2913	3.9201	5.4913	5.8594	5.9624	4.8515	5.9804	3.5901	6.0842	3.3238	6.3499	3.5737	5.2057	5.8650	5.8546	5.5364	5.2635
Wdr13	_YGPLS[Phospho (STY)]EPGSAR_.2	Q91V09_S70_M1_Wdr13	3.7650	9.2385	9.3107	9.3265	9.2163	9.1391	8.7474	9.4550	9.0205	9.6685	9.2657	9.0055	9.5288	9.2578	8.3910	9.0570	9.3954	8.8590	8.8092	8.9713
Wdr13	_LVVHEGSPVTSISAR_.3	#N/A	3.7114	4.2914	4.7187	4.3827	3.5407	3.3454	3.0804	4.6343	3.0502	3.9177	4.3185	4.1808	4.1288	5.6085	4.7667	4.0201	3.9668	4.3572	4.3221	4.6059
Wdr13	_LLWAGDDR_.2	#N/A	3.5475	4.4388	4.3208	4.0169	3.9718	3.8937	3.9498	5.3386	3.2139	4.6616	3.4265	3.5515	3.3793	4.5539	3.5773	3.1717	3.2704	3.6887	4.5873	4.3161
Wdr13	_VLALSFDAPGR_.2	#N/A	4.6052	4.2751	4.5807	3.9255	4.5107	7.0602	7.3951	3.7874	3.8967	3.5018	3.8405	3.6241	3.5742	7.4664	4.2703	4.0861	3.2662	4.1699	4.1823	4.2961
Wdr13	_[Acetyl (Protein N-term)]AAVWQQVLAVDAR_.2	#N/A	3.2628	3.2391	3.7991	4.4266	4.1014	4.3255	3.4397	4.4641	3.4988	3.4692	3.8731	3.1286	3.6068	3.7862	3.0169	3.6077	3.5009	4.3323	3.9809	3.7532
Acot7	_Y[Phospho (STY)]EAQKLER_.2	Q91V12_Y195_M1_Acot7	7.4172	7.5870	7.1661	7.2905	7.4547	7.3775	7.5626	7.7202	7.3463	7.5424	7.4082	7.7306	8.1019	7.5748	7.5768	7.5212	7.6100	7.6800	7.5886	7.2289
Acot7	_VLEVPPIVYLR_.2	#N/A	8.2503	8.0044	7.9188	8.1899	8.1050	8.3233	8.1878	7.6745	7.5637	7.7377	7.9426	7.8095	7.9170	7.6430	7.6528	7.7905	7.7741	8.0932	7.9739	8.0321
Acot7	_LM[Oxidation (M)]DEVAGIVAAR_.2	#N/A	7.3177	7.5059	7.7302	7.5113	7.2270	7.4197	7.4895	7.3901	7.5758	7.0695	7.0366	7.2143	7.2694	7.5418	7.2934	7.3244	7.3326	7.6335	7.4159	7.4783
Acot7	_SM[Oxidation (M)]EIEVLVDADPVVDNSQK_.2	#N/A	6.1484	5.4262	5.6937	4.8096	5.1591	5.1813	5.8755	4.3368	5.9416	5.8703	5.3935	5.1642	3.1563	3.6589	4.6081	5.7864	5.6853	6.1568	6.0272	4.9902
Acot7	_AASAFFTYVSLNQEGK_.2	#N/A	4.3134	5.2546	5.8140	4.7094	4.5174	5.3790	4.9147	3.7940	5.0465	5.5172	3.4275	4.5597	5.6651	5.0810	4.6299	3.6755	4.3316	4.0220	4.9234	5.3671
Acot7	_M[Oxidation (M)]IEEAGAIISTR_.2	#N/A	5.6980	6.1552	7.5585	4.9977	5.4571	5.9087	6.2614	5.6277	5.6958	5.6785	5.2941	5.4565	5.6982	5.0489	6.6295	6.1801	5.3298	6.0327	5.8122	6.3731
Acot7	_TNIVTASVDAINFHDK_.2	#N/A	6.0788	5.9558	5.9091	5.7177	5.5583	5.9523	5.8994	6.2931	6.0021	5.8772	5.7449	5.5849	5.9240	5.5939	5.3063	5.4114	5.9219	5.7353	5.9263	5.3058
Acot7	_M[Oxidation (M)]TFTSNK_.2	#N/A	4.2331	3.2125	3.6431	3.4777	3.5708	3.7949	3.9703	4.3322	4.0294	4.4394	2.9029	2.9386	4.1374	3.6812	3.5475	3.0771	3.8295	3.3200	3.6190	3.3585
Slc12a5	_EIQSITDESR_.2	#N/A	7.6515	7.8532	7.8236	7.5477	7.2395	7.6448	8.1973	7.9754	8.0521	7.4451	7.8655	7.6082	7.8701	7.4529	7.4711	7.2541	7.7911	7.9114	7.6105	7.3336
Slc12a5	_ENLWSSYLTK_.2	#N/A	7.6283	7.3791	7.0271	6.9418	7.1033	6.8864	7.2023	7.3947	6.9762	7.0192	7.1993	6.9145	7.0921	6.9113	6.4860	6.8411	7.5171	7.0696	7.2045	6.7100
Slc12a5	_LNEVIVNK_.2	#N/A	6.2942	6.2369	6.4284	6.0311	6.0396	6.5260	6.3054	6.5197	5.9588	6.2566	6.1527	5.7270	6.6221	5.5962	5.8187	6.0452	6.1915	6.1445	6.1952	5.3982
Slc12a5	_VDQDQNVVHPQLLSLTSQLK_.3	#N/A	5.1749	5.3608	4.4282	4.3728	4.5812	4.8850	5.0597	4.6182	5.4528	4.9694	5.1195	4.9304	5.1952	4.6223	4.0621	5.0823	5.3496	5.1656	4.6268	4.2135
Slc12a5	_LVLLNM[Oxidation (M)]PGPPR_.2	#N/A	7.3404	6.5304	6.2974	3.2905	6.5673	6.6222	6.3649	7.2115	6.6580	6.6823	6.5037	6.0880	6.9717	4.6924	6.1798	6.6721	6.8399	7.0210	6.7758	6.6153
Slc12a5	_GFC[Carbamidomethyl (C)]QVVISSNLR_.2	#N/A	6.0714	5.9919	6.2828	5.0892	5.5457	5.6280	6.6129	6.1889	6.2553	6.1236	5.9628	5.8866	5.6290	5.6971	4.8562	5.2452	6.0274	5.4751	5.5455	5.8701

Slc12a5	_NKGPS[Phospho (STY)]PVSSEGIK_.2	Q91V14_S1044_M1_Slc12a5	3.2340	3.7143	3.1414	3.9795	4.0726	4.2967	6.5366	3.8305	3.5276	3.4403	3.9020	3.4404	3.6356	3.1794	3.0457	3.5789	6.2193	3.8218	4.1208	4.3575
Slc12a5	_DLTTFLYHLR_.2	#N/A	5.5721	5.2890	5.1531	5.7966	5.2223	4.9147	5.7241	4.6781	5.4842	5.0945	4.4937	5.3222	5.6710	4.9745	3.7488	4.4260	4.8991	4.8561	5.1099	4.4575
Slc12a5	_HGFDVC[Carbamidomethyl (C)]AK_.2	#N/A	4.8740	5.2510	5.3595	4.1554	4.6137	4.8155	4.7727	5.1391	3.9611	3.7403	5.0720	4.9698	3.7802	3.9627	4.7303	3.7606	4.9272	5.0304	3.9733	5.0956
Slc12a5	_ESS[Phospho (STY)]PFINSTDTEK_.2	Q91V14_S49_M1_Slc12a5	10.5173	9.9271	10.0293	10.0623	9.5695	9.5691	9.8439	9.8640	9.4883	9.9050	9.6594	9.2456	9.7081	9.2378	8.1213	9.3737	9.9702	9.6939	9.2253	9.3309
Slc12a5	_EIQSITDES[Phospho (STY)]RGSIRR_.3	#N/A	5.5528	3.6174	6.0586	8.3233	3.8680	3.9791	4.2236	4.2796	6.2660	4.1036	6.7311	3.4400	4.3454	5.1919	3.3022	7.0845	3.5825	5.1937	3.9670	7.1700
Slc12a5	_EREIQSITDESRGS[Phospho (STY)]IR_.3	#N/A	7.6456	7.7520	5.8252	7.2828	3.9686	4.0019	3.0795	3.9874	2.9997	7.7947	3.9792	3.2835	3.1651	6.9383	3.1049	6.2923	3.4846	6.5436	6.0450	5.9883
Slc12a5	_EIQSITDESRGS[Phospho (STY)]IR_.2	#N/A	6.9032	6.7169	6.4775	6.9399	5.1243	5.5017	6.6741	5.1534	5.2512	7.0072	5.3306	5.7327	5.6801	5.8807	3.8296	5.8746	5.1055	5.9083	5.5788	5.8588
Slc12a5	_ESSPFINS[Phospho (STY)]TDTEKGR_.2	Q91V14_S54_M1_Slc12a5	3.4323	3.8868	4.4697	4.2156	3.6393	3.6726	3.4088	6.5997	3.3291	3.2429	4.3085	3.6129	3.4945	3.9972	3.4342	4.2953	3.7118	4.1213	3.7699	3.9642
Slc12a5	_GLTIVGSVLEGTFLDNHPQAQR_.3	#N/A	3.3509	4.2228	4.6539	4.7275	3.9632	3.8176	4.5280	3.6685	4.7900	4.8659	4.0782	3.6093	3.4737	4.5183	3.7578	4.3058	3.0271	2.9971	4.6743	5.0800
Slc12a5	_TLVMEQR_.2	#N/A	4.5393	5.4015	5.6149	5.8325	3.8771	4.6403	5.8233	3.0039	4.3946	3.6782	3.8733	3.3529	4.5601	5.6548	2.9989	3.3834	3.2766	3.0558	3.0183	3.5289
Slc12a5	_ITAEVEVVEM[Oxidation (M)]HESDISAYTYEK_.3	#N/A	6.0872	6.1040	4.4388	3.8265	5.2168	3.3634	3.7463	6.1255	4.5307	4.8006	4.5415	5.0116	3.8319	4.4563	3.7717	5.1850	5.0236	3.8788	4.4084	3.6244
Slc12a5	_NVSMFPGNPER_.2	#N/A	3.2039	3.5659	3.2898	3.8310	3.9601	4.4898	3.0880	3.9789	3.0082	3.5638	3.9877	3.2920	3.1736	3.3278	3.1133	3.1153	3.4761	3.6733	3.9724	3.0051
Slc12a5	_IFTVAQM[Oxidation (M)]DDNSIQM[Oxidation (M)]K_.2	#N/A	3.9717	3.3474	3.5083	3.6125	4.1787	4.2120	4.3703	4.1974	4.2905	3.7823	3.7691	3.0735	4.4559	3.4578	2.8948	3.3338	3.6946	3.1599	4.3093	3.4248
Nrbp2	_EDES[Phospho (STY)]EDESDILEESPC[Carbamidomethyl (C)]GR_.2	Q91V36_S20_M1_Nrbp2	3.3395	7.2966	6.8852	4.2447	3.5464	6.6811	7.0083	3.5652	6.7339	3.1501	2.9005	3.7057	3.5873	7.1180	3.5270	4.2024	3.0624	6.1177	4.3861	4.0923
Nrbp2	_AKT[Phospho (STY)]PT[Phospho (STY)]PEPFDSETR_.2	#N/A	8.9897	8.6008	7.7131	8.8321	3.5778	7.5810	8.2024	6.7556	7.4676	9.2536	7.0607	7.9489	3.9170	8.4176	5.6916	7.9279	7.7914	7.7869	7.6696	7.6066
Nrbp2	_AKT[Phospho (STY)]PT[Phospho (STY)]PEPFDSETRK_.3	#N/A	3.4860	4.7916	4.5234	4.0983	3.6929	4.0042	5.3616	3.7117	3.2754	6.6617	4.2549	3.5592	3.4408	3.9435	3.3806	4.3489	3.2089	4.1750	4.6332	3.9105
Rab14	_NLTNPNTVIILIGNK_.2	#N/A	6.5868	6.2785	6.5083	5.8050	6.3476	6.3739	5.9919	6.0125	6.4146	6.1336	5.9810	5.6732	6.4697	4.7447	5.7322	5.9191	6.0917	6.4732	6.2033	6.3248
Rab14	_IYQNIQDGSLDLNAAESGVQHKPSAPQGGR_.4	#N/A	4.3458	3.6570	4.9371	3.3435	4.0941	4.9765	5.4930	4.0462	5.9101	4.5522	5.1629	4.0939	4.0246	5.1910	4.3469	3.1899	3.0831	4.9231	4.8697	4.1358
Rab14	_STYNHLSSWLTDAR_.3	#N/A	5.9828	5.7904	5.6852	5.0867	5.4253	5.6603	5.5082	5.9581	5.2583	5.7515	5.5384	5.0788	5.5248	5.6410	4.5991	4.8520	5.6581	5.8458	4.1327	5.1038
Rab14	_QFAEENGLLFLEASAK_.2	#N/A	3.8376	4.4422	4.4754	4.4012	4.0741	3.5857	3.1419	3.8293	3.4159	3.7670	4.3954	4.3316	3.5814	4.0840	4.2199	3.9055	3.7987	4.0345	3.6831	3.9704
Rab14	_FM[Oxidation (M)]ADC[Carbamidomethyl (C)]PHTIGVEFGTR_.3	#N/A	3.0948	4.9912	4.8871	4.5945	3.9334	4.1575	3.6077	4.2961	3.6668	3.3012	4.0411	3.2966	3.7748	3.6182	3.1849	3.4397	3.3329	3.6826	3.9816	2.9959
Rab14	_TGENVEDAFLEAAKK_.3	#N/A	3.9914	4.0114	2.1568	3.6980	3.3291	1.6308	4.2119	3.6918	4.2710	4.1977	3.1446	3.9008	4.0121	4.5148	3.7891	4.3363	4.2295	3.6037	2.2276	2.9810
Sfxn3	_ELQVGIPVTDEAGQR_.2	#N/A	9.5570	9.3481	9.3716	9.3430	9.0067	9.1662	9.3182	9.5872	9.4233	9.1641	9.2412	8.9396	9.4066	9.0640	8.5514	9.0797	9.5272	9.2099	9.0927	9.0331
Sfxn3	_NLLLSGEQLEASR_.2	#N/A	8.2298	7.9260	7.7536	7.8769	7.5130	7.8330	8.1058	8.0965	7.7293	7.9382	7.7666	7.7775	7.8511	7.9310	7.3628	7.7045	7.8825	8.1009	7.5564	7.6557
Sfxn3	_NIVQNYR_.2	#N/A	7.9378	7.7140	7.2450	7.6714	7.6683	7.4242	8.1406	7.5685	7.8063	7.2664	7.6391	7.8293	7.8188	7.4086	7.8151	7.2935	7.4376	7.8129	7.5905	7.3738
Sfxn3	_HFFTVTDPR_.2	#N/A	7.1239	7.1079	6.3909	6.9106	6.7557	6.7110	6.8956	7.0224	6.6050	6.9786	6.7312	6.9220	5.8994	6.1953	6.3050	6.7323	6.8674	6.7394	6.6908	6.5884
Sfxn3	_[Acetyl (Protein N-term)]GDLPLNINIQEPR_.2	#N/A	6.7449	6.5803	6.5871	6.7662	6.5396	6.3887	6.7461	6.7963	6.5557	6.4154	6.6239	5.9517	5.4235	5.7140	6.7561	6.7679	6.9187	6.9997	6.1631	5.7358
Sfxn3	_SSIHVTR_.2	#N/A	5.9628	6.2060	4.7452	6.1143	5.5773	5.7430	6.2130	4.9077	5.6560	6.1679	6.2395	5.6327	5.4836	5.7858	5.8274	5.6126	5.8310	5.9709	5.5903	5.5300
Sfxn3	_AGVATPGLTEDQLWR_.2	#N/A	6.7073	6.7445	7.0190	6.7632	6.4331	6.5188	6.3187	6.7395	6.7424	5.6920	6.1880	6.1890	6.7042	6.7635	5.3598	5.5133	6.5413	6.3858	6.1339	5.3030
Sfxn3	_QGIFQVVISR_.2	#N/A	5.1359	5.0035	4.1599	4.4133	4.4399	4.6279	4.7991	5.0197	4.8586	4.5093	5.0125	3.5604	4.2974	3.6982	3.4487	4.1541	4.5445	5.0731	4.2836	4.0651
Sfxn3	_YVYDSAFHPDTGEK_.2	#N/A	7.1953	6.8287	6.8677	6.6970	6.6104	6.8377	7.0571	7.1061	7.0121	6.8310	6.9705	6.7996	7.2155	6.4256	6.1619	6.6397	7.1552	6.5459	6.6758	6.9557
Sfxn3	_AQIQAQNPSIDVVYYNK_.2	#N/A	5.8828	5.5690	5.8330	5.5709	5.3035	5.4482	6.1669	5.8562	5.4141	5.3424	5.5338	5.4706	5.9518	5.9884	5.0548	5.7036	5.9100	6.0447	5.5766	5.3294
Sfxn3	_SGDAPITVQQLGTAYVSATTGAVATALGLK_.3	#N/A	3.7998	4.1973	3.6314	3.6942	4.0970	3.1848	3.4786	4.2670	4.3721	2.8415	3.8508	3.4203	4.5376	4.3399	2.9765	3.2522	3.6130	2.5741	3.5023	2.3689
Acly	_TAS[Phospho (STY)]FSESR_.2	#N/A	12.1113	12.1306	11.9902	12.3101	11.4871	11.4532	11.8650	11.4327	11.5248	12.2580	11.7076	11.5594	11.7173	11.8985	10.1206	11.4914	11.4373	11.5996	11.5749	11.4736
Acly	_TAS[Phospho (STY)]FSESRADEVAPAKK_.3	#N/A	8.2237	8.4255	8.2112	8.4779	7.7526	7.2316	7.8638	8.3933	4.1932	8.3448	7.5356	7.9929	8.6331	8.2513	7.1800	8.1814	7.9516	7.7301	7.8716	7.8660
Acly	_EAGVFVPR_.2	#N/A	6.4379	6.3992	6.2563	6.6564	6.2469	6.8334	6.7293	6.5954	6.5384	6.2292	6.6150	6.6298	6.1840	6.4318	6.4187	6.0277	6.1527	6.9724	6.4081	6.5186
Acly	_TIAIIAEGIPEALTR_.2	#N/A	5.7968	6.0371	6.4488	5.7597	5.8671	5.8209	6.2149	6.2598	5.5674	5.6399	5.8728	5.9186	6.0586	6.1965	5.4821	6.0760	5.9439	5.8962	6.0498	5.8839
Acly	_LNTEDIKR_.2	#N/A	5.6482	6.0228	6.3588	5.1149	3.1033	6.0054	5.9756	6.0154	5.7426	5.6734	5.4896	5.5881	5.6086	5.9753	6.0619	3.7594	5.8464	5.8181	5.5225	5.4427
Acly	_DLVSSLTSGLLTIGDR_.2	#N/A	4.1374	2.6398	4.8654	3.8124	3.7542	3.9954	4.7548	4.4503	4.1418	4.0833	4.1058	3.3970	4.6226	3.2179	4.3328	4.2219	4.0294	3.7957	3.1968	4.1730
Acly	_DGVYILDLAAK_.2	#N/A	4.6810	4.3375	4.8400	4.2215	4.6265	5.1275	4.7864	3.8885	4.9487	4.7433	4.3691	4.4406	4.2089	4.9827	4.1486	4.9572	4.4262	4.4585	3.0556	4.6645
Acly	_TAS[Phospho (STY)]FSESRADEVAPAK_.2	#N/A	4.0169	3.9859	4.5532	3.6725	3.3546	3.5787	4.1865	3.7172	4.2456	7.5698	3.1191	3.8754	4.3536	4.5402	3.7637	4.3618	4.2549	3.5782	3.2268	3.0064
Acly	_LGLVGVNLSLDGVK_.2	#N/A	5.6203	4.1121	5.9564	5.2557	3.4139	5.3321	5.9308	3.4327	5.4853	3.8381	5.6166	4.8360	3.2379	4.2825	5.3061	4.0699	5.4023	5.3091	3.0178	3.9597
Acly	_SINNPDMR_.2	#N/A	4.1942	3.7901	3.2482	3.4767	2.9003	4.4345	4.1478	3.9131	4.0680	4.0048	3.5466	4.3518	4.2334	3.2353	4.1732	3.5563	4.4508	3.3824	3.0310	3.2023
Acly	_SAYDSTM[Oxidation (M)]ETM[Oxidation (M)]NYAQIR_.2	#N/A	3.8999	4.1029	4.4362	3.7895	3.2376	3.2465	3.8350	3.6003	3.7552	4.3176	3.2338	3.9924	3.9206	4.4233	3.8603	4.2448	4.1379	3.6952	3.3438	4.3903
Ppp1r14a	_GPGGS[Phospho (STY)]PSGLQK_.2	#N/A	10.3759	10.7799	11.0175	10.6698	10.2411	10.5382	10.1379	10.6916	10.7521	10.3435	10.2344	10.6386	10.7079	10.8842	10.9192	10.2941	10.9076	10.1708	10.2316	10.4590
Ppp1r14a	_QPGFPQPSPSDDPS[Phospho (STY)]LSPRQDR_.3	Q91VC7_S134_M1_Ppp1r14a	3.2230	3.2789	3.7593	3.9685	4.0616	5.9718	3.4795	3.8414	3.5386	3.4294	3.9129	3.1684	3.6466	3.1904	3.0567	3.5679	3.3387	3.8108	4.1098	4.3685
Ppp1r14a	_QPGFPQPSPSDDPSLS[Phospho (STY)]PR_.2	Q91VC7_S136_M1_Ppp1r14a	9.6126	9.6231	9.7851	9.5691	3.6774	9.4678	8.9266	9.0908	9.4315	9.6023	9.2641	9.3717	3.8796	9.8497	9.0106	9.4610	9.8071	9.0221	9.1461	9.4389
Ppp1r14a	_ARGPGGS[Phospho (STY)]PSGLQK_.2	#N/A	9.1310	9.4898	9.3005	4.5186	4.0093	8.8710	8.9116	9.6003	9.1939	3.3771	8.8035	9.4485	9.7610	9.5819	9.6170	9.1529	9.6046	3.7585	8.8074	9.1331
Ppp1r14a	_GPGGS[Phospho (STY)]PSGLQKR_.2	#N/A	7.6474	8.3049	3.1628	3.9580	7.7688	3.8665	3.2149	3.8519	8.0266	7.4904	4.1146	8.2669	8.5741	7.9695	8.4630	2.9883	3.3491	7.7594	7.3006	7.5992
Ndufs1	_VAVTPPGLAR_.2	#N/A	9.0232	9.2309	9.0247	9.0384	9.1904	8.7833	9.1307	9.1321	8.9929	8.8818	8.8182	9.0951	9.0322	9.0024	9.3686	8.8351	8.8066	9.0203	8.9080	8.9637
Ndufs1	_FEAPLFNAR_.2	#N/A	8.6594	8.8572	8.6207	8.9536	8.9307	8.7345	8.9002	9.1461	8.4125	8.7378	8.8564	9.0471	8.7362	8.9109	8.9823	8.8248	8.8460	9.0448	8.7744	8.8661
Ndufs1	_ILQDIASGR_.2	#N/A	8.3681	8.3965	8.2999	8.1272	8.5084	8.3760	8.4172	8.7858	8.4561	8.1665	8.2790	8.5318	8.8698	8.3287	8.6667	8.2816	8.3099	8.6576	8.4228	8.2116
Ndufs1	_FASEIAGVDDLGTTGR_.2	#N/A	8.0199	8.3134	7.8913	8.0820	7.6385	7.9730	8.0879	7.9292	8.0164	8.1879	7.9109	7.9870	8.6336	8.5725	8.2076	7.8342	8.3029	8.0194	7.9972	7.8635
Ndufs1	_GWNILTNSEK_.2	#N/A	7.9881	7.6703	7.9953	8.0968	7.9132	7.6975	7.9187	8.1239	7.7316	7.9647	7.8858	7.9325	7.9962	7.9319	7.8265	8.0768	8.2783	8.0102	8.0177	7.8693
Ndufs1	_AVTEGAQAVEEPSIC[Carbamidomethyl (C)]_.2	#N/A	7.1223	7.6392	7.7652	7.7219	7.0097	7.3523	7.1997	7.0180	7.3818	7.0481	7.2926	7.4027	7.3365	7.5448	7.3974	7.2885	7.7881	7.3036	7.2970	7.4597
Ndufs1	_VALIGSPVDLTYR_.2	#N/A	7.9026	7.4097	7.0305	7.8394	6.4875	7.1728	7.7504	7.6481	7.5041	6.8069	7.1499	7.8037	7.8711	7.8275	7.3497	7.1427	7.5653	7.8308	7.5157	7.2096
Ndufs1	_SATYVNTEGR_.2	#N/A	7.3704	7.2198	6.8387	7.3385	7.0082	7.0241	7.2233	7.3030	7.3863	6.9517	7.1465	7.2712	7.2455	7.5155	7.5829	7.3545	7.3487	7.3431	7.2618	7.2986
Ndufs1	_GNDM[Oxidation (M)]QVGTYIEK_.2	#N/A	7.6307	6.4643	6.5988	6.9437	7.2541	7.3237	6.4448	7.6130	7.2496	7.4061	7.1928	7.2911	7.1645	6.7851	7.1136	7.5105	7.3364	7.0244	7.1473	7.7050
Ndufs1	_YDHLGDSPK_.2	#N/A	7.2631	7.3267	7.5127	6.4944	6.8968	7.1144	7.3325	6.9511	7.1875	6.7500	7.1431	7.1845	6.7627	7.4324	7.6475	7.1986	7.2177	7.3797	6.9803	7.1069
Ndufs1	_ALSEIAGITLPYDTLDQVR_.2	#N/A	7.0436	7.0053	6.9769	7.2854	6.6287	7.2822	7.3064	7.0194	7.1915	7.1794	7.2375	7.2831	6.8788	7.5758	7.2064	7.4316	7.2407	7.3134	7.3850	7.1781
Ndufs1	_LSVAGNC[Carbamidomethyl (C)]R_.2	#N/A	6.9323	7.2098	6.9354	7.0658	7.0255	6.9224	7.3929	7.1201	7.0261	6.8184	6.7994	7.1032	7.0244	7.2299	7.7610	6.7480	7.2804	7.3142	7.3856	6.9692
Ndufs1	_GLLTYTSWEDALSR_.2	#N/A	6.5870	6.5370	7.4861	6.0782	6.7302	6.3680	6.1939	6.8749	6.4435	6.7186	6.4337	6.3783	6.9666	6.7900	6.8859	6.7942	6.9810	5.9186	7.0718	6.6851
Ndufs1	_SWLHNDLK_.2	#N/A	6.3503	6.5725	6.7529	6.5631	6.7842	6.3043	6.6318	6.4719	6.3629	6.2853	6.4259	6.7446	6.4053	6.5083	6.9787	6.6122	5.8367	6.9312	6.4830	6.1827
Ndufs1	_HSFC[Carbamidomethyl (C)]EVLK_.2	#N/A	6.4834	6.6420	6.4702	6.5408	6.8995	6.2932	5.8135	6.2251	5.6702	6.1347	6.4806	6.3045	6.0696	6.5756	6.6164	6.2260	6.1073	6.2124	6.4892	6.1547
Ndufs1	_IASQVAALDLGYKPGVEAIR_.3	#N/A	5.2876	5.1676	5.7376	5.3966	5.3982	5.4888	5.7181	5.5060	5.4446	5.5188	5.7533	5.6538	5.6505	6.0787	5.4690	5.3151	5.2351	5.6538	5.4793	5.6593
Ndufs1	_M[Oxidation (M)]LFLLGADGGC[Carbamidomethyl (C)]ITR_.2	#N/A	5.6085	5.5040	6.4440	6.3678	5.4730	5.4376	5.8136	5.6465	5.3350	5.5392	6.2202	5.0065	5.5955	5.9689	5.7334	5.4817	5.4670	4.7303	5.6926	5.5655
Ndufs1	_VTTGVAAEWK_.2	#N/A	3.5998	3.7193	6.7393	6.2069	3.8067	6.2128	6.6103	4.5076	6.4190	5.3902	6.1472	5.8002	3.3271	6.7841	6.5023	6.0875	6.5304	6.1436	6.2001	6.5471
Ndufs1	_MC[Carbamidomethyl (C)]LVEIEK_.2	#N/A	3.6678	5.7006	5.9060	5.8435	4.2926	5.5555	5.4732	3.3681	4.7850	4.5498	5.8785	4.8187	3.6885	6.6697	4.2793	4.1013	5.2467	4.3399	4.4851	4.9377
Ndufs1	_DFYM[Oxidation (M)]TDSISR_.2	#N/A	6.4339	4.7561	7.0826	6.8265	6.4806	6.4325	6.5677	6.8042	6.5523	6.6617	6.6817	6.6264	6.5965	6.0256	6.4562	6.6437	6.6143	6.5289	6.3582	6.9086
Ndufs1	_LTEPMVR_.2	#N/A	4.0213	4.0547	4.4125	5.8706	4.2282	4.2940	4.6356	4.0861	3.7249	3.8318	4.6926	3.5066	4.4064	5.0056	4.6650	3.3834	3.1229	3.2094	2.8514	3.5194
Ndufs1	_NRLEEVSPNLVR_.3	#N/A	5.7184	6.2603	6.1501	6.8036	6.5620	6.4206	6.3869	6.3936	6.2326	6.7213	6.8201	6.7571	6.9132	6.3774	6.9381	6.9978	6.3641	6.6449	6.8961	5.9794
Ndufs1	_M[Oxidation (M)]HEDINEEWISDK_.3	#N/A	3.2254	3.2766	3.7617	4.4640	4.0640	3.9210	3.1605	4.4266	4.4441	3.7577	3.5899	3.4935	3.2461	3.7488	3.1858	4.3691	3.4635	4.3756	4.3137	4.0962
Ndufs1	_VAGM[Oxidation (M)]LQNFEGNAVAAIAGGLVDAEALVALK_.3	#N/A	3.4198	4.5830	3.9561	4.2696	4.2583	2.9816	3.2827	1.1165	3.3418	3.6261	3.7162	2.9716	3.4499	4.4945	2.8599	3.7647	3.1419	4.0075	4.3066	4.1718
Ndufs1	_SNYLLNTTIAGVEEADVVLLVGTNPR_.3	#N/A	4.3192	3.9150	3.1232	3.6016	3.0253	3.0586	4.0228	3.7881	3.9430	4.1298	3.4217	3.8045	4.1085	4.6111	4.0482	3.6813	4.3258	3.5073	3.1559	3.0773
Rnh1	_[Acetyl (Protein N-term)]S[Phospho (STY)]LDIQC[Carbamidomethyl (C)]EQLSDAR_.2	Q91VI7_S2_M1_Rnh1	8.9469	9.9589	10.3759	9.8125	2.9548	9.3169	9.3742	3.4474	9.4550	9.9082	7.4925	8.8187	9.2098	10.1021	9.1536	9.5361	9.2763	9.2947	9.7164	9.6429
Rnh1	_ELSLASNELKDEGAR_.3	#N/A	3.2833	3.8723	3.5834	4.5569	4.1229	4.6346	4.2286	4.4845	5.2115	5.5606	5.4804	4.8337	3.5864	4.0726	5.1783	4.8287	3.5213	4.2585	5.0867	5.4174
Rnh1	_LLC[Carbamidomethyl (C)]EGLQDPQC[Carbamidomethyl (C)]R_.2	#N/A	3.0889	4.2302	5.1962	4.4953	3.2959	5.6552	5.8256	6.4739	3.6725	4.4004	3.1511	4.7590	3.1198	5.7245	6.0175	3.9519	4.3127	5.4862	5.0333	3.8417
Itm2c	_AAASGPASASAPAAEILLT[Phospho (STY)]PAR_.2	Q91VK4_T39_M1_Itm2c	5.0244	5.1132	4.8067	3.7150	3.8559	3.8049	4.7138	5.7821	3.7443	3.2237	4.1186	4.2469	3.8523	5.5109	3.2624	3.3622	3.2554	4.0232	3.9041	3.0734
Terf2ip	_YLLGNAPVS[Phospho (STY)]PSSQK_.2	Q91VL8_S200_M1_Terf2ip	8.6681	8.8381	8.6095	3.6013	8.6001	8.3420	4.0231	3.7884	8.2693	4.1295	8.5407	4.2272	9.1508	9.0353	8.1234	8.8747	4.3261	3.5071	3.1556	8.7237
Zbtb1	_LST[Phospho (STY)]PKER_.2	Q91VL9_T203_M1_Zbtb1	13.2071	13.3526	13.3052	13.3134	13.0184	13.0081	13.0580	13.2120	12.8724	13.5246	12.9191	13.1249	13.5920	13.6379	12.6964	13.3454	13.2154	13.1809	13.2611	13.3479
Rbmxl1	_DVYLS[Phospho (STY)]PR_.2	#N/A	8.7443	4.0801	9.7292	10.4890	8.7619	3.2237	10.4947	3.6231	3.7780	11.0885	3.2566	3.9696	3.9434	4.4460	10.4206	4.2676	7.5287	3.6724	3.3210	10.9602
Rbmxl1	_RDVYLS[Phospho (STY)]PR_.2	#N/A	7.8783	7.5728	7.3840	3.6384	7.2678	7.8746	7.6453	4.1715	7.4810	7.7029	4.2431	7.5125	3.9767	3.5205	7.3765	7.5584	7.8061	7.7139	7.8981	7.8925
Rbmxl1	_[Acetyl (Protein N-term)]VEADRPGK_.2	#N/A	6.5850	7.0009	7.1126	6.8103	6.8703	6.5694	6.8679	7.0527	7.0058	6.7825	6.9644	6.9705	6.5395	7.0917	7.3953	7.0213	6.7238	6.7129	7.1903	7.2787
Rbmxl1	_LFIGGLNTETNEK_.2	#N/A	5.5817	6.1134	6.4447	5.9717	5.9922	5.9123	5.7128	6.3630	5.4845	5.9179	5.8761	5.8773	5.9402	6.8486	6.1528	6.2111	5.7854	5.7998	5.6449	5.9406
Rbmxl1	_ST[Phospho (STY)]PSGPVR_.2	#N/A	3.4354	6.6055	4.2436	5.9106	3.9928	3.3755	3.3382	3.6433	3.3261	3.4576	4.0349	3.3201	3.4632	4.4931	3.1677	4.0621	3.1406	4.0090	6.3990	6.6892
Rbmxl1	_GFAFVTFESPADAK_.2	#N/A	5.2426	5.6007	5.9783	5.5335	5.6479	5.0184	5.1376	5.4983	6.0555	5.2434	5.7981	5.0536	6.3454	6.1613	6.2021	6.1652	5.6202	5.5987	5.8889	5.6507
Rbmxl1	_VEQATKPS[Phospho (STY)]FESGR_.2	Q91VM5_S88_M1_Rbmxl1	3.7745	6.1606	4.3108	3.9149	3.1122	5.9949	5.4183	3.4748	5.7680	5.9902	6.1512	5.3678	6.7118	4.1398	6.0567	6.1410	4.0125	4.3622	3.1604	3.8171
Rbmxl1	_DVYLS[Phospho (STY)]PRDDGYSTK_.2	#N/A	9.6034	9.6722	9.0483	9.5612	8.9308	9.1963	9.1836	9.6336	9.3882	9.5066	9.0534	9.7392	10.5016	10.0391	9.4541	9.7319	10.0430	9.7270	9.4732	9.7094
Rbmxl1	_REPLPSR_.2	#N/A	3.9827	4.7216	4.1019	4.5861	5.0063	4.4124	5.2319	4.8366	4.4506	4.2892	3.3242	4.8966	4.5587	4.3352	4.7527	4.6656	4.0499	5.1021	3.6667	4.0044
Rbmxl1	_DSYESYGNSR_.2	#N/A	4.3371	6.7108	3.5391	3.5817	6.0435	6.6399	6.5765	4.2282	6.8516	6.2085	6.3088	3.0427	6.5579	6.6429	6.5760	6.4704	6.2169	6.4274	6.6331	6.0707
Rbmxl1	_RST[Phospho (STY)]PSGPVR_.2	#N/A	9.9627	10.2043	9.9704	10.0766	9.8904	9.9497	9.8033	10.1989	9.8199	10.0449	9.7767	9.9750	10.5351	10.3656	9.9012	9.9743	10.0627	9.9636	9.9031	10.0328
Rbmxl1	_DYSHS[Phospho (STY)]SSRDDYPSR_.3	Q91VM5_S246_M1_Rbmxl1	3.4852	3.8631	3.8580	4.1283	3.6922	3.7808	3.3490	3.7109	3.4081	3.5891	3.7824	3.5892	3.5161	3.8961	3.3798	3.6836	4.0444	3.9706	3.6050	4.2503
Rbmxl1	_DRDYSDHPSGGSYR_.3	#N/A	3.7859	4.7494	3.8097	4.0167	3.4692	4.7435	5.3276	3.6801	4.9962	5.0514	5.0174	3.7776	3.7582	4.0014	5.6599	4.5521	4.5265	4.0430	5.1431	4.2114
Rbmxl1	_SDLYSSGR_.2	#N/A	3.4434	4.5594	3.9797	4.2459	4.2820	3.7029	3.2591	3.1438	3.2987	3.2732	4.2782	2.9480	3.4642	3.9668	2.8363	3.7883	3.6815	4.1517	3.8002	3.9338
Ppa2	_ILGTLALIDQSETDWK_.2	#N/A	4.4392	3.4878	4.2227	3.9411	4.4189	3.8823	3.7642	3.8943	3.6809	5.0681	4.5244	3.4531	3.9313	4.3113	3.8997	4.8573	3.4617	3.8460	3.6275	4.9531
Ppa2	_AFALDVINSAHER_.2	#N/A	3.8703	3.5726	5.1963	4.9500	4.8639	4.4673	3.7732	4.0129	4.0141	4.7411	3.8700	3.0780	4.4029	1.7745	5.1734	3.1864	3.9409	2.4576	3.7221	4.0071
Chchd6	_LSES[Phospho (STY)]VVNR_.2	Q91VN4_S33_M1_Chchd6	3.4572	5.7548	4.4946	4.1271	3.6641	4.4443	3.3209	3.6828	3.3800	5.8846	3.7544	3.5880	3.4880	4.5326	4.2635	5.2628	3.1801	5.3482	4.2684	4.2099
Chchd6	_KKAS[Phospho (STY)]LTHEQQQSAR_.3	#N/A	4.3396	4.4439	3.3751	3.3498	3.6773	4.9760	4.2218	5.0616	5.3594	5.1363	4.2972	5.1933	5.8444	5.7258	5.4318	5.2257	4.8300	5.8514	5.2576	5.4844
Chchd6	_AYQHC[Carbamidomethyl (C)]VSTAR_.2	#N/A	4.7572	1.2725	5.2175	3.3119	4.6732	4.7893	5.0339	4.8439	4.3725	4.9555	4.6523	4.8448	4.3982	5.3076	5.7120	4.9706	5.2475	5.2264	4.5471	3.8728
Chchd6	_KAS[Phospho (STY)]LTHEQQQSAR_.3	#N/A	4.5555	4.2644	4.0921	4.5296	3.2616	3.2949	3.7865	3.2803	3.0575	4.3661	3.1854	3.9906	3.8722	4.3748	1.9623	3.9176	3.5720	3.7437	3.3922	4.3418
Chchd6	_AS[Phospho (STY)]LTHEQQQSAR_.2	#N/A	3.8136	3.9253	4.3388	3.8386	3.0649	3.7381	3.7105	3.8220	3.7059	3.9895	3.7464	3.7605	3.8398	4.1898	2.9491	3.7398	3.7955	4.2687	4.0163	3.7641
Chchd6	_LSSQQFHEAASK_.2	#N/A	4.1843	4.9474	3.9759	3.4668	2.8904	4.4246	4.4697	3.9230	4.0779	4.8388	3.5565	4.9779	4.2433	5.6672	5.6893	3.5464	4.4607	4.9788	4.8258	4.9725
Atp5f1c	_HLIIGVSSDR_.2	#N/A	7.6893	8.7711	8.7032	8.4450	7.7988	8.2746	8.2806	8.1009	8.1906	8.1191	7.8291	8.2399	8.2565	8.8045	8.3760	8.5840	8.2036	8.6131	8.6458	8.3022
Atp5f1c	_ADIKAPEDK_.2	#N/A	7.3571	7.7426	7.0956	6.8237	7.5792	7.3951	7.8253	7.9221	7.4843	7.7911	7.7282	7.5211	8.0515	7.7421	7.9901	8.2028	7.4181	7.7705	7.8402	7.4180
Atp5f1c	_NEVAALTAAGK_.2	#N/A	6.6012	7.2452	6.1178	5.9744	6.7512	7.3527	7.5624	6.1130	5.8444	6.9052	7.3002	7.1697	6.9142	7.5391	6.5639	5.8556	6.5188	7.6842	6.7370	6.7053
Atp5f1c	_VYGTGSLALYEK_.2	#N/A	6.5677	6.9116	6.2921	6.0886	6.7705	6.8805	6.6226	6.6822	6.8393	6.3788	6.4682	6.4993	6.9017	6.4591	6.7836	7.0849	6.7037	6.5310	6.9657	6.2731
Atp5f1c	_ELIEIISGAAALD_.2	#N/A	6.3013	6.3195	6.4250	6.3888	5.9095	6.0965	5.2800	5.7844	5.1508	6.2960	5.6312	6.3392	6.5493	6.5048	5.6819	6.2814	5.4898	5.5438	5.6943	6.5614
Atp5f1c	_NASDM[Oxidation (M)]IDKLTLTFNR_.3	#N/A	6.3412	5.8744	6.3524	6.4291	6.0897	5.8183	5.7016	5.8490	6.5394	6.5615	5.9612	6.2982	6.5839	5.9496	6.3282	6.5076	5.7887	6.1541	6.4142	6.4687
Atp5f1c	_EVMIVGVGEK_.2	#N/A	3.1476	7.2472	8.3979	8.3754	5.4322	6.7188	7.7422	4.3489	6.6653	5.9145	7.0557	5.8092	3.1684	8.6240	6.0857	3.4925	5.9851	5.5144	5.6068	6.1142
Atp5f1c	_THSDQFLVSFK_.2	#N/A	6.7072	7.1932	6.9817	6.7703	6.6382	7.0049	6.6773	7.1385	6.4214	6.8490	6.7952	6.7658	6.9193	7.2305	6.8628	7.1705	6.8726	6.8326	6.6684	7.2208
Atp5f1c	_GLC[Carbamidomethyl (C)]GAIHSSVAK_.2	#N/A	3.6261	6.4650	6.3356	5.6847	2.9638	5.6700	5.6792	4.8496	6.0604	3.8324	3.5099	6.2953	3.2435	6.9141	6.2897	5.3481	5.0038	5.8699	3.0121	3.9654
Atp5f1c	_MTAMDNASK_.2	#N/A	4.3749	3.6279	6.5082	6.3561	3.7126	4.3629	3.8285	4.0752	3.8876	4.5812	4.6022	3.5174	3.9956	5.8877	3.4057	3.2189	3.1121	3.4618	3.7608	3.2167
Atp5f1c	_NASDMIDK_.2	#N/A	4.4027	4.4172	3.9393	3.1815	3.1088	3.4987	4.2664	3.1276	4.3255	4.1432	3.1991	4.1433	4.4336	3.9774	3.8436	3.7648	4.1256	3.0238	3.3229	3.6546
Ddx1	_ALIVEPSR_.2	#N/A	5.4965	5.4860	5.7481	5.7297	6.0487	6.3596	5.6965	6.2274	5.8012	5.5210	5.6627	5.5810	5.3961	6.0705	5.6683	5.9566	5.4027	5.6280	5.3371	5.1774
Ddx1	_ELAEQTLNNVK_.2	#N/A	6.9518	6.7203	5.6831	3.1610	6.4078	6.4619	5.8659	7.3054	5.0928	6.6698	7.0328	6.6828	7.3978	7.1070	6.6303	6.4926	7.0202	6.4359	6.1106	6.7137
Ddx1	_DQLSVLDNGVDIVVGTPGR_.2	#N/A	5.2331	3.9290	4.8261	3.7768	4.8579	3.9515	5.0878	4.1494	3.6910	4.1496	4.7805	3.1215	3.9384	5.3510	4.0881	3.2859	5.0748	4.5286	4.3590	4.4057
Ddx1	_DNTRPGANS[Phospho (STY)]PEM[Oxidation (M)]WSEAIK_.3	#N/A	5.5906	4.8826	4.6618	4.2193	4.4487	4.9117	4.0421	5.1103	5.3978	4.8856	4.0136	4.0294	5.5496	5.5829	4.5400	5.4382	5.2803	4.8056	4.4188	4.4127
Ddx1	_FLVLDEADGLLSQGYSDFINR_.2	#N/A	3.6507	1.4616	2.4507	3.9335	3.8576	1.6295	3.1905	3.8764	3.5735	3.3944	0.9569	3.3945	3.6816	2.8339	3.2158	3.0128	3.3736	1.8056	2.2732	4.4035
Map1lc3a	_IPVIIER_.2	#N/A	8.2127	8.3737	8.5170	8.7592	8.5734	8.6348	8.8529	8.1367	8.5818	8.6905	8.8070	8.6894	8.9929	8.3598	8.9362	8.8384	7.9753	8.8425	8.7904	8.7262
Map1lc3a	_QLPVLDK_.2	#N/A	6.9883	7.2637	6.6734	4.4251	7.6031	6.3547	7.4140	7.0029	6.9277	7.3277	6.9515	7.3709	6.8712	6.7837	7.6513	6.7558	7.0324	7.3902	7.6682	7.0787
Map1lc3a	_PS[Phospho (STY)]DRPFK_.2	Q91VR7_S3_M1_Map1lc3a	4.6448	5.0644	3.5288	4.9023	5.3232	5.2073	4.2567	4.2179	4.2700	5.6442	5.2120	4.6795	4.4355	4.9856	5.1588	4.9401	5.3680	5.2027	5.2842	5.4057
Map1lc3a	_FLVPDHVNM[Oxidation (M)]SELVK_.3	#N/A	4.8876	4.6941	4.8507	5.0067	4.7412	3.7994	4.9445	4.6481	3.8283	4.7316	5.3378	4.9291	5.0881	5.3053	5.1481	5.2523	4.4187	4.7985	4.6628	5.1594
Brk1	_EIHQDWANR_.2	#N/A	7.0950	7.0194	7.1381	7.1758	6.8992	6.9796	7.0223	7.3897	7.3407	7.2742	6.9685	7.0978	6.8595	7.3805	7.4599	7.3146	6.8575	6.9962	7.4454	6.9990
Brk1	_EYIEIITSSIK_.2	#N/A	5.9422	3.9306	6.5040	5.5609	5.5822	5.9586	5.3256	5.3365	5.4979	5.3384	5.4095	5.0190	5.7554	5.9533	6.1187	5.6443	5.1700	5.2634	6.1478	5.4524
Brk1	_RIEYIEAR_.2	#N/A	4.9484	4.5798	5.2163	5.3068	5.3090	5.0524	5.5737	5.3908	5.4450	4.4489	5.3974	4.9617	4.9876	5.4920	5.2814	5.5961	5.5978	5.1795	5.6742	4.6511
Brk1	_ISDFLNSFDM[Oxidation (M)]SC[Carbamidomethyl (C)]R_.2	#N/A	5.3512	2.8849	5.5790	4.1980	5.2129	5.7447	5.0569	5.0201	5.8444	5.3976	4.3546	5.2586	5.6197	4.0432	5.0578	5.1088	5.9954	5.5037	6.3587	4.7524
Brk1	_VTKGETLT[Phospho (STY)]_.2	Q91VR8_T75_M1_Brk1	4.5190	3.4985	3.3572	3.7636	3.8567	4.0808	3.6843	4.0463	3.7434	3.2245	4.1178	3.3732	3.8515	3.3952	3.2615	3.3631	3.5435	3.6059	3.9050	3.0725
Ptov1	_SRS[Phospho (STY)]WPGGPR_.2	Q91VU8_S36_M1_Ptov1	7.1638	4.5761	3.9630	4.2627	3.6863	3.7197	3.3618	3.1271	7.3732	7.6434	7.3320	7.0462	3.4474	3.9501	3.3871	4.3424	7.8895	7.6780	7.4724	3.9171
Sh3bgrl3	_SQQSEVTR_.2	#N/A	6.3103	6.9271	7.1107	6.4816	6.9312	6.4736	6.2459	6.4391	6.8015	6.8543	6.6890	6.8333	6.6850	7.0137	8.1341	7.0437	6.4212	6.6702	6.7128	7.0925
Sh3bgrl3	_VYSTSVTGSR_.2	#N/A	6.6017	8.0751	5.7175	3.9165	6.5901	8.0153	8.2827	6.1621	8.5761	8.3369	7.9343	8.1227	8.4649	8.5049	9.0828	8.5298	8.1782	8.2945	8.4149	8.2405
Sh3bgrl3	_IQYQLVDISQDNALRDEM[Oxidation (M)]R_.3	#N/A	5.6075	5.5359	6.8149	6.2582	4.9722	5.7929	5.9034	5.4093	5.6808	5.2808	6.3140	5.4970	5.3713	6.4812	6.0243	5.1626	6.1020	5.7028	5.5627	5.9094
Sh3bgrl3	_IQYQLVDISQDNALR_.2	#N/A	4.3489	4.4711	5.1043	4.5559	4.0802	4.2777	3.7036	3.4912	4.4842	4.1594	4.1992	3.1745	4.0788	4.8621	4.1032	4.3080	4.0718	3.5370	4.0114	4.9972
Golga4	_TSS[Phospho (STY)]FTDQLDDVTPNR_.2	Q91VW5_S41_M1_Golga4	3.8024	3.5764	3.4795	3.2630	3.7818	4.2684	3.7770	3.9730	6.6229	6.7558	4.2105	6.4603	4.0591	7.4075	5.4429	3.2881	4.0405	6.8149	6.7752	6.9418
Ubap2	_NS[Phospho (STY)]VEEWTTEDWTEDLSETK_.2	Q91VX2_S254_M1_Ubap2	4.0061	4.0987	4.2748	3.7852	3.7460	3.8352	3.8149	3.6315	3.7798	3.8270	3.6218	3.9881	3.8088	3.6721	3.6718	3.6916	3.4884	3.6910	4.0846	3.7407
Smap1	_LLYEANLPENFR_.2	#N/A	7.3575	7.1323	7.8605	7.4723	7.3608	7.1356	7.1067	7.2881	7.2940	7.5309	6.7388	7.1642	7.4220	7.5047	7.5312	7.4447	7.1497	7.1843	7.2101	7.1930
Ncoa5	_SS[Phospho (STY)]ADSLPGPISR_.2	Q91W39_S378_M1_Ncoa5	3.8794	9.2733	9.6182	3.9968	3.4357	8.9340	8.6006	3.7344	8.7233	9.4079	9.0763	3.9056	9.0918	4.0284	3.7717	9.0358	3.7431	8.7071	8.9894	8.9773
Ncoa5	_[Acetyl (Protein N-term)]M[Oxidation (M)]NTAPSRPS[Phospho (STY)]PTR_.2	#N/A	8.3596	8.0981	7.3891	7.4090	7.4962	7.8196	7.7092	8.5947	7.2764	8.1275	7.6975	3.0454	8.5185	8.3265	7.5381	3.3834	8.2414	8.1517	7.9552	7.5611
Ncoa5	_DRS[Phospho (STY)]PIRGS[Phospho (STY)]PR_.2	#N/A	9.7634	9.7457	10.0444	10.0312	9.7725	9.5934	9.4867	9.6777	9.7194	9.8962	9.6228	9.7796	9.8023	10.0833	9.5716	9.6932	9.7772	9.6915	9.5013	9.9256
Ncoa5	_LLRSS[Phospho (STY)]ADS[Phospho (STY)]LPGPISR_.3	#N/A	4.0404	6.3955	3.4290	3.3229	3.5868	4.2807	5.7433	4.0939	4.2488	3.8510	3.7274	3.9041	4.4142	3.4161	3.6934	3.4025	3.9710	3.2286	3.7175	3.3831
Csde1	_EAFGFIER_.2	#N/A	5.7493	5.6262	5.7107	4.6205	5.3465	5.8222	5.8852	5.7114	5.7146	5.4937	5.9847	6.3215	5.2139	5.3813	5.8943	5.5636	5.5545	5.9359	5.0025	6.3353
Csde1	_ATNIEVLSNTFQFTNEAR_.2	#N/A	4.6576	4.5134	4.6591	5.1538	4.9285	4.1744	4.3545	4.1746	4.9907	3.9155	3.9820	4.1610	3.6594	4.7278	4.6209	4.8872	4.7910	4.4392	4.6424	4.4797
Csde1	_LLGRNS[Phospho (STY)]NSK_.2	Q91W50_S514_M1_Csde1	3.9550	5.1843	4.4913	3.7344	5.5790	5.7848	4.2484	3.6554	3.8103	5.6313	3.2889	5.5704	3.9757	5.3602	3.8256	5.7947	4.1930	3.6401	3.2887	5.5805
Csde1	_SPAAPGQS[Phospho (STY)]PTGSVC[Carbamidomethyl (C)]YER_.2	Q91W50_S123_M1_Csde1	7.8601	7.9103	7.9390	7.9040	7.5346	7.3357	7.2684	7.8688	7.4560	7.8616	7.2980	7.7760	3.7793	8.2632	7.3186	3.7484	7.6407	7.5695	7.6854	3.7093
Ubl7	_DM[Oxidation (M)]PGGFLFDGLS[Phospho (STY)]DDEDDFHPSTR_.3	#N/A	3.8396	6.0799	5.9532	3.9430	3.5365	3.4941	4.2889	3.9798	6.0279	3.9225	3.2851	3.4040	3.7849	4.0634	6.3549	3.7150	4.2117	6.1444	5.6872	3.7407
Ube2e2	_VDDS[Phospho (STY)]PSTSGGSSDGDQR_.2	Q91W82_S11_M1_Ube2e2	4.4594	4.0316	3.7712	3.1796	3.5359	3.4518	3.8141	3.7452	4.1452	3.8294	3.8127	3.7915	4.2532	3.9319	3.6398	3.5899	4.0218	4.0567	3.4995	3.4680
Cdc42ep1	_RSDS[Phospho (STY)]LLSFR_.2	#N/A	8.4964	8.7734	8.1949	8.5464	3.7110	8.5334	8.0861	8.0586	8.1676	8.6156	8.3942	8.3098	8.9245	8.8125	7.8832	8.4591	8.8703	8.0639	8.1535	8.5905
Cdc42ep1	_AS[Phospho (STY)]WGASR_.2	Q91W92_S332_M1_Cdc42ep1	3.7481	4.2547	4.2844	3.9413	3.0858	3.3099	4.4552	3.3164	3.0135	3.9544	3.3879	4.1441	3.1215	4.1662	4.0324	4.0930	3.9862	4.3359	3.1341	3.8434
Cdc42ep1	_SDS[Phospho (STY)]LLSFR_.2	#N/A	4.5740	4.1698	4.3693	3.8564	3.2801	3.3134	3.7680	3.5333	3.6882	4.3846	3.1669	4.0593	3.8537	4.3563	3.7934	3.9361	4.0710	3.7621	3.4107	4.3233
Plpp7	_RQS[Phospho (STY)]QQLPEEDC[Carbamidomethyl (C)]M[Oxidation (M)]QLNPSFK_.3	#N/A	6.5773	6.0600	4.9870	5.9064	4.7036	4.8550	5.9514	5.6202	5.1866	5.8738	4.6798	4.9094	6.1633	6.3795	4.0218	4.4673	5.8516	5.6711	5.0111	3.8328

Setd3	_S[Phospho (STY)]GTGATATVSPK_.2	Q91WC0_S12_M1_Setd3	4.2496	6.8343	6.9125	3.2303	4.0105	4.1305	6.2713	5.6301	5.6644	3.5154	6.4910	3.2514	4.1998	3.4820	3.1104	6.0989	3.1967	6.2819	5.6573	6.4178
Acsl6	_LVILM[Oxidation (M)]EPFEDALR_.2	#N/A	3.2584	5.0712	4.2958	4.9989	5.6192	5.6764	5.2171	5.3250	5.9450	4.3007	4.6819	5.4110	5.0477	4.4204	4.8713	4.1214	5.7267	5.3471	5.2518	4.0112
Acsl6	_LPELSDLGQFFR_.2	#N/A	5.5115	5.5376	3.6634	5.3866	5.6404	4.4403	5.3025	4.8235	5.5750	5.0520	4.9017	5.3984	3.7426	4.6915	3.1527	3.4720	5.5561	5.8228	4.1166	4.7812
Acsl6	_VGFFQGDIR_.2	#N/A	5.7234	5.4840	3.9829	5.2252	4.7757	3.4992	5.6525	6.0037	4.2556	3.6529	3.6894	4.9788	3.4231	4.4677	3.6906	3.8715	5.1291	5.2287	5.0007	4.1450
Acsl6	_LVDAEELNYWTC[Carbamidomethyl (C)]K_.2	#N/A	3.7255	4.2773	5.1476	3.9639	4.8142	3.2873	5.4954	5.4832	3.0361	3.9318	4.6158	4.7983	3.1442	5.5751	4.0551	3.6039	3.9635	3.8696	3.5182	4.6966
Acsl6	_EALDSDGWLHTGDIGK_.3	#N/A	4.2394	3.7634	3.2749	3.4500	3.5771	3.8012	3.9640	3.9398	4.0230	4.4457	2.8966	3.6529	4.1311	3.2619	3.5412	3.0835	4.4775	3.3557	3.0043	3.2289
Ndufs2	_VLFGEITR_.2	#N/A	7.3279	7.1102	7.2355	6.9008	7.5635	7.2943	7.3880	6.9169	7.1397	7.0985	7.3063	7.2450	6.9325	7.0084	7.2333	6.9881	6.9126	7.3155	7.0833	7.1079
Ndufs2	_IIEQC[Carbamidomethyl (C)]LNK_.2	#N/A	6.7243	7.2249	7.3216	6.6143	7.0451	6.9306	6.9177	7.0751	6.5079	6.7868	6.7532	7.1642	6.9990	6.9742	7.2802	6.6833	6.8474	6.9763	6.8384	6.6445
Ndufs2	_TQPYDVYDQVEFDVPIGSR_.2	#N/A	6.4934	7.0287	6.9308	7.3972	6.7454	6.7794	7.1844	6.9866	6.8306	6.8577	6.9083	7.0589	6.4706	6.8158	6.6515	7.0887	6.4887	6.6132	6.6967	7.2599
Ndufs2	_TYLQALPYFDR_.2	#N/A	6.8437	6.9351	7.0453	6.9441	6.5219	7.1147	6.6516	7.1460	6.3149	6.7372	6.6072	6.5184	6.7732	6.7764	6.3272	6.4276	6.6899	7.0760	6.8302	6.8218
Ndufs2	_GSGIQWDLR_.2	#N/A	6.7932	6.3768	7.0611	6.3151	6.3786	5.8282	6.3949	6.3237	6.8812	5.9521	6.5942	6.6014	6.4201	6.6121	6.5790	6.5300	6.7481	6.6713	6.7682	6.0852
Ndufs2	_APGFAHLAGLDK_.2	#N/A	6.1057	6.4379	6.4470	6.4188	6.8194	5.8862	6.2850	6.3663	5.7935	6.0049	6.6503	6.5598	6.0914	6.1616	6.1172	3.6065	6.2155	6.5336	5.9096	6.5003
Ndufs2	_M[Oxidation (M)]FEFYER_.2	#N/A	4.9775	4.0466	3.2176	3.9032	4.6696	4.4385	4.5024	4.0615	5.1541	5.6393	4.7999	5.0481	5.4117	3.2556	3.9308	5.2028	5.3824	4.9566	5.8916	5.4111
Ndufs2	_TVDIGVVTAEDALNYGFSGVM[Oxidation (M)]LR_.3	#N/A	5.0951	4.5838	4.5688	5.2011	3.3762	4.6746	4.0127	5.3758	3.0228	5.1583	4.1130	4.5241	5.9633	5.2962	3.6760	4.2946	3.4056	4.4276	4.8885	4.8797
Ndufs2	_IDEVEEM[Oxidation (M)]LTNNR_.2	#N/A	8.5222	8.7748	9.2235	9.2253	8.4842	8.8320	8.5732	8.5398	8.6966	8.6705	8.9342	8.4869	9.0148	9.3523	7.9168	8.3490	9.0163	8.4553	8.3677	8.7481
Ndufs2	_AVTNM[Oxidation (M)]TLNFGPQHPAAHGVLR_.4	#N/A	4.2664	4.0700	4.1796	3.9339	4.5822	4.6306	3.9952	4.6605	4.5291	4.5319	4.4584	4.1192	3.8386	4.8310	4.0516	4.8510	3.7127	5.1730	4.9669	4.5495
Ndufs2	_TSM[Oxidation (M)]ESLIHHFK_.3	#N/A	3.4219	3.9008	4.4593	4.5326	3.6288	3.4740	3.3301	3.0916	3.2703	3.1273	4.1277	3.2424	3.4073	4.2010	3.1114	3.7793	3.1448	3.7372	4.1292	4.1846
Ndufs2	_LDYVSM[Oxidation (M)]M[Oxidation (M)]C[Carbamidomethyl (C)]NEQAYSIAVEK_.2	#N/A	3.5000	3.8191	4.5375	4.0842	2.6802	4.4014	1.1445	3.7257	3.2613	3.3106	4.2408	3.5452	3.5309	4.5755	3.3665	4.3630	3.2229	3.9265	4.2256	4.2528
Prkag2	_IYASSS[Phospho (STY)]PPDTGQR_.2	Q91WG5_S196_M1_Prkag2	11.3482	11.2834	11.3076	11.3673	11.1880	11.1426	10.9612	4.4653	10.9122	11.2650	4.0988	11.1098	11.5469	11.5761	10.5614	11.2301	11.5698	11.2076	11.1759	11.1844
Prkag2	_PSS[Phospho (STY)]PVS[Phospho (STY)]APVRPK_.2	Q91WG5_S87_M2_Prkag2	3.6308	3.2266	3.8116	6.6195	5.9444	3.8711	3.2104	4.4765	3.1306	3.4414	6.8437	3.1161	3.2960	6.5636	3.2358	6.4275	3.5134	4.3198	3.9684	3.7657
Prkag2	_SSS[Phospho (STY)]KESSPNSNPSTSPGGIR_.2	Q91WG5_S131_M1_Prkag2	4.5214	4.0451	4.0580	4.0853	3.2275	3.3800	3.8206	3.2463	4.4443	4.3320	3.3178	4.0246	4.5523	4.0961	3.8459	3.8836	4.2444	4.4059	3.2041	3.7734
Dgkg	_[Acetyl (Protein N-term)]S[Phospho (STY)]EEQWVSLSSEEFDQLQK_.2	Q91WG7_S2_M1_Dgkg	5.3196	4.4045	4.1346	4.0911	2.9360	3.1601	5.3413	3.2986	3.1633	6.2998	3.5377	4.2940	3.2714	4.3160	4.1823	3.9432	3.8363	4.1860	5.8599	3.9933
Dgkg	_EGASSSEPNVSDYNS[Phospho (STY)]DNAAK_.2	Q91WG7_S107_M1_Dgkg	3.7716	4.1632	5.6931	5.2509	4.4757	5.8282	3.8830	4.2402	4.9310	5.4709	3.6297	3.8894	4.0454	5.8379	3.7385	3.5158	3.7374	3.4519	3.7509	4.1711
Dgkg	_QET[Phospho (STY)]PDHPK_.2	Q91WG7_T87_M1_Dgkg	3.1079	3.2377	4.1453	4.4763	3.3149	3.5363	3.7332	3.4986	3.6535	4.4194	3.1321	3.9373	3.8189	4.3216	3.7586	3.9709	4.3317	3.7969	3.4455	4.2885
Itfg2	_DTGS[Phospho (STY)]PPASEEATGDSR_.2	Q91WI7_S219_M1_Itfg2	4.3047	8.7504	8.3865	8.7305	8.4228	8.7431	8.6200	8.9597	8.3133	8.4957	8.4240	8.2077	8.2706	8.8383	8.0234	8.4628	4.3403	8.5810	8.3640	8.4428
Lrrfip2	_SDKQYAENYTRPSS[Phospho (STY)]R_.3	Q91WK0_S92_M1_Lrrfip2	3.8100	3.5091	3.3466	4.5933	4.0169	4.0503	3.0312	4.2973	4.4522	3.6206	3.9309	4.6839	3.1168	3.6195	3.0565	3.1721	3.3342	4.7135	4.1476	3.5865
Eif3h	_DFS[Phospho (STY)]PEALKK_.2	#N/A	6.8607	6.6770	6.1268	5.8812	6.3227	6.2627	6.1703	6.5449	4.4682	6.6222	6.8197	6.7356	6.4185	6.3322	6.0070	6.5730	6.6814	6.7501	7.0255	6.7953
Eif3h	_LFM[Oxidation (M)]AQALQEYNN_.2	#N/A	6.0339	6.1901	5.1539	4.3023	3.7067	4.0406	6.0053	6.8963	4.4145	4.6688	3.6051	5.8092	6.2672	7.3004	4.4459	5.0044	5.2322	5.9541	5.1328	4.8131
Eif3h	_EKDFS[Phospho (STY)]PEALKK_.3	#N/A	3.5678	3.7513	4.6052	4.0165	3.7747	3.8080	2.7055	3.7934	3.1936	3.3783	4.1731	3.4775	3.3591	3.1424	3.2988	4.4307	3.2907	3.8588	4.1578	4.3205
Gcsh	_SC[Carbamidomethyl (C)]YEDGWLIK_.2	#N/A	4.7936	4.6271	4.6923	5.2151	4.7835	4.6330	4.9438	4.9979	4.4697	4.6103	4.6215	5.0028	4.3143	5.3791	5.3739	4.5964	4.0063	5.1789	4.6472	4.9964
Rrp9	_RLPVS[Phospho (STY)]PVAGS_.2	Q91WM3_S470_M1_Rrp9	7.4974	7.3516	7.2864	7.0621	7.3334	7.1122	6.8393	4.0664	7.2806	7.4518	7.4347	7.2018	8.0373	7.6679	6.8672	6.9916	7.6277	7.3928	7.1998	7.3848
Yars1	_APWELLELR_.2	#N/A	5.1470	5.4101	4.5215	4.9562	4.3530	4.9521	5.2277	4.3501	4.5389	4.3393	5.2478	4.5604	4.3853	5.2637	4.1748	5.1393	4.3725	5.1619	4.8868	4.3575
Yars1	_TSYYENVIK_.2	#N/A	4.7815	5.0609	3.9619	4.9765	4.7753	4.0467	5.4672	3.1260	5.5687	4.3338	5.0202	4.5719	4.7887	4.6924	5.3440	5.4895	4.9545	5.6092	5.0122	4.7755
Cisd1	_VVHAFDM[Oxidation (M)]EDLGDK_.2	#N/A	6.9776	6.8562	6.5322	6.5488	6.5917	6.9333	6.8317	6.8086	6.9304	6.5929	6.7861	6.7722	6.6497	6.6094	6.7036	7.1621	7.0677	6.9575	6.8744	7.1718
Cisd1	_AM[Oxidation (M)]VNLQIQK_.2	#N/A	5.2762	5.3720	5.9515	5.6909	4.7684	5.4116	6.0331	4.9702	4.5602	5.2550	4.8808	6.2691	3.9365	5.9670	5.0920	6.0636	5.3821	6.1258	5.3180	6.1477
Cisd1	_HNEETGDNVGPLIIK_.2	#N/A	6.0751	5.9079	5.6438	5.7851	5.5319	5.5857	5.8033	6.2773	5.9501	6.0665	5.8992	5.7056	6.1271	6.3091	5.7918	5.9076	6.2156	6.0075	5.9365	5.6402
Dtnbp1	_STPGVS[Phospho (STY)]DDSDQC[Carbamidomethyl (C)]DSTQDI_.2	Q91WZ8_S340_M1_Dtnbp1	3.5318	4.4710	4.0681	4.1576	2.8695	3.0936	3.1708	3.2321	3.2298	5.5054	3.6042	6.4214	3.3379	4.0551	4.2488	3.8767	3.7698	4.0633	3.7119	4.0221
S1pr5	_S[Phospho (STY)]PSAAGPSGGGLR_.2	#N/A	9.1028	9.1914	9.1016	8.9264	8.2969	9.8420	9.0454	8.8284	9.7213	8.2381	3.6458	8.3192	8.3657	8.3416	7.3351	8.2928	9.2330	3.2233	4.0031	8.0302
S1pr5	_S[Phospho (STY)]PSAAGPSGGGLRR_.2	#N/A	3.7668	4.0322	3.8042	7.0408	7.6649	7.0364	3.8873	7.2556	3.8522	3.7551	3.6937	3.8735	7.4005	6.3417	2.8281	3.6199	3.4259	3.7967	4.0958	3.7302
Zfand2b	_TLPSSSS[Phospho (STY)]PSR_.2	Q91X58_S173_M1_Zfand2b	4.2470	4.5729	3.7836	7.1181	2.9531	3.6544	5.1350	4.4727	4.1698	4.0576	3.0434	4.2990	4.2779	3.8217	4.1204	3.6091	3.9699	3.1795	3.4786	3.4989
Ncald	_IYGNFFPYGDASK_.2	#N/A	7.4252	7.4352	4.3791	8.0539	7.4901	7.7674	7.8198	7.5110	7.2825	7.6604	7.5852	7.6908	3.8635	7.4970	7.8379	7.2869	8.1409	7.6981	7.8245	7.4851
Ncald	_AEM[Oxidation (M)]LEIVQAIYK_.2	#N/A	5.4421	5.6792	7.2699	6.7774	5.4092	5.8311	5.3426	5.1558	5.6919	5.7138	5.7896	5.2865	5.5011	5.7727	5.7226	5.5681	5.4555	5.3066	6.3217	5.6513
Golm1	_LQEEVPS[Phospho (STY)]EEQM[Oxidation (M)]PQEK_.2	#N/A	7.0192	7.3021	7.2016	6.6719	6.6690	6.8423	6.7360	6.8971	6.2558	6.8445	6.5061	6.3347	6.9496	6.9964	5.7522	6.1826	6.8932	6.5577	6.2526	6.0050
Trex1	_AC[Carbamidomethyl (C)]S[Phospho (STY)]PGASEITGLSK_.2	Q91XB0_S78_M1_Trex1	8.4962	8.2462	8.4949	8.5209	8.2044	7.8719	8.0448	8.3387	8.1195	8.3737	8.2544	3.6171	8.2501	8.5579	8.1893	8.3505	8.7772	3.3200	8.2073	8.2979
Dap	_[Acetyl (Protein N-term)]SS[Phospho (STY)]PPEGKLETK_.2	Q91XC8_S3_M1_Dap	9.7994	10.1400	9.6678	3.6846	4.1066	4.1399	9.6855	10.0321	9.8171	3.7102	3.8412	10.3014	11.0349	10.3770	2.9669	10.3248	10.3556	9.8378	9.9955	10.0113
Pnpo	_S[Phospho (STY)]SQIGAVVSR_.2	Q91XF0_S164_M1_Pnpo	7.4634	4.3157	4.0408	4.5809	3.2103	3.2437	3.8378	3.6031	6.7852	7.5863	3.2366	4.0418	3.9234	4.4261	3.8632	7.1589	4.1408	6.3929	6.7742	6.5394
Pnpo	_FFTNYESR_.2	#N/A	3.7766	6.0438	5.4361	3.9128	5.8090	3.3384	6.1611	6.1910	6.2141	3.9829	6.4242	6.1561	5.7490	6.3870	6.4276	5.3812	5.5769	5.5875	5.9115	6.0304
Pnpo	_GLATGDS[Phospho (STY)]PLGPM[Oxidation (M)]THHGEEDWVYER_.4	#N/A	3.9530	3.8059	4.4030	3.8822	3.5996	3.3365	3.3799	3.8724	3.4023	3.5106	3.9566	3.5437	3.5252	4.1861	3.6425	3.4729	3.8113	4.4268	3.6387	3.6551
Osbpl1a	_ITM[Oxidation (M)]PVIFNEPLSFLQR_.2	#N/A	3.4452	3.4556	3.6701	4.5555	3.5014	3.1424	4.8542	3.7074	2.2355	4.4863	2.9330	3.9610	3.7358	3.6572	3.4626	3.9449	3.7925	4.5184	3.6625	4.0645
Osbpl1a	_M[Oxidation (M)]QC[Carbamidomethyl (C)]VAAFAVSAVASQWER_.2	#N/A	3.5886	3.7305	3.1252	3.9956	3.7955	4.3128	3.2526	3.8143	3.5114	4.0789	3.8858	3.4566	3.6195	3.1632	3.2779	3.8817	3.3115	3.8379	4.1370	4.3414
Dlg2	_AIS[Phospho (STY)]LEGEPR_.2	#N/A	12.2347	12.0289	11.7674	11.9946	11.6774	11.7337	11.8108	11.6887	11.4946	12.2587	12.1731	11.3517	12.5146	11.9730	10.4303	11.6190	11.8864	11.8623	11.8649	11.8210
Dlg2	_LC[Carbamidomethyl (C)]DKPAS[Phospho (STY)]PR_.2	Q91XM9_S360_M1_Dlg2	11.8619	11.8134	11.9665	11.8848	11.1777	11.3708	11.6285	11.8239	11.5049	11.5118	11.1812	11.3193	11.7466	11.3934	10.4693	10.8205	11.5280	11.4188	11.1666	11.0724
Dlg2	_QPS[Phospho (STY)]VTLQR_.2	Q91XM9_S406_M1_Dlg2	8.6201	12.0297	12.3125	9.8762	3.4080	3.4414	3.6401	10.8544	11.2224	10.8534	9.7168	9.3428	3.7257	9.9271	3.6655	4.0640	9.4165	11.3601	11.5538	11.3160
Dlg2	_GPELVHVS[Phospho (STY)]EK_.2	Q91XM9_S45_M1_Dlg2	10.0415	9.8478	9.6190	9.7854	9.1060	9.4990	9.5975	9.8212	9.4831	9.4815	9.6672	9.1126	9.7712	9.6219	8.3904	9.2436	9.8361	9.7151	9.4185	9.3363
Dlg2	_GQEDLILSYEPVTR_.2	#N/A	7.6206	7.4396	7.5454	7.4051	6.9982	7.1871	7.3509	7.5700	7.4109	7.3511	7.3410	7.1700	7.6491	7.4747	7.0034	7.1522	7.3581	7.1207	7.2888	7.1702
Dlg2	_LVIEEQSGPFIWIPSK_.2	#N/A	5.6601	6.2345	5.9207	5.3622	5.9301	6.0878	6.1997	6.0441	6.3355	5.0359	5.4975	6.0474	5.7666	5.8610	5.9616	5.8421	5.4906	5.4206	6.2057	5.9546
Dlg2	_AIS[Phospho (STY)]LEGEPRK_.2	#N/A	10.2318	10.4073	3.5035	10.2384	9.9246	9.1768	9.7980	10.2554	9.5486	9.8660	9.2971	9.9239	10.7849	9.9649	8.6843	9.9588	10.1174	9.9485	9.9499	9.9094
Dlg2	_DYHFVISR_.2	#N/A	6.5108	5.6518	6.3017	6.4000	5.9175	6.6319	6.1903	6.3993	6.0189	6.1921	6.1007	6.2316	6.3139	6.7133	6.0098	6.2247	6.4421	6.2732	6.2149	6.3076
Dlg2	_YQDEDGPHDHS[Phospho (STY)]LPR_.3	#N/A	9.7980	9.8766	9.3892	9.3948	9.1064	9.5259	9.5917	9.5364	9.6418	9.5569	9.4747	9.0244	9.9605	9.4796	8.4796	9.0767	9.6344	9.5214	9.4748	9.1070
Dlg2	_YGDILHVINASDDEWWQAR_.3	#N/A	3.9985	4.3035	3.3535	4.3947	4.5691	4.3535	4.5701	4.0426	4.0539	4.3322	4.4093	4.7218	4.8164	6.2018	4.5894	3.1791	5.1843	4.2357	4.5573	4.7937
Dlg2	_TSLPPIS[Phospho (STY)]PGR_.2	Q91XM9_S323_M1_Dlg2	10.1975	10.1264	9.2290	4.3770	10.5592	3.4475	8.0055	10.5716	8.2193	4.5187	10.0224	3.8380	10.4077	4.2827	3.6593	9.9259	9.5998	10.4975	9.2483	3.9600
Dlg2	_TSLPPIS[Phospho (STY)]PGRYS[Phospho (STY)]PIPK_.2	#N/A	3.7479	8.5605	3.7559	4.2213	3.9108	4.0549	4.0972	3.9750	4.1063	3.8001	3.6532	3.6268	4.2346	8.2920	3.8574	3.7552	4.1598	7.4600	3.9955	6.9619
Dlg2	_HYS[Phospho (STY)]PVEC[Carbamidomethyl (C)]DK_.2	Q91XM9_S365_M1_Dlg2	9.6526	9.6220	9.4319	9.6529	8.9304	9.4351	9.5580	9.6182	9.4297	9.6029	9.3551	9.3492	9.6564	9.5964	8.6289	9.0115	9.6205	9.5024	9.3677	9.2938
Dlg2	_NLSQIENVHGYVLQSHIS[Phospho (STY)]PLK_.3	Q91XM9_S65_M1_Dlg2	7.1758	8.2701	4.4048	6.2894	4.9287	5.6425	8.0620	8.8701	5.9384	3.1779	4.3735	7.0107	3.5595	8.7113	5.4226	5.6396	7.2857	7.8215	7.3620	4.0291
Dlg2	_FIEAGQYNDNLYGTSVQSVR_.2	#N/A	4.4659	3.5572	4.6619	4.5786	3.8044	4.4452	3.6522	4.4646	4.0452	4.3632	4.3937	4.3441	4.5209	3.1768	3.6950	4.2489	4.3733	3.7895	4.4085	4.4578
Dlg2	_DSGLPSQGLSFK_.2	#N/A	6.0443	6.8452	6.7817	6.5692	6.4654	6.6684	6.6197	6.7388	6.9385	6.7990	6.6883	6.4579	6.7901	6.5593	6.7337	6.8381	6.7945	6.6942	6.3660	6.5976
Dlg2	_SKDS[Phospho (STY)]GLPSQGLSFK_.2	Q91XM9_S553_M1_Dlg2	7.2002	7.3396	6.6880	6.7895	3.5553	6.9655	6.9026	7.1535	6.9496	6.9649	3.8271	3.6877	7.6840	7.2597	6.0703	3.7300	7.6400	6.8773	6.8000	6.9654
Mtmr4	_TSS[Phospho (STY)]DPNLNNHSQEVR_.2	Q91XS1_S629_M1_Mtmr4	4.4132	3.5896	5.3206	3.2762	3.7509	5.2776	5.8757	4.1135	4.2684	6.0168	3.7471	3.4791	5.5392	5.2408	3.3674	3.2572	3.1504	3.1819	4.3314	3.4027
Wrnip1	_AKGPS[Phospho (STY)]PPGAK_.2	Q91XU0_S65_M1_Wrnip1	8.4976	9.0933	9.0213	8.9572	8.7443	9.1462	8.9036	9.2737	8.9737	8.5641	8.7873	8.7994	9.1602	8.9009	7.9042	8.7159	9.0477	7.9639	8.1704	8.8016
Wrnip1	_RPAAAAAAGSAS[Phospho (STY)]PR_.2	#N/A	6.6887	8.1427	5.8359	4.0140	3.4270	5.9232	7.1755	8.0552	6.0013	3.9815	3.7953	7.2523	6.4248	8.3697	5.7860	6.7502	5.9057	6.2755	6.5483	6.5869
Wrnip1	_RLS[Phospho (STY)]ESSALK_.2	Q91XU0_S75_M1_Wrnip1	7.1200	7.1274	7.6118	4.4999	4.0280	6.9059	6.9029	6.9774	6.7960	3.3958	7.1183	6.6912	6.9700	3.7128	3.0902	6.7244	7.1836	6.5509	7.4031	7.0981
Pip4k2c	_FAIDDHDYLVSLTR_.3	#N/A	2.9272	4.8336	5.5437	4.9904	5.4950	5.3491	5.0859	5.3510	5.3005	5.4483	5.4547	5.0575	4.7264	5.3743	4.5806	5.2191	5.1772	5.5615	5.0368	5.2538
Pip4k2c	_ENLPSHFK_.2	#N/A	3.6375	4.1179	4.2387	5.2237	5.6454	5.5510	3.0650	5.2627	5.0881	3.8439	3.4984	5.2185	4.2569	5.7398	6.1592	3.9824	4.6634	4.2253	5.5434	3.9540
Basp1	_AEGAGTEEEGT[Phospho (STY)]PK_.2	#N/A	11.3835	11.3337	11.6619	11.6603	11.0503	11.4263	11.0645	11.3006	11.3152	11.4601	11.3664	11.2661	11.4231	11.8857	11.5975	11.3181	11.5389	11.1860	11.3637	11.4586
Basp1	_ETPAASEAPSSAAK_.2	#N/A	10.9577	10.6475	11.6449	11.3369	10.7449	10.6497	10.5317	10.9561	11.3713	11.3603	10.9727	10.8021	11.6162	11.1740	11.3033	11.0327	10.6624	10.9933	11.0403	10.9123
Basp1	_AGEAS[Phospho (STY)]AES[Phospho (STY)]TGAADGAAPEEGEAK_.2	Q91XV3_S131_M2_Basp1	10.4334	10.3329	10.5360	10.6133	9.3929	10.2213	9.2432	10.4354	10.3837	10.4101	10.0980	9.6202	10.7004	11.0927	10.5552	10.4247	10.6652	10.2274	10.3055	10.4336
Basp1	_ESEPQAAADATEVK_.2	#N/A	11.3659	11.1205	11.9437	11.4934	11.4239	11.4626	11.2533	11.2914	11.6508	11.3584	11.5077	11.4424	11.1699	11.8436	12.2459	11.5946	11.3457	11.2545	11.3758	11.5458
Basp1	_KTEAPAAAGPEAK_.2	#N/A	10.3177	10.1559	10.5697	10.4662	10.2425	10.1423	10.3798	10.4360	10.6100	10.3256	10.1646	10.4590	10.5297	10.6559	11.1394	10.7636	10.1212	10.2236	10.1931	10.4641
Basp1	_APAPAAPAAAEPQAEAPAAAASS[Phospho (STY)]EQSVAVK_.3	#N/A	4.4680	7.0218	7.2108	7.0948	6.9587	7.2223	6.1007	3.5833	6.2355	6.2363	6.2397	4.7180	6.6637	7.8955	6.3584	6.3770	4.7518	6.9041	6.2047	7.2377
Basp1	_GYNVNDEK_.2	#N/A	6.1454	7.2366	6.2688	7.3351	6.5730	7.2880	7.3477	6.3875	7.5776	7.5668	7.4908	7.2793	7.6932	7.3614	8.2874	7.1062	6.3494	7.1693	7.5151	7.6555
Basp1	_TEAPAAAGPEAK_.2	#N/A	6.5403	7.2874	7.6941	7.3745	7.3235	7.1719	7.1181	7.3089	7.3528	7.3643	7.2565	7.3047	7.1099	7.5347	8.0272	7.4100	7.1629	7.3520	7.3665	7.2446
Basp1	_DAADGEAKAEEK_.2	#N/A	6.3656	4.9137	6.8810	6.1530	6.1414	6.5213	6.5341	6.3059	6.4701	6.8033	6.5296	6.3513	5.9840	6.3768	7.9777	6.8086	6.1385	6.3427	6.4042	6.5791
Basp1	_AAAAKEEAPK_.2	#N/A	5.6897	4.9152	4.9159	5.4668	4.4818	4.9970	4.7518	4.8234	5.5371	5.0675	5.0364	4.6261	4.9172	5.3827	5.8854	5.3338	4.7999	4.5618	4.6013	5.8231
Basp1	_SDAAPAASDSKPSSAEPAPSSK_.3	#N/A	9.3897	9.8310	10.1730	9.8748	9.4484	9.7820	9.7682	9.6334	10.1159	9.9562	9.4986	9.8294	10.1214	10.1057	10.9557	9.8371	9.6210	9.6748	10.1249	10.1266
Basp1	_SDAAPAASDSKPSSAEPAPSSKET[Phospho (STY)]PAASEAPSSAAK_.4	Q91XV3_T183_M1_Basp1	4.3583	4.4304	4.8265	4.8827	3.9553	4.8552	4.6086	4.4818	4.5535	4.0389	4.4644	4.6376	4.0048	5.4058	5.0922	4.0735	4.2052	4.3843	4.4520	4.9755
Basp1	_APAPAAPAAAEPQAEAPAAAASS[Phospho (STY)]EQSVAVKE_.3	#N/A	10.6596	10.9074	11.7843	11.6325	11.6285	11.4620	10.4245	11.7225	11.4104	10.9707	11.1611	11.5399	11.0558	12.3679	11.0511	11.6277	11.6953	11.4545	11.5047	11.6094
Basp1	_GYNVNDEKAK_.2	#N/A	4.7848	5.5349	4.2116	5.9528	5.6773	5.1062	5.1289	5.5935	5.5401	5.4651	5.4177	6.2254	5.9072	5.9129	6.6079	5.9911	5.4035	5.6943	5.8240	6.0892
Basp1	_ESTEEKPKDAADGEAK_.2	#N/A	4.5409	4.0288	4.8095	4.5301	3.9967	4.3300	4.2142	3.8620	4.4170	4.8999	4.1697	4.5287	4.6037	4.0552	5.4628	4.7143	4.5771	3.9682	4.5444	4.4235
Basp1	_KAEGAGTEEEGT[Phospho (STY)]PKESEPQAAADATEVK_.4	#N/A	9.2699	9.7871	9.8378	9.8439	9.3004	9.6477	9.5402	9.1111	9.5296	10.0335	9.7477	9.5648	10.1472	10.3502	9.9999	8.0623	9.6176	9.7482	9.9020	9.9240
Basp1	_AEGAGTEEEGT[Phospho (STY)]PKESEPQAAADATEVK_.3	#N/A	11.4337	11.5037	11.6739	11.4506	10.7711	11.0346	10.9773	11.4432	11.2514	11.3127	11.0448	11.2788	11.9416	11.9292	11.2662	11.2575	11.5596	11.1935	11.3153	11.2367
Basp1	_AEPEKS[Phospho (STY)]EGAAEEQPEPAPAPEQEAAAPGPAAGGEAPK_.4	#N/A	11.0742	10.7728	11.3864	11.3693	10.4041	10.8719	10.2735	10.6901	10.8383	11.2726	10.8974	10.9004	11.4018	11.5507	11.2993	11.1933	11.3276	10.5899	10.9789	11.1376
Basp1	_DAADGEAKAEEKEADK_.3	#N/A	4.9583	5.1763	5.9409	5.7933	3.7148	5.5516	5.2326	3.6084	6.6689	5.6487	5.5663	5.2570	5.5432	5.7455	4.9495	5.8931	6.3526	5.7288	5.9124	5.8156
Basp1	_AGEASAESTGAADGAAPEEGEAKKTEAPAAAGPEAK_.4	#N/A	4.2053	4.7080	4.1076	3.3789	3.7513	3.6961	4.6304	4.4310	5.2427	4.7675	4.3383	4.0680	4.2362	4.6974	4.4817	4.7786	3.9282	3.6967	5.0052	5.0690
Basp1	_EADKAAAAKEEAPK_.2	#N/A	5.3174	5.2100	5.7000	5.2531	4.9730	5.5238	5.4220	5.4938	5.0116	5.7786	5.5141	5.7396	5.9240	6.0034	6.2293	5.8144	5.5550	5.5406	5.4821	5.8759
Basp1	_KAEGAGTEEEGT[Phospho (STY)]PK_.2	#N/A	8.1015	8.0116	8.4654	8.4626	8.0324	8.3109	7.7279	8.0898	7.9009	8.3855	8.1424	8.2058	7.9081	8.6757	8.7247	8.4225	8.2860	8.0784	8.2383	8.3986
Basp1	_AEEKEADKAAAAK_.2	#N/A	4.2011	4.9448	5.3998	4.2730	4.6799	4.6858	4.3313	5.7052	5.4556	4.7467	4.8509	4.6433	4.7703	4.3777	5.8894	5.6847	4.6949	4.4868	5.5505	4.9923
Basp1	_ESEPQAAADATEVKESTEEKPK_.3	#N/A	7.9844	8.3401	8.9566	8.5456	8.2756	7.8404	8.0789	7.6568	8.4694	8.1531	8.8919	8.3243	8.7595	9.5428	9.1280	8.4464	8.5751	8.1510	8.4181	8.2174
Basp1	_KKGYNVNDEK_.2	#N/A	3.1646	4.2614	4.2778	3.9479	4.6933	3.4049	3.6765	3.4418	3.5967	4.4761	3.7813	4.1508	3.7622	3.8896	5.2304	4.0276	4.9262	3.2266	3.5022	4.2318
Basp1	_DKKAEGAGT[Phospho (STY)]EEEGTPK_.2	#N/A	3.4906	3.4555	3.5767	4.1586	4.0249	4.0627	3.2855	3.4961	3.3825	3.3953	4.0621	3.2485	4.3145	4.2721	3.0986	3.3915	3.4754	3.6263	4.0042	4.1276
Basp1	_EEAPKAEPEKS[Phospho (STY)]EGAAEEQPEPAPAPEQEAAAPGPAAGGEAPK_.4	#N/A	3.5402	3.8088	4.0765	4.2405	3.6430	3.7493	3.8418	3.9963	4.5005	3.8460	3.6613	3.6982	3.5609	4.0635	3.5648	3.7675	3.7782	4.1462	3.7948	4.0305
Basp1	_SEGAAEEQPEPAPAPEQEAAAPGPAAGGEAPK_.3	#N/A	4.3714	2.2547	3.4069	3.3180	3.7092	4.2758	3.8319	4.0718	4.2267	4.5778	3.7053	3.5208	4.3922	3.3940	3.4091	3.2155	3.1087	3.2237	4.3731	3.3610
Mapk8	_TAGTSFM[Oxidation (M)]M[Oxidation (M)]T[Phospho (STY)]PY[Phospho (STY)]VVTR_.2	#N/A	3.8118	4.1787	3.8784	3.5711	3.7613	6.2539	7.1040	3.5421	4.0385	8.0873	3.2991	8.6528	4.1807	9.2591	3.9313	3.9842	4.2103	3.4610	3.2977	8.8117
Vill	_GSQDS[Phospho (STY)]PENELGLDLR_.2	Q91YD6_S766_M1_Vill	4.3441	3.9400	3.8807	3.6265	3.0502	3.0835	3.9979	3.7632	3.9181	4.1547	3.3968	4.2020	4.0836	4.5862	4.0233	3.7062	4.3009	3.5323	3.1808	3.0524
Wasl	_VIY[Phospho (STY)]DFIEK_.2	Q91YD9_Y253_M1_Wasl	3.6321	5.0969	5.3967	5.1238	3.8391	5.2364	5.4270	3.8578	5.6939	5.7378	4.1087	5.0444	5.5899	5.6024	4.3117	5.0414	4.6242	5.2917	5.2064	5.7867
Rbm5	_DYDS[Phospho (STY)]PERER_.2	#N/A	7.0478	4.5724	3.7841	3.3367	6.6516	3.6539	4.0944	4.4732	4.1704	4.0582	3.0439	4.2985	4.2784	3.8222	4.1198	3.6097	6.8262	3.1790	3.4780	6.6282
Rbm5	_YDDYRDYDS[Phospho (STY)]PERER_.3	#N/A	3.4221	4.5188	4.0203	4.2053	3.6290	4.7635	3.4191	3.1844	3.3393	3.2326	4.3188	4.4082	3.5048	4.0074	3.4445	4.2850	3.7221	4.1111	5.1878	3.9744
Rbm5	_GLVAAYSGDS[Phospho (STY)]DNEEELVER_.2	Q91YE7_S624_M1_Rbm5	3.7622	7.2015	6.4537	4.1281	3.8811	6.5789	3.9780	4.1087	3.9347	4.5575	3.7168	3.4508	4.0848	8.3908	6.0077	3.7928	3.8174	7.6629	3.7404	3.3075
Synpo2	_AQS[Phospho (STY)]PTPSLPASWK_.2	Q91YE8_S895_M1_Synpo2	8.1932	8.7689	3.4965	3.7057	3.7988	8.4495	7.8030	4.1042	7.9954	8.2790	4.1757	9.0035	9.0758	9.5213	8.3408	8.5989	3.6015	3.5480	8.5175	8.7328
Uckl1	_YFGTDAVPDGS[Phospho (STY)]DDDEAATVG_.2	Q91YL3_S539_M1_Uckl1	3.3917	10.8755	9.1170	4.1372	4.2303	10.5673	10.6975	3.6727	3.3699	7.3943	6.2028	3.5982	3.4779	11.4664	9.6034	3.7366	3.1700	10.9741	9.4033	7.2863
Arhgap35	_NEEENIY[Phospho (STY)]SVPHDSTQGK_.2	#N/A	7.3521	7.0755	7.1860	7.1225	7.1207	6.8492	7.0133	7.4136	7.0048	6.7809	7.5137	7.5578	8.0349	7.7670	6.5769	7.3613	7.5654	7.5169	7.5975	7.4205
Arhgap35	_TSFSVGS[Phospho (STY)]DDELGPIRK_.2	#N/A	4.3131	6.1009	3.3485	3.3767	3.6508	4.5469	5.5205	4.0135	3.9498	4.5194	4.3241	3.5796	4.0578	4.2302	3.4679	3.1571	3.9760	5.8965	3.3385	3.2663
Arhgap35	_NQKNS[Phospho (STY)]LSDLNIDR_.2	#N/A	4.1906	3.5008	3.4476	4.7237	3.9402	4.1510	4.1568	4.7557	4.3664	4.4979	4.0362	3.3136	4.5182	3.5200	3.1718	3.2644	4.5964	3.0989	4.5751	5.1623
Arhgap35	NIIEATHM[Oxidation (M)]YDNVAEAC[Carbamidomethyl (C)]STTEEVFNS[Phospho (STY)]PR_.:	#N/A	6.1625	7.0570	4.4799	4.4373	4.0488	4.6534	6.5822	5.9813	5.1525	5.4277	4.9878	6.3168	6.6206	6.6325	4.0232	4.7155	5.2445	6.5235	5.3149	4.6815
Arhgap35	_TSFSVGS[Phospho (STY)]DDELGPIR_.2	#N/A	8.8582	9.6193	9.3257	9.0405	3.5312	9.2387	9.3143	3.9929	9.3209	8.9182	3.5563	8.5677	8.5087	9.9995	8.2725	8.4926	8.8048	9.7248	9.0980	8.4030
Arhgap35	_AQSNGSGNGS[Phospho (STY)]DSEMDTSSLER_.2	Q91YM2_S1134_M1_Arhgap35	4.3883	3.9841	6.4125	3.6707	3.0944	3.1277	3.9537	3.7190	3.8739	4.1989	3.3526	3.8736	4.0394	4.5420	3.9791	3.7504	4.2567	3.5764	3.2250	3.0082
Nln	_EAAS[Phospho (STY)]PLQAM[Oxidation (M)]SSYTAAGR_.2	#N/A	4.9980	5.0741	4.5334	4.0864	3.5396	3.6547	5.5042	4.1548	4.2754	3.8020	4.3160	4.3570	4.6540	5.6195	3.6053	4.3509	3.8997	4.7213	3.6368	3.7515
Csdc2	_DLPS[Phospho (STY)]PLPTKR_.2	#N/A	9.9268	9.7043	4.2370	3.9887	3.0384	7.9488	8.5899	7.9550	3.0609	10.4696	3.4353	9.1596	9.1124	9.3475	4.0798	9.2313	8.4655	8.3749	8.3423	3.8908
Csdc2	_[Acetyl (Protein N-term)]TSESTLPPVVPPLHS[Phospho (STY)]PK_.2	Q91YQ3_S16_M1_Csdc2	6.9482	6.8138	7.3121	3.7360	3.2911	5.8253	6.3860	6.7964	4.3091	6.5267	3.1826	6.9730	6.2764	7.8137	5.8347	6.4988	5.7943	6.6921	6.1360	5.7401
Csdc2	_DLPS[Phospho (STY)]PLPTK_.2	#N/A	4.1690	3.8338	3.2045	3.5204	3.5067	3.7308	4.0343	3.8694	4.0934	4.3753	2.9670	3.7232	4.2015	3.1916	3.6115	3.0131	4.4071	3.4261	3.0747	3.1586
Csdc2	_AS[Phospho (STY)]AGPVFK_.2	Q91YQ3_S65_M1_Csdc2	3.5681	3.7510	4.6055	4.0161	3.7750	2.7514	3.2731	4.5392	3.1933	3.4652	4.1728	3.4771	3.3587	3.8614	3.2985	4.4310	3.5761	4.2571	3.9057	2.8489
Rpn1	_VTAEVVLVHPGGGSTSR_.2	#N/A	3.4301	3.9104	4.4461	3.7738	4.2687	3.8873	3.9098	3.7209	3.3315	3.6364	3.7059	3.2972	3.4396	4.4842	3.2936	3.7273	3.5363	4.1411	4.3169	4.1614
Rpn1	_NIQVDSPYDISR_.2	#N/A	3.5003	5.0062	5.1137	4.0839	4.2755	3.7406	3.3409	3.7260	5.2750	3.3109	4.2405	4.6658	3.4265	4.5758	4.3626	4.3633	3.2232	4.1893	3.8379	5.0471
Twf1	_SPLLEIVER_.2	#N/A	5.4182	6.0569	6.3092	6.5208	5.7747	6.3863	5.9735	5.9942	6.0688	6.0740	6.0613	5.8064	5.6841	5.7309	5.7969	6.1437	6.2069	5.9517	6.1835	5.7655
Twf1	_YLLSQSS[Phospho (STY)]PAPLTAAEEELR_.2	Q91YR1_S143_M1_Twf1	3.9880	3.6718	5.7426	5.6920	5.2706	5.3772	3.1684	5.8013	5.2621	5.9408	3.1098	3.8341	5.7208	6.2449	5.3675	2.8592	6.0994	5.6399	5.9922	5.6544
Ndufv1	_GPDWILGEM[Oxidation (M)]K_.2	#N/A	7.8228	7.8881	7.5624	7.4194	7.9090	7.7415	7.5214	8.4099	7.5409	7.9060	8.0293	8.4249	8.4427	7.6454	7.9527	8.1137	8.0307	8.0608	7.9773	7.7348
Ndufv1	_IFTNLYGR_.2	#N/A	7.7088	7.7916	7.5377	8.0376	8.0862	7.8910	7.7432	7.4533	7.7478	7.5452	7.7752	8.0285	7.8678	7.4386	7.9816	7.8656	7.3106	8.0082	7.9292	8.0146
Ndufv1	_LVEGC[Carbamidomethyl (C)]LVGGR_.2	#N/A	7.3593	7.0956	7.8680	7.6230	7.6483	7.4001	7.7252	7.3212	7.4364	7.6589	7.4430	7.2135	7.2628	7.6226	7.8022	7.3292	7.3465	7.4168	7.0560	7.3476
Ndufv1	_EAYEAGLIGK_.2	#N/A	7.3134	7.4619	7.1588	7.3675	7.8423	7.0817	7.4713	7.5763	7.8885	7.5718	6.9340	7.2157	7.6629	7.4740	7.5499	7.4135	6.9651	7.2577	7.3005	7.6283
Ndufv1	_NAC[Carbamidomethyl (C)]GSDYDFDVFVVR_.2	#N/A	7.2368	7.0375	7.5975	6.7650	6.4729	6.6404	6.3087	7.1754	7.2559	6.7466	6.7701	6.9496	7.4561	7.2870	6.6938	6.6940	7.0465	6.6683	6.7718	7.1166
Ndufv1	_YLVVNADEGEPGTC[Carbamidomethyl (C)]K_.2	#N/A	6.7144	6.2599	6.6139	6.7333	6.3111	6.4192	6.4050	6.5725	6.8708	6.4307	6.2055	6.4022	5.6764	7.1303	6.6296	6.7136	6.5536	6.5588	5.9640	6.2100
Ndufv1	_GAGAYIC[Carbamidomethyl (C)]GEETALIESIEGK_.2	#N/A	6.9248	6.7888	5.5445	7.0963	6.1240	6.0426	6.3195	6.9536	6.3494	6.8769	6.3606	6.4358	7.3108	7.5789	6.1320	6.4247	6.9127	6.5882	6.2619	6.5983
Ndufv1	_HFRPELEDR_.3	#N/A	3.1165	4.8046	6.2536	6.1776	7.1578	6.2890	6.1700	6.7496	6.3145	6.4479	7.5092	5.6294	6.7821	6.6861	6.8525	6.7778	7.0296	6.3705	6.7672	5.7410
Ndufv1	_GEFYNEASNLQVAIR_.2	#N/A	6.4760	6.5277	5.8414	6.0894	5.7685	6.0989	5.6764	6.3751	6.5756	6.2885	6.2901	2.7964	6.2346	5.9678	5.6840	5.7775	6.2892	5.4264	6.3895	6.1948
Ndufv1	_GDARPAEIDSLWEISK_.3	#N/A	4.6711	5.6935	5.5883	5.8266	5.5347	5.4561	5.4138	4.7411	5.3535	5.4623	6.1388	5.8594	6.0452	5.1667	5.5664	6.2039	3.9579	5.3984	5.6616	5.7923
Ndufv1	_YLVVNADEGEPGTC[Carbamidomethyl (C)]KDR_.2	#N/A	5.6422	5.3482	4.7645	5.8494	5.4089	5.4863	5.7342	5.8938	5.7074	5.8402	5.3840	5.6635	5.8524	6.0448	5.7840	6.0407	5.5784	5.9664	5.7196	5.6506
Ndufv1	_KTSFGSLKDEDR_.3	#N/A	3.2070	6.0128	6.1904	5.3514	3.4140	6.4890	6.1176	3.3994	5.7989	5.9865	6.6922	4.1932	3.7198	5.6554	5.9683	6.5476	5.7305	5.5970	5.9819	5.9961
Ndufv1	_EGVDWMNK_.2	#N/A	3.3356	3.1664	4.1455	5.2965	3.6580	4.3982	4.5529	4.5368	3.4261	3.5419	4.3099	4.1534	3.5341	4.8754	2.9442	3.6804	1.6971	3.9233	1.8977	4.2560
Ndufv1	_HESC[Carbamidomethyl (C)]GQC[Carbamidomethyl (C)]TPC[Carbamidomethyl (C)]R_.2	#N/A	3.0905	3.6893	4.3519	3.8738	2.4959	3.8977	2.7790	3.5160	3.6709	4.4019	3.1495	4.0766	3.8363	4.3390	3.7771	3.9534	4.0537	3.7795	3.4281	4.3060
Ndufv1	_HAGGVTGGWDNLLAVIPGGSSTPLIPK_.3	#N/A	3.8562	2.0525	4.3925	3.8332	0.6228	3.1663	3.7913	3.5566	1.5354	4.3613	3.1901	0.7747	3.8769	4.1697	1.7370	4.2011	4.0943	3.7389	3.3875	4.3466
Ndufv1	_TSFGSLKDEDR_.3	#N/A	4.1275	3.1916	3.6641	3.4567	4.3344	4.3678	4.2145	4.3532	4.1347	3.9381	3.6134	4.4186	4.3002	3.7021	4.2399	3.4896	3.8504	3.2990	3.5981	3.3794
Ndufv1	_QIEGHTIC[Carbamidomethyl (C)]ALGDGAAWPVQGLIR_.3	#N/A	4.5451	4.2748	4.0817	4.5399	3.2512	3.2846	3.7969	3.2700	3.7171	4.3557	3.1957	3.6541	3.8825	4.1198	3.8222	3.9072	4.2680	4.3822	3.1805	3.7971
Ythdf2	_DGLNDDDFEPYLS[Phospho (STY)]PQAR_.2	Q91YT7_S39_M1_Ythdf2	8.1714	8.3184	8.4655	7.9257	3.9753	7.9110	7.8899	3.9277	3.6248	3.3431	8.2050	3.2547	7.6472	3.2767	3.1430	7.5034	3.3748	3.7245	7.9222	2.9540
Ptbp2	_DFGNSPLHR_.2	#N/A	3.7110	3.6081	3.2476	3.8732	3.9179	3.9513	3.1302	3.9367	3.0504	5.2461	4.0298	4.8385	3.2158	5.6814	5.7455	3.0731	3.4339	3.7155	4.0146	2.9629
Ptbp2	_LALDGQNIYNAC[Carbamidomethyl (C)]C[Carbamidomethyl (C)]TLR_.2	#N/A	3.3882	3.9309	4.4256	4.1961	3.5951	3.6284	3.4530	3.5203	3.3732	3.1988	4.3527	3.6570	3.5386	4.4637	3.4784	4.5366	3.1111	4.0384	4.3374	4.0083
Ttc1	AEAHPQDDHVEEEC[Carbamidomethyl (C)]FHDC[Carbamidomethyl (C)]SASFEEEQPGAHVAGSK	#N/A	4.3403	4.4796	3.8769	3.2439	3.0464	3.5611	4.0017	3.0652	4.2631	6.7145	5.8717	4.2057	4.3712	3.9150	4.0271	3.7024	6.0663	3.0862	3.3853	3.5922
Ttc1	_QDSSTGSYSINFVQNPNNNR_.2	#N/A	3.1154	4.2036	4.1529	4.4688	3.3224	3.2851	5.3566	3.3411	3.0383	3.9297	3.4126	3.9298	3.1463	6.1489	4.0572	3.9784	4.3392	4.3111	3.1093	3.8682
Grhpr	_LLDAAGANLR_.2	#N/A	6.4461	6.6031	5.6197	6.5012	6.6128	6.1082	6.4710	6.7013	5.6612	6.7686	6.3215	6.4933	6.5457	6.3814	6.8058	6.1701	5.6592	6.4483	6.6833	5.9077
Grhpr	_VFVTGPLPAEGR_.2	#N/A	5.5696	6.1903	6.7144	6.6953	6.2535	5.6519	6.3349	6.4481	5.7787	5.9553	5.7489	6.5704	5.4211	5.5226	5.8331	5.8021	6.2785	5.4789	5.9646	6.0972
Grhpr	_NTM[Oxidation (M)]SLLAANNLLAGLR_.2	#N/A	5.3130	3.8480	4.8496	4.9321	5.0065	5.0408	4.3701	4.4119	5.0378	5.3973	5.0173	5.1286	5.3100	4.4212	5.1056	4.8749	5.0946	4.7228	4.4339	5.3075

Grhpr	_VISTLSVGVDHLALDEIK_.3	#N/A	3.2631	3.2389	3.7994	4.4263	4.1017	3.8833	0.7762	4.4643	3.0747	3.4694	4.0979	3.1283	3.2838	3.7865	3.0166	3.6080	3.5012	1.8081	3.9806	3.4980
Srgap2	_S[Phospho (STY)]PDSTANVR_.2	#N/A	9.8651	9.6500	9.7979	9.7547	9.2227	9.3494	9.4031	9.3052	9.1210	9.6985	9.3837	9.0777	9.0981	9.4564	2.8079	9.1372	9.3664	9.0000	9.1745	9.0999
Srgap2	_ELEGPIYS[Phospho (STY)]R_.2	Q91Z67_S691_M1_Srgap2	4.2953	4.5246	8.2723	8.7614	3.0014	8.4956	4.0467	4.5210	8.5316	8.5917	8.5065	8.0396	4.3262	8.6763	4.0720	8.1066	8.8106	3.1312	3.4302	8.2642
Srgap2	_QTPRS[Phospho (STY)]PDSTANVR_.3	#N/A	3.6563	3.6627	3.1929	3.9279	3.8633	3.8966	3.1848	3.8820	3.1050	3.4669	4.0845	3.3889	3.2705	3.2310	3.2102	3.0185	3.3793	3.7702	4.0692	4.4091
Srgap2	_KQDS[Phospho (STY)]SQAIPLVVESC[Carbamidomethyl (C)]IR_.2	Q91Z67_S500_M1_Srgap2	3.3585	3.1435	3.8948	4.3309	4.1970	4.4211	3.3440	3.0589	3.4031	3.5648	3.7775	3.0329	3.5112	6.7151	2.9212	3.7034	3.5965	6.2904	4.2453	4.2331
Srgap1	_GLNNDS[Phospho (STY)]PERR_.2	Q91Z69_S883_M1_Srgap1	5.5080	6.2686	3.9854	5.7017	5.6657	5.4775	5.8983	5.3217	3.8133	6.0803	5.8883	5.8592	3.9788	6.8196	3.9185	5.3725	4.1718	5.1023	3.2767	3.7008
Srgap1	_DMNS[Phospho (STY)]PTDR_.2	Q91Z69_S812_M1_Srgap1	3.8580	4.1448	4.3943	3.8314	3.1957	3.2884	4.3454	3.5583	3.7132	4.0643	3.1919	4.0343	3.8787	5.6353	3.9226	4.2029	4.0960	3.7371	3.3857	4.3483
Ugp2	_IYTFNQSR_.2	#N/A	5.4077	5.9324	6.4512	5.5423	5.6094	5.9059	6.1285	5.6654	5.9328	5.9665	5.7454	5.6320	5.0114	6.1612	6.0243	5.6736	5.5852	6.2439	5.8394	5.5440
Ugp2	_AM[Oxidation (M)]S[Phospho (STY)]QDGASQFQEVILQELELSVK_.3	Q91ZJ5_S13_M1_Ugp2	4.0152	3.3039	3.5518	3.5690	4.2222	4.2555	4.3268	4.2409	4.2470	3.8258	3.7256	3.0300	4.4124	3.5899	2.8513	3.3773	3.7381	3.2034	3.7104	3.3812
Ugp2	_NENTFLDLTVQQIEHLNK_.3	#N/A	3.8836	3.4355	3.4202	3.7007	4.0905	4.1238	4.4584	4.1093	4.3786	3.6942	3.8573	3.1616	4.5441	3.4582	2.9830	3.2457	3.6065	3.5430	3.8420	3.1355
Sh2b1	_ALANDGTS[Phospho (STY)]PGER_.2	Q91ZM2_S219_M1_Sh2b1	7.7045	8.2063	4.3864	8.3097	3.5559	3.0516	7.1665	3.5747	3.2718	8.2309	3.6462	7.4475	3.3799	7.7313	2.7899	4.2120	3.0719	4.0775	4.3766	4.1018
Sh2b1	_S[Phospho (STY)]SEDLAGPLPSSVPSSTTSSKPK_.3	Q91ZM2_S126_M1_Sh2b1	4.5166	4.3034	4.0531	8.1135	3.2227	7.3733	3.8254	3.2414	4.4394	8.8727	3.3129	4.0295	4.5474	4.0912	3.8508	3.8787	4.2395	7.8076	3.2090	3.7685
Ndfip2	_DDFS[Phospho (STY)]DVEQLR_.2	Q91ZP6_S195_M1_Ndfip2	3.8158	6.9960	7.1697	3.8736	3.1535	7.6155	6.3526	3.5162	6.8283	4.0221	3.3202	6.1528	4.5547	7.2013	5.0744	4.1607	4.0539	6.8947	6.7966	6.5627
Necab2	_TLS[Phospho (STY)]FDLQQR_.2	Q91ZP9_S271_M1_Necab2	3.7252	3.5939	3.2618	3.8590	3.9322	7.5844	3.1159	3.9509	7.3868	7.0532	7.3606	3.3200	3.2016	8.7129	7.5774	3.0873	8.2623	7.7270	8.0030	8.1304
Necab2	_VYEGGSNVDQFVTR_.2	#N/A	3.7819	4.4168	4.3181	3.9075	3.8664	3.3437	4.8499	3.4822	2.9797	4.3287	4.8177	4.1104	3.8026	4.3052	4.0397	4.1267	4.0199	3.8133	3.4618	4.2722
Dst	_SES[Phospho (STY)]NSSITATQPTLAK_.2	Q91ZU6_S7186_M1_Dst	9.2134	9.2252	9.2435	9.2162	8.3389	8.6685	8.8781	8.8465	8.5470	9.0097	8.7484	8.8627	9.1274	8.8946	7.2713	8.7191	9.4427	8.9469	8.8912	8.7561
Dst	_QKS[Phospho (STY)]FSEDVISHK_.3	#N/A	4.2620	6.5332	6.4662	6.3055	5.8721	6.0927	6.3904	6.0805	6.3517	6.6378	6.5287	3.7817	6.4026	3.9911	3.4592	3.8729	4.8300	6.2300	3.9737	3.9581
Dst	_LLDPEDVDVSS[Phospho (STY)]PDEK_.2	Q91ZU6_S237_M1_Dst	3.6679	10.8417	10.3296	4.1597	3.7179	8.3191	10.4648	4.0806	10.1695	3.8184	3.7165	3.5741	3.6886	10.3566	8.6880	4.0128	3.9059	10.5949	10.0857	9.7311
Dst	_AM[Oxidation (M)]VESQQKS[Phospho (STY)]PVKR_.3	#N/A	4.7697	5.6001	4.5778	5.1829	4.2750	4.2411	5.2673	4.9499	4.8471	5.1771	4.8816	5.0920	5.7713	4.9111	4.6483	5.0113	4.8891	5.2101	4.9997	5.0023
Dst	_AMVESQQKS[Phospho (STY)]PVK_.2	#N/A	3.1617	5.0362	5.7598	5.6727	4.0002	4.2243	5.0400	3.9028	3.5999	3.3680	5.0332	3.2297	3.7080	3.2518	3.1180	3.5066	3.4000	3.7494	4.0485	4.4299
Dst	_QLS[Phospho (STY)]LLDSR_.2	Q91ZU6_S5407_M1_Dst	3.4653	4.5620	3.9771	4.2486	3.6722	3.7056	3.3759	3.1412	3.2961	3.2759	4.2755	2.9506	3.4615	3.9642	3.4012	4.3283	3.6789	4.1543	3.8029	3.9312
Mia2	_DNLGNSNVPDS[Phospho (STY)]PIPAEC[Carbamidomethyl (C)]EAAGR_.2	Q91ZV0_S1271_M1_Mia2	3.5663	4.4365	4.1026	4.1231	2.9040	3.5801	6.6388	3.2667	3.4216	3.1504	2.9002	4.3259	3.5870	4.0897	4.2142	3.9112	3.8044	4.0288	3.6774	4.0567
Mia2	_SLS[Phospho (STY)]PPWEQDRR_.2	Q91ZV0_S1190_M1_Mia2	3.6586	3.6605	8.5302	8.5027	3.8655	4.2428	3.5223	9.3368	3.5814	9.1290	3.9558	3.3866	10.1685	3.2332	8.4247	3.0207	7.9860	3.7679	9.9351	9.3633
Lrp1	_IDLETGENR_.2	#N/A	3.6456	5.4290	5.3263	3.7604	5.5151	5.5071	4.9389	3.8713	4.9781	5.3482	5.2413	3.3996	5.4086	5.2132	4.7727	4.9128	4.9699	5.1290	4.9690	4.5637
Lrp1	_AVTDEEPFLIFANR_.2	#N/A	5.5571	5.1349	5.1522	5.6218	5.6472	5.7766	5.3357	5.9465	4.8603	4.9993	5.2367	5.4343	4.3588	3.9025	5.4593	5.8475	4.0508	5.4682	4.9410	3.5798
Lrp1	_AEGSEYQVLYIADDNEIR_.2	#N/A	5.6864	4.1687	3.5148	4.7651	4.1386	4.6468	4.3636	5.0402	5.0315	5.5655	4.7943	4.0525	5.2827	4.8828	3.9972	4.5622	5.2717	5.0211	3.9641	4.1403
Lrp1	_HS[Phospho (STY)]LAS[Phospho (STY)]TDEKR_.2	Q91ZX7_S4521_M2_Lrp1	6.7090	5.8621	6.4674	7.0172	6.2778	6.2731	5.8682	5.8360	5.9711	7.1170	6.3491	4.1319	4.8371	6.1525	4.6657	6.0006	6.1464	6.0490	6.1233	6.1356
Lrp1	_IFFSDIHFGNIQQINDDGSGR_.3	#N/A	3.9034	3.4157	3.4400	3.6808	4.1104	4.1437	4.4386	4.1291	4.3588	3.7140	3.8374	3.1418	4.5242	3.4781	2.9631	3.2655	3.6263	3.0916	3.8222	3.4821
Sncb	_EGVVAAAEK_.2	#N/A	8.4300	8.9357	8.7987	8.6587	8.5140	8.8431	9.3279	8.0711	9.3414	9.3129	9.0549	8.9298	9.3932	8.9541	9.3633	8.9333	7.7113	9.0449	9.0409	9.1689
Sncb	_TKEQASHLGGAVFSGAGNIAAATGLVK_.3	#N/A	7.0450	7.5088	7.0942	7.1347	7.0941	6.9509	7.1238	7.3780	7.2744	7.3666	6.8888	7.1737	7.8975	7.7147	7.4309	7.4331	7.2358	7.1354	7.5255	7.8340
Sncb	_QGVTEAAEK_.2	#N/A	8.1974	7.8048	8.2064	7.9579	8.1019	8.0470	8.0553	8.1923	8.2241	8.0411	8.0982	8.3058	8.3507	8.0550	8.5772	8.0182	8.0632	8.2508	8.0324	8.0764
Sncb	_TKQGVTEAAEK_.2	#N/A	6.7247	6.6423	7.2764	6.8254	7.2124	6.7492	6.7817	7.3504	7.1307	7.0841	6.9294	7.2631	7.0081	7.5460	8.2596	8.0465	7.2394	7.0999	7.0134	7.3932
Sncb	_TKEGVLYVGSK_.2	#N/A	8.2084	8.6991	8.6998	8.7680	8.5203	8.4807	8.4717	8.1794	8.4858	8.7187	8.3144	8.6155	8.8326	8.6159	9.1196	8.7236	8.6723	8.1641	8.8000	8.5180
Sncb	_EGVLYVGSK_.2	#N/A	9.4076	9.3779	9.4612	9.5385	9.3907	9.3395	9.5340	9.1578	9.7137	9.7514	9.8580	9.3984	9.8442	9.7003	9.4310	9.6138	9.1860	9.2635	4.1037	9.7499
Sncb	_EQASHLGGAVFSGAGNIAAATGLVK_.3	#N/A	6.3251	5.9869	5.8973	5.9293	6.6261	5.9769	5.5724	5.8853	5.8989	6.6103	6.6038	6.1412	6.6792	6.3913	5.6651	6.2858	6.0512	6.0035	6.2066	6.4177
Sncb	_TSGVVQGVASVAEK_.2	#N/A	8.6534	9.0644	9.3338	8.9978	8.4633	8.7037	8.8509	8.6815	9.0857	8.6712	8.2797	8.9324	8.5680	9.5282	9.5418	9.2157	8.7533	8.8908	9.0358	8.9520
Kcnh5	_KTDS[Phospho (STY)]C[Carbamidomethyl (C)]DSGITK_.2	Q920E3_S863_M1_Kcnh5	4.1204	3.2937	4.1111	5.4307	4.0468	4.0998	3.4943	4.4095	4.0573	3.4146	4.6596	4.5828	5.7826	3.7316	3.0715	3.5531	3.4463	4.3869	4.9848	3.7522
Kcnh5	_AGEARS[Phospho (STY)]PLEHS[Phospho (STY)]PSQADVK_.3	Q920E3_S883_M2_Kcnh5	3.2041	4.1150	4.2415	4.3801	3.4111	3.1965	3.6370	3.4298	3.1269	4.5156	3.5013	3.8411	3.2350	4.2796	3.6624	4.0671	3.0480	4.2224	3.0206	3.9569
Fdps	_YEEDSYNR_.2	#N/A	4.4982	11.1382	4.4450	3.9701	3.5270	4.2235	3.8438	3.6091	3.7640	4.2537	2.5280	2.8777	3.9294	3.7484	3.8692	3.8603	4.3173	3.6864	3.3350	4.0499
Wdr7	_TYQVPPVQPAS[Phospho (STY)]PGSHNALK_.3	#N/A	11.7433	11.8320	11.1088	10.5154	9.2366	11.4788	11.0964	9.2619	10.7819	3.7704	10.9933	10.6112	11.4364	11.9602	8.4093	10.3130	9.5724	12.2510	10.7388	9.8945
Wdr7	_GPITAVSFAPDGR_.2	#N/A	6.0394	6.1765	6.3190	6.1561	6.4591	5.8250	5.9069	5.8371	6.1790	5.7597	6.1532	5.4282	3.3626	5.6858	5.6786	5.7317	6.3215	6.1437	5.5788	5.6939
Wdr7	_ARDS[Phospho (STY)]PPPSSNIVQGQIK_.3	Q920I9_S935_M1_Wdr7	3.3893	4.6135	3.9256	4.3001	4.2278	3.7571	3.3243	3.0896	3.2445	6.8788	6.1584	3.0021	3.4100	3.9126	3.3497	3.7342	3.6273	5.5746	3.8544	3.8796
Wdr7	_LPASC[Carbamidomethyl (C)]LPASDSFR_.2	#N/A	3.7791	4.2238	6.0740	3.9103	3.1168	6.2446	4.4243	6.0200	6.1793	6.3349	5.8230	4.1132	4.5914	4.1352	5.8921	5.6405	6.1832	5.9939	5.9933	3.8125
Wdr7	_LAC[Carbamidomethyl (C)]THTGIQFYQFSVGNQQEGR_.3	#N/A	4.1155	3.8873	3.1509	3.5739	3.4532	3.0309	4.0505	3.3990	3.9707	4.1021	3.4494	3.7768	4.1362	3.1380	4.0759	2.9595	4.3535	3.4796	3.1282	3.1050
Wdr7	_RQS[Phospho (STY)]REDSDPEYR_.3	Q920I9_S772_M1_Wdr7	4.1784	3.7742	3.2640	3.4608	2.8845	4.4187	4.1636	3.9289	4.3726	3.9890	3.5625	3.6637	4.2493	3.2511	4.1890	3.5405	4.4666	3.3665	3.0151	3.2181
Syt17	_RIS[Phospho (STY)]SLDSR_.2	Q920M7_S106_M1_Syt17	6.8325	6.3099	3.7144	6.5890	4.4641	4.1787	3.9383	6.2574	3.9065	7.0788	3.7713	5.6010	6.7797	6.4561	5.0794	6.2721	6.4292	6.2319	6.3287	6.1090
Msi2	_[Acetyl (Protein N-term)]M[Oxidation (M)]EANGS[Phospho (STY)]PGTSGSANDSQHDPGK_.2	Q920Q6_S6_M1_Msi2	3.9884	4.0144	4.5247	3.7010	3.3261	3.5502	4.2150	3.6887	4.2741	4.1912	3.1476	3.9039	4.3821	4.5117	3.7922	4.3333	4.2264	3.6067	3.4751	3.6032
Ivns1abp	_NS[Phospho (STY)]PQSS[Phospho (STY)]PTSTPK_.2	Q920Q8_S322_M2_Ivns1abp	8.1305	5.8395	8.3357	8.2563	8.0861	6.0310	8.0153	8.1680	8.0501	5.0684	6.0974	7.6812	6.1376	6.5117	7.5103	5.8241	7.5034	5.7189	5.5418	8.0446
Als2	_QHKDS[Phospho (STY)]PEILSR_.3	#N/A	4.0943	3.6902	3.3481	5.8977	4.3012	5.3889	4.2477	5.8826	4.1679	3.9049	3.6465	5.2324	4.3333	5.4404	4.3051	3.4564	4.5507	3.2825	4.4319	3.3022
Als2	_QLGC[Carbamidomethyl (C)]ES[Phospho (STY)]PGQGEVWK_.2	Q920R0_S1302_M1_Als2	4.2653	5.8097	5.8151	5.6686	4.8382	5.3857	5.9753	6.1420	5.3459	5.3205	5.8843	5.7445	7.1120	6.1767	4.4216	4.9946	6.5348	5.7750	4.8410	4.4703
Als2	_RQHKDS[Phospho (STY)]PEILSR_.3	#N/A	3.6216	4.3812	4.1579	4.0678	2.9593	3.1834	3.0809	3.3219	5.7904	3.8279	3.5144	4.2707	3.2481	4.1449	4.1590	3.9665	3.8596	3.9735	3.6221	4.1119
Als2	_SSS[Phospho (STY)]LM[Oxidation (M)]DIREEELEGGSR_.3	#N/A	4.0574	4.0349	4.9117	3.6929	3.3951	3.6305	3.9189	4.4073	3.8168	3.8061	3.5272	3.8958	4.0781	4.6493	3.7841	3.9447	3.8378	3.9939	3.7969	3.5973
Als2	_RLS[Phospho (STY)]LPGLLSQVS[Phospho (STY)]PR_.2	Q920R0_S477_M2_Als2	4.5230	4.1188	4.4203	3.8054	3.2291	3.2624	3.8190	3.5844	3.7393	4.3335	3.2179	4.0231	3.9047	4.4074	3.8444	3.8851	4.1220	3.9192	3.3597	4.3744
Gjc3	_S[Phospho (STY)]KDTTDELSVVEAK_.2	Q921C1_S253_M1_Gjc3	3.5601	4.4427	4.0964	4.1293	2.8978	3.5862	3.1424	3.2605	3.2015	3.7664	3.5759	4.3321	3.5808	4.0835	4.2204	3.9050	3.7982	4.0350	3.6836	4.0505
Brwd1	_KS[Phospho (STY)]SSDKESNLQVTQK_.3	Q921C3_S1888_M1_Brwd1	3.7948	3.5242	3.3314	3.7894	4.0018	4.1066	3.0463	4.0205	4.4674	3.6054	3.9460	3.2504	3.8257	3.3695	3.0717	3.1570	3.5177	3.0396	3.9307	3.0468
Brwd1	_RGPEVEGS[Phospho (STY)]PVSEALR_.3	Q921C3_S1830_M1_Brwd1	3.8297	3.4894	3.3662	3.7546	4.0366	4.0699	3.0115	4.0553	4.4326	3.6403	3.9112	3.2156	4.5980	3.4043	3.0369	4.2344	3.5526	3.0178	4.1672	3.5668
Bicd2	_QTS[Phospho (STY)]LDNEKDR_.2	Q921C5_S411_M1_Bicd2	4.0737	4.6460	4.6100	3.6157	3.4115	3.6355	4.1296	3.7741	4.1887	4.1963	3.0622	3.8185	4.2968	4.5971	3.7068	4.4186	4.3118	3.5214	3.1700	3.0633
Bicd2	_S[Phospho (STY)]PVLLPK_.2	Q921C5_S578_M1_Bicd2	3.3468	3.1551	3.8831	4.3426	4.1854	3.7996	3.3557	3.0472	3.2021	3.5531	4.1816	3.0446	3.3675	3.8702	2.9329	3.6917	3.5849	4.2483	3.8969	3.8372
Tardbp	_LPNSKQS[Phospho (STY)]PDEPLR_.2	#N/A	7.8711	8.1114	3.6535	4.0731	8.0976	3.9315	7.5170	4.3286	7.6692	8.2979	4.1632	3.1227	3.6472	3.6621	3.2979	3.5154	8.0775	3.9542	7.8321	3.5127
Tardbp	_FGGNPGGFGNQGGFGNSR_.2	#N/A	3.5860	3.7331	3.1226	3.9982	3.7929	4.3154	6.0854	3.8117	3.5088	3.4591	3.8832	3.4592	3.6169	6.1439	3.2805	2.9481	3.3089	3.8405	4.1396	5.5506
Tardbp	_GISVHISNAEPK_.3	#N/A	3.3915	4.6113	3.9278	4.2979	4.2300	4.4541	3.3110	3.0919	3.3701	3.5978	3.7445	2.9999	3.4782	3.9148	2.8882	3.7364	3.6295	3.9792	3.8522	4.2001
Hnrnpll	_TEEGEIVYS[Phospho (STY)]AEESENR_.2	#N/A	3.5243	4.4785	4.0606	4.1651	2.8620	3.0861	3.1782	3.2247	6.4483	3.7306	3.6117	4.3680	3.3454	4.0476	4.2563	5.6800	3.7623	4.0708	3.7194	4.0673
Hnrnpll	_LKTEEGEIVYS[Phospho (STY)]AEESENR_.3	#N/A	4.0830	5.0987	4.3015	4.1571	3.2856	5.1768	5.1088	3.4769	3.8267	5.0607	3.3657	4.5699	3.5785	5.2535	4.0103	4.1180	4.2486	5.2402	2.9271	4.5553
Hnrnpll	_ITRPGNTDDPSGGNK_.2	#N/A	3.8791	3.9218	3.8903	3.9426	3.2168	4.2104	3.6722	3.7135	3.6827	5.0201	3.5582	3.7273	3.8273	3.9128	3.9728	3.5005	4.4740	3.8090	3.8913	3.8089
Sh3bp4	_SYS[Phospho (STY)]LSELSVLQAK_.2	Q921I6_S245_M1_Sh3bp4	3.7727	4.7676	3.3093	3.8115	3.9796	4.0130	3.0685	3.9984	5.0401	3.5833	3.9682	3.2725	3.1541	3.3473	3.0939	3.1348	5.4987	3.6539	3.9529	3.0246
Rheb	_IILEAEKIDGAAS[Phospho (STY)]QGK_.2	Q921J2_S175_M1_Rheb	4.5086	3.4942	3.5440	3.7532	3.8463	4.0704	4.0207	4.0567	3.7539	3.2141	4.1282	4.7100	3.8619	4.4632	3.2720	4.2119	3.5539	3.5955	3.8945	4.5083
Rheb	_ALAESWNAAFLESSAK_.2	#N/A	4.0359	3.9669	4.5722	3.6535	3.9401	3.5977	4.1675	3.7363	4.4605	4.2422	3.1001	3.8564	4.3346	4.5593	3.7447	3.9746	4.2739	3.5592	3.8097	3.0254
Cyrib	_INNVPAEGENEVNNELANR_.2	#N/A	8.5831	8.2408	8.4809	8.1755	8.2155	8.4251	8.2717	8.9196	8.1202	8.5642	8.3302	8.1683	8.3906	8.2383	7.2706	8.1475	8.7666	8.1911	8.1843	8.0218
Cyrib	_DAEGILEDLQSYR_.2	#N/A	7.9654	7.4222	8.0884	7.8156	7.3335	7.5922	7.5148	7.6806	7.6650	7.5708	7.3691	7.3221	7.3497	7.0461	6.9005	7.4003	7.7315	7.4629	7.2609	7.1910
Cyrib	_EAIQHPADEKLQEK_.3	#N/A	4.7414	3.5887	3.2669	3.8539	4.5282	3.9706	4.0379	3.9560	5.1735	4.6525	4.0767	3.3149	5.1163	3.3050	4.4973	3.8892	3.4533	5.0152	4.1154	4.4384
Rin1	_RLS[Phospho (STY)]ADGSLGR_.2	Q921Q7_S446_M1_Rin1	3.3563	5.3228	4.0861	4.1396	3.5632	3.5966	3.4849	5.9376	3.4051	3.1669	6.0177	4.3424	3.5705	4.0732	3.5103	4.2192	3.7879	5.8435	3.6939	4.0402
Rin1	_EKPSTDPLY[Phospho (STY)]DTPDTR_.2	#N/A	3.9114	3.5666	3.3818	3.5622	4.1183	7.9993	3.8227	4.0585	4.3590	3.7220	5.9344	7.4351	3.9083	6.9978	2.9633	7.0533	6.8550	7.4912	7.4584	6.4015
Rin1	_SM[Oxidation (M)]S[Phospho (STY)]SAFC[Carbamidomethyl (C)]SLLAPER_.2	#N/A	4.1161	3.5659	3.4004	3.5294	3.9129	4.1840	6.7795	7.7851	4.0024	3.8818	3.9665	3.3876	4.1414	7.8258	3.2494	3.2181	4.1431	3.4020	3.4234	3.2305
Rin1	_ALPLS[Phospho (STY)]PAQELQR_.2	Q921Q7_S590_M1_Rin1	3.7061	4.2967	4.2424	3.9833	3.0438	3.2679	3.8966	3.3584	3.0555	3.9124	3.4299	4.1862	3.1636	4.2082	4.0745	4.0510	4.3565	4.2938	3.0920	3.8855
Dars1	_NNAYLAQS[Phospho (STY)]PQLYK_.2	Q922B2_S249_M1_Dars1	7.8890	8.1390	7.6556	7.9279	2.8589	7.7355	7.3567	7.6597	7.7624	7.9018	7.6809	7.2086	7.7912	7.9539	6.7485	7.7423	7.9228	7.1653	3.7226	7.5684
Dars1	_FLEPTLR_.2	#N/A	4.8596	5.2908	4.2954	5.4983	5.4067	4.4666	6.0108	5.8157	4.9490	5.0400	4.8505	5.6335	5.2260	5.1661	5.2593	4.6798	3.8091	5.1931	4.8984	4.8851
Dars1	_IISAASEGGANVFTVSYFK_.2	#N/A	4.3448	4.0344	4.3221	4.2995	3.4916	3.1735	3.5747	4.7800	3.2075	3.0953	2.2473	3.6167	3.3156	4.3602	3.5818	4.1476	4.5084	4.1418	4.4409	2.6730
Ppp6r3	_C[Carbamidomethyl (C)]TAPLTPSSS[Phospho (STY)]PEQR_.2	Q922D4_S824_M1_Ppp6r3	4.4612	4.2525	4.1918	3.9428	3.1673	5.6201	3.8811	3.3702	6.4641	4.2718	3.2800	4.0852	3.9668	4.4694	3.9065	5.7814	4.1972	3.6494	6.0949	4.0808
Ppp6r3	_IQQFDDGGS[Phospho (STY)]DEEDIWEEK_.2	Q922D4_S588_M1_Ppp6r3	4.8208	8.9168	8.1625	6.1232	3.0020	8.3880	8.4328	4.2247	8.2471	7.2501	3.2944	7.0963	4.2475	9.0376	7.1234	6.0267	4.7050	8.7823	7.8886	7.2389
Mthfd1	_TPVPSDIAISR_.2	#N/A	5.6368	5.3027	5.4947	5.4539	5.9838	5.7213	5.4077	5.9221	5.6460	5.1093	5.4954	5.3137	6.3694	5.1472	5.8070	5.8221	5.3046	5.6784	5.1608	5.7141
Mthfd1	_LDIDPETITWQR_.2	#N/A	3.8353	4.3156	4.0409	4.5807	3.1730	3.3971	4.3681	3.2292	4.4272	4.0107	4.1243	4.0417	4.5352	4.4864	3.9453	4.1802	4.2273	4.4230	3.2212	4.1756
Clip1	_TASESISNLSEAGS[Phospho (STY)]VK_.2	Q922J3_S203_M1_Clip1	8.5743	8.6774	9.1089	8.9676	7.9365	7.9468	8.2886	7.9419	8.1024	8.9393	8.0672	8.4215	8.5025	8.8867	7.2947	8.4691	8.2988	8.1138	8.2126	8.5150
Clip1	_TASESISNLS[Phospho (STY)]EAGS[Phospho (STY)]VKKGER_.3	#N/A	4.9453	5.5293	4.1627	5.9283	3.3322	3.3655	4.7765	3.3509	3.6361	5.8446	3.9149	5.0180	3.8016	4.8426	3.7413	4.7319	4.3490	4.4425	3.0995	4.1191
Clip1	_S[Phospho (STY)]VEELQLK_.2	Q922J3_S910_M1_Clip1	4.2709	1.8440	4.6412	3.3134	2.9770	3.8671	4.0711	4.4965	3.9913	4.0815	3.4700	4.2752	4.1568	4.7734	4.0965	3.4991	3.9938	4.8773	5.0008	4.8165
Tspan2	_HVQSM[Oxidation (M)]YEEAYSDYLKDR_.3	#N/A	7.3686	6.0732	4.4067	5.2356	6.0070	6.5055	5.9442	6.9055	6.6003	5.4764	3.6664	6.1098	6.5292	4.4447	3.4973	4.8833	6.4163	5.9091	5.1991	4.1220
	KSPSGPVKS[Phospho (STY)]PPLS[Phospho (STY)]PVGTTPVK.3	Q922M7_S189_M2_	4.3994	6.4329	3.2196	3.5052	3.5219	4.8063	4.0192	3.8845	4.0783	4.3905	2.9518	3.7081	4.1863	7.0650	4.4520	3.0282	4.4222	5.3327	6.0431	3.4074
Glyr1	_KLS[Phospho (STY)]LSEGK_.2	Q922P9_S130_M1_Glyr1	5.5012	4.5710	3.9681	4.2576	4.7196	2.9936	4.1872	3.1322	3.3298	5.6943	4.5580	4.9712	4.7249	5.0729	2.8479	3.7767	5.1851	5.2424	4.3186	4.1597
Lrrc59	_LQQLPADFGR_.2	#N/A	3.3388	5.0429	4.3762	4.2454	4.9477	5.3259	5.6501	3.2677	3.4225	4.9977	4.6477	3.7064	3.5880	4.8288	5.0435	4.2018	5.5112	5.4439	3.6764	4.0576
Lrrc59	_LVNLQHLDLLNNR_.2	#N/A	3.4899	3.4014	4.5274	2.9057	3.6969	1.6485	3.3243	3.7156	2.9820	4.0350	3.7871	3.4885	3.5208	3.5235	1.5976	4.3529	4.0354	3.9366	3.0939	3.5262
Pdia6	_GSFSEQGINEFLR_.2	#N/A	5.8894	5.5125	5.9335	3.3665	5.7379	6.4477	6.0374	5.5697	5.8538	6.2388	5.8066	5.9792	6.2042	6.0927	6.1656	6.3190	5.8738	5.9198	6.0840	5.8138
Pdia6	_LAAVDATVNQVLASR_.2	#N/A	4.4530	4.0474	4.1148	4.9837	5.2463	3.2871	4.0290	3.5040	4.1091	3.6501	4.0449	4.1205	4.7882	4.7276	4.5586	4.9213	4.3428	4.2356	4.4748	4.6518
Pde2a	_VETVYTYLLDGESR_.2	#N/A	6.1133	5.2616	5.9022	5.4880	5.0654	5.8486	5.4761	5.9906	5.8170	6.0267	5.7640	5.3090	5.7001	3.6927	4.7218	5.5976	5.8031	6.0009	5.8247	5.9550
Pde2a	_DIILATDLAHHLR_.3	#N/A	3.0918	3.7743	4.4043	2.9226	3.7768	3.8936	3.5357	3.6147	3.9081	3.8787	4.3740	4.3213	3.5600	4.4423	3.5168	4.2298	3.0898	3.6444	3.7709	4.0771
Pde2a	_RPEAVQNTS[Phospho (STY)]VDASEDQKDEK_.3	Q922S4_S204_M1_Pde2a	3.6958	3.2702	3.6801	4.0671	4.1657	4.2918	4.8080	4.3563	3.9567	3.8507	4.8651	3.2263	4.0569	3.2850	2.9078	4.4747	4.4649	3.5587	4.2582	3.3655
Pde2a	_GLPS[Phospho (STY)]NNSLDFLDEEYEVPDLDGTR_.3	Q922S4_S908_M1_Pde2a	4.0623	4.0679	3.8194	3.8795	3.4000	3.6241	3.4892	3.8488	3.5483	3.4894	3.9227	3.9574	3.6564	3.9686	3.8457	3.6280	3.9795	3.7428	3.7883	3.7539
Pde2a	_LLHDGIQPVAAIDSNFANFTYTPR_.3	#N/A	4.1860	4.3178	3.9307	2.8805	3.2106	3.3993	3.8398	4.2404	4.2331	4.0438	3.2985	2.9276	4.5330	4.0768	3.8652	3.8643	4.2251	4.4252	3.5949	1.1670
Pde2a	_SVLAALYSSEGSVM[Oxidation (M)]ER_.2	#N/A	5.4329	4.3315	4.2076	5.4382	4.0871	4.6761	3.0312	4.7917	3.0903	3.8776	4.6798	4.3069	3.6920	6.1414	4.1093	4.0162	3.9093	3.9238	3.5724	4.1617
Pde2a	_SQQYPAAR_.2	#N/A	3.4183	3.8986	4.4579	4.1637	4.2568	4.4809	3.2842	4.2495	3.3433	4.8071	3.7177	3.6247	3.4514	4.4960	2.8614	4.0043	3.1434	4.0060	4.3051	4.1733
Prpf3	_WDEQTSNTKGDDDEES[Phospho (STY)]DEEAVKK_.3	#N/A	3.3739	3.9452	4.4113	4.2103	3.5808	3.0267	3.2376	3.5996	1.1387	3.6712	3.6711	3.6713	3.4048	4.4494	2.0988	4.2369	3.0968	4.0526	4.3517	4.1267
Prpf3	_GDDDEES[Phospho (STY)]DEEAVKK_.2	#N/A	4.4445	4.9787	4.5184	4.1657	4.3519	4.3927	4.5605	4.1260	3.5814	4.6777	4.0520	3.3914	4.2639	4.8728	3.3674	4.7779	4.5126	4.9088	4.0980	4.5279
Ubxn1	_SS[Phospho (STY)]PPATDPGPVPSSPSQEPPTKR_.3	Q922Y1_S188_M1_Ubxn1	8.9570	3.9588	3.8622	3.8681	3.6666	3.8727	3.8939	3.8010	3.9772	4.2171	3.4559	3.7951	4.1427	3.8688	3.6627	3.6772	3.7670	3.7467	3.7061	3.7305
Dbr1	_NQAIYAAVDDGDAS[Phospho (STY)]AE_.2	Q923B1_S548_M1_Dbr1	4.5640	5.6154	4.1006	3.8465	3.2701	5.1437	5.6557	3.5433	5.2487	4.3746	3.1768	3.9820	3.8636	4.3663	4.7096	3.9262	4.0810	5.1142	5.2349	4.5715
Dbr1	_C[Carbamidomethyl (C)]GETVES[Phospho (STY)]GDEKDLAK_.2	#N/A	3.7646	3.7162	4.0849	3.7442	3.9715	4.0048	3.9331	4.3489	3.8533	3.5752	4.3972	3.5195	4.0187	4.1068	3.1464	3.9387	3.1801	3.6098	4.2862	3.8813
Wbp11	_RRDEDM[Oxidation (M)]LYS[Phospho (STY)]PELAQR_.3	#N/A	4.0276	4.1440	5.2927	3.6112	3.5317	3.7992	3.6661	4.2533	6.6462	3.9429	5.7233	3.8702	3.8637	3.6023	3.6915	3.3897	6.9491	3.4535	3.7526	3.2796
Ppp1r16a	_SVS[Phospho (STY)]YHLSPEENSAPDALVR_.3	Q923M0_S432_M1_Ppp1r16a	3.6208	3.8277	3.7812	4.1532	4.2032	4.9259	3.6696	4.0283	3.6907	3.7874	5.1891	3.6825	3.6462	5.2892	3.1595	3.5949	3.6450	3.5509	4.9259	5.4489
Ppp1r16a	_RVS[Phospho (STY)]LTHR_.2	Q923M0_S353_M1_Ppp1r16a	3.8224	3.4967	3.3590	3.7618	4.0294	4.0627	3.0187	4.0481	4.4398	3.6330	4.3485	3.2228	4.6052	3.6070	3.0441	3.1845	3.5453	3.0106	4.0262	3.5740
Stard13	_KKGDDS[Phospho (STY)]DEEDLC[Carbamidomethyl (C)]ISNK_.3	Q923Q2_S133_M1_Stard13	3.2061	5.8374	5.2517	4.4833	4.0446	6.0154	4.9569	5.2744	5.9157	6.1219	5.2496	5.4990	3.6636	4.7143	4.4708	5.2074	5.3019	5.5656	4.0929	4.3855
Camk2g	_QET[Phospho (STY)]VEC[Carbamidomethyl (C)]LR_.2	Q923T9_T287_M1_Camk2g	7.9962	7.9966	8.6602	8.2765	7.6618	7.4994	7.3911	7.2447	7.0807	7.7463	7.6722	6.3243	6.3237	7.4198	6.2833	7.3386	7.4152	6.9429	7.1751	7.1337
Camk2g	_FTDDYQLFEELGK_.2	#N/A	6.9091	6.4837	6.8385	6.8056	6.3842	6.3839	6.5472	6.5896	6.9548	6.6139	6.6029	5.9848	6.5756	6.2940	6.0146	6.3501	6.8609	6.3331	6.4042	6.4755
Camk2g	_FYFENLLSK_.2	#N/A	5.8435	6.6154	5.7519	6.2385	6.1225	5.9423	6.6253	6.1825	6.0463	5.6638	5.5757	6.0212	3.3285	5.8891	5.4967	6.0434	6.0629	5.8780	5.5591	5.1135
Camk2g	_GST[Phospho (STY)]ESC[Carbamidomethyl (C)]NTTTEDEDLK_.2	Q923T9_T382_M1_Camk2g	3.8773	4.4558	4.0624	5.0654	4.8768	3.6180	3.9874	3.8285	4.0183	3.9532	3.6086	4.6807	4.1377	3.9321	3.7315	3.8756	3.9148	4.0413	3.5487	3.7111
Camk2g	_SLLNKKS[Phospho (STY)]DGGVK_.2	#N/A	5.3458	5.5454	4.1500	4.5829	3.0172	4.9117	5.1837	5.1982	3.8850	5.4432	4.9855	5.2016	5.4256	5.2084	4.0901	3.7258	4.5510	4.6168	5.0849	3.8654
Camk2g	_ITEQLIEAINNGDFEAYTK_.2	#N/A	5.5367	5.4447	4.8728	5.5041	4.9957	4.9934	5.2570	5.3658	4.9217	5.1238	5.1914	5.2423	4.9903	5.5578	4.7189	5.0567	4.9516	5.0250	5.4576	5.1825
Camk2g	_WLNVHYHC[Carbamidomethyl (C)]SGAPAAPLQ_.3	#N/A	4.9930	4.7542	5.4361	4.8187	5.0890	3.2059	3.8756	4.7151	5.0458	4.9079	3.9340	4.6234	3.9613	4.4639	4.5729	4.6433	5.0928	4.3195	4.7739	4.8407
Camk2g	_KTS[Phospho (STY)]TQEYAAK_.2	Q923T9_S36_M1_Camk2g	3.3967	4.6061	3.9330	4.2927	4.2353	3.5714	3.3058	3.0971	2.6731	3.6031	3.7392	2.9947	3.4729	4.5175	2.8830	3.7416	3.6348	3.9845	4.2835	4.1948
Camk2g	_S[Phospho (STY)]SSSVHLM[Oxidation (M)]PQSNNK_.2	#N/A	5.4552	4.1317	3.5905	3.5582	3.5987	4.2942	3.6538	4.2796	4.2362	3.8645	3.7148	3.8578	4.4389	5.8028	3.6791	3.4161	3.7769	3.4005	3.6995	3.3059
Camk2g	_GSTES[Phospho (STY)]C[Carbamidomethyl (C)]NTTTEDEDLKVR_.2	#N/A	3.5789	3.7632	4.2898	4.1440	3.7027	3.6500	3.6640	3.8250	3.4218	3.7066	3.9208	3.7586	3.5872	3.7872	3.4835	4.1050	3.9743	4.3000	3.7473	3.6892
Gprc5b	_SAVGFSNGS[Phospho (STY)]LEQR_.2	Q923Z0_S355_M1_Gprc5b	6.7666	6.0348	6.3901	6.0563	6.0626	6.2925	6.2411	6.6317	6.0864	6.4941	5.9359	5.9947	3.8445	6.3363	4.6064	5.0280	6.8935	5.6355	5.2868	5.9773
Cic	_KNS[Phospho (STY)]TDLDSAPEDPT[Phospho (STY)]SPK_.2	Q924A2_S2299_M2_Cic	3.9127	4.0901	4.4490	3.7767	7.4819	3.4745	4.2906	3.6131	8.2107	8.8149	3.2232	3.9795	8.4974	4.0015	7.9831	4.2576	4.1508	2.9996	3.2987	3.6788
Cic	_NSTDLDSAPEDPTS[Phospho (STY)]PK_.2	Q924A2_S2311_M1_Cic	4.4721	4.3478	4.0087	7.3480	3.1782	3.2115	3.8699	3.1969	3.7901	4.2827	3.2688	4.0740	7.2638	4.0467	7.5885	7.4225	4.1950	7.2472	7.2349	3.7240
Cic	_TQS[Phospho (STY)]LSALPK_.2	Q924A2_S1080_M1_Cic	5.4787	5.6467	4.5994	5.6189	3.7550	5.3280	3.2931	3.7738	5.3505	3.3587	4.1927	4.8480	3.3787	5.6525	3.3184	5.3811	4.9032	5.1534	5.4355	3.8484
Cic	_S[Phospho (STY)]SSEAKPASLGLAGGHK_.3	Q924A2_S1184_M1_Cic	3.6712	4.4156	4.0406	4.8633	3.4001	5.1571	3.9310	3.2156	3.9374	4.3973	3.0645	4.0867	4.0668	4.0509	3.7006	4.2337	3.9805	3.5795	3.4922	3.8922
Cic	_SS[Phospho (STY)]PPPPLPAEERPGTK_.2	Q924A2_S2148_M1_Cic	11.0333	10.9582	10.6183	10.8939	3.3073	3.3003	10.3970	3.3260	3.0231	3.9448	3.3975	10.5710	3.1312	4.1758	10.2883	3.9633	11.3997	4.3262	10.4707	10.1396
Cic	_EGS[Phospho (STY)]TEFDWGDETSR_.2	Q924A2_S553_M1_Cic	3.4295	4.5733	3.9658	6.2774	4.2680	6.4849	3.2730	3.1298	3.3321	3.6358	3.7065	5.7756	3.4402	3.9528	5.2043	3.7744	3.6675	6.3312	6.2373	4.1621
Cic	_AQSVS[Phospho (STY)]PVQATPSGGSAQLLPGK_.2	Q924A2_S1809_M1_Cic	3.7617	4.2411	4.2980	3.9276	3.0995	3.3846	4.4416	3.4621	2.9999	3.9681	3.3742	4.1305	3.7825	7.6485	4.0188	4.1066	3.9998	3.8334	3.4819	4.2521
Cic	_AAS[Phospho (STY)]EDMTS[Phospho (STY)]DEER_.2	#N/A	3.9730	4.5420	4.0892	6.2486	3.5015	3.6141	3.7414	3.7396	3.7107	3.6079	5.0460	3.9409	3.8555	5.9017	4.2745	3.8001	3.9075	3.7335	3.8408	3.4846
Wac	_QQSHDPVS[Phospho (STY)]PR_.2	#N/A	8.5525	8.7659	9.0556	8.4490	8.4636	8.8001	8.1782	8.6984	8.3699	8.5668	8.6208	8.3994	8.5343	8.9288	8.2863	8.5142	8.4965	8.4533	8.4310	8.3937
Wac	_DAADPS[Phospho (STY)]PPNK_.2	#N/A	8.9857	9.0284	7.8422	8.8820	8.6635	8.4022	8.6371	8.8798	8.4670	8.9515	8.7424	8.8421	8.9907	9.1716	8.5774	8.6399	8.9905	8.0301	8.8935	8.9823
Wac	_QGPVSHSATQQPVTADKQQSHDPVS[Phospho (STY)]PR_.4	#N/A	3.9298	6.3003	3.4664	3.6544	4.1367	4.1701	4.4122	4.1775	4.3324	3.7404	3.8111	3.1154	4.4979	3.4997	4.6724	3.2919	3.6527	3.1179	4.2674	3.4667
Wac	_MRDAADPS[Phospho (STY)]PPNK_.2	#N/A	3.5772	3.7419	7.9602	7.9822	3.7841	4.3243	3.2640	3.8029	3.5000	3.3878	3.8744	7.2185	3.6080	9.2963	3.2894	2.9393	3.3001	3.8494	4.1484	4.3299
Wac	_S[Phospho (STY)]NS[Phospho (STY)]PENKYSDSTGHNK_.2	Q924H7_S62_M2_Wac	3.5634	4.4498	4.0997	4.1364	3.7325	3.5934	3.1495	3.2638	3.4187	3.7698	3.7749	3.7160	3.5842	4.0868	3.6043	3.9083	3.8015	4.0421	3.6907	4.0538
Wac	_QGPVS[Phospho (STY)]HS[Phospho (STY)]ATQQPVT[Phospho (STY)]ADK_.2	Q924H7_S491_M3_Wac	3.7981	4.2047	4.3344	3.8913	3.1359	3.3599	4.4052	3.4985	4.4643	4.0045	8.5187	4.0941	4.5724	4.1161	3.9824	4.1430	4.0362	4.3859	3.1841	3.7934
Mta3	_HAELSGS[Phospho (STY)]PLK_.2	Q924K8_S516_M1_Mta3	7.5663	7.6834	7.4691	7.4430	7.3306	7.4610	7.4643	7.5985	7.4109	7.3791	7.3578	7.1515	7.8007	7.6601	6.3571	7.2337	7.4369	7.4514	7.2727	7.1912
Mta3	_S[Phospho (STY)]PPSLQTPATK_.2	Q924K8_S547_M1_Mta3	7.2059	4.2122	4.3269	3.8988	6.6775	6.9285	6.9791	6.9319	3.6459	6.9342	6.9995	4.1017	3.8113	4.3140	5.9059	6.7006	6.9324	6.5766	3.4531	6.2126
Mta3	_M[Oxidation (M)]PTQSDEEKS[Phospho (STY)]PS[Phospho (STY)]PTAEDPR_.2	#N/A	3.6824	4.1130	4.3767	5.1946	3.3193	4.8635	3.6915	3.5494	3.7136	3.7888	4.0690	3.9575	4.1878	5.7615	3.8284	4.1913	3.7943	3.9142	3.7935	4.7561
Mpi	_ISQQVFDGNNMEDIYGK_.2	#N/A	4.5403	4.2796	4.0769	4.9402	3.2464	3.2797	3.8017	3.2652	3.7219	4.3509	4.3243	4.0057	3.8873	5.6411	3.8271	3.9024	4.2632	4.3871	3.1853	3.7922
Slc12a6	_IDDIPGLSDTS[Phospho (STY)]PDLSSR_.2	Q924N4_S32_M1_Slc12a6	4.3359	10.0805	9.6714	9.3358	3.3809	9.4885	9.6691	3.6124	9.4927	4.3508	9.4853	8.7638	4.3619	3.6706	3.7591	3.4714	9.3212	3.1822	9.3244	8.7195
Slc12a6	_LTSIGS[Phospho (STY)]DEDEETETYQEK_.2	Q924N4_S1032_M1_Slc12a6	7.6312	8.5042	8.3629	8.3561	3.4123	8.2473	8.2278	6.5137	8.1527	7.7789	3.5628	7.8669	7.4185	8.2129	6.9706	7.9841	8.6594	8.4340	8.2366	8.0309
Spred2	_TISS[Phospho (STY)]PTSC[Carbamidomethyl (C)]EHR_.2	Q924S7_S168_M1_Spred2	8.5656	8.5775	7.7891	8.0609	4.2667	7.9277	8.3437	8.2001	7.9207	7.7828	7.9231	7.6341	4.3679	7.8581	6.6357	7.0634	8.1245	8.0914	7.7575	3.3367
Spred1	_HVS[Phospho (STY)]FQDEDEIVR_.2	Q924S8_S239_M1_Spred1	5.3783	5.6123	5.5192	5.2320	2.9067	5.5532	5.0288	3.4955	5.2584	5.6678	3.5670	4.2813	3.3007	4.3089	4.1077	4.2838	4.1406	5.0342	4.4205	5.1264
Trim66	_SVSS[Phospho (STY)]PGSGPAAESLGTR_.2	Q924W6_S767_M1_Trim66	8.2456	8.4978	3.8887	8.1470	3.0582	8.0332	3.9899	8.0017	8.0918	4.1627	7.6220	7.9292	8.3947	8.3540	7.2779	8.0186	4.0750	7.8411	7.9746	7.6534
Trps1	_GS[Phospho (STY)]SERGSPIEK_.2	Q925H1_S1061_M1_Trps1	3.3956	4.6072	3.9319	4.2938	4.2341	4.4582	3.3069	3.0959	3.3660	3.6019	3.7404	2.9958	3.4741	6.3578	2.8841	3.7405	3.6336	4.1995	3.8481	3.8859
Trps1	_HPNYS[Phospho (STY)]PPGS[Phospho (STY)]PIEK_.2	Q925H1_S1081_M2_Trps1	3.2600	4.3567	7.0203	4.0433	3.4669	3.5002	3.5812	3.3465	3.5014	6.7944	2.9800	3.7852	3.6669	4.1695	3.6066	4.1229	3.8842	3.9490	3.5976	4.1365
Atad3	_TAGTLLGEGFR_.2	#N/A	3.3615	5.5568	5.2044	4.3279	5.1623	5.9029	5.4128	4.9841	5.8060	4.6419	5.2084	5.3432	5.2941	5.3591	6.1173	4.9579	3.5996	4.9846	5.6664	5.2054
Med1	_SQT[Phospho (STY)]PPGVATPPIPK_.2	Q925J9_T1051_M1_Med1	9.4459	3.3274	8.9174	8.5591	8.7635	4.2372	8.5636	9.4292	3.5870	9.2011	8.8150	3.2169	9.1531	9.5402	3.1052	8.9266	9.4726	8.9663	4.0691	3.6649
Med1	_NSSQTGGKPGSS[Phospho (STY)]PITK_.2	Q925J9_S1158_M1_Med1	3.4279	4.5749	3.9642	4.2615	4.2665	2.9897	3.2746	6.2689	5.3098	5.6966	3.7081	5.6414	3.4417	3.9513	4.8945	5.4796	3.6660	4.0157	3.8158	4.1636
Med1	_HDGGS[Phospho (STY)]PSIK_.2	Q925J9_S1403_M1_Med1	5.3705	4.8378	4.4890	4.1326	3.6585	5.1053	3.3896	5.6860	3.3744	5.5291	3.7488	3.5936	3.4825	4.5271	4.8330	4.3145	5.7348	3.9749	4.2740	4.2043
Sfxn5	_EAVQLLEDYK_.2	#N/A	5.9766	5.9637	6.7840	6.4639	6.3753	6.3460	6.2508	5.4535	6.4502	6.3295	6.1612	5.7674	5.3779	5.6674	5.4575	5.7409	5.9210	6.0678	5.9418	6.0065
Sfxn5	_HALLETALTR_.2	#N/A	5.3285	5.4678	3.3099	5.6955	5.5823	5.3546	6.0457	5.0668	5.0822	5.1337	5.6306	5.5631	4.3269	5.0086	4.3809	4.7313	5.1577	5.0534	5.3692	4.6801

Sfxn5	_YGELEEGIDVLDADGNLVGSSK_2	#N/A	5.8746	5.1995	4.7659	4.1076	4.0112	5.2494	4.2155	5.1964	3.9319	4.4637	5.3144	4.8902	5.3428	5.5346	4.1111	4.3788	4.9295	5.5918	4.7444	3.9215
Sfxn5	_HFLDIDPR_2	#N/A	4.9946	5.9312	5.3793	5.6599	5.1537	5.6523	6.1428	5.7354	4.7185	5.5482	4.5204	5.5673	4.3405	5.4304	3.4808	4.5290	5.3085	5.5124	5.1548	5.1655
Sfxn5	_FQQTSFYGR_2	#N/A	6.8571	7.3547	6.9051	3.8685	6.6890	7.7509	3.6181	7.7523	6.9910	6.5781	6.5243	7.0512	6.9811	7.0840	3.9597	6.3175	4.0589	7.0559	6.5548	4.3511
Grip1	_RQS[Phospho (STY)]PEEFK_2	Q925T6_S43_M1_Grip1	9.3061	9.2537	9.3138	9.3702	9.0964	9.3400	9.0685	9.1990	8.8869	9.2778	9.1456	8.9480	9.5459	9.5759	8.1759	9.0433	9.5400	9.0291	9.0228	9.0123
Grip1	_SLYSTS[Phospho (STY)]FR_2	Q925T6_S432_M1_Grip1	3.5431	3.7760	7.8796	4.3264	3.7501	3.7834	7.3294	3.0634	7.9256	8.2457	4.1977	7.7538	3.3837	8.0028	3.3234	4.4061	7.6120	7.7381	3.8807	3.8533
Grip1	_TSLS[Phospho (STY)]PVPKPR_2	Q925T6_S846_M1_Grip1	4.3157	7.1808	3.3512	3.3737	6.5451	3.8775	3.8876	7.1635	6.3639	4.5220	5.9257	7.0182	4.0548	3.5986	3.4648	3.1598	4.5538	3.4026	3.7017	3.2758
Grip1	_QAS[Phospho (STY)]FQER_2	Q925T6_S952_M1_Grip1	4.8515	6.1330	5.9897	5.9308	5.6817	5.6642	5.6513	3.3778	5.5559	6.2120	5.3333	4.4860	5.0647	5.4135	4.9384	5.2822	4.6359	5.3628	5.3282	4.6704
Grip1	_QTDAQSASS[Phospho (STY)]PK_2	#N/A	3.9457	4.0571	4.4820	3.7436	3.2835	3.2006	3.8808	3.6461	3.8010	4.2718	3.2797	3.9465	3.9665	4.4691	3.9062	4.2906	4.1838	3.6494	3.2979	4.4361
Grip1	_KQTDAQSASS[Phospho (STY)]PK_2	#N/A	3.1271	3.3748	3.6634	4.5622	3.9657	4.0192	3.0622	4.3284	2.9824	3.5896	3.9619	3.2643	3.1479	3.6505	3.0876	3.4720	3.3652	4.4680	4.1166	3.6175
Taldo1	_SYEPQEDPGVK_2	#N/A	5.4976	5.3766	3.5584	5.6314	5.0769	5.3408	4.6734	5.4638	5.5429	5.8143	4.7774	5.5117	4.0527	5.5631	5.4513	5.5094	4.7078	4.9824	3.7038	5.5757
Taldo1	_WLHNEDQM[Oxidation (M)]AVEK_3	#N/A	4.6764	3.5320	4.6928	3.2186	3.8085	4.9116	5.1858	4.1712	5.3054	4.5628	3.5244	4.8094	4.1997	4.1997	5.4480	4.8533	3.2080	3.1243	4.7852	4.5219
Taldo1	_LSSTWEGIQAGK_2	#N/A	3.7550	6.3700	7.1524	3.8292	3.9619	6.6556	6.2420	6.7663	3.6778	6.6099	6.0552	6.1565	3.7859	6.3946	6.7776	6.8728	6.3395	5.9792	6.6615	6.5596
Taldo1	_M[Oxidation (M)]ESALDQLK_2	#N/A	5.4001	4.7404	4.6064	4.3497	5.0915	5.6544	5.2975	5.6350	5.6779	5.2622	5.0246	4.8333	5.3280	3.9294	5.1028	5.3933	4.0322	4.6276	5.0383	5.3521
Taldo1	_[Acetyl (Protein N-term)]SGS[Phospho (STY)]PVKR_2	#N/A	13.3969	13.5082	13.2724	13.4667	13.2160	13.2127	13.2208	13.3916	13.1784	13.4072	13.0636	13.2089	13.6700	13.3750	12.3082	13.0801	13.3037	13.1993	13.1349	13.1843
Taldo1	_TIVM[Oxidation (M)]GAS[Phospho (STY)]FR_2	Q93092_S237_M1_Taldo1	10.8839	10.9012	10.3573	10.6639	10.6692	10.9073	10.3546	11.1022	10.6163	10.9727	10.4668	10.3714	11.1734	10.5300	10.1182	10.7200	10.8553	10.5964	10.6834	10.6475
Hnrnpab	_IFVGGLNPEATEEK_2	#N/A	6.9049	7.2396	6.8452	6.7453	6.9500	6.9001	6.4094	6.4788	7.1584	7.0008	7.0638	7.0499	6.7346	7.1992	7.2786	6.2362	6.6901	6.7914	6.3781	6.7646
Hnrnpab	_EVYQQQQYGSGGR_2	#N/A	3.7485	3.5706	5.6151	5.6550	5.9164	5.6120	3.0927	5.9210	5.4940	5.7620	5.1571	4.8200	3.1783	3.6810	5.8140	5.4129	5.2214	4.6467	5.4949	5.4553
Hnrnpab	_EYFGQFGEIEAIELPIDPK_2	#N/A	4.2572	5.3111	4.6133	4.0700	5.1539	4.0505	4.5172	4.0386	3.1423	4.3521	4.2379	4.5522	5.5143	5.2019	4.1612	3.9642	4.0868	4.2210	4.1104	4.1362
Myo5a	_AIS[Phospho (STY)]PTSATSSGR_2	#N/A	14.4505	14.2081	14.4326	14.0811	13.7862	14.1978	14.0541	14.3267	14.0615	13.7985	14.0474	13.7083	14.1242	13.8788	12.5988	13.4055	14.4280	14.1461	13.8084	13.7384
Myo5a	_SAPEVTAPGAPAYR_2	#N/A	8.0901	7.7278	7.9782	7.8401	7.5273	7.5973	7.9993	8.1214	8.0586	6.9646	7.3005	7.5177	3.9451	7.1909	7.4305	7.2802	7.9052	7.4647	7.4436	7.2082
Myo5a	_YQNLLNEFSR_2	#N/A	6.3402	6.3651	5.4755	6.1835	6.2291	5.8809	6.4602	6.7508	5.9246	5.9598	6.2512	6.0667	5.9065	5.5884	5.1793	5.8421	5.9830	5.9548	5.0940	5.0956
Myo5a	_M[Oxidation (M)]LPELFQDDEKAIS[Phospho (STY)]PTSATSSGR_3	#N/A	8.9842	10.1035	6.7151	7.6060	6.7818	7.4674	9.6349	9.5092	7.3612	7.9743	7.3462	9.1119	9.2707	9.8413	6.6927	7.6191	8.7739	9.4473	8.0789	7.3023
Myo5a	_AC[Carbamidomethyl (C)]GVLETIR_2	#N/A	4.1717	5.5131	5.6423	5.8848	5.0887	5.2194	5.8235	3.7017	5.0266	5.6674	5.5163	5.2566	4.2743	4.3069	4.7979	4.3389	3.1989	5.5870	4.9234	5.0147
Myo5a	_QLELDLNDER_2	#N/A	4.5678	5.2924	4.9475	3.6733	3.3539	5.1527	5.2103	5.2795	5.2131	5.0308	5.4135	4.2712	4.0369	5.4505	3.9766	4.3610	5.1896	5.4392	4.6403	4.4933
Myo5a	_ETLEPLIQAAQLLQVK_2	#N/A	5.3256	4.3696	4.5637	4.5395	3.8390	4.5298	4.2904	4.5672	3.4885	3.5188	4.6448	4.7868	3.5571	3.7389	4.0349	3.3269	3.2492	4.1879	4.0486	4.2790
Myo5a	_TSS[Phospho (STY)]IADEGTYTLDSILR_2	Q99104_S1650_M1_Myo5a	8.5394	8.2998	8.0457	2.8749	3.3159	6.9244	7.9989	7.1871	7.3695	3.3904	7.3944	7.5608	4.1586	7.2067	3.3407	7.0385	4.0649	7.5842	3.2186	7.0341
Myo5a	_VLNLYTPVNEFEER_2	#N/A	6.7372	4.1958	6.9670	3.8824	6.4453	3.3394	6.2545	6.4071	5.6242	5.8858	6.2382	3.9461	6.2117	5.5469	3.7674	3.9620	6.2688	6.4018	5.4120	5.5808
Myo5a	_SHENEAEALRGEIQSLKEENNR_4	#N/A	5.9437	4.6092	4.8351	5.9567	5.8552	5.8945	4.8409	6.6643	5.7903	6.2108	5.3449	6.4999	7.1494	6.3900	3.9533	6.5178	6.8460	5.7706	6.4685	5.2862
Myo5a	_LHIGMENK_2	#N/A	6.3829	4.4926	4.5971	4.4588	5.9420	3.5897	3.6516	4.1920	4.3469	3.7259	3.8226	3.1299	6.1258	5.2140	2.9512	3.2775	3.2288	3.1035	4.2529	3.4811
Myo5a	_YNVSQLEEWLR_2	#N/A	4.5991	4.9866	4.1180	4.5036	4.3523	4.8103	4.8951	4.6305	4.3294	4.3920	4.6861	3.9646	3.1115	4.1561	3.7859	3.9436	4.0797	4.9598	4.9286	4.5961
Myo5a	_YFATVSGSASEANVEEK_2	#N/A	6.1764	5.7310	3.8925	4.3332	3.4959	4.4188	3.3463	5.0745	3.4054	3.5625	3.7798	3.0352	3.5135	4.5581	2.9235	3.7011	3.2055	4.6077	4.2430	4.2354
Myo5a	_IGELEVGQMENIS[Phospho (STY)]PGQIIDEPIRPVNIPR_3	Q99104_S1450_M1_Myo5a	4.2032	3.7991	3.2392	3.4857	2.9093	4.4435	1.0173	3.9041	4.0590	4.0138	3.5376	3.6885	4.2244	3.2262	4.1642	3.5653	4.4418	3.3914	3.0400	3.1932
Myo5a	_VLMEQLTSVSEELDVR_2	#N/A	3.6621	4.3407	4.1984	4.0273	2.9998	3.4843	3.5971	3.3625	3.5173	4.5555	2.9960	4.2302	3.6828	4.1854	4.1185	4.0070	3.9001	3.9330	3.5816	4.1524
Cacna1d	_SES[Phospho (STY)]GDEQFPTIC[Carbamidomethyl (C)]R_2	Q99246_S1833_M1_Cacna1d	3.7862	4.4055	3.9723	7.1233	3.6711	7.0007	7.0196	4.0119	3.9394	4.3550	3.0937	6.4702	4.0714	4.0104	5.1792	6.2656	6.9519	6.6345	3.4569	6.4733
Cacna1d	_QT[Phospho (STY)]VLSWQAAIDAAR_2	Q99246_T49_M1_Cacna1d	3.3845	4.6183	3.9208	4.3049	4.2230	3.7619	3.3195	3.0849	3.2398	3.3222	4.2192	3.0069	4.3052	3.9079	2.8952	3.7294	3.6225	4.2106	3.8592	3.8749
Cacna1d	_RQS[Phospho (STY)]S[Phospho (STY)]QDDVLPSPALPHR_3	Q99246_S1950_M2_Cacna1d	3.8082	6.7343	4.3445	3.8812	3.1460	5.3774	6.0337	3.5086	4.1978	4.0146	3.3278	4.0840	4.5623	4.1060	3.9723	4.1531	4.0463	3.8167	3.1942	3.7833
Cacna1d	_YS[Phospho (STY)]PSHSTR_2	Q99246_S1991_M1_Cacna1d	3.5101	3.8090	4.5475	4.0742	3.7170	4.3914	3.3311	3.7357	4.2126	3.5350	3.8073	3.5351	3.5409	4.5856	3.3565	4.3730	3.2330	3.9165	4.2155	4.2628
Cacna1d	_RIS[Phospho (STY)]ELNMK_2	Q99246_S869_M1_Cacna1d	4.1755	3.8273	4.1934	3.8875	3.5132	3.3534	4.0279	4.3898	4.0869	4.1918	2.9605	3.7168	4.1950	4.0558	3.6051	3.0195	3.8870	3.2624	3.5615	3.4161
Sec14l2	_MNQQGADT[Phospho (STY)]PK_2	Q99J08_T401_M1_Sec14l2	4.3286	3.4536	3.8652	5.8733	3.0347	5.1873	4.9327	3.7787	4.6871	4.8338	4.9512	4.2174	4.0990	6.7210	1.7680	3.6908	4.3163	4.2614	3.1654	2.7928
Wdr77	_KDT[Phospho (STY)]PPPLVPPAAR_2	#N/A	7.0304	7.9097	7.5098	7.2667	7.9549	7.8638	6.7885	7.9191	6.8788	7.5819	7.8413	8.0613	7.8344	8.3078	7.4908	8.0191	8.3972	7.4433	7.4843	8.0520
Wdr77	_YEHDDIVSTVTVLSSGTQAVSGSK_3	#N/A	4.5786	4.2414	4.1151	4.5065	3.2847	3.3180	3.7634	3.3034	3.6836	4.3892	3.1623	3.9675	3.1086	4.1532	3.2078	3.9407	4.3015	4.3488	3.1470	3.8305
Ctu1	_AGATC[Carbamidomethyl (C)]VSQC[Carbamidomethyl (C)]DLS[Phospho (STY)]PVAE_2	Q99J10_S416_M1_Ctu1	6.3679	7.1576	7.0574	7.1989	3.1780	6.1289	6.8097	3.1967	6.6103	6.8600	5.6305	6.5182	4.5028	6.8172	5.1590	6.2736	6.0552	6.4974	6.3106	6.2550
Ctu1	_AGATC[Carbamidomethyl (C)]VSQC[Carbamidomethyl (C)]DLS[Phospho (STY)]PGNGEDR_2	Q99J10_S378_M1_Ctu1	4.8581	4.0896	4.4495	3.7762	3.2509	4.3750	4.2902	3.1513	4.3493	4.1195	3.6138	3.9791	4.4573	4.0011	3.8674	4.2581	4.1494	3.0001	3.2991	3.6784
Mcoln1	_SELQAYIEQC[Carbamidomethyl (C)]QDS[Phospho (STY)]PTSGK_2	Q99J21_S547_M1_Mcoln1	10.0342	10.9413	10.8339	9.9024	5.9286	10.4544	10.2911	8.8842	10.4077	9.9002	3.3022	9.4887	4.5368	10.4936	8.9936	4.1786	10.2678	10.6111	10.1811	9.8037
Thumpd1	_[Acetyl (Protein N-term)]ATTAQQS[Phospho (STY)]PQPVAGKR_2	#N/A	8.7213	8.6907	7.9928	8.8875	8.4187	8.3082	8.3789	8.9183	8.1835	8.6082	8.0336	8.7312	9.4078	8.9564	8.1447	8.7859	8.7681	8.3645	8.4906	8.3797
Thumpd1	_FIDKDQQPS[Phospho (STY)]GS[Phospho (STY)]EGEDDDAEAALKK_3	#N/A	7.0152	9.4547	9.4211	9.5731	3.4880	9.2524	9.1419	3.6621	9.6615	8.7421	8.3290	9.4881	3.7382	10.0375	9.3405	9.2390	9.0251	9.5180	9.7475	9.0296
Thumpd1	_[Acetyl (Protein N-term)]ATTAQQS[Phospho (STY)]PQPVAGK_2	#N/A	12.7022	12.5438	12.8734	12.7915	12.4065	12.3254	12.2434	12.6320	12.4638	12.6459	12.2686	12.5185	12.9215	13.0334	12.2119	12.5551	12.8257	12.3756	12.4914	12.5403
Thumpd1	_DQQPS[Phospho (STY)]GS[Phospho (STY)]EGEDDDAEAALKK_3	#N/A	3.7791	3.9772	3.8796	4.0151	2.9516	4.6974	4.6113	3.6784	3.6609	3.9394	3.5097	3.7777	5.3262	4.8265	4.5903	4.7091	4.8399	3.8797	4.6137	4.4978
Thumpd1	_FIDKDQQPS[Phospho (STY)]GS[Phospho (STY)]EGEDDDAEAALK_3	#N/A	3.1107	3.5430	3.6470	4.5786	3.9493	2.8205	3.0458	4.3120	3.2344	3.6059	3.9455	3.2807	3.1315	3.4962	3.0712	3.4556	3.3488	3.5200	2.4658	3.6011
Nrbp1	_[Acetyl (Protein N-term)]S[Phospho (STY)]EGESQTVVSSGSDPK_2	Q99J45_S2_M1_Nrbp1	9.6973	9.2457	9.4626	9.6133	9.3365	9.0898	8.7290	9.1959	9.4192	9.2415	8.8931	8.9168	8.7755	9.8892	8.9868	9.1992	9.2023	9.1636	9.6018	9.0618
Nrbp1	_TPT[Phospho (STY)]PEPAEVETR_2	Q99J45_T433_M1_Nrbp1	3.6647	4.3381	8.3373	4.0247	8.0996	8.3129	3.5998	3.3651	3.5200	8.7171	2.9986	4.2275	3.6854	4.1881	3.6252	7.9189	8.5032	7.6868	3.5790	7.9000
Nans	_GSDHSASLEPGELAELVR_3	#N/A	4.7971	4.7406	3.4581	4.4618	4.4729	3.3708	4.6765	5.1023	4.4842	4.7875	4.8212	4.3644	3.7962	5.1189	4.7050	3.5899	4.3126	2.6594	3.3401	4.6157
Nptxr	_AELLLQSTAEQLR_2	#N/A	5.1637	4.0509	6.1134	4.8005	4.7243	5.2604	4.7212	5.0677	5.1898	5.4803	4.3357	5.0547	5.3726	5.0076	5.4393	3.1513	5.2852	5.3032	4.8861	4.7700
Nptxr	_FLC[Carbamidomethyl (C)]TPLAAAC[Carbamidomethyl (C)]PSGAEQGDAAGER_2	#N/A	3.7142	4.2886	4.2505	3.9751	3.0520	3.4321	2.9883	3.4146	3.5695	3.9206	3.0482	4.1780	3.7350	4.2376	4.0663	4.0591	3.9523	3.8809	3.5295	4.2046
Mpst	_HIPGAAFFDIDR_2	#N/A	4.9991	5.7937	5.6496	5.6018	3.3459	5.0828	5.7409	5.5778	5.3337	5.5067	5.2613	5.4559	6.8364	5.8496	5.8875	4.3530	4.7945	5.5343	5.7076	5.6626
Mpst	_ALVSAQWVAEALK_2	#N/A	4.1121	5.7195	5.9985	5.3624	5.2449	5.7124	5.5819	5.3003	4.5015	5.3305	2.9085	5.5163	4.0941	4.9804	5.0038	5.0526	4.8500	4.9959	5.4526	4.5676
Mpst	_AQPEHISEGR_3	#N/A	3.6477	2.4490	3.1843	3.9365	3.8547	0.3532	3.5116	3.8734	3.9459	3.3974	3.6595	3.9359	3.6786	3.2224	3.7760	3.8313	3.7706	3.7788	2.6757	4.3389
Mpst	_FQGTQPEPR_2	#N/A	2.6095	3.6232	5.9287	3.3098	6.6881	5.9736	6.3262	4.1857	6.3152	4.5859	4.2572	6.3204	3.9909	6.6893	6.6946	5.9217	3.6830	3.4665	3.7655	3.2120
Mpst	_THEDLENLDAR_3	#N/A	3.7609	3.3568	3.2975	3.6202	3.9678	4.2804	4.4239	4.3464	3.0005	3.5715	3.9800	3.2843	3.1659	3.6686	4.2237	4.5120	3.3833	3.4209	4.5713	3.6356
Mpst	_HWLNQNLPISSGK_2	#N/A	3.3120	3.9410	4.2496	4.3774	4.1506	3.8344	5.1720	4.5132	3.1673	3.4047	4.1467	3.0794	3.3327	3.8354	4.7505	3.6569	3.5501	4.2831	5.0980	3.8024
Mpst	_DGIEPGHIPGSVNIPFTEFLTNEGLEK_3	#N/A	4.3201	3.2565	4.0261	4.4584	4.1996	3.8638	3.2127	3.1902	3.2718	2.0475	3.6462	4.4025	3.3799	4.7260	2.7899	3.8347	2.8336	4.0775	3.7539	4.1018
Mpst	_AFGHHSVSLLDGGFR_3	#N/A	5.4705	4.5744	3.9647	4.2610	4.2670	6.1614	6.1496	5.5585	3.3332	3.6347	6.1551	2.9630	3.4413	3.9518	6.0146	3.7733	3.6664	4.1667	3.8153	4.1632
Stoml2	_ILEPGLNVLIPVLDR_2	#N/A	5.3373	4.6747	4.9544	4.9267	4.9936	5.1196	5.7029	4.7168	5.2047	5.1573	4.9477	5.4976	5.6268	5.3048	5.3165	5.2488	5.1379	4.5779	5.3412	4.9725
Stoml2	_NTVILFVPQQEAWVVER_2	#N/A	3.8482	4.3709	3.6031	4.4177	3.6374	3.6543	4.0945	4.0739	3.4951	3.1969	3.5748	4.2182	3.8791	3.4229	4.2199	2.5284	3.5711	3.8904	3.4615	3.1001
Stoml2	_APVPGAQNSSQSR_2	#N/A	3.9877	3.3314	3.5242	3.5966	4.1946	4.8855	4.6152	4.2133	4.2746	3.7982	3.7532	3.0576	4.4400	6.6489	4.8811	3.3498	3.7106	3.1758	3.7379	3.4088
Pacsin3	_DLQQSIEAAS[Phospho (STY)]DEEDLR_2	Q99JB8_S276_M1_Pacsin3	3.9513	5.4074	4.4876	3.7381	3.2890	3.5131	4.2520	3.6517	4.3111	4.1576	3.1847	3.9409	4.0192	4.4747	3.8292	4.2962	4.1894	3.6438	3.2924	4.4417
Pacsin3	_DGTAPPPQSPSSPGSGQDEDWS[Phospho (STY)]DEESPR_3	Q99JB8_S354_M1_Pacsin3	3.8797	3.4394	3.4162	3.7046	3.7977	4.0218	3.7434	4.1053	4.0883	3.1655	4.1769	3.1656	3.9105	5.4743	3.3206	3.9857	3.6026	3.5469	3.8459	3.1316
Pacsin3	_GGRS[Phospho (STY)]PDEVTLTSIVPTR_3	Q99JB8_S319_M1_Pacsin3	3.7912	4.1972	4.3275	3.8982	3.1289	3.3530	4.4122	3.4915	2.9704	3.9975	3.3448	4.1011	4.5793	5.7641	3.9894	4.1361	4.0292	3.8039	3.4525	4.2815
Pacsin3	_ALYDYAGQEADELS[Phospho (STY)]FR_2	Q99JB8_S383_M1_Pacsin3	3.6772	4.3256	4.2135	4.0122	3.0149	3.4692	3.0253	3.3776	3.5325	4.5403	3.0111	4.2150	3.6979	4.2006	4.1033	4.0221	3.9153	3.9179	3.5665	3.1676
Psip1	_QSNASS[Phospho (STY)]DVEVEEK_2	#N/A	8.9348	8.5615	9.7023	9.4177	8.8827	8.6779	8.5443	9.3103	8.7744	9.0760	10.4015	10.2991	10.6470	11.0462	9.8832	10.4086	10.7098	10.1835	10.1474	10.4779
Psip1	_NLAKPGVTSTS[Phospho (STY)]DS[Phospho (STY)]EDEDDQEGEKK_3	#N/A	4.3698	3.6330	4.6258	3.3196	3.7075	4.2774	3.8836	4.0702	4.2251	3.8477	3.7037	4.9893	4.3905	6.1732	3.5923	3.2138	3.1070	4.9795	4.7309	3.3593
Psip1	_FSSQQASTK_2	#N/A	3.4233	2.4882	3.8203	3.3005	2.9899	4.7311	5.1726	4.5094	4.4229	4.3623	3.0801	4.0614	4.3146	4.1804	5.5221	4.8448	4.7720	3.4836	5.1949	4.6203
Psip1	_QVDTEEAGM[Oxidation (M)]VTAATASNVKAS[Phospho (STY)]PK_3	#N/A	6.0507	6.9959	6.5734	6.0501	5.5816	5.9997	6.6069	6.3898	6.1713	3.9968	5.7213	5.8535	6.6094	7.3255	5.9686	6.1458	5.7896	6.5545	6.0310	5.6260
Psip1	_NLAKPGVTSTS[Phospho (STY)]DS[Phospho (STY)]EDEDDQEGEK_3	#N/A	5.6753	6.3146	5.8731	6.1267	2.9869	5.4095	5.9428	3.3496	5.9282	3.8555	3.4868	5.5186	3.2205	4.1726	6.0910	5.4272	3.8873	3.9459	5.3267	5.1497
Psip1	_QSNASS[Phospho (STY)]DVEVEEKETNVSK_2	#N/A	4.4963	4.3236	4.0329	4.5887	3.2024	3.4672	3.1578	4.4655	4.4191	4.2084	3.2927	3.7958	2.7781	4.0710	3.8711	3.8584	4.2192	4.4310	3.2293	3.7482
Psip1	_PGVTSTS[Phospho (STY)]DS[Phospho (STY)]EDEDDQEGEK_2	#N/A	4.5047	3.4981	6.5693	3.1847	3.8424	4.1425	5.7655	4.2051	5.5822	3.7128	3.8386	3.3876	4.5254	5.8930	4.4919	3.3487	3.2419	3.0904	4.2398	3.4942
Psip1	_QVDTEEAGM[Oxidation (M)]VTAATASNVKAS[Phospho (STY)]PKR_3	#N/A	5.6398	6.2620	3.3391	3.5842	4.6965	5.2833	5.6988	6.3797	5.0924	5.3756	4.7299	5.7677	6.5414	6.0937	4.8803	4.3628	4.5364	5.8961	5.2138	4.3539
Psip1	_ETNVSKEDT[Phospho (STY)]DQEEK_2	#N/A	3.7393	4.9208	4.2755	3.9501	4.3310	5.2317	3.6743	3.4396	3.5945	4.4783	5.7050	4.8566	3.7600	4.2626	4.0413	4.0841	4.7502	4.2424	5.3044	4.2296
Psip1	_EDTDQEEKAS[Phospho (STY)]NEDVTK_2	#N/A	3.7718	4.0645	5.4858	4.1713	2.9230	3.3134	4.0191	3.5276	3.8749	4.3366	3.3470	3.7607	4.1921	4.3133	3.6748	3.6808	4.0079	3.7186	3.5146	3.5126
Psip1	_NFQAAHR_2	#N/A	3.5496	4.4532	4.0859	4.1398	2.8874	4.0282	3.1529	3.2500	3.2120	4.3464	3.5863	4.3426	3.3200	4.9818	5.0699	3.8945	3.7877	4.3808	3.6941	4.0400
Psip1	_ETNVSKEDT[Phospho (STY)]DQEEKASNEDVTK_3	#N/A	3.9662	3.3897	3.5028	3.6372	4.1732	3.9494	3.5093	4.0689	3.9756	3.8440	3.7326	3.7335	3.7826	2.7975	3.9905	3.3283	3.8985	3.4978	3.2160	3.7609
Psip1	_QSNASS[Phospho (STY)]DVEVEEKETNVSKEDTDQEEK_4	#N/A	3.5519	4.4509	4.0882	4.1375	2.8896	3.1137	3.3035	3.5125	3.2097	3.7582	3.5841	3.3403	3.3177	3.3624	4.2286	3.8968	4.5106	4.5850	4.4387	4.0396
Gpr37l1	_LLGDVSC[Carbamidomethyl (C)]R_2	#N/A	7.0421	6.9765	5.9818	6.3455	6.2005	6.4602	7.4407	7.1003	6.5872	5.4841	6.5391	6.5687	6.6067	6.2143	6.2367	5.5272	6.5443	6.7029	6.1179	5.7543
Gpr37l1	_ES[Phospho (STY)]PPLPLGTPC[Carbamidomethyl (C)]_2	#N/A	9.9666	10.0892	10.3840	9.8708	9.0437	9.9561	9.9049	8.9340	9.9903	9.3097	9.8747	9.1773	9.1155	9.6670	8.5281	9.1806	10.1323	9.5966	9.5306	9.2834
Gpr37l1	_PRES[Phospho (STY)]PPLPLGTPC[Carbamidomethyl (C)]_2	#N/A	3.1291	3.6094	3.2463	3.8745	3.9676	6.6021	7.2647	7.1196	6.6124	3.3354	4.0069	6.7678	7.7406	7.4081	3.1506	3.4740	7.2260	7.1510	4.0159	2.9616
Klhl22	_SLLHEQPRGT[Phospho (STY)]PNR_2	#N/A	5.6893	4.4691	4.3866	5.1013	5.3449	4.5688	4.6282	5.7636	4.5570	4.1951	4.5706	5.4159	5.8738	3.9411	3.7578	3.7286	5.0650	4.8657	4.0714	4.7682
Cdc26	_EDVEGVGTS[Phospho (STY)]DGEGAAGLSSDPK_2	#N/A	3.8798	3.9446	3.9023	3.8265	3.7981	4.4339	3.6372	3.9173	3.7759	4.1206	3.8429	4.4403	3.9066	3.9114	3.9363	3.7181	3.8316	3.7083	3.6348	4.7214
Cdc26	_QKEDVEGVGTS[Phospho (STY)]DGEGAAGLSSDPK_3	#N/A	4.0610	3.2613	3.5975	3.5264	4.2679	3.8436	3.4438	4.2866	3.9838	4.4881	3.7621	3.8922	4.0918	3.6356	3.5019	3.3480	3.7839	3.3687	3.6678	3.3129
Homer3	_SQS[Phospho (STY)]ADTPGPTER_2	Q99JP6_S159_M1_Homer3	8.4282	8.5406	8.5963	8.6343	8.2749	8.5782	8.3994	8.3241	8.0822	8.7619	7.8816	8.1370	8.8086	8.7871	8.3750	8.2610	8.2571	7.8854	8.6816	8.1527
Ggt7	_LPSSSSEM[Oxidation (M)]GSQDGS[Phospho (STY)]PLRETR_3	#N/A	6.1329	3.8098	4.1621	6.7276	3.7812	5.2936	4.9225	6.0815	6.1289	6.3372	5.8810	6.5071	3.5476	4.2001	3.3261	6.4446	3.4522	3.9172	6.2151	6.6638
Ggt7	_LPSSSSEM[Oxidation (M)]GSQDGS[Phospho (STY)]PLR_2	#N/A	5.6118	3.8699	5.2748	7.0536	5.2232	5.7320	6.8779	5.6330	5.7219	5.4987	5.6606	5.7037	5.4747	6.4880	5.3212	5.4752	5.9338	5.5076	5.0362	5.4258
Ggt7	_KDEDAFLGDPDT[Phospho (STY)]DPDSFLK_3	Q99JP7_T56_M1_Ggt7	4.0054	3.3137	3.5420	3.5788	4.2123	3.8961	3.8691	4.2311	3.9282	4.5406	4.3026	3.0398	4.0363	3.5800	3.4463	3.3675	3.7283	3.4212	3.7202	3.2573
Sfxn1	_NILLTNEQLENAR_2	#N/A	5.8417	5.8696	5.7244	5.0082	5.4309	5.6739	5.8214	5.8649	5.2440	5.1549	5.3438	5.7237	5.9165	4.6685	5.1838	4.1951	5.3471	5.9339	5.1365	4.4626
Sfxn1	_QAITQVVISR_2	#N/A	4.5633	5.7097	4.5766	4.0693	4.8660	4.7511	5.5458	5.9353	5.3969	4.1772	4.6089	4.9425	4.0611	4.2623	3.9767	4.1682	5.0737	4.6810	5.0870	3.8488
Sfxn1	_ASHFFTVTDPR_3	#N/A	4.4081	3.5947	4.4435	3.2813	0.2258	3.9699	2.6138	4.1085	3.8544	3.1551	2.2729	3.4842	3.9624	3.4306	3.3725	3.2521	3.1453	3.4950	1.6696	3.1835
Gorasp2	_ADASSLTVDVTS[Phospho (STY)]PASK_2	Q99JX3_S418_M1_Gorasp2	9.6202	10.0489	10.1140	9.9134	8.7115	3.0692	9.3313	3.2078	9.1538	9.9735	9.2719	9.4554	9.5335	10.2776	8.7424	9.5439	9.5111	9.3453	9.3297	9.7264
Gorasp2	_LYVYNTDTDNC[Carbamidomethyl (C)]R_2	#N/A	4.2914	4.4224	5.6973	5.2737	4.8211	5.5265	4.8588	4.8355	5.5511	5.3794	5.0583	5.3602	4.1363	5.8341	4.9404	5.4074	5.3860	4.7288	4.6566	5.1417
Eif3m	_[Acetyl (Protein N-term)]SVPAFIDISEEDQAAELR_2	#N/A	5.8421	5.4668	5.9932	4.8545	4.1886	5.1752	5.3115	5.2855	5.4473	5.2826	5.3067	5.6085	6.4278	5.0136	4.2767	5.2335	5.3452	4.4203	5.8286	5.1255
Hadhb	_AALSGLLHR_2	#N/A	6.7055	7.1693	6.5342	7.2869	7.3829	6.8606	6.9527	7.3405	6.4740	6.2979	6.3622	6.8325	6.9761	6.8035	6.9635	6.4298	7.0804	6.9941	6.6274	6.9531
Hadhb	_DFIYVSQDPK_2	#N/A	5.9680	6.1226	6.3063	6.3911	6.0711	6.1661	5.9101	6.1289	5.6224	6.4416	6.6080	5.6646	6.0312	6.5820	5.9621	6.3851	6.2050	5.7300	5.5641	5.8480
Hadhb	_NIVVVEGVR_2	#N/A	3.5753	6.2950	6.1252	6.7257	6.4555	6.2650	6.2364	6.2471	6.8143	5.9345	6.2370	5.1549	6.3062	6.1918	6.0305	6.5739	5.4700	5.7792	6.4461	6.4197
Hadhb	_DVVDYIIFGTVIQEVK_2	#N/A	4.2200	4.5999	3.7566	3.3642	2.9261	2.2135	4.1220	2.6495	4.1428	4.0306	3.0163	4.3261	4.2509	2.9581	2.7252	3.5821	3.3929	3.2066	3.5056	3.4719
Plpp3	_EILSPVDIIDR_2	#N/A	6.1448	6.1147	6.0646	5.7949	6.1633	5.9858	6.3562	5.7409	6.0837	5.8270	5.4016	5.9776	5.3876	4.5553	5.0827	5.4449	6.2498	6.2107	5.7632	5.4418
Plpp3	_NGGS[Phospho (STY)]PALNNNPR_2	Q99JY8_S19_M1_Plpp3	7.7388	8.2566	7.3417	7.6080	7.2174	7.9090	7.6994	7.8632	7.4365	7.5860	9.1018	7.0763	7.9289	7.4669	5.9644	6.2220	8.9945	8.9096	6.5697	7.1828
Actr3	_DYEEIGPSIC[Carbamidomethyl (C)]R_2	#N/A	7.8723	7.4522	7.8352	7.6521	7.4006	7.7028	7.4200	7.7155	7.8410	7.7237	7.5417	7.3830	7.8052	8.0088	7.7863	7.6352	7.7983	7.2809	7.6438	7.7943
Actr3	_AEPEDHYFLLTEPPLNTPENR_3	#N/A	7.4800	8.0398	5.1446	6.8095	5.9365	6.6768	7.9061	7.6376	6.1278	7.1018	6.8619	7.9196	7.8635	7.9057	6.1153	6.6572	7.3284	7.7909	6.4941	5.6114
Actr3	_GVDDLDFFIGDEAIEKPTYATK_3	#N/A	5.4500	5.6158	5.6689	6.4020	4.9922	5.6188	5.4799	5.5460	6.1608	5.7742	4.4155	5.7147	5.8335	4.8490	5.5271	5.8079	5.3528	5.1123	6.1196	6.0270
Actr3	_YSYVC[Carbamidomethyl (C)]PDLVK_2	#N/A	7.1267	7.0990	7.1676	6.5181	7.1178	6.6963	6.9537	7.3221	6.6977	6.8251	6.8232	6.9327	6.7059	6.9645	6.9589	6.4020	6.7959	6.5277	6.8006	6.7124
Actr3	_QYTGVNAISK_2	#N/A	5.2481	5.4975	6.1857	5.4875	5.4350	6.0911	5.8973	5.0054	6.0467	5.8023	5.0025	5.4263	4.9020	6.1477	5.3698	5.3530	5.2362	5.1756	5.8105	5.9789
Actr3	_DITYFIQQLLR_2	#N/A	4.9724	5.9145	3.4905	5.3258	5.1506	5.3798	4.9453	5.8154	4.9960	5.6056	3.7889	5.0335	4.4757	5.8070	4.6154	5.3713	5.4045	5.1893	5.2748	5.1441
Actr3	_LPAC[Carbamidomethyl (C)]VVDC[Carbamidomethyl (C)]GTGYTK_2	#N/A	3.8653	4.1375	7.0246	6.5107	6.5739	6.7631	7.2598	3.5657	6.6454	6.7097	6.0077	6.7395	3.8860	6.3944	7.0652	4.2102	4.1033	6.6717	6.5285	5.8252
Actr3	_HNPVFGVM[Oxidation (M)]S[Phospho (STY)]_2	#N/A	11.8311	11.5210	11.2528	11.4321	11.2401	11.2160	10.9890	6.5774	7.0287	11.6106	11.3004	11.4417	12.0706	11.4050	10.7178	11.4531	11.8915	11.1140	11.5578	11.3769
Actr3	_TLTGTVIDSGDGVTHVIPVAEGYVIGSC[Carbamidomethyl (C)]IK_3	#N/A	4.2390	4.0784	3.8183	2.7755	3.4476	2.5211	3.3231	3.5652	3.6696	3.4224	2.9993	3.8045	4.0220	4.2278	3.7231	4.3247	4.4644	2.8278	2.1732	4.1558
Arfgap2	_VSNQS[Phospho (STY)]FTEIER_2	Q99K28_S239_M1_Arfgap2	3.1588	6.5720	7.8325	7.3200	3.9974	4.2215	5.6767	6.5577	6.5746	3.3651	3.9772	6.5836	7.6343	3.2546	5.9002	6.4887	3.3969	7.0958	6.8652	6.3241
Arfgap2	_AIS[Phospho (STY)]SDM[Oxidation (M)]FFGR_2	#N/A	5.2845	6.0388	5.5527	5.1238	4.4029	5.0943	5.2737	4.7917	4.4178	5.9048	4.4245	4.7018	5.1487	5.2704	3.8545	5.3846	5.6932	5.1157	5.0834	5.0501
Arfgap2	_HGTDLWDSM[Oxidation (M)]NSAPSHS[Phospho (STY)]PEKK_4	#N/A	4.2263	3.9784	3.5725	5.5367	3.7065	5.8058	5.1875	3.7629	4.2226	6.2522	5.3126	3.7976	6.7789	6.9991	4.9927	4.3388	4.0023	6.8524	5.9070	3.4270
Arfgap2	_[Acetyl (Protein N-term)]AAS[Phospho (STY)]PSKTEIQTIFK_2	Q99K28_S4_M1_Arfgap2	4.4567	4.0526	4.4865	3.7392	3.1628	3.1961	3.8853	3.6506	3.8055	4.2673	3.2841	4.0893	3.9709	5.8793	3.9107	3.8188	4.1883	4.7985	3.2935	4.4406
Arfgap2	_LAYQELQIDR_2	#N/A	3.6278	3.4269	3.4287	3.6921	4.0991	4.1324	4.4498	4.2152	4.3701	3.7027	3.8487	3.1531	4.5355	5.5373	2.9744	3.2543	3.6151	3.0803	4.2297	3.5043
Eps8l2	_YWGPAS[Phospho (STY)]PTHK_2	Q99K30_S573_M1_Eps8l2	3.0892	9.1379	3.6255	3.0994	3.9277	4.0572	3.6133	4.2904	3.6724	3.2955	4.0468	3.3022	3.1099	3.6125	8.6501	8.7324	3.3272	8.5171	8.7624	8.9335
Eps8l2	_RAEDSYTSQHTS[Phospho (STY)]PESEGAPHL_3	Q99K30_S720_M1_Eps8l2	5.0818	4.4938	3.8627	3.2581	3.3513	3.5753	4.1898	3.0509	4.2489	4.5231	3.1224	4.3078	4.3570	3.9007	3.7670	4.3584	4.0490	3.1004	5.0950	4.9804
Eps8l2	_RRES[Phospho (STY)]QDEEPR_2	Q99K30_S242_M1_Eps8l2	4.3189	4.5010	3.8555	3.2653	3.0250	3.0583	4.0231	3.0437	3.9433	2.9953	3.4220	4.2272	4.1088	3.8935	4.0485	3.6810	4.0418	3.4665	3.1556	3.0776
Usp11	_QSQTSSSDTPAS[Phospho (STY)]PVSSSTPNSDM[Oxidation (M)]DIN_3	#N/A	4.8647	3.8917	4.4744	4.1569	3.6440	3.6773	3.4138	3.5545	3.3340	3.2476	3.6925	3.6179	3.4995	4.3932	3.4392	5.1273	3.5785	3.9409	3.9369	3.9691
Nono	_AVVIVDDR_2	#N/A	6.6843	7.0124	7.4217	7.5896	7.1162	6.8577	7.3587	6.9769	7.4731	7.6825	7.3582	7.1530	7.6708	7.2662	7.3452	7.2465	6.1521	6.7993	7.3313	7.7938
Nono	_AGEVFIHK_2	#N/A	4.0586	5.4962	5.0428	3.9076	4.2690	4.7754	4.5947	4.2464	4.5387	4.8680	4.5988	4.5840	5.3642	4.3830	4.9567	4.0016	4.9262	4.4868	4.2198	4.2822
Nono	_VELDNMPLR_2	#N/A	4.5471	5.0425	6.2767	5.7179	3.8848	4.6448	4.4140	4.2475	2.4287	3.2526	4.0897	3.3452	3.8234	5.8426	4.4452	3.3912	3.2843	3.0480	4.1974	3.5404
Nono	_FAC[Carbamidomethyl (C)]HSASLTVR_3	#N/A	3.1812	3.6616	3.1941	3.9267	4.0198	1.8343	3.2349	3.8832	3.5804	3.3876	3.9547	3.3877	3.6884	3.2322	1.9817	3.5261	3.3804	3.7690	4.0680	4.4103
Rragc	_SC[Carbamidomethyl (C)]SHQTS[Phospho (STY)]APSLK_2	Q99K70_S381_M1_Rragc	6.3111	6.3083	6.3320	4.4500	5.9342	6.3918	3.1744	5.6160	5.6736	5.6230	5.6321	3.3785	3.2601	5.0914	4.6845	3.0288	5.9700	4.3557	5.3873	5.4078
Rragc	_[Acetyl (Protein N-term)]SLQYGAEETPLAGS[Phospho (STY)]YGAADSFPK_3	Q99K70_S15_M1_Rragc	3.1432	4.1759	4.1806	4.4410	4.4195	5.5173	5.5378	3.3689	3.0660	4.4546	3.4404	5.4255	3.1741	4.2187	3.7233	4.0061	4.3669	4.2833	3.0816	3.8960
Rragc	_MSPNETLFLEST[Phospho (STY)]NK_2	Q99K70_T95_M1_Rragc	4.0914	5.7177	4.3129	4.4372	3.4824	3.5770	3.9473	3.1714	3.9622	3.7190	3.8473	3.8115	4.0882	4.0085	3.6329	4.1384	3.9677	4.3083	3.3605	3.8301
Med24	_LLS[Phospho (STY)]SSDDDANLSSPTDR_2	Q99K74_S860_M1_Med24	3.8887	6.8102	3.7314	3.8501	3.8309	6.3458	6.9867	3.4485	6.7041	3.8152	3.6678	3.5876	3.7574	7.0044	4.5295	3.5505	3.7511	6.5740	7.0458	6.2209
Psat1	_ELLAVPNNYK_2	#N/A	8.3409	8.2380	8.4439	8.0462	8.2931	8.1917	8.1497	8.3756	7.9285	8.6202	8.2431	8.3168	8.1782	8.3025	7.7927	8.0135	8.5741	8.2295	7.9650	8.2491
Psat1	_ASLYNAVTTEDVEK_2	#N/A	7.2557	7.3837	7.1492	7.0933	6.4772	6.7814	6.6346	6.8889	7.2858	6.7830	6.7387	6.8013	7.1176	7.0930	6.6698	6.8414	6.9663	6.8931	6.2032	7.0483
Psat1	_QVVNFGPGPAK_2	#N/A	5.2991	5.9699	5.9131	6.6803	6.3982	6.1643	6.2493	5.9442	5.5306	6.6395	5.8810	6.3117	6.1872	6.1942	6.5035	5.6200	5.8683	6.2526	5.8203	6.0459
Psat1	_EC[Carbamidomethyl (C)]PSVLDYK_2	#N/A	5.8438	6.6684	6.5563	6.3310	6.9933	6.7589	5.9315	6.7727	6.1293	6.4455	6.1788	5.8278	3.8746	3.4184	6.6916	5.3256	6.4639	6.2640	6.6191	3.0957
Psat1	_DDLLGFSLR_2	#N/A	3.8782	5.4668	5.2206	5.7431	3.7424	4.5980	4.7146	2.8305	3.8578	5.2827	5.3066	5.1435	3.9658	3.5096	4.5192	5.0186	3.1419	4.6272	5.1286	4.9016
Psat1	_LPHSVLLEIQK_2	#N/A	4.9654	4.5384	4.3343	4.3758	5.0762	4.4606	4.5386	5.1094	4.5694	4.7976	3.8594	5.0216	4.5410	4.8934	4.5429	4.3175	4.4124	4.3368	3.6841	4.9601
Psat1	_SQM[Oxidation (M)]YEIIDNSQGFYVC[Carbamidomethyl (C)]PVER_3	#N/A	4.9796	4.0936	4.8013	4.8493	4.8546	4.2286	4.0424	5.2146	4.3532	5.2303	3.2268	3.9830	5.7168	5.7183	3.8713	4.2541	3.2815	2.9961	3.2952	5.3036
Psat1	_SADYVVTGAWSAK_2	#N/A	6.2094	3.7910	4.5656	4.0561	3.7351	5.5025	5.8392	3.7538	3.4510	3.3387	3.8253	3.5171	3.5590	5.4068	3.3384	4.3911	3.2510	3.8984	4.1975	4.2809
Hagh	_ALLEVLGR_2	#N/A	4.5106	4.2692	5.0563	6.1327	5.2934	4.9998	4.9418	1.6778	5.1264	5.9003	4.6670	6.2393	3.5652	5.1241	5.7921	5.3483	3.6382	5.2504	6.1420	5.6638
Hagh	_HVEPGNAAIQEK_2	#N/A	3.7563	5.9538	5.8931	5.9681	5.5887	5.6535	5.7191	5.7885	5.3178	5.8943	6.1221	5.4506	5.8173	5.7832	6.1901	6.3980	6.1643	4.9377	5.5105	5.5105
Hagh	_TVQQHAGETDPVTTM[Oxidation (M)]R_2	#N/A	3.4834	3.9637	3.2005	4.2288	4.9951	3.0452	3.2117	4.2830	4.3833	3.6897	3.6526	3.6291	2.3394	4.4309	4.0292	3.9167	3.4821	4.2759	3.6956	3.7471
Hagh	_VTHLSTLQVGSLSVK_3	#N/A	2.8630	2.4799	3.8634	2.1592	3.1853	3.3638	3.6887	3.3794	3.3249	2.2412	2.5893	3.8927	3.4672	4.2292	3.2954	3.3425	4.3774	4.1154	2.6398	4.0429
Rbm10	_GLVAAYSGES[Phospho (STY)]DS[Phospho (STY)]EEEQER_2	Q99KG3_S736_M2_Rbm10	3.4203	9.1639	9.4945	4.1639	3.6272	9.3141	8.8384	3.6460	9.0416	3.6248	3.7175	8.4557	3.4512	9.7313	8.8009	8.8315	6.2380	8.7512	8.9649	9.3631
Rbm10	_LASDDRPS[Phospho (STY)]PPR_2	Q99KG3_S723_M1_Rbm10	10.1167	4.6165	10.1172	10.5150	9.9376	3.7600	9.1138	10.3990	9.5704	3.3304	9.6941	10.0431	10.6898	10.5308	9.5926	9.8529	9.9963	9.7554	10.0514	10.0538
Stk24	_[Acetyl (Protein N-term)]AHS[Phospho (STY)]PVQSGLPGMQNLK_2	Q99KH8_S4_M1_Stk24	3.9792	3.3398	3.5158	3.6050	4.1862	3.9222	3.8430	4.2049	3.9021	4.5667	4.2764	3.0660	4.0101	9.1327	3.4202	3.3414	3.7022	3.4473	3.7463	3.2312
Aco2	_SQFTITPGSEQIR_2	#N/A	10.6040	10.5798	10.8048	10.7543	10.4854	10.5625	10.6476	10.7570	10.6976	10.4816	10.5834	10.6134	10.8563	10.9080	10.7978	10.7085	10.7909	10.5813	10.5566	10.5284
Aco2	_NAVTQEFGPVPDTAR_2	#N/A	10.6460	10.6773	10.6395	10.7345	10.7585	10.6225	10.6137	10.8167	10.6645	10.6561	10.7304	10.6356	10.9103	10.9011	10.6210	10.5757	10.8856	10.4947	10.6049	10.5394
Aco2	_VDVSPTSQR_2	#N/A	9.4963	9.5236	9.6938	9.6748	9.3743	9.5429	9.5565	9.7262	9.6231	9.6734	9.2592	9.5685	9.6431	9.6797	9.6541	9.4482	9.4533	9.4719	9.3526	9.5185
Aco2	_SDFDPGQDTYQHPPK_3	#N/A	9.5038	9.0815	9.5871	9.3167	9.1748	9.2700	9.2960	9.4032	9.3428	9.3077	9.3477	9.3377	9.1351	9.7163	9.4306	9.1946	9.3656	9.3025	9.1733	9.3486

Aco2	_NTIVTSYNR_.2	#N/A	9.8995	10.3476	10.2453	10.3170	10.0607	10.1998	10.4441	10.3868	10.2486	10.2795	10.0467	10.2095	10.3702	10.4210	10.5151	10.1746	10.1725	10.2695	10.3826	10.1959
Aco2	_FNPETDFLTGK_.2	#N/A	10.3023	10.1763	10.1338	10.1048	10.2849	10.0007	10.2211	10.3673	9.7227	10.2359	10.0855	10.2744	10.4391	10.2787	10.1411	10.1122	10.4019	10.2046	9.9341	10.1781
Aco2	_EHAALEPR_.2	#N/A	9.8092	9.6133	9.9157	9.5986	9.7132	9.7937	9.9516	9.8273	9.8635	9.8683	9.9489	9.8357	9.6833	9.9155	10.0967	9.8102	9.5549	9.7392	9.7321	9.8344
Aco2	_IVYGHLDDPANQEIER_.3	#N/A	9.0474	8.6964	8.7372	8.7376	8.7451	8.8342	8.8135	9.1617	8.7764	8.9832	8.9838	8.8475	8.9084	9.1491	8.7470	9.0089	8.8862	8.6901	8.5233	8.7628
Aco2	_DFAPGKPLK_.2	#N/A	9.6886	9.7398	9.9078	9.7639	9.7664	9.6683	10.0085	9.7891	9.5214	9.6456	9.6704	10.0001	9.3054	9.8197	10.1672	9.3549	9.7691	9.7375	9.8445	9.5892
Aco2	_FKLEAPDADELPR_.2	#N/A	8.5060	8.1999	8.3595	8.1952	8.3278	8.1472	8.2152	8.3674	8.4502	8.3172	8.3714	8.2554	8.2913	8.4724	8.4203	8.3642	8.4723	8.1601	8.2510	8.3424
Aco2	_LEAPDADELPR_.2	#N/A	9.2674	8.6008	9.4153	8.6863	9.1309	9.1142	8.8728	8.9333	9.1356	9.0198	9.2532	8.5191	8.4961	8.8132	9.0447	8.7463	9.0287	8.7177	9.7068	9.1283
Aco2	_LTGSLSGWTSPK_.2	#N/A	8.5397	8.7796	8.8175	8.8131	8.3892	8.5122	8.7548	8.6428	8.8398	8.5056	8.2789	8.5144	8.8053	9.0684	8.9105	8.7074	8.5880	8.7232	8.5538	8.7579
Aco2	_EGWPLDIR_.2	#N/A	8.4154	8.4533	8.7063	8.7589	8.2200	8.5282	8.7981	8.2510	8.5187	8.5125	8.4246	8.5125	7.7178	8.8205	8.0304	8.0011	8.5946	8.4563	8.1033	8.6817
Aco2	_IHETNLK_.2	#N/A	7.5986	7.4044	8.1213	7.6638	7.8052	7.5162	7.4904	7.5371	7.8260	7.8266	7.9962	7.5489	7.3558	7.5599	7.9536	7.6465	7.0165	7.8604	7.5566	7.7958
Aco2	_DVGGIVLANAC[Carbamidomethyl (C)]GPC[Carbamidomethyl (C)]IGQWDR_.2	#N/A	7.2459	7.0915	8.1460	7.4846	7.1598	7.5967	7.8889	7.1075	7.2419	7.2055	7.4526	7.5922	6.9482	7.6699	7.6550	7.1755	7.5968	7.2510	7.4217	6.6703
Aco2	_QGLLPLTFADPSDYNK_.2	#N/A	7.6938	7.3110	8.0328	7.5323	7.4982	7.5049	7.4969	7.7206	7.5220	7.7442	7.3814	7.4279	7.5140	7.5266	6.9230	7.1022	7.6947	7.2550	7.4772	7.4968
Aco2	_DGYAQILR_.2	#N/A	6.1470	6.4669	6.6991	7.7211	7.3525	7.3149	7.7797	6.3139	7.8105	7.7039	8.0151	7.2679	7.6508	7.0005	7.3427	7.6627	5.7255	7.6933	7.8524	8.0622
Aco2	_QALAHGLK_.2	#N/A	6.7962	6.3933	6.6711	6.5247	6.7248	6.3186	6.4835	6.2163	6.6191	6.1906	6.5144	6.2344	6.5006	6.6647	7.1469	6.4505	5.8744	6.7000	6.8376	6.8462
Aco2	_DVGGIVLANAC[Carbamidomethyl (C)]GPC[Carbamidomethyl (C)]IGQWDRK_.3	#N/A	5.7893	6.7539	6.3011	6.6884	5.8197	5.6548	6.0381	6.2809	6.0525	5.7801	5.6377	6.0360	6.6767	6.6617	6.6544	5.9697	5.7935	6.2456	6.1353	6.0839
Aco2	_TDIANLAEEFK_.2	#N/A	5.8877	5.2364	5.7934	6.1224	5.6884	6.0464	5.5052	5.3165	5.8939	6.1987	4.6609	5.5455	4.6814	6.3699	5.8053	6.1270	5.6901	5.7785	5.5242	6.1436
Aco2	_NDANPETHAFVTSPEIVTALAIAGTLK_.3	#N/A	5.2462	5.5968	6.0247	5.7755	6.3512	5.9845	6.0133	5.8635	5.2276	5.8160	6.3033	5.7390	5.7891	6.5547	5.1318	5.7157	5.6444	5.7585	5.7165	5.8349
Aco2	_WVVIGDENYGEGSSR_.2	#N/A	9.1206	9.1769	9.0353	9.1166	4.2068	8.4816	8.4959	9.0282	8.6793	8.8817	8.4496	8.5941	9.2285	9.5443	8.4440	8.8446	8.9563	8.8422	8.7589	8.7383
Aco2	_VAGILTVK_.2	#N/A	5.0868	5.5572	5.0736	5.9972	3.9091	4.6858	5.4271	3.4727	5.6349	5.9449	5.5717	4.7362	6.1568	5.2735	5.6744	6.0085	3.5551	6.0228	6.3518	6.0533
Aco2	_AKDINQEVYNFLATAGAK_.3	#N/A	6.5595	7.2279	6.8653	7.3380	6.8520	6.8738	6.9335	7.0966	6.9834	6.9680	6.9453	7.0326	7.4227	7.2738	6.6539	7.1106	7.0061	7.1818	7.0198	7.3376
Aco2	_GHLDNISNNLLIGAINIENGK_.3	#N/A	6.9184	6.9194	5.9350	6.6692	6.6369	6.6138	6.4640	7.0592	6.2148	6.9288	6.9463	6.4031	6.9048	7.4659	5.9924	6.4349	6.2900	6.5118	6.6468	6.9942
Aco2	_C[Carbamidomethyl (C)]TTDHISAAGPWLK_.2	#N/A	5.3110	5.2282	4.6677	6.0323	5.1062	6.0526	5.1939	5.7485	5.8425	5.4576	5.1049	5.6179	4.3460	6.0634	6.1470	5.9270	6.2057	5.8267	5.6816	5.9727
Aco2	_VAM[Oxidation (M)]SHFEPSEYIR_.3	#N/A	7.7932	7.4699	7.5273	7.4810	7.7814	7.3940	7.5792	7.9747	7.6050	7.8955	7.5128	7.7984	7.9327	7.7882	8.1212	7.5943	7.3701	7.8601	7.8381	7.6289
Aco2	_VGLIGSC[Carbamidomethyl (C)]TNSSYEDM[Oxidation (M)]GR_.2	#N/A	5.2481	5.2828	5.2380	4.4296	4.7164	5.3513	5.4734	5.1870	5.1423	4.9912	5.2592	5.2596	3.4401	5.6383	5.1149	4.9861	3.7290	5.3677	5.1363	5.0665
Aco2	_VAM[Oxidation (M)]QDATAQM[Oxidation (M)]AM[Oxidation (M)]LQFISSGLPK_.3	#N/A	5.5915	4.9955	3.7983	5.0217	5.4218	5.1187	4.8136	4.8273	4.9559	5.2431	4.8913	4.7122	5.6597	4.4877	4.9189	4.8487	5.5428	5.3068	5.0105	4.8983
Aco2	_SDFDPGQDTYQHPPKDSSGQR_.4	#N/A	4.3615	5.8814	3.8980	3.2228	3.0676	5.5364	6.0073	3.0863	5.3300	4.1721	3.3794	6.1245	4.0662	5.3746	5.8567	5.4518	4.0844	5.5803	5.6672	5.4945
Aco2	_DINQEVYNFLATAGAK_.2	#N/A	6.9329	6.3943	7.1202	7.0516	6.7105	6.4822	6.4379	6.6009	6.8071	6.9035	6.7643	6.5776	6.8642	6.8166	6.6716	6.9644	6.6932	6.2924	6.6575	6.7545
Aco2	_VAVPSTIHC[Carbamidomethyl (C)]DHLIEAQVGGEK_.3	#N/A	7.1302	6.0150	7.5890	6.7886	6.9623	6.5294	6.8053	7.1074	6.6072	7.0153	7.0933	6.7370	7.0839	6.4827	7.0374	6.5647	6.8907	6.2572	6.7486	6.7766
Aco2	_VAVPSTIHC[Carbamidomethyl (C)]DHLIEAQVGGEKDLR_.4	#N/A	4.0568	3.6527	3.5934	3.3393	4.2638	4.2971	4.2852	4.0505	4.2054	3.8674	3.6840	2.9884	4.3708	3.3726	2.8097	3.4189	3.0873	3.7742	4.3944	3.3396
Aco2	_HPNGTQETILLNHTFNETQIEWFR_.4	#N/A	4.1491	3.1700	3.6856	3.4352	2.8552	3.7524	4.0128	4.3747	4.0719	4.3969	2.9454	4.3970	4.1799	3.7237	4.2183	3.5112	3.8720	3.2775	3.5765	3.4010
Ubxn2a	_AKS[Phospho (STY)]VEVDNK_.2	Q99KJ0_S154_M1_Ubxn2a	6.3002	6.3664	6.2242	5.9110	5.6900	5.8464	5.3874	6.7188	5.5582	6.1696	5.9381	6.8521	6.4054	6.8698	5.4309	6.9455	6.7556	6.7069	6.0482	6.3064
Ubxn2a	_C[Carbamidomethyl (C)]LS[Phospho (STY)]PTEQKK_.2	Q99KJ0_S54_M1_Ubxn2a	3.3071	4.0120	4.3445	4.2772	5.2405	3.0935	3.1708	3.5328	3.2299	3.7380	3.6043	3.7381	3.3379	4.3826	4.2488	4.1700	4.5308	4.4325	4.4185	4.0598
Dctn2	_YADLPGIAR_.2	#N/A	7.4569	7.8661	8.0296	7.4781	7.9078	7.5166	7.9496	7.8921	7.6789	7.7178	7.4222	7.9130	7.7741	7.0319	7.8649	7.5405	7.3830	7.4957	7.6596	7.7672
Dctn2	_WSPVASTLPELVQR_.2	#N/A	7.3976	7.2193	7.5058	7.3924	7.2458	7.4093	7.4139	7.3228	7.2367	7.4640	6.7218	7.1420	6.9075	7.4278	7.4573	7.3852	7.4819	7.0571	7.1623	7.2216
Dctn2	_LLGPDAAINLADPDGALAK_.2	#N/A	6.4090	6.4085	5.3459	6.2680	6.1797	6.5037	6.6180	6.7368	6.2030	5.9891	5.9256	6.3150	6.6525	6.7689	6.6271	6.5196	6.3473	6.5361	6.3416	6.5973
Dctn2	_QQLVASHLEK_.2	#N/A	6.2677	6.1047	6.8021	6.2416	6.0387	6.0243	6.2140	6.0645	6.1390	6.2909	5.8769	6.4279	6.2250	6.1836	6.8064	6.3787	5.9811	6.1604	6.0329	6.0216
Dctn2	_ASVEDADTQNK_.2	#N/A	6.3831	5.9024	6.4094	6.2630	6.6183	6.4219	5.7502	6.2788	6.1461	6.1248	6.1070	5.7612	5.4032	6.1738	6.4759	5.4761	5.9973	5.4843	6.2450	6.2743
Dctn2	_VSALDLAVLDQVEAR_.2	#N/A	4.8595	4.8438	4.9348	5.2870	4.7356	5.2437	4.9707	4.4419	5.4388	5.5809	5.0232	4.7337	5.2965	3.7701	5.5733	5.0241	4.7020	4.2803	5.4883	5.2520
Dctn2	_LLHEVQELTTEVEK_.3	#N/A	3.1199	5.3423	5.5598	6.5929	5.8290	5.1238	5.8529	5.0865	6.1171	6.2326	6.1252	5.2884	3.8069	6.1267	6.2088	6.0005	4.5361	5.6545	5.7583	6.6687
Dctn2	_ENLATVEGNFASIDAR_.2	#N/A	6.1875	6.2749	6.4535	6.5960	5.8639	5.7627	5.7334	5.5620	6.0411	6.4414	6.0390	5.9259	6.4231	6.2905	6.0382	5.7572	6.2801	5.4935	5.7889	6.0203
Dctn2	_LTELEATVR_.2	#N/A	3.6731	5.1836	3.2097	3.9112	3.8800	4.8927	3.8490	3.8988	3.0883	3.4837	4.0694	3.3721	3.8352	4.1714	4.4358	4.8953	3.3960	5.3032	5.1163	5.5195
Dctn2	_DNTALLTQVQTTM[Oxidation (M)]R_.2	#N/A	6.0613	5.9198	6.5330	6.4105	6.2145	6.0437	5.8522	6.0154	5.7974	6.2813	5.6804	6.3540	6.3650	5.6209	5.9324	6.1404	6.4149	5.7069	6.0080	6.3095
Dctn2	_VHQLYETIQR_.2	#N/A	4.4868	3.3254	5.3098	4.6085	4.5391	4.9776	4.3639	4.4319	4.2163	3.6467	4.8109	4.1487	3.4425	4.8783	5.0335	4.6475	4.9727	4.8332	4.2409	4.7994
Dctn2	_GLDFSDR_.2	#N/A	4.5552	3.4476	3.5907	3.1342	3.8929	3.6626	2.9894	4.2556	4.5010	4.0554	3.4929	3.3370	4.4964	4.1591	4.1574	4.2575	3.2924	3.0399	4.7913	3.8402
Dctn2	_TGYES[Phospho (STY)]GYEM[Oxidation (M)]LGEGLGVK_.2	Q99KJ8_S83_M1_Dctn2	3.4650	4.5378	4.0013	4.2105	4.3036	3.0268	3.2375	3.5994	3.2966	3.6714	3.6709	2.9264	3.4046	5.2604	2.8147	3.8099	3.0967	4.0528	4.3518	4.1265
Hars2	_DLS[Phospho (STY)]PQQMVVR_.2	Q99KK9_S66_M1_Hars2	4.3396	9.1615	9.7068	9.6465	7.1329	8.5766	8.8454	4.0399	7.9123	7.7222	8.9779	7.1216	4.3603	10.1939	7.4630	3.1836	8.3685	6.7604	5.6158	7.8045
Clint1	_TIDLGAAAHYTGDKAS[Phospho (STY)]PDQNASTHTPQSSAK_.4	Q99KN9_S307_M1_Clint1	3.9199	4.9726	5.3619	4.2472	3.9195	4.4163	3.7098	4.0441	3.6886	3.9919	4.0041	3.5995	4.2133	4.1817	3.4557	3.8555	3.5413	4.2256	3.9691	4.0256
Cry1	_[Acetyl (Protein N-term)]ASPAAGGVVIVGSGLIGR_.2	#N/A	3.2174	2.5927	3.8837	2.4754	2.3944	3.4111	3.4000	4.3002	4.0635	3.1746	3.0226	3.6101	3.1559	3.4564	3.2859	3.0878	3.9205	1.5663	4.0019	4.1790
Nampt	_SYS[Phospho (STY)]FDEVRK_.2	Q99KQ4_S472_M1_Nampt	7.6994	7.8226	4.3486	4.2730	7.3450	3.0894	6.8710	7.8532	3.2340	7.7809	7.0200	7.5528	7.8284	7.9184	3.5553	7.4434	7.9795	7.5624	7.7431	7.5950
Rnf34	_GLGSGDDLDSSS[Phospho (STY)]LNSSR_.2	Q99KR6_S163_M1_Rnf34	4.5766	5.3515	5.1317	4.0391	4.0639	4.2880	3.4771	4.4266	5.1313	5.0527	3.9106	3.1660	3.6643	3.7487	3.8888	4.6784	5.3629	5.2018	4.5303	4.4643
Rnf34	_AS[Phospho (STY)]LSDLSSLEEVEGMSVR_.2	Q99KR6_S258_M1_Rnf34	3.0940	5.5932	4.1315	4.4902	3.3010	3.3066	5.3324	3.3197	3.0169	3.9511	3.3912	3.9512	3.1249	4.1695	4.4816	4.0896	4.3178	4.3325	3.1307	3.8468
Vmp1	_DQHNGS[Phospho (STY)]LTDPSSVHEK_.3	Q99KU0_S23_M1_Vmp1	3.7131	3.9168	5.9083	5.8618	3.3536	3.7902	5.7229	4.4900	3.3845	5.6947	5.7247	5.3881	5.6750	5.0001	5.1443	4.7668	4.8144	5.8817	3.6143	3.5055
Mtf2	_LAIQGPEDS[Phospho (STY)]PSR_.2	Q99KX1_S237_M1_Mtf2	12.6894	13.0967	7.4670	13.2170	12.8729	12.7824	12.5764	12.8681	12.8870	13.2211	12.8082	13.0776	13.7782	13.3905	13.0174	14.0935	13.3837	12.6342	13.1222	13.2428
Mtf2	_DSDS[Phospho (STY)]GLEQMSIGHHIR_.3	Q99KX1_S145_M1_Mtf2	3.8165	3.8649	4.1440	4.4461	3.4379	4.0766	3.8727	3.7508	3.6456	3.7558	3.6657	3.6420	3.7809	4.4937	3.4544	3.8655	4.2023	3.8360	4.0087	3.9075
Gak	_EPETGLDNTS[Phospho (STY)]PK_.2	Q99KY4_S809_M1_Gak	3.1332	7.0464	4.1706	7.3758	3.3401	7.6162	3.7080	3.3588	6.8727	6.7303	3.1068	3.9120	3.7936	6.0498	5.5499	3.9961	4.3569	4.2934	3.0916	6.8918
Znf639	_YFDNKDDS[Phospho (STY)]DPETANDLPK_.3	Q99KZ6_S60_M1_Znf639	4.5565	5.3387	4.0930	4.5286	3.2626	3.3450	3.7855	3.2813	2.9784	4.3671	3.3528	3.9896	4.5873	5.5173	3.8109	3.9186	4.2794	6.0822	3.1691	6.2417
Pagr1a	_DLFSLDSEGPSPTS[Phospho (STY)]PPLR_.2	Q99L02_S236_M1_Pagr1a	7.7539	8.0908	3.8079	7.4074	6.6236	7.3483	7.4909	7.2510	7.4129	7.7256	7.7150	7.9264	3.5981	8.4557	7.8844	7.6616	8.0621	7.5893	7.4048	7.5358
Dhrs1	_VLPSC[Carbamidomethyl (C)]DLAR_.2	#N/A	3.5478	3.1436	3.8946	5.0084	3.7547	5.0349	3.2934	5.3297	5.0704	3.3584	4.1931	3.0331	3.3790	4.2207	5.6614	5.0175	3.5964	4.2368	3.8854	5.2298
Dhrs1	_ATAQEAQSLGGR_.2	#N/A	3.8079	3.5112	3.3444	5.4964	4.0148	4.0936	3.6716	4.0335	3.7307	3.2372	5.5698	3.2373	3.8387	3.3825	5.4436	3.1700	3.5308	3.6187	3.9177	5.8159
Hibadh	_DLGLAQDSATSTK_.2	#N/A	5.7034	5.9025	6.6382	5.7101	6.3070	6.0628	6.0789	6.2876	6.4788	5.9128	6.0178	5.7319	6.3626	5.8312	6.2645	5.9850	6.5117	6.2120	6.1793	6.1184
Hibadh	_TPILLGSLAHQIYR_.2	#N/A	3.8618	3.9957	3.2144	4.9286	5.0245	4.1112	3.0158	3.7909	4.4074	4.2769	4.4194	3.2999	4.0955	3.8940	5.3233	4.7338	3.9362	4.6627	3.7879	3.9868
Gmpr2	_VTQQVNPIFSNS[Phospho (STY)]Q_.2	Q99L27_S347_M1_Gmpr2	5.2215	6.0298	3.8977	4.0187	3.5363	6.4145	5.4414	5.1754	4.7787	5.7592	5.5279	5.5415	3.5370	5.8035	4.8950	5.5525	5.8596	3.9492	5.4918	5.7373
Cds2	_AETAPLPTSVDDT[Phospho (STY)]PEVLNR_.2	Q99L43_T50_M1_Cds2	4.5546	8.0011	7.2714	3.1348	3.8923	7.6092	7.6302	4.2550	6.9806	3.2601	4.0822	3.3377	3.8159	7.9753	3.2260	3.3986	6.0252	7.7543	7.0311	3.0370
Cds2	_LDGETAS[Phospho (STY)]DSESR_.2	#N/A	10.2529	9.8365	10.3242	9.9236	9.5088	9.9347	9.8439	9.7044	9.7299	9.7276	9.8893	9.1983	9.3674	9.4785	8.6177	9.2038	9.6662	9.6115	9.6664	9.4791
Cds2	_ESESEAKLDGETAS[Phospho (STY)]DSESR_.2	#N/A	3.3927	3.5471	3.4365	4.2894	3.7038	3.9240	3.6329	4.1113	3.0819	3.4943	4.0614	3.3470	3.2474	3.4445	3.1871	4.1441	3.3554	4.3168	4.1100	3.7819
Cds2	_EDAPPEDKES[Phospho (STY)]ESEAK_.2	Q99L43_S20_M1_Cds2	3.7665	3.3526	3.3031	3.8177	3.9734	6.9241	3.0747	4.3408	2.9949	3.5771	3.9744	3.2787	3.1603	3.6630	3.1000	3.1286	3.4894	4.4555	4.1041	3.6300
Cds2	_ALS[Phospho (STY)]NLSSR_.2	Q99L43_S59_M1_Cds2	4.0806	3.2385	3.6172	5.8813	4.2875	6.6945	3.9443	4.3063	5.9062	6.6194	6.4903	5.7751	4.1114	3.6552	3.5215	6.1110	3.8035	6.1953	3.6450	3.3325
Cds2	_EDAPPEDKESESEAKLDGETAS[Phospho (STY)]DSESR_.4	#N/A	5.3904	5.5265	3.6207	3.3209	3.7765	5.8719	6.3831	4.0367	4.0831	3.9985	6.5530	3.9441	4.2486	3.2504	3.5700	3.5500	3.6225	5.9538	3.9072	3.2174
Eif2s2	_DYTYEELLNR_.2	#N/A	4.6293	5.7695	5.6857	4.4333	5.6099	4.7678	5.8967	5.1123	5.8179	5.7550	5.2742	5.3302	5.6622	5.2767	5.0661	5.7288	5.3112	5.4431	5.5099	5.6030
Eif2s2	[Acetyl (Protein N-term)]S[Phospho (STY)]GDEM[Oxidation (M)]IFDPTM[Oxidation (M)]SKK .	#N/A	9.3999	9.8719	8.3462	9.5357	8.6695	9.2795	9.3053	9.2793	9.2527	9.2585	9.1138	9.2760	10.2759	9.7187	9.1487	9.5420	9.6378	9.3616	9.4858	9.4305
Eif2s2	[Acetyl (Protein N-term)]S[Phospho (STY)]GDEM[Oxidation (M)]IFDPTM[Oxidation (M)]SK .	#N/A	14.3295	13.7773	13.5804	13.4350	13.6600	13.6706	13.2501	14.0127	13.8493	14.1626	13.4816	13.7975	14.5640	13.5273	13.4361	14.1901	14.3015	13.7249	13.9289	13.9174
Eif2s2	_KKDAS[Phospho (STY)]DDLDDLNFFNQK_.3	Q99L45_S67_M1_Eif2s2	4.5194	4.3005	4.0560	4.5657	3.2255	3.2588	3.8226	3.2442	3.7428	4.3300	3.2214	4.0266	3.9083	4.4109	3.8480	3.8815	4.2423	3.7076	3.3562	4.3779
Eif2s2	_IFDIDEAEEAIKDVK_.3	#N/A	2.3444	4.5927	3.9464	4.2793	4.2486	4.4727	3.2925	3.1104	3.3515	3.6164	3.7259	1.1705	3.4596	3.3320	2.8697	3.7550	3.6481	3.9978	3.8336	4.1815
Eif2s2	_EYVTC[Carbamidomethyl (C)]HTC[Carbamidomethyl (C)]R_.2	#N/A	3.7808	3.5383	3.3174	3.8034	3.9877	4.1206	3.6445	4.0065	3.7036	3.2643	4.0780	3.2644	3.8117	3.3554	3.2217	3.1429	3.5037	3.6457	3.9448	3.0327
Eif2s2	_IES[Phospho (STY)]DAQEPAEPEDDLDIMLGNK_.3	Q99L45_S105_M1_Eif2s2	3.8032	4.1996	4.3395	3.8861	3.1410	3.3651	4.4001	3.5036	4.4592	4.0096	3.3327	4.0890	4.5673	4.1110	3.9773	4.1481	4.0413	4.3910	3.1892	3.7883
St13	_AIEINPDSAQPYK_.2	#N/A	7.4390	7.2850	7.6006	7.6401	7.8368	7.7276	7.4933	7.7744	7.3906	7.6333	7.5765	7.7741	8.1667	7.4439	7.9307	7.4887	7.7885	7.5236	7.5417	7.6524
St13	_GAAIEALNDGELQK_.2	#N/A	5.7002	6.2722	5.5340	5.7936	5.8192	5.6027	5.4604	5.3104	5.7590	5.6544	5.4936	6.0334	5.6101	6.1904	5.6379	5.1777	5.2830	5.7896	5.0505	5.8538
St13	_AIDLFTDAIK_.2	#N/A	4.5072	5.6270	5.6999	6.0626	5.6352	5.6333	5.8921	4.5767	5.4485	6.0918	4.5844	5.9500	5.6282	6.0141	6.5741	6.5174	5.3842	5.9028	6.3585	6.2052
St13	_KGAAIEALNDGELQK_.3	#N/A	3.8095	3.9194	4.4371	4.8293	4.5555	4.4480	3.2634	3.6254	4.4595	3.8823	4.7333	4.4306	4.7073	5.2604	4.7118	3.7840	3.1226	4.5021	4.1731	4.9450
St13	_QDPSVLHTEEMR_.3	#N/A	3.4460	4.5568	3.9823	4.8161	4.2845	3.7004	3.2565	3.1463	3.3012	3.2707	4.2807	2.9454	3.4667	4.7698	2.8337	3.7909	3.6840	4.1491	2.5352	3.6764
Sntb1	_GSPVSEIGWETPPPES[Phospho (STY)]PR_.2	#N/A	7.5350	8.1138	7.1919	7.6264	6.5202	7.8492	7.7049	6.9321	4.1656	7.4940	7.0953	6.7066	7.5089	8.5612	6.4603	7.2600	7.3849	7.6194	3.7811	7.2349
Sntb1	_KGSPVSEIGWETPPPES[Phospho (STY)]PR_.3	#N/A	4.1880	4.0644	3.9118	4.0751	3.4928	5.2938	4.9933	3.7632	4.9947	3.6337	3.8298	5.3561	3.2780	5.4125	3.8077	4.6595	4.0981	5.5142	4.9278	5.4752
Mcrs1	_GDQVLNFS[Phospho (STY)]DAEDLIDDSK_.2	#N/A	3.2305	4.3272	4.2971	4.0138	3.4375	3.4708	3.6106	3.3760	3.5309	4.5420	3.0095	4.2167	3.6963	4.1190	3.6360	4.0935	3.9136	3.9195	3.5681	4.1660
Mcrs1	_GDQVLNFS[Phospho (STY)]DAEDLIDDSKLK_.3	#N/A	3.8869	4.1159	4.4232	3.8025	3.2246	3.4487	4.3164	3.1775	4.3755	4.0932	3.2491	4.0053	4.4836	4.0273	3.8936	4.2318	4.1756	2.9738	3.2729	3.7046
Dnttip1	_QAEEESHRGS[Phospho (STY)]PIPK_.2	Q99LB0_S160_M1_Dnttip1	4.8950	4.5637	3.3905	2.3562	3.9347	4.0629	3.0185	4.2847	3.6291	3.6332	3.9182	3.2226	3.9276	4.7862	2.6536	4.3678	1.8205	3.9305	3.6979	3.1431
Mat2b	_VLITGATGLLGR_.2	#N/A	6.1092	6.0113	5.7153	6.1970	5.6047	5.3196	5.9247	6.2667	5.5667	5.2087	6.1454	5.1862	6.0398	5.7814	5.9276	5.9716	6.2084	6.2151	5.8575	5.6493
Mat2b	_AVLENNLGAAVLR_.2	#N/A	3.6789	3.0206	4.6750	4.4243	4.5116	4.2628	3.0236	4.7494	3.8809	3.8852	4.0683	4.2133	5.0261	4.2553	5.3926	4.1852	4.3836	3.3602	4.6224	3.1808
Ndufa10	_LQSWLYASR_.2	#N/A	6.9655	7.4945	7.4510	6.9669	7.2942	7.4464	7.4681	7.4891	6.8179	7.4294	6.7248	7.1286	7.1509	7.8981	7.2334	7.4276	7.1383	7.4993	7.1970	7.3674
Ndufa10	_QDDWTFHYLR_.2	#N/A	6.2359	3.9606	5.7316	6.2792	5.9670	5.8153	6.0587	5.9687	5.7571	6.3563	5.5593	5.9289	6.1408	5.8331	5.7307	6.2503	6.4299	5.9401	6.1617	6.0868
Ndufa10	_VITVDGNIC[Carbamidomethyl (C)]SGK_.2	#N/A	4.5741	6.3265	4.1107	6.3086	6.2851	6.0909	6.1285	5.8712	6.1153	5.9665	6.0097	5.8299	5.4842	6.1777	6.3596	6.0094	6.2737	6.2191	6.2184	5.9592
Ndufa10	_VTSAYLQDIENAYKK_.3	#N/A	4.2221	4.5613	4.4195	5.1153	3.9739	4.1880	5.2416	3.9927	4.1445	5.5145	5.2045	4.4539	3.7979	4.3044	5.3503	5.1306	3.4899	5.3634	5.0861	5.2232
Ndufa10	_LLQYADALEHLLSTGQGVVLER_.3	#N/A	3.6118	3.7073	4.2157	3.9724	3.8187	4.1857	3.2294	3.3731	3.1496	4.7314	4.1290	3.4334	3.3150	3.8177	3.3832	2.9739	3.3347	4.3008	3.9494	3.7847
Ndufa10	_VTSAYLQDIENAYK_.2	#N/A	6.1801	3.4212	6.5012	3.1078	4.0323	5.4284	3.0158	5.6827	5.7851	3.6359	3.9155	3.2199	4.6023	3.6041	5.7303	3.1874	3.3188	3.0135	4.1629	3.5711
Ndufa10	_TEVLNYTTIPVYLPEITIGAHQGSR_.3	#N/A	4.5110	4.3089	4.0476	4.5741	3.2171	3.2504	3.8310	3.2358	3.7512	4.3216	3.2299	4.0351	3.9167	4.0856	3.8564	3.8731	4.2339	3.6992	3.2146	4.3863
Ndufa10	_YAPGYNAEVGDK_.2	#N/A	4.1338	7.2567	3.7424	3.3784	3.4715	3.6956	7.7345	4.4315	4.1286	4.3401	3.0022	3.7584	4.2367	3.7804	3.6467	2.9779	3.9287	3.2207	7.6790	3.4577
Etfa	_LNVAPVSDIIEIK_.2	#N/A	6.1934	6.6636	6.4692	6.6928	6.7049	6.7001	6.3457	6.7863	6.6917	6.2914	6.3753	6.6615	6.2940	6.9081	6.8057	6.8020	6.8562	6.4710	6.1888	6.8559
Etfa	_GLLPEELTPLILETQK_.2	#N/A	5.5725	5.3137	5.7309	5.9395	6.0969	5.6068	5.8129	5.7641	5.3248	5.1742	5.3223	5.6136	5.1959	5.8814	6.0452	5.4882	6.0878	6.3034	5.9906	6.1630
Etfa	_TIYAGNALC[Carbamidomethyl (C)]TVK_.2	#N/A	5.6812	5.4343	5.9915	5.6617	5.4485	5.5188	5.8556	5.3867	5.2695	4.9380	4.9476	5.5346	4.7920	4.0656	5.6078	5.5471	5.0054	5.2890	5.4309	5.4025
Etfa	_VLVAQHDAYK_.2	#N/A	6.0143	4.7930	5.3173	5.8898	6.0991	5.8561	3.2590	5.7034	5.6490	3.3927	4.8282	5.5326	4.9914	5.3168	6.1109	5.3447	4.8194	4.5715	4.1435	4.8170
Etfa	_APSSSSVGISEWLDQK_.2	#N/A	4.9984	3.5221	6.1443	5.0406	4.0402	3.9076	3.9821	3.1450	5.0608	4.8191	3.2165	4.7275	4.9420	4.6952	5.3080	5.0397	3.8776	3.0064	4.5380	4.9680
Etfa	_AAVDAGFVPNDM[Oxidation (M)]QVGQTGK_.2	#N/A	7.0074	6.3977	6.8841	7.0773	6.7307	6.8394	6.4999	6.8941	6.5049	6.8979	6.4208	6.8710	7.1335	6.9712	6.9121	7.0160	6.7895	6.5246	6.5839	6.9996
Etfa	_SPDTFVR_.2	#N/A	3.2478	3.9603	4.7013	4.1657	4.2412	2.9820	5.4282	3.6442	5.3391	4.7007	3.5211	4.5175	3.4494	4.6697	5.2578	4.8173	3.1415	4.1974	4.4689	4.1713
Etfa	_VVPEMTEILK_.2	#N/A	4.5052	5.3306	5.8684	5.9769	3.8429	4.1570	5.0187	4.0601	3.7573	3.2107	4.5110	4.0770	3.8653	5.8454	3.2754	3.3492	4.7905	3.5921	3.4260	3.0864
Etfa	_SDRPELTGAK_.2	#N/A	5.2449	4.9201	4.4099	3.4492	4.5621	5.1030	5.1264	3.7527	4.0954	3.9774	5.0622	5.2895	4.2609	5.1511	4.2006	3.5289	4.3360	4.0769	3.0035	5.2970
Etfa	_GTSFEAAATSGGSASSEK_.2	#N/A	5.9008	4.1062	4.2503	5.2531	3.4199	3.4532	3.6282	4.9948	5.1897	4.5244	3.0271	3.8323	3.7139	6.3367	3.6536	5.1896	4.4367	4.2136	3.0118	3.9657
Etfa	_LLYDLADQLHAAVGASR_.2	#N/A	3.3933	3.1048	4.4307	4.1910	3.6002	3.6335	3.4479	3.2132	3.3681	4.0894	4.3476	3.6520	3.5336	4.0362	3.4733	4.2562	3.7509	4.0823	3.7308	4.4333
Gps1	_SPPREGS[Phospho (STY)]QGELTPANSQSR_.3	#N/A	9.8133	10.0434	9.2297	9.7454	9.3944	9.7771	9.8765	9.7572	9.6537	9.8758	9.6221	9.6025	10.4519	9.6868	8.8137	9.4377	9.4610	9.8367	9.6769	9.6047
Gps1	_EGS[Phospho (STY)]QGELTPANSQSR_.2	#N/A	11.9455	11.4234	12.0198	11.8554	11.5820	11.4593	11.4284	11.7746	11.6023	11.4661	11.7168	11.2912	11.4535	11.9418	10.6766	11.4107	11.9113	11.5418	11.5488	11.5611
Gps1	_LFLELEPQVR_.2	#N/A	5.3997	5.7577	4.4919	5.2536	4.7724	4.7159	5.5543	5.8707	4.3121	5.2624	4.9947	5.2612	4.5909	4.6984	4.8888	5.1471	4.9978	5.3691	5.0538	4.7788
Gps1	_GERDS[Phospho (STY)]QTQAILTK_.2	Q99LD4_S220_M1_Gps1	3.6694	4.3334	4.2057	4.9012	3.0071	3.2312	4.7631	3.3950	3.0922	3.8757	3.4666	4.2228	3.2002	4.2449	4.1111	4.0143	4.3931	4.2572	3.0554	5.0159
Rtcb	_NLDFQDVLDK_.2	#N/A	4.4852	4.6511	5.3106	3.2042	4.3196	4.6777	4.4202	4.7398	4.7096	4.9324	5.2492	4.9148	4.5059	3.9559	4.9932	4.8009	4.8019	4.4157	3.6412	4.6534
Rtcb	_MLQADPNKVS[Phospho (STY)]PR_.2	Q99LF4_S208_M1_Rtcb	3.9584	4.0444	2.8369	3.7310	2.2606	4.2037	4.2449	3.6588	4.3040	4.1648	1.4981	3.9338	4.4121	4.4818	3.8221	4.3033	4.1965	3.6367	3.2853	4.4488
Tmem51	_AETETS[Phospho (STY)]PGHAPDR_.2	Q99LG1_S178_M1_Tmem51	3.7405	3.5785	3.2771	3.8437	3.9475	3.9808	3.7930	3.9662	3.0208	3.5511	4.0003	3.3047	3.1863	3.8602	3.1260	4.0150	3.4634	4.4295	4.0781	3.6559
Ptdss1	_TYSEC[Carbamidomethyl (C)]EDGTYS[Phospho (STY)]PEISWHHGK_.3	Q99LH2_S425_M1_Ptdss1	3.4438	3.8752	4.4813	4.1404	3.6508	4.4576	3.3076	3.6695	3.3667	3.6013	3.7410	3.6014	3.4747	4.5193	4.3095	4.3068	3.1668	3.9827	4.2817	4.1966
Sh3bp5l	_TVAS[Phospho (STY)]DLQK_.2	Q99LH9_S342_M1_Sh3bp5l	3.6479	4.3549	8.2416	8.8144	8.2407	3.2097	8.3051	8.2849	3.1137	9.0144	8.4968	4.2443	8.7987	8.9799	4.1326	8.2905	8.3000	3.9472	8.0857	8.5080
Sh3bp5l	_RGS[Phospho (STY)]DIGVR_.2	Q99LH9_S377_M1_Sh3bp5l	8.2558	8.2596	7.8983	8.2605	7.8217	7.9307	8.0805	7.7525	7.7771	8.4931	8.0055	7.8882	8.3727	8.3776	7.3756	8.1357	8.3926	8.1597	8.0771	8.1586
Sh3bp5l	_GLSDHAS[Phospho (STY)]LDGQELGAQSR_.2	#N/A	6.6981	6.0453	5.9424	5.7933	4.0958	4.0457	5.6186	6.6341	5.5850	6.7462	5.6513	4.6102	6.3447	6.4486	5.5813	3.6899	5.6649	5.7780	4.4251	6.6408
Sh3bp5l	_TVAS[Phospho (STY)]DLQKC[Carbamidomethyl (C)]DS[Phospho (STY)]VEHLR_.3	#N/A	3.6548	5.5705	5.1957	5.0495	4.1518	4.2862	3.9937	3.6900	3.9599	3.6391	3.8102	3.0907	4.1060	6.0943	2.9447	3.4979	3.6091	5.7488	5.3985	5.9947
Clcc1	_FHSGNKS[Phospho (STY)]PEVLR_.3	Q99LI2_S433_M1_Clcc1	6.4676	4.3933	6.8173	6.4687	3.1328	6.8463	6.5480	3.1515	4.3495	4.2373	3.2230	4.1194	4.4575	6.5172	3.9407	6.7825	6.6856	6.6106	6.8440	6.6214
Clcc1	_KEVSPDS[Phospho (STY)]SEAEELSDC[Carbamidomethyl (C)]LHR_.3	#N/A	4.4975	4.3224	4.0341	4.5875	3.2036	3.2370	6.4168	3.2224	3.7647	4.3081	3.2433	4.0485	4.5284	4.0722	5.6185	3.8596	4.2204	4.4299	3.2281	3.7494
Clcc1	_EVSPDS[Phospho (STY)]SEAEELSDC[Carbamidomethyl (C)]LHR_.3	#N/A	4.0726	3.9106	3.9100	3.8208	3.4103	6.3344	3.4776	3.8462	3.5367	4.2789	3.9111	3.7466	3.6647	3.9165	3.7880	3.6843	3.8140	3.6993	4.7794	4.6057
Cstf3	_RPNEDS[Phospho (STY)]DEDEEKGAVVPPVHDIYR_.4	#N/A	3.8456	8.9806	8.3108	3.8390	3.2983	9.1552	8.9053	4.0712	8.7765	3.6562	3.6383	4.0466	3.9022	9.5073	8.2821	8.1563	3.5685	9.1010	9.1319	8.9892
Hgs	_ALQNAVSTFVNR_.2	#N/A	5.4495	5.8468	6.4350	5.7098	6.1009	6.3420	6.3390	6.2126	4.4650	5.9621	5.5820	5.7689	5.1296	6.5902	6.3004	5.9613	5.7823	6.3648	6.1859	6.2435
Hgs	_ASSTTEELPPEYLTSPLSQQS[Phospho (STY)]QLPPK_.3	Q99LI8_S246_M1_Hgs	3.9933	3.3258	3.5299	3.5909	4.2002	4.2336	4.3487	4.2190	4.2689	3.8039	3.7475	3.0519	4.4344	3.4362	2.8732	3.3554	3.7162	3.1815	4.3309	3.4032
Cttnbp2nl	_FQSQADQDQQASGLQS[Phospho (STY)]PPSR_.2	Q99LJ0_S481_M1_Cttnbp2nl	4.5766	5.7107	6.9091	6.2565	6.4130	5.9892	4.5542	5.7943	2.9986	6.0965	6.2488	6.2682	6.1239	7.1857	6.4334	5.0005	7.0581	4.3507	5.1490	5.5303
Cttnbp2nl	_DLS[Phospho (STY)]PTLDNSAAK_.2	Q99LJ0_S488_M1_Cttnbp2nl	8.2705	9.2547	9.1110	8.8983	3.1576	8.6726	8.4643	8.2078	8.9044	8.9134	3.2894	8.3862	8.4392	9.5013	8.1406	8.6748	8.7406	8.8162	8.8288	8.8864
Pwp1	_GLSVSGPC[Carbamidomethyl (C)]GSRS[Phospho (STY)]PQQTPMES_.2	Q99LL5_S493_M1_Pwp1	4.1680	3.1511	3.7045	3.4163	2.8741	4.4082	6.7412	4.3936	4.0942	3.9786	6.0773	4.3781	4.2597	8.1606	4.1994	3.5301	3.8909	3.3561	3.0047	3.2285
Pwp1	_GKLQEEGGS[Phospho (STY)]EEEEAGNPSEDGM[Oxidation (M)]QSGPTQAPPR_.3	Q99LL5_S49_M1_Pwp1	3.2043	4.1148	4.2417	4.3800	3.4112	3.1963	6.3669	3.4299	3.1271	4.5157	3.5015	3.8409	3.2351	4.2798	3.6623	4.0672	4.4280	4.2223	3.0205	3.9570
Grpel1	_DLLEVADILEK_.2	#N/A	5.2688	5.9460	5.4618	5.7551	5.1740	5.5648	5.4280	5.7235	6.0024	5.6265	6.0636	5.9779	5.2414	5.7601	6.0273	5.8006	5.6373	5.1606	5.6828	5.7605
Park7	_VTVAGLAGKDPVQC[Carbamidomethyl (C)]SR_.3	#N/A	5.8621	6.3089	6.6148	6.2453	6.1136	5.9525	6.4808	6.3497	6.6477	6.1542	6.0721	6.5305	5.9078	6.3685	7.0597	6.2989	6.1560	6.1594	6.4572	6.1013
Park7	_VTTHPLAK_.2	#N/A	6.1028	7.1713	6.7005	6.4524	6.5099	7.2648	7.2806	6.7194	6.7179	6.7423	6.3708	7.2141	6.7914	6.8239	7.7952	6.8429	6.5121	7.4798	7.2133	7.2589
Park7	_DVMIC[Carbamidomethyl (C)]PDTSLEDAK_.2	#N/A	3.2627	6.1493	7.8086	7.2995	5.1719	5.7238	6.3914	3.3438	5.3348	4.5741	7.7848	4.3343	3.6641	7.3642	5.9332	4.1256	5.9105	3.6522	4.7160	5.6389
Park7	_VEKDGLILTSR_.2	#N/A	7.0371	7.2323	5.3746	6.6035	6.9365	6.8452	6.9914	7.1143	7.0419	6.8520	6.3924	6.9578	7.3689	6.8813	7.0422	7.1945	7.0732	6.7029	6.6813	6.9196
Park7	_TQGPYDVVVLPGGNLGAQNLSESPM[Oxidation (M)]VK_.3	#N/A	6.2012	6.0972	6.5715	6.1089	6.6517	5.9821	5.1892	6.3246	5.7422	6.3405	6.0359	5.7183	6.4643	5.9277	6.3168	6.4006	6.1578	6.1265	6.0090	6.1777
Park7	_GAEEM[Oxidation (M)]ETVIPVDVMR_.2	#N/A	4.1392	6.7022	7.0763	7.7985	5.6606	6.3184	6.9343	4.1786	5.5322	4.9468	7.7536	5.7346	4.3028	7.7734	6.4467	3.3173	5.2302	4.4641	4.8153	5.5925
Ndufs5	_PFLDIQK_.2	#N/A	6.0017	6.0768	6.4312	5.4955	6.3838	6.4970	6.2146	6.3648	5.6779	6.1952	6.2802	6.5524	6.4605	6.2240	7.0374	6.1886	6.3118	6.2644	6.4477	6.8390
Ndufs5	_IEFDDFEEC[Carbamidomethyl (C)]LLR_.2	#N/A	5.5790	5.8444	6.3767	6.9044	6.1416	5.6160	5.9538	6.0786	6.4976	6.1585	6.2173	5.9838	6.1917	6.3021	6.7366	6.1337	5.8553	5.8079	5.9769	6.7515
Ndufs5	_EC[Carbamidomethyl (C)]KIEFDDFEEC[Carbamidomethyl (C)]LLR_.3	#N/A	3.9633	5.6200	4.8299	5.2114	4.9596	4.0277	4.7662	3.9375	5.5411	4.6923	4.7678	4.5458	5.5888	6.6845	5.3594	5.5657	4.7929	4.6676	5.0740	5.8742
Ndufs5	_HFM[Oxidation (M)]FLSAEQPYK_.2	#N/A	5.9675	6.1108	3.9808	5.9717	6.3337	6.0922	3.3796	6.0006	6.1337	5.5660	4.2793	6.5442	7.0090	3.9679	5.8192	6.6395	6.3698	4.1506	6.2372	6.4862
Ndufs5	_EWIEC[Carbamidomethyl (C)]AHGIGGTR_.3	#N/A	4.7266	5.4075	5.8549	5.6444	4.2126	5.5819	5.1143	5.0757	5.3331	4.9465	5.1963	4.8754	4.6504	6.1190	6.3361	5.7940	5.2569	5.4028	5.6096	5.8096
	RGS[Phospho (STY)]FEAGR.2	#N/A	7.3221	6.8539	6.8656	6.4702	6.3759	6.9468	6.2234	6.6452	6.2492	6.7224	6.2026	6.4235	6.6052	6.4941	4.7883	5.7719	6.8085	6.6540	6.8100	6.3698
	RGS[Phospho (STY)]FEAGRR.2	#N/A	7.0730	6.4015	6.5038	6.5914	6.4743	6.0287	6.3606	6.9794	6.3193	6.6042	5.8685	6.6675	5.2458	6.2141	5.6517	6.0447	6.8125	6.7838	5.9845	6.2928
Rnps1	_S[Phospho (STY)]PS[Phospho (STY)]PKPTK_.2	#N/A	3.1075	6.9589	5.2776	8.3356	7.7064	5.9126	8.2220	7.0870	6.5444	8.2272	8.3518	3.2840	3.7622	7.1850	3.1723	6.1583	7.5703	7.9650	6.0767	7.6800
Rnps1	_RS[Phospho (STY)]PS[Phospho (STY)]PKPTK_.2	#N/A	6.5208	4.8158	3.9238	5.0348	4.5465	3.1267	5.5965	3.7201	3.8750	5.1617	3.3536	4.6049	4.0404	4.5431	3.9801	3.7494	4.2577	5.6298	3.2240	3.0092
Rnps1	_APS[Phospho (STY)]PTKR_.2	#N/A	4.4593	3.5435	3.4948	3.7039	3.7970	4.0211	3.7440	4.1060	3.8031	3.1648	4.1775	3.4329	3.9112	3.4549	3.3212	3.3034	3.6032	3.5462	3.8453	3.1322
Rnps1	_SSTRAPS[Phospho (STY)]PTK_.2	#N/A	3.8062	4.1966	4.3425	3.8832	3.1440	3.3680	2.5172	3.5066	4.4562	4.0126	3.3297	4.0860	3.8270	4.3296	3.9743	4.1511	4.0443	3.7889	3.4375	4.2966
Epdr1	_ALVSYDGLNQR_.2	#N/A	7.3878	7.3205	7.1358	6.9617	7.1974	6.6997	6.8118	7.4354	7.3003	7.3379	7.1313	7.1825	6.6918	7.2229	7.3378	7.1710	7.0671	7.3313	6.9246	6.8410
Epdr1	_DC[Carbamidomethyl (C)]YPVQETFIR_.2	#N/A	6.8920	6.9009	7.1507	7.1710	6.9948	6.9342	6.8986	6.3511	6.6505	7.5571	7.0627	6.6459	6.3569	7.1061	7.0339	6.9362	6.8138	6.6143	6.7811	6.8804
Epdr1	_SYETWIGVYTAK_.2	#N/A	3.2460	5.7053	3.7823	5.5222	4.0846	3.9004	6.0510	5.6460	5.7699	5.5904	5.3615	5.4670	3.2667	6.0696	5.4645	3.5909	6.1080	5.7181	5.4592	5.6668

Epdr1	_QVLYQQSSGHNNR_.2	#N/A	4.3870	5.4381	5.6174	3.2560	4.6686	3.9146	5.2838	5.0802	4.1871	5.2114	3.6657	5.0385	4.4124	5.4052	5.5333	3.4923	3.6607	5.2431	3.9054	3.5031
Scrt1	_GGASGPATPAPPQLS[Phospho (STY)]PVQA_.2	Q99M85_S344_M1_Scrt1	7.7849	8.2192	7.1001	7.4552	6.5298	7.2623	7.8223	7.6942	7.3506	7.2908	7.3553	7.5134	8.1696	8.1353	6.5377	4.1079	8.0937	7.6101	7.2149	7.2394
Dnaja3	_VTIPVPAGVEDGQTVR_.2	#N/A	3.4750	6.4734	6.6344	6.9784	6.3941	6.7288	6.3173	7.2055	6.7901	6.9117	6.2228	6.1632	6.7021	6.3278	7.1341	7.0836	6.4551	6.2752	6.5034	6.7754
Grk2	_NKPRS[Phospho (STY)]PVVELSK_.3	#N/A	6.6714	4.9908	5.9420	6.2307	5.4970	3.5914	5.7862	4.8306	5.4318	6.4127	5.8326	5.5746	3.6030	5.5590	4.7525	5.5345	5.6741	4.0401	3.6887	6.0737
Grk2	_SLLEGLLQR_.2	#N/A	4.4512	4.9490	4.6459	5.0593	5.3645	4.0502	5.2342	4.7361	4.5085	3.1939	4.2010	4.4432	3.8821	3.7250	4.4645	5.0633	3.2256	4.7385	4.0036	4.4849
Grk2	_GEVNAADAFDIGS[Phospho (STY)]FDEEDTK_.2	Q99MK8_S487_M1_Grk2	4.3455	3.7621	3.9438	3.4753	3.4998	4.0192	3.5568	3.9187	4.2238	4.4542	3.9902	3.4882	3.7239	4.1664	3.6029	3.6503	3.6965	3.8696	3.8389	3.6396
Grk2	_EAQQLVQR_.2	#N/A	3.3622	4.2642	4.2749	4.5266	3.0764	4.1587	4.4647	3.2507	4.1715	3.8597	3.3973	4.1536	3.7594	4.2620	4.0419	4.1569	2.6312	3.8565	2.5095	4.4053
Grk2	_S[Phospho (STY)]PVVELSK_.2	#N/A	3.8064	4.1964	4.6533	5.5504	3.1442	3.3683	4.3969	3.5068	3.7658	4.0128	4.3702	4.0858	3.8272	4.3298	3.9741	4.1513	4.0445	3.7887	3.4372	4.2968
Kars1	_ETAATTETPESTEASPS[Phospho (STY)]V_.2	Q99MN1_S594_M1_Kars1	3.5531	8.1233	4.3231	7.6727	2.8908	3.1149	7.7188	3.5113	7.8678	8.0843	3.5829	3.7596	3.3165	8.4927	4.2274	7.9727	4.5094	4.1409	4.4399	4.0384
Pccb	_DTSYLFITGPEVVK_.2	#N/A	4.4755	4.9234	5.4936	5.4701	5.0104	4.6541	5.3052	4.5057	5.0589	5.5442	5.2694	4.9683	3.3252	4.8875	5.5570	5.5166	4.3056	4.9126	5.0536	5.1373
Pccb	_EFFNFLPLSSQDPAPIR_.2	#N/A	4.7409	4.9918	4.8720	5.1345	4.6197	4.2278	4.5870	4.9456	3.8362	4.1128	4.3280	4.9876	4.4112	5.1109	4.3088	4.3208	4.2618	4.9589	5.1538	4.2198
Pccb	_IM[Oxidation (M)]DQAITVGAPVIGLNDSGGAR_.2	#N/A	4.4337	3.7789	3.6046	3.9867	3.5616	4.5045	3.9794	4.4921	4.4967	4.4302	4.0199	3.6683	4.5944	3.2465	4.0523	3.0680	4.0097	4.8414	3.0198	3.2135
Pccb	_IQEGVESLAGYADIFLR_.2	#N/A	4.2574	5.0262	3.2929	4.4320	3.5951	4.3898	4.1925	3.9578	4.1127	4.2369	3.6825	3.6349	4.2781	3.2799	3.3132	3.9347	4.4955	2.9025	2.9863	4.3164
Brap	_EQSESVNTAPESPS[Phospho (STY)]K_.2	Q99MP8_S118_M1_Brap	7.9343	7.8213	8.6266	8.4506	7.8289	7.8361	7.6884	7.8710	7.5657	8.0561	7.9032	7.1923	7.7424	8.0764	6.9726	7.5859	7.8119	7.3568	7.8034	7.6060
Brap	_SREQSESVNTAPES[Phospho (STY)]PSK_.2	#N/A	5.0059	4.8887	5.1304	3.7047	4.4436	3.7675	4.6976	3.8488	4.5059	4.6311	3.6632	3.9076	5.4945	3.9444	4.6171	3.6770	3.9492	3.7697	5.1588	4.6931
Col25a1	_GEPGS[Phospho (STY)]PGAAGQSGLPGPK_.2	Q99MQ5_S265_M1_Col25a1	8.4275	3.6523	3.3859	9.0354	8.4155	3.9123	8.3959	8.8531	3.9120	8.8294	4.2863	8.6297	4.0200	9.4212	4.3301	8.6051	8.8774	3.4374	8.4395	3.2411
Gigyf1	_S[Phospho (STY)]EEGDGAFGR_.2	Q99MR1_S137_M1_Gigyf1	9.7513	8.8507	9.7559	10.0940	9.0423	9.2122	9.3602	9.0713	9.2500	10.3857	9.4912	9.3212	9.8525	9.8685	8.7096	9.6116	9.0952	9.1802	9.5437	9.7066
Gigyf1	_ISS[Phospho (STY)]PPGPPGDLDEEGLK_.2	Q99MR1_S413_M1_Gigyf1	3.2159	3.3260	3.7522	4.4734	4.0545	9.9441	3.4866	4.4172	3.5457	3.4223	3.9200	3.1755	3.2367	10.6315	3.0638	3.5608	10.3099	9.7984	4.0277	3.7063
Gigyf1	_SQS[Phospho (STY)]WDDRGER_.2	Q99MR1_S157_M1_Gigyf1	4.0237	7.4908	7.0881	7.4232	3.3614	6.4230	6.8177	4.5416	4.2387	7.4191	6.4087	6.7696	7.4261	7.2948	3.7568	4.3686	6.5701	7.0345	6.7082	3.5678
Gigyf1	_STS[Phospho (STY)]PDGGPR_.2	Q99MR1_S228_M1_Gigyf1	4.5064	4.1023	4.0430	3.7889	3.2125	7.6646	3.8356	3.6009	3.7558	8.2964	3.2344	7.8695	3.9212	7.8276	3.8610	6.3545	4.1386	3.6946	3.3432	4.3909
Gigyf1	_GLEEEEEEEEEPS[Phospho (STY)]EGVDEERPEAGGK_.3	Q99MR1_S343_M1_Gigyf1	4.1706	3.1484	3.7072	3.4136	2.8767	4.4109	4.1714	4.3963	4.0916	3.9812	3.5702	4.3754	4.2570	3.7453	4.1967	3.5328	3.8936	3.3588	3.0074	3.2258
Slc12a9	_[Acetyl (Protein N-term)]ASESS[Phospho (STY)]PLLAYR_.2	Q99MR3_S6_M1_Slc12a9	9.6362	9.4769	9.4398	9.2533	3.6366	9.2368	9.1221	9.3575	9.2906	9.3861	9.1030	8.6256	9.3288	8.3942	7.6876	8.8626	4.5369	8.8925	8.9793	9.0119
Srrt	_[Acetyl (Protein N-term)]GDS[Phospho (STY)]DDEYDRR_.2	#N/A	9.1492	8.9648	9.1768	9.4578	9.0723	8.8123	8.7673	8.9395	8.7131	9.0041	8.6357	8.8322	9.4322	9.3019	8.6946	9.1241	9.2769	8.6592	8.9504	8.8475
Srrt	_ERFS[Phospho (STY)]PPRHELS[Phospho (STY)]PPQK_.3	#N/A	8.3640	7.5366	9.6333	9.0389	7.9289	9.4006	8.4506	8.6627	7.6250	8.9285	9.2856	6.6457	6.3469	9.4033	7.3959	7.3774	7.4775	9.2552	3.9156	8.5039
Srrt	_HELS[Phospho (STY)]PPQKR_.2	Q99MR6_S74_M1_Srrt	8.3492	8.3795	8.6179	8.0599	8.2324	8.8818	8.2428	8.7622	7.4097	8.3579	8.2055	8.0084	4.1148	8.7317	7.8110	7.5662	8.6254	7.9355	8.2990	7.5622
Srrt	_FS[Phospho (STY)]PPRHELS[Phospho (STY)]PPQKR_.3	#N/A	4.6680	7.0434	6.8227	5.9711	6.1029	5.2583	6.3686	6.9469	3.2952	3.6728	6.7397	5.5860	3.4032	5.6417	5.6672	5.8673	6.4599	4.0542	3.7772	5.7586
Srrt	_[Acetyl (Protein N-term)]GDS[Phospho (STY)]DDEYDR_.2	#N/A	6.8004	7.7888	6.6208	4.1632	5.7440	8.9315	7.5875	3.6467	7.0723	8.0870	6.6947	7.2384	3.4519	6.8918	7.3524	3.7627	6.7657	7.6623	4.3046	4.1738
Srrt	_ERFS[Phospho (STY)]PPR_.2	Q99MR6_S67_M1_Srrt	3.1812	8.4991	4.2186	8.0877	3.3881	9.1432	3.6600	8.0819	3.5802	4.4926	3.0588	3.8640	3.7456	8.8590	3.6853	8.0036	7.6121	7.1254	3.0435	8.0788
Srrt	_LREC[Carbamidomethyl (C)]ELS[Phospho (STY)]PGVNR_.3	Q99MR6_S492_M1_Srrt	4.5050	4.1008	4.4383	7.8874	3.2111	3.2444	3.8370	3.6023	3.7572	4.3156	3.2359	3.9903	3.9227	4.4253	3.8624	3.8671	4.1400	3.6931	3.3417	4.3923
Tmem121b	_GSWVS[Phospho (STY)]PSR_.2	Q99MX7_S521_M1_Tmem121b	3.7092	4.2936	7.6020	7.5250	3.0469	3.4371	2.9933	3.4096	7.4102	3.9155	3.4268	4.1830	8.2266	4.2326	4.0713	4.0541	3.9473	3.8859	7.9106	7.5437
Tmem121b	_GHLENEGGPHQYVNTLAVASQN_.3	#N/A	4.5388	3.4640	3.5742	3.1506	3.8765	4.1006	3.6546	4.2392	3.7237	3.2443	4.0980	3.3535	3.8317	3.5613	3.2418	3.3828	3.2760	2.9125	4.2057	3.5283
Cadm3	_GNPVPQQYVWVK_.2	#N/A	7.6129	7.7357	7.5584	7.9488	7.7009	7.5675	7.6424	7.4989	7.7190	7.2180	7.7226	7.0766	7.6647	7.3445	7.0162	7.2480	7.1343	7.4150	7.1991	7.3207
Cadm3	_LLLHC[Carbamidomethyl (C)]EGR_.2	#N/A	5.8089	5.9864	5.4244	3.1391	5.4801	5.7769	6.6277	6.4786	5.4181	5.5499	5.6044	6.1754	5.3669	4.3898	5.0913	5.3837	5.3069	6.3890	5.7368	6.2546
Cadm3	_GTYLTHEAK_.2	#N/A	6.0357	5.2077	5.3904	5.7351	6.0807	6.1273	6.3286	6.0243	5.7459	5.0501	4.9321	5.5549	5.3212	5.5306	5.4514	5.0332	5.3174	5.6527	5.5150	5.9900
Cadm3	_GTYLTHEAKGS[Phospho (STY)]DDAPDADTAIINAEGGQSGGDDKK_.4	#N/A	3.7748	6.0683	4.3111	3.9146	3.1125	3.3366	4.9853	3.2896	4.8644	3.9811	3.3612	4.1174	4.5957	4.1395	4.0057	4.1197	4.2877	4.3626	5.1410	3.8167
Cadm3	_GSDDAPDADTAIINAEGGQS[Phospho (STY)]GGDDKK_.3	Q99N28_S386_M1_Cadm3	3.4817	3.9064	4.0180	4.2191	3.6942	3.9183	3.5355	3.1821	3.2913	3.6881	3.6657	4.1723	3.3175	4.3560	3.6842	3.8266	3.7198	4.6559	3.7734	3.9143
Cadm3	_EDDGANIVC[Carbamidomethyl (C)]SVNHESLK_.2	#N/A	4.4784	4.5377	5.2815	4.2979	4.0715	4.5168	4.6779	4.4111	3.8115	4.0799	4.1304	4.0199	3.3164	3.8190	4.0825	3.8689	4.9702	4.4530	4.5988	4.3101
Cadm3	_GSDDAPDADTAIINAEGGQSGGDDKKEYFI_.3	#N/A	3.8064	4.1964	4.3427	3.8830	3.1441	3.3682	4.3969	3.5068	4.4560	4.0127	3.3296	4.0858	4.5641	4.3298	3.9741	4.1513	4.0445	4.3942	3.1924	3.7851
Sytl2	_LALQYVPEPS[Phospho (STY)]PGK_.2	#N/A	11.1006	10.9611	10.8679	10.9044	2.8639	10.5945	10.6585	11.0359	10.5536	10.8135	10.6841	10.4963	11.2867	11.0189	9.9699	10.6443	11.2181	10.5704	10.6151	10.4947
Sytl2	_LALQYVPEPS[Phospho (STY)]PGKK_.3	#N/A	3.2004	4.1187	4.2378	4.3838	3.4074	3.2002	3.6407	3.4261	3.1232	4.5119	3.4976	3.8448	3.2313	4.2759	3.6661	4.0634	4.4242	4.2261	3.0243	3.9532
Raf1	_SAS[Phospho (STY)]EPSLHR_.2	Q99N57_S621_M1_Raf1	9.2848	9.4950	4.0700	9.5168	9.2720	9.7578	8.8653	9.3224	9.4924	9.3363	8.6752	9.4265	8.3651	8.8699	3.4941	9.0041	3.7717	4.0614	9.2686	8.9737
Raf1	_AAHTEDINAC[Carbamidomethyl (C)]TLTTS[Phospho (STY)]PR_.2	#N/A	4.4339	5.1588	4.7572	4.1184	3.6787	4.0226	4.5230	4.1993	3.4361	4.1760	4.1342	3.5794	3.5721	3.9387	3.2674	3.9629	3.1947	3.9681	4.4790	4.1752
Raf1	_RAS[Phospho (STY)]DDGKLTDSSK_.2	#N/A	4.7481	4.9595	4.2050	5.0317	3.3504	4.4397	4.0166	3.5289	4.0417	4.8378	3.9106	3.9140	4.0782	4.6220	4.2592	4.1697	4.3177	4.3924	4.6442	4.0516
Sf3b1	_GGDSIGET[Phospho (STY)]PT[Phospho (STY)]PGASK_.2	Q99NB9_T326_M2_Sf3b1	7.9511	3.7902	4.5663	7.6998	3.7358	3.7692	7.5088	7.8211	3.2325	7.3672	7.5580	3.5163	7.4665	4.6044	3.3376	4.3919	3.2518	7.5834	7.0196	7.0876
Sf3b1	_AKGSET[Phospho (STY)]PGAT[Phospho (STY)]PGSK_.2	#N/A	4.3525	5.9452	3.8092	4.4165	4.1114	4.6281	5.0269	4.8571	5.4681	3.4792	4.1396	3.1185	3.2936	4.1886	3.0068	3.6178	3.5109	4.3222	4.0829	3.7633
Sf3b1	_WDETPASQMGGST[Phospho (STY)]PVLTPGK_.2	Q99NB9_T350_M1_Sf3b1	4.2340	3.7688	3.2695	3.4554	3.5717	4.4132	3.9694	3.9344	4.0893	3.9835	3.5679	2.1688	4.2547	3.2565	3.5466	3.0781	4.4720	3.3611	3.0097	3.2235
Sf3b1	_GSET[Phospho (STY)]PGAT[Phospho (STY)]PGSK_.2	#N/A	4.4611	4.3588	3.9977	3.1231	3.1672	3.4403	4.3249	5.2370	4.3840	4.0848	3.2575	4.0849	4.4920	5.0623	3.9021	3.8233	4.1841	4.4662	3.2644	3.7131
Sf3b1	_DT[Phospho (STY)]PGHGSGWAETPR_.2	Q99NB9_T303_M1_Sf3b1	6.1725	3.3384	3.6999	4.5258	4.0021	3.9828	4.4356	5.7387	3.0188	5.7974	4.6384	3.2279	3.1843	3.5195	3.1240	3.1047	3.4016	5.5791	4.0801	3.6539
Sf3b1	_TM[Oxidation (M)]IS[Phospho (STY)]PERLDPFADGGKT[Phospho (STY)]PDPK_.3	#N/A	5.5422	5.8909	4.0819	3.7458	4.1208	5.1680	5.2595	4.9678	5.1448	5.1377	4.2358	4.7225	5.5466	5.5510	3.8862	4.7421	4.3720	5.6878	4.7017	4.5639
Sf3b1	_KLSSWDQAET[Phospho (STY)]PGHT[Phospho (STY)]PSLR_.3	#N/A	4.3350	4.4849	3.8716	3.8279	3.0411	3.5664	4.0070	3.0599	4.2578	4.1456	3.1314	4.2110	4.3659	3.9097	1.0658	3.6971	4.0579	4.1483	3.3906	3.4638
Sf3b1	_RWDQTADQT[Phospho (STY)]PGAT[Phospho (STY)]PK_.2	Q99NB9_T207_M2_Sf3b1	3.5491	3.7700	4.5865	4.0352	3.7560	5.0176	3.2921	3.7747	3.2123	3.3597	4.1920	3.4961	3.5799	4.6245	3.3175	4.4120	3.2720	3.8775	4.1765	4.3018
Rims1	_QGS[Phospho (STY)]PTQSPPADTSFGSR_.2	Q99NE5_S1023_M1_Rims1	10.5609	10.1241	10.3606	9.0452	9.2151	10.1841	10.1172	10.2328	10.2916	10.1285	9.3886	10.0780	10.3769	10.3632	8.4265	10.0441	10.6721	10.2040	8.7671	9.9211
Rims1	_GRS[Phospho (STY)]QDYPDRLEK_.3	#N/A	11.5141	11.7247	11.2792	11.2109	10.7875	10.9393	11.2436	10.7219	10.8280	11.4849	10.7888	10.7951	11.3837	11.4524	10.2377	11.0369	10.8989	10.9155	11.0023	10.9119
Rims1	_S[Phospho (STY)]LDEHPTRR_.3	#N/A	11.4009	11.8056	11.2353	11.5190	10.5800	10.8780	11.3443	10.6951	10.9704	11.6121	11.0617	10.9358	11.6194	11.1881	10.1575	11.0707	10.6476	11.1112	11.1267	11.1240
Rims1	_S[Phospho (STY)]LDEHPTR_.2	#N/A	12.5918	12.4025	12.4306	12.1721	11.5338	11.7516	11.8778	11.6782	11.5396	12.2482	11.9120	11.4661	11.8311	12.1915	10.5327	11.4977	11.7817	11.6559	11.6781	11.7018
Rims1	_SGS[Phospho (STY)]EQASLVVEER_.2	Q99NE5_S1051_M1_Rims1	9.3147	9.4983	10.0757	9.0738	4.2489	8.4707	8.8171	7.7574	8.5058	9.0550	8.4735	8.0650	8.4161	8.8930	2.8706	8.3087	8.4612	8.7220	8.3890	8.3942
Rims1	_ETS[Phospho (STY)]PISSHPVTWQPSK_.2	#N/A	7.7342	7.6985	7.0752	7.3742	6.5948	7.1655	7.1636	7.5629	6.8748	6.9079	6.9896	7.3738	8.1081	8.1048	6.2766	7.1235	7.7066	7.4033	7.5911	6.8639
Rims1	_GRS[Phospho (STY)]QDYPDR_.2	#N/A	9.3441	9.0475	9.2077	9.0735	8.3236	8.6928	8.6922	8.2924	8.1450	8.8535	8.6474	8.1460	8.2581	8.8820	7.6821	8.4162	8.3543	8.4026	8.5576	8.3620
Rims1	_S[Phospho (STY)]QDYPDRLEK_.2	#N/A	9.0161	8.6266	8.8726	8.6854	7.8916	8.5554	8.5750	7.9034	7.9208	8.7826	8.4241	7.8968	7.5439	8.5565	4.3489	8.0861	7.9508	3.2535	8.1148	3.2499
Rims1	_ETS[Phospho (STY)]PISSHPVTWQPSKEGDR_.3	#N/A	3.9996	3.5955	3.4428	7.2000	4.2066	4.2399	4.3424	7.1402	6.6216	3.8102	7.3412	3.4850	8.2654	3.4298	6.2366	3.3617	3.1445	6.8978	6.6055	3.3968
Rims1	_IQIQHAS[Phospho (STY)]PENDR_.2	Q99NE5_S927_M1_Rims1	6.4167	4.1189	5.7728	6.5055	5.5329	5.6731	5.9137	6.5075	6.1931	4.4172	6.6205	6.2866	6.9693	6.7202	5.6322	6.7634	6.5099	6.5381	5.9328	6.4333
Rims1	_SS[Phospho (STY)]DSDVSDVSAISR_.2	Q99NE5_S1111_M1_Rims1	8.4949	3.9553	8.9291	8.1628	3.5038	8.9940	8.1918	3.9080	6.5807	7.4475	4.1484	8.1065	4.0418	3.7023	3.4130	8.3947	9.0945	8.5902	8.5562	5.8887
Rims1	_SASTNC[Carbamidomethyl (C)]LRPDTSLHS[Phospho (STY)]PER_.3	#N/A	5.5492	4.7946	4.1911	4.9349	4.2291	4.9423	5.2428	3.3793	3.7995	5.5638	3.4508	3.8916	5.6389	5.5181	3.2398	4.8388	4.7393	5.2504	4.3715	4.8897
Rims1	_ASQSS[Phospho (STY)]LESSSGPPC[Carbamidomethyl (C)]IR_.2	#N/A	9.9102	9.7799	9.7218	9.8005	9.2208	9.5650	9.5521	9.7906	9.5047	9.6783	9.7635	9.4251	10.2667	9.9714	8.7232	9.4860	10.2841	9.6389	9.5418	9.5399
Rims1	_RAS[Phospho (STY)]QSSLESSSGPPC[Carbamidomethyl (C)]IR_.3	#N/A	11.2150	11.1883	3.5527	5.6697	4.7392	10.6110	10.8653	4.3248	10.5569	4.6228	3.8086	5.5339	4.0894	3.6564	9.5413	4.6729	3.5409	4.7927	5.4598	10.6601
Rims1	_IPESSHPPLESSSS[Phospho (STY)]SFESQK_.3	Q99NE5_S547_M1_Rims1	3.7057	8.6249	8.6310	4.2624	7.6600	5.7585	4.1124	3.4558	7.8977	4.2722	3.3786	7.5270	4.0088	4.2956	7.0630	8.1360	4.2984	8.2497	3.1769	7.6825
Rims1	_SASTNC[Carbamidomethyl (C)]LRPDTSLHS[Phospho (STY)]PERER_.4	#N/A	4.1824	3.8204	3.2179	3.5070	2.9306	4.4648	6.2126	3.8828	4.0377	4.9675	3.5163	3.7098	4.2031	5.5775	4.7397	5.1790	4.4205	6.0900	3.0613	3.1720
Rims1	_S[Phospho (STY)]AEC[Carbamidomethyl (C)]LHM[Oxidation (M)]TSELQPSLDR_.3	#N/A	6.6293	4.3188	4.1514	5.3266	5.7385	5.8718	4.8447	3.3287	6.6507	6.8294	6.1577	6.2580	3.3893	7.4132	3.2733	6.3676	4.1369	6.9745	6.9625	6.7273
Rims1	_FKQTTGSGSS[Phospho (STY)]QELDHEQYSK_.3	Q99NE5_S1082_M1_Rims1	3.9006	3.4185	3.4372	3.6836	4.1075	4.0008	4.4414	4.1263	3.8234	3.7112	4.1978	3.1446	3.9315	3.4752	2.9659	4.3230	3.6235	5.0028	3.8250	3.1525
Rims1	_S[Phospho (STY)]TETGM[Oxidation (M)]AAEMR_.2	Q99NE5_S1240_M1_Rims1	3.9561	7.0453	3.7824	3.3703	5.2287	6.2187	7.2467	3.8023	4.3087	3.9699	5.5038	5.3274	4.4446	3.8204	3.4594	3.8719	5.7555	4.8358	3.5117	3.5477
Rims1	_STS[Phospho (STY)]QLS[Phospho (STY)]QTESGHKK_.2	Q99NE5_S1224_M1_Rims1	3.6763	3.2721	3.7661	4.4596	3.8832	3.9166	3.1649	4.4310	3.0851	3.4869	4.0646	3.1616	3.2505	3.7532	3.1903	3.0384	3.4679	4.3653	4.0139	3.7202
Rims1	_SC[Carbamidomethyl (C)]DNASAKS[Phospho (STY)]SDSVSDVSAISR_.3	#N/A	3.6789	3.6402	3.2155	3.9053	3.8858	4.2225	3.1623	3.9046	3.6017	3.3662	3.9761	3.3663	3.7098	5.9236	3.1876	3.0410	3.4018	3.7476	4.0467	4.4317
Rims1	_SRS[Phospho (STY)]SEHSSIR_.2	Q99NE5_S988_M1_Rims1	3.7016	3.6175	3.2382	3.8826	3.9085	3.9419	3.1396	3.9273	3.0598	3.5122	4.0392	3.4336	3.7325	3.2762	3.1649	3.0637	3.4245	3.7249	4.0240	2.9535
Rims1	_QPSRESTDGSINSY[Phospho (STY)]SSEGNLIFPGVR_.3	Q99NE5_Y1268_M1_Rims1	6.9606	4.4961	4.0430	4.8234	4.3452	3.6397	6.2342	6.5784	3.3619	4.6858	4.3414	4.3856	3.5274	3.7255	4.2739	3.8516	3.7447	4.0884	4.9092	3.9970
Rims1	_S[Phospho (STY)]VS[Phospho (STY)]PHRGDDQGRPR_.3	Q99NE5_S788_M2_Rims1	4.3608	2.6349	3.3962	2.8052	2.7964	3.2195	1.7662	4.2045	1.8460	4.5671	4.2761	2.8816	4.0097	2.6473	1.7253	3.2048	3.7018	3.4477	3.2311	3.2308
Rims1	_S[Phospho (STY)]RSVS[Phospho (STY)]PHR_.2	Q99NE5_S786_M2_Rims1	4.2054	3.7974	3.2408	3.4840	3.5431	4.4419	3.9980	3.9057	4.0606	4.4117	3.5393	3.6869	4.2261	3.2279	3.5752	3.0494	4.4434	5.3704	3.0383	3.1949
Rims1	_LQTHDESSLPLPQPS[Phospho (STY)]PFMPR_.3	Q99NE5_S716_M1_Rims1	4.0377	3.2814	5.5392	3.5465	4.2446	3.8637	4.3043	4.2634	3.9605	3.8483	4.3349	3.0075	4.0686	6.9196	2.8288	3.3998	3.7606	3.3888	3.6879	3.2896
Nsmf	_QGS[Phospho (STY)]EKC[Carbamidomethyl (C)]PGC[Carbamidomethyl (C)]AQLVPGPSSR_.3	#N/A	7.7913	8.1394	8.0655	7.8843	6.7471	7.0518	7.8170	7.2143	6.2135	6.3404	7.3823	7.2993	7.2229	7.8290	7.8476	6.1707	4.4261	6.7749	7.0539	7.1606
Nsmf	_M[Oxidation (M)]YS[Phospho (STY)]IVDGVSDDVPIR_.2	#N/A	9.1195	9.2479	8.9576	9.2423	3.4624	8.2393	8.6168	3.8250	8.2414	8.4008	6.9550	7.2246	4.1454	9.1924	5.9546	7.4796	3.6935	8.0436	8.0649	7.6598
Nsmf	_SWS[Phospho (STY)]DPTPMK_.2	Q99NF2_S294_M1_Nsmf	3.8159	3.4829	5.5632	5.9004	3.8700	4.9776	5.5588	4.1119	3.5784	3.9064	6.1105	3.3723	3.6994	5.6907	3.2494	3.2603	3.9001	3.9258	3.9483	4.6439
Nsmf	_ERS[Phospho (STY)]FS[Phospho (STY)]RS[Phospho (STY)]WSDPTPMK_.2	#N/A	3.3633	4.4933	4.0515	4.1799	3.9330	3.2901	3.4155	3.2156	3.4006	3.3933	3.3777	3.4073	3.5389	4.0386	3.2538	3.9798	3.7533	4.0856	3.7342	4.0056
Cep41	_LEDNDS[Phospho (STY)]AT[Phospho (STY)]SEADAEIAAK_.2	Q99NF3_T98_M2_Cep41	3.6973	3.7290	3.8817	4.0955	3.6064	3.8304	3.6003	3.5005	3.6594	4.6807	3.5063	5.3298	3.7675	5.8663	3.7199	3.5598	4.2134	5.6210	3.8989	3.7301
Cep41	_GGQNLPTSC[Carbamidomethyl (C)]PASHSS[Phospho (STY)]PR_.3	Q99NF3_S358_M1_Cep41	3.4060	4.5968	3.9423	4.2834	4.2446	4.4687	3.2965	3.6585	3.3556	3.6123	3.7300	2.9854	3.4636	4.5083	2.8737	3.7509	3.1557	3.9938	4.2928	4.1855
Ankrd17	_NS[Phospho (STY)]PLDC[Carbamidomethyl (C)]GSASPNK_.2	Q99NH0_S2055_M1_Ankrd17	6.4180	6.6072	7.0579	6.0138	6.0906	6.6122	5.6470	5.9202	6.3221	6.3381	6.3222	3.1484	3.2637	6.9842	5.6387	5.4039	6.1095	6.2113	4.0007	5.7953
Ankrd17	_EHYPVSSPSS[Phospho (STY)]PS[Phospho (STY)]PPAQPGGVSR_.3	Q99NH0_S2041_M2_Ankrd17	3.9285	9.2617	3.4651	3.6557	3.7488	9.0030	3.7922	4.1542	8.9342	8.9355	4.2257	8.8138	3.9594	8.9475	8.4281	8.9924	9.5064	8.9961	8.9099	9.0562
Pard3	_RSSDPALTGLS[Phospho (STY)]TSVSDNNFSSEEPSR_.3	Q99NH2_S152_M1_Pard3	4.2354	3.7674	3.2709	3.4539	5.4629	4.4118	3.9679	3.9358	4.0907	3.9821	3.5694	3.6568	4.2562	3.2580	3.5451	3.0795	4.4735	3.3597	3.0082	3.2250
Pard3	_NNYSPGRFS[Phospho (STY)]PDSHC[Carbamidomethyl (C)]VANR_.3	Q99NH2_S383_M1_Pard3	3.8995	4.1033	4.4358	3.7899	3.2372	4.2151	2.9159	3.5999	3.7548	4.3180	3.2334	3.9927	3.9202	4.4229	3.8810	4.2444	4.1376	4.6456	3.3442	4.3899
Hspbp1	_LLQTC[Carbamidomethyl (C)]FSS[Phospho (STY)]PTDDSM[Oxidation (M)]DR_.2	#N/A	8.4597	8.4717	7.6603	8.4260	7.1047	8.4763	8.5154	7.7985	5.3361	4.8757	3.1911	3.9963	8.3779	8.8224	7.2556	8.2801	8.7474	8.6789	8.3827	4.7930
Rtn4	_KAQIITEKTS[Phospho (STY)]PK_.2	#N/A	11.0727	10.9398	10.3509	10.6378	10.3362	10.4692	10.6604	11.1462	10.5174	10.2173	10.1267	10.6000	11.1046	10.6629	9.5945	10.2161	10.8161	10.7563	10.3255	10.0419
Rtn4	_GSGS[Phospho (STY)]VDETLFALPAASEPVIPSSAEK_.3	Q99P72_S167_M1_Rtn4	9.0040	9.1416	4.5159	4.1057	4.1989	4.4229	3.3422	3.7042	8.2980	3.5666	3.7757	3.5667	3.5094	4.5540	2.9194	3.7052	3.2014	3.9480	4.2471	4.2312
Rtn4	_LPS[Phospho (STY)]DTEKEDR_.2	#N/A	3.8864	4.1164	4.4227	8.3503	3.2241	8.8755	3.8214	3.5867	3.7416	4.4312	3.2203	8.1381	8.7669	4.4097	3.8468	8.2241	4.1244	8.7559	3.3573	4.3767
Rtn4	_HQAQIDHYLGLANK_.3	#N/A	7.6061	7.4672	3.3575	6.7746	7.3583	7.3771	7.3284	7.9062	7.0327	6.9222	7.2291	7.2201	7.6663	7.0350	6.7557	6.7702	7.4892	7.5919	7.3719	6.9918
Rtn4	_[Acetyl (Protein N-term)]M[Oxidation (M)]EDIDQSSLVSSSADS[Phospho (STY)]PPRPPPAFK_.3	#N/A	10.7669	11.1789	9.8832	9.8691	8.5848	9.9483	10.3714	11.0286	9.7867	10.2286	9.1904	7.1940	10.9590	11.3351	9.4408	9.4919	10.1633	10.6460	10.1812	9.3946
Rtn4	_TSNPFLVAIHDSEADYVTTDNLSK_.3	#N/A	5.5129	5.4026	4.5118	4.9615	4.5371	4.1466	4.6806	5.3540	4.6447	5.1701	3.5107	4.6332	4.7394	5.0059	4.6093	4.3357	4.9480	3.1106	4.8797	4.4025
Rtn4	_LSTEPS[Phospho (STY)]PEFSNYSEIAK_.2	Q99P72_S690_M1_Rtn4	9.1521	9.5498	9.2510	9.2173	7.9576	8.6946	8.8716	8.3478	8.6216	4.9909	8.0115	8.5590	3.3393	9.0540	5.0701	8.5852	8.8507	9.0201	8.6484	8.5977
Rtn4	_GPLPAAPPT[Phospho (STY)]APERQPSWER_.3	Q99P72_T98_M1_Rtn4	3.3314	8.5654	4.3688	4.2528	5.3590	5.2782	5.5097	3.5571	3.4299	8.0626	2.9086	3.7138	3.3623	8.3035	4.9248	4.1944	7.0183	4.0951	6.5822	5.5413
Rtn4	_EAEEKLPS[Phospho (STY)]DTEKEDR_.2	#N/A	8.4754	8.7524	6.1085	8.1485	7.9030	8.5727	8.4739	8.5012	6.7112	7.8420	6.6736	8.2250	8.9570	8.0174	6.6901	7.9395	8.5988	8.5859	8.1097	8.0805
Rtn4	_VVKEDGVM[Oxidation (M)]S[Phospho (STY)]PEK_.2	#N/A	10.1291	9.9058	10.1625	9.9331	9.6403	9.9135	9.6703	9.9269	9.6987	9.6299	9.8087	9.5615	9.5796	9.9943	8.7881	9.5091	10.1199	9.7866	9.4878	9.7116
Rtn4	_DDS[Phospho (STY)]PKEYTDLEVSNK_.2	#N/A	9.1127	9.4999	9.6096	9.1793	6.6170	9.2888	9.0855	7.9554	9.2606	8.8923	8.1851	8.9827	8.2612	9.5676	8.0201	8.9392	9.5272	9.3125	8.9441	8.9755
Rtn4	_RRGS[Phospho (STY)]GSVDETLFALPAASEPVIPSSAEK_.3	#N/A	9.4539	10.3149	6.1009	8.1067	3.7713	8.0384	9.7535	10.4136	7.7641	8.5730	7.0473	9.5529	9.6174	10.5700	7.4235	7.4637	8.9402	9.8161	8.8092	4.0172
Rtn4	_EHDYLGNLSAVASTEGTIEETLNEASR_.3	#N/A	3.8306	4.2023	3.0227	4.3116	2.3874	2.4906	2.6079	4.5315	4.1745	3.6285	4.1651	4.6153	4.3456	3.8537	3.0504	3.3291	3.5684	4.3808	3.6345	3.8207
Rtn4	_RGS[Phospho (STY)]GSVDETLFALPAASEPVIPSSAEK_.3	#N/A	10.3975	3.4307	9.5864	9.4985	4.0214	4.1397	10.3300	4.1668	9.2620	3.5284	5.1648	8.8983	4.1876	10.4366	3.1067	8.8933	3.6641	10.1501	9.4265	8.5806
Rtn4	_AQIITEKTS[Phospho (STY)]PK_.2	#N/A	10.4973	10.3731	10.1479	10.3126	10.1268	9.8802	10.1212	10.4266	3.6561	9.8729	4.2939	2.9910	10.5258	10.1600	9.1931	9.7894	10.3847	10.3314	3.7586	9.9714
Rtn4	_AYLESEVAISEELVQK_.2	#N/A	7.0870	6.8302	6.7695	6.7980	5.9866	7.6106	5.4112	6.8796	5.6324	6.0302	5.8495	6.1251	7.2816	6.5446	4.7551	6.6141	6.4576	5.6338	5.5894	6.0130
Rtn4	_ESLTEVSETVTQHK_.2	#N/A	5.0600	4.8430	5.2897	4.2958	4.6303	4.8856	4.4650	4.7083	4.2583	4.4804	4.6753	4.4974	4.7427	4.3835	3.5612	4.3963	4.3724	4.4040	3.9569	4.2831
Rtn4	_SKDKEDLVC[Carbamidomethyl (C)]SAALHNPQES[Phospho (STY)]PATLTK_.4	Q99P72_S329_M1_Rtn4	3.5416	3.1375	3.9008	4.3249	3.7485	3.7819	3.2996	3.0649	3.2198	3.3522	4.1993	3.2029	3.3852	3.8879	3.3249	4.4045	3.6026	4.2306	3.8792	3.8549
Rtn4	_EDGVMS[Phospho (STY)]PEK_.2	#N/A	4.1845	5.1480	6.4420	6.8594	3.5222	5.5952	5.6451	3.8849	4.0398	4.3908	6.4059	3.7077	4.2052	6.5037	3.5960	3.0286	4.4226	4.3106	3.0592	3.1741
Rtn4	_LSAS[Phospho (STY)]PQEVGKPYLESFQPNLHITK_.3	Q99P72_S768_M1_Rtn4	7.3766	4.0997	4.4394	3.7863	3.2408	3.4649	4.3003	3.6035	4.3594	6.7093	3.2329	3.9892	4.4674	4.4265	4.8775	4.2480	4.1411	2.9900	3.3406	3.6885
Rtn4	_ESAEFSVLEYSEM[Oxidation (M)]GSSFNGS[Phospho (STY)]PK_.3	Q99P72_S291_M1_Rtn4	4.6869	4.0574	4.2991	4.3225	2.9148	3.1389	3.1255	3.4874	3.1845	3.7834	3.5589	4.3152	3.2926	4.3372	4.2035	3.9220	4.4855	4.1648	2.9630	4.0145
Nup155	_AAPQS[Phospho (STY)]PSVPK_.2	Q99P88_S992_M1_Nup155	3.4930	9.8544	4.0293	4.1964	4.3316	3.6534	3.4281	3.1934	3.3483	3.2237	4.3278	4.3992	3.5137	4.0164	2.7862	3.8379	3.7311	4.1021	9.3021	3.9834
Ogfr	_IALNLEGC[Carbamidomethyl (C)]ALS[Phospho (STY)]PTSQEPR_.2	Q99PG2_S403_M1_Ogfr	4.3871	7.0426	6.4411	5.2624	3.0932	3.1265	6.4304	6.3292	5.7098	6.5365	5.0378	6.3940	6.6508	7.4131	5.1587	7.3492	6.6007	6.8605	3.3238	5.9087
Lpin2	_S[Phospho (STY)]DSELEVKPSESLLR_.2	#N/A	6.9341	7.6429	6.9591	4.4968	3.1531	7.2090	3.9245	4.0076	3.8447	4.2576	3.3234	4.1286	4.0414	7.5554	3.9499	6.2286	4.1699	7.1096	3.3081	3.9201
Ntm	_VTVNYPPYISEAK_.2	#N/A	8.5982	8.2439	7.8345	7.9245	8.4035	8.0744	8.4321	8.3731	7.9803	8.1174	8.3156	8.2260	8.2770	8.3792	8.1528	7.9937	8.0448	8.3382	8.1738	7.7839
Ntm	_AVGFVSEDEYLEIQGITR_.2	#N/A	6.7016	6.2913	5.8280	6.2605	6.0988	5.6733	6.1797	6.7915	5.9598	6.3549	5.8787	6.2794	7.2596	6.9473	5.5351	6.5105	6.2140	6.4165	5.8455	5.9158
Ntm	_EQSGEYEC[Carbamidomethyl (C)]SASNDVAAPVVR_.2	#N/A	6.8450	7.5314	7.0914	6.4894	6.3990	6.7208	6.8364	7.2309	7.1396	6.4151	6.2796	6.0997	6.6287	7.5314	6.2649	6.7819	6.3361	7.0725	6.1910	6.7363
Ntm	_GTGVPVGQK_.2	#N/A	6.4702	6.7568	5.5487	5.9952	6.3501	5.9956	6.5267	6.0799	6.3082	6.1276	5.9242	6.3530	7.6665	6.4708	6.2114	6.1536	6.2703	6.5403	6.0231	6.1815
Ntm	_VHLVQVSPK_.2	#N/A	5.8769	5.7937	3.6520	3.1396	6.3754	4.2484	5.1092	4.2502	4.6674	5.3192	6.1302	5.1508	6.1015	4.2109	4.2861	4.6877	6.2530	5.0811	5.4215	5.6908
Ntm	_STILYAGNDK_.2	#N/A	7.2095	7.0854	7.2272	6.9276	4.0693	7.3506	7.2545	6.4333	7.2149	7.0318	7.9979	6.8563	6.7683	7.3918	7.8476	7.0142	7.3033	7.4878	7.3527	7.4835
Ntm	_AMDNVTVR_.2	#N/A	3.5705	3.7486	5.7465	5.2301	3.7775	5.3071	5.7061	3.7962	3.4933	3.4746	5.8607	3.4747	3.6014	5.8571	3.2960	6.7017	3.2934	3.8560	4.1551	4.3233
Rrbp1	_LASS[Phospho (STY)]PKDR_.2	Q99PL5_S135_M1_Rrbp1	3.9193	4.0835	6.9125	7.1187	3.2571	6.8823	7.2105	7.3351	4.3431	4.1257	3.2166	3.9729	7.5877	4.4427	3.8612	4.2642	4.1574	7.3688	3.3244	6.5026
Ubxn6	_GKS[Phospho (STY)]PQLALR_.2	Q99PL6_S36_M1_Ubxn6	11.8638	11.7993	12.0561	12.0527	11.1886	10.6736	11.0826	10.9382	10.7193	12.0884	11.1017	11.3272	11.5523	11.6699	10.0556	11.2109	10.9285	10.9168	10.9955	11.0843
Ubxn6	_EALQNDWLPFELR_.2	#N/A	6.2179	5.7855	6.3275	6.2619	5.9972	5.9223	5.6256	5.7659	6.2718	6.1361	5.5244	5.6282	5.4902	4.0009	6.0042	5.7242	6.4919	5.6799	5.7273	5.3864
Ubxn6	_SVLRPELLAAIEQLS_.2	#N/A	3.9274	4.0754	4.4637	3.7619	3.2652	3.4893	2.5065	3.6278	4.3350	4.1338	3.2085	3.9648	4.4431	4.4508	3.8531	4.2723	4.1655	3.6677	3.3162	4.4178
Znf318	_RAS[Phospho (STY)]PS[Phospho (STY)]PPR_.2	#N/A	9.7407	9.3003	10.0297	9.7552	9.1288	9.7857	9.6366	9.4201	9.8267	9.7607	9.5114	9.5582	9.4394	10.0352	8.9871	9.4970	9.8596	9.3220	9.4507	9.4948
Znf318	_RGS[Phospho (STY)]PS[Phospho (STY)]PPR_.2	Q99PP2_S79_M2_Znf318	9.9625	10.0221	10.0017	9.9510	9.3932	9.6748	9.8937	9.8063	9.8664	10.0170	9.4759	9.6389	10.1433	10.1937	8.8187	9.4467	9.8435	9.6852	9.7048	9.5971
Znf318	_DQVVGGNVS[Phospho (STY)]PR_.2	Q99PP2_S2206_M1_Znf318	8.1760	8.2756	8.5719	8.1458	8.2195	8.0949	8.1563	8.3816	8.0946	8.2214	8.1944	7.9483	7.8936	8.8735	7.9750	7.9691	8.4826	8.1520	8.1190	8.1175
Znf318	_WAS[Phospho (STY)]PS[Phospho (STY)]PPR_.2	Q99PP2_S69_M2_Znf318	8.8748	8.3726	8.4839	9.4785	3.8875	8.8559	8.4221	6.7194	8.5983	8.2679	7.7025	8.6777	6.5276	9.1929	7.9767	8.3203	9.0311	7.4796	8.7935	8.6630
Znf318	_LGS[Phospho (STY)]PVDGLQDM[Oxidation (M)]DRDDLTDDSVFTR_.3	Q99PP2_S205_M1_Znf318	3.8426	3.4384	3.5998	3.1250	4.0495	4.0828	2.9986	4.2648	4.4197	3.6532	3.8983	3.3279	4.5851	3.5869	3.0240	3.2047	3.3016	2.1477	4.1801	3.5539
Znf318	_S[Phospho (STY)]PGLC[Carbamidomethyl (C)]SDSSLEESLR_.2	Q99PP2_S167_M1_Znf318	3.1404	3.3616	3.6767	4.5490	3.9790	4.2031	3.5621	4.3416	3.6212	3.3467	3.9956	3.2510	3.7292	3.6638	3.1393	3.4853	3.3785	4.4547	4.1033	3.6308
Znf318	_RAS[Phospho (STY)]PS[Phospho (STY)]PPRGR_.3	#N/A	3.6387	4.1191	4.2375	4.3842	2.9765	3.2006	3.0638	3.4257	3.1229	3.0912	3.4972	3.8452	3.2309	4.2755	4.1418	3.9836	4.4238	4.2265	3.0247	3.9528
Trim33	_LKS[Phospho (STY)]DERPVHIK_.3	Q99PP7_S1134_M1_Trim33	4.5638	3.2186	3.5993	3.1256	3.9015	5.3407	3.7334	4.2642	4.6291	3.6537	4.7632	4.2714	4.7033	4.2054	4.4344	3.4079	4.2054	3.0313	4.6451	4.7825
Arhgdia	_VAVSADPNVPNVVVTR_.2	#N/A	9.8296	9.7066	9.9242	9.8841	9.4895	9.6970	9.5406	9.8888	9.9102	9.7524	9.4338	9.6279	9.9235	9.8677	9.6989	9.8445	9.7015	9.5693	9.6917	9.6415
Arhgdia	_[Acetyl (Protein N-term)]AEQEPTAEQLAQIAAENEEDEHSVNY[Phospho (STY)]KPPAQK_.4	Q99PT1_Y27_M1_Arhgdia	6.8893	7.2496	5.5946	7.3134	6.3639	7.0838	7.8092	6.2010	7.0080	5.3455	5.4857	6.9133	6.9850	7.4064	6.5591	6.8481	6.4332	6.9593	6.7738	6.9645
Arhgdia	_TDYM[Oxidation (M)]VGSYGPR_.2	#N/A	7.8525	7.5898	7.9293	7.3101	7.9177	7.8571	7.7780	7.8284	7.9290	8.0229	7.8105	7.4537	7.5493	7.3614	8.0747	7.8865	7.7347	7.8142	7.7948	7.8880
Arhgdia	_S[Phospho (STY)]IQEIQELDKDDESLRK_.3	#N/A	8.6810	9.0501	8.7135	8.6081	7.9168	8.5821	8.6242	8.2071	8.1439	9.0373	8.3545	8.3706	8.8670	9.3878	8.0785	8.6114	8.4621	8.7863	8.4975	8.7421
Arhgdia	_LTLVC[Carbamidomethyl (C)]STAPGPLELDLTGDLESFKK_.3	#N/A	4.9036	4.4268	3.8677	4.7147	5.2330	3.6075	4.8277	3.0560	5.4693	4.9512	3.9602	3.9815	4.0965	4.7039	5.0218	4.1517	4.4496	5.0270	5.2556	4.8161
Arhgdia	_YIQHTYR_.2	#N/A	7.3864	7.3774	7.3360	7.0832	7.4173	7.5802	7.5069	7.2454	7.5402	7.3680	7.1327	7.3894	6.9878	7.1869	7.6032	6.9572	6.9444	7.3593	7.2515	7.0936
Arhgdia	_SIQEIQELDKDDESLR_.3	#N/A	5.9996	6.6345	6.4411	6.5151	4.7146	6.1309	6.3616	4.0304	6.1881	6.6839	6.3258	5.9947	6.4183	7.0434	5.7628	5.5790	5.9497	6.3922	6.0529	6.2327
Arhgdia	_IDKTDYM[Oxidation (M)]VGSYGPR_.3	#N/A	7.5408	8.0675	6.0866	5.5102	7.5305	7.9475	7.8598	7.7974	6.6185	7.3922	7.3335	7.8373	8.2627	7.2616	7.1300	6.9338	7.1575	7.9785	7.1224	7.2387
Arhgdia	_AEEYEFLTPM[Oxidation (M)]EEAPK_.2	#N/A	8.5469	8.5023	8.8887	8.1154	8.5423	8.4533	8.4314	8.5802	8.3674	8.6231	8.3211	8.1779	8.6426	8.5202	8.3262	8.6454	8.4063	8.0046	8.0928	8.4863
Acsbg1	_ES[Phospho (STY)]PSHGLELSAPEK_.2	Q99PU5_S53_M1_Acsbg1	3.7421	3.5770	3.2787	3.8421	5.3642	3.9824	3.0991	6.1084	3.0193	3.5527	3.9987	3.3031	3.1847	3.3168	3.1244	3.1042	3.4650	4.4311	3.9835	3.6544
Acsbg1	_AAS[Phospho (STY)]LDGAEEALWTTR_.2	Q99PU5_S70_M1_Acsbg1	3.4573	8.6774	7.9866	8.5066	3.6643	7.1309	7.9817	3.6830	4.3591	5.8036	4.2835	3.5879	4.8882	7.5224	3.4092	4.3203	7.4578	7.2323	5.0124	4.2101
Acsbg1	_DDQQQGASLGTSQDNSQTSSLIDGQTLSK_.3	#N/A	4.5022	4.9263	4.2482	4.4574	3.0496	3.4165	4.8077	4.7408	3.0497	3.9182	3.4241	4.2574	4.5852	6.1225	4.0686	3.5140	5.3486	4.7130	3.0979	3.8796
Dhx30	_GGS[Phospho (STY)]FEM[Oxidation (M)]TDDDSAIR_.2	#N/A	6.9337	7.3730	7.7945	7.4381	6.3090	6.5224	6.7966	6.1667	6.1315	6.5839	6.4125	5.6843	6.8418	6.8815	5.1851	5.7798	6.3276	6.0763	5.9067	6.2812
Bcl11b	_ELAGNSST[Phospho (STY)]PPPVS[Phospho (STY)]PGR_.2	Q99PV8_S381_M2_Bcl11b	9.5498	9.3168	9.3678	9.8322	7.9772	9.3318	8.3896	9.2159	8.8336	9.4524	8.6563	9.5680	8.8576	10.1574	9.1179	9.4545	9.4403	9.1580	9.1130	9.6512
Bcl11b	_FST[Phospho (STY)]PPGDLLDGGLSGR_.2	Q99PV8_T744_M1_Bcl11b	8.3174	9.0836	3.5919	3.5289	3.6220	6.8832	8.0851	8.9469	3.9781	4.4906	4.3525	8.6758	4.0862	9.8417	8.1634	7.9027	7.8856	9.1684	8.2452	7.5225
Bcl11b	_SDDGLSAAS[Phospho (STY)]SPEPGTSELPGDLK_.2	Q99PV8_S495_M1_Bcl11b	3.7763	3.5519	3.8321	4.1236	3.6337	3.6514	3.4683	4.1457	3.4898	3.9692	4.0484	5.9914	3.6130	8.3428	6.2690	6.0890	7.1355	7.8148	6.4522	6.3881
Bcl11b	_VLDKSS[Phospho (STY)]PPPSSR_.2	Q99PV8_S96_M1_Bcl11b	9.2869	9.5217	9.8092	3.4601	9.4995	4.4179	4.1644	9.9715	9.2859	3.9882	8.9982	3.6630	4.2500	3.2518	9.5323	3.0733	9.9643	3.3658	3.0144	3.2188
Bcl11b	_VSEPVEIGIQVTPDEDDHLLS[Phospho (STY)]PTK_.3	Q99PV8_S128_M1_Bcl11b	4.2944	3.7084	3.3298	3.3950	3.6321	3.8562	3.9090	4.2709	3.9681	4.5007	4.3424	3.5979	4.0761	3.6199	3.4862	3.1384	3.7682	3.3813	3.6803	3.2972

Bcl11b	_SPFLST[Phospho (STY)]PPLPPMPAGT[Phospho (STY)]PPPQPPAK_.3	Q99PV8_T406_M2_Bcl11b	3.9983	3.3208	3.5349	3.5859	4.2052	4.2386	4.3437	4.2240	4.2639	3.8089	3.7426	3.0469	4.4294	6.9407	2.8682	3.3604	3.7212	3.1865	3.7273	3.3982
Ndufa5	_TTGLVGLAVC[Carbamidomethyl (C)]DTPHER_.2	#N/A	4.6786	5.0984	5.2988	5.4406	4.7753	5.0474	5.2571	4.4081	5.0585	5.4421	4.9124	4.8466	4.9456	5.4483	5.4053	4.3849	5.5824	4.9304	4.5964	5.1368
Ndufa5	_KYTEQITNEK_.2	#N/A	4.4591	4.3608	4.8715	3.1251	3.1652	4.3888	3.6815	3.1840	4.8151	4.7647	4.2699	4.0869	3.9685	5.3840	4.6289	4.2843	4.1821	4.4682	3.7094	4.3332
Ndufa5	_LEALLQGGEVEEVILQAEK_.2	#N/A	3.3028	3.2428	3.2298	3.1227	3.9044	3.8871	3.6366	4.2671	3.6957	3.7939	3.6482	4.2581	3.5483	4.3484	3.2138	3.5714	3.3039	3.0284	4.1778	3.5562
Cox6c	_AGIFQSAK_.2	#N/A	3.6353	4.9469	3.2056	5.1514	4.9287	5.5385	6.4440	3.5622	5.3890	5.5263	5.6658	4.6114	5.9059	5.0930	5.4519	5.7279	4.0777	5.6713	5.6410	5.2789
Cox6c	_NYDSM[Oxidation (M)]KDFEEMRK_.3	#N/A	4.2016	4.4111	6.4218	6.5332	3.1826	3.3124	5.8201	3.2413	4.1433	4.1765	5.9626	4.0589	4.5474	5.2341	3.8981	4.0211	4.2394	4.4110	3.2092	3.7684
Cox6c	_AYAEFYR_.2	#N/A	3.5187	4.4841	4.0550	7.2379	2.8564	3.0805	7.9522	3.2190	6.9135	7.4536	3.6173	6.9162	3.3510	6.1734	4.2619	3.8636	3.2804	7.5512	7.3277	7.4276
Cox6c	_NYDSMKDFEEMR_.3	#N/A	4.0301	3.2890	3.5667	3.5541	4.2371	4.2704	4.3119	4.2558	4.2321	3.8407	3.7107	3.0151	4.0610	3.6048	2.8364	3.3922	3.7530	3.3964	3.6955	3.2820
Tomm22	_[Acetyl (Protein N-term)]AAAVAAAGAGEPLS[Phospho (STY)]PEELLPK_.2	#N/A	11.9046	12.6999	11.3897	11.1378	10.3321	11.5276	12.7128	12.1993	11.3468	11.6778	10.7653	12.2672	12.6897	13.0191	11.3005	11.2970	11.4892	12.4848	11.9533	11.0702
Tomm22	_LWGLTEM[Oxidation (M)]FPER_.2	#N/A	3.9024	4.6818	4.4387	3.7870	4.7721	5.6386	5.1765	3.6028	4.3600	5.8373	3.2336	5.1491	4.4681	4.4258	5.3196	5.2081	4.1405	5.4157	5.6642	5.1917
Tomm22	_LQM[Oxidation (M)]EQQQQLQQR_.2	#N/A	4.7758	4.4145	4.3830	4.1169	3.8911	4.5367	4.5202	3.2919	4.4968	4.4115	3.6889	4.4251	4.6753	4.6342	3.9773	4.2056	4.4596	4.5347	4.5120	4.2922
Tomm22	_AEAEKAEEELEEDDDDELDETLSER_.3	#N/A	4.4545	3.5483	3.4899	3.2349	3.7922	4.1805	3.7489	4.1548	3.8080	3.1599	4.1824	3.4378	3.9160	4.3032	3.3261	3.2985	3.1917	3.1406	4.2901	3.4440
Atp5mg	_VELVPPTPAEIPTAIQSVK_.2	#N/A	9.9375	10.1556	9.7549	9.9055	10.0518	10.0637	10.3492	10.3252	10.0349	9.8206	9.9745	9.9292	9.8853	10.0667	9.8061	9.8334	10.0734	10.1384	9.9445	9.7225
Atp5mg	_LATFWHYAK_.2	#N/A	8.0872	7.6335	7.6732	7.5679	7.8274	7.6468	7.6058	8.2291	7.4268	7.6593	7.8626	7.7985	7.9919	7.8371	6.9070	7.5705	7.7923	7.9200	7.7431	7.3725
	TPS[Phospho (STY)]PGEQQQVQEVK.2	#N/A	9.9451	10.5220	10.2962	10.3021	10.2018	10.4976	10.1980	10.4753	10.4348	10.3842	10.3239	10.4550	3.8517	10.9892	11.2248	10.5749	10.8789	10.4545	10.5389	10.6661
	TPS[Phospho (STY)]PGEQQQVQEVKK.3	#N/A	3.8296	4.1733	4.3658	3.8598	3.1673	3.3168	3.7646	3.5299	3.6848	4.3880	3.1635	4.0627	3.8503	4.3529	5.3472	4.1745	4.0676	3.7656	3.4141	4.3199
Glo1	_RFEELGVK_.2	#N/A	6.8361	7.2638	7.9504	6.6664	7.3766	7.1606	7.3937	7.3480	7.0573	7.4170	6.8239	7.7514	7.5497	7.4552	7.9966	7.2260	7.2746	7.2131	7.5726	7.0637
Glo1	_GLAFIQDPDGYWIEILNPNK_.2	#N/A	5.3985	5.9014	5.6399	5.9085	4.9237	5.1017	5.8485	6.3339	5.7753	5.1980	5.8318	6.1443	5.4117	6.6766	5.6059	5.7695	6.1012	5.8086	5.9200	5.3269
Glo1	_DFLLQQTM[Oxidation (M)]LR_.2	#N/A	6.9806	6.0207	7.2287	5.9015	6.1055	7.2172	6.5318	3.9047	3.0824	6.8802	6.5706	6.6998	5.7765	6.2918	6.9522	7.0673	6.5419	6.5320	6.7969	3.7175
Glo1	_FEELGVK_.2	#N/A	4.0475	3.1996	3.6561	4.6163	4.3970	3.8313	4.0629	4.3452	4.2118	4.3230	4.1657	4.8085	4.7066	2.9846	4.3485	4.5235	3.8424	4.3326	4.5587	4.3341
Glo1	_SLDFYTR_.2	#N/A	4.5234	4.2965	4.0600	4.4193	4.3031	3.2629	4.5019	3.3868	4.2895	4.3581	4.8716	4.0226	5.7640	4.2102	4.7162	5.0463	4.2463	4.3926	5.0015	4.8512
Glo1	_GFGHIGIAVPDVYSAC[Carbamidomethyl (C)]K_.2	#N/A	4.4248	4.3951	4.1586	4.6379	3.1309	3.1642	4.9705	3.1496	4.3641	5.3197	4.6138	3.6109	4.4557	3.6705	3.9426	3.7869	4.7976	2.0172	5.0722	3.6767
Mgst3	_IASGLGLAWIIGR_.2	#N/A	3.2184	4.9571	4.9003	3.2147	4.1537	4.3277	4.7470	4.4196	4.0084	3.4247	4.5948	4.2511	3.6512	3.7418	3.0613	3.9212	3.4565	3.8284	4.0012	4.3731
Mgst3	_VLYAYGYYTGDPSK_.2	#N/A	3.8397	4.1631	4.3760	3.8497	3.1775	4.0856	4.9591	3.5401	4.4227	4.0461	3.2962	4.0525	3.8605	4.3631	3.7835	4.1846	4.0778	4.0912	3.4040	3.9885
Glod4	_VTLAVSDLQK_.2	#N/A	6.5729	7.1130	6.9125	6.6262	6.7912	6.8511	7.0088	6.6207	6.5949	6.4585	6.4554	6.6653	6.7694	7.2459	6.7200	6.8702	6.9687	6.6859	6.6673	6.6975
Glod4	_VAEGIFETEAPGGYK_.2	#N/A	6.7015	6.5500	7.3738	6.6283	6.8377	6.6167	6.6569	6.6383	6.5369	6.8292	6.7030	6.6871	6.9201	7.3239	6.6587	7.1651	6.4096	6.7410	6.4306	6.8983
Glod4	_FQTVHFFR_.2	#N/A	6.8771	6.9782	6.8078	6.4365	6.6352	6.7844	6.0984	6.9573	6.4311	6.1435	6.2282	6.5922	6.6672	6.6075	6.2982	6.3628	6.6113	6.5317	6.1386	6.6337
Glod4	_SPSQSDPVLK_.2	#N/A	6.4547	6.0075	6.6483	5.4372	5.9887	6.3575	6.0118	5.9701	6.1431	5.8372	6.1940	6.1557	6.4415	6.5441	6.5450	6.4282	5.6845	6.4874	6.0207	6.0258
Glod4	_IYEQDEEKQR_.2	#N/A	4.8838	4.8641	4.8759	4.9882	3.7290	4.9225	5.2256	5.4690	4.6518	4.6538	4.4621	5.1900	5.3570	4.4929	4.8485	4.8224	4.0991	5.2663	5.0500	4.8583
Glod4	_ALLGYADNQC[Carbamidomethyl (C)]K_.2	#N/A	3.0887	2.3070	6.3992	1.9892	5.8494	5.0536	5.8684	5.5354	5.8245	6.4035	5.0712	5.6228	5.7994	5.9633	5.7449	5.2666	5.4090	5.8649	5.6118	5.6523
Glod4	_FYLQDRS[Phospho (STY)]PSQSDPVLK_.3	#N/A	4.7853	3.7333	5.0611	3.7970	4.6737	5.3877	4.9892	5.0022	5.1965	5.2714	4.0211	3.6967	4.0146	6.0423	4.5515	5.5904	5.3863	5.3387	3.7839	5.8070
Glod4	_ESHSILTPLVSLDTPGK_.3	#N/A	4.1650	4.9520	5.0225	5.5286	4.8372	5.0896	4.6133	4.7796	5.1466	5.3208	5.1809	5.0968	4.8876	5.5679	5.3987	5.5074	4.3605	4.9380	5.4359	5.5402
Glod4	_DVLGM[Oxidation (M)]QVLR_.2	#N/A	4.7755	5.1152	5.1344	6.1756	5.1335	4.4739	4.6056	4.8795	7.6277	8.1267	4.6367	4.2306	4.5951	5.0995	8.1277	7.7360	4.7450	5.2009	6.1929	4.8080
Glod4	_LELQGIQGAVDHAAAFGR_.3	#N/A	5.1891	5.1576	5.5865	5.5435	5.1636	5.0416	4.9048	4.7299	4.9628	5.0325	5.3242	5.1302	5.4100	5.5779	5.5282	5.4254	5.2074	5.0263	4.8756	5.5599
Glod4	_LLDDAMEADKSDEWFATR_.3	#N/A	3.3162	3.8296	4.7444	5.1089	3.5231	3.0844	3.1799	3.5419	3.2390	3.7289	3.6134	3.7290	3.3471	5.0667	4.2580	3.8675	4.5400	4.1103	4.4094	4.0690
Arpc5	_ALAAGGVGSIVR_.2	#N/A	6.4741	6.4817	7.0142	6.1341	6.9638	6.5917	6.6578	6.9782	6.6897	6.8631	6.3218	6.7963	6.4833	7.0643	6.9713	6.7000	6.3803	6.7064	6.9415	6.5037
Lap3	_QVIDC[Carbamidomethyl (C)]QLADVNNLGK_.2	#N/A	7.5322	7.4778	7.4297	7.7158	7.3136	7.3005	7.4771	7.3869	7.6046	7.8480	7.5096	7.2361	7.9821	8.0272	7.5473	6.7325	7.8963	7.4401	7.3640	7.4105
Lap3	_ANKPGDVVR_.2	#N/A	6.1598	5.4127	7.0705	6.5534	6.4609	6.7113	6.8024	6.3014	6.2458	5.7879	6.5349	6.3959	6.4931	6.7589	7.1011	6.9085	6.2647	6.5454	5.7852	6.3161
Lap3	_ADM[Oxidation (M)]GGAATIC[Carbamidomethyl (C)]SAIVSAAK_.2	#N/A	5.5477	5.3862	4.4959	5.3628	6.0181	5.1391	4.5382	4.9252	5.0597	5.9928	5.4225	5.3882	5.8541	5.2682	5.5068	5.4153	5.9181	4.8295	4.8081	6.3187
Lap3	_SAGAC[Carbamidomethyl (C)]TAAAFLR_.2	#N/A	4.7288	5.3956	5.7640	5.6646	4.9606	5.2694	5.4314	5.5011	5.2744	4.5879	5.0018	5.3899	4.0808	5.4928	5.8240	5.3476	5.0316	4.6705	5.2019	5.3421
Lap3	_DKDDDLPQFTSAGESFNK_.3	#N/A	4.5495	4.2705	6.1814	6.4320	3.2556	5.7423	6.1245	3.5578	6.1294	5.8038	3.1914	5.8667	3.8782	6.7147	5.9913	6.3461	4.0955	6.1148	5.6126	6.3123
Lap3	_TLIEFLLR_.2	#N/A	4.2384	4.6512	3.7750	4.6661	4.5634	4.9272	4.2997	4.4641	4.6518	4.9053	3.0485	5.2159	3.4045	4.1213	1.3138	3.6005	3.9613	4.6492	4.5490	4.6194
Lap3	_FAEIIEK_.2	#N/A	3.9252	4.0776	4.4615	3.7642	3.2629	3.2212	3.8602	3.6256	3.7804	4.2924	3.2591	3.9671	3.9459	4.4485	3.3513	4.2701	4.1632	2.9403	3.3185	2.1234
Lap3	_S[Phospho (STY)]WIEEQEMGSFLSVAK_.2	Q9CPY7_S238_M1_Lap3	3.3464	3.9727	4.3838	4.2378	3.5534	3.0542	3.4947	3.5721	3.2692	3.6987	3.6436	3.6988	3.3773	4.4219	3.5201	4.2094	3.0693	4.0801	4.3792	4.0992
Mid1ip1	_T[Phospho (STY)]TPAPS[Phospho (STY)]PGSANESFFAPSR_.2	Q9CQ20_S71_M2_Mid1ip1	4.2853	8.1451	3.8218	3.2990	2.9914	3.0247	4.0567	4.5109	3.9769	4.0959	3.4556	4.2608	4.3161	3.8599	4.0821	7.2382	4.0082	3.1413	3.4403	3.5372
Lamtor1	_LLLDPSST[Phospho (STY)]PTK_.2	#N/A	3.9003	4.2665	9.2767	3.7980	3.3400	3.6627	3.8038	9.1637	3.8118	3.8633	3.8098	3.9926	9.3641	9.3909	3.5679	8.8982	3.8479	8.9826	9.0315	3.7505
Lamtor1	_KLLLDPSST[Phospho (STY)]PTK_.2	#N/A	10.6103	10.4877	4.1008	10.2309	10.3703	10.1820	9.9747	10.7658	10.1883	10.2689	10.2622	10.0993	10.9114	10.4382	9.7171	10.0496	10.6258	10.2143	2.9505	10.1250
Ndufc2	_DHDMFGYIK_.2	#N/A	4.0665	3.1613	5.5259	3.9639	3.4042	3.7835	4.7653	3.7668	2.9754	4.2728	5.3477	2.6822	4.0872	4.3685	3.7141	4.4114	4.3045	3.5286	3.1772	1.4496
Ndufc2	_TYAEILEPFHPVR_.2	#N/A	5.9698	6.2482	5.9517	5.8244	5.9223	6.1106	5.9675	6.6267	5.7983	6.2453	6.4497	5.7745	6.1274	6.1605	5.7229	5.5805	6.4231	6.3691	6.4374	5.5720
Pgls	_DLPAAAAPAGPASFAR_.2	#N/A	6.7662	6.8571	7.1370	6.7984	7.1635	7.2009	7.2324	6.7682	7.0137	6.5704	6.8894	6.6364	6.4802	4.0793	7.4244	6.6647	6.8296	7.0477	6.6158	6.8152
Pgls	_LPIPDSQVLTINPALPVEDAAEDYAR_.3	#N/A	4.5700	3.4328	4.2105	3.8146	3.9077	4.2926	3.4721	4.5056	3.6925	3.2754	4.0669	3.7963	3.8005	3.3443	5.3944	4.6727	3.4926	3.6569	4.3880	3.0216
Pgls	_TGALC[Carbamidomethyl (C)]WFLDEAAAR_.2	#N/A	3.7140	4.2888	3.0084	3.9754	3.0517	3.4324	3.6491	3.6948	3.5693	4.5035	3.0479	4.1782	3.7347	4.2374	3.6744	4.0589	3.9521	2.6793	3.5297	4.2044
Decr1	_VAGHPDVVINNAAGNFISPSER_.3	#N/A	5.8750	5.3344	5.0195	5.7255	6.1275	5.5824	5.3576	6.2925	6.0768	5.3061	5.7378	5.9722	5.5630	5.6831	4.5792	5.0842	5.5945	5.0593	5.6246	5.7617
Uqcrq	_HVISYSLSPFEQR_.3	#N/A	5.3236	4.9215	5.6777	5.4504	4.9011	4.7893	5.1371	5.1799	5.0459	5.2331	4.5933	4.8297	4.5740	5.5831	5.2861	4.8602	4.3592	5.1937	5.5540	5.0720
Uqcrq	_EFGNLAR_.2	#N/A	3.7811	5.6827	5.4776	5.4524	5.7905	6.0029	6.6196	5.2131	6.1279	5.1732	6.2953	6.2576	4.4479	5.6621	5.6905	5.8853	4.8036	6.4316	6.1986	6.0674
Uqcrq	_AFPSYFSK_.2	#N/A	5.8192	5.8305	3.7255	6.5229	6.1327	5.8008	4.1531	5.6462	6.3054	3.9995	5.9347	6.3637	6.6058	5.7416	6.2999	6.0514	5.6814	6.5349	5.8465	3.4408
Uqcrq	_KNPAMYENDK_.2	#N/A	4.0476	4.8780	6.7098	6.4151	4.2545	5.3895	5.4833	4.2732	5.2415	4.4984	5.7706	4.6671	4.0784	6.3242	5.0679	3.4097	5.9706	3.3790	4.9702	3.2995
Ndufa2	_AHPNLPILIR_.2	#N/A	7.8054	7.8886	7.7171	7.8520	7.9143	7.5634	7.7628	7.9471	7.2537	7.5481	7.6639	8.0272	7.9979	8.0457	7.8567	7.3707	7.4969	7.9309	8.0331	7.8977
Ndufa2	_TVSLNNLSADEVTR_.2	#N/A	6.6811	6.9155	7.2387	7.7634	6.8799	6.6018	6.5288	7.0315	7.1099	6.9603	6.9740	6.4770	7.1397	7.0370	6.9921	7.2266	6.9084	6.8168	6.4773	6.7727
Ndufa2	_EC[Carbamidomethyl (C)]SEVQPK_.2	#N/A	5.1296	4.4024	4.2801	5.2375	5.3310	5.2657	5.1801	3.4441	4.8003	5.0433	5.1055	5.1554	4.4679	4.2671	5.7123	4.6205	3.9818	4.7714	5.1483	4.9505
Ndufa2	_YAFGQEK_.2	#N/A	3.4400	4.7867	4.4774	4.4922	4.9075	4.9866	5.6265	3.1665	5.4989	3.2506	5.2168	5.7933	5.1680	4.4106	5.0905	5.3065	4.1127	5.5873	5.3322	5.6952
Ndufa2	_AM[Oxidation (M)]QNVLSGK_.2	#N/A	5.0374	5.2077	5.3147	4.6796	3.6253	5.0629	5.3706	5.0585	5.8974	5.5668	5.1807	5.1142	4.9289	3.8498	5.8713	5.7845	5.4244	5.6636	5.3926	5.5074
Mien1	_RAS[Phospho (STY)]NGEPVEK_.2	Q9CQ86_S97_M1_Mien1	4.4843	4.1459	4.2106	3.7502	3.2148	4.1711	3.5714	3.2227	4.0586	3.7970	3.4704	2.1863	3.2041	3.7432	3.9752	4.0573	3.7730	3.5793	2.3409	3.3320
Cuta	_TQSSLVPALTEFVR_.2	#N/A	5.0630	3.4578	5.4658	5.4978	5.9962	5.9628	5.4880	5.1926	6.0213	5.1933	5.8112	4.9151	5.5863	4.9347	6.5560	6.0592	4.5236	4.9596	5.6463	5.7750
Fis1	_GLLQTEPQNNQAK_.2	#N/A	6.8933	6.8930	6.7695	6.8094	6.6502	6.4090	6.5105	6.7789	6.6783	6.7696	6.4761	6.1886	7.1333	4.0200	6.3591	3.8074	6.7394	6.6669	6.4567	6.3227
Fis1	_[Acetyl (Protein N-term)]M[Oxidation (M)]EAVLNELVSVEDLK_.2	#N/A	5.1542	4.4164	5.1399	4.8018	5.2268	5.2388	5.0263	5.2886	4.5533	4.8733	4.4521	4.8956	4.9223	4.8490	4.7222	5.3748	4.7340	4.7069	4.3419	5.1634
Fis1	_KFQSEQAAGS[Phospho (STY)]VSK_.2	Q9CQ92_S29_M1_Fis1	3.7692	4.2336	4.3055	3.9202	3.1069	3.3772	4.4342	3.4696	3.6245	4.4484	3.1031	4.1231	3.7899	4.2925	4.0114	3.9329	4.0072	3.8259	3.4745	4.2595
Fis1	_STQFEYAWC[Carbamidomethyl (C)]LVR_.2	#N/A	3.6453	3.6738	3.1819	4.5255	3.8523	4.4374	3.1958	3.8710	4.9825	3.4559	3.7135	3.3999	3.6762	3.2200	3.2212	3.0074	3.3682	3.7812	4.0803	4.3981
Sdhb	_LQDPFSVYR_.2	#N/A	8.3821	8.5415	8.3130	8.5762	8.4189	8.4124	8.3043	8.7674	8.0412	8.4390	8.4129	8.7792	8.5645	8.6782	8.7084	8.4963	8.6935	8.6233	8.6961	8.6281
Sdhb	_DLVPDLSNFYAQYK_.2	#N/A	8.3183	8.2232	8.3095	8.3765	8.1763	7.9715	8.0364	8.0124	7.9048	8.1142	7.8120	8.1917	8.4647	8.5158	8.5512	8.4440	8.3245	8.1235	8.1127	8.2381
Sdhb	_QQYLQSIEDR_.2	#N/A	7.7926	7.8427	8.0576	8.3544	8.0240	7.7589	7.7741	7.7361	7.8833	8.0817	8.1149	7.8272	8.0695	8.2905	8.1344	8.0084	8.0358	7.6442	7.8899	8.0166
Sdhb	_RIDTDLSK_.2	#N/A	5.4726	6.1967	5.5776	5.3080	5.6062	5.8829	5.7160	5.1780	6.3726	5.9911	6.1308	5.9793	6.0330	6.1075	6.6382	5.8924	6.0027	5.8607	5.5439	5.8525
Sdhb	_WDPDKTGDKPR_.2	#N/A	6.3696	6.1249	6.6572	6.3943	6.5512	6.2721	6.2669	6.7581	6.6364	6.5928	6.8633	6.2222	4.7693	6.6665	7.1439	6.8937	6.3609	6.1962	6.4465	6.5821
Sdhb	_MQTYEVDLNK_.2	#N/A	4.1981	5.8388	6.7177	6.4590	5.2295	5.9258	6.0700	4.4238	5.6201	4.3022	5.9389	4.5150	4.2290	6.8686	5.5699	3.5602	5.1451	4.6352	3.5275	4.8852
Sdhb	_SIEPYLK_.2	#N/A	3.6892	4.1695	4.1870	4.4347	3.0269	3.1194	3.6231	3.3752	3.6925	3.8955	4.1102	2.6590	3.1804	4.2250	3.0738	2.2084	4.3733	2.0227	3.3305	4.0830
Sdhb	_IKNEVDSTLTFR_.2	#N/A	3.7500	4.2528	4.2863	3.9394	3.0877	3.3118	4.4534	3.3145	4.7033	3.9563	3.3860	4.1423	3.1197	2.5279	4.0306	3.4003	4.3126	4.3377	3.1359	3.8416
Sdhb	_QQYLQSIEDREK_.3	#N/A	3.2817	3.2203	3.8180	4.4077	4.1203	3.8647	3.4208	4.4829	3.1370	3.4880	4.1164	3.1097	3.3024	3.8051	2.8408	2.3946	3.5198	1.4436	3.9620	3.7721
Cisd2	_VVNEINIEDLC[Carbamidomethyl (C)]LTK_.2	#N/A	3.6685	3.6506	3.2051	3.9157	3.8754	3.0064	3.1727	3.8942	3.0929	3.4791	4.0723	3.3767	3.2583	3.7610	3.1980	4.3991	3.3914	4.3575	4.0061	3.7280
Bzw1	_NAEEES[Phospho (STY)]ESEAEEGD_.2	Q9CQC6_S411_M1_Bzw1	7.3288	4.3412	4.1979	4.0278	2.9993	7.6607	3.0409	3.3620	3.1000	3.8679	7.2263	4.2307	3.2081	9.2148	7.1215	4.0065	9.5048	4.2493	3.5821	3.9300
Ndufb4	_VSHIEDPALIR_.2	#N/A	6.8587	6.8673	6.5305	6.2394	7.0862	6.4173	6.9128	7.1138	6.4804	6.9485	6.8328	6.7749	6.7072	7.1917	7.1409	6.6861	6.7579	6.8534	7.1624	6.6303
Ndufb4	_EYLLQYNDPK_.2	#N/A	6.8118	6.6828	6.9933	7.2580	6.7796	6.4606	6.8190	7.3722	7.3138	7.1447	7.1335	6.7187	7.0817	7.1636	6.9062	7.3301	7.0474	6.5929	6.4041	6.9689
Ndufb4	_EYLLQYNDPKR_.3	#N/A	6.6803	6.1713	5.7498	6.4715	6.1961	6.4091	6.2622	6.8660	6.4027	6.3643	6.4035	6.1150	6.7490	6.4273	6.4494	6.7474	6.3857	6.3462	5.9765	6.4279
Ndufb4	_YKPAPLATLPSTLDPAEYDVSPETR_.3	#N/A	5.2311	5.6470	5.4667	5.6241	4.9880	5.8142	5.4271	5.2973	5.2326	4.8983	5.1006	5.5232	5.9345	6.5290	5.5634	5.8066	5.8490	5.3159	5.2830	5.2336
Rab5a	_QASPNIVIALSGNK_.2	#N/A	5.6571	5.6813	4.4158	4.3253	4.2837	4.7571	4.7299	4.2394	4.7732	4.3931	4.9919	4.1574	5.2384	5.2479	4.3147	4.2973	4.9033	4.9354	4.9461	5.1885
Rab5a	_GAQAAIVVYDITNEESFAR_.2	#N/A	4.3511	3.6517	3.3865	3.3383	3.6888	3.9129	3.8523	4.7785	3.9114	4.5574	4.2857	3.5412	4.0194	4.5803	3.4295	3.1951	3.0883	3.2440	4.3934	3.2238
Rgs10	_WASS[Phospho (STY)]LENLLEDPEGVQR_.2	Q9CQE5_S41_M1_Rgs10	3.8742	6.3876	6.5628	6.7107	3.2119	6.6324	4.3292	5.9944	4.3883	4.0805	6.5663	4.0181	3.8949	7.1912	7.0082	7.0357	6.5935	6.7468	7.0080	4.3646
RTRAF	_INEAIVAVQAIIADPK_.2	#N/A	3.4051	3.3635	4.2325	3.9932	3.0339	3.2580	3.2990	3.3966	3.0654	3.9025	3.4398	5.0064	3.1734	4.2196	3.4968	4.0411	3.9343	3.2846	3.7907	4.1866
Nudt21	_LPGGELNPGEDEVEGLKR_.3	#N/A	6.4643	6.3480	6.0780	5.9853	5.5284	6.1867	6.5174	6.7613	5.9986	6.1901	5.9191	6.7459	7.0564	6.9121	6.2684	6.9095	6.5967	6.2423	6.2772	6.2369
Mtres1	_EADEEDS[Phospho (STY)]DEETSYPER_.2	Q9CQF4_S106_M1_Mtres1	11.6196	11.6106	11.8147	11.8032	11.3479	11.4805	11.1512	11.6565	11.4627	11.6745	11.3921	11.5212	11.6611	12.1987	11.4244	11.5669	11.8047	11.4246	11.4818	11.6926
Ndufb5	_NFYDGPEK_.2	#N/A	6.1667	6.1212	5.7384	5.9901	5.8691	6.7019	6.9248	5.7189	6.7260	6.3694	6.5057	6.4554	6.4451	5.7480	5.8345	5.7851	5.6905	6.9497	5.9780	6.5768
Ndufb5	_EFIDHSPK_.2	#N/A	3.1798	5.8332	5.8839	5.0605	4.8292	5.6247	5.4902	5.6631	5.5938	3.3861	5.8201	5.5799	4.7114	3.2336	5.6531	3.5247	5.4960	5.1206	4.6811	4.9315
Ndufb5	_TLAILQIESEKAELR_.2	#N/A	4.2085	4.6455	3.2498	3.6975	4.4991	3.8501	3.7634	4.5351	4.0262	4.1789	3.8378	4.2266	4.8916	3.8700	3.3593	3.7973	4.4548	4.1576	4.0903	4.0149
Gmfb	_NTEDLTEEWLR_.2	#N/A	6.9066	7.0349	7.2186	7.5764	6.8225	7.2304	6.9488	6.7447	7.1206	7.1156	7.2323	7.0716	7.0669	7.4029	7.1290	7.3860	6.9506	7.0787	7.0446	7.2716
Gmfb	_LVQTAELTK_.2	#N/A	5.4998	5.9637	6.5303	6.4763	5.7530	5.9695	6.1855	5.1641	6.5640	5.8812	5.7747	5.5316	6.4229	6.0662	6.8956	5.9157	5.3887	6.0424	6.2784	6.5023
Gmfb	_LVVLDEELEGVSPDELKDELPER_.3	#N/A	4.6234	5.2299	3.7968	4.0191	4.4417	4.2135	4.9003	4.3194	5.3293	4.9886	4.4342	4.9719	5.2421	5.2336	5.0581	4.2752	4.2784	5.3575	4.7189	4.6885
Gmfb	_ETHNAAIIM[Oxidation (M)]K_.2	#N/A	4.1328	4.9084	3.1682	3.5566	3.4705	3.6946	4.0706	3.8332	4.1297	4.1559	4.2032	3.7595	3.8287	3.1553	5.1691	5.3368	4.5495	4.0374	4.5692	3.1223
Gmfb	_[Acetyl (Protein N-term)]SESLVVC[Carbamidomethyl (C)]DVAEDLVEK_.2	#N/A	10.5508	10.2234	10.0204	10.4179	9.3735	9.4789	9.5507	10.2484	9.5185	10.2181	9.5182	10.0859	11.3560	11.0698	9.8425	10.6789	10.0441	9.8327	9.4893	9.9599
Cotl1	_KAGGANYDAQS[Phospho (STY)]E_.2	#N/A	3.4189	8.3002	8.0337	8.7427	7.7637	8.1186	8.4308	3.6456	8.0356	8.1486	7.8014	8.0317	8.1896	8.3287	8.1162	7.5739	8.4036	7.8487	8.2439	4.1727
Cotl1	_EFVISDRK_.2	#N/A	7.1740	7.6344	7.4091	7.4686	4.2858	7.4029	7.2504	7.5376	7.7105	7.4143	6.9083	7.3780	7.5138	7.3389	7.4361	7.4108	7.0562	7.1153	7.4706	7.1754
Cotl1	_ELEEDFIR_.2	#N/A	6.6275	6.6230	6.6183	7.1139	6.8936	6.8465	7.0935	6.0304	7.0700	7.2121	7.1235	6.6896	6.2941	6.7737	6.9907	7.3353	5.4415	6.9306	6.8176	7.1842
Cotl1	_EVVQNFAK_.2	#N/A	4.0424	5.1859	4.2376	5.5340	5.0047	5.7415	5.8724	3.2959	5.7153	5.2461	4.6480	5.7144	4.5964	5.0315	6.0330	5.2975	3.5755	5.7565	6.2340	4.6682
Cotl1	_EFVISDR_.2	#N/A	5.2227	5.3546	5.6492	6.2944	5.6628	3.1826	5.7465	3.6641	5.9306	6.4255	6.5393	5.6685	5.9140	5.6203	5.6873	6.1588	4.2018	5.8622	6.4665	5.9507
Cotl1	_YDGATIVPGDQGADYQHFIQQC[Carbamidomethyl (C)]TDDVR_.3	#N/A	4.3973	3.6055	3.4328	3.2921	1.4271	3.9591	3.8060	4.0977	3.8651	3.1028	1.4475	3.4949	3.9732	3.4199	3.3832	3.2414	3.1346	3.1978	4.3472	3.3869
Cotl1	_AGGANYDAQS[Phospho (STY)]E_.2	#N/A	9.4937	10.0814	10.3186	10.3378	8.6649	9.9666	9.7335	9.7669	9.9777	9.9826	9.8004	9.8166	9.8936	9.8938	9.2110	8.7629	9.9271	9.6436	9.6504	9.7043
Cotl1	_FALITWIGEDVSGLQR_.2	#N/A	4.5018	3.9270	4.7004	3.8252	4.6065	5.0778	4.0885	4.5013	4.4103	4.9765	4.9367	4.4688	3.7104	4.2796	4.2713	3.8884	4.4600	4.0201	5.0812	4.4942
Cotl1	_FTTGDAMSK_.2	#N/A	3.6833	4.3659	6.1342	4.0061	3.0211	3.2033	4.0085	3.3837	3.5386	3.8897	3.0172	4.2089	3.7040	4.6762	4.8441	4.0282	3.9214	3.9118	3.5604	4.1737
Denr	_LTVENS[Phospho (STY)]PK_.2	#N/A	6.5801	3.4807	7.1794	3.7458	6.9366	4.0787	6.8950	6.7655	6.8315	7.2529	7.0542	6.4970	4.5893	7.3101	5.9787	3.2005	6.9457	6.9001	4.1760	7.0312
Denr	_WPEVDDDSIEDLGEVKK_.3	#N/A	5.4791	5.8126	3.9297	5.2111	4.8167	4.9747	4.7162	5.0060	5.9661	5.2621	5.1810	5.1009	4.5531	6.1109	5.4150	5.9476	5.5057	5.0616	5.5879	5.4401
Denr	_LTVENS[Phospho (STY)]PKQETGITEGQGPVGEEEEK_.3	#N/A	4.4240	6.4059	3.4595	4.7289	3.7617	4.8274	5.3147	5.4042	3.8384	3.1295	4.2128	3.4682	3.9465	3.4466	3.3565	3.2681	3.1612	3.1711	4.3205	5.1719
Ndufb9	_HLESWC[Carbamidomethyl (C)]IHR_.3	#N/A	4.5842	5.5285	4.8640	5.3080	4.4856	5.5371	6.0385	5.7953	4.7836	5.0636	5.1175	5.2737	5.7348	6.3438	4.3925	5.3608	3.3763	6.1939	5.2012	5.6892
Ndufb9	_VPEWC[Carbamidomethyl (C)]LDYWHPSEK_.3	#N/A	4.0089	3.3102	5.1221	3.5754	4.7170	3.8926	3.8726	4.2346	5.1935	4.5371	3.8723	3.0363	4.0397	5.0724	5.2632	4.9349	3.7318	3.4177	3.7167	5.1894
Ndufb9	_ARFEEHKNEK_.3	#N/A	4.0415	3.2776	3.5780	3.3239	4.2484	4.2817	4.3005	4.0658	4.3385	5.2256	3.7919	3.0037	4.3862	3.3880	2.8251	3.9613	3.1027	3.2296	4.6518	3.3550
Ndufb9	_EAEEEFWQNQHPQPYIFPDSPGGTSFER_.3	#N/A	3.5346	3.7844	4.5721	4.0496	3.7416	4.3668	3.3065	3.7603	3.4575	3.5105	3.8318	3.5106	3.5655	4.6101	3.3319	4.3976	3.2576	3.8919	4.1909	4.2874
Nicn1	_S[Phospho (STY)]PSLAFPK_.2	Q9CQM0_S142_M1_Nicn1	3.2296	4.0895	4.2670	4.3546	3.4365	5.8553	3.6116	3.4553	3.5318	4.5410	6.0209	3.8156	3.6972	4.1715	3.6369	5.7507	4.4534	3.9186	5.9007	4.1669
Txndc17	_SWC[Carbamidomethyl (C)]PDC[Carbamidomethyl (C)]VEAEPVIR_.2	#N/A	6.0240	7.1583	6.8961	6.3208	6.1605	5.7948	6.7395	6.6621	6.3041	6.3282	6.4294	6.8774	6.7259	6.7345	7.2169	6.2777	6.8546	6.1507	6.4005	6.4972
Txndc17	_[Acetyl (Protein N-term)]ATFEEVSVLGFEEFDK_.2	#N/A	5.6267	5.3231	5.9426	6.2102	5.6899	5.3315	4.3396	6.4130	5.2289	5.7971	4.8957	5.7305	7.0620	6.8959	5.9054	6.4700	5.9003	4.2623	5.6923	5.3516
Txndc17	_LVESEC[Carbamidomethyl (C)]C[Carbamidomethyl (C)]QSSLVEM[Oxidation (M)]IFSED_.2	#N/A	5.8925	5.2694	5.6077	5.1425	4.6261	4.7499	5.4937	6.9090	5.2360	6.3061	5.3909	5.0820	5.9853	7.2033	5.1209	5.1274	5.4639	5.2366	4.6417	5.0900
Glrx3	_HNIQFSSFDIFSDEEVR_.3	#N/A	3.6064	3.7127	3.1429	4.8175	3.8133	5.2225	3.9810	4.1683	3.1550	5.0156	4.8656	3.4389	5.7577	5.2107	4.0354	4.8026	3.5378	4.1192	4.2558	5.3466
Glrx3	_[Acetyl (Protein N-term)]AAGAAEAGEAAVAVVEVGSAQQFEELLR_.3	#N/A	3.1044	3.3975	3.6407	4.5850	3.1012	4.1671	3.5981	4.3056	3.6572	4.1115	4.0316	3.2870	3.7652	4.4771	3.0926	3.4493	2.4278	2.1876	4.1393	3.5948
Trap1	_AFLEALQNQAETSSK_.2	#N/A	3.6455	3.6736	4.5874	3.9388	3.8524	4.2560	3.1957	3.8711	3.5683	4.0460	3.9427	3.6745	3.6763	3.2201	3.4168	3.0076	3.3684	4.2328	4.0801	4.3982
Trap1	_GVVDSEDIPLNLS[Phospho (STY)]R_.2	Q9CQN1_S403_M1_Trap1	3.1739	3.3280	3.7102	4.5155	4.0125	3.9724	3.5286	4.3751	3.0292	3.3803	4.0087	3.2175	3.1946	7.8853	3.1058	3.5188	3.4120	7.4911	4.0698	3.6643
Tomm6	_NLSDIDLM[Oxidation (M)]APQPGV_.2	#N/A	3.1347	5.0319	5.6584	3.9180	4.6544	4.3102	3.7065	3.4718	4.1237	4.4461	4.1822	3.9105	3.7921	4.2948	5.2417	5.5535	4.0095	4.5926	5.0309	5.0998
Trappc2	_FAMNPFYEPNS[Phospho (STY)]PIR_.2	Q9CQP2_S119_M1_Trappc2	3.4725	4.5303	4.0088	4.9236	4.3110	3.0343	3.2300	3.1729	3.2891	3.6788	3.6635	4.1713	3.3972	7.2592	4.1698	3.8174	3.7105	4.1226	3.7712	3.9628
Atp5pb	_HYLFDVQR_.2	#N/A	9.2992	9.2951	9.1167	8.9835	9.1079	9.1538	9.2310	9.3688	9.0150	9.1122	8.8468	8.9786	9.2050	8.8860	8.4613	8.8849	9.0692	9.1936	8.9657	8.6779
Atp5pb	_IAQLEEVK_.2	#N/A	7.6514	7.1087	7.6918	7.9623	7.5434	7.4141	7.8546	6.7848	7.7947	7.7290	7.5319	7.5616	7.6502	7.0355	7.5507	7.6416	6.7003	7.8326	7.8161	7.6530
Atp5pb	_AQQALVQK_.2	#N/A	5.9094	6.9303	6.9375	6.5480	6.8088	6.7802	7.0947	6.6053	7.0232	6.6716	6.5824	6.9641	6.8545	6.5288	6.7991	6.3245	6.1066	6.6833	6.8421	6.7930
Atp5pb	_QIQDAIDMEK_.2	#N/A	4.5539	5.7865	6.9057	6.5931	5.2220	5.1645	5.7702	3.3571	4.9847	4.7850	6.3530	4.8745	3.8738	6.6900	5.3842	3.9160	4.9894	3.3813	3.3906	5.2025
Atp5pb	_SISVQQEKETIAK_.2	#N/A	5.7502	5.9139	5.5831	5.9445	5.6854	5.2396	5.7121	6.2222	5.8379	5.6497	5.4786	5.8761	6.4696	5.8371	5.0524	5.7177	5.6715	5.6489	5.0106	4.6194
Atp5pb	_RKEEEHM[Oxidation (M)]IDWVEK_.4	#N/A	5.0332	5.1030	5.1961	4.5901	5.3284	5.4306	5.0302	6.8751	5.6949	6.1801	7.2057	5.1345	3.7746	4.1628	4.5217	5.9726	6.1892	5.5769	5.7090	5.4322
Atp5pb	_KEEEHMIDWVEK_.3	#N/A	3.2237	3.2783	3.7600	4.4657	4.0622	4.2863	3.4789	3.8408	3.5379	3.4300	3.9123	3.1678	3.6460	3.1898	3.0561	3.5686	3.3380	3.8114	4.1105	4.3679
Atp5pb	_LDYHISVQNM[Oxidation (M)]M[Oxidation (M)]R_.3	#N/A	3.4192	4.5159	4.0232	4.2025	3.6261	3.6595	3.4220	3.1873	3.3422	3.2298	4.3216	4.4054	3.5076	4.0103	2.1859	4.2822	3.7250	4.1082	3.7568	3.9773
Rps21	_TYGIC[Carbamidomethyl (C)]GAIR_.2	#N/A	6.1140	6.3086	6.4474	6.1893	6.1085	5.9657	6.1203	6.1810	6.3842	5.5866	5.6917	6.2900	5.4600	6.4489	6.7574	5.8681	5.8470	6.0221	6.2205	5.7370
Rps21	_[Acetyl (Protein N-term)]M[Oxidation (M)]QNDAGEFVDLYVPR_.2	#N/A	3.7741	3.2389	2.7038	3.4225	3.6085	3.3640	3.2504	3.9153	3.0843	4.2866	3.6008	3.7811	4.2498	4.2687	2.5947	4.3319	4.2526	3.6298	3.7924	3.9558
Rps21	_DHASIQM[Oxidation (M)]NVAEVDR_.2	#N/A	3.4929	4.6690	4.5848	3.8958	3.6781	3.8276	3.6420	3.7312	3.6321	5.0759	3.6405	3.6099	3.7683	3.8646	4.1881	4.8181	3.7438	3.7716	5.2036	4.5717
Acot13	_VTLVSAAPEK_.2	#N/A	7.1605	7.5755	6.5010	6.8906	6.9870	7.5784	7.9967	7.6843	7.0652	7.4860	7.6612	7.9905	8.3025	7.8757	7.9379	6.5246	6.5149	7.7288	6.8070	6.6354
Acot13	_TLAFASVDLTNK_.2	#N/A	6.5017	6.8805	6.7390	6.5011	6.3659	5.8490	6.1707	6.1200	5.8916	6.4713	5.9856	6.1771	5.7252	7.1932	6.6734	6.2069	6.2555	5.9725	5.7145	6.3342
Acot13	_[Acetyl (Protein N-term)]SSMTQNLR_.2	#N/A	4.4882	4.3317	4.0248	3.0960	3.1891	3.4132	4.3520	3.2131	4.4110	4.0577	3.2846	4.0578	4.5191	4.0629	0.3837	4.1963	4.2111	4.4391	3.2374	3.7402
Ppp6c	_APLDLDKYVEIAR_.3	#N/A	4.1451	3.1246	3.1806	3.3897	4.7574	3.7070	3.8548	4.4202	4.1173	4.8956	2.9908	3.0126	4.2254	2.6671	3.0196	2.9892	3.5580	3.2634	3.5311	3.2848
Slc25a46	_SFGSGTELGHWVTT[Phospho (STY)]PPDIPGSR_.3	Q9CQS4_T45_M1_Slc25a46	3.8694	6.3645	3.4059	3.7149	4.0763	4.0321	6.9083	4.0950	3.7922	4.8307	4.1666	5.8873	6.1636	7.5262	3.3103	3.3143	3.5923	3.5572	3.8562	4.4359
Sec61b	_PGPTPSGTNVGSSGRS[Phospho (STY)]PSK_.2	#N/A	6.8541	6.6941	6.5173	3.6800	5.6499	6.0928	6.4097	3.8653	5.9648	6.1382	6.6428	6.0439	6.8238	6.3348	5.2878	6.1741	6.5034	6.6043	6.7621	5.3965
Sec61b	_S[Phospho (STY)]PSKAVAAR_.2	#N/A	4.3377	3.9336	4.6055	3.6201	3.0438	4.9417	4.0043	3.7696	3.9245	4.1483	3.4032	3.8230	5.1029	3.6627	4.0297	3.6998	4.3073	3.5259	3.1744	3.0588
Rer1	_VDPSLM[Oxidation (M)]EDS[Phospho (STY)]DDGPSLPTK_.2	#N/A	3.6602	8.9281	4.0982	4.0391	3.5361	7.9730	7.7550	3.4863	3.6014	4.1475	3.6895	3.7188	3.7248	7.1983	3.5640	3.9209	4.1919	8.1112	3.4784	7.3977
Rer1	_[Acetyl (Protein N-term)]S[Phospho (STY)]EGDSVGDSVHGKPSVVYR_.3	Q9CQU3_S2_M1_Rer1	3.2429	4.0762	4.2803	4.3414	3.4498	3.4831	3.5983	3.4685	3.5185	4.5543	2.9972	3.8024	3.6840	4.3183	3.6237	4.1058	4.4666	4.1837	2.9819	4.1536
Zwint	_NQS[Phospho (STY)]YLQLLC[Carbamidomethyl (C)]SLQNK_.2	Q9CQU5_S216_M1_Zwint	3.3791	3.1229	3.9154	4.3103	4.2176	3.7673	5.4818	5.9348	3.2343	3.3376	4.2138	4.3233	3.3998	5.8936	2.9006	3.7240	3.6171	3.8899	3.8646	3.8694
Retreg3	_AM[Oxidation (M)]S[Phospho (STY)]GSWER_.2	#N/A	7.5046	7.2587	6.3397	6.6924	6.8154	7.0958	6.5953	7.0843	7.1254	7.1941	6.8391	6.6756	7.4307	6.5443	5.3787	6.8065	7.4582	7.3175	7.0968	6.9752
Retreg3	_GQTPLT[Phospho (STY)]EGSEDLDGHSDPEES[Phospho (STY)]FAR_.3	Q9CQV4_S325_M2_Retreg3	4.1311	4.5541	4.1817	4.2851	3.4914	3.2630	3.7936	3.8716	3.5995	4.0614	3.5694	3.7150	3.7649	5.4657	3.7064	3.7142	4.0535	5.0631	3.5440	3.5401
Map1lc3b	_S[Phospho (STY)]FEQRVEDVR_.2	Q9CQV6_S12_M1_Map1lc3b	7.2695	7.7653	6.9509	7.6448	6.9218	7.5610	7.5786	7.9455	7.4043	7.4675	7.5706	8.3899	8.6574	8.7436	8.5602	8.1738	8.5021	8.3161	8.4515	8.1407
Dnajc19	_EAALILGVS[Phospho (STY)]PTANK_.2	Q9CQV7_S70_M1_Dnajc19	3.9259	3.3932	3.4625	3.6583	4.1329	4.1662	4.4161	4.1516	4.3363	3.7365	3.8149	3.1193	4.5017	3.5006	2.9406	3.2880	3.6488	3.5006	3.7996	3.4705
Ywhab	_DSTLIM[Oxidation (M)]QLLR_.2	#N/A	9.8808	9.9456	10.7428	10.3858	9.9969	10.1788	9.9191	9.6685	9.8347	10.1254	10.2856	9.7964	9.5232	10.2517	9.9818	9.7059	10.0111	9.6807	9.7745	9.9140

Ywhab	_YLSEVASGENK_.2	#N/A	10.2871	10.2116	10.5150	10.5036	10.3336	10.4321	10.2374	10.4261	10.5865	10.4504	10.1674	10.2806	10.4125	10.5327	10.3377	10.4211	10.5455	10.3671	10.4084	10.3793
Ywhab	_LAEQAER_.2	#N/A	7.4898	8.8809	8.0545	8.7145	8.3062	8.8559	9.4301	6.7840	9.3689	9.3553	8.9345	8.8263	9.0034	8.5154	9.6819	8.7431	7.5253	9.0126	9.3538	9.2292
Ywhab	_YLILNATQAESK_.2	#N/A	8.1072	8.5394	9.0429	8.7143	7.9717	8.4697	8.3788	8.0970	8.4166	8.3620	8.1008	7.6517	8.0333	8.5883	8.2332	8.3738	8.0629	8.2312	8.4611	8.4004
Ywhab	_AVTEQGHELSNEER_.3	#N/A	7.0796	7.4367	6.9602	7.6024	6.7951	7.2694	7.5268	6.7885	7.2084	7.3885	7.3024	7.3264	7.6090	7.4270	7.8937	7.4126	6.7895	7.0956	7.3445	7.1658
Ywhab	_AKLAEQAER_.2	#N/A	7.7057	7.9415	7.9759	7.9050	8.0482	7.9506	7.7756	7.9728	7.7835	8.1407	7.8299	7.7440	8.2302	7.5000	7.8867	8.2066	7.9767	7.7502	7.9109	7.9802
Ywhab	_QTTVSNSQQAYQEAFEISKK_.3	#N/A	7.6325	7.2486	7.5758	7.3756	7.1840	6.8942	7.3106	7.5541	7.3708	7.5093	7.1583	7.4075	8.2703	7.5138	7.2334	7.3219	7.2475	7.1753	7.5963	7.3128
Ywhab	_QTTVSNSQQAYQEAFEISK_.2	#N/A	6.5947	5.8897	5.9332	5.8531	5.5939	4.8428	4.6181	6.7884	5.3836	6.2004	5.9217	5.5386	6.2958	6.1442	5.5808	5.7809	6.4545	5.4143	6.1787	5.9330
Ywhab	_DNLTLWTSENQGDEGDAGEGEN_.2	#N/A	13.8473	13.8215	14.0472	13.8793	12.5868	13.4882	13.3108	4.4402	13.5748	13.9448	12.9611	13.1466	14.0091	14.0034	13.2157	13.4572	13.8182	13.3885	13.5225	13.3661
Ywhab	_EM[Oxidation (M)]QPTHPIR_.2	#N/A	10.4353	10.7207	10.9305	10.6349	10.5374	10.5408	10.8333	10.6806	10.5896	10.4781	10.3622	10.7367	10.5716	10.8402	11.1492	10.4194	10.3569	10.7210	10.5469	10.4860
Ywhab	_M[Oxidation (M)]KGDYFR_.2	#N/A	8.2868	8.7658	8.9861	8.7211	8.5357	8.6942	8.9311	8.6272	8.5539	8.5978	8.5288	8.5751	8.4069	8.8874	9.0191	8.3076	8.4613	8.6229	8.5254	8.5465
Ywhab	_VISS[Phospho (STY)]IEQK_.2	Q9CQV8_S66_M1_Ywhab	3.1874	3.3146	3.7237	7.2978	7.1596	4.2500	3.5151	7.1653	7.4370	3.3937	3.9486	3.2040	8.4751	7.8809	7.7285	7.4265	7.4874	3.7751	4.0742	7.8308
Ywhab	_[Acetyl (Protein N-term)]T[Phospho (STY)]M[Oxidation (M)]DKSELVQK_.2	#N/A	6.7205	6.9013	5.9774	7.0898	7.0104	6.5187	6.6270	6.0319	6.5661	6.8422	6.8039	7.2033	7.4083	7.2465	6.8353	7.2223	7.1419	6.9147	7.0860	7.1072
Ywhab	_KEM[Oxidation (M)]QPTHPIR_.3	#N/A	7.4701	7.3799	7.7986	5.9371	7.2985	7.4708	8.0513	7.1460	7.9697	8.0072	7.2783	7.1990	7.2979	6.4856	8.6570	7.9255	7.0829	7.4422	8.2525	7.6460
Ywhab	_TAFDEAIAELDTLNEESYK_.2	#N/A	7.8016	7.5412	7.7686	7.4095	7.3024	7.4566	7.2946	7.3135	7.3134	7.6031	7.6299	7.3887	7.2563	8.1996	7.0329	7.5140	7.6944	7.1593	7.1169	7.4035
Ywhab	_YDDM[Oxidation (M)]AAAM[Oxidation (M)]K_.2	#N/A	4.4634	4.9337	5.1036	5.4210	5.0034	4.8320	4.5732	5.0993	5.0005	5.3959	4.8417	4.8061	4.4562	5.2841	6.0868	4.3209	4.7787	5.1558	5.6644	4.9442
Ywhab	_NLLSVAYK_.2	#N/A	9.3235	9.7008	9.8893	10.6136	3.7021	9.9988	10.5351	8.5813	10.3518	10.4868	10.2738	10.1138	10.4176	9.7717	10.4250	10.4692	8.4008	10.5754	10.6523	10.5957
Ywhab	_IEAELQDIC[Carbamidomethyl (C)]NDVLELLDK_.2	#N/A	3.9289	4.1335	2.8038	3.7605	3.2666	3.2175	3.8639	3.6292	3.7841	3.5175	3.2628	3.9634	3.9496	3.2566	3.8517	4.2738	4.1669	2.5284	0.9827	4.4192
Arl8b	_DLPNALDEK_.2	#N/A	7.4512	6.7387	6.9490	6.5383	6.3906	6.9954	6.9299	7.0469	6.7765	6.2184	6.4436	6.5107	6.6943	6.5455	5.7299	6.4556	6.6804	6.6070	6.5997	6.7483
Cyb5b	_[Acetyl (Protein N-term)]AT[Phospho (STY)]PEASGSGEKVEGSEPSVTYYR_.3	#N/A	7.3709	7.3200	6.8563	7.2577	6.4984	7.5672	7.1989	7.5158	6.9378	7.4108	6.8833	7.2454	7.8061	7.4717	6.8364	7.4831	7.3945	7.6307	7.2642	7.7463
Cyb5b	_RNS[Phospho (STY)]AEETWM[Oxidation (M)]VIHGR_.3	#N/A	5.9052	5.8759	4.3090	4.5953	3.4620	5.4901	5.3627	5.8173	5.5141	5.6401	4.8582	5.0594	6.0004	5.8521	4.9476	5.0125	5.6244	5.7111	5.2258	5.5532
Cyb5b	_FLSEHPGGEEVLLEQAGADATESFEDVGHSPDAR_.4	#N/A	6.8942	7.1403	6.7585	6.7077	6.1879	6.6687	6.2700	6.6015	7.0288	6.7538	5.4980	6.2254	6.7234	7.9388	5.8035	6.3886	6.4927	6.4435	6.1042	6.6913
Cyb5b	_[Acetyl (Protein N-term)]AT[Phospho (STY)]PEASGSGEK_.2	#N/A	9.0721	8.6142	9.2425	8.9907	8.8790	9.0647	3.3378	9.1444	8.9644	8.5509	8.9336	8.7137	8.6274	9.0030	7.9172	9.0551	8.9925	8.6826	3.8790	9.0217
Cyb5b	_NSAEETWM[Oxidation (M)]VIHGR_.2	#N/A	3.8653	3.7739	4.2539	4.1680	4.1934	4.2182	3.7063	4.9572	3.7597	4.1387	3.3684	4.0385	3.8856	3.8054	3.9874	4.5212	3.7649	3.6702	3.7630	4.0572
Mrps36	_LSASEALGSAALPSHSSAISQHSK_.3	#N/A	5.3363	5.7394	5.5445	6.0415	5.4528	5.0507	5.5422	5.7004	5.2571	5.3065	5.3205	5.5334	3.8581	5.9534	6.1986	5.5143	5.6428	5.3711	5.3390	5.4156
Ramac	_RPPES[Phospho (STY)]PPIVEEWNSR_.3	#N/A	6.8372	8.3043	7.9287	7.8262	3.5531	8.1374	8.3220	6.0804	7.9186	7.5138	6.1057	8.7095	6.8345	9.5349	8.5416	9.1123	6.7659	8.6948	8.2340	8.8387
Ramac	_[Acetyl (Protein N-term)]S[Phospho (STY)]DTSEEIPNFEEMFASR_.2	Q9CQY2_S2_M1_Ramac	3.8261	6.2785	3.5374	7.5630	3.8397	4.0637	3.7373	4.0993	3.7964	4.0325	4.1708	3.4262	3.9045	3.4482	3.3145	3.3460	3.5965	5.5630	3.8879	3.9506
Hsbp1	_TM[Oxidation (M)]QDITLVVETLLQQM[Oxidation (M)]QDK_.3	#N/A	4.4557	2.7313	4.4570	3.1285	3.1618	4.3836	3.8863	3.1806	3.8065	3.7521	3.1330	4.0903	4.4866	3.7872	4.1173	3.8178	4.1786	2.6092	4.3179	3.7077
Hsbp1	_NIADLM[Oxidation (M)]TQAGVEELDPENKIPTAQKS[Phospho (STY)]_.3	Q9CQZ1_S76_M1_Hsbp1	3.3569	5.3043	1.8433	4.2273	3.5639	3.5972	3.4842	3.5826	3.4044	3.1675	4.3839	4.2241	4.2809	3.8399	3.2038	4.2199	3.9300	3.7764	4.3687	4.1097
Hsbp1	_NIADLMTQAGVEELDPENKIPTAQK_.3	#N/A	3.5532	4.4496	4.0895	4.1361	2.8910	3.5931	3.4883	3.2536	3.4085	3.1634	3.4705	4.3390	3.5740	4.9219	3.5377	3.8981	3.7913	4.0419	3.6904	4.0436
Ndufa6	_QAAAAAASTSVKPIFSR_.2	#N/A	4.2572	4.3495	4.9965	4.8049	4.2700	4.4949	4.5313	3.8995	4.6266	4.5875	4.3788	4.0867	3.8421	5.2183	4.5831	4.4856	3.5519	4.0978	4.4429	4.1772
Ndufa6	_NAHVTDPR_.2	#N/A	4.3069	4.5130	5.7046	5.3274	5.4110	5.6446	5.3725	5.3859	5.8040	4.1175	5.6809	6.0338	4.3378	5.5202	6.7850	5.2754	5.4543	5.4575	3.4187	3.5588
Ndufa6	_MELQETIK_.2	#N/A	3.8014	3.3972	4.0838	4.2263	4.0083	4.0417	4.3848	4.3059	2.7452	3.6120	3.9912	3.2538	3.1254	4.3700	3.5995	3.1635	3.3428	2.9681	2.0806	3.5951
Ndufa6	_VVDLLVIK_.2	#N/A	3.3389	1.2804	3.5872	2.8420	4.1774	0.4173	3.3636	4.5401	3.4227	3.6706	2.0724	3.0525	3.5308	2.6611	1.9434	1.0399	3.5769	2.4387	1.6357	1.1262
Ndufa6	_EVPNTVHLM[Oxidation (M)]QLDITVK_.2	#N/A	3.2867	3.2152	3.8230	4.4026	4.1253	4.3494	3.4158	4.4880	3.4749	3.4931	3.8492	3.1047	3.5829	4.6275	2.9930	3.6316	3.5248	8.2416	4.1735	4.3048
Ndufb3	_MELPDYR_.2	#N/A	3.6094	5.8357	7.0095	6.4030	5.0736	5.5357	6.4378	3.4550	4.9106	5.3251	6.6322	4.8778	3.2602	6.7284	5.4551	3.9543	4.7839	4.5259	4.8703	3.9821
Psmd9	_[Acetyl (Protein N-term)]S[Phospho (STY)]GEDVPHR_.2	Q9CR00_S2_M1_Psmd9	8.1282	8.7211	8.6175	8.4820	8.3682	8.9432	8.4071	8.4739	8.7011	8.8344	8.8117	8.4097	8.4308	8.8832	8.6943	8.9205	8.5550	8.3061	8.7166	9.1032
Psmd9	_ADVDLYQVR_.2	#N/A	3.3225	5.4004	5.6470	5.4166	5.3465	5.7469	6.0413	5.9500	5.8265	6.0391	3.6197	6.1123	6.5557	5.5106	5.8448	5.7399	6.1580	5.4412	5.9584	6.0292
Psmd9	_LASNS[Phospho (STY)]PVLPQAFAR_.2	Q9CR00_S128_M1_Psmd9	7.2552	4.6150	5.8853	4.1332	4.2264	4.4504	3.3147	8.9369	3.3738	3.5941	3.7482	7.5308	3.4819	9.5317	2.8919	3.7327	3.1739	3.9755	4.2746	4.2037
Ppid	_VFFDVDIGGER_.2	#N/A	5.9767	5.5597	6.3012	5.4393	5.7923	5.9160	5.8446	5.4519	5.8862	5.8948	5.3303	5.3408	4.2948	6.2775	5.7668	4.7965	5.7399	5.7801	6.3645	5.9446
Ppid	_ILLISEDLK_.2	#N/A	4.3482	4.3431	4.6557	5.5017	4.3502	4.3064	4.1285	3.3496	5.1385	4.3739	4.6979	5.1423	3.8029	4.1994	3.8929	4.6947	4.3477	4.8177	4.6662	3.9037
Ppid	_AIQAELLK_.2	#N/A	4.2175	4.3274	4.0301	4.5507	4.3927	4.2137	4.9081	4.0595	4.6560	4.0525	3.3066	4.1632	5.0019	4.6726	5.2140	5.1408	4.2164	4.5843	4.4782	4.2623
Ppid	_IVLELFADIVPK_.2	#N/A	4.2973	3.7055	3.3328	3.3921	2.3839	3.8639	2.5427	3.9977	2.8844	3.9202	3.6312	3.5949	4.3180	3.3199	1.1320	3.1414	4.5354	3.2978	2.9464	3.2869
Ppid	_EYDQALADLKK_.3	#N/A	3.9279	4.0749	4.4642	3.1726	3.2657	3.4898	4.2754	3.1365	4.3345	4.1343	3.2080	3.9643	4.4425	3.9863	3.8526	4.2728	4.1346	3.0149	3.3139	3.6636
Ppid	_AQEIAPGDK_.2	#N/A	3.8690	3.4501	3.4056	3.7152	4.0760	5.4713	3.7328	4.0947	3.7919	3.1761	4.1662	3.1762	3.8999	3.4437	3.3100	3.3146	3.5919	3.5575	3.8565	3.1210
Ppid	_[Acetyl (Protein N-term)]SHASPAAKPSNSK_.3	#N/A	4.2667	3.7361	3.3021	3.4227	3.6044	4.3805	3.9367	3.9670	4.1219	4.4730	3.6006	3.6256	4.2874	3.2892	3.5139	3.1107	4.5047	3.3284	2.9770	3.2562
Ndufab1	_LYDKIDPEK_.2	#N/A	8.5776	8.6014	8.8928	8.5401	8.6015	8.6893	8.4835	8.8113	8.6426	8.4938	8.5604	8.8554	8.8197	9.1991	9.3909	8.9184	8.9171	8.9670	8.7572	8.8124
Ndufab1	_LM[Oxidation (M)]C[Carbamidomethyl (C)]PQEIVDYIADKK_.3	#N/A	8.2135	8.1852	8.2577	8.2083	8.2222	8.0656	8.0552	8.3427	7.9633	8.3994	8.1532	8.3745	8.0979	8.3653	8.4941	8.4088	8.1433	8.3207	8.3417	8.2456
Dph2	_PALPLPVGS[Phospho (STY)]PSSQPESLER_.2	Q9CR25_S188_M1_Dph2	3.1369	7.2706	4.1743	5.2077	3.3438	5.2667	6.3570	7.0756	3.0597	5.8854	5.7942	6.4906	3.1678	7.8393	4.7882	3.9999	4.3607	6.7175	3.0878	3.8897
Vta1	_YAGSALQYEDVGTAVQNLQK_.2	#N/A	4.2855	3.4185	3.8313	3.5077	3.5194	3.1746	4.2264	4.2380	4.0369	4.0248	4.6826	5.0123	5.5557	4.8440	3.7380	3.6582	2.3030	4.3406	3.8249	2.5832
Vta1	_LTPEDFAR_.2	#N/A	4.0806	3.2385	3.6172	3.5037	4.2875	4.3209	4.2614	4.0267	4.1816	3.7968	3.6603	2.9646	4.3471	3.3489	4.4857	3.4427	3.0636	3.2687	3.1317	3.3159
Ccdc43	_DSLRDES[Phospho (STY)]QR_.2	Q9CR29_S186_M1_Ccdc43	7.9859	4.3655	7.6472	7.8708	3.1606	3.4470	3.8875	3.1793	7.3354	8.1429	7.2427	7.4296	4.4853	7.8261	3.9129	3.8166	4.1774	7.7357	3.2711	7.0340
Ccdc43	_AALLAQYADVT[Phospho (STY)]DEEDEADKKDDAGASTANVSSDR_.4	Q9CR29_T137_M1_Ccdc43	4.0530	3.2661	3.5895	3.5313	4.2599	3.8485	4.2891	4.2786	4.6770	3.8635	4.3501	2.9923	4.0838	3.6276	2.8136	3.4151	3.7759	3.3736	3.6726	3.3049
Atp6v1g1	_[Acetyl (Protein N-term)]ASQSQGIQQLLQAEK_.2	#N/A	6.0980	5.9708	6.1865	5.9232	5.9646	5.9631	5.4509	6.1341	6.2867	6.6523	5.9768	6.2295	5.6739	6.2184	6.9313	6.4465	6.2736	5.5048	6.0667	6.2498
Atp6v1g1	_[Acetyl (Protein N-term)]ASQSQGIQQLLQAEKR_.3	#N/A	4.2441	4.5365	4.2523	3.4453	5.2726	7.0374	6.6356	4.7127	4.7587	4.4504	4.9469	5.0305	5.6061	5.3368	5.1443	5.5990	3.7473	4.4798	4.6193	3.3475
Rpl14	_AAILKAS[Phospho (STY)]PK_.2	Q9CR57_S139_M1_Rpl14	12.1861	12.3735	11.5214	11.7777	11.6051	11.9799	12.1927	12.3143	12.1286	11.7184	11.9201	12.0223	12.9079	12.1973	11.6790	11.8659	12.4402	12.6203	12.0304	11.9811
Rpl14	_LVAIVDVIDQNR_.2	#N/A	6.1758	6.5215	6.7700	6.6005	5.5632	6.0248	6.9342	6.5406	6.9048	6.3582	6.1537	5.9384	6.8819	6.4863	5.8161	5.9881	6.3626	6.2642	5.7562	5.8671
Rpl14	_AAIAAAAAAAAAK_.2	#N/A	4.5434	3.4594	3.5789	3.1460	3.8811	4.1052	3.6600	4.2438	3.7190	3.2489	1.4009	3.3489	3.8271	3.5659	3.2372	3.3875	3.2806	3.0517	1.6685	3.5329
Ndufb7	_DYC[Carbamidomethyl (C)]AHYLIR_.2	#N/A	7.0521	7.5738	7.3364	7.2246	7.2831	7.5441	7.6710	7.2766	7.2358	7.8093	7.4995	7.2349	7.7245	8.0978	7.6793	7.7640	7.5646	7.6776	7.1291	7.2445
Ndufb7	_DSFPNFLAC[Carbamidomethyl (C)]K_.2	#N/A	5.7955	5.8659	6.0770	6.0690	5.9390	5.9540	6.2813	4.6948	5.8266	5.5310	6.4330	6.0888	6.3110	6.4397	6.2346	5.6126	5.7563	6.2324	5.9055	6.3066
Ndufb7	_VAQGQGEGEVGPEVAL_.2	#N/A	6.3453	6.1334	6.0926	6.4874	3.3086	6.2925	5.9991	6.3043	6.4385	6.3102	3.1651	6.4947	6.0578	6.5765	6.2987	6.5599	4.2089	6.5092	5.8843	6.3955
Ndufb7	_HEQHDWDYC[Carbamidomethyl (C)]EHLDYVK_.4	#N/A	3.9305	3.8667	4.4898	5.0651	3.6593	3.6927	3.3888	3.1541	3.3090	4.3658	4.6455	4.0681	3.4744	3.9771	3.4141	4.3153	3.6918	4.1414	3.7900	5.0801
Slc25a11	_[Acetyl (Protein N-term)]AATASPGAGR_.2	#N/A	8.1729	7.7563	7.8472	7.9171	7.8551	7.7641	7.7239	8.1989	7.8298	6.9027	7.5064	8.0990	7.7999	7.6702	7.2507	7.6168	7.8303	7.9844	7.3259	7.6567
Slc25a11	_NVFNALVR_.2	#N/A	7.8939	7.6083	7.4935	7.7335	7.8517	7.8354	8.3400	7.7000	7.4152	7.7338	7.7118	7.6676	8.0342	7.0493	6.9575	7.3824	7.6138	8.0303	7.9137	7.9396
Slc25a11	_TSFHALTSILK_.2	#N/A	6.3224	6.0120	6.4624	6.0680	5.8090	6.5228	6.5504	6.7813	5.7913	5.4584	6.3203	6.1760	6.6832	5.6931	5.1945	5.6312	5.8249	6.0459	5.8529	5.9423
Slc25a11	_LTGADGTPPGFLLK_.2	#N/A	7.4482	7.1237	6.7641	7.0512	6.9731	7.1933	7.5222	7.2689	6.9665	3.8348	6.5631	7.1524	3.2412	5.6459	6.4541	6.2855	7.1300	7.2784	6.3552	6.2834
Slc25a11	_AVVVNAAQLASYSQSK_.2	#N/A	5.1618	4.9090	5.2203	5.7254	5.6704	5.3639	3.4908	4.4130	5.0329	5.5708	5.9363	3.1797	3.6579	3.2017	4.5367	5.5101	5.2741	4.4795	5.5726	5.2399
Slc25a11	_M[Oxidation (M)]IDGKPEYK_.2	#N/A	3.6472	4.0216	3.7164	3.8995	4.1324	4.7133	4.8032	4.8684	3.8365	4.4426	4.1274	4.4033	3.3030	2.3207	4.4729	4.5448	4.0279	4.7243	4.3133	4.5428
Slc25a11	_MQLSGEGAK_.2	#N/A	3.8342	5.8491	6.5229	6.9995	4.7489	5.4031	6.2373	4.0599	4.9422	4.5346	5.9924	4.1026	4.5934	6.6117	5.0605	3.1963	4.7740	5.0463	4.7190	4.3263
Slc25a11	_LGIYTVLFER_.2	#N/A	6.1142	5.4976	4.8562	4.2899	2.8810	4.5384	5.4449	5.4847	5.2123	3.9855	4.5492	5.2225	4.2057	3.7495	4.1925	3.5370	5.3061	5.7651	3.5507	4.5538
Slc25a11	_ALIGM[Oxidation (M)]TAGATGAFVGTPAEVALIR_.2	#N/A	4.3705	4.0715	3.8621	3.9424	3.5502	4.0087	4.0519	4.3495	3.1922	4.0676	3.6216	4.1760	4.7041	3.1304	3.8169	3.7866	4.4984	4.5559	3.3532	3.5108
Slc25a11	_QATYTTTR_.2	#N/A	4.7613	3.8214	3.2169	3.1501	3.5191	4.4094	3.9147	3.8818	3.5335	4.0361	3.5153	3.7108	4.2021	3.2039	3.5991	3.0255	4.4195	4.2628	3.0623	2.9958
Slc25a11	_FLFGGLAGM[Oxidation (M)]GATVFVQPLDLVK_.2	#N/A	4.2295	3.7733	1.1689	3.4599	3.5672	1.5105	1.1616	0.9317	4.0848	4.4358	3.5634	3.6627	4.2502	3.2520	3.5510	3.0736	2.2525	3.3656	3.0142	3.2190
Uqcrfs1	_SGPFAPVLSATSR_.2	#N/A	9.2512	9.5219	9.2765	9.4575	9.3090	9.2112	9.3767	9.3050	9.1521	9.2797	9.1546	9.3178	9.5526	9.4538	9.7004	9.4765	9.3388	9.3956	9.3168	9.3495
Uqcrfs1	_LSDIPEGK_.2	#N/A	7.0625	7.4073	7.7731	7.4456	7.4549	7.9612	8.0959	7.3115	7.6404	8.2033	7.8992	7.9855	8.1162	7.3818	8.3049	8.0441	6.6683	8.1727	8.1407	7.9739
Uqcrfs1	_GVAGALRPLLQGAVPAASEPPVLDVK_.3	#N/A	7.0264	7.3403	6.6610	6.9521	7.2261	7.3018	7.2724	6.9375	7.2183	7.3760	7.1643	7.3320	7.3708	7.3223	7.4547	7.4619	7.6620	7.2713	7.3418	7.2513
Uqcrfs1	_RAEVLDSTK_.2	#N/A	6.9004	6.8956	7.4232	6.6606	6.9558	6.8011	7.2773	7.3409	7.5345	6.9257	7.0164	7.3052	6.9369	7.0138	7.2855	7.2510	6.7501	7.2246	6.7537	7.0217
Uqcrfs1	_VPDFSDYR_.2	#N/A	6.4340	6.4361	6.7370	5.9682	6.3516	6.4433	6.8458	6.5163	6.4370	6.5825	7.0092	6.5021	6.4624	6.6745	6.7501	6.2364	6.6653	6.4281	6.8053	6.2837
Uqcrfs1	_DPQHDLDR_.2	#N/A	5.9555	6.2313	6.8782	6.2806	5.9896	6.5995	6.1375	6.2467	6.7806	6.3635	6.6982	6.0573	5.7988	6.0347	7.0424	6.0871	6.1913	6.1331	6.2012	6.1110
Uqcrfs1	_PLVATVGLNVPASVR_.2	#N/A	5.5372	4.7216	5.9388	4.8755	5.4856	5.1526	5.8003	4.7382	5.3614	5.4162	5.9692	3.7197	5.7590	4.6604	5.5231	5.4394	5.3804	5.0569	5.4912	5.4701
Uqcrfs1	_EIDQEAAVEVSQLRDPQHDLDR_.3	#N/A	6.6324	6.5277	6.5399	6.5424	6.2847	6.3437	6.5289	6.4751	6.7930	6.7534	6.6623	6.4685	7.2159	6.7858	6.2915	6.6960	6.5443	6.3937	6.7715	6.6965
Uqcrfs1	_NVVSQFVSSM[Oxidation (M)]SASADVLAM[Oxidation (M)]SK_.2	#N/A	6.1558	5.6669	6.5223	5.7139	5.3644	5.5349	4.9262	6.4052	5.4392	6.1448	5.2768	5.6943	6.4948	5.8439	5.8038	6.1693	5.7190	5.6776	6.6357	6.1066
Uqcrfs1	_GPAPLNLEVPAYEFTSDDVVVVG_.2	#N/A	4.0248	3.8424	4.2563	4.1386	3.4258	3.8240	3.6223	3.9517	3.8369	4.5882	4.1951	2.9534	3.7079	5.0403	4.3060	4.0818	4.4426	3.9052	4.1844	4.4292
Uqcrfs1	_EIDQEAAVEVSQLR_.3	#N/A	3.5375	4.4653	4.0738	4.1518	2.8753	3.6088	3.4726	3.2379	3.3928	3.1791	4.3723	3.5225	3.5583	4.0609	3.4980	3.8824	3.7756	4.0576	3.7061	4.0279
Carhsp1	_GNVVPS[Phospho (STY)]PLPTR_.2	Q9CR86_S42_M1_Carhsp1	12.2489	11.9820	12.0568	12.0053	11.5991	11.5679	11.3468	4.0646	3.8982	12.2050	11.8068	11.6047	11.8038	12.4317	10.9062	11.6921	3.1014	11.2418	11.4150	11.6729
Carhsp1	_TFS[Phospho (STY)]ATVR_.2	Q9CR86_S53_M1_Carhsp1	3.1638	7.8861	4.2012	8.6504	7.0134	7.8698	8.3485	3.3895	8.2210	9.3684	8.4737	7.3997	8.7268	4.2393	7.9019	4.0267	4.3875	8.3784	3.0610	8.7748
Carhsp1	_DRS[Phospho (STY)]PSPLR_.2	#N/A	10.9825	11.1416	11.1275	11.3037	10.6686	10.8138	10.6330	10.7445	10.6910	11.0860	10.6507	10.8636	11.1348	11.3689	10.6635	10.9475	10.9674	10.6011	10.6999	10.9010
Carhsp1	_TRDRS[Phospho (STY)]PSPLR_.2	#N/A	5.1977	5.5437	5.1933	5.1038	3.5844	5.3506	4.5455	4.7406	4.4186	5.2184	4.4396	5.0936	3.6899	5.7748	4.3386	5.2143	5.2870	3.6984	4.5208	5.4071
Carhsp1	_DRS[Phospho (STY)]PS[Phospho (STY)]PLRGNVVPS[Phospho (STY)]PLPTR_.3	Q9CR86_S31_M3_Carhsp1	3.7335	8.6885	4.4961	3.9432	3.4914	7.6352	8.4379	3.6722	4.7539	3.7908	3.8382	3.7682	4.0105	9.7140	6.3988	4.3132	3.7825	8.1215	5.1234	3.9357
Carhsp1	_HETWSGHVISN_.2	#N/A	3.0933	3.4086	3.6296	3.0952	3.9319	5.1757	3.6092	4.2945	3.6683	3.2996	4.0427	3.2981	3.7763	3.6167	3.1864	6.3054	3.3314	3.0009	4.1504	3.5837
Necap1	_ASGTGGLSLLPPPPGGK_.2	#N/A	8.5942	8.4349	9.0145	8.4223	8.9978	8.7440	8.2608	8.6815	8.8767	8.9783	8.6111	8.7979	8.9157	9.0429	9.0052	9.0580	8.6701	8.6829	8.6065	8.7001
Necap1	_LDQPDWTGR_.2	#N/A	7.7753	7.6511	8.3998	8.0045	8.0539	7.7906	7.8773	8.3788	8.0636	7.7835	7.9841	7.9459	7.6012	8.2185	8.4181	7.9304	7.7059	7.8960	7.8365	7.8949
Necap1	_LSIGNITAK_.2	#N/A	7.2971	7.5334	7.4640	7.6573	7.5152	7.4932	7.7402	7.6667	7.3422	7.6981	7.4892	7.6416	8.0357	7.7309	7.6437	7.7330	7.2764	7.7051	7.5321	7.4582
Necap1	_VSGELFAQAPVEQYPGIAVETVTDSSR_.3	#N/A	5.2149	5.1624	5.8370	5.1998	4.5414	5.2677	5.5289	5.4260	5.1635	5.4279	4.4163	4.4686	5.8940	5.0883	5.8253	5.0127	4.3537	4.5690	5.4507	5.0640
Necap1	_SAFIGIGFTDR_.2	#N/A	5.3157	5.1708	6.4116	5.5582	5.3815	5.1650	6.0867	6.0755	2.4045	6.1128	6.4371	4.9722	3.5753	5.8800	5.9816	5.9842	5.9189	5.4561	3.9494	6.1176
Necap1	_ASDWKLDQPDWTGR_.3	#N/A	4.8356	5.5560	4.1974	5.5161	5.2060	4.4843	5.1093	4.3387	5.4928	4.7839	4.8414	5.0002	5.8852	4.5293	5.4696	3.0469	4.4409	4.7607	5.4644	5.5595
Necap1	_ESQEMDNRPK_.2	#N/A	3.6666	4.5550	4.2029	4.0228	3.0043	3.3651	3.9687	3.3669	3.0950	3.8729	4.0583	4.2257	3.6873	5.2951	4.1140	4.0115	3.9046	3.9285	3.5771	4.1569
Fam136a	_C[Carbamidomethyl (C)]HAPLAQAQALVTSELER_.3	#N/A	3.7774	4.9799	3.3140	3.8068	3.9844	4.1240	4.0783	4.0031	3.7365	4.2494	4.0746	4.7928	3.8083	4.2982	5.1934	4.4768	4.5678	3.6491	5.1568	4.4790
Golph3	_AAGGGGGS[Phospho (STY)]GEDEAQSR_.2	Q9CRA5_S35_M1_Golph3	6.0768	7.3343	6.3449	6.4996	5.8632	6.5989	5.5052	6.7185	6.8751	6.3326	6.1664	7.2615	7.3028	7.2335	5.0259	4.6169	6.9706	6.1145	5.5661	6.9119
Exosc5	_ADANLLTDTGTESS[Phospho (STY)]PR_.2	Q9CRA8_S20_M1_Exosc5	3.9806	4.0222	4.5169	3.2252	3.3183	3.5424	4.2228	3.0839	4.2818	4.1869	3.1554	3.9117	4.3899	3.9337	3.8000	4.3255	4.0819	6.1590	3.3666	6.4644
Snx24	_AESC[Carbamidomethyl (C)]GS[Phospho (STY)]FDETESEESSK_.2	Q9CRB0_S116_M1_Snx24	4.0743	5.4684	3.7888	5.8182	5.5382	4.9150	4.9324	5.4902	5.0043	5.7070	5.0806	3.9051	4.6534	5.7874	4.6590	5.2124	5.1098	5.0813	5.2538	5.0803
Tppp3	_ALEELATKR_.2	#N/A	5.7750	6.1745	5.9379	6.5626	5.9420	5.8541	5.9880	5.9436	5.6049	6.0427	6.1027	6.6818	6.1681	5.4070	6.8533	6.6464	6.3675	5.8768	6.0755	5.7947
Tppp3	_[Acetyl (Protein N-term)]AASTDIAGLEESFRK_.2	#N/A	5.7237	6.0867	5.9113	6.5192	6.1716	5.9871	5.2677	5.8701	6.2876	5.6968	5.2743	6.0436	6.3347	6.3792	6.5104	6.4176	5.9821	5.9469	6.0543	6.3850
Tppp3	_AVTGTDVDIVFSK_.2	#N/A	4.4633	4.3566	3.9999	4.7166	3.1694	3.3924	3.6586	3.1882	4.5000	3.7868	4.3804	4.3213	4.2867	5.1021	3.9040	3.2809	4.1862	2.9933	3.2623	3.7766
Chchd3	_LSENVIDR_.2	#N/A	5.8911	5.5361	6.1134	5.9752	6.3905	5.5075	6.1632	5.9879	5.8228	6.0449	5.9989	6.2065	6.7404	5.9994	6.4599	5.8406	6.0397	6.0952	5.8466	5.7517
Chchd3	_YSSVYGASVSDEDLK_.2	#N/A	5.1760	6.6160	6.7482	4.5598	4.0807	6.6246	6.7740	5.2309	6.4321	5.2855	4.6943	5.8186	5.4648	7.3754	6.5281	6.4004	4.5485	6.8250	5.1454	5.7780
Chchd3	_YEYHPVC[Carbamidomethyl (C)]ADLQTK_.3	#N/A	3.5877	3.7862	5.6153	3.8214	3.6570	5.3106	3.6506	3.8654	3.6225	5.0662	5.4354	5.3620	3.2544	5.6902	5.6077	4.2143	3.1133	5.3497	4.3519	3.9362
Ociad1	_FDSNVSGQSSFGT[Phospho (STY)]SPAADNIEK_.2	Q9CRD0_T146_M1_Ociad1	3.4803	4.2194	4.0046	4.2229	3.2172	3.3172	4.4483	3.3095	3.0066	3.9617	3.3810	4.0299	4.0414	4.1593	4.0255	4.0125	4.3075	4.3431	3.1413	3.8365
Ociad1	_RSS[Phospho (STY)]PPGHYTQKPK_.3	Q9CRD0_S123_M1_Ociad1	3.5950	3.7626	4.1313	4.1406	3.7812	4.5533	3.5901	4.1048	3.5724	4.0171	3.5313	3.6520	3.6157	4.1184	3.4373	3.8047	5.0875	4.0464	3.6949	4.0854
Dbndd2	_TSS[Phospho (STY)]LSSDSSNLR_.2	Q9CRD4_S117_M1_Dbndd2	8.5182	8.6232	8.8431	8.5599	8.1302	8.2102	7.6371	8.5636	3.8664	9.0747	7.9288	8.3047	3.9868	8.7691	8.6118	6.7694	8.7686	8.5298	8.6184	8.4687
Filip1	_ENGPS[Phospho (STY)]APQEK_.2	#N/A	9.1079	8.9238	9.4551	9.2483	8.9914	9.1021	8.8896	9.0734	8.8834	9.2292	9.0632	8.9798	8.6534	9.5499	8.3867	9.0112	9.1685	8.8254	9.0115	9.1717
Pard3b	_ASS[Phospho (STY)]PEGEEPASPQQSK_.2	Q9CSB4_S338_M1_Pard3b	6.6718	3.5316	3.3241	3.7967	6.3599	6.4878	3.0537	5.9981	2.9739	6.3582	3.9534	3.2577	3.1393	3.3621	3.0790	3.1496	3.5104	3.6390	3.9381	3.6090
Snw1	_GPPS[Phospho (STY)]PPAPVM[Oxidation (M)]HS[Phospho (STY)]PSR_.2	#N/A	9.3120	9.5705	9.4546	9.2938	9.0621	9.2345	8.6319	9.4781	9.0720	9.3073	9.1139	9.2619	3.7367	10.1118	9.2167	9.3212	9.5779	8.9791	9.2089	9.2975
Snw1	_GPPS[Phospho (STY)]PPAPVM[Oxidation (M)]HSPS[Phospho (STY)]RK_.3	#N/A	8.2937	4.5039	3.8209	6.0469	4.5560	3.8567	6.6946	3.8965	4.0014	5.7666	6.9754	4.8617	4.1458	8.1310	5.7526	3.6384	3.6800	8.1275	3.7991	3.5507
Ranbp3	_TSS[Phospho (STY)]LTHSEEK_.2	#N/A	8.9748	9.3541	8.9474	8.7403	8.4974	3.0291	8.6770	8.7495	8.6382	8.9015	8.5606	8.8608	9.2959	9.2089	8.7158	8.9700	8.7970	8.6216	8.8247	8.7535
Ranbp3	_S[Phospho (STY)]PSESAEETHTLEEKVPQK_.3	#N/A	4.0281	6.1344	5.7643	3.5935	3.5569	4.9925	3.6731	4.4009	3.6923	3.8387	3.5744	3.8771	3.8182	3.7371	5.6265	3.3902	3.6970	3.9628	3.8330	6.0221
Ranbp3	_VLS[Phospho (STY)]PPKLNEANSDTSR_.3	Q9CT10_S257_M1_Ranbp3	4.4183	7.5787	7.3960	7.4104	7.3201	7.6581	7.0727	7.7819	7.2857	7.6648	8.1066	7.2572	3.9530	7.8140	7.2090	7.4544	7.7931	6.9579	7.9291	6.8989
Ranbp3	_S[Phospho (STY)]PSESAEETHTLEEK_.2	#N/A	6.3635	4.3116	7.0166	4.6096	6.1803	6.2589	4.7035	3.2013	5.8775	3.7507	4.8286	5.0362	3.9046	6.0756	5.6809	5.2653	5.4822	5.3086	6.2570	5.1427
Ranbp3	_ERTSS[Phospho (STY)]LTHSEEK_.2	#N/A	4.4569	4.3631	3.9935	3.1274	5.7875	3.1963	6.0638	3.6504	5.3265	4.2675	3.2840	4.0892	6.6870	4.4734	5.2714	3.8190	6.0479	5.7948	5.9469	4.4404
Ranbp3	_SAGSSSPEAGEDS[Phospho (STY)]DHEDGNYC[Carbamidomethyl (C)]PPVKR_.4	Q9CT10_S40_M1_Ranbp3	3.8342	4.1686	4.3705	3.8552	3.1719	3.9586	4.0949	3.5346	4.1004	4.2273	3.2313	4.0581	4.2302	4.2203	3.6809	4.1791	4.0723	4.1342	3.3232	4.0634
Arpc2	_ASHTAPQVLFSHR_.3	#N/A	7.4022	6.1809	7.2449	7.2265	7.3346	7.3183	6.6755	7.1271	6.6173	7.2880	7.2002	7.3247	7.3900	6.8137	7.4081	7.2041	7.3088	6.8990	7.6671	7.0889
Arpc2	_NC[Carbamidomethyl (C)]FASVFEK_.2	#N/A	5.9852	6.6030	6.4934	6.7319	6.3403	6.3707	6.7397	6.6928	6.1725	6.8927	6.5240	6.5124	5.5831	6.1128	6.3115	6.0327	5.8484	6.2177	6.1045	6.0806
Arpc2	_DYLHYHIK_.2	#N/A	6.5656	6.8683	6.4746	6.1270	6.6141	6.2519	6.3182	7.0423	6.4894	6.4042	6.3763	6.1036	7.0123	6.1088	5.7238	6.6677	5.9995	6.2791	6.4893	5.8465
Arpc2	_M[Oxidation (M)]ILLEVNNR_.2	#N/A	7.3288	7.8785	7.9854	7.8885	7.3355	7.6461	7.4296	8.0105	7.4900	7.4188	7.2896	7.4824	7.7917	8.1799	7.3732	7.6169	7.9348	7.7916	7.6307	7.5968
Arpc2	_IIEETLALK_.2	#N/A	4.1829	5.5221	5.6160	6.2993	3.8857	4.4643	5.6000	3.8833	5.1556	4.3790	3.2391	3.7093	4.2036	5.8036	5.0817	4.5422	4.4210	5.3651	5.7657	4.6223
Arpc2	_ELQAHGADELLKR_.3	#N/A	4.8929	5.3804	5.7855	3.8948	5.0330	5.3671	5.2691	5.0609	5.1878	4.8665	4.7921	5.0608	5.5663	5.1484	4.5433	5.0142	4.8083	5.2416	5.1429	5.1805
Arpc2	_VFM[Oxidation (M)]QEFK_.2	#N/A	4.1559	5.0237	4.0487	4.5921	4.7796	5.1302	5.3320	4.1073	3.4368	4.8613	3.9981	5.0240	5.1537	4.8209	4.5028	4.5837	3.4032	4.9656	5.1079	4.6675
Arpc2	_DNTINLIHTFR_.2	#N/A	7.0469	7.1113	6.9209	6.9949	6.9749	6.6957	6.9803	7.0963	7.1273	7.0509	6.6662	7.0205	7.2437	7.2381	6.7147	6.9720	6.9829	6.8969	7.3529	7.0569
Arpc2	_YFQFQEEGKEGENR_.2	#N/A	3.5202	4.4868	4.0565	4.1734	4.1031	3.3016	3.5620	3.2206	3.3883	3.4069	3.3636	3.7749	5.5219	4.3081	3.6632	3.8651	3.7583	4.0131	4.0265	4.1197
Smc3	_SLQSLEASLHAM[Oxidation (M)]ESTRES[Phospho (STY)]LK_.3	Q9CW03_S776_M1_Smc3	3.8938	8.9406	7.0274	3.7956	3.2315	3.4556	8.5496	7.3176	7.4750	7.4539	3.2422	8.1061	4.4767	4.4172	6.6661	7.5816	7.1621	8.6553	7.9146	3.6977
Smc3	_GDVEGSQS[Phospho (STY)]QDEGEGSGESER_.2	#N/A	3.4408	4.5620	3.9771	4.2486	4.2794	3.0026	3.2617	2.8589	3.3208	3.6471	3.6952	2.9506	3.4288	4.4735	2.8389	3.7857	3.1209	4.0286	4.3276	4.1507
Smc3	_KGDVEGSQS[Phospho (STY)]QDEGEGSGESER_.3	#N/A	3.7372	4.2175	4.1390	4.4826	3.0749	3.2990	4.4662	3.3273	3.0244	3.9435	3.3988	3.9436	3.1325	4.1771	4.0434	4.0821	4.3253	4.3249	3.1232	3.8544
Ppp1r3g	_SFS[Phospho (STY)]LPADPILQAAK_.2	Q9CW07_S81_M1_Ppp1r3g	3.2395	9.9627	8.9759	8.7958	7.8878	8.5975	9.6267	10.2150	8.3921	9.4930	7.7611	9.3060	9.6780	10.5150	8.4040	8.8488	8.8055	4.1871	8.8362	8.3523
Raver1	_LLS[Phospho (STY)]PIASNR_.2	Q9CW46_S576_M1_Raver1	5.8713	6.4043	5.0740	5.9102	5.0708	5.6503	6.1694	6.7157	5.3948	3.7830	2.9105	5.7861	5.1572	7.4734	4.5695	5.4185	6.1653	5.9960	5.2980	5.2585
Golga1	_ESVASM[Oxidation (M)]GADS[Phospho (STY)]GDDFASDGSSSR_.2	#N/A	4.0587	4.3990	4.4884	4.5409	3.9482	4.1602	3.9827	4.3669	3.9796	3.4090	4.0289	3.3073	4.1130	3.4167	3.1536	3.2171	3.4305	3.5884	4.3387	3.2269
Golga1	_GSIRPS[Phospho (STY)]IS[Phospho (STY)]NPR_.2	Q9CW79_S749_M2_Golga1	3.3685	11.5215	3.9048	4.3209	4.2070	3.7779	3.3340	3.0689	3.2238	3.3482	4.2032	3.0229	3.3892	3.8919	2.9112	3.7134	3.6065	4.2266	3.8752	3.8589
Mtfr1l	_NAS[Phospho (STY)]VPNLR_.2	Q9CWE0_S100_M1_Mtfr1l	9.1804	9.9850	10.0516	10.4803	9.7289	10.0787	9.3403	9.3532	9.8936	10.1803	9.4802	8.4646	9.7438	9.5290	8.7281	9.4936	9.2893	9.1574	9.7471	9.3299
Mtfr1l	_AS[Phospho (STY)]FETLPNISDLC[Carbamidomethyl (C)]LK_.2	Q9CWE0_S38_M1_Mtfr1l	6.1090	7.5587	6.4006	5.7258	4.8787	3.4533	6.9689	7.1810	5.9566	6.6331	3.0270	6.8686	6.6681	7.9265	6.1050	5.8768	6.4577	7.0746	6.5204	5.7975
Mtfr1l	C[Carbamidomethyl (C)]SS[Phospho (STY)]S[Phospho (STY)]EEDDC[Carbamidomethyl (C)]ISLS	Q9CWE0_S222_M2_Mtfr1l	4.3891	5.6580	5.1412	5.7058	3.8599	3.9581	3.9367	4.0689	3.8980	5.9257	3.7138	3.5186	4.0461	6.0073	5.4766	3.9801	5.1304	5.2120	3.7969	5.8096
Mtfr1l	_ASS[Phospho (STY)]FADM[Oxidation (M)]M[Oxidation (M)]GILK_.2	#N/A	8.9969	9.3508	7.2473	6.8586	3.3929	7.4475	8.4643	3.6632	7.4737	8.4221	6.6809	9.0786	4.1837	10.0351	7.3607	3.7762	3.8522	8.8889	3.7465	3.7075
Mtfr1l	_TTELQDELSHLR_.3	#N/A	4.0994	3.6953	3.3430	3.3819	4.3063	4.3397	4.2426	4.0079	4.1628	3.9100	3.6415	3.5847	4.3283	3.3301	4.2680	3.4615	4.5456	3.2876	4.4370	3.2971
Tubb2b	_INVYYNEATGNK_.2	#N/A	9.9705	10.8088	11.1151	10.8381	10.1950	10.9454	10.5924	10.4159	10.6811	10.6493	9.2233	8.8902	9.3199	9.5807	9.2913	9.1547	9.1205	9.0461	9.1330	9.3481
Cd2bp2	_GTGRPNS[Phospho (STY)]PQRLDR_.3	#N/A	7.5761	8.2673	7.6557	7.8365	7.5653	7.8636	7.8051	3.0595	8.0013	8.3581	7.9124	7.6331	3.5105	8.3923	8.3672	8.0403	7.8057	7.8335	7.8461	4.2324
Cd2bp2	_GTGRPNS[Phospho (STY)]PQR_.2	#N/A	6.7598	3.9112	7.2335	7.5233	6.7854	7.1728	6.3473	6.9013	6.6183	7.1866	6.7708	6.9756	5.9706	4.4834	6.6794	4.2708	7.1425	6.9564	6.7821	4.1606
Cd2bp2	_GKHSLDS[Phospho (STY)]DEEDDDEEGSSK_.2	Q9CWK3_S49_M1_Cd2bp2	3.1952	2.2949	4.2326	4.3890	3.4022	3.4355	3.6459	3.4209	3.5661	4.5067	3.0448	3.8500	3.7316	4.2707	3.6713	4.0582	4.4190	4.2313	3.0295	3.9480
Snx2	_SIS[Phospho (STY)]APVIFDR_.2	Q9CWK8_S119_M1_Snx2	3.7078	7.8556	7.0643	3.8764	3.9148	3.9481	6.8065	3.9335	3.0535	3.5184	6.7876	3.3374	7.4450	3.7216	3.1587	6.9103	7.1348	7.1025	4.0454	6.5719
Snx2	_ELILSSEPS[Phospho (STY)]PAVT[Phospho (STY)]PVTPTTLIAPR_.3	Q9CWK8_S97_M2_Snx2	3.6712	4.3316	4.2075	4.0182	3.0090	3.4751	3.6063	3.3716	3.5265	4.5463	3.0052	4.2210	3.6920	4.1946	3.6317	4.0161	3.9093	3.9239	3.5725	4.1616
Ctnnbl1	_EYAENIGDGRS[Phospho (STY)]PEFR_.3	Q9CWL8_S545_M1_Ctnnbl1	3.5591	3.7600	4.5965	4.0251	3.7661	4.8354	3.4229	3.7848	5.6837	3.4860	5.1306	3.4861	3.5900	3.1338	3.0001	4.4221	3.2820	5.4923	5.0211	4.6348
Pfdn1	_LADIQIEQLNR_.2	#N/A	6.0498	6.5366	6.8881	6.4579	6.9024	6.6512	6.5079	6.7413	6.5531	6.6910	6.7938	6.3751	6.4963	7.1287	7.0019	7.1630	6.6040	6.7308	6.5587	6.7410
Ddah1	_S[Phospho (STY)]QGEEVDFAR_.2	Q9CWS0_S33_M1_Ddah1	8.7018	8.8672	9.0199	8.8036	8.6316	8.5746	8.4929	8.6585	8.7330	8.9650	8.6923	8.6536	9.2845	9.1266	8.7169	9.0123	8.7720	8.4632	8.7945	8.8761

Ddah1	_[Acetyl (Protein N-term)]AGLGHPSAFGR_.2	#N/A	9.3078	9.1797	9.2842	9.2421	9.2428	9.0423	9.2293	9.3565	8.9019	9.0224	9.0290	9.2659	9.3210	9.1155	9.5415	8.8436	8.9856	9.2459	9.0449	9.0559
Ddah1	_GAEILADTFKDYAVSTVPVADSLHLK_.3	#N/A	6.0349	6.4333	6.2225	6.1260	5.7183	6.0600	5.6030	6.4980	6.1393	6.5319	5.8104	6.4487	7.3235	6.9625	5.6876	6.2743	6.2860	6.0117	6.2550	6.7446
Ddah1	_TPEEYPESAK_.2	#N/A	5.5976	6.4407	5.3648	5.9697	4.8639	5.6996	5.9067	6.0549	5.5523	6.4512	5.7731	5.3872	5.9303	5.2940	6.8656	5.1597	5.4076	5.6706	6.3664	5.2103
Ddah1	_GAEILADTFK_.2	#N/A	3.5557	5.8291	6.3884	5.7264	6.0313	5.9495	5.5261	4.4963	5.8173	3.7620	6.4053	5.6394	6.1551	5.8955	5.7859	6.1004	2.8838	6.0831	6.3069	6.1247
Ddah1	_EFFVGLSK_.2	#N/A	4.2013	5.1008	3.2368	5.5685	5.4636	5.5235	6.4408	4.0556	5.8907	5.1054	5.6437	6.1025	5.3411	6.2849	5.6938	5.5097	5.1968	6.1200	5.6967	6.1306
Ddah1	_LQLNIVEM[Oxidation (M)]KDENATLDGGDVLFTGR_.3	#N/A	7.1856	11.0605	5.4985	5.7744	5.9641	6.4212	6.1579	3.7183	5.6712	6.5351	5.6775	3.5526	3.5235	6.0256	5.3840	6.4757	5.7848	6.1107	6.6207	6.6635
Ddah1	_SFC[Carbamidomethyl (C)]SM[Oxidation (M)]AGPNLIAIGSSESAQK_.2	#N/A	4.1281	3.1910	3.6647	3.4561	5.7868	5.4218	5.9539	6.1751	4.0509	6.5907	6.0664	5.6134	6.1372	3.7027	6.2123	6.1619	5.8106	5.9323	3.5975	5.9855
Ddah1	_QHELYVGVLGSK_.2	#N/A	4.1847	3.8834	4.9596	3.5069	3.6464	4.4746	4.2903	4.6619	4.7233	4.5417	3.6541	4.1139	4.1147	3.6519	4.1750	3.9055	3.3666	3.5173	4.0235	3.9551
Ddah1	_DYAVSTVPVADSLHLK_.3	#N/A	4.3756	4.4443	3.9122	3.2086	3.0817	3.1150	3.9664	3.1004	3.8866	4.1862	3.3653	4.1705	4.0521	4.5547	3.9918	3.7377	4.0985	3.5637	3.2123	3.0209
	LLPEGEETVES[Phospho (STY)]DDDKDER.3	Q9CWU4_S156_M1_	4.3817	4.0062	3.7137	3.4381	3.4397	3.7553	4.0408	3.7351	4.0999	3.7322	3.8066	3.7323	4.2080	3.7517	6.4556	3.5064	3.9000	3.2804	3.5795	3.4290
Ddx47	_[Acetyl (Protein N-term)]AADEEPDSPS[Phospho (STY)]GALQTAAEEEETK_.3	Q9CWX9_S11_M1_Ddx47	3.2295	6.9400	7.3609	4.4599	4.0681	3.9169	6.2824	4.4307	3.0848	4.8365	4.0643	5.1148	3.2502	3.7529	6.3698	3.5744	3.4676	7.1410	6.5333	3.7199
Rpain	_[Acetyl (Protein N-term)]AESSGS[Phospho (STY)]PHR_.2	Q9CWY9_S7_M1_Rpain	3.9638	3.3553	3.5003	5.8884	4.1707	4.2040	4.3782	4.1894	4.2984	6.3156	3.7771	3.0814	4.4639	3.4657	2.9028	3.3259	3.6867	6.1787	4.3014	3.4327
Rbm8a	_GFGS[Phospho (STY)]EEGSR_.2	Q9CWZ3_S42_M1_Rbm8a	7.7397	8.1482	7.8238	8.2640	7.8714	7.6446	7.6949	7.4785	7.6819	3.4433	7.9799	7.6844	8.2845	7.9854	8.0237	3.5819	7.7913	3.8247	8.0216	8.1140
Rbm8a	_M[Oxidation (M)]REDYDS[Phospho (STY)]VEQDGDEPGPQR_.3	#N/A	4.7520	4.5321	4.6828	5.1956	3.9320	4.9142	4.7867	3.5226	4.8909	5.4439	3.2843	5.0149	4.1727	4.9654	5.1088	5.1060	4.9255	3.4692	5.1936	5.2833
Rbm8a	[Acetyl (Protein N-term)]ADVLDLHEAGGEDFAM[Oxidation (M)]DEDGDES[Phospho (STY)]IHK_.	Q9CWZ3_S24_M1_Rbm8a	3.9365	3.3826	3.4730	3.6478	4.1434	4.1767	4.4055	4.1621	4.3257	3.7471	3.8044	3.1087	3.9673	3.5111	2.9301	3.2986	3.6594	3.4901	3.7891	1.0628
Napg	_LIENVDPEK_.2	#N/A	7.4843	7.4341	7.6269	7.4193	7.8391	7.7205	7.9018	7.2018	7.8086	7.9729	7.5383	7.6354	7.9924	7.8031	8.0051	7.8827	6.9328	7.7209	7.9392	8.0632
Napg	_AVQLYQQTANVFENEER_.2	#N/A	6.9911	7.2076	6.8303	6.8353	6.6901	6.5281	6.6891	7.4537	7.2988	7.2176	6.9768	7.3959	7.4238	7.5914	6.5983	7.2539	7.3651	6.8906	6.9784	7.1037
Napg	_INEGLEHLAK_.2	#N/A	6.2419	7.0943	6.8504	6.7149	6.7746	6.3652	6.7557	6.7259	6.7284	6.4091	6.6199	6.7290	6.8614	6.9694	7.2350	6.6400	6.6229	6.9848	6.6738	6.0918
Napg	_FDEAALSIQK_.2	#N/A	5.3784	5.5630	3.7948	5.2429	5.3647	6.0011	5.6947	5.6584	5.3889	5.1653	5.2291	5.5428	5.1045	6.2662	6.0209	5.8779	5.5012	5.5622	5.8578	5.8399
Napg	_QAVELLGK_.2	#N/A	4.7202	5.0942	4.7796	5.1826	4.9616	4.9056	5.0107	3.7850	4.6004	5.1500	4.6753	4.7822	5.0930	4.8764	5.1666	5.0121	4.6184	5.4182	4.2478	5.0886
Napg	_LPEAVQLIEK_.2	#N/A	7.3453	6.8759	7.1222	7.1006	6.5413	7.3598	7.3351	6.9499	6.5832	7.2664	7.4695	7.0626	3.2482	7.3747	7.3285	7.0391	7.0668	7.4626	7.1678	6.9281
Napg	_TIAQVLVHLHR_.3	#N/A	6.2213	6.6720	6.9566	7.3028	7.4467	6.2156	6.3017	6.6896	6.6325	7.2278	5.9101	6.9803	7.0122	6.9585	7.3350	7.1330	6.7950	6.7909	7.2756	7.0728
Napg	_WKPDYDSAASEYGK_.2	#N/A	3.9773	5.4238	3.6288	5.7551	5.6488	3.9968	3.9890	4.2938	6.2051	4.7405	4.0951	5.6744	3.5924	4.3680	5.8317	4.7961	3.3306	4.2712	3.9596	5.6270
Napg	_YMDNDYAK_.2	#N/A	4.5476	3.4552	3.5831	3.1418	3.8853	4.0996	3.6558	4.2480	4.4029	3.2531	3.8815	3.3446	4.5683	4.1946	3.2329	3.3917	3.2848	3.0475	4.1969	3.5371
Napg	S[Phospho (STY)]PAT[Phospho (STY)]PQAKPDGAAGMAAEEEEDEYSGGLC[Carbamidomethyl (C)]	#N/A	4.5106	4.1064	4.0472	3.7930	3.2167	3.2500	3.8314	3.5967	3.7516	4.3212	3.2303	4.0355	3.9171	4.4197	3.8568	3.8727	4.1344	3.6987	3.3473	4.3867
Napg	_KKS[Phospho (STY)]PAT[Phospho (STY)]PQAK_.2	#N/A	3.7058	4.2970	4.2421	3.9836	3.0435	3.2676	2.9967	3.3587	3.0558	3.9121	3.4302	4.1865	3.1639	4.2085	4.0748	4.0507	4.3568	4.2935	3.0917	3.8858
Ist1	_IVADQLC[Carbamidomethyl (C)]AK_.2	#N/A	3.7333	3.5858	4.7103	4.5166	3.9403	4.6439	4.5145	5.4195	3.0280	4.1864	4.0075	3.3119	3.1935	3.6961	4.5175	3.0954	3.4108	4.4223	4.6141	3.6631
Ist1	_ELDSGLAESVSTLIWAAPR_.2	#N/A	3.5864	3.7326	5.3904	3.9978	3.7934	5.4471	3.2547	4.3067	3.1749	3.3970	4.1544	5.5828	5.9961	3.1611	3.2801	2.9486	3.3094	4.2755	5.4870	6.0326
Sugt1	_ALEQNPDDAQYYC[Carbamidomethyl (C)]QR_.2	#N/A	4.8282	4.7885	5.3243	4.1575	5.0848	5.6669	5.9714	5.5448	5.5762	5.3811	5.1600	4.9438	3.3378	5.4980	5.3045	5.4060	4.5307	5.0055	4.4199	5.3153
Sugt1	_[Acetyl (Protein N-term)]AAAAAGPASSQR_.2	#N/A	4.1370	3.2007	4.2234	4.3143	2.9066	3.1306	3.3185	4.5974	2.3763	3.4504	4.6538	4.5379	3.3008	4.3454	4.8107	5.2643	4.2676	4.1566	5.0941	5.1228
Sugt1	_LFQQIYSDGSDEVKR_.3	#N/A	4.2901	3.6641	3.5861	3.5347	3.6278	3.8519	3.9133	4.2752	3.9724	4.4964	4.3467	3.6022	4.0804	3.6242	3.9953	4.8874	3.7725	3.3770	3.6760	3.3015
D10Wsu102e	_IEILDS[Phospho (STY)]PASK_.2	Q9CX66_S177_M1_D10Wsu102e	4.4663	5.3373	3.4099	3.7109	3.8040	4.0281	5.0940	4.0990	5.5435	5.7133	4.1705	3.1719	3.9042	4.9624	3.3142	3.3104	4.7894	4.3944	3.8523	4.7003
D10Wsu102e	_SDS[Phospho (STY)]KEEDSPEESSR_.2	Q9CX66_S125_M1_D10Wsu102e	3.3874	3.9317	4.4248	4.1969	3.5943	3.6277	3.4538	3.6131	3.3740	3.1980	4.3535	3.6578	3.5394	3.5139	3.4792	4.2503	3.1103	4.0392	4.3382	4.1401
Rgs19	_LQPLPSC[Carbamidomethyl (C)]EVC[Carbamidomethyl (C)]TPPS[Phospho (STY)]PK_.2	Q9CX84_S80_M1_Rgs19	9.3815	9.3624	8.5604	3.2110	3.0793	8.5290	9.1605	3.0980	3.8890	8.3695	8.5050	9.0928	9.2393	3.9478	7.2323	3.7353	9.4675	9.3788	8.6847	3.0233
Rgs19	_LMHS[Phospho (STY)]PTGR_.2	Q9CX84_S97_M1_Rgs19	4.4010	3.6018	3.4364	6.1302	3.7387	4.2463	4.3360	4.1013	4.2562	3.8166	3.7349	3.4913	4.4217	3.4235	2.8606	3.3681	3.1382	3.1941	4.3436	3.3905
Hnrnpa0	_LFIGGLNVQTSESGLR_.2	#N/A	5.9208	5.2603	5.9230	5.7651	5.3625	5.6435	4.6072	5.6672	5.7551	6.3008	4.7155	5.5997	6.3641	5.2887	5.8108	6.0394	5.6412	5.2859	6.0192	5.7570
Hnrnpa0	_GDVAEGDLIEHFSQFGAVEK_.3	#N/A	3.6233	2.5704	4.9097	3.8892	4.1197	4.5689	3.0346	3.9316	4.6867	4.5797	4.4234	3.1226	4.1371	4.9937	2.2373	4.6888	3.7553	3.5449	4.6900	4.2128
Hnrnpa0	_SGGGGGGGGSWGGR_.2	#N/A	3.8409	4.1619	4.3772	5.4668	4.6454	4.6496	4.3625	3.5413	4.6764	4.0472	3.2951	4.0514	4.5296	4.3643	3.9397	4.1715	4.0790	3.7542	4.2170	4.3313
Bcl7a	_QENSSNSS[Phospho (STY)]PAPETNPPVPSDGTEAK_.3	Q9CXE2_S114_M1_Bcl7a	5.1266	3.6034	3.2523	3.8685	3.9226	4.1857	4.7730	3.9414	3.6385	3.3294	4.0129	3.3295	3.7466	3.2903	3.1509	3.0778	3.4386	3.7108	4.0099	2.9676
Bcl7a	_MKLEASQQNS[Phospho (STY)]EEM[Oxidation (M)]_.2	Q9CXE2_S207_M1_Bcl7a	3.6040	4.3988	4.1403	4.0854	2.9418	3.5423	4.7203	3.3044	3.4593	3.1127	2.9380	4.2882	3.6248	4.1274	4.7203	3.9489	3.8421	3.9911	3.6397	4.1523
Tbc1d15	_DDS[Phospho (STY)]PTQTLASPNAC[Carbamidomethyl (C)]R_.2	Q9CXF4_S655_M1_Tbc1d15	3.9522	7.1524	7.3637	7.1119	3.7842	7.0772	6.8846	6.9259	6.8286	6.6669	7.0745	6.3546	3.8107	7.0956	6.2418	3.7377	4.0280	6.8047	6.6763	3.5580
Rbm33	_SIVNTS[Phospho (STY)]PPC[Carbamidomethyl (C)]R_.2	Q9CXK9_S792_M1_Rbm33	4.0672	3.9356	4.6035	3.6222	5.9124	3.0792	7.7720	6.1831	3.9225	4.1503	8.0406	3.8250	4.0879	4.5906	7.4495	3.7018	4.3053	7.4374	3.1765	3.0567
Rbm33	_ANS[Phospho (STY)]GGGGDGPHVSSK_.2	Q9CXK9_S1032_M1_Rbm33	7.7503	8.5760	8.2659	8.2013	7.9232	7.8348	7.9525	8.4014	7.7849	8.3758	7.7461	8.1144	8.3040	8.8142	7.8288	8.1262	8.4004	8.0667	8.3486	8.0050
Rbm33	_DIKEES[Phospho (STY)]DEEDDDEESGR_.2	Q9CXK9_S243_M1_Rbm33	1.5143	4.4996	4.4524	3.9068	2.3192	3.0144	3.2432	3.9648	2.3029	2.8059	3.5569	4.0015	3.2449	3.1685	3.5350	2.3683	3.0256	3.0972	2.6670	4.2602
Gng10	_[Acetyl (Protein N-term)]SSGASVS[Phospho (STY)]ALQR_.2	Q9CXP8_S8_M1_Gng10	8.7260	8.0871	8.4332	8.4977	7.9670	8.5374	7.9325	8.6552	8.7954	8.4061	7.9095	7.9543	8.5998	8.1890	8.1109	8.1234	9.0213	7.8373	8.7108	8.0724
Cenpv	_LLLDTFEYQGLVK_.2	#N/A	4.1264	4.0133	4.3622	4.5893	4.4265	4.5404	4.1650	4.9894	5.1374	4.1251	4.3150	3.8144	4.4235	4.3923	4.6747	4.7625	4.9161	3.4239	3.3330	4.9314
Cenpv	_SVVTEEFNGS[Phospho (STY)]DWER_.2	Q9CXS4_S234_M1_Cenpv	3.8181	4.1847	4.3544	3.8713	3.1558	3.3799	4.3853	3.5184	4.4444	4.0244	3.3179	4.0742	4.5524	6.1650	4.2228	4.1630	4.0561	4.4863	3.2040	3.7735
Cenpv	_SNPGGFGIAPHC[Carbamidomethyl (C)]LDEGTVR_.2	#N/A	3.5439	4.2999	4.0600	3.9677	3.2623	3.3918	4.0602	3.3559	4.0649	4.2566	3.1993	3.9794	4.1951	4.1908	3.8352	4.1002	4.1151	3.4329	3.3534	4.0356
Cenpv	_QRLS[Phospho (STY)]FEGAAK_.2	Q9CXS4_S105_M1_Cenpv	4.3398	3.6630	3.3752	3.3496	3.6775	4.3074	4.2748	4.0402	4.1950	3.8778	3.6737	3.5525	4.3605	3.3623	2.7994	3.4293	3.0770	3.2553	4.4048	3.3293
Cacybp	_WDYLTQVEK_.2	#N/A	3.1386	4.2545	4.2673	4.8964	4.8586	4.2332	4.9270	4.9296	4.2680	4.5739	4.8009	5.1761	3.1593	5.1593	5.1813	4.8228	4.2892	4.4120	4.9185	5.0051
Cacybp	_[Acetyl (Protein N-term)]ASVLEELQKDLEEVK_.2	#N/A	1.2839	3.3469	3.1658	3.9550	1.5824	4.2722	3.2489	1.9376	2.5280	0.9415	3.9264	3.2192	3.6601	2.3579	1.3541	2.3888	3.5716	2.8033	2.2548	4.3820
Rpl11	_VLEQLTGQTPVFSK_.2	#N/A	6.6287	6.5225	6.4806	6.2940	6.1959	6.1527	6.3347	6.1950	6.5933	6.6280	6.3646	5.9336	6.5911	5.8450	6.2642	6.6490	6.1537	5.6636	6.3432	5.9850
Rpl11	_YDGIILPGK_.2	#N/A	5.3940	6.5758	5.8206	6.7603	5.5150	5.5558	6.7041	6.0475	6.3988	5.5075	5.6028	6.5248	6.5283	5.7855	5.7688	5.4020	6.3182	6.1439	6.2970	5.9010
Rpl11	_[Acetyl (Protein N-term)]AQDQGEKENPM[Oxidation (M)]R_.2	#N/A	4.4037	3.5991	4.0474	3.2857	5.0640	3.9655	3.7996	4.1616	3.8587	3.1092	3.2039	3.4885	3.9668	3.5106	4.2843	3.2478	3.6588	3.4906	3.7897	3.1878
Ilf2	_VLQSALAAIR_.2	#N/A	5.4146	5.2741	4.3888	4.2659	5.4444	4.5886	5.2063	5.4080	4.2880	4.4453	4.2804	5.3310	4.9330	5.2851	4.4642	4.4115	5.3058	5.3941	4.9629	4.9371
Ilf2	_NQDLAPNSAEQASILSLVTK_.2	#N/A	3.3875	3.9316	5.3502	5.4145	4.4099	5.7923	5.4864	5.3513	3.3739	5.9559	4.4407	3.6577	3.4184	4.8917	4.4218	5.3768	5.4547	4.0390	5.3705	4.3880
Ndufs4	_EDAIAFAEK_.2	#N/A	7.1259	6.4449	7.3887	7.0311	7.3947	7.0050	6.7679	6.7253	7.2108	7.2535	7.3711	6.9362	6.7129	7.2092	7.6889	7.6783	6.9124	6.9783	7.0258	7.5131
Ndufs4	_DTQLITVDEK_.2	#N/A	6.6319	6.7308	7.7632	7.1026	6.8260	6.3709	6.7373	6.3911	6.8325	7.0780	6.8420	6.4768	6.7398	7.4147	7.1098	6.9724	7.0176	6.4094	7.2941	6.9744
Ndufs4	_LDITTLTGVPEEHIK_.2	#N/A	5.5972	5.4447	5.6426	5.3815	5.3158	5.2255	5.4265	5.8464	5.5022	5.4674	5.6172	5.8317	5.1499	5.9377	5.6137	5.4802	5.9049	5.7569	5.6561	5.5992
Ndufs4	_NNMQSGVNNTK_.2	#N/A	3.4692	4.8546	5.3262	5.4491	3.8254	5.5462	4.1710	6.1141	5.7288	4.0602	4.6329	5.6929	5.2744	6.0794	4.2353	3.6117	4.1247	5.6637	3.8068	4.4716
Ndufs4	_DTQLITVDEKLDITTLTGVPEEHIK_.3	#N/A	3.2250	5.3871	4.2624	4.0083	5.1899	3.4653	3.6161	6.0512	3.5363	5.3324	4.8982	5.0413	3.7018	7.2126	4.7253	4.0880	5.5941	5.7152	5.1241	4.1714
Ndufs4	_NNMQSGVNNTKK_.2	#N/A	3.4162	4.5866	3.9525	4.2732	4.2548	3.7301	2.8911	3.1166	3.2715	3.3005	2.4598	2.9752	3.4369	4.5545	3.5027	3.7611	3.6543	4.1789	3.8275	3.9066
Tecr	_S[Phospho (STY)]LKDEDVLQK_.2	Q9CY27_S58_M1_Tecr	6.3514	6.2432	6.2530	6.4316	5.4553	5.7413	4.9705	5.0721	4.7606	6.2484	5.6106	5.5292	4.1518	5.4781	4.5764	5.2459	5.5816	6.0719	5.6840	4.6403
Chtop	_GHLDAELDAYM[Oxidation (M)]AQTDPETND_.2	#N/A	5.5592	5.8697	4.0640	4.2732	5.2368	6.1589	5.7969	6.5141	6.0321	5.8551	6.0142	5.5350	6.7758	4.3865	5.7338	5.7574	6.5997	6.2733	5.7603	6.0778
Serbp1	_SAAQAAAQTNSNAAGK_.2	#N/A	4.8152	4.3178	5.2351	5.6407	5.3354	5.6908	5.7134	5.2984	4.3300	4.6391	4.7004	5.4120	4.8711	5.9252	5.4239	5.4015	4.1605	4.3137	4.8635	5.1761
Serbp1	_GGSGSHNWGTVKDELTES[Phospho (STY)]PK_.3	#N/A	4.7271	3.3952	5.7590	3.6603	4.5403	4.1642	5.8333	4.1496	5.9274	5.5297	5.7161	5.1749	4.5037	6.3592	5.2539	3.2860	3.6468	5.4113	5.4460	3.4726
Serbp1	_EFDRHS[Phospho (STY)]GSDR_.2	Q9CY58_S197_M1_Serbp1	3.4527	3.8664	4.4901	4.1316	3.6596	3.6930	3.3885	3.6784	3.3087	3.9406	4.2882	4.0091	3.4741	4.5282	5.0204	4.3156	3.1756	4.1695	4.4547	3.9438
Serbp1	_TDKSSAS[Phospho (STY)]APDVDDPEAFPALA_.2	#N/A	3.9450	3.3741	3.4816	3.6392	4.1519	3.9564	4.3970	4.1707	3.8678	3.1001	4.2422	3.1002	6.7601	3.5196	2.9215	3.3071	3.6679	6.9648	3.7806	3.1969
Serbp1	_SSAS[Phospho (STY)]APDVDDPEAFPALA_.2	#N/A	3.6748	4.0573	6.3000	4.0821	3.3797	3.2568	5.6184	3.5818	3.7613	4.3702	3.2400	3.8616	3.9268	4.4294	3.7163	4.2232	4.3390	7.1273	3.3541	3.7576
Serbp1	_DELTES[Phospho (STY)]PKYIQK_.2	#N/A	3.5091	4.4937	4.0454	4.1803	4.3477	6.3617	5.9400	3.2095	3.2525	3.7154	3.6269	4.3831	3.5298	6.9980	4.2714	3.8540	6.1977	6.1046	3.7346	3.9995
Serbp1	_FDQLFDDES[Phospho (STY)]DPFEVLK_.2	Q9CY58_S25_M1_Serbp1	3.7805	2.7052	5.2617	3.9089	3.1182	5.6258	5.8953	3.4808	5.5134	4.4371	3.1144	4.1118	3.8012	4.3038	5.6841	4.1254	4.0185	4.4974	4.9123	4.2708
Serbp1	_GGSGSHNWGTVKDELTES[Phospho (STY)]PKYIQK_.4	#N/A	3.5961	4.4067	4.1324	4.0933	2.9338	3.1579	5.4889	3.2965	3.1655	4.9290	3.5399	4.2962	3.2736	4.3182	4.1845	3.9410	3.8341	4.1838	2.9820	3.9955
Serbp1	_SKS[Phospho (STY)]EEAHAEDSVM[Oxidation (M)]DHHFR_.4	#N/A	8.9283	9.0404	8.4040	8.7005	8.6775	3.5476	9.1598	8.9319	9.5504	3.2835	9.4532	9.0165	8.1794	10.0191	7.9030	3.9528	8.2276	9.2800	7.7467	8.5795
Serbp1	_S[Phospho (STY)]EEAHAEDSVMDHHFR_.3	#N/A	4.0538	4.4624	3.9920	4.4409	3.8212	4.3175	3.5943	3.1685	3.5722	4.0036	3.5029	3.6116	3.7054	3.8920	3.4548	3.8093	3.9629	3.9530	3.6016	4.1751
Blvra	_SGS[Phospho (STY)]LEEVPNVGVNK_.2	Q9CY64_S236_M1_Blvra	9.6671	9.6053	9.5976	3.7657	9.2729	9.1469	9.2016	9.6852	9.3587	9.5966	9.8646	9.7788	10.3835	10.2054	9.1569	9.7411	10.3571	9.6685	9.8082	9.8290
Blvra	_SQEVDVAYIC[Carbamidomethyl (C)]TESSSHEDYIR_.3	#N/A	7.6509	6.5282	6.4496	7.0121	6.3874	5.9794	6.1514	7.3082	6.1078	7.4779	6.6774	6.9158	8.0714	7.3955	5.3804	6.5112	7.2438	6.1848	6.4787	5.7559
Blvra	_QISLEDALR_.2	#N/A	3.8843	4.8453	3.4715	4.8922	4.1418	3.9375	4.8472	4.5587	4.3273	3.7455	4.7239	3.1103	4.4927	4.1551	4.7659	3.9850	3.6578	3.4916	3.7907	3.7219
Blvra	_DQDIFIQK_.2	#N/A	3.7149	4.6463	4.5166	4.4982	3.9219	3.5988	4.2881	3.5289	3.7680	3.5255	3.7700	3.6220	3.2119	4.5453	3.8348	4.7640	4.7317	4.4039	4.2934	3.6815
Tomm34	_VPS[Phospho (STY)]AGDVER_.2	Q9CYG7_S186_M1_Tomm34	6.7173	7.5653	7.3913	7.0651	7.4136	7.3166	6.6735	7.2999	5.5459	7.0166	7.1411	6.9785	6.3939	7.8140	6.8351	7.3565	7.4763	6.9036	6.6780	7.2372
Tomm34	_TVLQIDNSVASALEGINR_.2	#N/A	4.2598	4.6894	4.2796	4.8856	5.0134	4.8290	3.8926	4.4762	3.0519	3.9477	5.1617	4.4319	3.9977	4.0854	2.8917	4.2578	4.9228	3.1872	4.4735	4.2107
Tomm34	_VSDS[Phospho (STY)]VEQLR_.2	Q9CYG7_S8_M1_Tomm34	3.5232	3.7959	4.5606	4.0610	3.7302	5.7359	3.3179	3.7489	3.2381	5.1192	4.2176	3.5220	5.3470	4.5987	3.3433	4.3862	3.2461	5.5235	3.8608	3.8732
Tomm34	_SSLSDISSLLQIEPR_.2	#N/A	3.4733	4.5295	4.0096	4.2160	4.3119	3.0351	3.2292	3.1737	3.2883	4.0721	3.6626	4.4189	3.3963	3.9967	2.8064	3.8182	3.7114	4.1218	3.5681	4.1182
Tomm34	_YSESLLC[Carbamidomethyl (C)]SSLESATYSNR_.2	#N/A	3.5308	4.4720	4.0671	4.1585	2.8686	3.0926	3.1717	3.2312	3.2308	3.7372	3.6051	4.3614	3.3388	4.0542	4.2497	3.8757	3.7689	4.0643	3.7128	4.0607
Prxl2a	_LDELGVPLYAVVK_.2	#N/A	6.3440	6.7322	6.5713	6.3848	6.7105	6.8268	6.6315	6.5480	6.7560	6.3417	6.1323	5.9241	6.4392	6.0803	6.0494	5.5620	5.8169	6.2746	6.2648	5.7969
	LTQNRGDS[Phospho (STY)]DDGR.2	Q9CYI0_S55_M1_	3.7249	3.9073	4.2693	3.1376	3.1528	4.2448	3.8930	3.1715	3.8156	4.3030	3.2942	3.3645	4.4775	4.0213	3.9207	3.8088	3.4596	2.9799	3.2789	3.8373
	S[Phospho (STY)]LGNDVK.2	Q9CYI0_S186_M1_	4.4375	3.5653	3.4730	3.2519	3.7752	3.9993	1.7385	4.1379	3.2446	3.1430	4.1993	3.4548	3.9330	3.4600	3.3430	3.2816	3.1747	3.1576	4.3070	3.1540
Luc7l	_RS[Phospho (STY)]EEKEAGEI_.2	Q9CYI4_S363_M1_Luc7l	8.6477	8.4995	8.7053	8.0419	8.4454	8.3424	8.3140	8.6754	8.0558	8.6390	8.2611	8.3868	8.6556	8.7570	7.6254	8.2341	8.6583	8.1857	7.8783	8.3080
Luc7l	_ERS[Phospho (STY)]LREESWEYGR_.3	#N/A	4.0638	4.0921	4.0019	3.8552	3.6492	4.3969	4.2722	4.0534	4.2082	5.4512	3.6234	4.2227	4.3737	3.6343	3.2229	4.4858	4.1199	4.2225	3.7509	3.6506
Luc7l	_S[Phospho (STY)]LREESWEYGR_.2	#N/A	4.1152	3.8876	5.4708	3.5742	3.4529	3.0312	4.0882	3.8156	3.9705	5.2606	3.4491	3.7771	4.1359	5.3914	3.6654	5.1066	4.3532	3.4799	3.1285	3.1047
Atp6ap2	_QENTQSPYNLAYK_.2	#N/A	5.5630	5.4106	5.0340	5.2200	4.5819	5.8445	6.2178	5.1962	6.0750	4.7862	5.2190	4.9212	5.9283	5.0541	5.0392	5.5346	6.7111	5.7596	5.8063	5.6681
Ssbp1	_S[Phospho (STY)]GDSEVYQMGDVSQK_.2	Q9CYR0_S67_M1_Ssbp1	4.2138	3.8097	6.5223	7.0777	2.9199	5.5945	4.1282	3.8935	4.0484	4.0244	5.6536	4.3323	4.2139	3.2157	4.7427	3.5759	4.4312	3.4020	3.0505	3.1827
Cap2	_THTPSPTS[Phospho (STY)]PK_.2	#N/A	12.8301	12.5638	10.8148	12.6251	12.6583	12.6755	12.5542	12.8478	12.5391	12.7911	12.8597	12.5640	12.9822	13.2724	12.4953	12.5242	12.7955	12.6677	12.8635	12.9129
Cap2	_IQEIQTFR_.2	#N/A	7.2131	7.5333	7.3313	6.9579	7.6512	7.1180	7.3290	7.5177	7.1858	7.7679	7.6049	7.6525	7.6044	7.6154	7.6534	7.5023	7.4158	7.3744	7.5480	7.2646
Cap2	_LEQLSAGLDGPPR_.2	#N/A	6.7910	7.2376	7.3540	7.3644	6.8873	7.2023	7.0998	7.1875	7.0341	7.2462	6.8831	7.0137	7.1521	7.6318	7.3828	7.0110	7.3566	7.0431	7.4292	7.1940
Cap2	_TEGC[Carbamidomethyl (C)]HLYLSEDALDC[Carbamidomethyl (C)]EIVSAK_.3	#N/A	7.1763	6.9479	5.8486	6.9107	6.1050	6.3160	5.8134	7.1854	5.7543	6.5098	6.3756	6.6734	6.5573	7.6157	5.5413	5.8686	7.0565	5.8593	5.9309	6.0946
Cap2	_LINSM[Oxidation (M)]VAEFLK_.2	#N/A	5.1207	5.0716	4.4536	3.9420	5.3297	4.8841	4.3980	5.6326	4.6347	4.9696	4.5989	4.9476	4.9677	4.5339	4.6264	3.9403	5.1184	4.9913	5.0990	5.2289
Cap2	_SNS[Phospho (STY)]PQKHTPVLELEGK_.3	#N/A	8.7375	9.0045	8.1446	8.8673	8.4705	8.2492	8.4654	9.3938	8.2932	8.7087	8.6740	9.0479	10.0062	9.2616	8.5804	9.0021	9.0370	9.0382	8.7949	8.7969
Cap2	_S[Phospho (STY)]NSPQKHTPVLELEGKK_.3	#N/A	3.5926	3.8366	3.7886	7.2203	3.7726	4.3103	3.7974	4.0081	3.7872	3.6456	3.7931	3.6528	3.9235	3.6818	3.5260	3.6017	3.5042	3.7439	3.8567	3.5303
Cap2	_HTPVLELEGKK_.3	#N/A	4.1570	4.2890	3.2589	2.8958	3.0607	3.8029	4.4805	4.8353	3.1233	3.2711	3.2595	3.1874	4.1879	3.3124	4.2180	4.5602	2.6549	3.8489	3.9940	3.6561
Cap2	_AQGQIRS[Phospho (STY)]PTKTHTPSPTS[Phospho (STY)]PK_.3	#N/A	10.3978	3.8126	4.5439	4.0777	10.1175	4.3949	3.3702	10.6189	9.8801	3.5386	9.7686	10.5215	11.3998	10.7407	10.2689	10.5964	10.4463	10.5613	4.2191	4.2592
Cap2	_EM[Oxidation (M)]NDAATFYTNR_.2	#N/A	5.3968	5.4471	4.7170	5.8745	5.6861	5.2297	5.8125	6.9650	5.6527	5.2567	5.2559	4.7840	5.9445	5.3258	5.5334	5.2411	5.4117	5.4155	5.3410	5.2492
Cap2	_THTPS[Phospho (STY)]PTS[Phospho (STY)]PKSNSPQK_.2	#N/A	8.0352	7.0195	3.6456	7.4368	6.3643	5.9501	7.3797	6.7421	6.9973	4.1066	5.6696	7.4660	4.0729	6.7368	3.7104	6.6281	6.4786	6.9140	8.2050	7.7602
Cap2	_S[Phospho (STY)]PTKTHTPSPTS[Phospho (STY)]PK_.2	#N/A	8.0975	6.0836	4.0681	3.7834	5.7821	7.9942	3.7915	8.2142	5.9174	3.7382	8.0871	8.2998	4.0214	8.4140	7.9317	6.2433	6.5637	6.1633	6.0601	8.3910
Cap2	_VEYQEDRNDLVISETELK_.3	#N/A	6.6980	5.6561	6.9111	6.9296	5.5583	3.9200	5.2539	7.2689	6.2630	5.7995	5.3966	6.5092	8.0210	6.7293	6.0787	4.4289	5.8747	5.1629	5.9378	5.6156
Cap2	_SSEM[Oxidation (M)]NVLVPQDDDYREFPIPEQFK_.3	#N/A	5.4651	5.2429	4.5075	4.8494	4.9710	5.4659	5.5035	5.8087	5.5993	5.4931	5.2149	5.4273	5.6630	5.9504	4.7087	4.9798	5.7504	5.3127	5.1972	4.8084
Cap2	_AQGQIRS[Phospho (STY)]PTK_.2	#N/A	12.0273	12.1016	12.1213	12.0874	11.7705	11.8378	11.8018	12.1747	11.7821	11.9657	11.8500	11.9746	12.3847	12.3969	11.7672	11.9335	12.1577	11.9997	11.9125	11.8870
Cap2	_SALFAQLNQGEAITK_.2	#N/A	4.9991	4.6456	5.4661	4.7040	5.0245	4.8836	4.9867	5.4395	4.4586	5.4974	5.4344	4.3493	6.0328	4.8626	5.3359	4.8252	5.5276	5.3931	5.2921	4.7481
Cap2	_VLAGDVETHAEM[Oxidation (M)]VHGAFQAQR_.3	#N/A	3.4897	3.7717	4.0122	4.4246	6.3880	5.2292	3.2425	6.7528	5.0520	6.1265	3.6760	5.9764	3.4096	3.7889	5.3270	5.7376	3.9372	5.2425	3.7112	5.8819
Cap2	_TIWDGSK_.2	#N/A	3.1179	4.2012	4.1553	4.4664	3.0586	3.2827	2.9816	3.3435	3.0407	3.9272	3.4151	3.9273	3.1487	4.1934	4.0596	3.6033	4.3416	4.3087	3.1069	4.2920
Hdhd3	_DVVLHTFR_.2	#N/A	4.9348	5.3653	4.5167	5.4477	5.7240	5.5630	4.9918	6.2030	5.1188	5.4274	5.6050	4.8291	5.6098	5.3309	5.9883	5.6251	4.3278	5.8865	5.3848	5.4828
Tpd52l2	_NSATFKS[Phospho (STY)]FEDR_.2	#N/A	10.6326	10.7579	10.4099	10.3824	9.6781	9.9042	10.1328	10.0999	9.4022	10.5766	9.9425	9.9925	10.8255	10.8084	9.4767	10.1074	10.1390	9.9232	10.1625	10.1552
Tpd52l2	_S[Phospho (STY)]WHDVQVSTAYVK_.2	Q9CYZ2_S96_M1_Tpd52l2	3.4578	6.8195	4.4953	4.1264	3.6648	4.4436	3.3833	3.6835	3.3807	3.5873	3.7550	3.5874	3.4887	4.5333	5.8577	4.3208	3.1808	3.9687	5.9391	4.2106
Tpd52l2	ein N-term)]M[Oxidation (M)]DSASQDINLNSPNKGVLS[Phospho (STY)]DFM[Oxidation (M)]TDV	Q9CYZ2_S19_M1_Tpd52l2	3.7893	3.9950	3.8361	3.8898	3.6885	3.9482	4.8489	3.8148	4.1574	3.5185	4.1032	3.8350	4.0679	5.5210	3.2288	3.6515	3.7536	3.7700	3.7122	3.6984
Tpd52l2	_[Acetyl (Protein N-term)]MDSASQDINLNS[Phospho (STY)]PNK_.2	#N/A	3.4010	7.2206	8.7866	8.2403	5.9169	6.9303	7.0322	3.7507	5.9312	6.4087	7.3029	5.4129	3.5559	8.5334	5.6276	4.2784	6.7009	4.5140	5.2326	6.0073
Tpd52l2	_ENGSDNLPPS[Phospho (STY)]PGSGDQTLPDHAPF_.3	Q9CYZ2_S206_M1_Tpd52l2	9.6536	9.1660	3.6552	9.7172	3.3502	9.5720	9.6844	8.2943	8.9344	8.6732	3.4035	9.2104	9.5615	9.6727	8.2668	10.0985	9.7459	3.5345	8.8781	9.6904
Tpd52l2	_S[Phospho (STY)]FEDRVGTIK_.2	#N/A	3.5438	4.4590	4.0801	4.1456	2.8816	3.1056	3.1587	3.2442	3.2178	2.9157	3.5921	4.3484	3.3258	4.3704	4.2367	3.8887	3.7819	4.1316	4.4306	4.0477
Tpd52l2	_HSIS[Phospho (STY)]M[Oxidation (M)]PVM[Oxidation (M)]R_.2	Q9CYZ2_S168_M1_Tpd52l2	4.1783	3.8245	3.2138	3.5111	3.5160	3.7401	4.0250	3.8787	4.0841	4.3846	2.9577	3.7139	4.1922	3.2008	3.6022	3.0224	4.4164	3.4168	4.3273	4.9387
Dbndd1	_[Acetyl (Protein N-term)]M[Oxidation (M)]ES[Phospho (STY)]PEGAGPGEIVK_.2	#N/A	8.3848	9.1967	8.9681	8.9546	6.4838	8.8650	8.3631	7.5505	8.5807	9.1250	7.9069	8.7185	8.7596	9.4101	8.6792	8.9470	9.1194	8.4614	8.8848	9.0339
Uqcrc1	_VYEEDAVPGLTPC[Carbamidomethyl (C)]R_.2	#N/A	10.8311	10.6180	11.0284	10.8937	10.5730	10.5094	10.5602	10.8244	10.7293	10.7743	10.7192	10.6056	11.0548	11.0100	10.8317	10.6679	10.8852	10.4598	10.5241	10.5745
Uqcrc1	_TDLTDYLNR_.2	#N/A	10.7497	10.6582	10.4457	10.4067	10.6799	10.4375	10.4918	10.8849	10.4305	10.7760	10.7116	10.7232	10.9780	10.8574	10.3221	10.6792	10.8986	10.5549	10.5838	10.6271
Uqcrc1	_NALVSHLDGTTPVC[Carbamidomethyl (C)]EDIGR_.3	#N/A	7.6413	7.6889	8.4633	8.6659	7.8932	7.9773	7.3640	8.2606	8.4158	8.3268	7.5862	8.0315	8.4115	8.1656	8.7395	8.2114	7.7828	7.6417	8.3032	8.4308
Uqcrc1	_LC[Carbamidomethyl (C)]TSATESEVTR_.2	#N/A	7.9943	7.8835	8.3728	8.1060	7.9942	7.7810	7.9559	7.9755	8.0853	8.2825	7.4484	8.0739	8.2036	7.9739	8.1422	8.0167	7.7255	7.8818	7.8517	8.3015
Uqcrc1	_EHTAYLIK_.2	#N/A	7.6619	7.9336	7.5023	7.6416	7.9716	7.5888	7.9242	8.0239	7.8241	7.6835	7.9235	7.7475	8.3245	7.8153	8.0295	8.0240	7.7412	7.7985	7.7222	7.9291
Uqcrc1	_IPLAEWESR_.2	#N/A	7.4851	6.6715	7.1820	6.9208	7.3382	6.7254	7.2816	7.3711	6.6291	7.3087	7.1767	7.1089	7.0488	7.0063	7.5036	6.9035	6.9693	7.3231	6.9597	7.4264
Uqcrc1	_RIPLAEWESR_.2	#N/A	7.1933	7.2066	6.1071	7.5185	7.2106	6.7880	6.7996	7.5190	6.5325	7.0419	7.0492	7.2641	7.1254	6.6530	6.9440	7.1214	7.0580	6.9915	6.8094	7.3676
Uqcrc1	_VVELLADIVQNSSLEDSQIEKER_.3	#N/A	6.4007	6.9856	6.0520	6.3598	6.8288	6.6564	6.3350	6.8686	6.2954	6.8464	6.3792	6.7962	7.7351	7.8392	6.1135	7.1396	7.1877	6.4984	6.0947	7.3858
Uqcrc1	_NRPGNALEK_.2	#N/A	6.1678	6.2800	6.4403	6.6159	6.5583	6.5169	6.7401	6.3101	6.4701	6.2377	6.4563	6.5312	6.2367	6.5721	7.0719	6.1597	5.7631	6.1403	6.3927	6.2930
Uqcrc1	_NNGAGYFLEHLAFK_.2	#N/A	5.6291	5.5855	4.6659	4.8901	4.6765	4.9409	3.0078	4.3411	3.4988	4.8348	5.3549	5.0739	4.7243	4.3818	4.8216	4.0714	5.5127	5.5408	5.4650	5.6815
Uqcrc1	_IQEVDAQM[Oxidation (M)]LR_.2	#N/A	7.6686	8.5734	8.1737	8.2432	7.7761	8.7140	7.9128	7.9335	7.8116	7.8867	8.1742	8.6538	8.9058	8.3505	8.0130	8.7522	7.8225	7.5407	7.7353	7.7278
Uqcrc1	_SLLTYGR_.2	#N/A	4.6123	5.3078	5.2463	6.2697	5.9830	5.4650	6.8334	3.5353	5.5775	5.9732	6.8500	5.6883	4.0542	5.5702	6.5460	6.3202	3.2794	6.9136	6.8963	6.6380
Uqcrc1	_YFYDQC[Carbamidomethyl (C)]PAVAGYGPIEQLPDYNR_.3	#N/A	6.5587	7.1826	6.0286	6.5198	5.7306	6.0066	6.5509	6.4997	5.7192	6.4677	5.5313	7.0225	6.8526	7.5214	5.9112	6.1611	6.1686	7.0267	6.3398	5.9440
Uqcrc1	_SGM[Oxidation (M)]FWLR_.2	#N/A	4.2619	4.2192	4.2455	5.5251	6.6095	5.6533	5.8197	3.9661	5.5923	6.2676	5.9975	6.2342	5.7583	5.5201	5.6606	6.7892	3.4633	6.8354	5.5217	6.1047
Uqcrc1	_FTGSEIR_.2	#N/A	4.4673	6.5427	5.5335	6.9851	6.1557	6.3729	7.1809	4.1676	7.2738	6.7053	6.9115	6.9010	6.3959	5.8511	7.6357	6.8524	3.2045	7.1457	7.1898	7.4722
Uqcrc1	_M[Oxidation (M)]VLAAAGGVEHQQLLDLAQK_.3	#N/A	6.4197	6.0211	5.3331	5.7719	6.3222	5.5458	5.3032	6.5547	5.5549	6.2292	6.1381	6.0109	6.7458	5.8112	5.7851	6.0018	6.1216	5.7150	5.7893	6.0566
Uqcrc1	_VVELLADIVQNSSLEDSQIEK_.2	#N/A	4.8363	4.4411	4.4487	4.8803	4.5137	4.8873	4.8564	5.0624	4.4170	5.0859	4.3629	4.6683	5.1660	5.9262	4.2606	3.9188	5.3499	4.2911	4.5351	5.2351
Uqcrc1	_VASEQSSHATC[Carbamidomethyl (C)]TVGVWIDAGSR_.3	#N/A	5.6901	4.4620	3.4873	4.1714	4.7355	4.6532	3.7193	6.2193	3.3961	5.5823	4.1850	4.3515	3.5615	4.0642	4.7049	2.8119	3.6344	4.0543	3.7029	4.0312
Uqcrc1	_EVESIGAHLNAYSTR_.2	#N/A	4.1123	3.9844	4.0297	3.6995	3.0007	3.4502	3.6829	4.0465	3.7420	4.0146	4.0207	4.2409	4.2553	4.1195	4.0954	4.5532	3.9595	3.5813	3.5070	3.8861
Ola1	_IGIVGLPNVGK_.2	#N/A	6.6480	6.0017	6.1824	6.0065	6.1900	6.1457	6.1709	6.3908	5.9540	6.2227	5.8325	6.4391	6.2419	5.7686	6.3657	6.0026	6.0457	6.2619	5.3687	6.1314
Ola1	_IPAFLNVVDIAGLVK_.2	#N/A	3.6807	1.8985	2.3454	2.8434	3.3753	2.4293	3.8529	1.5146	4.3139	3.1497	3.4138	3.9438	4.4220	4.4719	3.2614	4.2258	2.8561	1.7571	2.3613	3.2716
Ola1	_NYIVEDGDIIFFK_.2	#N/A	5.3141	5.1104	5.8636	5.2811	4.6910	4.6233	4.3319	5.0868	5.3507	4.8526	3.7648	5.7002	3.4985	4.5431	3.8319	5.4181	5.0736	3.8764	4.5195	4.6276
Naxd	_DDLLLNNVR_.2	#N/A	6.8216	7.2117	7.1624	7.1603	7.0975	7.0438	6.9506	7.1891	7.0890	7.1773	7.5024	7.2932	7.4848	8.1036	6.9795	6.9294	6.6075	7.0514	7.4800	7.5093
Naxd	_SYSPELIVHPVLDSSNAVEEVEK_.3	#N/A	3.7435	5.1770	4.7943	4.6977	3.9864	3.0222	5.2230	4.6284	4.8886	4.3071	5.2833	4.1874	4.8843	5.4968	4.4511	4.7388	5.2990	5.0831	4.5103	5.0826
Naxd	_LSQALGNITVVQK_.2	#N/A	4.0265	4.3314	5.1501	5.0544	4.0977	5.0940	4.8785	4.6706	3.6539	4.6829	4.6507	4.4810	3.6105	4.6707	4.8612	5.2456	4.1726	4.3510	4.2241	4.2045
Naxd	_AILTPNHVEFSR_.3	#N/A	4.0100	4.8936	4.4989	3.7268	4.4643	4.5104	3.8917	2.8665	3.5566	4.2549	3.2965	3.9297	3.9833	5.0776	4.5831	4.3586	4.2006	4.4122	3.6584	4.0617
Naxd	_STTTTDMITEVGTAFSR_.2	#N/A	4.1004	3.2186	3.6370	4.2137	4.3074	4.3407	4.2416	4.3261	4.1618	3.9110	3.6404	2.9448	4.3272	5.9732	4.2669	3.4626	3.8233	3.2886	4.4380	3.2960
Nsfl1c	_S[Phospho (STY)]PGETSKPRPFAGGGYR_.3	#N/A	9.0219	9.1010	9.2534	9.1394	8.4164	8.4275	8.5160	8.3456	8.3984	9.1881	8.8690	8.6365	8.9116	9.2761	8.2289	8.8769	8.2795	8.6446	8.6042	8.8645
Nsfl1c	_EANLLNAVIVQR_.2	#N/A	6.0056	6.6723	6.7498	6.3251	6.2890	6.2456	6.0445	6.3013	6.2887	6.1226	6.6004	6.3753	6.1018	6.6717	6.8855	6.2865	6.5799	6.5148	6.4574	6.2337
Nsfl1c	_SYQDPSNAQFLESIR_.2	#N/A	6.6107	6.2072	6.5475	5.9861	5.8792	6.1941	6.2732	6.4087	6.3878	6.4349	6.1342	6.2667	6.3012	6.6165	6.7724	6.5221	5.7713	5.6647	6.0099	5.7008
Nsfl1c	_DLIHDQDEEEEEEEGQR_.3	#N/A	5.2024	5.1877	6.3239	5.7307	5.4009	5.9441	5.2845	5.6630	6.1373	6.3931	5.9241	5.5377	6.8830	6.7339	6.2797	5.9273	5.8155	5.9785	5.4911	6.5636

Nsfl1c	_ASSSILINEAEPTTNIQIR_.2	#N/A	5.6157	5.7312	5.8126	5.7191	5.4792	4.5676	5.5554	5.3465	6.0543	5.9444	6.0061	4.9754	5.9859	5.7406	6.2096	6.0452	5.1972	5.6006	5.9227	5.9750
Nsfl1c	_RHS[Phospho (STY)]GQDVHVVLK_.3	#N/A	9.4505	10.0070	10.0434	10.3928	9.0672	9.5714	9.5176	9.0067	9.8065	10.0978	9.2727	9.3892	9.8145	9.9570	9.6442	10.1921	9.6285	9.4468	9.8401	10.2984
Nsfl1c	_S[Phospho (STY)]PNELVDDLFK_.2	#N/A	6.7242	7.0288	7.2683	7.3005	6.3455	7.2107	6.6617	6.1228	7.0294	7.2233	7.2148	6.4973	6.6707	7.4662	7.1421	6.8354	7.2394	6.5559	6.8866	7.3620
Nsfl1c	_RRHS[Phospho (STY)]GQDVHVVLK_.3	#N/A	6.8300	9.6412	6.9817	7.7176	6.7358	8.1658	8.7314	8.3461	7.2140	8.4746	7.1573	8.4199	3.8364	10.2410	6.5640	7.5405	7.6997	8.5612	8.0562	7.6706
Nsfl1c	_VTKS[Phospho (STY)]PGETSKPR_.2	#N/A	5.3221	6.0348	5.9396	5.8621	5.1149	4.7576	4.8420	5.4308	4.5000	6.1530	5.2673	6.0449	5.6037	6.1336	4.4504	5.8492	5.1984	5.4150	4.0062	5.3734
Nsfl1c	_TGFSLDNGDLR_.2	#N/A	4.4442	3.4237	5.7986	5.7220	5.0218	5.3682	5.3654	4.1211	5.5440	3.1497	5.8182	5.8156	3.9263	6.2400	5.6118	3.2883	5.6485	5.2989	5.7920	5.5953
Nsfl1c	_FYAGGSER_.2	#N/A	5.0948	6.1398	3.3975	3.3273	4.6030	5.3422	5.5857	4.0624	5.7288	5.5300	5.8873	5.5981	5.2735	5.2199	6.3345	5.8337	3.0993	5.8562	5.9595	5.9556
Nsfl1c	_S[Phospho (STY)]PGETSKPR_.2	#N/A	3.9784	4.1109	5.0865	3.6058	4.1853	4.5822	4.3636	4.2041	4.9003	5.3646	4.2756	4.7615	4.0093	3.5530	2.8881	3.3405	4.9616	3.4481	3.7472	3.2303
Nsfl1c	_HS[Phospho (STY)]GQDVHVVLK_.2	#N/A	4.0183	4.5935	4.3460	3.6897	3.2751	3.6235	4.2265	3.7657	4.3343	4.7601	4.3592	4.5475	4.0810	4.8934	3.8494	4.8085	4.0948	3.8317	3.8285	4.6837
Nsfl1c	_KKS[Phospho (STY)]PNELVDDLFK_.2	#N/A	3.8705	3.9674	3.8470	3.9060	3.7187	4.4602	3.9690	3.5316	3.7395	3.8350	3.6350	3.8025	3.8829	11.2349	3.6435	3.6636	3.7989	3.8706	3.5192	3.7782
Antxr1	_EVPPPPVEES[Phospho (STY)]EEEDDDGLPK_.2	Q9CZ52_S360_M1_Antxr1	4.5783	9.6858	8.6882	4.5068	3.2844	7.9437	9.4634	3.3031	9.5222	8.8357	3.1626	3.9678	3.8494	8.9713	7.7331	7.9414	8.7729	9.4670	8.8945	9.1132
Cep97	_QLM[Oxidation (M)]SQS[Phospho (STY)]QDEELSPLAAVETR_.2	#N/A	3.9719	5.5328	5.2097	4.0492	3.3220	4.3822	5.0796	3.5831	3.6651	4.4265	3.1437	3.9489	3.8305	4.4187	3.7702	3.9780	4.3558	3.9171	3.1258	4.5526
Sdhc	_HLLWDLGK_.2	#N/A	5.0985	4.8374	4.1766	4.9271	4.9477	4.7273	5.4957	5.0100	3.0109	4.5943	5.4289	5.1301	3.7715	4.5973	4.4102	4.0956	5.1835	5.2539	4.5423	4.1690
Thumpd2	_LLQGS[Phospho (STY)]PEQGEAVTR_.2	#N/A	6.0923	6.1064	5.6942	5.7163	4.1629	6.1212	5.7095	5.7403	5.5289	3.5307	3.8116	3.5308	4.9538	5.7867	4.5923	3.6692	5.6189	5.5766	5.7927	5.2185
Thumpd2	_DHGESQEDKLLQGS[Phospho (STY)]PEQGEAVTR_.3	#N/A	4.0738	3.6697	3.3686	3.3563	4.2808	4.3141	4.2682	4.0335	4.1884	3.8844	3.6670	3.5591	4.3538	3.3557	2.7927	1.6025	3.0703	3.2620	2.6456	3.3226
Scrn1	_AVIES[Phospho (STY)]DEEQGR_.2	Q9CZC8_S360_M1_Scrn1	9.4092	9.3045	9.5655	9.6589	9.4282	9.1573	9.0194	9.6672	9.4362	9.4873	9.2748	9.5564	9.9600	9.7910	9.3568	9.4590	9.7173	9.3091	9.4070	9.7629
Scrn1	_THAIVISR_.2	#N/A	5.5321	6.1325	6.0134	6.0260	5.5697	5.7195	6.2152	5.9482	6.0975	6.2610	6.1698	5.3201	5.7133	5.6951	5.8699	6.1385	5.3702	5.7190	6.0159	4.9564
Scrn1	_FQEKPDR_.2	#N/A	7.0681	5.9178	7.5691	7.5192	7.8604	5.8249	6.2714	7.1318	7.4205	5.9803	6.6085	7.6711	6.4031	8.2104	7.3015	7.7924	6.9098	6.7808	6.4949	7.2046
Scrn1	_VEC[Carbamidomethyl (C)]TYISIDQVPR_.2	#N/A	4.8467	5.3167	6.8703	5.6909	5.3505	5.4821	5.2328	4.8528	6.0022	5.7591	5.7884	5.5488	3.6282	5.6648	5.7260	5.7707	5.1469	5.6985	5.1062	5.7205
Scrn1	_DKASGVC[Carbamidomethyl (C)]IDSESFLTTASIVSVLPQNR_.3	#N/A	6.8222	8.1093	7.4835	6.7697	6.4030	6.9323	7.6605	8.6309	6.8438	7.2748	6.4512	8.2265	8.3245	3.4571	7.2622	7.2830	7.9072	7.8085	7.9135	6.9734
Scrn1	_AQS[Phospho (STY)]PC[Carbamidomethyl (C)]FGDDDPAK_.2	#N/A	6.6247	6.5796	7.3028	6.9523	3.7011	6.6046	6.3355	6.5293	6.1804	7.5244	7.0525	6.1408	5.9784	7.3907	5.9412	6.3227	6.9396	6.6858	6.5728	7.0817
Scrn1	_ASGVC[Carbamidomethyl (C)]IDSESFLTTASIVSVLPQNR_.3	#N/A	4.8932	4.1153	3.6411	3.3869	4.3114	4.3156	4.0830	4.4407	4.1577	4.4744	3.6364	3.7919	4.3232	5.4940	4.2629	4.1402	5.1057	4.1211	4.4696	3.8318
Scrn1	_SSPC[Carbamidomethyl (C)]IHYFTGTPDPSR_.3	#N/A	6.4273	6.8794	6.4768	6.5089	6.8797	6.2696	6.7242	6.8839	6.4008	6.7254	6.7672	6.8711	6.9644	7.2817	6.8338	6.5618	6.8120	6.6221	6.7416	6.4629
Scrn1	_PAWLWGAEM[Oxidation (M)]GANEHGVC[Carbamidomethyl (C)]IANEAINAR_.3	#N/A	3.8742	4.3488	4.7951	4.9712	4.6430	4.3810	3.9869	4.1306	3.6147	4.7557	4.4031	4.2881	4.0856	5.5064	3.3604	3.8347	4.4829	4.5895	4.6033	4.3909
Scrn1	_AQS[Phospho (STY)]PC[Carbamidomethyl (C)]FGDDDPAKKEPR_.3	#N/A	5.4045	7.0344	6.3083	6.7446	6.7324	6.8706	7.1363	7.0119	6.6993	7.5017	6.9316	7.0958	6.9829	7.5630	6.9462	7.1279	7.2667	7.5645	7.2103	7.4399
Scrn1	_EPAAETEALLGM[Oxidation (M)]DLVR_.2	#N/A	6.7397	6.7041	7.0266	6.6838	6.4389	6.5593	6.6500	6.8031	6.7437	6.5035	6.5167	6.7466	6.8828	6.8440	6.8194	6.7370	7.0230	6.5576	6.4805	6.6878
Scrn1	_TMLELEK_.2	#N/A	3.8386	3.4344	3.6038	3.1210	4.0455	4.0788	3.0026	4.2687	4.4236	2.3762	3.9023	3.3239	4.5891	3.5909	3.0280	3.2007	3.3056	3.0267	2.5056	3.5579
Scrn1	_DEVQEVVYFPAVDHDAESK_.3	#N/A	3.4467	4.5561	3.9830	4.2427	4.2853	3.0085	3.2558	3.1471	2.7539	3.6530	3.6893	2.9447	3.4230	3.9701	2.8330	3.7916	3.6847	4.1484	3.7970	4.1449
Mxra7	_YS[Phospho (STY)]PGQLR_.2	Q9CZH7_S118_M1_Mxra7	3.9032	8.1284	7.9552	3.7862	3.2409	7.8703	7.9169	3.6035	7.6710	4.1095	3.2328	3.9891	8.1912	8.2743	3.8774	7.9208	4.1412	7.8903	7.9335	3.6884
Mxra7	_VAEPEES[Phospho (STY)]EAEEPAAEGR_.2	Q9CZH7_S79_M1_Mxra7	10.6370	10.6404	10.3972	10.6390	9.9690	10.4739	10.3781	10.4581	10.1631	10.3362	10.2828	10.1514	10.6478	10.6888	9.3963	10.1529	10.6438	10.2258	10.1800	10.0849
Hspa12b	_SPVPS[Phospho (STY)]PPGSPR_.2	Q9CZJ2_S25_M1_Hspa12b	10.1248	9.8440	9.1986	9.9994	9.8840	9.7879	9.8675	10.0413	9.6900	9.0750	4.0130	9.7342	10.5879	9.7574	7.8730	3.4679	3.3611	9.9106	9.7856	3.6134
Hspa12b	_RSS[Phospho (STY)]VNLVK_.2	Q9CZJ2_S434_M1_Hspa12b	7.4199	7.7716	3.4702	6.9865	7.0244	3.9678	3.7973	7.1407	6.4081	7.3225	4.2308	3.1116	7.2934	3.5082	5.9979	6.8046	6.9055	7.3487	7.2686	7.0893
Hspa12b	_TQESC[Carbamidomethyl (C)]GIAPLTPS[Phospho (STY)]QSPKPEAR_.3	Q9CZJ2_S44_M1_Hspa12b	6.2909	6.3448	7.4671	7.8594	3.3097	3.6242	7.0326	3.6176	4.2283	4.2687	6.8605	6.1339	4.3363	5.9535	5.0637	6.1634	6.6690	6.4796	6.1270	6.6922
Hspa12b	_RS[Phospho (STY)]IDSS[Phospho (STY)]FR_.2	#N/A	4.5734	4.5751	3.5571	3.6356	2.9321	5.1540	4.9717	4.6714	3.9709	4.0366	4.7361	4.0057	4.7263	3.8082	4.1426	3.5807	4.4202	4.2306	3.0628	3.1717
Hspa12b	_S[Phospho (STY)]IDSS[Phospho (STY)]FR_.2	#N/A	4.2321	3.8229	4.0687	4.4707	3.5698	4.2178	5.4692	3.9325	4.0874	4.0377	3.5660	3.7124	4.2528	4.0558	3.6007	3.8773	3.7705	5.0034	3.8650	4.0228
Rpl15	_SLQSVAEER_.2	#N/A	3.5165	6.0258	5.6582	5.3744	5.2759	5.5357	6.1996	5.7275	6.0194	5.1515	5.7994	5.9937	6.0660	4.9050	5.7341	5.9017	4.5460	5.8982	6.3451	5.5253
Rpl15	_NTLQLHR_.2	#N/A	5.4121	5.8996	5.6770	3.2553	5.5754	5.2721	5.7865	5.6085	6.0417	5.0669	4.8881	5.6373	4.4548	5.0846	5.9100	5.1615	4.5249	5.2143	5.2864	3.4236
Rpl15	_VLNSYWVGEDSTYK_.2	#N/A	3.1890	3.3129	5.8098	4.5003	4.0276	5.1785	3.5135	4.3903	3.0443	3.5276	4.0238	5.1764	3.2098	3.7124	3.0907	3.5339	3.4271	4.4061	5.2570	3.6794
Shisa9	_AVGNSDGDWAVATLKS[Phospho (STY)]PK_.3	Q9CZN4_S296_M1_Shisa9	5.1908	4.0923	4.4468	3.7788	4.5957	3.4724	4.2928	5.2017	4.3519	4.1169	5.2935	4.8021	4.4600	5.8152	3.8700	5.1680	4.1486	2.9975	3.2965	3.6810
Shisa9	_AFS[Phospho (STY)]PEHGPAQQNGQK_.2	Q9CZN4_S337_M1_Shisa9	8.0311	7.9956	7.5631	7.8958	6.8752	7.4816	7.6521	7.7026	3.7803	7.8012	7.5705	7.3508	3.8884	8.0906	6.6533	7.2515	7.9515	7.8811	7.3679	7.2697
Aldh1b1	_TFVEESIYR_.2	#N/A	4.1505	3.8523	3.1860	4.1706	3.4883	4.4590	4.2508	3.8509	4.3236	4.3569	2.9855	2.7357	4.2200	4.5270	3.6300	2.9946	4.3886	3.2374	3.5365	3.4410
Rab3b	_LLAEQLGFDFFEASAK_.2	#N/A	4.3014	4.3862	3.3877	4.6324	4.4911	4.0503	3.2955	3.5623	3.8310	3.8201	4.0392	3.0566	3.8820	4.0091	3.2920	3.3326	3.5740	4.5356	3.8745	4.0365
Rab3b	_M[Oxidation (M)]SDS[Phospho (STY)]M[Oxidation (M)]DTDPSVLGASK_.2	#N/A	3.9908	4.3249	4.3426	3.9766	4.0273	7.6299	3.5653	3.7475	7.7185	7.0637	6.6795	3.0270	7.7554	4.7852	3.1425	7.6859	8.1434	8.0632	3.7917	6.8392
Rab3b	IC[Carbamidomethyl (C)]DKM[Oxidation (M)]S[Phospho (STY)]DSM[Oxidation (M)]DTDPSVLG	Q9CZT8_S188_M1_Rab3b	3.2299	6.1195	4.2125	4.0132	3.4368	3.4702	3.6113	3.3766	4.7814	3.9327	3.0101	4.2161	3.6969	4.1996	3.6366	4.0929	3.9143	6.5082	3.5675	4.1666
Cs	_VVPGYGHAVLR_.2	#N/A	10.5888	10.6189	10.3739	10.4221	10.7630	10.5297	10.8379	10.9483	10.3056	10.2648	10.2946	10.6302	10.6596	10.4680	10.7158	10.3219	10.5691	10.8252	10.5306	10.3099
Cs	_GLVYETSVLDPDEGIR_.2	#N/A	10.5548	10.3308	10.7672	10.5335	10.1291	10.2348	10.2496	10.3610	10.4422	10.5221	10.3558	10.0257	10.6064	10.4698	9.9864	10.3859	10.4538	10.1187	10.1936	10.3010
Cs	_EGSSIGAIDSR_.2	#N/A	8.7415	8.8285	8.6819	8.8111	8.6552	8.8795	8.6690	8.6465	8.6658	8.8291	8.8601	8.7999	8.4185	8.7641	7.9482	8.2303	8.5485	8.4660	8.2506	8.8207
Cs	_ALGFPLERPK_.2	#N/A	10.4881	10.2911	10.2933	10.3404	10.3717	10.0779	10.2518	10.5971	10.0635	10.1604	9.9570	10.4155	10.4493	9.9821	10.1916	9.9219	10.2533	10.2550	10.2211	10.0817
Cs	_IVPNILLEQGK_.2	#N/A	9.8131	9.8616	9.5794	9.8825	9.7431	9.7393	9.9547	9.7211	9.4710	9.5532	9.5985	9.8044	9.8509	9.3773	9.5179	9.5359	9.7618	9.8950	9.7509	9.4126
Cs	_GYSIPEC[Carbamidomethyl (C)]QK_.2	#N/A	9.1435	9.1197	9.4200	9.2697	9.0865	9.1555	9.3583	9.1993	9.3760	9.2501	9.1683	9.2118	9.4526	9.2056	9.3377	9.1254	9.1355	9.1126	9.2311	9.2194
Cs	_DYIWNTLNSGR_.2	#N/A	9.3723	9.3414	9.4948	9.5171	9.1589	9.2337	9.4270	9.3335	9.1384	9.1798	9.3560	9.1223	9.1525	9.4109	8.9650	9.1150	9.1752	9.1917	9.2139	9.1771
Cs	_LVAQLYK_.2	#N/A	5.8161	6.8172	6.2355	7.1040	7.0513	6.3250	7.3312	5.2202	6.9149	7.1521	7.3908	7.1100	6.8496	6.6071	6.9053	7.0277	5.0849	7.4281	7.5000	7.2795
Cs	_EVGKDVSDEKLR_.3	#N/A	5.8444	6.2435	6.4969	6.1529	5.7260	6.5592	7.1324	6.4621	6.5901	7.0416	6.5227	6.4743	6.4007	6.0985	7.1301	6.7079	6.5674	6.5310	7.0854	6.9033
Cs	_EVGKDVSDEK_.2	#N/A	6.6144	6.3919	6.7036	6.6897	6.5040	6.5039	6.9322	6.5776	6.5763	6.5270	6.6723	6.4679	5.7907	6.1965	6.7785	6.4017	6.4812	6.5161	6.4199	6.5374
Cs	_ALGVLAQLIWSR_.2	#N/A	5.3566	5.6465	5.2632	5.2627	5.8501	5.6094	5.9075	5.5358	5.7775	5.7330	4.8065	5.5873	5.2674	5.2208	5.3922	6.3610	6.0724	6.2339	6.1567	5.6031
Cs	_AYAEGM[Oxidation (M)]NR_.2	#N/A	6.2031	7.2271	7.1647	7.5970	6.6866	7.1202	7.1365	6.6998	7.0887	6.9711	6.8870	6.9381	6.0146	7.1635	7.9510	7.0158	6.3643	6.6829	7.0440	6.7850
Cs	_LDWSHNFTNM[Oxidation (M)]LGYTDPQFTELM[Oxidation (M)]R_.3	#N/A	5.8540	4.8716	4.0867	4.3419	4.1143	4.8968	5.7394	5.6631	5.1492	4.3489	4.4699	4.0569	4.9448	4.6955	4.0187	4.7886	4.7404	4.9640	5.8088	3.5701
Cs	_AALPSHVVTM[Oxidation (M)]LDNFPTNLHPM[Oxidation (M)]SQLSAAITALNSESNFAR_.4	#N/A	5.4263	6.4032	4.7860	5.1418	5.9870	6.5044	6.8466	6.7165	4.5032	5.7226	5.6415	6.2013	5.6850	6.8177	4.7736	5.5093	6.8189	6.5426	6.1354	6.1349
Cs	_TVVGQITVDM[Oxidation (M)]M[Oxidation (M)]YGGM[Oxidation (M)]R_.2	#N/A	6.3159	6.3431	5.2547	5.3440	6.4056	5.9775	5.7815	7.1569	6.2195	6.8037	6.0078	6.1941	7.3846	5.4352	5.6601	6.2318	6.0025	6.4084	6.4150	6.0876
Cs	_DVLSNLIPK_.2	#N/A	4.0080	3.9948	4.5443	3.6813	3.3458	3.1383	3.9431	3.7084	2.2817	4.2095	3.3420	3.8842	4.0288	4.5314	3.9685	4.3529	4.2461	3.5871	3.2356	2.9976
Cs	_YWELIYEDC[Carbamidomethyl (C)]M[Oxidation (M)]DLIAK_.2	#N/A	4.4518	3.5510	3.4872	3.2376	3.7895	4.0136	3.2452	4.8721	3.8107	3.1572	4.1851	2.1926	3.9187	2.7102	3.3288	3.2958	3.1890	3.1433	4.2928	3.4932
Cs	_SMSTDGLMK_.2	#N/A	3.4288	3.9091	4.4474	4.1372	4.2674	2.9906	4.4061	3.6357	3.3328	3.6351	3.7072	3.6352	3.4409	4.4855	2.8509	3.7737	3.1329	4.0166	4.3156	4.1627
Tomm70	_NVDLSTFYQNR_.2	#N/A	7.5117	7.9412	7.4109	7.5455	7.2435	7.2356	7.4608	7.8437	7.2030	7.0976	7.3643	7.4791	7.7323	7.9114	7.4403	7.4706	7.6471	7.6565	7.5434	7.6236
Tomm70	_AAAFEQLQK_.2	#N/A	6.9486	7.0460	7.1298	6.7291	7.1241	7.0630	6.9637	7.2661	7.0889	6.8392	7.1029	7.2610	7.4424	7.1950	7.1799	6.8794	7.2352	6.9986	6.5910	6.7176
Tomm70	_SYFSSFTDDIISQPM[Oxidation (M)]LK_.2	#N/A	6.2835	6.5853	6.7465	6.2082	6.6118	6.5478	6.2069	6.7967	6.5579	6.4258	6.8333	6.6439	6.9891	6.7642	6.8942	6.9028	6.4730	6.7716	6.6777	6.7773
Tomm70	_FALAQAQK_.2	#N/A	5.5996	6.0700	5.8601	5.7546	5.8944	5.6526	6.2468	5.9614	5.4936	5.9790	6.2535	6.2673	6.0846	6.0808	6.3658	5.8089	5.5253	6.3704	6.1301	6.0649
Tomm70	_YEQAIQC[Carbamidomethyl (C)]YTEAISLC[Carbamidomethyl (C)]PTEK_.2	#N/A	5.6502	4.9976	5.1012	4.8480	3.3165	4.3406	4.6659	5.6321	3.6853	5.2660	5.1092	4.3288	5.4196	5.9632	4.7124	4.4871	4.6386	4.9603	4.4192	4.6633
Tomm70	_ATFYLLIGSANAAKPDLDK_.2	#N/A	5.4260	5.1711	4.8897	4.7806	3.6716	4.9833	5.1946	4.5699	4.4667	4.3214	4.6026	4.3333	5.5513	5.5074	4.8148	5.1547	5.3503	5.6763	4.3744	5.1988
Tomm70	_C[Carbamidomethyl (C)]IDLEPDNATTYVHK_.3	#N/A	3.6302	4.7336	5.1551	4.4288	4.2583	4.2817	4.1840	3.3306	4.3970	3.8858	5.8596	3.5581	3.2394	4.4343	5.6338	5.2263	3.8683	4.9976	3.6795	5.0239
Tomm70	_GDASGLKR_.2	#N/A	5.6821	5.2118	5.0765	3.9904	4.9786	4.7160	4.7116	5.0260	5.0469	5.1358	5.6062	3.4514	5.2086	4.8392	5.7145	5.4186	5.3442	5.2432	4.9722	5.2574
Tomm70	_YM[Oxidation (M)]AEALLLR_.2	#N/A	4.8022	5.3125	5.6088	5.4259	4.7959	5.0890	5.2957	4.9259	4.4374	5.1241	5.2917	5.5719	4.9051	5.9224	4.7044	5.0506	5.3216	5.4713	5.6192	4.9645
Tomm70	_C[Carbamidomethyl (C)]AEGYALYAQALTDQQQFGK_.2	#N/A	3.4804	3.4540	4.3566	4.2090	4.0667	4.1389	3.2221	3.8276	4.5674	2.8300	3.6556	3.4896	3.5011	4.0038	3.7514	4.1932	3.7185	4.1147	4.8191	4.6106
Tomm70	_AS[Phospho (STY)]PALGSGHHDGSGDSLEM[Oxidation (M)]SSLDR_.3	#N/A	9.7991	9.8887	9.4423	8.8870	8.8775	9.5622	9.4104	9.8556	9.5521	9.4108	9.7024	9.4879	9.5827	10.6679	9.0217	9.2871	9.5618	9.6438	9.6269	9.2562
Tomm70	_SDEDKDKEGEALEVK_.3	#N/A	4.3794	3.6234	3.4148	3.3100	3.7171	3.9412	3.8240	4.0797	4.4648	4.5857	4.2575	3.5129	3.9911	3.6761	3.4012	3.2234	3.1166	3.4663	4.5289	3.2122
Tomm70	_WKEVAQDC[Carbamidomethyl (C)]TK_.2	#N/A	3.7934	3.5257	3.3300	3.7908	4.0003	3.9382	5.4242	4.0191	4.4688	3.6040	3.9475	3.2518	3.1334	3.3680	5.2649	3.1555	3.5163	3.6331	3.9322	3.0453
Tomm70	_GFEEIIK_.2	#N/A	3.3442	0.2923	3.8805	4.3452	3.7688	3.8022	3.2793	3.0446	3.1995	3.3725	1.2679	3.0472	3.3649	3.8676	3.3047	4.4248	3.5823	4.2509	3.8995	3.8346
Tomm70	_QAYTANNSSQVQAAMK_.2	#N/A	4.4810	3.5218	3.5164	3.2084	3.8187	4.1662	4.4160	4.1813	4.3362	3.7366	3.8149	3.4113	4.5017	3.5035	3.2996	3.3250	3.2182	3.1141	4.2636	3.4705
Tomm70	_NSERKT[Phospho (STY)]PEGR_.2	Q9CZW5_T88_M1_Tomm70	4.3119	3.6909	3.3473	3.3775	3.6496	3.8737	3.8915	4.0122	3.9506	4.5182	4.3250	3.5804	4.0586	3.3344	3.4687	3.1559	4.5499	3.2832	4.4327	3.3014
Tomm70	C[Carbamidomethyl (C)]LEDVTAVC[Carbamidomethyl (C)]LLEGFQNEQSM[Oxidation (M)]LLAD	#N/A	4.2808	4.5391	3.8174	3.3034	2.9869	3.0203	4.0612	4.5065	3.9814	4.0914	3.4600	4.2652	4.1468	3.8555	4.0866	3.6429	4.0037	3.1457	3.4448	3.1157
Rps19	_DVNQQEFVR_.2	#N/A	7.5061	7.2874	7.2342	7.1305	7.3774	7.3506	7.3677	7.4794	7.1400	7.5146	7.3711	7.6472	7.4805	7.2345	7.5994	7.4121	6.8078	7.3889	7.3322	7.3100
Rps19	_ELAPYDENWFYTR_.2	#N/A	4.3772	6.8658	6.9967	6.7446	6.4299	6.6704	6.2829	6.3278	3.8850	4.1878	6.7985	6.5839	6.3347	6.3528	6.8535	6.3554	6.6343	6.4249	6.2669	6.3304
Rps19	_IAGQVAAANK_.2	#N/A	4.1891	4.5893	3.7256	3.3952	4.2921	4.4293	3.6019	3.7620	5.0559	3.9997	4.3235	4.3570	4.2386	3.4703	5.2723	3.8410	4.4613	3.3772	4.5364	2.9340
Rps19	_GGAGVGSMTK_.2	#N/A	4.4825	4.3374	4.0191	3.1017	3.1886	3.4189	3.6114	3.2073	4.4053	4.0635	3.2788	4.0636	4.5134	4.0571	4.4820	3.8446	4.2054	4.4449	3.2431	3.7344
Emc4	_S[Phospho (STY)]DRGSGQGDSLYPVGYLDK_.3	Q9CZX9_S32_M1_Emc4	4.0282	3.9746	4.5645	3.6612	3.3659	3.5900	4.1752	3.7286	4.2343	4.2345	3.1078	3.8641	4.3423	4.5516	3.7524	4.3731	4.2662	3.1151	4.6707	3.5634
Ube2v1	_LPQPPEGQC[Carbamidomethyl (C)]YS[Phospho (STY)]N_.2	Q9CZY3_S146_M1_Ube2v1	3.4413	3.9216	4.4349	9.1195	4.2799	3.0031	3.2612	3.6231	3.3203	3.6477	3.6946	2.9501	3.4283	4.4729	2.8384	8.4564	3.1204	4.0291	8.8742	4.1502
Ube2v1	_VNM[Oxidation (M)]SGVSSSNGVVDPR_.2	#N/A	4.1587	5.4265	4.9549	3.5983	4.3285	4.7107	4.1123	3.7629	5.3839	3.6459	3.8267	5.3254	4.2794	4.4218	5.4199	5.2434	3.6777	4.9509	4.4800	4.6891
Nt5c3a	_DNSNIILLGDS[Phospho (STY)]QGDLR_.2	Q9D020_S273_M1_Nt5c3a	4.1925	7.0401	3.7291	3.3917	2.8986	6.7497	6.3543	4.4182	4.0697	4.0031	3.5483	4.3535	4.2234	7.8558	6.4591	5.6811	3.9154	6.6516	5.5331	3.4444
Ssbp3	_NS[Phospho (STY)]PNNISGISNPPGTPR_.2	Q9D032_S347_M1_Ssbp3	7.1726	3.1236	7.0712	7.0845	2.9015	3.7060	6.9245	7.3559	4.1183	4.0060	2.9918	4.3506	4.2263	7.7712	4.1719	5.6287	3.9184	3.2311	5.7291	3.4474
Pdhb	_VLEDNSVPQVK_.2	#N/A	9.8710	9.5385	9.9233	9.8635	9.9689	9.5607	9.7712	9.9505	9.8994	9.7930	9.8564	9.7503	10.0507	9.9525	10.0461	9.7302	9.8185	9.6373	9.6367	9.8247
Pdhb	_VVSPWNSEDAK_.2	#N/A	9.4174	9.3163	9.1715	9.2902	9.3281	9.1841	9.1683	9.5088	9.1493	9.2984	9.1915	9.1730	9.3323	9.4464	9.4468	9.2395	8.9771	9.1228	9.1808	9.3083
Pdhb	_EGIEC[Carbamidomethyl (C)]EVINLR_.2	#N/A	8.8256	8.6302	8.5877	8.6472	8.4367	8.6288	8.4992	8.8699	8.6794	8.5995	8.6507	8.6408	8.2076	8.8542	8.1075	8.1897	8.6898	8.3373	8.4264	8.5504
Pdhb	_VFLLGEEVAQYDGAYK_.2	#N/A	8.5923	8.2254	8.5633	8.2334	8.1666	8.0807	8.0551	8.6880	8.3856	8.5786	8.3093	8.2374	8.6512	8.3946	8.2760	8.6561	8.5347	8.0404	8.3309	8.4494
Pdhb	_DFLIPIGK_.2	#N/A	5.2593	6.2705	6.7041	7.2878	6.3679	5.6583	6.6378	5.6645	7.0838	7.0017	6.5825	6.5425	5.7703	6.6271	6.9152	7.1446	5.6475	6.9537	7.0058	7.0497
Pdhb	_TIRPM[Oxidation (M)]DIEAIEASVM[Oxidation (M)]K_.3	#N/A	7.7785	7.3320	7.9725	7.8085	7.7212	7.6770	7.4024	7.6884	7.5653	7.9535	7.5186	7.8864	7.9920	7.6377	7.4645	7.7956	7.3314	7.7789	8.0182	8.0449
Pdhb	_IM[Oxidation (M)]EGPAFNFLDAPAVR_.2	#N/A	7.1172	6.6154	7.1056	6.8987	7.0571	6.6451	6.8910	6.9501	6.5628	6.6976	6.8198	7.0940	7.2722	7.3018	6.6319	6.8332	6.6667	6.9674	6.9787	6.8730
Pdhb	_TYYM[Oxidation (M)]SAGLQPVPIVFR_.2	#N/A	8.2244	8.0571	8.0634	8.2100	8.1297	8.0524	7.9145	8.0840	8.1007	8.4361	8.1594	8.1409	8.4530	8.2732	8.2283	8.2145	8.1352	8.0334	8.2022	8.2883
Pdhb	_EAINQGM[Oxidation (M)]DEELERDEK_.3	#N/A	5.9897	6.3950	6.3118	6.1287	6.2224	6.6531	6.9060	6.1600	6.4320	6.8684	6.3876	6.7304	6.9160	6.9838	6.8485	6.5522	5.8418	6.5632	6.7541	6.8482
Pdhb	_PVGHC[Carbamidomethyl (C)]LEAAAVLSK_.3	#N/A	3.4592	3.8599	4.4966	4.1251	3.3646	3.6994	3.3820	3.6848	3.3022	3.2698	2.7493	3.5861	3.4677	4.5346	4.5091	4.3221	3.1821	3.9674	4.2664	4.2119
Mdm1	_ASS[Phospho (STY)]QDGLETLKK_.2	Q9D067_S556_M1_Mdm1	8.1355	8.3250	3.7528	8.0086	7.8985	7.8012	7.4655	8.2583	4.1390	8.0718	8.0716	4.3298	4.2471	8.4981	7.8308	8.3763	8.3426	8.2641	8.1518	8.4371
Mdm1	_VQGTHFS[Phospho (STY)]R_.2	Q9D067_S357_M1_Mdm1	4.2797	4.7863	3.8163	3.3046	2.9858	3.0191	4.0623	3.8276	3.9825	4.0903	3.4612	4.2664	4.1480	3.1498	4.0877	3.6418	4.3844	3.4678	3.1164	3.1168
Mdm1	_RAS[Phospho (STY)]S[Phospho (STY)]QDGLETLKK_.3	Q9D067_S555_M2_Mdm1	4.1014	4.2633	3.1369	3.5880	3.4391	3.0450	4.0365	3.8018	3.9567	4.1161	3.4353	3.7909	4.1221	4.6248	4.0618	2.9455	4.3395	4.2321	3.1423	3.0909
Mgrn1	_TQSKS[Phospho (STY)]PDSTLR_.2	Q9D074_S428_M1_Mgrn1	8.6647	8.6331	8.6658	8.8511	8.5208	8.3449	4.2115	8.6767	8.3000	8.8441	3.1441	8.4976	8.7302	8.7443	8.0041	8.3338	8.5345	8.5349	8.3666	2.9814
Mgrn1	_ESSS[Phospho (STY)]PESFGTEEGDEPSLK_.2	Q9D074_S470_M1_Mgrn1	3.6199	3.6934	4.0328	3.9585	4.1295	4.2678	3.1760	3.8748	4.0045	3.6120	3.8374	3.4195	4.1516	4.0709	2.9717	3.4827	5.3783	3.8008	4.0999	3.7482
Mgrn1	_YKEDADS[Phospho (STY)]PTEDGEKPR_.2	#N/A	4.0069	3.8348	3.7617	4.5992	3.6988	4.1861	4.4065	3.8407	4.2240	4.0917	4.4136	4.4789	5.0813	4.3862	4.2240	3.5752	3.9526	4.4860	3.8760	3.7181
Srsf9	_GRDS[Phospho (STY)]PYQSR_.2	Q9D0B0_S205_M1_Srsf9	5.4311	6.2161	3.7943	4.4314	4.0966	6.2266	3.4445	4.4592	3.5036	3.4643	3.8780	3.1334	3.2787	3.7814	3.0217	3.6029	6.2449	4.3371	3.9857	3.7484
Srsf9	_GS[Phospho (STY)]PHYFS[Phospho (STY)]PFRPY_.2	#N/A	3.5603	7.2470	7.1114	7.0517	5.9353	4.3419	6.8420	3.7860	7.1860	7.3756	3.8575	6.5325	6.8152	7.5829	7.0307	6.7756	7.0421	6.7988	6.8676	6.9235
Srsf9	_STS[Phospho (STY)]YGYSR_.2	Q9D0B0_S190_M1_Srsf9	3.6858	4.3170	4.2221	4.0036	3.0235	5.0512	3.0167	3.3786	3.0758	3.8921	3.4502	4.2064	3.1838	4.2284	4.0947	4.0307	4.3767	4.2736	3.0718	3.9057
Tstd3	_EWVTYEISEEKQES_.2	#N/A	6.2412	6.1752	5.9539	5.7790	5.5879	5.1661	5.1060	6.1794	5.5687	6.1128	5.4613	5.6992	6.6721	6.8152	5.6109	6.7251	6.2157	4.3960	5.1804	5.3474
Pbdc1	_GADS[Phospho (STY)]GGEKEEGANR_.2	#N/A	3.1593	5.6913	4.1967	4.4249	3.3663	3.9042	4.2592	3.3850	3.0821	5.1849	4.4756	4.3647	3.1902	6.0642	5.5470	4.0223	4.3831	3.8183	3.0654	5.1708
Pbdc1	_GADS[Phospho (STY)]GGEKEEGANREGEK_.3	#N/A	3.2834	4.3801	4.1590	4.0666	3.4903	3.5236	3.5578	3.3231	3.4780	5.0986	2.9567	4.2695	3.6435	4.1461	3.5832	4.1463	3.9587	3.9724	3.8874	4.1131
Hnrnpm	_AFITNIPFDVK_.2	#N/A	6.2820	6.2287	6.3729	6.7974	6.6789	6.4507	6.6862	6.1671	5.5596	6.5799	6.7150	6.7132	6.3960	6.6710	6.0536	6.7082	6.4058	6.6433	6.2313	6.3660
Hnrnpm	_INEILSNALK_.2	#N/A	5.0802	5.5273	6.0323	5.8815	5.5050	5.7764	5.8116	5.0582	5.5711	5.5058	5.2229	5.7916	5.0145	5.3066	5.7793	5.7784	5.3643	5.4803	5.7404	5.3362
Hnrnpm	_MGLS[Phospho (STY)]MDR_.2	Q9D0E1_S527_M1_Hnrnpm	3.8399	3.4792	3.3765	3.7443	4.0468	4.0802	3.0013	4.0656	4.4223	3.6505	3.9010	3.2053	3.8708	3.4145	3.0266	3.2020	3.5628	3.5866	3.8857	3.0918
Hnrnpm	_MGLAMGGAGGAS[Phospho (STY)]FDR_.2	Q9D0E1_S617_M1_Hnrnpm	4.3658	3.6370	3.4012	3.3236	3.7035	3.9276	3.8376	4.0662	3.8967	4.5721	4.2710	3.5265	4.0047	3.3883	3.4148	3.2098	3.1030	3.2293	4.3788	3.2258
Hnrnpm	_VGS[Phospho (STY)]EIER_.2	Q9D0E1_S431_M1_Hnrnpm	4.3716	4.4483	3.9082	3.2126	3.0777	3.1110	3.9704	3.0964	3.8906	4.1822	3.3693	4.1745	4.0561	4.5587	3.9958	3.7337	4.0945	3.5598	3.2083	3.0249
Hnrnpm	_MGS[Phospho (STY)]GVER_.2	Q9D0E1_S512_M1_Hnrnpm	3.3932	3.9259	4.4306	4.1911	3.6001	3.6334	3.4480	3.6188	3.3682	3.2038	4.3477	3.6520	3.5336	4.4687	3.4734	4.2561	3.1161	4.0334	4.3324	1.0397
Lysmd1	_S[Phospho (STY)]YGSLVQSSC[Carbamidomethyl (C)]SPVR_.2	Q9D0E3_S23_M1_Lysmd1	3.8449	3.4408	3.5975	6.2442	4.0519	4.0852	2.9962	4.2624	4.4173	6.6034	3.8959	3.3303	5.6754	3.5845	3.0216	3.2071	3.2992	3.0331	4.1825	3.5515
Lysmd1	_DLFNGLDS[Phospho (STY)]EEENDGEEEVRPSKDEIGSSSGR_.4	Q9D0E3_S99_M1_Lysmd1	3.5983	3.1941	3.8441	4.3816	3.8052	3.8385	3.2429	4.5090	3.1631	3.4089	4.1426	3.4469	3.3285	3.8312	3.2683	2.9604	3.5459	4.2873	3.9359	3.7982
Pgm1	_YDYEEVEAEGANK_.2	#N/A	10.1261	10.0653	10.2417	10.3618	10.2328	10.2433	9.9529	10.5649	10.2803	10.3189	10.0391	9.8301	10.5818	10.0383	9.5837	10.0894	10.4530	9.7907	10.0508	10.1727
Pgm1	_ADNFEYSDPVDGSISK_.2	#N/A	7.2212	7.4719	7.6414	7.4414	7.3143	7.2309	7.3175	7.4379	7.6115	7.4783	7.0701	7.1155	7.6606	7.1911	6.7852	7.2896	7.2196	6.9329	7.2592	7.2094
Pgm1	_ELLSGPNR_.2	#N/A	6.7529	6.7094	7.2211	6.9051	6.9736	7.1789	7.3466	6.9250	7.1893	6.8418	6.5270	6.9325	6.8767	7.0250	6.8752	6.7584	6.5498	6.8119	6.8821	6.6898
Pgm1	_TQAYPDQKPGTSGLR_.3	#N/A	5.6263	6.3915	6.3481	6.2234	6.2710	6.8682	6.7351	5.8877	6.6068	6.7794	6.3836	5.8398	6.0290	6.1316	6.5587	6.0599	5.6533	6.3339	6.5820	6.4286
Pgm1	_IALYETPTGWK_.2	#N/A	6.6710	6.2899	6.2952	6.2619	6.4937	6.7856	7.1161	6.9295	6.1517	6.1913	6.6336	6.5315	6.6039	6.4040	6.3966	6.2913	6.4246	6.3981	5.9964	6.7827
Pgm1	_LIFADGSR_.2	#N/A	5.4951	4.4153	4.5244	5.3839	5.4569	5.6120	5.4393	5.6345	5.2297	5.8602	5.5628	5.3623	4.4182	5.5630	5.1744	5.2674	4.8360	6.1736	5.4024	5.6013
Pgm1	_QQFDLENK_.2	#N/A	4.8153	5.7448	4.1222	5.2411	4.6428	5.0276	5.1461	5.5921	4.9967	5.4625	5.0695	5.2759	4.5823	5.6025	4.7872	5.4617	5.0715	4.7237	5.3329	4.9328
Pgm1	_AIGGIILTAS[Phospho (STY)]HNPGGPNGDFGIK_.3	Q9D0F9_S117_M1_Pgm1	3.7195	7.7475	3.2561	3.8647	3.9264	4.3311	3.1217	8.9287	4.9536	3.3256	4.0167	3.3257	7.3056	9.2411	5.0540	3.0816	4.9775	7.7121	4.0061	2.9714
Pgm1	_VDLGVLGK_.2	#N/A	3.6858	4.0938	5.2808	3.5664	4.3441	4.0160	4.1170	3.2059	4.3634	4.6663	4.2863	4.3038	3.3641	3.7706	3.6684	3.9699	2.8120	4.0577	4.0911	5.0084
Pgm1	_INQDPQVM[Oxidation (M)]LAPLISIALK_.2	#N/A	4.7062	4.2761	4.5983	4.0262	3.4022	4.6230	3.4832	3.9727	4.7632	3.4807	3.2432	3.9150	3.2567	4.2430	4.0797	5.0191	4.3135	4.6228	4.5319	4.3877
Pgm1	_QEATLVVGGDGR_.2	#N/A	7.0419	3.8177	7.6221	7.5406	3.5228	7.0053	4.0183	6.6876	7.2748	6.6263	2.9509	6.3636	4.1854	3.2076	3.5955	3.0291	4.4231	3.4100	3.0586	6.7451
Pgm1	_FFGNLM[Oxidation (M)]DASK_.2	#N/A	4.0500	3.9334	4.4231	3.6727	4.3128	3.9643	4.2083	4.6611	2.7839	4.5542	4.3552	3.9110	3.4166	4.4612	4.8085	4.2486	4.1759	4.0409	4.4813	4.3077
Pgm1	_FNISNGGPAPEAITDK_.2	#N/A	4.3426	4.4773	3.8792	3.2416	3.0487	5.5136	4.8199	3.0675	3.9196	4.1532	4.8563	4.8228	4.0850	3.9173	5.5781	3.7047	4.0655	3.0839	3.3830	3.9409
Pgm1	_DLEALMLDR_.2	#N/A	3.3305	3.5999	3.8668	4.3589	4.1691	4.3932	3.3720	4.5318	3.4311	3.5369	3.8054	3.0609	3.5391	3.8539	2.9492	3.6754	3.5686	4.2646	3.9132	3.8209
Pgm1	_LYIDSYEKDVAK_.3	#N/A	4.5331	3.4697	3.5686	3.1563	3.8709	4.1141	3.6702	4.2335	4.3884	3.6844	3.8671	3.3591	4.5539	3.5557	3.2474	3.3772	3.2704	3.0620	4.2114	3.5227
Stx17	_LTSS[Phospho (STY)]C[Carbamidomethyl (C)]PDLPSQSDK_.2	Q9D0I4_S288_M1_Stx17	8.6808	9.5299	8.7691	3.9267	8.0425	8.0825	3.7129	3.9536	3.6915	3.8857	8.3684	7.9778	8.2004	8.5197	3.5715	3.6886	3.8444	8.0601	3.8114	8.0676
Ptms	_[Acetyl (Protein N-term)]S[Phospho (STY)]EKSVEAAAELSAK_.2	Q9D0J8_S2_M1_Ptms	4.4500	8.0975	8.0719	4.0189	3.8998	7.6333	7.5396	3.9672	6.5752	6.6472	3.6636	6.0096	6.6078	8.6408	7.6193	6.3259	6.6458	6.7547	7.3328	7.4548
Ptms	_TAEEEDEADPKR_.3	#N/A	3.5648	4.1169	4.3388	4.0933	3.3424	3.4969	3.9172	3.5154	4.8514	3.6136	3.8799	3.9350	3.5899	4.3525	3.5069	4.1562	3.6028	6.3498	3.9999	4.1742
Oxct1	_FYTDPVEAVK_.2	#N/A	8.4996	8.5661	8.8446	8.6645	8.7863	8.4372	8.4974	8.5251	8.5904	8.5048	8.6454	8.3186	8.2299	8.4729	8.7320	7.9729	8.5400	8.4798	8.3788	8.2924
Oxct1	_GVFDVDKK_.2	#N/A	8.1204	8.0718	8.4083	8.0940	8.0791	8.0917	8.2849	8.4805	8.0731	8.2109	8.2413	8.1174	8.5080	8.7204	8.8066	8.1781	8.2872	8.3503	8.4304	8.5796
Oxct1	_DGSVAIASKPR_.2	#N/A	8.0723	8.3180	8.4007	8.1947	7.9939	8.2357	8.4675	8.2299	8.1562	8.2495	8.0085	8.0401	8.3895	8.4104	8.2861	8.0976	7.9585	8.0523	7.9788	8.1395
Oxct1	_YGDLANWM[Oxidation (M)]IPGK_.2	#N/A	8.0159	7.3510	7.5908	7.5817	7.9801	7.9670	7.5416	7.9901	7.4769	7.9175	7.7815	7.9213	7.9357	7.2972	7.4725	7.6369	7.6728	8.1494	7.7310	7.8406
Oxct1	_AGGAGVPAFYTSTGYGTLVQEGGSPIK_.3	#N/A	4.7783	4.3671	4.3259	5.4726	4.2416	5.4030	4.9244	3.1295	4.1820	5.2633	4.5938	3.4361	5.5271	4.6174	4.2871	4.7978	5.0383	4.4333	4.2126	4.9909
Oxct1	_GM[Oxidation (M)]GGAM[Oxidation (M)]DLVSSSK_.2	#N/A	6.2897	6.1960	3.4704	5.5659	6.6737	6.5782	6.0354	7.0718	6.8956	7.0524	6.5143	6.7763	7.1043	5.4935	6.9116	6.7852	6.6648	6.6260	6.4337	6.7661
Oxct1	_C[Carbamidomethyl (C)]TLPLTGK_.2	#N/A	3.5565	4.4463	4.6796	4.1329	4.7347	5.1309	5.3325	3.2568	4.7686	3.7628	3.5795	5.1304	3.3132	4.0798	6.2680	4.2460	4.6653	5.0385	5.7032	5.2582
Oxct1	_QFLSGELEVELTPQGTLAER_.2	#N/A	3.2523	5.7445	4.1901	4.6404	4.7291	5.1810	5.3450	5.1409	5.2523	5.6162	2.9877	5.3894	5.5201	6.0688	5.2624	4.1153	3.8918	5.3388	5.5113	5.2055
Oxct1	_DLTAVSNNAGVDNFGLGLLLR_.2	#N/A	4.9309	5.6508	5.5422	4.9857	5.0066	5.6018	5.8453	5.5548	5.5654	5.5396	5.4850	5.3727	5.7268	6.2771	5.5053	5.4368	5.6687	5.6114	5.2349	5.7493
Oxct1	_ETVTVLPGASFFSSDESFAMIR_.2	#N/A	3.3446	3.3756	4.6581	4.6904	4.1832	3.0933	4.1188	3.7198	3.4170	3.5510	3.7913	3.0468	3.5250	6.3487	3.4468	3.6895	3.2171	3.9324	4.2314	3.1073
Oxct1	_STGC[Carbamidomethyl (C)]DFAVSPNLM[Oxidation (M)]PM[Oxidation (M)]QQIST_.2	#N/A	5.6810	5.5221	3.8955	4.8841	5.3544	5.4001	5.1958	5.5570	5.8719	5.7841	5.0383	5.5826	5.3174	4.6190	5.8967	5.8237	5.7707	5.5838	5.5527	5.6597
Oxct1	_VVVTMEHSAK_.2	#N/A	4.1525	3.1320	3.7237	5.4737	3.4902	3.7143	3.2150	4.4128	4.1099	4.3588	2.9835	4.3589	4.2180	5.1956	3.9224	2.9966	4.7023	3.2394	3.5385	2.7317
Oxct1	_M[Oxidation (M)]ISSYVGENAEFER_.2	#N/A	3.1115	4.2077	4.1489	4.2196	4.9702	3.3279	3.7297	3.4111	4.0693	5.3262	5.2202	4.7729	5.1920	4.2468	4.3465	3.9744	5.1995	4.0877	4.7286	4.6423

Armc10	_S[Phospho (STY)]AEDLTDGSYDDILNAEQLKK_.3	#N/A	3.3147	9.9392	8.9095	4.2695	3.5217	3.5550	9.0347	3.5404	9.5927	3.1253	2.9253	3.7305	9.7311	9.6273	3.5518	4.1777	4.5385	9.9221	3.6523	4.0817
Armc10	_S[Phospho (STY)]AEDLTDGSYDDILNAEQLK_.2	#N/A	9.2285	9.8164	9.7272	9.0609	7.4098	9.6240	9.6944	3.3753	9.5347	9.3757	8.8990	8.9782	9.0858	4.2251	3.7169	4.0126	9.5702	9.7365	9.4238	8.8927
Armc10	_LLYLLESTDDPVITEK_.2	#N/A	4.0922	3.8271	4.4967	1.5089	3.2982	3.1859	2.7289	3.1407	3.2463	4.1668	2.5236	1.7964	3.9812	4.4838	3.5772	4.3053	3.5250	2.9917	3.4063	4.4508
Rnmt	_ASVASDPES[Phospho (STY)]PPGGNEPAAASGQR_.2	#N/A	10.4337	10.5799	10.4999	10.5036	9.9561	10.3695	10.1599	10.5120	10.1120	10.5178	10.2404	10.0878	10.8305	10.9814	9.9918	10.4444	10.6780	10.3541	10.4584	10.2880
Rnmt	_GDVS[Phospho (STY)]EDEPSLGR_.2	Q9D0L8_S100_M1_Rnmt	11.8932	11.6688	11.9129	11.8596	11.1421	10.8797	11.2064	11.2186	10.8482	12.0971	11.2390	11.2223	11.7970	11.5471	10.3139	11.4177	11.2018	11.1227	11.2291	11.2078
Rnmt	_EFGEDLVEQNSSYVQDS[Phospho (STY)]PSKK_.3	#N/A	3.8952	6.5139	3.9810	3.1398	3.2329	3.4570	4.3082	3.1693	4.3673	6.8787	3.2408	3.9971	4.4753	4.0191	3.8854	6.1349	4.1674	6.2402	3.2811	3.6964
Rnmt	_EFGEDLVEQNSSYVQDS[Phospho (STY)]PSKKR_.3	#N/A	5.7242	4.7072	3.9555	6.3090	3.1250	3.1583	6.3652	4.0038	3.8606	4.2295	3.3393	6.3217	6.8091	6.3403	5.9501	3.7810	4.1418	3.8850	3.3240	5.8455
Rnmt	_EFGEDLVEQNSSYVQDS[Phospho (STY)]PSK_.2	#N/A	8.5818	8.9889	9.5786	8.6941	5.9050	8.9352	8.7753	5.5965	8.9492	9.5025	6.5771	3.6171	8.9081	9.2283	8.4752	9.0210	9.2047	8.7311	8.9829	8.6680
Rnmt	_LPENT[Phospho (STY)]PPC[Carbamidomethyl (C)]QQVDQPK_.2	Q9D0L8_T34_M1_Rnmt	3.4235	6.6604	5.5978	6.5954	4.2621	6.9961	6.3312	3.1239	3.3381	3.6298	3.7125	2.9679	3.4442	3.9469	6.1386	3.7684	3.6616	4.1716	3.8202	3.9139
Cyc1	_HLVGVC[Carbamidomethyl (C)]YTEEEAK_.2	#N/A	5.0275	5.0924	5.4466	5.2308	4.4343	4.9198	4.3774	4.2303	5.2156	4.8787	4.8727	4.9667	4.3514	5.5834	4.7991	4.9273	4.8463	5.1134	5.2508	5.1131
Cyc1	_LSDYFPKPYPNPEAAR_.3	#N/A	7.0602	7.8460	7.5123	7.2767	7.6416	7.0681	7.8791	7.6792	7.4688	7.6890	7.7527	7.2672	8.2134	7.7690	7.3448	7.9125	4.2157	7.9695	7.6371	7.4074
Cyc1	_HGGEDYVFSLLTGYC[Carbamidomethyl (C)]EPPTGVSLR_.3	#N/A	5.5035	4.7415	4.8243	5.3625	5.6313	4.7049	5.0480	4.4965	4.9578	5.0028	4.8830	5.1683	3.1822	5.3609	4.7044	5.2685	4.4909	5.3094	4.8883	4.5926
Cyc1	_GLLSSLDHTSIR_.2	#N/A	6.4891	6.5580	6.2215	6.6777	6.6972	6.5957	6.6035	6.5624	6.0356	6.6033	6.7132	6.6857	6.5288	6.5748	6.6574	6.5616	6.5876	6.5117	6.2010	6.7489
Cyc1	_DVATFLR_.2	#N/A	3.5076	4.7795	5.8151	6.9074	6.4954	5.5362	6.1784	3.2080	6.7259	6.5010	7.0623	5.9939	5.8628	4.0310	6.2449	6.5902	3.9809	6.0573	7.3011	6.8659
Cyc1	_ALAEEVEVQDGPNDDGEM[Oxidation (M)]FM[Oxidation (M)]RPGK_.3	#N/A	5.6382	5.3319	5.1654	4.2787	4.1724	5.1852	5.0482	5.4665	4.7168	5.4408	4.3329	5.0384	5.4754	4.3974	4.6658	5.1415	5.1970	4.6485	4.9990	4.5783
Cyc1	_WASEPEHDHRK_.3	#N/A	3.3383	3.1636	3.8746	4.3510	4.1769	4.4010	3.3642	3.7261	3.4233	4.3974	3.7976	3.0531	3.5313	4.5759	4.0275	3.6832	3.2233	3.9261	4.3290	4.2532
Dynll2	_DIAAYIKK_.2	#N/A	6.9155	7.3066	6.0108	6.9026	7.0749	6.9288	6.9449	7.5400	6.8474	6.3751	6.3860	7.3873	7.5512	6.8133	6.5574	7.0898	6.7961	6.9782	6.9447	6.9533
Dynll2	_NFGSYVTHETK_.2	#N/A	6.8652	6.6770	6.5282	6.3265	6.3114	6.2813	6.7572	6.5922	6.4930	6.5726	6.2487	6.1685	5.7044	6.3181	6.3439	5.6059	6.2841	6.3459	5.9387	5.8753
Dynll2	_YNPTWHC[Carbamidomethyl (C)]IVGR_.2	#N/A	9.0755	9.1841	8.9760	8.8290	9.1560	8.8229	9.1885	9.3877	8.7332	8.8617	8.9600	8.9515	8.8707	8.6954	8.6734	8.4765	9.1203	8.9196	8.6060	8.4950
Dynll2	_NADM[Oxidation (M)]SEDMQQDAVDC[Carbamidomethyl (C)]ATQAMEK_.3	#N/A	4.0414	3.6921	4.1126	3.7176	3.7452	4.0136	3.5681	3.6936	3.5705	3.2346	4.2160	3.4961	4.0139	3.6928	3.3507	3.9328	3.4075	3.7852	4.1109	3.6598
Hint2	_ISQAEEDDQQLLGHLLLVAK_.3	#N/A	4.3211	3.6817	3.3566	3.3682	3.6589	4.3261	3.8822	4.0215	4.1764	4.5275	3.6551	3.4544	4.3419	3.2685	3.6307	3.1652	4.5592	3.2740	4.4234	3.3107
Snu13	_QQIQSIQQS[Phospho (STY)]IER_.2	Q9D0T1_S122_M1_Snu13	3.9336	4.3385	3.8017	4.0725	3.2713	4.0458	3.6329	3.6144	4.2736	4.1399	3.2337	4.2511	3.8001	4.0120	4.8323	3.6103	4.3631	3.8532	4.4435	3.5555
Ebag9	_KLS[Phospho (STY)]GDQITLPTTVDYSSVPK_.2	#N/A	3.9139	4.0968	4.4502	3.7834	3.2516	3.4659	3.9795	3.6143	3.9815	4.3221	3.1279	3.9863	4.1128	7.2800	3.8746	4.2588	4.1520	6.8624	3.3377	3.7888
Fxyd6	_APGDEEAQVENLITTNAAEPQKAEN_.3	#N/A	11.2016	11.0888	10.6120	10.4717	10.1134	10.4670	10.7167	11.4152	10.5660	10.1816	10.1696	10.8017	11.9110	11.1283	9.1148	10.2686	11.1149	10.9237	10.4205	10.3998
Fxyd6	_APGDEEAQVENLITTNAAEPQK_.2	#N/A	3.3836	2.2399	2.3998	3.9826	3.0445	1.9873	3.4905	2.6202	4.1304	3.9131	2.2291	0.7633	3.1628	3.7463	4.0737	4.0517	1.5017	3.5336	1.7994	3.8847
Ints12	_SS[Phospho (STY)]PITVQTSK_.2	Q9D168_S127_M1_Ints12	8.2568	3.6641	8.3154	3.9292	4.0195	8.0663	7.9138	8.4990	7.9427	8.1123	7.8812	3.3902	8.6984	8.9232	8.2932	8.3379	3.3818	8.1985	4.0706	8.1897
Ints12	_SVS[Phospho (STY)]C[Carbamidomethyl (C)]DNVSK_.2	Q9D168_S377_M1_Ints12	6.3963	6.8352	4.3266	6.8768	6.6857	3.3521	6.1795	5.7903	5.7465	7.2345	6.4740	6.3022	6.0520	6.3277	5.9086	4.1352	4.2722	6.0356	5.9389	3.8013
Gatd3a	_GVTEAHVDQK_.2	#N/A	6.6269	6.5370	6.8000	6.5243	6.4647	6.3183	6.7716	6.9502	6.9222	6.8455	6.9300	6.9735	6.8915	6.9499	7.2945	7.0181	6.2545	6.7830	6.8367	6.5581
Gatd3a	_ITSLAQLNAANHDAAIFPGGFGAAK_.3	#N/A	6.5758	6.9190	5.9761	6.8454	6.9079	6.7566	6.5375	6.5265	6.5040	6.7196	6.0156	6.6871	6.3717	6.9649	6.8414	6.4801	6.9325	6.4487	6.8881	6.8194
Gatd3a	_NLSTFAVDGK_.2	#N/A	6.1195	6.2789	6.0361	6.6304	6.4281	6.1474	6.7654	5.4892	6.1977	6.9034	6.1589	6.7330	7.5736	6.4604	6.5461	6.5626	5.3753	6.7005	6.5568	6.2113
Gatd3a	_NVLELTGK_.2	#N/A	4.3215	4.4985	3.8581	5.4227	3.6201	4.5223	5.6163	3.0463	5.2414	4.1321	3.8675	4.5934	5.0507	4.8484	5.4217	5.4771	4.0444	5.6251	5.8248	5.7582
Gatd3a	_VNKEVER_.2	#N/A	3.5095	4.4238	4.0458	4.1799	3.5522	4.7469	4.9238	5.1390	3.8942	4.5041	3.9472	3.9535	3.3602	3.7825	5.3969	3.8544	3.7995	4.0367	4.2480	4.4143
Gatd3a	_NVLAESAR_.2	#N/A	3.7356	3.5835	3.2722	3.8486	3.9425	3.9918	3.5993	1.4790	3.5570	3.3095	4.4887	3.6393	4.2883	3.3102	5.4187	3.0977	3.4585	4.6653	4.6284	5.2108
Gatd3a	_GGAEVQIFAPDVPQMHVIDHTK_.3	#N/A	4.1230	4.6033	5.0611	4.5623	3.4607	3.6848	4.3539	4.4423	4.1394	4.3293	3.0130	3.6509	4.2475	6.0048	3.6575	2.9671	4.0117	3.2099	3.5090	3.4685
Cndp2	_TVFGVEPDLTR_.2	#N/A	7.0754	7.4452	6.9690	7.4125	7.5658	7.2557	7.0682	7.3158	7.1419	7.1216	7.0582	7.1524	7.3263	7.5299	7.5776	7.2965	7.4593	6.9846	6.9905	7.2184
Cndp2	_AVFQYIDENQDR_.2	#N/A	7.2647	7.3670	7.7142	7.3509	7.4920	7.2128	7.0830	7.0380	7.5954	7.3963	7.2911	7.4588	7.4608	7.4584	6.9935	7.5774	7.7464	6.9648	7.2288	7.3218
Cndp2	_TGQEIPVNLR_.2	#N/A	6.3792	6.7870	7.0358	7.4429	7.3014	6.8784	7.0543	7.0792	6.9413	6.8121	6.8443	7.1102	6.8297	7.0391	7.2757	6.6340	6.8015	6.8261	6.8600	6.9293
Cndp2	_LNYIEGTK_.2	#N/A	3.4756	6.0151	3.6971	4.2050	3.9088	4.4886	4.9326	4.8448	4.7554	6.4534	4.4219	3.1593	4.4315	3.7832	7.0763	4.7431	3.3219	6.5870	4.6535	4.0499
Cndp2	_FAELQSPNK_.2	#N/A	3.0896	5.9610	5.1676	5.8828	5.9566	5.5949	5.4742	6.2520	5.8921	6.2282	3.9243	6.2887	3.1103	6.1467	6.1673	6.1215	3.3276	5.5460	5.7426	5.9051
Cndp2	_EGGSIPVTLTFQEATGK_.2	#N/A	5.4647	4.4420	5.9115	6.3417	5.8123	5.5554	5.6525	5.5593	6.5818	6.2840	5.6169	5.4273	4.4088	6.2407	5.4188	5.7862	6.3765	4.9286	5.8414	6.0403
Tbcb	_YEIS[Phospho (STY)]PEAYER_.2	Q9D1E6_S110_M1_Tbcb	9.1250	9.3361	9.6063	9.3799	8.6225	9.1374	3.2760	8.3137	8.9825	9.5250	8.9310	8.7368	8.9141	9.2700	8.2139	8.7726	9.0354	8.8130	8.9368	8.7797
Tbcb	_AQQEAEAAQR_.2	#N/A	2.8590	4.3220	4.8904	3.7578	3.3048	5.0990	4.4014	3.8395	4.8026	4.2216	3.2655	4.3010	3.9523	4.1376	4.9306	4.2765	4.0437	4.6890	3.8491	4.2205
Tbcb	_AQASAISVGSR_.2	#N/A	3.8396	4.1632	5.8836	3.8498	3.1773	4.2805	4.3638	3.5399	4.3830	4.8493	3.1735	4.0527	3.8603	4.3629	3.8202	4.4547	4.0776	3.7555	4.7337	4.3299
Akt1s1	_SSDEENGPPSS[Phospho (STY)]PDLDR_.2	Q9D1F4_S213_M1_Akt1s1	7.8820	7.9464	8.8329	8.0955	7.6572	8.3047	7.3431	7.5504	7.4184	7.9458	7.3260	7.8671	7.9564	8.3084	7.4736	7.5974	8.1478	7.4811	8.1721	7.8246
Akt1s1	_EDEEEDEDEPTETETS[Phospho (STY)]GER_.2	Q9D1F4_S117_M1_Akt1s1	8.0398	7.7634	8.4286	8.1108	7.7439	7.7474	7.7381	8.0484	4.3464	7.6182	7.6884	8.0760	4.5118	8.8441	7.8525	7.9769	8.7057	3.5107	7.8360	7.8783
Akt1s1	_LNT[Phospho (STY)]SDFQK_.2	Q9D1F4_T247_M1_Akt1s1	4.4699	3.4494	3.4063	7.0606	5.2559	4.0317	3.7334	4.4596	5.0633	6.8014	6.1040	5.1761	5.6893	6.1825	3.3106	5.6318	5.1918	5.5320	5.4460	3.1216
Akt1s1	_TEARSS[Phospho (STY)]DEENGPPSS[Phospho (STY)]PDLDR_.3	#N/A	4.7471	3.9681	4.9223	5.5318	5.0655	5.0997	6.0706	3.8225	3.3972	3.4672	6.1113	5.5121	3.5239	5.9237	3.6767	5.6904	5.5755	5.4195	4.2881	4.2288
Akt1s1	_AATATRPPGPPPAPQPPS[Phospho (STY)]PAPS[Phospho (STY)]PPPRPALAR_.4	Q9D1F4_S88_M2_Akt1s1	3.4601	4.5427	3.9964	4.2293	4.2986	3.6863	3.3951	3.1604	3.3153	3.2566	4.2948	2.9313	3.4808	7.8386	3.4205	3.8050	3.6981	4.1350	3.7836	3.9504
Rab1b	_EFADSLGVPFLETSAK_.2	#N/A	7.4325	7.4452	7.5668	7.5631	7.0210	7.3465	7.1396	7.5665	7.5414	7.6877	7.2351	7.1271	7.1709	7.1852	6.5106	6.6103	7.5802	7.0857	6.7255	7.1845
Rab1b	_[Acetyl (Protein N-term)]M[Oxidation (M)]NPEYDYLFK_.2	#N/A	5.9653	4.5512	4.4858	5.0626	4.4948	5.2605	4.9322	5.3052	5.8413	4.3016	4.5533	5.4971	5.5982	3.8585	4.8164	5.3668	5.8926	5.5071	4.8663	5.4937
Rab1b	_GAHGIIVVYDVTDQESYANVK_.3	#N/A	6.3785	5.7293	4.1072	5.8108	3.2767	3.6652	4.7402	6.2322	5.1907	4.7045	3.3670	5.4728	4.6015	5.3147	3.7968	3.9327	4.2935	4.4908	4.2541	3.9297
Rab1b	_MGPGAAS[Phospho (STY)]GGERPNLK_.2	#N/A	4.0943	5.1950	5.2510	5.8429	3.4320	5.1941	5.4417	3.7552	3.7560	4.4959	5.1176	3.8702	3.9044	5.3143	3.7585	3.7147	3.8472	3.8712	3.8707	3.6351
Lrrc57	_SLSLNNNK_.2	#N/A	4.6428	4.4800	4.3039	7.2234	6.6957	3.5068	5.1983	3.4922	3.4949	4.5779	4.8858	6.6839	3.2974	7.0866	4.5644	6.7656	4.6433	4.8645	2.9582	4.0193
Atp6v1f	_DTTINEIEDTFR_.2	#N/A	8.5748	8.0950	8.9511	8.4092	8.3766	8.3374	7.9899	7.9515	8.7584	8.4172	8.3587	8.2059	8.2994	8.5798	8.8318	8.8661	8.6093	8.1624	8.4817	8.6714
Atp6v1f	_DDIGIILINQYIAEM[Oxidation (M)]VR_.2	#N/A	4.3361	4.6356	5.3593	4.6878	4.2611	4.8954	3.1162	4.3220	4.5228	4.6562	3.5497	3.9132	4.2201	4.0965	4.9423	4.2812	5.0374	4.0757	3.6574	3.9635
Atp6v1f	_GM[Oxidation (M)]FTAEDLR_.2	#N/A	6.5367	6.4577	6.9583	6.6855	6.7800	6.4165	6.8742	6.8645	6.5521	6.7347	6.7922	7.0306	7.4972	6.7302	7.4090	6.9546	6.2088	6.7367	6.6016	7.0457
Atp6v1f	_LIAVIGDEDTVTGFLLGGIGELNK_.3	#N/A	3.0857	4.7355	3.3824	4.4985	3.6992	3.5924	4.2845	3.8729	3.3670	4.3972	3.3829	2.9506	3.1166	3.1029	3.7808	3.9487	3.1938	3.7200	3.7592	3.8385
	LLSVTPTS[Phospho (STY)]EGYSIK.2	Q9D1K7_S99_M1_	4.3473	3.6555	3.3828	6.6290	3.6850	4.2999	3.8560	4.0477	4.2026	4.5536	3.6812	3.5449	4.3680	3.3698	3.4332	3.1914	3.0845	3.2478	7.1452	3.3368
Chchd2	_LC[Carbamidomethyl (C)]EGFNEVLR_.2	#N/A	4.3969	4.4230	6.0607	3.1873	3.1030	3.1363	3.6019	3.1217	3.8653	4.2075	5.6780	4.9948	4.0308	4.9580	5.0214	3.7590	4.1198	3.0296	5.1508	3.6488
Sec13	_GQGSVSASITEGQQNEQ_.2	#N/A	3.7862	6.5255	6.4624	6.7508	3.9931	6.3764	3.0550	4.0118	2.9752	6.3224	5.9168	3.2591	3.1407	7.3421	5.4188	5.9536	3.5091	6.8928	7.0981	3.0381
Kat8	_S[Phospho (STY)]PGRVS[Phospho (STY)]PPTPAR_.2	Q9D1P2_S37_M2_Kat8	9.8246	9.9420	10.2723	10.2268	2.9051	9.9759	9.7394	9.8563	9.9964	10.1046	9.6580	4.3248	9.0267	10.0158	4.2131	9.5602	10.2606	9.3041	2.9534	9.6051
Rpl34	_AFLIEEQK_.2	#N/A	5.4317	5.5024	5.8232	5.2674	5.5641	5.7355	6.2244	5.5363	5.7613	5.7921	5.2962	5.3432	6.0583	6.2188	5.9761	5.3418	5.9354	5.0595	5.0879	5.3587
Rpl34	_SAC[Carbamidomethyl (C)]GVC[Carbamidomethyl (C)]PGR_.2	#N/A	3.8980	3.4211	4.4035	3.6862	4.1049	4.9218	5.1497	5.1465	4.4627	4.3282	3.8429	4.7984	4.5297	5.3750	4.8962	4.9320	4.9614	4.8428	4.2356	3.8469
Rpl34	_LSYNTASNK_.2	#N/A	4.1834	3.1357	3.7199	3.4009	2.8894	3.7181	4.0471	4.4090	4.1062	4.3626	2.9797	4.3627	4.2142	3.7580	4.0934	3.0003	3.9063	3.2432	3.5422	3.4353
Lingo1	_S[Phospho (STY)]DAGISSADAPR_.2	#N/A	9.3664	9.1302	9.1537	9.4908	8.8159	8.9485	8.6341	8.9557	8.6604	8.8973	9.1451	8.8291	9.0601	9.2239	7.9151	8.8524	9.3595	9.1198	9.0106	9.1985
Lingo1	_HNIEIEYVPRKS[Phospho (STY)]DAGISSADAPR_.4	#N/A	7.2898	7.4502	4.0685	6.7421	3.1869	6.3526	7.5604	5.0593	3.9559	6.2060	3.0882	6.4141	7.2346	4.0783	6.5005	4.1940	6.3273	7.9544	7.3653	6.8499
Lingo1	_FVAVPEGIPTETR_.2	#N/A	6.3876	5.8199	5.5277	6.0407	4.1797	1.0684	6.1049	5.7728	5.8001	3.5475	6.2994	6.3181	6.7604	5.5931	5.7892	5.7672	6.1982	6.0986	5.9438	5.8417
Lingo1	_LTVFPDGTLEVR_.2	#N/A	3.5665	5.4289	3.4282	4.7173	3.7734	4.6936	5.2412	5.0963	3.1949	3.3771	4.1744	4.7809	5.7064	3.8630	3.3001	4.6972	5.1294	4.6098	5.1257	3.8300
Lingo1	_KS[Phospho (STY)]DAGISSADAPR_.2	#N/A	9.7152	9.2460	9.5158	9.3830	8.8784	9.0030	8.7918	9.3031	9.0674	8.7776	9.0692	9.0950	9.5315	9.4460	8.0967	9.1071	9.5585	9.3054	8.9646	9.0886
Nol3	_ASEQEEVGGPEGS[Phospho (STY)]EALQPR_.2	Q9D1X0_S142_M1_Nol3	5.6659	5.6742	6.1980	6.5913	4.9920	6.2242	5.7914	3.7813	5.4909	5.9476	5.1702	5.6752	5.3668	6.4563	4.9475	5.3388	3.2785	5.0126	5.7101	5.5397
Nol3	_LVETLQADSGLLLDALVAR_.2	#N/A	4.1690	2.8172	3.9231	3.6512	3.5067	3.7326	3.3830	3.8694	3.8881	3.5931	3.6868	3.5124	4.1897	3.9102	3.7424	3.7317	3.6248	3.5569	3.9242	3.8772
Bcl9	_IPVEGPLS[Phospho (STY)]PSR_.2	Q9D219_S686_M1_Bcl9	3.5028	4.5000	4.0391	8.3476	4.3414	8.4929	3.1997	3.2032	3.2588	8.4008	8.2714	4.3895	7.9123	4.0262	7.7824	3.8477	8.5816	7.6510	8.0663	8.5270
Bcl9	_SST[Phospho (STY)]PSHGQTPAPEPISAQK_.2	Q9D219_T155_M1_Bcl9	3.0807	3.4212	3.6170	3.1078	3.9193	4.0656	3.6218	4.2820	4.4369	3.2871	3.9155	5.0662	4.6023	5.6774	3.1990	3.4256	3.3188	3.0135	4.1629	3.5711
Bcl9	_FSNQGKPGGSASQSQPS[Phospho (STY)]PC[Carbamidomethyl (C)]DSK_.3	Q9D219_S62_M1_Bcl9	3.5238	3.7953	4.5612	4.0605	4.1536	4.3777	3.3875	3.7494	3.4466	3.5213	3.8210	3.5215	3.5546	4.5992	2.9647	3.6599	3.2467	3.9028	4.2018	4.2765
Toe1	_AADS[Phospho (STY)]DDDVPPVPTPSDGGVNK_.2	Q9D2E2_S5_M1_Toe1	3.2048	9.8914	9.6559	4.4846	4.0433	4.2674	9.4355	4.4060	3.5568	8.4057	3.9312	3.1866	3.6649	3.2086	8.3416	7.1204	3.4428	3.7925	4.0916	4.3868
Dlst	_VEGGTPLFTLR_.2	#N/A	8.2158	8.3220	8.1608	8.3777	8.4066	8.3588	8.3380	8.4300	8.0689	8.4264	8.2337	8.4357	7.7094	8.5269	8.5910	8.3741	8.5299	8.3893	8.3904	8.5634
Dlst	_AKPAETPAPAHK_.3	#N/A	4.1276	5.1639	5.0894	5.2546	5.3020	5.1654	5.4422	4.0017	5.8178	6.2004	5.9864	4.8513	5.2575	5.4075	6.6222	6.0323	5.7136	5.1368	5.6598	6.0360
Dlst	_NVETM[Oxidation (M)]NYADIER_.2	#N/A	3.9414	4.2716	5.7225	4.7237	4.6622	5.2568	5.5636	3.9602	5.5203	5.0379	4.9061	4.9539	4.0694	5.5989	5.3823	5.4260	4.9840	5.2079	5.1199	5.6552
Dlst	_ASAFALQEQPVVNAVIDDATK_.2	#N/A	6.7223	6.8683	6.2542	6.8342	6.4673	6.3721	6.6674	6.9096	6.3489	6.6025	6.4254	6.8302	6.6336	7.0926	6.9335	6.7251	6.8737	6.6043	6.6716	6.5944
Dlst	_TINELGEK_.2	#N/A	3.3284	3.9907	4.3658	4.2559	3.5353	5.5231	5.8905	3.5540	5.5976	5.4419	5.7581	5.6792	3.5984	4.8700	6.6094	6.1911	4.5521	5.9709	5.8236	5.5996
Dlst	_NDVITVQTPAFAESVTEGDVR_.2	#N/A	3.5430	5.0124	3.8994	4.3263	3.7499	3.7833	3.2982	3.0635	3.7815	4.2847	4.1978	3.0283	3.3838	5.2305	3.3235	4.4060	3.6011	4.2320	3.8806	3.8535
Ube2v2	_LPQPPEGQTYNN_.2	#N/A	11.4667	11.1846	11.8243	11.7213	11.6561	11.5244	10.7229	11.8788	11.3963	11.6962	11.6495	11.4717	12.0652	11.7636	11.3999	11.6211	11.9930	11.2917	11.5431	11.6905
Ube2v2	_LLEELEEGQK_.2	#N/A	8.6160	8.0214	8.6889	8.5149	8.6676	8.2458	8.3004	8.3811	8.5434	8.5462	8.4946	8.5474	8.2243	8.5132	8.5764	8.6416	8.4710	8.4032	8.4642	8.5595
Ube2v2	_VILQELR_.2	#N/A	7.5641	7.8361	8.3889	8.3503	8.2402	7.9882	8.2132	7.2982	7.8990	8.4373	8.2794	8.2318	7.6710	7.9239	8.5240	8.1693	7.0607	8.3250	8.4275	8.2255
Ube2v2	_WTGM[Oxidation (M)]IIGPPR_.2	#N/A	7.9583	7.4502	6.4101	6.8534	7.7175	7.2781	7.4849	7.8261	6.8804	7.8849	7.2777	7.5333	7.4952	6.8893	7.7156	7.9329	7.3470	7.5058	7.6680	7.5463
Ube2v2	_YPEAPPSVR_.2	#N/A	8.3911	8.2844	8.1108	7.9594	8.3572	8.5289	8.6252	8.4111	8.4354	8.4984	8.4502	8.6608	4.1122	8.3127	9.1431	7.5061	8.1187	8.5675	8.5667	8.5011
Ube2v2	_ENM[Oxidation (M)]KLPQPPEGQTYNN_.2	#N/A	9.1025	10.2290	8.5151	10.1981	9.8736	9.5992	9.7212	10.4924	9.7913	9.9292	9.6655	9.9000	10.7361	10.4046	9.6847	9.9904	10.1505	9.8464	9.8845	9.9016
Ube2v2	_VEC[Carbamidomethyl (C)]GS[Phospho (STY)]KYPEAPPSVR_.3	Q9D2M8_S71_M1_Ube2v2	4.2368	3.7660	3.2723	3.4814	3.5746	5.7665	4.2345	4.3285	4.0256	4.4432	2.8991	3.6554	4.1337	3.6774	3.5437	3.0809	3.8257	3.3238	3.6228	3.3547
Ube2v2	_GVGDGTVSWGLEDDEDM[Oxidation (M)]TLTR_.2	#N/A	6.3819	4.7265	4.7812	4.7996	4.7709	4.8059	5.0353	5.3431	5.3102	5.4819	5.0397	5.2087	5.5193	5.9152	5.0599	5.3062	5.3346	4.7820	4.4319	5.3597
Dtna	_SS[Phospho (STY)]PSHTISR_.2	Q9D2N4_S568_M1_Dtna	3.9432	3.7949	4.0064	3.8228	5.9788	5.4894	3.6681	3.9658	3.6699	4.0247	4.3179	3.5982	3.8013	3.8173	3.4526	3.8265	3.4630	3.7987	3.7328	3.6682
Mobp	_HQPAAS[Phospho (STY)]PVVVR_.2	Q9D2P8_S85_M1_Mobp	12.0733	12.2364	11.7753	11.8476	11.5620	11.9318	11.6279	12.1056	11.8420	11.4163	11.4783	11.5532	12.0878	11.4978	10.7044	11.2751	12.1809	11.8248	11.4996	11.3992
Mobp	_ATS[Phospho (STY)]PQRPK_.2	#N/A	10.1614	9.9896	9.5074	4.2353	9.2401	9.5892	9.3893	9.7478	3.3076	9.1320	9.3245	9.3820	9.5958	8.8213	2.8257	8.4324	9.9975	9.5996	4.3408	9.1241
Mobp	_GGS[Phospho (STY)]PTRAPR_.2	Q9D2P8_S162_M1_Mobp	9.6114	9.7507	8.7310	9.1988	9.1685	9.0084	9.1262	9.6895	9.2747	8.7295	8.3657	9.2783	9.9208	3.6362	7.7888	8.6466	9.3982	9.2456	8.6658	9.1563
Mobp	_S[Phospho (STY)]PPRPAKPR_.2	Q9D2P8_S107_M1_Mobp	9.2543	9.4913	8.8561	9.0938	8.5589	9.2499	8.6479	9.0723	8.8789	8.4864	8.7286	8.7236	8.5020	8.9947	8.1892	8.3973	9.3521	9.0884	8.7216	8.5843
Mobp	_RATS[Phospho (STY)]PQRPK_.2	#N/A	3.8727	8.5041	3.5698	8.0960	4.0796	8.3634	3.9004	4.2347	4.3896	3.6833	3.8683	8.1031	4.5551	3.5569	2.9939	3.2348	3.2716	3.0609	7.2435	3.5239
Mobp	_SS[Phospho (STY)]PLRGPGTSR_.2	Q9D2P8_S150_M1_Mobp	7.1106	3.9984	4.5413	3.6850	6.4539	3.3999	4.0448	7.2811	6.8742	6.4058	6.0910	6.4707	6.9311	6.6226	6.0575	4.3499	4.2431	6.5065	6.7407	6.2554
Mobp	_S[Phospho (STY)]PLM[Oxidation (M)]PAKPR_.2	#N/A	8.1666	8.4500	8.5465	8.5977	7.5703	8.8761	8.1518	7.7087	8.1305	7.7401	8.5046	7.9749	7.1351	8.7418	6.7595	7.3864	8.7713	7.8771	7.6930	7.9254
Hist3h2ba	_PEPSRST[Phospho (STY)]PAPK_.2	Q9D2U9_T8_M1_Hist3h2ba	8.1086	8.2469	8.1573	3.7058	8.0217	7.7911	3.8137	3.2199	7.7980	8.2368	7.7105	8.0416	8.5465	8.2255	7.7459	4.1094	8.2117	7.9609	3.1505	7.9569
Coro7	_IWDLQTGAER_.2	#N/A	4.1645	3.6623	3.7010	4.4972	2.8706	4.9935	3.9682	3.0432	4.0978	3.9751	4.4564	4.9474	4.1953	4.7964	4.2456	5.0501	4.6110	4.7642	3.7656	4.7738
Coro7	_S[Phospho (STY)]LQSLLGPSSK_.2	Q9D2V7_S459_M1_Coro7	3.6489	8.5562	8.1534	8.0811	3.8558	3.8892	7.6554	3.8746	7.8862	8.0500	4.0919	7.9369	3.2779	8.9491	3.2176	8.0392	8.2569	7.8220	7.7256	3.7476
Coro7	_EDPLPQDS[Phospho (STY)]FEGVDEDEWD_.2	#N/A	3.0909	3.4111	3.6272	3.0977	3.9295	4.0555	3.0260	4.2921	4.4470	3.6258	3.9257	3.3005	3.1116	3.6143	3.0513	3.4358	3.3290	3.0034	4.1528	3.5813
Coro7	_LGNREDPLPQDS[Phospho (STY)]FEGVDEDEWD_.3	#N/A	4.0965	3.9063	3.1320	3.5929	3.0165	3.0499	4.0316	3.7969	3.9518	4.1210	3.4304	3.7957	4.1172	4.6199	4.0570	3.6725	4.3346	3.4986	3.1472	3.0861
Acp1	_SPIAEAVFR_.2	#N/A	6.9879	7.4578	7.0927	7.6138	6.9101	7.0541	7.3603	6.4921	7.2327	7.2897	7.1951	7.4320	7.2275	6.7680	7.2355	7.6656	6.8988	7.4375	7.5067	7.4118
Acp1	_SVLFVC[Carbamidomethyl (C)]LGNIC[Carbamidomethyl (C)]R_.2	#N/A	5.7008	5.4962	5.9012	3.3703	5.6953	5.5640	5.3134	5.2546	5.6155	5.9544	5.7719	4.9765	4.3398	5.8543	5.9029	5.1878	6.0384	5.4127	5.7078	5.4122
Lamp5	_VHFVYDSSEK_.2	#N/A	5.5669	3.3439	3.5118	3.6090	4.1821	3.9262	4.4591	4.2009	3.8980	4.5589	5.1835	3.0700	4.0061	3.5498	5.1604	3.3373	3.6981	6.1208	3.7504	3.2271
Rufy3	_NYVEELNR_.2	#N/A	7.0811	7.4983	7.2320	7.2587	7.5320	7.4078	7.3399	7.5848	7.5734	7.4410	7.1050	7.4891	7.4276	7.6528	7.1350	7.3599	7.2903	7.4357	7.5196	7.3256
Rufy3	_TAEGQALSEAR_.2	#N/A	6.4874	6.7987	6.8758	6.3562	6.8541	6.7030	6.9474	7.2062	6.9854	6.5220	6.2616	6.4097	6.2427	6.9279	6.8218	6.8666	6.8114	6.4160	6.1824	6.5720
Rufy3	_GLIESALNLGR_.2	#N/A	5.6840	6.3793	6.4999	6.4003	6.8010	5.7512	6.1659	6.1359	6.2687	6.0372	6.1231	6.2312	5.8823	6.1629	6.0348	6.5718	6.1845	6.0800	6.5073	6.2578
Rufy3	_LQSSDLGVK_.2	#N/A	3.8411	4.1207	5.0884	4.4239	4.2408	4.0433	3.6863	4.1689	3.9741	4.7927	2.8975	4.4186	5.0901	4.2828	4.8224	4.1137	3.3363	4.1134	3.5080	4.8464
Rufy3	_VS[Phospho (STY)]M[Oxidation (M)]DGEWLC[Carbamidomethyl (C)]LR_.2	#N/A	7.2176	7.4924	6.8647	7.0634	5.9348	7.4287	7.2381	7.0912	6.7117	7.0643	6.6442	6.6554	7.0200	7.4917	6.2938	6.7748	6.9211	7.0662	7.0714	6.9833
Rufy3	_LT[Phospho (STY)]PPTDM[Oxidation (M)]PT[Phospho (STY)]PTTDKITQAAM[Oxidation (M)]ETIY	#N/A	4.2295	7.9277	3.2650	3.4741	3.5672	3.7913	3.9739	4.3358	4.0329	7.3198	2.9065	3.6628	4.1410	3.6848	3.5511	3.0736	3.8330	3.3164	3.6155	3.3621
Rufy3	_[Acetyl (Protein N-term)]SALT[Phospho (STY)]PPTDM[Oxidation (M)]PTPTTDK_.2	#N/A	11.3112	10.8886	11.1287	10.7590	10.9633	11.0928	10.4927	11.3195	10.9583	11.0453	11.1414	10.9433	11.4880	10.9881	10.4153	10.9937	11.6612	10.8263	11.0539	10.8784
Rufy3	_DGNS[Phospho (STY)]SKGSEGDGQITAILDQK_.3	Q9D394_S247_M1_Rufy3	3.3412	3.1608	3.8775	4.3482	4.1797	4.4038	4.6322	4.5424	3.4204	3.5475	3.7948	3.0503	3.5285	3.8645	2.9386	3.6861	3.5792	4.2539	3.9025	3.8315
Rufy3	_GEDLDSQVGVIDFSM[Oxidation (M)]YLK_.2	#N/A	4.2391	4.8357	3.6457	4.5800	3.9480	4.0370	3.5931	4.3401	4.4655	3.6073	3.9441	3.2820	3.1301	3.6328	3.1703	4.7617	3.7893	2.9849	4.1343	3.5998
Rufy3	_VKEESSYLLESNR_.3	#N/A	3.6461	3.2420	3.1827	4.4294	3.8531	3.8864	3.1950	4.4612	3.1152	3.4567	4.0947	3.3991	3.2807	3.7833	3.2204	3.0083	3.4980	4.3351	3.9837	3.7503
Atp5f1d	_IEANEALVK_.2	#N/A	8.8569	8.9224	9.1637	9.3023	9.3314	8.9720	9.1147	9.1716	9.2263	9.0492	9.0882	9.2059	9.3923	9.2771	9.8291	9.4122	8.9132	9.3054	9.3680	9.5743
Atp5f1d	_IEANEALVKALE_.2	#N/A	3.9952	4.2294	4.7371	4.9837	4.4008	5.1312	3.5975	4.6100	4.4949	3.6052	4.7560	5.0518	3.7646	5.0762	5.0375	4.4206	5.6879	4.8302	4.1900	5.1419
Atp5f1d	_AQSELSGAADEAAR_.2	#N/A	8.6623	8.6556	9.0894	8.9234	9.0274	8.6692	8.7298	8.8103	8.9860	8.8560	8.8048	8.8236	8.9721	9.2583	9.3864	9.2163	8.8389	8.8202	8.9263	9.1175
Mfap3l	_SES[Phospho (STY)]PTADSDASSLHEQPQQIAIK_.3	Q9D3X9_S298_M1_Mfap3l	3.8469	4.2254	3.6619	3.9120	3.9642	3.4087	3.7047	3.7062	5.4783	4.0532	3.3586	4.1149	3.8718	3.9212	3.2819	3.4705	4.0849	5.3564	3.8823	3.6897
Dlgap1	_GC[Carbamidomethyl (C)]S[Phospho (STY)]QDDEC[Carbamidomethyl (C)]VSLR_.2	#N/A	11.9740	12.1604	12.3556	12.0037	11.5209	11.8835	11.6587	11.9063	11.6861	12.2545	11.8835	11.7195	12.1762	12.2536	11.3587	11.5763	12.3462	11.6349	11.9265	11.9306
Dlgap1	_SHS[Phospho (STY)]LEGPSK_.2	Q9D415_S154_M1_Dlgap1	9.4356	9.7208	8.4922	9.4366	9.1250	9.1922	9.1408	9.1861	8.8946	9.8406	8.8037	9.1404	9.8200	9.5089	3.2665	9.1216	9.2295	9.1733	8.9568	9.4753
Dlgap1	_ERS[Phospho (STY)]LESSQR_.2	Q9D415_S947_M1_Dlgap1	10.3085	10.3242	10.4707	10.2051	9.6873	9.8245	9.9078	10.1341	9.7608	10.2922	9.6860	9.8482	10.2330	10.2233	9.6940	9.9649	9.8397	9.8517	9.9034	9.9729
Dlgap1	_SLDSLDPAGLLTS[Phospho (STY)]PK_.2	Q9D415_S437_M1_Dlgap1	6.8486	7.6014	8.9465	8.4708	7.3048	6.3302	8.6198	8.5183	8.4125	8.5405	4.1076	8.5822	8.8909	9.2950	3.0751	8.2719	8.3946	8.6340	8.2851	7.9103
Dlgap1	_AVSEVS[Phospho (STY)]INR_.2	Q9D415_S421_M1_Dlgap1	8.8334	8.9538	9.4798	9.1731	8.4340	8.4497	7.4371	7.1142	8.5752	9.1443	8.7327	8.4900	8.7495	8.9983	8.1427	8.6804	8.6507	8.4933	7.7689	8.7482
Dlgap1	_AS[Phospho (STY)]PDESQTLR_.2	#N/A	7.8775	7.5629	7.7850	8.0865	7.5093	4.0417	7.8028	4.1803	3.7825	8.0869	7.7651	3.4123	3.8906	8.0353	7.5176	7.6787	3.2171	7.8185	7.8917	7.4159
Dlgap1	_SRNES[Phospho (STY)]YM[Oxidation (M)]R_.2	#N/A	3.1719	4.7774	5.2614	5.5081	3.3789	4.1595	3.6694	3.3995	3.3465	4.4834	3.2658	4.3074	3.4884	4.9924	3.6948	4.0349	4.1827	4.0879	3.3159	4.0657
Dlgap1	_AM[Oxidation (M)]GDEDS[Phospho (STY)]GDS[Phospho (STY)]DTS[Phospho (STY)]PKPSPK_.2	#N/A	6.2689	5.8403	7.2991	3.8397	7.4336	6.2503	7.6279	6.5521	7.9827	7.9808	7.1612	6.1329	8.4687	7.5627	7.4826	7.8655	8.0710	8.0960	7.9284	6.0333
Dlgap1	_GSVNGGKAS[Phospho (STY)]PDESQTLR_.2	#N/A	5.1050	5.4003	4.1391	4.8170	4.6596	5.2756	5.1250	4.7738	4.9539	5.3083	3.7036	5.2400	3.8742	4.0125	4.8018	3.6428	5.3080	4.9181	4.7546	4.4438
Dlgap1	_AIEKGC[Carbamidomethyl (C)]S[Phospho (STY)]QDDEC[Carbamidomethyl (C)]VSLR_.3	#N/A	4.3523	3.6505	3.3878	3.3371	3.6900	4.2949	4.2874	4.0527	4.2076	3.8652	3.6862	3.5399	4.3730	3.3748	4.4819	3.1964	3.0895	3.2428	4.3922	3.3418
Dlgap1	_GDMISQSGLSNS[Phospho (STY)]TESLDSM[Oxidation (M)]K_.2	Q9D415_S605_M1_Dlgap1	3.6410	4.8534	3.2840	3.8483	4.0337	4.1349	4.5639	4.1655	3.6561	4.7310	3.9289	3.8513	3.8965	5.2790	3.7106	3.3183	4.1720	3.8601	4.1368	3.8991
Dlgap1	_QNS[Phospho (STY)]ATESAESIEIYIPEAQTR_.3	Q9D415_S973_M1_Dlgap1	4.2191	4.6008	3.7557	3.6551	2.9252	4.4594	4.1229	4.4448	4.0431	4.0297	3.5218	4.3270	4.2086	3.7937	4.1483	3.5812	3.9420	7.7268	3.0558	3.1774
Dlgap1	_C[Carbamidomethyl (C)]LS[Phospho (STY)]IGIQVDDAEEPEK_.2	Q9D415_S666_M1_Dlgap1	4.4380	4.3820	7.0336	3.1463	3.1441	3.1774	3.9040	3.1628	3.8242	4.2486	3.3029	4.1081	3.9897	4.0126	3.9294	3.8001	4.1609	3.6261	3.2747	2.9585
GOLGA7B	_RSAS[Phospho (STY)]LATK_.2	Q9D428_S16_M1_GOLGA7B	4.1936	5.7009	3.7301	3.3907	2.8997	4.4338	4.1484	4.4192	5.3130	4.0042	3.5473	4.3525	4.2341	3.7682	4.1738	3.5557	3.9165	3.2330	5.0473	3.2029
GOLGA7B	_DYS[Phospho (STY)]DGTIC[Carbamidomethyl (C)]QFQTK_.2	Q9D428_S28_M1_GOLGA7B	3.6292	4.3736	7.4536	7.0613	2.9670	7.4639	3.0733	3.3296	3.1324	3.8356	3.5067	7.7742	3.6500	4.1526	4.1513	3.9741	3.8673	7.4791	7.1836	7.0730
Clvs1	_SQS[Phospho (STY)]VVEAGTLK_.2	#N/A	8.2535	8.1640	8.4692	8.3060	7.6431	7.9225	3.2031	7.3427	3.1233	8.3653	7.7436	7.3068	6.8355	7.3606	6.0712	7.3808	3.5061	7.1519	7.5488	7.4826
Clvs1	_HTC[Carbamidomethyl (C)]S[Phospho (STY)]NLER_.2	Q9D4C9_S313_M1_Clvs1	3.7173	3.2386	3.7160	4.0223	5.2099	4.0702	3.8738	4.3919	3.7940	3.6405	3.9443	3.0459	3.7443	3.7263	2.9019	5.1324	4.4073	3.9055	3.8168	3.5670
Clvs1	_SQS[Phospho (STY)]VVEAGTLKHEEK_.2	#N/A	4.6910	4.4430	4.7660	4.4852	4.2500	3.8267	3.4247	3.4917	3.3449	5.2654	4.0655	4.4887	3.4767	4.3302	3.2538	4.2817	3.5209	3.8523	4.0001	4.5309
Clvs1	_EC[Carbamidomethyl (C)]S[Phospho (STY)]PKPMK_.2	#N/A	4.2655	4.2758	4.8780	4.9274	5.6437	4.3315	4.2084	4.4912	3.9967	4.0761	4.7631	2.5966	4.1622	5.0241	4.1019	3.6276	3.9884	3.1611	3.4601	3.5174
Clvs1	_EC[Carbamidomethyl (C)]S[Phospho (STY)]PKPMKR_.2	#N/A	3.2521	6.6387	7.0163	6.9101	5.3318	5.2961	5.9728	3.8124	3.4298	3.4584	5.8884	3.1393	3.6176	7.0540	3.0276	3.5970	3.3096	3.8398	4.1389	4.8968
Plpp6	_ASDS[Phospho (STY)]PVHR_.2	Q9D4F2_S60_M1_Plpp6	3.9738	4.0290	4.5101	3.7156	3.3115	3.5356	4.2296	3.6742	4.2887	4.1801	3.1622	3.9185	4.3967	4.4971	3.8068	4.3187	4.2118	4.2299	3.2699	3.6178
Exoc2	_ASNTADTLFQEVLGR_.2	#N/A	3.3330	5.1022	5.4731	4.2513	4.6338	4.2859	5.4618	4.9434	3.4284	4.1455	2.9071	3.7122	3.3638	5.1191	3.5336	4.1959	4.5567	4.0386	4.3926	4.0857
Exoc2	_GSS[Phospho (STY)]FQSGRDDTWR_.2	Q9D4H1_S432_M1_Exoc2	3.9936	4.0092	4.5299	3.6958	3.3313	3.5554	4.2098	3.6940	4.2689	4.1999	3.1424	3.8987	4.3769	4.5170	3.7870	4.3385	4.2316	3.0805	3.2501	3.5980
Phf14	_NSADDEELT[Phospho (STY)]NDS[Phospho (STY)]LTLSQSK_.2	Q9D4H9_S283_M2_Phf14	3.7438	5.7137	3.2804	3.8404	3.9508	3.9841	6.2367	4.3635	6.1030	3.5544	3.9970	3.3014	3.1830	5.8640	3.1227	3.1060	3.4003	4.4329	5.6824	3.6526
Wdr20	_SNS[Phospho (STY)]LPHSAVSNAASK_.2	#N/A	8.4983	8.3640	8.2180	8.3150	7.7505	7.7193	7.9774	8.3181	7.7463	8.2224	7.8173	8.0551	8.1708	8.4970	6.9992	7.7657	8.0729	7.7774	7.8081	7.7453
Atad1	_EYVNSTSEES[Phospho (STY)]HDEDEIRPVQQQDLHR_.4	Q9D5T0_S322_M1_Atad1	4.4006	3.6022	3.4361	3.2888	3.7384	4.2466	3.8027	4.1010	4.2559	3.1061	3.7345	3.4916	4.4213	3.4232	3.3799	3.2447	3.1378	3.1945	4.3439	3.3902
Rab11fip1	_ESS[Phospho (STY)]PSNSPSPQGFR_.2	Q9D620_S340_M1_Rab11fip1	4.3510	7.1311	3.8876	3.2333	3.0571	7.3210	6.8455	3.0758	4.2738	7.2832	6.7610	7.2467	4.3818	3.9256	4.0164	3.7131	7.7143	3.0756	3.3746	3.6029
Lratd1	_GPPGC[Carbamidomethyl (C)]SPC[Carbamidomethyl (C)]PES[Phospho (STY)]PSR_.2	Q9D650_S67_M1_Lratd1	4.3557	7.1350	4.5876	3.6381	3.0618	6.5717	6.0326	3.7516	5.8292	6.6018	6.4098	3.8410	4.0720	4.5746	4.0117	5.2783	6.3222	6.2897	3.1924	3.0408
Ttc9b	_C[Carbamidomethyl (C)]SLQREDS[Phospho (STY)]DGGTGGPAR_.2	#N/A	5.1300	5.4423	5.0749	5.3955	4.8402	4.7814	3.9378	5.0999	5.0105	5.2849	3.8709	4.8610	4.8744	5.0938	4.5786	5.3002	4.6516	4.5782	5.0997	4.8360
Ttc9b	_EDS[Phospho (STY)]DGGTGGPAR_.2	#N/A	4.5399	8.4733	8.6383	7.7892	8.2674	8.8056	3.6635	7.2473	3.7226	8.7553	5.9112	8.5444	4.5606	8.3446	3.2407	3.3839	6.9397	7.6497	4.2047	8.0232

Tubb4a	_IREEFPDR_.2	#N/A	10.8406	10.9953	11.1209	10.7015	10.7268	10.9452	10.9265	11.1282	10.9213	11.1117	10.3127	10.2970	10.2141	10.4762	10.4909	10.3009	10.2602	10.2000	10.2648	10.2231
Tubb4a	_GHYTEGAELVDAVLDVVR_.3	#N/A	8.6054	9.2470	9.2138	8.8271	8.1025	8.8753	8.4635	8.2725	8.7346	9.0515	8.0899	8.0943	8.0330	8.7346	7.8240	8.3427	8.0965	8.1333	8.4615	8.3208
Tubb4a	_GHYTEGAELVDAVLDVVRK_.3	#N/A	8.0821	8.6411	6.7999	8.1041	7.9180	7.6288	8.1278	8.2441	8.0630	8.6611	6.8696	7.7190	8.6573	8.3313	7.6041	7.5632	7.6238	8.0387	7.6730	7.6335
Tubb4a	_EVDEQM[Oxidation (M)]LSVQSK_.2	#N/A	9.8200	10.0865	10.2570	10.2410	9.8903	10.0508	10.0365	9.8551	10.1763	10.2493	9.3294	9.2745	9.2526	9.8219	9.1964	9.5723	9.3602	9.1532	9.4182	9.5266
Tubb4a	_M[Oxidation (M)]AATFIGNSTAIQELFK_.2	#N/A	9.9710	9.5910	9.7117	9.6139	9.4042	9.5792	8.9176	9.7409	9.3797	10.2990	8.7802	8.9121	9.9696	10.1986	8.9426	9.3699	9.1680	9.0075	9.0159	9.4635
Tubb4a	_INVYYNEATGGNYVPR_.2	#N/A	8.5500	8.5486	8.9240	8.4453	8.2879	8.3761	8.3783	8.5654	8.6141	8.6945	7.8482	7.8969	8.4493	8.0805	8.0335	8.0238	8.0628	7.7113	7.9029	7.8558
Lurap1	_RGS[Phospho (STY)]WDSLPDTSSTDR_.2	Q9D6I9_S126_M1_Lurap1	6.6329	5.2673	4.5284	4.4640	4.0218	3.9210	6.9603	6.1594	6.4830	7.1564	4.0614	3.2255	3.2474	7.1842	6.0750	3.3739	3.4647	6.6748	4.0183	6.8901
Ndufb8	_GGDPTKEPEPVVHYDI_.3	#N/A	6.7512	6.3549	6.2723	6.3891	6.1519	6.0719	6.4708	6.1590	6.6178	5.8158	6.5396	6.5958	6.7929	6.4621	5.9548	6.4476	6.2387	6.5516	6.1224	6.1233
Ndufb8	_QYPYNNLYLER_.2	#N/A	7.7509	7.1549	6.5868	6.0157	6.9650	6.7025	6.9125	7.1015	6.6551	6.5412	7.4258	7.4190	7.2947	6.9226	6.7035	7.2084	7.2881	7.0441	6.5830	6.7769
Ndufb8	_DPWYQWDHSELR_.2	#N/A	6.3303	5.7373	6.2061	5.6938	5.7690	4.6247	5.4206	3.9737	3.9891	5.5474	5.8065	5.0148	4.0971	3.2959	5.9949	4.3265	5.6698	5.8537	4.0690	5.4734
Ndufv2	_DSDSILETLQR_.2	#N/A	9.4508	9.5695	9.7090	9.5910	9.4774	9.5225	9.3044	9.6408	9.5201	9.5436	9.6659	9.7607	10.0163	10.0597	9.9487	9.8234	9.8546	9.5009	9.6139	9.7755
Ndufv2	_DIEEIIDELK_.2	#N/A	8.3976	7.9171	8.7117	8.2842	8.3915	8.1236	7.8869	8.3293	8.3378	8.2899	8.4086	8.4662	8.2695	8.4560	8.6847	8.3943	8.4332	8.1446	8.1486	8.7306
Ndufv2	_DTPENNPDTPFDFTPENYKR_.3	#N/A	7.8924	7.5060	7.2547	7.5726	7.5511	7.4423	7.1795	7.2312	7.5464	7.5521	7.6769	7.6678	7.8574	8.1265	7.8321	8.0519	7.8437	7.5067	7.4530	7.6081
Ndufv2	_DTPENNPDTPFDFTPENYK_.2	#N/A	6.8392	6.7339	6.6537	6.7512	6.0421	6.5279	6.1207	6.6232	6.4949	6.5533	6.9252	6.4526	6.1645	7.1991	6.9862	6.4021	6.7775	6.4576	6.4156	6.5937
Ndufv2	_NYPEGHQAAAVLPVLDLAQR_.3	#N/A	7.4153	7.6519	6.9180	7.2640	7.0769	7.3067	7.3384	7.3461	7.2488	7.2370	7.3976	7.6042	7.7923	8.1279	7.7862	7.7532	7.3814	7.3820	7.2863	7.4443
Ndufv2	_FC[Carbamidomethyl (C)]C[Carbamidomethyl (C)]EPAGGLTSLTEPPKGPGFGVQAGL_.3	#N/A	4.0037	4.0305	3.9494	3.6857	3.0449	3.9301	4.2633	3.7756	4.2588	4.2100	2.5169	3.7337	4.9695	3.9106	4.3480	4.3486	4.0589	3.0906	3.3896	4.7365
Ndufv2	_VYEVATFYTM[Oxidation (M)]YNR_.2	#N/A	5.8365	3.8493	3.1889	6.7347	3.4912	6.0215	4.0499	6.1402	6.1855	4.3598	2.9825	6.0790	6.7629	3.7608	6.3957	6.9141	3.9091	3.2404	5.5756	6.4543
Ndufv2	_YHIQVC[Carbamidomethyl (C)]TTTPC[Carbamidomethyl (C)]M[Oxidation (M)]LR_.3	#N/A	6.0204	4.0070	4.5321	4.8780	4.9873	5.5799	4.2075	3.6962	4.2666	5.8242	3.1401	3.8964	4.3747	4.5192	6.4699	4.3407	4.2339	3.0827	4.7134	5.9444
Slc25a22	_LAANDFFR_.2	#N/A	8.3227	8.5335	7.9205	8.5352	8.3731	8.4219	8.5085	8.7007	7.8552	8.0681	8.3501	8.4688	8.3459	7.9105	7.9385	7.9576	8.3589	8.6730	8.1383	7.9748
Slc25a22	_HEGPSAFLK_.2	#N/A	6.7908	7.0791	7.1183	7.0153	6.9734	6.9981	7.1337	7.2564	6.6014	6.3838	6.5921	7.3425	6.8577	6.6928	6.8149	6.5247	6.4464	7.4470	7.1415	6.7591
Slc25a22	_NHGIAGLYK_.2	#N/A	6.1303	6.9805	6.8495	6.7173	6.0478	6.6215	6.6044	6.9766	6.0304	6.6554	6.7377	6.5760	6.2997	6.2109	6.6226	6.1305	6.6065	6.8353	6.5251	6.4035
Slc25a22	_GVNEDTYSGFLDC[Carbamidomethyl (C)]AR_.2	#N/A	7.7713	7.8357	7.9583	7.6940	7.1415	7.2875	7.7316	8.1393	7.5392	7.3672	7.7972	7.3875	7.8917	7.6951	7.3926	7.2301	7.8941	7.5895	7.1718	7.1388
Slc25a22	_SEGYFGMYR_.2	#N/A	4.4977	6.9363	7.5560	7.4488	3.8354	5.7370	6.7336	4.0676	5.3011	3.2031	6.3892	5.4325	3.8728	6.7493	5.0084	3.3417	5.4243	4.2746	4.6119	2.4316
Slc25a22	_LINGGIAGLIGVTC[Carbamidomethyl (C)]VFPIDLAK_.2	#N/A	3.1684	4.2651	2.7481	3.9517	3.3753	1.6042	3.6728	1.1210	3.5930	4.4798	3.0716	3.8768	3.7584	4.2611	3.6981	4.0314	3.9758	3.8574	3.5060	4.2281
Slc25a22	_SPFYVSFLAGC[Carbamidomethyl (C)]VAGSAAAVAVNPC[Carbamidomethyl (C)]DVVK_.3	#N/A	3.2752	1.4215	4.3127	4.3090	3.4822	3.1253	3.1390	3.5009	3.1981	3.7699	3.5724	3.7700	3.3061	4.3507	3.5913	4.1382	4.4990	1.5902	2.9495	4.0280
Idh3a	_APIQWEER_.2	#N/A	9.6735	9.4429	10.3350	9.8649	9.5313	9.8685	9.4898	9.9391	9.5937	9.8702	9.9266	10.0669	9.5577	10.2437	10.1646	9.9329	10.0091	9.6899	9.7658	9.6953
Idh3a	_TPYTDVNIVTIR_.2	#N/A	8.8803	9.4017	9.5428	9.2640	8.8896	9.0480	9.0910	9.1133	9.0830	9.0303	8.9478	9.1798	9.4046	9.4742	9.2110	9.4007	9.3128	9.0851	9.1589	9.0044
Idh3a	_NVTAIQGPGGK_.2	#N/A	8.9999	9.1577	9.0953	9.2008	9.0920	9.0193	9.3973	9.0470	9.0258	9.0938	8.8781	9.0757	9.3608	9.2844	9.6029	9.0881	9.0370	9.1712	9.2229	9.0683
Idh3a	_LITEEASKR_.2	#N/A	8.7326	8.9992	9.0808	9.0748	9.0395	8.9452	9.0900	9.1087	9.0576	9.0039	8.7422	9.1649	9.1280	9.2134	9.3995	9.0776	8.9244	9.0399	9.1254	8.9952
Idh3a	_IAEFAFEYAR_.2	#N/A	8.8912	8.9990	8.8932	8.7991	8.8318	8.7219	8.8251	8.9928	8.8631	9.0843	8.7009	8.8891	8.8997	8.9910	8.9444	9.0548	9.1290	8.7398	9.0088	8.8891
Idh3a	_SNVTAVHK_.2	#N/A	7.8612	7.7903	8.0291	8.0414	8.0557	7.6066	8.0569	7.9711	7.8533	7.8998	7.9032	7.9636	7.3180	7.5524	8.4881	7.7609	7.5970	7.8472	7.8743	8.0278
Idh3a	_ENTEGEYSGIEHVIVDGVVQSIK_.3	#N/A	8.5863	8.2858	7.2050	8.5496	7.9246	7.8224	7.4963	8.1120	7.7647	8.0241	7.6792	7.8927	9.1954	9.4420	7.3956	7.7232	8.1208	7.8655	7.8429	8.8069
Idh3a	_C[Carbamidomethyl (C)]SDFTEEIC[Carbamidomethyl (C)]R_.2	#N/A	6.7322	6.8291	8.0416	7.6563	7.2204	7.1204	7.2563	7.1624	7.4975	7.3343	7.0706	6.7661	6.4656	7.3709	7.3996	7.4123	7.0165	6.9665	7.2057	7.5448
Idh3a	_IEAAC[Carbamidomethyl (C)]FATIK_.2	#N/A	5.3411	6.7470	6.4810	7.0885	5.9196	6.7596	7.0272	6.1067	7.1409	6.5070	6.4248	6.0243	5.7933	7.1863	7.0093	6.7080	5.8673	7.2011	6.5823	6.2733
Idh3a	_EVAENC[Carbamidomethyl (C)]KDIK_.2	#N/A	5.6863	5.9298	5.0928	5.5610	5.5535	5.8432	5.7208	6.3719	6.0900	6.0471	5.2416	5.6608	6.5147	5.9630	6.1918	5.4272	5.8243	6.0317	5.9404	5.5928
Idh3a	_M[Oxidation (M)]SDGLFLQK_.2	#N/A	7.2984	7.3650	7.3487	7.2533	7.4644	7.2811	7.2970	7.3456	7.1681	7.5470	7.3665	7.3226	7.4462	7.2489	7.5728	7.2668	7.3329	7.3367	7.1920	7.3585
Idh3a	_HM[Oxidation (M)]GLFDHAAK_.2	#N/A	6.8260	6.5294	5.7959	6.2667	7.2156	6.6397	6.4641	7.2598	6.3662	6.9542	6.4609	6.8166	6.7239	6.1834	7.4779	6.3497	6.5087	6.8336	6.2437	7.0393
Idh3a	_TPIAAGHPSM[Oxidation (M)]NLLLR_.2	#N/A	7.4806	7.3009	6.6750	6.8508	7.4933	7.0537	7.1504	7.7124	6.8042	7.3188	6.9475	7.5415	7.6967	6.8126	7.1778	7.2618	7.2980	7.4148	7.4874	7.3212
Idh3a	_C[Carbamidomethyl (C)]SDFTEEIC[Carbamidomethyl (C)]RR_.2	#N/A	3.8089	3.8877	4.0524	3.9201	3.2219	4.0492	3.8839	3.7200	3.8041	4.3264	3.2828	4.0880	3.8411	3.8975	4.0275	3.8779	4.4737	3.8184	3.6942	3.6725
Mrrf	_ALVEDIISLEEVDEDM[Oxidation (M)]K_.2	#N/A	5.4697	4.3172	5.2040	5.3459	3.1247	4.7320	3.4704	4.6437	4.6598	5.2878	4.2243	4.1304	5.5053	5.6748	5.0639	5.5649	4.0904	5.1881	4.9380	5.3435
Borcs6	_TLS[Phospho (STY)]GEEEAESVGVSSR_.2	Q9D6W8_S43_M1_Borcs6	9.1531	9.9099	9.7291	9.6660	8.2247	9.4406	9.3770	8.4837	9.3854	9.5982	8.4538	9.0302	9.2265	10.1753	8.6821	9.4427	9.2449	9.4352	9.3891	9.5267
Borcs6	_S[Phospho (STY)]LDGLSGAC[Carbamidomethyl (C)]GGGGSSSSGETGAGGGR_.2	#N/A	5.5552	5.7949	5.7782	5.7776	5.5833	5.8140	5.4639	4.4384	5.9351	5.9667	5.8175	5.5670	5.3399	5.4556	4.7179	5.4979	5.7482	5.4996	5.0615	5.8646
Borcs6	_S[Phospho (STY)]LDGLSGAC[Carbamidomethyl (C)]GGGGSSSSGETGAGGGRR_.3	#N/A	6.1991	6.5784	3.6758	6.3861	4.3462	6.0281	5.9161	4.3649	6.0900	6.5042	6.1444	4.4068	4.2884	3.2902	4.2281	5.8780	6.0667	6.0896	5.9681	3.2572
Borcs6	_RGS[Phospho (STY)]PGGVEMNVELPQQEGDDDDDEDEEAAAGR_.3	Q9D6W8_S130_M1_Borcs6	4.2803	3.7225	3.3157	3.4091	3.6180	3.8421	3.9231	3.9806	3.9822	4.4866	4.3566	3.6120	4.3010	3.3028	3.5003	3.1243	4.5183	3.3148	2.9634	3.2698
Nop56	_EEVAS[Phospho (STY)]EPEEAASPTTPK_.2	#N/A	8.3440	8.1226	8.5863	8.1901	5.7733	8.1840	7.9640	8.1434	8.2563	8.0754	7.7004	7.8916	7.6368	8.3473	7.0178	7.7119	8.4356	8.0548	7.9371	7.6514
Nop56	_SSPKEEVAS[Phospho (STY)]EPEEAASPTTPK_.3	#N/A	7.6384	7.9099	7.2133	7.0111	6.6020	7.4518	7.5338	7.5251	7.4295	6.9986	7.2281	7.2270	7.5002	7.4639	6.1575	7.3199	7.0900	7.7114	7.4400	5.7917
Nop56	_EELAS[Phospho (STY)]DLEEM[Oxidation (M)]ATSSAK_.2	#N/A	3.5905	7.4189	7.4584	5.3204	3.7975	7.7148	7.3697	3.9846	7.4934	5.7961	4.4865	5.3847	3.3723	7.6069	6.2845	6.1365	3.2117	7.6610	6.7518	7.2440
Nop56	_KSSPKEEVAS[Phospho (STY)]EPEEAASPTTPK_.3	#N/A	4.7728	3.5915	4.8415	4.4106	4.2255	3.9153	4.9980	4.4991	4.6482	4.6959	3.9440	3.4328	4.9357	3.8481	4.0498	4.8989	5.1160	5.1374	4.4328	4.0073
Nop56	_APKEELAS[Phospho (STY)]DLEEM[Oxidation (M)]ATSSAK_.2	#N/A	4.7944	3.5196	4.7377	3.4722	3.9803	4.1657	5.1882	4.1394	5.2808	3.9340	3.6184	3.3442	4.3052	5.2866	3.2072	3.2790	4.1847	6.2277	4.8271	5.4117
Nop56	_KAPKEELAS[Phospho (STY)]DLEEMATSSAK_.3	#N/A	3.6404	4.0475	4.4367	4.1772	3.4563	3.6342	3.4711	4.0827	3.3913	4.0508	3.7163	3.8727	3.5567	4.0594	3.7359	4.2548	3.7741	4.0829	3.7315	4.0264
Rpap3	_AVDNPPRGS[Phospho (STY)]PK_.2	Q9D706_S429_M1_Rpap3	8.7131	8.8661	8.4848	8.7666	8.8150	8.4561	8.3085	8.3665	8.3505	8.7636	8.5421	8.5724	8.5613	8.7773	8.3352	8.4959	8.6242	8.2189	8.4925	8.7654
Fam219a	_NGSM[Oxidation (M)]GS[Phospho (STY)]PVNQQPK_.2	#N/A	8.3752	7.6426	8.4841	8.6999	7.8949	7.6936	8.3435	7.9656	7.5270	8.2019	7.7793	7.9438	7.7829	7.6604	7.3050	7.8578	8.0007	7.7766	7.7345	8.0953
Prps1	_ENISEWR_.2	#N/A	6.0692	6.4589	6.8700	6.7331	6.6358	6.6457	7.0248	6.3679	6.6887	6.8154	6.7204	6.3816	7.0193	6.2443	7.0087	6.6348	5.9021	6.7918	6.9126	6.9305
Prps1	_VYAILTHGIFSGPAISR_.3	#N/A	4.0836	4.3590	4.1981	4.0555	3.4584	3.0203	4.4863	3.3237	3.6433	4.3180	3.5279	3.4455	3.8294	3.7114	3.0856	3.9805	3.2125	3.4929	4.1169	3.9392
Prps1	_INNAC[Carbamidomethyl (C)]FEAVVVTNTIPQEDK_.2	#N/A	5.9152	6.6453	5.2130	3.4434	4.3478	5.7570	6.2737	6.4911	3.4876	6.2826	3.6000	6.1559	4.2868	3.2886	4.2265	5.7658	6.4211	3.3290	5.8484	3.2556
Prps1	_IQVIDISM[Oxidation (M)]ILAEAIR_.2	#N/A	3.4301	4.5268	4.4675	4.2134	3.6370	3.5436	4.9453	3.1764	2.9661	4.0714	4.3107	3.6151	3.4967	3.9994	3.4364	4.2931	3.7140	2.9231	3.7677	3.9664
Prps1	_VAILVDDMADTC[Carbamidomethyl (C)]GTIC[Carbamidomethyl (C)]HAADK_.3	#N/A	3.5839	3.7352	3.1204	3.5175	2.0316	3.8776	2.5230	3.8095	3.1775	3.3945	4.1570	3.4613	3.3429	5.1787	3.2827	2.9460	3.3068	4.2729	3.9215	3.8126
Prps1	_IFSGSSHQDLSQK_.2	#N/A	4.0665	4.5468	3.8097	4.4560	3.4043	3.4560	4.1368	4.4988	4.1959	4.2786	3.0694	4.2343	4.3040	3.8477	4.1870	4.4114	3.9960	3.1535	3.4525	3.5250
Iscu	_ELS[Phospho (STY)]APAR_.2	Q9D7P6_S30_M1_Iscu	8.3384	9.1695	8.5804	9.4354	8.8359	9.3880	9.1623	8.1469	9.4826	9.7396	9.3418	9.0428	9.6299	9.4716	9.5571	9.4035	8.8353	9.2426	9.6572	9.5585
Iscu	_AAS[Phospho (STY)]ALLLRS[Phospho (STY)]PR_.2	#N/A	9.1342	9.8217	9.8098	9.9196	4.2364	9.7880	9.3871	6.5558	9.6876	9.9752	8.8040	9.5560	9.4274	10.3124	9.6413	9.8268	9.8903	9.6471	9.9408	9.8885
Iscu	_RAAS[Phospho (STY)]ALLLRS[Phospho (STY)]PR_.3	#N/A	3.5127	5.8771	5.2730	5.8043	3.2611	5.3852	6.1334	3.4511	4.9702	6.0121	3.2136	5.5226	4.2279	6.3694	5.4379	5.5561	5.6675	6.1699	5.9172	5.9853
Iscu	_RAAS[Phospho (STY)]ALLLR_.2	Q9D7P6_S15_M1_Iscu	4.4719	3.4513	3.4043	3.7165	3.8096	4.0337	3.7315	4.0934	3.7906	3.1774	4.1649	3.4204	3.8986	3.4424	3.3087	3.3159	3.5906	3.5588	3.8578	3.1197
Rpl22l1	_YFQISQDEDGS[Phospho (STY)]ESED_.2	Q9D7S7_S118_M1_Rpl22l1	3.4401	6.9696	8.7430	6.7677	4.2786	8.2789	6.4534	3.1405	3.3272	6.0962	4.0329	7.5084	3.4640	7.5710	6.0870	8.6203	6.2337	8.6704	8.3674	8.5403
Chmp5	_NKDGVLVDEFGLPQIPAS_.2	#N/A	4.1471	3.8557	3.1826	3.5423	3.4849	4.2505	4.0562	3.8475	4.1153	5.3191	2.9888	4.0372	4.1678	3.1697	4.3807	2.9912	4.3852	3.4480	4.5700	3.1367
Lysmd2	_[Acetyl (Protein N-term)]ADLS[Phospho (STY)]PAPALR_.2	Q9D7V2_S5_M1_Lysmd2	7.6861	7.8741	8.3945	8.1291	6.3898	6.6673	7.6537	6.6206	7.2172	8.7238	7.0133	7.5189	7.2967	7.7046	6.0615	7.4924	7.1101	6.9194	6.4034	7.4760
Lysmd2	_AHRPSAPS[Phospho (STY)]PPPR_.3	#N/A	6.8607	7.5166	4.0793	3.9780	5.5384	6.7775	6.3150	6.8870	6.3914	7.4338	6.6892	6.6669	7.5244	6.5559	3.3530	6.7909	3.3558	6.7835	7.2557	6.5694
Lysmd2	_STS[Phospho (STY)]EPEEAELSLSLAR_.2	Q9D7V2_S33_M1_Lysmd2	4.0902	6.4442	3.7860	3.3348	3.4279	3.6520	4.1131	4.4751	4.1722	4.2965	3.0458	3.8020	4.2803	3.8241	3.6903	2.9343	3.9723	5.8540	3.4762	3.5013
Kctd4	_HNS[Phospho (STY)]VEDAEQGK_.2	#N/A	7.2460	7.0217	7.1821	7.1462	6.6509	7.2942	7.0494	7.2054	6.9528	7.5611	7.3627	7.2266	7.4245	7.5624	6.8511	7.1777	7.4304	7.3069	7.2032	7.5161
Kctd4	_HNS[Phospho (STY)]VEDAEQGKNC[Carbamidomethyl (C)]K_.2	#N/A	4.0020	4.1695	3.5386	3.6373	3.5054	2.5019	3.4462	3.8840	3.8228	3.7546	3.2382	3.5108	3.4720	3.2199	3.7169	3.3642	3.5641	3.0889	3.6393	3.9345
Dusp3	_VYVGNASVAQDITQLQK_.2	#N/A	5.5628	5.0011	4.8351	5.4710	5.5436	5.2927	5.1874	5.8147	4.9656	4.5557	5.3065	4.8358	4.9884	5.3539	5.1594	5.4848	5.3304	4.9268	4.7286	4.9232
Dusp3	_ANDTQEFNLSAYFER_.2	#N/A	5.5764	5.6780	5.9279	5.2249	3.1534	5.0134	5.2578	5.9778	5.8295	5.5750	5.7675	4.0766	5.7991	5.1281	5.3515	5.3672	5.5020	5.2845	5.0433	5.4111
Dusp3	_EIGPNDGFLAQLC[Carbamidomethyl (C)]QLNDR_.2	#N/A	5.3332	5.3908	4.9976	5.2186	4.9266	4.4291	5.4208	6.2689	4.9988	4.7521	4.9531	4.9912	5.1524	5.6422	5.1981	4.7427	5.1344	5.4383	5.1904	4.6674
Dusp3	_LGITHVLNAAEGR_.3	#N/A	4.3190	4.3625	3.8555	4.9088	4.5581	4.4902	3.9217	4.4659	3.9432	4.1988	4.6611	4.5815	4.3498	4.0530	4.0484	4.0209	4.3691	3.2170	3.9869	3.5709
Ggct	_GKIS[Phospho (STY)]DEM[Oxidation (M)]EDIIK_.2	#N/A	3.5984	4.0187	3.7983	3.9973	4.1298	3.8798	3.2033	3.8397	3.6190	3.7719	3.5600	3.2482	3.9126	4.6208	3.6677	4.3751	4.3757	4.2305	4.1897	3.7905
Ppa1	_AAPFTLEYR_.2	#N/A	7.5493	7.5090	7.5932	7.8084	7.5943	7.3773	7.6083	7.1810	6.9952	7.7030	7.4662	7.7274	7.8523	7.4595	7.8692	7.6966	7.4765	7.4127	7.4168	7.4892
Ppa1	_VIAINVDDPDAANYKDISDVER_.3	#N/A	6.2675	6.9325	5.9155	6.0696	6.0838	6.1957	6.2462	6.7998	6.3991	6.0911	6.0308	6.0412	7.2550	6.6695	6.9095	6.1069	6.7047	5.7993	6.2823	6.1700
Ppa1	HTGC[Carbamidomethyl (C)]C[Carbamidomethyl (C)]GDNDPIDVC[Carbamidomethyl (C)]EIGSK	#N/A	5.8254	5.9955	6.8952	6.4800	6.1741	6.0485	5.6170	6.2997	6.3219	6.4135	6.8358	5.7839	5.8801	6.8952	6.3192	5.7500	6.3223	5.8620	6.1124	6.5672
Ppa1	_VLGILAM[Oxidation (M)]IDEGETDWK_.2	#N/A	4.3760	4.7925	5.4634	4.0904	5.2204	5.0905	4.6749	4.2292	4.7397	5.1251	4.9136	4.3473	3.6822	4.8773	4.5498	4.7618	4.7276	4.4051	4.1606	5.0214
Ppa1	_STHDYWK_.2	#N/A	4.6134	4.4186	3.3189	3.8615	4.7178	3.9985	4.9059	4.6021	5.0628	3.9341	4.3325	4.7001	4.3509	4.8471	5.6897	4.8857	3.7530	4.7395	4.1279	4.7855
Fip1l1	_ERDHS[Phospho (STY)]PTPSVFNSDEER_.3	#N/A	6.6570	6.9215	6.9362	3.9678	5.8734	7.1437	6.0757	6.7155	6.5964	6.9832	6.5541	6.1903	6.5784	7.4221	6.2751	6.4220	6.6242	6.7737	6.2884	6.2220
Fip1l1	_ERDHS[Phospho (STY)]PTPSVFNSDEERYR_.4	#N/A	4.0139	7.3003	4.1111	3.7163	4.0058	6.7679	3.5589	6.8842	3.6180	6.8436	6.7164	7.0472	7.7537	8.3530	6.6632	7.1219	6.8916	7.7060	7.1052	6.6433
Fip1l1	_AES[Phospho (STY)]PDLR_.2	Q9D824_S280_M1_Fip1l1	4.0649	3.2542	3.6015	3.5193	4.2718	4.3052	4.2771	4.2906	4.1973	3.8755	3.6759	2.9803	4.3627	3.6395	2.8016	3.4270	3.7878	3.3616	3.6607	2.2804
Aplf	_TTGASQLQGS[Phospho (STY)]PEITK_.2	Q9D842_S149_M1_Aplf	3.4596	6.1455	6.6719	6.5631	4.2981	6.3024	3.2430	6.9153	6.2649	3.6659	3.6764	3.6660	3.4101	6.8869	6.2100	3.8045	7.0665	6.3616	6.1758	4.1320
Aplf	_RLS[Phospho (STY)]PSGNSK_.2	Q9D842_S238_M1_Aplf	3.1126	2.4515	3.6489	4.5768	4.0005	4.0338	3.0476	4.3138	4.4687	3.6041	3.9473	3.2788	3.1333	3.6359	3.0730	3.1556	3.3506	2.9817	4.1311	3.6029
Uqcrb	_WYYNAAGFNK_.2	#N/A	7.8372	7.9147	7.9648	8.2129	7.7690	7.6585	7.5503	7.8566	7.5739	7.9315	7.7829	8.0561	7.7216	8.1090	8.2519	8.0425	7.9256	7.8714	8.0887	7.9466
Uqcrb	_LPEDLYNDR_.2	#N/A	7.4947	8.1035	8.0847	7.9470	8.1080	7.8489	7.8207	7.4240	7.9571	7.5860	8.0816	8.2625	8.2966	8.2557	8.5972	8.3476	8.1588	7.8346	8.0685	8.4448
Uqcrb	_DDTLHETEDVKEAIR_.3	#N/A	6.4812	6.9916	7.4861	6.6330	6.8494	6.8846	6.6623	7.0532	7.4999	7.0113	7.2454	6.8616	7.7942	7.3833	7.7530	7.3727	6.7937	6.6508	7.2904	6.7389
Uqcrb	_DDTLHETEDVK_.2	#N/A	6.7790	5.8365	7.4175	7.1488	6.7180	6.7129	6.2871	6.1193	6.9005	6.4924	6.7551	6.7246	6.4561	7.0692	7.2852	6.8788	6.5823	6.2373	6.2567	7.0355
Uqcrb	_RLPEDLYNDR_.2	#N/A	6.7850	6.9301	6.9090	6.7085	7.4120	5.3344	5.7758	6.8742	7.6674	5.5990	7.4787	6.9716	7.0575	6.4534	5.9893	7.5544	7.8158	7.5118	7.2488	8.0292
Uqcrb	_YEEDKFYLEPYLK_.2	#N/A	6.4124	5.8847	5.9293	6.0334	6.1436	6.2178	5.9707	6.0948	6.1990	6.4052	6.3823	6.2037	6.4397	6.9689	6.3577	6.5184	6.4885	5.9221	6.4994	6.4159
Fam241b	_SS[Phospho (STY)]SSQQGFFNR_.2	Q9D882_S27_M1_Fam241b	7.7549	4.0146	3.9537	3.6338	7.2353	3.6411	4.1139	3.7004	7.4456	3.7385	3.5791	6.9690	7.4331	4.0106	6.4123	3.9989	3.5406	3.4917	4.0545	3.7631
U2af1	_AVIDLNNR_.2	#N/A	8.2053	8.2493	8.2645	8.0951	8.0377	8.0799	7.9370	8.4369	7.8455	8.2266	8.0121	7.7856	8.5280	8.1632	7.6682	7.5751	8.4566	7.9179	7.8258	7.9780
Itpa	_IDLPEYQGEPDEISIQK_.2	#N/A	7.1894	7.0521	7.1704	7.5556	7.0081	6.9268	7.0279	6.9528	7.2493	7.1028	7.3657	7.2489	6.4675	7.4582	6.9979	7.0484	7.1954	6.7191	6.9130	6.9895
Itpa	_IVFVTGNAK_.2	#N/A	3.4670	3.0383	2.0238	3.3472	1.8570	2.9409	3.4133	3.6926	3.3898	2.9197	3.2261	2.3877	3.6293	4.5424	2.9079	2.8134	0.8946	3.9596	4.2586	3.5863
Itpa	_LQEYFSVAAGAGDH_.2	#N/A	4.3779	6.1487	3.4134	5.5926	6.0609	5.3589	3.8254	5.5750	3.8845	4.5842	4.2589	3.5143	3.9926	3.4004	3.4026	3.2220	5.7832	3.2172	5.6718	3.3674
Arpc5l	_ALAVGGLGSIIR_.2	#N/A	7.6379	7.7679	7.8583	7.9532	7.6960	7.4682	7.5344	7.7260	6.9550	7.9774	7.4512	7.9243	7.7598	7.8330	7.7830	7.8618	7.6156	7.9175	7.5809	7.7334
Arpc5l	_AFHAALRNS[Phospho (STY)]PINTK_.3	#N/A	5.0457	4.1125	3.7119	4.0155	3.9094	3.6517	3.4752	7.5545	3.5342	4.3799	3.9086	6.5686	3.6423	8.0119	5.0202	3.6056	3.6221	7.0064	4.1475	4.9539
Arpc5l	_NS[Phospho (STY)]PINTK_.2	#N/A	3.8902	4.1126	4.4265	7.2097	6.5392	3.4520	6.9814	3.5906	6.4413	4.0965	3.2458	7.0017	4.4803	4.4136	3.8903	7.0297	4.1283	7.3656	3.3535	7.8695
Arpc5l	_SSEIEQAVQSLDR_.2	#N/A	7.2737	7.2442	7.7200	7.3225	7.1682	7.3161	6.8516	7.1953	7.4141	7.3747	7.0320	7.1405	7.2101	7.3080	7.4910	7.3916	7.4977	6.9702	7.1930	7.3696
Chmp4b	_GGPTPQEAIQR_.2	#N/A	6.2568	5.9599	6.4753	6.3261	6.7605	6.4292	6.3359	6.0707	6.5477	6.9340	6.0681	6.5330	3.5040	6.8065	7.1382	6.4055	6.0149	6.0076	6.6147	6.7485
Chmp4b	_KIEQELTAAK_.2	#N/A	3.4000	3.8296	3.1758	5.0907	3.2616	4.6259	4.1768	3.8575	3.8941	3.2106	3.3784	3.7456	4.1852	4.5066	5.2556	4.4011	3.6196	3.7818	4.5555	3.5206
Chmp4b	_QLAQIDGTLSTIEFQR_.2	#N/A	3.2773	4.0418	4.6582	5.0031	3.4842	3.9856	3.5639	3.5030	4.1335	4.3574	4.1013	3.7679	5.2911	4.3528	4.3314	3.9838	4.5011	3.9663	4.1845	3.6761
Ndufa11	_FFESYHEVPDGTQC[Carbamidomethyl (C)]HR_.3	#N/A	7.0821	6.6920	6.5192	5.8708	6.8744	6.7060	6.7903	6.5777	6.5556	6.0652	6.7321	6.1957	3.3195	6.1450	6.2123	6.5101	6.7489	6.3048	6.4444	3.7892
Ndufa11	_EKPDDPLNYFIGGC[Carbamidomethyl (C)]AGGLTLGAR_.3	#N/A	4.7765	5.6057	5.2167	3.9579	4.5955	5.4124	4.7287	4.6418	4.5613	4.5097	5.5045	4.7646	4.0671	5.7115	4.2612	4.4976	5.3823	5.1828	4.8936	4.9359
Rpl4	_ILKS[Phospho (STY)]PEIQR_.2	Q9D8E6_S295_M1_Rpl4	7.0547	7.3108	6.5113	6.3610	6.1471	6.5073	6.5849	6.7151	6.4384	6.5749	6.7332	6.8785	7.3335	7.2203	6.6475	6.3130	7.3512	6.7220	6.5291	6.4156
Rpl4	_FC[Carbamidomethyl (C)]IWTESAFR_.2	#N/A	5.2089	5.8072	5.9506	5.2810	4.7875	5.0543	5.5898	5.1945	5.2527	4.5662	4.6777	5.8883	4.0140	3.5578	4.6398	4.8483	5.3195	5.2079	3.7425	4.9132
Rpl4	_QPYAVSELAGHQTSAESWGTGR_.3	#N/A	4.0020	2.5564	4.5383	3.6874	3.3397	3.1444	3.9371	4.2315	3.8573	4.2155	2.9758	3.8902	4.0227	4.5254	3.7785	4.3469	4.2401	3.5931	3.2417	2.9915
Rpl4	_SGQGAFGNM[Oxidation (M)]C[Carbamidomethyl (C)]R_.2	#N/A	3.1678	4.1513	4.2052	3.9511	3.3747	3.4081	3.6734	3.4387	3.5936	4.4792	4.8925	3.8774	3.7590	4.2617	3.6988	4.0307	3.9764	3.8568	3.5054	4.2287
P2yr13	_TAGSSEDHHS[Phospho (STY)]S[Phospho (STY)]QTDNITLA_.2	Q9D8I2_S328_M2_P2yr13	4.4285	3.4079	3.4477	3.6731	3.7662	3.9903	3.7749	4.1368	3.8340	3.1339	4.2084	3.1340	3.9420	3.4858	3.3521	3.2725	3.6341	3.5154	3.8144	3.1631
Eef1g	_STFVLDEFKR_.2	#N/A	7.7175	7.8224	7.3493	7.7147	7.8037	7.5966	7.9122	8.1719	7.5887	7.7662	7.2838	7.5053	7.6181	7.6548	7.1063	7.7708	7.9369	7.7403	7.7779	7.2329
Eef1g	_[Acetyl (Protein N-term)]AAGTLYTYPENWR_.2	#N/A	7.5966	7.4471	7.7068	7.5721	7.2555	7.2877	7.5021	7.6358	7.3881	7.3306	7.1445	7.1871	7.5518	7.1586	6.7602	7.0498	7.2116	7.2491	7.2123	6.7156
Eef1g	_TFLVGER_.2	#N/A	6.9286	6.3780	10.6866	6.4336	6.2089	6.4656	6.4288	6.8312	6.1808	6.3994	6.8371	5.9766	6.2000	5.4650	5.9740	5.9223	6.1478	6.7451	6.3818	6.4179
Eef1g	_QVLEPSFR_.2	#N/A	7.0837	6.8464	7.1253	6.8900	6.9405	7.2632	7.1953	7.1554	6.7842	6.5400	6.8137	7.3008	7.0329	6.1007	6.6677	6.4408	6.5300	6.8227	6.7298	6.3665
Eef1g	_ILGLLDTHLK_.2	#N/A	7.3145	7.1998	6.6776	7.0959	6.9556	6.9679	7.2392	7.1642	5.9823	6.3757	6.2900	7.1339	7.0981	6.9483	6.0610	5.8416	6.3578	7.0213	6.6588	6.7131
Eef1g	_WFLTC[Carbamidomethyl (C)]INQPQFR_.2	#N/A	6.3285	5.8088	6.4552	6.3022	6.2239	5.5299	6.1406	6.3940	6.0380	6.1616	6.3302	5.6416	3.2135	6.2911	5.5640	5.7151	6.1679	6.0449	5.8799	5.8932
Eef1g	_ALIAAQYSGAQVR_.2	#N/A	5.2233	4.2381	5.2987	5.0808	4.7950	4.9536	5.1839	5.1347	3.2458	4.7665	4.7923	4.5891	3.5465	5.6947	4.9069	4.4394	4.9529	5.0867	4.9921	4.7803
Eef1g	_KLDPGSEETQTLVR_.2	#N/A	4.8863	4.4900	4.4881	4.3495	4.7750	4.8127	4.5567	4.9835	4.6558	4.6715	4.7800	5.0926	4.9669	5.0330	4.4322	4.3901	4.7201	4.9800	4.4740	4.8212
Eef1g	_KFAESQPK_.2	#N/A	5.0660	4.6774	3.9850	3.1358	4.7811	4.4145	4.2814	3.1733	5.2311	3.7964	4.6957	4.0976	4.4793	4.0231	5.0055	3.8105	4.8613	4.7587	4.2141	5.1030
Eef1g	_QATENAKEEVKR_.3	#N/A	3.6638	3.2597	3.7786	4.4471	3.8707	3.9041	3.1774	4.4435	3.0976	3.4744	3.3138	2.7401	3.2630	3.7657	3.2027	3.3367	3.4804	3.0111	3.8753	3.7327
Arfgap3	_LTNTS[Phospho (STY)]FTEIEK_.2	Q9D8S3_S241_M1_Arfgap3	3.3001	4.0190	4.3375	4.2841	2.8764	3.1005	5.7512	3.5258	5.9245	7.0898	3.5973	6.0958	3.3310	4.3756	4.2419	6.3049	4.5239	4.1264	4.4255	4.0529
Arfgap3	_SSS[Phospho (STY)]FSSWDDGADSYWK_.2	Q9D8S3_S374_M1_Arfgap3	3.4828	5.8807	4.5202	5.9702	3.6897	5.2268	3.3584	3.7085	3.2786	3.2934	4.2581	3.5624	3.4440	5.6652	3.3838	4.3457	3.2057	5.1395	3.8204	4.9812
Bola1	_FEGM[Oxidation (M)]S[Phospho (STY)]PLQR_.2	#N/A	7.6931	7.9026	8.1264	8.8292	7.3859	6.9703	7.3593	7.0824	6.8320	7.5469	7.3696	7.2765	7.7119	8.2339	6.3183	7.3082	7.1524	6.7795	6.9835	7.4521
Bola1	_ENPQLDIS[Phospho (STY)]PPC[Carbamidomethyl (C)]LGGSK_.2	Q9D8S9_S123_M1_Bola1	4.0889	3.6848	3.3535	7.7013	4.2958	8.1033	4.2531	4.0184	4.1733	7.3307	3.6519	7.0747	4.3387	3.3406	4.2785	3.4510	4.5561	3.2771	7.7457	3.3076
Bola1	_AKLEQALS[Phospho (STY)]PEVLELR_.3	Q9D8S9_S45_M1_Bola1	3.1574	4.0423	4.1948	4.4268	3.3644	3.2432	3.6837	3.3831	3.0802	4.4689	3.4546	3.8878	3.1883	4.2329	3.7091	4.0204	4.3812	4.2691	3.0673	3.8089
Slirp	_ALHGAQTS[Phospho (STY)]DEER_.2	Q9D8T7_S105_M1_Slirp	6.8316	6.7332	6.6360	6.6806	6.5883	4.1161	6.5427	3.7675	6.5870	6.8572	6.6758	6.3706	4.3422	6.8704	6.8553	6.6738	4.1837	6.6152	6.6611	6.6972
Snx5	_[Acetyl (Protein N-term)]AAVPELLEQQEEDR_.2	#N/A	6.1561	6.2633	6.7367	6.6841	5.5728	6.4239	6.3360	5.5200	6.4870	6.6253	6.6198	6.3379	7.1179	6.4280	5.8442	6.3365	7.2270	5.9289	6.7041	6.6950
Snx5	_[Acetyl (Protein N-term)]AAVPELLEQQEEDRSK_.2	#N/A	3.7061	3.9124	3.2427	2.6112	4.0121	3.0315	3.5698	4.1153	4.5153	3.3390	2.9781	3.3391	4.3242	4.9262	4.3932	3.5855	2.6532	2.9593	4.0195	3.9300
Ociad2	_FHSFEDQLR_.2	#N/A	6.8357	6.5927	6.2334	6.8249	7.0061	6.4210	6.2124	3.5597	6.2728	5.9634	5.8465	5.9175	6.6759	6.6374	6.6765	6.2173	6.5384	6.6188	6.6710	6.4587
Ociad2	_[Acetyl (Protein N-term)]ASVSTHGNQEK_.2	#N/A	2.8662	4.2707	5.2674	4.6769	4.4587	3.5211	4.5039	3.5578	3.9439	4.2676	3.7428	3.8331	3.1376	4.3369	4.7960	3.9747	3.9372	3.0037	3.9960	4.0937
Ociad2	_GAGFGPEHNR_.2	#N/A	3.6239	6.1044	5.5314	6.1577	5.8967	6.1520	6.3843	6.2659	5.9686	6.0632	5.9829	5.9571	6.5920	5.9567	6.3159	6.4490	6.0327	6.5942	6.8905	5.5493
Efhd2	_ADLNQGIGEPQS[Phospho (STY)]PSR_.2	#N/A	10.4120	10.2289	10.7050	10.6490	9.6418	9.1594	9.4841	9.4125	9.2577	11.1501	9.8256	9.7485	9.9181	9.9727	9.0097	9.9677	9.6301	9.2770	6.8870	9.8453
Efhd2	_RADLNQGIGEPQS[Phospho (STY)]PSR_.3	#N/A	6.8331	7.3029	8.4476	5.2764	5.1927	5.0777	6.9276	5.8901	5.1117	8.5419	7.1957	5.0700	5.1250	7.1397	6.1799	5.4915	6.4780	4.9428	7.1177	5.1184
Efhd2	_VQAINVSSR_.2	#N/A	8.1925	8.4016	8.6456	8.4847	8.0681	8.2355	8.4133	8.4464	8.4257	8.2151	8.1415	8.5735	8.7149	8.5629	8.9617	8.5870	8.0637	8.2570	8.6035	8.2707
Efhd2	_LSEIDVSTEGVK_.2	#N/A	6.8359	7.0787	7.4554	6.8452	6.7065	6.5466	6.6502	6.3763	7.3945	6.6522	6.5018	6.9714	6.8262	7.0769	7.2263	7.3817	7.1959	6.5889	6.9682	7.0562
Efhd2	_RADLNQGIGEPQS[Phospho (STY)]PSRR_.3	#N/A	8.6432	7.8182	7.6907	7.4409	3.6275	3.8516	3.9136	4.1413	6.3123	8.7498	4.3471	7.2825	7.7326	7.5929	3.4908	7.1923	4.2005	6.8991	7.0786	3.2920
Efhd2	_ADLNQGIGEPQS[Phospho (STY)]PSRR_.3	#N/A	6.9615	6.9558	3.7447	8.0076	3.5685	6.2981	6.6293	5.3078	5.7292	7.8481	6.1227	6.5304	6.5965	3.7318	5.5359	6.1651	5.5010	3.4811	4.0987	6.3976
Efhd2	_[Acetyl (Protein N-term)]ATDELASKLS[Phospho (STY)]R_.2	Q9D8Y0_S11_M1_Efhd2	6.8757	7.0994	5.7052	6.9350	6.3739	7.0362	5.4910	6.2201	6.7088	7.6313	3.7798	6.6610	4.1480	7.3713	6.8065	6.8736	6.6186	5.3431	5.7464	6.2847
Efhd2	_ELQSTFK_.2	#N/A	2.2735	3.1492	3.8890	4.3366	3.7603	3.7936	3.2878	3.0531	3.2080	3.3639	4.1875	3.5879	3.3735	3.8761	3.6226	4.4163	3.5908	4.2424	3.8909	3.8431
Efhd2	_SMIQEVDEDFDSK_.2	#N/A	4.1355	4.6158	3.7407	3.3801	3.4732	3.6973	4.0679	4.4298	4.1270	4.3418	3.0005	4.3419	4.2350	3.7788	3.6451	2.9795	3.9271	3.2224	3.5214	3.4561
Borcs5	_GLLSGQTS[Phospho (STY)]PTNAK_.2	#N/A	9.8717	9.5419	10.1452	3.6571	9.7766	9.3753	9.0471	9.6814	9.3818	9.5371	3.5744	3.8194	10.0075	10.1248	9.2586	9.8572	10.0305	4.2007	9.7051	9.8012
Borcs5	_GLLSGQTS[Phospho (STY)]PTNAKLEK_.2	#N/A	6.5750	6.8267	4.2976	6.0299	6.2259	3.1404	3.5810	3.4859	5.2614	4.5716	3.5574	6.4625	3.2911	6.4370	5.7175	6.2760	6.3592	4.1663	2.9646	6.3672
Rwdd2a	_KLS[Phospho (STY)]DEPSVQAK_.2	#N/A	4.3856	4.4343	3.9222	5.7411	3.0917	6.6572	3.9564	3.1105	5.0264	4.1962	3.1820	4.1604	4.4165	4.7421	3.9817	3.7477	4.1085	3.0409	3.3400	3.6376
Rwdd2a	_KLS[Phospho (STY)]DEPSVQAKPVK_.2	#N/A	3.2560	3.2459	3.7923	4.4334	5.7753	3.8903	3.1911	6.0539	3.1113	3.4607	4.0908	5.7328	3.2767	6.4961	5.7354	3.6009	6.4490	4.3391	3.9877	3.7464
Fam135b	_SVSAPQIS[Phospho (STY)]SPEESAEGADTIK_.2	Q9DAI6_S775_M1_Fam135b	4.2990	6.6223	3.4838	3.3909	3.9202	4.9712	5.9153	4.1569	4.1723	3.8783	3.9543	4.5928	5.4992	6.3492	3.3063	3.2981	3.9432	5.8613	4.1519	3.3622
Fam135b	_NTAGFSEDLDPSSKENS[Phospho (STY)]PPR_.3	Q9DAI6_S805_M1_Fam135b	4.1707	3.8321	3.2062	3.4153	3.5084	3.7325	4.0326	4.3946	4.0917	5.0733	2.9653	3.7215	4.1998	5.6437	3.6098	3.0148	3.8918	3.2576	3.5567	3.4208
Gng12	_TASTNS[Phospho (STY)]IAQAR_.2	Q9DAS9_S10_M1_Gng12	8.1770	8.9988	8.7872	8.6213	8.1287	8.7193	8.4878	8.4170	8.6870	8.9371	8.5560	8.4510	9.0708	8.8349	8.4578	8.5973	8.7207	8.6084	8.6457	8.5808
Gng12	_SDPLLM[Oxidation (M)]GIPTSENPFK_.2	#N/A	7.7393	7.4661	6.9736	7.0710	7.0148	7.4147	7.4876	7.8713	7.0707	7.1331	7.1211	7.2694	7.4956	7.3558	6.9185	7.0135	7.2550	7.2661	7.3161	6.8880
Gng12	_LEASIER_.2	#N/A	5.5302	5.9151	5.6319	3.5208	5.3179	6.0312	6.1343	5.1456	6.4556	6.2089	5.8496	6.1869	6.1008	5.5896	6.0634	6.2799	4.9731	6.3405	6.2683	5.7791
Gng12	_ASADLM[Oxidation (M)]SYC[Carbamidomethyl (C)]EEHAR_.2	#N/A	4.4251	4.0827	3.7134	3.4954	3.5052	3.6643	3.8634	4.0324	3.8460	4.5871	3.8068	3.8643	3.9852	4.1851	3.7096	3.5313	3.8998	3.2956	3.7747	3.3216

Mrgbp	_VLTANSNPSS[Phospho (STY)]PSAAK_.2	Q9DAT2_S195_M1_Mrgbp	5.7947	4.1911	5.4604	3.5860	3.9385	3.5284	5.6038	5.6682	3.9032	3.8966	5.8914	5.6624	6.0422	4.0897	6.6436	3.6471	3.7608	5.7589	6.4929	6.0677
Zfyve19	_WS[Phospho (STY)]PPQNYK_.2	Q9DAZ9_S69_M1_Zfyve19	3.1932	7.7475	4.2306	6.6882	7.6031	7.4473	3.0569	7.3369	7.6258	3.8519	8.0657	3.8520	3.2241	4.2687	8.0284	8.2238	8.0962	7.8003	7.3383	3.9460
Zfyve19	_VTLQDYHLPDS[Phospho (STY)]DEDEETAIQR_.3	Q9DAZ9_S280_M1_Zfyve19	4.1356	4.6159	3.8963	3.3802	3.4734	3.6975	4.0677	4.4297	4.1268	4.3420	3.0003	4.3421	4.2349	7.8342	3.6449	2.9797	3.9269	6.7067	3.9301	3.4559
Zfyve19	_TNS[Phospho (STY)]QGQASQSLEEEKYK_.2	Q9DAZ9_S219_M1_Zfyve19	3.3940	3.9251	4.4314	4.1902	3.6009	3.6343	3.4472	3.6197	3.3674	3.2046	4.3469	2.9807	3.5328	4.1763	3.4725	4.2569	3.1169	1.9154	3.2943	4.0025
Zfyve19	_EHQTS[Phospho (STY)]PYHPR_.2	Q9DAZ9_S378_M1_Zfyve19	3.9656	3.5329	3.5545	4.0249	3.7160	3.8383	3.6379	4.2194	3.6474	4.0262	4.6204	3.4224	3.7756	3.5415	5.0676	3.0399	4.0616	4.1152	3.5792	3.5704
Napa	_YEELFPAFSDSR_.2	#N/A	8.3438	8.3942	8.3069	8.6183	8.1777	8.5256	8.4607	8.3887	8.6751	8.5333	8.5092	8.6016	8.2125	8.9428	8.5893	8.5547	8.4994	8.5537	8.3992	8.2836
Napa	_TIQGDEEDLR_.2	#N/A	7.3107	7.0278	6.8748	7.1171	7.2178	7.4356	7.4444	7.2726	7.3773	7.1605	7.1100	7.1504	7.2669	7.5122	7.3860	7.4204	7.2329	7.3137	7.1323	6.6942
Napa	_VAGYAAQLEQYQK_.2	#N/A	5.9126	6.7009	6.9687	6.3406	6.1090	6.4899	6.5986	7.6395	6.7228	6.5725	6.3997	5.8653	5.8226	7.5488	7.2077	6.2115	6.3773	6.6108	6.1248	6.3467
Napa	_NSQSFFSGLFGGSSK_.2	#N/A	5.7511	4.8664	5.6848	5.3638	4.6255	4.8438	5.4012	5.3943	5.0976	5.9373	5.0164	5.2321	5.1238	5.6512	5.3844	4.7288	5.0505	4.8985	5.6349	5.3134
Napa	_LLEAHEEQNVDSYTEAVKEYDSISR_.4	#N/A	5.3408	5.6561	4.6989	5.6056	4.4003	4.4211	4.9802	3.4911	5.0806	4.6506	5.0503	5.5687	6.0117	5.9107	4.6005	4.8385	4.9888	4.2542	5.3108	5.2726
Napa	_AIDIYEQVGTSAM[Oxidation (M)]DSPLLK_.2	#N/A	4.0552	4.6140	4.7407	4.1760	4.3619	4.6463	5.1093	3.8659	4.6822	4.5875	4.1285	4.2029	4.0898	5.6933	4.6667	4.7918	4.5515	5.1496	5.1491	4.5322
Napa	_LLEAHEEQNVDSYTEAVK_.3	#N/A	6.0104	4.8282	5.3595	5.3277	5.2193	5.0144	4.6594	4.8895	4.6505	5.4759	5.7138	4.8432	5.0770	5.4489	4.1012	4.7322	5.5418	4.2311	5.0074	4.8801
Napa	_AIEIYTDMGR_.2	#N/A	3.7124	3.3082	3.7300	4.4738	3.9193	3.9526	3.1288	4.3949	3.0490	3.5230	4.0285	3.1977	3.2144	4.6624	3.1542	3.0745	3.4318	4.4014	4.0500	3.6841
Napa	_HDAATC[Carbamidomethyl (C)]FVDAGNAFK_.2	#N/A	3.1594	3.3426	3.4886	4.5300	3.9979	4.2220	3.5431	4.3606	3.6022	4.4756	3.9766	3.2320	3.7103	5.3173	3.1203	3.5043	3.3974	3.7471	4.0462	4.4322
Mrpl12	_NYVQGINLVQAK_.2	#N/A	4.4001	3.5080	4.9972	5.2769	4.3276	4.6361	4.4252	4.9630	4.6397	4.7361	4.6641	4.5885	5.1014	5.8376	4.8279	4.5679	4.6691	4.9014	5.2004	4.5004
Mrpl12	_LTEAKPVDK_.2	#N/A	5.1646	5.3218	5.3785	5.3580	5.4310	5.4355	5.2739	5.4424	5.4240	5.3140	4.7652	5.3834	5.4118	5.3840	6.2252	5.8939	5.5203	5.1689	4.5281	5.3879
Atp5po	_LVRPPVQVYGIEGR_.3	#N/A	10.4490	9.9674	10.7490	10.9141	10.4966	10.1828	10.3221	10.7468	10.4343	10.5957	9.9627	10.4282	11.1191	10.5229	10.5260	10.4907	10.4341	10.2739	10.7213	10.6886
Atp5po	_VSLAVLNPYIK_.2	#N/A	9.1468	9.2729	8.9246	9.2735	9.2544	9.0675	9.2551	9.0779	8.9132	9.1793	9.0013	9.3813	9.2419	9.4259	9.2398	9.0044	9.1309	9.4715	9.2569	9.3454
Atp5po	_SFLSPNQILK_.2	#N/A	8.7932	8.9961	8.8606	8.9777	9.1045	8.7905	9.1307	9.0717	8.5888	8.9665	8.8267	9.1825	9.1213	9.1391	9.0783	8.7167	8.9783	9.3357	8.9375	9.1138
Atp5po	_YATALYSAASK_.2	#N/A	8.2224	8.5358	8.5729	8.8713	8.2190	8.1739	8.5928	8.4001	8.1339	8.0055	8.0891	8.7384	8.3177	8.5712	8.2903	8.1215	7.7899	8.3234	7.9995	8.1039
Atp5po	_SLNDITKR_.2	#N/A	7.3125	7.5376	7.3474	7.7471	7.8850	7.4347	7.7735	7.8301	7.3737	7.5177	7.4086	7.7146	7.9631	7.8368	8.1590	7.5413	7.4913	7.8035	7.8938	7.2441
Atp5po	_EKFSPLTANLM[Oxidation (M)]NLLAENGR_.3	#N/A	7.8019	7.8950	6.8475	7.2158	7.6362	7.6077	7.7031	7.8940	7.4564	7.6299	7.6525	7.8142	8.0594	7.6140	7.5741	7.8971	7.6674	7.8769	7.9475	7.8300
Atp5po	_FSPLTANLM[Oxidation (M)]NLLAENGR_.2	#N/A	6.3475	6.1134	5.5865	6.3200	6.3864	6.8429	6.0049	6.6081	6.0689	6.8265	6.5610	6.0685	6.0006	6.4587	6.3303	6.1964	6.3491	6.4993	6.2695	6.4195
Atp5po	_LGNTQGIISAFSTIM[Oxidation (M)]SVHR_.3	#N/A	5.8572	5.7900	6.0394	6.1999	5.5587	5.6108	5.5535	5.0958	5.5573	5.9696	6.0041	5.6462	6.2718	6.3427	5.2105	5.5980	5.5807	5.7671	5.5406	6.2516
Atp5po	_TDPSIMGGMIVR_.2	#N/A	4.1860	3.1330	7.4772	7.3834	2.8921	4.9346	6.6444	4.4117	4.0762	3.3966	3.5548	4.3600	4.2416	7.7201	4.4378	3.5482	3.2361	3.2405	3.5395	3.4380
Atp5po	_GEVPC[Carbamidomethyl (C)]TVTTASPLDDAVLSELK_.2	#N/A	6.4206	6.9004	6.4916	6.8540	6.3050	6.7129	6.8475	6.7545	6.7092	6.6780	6.8653	6.7176	6.6176	7.1529	7.1457	6.5459	6.7015	6.8090	6.6361	6.6398
Atp5po	_YVDMSAK_.2	#N/A	4.0983	3.2208	3.6349	3.4859	3.8031	4.2437	4.2417	4.3240	4.1639	3.9089	3.6426	2.9469	4.1292	3.6729	4.2691	3.4604	3.8212	3.3283	3.6273	3.3502
Slc25a18	_IQLQDAGR_.2	#N/A	8.3660	8.3774	8.3199	8.4985	8.3782	8.4015	8.7103	8.7857	8.6035	8.2598	8.3304	8.3876	8.1330	8.2463	8.3012	8.2789	8.3892	8.5308	8.4154	8.1658
Slc25a18	_GAAVNLTLVTPEK_.2	#N/A	7.7721	7.3218	7.2924	7.1323	7.1894	7.5102	7.5002	7.5971	7.2410	7.0000	7.2611	7.0021	7.1356	7.6709	6.8942	7.6574	7.2262	7.5500	7.3534	7.2159
Slc25a18	_LAANDFLR_.2	#N/A	5.9562	6.0803	5.0997	5.8011	6.1990	6.2038	6.2580	6.2929	5.6155	5.8993	5.2693	4.9243	5.6393	5.5793	5.2940	5.2006	5.6025	5.5063	5.7342	5.6409
Slc25a18	_ASFTHSFVAGC[Carbamidomethyl (C)]TAGSVAAVAVTPLDVLK_.3	#N/A	3.4168	3.9023	4.4542	4.1674	3.6237	3.6571	3.4244	3.6425	3.3446	3.2274	4.3241	3.6284	3.5100	4.4923	3.4498	4.2797	3.1397	4.1058	3.7544	3.9797
Pabir1	_VSTTTDSPVS[Phospho (STY)]PAQAASPFIPVDELSSK_.3	Q9DBS2_S267_M1_Pabir1	3.8212	6.1314	5.2840	4.5666	3.1589	3.3830	5.7367	6.2857	4.4413	4.0275	3.3148	5.7231	6.3263	6.7366	3.9594	4.1661	4.2413	5.7728	3.2071	4.9091
Pabir1	_RIDFIPVS[Phospho (STY)]PAPS[Phospho (STY)]PTR_.2	Q9DBS2_S140_M2_Pabir1	4.1321	8.3345	7.7889	3.4521	4.3390	3.7693	3.9958	4.3578	4.0549	4.4139	2.9284	4.4140	4.1630	8.9486	6.9334	3.4942	3.8550	7.4610	7.1888	3.3840
Pabir1	_RNS[Phospho (STY)]TTFPSR_.2	Q9DBS2_S59_M1_Pabir1	4.3742	3.6286	3.4097	3.3152	3.7119	3.9360	3.8291	4.0746	3.8882	4.5805	4.2626	3.5180	3.9963	3.3968	3.4063	3.2183	3.1114	3.2209	4.3703	3.3638
Pabir1	_SNS[Phospho (STY)]APLHGLSDSSPVFQAEAPSAR_.3	Q9DBS2_S34_M1_Pabir1	3.4811	3.8918	3.8167	4.1570	3.6881	3.6480	5.8137	3.7068	3.0629	3.7167	3.7640	3.6179	5.5120	6.9835	3.7067	3.6236	4.0030	3.9993	3.5964	3.5320
Prxl2b	_SILDQHDVR_.2	#N/A	5.5703	5.8956	5.4775	5.2064	5.6893	5.4022	6.3058	5.1053	5.8227	5.9755	5.5464	5.9183	5.8216	5.6595	6.2943	5.0645	5.2458	5.5044	5.5021	6.0254
Prxl2b	_WIAQDLSNLR_.2	#N/A	5.9601	5.4235	5.7426	5.5341	5.6964	6.0307	5.4909	5.5186	5.7342	5.5151	5.4260	5.8009	5.2016	5.4725	5.6644	5.6854	5.4542	5.4552	5.4549	5.3506
Fundc1	_NPPPQDYES[Phospho (STY)]DDESYEVLDLTEYAR_.3	Q9DB70_S13_M1_Fundc1	3.9545	6.3798	5.2454	5.8449	3.6795	6.9128	6.0049	3.5142	4.3181	4.0258	3.3987	3.7445	4.4434	3.8426	5.8443	4.0060	4.3135	3.7061	4.9691	4.9463
Cdip1	_[Acetyl (Protein N-term)]S[Phospho (STY)]NEPPPPYPGGPTAPLLEEK_.2	Q9DB75_S2_M1_Cdip1	4.2801	3.7227	3.3156	3.4092	3.6179	6.9635	3.9232	3.9805	4.1354	3.9374	3.6141	3.6121	4.3009	3.3027	3.5004	3.1242	4.5182	6.7837	2.9635	3.2697
Uqcrc2	_TSAAPGGVPLQPQDLEFTK_.2	#N/A	9.1244	9.1431	9.7306	9.2515	8.9326	9.2427	9.0737	9.0709	9.0941	9.3807	9.2038	9.0633	8.9442	9.1674	9.1388	9.1583	9.2671	9.0279	8.9697	8.8297
Uqcrc2	_AVAQGNLSSADVQAAK_.2	#N/A	7.3919	7.3715	7.7029	8.1086	8.0132	7.9554	7.5668	7.6639	7.3196	7.4597	7.2104	7.8985	7.6270	8.2664	7.6579	8.0663	8.1743	8.0087	8.0069	8.1814
Uqcrc2	_GNNTTSLLSQSVAK_.2	#N/A	8.8285	8.8496	9.1152	9.2261	8.9099	8.9398	8.8192	9.0394	9.0019	9.0073	8.8451	8.9339	8.8456	8.9009	8.9998	9.1538	9.1379	8.6628	9.0732	8.7463
Uqcrc2	_AVAFQNSQTR_.2	#N/A	8.4990	8.6764	8.6931	8.8272	8.3471	8.3912	8.6347	8.9013	8.6630	8.2734	8.2716	8.6241	8.6532	8.8656	8.8269	8.4957	8.3245	8.5191	8.3477	8.3689
Uqcrc2	_NALANPLYC[Carbamidomethyl (C)]PDYR_.2	#N/A	7.7205	7.7703	8.2639	7.9650	7.6250	7.6221	8.0452	7.8812	7.6074	7.7369	7.7860	7.8782	8.0103	7.8708	7.7492	7.5185	7.8651	7.5382	7.8472	7.5808
Uqcrc2	_YRGGEIR_.2	#N/A	6.1434	7.7167	5.7471	5.4375	6.4692	7.4728	6.8989	7.3644	6.2330	7.1769	7.3269	7.3484	6.0168	7.9018	6.3418	6.5821	6.9426	7.3358	6.4274	7.5392
Uqcrc2	_WEVAALR_.2	#N/A	5.5811	5.5177	4.6947	6.0805	5.1654	5.1049	6.1395	5.6167	5.4614	6.3766	5.8007	5.4149	5.6220	5.6437	5.5977	5.7541	4.2557	5.7408	6.2316	5.7016
Uqcrc2	_LPNGLVIASLENYAPLSR_.2	#N/A	4.9585	5.3453	6.0054	6.0744	5.9204	5.8208	6.0554	5.8342	5.5543	5.1642	5.8445	5.8527	4.5811	5.3462	6.0042	5.3015	5.3964	5.4432	5.8392	6.1334
Uqcrc2	_YEDSNNLGTSHLLR_.2	#N/A	7.6851	7.8204	7.6391	7.8240	7.6266	7.5020	7.8196	8.0403	7.6929	7.6128	7.6714	7.6875	7.7974	7.7788	7.6149	7.6274	7.6573	7.6870	7.7846	7.5112
Uqcrc2	_M[Oxidation (M)]ALVGLGVSHSVLK_.2	#N/A	7.7520	7.5314	7.6132	7.3120	7.5593	7.4345	7.7064	7.1562	7.3748	7.4078	7.2566	7.3576	7.8069	6.9546	6.9182	7.4142	7.5022	7.4902	7.5656	7.5042
Uqcrc2	_ENM[Oxidation (M)]AYTVEGIR_.2	#N/A	6.1614	7.3372	7.9749	7.9872	7.5110	7.1469	7.2161	7.2732	7.4699	6.6544	7.3552	7.0958	6.3387	8.0313	6.6039	7.1822	6.8664	7.3478	6.4151	6.7433
Uqcrc2	_IIENLHDVAYK_.2	#N/A	7.3507	7.3223	6.9870	7.4407	7.4820	7.1290	7.4440	7.6851	7.3849	7.3312	6.8930	7.5860	7.6385	7.3511	7.4291	7.1471	7.4663	7.2616	7.3041	7.1914
Uqcrc2	_SM[Oxidation (M)]AASGNLGHTPFLDEL_.2	#N/A	6.7592	6.3689	6.7918	7.0099	6.3488	6.6232	6.3119	6.6198	6.5406	6.6417	6.7095	6.2443	6.8214	6.6230	6.8303	6.8564	6.7671	6.2394	6.4295	6.5892
Uqcrc2	_QVAEQFLNM[Oxidation (M)]R_.2	#N/A	6.1684	6.5399	6.6759	6.8940	6.4106	6.5148	6.6178	6.3577	5.7873	6.5002	6.6235	6.3752	6.1961	6.6075	6.2098	6.6074	6.3423	5.9591	6.1435	6.2868
Uqcrc2	_SQLKIDK_.2	#N/A	5.0136	4.5478	4.5916	4.6854	4.3992	3.9891	4.9181	4.8063	5.4840	4.7152	4.6888	4.4622	5.4978	5.3926	4.0399	5.2759	4.8859	5.6220	5.1821	4.1222
Uqcrc2	_LASSLTTK_.2	#N/A	3.6463	3.7771	3.1828	3.9380	3.7691	4.2552	4.4559	3.8719	3.5691	3.3988	3.9435	4.0093	3.6771	3.2209	3.2203	3.0084	3.3692	4.3981	5.3375	4.4427
Uqcrc2	_ITSEELHYFVQNHFTSAR_.3	#N/A	7.3046	7.0946	6.9185	7.0023	6.6975	6.6621	6.8048	7.2131	6.1032	7.0048	6.7365	6.9804	7.3933	7.0860	5.6414	6.6540	6.6072	6.4771	6.7519	6.8793
Smg9	_WKEPGSSGPQNLS[Phospho (STY)]GPGGR_.3	Q9D890_S32_M1_Smg9	4.2434	5.3687	3.2788	3.4460	3.5811	3.8052	4.6553	3.9438	4.0191	4.4497	4.3934	3.6489	4.1271	3.2659	3.5372	3.0874	4.4814	3.3303	3.6293	3.3482
Selenoo	_LEQLS[Phospho (STY)]PSDLQR_.2	Q9D8C0_S544_M1_Selenoo	3.5659	7.2630	4.6034	4.0183	3.7729	3.8062	3.2752	3.7916	3.1954	3.3765	4.1749	3.4793	3.3609	3.1406	3.3006	4.4289	3.2888	3.8606	4.1597	3.8305
Cmtr1	_HLS[Phospho (STY)]STSDDEPLSSVNHAAK_.3	Q9DBC3_S27_M1_Cmtr1	4.2433	4.3480	4.0812	4.0103	3.1227	6.3392	3.8258	3.2604	6.1156	4.3408	3.2247	6.0873	4.1782	6.0282	4.9358	5.9435	4.1034	6.5236	3.3689	6.0967
Prkar1a	_EDEIS[Phospho (STY)]PPPPNPVVK_.2	#N/A	7.7047	7.4127	6.8153	7.6551	6.3590	7.8574	6.9567	7.1227	7.1579	7.4959	7.4044	7.2687	6.5432	7.9112	6.1709	7.4493	8.0919	7.1653	7.5667	7.2914
Prkar1a	_LTVADALEPVQFEDGQK_.2	#N/A	6.4140	6.4112	6.6297	6.6621	6.1834	6.0724	6.3731	6.5231	6.4757	6.3208	6.4298	6.5693	6.4289	6.6025	6.3417	6.6526	6.2179	6.0620	6.2995	6.3758
Prkar1a	_TDSREDEIS[Phospho (STY)]PPPPNPVVK_.3	#N/A	10.5302	10.6749	10.2187	10.4879	10.1249	10.9985	10.6873	10.4986	10.5992	10.4933	10.3752	10.9632	11.0693	11.5721	10.4937	10.6048	11.3039	10.4048	10.5639	10.3666
Aldh7a1	_FQDEEEVFEWNNEVK_.2	#N/A	5.6547	5.5467	5.4793	5.8368	5.2564	5.3303	5.4732	5.4830	6.1164	5.4524	4.2878	5.7174	5.8879	5.5524	5.2351	5.6115	4.5062	5.0235	5.2290	5.2852
Aldh7a1	_GEVITTYC[Carbamidomethyl (C)]PANNEPIAR_.2	#N/A	4.5132	5.2646	3.9954	5.4973	4.5620	5.4277	5.5138	6.0995	5.2279	5.2346	5.0171	5.0909	5.7615	5.3965	5.1249	5.3053	4.8834	5.1749	5.4767	4.8296
Aldh7a1	_VNLLSFTGSTQVGK_.2	#N/A	3.3961	4.6067	4.7430	4.2933	4.6955	3.7503	4.9169	3.0965	4.6135	3.3206	3.9469	2.9953	3.4168	3.9195	4.6521	3.7410	4.6095	4.1990	3.3372	4.2211
Ap2b1	_DIPNENELQFQIK_.2	#N/A	9.2347	9.1928	9.3579	9.1883	8.8164	9.2808	9.2075	9.3128	9.4432	9.0734	8.9053	8.6211	9.0485	8.7581	8.6858	8.9473	9.3035	9.2339	9.0970	8.8358
Ap2b1	_IQPGNPNYTLSLK_.2	#N/A	8.8640	8.6157	8.8783	8.6769	8.5396	8.5934	8.7578	8.8089	8.5784	8.5417	8.4602	8.3952	8.3307	8.4203	8.0068	8.1900	8.3363	8.6204	8.2763	8.1508
Ap2b1	_LLSTDPVTAK_.2	#N/A	7.9948	8.2814	8.2459	7.8600	8.3306	8.0911	8.4898	8.4062	7.9962	7.9777	7.9855	7.9626	8.0782	7.3345	7.8476	7.5599	7.9771	8.1220	7.9944	7.5509
Ap2b1	_LQNNNVYTIAK_.2	#N/A	7.7160	7.5172	7.6331	7.6386	7.4148	7.0383	7.5121	7.8793	7.5599	7.3850	7.3086	7.2279	7.0766	7.3427	6.9761	6.6235	7.2733	7.3376	7.5559	7.1781
Ap2b1	_QVFLATWK_.2	#N/A	6.5029	6.4018	5.9024	6.3736	6.3295	6.2378	6.5326	6.2279	6.1409	5.5964	6.0733	6.3586	6.5104	6.1376	5.5970	6.2625	5.5197	6.3532	5.9024	5.8186
Ap2b1	_VNYVVQEAIVVIR_.2	#N/A	6.7118	5.6783	6.6031	4.7624	5.8707	5.2934	6.4414	5.5704	5.7825	5.1779	5.1973	4.6874	5.4272	4.9489	4.1505	4.7389	5.4815	5.1938	5.1729	5.1794
Ap2b1	_APEVSQYIYQVYDSILKN_.3	#N/A	4.2894	4.3248	3.7151	4.2063	3.9439	4.0799	3.9373	4.5582	3.9264	4.0026	3.1408	3.6098	4.7268	5.0040	2.7015	3.8519	4.0694	3.9902	4.1253	4.4090
Ap2b1	_DSDYYNM[Oxidation (M)]LLK_.2	#N/A	6.6899	5.7951	6.5646	6.9076	6.6374	5.8173	6.0078	6.8313	7.0020	5.9660	6.4129	6.3548	6.4780	6.2905	6.0940	6.0612	6.6370	6.7020	6.3175	6.0057
Ap2b1	_KPSETQELVQQVLSLATQDSDNPDLR_.3	#N/A	5.0937	4.9717	4.3925	4.1689	4.2415	2.6943	5.1249	4.6768	3.9557	4.4304	4.8127	4.4237	4.5143	4.7084	1.2951	4.2011	5.1320	4.1925	3.2422	3.8982
Ap2b1	_LHDINAQM[Oxidation (M)]VEDQGFLDSLR_.3	#N/A	7.2640	6.4410	6.7417	5.9049	6.6370	6.7192	6.7357	7.1078	6.4718	7.0085	6.9169	6.4262	6.4594	6.4895	5.9624	6.3715	6.9606	6.8284	6.5991	6.6371
Ap2b1	_LAPPLVTLLSGEPEVQYVALR_.2	#N/A	5.4763	5.1294	5.2353	4.9551	5.3848	5.3682	4.9899	5.2295	4.8477	4.2866	5.2858	4.9954	5.1185	4.8342	4.6646	4.4976	3.6915	5.6202	5.0822	4.7413
Ap2b1	_GLEISGTFTHR_.2	#N/A	6.9794	4.4267	6.0009	6.3931	6.7277	6.8338	6.3429	6.8348	6.1072	6.9325	6.7385	5.9644	6.0213	5.6314	6.2880	3.7553	6.0520	5.4754	3.1095	3.0033
Ap2b1	_EC[Carbamidomethyl (C)]HLNADTVSSK_.2	#N/A	6.2958	5.7044	6.5211	5.4497	5.9138	5.7129	6.3716	6.3170	6.3223	6.1093	5.9663	5.7700	5.1614	5.3800	5.7045	5.8991	6.1327	5.8302	5.8662	6.3020
Ap2b1	_DLIADSNPM[Oxidation (M)]VVANAVAALSEISESHPNSNLLDLNPQNINK_.4	#N/A	3.6887	3.3352	3.2253	2.4203	3.8956	3.6896	3.2145	4.5752	3.0727	3.4993	4.0521	3.8108	3.7196	4.7428	3.1778	3.0508	4.2388	4.0010	4.0369	4.4415
Ap2b1	_APEVSQYIYQVYDSILK_.2	#N/A	4.1635	3.1556	3.7001	3.4580	2.8696	4.4038	4.1785	1.9729	4.0987	3.9741	3.5774	4.3826	4.1943	3.7381	4.2039	3.5256	3.8864	3.2631	3.5621	3.4154
Ap2b1	_LTNGIWILAELR_.2	#N/A	3.9643	4.1629	3.5009	3.2468	4.1712	4.3280	4.3777	4.1430	4.2979	3.7749	3.7765	2.9896	4.4633	3.4652	2.9022	3.3264	3.1798	3.2243	2.4319	3.4322
Rpn2	_YHVPVVVVPEGSTSDTQEQAILR_.3	#N/A	4.0514	3.9514	4.5874	5.3280	3.3892	4.7565	5.3071	4.5139	3.8588	5.2091	4.3935	4.8669	4.3191	3.8628	3.7291	3.9408	3.7723	4.6761	4.5156	3.5401
Wwp2	_TATAASEQS[Phospho (STY)]PGAR_.2	Q9D8H0_S211_M1_Wwp2	8.3637	8.4162	8.1783	8.3664	7.3029	7.3445	7.9687	7.7645	7.7875	8.3247	7.3822	7.8026	8.0956	8.0609	6.7974	7.3900	7.4388	7.6060	7.4069	7.4906
Pgam1	_ALPFWNEEIVPQIK_.2	#N/A	11.0433	11.1388	11.2395	11.1998	11.1944	11.1334	10.9763	11.1148	11.1470	11.1816	10.9051	11.1909	11.2480	11.0939	11.2049	11.0453	10.9893	10.9739	11.1032	11.0734
Pgam1	_VLIAAHGNSLR_.2	#N/A	10.1583	10.3087	10.4846	10.0597	10.3071	10.1236	10.2069	10.4576	10.0530	10.0445	9.6434	10.3307	10.0203	10.4293	10.2090	10.1035	10.1616	10.1797	10.0536	10.0833
Pgam1	_HYGGLTGLNK_.2	#N/A	8.7172	8.9843	8.7950	8.8218	8.7299	8.8392	8.7290	8.7694	8.7708	8.7389	8.4740	8.6735	8.8886	8.9696	9.0404	8.7889	8.6164	8.7593	8.8223	8.7446
Pgam1	_YADLTEDQLPSC[Carbamidomethyl (C)]ESLKDTIAR_.3	#N/A	8.6785	9.1645	8.9478	9.2502	8.5344	8.9824	8.9948	8.6634	9.1050	9.2429	8.6174	8.9152	9.0590	9.3270	9.3707	9.2404	9.2240	8.9167	8.9259	8.8597
Pgam1	_FSGWYDADLSPAGHEEAK_.2	#N/A	6.3951	6.4615	6.7322	6.2932	6.3698	5.8104	6.0839	5.6714	6.1233	6.4232	6.5928	6.2990	6.2682	5.9660	5.9964	6.6104	5.8094	5.9696	5.9765	6.4885
Pgam1	_AM[Oxidation (M)]EAVAAQGK_.2	#N/A	7.4393	7.6910	7.5070	7.4105	7.6100	7.6015	7.6447	7.1903	7.4485	7.5555	7.2369	7.4133	7.2554	7.6111	8.3411	7.4731	7.1738	7.6640	7.7015	7.6200
Pgam1	_YADLTEDQLPSC[Carbamidomethyl (C)]ESLK_.2	#N/A	6.7394	6.5363	6.6828	6.4721	6.1908	4.0186	6.5656	6.9099	6.4583	6.3064	6.5598	5.8852	6.4339	6.6926	6.6495	6.3491	6.0811	3.5437	6.1853	5.9008
Pgam1	_HGES[Phospho (STY)]AWNLENR_.2	Q9D8J1_S14_M1_Pgam1	8.2473	8.2006	8.3036	8.4366	7.8630	8.0813	7.8098	8.4061	7.9894	8.2383	8.1166	8.1603	8.7281	8.5188	7.7438	8.2656	8.3723	7.9323	8.0120	8.2203
Pgam1	_KAM[Oxidation (M)]EAVAAQGK_.2	#N/A	5.1199	5.6135	5.4921	5.5698	5.1976	5.1354	5.5155	5.2848	5.4273	4.7943	5.3128	5.2987	5.5976	5.3859	6.1791	5.6930	5.0801	4.6568	5.0801	5.0650
Pgam1	_SYDVPPPPM[Oxidation (M)]EPDHPFYSNISK_.3	#N/A	9.6037	9.6867	9.4953	9.6236	9.3428	9.5007	9.4724	9.9285	9.6833	9.8800	9.4717	9.5660	9.9332	9.8934	9.8590	9.7598	9.5680	9.3546	9.4770	9.2537
Pgam1	_DAGYEFDIC[Carbamidomethyl (C)]FTSVQK_.2	#N/A	3.7521	6.2187	4.2884	3.9373	3.0898	3.5651	4.9017	5.4614	3.0095	5.1766	4.7749	5.8038	3.7728	5.2103	4.0285	4.0970	4.6295	3.8430	4.1934	4.4247
Pgam1	_FSGWYDADLSPAGHEEAKR_.3	#N/A	7.0598	7.8756	8.1082	7.9230	6.9458	7.6893	7.6696	7.4211	7.9739	7.6817	7.0974	7.0869	7.4585	7.7677	8.0511	7.8406	7.3823	7.3632	8.0647	7.8124
Pgam1	_HLEGLSEEAIM[Oxidation (M)]ELNLPTGIPIVYELDK_.3	#N/A	3.8437	3.4342	3.5987	3.1262	4.0507	4.0840	2.9974	4.2636	4.4185	3.6543	3.8971	3.3290	4.5839	1.1721	3.0228	3.2058	3.3004	3.0319	4.1813	3.5528
Pgam1	_TLWTVLDAIDQMWLPVVR_.2	#N/A	4.3821	3.6207	3.4176	3.3072	3.7199	4.2651	4.3172	4.0825	4.2374	3.8354	3.7161	3.5101	4.4029	3.4047	2.8417	3.2262	3.1194	3.2130	4.3624	3.3717
Acadsb	_YAIGSLNEGR_.2	#N/A	4.1122	3.8906	6.0726	3.3568	3.4499	3.6740	4.0911	6.2342	6.0189	6.0467	5.5381	5.5063	4.2583	5.4604	5.4655	5.6927	3.9503	5.7142	6.0835	3.4793
Acadsb	_IFDFQGLQHQVAQVATQLEATR_.3	#N/A	3.9921	4.0107	4.5284	3.6973	3.3298	3.1543	4.2112	3.6925	3.8474	4.1984	3.3260	3.9001	4.0128	4.5155	3.7884	4.4370	4.2302	3.6030	3.2516	2.9816
Gdap2	_ENGPYFASYPPS[Phospho (STY)]PDL_.2	Q9DBL2_S495_M1_Gdap2	4.3356	9.7197	9.1528	3.5802	3.6734	9.6814	9.7326	7.6566	8.7332	9.1443	9.4952	8.8661	4.0349	8.8509	8.4820	3.1797	8.8225	3.4225	9.2595	8.7388
P33monox	_EEKHPASAQSTPSST[Phospho (STY)]PHAS[Phospho (STY)]PK_.3	#N/A	6.1976	4.9414	4.1497	6.4481	5.6483	6.1903	3.7720	5.8990	5.6500	4.1637	4.7174	3.6753	6.0662	3.8839	6.0613	6.1860	4.0321	5.7597	5.7192	6.3300
P33monox	_HPASAQSTPSSTPHAS[Phospho (STY)]PK_.3	#N/A	4.6255	5.8247	5.2309	6.3127	5.7688	5.9862	4.2545	4.9226	5.8156	6.1726	6.1089	4.9220	4.7027	5.0444	4.4594	5.7427	6.1446	5.4597	5.7645	6.0927
Ddx59	_EGEGSLRPSS[Phospho (STY)]PQR_.2	Q9DBN9_S150_M1_Ddx59	3.9487	4.0541	4.4850	3.7407	6.4449	3.5105	4.2546	3.6491	4.3137	4.1551	3.1872	3.9435	4.4218	3.9655	3.8318	4.2936	4.1868	3.0356	3.3347	3.6428
Cmpk1	_NQDNLQGWNK_.2	#N/A	8.2754	8.3958	8.7651	8.6105	8.6735	8.3496	7.8023	8.3796	8.4041	8.6642	8.3580	8.5784	8.9027	9.0104	8.7082	8.6159	8.7742	8.3449	8.4086	8.6610
Cmpk1	_FLIDGFPR_.2	#N/A	7.0500	7.4127	7.0665	7.3606	7.1066	7.0708	7.4911	7.0670	6.6143	6.8798	6.7468	7.9919	6.9876	7.7998	7.3573	6.9799	7.2961	7.6104	6.9882	6.9730
Cmpk1	_IVPVEITISLLK_.2	#N/A	5.5346	5.1327	5.5745	5.8765	5.1431	5.4532	4.9355	5.2300	5.7737	5.0780	5.6060	5.3488	5.0276	5.3696	5.3355	5.4920	5.5794	5.4131	5.8998	5.9461
Cmpk1	_IQTYLESTKPIIDLYEEM[Oxidation (M)]GK_.3	#N/A	5.0912	5.0334	5.4726	5.3148	5.0008	5.3139	3.3205	5.5013	4.9850	5.2178	4.2201	5.0972	5.9037	5.5061	5.3070	5.9375	3.2436	5.4773	5.4910	5.6092
Cmpk1	_SVDEVFGEVVK_.2	#N/A	6.3008	6.1324	6.5538	6.4582	5.9615	6.3936	6.2263	6.1294	6.3177	6.5641	6.0697	6.2103	6.7265	6.9173	6.4002	6.5122	6.4465	6.1959	6.5025	6.2112
Cmpk1	_YGYTHLSAGELLR_.3	#N/A	3.8978	3.7735	4.0341	3.6870	3.9395	4.0332	3.9881	3.1980	4.1575	4.1147	3.7518	3.6697	4.0470	5.1179	4.3498	4.2524	3.9619	3.5958	3.9085	4.0340
Cmpk1	_KIDASKS[Phospho (STY)]VDEVFGEVVK_.3	#N/A	3.7280	3.5910	3.2646	3.8562	3.9493	2.3865	3.5918	3.9537	3.6509	3.3171	4.0252	3.3172	3.7589	3.3303	3.1690	3.4556	3.4510	3.6985	3.9975	2.9800
Cmpk1	_SDDNRESLEK_.2	#N/A	3.6893	3.6298	3.2258	3.8950	3.8962	4.2122	2.0508	3.9149	3.6121	3.4999	3.9865	3.3559	3.7201	3.2639	3.1773	1.3343	3.4122	3.7373	4.0363	4.4420
Cmpk1	_KNPDSQYGELIEK_.3	#N/A	3.4693	4.5660	3.9731	4.2526	3.6762	3.7095	3.3719	3.1372	3.2921	3.2799	4.2716	2.9546	3.4576	3.9602	3.3973	3.4322	3.6749	4.1583	3.8069	3.9272
Akap8	_ADS[Phospho (STY)]DGDLS[Phospho (STY)]ENDDGAGDLR_.2	Q9DBR0_S320_M2_Akap8	4.4030	6.5229	6.4595	6.7786	3.2671	6.5012	6.0097	3.2397	6.5023	6.4795	4.3740	6.1360	3.5600	6.8199	6.2280	6.8712	6.9997	6.3033	6.2436	6.7378
Akap8	_S[Phospho (STY)]EDKTR_.2	Q9DBR0_S634_M1_Akap8	3.6406	3.6785	3.1771	3.9437	4.8773	3.8808	3.2006	3.8662	3.1208	3.4512	4.8700	3.4046	3.2862	3.2152	3.2260	3.0027	4.3924	3.7860	5.1327	3.7559
Akap8	_SNS[Phospho (STY)]STFIR_.2	Q9DBR0_S201_M1_Akap8	3.3247	3.1773	3.8610	4.3647	4.1632	4.3873	3.3779	4.5259	3.4369	3.5310	3.8113	3.0668	3.5450	3.8480	2.9551	3.6696	3.5627	4.2704	3.9190	3.8150
Xrn2	_AEDS[Phospho (STY)]DS[Phospho (STY)]EPEPEDNVR_.2	#N/A	8.2893	8.4739	8.1766	8.4554	8.2081	8.4067	8.0680	8.6141	8.1393	8.3955	8.0317	8.3090	7.9558	8.2671	7.4876	8.2271	8.6231	8.1279	8.2887	7.8174
Xrn2	_NS[Phospho (STY)]PGC[Carbamidomethyl (C)]QVASNPR_.2	Q9DBR1_S448_M1_Xrn2	8.0839	8.2663	8.3244	8.0111	7.6102	7.8797	8.1227	8.3196	8.1192	8.1255	7.6558	8.1357	8.0506	8.2668	7.6323	7.8694	7.9399	7.8614	7.8899	8.0651
Xrn2	_RKAEDS[Phospho (STY)]DS[Phospho (STY)]EPEPEDNVR_.2	#N/A	4.6737	5.0056	5.3483	5.1452	4.1376	4.5834	5.0491	4.6291	3.9894	4.9685	4.3900	4.4770	4.8963	5.2302	3.8755	4.5511	4.8113	4.9011	4.3738	4.5243
Xrn2	_KAEDS[Phospho (STY)]DS[Phospho (STY)]EPEPEDNVR_.2	#N/A	8.0750	7.8806	8.0922	8.2526	7.6898	7.7139	7.6152	8.1876	8.1073	7.9380	7.6625	7.8120	8.2732	8.1623	7.2286	7.7163	8.1302	7.9381	7.8769	7.7760
Fam13c	_AGTPAHES[Phospho (STY)]PQSSIFR_.2	#N/A	6.5044	7.2948	7.3784	5.6695	5.9912	6.2024	4.3245	6.5028	6.2926	6.1900	5.9698	6.2508	4.0055	7.2365	4.1536	6.4269	6.7799	6.1012	3.7343	5.7799
Fam13c	_SEAVGPEPGS[Phospho (STY)]S[Phospho (STY)]GEETSDAVVPEKR_.3	Q9DBR2_S405_M2_Fam13c	4.1889	6.5060	6.1921	6.4345	3.2418	6.6442	6.1431	4.1784	6.3516	4.3610	2.9816	6.1266	4.2147	6.7427	6.1281	6.3540	6.2257	6.1923	6.6207	6.3231
Ppp1r12a	_SAS[Phospho (STY)]YSYLEDR_.2	Q9DBR7_S909_M1_Ppp1r12a	8.4648	8.1907	4.4522	8.3959	8.3955	3.4777	7.8286	7.5401	7.6832	8.5738	7.9880	3.9764	8.6910	4.4392	7.2855	8.3837	4.1539	8.1418	7.9612	8.1948
Ppp1r12a	_T[Phospho (STY)]GSYGALAEISASK_.2	Q9DBR7_T443_M1_Ppp1r12a	3.9880	6.8565	6.8712	6.6240	3.5366	5.8971	5.9449	5.4553	5.6179	6.9026	5.8288	6.1270	6.0898	3.3096	5.1508	3.3501	5.8822	6.2858	6.0227	6.2169
Ppp1r12a	_DKKS[Phospho (STY)]PLIESTANMENNQPQK_.3	#N/A	3.7802	7.3668	4.3165	8.2245	4.7848	3.3420	7.1739	3.2843	5.1592	3.9865	7.0645	4.1121	4.5903	8.4480	4.0004	4.1251	5.4420	4.3679	3.1662	6.0481
Ppp1r12a	_S[Phospho (STY)]PKEEER_.2	Q9DBR7_S422_M1_Ppp1r12a	10.9310	3.4912	11.3095	3.7564	4.0348	10.8778	3.0133	10.7728	10.8943	11.0989	10.3804	10.5723	11.1759	3.4025	10.3899	10.4392	10.5499	10.6860	10.2328	10.4591
Ppp1r12a	_STGVSFWTQDS[Phospho (STY)]DENEQER_.2	Q9DBR7_S861_M1_Ppp1r12a	3.0901	4.2290	4.1275	4.4941	3.2971	3.3304	3.7510	3.3158	3.6713	4.4016	3.1499	3.9551	3.8367	4.3394	4.4028	3.9531	4.3139	6.4535	3.4277	4.3063
Ppp1r12a	_SPLIESTANM[Oxidation (M)]ENNQPQK_.2	#N/A	5.0635	4.9828	4.4165	3.7985	5.0047	4.5001	4.0539	4.9226	6.4764	4.6067	4.2697	5.0711	5.7755	6.0627	4.3074	5.0901	5.4880	4.6142	4.6691	4.0045
Ppp1r12a	_KTGS[Phospho (STY)]YGALAEISASK_.2	#N/A	4.0808	3.6767	3.3615	3.3633	4.2878	4.3211	4.2612	4.0265	4.1814	5.5246	3.6600	5.5662	4.3468	5.9634	2.7857	3.4430	3.0633	3.2690	4.4184	3.3156
Ppp1r12a	_STGVSFWTQDSDENEQERQS[Phospho (STY)]DTEDGSSKR_.4	Q9DBR7_S870_M1_Ppp1r12a	4.1307	6.4090	3.3673	4.4930	3.5524	5.9143	6.0316	3.9923	6.2455	3.7663	3.7695	5.0722	4.0501	7.1591	5.7157	3.4928	3.9200	6.6374	6.0378	5.9162
Ppp1r12a	_RIS[Phospho (STY)]EMEEELK_.2	Q9DBR7_S994_M1_Ppp1r12a	3.4552	3.8639	4.4926	4.1290	3.6621	4.4462	3.3860	4.0156	3.3780	3.5899	3.7524	3.5900	4.8461	4.1383	3.4113	4.3182	3.1781	3.9713	4.2704	4.2080
Tprg1l	_YDFISLQC[Carbamidomethyl (C)]QQVVR_.2	#N/A	5.4164	3.1999	3.6558	5.5918	5.2196	3.7822	3.9829	4.3449	5.4720	4.4268	5.4752	5.5170	4.1501	5.6335	5.1492	4.4918	5.5598	3.3074	4.7830	3.3711
Tprg1l	_DTVDSAGTSPT[Phospho (STY)]AVLAAGEDAGAGRPGAGTPLR_.3	Q9DBS2_T16_M1_Tprg1l	2.4675	3.7738	4.5828	4.0389	4.1320	4.3561	3.4091	3.7710	3.4682	3.4898	3.8425	3.0426	3.5762	4.6208	2.9863	3.6383	3.2682	3.8812	4.1802	4.2981
Klc4	_VSS[Phospho (STY)]PTVNTTLK_.2	Q9DBS5_S460_M1_Klc4	8.8164	8.7702	3.4192	3.3057	8.6450	8.3908	8.1748	8.5757	8.4948	9.0409	8.5441	8.3067	8.8536	8.6228	6.8600	8.8482	3.1209	8.1695	8.3977	8.4860
Klc4	_RSS[Phospho (STY)]ELLVR_.2	Q9DBS5_S566_M1_Klc4	3.5302	3.7889	4.5676	6.4546	3.7372	4.3712	5.5187	3.7559	4.4530	6.8214	5.6552	3.5150	3.5611	4.6057	3.3363	5.1578	3.2531	3.8963	4.1954	5.3712
Klc4	_S[Phospho (STY)]SELLVR_.2	Q9DBS5_S565_M1_Klc4	4.0563	3.9465	4.5926	3.6331	3.3940	3.6181	4.1471	4.5090	4.2062	4.2626	3.0797	3.8360	4.3142	3.8580	3.7243	4.4012	3.5482	3.4277	3.3422	3.5353
Klc4	_AAS[Phospho (STY)]LNYLNQPNAAPLQVSR_.2	Q9DBS5_S590_M1_Klc4	3.2033	3.2986	3.7396	4.4861	4.0419	4.2660	4.4992	4.4045	3.5583	4.3097	3.9327	3.1881	3.6663	7.1761	3.0764	3.5482	3.4414	4.3918	4.0404	4.3882
Osbpl3	_ALVHQLS[Phospho (STY)]NESR_.2	Q9DBS9_S405_M1_Osbpl3	8.7188	8.8470	8.5966	3.7182	8.3066	8.2252	8.4242	8.4523	3.7888	8.4043	8.5159	8.1785	8.8150	3.4407	7.4069	8.0820	8.7034	8.1817	8.2985	8.2532
Osbpl3	_QDS[Phospho (STY)]WEVVEGLR_.2	Q9DBS9_S33_M1_Osbpl3	4.1523	6.1867	6.2062	3.4348	2.8584	5.7723	5.0120	3.9550	4.6755	3.9629	3.5885	4.3937	4.2753	5.8871	3.4244	3.5144	4.4927	5.0017	5.1664	4.9436
Ampd2	_S[Phospho (STY)]LPGNAPC[Carbamidomethyl (C)]LK_.2	Q9DBT5_S19_M1_Ampd2	9.7252	9.6223	10.0115	9.7275	8.6665	7.9493	8.8782	7.6524	7.9279	10.0974	9.3321	8.3218	7.3524	8.9342	3.4332	8.7050	6.0820	8.5774	9.2909	9.0692
Ampd2	_QIS[Phospho (STY)]QDVKLEPDILLR_.3	#N/A	7.1983	7.6831	6.3274	6.3990	5.3003	6.1844	7.2791	7.0384	6.0436	6.8205	6.1094	6.7837	7.0457	7.4618	5.5571	6.2310	5.9416	6.8755	6.9061	6.0712
Ampd2	_S[Phospho (STY)]LAESELR_.2	Q9DBT5_S53_M1_Ampd2	6.5639	4.0189	7.1048	7.5291	3.5072	5.8498	6.9643	3.3062	3.4611	8.0745	2.9398	6.5104	3.6266	6.7415	3.5663	6.9144	3.8439	3.9892	7.0111	4.0962
Ampd2	_TDS[Phospho (STY)]DSLQLYK_.2	Q9DBT5_S109_M1_Ampd2	3.3981	3.9210	6.2940	4.1861	3.6050	3.6384	5.1146	3.6238	5.7574	3.2087	4.3428	3.6471	3.5287	4.4736	4.7416	5.5763	6.6732	5.5987	5.7523	6.3517
Ampd2	_SAPYEFPES[Phospho (STY)]PIEQLEER_.2	Q9DBT5_S70_M1_Ampd2	3.4613	3.8578	4.4987	4.1229	3.6683	3.3798	3.7016	3.6870	3.3001	3.2719	4.2795	3.5839	3.4655	3.9682	3.4052	4.3243	3.1842	4.1503	3.7989	3.9351
Ampd2	_QIS[Phospho (STY)]QDVK_.2	#N/A	4.0097	3.6056	5.5463	3.2922	4.2167	4.2500	4.3323	4.0976	4.2525	3.7311	3.0355	4.4179	3.4197	2.8568	3.3718	4.3197	3.1344	3.1979	4.3473	3.3867
Riok3	_AASFLKDDGS[Phospho (STY)]PPVLSAD_.2	Q9DBU3_S512_M1_Riok3	6.4221	6.6536	3.9404	6.5506	3.5194	6.2445	6.1806	5.6065	3.4287	6.3327	6.2873	6.2081	6.9835	6.9507	6.0972	6.5618	6.9542	6.2627	6.4908	6.3309
Pdcl	_NSATC[Carbamidomethyl (C)]HSEDS[Phospho (STY)]DLEID_.2	Q9DBX2_S296_M1_Pdcl	8.7741	9.3585	9.3591	9.6164	7.7242	9.0468	8.9490	7.7666	9.1450	9.8216	3.6784	9.2134	6.2300	9.7431	8.9190	9.2764	9.6896	9.1778	9.3321	9.4795
Nvl	_ESLPLDLS[Phospho (STY)]DDQSNSK_.2	Q9DBY8_S190_M1_Nvl	3.2806	9.7183	3.8169	4.4087	4.1192	9.2430	9.1696	4.4819	3.4810	3.4870	3.8553	3.1108	3.5890	9.9343	8.7888	3.3655	3.5187	9.2017	9.3671	8.7308
Nebl	_QSFTTVADTPENLR_.2	#N/A	5.7088	6.2118	6.2060	3.4693	6.3147	5.7332	5.8507	5.9380	6.5173	6.3090	5.9749	5.9714	6.6259	6.4561	6.8403	6.1703	6.3632	5.9043	6.0058	6.2486
Nebl	_GVQPHVVEMDR_.3	#N/A	3.7455	4.2573	4.2818	3.9439	3.0832	3.4009	4.4578	3.4459	3.6008	3.9518	3.0794	4.1467	3.7662	4.2689	4.0350	4.0904	3.9836	3.8496	3.4982	4.2359

Plxdc2	_RGS[Phospho (STY)]GHPAYAEVPVGEK_3	Q9DC11_S507_M1_Plxdc2	4.3163	4.5036	3.8529	3.2679	3.0224	3.0558	5.4655	3.7910	3.9459	6.1116	5.4809	4.2297	4.1113	4.6140	4.0510	3.6784	4.3287	3.5045	3.1531	3.0801
Dcaf6	_DGEQS[Phospho (STY)]PNVSLM[Oxidation (M)]QR_2	#N/A	7.6455	7.4789	7.8937	7.1040	6.8723	7.3279	7.5423	7.6764	7.4204	7.6735	7.1415	7.4435	8.0047	6.7041	6.3899	7.2424	7.8242	7.2551	7.4344	7.3141
Dcaf6	_DSALQDTDDS[Phospho (STY)]DDDPVLIPGAR_2	Q9DC22_S675_M1_Dcaf6	3.7850	3.5341	3.3216	3.7992	3.9920	4.1164	8.0574	4.0107	3.7079	3.2601	4.0822	3.2602	3.8159	3.3597	3.2260	3.1471	3.5079	8.8491	3.9406	3.0370
Csnk1d	_GAPVNVS[Phospho (STY)]SSDLTGR_2	Q9DC28_S382_M1_Csnk1d	4.5282	3.4746	3.5636	3.1612	3.8659	4.1190	7.7843	8.1388	4.3834	3.6894	3.8621	3.3641	4.5489	3.5507	3.2524	3.3722	3.2654	3.0669	7.9064	3.5177
Abcb6	_AIQASLAK_2	#N/A	3.3006	5.6158	4.6822	6.5072	4.6418	5.8841	6.5152	3.4709	6.1788	6.2663	6.1958	6.6328	6.7005	5.3253	6.8947	5.7196	3.8921	6.2105	6.6516	6.6247
Hmg20a	_S[Phospho (STY)]PLTGYVR_2	#N/A	6.8520	6.9467	7.4850	7.0889	4.1964	7.2429	6.5987	6.7094	7.2055	7.4062	7.5676	7.3212	6.4454	3.8812	7.2385	7.1053	6.9073	4.2373	3.8859	7.3210
Hmg20a	_DSNAPKS[Phospho (STY)]PLTGYVR_3	#N/A	4.5449	4.2750	4.0815	4.5401	3.2510	3.2844	3.7971	3.2698	3.7173	4.3555	3.1959	4.0011	3.8827	4.3854	3.8224	3.9070	4.2678	3.7331	4.7320	4.7330
Gnai3	_ISQTNYIPTQQDVLR_2	#N/A	6.9046	5.7407	5.5519	6.3015	6.0128	6.0631	5.7255	6.1079	6.2801	6.2390	5.8892	5.4999	5.5511	5.7249	5.1342	4.8485	6.2911	5.3672	5.4967	6.0521
Gnai3	_EYQLNDSASYYLNDLDR_2	#N/A	3.7579	6.4773	6.5269	3.9315	3.0956	6.6940	6.6460	5.5635	6.7173	5.7925	3.8917	4.1344	3.1118	6.2384	4.0227	4.1028	6.5665	3.4356	3.6708	3.8337
Ndufa9	_NFDFEDVFVNIPR_2	#N/A	6.8975	6.8088	6.8122	6.8577	6.5458	6.5740	6.8054	6.6528	6.8965	6.8025	6.9701	6.8055	6.6180	6.1438	6.3974	6.4291	6.7179	6.3081	6.3939	6.8305
Ndufa9	_QPVYVADVSK_2	#N/A	6.5511	6.7889	6.3973	6.0937	6.5867	6.6022	6.7067	6.6841	6.7308	6.5060	6.7927	6.4640	6.8408	6.6482	6.4959	6.4543	6.8092	6.5150	6.3301	6.4411
Ndufa9	_M[Oxidation (M)]GSQVIIPYR_2	#N/A	7.4981	6.8165	5.9430	6.2049	7.1561	6.8059	7.0576	7.3786	7.4172	7.0033	6.9157	6.7387	7.0097	5.3803	6.2288	6.4248	6.9633	7.6185	6.6695	6.5848
Ndufa9	_SVFPEAIIRPSDIFGR_3	#N/A	6.4098	5.6429	5.5073	5.7092	5.3338	5.7097	5.4651	4.8707	5.2898	5.3106	5.9398	5.1919	5.4608	5.3247	4.5283	5.8912	5.2290	5.4529	6.3100	5.5050
Ndufa9	_SSVSGVVATVFGATGFLGR_2	#N/A	0.8493	3.6128	3.2771	2.7231	2.7371	2.4815	1.1764	2.1202	3.3370	3.1610	4.3951	3.6506	4.1288	3.6726	3.5389	2.3689	1.8089	2.2037	2.4863	3.0732
Ndufa9	_AVQHSNVVINLIGR_3	#N/A	5.6664	5.4765	5.7125	5.5208	5.2621	4.9697	5.8233	5.7600	5.4830	5.1134	5.5400	5.5380	6.0802	5.2171	5.0480	5.3295	4.8652	5.3183	5.5136	5.0802
Ndufa9	_IHSDVM[Oxidation (M)]PTDLPGLEDLGVQPTPLELK_3	#N/A	4.1066	3.5887	4.3488	3.8769	3.4918	3.4544	4.3909	3.5129	3.8011	2.8792	3.3235	3.6482	4.6610	4.3359	2.4633	4.1574	4.0827	4.2817	3.2883	3.2027
Ndufa9	_GIVNATKDPDAVGK_2	#N/A	4.9296	5.2619	5.1200	4.5664	5.0068	5.0652	5.1935	5.8319	5.5499	5.1460	5.2926	5.3024	4.8399	4.8433	4.7525	4.8628	5.4171	5.1489	5.0060	4.8435
Ndufa9	_LM[Oxidation (M)]GDLGQLTFLEWDAR_2	#N/A	5.6485	3.6737	3.6144	3.3603	4.5031	4.9974	4.8437	4.8367	4.1844	3.8884	4.7717	2.9674	5.8049	3.3517	2.7887	4.8242	3.0664	4.2856	4.4154	4.1850
Ndufa9	_WLSSEIEETKPAK_3	#N/A	4.4544	3.5484	3.4898	3.2350	3.7921	4.0162	3.7490	4.1548	3.8081	3.1599	3.0372	3.4379	3.9161	3.4769	3.3262	3.2984	3.1916	1.9042	4.2901	3.4439
Ndufa9	_C[Carbamidomethyl (C)]DVYDIMHLR_3	#N/A	4.0045	3.9983	4.5408	3.2491	3.3422	3.5663	4.1989	3.0600	4.2580	4.2108	3.1315	3.8878	4.3660	3.9098	3.7761	4.3494	4.0580	3.0914	3.3904	3.5871
Ndufs7	_LDDLINWAR_2	#N/A	7.6121	8.0101	7.6184	7.6987	7.7243	7.3813	7.9952	7.9572	7.6853	7.4364	7.6642	7.8231	7.5556	7.4537	7.4396	7.6921	7.9362	7.7822	7.5761	7.4822
Ndufs7	_SRAEYVVTK_2	#N/A	5.9181	6.6311	6.2788	6.8296	6.2887	6.2965	7.0330	6.3204	6.8509	6.5176	6.1838	6.3703	6.4230	6.0848	7.1548	5.7915	6.4111	6.7454	6.4438	6.0869
Ndufs7	_KVYDQM[Oxidation (M)]PEPR_2	#N/A	5.7600	5.0838	4.5980	4.5559	3.9721	5.1533	4.7878	5.2528	5.1855	5.2725	5.0762	5.0704	5.2328	4.4478	5.5098	3.4784	5.0742	4.0835	5.0229	4.7285
Ndufs7	_QADVM[Oxidation (M)]VAGTLTNK_2	#N/A	6.1534	5.7911	6.2626	5.7743	5.3011	6.6461	6.4412	6.1390	6.6873	5.5012	5.3675	5.9974	7.0525	6.0885	6.0724	6.4169	5.5479	5.9626	5.6828	6.5456
Hmgn3	_TES[Phospho (STY)]IEKEGE_2	#N/A	3.6413	4.1251	3.7004	3.7810	4.1584	3.8879	3.1468	3.8313	3.9908	3.6381	3.8137	3.0958	3.3008	3.8034	2.9477	3.5156	3.5181	3.6867	3.9852	3.7704
Arpp21	_AWSS[Phospho (STY)]TDSDSSNR_2	Q9DCB4_S362_M1_Arpp21	8.1488	8.0043	8.2110	8.4901	7.7610	7.6167	7.4965	7.6656	7.3782	7.9652	7.4965	7.2705	7.8708	8.0112	6.9481	7.6306	7.5488	7.2423	7.4242	3.6975
Arpp21	_TGS[Phospho (STY)]ESSSSAGSSGSLSR_2	Q9DCB4_S408_M1_Arpp21	7.3253	7.2793	7.5201	7.6493	6.8571	6.6560	6.4638	6.2232	6.7636	7.3339	6.4658	6.5316	6.6820	7.0895	3.2535	6.5995	6.5475	6.6415	6.5820	6.6727
Arpp21	_SES[Phospho (STY)]LDEEEKLELQR_3	#N/A	4.0890	3.2301	3.6255	3.4953	4.2959	4.3292	4.2530	4.3146	4.1733	3.8995	3.6519	2.9563	4.3387	3.6636	4.2784	3.4511	3.8119	3.7976	3.6366	3.3075
Arpp21	_SES[Phospho (STY)]LDEEEKLELQR_2	#N/A	3.8735	3.4989	8.2859	8.2902	4.0804	7.8739	5.5770	4.4478	3.6828	3.9921	3.6783	4.0247	7.8198	9.1961	4.3382	7.9193	7.7324	8.5795	8.4951	7.8920
Arpp21	_QSS[Phospho (STY)]SETELRWPDHQR_3	Q9DCB4_S346_M1_Arpp21	4.4318	4.3881	3.9684	3.1524	3.1379	3.1713	3.9102	5.4552	3.8304	4.2424	3.3090	4.1142	3.9958	4.0065	3.9355	3.7939	4.1547	2.9947	3.2938	2.9646
Arpp21	_TAS[Phospho (STY)]FGGITVLTR_2	Q9DCB4_S381_M1_Arpp21	4.2542	3.7486	3.2897	3.4352	3.5919	4.3930	6.3379	6.4097	4.1095	5.5404	3.5881	3.6381	4.2749	7.4972	4.2146	3.0983	4.4923	3.3409	2.9895	3.2437
Arpp21	_DNS[Phospho (STY)]S[Phospho (STY)]DKEDNQNR_2	Q9DCB4_S256_M2_Arpp21	4.3760	4.4439	3.9126	3.2545	3.0821	3.1154	3.9660	3.7313	3.8862	4.1866	3.3649	4.1701	4.0517	4.5543	3.9914	3.7381	4.0989	3.5642	3.2127	3.0205
Tomm20	_IVS[Phospho (STY)]AQSLAEDDVE_2	Q9DCC8_S135_M1_Tomm20	7.0745	7.5795	7.6329	7.5421	5.4089	7.7931	7.3139	6.1179	5.5287	7.3503	5.7151	7.0542	4.5651	7.9354	5.0758	5.3889	7.8816	7.5726	7.7108	7.7665
Pgd	_LVPLLDTGDIIIDGGNSEYR_2	#N/A	6.0532	3.6857	5.6293	5.3830	5.3740	4.7484	5.4040	5.7060	5.4526	5.5919	5.4586	5.0242	5.4190	5.0566	4.3631	5.3047	4.5552	5.7307	3.7031	3.2744
Pgd	_VDDFLANEAK_2	#N/A	3.5052	5.3453	6.0360	5.7878	3.7121	5.7127	3.3360	6.2255	5.0228	5.3399	5.3143	5.3400	5.3156	5.4806	5.1433	4.3681	5.6898	5.5915	5.3277	5.6850
Pgd	_NPELQNLLLDDFFK_2	#N/A	3.5551	3.3257	4.0914	4.1343	4.2353	3.8970	4.0823	2.5599	3.2065	3.7614	3.5809	3.5717	3.3146	4.3592	2.0600	3.9000	3.7931	3.6423	4.4419	3.0176
Pgd	_GILFVGSGVSGGEEGAR_2	#N/A	4.5625	4.7004	5.3176	3.9782	3.8129	3.9130	3.2352	3.8317	4.6761	3.4166	4.2727	3.8426	3.6369	3.1806	3.9277	2.9681	3.3289	3.2105	4.1196	3.8663
Tjap1	_KDS[Phospho (STY)]LTQAQEGTVLS_2	Q9DC05_S527_M1_Tjap1	3.7998	6.7807	6.1409	3.7845	4.0067	6.0930	3.0414	6.0521	5.3814	6.5151	3.9411	5.9579	3.1270	6.0869	3.0668	5.6766	6.5874	2.9879	3.9258	6.1983
Ssr3	_KLS[Phospho (STY)]EADNR_2	#N/A	6.6335	6.4497	6.6491	6.1471	5.9944	6.7279	6.2856	6.4693	6.5948	6.3068	6.1591	6.1268	6.8681	6.0057	6.2282	5.7147	6.6028	6.7024	6.4380	3.5520
Ssr3	_KLS[Phospho (STY)]EADNRK_2	#N/A	4.0784	4.5988	3.5805	4.7263	2.4323	4.9659	4.2880	4.4293	4.7601	3.9949	4.2346	4.4412	4.8695	4.7858	2.8226	1.6652	4.9873	4.3805	4.7487	3.2419
Ssr3	_QQS[Phospho (STY)]EEDLLQDFSR_2	Q9DCF9_S11_M1_Ssr3	3.3758	5.9606	4.4132	4.2084	3.5828	6.4787	8.0046	6.9677	8.4827	7.9318	4.3650	7.5209	3.5510	7.9473	7.1654	4.2388	3.0987	4.0507	8.3491	4.1286
Eif3f	_TC[Carbamidomethyl (C)]FS[Phospho (STY)]PNR_2	Q9DCH4_S262_M1_Eif3f	9.9431	9.9537	10.1158	9.9747	9.5640	9.7356	9.5317	9.5833	9.5064	9.7947	9.7065	9.6909	9.7638	10.3713	9.6402	9.6784	9.8502	9.4556	9.5250	9.7738
Eif3f	_VIGLSSDLQQVGGASAR_2	#N/A	4.2884	3.7144	3.3238	3.4010	3.6261	3.8502	5.2043	3.9887	3.9741	4.4947	4.3485	3.6039	4.0821	3.6259	3.4922	3.1324	4.5264	3.3753	4.7941	3.3032
Ndufa8	_ARPEPNPVIEGDLKPAK_3	#N/A	9.0946	9.2579	9.4264	9.2449	9.0848	9.2196	8.9900	9.4645	9.1453	9.1222	9.3881	9.2431	9.7461	9.7491	9.4505	9.4501	9.5143	9.1823	9.2004	9.2558
Ndufa8	_TDRPLPENPYHSR_3	#N/A	7.0870	7.1163	7.4586	6.8109	6.9031	7.3017	7.2598	7.2756	7.5873	7.0249	7.5319	7.2108	6.8168	7.4941	8.2526	7.7407	6.9749	7.1477	7.5703	7.3800
Ndufa8	_FDQC[Carbamidomethyl (C)]VLDK_2	#N/A	6.0305	6.2137	6.4661	6.6853	6.8561	6.4142	6.6949	5.4010	6.1044	6.6788	6.5958	6.6953	6.2532	6.7274	7.0633	6.2242	5.2649	6.5575	5.7908	6.6650
Ndufa8	_PGIVELPTLEELKVEEVK_3	#N/A	4.9196	5.7689	5.3350	5.3062	4.9277	5.8843	5.3987	5.0666	4.8947	5.5725	5.8144	5.8830	5.3786	6.2983	6.1384	5.9197	5.1892	5.9697	5.5248	5.9462
Ndufa8	_LVNGC[Carbamidomethyl (C)]ALNFFR_2	#N/A	4.4498	5.6571	4.9902	5.7267	5.6794	5.1586	5.5979	5.1044	5.1247	5.8759	5.2210	5.4247	4.9312	5.6576	5.0694	5.1270	5.1870	5.7941	4.9702	5.5878
Ndufa8	_AAAHHYGAQC[Carbamidomethyl (C)]DK_3	#N/A	4.3517	3.9475	4.5916	3.6341	3.0578	3.0911	3.9903	3.7556	3.9105	4.1623	3.3892	4.1944	4.0760	4.5786	4.0157	3.7138	4.2933	3.5398	3.1884	3.0448
Ppp1r2	_TSAAS[Phospho (STY)]PPVVPSAEQPRPIVEEELSKK_4	#N/A	3.4141	3.9050	7.5787	4.1702	7.4542	7.1325	3.4271	8.4521	3.3473	3.2247	4.3268	3.6312	9.2773	4.4895	7.8315	4.2770	3.1370	8.3261	4.3115	4.1668
Ppp1r2	_TSAAS[Phospho (STY)]PPVVPSAEQPRPIVEEELSK_3	#N/A	7.8184	7.6666	6.1864	3.7446	3.2825	3.2016	7.0738	7.7425	3.8000	7.1323	7.1969	7.5567	3.9655	8.3292	6.9438	7.3347	7.6917	7.3518	7.3830	4.4351
Ppp1r2	_EQESS[Phospho (STY)]GEEDNDLSPEER_2	#N/A	8.8054	10.3819	10.0195	9.0901	8.8845	9.5001	9.7125	10.3219	10.0217	9.3478	7.3489	10.1868	10.3507	11.4736	10.3988	10.1598	10.2478	10.1945	9.1461	9.7660
Ppp1r2	_TREQES[Phospho (STY)]S[Phospho (STY)]GEEDNDLSPEER_3	#N/A	8.2627	8.6963	7.8147	8.6412	7.4256	8.2895	8.3924	8.0349	8.4715	8.1303	8.2244	8.6821	9.0334	9.4272	9.1511	8.7998	8.5057	8.6788	9.0295	8.4863
Paics	_EIVLADVIDNDSWR_2	#N/A	5.3973	5.5533	6.2571	5.5543	5.7563	5.6039	5.9537	5.7849	5.6399	5.5092	4.6664	5.5716	5.3546	5.7049	5.2174	5.4836	6.0204	4.8147	5.7289	4.6627
Paics	_DDANNDPQWSEEQLIAAK_2	#N/A	3.1800	4.1391	5.7400	4.7096	4.2778	5.2611	4.5229	4.8557	3.9446	4.9541	4.6797	4.1666	3.9424	5.4239	3.5850	3.7978	5.2110	4.3527	4.5658	4.6404
Slc38a3	_S[Phospho (STY)]PSKEPHFTDFEGK_3	#N/A	8.2229	8.5135	7.3661	7.0095	6.4740	7.2147	7.9475	7.8912	7.6735	7.8794	7.7934	6.0470	7.8113	7.5579	7.5395	7.3798	7.8181	8.2124	7.8065	5.9843
Slc38a3	_HLEGLLPVGVPTTDTQR_3	#N/A	6.9966	6.3737	5.8334	6.5296	6.3373	6.7496	6.5770	6.6807	6.8228	6.3074	6.4542	6.5756	6.2533	5.9784	5.3331	6.0370	6.7429	6.4967	5.9456	6.1942
Slc38a3	_AYEQLQYR_2	#N/A	5.0986	3.4302	4.8324	4.8532	4.9523	5.4577	5.4810	5.1020	5.5404	5.2597	4.9326	4.6793	4.7724	4.4670	3.0322	4.7296	4.8604	5.5580	4.1719	3.5622
Ap3s1	_FYQPY[Phospho (STY)]SEDTQQQIIR_2	Q9DCR2_Y23_M1_Ap3s1	3.9231	5.2774	4.0745	4.7538	4.5850	5.2980	5.5379	3.7688	4.6649	3.5151	4.4966	4.1332	3.9462	5.0815	4.2528	4.7315	4.4660	5.2175	3.5576	4.8924
Mettl26	_IS[Phospho (STY)]PQSNVDFDLTLR_2	Q9DCS2_S149_M1_Mettl26	9.3467	9.4145	9.0656	8.9423	9.0812	8.6029	9.0960	9.0576	9.0566	8.8897	8.4472	8.5009	9.1567	9.4698	8.4910	8.6408	8.9037	8.9508	8.9493	8.4803
Mettl26	_DTVLLEELGQASGLVLER_2	#N/A	3.5559	3.9879	4.0921	3.9301	3.7825	3.5974	3.1536	3.5801	3.5929	3.1113	3.9408	3.1410	3.5766	4.2335	3.0016	3.9008	3.7939	3.0594	3.6947	3.4698
Ndufb10	_PDSWDKDVYPEPPSR_3	#N/A	8.7581	9.0129	8.8755	8.8587	9.0228	8.9208	8.8742	9.1057	8.8093	9.0525	9.0334	8.9007	9.2502	9.1941	9.3697	9.1789	8.9587	8.8784	8.9502	9.0226
Ndufb10	_YLDLGAYYSAR_2	#N/A	6.8082	6.7827	6.9644	6.9699	6.8797	6.6445	6.7525	7.0693	7.1357	6.4800	6.9608	6.9613	6.6665	7.0670	7.1678	6.5553	6.9585	6.7148	7.1315	6.7546
Ndufb10	_AYDLVVDWPVTLVR_2	#N/A	6.2340	6.6690	7.2459	7.1950	6.6690	6.6247	6.4826	6.4809	6.0269	6.3213	6.8683	6.4389	6.5574	6.4635	7.0138	6.4229	6.4446	6.4519	6.8722	6.4742
Ndufb10	_RVPDITEC[Carbamidomethyl (C)]K_2	#N/A	5.2711	5.3178	5.8412	4.8711	5.8285	5.2944	5.4096	5.0953	5.3569	5.6974	5.8432	5.7012	4.8402	5.9021	6.5139	6.1080	6.0849	5.9605	5.2941	5.4788
Ndufb10	_EGDVLC[Carbamidomethyl (C)]IYEAEM[Oxidation (M)]QWR_2	#N/A	5.2813	4.9111	4.2085	3.8468	3.8969	4.5424	4.0968	5.2342	3.3549	3.7929	3.6337	4.5142	5.5867	5.0579	4.0207	5.2312	4.7792	3.9020	4.7239	3.4019
Ndufb10	_DFKVDQEM[Oxidation (M)]NIIQER_3	#N/A	5.1492	5.6422	4.6199	3.2227	5.7977	5.5477	5.3983	5.5029	5.8366	6.0397	6.1937	5.8426	6.8110	5.6633	5.9771	6.5478	5.4857	5.3072	6.0405	6.4204
Ndufb10	_VDQDIMNIQER_2	#N/A	3.3881	5.5119	6.8795	6.8128	6.6366	5.3230	5.3748	3.6137	4.3830	4.0275	5.4701	5.1294	3.5387	6.9015	5.5576	4.2510	3.1110	4.0385	4.5310	4.8713
Ndufs3	_SLADLTAVDVPTR_2	#N/A	8.5494	8.7439	8.8840	8.7365	8.5565	8.6830	8.5453	8.7622	8.7597	8.5612	8.6025	8.8201	8.7503	8.8745	8.7039	8.8059	8.6260	8.5671	8.5313	8.6189
Ndufs3	_VVAEPVELAQEFR_2	#N/A	8.4444	8.3439	8.5808	8.3840	8.3531	8.3796	8.4330	8.3945	8.3004	8.1232	8.3186	8.3075	8.6453	8.1221	8.5565	8.1941	8.5515	8.3393	8.5055	8.3693
Ndufs3	_DFPLTGYVELR_2	#N/A	7.7695	7.9877	8.1611	8.0666	7.6682	7.9506	7.7909	7.7994	7.6923	7.6610	7.7732	8.0553	7.8531	7.9651	8.0865	7.8390	7.7322	7.8372	8.1492	7.6674
Ndufs3	_YDDEVKR_2	#N/A	6.5495	6.4543	7.1810	6.8902	7.1769	7.0349	7.3532	6.8817	7.1762	7.0523	7.2504	6.9732	7.4791	7.4374	7.6648	7.2437	6.5699	6.6138	7.4368	7.3049
Ndufs3	_DHTNAQFK_2	#N/A	5.8848	5.0979	5.9053	4.7556	5.4796	6.0019	5.7666	5.6459	5.8465	6.0696	5.6230	5.0738	5.7407	5.0530	6.0433	5.3095	5.6801	5.5566	5.8887	5.3625
Ndufs3	_FEIVYNLLSLR_2	#N/A	4.6620	4.8193	5.3312	5.6268	4.9511	4.9895	5.0435	5.3036	5.0117	5.0816	5.6838	4.8154	5.1243	4.8089	3.9818	4.2487	5.2561	5.0622	4.8483	5.4139
Ndufs3	_LEAGDKKPETK_2	#N/A	5.6022	4.3489	4.7414	5.1265	4.9609	4.4309	4.1765	5.1262	5.6436	4.1703	5.3164	5.2072	5.2666	4.8172	5.2369	5.1918	5.0750	5.6085	4.9206	5.5218
Ndufs3	_QLSAFGEYVAEILPK_2	#N/A	4.2609	4.4279	4.8049	5.6823	4.9588	4.6402	4.0958	4.4930	4.6746	4.7935	4.4561	4.6928	4.4056	4.6812	5.4577	4.7952	5.1822	4.4279	5.1827	4.5742
Ndufs3	_ILTDYGFEGHPFR_2	#N/A	5.1063	5.6124	5.7469	5.7171	6.0005	5.6445	5.9196	5.9864	5.4159	5.9699	5.7025	5.4168	5.4572	6.1257	6.1898	5.8381	5.7265	5.8408	5.8811	5.9129
Ndufs3	_FDLNSPWEAFPAYR_2	#N/A	5.8004	5.6449	5.6110	5.0357	5.2316	5.1527	5.5265	4.9400	4.4763	3.3882	5.1556	5.4272	3.3492	3.1522	4.6521	4.8651	4.6795	5.1751	5.7170	4.5845
Bap18	_VYEDSGIPLPAES[Phospho (STY)]PK_2	Q9DCT6_S96_M1_Bap18	8.8923	8.7058	9.2895	9.0474	8.3245	8.7183	8.3153	8.1997	8.7676	9.0053	8.8816	8.3352	8.8393	9.1557	8.7020	9.0085	9.2800	8.5846	8.7545	8.9790
Crip2	_GVNTGAVGSYIYDKDPEGTVQP_2	#N/A	6.4222	6.2607	6.2087	6.2560	6.1714	6.3540	6.5560	6.7248	6.4699	6.1956	6.4879	6.4494	6.6779	6.5042	6.8940	6.9433	7.0063	5.9846	6.2670	6.7238
Crip2	_KTS[Phospho (STY)]GPPKGPSK_2	Q9DCT8_S104_M1_Crip2	6.0304	6.7196	5.9484	6.1062	5.9562	5.9119	4.8330	6.5106	6.0293	6.4303	3.7778	6.2365	6.0551	6.4848	6.2326	3.3251	5.9337	6.0194	5.9749	6.4416
Crip2	_TVYFAEK_2	#N/A	3.4431	3.2276	3.9794	4.3364	2.9641	3.5273	3.1166	3.1435	4.3922	3.6494	2.8122	3.6849	3.4265	3.2200	3.9136	4.4519	3.6812	3.0113	3.2660	3.6468
Rmdn1	_VEEILAQADYLYESGETEK_2	#N/A	6.3959	5.6862	5.1867	5.9262	4.8465	4.3990	5.3833	5.8390	5.3844	5.5882	5.1756	5.8569	6.0586	6.5806	4.8646	5.3273	5.1815	4.7510	4.8396	5.3759
Etfb	_EIDGGLETLR_2	#N/A	7.2750	7.0212	7.3745	7.3001	7.2164	6.9910	6.9690	6.7027	7.2531	6.9940	7.0728	6.9912	7.1796	7.0240	7.2323	6.8023	6.8234	7.0286	7.3440	7.2259
Etfb	_GIHVEIPGAQAESLGPLQVAR_3	#N/A	6.2414	6.6225	6.3186	6.0595	5.8444	6.1414	6.5516	6.5800	6.2190	6.4832	6.2650	6.8296	6.4689	6.9819	6.1114	6.3085	6.4961	6.9609	6.5019	6.7431
Etfb	_EIIAVSC[Carbamidomethyl (C)]GPSQC[Carbamidomethyl (C)]QETIR_2	#N/A	4.9180	5.1666	4.8007	4.4450	4.3115	5.1840	5.3642	5.5615	4.6976	5.3906	4.9948	5.2540	5.4334	5.4213	5.3228	5.7709	4.7867	5.1594	4.8228	4.0861
Etfb	_AGDLGVDLTSK_2	#N/A	3.9031	5.9695	5.9273	5.6995	4.7059	6.0745	5.7827	6.2751	6.2991	5.9116	5.6126	5.5530	4.4674	6.1837	5.8589	5.8251	4.1412	5.7257	3.2891	5.3671
Etfb	_TALAMGADR_2	#N/A	3.4862	4.5506	4.5156	4.9529	3.6931	3.3528	5.1311	3.7118	3.1752	3.2967	4.5948	0.9321	3.4407	4.9721	4.0796	3.4391	3.2091	2.6058	4.2394	3.9103
Atp5pd	_SC[Carbamidomethyl (C)]AEFVSGSQLR_2	#N/A	7.7326	8.0782	8.5118	8.3560	7.3423	8.1309	8.0744	8.1086	8.2824	7.8212	7.7806	8.0564	8.1092	8.7206	9.0469	7.9604	7.9240	8.0737	8.1393	7.9050
Atp5pd	_LASLSEKPPAIDWAYYR_3	#N/A	6.7212	7.3741	7.2962	7.5396	7.3321	6.9232	7.2594	7.0144	7.0027	7.2539	7.3673	7.4947	7.1249	7.8018	7.7400	7.5848	7.2471	7.4311	7.5653	7.5151
Atp5pd	_ANVAKPGLVDDFEK_2	#N/A	7.1554	7.2850	7.1641	6.9913	7.2327	7.1838	7.2037	7.4718	7.1329	7.3585	7.3207	7.2875	7.3793	7.6825	7.8372	7.4408	7.3998	7.2138	7.0679	7.1983
Atp5pd	_ANVAKPGLVDDFEKK_3	#N/A	8.0435	8.1576	8.0576	7.8565	8.0524	7.7923	8.0115	8.3442	7.6210	7.5366	8.1919	8.3362	8.5687	8.2992	8.6303	8.3612	8.0863	8.2372	7.9666	8.1724
Atp5pd	_SWNETFHAR_2	#N/A	7.8962	8.0216	8.0812	7.9187	7.9634	7.9805	7.9533	7.8471	7.7383	7.7322	7.5587	7.9520	7.7735	8.3577	8.4682	8.1780	7.6811	7.9563	7.7713	8.0684
Atp5pd	_IPVPEDKYTALVDQEEKEDVK_3	#N/A	8.2093	7.6778	8.0942	8.1117	7.9371	7.5194	7.4165	8.0049	7.9587	8.0098	7.7135	7.7575	8.6414	7.3492	8.1348	8.2037	8.3264	7.0710	7.5287	8.1341
Atp5pd	_YTALVDQEEKEDVK_2	#N/A	4.6760	4.4038	5.1412	5.1134	3.1068	4.3382	4.8961	4.1555	4.3273	4.4117	4.7145	5.0856	4.1837	4.6580	5.3154	3.9457	4.4993	4.4319	4.3825	4.7135
Atp5pd	_TIDWVSFVEVM[Oxidation (M)]PQNQK_2	#N/A	7.3535	7.6270	8.0835	7.9999	7.4512	7.5143	7.5349	7.6100	7.8639	7.7080	7.4095	7.5289	8.0901	7.7950	8.0605	7.8464	7.8497	7.4866	7.7723	7.9513
Atp5pd	_NIPFDQM[Oxidation (M)]TIDDLNEIFPETK_3	#N/A	3.4414	5.2142	4.3749	5.0604	4.2799	4.9989	4.9679	5.8049	5.0723	4.9253	4.5180	5.6339	4.3980	6.1539	5.0636	6.1831	5.2473	5.4626	5.0477	5.0378
Atp5pd	_PGLVDDFEK_2	#N/A	3.4878	3.7381	5.2917	3.8896	3.1375	4.5497	5.7785	3.9498	3.9573	6.0642	4.5137	4.0925	3.8205	3.7542	5.0822	4.1608	4.8954	4.0457	4.6644	4.2901
Apoo	_IDELSLYSVPEGQSK_2	#N/A	8.6434	8.3353	8.5870	8.4377	8.2939	8.2465	8.4027	8.5730	8.4339	8.5085	8.3601	8.3325	8.6696	8.5428	8.3521	8.6113	8.5317	8.2574	8.4143	8.2627
Apoo	_LYDWGLR_2	#N/A	6.8680	6.9038	6.8420	7.3377	7.0587	7.1570	7.1019	7.0703	7.2119	7.1427	7.1347	7.3382	7.3882	6.7371	7.0292	6.8178	6.8134	7.1535	7.3576	6.8499
Apoo	_TQLEENISQLR_2	#N/A	7.2914	7.3201	6.9597	7.1073	6.7919	6.8285	7.0722	6.9109	7.0498	6.8527	6.8034	6.9613	7.2739	6.8237	6.9999	6.7912	6.6166	6.8594	6.8048	6.6566
Apoo	_GYIVIEDLWK_2	#N/A	5.6538	5.1472	5.6870	6.2228	5.2906	5.7516	5.3000	5.7078	6.0957	5.5190	5.3114	5.7577	3.9850	5.8005	5.5896	5.5500	4.6962	5.7108	5.9860	5.9021
Dtd1	_SASS[Phospho (STY)]GAEGDVSSER_2	#N/A	7.1860	7.0571	7.0392	3.3573	6.8340	6.9547	6.8761	6.9385	6.7223	6.4637	7.0199	6.4869	6.2827	7.1867	6.4437	6.7263	6.6608	6.7029	6.9645	3.2595
Dtd1	_SASS[Phospho (STY)]GAEGDVSSEREP_2	#N/A	10.6966	10.6937	10.4074	10.9424	10.4032	10.4505	10.3234	10.7220	10.3622	10.7234	10.1635	10.4151	10.8406	10.8490	10.0591	10.4905	10.5967	10.3379	10.2872	10.3276
Dtd1	_ASVTVGGEQISAIGR_2	#N/A	4.5307	4.1266	4.8447	4.6935	3.2368	3.2702	4.7256	3.5766	3.7315	4.3413	5.0846	4.0160	3.8969	4.3996	4.7980	4.8154	4.9411	3.7189	3.3675	4.3666
Dtd1	_AKGPSESS[Phospho (STY)]KER_2	Q9DD18_S182_M1_Dtd1	4.1948	3.8080	3.2303	3.4946	3.5325	3.7566	4.0086	4.3705	4.0676	4.4011	2.9412	3.6975	4.1757	3.7195	3.5857	3.0389	3.8677	3.2817	3.5808	3.3967
Tsc1	_GGFDS[Phospho (STY)]PFYR_2	Q9EP53_S502_M1_Tsc1	4.5131	7.0032	3.5486	6.7225	3.8508	4.1341	6.6386	4.2135	4.3684	3.2186	3.8470	6.1637	4.5338	6.7603	3.2675	3.3572	3.2503	3.0820	6.4929	6.6714
Wdr4	_SPFPGS[Phospho (STY)]PEQTKK_2	Q9EP82_S397_M1_Wdr4	4.1743	3.8285	3.2097	3.5151	7.9989	7.8804	7.8085	8.2422	4.0882	7.6583	7.8058	3.7180	8.2966	8.2900	3.6063	3.0183	4.4123	3.4208	7.7574	3.1638
Senp3	_NHLS[Phospho (STY)]PQEGGATPQVPSPC[Carbamidomethyl (C)]C[Carbamidomethyl (C)]R_3	Q9EP97_S163_M1_Senp3	3.6453	7.3216	4.1816	4.0441	2.9830	6.5529	3.0572	3.4192	3.1163	3.8516	3.4907	4.2469	3.2244	4.2690	4.1352	3.9902	6.7453	4.2331	3.0313	6.0437
Senp3	_GS[Phospho (STY)]PPVPSGPPMEEDGLR_2	Q9EP97_S206_M1_Senp3	4.3132	3.6896	3.3486	3.3762	3.6509	3.8750	3.8902	4.0135	3.9493	4.5195	4.3237	3.5791	4.0573	3.3357	3.4674	3.1572	4.5512	3.2819	4.4314	3.3027
Pycard	_EIHPYLVM[Oxidation (M)]DLEQS[Phospho (STY)]_2	#N/A	7.3233	7.9006	7.3312	7.4625	6.6786	7.5021	7.5727	7.0911	8.5227	8.0110	6.9914	7.3900	7.8691	7.9827	8.4839	7.9817	7.5866	7.7757	7.9265	7.6598
Parva	_[Acetyl (Protein N-term)]ATS[Phospho (STY)]PQKSPLVPK_2	Q9EPC1_S4_M1_Parva	7.1468	5.7061	6.6071	6.8194	6.5895	5.0241	5.0631	5.1985	6.1531	5.4265	6.4995	5.1898	5.4989	5.4053	5.3821	5.3038	6.9899	4.9785	5.0858	5.8644
Parva	_SPTPKS[Phospho (STY)]PPSR_2	Q9EPC1_S19_M1_Parva	8.8074	8.3575	3.6548	8.4135	8.2329	7.9162	8.0666	3.9685	8.1893	3.6678	7.9594	8.8899	7.8691	3.3044	3.7901	7.6886	3.5137	7.9811	7.9287	3.8644
Atp13a1	_DS[Phospho (STY)]PVLSNSGPR_2	Q9EPE9_S896_M1_Atp13a1	8.4074	4.6079	3.9312	4.2945	7.8452	4.4575	3.3076	8.0840	3.3667	3.6012	7.9169	2.9965	3.4748	3.9183	2.8848	7.2254	8.1639	3.9826	7.1338	7.5026
Arfgap1	_S[Phospho (STY)]PSSDSWTC[Carbamidomethyl (C)]ADASTGR_2	#N/A	10.1190	9.7971	10.3331	10.5959	9.4788	9.3204	6.5825	9.2837	6.7472	10.3766	9.8322	9.7436	9.7639	7.5250	8.7906	9.6265	9.6328	9.3122	9.5659	7.2030
Arfgap1	_AAPSTADEGWDNQNW_2	#N/A	6.9747	6.9355	7.4813	7.5378	6.9506	7.3671	6.2908	7.3406	7.1509	7.3495	6.9393	6.9964	7.5480	7.7365	7.3794	7.5176	7.8976	7.1047	7.0908	7.6582
Arfgap1	_TLQFTAHR_2	#N/A	5.7563	6.3620	6.5450	6.3987	6.4843	6.8174	6.6749	6.9479	6.6115	6.7494	6.1310	6.9626	6.5811	7.0786	7.3046	6.9775	6.0415	6.8556	7.0999	6.8817
Arfgap1	_NSNSDGWESWEGASGEGR_2	#N/A	4.9188	7.4400	6.7163	7.0416	4.5398	4.4978	6.8150	4.6612	6.9858	5.1018	4.5910	7.7934	4.5127	7.9800	6.9221	7.1051	5.3230	7.1520	7.1258	6.9383
Arfgap1	_KAAPSTADEGWDNQNW_2	#N/A	7.0602	6.8598	6.8509	7.0462	6.4438	6.9811	6.7018	6.6900	6.2445	7.3989	6.9967	6.7768	6.9730	7.0332	6.1171	7.1087	7.7954	7.0611	7.1645	7.0653
Arfgap1	_AKS[Phospho (STY)]PSSDSWTC[Carbamidomethyl (C)]ADASTGR_3	#N/A	5.7265	5.6016	4.1797	8.1623	4.5272	5.2124	6.3111	5.1141	3.9487	7.2822	6.9541	6.6992	3.4835	6.0396	3.7602	5.5318	6.3680	6.7044	6.3980	7.2680
Arfgap1	_SS[Phospho (STY)]DSWDVWGSGSASNNK_2	#N/A	9.5256	10.6447	4.2968	10.8857	3.2498	9.9673	10.1422	3.4673	3.9643	4.1184	3.2355	3.9917	4.0723	10.9685	10.8923	4.2570	10.1315	10.0975	3.3399	3.5605
Arfgap1	_RS[Phospho (STY)]SDSWDVWGSGSASNNK_3	#N/A	3.4886	3.8820	5.1521	5.2266	3.5894	3.6366	3.6835	3.7032	3.5740	5.6314	3.5652	3.7079	3.6950	3.9595	3.5715	3.9907	4.0029	4.0491	3.5854	3.7210
Arfgap1	_AFEDWLNDDLGSYQGAQENR_2	#N/A	6.0158	5.9495	5.8101	6.0585	5.4865	6.0872	5.8907	6.1731	5.3299	5.7076	6.2969	5.6232	5.6396	6.8552	5.7549	5.5581	6.1647	5.8395	6.1049	6.5057
Arfgap1	_DVTTFFSGK_2	#N/A	3.9862	3.6398	3.5228	4.8871	4.3580	3.9152	4.3450	4.2119	3.8922	4.7533	4.3237	3.3883	4.9675	4.6019	4.7762	3.3483	3.7091	3.8015	4.7615	4.7437
Arfgap1	_AKS[Phospho (STY)]PSSDS[Phospho (STY)]WTC[Carbamidomethyl (C)]ADASTGRR_3	#N/A	3.5239	4.1671	4.3237	6.3537	3.3458	3.6442	3.4406	3.5015	3.3608	6.2960	3.7431	3.9933	5.3263	4.0289	3.4660	4.1419	3.5362	4.0929	3.7415	3.9959
Arfgap1	_GLGVHLSFVR_2	#N/A	6.5735	5.0079	5.0407	4.2905	5.0652	3.0138	3.2506	5.8641	3.3096	5.8709	5.6242	5.1922	4.3129	4.1600	5.7887	5.7468	4.5929	5.1499	5.6417	4.1396
Arfgap1	_S[Phospho (STY)]PSSDSWTC[Carbamidomethyl (C)]ADASTGRR_2	#N/A	4.0751	7.6058	6.8726	4.1976	7.0661	6.0203	7.0812	4.1462	6.3067	8.0181	3.3932	6.0816	4.1415	6.6416	3.4981	5.5139	5.9926	7.5714	6.2828	3.6733
Arfgap1	_FGSQASQK_2	#N/A	4.2308	3.7720	3.2663	3.4586	2.8822	4.4164	3.7484	3.0827	4.4647	3.7743	3.5647	3.6615	4.2515	3.6555	4.4114	3.5583	5.0624	4.4714	4.3599	4.4664
Arfgap1	_EWSLESSPAQNWTPPQPK_2	#N/A	5.1706	5.4246	5.0538	5.6455	5.2048	5.7382	5.1622	5.2383	5.4893	5.2480	5.4471	5.7597	5.3052	5.8974	5.7399	5.8485	5.8325	5.2545	5.3539	5.3588
Ipo7	_ETENDDLTNVIQK_2	#N/A	5.2720	5.5330	5.2265	5.8026	4.8416	5.5432	5.4084	5.5139	5.4036	5.6638	5.6390	5.1843	5.1249	3.5709	3.7444	5.2419	5.0513	5.6442	4.9116	4.3643
Nbea	_RQFS[Phospho (STY)]PGPR_2	#N/A	11.6094	11.4335	11.1050	11.2249	10.8332	11.0397	11.2226	11.3639	10.9998	11.0713	10.9270	10.7799	11.3828	10.6090	9.5373	10.4296	11.2666	11.2370	10.9138	10.5174
Nbea	_TPLENVPGNLS[Phospho (STY)]PIKDPDR_2	#N/A	9.2975	9.7519	8.6173	8.6498	7.6023	9.0249	9.3377	9.2596	8.4075	8.8074	8.4956	8.8828	9.4663	9.6237	7.2221	8.4912	9.1497	8.6505	8.7765	8.5875
Nbea	_QFS[Phospho (STY)]PGPR_2	#N/A	9.9056	9.9937	9.6924	9.6571	9.4522	9.7514	9.6503	9.5901	9.8578	9.7476	9.6139	9.9731	9.1953	9.7874	7.8852	9.3434	9.7872	9.6337	9.3884	9.3446
Nbea	_AVPNVDAGSIIS[Phospho (STY)]DTERSDDGKESGK_3	Q9EPN1_S1252_M1_Nbea	6.9717	9.4320	9.4443	6.2490	2.3209	9.2145	9.0691	9.3283	4.1755	7.3919	3.2798	6.2086	4.3029	3.6834	8.0404	8.4496	8.8027	10.4357	9.0526	5.8413
Nbea	_EIEDLSQSQS[Phospho (STY)]PES[Phospho (STY)]ETDYPVSTDTR_3	Q9EPN1_S1001_M2_Nbea	3.9853	3.6495	3.9321	3.5108	3.8504	3.9539	3.9529	3.9643	3.9109	3.8754	3.7031	3.4980	4.0604	3.8094	3.3607	3.5803	3.8105	3.5452	3.9526	3.7126
Nbea	_SQAIVNQNS[Phospho (STY)]ETELMLEGDDDAVSLLQEK_3	Q9EPN1_S2114_M1_Nbea	3.8469	4.2282	3.7324	3.9079	3.2396	3.5253	4.2297	4.0726	3.6805	4.0553	3.3299	4.0554	3.8778	4.6978	3.6556	3.8956	4.2564	3.7502	3.2350	3.7854
Tcf20	_AGSS[Phospho (STY)]PTQGAQNEAPR_2	Q9EPQ8_S588_M1_Tcf20	3.8104	8.5349	8.1083	4.0136	8.1134	4.0161	8.2818	4.4395	8.0816	8.2500	8.0919	8.2109	8.3566	8.7798	7.6124	8.0635	8.6237	8.2413	7.7470	8.1727
Tcf20	_LSTS[Phospho (STY)]PATR_2	Q9EPQ8_S603_M1_Tcf20	4.4421	5.3661	3.4776	3.6867	3.7798	4.0039	4.4272	4.1232	3.8203	3.1476	4.1947	3.4501	3.9284	3.4722	3.3384	3.2862	3.6204	5.6984	3.8281	3.1494
Tcf20	_GSQEDDPAASQRPPSNS[Phospho (STY)]GVK_3	Q9EPQ8_S671_M1_Tcf20	4.0286	4.4132	4.0640	3.6624	3.0298	3.1658	4.1288	3.6042	3.5895	4.0160	3.7242	4.2145	3.7360	3.7487	4.0680	3.8797	4.3477	3.9626	4.0949	3.5296
Tcf20	_LSTS[Phospho (STY)]PATRDEAAS[Phospho (STY)]PGAK_3	Q9EPQ8_S603_M2_Tcf20	3.4625	3.8566	4.4999	4.2458	3.6695	3.7028	3.3786	3.1440	3.2989	3.2731	4.2783	3.5827	3.4643	3.9669	3.4040	4.3255	3.6816	4.1515	3.8001	3.9339
Slc23a2	_SSLAETLDSTGS[Phospho (STY)]LDPQR_2	Q9EPR4_S81_M1_Slc23a2	8.9024	10.1283	8.8264	8.4592	3.2597	9.6352	9.7968	8.2475	8.6224	8.1963	7.7918	8.2483	8.9670	10.0759	8.6138	7.9143	8.6936	8.4406	8.9401	8.0180
Inpp4a	_AS[Phospho (STY)]PTSTEEEQVMLR_2	Q9EPW0_S488_M1_Inpp4a	3.2932	4.0259	4.3306	4.2911	3.5001	3.5334	5.8610	3.3133	3.4682	3.1038	2.9468	3.7520	3.6336	4.1363	3.5734	4.1561	3.8510	3.9822	3.6308	4.1033
Aldh6a1	_VNAGDQPGADLGPLITPQAK_2	#N/A	7.5404	7.4885	8.0117	7.4306	7.2892	7.3036	7.5182	7.5118	7.6162	7.4178	7.2340	6.8072	7.2366	7.4912	7.5693	7.4564	6.9274	7.0746	7.1554	6.9185
Aldh6a1	_TLADAEGDVFR_2	#N/A	6.8324	7.0663	7.0861	6.9810	6.7710	7.0108	6.6009	7.3682	7.3736	7.1970	6.8176	6.7554	7.3960	6.4505	6.8141	7.2529	6.9709	6.7887	6.5829	6.3969
Aldh6a1	_EGASILLDGR_2	#N/A	5.6761	5.9660	5.1555	5.5404	5.6671	6.0218	5.4735	5.1061	5.5702	6.1734	5.5515	2.2061	3.9616	4.8885	4.7238	5.0003	5.4363	5.6400	4.9277	5.2722
Aldh6a1	_AISFVGSNQAGEYIFER_2	#N/A	4.8782	4.0405	5.2555	4.9464	4.9969	5.0893	5.1728	3.8890	4.8702	5.8169	5.1330	5.3094	5.8447	4.4857	4.5938	5.0905	4.5975	3.6328	4.3536	5.1187
Aldh6a1	_DMDLYSYR_2	#N/A	3.2980	4.0211	4.3354	4.2862	3.5049	3.1026	3.5432	3.5237	3.4634	3.3086	2.9420	3.7472	3.3289	4.3735	3.5685	4.1610	4.5218	4.1285	4.4276	4.0508
Pold3	_VDLS[Phospho (STY)]DEEAKETEHLK_3	Q9EQ28_S306_M1_Pold3	3.4029	4.4996	4.0395	4.1862	3.6098	5.7866	5.4886	3.2036	5.7334	5.1977	4.3380	5.5405	3.5239	5.9265	5.5655	4.2658	3.7413	4.0919	5.6186	3.9936
Dpysl5	_IPHGVSGVQDR_2	#N/A	7.5681	7.9763	8.1115	8.0868	7.9384	7.9222	7.1494	8.2202	8.1854	7.7577	7.0064	7.3211	7.1328	7.5086	7.6994	7.0143	7.3725	7.1808	7.0854	7.1151

Dpysl5	_ILNLYPR_.2	#N/A	6.9082	7.7117	7.6264	7.5047	7.1492	7.7913	7.7789	7.7791	6.9619	7.3281	6.6473	7.1499	7.2338	6.5918	6.8214	6.0002	6.9722	7.1456	6.7664	6.8505
Dpysl5	_VVNDDC[Carbamidomethyl (C)]THEADVYIESGIIQQVGR_.3	#N/A	7.6504	7.8857	8.0203	7.6844	7.0381	7.3284	7.3143	7.7835	7.3249	7.6080	6.2215	6.7977	7.2725	7.2538	6.5153	6.6579	6.9770	6.1462	6.5642	6.6862
Dpysl5	_FVAVTSSNAAK_.2	#N/A	7.2218	7.4079	7.6301	7.6600	6.8434	7.4248	7.3315	7.1076	7.8180	7.3601	6.0152	6.6263	6.2011	6.7197	6.7975	6.5432	6.6086	6.5171	6.4152	6.4168
Dpysl5	_AEM[Oxidation (M)]ETLVR_.2	#N/A	6.4505	6.7876	5.8644	6.1942	6.2566	6.6119	6.4258	6.6282	6.9967	6.8238	5.7007	5.8755	5.4015	4.9022	6.5617	6.0822	5.2188	5.8131	6.0622	5.9498
Dpysl5	_C[Carbamidomethyl (C)]HGVPLVTISR_.2	#N/A	6.4814	7.5300	6.6382	6.7445	5.9285	6.9284	6.4393	6.6586	6.4396	7.5118	5.5993	6.1652	4.3829	6.4719	5.8978	6.0308	5.3374	6.0868	5.4069	5.4209
Dpysl5	_EM[Oxidation (M)]GT[Phospho (STY)]PLADT[Phospho (STY)]PTRPVTR_.2	#N/A	12.7761	12.8490	12.4266	12.6553	10.9319	12.4398	12.4362	12.7672	12.6220	12.9757	11.3656	11.8271	12.6257	12.0117	11.3556	12.0391	12.1443	11.8293	11.9270	11.9415
Dpysl5	_KEM[Oxidation (M)]GT[Phospho (STY)]PLADT[Phospho (STY)]PTRPVTR_.3	#N/A	7.7405	8.0671	7.9366	8.0305	7.5414	7.8365	8.2331	7.3431	7.3782	8.0414	6.5678	6.6747	3.9457	6.7121	5.6160	6.9198	6.8077	6.2226	6.6955	6.5841
Dpysl5	_DLHESSFSLSGS[Phospho (STY)]QIDDHVPKR_.4	#N/A	7.8805	8.7141	6.6516	7.0962	6.6941	3.5437	8.4196	3.4955	4.0483	7.6361	3.7068	7.1054	8.1182	8.1720	3.7559	6.7156	4.2949	6.8988	5.2544	6.5058
Dpysl5	_ELM[Oxidation (M)]IPGGAK_.2	#N/A	6.8336	6.8390	6.6675	7.4199	7.0767	7.2289	7.0527	7.1362	6.9129	7.2739	6.3736	6.5663	6.5030	6.1393	6.8052	6.1524	6.0158	6.2632	6.4349	6.2876
Dpysl5	_VVYENGVFM[Oxidation (M)]C[Carbamidomethyl (C)]AEGTGK_.2	#N/A	5.4199	5.6601	5.7917	5.6364	4.5595	5.9621	5.1169	3.0812	6.2390	5.3632	4.2155	5.1409	3.4015	5.1464	4.5779	5.3243	3.6188	5.3577	5.4060	4.9445
Dpysl5	_M[Oxidation (M)]SVVWER_.2	#N/A	5.7732	6.4676	4.8555	5.9719	5.3729	5.9724	6.4700	5.9900	6.5642	6.7623	4.9091	5.2231	5.9977	4.7611	5.3014	4.2774	4.6627	5.0739	4.8972	4.7854
Dpysl5	_DLHESSFSLSGS[Phospho (STY)]QIDDHVPK_.3	#N/A	7.8375	8.2053	8.5274	7.3771	7.0115	7.1134	7.7751	6.8750	7.5568	8.2327	6.0931	6.2032	6.5700	7.3945	5.9028	6.3383	6.2464	6.6903	5.9625	6.5746
Dpysl5	_EALDLGITGPEGIEISHPEELEAEATHR_.3	#N/A	5.0360	4.9086	4.8544	4.9634	4.1214	5.1416	5.6265	4.7975	4.9085	4.9569	3.7896	3.7110	4.7226	4.6099	3.7328	4.2075	3.7488	4.5315	3.8788	4.7187
Dpysl5	_LDTNTSTYLM[Oxidation (M)]SLLANDTLNIVASDHRPFTTK_.4	#N/A	3.6922	5.2400	4.3097	3.9972	3.6836	4.5559	3.3402	2.8929	3.0694	4.8725	3.4438	4.2001	3.1775	3.5296	4.0884	4.0126	3.9302	4.2799	3.1321	3.6534
Dpysl5	_DSELYQVFHAC[Carbamidomethyl (C)]R_.2	#N/A	5.9053	6.1681	6.0183	5.5670	5.7275	5.6707	5.8876	5.9078	5.7369	5.8904	4.9112	4.9543	3.9426	5.9670	5.2597	4.8080	5.0202	4.7566	5.1697	4.9891
Dpysl5	_TISASTQVQGGDFNLYENM[Oxidation (M)]R_.2	#N/A	3.5778	4.7218	4.6152	4.3611	4.1139	4.1851	3.2417	4.5295	3.1836	4.9985	4.1631	3.4674	3.3490	3.8517	3.2888	4.3513	3.9872	4.2668	3.5917	4.8066
Dpysl5	_AMGKEDFTK_.2	#N/A	4.1842	3.1637	5.0924	3.3429	3.5219	3.7460	4.8369	4.3811	4.0782	4.3905	2.9518	4.3906	4.1863	3.7301	3.5963	3.0283	3.8783	3.2711	3.5702	3.4073
Dpysl5	_THC[Carbamidomethyl (C)]PIYLVNVSSISAGDVIAAAK_.3	#N/A	4.5793	4.0946	4.1012	4.1245	2.9026	3.1267	3.1377	3.2652	3.1967	3.4025	3.5711	4.3274	3.3048	3.4101	4.2157	3.9098	3.8029	4.1526	2.9508	4.0267
Dpysl5	_TPYLGDVAIVVHPGK_.3	#N/A	4.1769	3.7727	3.2655	3.4593	2.8830	4.4172	4.1651	3.9304	4.0853	3.9875	3.5640	3.6622	4.2508	3.2526	1.1137	3.5390	4.4681	3.3650	3.0136	3.2196
Cert1	_FVQKPYS[Phospho (STY)]R_.2	Q9EQG9_S373_M1_Cert1	7.7843	4.5365	4.0026	4.2231	4.3049	3.0281	3.2362	3.1667	3.2953	3.6726	3.6697	4.4259	3.4033	7.5686	2.8134	3.8112	3.7044	4.1288	6.7403	3.9567
Cert1	_SSS[Phospho (STY)]M[Oxidation (M)]SS[Phospho (STY)]IDLVSASDDVHR_.2	Q9EQG9_S377_M2_Cert1	3.8957	3.7145	3.8186	3.6544	3.8867	3.6124	4.4040	3.9266	4.8329	3.8398	3.7994	3.5205	4.3380	3.7890	3.3759	3.6362	3.6325	3.5387	3.9279	3.6843
Vps35	_RES[Phospho (STY)]PESEGPIYEGLIL_.2	#N/A	9.9307	9.7860	10.2088	9.7742	8.8483	9.7981	9.8504	9.1012	9.9919	9.6422	9.5694	9.2969	9.6720	9.6515	8.7783	9.1602	9.8286	9.6931	9.5273	9.3001
Vps35	_SRRES[Phospho (STY)]PESEGPIYEGLIL_.3	#N/A	7.2928	7.5645	4.2885	6.9375	3.2724	9.1180	9.1215	3.5091	7.5514	6.9269	8.9092	3.9725	3.8294	8.7479	8.1419	4.1050	8.0678	9.4815	9.3422	8.7467
Vps35	_IREDLPNLESSEETEQINK_.3	#N/A	7.2322	6.7308	7.0068	7.1527	6.5159	6.9618	6.2058	6.9749	6.7382	6.9435	6.8585	6.3059	6.9057	6.4698	5.4746	6.3611	6.6433	6.6534	6.0864	6.5921
Vps35	_AELAELPLR_.2	#N/A	6.9920	7.1060	6.6301	6.7377	6.3234	6.7356	7.1223	7.3233	6.6119	6.3796	6.6839	6.7042	6.2806	5.9773	5.5996	5.6959	6.4785	6.8972	6.2303	6.3730
Vps35	_SDDPDQQYLILNTAR_.2	#N/A	6.7784	6.1283	6.7922	5.9532	6.2447	6.3695	6.4695	6.6424	6.7622	6.7962	6.1509	5.4342	6.4017	5.6064	5.8095	6.2472	6.3707	6.3426	5.9879	6.2135
Vps35	_LSQLEGVNVER_.2	#N/A	6.3555	5.7587	5.2747	5.5676	4.0653	5.5652	5.9337	6.5119	5.9867	6.0550	5.7927	5.2228	3.8892	5.9188	4.8952	3.2205	5.9974	5.5374	5.4306	4.9305
Vps35	_IPVDTYNNILTVLK_.2	#N/A	5.3712	5.8111	5.3217	5.7037	4.6133	5.7183	5.2548	5.5704	5.0491	4.5441	5.1937	5.1637	4.7666	5.1361	3.6691	5.0448	5.5088	5.3212	4.3846	5.4321
Vps35	_LFDIFSQQVATVIQSR_.2	#N/A	5.1227	4.0963	3.9817	4.3366	4.4241	5.1287	5.4130	5.1498	4.5549	5.0366	3.9467	4.1009	5.4225	4.0198	3.0546	4.6059	4.8013	4.7365	4.1057	4.7216
Vps35	_PTTQQS[Phospho (STY)]PQDEQEKLLDEAIQAVK_.3	#N/A	7.4475	7.8569	7.0761	4.2329	3.7940	6.7205	7.5456	7.2776	7.0351	6.8799	3.4568	7.1175	7.8361	7.4992	6.2819	3.8916	3.6351	7.6214	6.9894	6.5808
Vps35	_LLDEAIQAVK_.2	#N/A	3.7992	3.5199	3.3358	3.7850	3.5091	3.0349	4.3423	3.3595	5.0989	3.6098	4.5928	3.4422	3.1276	3.3886	4.5373	3.1613	3.5221	3.9561	4.1368	4.6046
Vps35	_PTTQQS[Phospho (STY)]PQDEQEK_.2	#N/A	9.5251	9.2534	9.7076	9.4217	8.9650	9.2988	9.3404	9.2367	9.2219	9.1896	9.1954	8.6862	8.7668	9.0281	8.2297	8.9466	9.3373	9.0228	9.0160	8.8854
Vps35	_LNLEHIATSSAVSK_.2	#N/A	4.1424	4.3177	4.3728	4.1600	3.3794	3.7392	3.8655	4.4510	4.5361	3.8881	3.3081	3.7438	4.2865	3.8302	3.6281	3.8528	3.8633	3.9543	4.0464	3.6508
Vps35	_ES[Phospho (STY)]PESEGPIYEGLIL_.2	#N/A	3.6249	3.4587	3.8992	3.8478	4.0393	3.1706	3.9583	3.7236	3.6446	3.5535	3.7907	3.2282	4.0439	3.7241	3.1612	3.9822	3.4388	3.7535	3.5608	3.6911
Vps35	_TQC[Carbamidomethyl (C)]ALAASK_.2	#N/A	4.1029	4.5832	3.7734	3.3475	3.4406	5.7532	5.9399	4.4625	4.1596	3.4422	3.0331	4.3093	4.2676	3.8114	3.6777	2.9469	3.9597	5.7657	3.4888	3.4887
Magi3	_SGS[Phospho (STY)]PKLDPSEVYLK_.2	#N/A	6.8743	3.7170	6.4199	6.7636	3.6682	3.7858	6.6486	4.0308	6.4929	4.0903	3.9164	6.6819	7.3662	7.2641	3.4947	6.4846	7.0509	6.8432	3.5224	6.1076
Magi3	_GGPC[Carbamidomethyl (C)]S[Phospho (STY)]PTK_.2	Q9EQJ9_S662_M1_Magi3	3.6630	4.3398	4.1993	4.0264	3.0007	3.4834	3.0395	3.3634	3.5183	4.5545	2.9969	4.2292	3.6837	4.1864	4.1175	4.0079	3.9011	3.9321	3.5807	4.1534
Magi3	_QPEDESHQAFSQNGS[Phospho (STY)]PR_.3	#N/A	3.3927	4.5094	4.0792	4.1492	3.9686	3.6062	3.4780	3.2433	3.8643	3.1765	3.7838	4.3521	3.5636	3.5681	3.5034	4.2289	7.1689	4.0549	3.7035	4.0333
Slc19a2	_KQEDPSSS[Phospho (STY)]PQASTS_.2	Q9EQN9_S492_M1_Slc19a2	7.4714	7.4162	7.4440	6.7283	4.0628	7.3886	7.5507	7.6366	7.4447	6.8880	4.1531	6.6888	7.4177	3.4306	3.0106	3.2180	7.5571	7.0859	7.1833	6.7272
Ehd4	_DKPVYDELFYTLS[Phospho (STY)]PINGK_.3	Q9EQP2_S459_M1_Ehd4	4.0264	4.5430	4.5627	3.6630	3.3642	4.9500	4.1769	3.7268	4.5555	4.4419	4.8488	4.8144	4.3441	4.5990	3.9984	4.7468	4.2645	4.6750	5.0931	4.3556
Oga	_LENEGS[Phospho (STY)]DEDIETDVLYSPQM[Oxidation (M)]ALK_.3	#N/A	3.2380	3.7732	5.9225	4.2992	3.8211	6.2881	3.5517	3.5347	3.5338	4.0549	3.4916	3.5543	3.6733	4.3845	3.3994	3.8432	3.8778	6.5710	3.7857	4.2614
Syt13	_PAVTAPEVINYADYTLETTEESAAPAS[Phospho (STY)]PQAQSDSR_.4	Q9EQT6_S113_M1_Syt13	4.0082	7.1886	7.1368	6.8107	5.8638	6.8541	6.5463	6.8755	6.2196	7.3317	6.2076	6.1843	4.4195	6.6014	5.9028	3.3703	3.7311	6.8107	6.4185	6.4826
Set	_IDFYFDENPYFENK_.2	#N/A	6.4909	5.7857	6.3901	6.6640	6.5770	6.3061	5.9138	6.3096	6.4247	6.4122	6.6347	6.6067	6.9955	6.3349	6.4090	6.8992	7.0699	6.3313	6.4464	6.5517
Set	_VEVTEFEDIK_.2	#N/A	5.6757	5.9732	3.6046	6.0401	5.6441	4.8631	5.7204	4.8199	5.5616	3.2746	5.7236	4.8106	5.9816	6.2508	5.9262	5.8518	5.9889	5.4810	5.1638	6.2296
Set	_EFHLNESGDPSSK_.2	#N/A	3.8832	5.0938	5.1662	5.2152	5.3194	5.4485	5.1511	3.8431	4.7964	5.4224	4.7039	5.2565	5.6853	5.9887	5.9111	5.9321	5.5768	5.3775	5.0619	5.5851
Rims2	_TGSVQTS[Phospho (STY)]PSSTPGTGR_.2	Q9EQZ7_S1095_M1_Rims2	9.5798	9.3214	9.2939	9.2831	8.9904	9.1795	9.2385	9.3397	9.1467	9.1113	9.1122	9.1835	9.4945	9.4190	8.1351	8.7213	9.5576	9.1176	8.4983	8.9397
Rims2	_TSNHS[Phospho (STY)]PPTPR_.2	Q9EQZ7_S472_M1_Rims2	10.0678	10.3179	9.9083	9.9908	10.0032	4.0276	10.0989	4.3200	2.9741	10.0728	9.8574	10.1462	10.4604	10.3457	9.6238	9.2740	10.2856	2.9755	10.0175	9.8775
Rims2	_SWS[Phospho (STY)]PSAPPPQR_.2	Q9EQZ7_S1039_M1_Rims2	3.7750	10.0797	3.3116	3.8092	9.8933	9.7568	9.7994	10.1754	9.9051	3.5856	9.8921	9.8817	3.1518	3.3496	3.0915	10.0056	3.4979	9.9115	9.7000	9.6482
Rims2	_ASQSS[Phospho (STY)]LESSTGPSYSR_.2	Q9EQZ7_S1518_M1_Rims2	8.4219	8.3942	8.0596	8.5092	8.0248	7.8645	8.0690	8.4356	8.2652	8.4445	8.2498	8.2933	8.3249	8.9068	3.8158	8.0841	8.8772	8.3491	7.8499	8.0505
Rims2	_RHS[Phospho (STY)]DVSLANAELEDSR_.3	Q9EQZ7_S409_M1_Rims2	8.0456	7.9981	8.4638	8.2223	6.3031	7.7367	7.3981	7.6861	7.8719	3.1706	7.2455	7.7058	7.7089	8.1325	7.2789	7.7891	7.8079	7.8467	7.8488	7.6047
Rims2	_YRS[Phospho (STY)]DPNLAR_.2	Q9EQZ7_S375_M1_Rims2	4.5148	3.4880	3.5503	8.5991	3.8526	8.1883	3.6885	4.2152	3.7476	3.2203	7.9955	3.3774	8.9472	3.5374	7.3298	3.3589	8.2532	3.6017	4.2297	3.0767
Rims2	_SPS[Phospho (STY)]DYADRR_.2	Q9EQZ7_S311_M1_Rims2	6.7013	6.7983	5.0933	6.5627	4.3382	6.1027	6.9579	6.2667	6.4503	6.6713	3.2040	6.3328	6.3469	3.9823	5.6495	5.9040	6.4294	6.1427	6.2696	3.6596
Rims2	_S[Phospho (STY)]ASQLSQTEGGGKK_.2	Q9EQZ7_S1285_M1_Rims2	3.4696	4.5332	4.0059	4.2198	4.3081	3.0314	3.2330	3.1699	3.2920	5.0222	3.6664	4.4227	3.4001	4.4447	2.8101	3.8145	3.7076	4.0573	4.3564	4.1220
Rims2	_NDGSQS[Phospho (STY)]DTAVGALGTSGK_.2	Q9EQZ7_S1251_M1_Rims2	7.6312	7.6619	4.0652	4.5565	3.2347	7.0634	7.3806	3.2535	7.0397	4.0173	6.7609	7.0379	7.2207	4.1033	3.8388	3.8907	7.4442	7.3893	3.1970	7.0083
Rims2	_IPDSTHAQLESSSSSFES[Phospho (STY)]QK_.3	Q9EQZ7_S788_M1_Rims2	4.3334	3.6694	3.3689	3.3560	3.6712	3.8952	3.8699	4.2319	3.9290	4.5398	4.3034	3.5588	4.0371	3.5808	3.4471	3.1775	3.7291	3.4203	3.7194	3.2581
Rims2	_RAS[Phospho (STY)]QSS[Phospho (STY)]LESSTGPSYSR_.2	Q9EQZ7_S1515_M2_Rims2	4.2793	3.6353	3.3386	5.3242	3.6171	3.8412	3.9348	4.4410	3.9938	3.8615	3.7441	4.9712	4.0744	3.3540	3.5120	4.5234	4.0705	3.4658	3.4804	4.3429
Rims2	_RADS[Phospho (STY)]LRK_.2	Q9EQZ7_S493_M1_Rims2	4.0214	4.6137	2.6956	4.3003	3.7239	1.5587	3.3242	3.0895	2.3154	1.9503	3.8056	3.5282	3.4099	3.9125	3.3496	3.1605	3.3575	1.3439	3.8546	2.7848
Rims2	_RQLHGES[Phospho (STY)]PTR_.2	Q9EQZ7_S965_M1_Rims2	5.3171	5.1114	5.0102	3.4899	3.6721	5.5763	5.5831	4.3423	3.9144	3.9272	3.8900	3.8003	4.0528	3.6913	3.6216	3.4787	3.8395	3.2555	3.6312	3.1451
Rims2	_NDS[Phospho (STY)]LSSDQSESVRPPPPRPHK_.3	Q9EQZ7_S522_M1_Rims2	4.5391	6.8963	5.6601	4.5459	3.2452	3.3623	3.8029	3.2640	4.4620	4.3497	3.3355	4.0069	4.5700	4.1138	3.8282	3.9012	4.2620	4.3882	3.1864	3.7911
Rims2	_RS[Phospho (STY)]PIPLDRPDMR_.3	Q9EQZ7_S479_M1_Rims2	4.4076	4.4124	3.9442	2.7450	3.1137	3.1470	2.6364	3.6997	3.8546	4.2182	2.4469	4.1385	4.0201	2.1526	3.9598	3.7697	4.2374	3.5957	3.2443	2.9889
Stx12	_ELGSLPLPLSASEQR_.2	#N/A	4.8254	4.0803	5.2252	4.9056	4.7747	4.9380	5.2886	4.7535	4.9861	4.4882	4.4309	4.5482	4.6509	4.8326	4.4466	4.5567	5.1456	4.6115	4.7669	4.3371
Stx12	_AGSRLS[Phospho (STY)]AEDR_.2	Q9ER00_S142_M1_Stx12	6.3852	6.5326	3.6289	6.5424	6.1930	6.3352	6.2941	6.4334	4.1699	3.9029	6.1823	2.9529	4.3353	3.3371	4.2750	3.4545	6.0159	5.8930	4.4299	5.5689
Stx12	_DFNSIIQTC[Carbamidomethyl (C)]SGNIQR_.2	#N/A	5.6930	6.0056	5.9756	5.4818	5.4256	5.2252	5.4435	5.6166	5.5830	5.3540	5.3246	5.1167	6.0422	4.5764	4.7505	4.9719	6.0303	4.1899	4.5391	3.8956
Stx12	_LQENLQQLQHSTNQLAK_.3	#N/A	4.7965	5.0806	3.7178	3.4637	5.0113	5.4297	5.4710	5.8307	5.1744	3.9918	4.8811	4.3648	4.2464	5.2361	4.9005	3.5434	4.7444	5.3001	4.8520	4.7739
Stx12	_ISQATAQIK_.2	#N/A	4.4351	4.2775	4.2641	4.5848	3.4297	4.3256	4.7814	3.4282	4.3687	3.6961	3.8053	2.8582	5.3785	2.6194	3.4489	4.0412	3.9659	4.5421	3.5159	4.2182
Stx12	_QLEADILDVNQIFK_.2	#N/A	4.3249	3.9208	3.1175	3.6074	3.0310	3.0644	2.8221	3.8773	3.2949	2.7123	3.4159	3.3464	4.1027	4.6054	4.0424	3.6870	4.3201	3.5131	3.1617	3.0715
Stx12	_LM[Oxidation (M)]NDFSSALNNFQVVQR_.2	#N/A	4.3819	3.0879	3.9185	2.8397	3.0880	2.5037	1.9698	3.1067	4.3047	4.1640	3.1783	2.7398	4.4128	3.9565	3.8228	3.7440	4.1048	2.2874	3.3437	1.8270
Stx12	_NPGPSGPQPR_.2	#N/A	3.4859	4.5169	3.6987	4.2035	4.3245	5.2070	3.4210	3.1863	3.3412	3.2308	4.3207	4.4064	3.5066	4.0093	3.4463	4.2832	3.7240	4.1092	3.7578	3.9763
Kcnh7	_HVS[Phospho (STY)]DPGLPGK_.2	Q9ER47_S1188_M1_Kcnh7	9.8759	10.1433	9.3192	9.5241	9.2645	9.3811	9.6469	9.7340	9.4686	9.6081	9.2029	9.1766	9.9605	9.2948	8.0527	9.1358	9.6408	9.5490	9.5317	9.1504
Kcnh7	_KQS[Phospho (STY)]LPQEDPDVVVIDSSK_.2	#N/A	7.1158	6.6367	6.5130	7.0176	5.6982	6.2855	6.5211	5.6334	6.3752	7.1210	6.7112	5.9880	6.4999	6.0941	4.2144	6.1010	6.5878	6.5197	6.2023	5.9439
Kcnh7	_SSLLGSTS[Phospho (STY)]DSNLNK_.2	Q9ER47_S319_M1_Kcnh7	4.5773	8.1260	7.6249	4.5078	3.2834	3.3242	4.4410	3.3021	2.9992	3.9687	3.3736	3.9688	3.1073	4.1519	3.7901	3.9394	7.3369	4.3501	3.1483	6.5438
Kcnh7	_SFS[Phospho (STY)]PSSQC[Carbamidomethyl (C)]PEFLDLEK_.2	Q9ER47_S1103_M1_Kcnh7	3.4765	4.5264	4.0127	4.2129	4.3150	3.6699	3.4115	3.1768	3.3317	3.2402	4.3112	4.4158	3.4972	3.9998	3.4369	3.8213	3.7145	4.1187	3.7672	3.9668
Kcnh7	_RLS[Phospho (STY)]FES[Phospho (STY)]EGEKDFSK_.3	#N/A	3.8974	4.2927	3.6410	3.8019	3.9432	4.0362	3.6565	3.8481	4.3844	4.1037	4.0900	3.3454	3.8237	4.0983	3.2337	3.4496	4.0060	3.6924	3.9915	3.8374
Kcnh7	_LNTAS[Phospho (STY)]EDNLTSLLK_.2	Q9ER47_S1135_M1_Kcnh7	3.6332	3.2291	3.1698	4.4165	3.8402	3.8735	3.2079	4.4741	3.1281	3.4438	4.1076	3.4120	3.2936	3.7962	3.2333	2.9954	3.5109	4.3222	3.9708	3.7632
Kcnh7	_SQSVNDS[Phospho (STY)]EGDTGK_.2	Q9ER47_S878_M1_Kcnh7	5.3086	3.3630	3.6753	4.5504	3.9775	4.0074	3.0741	4.3402	2.9943	3.5777	3.9737	3.2524	3.1597	3.6624	3.0994	3.4839	3.3770	4.4561	4.1047	3.6294
Osbpl5	_ETMSGQTTFLGS[Phospho (STY)]PDSR_.2	Q9ER64_S746_M1_Osbpl5	3.1115	4.2082	4.8824	3.8948	3.3184	3.3518	3.7297	3.4950	3.6499	4.4229	3.1285	4.0976	3.8153	5.4543	3.7551	3.9744	4.0327	3.8005	3.4491	4.2850
Osbpl5	_ENAEDS[Phospho (STY)]DAETQDHSR_.2	Q9ER64_S311_M1_Osbpl5	3.6828	4.3200	4.2191	4.0065	3.0206	3.4635	3.0197	3.3832	3.5781	4.5347	3.0168	4.2094	3.7036	3.9888	4.0977	4.0277	3.9209	3.9123	3.5608	4.1732
Wtap	_EGS[Phospho (STY)]TPEDDFPSSSGNGNK_.2	Q9ER69_S297_M1_Wtap	4.2590	4.5609	6.3986	3.3252	2.9651	2.9984	5.7417	3.8483	4.0032	4.0696	3.4818	4.2870	4.1686	6.7898	4.1084	3.6211	4.3860	3.4472	3.0958	3.1375
Cars1	_VQPQWS[Phospho (STY)]PPAGTEPC[Carbamidomethyl (C)]R_.2	Q9ER72_S102_M1_Cars1	9.4048	9.8569	3.5226	7.3925	4.1929	8.8433	4.3560	9.4077	4.2762	9.3240	3.7549	3.5418	9.5008	9.8795	2.8806	8.5932	9.5845	9.3153	9.2885	9.1773
Cars1	_ALQEGEGDLSIS[Phospho (STY)]ADR_.2	Q9ER72_S390_M1_Cars1	3.4866	3.9669	4.3897	4.2320	4.3251	3.0484	3.2160	3.5779	3.2750	3.6929	3.6494	3.6930	3.3831	4.4277	2.7932	3.8314	3.0751	4.0743	4.3734	4.1050
Tfip11	_TTQSLQDFPVADS[Phospho (STY)]EEEAEEEFQK_.3	Q9ERA6_S211_M1_Tfip11	4.1417	3.8611	3.1772	3.5477	3.4795	4.4252	4.0616	4.4236	4.1207	4.3481	2.9942	3.7505	4.2288	3.7725	3.6388	2.9858	3.9208	6.1064	3.5277	3.4498
Tfip11	_EEATYGVWAERDS[Phospho (STY)]DEERPSFGGK_.3	Q9ERA6_S60_M1_Tfip11	4.0895	3.9046	3.9562	3.8770	3.5141	3.5474	3.6012	4.1488	3.5214	3.9001	3.7824	3.7239	3.6868	4.1895	3.6265	3.4516	3.9041	4.3790	3.0403	4.1565
Tfip11	_GAAEEADS[Phospho (STY)]EDS[Phospho (STY)]DAEEKPVKQEDFPK_.3	Q9ERA6_S96_M2_Tfip11	3.4435	2.7653	4.4809	4.1407	3.6504	1.5348	1.6723	3.6692	2.9264	3.6016	3.7407	3.6017	3.4744	3.6872	2.8844	2.4059	3.1664	3.9830	4.2821	2.3367
Ralgps2	_EDLAGPDIGAS[Phospho (STY)]PQGGR_.2	Q9ERD6_S308_M1_Ralgps2	6.8117	6.0091	6.9519	6.1429	3.9456	6.1643	5.9867	5.9344	3.0227	7.3526	6.3386	6.2222	3.1882	6.4321	3.1279	6.1052	6.4048	4.4277	6.3651	5.8716
Tubb3	_YLTVATVFR_.2	#N/A	10.9651	11.4295	11.4531	11.3591	11.1332	11.1690	11.2408	11.0593	11.0784	11.5244	10.3435	10.6260	10.8861	10.7290	10.3605	10.5226	10.3034	10.6023	10.5880	10.5707
Tubb3	_VREEYPDR_.2	#N/A	9.3424	9.4964	9.6995	9.4474	9.4632	9.5475	9.6766	9.4818	9.6376	9.6300	9.0431	8.7669	8.6761	8.6617	9.1877	8.7364	8.6596	8.9332	8.7715	8.6776
Tubb3	_M[Oxidation (M)]SSTFIGNSTAIQELFKR_.3	#N/A	6.8942	7.3487	6.4607	7.0916	6.6517	6.8171	6.8515	7.7085	7.0525	7.8906	6.3583	6.7987	8.2216	6.7040	6.0950	7.0813	6.0475	6.3716	6.7202	7.3332
Tubb3	_EVDEQM[Oxidation (M)]LAIQSK_.2	#N/A	10.3566	10.4708	10.7157	10.3863	10.2043	10.4084	10.3548	10.5238	10.4881	10.6115	9.5651	9.7213	9.8353	9.9031	9.4616	9.8445	9.5268	9.5338	9.6722	9.8469
Tubb3	_LHFFM[Oxidation (M)]PGFAPLTAR_.3	#N/A	9.1333	9.6962	8.8887	9.0853	9.1819	9.0381	8.9019	9.1840	8.6379	9.2065	8.4655	8.6269	8.3565	7.9511	8.0850	8.3635	8.1993	8.5459	8.3978	8.1037
Tubb3	_MSSTFIGNSTAIQELFK_.2	#N/A	11.1576	10.8506	10.9622	10.9012	10.5150	10.8160	10.5312	11.0315	10.8361	11.6765	9.9466	10.0613	11.5864	10.7587	10.2401	10.7178	10.5380	10.2445	10.3600	10.9824
Tubb3	_ISVYYNEASSHK_.2	#N/A	7.2401	8.2376	7.9437	7.7728	7.0912	7.8973	8.0211	7.4132	7.8322	7.7462	6.7883	6.9800	6.6796	7.4923	6.9374	7.1220	6.6468	7.2431	6.9325	7.1842
Tubb3	_LATPTYGDLNHLVSATM[Oxidation (M)]SGVTTSLR_.3	#N/A	6.5655	6.7944	6.3220	6.4997	6.1429	6.5274	5.8439	6.1721	6.1338	6.9180	5.5512	5.6654	6.0619	5.9697	5.8079	6.2043	5.3040	5.9611	5.8149	6.2091
Tubb3	_ISVYYNEASS[Phospho (STY)]HKYVPR_.3	Q9ERD7_S56_M1_Tubb3	4.0666	3.9362	4.6029	3.6228	3.4044	3.0797	4.0017	3.7670	3.9219	4.1509	3.4005	3.8256	4.0874	4.5900	3.7139	4.4115	4.3047	3.5285	3.1771	3.0562
Tubb3	_VAVC[Carbamidomethyl (C)]DIPPR_.2	#N/A	3.5751	9.7230	10.0837	4.1143	2.9128	10.1521	11.1233	3.2755	9.8877	9.0936	2.9090	4.3171	3.5958	4.0985	9.4809	3.9200	3.8132	4.0200	8.9626	4.0655
Tubb3	_FWEVISDEHGIDPSGNYVGDSDLQLER_.3	#N/A	3.6032	3.7158	3.1398	3.9810	3.8102	4.2982	3.2379	3.8289	3.5261	3.4138	3.9004	3.4420	3.6341	3.1779	3.2633	2.9654	3.3262	3.8233	4.1223	4.3560
Tubb3	ethyl (C)]ENC[Carbamidomethyl (C)]DC[Carbamidomethyl (C)]LQGFQLTHSLGGGTGSGM[Oxidat	#N/A	4.1683	4.2802	4.1825	3.5489	3.1561	3.2594	3.8254	3.3571	3.7456	4.3305	3.2242	4.0850	3.9110	4.4137	3.8508	3.8820	4.1158	3.7081	3.3567	4.3807
Tlnrd1	_[Acetyl (Protein N-term)]ASGS[Phospho (STY)]AGKPTGEAASPAPGSAVGGASSQPR_.3	Q9ERE8_S5_M1_Tlnrd1	4.4675	4.3525	4.0040	3.7499	3.1736	3.2069	3.8745	3.6399	3.7948	4.2781	3.2734	4.0786	3.9602	4.4629	3.8999	3.8296	4.1775	3.6556	3.3042	4.4299
Mapk8ip2	_MISEGSS[Phospho (STY)]PIR_.2	Q9ERE9_S370_M1_Mapk8ip2	3.8898	7.2785	8.2537	3.6944	4.0968	6.4167	4.4522	4.1155	3.8127	3.1553	4.1870	5.7635	3.9207	3.4645	2.9767	3.2520	6.1393	3.5367	3.8357	3.1418
Mapk8ip2	_SSQELSS[Phospho (STY)]PGSDSEDAGGAR_.2	Q9ERE9_S254_M1_Mapk8ip2	4.4609	3.5419	3.4964	3.2285	3.7986	4.0227	3.7424	4.1613	3.8015	3.1664	4.1759	3.4313	3.9096	3.4534	3.3196	3.3050	3.1981	3.5478	3.8469	3.1306
Wdr61	_ENIETVVTGSLDDLVK_.2	#N/A	3.6984	3.6206	4.0408	4.4817	3.9054	4.1325	3.1427	4.4089	3.0629	4.2018	4.0424	3.3468	3.2284	4.6029	3.7941	3.0606	3.4457	4.3874	3.6259	4.4710
Lima1	_ELS[Phospho (STY)]VEEQIK_.2	Q9ERG0_S735_M1_Lima1	12.5624	10.7698	4.1523	4.0734	11.1569	3.1778	10.6214	11.7710	3.1456	11.4109	12.1265	10.0047	3.2537	8.7789	4.1646	8.2091	11.1534	10.1461	3.6277	3.9756
Lima1	_TSS[Phospho (STY)]LPESSPSK_.2	Q9ERG0_S367_M1_Lima1	3.2605	7.6700	4.6157	4.0059	4.0990	6.9700	3.4420	3.8040	3.5011	7.4339	6.6812	3.4669	3.6092	7.6004	3.0192	3.6054	3.3012	3.8482	4.1473	4.3311
Strn3	_SLLGLSNSEPNGS[Phospho (STY)]VEAK_.2	Q9ERG2_S214_M1_Strn3	7.6652	8.4799	8.3774	8.3715	3.5532	7.8834	7.7646	7.4071	7.6807	8.4883	7.8293	7.9264	8.3192	8.4825	7.4971	8.1882	8.4037	7.6936	8.1171	8.1593
Strn3	_NLEQILNGGES[Phospho (STY)]PKQK_.2	#N/A	4.2727	8.6014	8.5986	4.1669	3.7871	8.3782	8.2221	3.8059	7.7746	4.0833	3.5470	3.6278	4.4913	3.7988	3.4492	3.6348	9.1517	8.5466	8.7677	3.5962
Strn3	_VVSHPTLPVTITAHEDR_.4	#N/A	3.9746	4.0282	4.5109	3.7147	3.3124	3.1717	3.9097	3.6750	3.8299	2.6268	3.3086	3.9176	3.9954	2.6384	3.9351	4.3195	4.2127	3.6205	3.2690	2.9642
Strn3	_NLEQILNGGES[Phospho (STY)]PK_.2	#N/A	4.3949	4.4251	3.9314	3.1894	3.1010	3.1343	3.9471	3.1197	3.8673	4.2055	4.1258	4.1512	4.0328	8.9436	3.9725	3.7570	4.1178	3.5830	3.2316	3.0016
Cse1l	_LLQTDDEEEAGLLELLK_.2	#N/A	3.0921	4.2270	4.1295	4.4922	3.8154	3.3085	4.4566	3.3177	3.0149	3.9530	4.3708	0.3126	3.6875	4.1676	2.3515	3.9550	4.3158	3.3986	3.1327	3.2569
Cse1l	_IVEDEPNKIC[Carbamidomethyl (C)]EADR_.3	#N/A	3.5981	3.3693	2.7407	4.5567	3.9712	4.1812	3.3713	4.3339	2.9879	3.5840	3.3013	2.6707	3.1534	3.6560	3.0931	3.4776	3.3707	4.4624	4.1110	3.6230
Znf704	_VGESDC[Carbamidomethyl (C)]S[Phospho (STY)]DGEEDFYYTEIK_.2	Q9ERQ3_S381_M1_Znf704	4.1408	3.1783	3.6774	3.4434	4.3477	3.7606	4.2012	4.3665	4.0636	3.9514	2.9372	4.4052	4.1717	3.7155	4.2266	3.5029	3.8637	3.2857	3.5848	3.3927
Ndel1	_S[Phospho (STY)]APSS[Phospho (STY)]PTLDC[Carbamidomethyl (C)]EK_.2	#N/A	11.6967	12.0120	12.3277	12.1043	11.5310	11.7383	11.3659	12.0959	11.5197	12.0708	11.8477	12.0399	12.0056	12.2430	12.1060	11.9777	11.9543	11.7889	11.8994	12.1275
Ndel1	_GTENSFPS[Phospho (STY)]PK_.2	Q9ERR1_S231_M1_Ndel1	9.4490	9.8131	9.5969	9.6326	9.4557	9.3447	9.3243	9.6549	9.2939	9.7987	9.4393	9.8536	10.2571	10.2068	9.2728	9.7135	9.8430	9.4810	9.3873	9.6278
Ndel1	_KSAPS[Phospho (STY)]SPTLDC[Carbamidomethyl (C)]EK_.2	Q9ERR1_S197_M1_Ndel1	4.4114	4.4213	3.9480	4.0567	3.1175	3.1951	6.0192	4.0073	4.2477	4.2220	3.4062	4.1474	6.4327	3.9861	3.9688	3.7735	5.6889	3.8990	3.3270	3.6633
Ndel1	_M[Oxidation (M)]DSAVQASLS[Phospho (STY)]LPAT[Phospho (STY)]PVGK_.2	#N/A	7.4238	8.8761	8.4408	6.8832	3.5730	7.6927	8.2920	8.1530	7.5533	7.1933	6.8220	5.8210	7.9055	8.0640	8.2691	7.2158	7.3558	8.4450	8.5573	7.5424
Ndel1	_ISALNIVGDLLR_.2	#N/A	4.3043	4.3074	4.0307	4.3005	1.8291	3.8446	3.9453	4.1946	4.1004	3.1590	3.6361	4.7858	3.9170	3.4608	3.5776	3.2482	3.6978	4.0178	4.6217	3.1381
Ndufa13	_LLIEDLEAR_.2	#N/A	8.9156	8.8042	9.1146	8.9035	8.7728	8.8113	9.0272	8.7089	8.7928	8.8507	8.7840	8.7026	8.9390	8.6554	8.2020	8.5842	8.9971	9.0169	8.6833	8.7306
Ndufa13	_VGESVFHTTR_.2	#N/A	7.5779	7.2908	7.3360	6.8296	6.8223	7.1460	7.3476	7.1779	6.9821	6.3915	6.6836	6.8379	6.8517	7.0011	6.9147	6.8446	6.9035	7.0094	6.5444	6.6719
Ndufa13	_IALM[Oxidation (M)]PLFQAEK_.2	#N/A	6.6479	6.4652	5.9810	6.2046	6.2806	5.9362	6.5081	6.3114	5.7381	5.8771	6.5788	6.1724	6.0341	5.9038	5.5924	5.4972	6.0896	6.3988	6.0807	5.5429
Ndufa13	_ENLEEEAIIM[Oxidation (M)]K_.2	#N/A	5.9319	5.7398	6.1990	5.5395	4.1345	4.7009	6.4564	5.0879	5.7638	3.7381	5.4663	5.8284	3.9584	5.0205	5.6708	6.2998	3.6505	6.6763	4.9631	5.7854
Plekha3	_SAS[Phospho (STY)]HPGSC[Carbamidomethyl (C)]SSER_.2	Q9ERS4_S211_M1_Plekha3	4.2030	5.5435	3.2385	5.2451	3.5407	4.4442	4.1381	3.9034	4.0583	4.0145	3.5369	3.6892	4.2237	4.8142	4.1634	3.5660	4.4411	4.5304	3.0407	3.1925
Ppp1r1a	_STLSM[Oxidation (M)]S[Phospho (STY)]PR_.2	#N/A	11.0154	11.2646	11.4696	11.5827	10.6584	10.3848	10.6312	10.5337	10.5005	11.4843	10.5131	10.8129	11.0385	11.1645	10.6296	10.7597	10.5208	10.5545	10.5995	10.7444
Ppp1r1a	_[Acetyl (Protein N-term)]MEPDNS[Phospho (STY)]PRK_.2	#N/A	4.4849	6.9724	7.0514	7.8257	5.4693	5.7918	6.4365	3.2098	3.7773	5.3468	6.6682	4.6456	4.5158	8.3338	5.2974	3.8470	4.9417	4.4377	3.2407	5.6553
Ppp1r1a	_[Acetyl (Protein N-term)]MEPDNS[Phospho (STY)]PR_.2	#N/A	3.5783	5.7753	6.7002	7.0211	4.7326	4.8603	5.6786	3.6413	4.0398	3.7847	5.9596	3.8243	4.1479	7.0189	5.2932	3.9232	3.7065	4.1661	4.1531	4.7764
Ppp1r1a	_TEDSSAHMLPLDSQGASLV_.2	#N/A	4.4942	4.3257	4.0595	4.5909	3.2003	3.2336	4.3703	3.2190	3.7680	4.3048	3.2467	4.0519	4.5251	4.0688	3.1524	3.8563	4.2171	4.4332	3.2314	3.7461
Ppp1r1a	_EPDNS[Phospho (STY)]PR_.2	#N/A	3.5779	3.7412	4.6153	4.0063	3.7849	2.1109	3.2632	3.8036	3.5007	3.3885	3.8751	3.4673	3.6088	3.1526	3.2886	2.9400	3.3008	3.8486	4.1477	4.3307
Ranbp2	_SHETDGGS[Phospho (STY)]AHGDEEDDGPHFEPVVPLPDKIEVK_.5	Q9ERU9_S1154_M1_Ranbp2	4.3287	3.9246	4.6145	3.6112	3.0348	3.0682	4.0133	3.7786	3.9335	4.1393	3.4121	3.8140	4.0989	4.6016	4.0387	3.6908	4.3163	3.5169	5.5853	3.0678
Ranbp2	_YIASVQGSAPS[Phospho (STY)]PR_.2	Q9ERU9_S21_M1_Ranbp2	7.3080	7.0502	6.7891	6.4334	5.9262	6.4885	6.3067	3.9623	5.4320	6.8562	5.8942	5.9124	5.8722	6.6703	4.2224	5.5608	5.9446	3.3332	5.5914	6.4761
Ranbp2	_HSTPS[Phospho (STY)]PTKYSLS[Phospho (STY)]PSK_.2	Q9ERU9_S781_M2_Ranbp2	3.2829	4.0362	4.3203	4.3014	3.4898	3.1177	7.2126	3.5085	3.2057	3.0935	3.5801	7.0092	3.3137	4.3583	3.5837	4.1458	4.5066	4.1437	2.9419	4.0356
Ranbp2	_SALS[Phospho (STY)]PSKSPAK_.2	Q9ERU9_S2113_M1_Ranbp2	3.8570	6.0720	5.8346	6.0807	5.4756	3.7399	3.7459	4.4999	3.7568	5.5204	3.7089	5.2682	3.8844	3.8353	3.7674	5.2510	4.4831	5.8137	5.6168	3.6665
Ranbp2	_LNSNNSAS[Phospho (STY)]PHR_.2	Q9ERU9_S837_M1_Ranbp2	3.9115	3.5074	3.5309	3.1940	4.1184	4.1518	4.4305	4.9905	4.3507	3.7221	3.8293	3.3968	4.5162	4.2094	2.9550	3.2736	3.2327	4.7507	4.2491	3.4850
Ranbp2	_HSTPS[Phospho (STY)]PTK_.2	Q9ERU9_S781_M1_Ranbp2	4.4879	3.5149	3.5234	3.7325	3.8256	4.0497	3.7154	4.0774	3.7745	3.1934	4.1489	3.4043	3.8826	3.4263	3.2926	3.3320	3.5746	3.5748	3.8739	3.1036
Ranbp2	_NRPGYVS[Phospho (STY)]EEEEDDEDYEM[Oxidation (M)]AVK_.3	Q9ERU9_S2505_M1_Ranbp2	3.9255	3.3936	3.4621	3.6587	4.1324	3.9759	4.4165	4.1512	4.3367	4.0719	3.8154	3.1197	3.9564	3.5001	2.9410	3.2876	3.6484	3.5010	3.8001	3.1774
Mkrn2	_KQLS[Phospho (STY)]SEGTVR_.2	Q9ERV1_S365_M1_Mkrn2	3.7431	3.9967	4.2794	3.9463	3.0808	3.3049	4.4603	3.4434	3.0185	3.9494	3.3929	4.1492	3.1266	4.1712	4.0375	4.0880	4.2817	4.7620	4.6558	4.7877
Mkrn2	_VNNPGGC[Carbamidomethyl (C)]SDPQTS[Phospho (STY)]PEMKPHSYLDAIR_.4	Q9ERV1_S139_M1_Mkrn2	3.7919	4.2722	4.0843	4.5373	3.1296	3.3537	4.4115	3.2726	2.9697	3.9982	3.3441	3.9983	4.5786	5.9943	3.9887	4.1368	4.2706	4.3796	3.1779	3.7997
Chrm3	_SC[Carbamidomethyl (C)]SSY[Phospho (STY)]ELQQQGTKR_.2	Q9ERZ3_Y292_M1_Chrm3	4.4380	3.5648	3.4734	3.2514	3.7757	3.9998	3.7654	4.1383	3.8245	3.6444	4.1988	3.4543	5.2011	3.4763	3.3426	3.2820	4.6339	3.5249	3.8239	3.1536
Chrm3	_SC[Carbamidomethyl (C)]S[Phospho (STY)]SYELQQQGTK_.2	Q9ERZ3_S290_M1_Chrm3	3.2759	3.6940	4.0830	4.8907	3.8346	3.6961	3.4071	4.0229	4.6535	4.7485	3.6565	4.9629	3.4927	3.9954	3.4324	3.9015	5.4845	4.1448	3.7934	3.9624
Chrm2	_KEPVANQDPVS[Phospho (STY)]PSLVQGR_.3	#N/A	3.9958	8.1307	7.3327	3.6734	6.6606	4.2203	7.2831	3.9389	6.9180	7.1047	3.7966	6.6153	7.8906	7.0243	3.7475	6.6173	6.8994	7.4665	7.2052	5.9058
Chrm2	_EPVANQDPVS[Phospho (STY)]PSLVQGR_.2	#N/A	4.1711	8.8019	8.6227	3.4131	7.9777	8.2950	4.0348	8.6907	4.0939	4.3749	2.9674	8.2975	4.2020	3.7457	3.6120	8.1358	3.8940	8.5932	3.5545	3.4230
Chrm2	_EKKEPVANQDPVS[Phospho (STY)]PSLVQGR_.3	#N/A	8.8437	9.0386	3.7596	4.4661	4.0619	3.9231	3.1584	4.4245	3.0786	8.2178	4.0581	3.1681	3.2440	3.7467	3.1837	3.5682	3.4614	4.3718	4.0204	3.7137
Ube4b	_LAGGQTSQPTTPLTS[Phospho (STY)]PQR_.2	Q9ES00_S31_M1_Ube4b	3.6232	3.6959	3.1598	3.9610	3.8301	3.8635	6.8690	6.7536	3.1382	3.4338	6.2478	3.4220	3.3036	3.8063	3.2433	2.9853	3.3461	6.5090	6.5529	3.7733
Ube4b	_TVEDLKNNESQWKDS[Phospho (STY)]PLATR_.3	#N/A	3.5242	6.1092	4.0605	5.9443	2.8619	5.0917	5.1110	3.2246	5.4461	3.7305	4.2120	4.4357	3.5449	4.0475	4.2564	4.3296	3.7622	5.4343	3.7195	4.1927
Ube4b	_HLLNS[Phospho (STY)]PTDPFNR_.2	Q9ES00_S1136_M1_Ube4b	6.4569	6.3490	4.3235	4.2982	3.4930	5.5172	3.5551	3.3204	3.4753	3.0966	2.9540	3.7592	3.6408	6.6392	3.5805	4.1490	3.8581	3.9751	3.6236	4.1104
Ube4b	_SQSSEGVSSLSSS[Phospho (STY)]PSNSLETQSQSLSR_.3	Q9ES00_S88_M1_Ube4b	3.8804	3.5128	4.9281	4.7507	4.0874	4.1207	4.9743	4.1061	4.8269	3.6910	3.9346	3.2390	5.1390	3.8910	3.6456	4.6661	3.6033	4.8875	4.1841	3.6394
Ube4b	_SQS[Phospho (STY)]M[Oxidation (M)]DIDGVSC[Carbamidomethyl (C)]EK_.2	#N/A	7.4658	7.4668	7.0762	7.8787	6.7550	6.2883	7.1812	7.0637	6.6143	7.3842	6.8161	6.2202	7.1392	7.0044	5.4734	6.5657	7.0188	6.3663	6.4625	6.7058
Ube4b	_NNESQWKDS[Phospho (STY)]PLATR_.2	#N/A	5.9726	5.6590	5.9464	5.5628	4.9191	5.1697	3.9489	5.8398	5.3110	3.8558	3.4934	3.9630	3.2271	4.3194	4.5074	4.1234	5.9069	5.4512	5.6649	4.1439
Arhgef7	_KPS[Phospho (STY)]DEEFAVR_.2	#N/A	11.7551	11.5554	11.3567	11.5245	11.1445	11.1184	10.9919	11.3958	10.8390	11.8234	11.3586	11.2158	11.7667	11.8224	10.1464	11.2931	11.5387	11.2091	11.1997	11.2766
Arhgef7	_EDLSKS[Phospho (STY)]PK_.2	Q9ES28_S655_M1_Arhgef7	3.7409	9.3274	9.0163	9.1092	8.8420	8.5037	8.9343	8.8577	8.4445	9.6067	8.3834	9.0277	9.1589	9.3011	7.5927	8.8979	8.5893	8.9563	8.5894	8.6076
Arhgef7	_EIKPSEKPVS[Phospho (STY)]PK_.2	#N/A	10.9412	11.0242	10.8634	11.1025	10.7010	10.6699	10.6920	11.0000	10.6342	10.9238	10.5499	10.6885	11.3066	11.1799	10.3194	10.6777	10.9662	10.8657	10.7945	10.6433
Arhgef7	_M[Oxidation (M)]S[Phospho (STY)]GFIYQGK_.2	#N/A	9.5915	9.7513	9.8086	10.1518	9.8625	9.7960	9.0753	9.7624	9.8408	9.9578	9.7633	8.9869	10.3606	9.6804	9.5276	9.9093	10.0009	9.3576	9.7585	9.5734
Arhgef7	_SFDS[Phospho (STY)]LGSQSSHSR_.2	Q9ES28_S135_M1_Arhgef7	4.2776	6.9096	6.2403	6.7803	5.6655	3.8394	5.8327	3.9779	4.5860	7.0240	5.3166	3.6147	6.1157	6.1565	3.3628	6.0012	4.4597	5.6067	4.6685	4.5328

Arhgef7	_LETFDANDLYQGQNFNK_.2	#N/A	5.1027	4.8384	4.6618	4.8721	4.0341	3.9652	4.5355	4.2770	4.0856	3.4792	4.7915	3.9384	5.5303	4.6281	4.2185	3.6177	3.8011	4.7935	4.6374	4.7483
Arhgef7	_IKS[Phospho (STY)]FDSLGSQSSHSR_.3	Q9ES28_S132_M1_Arhgef7	6.2771	6.9602	6.6304	6.5520	3.7019	4.9832	6.0845	5.1581	4.6880	6.1630	4.8649	5.5506	5.0356	4.3861	3.8443	4.5512	3.9846	5.5546	3.7674	5.1708
Arhgef7	_S[Phospho (STY)]LDM[Oxidation (M)]TDNTNSQLVVR_.2	#N/A	8.2996	8.8348	8.9621	8.3504	6.4522	8.0692	8.0441	7.2436	8.0175	8.4794	6.9562	7.9838	6.5482	8.5824	7.0813	8.2971	7.8735	8.2578	8.0194	8.6176
Arhgef7	_KPS[Phospho (STY)]DEEFAVRK_.3	#N/A	8.3896	8.5543	7.2562	8.5617	8.1372	7.9109	8.3315	8.0534	7.8370	8.8414	8.0913	8.4660	9.1954	8.7944	7.4712	8.5758	8.2335	8.3257	8.3460	8.4629
Arhgef7	_S[Phospho (STY)]TAALEEDAQILK_.2	Q9ES28_S682_M1_Arhgef7	3.7191	4.2838	4.2553	3.9703	3.0568	3.4273	4.7773	3.4194	3.5743	4.4985	3.0530	4.1732	3.7398	4.2424	4.0615	4.0640	3.9571	3.8761	3.5246	4.2094
Arhgef7	_SGTLKS[Phospho (STY)]PPK_.2	Q9ES28_S236_M1_Arhgef7	3.2628	5.9880	3.7991	4.4266	4.1013	3.8836	3.4398	4.4640	3.1180	3.4691	4.0975	2.2931	3.2835	3.7861	3.0169	3.6077	3.5008	4.3323	3.9809	3.7531
Rtn3	_SEEGHPFK_.2	#N/A	7.6477	7.3822	6.9181	7.0268	6.8610	7.4088	7.2839	7.4505	7.1490	7.2100	7.2174	7.1886	6.8321	6.1909	7.0802	6.9746	7.0272	7.2319	6.7845	6.3408
Rtn3	tein N-term)]AESSAATQSPSVSSSSSGAEPSALGGGGGS[Phospho (STY)]PGAC[Carbamidomethyl (	Q9ES97_S31_M1_Rtn3	10.9359	11.1963	10.6686	10.2203	9.0020	10.3167	10.9153	11.0912	9.9564	10.6992	9.5341	10.5725	10.9396	11.1559	8.9861	9.8487	10.4944	10.6515	10.4157	9.6864
Rtn3	_SALDFGQSK_.2	#N/A	5.6123	5.0594	4.2821	4.3119	5.2937	5.7154	4.9961	5.4357	5.5155	3.5809	3.7614	5.5303	3.4951	5.5367	5.6033	5.4997	5.4115	5.8942	5.3765	4.9942
Rtn3	_ETTGGAPTM[Oxidation (M)]S[Phospho (STY)]PDLEQEQLTIR_.2	#N/A	9.6729	9.4809	9.3251	9.3573	8.3464	9.2696	9.3198	9.2310	9.0371	8.9609	9.1357	9.1816	9.7809	9.6749	8.0829	9.2925	9.7998	9.6024	9.2945	9.2776
Rtn3	_KLDS[Phospho (STY)]PQGTNK_.2	#N/A	6.8824	6.6765	6.5832	4.4988	6.0844	5.7486	6.7145	4.3918	6.1377	7.3179	4.0253	3.3297	3.2113	5.7598	4.5482	6.4363	3.4286	4.4045	5.8954	3.6810
Rtn3	_TQIDHYVGIAR_.2	#N/A	7.2229	7.2075	7.2512	6.5973	6.6978	6.8715	6.8727	7.2586	6.7299	6.4662	5.9282	6.5555	6.3820	6.7497	6.1725	6.2998	7.0195	6.5599	6.3602	6.3457
Rtn3	_GIVDKEEGDVLEAVLEK_.3	#N/A	3.6455	3.2413	3.7969	4.4288	3.8524	3.8858	3.1504	4.4618	4.3680	4.8548	4.6567	3.1308	4.8844	5.7006	3.2211	4.1021	4.6615	5.0186	4.4187	4.4983
Rtn3	_QTFAPQSGPQSSSDILEHTDVK_.3	#N/A	4.3671	4.7571	3.7164	4.5093	4.0186	4.1260	4.3890	4.3813	4.1009	3.3864	4.1830	3.2113	3.2008	3.7034	3.0996	3.5449	3.4181	4.4150	3.7823	4.2612
Rtn3	_KLDS[Phospho (STY)]PQGTNKDR_.2	#N/A	4.3923	3.7842	3.2541	3.3788	2.8944	4.4286	4.1537	3.9190	3.6926	3.9989	3.5526	4.3578	4.2394	4.8941	4.1791	3.5504	4.9658	5.4790	4.4881	3.2082
Rtn3	_DQTKSIVEK_.2	#N/A	3.5590	3.3212	4.5965	4.0252	3.7660	3.7993	3.2821	3.0474	3.2023	3.3696	2.2509	3.4862	3.3678	3.8704	3.3075	4.4220	3.8876	2.8024	3.8966	0.6863
Rtn3	_Y[Phospho (STY)]VEDTDGSSPEDLM[Oxidation (M)]AVLTGAEEK_.3	Q9ES97_Y589_M1_Rtn3	4.3295	3.6778	3.3650	3.3644	3.6672	4.2014	3.8784	4.0299	3.9833	3.6052	3.9406	3.5673	4.1227	3.3521	3.4556	3.1736	3.9650	5.7901	3.8169	3.3191
Rtn3	_S[Phospho (STY)]LEGEVASQVPNTLNEVTPEK_.2	Q9ES97_S529_M1_Rtn3	3.6159	4.3869	4.1522	4.0734	2.9537	3.1777	3.0866	3.3163	3.1457	3.8223	3.5200	4.2763	3.6367	4.1393	4.1646	3.9608	3.8540	5.2816	3.6277	4.1063
Rtn3	_SEMC[Carbamidomethyl (C)]ENSEQPQAQPET[Phospho (STY)]PTQK_.2	Q9ES97_T524_M1_Rtn3	4.3191	5.9094	3.3545	3.3703	3.6568	3.8809	5.9323	4.0194	3.9434	4.5254	4.3178	3.5732	4.0514	3.3416	3.4615	3.1631	4.5571	3.4060	4.4255	3.2725
Rtn3	_QFSHTTAALEEVSR_.3	#N/A	3.9737	4.5526	3.5103	3.6105	4.1806	3.9277	4.3683	4.1994	3.8965	3.7843	4.2709	3.0715	4.0046	3.5483	2.8928	3.3358	3.6966	3.4528	3.7519	3.3450
Nckipsd	_AAPTT[Phospho (STY)]PPPPVK_.2	#N/A	3.1432	3.3588	3.6795	4.5462	3.9817	4.0032	8.7254	4.3444	2.9984	3.5735	3.9779	3.2482	3.1639	3.6665	3.1365	3.4881	3.3812	4.4519	4.1005	3.6335
Nckipsd	_RAAPTT[Phospho (STY)]PPPPVK_.2	#N/A	3.9169	3.8899	9.1837	3.7740	3.2547	3.4787	3.6397	3.9623	3.6988	4.1233	3.2691	4.3197	3.8069	4.0474	3.9137	3.5651	3.8809	3.8079	3.3029	3.7247
Rb1cc1	_VSTSQAS[Phospho (STY)]PQSAASPR_.2	Q9ESK9_S646_M1_Rb1cc1	8.3390	7.9127	7.9073	7.9491	7.6796	7.8362	7.4295	8.1548	7.8870	7.7469	7.0022	7.7420	7.9108	7.8069	3.7821	7.0855	8.1928	7.8376	7.2533	7.4929
Rb1cc1	_STELVLS[Phospho (STY)]PDM[Oxidation (M)]PR_.2	#N/A	6.9819	7.0196	6.1618	6.1103	5.8838	6.8532	6.4313	6.3895	6.1265	6.3704	6.0656	6.1491	3.7829	5.8345	3.6915	6.5093	6.6929	6.5957	6.3087	6.3479
Rb1cc1	_S[Phospho (STY)]MEHVAPDPTGTER_.2	Q9ESK9_S261_M1_Rb1cc1	3.1064	5.1757	3.6427	4.5830	3.9450	4.0399	3.5961	4.3076	4.4625	3.6103	3.9412	3.2850	3.1271	3.6298	3.1733	3.4513	3.3445	2.9878	4.1373	3.5968
Trim2	_[Acetyl (Protein N-term)]ASEGASIPS[Phospho (STY)]PVVR_.2	Q9ESN6_S10_M1_Trim2	11.9436	12.1168	12.0903	11.9795	11.3951	11.4480	11.3381	11.5382	11.4757	12.2096	11.4916	11.6590	11.9590	12.0148	10.5578	11.6601	11.9176	11.3028	11.4788	11.6271
Trim2	_VKS[Phospho (STY)]PGSGHVK_.2	Q9ESN6_S440_M1_Trim2	10.3784	10.3910	10.4689	10.4770	10.1796	10.0016	10.1010	10.3808	10.0911	10.3523	9.9800	10.1234	10.4304	10.3496	9.1810	9.8492	10.2787	9.9607	9.6277	10.0797
Trim2	_VIRS[Phospho (STY)]ADVS[Phospho (STY)]PTTEGVKR_.3	#N/A	8.4800	8.8667	8.1556	9.0266	8.4121	8.3496	8.3087	8.6469	8.5389	8.8415	7.8154	8.5314	9.3127	8.6217	8.5833	8.4892	8.3551	7.9103	8.0652	8.3414
Trim2	_SADVS[Phospho (STY)]PTTEGVK_.2	#N/A	13.0964	12.9473	13.2664	13.3537	12.9453	12.8325	12.5445	13.1784	12.9304	13.1083	12.6216	12.7967	13.0801	13.2961	12.6136	12.8673	13.0888	12.6671	12.7154	12.9847
Trim2	_VLQSQLDTLLQGQESIK_.2	#N/A	4.1509	3.0829	4.9074	4.6713	4.7641	4.5397	4.1733	3.7195	4.4295	4.6881	4.4926	4.2408	3.5247	4.0500	4.3226	4.2562	3.2167	3.8452	4.4876	5.3279
Trim2	_EGDFTLSLR_.2	#N/A	4.5269	4.7304	4.0635	4.5582	3.9384	4.5577	4.0318	3.6741	3.7353	5.4544	4.8862	4.0002	3.9008	3.2872	3.7406	3.4443	4.1181	3.7150	4.4078	4.3304
Trim2	_LYDQHIR_.2	#N/A	3.9076	5.6052	4.5539	5.1817	5.0055	5.6589	4.9492	4.7434	4.9014	5.7829	4.7149	4.2820	3.9283	4.8666	4.7508	4.2525	4.1456	4.4845	3.3361	5.4657
Trim2	_VIRS[Phospho (STY)]ADVS[Phospho (STY)]PTTEGVK_.2	#N/A	9.7895	10.0846	9.5628	10.2283	6.5834	9.3967	9.5282	9.9742	9.8286	9.9445	8.6294	9.6182	9.7945	9.9514	9.4354	9.6601	9.8435	9.3230	9.4764	9.5810
Trim2	_ASIVDDIHSTFDELQK_.3	#N/A	3.6909	3.6282	3.2275	3.8933	3.8979	4.2796	5.0486	3.9166	4.3227	5.2271	3.9881	3.3543	3.7218	4.0405	3.6243	3.0530	3.4138	4.5768	4.1001	4.4437
Trim2	_LNELADQDFPLHPR_.2	#N/A	5.2962	3.8573	3.9434	4.4345	5.2006	4.4291	5.1966	3.6590	4.6280	4.2800	4.3803	4.8052	4.5275	5.2703	4.4440	3.8094	5.1042	3.7360	5.2769	4.7263
Trim2	_SADVS[Phospho (STY)]PTTEGVKR_.2	#N/A	10.3385	10.3708	10.3096	10.5578	10.0369	10.2071	10.0301	10.4055	10.0484	10.3538	9.8378	9.9388	10.2143	10.4461	9.8089	10.0708	10.2804	9.9014	9.8890	10.1714
Trim2	_TGNAYLTAELS[Phospho (STY)]TPDGSVADGEILDNK_.3	Q9ESN6_S370_M1_Trim2	3.7772	3.7509	6.5087	4.1597	3.5810	3.6167	3.5350	4.0790	3.4552	5.7542	3.7125	3.6404	3.6207	4.1233	3.5604	3.7489	3.8380	4.0654	3.7140	4.0903
Trim2	_RVKS[Phospho (STY)]PGS[Phospho (STY)]GHVK_.2	#N/A	4.5148	3.6374	3.3112	4.2115	4.6973	4.4998	4.2972	3.7541	4.0940	4.2381	4.4263	4.1901	4.0818	3.6060	2.9853	4.8813	4.9730	4.3815	4.7972	4.2635
Trim2	_RPASMYS[Phospho (STY)]TGK_.2	Q9ESN6_S459_M1_Trim2	3.1748	3.3272	5.6803	4.5146	4.0133	3.9716	3.5278	4.3760	3.0300	3.3811	4.0095	3.2167	3.1955	3.6981	3.1050	3.5197	3.4128	4.4204	4.0689	3.6651
Trim2	_VFNQEGEFMLK_.2	#N/A	4.3002	4.5197	3.8368	3.2840	3.0063	3.0396	4.0418	4.5259	3.9620	4.1108	3.4407	4.2459	4.1275	3.8748	4.0672	3.6623	4.0231	3.1263	3.4254	3.5868
Mapk8ip3	_LFSSSSS[Phospho (STY)]PPPAK_.2	#N/A	10.6968	10.4982	4.0148	4.2109	4.3171	3.6679	3.2240	10.2704	9.3200	10.5704	10.3238	4.4138	9.8471	4.0019	2.8012	8.7299	3.7166	4.1166	3.7652	3.9689
Mapk8ip3	_STHPS[Phospho (STY)]PEK_.2	Q9ESN9_S755_M1_Mapk8ip3	8.6268	8.2764	8.2038	7.6626	8.0170	8.0030	8.1023	8.2858	7.8703	8.0166	7.9872	7.5530	7.7683	7.6495	3.0099	6.2070	8.0465	3.0448	3.8690	7.8002
Mapk8ip3	_ANKTS[Phospho (STY)]PTSEGETRPGGIIHVYGDDSSDK_.4	Q9ESN9_S1192_M1_Mapk8ip3	3.3857	4.6171	3.9220	4.3037	4.2242	7.0634	3.3168	3.0861	3.3759	3.5920	3.7503	3.0057	3.4840	7.2773	2.8940	3.7306	3.6237	3.9734	3.8580	4.2059
Mapk8ip3	_QLS[Phospho (STY)]PNGGQEDTR_.2	#N/A	9.3017	8.8900	9.2076	8.8262	8.6702	8.6008	8.3541	8.7642	8.4984	8.8655	8.4779	8.2281	8.5558	8.3662	6.8965	8.2938	8.5044	8.3085	3.1937	8.2109
Mapk8ip3	_S[Phospho (STY)]PTAAGFSQR_.2	Q9ESN9_S603_M1_Mapk8ip3	3.9849	6.4222	8.1145	8.1979	4.1919	7.6558	7.7338	4.2106	7.4603	3.7955	7.3945	6.3867	4.4427	3.4445	2.8816	3.3470	7.1529	6.9774	4.3225	7.2361
Mapk8ip3	_TGSS[Phospho (STY)]PTQGIVNK_.2	Q9ESN9_S366_M1_Mapk8ip3	12.0793	11.8700	11.8911	11.9300	11.5333	11.4293	11.6028	12.0628	11.7330	11.7210	11.2412	11.3497	11.7937	11.5129	10.2893	11.1112	11.6514	11.4009	11.1745	11.2601
Mapk8ip3	_YKQLS[Phospho (STY)]PNGGQEDTR_.2	#N/A	7.0778	7.0486	6.5086	4.1461	3.1216	3.2472	6.7226	6.7629	6.5783	4.1206	5.6484	6.3151	6.3120	6.1716	4.8839	5.9932	4.2620	6.2499	5.8758	4.1682
Mapk8ip3	_AKET[Phospho (STY)]PEADATSSR_.2	Q9ESN9_T764_M1_Mapk8ip3	3.2763	4.0529	3.8126	4.0218	4.1149	4.3390	3.1405	3.7881	3.4853	3.4827	3.8596	3.1151	3.5933	3.1371	3.0034	3.6212	3.2854	3.8304	4.1631	4.3152
Mapk8ip3	_LFSSSSS[Phospho (STY)]PPPAKR_.2	#N/A	4.4628	9.9207	3.4983	3.2266	3.8005	4.0246	9.4728	4.1632	3.7996	3.1683	4.1740	3.4294	3.9077	3.4514	3.3177	3.3069	3.2000	3.5497	3.8488	3.1287
Mapk8ip3	_EYNALHQR_.2	#N/A	4.5736	3.4292	4.8835	3.1157	4.8930	4.6425	3.6063	4.2740	4.4289	3.8634	3.4594	4.4950	4.5944	3.5962	4.0231	3.4177	3.3109	3.8995	3.8591	3.5632
Mapk8ip3	_LMELQEAVR_.2	#N/A	4.2830	4.5369	3.8196	3.3012	3.3944	3.6184	4.1467	4.5087	4.2058	4.2630	3.0793	4.2631	4.3139	3.8576	3.7239	4.4015	4.0059	3.1435	3.4426	3.5349
Eif4enif1	_SS[Phospho (STY)]PVESLKK_.2	Q9EST3_S77_M1_Eif4enif1	8.1866	4.1033	3.8254	8.4463	4.1277	8.3619	3.4227	8.4647	9.1870	8.2186	7.9905	8.4124	8.9106	8.7686	8.3202	8.2160	8.4584	4.2237	8.3419	8.1689
Eif4enif1	_SSS[Phospho (STY)]PVGLAK_.2	Q9EST3_S949_M1_Eif4enif1	3.4292	7.3063	4.4666	7.6000	6.4030	7.0060	3.4120	3.6548	6.8769	3.2398	3.7264	3.6160	3.4600	7.5171	6.9876	6.9528	5.9706	6.8022	7.1932	4.1819
Eif4enif1	_SSS[Phospho (STY)]LGSTPHEELER_.2	Q9EST3_S352_M1_Eif4enif1	7.7742	7.8815	3.7851	7.4944	7.1972	7.3899	7.0558	7.5541	7.1674	7.4679	7.1434	7.5616	8.0297	8.2817	7.2459	7.5508	8.0052	7.4466	7.6595	7.7301
Brd4	_IHS[Phospho (STY)]PIIR_.2	Q9ESU6_S1153_M1_Brd4	8.2734	8.3931	8.0532	7.8374	7.8050	7.9116	8.1351	8.1087	7.7437	8.2370	7.9370	7.9680	8.2973	8.4389	7.3315	7.9873	8.1525	8.0814	7.9057	8.1017
Brd4	_MPDEPEEPVVTVSS[Phospho (STY)]PAVPPPTK_.2	Q9ESU6_S471_M1_Brd4	3.7893	4.2135	4.3256	3.9001	3.1270	3.3511	4.4141	3.4897	2.9723	3.9956	3.3467	4.1030	4.5812	4.1250	3.9913	4.1342	4.0273	4.3770	3.1752	3.8023
Ddx24	_AQAVS[Phospho (STY)]EEEEEEEGQSSSPK_.2	Q9ESV0_S80_M1_Ddx24	5.4821	4.9587	5.2699	5.4810	4.7045	5.1578	4.8890	5.6137	4.9090	4.6375	3.8113	5.3418	5.4224	4.7999	3.3709	4.7560	5.7209	4.7347	4.5510	5.0243
Agk	_LASFWAQPQDASSR_.2	#N/A	6.2530	6.8901	6.7648	6.7577	6.7586	6.6493	4.0945	7.0017	6.3128	6.7908	6.8584	6.3375	4.2783	3.8221	6.5325	6.7084	6.0914	6.2865	6.8522	6.4406
Agk	_IPIGFIPLGQTSSLSHTLFAESGNK_.3	#N/A	3.1151	3.5137	3.5246	3.2002	3.8269	4.0509	3.7142	4.1895	4.1388	3.1946	4.1477	2.0446	3.8814	3.5117	3.2914	3.3332	2.8728	3.5761	2.9783	2.9761
Dkc1	_TVLES[Phospho (STY)]GGETGDGDNDTTK_.2	#N/A	7.9044	8.1452	7.8698	7.7596	7.7974	7.4828	7.7880	8.1064	7.3888	7.6474	7.4903	7.5383	8.1272	7.7332	6.6341	7.6315	8.2491	7.8837	7.6229	7.4565
Dkc1	_KRDSES[Phospho (STY)]ES[Phospho (STY)]DETPTVPQLK_.3	#N/A	4.1418	7.3974	6.7362	6.6354	3.7014	8.1172	7.2771	3.9943	7.7717	6.2926	3.8513	3.6403	4.1249	8.0295	7.1172	6.6973	3.6468	8.1911	8.0031	7.0223
Dkc1	_VKVVEEM[Oxidation (M)]S[Phospho (STY)]E_.2	#N/A	5.3834	4.6177	4.4760	4.8272	5.3307	5.1062	4.5743	5.1873	5.1771	4.2934	4.5054	4.7850	5.5664	4.6379	4.6612	5.0501	5.2957	5.0138	4.5036	4.7395
Dkc1	_TVLES[Phospho (STY)]GGETGDGDNDTTKK_.2	#N/A	3.7995	3.5700	3.2857	3.8351	3.9282	4.1523	3.7368	3.9748	3.6719	3.8860	2.3116	3.2961	3.9061	3.3238	3.1901	3.4346	3.4720	3.3969	3.9765	3.0011
Gtf2i	_ESTSSKS[Phospho (STY)]PPR_.2	Q9ESZ8_S823_M1_Gtf2i	7.5079	7.7884	7.3662	3.7037	6.9314	4.1208	4.4615	7.9247	7.5258	7.7257	3.8603	3.1647	4.5471	7.4290	2.9860	3.2426	3.6034	3.0687	6.8975	7.1447
Jph3	_TS[Phospho (STY)]INSLR_.2	Q9ET77_S163_M1_Jph3	4.3045	4.5154	3.8411	3.2797	3.0106	3.5969	4.0375	4.5302	6.5517	6.8192	3.1008	4.2416	4.3354	3.8791	4.0629	6.6067	4.0274	6.8331	6.5496	3.5564
Jph3	_TES[Phospho (STY)]PTTFSWTSHHR_.3	Q9ET77_S578_M1_Jph3	6.7658	6.1009	6.1812	5.7776	3.4711	5.5020	6.4378	4.4320	4.1291	7.0145	6.2932	5.2577	4.2372	5.9415	3.6472	2.9774	5.8702	3.2203	5.5264	6.2140
Jph3	_SLPVALES[Phospho (STY)]DEETGDELK_.2	Q9ET77_S706_M1_Jph3	9.4510	10.4905	9.9691	10.1296	3.4110	10.1924	10.2009	3.7737	10.1113	9.8011	8.7856	9.5091	9.9101	10.5063	8.8341	9.6411	9.9025	10.2172	9.8403	9.7730
Jph3	_S[Phospho (STY)]PLRTS[Phospho (STY)]INSLR_.2	Q9ET77_S158_M2_Jph3	3.3041	4.0150	4.3415	4.2802	3.5110	3.0965	3.1678	3.5297	3.2269	3.7411	3.6013	3.7412	3.3349	4.3795	4.2458	6.3722	4.5278	4.1225	4.4215	4.0568
Jph3	_QVS[Phospho (STY)]VDEER_.2	#N/A	9.7252	9.6192	9.5608	9.4807	8.9708	9.1348	9.2420	9.2047	9.0021	9.4637	8.8789	8.6896	9.0190	9.0138	8.0578	8.8522	8.9813	8.9702	8.5807	8.8950
Jph3	_EFS[Phospho (STY)]PSFQHR_.2	Q9ET77_S420_M1_Jph3	12.5507	12.4714	12.2241	12.2518	11.9613	12.1477	12.0771	12.5967	12.0711	12.0713	12.1224	12.0010	12.7567	12.2831	11.2868	11.8074	12.6155	12.2383	12.0180	11.9466
Jph1	_VLEKPPS[Phospho (STY)]PKES[Phospho (STY)]PHFYR_.3	#N/A	10.1269	10.0097	9.7974	9.8650	9.1358	9.6660	9.7282	9.7919	9.6525	9.9373	9.8489	9.6964	10.0028	10.0260	8.8834	9.4896	10.2541	9.9596	9.6742	9.7684
Jph1	_QNPS[Phospho (STY)]PGAR_.2	#N/A	8.8404	8.6707	8.4024	8.6013	8.1667	8.0106	8.2960	8.2242	7.7521	8.7123	7.8573	8.0287	8.1223	8.1401	6.3380	6.9300	8.1720	8.0856	7.7509	7.8781
Jph1	_KQNPS[Phospho (STY)]PGAR_.2	#N/A	5.7604	5.3450	6.1222	4.0060	4.6083	4.6590	4.4450	5.0594	3.8218	4.9205	4.8400	5.0252	4.8876	4.9450	2.9696	3.2591	3.6199	4.4004	4.4331	4.4955
Jph1	_QSHS[Phospho (STY)]PQPSS[Phospho (STY)]PEPSK_.2	Q9ET80_S475_M2_Jph1	4.4363	3.5665	3.4718	3.2531	3.7740	3.9981	3.7670	4.1367	3.8261	3.1418	3.4274	3.4559	3.9342	3.4589	3.3442	3.2804	3.1735	3.1588	4.3082	3.4259
Jph1	_TS[Phospho (STY)]LASLR_.2	Q9ET80_S162_M1_Jph1	3.5685	4.4343	4.1048	4.1209	2.9062	3.5779	3.5035	3.2688	3.4237	5.0501	2.9024	4.3238	3.5892	4.0918	3.5289	4.2006	3.8065	4.0266	3.6752	4.0588
Jph1	_QS[Phospho (STY)]HSPQPSS[Phospho (STY)]PEPSKK_.3	Q9ET80_S473_M2_Jph1	4.5162	4.1121	4.4270	3.7986	3.2223	3.2556	2.4941	3.5911	3.7460	4.3268	3.2247	4.0299	3.9115	4.4141	3.8512	3.8783	4.1288	3.7044	3.3529	4.3811
Jph1	_RGS[Phospho (STY)]LLGS[Phospho (STY)]MK_.2	Q9ET80_S216_M2_Jph1	3.8841	4.5758	5.8241	3.1287	3.2219	3.6327	4.4412	3.1803	4.3783	4.0905	3.2518	4.0081	4.4864	4.0301	3.8964	4.2290	4.1784	2.9710	3.2701	3.7074
Rcan1;Rcan3	_QFLIS[Phospho (STY)]PPAS[Phospho (STY)]PPVGWK_.2	Q9JHG6_S161_M2_Rcan1;Rcan3	6.7648	8.3344	8.0918	3.7349	3.0001	6.3044	8.1430	8.0283	6.9745	7.0959	3.0472	7.8561	4.0600	8.9164	7.0196	3.8308	3.9637	7.7621	7.3584	3.8612
Ivd	_FLQENLAPK_.2	#N/A	5.5599	4.7240	4.2240	5.4818	5.6071	5.2201	5.7760	5.1682	4.3653	5.5014	4.9418	5.2092	4.8755	5.5079	5.4609	5.0340	4.9843	5.6062	4.1786	5.5434
Ivd	_LYEIGAGTSEVR_.2	#N/A	3.9836	4.0192	4.5199	5.5809	5.0201	3.5454	4.2198	5.4324	5.0631	4.1899	5.5571	3.9087	4.3869	4.5069	5.8757	5.1243	4.2216	5.6503	5.1145	4.9004
Rabggta	_ELLLC[Carbamidomethyl (C)]NNR_.2	#N/A	3.8930	5.5301	5.8562	5.6247	5.7815	6.0129	5.7819	5.9036	4.7812	3.7036	5.2632	5.2091	5.5809	6.2386	5.2894	5.5742	5.6723	4.9294	5.8087	5.3828
Rabggta	_LPENVLLR_.2	#N/A	5.5075	5.5260	4.8478	3.5471	5.5035	5.1618	5.3727	5.7808	4.8234	5.1019	5.3673	5.8684	4.6625	4.9841	5.0767	3.6268	4.9841	5.0811	5.2015	5.2620
Lamtor2	_AQALVQYLEEPLTQVAAS_.2	#N/A	3.8450	3.9495	4.5896	3.6201	3.3911	3.6151	3.2184	3.4551	3.3896	3.3725	3.0826	3.1752	4.3172	3.8609	1.1250	4.3982	4.0092	3.1403	1.4307	3.5382
Palmd	_SGPQC[Carbamidomethyl (C)]SS[Phospho (STY)]PTC[Carbamidomethyl (C)]QEETEDVR_.2	Q9JHU2_S385_M1_Palmd	6.4344	6.4206	3.7180	6.3482	5.7428	6.0843	5.8645	5.3690	5.9515	6.4331	6.2783	3.2097	6.3896	6.6370	4.7750	3.5266	3.4197	5.7725	5.5916	3.6720
Palmd	_SNGPTHTS[Phospho (STY)]PTRPTPQPR_.3	Q9JHU2_S321_M1_Palmd	4.3732	3.6296	3.4087	3.3162	3.7110	3.9350	5.8687	4.0736	3.8892	5.3528	5.8618	3.5190	3.9973	6.8293	5.6971	3.2173	3.1105	6.0002	5.7872	3.2183
Palmd	_MELS[Phospho (STY)]PSRAS[Phospho (STY)]PGK_.2	Q9JHU2_S370_M2_Palmd	3.5061	6.3853	3.6607	6.9209	5.8195	6.2990	6.7663	4.3330	4.9563	5.0963	6.5005	3.1568	3.9815	7.1456	5.4111	4.8310	5.5097	4.7459	4.8595	5.6349
Dync1h1	_LVQT[Phospho (STY)]PLTDR_.2	Q9JHU4_T1880_M1_Dync1h1	10.9509	10.5591	5.8206	11.0348	6.3998	9.4924	10.0606	5.8721	9.7251	11.2207	10.3163	9.8952	10.1040	5.1737	8.1134	9.8305	5.3047	9.9513	5.5590	9.9904
Dync1h1	_VTDFGDKVEDPTFLNQLQSGVNR_.3	#N/A	7.4059	7.7001	7.3581	7.2108	6.9345	7.4344	7.4402	7.8564	7.6235	7.5692	7.3421	7.2953	7.8442	7.1290	5.8315	6.7431	7.6820	7.6370	7.0921	6.9107
Dync1h1	_VAAPDVVVPTLDTVR_.2	#N/A	7.2289	7.2050	7.0115	6.7756	6.8331	6.9630	7.2666	7.1885	7.4447	6.8396	6.4624	6.4772	6.8542	6.3646	6.3819	6.2139	7.3220	7.1213	6.4356	6.3601
Dync1h1	_EQPWVSVQPR_.2	#N/A	7.3233	7.2548	6.9082	6.4404	7.0971	7.1747	7.0533	7.0629	6.9034	6.6571	6.4479	6.6345	6.5054	5.8695	5.9208	6.1765	7.0415	7.0181	6.2102	6.3406
Dync1h1	_EYQTQLIQR_.2	#N/A	7.7254	6.5437	6.6253	6.6972	6.9754	6.9524	7.0337	7.3626	7.2674	6.4275	7.0276	6.4371	6.6446	6.1414	6.2420	6.4021	7.0967	6.5926	6.8559	6.0126
Dync1h1	_SLLQALNEVK_.2	#N/A	6.2850	6.0845	6.2826	6.1672	6.3534	6.6249	6.3066	6.0458	5.8593	6.0839	5.9361	6.3973	6.7717	5.8916	5.3446	5.5016	5.5871	6.4192	5.9542	6.0624
Dync1h1	_LLNTFLER_.2	#N/A	6.5518	6.4498	4.9710	6.5551	6.0324	6.1921	6.4801	6.4061	6.3011	6.1811	5.6918	6.2417	6.2210	5.3865	4.5239	5.6923	6.3422	6.2087	6.0236	5.4918
Dync1h1	_VTFVNFTVTR_.2	#N/A	6.2351	6.6608	6.5541	6.5166	5.9280	6.1012	5.9172	5.4218	6.4676	5.9755	5.7177	5.9349	6.0588	5.2745	5.5063	5.8193	5.7632	6.0143	6.2428	6.2255
Dync1h1	_QNLDGLLNQLK_.2	#N/A	5.3417	5.0334	4.5021	5.4591	5.4182	4.9257	5.4565	5.4949	4.5896	5.0966	5.2075	5.0769	3.9529	4.2451	3.5052	4.5032	5.2730	5.0248	4.3547	3.8066
Dync1h1	_EWTDGLFTHVLR_.2	#N/A	5.1387	5.0689	3.8877	4.1873	3.8480	4.1662	3.6694	4.8617	4.7894	3.6323	4.8296	4.0871	5.0911	3.6115	3.6811	3.7362	4.3474	4.9016	3.6897	3.9590
Dync1h1	_TFSEILNR_.2	#N/A	6.1660	5.5185	4.5295	5.7212	5.6286	6.2643	6.4995	5.8427	5.1130	6.0775	5.1775	5.8383	5.8598	4.5676	4.9755	4.9743	5.6353	5.9791	5.5002	5.4521
Dync1h1	_LNTQEIFDDWAR_.2	#N/A	5.5320	5.4380	5.8692	5.2395	5.5698	4.7556	5.1642	5.2488	5.6098	5.5381	5.4120	4.8540	3.8681	5.5486	4.8585	3.3465	5.8875	4.6854	4.8135	3.0891
Dync1h1	_LSLSNAISTVLPLTQLR_.2	#N/A	5.3172	4.0540	4.6784	3.7911	3.7954	5.2576	5.2166	4.7087	3.1163	4.1599	4.6274	4.3607	4.2647	3.9418	2.5758	3.7072	4.4895	4.5718	4.5768	4.1717
Dync1h1	_FLSDPQVHTVLVER_.3	#N/A	5.5406	4.0664	5.6414	5.7556	4.8058	5.2841	6.0754	3.7781	5.3719	5.6308	5.7053	5.4730	4.4410	3.8366	4.4241	5.0260	5.4267	5.7527	5.2831	4.9468
Dync1h1	_IQGLTVEQAEAVVR_.2	#N/A	5.7280	5.1181	5.0148	4.7702	5.3744	4.9681	5.0177	5.7877	4.5156	4.1650	3.8533	3.1190	5.1872	3.5033	2.9404	3.4771	5.2125	4.4663	3.1475	3.6265
Dync1h1	_LQGATC[Carbamidomethyl (C)]SNNK_.2	#N/A	5.2299	5.2820	5.3604	4.8308	5.1516	4.6606	5.1529	4.7826	4.9685	4.4725	4.4588	4.0300	3.3390	4.6090	4.2178	3.9967	4.5884	5.4020	4.4670	4.2572
Dync1h1	_SAC[Carbamidomethyl (C)]DTVDTWLDDTAK_.2	#N/A	5.0802	4.9009	5.0788	4.8729	4.5505	5.3726	4.6908	5.3609	5.3898	4.5719	4.6527	4.2015	3.8026	4.3052	4.3683	3.6686	4.0199	4.3072	5.1874	4.2722
Dync1h1	_EVWNTYELDLVNYQNK_.2	#N/A	5.3313	5.3156	3.2610	5.2638	4.6332	5.1875	5.5449	4.8683	3.0370	5.0164	5.3600	4.3755	5.4380	4.7243	3.1422	3.0865	5.3915	4.8327	5.0776	4.4459
Dync1h1	_VQVALEELQDLK_.2	#N/A	4.7096	4.2348	4.8711	5.3928	4.5954	4.1892	4.9115	3.4238	5.3310	3.9297	3.4126	4.3802	3.7441	4.2468	3.9780	4.0683	3.9615	4.6601	4.9107	4.2138
Dync1h1	_FNYGFEYLGVQDK_.2	#N/A	5.1681	5.0207	5.2272	4.3337	3.4574	3.7318	5.1268	5.0125	5.0016	4.9927	4.4424	3.7947	3.6763	4.3260	3.3143	4.5557	5.0285	4.9029	4.6492	4.4463
Dync1h1	_ESPEVLLTLDILK_.2	#N/A	3.2756	4.0687	4.2814	3.9443	3.7200	3.9940	5.1930	3.4455	4.4626	3.9514	4.6263	3.4126	3.1246	3.7926	4.0355	3.8191	4.7370	4.2133	4.2744	4.3619
Dync1h1	_LKQDGDSFR_.2	#N/A	4.0405	5.3144	6.0859	5.9562	5.2707	5.7550	6.5507	5.6723	4.2217	3.8511	5.9615	3.0047	5.7507	5.0505	4.8210	5.5230	5.9875	5.7861	3.6851	5.3739
Dync1h1	_LQGEFQLR_.2	#N/A	5.5098	5.1443	4.5712	3.6545	3.3727	5.4274	5.7551	5.6160	4.7680	4.5781	4.7556	4.5950	4.9855	4.5583	3.7456	4.7487	4.8631	5.9914	4.9069	4.5575
Dync1h1	_IFTIESAR_.2	#N/A	5.9058	5.7323	3.2638	5.6728	5.6583	5.6182	6.3966	6.0955	5.8107	5.5959	2.2952	3.2799	3.7581	3.3019	5.1501	5.5201	3.4502	3.6993	3.9983	4.7664
Dync1h1	_ENFIPTIVNFSAEEISDAIR_.2	#N/A	4.6676	4.8729	5.1064	4.3296	3.2425	4.1315	5.0189	4.6978	4.4952	3.6512	4.0305	3.2859	3.7642	4.8156	3.1742	3.4504	3.3436	4.3698	4.5327	2.9852
Dync1h1	_M[Oxidation (M)]LSAVSQQVQC[Carbamidomethyl (C)]IQEALR_.2	#N/A	4.2843	4.6520	3.8700	4.0559	4.3978	3.8602	3.8009	4.1479	3.8125	4.2105	4.0080	4.0003	5.0329	3.4150	3.6768	3.6209	4.2740	4.5749	4.2445	4.1928
Dync1h1	_TDS[Phospho (STY)]TSDGRPAWMR_.2	#N/A	3.6258	3.9507	8.9524	8.8596	3.8327	8.2033	8.1258	3.7573	3.9872	7.3777	8.3375	3.7435	4.1527	8.6415	3.3853	3.6950	3.8421	3.8761	3.8670	3.6326
Dync1h1	_SPLVM[Oxidation (M)]DVLNIQGVQR_.2	#N/A	4.5004	4.6234	2.7933	3.5123	4.2609	4.3057	4.5827	4.6869	3.5492	3.9716	3.6754	4.1719	4.4216	3.3640	2.0979	4.1225	5.2382	4.2530	4.0853	3.4905
Dync1h1	_IAFIM[Oxidation (M)]DESNVLDSGFLER_.2	#N/A	3.1669	3.3351	3.7032	3.8053	4.0054	2.4524	3.5150	3.6311	3.5947	3.3732	3.9691	3.6236	3.9962	3.6902	3.1129	3.5118	4.3801	3.4614	3.0531	4.4247
Dync1h1	_DHLYGTLDPNTR_.2	#N/A	6.0167	3.8999	4.4567	4.1650	3.6262	4.8853	5.2071	5.9272	3.3421	5.4957	5.7468	3.6260	3.4501	4.4947	3.4473	4.2822	5.0433	5.3895	4.3064	5.2084
Dync1h1	_DVLLVAQGEM[Oxidation (M)]ALEEFLK_.2	#N/A	4.5350	4.4325	3.7216	3.1544	3.8727	3.5410	3.6683	4.2354	3.1998	3.2405	4.1018	3.3572	3.8355	3.5575	3.2455	3.3791	3.2722	3.1026	4.2095	3.0565
Dync1h1	_QLQNISQAAASGGAK_.2	#N/A	5.0617	4.4742	3.8815	4.3442	4.9490	5.3648	5.3864	3.0456	4.8779	3.3715	4.9698	3.0462	3.3659	3.8686	4.2966	4.8093	3.5832	4.5634	3.8985	3.8356
Dync1h1	_IQFVGAC[Carbamidomethyl (C)]NPPTDPGR_.2	#N/A	3.9307	3.3884	3.4673	6.0609	4.1376	6.8860	6.9607	4.1564	6.4580	3.7413	3.8102	3.1145	4.4970	3.5053	2.9359	3.2928	3.6536	6.3296	3.7949	3.1826
Dync1h1	_FTQDTQPHYIYSPR_.3	#N/A	3.6933	4.3064	3.2299	3.8909	3.9002	4.2081	4.6093	4.8491	3.6161	4.0495	3.9905	3.3519	3.7242	3.2679	3.1732	4.4873	3.4162	4.0894	3.9261	4.4461
Dync1h1	_LEDDAKDNQQK_.2	#N/A	3.9444	4.0585	4.4806	3.7450	5.3471	3.5062	4.2590	3.8688	4.3181	4.1507	3.1916	3.9479	4.4261	3.9699	3.6993	4.2893	4.1824	3.0313	3.3303	3.6472
Dync1h1	_VAEVLFDAADANAIEEVNLAYENVK_.3	#N/A	4.4199	3.5829	3.4553	3.6645	3.7576	2.6428	3.7835	4.1454	3.8425	3.1254	4.2169	3.4724	3.9506	3.4944	3.3607	3.2639	3.6426	3.5068	3.8059	3.1717
Dync1h1	_TFSSIPVSRIC[Carbamidomethyl (C)]K_.2	#N/A	4.5669	4.2531	4.1035	4.5182	3.2730	3.3346	3.7751	1.8224	3.6953	4.3775	3.1740	3.9792	4.5977	4.1415	3.8005	3.9290	4.2898	4.3605	1.6333	1.7584
Dync1h1	_EAIVNSC[Carbamidomethyl (C)]VFVHQTLHQANAR_.3	#N/A	3.1279	4.1912	3.7494	4.4563	3.3349	3.2727	2.9917	4.3025	3.0508	5.0521	4.1800	3.9173	4.1618	4.2034	4.0697	3.9909	5.2890	4.2986	3.0968	3.8807
Dync1h1	_LALESIC[Carbamidomethyl (C)]LLLGESTTDWK_.2	#N/A	4.1515	3.8513	3.1655	3.3961	3.4892	3.7133	4.0519	4.4138	4.1110	4.3578	2.9845	3.7408	4.2190	3.7628	3.6291	2.9955	3.9111	3.2384	3.5374	3.0267
Isyna1	_SC[Carbamidomethyl (C)]IENIFR_.2	#N/A	4.0048	4.8494	5.0642	6.0382	3.3425	4.9400	4.8810	4.4389	4.2576	5.2458	5.2040	3.3476	4.3172	4.5282	5.0921	4.3497	6.5104	6.4847	4.6077	4.7277
Isyna1	_VIVLWTANTER_.2	#N/A	3.4558	4.5525	3.9866	5.1875	3.6627	3.3019	2.8409	3.1507	3.3056	4.4009	4.2850	2.9411	3.4710	3.9737	4.5746	4.3188	3.6883	4.2513	4.3480	3.9407
Isyna1	_HVFVGGDDFK_.2	#N/A	3.6129	4.3899	4.1492	4.0765	5.1394	4.4174	3.0896	3.3133	3.8542	3.8192	4.6799	4.4609	3.2567	4.3479	4.3081	4.8907	3.8510	4.2007	2.9989	4.2071
Nit2	_AVDNQVYVATASPAR_.2	#N/A	4.5052	3.4976	5.0136	4.1417	2.6475	3.3351	3.6982	3.9883	5.2878	3.2106	3.5879	3.3871	3.8653	3.4091	4.7128	3.8901	3.5574	3.9807	3.8506	3.0864
Nit2	_TLS[Phospho (STY)]PGDSFSTFDTPYC[Carbamidomethyl (C)]K_.2	Q9JHW2_S133_M1_Nit2	3.2761	4.0430	4.3135	4.0594	3.4830	3.5163	3.5651	3.3304	3.4853	4.5875	2.9639	3.7691	3.6507	5.3485	3.5905	4.1390	3.8681	4.1235	3.6137	4.1204
Nit2	_FAELAQIYAQR_.2	#N/A	3.4513	3.9316	4.4249	4.1967	4.2898	3.0131	3.2513	3.6132	3.3103	4.3707	3.6847	2.9402	3.4184	4.4630	2.8284	3.7962	3.1104	4.0390	4.3381	4.1403
Stk3	_ELEEEEENS[Phospho (STY)]DEDELDSHTM[Oxidation (M)]VK_.3	#N/A	3.2727	5.1677	4.2048	4.4959	3.8521	4.8533	4.5396	3.2405	4.7304	5.3839	3.4595	4.1436	3.6355	5.2598	4.0454	4.5230	4.3220	5.2433	4.6234	5.3717
Nudt3	_LVGIFENQER_.2	#N/A	7.7366	7.6902	7.2431	7.7113	7.4432	7.3132	7.4791	7.6635	7.2554	7.6535	7.5418	7.6002	7.8605	7.5100	7.2445	7.6677	7.4797	7.4151	7.4748	7.3868
Nudt3	_SESEEEVLLVSSSR_.2	#N/A	7.6780	8.4282	8.3912	7.7911	5.3080	7.7527	7.6678	6.9511	7.7470	7.6209	5.2003	7.6231	8.1067	8.8064	7.6147	7.6968	7.9670	5.3790	8.0289	7.9923
Nudt3	_WIVPGGGM[Oxidation (M)]EPEEEPSVAAVR_.2	#N/A	5.8727	5.3223	5.3336	5.5516	5.4575	5.2220	5.4064	5.8281	5.2415	5.4910	5.2299	4.9786	5.6673	5.5807	5.0864	5.5289	5.4810	5.3656	4.9728	5.2423
Rnf14	_LTYHGLS[Phospho (STY)]PC[Carbamidomethyl (C)]K_.2	#N/A	8.4333	8.3856	8.1348	7.7017	8.3656	8.4974	8.3921	8.8459	7.6236	8.4724	8.7818	8.4082	8.6227	8.6446	7.4005	8.0316	9.0657	8.4154	8.2260	8.1502
Rnf14	_LTYHGLS[Phospho (STY)]PC[Carbamidomethyl (C)]KVTAEK_.3	#N/A	3.4154	4.5874	3.9517	4.2740	4.2540	3.7310	3.2871	3.1158	1.5254	3.6217	4.2502	2.9760	3.4361	3.9388	2.8643	3.7603	3.6535	4.1797	3.8283	3.3438
Actn2	_ETADTDTAEQVIASFR_.2	#N/A	5.3856	3.9363	5.6836	4.2014	4.7107	4.7187	4.5572	4.2441	3.7423	5.1595	3.6090	5.0514	5.1829	4.6773	4.7069	5.2821	5.4797	4.8228	4.8616	5.5393
Actn2	_ISSSNPYSTVTM[Oxidation (M)]DELR_.2	#N/A	5.8541	4.8258	3.4412	3.2836	4.5312	5.2934	5.4533	5.7405	5.4202	5.3367	5.1522	6.0042	6.0979	4.8495	5.2807	5.8498	5.8557	5.9040	5.8282	5.5417
Actn2	_ATLPEADGER_.2	#N/A	3.2219	5.9190	5.4344	5.6071	5.7993	6.0128	6.2084	5.3097	6.0753	3.4282	6.3824	3.1695	3.2426	6.8357	6.5286	6.2433	3.4600	6.8628	6.4053	6.1898
Actn2	_AC[Carbamidomethyl (C)]LISM[Oxidation (M)]GYDLGEAEFAR_.2	#N/A	3.0940	4.2251	4.1314	4.4903	4.9456	5.0241	3.7472	5.6577	3.0168	6.1946	6.1463	5.6856	6.2380	4.1694	5.1414	4.3921	6.3307	5.5676	5.4096	5.5026
Actn2	_HTNYTMEHIR_.3	#N/A	4.4502	3.5526	3.4856	3.2392	3.7879	4.1970	3.7532	4.1505	2.0146	3.7674	3.4715	3.4421	4.4709	4.5084	3.8028	3.2942	3.1874	3.1449	1.0356	3.4397
Actn2	_VEQIAAIAQELNELDYHDAVNVNDR_.3	#N/A	3.1635	3.3384	3.6998	4.5259	4.0021	4.2262	3.5390	4.3647	3.0892	3.3698	3.9725	3.2279	3.7061	3.6869	3.1162	3.5084	3.4016	4.4316	4.0802	4.4280
Sgpp1	_NS[Phospho (STY)]LTGEEGELVK_.2	#N/A	3.9303	8.0880	4.4666	3.7590	3.1827	3.2160	3.8654	3.6307	3.7856	4.2872	3.2643	3.9619	3.9511	4.4537	3.8908	7.6952	4.1684	3.6648	3.3133	4.4207
Sgpp1	_RNS[Phospho (STY)]LTGEEGELVK_.2	#N/A	7.7505	7.4970	4.0855	7.3554	3.2551	7.3527	7.2095	3.2738	7.5266	7.0296	6.9045	7.0430	7.4996	4.1236	3.8184	6.9614	4.2719	4.3784	3.1766	6.9073
Lgi1	_DNALC[Carbamidomethyl (C)]ENAR_.2	#N/A	7.2271	7.3571	7.9374	7.8224	7.2714	7.4618	7.1502	7.6313	7.7395	7.2861	7.4128	7.6211	7.3425	7.9157	7.6917	7.7080	7.6049	7.5877	7.7317	7.7791
Lgi1	_FQELNVQAPR_.2	#N/A	6.8321	6.8739	7.2658	7.1397	6.9659	6.7310	6.8968	6.9493	6.9474	6.5961	7.1362	6.6707	7.2836	6.9940	6.3859	6.5077	6.9157	6.7286	6.9828	6.7429
Lgi1	_GLDSLTNVDLR_.2	#N/A	6.4171	6.4913	6.4217	6.5529	6.2134	6.1455	6.3898	6.2724	6.1543	6.4465	6.5564	6.1649	6.4654	6.1257	6.3121	6.4852	6.8134	6.1982	6.4099	6.4244
Lgi1	_DFDC[Carbamidomethyl (C)]IITEFAK_.2	#N/A	5.9394	5.7362	6.6598	6.5684	6.2930	6.0038	6.4512	6.3799	5.9993	5.9570	6.2157	6.2149	6.7111	6.2800	6.0332	6.2557	6.5954	6.5548	6.1622	6.3375

Lgi1	_SLIHLSLANNNLQTLPK_.3	#N/A	5.2817	4.6945	4.2081	4.1274	4.1485	3.9843	4.4206	4.3496	4.7383	5.0371	5.1033	4.7411	5.3148	5.1452	3.2619	4.1963	4.3362	4.4509	4.9353	5.2297
Lgi1	_IQDIEVLK_.2	#N/A	5.5361	5.2483	5.8611	5.2935	5.1785	5.0439	5.0310	5.7474	5.1367	5.6499	5.6570	4.9955	5.7301	5.5462	5.3798	5.6691	4.3303	5.5236	4.9604	5.3409
Lgi1	_GNAFNC[Carbamidomethyl (C)]DC[Carbamidomethyl (C)]K_.2	#N/A	3.5610	3.4374	3.4183	4.6078	3.9826	5.1834	4.7391	4.5288	4.3080	3.6923	4.7118	4.8046	4.5459	3.5478	4.5172	4.4878	4.6331	4.9482	5.1101	4.6498
Lgi1	_KPNDIETFK_.2	#N/A	4.2800	3.0595	4.3276	2.8358	2.9861	3.4233	3.8941	3.5384	3.9822	3.1181	3.6716	4.2889	3.8631	4.0347	3.8694	3.6421	4.0029	4.0186	3.1310	3.1041
Lgi1	_WGGSSFQDIQR_.2	#N/A	3.6905	4.4995	5.8667	3.9989	4.9687	4.9098	5.4248	3.3908	4.9378	3.8968	3.0244	5.1397	3.7112	5.5013	4.0901	4.0354	3.9285	4.7826	5.0047	4.1808
Lgi1	_DTDVEYLEIARPPLALR_.2	#N/A	2.7683	2.9973	3.8666	3.7633	3.8491	4.3669	3.2260	3.8679	3.9865	3.4528	3.4845	3.4136	4.1519	4.0572	3.2513	3.8446	3.3651	3.8834	3.4909	3.1321
Lgi1	_C[Carbamidomethyl (C)]PAVC[Carbamidomethyl (C)]TC[Carbamidomethyl (C)]SK_.2	#N/A	4.5043	3.4985	3.5398	3.1851	3.8421	4.0661	3.6990	4.2047	3.7581	3.2098	4.1325	3.3879	3.8662	3.5269	3.2762	3.3484	3.2416	3.0908	4.2402	3.0872
Sh2b2	_S[Phospho (STY)]SEDVSAHAATK_.2	Q9JID9_S106_M1_Sh2b2	6.4479	6.0005	6.1089	6.1986	4.0295	4.1609	5.6902	4.2296	5.6441	3.5206	3.9318	3.2116	5.7323	3.5637	4.9277	3.3701	3.4887	5.7878	5.8323	3.4081
Praf2	_ALDDFVLGSAR_.2	#N/A	5.6750	6.0165	5.9363	5.8498	4.7348	5.9119	5.9706	5.9290	5.2931	4.8410	5.6916	5.2850	5.7149	4.7971	5.3180	5.4377	5.9562	6.0792	5.3861	5.4771
Praf2	_TPMGLLLEALGQEQEAGS_.2	#N/A	4.3924	4.4275	3.6882	3.5395	3.0985	3.1319	4.4374	3.1173	3.8698	4.2030	3.3484	4.1536	4.0352	4.6166	3.9750	3.7545	4.1153	3.5806	3.2292	3.0040
Inpp5e	_TLS[Phospho (STY)]LDDKGWR_.2	Q9JII1_S89_M1_Inpp5e	3.7632	8.4620	4.2995	3.9262	6.8241	7.8231	4.4402	3.4636	7.8347	7.7230	3.3728	4.1291	3.1065	4.2866	6.1505	4.1081	4.0012	7.8181	3.4805	7.3952
Inpp5e	_FRGS[Phospho (STY)]QEDLTVQNGASPC[Carbamidomethyl (C)]R_.3	Q9JII1_S103_M1_Inpp5e	3.2124	4.1067	4.2498	4.3719	3.4193	4.2502	3.6288	3.4380	3.1352	3.8327	3.5096	3.8328	3.2432	5.8335	3.6542	4.0753	4.4361	4.2142	3.0124	3.9651
Dazap1	_LFVGGLDWSTTQETLR_.2	#N/A	4.5557	4.0724	4.2676	3.2717	3.2618	4.1410	3.7863	3.2805	4.1403	3.9903	4.4023	3.9904	4.5865	4.1303	4.1269	4.3434	4.3352	3.6392	4.4912	3.8076
Dazap1	_IFVGGIPHNC[Carbamidomethyl (C)]GETELR_.3	#N/A	3.2111	3.2909	3.7474	4.4783	4.0496	4.6038	4.6790	4.4123	2.8198	4.2602	4.0458	4.8588	6.0626	5.0419	4.2940	4.5589	3.4491	4.0160	3.0189	4.6619
Dazap1	_SYFSQYGEVVDC[Carbamidomethyl (C)]VIM[Oxidation (M)]K_.2	#N/A	3.6220	4.8395	4.0261	3.4369	2.9597	4.8010	3.0805	4.4876	4.4490	4.8041	3.5140	3.8285	5.1674	5.4012	3.5218	3.5759	4.4405	4.2098	3.3259	4.0591
Akr1a1	_DAGHPLYPFNDPY_.2	#N/A	7.1800	7.4811	7.5747	7.6209	7.0036	7.2309	7.3027	7.2723	7.6082	7.7920	7.2025	7.1360	7.4136	7.7407	7.2505	7.5860	7.5647	7.4634	7.4422	7.9663
Akr1a1	_[Acetyl (Protein N-term)]TASSVLLHTGQK_.2	#N/A	6.9014	7.4921	7.3050	7.4212	7.4638	7.5791	7.5267	7.5927	7.5247	7.1531	6.9127	7.5984	7.4597	7.6367	7.5536	7.4285	7.2320	7.2587	7.1443	7.1910
Akr1a1	_ALGLSNFNSR_.2	#N/A	7.5141	7.1407	7.3443	7.3308	7.4805	7.2056	7.2607	7.6802	7.1500	7.1905	7.1877	7.2559	7.4438	7.0140	7.1311	7.2910	7.1445	7.2061	7.0455	7.0574
Akr1a1	_GLEVTAYSPLGSSDR_.2	#N/A	6.6247	6.8509	6.8812	6.7589	6.7088	6.4260	6.2919	6.7613	6.8179	7.2023	6.3090	6.5519	6.7495	7.3350	6.8497	6.8257	6.4861	6.4636	6.4186	6.8934
Akr1a1	_ILQNIQVFDFTFSPEEM[Oxidation (M)]K_.2	#N/A	4.0187	4.5261	4.3717	4.4596	4.5582	4.1647	4.2025	4.5286	4.3280	4.0732	4.1636	3.7080	4.5843	4.3630	4.0711	4.1240	4.3870	3.9300	4.7345	4.5635
Akr1a1	_HALSAGYR_.2	#N/A	6.3758	6.3421	6.5213	6.8571	5.6153	6.2868	6.5222	6.3269	6.2669	5.9186	6.1097	6.3112	6.2168	6.6437	6.7558	6.2152	5.8784	5.9518	6.1931	5.9755
Akr1a1	_HPDEPVLLEEPVVLALAEK_.3	#N/A	4.1529	4.0534	4.1527	3.3830	2.6381	3.9498	3.3142	3.9709	4.2449	4.3320	4.1765	3.7945	4.3830	4.4215	3.8424	4.3017	4.0484	3.5815	4.0755	4.0089
Akr1a1	_EELFVTSK_.2	#N/A	4.2709	3.7319	3.3064	3.4185	3.6087	3.8327	2.8755	3.9713	3.9193	4.4773	1.8187	3.6213	4.0996	3.6433	2.3632	2.8985	4.5090	3.3578	3.6569	3.3206
Akr1a1	_YDSTHYK_.2	#N/A	4.3188	3.6840	3.3543	3.3705	3.6566	3.8807	3.8845	4.0192	3.9436	4.5252	4.3180	3.5734	4.0517	3.5954	3.4617	3.1629	4.5569	3.4058	3.7048	3.2727
Ddx21	_EIITEEPS[Phospho (STY)]EEEADM[Oxidation (M)]PKPK_.3	#N/A	3.7353	3.6142	4.3109	3.9508	3.8389	3.9026	4.6088	3.7273	3.6733	3.9416	3.5945	3.5036	3.8151	4.5636	5.1563	4.0802	3.8408	5.6433	3.8108	3.7592
Sv2a	_GGLS[Phospho (STY)]DGEGPPGGR_.2	#N/A	14.6118	14.3617	14.4593	14.4443	14.0056	14.1696	14.2225	14.5042	14.2062	14.1390	14.1870	14.0115	14.4018	14.3173	13.0765	13.8440	14.5697	14.2706	14.0778	13.9971
Sv2a	_HLQAVDYAAR_.2	#N/A	7.7339	7.5029	7.2549	7.8794	7.6298	7.5098	7.9602	8.0034	7.6269	7.3564	7.4383	7.3692	7.7324	7.7459	7.5076	7.1565	7.5418	7.7703	7.4180	7.3626
Sv2a	_GGLS[Phospho (STY)]DGEGPPGGRGEAQR_.2	#N/A	8.7679	8.8874	7.9306	8.7360	8.1617	8.3116	8.6564	9.1110	8.5058	8.4852	8.2803	8.4078	9.2801	8.4840	7.7690	8.3339	8.5399	8.6861	8.5371	8.3999
Sv2a	_M[Oxidation (M)]ADGAPLAGVR_.2	#N/A	7.9874	8.2687	8.0603	8.0412	7.9270	7.9575	8.3566	8.0597	7.7395	7.2992	7.7171	7.9907	7.5640	7.4386	7.7226	7.2138	7.7942	8.1213	7.7123	7.4071
Sv2a	_FEEEDDDDFPAPADGYYR_.2	#N/A	7.9762	7.9890	7.9304	7.2197	6.1059	7.8959	8.1247	3.9305	7.9584	7.0769	7.2031	7.0572	4.2508	7.9284	6.9443	7.0355	7.4915	8.0614	7.7175	7.4353
Sv2a	_VFSVTHIK_.2	#N/A	6.7160	6.8428	6.6531	6.6443	7.0138	6.8332	7.2841	7.4681	6.2836	6.7571	6.8593	6.4962	6.8111	6.1775	5.7704	6.0829	6.8124	6.9803	6.4269	5.8003
Sv2a	_DREELAQQYETILR_.3	#N/A	5.6302	5.4711	5.4906	6.0206	5.4797	5.8961	5.6982	4.6835	5.7227	6.4244	6.2051	5.8057	5.5006	5.2452	4.7681	5.6761	5.4984	5.4358	5.3016	6.0234
Sv2a	_[Acetyl (Protein N-term)]MEEGFRDR_.2	#N/A	4.4402	4.0360	4.6649	3.7557	3.1463	4.3167	5.1862	3.6671	4.4410	4.2508	4.8039	3.3079	3.9875	5.4085	3.7652	3.8023	3.1259	3.6283	3.2769	4.1397
Sv2a	_GGQYFNDK_.2	#N/A	3.1338	3.3682	3.6701	4.5556	3.9723	4.1964	4.7777	4.3350	4.4175	3.7994	4.4496	3.7037	3.1545	3.6572	3.9515	3.6428	3.3718	4.5946	4.4234	3.8840
Sv2a	_VQDEYSR_.2	#N/A	4.4142	4.4057	3.9508	3.8732	3.1203	3.1536	4.0694	3.1390	3.3853	1.9512	3.4394	3.3861	4.0135	3.9888	3.5204	3.7763	4.1371	2.8830	3.3114	3.6661
Sv2a	_TIHQEDELIEIQSDTGTWYQR_.3	#N/A	8.2551	7.9127	7.3279	7.5324	6.3871	6.5930	7.3186	8.2389	6.9673	7.3978	7.0015	6.9718	8.1050	8.0385	5.1462	7.1761	7.6471	7.1397	6.1904	6.4934
Acin1	_TAQVPS[Phospho (STY)]PPR_.2	#N/A	13.6478	13.6427	13.6538	13.5184	13.4182	13.5388	13.2780	13.6595	13.2686	13.6546	13.3880	13.2341	13.7056	13.7508	12.6658	13.1832	13.6337	13.3224	13.2599	13.3042
Acin1	_TAQVPS[Phospho (STY)]PPRGK_.2	#N/A	11.9157	12.3701	10.7403	11.7369	10.8972	11.4324	11.7225	12.3210	11.5060	11.5295	10.9096	11.9637	12.9511	12.2037	11.2944	11.8187	11.7917	11.9419	11.4662	11.5130
Acin1	_LQPEQGS[Phospho (STY)]PK_.2	Q9JIX8_S729_M1_Acin1	11.7932	11.9127	11.6192	11.7656	11.4346	11.7116	11.7780	11.7554	11.6959	11.5929	11.4489	11.5154	11.9447	11.6828	10.9518	11.4110	11.5903	11.6906	11.5309	11.4047
Acin1	_SLS[Phospho (STY)]PLSGTTDTK_.2	#N/A	10.7623	10.6285	10.7550	10.6457	10.0523	10.4633	10.1149	10.4581	10.1329	10.6013	10.2866	10.1113	10.5219	10.6148	9.4113	10.2076	10.5872	10.2248	10.0548	10.2387
Acin1	_GVQAGNS[Phospho (STY)]DTEGGQPGR_.2	#N/A	8.3937	8.6281	8.5046	8.4638	4.0505	8.3678	8.2797	8.4062	8.1658	7.6299	7.9430	3.2017	8.4674	8.5124	7.6196	8.2390	8.1732	8.1443	3.8821	8.1757
Acin1	_SSSFSEEKGES[Phospho (STY)]DDEKPR_.3	#N/A	6.1563	6.5028	5.7679	5.6908	5.2046	6.1668	6.0068	6.0965	5.4665	6.2011	5.5630	5.3714	6.3907	6.0367	5.9700	6.0326	5.5791	6.3787	6.1766	5.8414
Acin1	_KS[Phospho (STY)]LS[Phospho (STY)]PGVSR_.2	Q9JIX8_S655_M2_Acin1	6.6163	6.2464	6.7426	6.4431	6.3717	6.7860	6.6807	6.4028	6.1897	3.7009	6.0339	6.1958	6.4666	6.8973	5.8442	6.1235	6.6834	6.3820	4.2279	3.5061
Acin1	_S[Phospho (STY)]RST[Phospho (STY)]PVRDR_.2	Q9JIX8_S1326_M2_Acin1	5.6874	5.1090	6.0661	5.7928	5.0539	5.2752	5.7708	4.8846	5.1839	6.3575	5.1613	4.6151	5.2067	5.3460	3.6477	4.9208	3.9993	4.4789	5.4573	5.0615
Acin1	_KIS[Phospho (STY)]VVSATK_.2	Q9JIX8_S825_M1_Acin1	4.3937	5.1066	5.9103	5.6188	5.1324	4.7609	3.8600	4.0941	4.6542	5.7760	4.3376	4.3826	4.5380	4.9132	3.3869	4.8495	3.1309	4.6078	4.3508	4.0018
Acin1	_SLS[Phospho (STY)]PLSGTTDTKAES[Phospho (STY)]PAGR_.2	#N/A	3.6372	7.1168	7.0648	7.7510	3.5043	4.8351	7.3686	3.6427	6.7309	6.5003	4.7649	6.2968	5.5814	6.6828	6.1090	6.9193	4.1116	3.9245	4.0891	6.2992
Acin1	_SQS[Phospho (STY)]PSPPPLPEDLEK_.2	Q9JIX8_S389_M1_Acin1	4.1278	3.3410	3.6071	3.9542	4.0308	4.1209	3.7730	4.1688	6.4745	3.5714	3.9569	3.2205	3.8858	9.6478	8.2955	3.2946	3.9456	9.1289	7.8438	3.3792
Acin1	_HLS[Phospho (STY)]HPEPEQQHVIQR_.3	#N/A	6.2277	6.5386	6.1382	5.9545	4.7116	5.6166	5.4527	5.2662	6.0153	5.8329	5.7693	6.1700	6.3744	5.8988	5.0835	5.4343	5.8793	5.8120	5.3940	5.7456
Acin1	_GVQAGNS[Phospho (STY)]DTEGGQPGRK_.2	#N/A	3.2584	3.7387	3.1170	4.0038	4.0969	4.3210	3.4441	3.8061	4.8220	3.4647	3.8776	3.1330	3.6113	3.1551	3.0213	3.6033	4.7303	3.8461	4.1452	4.3332
Plekho1	_AKDPPQS[Phospho (STY)]PPDSESEQLLLETER_.3	Q9JIY0_S341_M1_Plekho1	4.4020	3.6008	3.4375	3.2874	4.2119	6.1939	4.3371	4.1024	4.2573	3.8155	3.7359	3.4903	4.4227	3.4245	2.8616	3.3671	3.1392	3.1931	4.3425	3.3915
Kcnip2	_DLDGS[Phospho (STY)]YDQLTGHPPGPSKK_.3	#N/A	3.2580	8.9727	4.2955	9.1011	7.6655	8.2528	8.1219	7.6384	8.6339	9.0464	8.4728	8.5471	9.1257	9.4860	8.5723	9.1570	8.8610	8.8473	2.9667	9.1647
Kcnip2	_DLDGS[Phospho (STY)]YDQLTGHPPGPSK_.2	#N/A	4.4312	8.1614	3.7435	7.9572	3.4817	6.0259	7.7464	7.3997	7.9794	8.2421	3.5286	7.5473	7.4532	8.8760	7.4344	8.3301	8.2174	7.9868	7.9233	8.2040
Rprm	_RPS[Phospho (STY)]KEVEAVVVGPY_.2	Q9JJ72_S98_M1_Rprm	8.7848	9.0432	8.7412	8.8149	8.0590	9.3123	8.6608	8.6102	8.7517	8.6274	8.5554	8.6656	8.7873	9.4531	8.0946	9.1050	9.2551	9.2693	8.9795	8.8529
Tamalin	_NFTQS[Phospho (STY)]PEQQR_.2	#N/A	3.7808	8.9801	9.2515	9.6434	9.4111	9.6226	9.0936	9.7520	8.6484	9.8215	9.5354	9.5724	9.9441	10.0558	9.3011	9.6211	9.8504	9.7425	9.6784	9.7003
Tamalin	_FIPGLNRS[Phospho (STY)]LEEEESQL_.2	#N/A	8.8981	9.3161	8.6022	8.7260	4.0032	8.3215	8.9796	8.9105	8.7223	9.0177	8.4624	9.0239	9.9449	9.1353	8.7318	8.9328	9.1848	9.3849	8.8015	8.7209
Tamalin	_NFTQS[Phospho (STY)]PEQQRK_.2	#N/A	3.6013	4.0816	4.2749	6.6278	2.9390	3.1631	3.1012	3.4632	7.0431	3.8076	3.5347	4.2910	3.2684	4.3130	4.1793	3.9462	7.2081	4.1890	6.8148	3.9903
Tamalin	_S[Phospho (STY)]LEEEESQL_.2	#N/A	4.5412	6.2187	6.7483	6.5680	3.8789	6.4145	6.6601	4.2415	6.9976	7.2741	3.8751	6.1191	4.5619	6.8860	5.9987	7.0736	6.8141	6.9198	6.9971	6.9193
Rilpl1	_T[Phospho (STY)]SPQPESGIKR_.2	Q9JJC6_T348_M1_Rilpl1	4.4862	4.3337	4.0228	3.0980	3.1923	3.4152	4.3499	3.2110	4.4090	4.0598	3.2825	4.0599	4.5171	4.0608	3.9271	3.8483	7.5429	4.4412	3.2394	3.7381
Rilpl1	_[Acetyl (Protein N-term)]M[Oxidation (M)]EEPLGS[Phospho (STY)]PPAALSALEK_.2	#N/A	9.4062	9.4899	9.3823	8.0129	4.0348	9.4480	9.3788	9.4071	9.5146	9.5700	9.5986	9.8426	10.3473	10.3709	9.6253	9.9783	11.1608	9.8048	9.9854	10.0108
Tacc2	_VQNS[Phospho (STY)]PPVGR_.2	Q9JJG0_S419_M1_Tacc2	11.7037	11.8741	11.6340	11.9130	11.5147	11.7090	11.4778	11.7203	11.5290	11.7569	11.5498	11.7336	12.0411	12.3365	11.5569	11.8189	11.8295	11.6038	11.6298	11.8609
Tacc2	_FNS[Phospho (STY)]PSEELDYR_.2	Q9JJG0_S714_M1_Tacc2	7.9342	7.8787	8.4698	8.2302	6.9024	7.8258	7.6263	7.4792	3.3125	8.3731	7.6047	8.0144	7.9836	8.6492	7.4224	8.0779	8.7452	7.9458	8.0308	8.2393
Tacc2	_TRPPS[Phospho (STY)]LK_.2	#N/A	3.9785	8.0290	4.5148	7.3826	7.6277	7.4372	4.2249	7.6806	4.2840	7.9304	7.3552	7.9926	8.0884	8.1749	7.3088	4.3234	4.2165	7.6545	7.5659	7.7474
Tacc2	_KTRPPS[Phospho (STY)]LK_.2	#N/A	5.8410	3.5576	3.2981	5.0401	3.9159	4.1399	5.0431	3.9872	3.6843	3.2836	5.0902	3.3141	3.7924	3.3361	3.2024	5.4637	3.4844	3.6650	3.9641	5.1986
Tacc2	_LDNTPASPPRS[Phospho (STY)]PT[Phospho (STY)]EPSDTPIAK_.3	Q9JJG0_T516_M2_Tacc2	3.3740	6.3204	4.1400	4.0908	3.1670	3.2801	5.5136	5.3799	3.4027	4.1813	3.3525	4.7173	3.5426	6.6499	3.9900	3.9746	3.8418	3.9966	6.8580	6.3367
Tacc2	_ETQQEPGEES[Phospho (STY)]PVPSEEHLAPETK_.3	Q9JJG0_S354_M1_Tacc2	5.4465	5.4344	4.9746	4.2622	3.5793	3.6152	5.7348	3.5980	3.0737	3.9867	4.9148	5.4626	5.8117	7.0872	4.8597	4.2864	3.8990	5.5515	5.7594	3.8154
Lancl2	_SGNYPSSLSNETDR_.2	#N/A	5.8509	6.5444	5.4739	5.9100	5.2660	5.5541	6.5803	6.7725	5.5195	6.1393	6.4512	6.2625	5.8891	6.2415	5.0488	5.7741	5.6643	6.5278	5.1303	5.3344
Lancl2	_SFPNPFPDYEAAASAAGLAAGSAEETGR_.3	#N/A	4.8061	4.0446	2.7676	3.7467	3.0516	3.9285	4.1426	4.8188	3.0477	3.7317	4.7680	4.1784	3.1558	4.6719	2.3380	3.5969	2.5669	3.5365	4.4280	3.8777
Sh3bgrl	_GDYDAFFEAR_.2	#N/A	5.4469	5.8623	6.0545	6.2563	6.2366	6.1748	6.1274	6.0115	5.7301	6.7160	5.1425	5.7858	6.2303	6.5519	6.1742	5.6555	4.7345	5.9649	6.1892	5.7892
Sh3bgrl	_QQDVLC[Carbamidomethyl (C)]FLEANK_.2	#N/A	4.3112	4.5562	4.8956	4.6861	4.2019	5.1924	4.9265	3.9723	3.9610	4.9193	4.0054	4.6223	4.1165	5.1984	5.0834	4.2887	5.0155	4.7319	4.4722	4.5130
Pfn2	_SQGGEPTYNVAVGR_.2	#N/A	9.4007	9.2931	9.8049	9.4742	9.4955	9.4333	9.5332	9.4760	9.7522	9.4644	9.3894	9.2270	9.6868	9.7602	9.8796	9.3607	9.4683	9.3931	9.4206	9.4699
Pfn2	_DSLYVDGDC[Carbamidomethyl (C)]TM[Oxidation (M)]DIR_.2	#N/A	8.3472	8.5442	7.3441	7.7668	7.8399	7.8518	7.9234	8.1960	8.5386	8.3648	7.3081	7.7852	8.3623	8.2331	8.1181	8.3396	7.7124	8.0803	7.9781	7.6690
Pfn2	_EGVHGGGLNKK_.2	#N/A	5.7797	5.8671	6.6940	6.4204	6.4363	6.4086	5.8661	6.4001	6.7974	6.6106	6.8420	6.1611	5.1478	5.9618	5.1641	6.3799	6.5208	6.1050	6.0449	6.2113
Pfn2	_VLVFVMGK_.2	#N/A	3.5638	4.2367	4.7640	5.6962	2.9950	3.1692	4.2377	3.2642	3.1693	3.7701	4.5674	2.6019	3.3058	4.7367	3.8905	3.9087	3.5988	4.0313	2.6426	4.0542
Pfn2	M[Oxidation (M)]C[Carbamidomethyl (C)]DGC[Carbamidomethyl (C)]C[Carbamidomethyl (C)]	#N/A	4.3654	3.6374	3.4008	3.3240	3.7031	4.2818	3.2982	2.5242	4.2207	3.8521	3.6993	2.6102	4.3861	4.4529	2.8250	3.2094	3.1026	3.2297	4.3791	1.3603
Cacng4	_ASSSS[Phospho (STY)]PYAR_.2	Q9JJV4_S228_M1_Cacng4	3.9824	4.0204	4.5187	3.7069	3.3202	3.1639	4.2209	3.6828	3.8377	4.1888	6.1294	3.9098	6.5199	4.5058	3.7981	4.3273	4.2205	3.6127	3.2612	2.9720
Cacng4	_STEAS[Phospho (STY)]PSRDAS[Phospho (STY)]PVGLK_.2	#N/A	4.9615	3.8281	4.1270	4.0933	5.1348	4.9793	4.5800	4.6663	3.5493	4.7203	4.5251	4.0134	5.1061	5.6030	4.5894	5.6765	5.7877	5.7465	5.0598	3.7796
Cacng4	_DAS[Phospho (STY)]PVGLK_.2	Q9JJV4_S259_M1_Cacng4	3.0827	4.8417	4.1201	4.5016	4.2745	3.3230	5.2703	3.3084	3.6787	5.6145	6.4154	5.4418	3.8441	4.3468	3.7839	3.9456	4.8239	3.7717	3.4203	4.3138
Cacng3	_SRDLS[Phospho (STY)]PISK_.2	#N/A	10.7866	10.6010	10.3160	10.4297	10.0588	10.0482	10.3519	10.6490	10.3101	10.2570	10.3259	10.3431	10.8811	10.4423	9.3957	10.1363	10.9637	10.6721	10.4852	10.2059
Cacng3	_S[Phospho (STY)]HSELLK_.2	Q9JJV5_S211_M1_Cacng3	5.9465	3.9573	4.5818	5.6513	3.0675	6.2584	3.9806	5.1359	3.9008	4.1720	6.6670	5.7106	5.7592	4.5689	4.0059	3.7235	4.2836	6.3198	3.1982	6.0795
Cacng3	_DLS[Phospho (STY)]PISK_.2	#N/A	3.8982	3.4941	3.5442	6.5619	4.1051	5.4767	4.4438	4.2091	5.2501	6.4480	3.8426	3.1470	4.5295	3.5313	2.9683	5.9140	3.2460	3.0864	6.8271	3.4983
Smpd3	_DGDSGS[Phospho (STY)]LGSPSASR_.2	Q9JJY3_S289_M1_Smpd3	9.9287	9.5915	7.9021	9.8665	7.0950	7.0401	9.2151	7.4693	8.8883	7.9695	9.0187	3.9410	7.5746	4.0190	5.4880	8.1735	8.6857	7.6364	6.9716	8.9096
Smpd3	_TAS[Phospho (STY)]VEYKGDGGR_.2	Q9JJY3_S208_M1_Smpd3	7.4937	7.5853	7.4324	7.0122	6.0475	6.8326	6.6294	4.0384	6.3191	3.8796	3.6719	6.5537	4.3587	6.1404	5.5372	3.4311	3.0752	6.9944	6.4965	3.3275
Smpd3	_IY[Phospho (STY)]IDSPTNTSISAASFSSLVSPQGGDGSR_.3	Q9JJY3_Y170_M1_Smpd3	4.2562	6.7868	3.1862	3.5387	3.7033	2.9957	4.0858	6.5545	4.0060	5.3118	3.4846	6.0724	4.1714	7.2753	4.4388	3.6184	4.3887	6.3846	3.0930	3.1402
Ddx20	_DSS[Phospho (STY)]PGSLPK_.2	Q9JJY4_S569_M1_Ddx20	6.0695	3.3472	3.6911	4.5346	3.9933	4.2174	3.5478	4.3560	3.6068	3.3611	3.9812	5.7608	3.1755	3.6781	3.1250	3.4997	3.3928	4.4403	6.2512	3.6451
Ddx20	_AAMHTYSSPTVATQS[Phospho (STY)]PK_.2	Q9JJY4_S472_M1_Ddx20	3.5732	3.1691	5.9615	4.3565	3.7801	3.8135	3.2680	4.5341	3.1882	3.3838	4.1676	3.0585	3.3536	3.8563	3.2933	2.9353	5.3595	4.2622	3.9108	3.8233
Tuba8	_ES[Phospho (STY)]IDLVLDR_.2	Q9JJZ2_S114_M1_Tuba8	6.2723	6.0184	6.5399	5.9371	5.8467	6.4782	5.9569	6.0869	5.6203	6.4193	5.3511	4.8911	3.4446	5.0404	5.1165	5.4596	5.9597	5.3350	5.4684	5.4792
Ube2j1	_NTSMS[Phospho (STY)]PR_.2	Q9JJZ4_S251_M1_Ube2j1	3.2876	3.2143	3.8239	4.4018	4.1262	4.3503	3.4149	4.4888	3.4740	3.4939	3.8484	3.1038	3.5820	6.3374	2.9921	3.6325	3.5257	4.3075	4.2381	3.7780
Klf13	_ADLES[Phospho (STY)]PQRK_.2	#N/A	3.4036	3.9155	4.4410	6.2814	3.6105	5.4371	5.7077	5.7036	3.3578	3.2142	5.8723	4.8828	3.5232	6.4895	3.4630	6.0296	5.9960	4.0230	6.1603	5.9621
Klf13	_SRADLES[Phospho (STY)]PQRK_.3	#N/A	3.6386	3.2345	3.8037	4.4219	3.8456	3.8789	3.2025	4.4687	3.1227	3.4492	4.1022	3.1240	3.2882	3.7908	3.2279	3.0008	3.5055	4.3277	3.9762	3.7578
Pdk2	_TLSQFTDALVTIR_.2	#N/A	3.2707	1.5541	4.6268	4.0540	3.7752	3.6978	4.3716	3.3358	4.2317	4.5821	3.8446	3.8954	3.6561	4.1588	3.8997	4.1337	4.1306	3.9597	3.6083	3.7334
Kcnq5	_SPSTDITAEGS[Phospho (STY)]PTK_.2	#N/A	7.9619	7.6249	7.6878	7.6578	7.1823	7.4413	7.2873	7.6005	3.8437	3.7293	7.4312	3.4377	7.6733	7.3048	6.1253	3.3385	7.9738	7.5405	7.1027	6.7900
Kcnq5	_SWS[Phospho (STY)]FNDR_.2	Q9JK45_S467_M1_Kcnq5	4.4954	4.3245	4.0320	8.1240	6.5084	3.4060	3.8466	3.2203	4.4182	7.7979	3.2918	4.0506	4.5263	7.1916	3.8719	7.2953	4.2183	4.4319	3.2302	3.7474
Kcnq5	_RSPSTDITAEGS[Phospho (STY)]PTK_.2	#N/A	3.5798	5.9492	4.7056	5.3756	4.6415	3.5666	3.1227	3.2802	3.4351	3.1369	2.9137	4.3237	4.7467	4.1032	4.2007	4.1762	6.1180	4.0153	3.6639	4.6437
Uchl3	_FLENYDAIR_.2	#N/A	6.1991	6.3494	6.0545	6.5166	6.4807	6.3770	6.0846	5.9223	6.0260	6.3923	6.2632	5.8547	5.5570	6.2727	6.5232	5.9956	6.4606	6.6125	5.8663	6.4787
Uchl3	_WLPLEANPEVTNQFLK_.2	#N/A	4.9648	4.7964	5.5187	5.8826	5.4106	5.3985	5.0550	5.4904	5.5979	5.5431	5.2090	5.5016	4.5286	4.8833	5.7765	6.0797	5.6889	5.0802	5.4749	6.1013
Uchl3	_FLEESVSM[Oxidation (M)]S[Phospho (STY)]PEER_.2	#N/A	8.0766	7.9030	7.7141	7.4633	3.9254	7.7648	7.4314	3.9079	7.9178	8.0865	7.0504	7.9967	8.3283	8.0470	7.6994	8.1947	8.1968	8.0730	7.8894	7.9995
Uchl3	_TSDETLLEDAIEVC[Carbamidomethyl (C)]KK_.3	#N/A	3.8372	4.1656	4.4735	3.8522	3.1750	3.3990	4.3661	3.5376	4.4252	4.0436	3.2987	4.0550	3.8580	4.3606	3.9433	3.6814	4.0753	3.7579	3.4065	4.3276
Ybx3	_S[Phospho (STY)]RPLNAVSQDGK_.2	Q9JKB3_S328_M1_Ybx3	7.9252	8.0122	8.2574	8.1991	7.3335	7.6823	7.4364	7.2811	7.1506	8.1025	7.4410	7.1673	8.1299	8.5244	7.2831	7.7842	7.7752	7.4794	7.5102	7.9702
Ybx3	_[Acetyl (Protein N-term)]S[Phospho (STY)]EAGEATTGGTTLPQAAADAPAAAPPDPAPK_.3	Q9JKB3_S2_M1_Ybx3	3.8379	4.1649	4.3742	3.8515	3.1756	3.3997	4.3655	3.5382	4.4246	4.0442	3.2981	4.0544	4.5326	4.3612	3.9427	4.1828	4.0759	3.7572	3.4058	4.3282
Cend1	_PAPTVPAAPSS[Phospho (STY)]PDATSEPK_.2	#N/A	9.5277	3.6077	9.5509	9.2061	8.9278	9.1007	3.8760	9.2588	8.9635	9.1484	9.3153	9.0145	3.9617	9.5779	8.2306	3.9205	9.7386	9.1731	8.9775	3.8103
Cend1	_DTQAEKQEQAAAPGPAATK_.3	#N/A	7.8665	8.1118	7.5671	8.0419	7.5784	7.7014	8.0076	8.1577	8.1474	7.6440	7.6326	8.2360	8.4866	7.9782	8.2046	7.7902	7.9393	8.1767	7.8782	7.8707
Cend1	_QEQAAAPGPAATK_.2	#N/A	7.8302	7.2840	8.2109	7.5942	7.5894	7.7567	7.7592	7.7252	7.8923	7.7047	7.8993	7.6674	7.0493	7.6575	8.2391	7.7345	7.5500	7.4722	7.6376	7.6840
Cend1	_DTQAEKQEQAAAPGPAATKK_.3	#N/A	6.8317	6.8571	6.6336	7.0410	6.4741	6.8486	7.0407	7.2002	6.9394	6.5990	6.6595	6.8626	7.0947	6.4168	6.7854	7.1925	6.9404	6.8651	6.5675	6.6422
Cend1	_VPQATAEAK_.2	#N/A	6.4913	6.1541	5.6248	7.0229	6.1977	5.9595	6.7187	5.0026	7.0351	6.4385	7.0403	6.2867	6.0890	5.9704	6.8850	6.6571	5.7673	6.3532	6.5865	6.9510
Cend1	_SASS[Phospho (STY)]PKPDTK_.2	#N/A	9.1662	8.3367	8.7727	8.7526	8.3915	7.8912	8.2156	7.5964	7.9965	8.2487	8.2546	7.6164	8.1064	7.8001	7.3059	7.5862	8.3036	8.1821	7.5013	8.1311
Cend1	_SASS[Phospho (STY)]PKPDTKVPQATAEAK_.3	#N/A	12.5243	12.8647	11.8628	8.9248	12.1733	11.9155	12.3318	12.6835	12.1583	12.4424	11.7069	12.4127	13.6052	12.4232	12.1380	12.4989	12.4082	12.3792	12.2947	8.3089
Cend1	_GPGDGAEEDESNTGGR_.2	#N/A	4.9084	5.2396	4.9165	4.9948	5.2590	5.2506	5.0741	5.3590	5.1883	5.3268	5.0731	4.9669	4.8425	4.9950	4.5959	4.6400	5.0746	5.3664	5.1808	5.2424
Cend1	_ADPVLLNNHSNLKPAPTVPAAPSS[Phospho (STY)]PDATSEPKGPGDGAEEDESNTGGR_.5	#N/A	3.9112	8.7887	7.5953	7.4467	3.2847	7.4631	8.1813	8.2768	7.1249	3.9801	6.7702	8.3993	9.1748	9.3652	7.0555	7.5046	4.3015	7.9859	8.0372	7.3836
Cend1	_ADPVLLNNHSNLKPAPTVPAAPSS[Phospho (STY)]PDATSEPK_.4	#N/A	3.6392	9.9445	8.5216	10.2765	3.8462	10.0976	9.6772	10.4509	9.9297	3.4059	3.9364	9.8158	9.8820	11.0994	9.4151	7.9536	3.3621	9.7872	8.2698	9.9328
Cend1	_GKS[Phospho (STY)]ASSPKPDTK_.2	Q9JKC6_S7_M1_Cend1	4.0384	3.2996	3.5560	3.5648	4.2264	3.2643	3.6043	4.0878	4.2427	3.8301	3.7214	3.0257	4.4082	3.7260	2.8471	3.3816	3.1247	3.2076	4.3571	3.3770
Scamp5	_AQEEWTTGAWK_.2	#N/A	8.4796	8.4078	8.2643	8.1780	8.0810	8.0681	8.4038	8.5246	8.4212	7.9967	8.2930	7.8541	8.5745	7.8453	7.4413	7.8965	8.5554	8.2070	8.1445	7.9741
Scamp5	'QQAAQNAAM[Oxidation (M)]GAAQGAM[Oxidation (M)]NQPQTQYSATPNYTYSNEM[Oxidation	#N/A	4.4210	3.5818	3.4565	3.2684	3.7587	4.2262	4.3561	4.1214	4.2763	3.7965	3.7549	3.4712	4.4417	3.4435	2.8806	3.2651	3.1582	3.1741	4.3235	3.4105
Nectin1	_AGPLGGSS[Phospho (STY)]YEEEEEEEGGGGGER_.3	#N/A	4.1049	3.7359	3.8480	3.5943	3.7640	3.9624	6.2035	3.9849	4.1237	3.8514	3.6023	3.5852	4.2891	4.3441	3.4580	3.6610	3.6931	6.5659	4.0597	3.7973
Nectin1	_AGPLGGSS[Phospho (STY)]YEEEEEEEGGGGGERK_.3	#N/A	3.7019	3.6281	4.1148	4.8633	3.9088	3.9422	5.7547	3.9276	3.9467	5.4649	4.0498	3.3542	4.1895	3.2765	3.1755	3.9403	3.4248	3.4938	4.0346	3.9973
Nectin1	_AGIPQHHPPM[Oxidation (M)]AQNLQYPDDS[Phospho (STY)]DDEKK_.4	#N/A	3.5733	3.7664	4.2901	4.2249	3.7015	3.8637	3.7692	3.7961	3.7956	9.0544	3.8563	3.6021	3.9169	3.9474	8.3924	9.1874	3.7705	9.7306	3.7782	4.0206
Stx6	_[Acetyl (Protein N-term)]S[Phospho (STY)]M[Oxidation (M)]EDPFFVVK_.2	#N/A	7.6385	7.8642	7.6110	7.5382	6.2430	7.2974	7.5987	6.3361	7.4734	7.8796	7.6484	7.3031	7.4003	6.9946	5.8362	7.5039	6.8619	7.5105	7.3639	7.5412
Tmod2	_ANDPSLQEVNLNNIK_.2	#N/A	9.6035	9.8044	9.2657	9.3579	9.0812	9.1427	9.2515	10.0139	8.8555	9.4009	9.1740	9.6330	10.1754	10.3315	9.2964	9.4593	9.6440	9.4281	9.3321	9.3426
Tmod2	_SLETNTHVK_.2	#N/A	6.2437	5.7161	6.4477	6.5078	6.3487	7.0378	5.7879	6.4009	6.3818	5.8693	6.1801	6.2390	6.1084	6.2784	7.0509	6.5835	5.7797	5.6517	5.9947	6.1311
Tmod2	_SNDPVALAFAEM[Oxidation (M)]LK_.2	#N/A	5.3473	4.9353	4.9702	4.2315	5.2990	5.2728	4.9917	5.2278	4.3946	5.5141	4.6820	5.2662	4.8189	4.7348	5.2798	5.3364	5.7327	5.1869	5.1143	5.0959
Tmod2	_NIDEDELLGK_.2	#N/A	5.0489	5.5415	4.9882	6.0177	5.5303	5.5551	4.9225	4.9632	5.5359	5.7397	5.7373	5.0587	3.4742	5.6628	5.6604	5.7971	4.5083	4.8699	5.4785	5.3242
Tmod2	_QLENVLDDLDPESATLPAGFR_.2	#N/A	3.5137	5.5695	5.4702	5.3735	3.9716	5.5265	4.5843	5.1141	5.6496	5.6723	4.7872	5.3490	4.7145	5.8867	5.8646	5.2580	5.3812	5.3436	5.1603	5.3402
Tmod2	_ENDTLTEIK_.2	#N/A	6.0364	3.2312	5.9733	3.4963	6.1721	6.0420	6.0759	5.1369	6.2477	6.2984	6.3643	6.2061	4.3397	6.1684	6.6802	6.1764	5.2997	5.8367	5.9697	6.1742
Tmod2	_DREDFVPFTGEK_.2	#N/A	4.8694	5.7816	5.7226	5.7391	5.4127	5.8194	5.6183	6.1329	6.2633	5.9558	5.9852	5.8736	5.6415	6.0995	6.1859	5.9219	5.9913	5.8228	6.1923	6.1478
Tmod2	_SLNVESNFITGTGILALVEALR_.2	#N/A	4.2100	3.7928	3.2455	3.4794	3.5477	3.7718	3.9934	3.9104	5.1326	4.4163	2.9260	3.6823	4.1605	3.2325	3.5706	3.0541	4.4481	1.0231	3.0337	3.1995
Ndufaf3	_VTGAALIPPPGETALASLGQASE_.2	#N/A	3.7738	4.7665	5.1421	3.9156	5.1566	5.2545	5.4344	5.5641	5.7165	5.3483	5.1861	5.9964	4.5967	6.1267	4.0068	4.1187	5.7912	3.8213	5.4878	6.0816
Nova1	_QTLPSSPT[Phospho (STY)]TTK_.2	Q9JKN6_T156_M1_Nova1	7.9989	7.9350	4.0844	8.0049	2.9921	3.7684	7.6630	3.8596	7.7870	3.9393	7.9419	8.0852	8.5318	8.7374	8.1107	3.8975	8.3882	7.9362	8.2531	8.4103
Nova1	_IKQTLPSS[Phospho (STY)]PTTTK_.2	#N/A	4.3641	3.6387	3.3995	3.3253	3.7018	4.2831	4.2991	4.0645	4.2194	3.8535	3.6980	3.5282	8.2455	3.3866	3.4165	7.6336	3.1013	3.2310	7.5391	3.3536
Nova1	_TLVEYQELTGAR_.2	#N/A	4.0599	3.4600	3.3825	4.2610	4.2668	4.3002	4.2821	4.0474	4.2023	3.8705	3.6810	3.5452	4.3678	3.3696	4.5153	3.4220	3.0843	4.1184	4.3975	3.3366
Chrac1	_REEEEDNEDDGS[Phospho (STY)]DLGEALA_.2	Q9JKP8_S122_M1_Chrac1	4.2221	7.3156	6.8637	6.0069	2.9282	7.1376	6.4884	3.8852	7.3607	4.0327	3.5188	4.3240	4.2056	7.1058	6.3294	6.9887	4.4229	6.3458	7.1003	6.6294
Hyou1	_DAVIYPILVEFTR_.2	#N/A	3.5527	4.2685	4.1823	4.1367	4.6650	4.4502	4.8418	4.8971	3.4079	4.8898	5.0396	4.6216	3.5734	4.5483	5.0617	4.5150	4.6129	3.8042	4.6216	4.6224
Hyou1	_VLQLINDNTATALSYGVFR_.2	#N/A	4.8213	4.7778	3.6843	4.5593	3.9866	4.2107	4.1895	2.9075	3.6136	3.4376	3.9328	4.6464	5.5360	5.1024	3.5936	3.8200	3.6214	3.3646	4.6431	4.3930
Hyou1	_VESVFETLVEDSPEEESTLTK_.2	#N/A	3.6552	3.2511	3.7872	4.4385	3.8621	3.8955	3.1860	4.4521	3.1062	3.4658	4.0856	2.8578	3.2716	3.6003	3.2113	3.0173	3.4890	4.3442	3.9928	3.7413
Ldb3	_ASSEGAQGSVS[Phospho (STY)]PK_.2	Q9JKS4_S179_M1_Ldb3	3.9333	4.0695	5.6230	3.1779	5.8505	4.9457	4.3119	4.3435	4.1388	4.1396	5.1265	4.8409	5.2339	5.8099	4.8069	4.2782	5.5338	5.5590	3.3193	4.7920
Habp4	_SLAASGAQQPDS[Phospho (STY)]PGGPQPPGQK_.2	#N/A	9.4864	9.4074	9.5745	9.2301	9.2288	9.0631	8.7914	9.2601	8.9773	9.4628	9.1809	9.1010	9.5059	9.9323	8.9104	9.3720	9.6390	9.1195	9.1302	9.4105
Habp4	_AFDS[Phospho (STY)]FDQR_.2	Q9JKS5_S206_M1_Habp4	7.3766	7.7354	7.9057	7.5613	4.1538	6.0563	6.5049	4.1605	5.7214	7.6669	5.6804	5.7553	6.1399	7.1932	4.7018	6.6895	5.7344	6.3402	6.4876	6.7955
Habp4	_KSLAASGAQQPDS[Phospho (STY)]PGGPQPPGQK_.3	#N/A	8.9486	9.1794	8.6695	8.7979	8.5442	8.3238	8.7547	9.1515	8.2911	4.2539	8.5366	8.8173	9.1755	9.6605	8.4968	8.6737	8.6322	8.6728	8.5519	8.5640
Habp4	_S[Phospho (STY)]PAVASGHRPGAGGR_.3	Q9JKS5_S74_M1_Habp4	3.5044	3.8146	4.5419	4.0798	3.7114	4.3970	3.3367	3.7301	3.4273	3.5407	3.8016	3.5408	3.5353	4.5799	3.3621	4.3674	3.2274	3.9221	4.2211	4.2572
Habp4	_RGEQQGWNDNR_.3	#N/A	4.3737	3.3532	3.5025	3.6183	3.7114	3.9355	3.8297	4.1916	3.8888	4.5800	2.3204	3.0793	3.9968	3.5406	2.4478	3.2177	3.6888	3.4606	3.7597	3.2179
Adrm1	_DKKDEEEDM[Oxidation (M)]S[Phospho (STY)]LD_.2	#N/A	6.5896	7.5581	8.1923	8.4759	7.1788	7.0983	7.3244	6.9944	7.1855	7.0309	7.6244	7.1937	6.5711	8.4560	7.3736	6.9825	7.2988	6.9335	6.3605	6.9639
Adrm1	_SQS[Phospho (STY)]AAVTPSSSTSSAR_.2	Q9JKV1_S213_M1_Adrm1	6.2432	5.0355	5.4343	4.0269	5.9545	6.2521	5.6401	4.9713	6.3586	6.0433	5.8399	5.7965	5.0369	4.1168	5.3747	5.9975	5.1629	3.9897	5.4207	5.7893
Nudt5	_ESTESS[Phospho (STY)]PGKHLVTSEELISEGK_.3	Q9JKX6_S10_M1_Nudt5	3.8671	6.1700	4.6640	3.1496	4.0741	5.2390	4.4749	4.2402	4.3951	5.7361	5.6371	3.1781	4.5605	3.5623	2.9994	5.9160	3.2770	3.0553	4.2047	3.5293
Fmn2	_GATADDSGGGS[Phospho (STY)]PVLAAK_.2	Q9JL04_S493_M1_Fmn2	9.0553	8.9817	9.0014	9.1127	8.4348	8.5379	8.6198	9.1382	8.6011	9.0763	8.6163	8.5912	9.2426	8.8145	8.0125	8.6851	9.0033	8.4971	8.7165	8.3918
Fmn2	_SSILES[Phospho (STY)]PKK_.2	Q9JL04_S629_M1_Fmn2	10.2325	10.3016	10.0559	10.2887	9.9891	9.9482	10.0371	10.5338	9.9489	10.1943	10.1504	9.9971	10.5846	10.4936	9.6574	10.0304	10.5820	10.3081	10.2171	10.1837
Fmn2	_LPEEEPS[Phospho (STY)]PKDVDTEPK_.2	Q9JL04_S614_M1_Fmn2	6.8625	6.7781	3.6353	7.0135	4.3056	4.3390	6.4934	4.0086	4.1635	6.7478	3.6421	2.9465	7.5853	7.3827	6.3595	3.4608	6.9682	7.3118	6.8844	7.1000
Fmn2	_GS[Phospho (STY)]PGEEWVPEVEEASQR_.2	#N/A	10.2772	10.9043	10.6441	10.3851	9.4235	10.5784	10.4919	9.7552	10.4946	10.3412	10.2024	10.0174	10.4067	11.2444	10.1119	10.3816	10.6615	10.7142	10.5972	10.4502
Fmn2	_SIQTS[Phospho (STY)]PTEEGR_.2	Q9JL04_S724_M1_Fmn2	11.3853	11.2974	11.0877	11.1393	11.0347	11.0168	11.0900	11.4950	11.0383	11.0967	10.8933	11.1398	11.6521	11.4586	10.4904	11.0011	11.3714	11.1309	11.0482	11.1226
Fmn2	_S[Phospho (STY)]ADWTEELGVR_.2	Q9JL04_S459_M1_Fmn2	4.2274	8.5640	3.2628	3.4620	3.5651	3.7892	3.9760	3.9277	4.0351	4.4337	2.9086	3.3649	4.2481	3.2499	3.5532	3.0714	4.4654	3.3677	3.0163	3.2169
Fmn2	_QLSSPNHSPS[Phospho (STY)]QSPNQSPR_.3	Q9JL04_S411_M1_Fmn2	4.0329	3.6705	4.1484	3.9080	3.8967	3.9972	4.2291	3.8286	4.2196	4.5048	4.0123	3.5022	4.3556	4.1157	4.0503	3.9630	3.9507	3.1849	3.5089	3.4581
Fmnl1	_SKPLDQSVEDLS[Phospho (STY)]K_.2	Q9JL26_S189_M1_Fmnl1	8.7538	8.6004	8.6014	8.6432	8.3889	7.4589	8.1018	7.8520	3.8360	8.4842	3.6209	7.8929	4.5598	7.9633	6.2239	7.8152	3.8520	7.7453	7.5810	7.6452

Fmnl1	_EAAADTSGREEPPTPKS[Phospho (STY)]PPK_.3	Q9JL26_S1021_M1_Fmnl1	7.2979	4.4096	4.0621	6.8309	6.6273	3.4669	7.2201	7.1428	7.0639	4.0185	6.8173	6.8491	7.4737	7.1735	4.0660	6.7058	7.4222	7.4259	7.2581	7.2117
Fmnl1	_GPGDVVSIEILPGAAAT[Phospho (STY)]PSGDDAQAPR_.3	Q9JL26_T509_M1_Fmnl1	5.6323	5.6381	3.9558	3.1650	3.1253	3.1586	3.9228	3.1440	3.8430	4.2298	3.3216	4.1268	4.4501	5.7593	3.9482	3.7813	4.1421	5.2274	4.9312	3.6711
Gltp	_[Acetyl (Protein N-term)]ALLAEHLLKPLPADR_.3	#N/A	5.8248	5.9827	5.3907	5.5303	5.7869	5.5387	6.0585	5.3747	5.4633	5.6580	4.1797	5.4801	5.5768	5.4492	5.3592	4.0572	5.3118	5.9261	5.7197	5.3415
Mpp6	_IDSILEVVQTGR_.2	#N/A	5.8978	6.4755	6.1021	5.9480	6.2914	5.7789	5.8646	5.1996	6.0938	6.1416	6.4474	5.5383	6.1841	6.4648	6.3700	6.1465	6.0216	6.0128	5.9745	5.7780
Mpp6	_VENNDLVIAR_.2	#N/A	5.5450	5.3317	5.7282	5.3800	5.5624	5.6898	4.9211	5.6043	5.9599	5.5220	5.3750	5.5835	5.3946	5.1633	5.8660	5.7064	5.0082	5.2307	5.4617	4.9763
Mpp6	_NAEFDRHEIQIYEEVAK_.3	#N/A	3.9367	3.6547	4.4730	3.7527	3.2744	3.8168	2.7064	3.9708	4.3257	4.1430	3.1993	3.9617	4.4338	4.5001	3.8439	4.2816	3.5283	4.1278	3.9465	3.4937
Mpp6	_YLEHGEYEGNLYGTK_.3	#N/A	4.1938	3.8090	3.2293	3.4955	3.5316	3.7557	4.0095	3.8942	4.0686	4.4002	2.9421	3.6984	4.1766	3.2164	3.5867	3.0379	4.4319	3.4013	3.0498	3.1834
Mpp5	_NLDLAS[Phospho (STY)]PEEYPK_.2	Q9JLB2_S25_M1_Mpp5	4.3008	8.7773	8.5980	8.1082	7.3253	8.6387	8.3976	7.9422	8.2946	8.7209	8.4323	8.6610	8.6691	9.3059	8.0424	8.6392	9.0395	8.5873	8.6330	8.6497
Cubn	_VNVT[Phospho (STY)]NEFKSSGNTM[Oxidation (M)]K_.3	Q9JLB4_T2533_M1_Cubn	3.5741	9.3518	8.8119	9.3023	3.3515	9.2373	9.3443	8.3596	9.0588	9.6457	3.9641	8.6316	3.6782	9.0596	3.7860	9.3482	3.6122	10.2304	9.6620	4.1478
Sacs	_DSAPTTPTS[Phospho (STY)]PTEFLTPGLR_.2	Q9JLC8_S4266_M1_Sacs	5.4915	6.1259	4.2345	3.8431	3.2050	3.3380	5.7859	3.9474	3.7865	4.4578	3.3244	4.0133	5.5633	6.1656	4.3344	3.8610	4.2436	3.7050	4.7451	3.5610
Dclk1	_SPSPSPTS[Phospho (STY)]PGSLR_.2	#N/A	12.0605	12.0672	12.4637	12.1949	11.4179	11.4778	11.5073	11.8043	11.5480	12.0089	3.3314	11.2311	11.8853	11.6510	10.5375	11.3105	11.6252	11.2934	11.3667	11.2776
Dclk1	_DIKPENLLVYEHQDGSK_.3	#N/A	7.6701	7.7396	7.1533	7.5139	7.5717	7.3664	7.6153	7.5678	7.3115	8.0002	7.2288	7.2765	7.7058	7.3469	6.3575	7.4570	7.7542	7.1414	7.2098	6.8605
Dclk1	_SFEALLADLTR_.2	#N/A	6.5427	6.4151	6.0792	5.7347	6.0454	6.4048	6.6929	6.5519	6.3043	5.6970	6.1728	6.0983	6.1883	5.9796	6.1084	5.3274	6.2381	6.3972	6.4923	6.0762
Dclk1	_S[Phospho (STY)]PASTSSVNGTPGSQLSTPR_.2	#N/A	3.6682	4.3346	4.2045	4.0212	3.0059	3.4782	5.8928	3.3685	3.5234	4.5494	3.0021	4.2241	3.6889	4.1915	3.6286	4.0131	3.9062	3.9269	3.5755	4.1585
Dclk1	_TLSDNVNLPQGVR_.2	#N/A	7.6174	7.9655	7.2461	7.5175	7.3247	7.4715	7.6298	7.5862	7.3100	7.5534	7.5192	7.2314	7.5523	7.4057	7.3770	7.2513	7.4468	7.1882	7.5520	7.1055
Dclk1	_NVNPNWSVNVK_.2	#N/A	6.2577	5.9073	6.5028	5.8045	3.1014	6.4392	6.1091	6.3305	5.8387	5.7812	5.9333	5.1242	6.2307	5.8934	5.6231	5.5660	5.9724	6.1407	5.0466	6.0890
Dclk1	_REESEEGFQIPATITER_.3	#N/A	8.8126	9.9943	9.8100	9.4519	5.5384	9.4259	9.4539	7.0859	9.2807	9.6871	6.9150	8.8908	8.6217	9.8741	8.4829	9.0920	8.1104	9.7274	9.5412	9.2717
Dclk1	_TIGDGNFAVVK_.2	#N/A	6.6196	6.8513	6.4579	6.3160	6.0439	6.5054	6.3496	6.7831	6.3344	5.8119	5.6675	6.0820	6.3275	6.1463	6.1149	6.2276	6.3111	6.2383	5.7058	5.8426
Dclk1	_GIVYAISPDR_.2	#N/A	6.7254	3.8071	6.2346	6.6667	3.5334	6.1549	6.4474	6.7297	6.1355	4.4020	6.0524	5.7059	4.1748	5.7583	6.2061	6.0647	6.2920	6.0460	6.1029	5.5336
Dclk1	_SKS[Phospho (STY)]PASTSSVNGTPGSQLSTPR_.3	#N/A	8.9742	3.9978	4.0314	4.0878	3.5204	8.6254	3.7923	3.4869	5.5000	4.0255	3.5039	3.7826	4.0340	3.9321	3.6975	4.9477	3.9877	8.5871	8.0118	4.1805
Dclk1	_SPSPSPTS[Phospho (STY)]PGSLRK_.2	#N/A	11.8235	11.7417	11.8290	11.8554	11.0577	11.0213	11.2515	11.7132	11.1229	11.8939	10.8768	11.3698	11.8050	11.5482	10.6082	11.1008	11.3656	11.2629	11.0354	11.0117
Dclk1	_SGKS[Phospho (STY)]PSPSPT[Phospho (STY)]SPGSLR_.2	#N/A	3.8585	4.1004	4.2934	4.3655	3.4630	3.7195	3.4067	3.4817	3.3629	3.9251	6.1795	3.8265	3.5840	4.0139	3.6478	4.1190	4.5561	3.9797	3.6148	4.2061
Dclk1	_DM[Oxidation (M)]ELEHFDERDK_.3	#N/A	4.4055	3.9207	4.7726	3.9278	3.2718	3.4193	4.5276	3.1304	4.7644	5.2962	3.2019	3.2653	4.4364	4.0025	4.3795	4.6065	4.2091	4.1366	4.7104	3.9366
Dclk1	_AQPAPPELNSESEDYS[Phospho (STY)]PSSSETVR_.3	Q9JLM8_S742_M1_Dclk1	8.9545	9.1941	8.6174	9.0204	7.4008	8.3758	8.5394	8.5568	8.8574	8.4941	7.9326	8.5196	9.1134	9.3835	8.0480	8.5033	8.9178	8.6745	9.2393	7.7692
Dclk1	_ISQHGGS[Phospho (STY)]STSLSSTK_.2	#N/A	9.8288	9.7465	9.6957	10.2449	9.4200	9.1710	9.3896	9.5172	9.4448	9.7266	9.5478	9.2147	10.0867	9.9089	8.7319	9.6213	9.4061	9.3064	9.1584	9.2257
Dclk1	_VC[Carbamidomethyl (C)]SS[Phospho (STY)]M[Oxidation (M)]DENDGPGEGDELGRR_.3	#N/A	4.1120	6.8827	5.6869	3.6529	3.4623	5.5358	6.3622	5.9648	5.8607	3.7242	3.8484	3.5180	4.0144	5.4786	3.7320	5.7126	5.3979	6.2567	5.9972	5.9721
Dclk1	_VC[Carbamidomethyl (C)]SS[Phospho (STY)]M[Oxidation (M)]DENDGPGEGDELGR_.2	#N/A	8.3556	7.7640	8.5706	8.4684	6.6976	7.4425	8.0347	7.3871	6.5831	8.1687	6.6734	7.0204	7.3949	8.2104	3.7951	7.2427	7.2671	7.2519	3.4929	6.7955
Dclk1	_SGKS[Phospho (STY)]PSPSPTS[Phospho (STY)]PGSLRK_.3	#N/A	5.4606	3.7831	3.7199	3.8916	4.6436	4.7314	5.5339	3.8919	3.8657	5.2720	3.8981	3.5621	3.7848	5.1445	4.0966	5.0115	4.2446	4.8933	5.5353	3.6940
Dclk1	_TLQTLSSEK_.2	#N/A	3.8822	3.7557	3.4188	3.7020	2.3459	4.6073	4.8235	4.1079	3.8050	4.3445	4.1794	3.0059	4.2101	4.0350	4.3514	3.2443	3.6051	4.8917	4.8001	4.6467
Dclk1	_EES[Phospho (STY)]EEGFQIPATITER_.2	#N/A	3.7276	3.5915	3.2642	3.8566	3.9345	4.1738	3.5913	3.9533	3.6504	3.3175	4.0248	3.3176	3.7585	9.8520	3.1389	3.0897	3.4505	9.5211	3.9980	2.9795
Dclk1	_[Acetyl (Protein N-term)]S[Phospho (STY)]FGRDMELEHFDERDK_.3	#N/A	4.3828	4.8938	3.9274	3.6652	3.0889	3.1222	3.9592	3.7246	3.8795	4.1933	3.3581	3.8681	4.0449	4.0599	3.9846	3.7449	4.2622	3.5709	3.2195	3.0137
Dclk1	_[Acetyl (Protein N-term)]S[Phospho (STY)]FGRDMELEHFDER_.3	#N/A	4.3000	3.7028	3.3354	3.3894	3.6377	4.3472	3.9034	4.0003	4.1552	4.5063	3.6339	3.5923	4.3207	3.3225	3.4806	3.1440	4.5380	3.2951	2.9437	3.2895
Cd2ap	_S[Phospho (STY)]VDLDAFVAR_.2	Q9JLQ0_S458_M1_Cd2ap	4.0467	7.3017	7.2183	5.5060	4.2536	6.1414	6.4198	4.2724	6.4245	4.4992	4.3439	6.0465	4.0776	7.6514	5.8660	5.8013	3.7696	7.0206	5.6920	6.3984
Higd1a	_[Acetyl (Protein N-term)]STNTDLSLSSYDEGQGSK_.2	#N/A	5.0227	4.6160	5.0286	4.1523	3.6674	4.9890	5.0948	3.2375	5.6019	4.6599	4.0168	4.5470	5.0788	4.8235	3.4747	4.4157	4.5479	4.8770	4.2691	4.3704
Bag3	_SQS[Phospho (STY)]PAASDC[Carbamidomethyl (C)]SSSSSSASLPSSGR_.2	Q9JLV1_S179_M1_Bag3	4.0417	3.7937	5.7920	5.7472	5.4198	5.5970	5.5434	5.7790	3.8481	6.2959	5.4528	5.8382	5.2930	4.0342	3.6532	5.9827	6.0922	5.3989	3.5399	5.8150
Bag3	_AASPFRS[Phospho (STY)]PVR_.2	Q9JLV1_S274_M1_Bag3	3.1573	3.3447	3.6936	4.6992	3.9958	4.2199	3.5453	4.3585	3.6043	3.3636	4.5131	3.2342	3.7124	3.2562	3.1225	3.5022	3.3953	3.7450	4.0441	4.4343
Cul3	_FLQESFNNDR_.2	#N/A	4.3562	7.0455	3.3916	6.7851	3.6939	6.9768	7.0433	4.0565	3.9063	4.5625	4.2807	3.5361	4.0143	3.3787	3.4244	2.8903	7.3545	6.8742	4.3884	3.3457
Auh	_LAIIPGGGGTQR_.2	#N/A	8.1780	8.7033	7.9745	8.0145	8.2644	8.1227	8.3574	8.3480	7.7797	8.1423	7.9513	8.0864	8.5220	8.5560	8.1558	8.1291	7.9807	8.4295	7.9321	8.0292
Auh	_HLEEENR_.2	#N/A	6.5595	4.5785	5.5357	5.5884	6.1658	4.8004	5.0253	5.6907	5.2947	5.2015	5.3428	6.9276	4.3172	7.0100	7.6767	6.8782	5.1995	4.9390	5.6125	5.2307
Auh	_EFLPQGPVAM[Oxidation (M)]R_.2	#N/A	7.3399	7.2702	7.0546	7.0462	7.5363	7.1037	6.9622	7.0621	6.7724	7.4267	6.8296	7.0295	6.9662	6.8512	7.1857	7.1459	7.3432	7.2130	6.9916	7.3467
Auh	_SEVPGIFC[Carbamidomethyl (C)]AGADLK_.2	#N/A	4.4506	5.2678	4.3644	3.7331	3.1567	4.2818	3.8914	3.6567	4.6096	4.2612	3.2902	4.0954	3.9770	5.3924	4.9965	5.6284	4.1943	5.1075	5.1866	4.7442
Auh	_AVGLISHVLEQNQEGDAAYRK_.3	#N/A	5.1440	5.1033	3.7639	5.3699	4.4707	4.4396	4.4829	5.4487	4.1502	5.2145	4.8006	4.8291	5.0465	3.8020	4.9170	5.0352	4.9835	4.5497	5.4318	5.0901
Auh	_AVGLISHVLEQNQEGDAAYR_.3	#N/A	3.8195	6.7117	6.7589	3.1020	4.1478	7.2925	4.4321	4.2878	4.4427	4.8625	3.6473	3.8531	7.4523	4.7648	2.7734	4.0843	4.4805	4.2362	4.9441	4.1825
Rabgef1	_QTS[Phospho (STY)]IETDR_.2	Q9JM13_S132_M1_Rabgef1	3.2433	10.8516	11.0048	10.8797	10.5723	10.6247	10.4631	10.5846	10.3157	10.8518	10.5538	10.6312	3.6835	11.0019	9.9674	10.7817	10.7220	10.6403	10.5698	10.7083
Rabgef1	_YM[Oxidation (M)]SGQTS[Phospho (STY)]PR_.2	#N/A	7.8448	8.2216	8.3179	8.4528	7.8353	8.0275	8.1143	7.8256	7.6387	7.9929	8.2200	7.9685	7.8681	8.4173	8.3117	7.9399	7.8914	7.7861	8.1298	7.9181
Rabgef1	_LDAQSLNLSQEDFDRYM[Oxidation (M)]S[Phospho (STY)]GQTSPR_.3	#N/A	3.7697	5.0310	4.0038	3.9298	3.9470	3.6751	3.9027	3.6918	3.6509	3.8602	3.8130	3.4365	3.7943	4.0029	3.3075	3.6799	3.8339	3.8186	3.6684	3.9056
Nt5c	_DTEVFIC[Carbamidomethyl (C)]TT[Phospho (STY)]PLLK_.2	#N/A	4.1002	5.8435	6.5608	5.6969	3.0129	5.3285	4.0352	5.5408	3.9555	4.1174	3.4341	3.7921	5.4558	4.6236	4.0606	6.0900	6.2720	5.3355	3.1435	3.0897
Nt5c	_WVEQNLGPEFVER_.2	#N/A	6.0786	6.3640	5.7868	6.3870	5.9038	6.2092	5.9201	6.5422	5.9378	6.0009	5.7617	5.7218	4.5472	6.7492	6.3331	6.1391	6.0390	5.9720	6.4004	5.1982
Nt5c	M[Oxidation (M)]NDM[Oxidation (M)]KDTEVFIC[Carbamidomethyl (C)]TT[Phospho (STY)]PLLK	#N/A	5.1758	3.8572	3.8631	4.3462	3.9973	4.2581	3.8949	5.2798	3.8897	4.3891	4.1486	5.3599	5.5166	5.6259	4.7710	3.5376	5.0785	4.7987	4.8620	4.8910
Nt5c	_VLVDM[Oxidation (M)]DGVLADFESGLLQGFR_.2	#N/A	3.9287	4.0741	4.4650	3.7606	3.2665	3.4905	4.2746	3.6291	4.3337	4.1351	3.2072	3.9635	4.4418	4.4521	3.8518	4.2736	4.1668	2.8614	3.3149	3.6628
Mink1	_SQS[Phospho (STY)]LQDQPTR_.2	Q9JM52_S603_M1_Mink1	12.3546	12.2007	12.3928	12.4588	11.4173	11.2940	11.7408	11.5376	11.4375	12.4074	11.4659	11.4134	11.6088	11.5689	9.7649	11.4364	11.3278	11.5229	11.4778	11.3816
Mink1	_LDSS[Phospho (STY)]PVLSPGNK_.2	Q9JM52_S746_M1_Mink1	12.6188	12.4183	12.2737	12.1987	11.1622	11.6189	11.9965	11.6637	11.6000	12.3183	11.5055	11.7373	11.8722	11.9131	10.1916	11.5436	11.9226	11.7999	11.5024	11.4841
Mink1	_SDSVLPASHGHLPQAGS[Phospho (STY)]LER_.3	#N/A	12.6348	12.4246	11.7604	11.8240	10.9255	11.2094	11.9141	12.0415	10.9699	12.1774	11.3403	11.6396	11.9093	12.0198	9.2883	11.3115	11.4510	11.6935	11.3914	11.1043
Mink1	_S[Phospho (STY)]DPGWER_.2	Q9JM52_S706_M1_Mink1	6.5158	6.1835	5.7858	6.7787	4.1165	5.7559	6.6994	4.8785	5.3484	5.9295	6.1609	5.0864	5.9582	6.2137	2.9569	3.2717	5.3734	6.0249	6.2333	6.3130
Mink1	_QQNS[Phospho (STY)]PLAK_.2	Q9JM52_S537_M1_Mink1	8.4657	8.1696	3.9243	8.2759	7.2589	7.3114	3.3230	7.2685	7.3931	3.3287	4.2227	7.3949	7.4304	6.9375	4.8352	6.9788	3.6260	4.2071	3.8557	6.5665
Mink1	_GQS[Phospho (STY)]PPTKDGGSDYQSR_.2	Q9JM52_S903_M1_Mink1	8.2617	4.5295	4.0096	8.1965	4.3118	3.0351	3.2292	8.5373	3.2883	3.6796	7.6711	4.4190	8.7833	7.8880	7.5317	3.8182	8.1192	8.5684	7.9499	7.7390
Mink1	_QQQQLQQQQQR_.2	#N/A	4.1229	3.8468	3.3195	3.4054	4.3299	4.1188	4.2191	4.6095	2.9639	3.4912	2.7038	3.6710	4.3047	3.3065	3.5412	3.4851	3.9834	4.3124	2.9597	3.2735
Mink1	_VGASTKLDSS[Phospho (STY)]PVLS[Phospho (STY)]PGNK_.2	#N/A	4.4867	6.3709	4.0233	3.0975	3.1928	3.2261	3.8553	3.6206	3.7755	4.2973	3.2542	5.4437	3.9410	4.4436	3.8807	3.8488	4.1583	5.5494	3.6683	4.6471
Kcnj10	_VAS[Phospho (STY)]PSGLR_.2	Q9JM63_S338_M1_Kcnj10	7.8406	8.0859	4.1496	4.0761	7.8479	3.5331	8.4350	7.5199	8.3153	3.1034	2.9472	4.2790	3.6340	7.4973	4.1673	3.9582	3.8513	3.9818	3.6304	7.6597
Arpc3	_AYLQQLR_.2	#N/A	7.0938	7.4689	7.5329	7.5494	7.6229	7.4645	7.7234	7.7702	7.1711	7.5180	7.3754	7.5668	7.9140	7.1559	7.5802	7.0911	7.0327	7.6759	7.4525	7.5193
Arpc3	_DTDIVDEAIYYFK_.2	#N/A	5.9976	4.5320	6.4822	6.0384	5.8755	5.9240	4.6946	5.4831	5.9364	6.3904	6.2152	5.3566	5.4138	6.1455	6.1845	6.0433	6.0908	5.8978	5.9241	5.9464
Arpc3	_LIGNMALLPLR_.2	#N/A	3.3562	5.7165	5.2034	6.2599	4.7446	3.4152	5.6157	3.5819	4.6833	3.1668	5.7573	4.4098	3.5706	6.6308	4.8771	4.2192	4.5631	3.9936	2.9911	4.3749
Arpc3	_ETKDTDIVDEAIYYFK_.3	#N/A	4.4824	4.0149	3.9755	4.1996	3.5320	3.4654	3.2792	4.0839	3.9097	4.1530	3.8129	4.1034	4.5658	3.8088	3.3651	2.8674	3.1913	4.0318	3.3458	3.4091
Arpc3	_PAYHSSLM[Oxidation (M)]DPDTK_.3	#N/A	3.5680	3.7809	4.1043	4.2190	3.6550	3.2424	5.4959	5.1319	5.4030	3.7743	4.9465	3.6703	3.9623	4.0914	5.6292	4.8356	3.8061	4.1247	3.7733	5.6839
Arpc3	_VFDPQSDKPSK_.3	#N/A	3.4852	3.8339	4.5226	4.0991	3.6921	3.7254	3.3560	3.7108	3.2762	3.2958	4.2557	3.5600	3.4417	3.9443	3.3951	4.3481	3.2081	4.1742	3.7683	3.9113
Arl6ip4	_S[Phospho (STY)]AGEDNDGPVLTDEQK_.2	Q9JM93_S140_M1_Arl6ip4	8.4049	7.9576	7.9037	7.8341	6.7554	7.9674	7.7158	8.0519	7.7808	7.4943	7.3196	7.6920	8.1611	7.8286	6.3960	7.5510	8.3019	7.6527	7.7587	7.4708
Cdc42ep4	_SLSSS[Phospho (STY)]PVKK_.2	Q9JM96_S140_M1_Cdc42ep4	9.6382	9.9148	9.5093	9.7524	9.5890	9.4263	9.3319	9.6344	9.4445	9.6977	9.2496	9.4830	9.9225	9.8838	9.5531	6.7546	9.5874	9.3400	9.3778	9.4456
Cdc42ep4	_EADDES[Phospho (STY)]LDEQASASK_.2	#N/A	9.4461	9.2258	8.0876	9.0257	9.1703	8.5629	8.8355	8.1637	7.8952	9.9108	8.2742	7.9224	8.4228	9.1689	7.0552	9.0749	8.1637	8.5056	7.8128	8.5180
Cdc42ep4	_DQWGS[Phospho (STY)]EEEEEAGGYR_.2	#N/A	3.5998	7.3966	8.0494	7.2487	2.9375	7.1076	6.8999	3.3002	6.6578	7.0759	3.5362	5.9156	3.2699	7.7375	6.2712	6.8483	6.9786	6.9783	7.3079	7.5216
Cdc42ep4	_AREADDES[Phospho (STY)]LDEQASASK_.2	#N/A	8.5472	8.3694	3.8807	8.1080	8.3437	7.9640	8.2506	3.8716	7.1087	9.2720	7.6632	3.9087	7.5781	8.2453	3.3048	8.2406	7.2082	3.6115	7.2759	3.2543
Cdc42ep4	_DQWGS[Phospho (STY)]EEEEEAGGYRDK_.3	#N/A	4.0094	4.4324	4.0449	3.6815	3.0107	3.5419	3.9018	4.0406	3.9047	4.2830	3.0526	4.2337	4.0361	4.0533	3.9272	5.7357	3.9679	6.7526	5.8160	3.9046
Cdc42ep4	_NAMS[Phospho (STY)]LPQLNEK_.2	Q9JM96_S116_M1_Cdc42ep4	3.1227	3.3793	3.6590	4.5667	3.9612	2.7008	3.5798	4.3239	3.6389	3.3290	4.0133	3.2687	3.1434	3.6460	3.1570	3.4676	3.3607	2.9716	4.1210	3.6130
Usp14	_VNQQPNANDKNS[Phospho (STY)]PPKEIK_.3	Q9JMA1_S393_M1_Usp14	9.4020	9.7091	9.2310	9.4842	9.3551	9.5785	9.4914	9.7661	9.2750	9.5533	9.3076	9.1685	9.9315	10.0282	9.2093	9.4566	9.6312	9.6887	9.3405	9.3551
Usp14	_AS[Phospho (STY)]GEM[Oxidation (M)]ASAQYITAALR_.2	#N/A	8.9701	9.0571	8.2834	8.2594	3.1077	7.8542	8.6024	9.1975	7.8066	8.5049	5.4297	8.4626	8.8564	9.4421	3.9070	7.8704	8.3181	8.5733	8.1936	3.9998
Usp14	_QSPTLQR_.2	#N/A	2.4957	3.7249	4.1279	4.8913	3.0739	3.8397	3.9255	3.9783	4.9367	4.9063	4.4952	4.6428	2.7339	3.7667	5.6376	3.1219	4.7954	3.3648	3.6638	3.3137
Usp14	_LEAIEDDSGRETDSSSAPAVT[Phospho (STY)]PSK_.3	Q9JMA1_T235_M1_Usp14	4.5398	5.2456	3.5753	3.1496	4.0740	4.1074	4.7874	4.2402	4.3951	3.6777	3.8737	3.3524	4.5606	4.9879	2.9994	3.2292	3.2771	4.2680	4.2047	3.5294
Qtrt1	_[Acetyl (Protein N-term)]AAVGS[Phospho (STY)]PGSLESAPR_.2	Q9JMA2_S6_M1_Qtrt1	8.4134	7.8448	7.8344	7.9432	7.5855	7.8852	3.7729	7.8342	8.0583	7.9265	7.7421	7.7075	7.9928	7.9300	7.2082	3.2745	8.4569	7.2407	8.0160	3.4200
Dnaja4	_LISQAYEVLSDPK_.2	#N/A	3.6296	3.6895	3.1662	3.9546	3.8366	4.2718	3.4934	3.8553	3.5524	3.4155	3.9268	3.4156	3.6605	4.4679	3.2369	2.9917	3.3525	4.7524	4.8143	4.3824
Stard10	_AGGAGGEGS[Phospho (STY)]DDDTSLT_.2	Q9JMD3_S284_M1_Stard10	11.7236	11.3660	11.7388	11.8534	11.3166	11.2909	11.0830	11.8294	11.5165	11.6462	11.2867	11.5021	11.6702	11.9826	10.8637	11.4593	11.7960	11.2709	11.3259	11.5686
Ap3b2	_AFYGS[Phospho (STY)]EEDEAK_.2	#N/A	9.2636	4.2393	8.8263	9.2170	8.5204	3.3828	3.6986	8.8765	3.6188	4.4540	8.9613	8.5790	8.8887	4.2869	7.4899	4.0055	4.0016	3.8316	3.4802	4.2539
Ap3b2	_QLIVPSEQGGALSR_.2	#N/A	5.6185	5.0817	4.8653	5.2455	5.3914	5.5848	5.8364	6.0856	5.9345	5.6773	5.3071	5.0553	5.2450	4.9788	5.4618	3.4236	5.2703	5.6285	4.7412	4.3548
Ap3b2	_ATGYQELPDWPEEAPDPSVR_.2	#N/A	5.1932	5.5460	4.0422	4.9538	5.5231	5.2928	5.7198	6.0244	5.4042	5.5538	5.1138	5.1287	3.3637	4.4084	4.9323	4.9681	5.3810	5.2248	5.2647	4.8814
Ap3b2	_AFYGS[Phospho (STY)]EEDEAKGPGSEEAATAALPAR_.3	#N/A	9.3625	10.4166	9.4355	9.4404	6.3551	9.4443	9.8688	8.2028	9.1371	9.3515	8.9299	8.8363	9.3784	10.2538	8.2512	8.6914	8.8390	10.1298	8.8136	8.3418
Ap3b2	_TQFLS[Phospho (STY)]PTQNESLLEENPEK_.2	Q9JME5_S253_M1_Ap3b2	4.4624	4.3576	3.9990	3.1219	3.1685	3.2018	3.8796	3.1872	3.7998	4.2730	3.2785	4.0837	3.9653	4.4679	3.9050	3.8245	4.1853	3.6505	3.9123	4.4349
Ap3b2	_LEVLTNLANETNIPTVLR_.2	#N/A	3.2855	3.2165	3.8218	3.6610	4.1617	4.6246	3.7329	3.9624	3.7153	4.8456	4.0525	3.8258	3.3062	3.3481	3.9012	2.9828	4.0265	4.8679	3.9582	4.1810
Ap3b2	_TTLVAGSVVM[Oxidation (M)]AFEEVC[Carbamidomethyl (C)]PER_.2	#N/A	4.8874	3.8476	4.4512	4.2548	3.6785	3.7118	3.3696	3.1349	3.2898	3.2821	4.2693	3.5737	3.4553	3.9579	3.3950	4.3345	3.6726	4.1605	3.8091	3.9249
Ap3b2	_GGGS[Phospho (STY)]ES[Phospho (STY)]EQS[Phospho (STY)]EEEDEKKK_.2	Q9JME5_S731_M3_Ap3b2	3.5213	1.6658	4.5588	4.0629	3.7283	3.7616	3.3198	3.7470	3.2400	3.3319	4.2195	3.5239	3.4055	3.9081	3.3452	4.3843	3.2443	4.2103	3.8589	3.8751
Tmub1	_EEAPGAES[Phospho (STY)]PSLR_.2	Q9JMG3_S71_M1_Tmub1	4.3981	3.6047	8.2458	3.2913	3.7359	3.9599	3.8052	4.0985	3.8643	3.1036	4.2387	3.4941	7.9923	3.4207	3.3824	3.2422	3.1353	3.1970	4.3464	3.1934
Hdgfl3	_GFNEGLWEIENNPGVK_.2	#N/A	5.9174	5.9662	6.1486	5.3148	5.0394	5.2087	5.7120	6.0118	6.1779	5.6783	5.5689	5.3615	6.2794	5.9854	5.8707	5.9357	6.4527	5.5689	5.6609	5.2759
Hdgfl3	_S[Phospho (STY)]PGDEDKDC[Carbamidomethyl (C)]KEEENK_.2	#N/A	3.1604	3.9740	3.7987	2.4890	3.7842	3.3548	4.1169	3.7604	4.0371	4.0891	3.5157	3.7640	4.2025	3.8167	2.6881	3.6407	3.8112	3.9077	3.8584	3.6138
Hdgfl3	_FTGYQTIQQQSSSETEGEGGNTADAS[Phospho (STY)]S[Phospho (STY)]EEEGDRVEDGKGK_.4	#N/A	3.8130	4.1898	4.3493	4.5585	3.1507	3.3748	4.3903	3.2514	4.4494	4.0193	3.3230	4.0792	4.5575	4.1012	3.9675	4.1579	4.2495	4.4008	3.1990	3.7785
Hdgfl3	_FTGYQTIQQQSSSETEGEGGNTADAS[Phospho (STY)]S[Phospho (STY)]EEEGDRVEDGK_.4	#N/A	3.2018	3.3002	3.7381	4.4876	4.0403	3.9446	3.5007	4.4030	3.0571	3.5149	4.0365	3.1896	3.2225	3.7252	3.0779	3.5467	3.4398	4.3933	4.0419	3.6922
Txnrd1	_VLVLDFVTPTPLGTR_.2	#N/A	3.6741	5.5389	6.4714	5.6328	5.2718	4.8844	4.4608	4.7862	5.2104	5.4147	4.8084	4.8672	3.2528	4.2689	5.0398	5.7094	4.6977	4.6107	5.3199	5.2768
Txnrd1	_VTAQSTNSEETIEGEFNTVLLAVGR_.3	#N/A	4.3112	1.5469	3.6511	2.8072	2.5534	3.8730	1.9386	2.7841	3.6022	4.6714	4.3256	2.4516	4.0593	4.1372	3.4693	3.1660	3.7513	2.6301	3.6972	3.3701
Txnrd1	_VVYENAYGR_.2	#N/A	4.2931	4.5268	3.8297	3.2911	2.9992	3.6083	4.0489	4.5188	4.2159	4.2528	3.0895	4.2529	4.3240	3.8678	4.0743	3.6552	4.0160	3.1334	3.4325	3.5450
Myo18a	_RFS[Phospho (STY)]FSQR_.2	Q9JMH9_S140_M1_Myo18a	8.1947	7.9575	3.1284	7.7406	7.3148	7.1397	7.2291	7.9788	6.9083	7.7570	7.3441	7.2754	3.6227	7.2245	6.0392	2.9539	3.3147	7.5333	7.3057	7.2745
Myo18a	_S[Phospho (STY)]LAHADEAR_.2	Q9JMH9_S865_M1_Myo18a	8.1517	7.5548	6.8271	7.0559	6.9922	7.2743	7.0134	6.9856	6.2877	7.8705	7.4209	6.7551	7.2186	7.1049	5.4146	6.3379	6.9031	7.0832	7.4013	6.8603
Myo18a	_FSHSYLSDS[Phospho (STY)]DTEAK_.2	#N/A	8.5881	9.2796	9.7003	9.3942	6.2230	9.0855	9.0167	8.4523	9.2468	9.1776	8.3400	8.7721	6.7233	9.1699	7.8354	8.7281	9.5389	8.9990	8.9532	8.8004
Myo18a	_SSSPTSHWKPLAPDPS[Phospho (STY)]DDEHDPVDSISRPR_.5	Q9JMH9_S2016_M1_Myo18a	3.4555	4.5473	3.9918	4.2339	4.2941	3.6909	3.3906	3.1559	3.3108	3.2612	4.2903	2.9359	3.4762	3.9789	3.4160	4.3135	3.6936	4.1396	3.7882	3.9459
Myo18a	_NELRLS[Phospho (STY)]SDRLETR_.3	Q9JMH9_S1279_M1_Myo18a	3.2394	4.3255	4.2030	4.0227	3.4464	3.4797	3.6017	3.3671	3.5220	4.5509	3.0006	3.2331	3.6874	4.1901	3.6271	4.1024	3.9047	3.9284	3.5770	4.1571
Myo18a	_VASGSDLHLTDIDSDSNR_.3	#N/A	4.5231	4.9526	5.2389	4.9621	3.6691	4.4958	4.6509	3.5930	3.5355	4.9104	4.9008	4.2212	3.6769	4.4922	3.9697	4.4765	3.6989	4.7303	4.5085	4.8963
Myo18a	_NKGPSKAPS[Phospho (STY)]DDGS[Phospho (STY)]LK_.2	Q9JMH9_S1994_M2_Myo18a	6.4464	5.8561	3.5941	3.5317	5.8638	5.6615	3.9212	4.2832	3.9803	4.4934	3.7502	3.4165	6.0111	3.6322	3.4984	3.1312	3.7804	6.1403	5.6611	3.3094
Myo18a	_FSHSYLSDS[Phospho (STY)]DTEAKLTETSA_.3	#N/A	3.7928	3.8844	3.7278	3.7594	3.6111	3.8352	4.0033	3.7862	4.0624	3.9992	3.4631	3.7295	4.1704	3.9027	3.5805	3.6675	4.0287	4.4324	3.5599	4.1411
Myo18a	_NKLEGDS[Phospho (STY)]DVDS[Phospho (STY)]ELEDRVDGVK_.3	Q9JMH9_S1966_M2_Myo18a	4.2811	3.8769	3.1613	3.5635	2.9872	3.0205	4.0609	3.8262	3.9811	4.0917	3.4598	4.2650	4.1466	3.1484	4.0863	3.6432	4.3639	4.1419	3.1178	3.1154
Myo18a	_APS[Phospho (STY)]DDGSLK_.2	Q9JMH9_S1994_M1_Myo18a	3.1540	4.1651	4.1915	4.4302	6.0441	3.2466	7.0314	3.3797	3.0769	4.4655	3.4512	3.8912	3.1849	4.2295	3.7125	4.0170	4.3778	4.2725	3.0707	6.4720
Csnk1e	_GAPANVS[Phospho (STY)]SSDLTGR_.2	Q9JMK2_S389_M1_Csnk1e	3.2509	9.4492	4.2884	4.3333	9.5783	9.4181	3.5902	9.2597	3.1738	4.5624	3.5481	3.7943	3.2818	4.3264	3.6156	4.1139	4.4747	4.1756	2.9738	4.0037
Csnk1e	_IQQTGNTS[Phospho (STY)]PR_.2	Q9JMK2_S363_M1_Csnk1e	9.2540	9.1645	9.1283	9.1451	8.7735	8.6420	8.8166	8.9766	8.8482	9.3564	8.6299	8.7800	9.0332	8.8304	7.7173	8.5467	8.8070	8.7098	10.0894	8.5635
Glrx	_[Acetyl (Protein N-term)]AQEFVNC[Carbamidomethyl (C)]K_.2	#N/A	5.3496	6.0814	6.4857	5.8960	5.8464	5.8629	5.9542	6.0729	6.1409	5.4287	5.5678	6.2069	6.6599	6.2359	6.2890	5.1840	5.7874	6.0147	5.7515	5.4873
Rhoa	_LVIVGDGAC[Carbamidomethyl (C)]GK_.2	#N/A	7.9904	8.0450	8.2229	8.2225	7.7335	8.2596	8.1737	8.3351	7.7131	7.8019	8.0112	8.0588	8.3384	7.8049	7.8976	7.6438	7.9404	7.9985	7.6442	7.5594
Rhoa	_TC[Carbamidomethyl (C)]LLIVFSK_.2	#N/A	5.3134	5.3108	5.5959	5.5724	4.2693	5.3759	5.0979	5.5493	5.5213	4.8008	4.5225	5.4259	4.4979	4.3039	5.4268	4.9288	5.7376	5.5826	5.1867	4.7005
Rhoa	_M[Oxidation (M)]KQEPVKPEEGR_.3	#N/A	6.2678	3.9243	5.8429	5.2650	5.7456	5.9532	6.4624	6.1038	6.7212	6.6436	5.9270	5.9445	7.0116	4.5461	6.4403	6.8972	5.7753	6.3218	6.8205	6.3386
Rhoa	_EVFEMATR_.2	#N/A	2.7575	5.9580	6.4737	6.7279	5.0404	4.8621	6.3148	3.8977	4.9689	4.0672	6.1152	4.5557	3.7029	6.8801	5.1312	3.0342	4.8730	3.8361	4.1670	4.8355
Rhoa	_QVELALWDTAGQEDYDR_.2	#N/A	6.9144	6.7334	6.6129	6.5124	3.8122	6.2954	6.5316	6.1187	6.7613	4.6959	6.6595	6.3939	6.8588	6.6866	5.7949	6.3807	7.1732	6.4962	6.5883	6.5297
Psma6	_AINQGGLTSVAVR_.2	#N/A	6.0265	6.8257	6.7924	5.7814	5.6995	6.4563	6.3791	6.1069	6.6075	6.5179	5.8316	6.1629	5.6490	6.3213	6.3963	5.7512	5.5313	6.3237	6.0683	6.3766
Psma6	_LLDSSTVTHLFK_.2	#N/A	4.5638	3.8407	5.2873	5.5451	5.2996	4.8602	5.3204	5.2505	4.6420	5.0817	4.8328	5.4284	5.1414	5.0241	5.0180	5.5040	5.5913	4.5729	5.0770	4.5481
Psma6	_HITIFSPEGR_.2	#N/A	3.0801	6.6039	3.6164	3.1084	6.6045	6.5971	6.8908	6.9531	6.3848	6.5237	6.8588	6.5554	6.3530	6.5615	6.1649	6.1857	6.6754	6.2366	6.1764	5.6058
Psma6	_YEAANWK_.2	#N/A	4.4266	4.3389	5.2105	4.0254	4.0713	5.0156	4.6482	3.7395	2.9599	3.5682	4.4318	4.5507	3.1901	4.3705	5.2199	4.5723	3.9020	4.7002	4.6023	4.2914
Psma6	_ITESIGC[Carbamidomethyl (C)]VM[Oxidation (M)]TGM[Oxidation (M)]TADSR_.2	#N/A	4.5678	4.2522	4.1044	4.5173	3.2739	3.3072	3.7742	3.2926	3.6944	4.3784	5.2411	3.9783	3.8599	4.1424	3.7996	3.9299	4.2907	4.3596	3.1578	4.3295
Psma6	_ILTEAEIDAHLVALAERD_.3	#N/A	4.2586	4.5613	3.7118	3.3256	2.9647	2.9981	4.0834	4.4843	4.0036	4.0692	3.4822	4.2874	4.1690	3.1708	4.1087	3.6208	3.9815	3.4468	3.0954	3.1378
Dkk3	_PAFVGSHDHS[Phospho (STY)]EESQLPR_.3	#N/A	8.0239	7.5261	8.5499	8.4974	7.5697	7.7425	7.0813	7.3076	7.8473	8.2256	7.3064	7.6677	7.1601	8.3373	8.2880	7.9714	7.9690	7.1286	8.0438	8.1830
Hapln1	_LLVEAEQAK_.2	#N/A	6.8431	6.5567	6.7335	6.5646	6.7896	6.6826	6.9030	6.8707	7.0402	7.4399	6.7236	6.6847	6.8972	7.0270	6.9847	6.7595	6.8735	6.4074	6.7465	7.2692
Hapln1	_GGSDNDASLVITDLTLEDYGR_.2	#N/A	5.4836	5.2896	4.8534	5.9233	5.1425	4.9667	5.3396	5.4707	5.1334	5.5737	5.4137	5.3509	5.0660	6.6620	5.2212	5.5898	5.7429	5.1359	3.5815	4.8975
Hapln1	_EPC[Carbamidomethyl (C)]GGQNTVPGVR_.2	#N/A	3.3807	7.7823	7.0505	7.3806	7.7493	7.7795	8.0455	7.4172	8.0152	7.9157	7.5323	6.9042	3.4116	8.0463	7.8695	6.3569	7.7025	7.7968	7.3816	7.6011
Hapln1	_DPTAFGSGIHK_.2	#N/A	6.4056	5.7728	4.6110	6.1912	6.1401	6.4017	4.1286	6.5068	6.2665	6.1210	6.1423	5.7098	6.3665	6.4526	6.8344	5.7571	6.1396	6.1032	6.7295	6.1693
Hapln1	_YNLNFHEAR_.2	#N/A	4.5525	6.5447	6.4761	5.8861	6.1604	6.0150	6.2595	5.7981	6.0970	6.0643	5.6212	5.5216	6.6703	6.1006	6.3158	3.3966	2.4541	5.7989	6.3941	6.6739
Hapln1	_C[Carbamidomethyl (C)]SPTEAAVR_.2	#N/A	4.2689	4.0789	6.3597	3.7655	3.2617	5.3710	5.1904	4.2863	5.5540	5.2829	5.6847	5.4283	4.4466	5.2139	5.7173	5.5586	5.5997	5.2092	5.5230	5.5989
Hapln1	_C[Carbamidomethyl (C)]DAGWLADGSVR_.2	#N/A	3.1004	4.2186	5.8815	4.4838	5.5167	5.0948	5.4933	3.5060	5.3090	4.4119	5.3863	4.9686	3.8264	5.0488	5.5272	5.2753	5.1362	3.7894	4.9149	5.5472
Hapln1	_LTSDYLR_.2	#N/A	4.2150	4.4379	3.2504	5.1397	5.0612	4.5689	5.3622	3.9153	4.7742	4.8584	4.8968	4.6211	5.1598	3.2201	5.1619	4.8619	4.4530	4.8138	4.7843	5.0718
Prep	_VFLDPNTLSDDGTVALR_.2	#N/A	3.4506	4.5522	6.3674	4.2388	4.2892	4.1537	4.6271	3.1510	5.6822	3.2661	4.2854	2.9408	3.4713	3.9740	5.1476	3.7955	3.6887	5.4493	3.7931	3.9410
Pin1	_SGEEDFESLASQFSDC[Carbamidomethyl (C)]SSAK_.2	#N/A	6.7794	5.2696	5.1028	5.8111	5.4779	4.9491	5.0225	6.0301	5.5531	5.3547	5.5435	6.0117	5.6106	6.6329	4.5775	5.4246	6.0861	5.5015	5.0371	5.5593
Pin1	_TGEM[Oxidation (M)]SGPVFTDSGIHIILR_.3	#N/A	4.0998	3.7469	4.1931	4.1418	4.3552	3.9961	4.0231	3.8919	4.6265	4.6525	4.2959	2.5499	4.1205	3.7812	3.9409	3.6028	3.9698	3.1045	4.5069	3.6857
Ptk2b	_RNS[Phospho (STY)]LPQIPTLNLEAR_.3	#N/A	3.2623	8.4160	5.7859	5.4024	4.1009	5.8063	8.5676	8.7137	5.4908	6.1393	5.4734	7.9205	7.6508	9.5120	4.8395	3.6072	6.7482	8.2391	7.0508	5.0938
Ptk2b	_NLLDAVDQAK_.2	#N/A	6.0245	5.1506	4.4706	5.5042	5.3976	5.6408	5.3474	5.1979	5.2972	5.0943	5.4835	5.0634	5.3590	4.8299	5.2647	4.7948	4.6343	5.0460	4.2924	3.8480
Ptk2b	_MSGVSEPLSRVK_.2	#N/A	5.1357	5.5596	6.5961	5.3924	5.2717	3.7844	5.2032	3.7698	4.6578	5.6856	5.3982	5.5731	5.9482	4.5257	4.4516	4.7748	5.1788	4.7468	5.1657	3.8523
Ptk2b	_SHLSESC[Carbamidomethyl (C)]SIES[Phospho (STY)]DIYAEIPDETLR_.3	Q9QVP9_S399_M1_Ptk2b	4.1199	3.8829	3.1554	3.5695	3.4576	5.2057	4.0834	3.8203	4.1425	6.1543	3.0161	3.7723	4.2506	3.1425	3.6606	2.9640	4.3580	3.4752	3.1238	3.1095
Ptk2b	_NS[Phospho (STY)]LPQIPTLNLEAR_.2	#N/A	3.1566	3.3454	3.6929	4.5328	3.9952	4.2193	3.5459	4.3578	3.6050	3.3629	3.9794	3.2348	3.7130	3.2568	3.1231	3.5015	3.3947	3.7444	4.0434	4.4349
Srcin1	_LPPSS[Phospho (STY)]PQK_.2	Q9QWI6_S540_M1_Srcin1	12.9567	13.0809	13.2566	13.1359	12.7274	12.7429	12.7815	12.8397	12.8457	13.2056	12.8144	3.2364	12.9545	13.1776	12.5736	12.8259	12.9592	12.7436	12.8152	12.8977
Srcin1	_KAES[Phospho (STY)]EELEVQKPQVK_.3	#N/A	9.9080	10.0674	9.6349	10.1543	9.1719	9.4482	9.6408	9.3950	9.3512	10.3551	9.6690	9.7858	10.4554	10.5080	9.2312	10.1072	9.6916	9.9809	9.9415	10.1168
Srcin1	_AES[Phospho (STY)]EELEVQKPQVK_.2	#N/A	10.1985	10.0477	10.1150	10.2400	9.1007	9.7049	9.8182	9.4929	9.4083	10.4005	9.6760	9.7095	9.9196	10.1850	9.0537	10.0128	10.1348	9.7828	9.9106	10.1108
Srcin1	_RFS[Phospho (STY)]NVGLVHTSER_.3	#N/A	7.6293	8.7298	3.3463	7.5785	6.6875	7.3938	8.3760	8.7405	6.8466	8.1416	6.8765	7.4566	7.9643	9.3275	3.2507	6.8099	7.6479	8.3663	7.4700	7.4581
Srcin1	_AVS[Phospho (STY)]VEAAERDWEEKR_.3	#N/A	7.2140	7.0601	8.1866	8.0499	6.6482	6.7823	6.9794	7.1526	7.1543	8.0573	7.2771	6.9548	7.6522	7.4892	6.0726	6.9929	7.3430	7.2984	7.4060	6.8960
Srcin1	_AVS[Phospho (STY)]VEAAER_.2	#N/A	7.0786	6.7510	7.8269	7.0153	6.6037	7.0983	6.3730	6.3567	6.6288	8.0867	6.8669	6.2226	5.6080	6.6512	6.3164	6.5454	6.5417	6.6023	6.7452	6.9137
Srcin1	_GVTDTLAQIR_.2	#N/A	6.7437	6.7270	6.8316	6.9540	6.2975	6.7406	6.9415	6.9055	6.9993	6.5654	6.5355	6.6281	5.7729	6.9045	6.6139	6.6985	6.6716	6.4504	6.1067	6.6464
Srcin1	_TLGGGGGGGS[Phospho (STY)]GGRR_.2	Q9QWI6_S73_M1_Srcin1	6.5650	6.9217	5.9927	6.3826	6.6295	5.5776	6.0686	6.6456	6.1984	6.8649	6.1541	6.7323	5.3572	6.5849	5.5557	7.1894	6.3656	6.4838	5.8429	6.0439
Srcin1	_YLNDEELITQQLNDLEK_.2	#N/A	5.5529	5.4050	4.5666	5.4315	4.7652	4.8460	5.7544	5.1525	5.1261	5.0348	5.4625	5.4447	5.4239	5.7081	5.5129	5.5774	5.9655	5.1078	4.3992	4.9486
Srcin1	_AAQGPAGS[Phospho (STY)]PDKGK_.2	#N/A	8.9391	9.0040	9.1112	8.9684	8.6775	8.4672	8.6695	8.9820	8.2536	9.0748	8.4539	8.7471	9.0603	9.0592	7.4414	8.4199	8.7496	8.7520	8.1606	8.6149
Srcin1	_FSNVGLVHTS[Phospho (STY)]ERR_.3	#N/A	3.8995	3.4196	3.4361	3.6847	4.1064	4.0019	4.4425	6.9230	3.8223	7.2546	4.1967	6.6268	3.9304	7.4302	5.5424	3.2616	3.6224	6.7748	6.2334	6.1507
Srcin1	_EVSGPNETSS[Phospho (STY)]PGSEKPSGSR_.3	#N/A	6.6043	7.7218	7.7028	6.6297	6.1640	7.2161	7.4250	6.9336	7.2426	3.5820	6.7227	6.4293	7.4424	8.0176	6.5162	7.7719	3.7065	7.6265	7.1073	3.9059
Srcin1	_QLGETLTELK_.2	#N/A	5.3441	5.3393	6.0952	5.5206	5.5859	5.6017	5.5846	3.3174	5.3829	6.0144	5.6810	5.9657	3.1226	6.0860	6.0231	5.1299	5.5434	5.0661	5.1274	5.3926

Srcin1	_GEGLYADPYGLLHEGR_.3	#N/A	5.0221	4.9178	4.1321	5.3318	5.6727	5.3274	5.6532	3.3203	5.4848	5.8688	5.7324	6.0288	5.5432	5.4771	5.4454	5.6999	5.0366	5.6017	5.5494	5.5598
Srcin1	_QIASLTGLVQSALLR_.2	#N/A	3.4900	3.4404	3.7563	3.1149	4.0969	4.1905	3.9503	3.9390	3.2422	3.6053	4.3878	3.7738	4.1910	4.0747	4.1641	4.0873	3.7879	3.9440	2.4146	4.1960
Srcin1	_DSGSSSVFAES[Phospho (STY)]PGGK_.2	#N/A	12.1346	11.9756	12.3926	12.2209	11.6763	11.6747	11.6530	12.1467	11.8634	12.2100	11.7521	11.9224	12.5026	12.1821	11.6536	11.9277	12.2867	11.5757	11.6924	11.8659
Srcin1	_KDS[Phospho (STY)]GSSSVFAES[Phospho (STY)]PGGK_.2	Q9QWI6_S588_M2_Srcin1	10.3904	10.3414	10.4728	10.3766	9.4552	8.9431	9.8322	10.4213	8.8937	10.4576	9.9644	10.1921	10.5922	9.7873	9.7263	10.2098	10.6203	10.0314	10.1216	10.1088
Srcin1	_LSYAGGRPPSYAGS[Phospho (STY)]PVHHAAER_.3	#N/A	6.8447	7.8590	7.3192	7.3073	5.9791	6.9384	7.5878	7.4272	6.2248	7.6679	6.7169	7.5512	8.3704	8.8765	6.6927	6.4481	3.5035	7.5193	7.2720	7.0013
Srcin1	_HTQGAQPGLADQAAK_.2	#N/A	2.7482	4.1884	3.5277	5.2563	4.0158	4.5400	4.8273	5.2316	3.0763	4.5973	4.2456	4.6411	3.1844	4.1283	4.6079	4.0748	4.9443	3.7995	3.7283	4.3566
Srcin1	_S[Phospho (STY)]SPPM[Oxidation (M)]LSADDAEYPR_.2	#N/A	3.1745	3.7334	6.9015	4.2841	3.8331	3.9783	5.9367	3.9692	3.4355	3.5708	3.8099	4.6230	3.5435	7.4563	4.7277	3.7122	7.6442	3.9522	6.9730	4.2654
Srcin1	_AALTQYSAK_.2	#N/A	3.3950	4.6078	3.9313	5.1121	4.6398	4.9225	4.1799	4.1710	4.9796	3.6013	4.4975	2.9964	3.4157	4.1933	5.4127	4.3481	3.6330	4.6441	4.0919	4.9459
Srcin1	_GS[Phospho (STY)]DELTVPR_.2	#N/A	6.9169	7.0554	4.0895	3.8892	3.2591	7.3179	3.8876	4.0943	3.8927	4.4325	3.0250	6.9672	7.8374	7.9038	7.6739	3.9151	4.2759	7.2840	7.0870	7.6444
Srcin1	_GLQNSASDLR_.2	#N/A	3.3505	5.7078	6.3192	4.2337	6.1693	6.1567	5.9098	3.5762	6.5356	6.1684	4.3903	6.3313	3.3814	6.4196	6.6788	6.6138	3.0734	6.4048	6.6019	6.3759
Srcin1	_RQEDPLQR_.2	#N/A	6.3393	6.1182	4.3717	6.5932	6.5358	6.2411	6.1733	5.9913	6.1235	5.6417	6.1360	4.0568	3.8562	6.3269	6.3454	6.3727	4.0735	6.5215	5.8633	6.2108
Srcin1	_RGS[Phospho (STY)]DELTVPR_.2	#N/A	11.2170	11.1815	11.0843	11.1354	10.7131	10.7592	10.7354	11.0830	10.8156	11.1113	10.8307	10.7871	11.4706	11.2937	10.1763	10.7922	11.2941	10.6724	10.8570	10.9152
Srcin1	_S[Phospho (STY)]PGVLFLQFGEETR_.2	Q9QWI6_S247_M1_Srcin1	4.4143	4.7376	5.4689	4.7591	3.7520	4.2263	4.9132	4.1147	5.1853	3.1198	4.2225	3.4779	4.4350	3.4368	2.5389	3.2584	3.1515	3.2828	4.3302	4.2447
Srcin1	_S[Phospho (STY)]FPSSHGLTTTR_.2	Q9QWI6_S1079_M1_Srcin1	3.9006	4.1022	4.4369	8.9407	6.6715	3.2458	3.8356	3.6009	3.7558	4.3170	8.4638	3.9917	3.9213	8.4446	3.8800	4.2455	4.1386	3.6945	3.3431	4.3909
Srcin1	_GEPDPERS[Phospho (STY)]SPPM[Oxidation (M)]LSADDAEYPR_.3	#N/A	3.7170	6.6353	3.5529	6.8945	4.0960	5.9183	6.5296	4.3403	3.5757	8.9382	4.1086	5.2225	3.6967	3.5238	3.0050	3.3721	3.5421	3.9350	3.9592	8.5091
Srcin1	_RFS[Phospho (STY)]NVGLVHT[Phospho (STY)]SERR_.3	Q9QWI6_T86_M2_Srcin1	4.6068	5.1854	5.7745	6.0061	4.0218	5.1162	3.8911	4.3227	4.4023	5.6633	3.9562	3.0564	3.9768	5.4274	3.0819	4.6624	3.3595	5.2192	4.9751	5.3254
Srcin1	_QVDEGM[Oxidation (M)]WPPPNNLLNQS[Phospho (STY)]PK_.2	#N/A	8.4949	9.2271	7.9810	7.0305	7.0059	7.6334	8.7460	8.9751	7.4730	8.1659	7.3989	8.5545	8.7348	10.1097	7.1661	7.8173	7.9598	8.6984	8.1348	7.4706
Srcin1	_LKAAQGPAGS[Phospho (STY)]PDKGK_.3	#N/A	4.6304	4.2539	3.4851	4.8054	3.9937	4.0841	4.0629	3.8364	5.3258	4.7721	4.1059	5.0607	4.9675	4.8756	4.6964	3.6452	5.0727	3.4348	5.5157	4.6079
Srcin1	_S[Phospho (STY)]PNTAILIKDEAR_.2	#N/A	8.1247	8.2723	8.4681	8.3157	7.8941	8.1676	7.6437	8.0531	7.9643	8.2369	8.3235	7.8745	7.8303	8.7558	7.4534	8.0450	8.5943	8.1270	8.2690	8.3005
Srcin1	_AAQGPAGS[Phospho (STY)]PDK_.2	#N/A	5.2588	4.5485	6.0861	4.2351	4.2928	5.4272	3.2482	3.1547	3.3096	5.7324	6.0013	5.1355	3.4750	3.9777	2.8254	3.7992	3.6923	4.1408	3.7894	3.9447
Srcin1	_LGGAPTGQGVSPSPSAILER_.2	#N/A	4.6765	5.7439	4.9831	4.3967	4.1533	3.4388	4.2221	3.4512	3.9497	3.9668	4.1042	4.0903	4.0531	4.5933	3.8179	3.9506	4.6797	4.3287	3.4821	4.0831
Srcin1	_VEGSNGAAT[Phospho (STY)]PSAPVC[Carbamidomethyl (C)]GSGSK_.2	Q9QWI6_T675_M1_Srcin1	5.0964	5.6454	3.9611	5.2081	3.6440	3.6773	4.2028	3.7950	4.7956	4.0033	3.6499	3.6956	3.5811	3.9644	3.5208	4.7790	3.8336	5.4832	3.7747	6.0151
Srcin1	_S[Phospho (STY)]S[Phospho (STY)]GATPVSGPPPPSASSTPAGQPTAVSR_.3	Q9QWI6_S687_M2_Srcin1	3.8844	3.8698	3.9218	3.9051	3.5735	3.5298	3.5815	3.7820	3.6406	4.1743	4.0149	3.6710	3.9085	7.4834	3.9117	4.2475	4.5306	5.4811	3.8669	3.7210
Srcin1	_TEKPSKS[Phospho (STY)]PPPPPPR_.3	Q9QWI6_S1070_M1_Srcin1	4.0325	3.9703	4.5688	3.6568	3.3703	5.8426	4.1708	3.7329	4.2299	6.1270	3.1034	3.8597	4.3380	3.8817	3.7480	5.6329	4.2706	3.1195	3.4185	3.5590
Srcin1	_S[Phospho (STY)]PNTAILIK_.2	#N/A	4.0901	3.9127	6.6918	3.5993	3.4279	3.6519	4.1132	3.7905	4.1723	4.2965	3.0458	3.8021	4.2804	4.6135	3.6904	2.9342	6.1818	3.5050	3.1536	3.0797
Srcin1	_FRQS[Phospho (STY)]LPLSR_.2	Q9QWI6_S233_M1_Srcin1	4.3902	3.6126	3.4256	3.2992	3.7279	3.9520	5.1425	6.5364	3.8723	3.0957	4.2466	3.5021	3.9803	3.4127	3.3904	3.2342	3.1274	3.2049	4.3543	3.2014
Srcin1	_DAFMDHLK_.2	#N/A	3.9962	3.3229	4.3915	5.0798	4.2031	4.2365	3.6713	4.2219	2.9839	3.8068	3.7447	3.0490	4.4315	5.2821	3.4030	3.3583	3.7191	3.4304	3.7294	3.2481
Srcin1	_HTVIAAQS[Phospho (STY)]LEALSGLQK_.3	#N/A	4.5466	4.7939	3.6162	3.9330	4.0223	3.8509	3.9188	4.9840	3.7740	4.0326	4.1484	4.0336	4.3065	5.1604	3.2553	2.7510	3.8144	4.3370	3.8184	3.6895
Srcin1	_EMVYASRES[Phospho (STY)]S[Phospho (STY)]PTR_.2	Q9QWI6_S356_M2_Srcin1	3.3957	4.6071	3.9320	4.2937	4.2343	4.4583	3.3068	3.6688	3.3659	3.6020	3.7403	2.9957	3.4740	4.5186	2.8840	3.7406	3.1660	3.9834	4.2825	4.1959
Srcin1	_FSNVGLVHTSER_.2	#N/A	4.5657	4.6119	4.3859	4.1977	3.6048	4.2755	4.1795	4.4971	4.0570	4.1540	3.5593	3.8878	4.1804	3.9259	4.2751	4.1820	4.3662	3.8895	3.8397	4.0358
Srcin1	_KVAAETDFSK_.2	#N/A	4.3248	3.6780	4.5337	3.3646	3.6625	3.8866	3.8786	2.0311	3.9377	4.5311	4.3120	3.5675	4.3455	3.3473	5.5471	3.1688	3.0620	3.2703	2.5479	2.2328
Srcin1	_LADVSAPSGGPPPPHSPY[Phospho (STY)]SGPPSRGSPVR_.3	Q9QWI6_Y561_M1_Srcin1	3.6047	4.4037	4.1410	4.1331	3.8361	3.2764	3.1034	4.2490	3.4063	3.8110	3.3559	4.2932	3.5463	5.3472	4.1815	3.9496	4.2736	4.0046	4.0363	4.1321
Srcin1	_QVDEGMWPPPNNLLNQS[Phospho (STY)]PKK_.3	#N/A	3.3329	3.8132	4.5433	4.0783	4.1715	4.3955	3.3696	3.7316	3.4287	3.5392	3.8031	3.5393	3.5368	6.7079	2.9468	3.6778	3.2288	3.9206	4.2197	4.2586
Srcin1	_RQVDEGMWPPPNNLLNQS[Phospho (STY)]PK_.3	#N/A	3.7896	4.2132	4.3259	3.8998	3.1274	3.3514	4.4137	3.4900	2.9720	3.9959	3.3464	4.1026	4.5809	9.8177	3.9909	4.1345	4.0277	3.8055	3.4541	4.2800
Asap1	_SHTGDLS[Phospho (STY)]PNVQSR_.2	Q9QWY8_S1045_M1_Asap1	7.6142	4.4971	4.0420	7.9366	4.3442	3.6407	3.4407	3.2060	7.1142	3.2110	6.1843	4.3866	7.1918	4.0290	3.4661	3.8506	3.7437	6.6317	3.7380	7.0232
Asap1	_TLS[Phospho (STY)]DPPSPLPHGPPNK_.2	#N/A	9.8495	10.0878	9.5377	9.3659	3.7892	9.0887	9.3566	9.7789	9.2524	9.5114	9.3202	9.7339	10.2158	10.4059	8.8408	9.4424	9.9981	9.8649	9.6251	9.5310
Asap1	_SPRPQSFC[Carbamidomethyl (C)]HSS[Phospho (STY)]S[Phospho (STY)]ISPQDK_.2	#N/A	3.6678	4.1348	9.8654	4.0009	3.3211	8.3183	8.1101	3.5444	3.4054	3.8826	3.5010	9.0426	3.5847	9.6567	3.8297	9.5439	3.7717	3.8845	7.7296	3.8975
Asap1	_QDEM[Oxidation (M)]DES[Phospho (STY)]DDDLDDKPSPIKK_.3	#N/A	4.9181	6.0559	5.3505	5.8082	4.1403	5.6561	5.8015	5.4722	4.9665	5.2993	4.3377	5.1903	4.7927	7.2152	4.8050	6.4847	5.4696	6.6658	6.7437	5.5170
Asap1	_QDEMDES[Phospho (STY)]DDDLDDKPSPIK_.2	#N/A	3.4587	4.2604	4.2515	4.1285	3.6657	5.2431	3.3825	3.1478	3.3027	3.2693	4.2822	3.3118	3.4681	4.8839	3.4079	4.3217	3.6855	4.1478	3.7963	4.8942
Son	_ESAQAVAVALS[Phospho (STY)]PK_.2	#N/A	9.1485	9.5075	9.5868	9.3848	8.3718	9.4225	9.0568	8.8864	9.2079	8.9372	8.5972	8.8748	8.6570	9.7925	8.1392	8.8459	9.5715	9.0757	9.1243	9.2439
Son	_S[Phospho (STY)]RT[Phospho (STY)]PLRR_.2	Q9QX47_S2038_M2_Son	8.1901	8.5458	8.4639	8.2193	8.0909	8.3184	8.3333	8.5351	8.1791	8.3061	8.1988	7.9415	8.3443	8.2565	7.7280	8.3113	8.5018	8.2087	8.3704	8.2090
Son	_SAAS[Phospho (STY)]PVVISIPER_.2	Q9QX47_S1794_M1_Son	8.1646	8.0114	4.3757	4.2459	7.5881	7.7190	7.3756	7.7713	3.4231	7.8046	2.9017	7.2950	7.8967	7.4697	6.4583	7.2227	8.2538	7.4542	7.3611	7.3694
Son	_FS[Phospho (STY)]RS[Phospho (STY)]PIR_.2	#N/A	5.4607	5.4106	6.0066	5.5493	5.2592	5.7936	6.1036	5.5066	5.5255	6.1055	6.2121	5.8204	3.5218	6.4503	4.8194	5.8214	5.9167	4.1548	6.1948	5.3236
Son	_RFS[Phospho (STY)]RS[Phospho (STY)]PIR_.2	#N/A	7.4166	6.2270	7.4889	7.1510	7.0317	5.8869	4.0763	7.1463	6.6225	4.0763	5.6974	6.8471	4.1620	7.4130	4.1017	6.5754	7.0720	6.2314	3.1024	5.9246
Son	_DVEDSLPIKESAQAVAVALS[Phospho (STY)]PK_.3	#N/A	4.7507	5.8551	3.9284	4.2973	4.2306	4.5208	5.8229	5.8476	3.3695	3.5984	3.7439	5.3119	4.6640	5.7260	4.9486	4.1331	3.6301	5.7826	4.6448	4.2890
Son	_RRS[Phospho (STY)]FS[Phospho (STY)]IS[Phospho (STY)]PVR_.2	#N/A	4.4916	8.6361	7.7270	7.9513	3.1977	8.3608	8.3902	3.2164	8.2140	4.0544	3.2879	7.9475	4.5224	8.5956	7.6361	7.9492	5.7526	8.4503	8.4872	8.5153
Chm	_ESTVLGDSEEPS[Phospho (STY)]E_.2	Q9QXG2_S664_M1_Chm	10.5217	10.1690	10.4046	10.2944	3.1543	10.1143	9.8198	10.0126	10.0799	10.1223	9.7107	9.9547	10.1113	10.3260	9.5344	10.0869	10.6701	9.8977	10.0908	10.0070
Chm	_VQDNTET[Phospho (STY)]PKR_.2	#N/A	3.7636	3.7238	3.9802	3.9889	3.3554	3.5794	3.5332	3.8952	3.5923	4.2240	3.9667	3.4499	3.7004	4.0183	3.8846	3.5884	4.1665	3.8312	4.1303	3.6956
Acss2	_GWS[Phospho (STY)]PPPEVR_.2	#N/A	12.9472	12.7252	12.8509	12.6245	12.4439	12.7063	12.3841	12.6948	12.4496	12.5429	12.8352	12.1974	12.5757	12.4256	11.1826	12.0819	13.1047	12.3666	12.3467	12.3038
Acss2	_VRGWS[Phospho (STY)]PPPEVR_.2	#N/A	9.7774	9.7899	8.7535	9.0946	9.1299	9.1635	9.2937	10.0162	8.7624	9.2321	9.1869	9.1220	9.8424	9.3276	7.7268	8.7166	9.7923	9.5301	9.1675	9.1304
Acss2	_AELGM[Oxidation (M)]NDS[Phospho (STY)]PSQSPPVK_.2	#N/A	9.6577	9.0972	8.9367	9.0589	8.7823	8.9248	8.9447	9.4610	9.0153	8.9863	8.6518	6.4529	9.2037	8.2028	7.7578	6.8448	9.5672	9.1706	7.2211	8.7824
Kif21b	_GATDTS[Phospho (STY)]PLTR_.2	Q9QXL1_S1217_M1_Kif21b	6.8977	6.8684	4.1631	6.8527	2.9645	3.1886	3.0757	3.3272	5.9437	6.3765	6.4271	5.7876	3.2429	4.1501	5.0269	3.9717	3.8648	6.7386	3.0127	3.9648
Kif21b	_AVS[Phospho (STY)]AEC[Carbamidomethyl (C)]LGPPLDSSTK_.2	Q9QXL1_S1150_M1_Kif21b	9.3205	8.8984	8.3244	8.6210	7.8585	8.1938	8.6140	8.6484	7.9249	8.8944	7.8511	7.9189	8.4043	8.0813	3.7743	7.9201	7.9620	8.3560	7.5836	7.6005
Kif21b	_TVS[Phospho (STY)]LPTR_.2	Q9QXL1_S1198_M1_Kif21b	3.3837	3.1183	3.9200	4.3057	5.7776	5.8204	5.9240	3.0840	3.2389	6.4747	4.2184	3.0077	3.4044	3.9070	2.8960	3.7286	4.5224	4.2114	3.8600	3.8740
Kif21a	_VC[Carbamidomethyl (C)]PQTDAENATDNK_.2	#N/A	8.3425	8.1349	8.7180	8.4526	8.1119	8.2730	8.0496	8.4927	8.3665	8.2712	8.0679	7.8420	8.2574	8.2629	7.6300	7.7889	8.4117	8.0209	8.1908	8.0330
Kif21a	_DQVLQNLGSVESYSEEK_.2	#N/A	7.4015	6.7304	6.4876	6.3740	6.3185	6.8902	6.5730	6.4647	6.6475	6.5871	6.5551	6.5713	7.4627	6.6289	5.6572	6.4619	7.0103	6.2620	6.2831	6.2915
Kif21a	_ITQLVSEQANQVLAR_.2	#N/A	6.0168	5.2880	5.2032	4.6652	5.4320	5.3617	4.9447	6.0619	5.9969	5.5052	5.4991	5.8358	5.4719	4.9875	5.2577	5.1543	5.5322	5.2486	5.0253	5.2136
Kif21a	_HSDSGASETSLS[Phospho (STY)]PPSS[Phospho (STY)]PPSRPR_.3	Q9QXL2_S1273_M2_Kif21a	8.8975	8.8342	6.5371	3.4501	4.3411	3.7673	8.9433	4.3598	4.0570	4.4118	2.9305	4.4119	4.1650	3.7088	3.5751	3.4963	3.8571	3.2924	3.5914	3.3861
Kif21a	_TTASTQM[Oxidation (M)]NVQSS[Phospho (STY)]R_.2	Q9QXL2_S224_M1_Kif21a	6.3342	6.4135	5.6891	5.9124	5.2864	5.8210	6.0314	5.5886	5.7607	3.5496	4.9999	5.9140	6.0963	5.4395	5.5450	5.5805	5.9655	5.8962	6.5202	5.3491
Kif21a	_AHNLQDGQLSDTGDLGEDIAS[Phospho (STY)]N_.2	#N/A	3.6836	6.0897	6.2801	5.1331	3.3706	6.8819	5.8293	3.9093	7.2123	5.9021	4.3409	5.5519	4.7816	7.6524	6.4022	4.0861	5.1226	7.6149	6.8988	6.2308
Kif21a	_LISESS[Phospho (STY)]PM[Oxidation (M)]NEFETLTAK_.2	#N/A	6.9060	7.2543	7.1853	7.2501	5.7072	5.8012	7.0947	6.3285	6.4803	6.6856	6.9302	5.9338	6.4970	7.4459	4.8750	5.7737	6.9539	6.4460	6.3464	6.1000
Kif21a	_MTISNMEADMNR_.2	#N/A	4.0937	3.9091	3.1292	3.5957	3.4315	3.0526	4.0288	3.7941	3.9490	4.1238	3.4276	3.7985	4.1144	4.6171	3.6868	2.9378	4.3318	3.5014	3.1500	3.0833
Jmy	_KSQAWAEGGS[Phospho (STY)]PR_.2	Q9QXM1_S108_M1_Jmy	7.3166	7.5810	7.4628	7.1040	6.9480	6.8288	7.2615	6.5567	7.0076	7.2950	6.5508	6.5881	7.4852	6.6784	5.9617	6.9469	7.0395	6.6467	6.3814	6.4931
Jmy	_GPES[Phospho (STY)]PLRS[Phospho (STY)]PAR_.2	Q9QXM1_S130_M2_Jmy	6.6434	6.5098	6.5032	6.7612	4.9916	5.6562	5.9714	5.6893	5.5043	7.1083	5.8973	5.8163	4.0870	3.9488	3.7035	5.6307	5.8809	5.5004	5.1513	5.3474
Jmy	_IKEASPES[Phospho (STY)]EDEEEALPC[Carbamidomethyl (C)]TDWEN_.3	Q9QXM1_S969_M1_Jmy	3.3638	3.9553	4.4012	4.2204	3.5708	3.6041	3.4773	3.5895	3.3975	3.1744	4.3770	3.6814	3.3947	4.4393	3.5027	4.2268	3.0867	4.0627	4.3618	4.1166
Pde7b	_RGS[Phospho (STY)]GQDLAGPAPETLEQTEGATP_.3	#N/A	7.3375	7.4729	7.2685	7.3388	5.7916	7.0223	6.6883	3.6891	6.8766	7.6222	6.9923	6.6913	7.1829	7.1663	3.7918	7.0580	7.0213	6.8003	6.7010	7.0650
Pde7b	_GSGQDLAGPAPETLEQTEGAT[Phospho (STY)]P_.2	Q9QXQ1_T445_M1_Pde7b	4.2278	3.2072	3.6484	3.4724	3.5655	6.4576	5.8774	4.3375	6.1293	4.4341	2.9082	4.5103	4.1427	6.3576	5.5464	5.8226	3.8348	6.7400	6.4644	6.3343
Pde7b	_RRGS[Phospho (STY)]GQDLAGPAPETLEQTEGATP_.3	#N/A	3.3213	4.8681	4.3587	4.2629	3.5282	3.5616	3.5199	3.5470	4.0036	3.1319	2.9187	4.5167	3.6055	4.3968	3.5452	4.1842	4.5450	4.1052	4.6469	4.0741
Plec	_AQLEPVAS[Phospho (STY)]PAK_.2	#N/A	12.6858	12.3821	12.3419	12.2367	11.9730	12.0922	12.0812	12.4950	12.0375	12.0896	12.1805	11.9958	12.5679	12.0616	10.7703	11.7474	12.7979	12.2860	12.0332	11.9029
Plec	_NDESQLS[Phospho (STY)]PATR_.2	#N/A	10.3530	4.3166	4.2225	9.9499	9.4735	3.2480	9.9472	10.0671	9.8823	9.7596	9.9006	9.5951	9.9078	4.2281	4.0944	4.0311	10.1249	9.8887	9.7723	9.6065
Plec	_RAS[Phospho (STY)]FAEK_.2	Q9QXS1_S1729_M1_Plec	8.5480	8.6401	7.6940	8.0843	7.7479	8.0803	8.1393	7.9080	7.8491	8.1517	7.6443	7.7272	8.1897	7.5289	5.6818	7.2904	7.7968	8.2031	7.9568	7.4513
Plec	_AQLEPVAS[Phospho (STY)]PAKKPK_.2	#N/A	7.7261	8.0476	6.3187	7.2090	6.9618	6.9088	7.6720	7.9348	7.5581	7.1226	6.9105	7.6053	8.8927	7.0715	6.2261	7.6085	7.4318	7.9277	7.4824	6.9870
Plec	_LPVDVAYQR_.2	#N/A	6.0961	6.1777	5.7766	3.9565	5.3308	5.7959	6.2364	5.7153	5.2250	5.8758	5.6140	5.5247	3.7536	5.3581	5.8020	4.9972	5.8186	6.0228	5.0141	5.5401
Plec	_ARNDESQLS[Phospho (STY)]PATR_.2	#N/A	8.8757	8.7574	8.5582	8.7577	4.0174	8.4669	8.5337	8.9518	8.4467	4.1858	8.2536	8.3544	8.9521	8.4387	7.5248	8.3838	8.9448	8.6612	4.1175	8.3485
Plec	_SSS[Phospho (STY)]VGSSSYPISSAGPR_.2	Q9QXS1_S4393_M1_Plec	11.3304	11.0683	11.2208	11.1425	10.5836	10.7048	10.6200	10.9310	10.7058	10.8637	10.4942	10.1565	11.0165	10.3946	9.0782	10.2355	10.9055	10.4058	10.4085	10.2867
Plec	_WQAVLAQTDVR_.2	#N/A	4.6725	4.2990	5.0170	4.2895	4.3487	4.2811	4.5233	3.2106	5.0697	3.7165	4.1038	4.6124	3.5309	4.0335	3.4749	3.8551	3.7482	4.3972	4.1091	4.0005
Plec	_DAPDGPSVEAEPEYTFEGLR_.2	#N/A	3.3788	4.7691	4.4162	5.1955	4.6426	4.8633	4.5256	3.6044	4.9498	5.6929	5.5267	3.6664	3.4096	4.4542	4.3840	4.2417	5.6712	5.5964	4.0600	4.1839
Plec	_LTVDEAVR_.2	#N/A	3.3621	5.5193	5.8647	4.3273	5.4557	4.4247	6.1927	3.7024	5.3391	5.3890	5.7400	3.0293	5.6276	5.4794	2.9176	3.7070	3.6002	6.4260	2.3220	4.9091
Plec	_GYYSPYSVSGSGSTAGS[Phospho (STY)]R_.2	Q9QXS1_S4633_M1_Plec	4.1432	3.1759	3.6798	5.8641	4.3501	3.7582	4.0069	4.3689	6.3636	4.4027	5.8452	4.4028	4.1741	3.7179	3.5841	3.5053	3.8661	3.2833	3.5824	3.3951
Plec	_VLALPEPSPAAPTLR_.2	#N/A	4.4096	5.1922	3.9462	4.8563	5.7238	5.6078	5.5071	5.0663	4.9165	5.3139	5.3124	4.9428	4.4405	3.9843	3.9578	3.7717	5.0417	3.0169	4.8012	5.0785
Plec	_LLDAQLATGGIVDPR_.2	#N/A	4.3997	4.4202	4.9929	3.1845	3.1058	3.3561	3.9423	3.1246	4.3226	3.0355	3.1961	4.1463	4.4306	3.5513	1.1345	3.7619	4.1227	4.3102	2.5937	3.4414
Plec	_FLEVQYLTGGLIEPDTPGR_.2	#N/A	4.8700	4.6661	3.5552	3.5656	4.2813	4.2589	4.0910	5.1036	4.2436	4.1293	3.9354	4.0337	4.4090	3.4108	4.3905	3.3807	3.7415	3.2068	4.3562	4.5601
Plec	_VYHDPSTQEPVTYSQLQQR_.3	#N/A	4.5033	4.5063	4.0399	4.5817	3.2094	3.2428	3.8387	5.2893	3.7589	4.3139	3.2375	4.0427	3.9243	4.1533	4.3058	3.8654	4.2262	3.6915	4.3819	4.7539
Plec	_LLDAQLSTGGIVDPSK_.2	#N/A	4.3815	4.6477	3.4947	3.6262	3.7193	3.9434	3.8218	4.1838	3.8809	4.5879	4.2553	3.0871	3.9889	3.5327	3.3990	3.2256	3.6810	3.4685	3.7675	3.2100
Plec	_FLEGTSC[Carbamidomethyl (C)]IAGVFVDATK_.2	#N/A	3.2300	4.3267	4.2124	4.0133	3.4369	3.4703	3.6112	3.3765	3.5314	4.0975	3.0100	3.9026	3.6968	3.9953	3.6365	4.6013	3.9141	3.9190	3.8563	3.2875
Plec	_AQAEVEGLGK_.2	#N/A	5.4709	3.1281	3.7276	3.3932	3.4863	4.1544	4.4265	4.4167	4.1138	4.3549	2.9874	3.7436	4.2219	3.7657	3.6319	4.4174	3.9139	3.2355	3.5346	3.4429
Plec	_TEVVETTQVYTEEETRR_.3	#N/A	3.2375	3.2644	3.7738	4.4519	4.0761	4.3002	4.9276	4.4388	3.5241	3.4439	3.8984	3.1539	3.6321	3.1759	3.0422	3.5824	3.4756	4.6539	4.1243	4.3540
Plec	_EEELQQTLQQEQNMLDR_.2	#N/A	3.3301	4.4268	4.1123	4.1134	3.5370	3.5704	3.5111	3.2764	3.4313	3.1407	2.9099	4.3163	3.5967	4.0994	3.5364	4.1931	3.8140	4.0191	3.6677	4.0664
Dbn1	_[Acetyl (Protein N-term)]AGVSFSGHR_.2	#N/A	7.7658	6.6842	7.7396	7.7678	6.8565	6.6093	7.8623	7.4863	6.7983	6.8321	7.5946	7.6886	6.9516	8.0923	7.9863	6.7322	7.8438	6.8681	6.7584	6.8862
Dbn1	_YVLINWVGEDVPDAR_.2	#N/A	6.8811	6.5265	7.4507	7.4665	7.1860	6.8251	7.0275	6.5423	6.9811	7.4987	7.0755	6.3908	6.9488	6.9960	7.1933	7.1816	6.9686	6.7746	6.8108	6.9605
Dbn1	_KQQS[Phospho (STY)]LEAEEAK_.2	#N/A	5.7368	6.5967	5.8532	4.6721	6.0672	6.2807	5.6064	6.4005	6.0134	5.9770	6.3488	5.8716	6.4286	7.0778	5.8993	5.8289	6.7100	6.4374	6.1140	6.7027
Dbn1	_EESAADWALYTYEDGSDDLK_.2	#N/A	5.7486	5.0741	5.9497	5.6722	5.3585	4.9265	4.9617	5.5875	5.3587	5.5659	4.6390	5.6660	5.1708	6.8816	5.1551	5.4715	5.5490	5.0689	5.3563	4.8435
Dbn1	_LSS[Phospho (STY)]PVLHR_.2	Q9QXS6_S142_M1_Dbn1	13.9511	13.9729	13.9338	13.9443	13.6698	13.4735	13.5151	13.9116	13.5205	13.9978	13.5039	13.7226	14.2751	14.0479	13.3138	13.6949	13.8122	13.6696	13.6583	13.6741
Dbn1	_EGTQASEGYFSQS[Phospho (STY)]QEEEFAQSEEPC[Carbamidomethyl (C)]AK_.3	Q9QXS6_S658_M1_Dbn1	6.8128	6.5013	4.1845	5.1970	4.8640	5.7270	4.6285	5.5193	5.0003	7.0304	5.1186	5.1957	4.9448	7.3907	4.5960	5.9016	6.8519	4.7465	4.8749	4.4939
Dbn1	_ASDSGPSSSSSSSSS[Phospho (STY)]PPR_.2	#N/A	11.6872	11.6019	12.0322	11.7549	11.4846	11.4324	11.2301	11.6403	11.3044	11.5754	11.5485	11.4536	11.6494	12.0368	11.2954	11.5120	11.6803	11.4147	11.4788	11.6687
Dbn1	_EQFWEQAK_.2	#N/A	4.2757	7.4487	3.8123	7.5956	7.1787	7.0914	7.2441	7.8620	4.1986	7.4810	7.3600	7.3304	7.4880	7.5472	7.5709	7.3756	7.1246	7.3375	7.1746	7.3450
Dbn1	_LREDENAEPVGTTYQK_.3	#N/A	5.7177	5.9187	6.1991	5.9236	5.7568	6.0727	6.3428	5.9228	6.4075	6.1517	6.2274	6.5175	6.4287	6.0769	6.4218	6.3825	5.5795	5.8411	5.8855	6.4594
Dbn1	_M[Oxidation (M)]APTPIPTR_.2	#N/A	6.8745	4.3893	3.9672	6.8351	7.4413	6.9801	7.0977	7.1092	7.1902	7.2123	3.3102	7.5046	7.4063	4.0053	7.9259	7.4664	7.2040	7.5677	7.6252	7.2306
Dbn1	_SPS[Phospho (STY)]DSSTASTPIAEQIER_.2	Q9QXS6_S387_M1_Dbn1	8.1799	8.7168	8.4773	8.5744	6.2945	7.9487	8.0626	7.0350	7.8249	8.8122	5.9383	8.1719	8.2098	9.0550	7.3487	8.2883	8.2513	7.9517	8.2384	8.4864
Dbn1	_ASDSGPSSSSSSSSS[Phospho (STY)]PPRTPFPYITC[Carbamidomethyl (C)]HR_.4	#N/A	3.3650	3.1369	3.9013	4.3243	4.2036	4.4277	3.3375	3.0654	3.3966	3.5714	3.7709	3.0264	3.5046	3.8884	2.9147	3.7099	3.6031	4.2301	3.8786	3.8554
Dbn1	_LELLAAYEEVIR_.2	#N/A	4.1568	3.1623	5.7666	3.4274	5.0409	4.1910	3.5170	4.1542	5.0756	4.9422	4.7819	4.3892	4.2709	3.7314	4.5038	5.0256	4.4831	3.2697	4.9952	4.3374
Dbn1	_SESEVEEAAAIIAQRPDNPR_.3	#N/A	4.4699	3.6594	4.4504	4.6394	4.3312	4.2423	5.0029	3.6374	4.5808	4.2805	3.2709	4.9232	3.9577	4.4604	3.8975	4.6498	4.1751	4.9649	4.4215	4.1386
Dbn1	_LAASGEGGLQELSGHFENQK_.3	#N/A	5.2417	5.3247	6.3160	6.2647	5.9524	5.7357	5.3124	5.4808	5.8745	6.1076	5.9493	5.1945	5.9680	5.7092	6.2712	6.0126	5.6542	5.8763	5.8259	6.0080
Dbn1	_KSESEVEEAAAIIAQRPDNPR_.3	#N/A	4.1875	4.2373	3.2230	4.1679	4.5453	4.3920	3.9657	4.1926	3.9180	4.9547	3.8484	3.6146	4.7121	4.2108	4.4057	4.1265	4.8264	3.9168	4.1683	4.3481
Dbn1	_KQQS[Phospho (STY)]LEAEEAKR_.3	#N/A	3.7064	2.5424	3.8782	3.8421	3.9697	4.1496	3.9334	3.4135	3.8721	3.7163	3.7848	3.2263	4.3883	2.9158	2.5847	3.4732	3.0561	3.8038	3.8263	3.6543
Dbn1	_EREQQIEEHR_.3	#N/A	3.6906	2.7499	3.2272	3.8936	3.8975	4.2108	3.1506	3.9163	3.6134	3.5012	2.5431	3.3546	3.7215	1.5380	3.8206	3.9315	3.4135	3.7359	4.0350	2.6142
Dbn1	_VAEFFQGVDVIVNASSVEDIDAGAIGQR_.3	#N/A	3.0984	4.2207	4.1358	4.4859	3.3053	3.3022	4.4629	3.3241	3.0212	3.9467	3.3956	3.9468	3.1292	4.1739	4.0401	3.9613	4.3221	4.3282	3.1264	3.8511
Cnpy2	_NGES[Phospho (STY)]SELDLQGIR_.2	Q9QXT0_S115_M1_Cnpy2	4.2321	3.4504	3.5026	3.4298	4.0051	3.9473	4.0844	4.1799	5.1736	4.0061	4.2528	3.2676	3.9865	3.5160	3.1277	3.3199	3.4728	3.3006	4.0488	3.3393
Cnpy2	_SEAHLTELLEEVC[Carbamidomethyl (C)]DR_.3	#N/A	3.6792	4.5749	3.2158	3.9050	3.8861	3.3805	3.9519	3.7225	3.5026	3.4898	4.3486	4.0243	3.7101	5.0211	4.0709	4.4079	4.1245	3.4159	3.3323	4.4319
Cnpy2	_SQDLHC[Carbamidomethyl (C)]GAC[Carbamidomethyl (C)]R_.3	#N/A	3.2796	3.7599	4.5966	4.0251	4.1182	4.3423	3.4229	3.7849	3.4820	3.4859	3.8564	3.4860	3.5900	3.1338	2.0116	3.6245	3.2821	3.8674	4.1664	4.3119
Pcsk1n	_ILTGS[Phospho (STY)]SEPEAAPAPR_.2	Q9QXV0_S204_M1_Pcsk1n	11.2833	11.3793	11.3178	11.3965	11.1612	11.2725	11.0407	11.4360	11.3469	11.3520	11.0686	11.3036	11.8749	11.5012	11.4263	11.4939	11.4965	10.9171	11.3059	11.2285
Pcsk1n	_ALAHLLEAER_.2	#N/A	7.8383	7.9614	8.0527	7.7395	8.3377	8.0792	8.1482	8.5164	8.3672	7.3879	7.6802	8.2176	7.9128	7.7235	8.4897	8.0693	8.0043	7.7518	7.7664	7.6715
Pcsk1n	_VLAQLLR_.2	#N/A	6.8756	6.7408	5.9816	7.0159	7.2058	6.9728	7.8252	6.9970	6.7600	7.3840	7.2454	7.5704	8.1540	6.9170	6.3691	7.1918	6.4726	7.1404	6.7022	7.3425
Pcsk1n	_LDPAALAAQLVPAPAAAPR_.2	#N/A	6.1509	5.9741	6.5861	6.6094	6.6987	5.5836	6.6006	6.0539	6.5933	6.1467	6.4596	6.4109	6.1407	5.9748	6.3919	6.5519	6.1514	5.9042	6.1243	6.2565
Pcsk1n	_AEAQEAEDQQAR_.2	#N/A	5.3698	5.6138	6.6550	6.0539	5.9388	6.0618	6.6326	5.8565	6.4142	5.8458	5.3099	6.2513	5.1613	6.0708	6.8285	6.6188	5.3124	5.4314	5.8908	6.2930
Pcsk1n	_ASDPPLAPDDDPDAPAAQLAR_.2	#N/A	7.0387	7.1849	7.4393	7.2124	7.0501	7.3310	7.4932	7.2492	7.9950	7.3842	7.1928	7.5636	7.4073	7.3544	7.7905	7.7117	7.3356	6.8237	7.2748	7.4351
Pcsk1n	_ARAEAQEAEDQQAR_.2	#N/A	3.6917	5.5990	3.2283	3.8925	3.8986	4.2097	3.1495	3.9174	3.6145	3.3534	2.7921	6.2919	3.7226	3.2663	3.1748	3.0538	5.8046	3.7348	4.0339	4.4445
Cbx8	_VDDKPSS[Phospho (STY)]PGDSSK_.2	Q9QXV1_S164_M1_Cbx8	3.1336	4.2303	4.3088	3.9169	3.3405	3.3739	6.0323	3.4729	3.6278	4.4450	3.1064	4.1197	6.1164	4.2959	3.7329	3.9965	5.8379	5.9230	6.0987	4.2629
Cbx8	_RQDS[Phospho (STY)]DLVQYGVTSPSSAEASSK_.3	Q9QXV1_S229_M1_Cbx8	4.2823	3.7205	3.3177	3.4071	3.6200	3.8441	3.9211	3.9827	3.9802	4.4886	4.3545	5.1053	4.0882	3.6320	5.0921	3.1263	4.5203	3.3692	5.7775	3.3093
Slc7a8	_PTPVKDPDS[Phospho (STY)]EEQP_.2	Q9QXW9_S527_M1_Slc7a8	8.4056	3.6720	3.1837	8.8138	3.8540	8.3474	8.2744	8.0304	8.1104	8.5363	8.3316	7.9209	3.2797	3.7824	7.3148	3.0092	9.0380	8.4376	8.1454	3.7494
Slc7a8	_NHPGSDTS[Phospho (STY)]PEAEASSGGGGVALKK_.3	#N/A	10.0319	10.3546	9.5627	10.0120	8.9266	9.7491	10.0198	9.6443	9.6693	9.6700	9.4197	9.7369	10.0888	10.1188	8.7574	9.2041	8.3899	10.1197	9.6697	9.6352
Slc7a8	_NHPGSDTS[Phospho (STY)]PEAEASSGGGGVALK_.3	#N/A	3.3478	6.6069	6.5835	6.7482	3.7654	3.9580	3.3829	3.8235	6.6130	3.5542	6.6334	3.7874	3.3202	5.7170	3.5541	3.6927	3.4793	6.3853	3.8860	3.9741
Ehd3	_SKLPNS[Phospho (STY)]VLGK_.2	Q9QXY6_S479_M1_Ehd3	4.5143	9.0764	3.5498	8.8304	8.7782	8.4893	8.6403	9.0323	8.3598	8.5990	8.5526	8.8640	9.5088	9.3233	8.0337	8.4227	8.8520	8.6342	8.4813	8.6806
Ehd3	_LLPLEEYYR_.2	#N/A	6.0421	6.5579	6.2747	6.2504	6.5422	6.2613	6.1223	6.2039	5.9700	6.1663	6.0000	6.1154	5.8056	6.2480	6.2557	5.8852	6.2277	6.0863	6.1857	6.4773
Ehd3	_ELVNNLAEIYGR_.2	#N/A	5.7958	5.4847	5.7930	5.7189	5.5942	5.8914	5.8160	6.0634	5.1515	5.9071	5.4767	5.4772	5.2583	5.8337	5.9343	3.4154	5.9801	6.0001	5.2975	5.8517
Ehd3	_IVNTPEVIR_.2	#N/A	6.3449	3.4897	6.4576	6.1564	6.3477	6.2396	3.0117	6.2065	5.9277	6.1381	6.4338	6.3832	6.0246	5.8119	6.7942	6.3827	5.5467	6.2342	6.4358	5.8617
Ehd3	_DKPM[Oxidation (M)]YDEIFYTLS[Phospho (STY)]PVDGK_.3	#N/A	7.4821	8.6803	8.2050	8.1675	6.9431	8.3254	8.2213	7.0471	8.6816	8.6022	7.3734	8.3837	8.0478	7.6666	7.2796	7.8117	8.4678	7.9557	7.8800	8.3368
Ehd3	_EHQIS[Phospho (STY)]PGDFPNLKR_.3	#N/A	3.2543	7.6542	6.3408	4.4351	4.0929	7.0766	7.4343	4.4555	6.3236	3.4606	3.8817	6.8731	3.6153	8.0930	5.8937	6.2445	6.5066	7.1771	6.7814	6.6200
Ehd3	_LNAFGNAFLNR_.2	#N/A	3.3832	5.4913	4.0607	4.8556	4.6450	5.3142	5.7656	5.2898	3.3060	4.4227	3.6804	5.1208	3.4141	4.7252	3.9069	4.4611	4.5561	5.4723	4.5714	4.6850
Ehd3	_DIQS[Phospho (STY)]LPR_.2	Q9QXY6_S284_M1_Ehd3	3.1127	3.3893	3.6490	4.5767	3.9513	4.0337	3.5898	4.3139	4.4688	3.3190	3.9475	3.2787	3.1334	3.6361	3.1670	3.4576	3.3508	3.9979	4.8520	4.8127
Ehd3	_EHQIS[Phospho (STY)]PGDFPNLK_.3	#N/A	3.9580	4.0448	4.4943	3.7314	3.2957	3.1883	3.8931	3.6584	3.8133	4.2595	3.2919	3.9342	3.9787	3.3175	3.9185	4.3029	4.1961	3.6371	3.2857	4.4484
Ehd3	_IGPEPTTDSFIAVM[Oxidation (M)]QGDVEGIIPGNALVVDPK_.3	#N/A	3.9603	3.3588	3.4969	3.6239	4.1673	4.2006	4.3817	4.1860	4.3019	3.7709	3.7805	5.8121	4.4673	3.5350	2.9062	3.3224	3.6832	5.0340	3.7652	3.2123
Macf1	_LLDAEDVDVPS[Phospho (STY)]PDEK_.2	Q9QXZ0_S280_M1_Macf1	12.0164	12.8751	12.6154	12.2597	8.6955	12.6762	12.5047	11.2861	12.5564	11.9111	11.1491	12.2403	11.9129	12.2895	11.0749	11.9172	12.6766	12.5104	12.2899	12.1776
Macf1	_S[Phospho (STY)]QDSVLDPAER_.2	#N/A	4.1401	9.2589	9.5654	9.1111	4.3471	8.9725	9.0445	9.0724	8.9990	9.3225	3.6007	4.4059	4.2875	8.7844	4.2272	8.4838	4.5049	8.8674	8.7425	8.7177
Macf1	_AFVESQQKS[Phospho (STY)]PGKR_.3	#N/A	7.6444	7.8998	7.2930	7.5450	7.2576	7.0172	7.7482	7.5319	7.4836	7.5129	7.1195	7.5308	7.8841	6.8707	5.4332	6.9187	7.0705	7.4618	6.8560	6.7652
Macf1	_DTQDIVHDLES[Phospho (STY)]PGIDPSIIK_.3	#N/A	6.9427	7.3139	6.4641	6.5179	5.8868	6.7198	7.1619	6.1532	6.7629	6.2269	6.6823	5.6660	6.4579	7.0847	4.4777	5.2171	7.0154	7.2154	6.4167	5.9179
Macf1	_RQGS[Phospho (STY)]FSEDVISHK_.3	#N/A	8.4405	8.2428	7.6955	8.0095	3.7239	3.9480	7.6550	8.1372	7.8127	7.7072	7.7060	3.5061	8.5561	7.6068	3.3944	3.2302	8.0206	7.8088	3.7721	3.2054
Macf1	_AFVESQQKS[Phospho (STY)]PGK_.2	#N/A	7.0736	7.3694	7.0135	6.8092	6.4221	6.3978	6.9498	7.0884	6.2478	6.7591	6.7671	6.4799	6.9896	6.9954	4.9034	6.2039	6.5923	6.9051	6.3380	6.3067
Macf1	_EAEEELAASGGQSPT[Phospho (STY)]GEQIPQFQQR_.3	Q9QXZ0_T5553_M1_Macf1	3.1000	4.2192	4.9401	4.4844	3.3070	3.3403	3.7413	5.1893	5.1104	4.4115	3.1401	3.9453	3.8269	4.2396	3.7667	3.9630	4.1758	4.0976	3.3045	4.2966
Macf1	_AFLAELEQNS[Phospho (STY)]PK_.2	Q9QXZ0_S4483_M1_Macf1	9.5273	9.6923	9.4123	9.4389	4.2563	9.5736	9.4499	4.2750	9.6677	9.1865	3.6915	8.9504	9.7322	9.4493	8.0804	9.3113	10.1235	9.7342	9.5195	9.2270
Macf1	_RKS[Phospho (STY)]QDSVLDPAER_.3	#N/A	3.5576	4.4452	4.0939	4.3030	2.8953	3.1194	6.7851	3.5069	3.2040	3.7639	3.5784	4.3347	6.8655	4.3567	4.2230	5.9981	4.5049	4.1453	2.9436	4.0340
Macf1	_QGS[Phospho (STY)]FSEDVISHK_.2	#N/A	8.6716	8.3874	8.2430	7.8759	7.9740	7.9456	8.0567	8.1790	8.1821	8.1902	7.9670	7.8911	8.6015	8.0495	3.8968	7.6460	8.7059	8.2269	8.0795	8.0552
Macf1	_AM[Oxidation (M)]DATLS[Phospho (STY)]PTKTETVK_.2	#N/A	3.9536	7.1190	7.2045	7.1216	6.4235	6.8465	6.7012	3.6877	4.5617	4.3044	6.6899	6.6102	3.6250	5.5750	5.5864	6.1776	7.4269	3.5732	3.7091	6.2851
Macf1	_LNSQES[Phospho (STY)]DEEPQISDVPHISK_.3	Q9QXZ0_S3085_M1_Macf1	4.1840	6.0135	5.6075	4.4968	3.6088	5.5086	5.7001	3.9645	5.4432	3.9946	3.6876	5.2232	4.0671	5.2633	4.4347	5.2265	3.9282	5.8335	5.0316	3.7626
Ulk2	_TPLPTIIGS[Phospho (STY)]PTK_.2	Q9QY01_S600_M1_Ulk2	6.2436	6.8785	4.3219	3.9038	3.1234	5.5187	3.7207	3.4860	3.6409	4.4319	5.6347	4.1066	3.8063	4.3090	3.7461	3.9834	5.9870	6.3205	5.2545	4.2760
Myo9b	_AQDKPES[Phospho (STY)]PSGSTQIQR_.2	Q9QY06_S1264_M1_Myo9b	4.0849	3.9179	3.1204	3.6044	3.4227	3.6467	4.1184	3.7853	4.1775	4.2913	3.0510	3.8073	4.1057	4.6083	3.6956	4.4298	4.3230	4.2171	3.1587	3.0745
Plxnb3	_TNS[Phospho (STY)]LLLR_.2	Q9QY40_S1747_M1_Plxnb3	3.7346	4.2682	4.2709	3.9548	3.0723	3.2964	4.4688	3.4350	3.0270	3.9409	3.4014	4.1577	3.1351	4.2579	4.0460	4.0795	3.9726	3.8605	3.5091	4.2249
Vapb	_SLTSPLDDTEVKK_.2	#N/A	6.0958	5.2744	4.9284	5.8819	5.0253	5.4713	5.3352	5.8039	4.7814	4.9286	4.5925	4.5060	4.7238	5.6664	4.8009	4.7843	5.8861	4.9112	4.4925	4.9836
Vapb	_SLTS[Phospho (STY)]PLDDTEVK_.2	#N/A	3.4474	4.5554	3.9837	4.2420	4.2860	3.6989	3.3825	3.1478	3.3027	3.2693	4.2822	2.9440	3.4682	3.9708	3.4079	3.7923	3.6855	4.2497	3.7963	3.9378

Nup210	_ELAS[Phospho (STY)]PLALT[Phospho (STY)]PR_.2	Q9QY81_S1839_M2_Nup210	3.4450	7.5820	7.3471	4.1392	3.6520	7.4236	7.0628	3.6707	7.1100	5.7592	3.7422	3.6002	3.4759	6.9375	6.0879	4.3080	3.1679	7.3349	6.8893	4.1978
Tomm40	_FVNWQVDGEYR_.2	#N/A	4.0658	4.5461	3.8104	3.3104	3.4035	4.8690	5.0366	4.4995	4.1967	6.0063	3.0702	4.2722	4.3047	3.8485	3.7148	4.3718	3.9968	5.4943	4.9622	3.5258
Tomm40	_MAIQTQQSK_.2	#N/A	3.2498	4.0693	3.5393	5.1040	3.4567	3.4901	3.5914	3.4755	3.5116	4.5612	3.7378	3.7954	3.6770	4.7396	3.6168	4.1127	4.4735	3.9388	3.5874	4.1467
Add3	_TEEVLS[Phospho (STY)]PDGSPSK_.2	#N/A	9.5261	8.5725	8.9028	9.4930	9.4150	9.7786	9.0953	8.9814	9.5161	9.3812	9.4698	9.2885	9.6370	8.5974	9.1067	8.3967	9.0946	8.5123	8.5658	9.4585
Add3	_EQDHIIIIPR_.3	#N/A	3.7653	3.7001	4.8177	4.7289	4.5108	4.9046	3.8333	5.0548	4.9591	5.2495	4.7986	4.5371	3.1449	4.1287	3.6374	4.2085	4.0737	4.1500	4.0464	4.5228
Add3	_TLDNLGYR_.2	#N/A	5.7248	6.0071	3.9875	6.1816	6.3556	6.1417	6.4762	6.3666	5.9828	5.7894	5.7248	5.4382	3.4719	4.7819	6.3398	5.5060	5.4582	6.1262	6.8465	5.5318
Add3	_TEEVLSPDGSPSKS[Phospho (STY)]PSKK_.3	#N/A	6.6535	7.2816	8.8775	8.9086	6.3889	8.8986	7.1859	3.8269	8.3746	8.8691	6.9939	8.5343	8.9945	8.8729	7.8716	8.6569	8.5924	6.6059	8.4372	8.7477
Add3	_TEEVLSPDGSPSKS[Phospho (STY)]PSK_.2	#N/A	10.7797	10.9055	9.7434	10.9700	10.4020	9.8006	9.3565	9.7762	3.7063	10.7347	9.1473	9.4024	9.8951	10.2632	8.7725	9.5198	9.6274	10.3044	10.5357	3.8511
Add3	_GLSFSEATASTLVK_.2	#N/A	4.4763	3.4526	3.9153	4.7008	4.5410	4.3433	4.1003	4.9413	4.3957	4.7866	3.8743	1.6650	4.5611	4.4563	3.0571	4.1364	3.5894	4.1821	3.4695	3.5300
Add3	_SPERTEEVLSPDGSPSKS[Phospho (STY)]PSK_.3	#N/A	7.2192	3.9214	6.2167	7.3476	6.9038	6.9984	7.1864	7.1975	6.9939	7.5846	6.7639	7.6476	8.5983	6.2089	3.4615	7.4173	7.1641	7.2010	7.4599	3.8207
Add3	_[Acetyl (Protein N-term)]SSDTSPAVVTT[Phospho (STY)]PPPPSM[Oxidation (M)]PHK_.2	#N/A	3.3390	4.2304	4.2080	6.9001	7.0832	3.5369	3.4774	3.2924	3.4718	3.8773	7.1278	4.0240	3.6372	7.6241	6.3484	4.0285	4.0106	7.6288	3.5182	4.1069
Add3	_SDVEIPATVTAFSFEDDSAPLS[Phospho (STY)]PLK_.3	#N/A	4.0488	3.9540	5.3736	3.6406	3.3865	3.6106	5.0121	3.7492	5.0348	4.2551	3.0872	3.8434	4.3217	6.3184	3.7317	4.3937	4.2869	3.5463	4.4629	3.0383
Add3	_HKSDVEIPATVTAFSFEDDSAPLS[Phospho (STY)]PLK_.3	#N/A	4.1196	5.8772	3.9708	3.6117	3.4873	3.2540	4.9756	5.3069	4.2426	4.4779	3.7417	4.5210	4.6812	3.9579	3.6362	3.5985	3.6725	5.3112	4.9143	4.4047
Add3	_SPERTEEVLS[Phospho (STY)]PDGSPSK_.2	#N/A	5.1430	5.0422	3.9789	3.8120	5.1370	4.8269	3.5251	5.5344	3.4453	3.9736	3.6759	3.6818	5.9417	5.6749	3.5446	4.9330	5.4936	5.4157	4.2233	4.0804
Add3	_NMS[Phospho (STY)]PDLR_.2	#N/A	4.0573	4.4063	5.4818	6.1145	3.3998	4.5700	5.0429	3.5654	4.1521	4.6190	5.3056	3.8677	3.6520	5.8395	2.6481	4.0559	4.3508	3.5283	3.9701	4.2304
Add2	_STPAS[Phospho (STY)]PVQS[Phospho (STY)]PSK_.2	#N/A	14.4886	14.5181	14.8035	14.7020	14.4281	14.6048	14.2263	14.9090	14.7041	14.1810	14.4161	14.5442	14.9116	15.0907	14.6274	14.5721	15.0871	14.6016	14.5592	14.7204
Add2	_DIATEKPGS[Phospho (STY)]PVK_.2	#N/A	8.7889	8.6753	9.1150	8.7474	8.7580	9.2126	8.4812	9.0214	9.0140	8.8315	9.0636	8.5430	8.3029	9.6305	8.8520	8.6864	9.3678	8.8151	8.8163	9.0953
Add2	_VNVADEVQR_.2	#N/A	8.2864	8.0488	8.2788	8.4432	8.1508	7.9554	8.1064	8.1368	8.2686	7.8120	8.1040	8.2861	7.6096	8.1503	8.3204	8.3172	8.0184	8.1281	8.0671	8.1559
Add2	_FSEDDPEYLR_.2	#N/A	7.9239	8.5497	8.1880	7.8293	7.8149	7.8487	7.7528	8.1192	7.8635	7.8458	8.1482	8.1601	7.7745	8.1035	8.1011	7.8398	7.8579	7.6835	7.9445	8.2033
Add2	_WLNTPNTYLR_.2	#N/A	6.8465	7.0948	6.6977	7.1198	6.9520	6.9855	7.2091	7.6482	6.9121	7.3414	7.0163	7.2047	7.1064	7.4888	7.0856	6.9387	6.9685	7.5107	7.2304	7.0993
Add2	_VNILGEVVEK_.2	#N/A	6.7635	6.8906	7.4629	6.8832	6.6824	6.5351	6.7711	6.6881	7.3684	6.5446	6.6072	6.5304	6.6363	7.2710	7.3067	6.8611	6.9656	7.0552	7.0787	6.7843
Add2	_GVSC[Carbamidomethyl (C)]SEVTASSLIK_.2	#N/A	5.9610	5.8420	5.9017	6.3832	5.5342	6.0975	6.5313	5.9198	6.3667	5.5654	5.5694	5.4000	6.6146	6.5451	6.2475	6.1539	5.4755	5.9030	5.9902	5.8323
Add2	_EQDHFLISPK_.2	#N/A	6.2934	5.6068	6.5608	6.5955	6.5698	5.8540	5.9748	6.0475	5.7249	5.4304	6.5216	6.1120	6.0094	6.2494	6.2160	6.2302	6.3682	5.8767	5.9139	6.1807
Add2	_SAGPQSQLLASVIAEK_.2	#N/A	6.4448	5.6653	5.9471	6.0708	5.7683	6.0535	5.8446	5.3962	6.1281	5.5133	6.0617	6.0128	6.2638	5.6111	5.9795	6.0140	6.0696	5.6481	5.7297	5.7137
Add2	_[Acetyl (Protein N-term)]SEDTVPEAAS[Phospho (STY)]PPPSQGQHYFDR_.3	Q9QYB8_S11_M1_Add2	15.5945	15.5174	15.5993	15.5732	14.9412	15.3705	15.1180	15.2736	15.2359	15.5869	15.4301	15.3583	15.9144	15.9186	14.8555	15.5337	15.8619	15.3105	15.4807	15.5170
Add2	_TESVTSGPLSPEGS[Phospho (STY)]PSKS[Phospho (STY)]PSK_.2	#N/A	7.2347	6.9081	5.3521	7.5500	3.8748	5.5680	5.0509	6.2203	7.2369	7.4281	4.5742	5.2897	3.5163	6.3286	7.2681	5.4232	6.4274	5.5524	7.3854	7.5264
Add2	_TESVTSGPLS[Phospho (STY)]PEGSPSK_.2	#N/A	7.0150	6.6770	7.0063	6.5122	6.3826	3.3904	6.4333	6.7831	6.8233	5.9855	7.2291	6.4352	6.9686	7.7796	6.9845	6.5440	7.6615	6.8252	7.2884	6.8023
Add2	_LEQEQEGEKDIATEKPGS[Phospho (STY)]PVK_.3	#N/A	3.2339	3.2690	7.7924	7.1900	6.4531	7.3163	3.3628	4.2003	7.1154	3.4255	7.4792	6.6573	3.4939	3.5332	7.3475	6.6896	7.6510	4.0804	7.0336	7.1831
Add2	_HPFVQEK_.2	#N/A	5.3768	5.4877	4.6468	3.8205	3.2066	3.2775	5.6080	4.4114	5.4643	4.5714	4.9166	5.9387	3.8896	5.6413	5.9439	4.9993	5.7077	5.4691	5.3487	5.4164
Add2	_TESVTSGPLSPEGSPS[Phospho (STY)]KS[Phospho (STY)]PSKK_.3	#N/A	4.0814	7.4493	7.4139	8.5462	3.5193	8.3263	7.9408	8.0399	7.7605	4.0411	7.8869	3.6664	7.5049	8.2330	7.9936	3.2681	7.5340	8.0306	7.5595	7.7978
Add2	_SPAVS[Phospho (STY)]PSKTSEDTK_.2	#N/A	10.1580	10.1780	10.3810	10.2760	9.8875	10.0052	9.8754	10.3199	10.0845	10.2049	10.0076	10.0527	10.4338	10.5997	9.9630	10.0803	10.4156	10.0569	10.1217	10.1572
Add2	_NMGS[Phospho (STY)]PRPK_.2	#N/A	10.0378	10.7516	10.3411	10.0272	7.9443	9.9218	10.4164	9.9896	9.6594	10.1559	10.0022	7.9830	10.2133	10.9967	7.4175	5.2727	9.8373	7.1432	7.5645	10.0732
Add2	_AGTKS[Phospho (STY)]PAVS[Phospho (STY)]PSK_.2	#N/A	12.0491	11.6834	12.2789	12.1556	11.5438	11.5581	11.5159	11.6566	11.6460	11.9475	11.7048	11.6002	11.5148	12.1576	10.3736	11.4494	11.8872	11.5833	11.4901	11.7573
Add2	_IENPNQFVPLYTDPQEVLDM[Oxidation (M)]R_.3	#N/A	5.0360	5.0267	3.3777	3.3472	3.6799	4.3050	4.6683	4.6452	4.7693	3.8753	3.6761	5.0525	4.3629	4.2970	4.6568	4.7808	4.4091	4.7959	4.4023	4.9531
Add2	_AGTKS[Phospho (STY)]PAVS[Phospho (STY)]PSKTSEDTK_.2	#N/A	10.4890	10.2705	6.7698	10.6924	9.9494	9.9603	10.1578	10.0987	10.1319	10.4957	10.0630	10.1119	10.5040	10.5688	9.4187	10.2151	10.2679	10.0970	10.1777	10.2904
Add2	_STPAS[Phospho (STY)]PVQS[Phospho (STY)]PSKAGTK_.2	#N/A	9.8113	10.4671	9.4353	9.9794	10.2137	10.1950	10.0263	11.0821	10.4749	9.5897	9.6058	10.7832	11.4727	10.6369	10.9766	10.5405	10.9013	10.5553	10.3749	10.4563
Add2	_SPS[Phospho (STY)]TESQLMSK_.2	#N/A	4.1494	7.6093	8.6063	8.9212	7.0356	7.1764	7.5030	5.8301	3.7394	3.9222	8.0743	6.8099	4.1701	8.9788	6.9478	3.7708	7.3582	6.1022	3.9431	7.2098
Add2	_S[Phospho (STY)]PAVS[Phospho (STY)]PSKTSEDTKK_.2	#N/A	8.3453	8.6739	8.4054	8.6202	7.5334	8.5395	8.5871	8.5240	8.2689	8.5056	8.4772	8.4762	8.5033	9.0722	8.6368	8.6541	8.7530	8.7102	8.8034	8.6421
Add2	DIATEKPGS[Phospho (STY)]PVKS[Phospho (STY)]TPAS[Phospho (STY)]PVQS[Phospho (STY)]PSK	#N/A	4.0089	3.9012	3.3151	3.8269	3.3466	3.5707	4.0442	3.7653	3.8370	4.2152	3.1843	3.7323	3.9450	3.8258	3.8289	4.1963	4.4451	4.3223	3.3948	4.2395
Add2	_DKTESVTSGPLS[Phospho (STY)]PEGS[Phospho (STY)]PSK_.2	#N/A	3.4531	4.0597	3.9967	4.7640	3.6600	3.6496	3.6927	3.6788	3.6624	4.1689	4.3737	4.5352	3.7065	4.0348	3.4926	4.6340	4.9119	4.7245	4.1280	4.3725
Add2	_DKTESVTSGPLSPEGSPS[Phospho (STY)]KSPSK_.3	#N/A	6.1670	3.7050	3.8922	6.3212	5.3559	3.9649	5.6953	3.7648	3.4619	3.5765	3.8363	5.9050	3.5700	6.7650	5.6952	5.7617	6.1192	3.9580	3.7890	5.6919
Add2	_SRS[Phospho (STY)]PSTESQLM[Oxidation (M)]SK_.2	#N/A	8.2032	8.6215	8.5368	9.2279	8.0334	8.5044	8.1791	3.5384	5.6397	8.5130	8.1818	8.6472	9.0625	9.3906	7.3769	8.0505	8.4010	8.5988	7.9727	7.9375
Add2	_GLGQM[Oxidation (M)]TTNADTDGDSYKDKTESVTSGPLS[Phospho (STY)]PEGSPSK_.4	#N/A	3.9567	4.8315	4.4930	3.7407	3.1191	4.4947	4.1883	3.6571	4.2298	4.1052	3.5575	3.9436	3.9774	5.3480	4.6332	3.9693	4.7613	3.6464	4.5408	4.6779
Add2	_C[Carbamidomethyl (C)]LGPTC[Carbamidomethyl (C)]K_.2	#N/A	3.1744	4.2462	4.2118	4.4098	3.6675	3.2262	4.0343	4.2340	3.0972	4.1607	3.9159	3.8708	3.2053	4.2499	4.4052	4.0374	4.3982	2.9984	3.0503	4.6600
Add2	_NHGM[Oxidation (M)]VALGDTVEEAFYK_.3	#N/A	5.1789	3.6330	5.1319	3.3196	4.9517	4.2774	4.3049	4.0702	4.5645	5.8689	3.9659	5.0620	6.1116	4.3410	4.7258	3.2139	3.1070	3.2253	4.2297	4.8196
Add2	_SPAVS[Phospho (STY)]PSK_.2	#N/A	3.6371	5.4798	4.3885	5.5527	4.8917	3.1747	3.5450	3.5640	3.5189	4.0543	3.4227	3.8723	5.3326	4.3999	4.8229	5.5209	4.3393	3.9325	5.6292	4.2348
Add2	_SSSGMPIR_.2	#N/A	4.2570	3.7458	3.2924	4.6134	3.5947	4.3902	4.1920	3.9574	4.1123	3.9606	3.5909	3.6353	4.2777	3.2795	4.2174	3.5121	4.4950	3.3381	2.9867	3.2465
Add2	_LGEHEFEALMR_.3	#N/A	4.0797	3.9231	4.6160	3.0648	3.4175	3.0666	4.1236	3.7801	3.9350	4.1378	3.4136	1.9426	4.1004	4.6031	3.7008	4.4246	4.3178	3.5154	3.1640	3.0693
Add2	_SAGPQSQLLASVIAEKS[Phospho (STY)]R_.3	#N/A	3.4893	4.5135	4.0256	4.2001	4.3278	3.0511	3.2132	3.5752	3.2723	3.6956	3.6467	4.4030	3.3804	6.2947	2.7904	3.8342	3.0724	4.0770	4.3761	4.1023
Add1	_AAVVTS[Phospho (STY)]PPPTTAPHK_.2	#N/A	14.0680	14.2493	14.0190	14.0297	13.7265	13.6082	13.5719	14.1503	13.8334	14.1055	13.8570	14.0752	14.5332	14.4064	13.5106	13.8893	14.3193	13.7176	13.9530	13.8320
Add1	_TPS[Phospho (STY)]FLKK_.2	Q9QYC0_S724_M1_Add1	13.3009	13.3456	13.3208	13.3883	13.0220	12.8118	12.7456	13.0370	12.4901	13.5371	12.8762	12.8345	13.2135	13.1623	11.9201	12.7131	12.9303	12.7496	12.8419	12.9421
Add1	_AAVVTS[Phospho (STY)]PPPTTAPHKER_.3	#N/A	10.3484	11.1674	8.8900	10.3503	10.0761	10.1778	10.5294	11.0669	10.1507	10.4802	9.8676	10.8501	11.9712	10.7790	10.3156	10.7124	10.5699	10.8603	10.6814	10.4497
Add1	_GS[Phospho (STY)]EENLDETR_.2	#N/A	8.9422	9.2018	9.2439	9.1877	8.9559	9.0948	8.9895	8.8446	8.8808	9.3668	9.1450	8.9881	9.2630	9.3162	8.4883	8.9154	9.0259	8.9612	8.9466	9.2866
Add1	_LEEDLPQEPTS[Phospho (STY)]R_.2	Q9QYC0_S628_M1_Add1	9.9221	3.1945	3.8437	4.3820	4.1460	4.3701	3.3951	4.5086	3.4542	3.5138	9.7857	3.0840	3.5622	3.8308	2.9723	9.1777	3.5455	3.8952	4.1942	9.4556
Add1	_VNLQGDIVDR_.2	#N/A	8.4428	8.0897	8.6058	8.3120	8.0971	8.2052	7.9916	7.9344	8.3897	8.1997	8.1251	8.2299	7.9372	8.0460	7.9780	8.1935	8.0960	8.0877	8.2469	8.2187
Add1	_TSTSAVPNLFVPLNTNPK_.2	#N/A	7.6746	7.6842	7.3296	7.5955	7.8249	7.8039	7.8028	7.3276	7.7298	7.8219	7.9333	7.4548	7.8954	7.3955	7.7309	7.6316	7.5728	7.8943	7.9586	7.7694
Add1	_TLASAGGPDNLVLLDPGK_.2	#N/A	7.3936	7.3685	7.6556	7.7406	7.1391	7.4661	7.7176	7.2835	7.5625	7.6505	7.5777	7.1937	6.6516	7.4282	7.4404	7.5888	7.4224	7.1741	7.4510	7.5250
Add1	_YFDRVDENNPEYLR_.3	#N/A	7.9799	7.4363	7.0593	7.5416	7.4212	7.6686	7.4016	7.7885	7.6179	7.7311	7.8444	7.7305	8.1119	7.7831	7.3768	7.8523	7.6762	7.5183	7.7446	7.7819
Add1	_GSDSIAYDKGEK_.2	#N/A	7.1286	7.3128	6.9843	7.6508	6.8491	7.0184	7.1964	7.4221	7.4468	7.2463	6.9771	7.1739	7.1932	7.3940	7.2685	7.3092	7.0959	7.0277	7.0099	7.1116
Add1	_SLVQGELVTASK_.2	#N/A	6.9694	6.8328	7.0398	7.2175	6.1608	7.0956	6.7871	6.7762	6.8088	6.7031	6.6715	6.9080	6.8237	6.8954	7.0752	6.9105	6.8877	6.8323	7.0480	6.8016
Add1	_SPGT[Phospho (STY)]PAGEGSGSPPK_.2	#N/A	12.7473	12.6685	12.8822	12.8429	12.5016	12.7229	12.4547	12.7520	12.6040	12.6387	12.5658	12.4218	12.6956	12.8874	12.1314	12.5645	12.8234	12.5289	12.5596	12.6348
Add1	_EKS[Phospho (STY)]PPDQSAVPNT[Phospho (STY)]PPSTPVK_.3	#N/A	8.2005	8.3436	9.7813	9.3206	7.1412	3.6318	3.7323	7.1816	9.4830	7.7351	8.2723	7.8603	3.8593	9.0266	9.3070	8.3224	9.4905	9.2170	8.0144	9.9308
Add1	_EYQPHVIVSTTGPNPFNTLTDR_.3	#N/A	5.5906	5.3856	5.0696	5.2212	4.5946	5.3424	5.5479	5.1606	5.5667	5.1203	5.1048	4.3437	4.3769	5.3830	5.1079	4.8826	5.0354	5.2519	4.9989	5.1934
Add1	_M[Oxidation (M)]LDNLGYR_.2	#N/A	5.3854	6.9963	6.8433	7.4082	6.4070	6.7195	7.0661	6.0841	6.0646	5.8056	6.3454	6.7595	5.7428	7.0677	6.7954	5.9249	6.0566	6.3024	6.7510	6.5084
Add1	_KQKGS[Phospho (STY)]EENLDETR_.3	#N/A	8.4419	9.1722	8.7512	8.8283	8.2206	8.8109	9.0056	8.4579	8.8996	9.1696	8.7874	8.5017	9.3644	8.8075	8.8201	8.7807	8.5739	8.7635	9.1687	8.8306
Add1	_SPPDQSAVPNT[Phospho (STY)]PPSTPVK_.2	#N/A	9.9058	9.7956	9.6635	9.5342	9.4865	9.9853	9.4408	10.1965	9.7004	3.8725	7.5757	9.6829	9.8323	10.5193	9.2701	3.7223	7.6606	9.7784	10.0388	9.6954
Add1	_SRS[Phospho (STY)]PGT[Phospho (STY)]PAGEGSGSPPK_.2	#N/A	11.7658	11.7392	12.0556	12.0191	11.5276	11.6052	11.3872	11.7472	11.7438	11.4350	11.4576	11.6716	11.9876	12.1798	11.4249	11.5682	11.9420	11.7132	11.5081	11.6166
Add1	_WQIGEQEFEALM[Oxidation (M)]R_.2	#N/A	5.9229	5.6452	5.3404	4.6816	5.2520	4.9510	5.0964	5.2411	5.1615	4.6557	5.5242	5.3350	4.4492	3.4510	4.2471	3.2725	5.1267	4.5291	5.4871	4.6210
Add1	_YSDVEVPAS[Phospho (STY)]VTGHSFASDGDSGTC[Carbamidomethyl (C)]SPLRHSFQK_.4	Q9QYC0_S415_M1_Add1	7.6274	4.5170	4.0221	4.2036	5.7544	3.6605	3.2167	3.1862	3.3411	3.6922	4.3206	4.4064	3.5065	4.0092	2.7939	3.8307	3.7239	4.1093	3.7579	3.9762
Add1	_GDDASEEGQNGSS[Phospho (STY)]PK_.2	#N/A	7.9321	9.0963	9.8401	9.3297	9.4919	9.2008	7.2199	7.3508	9.0750	7.4076	9.0922	8.3534	8.0335	8.4963	7.0401	7.8060	9.0295	8.5299	7.4663	9.9225
Add1	_WLHSGRGDDASEEGQNGSS[Phospho (STY)]PK_.3	#N/A	7.5213	8.5243	3.3317	7.8243	6.6813	7.6003	7.9973	7.4601	7.2661	8.1162	7.3002	7.5663	8.4745	7.7805	7.7296	8.0504	7.4541	7.7021	7.9910	7.6193
Add1	_QKGS[Phospho (STY)]EENLDETR_.2	#N/A	10.1236	10.4634	10.2412	10.3651	9.8457	10.0944	10.1818	10.1454	10.1969	10.1576	9.9036	10.0688	10.6796	10.5081	9.8266	10.2070	10.3070	10.2309	10.1152	10.0553
Add1	_YSDVEVPASVTGHSFASDGDSGTC[Carbamidomethyl (C)]S[Phospho (STY)]PLR_.3	#N/A	11.0738	11.4831	10.9021	10.8295	3.4477	10.2392	10.7860	10.8649	10.3421	10.9031	10.2912	10.6019	11.3238	11.6253	10.2181	10.4090	10.7583	10.7451	10.7924	10.2867
Add1	_SPPDQSAVPNT[Phospho (STY)]PPST[Phospho (STY)]PVKLEEDLPQEPTSR_.4	#N/A	6.7141	7.5245	8.4369	3.7634	3.4744	6.7260	6.9246	4.8505	7.6886	3.9463	6.0026	7.3204	6.5335	7.8396	6.1237	4.3596	4.0308	7.5514	7.1910	7.0492
Add1	_NMAPDLR_.2	#N/A	4.0600	3.2590	3.3790	3.5242	4.2670	3.8414	4.8296	4.2857	3.9829	4.4859	3.9251	1.3672	4.0909	2.8073	2.8065	3.4222	3.7830	3.3665	3.6655	3.3120
Add1	_KYSDVEVPASVTGHSFASDGDSGTC[Carbamidomethyl (C)]S[Phospho (STY)]PLR_.4	#N/A	4.0533	5.6782	5.6976	3.6361	3.3910	3.6151	4.1501	3.7536	4.2092	5.6758	3.0827	4.8227	4.3172	4.5766	3.7273	4.1195	4.2913	3.5419	5.1498	3.0428
Add1	_EKSPPDQS[Phospho (STY)]AVPNT[Phospho (STY)]PPSTPVKLEEDLPQEPTSR_.4	Q9QYC0_S605_M2_Add1	3.3732	3.9760	4.4106	3.9755	3.5801	3.9380	3.4981	3.6347	3.5523	4.0515	3.5287	3.7022	3.6905	3.8205	3.5235	4.2361	3.6850	4.7095	3.8615	4.2839
Add1	_IREQNLQDIK_.3	#N/A	3.0823	4.2368	4.1197	4.5019	3.2893	3.3183	3.7588	3.3080	3.0051	4.3938	3.3795	3.9629	3.1132	4.1578	3.7842	3.9453	4.3061	4.3442	3.1424	3.8351
Pcnx1	_SSS[Phospho (STY)]LIHR_.2	Q9QYC1_S551_M1_Pcnx1	3.8969	4.1059	5.9853	5.7014	3.2346	3.4587	5.9992	3.5972	4.3656	4.1032	5.1746	3.9954	5.4854	4.5459	3.7476	4.6958	4.1349	3.6982	3.3468	3.6947
Bcl11a	_GC[Carbamidomethyl (C)]S[Phospho (STY)]PGESASGGLSK_.2	#N/A	9.7871	9.9843	10.1875	10.1892	9.6100	9.8054	9.5855	9.8994	9.7639	10.0112	9.6477	9.6305	10.1714	10.4402	9.6504	9.6040	10.0125	9.7089	9.7609	9.7685
Bcl11a	_GC[Carbamidomethyl (C)]S[Phospho (STY)]PGESASGGLSKK_.2	#N/A	7.1921	4.3756	4.1635	4.0622	3.4858	6.9447	3.5623	7.5146	3.4825	7.3208	6.8571	4.2650	3.6479	6.9637	3.5877	7.1049	7.3291	3.9679	3.6165	4.1176
Bcl11a	_IYLESEHGS[Phospho (STY)]PLTPR_.3	#N/A	6.2552	5.5964	5.6059	4.1537	5.8596	4.5324	5.2349	5.0563	5.0149	4.0035	5.7250	5.6472	6.1043	4.3728	5.6248	6.2573	5.2995	5.9468	5.6179	5.7619
Bcl11a	_FST[Phospho (STY)]PPGELDGGISGR_.2	Q9QYE3_T701_M1_Bcl11a	3.2214	3.2806	8.7276	9.5091	4.0600	9.0712	3.4811	4.4226	3.5402	8.4390	8.8974	3.1700	3.2421	9.1790	3.0583	8.7730	9.0183	8.3030	4.0223	3.7118
Bcl11a	_ELAGNTSS[Phospho (STY)]PPLS[Phospho (STY)]PGRPSPMQR_.3	Q9QYE3_S328_M2_Bcl11a	3.3360	3.1660	3.8723	4.3534	4.1745	4.3986	3.3666	4.5372	3.4256	3.5423	3.8000	3.0555	3.5337	4.5783	2.9438	3.6809	3.5740	3.9237	4.2228	4.2556
Bcl11a	_GVDKPPS[Phospho (STY)]PSPIEMK_.2	Q9QYE3_S86_M1_Bcl11a	4.4894	3.5134	3.5249	3.2000	3.8271	4.0512	3.7140	4.1898	3.7730	3.1949	6.4272	3.4029	3.8811	3.4249	3.2912	3.3335	3.2266	3.5763	3.8754	3.1021
Bcl11a	_LLLGS[Phospho (STY)]PSSLS[Phospho (STY)]PFSK_.2	Q9QYE3_S625_M2_Bcl11a	3.1939	3.3080	3.7302	4.4954	4.0325	3.9524	3.1290	4.3952	3.0492	3.5227	4.0287	3.1975	3.2147	3.7173	3.1544	3.0743	3.4320	4.4012	4.0497	3.6843
Ndrg3	_LDPINTTLLK_.2	#N/A	7.0911	7.1167	6.1289	6.9912	6.8734	6.5570	7.1711	7.1440	7.0935	6.4298	6.6777	6.6266	6.1365	6.4283	6.9181	6.9050	6.4280	7.2454	6.8021	6.7160
Ndrg3	_DLEIERPILGQNDNR_.3	#N/A	9.8331	9.8863	9.7043	10.1315	4.2939	9.7710	9.4278	9.9634	9.8204	9.8215	9.8750	9.6383	10.3360	10.0872	9.2088	9.7345	10.2651	9.6418	9.7587	9.9498
Ndrg3	_THSTSSS[Phospho (STY)]IGSGESPFSR_.2	#N/A	7.2666	7.3775	6.8318	7.0614	6.9765	7.2949	6.9183	6.7144	6.9082	7.8326	7.6501	6.5694	6.9821	7.2452	5.5986	3.7223	7.6263	7.0268	7.0726	7.0951
Ndrg3	_S[Phospho (STY)]RT[Phospho (STY)]HSTSSSIGSGESPFSR_.3	Q9QYF9_T329_M2_Ndrg3	3.9355	3.6156	3.9291	4.2469	3.8006	5.1489	4.6742	4.1511	4.5871	4.2057	4.2226	3.4039	3.9563	4.3642	3.3664	3.3070	3.6483	3.5498	3.8489	3.7259
Ndrg3	_HQTMEVS[Phospho (STY)]C[Carbamidomethyl (C)]_.2	Q9QYF9_S374_M1_Ndrg3	3.3196	6.2018	6.6908	7.1778	5.2985	6.0461	5.8262	3.2869	4.6168	3.1302	5.9071	4.3058	3.6072	6.5269	3.5469	4.1826	5.2236	4.0086	3.6572	4.2131
Ndrg3	_LHIAQDINQENLQLFLGSYNGR_.3	#N/A	3.4497	3.8694	4.4871	4.1345	3.6567	3.6900	3.3914	3.1567	3.3116	3.2603	4.2911	3.8583	3.4771	3.9797	2.6846	4.3127	3.6944	4.1387	3.7873	4.1354
Ndrg3	_SVTSNQSDGTQESC[Carbamidomethyl (C)]ES[Phospho (STY)]PDVLDR_.3	Q9QYF9_S361_M1_Ndrg3	3.7540	4.2488	4.2903	3.9354	3.0918	3.3923	4.4493	3.4544	3.6093	3.9604	3.0879	4.1382	3.7748	2.1393	4.0265	4.0989	3.9921	3.8411	3.4897	4.2444
Ndrg3	_C[Carbamidomethyl (C)]STLLVVGDNSPAVEAVVEC[Carbamidomethyl (C)]NSR_.3	#N/A	4.2782	4.5417	3.8148	3.3060	2.9843	3.6232	4.0638	4.5039	4.2010	4.0888	3.0746	4.2678	4.3091	3.8529	4.0892	3.6403	4.0011	3.1483	3.4474	3.5301
Ndrg2	_SC[Carbamidomethyl (C)]FQPLFR_.2	#N/A	8.2098	8.4211	8.6406	8.9001	8.7929	8.5705	8.5125	8.8218	8.3174	8.4751	8.3448	8.7140	8.4008	8.2741	8.5750	8.2361	8.2772	8.6321	8.3901	8.4221
Ndrg2	_[Acetyl (Protein N-term)]AELQEVQITEEKPLLPGQTPETAK_.3	#N/A	6.3075	6.0923	7.1381	6.8623	6.2662	6.4282	6.5087	6.3837	6.8466	6.8922	5.8671	6.0542	6.4792	6.2589	6.4137	6.7216	6.0233	6.0779	6.0933	6.3367
Ndrg2	_GIIQHAPNLENIELYWNSYNNR_.3	#N/A	7.8997	7.7736	8.0022	7.9389	6.9435	7.7515	7.6830	8.1451	7.5477	7.7501	7.2740	7.6037	7.5235	8.4104	7.6027	7.4618	7.5053	7.6188	7.6430	7.6713
Ndrg2	_LDPTQTSFLK_.2	#N/A	7.6903	7.8107	7.7357	7.8691	7.8011	7.6291	7.5678	7.9736	7.5989	7.7681	7.4287	7.5664	7.7778	7.5101	7.9583	7.2745	7.8359	7.4855	7.5664	7.6257
Ndrg2	_GWM[Oxidation (M)]DWAAHK_.2	#N/A	6.6715	6.6926	5.9080	5.7722	6.6554	6.4991	6.1705	6.9223	6.4643	6.4574	6.0918	7.0123	7.0097	5.7785	6.1192	6.3936	6.5876	6.7802	5.9435	6.5042
Ndrg2	_YALNHPDTVEGLVLINIDPNAK_.3	#N/A	5.7985	6.3925	6.7896	6.4566	6.3821	6.1895	5.9498	6.7596	6.2760	6.0619	6.2475	6.4270	5.9503	6.1664	6.0600	6.2771	6.3239	6.0878	6.3985	6.4607
Ndrg2	_M[Oxidation (M)]ADSGGQPQLTQPGK_.2	#N/A	4.1355	6.1052	7.8614	8.3620	7.8235	7.2151	7.7454	7.3371	7.3810	6.7471	7.3545	7.8366	7.3738	7.3390	7.9396	7.6756	7.5100	7.6470	7.6794	7.7039
Ndrg2	_TAS[Phospho (STY)]LTSAASIDGSR_.2	Q9QYG0_S332_M1_Ndrg2	12.9498	13.4575	13.6874	13.6503	12.4222	13.1005	13.0252	12.6702	13.2810	13.0605	12.0632	12.7060	12.7895	13.4332	12.6559	12.9932	13.0121	12.9894	12.7989	12.9702
Ndrg2	_FGDM[Oxidation (M)]QEIIQNFVR_.2	#N/A	6.7863	6.6766	6.0431	7.2396	6.3306	6.2881	6.3936	6.8825	6.4787	7.4309	6.0491	6.5117	6.5428	7.0012	5.9333	5.9077	7.4775	6.3941	6.0303	7.1965
Ndrg2	_TASLTSAAS[Phospho (STY)]IDGS[Phospho (STY)]RSR_.2	#N/A	3.7422	3.6059	3.9906	3.9626	3.9010	3.9730	6.6306	3.6553	6.4355	3.8729	3.7810	3.4481	3.8012	4.8638	5.4269	4.9117	3.8143	3.8506	6.3493	3.8952
Ndrg2	_SRT[Phospho (STY)]AS[Phospho (STY)]LTSAASIDGSR_.2	#N/A	8.2779	9.4132	9.8953	9.7033	4.0194	9.2739	9.0817	4.1534	9.0459	9.6092	5.4044	7.8620	6.8695	9.6368	8.4757	9.2390	8.9174	9.0344	8.8925	9.2709
Ndrg2	_TLSQSS[Phospho (STY)]ESGTLPSGPPGHTM[Oxidation (M)]EVSC[Carbamidomethyl (C)]_.3	#N/A	7.9462	8.0010	7.6427	3.5703	8.9004	7.9416	3.9747	8.6618	7.4254	8.8992	8.7732	3.8318	4.0456	7.8704	8.1100	8.3049	8.9969	8.0737	8.8317	8.0735
Ndrg2	ospho (STY)]RT[Phospho (STY)]LSQSSESGTLPSGPPGHTM[Oxidation (M)]EVSC[Carbamidomethyl	Q9QYG0_S346_M2_Ndrg2	3.6848	4.0512	3.9621	7.4839	3.3882	6.5103	3.0648	3.6105	3.0783	3.7650	3.6739	3.8465	3.2048	4.0937	3.8641	3.7567	7.2362	7.4983	7.1010	5.8384
Ndrg2	_LSRS[Phospho (STY)]RT[Phospho (STY)]AS[Phospho (STY)]LTSAASIDGSR_.3	#N/A	3.6226	3.6054	4.1241	3.8705	4.1135	4.1877	3.5977	3.9597	3.6568	3.3314	4.0312	3.3315	3.7649	4.1621	3.1749	3.4563	3.4569	5.4569	4.0119	3.8394
Ndrg2	_LTGLTSSIPDM[Oxidation (M)]ILGHLFSQEELSGNSELIQK_.3	#N/A	3.9701	3.3490	3.5067	3.6141	4.1771	4.2104	4.3719	4.1372	4.2921	3.7807	3.7707	3.0751	4.4575	3.4593	2.8964	3.3323	3.1740	3.1583	4.3077	3.4263
Ndrg2	_C[Carbamidomethyl (C)]PVMLVVGDQAPHEDAVVEC[Carbamidomethyl (C)]NSK_.3	#N/A	3.2246	4.0945	4.2620	4.0078	3.4315	3.4648	3.6166	3.3819	3.5368	4.5360	3.0155	3.8207	3.7023	4.2049	3.6420	4.0875	3.9196	3.9136	3.5621	4.1719
Dnajb12	_SAS[Phospho (STY)]DEDLKK_.2	Q9QYI4_S123_M1_Dnajb12	3.8213	3.4978	3.3579	3.7629	4.0283	4.0616	3.0198	4.0470	4.4409	3.6319	3.9195	3.2239	3.8522	3.3960	3.0452	3.1834	3.5442	3.6052	3.9042	3.0733
Dnajb2	_GEAAKLS[Phospho (STY)]PSSEEK_.2	#N/A	8.7566	9.0386	8.6813	8.9705	8.6696	8.9848	8.3979	8.9459	8.6675	8.8714	8.4524	8.8417	9.2880	9.5339	8.8288	8.8379	8.9960	8.8808	8.8639	8.9681
Dnajb2	_SAS[Phospho (STY)]PDDIKK_.2	Q9QYI5_S15_M1_Dnajb2	6.6378	4.1053	6.9026	6.6031	3.2352	6.8076	4.3059	7.2176	3.7528	4.1038	6.5739	3.9948	7.4955	4.4209	3.8831	4.2424	4.1356	6.8511	6.8043	6.8343
Dnajb2	_LS[Phospho (STY)]PSSEEK_.2	#N/A	4.5744	4.2455	4.1110	5.2983	5.8044	5.9073	5.6852	3.2992	2.9964	5.6470	6.0787	3.9717	4.6053	5.6891	6.0922	3.9365	5.6612	5.0916	5.1261	3.8263
Dnajb2	_GEAAKLS[Phospho (STY)]PS[Phospho (STY)]SEEKASR_.2	#N/A	3.3516	7.8380	4.5246	4.0970	4.1902	4.4142	3.3509	3.7129	3.4100	7.4315	3.7844	3.5580	7.8533	4.5627	2.9281	3.6965	3.2101	7.0481	4.2384	4.2400
Dnajb2	_S[Phospho (STY)]PEEVFR_.2	Q9QYI5_S98_M1_Dnajb2	4.5054	4.1013	4.4378	3.7879	3.2115	3.2448	3.8366	3.6019	3.7568	4.3160	3.2354	3.9907	3.9222	4.4249	3.8620	3.8675	4.1396	2.6484	3.3422	4.3919
Dnaja2	_VIEPGC[Carbamidomethyl (C)]VR_.2	#N/A	6.1190	7.3534	7.0018	7.1804	7.3094	7.0073	7.1339	6.5479	6.9973	6.9741	6.8711	7.0333	6.9721	7.4502	8.0272	6.8226	6.7229	7.2548	7.2307	7.2618
Dnaja2	_EHEVFQR_.2	#N/A	5.1967	5.8542	6.2840	5.9953	5.7825	5.9687	5.4807	5.4164	6.1679	5.2183	6.5295	5.6337	5.1570	6.4973	6.2722	6.0042	5.9566	5.6081	5.6701	6.5241
Dnaja2	_VSLEDLYNGK_.2	#N/A	5.9687	5.7124	6.1756	6.4102	6.3691	6.2457	6.1027	5.5715	5.7678	5.8488	6.1931	5.6356	6.4242	6.2849	5.9854	6.7022	6.0274	5.8764	6.2402	6.5625
Dnaja2	_YGEQGLR_.2	#N/A	3.4160	6.1830	5.9100	5.9381	6.0826	5.7120	6.3875	5.7639	6.5919	6.0655	6.2038	6.2822	3.5108	5.8211	6.2731	6.0942	5.5784	6.2725	5.9731	5.8695
Dnaja2	_NVLC[Carbamidomethyl (C)]SAC[Carbamidomethyl (C)]SGQGGK_.2	#N/A	4.6831	4.3045	4.9234	4.7109	3.7683	3.6682	3.6186	4.5138	4.4621	3.9837	4.4773	5.0597	3.7393	5.2221	5.0680	4.5937	4.5732	3.8125	4.1179	5.3919
Dnaja2	_DGNDLHM[Oxidation (M)]TYK_.2	#N/A	4.2595	7.1302	5.0811	3.5041	3.5972	5.9410	5.5825	6.1003	6.2979	7.0481	6.7303	5.9391	5.4099	3.6548	5.6323	6.1645	5.8938	6.5200	4.9297	3.3321
Dnaja2	_ITFTGEADQAPGVEPGDIVLLLQEK_.3	#N/A	4.2122	3.7906	3.2477	3.4772	3.5499	1.8785	4.1473	3.9126	4.0675	4.0053	3.5461	3.6800	4.2329	3.2347	3.5683	3.0563	4.4503	3.3829	3.4917	3.2017
Dnaja2	_IGLVEALC[Carbamidomethyl (C)]GFQFTFK_.2	#N/A	4.4036	3.5992	3.4390	3.2858	3.7413	3.9654	3.7998	4.1039	3.8589	3.1091	4.2333	3.4887	3.9669	3.4261	3.3770	3.2476	3.1408	3.4905	4.3410	3.1880
Dnajb1	_GAS[Phospho (STY)]DDEIKR_.2	Q9QYJ3_S16_M1_Dnajb1	7.6347	8.1579	7.5925	7.9557	7.7983	7.3798	7.6389	7.9625	7.6304	7.4163	7.6793	7.8484	7.8844	8.4755	7.9665	7.9253	7.4574	7.7657	7.9317	7.7764
Pnck	_HSHPGLGTSQS[Phospho (STY)]PKW_.3	#N/A	4.1652	3.1539	4.4896	3.4190	3.5122	3.7362	4.0289	4.3909	5.4158	4.3808	2.9615	4.3809	4.1961	3.7398	3.6061	3.0185	3.8881	3.2613	5.1312	3.4171
Pnck	_KLGQS[Phospho (STY)]PEGEEASR_.2	Q9QYK9_S316_M1_Pnck	6.2556	6.2840	3.8816	6.8980	5.7361	6.3900	6.4725	6.1383	4.2678	4.2009	3.1414	4.2010	6.1842	6.2118	5.6481	6.4133	4.0679	6.1499	6.7800	3.5970
Pnck	_HSHPGLGTSQS[Phospho (STY)]PK_.3	#N/A	3.1631	4.1560	4.2005	4.4212	3.3700	3.2375	3.0268	3.3887	3.0859	3.8820	3.4603	3.8821	3.1939	4.2386	4.1048	4.0260	4.3868	4.2635	3.0617	3.9158
Map1a	_VPSAPGQES[Phospho (STY)]PVPDTKST[Phospho (STY)]PPTR_.3	#N/A	3.6306	10.7375	10.5435	4.0727	9.5630	10.2990	7.0713	3.5723	10.3092	6.6693	4.1347	3.6465	10.0270	11.2490	9.1943	7.3207	9.6283	7.8112	10.7558	3.7903
Map1a	_GEPVPAWEGKS[Phospho (STY)]PEQEVR_.2	#N/A	10.5798	10.4943	10.8047	10.8807	10.0467	10.5766	10.0876	9.8822	10.2044	10.9455	10.9583	10.1996	10.6156	11.0398	9.8348	10.7967	10.4796	10.4647	10.7941	10.9836
Map1a	_EAELT[Phospho (STY)]PENIAAAR_.2	Q9QYR6_T633_M1_Map1a	9.6649	9.6517	9.5758	9.5602	9.1179	9.5234	9.2346	9.3425	9.5790	9.5406	9.5609	9.6309	9.7033	10.0393	9.3642	9.6356	10.0221	9.5510	9.6663	9.7434
Map1a	_EQDVVQGWR_.2	#N/A	8.2377	8.2458	8.5415	8.2755	8.3873	8.1936	8.3821	8.4768	8.5205	8.3548	8.3682	8.5298	8.7520	8.6651	8.6901	8.0848	8.5524	8.3234	8.3761	8.5509
Map1a	_VLFPGNAPQNK_.2	#N/A	8.5333	8.3800	8.2028	8.4357	8.3502	8.3295	8.3987	8.5967	8.0203	8.2396	8.5505	8.6987	8.8587	8.6911	8.7621	8.2617	8.5219	8.7507	8.5282	8.0913
Map1a	_ASYYVVSGNDPANGEPSR_.2	#N/A	8.2434	8.1419	7.9963	8.0062	8.0144	7.8825	7.9342	8.3816	8.2062	8.0365	8.0857	7.7159	8.6410	8.4399	8.3150	7.0387	8.2282	8.0851	8.2274	7.4951
Map1a	_GVEQQDGAVPEK_.2	#N/A	8.2469	7.7379	7.8143	8.0988	7.9372	7.8786	7.7348	7.9365	8.1090	7.9377	7.9350	7.9752	8.3223	7.9527	8.2650	8.1274	7.9701	7.6741	7.6129	7.9836
Map1a	_DLAAGAVPANLKPSK_.2	#N/A	8.1223	7.7288	8.1679	7.5239	7.9030	7.5047	7.9573	8.2150	8.0160	8.0754	7.8703	8.1869	7.7135	8.1047	8.3693	8.3308	8.0245	7.8361	8.0325	8.1008
Map1a	_SSRPDTLLSSEQRPGK_.3	#N/A	7.4310	7.9642	7.6961	7.8945	7.8647	7.3354	7.7892	7.8266	7.8990	7.3731	7.6407	7.7024	7.7788	8.1406	8.6369	8.0115	7.6431	7.6669	7.9405	7.6677
Map1a	_GAALQQTQAPEPR_.2	#N/A	7.6795	7.2827	7.8379	7.5224	7.5703	7.3302	7.1164	7.8930	7.7629	7.4023	7.1471	7.4844	7.5269	7.8828	7.9368	7.4775	7.5175	7.4514	7.6180	7.7967
Map1a	_GAAPPPAASGHR_.2	#N/A	7.0941	7.2743	7.0522	6.9066	6.7480	7.1251	7.2265	7.2891	7.0116	5.9256	6.7009	7.7442	7.4728	7.6471	7.9655	7.4574	7.1549	7.4435	7.1349	7.4652
Map1a	_EVVPDFPEDK_.2	#N/A	7.2955	7.1855	7.3058	7.0926	7.1309	7.2185	6.5638	7.1662	7.4845	6.5251	7.0523	7.2765	6.9749	7.4365	7.5550	7.4975	7.5685	7.2885	7.0596	7.3149
Map1a	_DTAVELGKGPEPK_.2	#N/A	6.6200	7.0170	6.2047	6.7244	6.9440	6.6501	7.1571	7.2703	6.6759	6.8219	7.2750	7.0784	7.4756	7.6750	7.3745	7.3095	7.3309	6.9992	7.2676	7.1347
Map1a	_NRAPDS[Phospho (STY)]GAEVER_.2	#N/A	7.0440	7.2584	7.1389	7.0622	6.7775	7.1626	7.0898	7.0696	7.0248	7.0483	6.5263	6.7833	7.0156	7.2622	6.6358	7.0441	7.3599	7.0564	6.6087	7.0149
Map1a	_DLEQKDEHLELR_.3	#N/A	6.4388	6.7255	5.7765	6.8160	7.0309	6.9507	6.7091	6.1283	6.5127	6.9047	7.0867	6.6899	7.4128	7.0661	7.4348	7.3158	6.2043	6.7837	6.7577	7.3764
Map1a	_LGIQAEPLYR_.2	#N/A	7.3305	7.0269	6.8544	7.0301	6.8700	6.7288	6.8536	6.9377	6.6105	6.2432	7.0251	7.2586	7.1758	7.1392	6.2747	6.6901	6.8346	7.2730	6.8013	7.2478
Map1a	_LDM[Oxidation (M)]YVLNPVK_.2	#N/A	6.5749	6.4159	6.3219	6.6661	6.9258	6.6233	6.1535	6.5090	6.6806	6.4486	6.7966	6.7353	6.2223	6.0505	6.8035	6.8047	6.9933	6.9446	6.8436	6.9990
Map1a	_EGEGGAGAPDSSS[Phospho (STY)]FSSK_.2	Q9QYR6_S1900_M1_Map1a	6.3021	5.8065	4.0233	4.8051	5.1527	3.5865	5.2250	5.7640	3.6524	6.1100	5.2815	4.9993	3.7726	5.9684	3.7976	4.7725	5.6563	6.0652	3.5660	6.1420
Map1a	_DFQEPLPQK_.2	#N/A	6.1947	6.0278	6.4648	6.4895	5.5578	5.7019	5.9760	5.7322	5.9020	5.9616	6.4526	6.0365	6.0643	6.6415	6.3366	6.6809	6.0487	6.1758	6.6574	6.8041
Map1a	_NEPTTPSWLAEIPPWVPK_.2	#N/A	5.5272	6.5614	6.1819	6.0867	5.3883	5.9156	5.8273	6.2269	5.9454	6.6975	5.9822	6.7480	6.2645	6.5586	6.5231	6.0317	6.3633	6.2215	5.9865	6.6351
Map1a	_DAEQTEPEQREPTPYPDER_.3	#N/A	5.6416	5.8854	5.9377	5.9304	5.9495	5.9881	6.1779	5.3413	6.1307	5.1719	5.8885	6.5490	6.6382	6.6209	6.3969	6.5997	6.0133	6.3997	6.0554	6.5347

Map1a	_VRTES[Phospho (STY)]SEALK_.2	#N/A	4.1783	3.8245	3.2137	3.4229	3.5160	6.7216	4.0251	4.3870	4.0842	6.7748	7.0787	6.8116	6.9431	3.7360	3.6023	3.0223	7.0990	7.0375	3.5642	3.4133
Map1a	_AQWGENLQVTLIPTHDTEVTR_.3	#N/A	5.3006	4.9925	5.8483	5.9356	5.4274	5.3058	4.3862	5.3040	4.7641	5.6921	5.2903	4.7133	6.0293	5.5127	5.7791	5.3958	5.6197	5.4359	4.7581	6.3433
Map1a	_S[Phospho (STY)]PTPGKGPVDR_.2	#N/A	13.4460	13.5587	13.3547	13.5414	13.2030	13.1202	13.0984	13.4379	13.1504	13.4125	13.1724	13.4185	13.7695	13.7282	13.3738	13.5366	13.5390	13.4081	13.3594	13.4324
Map1a	_ELSSEPRT[Phospho (STY)]PPAQK_.2	#N/A	11.1855	11.4340	11.2536	11.0814	10.8520	10.8072	10.9183	11.2266	10.7507	11.3183	10.8680	11.2012	11.6091	11.5703	10.5963	11.1480	11.4240	11.1414	11.1062	11.1136
Map1a	_ALGLEESPEEEGK_.2	#N/A	10.7976	11.0289	11.0790	10.9901	6.7553	10.9882	10.3558	10.0764	10.7482	10.8658	10.7285	11.0774	11.0041	11.5976	10.6703	10.9897	11.5764	10.9824	11.0921	11.2263
Map1a	_ADS[Phospho (STY)]KESLK_.2	#N/A	10.4529	10.2611	10.1720	10.0617	10.0645	10.0944	10.0011	10.0917	9.9375	10.4568	10.3444	10.3454	10.8351	10.6623	10.0471	10.5094	10.6490	10.3876	10.4502	10.5903
Map1a	_ALGLEES[Phospho (STY)]PEEEGKAR_.2	#N/A	9.5025	9.6748	8.9539	9.5534	8.1710	9.2968	9.2185	9.7343	9.4686	9.2437	9.1796	9.9480	10.4800	10.1567	9.5260	9.8542	10.1082	9.7829	9.7773	9.8470
Map1a	_ALALVPGTPTR_.2	#N/A	9.9120	9.9989	7.5852	9.5294	7.4440	9.0225	9.4346	10.0903	7.2738	9.8883	7.2496	9.7996	7.5583	10.3134	8.5691	9.3518	9.7962	9.6384	9.4431	9.3384
Map1a	_GEKELSSEPRT[Phospho (STY)]PPAQK_.3	#N/A	7.2367	7.6512	6.8403	7.3957	7.7069	6.6713	7.2319	7.2184	7.0151	4.2065	7.0460	7.2715	8.2275	3.9219	7.0689	8.2246	8.2837	7.1505	7.6181	8.0941
Map1a	_SHWDDGTNDS[Phospho (STY)]DLEKGAR_.3	#N/A	7.2525	7.7294	5.1978	6.8353	6.4263	6.6637	7.2060	7.3474	6.4981	6.4993	6.2570	7.5881	8.9436	7.8109	7.2816	8.0179	7.2113	7.4282	6.9007	7.2689
Map1a	_APEHIIPQPTQTDR_.2	#N/A	7.0493	6.6737	7.2535	6.5112	6.3761	6.8842	7.0319	6.8843	6.7843	6.4063	6.9168	7.1073	6.1371	7.1403	7.4866	6.9299	7.0395	6.9818	7.0146	6.8107
Map1a	_AKPAS[Phospho (STY)]PARR_.2	#N/A	8.6168	7.8955	7.7626	7.7945	8.3839	7.1524	7.7292	6.9499	7.1781	7.4896	7.6043	3.2918	8.0061	7.0657	7.2102	6.9909	8.0858	7.8270	6.9475	7.7063
Map1a	_QLS[Phospho (STY)]PESLGTLQFGELSLGK_.2	#N/A	7.5100	8.2155	6.7728	6.1156	4.8675	3.1293	8.2053	8.7490	3.8723	6.4585	5.5124	8.5168	8.1947	9.6151	6.7536	6.5298	7.5372	8.0968	7.3875	5.5857
Map1a	_ESTFLDEGPNEQEITPLQHTPR_.3	#N/A	6.8486	6.9851	6.0028	6.0552	5.8066	5.4751	6.3219	6.3185	5.5832	6.6267	6.4432	6.7082	6.4222	6.7226	5.6234	5.6108	6.7230	6.2279	6.0156	6.1670
Map1a	_VLVLEDR_.2	#N/A	6.2083	6.4558	6.5966	6.6237	6.2542	6.6592	6.8707	6.7083	6.2307	6.8102	6.7969	6.5996	6.9309	6.8756	6.5774	6.6322	6.3199	6.9638	6.7238	6.9580
Map1a	_EKETWEER_.2	#N/A	6.1875	6.1347	6.1738	4.0673	6.1324	5.6156	5.8899	6.4510	6.3890	5.8078	6.0520	6.1435	6.0448	6.1722	6.3405	6.2530	6.4122	6.0159	5.6996	5.8419
Map1a	_VVSNTIEPLTLFHK_.2	#N/A	3.9712	4.5207	4.5601	5.5541	5.0210	4.3867	4.4622	3.8677	4.5444	5.2068	4.7185	4.4093	4.7066	4.7489	4.9654	4.8803	4.0028	4.8391	5.0359	5.1493
Map1a	_APSLDSSLPQLPSPSSPGAPLLSNLPR_.3	#N/A	4.8479	4.7785	3.8029	5.0474	4.5439	4.6759	5.4444	5.3833	5.2746	4.3000	5.2696	5.3715	3.4458	5.4430	5.6623	5.3560	5.8693	4.8633	5.0821	5.7113
Map1a	_SPQAQDTLGSLAGGQTGC[Carbamidomethyl (C)]TIQLLPEQDK_.3	#N/A	9.9158	10.0710	9.0153	8.6096	4.9466	8.1411	9.8491	9.9313	7.9665	8.6521	5.8583	9.8070	9.3599	11.0272	7.9356	8.2064	8.9991	9.6191	9.1261	7.8560
Map1a	_IDSVLLTHIGADNLPGINGLLQR_.3	#N/A	5.3878	5.1475	5.6450	5.1262	3.3229	4.9492	4.8874	4.7364	5.3804	4.6099	5.6104	5.3277	5.6140	5.5907	5.1390	4.9867	4.4933	5.1685	5.9228	5.0755
Map1a	_C[Carbamidomethyl (C)]LS[Phospho (STY)]PDDSTVK_.2	Q9QYR6_S981_M1_Map1a	11.7791	11.9336	11.9513	11.8128	11.7592	11.6425	11.5952	12.0562	11.5195	11.8326	11.6998	12.0054	12.7362	12.3627	11.7351	12.0156	12.2283	11.9629	11.9471	11.9342
Map1a	_SEPQDFQEDS[Phospho (STY)]WGDTK_.2	Q9QYR6_S1023_M1_Map1a	10.3234	12.0800	12.0434	12.0068	8.3874	11.8265	11.5039	9.6730	11.7935	11.8666	10.1452	11.8626	11.5670	12.5949	11.6242	11.9464	12.3336	11.9430	11.9299	12.1181
Map1a	_AKPAS[Phospho (STY)]PAR_.2	#N/A	11.3743	10.7236	11.0176	10.8487	10.9470	10.3942	10.5373	10.2851	10.4576	10.6748	10.8123	3.2539	10.8576	10.3294	3.7170	10.2988	11.0253	10.5746	3.1801	10.6680
Map1a	_YPVATQK_.2	#N/A	5.0596	3.6232	3.4151	4.6369	5.6123	5.0828	5.7355	4.0800	5.7589	4.9417	5.6270	5.5407	4.7364	5.7418	6.1060	5.5125	3.5315	5.6683	5.5824	5.6759
Map1a	_TQEPAEPQKDELLGFTDQSFS[Phospho (STY)]PEDAESLSVLSVVSPDTAK_.4	Q9QYR6_S1133_M1_Map1a	4.0255	3.6558	3.3056	3.6241	3.8151	4.3075	6.5667	3.9834	3.9112	3.9054	3.8679	4.0514	3.9759	4.8955	3.4594	3.1232	4.4942	3.4961	3.7595	4.2870
Map1a	_NLISPELGVVFFNVPDK_.2	#N/A	4.7576	5.4160	5.6011	4.9280	4.0675	4.0966	3.9235	3.7591	4.6855	4.8539	4.9061	4.9575	4.9862	5.2314	4.6268	3.6502	5.3645	4.5825	4.1921	4.4366
Map1a	_ETS[Phospho (STY)]PTRGEPVPAWEGK_.3	#N/A	12.7856	12.7003	12.7300	12.9351	11.8235	12.7192	12.6889	12.8210	12.4826	12.8241	12.9766	12.7130	13.9813	13.4554	12.4184	12.8443	12.8101	13.1192	12.8704	12.9583
Map1a	_ELALSS[Phospho (STY)]PEDLTQDFEELKR_.3	#N/A	6.8211	7.4782	7.4237	6.8768	5.2288	7.0710	7.5945	5.9961	7.5573	7.3413	4.8793	7.2991	7.7416	6.2467	7.1743	4.1245	6.2612	7.7813	7.6788	6.0832
Map1a	_EM[Oxidation (M)]TLDQKS[Phospho (STY)]PEKAK_.2	#N/A	9.6449	9.9669	9.5083	9.9694	9.4094	9.4723	9.7064	9.8365	9.4967	9.8477	9.5234	9.9866	10.6155	10.3986	9.6706	9.7859	9.9803	9.9992	9.9211	9.8719
Map1a	_ETS[Phospho (STY)]PTRGEPVPAWEGKSPEQEVR_.4	#N/A	3.5680	4.2307	4.2511	4.1220	3.9847	3.2578	7.5184	7.5989	3.0682	3.9023	6.4097	4.0716	9.5960	8.5236	3.9411	4.0653	4.1313	8.4871	7.7351	7.6218
Map1a	_HRADS[Phospho (STY)]KESLK_.2	#N/A	7.6805	7.6779	7.0440	7.4171	7.5589	7.3851	7.1363	7.4337	7.0887	7.5406	7.7069	5.0933	8.2699	7.9244	6.0096	7.3318	7.9931	7.7756	7.1040	7.9777
Map1a	_TES[Phospho (STY)]SEALKAEK_.2	#N/A	5.9741	6.1585	5.7663	6.0855	5.9416	6.1863	6.1420	6.1321	5.8233	6.3079	6.3017	6.2636	6.5291	6.8891	6.1655	6.2910	6.6478	3.9656	6.3635	6.5212
Map1a	_ADS[Phospho (STY)]KES[Phospho (STY)]LKAAPK_.2	#N/A	11.0217	10.9352	11.0360	11.2106	10.7253	10.4251	10.6261	10.9478	10.6422	11.0730	10.6744	10.9411	11.5037	11.3783	10.5911	11.0279	11.1909	10.9513	10.8152	10.9436
Map1a	_S[Phospho (STY)]PPC[Carbamidomethyl (C)]EDFSVTGESEK_.2	#N/A	3.1763	6.3613	6.6564	6.3814	3.3832	6.2196	5.4414	3.4020	5.0663	4.4877	6.0099	5.7886	3.7505	7.7045	6.0297	6.3986	4.4000	6.4726	6.1127	6.5423
Map1a	_STPSQVTSEEKDGHS[Phospho (STY)]PM[Oxidation (M)]SK_.3	#N/A	10.6206	10.9460	8.5055	8.2068	3.2679	10.3835	8.6990	10.6235	10.2705	10.6850	10.4417	7.4210	11.1447	11.1772	10.3584	10.6915	10.7635	10.5192	6.9469	3.4828
Map1a	_GFKS[Phospho (STY)]PPC[Carbamidomethyl (C)]EDFSVTGESEK_.3	#N/A	5.7836	7.7415	7.0701	7.0266	3.6372	7.4637	7.0772	4.0442	6.9984	7.0305	7.0274	6.4733	6.4461	8.5525	6.4442	6.6561	6.7195	7.3432	7.3603	7.4709
Map1a	_LSSFATSVAEDQSVASLTAPQTEETGK_.3	#N/A	6.0440	5.4332	5.2092	5.2218	4.9762	4.9895	5.7139	4.5936	5.0458	5.8741	5.4635	5.2766	5.7976	6.6701	5.4675	4.9577	5.2274	5.0558	5.7076	5.7778
Map1a	_TPS[Phospho (STY)]PKEAGR_.2	Q9QYR6_S2066_M1_Map1a	4.3975	7.7174	5.0809	6.6596	5.3052	5.8093	5.8624	7.1411	5.7989	7.3594	5.3206	7.3509	7.2347	5.3962	4.4451	7.4273	6.0483	7.0452	7.0502	6.3821
Map1a	_NRAPDS[Phospho (STY)]GAEVEREK_.3	#N/A	3.8552	5.4359	4.7719	5.2306	5.0447	4.3691	5.2393	5.4634	5.2179	6.0134	5.2688	5.1085	4.5153	5.4443	5.5767	5.3551	5.0478	5.9752	5.8228	5.3353
Map1a	_SSLLLDTVTSIPSSR_.2	#N/A	7.3772	6.1999	6.6293	6.6076	6.0610	6.6251	6.0063	6.3164	6.5922	5.9382	6.3809	6.4931	6.7517	6.7123	7.5376	6.4145	6.5946	8.0961	6.4420	6.4030
Map1a	_VPSAPGQES[Phospho (STY)]PVPDTK_.2	#N/A	13.7401	13.7477	13.5775	13.6780	13.1649	13.2559	13.2326	13.5428	13.1363	13.8069	13.3662	13.4840	14.1270	14.0449	12.8859	13.4952	13.8876	13.3990	13.4483	13.5208
Map1a	_SHWDDGTNDS[Phospho (STY)]DLEK_.2	#N/A	9.3698	9.1425	8.9307	9.3295	8.6199	8.9331	8.7574	9.2000	8.6852	9.3336	9.0416	9.0417	9.1893	9.6493	8.2567	9.1458	9.5045	8.8957	9.0625	9.2103
Map1a	_VRTES[Phospho (STY)]SEALKAEK_.3	#N/A	8.1348	8.5118	7.3949	5.9256	7.9714	3.8902	8.0354	7.9428	3.7531	8.5019	4.9333	8.4153	8.8186	8.8988	8.1958	8.3125	8.6405	8.4452	5.6804	8.5169
Map1a	_APDS[Phospho (STY)]GAEVEREK_.2	#N/A	5.8579	6.6831	3.8905	3.2303	3.0600	6.0735	6.0141	3.7534	3.9083	4.1645	4.9689	5.9186	6.2059	6.3197	4.0135	5.6029	6.3165	5.5796	5.6157	6.0251
Map1a	_AELEEM[Oxidation (M)]EEVHPS[Phospho (STY)]DEEEEETK_.3	#N/A	3.5263	4.0117	3.8672	3.8895	3.7340	3.9044	4.3323	3.9567	3.7158	3.8579	3.6154	3.7761	3.8589	6.0196	3.6174	3.6839	3.8182	3.7084	2.9371	3.7118
Map1a	_EM[Oxidation (M)]TLDQKS[Phospho (STY)]PEK_.2	#N/A	11.8280	11.8564	11.9992	11.8808	11.4232	11.6952	11.5217	11.6779	11.5286	11.6696	11.6592	11.5739	11.8222	12.2966	11.3528	11.5741	11.9711	11.7079	11.6212	11.7160
Map1a	_S[Phospho (STY)]PC[Carbamidomethyl (C)]T[Phospho (STY)]PKEQQLHK_.2	#N/A	8.5634	8.8112	8.2109	8.6466	7.9474	7.8423	8.1644	8.1261	7.5423	9.2121	8.1109	8.5042	8.4250	8.8528	7.3875	8.3269	8.2509	8.1808	8.3028	8.3088
Map1a	_WLAES[Phospho (STY)]PVGLPPEEEDK_.2	#N/A	3.6330	8.8840	8.0767	9.7292	3.8400	8.4208	9.0203	3.8587	8.5422	9.6310	8.8615	3.4122	3.2938	9.1381	3.2335	2.9951	8.8751	8.7430	8.7874	9.0757
Map1a	_TRHDEYLEVTK_.3	#N/A	4.1718	4.6110	3.7455	3.3753	3.7204	3.5894	4.1330	4.4346	4.0532	3.2839	5.5525	4.6698	4.2187	5.1372	5.1933	5.0791	3.9319	4.2972	4.6950	3.1875
Map1a	_SPFEIIS[Phospho (STY)]PPAS[Phospho (STY)]PPEMTGQR_.2	#N/A	12.7077	13.3488	11.7377	11.6998	11.0932	11.0411	13.0759	12.3995	10.7019	12.3522	12.7814	12.8743	11.0459	14.2081	11.3388	12.3570	12.9602	13.3801	11.8547	11.6282
Map1a	_QLS[Phospho (STY)]PESLGTLQFGELSLGKEEK_.3	#N/A	6.8459	7.3538	6.8073	6.4504	5.7125	6.4440	6.8185	7.4483	6.4020	6.5351	6.1070	7.4496	8.1763	7.9696	6.8931	6.7542	6.4573	7.2833	7.0781	6.6056
Map1a	_S[Phospho (STY)]PESLSSPAM[Oxidation (M)]EDLAM[Oxidation (M)]EWGGK_.2	#N/A	6.2733	5.8828	3.7764	4.7526	5.0860	5.5750	5.1718	6.4243	5.6092	5.6885	5.2211	5.9092	5.9255	5.9198	3.7332	6.3872	6.1375	7.0574	5.9311	6.4166
Map1a	_EKEGAALDQDNR_.2	#N/A	6.1823	6.1491	6.1293	6.2848	5.9787	5.9671	5.9811	5.8621	6.0773	6.1001	6.0256	6.4436	6.1673	6.5816	5.8059	5.9720	6.5392	6.1506	6.3104	6.4904
Map1a	_GKDLYLEDQGLAEKDK_.3	#N/A	6.0015	6.5648	5.7633	5.1966	5.9495	5.6485	6.1458	6.1457	5.7432	5.1708	5.5870	5.5648	6.8063	6.3352	5.8956	6.0979	5.7686	5.8131	5.8578	5.5363
Map1a	_KVAELEEEQS[Phospho (STY)]QGSSSYSDWVK_.3	#N/A	7.4123	7.3434	5.9645	6.5839	6.5351	6.0810	6.7635	6.9349	6.7189	7.2591	6.7604	7.2391	8.5624	7.9319	6.8735	7.4027	6.9841	7.2156	7.1449	7.2577
Map1a	_VAELEEEQS[Phospho (STY)]QGSSSYSDWVK_.2	#N/A	9.0443	8.9311	9.1486	9.2056	7.8717	8.5530	8.4407	8.6151	8.6272	9.2326	8.9339	8.8790	9.6754	9.6309	8.7237	9.1475	9.4754	8.6799	8.9734	9.0407
Map1a	_M[Oxidation (M)]AS[Phospho (STY)]PPPSGPPSAAHTPFHQSPVEEK_.4	#N/A	9.3640	9.5369	7.0538	7.8187	5.5517	8.6081	9.1550	10.0351	6.4241	9.2268	8.7955	9.5213	10.4649	9.9699	9.0745	9.3200	9.1898	10.1562	9.9627	9.6497
Map1a	_PASPALSEGS[Phospho (STY)]S[Phospho (STY)]SEATTPVISSVAER_.3	#N/A	3.6128	4.1065	6.3763	3.9586	3.9487	7.5279	3.6870	3.6271	6.8816	3.8191	3.6955	3.4364	3.7551	4.2100	7.1663	3.9577	3.8369	4.7209	6.6309	5.2378
Map1a	_QEPEPGPNVEPSFT[Phospho (STY)]PPAVPPR_.2	Q9QYR6_T2031_M1_Map1a	8.1312	8.9240	8.1481	4.0517	8.2901	8.7735	8.7271	9.0278	3.6750	7.1580	8.6635	4.2865	3.8223	10.2903	6.7072	3.8198	4.4185	9.0936	7.9254	7.0044
Map1a	_VPEVTESHTTR_.2	#N/A	7.4452	7.1504	7.2608	6.8924	7.1950	7.2986	7.1960	7.3130	7.1809	7.3457	7.1160	7.3023	7.4393	7.4325	7.7201	7.4752	7.2959	7.3961	7.2168	7.4930
Map1a	_APDS[Phospho (STY)]GAEVER_.2	#N/A	6.6046	6.6541	6.7595	7.3254	6.8243	6.9982	6.6421	6.3499	6.7995	7.2058	7.1145	6.3968	6.7297	7.1871	6.5379	6.8916	6.8449	6.6005	7.1345	6.9325
Map1a	_DEALDEENKPGGQQDK_.2	#N/A	4.0895	4.5534	3.5960	3.8155	4.0776	4.7001	4.0272	4.3607	3.6948	3.6594	4.0239	4.1386	4.3642	5.4021	3.6820	3.6714	4.2587	4.5951	4.3836	4.7688
Map1a	_GKDLYLEDQGLAEK_.3	#N/A	4.8962	4.0303	4.3262	4.2954	3.4957	4.4122	3.1525	3.5145	4.8086	3.7563	4.3229	4.5418	3.3197	4.9803	4.8562	4.9740	4.5125	5.0877	3.9117	4.2442
Map1a	_S[Phospho (STY)]APC[Carbamidomethyl (C)]GSLAFSGDR_.2	Q9QYR6_S2162_M1_Map1a	9.5705	10.4777	10.5174	9.8802	8.5355	9.5416	9.6694	9.4600	9.4715	10.0784	9.6527	9.7548	9.7148	10.3740	9.1252	9.6454	9.9240	9.7308	9.9618	9.7846
Map1a	_EES[Phospho (STY)]EPEVKEDVIEK_.2	#N/A	7.6726	7.9825	7.8935	7.9225	4.8619	7.5867	7.3870	6.8808	7.1654	7.8768	6.9915	6.7818	6.6491	8.0042	6.5524	7.2270	7.6093	7.2685	7.3009	7.2461
Map1a	_QEATPRS[Phospho (STY)]PC[Carbamidomethyl (C)]T[Phospho (STY)]PKEQQLHK_.3	#N/A	3.8043	7.5026	3.3409	7.1939	6.9481	4.0446	6.9556	6.5952	4.4579	7.8718	3.9365	6.8236	3.1225	7.3427	6.1309	7.1489	6.7848	6.8372	4.1419	6.1195
Map1a	_WLAESPVGLPPEEEDKLTR_.3	#N/A	5.3479	5.4793	4.2251	3.8331	3.2691	5.3922	5.0934	6.0858	5.3865	6.1724	4.9709	6.1514	3.9022	10.7186	5.9168	6.3501	5.5249	10.1256	5.7962	5.5516
Map1a	_EKEVVPDFPEDK_.2	#N/A	7.0438	6.3892	3.4653	6.7591	6.6055	6.7009	6.3221	6.7871	6.6846	6.7904	6.7704	6.5518	6.8870	6.8450	6.6533	6.9300	6.7624	6.5469	6.5597	6.5975
Map1a	_HMQEALK_.2	#N/A	3.2940	3.7036	4.5671	4.3954	4.1326	4.3567	3.7714	4.4952	3.4676	3.5003	4.9338	3.0974	3.5757	3.9203	2.9580	3.6389	3.8479	3.8818	4.1808	4.2975
Map1a	_TEATQGLDYVPSAGTIS[Phospho (STY)]PTSSLEEDK_.3	#N/A	6.3512	6.9013	6.7741	6.5623	3.6233	6.2471	6.2109	3.9375	6.0532	6.0487	6.6419	5.9601	6.1243	7.4313	6.5462	6.5225	6.7488	6.5581	6.6241	6.3776
Map1a	APISLSQDPSPPLNGSTTSC[Carbamidomethyl (C)]GPDRRT[Phospho (STY)]PS[Phospho (STY)]PK	#N/A	4.2480	7.9191	9.0812	3.3362	2.9541	9.5548	8.1302	6.9441	4.1708	9.9138	3.0444	4.2980	4.2789	3.8227	6.2670	3.6101	3.9709	10.4242	6.8993	9.5205
Map1a	_RS[Phospho (STY)]PT[Phospho (STY)]PGKGPVDR_.2	#N/A	5.9494	5.5682	6.3637	5.7371	6.0975	5.9598	5.4598	4.1926	5.6482	6.3572	5.9364	5.1886	5.4831	6.1316	6.2744	5.5122	5.9967	5.0873	5.1390	5.3499
Map1a	_EAELTPENIAAAREES[Phospho (STY)]EPEVKEDVIEK_.4	#N/A	3.9628	3.9398	3.7808	3.3700	3.8169	3.6872	4.3111	3.8041	4.1670	3.9767	3.8756	3.6659	4.2751	3.8189	3.4525	3.8788	3.9671	8.1072	3.5113	3.4961
Map1a	_KHS[Phospho (STY)]PGEITGPGGHFM[Oxidation (M)]TSDSSLTK_.4	#N/A	5.0570	6.2281	4.0921	5.4835	3.7849	4.5832	6.2235	4.9447	4.3045	5.6267	3.5196	5.7069	4.2812	7.2412	5.1406	5.0930	3.9684	6.5599	5.9629	5.6673
Map1a	_S[Phospho (STY)]IEEAC[Carbamidomethyl (C)]LTLQHLNR_.2	Q9QYR6_S155_M1_Map1a	4.2607	4.2190	3.6005	4.1324	4.0507	4.1953	5.1749	3.5281	3.9079	3.5253	4.0806	3.2182	3.3864	5.4888	4.1305	3.3655	3.5017	4.4104	4.7792	4.3665
Map1a	_EAELTPENIAAAREES[Phospho (STY)]EPEVK_.3	#N/A	3.3222	3.1798	3.8584	4.3672	4.1607	4.3848	3.3804	4.5234	3.4395	3.5285	3.8138	3.0693	3.5475	4.5921	2.9576	3.6670	3.5602	3.9099	5.8139	4.2694
Map1a	_GDSALFAVNGFNILVDGGSDR_.2	#N/A	4.1592	4.5715	4.0457	3.7916	3.2152	3.8957	3.8329	3.5982	3.7531	2.7797	3.2318	4.0369	3.9186	5.1591	3.8583	3.8712	4.1359	4.3614	2.2855	3.3362
Map1a	_QEATPRS[Phospho (STY)]PC[Carbamidomethyl (C)]T[Phospho (STY)]PK_.2	#N/A	7.2777	7.9352	8.0287	8.2383	7.2814	6.7304	7.3647	6.8565	7.0702	8.5019	7.2505	7.9147	7.4058	7.6785	6.2834	7.3356	7.3939	7.3160	7.4835	7.4813
Map1a	_DQILSEK_.2	#N/A	4.0357	3.2834	3.5723	3.5485	4.2427	1.8678	4.3063	4.2614	3.2872	3.8463	3.1435	3.0831	4.0666	3.6104	7.4169	3.3978	3.7586	3.3908	3.0619	3.2877
Map1a	_PRYS[Phospho (STY)]EER_.2	Q9QYR6_S1694_M1_Map1a	3.3085	3.1934	3.8448	4.3809	4.1471	4.3712	3.3940	4.5097	3.4531	3.5788	3.8275	3.0829	3.3292	3.8319	2.9712	3.6534	3.5466	4.2866	3.9352	3.7989
Map1a	_ELALS[Phospho (STY)]S[Phospho (STY)]PEDLTQDFEELK_.2	#N/A	4.4825	4.3374	4.0191	3.1017	3.1886	3.2220	3.8595	3.2074	3.7797	4.2931	3.2583	4.0635	3.9451	4.0572	3.8848	3.8446	4.2054	3.6707	3.3193	4.4148
Map1a	_SPEQEVR_.2	#N/A	4.3002	4.0311	4.1604	3.8598	3.2834	3.2689	3.7697	3.5350	3.9265	4.3879	3.1686	4.1016	3.8554	4.3580	4.4414	3.9394	4.0727	4.0895	3.6680	4.1803
Map1a	_APDS[Phospho (STY)]GAEVEREKETWEER_.3	#N/A	3.7894	3.5297	3.3260	3.7948	3.9963	4.1120	3.0518	4.0151	3.7122	3.6000	4.0866	3.2558	3.8203	4.5613	3.0771	3.1515	3.5123	3.6371	3.9362	3.0413
Map1a	_AELEEM[Oxidation (M)]EEVHPS[Phospho (STY)]DEEEEETKAESFYQK_.4	#N/A	3.5863	6.4488	3.8954	4.0735	3.7933	6.4273	6.1715	3.8120	4.0232	4.1695	3.5018	5.3788	6.4691	3.9188	6.1110	3.7210	4.0818	3.5470	3.9239	3.8858
Map1a	_DSVMHQKDEALDEENKPGGQQDK_.4	#N/A	3.3164	3.6289	4.1438	4.8030	3.8493	3.8113	3.2839	4.0742	3.2041	3.3816	4.3281	3.4223	3.3695	4.9386	3.7950	3.9630	3.3000	4.9508	3.9086	3.8392
Map1a	_TEATQGLDYVPSAGTIS[Phospho (STY)]PTSSLEEDKGFK_.4	#N/A	4.0124	5.5249	4.2053	3.7847	3.3749	3.1331	3.5799	3.6058	3.5554	4.0176	3.4697	5.1979	5.8626	4.4420	3.4481	3.8340	3.7271	5.5250	3.9405	4.2735
Map1a	_DEAS[Phospho (STY)]EEKEQVLEQK_.2	#N/A	4.1873	3.1318	3.7239	3.3969	2.8934	3.7141	4.0510	4.4130	4.1101	4.3586	2.9837	4.3587	4.2182	4.9824	4.1801	3.5494	3.9102	3.2392	6.3541	2.5056
Map1a	_FPTSTYDLSGPEGPGPFEASQSAESAVPASSSK_.3	#N/A	4.4204	3.5824	3.4558	3.2690	3.7581	3.9822	3.7830	4.1208	3.8421	3.1259	4.2164	2.6400	3.9501	3.4429	3.3602	3.2644	3.1576	1.5439	4.3242	3.4099
Map1a	_SSFLEDK_.2	#N/A	3.2696	4.0495	4.3070	4.3147	3.4765	3.1310	3.5716	3.4952	3.4918	4.5810	2.9704	3.7756	3.3004	4.3450	3.5970	4.1325	4.4933	4.1570	2.9552	4.0223
Map1a	_DRDITLQQDAYWK_.3	#N/A	4.4593	4.3606	3.9959	3.1249	3.1654	3.1988	3.8827	3.1842	3.8029	4.2699	3.2815	4.0867	3.9683	4.4710	3.9080	3.8214	4.1822	3.6475	3.2961	4.4380
Map1a	_EQKDEAS[Phospho (STY)]EEKEQVLEQK_.3	#N/A	3.9659	3.3532	3.5025	3.6183	4.1728	4.2062	4.3761	4.1916	4.2963	3.7765	3.7749	3.0793	4.4617	3.5405	2.9006	3.3280	3.6888	3.4606	3.7597	3.2178
Map1a	_DKT[Phospho (STY)]PEEKDK_.2	Q9QYR6_T1472_M1_Map1a	3.5509	3.7682	4.5883	4.0333	3.7579	3.7912	3.2902	3.7766	3.2104	3.3615	4.1899	3.4943	3.3759	4.6264	3.3156	4.4139	3.2738	3.8756	4.1747	3.8455
Pclo	_EHKENPELVDDLS[Phospho (STY)]PR_.3	#N/A	9.3971	9.4522	9.6694	9.9742	7.1826	9.0095	8.9283	8.6489	9.1757	9.7441	8.7552	9.5485	9.7557	9.6130	8.7679	9.5559	9.6889	9.3037	9.3787	9.3913
Pclo	_ENPELVDDLS[Phospho (STY)]PR_.2	#N/A	9.7559	10.5666	10.7937	10.6117	6.4177	10.2862	10.0839	8.2044	10.1689	10.6709	9.1957	10.1332	9.9310	10.8495	9.3971	10.3267	10.6085	10.1819	10.3256	10.4826
Pclo	_TQPQKVS[Phospho (STY)]PEQPQDQQK_.2	#N/A	8.8794	8.7885	9.0446	8.5865	8.2766	3.4180	9.0076	8.6491	8.6758	8.4697	8.1944	8.5742	9.4012	9.1098	8.8124	8.8116	9.3001	8.6301	9.0614	9.0887
Pclo	_SPVPSQQAS[Phospho (STY)]PK_.2	#N/A	9.8146	9.5417	10.2478	10.1583	9.4622	9.4169	9.3909	9.2897	9.3174	9.8989	9.6110	9.3066	9.2909	9.9015	8.9905	9.4465	9.7250	9.1753	9.2627	9.7012
Pclo	_SHSSQNSQQS[Phospho (STY)]PKPSVIK_.3	#N/A	7.6675	7.6865	7.6429	7.7663	7.1128	7.1498	7.3472	7.5248	7.3228	7.8556	7.5665	7.0188	7.9721	7.7979	6.7590	5.4980	7.4224	7.3178	7.3408	7.4670
Pclo	_AKS[Phospho (STY)]PGVDPK_.2	Q9QYX7_S4544_M1_Pclo	8.2810	8.2512	8.1238	8.7335	7.9034	7.4821	8.3312	7.8286	7.4511	8.9271	7.9512	8.3162	8.5991	8.4043	6.4821	8.1093	8.0761	7.9256	7.7159	8.0144
Pclo	_SLVSVLPEKKPS[Phospho (STY)]EEEK_.3	Q9QYX7_S1149_M1_Pclo	7.2634	7.7077	7.5580	4.3538	6.3500	7.4282	7.2912	7.7807	7.2955	7.8509	7.5336	7.7358	8.0650	8.5603	7.0164	7.4733	8.0614	7.9336	7.7099	7.6672
Pclo	_SPVPSQQAS[Phospho (STY)]PKKELPSK_.3	#N/A	7.8499	8.1677	4.4800	7.7800	7.3945	7.3478	7.8550	3.6441	7.3591	7.8886	7.7704	7.5989	8.2534	8.7889	7.3752	4.2886	4.1818	7.8287	7.3977	7.9808
Pclo	_S[Phospho (STY)]QEVTDFLAPLQTSSR_.2	Q9QYX7_S4016_M1_Pclo	4.4924	8.3289	8.2435	7.4029	3.8301	6.7188	7.8179	4.1927	7.3699	7.5975	6.6697	7.7817	3.8781	8.9711	6.7931	6.2177	7.4361	8.1941	3.8783	6.8760
Pclo	_KSS[Phospho (STY)]TS[Phospho (STY)]FDDDAGR_.2	#N/A	8.0829	8.0782	6.1155	8.0818	7.0834	3.6452	7.0195	7.4566	6.6617	8.0949	7.2738	7.0907	7.1830	7.5656	5.8481	6.9480	6.9650	7.0546	7.1842	7.3398
Pclo	_SEGITKPSLQQPS[Phospho (STY)]PK_.2	#N/A	9.4019	9.3509	9.6879	9.5336	8.7624	8.4882	8.8342	8.8312	8.7927	9.5981	9.0681	9.0094	9.7032	9.5781	8.4034	9.2057	8.9538	8.8368	8.8777	9.0771
Pclo	_SQPTT[Phospho (STY)]PQETVTGK_.2	Q9QYX7_T860_M1_Pclo	3.1894	9.5633	10.4098	7.7409	9.4863	3.9583	9.6893	9.7231	9.2344	10.3609	9.3178	3.2033	3.2101	8.9708	3.1633	10.0072	8.9515	8.4723	8.7357	10.1771
Pclo	_ITQHFSTEEFEDEYVTDYTR_.3	#N/A	7.5405	7.9842	7.1338	7.7036	6.0417	6.6936	7.0701	7.9183	6.7239	7.3416	3.1663	7.4286	8.0696	8.5956	6.7507	7.4573	7.6342	7.2256	6.8398	7.4848
Pclo	_ELPSKQDS[Phospho (STY)]PKAPESK_.2	#N/A	3.7280	4.3733	4.5020	4.0599	3.5056	3.5169	3.8868	3.3769	3.5318	5.6501	3.8187	3.7718	3.6972	6.6664	3.6321	4.3194	3.9146	6.0218	3.6142	4.1669
Pclo	_YNS[Phospho (STY)]LDLR_.2	Q9QYX7_S3936_M1_Pclo	4.1178	6.4443	3.6544	6.9458	3.5595	3.7836	3.9816	4.3435	4.0407	4.4281	2.9142	2.9274	4.1487	3.6925	6.5016	3.0658	3.8407	3.3087	3.6077	6.8362
Pclo	_SSTS[Phospho (STY)]FDDDAGRR_.2	#N/A	3.1064	4.2127	4.1438	6.3060	3.3133	3.3467	6.8554	3.3321	3.6550	4.4178	3.1336	3.9388	3.1373	4.1819	3.7601	3.9694	4.3302	4.3201	3.1183	3.8592
Pclo	_KTQPQKVS[Phospho (STY)]PEQPQDQQK_.3	#N/A	7.2251	7.6261	7.1371	7.4562	7.0583	7.3133	7.5007	7.2971	7.1199	7.3371	6.9899	7.1568	7.7881	7.7993	7.1344	7.5179	7.7879	7.4277	7.4777	7.2294
Pclo	_DSFSQESSPSS[Phospho (STY)]PSDLAK_.2	#N/A	10.2746	10.1028	10.4915	10.3467	9.5837	9.8527	9.7557	10.1052	9.9504	10.1939	9.8982	10.1680	10.6031	10.4108	9.6599	10.1969	10.5454	9.8429	10.0592	10.1484
Pclo	_KIVDSGVQT[Phospho (STY)]DDEETADR_.2	#N/A	5.8835	5.5619	5.5136	5.6303	5.1027	5.3958	6.1397	5.6593	5.3956	6.1588	5.2119	3.7032	5.1087	6.4405	3.6429	3.8292	5.8156	3.6885	5.5625	3.8280
Pclo	_FS[Phospho (STY)]PIQESR_.2	#N/A	6.1221	6.2046	6.4770	6.3808	6.0801	6.1914	6.2942	6.2562	6.3363	6.0985	6.5083	5.7331	6.4378	6.6483	6.2320	5.9802	6.6664	6.0671	6.1322	6.4606
Pclo	_VYKLPTAVSLYS[Phospho (STY)]PTDEQSVM[Oxidation (M)]QK_.3	#N/A	3.5930	3.7261	3.1296	5.7994	3.7999	3.8333	6.5876	3.8187	3.1684	3.4036	4.1479	3.4522	7.2492	3.8365	5.3502	2.9551	3.3159	7.1988	6.2834	4.6278
Pclo	_SSTS[Phospho (STY)]FDDDAGR_.2	#N/A	3.9976	3.3215	3.5342	3.2800	4.2045	4.2379	4.3444	8.7430	4.2646	3.8082	3.7433	3.0476	9.0541	3.4319	2.8689	3.3597	3.1466	3.1858	4.3352	3.3989
Pclo	_ASYDSVEDSSESENS[Phospho (STY)]PVAR_.2	#N/A	6.2929	5.8728	7.2759	6.1816	5.6048	6.9183	5.7625	6.4054	6.5678	5.4510	6.1229	6.7627	6.0613	7.3098	7.1031	6.2072	7.3249	5.8324	6.0386	5.9829
Pclo	_TQT[Phospho (STY)]PSETR_.2	Q9QYX7_T1390_M1_Pclo	3.6523	8.0610	4.1886	8.5681	7.8046	3.4941	3.0503	8.0581	3.5075	7.8501	2.9862	4.2400	3.6730	4.1756	4.1283	8.2590	3.8903	8.1435	3.5914	7.9560
Pclo	_AMPRPAS[Phospho (STY)]DSK_.2	Q9QYX7_S769_M1_Pclo	3.7411	7.4406	4.1351	4.4865	3.0788	3.3029	7.5532	3.3234	3.0205	3.9474	3.3949	3.9475	3.1286	8.4706	4.0395	4.0860	4.3215	4.3288	3.1270	3.8505
Pclo	_DQFGSSHS[Phospho (STY)]LPEVQQHM[Oxidation (M)]R_.3	#N/A	6.8133	7.7862	8.8164	8.8807	6.9421	8.7336	8.7707	7.9089	8.3990	9.5873	6.9376	8.4645	8.2730	7.6684	8.1730	9.1288	8.8194	9.1549	8.8398	8.8356
Pclo	_LPAQQPGPQTAAK_.2	#N/A	4.0784	3.2407	3.6150	3.3609	4.2854	5.5381	6.1916	4.7938	5.1946	5.6588	5.9489	5.2282	4.3492	6.4023	6.9070	6.6376	5.6334	6.1202	6.0710	6.3038
Pclo	_SHGPLLPTIEDSS[Phospho (STY)]EEEELREEEELLKEQEK_.4	Q9QYX7_S1709_M1_Pclo	3.6677	7.3850	4.0067	3.7729	3.4426	3.5596	3.9455	3.1829	3.9586	3.7177	3.8745	4.8594	4.0853	4.0196	3.6921	4.2575	3.9723	7.1655	5.6185	5.9613
Pclo	_SSS[Phospho (STY)]EGHLR_.2	Q9QYX7_S4814_M1_Pclo	4.1772	6.1560	3.7138	3.4070	2.8833	4.4175	4.1648	3.9301	4.0850	3.9878	3.5637	4.3688	4.2505	3.2523	4.1902	3.5393	4.4678	3.3654	3.0140	3.2193
Pclo	_DQGKSEGITKPSLQQPS[Phospho (STY)]PK_.3	#N/A	5.3783	5.1959	4.0404	4.6507	4.7449	3.3976	4.5081	3.2286	4.4266	5.4628	3.3002	4.0422	4.5347	4.0784	3.8636	3.9647	4.2267	4.4236	3.2218	5.1482
Pclo	_SM[Oxidation (M)]S[Phospho (STY)]DPKPLSPTADESSR_.2	#N/A	8.6374	8.3235	7.4199	5.3646	8.5825	8.5673	8.2619	8.9463	8.3924	8.3219	8.5728	8.7150	9.7806	9.1841	8.8599	8.8486	8.9150	8.8571	8.7959	8.5685
Pclo	_SPVPSQQAS[Phospho (STY)]PKK_.2	#N/A	9.5495	9.2687	9.6611	9.6702	9.2344	8.9008	8.8999	8.9041	8.9915	9.3633	9.1455	9.0156	8.8989	9.6172	8.7260	9.1599	9.1533	8.8459	8.8932	9.3314
Pclo	_KDS[Phospho (STY)]FSQESSPSS[Phospho (STY)]PSDLAK_.2	Q9QYX7_S1332_M2_Pclo	3.8463	3.8876	3.6618	3.9169	3.7927	6.0123	6.2517	4.1553	3.6978	3.7792	3.9487	3.6516	3.8425	3.4864	3.4894	6.1654	3.8927	3.8457	3.6966	6.1525
Pclo	_GSVPAAAAES[Phospho (STY)]PSM[Oxidation (M)]HR_.2	#N/A	9.9865	9.6431	9.8609	9.8556	5.9253	3.5493	9.0843	9.2977	8.7729	10.2486	9.0278	9.2136	9.3014	9.4273	7.7965	9.1999	8.8935	8.9249	8.7535	8.9123
Pclo	_DISISEEEIKESQEK_.2	#N/A	2.6475	3.6912	3.5304	3.5904	3.6835	2.5887	3.8575	2.5022	3.9166	4.5521	4.2910	2.4380	1.9634	1.6374	3.4347	3.1899	2.8283	2.2622	3.7318	1.7489
Pclo	_TPPSNLSPIEDAS[Phospho (STY)]PTEELR_.2	Q9QYX7_S1772_M1_Pclo	7.7334	10.5317	10.0314	3.9665	7.6243	10.0036	10.1961	8.2069	10.3310	5.8590	7.6705	8.0602	7.8055	12.1549	10.0744	8.1060	7.1698	11.3113	10.1480	9.5586
Pclo	_M[Oxidation (M)]SKFS[Phospho (STY)]PIQESR_.2	#N/A	3.6963	7.8277	7.7790	7.7459	7.2413	6.7963	5.8739	7.6700	7.2632	7.5159	5.8496	7.3718	8.0630	8.0300	6.8904	7.3816	7.8157	7.0084	7.3914	7.3057
Pclo	_HSWHDEDDETFDES[Phospho (STY)]PELK_.3	#N/A	3.9771	7.4349	6.0748	6.4708	4.0053	6.9719	7.2841	3.6775	6.2028	5.2951	4.0148	5.9760	3.7293	7.8513	6.1461	6.5933	5.1518	7.4044	6.5801	7.0100
Pclo	_ADPTVQLAPS[Phospho (STY)]PPKS[Phospho (STY)]PK_.2	#N/A	9.6551	9.7122	9.4338	9.7865	7.0853	9.0531	9.1679	8.5979	9.3472	9.7791	8.6709	9.4060	9.5248	9.7175	8.4758	9.2887	9.8079	9.1454	9.3288	9.4816
Pclo	_IVDSGVQT[Phospho (STY)]DDEETADR_.2	#N/A	9.9314	10.0106	9.5939	9.9876	9.2013	9.4738	9.5786	9.8319	9.3369	10.2088	9.5870	9.4551	9.9672	10.3241	8.8433	9.5541	9.7994	9.6475	9.6258	9.6334
Pclo	_LPTAVSLY[Phospho (STY)]SPTDEQSVM[Oxidation (M)]QK_.2	#N/A	7.9128	3.8033	7.1615	6.7557	6.2440	7.3611	7.6229	7.1458	6.9801	7.6426	6.5767	8.7493	4.0815	8.5739	6.5856	7.7192	7.2686	8.1879	7.4991	7.5649
Pclo	_ERPKT[Phospho (STY)]PPS[Phospho (STY)]NLSPIEDAS[Phospho (STY)]PTEELR_.3	#N/A	3.5058	6.4653	4.4333	4.0731	3.7216	4.6596	4.5473	3.6152	3.3460	3.3724	3.7082	3.9390	3.4917	7.6243	5.1656	3.5766	3.4670	4.0818	4.9563	4.0520
Pclo	_KSS[Phospho (STY)]TS[Phospho (STY)]FDDDAGRR_.2	#N/A	6.9277	7.0256	7.2487	6.8413	6.2612	6.2303	6.4669	6.2979	6.1384	7.1416	6.1349	6.1148	5.9330	3.8254	3.4637	3.5497	6.2354	6.1356	5.9774	6.1011
Pclo	_T[Phospho (STY)]PPAVATDQK_.2	Q9QYX7_T1120_M1_Pclo	4.2976	8.5471	7.8197	3.5801	8.3468	8.8564	4.0444	3.8097	3.9646	7.0418	3.4432	4.2484	4.1300	3.1319	4.0698	3.6597	4.3474	3.4858	3.1344	3.0989
Pclo	_GAHAHSGPTSAGSSSVPS[Phospho (STY)]PGQPGS[Phospho (STY)]PSVSK_.3	Q9QYX7_S4592_M2_Pclo	5.8517	7.8009	4.2040	6.1552	3.7045	5.8843	6.8143	7.6506	5.2240	6.4493	4.2452	6.7419	6.4197	8.8382	5.2859	4.8350	6.0996	7.1299	5.7087	4.0502
Pclo	_TSQS[Phospho (STY)]FPPTGDTITPLDSK_.2	Q9QYX7_S748_M1_Pclo	7.5777	3.6249	8.0253	3.8900	3.9831	7.1515	8.0637	7.5335	8.0687	8.3648	3.9914	7.3107	3.7251	3.2688	3.1351	3.4895	9.3168	8.0307	8.0215	8.6640
Pclo	_ETGDGIILEVLDAYK_.2	#N/A	3.0835	2.4886	4.2318	3.1050	3.9221	4.1462	3.6190	4.2847	3.6781	3.2898	4.0758	3.3079	4.6051	2.4570	3.1962	3.4284	3.3216	3.8589	4.1602	3.5739
Pclo	_AYLQGVAEDRDY[Phospho (STY)]M[Oxidation (M)]SDSEVSSTRPSR_.3	#N/A	7.1477	7.3417	7.1774	4.0290	3.8843	6.7578	6.7602	6.8278	7.1778	7.0160	3.9470	7.1446	3.4939	7.1727	6.4748	7.1948	7.3126	7.0328	6.9488	3.6788
Pclo	_S[Phospho (STY)]RSHGIFPDPSK_.3	Q9QYX7_S4782_M1_Pclo	3.6206	3.6985	3.1572	3.9636	3.8276	3.8609	3.2205	3.8463	5.4647	3.4312	4.1202	3.4246	3.3062	3.8088	3.2459	2.9827	3.3435	4.3096	3.9582	3.7758
Pclo	_GSVPAAAAES[Phospho (STY)]PSMHRK_.2	#N/A	4.5054	3.4974	5.3047	5.3011	3.8431	4.0672	3.6979	4.0599	3.7570	3.2109	2.9646	2.9869	3.8651	5.2917	3.2751	3.3495	3.5571	3.5923	3.8914	3.0861
Pclo	_S[Phospho (STY)]RTDFKEEYK_.2	#N/A	3.7368	4.3828	4.2806	4.4649	3.9121	4.3635	4.5439	3.6841	3.6300	4.3034	3.5050	4.2663	3.8393	4.0457	3.6812	3.5690	3.9265	3.7806	3.6923	3.9345
Pclo	_KTTLYFDEEPELEM[Oxidation (M)]ESLTDS[Phospho (STY)]PEDR_.3	#N/A	4.1087	7.1458	3.6453	3.4755	4.3157	5.3409	6.5682	4.3344	4.0316	4.6045	2.9051	2.9365	4.1396	6.6597	5.4754	3.4709	3.7900	7.0947	6.2506	3.3607
Pclo	_QAELDEEEKEIDAK_.2	#N/A	3.3832	3.9358	4.4207	4.2010	3.5902	3.0173	3.4579	3.6089	3.3061	3.6619	1.2139	3.6620	3.4141	4.4587	1.7403	4.2462	3.1061	4.0433	0.8625	4.1360
Pclo	_RASYDSVEDSSESENS[Phospho (STY)]PVAR_.3	#N/A	3.9817	4.2553	4.4555	4.5204	3.2720	4.0344	3.7774	3.3784	4.2824	4.3765	3.1762	3.9814	5.1908	4.2137	4.7413	3.9280	4.1446	4.1328	4.2179	4.0543
Pclo	_SIS[Phospho (STY)]M[Oxidation (M)]EQLMK_.2	Q9QYX7_S4706_M1_Pclo	4.2446	3.7582	3.2801	5.9548	3.5823	4.4026	4.1797	3.9450	4.0999	3.9729	3.5785	4.4436	4.2653	5.7495	4.2050	3.0887	4.6855	3.3505	2.9991	3.2341
Pclo	_DYMS[Phospho (STY)]DSEVSSTRPSR_.2	#N/A	3.5797	3.5750	4.6093	4.2842	3.7866	4.1827	3.2655	4.1223	3.2610	3.5181	4.4119	3.3691	3.3950	5.1718	3.2924	3.8357	3.3501	4.1624	4.1260	4.0074
Pclo	_DIAFIMDDFQHAM[Oxidation (M)]S[Phospho (STY)]DSEAYHLR_.3	Q9QYX7_S4358_M1_Pclo	4.0107	3.9921	4.5470	3.6787	3.3484	3.5725	4.1927	3.0538	4.2518	4.2170	3.1253	3.8816	4.3598	5.9443	3.7699	4.3556	4.0519	3.0976	3.3966	3.5809

Pclo	_AEEDS[Phospho (STY)]MEDPYELK_.2	Q9QYX7_S4042_M1_Pclo	3.7152	3.3111	3.7272	4.4985	3.9222	3.9555	5.3635	4.3921	3.0461	3.5258	4.0256	3.2005	3.2116	3.7142	3.1513	3.0773	3.4289	4.4042	4.0528	3.6812
Pclo	_RHS[Phospho (STY)]WHDEDDETFDESPELK_.4	Q9QYX7_S1563_M1_Pclo	4.1591	4.4677	3.6957	3.4681	3.7479	3.8536	4.3561	3.6981	4.3426	5.0573	3.5568	3.7432	4.4802	3.7670	3.4651	3.8666	3.8820	4.5511	3.3375	5.3993
Pclo	_SPSTIS[Phospho (STY)]LK_.2	Q9QYX7_S131_M1_Pclo	3.5390	3.7800	4.5765	5.2973	4.2418	4.5286	3.3021	3.7647	3.4619	3.3496	3.8362	3.5062	3.5699	4.6145	3.3275	4.4020	3.2620	3.8875	4.1865	4.2918
Pclo	_TSVAQTHLEDAGAAIAAAEAAVQQLR_.3	#N/A	4.2100	4.6099	2.8411	3.3742	2.9161	3.6914	4.1320	4.4357	4.1328	4.3359	3.0064	4.3361	4.2409	3.7846	4.1574	3.5721	2.6738	2.2774	3.5156	1.2280
Pclo	_TTLYFDEEPELEM[Oxidation (M)]ESLTDS[Phospho (STY)]PEDR_.3	#N/A	3.4340	4.5688	3.9703	4.2554	4.2726	2.9958	3.2685	3.1344	3.3276	3.6403	3.7020	2.9574	4.4356	3.9574	2.8457	3.7789	3.6721	4.1611	3.8097	3.9244
Pclo	_SPS[Phospho (STY)]TIS[Phospho (STY)]LKESK_.2	Q9QYX7_S128_M2_Pclo	4.0827	3.2364	3.6193	3.5015	4.2896	4.3230	4.2593	4.3084	4.1795	3.8933	3.6582	2.9625	4.3450	3.6573	2.7838	3.4448	3.8056	3.3438	3.6429	3.3346
Pclo	_GGSLGC[Carbamidomethyl (C)]QTETDPDTQS[Phospho (STY)]PPYMGATSPPK_.3	Q9QYX7_S3506_M1_Pclo	3.5968	3.7222	3.1334	4.8840	3.8038	3.6935	3.2443	3.8225	3.1645	3.4074	4.1440	3.4484	3.3300	4.9191	3.2697	2.9590	3.3198	3.8297	4.1287	4.3496
Pclo	_KSVDTSVQT[Phospho (STY)]DDEDQDEWDM[Oxidation (M)]PSR_.3	#N/A	4.0241	3.4015	3.6373	4.0112	3.9396	4.1637	3.0488	4.3023	3.6612	3.3074	4.0356	3.2910	3.5109	3.4484	3.1793	3.4459	3.3391	4.1173	3.5920	3.3513
Pclo	_LPS[Phospho (STY)]PT[Phospho (STY)]SPLSPHSNK_.2	Q9QYX7_S2489_M2_Pclo	4.1705	3.8323	7.8576	3.5189	3.5083	3.7323	4.0328	3.8709	4.0919	4.3769	2.9654	3.7217	4.2000	3.7437	3.6100	3.0146	4.4086	3.2574	3.5565	3.4210
Pclo	_LPSPTSPLSPHSNKS[Phospho (STY)]S[Phospho (STY)]PR_.3	Q9QYX7_S2501_M2_Pclo	3.3068	4.0123	4.3443	4.2774	3.5138	3.0938	3.5343	3.5325	3.4545	5.8147	2.9332	3.7384	3.3377	4.3823	3.5597	4.1698	4.5306	4.1197	4.4188	4.0596
Pclo	_TPPSNLS[Phospho (STY)]PIEDAS[Phospho (STY)]PTEELRQAAEM[Oxidation (M)]EELHR_.4	#N/A	3.3556	3.9635	4.3930	4.2286	3.5625	3.5959	3.4856	3.5813	3.4058	3.1662	4.3852	3.6896	3.5712	4.4311	3.5109	4.2186	3.0785	4.0709	4.3700	4.1084
Pclo	_KQIIEM[Oxidation (M)]SADEDAS[Phospho (STY)]GSEDEEFIR_.3	Q9QYX7_S1505_M1_Pclo	3.9927	4.0101	4.5290	3.6967	3.3304	3.1537	4.2106	3.6931	3.8480	4.1990	3.3266	3.8995	4.0134	4.5161	3.7878	4.3376	4.2308	3.6024	3.2510	2.9823
Pclo	_SSSEGHLRS[Phospho (STY)]HGPS[Phospho (STY)]R_.3	Q9QYX7_S4820_M2_Pclo	3.9682	3.3509	3.5048	3.6160	4.1751	3.9332	4.3738	4.1939	3.8910	3.7788	4.2654	3.0770	3.9991	3.5428	2.8983	3.3303	3.6911	3.4583	3.7574	3.2201
Gab1	_SYS[Phospho (STY)]HDVLPK_.2	Q9QYY0_S266_M1_Gab1	6.1525	4.5129	4.0262	4.1994	6.1777	3.6564	5.4093	3.1903	5.2810	3.2267	4.3247	4.4023	3.5107	4.0133	3.4504	4.2791	3.7280	6.6969	3.7537	3.9803
Gab1	_SSS[Phospho (STY)]LEGFHSQYK_.2	Q9QYY0_S420_M1_Gab1	7.4876	7.6022	3.2819	3.4429	3.5842	7.2753	4.1815	7.2096	4.1017	7.8852	6.7691	3.6458	4.2672	3.2690	4.2069	3.0905	4.4845	3.3486	2.9972	3.2360
Gab1	_SVLTAGGVSGEELDENYVPM[Oxidation (M)]NPNS[Phospho (STY)]PPR_.3	#N/A	3.4680	4.2463	6.7730	6.1069	5.7897	3.3898	6.4335	7.2482	3.3568	6.9963	3.2589	4.1357	3.7774	5.6254	3.8954	4.0409	3.9948	3.8386	3.4872	5.8376
Spast	_SVS[Phospho (STY)]PQTLEAYIR_.2	Q9QYY8_S595_M1_Spast	8.5248	3.1560	8.3525	4.3434	8.1299	8.5242	3.2811	3.0464	3.2013	8.4847	8.5076	8.6551	8.8694	3.8694	8.1335	8.5355	8.9622	8.6386	8.5704	8.3840
Spast	_SSGTAPAPAS[Phospho (STY)]PS[Phospho (STY)]PPEPGPGGEAESVR_.3	Q9QYY8_S101_M2_Spast	3.1895	3.3124	3.7258	5.1333	4.0281	3.9568	3.1246	4.3907	3.0448	3.5272	4.0243	3.2019	3.2102	5.6079	3.1500	3.5344	4.7756	4.4056	4.0542	3.6799
Tollip	_GPVYIGELPQDFLR_.2	#N/A	6.7362	6.4115	6.5694	6.3126	6.4864	6.1852	5.9483	6.4621	6.2910	6.6183	6.4832	6.5599	6.4715	6.5815	6.5692	6.4257	6.8764	6.6530	6.6676	6.6197
Tollip	_LSITVVQAK_.2	#N/A	5.2080	5.5186	4.5717	5.4831	5.2089	5.3053	4.7297	4.4188	5.2025	4.5331	4.7305	4.5538	5.3794	5.8239	5.4727	5.0984	4.0092	5.2585	4.3617	5.4178
Tollip	_QGQVEDEWYSLSGR_.2	#N/A	6.3146	6.1840	3.9932	4.2324	3.6561	3.6894	6.0170	3.1573	6.1464	4.4786	4.5648	5.9098	3.4777	3.9803	3.4174	6.0741	3.6950	6.4128	3.7867	3.9473
Nagk	_[Acetyl (Protein N-term)]AALYGGVEGGGTR_.2	#N/A	4.2326	3.2120	3.6436	3.4772	3.6763	3.7944	4.7192	4.3327	5.3824	4.4389	2.9034	3.6597	4.1379	4.4042	4.4358	4.9151	3.8300	3.3195	3.6185	4.6838
Nagk	_LLIEELR_.2	#N/A	3.6281	4.3747	2.9679	4.0612	2.9659	3.1748	4.1558	3.3285	4.9218	3.0885	3.1021	2.4328	3.6489	4.1515	3.4336	3.8274	3.8662	3.9670	3.0981	4.1185
Nagk	_SLGLSLS[Phospho (STY)]GGEQEDAVR_.2	Q9QZ08_S76_M1_Nagk	4.5072	3.4956	3.5427	3.7518	3.8449	4.0690	3.6962	4.0581	3.7552	3.2127	4.1296	3.3851	3.8633	3.4071	3.2734	3.3513	3.5553	3.5941	3.8932	3.0843
Nfu1	_IRPTVQEDGGDVIYR_.3	#N/A	4.4972	4.8300	4.0338	4.5879	3.2033	3.4042	3.8448	3.2220	4.4200	4.1849	3.2936	4.0488	4.5281	4.9229	4.7003	3.4055	4.2201	2.7536	3.2284	3.6348
Nfu1	_GFEDGIVR_.2	#N/A	3.9810	4.4614	3.8952	3.2256	3.3188	3.5429	4.2223	3.0834	4.2814	4.1874	3.1549	4.1875	4.3895	3.9332	3.7995	4.3259	4.0815	3.0680	3.3670	3.6105
Vps29	_IQHILC[Carbamidomethyl (C)]TGNLC[Carbamidomethyl (C)]TK_.3	#N/A	5.5856	6.1164	5.9208	5.8101	5.3194	6.1263	5.7185	5.8452	6.1504	6.0874	5.4368	5.7741	5.6639	6.1278	5.3412	5.7895	5.0002	6.0832	5.8404	5.7723
Vps29	_VVTVGQFK_.2	#N/A	5.9675	4.7250	5.7791	5.5912	5.6638	5.2544	5.6671	5.8429	5.2505	5.1848	5.5873	5.9002	5.9343	6.2209	5.9285	5.9127	4.7651	5.9758	5.9065	5.4577
Vps29	_TLAGDVHIVR_.2	#N/A	6.2920	6.8194	6.3583	6.2739	6.3607	6.5352	6.6028	6.4180	6.5584	6.4551	5.3931	5.7080	5.3061	6.2797	6.3629	6.1299	5.9729	6.2682	6.0858	6.2719
Vps29	_FEAFEHENK_.2	#N/A	5.7911	5.5436	6.3926	3.4242	4.7557	4.5743	5.0695	5.1747	4.9996	5.5799	5.7289	4.5364	4.1909	5.9386	5.7450	5.4230	4.8072	5.1978	5.0869	3.4119
Rgs20	_ARAS[Phospho (STY)]PSASPADPGLPEGSER_.3	Q9QZB1_S13_M1_Rgs20	6.2746	6.1520	3.5317	3.9267	3.8064	6.0459	3.6257	4.3946	6.2269	4.3100	6.5160	6.1794	3.7745	3.5897	3.3242	5.8902	6.0016	6.4856	4.9173	5.8130
Rgs20	_NQEDQRPQRAS[Phospho (STY)]HEIR_.3	Q9QZB1_S86_M1_Rgs20	3.5121	4.1212	4.0484	2.0114	1.7970	3.0739	1.2609	1.0955	1.1196	1.5536	2.7340	4.3801	3.5684	2.6877	2.9245	3.8570	4.5504	3.5459	3.3637	2.3553
Actr10	_RAFS[Phospho (STY)]TEK_.2	Q9QZB7_S414_M1_Actr10	7.8136	8.7411	4.1015	4.0532	3.0660	3.2027	4.2408	4.0176	4.2404	7.6187	3.3975	4.1794	4.4484	3.9922	4.0327	3.9171	4.1405	3.8955	7.1481	3.6695
Actr10	_DSVVEILFEQDNEEK_.2	#N/A	6.2427	5.1850	6.5107	5.3186	4.2491	5.8030	4.3866	5.6495	6.4908	5.5612	5.4696	3.0031	6.1933	3.3873	5.0417	5.6084	6.0231	4.7208	5.1809	5.1167
Plxnc1	_KQS[Phospho (STY)]QQLELLESELRK_.3	Q9QZC2_S984_M1_Plxnc1	5.9380	6.5027	5.6340	3.5808	4.1998	5.9394	6.2101	5.8568	3.9638	5.3781	3.4425	5.3676	6.2739	6.3826	3.6720	4.7950	5.3601	6.2932	5.7846	3.0981
Ercc4	_TSS[Phospho (STY)]PEVQETKK_.2	Q9QZD4_S363_M1_Ercc4	7.2254	7.0877	6.3540	3.7936	6.2653	6.3322	6.6905	6.5357	6.5597	6.7938	6.9753	3.9964	6.8543	4.4192	5.6306	6.4814	6.9349	6.9338	6.3850	6.6927
Chml	_S[Phospho (STY)]SPLEASATPADSLDSASLPK_.2	Q9QZD5_S122_M1_Chml	3.2142	3.2878	3.7505	4.4752	4.0527	3.9322	3.4883	4.4154	3.0695	3.4205	4.0489	3.1772	3.2349	5.5097	3.0655	3.5591	3.4522	4.3809	4.0295	3.7046
Chml	_LVVQDS[Phospho (STY)]IEQPKR_.2	#N/A	6.5422	6.5581	6.5215	6.9968	5.3473	5.8206	5.9870	5.7908	5.2634	6.5457	5.6430	5.8923	5.6784	6.4423	4.3266	6.0181	5.9366	5.8624	6.0936	5.9888
Serinc1	_LTLTS[Phospho (STY)]DESTLIEDGNGR_.2	Q9QZI8_S348_M1_Serinc1	3.7714	3.6620	3.8894	4.1186	3.7811	4.0024	3.8980	3.8462	3.8562	3.4237	4.8482	3.4841	4.0056	3.6800	3.3460	3.7058	3.5164	6.1341	4.0010	4.0588
Serinc1	_SDGS[Phospho (STY)]LDDGDGIHR_.2	Q9QZI8_S364_M1_Serinc1	10.8415	10.4249	10.6087	10.4777	10.1549	10.1754	10.5078	10.7646	10.5534	10.1448	10.2123	10.0879	10.7307	10.1444	9.0452	9.8907	10.6523	10.4026	10.0616	9.9400
Dkkl1	_GGAREDS[Phospho (STY)]LEDGVHIPQHAK_.4	Q9QZL9_S200_M1_Dkkl1	4.3199	4.5000	3.8565	3.2643	3.0260	3.5815	4.1837	3.0448	4.2427	2.4861	3.1163	3.1058	4.3508	3.8946	3.7609	3.3624	4.0428	3.1066	1.6636	2.9579
Ubqln2	_GPAAAPGAAS[Phospho (STY)]PPAEPK_.2	Q9QZM0_S25_M1_Ubqln2	13.1076	13.3102	13.2181	13.3405	13.0802	13.0949	12.8216	13.4692	13.0860	13.2967	13.0367	13.3551	13.9724	13.8035	13.2311	13.3503	13.5576	13.0565	13.1564	13.3786
Ubqln2	_EKEEFAVPENSTVQQFK_.3	#N/A	6.6087	6.4542	7.4088	7.2875	6.8794	6.6666	6.4527	6.4283	6.8201	7.1709	7.0046	7.0959	7.2969	7.0496	7.4213	7.1862	6.8864	6.8554	6.8855	7.4884
Ubqln2	_SQTDQLVLIFAGK_.2	#N/A	4.3211	4.8275	5.7147	4.9452	5.2902	5.2458	5.0637	4.2359	4.8958	4.7570	4.7407	4.9710	4.1462	5.2082	5.3911	4.9315	5.1428	4.8212	4.9124	4.4518
Ubqln2	_NQDLALSNLESIPGGYNALR_.2	#N/A	5.6251	5.8991	5.4003	5.6092	5.8697	5.4052	4.9172	6.1688	5.4023	5.8595	5.2949	6.0731	5.2866	6.1559	6.2059	6.2786	5.0530	6.0175	5.8481	5.5746
Ubqln2	_NPEISHLLNNPDIM[Oxidation (M)]R_.3	#N/A	3.8105	3.9787	4.1074	4.6865	4.1932	3.8769	4.5547	5.3351	3.8189	3.8936	4.1544	3.0711	3.7151	3.7022	4.1981	4.3616	4.9134	4.5975	4.6420	4.1096
Afdn	_YVLSSQHRPDIS[Phospho (STY)]PTER_.3	Q9QZQ1_S655_M1_Afdn	9.8461	3.8371	9.1021	9.4929	3.6890	9.5379	9.6709	9.8413	9.3639	3.2926	9.4026	9.6265	9.9860	9.6496	8.4208	9.1701	9.9819	9.8037	3.8196	9.3801
Afdn	_S[Phospho (STY)]QEELREEK_.2	#N/A	9.3478	8.9270	9.0890	9.1784	8.5144	8.5893	8.7920	8.3272	8.5343	9.0830	8.7712	8.3994	8.8328	8.6300	7.2808	8.3165	8.9329	8.5870	8.5286	8.4059
Afdn	_S[Phospho (STY)]QDADLPGSSGAPENLTFK_.2	Q9QZQ1_S1770_M1_Afdn	8.3951	9.1179	8.8872	8.7313	6.0427	8.0092	8.2196	5.9429	7.3595	8.9310	2.9787	3.7350	7.7343	9.0296	6.8963	8.2819	7.7278	8.5964	8.4595	8.1436
Afdn	_TSS[Phospho (STY)]VVTLEVAK_.2	Q9QZQ1_S1083_M1_Afdn	8.3697	8.5565	8.5005	8.4397	7.9876	8.3929	4.1134	4.4543	8.3983	8.3609	8.2735	4.3174	8.4948	7.9316	7.1190	8.2669	8.6556	8.1687	8.2429	8.2791
Afdn	_TQVLS[Phospho (STY)]PDSLFTAK_.2	Q9QZQ1_S1719_M1_Afdn	4.3342	8.1709	7.6732	7.6818	6.9667	7.5102	7.8974	4.2311	7.2781	7.4404	7.2737	3.0398	4.0363	3.5800	6.0784	7.5935	7.8549	3.4211	7.1653	7.1337
Afdn	_SSPNVANQPPS[Phospho (STY)]PGGKGPYTSGTAAK_.3	#N/A	4.4082	4.0041	4.5350	7.9138	6.4603	3.1476	8.3992	6.3077	3.8540	4.2188	7.4008	8.0523	4.0194	4.5221	6.7420	7.3357	7.3513	8.6121	7.8148	7.1842
Afdn	_ADHRS[Phospho (STY)]SPNVANQPPS[Phospho (STY)]PGGK_.3	#N/A	4.1725	5.4560	3.7167	6.0027	4.8367	5.2517	5.1192	3.7845	4.4727	4.9551	6.2114	5.7821	5.4919	4.2074	3.5667	4.6143	4.5887	3.6439	3.5499	4.8550
Afdn	_SSPNVANQPPS[Phospho (STY)]PGGK_.2	#N/A	10.9393	10.4575	11.1462	10.7523	9.3564	9.3542	10.3001	10.7222	10.4046	10.3251	10.5194	10.2847	10.8053	10.7706	9.3525	10.2261	10.9082	10.6183	10.3412	10.4261
Afdn	_VTRS[Phospho (STY)]QEELREEK_.3	#N/A	4.2772	6.4647	3.8138	5.0968	2.9833	3.0166	6.0341	4.5029	3.9850	4.0878	3.4636	4.2688	6.3740	5.3345	4.0902	5.0269	4.0001	5.8487	5.4229	3.5291
Afdn	_LPYLVELS[Phospho (STY)]PGR_.2	Q9QZQ1_S391_M1_Afdn	4.0971	5.5033	5.6519	3.5923	3.4348	4.9990	4.8375	3.7974	3.9523	4.1205	3.4310	3.7952	4.1178	4.6204	2.9189	2.9411	4.3351	5.4563	3.1466	3.0866
Afdn	_TIS[Phospho (STY)]NPEVVMK_.2	Q9QZQ1_S216_M1_Afdn	3.7045	3.3003	3.2411	7.3511	3.9114	3.9448	5.8489	4.4028	3.0569	3.5151	5.7437	3.3407	3.2223	6.6174	3.1621	3.0666	3.4397	4.3935	4.0421	4.6378
Afdn	_EYFTFPASKS[Phospho (STY)]QDR_.2	#N/A	7.3601	7.6294	4.1446	6.8666	5.8675	6.1198	6.8861	3.6028	3.7577	7.2659	6.1815	6.1057	3.9232	4.4258	3.8629	3.9606	6.3188	6.6573	6.4693	6.2091
Macroh2a1	_EFVEAVLELR_.2	#N/A	3.2386	5.1262	3.7749	5.4505	4.4787	4.4592	4.2778	4.1835	5.1538	3.4781	4.9735	3.1528	3.2593	4.2048	4.8612	4.6871	4.3849	4.3565	4.0073	3.7289
Macroh2a1	_AASADSTTEGT[Phospho (STY)]PTDGFTVLSTK_.2	Q9QZQ8_T178_M1_Macroh2a1	4.3445	4.8834	4.6356	5.0486	3.1481	4.8172	5.0663	3.4940	4.6901	4.2732	3.4160	3.9973	4.0173	5.1757	3.6277	4.6293	4.2530	3.9066	3.7584	4.2354
Hipk2	_AVC[Carbamidomethyl (C)]STY[Phospho (STY)]LQSR_.2	Q9QZR5_Y361_M1_Hipk2	7.5666	7.6790	7.8456	7.5198	7.3930	7.5934	7.3844	7.8486	7.6275	7.8873	7.5849	7.2803	7.6016	7.3305	6.8735	7.0022	7.8720	7.2857	3.0693	7.6362
Hipk2	_NVSTC[Carbamidomethyl (C)]EVTSSQAISS[Phospho (STY)]PQR_.2	Q9QZR5_S827_M1_Hipk2	3.2523	4.0668	4.2897	4.3320	4.4592	3.4925	3.5889	3.4779	3.5091	4.5637	2.9877	3.7929	3.6745	4.1772	3.6143	4.1152	4.4760	3.9413	3.5899	4.1442
Rnf4	_LRQDHADSC[Carbamidomethyl (C)]VVS[Phospho (STY)]S[Phospho (STY)]DDEELSR_.3	Q9QZS2_S98_M2_Rnf4	4.0208	3.9820	4.5571	3.6685	3.3586	3.1255	3.9559	3.7212	3.8761	4.1967	3.3548	3.8714	4.0416	4.5442	3.9568	3.7482	4.0395	3.5743	3.2228	3.0104
Numb	_TNPS[Phospho (STY)]PTNPFSSDLQK_.2	Q9QZS3_S636_M1_Numb	10.0354	9.7289	9.8260	9.9785	9.0862	8.5470	8.9125	8.9052	8.7118	10.2203	9.3498	9.2815	9.5524	9.5626	8.1523	9.3976	9.1668	9.0399	9.0099	9.1131
Numb	_RTPS[Phospho (STY)]EADR_.2	Q9QZS3_S440_M1_Numb	3.2963	4.0228	4.3337	4.2879	3.5032	3.7620	3.5449	3.5220	3.2191	3.1069	3.5935	3.7489	3.3272	4.3718	3.5702	4.1593	4.5201	4.1302	4.4293	4.0491
Sh2d3c	_TASPAS[Phospho (STY)]PSGSK_.2	Q9QZS8_S341_M1_Sh2d3c	3.3339	7.5256	4.3713	6.9464	3.5408	3.5742	6.8852	3.2726	3.4275	3.1445	2.9061	3.7113	3.5929	4.0956	3.5327	4.1968	4.5576	4.0229	3.6715	4.0626
Sh2d3c	_VHAPSATPSTSAQPAS[Phospho (STY)]PVAR_.3	Q9QZS8_S435_M1_Sh2d3c	3.4700	6.3965	4.0063	4.2155	4.3086	3.0318	6.2830	6.0619	3.2916	3.6763	5.2276	4.4222	3.3996	4.4443	2.8097	3.8149	3.0917	4.0578	4.3568	4.1215
Sh2d3c	_S[Phospho (STY)]ITMTDGLTTDK_.2	Q9QZS8_S354_M1_Sh2d3c	3.9262	4.4065	3.9500	5.2556	3.2639	3.4880	4.2772	3.1383	4.3362	4.1325	3.2098	4.1326	4.4443	3.9881	3.8544	4.2711	4.1363	3.0131	3.3122	3.6654
Atp11c	_RAS[Phospho (STY)]DSLSAR_.2	Q9QZW0_S1105_M1_Atp11c	3.5800	3.7391	3.1165	4.0043	3.7869	4.3215	2.9737	3.8056	3.5028	3.4651	3.8772	3.4652	3.6108	3.1546	3.0209	2.9421	3.3029	3.8466	4.1456	4.3327
Srr	_GLIPDT[Phospho (STY)]PEEKPK_.2	Q9QZX7_T71_M1_Srr	9.2546	9.8406	9.6196	9.9421	7.5111	9.5006	9.1722	8.6938	9.3118	9.8947	8.8021	9.6329	9.5975	10.0784	9.1705	9.8362	10.2284	9.6913	9.8429	10.0059
Srr	_DLVDDVFTVTEDEIK_.2	#N/A	7.5241	7.2853	7.5991	7.6313	7.2199	7.0195	6.7336	7.1625	7.7732	7.4937	7.6919	6.9293	8.1104	7.7595	7.1325	7.9185	7.3013	6.8408	7.0218	7.4595
Srr	_VYAAEPSNADDC[Carbamidomethyl (C)]YQSK_.2	#N/A	6.4417	6.6597	6.5638	6.4896	6.0397	6.1659	6.4405	6.3338	6.6125	6.4019	6.1756	4.0352	6.1878	7.1208	6.3486	6.1023	6.7979	6.6635	6.2422	6.2242
Srr	_LAIQAYGASIVYC[Carbamidomethyl (C)]DPSDESR_.2	#N/A	4.3831	4.4368	3.9197	3.2011	3.0892	3.5183	3.9589	3.1079	3.8791	4.1937	2.9496	2.4777	4.4140	3.9577	3.9843	3.9336	4.1060	3.3862	3.3425	3.6350
Zranb2	_S[Phospho (STY)]RPSS[Phospho (STY)]PAVR_.2	#N/A	8.9189	10.3043	10.3185	10.1543	9.2793	10.1846	8.8051	10.3831	10.1175	8.5884	10.0636	9.9473	10.4084	10.1522	9.2820	9.8908	10.3177	10.8175	9.9515	9.8881
Zranb2	_YNLDAS[Phospho (STY)]EEEDSNK_.2	#N/A	6.9860	6.5268	6.8480	6.4621	3.3966	6.6151	6.0779	6.1694	5.8155	6.1808	6.6574	5.8029	6.0914	6.7131	4.7260	5.9505	4.0037	6.1510	5.9184	5.6134
Zranb2	_EES[Phospho (STY)]DGEYDEFGR_.2	#N/A	3.1540	8.1734	9.3048	9.4072	3.3609	9.2202	8.1787	3.4525	9.0002	9.3766	3.0860	8.5676	3.7728	9.0081	3.7125	8.8250	9.3677	8.7990	9.2635	9.0135
Zranb2	_YNLDAS[Phospho (STY)]EEEDSNKKK_.2	#N/A	5.2622	4.8443	4.3046	4.2506	4.2548	4.9475	5.3742	4.1971	4.0673	5.3239	4.4555	4.9921	4.7306	4.7040	4.0221	4.7363	4.4741	4.8592	4.9509	4.5130
Zranb2	_S[Phospho (STY)]RPSS[Phospho (STY)]PAVRK_.2	#N/A	3.3908	4.1528	4.2966	4.1516	5.6811	6.1438	3.4494	3.4723	5.3681	6.5118	3.3339	3.9646	3.5667	6.2672	3.9705	3.9681	4.2506	5.4473	3.2392	6.2327
Zranb2	_ENVEYIEREES[Phospho (STY)]DGEYDEFGR_.3	#N/A	3.4429	5.3444	3.9792	4.2465	4.2814	3.7035	4.1497	3.1432	3.2981	3.2738	4.2776	2.9486	3.4636	6.3725	3.4033	3.7878	3.6809	4.9039	3.2328	3.9332
Zranb2	_SRS[Phospho (STY)]RPSS[Phospho (STY)]PAVR_.2	#N/A	6.5012	4.3552	3.2601	3.7440	3.8149	4.1862	4.2645	6.1335	3.6647	3.6270	3.8922	3.4439	3.8117	3.4459	3.6038	3.2283	3.8790	3.8831	5.7771	3.3326
Zranb2	_ENVEYIEREES[Phospho (STY)]DGEYDEFGRK_.3	#N/A	4.5504	8.9416	7.9532	7.3276	3.2565	8.3285	8.1720	3.2753	7.8515	4.3610	3.1904	6.7929	4.5813	8.8978	7.2930	6.9112	6.5414	8.4653	7.9250	7.2973
Zranb2	_YNLDAS[Phospho (STY)]EEEDSNKK_.2	#N/A	6.6555	6.4852	6.4901	4.3164	4.2115	5.8241	6.2547	6.9053	6.0974	3.5793	3.7630	6.0381	5.7086	7.2324	2.9068	5.8982	6.2691	6.3312	6.0217	3.8633
Zranb2	_PS[Phospho (STY)]S[Phospho (STY)]PAVRK_.2	#N/A	2.9453	4.1703	4.1863	4.4354	3.3558	3.3891	3.6923	3.3745	3.6125	4.4603	3.4996	3.8964	3.7780	4.2243	3.7177	4.0118	4.3726	4.2822	3.0759	4.2476
Prkab1	_S[Phospho (STY)]QNNFVAILDLPEGEHQYK_.3	#N/A	7.5681	7.9144	7.5436	7.3714	6.6481	7.1555	7.6825	7.1462	7.3226	7.4252	7.2666	7.3298	7.7494	8.1738	6.9205	7.2828	7.2878	7.6581	7.5380	7.2937
Pex14	_[Acetyl (Protein N-term)]ASSEQAEQPNQPSS[Phospho (STY)]PPGSENVVPR_.3	Q9R0A0_S15_M1_Pex14	3.6615	8.2931	7.7077	7.9161	4.1200	8.0772	7.4157	4.3041	7.4757	7.6332	4.1686	3.1338	3.6889	8.1504	7.0460	7.5501	7.6474	7.7025	7.7701	7.6475
Zeb2	_AYLQSITPQGYSDS[Phospho (STY)]EER_.2	Q9R0G7_S1124_M1_Zeb2	4.0965	4.0695	3.6331	3.5831	3.5542	5.3592	3.5916	3.5729	3.8483	3.9071	3.7028	3.7956	3.9735	3.6712	3.6169	3.4586	3.8194	3.4254	3.7244	4.1000
Zeb2	_SNT[Phospho (STY)]PS[Phospho (STY)]PLNLSSTSSK_.2	Q9R0G7_T782_M2_Zeb2	3.2022	6.7186	3.8327	4.1511	3.7528	6.1726	3.5705	3.6914	3.5599	4.0753	3.5639	6.0293	3.6963	6.6042	3.3951	3.8346	4.0190	3.9934	6.1270	6.5407
Zeb2	_S[Phospho (STY)]DHEEDNMEDGME_.2	#N/A	3.8837	4.4809	4.3768	3.9924	3.8271	3.4295	3.6350	3.1443	3.6093	3.9637	3.5157	4.2940	3.7886	4.1985	3.5068	3.7852	3.9262	3.6801	3.5700	3.5602
Zeb2	_VYQYSNSRS[Phospho (STY)]PSLER_.3	Q9R0G7_S705_M1_Zeb2	3.1969	3.3051	3.7332	4.4925	4.0354	4.2595	3.5056	4.3981	3.5647	3.4032	3.9391	3.1945	3.6728	3.7202	3.0828	3.5418	3.4349	4.3982	3.7938	3.6872
Atp2b2	_TSPVEGLPGTAPDLEK_.2	#N/A	9.2601	9.0560	8.9723	8.5659	8.2978	8.6892	9.1131	9.0280	8.9234	8.5616	8.8747	8.7106	9.1054	8.8706	8.5568	8.5008	9.1568	8.8744	8.6720	8.4118
Atp2b2	_EIPDPSSINAK_.2	#N/A	8.8864	8.3788	8.1721	8.2252	8.2589	8.1911	8.5521	8.7421	8.4991	8.0606	8.4373	8.1484	8.8688	7.6035	8.0711	7.7336	8.4897	8.3708	8.3684	8.1579
Atp2b2	_ILSGAGEAR_.2	#N/A	7.3504	7.2528	7.3538	6.6566	7.1376	7.1616	7.7781	7.2962	7.0231	6.6829	6.9819	7.3219	7.0153	6.5364	6.9471	6.4801	7.1546	7.6777	7.2549	6.9979
Atp2b2	_GIIDSTHTEQR_.2	#N/A	7.4094	7.1111	7.4797	7.0313	7.1219	6.9889	7.1060	7.3483	7.2434	6.8588	7.1324	6.7232	7.4028	6.5204	6.8164	6.9439	7.3779	7.3482	7.2476	7.0268
Atp2b2	_GASEIVLKK_.2	#N/A	5.2404	4.5132	3.5857	4.1967	4.6367	4.7805	4.7157	4.9892	4.7260	5.5570	4.8464	4.5668	4.5500	3.3029	3.7652	5.5640	5.2272	4.8037	5.0268	3.7263
Atp2b2	_YGDLLPADGLFIQGNDLK_.2	#N/A	6.7402	6.4073	3.8368	6.2415	6.1623	5.9349	6.8665	6.8490	6.8773	6.2534	6.0831	6.2736	7.0606	6.5446	5.4932	5.7881	7.0014	7.0967	6.5606	6.3183
Atp2b2	_NNSAIDS[Phospho (STY)]GINLTTDTSK_.2	Q9R0K7_S1170_M1_Atp2b2	6.8494	6.8847	6.3499	6.9365	5.4746	6.0902	6.6856	6.4044	6.2129	6.7867	6.1753	6.0366	5.8668	6.4569	4.6766	5.9000	6.3535	6.5487	5.4170	6.0980
Atp2b2	_AFRSS[Phospho (STY)]LYEGLEKPESR_.3	Q9R0K7_S1105_M1_Atp2b2	6.4970	4.0196	4.5195	3.7062	3.1299	5.8448	5.9585	3.6836	6.2705	4.2343	3.3171	3.9091	4.0039	4.5066	3.9436	3.7859	4.2212	6.6859	3.2605	2.9727
Atp2b2	_AGQVVQIPVAEIVVGDIAQIK_.2	#N/A	5.5411	5.0288	5.2436	3.8583	5.0061	4.9268	5.4874	5.3643	5.1780	5.0176	5.6743	4.7990	3.7285	4.4361	4.1819	4.2318	5.4947	5.2376	6.7545	4.8599
Atp2b2	_IDESSLTGESDQVR_.2	#N/A	3.0818	7.5135	7.9160	7.7105	7.7830	7.3777	7.7363	7.6646	7.7855	7.2294	7.9380	7.5129	3.7879	7.0605	7.1318	6.8790	3.4799	3.6695	7.2695	7.4789
Atp2b2	_MPDESFR_.2	#N/A	3.8674	4.4847	5.8477	5.7628	3.6035	5.1065	6.3477	3.5677	4.1935	4.0737	5.7066	3.1592	4.5031	5.2295	4.6071	4.2123	3.7269	4.9879	3.3499	4.2734
Atp2b2	_EASDIILTDDNFSSIVK_.2	#N/A	7.4155	7.1324	6.9967	6.8780	6.6896	6.7766	6.9349	7.1376	6.7864	6.9800	7.0466	6.8875	7.4084	7.3063	5.8115	6.8610	7.4898	6.9300	6.7711	6.9571
Atp2b2	_NES[Phospho (STY)]SHGGEFGC[Carbamidomethyl (C)]TM[Oxidation (M)]EELR_.3	#N/A	4.1869	6.5617	7.1254	6.3273	3.7485	4.8669	3.7957	5.1903	3.7929	4.7919	3.8439	3.4781	3.9261	6.9958	5.0193	5.5130	3.8751	5.9947	5.9398	5.3258
Atp2b2	_T[Phospho (STY)]SIHNFM[Oxidation (M)]AHPEFR_.3	#N/A	8.4603	8.7819	4.4333	4.7763	6.5852	6.9090	8.3010	9.2171	6.6816	7.6783	5.1902	7.9182	5.0728	9.0819	5.1354	4.9302	7.8869	5.5952	7.7871	6.9078
Atp2b2	_QNSS[Phospho (STY)]PPSSLNK_.2	#N/A	11.2419	10.8564	11.3528	10.8429	10.6274	10.7991	10.7742	10.8975	10.6712	10.7067	10.9993	10.4453	10.8148	10.8958	9.7126	10.5227	11.2873	10.7515	10.7309	10.6227
Atp2b2	_IDESSLTGESDQVRK_.2	#N/A	5.3095	4.6892	4.7437	4.8659	5.0781	4.9546	5.0403	5.6839	5.1609	4.1686	4.8777	4.9631	5.7229	4.5569	4.3753	4.7150	5.1322	5.0001	4.7682	5.1532
Atp2b2	_TLELLVNAIAINSAYTTK_.2	#N/A	4.7373	2.5073	4.3264	4.4398	3.5366	3.4648	4.4132	3.5990	3.2904	1.9878	3.8075	4.1021	3.7485	4.3135	1.9000	4.1350	3.3886	3.8050	4.2001	4.2805
Atp2b2	_VIEPMAC[Carbamidomethyl (C)]DGLR_.2	#N/A	3.5452	6.3429	7.1330	6.3380	2.8829	5.5293	6.2001	3.5193	4.4423	3.7515	6.2584	4.3471	3.3245	6.0448	4.2354	3.8901	4.5173	4.1329	4.4320	4.0464
Atp2b2	_IEDSQPHIPLIDDTDLEEDAALKQNSS[Phospho (STY)]PPSSLNK_.4	#N/A	9.5153	10.1888	8.5378	3.7767	3.2504	8.6982	9.8996	9.2533	8.8394	9.3223	8.8108	3.9796	4.4578	9.6513	3.8679	8.6452	9.2673	9.9505	9.2289	8.5768
Atp2b2	_TEC[Carbamidomethyl (C)]GLLGFVLDLR_.2	#N/A	4.2148	4.6051	3.7514	3.3694	2.9209	4.0424	4.1272	4.4405	3.9473	4.0254	3.5260	4.3312	4.2128	3.7895	4.1526	3.5769	4.5747	3.4643	3.0516	3.1816
Atp2b2	_EEIPEEELNEDVEEIDHAER_.3	#N/A	4.5570	3.4458	3.5924	3.1324	3.8947	4.0902	3.6464	4.2574	4.4123	3.2625	3.8909	3.3353	4.5777	7.0784	3.2236	3.4010	3.2942	6.4434	4.1875	3.5465
Atp2b2	_LTQKEEIPEEELNEDVEEIDHAER_.4	#N/A	4.0519	4.5998	5.1027	3.6496	3.7901	3.9151	3.5501	3.9788	3.5578	4.1176	4.1794	3.0844	3.6867	4.5098	3.3149	3.1190	3.2531	5.1443	4.2319	3.9597
Atp2b2	_M[Oxidation (M)]TVVQAYVGDVHYK_.3	#N/A	3.7685	3.5506	3.3051	3.8157	3.9755	4.0088	3.0726	3.9942	3.4703	3.5791	3.9723	2.0395	3.1583	3.6609	2.2873	3.1306	3.4914	4.4575	4.1061	2.3462
Pcm1	_SNRPVS[Phospho (STY)]ADGNYRPLAK_.3	Q9R0L6_S957_M1_Pcm1	7.7744	7.4898	7.0729	7.2387	7.1343	7.4437	7.1486	3.3595	7.0911	7.7896	7.0477	7.1828	7.8276	7.9990	6.7574	6.8927	7.4634	7.2666	7.2000	7.6289
Pcm1	_VTNAIS[Phospho (STY)]PESSPGVGR_.2	Q9R0L6_S65_M1_Pcm1	9.0743	8.6188	8.5597	8.4706	8.4172	8.8962	8.5984	8.9170	8.4030	8.9742	8.6178	8.1651	9.0578	8.5936	8.0421	8.6737	8.9389	8.2802	8.5519	8.6702
Pcm1	_ILEGDHGS[Phospho (STY)]PAGEIDDEDKDKDETETVK_.4	Q9R0L6_S1729_M1_Pcm1	3.9962	3.3229	3.5327	3.5881	4.2031	4.2364	4.3458	4.2218	4.2660	3.8068	3.7447	3.0490	4.4315	3.5708	2.8704	3.3583	3.7191	3.4304	3.7294	5.0182
Pcm1	_RS[Phospho (STY)]S[Phospho (STY)]LVDEAPEDEEFEQK_.2	Q9R0L6_S644_M2_Pcm1	3.9633	5.1882	5.3507	3.8407	3.3212	5.7289	5.0321	3.3399	4.8797	4.0174	3.4547	3.9534	3.6998	5.7327	4.7862	3.9772	4.3380	4.7438	5.0929	5.2175
Pcm1	_HIS[Phospho (STY)]ESDER_.2	Q9R0L6_S1432_M1_Pcm1	4.1519	3.1672	3.6885	3.4323	2.8580	3.7495	4.0156	4.3776	4.0747	4.3941	2.3005	4.3942	4.1828	3.7265	3.5928	3.5140	3.8748	3.2746	3.5737	3.4038
Pcm1	_NVRS[Phospho (STY)]DVS[Phospho (STY)]DQEEDEESER_.2	Q9R0L6_S1766_M2_Pcm1	4.3598	3.6430	3.3953	3.3296	3.6975	2.4126	2.0350	4.0602	4.0766	4.5661	2.8165	3.3927	4.0107	3.1821	2.3273	3.2039	3.0970	3.2353	4.3847	3.3494
Pcm1	_KAS[Phospho (STY)]AQASLASK_.2	Q9R0L6_S1280_M1_Pcm1	4.2879	3.7149	3.3234	3.4014	3.6257	3.8497	3.9154	4.2774	3.9745	4.4943	4.3489	3.6043	4.0826	3.6263	3.4926	3.1320	3.7746	3.3749	3.6739	3.3036
Celsr2	_STLTSSYNC[Carbamidomethyl (C)]PS[Phospho (STY)]PYADGR_.2	Q9R0M0_S2648_M1_Celsr2	4.0558	3.2633	3.5924	3.5284	4.2628	4.6243	5.1731	4.2815	3.9786	4.4901	4.3530	2.9894	4.0867	3.6305	3.4968	3.4179	3.7787	3.3707	3.6698	3.3078
Syt11	_DYGEELRS[Phospho (STY)]PM[Oxidation (M)]TSLT[Phospho (STY)]PGESK_.3	Q9R0N3_T139_M2_Syt11	4.0435	4.3290	3.8766	3.7241	3.5755	3.8365	4.0954	3.9526	5.8203	3.9544	3.5390	4.3270	3.9862	3.5150	3.7897	3.6959	4.0747	3.6956	3.3442	3.4820
Syt7	_NSLETVGT[Phospho (STY)]PDSGR_.2	#N/A	9.7964	9.6876	9.7111	9.5358	9.1862	9.6134	9.3037	9.6070	9.3115	9.3708	9.4825	9.1667	9.6457	9.5398	8.3844	9.0876	9.8665	9.4778	9.2808	9.1661
Syt7	_NSLETVGT[Phospho (STY)]PDS[Phospho (STY)]GRGRGEK_.3	#N/A	4.4631	5.5723	4.8288	5.3199	5.1481	5.8464	5.7231	4.3222	5.7617	5.7236	5.6959	5.6155	6.3130	6.0669	5.8191	6.1918	5.5240	6.2949	6.2443	5.6656
Syt7	_YKNSLETVGT[Phospho (STY)]PDS[Phospho (STY)]GRGR_.3	#N/A	6.5609	6.6140	5.9793	6.1841	5.9457	6.7153	6.6662	6.3613	6.4231	6.9231	6.6141	6.2767	6.0822	6.3318	5.8290	6.5478	7.1626	7.1959	6.8633	6.4813
Syt7	_YKNSLETVGT[Phospho (STY)]PDSGR_.2	#N/A	6.8037	6.2974	5.9384	6.8859	5.6172	5.6467	6.9051	6.4876	6.0833	4.9857	5.1464	5.9024	5.1589	5.9447	6.0805	5.0428	6.2940	7.1787	5.5098	4.3004
Syt7	_VLYLQVLDYDR_.2	#N/A	4.6946	3.3723	3.6660	5.0859	3.9833	4.1502	3.0648	4.3309	4.5070	3.5870	4.2460	2.8586	5.0350	3.6531	3.0902	3.1385	5.4582	4.2403	4.1140	2.1471
Syt7	_NSLETVGT[Phospho (STY)]PDS[Phospho (STY)]GRGR_.2	#N/A	8.8450	8.8026	8.8283	8.4977	8.1335	8.5260	8.3167	8.9014	8.5197	8.6224	8.4827	8.2488	8.3391	8.5626	7.4673	8.1777	9.0053	8.7616	8.5381	8.5032
Syt6	_S[Phospho (STY)]VDGDDAKSEAAK_.2	#N/A	7.2002	6.9835	6.5791	6.7559	5.8726	6.5254	6.7758	6.5228	6.5358	6.6953	6.2437	4.1735	7.2544	7.3922	6.6342	6.5494	6.8040	6.8003	3.2093	6.7366
Syt6	_EASSPSSANPASETLQS[Phospho (STY)]PSSR_.2	Q9R0N8_S108_M1_Syt6	3.7635	6.2640	6.6622	3.8208	6.1372	5.7439	5.9004	3.9892	5.9536	3.2816	4.0607	3.2817	6.7304	3.3381	3.2044	3.1256	6.7433	6.4685	3.9621	5.9652
Syt6	_QKS[Phospho (STY)]VDGDDAKSEAAK_.2	#N/A	4.7793	4.4369	4.8851	4.8494	4.7071	4.3617	3.6266	4.7173	4.1477	4.7651	4.3544	4.6301	5.9244	5.1822	5.5051	4.7694	4.0735	4.3821	5.1737	5.0233
Syt6	_QKS[Phospho (STY)]VDGDDAK_.2	#N/A	3.9161	3.4030	3.4527	3.6681	4.1230	3.9853	3.7798	4.1418	3.8389	5.5885	4.2133	3.1291	3.9470	3.4908	3.3570	3.2782	3.6390	3.5104	3.8095	6.8438
Esd	_AFSGYLGPDESK_.2	#N/A	7.3997	7.0931	7.5492	7.2022	6.8604	7.4125	7.3180	7.7134	6.9778	7.1629	7.2629	7.0454	3.7779	7.1855	7.1213	7.0267	7.3688	7.1673	6.6012	7.3547
Esd	_M[Oxidation (M)]YSYVTEELPQLINANFPVDPQR_.3	#N/A	4.1971	4.4518	4.0834	4.6107	3.5987	4.7650	4.3815	4.7422	4.0946	5.7140	3.5732	3.5958	4.2600	3.7423	4.4346	4.9905	3.8905	3.2589	4.4933	5.3150
Esd	_AYDATC[Carbamidomethyl (C)]LVK_.2	#N/A	4.0181	3.3009	3.5547	3.5661	4.2251	4.2584	5.3103	4.0892	4.8397	5.6918	3.7227	3.0271	4.4095	3.4113	5.2686	3.3803	3.1260	3.2063	4.3557	5.1061
Smap	_SAS[Phospho (STY)]PDDDLGSSNWEAADLGNEERK_.3	#N/A	3.2280	3.2739	3.7643	4.4613	4.0666	3.9183	3.1631	4.4293	3.0833	3.4886	4.0628	3.1634	3.2488	3.7514	3.1885	3.5729	3.4661	4.3671	4.0156	3.7184
Smap	_SAS[Phospho (STY)]PDDDLGSSNWEAADLGNEER_.3	#N/A	3.2459	4.3426	4.1965	4.0292	3.4529	3.4862	3.5952	3.3605	3.5154	4.5574	2.9941	4.2321	3.6809	4.1835	3.6206	4.1089	3.8982	3.9349	3.5835	4.1505
Dstn	_[Acetyl (Protein N-term)]AS[Phospho (STY)]GVQVADEVC[Carbamidomethyl (C)]R_.2	Q9R0P5_S3_M1_Dstn	12.8300	13.1189	12.8459	13.1942	12.6098	12.5015	12.4496	12.5645	12.7424	13.4452	12.5076	12.3686	13.0546	12.9872	12.0790	12.7403	13.1143	12.3693	12.6750	12.7158
Dstn	_YALYDASFETK_.2	#N/A	7.5283	7.6177	8.1675	8.0485	7.6565	7.6211	7.6802	7.6279	7.8711	7.6668	7.3883	7.2945	7.2319	7.8378	7.9703	7.6111	6.9233	7.4617	7.7511	7.5169
Dstn	_HEYQANGPEDLNR_.2	#N/A	6.2089	6.7892	6.5384	6.5362	3.0886	6.7459	6.3670	6.2057	5.8670	7.0495	6.6476	6.7929	6.7512	7.1328	6.7733	6.6163	6.8120	6.6762	6.8233	6.6276
Dstn	_EILVGDVGATITDPFK_.2	#N/A	5.8119	4.9435	5.8073	3.9893	5.5822	5.4708	4.4421	3.3731	5.6195	5.2581	5.4860	4.8403	3.1783	5.2115	5.0081	5.4892	5.4399	5.0053	4.9513	5.6702
Dstn	_HFVGM[Oxidation (M)]LPEKDC[Carbamidomethyl (C)]R_.3	#N/A	5.3087	5.2710	5.2481	3.8525	6.8529	6.3017	5.9812	3.9574	6.0809	6.6573	5.7768	5.6644	3.7626	5.9724	6.3531	5.9961	7.0093	5.4053	6.4427	5.5700
Dstn	_C[Carbamidomethyl (C)]IVVEEGKEILVGDVGATITDPFK_.3	#N/A	4.2535	1.8418	3.7901	3.3308	2.9596	3.6480	4.1172	4.4792	2.2124	4.2925	3.0498	4.2926	3.9514	3.8281	3.6944	4.4310	4.0962	3.1731	3.4721	3.5054
Uchl1	_M[Oxidation (M)]PFPVNHGASSEDSLLQDAAK_.3	#N/A	9.2196	9.2968	9.0786	9.2910	9.3898	9.1089	9.0458	9.4283	9.0063	9.3829	9.2274	9.2710	9.3282	9.5767	9.3603	9.2942	9.1155	9.0305	8.9272	9.0053
Uchl1	_LGVAGQWR_.2	#N/A	9.7598	9.9802	9.6225	9.8435	9.7216	9.6535	10.0053	10.0734	9.4579	9.6803	9.4209	10.0374	9.7017	9.6704	9.4471	9.4153	9.6889	9.8467	9.3983	9.6736
Uchl1	_QFLSETEK_.2	#N/A	9.3646	9.3802	9.9006	9.4094	9.5365	9.4063	9.5470	9.3974	9.5901	9.3929	9.0897	9.4626	9.6195	9.3835	10.0681	9.4505	9.1953	9.2763	9.4701	9.5922
Uchl1	_NEAIQAAHDSVAQEGQC[Carbamidomethyl (C)]R_.3	#N/A	7.6910	7.9702	8.5856	8.1039	7.8735	8.0344	8.1098	7.8589	7.9961	8.1388	7.8654	7.8370	8.0812	8.4587	8.5275	7.7911	7.9295	7.6055	7.9030	8.0838
Uchl1	_FSAVALC[Carbamidomethyl (C)]K_.2	#N/A	7.0532	7.1816	7.8417	7.8842	7.8414	7.5593	7.4593	7.3855	7.6012	7.7695	7.8111	7.6677	7.5323	7.4694	7.8426	7.6713	7.3383	7.3429	7.8649	7.8906
Uchl1	_QFLSETEKLS[Phospho (STY)]PEDR_.2	#N/A	7.1210	7.8260	7.7316	7.6418	6.8105	7.4768	7.2600	7.2670	7.5759	7.5816	7.0376	7.5032	8.1532	7.9940	7.7312	7.8638	7.4197	7.2077	7.5128	7.7995
Uchl1	_QTIGNSC[Carbamidomethyl (C)]GTIGLIHAVANNQDKLEFEDGSVLK_.4	#N/A	4.6283	5.1380	5.2122	4.9477	4.8884	4.7013	3.9001	4.6131	4.8849	4.7031	5.1001	4.5329	5.7502	5.0466	4.2484	3.7025	4.1084	4.6699	5.0351	4.9286

Uchl1	_C[Carbamidomethyl (C)]FEKNEAIQAAHDSVAQEGQC[Carbamidomethyl (C)]R_.4	#N/A	5.9177	6.3269	6.3036	6.5585	6.0019	6.0750	6.3509	6.0102	6.4706	6.3670	6.2205	6.6997	6.4931	6.5993	7.1287	6.2238	6.1673	6.2550	6.2762	6.5004
Uchl1	_QIEELKGQEVSPK_.2	#N/A	7.1396	7.2007	7.7700	7.4773	7.2761	7.2832	7.1451	7.3829	7.2983	7.1854	7.1227	7.4423	7.7063	7.6595	7.8849	7.7614	7.0467	7.1683	7.2429	7.4952
Arpc1a	_TLESSIQGLR_.2	#N/A	8.1646	8.4859	8.4057	8.5034	8.4511	8.1307	8.5116	8.4604	8.0242	8.0406	8.1522	7.8928	8.6315	8.4626	8.2723	8.0406	7.7689	8.3053	8.4229	7.7230
Arpc1a	_WSPLENK_.2	#N/A	5.9624	5.9018	5.3033	5.9594	5.9638	5.8321	5.6965	5.5992	5.6655	5.7791	5.7649	6.3572	5.9003	5.6831	5.5980	5.9930	6.0400	6.7310	6.1781	5.5309
Arpc1a	_[Acetyl (Protein N-term)]SLHQFLLEPITC[Carbamidomethyl (C)]HAWNR_.3	#N/A	3.9058	5.5398	5.4252	5.2625	5.5869	5.5993	5.5349	5.2635	3.9113	4.9140	4.3615	4.9409	4.9220	5.8769	4.8112	4.9578	4.1520	5.4360	4.9674	4.7418
Arpc1a	_FC[Carbamidomethyl (C)]TTGIDGAM[Oxidation (M)]TIWDFK_.2	#N/A	5.8405	5.9101	3.6804	5.0778	5.4949	5.4325	6.0228	5.6765	5.3490	6.0500	5.8998	6.0537	6.1362	5.8091	5.5840	5.4590	6.5367	5.9689	5.9142	5.8325
Arpc1a	_NAYVWSQK_.2	#N/A	4.9915	5.5593	5.1832	4.8496	5.4532	5.5970	5.3303	5.3388	5.0958	4.4264	4.7148	5.2695	5.5688	3.1611	5.3151	5.0666	4.3766	5.4746	5.4042	5.4424
Arpc1a	_IVTC[Carbamidomethyl (C)]GADR_.2	#N/A	4.3510	5.8337	4.5922	5.3725	5.2378	5.6715	5.9044	3.7563	6.1211	4.1616	5.1282	3.8363	4.0767	4.5793	5.9862	3.7131	5.1994	5.6356	5.8877	4.8174
Arpc1a	_EVDEKPASTPWGSK_.2	#N/A	5.8808	6.2736	5.7122	5.7414	5.9750	6.0329	5.6235	6.2940	6.3172	6.0597	5.8811	5.9347	6.0794	6.0517	5.6973	5.9471	6.3219	5.9466	5.8752	5.7688
Arpc1a	_SVQVSTLR_.2	#N/A	3.4622	4.0545	3.8927	3.9101	3.9983	3.9825	3.9073	3.0567	3.9904	4.1751	4.2365	8.7917	3.3771	3.8797	3.7980	4.1063	3.5944	4.2388	4.1669	3.9904
Arpc1a	_NM[Oxidation (M)]S[Phospho (STY)]AMER_.2	#N/A	3.8203	4.5379	5.0797	4.6707	3.1580	4.4980	4.5756	3.2738	3.9648	4.0266	3.3248	4.6208	4.1251	4.1090	3.6345	4.1652	4.0387	3.5214	4.0368	3.9504
Arpc1a	_LAWVSHDSTVSVADASK_.2	#N/A	3.9314	4.0799	3.9549	4.1773	2.7178	3.2496	4.1948	3.5414	4.1960	4.1033	2.7162	3.9694	4.3276	4.3481	3.9814	4.2763	4.2612	3.1757	3.3685	3.4492
Arpc1a	_EHNGHITGIDWAPK_.3	#N/A	3.6618	3.6573	3.1984	3.9224	3.8687	3.9021	2.5843	3.8875	3.0996	3.6553	4.0791	3.3834	3.2650	3.7677	3.2048	3.0239	3.3847	4.3508	3.9994	3.7347
Arpc1a	_LISVC[Carbamidomethyl (C)]YFESENDWWVSK_.2	#N/A	3.0942	4.1909	4.3482	3.8775	3.3011	3.3345	3.7470	3.5123	3.6672	4.4056	3.1458	4.0803	3.8326	4.3353	3.7723	3.9571	4.0500	1.8406	4.3318	4.3023
Ptges3	_HLNEIDLFHC[Carbamidomethyl (C)]IDPNDSK_.3	#N/A	7.5540	7.9047	8.1981	7.7955	7.5924	7.4182	7.5390	7.8426	7.5559	7.9745	7.7046	7.7366	7.8342	8.7044	7.8310	7.7758	8.0350	7.7804	7.7469	7.8995
Ptges3	_DWEDDSDEDM[Oxidation (M)]SNFDR_.2	#N/A	8.8950	12.2641	13.1109	12.0686	4.9066	12.7581	12.4179	4.0388	12.2679	12.5411	7.2207	12.2057	8.5217	12.5189	12.0636	12.3929	12.7491	12.2232	12.2751	12.4052
Ptges3	_SILC[Carbamidomethyl (C)]C[Carbamidomethyl (C)]LR_.2	#N/A	4.5204	6.9551	7.3467	4.4410	7.0334	4.0822	7.4422	4.0449	6.7769	7.4135	6.9001	3.3718	7.1296	3.3938	7.8345	3.3645	5.9595	7.4682	7.4120	7.3390
Ptges3	_LTFSC[Carbamidomethyl (C)]LGGSDNFK_.2	#N/A	5.8062	6.0219	6.0185	5.4936	5.4520	6.1193	5.6548	5.9936	5.6312	5.3556	5.5018	5.9366	5.5293	6.7059	6.4094	5.9130	6.0118	5.8393	5.9604	5.7633
Ptges3	_KGESGQSWPR_.2	#N/A	4.9107	4.6441	4.9065	3.5103	4.9781	5.0362	5.3352	5.6059	5.0562	4.9272	4.8516	4.8483	4.0661	5.1680	4.8677	4.7930	5.5527	5.1918	4.1334	5.2727
Srsf10	_S[Phospho (STY)]RS[Phospho (STY)]FDYNYRR_.3	#N/A	8.2215	8.6864	8.2114	8.6761	7.9838	8.3489	8.4806	8.3061	8.4556	8.4495	8.0969	8.1684	7.8793	8.2795	7.9383	8.3308	8.3241	8.3039	8.4945	8.1043
Srsf10	_S[Phospho (STY)]RS[Phospho (STY)]FDYNYR_.2	#N/A	10.1507	10.0806	9.8612	9.6539	3.8090	9.9874	9.8574	8.7032	9.5394	10.1161	8.5441	9.5769	8.0631	9.9902	8.7555	9.4376	10.0415	9.6793	9.6200	9.6625
Srsf10	_S[Phospho (STY)]FDYNYR_.2	#N/A	3.4076	5.7093	6.3244	6.5240	3.6145	6.2216	6.3960	3.6332	6.1846	6.8180	3.7048	3.6376	3.4384	6.4423	5.1940	6.1792	3.1305	6.6783	6.9578	6.3489
Srsf10	_SRS[Phospho (STY)]WT[Phospho (STY)]SPK_.2	Q9R0U0_S253_M2_Srsf10	3.9068	3.4388	3.4434	3.7039	4.1137	4.1471	3.8021	4.1325	3.7223	3.7174	3.8606	3.1649	3.7425	3.4815	3.8275	3.2689	4.0864	3.9553	3.7981	3.5311
Srsf10	_S[Phospho (STY)]RS[Phospho (STY)]RS[Phospho (STY)]FDYNYR_.2	#N/A	7.0145	6.8199	3.5430	6.9496	4.1440	6.6959	6.3800	4.5583	4.1647	7.0746	4.0882	6.3159	4.3064	7.0525	5.6413	6.5461	6.5541	6.4938	6.5467	3.3840
Srsf10	_S[Phospho (STY)]RS[Phospho (STY)]RS[Phospho (STY)]YER_.2	Q9R0U0_S119_M3_Srsf10	4.4306	6.2608	3.4456	3.6752	3.7683	3.9924	3.7728	4.1347	6.4320	5.1392	4.2062	3.1362	6.5516	3.4837	3.3499	3.2747	5.6455	3.5175	3.8166	3.1609
Srsf10	_S[Phospho (STY)]RS[Phospho (STY)]HSDNDRFK_.3	Q9R0U0_S156_M2_Srsf10	3.9337	3.3854	3.4702	3.6506	4.1406	3.9678	3.7974	4.1593	3.8565	3.1114	4.2309	3.1115	3.9645	3.5083	3.3746	3.2500	3.6566	3.4929	3.7919	3.1856
Rpgr	_SS[Phospho (STY)]SSEILGGNDPTSK_.2	Q9R0X5_S913_M1_Rpgr	3.1285	3.6088	3.2468	3.8740	3.9671	4.1912	3.5740	3.9359	3.6331	3.3349	4.0074	3.2629	3.7411	7.8189	3.1512	3.4734	3.4331	3.7163	4.0153	2.9622
Ak1	_IIFVVGGPGSGK_.2	#N/A	8.8953	9.0609	9.2440	9.4006	9.0440	8.9550	9.1996	9.1808	9.1425	9.1970	8.8298	9.2638	9.4723	9.4455	9.4664	9.3970	9.2517	9.0775	9.3056	9.3381
Ak1	_VDSSNGFLIDGYPR_.2	#N/A	6.7837	7.5557	7.2480	6.9066	6.8697	7.2481	7.3072	7.0912	7.2091	7.0431	7.2207	7.3605	6.3312	7.6447	7.5699	7.0199	7.3519	7.2084	7.4405	6.7294
Ak1	_GELVPLDTVLDM[Oxidation (M)]LR_.2	#N/A	7.5307	6.4607	6.1031	6.2348	7.0869	6.8482	6.6109	6.4833	6.4148	7.0349	6.0657	6.6303	6.0960	6.5578	7.2287	6.9139	6.4615	6.9221	6.9118	6.5556
Ak1	_RLETYYNATEPVISFYDKR_.3	#N/A	6.5310	6.5710	6.0914	6.4346	5.6778	6.3048	6.1167	6.4843	6.5037	6.5619	6.0497	6.5920	6.7673	7.3570	6.9008	6.6943	6.7653	6.4732	6.6131	6.4581
Ak1	_VDDNEETIKK_.2	#N/A	5.7697	5.8392	5.0708	5.4543	5.6913	5.2381	5.5294	5.8680	6.0246	5.7803	5.6864	5.6275	5.9538	5.7574	6.2926	5.7560	5.8879	5.0971	5.1415	5.5830
Ak1	_LETYYNATEPVISFYDKR_.3	#N/A	5.1620	5.4136	5.9456	5.6974	5.2224	5.2155	5.3117	4.7574	4.8329	4.6941	5.2269	4.6153	5.1294	5.0117	5.5283	5.5598	5.0098	5.2588	5.4433	5.1852
Ak1	_YGYTHLSTGDLLR_.3	#N/A	5.7985	5.4160	4.1253	5.4087	5.3222	5.1759	5.6284	5.2113	5.7636	5.1123	5.3142	5.2160	5.1389	4.4285	5.3048	5.2623	5.5490	5.0654	5.0264	4.9949
Ak1	_AEVSSGSER_.2	#N/A	3.8114	4.6881	4.3477	3.8780	5.9600	5.7686	6.1446	5.8771	5.7117	5.2694	5.7596	4.0809	3.8321	5.1594	3.9692	4.6410	5.8281	5.3608	3.4323	5.8808
Dlc1	_SEDS[Phospho (STY)]DEDEPC[Carbamidomethyl (C)]AISGK_.2	#N/A	8.3068	3.4325	8.0195	7.9218	8.0492	7.6098	7.9127	8.0605	7.8634	8.0690	7.8905	7.6109	8.3588	8.5094	7.2614	7.7089	8.2181	7.9573	7.8973	7.7823
Dlc1	_QDPIPGS[Phospho (STY)]PDNSR_.2	Q9R0Z9_S129_M1_Dlc1	9.2024	8.9625	8.8820	3.3817	3.6454	8.6370	8.8045	4.0081	4.1629	4.5140	3.6416	3.5846	4.3284	3.3302	7.9579	8.5909	8.9238	3.2874	8.6471	8.3780
Dlc1	_FSDEGDSDSALDSVSPC[Carbamidomethyl (C)]PSS[Phospho (STY)]PK_.2	Q9R0Z9_S510_M1_Dlc1	4.1664	3.5153	3.4494	3.5572	3.9610	4.0737	5.5797	4.2528	4.1100	4.2076	4.0615	4.9096	4.0580	3.6018	4.4971	3.2671	3.7500	3.3995	3.6985	3.2791
Dlc1	_RSEDS[Phospho (STY)]DEDEPC[Carbamidomethyl (C)]AISGK_.2	#N/A	3.5674	3.7517	4.6048	4.0168	3.7743	3.8077	3.2738	3.7931	3.1940	3.3780	4.1735	1.2150	3.3594	3.8621	3.2991	4.4304	3.2903	4.2564	3.9050	3.8291
Dlc1	_KRSEDS[Phospho (STY)]DEDEPC[Carbamidomethyl (C)]AISGK_.3	#N/A	3.5774	4.1045	4.3618	4.0688	3.3730	3.4855	3.2162	3.5389	3.1976	3.6182	3.0019	3.9259	3.3374	4.1207	3.7876	3.3254	3.6051	4.0074	3.6560	3.9240
Gda	_YTFPTEQR_.2	#N/A	8.5014	8.1613	8.7047	8.3800	8.7142	8.7908	8.6346	8.6230	8.7035	8.7413	8.6609	8.7222	8.6461	8.6904	8.7328	8.8385	8.4241	8.6785	8.6678	8.8023
Gda	_TPPLALVFR_.2	#N/A	8.2222	8.4759	8.3770	8.5879	8.4503	8.5900	8.5806	8.2767	8.0911	8.6525	8.4696	8.8672	8.1762	8.3038	8.3662	8.4515	8.3831	8.7404	8.7614	8.5385
Gda	_DHLLGVSDSGK_.2	#N/A	8.4776	8.0859	8.3195	8.3055	8.3641	8.4854	8.2067	8.5795	8.4406	8.5216	8.5733	8.4229	8.6801	8.5502	8.6782	8.5326	8.3754	8.2757	8.6615	8.5447
Gda	_STDVAEEVYTR_.2	#N/A	7.3600	7.6157	7.9412	8.1665	7.6380	7.7795	7.6846	7.9686	8.1272	7.8822	7.7871	7.7002	7.8981	7.8909	8.1101	8.0378	7.6496	7.9410	7.9815	8.0376
Gda	_IVFLEESSQQEK_.2	#N/A	7.0982	7.6516	7.8171	7.3480	6.6297	7.8868	7.1520	7.2586	7.2621	7.4327	6.9514	7.2194	7.0217	8.0143	7.4173	7.7374	7.3223	7.4477	7.0050	7.8489
Gda	_NIEEVYVGGK_.2	#N/A	6.8236	6.8593	7.4464	6.8750	6.7549	6.8495	6.7970	6.1402	6.5625	7.1584	7.0838	6.8539	6.3099	7.2827	6.8927	7.0967	7.1187	6.1672	6.9571	7.2594
Gda	_EFDALLINPR_.2	#N/A	6.0059	6.4540	6.5720	6.6594	5.7826	6.4664	6.3445	5.8895	6.0420	6.4363	6.3518	6.3556	6.0627	6.7335	6.4861	6.3361	6.5442	6.4885	6.6074	6.2876
Gda	_FLYLGDDR_.2	#N/A	6.3458	5.3334	6.1003	6.2750	6.6474	6.5381	5.8947	6.0810	6.1661	6.6956	6.4761	5.9720	5.7767	6.2540	5.6987	6.3449	6.4606	6.3767	6.1804	6.6521
Gda	_ETTEESVKETER_.2	#N/A	4.1770	4.5383	4.6654	4.3258	4.1383	4.6897	4.8024	3.1156	4.7204	5.0756	3.9394	4.2354	4.0480	4.8440	5.1248	5.5048	4.6177	4.0612	5.0877	4.5878
Gda	_GTFVHSTWTC[Carbamidomethyl (C)]PM[Oxidation (M)]EVLR_.3	#N/A	5.4169	3.5858	3.2699	5.3396	6.2417	6.4363	5.2426	6.5816	4.3355	5.7345	6.1183	5.8583	3.1935	6.2488	6.3170	6.2571	5.8016	6.7213	6.2554	6.0221
Gda	_FVSEM[Oxidation (M)]LQK_.2	#N/A	4.6195	4.8869	4.5864	4.0353	4.3081	4.3479	4.3183	5.0723	2.9682	4.1303	3.2438	4.9677	4.0341	4.6244	5.0887	4.2687	4.4209	4.6891	4.4963	4.0886
Gda	_NYTDVYDKNNLLTNK_.2	#N/A	4.6368	4.5444	5.1562	5.4072	5.0146	4.9246	4.6858	5.8841	4.7534	4.7352	5.2909	4.7507	4.6977	5.3416	5.3700	5.0386	5.2413	5.3751	5.2691	5.3779
Gda	_TVM[Oxidation (M)]AHGC[Carbamidomethyl (C)]YLSEEELNIFSER_.3	#N/A	6.1925	6.0147	5.4054	5.4745	4.9476	5.4543	3.6946	6.0978	5.4586	5.8160	5.4393	5.8076	6.1163	6.1179	5.3070	5.7612	5.5690	5.3236	5.3036	5.7079
Gda	_FTLSC[Carbamidomethyl (C)]TETLM[Oxidation (M)]SELGNIAK_.2	#N/A	3.9830	4.5439	3.5217	3.6641	3.1256	3.6802	3.5736	3.5419	4.0758	4.1170	3.8497	2.8987	4.6989	4.3146	3.7843	3.8396	3.8874	4.4194	3.6157	4.0945
Gda	_AVM[Oxidation (M)]VSNVLLINK_.2	#N/A	3.7300	3.5891	3.2666	3.8543	3.9474	4.7516	3.5937	5.2706	5.1030	5.9516	4.4590	3.3152	3.7608	3.3046	3.1709	3.4537	3.4529	3.6966	5.4163	2.9819
Gda	_ASDSPIDLFYGDFVGDISEAVIQK_.3	#N/A	3.1351	4.1840	4.1725	4.4491	3.3420	3.1385	3.7061	3.4714	3.3799	4.4465	3.1049	3.9101	3.8683	5.2546	3.7314	4.3335	4.3589	3.8241	3.4727	4.1968
Gda	_IGLGTDVAGGYSYSM[Oxidation (M)]LDAIR_.2	#N/A	4.5603	4.2596	4.0969	3.8428	3.0197	3.4893	3.7817	4.6814	3.7019	4.5971	3.4516	4.0632	3.8673	4.3700	3.8070	3.7691	4.0847	3.7485	3.3971	3.8088
Gda	_THDLYIQSHISENREEIEAVK_.4	#N/A	4.4074	4.4125	3.9440	3.1768	3.1135	3.4940	3.9346	3.1322	3.8548	4.4432	3.3334	4.1386	4.4383	3.9820	3.9600	3.7695	4.1303	3.0191	3.3182	3.6593
Foxo1	_ASLQSGQEGPGDS[Phospho (STY)]PGSQFSK_.2	Q9R1E0_S284_M1_Foxo1	5.4013	5.4813	3.4487	5.3116	4.2007	4.6694	4.9624	4.1136	4.2685	3.8043	4.7635	3.4791	5.8676	5.6493	5.1481	5.2650	5.3009	5.0888	5.5918	5.7057
Cetn2	_M[Oxidation (M)]S[Phospho (STY)]PKPELTEDQKQEIR_.3	Q9R1K9_S20_M1_Cetn2	4.4259	4.3940	3.9625	3.1583	3.1320	4.8458	4.2896	3.1507	4.3864	4.1200	3.2223	5.0770	4.4568	4.0005	3.8668	4.2586	5.6861	3.0006	5.4404	4.5291
Mast1	_SRS[Phospho (STY)]LS[Phospho (STY)]PGR_.2	Q9R1L5_S161_M2_Mast1	10.9182	10.7606	11.1423	10.8413	10.3438	10.5693	10.7400	10.6439	10.8543	10.7791	10.6758	10.5579	10.8000	10.6588	9.7036	10.5436	10.8813	10.8545	10.7000	10.6333
Mast1	_HFGS[Phospho (STY)]TESITDEDGGRR_.3	#N/A	6.4285	4.4593	4.0798	4.1458	2.8813	3.1053	3.1590	3.2439	6.6550	3.7499	3.5924	4.3487	3.5643	4.0669	4.2370	3.8884	3.7816	4.0516	6.8762	4.0339
Mast1	_LEEQDS[Phospho (STY)]GGSNTPEQDDTSEGR_.2	Q9R1L5_S346_M1_Mast1	3.1614	6.1224	5.8290	5.6761	3.3683	4.8644	4.9535	5.3362	5.3069	5.3730	5.0953	5.0519	3.1922	4.8263	3.2018	4.0243	5.2595	5.1040	4.1803	4.3004
Mast1	_GGS[Phospho (STY)]PEIKR_.2	Q9R1L5_S772_M1_Mast1	5.5138	6.1888	5.7067	6.3465	5.0339	5.1904	6.0236	3.3191	5.2235	6.0297	4.8431	4.8883	6.1070	4.1689	3.7732	4.7626	5.5468	5.3057	4.7665	3.8461
Mast1	_DSS[Phospho (STY)]PSRDYSPAVSGLR_.3	Q9R1L5_S954_M1_Mast1	3.8625	3.4566	3.3991	3.7217	4.0695	4.0389	3.7263	4.0882	3.7853	3.1826	4.1597	3.1827	3.8934	3.4372	3.3034	3.2246	3.5854	3.5640	3.8631	3.1144
Mast1	_AALS[Phospho (STY)]PVQEHETGRR_.3	#N/A	5.8682	6.5192	3.4950	5.9229	4.0058	5.3716	6.1079	5.3912	3.3546	5.8427	5.3286	5.3837	5.9753	5.0160	3.1127	3.5122	5.6452	5.8827	5.7438	5.7379
Mast1	_AALS[Phospho (STY)]PVQEHETGR_.2	#N/A	5.9600	5.4237	3.7396	6.6533	5.2997	5.7445	5.8394	5.7670	4.0592	5.1807	4.5332	5.1582	5.1619	4.1485	4.2272	5.1514	4.5970	5.6159	3.8354	4.6231
Mast1	_HFGS[Phospho (STY)]TESITDEDGGR_.2	#N/A	3.8931	3.4260	3.4297	3.1755	4.1000	4.1334	4.4489	4.8090	4.3691	3.7037	3.8478	3.1521	4.5346	3.5364	2.9735	3.2552	3.2511	3.0813	4.2307	3.5034
Mast1	_S[Phospho (STY)]PITIQR_.2	Q9R1L5_S968_M1_Mast1	3.9844	3.3346	3.5210	3.5998	4.1914	4.2247	4.3576	4.1229	4.2778	3.7950	3.7564	7.6965	4.4432	3.4450	2.8821	3.3466	3.1597	3.1726	7.8413	8.0112
Mast1	_FSASEASFLEGEAS[Phospho (STY)]PPLGAR_.2	Q9R1L5_S791_M1_Mast1	3.9518	8.0675	3.9244	3.1964	3.2895	3.5136	7.2956	3.1127	4.3107	4.1581	3.1842	4.1582	4.4187	7.1497	3.8288	4.2967	4.1108	7.3034	3.3377	3.6398
Psma4	_TTIFSPEGR_.2	#N/A	7.4185	7.4007	7.6096	7.1473	7.5078	7.3599	7.5988	7.5885	7.2579	7.6845	7.3352	7.4522	7.7170	7.4916	7.6065	7.5915	7.1323	7.5430	7.3775	7.5840
Psma4	_LLDEVFFSEK_.2	#N/A	5.7913	6.3062	5.8129	6.0125	5.9687	6.3156	5.6208	6.0687	6.1577	6.2538	5.9347	5.6164	6.5701	6.2775	5.8225	6.3014	5.9944	6.1315	5.8716	6.1488
Psma4	_QAYTQFGGK_.2	#N/A	3.7387	3.5804	3.2753	3.8455	3.9456	4.1627	3.3950	3.9644	3.6615	3.3064	4.0359	3.3065	3.7696	3.3133	2.6695	3.1008	3.4616	3.6878	3.0093	5.2386
Psmb3	_LYIGLAGLATDVQTVAQR_.3	#N/A	3.8421	3.8453	3.9327	4.0104	3.5995	3.4716	3.7633	3.4662	3.7441	4.9162	4.3448	3.6596	3.7866	4.8055	4.0522	4.2503	4.0039	4.2472	3.5689	4.2998
Psmb3	_DAVSGM[Oxidation (M)]GVIVHVIEK_.3	#N/A	4.1804	3.8224	3.2159	3.5090	3.5182	3.7422	4.0229	3.8808	4.0820	4.3868	2.9555	3.7118	4.1901	3.2030	3.6001	1.4482	4.4185	3.4147	3.0633	3.1700
Psmb2	_FILNLPTFSVR_.2	#N/A	6.2895	6.7050	6.0111	7.1062	6.5551	6.6012	6.4722	6.6212	6.3349	6.5048	6.4103	6.4871	6.3059	6.3590	6.6650	6.3206	6.7476	6.8363	6.7474	6.5569
Psmb2	_YYTPTISR_.2	#N/A	3.1656	7.1489	7.3194	7.2977	6.9974	6.4239	7.2720	3.8989	6.4534	6.5326	6.6299	6.8907	3.7040	7.2910	6.8433	6.6391	6.5670	6.7786	6.7805	7.3171
Psmb2	_[Acetyl (Protein N-term)]M[Oxidation (M)]EYLIGIQGPDYVLVASDR_.2	#N/A	2.6664	3.6544	3.2013	3.9195	4.0126	4.2367	3.5284	3.8904	3.5875	3.3804	3.9619	3.3805	3.6956	3.2394	3.1056	4.2653	3.3876	3.8408	4.0609	4.4175
Psma1	_NQYDNDVTVWSPQGR_.2	#N/A	7.9295	8.0178	8.0540	8.1963	7.7870	8.0890	7.7586	7.9638	8.0747	8.0487	7.8867	7.7870	8.0820	8.4097	7.8057	7.9590	8.0434	7.8228	7.7584	7.9524
Psma1	_ETLPAEQDLTTK_.2	#N/A	7.7642	7.3654	7.1056	7.2051	7.6592	7.5371	7.3629	7.7388	7.6772	7.6670	7.5615	7.3238	7.5952	7.6954	7.8449	7.5232	7.8163	7.2565	7.3873	7.8323
Psma1	_TQIPTQR_.2	#N/A	6.4629	6.4164	4.1286	5.9790	5.8473	5.8994	6.1856	6.5763	6.7645	6.8857	3.6442	6.7285	6.4215	6.4310	6.8366	6.2863	5.7438	6.2009	6.5437	6.6735
Psma1	_AQPS[Phospho (STY)]QAAEEPAEK_.2	#N/A	3.2764	2.6322	5.4304	4.4130	4.1150	4.2110	3.4261	6.6556	3.4852	4.3935	3.8596	3.1150	3.2971	2.2770	3.0033	2.8433	3.5145	4.3187	3.3122	3.7668
Psma1	_AQPS[Phospho (STY)]QAAEEPAEKADEPMEH_.3	#N/A	4.1894	3.4983	3.4846	7.1102	4.0050	4.3715	4.0920	4.1632	4.1312	4.2213	3.6098	3.3234	4.2966	3.5006	3.9322	3.3028	4.2055	3.3232	3.3308	3.3096
Psma1	_IHQIEYAMEAVK_.2	#N/A	4.4692	4.3507	7.2302	3.1150	3.1753	3.4322	3.8728	3.1941	4.3920	4.2798	3.2656	4.0768	4.5001	4.0439	3.8982	3.8313	4.1921	4.4581	3.2564	3.7211
Tagln3	_GFS[Phospho (STY)]EEQLR_.2	#N/A	8.2367	8.5871	8.3804	8.9949	8.3232	8.3406	8.5039	7.4530	8.3633	9.1850	8.7825	8.1756	8.7840	8.6443	8.0704	8.6967	7.9041	8.6310	8.8384	9.0040
Tagln3	_LINSLYPPGQEPIPK_.2	#N/A	9.7192	9.2311	10.1307	9.6173	9.9679	9.4683	9.5403	9.8479	9.4108	9.3958	9.4516	9.3059	9.7535	9.3590	9.8625	9.2381	9.4584	9.4479	8.1674	9.5595
Tagln3	_RGFS[Phospho (STY)]EEQLR_.2	#N/A	9.3961	9.2148	8.9435	8.9095	8.7215	8.6951	8.7484	8.9175	8.2340	9.2264	8.7997	8.5804	9.1973	9.2049	7.3583	8.7332	9.0307	8.7487	8.6762	8.9806
Tagln3	_GEPSWFHR_.2	#N/A	8.3455	7.9308	8.1939	8.2449	8.1460	8.4603	8.0707	7.8777	7.7560	8.3195	7.7133	7.6226	7.2068	8.2740	8.0040	8.1717	7.7669	7.8247	8.2209	8.3499
Tagln3	_TTDIFQTVDLWEGK_.2	#N/A	7.6617	7.2299	8.1547	7.7742	7.4323	7.4261	7.1392	7.2734	7.5737	7.4019	7.2949	6.9853	8.2020	7.6133	7.3409	7.3902	7.4715	7.2804	7.5707	7.1445
Tagln3	_GPSYGLSR_.2	#N/A	6.7370	6.4161	6.6521	7.3554	6.9953	7.1084	7.5258	6.3377	7.2071	7.0590	7.1870	7.2790	6.5097	7.4318	7.8213	7.2865	6.3933	7.3985	7.3318	7.2058
Tagln3	_TLM[Oxidation (M)]ALGSVAVTK_.2	#N/A	6.3808	6.8242	6.7536	6.5884	6.5015	6.0616	6.3248	6.6223	6.9305	6.4764	5.9440	6.5279	6.0271	6.1620	6.7933	6.4162	5.8541	6.4548	6.7241	6.3022
Tagln3	_AAEVYGVR_.2	#N/A	6.1527	6.6094	5.7654	6.8620	6.3954	6.3399	6.9541	3.6908	7.4890	7.1320	6.7366	6.5325	7.0633	6.7631	7.5543	6.9726	5.2362	7.1557	7.0839	7.1327
Tagln3	_WLM[Oxidation (M)]DGTVLC[Carbamidomethyl (C)]K_.2	#N/A	6.2761	6.9822	5.8201	5.7182	6.6662	6.7023	6.8422	7.2390	6.4803	6.3753	6.1756	6.9419	3.6709	6.6184	6.7350	6.7863	6.6293	7.0373	6.9737	6.8281
Tagln3	_LVDWIILQC[Carbamidomethyl (C)]AEDIEHPPPGR_.3	#N/A	4.0769	5.5814	6.3465	5.8741	5.3031	4.9711	4.6315	4.9953	5.4440	5.9362	5.4303	5.2563	3.5517	5.6278	5.1340	3.9385	5.5157	5.1388	4.9689	5.7962
Tagln3	_QM[Oxidation (M)]EQISQFLK_.2	#N/A	6.8678	6.3200	6.4689	6.7919	7.0361	6.5219	6.6255	6.8838	6.6321	6.3825	6.0916	6.5457	6.8495	6.7087	7.1439	6.7075	6.3996	6.7056	6.8740	6.5630
Tagln3	_QGQNVIGLQMGSNK_.2	#N/A	3.1133	5.4440	4.1507	6.3784	3.3202	5.4297	3.7279	3.3390	3.6481	4.4247	3.1267	4.4693	3.8135	6.1866	3.7533	3.9762	4.3370	3.8023	3.1115	4.2832
Atp6ap1	_FY[Phospho (STY)]PVSAR_.2	Q9R1Q9_Y326_M1_Atp6ap1	7.6559	7.6842	7.4029	7.1853	7.1522	7.5287	7.4253	7.4938	7.3777	7.3445	7.5853	7.1850	7.9667	7.6034	6.4660	7.0402	7.6546	7.6052	7.5110	7.5221
Atp6ap1	_Y[Phospho (STY)]WFAMER_.2	Q9R1Q9_Y332_M1_Atp6ap1	4.4807	3.5221	3.5162	3.2087	3.8184	4.1665	3.7226	2.6655	3.7817	3.1862	4.1561	3.4115	4.5014	5.0846	3.2998	3.3248	3.2179	3.1144	4.2638	3.4702
Trim3	_S[Phospho (STY)]PGGPGSHVR_.2	#N/A	9.9578	9.9145	9.9392	9.9886	9.5837	9.5845	9.6145	9.6189	9.4717	10.0479	9.4883	9.5754	9.7708	9.9557	9.0084	9.5955	9.6542	9.5388	9.5170	9.5799
Trim3	_TGS[Phospho (STY)]AELC[Carbamidomethyl (C)]AEITGPDGVR_.2	Q9R1R2_S359_M1_Trim3	6.6490	3.2664	7.0300	6.6471	3.8964	7.1680	6.2459	4.2829	6.7050	4.4932	3.7518	3.8907	6.7284	7.1400	6.0698	6.1074	3.7801	3.3738	3.6729	6.5965
Trim3	_EDS[Phospho (STY)]PGPEVQPMDK_.2	#N/A	3.9879	8.1917	8.8433	8.8825	6.9550	7.7471	8.0739	4.2136	6.7987	6.9756	8.1995	6.7811	4.0188	9.3182	6.7646	3.3500	7.6563	3.4387	6.5381	7.1402
Trim3	_ALRPGDLPPS[Phospho (STY)]PDDVKR_.3	#N/A	3.7134	7.5405	6.9215	6.2965	3.9203	7.5449	6.9786	3.9391	6.7315	6.6862	4.0275	7.0561	3.2134	8.6821	5.5842	6.2103	7.6299	8.3257	6.8843	7.6150
Trim3	_VKS[Phospho (STY)]PGGPGSHVR_.2	#N/A	9.2331	8.9131	8.9299	9.0807	8.8671	8.6773	8.6061	8.9137	8.4173	8.9169	8.5561	8.7232	8.9643	9.0993	8.1887	8.7069	8.7152	8.5856	8.5340	8.6918
Trim3	_REDS[Phospho (STY)]PGPEVQPMDK_.2	#N/A	4.0182	7.6068	8.1710	8.3629	6.1132	6.9676	7.3813	4.8172	6.2066	6.3830	7.3509	6.5796	4.5979	9.0579	6.1013	3.3629	6.6552	5.3146	5.5996	6.3879
Trim3	_ALRPGDLPPS[Phospho (STY)]PDDVK_.2	#N/A	3.7266	6.9481	3.7150	6.0574	4.1991	4.3260	3.9108	4.3911	3.8819	3.6957	3.7667	3.0453	4.0259	3.6028	2.8791	3.5314	5.6865	5.4762	5.4598	3.5698
Septin6	_RNEFLGELQK_.2	#N/A	7.3064	6.9160	7.2140	6.8495	7.0613	7.1440	7.0779	7.2177	6.9915	6.9664	7.2796	6.7525	7.1370	6.8970	7.1119	7.0133	7.1989	7.0360	7.1018	6.9561
Septin6	_SDAISKS[Phospho (STY)]ELAK_.2	Q9R1T4_S192_M1_Septin6	7.0992	7.0910	6.8967	7.0575	7.2293	6.7337	7.1367	7.2373	6.6289	6.8632	6.8440	3.3234	7.5600	7.5246	6.6493	7.3773	7.4558	7.0761	7.1100	6.8381
Septin6	_EAELKEAEKELHEK_.3	#N/A	6.6446	5.8416	5.6725	6.6448	6.7022	6.1852	6.1125	6.4609	6.4918	6.4888	6.8101	6.0369	7.3699	6.5564	6.5451	7.1748	7.5111	6.2934	6.7796	5.8997
Septin6	_NEFLGELQK_.2	#N/A	6.1732	5.5170	5.7480	6.4123	6.0364	6.2151	6.2423	5.7752	5.9742	6.2288	6.6392	5.9333	5.5147	6.2605	6.2010	6.1749	6.4142	6.2795	5.8424	6.1891
Septin6	_AAAELLQSQGSQAGGS[Phospho (STY)]QTLK_.2	Q9R1T4_S416_M1_Septin6	5.5555	5.7167	4.5157	4.3045	4.9661	4.7341	5.2613	4.5111	3.7700	4.4805	4.7832	5.3145	3.5377	5.6702	4.3043	4.9567	4.5197	4.3549	4.8462	4.7843
Septin6	_STLM[Oxidation (M)]DTLFNTK_.2	#N/A	6.4570	6.7149	6.6465	6.8479	6.1520	6.5290	6.6008	6.1875	6.2268	6.4586	6.7653	6.1199	6.1507	6.9815	6.1288	6.5625	6.4826	5.9999	5.9912	6.5318
Septin6	_VNMEDLR_.2	#N/A	3.3796	5.5018	5.5304	6.1216	4.1195	4.5021	5.8607	3.0799	5.5447	3.8040	6.1911	4.7122	3.4901	6.3605	5.8478	3.7245	3.6176	3.4013	4.2391	4.1514
Septin6	_SVSQGFC[Carbamidomethyl (C)]FNILC[Carbamidomethyl (C)]VGETGLGK_.2	#N/A	4.0016	5.2566	3.5382	3.5826	4.2086	4.7701	4.6805	4.9726	5.2307	4.5443	4.7672	3.0436	4.0325	6.2617	4.4262	4.6939	5.9090	4.7541	4.7956	5.7738
Septin6	_TVPLAGHVGFDSLPDQLVNK_.3	#N/A	6.5885	7.3972	5.7598	7.0825	6.3856	5.8110	7.4011	7.6057	5.7853	6.1988	6.9729	7.3870	7.3714	7.4788	6.1938	5.8417	6.5683	7.2001	6.8201	6.6642
Septin6	_DTDPDSKPFSLQETYEAK_.3	#N/A	4.4428	4.4988	4.7436	4.9557	4.0675	4.7084	3.8310	4.2709	4.1610	3.4352	4.5865	4.3655	3.6489	5.0001	4.6684	4.4582	3.5097	4.1945	4.1902	5.0160
Septin6	_QVGEDC[Carbamidomethyl (C)]R_.2	#N/A	4.1861	3.8167	3.2216	3.5033	3.5238	3.7479	4.0172	4.1148	4.0506	4.3924	2.9499	4.1203	3.5674	3.2086	5.2932	3.0302	4.4242	3.4090	3.0576	3.4054
Adam22	_SNS[Phospho (STY)]TETLSPAK_.2	Q9R1V6_S855_M1_Adam22	10.1721	9.4327	10.2630	9.9785	9.8565	3.7039	9.4839	10.3939	9.6987	9.5414	10.0385	10.1152	10.4366	10.3049	9.7026	9.9390	10.5766	10.3678	10.1077	10.2822
Adam22	_SPSSS[Phospho (STY)]TGSIASSR_.2	Q9R1V6_S868_M1_Adam22	9.1714	8.5215	8.1574	9.1041	8.9825	9.0260	8.3598	9.5840	9.0309	7.9893	8.8755	9.0965	9.4640	9.2437	8.7715	3.8306	9.6605	8.5634	8.9816	7.5613
Adam22	_FAISENPLITLR_.2	#N/A	5.3800	5.4760	5.1940	5.7005	5.1946	5.6687	5.7945	5.5544	4.9974	4.7458	5.7374	4.9579	4.9362	5.0090	5.1061	4.4292	6.0773	5.8599	5.2231	5.0308
Adam22	_FQPLGTVC[Carbamidomethyl (C)]R_.2	#N/A	6.4287	6.6023	6.5051	3.2993	6.2129	6.3462	6.7732	6.2804	6.3674	5.6458	6.6246	4.2612	4.3158	6.4980	6.4046	6.5729	6.3424	6.4746	3.4407	6.0411
Adam22	_KLAS[Phospho (STY)]GEC[Carbamidomethyl (C)]K_.2	Q9R1V6_S387_M1_Adam22	5.8646	6.1902	4.6013	5.6417	5.5827	6.5160	6.5839	6.3099	6.2034	5.9161	6.5348	6.4845	6.1949	6.3683	4.0255	4.4099	6.8951	6.3205	6.2986	3.0546
Adam22	_S[Phospho (STY)]NSTETLSPAKS[Phospho (STY)]PSSSTGSIASSR_.3	Q9R1V6_S853_M2_Adam22	4.2313	4.1606	3.9172	3.5139	3.3824	3.5608	3.6272	3.8878	3.6249	3.8020	3.6793	3.8544	3.7579	4.0299	3.8274	3.7060	5.5428	3.9203	3.6658	3.3905
Adam22	_DVLC[Carbamidomethyl (C)]GYLLC[Carbamidomethyl (C)]TNIGNIPR_.2	#N/A	4.6240	3.0305	2.4298	3.3062	3.2994	3.1007	3.6844	4.5037	4.0224	4.0886	3.0744	4.2680	4.3089	3.8526	2.8037	3.6401	3.9837	3.3235	3.4476	3.5299
Adam22	_LSVVYTNTYAK_.2	#N/A	3.3524	3.0759	5.3284	4.3370	4.1909	5.2981	5.5552	3.0527	5.4149	3.5587	3.7836	3.1647	3.5173	5.2594	4.6025	4.0550	3.5904	5.0605	3.8913	5.2500
Adam22	_SNS[Phospho (STY)]WQGNMGGNK_.2	#N/A	4.0921	11.9158	12.4973	12.7723	10.4672	11.3294	11.8448	9.4008	10.7428	10.5535	11.8630	10.5050	8.8241	12.5664	10.1484	7.4446	11.3248	9.9910	10.1528	10.6374
Adam22	_SNGLSHS[Phospho (STY)]WSERIPDTK_.3	#N/A	3.2279	5.7269	4.2653	4.3563	3.4348	3.1727	3.6133	3.4536	3.5335	4.5393	3.0121	3.8173	5.4997	5.8765	3.6387	4.0908	4.4516	4.1987	2.9969	3.9806
Adam22	_SNS[Phospho (STY)]WQGNM[Oxidation (M)]GGNKK_.2	#N/A	7.9877	9.0718	7.9381	8.4235	8.0210	8.5428	8.6986	8.3803	8.3748	8.0334	8.4825	8.1534	8.6152	9.1085	7.4342	3.4079	8.7416	8.4579	8.0428	8.1368
Adam22	_ADAVHLFSGSQFESSR_.3	#N/A	3.2250	4.5787	4.2624	4.0083	3.8351	3.4653	3.6162	3.3815	5.3114	4.8920	3.0150	3.6220	3.7018	4.2045	3.6415	4.0879	3.9192	3.9140	3.5626	4.1185
Adam22	_SNGLSHS[Phospho (STY)]WSER_.2	#N/A	7.6773	7.1878	7.2446	3.5421	6.9357	7.5474	7.1676	7.5936	7.2266	6.7360	8.2773	7.2910	8.1080	7.2823	5.9120	7.7118	7.4567	7.1664	8.0749	6.8025
Adam23	_DNQC[Carbamidomethyl (C)]QYIWGTK_.2	#N/A	4.2289	5.9020	6.3684	6.5544	6.2757	6.2728	5.3228	6.5333	6.5187	6.7036	6.4756	5.8692	6.1893	6.2551	5.6403	5.7155	6.7377	6.0216	5.9563	6.1111
Adam23	_QDGYSC[Carbamidomethyl (C)]NQNQGR_.2	#N/A	4.5483	4.2716	4.0849	4.5368	3.2544	3.3531	4.4120	3.2731	2.9703	3.9976	3.3447	3.9977	4.5792	4.1229	3.9892	3.9104	4.2712	4.3791	3.1773	3.8002
Abcc5	_S[Phospho (STY)]LEC[Carbamidomethyl (C)]QDALETAAR_.2	Q9R1X5_S43_M1_Abcc5	6.7604	6.7214	6.7163	6.8921	6.1971	7.3407	6.7384	5.9946	6.5115	6.5599	6.7788	6.7622	5.7412	6.4487	5.3423	6.1761	7.2889	6.4837	6.9065	6.9043
Abcc5	_NATLAWDSSHSSIQNS[Phospho (STY)]PKLT[Phospho (STY)]PK_.3	Q9R1X5_S509_M2_Abcc5	5.7251	4.4812	3.8753	3.2455	3.0448	4.9590	5.6562	3.0635	3.9235	4.3407	3.4022	4.2074	4.0890	3.9133	4.0287	3.6779	4.9079	6.4443	3.3869	3.5906
Abcc5	_T[Phospho (STY)]RSLEC[Carbamidomethyl (C)]QDALETAAR_.3	Q9R1X5_T41_M1_Abcc5	3.0972	4.3723	3.6335	4.5922	3.9358	4.1599	4.8654	3.9673	3.6644	3.3035	4.0388	3.2942	3.7724	3.3162	3.1825	3.4421	3.4645	3.6850	3.9840	2.9935
Pts	_LHS[Phospho (STY)]PSLSDEENLR_.3	Q9R1Z7_S27_M1_Pts	6.0257	5.9321	5.6170	4.2096	5.2656	3.6149	5.4426	3.2318	5.2902	3.1853	4.3662	5.4375	3.5522	4.0548	5.9966	6.6163	3.0976	5.7626	6.1400	6.3803
Pts	_VFETDNNIVVYKGE_.2	#N/A	5.6536	4.3190	6.6595	6.0132	5.8784	5.5627	3.8411	5.0804	5.5996	6.0221	3.2400	4.9669	4.5318	4.0755	5.9128	5.1693	4.2238	4.4265	5.6169	6.3710
Tfeb	_RS[Phospho (STY)]SFSM[Oxidation (M)]EEGDVL_.2	Q9R210_S465_M1_Tfeb	3.3834	3.1186	5.5849	4.3060	4.2220	4.4461	3.3191	5.7228	6.1795	5.9228	3.7526	3.0080	6.4156	5.9372	5.2104	3.7283	6.4037	5.7550	6.1610	4.2081
Khdrbs3	_FLIPDYNDEIR_.2	#N/A	6.0817	5.8960	3.8387	3.2821	3.0082	3.0416	4.0399	4.5278	5.5955	4.1127	5.6164	5.7290	4.3330	6.3971	5.8201	6.3219	5.3917	3.1244	3.4234	3.5541
Hebp1	_FATVEVTDKPVDEALR_.3	#N/A	6.0354	5.8330	6.5769	6.7255	6.2538	6.5565	6.2579	6.1534	6.5069	6.6686	5.7415	6.5185	6.0803	6.2068	6.8364	6.6529	6.1207	5.8224	6.4034	6.7337
Hebp1	_IPNQFQGS[Phospho (STY)]PPAPSDESVK_.2	#N/A	3.6077	4.3951	5.9250	4.0817	6.0323	3.1695	3.0948	3.4568	3.1539	3.8140	6.8178	5.8471	3.2620	7.5598	4.1728	6.4144	6.8330	4.1955	2.9937	3.9838
Hebp1	_IPNQFQGS[Phospho (STY)]PPAPSDESVKIEER_.3	#N/A	3.7521	4.0164	3.7688	4.2656	3.4323	3.4003	3.8733	3.9979	3.8719	4.0549	3.0335	3.8447	4.0045	4.0436	3.6980	3.5885	3.9483	8.3217	3.2564	3.9011
Hebp1	_EADYVAHATQLR_.2	#N/A	5.4762	5.8860	5.3955	6.2217	5.1981	5.8633	5.3800	5.6919	5.7878	5.7241	4.9646	6.1787	5.7367	5.6736	6.0229	5.9708	5.7211	5.3225	5.6126	5.7378
Myo1c	_HLGY[Phospho (STY)]KPEEY[Phospho (STY)]K_.2	Q9WTI7_Y703_M2_Myo1c	4.2377	6.2882	3.2731	3.4517	6.1289	5.9780	5.1513	6.9250	6.5186	4.5521	6.0393	6.5518	7.2415	6.8649	6.8053	7.2428	6.9744	6.4533	6.3306	6.5443
Myo1c	_DAES[Phospho (STY)]PSWR_.2	Q9WTI7_S408_M1_Myo1c	4.2043	3.7985	3.2398	3.4851	3.5420	4.4429	3.9991	3.9047	4.0596	4.0132	3.5382	3.6880	4.2250	3.2268	3.5763	3.0484	4.4423	3.3908	3.0394	3.1938
Stk11	_IDS[Phospho (STY)]TEVIYQPR_.2	Q9WTK7_S31_M1_Stk11	12.0418	11.9751	12.2908	12.4447	11.4463	11.4505	11.3729	11.5935	11.7009	12.4601	11.6317	11.5990	11.9441	11.9166	10.7982	11.8367	11.9186	11.3692	11.7886	11.8897
Lypla2	_DLAILQC[Carbamidomethyl (C)]HGELDPM[Oxidation (M)]VPVR_.3	#N/A	4.9584	4.5248	4.8265	4.9776	4.7967	4.9463	4.3588	4.7092	5.2364	5.2153	3.7529	4.8991	5.0123	4.9455	4.8114	5.1146	4.6214	4.2392	4.9627	5.0844
Cdh22	_GPSS[Phospho (STY)]PEPDFSVFR_.2	Q9WTP5_S724_M1_Cdh22	3.8281	4.3084	4.0481	4.5736	3.1658	3.3899	4.3752	3.2363	4.4343	4.0344	3.3079	4.0641	4.5424	7.2630	3.9524	4.1730	4.2344	4.4159	3.2141	3.7634
Ak3	_TVGIDDLTGEPLIQR_.2	#N/A	6.4191	6.5187	6.7414	7.1402	6.7043	6.3966	6.5643	6.6899	6.4877	6.5543	6.4346	6.5524	5.8941	6.7866	6.7084	6.9172	6.5565	6.3379	6.4636	6.5334
Ak3	_HLSSGDLLR_.2	#N/A	5.2638	5.0785	5.4250	5.3133	5.2256	5.2862	5.4251	5.4206	4.7579	3.3438	5.2464	4.7956	3.5641	5.3414	6.1273	5.1031	3.5944	5.4996	4.7931	5.3906

Ak3	_EDDKPETVIKR_.3	#N/A	3.3775	4.7049	4.5464	4.1229	4.4524	4.0455	4.8011	3.6870	4.5782	4.0794	4.7474	4.0800	3.4922	4.5368	4.9050	3.9109	2.1536	4.1064	4.6807	4.2141
Ak3	_TLTQC[Carbamidomethyl (C)]SWLLDGFPR_.2	#N/A	4.4821	5.3576	4.7839	3.1021	3.1882	4.9980	4.5422	3.2070	3.7801	4.2927	5.6624	4.8956	3.9455	4.0568	4.6959	3.8442	4.2050	4.4035	3.7532	3.7340
Ak3	_GVLETFSGTETNK_.2	#N/A	5.6996	5.0350	5.8433	6.2886	5.2243	5.7505	3.6079	3.4589	3.1560	4.5447	3.5304	4.5853	3.2641	4.3087	5.5990	5.6412	4.4570	4.1933	2.9915	5.6375
Akap12	_EGITPWAS[Phospho (STY)]FK_.2	#N/A	7.2911	7.5947	7.2466	7.2651	4.0021	7.3025	6.9175	6.3867	6.6001	3.3698	7.4094	7.0418	6.5998	7.1709	5.2367	3.5084	7.4263	6.4110	6.3816	4.4280
Akap12	_GPSEAPQEAEAEEGAT[Phospho (STY)]SDGEKKR_.3	Q9WTQ5_T583_M1_Akap12	7.6172	7.3738	4.0688	5.0528	6.8745	3.6333	7.7024	8.1486	7.6453	3.7970	4.8930	8.8966	9.1919	8.3398	7.7925	7.6078	3.7483	8.1785	5.1090	7.8724
Akap12	_GPSEAPQEAEAEEGATS[Phospho (STY)]DGEK_.2	#N/A	7.4411	7.2366	4.0725	7.1414	6.9494	7.3324	6.9139	7.1901	7.2833	7.1114	7.3043	7.3333	3.5569	7.9405	7.2296	7.3317	7.3997	6.9638	7.0354	7.1970
Akap12	_AEADASGNLTKES[Phospho (STY)]PDTNGPK_.2	#N/A	4.6462	5.4974	5.5371	5.5075	5.6933	4.9442	6.4090	4.7448	5.0064	5.0849	5.7533	6.1959	5.6372	5.4818	5.0974	6.1367	5.7749	5.0522	5.0078	5.9827
Akap12	_EGITPWAS[Phospho (STY)]FKK_.2	#N/A	8.5941	8.4227	7.9850	8.2650	3.8508	8.2869	8.2586	8.4301	7.5156	8.6487	4.1237	8.2312	8.5260	8.6696	3.2674	3.3572	8.6601	8.0745	7.5263	8.2903
Akap12	_EVC[Carbamidomethyl (C)]VSGGDHTQLTDLS[Phospho (STY)]PEEK_.3	#N/A	3.6749	6.5139	4.4554	3.9752	3.4637	3.9801	6.1132	3.6323	4.0168	3.8088	3.5754	3.8265	3.7001	7.5424	3.5349	5.8144	3.7065	6.8108	6.0871	4.7598
Akap12	_EKEPTKPLES[Phospho (STY)]PTS[Phospho (STY)]PVSNETTSSFK_.3	#N/A	3.9938	4.4477	4.0309	3.6953	2.9961	6.7599	3.9090	4.0241	3.8292	4.2804	3.3078	4.2487	3.9946	7.1704	3.9344	3.8465	3.9531	6.4099	3.5121	3.8910
Akap12	_VELPLEDQVGDLEALS[Phospho (STY)]EK_.2	#N/A	3.3902	5.6908	5.9670	4.1735	3.5971	5.9636	5.7958	3.2163	4.9045	4.9638	4.3507	4.3763	3.5367	6.1455	5.5280	4.2531	3.7540	5.5445	5.6957	4.0063
Akap12	_EPTKPLES[Phospho (STY)]PTS[Phospho (STY)]PVSNETTSSFKK_.3	#N/A	4.0286	3.9742	4.5649	3.6608	3.3663	3.5904	4.1748	4.5367	5.2258	4.2349	3.1074	3.8637	4.3419	3.8857	3.7520	4.3735	4.0340	3.1155	3.4145	3.5630
Akap12	_SATLSSTESTAS[Phospho (STY)]GM[Oxidation (M)]QDEVR_.2	#N/A	3.9498	3.9064	3.7027	3.7372	4.8054	6.2354	5.7603	3.8616	3.9165	5.4296	3.3952	3.7626	4.0820	6.8144	6.5701	6.6129	4.0232	6.0314	6.0894	3.5951
Akap12	_EPT[Phospho (STY)]KPLES[Phospho (STY)]PTSPVSNETTSSFK_.3	Q9WTQ5_T265_M2_Akap12	3.9874	3.9837	3.9599	4.2488	3.6596	3.6101	3.7707	3.6745	4.8001	4.4743	3.6174	3.7098	4.1295	3.9818	3.6558	3.7855	3.9733	3.9746	3.5206	3.7810
Akap12	_LS[Phospho (STY)]ADYEKVELPLEDQVGDLEALSEK_.3	Q9WTQ5_S350_M1_Akap12	3.4060	5.3036	4.7044	4.1709	3.9563	4.1066	3.2193	3.4531	3.3562	3.4238	4.0581	3.3428	3.4926	3.6822	3.1912	4.0386	4.2175	4.0464	4.0383	4.0941
Akap12	_C[Carbamidomethyl (C)]QETESNEEQSIS[Phospho (STY)]PEKR_.2	#N/A	3.9401	3.7846	4.0201	3.8101	3.9847	4.2088	3.6895	4.4346	4.1599	3.9442	3.6188	3.5886	3.7812	4.6781	3.4060	3.4910	4.2936	4.2424	4.6763	3.7963
Akap12	_TKEVC[Carbamidomethyl (C)]VSGGDHTQLTDLS[Phospho (STY)]PEEK_.3	#N/A	3.7173	3.6018	3.2538	3.8670	3.9242	4.1842	3.1239	3.9429	3.6401	3.3278	4.0145	3.3279	3.7481	3.2919	3.1493	3.0794	3.4402	3.7093	4.0083	2.9692
Akap12	_AEDSGAEQLASEIEPSREESWVS[Phospho (STY)]IKK_.4	#N/A	3.6851	4.3023	4.0859	3.8985	3.4070	3.3838	3.9182	3.2742	3.8032	4.0284	3.3457	3.7837	3.9113	4.7277	3.6913	4.2435	4.2723	3.7408	3.2080	3.8013
Akap12	_HPEGIVSEVEM[Oxidation (M)]LSS[Phospho (STY)]QER_.3	#N/A	4.3038	3.7514	4.1402	3.4380	3.6415	3.7100	3.9520	4.0042	3.7849	3.8152	3.7575	3.6409	3.9234	4.0574	3.5292	3.9488	3.8419	3.6170	3.6324	3.0149
Akap12	_RPS[Phospho (STY)]ES[Phospho (STY)]DKEEELDK_.2	Q9WTQ5_S613_M2_Akap12	3.3903	3.9288	4.4277	4.1939	3.5972	3.6306	3.4509	3.2162	3.3711	3.2009	4.3506	3.6549	3.5365	4.0392	3.4762	4.2532	3.7539	4.0793	3.7279	4.0062
Trpv2	_LETS[Phospho (STY)]DGDEEGSAEVNK_.2	Q9WTR1_S15_M1_Trpv2	9.1159	8.7814	8.7775	8.7258	7.9131	8.3622	8.2554	8.7926	8.4417	3.6093	8.4352	8.4258	8.7338	8.5339	7.1321	8.0934	9.1178	8.8143	8.2988	8.3576
Trpv2	_NFS[Phospho (STY)]PQIK_.2	Q9WTR1_S47_M1_Trpv2	5.5468	6.5107	5.1634	5.3662	6.0590	5.0238	6.1388	4.6308	5.5824	6.3176	6.1184	5.6476	3.8870	4.9390	3.8268	5.6473	5.7167	5.9548	5.3608	5.4748
Trpv2	_NSAS[Phospho (STY)]EEDHLPLQVLQSH_.3	Q9WTR1_S743_M1_Trpv2	3.6240	3.6951	3.1606	3.9602	3.8310	3.8643	3.2171	3.8497	3.1374	3.4346	4.1168	3.4212	3.3028	4.5048	3.2425	2.9861	3.3469	3.8800	3.9616	3.7724
Cdh13	_INENTGSVSVTR_.2	#N/A	3.9589	4.2418	3.4955	5.6928	4.1259	3.7846	4.1399	4.6133	6.2559	3.7695	4.5563	3.8409	4.7332	3.5336	7.3811	4.6188	3.6818	3.7334	4.6853	5.1437
Cdh13	_ETIATYQLYVETTDASGK_.2	#N/A	3.3330	3.9861	4.3704	4.2513	3.5399	3.0676	3.1967	3.5587	3.2558	3.7121	3.6302	6.2982	3.3638	4.4085	4.2747	6.1883	7.1406	4.0936	4.3926	4.0857
Tenm1	_ANS[Phospho (STY)]ALS[Phospho (STY)]LTDTDHER_.2	Q9WTS4_S142_M2_Tenm1	4.4507	5.4336	4.5139	6.0661	3.1568	3.4507	4.8913	3.1756	4.3736	5.6133	3.2471	4.0953	4.4816	4.0254	3.9166	3.8129	4.0178	2.9758	3.2748	3.7027
Atp6v1g2	_[Acetyl (Protein N-term)]ASQTQGIQQLLQAEK_.2	#N/A	8.4470	7.9403	8.4259	8.4195	8.4741	8.1189	8.1694	8.3392	8.4102	8.2540	8.6272	8.3086	8.4753	8.6692	8.5935	8.2940	8.6201	8.0969	8.2625	8.4247
Atp6v1g2	_[Acetyl (Protein N-term)]ASQTQGIQQLLQAEKR_.3	#N/A	6.5683	6.8613	6.5296	6.9174	6.9719	6.9459	7.1342	7.6082	6.6992	6.8462	6.8517	7.2611	7.4109	7.3360	7.0200	7.0721	6.9444	6.7965	6.9068	7.0463
Atp6v1g2	_EREQEFQSK_.2	#N/A	6.2770	6.0095	6.1478	6.4521	6.2231	6.0079	6.1387	6.0037	5.9936	6.2029	6.3391	6.6596	7.1020	6.4692	6.8478	6.4472	5.5958	6.2913	5.7588	6.5566
Atp6v1g2	_VLAQLLGM[Oxidation (M)]VC[Carbamidomethyl (C)]EVRPQVHPNYR_.3	#N/A	6.6837	6.5417	6.1229	6.0898	5.9915	6.0787	6.1281	6.1201	6.2861	5.5048	6.3507	5.7427	6.7014	6.4420	5.6334	5.8686	6.1029	6.0748	6.1154	6.0256
Atp6v1g2	_QVQGM[Oxidation (M)]QSSQQR_.2	#N/A	3.9915	4.7046	5.8134	3.5237	4.1872	5.1868	4.8857	4.3454	4.3667	4.0886	5.4410	3.8180	4.2888	5.5334	5.6514	5.3639	4.0233	4.2913	5.0000	3.8809
Atp6v1g2	_EEAQM[Oxidation (M)]EVEQYRR_.3	#N/A	6.3114	6.3263	6.1553	6.8162	6.5124	6.7313	6.9854	6.4618	6.6616	7.1430	6.5840	6.7084	7.4398	6.3556	8.0557	7.4391	6.3174	7.4125	6.6346	7.0749
Atp6v1g2	_QQAAM[Oxidation (M)]GS[Phospho (STY)]QGNLSAEVEQATR_.2	#N/A	3.6767	4.2334	4.2951	4.3231	4.5736	3.7842	4.6811	5.0326	4.8761	5.5435	3.8263	4.3935	3.6249	5.1427	3.9323	4.6953	3.9837	4.1614	4.5472	4.4928
Atp6v1g2	_QQAAM[Oxidation (M)]GSQGNLSAEVEQATRR_.3	#N/A	3.4891	3.6806	3.9076	4.1042	3.6960	4.4425	3.8807	3.7147	3.2813	3.2997	4.2608	4.2846	5.0558	3.9457	4.5554	4.1682	3.2120	4.5629	4.7734	3.8730
Atp6v1g2	_EEAQMEVEQYR_.2	#N/A	3.4629	3.8562	4.5003	4.1214	3.6698	4.4386	3.3266	3.6885	3.3857	3.5822	3.7601	3.5823	3.4937	4.5383	3.4037	4.3258	3.1858	3.9637	4.2627	4.2156
Phf2	_RPSASS[Phospho (STY)]PNNTAAK_.2	Q9WTU0_S1060_M1_Phf2	3.2038	3.2982	3.7401	6.0867	4.0423	5.7880	3.4988	4.4050	3.5578	3.4101	3.9322	3.1877	3.6659	3.2097	3.0760	3.5487	3.4418	3.7915	4.0906	4.3878
Phf2	_DSDYVYPSLES[Phospho (STY)]DEDNPVFK_.2	Q9WTU0_S876_M1_Phf2	3.7119	3.9797	5.5944	3.9257	3.5348	3.8745	3.6129	3.6901	3.6002	4.0186	3.5910	3.8061	3.7375	3.8525	3.4442	3.6006	3.7236	3.8002	5.1373	4.3351
Phf2	_RKGS[Phospho (STY)]DDAPYSPTAR_.3	Q9WTU0_S893_M1_Phf2	4.4606	4.3593	3.9972	3.1236	3.1667	3.2000	4.3724	3.1854	3.8016	4.2712	3.2802	4.0854	3.9670	4.0352	3.9068	3.8227	4.1835	3.6488	4.6041	4.4367
Scn8a	_ELS[Phospho (STY)]EGEEKGDPEK_.2	Q9WTU3_S504_M1_Scn8a	7.2627	6.7047	6.8072	6.3151	6.3918	6.8623	6.5957	5.4859	6.2889	6.9441	2.9932	6.1028	3.6800	6.5033	3.6197	5.2148	6.6921	6.3547	6.4384	5.8548
Scn8a	_VFKS[Phospho (STY)]ES[Phospho (STY)]EDGMR_.2	#N/A	3.4999	5.6746	6.6954	6.2059	3.7068	4.5082	5.5704	3.1066	4.0240	3.3105	4.9382	3.5453	3.4269	5.8892	3.3667	4.3628	3.6443	4.1889	3.8375	3.8966
Scn8a	_FRDPGSENEFADDEHS[Phospho (STY)]T[Phospho (STY)]VEESEGRR_.4	Q9WTU3_S589_M2_Scn8a	3.2334	3.2685	3.7697	4.4560	4.0720	3.9130	3.6111	4.4346	4.6549	3.4833	4.0682	3.1580	3.2541	3.7568	3.0463	5.0595	3.4715	4.3617	4.0103	3.7238
Scn8a	_VFKS[Phospho (STY)]ES[Phospho (STY)]EDGMRR_.3	#N/A	4.0191	3.9837	4.5554	3.6703	3.3568	3.1273	3.9541	3.7195	3.8744	4.1984	3.3530	3.8732	4.0398	4.5425	3.9795	3.7500	4.2571	3.5760	3.2246	3.0086
Mapk9	_NYVENRPK_.2	#N/A	5.7751	3.2729	5.7976	5.3681	3.8839	5.4440	5.7969	5.9595	5.2103	5.4888	5.6012	3.1623	3.2498	5.2819	5.6068	5.2519	5.4851	5.7345	5.2879	5.7363
Mapk9	rbamidomethyl (C)]TNFM[Oxidation (M)]M[Oxidation (M)]T[Phospho (STY)]PY[Phospho (STY)	#N/A	3.8815	6.3163	3.4180	3.7166	3.8982	4.6756	3.6404	4.1071	5.5020	3.8410	4.1992	3.1776	3.7885	5.5567	3.3375	5.1618	3.6044	5.4680	3.8580	3.7499
Mapk9	_DYIDQWNK_.2	#N/A	3.8645	3.4546	3.4482	3.7198	4.0714	4.1047	4.0483	3.6692	4.3978	3.6750	3.8764	3.1808	3.8953	3.4391	3.0021	3.2266	3.5874	3.5621	3.8611	3.1164
Prkra	_EDS[Phospho (STY)]GTFSLGK_.2	#N/A	9.6336	9.2272	9.7723	9.8436	9.0954	9.6197	9.2683	3.5994	9.5367	10.2509	9.8878	9.3707	10.0117	9.9603	8.9317	9.6973	9.5244	9.4592	9.7138	9.8277
Prkra	_AEAPPLQREDS[Phospho (STY)]GTFSLGK_.3	#N/A	3.8825	4.1483	3.9936	4.1286	3.2202	3.4443	3.9322	3.4863	6.3638	6.5820	5.6654	4.0377	4.1866	4.0148	3.9260	3.8022	4.1671	3.9508	5.9152	3.8057
Prkra	_HRAEAPPLQREDS[Phospho (STY)]GTFSLGK_.4	#N/A	3.3772	3.1248	3.9135	4.3122	4.2158	4.4398	3.3253	3.0776	3.3844	3.5835	3.7588	6.5155	3.4925	8.3783	2.9025	3.7221	3.6152	3.9649	4.2640	4.2144
Skp1	_GKT[Phospho (STY)]PEEIRK_.2	#N/A	11.6698	11.7964	11.7782	11.7546	11.5453	11.3954	11.3780	11.9356	11.5072	11.6035	11.3845	11.6140	12.1956	12.1156	11.7864	11.7816	11.8637	11.5820	11.5881	11.7308
Skp1	_GKT[Phospho (STY)]PEEIR_.2	#N/A	9.8717	9.7733	10.0022	9.9144	9.7078	9.6633	9.6202	9.8676	9.5935	9.8006	9.6518	9.5191	9.9364	9.7207	9.6554	9.7257	9.7704	9.5510	9.6282	9.6790
Skp1	_TDDIPVWDQEFLK_.2	#N/A	8.2519	8.1594	8.7125	8.3523	8.2870	8.2497	8.0297	8.0590	8.4259	8.3905	8.4739	8.0762	8.0585	8.3251	8.6415	8.4288	8.3680	7.6958	8.1929	8.3318
Skp1	_T[Phospho (STY)]PEEIRK_.2	#N/A	6.5080	7.1829	6.9584	7.2510	7.3787	7.4418	6.9772	6.4171	6.7666	7.2863	7.2152	6.8828	7.2182	7.5795	6.8612	6.7297	7.1250	6.7325	6.7524	7.1402
Skp1	_RTDDIPVWDQEFLK_.3	#N/A	6.0136	6.5955	6.8575	5.9260	5.2271	4.5895	6.1837	6.6190	5.2296	4.1762	4.9363	6.8096	6.0465	7.3919	6.5367	4.9418	5.8073	6.1691	5.5791	5.0353
Skp1	_LQSSDGEIFEVDVEIAK_.2	#N/A	8.0506	7.7602	6.8512	6.8168	7.2381	6.2076	6.8298	7.4143	6.6543	7.7054	6.8339	6.8337	8.0386	8.0417	6.9278	7.4803	7.4827	6.3296	6.8980	7.4883
Ptpn4	_S[Phospho (STY)]PSKPLAR_.2	Q9WU22_S358_M1_Ptpn4	8.8283	3.2906	8.4571	3.5558	7.3346	7.3759	8.0559	4.2542	7.0962	8.6445	7.9581	6.9220	4.3992	7.5850	2.8381	7.4023	7.1727	7.7973	3.6971	7.4337
Ptpn4	_NSS[Phospho (STY)]FTQEATR_.2	Q9WU22_S403_M1_Ptpn4	7.4399	7.3546	7.9838	7.6575	6.4150	6.2853	6.4734	7.2288	6.9502	7.7333	4.1342	6.4794	3.8679	6.8559	5.5200	6.9480	6.7212	6.4968	6.4166	3.0889
Pfdn5	_IQPALQEK_.2	#N/A	3.4520	3.8671	5.8566	5.3432	6.0178	4.7732	5.5059	5.3524	5.9321	5.6673	5.3135	6.0025	5.7567	5.2260	6.6896	5.6336	5.0125	5.4549	5.7863	3.9445
Pfdn5	_NQLDQEVEFLSTSIAQLK_.3	#N/A	3.1552	3.3468	3.6915	4.5342	3.9937	3.9912	3.5474	4.3564	3.0104	3.3615	3.9808	3.9988	3.1759	3.6785	2.8427	3.5001	3.3932	4.4399	4.0885	3.6455
Lemd3	_VLLGFSSDES[Phospho (STY)]DVEASPR_.2	Q9WU40_S140_M1_Lemd3	3.7165	3.7073	3.9954	3.8166	3.9234	3.9568	3.9718	3.8836	3.8920	4.2694	3.3706	3.4334	4.0574	3.9170	3.2548	3.8210	3.8254	3.6967	3.6408	3.7723
Ncor2	_EGT[Phospho (STY)]PPPPPPPR_.2	Q9WU42_T1350_M1_Ncor2	9.4729	9.7138	8.9314	9.5453	9.4840	9.7094	9.3222	9.3797	9.5095	9.7066	9.2287	9.5198	9.4688	10.2037	8.8397	9.5059	9.8425	9.7404	9.4635	9.4649
Ncor2	_S[Phospho (STY)]PGNTSQPPAFFSK_.2	Q9WU42_S2215_M1_Ncor2	8.5137	8.4843	8.6540	8.6752	7.9219	8.4109	7.7487	8.0446	8.4160	8.6599	8.6164	7.9587	7.7968	8.8595	7.4464	8.1574	8.8755	8.0382	8.2541	8.3970
Ncor2	_SDHALTS[Phospho (STY)]PGGGGK_.2	Q9WU42_S2351_M1_Ncor2	8.4218	8.8756	3.4925	8.3753	3.7947	8.4160	3.7464	8.8411	8.3809	8.5184	8.0673	3.4353	8.7582	9.0535	8.2348	8.7337	8.6621	8.5568	8.4264	3.4465
Ncor2	_TDDT[Phospho (STY)]S[Phospho (STY)]GEDNDEKEAVASK_.2	#N/A	3.8138	3.5052	3.3504	3.0963	4.0208	4.1675	5.0340	4.2935	4.2266	3.6244	3.9270	3.2314	3.1130	5.0551	4.7641	3.1760	3.3303	4.8715	4.3769	3.9701
Ncor2	_PASHTHQHS[Phospho (STY)]PIS[Phospho (STY)]PR_.3	Q9WU42_S1816_M2_Ncor2	4.1710	5.0376	4.0822	4.5395	3.1318	4.5045	4.3320	3.2704	4.8299	5.3138	3.5189	4.8339	4.5765	5.9661	3.9865	4.7712	5.2225	4.3818	5.0650	4.7084
Ncor2	_LEPVS[Phospho (STY)]PPS[Phospho (STY)]PPHADPELELAPSR_.3	Q9WU42_S149_M2_Ncor2	4.0984	4.5787	3.7778	3.3430	3.4361	3.6602	4.1050	4.4669	8.4126	4.3047	3.0376	3.7939	4.2721	9.9997	3.6822	2.9425	3.9641	9.5132	3.4844	3.4932
Ncor2	_EKTDDT[Phospho (STY)]S[Phospho (STY)]GEDNDEKEAVASK_.2	#N/A	5.6927	3.2804	3.6779	5.3475	3.9385	3.8925	4.9882	5.7954	5.7385	4.1127	4.1995	5.3988	3.6975	5.9573	3.2110	3.4448	3.6146	5.3363	3.8557	3.5310
Pdcd6ip	_LLDEEEATDNDLR_.2	#N/A	3.2553	6.2125	6.4134	6.8013	5.6376	6.2197	6.0655	6.3741	6.0541	6.5384	6.4368	6.0769	6.1723	6.2737	6.3015	6.3198	6.5647	5.8913	6.1623	6.5947
Pdcd6ip	_STSVVEQGGIQTVDQLIK_.2	#N/A	3.4224	3.4950	3.3782	4.3623	4.5663	4.3898	1.4629	5.3418	3.4345	4.1405	3.3036	4.2512	5.5552	4.0330	3.5917	4.4626	4.6358	4.1006	4.1822	4.6414
Pdcd6ip	_LANQAADYFGDAFK_.2	#N/A	3.8407	3.2304	4.3340	3.1986	3.0167	4.3330	3.7929	4.1911	3.6552	3.7268	3.8247	4.7531	4.5115	3.5133	3.3552	3.3348	4.1407	3.1044	4.5220	3.9936
Pdcd6ip	_FYNELTEILVR_.2	#N/A	3.1402	3.3618	3.6765	4.4264	3.9788	3.6513	3.5623	3.9243	3.9520	3.3709	3.9958	4.3742	3.7295	2.4826	3.1395	3.4851	3.4215	4.5501	3.5506	4.4514
Pdcd6ip	_NIQVSHQEFSK_.2	#N/A	4.4977	3.5051	4.2837	4.5158	4.0409	4.1495	3.4290	4.1981	4.3530	3.7198	1.1068	4.5600	4.5184	3.5203	2.9573	3.3418	3.2349	3.5550	4.2468	3.4873
Pfkp	_[Acetyl (Protein N-term)]S[Phospho (STY)]DLDSSSSSAYPK_.2	Q9WUA3_S2_M1_Pfkp	9.0770	8.7884	8.5185	8.7988	5.5800	8.3680	3.4405	8.0191	8.5128	8.9552	8.0276	8.0128	8.1200	8.3080	7.4238	8.6457	8.9141	8.3752	8.3824	8.4214
Pfkp	_DLLFQPVAELK_.2	#N/A	6.8382	6.9035	7.1802	6.9064	6.8904	6.9370	6.9068	7.1929	6.7609	6.3452	6.6825	6.0783	6.9982	6.1932	6.8146	6.3994	6.5058	6.8820	6.7992	6.3483
Pfkp	_YLEHLSGDGK_.2	#N/A	7.0290	6.5251	6.8079	6.5105	6.5259	6.8919	6.8816	7.2079	6.7201	6.5342	6.4715	6.3871	7.2291	6.2226	6.1823	6.4863	6.2367	6.5997	6.6754	6.3429
Pfkp	_NVLGHM[Oxidation (M)]QQGGAPSPFDR_.3	#N/A	7.1240	6.8930	6.2185	6.1838	6.9978	6.7047	6.2041	7.1599	7.0028	7.1470	6.6672	6.9400	6.6935	6.1043	6.7977	6.9112	7.0031	6.6634	6.7027	6.6712
Pfkp	_EIGWADVGGWTGQGGSILGTK_.2	#N/A	5.4087	5.3533	4.8772	5.5033	4.9768	5.1615	5.1809	4.4395	4.9569	5.0393	5.3169	5.0651	5.4157	5.6191	4.8535	4.8976	5.9482	4.9610	4.8086	4.8638
Pfkp	_FVSDDSIC[Carbamidomethyl (C)]VLGIC[Carbamidomethyl (C)]K_.2	#N/A	6.5030	5.7951	6.7025	5.4077	5.9518	6.2744	6.0220	6.0443	3.4690	6.4480	5.9765	5.7519	3.6345	5.0762	5.8376	2.1866	5.6778	5.8967	5.8404	5.4310
Pfkp	_LNIIIVSEGAIDM[Oxidation (M)]QNKPITSEK_.3	#N/A	5.3819	5.2946	6.1134	5.2755	5.5258	5.3805	4.4161	5.5897	4.9393	5.5528	4.5130	3.6091	5.6911	4.6090	4.1486	4.3407	5.9546	5.1904	4.6412	4.3511
Pfkp	_KVTDFEHR_.2	#N/A	3.4774	5.6723	7.2517	4.2119	5.8101	6.3162	6.0925	5.6952	6.0683	6.4113	6.4562	4.4148	3.3922	5.4230	5.1519	5.8880	6.4886	6.0570	6.1076	5.3970
Pfkp	_IIEVVDAIM[Oxidation (M)]TTAQSHQR_.2	#N/A	4.5797	3.9371	3.7325	4.0306	3.7196	4.0364	3.4079	4.1449	3.2698	4.5230	4.4041	4.1034	4.2185	4.4919	3.8176	3.8787	3.3501	3.8322	3.4762	4.1068
Pfkp	_LGITNLC[Carbamidomethyl (C)]VIGGDGSLTGANLFR_.2	#N/A	2.8117	4.1937	4.3454	3.8802	2.7094	3.3709	0.7160	1.7529	4.4533	4.0155	2.4482	1.8401	4.5614	2.3208	0.3985	1.2830	4.2534	1.2167	3.1951	2.4467
Pfkp	_WDC[Carbamidomethyl (C)]VSSILQVGGTIIGSAR_.2	#N/A	3.9979	3.2802	3.5345	3.6133	3.5443	3.8417	3.7105	4.1064	3.6308	3.6685	3.7699	3.9146	3.7962	3.7450	3.7359	3.3600	3.7964	3.2259	3.3891	3.4256
Rbck1	_ALLS[Phospho (STY)]PEDYQR_.2	Q9WUB0_S344_M1_Rbck1	8.1918	8.5155	4.4026	8.6821	3.5721	8.7625	8.2700	3.2413	3.3962	3.1758	8.2114	8.5903	8.7609	9.1701	3.5014	8.4247	8.6875	8.5418	8.4406	8.6515
Pygm	_LITAIGDVVNHDPAVGDR_.3	#N/A	4.8014	6.0189	3.3862	6.7757	4.4371	5.6703	5.3319	5.9689	5.4928	5.6745	5.1272	5.4862	4.5781	4.5945	4.2281	5.4050	5.3497	5.3478	4.8565	5.1284
Pygm	_IGEDYISDLDQLRK_.3	#N/A	4.9519	5.2507	2.7976	4.8078	4.5055	4.5551	5.0414	4.0535	5.3166	3.0987	4.2436	3.9473	3.9773	5.1238	3.1934	3.2373	4.5410	4.7477	3.8041	3.5230
Pygm	_[Acetyl (Protein N-term)]SRPLSDQDKR_.3	#N/A	4.1718	1.7739	3.2072	3.4164	3.5095	3.7336	4.0316	4.3935	4.0907	3.2731	2.3209	3.7205	4.1987	3.7425	3.6088	3.0158	3.8908	3.2587	3.5577	2.7785
Ly6h	_QC[Carbamidomethyl (C)]QPTDTVC[Carbamidomethyl (C)]ASVR_.2	#N/A	6.3884	6.6044	6.8376	6.7933	6.5073	6.8563	6.6239	6.7094	7.0426	3.8775	6.5889	6.8455	6.5711	6.4272	7.0291	6.1683	6.4942	6.2658	6.6482	6.1128
Stub1	_WNS[Phospho (STY)]IEER_.2	Q9WUD1_S150_M1_Stub1	4.2907	5.6665	3.3261	5.1037	4.9591	5.8641	5.4233	5.8533	5.8523	5.6741	6.5224	6.1541	6.2576	6.4393	6.0125	6.4490	6.0461	5.9709	6.1461	6.4912
Stub1	_LGTGGGS[Phospho (STY)]PDKSPSAQELK_.2	#N/A	7.7937	8.1149	7.9197	8.2422	7.1693	7.8155	7.9226	7.8753	7.8726	8.2123	7.8831	8.0674	8.4191	8.6360	7.1074	7.3996	8.2904	7.9783	7.8490	8.1235
Casp8ap2	_KNS[Phospho (STY)]PGVSHSSQK_.2	Q9WUF3_S1818_M1_Casp8ap2	9.9580	9.9108	9.8256	9.7289	9.4260	9.3087	9.6945	9.7056	9.4151	9.6017	9.3343	9.3811	9.9373	9.6533	8.2508	9.2518	9.7192	9.6118	9.3272	9.2267
Tmem115	_TDS[Phospho (STY)]PLPLEEASTPPGK_.2	Q9WUH1_S320_M1_Tmem115	6.5575	7.5755	6.7692	4.1325	4.0199	6.2529	7.0664	6.7793	6.2869	3.4875	6.9929	5.4538	3.4286	7.0296	3.1268	4.1030	7.4181	4.1105	6.7637	6.0884
Eif4h	_EALTYDGALLGDR_.2	#N/A	6.8439	6.4883	7.0287	6.9471	6.6740	6.8611	6.4579	6.5111	6.7650	6.6752	6.9780	6.2935	6.2919	7.0165	7.0863	6.2251	6.5389	6.2405	6.1470	6.8599
Eif4h	_[Acetyl (Protein N-term)]ADFDTYDDR_.2	#N/A	6.6986	6.5976	6.9748	6.6686	5.7955	6.5069	6.2317	5.5248	6.6223	6.7697	5.9488	6.8127	6.8701	6.6532	6.9351	6.8671	7.0349	5.6759	6.4648	6.6255
Eif4h	_TVATPLNQVANPNSAIFGGAR_.2	#N/A	4.6947	4.3966	4.0937	5.2679	4.3514	4.9419	4.5803	4.2257	4.8224	4.7928	4.5034	4.7483	4.5460	4.8828	5.0122	4.4149	3.7657	4.5349	4.0185	3.5962
Eif4h	_GSNM[Oxidation (M)]DFREPTEEER_.3	#N/A	4.4323	4.6721	4.4763	4.5092	4.5238	5.1630	4.9524	4.5160	4.3143	5.2643	5.0601	4.3637	5.5876	5.2760	6.0558	5.2878	3.6803	4.3947	4.9779	5.2013
Eif4h	_DKDTDKFK_.2	#N/A	3.1380	3.3639	3.6743	5.1247	4.5699	4.1806	4.9821	4.3393	5.1819	4.9797	4.8285	4.6699	3.1588	4.0895	5.4959	5.1139	4.9369	4.0953	4.1056	5.0187
Eif4h	_AYSSFGGGR_.2	#N/A	4.3829	3.9787	4.5604	3.6653	3.0890	3.1223	4.7542	3.7244	3.8793	4.1935	4.1720	3.8682	4.0448	4.5474	3.9696	3.7450	4.2621	3.5710	4.5575	4.2659
Zbtb18	_TVAS[Phospho (STY)]PC[Carbamidomethyl (C)]SSTESLSQR_.2	Q9WUK6_S214_M1_Zbtb18	4.0226	7.6528	3.5592	3.5616	4.2295	4.2629	6.6939	4.2483	4.2396	7.5456	6.9143	3.0226	4.4050	3.4069	2.8439	3.3847	7.3257	3.2108	7.0324	3.3739
Arl3	_ILLLGLDNAGK_.2	#N/A	6.1422	6.4403	6.2536	6.5606	6.0297	6.3778	6.2422	5.9629	6.0909	6.2796	6.1160	6.6995	6.1587	6.6452	5.9942	6.1768	6.4112	6.5037	6.2991	5.8491
Arl3	_LNVWDIGGQR_.2	#N/A	3.7157	5.9100	6.1277	5.8922	3.0534	5.8970	6.2257	6.3128	5.7223	4.8805	5.5137	6.0074	5.7171	6.3339	6.5228	5.4719	6.1180	5.6282	5.6381	5.9552
Arl3	_QDLLTAAPASEIAEGLNLHTIR_.3	#N/A	3.9584	4.0444	4.4947	3.7310	3.2961	4.8890	4.2450	3.1061	4.3040	4.1647	3.1776	3.9339	4.4121	4.9718	3.8222	4.5649	4.1964	3.0453	3.8881	3.6332
Coro1b	_LEEQLGR_.2	#N/A	6.0770	5.7678	5.3105	4.9372	5.2261	4.8081	5.0920	5.1304	4.8227	4.8161	4.7783	4.7483	3.9773	4.7164	5.9120	5.3754	4.9980	5.5539	4.8889	4.8700
Coro1b	_VTWDSTFC[Carbamidomethyl (C)]AVNPK_.2	#N/A	4.3878	4.4322	3.9244	3.1965	3.0939	3.3063	3.9542	3.7195	4.4158	3.6613	3.3531	4.1583	4.0399	4.5425	3.9796	3.7499	4.2572	3.5759	3.5755	3.0087
Coro1c	_NDQC[Carbamidomethyl (C)]YDDIR_.2	#N/A	3.1907	4.9002	4.2358	6.0579	4.9494	5.3075	5.0527	5.0114	5.0626	4.5881	5.1370	5.1266	5.2451	4.9163	4.5555	5.6056	4.4803	5.0825	5.0825	4.4127
Coro1c	_VTWDSSFC[Carbamidomethyl (C)]AVNPR_.2	#N/A	3.3394	4.0906	4.5368	4.0848	4.5688	3.7899	4.3460	4.5668	3.0242	4.6468	3.7966	3.5458	3.5303	2.5809	3.6743	3.6843	4.6413	3.9745	4.2262	4.2120
Suclg1	_LIGPNC[Carbamidomethyl (C)]PGVINPGEC[Carbamidomethyl (C)]K_.2	#N/A	9.2041	9.4516	9.1875	9.4200	8.9068	9.0014	9.2928	9.2225	9.1482	9.1241	9.0606	9.3616	9.5916	9.5669	9.1658	8.9723	9.2314	9.0328	9.1024	8.9219
Suclg1	_HLGLPVFNTVK_.2	#N/A	7.4675	8.0379	7.7029	8.2490	8.3268	7.8336	7.8976	7.7793	7.3093	7.9783	7.7781	8.0337	8.2349	8.1186	8.0886	8.0720	7.5143	8.2804	7.9154	8.1255
Suclg1	_LVGGTTPGK_.2	#N/A	6.6499	6.6926	6.5750	6.5404	7.3195	6.6805	7.2892	6.9975	6.9934	6.8192	6.5046	7.2843	6.8546	6.9331	7.6726	6.9872	6.0679	7.1165	7.3685	7.2399
Suclg1	_IIC[Carbamidomethyl (C)]QGFTGK_.2	#N/A	5.6221	6.2036	6.8675	7.1242	6.4177	6.4219	6.7957	5.3828	6.4384	6.5180	6.5173	6.3300	6.6009	6.8096	7.4299	6.5903	5.7347	6.5306	7.0000	6.9280
Suclg1	_MGHAGAIIAGGK_.2	#N/A	5.2802	5.9985	6.0569	4.8057	5.6973	5.5991	5.1929	5.0189	4.6121	5.4198	5.6397	5.2198	4.4611	6.1567	4.7523	3.4249	5.4804	4.7619	6.0264	5.6656
Suclg1	_ISALQSAGVVVSM[Oxidation (M)]SPAQLGTTIYK_.3	#N/A	3.1290	3.0523	4.1665	4.4552	3.3360	3.3693	3.7121	3.3547	2.9973	3.4063	3.1110	3.9162	3.1599	3.6222	3.7375	3.9920	4.3528	4.2975	3.0957	3.7275
Suclg1	_QGTFHSQQALEYGTK_.3	#N/A	4.2217	4.5982	3.7583	3.3625	2.9278	4.4620	4.3740	4.4474	4.0405	4.0323	4.7949	4.3243	4.2059	4.2826	4.1456	3.5839	3.9447	3.4099	3.0585	3.9870
Eci2	_ASQQDFENALNQVK_.2	#N/A	4.3818	5.1266	4.7262	4.4435	4.1949	3.1062	3.8698	3.1066	3.7586	4.3400	3.7400	4.3539	4.0458	3.1669	4.7523	2.6712	4.1047	3.4943	3.5907	3.6337
Ak4	_IAQNFGLQHLSSGHLLR_.3	#N/A	4.1513	3.8618	5.0190	3.3856	5.6073	4.6495	4.0624	4.4243	4.1214	4.3473	5.1512	3.7513	4.2295	4.0712	4.3902	4.6492	5.2929	5.7177	3.5270	5.3126
Tnip1	_YLQDQLS[Phospho (STY)]PLTR_.2	Q9WUU8_S416_M1_Tnip1	6.8952	7.1025	6.2150	6.7475	6.0936	6.6209	6.8231	7.1435	4.2703	6.9227	6.8057	7.1905	4.3784	3.9222	6.7912	6.8555	7.3930	6.9499	6.7807	6.9218
Gabbr1	_HPPT[Phospho (STY)]PPDPSGGLPR_.2	#N/A	3.4700	3.4719	3.5012	4.2257	8.5128	3.9585	3.3393	4.1763	3.0432	3.4558	8.8914	3.2738	9.5523	4.2831	3.1276	8.4317	3.4263	5.6084	8.7602	4.1520
Gabbr1	_RHPPT[Phospho (STY)]PPDPSGGLPR_.3	#N/A	3.7899	6.3952	4.9563	4.5353	3.1276	4.2954	6.8122	6.9897	3.7206	5.5163	3.3461	7.0093	4.5806	6.8034	3.9907	4.1348	6.6202	4.3776	3.1758	4.0466
Nfat5	_RSS[Phospho (STY)]PIFQTTK_.2	Q9WV30_S618_M1_Nfat5	6.3197	4.4987	4.0404	4.1853	5.7903	3.6423	3.4392	3.2045	3.3594	3.2126	6.0814	4.3881	3.5248	4.0275	4.8398	4.2649	6.0919	4.0910	3.7396	3.9945
Mpp2	_TLVLIGAQGVGR_.2	#N/A	5.6923	5.9044	5.7896	5.8965	5.8798	6.3372	5.9572	5.4479	5.9133	5.8451	5.8393	5.6268	5.6013	6.1323	6.0431	5.6883	5.0503	6.0144	5.7881	6.0275
Mpp2	_VEGGELVIAR_.2	#N/A	5.4947	5.7500	5.9480	6.2743	5.5454	5.9888	5.9285	6.3369	5.8960	6.3193	5.8842	5.5087	4.7268	5.8769	5.9824	5.8612	5.5863	5.7945	6.0348	5.8493
Mpp2	_DNNLELVQEILR_.2	#N/A	5.4880	5.7987	6.6766	6.3256	6.0580	6.2311	5.7931	5.9982	5.6459	5.7979	5.5230	5.3966	5.5724	6.0212	6.4027	5.5457	5.9733	5.4071	4.7336	5.8081
Mpp2	_GIM[Oxidation (M)]ES[Phospho (STY)]PIVR_.2	#N/A	10.1685	10.1052	9.4765	10.6543	9.1673	8.6687	9.5038	8.7649	7.8862	10.6933	9.5971	8.7866	9.7592	10.0903	6.4957	9.2529	8.7757	9.1901	9.0147	9.3220
Mpp2	_DLAELAEQSS[Phospho (STY)]TAAELAR_.2	Q9WV34_S86_M1_Mpp2	3.2242	3.2777	3.7605	4.4652	4.0628	4.2869	3.4783	4.4255	3.5374	3.4306	3.9117	3.1672	3.6454	3.7476	6.0254	3.5691	6.8132	3.8120	4.1110	4.3673
Mpp2	_EGQGYSFVSR_.2	#N/A	6.5045	6.3986	6.3981	5.9441	6.6226	6.2942	6.2427	6.9964	6.3931	6.6659	6.7289	6.1274	3.6485	6.3063	6.2462	6.1047	6.5604	5.9007	6.0763	6.3876
Mpp2	_DLELT[Phospho (STY)]PTSGTLC[Carbamidomethyl (C)]GSLSGK_.2	Q9WV34_T303_M1_Mpp2	3.1799	4.1392	4.2173	4.4043	3.3868	3.4202	4.8616	3.4056	3.5815	4.4913	3.0601	3.8653	3.7469	4.2554	3.6867	4.0428	4.4036	6.2934	3.0449	3.9326
Mpp2	_ALQELLR_.2	#N/A	3.3949	3.9242	2.4113	5.0762	3.6018	4.5638	4.1059	3.6206	2.6853	4.2320	4.6881	4.1132	3.5319	4.6140	4.2827	4.3306	3.1178	4.8703	4.6327	4.4677
Mpp2	_TYET[Phospho (STY)]PPPS[Phospho (STY)]PGLDPTFSNQPVPPDAVR_.3	Q9WV34_T117_M2_Mpp2	6.7454	10.3024	8.7342	8.3733	3.9092	7.9155	9.6330	3.4994	3.3109	3.4218	3.6408	3.3937	3.4562	3.8160	3.2328	7.6868	3.6456	9.7963	3.8414	4.0186
Mpp2	_ILPSYQEPHLPR_.3	#N/A	5.0494	5.8235	4.1186	3.6185	4.8547	5.4087	5.0108	5.3396	4.9230	4.0379	3.4279	5.3879	5.7904	4.9047	5.2754	4.9486	4.3591	5.0173	5.3385	3.4810
Mpp2	_NAEFDRHELLIYEEVAR_.4	#N/A	3.6733	4.3295	4.2096	4.4425	3.0110	3.2351	4.1934	3.3737	3.0883	3.8796	3.4627	4.2190	3.1964	4.2410	4.1073	4.0182	3.9113	3.5859	3.6072	3.9183
Mpp2	_YLEHGEYEGNLYGTR_.3	#N/A	4.3031	3.7426	4.6139	5.7882	3.7834	3.8168	3.2647	3.8022	3.1849	3.3871	4.1644	3.4687	3.3503	3.1511	3.2900	2.9386	3.2994	3.8500	4.1491	4.3293
Vapa	_VAHSDKPGS[Phospho (STY)]TSAVSFR_.3	Q9WV55_S214_M1_Vapa	3.7956	4.2072	4.3319	3.8938	3.1333	5.2132	5.4024	3.4960	4.4668	4.0019	3.3404	4.0967	4.5749	5.5624	3.9850	4.1405	4.4673	4.6264	3.4481	3.7960
Gsk3b	_IQAAAS[Phospho (STY)]PPANATAASDTNAGDR_.2	#N/A	9.9314	9.7920	9.7162	9.2659	9.0688	9.4087	9.5586	9.6330	9.3732	9.6324	8.9528	8.9622	9.4645	9.5681	7.7608	8.6947	9.5177	9.4802	9.0424	8.7929
Gsk3b	_GQTNNAASASASNST_.2	#N/A	5.3926	5.3141	5.6393	4.7951	5.3388	6.4290	4.9053	4.6567	4.6847	6.3098	4.9362	5.9035	4.3967	4.9518	3.7690	5.0683	5.1271	5.9653	5.1539	6.0057
Gsk3b	_VTTVVATPGQGPDRPQEVSYTDTK_.3	#N/A	5.8796	4.6285	5.2850	5.8679	5.1237	4.9029	5.1511	5.9595	5.4062	4.2142	4.2284	4.1424	4.0241	4.5041	4.9209	4.9762	4.5538	4.0659	5.0206	4.7405
Dmtn	_GNS[Phospho (STY)]LPC[Carbamidomethyl (C)]VLEQK_.2	#N/A	14.1893	14.0496	14.2953	14.3282	13.5959	13.6186	13.4892	13.6546	13.4053	14.4541	14.0214	13.6430	13.9746	14.2792	12.9761	13.7851	14.0740	13.4144	13.6539	13.8576
Dmtn	_ESVGGS[Phospho (STY)]PQSK_.2	#N/A	11.7477	12.0681	11.7813	11.8866	11.5435	11.1022	11.2874	11.4954	11.1879	11.8047	7.4739	11.5273	11.8058	11.7312	11.0155	11.5517	11.3997	11.2582	11.0189	11.4616
Dmtn	_HLS[Phospho (STY)]AEDFSR_.2	Q9WV69_S372_M1_Dmtn	11.0987	11.5323	10.7502	11.2764	10.4896	10.7058	10.7041	11.0266	10.6363	11.4081	10.9599	11.1226	11.5495	11.7756	10.3109	11.0749	11.2583	10.9844	11.0669	11.3954
Dmtn	_GAEEEEEEDDDS[Phospho (STY)]EEEIK_.2	#N/A	11.3499	11.2713	11.2620	11.3710	11.0713	11.0534	10.7829	11.4403	11.0719	11.4239	11.0424	11.3079	11.4506	12.0751	11.1770	11.3468	11.6118	11.2426	11.0448	11.4045
Dmtn	_QPLTS[Phospho (STY)]PGSVSSSR_.2	Q9WV69_S11_M1_Dmtn	10.1612	10.3005	10.2943	10.3878	9.8181	9.7856	9.7011	9.8552	9.6540	10.5702	9.9868	10.1163	10.5503	10.7087	9.8220	10.2557	10.1932	9.7833	10.0531	10.2150
Dmtn	_SREC[Carbamidomethyl (C)]SLS[Phospho (STY)]PK_.2	#N/A	8.8744	9.0220	8.6927	9.2501	8.6379	7.8275	8.6763	8.6891	7.8846	9.0969	8.3047	8.8186	8.9230	8.9921	8.0240	8.5713	8.7242	8.1131	8.5592	8.8180
Dmtn	_TLGIISQASTPR_.2	#N/A	7.1458	7.0544	6.5441	7.1073	6.8751	6.8973	6.7836	6.6333	6.8559	6.8638	6.9761	6.9435	6.8339	7.1887	7.0799	7.0062	6.5474	6.8086	6.5486	7.4099
Dmtn	_DSSVPGS[Phospho (STY)]PSSIVAK_.2	Q9WV69_S26_M1_Dmtn	7.2633	13.3058	10.3920	13.3878	12.5797	12.5899	12.5070	12.9755	12.7429	13.4354	12.8467	9.3038	13.5033	13.3850	8.8741	13.0656	13.2455	12.7329	13.0005	12.9962
Dmtn	_SSS[Phospho (STY)]LPSYGR_.2	Q9WV69_S289_M1_Dmtn	11.7481	6.8134	11.8423	12.5454	12.0300	12.2978	12.1397	11.6342	12.1985	12.5842	12.3266	12.3270	13.0049	6.5665	7.3156	12.5756	12.1177	12.1963	12.4793	12.5771
Dmtn	_M[Oxidation (M)]DRGNS[Phospho (STY)]LPC[Carbamidomethyl (C)]VLEQK_.3	#N/A	8.6433	8.7277	8.1498	9.0720	8.1003	8.0583	8.6226	8.3186	7.9724	9.2656	8.5645	8.6368	9.5111	9.0072	8.0833	8.9838	8.0663	8.8290	8.6148	8.8955
Dmtn	_EC[Carbamidomethyl (C)]SLS[Phospho (STY)]PK_.2	#N/A	8.4370	3.2706	8.4172	9.7901	8.7988	8.9621	3.1665	4.4326	8.9657	10.0247	9.4684	3.1600	9.5233	3.7548	8.9825	9.0862	7.8620	4.3637	9.4584	9.5607
Dmtn	_STS[Phospho (STY)]PPPSPEVWAESR_.2	Q9WV69_S92_M1_Dmtn	15.7525	15.7997	15.7933	15.8914	15.3154	15.5305	15.2360	15.7505	15.5758	15.8597	15.6680	15.6669	16.4255	16.1770	15.5099	15.8985	16.2225	15.5322	15.7333	15.9052
Dmtn	_KGAEEEEEEDDDS[Phospho (STY)]EEEIK_.2	#N/A	7.5191	7.6006	7.4635	7.7546	7.1143	7.4941	7.5753	7.8244	7.7042	7.9788	7.6322	7.5041	7.7420	8.2299	7.3778	7.6365	7.6077	7.5231	6.5265	7.7525
Dmtn	_GAEEEEEEDDDS[Phospho (STY)]EEEIKAIR_.3	#N/A	3.4047	5.5550	4.4421	4.7695	3.6116	3.5877	4.8310	3.6303	4.8536	4.6044	4.3362	5.1044	3.5221	5.6581	5.2698	6.1493	3.1276	4.6897	5.2091	5.0102
Dmtn	_QRESVGGS[Phospho (STY)]PQSK_.2	#N/A	6.5004	6.5291	8.3139	6.8928	7.1038	6.1087	5.9114	6.5048	6.3134	8.5419	6.1595	6.3591	6.8139	9.3174	8.6065	6.7083	6.7753	8.5819	6.5954	6.7456

Dmtn	_TRS[Phospho (STY)]LPDR_.2	#N/A	9.5561	9.6859	4.0412	9.6297	9.3945	9.4825	9.2855	9.7097	9.3258	9.5797	9.3728	9.5723	9.9278	4.0793	9.6527	9.6169	9.7664	9.5890	9.4791	9.7281
Dmtn	_S[Phospho (STY)]LPDRTPFHTSLHSGTSK_.3	#N/A	3.9103	3.6589	4.2296	4.0109	3.6962	3.8108	3.7102	6.6555	3.7248	3.9555	3.6150	6.0311	3.8509	7.3850	3.4514	3.9011	4.5494	5.8727	3.7936	3.5959
Dmtn	_VFAM[Oxidation (M)]S[Phospho (STY)]PEEFGK_.2	Q9WV69_S383_M1_Dmtn	5.7521	5.1824	5.3941	5.6974	6.3809	6.3528	5.4143	6.2715	5.6875	5.4065	5.1774	5.4830	5.7879	5.9250	5.6966	5.4219	5.4870	6.1527	6.5515	6.6511
Dmtn	_MDNQVLGYK_.2	#N/A	4.3057	3.9016	3.8106	3.7415	3.0118	3.0452	2.3609	3.5162	3.6724	4.1163	3.4351	3.7911	4.1219	4.8965	4.0616	3.6679	4.3393	3.4939	3.1425	3.1640
Dmtn	_LQS[Phospho (STY)]TEFSPSGSEAGSPGLQNGEGQR_.3	Q9WV69_S303_M1_Dmtn	3.8388	4.3646	4.0044	4.0007	3.1739	4.2413	3.8867	3.1927	3.9874	4.3158	3.1342	4.0907	4.1187	4.0477	3.9120	3.8300	4.1908	3.4821	3.4157	3.9849
Dmtn	_KGAEEEEEEEDDDS[Phospho (STY)]EEEIKAIR_.3	#N/A	3.6772	2.6833	3.2138	3.9070	3.8842	4.2242	2.9065	2.7748	1.6345	3.3679	3.9744	2.2288	3.7081	3.0753	3.1181	3.0393	3.4001	1.9348	4.0484	4.4300
Asb3	_S[Phospho (STY)]VDVADNR_.2	#N/A	7.3021	7.4341	6.5608	7.5719	6.9126	7.0836	7.4552	6.7720	7.1489	7.7080	7.5448	7.2594	7.5471	7.4274	5.9535	7.1822	6.6630	7.3149	7.2212	7.0302
Asb3	_GRS[Phospho (STY)]VDVADNR_.2	#N/A	5.8096	5.9104	5.6922	6.0286	5.4162	5.5658	6.0572	5.0019	4.0684	5.9176	5.1032	5.1084	5.7034	5.8094	4.1955	4.8839	5.7334	5.4224	5.7401	5.2050
Stxbp4	_SSS[Phospho (STY)]PLDRDPAFR_.2	Q9WV89_S12_M1_Stxbp4	4.0296	3.2895	3.5661	3.5547	4.2365	4.2698	4.3124	4.2552	4.2327	3.8402	3.7113	3.0157	4.3981	3.6042	2.8370	3.3917	3.7525	3.3970	3.6960	3.2815
Epb41l3	_VESTSVGSIS[Phospho (STY)]PGGAK_.2	Q9WV92_S804_M1_Epb41l3	11.6729	11.2970	11.7680	11.8267	11.0152	10.6780	10.7408	10.8946	10.7996	11.6897	10.9193	10.9245	11.3085	11.1935	9.7673	11.0636	11.0311	10.6459	10.7278	10.9106
Epb41l3	_ETEIT[Phospho (STY)]PEDGED_.2	#N/A	4.3704	9.8649	9.6517	10.0218	9.5754	9.4922	9.3722	9.6858	9.3539	9.6611	9.5591	9.6981	9.9639	10.3405	9.3583	9.7859	10.0037	9.6459	3.2072	9.6807
Epb41l3	_DSVS[Phospho (STY)]AAEVGTGQYATTK_.2	Q9WV92_S470_M1_Epb41l3	7.7993	7.3892	7.9503	7.9488	6.7504	7.1804	6.8633	6.9030	6.9596	7.6305	7.0900	6.8860	6.6356	6.7649	6.4996	7.1483	7.4229	7.0314	7.1530	7.3284
Epb41l3	_KKAEEAT[Phospho (STY)]PVTALR_.3	#N/A	8.2085	7.9941	8.1298	8.3299	7.6086	7.3631	7.6382	7.4069	7.6642	8.5533	7.7850	7.6488	8.5231	7.6563	8.3382	8.1971	7.5762	7.5388	7.6578	7.1729
Epb41l3	_RLS[Phospho (STY)]TSPVR_.2	Q9WV92_S599_M1_Epb41l3	3.9634	9.8268	4.0939	3.6738	8.1074	3.7737	8.0869	3.7179	7.5990	9.1755	7.5295	8.4616	7.9964	3.7319	3.5982	3.8917	8.1935	3.2988	8.8795	7.4975
Epb41l3	_EVPVVHTETK_.2	#N/A	7.3899	7.6572	7.8102	7.7717	7.3092	7.4450	7.4802	7.3680	7.8122	7.3249	7.4900	7.2388	7.5398	7.3824	7.8287	7.2196	7.2946	7.1310	7.4813	7.4469
Epb41l3	_AEEAT[Phospho (STY)]PVTALR_.2	#N/A	8.2523	4.2332	8.6924	8.6607	3.2928	3.3147	7.8555	3.3115	3.0087	3.9593	8.0574	3.9594	3.1167	4.1613	4.0276	7.8086	4.3096	7.1403	7.4906	7.8740
Epb41l3	_ASALIDRPAPYFER_.3	#N/A	7.0200	7.2404	7.9236	7.6286	6.9968	7.2127	6.9219	6.9112	7.1983	7.8211	7.4124	7.1647	7.1973	7.3426	7.0826	7.1571	7.6524	7.2780	7.7958	7.2898
Epb41l3	_SHFESSTVR_.2	#N/A	6.8327	6.7068	6.7849	6.4290	6.5879	6.5717	7.0600	6.2904	6.9366	6.5411	6.8801	6.5881	6.7611	6.6292	7.2272	6.7452	6.4019	6.4876	6.8090	6.5192
Epb41l3	_LM[Oxidation (M)]DGSEILSLLESAR_.2	#N/A	6.4815	5.8707	6.3294	6.6114	6.1142	6.4202	6.1094	6.3972	6.4932	6.6688	6.2338	6.0442	6.8883	5.8301	6.3237	6.5333	6.2948	6.1218	6.3587	6.5883
Epb41l3	_KAEEAT[Phospho (STY)]PVTALR_.2	#N/A	8.5189	8.1396	3.7414	3.3794	2.9109	4.4451	4.1372	8.1186	7.5357	4.0154	5.3360	7.7951	4.2228	3.7795	4.1625	3.5670	3.9278	7.6472	3.5207	3.1916
Epb41l3	_DEVDAAEST[Phospho (STY)]PTDR_.2	#N/A	7.2101	4.2763	7.4501	7.0825	3.2498	6.4877	3.7983	5.9400	7.3186	4.3543	6.8057	4.0024	3.8840	4.3867	3.8237	3.9058	4.1013	3.7318	6.5844	4.3536
Epb41l3	_TEPVEAEVESTPHPQPLS[Phospho (STY)]TEK_.3	Q9WV92_S686_M1_Epb41l3	3.5955	4.4073	4.1318	8.6737	2.9333	3.1574	3.1070	3.2959	3.1661	3.8019	8.4177	4.2967	3.6163	8.5825	4.1850	3.9404	3.8336	3.9996	3.6481	4.0859
Epb41l3	_QLEYQQFEDKKLS[Phospho (STY)]QR_.3	#N/A	7.6737	7.6155	7.8174	7.7879	5.8247	6.4049	7.4402	6.8944	6.1906	8.4203	7.2654	6.7358	6.7947	7.8775	4.9381	7.2121	6.4552	7.1112	6.8832	7.2448
Epb41l3	_TFLETSTETALTNEWEK_.2	#N/A	4.6492	4.5094	4.3000	5.7627	4.8498	4.1609	3.9902	4.2896	4.0092	4.8138	5.0567	4.5398	4.3923	4.6983	4.1019	3.9219	4.7977	4.9491	3.5962	4.0345
Epb41l3	_VLQETILVEER_.2	#N/A	5.4815	5.9515	6.0812	6.6770	6.1327	5.7773	5.2058	6.0382	5.6122	5.2561	6.1812	5.9186	6.4799	5.7846	5.1808	5.7060	5.9264	5.7928	5.9395	5.9193
Epb41l3	_ELEDKVIEHK_.2	#N/A	4.7949	4.6640	4.5110	3.8445	5.0348	4.1581	4.9731	5.3113	4.9720	5.1515	4.8874	4.4467	5.2041	4.8217	5.1555	5.1613	4.8145	5.0608	5.0339	5.2470
Epb41l3	_LSTS[Phospho (STY)]PVR_.2	Q9WV92_S601_M1_Epb41l3	4.3510	3.6518	3.3865	3.3384	3.6887	3.9128	6.3705	4.0514	3.9114	4.5573	4.2858	3.5412	4.0195	3.5632	3.4295	3.1951	3.0882	3.4379	3.7370	3.2405
Epb41l3	_EIGDKDRDFAAAAAK_.3	#N/A	5.8251	6.0284	5.9442	5.1428	5.6224	5.7464	5.8103	5.9127	5.6722	5.3007	4.2809	5.9135	6.1855	6.3257	5.9871	6.2387	5.1249	6.3608	6.0068	3.2357
Epb41l3	_VVVHKETEIT[Phospho (STY)]PEDGED_.2	#N/A	8.8708	9.2817	7.3710	8.8885	8.6347	8.2020	8.7400	9.5300	8.6650	8.5057	8.2557	9.0116	10.4008	9.2815	8.7272	9.0248	9.0009	9.0565	8.8878	8.6714
Epb41l3	_S[Phospho (STY)]SSS[Phospho (STY)]KLSR_.2	#N/A	6.0596	3.8028	3.8563	3.6709	5.6275	3.8677	5.2118	4.0500	4.1264	3.7481	5.8340	3.7931	3.7080	3.8515	3.9435	4.0290	4.0710	4.6885	3.9100	5.3467
Epb41l3	_FAPNHTK_.2	#N/A	4.1817	3.6336	4.6612	3.4025	5.3097	5.0790	4.7736	5.1571	4.8087	3.9444	4.3106	5.1564	4.2459	4.3459	4.7181	3.5438	5.1555	4.7035	4.5454	5.1197
Epb41l3	_GKEGSS[Phospho (STY)]VTEAAK_.2	Q9WV92_S740_M1_Epb41l3	4.0935	3.5650	3.4733	3.2516	3.7755	3.9222	3.7655	4.1489	3.8246	3.1433	4.1990	3.4544	3.9327	3.4765	3.3567	3.1666	3.8776	3.5247	3.8238	3.1537
Epb41l3	_QQPALEQFPEAAAHST[Phospho (STY)]PVKR_.4	Q9WV92_T64_M1_Epb41l3	3.7576	4.2452	4.2939	3.9318	3.0953	3.3194	4.4458	3.4579	3.0040	3.9639	3.3784	4.1347	3.7783	4.2809	4.0230	4.1025	3.9956	4.8984	4.5310	4.2479
Epb41l3	_TITYESSQVDPGADLEPGVLM[Oxidation (M)]SAQTITSETTSTTTTTHITK_.4	#N/A	3.7744	4.3139	5.2500	4.5577	3.9813	4.6783	3.0668	5.5201	2.9870	5.4558	4.2203	4.7403	5.8969	5.7254	4.5771	4.8581	6.0178	3.7488	4.1120	4.6670
Epb41l3	_GIS[Phospho (STY)]QTNLITTVT[Phospho (STY)]PEKK_.2	#N/A	10.0807	9.7545	9.8088	9.8757	8.8119	8.7765	9.3928	8.2522	8.9271	10.1368	9.1481	9.2084	9.6810	9.8437	8.7061	9.2953	9.4254	9.2728	9.2252	9.1123
Epb41l3	_SSSKLS[Phospho (STY)]RS[Phospho (STY)]PLK_.2	#N/A	6.5470	6.7141	6.7016	6.6018	6.5539	6.2523	6.3468	6.3736	6.0160	6.0576	6.2456	6.0884	6.3206	6.7101	6.2515	6.2573	6.2515	6.3169	6.6796	6.1402
Epb41l3	_VTLLDGSEYGC[Carbamidomethyl (C)]DVDKR_.3	#N/A	4.4960	4.8582	5.8874	6.0622	4.8789	5.2881	5.2027	4.5535	5.4298	5.7731	5.9660	5.2434	6.0052	5.3164	5.6158	5.8286	5.1759	5.4124	5.7394	5.7693
Epb41l3	_LSRS[Phospho (STY)]PLK_.2	Q9WV92_S107_M1_Epb41l3	7.8639	8.1181	7.9505	8.0736	7.7045	7.3394	7.7232	7.9115	7.6069	7.5362	7.4034	7.6863	7.9609	7.5923	6.9188	7.5465	7.8194	7.5292	7.3846	7.6788
Epb41l3	_GISQTNLITTVT[Phospho (STY)]PEK_.2	#N/A	7.1231	7.2633	6.7640	6.9279	6.4913	6.6951	6.6035	7.0037	6.6282	7.0632	6.8436	6.4681	6.5849	10.3223	5.9226	6.8240	7.0772	9.0658	6.8247	6.7200
Epb41l3	_QLEYQQFEDKLSQRS[Phospho (STY)]S[Phospho (STY)]SSK_.3	#N/A	3.3591	5.9907	4.3965	4.1423	3.5660	3.5993	3.4821	3.2474	3.4023	3.1697	4.3818	3.6862	3.5678	2.6668	3.5075	4.2220	3.7851	4.0481	3.6966	4.0374
Epb41l3	_DEVDAAEST[Phospho (STY)]PTDRR_.2	#N/A	3.7903	3.1731	3.7779	3.9669	4.2623	4.1138	5.6441	3.8445	3.7401	3.6841	5.5690	3.8880	3.9056	3.7888	3.8453	3.5944	3.7243	3.8507	3.7551	3.6264
Epb41l3	_NWLDPAK_.2	#N/A	1.5589	3.6303	3.4080	3.1586	3.7103	3.2668	3.5131	4.1928	3.8899	4.2643	3.5197	3.3880	3.9980	3.5417	3.4080	3.6928	3.6900	3.7785	4.8017	3.2190
Epb41l3	_EQHPDMSVTK_.2	#N/A	4.3344	3.9302	4.7796	5.5666	3.0405	3.0738	5.1494	3.7729	4.0743	4.1450	4.7725	4.2117	4.0933	4.5959	4.4607	3.6965	4.3106	3.5225	3.1711	3.0621
Epb41l3	_IVITGDADIDHDQALAQAIK_.3	#N/A	4.2571	3.8129	4.4972	3.4995	3.5276	3.7517	4.2386	3.8902	4.0726	5.2874	2.9461	3.0287	4.2106	3.2124	4.4468	5.0844	4.4279	3.4052	4.5574	4.2909
Epb41l3	_S[Phospho (STY)]LDGASVSENHEIYM[Oxidation (M)]K_.2	#N/A	8.7738	9.3711	9.0974	9.0250	7.6823	9.0600	8.7914	8.4363	8.7174	8.8162	8.1031	8.6195	8.4363	9.4437	8.2403	8.9721	8.7686	9.1595	9.1466	8.9177
Epb41l3	_AEEERVEEEDRR_.3	#N/A	4.4399	3.5629	3.4753	3.2495	3.7776	4.0017	2.7899	4.1403	3.8226	3.1454	1.0725	3.4524	3.9306	3.4744	2.9742	3.2839	3.1771	3.5268	3.8258	1.8942
Epb41l3	_TFLETSTETALTNEWEKR_.3	#N/A	3.7160	3.6031	3.2526	3.8682	3.9229	3.9563	3.1252	3.9417	3.0454	3.5266	3.4659	3.3292	3.0671	3.2906	3.1505	3.0781	3.4389	3.7105	3.4314	4.8188
Epb41l3	_VC[Carbamidomethyl (C)]VEHHTFFR_.3	#N/A	4.4074	3.5954	3.4428	3.2820	4.2065	2.4576	4.3424	4.1077	4.2626	3.8102	2.7829	3.4849	4.4281	3.4299	2.8670	2.9585	3.1446	3.1877	2.9974	3.3969
Timm9	_LTETC[Carbamidomethyl (C)]FLDC[Carbamidomethyl (C)]VK_.2	#N/A	5.1560	6.4807	6.4935	6.1479	5.7279	6.3071	6.0691	6.2082	5.9702	5.7729	6.0339	6.0887	5.3264	6.3527	6.5246	6.0771	5.3866	6.2542	5.8175	6.1493
Timm9	_EFLGTYNK_.2	#N/A	3.9183	4.0845	4.7745	5.4609	3.2560	4.8540	5.1887	3.6187	5.2553	5.1801	5.4087	5.1780	5.7881	5.0316	5.7844	5.9683	4.1564	5.4387	5.9065	5.6018
Timm9	_EVKPEEVTC[Carbamidomethyl (C)]SEHC[Carbamidomethyl (C)]LQK_.3	#N/A	4.9639	5.4450	5.4311	4.8877	5.1419	5.3483	5.1881	5.4886	5.8080	5.2087	5.5248	5.4670	4.3274	5.9542	6.0197	5.3879	5.4483	5.4628	5.1165	5.2008
Timm9	_FQEYHIQQNEALAAK_.2	#N/A	2.1879	4.3344	3.4213	3.6995	2.7662	4.0167	3.7484	4.1104	3.8075	1.8985	4.1819	3.1605	3.9556	3.4593	3.3256	3.2990	3.6076	3.5418	3.8409	3.1366
Timm8a1	_FIDTSQFILNR_.2	#N/A	5.3062	5.5084	5.2085	5.6626	5.8048	5.3357	5.0612	5.4204	5.0925	5.2321	5.4221	5.2579	6.1978	6.1328	6.0373	5.5498	6.0379	4.2211	5.3581	4.9661
Timm8a1	_SKPVFSESLS[Phospho (STY)]D_.2	Q9WVA2_S96_M1_Timm8a1	4.3970	6.2724	7.2297	6.7433	7.7347	6.6155	5.7895	4.1683	3.8655	6.1234	4.2398	5.7535	5.9735	6.8948	6.6973	3.2410	6.7369	3.4839	3.7829	6.7016
Timm8a1	_LDS[Phospho (STY)]RAEAC[Carbamidomethyl (C)]FVNC[Carbamidomethyl (C)]VER_.3	Q9WVA2_S57_M1_Timm8a1	3.8362	3.4321	3.6062	3.1186	4.0431	4.0765	3.0050	4.2711	4.4260	3.6468	3.9047	3.2090	4.5915	3.5933	3.0304	3.1983	3.3080	3.5068	2.4281	3.5603
Bub3	_VAVEYLDPS[Phospho (STY)]PEVQK_.2	Q9WVA3_S211_M1_Bub3	3.8999	8.5661	4.4362	3.7895	3.2376	8.3090	4.3034	3.6003	4.3625	4.1062	3.2361	8.2540	4.4706	8.6285	7.9266	8.3823	8.6002	3.6952	3.3438	8.2443
Tagln2	_NFS[Phospho (STY)]DNQLQEGK_.2	Q9WVA4_S163_M1_Tagln2	6.3128	7.1405	7.6270	7.3847	6.8262	7.2014	6.2819	6.9750	7.0471	6.5075	6.4117	6.8906	7.0236	6.3012	7.2174	7.0155	7.2585	6.5510	6.6616	6.8438
Pacsin2	_LDSGQVGLYPANYVEAIQ_.2	#N/A	5.0773	5.1051	4.8038	3.3303	4.3347	5.0900	4.8700	5.6177	4.1767	4.8006	4.7911	4.9581	4.2848	5.2508	5.3039	5.1725	3.9768	4.9462	5.1209	5.3058
Pacsin2	_AYAQQLTEWAR_.2	#N/A	5.7136	5.8199	5.4599	5.5352	5.0224	5.6671	5.9039	5.4840	5.3327	4.2444	5.5321	5.3187	6.1127	4.4965	5.9157	5.2184	4.2112	5.8515	3.2705	2.9627
Mcl3	_SSS[Phospho (STY)]PIPLTPSK_.2	Q9WVG9_S404_M1_Mcl3	7.9209	7.3989	4.1610	4.4606	8.0939	3.2770	2.9873	8.5156	7.4892	8.3951	8.0793	7.4280	3.1545	7.9733	4.0654	7.5471	4.3474	7.9023	3.1011	3.8764
Foxo3	_WPGS[Phospho (STY)]PTSR_.2	Q9WVH4_S293_M1_Foxo3	3.6637	8.1077	4.2000	4.0257	3.0014	3.2255	3.0388	3.3641	3.0979	3.8700	3.4723	4.2285	3.2059	7.9136	4.1168	4.0086	3.9018	7.4262	7.3871	3.9278
Mapk8ip1	_PHPSISEEDEGFDC[Carbamidomethyl (C)]LSS[Phospho (STY)]PER_.3	#N/A	3.6787	3.8313	4.9119	4.4443	3.5418	3.6401	4.7274	3.9734	4.5727	3.6882	3.7781	3.5574	3.2238	5.4724	3.7494	4.3466	4.0300	4.3211	3.6462	4.4289
Psmd13	_GSIDEVDKR_.2	#N/A	4.2409	4.9136	5.4291	4.5927	2.9470	4.7415	5.4299	6.0384	5.4834	4.8032	3.5000	4.3052	5.2144	3.1886	4.2941	4.5514	4.4041	4.4290	4.5923	5.3801
Ehd1	_DKPTYDEFYTLS[Phospho (STY)]PVNGK_.3	#N/A	4.3095	7.1108	5.7414	6.4256	3.3225	5.3669	5.3766	5.3372	5.6296	6.6617	3.1704	3.9290	5.8001	7.3567	6.4395	5.9732	6.9835	7.2057	6.6407	6.5180
Magi2	_S[Phospho (STY)]SFPDSTEAFDPR_.2	Q9WVQ1_S725_M1_Magi2	9.6806	4.0121	4.5270	10.3490	3.3284	3.5525	9.4250	8.6182	4.2718	4.1970	9.2157	9.9049	4.3798	3.9236	3.7899	4.3356	4.0719	3.0776	3.3766	3.6009
Magi2	_GGFFS[Phospho (STY)]PWK_.2	Q9WVQ1_S685_M1_Magi2	6.6262	7.0292	7.1997	7.4598	5.9171	6.2464	6.9777	6.4142	6.5279	7.2225	7.0127	7.1892	6.8057	7.2372	6.0430	6.9934	6.9481	7.1185	7.0444	6.8615
Magi2	_IIPQEELNS[Phospho (STY)]PTSAPSSEK_.2	Q9WVQ1_S1013_M1_Magi2	11.4526	11.5824	11.4554	11.4562	10.9800	11.3039	11.1207	11.5735	11.1786	4.2435	11.3375	11.4983	12.0767	11.9020	11.2600	11.4375	11.9294	11.3803	11.4942	11.5832
Magi2	_M[Oxidation (M)]DS[Phospho (STY)]SGPDYKEDLVHLR_.3	Q9WVQ1_S768_M1_Magi2	3.8474	4.1554	4.3837	3.8419	3.1852	4.7084	4.3559	3.5478	4.4150	4.0538	3.2885	4.0448	4.5231	5.8041	4.4486	4.1923	4.0855	3.7477	5.2589	4.3378
Magi2	_GAQGFGFTIADS[Phospho (STY)]PTGQR_.2	Q9WVQ1_S621_M1_Magi2	4.1234	6.6421	6.0728	3.4059	4.3304	6.2252	4.2186	3.9839	4.4188	3.9340	3.6174	4.3661	4.3042	7.9231	4.2439	3.4856	4.5216	6.9436	2.9602	6.7179
Magi2	_SPGSVSTHHS[Phospho (STY)]PR_.2	#N/A	4.4164	5.6494	5.1290	5.8472	5.3866	5.6494	4.3177	5.6536	5.4246	5.3765	5.3557	5.0914	4.9836	5.8826	4.4958	5.4496	6.0268	5.3317	4.4297	5.7542
Magi2	_NKS[Phospho (STY)]VTNMEK_.2	Q9WVQ1_S219_M1_Magi2	3.7973	4.0743	3.6109	6.3529	4.2813	4.3146	4.2677	4.3000	4.1879	3.8849	3.6665	2.9709	4.3533	3.6490	2.7922	3.4365	3.7973	3.3522	3.6012	3.3263
Fxr2	_AGPGDRDPPS[Phospho (STY)]RGEESR_.3	Q9WVR4_S477_M1_Fxr2	4.0979	3.2212	3.6344	3.4864	4.3048	4.3381	6.2309	4.3235	4.1644	3.9085	3.6430	3.3606	4.7025	3.6725	4.2695	4.2801	3.8208	3.3287	3.6277	3.3498
Fxr2	_TDGS[Phospho (STY)]SGDRQPVTVADYSR_.3	Q9WVR4_S602_M1_Fxr2	4.4975	4.7729	4.1442	5.1027	3.6872	3.9001	6.3499	3.6440	3.5302	3.7772	3.8731	4.7718	5.0926	5.2643	3.4583	4.9324	3.7112	4.7103	4.5402	4.2810
Ncdn	_EAEPDLLAVLR_.2	#N/A	8.0879	7.8361	7.5817	7.8462	7.9191	7.8123	8.1145	8.1843	7.3280	7.7388	7.8792	7.7453	7.4356	7.3392	7.1298	7.6249	7.8070	7.9837	7.7638	7.5310
Ncdn	_IFDAVGFTFPNR_.2	#N/A	7.8831	7.9899	7.4942	7.6943	7.5915	8.1364	7.6869	8.1383	7.4750	7.6928	7.5556	7.5805	7.4060	6.5325	6.9660	7.3151	7.8512	7.4901	7.3309	7.3307
Ncdn	_IPLSTFLTAR_.2	#N/A	7.9600	7.8083	7.2388	7.5167	7.4786	7.7517	7.9843	7.8352	7.4644	7.5283	7.3043	7.6345	7.5664	6.8133	6.9371	7.1721	7.3649	7.8047	7.3938	7.2232
Ncdn	_GLSEDFQR_.2	#N/A	7.0307	7.0938	7.3531	7.3458	7.0825	7.4307	7.6173	7.0507	7.6193	6.9576	7.0868	6.8804	7.4722	6.2226	7.0203	7.0289	6.3783	7.3193	7.2471	6.9892
Ncdn	_EVC[Carbamidomethyl (C)]QLLPFLVR_.2	#N/A	7.0882	6.9147	6.6529	6.6767	6.7758	7.4392	7.3924	7.1694	6.4389	6.9997	7.0639	6.9464	6.7419	6.5036	6.4693	6.7345	6.9610	7.2528	6.7120	6.4684
Ncdn	_EGAPSLLC[Carbamidomethyl (C)]K_.2	#N/A	6.8239	6.5152	6.6002	6.7162	6.3363	6.6321	7.1325	7.0037	6.4837	6.7051	6.7472	6.8688	6.7634	5.7055	6.0648	5.1859	7.0171	6.6013	6.3422	6.4927
Ncdn	_DLQAGLAR_.2	#N/A	4.4582	5.9794	5.4451	4.7126	5.5443	5.5829	6.6219	3.1582	5.6732	5.4335	5.3840	4.9311	5.4654	5.0135	5.5732	5.5778	4.1562	5.9777	5.5707	6.0885
Ncdn	_TLYEEAEEASDSQDVANLAISPTT[Phospho (STY)]PGPSWPGDALR_.4	Q9Z0E0_T451_M1_Ncdn	4.3480	5.7909	3.6549	3.2896	3.4052	3.7471	4.0400	3.6430	4.0991	4.3916	3.9089	3.8227	4.2071	3.7509	3.6172	3.4734	3.6591	5.7664	3.5713	3.4282
Ncdn	_NDSEQFAALLVTK_.2	#N/A	3.3320	4.3212	4.6323	4.3924	4.4787	3.6285	4.0641	4.4940	4.0713	4.4570	3.9335	3.6999	3.1194	4.0274	3.7939	4.3971	3.3368	3.6580	4.7905	4.1000
Ncdn	_EAPDGC[Carbamidomethyl (C)]PDHVLR_.2	#N/A	7.0099	7.3232	7.2303	6.8951	7.1753	7.1610	7.3267	7.2807	7.0237	7.2548	7.1203	6.9002	7.3541	6.6560	6.7281	6.3799	7.0331	7.3389	6.8244	6.9157
Ncdn	_SM[Oxidation (M)]DDTYQC[Carbamidomethyl (C)]LTAVAGTPR_.2	#N/A	5.4513	4.6383	3.9110	4.6792	4.8146	4.8215	5.0220	5.4034	4.9940	5.0000	4.8077	5.0089	5.4391	4.8082	4.1577	4.8074	4.9151	4.9253	4.9265	4.1692
Ncdn	_LQAGEETASHYR_.2	#N/A	4.3806	4.5731	4.7129	5.2176	4.7968	4.5303	4.5864	4.7132	4.0460	5.0600	4.3599	3.7604	4.6645	4.2747	4.2439	4.3291	4.4333	4.2331	4.4109	4.2399
Ncdn	_LLSTS[Phospho (STY)]PALQGTPASR_.2	Q9Z0E0_S583_M1_Ncdn	4.3308	3.9267	4.6124	3.6132	3.0369	3.0702	4.0112	3.7765	3.9314	4.1414	3.4101	3.8161	4.0969	4.5995	4.0366	3.6929	4.3142	6.7466	3.1675	3.0657
Ncdn	_EAIGAVIHYLLQVGPEK_.2	#N/A	3.5936	4.4092	4.1299	4.0958	2.9313	3.5527	3.5287	3.2940	2.2312	3.1231	2.9275	4.2986	3.6143	4.1170	4.1869	3.9385	1.2833	4.0015	3.6501	4.0840
Slc22a5	_DGEES[Phospho (STY)]PTVLK_.2	Q9Z0E8_S548_M1_Slc22a5	3.6889	4.3139	4.2252	4.0005	3.0266	3.2507	3.0136	3.3893	3.0727	3.8952	3.4471	4.2033	3.1807	4.2254	4.0556	4.0338	3.9270	[illegible]	3.0749	3.9026
Adam17	_S[Phospho (STY)]FEDLTDHPVTR_.2	Q9Z0F8_S794_M1_Adam17	3.5429	5.7641	7.4033	6.1504	3.7499	6.3830	5.4931	6.0579	6.3579	6.8610	4.1979	5.4501	3.3839	6.7091	[illegible]	6.2070	6.5556	5.9431	6.3164	5.9758
Zbtb22	_ASENQS[Phospho (STY)]PSSNYFSPR_.2	Q9Z0G7_S203_M1_Zbtb22	7.6470	6.7171	3.4573	7.0083	6.6945	6.6807	6.2863	6.5720	4.3415	3.7313	6.6820	6.3297	4.5069	3.4954	6.5726	3.2829	6.9719	3.5058	6.6786	7.7866
Celf2	_ELFEPYGAVYQINVLR_.2	#N/A	5.8099	5.1356	4.9542	5.2705	5.3176	5.6623	5.3977	5.6935	5.3354	5.0954	4.2177	4.4310	3.9682	5.7582	5.5261	5.4061	5.7367	5.2554	5.2615	6.0709
Celf2	_SWS[Phospho (STY)]EKELK_.2	Q9Z0H4_S52_M1_Celf2	3.6134	6.9412	4.1497	4.0759	2.9512	3.5329	3.5485	7.4612	7.1238	7.5312	7.2326	4.2788	7.4540	7.8701	7.0826	7.0690	7.3694	7.1446	3.6302	7.3377
Celf2	_VM[Oxidation (M)]SPFGQIEEC[Carbamidomethyl (C)]R_.2	#N/A	6.0665	5.6072	5.9768	5.2379	4.9279	5.3877	5.1607	5.2131	5.7837	5.3809	4.8836	5.3798	5.7710	5.1980	5.3695	5.3845	5.4764	5.5284	5.4442	5.4542
Clip2	_IGFPSTS[Phospho (STY)]PAK_.2	Q9Z0H8_S295_M1_Clip2	10.8106	10.4989	10.3117	10.4737	10.1280	9.9469	10.0854	10.4003	10.0641	10.5872	10.3557	10.3235	10.6570	10.4053	9.8918	9.9247	10.5132	10.0864	9.9401	10.2736
Clip2	_QAS[Phospho (STY)]GPSSGAATTVSEKPGPK_.3	#N/A	6.7857	7.2221	6.8265	7.1995	7.0347	6.9062	7.0903	6.9024	6.9318	7.8521	6.9022	6.4738	7.7245	7.3536	6.6273	6.7329	6.5073	7.1214	6.6877	6.7900
Clip2	_YSLIDPAS[Phospho (STY)]PPELLK_.2	Q9Z0H8_S980_M1_Clip2	4.1354	3.8675	3.1708	3.5540	3.4731	3.6972	4.0680	3.8357	4.1271	4.3417	6.7233	3.7569	6.6911	3.1579	3.6452	2.9794	4.3734	3.4598	3.1083	3.1249
Clip2	_TGNESGSNLS[Phospho (STY)]DSGSVK_.2	Q9Z0H8_S208_M1_Clip2	7.6903	7.6359	7.4251	7.7583	7.6930	7.5060	7.3234	7.6169	7.1324	7.3765	7.1644	6.9926	7.6331	7.9206	6.4716	6.9781	7.3317	7.1528	7.1077	6.9305
Clip2	_EGS[Phospho (STY)]PLHK_.2	Q9Z0H8_S43_M1_Clip2	4.4788	5.4167	3.8846	4.4551	3.8836	4.6342	4.6419	4.7564	4.3280	5.2295	3.3682	4.4296	4.6787	4.2046	3.7375	3.9920	4.2762	4.3675	5.3783	4.2674
Clip2	_VLLLEANRHS[Phospho (STY)]PSPER_.3	#N/A	8.4739	8.7446	8.1099	8.3458	7.8359	7.7384	8.2543	8.6732	8.0720	8.1303	7.5962	8.0993	8.7128	7.8814	9.0178	7.8073	8.0071	8.2118	8.0379	7.7907
Clip2	_QQHIEQLLAER_.3	#N/A	4.0360	3.9668	4.4723	3.6534	3.3738	3.5978	4.1673	3.7364	3.9262	3.1927	3.1000	3.2899	4.3345	3.7093	4.0662	4.4591	4.2741	3.1229	3.4220	4.0751
Clip2	_TGNESGSNLS[Phospho (STY)]DSGS[Phospho (STY)]VKR_.2	#N/A	3.8203	3.4987	3.3569	3.7639	4.0273	4.0811	3.0208	4.0460	3.7432	3.2248	4.1175	3.2249	3.8512	3.3950	3.0462	3.1825	3.5433	3.6062	3.9052	3.0723
Nos1	_VTQPLGT[Phospho (STY)]PTK_.2	Q9Z0J4_T128_M1_Nos1	9.0618	9.1986	8.7401	8.3902	8.6751	8.8813	8.7603	8.9310	9.3168	8.7635	9.3912	8.4368	8.7249	8.7590	9.1349	8.4897	9.4169	8.7325	9.0316	9.4468
Nos1	_KSS[Phospho (STY)]QDGPDLR_.2	Q9Z0J4_S858_M1_Nos1	7.2626	7.1977	5.9516	5.3929	6.8506	5.5286	5.0680	5.2791	5.4152	5.7041	7.3679	6.9610	4.1225	7.3824	4.5011	7.0524	7.4407	5.2604	4.9291	6.9215
Nos1	_GNVPVVLNNPYSENEQS[Phospho (STY)]PASGKQS[Phospho (STY)]PTK_.3	Q9Z0J4_S287_M1_Nos1	4.5154	3.4874	3.5509	3.1740	3.8532	4.1318	4.4505	4.2158	4.3707	3.7021	3.8494	3.3768	4.5362	3.5580	2.9750	3.3595	3.2527	4.7956	4.2291	3.5050
Palm	_SETLVNAQQT[Phospho (STY)]PLGT[Phospho (STY)]PKENR_.2	#N/A	10.0419	10.2624	10.4070	10.6014	8.1242	9.2399	9.6757	9.2667	9.4876	10.6222	9.2213	9.9439	10.4139	10.4583	9.3186	10.3203	10.0457	9.8789	9.7646	9.9923
Palm	_EPAPLNGS[Phospho (STY)]AAELPATK_.2	Q9Z0P4_S345_M1_Palm	10.3455	10.1207	10.6073	10.1975	2.8968	9.7301	9.6140	9.1655	4.1135	4.3552	9.8508	4.3553	9.4551	10.2131	3.6316	9.7697	3.9136	3.2358	9.7741	9.7339
Palm	_VLGLQDTK_.2	#N/A	7.0037	7.1009	7.4685	7.6906	7.3203	7.1953	7.3610	7.6571	7.2763	7.2051	7.1316	7.4766	7.1149	7.4386	7.4628	7.2938	6.9770	7.4391	7.0251	7.2266
Palm	_GLAEDVTR_.2	#N/A	4.3551	6.7366	5.8044	6.8408	6.0474	5.9758	6.8417	7.3194	6.6171	6.8166	6.0494	6.6468	7.1493	6.4600	6.9121	6.0075	4.4175	6.1877	6.4834	6.6318
Palm	_GLEESITR_.2	#N/A	4.7479	5.2636	4.8964	6.3862	5.0928	5.9156	5.9552	4.6078	6.0488	5.7878	5.7489	5.7947	6.1562	6.0058	6.3866	6.7410	3.3320	6.3389	6.0645	6.4013
Palm	_ADEVTLS[Phospho (STY)]EAGSTAGPAEPR_.2	Q9Z0P4_S244_M1_Palm	4.6256	7.0078	6.1201	6.9317	4.1734	6.5735	4.3689	4.2925	5.9986	6.3361	5.7855	5.9901	5.9133	7.4760	5.5771	6.7994	4.4198	6.4625	6.1331	6.5286
Palm	_SETLVNAQQT[Phospho (STY)]PLGT[Phospho (STY)]PK_.2	#N/A	12.7438	12.5000	13.2763	12.9563	11.8540	12.2732	12.0620	11.7496	12.2442	13.0453	12.5100	12.1088	12.1618	12.8797	11.6027	12.3963	12.6610	11.9982	12.2668	12.5119
Palm	_EIDVLEFGESAPAASK_.2	#N/A	6.7865	7.0850	7.3195	6.8486	6.4842	6.8036	6.8914	6.7278	6.9383	6.9990	6.8641	6.3617	7.1386	7.4355	6.8535	6.7955	7.1789	6.5306	6.8268	7.0998
Palm	_EENQTGPTTT[Phospho (STY)]PS[Phospho (STY)]TDQDLDM[Oxidation (M)]K_.2	#N/A	9.5046	9.2307	9.8237	9.6494	9.5807	9.4221	8.8221	9.1485	9.1812	9.5869	8.5874	9.2784	7.1165	9.9630	9.2148	9.2948	9.8236	8.9578	9.0803	9.4593
Palm	_[Acetyl (Protein N-term)]M[Oxidation (M)]EVLATDTAS[Phospho (STY)]QQER_.2	#N/A	9.0191	8.8915	8.8095	8.9336	8.5091	8.7296	8.3723	8.9504	8.9858	9.1227	8.3105	9.0181	9.4428	8.8259	8.8429	9.3640	9.0495	8.7979	8.8079	8.9478
Palm	_AELVVIEDAATPR_.2	#N/A	6.7544	7.0125	7.5700	6.7033	6.4133	6.7728	6.8559	6.7169	6.6111	6.6992	6.6339	6.4669	6.7917	6.9555	6.7773	6.8225	6.8365	6.8615	6.6854	6.9261
Palm	_EENQTGPTTTPS[Phospho (STY)]DT[Phospho (STY)]QDLDM[Oxidation (M)]KPR_.3	Q9Z0P4_T367_M2_Palm	3.5885	4.4143	4.1248	4.1009	2.9262	3.1503	3.1140	3.4760	3.1731	3.7948	3.5475	4.3038	3.2812	4.3258	5.4328	3.9334	3.8265	4.1762	2.9744	4.0031
Palm	_ENSAAPSPGRPQSAS[Phospho (STY)]PAKEEQK_.3	#N/A	10.2337	10.4834	11.1770	10.8957	9.8214	10.3777	10.0488	10.7045	10.2389	10.2707	10.2803	10.1323	10.5314	10.9530	10.4613	10.2067	10.5473	10.3317	10.2629	10.3738
Palm	_TST[Phospho (STY)]PVRS[Phospho (STY)]PGGSTMM[Oxidation (M)]K_.2	#N/A	4.2409	7.4401	10.6203	10.8447	9.0523	5.7831	9.8157	3.7813	8.9476	9.3498	9.7447	9.1090	3.7889	8.0360	8.4797	6.0746	9.1294	5.2204	3.6600	9.2367
Palm	_WLLEGTPSSASEGDEDMRK_.3	#N/A	4.5452	4.2748	6.1895	6.7786	3.2513	3.3563	3.7668	3.7200	3.4680	4.3558	4.3415	4.0009	4.5761	7.0888	3.8222	3.9073	4.2681	4.3822	3.1804	3.7971
Palm	_PQSAS[Phospho (STY)]PAKEEQK_.2	#N/A	4.0470	4.4492	4.0010	3.7728	3.8481	3.1175	3.5945	3.1772	3.5702	4.0029	3.4565	4.2640	3.7123	4.2541	3.4619	3.8181	3.5186	3.8674	3.5524	4.2887
Palm	_EEQKSETLVNAQQT[Phospho (STY)]PLGT[Phospho (STY)]PKENR_.3	#N/A	3.3180	3.1840	3.8543	2.6396	4.1566	1.8405	3.4579	4.5192	3.4436	3.5243	3.8180	2.2417	3.3387	3.6027	2.9617	2.2237	3.5561	4.2771	3.9257	2.1514
Palm	_ENSAAPS[Phospho (STY)]PGRPQSASPAK_.2	Q9Z0P4_S116_M1_Palm	4.5861	4.8788	3.6302	3.8195	4.3145	4.0448	4.0334	4.3900	3.8351	5.1498	4.2292	4.7771	4.3640	4.3528	4.6346	4.1099	3.6205	3.1753	4.0913	3.4568
Palm	_S[Phospho (STY)]PGGSTMMK_.2	#N/A	3.9319	3.3872	5.8313	3.6523	4.1388	3.9695	3.7956	4.1576	3.8547	3.1132	4.2291	3.1133	3.9628	3.5066	3.3728	3.2940	3.6548	3.4946	3.7937	3.1838
Twf2	_ETIELVHTEPTNVAQLPSR_.3	#N/A	5.2661	5.8653	6.3350	6.1034	5.2209	5.7273	6.2675	5.5975	5.5647	5.5917	5.8048	5.4643	5.8696	6.2688	5.8516	5.6933	5.8280	5.7221	5.9337	5.9131
Twf2	_VIIEDEQLVLGASQEPVGR_.2	#N/A	3.4240	4.3179	3.0123	4.6027	4.7392	4.4219	2.8337	4.8587	4.6024	3.2346	3.9537	3.7615	3.5028	4.0055	2.7196	4.2445	5.0805	4.3108	3.0794	4.0143
Twf2	_AVLPLLDAQEPC[Carbamidomethyl (C)]YLLFR_.2	#N/A	4.3037	4.2545	3.8403	3.9499	3.9763	1.7927	4.3449	5.4294	3.9277	4.1143	3.8023	5.0252	3.8549	4.0488	2.9533	3.6658	4.0266	3.6532	3.5257	3.0928
Sufu	_KDS[Phospho (STY)]LGSDSTAIIPHELIR_.3	Q9Z0P7_S346_M1_Sufu	4.4724	3.5304	3.5079	3.2170	3.8101	4.1748	5.3687	4.1728	4.3277	3.7451	3.8063	3.4198	4.4931	3.4950	3.3081	3.3165	3.2096	3.1127	4.2721	3.4619
Sufu	_RLS[Phospho (STY)]GKDTEQR_.3	Q9Z0P7_S301_M1_Sufu	4.0317	3.2874	3.5683	3.5525	4.0539	3.8697	4.3103	4.2574	3.9545	3.8423	4.4289	3.0135	4.0626	3.6064	2.8348	3.3938	3.7546	3.3948	3.6939	3.2837
Itsn1	_LPEEPSS[Phospho (STY)]EDEQQPEK_.2	#N/A	9.6065	9.1367	9.6333	9.6122	9.1680	9.5759	9.4017	9.5763	9.3276	9.3881	9.6712	9.5188	9.6996	10.1348	9.8748	9.5095	9.7457	9.1050	9.3772	9.4781
Itsn1	_STS[Phospho (STY)]DTGFTESPASLK_.2	#N/A	9.2932	9.1617	8.9469	9.3113	8.7173	8.7747	8.4208	7.8625	7.4868	9.2049	8.3216	8.3594	8.4895	9.1739	6.7081	8.4824	8.2914	8.5428	8.5347	8.3986
Itsn1	_QRS[Phospho (STY)]LEAAR_.2	Q9Z0R4_S623_M1_Itsn1	7.6370	7.8317	7.5135	7.7210	7.2330	7.7602	7.6468	7.7148	7.4216	7.4503	7.6427	7.2985	8.5309	8.0026	6.9313	7.6020	7.5913	7.5776	7.3981	7.7725
Itsn1	_QVQQNS[Phospho (STY)]LHR_.2	Q9Z0R4_S558_M1_Itsn1	7.5100	7.7390	7.6340	7.7175	7.4602	7.3637	7.2018	7.3931	7.2800	7.4193	7.5426	7.4555	7.8426	7.8995	7.2590	7.7541	7.4956	7.1431	7.3713	7.0581
Itsn1	_STS[Phospho (STY)]DTGFTESPASLKR_.2	#N/A	6.9603	6.8134	6.0047	6.8991	6.2437	6.3039	6.5378	6.1830	6.1372	7.2209	6.1150	6.3052	6.7600	6.9480	4.4435	6.7762	6.3233	6.5240	6.4849	6.5856
Itsn1	_KSTS[Phospho (STY)]DTGFTESPASLKR_.3	#N/A	3.5122	6.6819	4.5497	4.0720	3.7192	4.3892	6.2245	3.7379	3.4351	3.7648	3.8094	3.5330	3.5431	4.5877	3.3543	4.3752	3.2352	3.9143	4.2133	4.2650
Itsn1	_SAFTPATATGSSPS[Phospho (STY)]PVLGQEEK_.2	Q9Z0R4_S897_M1_Itsn1	7.3445	7.6451	7.7876	7.1272	6.1069	7.8278	7.5004	7.0822	6.9766	6.6440	6.6594	6.7541	7.4659	8.1101	7.4050	6.6863	6.8573	6.5495	6.4294	6.1918
Itsn1	_LLS[Phospho (STY)]PGTSKIT[Phospho (STY)]PTELPK_.2	#N/A	7.4916	7.7928	7.3801	7.6845	7.3645	7.0361	7.2659	7.5637	7.0301	7.2681	6.7073	7.1949	7.4165	7.7024	6.1427	7.3198	7.2642	7.2220	7.2379	6.9319
Itsn1	_LPEEPSS[Phospho (STY)]EDEQQPEKK_.2	#N/A	8.0197	3.7629	3.2754	3.4495	3.5776	4.4073	4.1750	3.9403	4.0952	8.2002	7.6836	7.9781	8.1101	3.2624	7.1768	3.0840	7.9356	3.3552	3.0038	3.2294
Itsn1	_SGS[Phospho (STY)]GM[Oxidation (M)]SVISSSSVDQR_.2	#N/A	9.0561	8.8865	9.2113	9.1297	8.3049	8.1405	8.1488	8.5179	8.5469	9.5890	8.0030	8.5245	9.9788	8.8612	7.3210	8.4990	8.6524	8.2709	8.1662	8.4405
Itsn1	_LLS[Phospho (STY)]PGTSK_.2	Q9Z0R4_S1130_M1_Itsn1	3.1021	5.4980	3.6384	4.5873	5.2783	4.0443	5.7755	4.3033	6.2674	6.3844	6.2136	3.2893	6.4604	3.6254	5.2561	3.4470	3.3401	2.9922	4.1416	3.5924
Itsn1	YC[Carbamidomethyl (C)]EVTM[Oxidation (M)]GS[Phospho (STY)]QC[Carbamidomethyl (C)]H	#N/A	4.2065	4.2338	4.2548	3.4711	4.7149	3.5163	4.0896	4.1577	4.3082	4.1990	4.0442	4.0330	4.1163	4.2626	3.8856	4.0702	5.3922	3.0414	3.2944	4.8109
Itsn1	_DQFS[Phospho (STY)]PDDFLGR_.2	Q9Z0R4_S1663_M1_Itsn1	4.2495	3.7533	3.2850	3.4399	3.5872	3.8113	3.9538	4.3158	4.0129	4.4558	4.3873	3.6427	4.1210	3.6647	3.5310	3.6527	3.8130	3.3364	3.6355	3.3420
Itsn1	_AQS[Phospho (STY)]FDVASAPPAAEWAVPQSSR_.2	Q9Z0R4_S203_M1_Itsn1	3.8625	3.4566	3.3991	3.7217	4.0694	4.1028	4.4795	4.0882	4.3997	3.6731	3.8783	3.1827	4.5651	3.4371	3.0040	3.2246	3.5854	3.5640	3.8631	3.1144
Bpnt1	_NYEYASHVPEEVK_.2	#N/A	5.9949	6.4504	6.4941	5.3648	6.0128	6.2044	5.8235	6.4444	6.0148	6.1396	6.0940	5.8158	6.0992	6.3224	6.1019	6.3936	6.1692	6.4372	5.8912	6.2712
Bpnt1	_IIQLIEGK_.2	#N/A	4.5874	2.9006	3.8674	4.7600	3.7131	4.3060	4.8708	2.7621	4.3525	4.9070	3.7476	3.0603	3.3519	4.2663	3.7287	4.7150	3.5692	3.8453	5.1899	4.1443
Bpnt1	_LTDIHGNALQYNK_.3	#N/A	4.4596	5.5443	3.2689	3.8519	5.0179	4.8346	5.3641	3.9580	5.3190	3.3128	4.0295	4.5890	4.3687	3.3070	5.2273	5.4786	3.4552	4.1442	5.6232	2.9843
Bpnt1	_SHSNQLVTDC[Carbamidomethyl (C)]SAMNPDTVLR_.3	#N/A	4.2151	3.4704	3.4794	3.6656	3.9773	3.9828	4.0959	4.1446	3.8418	3.1265	3.8237	3.2856	3.9498	3.4936	4.1764	3.2965	3.6419	3.5079	4.6445	4.6007
Bpnt1	_LVQM[Oxidation (M)]SVC[Carbamidomethyl (C)]SSLAR_.2	#N/A	3.6954	3.6237	3.2320	3.8888	3.9023	4.2061	3.5591	3.9211	4.7036	3.3497	3.9926	3.3498	3.7263	3.2700	3.1363	3.4883	3.4183	3.7312	4.0302	4.4481
Bpnt1	_[Acetyl (Protein N-term)]ASSHTVLMR_.2	#N/A	3.2375	4.0816	4.2749	2.7683	3.4444	3.1631	3.6037	3.4632	3.1603	3.8076	3.5347	3.8077	3.2684	2.1585	3.6290	4.1005	4.4613	4.1890	2.9872	3.9903
Xpr1	_VLIEDT[Phospho (STY)]DDEANT_.2	Q9Z0U0_T689_M1_Xpr1	3.1936	6.9401	6.3324	6.4670	4.0322	6.8036	6.2865	3.8708	5.8689	5.9221	3.9423	5.4308	3.6760	6.1932	4.0628	5.9453	7.0939	6.2736	4.0804	5.8955

Tjp2	_GSYGS[Phospho (STY)]DPEEEEYR_.2	#N/A	8.4314	8.2302	8.5310	8.6573	8.2165	8.4100	7.8146	8.1214	8.2237	8.3111	8.1835	7.8797	7.7718	8.7760	7.8826	8.0834	8.6756	7.7458	7.7534	8.2106
Tjp2	_AAS[Phospho (STY)]RDQLR_.2	Q9Z0U1_S960_M1_Tjp2	5.3728	4.6382	4.6454	5.0019	5.0126	5.2485	4.6200	5.0295	5.1849	5.0980	4.0739	4.0042	5.2362	5.3927	4.9660	4.6864	5.3740	4.3592	5.0751	5.6309
Tjp2	_SNLPATAGSEIPGGST[Phospho (STY)]K_.2	Q9Z0U1_T1009_M1_Tjp2	6.6509	6.7740	3.7685	7.1396	3.0234	3.1548	5.7877	6.5163	3.5729	4.0153	5.9955	4.0255	3.7182	4.4183	4.0728	6.2046	6.8657	3.7284	6.2284	3.7226
Tjp2	_S[Phospho (STY)]IDRDYDR_.2	#N/A	4.4504	5.3343	5.5627	3.6950	3.7881	5.7824	4.7218	4.1149	4.8994	5.2895	4.1864	3.1560	3.9201	5.7124	3.7643	5.3636	3.6122	3.5373	4.4331	5.1600
Tjp2	_S[Phospho (STY)]IDRDYDRDYER_.3	#N/A	5.1200	7.1585	6.1271	6.9809	6.0958	5.8120	6.0832	5.1693	5.9509	6.6443	6.4914	6.7013	6.1623	5.8754	3.1628	6.1660	6.7023	6.5977	6.1099	6.5634
Tjp2	_S[Phospho (STY)]QNREDSFDYSK_.2	Q9Z0U1_S982_M1_Tjp2	4.0060	4.1604	6.5106	3.6883	6.4383	3.4827	3.6825	6.3812	5.5529	5.6787	3.8450	6.3961	5.8151	4.3179	6.0408	6.3349	6.2117	6.5048	3.8297	6.3076
Tjp2	_S[Phospho (STY)]RGRS[Phospho (STY)]LER_.2	Q9Z0U1_S169_M2_Tjp2	4.3497	3.7210	3.0399	3.5943	4.0729	3.5087	3.9554	4.2156	4.0325	3.3517	3.7940	3.6668	4.7089	4.2328	3.8931	3.8669	3.5543	3.3974	3.4793	3.0664
Tjp2	_GRS[Phospho (STY)]IDRDYDR_.3	#N/A	3.8051	3.9957	4.3608	4.2609	3.9034	3.7394	3.5178	3.5490	3.4043	3.1641	2.9167	3.7219	3.6035	4.3988	4.0636	3.6284	2.4425	3.3040	4.4022	3.2583
Tjp2	_KVQVAPLQGS[Phospho (STY)]PPLSHDDR_.3	#N/A	8.6870	8.8920	8.4464	8.1414	6.9095	8.0914	8.4555	9.1291	7.6529	8.1929	7.0798	8.5850	8.8275	9.7469	7.1973	7.6340	8.2397	8.3058	8.0520	7.7496
Tjp2	_EHLRS[Phospho (STY)]RS[Phospho (STY)]PS[Phospho (STY)]PESR_.3	#N/A	5.4230	5.2317	3.5542	5.1900	5.9415	5.6910	5.7068	4.3696	5.9087	5.3950	4.8384	4.5678	6.4869	4.8904	3.2904	4.9163	5.3537	6.2939	5.7181	5.2118
Tjp2	_DAS[Phospho (STY)]PPPAFKPEPPK_.2	#N/A	11.4138	11.4051	11.3725	11.4205	11.0849	11.2992	10.8721	11.3757	10.9691	11.5344	11.0871	11.1390	11.5484	11.7615	10.8450	11.2020	11.5801	11.0587	11.0929	11.1493
Tjp2	_SRS[Phospho (STY)]PS[Phospho (STY)]PESR_.2	Q9Z0U1_S265_M2_Tjp2	3.7770	6.8830	7.2109	5.2852	6.8820	7.6379	6.7958	7.1440	7.4561	7.1599	6.7756	6.9672	6.6677	3.7620	3.1325	3.9532	7.1978	6.8034	3.7832	7.2061
Tjp2	_RQQYS[Phospho (STY)]DQDYHS[Phospho (STY)]S[Phospho (STY)]TEKLK_.3	Q9Z0U1_S404_M3_Tjp2	4.0177	4.5036	3.9479	3.6770	3.3554	3.2832	4.0826	3.7181	4.1591	4.1240	3.3274	3.8799	4.0384	5.0464	3.7682	3.7565	4.2558	3.5827	3.2313	3.9020
Tjp2	_VQVAPLQGS[Phospho (STY)]PPLSHDDR_.3	#N/A	4.0183	3.6476	6.7868	3.3342	3.8063	7.2778	3.6574	5.4796	4.2366	3.8624	6.6694	3.5371	6.1210	6.5434	5.3402	6.4938	3.9604	6.5928	6.6092	6.6198
Tjp2	_GSYGS[Phospho (STY)]DPEEEEYRQQLAAHSK_.3	#N/A	9.5055	9.9241	9.2397	9.4840	8.5479	9.5137	9.2505	9.0403	9.4566	9.8169	8.4520	9.4648	9.8006	10.0838	9.2570	9.6893	9.6591	9.5705	9.5137	9.7104
Tjp2	M[Oxidation (M)]SYLTAM[Oxidation (M)]GADYLS[Phospho (STY)]C[Carbamidomethyl (C)]DSR	#N/A	3.9585	4.5475	3.7953	3.9237	3.8170	4.0009	3.9734	3.9325	3.9343	4.9620	3.7470	3.4621	4.0244	4.2838	4.0491	3.6406	3.9816	3.7660	4.0651	3.5106
Tjp2	_SYHEAYEPDYGGGYS[Phospho (STY)]PSYDR_.3	Q9Z0U1_S239_M1_Tjp2	3.7693	4.7449	4.0325	3.7945	3.9762	3.6541	3.9202	3.9291	3.7928	3.5799	2.9402	4.7576	3.9241	3.8779	3.9456	3.8580	3.8554	3.6755	3.6097	3.7915
Tjp2	_ERPS[Phospho (STY)]SREETSGR_.3	Q9Z0U1_S420_M1_Tjp2	4.1178	3.5665	3.4075	3.5237	3.9240	4.0342	4.1690	4.2927	4.1520	4.1538	3.3808	3.3898	4.2911	3.6417	3.2523	3.2255	3.7900	3.3616	3.6607	3.3190
Tjp2	_HSSHDMLSHS[Phospho (STY)]WEGNRER_.4	#N/A	3.2579	3.2441	3.7942	4.4315	4.0964	3.8885	3.4447	4.4591	3.1131	3.4642	4.0926	3.1336	3.2786	3.7812	3.0219	3.6028	3.4959	4.3372	3.5327	3.7482
Tjp2	_HSSHDMLSHS[Phospho (STY)]WEGNR_.3	#N/A	4.5613	4.7115	4.0979	4.5237	3.2674	3.3401	3.7807	3.2862	2.9833	3.9846	3.3577	3.9847	4.5922	4.1360	3.8060	3.9235	4.2843	4.3660	3.1642	3.8133
Tjp2	_RQQYS[Phospho (STY)]DQDYHSSTEK_.3	Q9Z0U1_S404_M1_Tjp2	3.8208	4.1820	4.3571	3.8685	3.1586	0.3061	4.3825	3.5212	3.6761	4.3967	3.1548	4.0714	3.8416	4.3442	3.9597	4.1657	4.0589	3.7743	3.4228	4.3112
Tjp2	_SRHS[Phospho (STY)]S[Phospho (STY)]HDMLSHSWEGNR_.4	Q9Z0U1_S139_M2_Tjp2	3.8533	3.4492	3.5891	3.1357	4.0602	4.0936	2.9879	4.2540	4.4089	3.6639	3.8876	3.3386	4.5744	3.5762	3.0132	3.2154	3.2909	3.0415	4.1909	3.5432
Tjp2	_LAGGNDVGIFVAGIQEGTSAEQEGLQEGDQILK_.3	#N/A	3.8730	4.1298	4.4093	3.8164	3.2107	3.4348	4.3303	3.5734	4.3894	4.0793	3.2630	4.0192	4.4975	4.3964	3.9075	4.2179	4.1111	4.4608	3.2590	3.7185
Kcnd2	_GS[Phospho (STY)]VQELSTIQIR_.2	Q9Z0V2_S552_M1_Kcnd2	8.9462	8.9540	9.2898	8.6937	3.4152	8.8179	8.4405	8.2646	8.6719	4.2838	8.6235	7.8956	8.4088	8.5752	7.3519	8.0847	9.1399	8.9363	8.4833	8.1613
Kcnd2	_TPLSNS[Phospho (STY)]RSS[Phospho (STY)]LNAK_.2	Q9Z0V2_S575_M2_Kcnd2	8.4301	7.9901	8.1222	3.6712	3.7507	3.7227	3.8055	4.0062	3.8791	8.3669	3.7339	8.2324	4.1364	3.7616	3.9999	3.5725	3.6279	7.3166	3.7872	7.0425
Kcnd2	_IPNANVS[Phospho (STY)]GSHR_.2	Q9Z0V2_S546_M1_Kcnd2	4.3790	4.4410	3.9155	3.2053	3.0850	7.1274	3.9631	3.1038	3.8833	4.1895	3.3619	7.0527	8.1280	3.9536	3.9884	3.7411	4.1019	7.7791	3.3466	3.6309
Ngfr	_ADIVESLC[Carbamidomethyl (C)]SESTATS[Phospho (STY)]PV_.2	Q9Z0W1_S425_M1_Ngfr	3.1324	4.2291	4.3100	3.9157	3.3393	3.3726	3.7088	3.4741	3.6290	4.4438	3.1077	4.1185	3.7945	4.2971	3.7342	3.9953	4.0118	4.1268	3.4700	4.2641
Aifm1	_AIASATEGGSVPQIR_.2	#N/A	3.9152	4.5387	5.6147	3.6691	4.1221	4.8158	4.6672	4.1408	4.9083	3.7257	4.2124	4.3697	3.9460	5.3452	4.6686	3.2773	3.6381	4.7539	3.8104	4.8458
Aifm1	_SITVIGGGFLGSELAC[Carbamidomethyl (C)]ALGR_.2	#N/A	4.3606	4.4593	3.8972	3.2236	3.0667	3.5408	4.2244	3.0855	2.8550	1.4942	3.1570	4.1854	4.3915	3.9353	3.8016	4.3239	4.0835	3.0659	2.6539	3.6126
Dctn3	_[Acetyl (Protein N-term)]AALTDVQR_.2	#N/A	6.9121	7.2062	7.0669	7.2381	7.2123	6.9832	7.0813	6.5032	7.1702	7.1221	6.9666	6.7746	6.4002	6.3925	6.9536	6.7152	6.5945	6.8697	6.6486	6.9415
Dctn3	_VQVALGNIASK_.2	#N/A	5.0137	5.3075	5.6468	4.9513	4.6255	5.7215	4.8902	4.7465	5.6094	5.6481	4.7286	4.6892	5.0289	5.6153	4.9958	5.0460	5.7672	4.8971	4.6811	5.1052
Dctn3	_LAQIHIQQQDQC[Carbamidomethyl (C)]VAITEESK_.3	#N/A	3.2767	5.4966	4.9298	6.5709	4.1153	5.8232	5.2156	6.7406	5.3504	5.8376	4.1115	5.5456	3.2974	3.8001	3.2371	4.5954	3.5148	4.3184	3.9670	3.7671
Pard6a	_LTGPSSVGPGPTDPDS[Phospho (STY)]DDDSSDLVIENR_.3	Q9Z101_S278_M1_Pard6a	3.9532	3.3659	3.4898	3.6310	4.1601	3.9482	3.8169	4.1789	3.8760	3.0919	4.2504	3.0920	3.9841	9.0492	2.9133	3.3153	3.6761	8.3363	3.7724	3.2051
Adnp	_LM[Oxidation (M)]HDAS[Phospho (STY)]DS[Phospho (STY)]EVDQDDVVEWK_.3	#N/A	3.7901	5.0127	4.1434	3.9498	3.7301	5.1496	4.7795	3.9025	3.5860	3.6906	3.8604	3.6856	3.7341	5.8147	4.2139	3.9642	3.6587	5.3774	3.7953	3.8305
Hnrnpdl	_VFVGGLS[Phospho (STY)]PDTSEEQIK_.2	Q9Z130_S122_M1_Hnrnpdl	3.5059	3.9862	4.3703	6.1555	4.3445	3.0677	3.1966	3.5585	3.2557	3.7122	3.6301	4.3863	3.3637	4.4083	4.2746	6.0061	4.5566	4.9237	4.3927	4.0856
Hnrnpdl	_DLTEYLSR_.2	#N/A	4.8337	3.0302	4.0783	4.8393	3.6227	4.7144	4.8438	4.2804	5.1568	5.4867	4.6556	3.5326	5.5164	4.7041	5.3681	5.9459	3.7776	5.0140	5.1388	4.9982
Hnrnpdl	_YHQIGSGK_.2	#N/A	4.0282	4.5775	3.1913	4.1963	4.2775	3.7482	4.3423	3.9488	4.5356	5.2209	4.3558	4.6780	3.4188	3.4986	4.9314	5.1706	3.6744	4.0243	4.4239	4.4443
Hnrnpdl	_[Acetyl (Protein N-term)]MEDM[Oxidation (M)]NEYSNIEEFAEGSK_.2	#N/A	3.8489	4.0199	4.1345	4.7969	4.2288	4.0507	3.8444	4.1202	3.8456	4.0837	3.9205	3.6669	3.9772	5.8177	3.4607	3.5055	3.6175	3.5879	3.7339	3.6684
Cpne6	_NNLNPSWEPFR_.2	#N/A	6.7264	6.8072	6.9418	6.8166	6.8921	6.6958	6.1621	6.8005	6.7558	6.9485	6.6658	6.7240	6.4834	6.4552	6.6837	6.7553	6.9519	6.6597	6.7891	6.9320
Cpne6	_LLDGDDGPLR_.2	#N/A	6.6876	6.1882	6.9397	6.9599	6.0792	7.2145	6.8015	6.5132	5.9794	6.3449	6.5393	6.9574	6.6281	6.5181	6.9436	6.1587	7.2763	6.1693	6.5816	6.7449
Cpne6	_SC[Carbamidomethyl (C)]SS[Phospho (STY)]PVFSR_.2	Q9Z140_S68_M1_Cpne6	12.4674	12.6347	12.5502	12.6320	12.4686	12.5775	12.3550	12.7035	12.4977	12.5382	12.4192	12.5397	13.0212	13.0319	12.2037	12.4111	12.8379	12.5133	12.4896	12.5850
Cpne6	_C[Carbamidomethyl (C)]VLAEVPR_.2	#N/A	4.2091	4.9636	4.4870	3.4802	3.5469	4.4814	4.6008	4.4157	5.4221	4.4155	3.5431	5.3524	4.0920	3.9025	4.4470	4.7784	4.2354	5.4380	4.6203	3.1987
Cpne6	_PSTPAM[Oxidation (M)]TPSPS[Phospho (STY)]P_.2	#N/A	11.8445	11.6201	11.7258	11.4549	11.5387	11.9850	11.3506	11.9818	11.7929	11.6436	11.6505	11.6385	12.2130	11.9998	11.6839	11.8125	12.2757	11.7175	11.9113	11.8323
Cpne6	_SSQSLHC[Carbamidomethyl (C)]LS[Phospho (STY)]PR_.2	#N/A	10.4205	10.5402	10.3917	10.5453	10.1092	10.6644	10.2332	10.7360	10.4297	10.6021	10.2439	10.3932	10.9833	10.7393	10.2512	10.4375	10.8032	10.5849	10.5621	10.4096
Cpne6	etyl (Protein N-term)]S[Phospho (STY)]DPEM[Oxidation (M)]GWVPEPPAM[Oxidation (M)]TLGA	#N/A	6.5002	8.4859	6.0170	3.9115	3.1159	6.5871	8.2284	7.3741	6.2949	6.7492	3.2180	7.5254	7.8018	8.8403	6.5338	6.2115	6.5430	9.4749	7.4385	6.3595
Cpne6	_LYSDEQWVEVER_.2	#N/A	3.3269	6.3569	4.5493	4.0724	4.1655	4.3896	3.3756	5.8072	6.1476	3.5333	5.9442	3.5334	3.5427	4.5873	2.9528	5.1076	3.2347	5.6114	6.4611	5.7683
Cpne6	_YSVLLVLTDGVVSDM[Oxidation (M)]AETR_.2	#N/A	3.8623	3.4568	2.8725	3.7220	4.0692	4.1025	4.4797	4.2451	4.3999	3.6729	3.8786	3.1829	4.5654	3.5672	3.0043	4.7543	3.2819	3.0504	4.1999	4.2239
Cpne6	_TEVLRSC[Carbamidomethyl (C)]S[Phospho (STY)]S[Phospho (STY)]PVFSR_.2	Q9Z140_S67_M2_Cpne6	4.4704	3.5324	3.5059	3.2190	3.8081	4.0322	3.7329	4.1708	3.7920	3.1759	4.1664	3.4218	3.9001	3.4929	3.3101	3.3145	3.2076	3.1247	5.3262	3.4599
Cpne6	_VLAIEYFFEEK_.2	#N/A	4.0399	3.2792	3.5765	3.5443	4.2468	4.2802	4.3021	4.2656	4.2223	3.8505	3.7010	3.0053	4.3878	3.6145	2.8267	3.4020	3.7628	3.3866	3.6857	3.7537
Sumo3	_VAGQDGSVVQFK_.2	#N/A	6.4002	7.1726	6.9138	7.1992	6.5480	6.7086	6.6598	6.6648	6.8259	6.9289	6.5102	6.9497	6.9453	7.1878	7.3949	7.0111	6.6035	6.3353	6.9951	6.5940
Sumo3	_[Acetyl (Protein N-term)]S[Phospho (STY)]EEKPKEGVK_.2	Q9Z172_S2_M1_Sumo3	5.1279	5.2485	4.8233	5.1682	4.7582	4.8227	4.4365	5.6532	4.8984	5.4829	4.6040	5.4090	6.2811	5.1671	5.3116	5.1572	5.6883	5.2464	5.7210	5.3389
Eya4	_NNPS[Phospho (STY)]PPPDSDLER_.2	Q9Z191_S338_M1_Eya4	9.0506	9.3055	3.2015	9.3019	8.1050	9.3546	8.9328	8.3906	3.5878	3.3802	8.4250	3.3803	3.6958	9.8236	3.1059	3.5187	3.3879	3.7616	4.0606	8.9778
Plcb1	_NDSIPQEDFTPDVYR_.2	#N/A	8.5130	7.9917	8.4412	8.6038	8.0130	8.1472	8.0776	8.5001	8.5166	8.5128	8.1950	8.2283	8.8425	8.4972	8.1617	8.4203	8.7450	8.1481	8.1544	8.5199
Plcb1	_YNEIQNDYLR_.2	#N/A	7.2150	6.9743	7.0368	7.3306	7.1318	7.1636	7.0558	6.9484	7.5325	6.9804	7.0186	7.0197	7.5968	7.2800	7.0836	7.3232	7.4090	7.2523	7.1409	7.0882
Plcb1	_WDDDSTIVTPIILR_.2	#N/A	6.8907	6.4722	7.5568	7.1728	6.7444	6.7432	6.3617	6.4028	6.7877	6.9560	6.9386	6.3123	6.8758	6.5284	6.7262	7.4207	6.6756	6.2530	6.6260	6.7503
Plcb1	_SYIQEVVQYIK_.2	#N/A	5.2276	4.9419	5.1568	5.0924	5.1558	5.1183	5.2400	4.7450	4.7280	4.4750	5.2968	5.1912	4.3372	4.6669	4.9821	5.1458	5.0117	4.9022	5.1157	5.7470
Plcb1	_SPVEFVEYNK_.2	#N/A	7.0267	6.9483	7.8127	7.2790	6.6913	7.1118	6.6604	6.5205	7.3112	6.9334	7.2255	6.9568	5.8467	7.3956	6.7496	6.7575	7.5389	7.0109	7.0325	7.3869
Plcb1	_EVIEAIAEC[Carbamidomethyl (C)]AFK_.2	#N/A	5.3564	5.9151	5.6089	6.1354	5.5843	5.8180	5.8603	6.2877	6.2701	5.6564	5.9987	6.0420	4.0320	6.4427	5.7152	5.4962	6.4883	5.7303	5.6706	5.4699
Plcb1	_LQVTPEGR_.2	#N/A	3.5563	5.7872	5.6564	5.7284	5.2830	5.9866	5.7763	5.8220	6.1949	5.9635	5.1485	5.0270	5.7931	5.6270	5.8149	5.8362	5.7489	6.1407	5.6029	5.4609
Plcb1	_LIFGDALLM[Oxidation (M)]EPLEK_.2	#N/A	4.8560	4.2703	5.4295	3.8549	4.9198	5.4450	4.0267	4.3864	4.8340	4.1360	5.0856	4.9459	3.7602	2.8392	4.8310	5.3066	4.7591	4.9858	4.5432	4.6838
Plcb1	_EADPGETS[Phospho (STY)]SEAPSETR_.2	Q9Z1B3_S848_M1_Plcb1	8.2799	8.1451	4.1093	8.1268	7.5719	8.1046	7.8769	3.8282	7.5950	8.0623	7.7867	7.5721	7.4984	8.4002	6.9928	7.5542	3.7842	7.8267	7.7242	3.8247
Plcb1	_VGTYVEVDM[Oxidation (M)]FGLPVDTR_.2	#N/A	6.1361	3.3557	4.6414	4.5431	4.9618	5.6649	5.6233	4.3475	5.3069	4.8320	5.9132	5.8413	3.1670	3.6696	5.2377	5.0108	3.3843	4.6787	5.6403	5.4255
Plcb1	_TSPFPILLSFENHVDSPK_.2	#N/A	4.7777	3.6554	4.9996	2.8507	4.4841	4.8243	3.8990	5.2534	4.2026	3.8702	5.3387	4.5656	4.3684	4.4106	4.6709	4.5007	4.2043	4.6661	4.3807	3.3369
Plcb1	_SEPSS[Phospho (STY)]PDHGSSAIEQDLAALDAEM[Oxidation (M)]TQK_.3	#N/A	11.4895	11.2393	10.7457	7.7813	10.6541	10.8108	10.8096	11.0085	10.8370	11.3844	11.1182	11.1072	11.4587	8.6186	10.5347	11.1598	11.4004	11.2333	11.1691	11.1443
Plcb1	_VNLKS[Phospho (STY)]PSSEEIERENPGR_.3	#N/A	3.7759	4.7704	3.8614	3.7671	3.9829	3.9645	4.1520	5.3500	5.2053	6.1744	4.0139	5.6256	4.0264	3.6888	3.1396	5.8213	3.3183	3.6328	3.9744	3.5781
Plcb1	_YVNLM[Oxidation (M)]EQR_.2	#N/A	3.7771	5.8073	4.3134	6.1994	3.1148	5.5585	5.0441	5.9754	5.6867	5.3056	3.3589	6.3440	4.5934	5.7501	6.1287	5.8204	5.2596	6.3216	5.5996	5.9791
Plcb1	_SPS[Phospho (STY)]SEEIER_.2	#N/A	9.3096	8.8135	9.1693	9.3983	3.1921	8.5081	8.6100	7.9987	3.7059	9.4505	8.8843	8.0750	3.8713	8.9430	7.1844	8.3168	8.2574	8.3674	3.4035	8.7588
Plcb1	_ELLDVGNIGHLEQR_.2	#N/A	3.7706	4.3185	3.7784	3.7470	4.3498	3.6589	3.2093	3.6720	3.4075	4.4707	4.0121	3.0911	3.9974	3.2117	3.8178	3.8731	3.7172	3.5223	4.7152	4.3368
Plcb1	_KSEPSSPDHGSS[Phospho (STY)]AIEQDLAALDAEM[Oxidation (M)]TQK_.4	#N/A	5.0990	4.9914	3.8883	4.1228	3.4213	4.0648	3.6454	4.1776	3.6394	6.4877	3.6712	4.0382	7.3014	3.9786	3.8986	3.7019	4.0129	7.2228	6.8430	3.6986
Plcb1	_FESFEISK_.2	#N/A	4.1515	4.3703	4.6849	3.3961	3.4892	3.5749	4.0518	4.4138	5.0633	4.3578	4.5056	4.1322	4.2190	5.3417	4.8371	2.9956	3.9110	3.4086	4.9595	5.3963
Plcb1	_C[Carbamidomethyl (C)]VELDC[Carbamidomethyl (C)]WK_.2	#N/A	3.7109	4.3410	3.8054	4.2252	2.9996	3.4845	4.4612	3.3622	3.5171	3.8682	5.0373	4.0054	3.6826	4.1852	4.4631	2.9853	3.5964	3.9228	4.7533	4.1522
Plcb1	_VNLKS[Phospho (STY)]PSSEEIER_.2	#N/A	8.6116	9.3456	8.0795	6.8527	7.8521	8.5509	8.7323	7.8591	8.6439	9.6305	7.9784	8.5527	8.1742	9.4400	7.5034	9.1890	9.2573	8.8327	9.0656	9.1736
Plcb1	_LTDVAEEC[Carbamidomethyl (C)]QNNQLKK_.2	#N/A	3.8878	1.9753	3.5837	3.2103	3.6959	3.7737	3.8447	4.1854	3.4278	3.7456	3.2492	3.6848	3.4502	3.9003	3.4194	2.6165	3.5260	3.3229	3.1195	3.9367
Plcb1	_KKSEPSSPDHGS[Phospho (STY)]S[Phospho (STY)]AIEQDLAALDAEM[Oxidation (M)]TQK_.4	#N/A	9.7143	4.3131	4.2221	7.2437	3.8994	7.4247	7.3116	3.3961	7.8429	3.5855	8.9396	7.7877	3.3960	4.2800	7.3870	3.8034	7.0167	4.1236	3.7867	7.8533
Plcb1	_SPS[Phospho (STY)]SEEIERENPGR_.2	#N/A	4.4477	3.8943	3.8148	4.1594	3.4861	3.6331	3.8274	3.8041	3.8193	4.4584	3.8388	3.7945	4.3091	3.8528	3.6440	3.5884	4.0011	4.0017	3.4997	3.5301
Plcb1	_DYVPDTYADVIEALSNPIR_.2	#N/A	4.0882	5.1904	4.3777	4.5569	4.2131	4.4469	4.6103	4.6391	5.2660	4.6449	4.7858	4.7551	3.7504	4.8023	4.5258	4.3923	3.9677	4.6943	4.4450	4.1284
Plcb1	_SPS[Phospho (STY)]SEEIERENPGREFDTPL_.3	#N/A	3.6864	4.3164	4.2227	4.0030	3.0241	5.7502	3.0161	3.3868	5.6294	3.8927	3.4496	4.2059	3.1833	4.2098	4.0942	4.0313	3.9245	4.2741	3.0724	3.9051
Plcb1	_VFLNNLC[Carbamidomethyl (C)]PRPEIDNIFSEFGAK_.3	#N/A	3.8262	3.8926	4.3625	3.8632	3.1639	3.3202	3.5209	4.2558	3.6814	4.3914	3.1601	4.0661	2.5676	3.5863	3.1804	3.6429	3.8841	3.9323	3.4175	3.5306
Eif3g	_GIPLPTGDTS[Phospho (STY)]PEPELLPGDPLPPPK_.3	Q9Z1D1_S42_M1_Eif3g	3.5545	4.4549	6.5777	5.8403	3.5407	6.6347	7.6126	7.1556	3.2116	6.7528	3.8313	3.9660	7.2097	4.2018	3.9671	3.8994	4.0973	4.1763	3.9657	3.9752
Gys1	_HSS[Phospho (STY)]PHQS[Phospho (STY)]EDEEEPR_.2	#N/A	3.6658	3.4931	3.4676	4.2006	3.4899	3.9061	3.8651	4.0870	3.0979	3.4764	4.0774	3.3818	3.9118	3.4839	3.2031	3.5735	3.4639	3.9404	3.9984	3.8440
Atp6v1c1	_GVTQIDNDLK_.2	#N/A	8.1109	8.0234	7.9443	8.2207	7.9562	8.0522	8.0364	8.2892	7.8871	7.8842	7.9374	7.8914	8.2773	7.6094	7.3904	7.5918	7.7171	7.9782	7.8116	7.9053
Atp6v1c1	_[Acetyl (Protein N-term)]TEFWLISAPGEK_.2	#N/A	7.0363	7.3382	7.1015	7.6376	7.0844	7.0961	7.4481	7.0984	7.2924	7.3249	7.3000	7.0965	7.3742	6.3326	6.6390	7.0545	7.4905	7.0183	7.1994	7.1045
Atp6v1c1	_GNLQNLER_.2	#N/A	7.0213	7.4879	6.5778	6.8668	7.0961	7.0488	7.5439	7.3637	6.9034	6.9207	6.5880	7.0330	7.4223	6.6269	6.7307	6.8082	6.9149	7.4075	6.9629	7.0590
Atp6v1c1	_VGTLDVLVGLSDELAK_.2	#N/A	6.4295	5.9709	5.8470	5.8681	6.2065	5.9301	5.5384	6.0264	5.9223	5.9520	6.4156	5.9043	5.3161	4.8098	3.7825	4.9700	5.7366	5.8508	5.8739	5.7429
Atp6v1c1	_IDC[Carbamidomethyl (C)]NLLEFK_.2	#N/A	5.3490	5.1503	4.1784	5.3546	5.3712	5.8064	5.7144	5.6841	5.1636	5.4176	5.0943	5.3866	4.2699	4.9445	5.1049	4.4132	5.7539	5.3581	5.1055	5.0257
Atp6v1c1	_SSNVLSEDQDSYLC[Carbamidomethyl (C)]NVTLFR_.2	#N/A	5.7557	5.7004	4.7581	4.0871	4.5448	4.5419	5.5770	4.8276	4.0227	5.3761	4.8960	4.7202	3.9342	5.4527	4.4331	4.2780	5.0944	5.2291	4.7881	5.5795
Atp6v1c1	_ASAYNNLK_.2	#N/A	5.5600	5.9423	5.9397	5.8691	5.9838	6.0878	6.5188	4.8078	5.9860	5.9013	5.5920	5.6333	6.0361	4.7529	5.5031	5.8361	6.2726	6.0473	5.5775	5.3780
Atp6v1c1	_NNNLAVSSK_.2	#N/A	4.7326	5.1797	4.3874	5.1137	5.0003	5.3608	4.7415	5.0184	5.0676	5.4483	4.9593	5.0039	4.9565	5.3463	4.7914	4.3037	4.6087	5.2019	4.4567	5.0502
Atp6v1c1	_KAVDDFR_.2	#N/A	6.7924	5.8571	6.6189	5.8759	5.9580	6.0571	6.1677	6.2437	6.2009	6.4256	6.1318	6.0836	3.6871	5.4316	5.5140	5.7149	6.1725	6.3737	5.7930	5.9572
Atp6v1c1	_VQENLLASGVDLVTYITR_.3	#N/A	4.8899	4.9736	4.6471	4.4048	3.3972	4.8213	3.9634	4.0496	4.6808	4.8667	4.7128	4.1615	5.1172	4.8387	3.7191	4.6060	4.1804	4.2463	4.6030	4.3223
Atp6v1c1	_FNIPDLK_.2	#N/A	4.3439	2.5669	3.8804	3.7105	3.3154	4.2826	4.1978	3.0687	2.2009	3.5268	3.1402	4.6384	3.6880	3.1569	2.4028	3.0772	4.0668	2.4815	4.3363	3.6961
Atp6v1c1	_NISEIIAK_.2	#N/A	3.3118	0.5285	4.3492	2.7174	3.5188	3.5521	3.9777	3.5375	1.7589	3.1224	2.9282	3.7334	3.3427	1.5110	3.5547	1.7182	4.5356	2.9479	2.3942	2.3204
Atp6v1c1	_TC[Carbamidomethyl (C)]QQTWEK_.2	#N/A	4.3746	4.4453	3.9112	3.2096	3.0807	3.1140	3.9674	3.0994	3.8876	4.1852	3.3663	4.5062	4.0531	3.9492	6.6357	3.7367	4.0975	3.0520	3.3510	3.0219
Atp6v1c1	_SRAS[Phospho (STY)]AYNNLK_.2	Q9Z1G3_S141_M1_Atp6v1c1	3.5033	3.8157	4.5408	4.0809	3.7103	3.7436	3.3378	3.1031	3.2580	3.3139	4.2375	3.5419	3.4235	3.9261	3.3632	4.3663	3.6408	4.1923	3.8409	3.8931
Atp6v1c1	_LDAFVEGVVK_.2	#N/A	4.1628	3.1563	3.6994	3.4214	2.8689	4.4031	4.1792	4.3885	4.0994	3.9734	3.5781	2.5719	4.1937	3.7374	4.2046	3.5249	3.8857	3.2638	3.5628	3.4147
Atp6v1c1	_VAQYMADVLEDSKDK_.3	#N/A	4.0880	3.2310	3.6246	3.4962	4.2950	4.3283	4.2540	4.0193	4.1742	3.8986	3.6528	2.9572	4.3396	3.3414	4.2793	3.4502	4.5569	3.2762	4.4256	3.3084
Atp6v0a1	_DLNPDVNVFQR_.2	#N/A	8.9967	8.6931	8.4955	8.3177	8.6139	8.7135	8.7831	8.9125	8.5661	8.5827	8.6112	8.5922	8.7496	8.1517	8.1666	8.1874	8.9666	8.6846	8.4510	8.3103
Atp6v0a1	_FLPFSFEHIR_.2	#N/A	7.2635	7.6238	6.8154	6.6127	6.4621	7.0685	7.3443	6.9337	6.3537	6.9373	6.5799	7.2834	6.6231	6.3273	6.2328	6.3564	7.4183	7.6041	6.9790	6.5708
Atp6v0a1	_HLGTLNFGGIR_.2	#N/A	7.7324	7.5688	6.2513	6.8875	7.0011	7.3040	7.4590	7.3576	6.4744	6.9702	7.1522	7.2549	7.1871	6.4849	6.2641	6.7327	7.5271	7.2910	6.6412	6.8033
Atp6v0a1	_LGFVAGVINR_.2	#N/A	6.9626	6.9381	6.2321	6.2868	6.7683	6.6469	7.1369	6.7536	6.6994	6.0602	6.7746	6.2557	6.3909	6.6837	5.6147	5.9608	6.5726	7.1259	6.3372	6.5439
Atp6v0a1	_NFLELTELK_.2	#N/A	5.8033	6.5913	6.2539	6.8293	6.2005	5.8455	6.3782	3.5419	6.5881	6.2547	6.2041	6.3312	6.2903	5.8553	5.6152	5.9026	5.8232	6.8494	6.8373	6.6946
Atp6v0a1	_FYTGTGFK_.2	#N/A	5.4092	6.0968	4.4188	6.2502	6.2680	5.3925	6.8311	4.8183	6.1760	6.2262	6.4597	6.1114	6.0767	5.3338	5.5925	5.5443	4.7819	6.7206	6.7188	6.0983
Atp6v0a1	_GTEHSGSTVPSILNR_.2	#N/A	6.0730	5.6547	5.9232	5.0758	5.5114	5.6084	5.9984	5.7926	6.0521	5.2048	5.0526	5.3043	4.3242	4.9117	5.1254	5.0076	5.3271	5.5426	5.3994	4.4515
Atp6v0a1	_KANIPIMDTGENPEVPFPR_.3	#N/A	4.4826	6.2271	6.4627	6.8542	5.2895	6.0813	6.8515	4.0827	4.2653	3.1881	5.6805	4.5292	3.8879	6.3181	4.8357	3.3267	5.2170	4.4096	3.7070	4.0698
Atp6v0a1	_ANIPIM[Oxidation (M)]DTGENPEVPFPR_.2	#N/A	7.0020	7.1667	6.6897	6.2446	7.0099	6.9743	6.9904	7.2490	6.9803	6.8725	6.8607	6.9792	6.6165	6.1786	6.1007	6.7221	6.7591	6.9906	6.5553	6.1607
Atp6v0a1	_LHWVEFQNK_.2	#N/A	6.1891	4.2168	4.3223	5.4301	5.8810	6.0179	5.6974	3.4864	5.6786	5.8404	3.1199	5.4964	3.8067	5.5935	4.5087	3.9831	6.1513	5.6185	4.4176	5.0438
Atp6v0a1	_IDDLQM[Oxidation (M)]VLNQTEDHR_.3	#N/A	5.3270	5.4405	4.0750	4.7988	5.0888	4.9139	4.8769	4.7358	4.3355	4.6766	5.0883	5.0209	4.7288	4.2499	3.5032	4.8723	4.8726	5.9798	5.2226	5.2801
Atp6v0a1	_QAEIENPLEDPVTGDYVHK_.3	#N/A	7.0173	6.7809	6.9226	7.0111	6.5539	6.8515	7.0442	7.1529	6.9879	6.7997	7.0182	6.4227	6.7951	6.5440	6.3286	6.9420	6.7191	6.7536	6.5449	6.3214
Atp6v0a1	_FTHGFQNIVDAYGIGTYR_.3	#N/A	7.0176	7.0321	5.0774	6.2037	6.4322	6.5278	6.5109	6.7986	6.2313	6.1952	6.1512	6.3529	6.9131	6.2651	5.0315	6.0695	6.6112	6.9723	6.1890	6.2343
Atp6v0a1	_AIYHTLNLC[Carbamidomethyl (C)]NIDVTQK_.2	#N/A	3.6200	2.5270	3.1566	1.5818	3.8269	2.4528	3.3744	4.8355	3.1414	3.4306	3.5826	3.6089	5.0077	3.4921	2.6305	2.9821	3.1567	2.4535	3.5658	3.7765
Atp6v0a1	_ASLYPC[Carbamidomethyl (C)]PETPQER_.2	#N/A	8.5022	8.5585	8.0733	8.0843	8.3062	8.3438	8.5926	8.3755	8.4163	7.9860	8.1409	8.3830	8.3650	7.7674	7.7532	7.6856	8.1276	8.2590	8.0218	7.9497
Atp6v0a1	_EINTNQEALKR_.2	#N/A	5.1018	5.0246	5.5642	5.2531	5.3364	5.0834	4.7441	5.3841	5.0021	4.9856	5.1551	5.0394	4.6375	5.1261	4.8595	5.0188	5.5828	5.2951	3.9411	5.3887
Atp6v0a1	_DM[Oxidation (M)]IDLEANFEKIENELK_.3	#N/A	4.0148	4.4951	3.9913	3.2594	3.3525	3.5766	5.3816	3.0497	4.5012	3.5532	3.1212	4.0769	4.3557	3.8995	3.1147	4.3596	4.0478	4.2529	4.3880	4.6177
Atp6v0a1	_M[Oxidation (M)]QTNQTPPTYNK_.2	#N/A	6.3546	6.5720	6.2635	3.5626	6.4375	5.8728	6.5631	4.0781	7.2017	6.1790	6.0734	4.9197	4.1839	6.4630	4.5720	6.2753	6.5002	7.3649	4.1306	3.2522
Atp6v0a1	_DMIDLEANFEK_.2	#N/A	3.5458	3.7733	4.5832	4.0384	3.7527	3.6671	5.0140	3.7715	3.4686	3.3564	3.8430	3.4994	3.5767	4.6213	3.3207	4.4087	3.2687	3.8807	4.1798	4.2986
Atp6v0a1	_ERIPTFER_.2	#N/A	3.4125	3.8928	4.4637	4.1580	7.4079	4.4752	3.2900	6.5131	3.3491	3.6189	6.4402	3.6190	3.4571	4.5017	2.8672	6.2387	7.2068	4.0003	4.2993	4.1790
Atp6v0a1	_SVFIIFFQGDQLK_.2	#N/A	3.5591	3.7600	4.5965	4.0252	3.7660	3.7936	3.4228	3.7847	3.4819	3.4860	3.8563	3.4861	3.5899	3.1337	3.0000	4.4220	3.2820	3.8675	1.7642	4.3118
Nek4	_SPAS[Phospho (STY)]LEGHT[Phospho (STY)]GK_.2	Q9Z1J2_S343_M2_Nek4	13.0958	12.8638	10.3074	13.4044	13.0604	8.1996	12.7230	12.5037	10.1903	12.7347	9.3694	9.7225	4.0233	10.2262	9.7286	5.0705	6.2350	3.7414	3.7834	5.5659
Nek4	_RSSDGGDGEGS[Phospho (STY)]ELVKPLYPSNK_.3	Q9Z1J2_S438_M1_Nek4	3.3664	3.5753	3.3985	4.9100	3.8663	4.9551	4.9571	4.0736	4.8580	3.3652	3.9086	3.3771	4.1981	3.4071	3.2309	3.2142	3.3231	5.4344	4.1422	4.5538
Apc2	_VSS[Phospho (STY)]PEDSPAGTPQR_.2	Q9Z1K7_S2143_M1_Apc2	8.0472	7.9926	7.5939	7.5027	7.1177	7.6079	7.8233	7.9844	7.7551	7.4009	7.4756	7.3403	4.1276	7.8753	3.6736	2.9510	7.9898	7.9802	7.4236	7.2263
Apc2	_RGS[Phospho (STY)]DGEARPLPR_.3	Q9Z1K7_S2099_M1_Apc2	5.6541	6.3416	4.0943	4.1314	2.8957	5.7136	5.5999	3.2584	5.8314	6.4301	5.4296	4.8845	3.5787	4.0814	3.5184	5.5851	5.9502	5.5471	5.7912	5.4878
Apc2	_APS[Phospho (STY)]PEQQR_.2	Q9Z1K7_S1805_M1_Apc2	4.3758	6.2219	6.2686	6.2388	3.7135	6.9119	6.3985	4.0761	6.3051	5.2558	6.8437	3.5165	4.3965	3.3983	3.4048	3.2198	6.4720	7.1520	4.3688	5.9103
Apc2	_FHAPALGPEPAART[Phospho (STY)]PEGS[Phospho (STY)]PVHGSGPSK_.4	Q9Z1K7_S108_M2_Apc2	3.6670	6.6881	5.6166	7.0350	3.0047	6.2575	7.0393	3.3975	6.0964	6.7174	3.4690	6.8524	3.2027	6.5693	5.8290	6.3585	6.9131	7.0640	6.9501	6.6536
Ddx39b	_GLAITFVSDENDAK_.2	#N/A	5.2856	5.6486	5.9177	5.2937	4.8273	5.2548	5.2200	4.8296	5.7270	4.6910	4.3720	4.8532	3.5579	5.1460	5.2343	4.5226	5.9583	5.2052	5.1790	5.0330
Ddx39b	_DVQEIFR_.2	#N/A	4.0739	3.2451	3.6105	3.5103	3.9400	3.8275	4.4500	4.2996	3.9968	3.3458	3.4200	3.9985	4.5391	3.6486	3.4140	3.4361	3.7969	4.7358	4.4484	3.3259
Ndufa7	_NWASGQDLQAK_.2	#N/A	7.8661	7.4349	7.5591	7.7249	8.0331	7.8858	7.6113	8.0639	7.8372	7.9535	8.0078	7.9107	7.8370	7.9302	8.2922	7.9462	7.9500	7.7131	8.0058	8.0716
Ndufa7	_LSNNYYC[Carbamidomethyl (C)]TR_.2	#N/A	6.2961	6.4714	6.6620	6.8199	6.4882	6.5848	6.9579	6.9250	6.2841	6.5448	6.4110	6.7282	5.9343	6.6149	7.2167	6.4963	6.3018	6.9600	6.5090	6.6542
Ndufa7	_AAESSAM[Oxidation (M)]AATEK_.2	#N/A	6.2897	5.2936	5.6271	5.5724	6.4088	5.8163	5.5865	6.2371	5.7427	5.5699	5.6388	6.4924	6.1585	6.0917	6.7388	6.6486	6.2057	5.9229	6.4208	5.9436
Ndufa7	_YQEIAKR_.2	#N/A	5.5662	6.4232	6.4446	6.0895	6.0585	5.5159	5.8625	5.9825	5.8933	5.4490	5.6348	6.4153	6.2033	5.5761	6.5659	6.1034	5.1477	6.1669	5.7309	5.6166
Vars1	_DINLDVNR_.2	#N/A	3.8576	3.4615	3.3942	3.7266	3.4765	3.9475	4.0437	4.0833	4.9721	4.7307	3.8832	3.1876	3.8885	3.4323	3.0089	3.2197	3.5805	4.1227	4.1166	3.1096
Vars1	_[Acetyl (Protein N-term)]SILYVSPHPDAFPSLR_.2	#N/A	3.9945	5.1943	3.5311	4.4153	4.6587	4.2348	4.6484	5.5054	4.2400	3.8051	3.7463	4.9206	4.4331	3.5692	2.8720	3.3566	3.7174	5.0350	3.7311	3.2465
Vars1	_LSATVTEAFVR_.2	#N/A	3.9328	3.3863	3.7014	3.6514	4.1397	4.1731	4.4092	4.1745	4.3294	3.7434	3.8081	3.1124	4.4949	3.4967	2.9338	3.2949	3.2114	3.1209	4.2704	3.4637
Bag6	_PLTS[Phospho (STY)]PESLSR_.2	#N/A	8.7632	8.6290	8.7294	4.5916	8.1656	8.8132	8.3276	8.5122	8.3129	4.3040	8.9881	8.0964	8.4799	4.0681	3.8739	8.4128	8.8960	4.4339	8.5867	8.6755
Bag6	_LQEDPNYS[Phospho (STY)]PQR_.2	#N/A	3.6646	6.3136	7.7740	6.8732	3.8715	6.9953	3.1766	3.8902	3.0968	3.4752	4.0763	6.1960	5.9899	6.6199	3.2020	3.0267	7.0038	4.3536	6.9176	6.8117
Bag6	_ESFSLVQVQPGVDIIR_.2	#N/A	4.2619	3.8268	3.9557	4.7145	4.4890	4.1072	3.8801	3.1440	4.5031	4.6222	3.3217	3.7503	4.0085	4.3162	4.1013	3.7813	3.5999	3.6073	3.7611	2.9773
Bag6	_TSPEPQRENAS[Phospho (STY)]PAPGTTAEEAM[Oxidation (M)]SR_.3	#N/A	7.2689	7.2597	6.9694	7.6428	5.8862	6.8671	7.1916	3.8806	6.5909	7.0425	6.6018	6.9266	7.8753	7.4552	6.4546	6.5959	7.0490	7.1386	6.9995	4.3195
Bag6	_LQEDPNYS[Phospho (STY)]PQRFPNAHR_.3	#N/A	10.0889	9.9944	9.7790	9.8381	3.2046	9.4051	9.4643	9.9968	3.7222	4.3506	9.8335	9.5032	10.4344	4.3903	8.9492	4.2118	9.9196	3.7282	9.7359	4.3573
Bag6	_AAGARPLTS[Phospho (STY)]PESLSR_.2	#N/A	3.3030	3.9150	4.1305	4.3475	3.8358	5.8585	6.2109	3.7616	3.4587	3.5193	3.8331	3.4357	3.5668	3.9892	3.2859	3.9496	3.2865	3.9007	4.1998	4.9962
Bag6	_ENAS[Phospho (STY)]PAPGTTAEEAMSR_.2	#N/A	3.7190	5.9554	7.0968	7.2476	3.5532	5.3192	6.0969	3.6669	4.8103	5.3002	6.1772	4.8454	3.1797	6.9198	4.6545	3.6576	5.2354	4.0470	3.6341	4.7155
Rasgrp1	_SNHVLAQMDHGDS[Phospho (STY)]A_.2	#N/A	3.9925	7.7873	7.3663	7.5501	3.9818	6.2141	6.5885	4.0653	5.4789	4.0316	6.5690	4.0097	4.1977	3.9186	4.6296	3.7354	6.3279	5.4013	5.1270	5.3797
Septin3	_VVNIIPVIAK_.2	#N/A	8.0821	8.4886	8.1879	8.7104	8.6873	8.4178	8.6629	7.9337	8.2529	8.4633	8.4468	8.5969	8.0297	8.2352	8.4100	8.2178	8.1390	8.4446	8.6397	8.4805
Septin3	_STLVNTLFK_.2	#N/A	7.3345	7.5991	7.4002	8.0283	7.8609	7.5391	7.7983	7.3793	7.4034	7.7601	7.5041	7.9105	7.8261	7.4825	7.5449	7.5018	7.3395	7.7879	7.5929	7.7456
Septin3	_KASS[Phospho (STY)]WNR_.2	Q9Z1S5_S92_M1_Septin3	7.8342	7.7695	3.6854	4.5403	3.9640	7.7034	7.2921	7.2275	3.0044	3.5676	3.9838	3.2882	3.1698	3.6725	7.0260	3.1191	3.3871	7.4238	7.3773	7.1399
Septin3	_THLQDLK_.2	#N/A	6.4505	6.0580	6.4457	7.0072	6.5398	6.6261	6.6110	6.2557	7.0115	6.3225	6.6822	6.8279	6.2555	7.2630	6.8514	6.1818	6.2700	6.5675	6.7998	6.7997
Septin3	_YINEQYEK_.2	#N/A	5.0912	3.3223	5.8379	5.4983	5.8692	6.2523	6.0501	5.0287	6.6331	6.5593	6.1982	6.1962	5.8974	5.8613	6.7371	5.8205	4.9946	6.5082	5.7216	6.2343
Septin3	_EVTHNIHYETYR_.3	#N/A	5.7470	5.8093	6.4998	5.7809	5.6875	5.8115	5.8721	4.3837	6.3303	6.0087	5.0461	5.2084	5.1777	5.1119	6.0807	6.5971	6.0654	5.6621	6.3054	6.2039
Septin3	_LTVIDTPGFGDQINNENC[Carbamidomethyl (C)]WEPIEK_.3	#N/A	3.8331	4.1356	3.6502	3.8563	2.8343	3.2801	4.4594	4.5688	3.3281	4.3133	3.1670	3.9058	4.6692	4.9947	3.6098	3.1013	2.8114	4.3378	2.6560	4.3235
Septin3	_ADTM[Oxidation (M)]TLEEK_.2	#N/A	5.6666	5.6984	5.4581	5.3276	5.7853	5.6576	5.4319	5.7257	6.1680	5.5077	5.3986	5.7227	5.6693	5.6925	5.6576	6.1345	5.4885	5.8940	5.6438	5.4527
Septin3	_AIGHVIEEGGVK_.2	#N/A	6.0367	6.4207	6.4449	6.1376	6.4077	6.5120	6.2628	5.6343	6.3124	5.7422	5.7297	6.2019	6.2018	6.6632	6.4778	6.4921	6.4328	6.1544	5.9801	6.1727

Septin3	_ASSWNREEK_.2	#N/A	4.4348	3.5680	3.4703	4.2927	3.7725	3.6729	3.7685	4.1352	3.8276	3.3691	3.3442	3.4574	3.9357	3.4795	3.3457	3.2789	3.1720	2.8963	3.8208	3.6823
Gab2	_ASS[Phospho (STY)]C[Carbamidomethyl (C)]ETYEYPAR_.2	Q9Z1S8_S402_M1_Gab2	10.0401	10.0685	10.3876	10.3444	9.6434	9.3482	9.4739	3.5235	9.4136	10.1059	9.4084	9.6501	10.1426	10.0834	8.9986	9.4166	9.8159	9.3965	9.5853	9.6864
Gab2	_SAS[Phospho (STY)]FSQGTR_.2	Q9Z1S8_S211_M1_Gab2	5.7319	6.0498	6.4412	6.3075	3.3368	5.9958	3.7113	3.3556	3.6315	6.5839	5.1236	4.9401	3.7969	4.2996	3.7366	6.0591	4.3537	3.8189	5.7412	5.7143
Gab2	_SSLTGS[Phospho (STY)]ETDNEDVYTFK_.2	Q9Z1S8_S282_M1_Gab2	4.4316	3.4111	3.4446	3.6762	3.7693	3.9934	3.7717	4.1337	3.8308	3.1371	4.2052	3.1372	3.9389	5.2845	3.3489	5.0304	3.6309	3.5185	3.8176	3.1599
Ap3b1	_EDGGLEDNEKNFYES[Phospho (STY)]EEEEEEKEK_.4	#N/A	4.3679	4.4520	3.9045	3.2163	3.0740	3.1073	3.9741	3.0927	3.8943	4.1785	3.3730	4.1782	4.0598	3.9425	3.9995	3.7300	4.0908	3.0586	3.3577	3.6198
Ap3b1	_NFYES[Phospho (STY)]EEEEEEKEK_.2	#N/A	3.1451	5.3878	3.6814	4.5442	3.9837	4.0012	3.5574	4.3464	3.6165	3.3515	3.9908	4.6637	3.1659	4.6604	3.1346	3.4900	3.3832	4.8108	4.9216	3.8006
Pikfyve	_SAS[Phospho (STY)]ITNLSLDR_.2	Q9Z1T6_S307_M1_Pikfyve	8.6579	8.8088	8.8599	8.5832	8.4026	3.3302	4.4350	8.7401	8.3230	8.7930	8.1106	7.5518	8.2868	4.1459	6.1903	7.8752	8.6777	8.0899	7.8795	8.0683
Pikfyve	_[Acetyl (Protein N-term)]ATDDKSS[Phospho (STY)]PTLDSANDLPR_.2	Q9Z1T6_S8_M1_Pikfyve	4.2918	6.5067	4.1884	5.9015	2.9979	3.9149	6.0472	7.1933	6.4498	6.2975	5.8181	5.9917	6.5007	6.6941	5.7188	3.6539	4.0837	6.3663	6.1942	6.1670
Pikfyve	_SGSPMVPSYETSVS[Phospho (STY)]PQANR_.2	Q9Z1T6_S329_M1_Pikfyve	3.6896	3.6295	3.2261	3.8947	3.8965	3.9298	4.7750	3.9152	3.0718	3.5002	4.0513	3.3556	3.2372	3.2642	3.1770	3.0517	3.4125	3.7370	4.0360	4.4423
Stk39	_T[Phospho (STY)]PDIAQR_.2	#N/A	12.1543	12.1206	12.0160	12.4021	11.6791	11.8611	11.9378	11.1602	11.5569	12.2634	12.2018	11.6643	11.6508	12.0186	10.1585	11.6473	11.3903	11.5707	11.8539	11.9713
Stk39	_RVPGSS[Phospho (STY)]GHLHK_.3	Q9Z1W9_S383_M1_Stk39	3.9617	4.3971	4.6140	3.9092	3.7596	4.3603	4.5020	4.2308	4.5912	6.7054	4.2035	4.1379	4.0427	5.5052	5.0398	3.7280	4.0613	3.3001	5.9954	5.9049
Stk39	_TEDGDWEWS[Phospho (STY)]DDEM[Oxidation (M)]DEKSEEGK_.3	#N/A	3.9252	5.3524	4.0880	3.9189	3.9595	5.0227	4.7332	4.2300	3.6787	4.4254	4.0254	4.3990	3.8934	6.0486	4.1838	5.3067	3.3939	4.7522	5.4243	4.3022
Stk39	_TEDGDWEWS[Phospho (STY)]DDEM[Oxidation (M)]DEK_.2	#N/A	3.5800	8.7150	8.7558	4.0860	3.3484	8.9320	8.3399	3.5251	8.6112	3.8632	3.4931	3.9199	4.0947	9.3167	7.4851	5.9852	4.3163	8.3689	8.2524	8.2138
Stk39	_LLTRT[Phospho (STY)]PDIAQR_.2	#N/A	6.1788	4.2911	4.2007	3.8630	3.8782	4.6346	3.7150	3.9245	3.7343	3.7297	3.3277	3.9472	6.3582	5.8753	4.3399	4.2883	3.7203	5.8182	3.8040	4.1947
Ptdss2	_VAGGSGSES[Phospho (STY)]PLLK_.2	#N/A	9.2798	8.9481	8.9969	8.6427	8.8512	8.5759	8.3223	8.9695	8.8780	8.6968	8.8234	8.2258	9.0584	8.0801	7.2039	8.4279	9.1439	8.7640	8.3772	8.4340
Ptdss2	_RVAGGSGSES[Phospho (STY)]PLLK_.2	#N/A	3.4148	4.5880	3.9511	4.2746	4.2534	3.7316	3.2877	3.1152	3.2701	3.6211	4.2496	2.9766	3.4355	3.9382	2.8649	3.7597	3.6529	4.1803	3.8289	3.9052
Ilf3	_RPM[Oxidation (M)]EEDGEEKS[Phospho (STY)]PSK_.3	#N/A	3.9443	3.8202	4.0047	3.7224	3.8326	3.5709	4.2329	3.9147	3.6119	4.2154	3.1656	3.6583	3.7199	3.9438	4.1453	3.8200	4.0921	3.9167	3.3950	3.6211
Strap	_TVDFTQDSNYLLTGGQDK_.2	#N/A	5.3885	5.5605	5.7456	5.5701	5.7533	5.7416	5.9088	5.3678	5.6948	5.1739	5.6294	5.6294	6.1209	6.3230	5.5926	6.3489	5.5842	4.1774	5.4507	5.1183
Strap	_C[Carbamidomethyl (C)]VLPEEDS[Phospho (STY)]GELAKPK_.2	#N/A	5.7888	6.0278	5.9038	5.8508	5.3187	5.3909	5.3712	5.3077	5.6651	5.4096	5.1455	5.6459	4.1760	6.4932	5.4541	6.0166	6.4058	5.6987	5.6460	6.2422
Hnrnpc	_GFAFVQYVNER_.2	#N/A	6.8491	6.5125	7.2864	6.6243	6.7785	6.4747	6.3778	6.9773	6.9222	6.6648	6.5838	6.2605	7.1438	6.3819	6.5365	6.4923	6.8593	6.5847	6.2655	6.7652
Hnrnpc	_VFIGNLNTLVVK_.2	#N/A	5.7013	6.0800	5.3112	5.7041	6.0397	4.8990	5.7613	5.6373	5.7376	6.0046	6.0424	6.0055	6.1917	5.8956	6.1753	5.4767	5.6117	5.6249	5.6728	6.4975
Hnrnpc	_M[Oxidation (M)]IAGQVLDINLAAEPK_.2	#N/A	4.0788	4.5049	3.8516	3.5975	3.6916	3.3004	3.8600	2.6336	3.8106	4.1256	3.4258	4.2310	3.5140	4.6153	3.7774	3.8692	4.3300	2.9227	3.3107	2.9954
Hnrnpc	_SEEEQSSASVK_.2	#N/A	3.4704	3.6541	4.0067	4.2189	4.3090	3.9374	3.1076	3.5940	3.2912	3.6768	3.6655	4.4860	3.3992	3.4173	4.6097	4.5823	3.7085	3.1620	3.5704	4.2389
Hnrnpc	_QADLSFSS[Phospho (STY)]PVEM[Oxidation (M)]K_.2	#N/A	4.0661	4.1009	3.6027	3.5960	5.2658	6.6042	5.9618	6.4712	5.8218	6.2129	6.8014	6.4100	3.7086	3.6408	3.6483	6.8531	7.1496	5.8391	3.7374	6.9390
Hnrnpc	_NEKS[Phospho (STY)]EEEQSSASVKK_.3	#N/A	4.2567	3.7462	3.2921	3.4327	3.5944	3.8185	3.9467	3.9570	4.0058	4.4630	4.3802	3.6356	4.1138	3.9545	3.5239	3.1007	4.4947	3.3436	2.9870	3.2463
Hnrnpc	_NEKS[Phospho (STY)]EEEQSSASVK_.2	#N/A	5.9783	6.1003	6.0793	6.3294	5.4324	5.9053	5.7816	6.1215	6.0355	6.4070	5.6579	5.9035	6.4473	6.5497	6.0503	6.1509	6.3109	6.1028	5.6574	5.8494
Hnrnpc	_SEEEQSSAS[Phospho (STY)]VKKDETNVK_.3	#N/A	3.3486	3.1534	3.8849	4.3408	4.1871	4.4112	3.9307	3.7159	3.4130	3.5549	3.7874	3.0428	3.5211	4.4657	2.9311	3.6935	3.5866	3.9363	4.2354	3.7145
Hnrnpc	_SDVEAIFSK_.2	#N/A	3.4705	4.5672	3.9719	4.2538	3.6775	3.6867	3.3706	3.1359	4.7440	3.2811	4.2703	3.5747	3.4563	2.7307	3.3960	4.0131	3.6736	3.6358	4.0451	4.3255
Dpp6	_KHEDESEAWLHR_.3	#N/A	7.2723	6.5788	6.9116	7.3002	6.8128	7.2869	6.9746	7.6543	7.8335	7.2836	7.8897	4.4895	6.9660	5.7631	6.4539	7.5938	8.2731	6.0127	6.2218	6.7581
Dpp6	_IYFLSTEDLPR_.2	#N/A	5.9521	5.2878	5.7324	5.2045	5.6686	5.7480	5.6014	5.9480	5.7972	5.3644	5.6836	5.2204	6.1163	4.4025	4.1795	5.4920	5.7451	6.0189	5.4088	5.0965
Dpp6	_HLYSANTVDDFNR_.2	#N/A	4.2552	3.9243	3.2907	3.4342	3.5929	4.2799	3.9482	4.9853	4.4059	4.4615	4.3156	4.4379	4.2759	4.3608	3.3419	3.0993	4.9355	4.5700	4.6111	3.9140
Fxyd1	_TGEPDEEEGTFRSS[Phospho (STY)]IR_.3	Q9Z239_S83_M1_Fxyd1	4.5246	3.8342	4.1270	3.8432	3.8032	6.1898	6.5765	3.8807	3.7732	3.9168	3.7677	3.5603	3.9386	4.2381	3.3817	3.9525	3.7310	3.6742	3.8127	4.0343
Snapin	_[Acetyl (Protein N-term)]AAAGSAAVSGAGT[Phospho (STY)]PVAGPTGR_.2	Q9Z266_T14_M1_Snapin	6.0129	8.1448	8.2792	8.5928	3.7526	7.4694	7.7305	3.7713	7.5716	7.9536	3.5614	7.5671	4.7524	8.3519	6.5795	7.7810	7.1830	7.4345	7.1118	7.6385
Snapin	_AMLDSGVYPPGS[Phospho (STY)]PSK_.2	Q9Z266_S133_M1_Snapin	4.4131	8.4429	9.5866	3.2763	3.7508	7.6172	5.4999	4.1134	3.8494	3.1185	8.3956	3.4792	3.9574	9.3166	3.3675	3.2571	3.1503	3.5000	4.3315	3.1785
Snapin	_VVLVNNILQNAQER_.2	#N/A	4.8439	4.0569	4.7578	3.7435	4.3252	3.2005	3.8810	3.6463	3.8012	4.2716	3.3438	4.3396	3.9666	4.4693	4.8088	4.2908	4.1839	3.6492	4.0351	4.4363
Rasal1	_RIS[Phospho (STY)]FKGT[Phospho (STY)]PTEEQVR_.3	Q9Z268_S396_M2_Rasal1	3.3807	4.7817	4.4853	4.3723	4.2192	4.1859	3.8645	3.0810	4.2512	5.0888	3.7553	4.2255	3.4890	3.9040	2.7182	3.5985	3.6187	3.9684	5.0112	4.2109
Rasal1	_LLEDSS[Phospho (STY)]LDTEVDPGR_.2	Q9Z268_S749_M1_Rasal1	4.0812	3.2379	3.6178	3.5030	4.2882	3.8202	3.9450	4.3069	4.0041	4.4647	4.3784	2.9640	4.1121	3.6559	2.7853	3.4433	3.8041	3.3453	3.6443	3.3332
Baz1b	_LADDEGDS[Phospho (STY)]DSESVGQSR_.2	Q9Z277_S1464_M1_Baz1b	8.0964	7.1484	3.9072	7.5051	6.8054	7.5031	7.8081	7.4734	7.0986	6.8281	7.6556	7.4265	8.2385	3.9165	6.3150	6.1527	8.4491	8.0243	7.4939	3.7967
Baz1b	_EENVLGKVDR_.2	#N/A	7.6965	7.4586	7.5437	7.0806	7.4594	7.5814	7.3278	7.6110	7.5285	7.6218	7.5030	7.3055	7.2764	7.3490	6.7686	7.3279	7.6750	7.5212	7.3993	7.2460
Pdpk1	_ANS[Phospho (STY)]FVGTAQYVSPELLTEK_.2	#N/A	10.2623	10.6540	9.7752	9.4754	8.9410	9.5753	10.2416	10.5088	9.4078	9.7891	9.3443	10.1468	10.4137	10.8545	9.1279	9.4681	9.7841	10.2386	9.8693	9.2296
Ate1	_KEGS[Phospho (STY)]KEFIHPQSIEEK_.3	#N/A	6.9833	7.0456	3.3318	6.6556	6.1289	6.5711	6.4750	4.0209	3.7181	6.7383	6.2217	3.2499	7.2160	7.0024	5.0187	6.1260	6.6874	6.6696	6.5024	3.0472
Ate1	_EFIHPQS[Phospho (STY)]IEEK_.2	Q9Z2A5_S174_M1_Ate1	6.1785	6.1584	6.1471	6.5310	3.4620	6.8601	5.7837	6.0885	5.6187	5.8877	6.5561	6.0420	6.0863	6.3338	4.9861	5.5318	6.6119	6.5143	5.8414	5.2561
Ate1	_EGS[Phospho (STY)]KEFIHPQSIEEK_.3	#N/A	7.0422	7.1377	4.2066	6.1987	6.2619	6.0344	6.2894	6.7235	3.6324	7.0954	6.4424	5.8528	6.5731	6.9289	4.8710	6.2865	6.3685	6.3584	5.8724	6.0541
Rps6ka4	_LEPVYSPAGS[Phospho (STY)]PPPGDPR_.2	Q9Z2B9_S347_M1_Rps6ka4	3.4777	3.8414	4.5151	4.1065	3.6846	3.7180	3.3635	3.7034	3.2837	3.2883	4.2632	3.5675	3.4491	3.9518	3.3888	4.3406	3.2006	4.1667	3.8153	3.9188
Rps6ka4	_RGS[Phospho (STY)]PVPASSGR_.2	Q9Z2B9_S745_M1_Rps6ka4	3.7998	4.2030	4.3361	3.8896	3.1375	8.3334	4.4036	3.5002	4.4627	4.0061	3.3362	7.0019	4.5707	4.3232	3.9808	4.1447	4.0378	7.1245	3.4439	3.7918
Mtmr1	_GSS[Phospho (STY)]PTHSATPVHTSV_.2	Q9Z2C4_S657_M1_Mtmr1	12.1864	12.2022	11.8576	12.0411	11.6665	11.7925	11.8655	12.2668	11.8078	11.8339	11.7191	11.7750	12.3536	11.9452	10.9393	11.6108	12.2149	11.9711	11.8278	11.6629
Mtmr1	_HPSIETLDS[Phospho (STY)]PTGSHVEWC[Carbamidomethyl (C)]K_.3	Q9Z2C4_S53_M1_Mtmr1	5.5335	5.7066	5.2588	3.6894	3.4783	4.9892	5.6971	4.0437	3.8552	4.1171	4.8483	3.6981	3.9796	3.8709	4.5721	5.5361	3.8438	4.4801	6.9112	3.6831
Mtmr7	_SPS[Phospho (STY)]QGDEDSALILTQDNLK_.2	#N/A	3.9611	7.6183	7.6087	3.7981	3.7037	3.5925	3.9757	3.7878	7.1620	6.9875	4.0574	3.5158	6.8791	7.9852	3.5291	3.8364	3.8170	7.7613	3.5827	3.4771
Mtmr7	_S[Phospho (STY)]PSGGEHAPSEDSGKDR_.3	#N/A	3.5059	4.4969	4.0422	4.1835	4.3445	3.0677	3.1966	3.2063	3.2557	3.7122	3.6301	4.3863	3.3637	4.0293	4.2746	3.8508	3.7440	4.0892	3.7378	4.0856
Mtmr7	_S[Phospho (STY)]PSGGEHAPSEDSGK_.2	#N/A	6.3461	3.1710	3.8673	4.3584	3.7820	3.8154	3.2661	4.5322	3.1863	3.3857	6.0155	3.4701	3.3517	3.8544	3.2914	5.9653	3.5690	4.2641	3.9127	3.8214
Mtmr2	_AS[Phospho (STY)]SPAQC[Carbamidomethyl (C)]VTPVQTVV_.2	Q9Z2D1_S630_M1_Mtmr2	4.5788	3.4240	3.6142	8.5712	3.9165	4.1406	3.6246	4.2792	3.6837	3.2843	8.2294	3.3135	3.7917	8.4237	7.1541	3.4228	3.3160	8.3558	4.1657	3.0128
Mtmr2	_SSS[Phospho (STY)]C[Carbamidomethyl (C)]ESLGAQLPAAR_.2	Q9Z2D1_S6_M1_Mtmr2	10.7549	10.9345	10.9525	10.6711	9.9389	10.2695	10.2520	9.9669	10.1249	10.4791	10.2219	10.0592	10.5696	10.3426	8.9601	9.9512	10.7073	10.1991	10.3777	10.3231
Mtmr2	_SASAISSDSISTSADNFS[Phospho (STY)]PDLR_.2	Q9Z2D1_S58_M1_Mtmr2	3.7864	4.2164	4.3227	3.9030	3.1241	3.3482	4.4170	3.4867	2.9752	3.9927	3.3496	4.1059	4.5841	4.1279	3.9942	4.1313	4.0244	4.3741	3.1723	3.8052
Mecp2	_AETSESSGSAPAVPEASAS[Phospho (STY)]PK_.2	#N/A	12.6584	12.5929	12.6821	12.5977	12.0096	12.4437	12.2188	12.4465	12.5016	12.3697	12.3171	12.1682	12.7861	12.9492	12.0902	12.3589	12.9040	12.3041	12.1435	12.4008
Mecp2	_VLEKS[Phospho (STY)]PGK_.2	Q9Z2D6_S216_M1_Mecp2	2.5603	3.7988	3.9911	4.1392	3.6058	4.4257	2.8802	4.6912	2.7495	4.1489	4.1849	4.3466	3.0576	3.7754	3.1758	3.5777	4.1604	3.6666	3.2142	2.8273
Mecp2	_GGS[Phospho (STY)]LESDGC[Carbamidomethyl (C)]PKEPAK_.2	Q9Z2D6_S421_M1_Mecp2	4.9164	4.7653	4.9338	6.0677	4.6492	6.3265	8.2589	9.6553	8.8133	5.9461	6.1728	6.4626	6.1920	7.0219	6.4854	6.2667	6.7386	4.7826	4.4155	6.3474
Mecp2	_EGKHEPLQPSAHHSAEPAEAGKAETSESSGSAPAVPEASAS[Phospho (STY)]PK_.5	#N/A	4.2882	3.7146	3.3236	3.4012	3.6259	6.7967	3.9152	3.9885	6.2671	4.4945	4.3487	5.0860	4.3089	3.3107	3.4924	3.1322	4.5262	5.0569	4.8758	3.2777
Mecp2	_VGDTSLDPNDFDFTVTGR_.2	#N/A	3.5221	5.3161	4.0584	5.6180	2.8598	4.5988	5.3289	3.2225	5.4139	5.2921	4.5713	4.5598	3.3476	4.8882	4.8291	3.8670	3.7602	5.1187	4.4089	4.0694
Mecp2	_MPFQAS[Phospho (STY)]PGGK_.2	Q9Z2D6_S229_M1_Mecp2	4.1586	3.1605	5.7514	3.4257	2.8646	4.3988	5.4194	4.3842	4.1037	3.9691	5.7436	4.3875	4.2691	6.2806	5.1006	3.5207	3.8815	3.2680	3.5670	3.2379
Mbd3	_VRYDS[Phospho (STY)]SNQVK_.2	Q9Z2D8_S85_M1_Mbd3	3.7906	3.5284	3.3272	3.7936	3.9976	4.1108	3.6544	4.0163	3.7135	3.2545	4.0878	3.2546	3.8215	3.3653	3.0759	3.1528	3.5136	3.6359	3.9349	4.4967
Mbd3	_YLGGS[Phospho (STY)]M[Oxidation (M)]DLSTFDFR_.2	#N/A	4.2073	6.1284	4.3088	3.9194	3.1701	3.3764	3.4302	3.4728	3.6277	4.1709	3.1064	4.1223	3.7932	4.2958	3.9372	3.9982	4.0105	3.8251	3.4737	4.2628
Mbd2	_AADTEEVDIDM[Oxidation (M)]DS[Phospho (STY)]GDEA_.2	#N/A	3.9379	9.3420	9.0943	8.7574	4.1449	9.1929	8.8636	4.1636	9.4319	9.7460	4.2351	9.4996	3.9688	9.6089	9.3318	9.8069	9.7055	9.3255	9.3677	9.5525
Bnip3l	_ILLDAQHES[Phospho (STY)]GQSSSR_.2	#N/A	4.9263	5.2993	3.7920	4.1372	5.6302	3.5942	4.9577	6.0032	5.2199	3.8767	5.2241	3.7989	3.7112	5.6211	5.8110	5.3803	5.6843	5.5054	5.7813	5.5144
Bnip3l	_DHSSQS[Phospho (STY)]EEEVVEGEKEVEALKK_.4	#N/A	3.8025	3.9660	4.1991	3.8471	3.7943	4.0746	3.6388	3.7324	6.3071	3.7862	3.6467	4.0977	3.7658	4.1503	3.8757	6.5156	3.9917	3.8727	6.5932	3.8860
Rgs6	_SVYGVTDETQSQS[Phospho (STY)]PVHIPSQPIR_.3	#N/A	7.9996	7.7209	7.2634	7.4137	6.7248	7.3051	7.6717	3.8212	6.6527	8.0822	7.6565	7.3582	7.8615	8.5434	5.4577	7.1917	7.1482	7.2720	7.4685	7.1636
Rgs6	_KSVYGVTDETQSQS[Phospho (STY)]PVHIPSQPIRK_.4	#N/A	7.0724	7.0656	4.1313	3.8772	3.3009	4.7873	7.3558	7.6762	3.6674	4.0554	3.1461	3.9513	3.8329	8.1515	5.1231	3.9569	4.0502	7.3311	3.4315	4.5846
Rgs6	_SVYGVTDETQSQS[Phospho (STY)]PVHIPSQPIRK_.3	#N/A	7.2306	7.6685	4.2185	7.0695	3.3880	6.2619	7.1825	8.4739	6.5135	7.5272	3.8351	7.5371	8.4079	5.1173	3.7449	6.9898	7.1521	3.6975	7.5148	6.9933
Rgs6	_KSVYGVTDETQSQS[Phospho (STY)]PVHIPSQPIR_.3	#N/A	4.5000	4.0959	4.0366	3.7825	3.2061	3.2394	3.8420	2.4084	3.7622	4.3106	3.2408	4.0460	3.9276	4.4303	3.8674	3.8621	4.1450	3.6882	3.3368	4.3973
Epb41l1	_ETDPS[Phospho (STY)]PEER_.2	#N/A	13.4300	13.5023	13.2962	13.4486	12.9123	13.1402	13.2211	13.2678	13.0806	13.4137	13.0175	13.0603	13.4968	13.2279	12.3831	13.1164	13.0881	13.1087	13.0879	12.9509
Epb41l1	_LVS[Phospho (STY)]PEPPPK_.2	Q9Z2H5_S378_M1_Epb41l1	11.7989	11.8038	11.7968	11.7230	11.3964	11.8569	11.5078	11.9569	11.4777	11.6687	11.8412	11.4987	11.8095	11.8715	10.9994	11.5931	11.9658	11.6905	11.5933	11.6354
Epb41l1	_S[Phospho (STY)]LPELDR_.2	#N/A	11.0338	10.6754	11.8038	11.4769	9.8581	10.1016	10.9700	9.6721	10.5458	11.7008	11.0361	10.3492	10.7225	10.8537	8.8631	10.5434	9.8727	10.6621	10.7975	10.6931
Epb41l1	_S[Phospho (STY)]LDGAEFSR_.2	#N/A	9.8041	9.2462	10.2423	10.2207	8.9723	9.9167	9.2794	9.2588	9.8748	10.2195	10.0242	9.4247	9.4669	9.7713	9.0930	9.7653	9.5842	9.6677	9.9832	9.9777
Epb41l1	_IKELKPEQETT[Phospho (STY)]PR_.2	#N/A	8.4387	8.5717	8.5975	8.6129	6.4192	7.7985	8.2592	6.5782	7.9633	8.6089	8.0779	3.8762	7.4169	7.2826	7.0764	8.1481	8.1127	8.2049	6.9280	6.8834
Epb41l1	_S[Phospho (STY)]LPELDRDK_.2	#N/A	9.5664	9.8744	9.6296	9.5477	7.7758	9.1964	9.5348	9.0948	8.6497	9.7205	9.2264	9.1993	9.1673	9.5417	7.5135	8.9944	9.1884	9.4425	8.7077	9.0313
Epb41l1	_AGLREGS[Phospho (STY)]EEK_.2	Q9Z2H5_S564_M1_Epb41l1	8.1301	8.1978	7.8354	8.2855	7.6206	8.0132	7.9524	7.8216	8.0700	8.3849	8.0576	7.8581	7.7825	8.1624	6.7811	7.6740	7.9724	7.9534	7.9135	7.8619
Epb41l1	_VTLLDASEYEC[Carbamidomethyl (C)]EVEK_.2	#N/A	7.3148	7.2021	7.2644	7.1620	6.6430	6.2760	5.9652	7.0853	6.6731	7.0127	6.7154	6.3199	7.3206	7.3581	6.1410	6.7978	6.9975	6.5103	6.5337	6.9879
Epb41l1	_ETDPS[Phospho (STY)]PEERDK_.2	#N/A	3.3232	3.9959	4.3606	4.2610	3.5301	3.5635	7.9242	3.5489	7.9934	3.1338	2.9168	7.9208	3.6036	4.3987	7.8220	8.1859	7.8915	4.1033	4.4024	7.7260
Epb41l1	_DNHLAIER_.2	#N/A	6.0274	6.4867	6.3264	6.6580	6.4273	6.3575	6.5616	6.3876	6.6713	6.4260	6.6482	6.2538	6.4153	6.5565	6.0782	6.6419	6.3066	6.5627	6.4842	6.2737
Epb41l1	_FAPNQTR_.2	#N/A	6.0407	5.6799	6.3326	5.5195	6.0633	5.8948	6.6671	6.2292	5.9806	6.0510	5.8355	6.2364	6.1803	5.9711	6.1905	5.9501	5.5068	6.0083	6.2975	6.1490
Epb41l1	_KAQEETPQQPEAAAAVTT[Phospho (STY)]PVT[Phospho (STY)]PAGHSHPETNSNEK_.4	#N/A	6.7434	3.4331	3.6051	3.1197	3.9074	4.1315	3.6337	4.2700	3.6928	6.6442	4.0672	3.3226	3.8008	3.3446	3.2109	3.4137	3.3069	3.6566	3.9556	3.0219
Epb41l1	_HQAS[Phospho (STY)]INELKR_.2	#N/A	8.2848	8.7854	8.2756	8.4696	7.3496	7.7821	7.0011	7.5897	7.4278	8.2744	7.9210	7.3253	8.3098	7.8986	6.5333	7.7302	7.3945	8.0041	8.0019	7.6870
Epb41l1	_S[Phospho (STY)]EAEEGEVR_.2	#N/A	7.8523	7.2802	7.8150	7.7617	7.7558	8.1549	7.7215	8.7473	8.0093	7.1072	8.0962	7.4925	7.5414	7.8056	7.4695	7.6165	8.1013	7.6070	7.6944	7.9140
Epb41l1	_S[Phospho (STY)]EAEEGEVRT[Phospho (STY)]PTK_.2	Q9Z2H5_T475_M2_Epb41l1	6.1809	7.0320	7.3374	7.5102	6.6799	5.3489	7.0572	7.2879	5.7716	7.3791	6.2866	6.4501	6.0701	6.8997	5.8708	6.0600	3.1190	6.9250	6.0613	6.8774
Epb41l1	_AQEETPQQPEAAAAVT[Phospho (STY)]T[Phospho (STY)]PVTPAGHSHPETNSNEK_.4	#N/A	6.5918	6.2831	6.4429	3.9089	4.6358	5.2744	5.0666	4.1159	4.8989	7.0447	4.0001	6.3979	5.3596	6.6325	5.3774	6.6686	4.7530	6.4059	3.8396	5.3676
Epb41l1	_RLPSSPAS[Phospho (STY)]PS[Phospho (STY)]PKGT[Phospho (STY)]PEK_.2	#N/A	8.8524	8.2639	8.7823	8.6639	7.6035	7.5591	7.8854	7.6813	7.4252	8.8710	7.9752	7.5376	6.3318	8.1943	6.0296	7.7947	7.7245	7.7289	7.6126	7.6918
Epb41l1	_GAC[Carbamidomethyl (C)]ST[Phospho (STY)]PEM[Oxidation (M)]PQFESVK_.2	#N/A	9.3874	9.1677	9.3722	9.4494	8.4557	8.6568	6.9310	9.6081	8.1523	9.5357	9.0575	8.3809	8.7251	9.0849	3.5728	8.4989	9.1618	8.4957	8.5530	8.4992
Epb41l1	_GAAAM[Oxidation (M)]IPGPQTVATEIR_.2	#N/A	6.6207	5.5707	4.6856	3.2314	3.7957	6.0640	6.4159	5.3095	5.8679	6.7281	6.2074	6.7118	6.7810	3.4805	6.1785	6.3244	3.1952	6.1214	6.7195	6.4309
Epb41l1	_LPSSPAS[Phospho (STY)]PS[Phospho (STY)]PK_.2	#N/A	8.5256	8.8549	8.9078	8.7891	8.4217	8.4740	8.3531	8.8865	8.4319	8.0555	8.2682	8.4715	8.7324	8.8968	8.1310	8.4236	8.7529	8.4400	8.5888	8.4695
Epb41l1	_IIITGDEDVDQDQALALAIK_.2	#N/A	4.7086	4.0857	3.8422	4.3896	4.4075	4.2347	4.7723	5.3231	4.7562	4.7446	4.1131	4.6567	4.6101	5.3303	3.3227	4.4271	3.6825	4.4269	4.3439	4.1581
Epb41l1	_GPSSQEDESGGLEDS[Phospho (STY)]PDR_.2	Q9Z2H5_S677_M1_Epb41l1	7.9886	7.7891	8.0872	7.6470	7.4727	7.9432	7.6726	7.8958	7.5766	7.5836	7.7305	6.8863	7.2332	7.5891	7.1156	7.5694	7.9492	7.5888	7.7802	7.5538
Epb41l1	_SLS[Phospho (STY)]PIIGK_.2	Q9Z2H5_S782_M1_Epb41l1	6.9638	7.1462	4.4700	3.7557	3.2714	6.0418	6.7491	3.6341	4.3287	8.6502	7.1104	6.5397	3.9544	6.5189	3.8468	4.2786	4.1718	3.6614	3.3100	4.4241
Epb41l1	_RLPSSPAS[Phospho (STY)]PS[Phospho (STY)]PK_.2	#N/A	11.2952	11.5672	11.6084	11.3228	11.1323	11.2949	11.2940	11.5897	11.3380	11.1488	11.1912	11.2801	11.5178	11.6829	10.6701	11.1553	11.6193	11.4313	11.4074	11.2340
Epb41l1	_EDDAES[Phospho (STY)]GGRRS[Phospho (STY)]EAEEGEVR_.3	#N/A	4.8294	4.2049	5.2919	5.1266	4.2950	4.8340	4.7513	5.1122	4.6505	4.1379	4.9474	4.1938	3.9457	4.9090	4.0319	3.8937	4.7458	4.9579	5.0703	3.9775
Epb41l1	_DVLTSTYGATAETLSTSTTTHVTK_.3	#N/A	4.0624	4.0737	5.1679	4.9323	3.7261	4.1473	4.2697	4.0888	4.6276	3.0939	2.8451	3.9359	3.9254	4.1069	3.9010	4.9976	3.1256	4.3338	4.2854	4.4752
Epb41l1	_RS[Phospho (STY)]EAEEGEVR_.2	#N/A	4.1800	5.2277	6.9192	6.0170	6.2867	5.9953	5.8095	6.7524	5.4994	5.8555	6.5333	4.3660	4.2109	3.7547	5.4559	6.4638	5.6229	6.6713	3.5456	5.9644
Epb41l1	_SLDGAEFSRPAS[Phospho (STY)]VSENHDAGPEGDKR_.4	Q9Z2H5_S441_M1_Epb41l1	3.3614	7.0402	4.0024	6.6813	3.5641	3.7298	5.3397	3.5727	6.5473	3.4526	3.5356	3.8361	3.5400	4.1614	3.7037	6.6102	3.9844	6.8043	4.0943	4.1776
Epb41l1	_LPSSPASPS[Phospho (STY)]PKGT[Phospho (STY)]PEK_.2	#N/A	6.3024	5.3641	6.2830	6.1201	5.2403	4.9817	5.4461	4.8941	4.8431	6.5222	3.7530	4.4781	3.6263	5.5269	3.5246	5.0714	4.8008	3.9105	4.0519	4.9477
Epb41l1	_RSEAEEGEVRT[Phospho (STY)]PTK_.3	#N/A	5.9260	6.9760	6.5679	6.1141	3.9908	4.7285	3.7199	5.9200	6.4949	3.9588	6.8482	5.9830	3.9239	6.5234	6.0822	4.7549	4.1412	6.4183	7.0170	3.6427
Epb41l1	_DYSEADGLS[Phospho (STY)]ERTT[Phospho (STY)]PSK_.2	Q9Z2H5_S75_M2_Epb41l1	3.3118	4.0073	4.3492	4.2724	3.5187	3.5521	5.3973	3.5375	3.4496	3.1224	2.9282	3.7334	3.6150	4.1177	3.5547	4.1747	4.5355	4.0008	3.6494	4.0847
Epb41l1	_ELKPEQETT[Phospho (STY)]PR_.2	#N/A	12.6482	12.5591	12.5015	12.4686	11.9763	11.8226	12.2089	12.2278	11.7887	12.6985	11.9986	11.9477	12.3495	12.2427	10.5471	11.8469	11.9708	11.9902	11.9564	11.9052
Epb41l1	_AETMTVSSLAIR_.2	#N/A	4.4779	4.0845	5.9341	4.6674	3.1840	4.2600	5.3702	3.2027	3.7843	4.2885	3.2630	4.0682	3.9498	5.9384	3.8895	3.8400	4.2008	4.4495	3.2477	4.4194
Epb41l1	_LPSSPASPS[Phospho (STY)]PKGT[Phospho (STY)]PEKASER_.3	#N/A	3.2644	7.4262	7.7799	7.5824	6.2720	3.1362	3.1281	3.4900	3.1872	3.7807	3.5616	3.7808	6.7783	6.7853	4.2061	3.9193	4.4881	5.9646	2.9604	4.0171
Epb41l1	_SLPELDRDKS[Phospho (STY)]DSETEGLVFAQDLK_.3	#N/A	3.9506	3.9805	4.0019	3.7305	3.6706	3.7039	4.0006	3.7231	4.7445	3.7612	3.8871	3.7066	4.2014	4.0506	3.5280	3.8274	3.5596	5.2855	4.0208	3.8004
Epb41l1	_DKS[Phospho (STY)]DS[Phospho (STY)]ETEGLVFAQDLK_.2	#N/A	3.8299	3.9835	5.3951	3.8914	3.4234	3.6612	3.9896	3.6718	3.9001	4.0835	3.4227	3.8201	4.0283	4.3524	3.6878	3.9260	4.3666	7.6542	3.7413	3.6558
Epb41l1	_APES[Phospho (STY)]DMGDEDQDQER_.2	Q9Z2H5_S578_M1_Epb41l1	3.8669	3.4628	3.5755	6.6318	4.0739	4.1072	4.4751	4.2404	4.3953	3.6775	3.8739	3.3523	4.5607	3.5625	2.9996	3.2291	3.2772	3.0551	4.2045	3.5295
Epb41l1	_VSAADSTQVDGGTPMVK_.2	#N/A	3.6791	4.3238	4.2154	4.0103	3.0168	3.2409	3.0235	3.3794	3.0825	3.8854	3.4569	4.2132	3.6998	4.2024	4.1015	4.0240	3.9171	5.6174	3.5646	4.1694
Epb41l1	_PASVSENHDAGPEGDKREDDAES[Phospho (STY)]GGR_.4	#N/A	3.8613	4.1415	4.3976	3.8281	3.1990	3.2851	3.7963	3.5617	3.7166	4.3562	3.1952	4.0310	3.8820	4.3847	3.8217	4.2062	4.0993	3.7338	3.3824	4.3517
Letm1	_LLELQSIGTNNFLR_.2	#N/A	6.3652	5.7869	5.7892	6.1002	5.8018	5.9468	5.9883	6.2586	6.4444	6.0816	6.0325	5.8901	6.3765	6.0861	5.6327	6.0239	6.0943	5.8494	5.7636	5.7719
Letm1	_VAEVEGEKVDNK_.2	#N/A	6.3065	5.7066	6.2092	6.2107	5.0620	5.6590	5.9746	6.0932	6.1721	5.8084	6.0740	5.7429	6.1202	5.7801	5.6229	5.8763	6.5041	5.6351	5.2129	5.2517
Letm1	_LISLTSALDDNKDGNINIDDLVK_.3	#N/A	4.6304	3.9546	4.6684	4.6862	4.7309	4.2800	5.0285	4.4891	3.8953	3.5247	3.9635	2.5562	3.1932	3.9945	4.0713	4.6483	3.2657	5.1169	3.8934	3.6628
Letm1	_FLQDTIEEMALK_.2	#N/A	3.6275	3.2233	3.8149	4.4107	3.8344	3.8677	3.2137	4.4799	3.1339	3.4381	4.1134	3.1128	3.2994	3.8020	3.2391	2.9896	3.5167	4.3165	3.9650	3.7690
Sucla2	_INFDS[Phospho (STY)]NSAYR_.2	Q9Z2I9_S279_M1_Sucla2	7.1269	9.0665	9.1642	9.2018	9.0584	8.9087	8.7911	9.1669	8.9627	9.2064	8.9876	8.9035	9.5845	9.1864	8.5720	9.0797	9.3776	8.8214	9.1027	8.9199
Sucla2	_EQAVTLAQK_.2	#N/A	7.8342	7.5103	8.0548	7.8286	7.9246	7.8635	7.8316	7.8987	7.9013	7.7695	7.7866	7.6526	7.5256	7.7679	8.1439	8.0982	7.7467	7.7443	7.8148	7.9033
Sucla2	_IVFSPEEAK_.2	#N/A	7.0603	7.0253	7.3994	7.6734	7.6817	6.9514	7.8022	7.3500	7.8489	8.0959	8.0318	7.2915	7.5425	7.2009	7.4953	7.4341	6.9486	8.0748	7.8433	7.9258
Sucla2	_ILAC[Carbamidomethyl (C)]DDLDEAAK_.2	#N/A	7.3377	7.1774	7.8177	7.3685	7.2419	7.1273	7.7498	7.2213	7.7241	6.9308	7.2477	7.3608	7.2135	7.8190	7.1841	7.4001	7.4099	7.0506	7.2268	7.3764
Sucla2	_IC[Carbamidomethyl (C)]NQVLVC[Carbamidomethyl (C)]ER_.2	#N/A	6.6480	7.2432	6.8171	6.5750	6.7852	6.8305	7.1504	6.4313	7.3212	7.1260	6.6420	6.6401	5.8779	7.3855	6.9916	6.7144	6.5237	7.0398	6.7843	6.9928
Sucla2	_LSEIVTLAK_.2	#N/A	4.7013	5.7291	6.3526	5.5890	5.8584	6.0029	6.1935	4.4678	6.2947	5.8800	6.0027	5.4219	5.9751	6.2450	5.9211	6.0536	5.4321	6.6297	5.9670	6.4838
Sucla2	_LHGGTPANFLDVGGGATVQQVTEAFK_.3	#N/A	6.0689	6.2363	5.8179	5.9325	5.6560	6.0654	4.7718	5.9501	6.0433	6.0529	5.6622	5.5658	6.7126	6.5078	5.8514	6.0187	6.3361	5.9487	5.9155	6.5331
Sucla2	_SSDEAYAIAK_.2	#N/A	3.1045	4.6360	4.5242	5.0871	4.4464	5.2110	5.3278	3.5020	5.6121	5.7674	5.4826	4.5682	3.8223	5.7071	5.9071	5.1611	4.1242	5.4231	5.1455	5.3170
Sucla2	_IFDLQDWSQEDER_.2	#N/A	4.1857	5.3315	5.8803	5.2169	5.1976	5.6431	5.4221	5.5577	5.1757	5.7118	5.8341	5.6060	4.2420	3.2438	5.1661	3.5478	6.3117	5.3986	5.5069	5.6121
Sucla2	_VQAILVNIFGGIM[Oxidation (M)]R_.2	#N/A	3.1401	3.0033	3.2281	3.8927	3.8117	3.7440	3.1578	3.3302	2.9789	4.0714	4.1301	4.0340	3.7224	3.0164	3.1750	3.2845	3.4144	3.7350	4.6775	4.1223
Sucla2	_AVSSQM[Oxidation (M)]IGQK_.2	#N/A	4.9949	5.1122	4.9186	5.1630	5.4326	4.7893	5.0929	5.0411	5.0564	4.6176	4.7733	4.9291	5.4133	4.1620	5.3337	4.8161	4.6384	5.1566	4.7099	4.6565
Sucla2	_C[Carbamidomethyl (C)]DVIAQGIVM[Oxidation (M)]AVK_.2	#N/A	5.3359	5.1962	5.0230	5.0934	5.0269	5.3330	4.6669	5.2657	5.2160	5.0704	5.0213	5.3650	4.1447	4.1068	5.1548	5.3312	5.3488	5.2510	5.0633	5.3945
Sucla2	_YDATMVEINPM[Oxidation (M)]VEDSDGK_.2	#N/A	3.5916	3.3550	3.6082	4.5328	4.0818	4.1809	3.5977	4.2836	3.6568	3.3246	3.7601	3.1594	3.7649	4.0391	3.1749	3.4632	3.5404	3.7060	3.9272	3.8552
Sucla2	_VLC[Carbamidomethyl (C)]MDAK_.2	#N/A	3.5163	4.4865	4.0526	4.1731	2.8540	3.6301	3.1863	3.2166	3.2453	3.7226	3.6197	4.3760	3.5370	4.0396	4.2643	3.8612	3.7543	4.0788	3.7274	4.0066
Ubl3	_EFLFS[Phospho (STY)]PNDSASDIAK_.2	Q9Z2M6_S27_M1_Ubl3	4.4901	4.3298	4.0267	3.0941	3.1962	3.2295	3.8519	3.2149	3.7721	4.3007	3.2507	4.0559	3.9375	4.4402	3.8773	3.8522	4.2130	5.8823	3.3269	4.4072
Actl6a	_EGS[Phospho (STY)]PANWK_.2	#N/A	6.4118	6.6436	6.4632	4.0461	6.3666	6.7305	6.4199	5.9541	6.0888	6.8799	6.8076	6.5146	6.5118	6.6003	5.8073	4.4011	6.8123	6.4990	6.5943	7.4997
Actl6a	_EGS[Phospho (STY)]PANWKR_.2	#N/A	5.7921	5.1578	5.2008	4.3238	5.5935	4.9462	4.9161	4.8082	5.3591	4.5095	4.5411	4.8790	4.1672	5.2956	5.0812	4.3839	4.7147	5.5626	5.5878	5.1318
Knop1	_ATS[Phospho (STY)]PSNNVDEVQIPEISLSK_.2	Q9Z2Q2_S57_M1_Knop1	3.2893	6.9405	5.9402	4.2949	3.4963	3.5296	3.5518	6.1389	3.4720	3.0999	2.9507	3.7559	3.6375	4.1401	3.5772	4.1523	4.5131	6.2641	3.6269	4.1071
Septin5	_ADC[Carbamidomethyl (C)]LVPSEIR_.2	#N/A	9.0555	9.0321	9.6239	9.4299	9.2369	9.2147	9.2368	9.4522	9.3319	9.2082	9.3890	9.2306	9.2494	9.0818	9.4597	9.1240	9.3812	9.0855	9.2251	9.4289
Septin5	_IREEIDK_.2	#N/A	8.6560	8.5481	8.8223	8.5850	8.4990	8.8501	8.8878	8.6557	8.9167	8.6766	8.9494	8.4548	8.4032	8.9173	9.1700	8.7801	8.6564	8.5822	8.6996	8.6285
Septin5	_VNIVPLIAK_.2	#N/A	8.0454	7.9175	8.1710	8.0430	7.9899	7.7040	8.2938	7.9587	7.3480	7.8422	7.6594	8.2070	7.7611	7.8504	8.1115	7.8290	7.7187	8.3346	8.2123	7.9087
Septin5	_KLLSAEER_.2	#N/A	7.5341	7.8100	7.8936	7.5831	7.6081	7.6792	8.2520	7.8567	7.6149	7.7625	7.7813	7.7691	7.9573	7.9909	8.2355	7.7477	7.7746	7.9183	7.8113	7.8445
Septin5	_INQTVEILK_.2	#N/A	7.7153	7.6088	7.5300	7.8844	7.7731	7.3335	7.5845	7.8431	7.7184	7.8534	7.8183	7.8963	7.5290	7.8228	7.6995	7.5988	7.6132	7.6202	7.6021	7.6658
Septin5	_HTVDIEEK_.2	#N/A	7.2994	7.2864	7.2627	6.9282	7.0513	7.2894	7.6805	7.3614	7.5778	7.4428	7.4814	7.3342	7.2815	7.3767	7.7658	7.4876	7.3355	7.2134	7.2278	7.7314
Septin5	_DVTC[Carbamidomethyl (C)]DVHYENYR_.2	#N/A	6.7547	6.8274	7.4298	7.1940	7.0070	7.2358	6.9336	7.4077	7.3568	6.8193	7.1086	6.4542	6.5730	7.3231	7.2978	7.1970	7.5052	6.9452	6.8796	7.2811
Septin5	_M[Oxidation (M)]KDEELR_.2	#N/A	6.7128	5.9853	6.1713	5.9704	6.3470	5.9529	5.7144	6.6888	6.6813	6.6073	6.4594	6.4253	6.9730	6.1875	7.3586	7.0322	6.5166	6.8086	6.6723	6.9302
Septin5	_LATPEDKQDIDKQYVGFATLPNQVHR_.4	#N/A	7.0709	8.6060	6.6068	7.2978	7.0594	7.2631	6.8596	7.3634	6.8499	7.5359	6.7791	8.2027	8.5482	7.3921	6.9868	7.0345	7.1212	7.1481	7.9616	6.8827
Septin5	_QYVGFATLPNQVHR_.2	#N/A	6.4767	6.1769	7.1069	6.6530	6.2993	6.2193	6.3476	6.4893	6.6779	6.6547	6.4576	6.3497	6.1426	6.6846	6.6792	6.6762	6.3663	6.0463	6.9077	6.6500
Septin5	_SKLAT[Phospho (STY)]PEDKQDIDK_.2	#N/A	5.7284	4.2055	4.2437	6.3160	3.2361	3.2703	3.9146	3.3569	3.0541	3.9148	3.4284	5.8274	3.1621	4.2067	4.0730	4.0606	4.3550	4.2962	3.0945	3.8840
Septin5	_STLVHSLFLTDLYKDR_.3	#N/A	4.8716	6.0666	4.3085	5.6543	6.1194	4.2816	5.9121	5.5536	5.1974	5.2242	5.2613	5.8719	4.4387	5.9749	5.3198	6.2981	4.5010	5.2309	5.3207	5.8500
Septin5	_M[Oxidation (M)]ES[Phospho (STY)]PIPILPLPTPDAETEK_.2	#N/A	10.3417	10.2363	9.5987	9.9346	9.3802	9.4602	9.9570	9.9367	9.5891	10.3007	9.5252	10.0566	10.3196	10.3872	8.9853	9.8932	9.9015	9.9607	9.6439	9.5277
Septin5	_STLVHSLFLTDLYK_.2	#N/A	4.6750	4.7528	5.5002	4.7286	4.4210	4.7226	4.1154	4.6311	3.9472	4.9720	4.6451	4.4092	3.4947	4.9832	4.4124	4.7090	4.6722	4.8097	4.5455	4.6449
Septin5	_LYPWGIVEVENQAHC[Carbamidomethyl (C)]DFVK_.2	#N/A	4.4860	5.0778	3.9939	6.3255	5.1368	5.0657	5.1020	6.2694	4.2981	6.2390	5.8586	4.2764	4.9560	5.3215	4.7493	3.8944	5.0948	4.9429	6.3188	6.6920
Septin5	_FGIHVYQFPEC[Carbamidomethyl (C)]DSDEDEDFKQQDR_.4	#N/A	7.7516	11.0907	10.8633	7.6050	6.5842	10.6085	10.5179	5.5272	10.8325	7.5012	7.5466	6.8959	8.9423	6.0049	10.4102	7.3168	7.6951	11.0651	10.7706	10.1466
Septin5	_ESAPFAVIGSNTVVEAK_.2	#N/A	7.3073	7.3680	8.2267	7.8177	7.3243	7.4373	7.1290	7.4013	7.8027	7.7197	7.3498	7.1116	7.5784	7.7486	7.2023	7.2529	7.2903	6.9521	7.3717	7.4529
Septin5	_LAT[Phospho (STY)]PEDKQDIDK_.2	#N/A	9.2146	9.2211	9.4920	9.1982	8.6263	8.6162	8.7251	9.0766	8.7260	9.2067	8.7167	8.7281	8.9773	9.4299	8.1841	8.7751	9.0040	8.6265	8.6093	8.8146
Septin5	_AHC[Carbamidomethyl (C)]IQQM[Oxidation (M)]TSK_.2	#N/A	5.1535	6.2098	6.6796	6.7039	5.4625	5.7880	6.2290	5.3726	5.3009	5.0961	6.1564	5.3926	5.5423	6.6665	6.7784	5.7049	5.8181	5.3842	6.1604	5.5665
Septin5	_ADC[Carbamidomethyl (C)]LVPSEIRK_.3	#N/A	3.8797	4.3600	3.5032	4.6399	3.2174	3.4415	3.9106	3.1848	5.8847	5.1270	3.2563	5.9670	4.4908	4.0346	4.0456	4.2245	4.1829	4.8155	5.9326	5.2061

Septin5	_THMHDLK_.2	#N/A	3.5907	3.7284	4.5440	3.9935	3.7976	5.9303	3.2505	4.5166	1.6578	3.4013	4.3479	3.4545	3.3361	3.8910	3.4023	2.9528	3.5535	4.2797	3.9283	5.3600
Septin5	_THM[Oxidation (M)]HDLKDVTC[Carbamidomethyl (C)]DVHYENYR_.4	#N/A	3.8612	3.4579	3.3978	3.7231	6.1503	4.1015	2.9800	6.6974	4.4010	5.7766	5.4740	6.3197	7.6284	3.4358	6.0890	6.7692	3.5841	6.9819	6.5707	6.4686
Septin5	_FGIHVYQFPEC[Carbamidomethyl (C)]DS[Phospho (STY)]DEDEDFK_.3	#N/A	3.2605	4.3572	4.1819	4.0438	3.4674	3.5008	3.5807	3.3460	3.6975	4.5719	2.9795	4.2466	3.6663	4.1690	3.6061	4.1234	3.8837	3.9495	3.5981	4.1360
Septin5	_MKDEELRR_.2	#N/A	4.2682	4.5517	3.8048	1.1345	2.9743	4.3003	3.1971	4.4939	2.2874	3.5357	3.5063	4.2779	4.2991	3.5210	4.0992	3.6303	4.1273	3.1583	3.4574	3.5201
Tsc22d3	_LSPEEPAPEAPET[Phospho (STY)]PETPEAPGGSAV_.3	Q9Z2S7_T125_M1_Tsc22d3	3.8035	3.6423	4.0623	3.9074	3.2318	3.4460	3.6672	4.0292	3.3768	4.0905	3.3221	4.0906	3.8344	7.3937	4.0490	3.8878	4.2486	6.7261	4.3437	3.7776
Psma7	_ALLEVVQSGGK_.2	#N/A	6.5554	6.8115	6.8653	6.7927	6.9176	6.6457	6.7165	6.5822	6.7074	6.4816	6.2054	6.5820	6.6184	6.5990	6.8687	6.9292	6.4830	6.9037	6.7917	6.4830
Psma7	_ILNPEEIEK_.2	#N/A	7.2566	6.8258	6.9517	6.9828	7.6272	7.1777	7.1844	7.5792	7.5109	7.0612	7.3091	6.8042	3.9253	7.2721	7.2140	7.2918	7.2831	7.3910	7.0145	7.5358
Psma7	_NYTDDAIETDDLTIK_.2	#N/A	6.9879	3.1733	6.8458	6.8073	4.3527	6.1364	5.9793	4.3715	6.5460	6.8702	2.9421	6.4247	4.1767	6.5532	5.9333	6.3386	6.2470	5.7976	6.1500	6.4030
Psma7	_YVAEIEKEKEENEK_.3	#N/A	6.8063	6.8255	6.7388	3.6363	3.7295	7.0195	6.5290	6.9945	3.8707	3.0972	6.9573	6.9337	7.2168	6.8087	7.1834	6.7299	7.1213	6.3771	6.7793	7.1884
Psma7	_AITVFSPDGHLFQVEYAQEAVKK_.3	#N/A	4.8863	5.8871	4.9697	5.4573	4.7386	4.4527	4.8681	3.7310	4.7682	5.6249	4.4571	4.9667	5.1254	4.7841	5.1279	5.0733	5.6183	5.0135	5.1965	4.2580
Psma5	_GPQLFHM[Oxidation (M)]DPSGTFVQC[Carbamidomethyl (C)]DAR_.3	#N/A	6.1537	5.8077	5.6239	5.1471	6.0224	5.8227	5.4785	6.1679	5.5319	6.1444	5.7730	5.7325	5.6990	5.6825	5.8999	6.5312	5.8288	5.5463	5.7481	6.1174
Psma5	_GVNTFS[Phospho (STY)]PEGR_.2	Q9Z2U1_S16_M1_Psma5	4.1500	5.7338	7.5997	6.4915	6.4345	6.0378	7.2546	6.1437	6.0566	6.3453	7.1769	6.2689	7.3664	6.4369	6.9315	7.2397	6.2442	6.3391	6.4379	6.2934
Psma5	_ITS[Phospho (STY)]PLM[Oxidation (M)]EPSSIEK_.2	#N/A	9.9598	10.0473	10.0437	10.1344	9.4253	9.7874	9.4404	9.9876	9.4690	9.8553	9.6237	9.5544	10.1774	10.0778	9.2122	9.6778	9.9598	9.2269	9.3912	9.6453
Psma5	_LFQVEYAIEAIK_.2	#N/A	5.9836	5.5618	6.0274	5.8744	5.1747	5.4159	4.8781	5.6131	5.1916	5.5243	3.6325	4.9529	3.3661	4.4107	5.1187	5.0625	5.5752	5.2989	5.2141	5.8716
Psma5	_EELEEVIKDI_.2	#N/A	4.0989	5.5057	5.4774	3.4853	4.3058	4.3392	4.6858	4.8067	5.8430	3.9095	3.6420	2.9463	4.9953	4.9440	5.2755	4.5267	5.0828	4.3917	5.2646	3.2976
Psma5	_AIGSASEGAQSSLQEVYHK_.3	#N/A	6.9987	4.2587	3.6235	3.8521	3.3007	3.5403	3.9807	3.3195	3.9297	6.5917	2.2401	3.7900	3.9538	4.1693	5.3613	3.7528	4.3175	6.0862	3.3133	3.2116
Psma5	_PFGVALLFGGVDEK_.2	#N/A	4.3310	3.6718	3.3664	2.7874	3.6687	4.3162	4.2660	4.0313	4.1862	3.8866	3.6649	3.5613	4.3517	3.3535	2.7906	3.1750	3.0682	3.2641	4.4136	3.3205
Hdac6	_AVLAQGQS[Phospho (STY)]SEQAAK_.2	Q9Z2V5_S909_M1_Hdac6	3.5240	6.7857	6.3065	6.5878	2.8617	3.0858	3.1785	5.4390	4.6701	7.9530	6.5981	4.3682	3.5447	6.0628	4.2565	3.8689	3.7621	4.0711	3.7197	4.0144
Hdac6	_HNPQS[Phospho (STY)]PLQESSATLK_.2	Q9Z2V5_S21_M1_Hdac6	7.1131	6.5716	6.7073	4.0061	6.1225	6.1703	3.0192	6.7150	6.6847	6.3859	6.5500	4.2089	3.1863	4.2309	4.0972	4.0282	4.3792	6.1148	3.0693	3.9082
Hdac5	_KTAS[Phospho (STY)]EPNLK_.2	Q9Z2V6_S250_M1_Hdac5	4.2928	4.5272	3.8293	3.2915	2.9989	3.6087	7.8780	4.5184	3.9694	8.4291	7.6536	7.5581	4.3236	7.7472	7.3661	7.7259	4.0157	3.1338	3.4328	3.5447
Hdac5	_DEDGES[Phospho (STY)]GPDEGPDLEESSAGYKK_.3	#N/A	4.1231	3.8797	3.1586	5.7154	3.4608	3.0233	4.0582	3.8235	4.6688	5.3671	3.4570	3.7691	4.1438	3.1457	4.0836	4.7803	4.1190	5.0420	3.1206	3.1126
Hdac5	_DEDGES[Phospho (STY)]GPDEGPDLEESSAGYK_.2	#N/A	3.2046	5.6802	5.1309	5.2392	4.0432	4.6690	4.7496	3.8598	4.1457	3.4110	3.9313	3.4111	3.6650	5.3929	4.1213	3.5495	3.3570	5.1779	4.7123	4.3869
Hdac5	_RSS[Phospho (STY)]PLLR_.2	Q9Z2V6_S270_M1_Hdac5	3.4196	3.8994	4.4571	4.1646	7.1078	2.9810	3.4215	3.6453	7.0729	3.6255	7.3951	7.5182	3.4505	4.4951	3.4469	7.6161	7.2522	4.0069	6.8818	4.1724
Hdac5	_VAERRS[Phospho (STY)]S[Phospho (STY)]PLLR_.3	Q9Z2V6_S269_M2_Hdac5	4.2837	5.4333	3.8203	5.9827	2.9898	3.0231	5.8088	3.8236	5.8962	6.2677	5.8796	5.1168	4.1440	5.0791	5.3507	5.8204	4.3613	5.7736	3.1204	5.8458
Hdac5	_TQS[Phospho (STY)]SPAAPGSMK_.2	Q9Z2V6_S650_M1_Hdac5	4.1353	3.8675	3.1708	3.5541	3.4730	3.6971	4.0680	3.8357	4.1271	4.3416	3.0007	3.7569	4.2352	3.7789	3.6452	2.9794	4.3734	3.2222	3.5213	3.4562
Dnpep	_ETAC[Carbamidomethyl (C)]TTGVLQTLTLFK_.2	#N/A	4.0823	4.2269	4.3122	3.9135	2.9954	2.4892	4.4275	1.8184	2.9857	3.6058	3.3601	4.1164	4.5946	4.2993	2.6324	4.1208	4.0139	2.6060	3.4678	3.8157
Dnpep	_SPSPFHVVAEC[Carbamidomethyl (C)]R_.3	#N/A	4.1428	3.8600	3.1783	3.2424	3.4806	3.7046	4.0605	3.8432	4.1196	4.3492	2.9932	3.7494	4.2277	3.7714	3.6377	2.9869	4.3809	3.2297	3.5288	3.4487
Hnrnpf	_ITGEAFVQFASQELAEK_.2	#N/A	3.8839	2.5742	4.0127	4.3276	4.3822	4.2987	3.3324	4.4384	4.1355	4.3333	4.4093	3.7650	5.1701	3.2396	4.1094	2.9710	3.9356	3.2138	3.0331	4.7041
Homer1	_LTAALLESTANVK_.2	#N/A	6.0826	5.9408	6.3297	6.5848	6.2426	6.4903	6.2156	6.5563	6.6127	6.8653	6.6261	6.1368	7.0283	6.9262	6.8812	6.2487	6.7066	6.7007	6.7097	6.7626
Homer1	_LQEVEIR_.2	#N/A	6.2459	6.3348	6.7276	6.1519	6.4575	6.1845	6.2703	5.5952	6.7023	6.9391	6.8362	6.3640	7.1819	6.9792	6.9315	6.6544	5.9646	6.6405	6.9079	7.0166
Homer1	_FGQWADSR_.2	#N/A	5.8642	6.0968	5.9214	5.7939	5.9264	6.3373	6.2997	6.1449	6.1010	5.9561	6.0335	6.0902	6.5967	6.0514	6.4819	5.6914	6.2366	6.0517	6.1566	6.4473
Homer1	_IFELTELR_.2	#N/A	4.6853	5.4584	6.0980	6.6875	5.5796	6.0753	5.8272	5.2570	5.6727	6.3220	5.8265	6.0121	4.8011	6.7190	5.9352	6.5330	4.8548	6.1944	6.2480	6.3945
Homer1	_TELNQTVQELEETLK_.2	#N/A	4.8327	5.1196	5.4261	5.8030	5.4065	4.5328	4.5508	3.8391	5.3176	4.9217	5.5408	4.6363	3.7010	5.7702	5.4121	5.3955	5.2137	5.2183	5.2137	5.6906
Homer1	_[Acetyl (Protein N-term)]GEQPIFSTR_.2	#N/A	6.5143	6.7222	7.1604	7.2764	7.0803	7.0946	7.1316	6.7842	6.9328	7.1961	7.4104	7.0540	4.5787	7.3294	7.5961	7.3233	7.1512	7.3665	7.3360	7.0410
Homer1	_TLLEILDGK_.2	#N/A	5.4595	6.2425	3.4534	6.2212	5.8979	5.8316	5.7399	5.4476	5.6289	6.3302	6.3504	6.2817	4.5108	5.8377	6.1540	6.1576	5.8734	6.4698	5.9518	6.0054
Homer1	_AIINSTITPNM[Oxidation (M)]TFTK_.2	#N/A	6.6855	6.0759	5.2026	2.9947	5.8561	5.7436	4.3814	6.7050	5.3956	4.7245	5.5031	5.9879	6.6014	3.2326	6.7073	5.4948	6.0249	6.0552	6.6708	6.6429
Homer1	_TQALSHASS[Phospho (STY)]AISK_.2	Q9Z2Y3_S189_M1_Homer1	3.5223	5.5976	5.1324	4.2483	4.8222	4.3648	3.7301	4.3485	4.4058	4.3474	4.8663	4.6189	4.4653	4.3941	4.7971	4.5338	4.6999	5.4424	4.2246	4.3464
Homer1	_IISLDGSK_.2	#N/A	3.5570	3.7621	4.5944	4.3403	4.5020	4.8505	5.4335	3.8382	3.2044	3.3676	3.1455	3.7405	5.8713	4.2092	4.2903	4.2144	3.5872	4.8853	4.2713	4.7679
Homer1	_NKDLEGQLSDLEQR_.3	#N/A	5.8785	6.2444	6.1812	5.7101	5.4960	6.1099	6.0635	5.2801	6.1247	5.6395	5.7084	6.2036	5.7626	6.8881	6.2581	6.3044	6.2905	6.1719	6.2907	6.2843
Homer1	_AEPTQNALPFPHSAGDR_.3	#N/A	5.5982	6.2866	5.9855	5.7431	6.2848	5.8117	6.2711	5.9552	6.4647	4.1026	5.9155	6.4444	5.8375	6.5824	6.6927	5.7966	6.0587	5.9000	5.8189	6.4601
Homer1	_HWEAELATLK_.2	#N/A	4.4004	4.4355	3.4779	3.3087	4.1482	4.3586	3.1388	3.8740	4.3209	4.6935	4.1275	4.0900	4.2522	4.7703	2.9252	3.7950	3.2029	3.9789	4.2789	4.3603
Homer1	_DLEGQLS[Phospho (STY)]DLEQR_.2	#N/A	3.9794	4.0234	4.5157	3.7100	3.3171	3.1670	4.7132	3.6798	3.8346	4.1857	3.3133	3.9129	4.0001	5.7020	3.8012	4.3243	4.2174	3.6157	3.2643	2.9689
Homer1	_NKDLEGQLS[Phospho (STY)]DLEQRLEK_.3	#N/A	3.8223	3.4968	3.3589	3.7619	4.0292	4.0791	3.0189	4.0480	3.7451	3.8673	4.1195	3.2229	3.8532	3.3969	3.0442	3.1844	3.5452	3.4062	3.9033	3.0742
Homer1	_ANTVYGLGFSSEHHLSK_.3	#N/A	4.0055	3.9973	4.5418	3.6838	5.3000	3.5674	4.1978	3.7059	4.2569	4.2119	5.0406	3.8867	4.3650	4.5289	3.7750	4.3504	7.3392	3.5896	3.2381	2.9951
Homer1	_HAVTVSYFYDSTR_.2	#N/A	3.6132	3.7059	3.1498	3.9710	4.0641	4.2882	3.4770	3.8389	3.5360	3.4319	3.9104	3.4320	3.6441	3.1879	3.0541	1.3449	3.3361	3.8133	4.1124	4.3660
Plpbp	_IGS[Phospho (STY)]TIFGER_.2	Q9Z2Y8_S244_M1_Plpbp	3.9918	4.6708	3.6493	4.8151	4.7689	3.4038	4.5424	4.4130	4.3272	4.0483	4.4600	5.2517	4.2144	5.4069	5.3452	4.4863	4.0800	5.1225	4.4981	5.0083
Plpbp	_KGPTEPLK_.2	#N/A	3.7130	3.6061	4.5004	3.8712	3.9199	3.9882	2.6666	3.9387	3.6367	3.5245	3.8579	4.2439	3.7439	3.0222	4.1111	3.0751	3.4359	3.4426	4.2544	2.9649
Kcnq2	_GKGS[Phospho (STY)]PQAQTVR_.2	Q9Z351_S429_M1_Kcnq2	9.1490	8.9003	8.5684	8.4926	8.2250	8.5794	8.7885	8.5717	8.5639	8.4335	8.1926	8.4584	8.6869	8.4490	7.0248	7.9995	8.8961	8.7435	8.2721	8.3067
Kcnq2	_RSPSADQSLDDS[Phospho (STY)]PSKVPK_.3	#N/A	6.7869	6.5131	5.8314	6.0581	5.5111	3.7453	5.5159	6.4227	5.8760	5.7010	5.7658	6.1205	6.6049	6.8267	5.1810	6.4105	6.4643	6.3053	5.8892	3.7920
Kcnq2	_SPS[Phospho (STY)]ADQSLDDSPSK_.2	Q9Z351_S440_M1_Kcnq2	6.7138	6.0448	5.9674	6.5719	3.2473	5.6761	5.5693	6.3963	5.8864	3.5046	6.2643	6.0874	3.4612	6.1830	4.1140	5.5811	6.8177	6.1484	5.5523	5.7496
Kcnq2	_SWS[Phospho (STY)]FGDR_.2	Q9Z351_S457_M1_Kcnq2	4.4670	4.3529	4.0036	8.8541	3.1731	3.4344	8.3766	3.1918	4.3898	4.0790	3.2633	7.5405	4.4979	4.0416	5.8470	8.1324	4.1899	4.4604	7.8968	7.9619
Kcnq2	_YPQT[Phospho (STY)]WNGR_.2	Q9Z351_T287_M1_Kcnq2	4.2108	3.7920	3.2463	3.4785	2.9022	4.4364	4.1459	3.9112	4.0661	4.0067	3.5448	3.6814	4.2316	3.2334	4.1713	3.5582	4.4489	3.3843	3.0328	3.2004
Kcnq2	_S[Phospho (STY)]PSADQSLDDSPSKVPK_.2	#N/A	6.8222	6.6165	6.4674	3.8464	5.9127	5.2060	4.9939	5.0281	6.5275	5.8524	6.1610	7.1629	4.9541	3.7634	3.4039	6.9663	7.6653	7.4175	6.0307	3.5543
Kcnq2	_QNS[Phospho (STY)]EEASLPGEDIVEDNK_.2	Q9Z351_S479_M1_Kcnq2	4.5189	8.0970	8.1680	3.1185	3.5822	7.8109	7.5347	3.8232	7.7417	7.3018	5.9007	3.7452	6.4956	7.4980	5.6365	3.6391	7.3483	7.4449	7.5586	7.2515
Kcnq2	_EPEPAPPYHS[Phospho (STY)]PEDSR_.2	#N/A	3.6141	3.8916	4.1254	5.4830	4.6100	6.1304	5.2894	5.6498	5.2540	3.9596	4.9469	5.5990	3.8856	5.4271	3.8511	3.9284	4.1514	3.7982	3.9025	4.7102
Kcnq2	_EPEPAPPYHS[Phospho (STY)]PEDSRDHADK_.3	#N/A	3.7217	4.2811	4.2580	4.4672	3.0594	5.8403	2.9808	3.3428	3.0399	3.9280	3.4143	4.1705	3.1479	6.4207	4.0588	4.0666	4.3408	4.3095	3.1077	3.8698
Kcnq2	_RS[Phospho (STY)]PSADQSLDDSPSK_.2	#N/A	6.0657	3.5931	3.4763	4.1127	3.7785	4.3214	3.7936	4.1412	3.8527	3.9794	5.5738	3.4825	4.0051	6.0385	3.3708	3.2849	6.0367	4.0184	5.5348	3.0838
Osbpl10	_SSPGSVAAS[Phospho (STY)]PSGGGGR_.2	S4R1M9_S66_M1_Osbpl10	8.9820	4.3107	8.6616	9.0548	3.1309	7.8063	7.8545	8.7666	3.6012	8.6219	8.3483	7.5725	3.7538	8.3058	3.9709	8.3871	8.0125	8.0545	7.4699	3.8350
Osbpl10	_ATSAGSS[Phospho (STY)]PSC[Carbamidomethyl (C)]SLAGR_.2	S4R1M9_S32_M1_Osbpl10	5.8021	6.1154	6.2746	5.5382	5.3003	5.4545	6.3882	5.6826	4.1545	6.0369	5.4915	5.2162	4.3047	3.5335	5.0463	6.0855	6.2198	3.3583	3.7677	5.1516
