# Supplementary material for: The Chronic Effects of a Single Low-Intensity Blast Exposure on Phosphoproteome Networks and Cognitive Function Influenced by Mutant Tau Overexpression
Source: Int J Mol Sci. 2024 Mar 15;25(6):3338. doi: 10.3390/ijms25063338 (PMC10970429; doi:10.3390/ijms25063338)
Supplement: Supplementary file 1 [file ijms-25-03338-s001.zip › Supplementary Table S1a- MS2 raw data_non-carriers.pdf]

Supplementary Table S1a- MS2 raw data (sheet = 'MS2 raw_non-carrier')

PG.Genes	PhosName	Phos site	Blast NC 3	Blast NC 4	Blast NC 5	Blast NC 6	Blast NC 1	Blast NC 2	Sham NC 3	Sham NC 4	Sham NC 1	Sham NC 2	Expression Ratio	LOG2FC	pvalue	(-) Log10 p value
Tbc1d25	_QAS[Phospho (STY)]LDGLQQLR_.2	A1A5B6_S560_M1_Tbc1d25	8.9584	8.9600	9.4783	9.2316	8.3473	9.0666	9.2384	8.8514	9.0263	8.9794	0.9981	-0.0027	0.9360	0.0287
Arfgef2	_GQSQLS[Phospho (STY)]NPTDDSWK_.2	A2A5R2_S1535_M1_Arfgef2	8.5675	8.4948	8.8466	8.3379	5.8936	8.5683	9.0935	8.6862	8.4371	8.4545	0.9366	-0.0945	0.3673	0.4350
Arfgef2	_ERGS[Phospho (STY)]PVSGR_.2	A2A5R2_S276_M1_Arfgef2	8.6389	8.4976	9.1067	8.1306	2.8533	7.9172	9.0192	9.0525	8.2180	8.2993	0.8701	-0.2007	0.3766	0.4241
Arfgef2	_PQSPVIQATAGS[Phospho (STY)]PK_.2	#N/A	6.0610	5.4814	5.6884	5.8783	4.8684	5.6602	6.5364	5.9357	5.8414	6.0118	0.9219	-0.1173	0.0872	1.0595
Arfgef2	_QSLS[Phospho (STY)]SADNLEPDVQGHQVAAR_.3	A2A5R2_S355_M1_Arfgef2	4.6102	4.0705	3.9502	4.0384	3.8918	6.2819	3.9997	3.6840	3.7655	3.8093	1.2515	0.3236	0.1633	0.7869
Arfgef2	_ELEKPM[Oxidation (M)]QSKPQS[Phospho (STY)]PVIQAT[Phospho (STY)]AGSPK_.3	A2A5R2_T224_M2_Arfgef2	5.7288	4.8657	5.1527	6.2437	3.8309	4.7142	6.8651	5.1759	4.8042	4.5822	0.9501	-0.0739	0.6635	0.1782
Arfgef2	_HLDVDLDRQS[Phospho (STY)]LSSIDR_.3	A2A5R2_S1518_M1_Arfgef2	3.8739	3.1782	3.8979	3.4416	2.9995	3.2291	3.5899	3.5595	3.8586	3.1189	0.9731	-0.0394	0.6860	0.1637
Tanc2	_FS[Phospho (STY)]PPPVGGQSK_.2	A2A690_S1521_M1_Tanc2	9.8002	9.3381	9.3071	3.8682	3.7345	9.1769	10.2135	9.6977	9.5116	9.3697	0.7772	-0.3636	0.1846	0.7338
Tanc2	_QGQTS[Phospho (STY)]PIKPK_.2	A2A690_S1981_M1_Tanc2	4.3009	3.0415	4.0346	6.3636	4.2184	3.3658	3.7266	3.4229	3.7219	3.2556	1.1951	0.2571	0.2841	0.5465
Tanc2	_SC[Carbamidomethyl (C)]DELSPVS[Phospho (STY)]PTQGGYPSEPTR_.2	A2A690_S1831_M1_Tanc2	2.9332	6.1858	6.0444	5.6663	5.2256	5.9082	6.4362	6.2120	3.6167	6.2782	0.9453	-0.0812	0.7165	0.1448
Tanc2	_EYPS[Phospho (STY)]PPPSPLR_.2	A2A690_S1534_M1_Tanc2	10.7981	10.6644	4.0266	10.5299	9.2770	10.6189	11.4241	10.7681	10.5462	10.6853	0.8584	-0.2202	0.2919	0.5348
Tanc2	_AS[Phospho (STY)]PPAESMSVYR_.2	A2A690_S1549_M1_Tanc2	9.7430	3.2035	3.6818	9.8563	3.0918	3.5328	8.6278	7.2940	7.0406	4.4037	0.8066	-0.3101	0.4912	0.3087
Tanc2	_QIASDS[Phospho (STY)]PHAS[Phospho (STY)]PK_.2	A2A690_S400_M2_Tanc2	7.7871	7.5338	8.3217	7.6900	6.4591	7.3309	8.1265	8.2614	8.0020	7.6722	0.9382	-0.0920	0.1724	0.7636
Tanc2	_DC[Carbamidomethyl (C)]SYGAVTS[Phospho (STY)]PTSTLESR_.2	A2A690_S169_M1_Tanc2	10.3597	10.2530	10.7540	10.3549	3.9047	10.1013	10.7762	10.3510	3.3069	10.0826	1.0763	0.1061	0.7442	0.1283
Tanc2	_SQS[Phospho (STY)]ASYYPVC[Carbamidomethyl (C)]HSK_.2	A2A690_S1726_M1_Tanc2	5.8179	3.8045	3.9916	6.0664	3.6727	4.8121	6.0123	3.7661	4.9443	3.7360	1.0172	0.0247	0.9106	0.0407
Tanc2	_SNSSVGS[Phospho (STY)]PTR_.2	A2A690_S1480_M1_Tanc2	9.3448	9.1354	9.5684	3.4529	3.3502	9.1198	9.5525	3.5173	9.1474	9.0411	0.9378	-0.0926	0.8071	0.0931
Tanc2	_SSS[Phospho (STY)]QLGSPDVSHLIR_.2	A2A690_S1744_M1_Tanc2	5.6003	6.3894	4.0499	6.9786	3.4600	4.9720	3.7420	6.5076	6.1604	3.2710	1.0653	0.0913	0.7436	0.1287
Tanc2	_PSQGLPVIQS[Phospho (STY)]PPSS[Phospho (STY)]PAHR_.3	#N/A	4.1279	5.5054	3.5043	6.8161	3.9697	4.7966	3.5381	4.1346	5.4118	5.2777	1.0427	0.0604	0.7911	0.1018
Tanc2	_[Acetyl (Protein N-term)]MFRNS[Phospho (STY)]LK_.2	A2A690_S5_M1_Tanc2	3.2114	4.1310	4.4459	3.9897	3.9524	3.7771	4.1379	3.0115	3.3106	3.6669	1.1093	0.1497	0.2118	0.6740
Fam171a2	_S[Phospho (STY)]VEGPGGLEPSLDEYR_.2	#N/A	3.1998	4.1426	4.4343	7.3000	3.8443	5.9759	4.1263	6.9742	7.2118	3.6553	0.8770	-0.1894	0.5460	0.2628
Fam171a2	_S[Phospho (STY)]VEGPGGLEPSLDEYRR_.3	#N/A	3.7736	5.4633	4.3062	6.3417	3.6490	6.3578	6.5094	4.4610	6.6912	6.5408	0.7605	-0.3949	0.0383	1.4173
Fam171a2	_TGLC[Carbamidomethyl (C)]SPEDNS[Phospho (STY)]LTPLLDEVVAPEGR_.3	A2A699_S744_M1_Fam171a2	3.9293	3.2969	3.1152	3.6179	3.1852	3.4394	3.3326	2.9997	4.1492	3.5849	0.9756	-0.0357	0.7364	0.1329
Fam171a2	_RDS[Phospho (STY)]LTSPEDELGAEVGDEAGDKK_.3	#N/A	4.0823	3.8676	3.8609	3.7275	3.7559	4.5483	3.7122	5.6597	4.5657	3.5315	0.9099	-0.1362	0.3711	0.4305
Fam171a2	_RDS[Phospho (STY)]LTSPEDELGAEVGDEAGDK_.3	#N/A	3.6695	4.4257	3.4031	4.4477	2.8132	3.8114	3.7046	4.0543	4.3533	4.1250	0.9267	-0.1098	0.4017	0.3961
Fam171a2	_DS[Phospho (STY)]LTSPEDELGAEVGDEAGDKK_.3	#N/A	3.3872	4.1634	3.5824	4.3454	3.3158	3.9357	3.5949	3.9714	4.0064	4.1669	0.9627	-0.0548	0.5528	0.2574
Gpatch8	_IYRS[Phospho (STY)]QS[Phospho (STY)]PHYFQSGR_.3	A2A6A1_S1037_M2_Gpatch8	4.2652	3.5695	3.4511	3.9538	3.3909	4.3386	3.6685	4.1647	3.8133	3.9208	0.9837	-0.0238	0.7874	0.1038
Gpatch8	_SEPPGSGS[Phospho (STY)]PAPPR_.2	A2A6A1_S735_M1_Gpatch8	7.7569	8.4479	4.1049	4.3783	7.6079	4.1851	4.2979	9.8876	9.4158	3.9733	0.8820	-0.1812	0.6335	0.1982
Gpatch8	_SQS[Phospho (STY)]PHYFQSGR_.2	#N/A	7.4257	7.6571	8.0181	8.0143	7.2241	7.2252	8.1066	7.7838	7.4469	7.4588	0.9864	-0.0198	0.6507	0.1866
Gpatch8	_KGS[Phospho (STY)]WGHES[Phospho (STY)]PEERR_.3	A2A6A1_S1013_M2_Gpatch8	3.2987	4.0550	4.5332	4.3607	3.9433	4.1822	4.0753	3.7578	3.4064	4.3277	1.0438	0.0618	0.5457	0.2631
Cdr2l	_SC[Carbamidomethyl (C)]S[Phospho (STY)]DTALNAIVAK_.2	A2A6T1_S318_M1_Cdr2l	4.0267	3.1995	3.2127	7.3691	3.1524	3.0763	6.7927	4.4032	6.2463	6.4978	0.6694	-0.5792	0.0730	1.1365
Cdr2l	_DLLGGEES[Phospho (STY)]PGEGK_.2	A2A6T1_S407_M1_Cdr2l	4.0422	6.6549	3.7758	6.9316	6.0446	6.8828	7.1759	6.5723	6.1005	6.6086	0.8651	-0.2091	0.2718	0.5657
Zmym4	_AAHQES[Phospho (STY)]DNENEIQIQNQLKK_.3	#N/A	4.0002	5.0537	4.6440	5.6004	4.4326	4.6454	5.0792	5.0244	4.8614	5.1167	0.9420	-0.0862	0.3336	0.4768
Zmym4	_ENS[Phospho (STY)]KETFSGK_.2	A2A791_S161_M1_Zmym4	5.0220	3.9049	5.4677	4.0496	4.8341	4.3323	4.0751	3.0743	3.3734	5.3856	1.1571	0.2105	0.2565	0.5910
Zmym4	_AAHQES[Phospho (STY)]DNENEIQIQNQLK_.3	#N/A	4.1640	4.2622	3.6587	4.5075	3.9097	4.1375	3.5789	5.2060	4.6590	4.6842	0.9061	-0.1422	0.2056	0.6871
Kiaa1522	_GS[Phospho (STY)]PSGGSTAETSDTASIR_.2	A2A7S8_S402_M1_Kiaa1522	6.5737	6.1312	3.8975	7.0939	3.3076	3.2288	6.8010	3.5599	3.8589	3.1186	1.1625	0.2172	0.5442	0.2642
Kiaa1522	_MGS[Phospho (STY)]PGSDPQKK_.2	#N/A	3.7447	3.1690	6.2206	5.6176	3.2737	3.4075	6.3304	5.7197	3.9427	5.0019	0.8076	-0.3083	0.2373	0.6247
Kiaa1522	_GLAVAPAS[Phospho (STY)]PGK_.2	A2A7S8_S543_M1_Kiaa1522	3.0792	3.4207	3.2655	3.2360	3.2052	4.9074	3.3842	4.3679	4.2836	4.4916	0.8517	-0.2316	0.1682	0.7742
Kiaa1522	_LQLERPVS[Phospho (STY)]PEAQADLQR_.3	A2A7S8_S909_M1_Kiaa1522	4.4090	4.6156	4.4164	4.3182	3.3849	3.2397	3.1329	4.8927	4.5833	4.6480	0.9072	-0.1405	0.3995	0.3985
Kiaa1522	_M[Oxidation (M)]GS[Phospho (STY)]PGSDPQK_.2	#N/A	3.9250	3.4174	3.6587	3.2024	3.2388	2.9899	3.3507	3.7988	4.0978	4.3805	0.8716	-0.1982	0.0754	1.1229
Hivep3	_GSYS[Phospho (STY)]FDDHVADPEVPSR_.2	#N/A	3.3715	3.6104	3.7057	4.7701	3.6692	3.4503	3.7371	3.9848	4.2763	4.2790	0.9247	-0.1130	0.3056	0.5149
Hivep3	_RSS[Phospho (STY)]VESPK_.2	A2A884_S489_M1_Hivep3	3.5786	4.3838	4.1931	3.7369	4.2052	3.5243	3.8851	3.2643	3.5634	3.4141	1.1147	0.1567	0.0961	1.0172
Kiaa1109	_VVFENEQDNNS[Phospho (STY)]LTK_.2	A2AAE1_S2755_M1_Kiaa1109	8.4093	8.3601	9.0069	8.3559	6.8427	8.0862	9.0025	8.4516	8.4025	8.1890	0.9607	-0.0578	0.4188	0.3780
Kiaa1109	_SDENVLDS[Phospho (STY)]PK_.2	#N/A	7.1435	6.8616	7.4928	7.0079	5.1009	6.8274	7.5410	6.9799	6.3634	6.9452	0.9686	-0.0460	0.6532	0.1849
Kiaa1109	_TLKTES[Phospho (STY)]PNASR_.2	A2AAE1_S3894_M1_Kiaa1109	6.9155	4.2841	7.6270	4.3062	4.1724	3.9530	7.1909	4.1959	2.9941	3.9834	1.1348	0.1824	0.5867	0.2316
Kiaa1109	_ALETIPIT[Phospho (STY)]PIER_.2	A2AAE1_T3996_M1_Kiaa1109	3.6615	3.5647	4.3483	3.3501	2.7872	3.4415	3.0648	3.2675	4.4169	3.3171	1.0025	0.0037	0.9804	0.0086
Kiaa1109	_YTAGSAS[Phospho (STY)]PTPTFK_.2	A2AAE1_S2603_M1_Kiaa1109	7.5463	7.4177	8.2006	6.9835	5.5678	6.9470	3.3747	8.3838	8.1230	3.3703	1.2232	0.2907	0.3119	0.5059
Kiaa1109	_SLDQDS[Phospho (STY)]PSKK_.2	A2AAE1_S3835_M1_Kiaa1109	3.7780	3.0823	3.4648	3.5375	2.9037	3.3250	3.6858	3.1510	3.4005	3.4336	0.9650	-0.0514	0.7215	0.1418
Kiaa1109	_SSETFGPAGVRS[Phospho (STY)]PTEPTC[Carbamidomethyl (C)]R_.3	#N/A	6.2630	5.7464	6.6138	6.2016	3.4163	4.3132	5.9110	6.2625	3.7878	6.2811	0.9757	-0.0354	0.8707	0.0601
Kiaa1109	_AS[Phospho (STY)]PGSSSYLK_.2	A2AAE1_S3196_M1_Kiaa1109	3.0453	5.9678	3.7321	6.1030	3.6718	5.7556	6.2205	6.5580	6.5001	4.2017	0.8028	-0.3169	0.2002	0.6985
Kiaa1109	_S[Phospho (STY)]PTEPTC[Carbamidomethyl (C)]R_.2	#N/A	6.2231	3.5477	4.3653	6.1893	3.4360	5.2287	6.5130	5.6121	4.4000	3.3341	0.9732	-0.0392	0.8780	0.0565
Kiaa1109	_SDENVLDS[Phospho (STY)]PKQR_.2	#N/A	6.3750	7.1249	7.2110	7.1989	5.7553	6.7225	7.2582	7.5775	6.6980	6.6912	0.9539	-0.0680	0.3710	0.4306
Kiaa1109	_MVPETGIPAEC[Carbamidomethyl (C)]QS[Phospho (STY)]GQK_.2	A2AAE1_S628_M1_Kiaa1109	3.7783	3.0826	3.9935	8.7380	2.9040	3.3247	3.6855	3.4640	3.7630	3.2145	1.2185	0.2851	0.5148	0.2883
Kiaa1109	_RDDGQASVPTEISGTS[Phospho (STY)]PVS[Phospho (STY)]PNTQDK_.3	A2AAE1_S1287_M2_Kiaa1109	4.1506	3.4549	3.6211	3.1649	3.2763	2.9524	3.3132	3.8363	4.1353	4.3430	0.8796	-0.1850	0.1286	0.8909
Sh3pxd2b	_KES[Phospho (STY)]IIKSEEELLER_.3	A2AAY5_S528_M1_Sh3pxd2b	3.8579	4.1456	4.5447	4.3362	2.9836	3.9642	3.2612	4.2669	4.2206	3.0571	1.0731	0.1018	0.4894	0.3103
Sh3pxd2b	_LRPAKS[Phospho (STY)]QEK_.2	A2AAY5_S672_M1_Sh3pxd2b	3.3334	4.1386	4.0202	4.5228	3.9599	3.7696	4.2375	3.5956	1.4932	2.9890	1.2854	0.3622	0.1206	0.9185
Arhgap27	_AES[Phospho (STY)]PKQVDDPPEPVYANVER_.3	A2AB59_S215_M1_Arhgap27	3.2191	4.0243	3.9059	4.4086	3.8456	3.8839	6.3067	3.7099	3.3585	4.3756	0.8746	-0.1933	0.3460	0.4609
Arhgap27	_KSS[Phospho (STY)]QDS[Phospho (STY)]DTPAQASPPEEK_.2	A2AB59_S462_M2_Arhgap27	4.2852	3.1843	3.4610	3.7368	3.1901	3.5583	3.4514	4.0094	4.0365	3.7038	0.9392	-0.0905	0.3575	0.4467
Ddi2	_TQAQHSSPGEM[Oxidation (M)]ASS[Phospho (STY)]PQGLDNPALLR_.3	#N/A	4.0005	5.6251	3.7058	5.4411	3.6676	5.0913	3.9232	5.0673	5.3762	5.3421	0.9313	-0.1027	0.5420	0.2660
Ccdc120	_SNS[Phospho (STY)]SEALLVDR_.2	A2AEV7_S357_M1_Ccdc120	9.0321	8.4479	9.0231	8.7397	7.9141	8.6688	8.7882	8.3940	8.6549	8.8243	0.9968	-0.0046	0.9058	0.0430
Tmem185a;Tmem18!	_VVITQS[Phospho (STY)]PGK_.2	A2AF53_S333_M1_Tmem185a;Tmem185b	7.7450	7.1800	4.2149	7.5867	3.6249	2.9997	4.3937	7.9685	7.7096	3.4359	0.9458	-0.0804	0.8299	0.0810
Map7d2	_EES[Phospho (STY)]LEKR_.2	#N/A	7.3636	7.3331	7.6860	7.9785	7.1294	7.2927	7.5311	7.4952	7.6151	7.7443	0.9825	-0.0254	0.4434	0.3532
Map7d2	_RLSS[Phospho (STY)]PVK_.2	A2AG50_S332_M1_Map7d2	4.3047	3.6090	3.4670	4.5116	3.4304	4.2991	6.5683	7.0942	4.2894	7.3063	0.6235	-0.6816	0.0042	2.3761
Map7d2	_VATSAASGGHGS[Phospho (STY)]PLR_.2	A2AG50_S315_M1_Map7d2	5.4040	5.6584	5.1384	5.4620	3.3436	5.4315	5.2266	4.8774	5.3677	5.4432	0.9702	-0.0436	0.7391	0.1313
Map7d2	_EGAPGQQSTVLPREES[Phospho (STY)]LEKR_.3	#N/A	3.6345	3.7079	8.8031	4.4128	4.2791	3.8464	4.5610	4.0892	4.3883	4.0901	1.1164	0.1589	0.6389	0.1945
Map7d2	_SSYKSS[Phospho (STY)]PTR_.2	A2AG50_S264_M1_Map7d2	5.5922	6.0678	6.6281	4.4313	2.7967	3.8279	3.0787	4.0708	4.3698	4.1085	1.2518	0.3240	0.2411	0.6178
Map7d2	_S[Phospho (STY)]NSLDDSTEDVQSM[Oxidation (M)]DVSPVSK_.2	#N/A	3.9955	7.0669	4.1494	7.2736	6.1991	7.1436	5.2909	3.2481	7.1116	7.5173	1.0310	0.0440	0.8739	0.0585
Map7d2	_NLIEGFNS[Phospho (STY)]PGQETTLNTFC[Carbamidomethyl (C)]_.2	#N/A	4.7792	4.8363	4.9687	5.1726	3.8517	4.3149	5.3277	4.8454	3.8293	4.0962	1.0286	0.0406	0.7340	0.1343
Map7d2	_TAKPTYLGS[Phospho (STY)]PVK_.2	A2AG50_S357_M1_Map7d2	9.6379	9.6652	9.7648	10.2519	8.3457	9.5338	9.6272	9.4714	9.2732	9.6186	1.0038	0.0054	0.9167	0.0378
Map7d2	_HLSSSTVAISYS[Phospho (STY)]PDR_.2	A2AG50_S248_M1_Map7d2	4.2921	2.9341	3.4780	3.9807	2.8224	3.8022	3.6954	6.3876	3.7864	3.9477	0.7973	-0.3267	0.1659	0.7803
Bclaf3	_SYVNRS[Phospho (STY)]PS[Phospho (STY)]PNVKPVEK_.3	#N/A	6.8173	7.1007	6.9599	7.6778	6.6115	7.0315	6.9561	7.0230	6.9493	7.2032	1.0000	0.0000	0.9992	0.0004
Med12	_EVKPPAKEK_.2	#N/A	11.8141	11.4788	11.9209	11.8250	12.0274	11.8257	11.0835	11.0822	12.3209	11.8507	1.0199	0.0285	0.3982	0.3999
Ambra1	_TSS[Phospho (STY)]PQPSTSR_.2	A2AH22_S1206_M1_Ambra1	7.1662	7.3245	3.8508	3.3945	6.0854	3.1820	3.5428	7.4284	3.9057	6.8638	0.9507	-0.0729	0.8363	0.0776
Camsap1	_S[Phospho (STY)]PSDPGGELPEK_.2	A2AHC3_S1133_M1_Camsap1	3.3138	3.9817	4.2981	4.3139	3.9493	4.2597	4.2291	4.0698	3.2361	7.7308	0.8345	-0.2610	0.3544	0.4505
Camsap1	_VES[Phospho (STY)]LEALPILSR_.2	A2AHC3_S1407_M1_Camsap1	3.5277	3.6985	4.2145	6.9817	3.5868	5.8623	5.7318	6.7252	6.1527	6.2169	0.7484	-0.4181	0.0719	1.1435
Camsap1	_TDVS[Phospho (STY)]PPSPQMPR_.2	A2AHC3_S550_M1_Camsap1	6.4393	3.6430	3.6380	7.7031	3.4243	3.5253	3.6826	3.9993	3.8770	3.7637	1.2345	0.3039	0.3724	0.4290

Camsap1	_SVHREES[Phospho (STY)]YSDSGTK_.3	A2AHC3_S1359_M1_Camsap1	3.2802	3.9460	3.9670	4.4696	3.9067	3.8228	4.1843	3.6488	3.2974	4.4366	1.0018	0.0026	0.9810	0.0083
Lzts3	_LERIES[Phospho (STY)]TEI_.2	A2AHG0_S697_M1_Lzts3	5.4311	4.3743	4.2026	6.9203	3.6126	6.6880	3.8946	6.2654	6.8824	3.4236	1.0173	0.0247	0.9300	0.0315
Ano3	_VHHSGS[Phospho (STY)]IQS[Phospho (STY)]FKQQK_.3	A2AHL1_S10_M2_Ano3	4.3685	4.3227	5.0130	4.0930	4.2110	3.9145	3.8076	4.0255	4.0023	4.0220	1.0898	0.1241	0.1080	0.9666
Ano3	_S[Phospho (STY)]LPC[Carbamidomethyl (C)]LAQSYAHSK_.2	#N/A	8.4752	8.5815	8.9266	8.6046	6.7213	7.9036	8.9071	8.6742	8.4646	8.1506	0.9594	-0.0598	0.4397	0.3568
Ano3	_VHHSGS[Phospho (STY)]IQSFK_.3	A2AHL1_S7_M1_Ano3	3.2932	4.0492	4.5278	4.0715	3.9378	3.8590	4.2198	4.4305	3.2287	3.7488	1.0127	0.0182	0.8704	0.0603
Virma	_SFLSEPSS[Phospho (STY)]PGR_.2	A2AIV2_S1578_M1_Virma	8.5978	4.0195	9.0951	8.2650	7.2604	7.9960	8.9798	8.4236	8.3261	8.1154	0.8910	-0.1665	0.3578	0.4464
Virma	_HADGEKEDQFNGS[Phospho (STY)]PPRPQPR_.4	A2AIV2_S173_M1_Virma	3.7510	3.0553	4.4378	3.5645	5.4905	3.3520	3.7128	5.7431	3.7357	3.2418	0.9595	-0.0597	0.7989	0.0975
Pnpla7	_SC[Carbamidomethyl (C)]S[Phospho (STY)]VPLPSNHGEVDELR_.3	A2AJ88_S379_M1_Pnpla7	3.6239	3.7185	3.3576	4.4022	3.5398	4.1897	4.5505	5.8029	4.3989	4.0795	0.8083	-0.3071	0.0377	1.4236
Ajm1	_S[Phospho (STY)]PAQAPEPAAEGLGR_.2	A2AJA9_S593_M1_Ajm1	10.0236	9.5877	9.9652	10.1084	8.9678	9.6698	10.2135	9.7681	9.8626	9.8765	0.9789	-0.0308	0.3856	0.4139
Ajm1	_PYLSEEPPRPS[Phospho (STY)]PR_.2	#N/A	8.4643	7.8398	8.2041	7.8477	7.1271	8.1302	7.8778	7.2224	7.9918	7.9408	1.0229	0.0326	0.5367	0.2702
Ajm1	_ELATHAAPLGS[Phospho (STY)]YAR_.3	#N/A	7.3553	7.1531	7.6817	3.2602	6.4716	7.2657	7.5195	7.4291	6.7064	6.9922	0.9120	-0.1330	0.4824	0.3166
Ajm1	_GRS[Phospho (STY)]YENLLGR_.2	#N/A	7.2173	7.2015	7.9122	7.4473	6.1109	7.1472	7.7791	7.0614	7.3853	6.6839	0.9924	-0.0110	0.8811	0.0550
Ajm1	_S[Phospho (STY)]WDNILAPGPR_.2	A2AJA9_S448_M1_Ajm1	7.6168	9.6928	9.0626	10.6261	9.0379	9.8441	10.0118	10.3246	9.9327	9.9807	0.9256	-0.1116	0.1903	0.7206
Ajm1	_S[Phospho (STY)]YENLLGR_.2	#N/A	8.8599	8.5676	8.7947	9.2131	7.7805	8.6624	8.9948	8.8493	8.6963	8.5816	0.9847	-0.0222	0.6123	0.2130
Ajm1	_ASQGLATAPAS[Phospho (STY)]PPVLQR_.2	A2AJA9_S109_M1_Ajm1	8.8798	10.4566	10.7692	11.4913	8.1039	8.7158	10.4034	10.3340	9.7561	8.7422	0.9926	-0.0107	0.9253	0.0337
Ajm1	_YAALSLSETSLT[Phospho (STY)]EK_.2	A2AJA9_T514_M1_Ajm1	3.4057	4.4505	3.9607	4.3360	4.5778	3.9604	3.9848	5.7613	4.5024	5.6841	0.8258	-0.2761	0.0680	1.1678
Ajm1	_S[Phospho (STY)]SPPWADWGPR_.2	A2AJA9_S397_M1_Ajm1	3.0570	4.0086	3.9263	8.3134	3.6926	4.2465	4.1437	8.0990	3.3600	3.7453	0.9387	-0.0912	0.8253	0.0834
Ajm1	_RYAALSLSET[Phospho (STY)]S[Phospho (STY)]LTEK_.2	#N/A	3.9924	3.2684	4.2020	3.5375	3.1286	3.3204	3.4744	3.4652	3.7643	3.2148	1.0274	0.0389	0.6983	0.1559
Ajm1	_VALAAGS[Phospho (STY)]PTRPPPAR_.2	A2AJA9_S854_M1_Ajm1	3.2146	7.5653	8.8143	10.2166	3.8411	3.8883	8.6557	3.7144	3.3630	4.3711	1.2449	0.3160	0.5152	0.2881
Ajm1	_HVALS[Phospho (STY)]RT[Phospho (STY)]PT[Phospho (STY)]PSDLYC[Carbamidomethyl (C)]TDPR_.3	A2AJA9_S236_M3_Ajm1	4.0246	3.2800	3.7583	3.3020	3.1683	3.4563	3.4503	3.6991	3.9982	2.9793	0.9905	-0.0137	0.8922	0.0496
Map7d1	_RSS[Phospho (STY)]QPS[Phospho (STY)]PTTVPASDSPPAK_.2	#N/A	8.7442	9.0621	6.5695	7.0146	8.2488	9.2748	8.4858	9.4119	8.2240	9.4245	0.9174	-0.1244	0.2713	0.5666
Map7d1	_SSQPS[Phospho (STY)]PTTVPASDSPPAK_.2	A2AJI0_S118_M1_Map7d1	7.7505	7.7488	8.1514	8.1830	8.2754	7.6740	8.2182	8.2229	3.7645	6.7968	1.1797	0.2384	0.1874	0.7273
Map7d1	_SSATLWNS[Phospho (STY)]PSR_.2	A2AJI0_S282_M1_Map7d1	3.6116	7.0713	7.7488	6.7622	6.9379	7.9278	7.9505	7.1156	7.5862	7.8786	0.8747	-0.1931	0.2748	0.5610
Map7d1	_RKPGAGGS[Phospho (STY)]PALAR_.3	#N/A	7.9309	8.2943	8.6772	8.6740	7.5502	7.9705	7.8160	8.5546	7.9607	7.6934	1.0221	0.0315	0.5374	0.2697
Map7d1	_KPGAGGS[Phospho (STY)]PALAR_.2	#N/A	7.4064	7.8871	7.9494	8.1259	7.2132	7.8664	8.3073	7.9643	7.2680	7.8475	0.9866	-0.0195	0.6821	0.1661
Map7d1	_SAS[Phospho (STY)]ASPLTPC[Carbamidomethyl (C)]SAPR_.2	A2AJI0_S368_M1_Map7d1	7.2405	7.0009	7.9871	7.5001	6.4262	7.4743	7.5177	6.9838	6.9639	7.0437	1.0202	0.0289	0.6308	0.2001
Map7d1	_S[Phospho (STY)]RGPT[Phospho (STY)]PTATGPR_.2	A2AJI0_S95_M2_Map7d1	7.3881	7.2156	7.0878	4.3486	6.2286	7.4516	7.2548	7.1663	7.2193	7.5529	0.9071	-0.1407	0.3020	0.5200
Map7d1	_AAEEKEPAAPAS[Phospho (STY)]PAPSPVPSPTPAQPQK_.3	A2AJI0_S544_M1_Map7d1	3.8060	5.9400	3.9408	6.4835	4.8371	4.8907	4.4540	5.4424	6.0565	5.0660	0.9483	-0.0766	0.6652	0.1771
Map7d1	_GPT[Phospho (STY)]PTATGPR_.2	#N/A	3.0968	4.1294	3.7836	4.2862	3.7233	4.1078	4.0009	3.8322	3.4808	4.2532	0.9904	-0.0139	0.8868	0.0522
Map7d1	_LSTGSELS[Phospho (STY)]PK_.2	A2AJI0_S460_M1_Map7d1	3.7233	3.0277	4.9478	3.5922	2.8490	3.3797	3.7404	3.2057	4.3551	3.3789	0.9773	-0.0332	0.8509	0.0701
Pnisr	_QRS[Phospho (STY)]PIALPVK_.2	A2AJT4_S211_M1_Pnisr	5.6280	4.9723	5.3550	6.3403	3.9651	5.0721	5.9842	5.6952	4.7429	4.9358	0.9780	-0.0321	0.8072	0.0930
Pnisr	_SNRSS[Phospho (STY)]IER_.2	A2AJT4_S601_M1_Pnisr	5.0836	3.8904	5.4984	4.5252	3.7787	4.3467	4.2399	5.6515	5.1785	4.2896	0.9340	-0.0985	0.4869	0.3125
Ptpn3	_SSSSVSPSSNAPGS[Phospho (STY)]C[Carbamidomethyl (C)]SPDGVDQR_.2	A2ALK8_S467_M1_Ptpn3	3.3810	3.6533	3.5316	4.4693	3.4746	4.2567	3.1167	4.0346	4.3336	4.1466	0.9710	-0.0425	0.7285	0.1376
Ptpn3	_S[Phospho (STY)]LS[Phospho (STY)]VERLETK_.2	#N/A	3.7516	4.1232	3.4853	5.3850	4.9818	6.0335	3.1773	4.2372	5.6765	4.2072	1.0699	0.0974	0.6555	0.1834
Ptpn3	_RS[Phospho (STY)]LS[Phospho (STY)]VER_.2	#N/A	5.4121	4.3814	3.1081	5.4749	4.4885	3.1808	5.5716	3.0068	5.5241	3.5778	0.9821	-0.0260	0.9177	0.0373
Ptpn3	_RS[Phospho (STY)]LS[Phospho (STY)]VERLETK_.2	#N/A	3.9733	3.9069	3.7070	3.7687	3.6851	3.5651	4.3715	3.8080	4.1070	3.6463	0.9459	-0.0802	0.1849	0.7331
Rap1gap	_RES[Phospho (STY)]PPAGQK_.2	#N/A	10.1122	10.0074	10.1182	10.4890	8.5755	9.4801	10.2639	10.0604	9.2313	10.0586	0.9892	-0.0156	0.7927	0.1009
Rap1gap	_DDVPFFGPPLPDPAVFR_.2	#N/A	5.9173	4.8792	4.1070	5.6419	5.2539	5.4582	5.4621	5.4232	5.5389	5.5839	0.9469	-0.0788	0.4016	0.3963
Rap1gap	_RSS[Phospho (STY)]AIGIENIQEVQEK_.2	#N/A	3.8614	3.8357	3.8316	5.4086	4.8874	3.6155	3.7561	5.7741	5.7770	3.6416	0.8950	-0.1600	0.4331	0.3634
Rap1gap	_TEVLQGFS[Phospho (STY)]R_.2	A2ALS5_S566_M1_Rap1gap	6.8984	3.3612	4.5517	7.0646	5.6255	7.0277	7.2955	6.3919	7.1781	6.9387	0.8279	-0.2725	0.1737	0.7602
Rap1gap	_SENSSTQS[Phospho (STY)]S[Phospho (STY)]PEM[Oxidation (M)]PTTK_.2	#N/A	10.8562	10.7310	10.7253	12.0610	10.5995	10.5928	11.0297	10.6233	10.6306	10.8694	1.0129	0.0185	0.6525	0.1854
Rap1gap	_S[Phospho (STY)]SAIGIENIQEVQEK_.2	A2ALS5_S498_M1_Rap1gap	3.9834	3.2389	3.7171	3.2609	3.1272	3.4974	3.4092	3.7403	4.0393	4.4390	0.8884	-0.1708	0.1081	0.9662
Rap1gap	_LPYTEGDAQQLQR_.2	#N/A	3.8356	4.0435	3.4183	4.5782	3.2288	4.3255	3.2315	4.3809	3.3508	4.3275	1.0215	0.0307	0.8249	0.0836
Rap1gap	_SSAIGIENIQEVQEKRES[Phospho (STY)]PPAGQK_.3	#N/A	6.4067	8.0145	9.1218	8.7764	7.5233	7.7757	8.0317	8.4828	7.8100	7.4938	0.9977	-0.0033	0.9729	0.0119
Rap1gap	_TPDS[Phospho (STY)]GHVSQEPK_.2	A2ALS5_S525_M1_Rap1gap	4.2654	5.8053	3.5063	4.9866	3.3911	4.3384	3.1983	3.9511	4.2501	4.2282	1.1216	0.1656	0.3735	0.4277
Rap1gap	_SQS[Phospho (STY)]M[Oxidation (M)]DAM[Oxidation (M)]GLSNK_.2	#N/A	6.7027	6.8425	6.7642	7.6595	3.1870	6.9325	6.6219	6.5383	6.9497	6.7943	0.9438	-0.0834	0.6545	0.1841
Rap1gap	_RES[Phospho (STY)]PPAGQKT[Phospho (STY)]PDSGHVSQEPK_.3	A2ALS5_S515_M2_Rap1gap	4.9910	4.2910	3.7650	3.6280	4.4036	3.4495	3.3427	5.3621	4.1391	3.5950	0.9947	-0.0076	0.9637	0.0160
Shroom2	_DSC[Carbamidomethyl (C)]SS[Phospho (STY)]PPSLNYGK_.2	A2ALU4_S1165_M1_Shroom2	13.3082	13.3565	13.8651	14.0161	13.2100	13.3666	13.7729	13.2772	13.3341	13.4417	1.0048	0.0068	0.7470	0.1267
Shroom2	_IVHSES[Phospho (STY)]QPEKESR_.2	#N/A	5.3250	5.4330	5.9513	5.9007	4.1605	6.0979	5.4644	6.0534	5.6891	5.9865	0.9448	-0.0820	0.4238	0.3729
Shroom2	_VENALNNLDDNPSPGDR_.2	#N/A	7.0123	7.0560	7.3869	7.7605	6.9108	7.1670	7.7008	6.9372	7.1441	7.4237	0.9882	-0.0171	0.6887	0.1620
Shroom2	_SPS[Phospho (STY)]PQFAPQK_.2	A2ALU4_S1043_M1_Shroom2	10.7037	10.8571	11.0674	11.3907	10.4459	10.9138	10.8118	10.5654	10.7144	10.9223	1.0133	0.0191	0.4362	0.3603
Ubr4	_HVTLPSS[Phospho (STY)]PR_.2	A2AN08_S2716_M1_Ubr4	10.9185	10.8637	11.3846	3.5896	9.5034	10.5822	11.3629	11.2128	10.9737	10.7022	0.8563	-0.2237	0.3233	0.4904
Ubr4	_TKEGVGS[Phospho (STY)]PK_.2	A2AN08_S457_M1_Ubr4	6.4829	6.5141	6.8892	5.8975	4.4236	5.7913	7.0167	6.7625	6.1187	6.0383	0.9253	-0.1120	0.3475	0.4590
Ubr4	_TLSDVEDQKELAS[Phospho (STY)]PVSPELR_.3	#N/A	6.0780	5.8463	6.4903	5.9679	4.7940	5.4000	6.6037	6.8775	5.8399	5.8830	0.9146	-0.1288	0.1786	0.7480
Ubr4	_T[Phospho (STY)]GSTSSKEDDYESDAATIVQK_.3	A2AN08_T360_M1_Ubr4	3.7925	3.5499	3.5262	4.5708	2.9363	3.6883	3.2183	3.9312	4.2302	4.2481	0.9412	-0.0874	0.5068	0.2952
Plch2	_GTKPGS[Phospho (STY)]LDSHAAGQPLPR_.3	A2AP18_S987_M1_Plch2	6.3363	6.2721	6.5911	7.0346	5.2400	6.0471	6.1661	6.3742	6.1305	5.6854	1.0270	0.0385	0.6285	0.2017
Plch2	_VAS[Phospho (STY)]VEEGDETLDSPGSQSR_.2	#N/A	9.1094	9.6439	9.9499	9.9362	8.5063	9.4580	9.9865	9.6380	9.4814	9.4429	0.9789	-0.0307	0.5155	0.2878
Plch2	_KVAS[Phospho (STY)]VEEGDETLDSPGSQSR_.3	#N/A	6.3102	6.3589	6.8376	7.3604	5.4943	7.2512	6.2678	7.2424	7.2861	7.3351	0.9388	-0.0912	0.3228	0.4911
Plch2	_IRDC[Carbamidomethyl (C)]EDPNDFSVSTLS[Phospho (STY)]PSGK_.3	A2AP18_S619_M1_Plch2	3.7889	3.0933	4.4757	3.4775	2.9146	3.3141	3.6748	3.1401	4.2895	3.4445	0.9652	-0.0511	0.7234	0.1406
Plch2	ANISEDAEEGEVS[Phospho (STY)]DEDS[Phospho (STY)]ADEM[Oxidation (M)]EDDC[Carbamidomethyl (C	A2AP18_S561_M2_Plch2	4.0360	3.3064	3.7697	3.3134	3.1797	3.4449	3.4617	3.6877	3.9868	2.9907	0.9934	-0.0096	0.9234	0.0346
Fmnl2	_S[Phospho (STY)]IEDLHR_.2	A2APV2_S171_M1_Fmnl2	11.2913	11.1179	11.7389	11.2720	9.7226	11.0278	11.4328	11.3794	11.2371	11.0851	0.9774	-0.0330	0.4927	0.3074
Fmnl2	_LLEQEALM[Oxidation (M)]EQQDAKS[Phospho (STY)]PSHK_.3	#N/A	3.2705	5.6426	6.4886	5.2663	3.9017	3.6154	4.1684	5.5045	3.3336	5.6661	1.0063	0.0091	0.9712	0.0127
Scn1a	_QAS[Phospho (STY)]FTYNK_.2	A2APX8_S1939_M1_Scn1a	5.3838	5.4982	3.6228	4.1255	3.1533	5.4562	5.1206	5.6755	5.1573	5.7955	0.8350	-0.2602	0.1403	0.8529
Scn1a	_FS[Phospho (STY)]IEGNR_.2	A2APX8_S537_M1_Scn1a	4.6056	4.6616	3.7927	3.8375	3.7324	4.3829	4.5023	4.6899	4.4563	2.8954	1.0079	0.0114	0.9360	0.0287
Scn1a	_NSNLS[Phospho (STY)]QTSR_.2	A2APX8_S623_M1_Scn1a	3.0810	4.2614	4.3155	3.8593	3.7256	4.3998	5.5263	3.1419	3.4409	3.5366	1.0074	0.0107	0.9551	0.0199
Scn1a	_RNS[Phospho (STY)]NLSQTSR_.2	A2APX8_S620_M1_Scn1a	3.0125	3.8177	3.6993	4.3030	3.6390	4.0905	4.4512	3.9165	3.5651	3.8591	0.9525	-0.0703	0.5058	0.2961
Scn1a	_RDS[Phospho (STY)]LFVPR_.2	A2APX8_S607_M1_Scn1a	4.8594	4.7694	4.1073	3.6511	3.6932	3.2995	4.2899	3.3501	3.6491	3.3284	1.1119	0.1530	0.3011	0.5214
Scn1a	_QQEAAQQAAATTAS[Phospho (STY)]EHSR_.3	A2APX8_S467_M1_Scn1a	3.2335	4.1089	4.4680	4.0118	3.8780	4.2474	4.1600	2.9894	3.2885	3.6890	1.1301	0.1765	0.1575	0.8028
Scn1a	_RYS[Phospho (STY)]S[Phospho (STY)]PHQSLLSIR_.3	A2APX8_S550_M2_Scn1a	3.8137	3.4124	4.5005	3.5023	2.9394	3.2892	3.2170	3.1153	4.2647	3.4693	1.0170	0.0243	0.8657	0.0626
Skt	_VSS[Phospho (STY)]EDPGPTPQTR_.2	A2AQ25_S1087_M1_Skt	9.9334	9.5789	10.0922	10.2011	9.4623	10.0274	9.8405	9.8090	10.2097	9.9826	0.9922	-0.0113	0.6540	0.1844
Skt	_NRPGS[Phospho (STY)]LDK_.2	A2AQ25_S1762_M1_Skt	9.2177	9.2986	9.6139	9.9710	8.4112	9.2770	9.0560	9.4293	9.2734	9.0638	1.0101	0.0144	0.7442	0.1283
Skt	_IPALS[Phospho (STY)]PSSGK_.2	A2AQ25_S1808_M1_Skt	7.6502	7.8359	3.8866	3.6598	3.6034	7.4000	6.6795	7.2068	3.4544	7.6099	0.9094	-0.1370	0.6817	0.1664
Skt	_NVY[Phospho (STY)]YELNDVR_.2	A2AQ25_Y244_M1_Skt	2.9862	7.4671	7.2826	3.7644	7.0733	7.4665	3.9127	3.2367	3.5358	7.8382	1.2971	0.3753	0.3388	0.4700
Skt	_IGGKS[Phospho (STY)]PPPPPPPPR_.3	#N/A	7.2386	7.0608	6.7406	8.2701	5.0039	5.6349	7.1510	7.3447	6.2807	6.3161	0.9830	-0.0247	0.8614	0.0648
Skt	_KYPDSHLPTLGSKT[Phospho (STY)]PPAS[Phospho (STY)]PHR_.4	A2AQ25_S474_M2_Skt	6.3044	7.0012	6.6685	7.1984	6.6777	7.1422	6.2703	6.4306	6.8946	3.6441	1.1759	0.2338	0.1302	0.8855
Skt	_RQEQLS[Phospho (STY)]PEGTLPASR_.2	#N/A	5.4525	5.4639	6.7548	5.8261	5.7486	5.4998	4.7969	5.7348	5.1872	6.6403	1.0360	0.0510	0.6330	0.1986
Skt	_HQEETAHAPGQPLHC[Carbamidomethyl (C)]STGS[Phospho (STY)]PGDVK_.4	#N/A	7.6316	6.9251	4.0916	7.2428	3.7709	6.6832	4.1396	3.8647	4.1638	3.6687	1.5300	0.6135	0.0410	1.3867
Skt	_KAGGDC[Carbamidomethyl (C)]KPTS[Phospho (STY)]PSLPASK_.3	A2AQ25_S1796_M1_Skt	3.2847	4.0899	4.4871	4.0308	3.9112	3.8183	4.1791	2.9703	3.2694	3.7081	1.1147	0.1567	0.1991	0.7008
Skt	_AAPTTSSSSSPPS[Phospho (STY)]PAS[Phospho (STY)]PTSLNQGAR_.3	A2AQ25_S1902_M2_Skt	3.3604	6.2663	4.0472	3.9551	5.4614	3.7425	4.1033	3.0461	6.7720	3.6324	1.0191	0.0273	0.9258	0.0335

Skt	_T[Phospho (STY)]PPASPHR_.2	A2AQ25_T470_M1_Skt	6.7799	4.8085	4.2148	6.8772	4.1545	6.3530	7.0865	3.4011	3.0496	3.1836	1.3232	0.4040	0.2171	0.6634
Skt	_SIS[Phospho (STY)]PSPSAILER_.2	A2AQ25_S361_M1_Skt	9.0040	9.3908	9.3464	3.6985	8.2129	3.0931	9.3640	8.9732	4.0686	9.0927	0.9047	-0.1445	0.6877	0.1626
Skt	_S[Phospho (STY)]PPPPPPPPRR_.2	#N/A	6.4551	6.0825	4.2578	6.7367	5.8631	6.3832	6.0763	5.8837	5.5359	3.4789	1.1372	0.1855	0.3044	0.5165
Skt	_FQS[Phospho (STY)]PPHAGK_.2	A2AQ25_S1868_M1_Skt	3.5481	3.6781	4.2349	6.2308	6.7194	3.0582	4.4522	3.3809	3.0295	3.2037	1.3019	0.3806	0.2305	0.6374
Skt	_GDAAGDKLELSEDS[Phospho (STY)]PNS[Phospho (STY)]EQELDK_.3	A2AQ25_S1035_M2_Skt	4.0505	3.3059	3.7841	3.3279	3.4228	3.4304	3.4762	3.6733	3.9723	3.3052	1.0062	0.0089	0.9237	0.0345
Skt	_NEGFYADPYLYHEGR_.3	#N/A	3.6431	3.5831	4.3299	3.3317	3.4714	3.1533	4.5473	3.2859	4.4353	3.2987	0.9213	-0.1183	0.3984	0.3996
Ppig	_ADREQS[Phospho (STY)]PVSK_.2	#N/A	3.8495	3.3767	6.0881	3.5381	3.2650	3.3596	3.2528	3.0795	4.2290	3.5051	1.1127	0.1540	0.5200	0.2840
Ppig	_FRRS[Phospho (STY)]ET[Phospho (STY)]PPHWR_.3	#N/A	10.5099	10.6495	10.8615	10.8675	9.9738	10.5815	11.3683	10.8309	10.7138	10.4759	0.9748	-0.0368	0.2579	0.5886
Ppig	_S[Phospho (STY)]ET[Phospho (STY)]PPHWR_.2	#N/A	8.6709	7.3053	3.9072	8.0713	7.2168	8.1148	8.6403	7.9537	8.0936	8.5170	0.8691	-0.2024	0.2529	0.5971
Ppig	_SELNEIKENQRS[Phospho (STY)]PVR_.3	#N/A	7.3060	7.0586	7.8121	7.7249	6.2224	6.8332	7.2568	7.4433	7.0070	7.0984	0.9942	-0.0084	0.8967	0.0474
Ppig	_HM[Oxidation (M)]S[Phospho (STY)]ES[Phospho (STY)]PNRKVEK_.3	#N/A	4.1911	4.0140	4.3776	4.9112	4.7099	4.3423	3.8270	4.2207	3.6473	3.7591	1.1452	0.1956	0.0211	1.6765
Ppig	_HMS[Phospho (STY)]ES[Phospho (STY)]PNR_.2	#N/A	5.0432	3.7960	3.9287	5.3511	3.5546	3.5008	3.7336	3.6859	3.9672	4.1054	1.0833	0.1155	0.4584	0.3387
Ppig	_KADREQS[Phospho (STY)]PVSK_.2	#N/A	5.4030	6.0845	3.1172	5.1570	4.7961	3.4414	3.3345	6.3922	4.1472	5.8137	0.9481	-0.0769	0.7623	0.1179
Ppig	_ENQRS[Phospho (STY)]PVR_.2	#N/A	5.1317	2.9902	4.0859	4.5064	2.8115	3.4171	4.8391	3.3715	3.6706	3.3069	1.0071	0.0101	0.9619	0.0169
Ppig	_RS[Phospho (STY)]ET[Phospho (STY)]PPHWR_.2	#N/A	9.5046	9.3516	9.7182	9.8089	8.6663	9.2695	9.8695	9.4996	9.7139	9.4279	0.9749	-0.0366	0.3117	0.5063
Ppig	_S[Phospho (STY)]PPKADDKER_.2	A2AR02_S288_M1_Ppig	5.9013	5.5595	5.8641	6.0086	3.9549	4.1705	5.9216	5.5064	3.2116	5.0233	1.0666	0.0930	0.6376	0.1954
Ppig	_T[Phospho (STY)]RSPVEKENQK_.2	A2AR02_T712_M1_Ppig	3.0896	4.1365	3.7765	4.2791	3.7162	4.1006	3.9938	3.8394	3.4880	4.2461	0.9892	-0.0157	0.8723	0.0593
Ppig	_EQS[Phospho (STY)]PVSK_.2	#N/A	3.6546	3.5715	4.3414	3.3432	3.4598	3.1648	4.5588	3.2744	4.4238	3.3102	0.9223	-0.1168	0.4050	0.3925
Ppip5k1	_ALQTS[Phospho (STY)]PQPVEGTGLPR_.2	A2ARP1_S959_M1_Ppip5k1	8.1488	8.1128	8.7752	3.1349	6.1463	8.0858	8.3217	8.2762	7.8536	4.3130	0.9828	-0.0251	0.9278	0.0325
Ppip5k1	_TDPGS[Phospho (STY)]IENLC[Carbamidomethyl (C)]PGK_.2	A2ARP1_S939_M1_Ppip5k1	8.7017	3.4845	3.5915	3.1353	3.3059	4.4236	3.2836	3.8659	4.1649	4.3134	1.1365	0.1847	0.6419	0.1925
Ppip5k1	_TLHS[Phospho (STY)]PPLQLR_.2	A2ARP1_S1147_M1_Ppip5k1	4.1801	3.1623	3.9138	9.5191	2.9836	3.2450	3.6058	3.5436	3.8427	3.1348	1.2743	0.3498	0.4715	0.3265
Ankrd63	_GSNSDS[Phospho (STY)]PPGHPAPAPS[Phospho (STY)]PERR_.3	#N/A	7.0430	7.0306	6.0299	7.2300	6.5483	7.3064	7.1216	7.2862	6.8377	6.8530	0.9772	-0.0332	0.5599	0.2519
Ankrd63	_GSNSDS[Phospho (STY)]PPGHPAPAPSPER_.2	#N/A	9.6126	9.3791	10.0744	8.4462	9.1815	9.5798	9.5122	9.7098	9.6941	9.5703	0.9748	-0.0369	0.4128	0.3843
Rabgap1	_KTTAS[Phospho (STY)]PSVR_.2	#N/A	3.5175	3.7086	7.0199	5.9256	4.1441	3.5854	4.4217	3.4115	5.7463	5.9949	0.9503	-0.0736	0.7911	0.1018
Rabgap1	_GVSDEDT[Phospho (STY)]DEEKETLK_.2	A2AWA9_T991_M1_Rabgap1	4.0632	3.0164	4.2540	4.6421	4.0056	4.4035	4.7710	4.0085	3.7367	3.6224	1.0073	0.0105	0.9354	0.0290
Rabgap1	_QGDETPSTNNGS[Phospho (STY)]DDEKTGLK_.2	#N/A	3.6947	3.8426	4.4828	4.3652	3.8487	4.2198	4.6169	4.2347	4.3316	3.6296	1.0245	0.0349	0.7290	0.1373
Rabgap1	_TTAS[Phospho (STY)]PSVR_.2	#N/A	3.3768	3.9656	3.1105	4.1551	4.0214	4.1041	4.3034	4.3469	3.1451	3.8324	0.9698	-0.0443	0.7162	0.1450
Atxn7l3	_LQWDGSSDLS[Phospho (STY)]PSDSGSSK_.2	A2AWT3_S281_M1_Atxn7l3	3.2872	4.0924	3.9740	6.2952	3.9137	5.7414	4.2577	3.7393	4.0383	3.7092	1.1561	0.2093	0.3453	0.4618
Arid1a	_PYVPC[Carbamidomethyl (C)]PTPPRK_.2	#N/A	10.4248	11.3359	11.5233	10.4786	11.0066	11.0356	11.3734	10.9618	10.8954	10.9059	0.9940	-0.0087	0.7913	0.1016
Arid1a	_IELLPS[Phospho (STY)]R_.2	A2BH40_S1866_M1_Arid1a	10.9979	10.6269	11.0896	11.3273	10.6427	11.1178	10.8341	10.4835	10.9453	11.1793	1.0098	0.0141	0.5762	0.2395
Arid1a	_SNS[Phospho (STY)]VGIQDAFPDGSDPTFQK_.2	A2BH40_S1185_M1_Arid1a	3.9932	3.2486	3.7269	3.2706	3.1369	3.4877	6.7005	6.4379	4.0296	5.7448	0.6071	-0.7201	0.0021	2.6798
Arid1a	_SHHAPM[Oxidation (M)]S[Phospho (STY)]PGSSGGGGQPLAR_.3	#N/A	4.6107	5.8763	3.8910	6.7967	4.8242	5.7378	5.7392	5.7749	5.7541	3.7343	1.0074	0.0106	0.9549	0.0200
Arid1a	_GPS[Phospho (STY)]PSPVGSPASVAQSR_.2	A2BH40_S697_M1_Arid1a	5.9699	6.7593	6.8930	4.1782	5.7521	6.2583	6.9437	6.6586	3.3354	7.1482	0.9912	-0.0128	0.9529	0.0209
Arid1a	_NPQMPQYTSPQPGS[Phospho (STY)]ALSPR_.2	A2BH40_S770_M1_Arid1a	3.1552	3.9604	3.8420	6.2724	3.7817	3.9478	4.0593	3.7738	3.4224	4.3116	1.0689	0.0961	0.6507	0.1866
Kalrn	_SC[Carbamidomethyl (C)]S[Phospho (STY)]WHTLR_.2	A2CG49_S2374_M1_Kalrn	3.0476	5.7097	3.7344	4.2371	3.6741	4.3553	3.9517	3.8814	3.5300	4.2041	1.0603	0.0844	0.6345	0.1976
Kalrn	_ATAAAESSDGS[Phospho (STY)]IKK_.2	A2CG49_S2368_M1_Kalrn	3.6024	3.0489	3.5272	4.5718	2.9372	4.2437	4.0062	3.8859	1.0885	4.2491	1.1052	0.1443	0.6198	0.2078
Arid4b	_TVSKS[Phospho (STY)]PER_.2	A2CG63_S778_M1_Arid4b	4.0286	3.2841	3.7623	3.6307	3.1724	3.4522	3.3454	2.9869	4.1363	3.5977	1.0109	0.0157	0.8817	0.0547
Uhrf1bp1l	_SAS[Phospho (STY)]DTNLQK_.2	A2RSJ4_S952_M1_Uhrf1bp1l	6.1002	5.5342	5.9986	4.2809	3.7180	4.1024	3.9956	5.7800	3.4861	4.2479	1.1321	0.1790	0.4067	0.3908
Uhrf1bp1l	_SS[Phospho (STY)]PEISLR_.2	A2RSJ4_S1250_M1_Uhrf1bp1l	4.2613	3.0811	3.9950	3.5387	2.9025	3.3262	3.6870	5.4751	3.7615	3.2160	0.8718	-0.1980	0.3075	0.5122
Uhrf1bp1l	_DVASTDS[Phospho (STY)]DSVLNYR_.2	A2RSJ4_S1006_M1_Uhrf1bp1l	3.2561	4.0124	4.4906	4.4032	3.9007	4.2247	4.1179	3.7153	3.3638	4.3702	1.0401	0.0568	0.6025	0.2200
Uhrf1bp1l	_SGS[Phospho (STY)]GEMTSEGSHTK_.2	A2RSJ4_S988_M1_Uhrf1bp1l	3.7267	3.7564	3.8823	5.7547	3.6438	3.8170	4.1686	3.6561	3.6644	4.2749	1.0395	0.0559	0.7301	0.1366
Uhrf1bp1l	_NLGS[Phospho (STY)]PPKSPT[Phospho (STY)]HASPQHTQTEK_.3	A2RSJ4_T420_M2_Uhrf1bp1l	3.4735	3.7529	4.1603	3.1622	3.9076	3.3235	4.3777	4.3761	4.8464	3.1292	0.8679	-0.2043	0.1520	0.8181
Dennd5b	_EEKQEHLTDS[Phospho (STY)]PVALGPER_.3	#N/A	4.1208	5.0340	3.3068	3.8094	2.9936	3.6310	3.5241	4.3090	3.9576	3.7764	0.9805	-0.0284	0.8494	0.0709
Dennd5b	_QEHLTDS[Phospho (STY)]PVALGPER_.2	#N/A	3.4827	4.6930	3.7832	4.0946	3.5804	3.8962	4.0786	4.9168	3.5034	4.0453	0.9482	-0.0768	0.5228	0.2817
Dennd5b	_QC[Carbamidomethyl (C)]RT[Phospho (STY)]PPQQKS[Phospho (STY)]PTTTR_.3	A2RSQ0_S1068_M2_Dennd5b	2.9741	4.2521	3.6609	4.1636	3.6006	4.1289	3.8782	4.2316	3.9208	4.1305	0.9397	-0.0897	0.3715	0.4301
Dennd5b	_LQSDVLATGPANNNR_.2	#N/A	3.1529	3.9091	4.3874	3.9510	3.7974	3.7342	4.9279	2.8061	4.2096	3.6084	0.9830	-0.0247	0.8762	0.0574
Kansl3	_GSRPAS[Phospho (STY)]PAAR_.2	A2RSY1_S515_M1_Kansl3	3.5739	4.3791	4.1978	3.7416	4.2004	3.5291	3.8899	3.2596	3.5586	3.4189	1.1147	0.1567	0.0958	1.0186
Palm3	_TSGAKDDVS[Phospho (STY)]PEEQGK_.2	A2TJV2_S601_M1_Palm3	3.9153	3.2197	4.6021	6.2657	3.0410	3.1876	3.3186	5.4969	4.1631	6.1796	0.8432	-0.2460	0.3823	0.4176
Palm3	_TGGEGS[Phospho (STY)]LDEEAEGSKK_.2	A2TJV2_S565_M1_Palm3	4.3716	3.6270	4.1053	3.6490	3.5153	3.1093	3.7973	3.3521	3.6512	3.3263	1.0560	0.0786	0.4442	0.3525
Palm3	_QGTSS[Phospho (STY)]PELPTWVK_.2	A2TJV2_S270_M1_Palm3	3.8614	3.1169	3.5951	3.1389	3.0052	3.6195	3.2871	3.8623	4.1614	4.3170	0.8676	-0.2050	0.0743	1.1287
Ralgapa2	_RSS[Phospho (STY)]S[Phospho (STY)]PAELELKDDLQQAHGR_.4	A3KGS3_S819_M2_Ralgapa2	3.7168	3.0212	4.4036	4.9771	2.8425	3.3861	3.1201	3.2122	4.3516	3.3724	1.0591	0.0829	0.6755	0.1704
Exoc6b	_DVYTIFDAEVESTS[Phospho (STY)]PK_.2	A6H5Z3_S261_M1_Exoc6b	3.2738	3.9523	3.9606	4.4633	3.9003	3.8291	4.1780	3.6552	3.3038	4.4303	1.0012	0.0018	0.9867	0.0058
Phrf1	_EVS[Phospho (STY)]PAPATQGESR_.2	A6H619_S1201_M1_Phrf1	9.5068	9.2042	10.2017	10.0638	9.1302	9.2187	10.0777	9.6462	9.5898	9.4317	0.9864	-0.0198	0.6286	0.2016
Phrf1	_S[Phospho (STY)]RS[Phospho (STY)]PS[Phospho (STY)]LEHRPR_.3	A6H619_S1169_M3_Phrf1	3.9279	3.4145	3.6616	3.2053	3.2358	2.9928	3.3536	3.7958	4.0949	4.3835	0.8719	-0.1978	0.0759	1.1197
Armt1	_[Acetyl (Protein N-term)]AES[Phospho (STY)]PAFLSAK_.2	A6H630_S4_M1_Armt1	4.0558	3.4775	3.2417	3.7444	3.1815	3.0472	3.4591	4.1831	4.0227	4.8688	0.8366	-0.2574	0.0552	1.2579
Kcnb2	_DSVDDNHLS[Phospho (STY)]PSR_.2	A6H8H5_S488_M1_Kcnb2	7.3852	7.2133	7.5827	3.6660	1.6606	3.1256	8.1697	7.6978	7.3450	7.0682	0.7735	-0.3705	0.1330	0.8761
Kcnb2	_S[Phospho (STY)]M[Oxidation (M)]ELIDVAVEK_.2	#N/A	3.8121	5.7514	4.4989	6.0620	5.0606	6.0211	6.1773	6.6794	6.4294	6.3981	0.8100	-0.3040	0.0325	1.4882
Arhgap31	_RNS[Phospho (STY)]APVSVSAVR_.2	A6X8Z5_S1163_M1_Arhgap31	4.2648	3.0776	3.9985	3.5423	2.8989	3.3297	3.6905	3.4589	3.7580	3.2195	0.9963	-0.0054	0.9647	0.0156
Arhgap31	_S[Phospho (STY)]MDSLC[Carbamidomethyl (C)]SVPVEGK_.2	A6X8Z5_S343_M1_Arhgap31	3.4513	4.2565	4.1381	3.8642	4.0778	3.6516	4.0124	3.1370	4.4360	3.5415	1.1061	0.1455	0.1173	0.9308
Dpy19l1	_SS[Phospho (STY)]PPPLSGASEVDAGELGSER_.2	A6X919_S23_M1_Dpy19l1	8.2593	8.4848	3.3162	8.5792	3.7654	3.7452	4.1120	8.6839	8.3270	3.9201	0.9623	-0.0554	0.8931	0.0491
Vwa5b1	_ETS[Phospho (STY)]LDLEPMAESEEQANPC[Carbamidomethyl (C)]R_.2	A9Z1V5_S782_M1_Vwa5b1	4.1068	3.3622	3.8404	3.3842	3.2505	3.3741	3.5325	3.6170	3.9160	3.0615	1.0060	0.0087	0.9262	0.0333
Ptprs	_SPQGLGAFTAVVR_.2	#N/A	3.2302	3.9959	3.9170	4.9418	4.2850	4.0417	4.1344	3.6988	3.3474	4.3867	1.0454	0.0641	0.6139	0.2119
Uprt	_QVNSTSSPS[Phospho (STY)]PEHLLAEDR_.3	B1AVZ0_S25_M1_Uprt	3.6549	3.9586	4.2646	4.7403	4.4273	3.6225	4.0124	3.3943	4.0733	3.2334	1.1177	0.1606	0.1643	0.7844
Scn2a	_SAS[Phospho (STY)]EDS[Phospho (STY)]IRKK_.2	#N/A	6.9850	7.2688	7.5648	6.9206	6.3225	7.2593	7.4943	7.7211	7.4360	6.8127	0.9576	-0.0625	0.2742	0.5620
Scn2a	_KVSS[Phospho (STY)]IYKK_.2	B1AWN6_S1931_M1_Scn2a	3.2882	4.0934	3.9751	4.0272	3.9148	3.8147	4.1755	2.9739	3.2730	3.7045	1.0908	0.1253	0.2445	0.6118
Scn2a	_KSAS[Phospho (STY)]EDSIR_.2	#N/A	7.9327	7.8155	8.3764	5.9726	6.8501	7.3391	8.1010	8.0783	7.5351	7.3509	0.9504	-0.0734	0.4333	0.3632
Scn2a	_FS[Phospho (STY)]LEGSR_.2	#N/A	7.5877	6.9202	7.0939	6.9703	2.7933	6.3947	7.2471	7.1338	6.9797	6.6918	0.8974	-0.1562	0.4477	0.3490
Scn2a	_S[Phospho (STY)]AS[Phospho (STY)]EDSIRK_.2	#N/A	5.9379	4.6984	6.7287	5.8419	5.5076	6.0666	7.3680	7.1054	6.5443	5.5448	0.8729	-0.1960	0.1092	0.9618
Scn2a	_QQEEAQAAAAAASAES[Phospho (STY)]R_.2	#N/A	7.8210	7.6178	7.0851	7.7923	5.4086	7.5234	8.1061	8.6608	6.7059	6.7640	1.0139	0.0199	0.8590	0.0660
Scn2a	_LSSKS[Phospho (STY)]EKELK_.2	B1AWN6_S497_M1_Scn2a	6.4535	6.5232	6.9723	6.2658	5.4965	6.0443	6.4987	6.8710	6.2896	6.2525	0.9714	-0.0419	0.5230	0.2815
Scn2a	_GSLFS[Phospho (STY)]PR_.2	B1AWN6_S568_M1_Scn2a	3.5096	4.3148	5.0408	3.1982	4.1362	5.5933	4.4138	3.4194	3.0680	3.1652	1.1277	0.1733	0.3160	0.5003
Scn2a	_[Acetyl (Protein N-term)]AQSVLVPPGPDSFR_.2	#N/A	4.2940	3.0484	4.0277	3.5715	5.9877	3.3590	3.7198	1.6671	3.7287	5.0762	0.8663	-0.2071	0.4054	0.3921
Scn2a	_KQQEEAQAAAAAASAES[Phospho (STY)]R_.3	#N/A	4.0753	4.1508	3.8011	4.0091	4.1306	3.8162	4.6145	3.6071	4.4321	3.8598	0.9682	-0.0466	0.5361	0.2707
Scn2a	_GFRFS[Phospho (STY)]LEGSR_.2	#N/A	3.9335	5.4109	3.1195	3.6221	3.0592	3.1694	3.3368	4.1451	4.1449	3.5891	0.9777	-0.0326	0.8653	0.0628
Scn2a	_VKDIGS[Phospho (STY)]ENDFADDEHSTFEDNDSRR_.4	B1AWN6_S589_M1_Scn2a	2.9089	3.5152	4.1434	3.2496	3.5535	3.0712	4.4652	5.1905	3.6131	3.3644	0.8193	-0.2875	0.0961	1.0174
Scn2a	_KSAS[Phospho (STY)]EDS[Phospho (STY)]IRK_.2	#N/A	6.4145	5.1023	5.9020	5.7068	4.0861	4.8828	5.6044	5.3273	4.8968	3.9640	1.0810	0.1124	0.4542	0.3428
Scn2a	_DFSGAGGIGVFS[Phospho (STY)]ESSSVASK_.2	B1AWN6_S484_M1_Scn2a	3.2091	3.9653	4.4436	3.9873	3.8536	4.2127	5.6791	3.0138	5.4934	3.6646	0.8841	-0.1778	0.3873	0.4119
Scn2a	M[Oxidation (M)]SM[Oxidation (M)]ASILT[Phospho (STY)]NT[Phospho (STY)]M[Oxidation (M)]EELEESR	B1AWN6_T713_M2_Scn2a	2.9191	3.6754	4.1536	3.2394	3.5637	3.0609	4.4549	7.0087	5.9138	5.8215	0.5923	-0.7555	0.0011	2.9697
Scn2a	_RPS[Phospho (STY)]NVSQASR_.2	B1AWN6_S623_M1_Scn2a	3.0593	4.1668	3.7461	4.2488	4.0551	4.0703	3.9635	3.8697	3.5183	4.2158	0.9998	-0.0003	0.9978	0.0009

Scn2a	_SSSYHVS[Phospho (STY)]M[Oxidation (M)]DLLEDPTSR_.3	#N/A	3.9474	3.3387	4.2036	3.4312	3.0827	3.3988	3.2920	3.4132	4.1119	3.2714	1.0128	0.0183	0.8700	0.0605
Scn2a	_RFS[Phospho (STY)]S[Phospho (STY)]PHQSLLSIR_.3	B1AWN6_S553_M2_Scn2a	4.0009	3.3052	3.1868	4.7810	3.1265	3.1021	3.4042	3.8560	4.0776	3.6565	0.9560	-0.0649	0.6619	0.1792
Akirin2	_TLDFDPLLSPAS[Phospho (STY)]PK_.2	B1AXD8_S21_M1_Akirin2	3.5360	4.9413	5.7454	3.7795	4.9693	5.1363	4.4992	4.7164	3.5208	5.0279	1.0548	0.0770	0.6421	0.1924
Nhsl2	_IIAQFS[Phospho (STY)]KDYEPTDNPST_.2	#N/A	7.3216	6.5557	7.6614	8.0652	3.6155	7.7804	7.6476	7.4878	7.7972	7.9688	0.8845	-0.1770	0.3250	0.4881
Nhsl2	_SKS[Phospho (STY)]IS[Phospho (STY)]LKK_.2	B1AXH1_S557_M2_Nhsl2	4.8771	5.6433	5.9008	5.8565	5.7360	6.3950	6.2929	5.3828	5.9555	3.9164	1.0646	0.0903	0.4942	0.3061
Nhsl2	_ATTPSQLS[Phospho (STY)]IEVEAR_.2	B1AXH1_S690_M1_Nhsl2	4.1192	3.1069	3.3052	7.7425	3.2449	7.3730	3.5225	4.3106	3.9592	7.7406	0.9861	-0.0202	0.9608	0.0174
Nhsl2	_RIIAQFS[Phospho (STY)]KDYEPTDNPST_.3	#N/A	3.7868	3.8303	3.5205	5.8161	3.9248	5.1731	3.9037	4.7437	4.3286	4.2424	1.0087	0.0125	0.9413	0.0263
Nhsl2	_S[Phospho (STY)]LSVPTDSGVTSVDYDEEQK_.2	B1AXH1_S497_M1_Nhsl2	3.5757	3.5250	3.9049	3.8611	3.3463	3.7328	4.0936	4.0575	3.8346	3.6498	0.9357	-0.0958	0.1032	0.9865
Nhsl2	_DQGHSSS[Phospho (STY)]PTGSLAR_.2	B1AXH1_S301_M1_Nhsl2	4.0449	7.7251	8.0259	8.0536	7.3662	7.4338	7.8200	7.6202	4.1014	7.7490	1.0419	0.0592	0.7944	0.1000
Frrs1l	_NPARDEEGVFENNR_.3	#N/A	5.2673	5.4361	5.5620	5.6977	4.6336	5.5770	5.7619	6.0432	5.7645	6.1163	0.9056	-0.1431	0.0287	1.5423
Nfx1	_S[Phospho (STY)]PQGFFQSSNK_.2	B1AY10_S81_M1_Nfx1	7.2977	6.5920	6.6651	7.1606	6.0477	6.7779	7.0751	6.1072	6.4997	6.9317	1.0155	0.0223	0.7271	0.1384
Nfx1	_EHS[Phospho (STY)]PSESEKEVVIADPR_.3	B1AY10_S147_M1_Nfx1	3.2965	4.0723	3.4840	4.2514	3.9340	4.0329	3.8315	4.0652	3.3246	4.0706	1.0058	0.0084	0.9270	0.0329
Nfx1	_AQGLSEQT[Phospho (STY)]SDTSSLESVAR_.2	B1AY10_T125_M1_Nfx1	3.6069	3.7881	4.5471	3.9740	3.5610	3.7702	3.6886	3.8789	3.9022	3.6500	1.0250	0.0357	0.6337	0.1981
Nfx1	_NYSSS[Phospho (STY)]PPC[Carbamidomethyl (C)]HLPR_.2	B1AY10_S51_M1_Nfx1	3.8674	4.4708	3.9524	4.4551	3.8921	3.8373	4.1698	3.6634	3.3120	4.4221	1.2237	0.2913	0.3762	0.4245
Usp24	_VSDQNS[Phospho (STY)]PVLPK_.2	#N/A	10.9816	10.8012	11.4369	10.9645	9.5957	10.5485	11.6124	11.0973	10.7645	10.7476	0.9698	-0.0443	0.3756	0.4253
Usp24	_NMDQGGGGS[Phospho (STY)]PR_.2	B1AY13_S1940_M1_Usp24	7.3331	5.1947	4.6040	7.3798	3.1972	3.4274	6.2745	3.9443	4.7514	6.1685	0.9820	-0.0263	0.9290	0.0320
Usp24	_TIS[Phospho (STY)]AQDTLAYATALLNEK_.2	B1AY13_S2558_M1_Usp24	3.9336	3.6078	4.1029	3.3135	3.8223	3.1351	3.9185	4.7246	3.8495	3.2805	0.9263	-0.1105	0.3643	0.4385
Usp24	_VSDQNS[Phospho (STY)]PVLPKK_.2	#N/A	8.7138	8.9779	9.6214	8.6073	7.2846	8.4574	9.2946	9.0244	8.8367	8.5016	0.9659	-0.0500	0.4833	0.3158
Tmem245	_SSPSSPS[Phospho (STY)]PTLGR_.2	B1AZA5_S329_M1_Tmem245	7.9446	8.0079	8.5739	8.0953	6.9651	7.6403	7.1208	8.4020	7.9034	7.6433	1.0134	0.0192	0.7717	0.1126
Tmem245	_GGPAEAPS[Phospho (STY)]PR_.2	B1AZA5_S12_M1_Tmem245	9.0923	8.8555	8.9260	8.4745	7.3831	8.1939	9.5864	8.9648	8.4940	8.6496	0.9511	-0.0723	0.2783	0.5555
Tmem245	_GGPAEAPS[Phospho (STY)]PRGS[Phospho (STY)]PRPESR_.3	#N/A	7.3727	7.1515	7.9107	6.9294	6.3767	7.4075	7.8355	7.8347	7.2386	6.7254	0.9707	-0.0429	0.5383	0.2690
Thoc2	_KIDSHPS[Phospho (STY)]PSHSSTVK_.3	B1AZI6_S1417_M1_Thoc2	4.3902	3.6946	3.5762	4.0789	3.5159	4.2136	3.0735	4.0396	3.6882	4.0459	1.0538	0.0756	0.4633	0.3342
Dlgap4	_ATQQS[Phospho (STY)]LGEQSNPR_.2	#N/A	10.4306	10.1022	10.5518	10.8584	9.9329	10.4516	10.5852	10.3605	10.6164	10.6218	0.9850	-0.0218	0.3932	0.4053
Dlgap4	_QNS[Phospho (STY)]ATESADSIEIYVPEAQTR_.2	#N/A	8.2161	8.0102	4.2463	8.9664	7.9817	8.4109	10.9604	8.4047	8.3338	8.4749	0.8447	-0.2436	0.1994	0.7002
Dlgap4	_ANSWQLVET[Phospho (STY)]PEK_.2	#N/A	7.5758	7.8790	7.8472	9.1003	7.7773	8.2359	8.7389	8.3036	8.4814	8.5887	0.9462	-0.0798	0.1513	0.8203
Dlgap4	_SNISGWWS[Phospho (STY)]SDDNLDGEGGAFR_.2	B1AZP2_S206_M1_Dlgap4	4.3359	6.8521	4.0695	7.4740	3.4796	3.2622	6.6694	6.9287	3.6871	6.1529	0.8383	-0.2544	0.4095	0.3878
Dlgap4	_ANSWQLVET[Phospho (STY)]PEKR_.2	#N/A	7.0549	7.4446	7.9022	8.2449	6.9073	7.5109	8.0607	7.7948	7.6761	7.5130	0.9677	-0.0473	0.3855	0.4140
Dlgap4	_APS[Phospho (STY)]M[Oxidation (M)]EGTAGK_.2	#N/A	4.5093	6.5924	4.0026	7.7035	6.0794	5.9651	6.5615	6.2053	6.1815	6.3382	0.9189	-0.1221	0.4832	0.3159
Dlgap4	_ATQQS[Phospho (STY)]LGEQS[Phospho (STY)]NPR_.2	B1AZP2_S405_M2_Dlgap4	4.4893	5.3918	5.1496	3.9478	5.1310	5.0781	5.0609	5.1327	5.7801	4.7025	0.9411	-0.0876	0.3799	0.4203
Dlgap4	_SLPDC[Carbamidomethyl (C)]TSHPNSIS[Phospho (STY)]IDAGPR_.3	#N/A	5.4349	5.7308	4.3122	6.8817	5.7695	3.4223	5.2756	6.3687	5.1619	6.2384	0.9128	-0.1317	0.4744	0.3238
Dlgap4	_AM[Oxidation (M)]GDEDS[Phospho (STY)]DESGGGS[Phospho (STY)]PKPSPK_.2	#N/A	4.9839	5.4937	5.1884	5.1183	4.6436	5.4006	5.9413	4.8133	5.6794	5.2275	0.9488	-0.0758	0.3016	0.5206
Dlgap4	_S[Phospho (STY)]LDRLDSVDM[Oxidation (M)]LLPSK_.2	#N/A	4.1120	6.3746	6.4433	7.7499	7.3436	5.9594	5.7691	7.8486	6.2206	5.6802	0.9923	-0.0112	0.9502	0.0222
Dlgap4	_RS[Phospho (STY)]LDRLDS[Phospho (STY)]VDM[Oxidation (M)]LLPSK_.3	#N/A	3.8622	3.4802	3.5959	6.0930	4.8933	4.4280	3.2879	6.4837	4.9463	4.3178	0.9229	-0.1157	0.6293	0.2011
Dlgap4	_RDT[Phospho (STY)]DS[Phospho (STY)]DTQDANDSSC[Carbamidomethyl (C)]K_.2	B1AZP2_S716_M2_Dlgap4	3.1685	4.1739	4.4030	3.9468	3.8130	4.3124	4.0950	3.0544	3.3535	3.6240	1.1240	0.1686	0.1677	0.7754
Cad	_IHRAS[Phospho (STY)]DPGLPAEEPK_.3	B2RQC6_S1859_M1_Cad	7.5961	7.2058	7.7295	7.5663	6.3176	7.0628	7.4060	7.8822	7.5989	7.5200	0.9532	-0.0691	0.2363	0.6265
Ythdc2	_ADGIPNDSS[Phospho (STY)]DSEM[Oxidation (M)]EDR_.2	#N/A	7.7902	7.9742	3.7071	7.4008	3.9599	3.9644	3.8863	8.3035	8.1038	7.9685	0.8208	-0.2849	0.3817	0.4183
Ythdc2	_STDSSSYPSPC[Carbamidomethyl (C)]AS[Phospho (STY)]PS[Phospho (STY)]PSSGK_.2	B2RR83_S1282_M2_Ythdc2	3.5354	3.8070	3.2691	4.1355	3.6284	4.1011	4.4619	4.3539	2.9866	4.8946	0.8974	-0.1561	0.2791	0.5542
Otud4	_TAADVVS[Phospho (STY)]PGANSVDR_.2	B2RRE7_S1000_M1_Otud4	7.1707	7.3674	7.8637	7.8716	6.5693	7.3239	7.6602	3.1778	7.4850	7.3303	1.1478	0.1989	0.3177	0.4980
Otud4	_EES[Phospho (STY)]S[Phospho (STY)]EDENEVSNILR_.2	#N/A	3.0075	4.3349	4.2420	8.7471	4.1563	3.5732	3.9340	7.3080	3.5145	3.4630	1.0268	0.0381	0.9264	0.0332
Otud4	_LQRPKEES[Phospho (STY)]S[Phospho (STY)]EDENEVSNILR_.3	#N/A	3.8574	3.1618	4.5442	3.5936	2.9831	3.2456	3.6064	3.0716	4.2210	3.5130	0.9892	-0.0156	0.9140	0.0390
Rrn3	_LQDPNNPAIIR_.3	#N/A	6.6560	6.5589	6.2187	6.8317	6.5805	6.6035	5.5153	6.6327	6.7088	6.4657	1.0386	0.0546	0.3410	0.4673
Gnai1	_DSGVQAC[Carbamidomethyl (C)]FNR_.2	#N/A	8.7651	8.4475	8.5906	8.3761	7.7647	8.3226	8.9366	8.4914	8.4326	8.5851	0.9729	-0.0397	0.2656	0.5757
Gnai1	_IAQPNYIPTQQDVLR_.2	#N/A	8.7876	8.5865	8.9803	8.5654	8.1609	8.5157	8.7064	8.6035	8.6276	8.4650	0.9999	-0.0002	0.9937	0.0027
Gnai1	_EYQLNDSAAYYLNDLDR_.2	#N/A	6.9271	6.8983	7.0638	6.5054	5.9043	6.5103	6.8676	6.7545	6.4993	6.2734	1.0055	0.0079	0.8846	0.0533
Gnai1	_IDFGDSAR_.2	#N/A	6.7440	6.3064	6.1839	5.9972	5.6695	5.9623	5.8031	6.3287	6.7698	6.2009	0.9790	-0.0306	0.6040	0.2190
Gnai1	_SREYQLNDSAAYYLNDLDR_.3	#N/A	6.1989	5.3157	6.7836	6.3800	5.3260	5.8934	6.7293	6.2604	6.1223	6.1200	0.9485	-0.0763	0.3410	0.4672
Gnai1	_AVVYSNTIQSIIAIIR_.2	#N/A	3.5739	3.7299	3.8947	3.8694	3.5986	3.7517	4.1209	2.6076	3.6539	3.6555	1.0646	0.0904	0.4117	0.3854
Apba1	_VVGT[Phospho (STY)]PGGSEVGQR_.2	B2RUJ5_T333_M1_Apba1	7.4222	7.4126	7.3834	7.4244	4.2484	7.7327	4.5261	7.0091	7.2485	7.4343	1.0584	0.0819	0.6695	0.1742
Apba1	_QDIS[Phospho (STY)]PTR_.2	B2RUJ5_S391_M1_Apba1	7.0169	3.4179	3.8961	3.4399	3.3062	3.3184	3.5882	3.5613	6.0755	3.1172	0.9952	-0.0069	0.9834	0.0073
Apba1	_APT[Phospho (STY)]PGGGHPDS[Phospho (STY)]PGLPAPAGQQQR_.3	#N/A	9.8928	9.4289	9.3078	10.2505	9.2004	9.6311	9.9961	9.3647	9.6747	9.7041	0.9932	-0.0099	0.7769	0.1097
Apba1	_M[Oxidation (M)]DS[Phospho (STY)]YEQEEDIDQIVAEVK_.2	#N/A	3.5116	3.8015	3.8629	4.1550	7.9799	3.8596	7.0559	3.7168	3.6379	4.0220	0.9827	-0.0252	0.9432	0.0254
Apba1	_QSM[Oxidation (M)]SSQS[Phospho (STY)]LDK_.2	#N/A	6.1512	6.4668	6.1800	6.4283	5.9785	5.6529	6.1664	6.2782	5.9936	6.8218	0.9728	-0.0399	0.4342	0.3623
Apba1	_SNS[Phospho (STY)]QENVEASHPSQDGKR_.3	#N/A	7.6955	7.4476	8.3181	8.0935	7.6092	7.8302	7.6683	7.8985	8.0330	7.8359	0.9966	-0.0049	0.8832	0.0539
Apba1	_SNS[Phospho (STY)]QENVEASHPSQDGK_.2	#N/A	7.1166	6.8576	6.6266	7.4695	6.4707	7.2777	7.1700	7.1186	7.1177	6.9945	0.9816	-0.0267	0.5311	0.2748
Apba1	_SDGES[Phospho (STY)]DSPEKEAEFAPYPR_.3	#N/A	3.9726	4.6155	3.7062	5.7579	3.2734	4.8078	3.9018	3.8126	5.9179	6.1589	0.8803	-0.1839	0.4064	0.3911
Apba1	_SAS[Phospho (STY)]TESGFHNHTDTAEGDVLAAAR_.3	B2RUJ5_S84_M1_Apba1	6.1698	3.6666	6.7782	3.9768	5.5022	3.3975	6.4037	6.3528	6.2347	3.8271	0.8616	-0.2149	0.3956	0.4028
Apba1	_LHHY[Phospho (STY)]DERSDGESDS[Phospho (STY)]PEKEAEFAPYPR_.4	#N/A	3.7066	3.8146	4.3957	3.5789	5.3918	4.9403	3.4727	3.1739	4.0790	3.4433	1.2152	0.2812	0.0969	1.0137
Apba1	_AAEDM[Oxidation (M)]PEAEQDLERAPT[Phospho (STY)]PGGGHPDS[Phospho (STY)]PGLPAPAGQQQR_.	#N/A	3.5919	7.0027	6.6454	7.7533	7.6843	7.8799	6.8317	8.4045	8.2526	8.1525	0.8545	-0.2268	0.2261	0.6456
Apba1	_LHHYDERS[Phospho (STY)]DGESDSPEK_.3	#N/A	3.6535	2.9579	4.3403	3.3422	4.2801	3.4494	4.5577	3.2755	4.4249	3.3092	0.9432	-0.0844	0.5868	0.2315
Cspp1	_[Acetyl (Protein N-term)]ADS[Phospho (STY)]LDEFIEEQK_.2	B2RX88_S4_M1_Cspp1	3.2487	3.9775	3.9355	4.4382	4.0185	4.2597	4.1528	3.6803	3.3289	4.4052	1.0226	0.0322	0.7628	0.1176
Dock5	_S[Phospho (STY)]LQLVDSR_.2	B2RY04_S1765_M1_Dock5	3.3445	5.4897	4.5790	5.7582	3.9891	3.9102	4.2710	5.6126	5.0046	3.8001	0.9657	-0.0504	0.7888	0.1030
Rbm25	_LGASNS[Phospho (STY)]PGQPNSVK_.2	#N/A	10.7133	10.7882	11.1195	11.2071	9.4864	10.4243	11.0542	10.7875	10.5078	10.5859	0.9897	-0.0150	0.7483	0.1259
Rbm25	_LGASNS[Phospho (STY)]PGQPNSVKR_.2	#N/A	7.7014	7.8441	8.4991	8.2005	7.0645	7.8147	8.1835	8.0507	7.7494	7.7878	0.9888	-0.0162	0.7432	0.1289
Ildr2	_GWTYS[Phospho (STY)]PAPAR_.2	B5TVM2_S511_M1_Ildr2	8.4731	8.0206	3.5482	4.5928	3.3492	4.3802	3.2402	3.9092	4.2083	4.2701	1.3806	0.4653	0.2396	0.6205
Ildr2	_GGS[Phospho (STY)]LETPSK_.2	B5TVM2_S562_M1_Ildr2	3.1290	5.9433	6.6204	4.3184	3.7555	3.9740	4.0331	3.8000	3.4486	4.2854	1.1880	0.2485	0.3313	0.4797
Ildr2	_AHGAFYQDGS[Phospho (STY)]LDEYYGR_.2	#N/A	4.1063	3.6597	3.8400	3.7923	3.7012	3.5249	4.1213	4.3832	3.2998	3.5122	0.9848	-0.0222	0.8015	0.0961
Clip3	_SPSS[Phospho (STY)]PSLGSLQQR_.2	B9EHT4_S402_M1_Clip3	3.9284	3.4884	3.9423	6.7380	5.5749	6.5445	7.2200	6.4264	7.3486	6.7301	0.7266	-0.4608	0.0356	1.4481
Clip3	_KKS[Phospho (STY)]PS[Phospho (STY)]SPSLGSLQQR_.3	#N/A	5.6620	3.6072	3.8119	4.0608	3.4617	5.4258	3.7013	5.3026	3.6776	5.6631	0.9459	-0.0802	0.7087	0.1495
Pdzd8	_VQTELKEETQPLSHS[Phospho (STY)]PK_.3	#N/A	7.3517	7.1380	7.0899	7.4129	2.8539	7.3484	7.7973	7.3854	7.5309	7.2746	0.8713	-0.1987	0.3282	0.4839
Pdzd8	_VQTELKEETQPLSHS[Phospho (STY)]PKR_.4	#N/A	3.4413	3.6131	3.1750	4.2196	3.7224	4.0070	4.3678	3.8492	3.0807	2.2129	1.0944	0.1301	0.4681	0.3297
Osbpl8	_KES[Phospho (STY)]LKVQK_.2	B9EJ86_S120_M1_Osbpl8	5.3210	6.1742	6.9261	5.4734	4.8476	5.3967	5.9911	6.3996	5.8127	5.5625	0.9576	-0.0624	0.5494	0.2601
Osbpl8	_GYSSPEPDIQDS[Phospho (STY)]SGSEAQSVKPSTR_.3	B9EJ86_S807_M1_Osbpl8	3.6467	4.5137	3.6533	4.2596	5.0518	5.3255	5.0686	6.0396	3.6548	4.1230	0.9337	-0.0990	0.5844	0.2333
Cttnbp2	_DVS[Phospho (STY)]PTSRDNLVAK_.2	#N/A	3.8103	7.7037	8.7856	3.8490	7.0741	7.8454	3.5637	4.2694	3.9180	7.2584	1.3701	0.4543	0.2082	0.6815
Cttnbp2	_DVS[Phospho (STY)]PTSR_.2	#N/A	3.4620	7.0974	4.1488	3.1506	4.0885	3.6410	6.7196	3.4671	3.1156	3.1176	1.0389	0.0551	0.8781	0.0564
Cttnbp2	_QSSHS[Phospho (STY)]DLGPSPPTALPSSANR_.3	B9EJA2_S362_M1_Cttnbp2	3.8171	3.6928	3.9124	4.1597	3.5491	3.7637	6.4638	3.7254	3.9876	3.7447	0.8517	-0.2316	0.2497	0.6026
Cttnbp2	_LSLGS[Phospho (STY)]DDEADLVK_.2	B9EJA2_S1510_M1_Cttnbp2	3.8836	3.3426	4.5704	3.5722	3.2309	3.3937	3.2869	3.0454	4.1949	3.5392	1.0424	0.0599	0.6549	0.1838
Cttnbp2	_FQGNANDPDQNGNNTQS[Phospho (STY)]PPSR_.2	B9EJA2_S465_M1_Cttnbp2	3.7417	3.6072	3.9005	4.2618	3.5205	3.8414	3.6511	3.9848	3.6876	3.9590	0.9978	-0.0032	0.9566	0.0193
Cttnbp2	_LASISVATEGPVTR_.2	#N/A	4.2445	3.0979	4.3046	3.5219	3.9883	3.2364	3.7505	4.6670	3.7783	3.8255	0.9318	-0.1019	0.4147	0.3823
ATp6v1fnb	_YQY[Phospho (STY)]LNTR_.2	B9EJX3_Y114_M1_ATp6v1fnb	3.3617	4.1180	6.4067	5.3104	5.7777	5.9672	4.2882	4.3620	5.5706	3.8173	1.1436	0.1935	0.3619	0.4415

Tmf1	_SVSEINS[Phospho (STY)]DDELPGK_.2	B9EKI3_S340_M1_Tmf1	4.1123	9.1192	4.5452	9.8887	8.9033	9.2524	9.8583	9.1854	9.2427	9.5352	0.8077	-0.3082	0.2074	0.6832
Ptprz1	_EVSADVS[Phospho (STY)]EEENFLTDFK_.2	B9EKR1_S576_M1_Ptprz1	11.7956	11.8139	12.1736	12.6500	12.1050	12.3520	12.2121	11.6887	12.0608	12.1609	1.0098	0.0140	0.5546	0.2560
Ptprz1	_FAVLYQPLAGNDQAK_.2	#N/A	8.7068	8.5966	9.1050	9.0485	8.6095	8.9649	8.9025	8.3093	8.7167	8.8084	1.0178	0.0254	0.3499	0.4560
Ptprz1	_FSSFEEAVK_.2	#N/A	6.9959	6.8177	6.5637	6.7280	6.7271	6.1482	6.4457	6.3103	6.1307	6.7655	1.0390	0.0553	0.2054	0.6875
Ptprz1	_FSEQGLLK_.2	#N/A	5.4809	5.4857	4.6827	6.2081	5.7711	6.1401	5.3592	6.0553	5.7784	5.7730	0.9803	-0.0288	0.7209	0.1421
Ptprz1	_QSPINIDEDLTQVNVNLKK_.3	#N/A	4.9529	5.3358	6.3162	5.6235	5.9473	5.8525	4.7571	5.5965	5.8975	5.8187	1.0279	0.0397	0.6442	0.1910
Ptprz1	_QSPINIDEDLTQVNVNLK_.2	#N/A	5.2661	4.8248	5.5419	5.9080	4.8915	5.1940	4.9993	5.5241	4.6702	4.4163	1.0752	0.1046	0.2257	0.6464
Ptprz1	_DTDGVLETDDTGIAPGSPR_.2	#N/A	6.5407	6.6218	7.0010	7.6276	7.1176	7.0823	7.0078	6.9649	6.6427	6.6344	1.0273	0.0389	0.4128	0.3843
Ptprz1	_ASLENTFIHNTGK_.2	#N/A	5.6489	6.2718	6.9961	7.2247	6.4949	6.3377	6.9089	6.0121	6.1636	6.6043	1.0114	0.0164	0.8289	0.0815
Ptprz1	_C[Carbamidomethyl (C)]MSC[Carbamidomethyl (C)]LPYRES[Phospho (STY)]R_.2	#N/A	6.1295	5.2968	4.0344	7.0798	4.9678	3.3738	3.9144	3.4995	4.7831	5.8098	1.1433	0.1933	0.4430	0.3536
Ptprz1	_C[Carbamidomethyl (C)]MSC[Carbamidomethyl (C)]LPYRES[Phospho (STY)]REK_.3	#N/A	2.9522	4.3902	4.1867	6.2038	3.5967	3.0279	3.8787	3.2707	3.5698	3.4077	1.1495	0.2010	0.4209	0.3758
Mfap1a;Mfap1b	_IVEPEVVGES[Phospho (STY)]DSEVEGDAWR_.3	C0HKD8_S116_M1_Mfap1a;Mfap1b	3.0662	3.7285	4.0739	4.0484	3.7955	3.9636	3.9824	6.2168	4.4790	3.8868	0.8143	-0.2964	0.1032	0.9865
Mfap1a;Mfap1b	_SLAALDALNTDDENDEEEY[Phospho (STY)]EAWK_.3	C0HKD8_Y276_M1_Mfap1a;Mfap1b	3.5120	4.3172	4.1988	3.4953	4.1385	3.5920	4.1643	3.3497	3.3353	3.1676	1.1060	0.1453	0.1955	0.7089
Mfap1a;Mfap1b	_KIVEPEVVGES[Phospho (STY)]DS[Phospho (STY)]EVEGDAWR_.3	#N/A	4.3324	3.5879	4.3251	3.3269	3.4762	3.1485	4.5425	3.2907	4.4401	3.2939	0.9506	-0.0731	0.6244	0.2045
Mfap1a;Mfap1b	_RPDYAPM[Oxidation (M)]ES[Phospho (STY)]S[Phospho (STY)]DEEDEEFQFIK_.3	C0HKD8_S52_M2_Mfap1a;Mfap1b	3.0876	3.8439	4.0691	4.5718	3.7322	4.3933	4.2864	3.5467	3.1953	3.0379	1.1231	0.1675	0.2486	0.6044
H2ac4;H2ac6;H2ac7;I	_NDEELNKLLGR_.2	#N/A	6.3868	7.0851	7.8793	6.8280	6.7741	6.9857	6.5442	6.9233	6.3749	6.7505	1.0514	0.0723	0.2427	0.6150
Slc35g2	_REDYQEILDS[Phospho (STY)]PIK_.2	#N/A	4.0672	7.2888	7.5886	7.5538	6.3500	7.3848	7.7060	7.6477	7.6143	7.0188	0.8945	-0.1609	0.2979	0.5259
Slc35g2	_EDYQEILDS[Phospho (STY)]PIK_.2	#N/A	3.0280	8.0744	7.7588	8.9239	7.5753	8.3221	8.7262	9.0975	8.6004	8.3333	0.8379	-0.2552	0.2351	0.6288
Akap5	_GPVAAS[Phospho (STY)]PQK_.2	#N/A	9.9421	9.1732	8.8120	9.8812	8.9522	9.3397	9.2137	9.4295	9.5253	9.8555	0.9836	-0.0239	0.5693	0.2446
Akap5	_VTVDHAEEATVGQAEEATVGQAEK_.3	#N/A	9.2418	9.4484	9.8008	10.0198	7.7766	8.7787	9.4187	8.7003	8.7311	9.0319	1.0231	0.0329	0.6465	0.1894
Akap5	_VTVDQAEEATVGQAEEATVGHTEK_.3	#N/A	7.5359	7.3141	8.0903	7.7901	6.9190	7.4476	7.8943	7.1165	7.1968	7.6525	1.0069	0.0098	0.8443	0.0735
Akap5	_VQGEADDLEIK_.2	#N/A	6.7690	6.2707	6.3924	7.1115	6.9542	6.5892	6.7288	6.5955	6.4550	6.8412	1.0039	0.0056	0.8876	0.0518
Akap5	_LSQAEETTVAQAK_.2	#N/A	5.7398	5.6483	5.8528	6.0643	5.9125	6.2849	5.3543	6.0488	5.7260	6.0932	1.0192	0.0275	0.5510	0.2589
Akap5	_ASLLESSAAGS[Phospho (STY)]PR_.2	D3YVF0_S251_M1_Akap5	13.3581	13.4088	14.0096	13.6564	12.8729	13.5381	13.6942	13.0656	13.2665	13.5065	1.0068	0.0098	0.6908	0.1607
Akap5	_ALSQAGEATAGHPEEATVIQAESQAK_.3	#N/A	4.2770	4.6315	3.9782	3.4148	4.1848	4.2219	3.9154	4.2957	3.6459	4.6820	0.9960	-0.0058	0.9527	0.0210
Akap5	_QPAGAWAS[Phospho (STY)]IK_.2	D3YVF0_S79_M1_Akap5	8.2836	7.8976	8.3988	8.2914	7.1287	8.4651	8.0016	8.4077	8.4118	8.5282	0.9688	-0.0457	0.3696	0.4323
Akap5	_SSAIQM[Oxidation (M)]GT[Phospho (STY)]PELEKETK_.2	#N/A	9.1122	9.2745	10.0875	9.7198	8.3757	9.3089	9.3638	9.0500	9.0087	9.2898	1.0147	0.0211	0.6689	0.1746
Akap5	_DEKGPVAAS[Phospho (STY)]PQKER_.2	#N/A	10.2054	10.3891	10.8944	10.9552	10.3284	10.4603	10.4731	10.3885	10.4125	10.5506	1.0079	0.0114	0.6224	0.2060
Akap5	_AQTQPDDQAIQAGS[Phospho (STY)]TQGLQEGVLVR_.3	D3YVF0_S171_M1_Akap5	5.5792	5.8974	6.2878	6.7417	5.0699	5.8775	5.8271	5.6287	5.1859	5.3638	1.0741	0.1031	0.2303	0.6377
Akap5	_GPVAAS[Phospho (STY)]PQKER_.2	#N/A	11.7720	11.8775	12.0981	12.4338	11.6641	11.9019	12.0246	11.9011	11.7883	11.9804	1.0029	0.0041	0.8203	0.0860
Akap5	_KATVGQAEEATIDHTEK_.3	#N/A	4.7828	4.1583	4.7759	3.8784	5.6198	4.7274	4.0266	3.1228	3.4218	3.5557	1.3186	0.3991	0.0110	1.9600
Akap5	_TSEQYETLLIETASSLVK_.2	#N/A	5.0443	5.4103	4.7684	6.2727	4.8860	4.8436	4.8944	4.5875	4.3313	5.6797	1.0679	0.0948	0.3995	0.3984
Akap5	_DEKGPVAAS[Phospho (STY)]PQK_.2	#N/A	10.4011	9.9815	10.0622	10.8338	9.8667	10.1983	10.5286	10.0733	10.2164	10.4254	0.9916	-0.0122	0.6698	0.1740
Akap5	_SSAIQMGT[Phospho (STY)]PELEK_.2	#N/A	9.1748	7.2922	3.5242	10.1268	7.3898	3.8136	7.9287	6.2781	6.5557	7.6957	0.9680	-0.0469	0.8769	0.0570
Akap5	_HTPEAGGS[Phospho (STY)]GQR_.2	D3YVF0_S68_M1_Akap5	6.0273	5.4239	4.4965	5.3857	3.9065	3.8277	4.1885	5.7050	3.2600	3.7175	1.1486	0.1999	0.3435	0.4641
Akap5	_EATVVGQAEEATVDRAEEATVGQAEEATVGHTEK_.3	#N/A	4.1242	5.1513	5.8254	8.2909	3.2499	2.9788	5.7818	8.2218	4.1089	4.2385	0.8835	-0.1787	0.6189	0.2084
Akap5	_METS[Phospho (STY)]VSEIQVETK_.2	D3YVF0_S4_M1_Akap5	3.6893	2.9937	4.0824	3.6262	2.8150	3.4136	3.7744	3.3750	3.6741	3.3034	0.9731	-0.0394	0.7198	0.1428
Akap5	_TAEETKKHT[Phospho (STY)]PEAGGSGQR_.3	D3YVF0_T62_M1_Akap5	3.2740	4.0792	3.9608	4.4634	3.9005	3.8290	4.1781	3.6550	3.3036	4.4304	1.0067	0.0096	0.9287	0.0321
Akap5	_VTVDQAEETTVGQAEEATVGQAGEAILSQAK_.3	#N/A	4.1453	3.1971	3.8790	3.4227	3.0184	3.2102	3.5710	3.5784	3.8775	3.1000	0.9850	-0.0218	0.8427	0.0743
Akap5	_METSVSEIQVETKDEKGPVAAS[Phospho (STY)]PQK_.3	#N/A	3.6413	4.3975	3.3749	4.4195	4.7043	3.8396	3.0670	4.0825	4.3815	4.0968	1.0399	0.0565	0.6676	0.1755
Akap5	_SGETALGQAEEAAVGQADKR_.2	#N/A	3.7410	3.4852	4.4278	3.4296	0.4072	3.2511	3.1443	0.8036	4.3375	1.0043	1.3450	0.4276	0.4378	0.3588
Akap5	_QFLISM[Oxidation (M)]ENEQVGVFANDSDFEGR_.3	#N/A	4.2844	3.5887	3.4873	4.5319	3.4101	4.3194	3.3104	3.9701	4.2691	4.2092	0.9993	-0.0010	0.9931	0.0030
Dgki	_VKKPQNS[Phospho (STY)]LK_.2	D3YWQ0_S328_M1_Dgki	5.8740	6.4815	6.5935	6.6344	5.5754	5.8991	5.9081	6.2755	6.4381	5.6182	1.0192	0.0274	0.6795	0.1678
Dgkh	_VPLHLES[Phospho (STY)]PHEER_.3	D3YXJ0_S1067_M1_Dgkh	8.0795	7.9426	8.2699	8.1051	7.3784	7.8691	7.5288	8.1723	7.8474	7.8563	1.0114	0.0164	0.6477	0.1887
Dgkh	_KVS[Phospho (STY)]TSGQMR_.2	D3YXJ0_S53_M1_Dgkh	3.9887	3.3537	3.7224	3.2662	3.1750	3.0537	3.4145	3.7350	4.0340	4.4443	0.8771	-0.1893	0.0925	1.0339
Samd1	_AAAATAPPS[Phospho (STY)]PGPAQPGPR_.2	D3YXK1_S150_M1_Samd1	3.1675	3.9727	3.8543	4.3569	3.7940	3.9355	4.2963	3.7615	3.4101	7.0486	0.8310	-0.2671	0.2850	0.5451
Samd1	_YLGGS[Phospho (STY)]GGASGR_.2	D3YXK1_S242_M1_Samd1	4.0627	3.3181	3.7964	3.3401	5.8123	3.4182	3.4884	3.6610	3.9601	3.0174	1.1207	0.1644	0.4289	0.3676
Safb	_APTAALS[Phospho (STY)]PEPQDSK_.2	#N/A	9.6856	9.6410	9.6917	10.5039	9.4132	9.5438	10.1052	9.5515	9.6494	9.6755	1.0001	0.0002	0.9960	0.0017
Safb	_APTAALS[Phospho (STY)]PEPQDSKEDVK_.2	#N/A	7.9860	7.8991	8.4503	8.3825	7.6386	7.9080	8.2495	7.7403	7.9628	8.0277	1.0061	0.0088	0.7917	0.1014
Safb	_SVVS[Phospho (STY)]FDKVK_.2	D3YXK2_S626_M1_Safb	8.4315	9.1375	9.4680	8.9913	8.0241	8.8725	8.7863	8.3927	8.2066	8.3688	1.0453	0.0639	0.2112	0.6752
Safb	_APTAALS[Phospho (STY)]PEPQDSKEDVKK_.3	#N/A	10.1685	10.4911	11.2994	11.0128	10.4266	10.4620	10.7270	10.5397	10.5009	10.5131	1.0069	0.0100	0.7479	0.1262
Safb	_[Acetyl (Protein N-term)]AETLSGLGDASAAGAAAVSSAAS[Phospho (STY)]ETGTRR_.3	#N/A	3.7418	4.9165	4.2370	3.1052	3.3016	3.4720	3.0666	2.9850	3.4208	3.2742	1.1911	0.2523	0.1231	0.9099
Safb	_[Acetyl (Protein N-term)]AETLSGLGDASAAGAAAVSSAASETGTR_.3	#N/A	4.0229	3.2737	3.4488	4.5589	2.3172	3.8648	3.6787	4.0091	3.7994	3.5197	0.9545	-0.0672	0.6806	0.1671
Safb	_AIEDEGGNPDEIEVTSEC[Carbamidomethyl (C)]NK_.2	#N/A	4.1498	3.1926	3.8835	3.4273	3.2936	5.4317	3.5755	3.5739	5.7732	3.1046	0.9724	-0.0403	0.8669	0.0621
Safb	_MSS[Phospho (STY)]VEEDSDTKR_.2	D3YXK2_S406_M1_Safb	4.2623	3.5177	4.3952	3.3971	3.4060	3.2186	3.1117	3.2206	4.3700	3.3641	1.0520	0.0731	0.6074	0.2166
Safb	_SEPVKEEGS[Phospho (STY)]ELEQPFAQATSSVGPDR_.3	D3YXK2_S235_M1_Safb	3.6723	3.5539	4.3591	3.3609	3.4422	3.1824	3.0756	4.2640	4.4062	3.3279	0.9540	-0.0679	0.6199	0.2077
Safb	_RPYEVDGR_.2	#N/A	4.3506	4.3764	3.5366	4.0392	3.4763	3.8607	3.7539	4.0793	3.7278	4.0062	1.0124	0.0177	0.8244	0.0839
Ccdc6	_SSS[Phospho (STY)]PDKFK_.2	#N/A	8.2217	7.9085	8.1538	8.5360	4.1450	8.2007	8.0636	8.0655	7.9077	8.3904	0.9286	-0.1069	0.5175	0.2861
Ccdc6	_RSSS[Phospho (STY)]PDKFK_.2	#N/A	3.7014	6.6513	6.9463	7.1176	3.2673	6.4523	3.8975	6.5502	4.2312	3.5504	1.2484	0.3201	0.3049	0.5159
Ccdc6	_LDQPVSAPPS[Phospho (STY)]PR_.2	D3YZP9_S237_M1_Ccdc6	10.3526	10.6017	10.7240	10.9503	9.7200	10.6543	10.6212	8.8069	10.3066	10.6691	1.0396	0.0560	0.3584	0.4457
Ccdc6	_QLS[Phospho (STY)]ESESSLEM[Oxidation (M)]DDER_.2	#N/A	5.4181	6.0101	3.3117	6.2066	4.9846	6.0320	5.9537	5.6307	5.7953	6.2909	0.9002	-0.1516	0.3278	0.4844
Shank1	_EQDVVPFQES[Phospho (STY)]PK_.2	#N/A	12.5969	12.3247	12.4964	13.0668	11.9090	12.5065	13.1898	12.5575	12.7775	12.6634	0.9755	-0.0358	0.1938	0.7126
Shank1	_S[Phospho (STY)]IDEGM[Oxidation (M)]FSAEPYLR_.2	#N/A	11.4673	10.9978	11.6560	11.4322	10.9145	11.4485	11.6641	10.6726	11.4745	11.3428	1.0027	0.0039	0.8952	0.0481
Shank1	_GSST[Phospho (STY)]EDGPGVPPPSPR_.2	D3YZU1_T1518_M1_Shank1	3.9417	3.2983	12.7627	3.5475	10.1655	11.8280	3.3845	4.2445	11.5439	9.9319	1.0432	0.0610	0.9129	0.0396
Shank1	_EQDVVPFQES[Phospho (STY)]PKYAAR_.2	#N/A	3.7175	8.6725	10.6421	9.8022	8.9283	8.5766	9.2954	9.6028	8.7898	9.4577	0.9035	-0.1465	0.4914	0.3086
Shank1	_DGPAGGTGGSGGPGGS[Phospho (STY)]LGSR_.2	D3YZU1_S540_M1_Shank1	6.5223	6.1989	6.9025	6.9981	3.8670	6.2189	6.6578	5.7263	5.9525	6.4001	0.9893	-0.0155	0.9164	0.0379
Shank1	_NHREQDVVPFQES[Phospho (STY)]PK_.3	#N/A	3.7403	6.6324	8.3831	7.1577	6.3751	7.3944	6.9151	7.1571	6.4670	6.6557	0.9728	-0.0398	0.8253	0.0834
Shank1	_GFFATESS[Phospho (STY)]FDPHHR_.2	#N/A	3.6599	3.7546	4.2102	3.9466	3.6208	3.7111	3.6612	5.7900	5.5528	3.9136	0.8071	-0.3091	0.0763	1.1175
Shank1	_ALDPASPLGLALAAR_.2	#N/A	4.2983	4.6756	4.5151	4.3862	4.0222	4.4355	5.2528	4.8611	4.5406	5.1565	0.8861	-0.1744	0.0105	1.9793
Shank1	_QKS[Phospho (STY)]IGAAEDDRPYLAPPAMK_.3	#N/A	6.9370	3.0875	3.5658	7.8862	2.9758	3.6488	3.2578	3.8916	4.1907	4.2877	1.1988	0.2616	0.5050	0.2967
Shank1	_AGLGS[Phospho (STY)]QEK_.2	D3YZU1_S1434_M1_Shank1	7.0133	5.6934	6.6792	6.5571	3.2756	6.1614	3.2422	6.8780	7.2479	6.8478	0.9740	-0.0380	0.8811	0.0550
Shank1	_GGSGGS[Phospho (STY)]TDSHHGGASYIPER_.3	D3YZU1_S1907_M1_Shank1	7.1753	3.6025	7.1493	3.8603	5.4211	6.8104	6.1597	7.0182	7.0541	3.5375	0.9541	-0.0677	0.8036	0.0949
Shank1	_GRPSS[Phospho (STY)]SGTPR_.2	D3YZU1_S519_M1_Shank1	4.0829	3.1433	3.2688	3.7715	3.0316	3.5930	3.4862	4.3470	3.9956	3.7385	0.8947	-0.1606	0.1434	0.8434
Shank1	_S[Phospho (STY)]IGAAEDDRPYLAPPAM[Oxidation (M)]K_.3	#N/A	6.7335	6.3069	6.0846	7.0335	6.0225	6.8287	6.5051	6.8339	7.1164	6.9864	0.9477	-0.0775	0.1710	0.7669
Shank1	_THS[Phospho (STY)]PATS[Phospho (STY)]EDEER_.2	D3YZU1_S4_M2_Shank1	3.8553	3.1107	3.3014	3.8041	2.9990	3.6256	3.5188	4.3144	3.9630	3.7711	0.8863	-0.1741	0.0883	1.0540
Shank1	_HS[Phospho (STY)]KSIDEGMFSAEPYLR_.3	D3YZU1_S1289_M1_Shank1	3.7696	3.6397	3.7858	6.1156	3.4554	3.3691	3.7299	4.1635	3.8268	3.7979	1.0369	0.0522	0.7962	0.0990
Shank1	_VGWFPSDC[Carbamidomethyl (C)]LEEVANRS[Phospho (STY)]QEGR_.3	D3YZU1_S615_M1_Shank1	3.7347	3.2886	3.9804	3.6696	2.8696	3.5666	3.3843	6.2081	4.1409	3.6366	0.8102	-0.3036	0.1687	0.7729
Shank1	_LESGGS[Phospho (STY)]SGGYGAYAAGSR_.2	D3YZU1_S1310_M1_Shank1	4.2241	3.1183	3.9578	3.5015	3.3678	3.2890	3.6498	3.4996	3.7987	3.1788	1.0127	0.0181	0.8577	0.0667
Selenon	_RPHS[Phospho (STY)]PDPGAQPAPPR_.3	D3Z2R5_S13_M1_Selenon	6.5275	6.1630	6.3883	6.1905	5.4388	6.5945	6.7271	6.1285	5.6839	6.2755	1.0022	0.0031	0.9621	0.0168

Bin2	_ASGSGSC[Carbamidomethyl (C)]NAPGS[Phospho (STY)]PEGSSQLC[Carbamidomethyl (C)]SPR_.2	D3Z6Q9_S430_M1_Bin2	3.6820	2.9863	4.0897	3.6335	5.0666	3.4210	3.7818	5.0041	3.6667	4.7969	0.8842	-0.1775	0.3029	0.5187
Bin2	_AS[Phospho (STY)]PDASSNPEPAETR_.2	D3Z6Q9_S443_M1_Bin2	6.3510	5.9415	3.3962	3.8988	5.8132	5.5511	6.1912	6.1878	6.2263	6.0532	0.8368	-0.2570	0.1421	0.8474
Gsg1l	_IEKGDVS[Phospho (STY)]EEEDFR_.2	D3Z7H4_S274_M1_Gsg1l	6.2372	6.3018	6.3874	6.2469	3.2641	5.8439	5.9421	5.7639	6.3397	5.6308	0.9653	-0.0510	0.7532	0.1231
Gls	_VADYIPQLAK_.2	#N/A	8.2094	8.0834	7.6916	7.5635	8.4271	8.1490	8.1537	8.0563	8.2546	8.1509	0.9837	-0.0238	0.4572	0.3399
Gls	_VLSPEAVR_.2	#N/A	7.8055	8.1473	7.7799	7.5063	8.0683	7.7199	7.2243	8.0567	7.9120	7.6972	1.0149	0.0214	0.5552	0.2555
Gls	_LTLQTTSDGVM[Oxidation (M)]LDKDLFK_.3	#N/A	5.7027	6.0457	6.1718	5.4945	5.9775	6.0504	6.2023	6.0885	5.7853	5.9274	0.9844	-0.0227	0.5470	0.2620
Gls	_DSPGETDAFGNSEGK_.2	#N/A	8.0331	8.3174	4.0209	8.5178	8.3128	8.1900	8.6437	8.3445	8.3001	8.2944	0.9011	-0.1502	0.3793	0.4210
Gls	_C[Carbamidomethyl (C)]VQSNIVLLTQAFR_.2	#N/A	3.4329	3.2243	4.4461	4.0660	3.5459	4.0792	3.5832	4.0762	3.9322	3.7457	0.9908	-0.0133	0.8930	0.0492
Gls	_YAIAVNDLGTEYVHR_.2	#N/A	5.8511	4.9814	6.3206	5.2270	5.1768	5.6580	6.0564	5.0294	5.4159	5.4209	1.0101	0.0144	0.8617	0.0646
Gls	_M[Oxidation (M)]AGNEYVGFSNATFQSER_.2	#N/A	3.6646	4.5182	5.0254	3.2312	3.7070	4.4084	4.8297	4.7933	4.4439	4.3294	0.8898	-0.1684	0.1908	0.7194
Gls	_ILQEYQVQYTPQGDSDDGKGNQTVHK_.3	#N/A	3.8147	3.4066	3.2970	4.0241	4.1080	4.6177	3.6441	4.5508	3.8393	4.2160	0.9546	-0.0671	0.5492	0.2603
Gls	_TALHVAAAEGHVEVVK_.2	#N/A	2.9711	3.7887	3.9480	4.2497	3.7047	2.8967	3.6196	2.8215	4.4944	3.8530	0.9719	-0.0412	0.7962	0.0990
Gls	_FALSAMDMEQR_.2	#N/A	2.9461	3.7024	4.1806	3.7244	3.5907	3.0339	3.8727	3.2768	3.5758	3.4017	0.9994	-0.0008	0.9939	0.0027
Gls	_SVINLLFAAYTGDVSALR_.3	#N/A	3.6521	3.3665	3.9117	4.0170	3.6782	3.6461	4.1109	3.7101	3.7865	3.7857	0.9646	-0.0521	0.3466	0.4601
Gls	_ILQEYQVQYTPQGDSDDGK_.2	#N/A	3.5510	4.3562	4.2378	3.7645	4.1775	3.5520	3.9128	3.3780	3.0266	3.2066	1.1653	0.2207	0.0470	1.3279
Prrt2	_AS[Phospho (STY)]PSPSPSEAR_.2	#N/A	10.6345	10.4949	10.1956	10.7910	9.0860	10.1706	10.7687	10.8157	10.5022	10.3383	0.9644	-0.0523	0.2775	0.5568
Prrt2	_AHGGHPGS[Phospho (STY)]PRGS[Phospho (STY)]LSR_.3	#N/A	8.6776	8.5828	8.8747	8.4741	7.0115	8.3638	8.5127	8.7735	8.7077	8.5740	0.9640	-0.0529	0.3933	0.4053
Prrt2	_HPS[Phospho (STY)]SQLAGPGVEGGEGTQKPR_.3	E9PUL5_S254_M1_Prrt2	4.5230	5.2538	4.7483	4.2025	3.6230	3.6778	4.3652	4.2837	4.3939	4.3493	0.9977	-0.0033	0.9762	0.0104
Prrt2	_T[Phospho (STY)]PPANGAPPR_.2	E9PUL5_T214_M1_Prrt2	3.5011	3.7251	6.4899	3.1897	5.2006	9.5059	4.4052	9.6542	3.0765	3.1567	1.0385	0.0546	0.9135	0.0393
Prrt2	_QENGAVVPLQAGDGEEGPAPQPHS[Phospho (STY)]PPSTK_.3	E9PUL5_S208_M1_Prrt2	3.3102	6.9155	7.8233	8.4437	5.1830	3.8760	7.3559	4.4135	3.2117	3.7658	1.2643	0.3383	0.3706	0.4311
Prrt2	_[Acetyl (Protein N-term)]AASS[Phospho (STY)]SQVSEMK_.2	E9PUL5_S5_M1_Prrt2	3.5735	3.8734	3.3925	5.4198	3.7271	3.7796	3.9666	4.2328	4.1141	3.9418	0.9747	-0.0370	0.7928	0.1008
Prrt2	_AELAPETTETPVETPETVQATDLSLNPEEGSKAS[Phospho (STY)]PSPSPSEAR_.4	#N/A	8.6924	3.9008	9.4700	9.4698	7.9392	8.7011	9.2002	9.3295	8.8019	8.6441	0.8927	-0.1638	0.3978	0.4004
Prrt2	_AHGGHPGS[Phospho (STY)]PR_.2	E9PUL5_S244_M1_Prrt2	3.6931	3.5330	4.3799	3.3818	2.8188	3.2033	3.0964	4.8054	4.3853	3.3487	0.8958	-0.1588	0.3603	0.4433
Prrt2	_AELAPETTETPVETPETVQATDLSLNPEEGSK_.3	#N/A	2.5514	2.9835	4.2744	4.0367	3.6845	3.8582	5.3388	3.0470	3.1476	3.9123	0.9232	-0.1153	0.5962	0.2246
Fam53a	_SLS[Phospho (STY)]EPEELAR_.2	E9PV82_S119_M1_Fam53a	3.2509	4.0561	3.9377	4.4404	3.8775	3.8520	4.1551	7.6737	3.3267	4.4074	0.7979	-0.3257	0.2413	0.6175
Mctp1	_AAS[Phospho (STY)]PSFQAR_.2	E9PV86_S16_M1_Mctp1	7.0140	6.6132	6.9215	6.4927	3.3029	4.4266	6.5989	3.8629	4.1619	4.3164	1.2239	0.2915	0.2890	0.5391
Mctp1	_GSSTATGTGGVT[Phospho (STY)]PPGGR_.2	E9PV86_T145_M1_Mctp1	3.2457	3.9805	3.9325	4.4351	3.8688	4.2567	4.1498	3.6834	3.3319	4.4021	1.0158	0.0226	0.8326	0.0796
Setd1a	_S[Phospho (STY)]GS[Phospho (STY)]PAPETTNESVPFAQHSSLDSR_.3	E9PYH6_S481_M2_Setd1a	4.0084	3.3340	3.7420	4.3075	3.1554	3.0733	3.4341	3.7154	4.0144	2.9631	1.0203	0.0290	0.8223	0.0850
Prr12	_TSS[Phospho (STY)]FHLLR_.2	E9PYL2_S1070_M1_Prr12	6.9217	3.2059	3.8702	7.2271	3.2802	3.3444	7.0293	3.5872	6.8249	7.0379	0.7585	-0.3989	0.2451	0.6107
Prr12	_TEDEEFLIQHLLQAPS[Phospho (STY)]PPR_.3	E9PYL2_S648_M1_Prr12	3.8214	4.2738	4.2023	5.1224	3.9748	3.4869	3.2471	5.3102	3.9456	3.9956	1.0054	0.0078	0.9611	0.0172
Prr12	_ASLAC[Carbamidomethyl (C)]SPLGGGEPS[Phospho (STY)]PGAGEPSK_.2	E9PYL2_S338_M1_Prr12	3.7806	4.3980	4.1012	4.0763	3.2698	3.8759	3.1944	4.2161	4.2759	3.8624	1.0077	0.0110	0.9169	0.0377
Prr12	_NLETLPSFS[Phospho (STY)]S[Phospho (STY)]DEEDSVAK_.2	E9PYL2_S1372_M2_Prr12	4.0282	3.3325	3.2141	3.7168	3.1538	3.0748	3.4315	4.4017	4.0503	3.6838	0.8788	-0.1864	0.1003	0.9988
Igsf9b	_TGS[Phospho (STY)]PELAAR_.2	E9PZ19_S1215_M1_Igsf9b	7.9512	7.4792	4.2996	7.2929	6.6622	7.3871	3.8509	8.0515	7.5496	3.3799	1.1993	0.2621	0.3603	0.4433
Igsf9b	_AEAEAEATT[Phospho (STY)]PIELISR_.2	E9PZ19_T845_M1_Igsf9b	4.1342	3.0919	3.3202	7.2993	5.6732	2.9687	7.0435	7.2442	3.9442	6.8024	0.7054	-0.5036	0.1252	0.9025
Igsf9b	_SLES[Phospho (STY)]PLSSGKVS[Phospho (STY)]PESIR_.2	E9PZ19_S775_M2_Igsf9b	3.5368	3.3420	4.2236	5.9275	4.1633	3.5662	3.9270	3.2225	3.5215	5.4834	1.0630	0.0882	0.6807	0.1671
Igsf9b	_APS[Phospho (STY)]ESS[Phospho (STY)]DDQGQPAAK_.2	E9PZ19_S794_M2_Igsf9b	4.3087	3.5642	4.0424	3.3506	3.4525	3.1721	3.0653	3.2670	4.4164	3.3176	1.0375	0.0531	0.6984	0.1559
Chd2	_LKDEHGLEPAS[Phospho (STY)]PR_.3	E9PZM4_S1365_M1_Chd2	6.5389	5.3061	6.4454	5.8639	4.8222	5.6722	6.4524	6.2972	6.4039	5.4927	0.9372	-0.0935	0.3399	0.4687
Chd2	_KQES[Phospho (STY)]S[Phospho (STY)]DDDDDDDEAPKR_.2	E9PZM4_S207_M2_Chd2	3.2577	3.9685	3.9445	4.4472	3.8568	4.2687	2.0567	3.6713	3.3199	4.4142	1.1758	0.2337	0.2171	0.6633
Chd2	_SPLDHRS[Phospho (STY)]PLER_.3	E9PZM4_S1806_M1_Chd2	3.5796	4.3848	4.2664	3.2682	4.2061	3.5234	3.8842	3.3494	2.9980	3.2352	1.1499	0.2015	0.1109	0.9550
Irf2bp2	_LEEPPELNRQS[Phospho (STY)]PNPR_.3	#N/A	8.9434	8.9126	9.1122	9.5064	8.7466	9.1057	8.9857	8.5590	8.8744	9.2254	1.0161	0.0230	0.4284	0.3681
Irf2bp2	_S[Phospho (STY)]PTGAQPAAAKPPPLSAK_.2	E9Q1P8_S71_M1_Irf2bp2	7.3308	7.7910	7.9788	8.6043	4.0650	7.5343	7.8322	7.5438	7.4680	7.5937	0.9485	-0.0763	0.6460	0.1898
Irf2bp2	_RKPS[Phospho (STY)]PEPEGEVGPPK_.3	E9Q1P8_S343_M1_Irf2bp2	5.9679	5.5601	5.5060	6.5525	5.7508	5.9328	4.1109	5.4587	6.0567	5.8010	1.0974	0.1340	0.2216	0.6545
Irf2bp2	_S[Phospho (STY)]PADSLSSAAGASELSAEGAGK_.2	E9Q1P8_S250_M1_Irf2bp2	3.0164	4.3260	4.2509	4.9458	3.6610	2.9637	3.9429	3.2065	4.7073	3.4720	1.0074	0.0107	0.9541	0.0204
Irf2bp2	_DTLLALHQHGHS[Phospho (STY)]GPFESK_.3	E9Q1P8_S300_M1_Irf2bp2	3.4741	6.2960	4.1609	3.1627	4.1007	2.9843	4.3783	3.4549	3.1035	3.1297	1.1459	0.1965	0.4596	0.3376
Irf2bp2	_NSSS[Phospho (STY)]PPSPSSMNQR_.2	E9Q1P8_S440_M1_Irf2bp2	5.8185	3.9679	3.9451	4.4477	3.8848	4.2693	4.1624	3.6707	3.3193	4.4147	1.1277	0.1734	0.2723	0.5650
Gpr137c	_RYDS[Phospho (STY)]DDDLPR_.2	E9Q343_S351_M1_Gpr137c	6.7632	6.7407	3.2405	7.0461	4.1514	6.7000	3.8672	3.9660	6.7361	6.7329	1.0841	0.1166	0.6823	0.1660
Pi4ka	_TSSVSSISQVS[Phospho (STY)]PER_.2	#N/A	12.0252	11.8288	12.5571	11.6455	10.2541	11.6289	12.3298	11.9641	11.7081	11.7074	0.9773	-0.0331	0.5261	0.2789
Pi4ka	_KTSSVSSISQVS[Phospho (STY)]PER_.2	#N/A	3.7152	6.0281	6.2747	6.0577	3.6525	6.0127	5.9988	6.0555	3.6803	3.8952	1.0780	0.1083	0.6521	0.1857
Ryr2	_EAAVPEEEGGT[Phospho (STY)]PEKEISIEDAK_.3	#N/A	3.0670	3.8722	8.4083	4.2565	3.6935	4.0360	4.3968	3.8620	3.5106	4.2235	1.1394	0.1883	0.5915	0.2280
Ryr2	_EAAVPEEEGGT[Phospho (STY)]PEK_.2	#N/A	7.5180	7.4498	7.5427	7.3112	6.2324	6.6829	8.2476	7.3296	7.2465	7.3968	0.9428	-0.0850	0.2287	0.6407
Ryr2	amidomethyl (C)]YM[Oxidation (M)]VC[Carbamidomethyl (C)]AGESM[Oxidation (M)]S[Phospho (STY)]	#N/A	6.1329	5.9116	5.5045	6.6745	5.0629	5.5527	6.7218	6.5095	6.2580	5.7001	0.9221	-0.1171	0.1818	0.7405
Ryr2	_IAEDPSRDGPS[Phospho (STY)]PTSGSSK_.2	#N/A	4.5141	3.9750	5.9615	4.3028	3.9208	4.1094	5.4525	4.0609	3.2420	4.1487	1.0563	0.0790	0.6667	0.1761
Ryr2	_DGPS[Phospho (STY)]PTSGSSK_.2	#N/A	3.2945	4.0508	3.8482	3.9903	3.9391	3.5446	3.9132	3.6319	3.5544	3.8361	1.0118	0.0169	0.7966	0.0988
Ryr2	_ISQTSQVS[Phospho (STY)]IDAAHGYSPR_.3	E9Q401_S2813_M1_Ryr2	3.0259	4.3165	4.2605	3.8042	4.1378	3.5917	3.9525	5.3670	3.4960	3.4815	0.9465	-0.0794	0.6274	0.2024
Arid1b	_APS[Phospho (STY)]PASFQR_.2	E9Q4N7_S1550_M1_Arid1b	8.0991	3.8099	9.2477	4.3108	4.1771	8.1904	8.8340	4.1912	8.0193	8.4157	0.8562	-0.2240	0.5036	0.2979
Arid1b	_SS[Phospho (STY)]PALAAPDASVDPK_.2	E9Q4N7_S1757_M1_Arid1b	6.9052	4.1295	6.6817	4.3319	4.0178	4.1534	4.0465	6.4988	6.3711	3.6646	0.8654	-0.2086	0.3780	0.4225
Pde8b	_RLS[Phospho (STY)]GNEYVFTK_.2	E9Q4S1_S497_M1_Pde8b	4.9251	5.4049	3.8150	4.3177	3.7547	5.5056	4.0324	5.7950	5.1651	4.7997	0.9338	-0.0988	0.5225	0.2819
Pde8b	_IHRDS[Phospho (STY)]GDNSQTEPHSFR_.3	#N/A	3.7909	3.8571	5.9712	6.2207	3.7454	3.7353	3.6708	3.8415	3.8213	3.6557	1.2151	0.2811	0.2247	0.6483
Pde8b	_FVNSINKPLAAESEGS[Phospho (STY)]DC[Carbamidomethyl (C)]EC[Carbamidomethyl (C)]NPTGK_.3	E9Q4S1_S734_M1_Pde8b	3.3957	4.8197	3.1294	4.1740	4.0403	4.0852	4.6630	5.5459	3.1263	3.8513	0.9172	-0.1247	0.5084	0.2938
Nolc1	_ASS[Phospho (STY)]PFRR_.2	E9Q5C9_S626_M1_Nolc1	3.1355	7.6481	3.8223	7.5256	3.7620	3.9675	4.3283	3.7935	3.1202	4.2919	1.2815	0.3579	0.3349	0.4750
Nolc1	_GGSISVQVNSVKFDS[Phospho (STY)]E_.2	E9Q5C9_S701_M1_Nolc1	3.5844	4.3896	4.2712	3.2731	4.2110	3.5185	4.4886	5.5548	2.9932	3.2401	0.9522	-0.0707	0.7218	0.1416
Nolc1	_AAKES[Phospho (STY)]EEEEEEETEEK_.2	#N/A	3.2552	4.2367	4.1366	4.2943	3.2542	3.9632	3.6750	4.3794	3.6862	3.6255	1.0039	0.0057	0.9586	0.0184
Setd2	_TGS[Phospho (STY)]PTPSNQLNDSPTFK_.2	E9Q5F9_S614_M1_Setd2	3.9685	5.4501	4.8440	4.1421	3.8097	4.1508	5.4732	4.4982	4.3231	4.1091	0.9551	-0.0663	0.6183	0.2088
Setd2	_RGS[Phospho (STY)]LS[Phospho (STY)]PPSSAYER_.2	#N/A	3.7411	8.3802	4.4279	3.4297	6.3521	8.0045	8.7406	7.0159	4.3374	6.9971	0.8449	-0.2431	0.4491	0.3476
Setd2	_RRGS[Phospho (STY)]LS[Phospho (STY)]PPSSAYER_.3	#N/A	3.8040	3.1084	4.4908	3.4926	3.2997	3.2990	3.0246	3.1250	4.2744	3.4596	1.0144	0.0206	0.8948	0.0483
Ythdc1	_AKS[Phospho (STY)]PT[Phospho (STY)]PDGSER_.2	#N/A	6.3265	6.2349	6.5768	6.1455	3.2858	3.7941	6.6804	6.9395	4.6688	4.3236	0.9542	-0.0677	0.7837	0.1059
Ythdc1	_RAKS[Phospho (STY)]PT[Phospho (STY)]PDGSER_.2	#N/A	5.7774	3.8554	4.0575	5.3836	3.7437	4.3817	5.5495	6.3938	5.8402	5.2274	0.7880	-0.3437	0.0334	1.4766
Ythdc1	_GIS[Phospho (STY)]PIVFDR_.2	E9Q5K9_S309_M1_Ythdc1	3.1244	3.8807	4.3589	4.5349	3.7690	4.3564	4.2496	3.0985	3.2321	3.5800	1.1311	0.1777	0.2049	0.6884
Ccdc85c	_S[Phospho (STY)]LDDLSAPPHHR_.2	#N/A	6.0810	9.1298	3.5810	9.6246	4.9593	4.1061	8.0172	8.6725	8.7217	9.2168	0.7216	-0.4707	0.1069	0.9709
Bod1l	_EVS[Phospho (STY)]PPGAR_.2	E9Q6J5_S3010_M1_Bod1l	8.4050	8.4441	8.3523	8.2823	7.0318	7.9499	8.4605	8.1512	8.0261	7.6577	1.0005	0.0007	0.9907	0.0041
Bod1l	_TETDITTVEQSS[Phospho (STY)]PSGK_.2	E9Q6J5_S2841_M1_Bod1l	3.6305	3.6461	3.5277	5.8208	3.4674	3.6120	5.7096	4.1493	4.4967	3.9973	0.8611	-0.2158	0.2881	0.5404
Bod1l	_YYS[Phospho (STY)]DSDDELTVEQR_.2	E9Q6J5_S480_M1_Bod1l	3.3810	4.1862	4.0678	3.9345	4.0075	3.7220	4.0828	3.0666	3.3657	3.6118	1.0995	0.1369	0.1577	0.8021
Bod1l	_AQLS[Phospho (STY)]PSVKR_.2	E9Q6J5_S3000_M1_Bod1l	3.2748	7.3530	3.8819	4.3845	7.2562	7.5456	7.5850	3.7340	7.4857	7.3663	0.8584	-0.2204	0.4795	0.3192
Bod1l	_KLS[Phospho (STY)]SQPSTDVSTDKER_.3	E9Q6J5_S236_M1_Bod1l	3.6051	3.9268	3.7976	4.0739	3.8151	3.8955	3.7886	3.6297	3.7562	4.8878	0.9594	-0.0599	0.5237	0.2810
Bod1l	_GSDDVLVSGEVPEC[Carbamidomethyl (C)]EVGHM[Oxidation (M)]S[Phospho (STY)]PR_.3	#N/A	3.6242	5.3535	5.7646	5.7683	3.9856	5.5227	5.3824	3.7319	5.7960	5.2908	0.9845	-0.0226	0.9025	0.0446
Ttc7b	_LPVSSS[Phospho (STY)]TSNLHVDR_.2	#N/A	6.9464	4.9857	3.7710	7.3898	3.2108	3.2262	7.0293	7.4452	7.2288	3.9020	0.7688	-0.3792	0.2371	0.6251
Ttc7b	_SC[Carbamidomethyl (C)]YNLTNPSDSGRGS[Phospho (STY)]SLLDR_.3	#N/A	3.4305	3.8297	4.0098	6.4508	3.5883	5.3164	3.8687	6.2806	3.7055	5.6250	0.9112	-0.1341	0.5999	0.2219
Ttc7b	_GS[Phospho (STY)]SLLDR_.2	#N/A	3.4226	3.8081	4.1094	3.8379	4.7360	3.6694	3.5526	3.6281	4.0033	3.8049	1.0489	0.0689	0.4755	0.3229
Ttc7b	_VEQALSEVASS[Phospho (STY)]LQSSAPK_.2	E9Q6P5_S678_M1_Ttc7b	8.1698	8.7326	9.4285	10.3087	8.1448	8.3512	9.3107	10.3313	9.1800	8.4590	0.9502	-0.0737	0.4096	0.3876

Cfap251	_[Acetyl (Protein N-term)]S[Phospho (STY)]DTEENPLEESR_.2	E9Q743_S2_M1_Cfap251	3.6745	7.7489	6.6568	7.9885	7.3438	7.8953	8.2685	7.4592	7.8991	4.1301	0.9921	-0.0114	0.9626	0.0166
Zc3h13	_NTEEPSS[Phospho (STY)]PVRK_.2	E9Q784_S110_M1_Zc3h13	8.2806	8.3508	8.9578	8.9740	8.0196	8.3141	8.5631	8.6371	8.3440	8.3228	1.0019	0.0027	0.9408	0.0265
Zc3h13	_SKLS[Phospho (STY)]PS[Phospho (STY)]PSLR_.2	#N/A	9.1649	9.2027	9.6570	9.7075	3.7690	9.1487	4.0466	9.1043	9.4107	4.2989	1.2571	0.3301	0.3267	0.4858
Zc3h13	_S[Phospho (STY)]PERPTGDLR_.2	#N/A	8.6665	8.7479	8.9962	9.5610	8.3905	8.9877	9.0436	8.8844	8.7713	8.6875	1.0051	0.0073	0.8374	0.0771
Zc3h13	_EVS[Phospho (STY)]PEVVR_.2	E9Q784_S198_M1_Zc3h13	7.4036	7.5308	7.9380	7.7291	6.9100	7.8400	7.5360	7.4839	7.4670	7.7038	1.0014	0.0021	0.9568	0.0192
Zc3h13	_DQRPSS[Phospho (STY)]PIR_.2	E9Q784_S704_M1_Zc3h13	3.4787	7.0088	7.3340	4.2423	6.6600	4.0639	3.9570	7.2001	3.5865	7.3168	0.9908	-0.0133	0.9670	0.0146
Zc3h13	_AAVVAS[Phospho (STY)]PLLDQQR_.2	E9Q784_S242_M1_Zc3h13	5.4475	4.2069	5.5061	5.7794	5.2413	5.7178	6.2955	5.5107	5.4523	5.2742	0.9438	-0.0835	0.3850	0.4145
Zc3h13	_SLS[Phospho (STY)]PSHLTEDR_.2	E9Q784_S953_M1_Zc3h13	8.2407	8.5562	9.1404	9.1470	8.0251	8.5707	8.9449	8.5704	8.5688	8.5315	0.9953	-0.0068	0.8735	0.0588
Zc3h13	_S[Phospho (STY)]KGDS[Phospho (STY)]DVS[Phospho (STY)]DEEAAPQNK_.2	E9Q784_S1086_M3_Zc3h13	4.7782	4.4730	4.9958	4.4679	4.7200	4.5795	5.1241	4.8095	3.4110	4.0337	1.0747	0.1039	0.3442	0.4632
Zc3h13	_RS[Phospho (STY)]PERPTGDLR_.3	#N/A	5.2108	4.1972	5.8332	3.5990	3.9698	3.9788	3.8720	4.3771	4.5860	3.9576	1.0635	0.0888	0.5788	0.2375
Zc3h13	_SSS[Phospho (STY)]PGQHHS[Phospho (STY)]PLSSR_.2	#N/A	6.2319	3.0644	5.3903	3.5065	6.2048	3.2711	3.4622	6.4719	6.3603	6.2682	0.9149	-0.1284	0.6682	0.1751
Zc3h13	_SKLS[Phospho (STY)]PS[Phospho (STY)]PSLRK_.2	#N/A	7.1145	7.4636	7.5670	4.5617	3.9988	7.4318	3.2764	7.4053	7.6085	3.0279	1.1392	0.1880	0.5441	0.2643
Zc3h13	_LRS[Phospho (STY)]PSNDSAHR_.2	#N/A	4.7886	4.6660	4.5115	5.1560	4.1169	4.4164	4.2580	4.9139	4.7882	4.5114	0.9981	-0.0027	0.9688	0.0138
Zc3h13	_S[Phospho (STY)]AS[Phospho (STY)]PYPTHC[Carbamidomethyl (C)]LSS[Phospho (STY)]PQR_.2	E9Q784_S369_M3_Zc3h13	3.4117	4.0530	4.0985	4.2708	4.0383	3.6916	4.1666	3.3460	3.3091	3.0674	1.1311	0.1777	0.1044	0.9813
Rnf169	_SQS[Phospho (STY)]C[Carbamidomethyl (C)]SDTVQDR_.2	E9Q7F2_S279_M1_Rnf169	5.2391	4.3550	5.1189	4.2364	4.5710	4.0238	4.3846	4.8968	3.0639	4.2356	1.1075	0.1473	0.2925	0.5338
Numa1	_TQPDGTSVPGEPAS[Phospho (STY)]PISQR_.2	E9Q7G0_S1739_M1_Numa1	9.9929	10.1327	10.7810	10.3287	9.1895	10.0461	10.7436	10.2385	10.2063	9.8847	0.9815	-0.0269	0.5455	0.2632
Numa1	_VSSETHQGPGT[Phospho (STY)]PESK_.2	E9Q7G0_T1982_M1_Numa1	3.0326	4.3098	4.2671	3.8109	3.6772	2.9474	3.9591	3.1903	3.4893	3.4882	1.0403	0.0570	0.6720	0.1726
Numa1	_LQES[Phospho (STY)]PAPEKGEVLGDALQLDTLK_.3	E9Q7G0_S398_M1_Numa1	3.3983	3.9441	3.1320	5.1388	4.0429	4.0825	4.3249	4.3185	3.1236	3.8539	1.0131	0.0188	0.9057	0.0430
Dnajc21	_YEKEFGDGS[Phospho (STY)]DENEVEDQEPR_.3	E9Q8D0_S283_M1_Dnajc21	3.5791	4.3843	4.2659	5.0743	4.7530	4.5214	4.1514	3.3499	2.9985	3.2347	1.2901	0.3675	0.0155	1.8085
Fry	_SFS[Phospho (STY)]VPKK_.2	E9Q8I9_S1991_M1_Fry	7.9021	7.9635	8.2885	7.2422	2.8010	7.5497	8.1638	8.1295	7.6051	7.5315	0.8855	-0.1754	0.4222	0.3744
Fry	_NSDLFTVLS[Phospho (STY)]R_.2	E9Q8I9_S1936_M1_Fry	5.8122	5.4591	5.5668	5.7684	3.2651	3.3595	5.8510	6.2305	5.2171	5.9109	0.8396	-0.2522	0.1855	0.7316
Fry	_AT[Phospho (STY)]FAGGSRDGVITC[Carbamidomethyl (C)]QPGDSEEK_.3	E9Q8I9_T2810_M1_Fry	5.1074	4.4196	5.8734	5.2106	2.8071	4.4562	5.6666	5.5656	5.5836	5.2658	0.8416	-0.2489	0.1440	0.8417
Fry	_LLLPGSSPS[Phospho (STY)]S[Phospho (STY)]PEDEVKDR_.2	E9Q8I9_S1382_M2_Fry	3.7493	3.3845	3.5320	4.0346	3.4717	4.2611	3.7493	5.1407	3.7357	4.0016	0.8994	-0.1529	0.2245	0.6488
Fry	_SSS[Phospho (STY)]PDLSSSSK_.2	E9Q8I9_S1940_M1_Fry	9.3300	3.0768	9.7067	8.9742	3.4093	3.2153	9.7165	9.8242	9.0082	3.2203	0.7914	-0.3376	0.4570	0.3400
Fry	_S[Phospho (STY)]LDSLDKC[Carbamidomethyl (C)]DM[Oxidation (M)]QILEER_.3	#N/A	4.1530	3.8041	3.8143	4.0738	3.8976	3.6698	4.2220	5.3983	4.9898	3.7510	0.8501	-0.2343	0.0556	1.2547
Fry	_EQENMDDTNS[Phospho (STY)]EQQFR_.2	E9Q8I9_S2459_M1_Fry	3.7792	3.5632	3.5129	4.5575	2.9229	3.7017	3.2049	3.9445	4.2436	4.2348	0.9401	-0.0891	0.5002	0.3009
Rabl2	_NFS[Phospho (STY)]FAKK_.2	E9Q9D5_S147_M1_Rabl2	5.1008	4.7400	6.4546	5.4328	5.3050	5.5086	5.5431	5.5646	4.3606	4.2947	1.0977	0.1345	0.2682	0.5716
Sec16a	_STHS[Phospho (STY)]LPSR_.2	E9QAT4_S1377_M1_Sec16a	7.0211	3.9785	4.5984	7.5035	6.6151	7.3446	6.9336	4.3598	7.3957	7.3300	0.9496	-0.0746	0.7409	0.1303
Sec16a	_APS[Phospho (STY)]LTSDSEGKKPAQAVK_.3	E9QAT4_S2101_M1_Sec16a	3.8066	8.4541	8.5660	8.8940	2.9323	3.2964	3.6572	3.4923	3.7913	3.1862	1.6965	0.7626	0.1377	0.8610
Sec16a	_ASHYS[Phospho (STY)]DQLAPR_.2	E9QAT4_S1245_M1_Sec16a	6.7049	3.4959	3.9742	3.5179	3.3842	3.2404	7.2721	6.4533	3.7823	3.1952	0.7831	-0.3528	0.3103	0.5082
Sec16a	_S[Phospho (STY)]IHS[Phospho (STY)]EHSAR_.2	E9QAT4_S1362_M2_Sec16a	4.5441	3.7667	3.3093	4.4966	3.5881	4.1414	4.9574	3.7486	2.9463	4.0312	1.0136	0.0196	0.9009	0.0453
Sec16a	_FTGS[Phospho (STY)]FDDDAEIHRDPYGEEADRR_.4	E9QAT4_S1342_M1_Sec16a	3.4962	4.3014	4.1830	3.1848	4.1227	3.6068	4.4003	3.4328	3.0814	3.1518	1.0851	0.1178	0.3941	0.4044
Sec16a	_RSS[Phospho (STY)]LS[Phospho (STY)]SHSHQSQIYR_.3	E9QAT4_S1384_M2_Sec16a	3.6861	2.9415	3.4198	3.9733	2.8298	3.7948	3.6879	4.1452	3.7938	4.1417	0.8728	-0.1962	0.0860	1.0653
Syngap1	_LPS[Phospho (STY)]PTKEKPPPPPPGGGK_.3	F6SEU4_S788_M1_Syngap1	11.1021	3.7028	4.1810	3.7248	3.5911	3.0335	11.6613	3.2764	3.5754	3.4021	0.8924	-0.1643	0.8007	0.0965
Syngap1	_S[Phospho (STY)]QPKLDR_.2	#N/A	10.2846	9.7750	10.4107	10.0313	8.7813	9.9861	9.7037	10.4396	10.3850	10.0220	0.9744	-0.0374	0.4503	0.3465
Syngap1	_TKS[Phospho (STY)]QPKLDR_.2	#N/A	10.1156	9.8236	10.6747	10.2487	8.7457	9.9197	9.9086	10.6038	10.3783	10.0104	0.9703	-0.0435	0.4165	0.3803
Syngap1	_FRS[Phospho (STY)]ADHDR_.2	F6SEU4_S165_M1_Syngap1	7.7174	7.7020	8.2103	8.2201	5.6807	7.2245	6.7882	8.1764	7.5707	7.3928	0.9969	-0.0044	0.9669	0.0146
Syngap1	_LLNDISTALR_.2	#N/A	6.8486	6.9305	6.5612	6.4875	6.5054	6.7732	7.4630	7.3272	6.7082	6.6978	0.9483	-0.0766	0.0867	1.0621
Syngap1	_ALYESEENC[Carbamidomethyl (C)]EVDPIK_.2	#N/A	5.2994	5.2561	5.5942	6.0081	5.6362	5.7782	6.3468	5.6315	5.9918	5.9331	0.9363	-0.0949	0.0754	1.1224
Syngap1	_GKS[Phospho (STY)]QQLTVSAAQKPR_.3	#N/A	6.0166	5.5056	6.6431	7.7727	3.6167	4.9837	4.4736	6.8314	7.0596	5.5231	0.9639	-0.0530	0.8106	0.0912
Syngap1	_EFAEYVTNHYR_.2	#N/A	5.2602	4.9700	5.6176	5.7387	4.4687	5.3744	5.8609	5.8304	5.4114	5.2740	0.9364	-0.0948	0.2149	0.6679
Syngap1	_QQS[Phospho (STY)]LSKEGS[Phospho (STY)]IGGSGGSGGGGGGGLKPSITK_.3	F6SEU4_S1111_M2_Syngap1	3.9357	4.2465	4.0949	4.9900	3.6911	5.0959	3.8809	5.5299	4.8778	4.2872	0.9351	-0.0969	0.4809	0.3180
Syngap1	_EYEEEIHS[Phospho (STY)]LKER_.2	#N/A	5.6060	5.3339	6.4540	5.5632	3.6573	4.8843	6.0387	5.3167	5.3426	5.6633	0.9391	-0.0907	0.5105	0.2920
Syngap1	_SSPAYC[Carbamidomethyl (C)]TSSS[Phospho (STY)]DITEPEQK_.2	F6SEU4_S825_M1_Syngap1	10.3027	3.4488	10.4717	11.2217	9.3414	9.8792	11.3096	11.1415	3.6659	10.8184	0.9867	-0.0193	0.9540	0.0205
Syngap1	_TVS[Phospho (STY)]VPVEGR_.2	#N/A	4.3402	6.3474	3.9567	5.0246	5.7111	6.5033	4.5217	6.9851	4.5327	4.5786	1.0309	0.0439	0.8306	0.0806
Syngap1	_DLNSS[Phospho (STY)]IDLQSFM[Oxidation (M)]AR_.2	#N/A	7.2392	7.7763	8.5273	9.0110	6.1789	3.0634	7.3218	8.6795	3.6487	7.1313	1.0404	0.0572	0.8503	0.0704
Syngap1	_ILM[Oxidation (M)]QYQAR_.2	#N/A	5.2495	5.5480	5.8525	5.6491	5.8465	5.2496	6.3509	6.5672	5.5651	4.8393	0.9546	-0.0670	0.4601	0.3372
Syngap1	_EGS[Phospho (STY)]IGGSGGSGGGGGGGLKPSITK_.3	#N/A	7.8609	6.6434	8.2288	7.5929	6.5576	7.5030	7.0949	7.8651	7.6349	7.7642	0.9747	-0.0370	0.6148	0.2113
Syngap1	_GKS[Phospho (STY)]QQLTVSAAQK_.2	#N/A	4.8567	3.2225	4.5652	3.3974	3.0438	5.1787	4.3084	3.6038	4.6159	3.5737	1.0046	0.0066	0.9722	0.0122
Syngap1	_QY[Phospho (STY)]SMEAAPAAPFR_.2	F6SEU4_Y116_M1_Syngap1	3.9961	3.8029	3.6845	5.9907	3.6242	3.7533	3.6185	3.8782	7.4186	3.8744	0.8818	-0.1816	0.5350	0.2716
Syngap1	_VKEYEEEIHS[Phospho (STY)]LKER_.3	#N/A	7.4602	7.5598	8.1344	8.1228	7.0606	7.5293	7.8564	7.8821	7.7433	7.3022	0.9933	-0.0097	0.8338	0.0790
Syngap1	_LSQM[Oxidation (M)]GVTTDGVPAQQLR_.2	#N/A	5.9716	6.2370	6.8509	5.8879	6.1559	6.3726	6.3552	6.4305	6.1850	5.9920	1.0008	0.0012	0.9787	0.0093
Syngap1	_S[Phospho (STY)]VSM[Oxidation (M)]LDLQGDGPGGR_.2	#N/A	3.3935	3.5056	4.0803	3.8899	4.0200	3.7569	6.3500	6.4420	6.6472	3.8569	0.6481	-0.6258	0.0052	2.2799
Syngap1	_DAIGEFIR_.2	#N/A	4.7790	4.8394	3.2680	4.3126	1.0007	4.1000	4.4608	4.1894	4.6981	5.1341	0.8044	-0.3141	0.2655	0.5759
Syngap1	_RLLSQEEQTSK_.2	#N/A	2.5304	2.7748	4.0451	3.6871	2.9831	4.0829	2.8927	3.4123	3.7114	2.7797	1.0474	0.0668	0.7043	0.1522
Syngap1	_GPS[Phospho (STY)]AEM[Oxidation (M)]QGYMMR_.2	#N/A	7.5649	3.9223	3.6575	7.9873	3.7436	4.0324	4.8191	3.9091	3.4422	3.8302	1.2878	0.3649	0.3121	0.5057
Syngap1	_GLNSS[Phospho (STY)]M[Oxidation (M)]DMAR_.2	#N/A	5.6800	4.1665	4.1690	5.8572	3.8310	3.7452	4.6179	3.4028	4.6648	4.0693	1.0922	0.1272	0.4905	0.3094
Syngap1	_C[Carbamidomethyl (C)]TAS[Phospho (STY)]SLAEHQANLR_.2	F6SEU4_S534_M1_Syngap1	3.5730	3.9509	3.6239	4.1266	3.5636	4.1742	4.1681	4.0003	3.6489	4.0936	0.9642	-0.0526	0.4282	0.3683
Syngap1	_QHS[Phospho (STY)]QTPSTLNPTM[Oxidation (M)]PASER_.3	#N/A	4.0226	3.3333	6.4889	3.9132	3.2247	3.7007	3.4483	3.7146	3.6130	5.9485	0.9839	-0.0234	0.9328	0.0302
Syngap1	_S[Phospho (STY)]MDESRLDR_.2	#N/A	8.3178	7.4077	4.5180	9.2881	6.3928	3.2717	7.5668	6.5129	6.7548	7.3946	0.9257	-0.1114	0.6700	0.1739
Syngap1	_TVS[Phospho (STY)]VPVEGRPHGEHEYHLGR_.4	#N/A	3.5941	3.6321	4.2809	3.2827	4.2206	3.5089	4.4983	3.3349	2.9835	6.1055	0.8872	-0.1727	0.4445	0.3522
Syngap1	_EYSKS[Phospho (STY)]M[Oxidation (M)]DESRLDR_.3	#N/A	3.8507	3.8920	3.5844	4.1192	2.9944	3.6366	3.4324	4.1255	4.1495	4.0681	0.9330	-0.1001	0.3016	0.5205
Syngap1	_EYSKS[Phospho (STY)]MDESR_.2	#N/A	3.5747	3.6515	4.2615	6.1560	4.2012	3.5283	4.4788	3.3544	3.0029	3.2303	1.2025	0.2661	0.2477	0.6060
Syngap1	_LS[Phospho (STY)]QGSGSSITAAGMR_.2	F6SEU4_S892_M1_Syngap1	3.6433	3.7214	3.8644	6.2499	3.5933	3.5205	3.5454	3.9304	4.0261	3.7783	1.0730	0.1016	0.6243	0.2046
Syngap1	_VKEYEEEIHS[Phospho (STY)]LK_.3	#N/A	3.8456	3.7417	4.3295	3.7132	4.0253	3.8111	4.3047	3.2105	4.1660	4.3090	0.9784	-0.0316	0.7283	0.1377
Syngap1	_TVAWVSNM[Oxidation (M)]PHLS[Phospho (STY)]ADIESAHIER_.3	F6SEU4_S1162_M1_Syngap1	3.4319	5.3076	4.1187	6.5899	4.0584	3.6756	4.1110	5.7951	5.1951	3.0875	0.9963	-0.0054	0.9832	0.0074
Syngap1	_S[Phospho (STY)]QPMVLR_.2	F6SEU4_S743_M1_Syngap1	3.8192	3.0746	3.5529	3.8401	2.9629	3.6617	3.5548	4.2783	3.9269	4.2748	0.8694	-0.2019	0.0575	1.2404
Tpr	_QTPQAPQS[Phospho (STY)]PR_.2	F6ZDS4_S2141_M1_Tpr	11.7125	11.9380	12.2989	12.4385	11.7733	12.1742	12.1719	11.6993	11.9200	12.1018	1.0069	0.0099	0.6429	0.1918
Tpr	_TDGFAEAIHS[Phospho (STY)]PQVAGVPR_.3	F6ZDS4_S2223_M1_Tpr	3.9954	5.2081	3.7290	6.3770	3.1684	3.5520	4.8619	4.8299	4.0274	5.6730	0.8949	-0.1603	0.4717	0.3264
Tpr	_GAILSEEELAAM[Oxidation (M)]S[Phospho (STY)]PTAAAVAK_.2	#N/A	5.3882	5.8699	6.7577	6.7098	5.3917	5.7675	6.6579	6.2170	5.4390	5.5133	1.0040	0.0058	0.9524	0.0212
Farp1	_LGAPENSGIST[Phospho (STY)]LER_.2	F8VPU2_T24_M1_Farp1	10.1253	9.7108	10.5247	10.0493	8.0087	9.6217	10.5809	10.2546	9.8609	9.9615	0.9517	-0.0714	0.3223	0.4917
Farp1	_AC[Carbamidomethyl (C)]TLELGPHQS[Phospho (STY)]PALPK_.2	#N/A	6.1018	6.3304	6.9982	6.7943	5.0380	6.3029	7.0095	6.8904	6.1315	5.9568	0.9637	-0.0534	0.5788	0.2375
Farp1	_SPDEATAADQES[Phospho (STY)]EDDLSASR_.2	F8VPU2_S892_M1_Farp1	6.4497	6.3487	6.2546	6.6267	5.5123	5.9302	7.5466	7.0799	7.0237	5.6973	0.9049	-0.1441	0.1220	0.9138
Farp1	_QSPQSASLTFGEGTES[Phospho (STY)]PGGQSC[Carbamidomethyl (C)]QQAK_.3	F8VPU2_S403_M1_Farp1	3.3591	5.2391	4.0459	5.3099	3.7824	3.7095	5.3586	5.3667	5.0519	4.4644	0.8381	-0.2549	0.1098	0.9595
Farp1	_SNGPTPELLASS[Phospho (STY)]PPDNK_.2	F8VPU2_S875_M1_Farp1	2.9562	6.5263	4.1907	3.7345	3.6008	3.0238	3.8828	6.9785	6.5538	3.4118	0.7693	-0.3784	0.2572	0.5897
Farp1	_AC[Carbamidomethyl (C)]TLELGPHQS[Phospho (STY)]PALPKS[Phospho (STY)]PPGSK_.3	F8VPU2_S427_M2_Farp1	7.0704	8.2397	8.2080	8.6196	5.6190	7.4909	7.4876	8.1810	7.2145	6.4491	1.0284	0.0404	0.7484	0.1259
Bicra	_GAGS[Phospho (STY)]PTPLPTK_.2	F8VPZ9_S1427_M1_Bicra	6.5394	4.3909	6.7289	7.0620	6.3443	6.2568	6.5935	6.2071	6.2222	6.4186	0.9780	-0.0321	0.7807	0.1075
Sipa1l3	_TLS[Phospho (STY)]DESLC[Carbamidomethyl (C)]SGR_.2	G3X9J0_S1538_M1_Sipa1l3	8.3914	8.4229	8.6907	8.9849	7.8962	8.3733	8.6740	8.4352	8.4692	8.6622	0.9883	-0.0170	0.6157	0.2106
Sipa1l3	_SSS[Phospho (STY)]EITLSEC[Carbamidomethyl (C)]DVEEPGDPR_.2	G3X9J0_S166_M1_Sipa1l3	2.9131	3.6694	4.1477	3.6914	3.5577	3.0669	3.8397	3.3097	3.6088	3.3687	0.9932	-0.0098	0.9257	0.0335

Sipa1l3	_EQSNPS[Phospho (STY)]PSQDTDGVK_.2	G3X9J0_S94_M1_Sipa1l3	3.3002	4.0422	4.5348	4.0785	3.9448	3.8660	4.2268	5.8864	3.2217	5.6890	0.8329	-0.2638	0.1785	0.7483
Sipa1l3	_S[Phospho (STY)]KDVEFQDGWPR_.2	G3X9J0_S140_M1_Sipa1l3	6.5329	3.1179	3.9581	7.4810	5.5918	6.8579	3.6502	6.0320	6.9667	6.9128	0.9490	-0.0755	0.7865	0.1043
Sipa1l3	_QDAAGKDS[Phospho (STY)]PNR_.2	G3X9J0_S1248_M1_Sipa1l3	3.8064	3.0618	3.3503	3.8530	2.9501	3.6745	3.5677	4.2655	3.9141	3.8200	0.8863	-0.1741	0.0886	1.0525
Arfgef1	_LSVSS[Phospho (STY)]NDTQESGNSSGPSPGAK_.2	G3X9K3_S394_M1_Arfgef1	3.7330	3.4932	4.4198	3.4216	3.3815	3.2432	3.1363	3.1960	4.3454	3.3886	1.0281	0.0400	0.7589	0.1198
Arfgef1	_GREGS[Phospho (STY)]LTGTK_.2	G3X9K3_S1076_M1_Arfgef1	5.7999	3.4249	4.4881	3.4899	3.3132	3.3114	4.9175	3.1277	4.2772	3.4569	1.0067	0.0096	0.9661	0.0150
Arfgef1	_S[Phospho (STY)]VDIHDSIQPR_.2	G3X9K3_S1566_M1_Arfgef1	4.3652	2.9772	4.0988	3.6426	3.5089	7.0243	3.7909	3.3586	7.0864	3.3199	0.9728	-0.0398	0.9100	0.0410
Arfgef1	_QQQHLLQS[Phospho (STY)]PVSHHEPESPHLR_.5	G3X9K3_S234_M1_Arfgef1	4.6949	3.1682	3.2439	3.7466	3.0565	3.5681	3.4613	4.3719	4.0205	3.7136	0.9198	-0.1206	0.3927	0.4059
Rab11fip2	_TLS[Phospho (STY)]FDTSK_.2	G3XA57_S277_M1_Rab11fip2	6.0360	3.7185	4.1967	6.7361	5.6749	3.0178	4.4118	6.4009	6.9506	3.1633	0.9360	-0.0955	0.7510	0.1244
Atp2b1	_NSS[Phospho (STY)]PPPSPNK_.2	G5E829_S1178_M1_Atp2b1	11.6867	11.6069	12.0028	11.6600	10.0504	11.3250	3.8477	11.9359	11.6390	11.4420	1.1721	0.2291	0.3231	0.4907
Atp2b1	_SATSSSPGS[Phospho (STY)]PLHSLETSL_.2	G5E829_S1211_M1_Atp2b1	11.9535	11.6394	12.5569	11.7573	10.3235	11.7845	12.3511	11.9221	11.7805	11.5852	0.9798	-0.0294	0.5609	0.2511
Atp2b1	_EASDIILTDDNFTSIVK_.2	#N/A	8.2321	8.3480	9.0009	8.7663	7.1811	8.4153	8.9441	8.5566	8.1189	8.3247	0.9809	-0.0278	0.6556	0.1834
Atp2b1	_VYTFNSVR_.2	#N/A	8.3760	8.2168	8.5262	7.2356	8.0143	7.6125	8.3811	8.6576	8.3453	7.8083	0.9637	-0.0533	0.3242	0.4892
Atp2b1	_TSPNEGLSGNPADLER_.2	#N/A	8.3297	7.8382	7.7237	7.5151	7.5006	7.5762	8.1321	8.2221	8.0704	7.6019	0.9676	-0.0475	0.2178	0.6619
Atp2b1	_GIIDSTVSEQR_.2	#N/A	7.8715	7.6583	8.4225	7.8348	7.5167	7.5028	8.1018	8.1101	7.6200	7.4741	0.9968	-0.0047	0.9097	0.0411
Atp2b1	_QVVAVTGDGTNDGPALK_.2	#N/A	7.2245	7.4052	8.0554	7.5850	6.8828	7.3618	7.6767	7.8975	7.7289	7.3869	0.9670	-0.0484	0.2748	0.5610
Atp2b1	_YGDLLPADGILIQGNDLK_.2	#N/A	7.4077	7.5156	7.8475	7.7736	6.8170	6.8713	7.7472	8.0279	7.3375	7.1096	0.9757	-0.0355	0.5265	0.2786
Atp2b1	_IDESSLTGESDHVKK_.3	#N/A	5.6457	5.3478	5.5018	4.8030	4.8569	5.4612	5.6480	5.4438	5.4811	5.2632	0.9653	-0.0510	0.3501	0.4558
Atp2b1	_ADVGFAM[Oxidation (M)]GIAGTDVAK_.2	#N/A	5.9456	6.2350	6.1441	5.2335	5.6147	6.0801	6.5839	6.9126	6.1611	6.2170	0.9083	-0.1388	0.0380	1.4203
Atp2b1	_EANHDGDFGITLTELR_.3	#N/A	6.0136	5.6423	5.9257	5.5581	5.1401	5.6447	6.4294	5.8057	5.5799	6.0991	0.9457	-0.0805	0.1688	0.7726
Atp2b1	_KQDGAIENR_.2	#N/A	5.6464	5.1388	5.5432	4.5947	6.0408	5.5103	5.7054	6.0978	5.8894	5.4010	0.9375	-0.0932	0.2300	0.6383
Atp2b1	_NS[Phospho (STY)]LKEANHDGDFGITLTELR_.3	#N/A	4.2663	3.5218	5.0553	3.3930	4.2805	4.9442	5.5859	6.4175	4.3740	3.2211	0.8661	-0.2074	0.3466	0.4602
Atp2b1	_TIC[Carbamidomethyl (C)]LAFR_.2	#N/A	5.3913	3.2210	5.1654	5.3967	5.1669	5.0044	3.8979	6.4249	5.6438	5.2859	0.9205	-0.1194	0.4985	0.3023
Atp2b1	_M[Oxidation (M)]VTGDNINTAR_.2	#N/A	7.1513	7.0414	7.0590	6.9312	7.0602	6.5761	7.3624	7.3302	7.0645	7.4405	0.9549	-0.0666	0.0280	1.5525
Atp2b1	_TVIEPM[Oxidation (M)]ASEGLR_.2	#N/A	7.3381	7.4815	7.4964	6.8901	7.1051	6.8004	7.4833	7.6812	7.2213	7.0929	0.9750	-0.0366	0.3484	0.4579
Atp2b1	_TGTLTMNR_.2	#N/A	4.3889	7.0143	4.1226	7.3415	6.0010	3.4538	3.8146	6.3829	6.9153	6.5538	0.9105	-0.1353	0.6109	0.2140
Atp2b1	_KADVGFAM[Oxidation (M)]GIAGTDVAK_.3	#N/A	5.4724	5.2885	6.1103	5.1872	5.4211	5.7033	5.2993	5.8869	5.9446	5.7640	0.9662	-0.0496	0.3757	0.4252
Atp2b1	_FFDIDSGR_.2	#N/A	2.9138	5.4865	3.6006	4.4017	4.2307	3.8446	4.3288	4.7434	5.0434	4.2110	0.8904	-0.1674	0.3139	0.5032
Atp2b1	_QVVAVTGDGTNDGPALKK_.2	#N/A	4.3028	3.3875	4.2472	3.8894	4.0475	3.7512	4.7835	4.2408	4.1799	3.5054	0.9426	-0.0853	0.4011	0.3968
Atp2b1	_IQESYGDVYGIC[Carbamidomethyl (C)]TK_.2	#N/A	5.3438	3.3189	4.7691	3.3409	3.2072	5.6274	3.4891	3.6603	3.9594	5.0790	1.0546	0.0767	0.7358	0.1332
Atp2b1	_SSLYEGLEKPES[Phospho (STY)]R_.2	#N/A	5.8866	5.8551	5.3485	6.2201	4.6480	5.7632	6.7404	6.0673	6.2891	5.7101	0.9062	-0.1420	0.1155	0.9374
Atp2b1	_EEIPEEELAEDVEEIDHAER_.3	#N/A	3.7656	6.6560	3.4993	6.0085	6.5596	6.1714	6.7444	6.7824	6.5605	6.6426	0.8146	-0.2959	0.1274	0.8948
Atp2b1	_SSPTDKHTLVK_.2	#N/A	5.8951	6.2094	5.9651	5.8468	6.1147	5.5823	6.3999	6.3946	5.8324	5.7582	0.9736	-0.0385	0.3925	0.4061
Atp2b1	_NVYDSISK_.2	#N/A	3.8892	3.1938	3.8822	3.4260	2.8243	2.9617	3.5743	3.5752	3.4844	3.1033	0.9792	-0.0303	0.7808	0.1075
Atp2b1	_[Acetyl (Protein N-term)]GDMANNSVAYS[Phospho (STY)]GVKNSLK_.2	G5E829_S12_M1_Atp2b1	3.7694	3.2422	4.0617	3.7048	3.1305	3.5264	3.4195	3.7761	4.0944	3.8758	0.9422	-0.0858	0.3257	0.4872
Atp2b1	_HLDAC[Carbamidomethyl (C)]ETM[Oxidation (M)]GNATAIC[Carbamidomethyl (C)]SDK_.3	#N/A	3.6421	2.9464	4.3289	3.3307	4.6489	3.4609	4.1895	3.2870	4.8013	4.8636	0.8695	-0.2017	0.2365	0.6263
Atp2b1	_MTVVQAYINEK_.2	#N/A	3.1969	3.5765	3.8837	4.3864	3.8235	3.9060	4.1011	3.7321	3.3807	4.3534	0.9753	-0.0361	0.7229	0.1409
Atp2b1	_IEDS[Phospho (STY)]EPHIPLIDDTDAEDDAPTKR_.3	G5E829_S1155_M1_Atp2b1	3.5736	3.5657	3.8984	4.1965	3.4540	4.0180	3.9111	4.0327	3.6813	3.3993	1.0075	0.0108	0.8837	0.0537
Atp2b1	_AQDGAAMEMQPLKS[Phospho (STY)]EEGGDGDEKDK_.3	G5E829_S338_M1_Atp2b1	3.0431	4.1831	3.7299	4.2326	3.6696	4.0541	3.9472	3.8859	5.3545	4.1996	0.9812	-0.0273	0.7790	0.1085
Trip12	_SES[Phospho (STY)]PPAELPSLR_.2	G5E870_S312_M1_Trip12	11.2571	10.7696	11.4771	4.1561	3.5932	10.7703	11.6517	11.0768	10.8774	10.8898	0.7795	-0.3595	0.2346	0.6297
Trip12	_AQTAPTKTS[Phospho (STY)]PR_.2	G5E870_S1460_M1_Trip12	7.7075	7.6115	8.1291	7.3523	6.2487	7.6961	8.0478	8.0201	7.7822	7.5130	0.9511	-0.0723	0.2956	0.5293
Trip12	_DDSLDLS[Phospho (STY)]PQGR_.2	G5E870_S1024_M1_Trip12	4.0583	7.8252	3.2442	3.7469	6.7081	7.9358	8.5494	8.0635	4.0202	7.6479	0.7901	-0.3398	0.3089	0.5102
Trip12	_AAS[Phospho (STY)]KDTISNNR_.2	G5E870_S1111_M1_Trip12	3.7473	4.6646	4.4341	3.4359	2.8730	3.3556	3.1506	3.1817	4.3311	3.4029	1.0669	0.0934	0.5833	0.2341
Trip12	_SAS[Phospho (STY)]PDYNR_.2	G5E870_S77_M1_Trip12	3.5611	3.6651	4.2479	3.2497	3.5534	3.0712	4.4652	5.4172	3.0165	3.2167	0.8831	-0.1793	0.3653	0.4373
Ank3	_DFPQYFAVVSR_.2	#N/A	7.8731	8.0230	7.9719	7.9875	7.3605	7.3894	7.9918	7.9895	7.6786	7.6559	0.9922	-0.0114	0.7330	0.1349
Ank3	_EFDS[Phospho (STY)]DSLR_.2	G5E8K5_S958_M1_Ank3	4.0989	3.4033	3.2849	3.7875	3.2246	7.0694	6.0617	4.3309	6.5999	6.9894	0.6913	-0.5326	0.0692	1.1599
Ank3	_TEEPVS[Phospho (STY)]PLTAYQK_.2	G5E8K5_S1883_M1_Ank3	7.9964	7.9069	4.2642	3.1288	3.6742	8.1528	4.3444	7.2385	7.9258	7.9188	0.8537	-0.2282	0.4935	0.3067
Ank3	_NEDLAELLNGM[Oxidation (M)]DEELDS[Phospho (STY)]PEELGTKR_.3	#N/A	4.2612	3.5656	6.0458	3.9499	3.3869	4.3426	3.6646	4.1686	5.3101	3.9169	0.9985	-0.0022	0.9913	0.0038
Ank3	_YSYLTEPSMS[Phospho (STY)]PQSPC[Carbamidomethyl (C)]ER_.2	G5E8K5_S1470_M1_Ank3	3.9483	3.2527	3.8234	3.3672	3.0740	3.1546	3.5154	3.6340	3.9331	3.0445	0.9731	-0.0394	0.6979	0.1562
Ank3	_IRDTS[Phospho (STY)]QEPC[Carbamidomethyl (C)]GR_.2	G5E8K5_S1406_M1_Ank3	3.9127	3.2170	4.5995	3.6013	3.0383	3.1903	3.5511	3.0164	4.1658	3.5683	1.0050	0.0072	0.9610	0.0173
Ank3	_NEDLAELLNGM[Oxidation (M)]DEELDS[Phospho (STY)]PEELGTK_.3	#N/A	3.9650	3.2204	3.1917	3.6944	3.1087	3.5159	3.4091	4.4241	4.0727	3.6614	0.8863	-0.1741	0.1105	0.9567
Ank3	_GETALHM[Oxidation (M)]AAR_.2	#N/A	3.5161	3.5059	3.8733	3.8122	3.3942	4.5832	3.7627	5.2002	3.7436	3.6397	0.9252	-0.1122	0.4311	0.3654
Ank3	_RQS[Phospho (STY)]FASLALR_.2	G5E8K5_S1451_M1_Ank3	3.6214	3.7210	3.3550	6.1980	3.5424	4.1871	4.5479	4.1024	4.4014	4.0769	0.9584	-0.0613	0.7545	0.1223
Ank3	_ADIVQQLLQQGASPNAATTSGYTPLHLAAR_.3	#N/A	3.6505	3.6919	3.3842	4.4288	2.7943	4.2163	3.0762	4.0732	4.3723	4.1061	0.9456	-0.0807	0.5859	0.2322
Ank3	_VALLLLDQGAS[Phospho (STY)]PHAAAK_.2	G5E8K5_S606_M1_Ank3	3.2248	3.9810	4.4593	4.0030	3.8693	4.2561	4.1493	1.5375	3.2972	3.6803	1.2525	0.3248	0.1481	0.8296
Brd1	_VHGEPAS[Phospho (STY)]DLS[Phospho (STY)]DID_.2	G5E8P1_S1052_M2_Brd1	3.4511	4.2281	3.6631	8.6127	7.5473	3.8602	3.6843	8.3959	8.5370	8.1996	0.7256	-0.4628	0.2175	0.6625
Cacna2d1	_FVVTDGGITR_.2	#N/A	6.8146	6.4518	6.7591	6.9293	6.3351	6.2980	6.2580	7.1415	6.7549	6.4900	0.9905	-0.0137	0.7644	0.1167
Cacna2d1	_SYDYQSVC[Carbamidomethyl (C)]DPGAAPK_.2	#N/A	4.9753	4.6817	4.9317	4.9869	5.0412	4.8955	4.2976	5.2339	3.1509	5.1417	1.1038	0.1425	0.2667	0.5740
Cacna2d1	_IIM[Oxidation (M)]LFTDGGEER_.2	#N/A	4.6909	4.5330	3.4187	4.0751	4.9489	4.6810	3.9904	4.2824	3.8457	4.9675	1.0280	0.0399	0.7380	0.1319
Cacna2d1	_QLVEIAAR_.2	#N/A	3.4246	4.2661	3.2435	4.2881	2.0805	3.9710	4.4364	4.6083	4.7900	3.9654	0.8442	-0.2443	0.1871	0.7280
Cacna2d1	_VFTFSVGQHNYDR_.3	#N/A	3.0027	3.4113	4.1388	3.4340	3.5912	2.2589	4.1201	3.3993	2.6099	3.2379	0.9893	-0.0155	0.9320	0.0306
Bin1	_VNHEPEPASGAS[Phospho (STY)]PGATIPK_.2	#N/A	8.5874	8.9062	9.2760	9.1959	8.7085	8.8725	8.7847	8.6687	8.5209	8.8006	1.0265	0.0378	0.1534	0.8142
Bin1	_GVFPENFTER_.2	#N/A	8.2766	8.6268	8.4541	8.5828	8.7869	8.7485	8.4644	8.4850	8.2336	8.5605	1.0170	0.0243	0.2364	0.6264
Bin1	_LNQNLNDVLVSLEK_.2	#N/A	5.3206	6.1270	6.3279	6.1805	6.0128	5.8930	5.4519	5.7579	5.7337	6.1703	1.0343	0.0487	0.3833	0.4165
Bin1	_VQAQHDYTATDTDELQLK_.3	#N/A	5.3084	5.3088	6.4106	6.1163	5.5538	4.9676	5.6745	5.8482	5.4017	5.4825	1.0016	0.0024	0.9755	0.0108
Bin1	_GNKS[Phospho (STY)]PS[Phospho (STY)]PPPDGSPAATPEIR_.3	#N/A	12.4848	13.0937	12.5310	13.8032	13.2354	13.3188	13.4555	12.9660	13.3057	13.4923	0.9829	-0.0248	0.4299	0.3666
Bin1	_NQAEEELIK_.2	#N/A	7.2018	7.0342	4.3995	7.5807	7.5372	7.6456	7.3947	7.1383	6.6171	7.6237	0.9592	-0.0601	0.6679	0.1753
Bin1	_ADETKDEQFEQC[Carbamidomethyl (C)]VQNFNK_.3	#N/A	8.5852	8.4735	9.2404	9.2173	8.3552	8.4916	9.0376	8.2536	8.4849	8.6670	1.0135	0.0194	0.6414	0.1929
Bin1	_SPS[Phospho (STY)]PPPDGSPAATPEIR_.2	#N/A	13.5943	13.6028	14.0283	14.1634	13.5447	13.6406	14.1456	13.3738	13.6292	13.7200	1.0033	0.0047	0.8129	0.0899
Bin1	_LVDQALLTM[Oxidation (M)]DTYLGQFPDIK_.3	#N/A	5.2542	4.2701	4.7345	4.7323	4.7495	5.8013	4.5107	4.7309	5.2691	4.7109	1.0246	0.0351	0.7036	0.1527
Bin1	_VNHEPEPASGASPGATIPKS[Phospho (STY)]PSQLR_.3	O08539_S332_M1_Bin1	3.0940	3.8992	3.1769	4.2215	3.7205	4.0090	4.3698	4.2805	5.1453	3.8988	0.8335	-0.2628	0.0458	1.3394
Bin1	_AGDVVLVIPFQNPEEQDEGWLM[Oxidation (M)]GVK_.3	#N/A	3.7524	4.1073	4.4392	3.4411	2.8781	3.3249	5.1389	3.9823	3.3011	4.2630	0.8767	-0.1898	0.2517	0.5991
Bin1	_AQPSDNAPEKGNKS[Phospho (STY)]PS[Phospho (STY)]PPPDGSPAATPEIR_.4	#N/A	9.3485	9.9657	10.1199	10.5859	9.5559	9.3303	8.4340	10.1502	8.2354	9.6716	1.0762	0.1059	0.1592	0.7980
Bin1	_HHYESLQTAK_.2	#N/A	5.8110	5.8084	5.6600	6.1337	6.4498	6.6404	6.4848	3.2999	5.4213	5.9588	1.1498	0.2014	0.2142	0.6691
Bin1	_AAPQWC[Carbamidomethyl (C)]QGK_.2	#N/A	3.6655	4.4217	3.3992	4.4438	6.2735	3.8154	4.6576	5.5161	5.3967	4.1210	0.8809	-0.1830	0.3487	0.4576
Bin1	_IAENNDLLWM[Oxidation (M)]DYHQK_.3	#N/A	3.4619	4.2722	4.4736	4.2393	4.2222	4.0462	4.1568	3.3895	4.2378	4.4450	1.0153	0.0219	0.8143	0.0892
Bin1	_QHGSNTFTVK_.2	#N/A	3.6542	3.6882	3.3879	4.4325	4.5345	4.2200	3.0800	4.0695	4.7508	4.3447	0.9815	-0.0269	0.8445	0.0734
Bin1	_LQAHLVAQTNLLR_.3	#N/A	3.2761	3.5097	4.5828	3.6524	3.3722	4.7379	3.8007	3.1329	3.7864	3.4996	1.0845	0.1170	0.4140	0.3830
Bin1	_ESDWNQHKELEK_.3	#N/A	2.9276	3.4756	4.1621	3.7059	4.2361	3.4934	3.8542	3.8941	5.5943	3.3832	0.9960	-0.0057	0.9574	0.0189
Bin1	_VGFYVNTFQSIAGLEENFHK_.3	#N/A	3.4206	4.1769	3.7361	4.2387	4.0652	4.0603	3.9534	3.8797	1.9944	4.2057	1.1258	0.1709	0.3380	0.4711

Sec22b	_NLGS[Phospho (STY)]INTELQDVQR_.2	O08547_S137_M1_Sec22b	9.6589	9.0786	9.8108	9.7422	8.1728	9.4793	9.7687	9.5412	9.7514	9.4776	0.9677	-0.0473	0.3634	0.4396
Sec22b	_DLQQYQSQAK_.2	#N/A	5.6589	6.1467	4.2874	5.9319	5.7706	6.0517	5.9294	5.3668	5.5159	5.6424	0.9621	-0.0558	0.5753	0.2401
Dpysl2	_GSPLVVISQGK_.2	#N/A	11.4550	11.6377	11.6812	11.7172	11.4028	11.5199	11.4948	11.3523	11.3921	11.5068	1.0116	0.0166	0.1033	0.9859
Dpysl2	_M[Oxidation (M)]VIPGGIDVHTR_.2	#N/A	10.5249	10.9339	11.1663	10.8472	10.8976	10.8287	10.8532	10.7658	10.7325	10.8250	1.0067	0.0096	0.5211	0.2831
Dpysl2	_SAAEVIAQAR_.2	#N/A	11.8538	12.3772	12.2340	12.5495	12.4380	12.0339	12.2098	12.1789	12.1538	12.2210	1.0026	0.0038	0.8191	0.0867
Dpysl2	_QIGENLIVPGGVK_.2	#N/A	11.9634	12.2863	12.4176	12.3983	12.4260	12.1407	12.1810	12.1418	12.1546	12.2030	1.0084	0.0120	0.3178	0.4978
Dpysl2	_VFNLYPR_.2	#N/A	11.0445	11.2047	10.9714	10.5140	11.0580	11.0906	10.7712	10.9385	11.1953	11.2545	0.9946	-0.0078	0.7063	0.1510
Dpysl2	_KPFPDFVYK_.2	#N/A	10.6591	10.7557	10.5417	10.7213	10.6422	10.4850	10.5629	10.5329	10.5507	10.6940	1.0046	0.0067	0.4405	0.3561
Dpysl2	_IVLEDGTLHVTEGSGR_.3	#N/A	9.0247	8.8536	9.2892	9.3163	9.1671	9.0714	8.8155	8.7466	9.0444	9.0959	1.0218	0.0311	0.1190	0.9244
Dpysl2	_ILDLGITGPEGHVLSRPEEVEAEAVNR_.4	#N/A	8.5599	8.8304	8.9099	9.5409	8.7727	8.7505	8.7731	8.9010	8.8416	9.0130	1.0013	0.0019	0.9481	0.0231
Dpysl2	_GTVVYGEPITASLGTDGSHYWSK_.3	#N/A	9.5293	9.2853	9.3689	9.5735	9.6090	9.6454	9.5345	8.9338	9.4324	9.8041	1.0080	0.0115	0.6513	0.1862
Dpysl2	_SITIANQTNC[Carbamidomethyl (C)]PLYVTK_.2	#N/A	8.6866	9.7249	9.2931	9.6757	9.3643	9.5184	9.4829	9.3225	8.9692	9.1053	1.0170	0.0244	0.4813	0.3176
Dpysl2	_AVGKDNFTLIPEGTNGTEER_.3	#N/A	8.0804	8.6138	9.4318	8.4380	8.0696	8.9759	8.6400	8.4292	8.4221	8.4996	1.0122	0.0175	0.7145	0.1460
Dpysl2	_TIEAHSR_.2	#N/A	8.2394	7.5014	6.9985	6.7049	8.4372	7.3810	7.8603	7.3279	7.2537	8.0382	0.9900	-0.0145	0.8457	0.0728
Dpysl2	_ITS[Phospho (STY)]DRLLIK_.2	O08553_S14_M1_Dpysl2	7.6959	8.0718	3.7242	8.6048	7.5994	7.9764	8.1204	7.9489	7.5978	8.0435	0.9181	-0.1232	0.4967	0.3039
Dpysl2	_NIPRIT[Phospho (STY)]SDR_.2	O08553_T13_M1_Dpysl2	6.5992	7.3267	4.2302	3.7740	3.6403	2.9843	7.2771	7.0442	3.5262	7.0166	0.7656	-0.3853	0.2402	0.6194
Dpysl2	_TS[Phospho (STY)]PAKQQAPPVR_.2	#N/A	12.0697	13.0298	13.9460	13.1749	12.7743	13.0395	3.6274	12.8480	12.7177	12.7794	1.2394	0.3097	0.2083	0.6813
Dpysl2	_DNFTLIPEGTNGTEER_.2	#N/A	7.5493	12.5580	12.5706	13.0454	12.6634	12.8070	12.9732	12.3885	12.6064	12.9019	0.9330	-0.1000	0.4563	0.3407
Dpysl2	_ISVGSDADLVIWDPDSVK_.2	#N/A	9.1054	9.4605	9.5270	9.4656	9.6977	9.7520	9.7325	9.3402	9.5288	9.7250	0.9916	-0.0121	0.5771	0.2388
Dpysl2	_M[Oxidation (M)]SVIWDK_.2	#N/A	7.2099	7.1812	6.3342	7.6857	7.5792	6.5311	5.0225	6.7434	7.4469	7.0952	1.0775	0.1077	0.3456	0.4614
Dpysl2	_GIQEEM[Oxidation (M)]EALVK_.2	#N/A	7.2240	7.2316	6.7201	7.7341	7.6126	7.2470	7.5274	7.5313	7.1190	7.6845	0.9771	-0.0334	0.4307	0.3658
Dpysl2	_TVT[Phospho (STY)]PASS[Phospho (STY)]AKTS[Phospho (STY)]PAK_.2	#N/A	12.3101	13.7280	14.0251	13.4045	13.2512	13.6004	13.6576	13.3479	13.3283	13.6284	0.9923	-0.0112	0.7456	0.1275
Dpysl2	_TVT[Phospho (STY)]PASS[Phospho (STY)]AK_.2	#N/A	10.9995	10.9843	11.1561	11.2299	9.7553	10.4081	11.0092	10.8553	10.6145	11.1745	0.9855	-0.0210	0.6190	0.2083
Dpysl2	_TVT[Phospho (STY)]PASS[Phospho (STY)]AKTS[Phospho (STY)]PAKQQAPPVR_.3	#N/A	9.8575	10.9701	11.8250	11.6662	11.3911	11.0398	11.3935	11.2534	11.2604	10.7515	0.9964	-0.0051	0.9187	0.0368
Dpysl2	_FQM[Oxidation (M)]PDQGM[Oxidation (M)]TSADDFFQGTK_.2	#N/A	7.4751	8.1441	8.5886	7.2417	8.4665	8.5029	8.2867	8.0264	8.0789	8.0656	0.9945	-0.0079	0.8847	0.0532
Dpysl2	_DHGVNSFLVYM[Oxidation (M)]AFK_.2	#N/A	5.9982	5.7723	6.3121	5.8420	5.9645	6.2753	6.1822	5.9840	6.0497	6.0550	0.9934	-0.0096	0.7415	0.1299
Dpysl2	_NLHQSGFSLSGAQIDDNIPR_.3	#N/A	8.5130	8.9606	9.3503	9.8875	8.7641	8.7940	8.9135	8.8242	8.8619	8.7507	1.0235	0.0335	0.4398	0.3567
Dpysl2	_THNSALEYNIFEGM[Oxidation (M)]EC[Carbamidomethyl (C)]R_.3	#N/A	8.6658	8.7155	9.3118	8.9592	8.6848	8.6344	8.7561	8.2920	8.7063	8.9083	1.0188	0.0269	0.3667	0.4357
Dpysl2	_FQLTDSQIYEVLSVIR_.3	#N/A	5.7011	5.4901	6.7395	6.2965	5.3348	5.5428	5.1550	5.2921	5.7712	5.8816	1.0590	0.0827	0.3283	0.4838
Dpysl2	_M[Oxidation (M)]DENQFVAVTSTNAAK_.2	#N/A	9.7116	10.1339	10.1926	10.3586	10.2193	10.2319	10.0780	9.9822	10.1541	10.2508	1.0025	0.0036	0.8431	0.0741
Dpysl2	_IVNDDQSFYADIYM[Oxidation (M)]EDGLIK_.2	#N/A	7.3507	7.8773	8.2640	8.3819	7.4742	7.8634	7.6150	7.6264	7.2626	7.6585	1.0435	0.0614	0.1781	0.7493
Dpysl2	_NLHQSGFSLSGAQIDDNIPRR_.4	#N/A	7.3093	7.5940	8.1928	6.9044	7.3004	7.4510	7.2958	6.3698	6.7215	7.3295	1.0764	0.1062	0.1005	0.9977
Dpysl2	_GLYDGPVC[Carbamidomethyl (C)]EVSVT[Phospho (STY)]PK_.2	#N/A	9.7579	9.8714	10.0843	11.0986	12.5638	9.9243	13.1528	10.3866	10.2587	12.9558	0.9026	-0.1478	0.2123	0.6731
Dpysl2	_GLYDGPVC[Carbamidomethyl (C)]EVSVTPKT[Phospho (STY)]VT[Phospho (STY)]PASSAK_.3	#N/A	3.6234	3.6567	4.2389	10.3311	8.5701	5.7348	3.6645	9.5636	5.5092	5.2510	1.0048	0.0069	0.9873	0.0056
Dpysl2	_DIGAIAQVHAENGDIIAEEQQR_.3	#N/A	9.0218	9.0258	8.6823	9.3508	8.3874	8.7144	9.2989	8.0294	8.7058	9.1476	1.0078	0.0112	0.8156	0.0885
Dpysl2	_DRFQLTDSQIYEVLSVIR_.3	#N/A	4.1922	3.4477	3.9259	3.4697	3.7376	3.2886	3.4967	3.1796	3.8305	3.1470	1.0772	0.1073	0.2544	0.5945
Dpysl2	_SC[Carbamidomethyl (C)]C[Carbamidomethyl (C)]DYSLHVDITEWHK_.3	#N/A	6.6940	6.8769	7.7752	8.3852	4.8592	7.1747	7.5545	5.4620	6.7553	7.3455	1.0268	0.0381	0.8067	0.0933
Dpysl2	idomethyl (C)]EVSVT[Phospho (STY)]PKT[Phospho (STY)]VT[Phospho (STY)]PAS[Phospho (STY)]S[Phosp	#N/A	3.4251	3.8020	3.8570	4.0031	3.5088	4.0534	4.0488	4.0034	4.0424	4.0620	0.9346	-0.0976	0.0794	1.1003
Dpysl2	_GRIS[Phospho (STY)]VGSDADLVIWDPDSVK_.3	#N/A	3.6813	2.9856	4.3681	3.3699	2.8070	3.4217	3.7825	3.2477	4.3972	3.3369	0.9317	-0.1021	0.4926	0.3075
Dpysl2	_AALAGGTTM[Oxidation (M)]IIDHVVPEPGTSLLAAFDQWR_.3	#N/A	3.2845	4.0897	3.9713	2.2454	3.9110	3.8185	4.1793	2.9702	3.2692	3.7083	1.0061	0.0088	0.9595	0.0180
Dpysl2	AAFVTSPPLSPDPTTPDFLNSLLSC[Carbamidomethyl (C)]GDLQVTGSAHC[Carbamidomethyl (C)]TFNTAQK	#N/A	3.2865	4.0559	4.5210	4.0648	3.9311	4.1944	4.2131	4.4372	3.2354	3.7421	1.0261	0.0372	0.7393	0.1312
Rundc3a	_FTQSYDYLT[Phospho (STY)]DEEER_.2	O08576_T215_M1_Rundc3a	3.3999	5.1682	3.1336	7.0091	5.6348	4.0810	5.0906	6.8554	6.8276	6.6785	0.7446	-0.4255	0.0849	1.0713
Rundc3a	_SNEC[Carbamidomethyl (C)]LVSDSPEGSPALS[Phospho (STY)]PS_.2	O08576_S444_M1_Rundc3a	7.5305	7.8828	3.2417	8.7040	6.2881	3.9139	7.3157	3.8655	8.7186	3.8793	0.8855	-0.1755	0.5878	0.2308
Emd	_DDIFSS[Phospho (STY)]LEEEGKDR_.2	O08579_S142_M1_Emd	3.9538	3.2723	3.1398	3.6425	3.1606	3.4640	3.3571	2.9752	4.1246	3.6095	0.9779	-0.0323	0.7636	0.1171
Kcnk1	_QSEPFVASQSPPY[Phospho (STY)]EDGSADH_.2	O08581_Y329_M1_Kcnk1	7.0680	7.0991	3.7294	7.1916	3.3216	3.6914	9.3649	8.1576	7.5011	7.1388	0.6654	-0.5877	0.0354	1.4506
Gtpbp1	_LVLVS[Phospho (STY)]PTSEQYDSLLR_.2	O08582_S69_M1_Gtpbp1	3.8854	4.7742	5.1188	5.6295	3.5439	3.3955	4.6643	5.3747	4.1931	5.0506	0.9109	-0.1346	0.4224	0.3743
Gtpbp1	_SRS[Phospho (STY)]PVDSPVPASM[Oxidation (M)]FAPEPSS[Phospho (STY)]PGAAR_.3	O08582_S25_M2_Gtpbp1	3.3344	4.1396	4.0212	3.9811	3.9609	3.7686	4.1294	3.0200	3.3191	3.6584	1.0951	0.1311	0.1970	0.7056
Gtpbp1	_SPVDSPVPASM[Oxidation (M)]FAPEPSS[Phospho (STY)]PGAAR_.3	O08582_S25_M1_Gtpbp1	3.6988	3.0032	4.3856	6.8819	2.8245	3.4041	3.1021	3.2302	4.3796	3.3544	1.1469	0.1977	0.5367	0.2702
Gtpbp1	_LLQTTNNS[Phospho (STY)]PMNSKPQQIK_.3	O08582_S580_M1_Gtpbp1	3.0510	3.8561	3.2199	5.9579	3.6775	4.0520	4.4128	4.2375	3.0357	3.9418	1.0159	0.0228	0.9182	0.0371
Alyref	_QQLS[Phospho (STY)]AEELDAQLDAYNAR_.2	#N/A	4.1813	5.7988	5.2103	6.6201	5.2698	5.8350	5.3477	6.0758	5.7337	5.9836	0.9483	-0.0766	0.5117	0.2909
Clta	_LQS[Phospho (STY)]EPESIR_.2	#N/A	9.3605	9.2549	9.2286	9.8387	9.5132	9.4256	9.4398	9.0512	9.2417	9.4188	1.0160	0.0230	0.3005	0.5221
Clta	_LEALDANSR_.2	#N/A	9.9447	10.2029	10.3305	10.3556	10.6648	10.1297	9.9802	9.9481	10.0840	10.1405	1.0232	0.0331	0.1090	0.9626
Clta	_ELEEWYAR_.2	#N/A	8.8376	8.1691	8.9036	8.7535	9.0396	8.8461	8.0573	8.5227	8.7492	8.8846	1.0239	0.0341	0.3605	0.4431
Clta	_VADEAFYK_.2	#N/A	6.7885	6.5498	7.0333	6.8769	7.5461	7.0984	5.5334	7.0481	7.3862	7.4405	1.0190	0.0271	0.7497	0.1251
Clta	_LC[Carbamidomethyl (C)]DFNPK_.2	#N/A	6.1685	7.2260	6.7446	7.1218	7.3649	6.3893	6.1320	6.5109	6.9144	6.3736	1.0545	0.0765	0.2404	0.6190
Clta	_KQEAEWK_.2	#N/A	6.6285	5.5769	6.2757	5.9518	6.9023	6.3026	6.2115	5.5962	6.0103	6.5313	1.0305	0.0433	0.5344	0.2721
Clta	_QDEQLQK_.2	#N/A	4.7862	4.3689	5.2548	5.7701	6.2129	5.3917	4.4229	5.4810	5.5986	5.2058	1.0232	0.0332	0.7698	0.1136
Clta	_WREEQTER_.2	#N/A	5.2076	5.5707	4.0579	5.4808	5.8836	5.6952	4.7374	5.2715	4.8332	5.4722	1.0467	0.0659	0.5308	0.2751
Clta	_AIKELEEWYAR_.2	#N/A	4.2166	6.9237	7.4244	6.4333	6.4140	6.8717	6.5178	2.3349	6.0754	6.0045	1.2193	0.2860	0.2659	0.5754
Clta	_LQS[Phospho (STY)]EPESIRK_.2	#N/A	9.9846	10.0950	10.4332	10.6195	10.3576	10.2516	10.2029	10.0469	10.1850	10.3584	1.0090	0.0130	0.4933	0.3068
Pten	_NNIDDVVR_.2	#N/A	3.9025	4.7789	3.2716	5.3279	4.5027	4.1037	4.9486	4.4143	4.9477	4.7160	0.9071	-0.1407	0.2782	0.5557
Pten	_VENGS[Phospho (STY)]LC[Carbamidomethyl (C)]DQEIDSIC[Carbamidomethyl (C)]SIER_.2	O08586_S294_M1_Pten	3.1762	3.9324	4.4107	3.9544	3.8207	4.3047	4.1027	3.0467	3.3458	3.6317	1.1137	0.1553	0.1964	0.7068
Stxbp1	_KLNKTDEEISS[Phospho (STY)]_.2	#N/A	7.0925	11.5960	12.3772	6.7740	7.0361	7.4656	11.8818	6.9332	11.7126	11.5677	0.8289	-0.2707	0.2958	0.5290
Stxbp1	_SSAS[Phospho (STY)]FSTTAVSAR_.2	O08599_S509_M1_Stxbp1	10.2878	9.4404	10.1614	9.5149	9.5437	9.3877	10.6476	9.3899	9.3770	9.3175	1.0041	0.0059	0.9058	0.0430
Stxbp1	_LNKTDEEISS[Phospho (STY)]_.2	#N/A	7.6797	7.4076	7.0701	8.4924	7.7232	12.1835	7.8220	7.1477	7.6239	8.7602	1.0750	0.1043	0.5751	0.2402
Stxbp1	_SQLLILDR_.2	#N/A	10.7390	10.6063	10.9058	10.5541	10.0858	10.6053	10.3061	10.6017	10.7580	10.6239	1.0010	0.0014	0.9500	0.0223
Stxbp1	_HYPYISTR_.2	#N/A	10.3667	10.3821	10.6197	10.2335	10.4587	10.3096	10.3755	10.5319	10.0641	10.3371	1.0066	0.0095	0.5270	0.2782
Stxbp1	_ISEQTYQLSR_.2	#N/A	10.2427	10.2466	10.5994	10.2015	10.1178	10.0973	10.3679	10.2932	10.3149	10.0341	0.9998	-0.0002	0.9883	0.0051
Stxbp1	_NGITEENLNK_.2	#N/A	9.6405	9.5139	9.4889	10.0000	8.7870	9.1991	9.8429	9.5819	9.5526	9.5793	0.9792	-0.0304	0.3810	0.4191
Stxbp1	_YETSGIGEAR_.2	#N/A	8.9451	8.7953	8.7782	9.0657	9.2170	8.8344	8.6795	9.0314	9.0124	8.9126	1.0034	0.0049	0.7885	0.1032
Stxbp1	_HIAEVSQEVTR_.2	#N/A	8.4071	8.7503	8.2536	8.7311	8.6268	8.6997	8.6443	8.7307	8.7538	8.4101	0.9934	-0.0095	0.6509	0.1865
Stxbp1	_DNALLAQLIQDK_.2	#N/A	8.8124	8.3698	8.0631	8.8448	8.2786	8.1008	8.8446	8.3394	8.6019	8.4348	0.9832	-0.0244	0.4833	0.3157
Stxbp1	_AIVPILLDANVSTYDK_.2	#N/A	8.1496	8.0839	7.5954	7.9870	7.5802	7.2439	8.1833	7.9231	8.0500	8.2822	0.9585	-0.0611	0.1177	0.9293
Stxbp1	_NKAPGEYR_.2	#N/A	8.1154	7.9194	8.2876	7.9469	8.5229	7.8957	7.5549	8.3772	8.0804	7.9082	1.0168	0.0241	0.4904	0.3094
Stxbp1	_LLDTLKK_.2	#N/A	7.5360	7.9851	7.7740	7.4338	8.2110	7.7750	7.7477	7.7939	7.9623	7.6507	0.9996	-0.0005	0.9858	0.0062
Stxbp1	_EPLPSLEAVYLITPSEK_.2	#N/A	7.5698	7.5032	7.8091	7.1191	7.7840	7.6207	7.8496	7.5074	7.5206	7.3397	1.0018	0.0025	0.9329	0.0302
Stxbp1	_ADDPTM[Oxidation (M)]GEGPDK_.2	#N/A	6.6858	6.8577	6.3697	6.4028	7.2200	6.8867	6.7329	6.9150	6.5735	6.8069	0.9970	-0.0043	0.9115	0.0402
Stxbp1	_EVLLDEDDDLWIALR_.2	#N/A	6.6568	6.3924	6.3366	7.0715	5.5094	6.5079	6.8982	6.3579	6.8818	6.9924	0.9454	-0.0810	0.2320	0.6345
Stxbp1	_IM[Oxidation (M)]HDVIKK_.2	#N/A	6.4210	7.2174	6.8798	6.0213	7.2023	6.8792	7.0054	7.0667	6.9818	6.8101	0.9719	-0.0411	0.4427	0.3539

Stxbp1	_SLKDFSSSK_.2	#N/A	6.3178	5.9108	6.7027	6.0041	6.4044	6.2995	5.5816	6.4198	6.1080	6.1398	1.0348	0.0493	0.3245	0.4888
Stxbp1	_LAEQIATLC[Carbamidomethyl (C)]ATLK_.2	#N/A	5.9238	6.2474	5.7393	5.3490	5.5168	5.5288	5.8709	6.0395	6.3097	5.9572	0.9459	-0.0802	0.1121	0.9504
Stxbp1	_DNALLAQLIQDKLDAYK_.2	#N/A	5.8607	6.1756	5.4457	6.3430	5.1233	6.1537	5.9839	6.0277	6.1589	5.4698	0.9899	-0.0147	0.8310	0.0804
Stxbp1	_AAHVFFTDSC[Carbamidomethyl (C)]PDALFNELVK_.3	#N/A	7.0601	7.1131	7.6118	7.5679	6.7780	7.2723	7.2523	7.5450	7.4682	7.3052	0.9785	-0.0313	0.3815	0.4185
Stxbp1	_HYQGTVDK_.2	#N/A	5.3538	4.3109	4.0609	4.7940	5.6533	5.3184	4.6217	4.5436	4.7737	5.0786	1.0338	0.0480	0.6457	0.1899
Stxbp1	_VLVVDQLSM[Oxidation (M)]R_.2	#N/A	9.4705	9.7145	10.1236	9.4526	8.8930	9.5410	9.8011	9.7201	9.6029	9.4678	0.9880	-0.0174	0.6014	0.2208
Stxbp1	_REPLPSLEAVYLITPSEK_.3	#N/A	7.1029	6.8109	7.5521	7.3819	6.6565	7.0512	7.5177	7.5082	7.6154	7.3148	0.9471	-0.0785	0.0574	1.2408
Stxbp1	_VEQDLAM[Oxidation (M)]GTDAEGEK_.2	#N/A	7.9359	7.7703	7.9595	7.9993	7.8625	7.9274	8.2940	8.1525	8.1274	8.1053	0.9681	-0.0468	0.0012	2.9097
Stxbp1	_SVHSLISDFKDPPTAK_.3	#N/A	8.6660	8.7800	9.0882	8.7234	8.4647	8.6917	9.0221	8.7352	8.7886	8.5581	0.9954	-0.0066	0.7614	0.1184
Stxbp1	_M[Oxidation (M)]LSSC[Carbamidomethyl (C)]C[Carbamidomethyl (C)]K_.2	#N/A	5.5019	4.4771	4.6507	5.6070	5.9818	4.5621	4.6591	4.4551	4.7469	4.5396	1.1152	0.1573	0.1494	0.8257
Stxbp1	_DFSSSKR_.2	#N/A	6.2485	5.7681	4.5471	5.0331	3.9571	5.0493	4.3785	5.4699	3.2094	5.7689	1.0837	0.1160	0.5447	0.2639
Stxbp1	_KM[Oxidation (M)]PQYQK_.2	#N/A	6.2751	4.9485	3.8131	6.7125	6.1029	5.7030	5.3704	5.8898	5.0303	5.2736	1.0374	0.0529	0.7271	0.1384
Stxbp1	_IM[Oxidation (M)]HDVIK_.2	#N/A	6.0710	5.9041	5.6048	6.1794	6.1169	5.8599	5.7509	6.1657	6.1635	5.5348	1.0089	0.0127	0.7588	0.1199
Stxbp1	_C[Carbamidomethyl (C)]AYEVTQANGK_.2	#N/A	5.4517	5.3614	4.4159	5.0994	3.8260	5.3713	5.4015	5.5346	3.3405	5.5314	0.9937	-0.0091	0.9557	0.0197
Stxbp1	_M[Oxidation (M)]TDIM[Oxidation (M)]TEGITIVEDINKR_.3	#N/A	7.1683	7.3890	8.4485	7.5603	6.5623	7.5834	7.3403	7.5716	7.5886	7.1846	1.0041	0.0060	0.9268	0.0330
Stxbp1	_WEVLIGSTHILTPQK_.2	#N/A	6.8718	6.4026	6.6742	6.5566	5.8754	6.4579	6.9193	6.4828	6.7250	6.7551	0.9632	-0.0541	0.2206	0.6565
Stxbp1	_M[Oxidation (M)]TDIM[Oxidation (M)]TEGITIVEDINK_.2	#N/A	6.1010	5.7670	6.4564	6.2382	5.5849	6.3545	6.1199	5.8346	5.8173	5.9954	1.0239	0.0340	0.4619	0.3355
Stxbp1	_DIM[Oxidation (M)]EDTIEDKLDTK_.2	#N/A	7.6600	7.4626	7.6731	7.6210	7.1745	7.4147	7.8809	7.2468	7.4337	7.6935	0.9917	-0.0120	0.6823	0.1660
Stxbp1	_DIMEDTIEDK_.2	#N/A	5.6890	3.4381	3.3197	6.4496	5.7159	2.9692	3.3300	3.8194	4.1185	4.3599	1.1766	0.2346	0.4099	0.3873
Stxbp1	_YSTHLHLAEDC[Carbamidomethyl (C)]M[Oxidation (M)]K_.3	#N/A	4.2700	4.7669	4.0108	5.0584	4.8927	4.6076	3.8162	5.4037	4.7756	3.7781	1.0355	0.0503	0.6824	0.1660
Stxbp1	_LIQHAQIPPEDSEIITNMAHLGVPIVTDSTLR_.4	#N/A	4.1003	3.4758	4.2406	4.8697	2.7848	5.1891	3.5181	3.8035	3.9445	3.3449	1.1252	0.1702	0.3534	0.4517
Stxbp1	_VEQDLAMGTDAEGEKIKDPMR_.3	#N/A	1.4262	3.3399	3.7362	4.2439	3.1462	3.4784	3.4282	3.7212	4.0203	2.9572	0.9141	-0.1295	0.5773	0.2386
Stxbp1	_GFDPSSPVLHELTFQAM[Oxidation (M)]SYDLLPIENDVYK_.3	#N/A	3.1067	0.6207	4.3413	3.8850	4.0570	3.6725	4.0333	3.1161	3.4152	3.5623	0.9289	-0.1064	0.7341	0.1342
Stxbp1	_LDAYKADDPTMGEGPDK_.3	#N/A	2.9125	3.7177	3.3584	4.4030	3.5390	4.1905	4.5513	4.0990	4.3980	4.0803	0.8610	-0.2160	0.0773	1.1119
Stxbp1	_DLS[Phospho (STY)]QMLKK_.2	O08599_S328_M1_Stxbp1	3.7302	3.0346	4.4170	3.5853	2.8559	3.3728	3.7336	3.1988	3.7149	3.3858	0.9974	-0.0037	0.9770	0.0101
Myh11	_VIENTDGS[Phospho (STY)]EEEM[Oxidation (M)]DAR_.2	#N/A	8.4610	8.2473	8.6405	8.4578	3.8615	4.0737	8.5669	8.9119	3.2754	8.2220	0.9604	-0.0583	0.8606	0.0652
Ephb6	_GTGYTEQLQQYSS[Phospho (STY)]PGLGVK_.2	O08644_S630_M1_Ephb6	3.2210	4.6137	5.0517	4.2775	3.4997	4.0990	4.7260	3.8413	4.6164	4.5737	0.9297	-0.1052	0.4387	0.3578
Map3k4	_ELESGTEES[Phospho (STY)]DEEPTPSPR_.2	O08648_S454_M1_Map3k4	3.4299	5.7482	4.4467	5.8155	5.2763	5.3471	6.5745	5.8153	5.9095	5.5616	0.8400	-0.2516	0.0913	1.0396
Map3k4	_LES[Phospho (STY)]EEDSIGWGTADC[Carbamidomethyl (C)]GPEASR_.2	O08648_S492_M1_Map3k4	4.2521	2.9740	3.4381	5.0065	3.3778	3.6742	3.6554	4.1777	3.8263	4.1770	0.9566	-0.0641	0.6683	0.1750
Kifc2	_AGVAS[Phospho (STY)]PVQR_.2	O08672_S643_M1_Kifc2	7.9782	7.9027	8.3076	7.5990	6.6223	7.2525	8.2333	8.0838	7.6072	7.7249	0.9618	-0.0561	0.3849	0.4146
Prdx6	_LSILYPATTGR_.2	#N/A	9.8007	10.2212	10.4229	10.3237	10.3009	10.1052	10.1006	10.1589	10.0873	10.0539	1.0095	0.0136	0.4262	0.3703
Prdx6	_LPFPIIDDK_.2	#N/A	8.5375	8.7174	8.1480	9.0079	9.1299	8.4214	8.5724	8.6940	8.6168	8.5629	1.0057	0.0082	0.8033	0.0951
Prdx6	_VVFIFGPDKK_.2	#N/A	8.0654	8.6110	8.6381	8.8932	8.6684	8.3215	8.3712	8.5359	8.3801	8.5328	1.0092	0.0132	0.6258	0.2036
Prdx6	_DINAYNGETPTEK_.2	#N/A	7.9732	8.1144	7.8011	8.3685	8.6747	8.0618	7.8663	7.7968	8.0321	8.1506	1.0256	0.0365	0.2664	0.5745
Prdx6	_VVDSLQLTGTKPVATPVDWK_.3	#N/A	6.5019	6.4124	6.9053	6.8114	6.8704	6.6295	5.9975	6.3190	6.6552	6.5281	1.0492	0.0693	0.0767	1.1154
Prdx6	_DLAILLGM[Oxidation (M)]LDPVEK_.2	#N/A	6.6637	6.9359	6.1755	5.9234	7.4346	6.5027	7.0959	6.8802	6.8777	7.4795	0.9326	-0.1007	0.1475	0.8313
Prdx6	_NFDEILR_.2	#N/A	6.1885	6.2235	7.0166	6.7586	7.0307	5.8936	4.5404	7.1442	6.4689	6.3171	1.0655	0.0916	0.4485	0.3482
Prdx6	_DFTPVC[Carbamidomethyl (C)]TTELGR_.2	#N/A	7.5724	7.0853	7.2427	8.0711	7.8639	6.6436	8.0451	7.2417	7.0284	7.1689	1.0057	0.0082	0.8998	0.0458
Prdx6	_PGGLLLGDEAPNFEANTTIGR_.2	#N/A	5.9287	5.9007	6.2718	6.3157	6.4360	6.0663	5.3593	5.7913	5.6181	5.9419	1.0838	0.1160	0.0130	1.8852
Prdx6	_VVFIFGPDK_.2	#N/A	5.5337	5.2776	5.7841	5.9339	5.8632	6.0819	4.9494	5.0315	5.4457	5.7051	1.0876	0.1212	0.0539	1.2682
Prdx6	_LIALSIDSVEDHLAWSK_.2	#N/A	4.2373	4.6286	4.1354	5.6053	4.6500	4.2609	4.5041	5.0843	4.5677	5.5546	0.9307	-0.1036	0.3428	0.4649
Prdx6	_KGESVM[Oxidation (M)]VVPTLSEEEAK_.3	#N/A	6.9873	6.9431	7.3552	7.3997	7.2466	6.8377	6.5220	7.1462	7.0554	7.1276	1.0238	0.0339	0.3532	0.4519
Prdx6	_LPFPIIDDKGR_.2	#N/A	6.5260	6.7858	7.4187	7.0950	7.0843	7.0583	6.9327	6.9301	6.7670	7.0303	1.0115	0.0165	0.6352	0.1971
Prdx6	_FHDFLGDSWGILFSHPR_.3	#N/A	3.0458	4.1803	3.7326	4.2353	3.6724	4.0571	3.9500	3.8832	3.5318	4.2023	0.9817	-0.0266	0.7845	0.1054
Akap1	_RRS[Phospho (STY)]ESSGNLPSVADTR_.3	#N/A	8.7867	8.3869	9.1896	8.8508	8.0015	8.4784	8.2756	8.4586	8.6698	3.1300	1.2078	0.2723	0.2064	0.6852
Akap1	_S[Phospho (STY)]ESSGNLPSVADTR_.2	#N/A	7.4203	4.3734	3.5396	4.0422	3.4793	4.2502	3.7569	4.0762	3.7248	4.0092	1.1608	0.2151	0.4298	0.3667
Dld	_NETLGGTC[Carbamidomethyl (C)]LNVGC[Carbamidomethyl (C)]IPSK_.2	#N/A	8.3498	8.5803	8.5553	8.7122	8.5432	8.3959	8.5777	8.2281	8.3156	8.6882	1.0083	0.0120	0.5358	0.2710
Dld	_GIEIPEVR_.2	#N/A	8.5495	7.8440	7.8491	7.6707	8.1629	7.5508	7.0065	8.0448	7.7854	8.3179	0.9875	-0.0182	0.7812	0.1072
Dld	_IPNIYAIGDVVAGPM[Oxidation (M)]LAHK_.3	#N/A	6.8664	7.2204	6.8017	7.1360	6.9609	7.1825	6.2688	7.4583	7.2645	7.0224	1.0035	0.0050	0.9159	0.0381
Dld	_ADGSTQVIDTK_.2	#N/A	7.6181	7.8030	7.6445	7.8800	7.9411	7.7255	7.4556	7.5616	7.7037	7.8530	1.0164	0.0234	0.2233	0.6511
Dld	_EANLAAAFGKPINF_.2	#N/A	7.1817	7.2688	7.4251	7.4931	7.5439	7.6158	7.4756	7.3033	7.4042	7.4282	1.0025	0.0036	0.8411	0.0752
Dld	_IDVSVEAASGGK_.2	#N/A	5.4830	5.9876	5.0356	6.5069	6.0536	4.1759	4.5366	5.9363	6.0408	5.9058	0.9885	-0.0167	0.9031	0.0443
Dld	_VC[Carbamidomethyl (C)]HAHPTLSEAFR_.3	#N/A	6.7515	5.4649	6.7659	7.2852	7.0639	6.8053	6.9107	6.4390	6.5689	6.5879	1.0095	0.0136	0.8555	0.0678
Dld	_ALTGGIAHLFK_.2	#N/A	3.9073	3.3189	4.5941	1.9133	2.1693	3.1957	3.3106	3.0217	4.1712	2.4903	0.9799	-0.0293	0.9144	0.0389
Dld	_SEEQLKEEGIEFK_.2	#N/A	5.9982	5.3385	5.7347	6.2474	5.6002	5.9258	5.5656	5.7909	5.4914	5.8343	1.0241	0.0344	0.4596	0.3377
Dld	_RPFTQNLGLEELGIELDPK_.3	#N/A	3.0879	4.2545	4.3224	3.5734	3.7164	3.6537	4.0145	4.4684	4.3340	3.5435	0.9749	-0.0367	0.7552	0.1220
Dld	_LGADVTAVEFLGHVGGIGIDM[Oxidation (M)]EISK_.3	#N/A	3.6920	2.9964	4.3788	3.3806	2.8177	5.5070	3.7717	3.2370	4.3864	3.3476	1.0298	0.0423	0.8475	0.0719
Dld	_AEVITC[Carbamidomethyl (C)]DVLLVC[Carbamidomethyl (C)]IGR_.2	#N/A	4.1496	3.1928	3.8833	3.4271	3.2934	3.3312	3.5754	3.5741	3.8731	3.1044	1.0041	0.0059	0.9516	0.0215
Dld	_VLGAHILGPGAGEM[Oxidation (M)]VNEAALALEYGASC[Carbamidomethyl (C)]EDIAR_.3	#N/A	3.4091	4.1654	3.1428	4.2502	4.0537	4.0717	3.9649	3.8683	3.5168	3.8647	1.0119	0.0170	0.8589	0.0661
Hsd17b10	_NFLASQVPFPSR_.2	#N/A	7.0426	7.2074	6.7288	7.4791	6.8867	7.2071	7.1410	6.9425	7.2442	7.0474	0.9997	-0.0004	0.9903	0.0042
Hsd17b10	_VVTIAPGLFATPLLTTLPEK_.2	#N/A	5.5818	5.3289	6.0629	5.2994	4.8945	5.5358	5.2934	4.6606	5.3255	5.1788	1.0657	0.0918	0.1854	0.7318
Hsd17b10	_LVAGEM[Oxidation (M)]GQNEPDQGGQR_.2	#N/A	5.9333	5.6568	5.9835	6.9134	5.3679	5.8504	5.6783	5.8033	5.6079	5.6525	1.0467	0.0658	0.3521	0.4533
Hsd17b10	_GVIINTASVAAFEGQVGQAAYSASK_.3	#N/A	3.0611	3.3144	3.1811	4.2119	1.9566	4.0334	3.4811	1.5531	3.2607	4.1892	1.0551	0.0774	0.7828	0.1064
Ube3a	_S[Phospho (STY)]PGESQSEDIEASR_.2	O08759_S8_M1_Ube3a	7.7227	7.6639	7.9570	7.9500	6.9810	7.9281	8.0924	7.7974	7.7563	7.7799	0.9801	-0.0289	0.4596	0.3377
Ube3a	_NLDFQALEETTEYDGGYTR_.2	#N/A	3.0898	3.8950	3.7766	4.2257	3.7164	4.0131	4.3739	4.2764	3.0746	4.2463	0.9482	-0.0767	0.5274	0.2778
Tcof1	_AASAPAKES[Phospho (STY)]PK_.2	#N/A	7.3511	7.3660	7.7056	7.6034	5.7022	6.7172	7.9060	7.6455	6.7165	7.2357	0.9591	-0.0603	0.5090	0.2933
Tcof1	_NSS[Phospho (STY)]PAVPAPTPEGVQAVNTTK_.2	O08784_S853_M1_Tcof1	7.8757	8.2291	3.2924	4.3370	4.2033	3.9222	4.4853	8.3209	2.9632	4.0143	1.0736	0.1025	0.8065	0.0934
Tcof1	_KLS[Phospho (STY)]GDLEAGAPK_.2	O08784_S1191_M1_Tcof1	4.8269	3.6445	4.1918	5.4620	4.2251	4.7329	5.3472	5.0768	4.6998	4.9192	0.9008	-0.1507	0.1812	0.7418
Tcof1	_AASAPAKES[Phospho (STY)]PKK_.2	#N/A	3.1887	4.2559	4.9871	4.2653	3.3442	4.3853	4.5694	3.7886	3.8600	3.8209	1.0153	0.0219	0.8749	0.0581
Tcof1	_DSAS[Phospho (STY)]PIQKK_.2	O08784_S1303_M1_Tcof1	5.0329	4.0124	4.4907	4.0344	3.9007	4.2247	4.1179	4.4676	3.2658	3.7117	1.1007	0.1385	0.2239	0.6499
Tcof1	_SAEPLANTVLASET[Phospho (STY)]EEEGNAQALGPTAK_.3	O08784_T171_M1_Tcof1	3.4307	3.9970	3.9263	4.2090	3.5573	3.7805	3.6183	3.6394	3.9385	3.8863	1.0122	0.0176	0.7817	0.1070
Tcof1	_ASAVS[Phospho (STY)]PEKAPMTSK_.2	O08784_S1216_M1_Tcof1	4.2557	3.5600	3.4416	5.8552	3.3814	4.3481	3.2081	3.9413	4.2404	4.2379	1.0597	0.0837	0.6630	0.1785
Dctn1	_LDETQTLLR_.2	#N/A	5.5232	5.8882	5.4931	5.0650	5.5389	5.8141	4.8054	5.5804	6.1367	5.5455	1.0021	0.0031	0.9656	0.0152
Dctn1	_ASEQIYGSPSSSPYEC[Carbamidomethyl (C)]LR_.2	#N/A	4.5869	4.4109	3.8126	5.6162	3.0909	3.7895	4.1434	3.9363	4.9053	3.9697	0.9951	-0.0071	0.9662	0.0149
Dctn1	_QLTAHLQDVNR_.3	#N/A	1.9938	4.3910	3.0915	2.5176	3.3106	4.2678	3.7393	3.1616	3.2522	0.3338	1.2442	0.3153	0.4349	0.3616
Dctn1	_QSQIQVFEDGADTTS[Phospho (STY)]PETPDSSASK_.3	O08788_S105_M1_Dctn1	4.0441	3.0210	7.2959	8.0624	6.9925	7.7042	7.5097	7.7939	7.4309	7.2762	0.8246	-0.2782	0.2583	0.5878
Dctn1	_AFLQGGQEATDIALLLR_.2	#N/A	3.5620	4.3672	3.9048	3.7535	4.1885	3.5410	3.3333	3.6711	3.5467	3.4348	1.1115	0.1525	0.0634	1.1981
Dctn1	_LVLTQEQLHQLHSR_.3	#N/A	3.7272	3.0226	4.1613	3.6015	3.8914	3.3890	3.3580	3.4584	4.1476	3.9353	0.9751	-0.0363	0.7223	0.1413
Dctn1	_LHISQLQHENSILR_.3	#N/A	5.8998	3.4599	3.9382	3.4549	3.3482	3.2764	3.1696	4.3945	4.3122	3.1592	1.0365	0.0518	0.8193	0.0865

Dctn1	_LNQLSTHTHVVDITR_.3	#N/A	3.5752	4.3804	4.1965	3.7403	4.2017	3.5278	3.8886	3.2609	3.5599	3.4176	1.1147	0.1567	0.0959	1.0183
Dctn1	_VTFSC[Carbamidomethyl (C)]AAGLGQR_.2	#N/A	3.8321	3.5104	3.5657	4.6103	2.9758	3.6488	3.2578	3.8917	4.1907	4.2876	0.9446	-0.0822	0.5287	0.2768
Dctn1	_VEAAQETVADYQQTIK_.2	#N/A	3.2760	4.0812	3.9628	4.4654	3.9025	3.8270	4.1801	3.6530	3.3016	4.4324	1.0070	0.0101	0.9252	0.0338
Prkcsh	_SLEDQVETLR_.2	#N/A	6.7264	5.9328	6.1032	6.3042	6.5415	6.2089	6.0361	5.4469	5.8960	6.4533	1.0579	0.0812	0.1575	0.8026
Prkcsh	_YEQGTGC[Carbamidomethyl (C)]WQGPNR_.2	#N/A	5.3838	5.9918	3.3802	6.5930	6.0636	6.8399	6.6600	4.2356	6.1794	5.5312	0.9806	-0.0282	0.8812	0.0549
Eef2k	_RHES[Phospho (STY)]DEDS[Phospho (STY)]LGSSGR_.2	O08796_S469_M2_Eef2k	3.6984	3.5278	4.3852	3.3870	2.6479	3.2085	3.1017	3.2307	4.3801	1.7386	1.1166	0.1591	0.5033	0.2982
Aip	_VES[Phospho (STY)]PGTYQQDPWAMTDEEK_.2	O08915_S159_M1_Aip	3.4540	3.7721	4.1408	5.0078	3.6604	2.9642	4.3582	3.4750	3.1236	3.1096	1.0900	0.1244	0.4756	0.3227
Flot1	_VTGEVLDILSR_.2	#N/A	5.0585	5.6465	5.5429	5.6871	5.1358	5.4547	5.3699	5.5046	5.2611	5.2900	1.0120	0.0173	0.6613	0.1796
Flot1	_DIHDDQDYLHSLGK_.3	#N/A	5.9827	4.2240	3.6941	5.4973	4.8922	5.3769	3.9281	4.3923	4.9445	5.6877	1.0436	0.0615	0.7067	0.1508
Flot1	_VASSDLVNM[Oxidation (M)]GISVVSYTLK_.2	#N/A	3.9157	5.7275	6.2212	3.1932	3.0595	6.3303	5.8341	6.0794	5.6119	7.2282	0.7661	-0.3843	0.1184	0.9266
Flot1	_S[Phospho (STY)]PPVMVAGGR_.2	O08917_S19_M1_Flot1	6.0081	4.2774	3.2549	6.5692	4.1657	3.9597	3.8529	4.9365	3.0008	3.9767	1.1939	0.2556	0.3249	0.4883
Flot1	_ITLVSSGSGTM[Oxidation (M)]GAAK_.2	#N/A	3.9390	3.2434	3.1250	3.3765	5.9691	3.1639	3.5247	3.6247	3.9238	3.0537	1.0768	0.1067	0.6528	0.1852
Numbl	_RTPS[Phospho (STY)]EAER_.2	#N/A	7.6336	7.9992	7.9536	4.2226	6.2373	4.0365	7.7368	7.7001	3.0776	3.8999	1.1327	0.1798	0.5972	0.2239
Numbl	_AEAAAAPAVAPGPAQPGHVS[Phospho (STY)]PTPATTS[Phospho (STY)]PGEKGEAGTPVAAGTTAAAIPR_.4	#N/A	4.9717	3.2540	3.7958	7.9931	2.9816	3.5470	3.5030	8.6710	3.9916	3.8170	0.8855	-0.1754	0.6864	0.1634
Numbl	_SVLWVSADGLR_.2	#N/A	5.7926	5.9541	4.4348	5.5661	5.5895	5.2144	5.0916	5.7178	4.3305	6.0428	1.0245	0.0349	0.7590	0.1198
Numbl	_KAEAAAAPAVAPGPAQPGHVS[Phospho (STY)]PT[Phospho (STY)]PATTSPGEK_.4	#N/A	4.1875	3.2425	3.6709	3.6869	3.3222	6.3237	3.6020	3.9291	3.8632	3.5378	1.0909	0.1255	0.5830	0.2343
Numbl	_KAEAAAAPAVAPGPAQPGHVS[Phospho (STY)]PT[Phospho (STY)]PATTSPGEKGEAGTPVAAGTTAAAIPR_.	#N/A	3.1629	4.7352	3.8497	4.3524	3.7894	3.9401	4.0671	3.7661	3.4147	4.3194	1.0205	0.0293	0.8055	0.0939
Numbl	_TPS[Phospho (STY)]EAER_.2	#N/A	5.4254	3.8308	4.0822	4.5848	3.7191	4.4064	4.2995	4.6068	3.1822	4.8611	1.0245	0.0350	0.8161	0.0883
Numbl	_EGS[Phospho (STY)]FRLS[Phospho (STY)]GGGRPAER_.3	O08919_S224_M2_Numbl	3.8953	3.1997	4.5821	3.4201	3.0210	3.2076	3.5684	3.5810	3.8801	3.0974	1.0064	0.0092	0.9464	0.0239
Sdcbp	_[Acetyl (Protein N-term)]SLYPS[Phospho (STY)]LEDLKVDK_.2	O08992_S6_M1_Sdcbp	4.1806	3.0456	3.3666	3.8692	2.9339	3.6907	7.2430	7.9731	3.8978	3.8362	0.6125	-0.7071	0.0382	1.4181
Atox1	_AVSYLGPK_.2	#N/A	4.8830	5.0150	6.1068	4.1895	6.3390	5.1747	3.9400	4.7647	5.4291	4.8044	1.1162	0.1586	0.2812	0.5509
Atox1	_VC[Carbamidomethyl (C)]IDSEHSSDTLLATLNK_.3	#N/A	3.6116	3.8041	3.8755	3.6487	3.6442	3.6868	4.1136	3.8158	3.4644	3.6157	0.9892	-0.0157	0.7501	0.1249
Sh2b3	_S[Phospho (STY)]AGELPGATSDTNDIDTTAASRPGPAR_.3	O09039_S129_M1_Sh2b3	3.9564	3.3860	3.6901	7.3997	3.2074	3.0213	3.3821	3.7673	4.0664	7.1126	0.8970	-0.1568	0.6731	0.1719
Snap23	_ATWGDGGDNS[Phospho (STY)]PSNVVSK_.2	O09044_S110_M1_Snap23	9.4348	9.8206	10.3525	9.8870	8.9503	9.6361	3.1084	4.0745	9.8436	9.6480	1.4516	0.5376	0.0689	1.1618
Psmb1	_NM[Oxidation (M)]QNVEHVPLTLDR_.3	#N/A	6.9459	6.9331	6.6985	6.0053	7.1040	7.2335	6.4832	6.8304	7.1967	6.8123	0.9984	-0.0022	0.9674	0.0144
Psmb1	_GAVYSFDPVGSYQR_.2	#N/A	6.2956	6.1439	5.9547	5.8743	6.0449	5.8874	6.6232	6.3508	6.3498	6.3288	0.9408	-0.0880	0.0053	2.2722
Psmb1	_AGGSASAM[Oxidation (M)]LQPLLDNQVGFK_.2	#N/A	4.4640	4.7294	4.4843	4.0281	4.0478	4.5531	4.1763	4.7774	3.2722	4.4296	1.0530	0.0745	0.4729	0.3252
Psmb1	_DVYTGDALR_.2	#N/A	5.9812	6.5530	4.2030	5.8397	7.3649	6.9357	3.8950	6.7884	6.7369	6.0041	1.0495	0.0698	0.7196	0.1429
Psmb1	_DVFISAAER_.2	#N/A	2.9243	3.6806	4.2324	4.8493	4.8269	5.1063	4.4497	5.7952	5.7744	5.3524	0.7992	-0.3234	0.0618	1.2090
Psmb1	_LSEGFSIHTR_.2	#N/A	4.0753	3.3307	4.5822	3.5840	3.2190	3.4056	3.2987	3.0336	4.1830	3.5510	1.0520	0.0731	0.5965	0.2244
Hdac1	_IAC[Carbamidomethyl (C)]EEEFS[Phospho (STY)]DS[Phospho (STY)]DEEGEGGRK_.2	#N/A	5.1168	5.4948	4.4916	6.1738	5.3965	6.0706	6.0983	6.1145	6.0272	5.7968	0.9082	-0.1390	0.1263	0.8986
Hdac1	_MLPHAPGVQM[Oxidation (M)]QAIPEDAIPEES[Phospho (STY)]GDEDEEDPDKR_.4	#N/A	3.7706	3.4441	4.4574	8.0110	4.5426	3.4056	3.7647	3.1816	3.9050	3.4262	1.2902	0.3676	0.2812	0.5509
Ndufb11	_AVIAPSGVEK_.2	#N/A	7.9923	8.0243	8.1932	7.9818	8.2339	7.8995	7.7827	8.3128	8.1913	8.0627	0.9959	-0.0059	0.7745	0.1110
Ndufb11	_QREPTMQWQEDPEPEDENVYAK_.3	#N/A	2.7481	3.5560	3.4376	3.9402	3.3773	4.3522	3.2122	4.1783	3.8268	3.9072	0.9438	-0.0835	0.5265	0.2786
Ptgds	_AQGLTEEDIVFLPQPDK_.2	#N/A	6.9781	6.5269	6.2369	6.8425	6.8070	6.9624	6.4290	6.4204	6.5783	6.2225	1.0488	0.0688	0.0837	1.0775
Sema4d	_SDFS[Phospho (STY)]DLEQSVK_.2	O09126_S782_M1_Sema4d	6.7580	8.7585	3.4851	9.7000	8.4197	8.6706	9.2671	9.7402	9.3367	9.1857	0.8134	-0.2979	0.1664	0.7787
Rpl21	_VYNVTQHAVGIIVNK_.3	#N/A	3.7404	3.5691	4.7880	4.3348	3.4036	4.0687	3.6283	3.5451	3.3892	3.4810	1.1348	0.1824	0.1151	0.9390
Gclm	_TLNEWSSQISPDLVR_.2	#N/A	4.2580	4.0321	4.4601	7.5267	3.8620	4.4442	3.8411	6.7067	4.1568	4.2207	1.0069	0.0099	0.9713	0.0127
Kif1c	_LYADS[Phospho (STY)]DS[Phospho (STY)]GEDSDKR_.2	O35071_S674_M2_Kif1c	5.8731	5.4733	3.3186	6.0710	5.3016	5.3034	5.8066	5.6315	5.7930	5.4978	0.9193	-0.1214	0.3907	0.4081
Kif1c	_RNS[Phospho (STY)]LDGGSR_.2	O35071_S1031_M1_Kif1c	4.3381	3.5935	4.0718	3.3212	3.4818	3.1428	4.5368	3.2964	2.9450	4.8353	0.9372	-0.0936	0.5871	0.2313
Dpysl4	_VVLEDGNLLVTPGAGR_.2	#N/A	8.0735	8.0023	8.4183	8.5549	8.1473	8.4079	8.0199	8.1185	8.0409	8.0559	1.0259	0.0369	0.1067	0.9719
Dpysl4	_GVPTVVISQGR_.2	#N/A	7.2188	7.1227	7.7806	7.7135	7.4816	7.2742	7.1660	7.0620	7.2241	7.2284	1.0365	0.0517	0.1025	0.9891
Dpysl4	_QIGENLIVPGGIK_.2	#N/A	6.3578	6.7142	6.1435	6.5043	6.8590	6.2148	6.3332	6.7994	6.6184	6.6080	0.9812	-0.0274	0.4669	0.3307
Dpysl4	_M[Oxidation (M)]DENEFVAVTSTNAAK_.2	#N/A	9.8284	10.1281	3.8995	10.6388	10.2939	10.2092	10.1874	10.0281	7.9571	10.3463	0.9519	-0.0712	0.7484	0.1259
Dpysl4	_QANC[Carbamidomethyl (C)]PLYVTK_.2	#N/A	4.9901	5.1133	4.0421	3.9238	5.3385	4.5750	4.9970	4.4419	4.3600	4.8211	1.0019	0.0027	0.9788	0.0093
Dpysl4	_VAVGSDADLVIWNPR_.2	#N/A	6.4168	6.2864	4.0998	3.6436	6.7203	6.2513	4.4562	6.0767	5.8964	6.5273	0.8927	-0.1637	0.3609	0.4426
Dpysl4	_DLGAVAQVHAENGDIVEEEQKR_.3	#N/A	6.1365	5.8011	7.1801	4.1386	5.5645	5.5902	6.9241	3.9799	6.1210	6.1105	0.9916	-0.0122	0.9468	0.0237
Dpysl4	_TIDAHGLM[Oxidation (M)]VLPGGVDVHTR_.3	#N/A	5.2223	5.3807	6.3997	5.5657	6.0809	6.0442	6.0894	5.7850	6.0933	6.0160	0.9644	-0.0524	0.4029	0.3948
Dpysl4	_NLHQSGFSLSGS[Phospho (STY)]QADDHIAR_.3	O35098_S544_M1_Dpysl4	3.7336	5.5520	5.9607	6.7390	4.4381	4.6866	4.0658	5.8961	3.4692	4.7678	1.1396	0.1886	0.3887	0.4104
Dpysl4	_GAADMVAQAK_.2	#N/A	5.1665	3.6544	3.5360	4.9365	4.4650	4.2537	4.4942	4.0798	4.3348	4.0057	1.0252	0.0360	0.7672	0.1151
Dpysl4	_[Acetyl (Protein N-term)]S[Phospho (STY)]FQGKKS[Phospho (STY)]IPR_.2	O35098_S2_M2_Dpysl4	3.8587	2.7532	4.4696	3.5474	2.7275	2.8188	3.2620	3.0703	4.2197	2.5301	1.0281	0.0400	0.8465	0.0724
Dpysl4	_C[Carbamidomethyl (C)]QC[Carbamidomethyl (C)]TDGQIYEIFSLIR_.2	#N/A	2.2735	3.0609	3.3512	3.6935	2.9492	3.6754	3.5686	4.2646	3.9132	2.7911	0.8715	-0.1984	0.2414	0.6172
Dpysl4	_SHNLNVEYNIFEGVEC[Carbamidomethyl (C)]R_.3	#N/A	3.3453	4.1016	4.5799	3.3639	4.4504	4.1355	4.2719	4.2763	3.1766	3.8009	1.0295	0.0420	0.7442	0.1283
Map3k5	_TLFLGIPDENFEDHS[Phospho (STY)]APPSPEEK_.3	O35099_S1036_M1_Map3k5	3.8289	4.8339	3.6979	5.0455	3.4466	4.0221	4.3141	5.0614	4.6907	4.1676	0.9095	-0.1369	0.2922	0.5343
Atn1	_TAS[Phospho (STY)]PPGPPQYSK_.2	O35126_S617_M1_Atn1	9.2144	10.0105	4.0164	3.5601	2.8811	3.3476	3.7084	9.8190	9.9116	10.1643	0.6553	-0.6098	0.1963	0.7071
Atn1	_RAPS[Phospho (STY)]PGSYK_.2	O35126_S630_M1_Atn1	3.7453	3.0008	3.4790	4.5236	5.6604	6.5395	3.6287	3.9784	4.2774	4.2009	1.1542	0.2069	0.4625	0.3349
Atn1	_SEEIS[Phospho (STY)]ESESEETSAPK_.2	O35126_S77_M1_Atn1	4.9848	5.9505	5.5145	6.1104	5.1262	3.9933	5.6933	5.5707	5.5857	5.2786	0.9544	-0.0673	0.5449	0.2636
Atn1	_GRAS[Phospho (STY)]PGGVSTSSSDGKAEK_.3	#N/A	3.0754	4.2670	4.2380	3.8537	4.8378	3.6411	3.9340	4.3400	4.5924	3.5310	0.9722	-0.0406	0.7598	0.1193
Atn1	_TEQELPRPQS[Phospho (STY)]PSDLDSLDGR_.3	O35126_S101_M1_Atn1	2.9778	3.7830	3.6646	7.3552	5.8389	4.1252	4.4860	6.9763	6.1042	6.4869	0.7690	-0.3790	0.1782	0.7490
Atn1	_TGT[Phospho (STY)]PPGYR_.2	O35126_T654_M1_Atn1	5.7619	2.9809	3.4592	3.9339	2.8692	3.7554	3.6486	3.9983	3.8332	6.1838	0.8590	-0.2192	0.4078	0.3896
Atn1	_GRAS[Phospho (STY)]PGGVSTSSSDGK_.2	#N/A	6.0970	3.8733	6.3157	6.3352	5.3985	5.9322	6.2165	5.8405	5.6611	5.5727	0.9718	-0.0412	0.7476	0.1264
Atn1	_PHVMS[Phospho (STY)]PGNR_.2	O35126_S881_M1_Atn1	5.9027	3.0604	3.5387	6.6339	2.9487	3.6759	3.5691	4.2641	3.9127	3.8214	1.1032	0.1417	0.6334	0.1983
Atn1	_QEPAEEYEPPES[Phospho (STY)]PVPPARS[Phospho (STY)]PS[Phospho (STY)]PPPK_.3	O35126_S724_M3_Atn1	3.0074	3.7637	4.2419	3.1511	3.6520	2.9727	4.3667	3.2155	3.5146	3.4630	0.9519	-0.0711	0.6051	0.2182
Phb2	_VLPSIVNEVLK_.2	#N/A	7.5513	7.9764	7.9200	7.8542	7.2824	8.0303	7.7958	8.1686	7.8480	7.6776	0.9869	-0.0191	0.5609	0.2511
Phb2	_ESVFTVEGGHR_.2	#N/A	7.6154	7.1448	7.0131	7.6295	7.1562	7.4084	7.1559	7.4676	7.1740	7.4683	1.0016	0.0023	0.9412	0.0263
Phb2	_FNASQLITQR_.2	#N/A	7.2106	7.1740	7.5672	7.4475	7.3649	7.2221	7.6731	7.3637	7.4534	6.9007	0.9977	-0.0033	0.9149	0.0386
Phb2	_LGLDYEER_.2	#N/A	6.8460	6.7021	6.1542	6.6248	6.1070	6.3650	6.7865	6.6981	6.3501	6.5147	0.9817	-0.0267	0.5048	0.2969
Phb2	_LLLGAGAVAYGVR_.2	#N/A	5.6253	5.5720	5.6345	4.9991	5.0626	4.8585	4.6014	5.5987	5.2033	4.9849	1.0382	0.0541	0.4496	0.3472
Phb2	_IGGVQQDTILAEGLHFR_.3	#N/A	5.8151	5.9170	6.2224	5.5119	5.1746	5.6183	5.4992	5.4784	5.2389	5.2213	1.0654	0.0914	0.1076	0.9682
Phb2	_DLQM[Oxidation (M)]VNISLR_.2	#N/A	5.1704	5.9400	6.5701	3.9962	6.6802	7.4746	7.5631	7.1974	6.7948	7.3072	0.8827	-0.1800	0.2834	0.5475
Phb2	_QVAQQEAQR_.2	#N/A	4.6540	5.0518	4.3690	4.3778	4.8027	4.8513	5.0852	5.3759	3.8085	3.7587	1.0394	0.0557	0.6368	0.1960
Phb2	_AQVSLLIR_.2	#N/A	4.6260	4.2076	4.9991	4.2080	4.0959	4.0295	3.9227	4.3296	4.6460	4.6367	0.9948	-0.0075	0.9252	0.0338
Phb2	_IYLTADNLVLNLQDESFTR_.2	#N/A	3.8720	4.0306	4.4749	5.1464	3.7956	4.2850	4.5332	3.9614	4.8400	4.2408	0.9712	-0.0421	0.6803	0.1673
Phb2	_IPWFQYPIIYDIR_.2	#N/A	3.0477	4.0148	4.2447	3.3393	3.9784	3.5760	3.9368	3.2127	3.5117	3.4072	1.0521	0.0732	0.5057	0.2961
Phb2	_AQFLVEK_.2	#N/A	3.2111	3.7140	2.5448	3.7360	3.6023	3.0223	4.4163	4.2625	3.5642	3.6750	0.8305	-0.2679	0.0501	1.3002
Ncam2	_ASGSPDPTISWFR_.2	#N/A	5.3938	5.7172	5.8969	4.5533	5.1642	5.7783	6.4349	6.0379	5.5903	5.2123	0.9310	-0.1032	0.2603	0.5846
Ncam2	_AAYLKDGS[Phospho (STY)]KEPIVEM[Oxidation (M)]R_.3	#N/A	9.2189	9.1991	9.9318	9.2858	8.4110	9.4237	9.8856	9.2783	9.6394	9.3954	0.9681	-0.0468	0.2954	0.5295
Ncam2	_IEIFQTLPVR_.2	#N/A	5.1599	4.1387	3.6580	4.2865	4.5559	3.5566	5.4445	5.1251	4.3221	4.6750	0.8639	-0.2111	0.1019	0.9916
Ncam2	_ITNHEDGS[Phospho (STY)]PVNEPNETTPLTEPEKLPLKEENGK_.4	#N/A	3.6674	3.5588	4.3542	3.3560	2.7931	3.4356	7.3229	3.2616	7.0684	3.3230	0.6727	-0.5720	0.1017	0.9928

Ncam2	_DGS[Phospho (STY)]KEPIVEMR_.2	#N/A	10.0865	7.9705	3.4038	10.7071	7.0840	3.8107	8.9918	7.4786	7.5473	8.2686	0.8892	-0.1695	0.5895	0.2295
Ncam2	_LTIYNANIEDAGIYR_.2	#N/A	6.9469	3.3666	3.8449	3.5482	4.1429	3.3697	3.2628	3.0695	5.0973	3.5152	1.1250	0.1699	0.5719	0.2427
Ncam2	_ITNHEDGS[Phospho (STY)]PVNEPNETTPLTEPEK_.3	#N/A	3.8246	3.1038	3.8188	3.6687	2.9597	3.4747	3.5986	3.6650	3.8744	3.8813	0.9255	-0.1117	0.1931	0.7143
Ncam2	_GSNTELTVR_.2	#N/A	4.0442	3.2996	3.7778	3.6152	3.1879	5.8190	3.3299	3.6796	4.1519	5.7248	0.9374	-0.0933	0.6930	0.1593
Atp5if1	_TKEQLAALR_.2	#N/A	9.2344	9.9851	9.7140	9.9717	10.6843	9.7327	9.3990	9.7897	9.7649	9.6753	1.0238	0.0339	0.3901	0.4088
Atp5if1	_EAGGAFGKR_.2	#N/A	7.5896	7.9969	8.2152	7.5026	8.6542	8.1711	7.7257	7.9981	8.1047	7.9399	1.0100	0.0144	0.7357	0.1333
Atp5if1	_EQLAALR_.2	#N/A	5.7284	6.1988	6.6156	5.9574	7.2945	6.2076	4.0522	6.7173	6.3296	6.0812	1.0930	0.1282	0.3558	0.4488
Atp5if1	_EKAEEDRYFR_.3	#N/A	6.0485	5.9013	6.4459	6.2314	7.1063	6.8617	5.6682	5.7863	6.1914	6.2921	1.0749	0.1042	0.1339	0.8733
Atp5if1	_AEEDRYFR_.2	#N/A	4.7843	3.0411	3.9065	3.8737	4.8312	4.7768	3.6594	4.2448	3.8934	3.8407	1.0749	0.1042	0.4645	0.3330
Atp5if1	_KHHEDEIDHHSKEIER_.4	#N/A	9.8729	7.7960	10.1685	8.8302	12.1845	11.4137	11.1308	7.9960	11.2616	10.8707	0.9738	-0.0383	0.7991	0.0974
Atp5if1	_KHHEDEIDHHSK_.3	#N/A	10.9417	5.5365	8.1126	7.0337	3.4775	8.0768	11.2997	5.5117	7.7583	9.8524	0.8363	-0.2580	0.4140	0.3830
Atp5if1	_HHEDEIDHHSK_.3	#N/A	3.0321	4.1941	3.7189	4.2215	3.6586	4.0709	3.9362	3.8969	3.5455	4.1885	0.9805	-0.0284	0.7723	0.1122
Terf2	_NS[Phospho (STY)]PMTISR_.2	O35144_S399_M1_Terf2	7.2673	3.3288	4.5842	8.4913	3.0230	3.2056	3.3007	3.0317	4.1811	6.0762	1.2016	0.2649	0.5422	0.2659
Terf2	_DLVLANLASPSS[Phospho (STY)]PAHK_.2	#N/A	5.2561	5.8444	6.0973	6.3275	3.6672	5.3915	4.2039	5.5780	5.6201	5.3657	1.0460	0.0649	0.6780	0.1687
Cx3cl1	_SLGS[Phospho (STY)]EEINPVHTDNFQER_.3	#N/A	8.1452	7.2630	7.3982	8.0322	7.9087	8.3549	6.4610	7.2471	8.4479	8.6738	1.0185	0.0265	0.7670	0.1152
Ddt	_IPAGLENR_.2	#N/A	7.9711	8.5941	8.8348	8.5912	9.3593	8.6289	8.3191	8.5147	8.5884	8.6425	1.0173	0.0247	0.5497	0.2599
Ddt	_FFPLEAWQIGK_.2	#N/A	7.0161	7.8462	7.2359	7.2608	7.3789	7.3571	7.2099	7.1696	7.0937	7.3990	1.0182	0.0260	0.4066	0.3908
Ddt	_PFVELETNLPASR_.2	#N/A	6.0801	6.3148	6.5791	6.3097	7.1699	6.8743	6.8784	6.6623	6.7181	6.8569	0.9669	-0.0485	0.3195	0.4955
Ddt	_STEPC[Carbamidomethyl (C)]AHLLVSSIGVVGTAEQNR_.3	#N/A	6.2140	6.2512	6.9167	6.4562	5.7599	6.6769	6.0972	5.4702	5.9592	6.3229	1.0699	0.0975	0.1342	0.8722
Ddt	_THSASFFK_.2	#N/A	4.7797	5.7646	5.9421	5.6048	6.7021	5.7761	5.7892	5.1984	6.0556	6.1210	0.9949	-0.0074	0.9361	0.0287
Ddt	_LC[Carbamidomethyl (C)]AATATILDKPEDR_.3	#N/A	4.6778	3.5928	5.5320	4.3442	5.1378	4.9526	4.3090	4.7843	4.3289	4.1750	1.0698	0.0973	0.4208	0.3759
Ddt	_FLTEELSLDQDR_.2	#N/A	6.0901	6.2165	6.3837	6.7123	6.8672	6.2750	6.2245	6.3541	6.0576	6.5621	1.0198	0.0282	0.4989	0.3020
Kcnh2	_SGLLNSTS[Phospho (STY)]DSDLVR_.2	O35219_S322_M1_Kcnh2	3.0823	4.0504	3.7691	4.2236	3.9085	4.0284	5.3200	3.8473	3.3260	5.0896	0.8744	-0.1936	0.2377	0.6240
Kcnh2	_DTNM[Oxidation (M)]IPGS[Phospho (STY)]PGS[Phospho (STY)]AELESGFNR_.2	#N/A	3.8467	3.1874	4.1618	6.1922	2.9724	3.3396	3.5523	3.0897	4.1651	3.5023	1.1042	0.1430	0.5695	0.2445
Kcnh2	_RAS[Phospho (STY)]S[Phospho (STY)]ADDIEAMR_.2	O35219_S285_M2_Kcnh2	3.6121	4.3683	3.3457	4.3904	4.2566	3.8688	4.5386	4.1117	4.4107	4.0676	0.9280	-0.1079	0.2340	0.6307
Psmd4	_VNVDIINFGEEEVNTEK_.2	#N/A	4.4568	4.5849	5.1179	4.3802	4.3034	4.1853	4.8062	3.7006	4.6963	3.8813	1.0547	0.0768	0.4256	0.3710
Psmd4	_EKVNVDIINFGEEEVNTEK_.3	#N/A	4.7687	5.5111	5.9711	5.5914	4.1794	4.7392	5.2906	4.7023	4.9871	4.7768	1.0380	0.0538	0.6144	0.2115
Psmd4	_AAAASAAEAGIAT[Phospho (STY)]PGTEDSDDALLK_.2	#N/A	3.3688	5.2124	3.5414	7.1991	6.0459	4.1891	6.0828	4.0011	5.8000	5.7703	0.9100	-0.1361	0.5852	0.2327
Psmd4	_RAAAASAAEAGIAT[Phospho (STY)]PGTEDSDDALLK_.3	#N/A	4.3247	4.4024	3.5106	5.8026	3.4503	4.2791	3.7280	5.4815	3.7538	3.9803	1.0139	0.0200	0.9165	0.0379
Pkd2	_SLDDS[Phospho (STY)]EEEDDEDSGHSSR_.2	O35245_S810_M1_Pkd2	2.0045	3.1767	3.7368	4.0765	1.9393	3.0353	3.9542	3.4374	3.0705	2.5157	0.9231	-0.1155	0.6363	0.1963
Exoc7	_QYS[Phospho (STY)]QHGLDGK_.2	O35250_S283_M1_Exoc7	6.0186	3.8320	4.3102	6.3550	4.8456	6.0176	4.2983	5.7558	3.4462	5.6958	1.0898	0.1240	0.5522	0.2579
Exoc7	_AVEYFQDNSPDS[Phospho (STY)]PELNK_.2	O35250_S133_M1_Exoc7	4.1030	3.4074	3.2890	5.5158	4.6866	5.5599	3.3607	5.4757	5.6655	5.1581	0.9007	-0.1509	0.4799	0.3189
Purb	_GGGSGGGDES[Phospho (STY)]EGEEVDED_.2	#N/A	11.5133	11.6700	11.8260	12.0748	11.1817	11.7308	11.9084	11.6647	11.4053	11.8114	0.9973	-0.0039	0.8634	0.0638
Purb	_VSEVKPSYR_.2	#N/A	6.9796	7.1110	6.5593	7.2356	7.7799	7.3482	7.0718	7.0155	6.9415	6.9007	1.0267	0.0380	0.3980	0.4001
Purb	_RGGGSGGGDES[Phospho (STY)]EGEEVDED_.2	#N/A	11.8313	12.0060	12.3355	12.3129	11.5784	12.1749	12.1760	11.8057	11.9764	11.9924	1.0044	0.0063	0.7560	0.1215
Purb	_GGGGGGGGPGGFQPAPR_.2	#N/A	6.2681	6.6463	6.9339	6.5735	6.9250	6.6104	6.1290	6.2654	6.5621	6.5558	1.0441	0.0623	0.1030	0.9871
Purb	_DSLGDFIEHYAQLGPSS[Phospho (STY)]PEQLAAGAEEGGPRR_.4	#N/A	3.9721	3.3703	3.7058	3.2496	5.6717	3.0370	3.3978	3.7516	4.0507	4.4277	0.9814	-0.0270	0.8924	0.0494
Purb	_[Acetyl (Protein N-term)]ADGDSGS[Phospho (STY)]ERGGGGGGGGGPGGFQPAPR_.3	O35295_S8_M1_Purb	3.8777	8.6402	4.1221	9.1286	4.8603	4.8187	9.2403	8.1670	8.5493	8.4818	0.6862	-0.5433	0.0558	1.2531
Purb	_DSLGDFIEHYAQLGPSS[Phospho (STY)]PEQLAAGAEEGGPR_.4	#N/A	4.2515	3.5558	7.5078	7.9369	3.3772	4.3523	6.8747	3.9372	7.4272	7.2886	0.8091	-0.3056	0.3473	0.4593
Purb	_GGGGGGGGPGGEQETQELASK_.2	#N/A	3.8184	7.3542	4.5052	3.5070	3.7973	3.2846	3.2217	3.8642	4.2600	4.3661	1.1145	0.1564	0.5893	0.2297
Purb	_[Acetyl (Protein N-term)]ADGDS[Phospho (STY)]GSER_.2	#N/A	4.1015	3.3569	4.5560	3.5579	3.2452	3.3794	3.2725	3.0598	4.2092	3.5248	1.0520	0.0731	0.5945	0.2259
Srsf5	_S[Phospho (STY)]GS[Phospho (STY)]RS[Phospho (STY)]PVPEK_.2	O35326_S226_M3_Srsf5	9.7665	8.9892	9.0831	9.4003	7.7254	8.8663	9.9099	9.5247	9.3788	9.0874	0.9469	-0.0788	0.2196	0.6583
Srsf5	_SKS[Phospho (STY)]PASVDR_.2	O35326_S247_M1_Srsf5	4.2676	3.5230	4.0013	6.5889	3.6088	3.2133	3.1064	6.3209	3.7552	5.5908	0.8950	-0.1601	0.5847	0.2331
Srsf5	_VS[Phospho (STY)]WQDLK_.2	O35326_S120_M1_Srsf5	4.2858	2.9404	3.4718	3.9744	3.4400	3.7959	3.2910	3.5644	0.9523	3.9414	1.2431	0.3140	0.2572	0.5898
Kpna4	_NVPQEDIC[Carbamidomethyl (C)]EDS[Phospho (STY)]DIDGDYR_.2	O35343_S60_M1_Kpna4	3.9481	3.2525	3.1341	3.3674	3.0738	3.1549	3.5157	3.6338	3.9328	3.6037	0.9047	-0.1444	0.0880	1.0555
Kpna4	_EAAWAISNLTISGR_.2	#N/A	4.2195	3.1229	3.9531	3.4969	2.9443	3.2844	3.6452	3.5043	3.8033	3.1742	0.9920	-0.0116	0.9203	0.0361
Kpna4	_VQNTSLEAIVQNASSDNQGIQLSAVQAAR_.3	#N/A	3.1625	3.9677	3.8493	4.1530	3.7891	3.9404	4.3012	4.3491	3.1473	3.8302	0.9753	-0.0361	0.7397	0.1309
Kpna3	_NVPQEESLEDS[Phospho (STY)]DVDADFK_.2	O35344_S60_M1_Kpna3	3.7704	3.5534	4.0965	10.7008	5.7626	3.5610	8.6708	12.0921	3.9037	3.7100	0.7387	-0.4368	0.4127	0.3844
Kpna6	_[Acetyl (Protein N-term)]M[Oxidation (M)]ETM[Oxidation (M)]AS[Phospho (STY)]PGKDNYR_.2	#N/A	5.5181	5.3500	5.6202	5.4991	3.1235	5.2118	5.3569	5.2425	5.3171	5.0748	0.9630	-0.0544	0.7028	0.1532
Abcc1	_GSS[Phospho (STY)]QLDVNEEVEALIVK_.2	O35379_S290_M1_Abcc1	5.7213	5.8287	6.4985	5.7359	3.7581	5.6574	4.0357	6.4758	3.4460	4.2880	1.2131	0.2787	0.2049	0.6885
Anp32a	_KLELSENR_.2	#N/A	7.9259	7.9578	7.9469	8.3140	7.9692	7.9493	7.8609	7.6446	8.0130	7.8745	1.0207	0.0295	0.1332	0.8756
Anp32a	_ELVLDNC[Carbamidomethyl (C)]K_.2	#N/A	6.3409	6.2383	6.5129	5.9285	6.5201	5.7927	6.2778	6.4179	5.9051	6.1214	1.0067	0.0097	0.8201	0.0861
Anp32a	_NRT[Phospho (STY)]PSDVK_.2	O35381_T15_M1_Anp32a	3.3928	4.1491	3.1265	4.2666	4.0374	4.0881	7.4088	7.3163	3.1292	7.0492	0.6173	-0.6959	0.0233	1.6317
Anp32a	_SLDLFNC[Carbamidomethyl (C)]EVTNLNAYR_.2	#N/A	4.6578	5.3345	6.3435	6.0293	5.2868	5.7527	5.8960	5.1446	4.8483	4.7653	1.0782	0.1087	0.3048	0.5160
Anp32a	_ISGDLEVLAEK_.2	#N/A	5.2802	4.2339	5.5111	5.8188	5.7159	4.1152	5.2612	5.1643	5.6313	4.8774	0.9769	-0.0338	0.7712	0.1128
Anp32a	_HLNLSGNK_.2	#N/A	4.8361	4.9547	4.6687	5.4209	5.8479	5.6033	4.3439	5.7479	5.1736	5.4331	1.0091	0.0131	0.8920	0.0496
Anp32a	_LLPQVM[Oxidation (M)]YLDGYDR_.2	#N/A	2.9892	4.2370	3.6760	4.1787	4.9819	4.1138	6.4745	5.2839	5.2148	4.1457	0.7632	-0.3899	0.0385	1.4148
Anp32a	_KREPDDEGEEDD_.2	#N/A	4.2708	3.5262	4.0044	3.5482	5.8258	3.2101	5.2714	4.7431	3.7520	3.2255	0.9567	-0.0638	0.7689	0.1141
Exoc4	_S[Phospho (STY)]GELQGGPDDNLIEGGGTK_.2	O35382_S469_M1_Exoc4	3.6495	5.7275	6.0658	6.2028	4.5852	5.4513	7.1622	6.6004	6.2218	5.3323	0.8343	-0.2614	0.1117	0.9519
Efnb3	_GGS[Phospho (STY)]LGLGGGGMGPR_.2	O35393_S274_M1_Efnb3	3.8514	3.4910	3.5850	7.3350	6.2366	3.6295	6.5793	3.8724	4.1714	4.3069	0.9906	-0.0136	0.9650	0.0155
Pld3	_LFVVPTDESQAR_.2	#N/A	7.8620	7.6749	7.9516	7.6710	7.3575	7.7568	8.0973	7.5826	7.7139	7.3972	1.0019	0.0027	0.9284	0.0323
Pld3	_ALLNVVDSAR_.2	#N/A	6.6094	6.9561	7.1676	6.6150	6.2000	6.6232	6.7850	6.4380	6.6636	6.7914	1.0039	0.0056	0.8912	0.0500
Pld3	_SQLEAVFLR_.2	#N/A	5.6071	5.7283	3.6765	5.9150	5.4767	4.6316	4.4740	5.8882	5.3600	5.6033	0.9702	-0.0436	0.7587	0.1199
Pdyn	_S[Phospho (STY)]QENPNTYSEDLDV_.2	O35417_S235_M1_Pdyn	4.1816	3.0445	3.3676	6.6490	6.5697	6.5060	5.6463	6.2364	6.4487	6.5893	0.8111	-0.3021	0.2212	0.6552
Clcn6	_SYPS[Phospho (STY)]SELR_.2	O35454_S684_M1_Clcn6	3.2795	3.9467	3.9663	4.4690	3.8350	4.2905	4.1836	3.6495	3.2981	4.4360	1.0187	0.0267	0.8099	0.0916
Clcn6	_ES[Phospho (STY)]RTPEELTILGETQEEEDEILPR_.3	O35454_S23_M1_Clcn6	4.2813	3.5368	4.0150	3.3780	3.4251	3.1995	3.0927	3.4424	4.3890	3.2361	1.0280	0.0399	0.7601	0.1191
Fkbp8	_VLAQQGEYSEAIPILR_.2	#N/A	4.5182	4.9858	3.4023	4.6000	4.9720	4.3875	5.5108	5.2534	4.7680	4.7262	0.8841	-0.1777	0.1154	0.9379
Clk3	_S[Phospho (STY)]RS[Phospho (STY)]HDRIPYQR_.3	O35492_S197_M2_Clk3	3.7128	2.9840	4.3665	3.3683	2.8054	3.4233	3.0830	3.9530	4.3988	3.3353	0.9325	-0.1008	0.5186	0.2851
Clk3	_YRS[Phospho (STY)]PEPDPYLSYR_.3	O35492_S157_M1_Clk3	3.4585	4.2148	3.1922	4.2008	4.1031	4.0223	3.9155	3.9177	3.5662	4.1678	0.9932	-0.0099	0.9152	0.0385
Cdk14	_ADS[Phospho (STY)]YEKLEK_.2	#N/A	10.6111	10.5411	11.0248	10.8788	9.9231	10.5058	10.9024	10.7001	10.6512	10.6082	0.9874	-0.0183	0.5221	0.2823
Cdk14	_SSSAGKES[Phospho (STY)]PKVR_.2	#N/A	4.5904	4.8015	5.8591	4.7935	3.6206	4.8478	4.5020	5.1225	4.5442	4.7938	1.0024	0.0035	0.9766	0.0103
Cdk14	_HSSPSS[Phospho (STY)]PTS[Phospho (STY)]PK_.2	#N/A	5.7570	3.7190	4.8096	4.9396	3.5548	3.8759	4.1163	4.0403	5.0876	3.6696	1.0507	0.0713	0.6809	0.1669
Cdk14	_RVHS[Phospho (STY)]ENNAC[Carbamidomethyl (C)]INFK_.3	#N/A	5.0621	5.4784	5.6141	6.3838	4.6593	5.9601	5.6538	5.9208	6.0922	5.3730	0.9594	-0.0597	0.5094	0.2930
Cdk14	_VHS[Phospho (STY)]ENNAC[Carbamidomethyl (C)]INFK_.2	#N/A	7.8444	7.7714	8.1701	8.3376	6.8913	7.8839	7.9215	7.5495	7.9228	7.8737	0.9999	-0.0001	0.9988	0.0005
Cdk14	_FGKADS[Phospho (STY)]YEKLEK_.2	#N/A	3.7852	3.5572	3.5189	4.5635	3.3785	4.3510	3.2110	3.9385	4.1000	4.2408	0.9965	-0.0050	0.9660	0.0150
Cdk14	_RHS[Phospho (STY)]SPSS[Phospho (STY)]PTSPK_.2	#N/A	3.5682	4.0054	4.4205	3.6319	3.7606	3.5470	4.0398	5.4981	3.6094	3.4809	0.9195	-0.1211	0.4318	0.3647
Cdk14	_TLS[Phospho (STY)]ESFSR_.2	O35495_S24_M1_Cdk14	3.8752	3.1795	4.5620	3.4403	3.0008	3.2278	3.5886	3.7556	3.8599	3.5308	0.9630	-0.0543	0.6643	0.1776
Cdk14	_HSSPSSPT[Phospho (STY)]S[Phospho (STY)]PKFGK_.3	#N/A	4.3611	3.6655	3.4106	4.4552	3.4868	4.2427	3.1027	4.0468	4.3458	4.1325	1.0077	0.0110	0.9282	0.0323
Stx1a	_HSAILAS[Phospho (STY)]PNPDEK_.2	#N/A	10.0631	9.7185	10.0597	9.8340	8.5268	9.3614	10.3974	10.0961	9.7667	9.6966	0.9604	-0.0583	0.2573	0.5896

Stx1a	_QALSEIETR_.2	#N/A	7.7115	6.8873	7.4533	7.0494	6.7884	6.9200	7.6635	7.6137	7.1174	6.8790	0.9749	-0.0366	0.4672	0.3305
Stx1a	_IEYNVEHAVDYVER_.2	#N/A	6.5074	6.1959	6.1321	4.6988	4.3700	4.7793	6.0269	5.9134	5.6125	6.2426	0.9157	-0.1271	0.3318	0.4791
Stx1a	_FM[Oxidation (M)]DEFFEQVEEIR_.2	#N/A	6.8812	6.6361	7.4480	7.2586	6.2379	6.9077	7.3226	7.0582	6.8789	6.8088	0.9826	-0.0253	0.6226	0.2058
Stx1a	_TAKDS[Phospho (STY)]DDDDDVTVTVDR_.2	#N/A	10.6802	10.6371	10.2029	11.1705	9.4718	10.5702	11.1834	10.8519	10.7766	10.6509	0.9622	-0.0555	0.2165	0.6646
Stx1a	_KHSAILAS[Phospho (STY)]PNPDEK_.3	#N/A	8.4941	7.9298	8.3712	8.1266	6.6473	6.9825	8.5662	8.5418	8.3413	7.8784	0.9312	-0.1029	0.1992	0.7006
Stx1a	_TAKDS[Phospho (STY)]DDDDDVTVTVDRDR_.3	#N/A	11.1248	11.4642	11.5387	12.1760	10.6627	11.4252	11.7404	12.0353	11.5240	11.4620	0.9750	-0.0365	0.3187	0.4966
Stx1a	_SIEQSIEQEEGLNR_.2	#N/A	9.8819	9.4883	10.4526	9.5645	8.6771	9.5236	10.4420	9.9363	9.7442	9.6441	0.9654	-0.0508	0.3243	0.4891
Stx1a	_DS[Phospho (STY)]DDDDDVTVTVDRDR_.2	#N/A	3.1956	5.5494	4.4301	5.3807	3.8401	4.2853	4.1221	5.7163	3.3264	5.0010	0.9792	-0.0304	0.8818	0.0546
Stx1a	_TKEELEELMSDIKK_.3	#N/A	6.1922	4.5701	4.4581	6.5691	3.9401	3.7894	4.9738	2.9993	4.2991	4.8171	1.1516	0.2036	0.3799	0.4203
Stx1a	_LKS[Phospho (STY)]IEQSIEQEEGLNR_.3	O35526_S95_M1_Stx1a	4.3844	3.9748	4.5648	4.4203	3.7945	3.6817	3.7793	4.4854	3.7455	4.2573	1.0172	0.0246	0.7749	0.1107
Stx1a	_KFVEVM[Oxidation (M)]SEYNATQSDYR_.3	#N/A	4.4199	3.9938	5.1368	3.8845	3.8151	4.1886	4.7587	4.4698	4.4497	3.7199	0.9748	-0.0369	0.7290	0.1373
Stx1a	_FVEVM[Oxidation (M)]SEYNATQSDYR_.2	#N/A	4.5606	3.3640	3.5535	3.7556	3.2168	3.2361	3.4703	5.1287	4.0177	3.4485	0.8999	-0.1521	0.3495	0.4565
Rabep1	_AQS[Phospho (STY)]TDSLGTSSSLQSK_.2	#N/A	9.0675	9.0160	9.1446	9.6830	7.9090	9.2149	9.0980	8.7827	9.0486	8.8502	1.0068	0.0098	0.8471	0.0721
Rabep1	_RAQS[Phospho (STY)]TDS[Phospho (STY)]LGTSSSLQSK_.2	#N/A	4.4666	4.1285	4.4623	4.8399	4.0138	4.0407	4.1470	4.0372	3.8397	4.0786	1.0744	0.1036	0.1209	0.9176
Rabep1	_QNAVLQAAQDDLGHLR_.3	#N/A	2.9529	3.7581	3.6397	4.3626	3.5794	4.1501	4.5109	4.1394	4.4385	4.1094	0.8700	-0.2010	0.0670	1.1740
Fyb1	_VAGQSS[Phospho (STY)]PSGIQSR_.2	O35601_S28_M1_Fyb1	6.8605	7.1701	7.5683	4.4629	2.8284	7.1808	3.6894	7.1827	4.3382	7.2787	1.0693	0.0967	0.7591	0.1197
Fyb1	_TTAVEIDYDS[Phospho (STY)]LKR_.2	O35601_S561_M1_Fyb1	3.1228	2.6417	3.8096	4.7087	3.5085	3.9802	4.0269	4.9861	3.8843	3.8894	0.8646	-0.2098	0.2147	0.6682
Bmpr2	_LNINSS[Phospho (STY)]PDEHEPLLR_.2	#N/A	3.7494	6.7635	3.9460	6.9542	4.8733	6.1625	3.6779	6.6389	6.2949	3.9302	1.0531	0.0746	0.7803	0.1077
Bmpr2	_LNINSS[Phospho (STY)]PDEHEPLLRR_.3	#N/A	3.8085	3.8737	3.7553	4.2580	5.3200	3.3808	6.4528	4.0002	2.6212	3.5432	0.9787	-0.0310	0.9071	0.0424
Bmpr2	_SVS[Phospho (STY)]PTVNPMSTAMQNER_.2	O35607_S515_M1_Bmpr2	2.9235	3.7287	3.6103	7.7537	3.5501	4.1794	5.4502	4.0055	4.4091	4.0800	1.0076	0.0109	0.9721	0.0123
Bmpr2	_NLKES[Phospho (STY)]SDENLMEHSLK_.3	O35607_S680_M1_Bmpr2	3.5587	3.7837	3.2924	4.3370	4.2033	3.9222	4.4853	4.1650	2.9632	4.0143	0.9853	-0.0214	0.8656	0.0627
Bmpr2	_EVDKNLKES[Phospho (STY)]S[Phospho (STY)]DENLMEHSLK_.3	O35607_S680_M2_Bmpr2	3.9188	3.2231	4.6056	3.6074	3.0445	3.1842	3.5450	3.0103	4.1597	3.5744	1.0070	0.0100	0.9455	0.0243
Scamp3	_KLS[Phospho (STY)]PTEPR_.2	#N/A	9.9693	10.0764	10.7453	9.9186	8.7652	9.5539	10.3180	10.5394	9.9002	9.7875	0.9706	-0.0431	0.4333	0.3632
Scamp3	_LS[Phospho (STY)]PTEPR_.2	#N/A	6.0655	3.1591	5.8700	6.1396	2.9804	6.4006	3.6090	3.5404	3.8394	3.1381	1.4448	0.5308	0.0907	1.0423
Scamp3	_TAAANAAAGAAENAFR_.2	#N/A	3.1464	3.9516	3.1245	4.1691	3.7729	3.9566	4.3174	4.3329	5.5925	3.8464	0.8153	-0.2947	0.0554	1.2562
Zfpm1	_AGS[Phospho (STY)]PVSATEEKPK_.2	O35615_S286_M1_Zfpm1	4.1602	3.4646	3.3462	3.8489	3.2859	2.9427	3.5636	4.2696	5.9193	3.8159	0.7987	-0.3242	0.0989	1.0049
Zfpm1	_GSEGSQS[Phospho (STY)]PGSSVDDAEDDPSR_.2	O35615_S681_M1_Zfpm1	3.6212	4.2640	3.3549	4.0381	4.2658	4.3889	3.7528	4.0803	3.7289	4.0051	1.0249	0.0355	0.6737	0.1716
Zfpm1	_APRS[Phospho (STY)]PS[Phospho (STY)]PAPENTPSDPADQGAR_.3	O35615_S925_M2_Zfpm1	4.7397	5.7777	5.6249	5.9576	3.6841	3.3919	5.6246	5.0015	5.3765	5.5827	0.9011	-0.1502	0.3833	0.4164
Mdm4	_TIS[Phospho (STY)]APVVRPK_.2	O35618_S367_M1_Mdm4	4.0982	6.7848	6.7561	3.2172	5.7831	3.0047	3.3655	6.5492	6.6698	4.3954	0.9420	-0.0862	0.7872	0.1039
Pmm1	_TVGHSVVS[Phospho (STY)]PQDTVQR_.2	#N/A	8.3708	8.4033	8.9870	9.1177	8.0166	8.4302	8.6567	8.5153	8.4034	8.4582	1.0054	0.0078	0.8376	0.0769
Bet1	_SLS[Phospho (STY)]IEIGHEVK_.2	O35623_S50_M1_Bet1	3.2311	5.3321	3.9179	6.3404	3.8576	3.8719	5.6580	6.1777	5.9935	5.2804	0.7659	-0.3847	0.0592	1.2275
Slc32a1	_GGAPLPPSGS[Phospho (STY)]KDQAVGAGGEFGGHDKPK_.3	#N/A	3.8482	7.5251	9.5672	4.0952	5.9600	3.8684	9.5078	3.5055	8.4022	9.1524	0.7604	-0.3952	0.2941	0.5314
Slc32a1	_SLFQEGSR_.2	#N/A	6.4360	6.4038	5.9237	4.9001	5.6843	6.1253	6.0391	6.2331	5.7371	5.8941	0.9894	-0.0154	0.8393	0.0761
Slc32a1	_GGAPLPPSGS[Phospho (STY)]K_.2	#N/A	4.3821	8.5630	3.5681	7.9177	4.2332	3.8923	3.7854	4.0477	3.6963	8.0320	1.1095	0.1500	0.7118	0.1476
Slc32a1	_LTNVATSVSNK_.2	#N/A	4.5515	5.3346	4.5574	4.1011	5.1724	4.1580	4.9413	5.5819	5.5008	4.7941	0.8927	-0.1638	0.1031	0.9866
Slc32a1	_ILIAC[Carbamidomethyl (C)]LYEENEDGEVVR_.2	#N/A	4.1348	4.3158	5.0421	3.9360	2.9645	4.2105	3.9339	4.6830	4.2020	3.7874	0.9877	-0.0178	0.8958	0.0478
Ap1b1	_DC[Carbamidomethyl (C)]EDPNPLIR_.2	#N/A	9.6800	9.2866	9.4771	9.2455	9.1053	9.1026	9.6123	9.4742	9.3386	9.2978	0.9879	-0.0176	0.3974	0.4008
Ap1b1	_DC[Carbamidomethyl (C)]PLNTEAASNK_.2	#N/A	8.7894	8.8091	8.4623	8.7321	7.9137	8.3816	9.0732	8.8064	8.6787	8.7168	0.9655	-0.0506	0.1464	0.8346
Ap1b1	_VDKITEYLC[Carbamidomethyl (C)]EPLRK_.3	#N/A	7.5379	7.0619	7.3837	6.8981	6.7420	7.5708	7.8187	7.3903	7.4786	7.0479	0.9684	-0.0463	0.3113	0.5068
Ap1b1	_YNDPIYVK_.2	#N/A	7.1500	6.7595	6.8307	6.9944	6.8944	7.2484	6.1966	7.2632	7.3953	7.1943	0.9953	-0.0068	0.8939	0.0487
Ap1b1	_KGEIFELK_.2	#N/A	6.5145	6.6835	6.0950	6.3222	5.7272	5.9326	7.0556	6.4754	6.2716	6.4300	0.9473	-0.0781	0.1694	0.7711
Ap1b1	_LASQANIAQVLAELK_.2	#N/A	6.2191	5.8509	5.5099	5.0283	5.4204	5.1961	6.2553	5.8870	5.9930	6.0451	0.9160	-0.1265	0.0593	1.2272
Ap1b1	_VIASMTVGK_.2	#N/A	5.8032	5.8274	4.0666	5.9669	6.1724	6.2056	5.7696	6.3088	6.7609	6.4751	0.8965	-0.1576	0.1773	0.7514
Ap1b1	_NINLIVQK_.2	#N/A	5.6737	4.7446	5.4769	3.3495	3.6417	3.9356	3.0094	5.6219	4.5805	5.0443	0.9795	-0.0299	0.8917	0.0498
Ap1b1	_EYATEVDVDFVR_.2	#N/A	5.3749	3.9092	3.7908	4.8647	4.5505	5.4111	5.3769	5.1673	4.4472	4.9590	0.9324	-0.1011	0.4125	0.3846
Ap1b1	_LSHANSAVVLSAVK_.2	#N/A	4.7651	4.9587	3.6814	5.1813	4.8646	5.2279	4.9784	5.0652	5.1114	4.8778	0.9544	-0.0673	0.4570	0.3401
Ap1b1	_LQSSNIFTVAK_.2	#N/A	3.6310	4.9703	3.3068	4.2451	4.6500	2.9821	3.5242	5.9413	4.2524	4.6053	0.8654	-0.2086	0.3075	0.5121
Ap1b1	_LVYLYLM[Oxidation (M)]NYAK_.2	#N/A	4.5370	3.1806	5.1488	5.0830	3.3055	3.3191	4.7453	5.4976	5.1534	5.1548	0.7972	-0.3270	0.0665	1.1769
Ap1b1	_AAM[Oxidation (M)]IWIVGEYAER_.2	#N/A	6.5312	3.2862	7.2047	3.3082	6.0338	6.7260	6.8671	3.6930	6.3448	6.5920	0.9389	-0.0910	0.7455	0.1275
Ap1b1	_NVEGQDM[Oxidation (M)]LYQSLK_.2	#N/A	4.6289	4.5992	5.2370	5.9326	4.8284	4.7411	4.7669	4.8731	5.1891	4.6265	1.0269	0.0382	0.6519	0.1858
Ap1b1	_APEVSQHVYQAYETILKN_.3	#N/A	3.4828	4.2391	4.3773	3.9304	4.1274	3.0757	4.4094	3.2219	3.6904	3.9384	1.0150	0.0214	0.8633	0.0638
Ap1b1	_ITEYLC[Carbamidomethyl (C)]EPLRK_.2	#N/A	5.8233	5.7246	5.1613	5.6726	5.0944	5.0173	6.1237	5.8799	5.6070	5.5555	0.9351	-0.0968	0.1140	0.9431
Ap1b1	_TAAVC[Carbamidomethyl (C)]VAK_.2	#N/A	3.5356	4.0929	3.9745	4.4772	3.2660	3.7028	4.1918	4.4186	3.2899	4.4442	0.9401	-0.0891	0.4489	0.3478
Ap1b1	_DDREAQSIC[Carbamidomethyl (C)]ER_.3	#N/A	4.0521	3.5256	4.3388	3.7267	3.6537	3.0513	4.0912	3.8787	4.1608	3.5960	0.9474	-0.0780	0.4273	0.3693
Ap1b1	_C[Carbamidomethyl (C)]LKDEDPYVRK_.3	#N/A	3.8598	3.1641	4.5466	3.5484	2.9855	3.2432	3.6040	3.0692	4.2187	0.3450	1.2665	0.3409	0.3390	0.4698
Ap1b1	_KPTETQELVQQVLSLATQDSDNPDLR_.3	#N/A	4.1277	3.4321	3.3137	3.8163	3.2534	2.9752	3.3360	2.5756	3.9507	3.7833	1.0220	0.0314	0.8222	0.0850
Ap1b1	_C[Carbamidomethyl (C)]LKDEDPYVR_.3	#N/A	4.1345	3.4388	3.3204	3.8231	3.2602	2.9685	3.3293	4.2954	3.9440	3.7901	0.9092	-0.1374	0.2270	0.6439
C1qbp	_AFVEFLTDEIKEEK_.2	#N/A	4.7334	3.8034	3.8832	4.9018	3.5233	4.1125	4.7325	4.4354	4.4293	4.2009	0.9348	-0.0972	0.3493	0.4568
Hap1	_ATHS[Phospho (STY)]PSAREEEGPSGAT_.2	O35668_S615_M1_Hap1	4.8727	6.6012	4.3855	6.0381	5.2985	5.9691	5.4596	3.2303	5.3198	3.3544	1.2733	0.3486	0.0999	1.0005
Mgll	_NKS[Phospho (STY)]EVDLYNSDPLVC[Carbamidomethyl (C)]R_.2	#N/A	7.2804	7.0840	7.2563	7.9546	6.4537	7.3928	7.4920	7.3310	7.6988	7.6037	0.9609	-0.0575	0.2824	0.5491
Mgll	_LTLPFLLLQGSADR_.2	#N/A	4.5838	5.3161	4.1887	4.8758	3.8396	4.5558	4.3622	4.3482	4.8307	4.3584	1.0190	0.0272	0.7684	0.1144
Mgll	_IDSSVLSR_.2	#N/A	5.5440	5.1009	3.6806	2.4944	5.0072	4.7596	3.3401	5.4476	4.8960	5.1411	0.9416	-0.0869	0.6997	0.1551
Mgll	_MPEASS[Phospho (STY)]PR_.2	O35678_S6_M1_Mgll	4.2621	3.5176	3.9958	6.4534	3.4059	3.2187	3.1119	3.4616	3.7606	3.2169	1.2227	0.2901	0.2592	0.5863
Mgll	_VC[Carbamidomethyl (C)]FGIQLLNAVAR_.2	#N/A	4.1187	3.3741	4.5388	3.5407	3.2624	3.5160	3.2553	3.0770	3.8097	3.5077	1.0916	0.1265	0.3011	0.5214
Mgll	_ALIFVSHGAGEHC[Carbamidomethyl (C)]GR_.3	#N/A	4.2088	3.0174	3.3947	3.8974	2.9057	3.7189	3.6121	4.2211	3.8697	3.8644	0.9054	-0.1433	0.2230	0.6516
Syt3	_GLSEKENS[Phospho (STY)]E_.2	O35681_S586_M1_Syt3	9.6046	9.4208	10.1768	9.3851	8.7510	9.2833	9.7681	9.8967	9.6412	9.5585	0.9713	-0.0421	0.2846	0.5457
Syt3	_KLHFS[Phospho (STY)]VYDFDR_.3	#N/A	6.3384	6.2686	3.5922	7.1827	3.0022	3.6224	3.5156	7.4514	7.1408	3.7679	0.9145	-0.1290	0.7155	0.1454
Syt3	_LHFS[Phospho (STY)]VYDFDR_.2	#N/A	3.9306	3.2956	5.6308	5.7404	4.9741	3.1724	6.3353	2.9984	4.1479	3.5862	1.0446	0.0630	0.8224	0.0849
Syt3	_GGGS[Phospho (STY)]GEAGAPC[Carbamidomethyl (C)]GR_.2	O35681_S290_M1_Syt3	5.7599	5.2895	3.8240	5.4276	3.7637	6.8797	5.5156	6.2259	3.4404	5.7089	0.9875	-0.0182	0.9352	0.0291
Nudc	_LQLEIDQK_.2	#N/A	2.9553	3.7605	3.6421	4.8450	3.9427	3.7539	3.8595	4.6435	3.6223	4.9138	0.8960	-0.1585	0.2943	0.5311
Nudc	_WTQTLAELDLAVPFR_.2	#N/A	4.1194	2.5431	3.9297	4.3322	4.2856	4.2241	3.6217	3.5277	3.2163	3.8842	1.0963	0.1327	0.3746	0.4264
Pnn	_GFS[Phospho (STY)]DSGGGPPAK_.2	#N/A	3.2282	4.1142	4.4627	4.0065	3.8727	4.2527	4.1547	2.9947	3.2938	3.6837	1.1296	0.1758	0.1580	0.8014
Pnn	_RGFS[Phospho (STY)]DSGGGPPAK_.2	#N/A	4.2729	2.9533	3.4588	3.9615	3.3985	4.3309	6.6459	4.1570	3.8056	3.9285	0.8047	-0.3134	0.1711	0.7667
Pnn	_SLS[Phospho (STY)]PGKENINSQEVEK_.2	#N/A	6.0576	5.8775	3.5410	6.6316	5.8030	5.7185	6.7155	6.3182	3.3275	5.8716	1.0084	0.0121	0.9555	0.0197
Pnn	_QES[Phospho (STY)]DPEDDDVKKPALQSSVVATSK_.3	#N/A	3.9742	4.7764	3.4385	5.5645	3.9693	4.6592	4.7273	4.7830	4.5163	4.6191	0.9433	-0.0842	0.5154	0.2879
Ppfibp2	_C[Carbamidomethyl (C)]VDGNQLS[Phospho (STY)]PVGEPK_.2	O35711_S405_M1_Ppfibp2	5.0146	5.7801	3.9532	4.8074	3.8930	4.4072	5.3888	4.5826	3.2512	4.4229	1.0524	0.0737	0.6588	0.1813
Ppfibp2	_T[Phospho (STY)]QSGNFNTDAPGM[Oxidation (M)]AEFR_.2	O35711_T516_M1_Ppfibp2	4.5269	3.7890	3.4245	3.7506	3.6773	3.8158	3.7089	3.8993	3.8993	4.1464	0.9788	-0.0309	0.6916	0.1601
Hnrnph1	_VHIEIGPDGR_.2	#N/A	8.3074	8.2994	8.4527	8.7338	8.5261	8.3345	8.4855	8.3552	8.4736	8.2870	1.0050	0.0072	0.6669	0.1759
Hnrnph1	_GLPWSC[Carbamidomethyl (C)]SADEVQR_.2	#N/A	8.2764	8.1838	8.5104	8.5086	8.4041	8.3040	8.2991	8.0433	8.3076	8.1763	1.0192	0.0275	0.0953	1.0207

Hnrnph1	_STGEAFVQFASQEIAEK_.2	#N/A	7.2458	7.2530	8.3231	8.1217	6.9472	7.5513	7.5634	6.9063	7.1180	7.8032	1.0308	0.0437	0.5002	0.3008
Hnrnph1	_ATENDIYNFFSPLNPVR_.2	#N/A	5.9273	5.6398	5.9436	5.8100	5.9547	5.8410	6.0383	5.2056	5.2879	5.9128	1.0431	0.0608	0.2136	0.6704
Hnrnph1	_HTGPNS[Phospho (STY)]PDTANDGFVR_.2	#N/A	9.4193	9.6978	10.3475	10.3712	9.5484	9.7380	9.8100	9.6597	9.7415	9.8076	1.0102	0.0146	0.6497	0.1873
Hnrnph1	_THYDPPRK_.2	#N/A	6.4830	5.4742	3.7990	5.2643	6.3581	3.1302	6.2161	3.6584	4.5651	6.2920	0.9811	-0.0276	0.9121	0.0400
Hnrnph1	_THYDPPR_.2	#N/A	5.3367	3.7585	4.1544	3.5994	5.2308	2.9778	3.4197	4.1166	4.5774	5.4413	0.9516	-0.0716	0.7257	0.1393
Hnrnph1	_DLNYC[Carbamidomethyl (C)]FSGM[Oxidation (M)]SDHR_.2	#N/A	3.9446	5.2722	3.3433	5.5506	4.4993	5.1190	6.5130	5.1028	5.2844	3.9279	0.8876	-0.1721	0.3606	0.4430
Hnrnph1	_EGRPSGEAFVELES[Phospho (STY)]EDEVK_.3	O35737_S63_M1_Hnrnph1	4.3875	3.6919	3.5735	4.0761	3.5132	4.2163	3.7908	4.0423	3.6909	4.0431	1.0046	0.0066	0.9305	0.0313
Api5	_TSEDTSSGS[Phospho (STY)]PPK_.2	#N/A	8.1691	7.6564	7.8864	7.7199	3.1768	7.7610	8.6123	8.0635	8.1975	7.8748	0.8625	-0.2133	0.2855	0.5443
Api5	_TSEDTSSGS[Phospho (STY)]PPKK_.2	#N/A	6.9131	4.8937	7.2431	7.2885	5.6364	6.2993	7.3477	7.4113	7.1898	6.2750	0.9041	-0.1455	0.2401	0.6197
Api5	_TSEDTSSGS[Phospho (STY)]PPKKSPGGPK_.3	#N/A	3.7604	3.0647	4.4472	3.5551	2.8860	3.3426	3.7034	3.4460	3.6188	3.2324	1.0026	0.0038	0.9761	0.0105
Slc1a4	_SEEETS[Phospho (STY)]PLVTHQNPAGPVAIAPELESK_.3	O35874_S507_M1_Slc1a4	7.9688	7.9258	8.5959	8.6603	6.7076	7.8949	8.6974	8.5936	7.9097	7.5668	0.9716	-0.0416	0.5926	0.2273
Calu	_EQFVEFR_.2	#N/A	4.5532	4.8745	5.6483	5.6016	5.7037	5.5268	3.9587	5.1008	5.3660	5.6444	1.0599	0.0839	0.4544	0.3426
Ctnnd2	_GGSPLTTTQGGS[Phospho (STY)]PTK_.2	O35927_S273_M1_Ctnnd2	12.2310	12.3910	12.7699	12.7367	11.8111	12.3067	12.7678	12.3899	12.2947	12.3665	0.9936	-0.0093	0.6981	0.1561
Ctnnd2	_GGS[Phospho (STY)]APEGAAYAAPR_.2	O35927_S282_M1_Ctnnd2	10.3136	10.2338	10.7238	10.7726	2.8422	10.1956	10.5943	10.3497	10.3851	10.2294	0.8836	-0.1785	0.4689	0.3289
Ctnnd2	_SPS[Phospho (STY)]IDSIQK_.2	#N/A	10.3687	9.5159	3.5949	10.1108	8.3816	3.6197	10.0034	9.8111	9.7770	4.3168	0.8964	-0.1578	0.6648	0.1773
Ctnnd2	_SASAPAS[Phospho (STY)]PR_.2	O35927_S1098_M1_Ctnnd2	10.8684	10.9556	11.2774	11.0726	9.7705	10.7748	11.1161	11.0200	10.8334	10.8977	0.9836	-0.0239	0.5287	0.2768
Ctnnd2	_ALQS[Phospho (STY)]PEHHIDPIYEDR_.3	#N/A	10.9511	10.8315	11.5737	11.2731	10.1779	10.9360	11.2900	10.9809	11.0444	10.8980	0.9913	-0.0126	0.7094	0.1491
Ctnnd2	_T[Phospho (STY)]GSQHGPQNAAAATFQR_.3	O35927_T469_M1_Ctnnd2	6.8581	6.9513	4.1214	3.3676	3.5314	3.1021	5.9982	7.1672	3.4647	3.1777	0.9401	-0.0891	0.8091	0.0920
Ctnnd2	_TSTAPSS[Phospho (STY)]PGVDSVPLQR_.2	O35927_S458_M1_Ctnnd2	11.8892	11.9818	12.7168	12.2352	10.9599	11.7341	12.4276	11.8748	11.7646	11.6899	0.9983	-0.0024	0.9531	0.0209
Ctnnd2	_NS[Phospho (STY)]YGAPAEDIK_.2	O35927_S1149_M1_Ctnnd2	3.9377	3.1931	3.6714	3.2151	3.0814	3.5432	3.4364	3.7861	4.0851	4.3932	0.8765	-0.1902	0.0724	1.1403
Ctnnd2	_TPSIS[Phospho (STY)]PVR_.2	O35927_S1082_M1_Ctnnd2	8.9412	8.8328	9.4253	9.1373	8.2412	8.9671	9.3802	9.2717	9.1303	9.2834	0.9631	-0.0543	0.1332	0.8754
Ctnnd2	_NSSLS[Phospho (STY)]PGLNTSNGDGSETETTSAILASVK_.3	O35927_S32_M1_Ctnnd2	3.6574	3.6589	3.5405	4.0011	3.4149	3.6025	3.9633	4.3288	3.6261	4.0101	0.9156	-0.1273	0.0573	1.2420
Ctnnd2	_TPSIS[Phospho (STY)]PVRVS[Phospho (STY)]PNNR_.2	#N/A	8.7067	8.7655	8.8922	9.1491	8.1205	8.8321	9.1483	8.4830	8.7225	8.8782	0.9928	-0.0105	0.7656	0.1160
Ctnnd2	_QLQYC[Carbamidomethyl (C)]ASVDS[Phospho (STY)]PYSK_.2	O35927_S511_M1_Ctnnd2	3.6398	3.8122	6.6431	6.7371	3.7005	6.5357	6.9464	5.1585	5.5418	6.3634	0.8627	-0.2131	0.3749	0.4261
Ctnnd2	_SLSQS[Phospho (STY)]QGDPLPPAHTGTFR_.2	O35927_S437_M1_Ctnnd2	4.7976	3.4865	3.5350	3.9551	3.3556	3.7693	4.5870	3.8975	3.7634	4.0046	0.9393	-0.0903	0.4413	0.3552
Ctnnd2	_SPS[Phospho (STY)]IDSIQKDPR_.2	#N/A	9.8182	9.3527	9.8613	9.8854	8.2272	9.3435	10.2498	9.6160	9.5441	9.7372	0.9620	-0.0559	0.3145	0.5024
Ctnnd2	_SGPALPPEGTLARS[Phospho (STY)]PS[Phospho (STY)]IDSIQK_.3	#N/A	3.4252	3.6348	3.8462	9.1256	3.9140	3.8604	4.2212	7.9527	3.2722	3.7502	0.9657	-0.0504	0.9098	0.0411
Ctnnd2	_AS[Phospho (STY)]YSSQHGHLAPELR_.3	O35927_S395_M1_Ctnnd2	3.8362	3.3504	4.1286	4.0364	3.1717	3.9278	3.6499	3.7317	4.1559	3.9423	0.9669	-0.0486	0.5730	0.2418
Ctnnd2	_SGPALPPEGTLARS[Phospho (STY)]PS[Phospho (STY)]IDSIQKDPR_.3	#N/A	3.6488	3.7693	3.7498	8.1795	6.6675	3.8712	4.0376	10.0670	5.9882	3.8486	0.8322	-0.2650	0.5261	0.2789
Ctnnd2	_AAGQSFSQGTTGR_.2	#N/A	5.2866	3.8433	5.8743	3.4730	5.3201	3.2945	3.1877	3.1446	5.1604	5.3387	1.0731	0.1017	0.6882	0.1623
Pitpnm1	_C[Carbamidomethyl (C)]NTGSEGPEAQT[Phospho (STY)]PGK_.2	O35954_T270_M1_Pitpnm1	5.6465	3.8063	5.0527	4.6093	4.0464	4.6018	5.9067	3.9752	5.3775	4.6333	0.9304	-0.1040	0.4894	0.3104
Pitpnm1	_PGTSTAGT[Phospho (STY)]PDGPEAPPGPDASPDAS[Phospho (STY)]FGK_.3	O35954_S304_M2_Pitpnm1	3.7331	3.7634	3.6094	3.5619	3.5800	3.2585	3.5720	3.3608	3.9292	3.8929	0.9717	-0.0414	0.4806	0.3182
Pitpnm1	_SSYSSQHGGGVS[Phospho (STY)]PQS[Phospho (STY)]LSEWR_.3	O35954_S326_M2_Pitpnm1	2.9728	4.3696	4.2074	3.2142	4.1909	3.5386	3.8994	4.9905	3.5491	3.4284	0.9451	-0.0815	0.6101	0.2146
Pitpnm1	_RGS[Phospho (STY)]M[Oxidation (M)]NNEM[Oxidation (M)]LSPEVGPVRDPLADGVEVLGR_.4	O35954_S593_M1_Pitpnm1	3.8899	3.3363	4.5767	3.5785	3.2246	3.4000	3.2932	3.0391	4.1885	3.5455	1.0430	0.0607	0.6516	0.1861
Rab33b	_S[Phospho (STY)]M[Oxidation (M)]VQHYYR_.2	#N/A	4.3225	4.3086	3.5686	5.1696	3.9686	4.5552	4.3541	5.1708	4.5805	4.9996	0.9035	-0.1463	0.1799	0.7450
Gamt	_[Acetyl (Protein N-term)]SSSAAS[Phospho (STY)]PLFAPGEDC[Carbamidomethyl (C)]GPAWR_.2	O35969_S7_M1_Gamt	3.1967	4.0019	3.8835	4.3862	3.8233	3.9062	4.1009	3.7323	4.8298	4.3532	0.9089	-0.1379	0.1858	0.7309
Pbx2	_TAVSVAQGGHS[Phospho (STY)]R_.2	O35984_S330_M1_Pbx2	4.9352	5.0972	5.4645	5.0790	4.1228	4.4968	4.6495	5.1083	5.3044	3.0617	1.0739	0.1029	0.4970	0.3037
Cavin1	_ATEEPSGTGS[Phospho (STY)]DELIK_.2	O54724_S42_M1_Cavin1	7.3485	7.7771	3.9203	8.5834	7.3590	7.6481	8.3637	7.6632	7.5982	7.8141	0.9041	-0.1454	0.3960	0.4023
Cavin1	_IIGAVDQIQLTQAQLEER_.2	#N/A	4.0629	2.8723	3.2489	3.7515	3.0516	3.5730	3.4662	3.0389	4.0155	2.6942	1.0372	0.0527	0.7121	0.1475
Ddost	_TLVLLDNLNVR_.2	#N/A	3.8867	4.7638	5.0209	4.6590	4.4347	4.3902	4.9680	4.5084	4.7280	4.1183	0.9880	-0.0174	0.8282	0.0819
Ap3d1	_MVKGS[Phospho (STY)]IDR_.2	#N/A	9.2832	8.6773	8.6273	9.5042	5.9261	8.2412	8.5260	8.7694	8.5360	8.5342	0.9750	-0.0365	0.7523	0.1236
Ap3d1	_NAEAVKS[Phospho (STY)]PEKEGVLGVEK_.3	#N/A	8.2538	7.7584	7.8566	8.1500	6.5110	7.8449	8.0551	8.1620	8.0806	7.6475	0.9678	-0.0472	0.4619	0.3355
Ap3d1	_RHSSLPTES[Phospho (STY)]DEDIAPAQR_.3	#N/A	8.5230	8.8985	9.1228	8.7137	8.1556	9.4123	9.7161	9.4341	9.5412	9.0737	0.9325	-0.1008	0.0348	1.4585
Ap3d1	_HSSLPTES[Phospho (STY)]DEDIAPAQR_.3	#N/A	11.3297	11.1896	11.9951	11.3957	10.2398	11.3577	11.5812	11.8263	11.6231	11.4202	0.9689	-0.0456	0.2594	0.5859
Ap3d1	_NAEAVKS[Phospho (STY)]PEK_.2	#N/A	5.3506	3.9838	3.6068	5.7550	3.2906	2.9381	5.5167	5.1566	5.3667	4.9840	0.7904	-0.3394	0.0994	1.0028
Ap3d1	_DLILQC[Carbamidomethyl (C)]LDDKDESIR_.3	#N/A	5.6914	4.8826	4.6082	3.2136	4.5806	3.5447	5.3392	5.5370	4.7343	4.3917	0.8839	-0.1780	0.2864	0.5430
Ap3d1	_EQAADTEAAQEVTQLLVER_.2	#N/A	3.8591	4.0971	4.5934	4.6388	3.6141	4.5435	4.3998	4.3653	4.4489	3.7955	0.9934	-0.0095	0.9139	0.0391
Ap3d1	_NLQDLVR_.2	#N/A	3.1591	3.9154	3.9976	4.5003	3.8037	3.5646	4.2149	3.6182	3.2668	2.9664	1.0873	0.1207	0.3541	0.4508
Ap3d1	_LEDPDPGVQSAAVNVIC[Carbamidomethyl (C)]ELAR_.2	#N/A	2.9934	4.2328	3.6802	4.1829	3.6199	4.1096	3.8975	1.4043	3.5842	4.1499	1.1670	0.2228	0.3540	0.4510
Ap3d1	_VDIITEEMPENALPS[Phospho (STY)]DEDDKDPNDPYR_.4	O54774_S784_M1_Ap3d1	3.7548	3.0102	3.4885	4.5331	2.8985	3.7261	3.6193	3.9689	4.2680	4.2103	0.8884	-0.1707	0.2042	0.6899
Srpk2	_SSS[Phospho (STY)]SERPEPQQK_.2	O54781_S10_M1_Srpk2	7.2026	4.2096	4.3771	7.7465	6.8567	7.5561	7.3976	7.4994	7.5215	7.2826	0.8518	-0.2314	0.2161	0.6653
Srpk2	_TVSASS[Phospho (STY)]TGDLPK_.2	O54781_S490_M1_Srpk2	4.0705	3.2290	3.6827	6.8939	5.0041	6.3764	3.5439	3.9285	4.6629	4.3921	1.1801	0.2389	0.3714	0.4301
Srpk2	_AADLEDT[Phospho (STY)]T[Phospho (STY)]EEETAK_.2	O54781_T331_M2_Srpk2	5.5927	4.8299	4.7477	4.9938	4.4058	4.8533	5.2579	4.3429	4.6585	3.6048	1.0980	0.1349	0.2304	0.6375
Srpk2	_[Acetyl (Protein N-term)]S[Phospho (STY)]VNS[Phospho (STY)]EKSSSSERPEPQQK_.2	O54781_S2_M2_Srpk2	4.2201	3.4073	3.8660	3.5431	3.2956	3.3646	3.6810	3.9356	3.5842	3.5101	0.9832	-0.0244	0.7624	0.1178
Dapk3	_NTSYAS[Phospho (STY)]FER_.2	O54784_S316_M1_Dapk3	3.6267	3.7157	8.5479	4.4050	4.2712	3.8542	8.2277	7.9950	8.0150	7.9964	0.5878	-0.7666	0.0089	2.0518
Dffa	_RS[Phospho (STY)]PLPGEPQRPK_.3	O54786_S314_M1_Dffa	6.2098	4.9915	7.0862	6.6711	3.3567	5.9364	5.6444	5.8719	6.2162	6.3393	0.9486	-0.0761	0.6714	0.1730
Tpd52l1	_S[Phospho (STY)]WHDM[Oxidation (M)]QTTTAYK_.2	#N/A	6.0474	5.7513	6.4288	4.3263	3.5703	6.8156	4.0068	6.0707	3.5478	6.3671	1.0984	0.1354	0.5827	0.2346
Tpd52l1	_S[Phospho (STY)]WHDM[Oxidation (M)]QTTTAYKK_.3	#N/A	5.1614	5.2258	4.0797	4.8240	4.7724	3.8809	4.4912	4.2429	3.4814	4.9596	1.0847	0.1173	0.3606	0.4429
Tpd52l1	_NSSTFKS[Phospho (STY)]FEER_.2	O54818_S149_M1_Tpd52l1	8.6026	8.5217	8.6543	9.0190	7.9466	8.8403	8.8888	8.3813	8.6807	8.7490	0.9911	-0.0129	0.7152	0.1456
Rgs9	_GC[Carbamidomethyl (C)]LAS[Phospho (STY)]PVFAR_.2	O54828_S588_M1_Rgs9	9.6623	9.4304	10.1033	9.7349	8.1501	9.2181	9.8453	9.3389	9.0124	9.2805	1.0015	0.0021	0.9709	0.0128
Rgs7	_FLESEFSSENLR_.2	#N/A	6.6925	6.4557	7.0673	7.0101	6.6684	6.4127	6.7718	6.2799	6.2157	6.5495	1.0408	0.0577	0.1645	0.7837
Rgs7	_SHS[Phospho (STY)]PTHTPTPETKPPTEDELHQQIK_.4	#N/A	8.3101	11.0158	12.0172	11.4083	8.4968	11.1256	3.8493	7.7147	11.3321	11.2323	1.2184	0.2850	0.2826	0.5488
Rgs7	_SSAYQELLQAK_.2	#N/A	4.9897	5.9600	6.3577	6.0691	5.8201	5.4707	5.7404	5.6295	5.6754	5.1509	1.0412	0.0583	0.4197	0.3770
Rgs7	_YTFEDAQEHIYK_.2	#N/A	4.6622	4.1375	4.4775	5.2529	4.4494	4.0878	5.0966	3.8151	4.9171	4.5262	0.9831	-0.0246	0.8098	0.0916
Rgs7	_S[Phospho (STY)]VYGLQNDIR_.2	O54829_S229_M1_Rgs7	4.0478	3.3522	3.2338	3.7364	3.1735	3.0551	3.4159	4.3820	4.6770	3.7034	0.8488	-0.2365	0.0775	1.1105
Cd200	_EVIC[Carbamidomethyl (C)]QVLYLGNVIDYK_.2	#N/A	4.2976	3.0448	4.0313	3.5750	3.4413	3.1833	4.3524	3.6152	3.7252	4.3928	0.8941	-0.1615	0.1880	0.7259
Reps1	_RQS[Phospho (STY)]SSYEDPWKITDEQR_.3	#N/A	3.3883	3.9603	4.0135	4.1665	5.2783	6.0936	4.3148	5.7729	5.9268	5.4375	0.8360	-0.2584	0.1722	0.7640
Reps1	_RQS[Phospho (STY)]SSYEDPWK_.2	#N/A	3.6477	8.1989	7.9290	9.0195	6.9240	7.6754	8.1689	7.7423	8.4090	7.6006	0.9063	-0.1419	0.4642	0.3333
Reps1	_SSS[Phospho (STY)]LDMNR_.2	O54916_S561_M1_Reps1	3.8439	3.0993	3.5775	8.5973	2.9876	3.6370	3.2696	6.3456	6.1724	4.2994	0.8544	-0.2271	0.5713	0.2432
Reps1	_LKS[Phospho (STY)]EDELRPDVDEHTQK_.3	#N/A	5.0507	5.2745	5.9967	5.6270	4.2853	5.2191	5.5181	5.2490	5.0248	5.4063	0.9892	-0.0157	0.8564	0.0673
Reps1	_KGPGS[Phospho (STY)]HDAVQPR_.3	O54916_S145_M1_Reps1	2.6734	3.6937	4.2193	3.3972	3.5820	3.0426	4.4366	3.8184	3.8824	2.8816	0.9148	-0.1285	0.4171	0.3798
Reps1	_LIDLEDSADVGEQPGEVGY[Phospho (STY)]SGSPAEAPPSK_.3	O54916_Y389_M1_Reps1	2.9338	3.7390	3.6206	4.3817	3.5603	4.1692	4.5300	3.9953	4.4193	4.0902	0.8768	-0.1897	0.0964	1.0160
Akap2	_TLS[Phospho (STY)]M[Oxidation (M)]IEEEIR_.2	#N/A	3.8831	3.9029	3.2965	6.6281	3.7659	4.2207	3.7810	5.6283	3.7169	4.0333	0.9984	-0.0024	0.9923	0.0034
Bnip2	_KGS[Phospho (STY)]ITEYTATEEK_.2	O54940_S114_M1_Bnip2	5.1877	3.0266	4.4090	5.6541	2.8479	3.3807	5.0830	5.3791	4.6118	4.9292	0.8167	-0.2921	0.1738	0.7599
Dnajb6;Dnajb7	_VDYYEVLGVQR_.2	#N/A	5.6003	5.2567	5.6542	5.7291	5.0075	5.2230	6.1923	5.6043	5.3937	5.3770	0.9592	-0.0600	0.3072	0.5125
Banf1	_DC[Carbamidomethyl (C)]FGC[Carbamidomethyl (C)]LR_.2	#N/A	4.0869	5.0572	5.6726	5.2910	4.6954	4.1559	3.5126	4.5465	4.8370	4.4780	1.1112	0.1521	0.2560	0.5918
Crym	_SSSLLIPPLEAALANFSK_.2	#N/A	7.5921	7.5270	7.2510	7.3446	7.8055	7.4257	6.9360	7.0104	7.5339	7.5316	1.0328	0.0466	0.1821	0.7397
Crym	_LVYDSWSSGK_.2	#N/A	7.6503	7.6571	8.0038	7.5830	7.6454	7.5821	7.3981	7.3903	7.6614	7.3614	1.0314	0.0446	0.0442	1.3551
Crym	_QAVLYVDSR_.2	#N/A	6.8508	7.0214	7.3682	7.1332	6.9646	7.1251	6.9520	6.4794	6.9561	6.9552	1.0353	0.0501	0.1011	0.9953

Crym	_FASTVQGDVR_.2	#N/A	5.8179	6.6603	6.4340	7.0737	7.0669	6.9343	6.5116	6.3996	6.7747	6.5633	1.0156	0.0223	0.6988	0.1557
Crym	_SLGM[Oxidation (M)]AVEDLVAAK_.2	#N/A	6.6158	6.9329	7.6653	6.5462	6.3923	7.1650	6.8714	7.1188	7.1713	6.8203	0.9844	-0.0227	0.6753	0.1705
Crym	_GFLGVM[Oxidation (M)]PAYSAAEDALTTK_.2	#N/A	6.0931	6.6085	6.3143	6.3119	6.1779	6.8147	6.2136	6.6295	6.3236	6.4316	0.9980	-0.0029	0.9363	0.0286
Crym	_TAAVSAIATK_.2	#N/A	6.1019	6.0436	5.0752	5.5753	6.2944	6.3840	5.1825	5.7535	6.4152	5.6476	1.0283	0.0403	0.6287	0.2015
Crym	_RAPAFLSAEEVQDHLR_.3	#N/A	7.1148	7.1827	7.4368	7.2383	7.0328	7.1984	7.0311	6.9004	7.5707	7.1208	1.0063	0.0090	0.7471	0.1266
Crym	_GPDGGVM[Oxidation (M)]QPVR_.2	#N/A	6.0165	6.0115	6.0747	6.4259	6.6550	5.9948	6.0213	6.2413	6.0057	6.1169	1.0164	0.0235	0.5176	0.2860
Crym	_APAFLSAEEVQDHLR_.2	#N/A	5.3922	4.3630	5.4178	4.6506	4.6364	4.6626	5.4117	4.6914	5.4192	5.3480	0.9303	-0.1043	0.2067	0.6846
Crym	_ELDDELMR_.2	#N/A	4.8137	3.8857	3.1735	5.7237	4.7982	3.1154	3.4762	4.4423	4.0909	3.6432	1.0865	0.1197	0.5597	0.2520
Get3	_LEETLPVIR_.2	#N/A	6.0748	6.2248	5.8359	5.7957	6.4092	6.1554	6.2425	5.3842	6.3578	5.8744	1.0198	0.0283	0.5909	0.2285
Get3	_LLNFPTIVER_.2	#N/A	6.6965	6.4529	6.3704	6.8851	6.3857	6.4474	4.4844	6.4901	6.2703	6.5771	1.0981	0.1350	0.1871	0.7279
Get3	_ESVLIISTDPAHNISDAFDQK_.3	#N/A	5.3660	4.1811	3.3936	4.4382	4.1510	3.8209	3.0857	4.6767	5.1546	5.2256	0.9315	-0.1023	0.5669	0.2465
Slk	_VITSDRSS[Phospho (STY)]EVGTDEALDDTQK_.3	O54988_S543_M1_Slk	3.7468	3.0023	3.4805	6.0470	2.8906	5.9949	3.6272	4.2059	3.8545	3.8795	1.0776	0.1078	0.6962	0.1573
Slk	_ATLEQPETDEVEQVSESNS[Phospho (STY)]IEELER_.3	O54988_S647_M1_Slk	4.3801	6.6556	3.5660	4.0687	6.1884	4.2237	3.0837	4.0498	6.6510	5.8859	0.9857	-0.0208	0.9403	0.0267
Slk	_LENLPDTQDQQTVDVNSVSEENENNR_.3	#N/A	3.0544	4.1718	3.7412	6.9731	3.6809	5.5692	5.9755	6.3581	3.5232	4.2108	0.9033	-0.1467	0.6127	0.2127
Cntnap1	_DQNLPQILEESRS[Phospho (STY)]E_.2	O54991_S1384_M1_Cntnap1	12.1773	12.5295	12.9586	12.9788	11.7242	12.5521	13.2222	12.9358	12.8736	12.6681	0.9661	-0.0498	0.1313	0.8816
Cntnap1	_AVATQGAFNSWDWVTR_.2	#N/A	3.5048	3.8376	3.2385	4.2831	4.1494	3.9761	4.4313	4.2189	3.0171	3.9604	0.9807	-0.0281	0.8190	0.0867
Bnip3	_NSTLS[Phospho (STY)]EEDYIER_.2	O55003_S88_M1_Bnip3	9.3837	9.4983	9.7889	10.0130	8.6175	9.8544	10.0294	9.6279	9.5462	9.5207	0.9840	-0.0233	0.5853	0.2326
Bnip3	_SSHC[Carbamidomethyl (C)]DS[Phospho (STY)]PPR_.2	O55003_S60_M1_Bnip3	8.2967	8.0191	3.7354	8.1333	7.4590	6.8404	8.6887	8.1027	8.4509	8.1646	0.8478	-0.2382	0.1894	0.7226
Bnip3	_AEIDSHS[Phospho (STY)]FGEK_.2	O55003_S79_M1_Bnip3	5.8091	6.0474	6.4936	6.3546	4.0215	6.6170	4.2992	5.7361	5.5790	5.8306	1.0987	0.1358	0.3777	0.4229
Bnip3	_SQT[Phospho (STY)]PQDTNRAEIDSHSFGEK_.3	O55003_T66_M1_Bnip3	3.1131	5.0625	5.2486	3.8914	4.4495	5.5078	5.7876	3.1097	3.4088	5.8504	1.0014	0.0020	0.9934	0.0029
Bnip3	_ILLDAQHES[Phospho (STY)]GR_.2	O55003_S48_M1_Bnip3	7.4720	6.8018	7.1949	7.3412	6.1467	3.8970	6.9622	6.5325	7.0978	7.1906	0.9323	-0.1011	0.5201	0.2839
Cacna1b	_ESLS[Phospho (STY)]WGTQR_.2	O55017_S1929_M1_Cacna1b	7.6509	7.1138	4.3465	3.8903	4.8169	7.0709	4.0386	3.1109	6.6921	3.5676	1.3361	0.4180	0.2016	0.6955
Cacna1b	_SHS[Phospho (STY)]KETPGADTQVR_.3	#N/A	5.4594	5.2947	5.4237	5.5785	3.5413	5.1423	4.7760	5.2078	5.3600	5.0335	0.9959	-0.0060	0.9597	0.0179
Cacna1b	_AS[Phospho (STY)]C[Carbamidomethyl (C)]EALYSEM[Oxidation (M)]DPEER_.2	#N/A	8.4447	8.0834	7.9201	7.7343	6.4147	7.7965	8.6440	8.6969	8.2147	8.0800	0.9195	-0.1210	0.1083	0.9654
Cacna1b	_GLS[Phospho (STY)]EHNALLQK_.2	O55017_S2221_M1_Cacna1b	6.9219	5.4953	5.9999	5.3698	4.1145	5.9163	6.6787	6.3514	6.0120	5.5904	0.9153	-0.1277	0.3329	0.4777
Cacna1b	_RGS[Phospho (STY)]PEEATER_.2	#N/A	5.9413	4.5460	3.9539	4.2038	3.3639	3.2607	6.2674	6.0579	5.7603	3.1749	0.7924	-0.3357	0.1835	0.7364
Cacna1b	_IGS[Phospho (STY)]DPYLGQR_.2	O55017_S2244_M1_Cacna1b	9.0519	8.7365	9.4374	8.7693	6.8893	8.7225	9.2911	9.2664	8.8708	8.6617	0.9533	-0.0690	0.3928	0.4058
Cacna1b	_RGS[Phospho (STY)]PEEATEREPR_.3	#N/A	6.7489	6.0215	4.4189	5.7741	5.2448	5.8971	6.5267	6.5144	6.5822	6.3559	0.8752	-0.1923	0.0793	1.1007
Cacna1b	_RSHS[Phospho (STY)]KETPGADTQVR_.3	#N/A	6.0515	6.3139	6.9393	6.1722	3.6075	5.5158	3.9805	6.5829	2.5706	3.8180	1.3607	0.4444	0.1237	0.9075
Cacna1b	_S[Phospho (STY)]LEKGPSLSVDPEGAPSTAAGPGLPHGEGSTAC[Carbamidomethyl (C)]R_.4	O55017_S2056_M1_Cacna1b	4.3660	4.3610	3.5520	6.5040	4.2493	3.8762	3.7693	5.9972	3.7124	4.0216	1.0250	0.0357	0.8766	0.0572
Cacna1b	_EVAEVS[Phospho (STY)]PM[Oxidation (M)]S[Phospho (STY)]AANISIAAR_.2	#N/A	3.7147	3.6533	3.6100	7.0840	3.5416	3.9342	3.8273	7.2316	3.7141	3.4512	0.9342	-0.0982	0.7726	0.1121
Cacna1b	_LQNLRAS[Phospho (STY)]C[Carbamidomethyl (C)]EALYSEM[Oxidation (M)]DPEER_.3	#N/A	4.3871	3.6426	4.2704	3.2722	3.5309	3.0938	4.4878	2.3820	2.9940	3.2392	1.1294	0.1755	0.3644	0.4384
Pgrmc1	_GDQPGASGDNDDDEPPPLPR_.2	#N/A	8.5560	8.0295	7.7504	8.9514	8.5285	8.6937	8.9232	7.9138	8.4730	8.3198	1.0013	0.0019	0.9702	0.0131
Pgrmc1	_RFDGVQDPR_.2	#N/A	7.6113	7.7477	7.9013	7.6164	8.3973	7.9907	7.6401	7.9060	7.8451	7.4500	1.0217	0.0309	0.3598	0.4439
Pgrmc1	_FYGPEGPYGVFAGR_.2	#N/A	7.0175	7.1605	6.8716	7.0795	7.0782	7.0441	7.3594	7.6260	7.0333	7.1987	0.9641	-0.0528	0.0460	1.3375
Pgrmc1	_EGEEPTVYS[Phospho (STY)]DDEEPKDETAR_.3	#N/A	11.6277	11.3913	11.7904	12.0218	11.0030	11.4171	11.3794	11.5384	11.1949	11.1582	1.0198	0.0283	0.2808	0.5517
Pgrmc1	_LLKEGEEPTVYSDDEEPKDETAR_.4	#N/A	6.6450	9.5589	7.4186	9.7908	9.4053	9.8342	6.7780	9.6417	9.6144	9.7775	0.9802	-0.0289	0.8501	0.0705
Pgrmc1	_DFTPAELR_.2	#N/A	5.4704	3.2925	3.7836	5.2116	5.0060	3.4310	3.4756	5.1070	4.4224	5.0617	0.9666	-0.0490	0.8012	0.0962
Pgrmc1	_EGEEPTVYS[Phospho (STY)]DDEEPKDETARK_.4	#N/A	4.2905	3.5459	4.0242	3.5679	3.4342	3.1904	3.7162	3.4332	3.7323	3.2452	1.0407	0.0576	0.5438	0.2645
Impa1	_SLLVTELGSSR_.2	#N/A	7.6723	8.1217	8.2481	8.3932	7.9657	7.8220	8.0789	7.8159	7.8597	8.0587	1.0105	0.0151	0.5850	0.2328
Impa1	_SS[Phospho (STY)]PADLVTVTDQKVEK_.2	#N/A	5.9018	5.9985	5.4018	4.9705	6.0033	6.3506	6.6511	6.0548	6.3273	6.3637	0.9089	-0.1377	0.0658	1.1820
Impa1	_EIEIIPLQR_.2	#N/A	7.5667	7.2518	6.9230	8.1057	7.4111	7.3240	7.5083	7.7348	7.3659	7.4624	0.9884	-0.0169	0.6884	0.1622
Impa1	_LC[Carbamidomethyl (C)]SIPIHGIR_.2	#N/A	7.4388	7.4617	7.2116	7.2340	7.3231	6.7576	7.7672	7.1725	7.2391	6.3996	1.0130	0.0187	0.7276	0.1381
Impa1	_LQVSQQEDITK_.2	#N/A	4.9214	5.3546	4.2445	5.2095	5.5052	5.4529	4.8042	5.4933	5.0632	5.1618	0.9969	-0.0045	0.9538	0.0205
Impa1	_S[Phospho (STY)]SPADLVTVTDQK_.2	#N/A	5.9080	6.3708	4.2986	5.7737	5.7748	6.7515	6.6210	5.0176	5.6834	7.3215	0.9435	-0.0839	0.5690	0.2449
Impa1	_IIAANSITLAK_.2	#N/A	4.6489	3.1548	4.5146	5.7295	5.8844	5.5011	4.2703	5.8535	4.4095	5.0536	1.0018	0.0026	0.9886	0.0050
Impa1	_[Acetyl (Protein N-term)]ADPWQEC[Carbamidomethyl (C)]M[Oxidation (M)]DYAVILAR_.2	#N/A	4.2566	5.1163	3.3820	3.8846	4.2934	4.2335	3.2678	3.0092	4.3291	4.9390	1.0793	0.1101	0.5225	0.2819
Bckdk	_S[Phospho (STY)]TSATDTHHVELAR_.3	O55028_S31_M1_Bckdk	7.8623	3.1909	8.0232	4.1017	7.8029	3.9052	7.7971	3.8715	8.3989	3.8688	0.9716	-0.0415	0.9140	0.0390
Copb2	_DNNQFASASLDR_.2	#N/A	7.0153	6.9360	7.0332	7.1509	6.3604	6.6191	7.1315	7.0317	6.9903	6.5790	0.9884	-0.0169	0.6679	0.1753
Nck2	_DSESS[Phospho (STY)]PSDFSVSLK_.2	O55033_S316_M1_Nck2	3.5364	4.2926	3.6203	4.1230	4.1809	3.9445	3.8377	3.9955	3.6441	5.6175	0.9242	-0.1138	0.4324	0.3641
Nck2	_DAS[Phospho (STY)]PTPSTDAEYPANGSGADR_.2	O55033_S90_M1_Nck2	3.5135	3.7126	4.2003	3.2021	5.0080	3.0237	4.4177	3.4155	3.0641	3.1691	1.0740	0.1030	0.5755	0.2399
Snca	_EQVTNVGGAVVTGVTAVAQK_.2	#N/A	8.6941	8.6482	9.0816	9.1800	8.4877	8.8233	8.9066	8.2103	8.7096	8.8691	1.0167	0.0240	0.4567	0.3403
Snca	_AKEGVVAAAEK_.2	#N/A	8.5664	8.7611	9.2302	8.9505	9.5421	9.3328	8.8040	8.7996	9.0058	9.0594	1.0164	0.0235	0.4751	0.3232
Snca	_TVEGAGNIAAATGFVK_.2	#N/A	8.4148	8.3565	8.9930	9.1402	9.6023	8.8632	8.3594	8.7061	8.8283	8.8531	1.0240	0.0342	0.4375	0.3591
Snca	_EGVLYVGSKT[Phospho (STY)]K_.2	O55042_T44_M1_Snca	3.8627	3.1181	3.5964	3.1402	3.0064	7.2351	3.5113	3.8610	6.6512	4.3183	0.8708	-0.1995	0.5697	0.2444
Snca	_TKEGVVHGVTTVAEK_.3	#N/A	10.0378	9.8945	10.6769	9.9964	7.9978	7.3655	10.1898	10.0352	7.6205	6.8140	1.0765	0.1064	0.5061	0.2958
Snca	_TKEQVTNVGGAVVTGVTAVAQK_.3	#N/A	9.1858	9.3829	10.1392	9.6541	8.9385	9.1305	9.3721	8.7746	9.4073	9.5483	1.0140	0.0200	0.6311	0.1999
Snca	_TVEGAGNIAAATGFVKK_.2	#N/A	5.0936	5.8131	5.9418	6.1532	6.2026	6.0938	5.6659	5.3979	5.9811	6.0077	1.0208	0.0297	0.6302	0.2005
Snca	_EGVVHGVTTVAEK_.2	#N/A	6.7313	6.9952	6.8249	7.3788	6.9664	6.5222	6.8324	6.6855	6.5659	6.8709	1.0244	0.0348	0.3280	0.4841
Tpmt	_[Acetyl (Protein N-term)]S[Phospho (STY)]LDM[Oxidation (M)]KEHPDAEVQK_.2	#N/A	4.3313	4.2772	5.0290	5.2629	4.4847	4.9196	5.0238	4.8673	4.9169	4.3864	0.9831	-0.0246	0.7401	0.1307
Akap7	_AVQQYLEETQNK_.2	#N/A	7.3562	7.6223	7.8409	7.4583	7.0657	7.7111	8.0017	7.7142	7.6420	7.6593	0.9684	-0.0464	0.1560	0.8069
Impact	_TEEVEVES[Phospho (STY)]EEDPILEHPPENPVK_.3	#N/A	3.2671	4.0723	3.9539	10.3709	3.8936	8.8851	8.4298	9.6682	9.1535	8.7709	0.6374	-0.6496	0.0720	1.1426
Impact	_KKTEEVEVES[Phospho (STY)]EEDPILEHPPENPVK_.4	#N/A	3.3598	8.7395	9.4915	10.5446	8.6964	4.1211	4.2863	9.8680	8.7507	8.0388	0.9685	-0.0462	0.8957	0.0478
Impact	_KTEEVEVES[Phospho (STY)]EEDPILEHPPENPVK_.3	#N/A	4.0338	3.5554	4.2190	3.3595	3.1704	3.1810	4.0047	3.3290	4.4077	3.3053	0.9534	-0.0688	0.5892	0.2297
Syngr1	_AGGAFDPYTLVR_.2	#N/A	9.0002	8.6502	8.5383	8.5572	8.1526	8.2691	9.0560	8.7856	8.5402	8.6600	0.9735	-0.0388	0.2226	0.6526
Strn	_LPEQELSR_.2	#N/A	5.8572	6.1103	4.9629	5.5724	6.4100	5.9825	5.0061	5.8117	5.3519	5.4707	1.0750	0.1043	0.1959	0.7080
Strn	_FLESAAADVS[Phospho (STY)]DEDEDETDGR_.2	O55106_S245_M1_Strn	3.4260	5.5376	4.3902	9.0250	6.7504	5.9774	4.3405	8.2173	6.8314	7.2010	0.8802	-0.1841	0.5218	0.2825
Strn	_EALKEFDFLVTSEEGDNESR_.3	#N/A	4.7470	4.6881	4.2458	4.6588	3.7691	4.0911	4.3751	4.5380	4.4237	4.5968	0.9740	-0.0381	0.5858	0.2322
Strn	_FYDNNTGK_.2	#N/A	3.9759	4.7965	3.1618	3.6645	3.1016	3.1271	3.3792	4.4540	4.1026	3.6315	0.9348	-0.0973	0.5335	0.2729
Strn	_VISHPTLPISITAHEDR_.3	#N/A	3.2428	4.0996	4.4773	4.0211	5.1631	4.2381	4.1694	2.9801	3.2791	3.6984	1.1912	0.2524	0.1131	0.9464
Nipsnap1	_AGPNIYELR_.2	#N/A	8.5311	8.7888	8.7317	8.5466	8.4877	8.1195	8.8623	8.6066	8.5872	8.3522	0.9921	-0.0114	0.6543	0.1842
Nipsnap1	_GWDENVYYTVPLVR_.2	#N/A	7.9818	7.9074	7.9191	7.9557	8.0374	8.3041	8.2172	8.0085	8.0750	7.9575	0.9942	-0.0084	0.6068	0.2170
Nipsnap1	_LKPGTM[Oxidation (M)]IEWGNNWAR_.3	#N/A	6.3138	7.5448	7.4310	6.1442	7.5147	7.6679	6.9975	7.2577	7.2068	6.9222	1.0009	0.0014	0.9854	0.0064
Nipsnap1	_KETSNLYK_.2	#N/A	4.5844	4.4853	4.2063	5.4875	4.6758	5.5531	4.6486	4.6761	5.4916	5.2184	0.9647	-0.0518	0.6048	0.2184
Nipsnap1	_DSEGSWFR_.2	#N/A	5.9888	5.1365	3.5870	5.7991	6.1297	3.9112	3.8043	5.8900	5.1379	5.6488	0.9945	-0.0080	0.9675	0.0143
Nipsnap1	_IQFHNVKPEC[Carbamidomethyl (C)]LDAYNSLTEAVLPK_.4	#N/A	3.2907	3.0288	4.4113	3.4131	2.8501	3.3785	3.7393	3.2046	4.3540	4.5574	0.8566	-0.2233	0.1615	0.7919
Nipsnap2	_PGPNIYELR_.2	#N/A	8.0624	8.2148	7.9658	7.8823	7.8530	7.7313	7.9510	8.0635	7.9332	7.8278	1.0010	0.0014	0.9371	0.0282
Nipsnap2	_LKENQEFVNFR_.3	#N/A	6.1384	4.7559	6.0687	5.5154	5.8371	5.8768	4.8750	5.6643	5.2321	5.7767	1.0579	0.0811	0.3408	0.4675
Nipsnap2	_IC[Carbamidomethyl (C)]QEVLPK_.2	#N/A	5.3067	5.4460	5.2539	5.6543	6.2254	5.8742	4.5878	5.4449	5.6662	6.3192	1.0222	0.0317	0.7290	0.1372

Nipsnap2	_KETSSLYK_.2	#N/A	5.5914	5.1185	4.7511	5.4446	5.6086	3.2555	3.2508	5.2267	5.3223	5.3158	1.0382	0.0541	0.7722	0.1123
Nipsnap2	_LQFHNVKPEC[Carbamidomethyl (C)]LDAYNK_.3	#N/A	5.1616	6.1719	5.7011	3.6441	5.5908	4.9261	5.7020	5.6132	5.7616	5.3985	0.9253	-0.1120	0.3804	0.4198
Nipsnap2	_SDMLLSR_.2	#N/A	4.0090	2.0105	3.1950	3.6976	3.1347	3.5191	3.4123	4.4209	4.0694	3.6646	0.8379	-0.2551	0.1488	0.8273
Septin7	_ILEQQNSSRT[Phospho (STY)]LEK_.2	#N/A	8.7109	8.6421	8.8287	9.1454	7.3614	8.6201	8.6451	8.2603	8.6611	8.9047	0.9923	-0.0112	0.8458	0.0727
Septin7	_LKDSEAELQR_.2	#N/A	9.1539	9.1381	9.0190	9.2535	9.1603	9.1546	9.1181	9.0154	9.2093	9.2216	1.0006	0.0009	0.9218	0.0354
Septin7	_FEDYLNAESR_.2	#N/A	8.8254	8.2076	8.2858	8.8925	8.8209	8.6512	8.7734	8.5102	8.5479	8.5226	1.0030	0.0043	0.8773	0.0568
Septin7	_NLEGYVGFANLPNQVYR_.2	#N/A	8.6147	8.4454	8.5413	8.8411	8.4820	8.8110	8.5105	8.4808	8.7244	8.5850	1.0055	0.0079	0.6345	0.1976
Septin7	_ANWEAQQR_.2	#N/A	8.2541	8.1866	8.3046	8.4701	8.6089	8.3643	8.3296	8.2220	8.3884	8.3749	1.0043	0.0062	0.6791	0.1681
Septin7	_ADTLTPEEC[Carbamidomethyl (C)]QQFK_.2	#N/A	8.1547	7.6706	7.4893	7.8295	7.9637	7.7853	7.9162	7.8148	7.5345	7.6784	1.0103	0.0148	0.5710	0.2433
Septin7	_ADTLTPEEC[Carbamidomethyl (C)]QQFKK_.2	#N/A	7.2978	7.5008	7.6897	7.2671	7.7680	7.7106	7.6387	7.1201	7.1249	7.7300	1.0183	0.0262	0.4494	0.3474
Septin7	_LAAVTYNGVDNNK_.2	#N/A	6.8236	6.7602	7.1646	7.0013	6.5056	6.5827	7.1155	6.3976	7.0297	6.9873	0.9889	-0.0161	0.6855	0.1640
Septin7	_STLINSLFLTDLYSPEYPGPSHR_.3	#N/A	4.7643	6.3777	6.5058	7.7636	6.1616	5.5475	5.4188	6.3818	5.4722	5.9695	1.0647	0.0905	0.5087	0.2936
Septin7	_IYEFPETDDEEENKLVK_.3	#N/A	6.0672	5.8878	6.4504	6.4760	6.8193	6.2794	5.8744	5.7318	5.8561	5.2718	1.0668	0.0933	0.0727	1.1386
Septin7	_DVTNNVHYENYR_.2	#N/A	6.9587	6.8458	7.0964	7.1471	7.1746	7.1388	6.8072	6.7117	7.2633	7.2639	1.0069	0.0100	0.7249	0.1397
Septin7	_GQLTKS[Phospho (STY)]PLAQM[Oxidation (M)]EEERR_.3	#N/A	5.9800	7.0594	7.8003	3.4755	5.9379	6.6120	6.3082	6.8203	6.6024	6.5031	0.9368	-0.0941	0.6011	0.2210
Septin7	_IYEFPETDDEEENK_.2	#N/A	6.6312	6.1021	4.1389	9.0034	6.4056	6.0074	6.7226	5.9583	6.2885	6.7310	0.9932	-0.0098	0.9583	0.0185
Septin7	_S[Phospho (STY)]PLAQM[Oxidation (M)]EEER_.2	#N/A	9.0116	7.9937	7.5165	8.8223	7.6699	8.1179	8.1200	7.9747	8.4109	8.1246	1.0038	0.0055	0.9245	0.0341
Septin7	_NLEAQHKELEEK_.2	#N/A	4.7883	5.4262	5.1037	4.2905	4.2669	4.9863	4.1070	4.7908	4.0019	5.3939	1.0518	0.0729	0.5154	0.2878
Septin7	_KTVQVEQSK_.2	#N/A	3.1994	1.9263	3.5925	2.5508	3.6332	3.7683	3.2845	3.6990	3.5123	3.6946	0.8771	-0.1891	0.2849	0.5454
Septin7	_THMQDLK_.2	#N/A	4.1438	3.3370	3.8924	5.4026	4.7548	3.3221	3.2153	3.1170	4.2665	3.9014	1.1426	0.1924	0.3043	0.5167
Septin7	_S[Phospho (STY)]PLAQM[Oxidation (M)]EEERR_.2	#N/A	10.5858	10.0524	9.7924	11.1053	9.5364	9.8527	10.3507	10.0369	10.0766	10.2073	0.9987	-0.0019	0.9651	0.0154
Septin7	_ILEQQNSSR_.2	#N/A	6.8522	7.1607	7.1715	7.2769	7.3877	7.2754	6.9748	7.0613	6.9079	6.8185	1.0356	0.0504	0.0424	1.3730
Septin7	_QFEEEKANWEAQQR_.2	#N/A	3.5541	3.5741	3.9817	3.2049	3.3918	3.0091	3.9716	3.5237	3.6584	4.0097	0.9108	-0.1348	0.1224	0.9122
Acot1	_YRADS[Phospho (STY)]HGELDLAR_.3	O55137_S56_M1_Acot1	3.6268	3.5993	4.3136	3.3155	4.2534	3.1370	5.9900	3.3022	2.9508	3.2825	0.9552	-0.0661	0.7834	0.1060
Acot1	_QLGSEC[Carbamidomethyl (C)]LHVS[Phospho (STY)]PK_.2	#N/A	4.9308	4.6228	3.8435	5.1171	4.1163	4.9740	4.9148	4.6630	5.1015	4.5514	0.9570	-0.0635	0.4815	0.3174
Rpl35a	_IEGVYAR_.2	#N/A	5.1865	5.2620	4.3490	5.2832	4.4669	4.9514	4.1729	5.8393	5.0164	5.2506	0.9698	-0.0443	0.6685	0.1749
Rpl35a	_NNTVTPGGKPNK_.2	#N/A	5.2920	5.7875	5.1297	5.3563	5.9843	5.3127	5.6597	5.3383	5.0311	5.0633	1.0387	0.0547	0.3488	0.4574
Atp2a2	_EFDELSPS[Phospho (STY)]AQR_.2	O55143_S663_M1_Atp2a2	12.2176	12.0924	12.6725	11.9788	11.1335	11.8866	12.7909	12.2053	12.1555	11.9503	0.9773	-0.0331	0.3716	0.4300
Atp2a2	_WGS[Phospho (STY)]NELPAEEGK_.2	O55143_S38_M1_Atp2a2	8.2114	7.8609	8.1931	7.8634	7.0975	7.9228	8.5805	7.9188	8.0677	7.9907	0.9654	-0.0507	0.2718	0.5657
Atp2a2	_VGEATETALTC[Carbamidomethyl (C)]LVEK_.2	#N/A	6.7258	6.0947	6.7929	5.9400	6.4280	6.2426	6.8104	6.5504	6.9669	6.1568	0.9622	-0.0556	0.2956	0.5293
Atp2a2	_VDQSILTGESVSVIK_.2	#N/A	6.5091	6.0510	5.6249	6.1612	5.1997	5.6927	5.9363	6.1398	6.0033	6.0437	0.9739	-0.0382	0.5265	0.2786
Atp2a2	_NM[Oxidation (M)]LFSGTNIAAGK_.2	#N/A	6.0655	6.4199	6.7073	4.3279	4.6075	5.9814	5.5914	6.5832	6.7074	6.1201	0.9095	-0.1368	0.3252	0.4878
Atp2a2	_EWGS[Phospho (STY)]GSDTLR_.2	O55143_S553_M1_Atp2a2	3.5777	3.6484	4.2645	3.2664	4.2043	6.9221	4.4819	3.3513	2.9999	7.5230	0.9401	-0.0892	0.8018	0.0959
Atp2a2	_VIM[Oxidation (M)]ITGDNK_.2	#N/A	5.9268	5.3303	5.9377	5.1448	4.7575	6.0719	5.8597	5.7959	5.6989	5.2678	0.9775	-0.0329	0.6722	0.1725
Atp2a2	_SLPSVETLGC[Carbamidomethyl (C)]TSVIC[Carbamidomethyl (C)]SDK_.2	#N/A	6.5307	6.6515	3.8955	6.2502	6.1428	6.2980	7.0697	6.1799	6.5927	6.3255	0.9113	-0.1341	0.3200	0.4948
Atp2a2	_ANAC[Carbamidomethyl (C)]NSVIK_.2	#N/A	6.0398	6.3691	5.6882	3.4673	6.2009	5.9336	6.1931	6.9399	6.5861	6.6594	0.8517	-0.2316	0.1208	0.9179
Atp2a2	_IGIFGQDEDVTSK_.2	#N/A	6.3757	5.7876	3.1954	6.4305	4.7290	5.3358	6.7974	6.6416	6.0584	6.1222	0.8289	-0.2707	0.1247	0.9042
Atp2a2	_DIVPGDIVEIAVGDKVPADIR_.3	#N/A	5.9060	5.6179	5.6636	4.4760	4.8582	5.7330	6.0340	5.2923	4.7017	5.0959	1.0180	0.0257	0.8018	0.0959
Atp2a2	_IEVASSVK_.2	#N/A	4.9213	4.0743	3.8471	4.3898	4.0566	4.1124	4.3034	4.5611	4.4170	4.4012	0.9577	-0.0624	0.3721	0.4294
Atp2a2	_MNVFDTELK_.2	#N/A	3.8591	3.6643	3.8877	4.8579	3.7575	4.2119	4.1761	3.3092	3.3767	4.3573	1.0617	0.0864	0.4706	0.3273
Atp2a2	_NYLEQPGKEC[Carbamidomethyl (C)]VQPATK_.3	#N/A	5.2111	5.0920	3.9994	5.0289	4.7112	4.7927	4.6921	5.4724	4.8570	4.1107	1.0048	0.0069	0.9439	0.0251
Atp2a2	_GTAVAIC[Carbamidomethyl (C)]R_.2	#N/A	2.9151	4.0027	4.0179	3.6934	3.5597	4.1235	3.3219	4.1685	4.0674	3.9311	0.9604	-0.0584	0.5901	0.2290
Atp2a2	_TASEM[Oxidation (M)]VLADDNFSTIVAAVEEGR_.3	#N/A	5.5345	5.5671	6.3412	5.9564	4.5627	6.0977	5.5508	5.5534	5.3563	5.6298	1.0079	0.0113	0.8946	0.0484
Atp2a2	_TGTLTTNQMSVC[Carbamidomethyl (C)]R_.2	#N/A	3.2709	7.6895	8.3535	8.2360	7.3537	7.6358	7.8617	7.5594	7.4270	7.6973	0.9284	-0.1071	0.5912	0.2282
Atp2a2	_IVEFLQSFDEITAM[Oxidation (M)]TGDGVNDAPALK_.3	#N/A	3.5629	4.3191	3.2965	4.0965	4.2074	3.9180	3.8112	4.0220	3.6706	4.0635	1.0021	0.0031	0.9703	0.0131
Atp2a2	_IVEFLQSFDEITAM[Oxidation (M)]TGDGVNDAPALKK_.3	#N/A	3.4552	4.2604	4.3166	3.8603	4.0817	3.6478	4.0086	3.1408	3.4399	3.5376	1.1147	0.1567	0.1106	0.9563
Atp2a2	_YETNLTFVGC[Carbamidomethyl (C)]VGM[Oxidation (M)]LDPPR_.2	#N/A	2.9476	4.2786	3.6344	4.1370	3.5741	4.1554	3.8517	3.9814	3.6300	4.1040	0.9733	-0.0391	0.7099	0.1488
Atp2a2	_IRDEMVATEQER_.3	#N/A	2.9027	3.6590	4.1372	3.2558	3.5473	3.0773	4.4713	3.3618	3.0104	3.2228	0.9753	-0.0360	0.8076	0.0928
Pja1	_SRS[Phospho (STY)]PFASTR_.2	O55176_S32_M1_Pja1	6.9584	7.1567	6.7995	7.1402	3.5899	4.1396	7.0778	6.9832	7.0058	6.5688	0.8632	-0.2122	0.2944	0.5310
Pja1	_EQPQSSS[Phospho (STY)]GESWELLPGKEELER_.3	O55176_S341_M1_Pja1	3.9826	3.3598	3.7162	3.2600	3.1263	3.0475	3.4083	3.7412	4.0402	4.4381	0.8742	-0.1940	0.0885	1.0528
Pja1	_FTSVNHDAKEEC[Carbamidomethyl (C)]GKVES[Phospho (STY)]PPAAR_.4	O55176_S137_M1_Pja1	3.3372	6.6536	4.5718	7.7502	7.0531	7.3170	6.5079	7.0890	5.4684	7.1253	0.9337	-0.0989	0.6587	0.1813
Pja1	_VFFDT[Phospho (STY)]DDDDVPHSTSR_.2	#N/A	3.7237	3.6901	4.1915	8.3740	5.9971	3.7792	3.8836	7.4085	6.8521	6.7654	0.7964	-0.3285	0.3025	0.5192
Pja1	_YYEEDS[Phospho (STY)]DSDKEWM[Oxidation (M)]AALR_.3	O55176_S317_M1_Pja1	4.2206	3.4760	3.9543	3.4388	3.3643	3.2603	3.1534	3.1789	4.3283	3.1753	1.0463	0.0653	0.6082	0.2159
Pja1	_GNNS[Phospho (STY)]LER_.2	O55176_S175_M1_Pja1	3.0991	4.1271	3.7859	4.2886	4.0154	4.3172	4.0032	3.8299	3.4785	4.2556	1.0121	0.0173	0.8638	0.0636
Psmb5	_GPGLYYVDSEGNR_.2	#N/A	7.7982	7.9051	8.1633	7.6265	7.0185	7.6972	8.0024	7.4009	7.9094	7.7982	0.9902	-0.0142	0.7400	0.1308
Psmb5	_RGPGLYYVDSEGNR_.3	#N/A	7.3648	7.0203	7.7283	6.7316	5.9688	7.1787	6.9554	7.3335	7.3145	6.8857	0.9827	-0.0252	0.7117	0.1477
Psmb5	_AIYQATYR_.2	#N/A	5.4262	5.2835	3.3583	4.9265	5.7223	6.0243	6.1289	5.8124	5.8806	5.9004	0.8639	-0.2110	0.1340	0.8728
Psmb5	_ATAGAYIASQTVK_.2	#N/A	5.0267	5.2941	3.5113	5.5985	5.1648	4.8392	4.7892	5.3988	5.5869	5.6091	0.9176	-0.1240	0.3052	0.5154
Psmb5	_VEEAYDLAR_.2	#N/A	5.0262	4.5661	3.7738	4.2563	5.2837	5.4648	3.9911	5.4601	5.2751	4.4456	0.9865	-0.0196	0.8843	0.0534
Psmb5	_DAYSGGAVNLYHVR_.2	#N/A	4.0557	3.7125	3.3636	4.4082	3.3330	4.2504	3.6681	4.0938	4.3929	4.7977	0.9093	-0.1371	0.2351	0.6287
Psmb5	_FLHGVIVAADSR_.3	#N/A	2.9157	3.7209	3.6025	4.1052	3.5422	4.1873	3.8198	4.0133	3.5285	3.8137	0.9697	-0.0444	0.6538	0.1845
Dhx9	_AEENNSGVESSGYGS[Phospho (STY)]PGPTWDR_.2	O70133_S136_M1_Dhx9	3.9362	3.1916	3.2205	3.7231	3.0799	3.5447	3.4378	6.6590	4.0439	3.6901	0.7738	-0.3700	0.1388	0.8577
Dhx9	_DFVNYLVR_.2	#N/A	4.4557	3.2902	3.7684	3.6470	3.1785	3.4461	3.3393	4.1617	4.1425	3.5916	0.9533	-0.0690	0.5499	0.2597
Dhx9	_YTQVGPDHNR_.2	#N/A	4.9401	5.4358	3.3830	3.8857	3.3227	4.4067	3.6004	5.4947	4.7582	4.6464	0.9144	-0.1291	0.4796	0.3191
Tesk1	_S[Phospho (STY)]LPSSPELPR_.2	O70146_S439_M1_Tesk1	3.6640	2.9683	4.3508	3.3526	2.7897	3.4390	3.0673	7.4068	7.5305	7.6419	0.5346	-0.9036	0.0122	1.9135
Pip5k1c	_SSSLKSS[Phospho (STY)]PSK_.2	O70161_S453_M1_Pip5k1c	3.4548	3.6124	6.4160	6.0665	3.4793	5.5654	6.3031	5.9601	5.5249	3.9231	0.8780	-0.1877	0.4457	0.3510
Pip5k1c	_SPS[Phospho (STY)]DTSEQPR_.2	O70161_S540_M1_Pip5k1c	6.5128	5.8904	4.3997	6.0671	4.8945	3.3900	3.7508	5.7830	6.1014	6.1017	0.9555	-0.0657	0.7555	0.1218
Pip5k1c	_ITVQVEPVC[Carbamidomethyl (C)]GVGVVPK_.2	#N/A	5.7214	5.4115	5.3525	6.0370	5.3275	5.8536	5.2836	5.4992	5.2751	5.1494	1.0595	0.0834	0.0870	1.0604
Pip5k1c	_RTQS[Phospho (STY)]SGQDGRPQEEPHAEDLQK_.4	#N/A	7.7702	3.8455	8.4066	8.2826	7.3536	8.2235	3.6962	3.8159	7.8754	3.6799	1.5346	0.6179	0.0683	1.1657
Pip5k1c	_SSS[Phospho (STY)]LKSS[Phospho (STY)]PSKK_.2	#N/A	3.2929	4.0495	4.5274	4.0712	3.8708	3.8587	4.2195	3.3805	3.2290	3.7485	1.0825	0.1144	0.2958	0.5290
Pip5k1c	_TQS[Phospho (STY)]SGQDGRPQEEPHAEDLQK_.3	#N/A	6.8720	6.3433	7.8189	7.2244	6.1121	7.0290	6.5148	7.1151	7.0848	6.4238	1.0170	0.0243	0.7476	0.1264
Pip5k1c	_DC[Carbamidomethyl (C)]LVLESFK_.2	#N/A	4.8661	5.0658	3.1199	3.6225	3.1806	3.4441	3.3372	3.7898	4.3623	4.5409	0.9690	-0.0455	0.8060	0.0937
Pip5k1c	_IM[Oxidation (M)]DYSLLLGVHNIDQQER_.3	#N/A	3.5376	4.3428	4.2244	3.7779	4.1641	3.5654	3.9262	3.2233	3.5223	3.4552	1.1143	0.1561	0.0959	1.0183
Pip5k1c	_SWVYSPLHYSARPASDGESDT_.3	#N/A	4.1986	3.2130	3.3846	5.8005	3.9739	4.1981	4.4986	4.2348	4.5597	4.2046	0.9437	-0.0836	0.6176	0.2093
Stmn3	_DAS[Phospho (STY)]LEELQKR_.2	#N/A	11.8231	12.0889	12.6402	12.6030	11.5431	11.9130	12.4436	12.1596	11.9872	12.0412	0.9954	-0.0067	0.8202	0.0861
Stmn3	_DAS[Phospho (STY)]LEELQK_.2	#N/A	8.8809	9.7961	10.1131	10.1362	8.9966	8.6059	8.9539	9.7417	8.6876	8.7363	1.0434	0.0612	0.3496	0.4564
Stmn3	_ALEENNNFSR_.2	#N/A	6.5932	6.4563	7.2648	6.3658	6.3954	6.6343	6.4984	6.1622	6.6523	6.4175	1.0289	0.0411	0.3542	0.4508
Stmn3	_KDAS[Phospho (STY)]LEELQK_.2	#N/A	5.3107	4.6141	4.7316	5.2281	4.1946	5.0233	4.9865	5.1246	4.3297	4.7209	1.0125	0.0179	0.8203	0.0860
Stmn3	_SPSDLSPES[Phospho (STY)]PVLSSPPK_.2	O70166_S68_M1_Stmn3	8.4925	9.1364	9.7957	9.3849	8.8363	9.1357	9.2127	8.7855	8.7210	9.1135	1.0192	0.0274	0.5066	0.2954

Stmn3	_ASGQS[Phospho (STY)]FEVILK_.2	O70166_S53_M1_Stmn3	3.6767	3.4526	3.9822	5.2772	3.3037	4.3044	4.2118	5.4417	3.8849	4.4942	0.8872	-0.1727	0.2953	0.5298
Stmn3	_RAS[Phospho (STY)]GQS[Phospho (STY)]FEVILK_.2	#N/A	3.5574	3.7109	3.7176	4.7486	3.5992	3.8795	3.7113	4.7803	3.8329	4.3115	0.9302	-0.1043	0.3591	0.4448
Stmn3	_RKDAS[Phospho (STY)]LEELQK_.2	#N/A	4.3109	4.6030	4.6856	4.4939	4.4759	3.8919	4.3537	5.0308	3.3416	4.1193	1.0472	0.0666	0.5407	0.2670
Stmn3	_KDAS[Phospho (STY)]LEELQKR_.3	#N/A	4.0210	3.8380	3.4640	4.2827	3.6594	4.0701	4.4309	2.9892	3.4072	4.1782	1.0367	0.0521	0.6632	0.1783
Stmn3	_NKEQREEMS[Phospho (STY)]G_.2	O70166_S179_M1_Stmn3	3.3813	4.1865	4.3904	3.9342	4.0079	3.7216	4.0824	3.0670	3.3661	3.6114	1.1147	0.1567	0.1409	0.8510
Pip4k2a	_IYIDDNNKK_.2	#N/A	6.5760	6.4527	6.4374	7.0826	6.4857	6.5602	6.9170	6.9888	6.4560	6.8363	0.9705	-0.0432	0.2339	0.6309
Pip4k2a	_DVEFLAQLK_.2	#N/A	3.9282	3.0862	4.4686	3.4704	4.1164	4.1757	3.1851	4.4546	4.6006	4.9279	0.9027	-0.1477	0.3245	0.4888
Pip4k2a	_FGIDDQDFQNSLTR_.2	#N/A	3.0246	5.4596	3.7114	4.2909	5.0750	4.7406	5.4677	5.2802	5.2133	4.1810	0.8705	-0.2000	0.2406	0.6186
Chrna4	_C[Carbamidomethyl (C)]TC[Carbamidomethyl (C)]KEPS[Phospho (STY)]PVS[Phospho (STY)]PITVLK_.3	O70174_S540_M2_Chrna4	4.3544	4.3726	3.5403	4.0430	4.2609	3.8645	3.7577	4.0755	3.7241	4.0100	1.0465	0.0655	0.3460	0.4609
Eif3d	_IFHTVTTTDDPVIR_.3	#N/A	5.1152	4.7267	4.0388	3.3542	4.5229	3.1758	4.2207	4.4520	5.0438	4.7928	0.8981	-0.1551	0.2944	0.5310
Eef1b	_TPAGLQVLNDYLADK_.2	#N/A	5.8797	6.0685	5.8737	6.2794	6.6386	6.2981	6.4242	6.2193	6.3628	6.2474	0.9778	-0.0324	0.3900	0.4090
Eef1b	_SIQADGLVWGSSK_.2	#N/A	3.5834	5.2512	5.5796	4.2426	5.7317	4.7781	4.4876	4.0417	2.9942	3.2390	1.3172	0.3974	0.0491	1.3092
Eef1b	_DDDDIDLFGS[Phospho (STY)]DDEEESEEAKK_.3	#N/A	4.3824	4.3446	3.5684	9.5124	5.8975	3.8926	3.7857	8.0697	3.6960	4.0380	1.0753	0.1048	0.8006	0.0966
Eef1b	_YGPSS[Phospho (STY)]VEDTTGSGAADAK_.2	O70251_S83_M1_Eef1b	3.9078	3.0373	3.9179	3.7338	3.0335	3.5393	3.6701	5.8407	3.8104	3.5708	0.8355	-0.2593	0.1822	0.7395
Eef1b	_YGPSSVEDTTGSGAADAKDDDDIDLFGS[Phospho (STY)]DDEEESEEAK_.4	#N/A	4.3798	2.9626	4.1135	3.6573	3.5236	3.1010	3.8056	3.3439	3.6429	3.3346	1.0258	0.0368	0.7666	0.1154
Eef1b	_DDDDIDLFGS[Phospho (STY)]DDEEESEEAK_.2	#N/A	3.5329	4.2892	3.2666	9.3351	4.1775	3.9480	4.4595	9.0104	2.9890	3.9885	0.9308	-0.1034	0.8274	0.0823
Grk6	ɪidomethyl (C)]C[Carbamidomethyl (C)]GNC[Carbamidomethyl (C)]S[Phospho (STY)]DS[Phospho (STY)	O70293_S566_M2_Grk6	4.1727	3.0534	3.3587	3.8614	2.9417	3.6829	3.5760	4.2571	3.9057	3.8283	0.9024	-0.1482	0.1947	0.7106
Grk6	_DVLDIEQFS[Phospho (STY)]TVK_.2	O70293_S484_M1_Grk6	3.2252	4.1172	4.4597	4.0035	3.8698	4.2557	4.1518	2.9977	3.2967	3.6808	1.1293	0.1755	0.1583	0.8006
Atxn2	_DNS[Phospho (STY)]EEFLKR_.2	O70305_S400_M1_Atxn2	9.5986	9.4772	9.7870	10.2941	8.4958	9.5007	9.8444	9.5169	9.3838	9.7631	0.9895	-0.0153	0.7531	0.1232
Atxn2	_VALENDDRS[Phospho (STY)]EEEKYTAVQR_.3	O70305_S435_M1_Atxn2	3.7895	4.2598	4.1414	3.1432	4.0811	4.3355	4.3587	4.7371	3.1230	3.1102	1.0329	0.0467	0.7614	0.1184
Atxn2	_ASETS[Phospho (STY)]PSFSK_.2	O70305_S741_M1_Atxn2	6.0586	3.7701	3.3060	4.3506	4.2169	4.1380	4.4988	5.4583	6.0610	4.0278	0.8594	-0.2187	0.2744	0.5616
Atxn2	_QNS[Phospho (STY)]PAGSKENVK_.2	O70305_S727_M1_Atxn2	5.3861	3.2848	3.7630	4.4200	3.1731	3.4515	3.4551	3.6944	4.7759	2.9841	1.0498	0.0702	0.7336	0.1345
Atxn2	_TNS[Phospho (STY)]PSIS[Phospho (STY)]PSMLSNAEHKR_.3	#N/A	4.2717	3.5271	4.0053	3.3877	3.4154	3.2092	3.1024	3.2300	4.3794	3.3547	1.0340	0.0482	0.7118	0.1477
Atxn2	_TNS[Phospho (STY)]PSIS[Phospho (STY)]PSMLSNAEHK_.2	#N/A	3.9149	3.3740	3.9114	5.4330	3.4182	4.0657	3.7525	3.6912	3.7315	3.7080	1.0803	0.1114	0.4568	0.3402
Atxn2	_MSSEGPPRM[Oxidation (M)]S[Phospho (STY)]PK_.2	O70305_S593_M1_Atxn2	4.2392	3.5435	3.4251	4.5772	3.3649	4.3646	3.2246	4.1907	4.2239	3.8948	1.0092	0.0132	0.9164	0.0379
Atxn2	_DSFIDSSSSSSNC[Carbamidomethyl (C)]TSGSSK_.2	#N/A	3.8251	3.0805	3.5588	4.4209	2.9688	3.6558	3.2508	3.8986	4.1977	4.2806	0.9176	-0.1241	0.3534	0.4517
Atxn2	_GM[Oxidation (M)]S[Phospho (STY)]PVVSEHRK_.3	O70305_S753_M1_Atxn2	3.3563	4.1125	4.5908	4.1345	4.0008	4.1246	4.0178	4.3675	3.1657	3.8118	1.0553	0.0777	0.4769	0.3216
Nmt1	_SGLS[Phospho (STY)]PANDTGAK_.2	#N/A	10.4469	10.6201	10.9565	11.0749	9.2975	10.5796	10.6986	10.6946	10.3395	10.5176	0.9937	-0.0091	0.8451	0.0731
Nmt1	_SGLS[Phospho (STY)]PANDTGAKK_.2	#N/A	6.4042	6.5999	7.5438	6.6822	5.9926	3.6157	7.2228	3.1729	6.3754	6.7156	1.0457	0.0644	0.7944	0.0999
Nmt2	_GS[Phospho (STY)]PGGDLGAK_.2	O70311_S38_M1_Nmt2	7.7682	6.8675	6.7699	7.7706	7.1699	6.6364	7.4792	3.1206	7.2928	7.1620	1.1437	0.1937	0.3302	0.4812
Nmt2	_EKPNSGGTKS[Phospho (STY)]DS[Phospho (STY)]AS[Phospho (STY)]DSQEIK_.2	O70311_S66_M3_Nmt2	3.9958	3.7523	3.7295	3.9256	3.6213	3.3659	3.8344	5.2590	4.2550	3.7804	0.8714	-0.1985	0.0981	1.0081
Epb41l2	_QRS[Phospho (STY)]YNLVVAK_.2	#N/A	6.8537	3.8464	4.0666	4.5692	3.7347	4.3908	4.2839	3.5492	3.1978	3.0354	1.3015	0.3802	0.1314	0.8814
Epb41l2	_[Acetyl (Protein N-term)]TTEVGS[Phospho (STY)]ASEVKK_.2	O70318_S7_M1_Epb41l2	5.6786	6.0861	3.7210	4.2237	4.0802	6.1311	5.8570	6.3571	6.2664	4.1907	0.8798	-0.1847	0.3504	0.4555
Epb41l2	_LVS[Phospho (STY)]PEQPPK_.2	O70318_S492_M1_Epb41l2	6.3556	5.8055	3.7934	6.0728	3.7331	5.3327	6.5327	5.6193	5.8721	6.1837	0.8563	-0.2238	0.1899	0.7215
Epb41l2	_EVQTSELKAEVAS[Phospho (STY)]QK_.2	#N/A	4.9804	6.2093	5.8704	5.7329	5.6850	6.4431	6.4298	6.0611	5.5353	5.8356	0.9756	-0.0356	0.6385	0.1949
Epb41l2	_EM[Oxidation (M)]S[Phospho (STY)]PGHGPGETR_.2	#N/A	6.4730	5.5489	6.2349	6.9475	5.0813	5.8182	5.7868	5.3897	5.9234	5.4628	1.0668	0.0933	0.3226	0.4913
Epb41l2	_EPTQADVEDQILGKEES[Phospho (STY)]LPEEESR_.3	#N/A	2.9875	3.7927	3.6743	5.2116	3.6140	4.1154	4.4762	4.8030	3.5901	4.1440	0.9168	-0.1254	0.4340	0.3625
Epb41l2	_S[Phospho (STY)]YNLVVAK_.2	#N/A	3.9509	3.2753	3.1368	3.6395	3.0765	3.1521	3.3542	2.9782	4.1276	3.6065	0.9588	-0.0606	0.5939	0.2263
Epb41l2	_IVITGDAALDHDQALAQAIR_.3	#N/A	4.8323	3.2618	3.1434	3.3581	2.6138	3.1455	3.5063	3.6431	4.2274	3.6130	0.9053	-0.1436	0.4055	0.3920
Epb41l2	_AKEVENEQTPVS[Phospho (STY)]EPEEEKGSQPGPPVER_.4	O70318_S38_M1_Epb41l2	3.4666	4.2229	3.2003	4.2449	4.1112	4.0142	4.3932	4.2571	3.0553	3.9222	0.9923	-0.0112	0.9284	0.0323
Epb41l2	_GLSPAQADSQFLENAK_.2	#N/A	3.2740	6.4647	3.3400	7.0427	3.9116	6.0508	5.7977	4.1181	6.2076	6.0020	0.9065	-0.1417	0.5983	0.2231
Epb41l2	_VTEGTIREEQEEC[Carbamidomethyl (C)]DEELEEEPGQGAK_.3	#N/A	2.9967	3.7530	4.2312	3.7750	3.6413	2.9833	3.9233	3.2262	3.5252	3.4523	1.0090	0.0129	0.9108	0.0406
Epb41l2	_KEPTQADVEDQILGKEES[Phospho (STY)]LPEEESR_.4	#N/A	3.8110	3.4152	4.4978	3.4996	3.3035	3.3211	3.2143	3.1180	4.2675	3.4666	1.0355	0.0503	0.6995	0.1552
Gpx4	_ILAFPC[Carbamidomethyl (C)]NQFGR_.2	#N/A	6.3990	6.3933	6.6651	6.3824	6.4503	6.6162	6.2786	6.9493	6.0390	6.3490	1.0126	0.0180	0.6404	0.1936
Gpx4	_YAEC[Carbamidomethyl (C)]GLR_.2	#N/A	5.4148	4.9274	4.8154	4.7726	5.1783	4.4209	4.4022	5.5498	4.5067	4.4893	1.0390	0.0551	0.5250	0.2799
Gpx4	_IC[Carbamidomethyl (C)]VNGDDAHPLWK_.3	#N/A	3.3775	4.5661	4.2354	3.2372	3.6933	3.0587	4.4527	3.7124	3.0290	3.8135	0.9848	-0.0222	0.8843	0.0534
Gpx4	_TDVNYTQLVDLHAR_.2	#N/A	3.0301	3.7863	4.2782	3.5887	3.6746	3.3985	3.9131	3.2673	3.2448	3.3845	1.0503	0.0708	0.4992	0.3017
Cbfa2t2	_QHS[Phospho (STY)]PGSTDSLSNDSQR_.2	O70374_S400_M1_Cbfa2t2	3.3371	3.8890	4.0239	4.5266	1.8146	4.3481	4.2413	3.5919	3.2405	2.9928	1.0398	0.0563	0.8054	0.0940
Ulk1	_IEQNLQS[Phospho (STY)]PTQQTAR_.2	O70405_S450_M1_Ulk1	3.2598	7.4309	7.3382	7.3238	5.3294	3.8432	7.6453	7.6361	3.3178	6.8605	0.9041	-0.1455	0.6417	0.1927
Ulk1	_VPS[Phospho (STY)]PQGADVR_.2	O70405_S521_M1_Ulk1	3.7478	3.0032	3.4814	4.5261	2.8915	3.7331	3.6263	3.9760	4.2750	4.6147	0.8644	-0.2103	0.1445	0.8402
Psma3	_DIREEAEK_.2	#N/A	5.7141	5.7815	5.9124	6.1377	6.0445	5.6929	5.3866	5.7500	5.2876	5.5648	1.0697	0.0972	0.0144	1.8408
Psma3	_SNFGYNIPLK_.2	#N/A	5.9834	6.7806	6.6690	6.4978	6.8840	6.5616	6.3692	6.6071	6.7487	6.4827	1.0017	0.0024	0.9519	0.0214
Psma3	_[Acetyl (Protein N-term)]SSIGTGYDLSASTFSPDGR_.2	#N/A	5.0831	5.3343	3.8105	4.6022	5.5073	5.8716	5.4559	4.3791	4.6941	4.9593	1.0334	0.0474	0.7060	0.1512
Psma3	_AVENSSTAIGIR_.2	#N/A	3.6441	4.7625	4.3309	4.9298	5.1499	4.1506	3.6049	3.2849	3.9008	4.2694	1.1938	0.2556	0.0578	1.2378
Psma3	_ESLKEEDES[Phospho (STY)]DDDNM[Oxidation (M)]_.2	#N/A	12.1506	12.2440	12.6894	12.8730	12.2021	12.4247	12.4828	12.0563	12.2228	12.2891	1.0137	0.0196	0.3382	0.4709
Psma3	_YAKESLKEEDES[Phospho (STY)]DDDNM[Oxidation (M)]_.2	#N/A	4.1377	5.0967	5.1163	3.9903	4.3593	4.4082	4.9942	4.2497	4.4425	3.1987	1.0703	0.0980	0.4629	0.3345
Stx7	_EFGSLPTT[Phospho (STY)]PSEQR_.2	O70439_T79_M1_Stx7	10.7139	10.4534	11.1909	10.5643	9.8196	10.6214	11.2733	10.6486	10.7741	10.5270	0.9773	-0.0331	0.3749	0.4260
Stx7	_TLNQLGT[Phospho (STY)]PQDSPELR_.2	O70439_T41_M1_Stx7	4.7962	7.2650	7.5506	7.5043	6.6395	7.2692	7.6277	7.2897	7.7030	6.5990	0.9360	-0.0954	0.4378	0.3587
Stx7	_[Acetyl (Protein N-term)]S[Phospho (STY)]YTPGIGGDSAQLAQR_.2	O70439_S2_M1_Stx7	4.9319	4.9920	5.0148	4.9371	4.8999	4.6303	5.2746	4.7896	4.7556	6.2569	0.9301	-0.1045	0.2333	0.6320
Stx7	_ITQC[Carbamidomethyl (C)]SVEIQR_.2	#N/A	3.4569	4.2132	3.1906	4.2352	4.1015	4.0239	4.3835	4.2668	3.0650	3.9125	0.9906	-0.0136	0.9128	0.0396
Gnaz	_SSEYHLEDNAAYYLNDLER_.3	#N/A	7.4403	7.2822	7.9536	7.1758	5.9419	6.9171	7.6161	7.1647	7.0102	6.8190	0.9952	-0.0069	0.9287	0.0321
Gnaz	_QFEDLNR_.2	#N/A	6.0184	5.8698	5.5884	5.6618	5.6605	5.4955	6.4704	5.6120	6.0592	5.7503	0.9569	-0.0635	0.1904	0.7203
Gnaz	_IAAPDYIPTVEDILR_.2	#N/A	7.1478	6.4304	6.4917	3.9291	6.1255	6.6117	6.9373	6.9352	6.8653	6.6965	0.8927	-0.1637	0.2373	0.6248
Gnaz	_GQNTYEEAAVYIQR_.2	#N/A	5.1324	4.3477	6.0974	4.0679	4.2360	3.8894	5.5443	4.0506	3.6992	4.0349	1.0684	0.0954	0.5964	0.2244
Gnaz	_LWADPGAQAC[Carbamidomethyl (C)]FGR_.2	#N/A	3.3581	4.1633	4.0449	3.9574	3.9846	3.7448	4.1056	3.0438	3.3429	3.0137	1.1478	0.1989	0.0800	1.0971
Slc16a7	_VGS[Phospho (STY)]RHDS[Phospho (STY)]TLKK_.2	#N/A	5.3832	5.8328	5.7122	5.5123	3.0995	5.6662	5.4934	5.5561	4.1070	3.9774	1.0873	0.1207	0.5264	0.2787
Slc16a7	_VGS[Phospho (STY)]RHDS[Phospho (STY)]TLK_.2	#N/A	2.9943	3.7995	3.2766	4.3212	3.6208	4.1087	4.4695	4.1808	3.9324	3.9985	0.8894	-0.1691	0.1302	0.8855
Vamp4	_NLLEDDS[Phospho (STY)]DEEEDFFLR_.2	#N/A	6.2770	6.9619	7.4532	7.4393	7.9191	6.3066	8.7731	8.6857	8.3102	6.1513	0.8846	-0.1768	0.1607	0.7940
Vamp4	_RNLLEDDS[Phospho (STY)]DEEEDFFLR_.3	#N/A	3.5829	3.8788	3.8240	3.5998	3.4557	3.0360	3.7481	4.7887	3.1847	3.5658	0.9322	-0.1012	0.4308	0.3657
Ubr1	_MDVS[Phospho (STY)]PEPPLAPQR_.2	O70481_S15_M1_Ubr1	3.3682	3.9742	4.6027	4.1465	3.7955	3.9339	4.2947	4.3555	6.2926	3.8238	0.8462	-0.2409	0.1707	0.7677
Snx3	_FTTYEIR_.2	#N/A	5.6379	4.5060	5.4901	5.2345	4.9636	5.2122	4.9252	5.3917	4.6881	5.0104	1.0340	0.0483	0.4909	0.3090
Snx3	_YSDFEWLR_.2	#N/A	4.3929	4.5506	3.8568	4.9920	4.4877	4.3794	3.5488	4.7025	4.7355	4.6597	1.0072	0.0103	0.9170	0.0376
Snx3	_[Acetyl (Protein N-term)]AETVADTRR_.2	#N/A	4.2908	4.5112	5.1649	3.9651	5.5474	4.0551	3.9483	3.8868	4.2384	4.1587	1.1309	0.1774	0.1473	0.8318
Snx12	_[Acetyl (Protein N-term)]S[Phospho (STY)]DTAVADTRR_.2	O70493_S2_M1_Snx12	3.6152	4.3715	3.3489	4.0441	6.6131	7.2470	7.2378	6.9144	6.5398	4.0111	0.7891	-0.3417	0.2377	0.6240
Snx12	_GDEGIFEESFIEER_.2	#N/A	5.2653	5.6354	6.6309	6.9862	5.1659	6.0603	6.1452	4.6064	5.6441	5.1685	1.1050	0.1441	0.2515	0.5994
Snx12	_FTTYEVR_.2	#N/A	5.1442	5.6574	4.5910	5.5235	5.7066	6.0698	3.7252	5.3798	5.4979	5.9559	1.0601	0.0842	0.5265	0.2786
Snx12	_RYS[Phospho (STY)]DFEWLK_.2	O70493_S73_M1_Snx12	3.6676	3.5585	4.3544	3.3562	3.4468	3.1778	3.0709	5.6523	4.4108	3.3232	0.8734	-0.1952	0.3376	0.4716
Snx12	_IAGHPLAQNER_.3	#N/A	3.1268	3.9320	3.8136	4.3162	3.7533	3.9762	4.0309	3.8022	3.4508	4.2832	0.9815	-0.0270	0.7751	0.1106
Hcn4	_S[Phospho (STY)]PQAAQPPPPLPGAR_.2	O70507_S921_M1_Hcn4	8.8582	8.9102	7.3139	8.9362	6.3707	8.5690	9.3187	9.0319	8.3863	8.5857	0.9240	-0.1140	0.2743	0.5618

Hcn4	_GGLS[Phospho (STY)]PPGHSPGPPR_.2	#N/A	3.8938	6.8575	4.0740	4.0227	3.6886	4.2532	7.6801	3.6386	3.5281	3.8532	0.9551	-0.0663	0.8386	0.0764
Hcn4	_RAS[Phospho (STY)]PHS[Phospho (STY)]S[Phospho (STY)]GESVAAFSLYPR_.3	O70507_S1090_M3_Hcn4	4.1674	3.4228	3.9011	3.4920	3.3111	3.3135	3.2066	3.1257	4.2751	3.4590	1.0241	0.0343	0.7657	0.1160
Srpk1	_ADT[Phospho (STY)]PS[Phospho (STY)]GDEQEPNGALDSK_.2	O70551_S450_M2_Srpk1	5.8768	6.1697	3.6829	3.3566	3.3229	4.8214	6.0726	5.3509	5.7690	4.9374	0.8203	-0.2857	0.1818	0.7404
Cask	_EINGISVANQTVEQLQK_.2	#N/A	3.1901	3.6947	4.4246	3.9684	3.1778	3.8010	3.6137	3.0534	2.9788	3.6549	1.1156	0.1578	0.2099	0.6779
Cask	_TQSSSC[Carbamidomethyl (C)]ERDS[Phospho (STY)]PSTSR_.2	O70589_S582_M1_Cask	5.0657	4.7201	5.5673	3.8637	4.1990	4.4287	5.3720	5.3970	5.5397	4.8365	0.8779	-0.1879	0.0914	1.0389
Pfdn2	_GAVSAEQVIAGFNR_.2	#N/A	5.9132	5.7831	6.4495	4.5772	6.2111	5.4278	6.1330	5.2617	6.0440	5.6479	0.9923	-0.0112	0.9079	0.0420
Pfdn2	_IIETLSQQLQAK_.2	#N/A	3.4421	3.6533	3.2932	3.8319	4.2347	3.5301	4.4770	3.2353	4.3801	3.2285	0.9567	-0.0639	0.6205	0.2073
Rtn2	_EFS[Phospho (STY)]EDEEEETTSQDWGTPR_.2	O70622_S44_M1_Rtn2	3.5499	3.7925	3.2835	6.0661	4.3960	3.9310	4.4764	6.1919	4.7690	4.6759	0.8293	-0.2701	0.1891	0.7234
Ttc3	_SSQS[Phospho (STY)]PPLAAAGPSAR_.2	O88196_S1903_M1_Ttc3	9.2414	9.4718	9.7846	9.7196	8.7780	9.2729	9.9940	9.4347	9.2500	9.1252	0.9923	-0.0112	0.7706	0.1132
Ttc3	_RNSDS[Phospho (STY)]AGPFAVPDHLR_.3	O88196_S1062_M1_Ttc3	4.0906	3.2433	4.2820	8.8247	6.2240	3.2015	3.5074	3.2695	7.6949	3.3667	1.1162	0.1586	0.7214	0.1418
Ttc3	_GASQVS[Phospho (STY)]PSEQS[Phospho (STY)]PEADEK_.2	O88196_S1794_M2_Ttc3	4.4452	3.1998	3.9708	3.5805	3.3809	3.2523	3.5344	4.6631	3.9799	4.5233	0.8714	-0.1986	0.1331	0.8758
Ttc3	_KQDS[Phospho (STY)]DEM[Oxidation (M)]PFGC[Carbamidomethyl (C)]IK_.2	#N/A	4.7394	7.1721	7.0075	6.5993	5.9833	6.9969	7.7965	7.4637	7.1116	6.8760	0.8775	-0.1885	0.1104	0.9571
Ttc3	_IIIYSSGGQVK_.2	#N/A	4.2679	3.0745	4.0016	3.5453	2.8959	3.3328	3.6936	3.4558	3.7549	3.2226	0.9966	-0.0049	0.9676	0.0143
Grem2	_EVLASS[Phospho (STY)]QEALVVTER_.2	O88273_S56_M1_Grem2	7.5507	7.6833	8.0476	4.2551	7.4605	7.5725	7.6599	7.6375	7.5519	7.4189	0.9376	-0.0929	0.5298	0.2759
Znf326	_MNLSKS[Phospho (STY)]PTK_.2	#N/A	3.0852	3.8415	4.3197	3.8635	3.7298	4.3957	4.7173	3.1377	3.4367	5.3454	0.9311	-0.1030	0.5657	0.2474
Znf326	_S[Phospho (STY)]PTKTDPKNEEEEK_.2	#N/A	3.3031	4.1083	3.9899	4.0666	3.9297	3.8284	3.9805	3.5488	3.4846	3.9183	1.0370	0.0524	0.4674	0.3303
Znf326	_M[Oxidation (M)]NLSKS[Phospho (STY)]PTKTDPK_.2	#N/A	4.1388	3.3988	3.3248	5.7839	3.2645	3.8521	3.3139	4.2007	4.1575	3.7944	1.0243	0.0346	0.8598	0.0656
Ctnnal1	_S[Phospho (STY)]AAEELELTVLK_.2	O88327_S373_M1_Ctnnal1	4.3107	3.5661	4.0443	4.2999	3.4544	3.1702	3.0634	3.4131	3.7121	3.2654	1.1320	0.1789	0.1343	0.8719
Wdr1	_VFASLPQVER_.2	#N/A	8.2448	8.4821	8.2759	8.3180	8.4136	8.1564	7.9214	8.2267	8.1366	8.0481	1.0287	0.0408	0.0189	1.7229
Wdr1	_YAPSGFYIASGDISGK_.2	#N/A	7.9192	7.5188	8.4908	7.7341	7.7088	8.0579	8.1502	7.8598	7.9001	7.8962	0.9941	-0.0085	0.8043	0.0946
Wdr1	_YEYQPFAGK_.2	#N/A	7.1135	7.3551	7.5338	7.0038	7.4796	7.2062	7.2365	7.4166	7.3772	7.1988	0.9965	-0.0050	0.8313	0.0803
Wdr1	_IAVVGEGR_.2	#N/A	6.5902	6.5917	6.2842	6.3937	7.1657	6.6811	6.4364	6.5759	6.7377	6.9956	0.9897	-0.0149	0.7167	0.1447
Wdr1	_SIQC[Carbamidomethyl (C)]LTVHR_.2	#N/A	6.3684	6.5421	6.4213	6.7026	6.3565	6.4871	6.2301	6.6166	6.6958	6.6295	0.9903	-0.0140	0.5694	0.2446
Wdr1	_DYSGQGVVK_.2	#N/A	6.8746	6.6472	7.0006	6.3519	6.9655	6.5252	5.7430	6.5880	6.5623	6.4508	1.0618	0.0865	0.0949	1.0225
Wdr1	_DIAWTEDSKR_.2	#N/A	6.4770	6.0542	5.5204	6.3744	6.0973	6.4288	6.2272	6.0470	6.2106	6.2786	0.9948	-0.0075	0.8679	0.0615
Wdr1	_GPVTDVAYSHDGAFLAVC[Carbamidomethyl (C)]DASK_.3	#N/A	6.4697	6.5301	7.8240	7.3447	5.7069	6.5334	7.4120	5.9196	6.6170	6.3445	1.0246	0.0350	0.7311	0.1360
Wdr1	_VINSVDIK_.2	#N/A	5.2164	4.9319	4.7824	3.9129	5.5825	4.7948	3.1610	5.0966	5.2126	4.6752	1.0736	0.1024	0.4978	0.3029
Wdr1	_LATGSDDNC[Carbamidomethyl (C)]AAFFEGPPFK_.2	#N/A	5.7910	5.9363	6.1247	6.1149	5.6116	4.0383	6.0496	4.2512	6.1603	5.5605	1.0177	0.0253	0.8592	0.0659
Wdr1	_VC[Carbamidomethyl (C)]ALGESK_.2	#N/A	4.5232	4.5996	5.2268	4.5736	5.6850	5.2929	4.2883	4.7795	4.6601	4.5547	1.0903	0.1248	0.1521	0.8180
Wdr1	_VYSILASTLKDEGK_.2	#N/A	5.7557	6.1847	6.2210	6.4609	5.8325	6.2529	6.1670	6.0920	5.9745	5.8779	1.0149	0.0214	0.5558	0.2551
Wdr1	_AHDGGIYAISWSPDSTHLLSASGDK_.3	#N/A	3.5114	3.2398	4.0298	3.5568	4.3492	4.3995	4.2557	3.5111	4.5827	4.4924	0.9138	-0.1300	0.2781	0.5558
Wdr1	_VVTVFSVADGYSENNVFYGHHAK_.3	#N/A	4.4908	4.0205	5.3082	4.5107	3.5187	4.2476	4.1145	3.8928	4.1807	4.4956	1.0428	0.0605	0.5917	0.2279
Wdr1	_GDHFLYTNGK_.2	#N/A	4.2446	3.8267	3.2494	4.2940	3.9871	3.9652	4.4422	4.4516	3.0063	4.8452	0.9382	-0.0920	0.5080	0.2942
Wdr1	_M[Oxidation (M)]TVNESEQLVSC[Carbamidomethyl (C)]SMDDTVR_.2	#N/A	3.7844	3.5149	4.2689	4.7230	3.3873	3.7495	4.0269	3.1813	4.1082	3.4007	1.0613	0.0858	0.4931	0.3070
Wdr1	_SYIYSGSHDGHINYWDSETGENDSFSGK_.4	#N/A	5.5449	4.6696	6.1612	5.1399	4.5553	3.5113	5.2491	5.8065	5.0344	3.1703	1.0240	0.0342	0.8633	0.0638
Wdr1	_NIDNPAIADIYTEHAHQVVVAK_.3	#N/A	3.8962	5.8872	4.5830	3.5848	5.7472	5.4326	5.7782	3.0329	5.9030	5.7844	0.9474	-0.0779	0.7268	0.1386
Slc4a4	_GSLDS[Phospho (STY)]DNDDSDC[Carbamidomethyl (C)]PYSEK_.2	O88343_S1029_M1_Slc4a4	6.3860	5.9402	6.1560	6.2899	4.6232	5.9558	7.0555	6.4156	3.0442	6.1382	1.0403	0.0571	0.7770	0.1096
Slc4a4	_NLTSSS[Phospho (STY)]LNDISDKPEKDQLK_.3	#N/A	10.1344	10.7837	10.8349	10.6843	9.0846	10.6000	11.2719	10.9104	10.5946	10.5894	0.9550	-0.0664	0.2182	0.6611
Slc4a4	_LQQAVM[Oxidation (M)]LGALTEVPVPTR_.2	#N/A	4.3645	4.2516	4.9913	4.4966	1.8338	3.7432	4.7022	4.9002	3.6038	4.4602	0.8936	-0.1622	0.4634	0.3341
Slc4a4	_NLTSSS[Phospho (STY)]LNDISDKPEK_.2	#N/A	10.9259	10.7434	3.6713	11.0856	3.3906	10.5866	11.6093	11.1292	10.6087	10.5348	0.7657	-0.3851	0.2213	0.6550
Slc4a4	_LADYYPINSDFK_.2	#N/A	3.0296	6.3111	4.2641	6.1746	3.6742	5.3462	6.6874	6.4444	6.2520	6.0484	0.7549	-0.4056	0.0564	1.2486
Slc4a4	_MFSNPDNGS[Phospho (STY)]PAM[Oxidation (M)]THR_.2	#N/A	7.5429	6.7369	5.6957	7.9719	5.8008	4.6752	7.5188	6.8891	4.5704	6.6636	0.9990	-0.0015	0.9937	0.0027
Slc4a4	_ISENYSDKS[Phospho (STY)]DVENADESSSSILKPLISPAAER_.4	O88343_S68_M1_Slc4a4	3.3157	4.3768	3.8241	3.9004	3.9603	3.5127	3.8239	3.7556	3.6440	3.7713	1.0177	0.0253	0.7377	0.1321
Slc4a4	_KGS[Phospho (STY)]LDS[Phospho (STY)]DNDDSDC[Carbamidomethyl (C)]PYSEK_.2	#N/A	4.4680	3.4530	3.3346	3.1669	3.2743	2.9544	3.3152	3.8343	3.5711	3.8042	0.9479	-0.0773	0.5279	0.2775
Slc4a4	_KKGS[Phospho (STY)]LDS[Phospho (STY)]DNDDSDC[Carbamidomethyl (C)]PYSEK_.3	#N/A	3.0357	3.7920	4.2703	4.6236	3.6803	2.9443	4.3383	3.4949	3.1435	3.0898	1.0591	0.0828	0.6241	0.2047
Mtx2	_TLDQVLEDVDQC[Carbamidomethyl (C)]C[Carbamidomethyl (C)]QALSQR_.3	#N/A	4.6979	5.0765	3.9810	5.9561	4.9556	4.7512	5.3923	4.8885	5.1782	5.3196	0.9439	-0.0833	0.4147	0.3823
Mtx2	_[Acetyl (Protein N-term)]SLVAEAFVSQIAATEPWPENATLYQQLR_.3	#N/A	3.5721	4.3773	4.2589	3.2607	4.1987	3.5308	4.4763	3.3569	3.0055	3.2277	1.0995	0.1368	0.3497	0.4562
Klc1	_VDS[Phospho (STY)]PTVTTLK_.2	#N/A	10.1141	9.9825	10.1577	10.3661	9.4491	10.0142	10.5233	9.9573	9.9935	10.2226	0.9843	-0.0229	0.4189	0.3779
Klc1	_DAANLLNDALAIR_.2	#N/A	5.5639	5.0245	4.3386	5.6203	5.5753	4.8027	4.0121	5.4555	5.6515	5.6728	0.9916	-0.0122	0.9182	0.0371
Klc1	_S[Phospho (STY)]RESLNM[Oxidation (M)]DVVK_.2	#N/A	8.1070	7.7903	8.2620	8.7993	7.2394	8.1371	8.3059	7.8950	7.8460	8.3392	0.9950	-0.0073	0.8897	0.0507
Klc1	_AC[Carbamidomethyl (C)]KVDS[Phospho (STY)]PTVTTTLK_.2	#N/A	4.3726	8.1304	8.3685	8.4683	2.7911	3.4375	8.5128	3.3511	7.6400	8.0956	0.8592	-0.2190	0.5744	0.2408
Klc1	_S[Phospho (STY)]RES[Phospho (STY)]LNMDVVKYESGPDGGEEA_.3	#N/A	3.0894	4.1368	3.7762	4.2788	3.7159	4.0136	3.9935	3.8396	3.4882	4.2458	0.9854	-0.0212	0.8259	0.0831
Klc1	_RS[Phospho (STY)]RES[Phospho (STY)]LNMDVVK_.3	#N/A	2.9885	4.3539	4.2230	3.7667	3.6330	2.9916	3.9150	3.2344	3.5335	3.4440	1.0362	0.0513	0.6992	0.1554
Klc2	_VDS[Phospho (STY)]PTVNTTLR_.2	#N/A	9.7952	9.6910	4.4324	10.0159	6.6180	9.6887	9.1787	9.2135	9.3674	9.6679	0.8949	-0.1602	0.4306	0.3659
Klc2	_ASS[Phospho (STY)]LNFLNK_.2	O88448_S575_M1_Klc2	9.7473	9.0976	9.8404	9.9145	9.0258	9.5413	9.0842	9.2878	9.6900	9.6751	1.0099	0.0142	0.6927	0.1594
Klc2	_YEVAVPLC[Carbamidomethyl (C)]K_.2	#N/A	5.8302	5.5069	5.8524	5.7793	6.2039	5.5467	4.9355	5.8357	6.0955	5.5249	1.0156	0.0223	0.7237	0.1405
Klc2	_S[Phospho (STY)]GS[Phospho (STY)]FGKLR_.2	O88448_S537_M2_Klc2	5.1633	5.1352	6.3812	5.2839	4.3564	6.0138	4.7541	4.8413	4.1987	5.4921	1.1177	0.1605	0.2151	0.6674
Klc2	_AC[Carbamidomethyl (C)]KVDS[Phospho (STY)]PTVNTTLR_.2	#N/A	3.3519	3.9905	7.6468	7.6189	3.9965	4.1290	6.0150	4.3718	6.7534	3.8075	0.9781	-0.0319	0.9224	0.0351
Klc2	_TLHNLVIQYASQGR_.2	#N/A	4.6852	4.7392	5.0049	3.8330	3.6993	5.3102	5.1389	4.3819	5.1108	5.8686	0.8869	-0.1732	0.1922	0.7162
Klc2	_RSS[Phospho (STY)]EMLVR_.2	O88448_S551_M1_Klc2	2.9184	3.7236	3.6052	4.1079	3.5449	4.1846	4.5454	4.0106	3.6592	4.0749	0.9038	-0.1459	0.1907	0.7197
Capns1	_YADESGNM[Oxidation (M)]DFDNFISC[Carbamidomethyl (C)]LVR_.2	#N/A	2.9216	4.3045	3.6084	4.1111	4.1928	1.7600	3.8258	1.3896	3.6560	3.5647	1.1203	0.1639	0.5967	0.2243
Capns1	_ILGGVISAISEAAAQYNPEPPPPR_.3	#N/A	4.2909	3.0515	4.0246	3.5683	4.0336	3.1900	3.7166	3.4328	3.6772	3.2456	1.0498	0.0701	0.5363	0.2706
Znf106	_ATHVPENS[Phospho (STY)]DTEQDVFTAKPAR_.3	O88466_S1381_M1_Znf106	3.9834	3.2877	3.1693	3.6720	3.1090	3.1196	3.3867	4.4465	4.0951	3.6390	0.8711	-0.1991	0.0911	1.0403
Dync1i1	_IGHDS[Phospho (STY)]ELENQEK_.2	O88485_S180_M1_Dync1i1	8.0865	8.1068	8.7179	8.8930	7.7385	8.1998	8.4390	8.2653	8.2083	8.4960	0.9926	-0.0107	0.7927	0.1009
Dync1i1	_VTQVDFLPR_.2	#N/A	6.5233	6.6542	6.3250	7.0213	6.5777	6.1792	6.1285	6.1527	6.5794	6.4153	1.0361	0.0511	0.2203	0.6571
Dync1i1	_ANRADS[Phospho (STY)]EEEGAVELAA_.2	O88485_S618_M1_Dync1i1	9.9798	12.1249	11.9880	12.5946	11.9378	12.3388	12.2047	12.1011	12.1842	12.3476	0.9687	-0.0459	0.4489	0.3478
Dync1i1	_KEPVQDDS[Phospho (STY)]DLDR_.2	O88485_S50_M1_Dync1i1	7.1070	7.2413	7.0548	7.9919	6.7738	7.7118	7.8841	7.5803	6.7987	7.3805	0.9869	-0.0191	0.7484	0.1259
Dync1i1	_ALAEDSDIFFDYSGR_.2	#N/A	6.1335	2.9438	6.9793	5.8761	6.1510	5.8903	3.8243	3.2896	5.4482	5.7599	1.2362	0.3059	0.2418	0.6165
Dync1i1	_ETQTPLATHQS[Phospho (STY)]EEDEEDEEMVEPK_.3	#N/A	3.8635	3.5912	5.7325	8.0135	5.2532	7.0785	3.6817	6.0322	3.8396	5.7045	1.1608	0.2151	0.4667	0.3310
Dync1i1	_SVSTPSDAGS[Phospho (STY)]QDSGDLGPLTR_.2	O88485_S94_M1_Dync1i1	6.3307	6.8377	7.2905	7.7079	6.1690	7.3203	7.1944	7.0132	6.8888	7.3599	0.9759	-0.0352	0.6064	0.2172
Dync1i1	_[Acetyl (Protein N-term)]SDKSDLKAELER_.3	#N/A	5.4822	5.4663	5.4858	4.8509	5.1150	3.5599	3.4530	5.5584	4.0287	5.3342	1.0870	0.1204	0.4912	0.3088
Dync1i1	_TLQWDTDPSVLQLQSDSELGR_.2	#N/A	3.3981	4.0382	3.9052	4.3249	3.9061	3.8135	4.2578	3.9018	3.2177	3.7868	1.0281	0.0400	0.6555	0.1834
Dync1i1	_LDLWNLNSDTEVPTASVAIEGASALNR_.3	#N/A	3.1629	3.9681	3.8497	4.1526	3.7894	3.2635	4.3009	3.0900	3.1476	3.8299	1.0294	0.0418	0.7387	0.1315
Dync1i2	_SVSTPSEAGS[Phospho (STY)]QDSGDGAVGSR_.2	O88487_S95_M1_Dync1i2	7.4602	7.8098	6.8732	8.5369	6.4559	7.7592	8.2880	7.9685	6.1419	6.0355	1.0526	0.0740	0.5511	0.2588
Dync1i2	_TLAEINANR_.2	#N/A	6.5744	6.5988	6.9724	6.8060	6.6929	3.1803	6.6082	6.2822	7.0713	7.0132	0.9101	-0.1359	0.4465	0.3501
Dync1i2	_EIAVGDSEGQIVIYDVGEQIAVPR_.3	#N/A	5.2494	5.4447	5.8830	6.3841	4.2149	5.4016	5.7962	4.9800	5.1409	5.6389	1.0075	0.0108	0.9217	0.0354
Dync1i2	_DLEDKEGEIQAGAK_.2	#N/A	4.8513	4.5258	5.5666	4.8261	4.8849	4.2072	5.0858	4.3835	4.1495	4.0667	1.0880	0.1216	0.2233	0.6510
Dync1i2	_EAEALLQSM[Oxidation (M)]GLTTDSPIVPPPM[Oxidation (M)]S[Phospho (STY)]PSSK_.3	#N/A	4.1298	3.3473	4.0705	3.9675	3.1399	3.7047	3.3955	3.5411	4.0858	6.1253	0.8693	-0.2021	0.3298	0.4817
Nsd1	_AAS[Phospho (STY)]PQEVTPQADEK_.2	O88491_S2369_M1_Nsd1	8.3502	4.2613	4.1429	3.1447	8.0460	3.6469	8.7966	8.3705	3.1215	3.1117	0.9000	-0.1519	0.7422	0.1295

Pde8a	_RFS[Phospho (STY)]GNEYILATK_.2	O88502_S452_M1_Pde8a	2.9351	3.7403	3.6219	4.1245	3.5616	4.1679	3.8392	3.9940	3.6425	4.0915	0.9486	-0.0761	0.4329	0.3636
Dnmt3a	_SEPQPEEGS[Phospho (STY)]PAAGQK_.2	O88508_S102_M1_Dnmt3a	9.0766	2.9517	9.3707	9.4141	9.3834	9.2691	9.6069	9.1252	9.3473	9.0889	0.8872	-0.1726	0.4529	0.3440
Ikbkg	_S[Phospho (STY)]PPEEPPDFC[Carbamidomethyl (C)]C[Carbamidomethyl (C)]PK_.2	#N/A	9.4675	9.3930	9.0500	10.0842	9.1356	9.0680	10.1141	9.2591	9.3043	9.5235	0.9807	-0.0280	0.4887	0.3109
Ikbkg	_RS[Phospho (STY)]PPEEPPDFC[Carbamidomethyl (C)]C[Carbamidomethyl (C)]PK_.2	#N/A	3.9417	3.2460	3.1276	3.6303	3.0673	3.1613	3.3450	2.9874	4.1368	3.5973	0.9561	-0.0647	0.5703	0.2439
Ppt1	_ETIPLQESTLYTEDR_.2	#N/A	7.2018	7.0769	7.5657	7.2010	7.1716	7.3863	7.2155	6.5118	7.2663	7.1751	1.0320	0.0454	0.2151	0.6673
Zfr	_RDS[Phospho (STY)]DGVDGFEAEGKK_.3	#N/A	7.0107	6.8081	7.7368	7.1703	6.7326	6.9220	7.4305	6.8517	7.2239	7.0587	0.9891	-0.0158	0.7202	0.1426
Zfr	_RRDS[Phospho (STY)]DGVDGFEAEGK_.3	#N/A	5.6322	5.1343	5.8475	5.3691	4.8401	5.1361	6.3504	6.4162	5.4132	6.0904	0.8779	-0.1879	0.0218	1.6616
Zfr	_RDS[Phospho (STY)]DGVDGFEAEGK_.2	#N/A	3.7187	3.6237	3.4524	4.4970	5.7578	4.2845	3.1444	7.0071	5.6394	4.1743	0.8459	-0.2414	0.3630	0.4401
Zfr	_DS[Phospho (STY)]DGVDGFEAEGK_.2	#N/A	3.8199	3.4063	4.5067	3.5085	3.2946	3.3300	6.7926	6.3830	5.9841	6.2925	0.5727	-0.8041	0.0000	5.0884
Cops3	_[Acetyl (Protein N-term)]ASALEQFVNSVR_.2	#N/A	5.5346	5.7142	4.6938	4.9463	4.7633	4.7911	4.8702	5.4386	5.4219	5.5679	0.9529	-0.0696	0.3540	0.4510
Cops3	_YTSQIVGR_.2	#N/A	4.9145	3.2002	4.7439	5.0777	4.2915	5.6936	5.1438	4.9793	4.6388	3.5515	1.0164	0.0235	0.8878	0.0517
Cops3	_SM[Oxidation (M)]GS[Phospho (STY)]QEDDSGNKPSSYS_.2	#N/A	7.1634	6.8888	7.6386	7.1876	6.2056	7.0109	7.3035	7.3618	7.2136	6.9516	0.9734	-0.0389	0.4669	0.3308
Cops3	_YATDTFAGLC[Carbamidomethyl (C)]HQLTNALVER_.3	#N/A	3.2217	4.0269	3.9085	4.0938	3.8482	3.8813	4.2421	4.4082	3.2064	3.7711	0.9803	-0.0287	0.7800	0.1079
Cops4	_NAAQVLVGIPLETGQK_.2	#N/A	5.9000	5.7465	6.1490	6.0526	5.6555	6.3987	6.2638	5.9240	6.2011	6.0136	0.9808	-0.0279	0.4672	0.3305
Cops4	_ATTADGSSILDR_.2	#N/A	6.4935	6.1306	4.1004	6.5634	6.6062	6.5024	5.9227	6.2629	5.6686	6.2841	1.0052	0.0075	0.9525	0.0212
Cops4	_LYLEDDDPVQAEAYINR_.2	#N/A	5.3762	4.4332	4.4454	4.6628	4.1164	4.8975	4.4685	4.9672	4.6009	4.4876	1.0052	0.0075	0.9227	0.0350
Cops4	_VISFEEQVASIR_.2	#N/A	3.8611	4.9389	5.4532	5.2840	5.2605	3.6198	5.2939	4.8909	5.5277	4.5907	0.9331	-0.0999	0.4593	0.3379
Cops4	_ISQTAPEWTAQAM[Oxidation (M)]EAQM[Oxidation (M)]AQ_.2	#N/A	4.1475	4.8475	4.8133	5.1411	4.9919	4.9726	5.1576	5.3529	4.9790	4.9784	0.9418	-0.0866	0.1584	0.8003
Cops4	_IASQMITEGR_.2	#N/A	3.9481	3.2036	3.6818	3.7112	3.9743	3.5328	3.4259	4.4072	4.0558	4.4037	0.9023	-0.1483	0.1270	0.8962
Hnrnpa2b1	_GGGGNFGPGPGSNFR_.2	#N/A	9.1172	9.4002	9.7872	9.5823	9.6528	9.1056	9.2798	9.0217	9.0994	8.8979	1.0403	0.0571	0.0493	1.3069
Hnrnpa2b1	_GGNFGFGDSR_.2	#N/A	9.0503	9.1077	9.5078	9.1501	9.2489	8.9606	8.8156	8.9536	8.9729	8.9861	1.0267	0.0381	0.0480	1.3190
Hnrnpa2b1	_IDTIEIITDR_.2	#N/A	8.7801	8.6542	8.6000	9.1184	8.4937	8.5820	8.7475	8.5146	8.4562	8.6375	1.0135	0.0193	0.3818	0.4182
Hnrnpa2b1	_EDTEEHHLR_.2	#N/A	7.0832	6.7010	5.7866	7.1780	7.6331	7.1345	7.2421	7.0492	6.2545	6.9754	1.0057	0.0082	0.9171	0.0376
Hnrnpa2b1	_GFGFVTFDDHDPVDK_.2	#N/A	6.7639	6.5631	6.8626	7.2184	6.3602	6.4034	6.6321	6.1767	6.3693	6.6844	1.0355	0.0503	0.2603	0.5846
Hnrnpa2b1	_NYYEQWGK_.2	#N/A	7.6835	7.5270	7.4598	7.4183	7.9029	7.5484	5.9702	7.4402	7.8391	7.9428	1.0400	0.0566	0.4547	0.3422
Hnrnpa2b1	_PHS[Phospho (STY)]IDGR_.2	O88569_S85_M1_Hnrnpa2b1	4.2811	4.6225	4.3194	5.7307	3.4818	5.1431	5.2684	4.3891	4.6416	5.2991	0.9381	-0.0921	0.5053	0.2965
Hnrnpa2b1	_YHTINGHNAEVR_.3	#N/A	5.9466	5.3259	4.4730	4.0168	6.3394	5.9884	5.2185	5.0109	5.6827	5.0577	1.0202	0.0289	0.8336	0.0790
Hnrnpa2b1	_QEMQEVQSSR_.2	#N/A	6.9366	5.5361	3.5133	7.7990	6.6587	4.2765	3.1364	5.4292	4.6656	5.3358	1.2467	0.3181	0.2585	0.5875
Hnrnpa2b1	_LTDC[Carbamidomethyl (C)]VVMR_.2	#N/A	4.5187	4.2788	4.2981	5.3542	4.3552	3.6293	3.9901	3.1593	3.4584	3.5191	1.2475	0.3190	0.0240	1.6202
Hnrnpa2b1	_DYFEEYGKIDTIEIITDR_.3	#N/A	6.4705	7.2864	8.3907	8.0432	7.1315	7.6378	6.6203	6.7358	6.4461	6.9246	1.1215	0.1654	0.0535	1.2719
Hnrnpa2b1	_DYFEEYGK_.2	#N/A	3.9849	3.9449	4.4231	4.5271	3.8747	4.5651	4.1854	3.9715	3.3333	3.6442	1.1153	0.1575	0.0818	1.0870
Hnrnpa2b1	_EES[Phospho (STY)]GKPGAHVTVK_.2	O88569_S102_M1_Hnrnpa2b1	6.9366	7.9957	8.5332	7.9669	7.4091	7.9785	8.1043	3.0228	3.8494	3.7149	1.6699	0.7398	0.0114	1.9430
Hnrnpa2b1	_LFIGGLSFETTEESLR_.2	#N/A	4.3453	4.1893	5.0870	5.1437	3.5107	4.6654	4.9562	4.0171	4.6221	4.8426	0.9741	-0.0378	0.7451	0.1278
Hnrnpa2b1	_EESGKPGAHVTVKK_.3	#N/A	4.3150	2.8065	4.4728	4.5013	4.7447	4.2888	3.1487	4.0007	5.3827	4.1014	1.0072	0.0103	0.9548	0.0201
Hnrnpa2b1	_GFGDGYNGYGGGPGGGNFGGS[Phospho (STY)]PGYGGGR_.3	O88569_S259_M1_Hnrnpa2b1	3.6965	3.6459	3.4302	4.4748	2.8402	3.7844	3.1222	4.0272	4.3263	4.1521	0.9330	-0.1000	0.4686	0.3292
Hnrnpa2b1	_NM[Oxidation (M)]GGPYGGGNYGPGGSGGS[Phospho (STY)]GGYGGR_.3	#N/A	3.4697	3.7137	3.9204	4.5958	3.6046	3.7347	4.1015	3.8535	3.3959	3.9486	1.0039	0.0056	0.9512	0.0217
Hnrnpa2b1	_GGSDGYGSGR_.2	#N/A	3.9680	3.3745	3.7016	3.2454	3.1958	3.0329	3.3937	3.7558	4.0548	4.4235	0.8753	-0.1922	0.0857	1.0669
Comt	_AVYQGPGSS[Phospho (STY)]PVKS_.2	O88587_S261_M1_Comt	11.7758	11.9327	12.5741	12.1576	11.3748	11.8272	12.3752	11.9356	11.8870	11.8555	0.9939	-0.0088	0.7558	0.1216
Cacng2	_DAS[Phospho (STY)]PVGVK_.2	O88602_S253_M1_Cacng2	8.4610	7.3813	7.7348	7.4636	6.7136	7.4454	6.8939	3.7890	8.1504	8.0795	1.1197	0.1631	0.3754	0.4255
Cacng2	_STEPSHS[Phospho (STY)]RDAS[Phospho (STY)]PVGVK_.3	O88602_S249_M2_Cacng2	4.8092	4.1667	5.6914	4.5510	3.9880	3.7414	4.2657	5.9869	4.7954	3.0171	0.9945	-0.0080	0.9680	0.0141
Pak1	_LLQTSNITK_.2	#N/A	7.4110	7.3897	7.6764	7.5489	7.8028	7.3241	7.3022	7.2382	7.4559	7.0019	1.0381	0.0539	0.0514	1.2892
Pak1	_STM[Oxidation (M)]VGTPYWM[Oxidation (M)]APEVVTR_.2	#N/A	7.0704	7.5722	7.5297	7.0372	7.2819	7.6052	7.2618	7.4466	7.2574	7.0584	1.0129	0.0184	0.5372	0.2699
Pak1	_ALYLIATNGTPELQNPEK_.2	#N/A	5.7763	5.5204	6.2295	6.2742	6.3124	6.3165	6.0081	6.0260	5.8671	6.0303	1.0148	0.0212	0.6276	0.2023
Pak1	_EC[Carbamidomethyl (C)]LQALEFLHSNQVIHR_.3	#N/A	6.3248	6.0993	6.5556	6.3707	5.8365	5.9011	6.3009	5.7441	5.8631	6.4798	1.0138	0.0198	0.6842	0.1648
Pak1	_SAEDYNS[Phospho (STY)]SNTLNVK_.2	O88643_S155_M1_Pak1	4.5119	6.2681	5.9350	3.8259	5.8705	4.5870	4.2703	4.1409	4.1619	4.2282	1.2300	0.2987	0.0914	1.0390
Pak1	_DVATSPIS[Phospho (STY)]PTENNTTPPDALTR_.2	#N/A	9.5005	13.3047	13.8696	13.9094	12.5771	13.2727	13.4996	13.0331	13.1162	13.0736	0.9665	-0.0492	0.6178	0.2091
Pak1	_MSDEEILEK_.2	#N/A	4.4084	4.7126	3.6710	5.2205	4.6960	4.1187	4.4795	4.1708	2.9792	4.0085	1.1437	0.1937	0.1730	0.7618
Pak1	_ELIINEILVM[Oxidation (M)]R_.2	#N/A	3.3132	4.5667	4.5477	4.8127	4.3050	3.8789	4.9653	4.5399	4.4595	3.9920	0.9439	-0.0833	0.4577	0.3394
Pak1	_SDNILLGM[Oxidation (M)]DGSVK_.2	#N/A	3.9621	6.8958	6.1495	3.6973	6.8106	6.1344	6.8071	6.3524	7.1119	6.6696	0.8327	-0.2642	0.1630	0.7877
Pak1	_LTDFGFC[Carbamidomethyl (C)]AQITPEQSK_.2	#N/A	3.4407	4.2459	4.1275	3.1293	5.8655	3.6623	4.3448	3.4883	3.1369	3.0963	1.1598	0.2139	0.3330	0.4775
Pak1	_SVIEPLPVTPTR_.2	#N/A	8.3871	8.3011	8.6251	8.5369	8.6508	8.3223	8.0618	8.5562	8.4010	8.4957	1.0110	0.0157	0.4565	0.3406
Pak1	_LLQTSNITKS[Phospho (STY)]EQK_.2	O88643_S115_M1_Pak1	6.5028	6.5692	7.3899	7.4145	6.8818	6.8687	7.1959	6.7798	6.8327	7.0832	0.9950	-0.0073	0.8741	0.0584
Pak1	_[Acetyl (Protein N-term)]S[Phospho (STY)]NNGVDIQDKPPAPPMR_.2	O88643_S2_M1_Pak1	3.0759	4.2665	4.3104	6.4244	2.9193	3.6416	5.4306	3.1470	3.4461	3.5314	1.0559	0.0785	0.7846	0.1053
Pak1	_IGQGASGTVYTAMDVATGQEVAIK_.3	#N/A	3.0367	3.8419	3.2342	4.2788	3.6632	4.0663	4.4271	4.2232	3.0214	3.9561	0.9437	-0.0837	0.5429	0.2653
Psmc3	_VDILDPALLR_.2	#N/A	6.5462	6.4838	6.0381	7.0358	6.7601	6.0829	6.2818	7.0296	6.7411	6.5475	0.9761	-0.0349	0.5147	0.2885
Psmc3	_DSYLILETLPTEYDSR_.2	#N/A	7.0006	5.8925	6.4669	6.1991	6.7574	6.4747	6.6224	6.1096	6.1410	7.0045	0.9994	-0.0009	0.9877	0.0054
Psmc3	_QTYFLPVIGLVDAEK_.2	#N/A	5.4553	4.8928	4.1836	5.5810	5.3765	4.7919	5.2075	4.8289	4.1766	5.7749	1.0100	0.0143	0.8983	0.0466
Psmc3	_M[Oxidation (M)]ATVWDEAEQDGIGEEVLK_.2	#N/A	4.3493	5.2613	6.0869	6.1678	4.0755	5.3947	5.4028	5.1799	5.1741	4.5186	1.0303	0.0431	0.7505	0.1247
Psmc3	_[Acetyl (Protein N-term)]M[Oxidation (M)]QEMNLLPTPES[Phospho (STY)]PVTR_.2	#N/A	7.3235	3.2764	3.5237	9.3587	3.2773	3.9230	3.4568	6.8746	4.0150	3.6199	1.1385	0.1872	0.6827	0.1658
Hcn2	_TSPYGVPGS[Phospho (STY)]PATR_.2	O88703_S771_M1_Hcn2	10.2909	9.3380	9.5097	10.0341	7.5736	3.9211	11.0213	10.2502	9.7744	10.5232	0.8126	-0.2994	0.1572	0.8035
Hcn2	_GEPQC[Carbamidomethyl (C)]S[Phospho (STY)]PEGPAR_.2	O88703_S90_M1_Hcn2	11.9864	11.6529	11.7096	11.8967	10.6025	11.4381	12.4036	11.9400	11.8058	11.6311	0.9667	-0.0488	0.2040	0.6904
Hcn2	_PLVGPLALGS[Phospho (STY)]PR_.2	O88703_S726_M1_Hcn2	5.2905	7.6889	5.9885	8.5723	3.8655	4.6349	6.5495	7.1833	5.6170	4.4521	1.0095	0.0136	0.9580	0.0186
Hcn2	_GPKVS[Phospho (STY)]FSC[Carbamidomethyl (C)]R_.2	O88703_S101_M1_Hcn2	3.0496	4.1765	3.7364	6.4082	3.6762	4.0606	3.9538	3.8794	3.5280	4.2061	1.0752	0.1046	0.6394	0.1942
Hcn2	_GSQAS[Phospho (STY)]FLQR_.2	O88703_S134_M1_Hcn2	8.0894	7.6403	7.6560	6.2291	4.6183	7.4565	8.3604	8.2747	8.2328	8.0376	0.8446	-0.2436	0.0916	1.0379
Hcn2	_TAAPS[Phospho (STY)]PDRR_.2	O88703_S834_M1_Hcn2	7.5071	7.5125	7.9501	7.6769	6.1854	7.4113	8.0643	7.9272	7.7751	7.4373	0.9452	-0.0812	0.2319	0.6347
Hcn2	_DSAS[Phospho (STY)]PGAASGLDPLDSAR_.2	#N/A	10.6065	10.4433	11.2145	10.5582	9.3241	10.3952	11.2015	10.5684	10.4790	10.3851	0.9780	-0.0321	0.5163	0.2871
Hcn2	_LC[Carbamidomethyl (C)]S[Phospho (STY)]RDS[Phospho (STY)]AC[Carbamidomethyl (C)]TPGAAK_.2	O88703_S64_M2_Hcn2	5.8904	3.9724	5.6978	5.7045	4.2087	5.1469	3.8513	3.4763	3.8304	5.5645	1.2207	0.2878	0.1390	0.8569
Hcn2	_GAASGPSAAEEAGS[Phospho (STY)]EEAGPAGEPR_.2	O88703_S119_M1_Hcn2	8.6231	8.6508	9.1811	8.6917	7.4783	8.4969	9.3528	8.8461	8.6327	8.4099	0.9671	-0.0483	0.4025	0.3952
Hcn2	_RDS[Phospho (STY)]ASPGAASGLDPLDSAR_.3	O88703_S840_M1_Hcn2	3.8022	3.0795	3.8635	3.5233	3.7908	3.4992	3.6410	3.9629	3.6115	3.4903	0.9773	-0.0331	0.6374	0.1956
Hcn2	_TAAPS[Phospho (STY)]PDRRDS[Phospho (STY)]AS[Phospho (STY)]PGAASGLDPLDSAR_.3	O88703_S834_M3_Hcn2	3.3527	3.7930	3.8970	3.6398	3.6491	4.2857	4.4110	3.7262	4.0252	3.8083	0.9441	-0.0830	0.2974	0.5267
Hcn2	_RAPPGPLPPAAS[Phospho (STY)]PGPPAAS[Phospho (STY)]PPAAPS[Phospho (STY)]SPR_.3	O88703_S743_M3_Hcn2	3.8917	3.1471	3.2650	3.7677	3.0354	3.5892	3.4824	6.8306	3.9994	3.7347	0.7645	-0.3874	0.1366	0.8645
Hcn1	_LGT[Phospho (STY)]PPGGGAAGK_.2	#N/A	11.4549	11.2703	11.6757	11.3431	10.2648	10.8158	11.7445	11.6035	11.2767	11.1434	0.9734	-0.0389	0.3149	0.5018
Hcn1	_RLGT[Phospho (STY)]PPGGGAAGK_.2	#N/A	8.7290	8.7777	8.8083	9.0156	7.9186	8.6068	9.2895	8.9095	8.6130	8.6392	0.9752	-0.0363	0.3663	0.4362
Hcn1	_VDGGGGEEPAGS[Phospho (STY)]FEDAEGPR_.2	#N/A	6.0008	7.1713	7.7335	7.1765	5.6918	7.0235	8.0041	7.5353	7.2412	6.8973	0.9165	-0.1259	0.1971	0.7053
Hcn1	_KNS[Phospho (STY)]ILLQK_.2	O88704_S588_M1_Hcn1	4.9655	4.0536	4.2600	3.7737	2.4948	3.9913	5.2987	4.9805	4.6749	4.5198	0.8058	-0.3115	0.0617	1.2094
Hcn1	_VDGGGGEEPAGS[Phospho (STY)]FEDAEGPRR_.3	#N/A	3.7951	4.7842	4.2192	5.2614	3.9894	4.4118	5.0054	5.3562	5.4318	4.6100	0.8646	-0.2099	0.0584	1.2337
Hcn1	_QMSS[Phospho (STY)]GAIPPNR_.2	O88704_S868_M1_Hcn1	3.8809	3.1853	4.5677	3.5696	3.0066	3.2220	3.2842	3.0481	4.1975	3.5366	1.0158	0.0226	0.8797	0.0557
Hcn3	_KRS[Phospho (STY)]EPS[Phospho (STY)]PGSSGGVM[Oxidation (M)]EQHLVQHDR_.4	#N/A	3.0666	4.2787	4.3012	3.8449	3.8572	4.0113	4.9833	3.1591	3.4582	3.5222	1.0298	0.0423	0.7836	0.1059
Ctbp1	_PLVALLDGR_.2	#N/A	7.6145	7.6466	7.3513	7.7954	7.4895	7.4766	7.3218	7.6671	7.7919	7.5871	0.9961	-0.0056	0.7975	0.0983

Ctbp1	_IGSGFDNIDIK_.2	#N/A	5.1878	5.7224	5.7806	4.7124	4.4578	4.0902	5.0776	5.3174	4.9507	5.0601	0.9785	-0.0313	0.7665	0.1155
Ctbp1	_GAALDVHESEPFSFSQGPLK_.3	#N/A	5.6871	5.3932	5.8704	5.8028	5.7750	5.4569	5.2138	4.9900	5.6131	5.0210	1.0873	0.1207	0.0168	1.7757
Ctbp1	_VGQAVALR_.2	#N/A	5.2885	5.0545	5.5751	5.2621	5.4285	4.8699	4.7434	5.2479	5.4872	5.2225	1.0137	0.0197	0.7009	0.1543
Ctbp1	_TTWLHQALR_.2	#N/A	6.2086	6.9788	6.9287	6.5529	6.7517	6.6631	7.3883	6.9131	6.9376	6.5020	0.9633	-0.0540	0.2443	0.6120
Ctbp1	_VQSVEQIR_.2	#N/A	6.7654	6.7602	6.8244	7.1255	6.9224	6.6964	6.6221	6.6782	6.8811	6.7850	1.0159	0.0228	0.2734	0.5632
Ctbp1	_DVATVAFC[Carbamidomethyl (C)]DAQSTQEIHEK_.3	#N/A	7.1584	6.8382	8.1853	7.8206	6.1338	7.0168	7.3303	6.5949	6.5993	6.7300	1.0556	0.0780	0.3683	0.4338
Ctbp1	_VLNEAVGALM[Oxidation (M)]YHTITLTR_.2	#N/A	3.3166	3.0501	3.1173	4.0705	3.0532	3.4523	3.7852	4.0490	2.8832	4.0375	0.9064	-0.1418	0.2755	0.5598
Map7	_LSSSSATLLNS[Phospho (STY)]PDR_.2	O88735_S209_M1_Map7	10.2649	9.9692	10.4756	10.7407	9.4296	10.1575	10.4887	9.9587	9.8935	10.3152	1.0009	0.0013	0.9732	0.0118
Bsn	_SLS[Phospho (STY)]DPKPLS[Phospho (STY)]PTAEESAKER_.3	#N/A	6.3251	8.5455	8.1678	8.5003	7.5010	8.6977	8.1821	7.8736	7.9086	8.6482	0.9759	-0.0353	0.6946	0.1583
Bsn	_GEEES[Phospho (STY)]EEDSYDPR_.2	O88737_S3301_M1_Bsn	10.4232	10.5324	10.9606	11.3352	9.7988	10.6358	10.7462	10.6136	10.4911	10.7857	0.9958	-0.0061	0.8724	0.0593
Bsn	_DYLS[Phospho (STY)]DSELNQLR_.2	#N/A	3.8523	8.5950	4.5391	11.1581	8.5512	9.4155	3.2556	10.0938	9.2499	9.6937	0.9519	-0.0711	0.8467	0.0723
Bsn	_DAC[Carbamidomethyl (C)]EPES[Phospho (STY)]GPDPSTVR_.2	#N/A	9.4514	8.9790	9.1810	10.2052	8.7684	9.5005	9.6884	9.1068	9.3985	9.3939	0.9948	-0.0076	0.8616	0.0647
Bsn	_DST[Phospho (STY)]PTASESQR_.2	O88737_T3254_M1_Bsn	3.7410	3.4852	4.4278	9.6339	2.8666	8.5270	3.1443	9.0754	9.0085	9.0841	0.7188	-0.4764	0.2892	0.5388
Bsn	_ATSVPGPTQATAPPEVGR_.2	#N/A	8.0229	8.4291	8.9584	8.7093	8.6590	8.4523	8.6422	8.2487	8.4324	8.3186	1.0152	0.0218	0.4875	0.3120
Bsn	_DPEPPEPLTFR_.2	#N/A	7.3640	7.1296	7.0541	7.2038	7.0832	7.0977	7.1492	7.0354	6.9841	7.6920	0.9917	-0.0120	0.6833	0.1654
Bsn	_AS[Phospho (STY)]PQAKPLR_.2	#N/A	6.5782	6.2371	6.8594	6.7062	5.9745	6.7425	6.5620	6.5256	6.5720	6.2389	1.0064	0.0093	0.8271	0.0825
Bsn	_ELQELQTIK_.2	#N/A	5.5981	6.4090	6.5950	6.1203	6.1576	5.6603	5.1647	5.4740	5.9208	5.9729	1.0811	0.1125	0.1089	0.9631
Bsn	_AQGPSGQEAES[Phospho (STY)]PR_.2	#N/A	5.5377	6.0789	5.9484	6.5546	3.0525	6.2601	5.9005	5.7843	5.5311	6.2337	0.9505	-0.0733	0.6730	0.1720
Bsn	_YLELGITQR_.2	#N/A	4.9723	5.7188	5.6812	5.6481	4.9950	5.4730	5.4939	5.4466	5.3178	5.3869	1.0006	0.0009	0.9852	0.0065
Bsn	_SQASEEES[Phospho (STY)]PVS[Phospho (STY)]PLGRPR_.2	#N/A	8.9323	9.5279	9.8516	10.2336	9.4117	9.7096	9.7726	9.5262	9.7310	9.7175	0.9922	-0.0113	0.7484	0.1259
Bsn	_QTS[Phospho (STY)]LADLEQK_.2	O88737_S3158_M1_Bsn	2.9811	4.3613	4.2156	8.4409	4.1827	3.5468	3.9076	3.2418	3.5409	3.4366	1.3085	0.3879	0.3063	0.5138
Bsn	_SHGPLLPTIEDSS[Phospho (STY)]EEEELREEEELLREQEK_.4	#N/A	3.4238	3.8873	3.6387	4.3905	5.9210	4.0649	4.3409	3.9303	3.9782	4.2180	1.0253	0.0361	0.8282	0.0819
Bsn	_LRDAC[Carbamidomethyl (C)]EPES[Phospho (STY)]GPDPSTVR_.3	#N/A	4.0813	2.9802	5.4460	3.5304	3.2182	5.3247	4.6060	3.2865	4.0570	5.3580	0.9468	-0.0788	0.7302	0.1366
Bsn	_SQAS[Phospho (STY)]EEESPVSPLGR_.2	#N/A	12.1586	12.0561	12.6443	12.7590	11.6427	12.2531	12.5375	11.9312	12.1509	12.3502	1.0008	0.0012	0.9671	0.0145
Bsn	_IADSSVQT[Phospho (STY)]DDEEGEGR_.2	#N/A	9.3918	9.7547	10.0452	10.6012	9.0538	9.7567	9.8586	9.6441	9.4975	9.7771	1.0075	0.0108	0.8007	0.0965
Bsn	_EKPLSGGDSEVGAPQPSR_.3	#N/A	7.0014	6.9220	6.8045	7.2758	7.0854	6.8090	6.2662	6.8647	6.8119	6.5486	1.0544	0.0764	0.0353	1.4525
Bsn	_ASPQTTKAS[Phospho (STY)]PQAK_.2	#N/A	5.2035	4.0985	3.8145	6.2303	3.9868	4.1386	5.3078	3.8014	3.4499	5.8390	0.9955	-0.0065	0.9758	0.0107
Bsn	_SLS[Phospho (STY)]DPKPLSPTAEESAK_.2	#N/A	13.1305	13.2754	13.8438	13.7764	12.6337	13.3149	13.6239	13.0601	13.2207	13.4276	0.9997	-0.0004	0.9876	0.0054
Bsn	_SADC[Carbamidomethyl (C)]SVQT[Phospho (STY)]DDEDNADWEQPVRR_.3	O88737_T2622_M1_Bsn	3.7256	3.6168	3.4593	7.6901	2.8693	4.2914	3.1513	3.9981	4.2972	7.2487	0.9148	-0.1285	0.7339	0.1344
Bsn	_AQGPSGQEAES[Phospho (STY)]PRR_.2	#N/A	7.4976	7.9907	8.3197	8.3684	7.6304	8.0588	7.9280	7.8977	7.8769	7.9314	1.0087	0.0125	0.7127	0.1471
Bsn	_YLGQGLQYGS[Phospho (STY)]FTDLR_.2	O88737_S2046_M1_Bsn	4.3164	3.7884	4.0501	4.4545	3.7060	4.0910	4.5354	4.9287	4.8366	4.8588	0.8492	-0.2358	0.0022	2.6533
Bsn	_QVEQAVQTAPYR_.2	#N/A	4.5388	5.1052	4.0410	3.5848	5.4968	5.1409	5.4635	5.3679	5.6040	4.7608	0.8778	-0.1881	0.1455	0.8373
Bsn	_GLGEHRDYLS[Phospho (STY)]DSELNQLR_.3	#N/A	3.3790	4.0687	3.1127	7.0456	4.0236	4.1018	3.9950	6.2532	4.6058	3.8346	0.9179	-0.1236	0.6610	0.1798
Bsn	_RIADSS[Phospho (STY)]VQTDDEEGEGR_.2	O88737_S2592_M1_Bsn	3.7526	3.7376	5.5707	5.8255	4.0992	5.2378	4.7778	4.9076	3.8939	5.0824	1.0082	0.0119	0.9436	0.0252
Bsn	_EQQDTAESS[Phospho (STY)]DDFGSQLR_.2	#N/A	4.4256	3.7484	4.1610	4.3304	3.9831	3.8544	3.9208	3.4678	4.5481	3.9403	1.0289	0.0411	0.6201	0.2075
Bsn	_LLEGSLGEPAPLPLPTPQQPPAGVPHR_.3	#N/A	4.6799	3.9036	3.1725	4.1343	3.6754	4.2545	4.3653	4.1652	4.2949	5.3313	0.8746	-0.1933	0.1309	0.8830
Bsn	_SQQQLHS[Phospho (STY)]PALS[Phospho (STY)]PAHSPAK_.3	#N/A	4.9742	5.2020	3.9929	4.7412	3.4911	4.4918	3.9956	4.9469	5.0173	5.4827	0.9222	-0.1169	0.3823	0.4176
Bsn	_SSC[Carbamidomethyl (C)]SEYS[Phospho (STY)]PSPSLDSEAETLDGGPTR_.3	O88737_S1137_M1_Bsn	7.8925	3.7650	3.6466	4.1493	3.5864	4.1431	3.8640	3.9692	3.6178	8.2199	0.9213	-0.1183	0.7586	0.1200
Bsn	_EPELEM[Oxidation (M)]ESLTGS[Phospho (STY)]PEDR_.2	#N/A	4.6763	7.6720	6.3691	8.1139	7.4404	7.9050	8.2393	8.0075	8.4028	8.3829	0.8512	-0.2324	0.1037	0.9844
Bsn	_SQGS[Phospho (STY)]FEYQDTQDHDYGGR_.3	#N/A	8.2970	7.6866	8.1489	8.5187	6.6123	8.3616	7.0503	7.8181	8.2167	8.3074	1.0114	0.0163	0.8393	0.0761
Bsn	_SHGPLLPTIEDSS[Phospho (STY)]EEEELREEEELLR_.4	#N/A	4.5775	3.3996	4.0543	3.6849	3.1219	3.1192	3.3995	3.8173	4.0946	3.6519	0.9783	-0.0317	0.8028	0.0954
Bsn	_SSS[Phospho (STY)]SAVEDQKR_.2	O88737_S767_M1_Bsn	4.5780	3.4891	3.8796	3.6304	4.5178	3.8464	4.7018	3.2902	3.8598	3.0944	1.0679	0.0948	0.5097	0.2927
Bsn	_MASAYS[Phospho (STY)]GEK_.2	O88737_S3460_M1_Bsn	3.5550	3.6711	4.2419	3.2437	3.5594	3.0652	4.4592	3.3740	3.0226	3.2107	1.0112	0.0161	0.9073	0.0423
Bsn	_LQPQSQPTTR_.2	#N/A	3.3993	6.1121	3.1329	6.5392	6.9013	5.4386	5.8550	5.9562	5.6835	5.7762	0.9031	-0.1471	0.5144	0.2887
Bsn	_QASATAPGRES[Phospho (STY)]PR_.2	O88737_S105_M1_Bsn	7.7698	7.8067	7.6520	8.3158	7.3584	7.7069	8.2045	7.6453	7.7262	7.8621	0.9884	-0.0168	0.6381	0.1951
Bsn	_ASPQATKAS[Phospho (STY)]PQTTK_.2	O88737_S593_M1_Bsn	7.1442	3.6855	3.7160	8.2326	7.0480	3.7937	4.1010	7.4502	7.5600	3.6300	0.9856	-0.0209	0.9533	0.0208
Bsn	_GYM[Oxidation (M)]TPT[Phospho (STY)]SPAGSER_.2	#N/A	3.8292	6.4951	3.5296	3.9544	3.3551	3.9827	4.2850	4.0047	4.0914	3.7335	1.0403	0.0570	0.7924	0.1011
Bsn	_LLDTSFAS[Phospho (STY)]SER_.2	O88737_S2822_M1_Bsn	8.9684	8.8455	4.9249	9.2424	7.9627	8.9159	9.0769	8.4766	5.0175	9.0368	1.0305	0.0434	0.8368	0.0774
Bsn	_GPGATQSGPR_.2	#N/A	5.3883	3.0614	4.0147	5.8202	6.6565	4.5570	3.7067	5.2073	5.5111	5.7357	0.9754	-0.0359	0.8739	0.0585
Bsn	_DSAVDLSSLK_.2	#N/A	5.0637	4.3232	3.4612	4.5058	5.2590	4.7440	3.1532	4.3660	5.1639	5.7372	0.9901	-0.0144	0.9360	0.0287
Bsn	_SKTPPSNLSPIEDAS[Phospho (STY)]PTEELR_.3	O88737_S1114_M1_Bsn	7.0123	7.5993	7.5601	7.6961	8.1390	7.9100	6.8831	8.1870	8.3502	8.4088	0.9617	-0.0563	0.4038	0.3939
Bsn	_DAC[Carbamidomethyl (C)]EPES[Phospho (STY)]GPDPSTVRR_.3	#N/A	3.6128	3.6133	4.2996	3.3015	4.2394	3.4901	4.5170	3.3162	2.9648	4.5465	0.9800	-0.0291	0.8471	0.0720
Bsn	_LHSS[Phospho (STY)]PVSSTLTSK_.2	O88737_S1370_M1_Bsn	3.0546	4.1715	3.7414	4.2441	3.6812	4.0483	3.9588	3.8744	3.5230	4.2111	0.9825	-0.0255	0.7925	0.1010
Bsn	_HSYS[Phospho (STY)]LGFADGR_.2	O88737_S2029_M1_Bsn	3.6084	3.7267	3.3421	4.8404	4.1860	3.8886	3.6003	6.5077	3.9565	4.1936	0.8614	-0.2152	0.3121	0.5057
Bsn	_QPQTQQQQQQQQQQGLGQQAPQQAPSQAR_.3	#N/A	3.5590	3.5838	4.0591	4.9699	3.4951	3.9936	4.1848	3.4732	4.3276	4.0251	0.9852	-0.0215	0.8577	0.0666
Bsn	_SPQVLYS[Phospho (STY)]PVS[Phospho (STY)]PLSPHR_.2	#N/A	3.7706	3.9803	3.8576	7.4902	4.9900	4.5433	3.8215	6.0585	4.6877	5.9269	0.9314	-0.1026	0.6844	0.1647
Bsn	_QAAPGPQQSQPPSSR_.2	#N/A	6.4957	6.7175	6.7543	7.2409	6.9911	3.8888	6.8476	6.8008	4.4307	4.0477	1.1476	0.1986	0.3725	0.4289
Bsn	_YSS[Phospho (STY)]VSNIYSDHR_.2	O88737_S2068_M1_Bsn	6.2024	3.9529	4.6116	6.5421	6.0834	4.9279	3.8257	3.4403	6.0999	6.5858	1.0800	0.1110	0.6395	0.1942
Bsn	_AS[Phospho (STY)]GAGGPPR_.2	O88737_S2295_M1_Bsn	4.2197	3.2189	3.1932	3.6959	3.1330	3.9787	3.4106	4.4226	4.0712	3.6629	0.9181	-0.1232	0.3100	0.5086
Bsn	_LQLEQIQQLQQQLQLQLEEQK_.3	#N/A	3.5523	2.7034	4.2391	2.8371	2.2642	3.8292	3.4067	3.3767	3.4610	3.2079	0.9627	-0.0549	0.7542	0.1225
Bsn	_ISKFS[Phospho (STY)]PIEEAK_.2	#N/A	8.9294	3.7510	8.5833	9.2536	7.9840	8.7359	9.0258	8.4364	8.7956	9.0054	0.8930	-0.1632	0.3989	0.3991
Bsn	_ASPQATKAS[Phospho (STY)]PQATK_.2	O88737_S579_M1_Bsn	10.0866	10.5797	10.9614	10.9619	9.8766	10.5727	10.4842	10.3368	10.1690	10.4203	1.0149	0.0213	0.5295	0.2761
Bsn	_QREQQDTAESS[Phospho (STY)]DDFGSQLR_.3	#N/A	2.5353	4.2696	3.7663	3.9799	3.3434	4.6799	3.5635	3.8845	3.3526	3.6572	1.0409	0.0579	0.7167	0.1447
Bsn	_LYSSM[Oxidation (M)]SDT[Phospho (STY)]NLAEAGLNYHAQR_.3	O88737_T1992_M1_Bsn	3.2614	4.0666	3.9482	4.4508	3.8879	3.8416	4.2024	3.6677	3.3162	4.4178	1.0021	0.0031	0.9768	0.0102
Bsn	_HREEEQLLVQR_.3	#N/A	4.0932	3.5008	3.7983	4.5453	4.1312	4.0839	4.7762	3.7786	4.8999	3.7378	0.9366	-0.0945	0.3980	0.4001
Bsn	_GYSYFTGSS[Phospho (STY)]PPLSPSTPS[Phospho (STY)]ESPTFSPGK_.3	#N/A	3.2747	4.1212	4.1636	7.5310	3.9194	3.9133	4.1037	3.3169	3.3576	3.9114	1.2219	0.2891	0.3346	0.4754
Bsn	_VTQHFAK_.2	#N/A	2.9047	3.7099	3.5915	4.0941	4.5725	3.8983	4.5591	3.3863	3.6729	4.0611	0.9682	-0.0466	0.7291	0.1372
Bsn	_SQQQLHS[Phospho (STY)]PALS[Phospho (STY)]PAHS[Phospho (STY)]PAKQPLGKPEQER_.4	O88737_S241_M3_Bsn	3.1734	4.1742	4.1933	4.1462	4.0625	4.0667	4.0902	4.0716	3.4028	3.9780	1.0215	0.0308	0.7347	0.1339
Bsn	_SPSVS[Phospho (STY)]PDR_.2	O88737_S142_M1_Bsn	4.8236	3.8467	4.3249	4.5689	3.7350	4.3904	4.2836	3.5496	3.1981	3.0351	1.2175	0.2840	0.0371	1.4312
Bsn	_SSVSQSPAPTYPSDSHYTSLEQNVPR_.3	#N/A	3.4499	3.6978	5.0578	4.4229	3.4061	4.2103	3.0703	2.4856	4.3782	4.1002	1.1517	0.2038	0.2997	0.5233
Bsn	_HDY[Phospho (STY)]VEDSS[Phospho (STY)]EGGLSPLPPQPPAR_.3	O88737_Y829_M2_Bsn	3.4023	3.6977	3.8338	4.5016	3.5677	4.0092	3.9291	3.7542	3.6866	3.9872	0.9990	-0.0015	0.9854	0.0064
Bsn	_GEHSSTLPASTPSYTSGTSPTSLSSLEEDSDSSPS[Phospho (STY)]RR_.4	O88737_S1021_M1_Bsn	3.5562	3.7862	3.2899	4.3345	3.6075	4.1220	4.4828	4.1675	2.9657	4.0118	0.9682	-0.0466	0.7131	0.1468
Bsn	_FS[Phospho (STY)]PIEEAK_.2	#N/A	3.3283	4.0846	4.5628	3.0597	3.9729	4.1526	4.0457	4.3954	3.1936	3.7839	1.0014	0.0020	0.9879	0.0053
Bsn	_ASQPVAESTPAGLGAAVYEEILQTSQSIAR_.3	#N/A	3.7515	3.0069	3.4851	3.9079	2.8952	3.7294	3.6226	4.2106	3.8592	3.8749	0.8897	-0.1686	0.1050	0.9788
Bsn	_GPANYNTC[Carbamidomethyl (C)]TAC[Carbamidomethyl (C)]K_.2	#N/A	3.2467	4.0030	4.4812	4.0250	3.8913	4.2342	4.1733	2.9762	3.2752	3.7023	1.1270	0.1725	0.1678	0.7751
Bsn	_S[Phospho (STY)]KSQQQLHSPALSPAHSPAK_.4	O88737_S233_M1_Bsn	3.0257	3.8309	3.7125	4.2151	3.6522	4.0773	3.9298	3.9033	3.5519	4.1821	0.9641	-0.0527	0.5703	0.2439
Bsn	_ADTQAQPQMQGR_.2	#N/A	4.1274	3.3828	3.8610	5.4642	3.2711	3.3535	3.2467	3.0856	4.2351	3.0821	1.1458	0.1964	0.3280	0.4841
Birc6	_SDS[Phospho (STY)]VTGHTSQK_.2	O88738_S493_M1_Birc6	6.0877	4.9628	5.4657	5.2146	4.1036	3.6259	6.1328	6.2049	3.1005	5.6932	0.9294	-0.1056	0.6293	0.2012
Scrg1	_ISFVIPC[Carbamidomethyl (C)]NNH_.2	#N/A	6.6591	7.4104	8.0256	8.6499	6.9750	6.5705	7.1969	6.7689	6.8200	7.5577	1.0418	0.0590	0.5234	0.2812

Tom1	_QQS[Phospho (STY)]TGAIPATQAR_.2	O88746_S405_M1_Tom1	4.2212	7.5156	7.6619	3.9098	3.3469	4.3826	3.6245	4.2086	7.3924	3.8768	1.0832	0.1153	0.7481	0.1260
Tom1	_QNS[Phospho (STY)]VSSNTSQR_.2	O88746_S180_M1_Tom1	7.0260	7.8432	7.7214	8.2735	3.1724	3.5568	3.4500	7.7309	4.0317	3.7023	1.3250	0.4060	0.3083	0.5110
Tom1	_GSS[Phospho (STY)]LADQRK_.2	O88746_S376_M1_Tom1	3.1249	3.9301	3.8117	4.3144	3.7515	3.9780	4.0291	3.8041	4.4311	4.2814	0.9231	-0.1154	0.2009	0.6971
Tom1	_AADRLPNLAS[Phospho (STY)]PSAEGPPRPSPGTAPR_.4	O88746_S462_M1_Tom1	3.9242	3.1797	3.6579	7.0289	3.0680	3.5566	3.4498	5.3428	4.0320	4.3798	0.9461	-0.0800	0.7844	0.1055
Dcx	_SKQS[Phospho (STY)]PISTPTS[Phospho (STY)]PGSLRK_.3	O88809_S332_M2_Dcx	3.5398	3.6864	4.2266	3.2284	3.5747	3.0499	4.4439	3.3892	3.0378	3.1954	1.0098	0.0140	0.9186	0.0369
Dcx	_GNPSAAAGPKAS[Phospho (STY)]PTPQK_.2	O88809_S287_M1_Dcx	3.8403	3.1446	4.5271	3.5289	2.9659	3.2627	3.2436	3.0888	4.2382	3.4959	1.0081	0.0116	0.9382	0.0277
Idh1	_ATDFVVPGPGK_.2	#N/A	6.5729	6.9855	6.2224	7.1054	7.3231	7.0544	6.4195	6.8634	6.8315	6.9599	1.0161	0.0230	0.6444	0.1908
Idh1	_ALEDVC[Carbamidomethyl (C)]IETIEAGFM[Oxidation (M)]TK_.2	#N/A	4.7108	5.2850	5.8699	6.1815	4.9372	5.6840	5.0957	5.5560	5.1872	5.0891	1.0407	0.0575	0.5008	0.3003
Idh1	_TVEAEAAHGTVTR_.2	#N/A	4.2814	4.0885	4.1875	4.0844	4.3473	3.9578	4.1701	4.0290	3.9649	3.8802	1.0366	0.0518	0.1322	0.8788
Idh1	_HAYGDQYR_.2	#N/A	5.2067	4.5829	3.7924	5.0566	5.7953	3.9973	4.3581	4.2450	3.4720	4.6552	1.1329	0.1801	0.2391	0.6213
Akap10	_QSSLAEPVS[Phospho (STY)]PSKR_.2	O88845_S187_M1_Akap10	3.6076	4.4128	4.2944	3.2962	4.2341	6.7649	3.8562	3.3214	2.9700	6.3565	1.0749	0.1042	0.7314	0.1358
Akap10	_ASVAVHS[Phospho (STY)]PQK_.2	O88845_S52_M1_Akap10	6.6058	6.5224	3.2256	3.7283	5.4696	3.5498	6.2281	4.3902	4.0388	3.6953	1.0571	0.0802	0.7791	0.1084
Akap10	_NSC[Carbamidomethyl (C)]SS[Phospho (STY)]PLRELSEK_.2	O88845_S281_M1_Akap10	2.8956	3.7008	3.5824	4.0850	3.5221	4.2074	3.7997	4.0334	3.6820	4.0520	0.9419	-0.0864	0.3899	0.4091
Tspyl1	_[Acetyl (Protein N-term)]SS[Phospho (STY)]PERDEGTPVPDSR_.2	O88852_S3_M1_Tspyl1	3.5493	4.3545	4.2361	3.7661	4.1759	3.5536	3.9144	6.1528	3.5341	6.6587	0.7777	-0.3626	0.1201	0.9205
Ccnk	_KPS[Phospho (STY)]PQPS[Phospho (STY)]PPR_.2	O88874_S325_M2_Ccnk	9.4739	9.5617	10.0494	10.0123	8.9025	9.7018	9.8151	10.0152	10.0256	9.4261	0.9793	-0.0302	0.4237	0.3729
Ccnk	_AVVVS[Phospho (STY)]PKEENK_.2	O88874_S341_M1_Ccnk	5.6634	5.7603	5.9906	5.8338	5.4298	5.6272	6.2999	6.0728	6.0600	6.0536	0.9340	-0.0985	0.0059	2.2319
Zfand5	_M[Oxidation (M)]S[Phospho (STY)]PMGTASGSNSPTSDSASVQR_.2	#N/A	3.7686	3.7394	4.2541	4.1487	3.6042	3.7261	3.7945	4.0503	3.6003	3.4751	1.0385	0.0544	0.4140	0.3830
Mpp3	_TTGTLPS[Phospho (STY)]PQNFK_.2	O88910_S307_M1_Mpp3	12.4398	12.3490	12.7708	12.9848	11.6402	12.4159	12.8204	12.0779	12.1734	12.4504	1.0043	0.0062	0.8489	0.0711
Syn1	_QAS[Phospho (STY)]ISGPAPTK_.2	O88935_S568_M1_Syn1	12.5073	11.7252	12.4779	12.8166	11.6144	12.4583	12.0395	11.6247	12.1069	11.9287	1.0287	0.0408	0.2250	0.6478
Syn1	_SLKPDFVLIR_.2	#N/A	11.2856	11.3783	11.5917	11.7827	11.1499	11.3461	11.4356	11.3587	11.2680	11.3540	1.0060	0.0087	0.5826	0.2346
Syn1	_TYATAEPFIDAK_.2	#N/A	11.0154	11.0538	11.1929	11.1389	11.1156	11.0689	11.0542	10.9267	10.8828	11.0782	1.0102	0.0147	0.0550	1.2597
Syn1	_ASGAPPGGQQR_.2	#N/A	9.8663	10.0476	9.5665	10.1840	10.8963	9.8743	9.6975	9.8753	9.6422	10.0325	1.0266	0.0378	0.3129	0.5046
Syn1	_VLLVIDEPHTDWAK_.2	#N/A	8.5472	8.7237	9.0408	9.3713	8.5375	8.6492	8.9695	8.5686	8.7081	8.5948	1.0116	0.0167	0.5953	0.2252
Syn1	_TGPPTTQQPR_.2	#N/A	9.9012	9.5931	9.4931	9.9299	10.3077	9.7107	9.6254	9.8665	9.6677	9.7605	1.0095	0.0137	0.5666	0.2467
Syn1	_VDNQHDFQDIASVVALTK_.3	#N/A	7.9306	7.6196	7.9185	8.4622	7.6463	7.8771	7.8617	7.7193	7.9121	8.0317	1.0035	0.0051	0.8686	0.0612
Syn1	_KLGTEEFPLIDQTFYPNHK_.3	#N/A	8.3492	8.2280	8.3898	8.3973	8.2132	8.6470	8.4015	8.2615	8.1258	8.3772	1.0096	0.0137	0.4251	0.3715
Syn1	_IHGEIDIK_.2	#N/A	7.8392	6.6841	7.2372	8.1235	7.1939	7.4833	7.5687	7.6155	7.7498	7.7940	0.9668	-0.0487	0.3616	0.4418
Syn1	_QGPPQKPPGPAGPTR_.3	#N/A	7.1802	6.7997	7.4133	6.7585	8.1371	7.3623	7.0924	7.0285	7.4885	7.0763	1.0145	0.0207	0.7116	0.1478
Syn1	_EM[Oxidation (M)]LSSTTYPVVVK_.2	#N/A	9.1611	9.1147	9.4971	9.3304	9.2353	9.3036	9.1690	9.0349	9.1677	9.1191	1.0166	0.0237	0.0758	1.1201
Syn1	_QAGAPQATR_.2	#N/A	6.8047	3.3184	7.2084	7.0928	7.9394	7.2924	6.6246	7.3492	7.1570	6.9064	0.9382	-0.0920	0.6409	0.1932
Syn1	_M[Oxidation (M)]TQALPR_.2	#N/A	7.9914	8.2938	8.2234	8.3751	8.7354	8.0258	7.6715	8.3835	8.4250	8.2951	1.0098	0.0141	0.6929	0.1593
Syn1	_QPQRDAS[Phospho (STY)]PGR_.2	#N/A	7.3914	7.6111	7.2232	7.5813	3.9672	6.4942	7.5383	7.1190	6.8198	7.1519	0.9377	-0.0928	0.5564	0.2546
Syn1	_LGTEEFPLIDQTFYPNHK_.3	#N/A	7.2286	7.2192	7.3862	7.4236	7.0609	7.2173	6.8335	7.2787	7.2946	7.5788	1.0013	0.0019	0.9470	0.0237
Syn1	_ASTAAPVASPAAPSPGSSGGGGFFSSLSNAVK_.3	#N/A	5.3650	6.9196	6.8360	6.8815	5.5370	4.8643	5.2728	6.3833	5.0129	5.2915	1.1506	0.2024	0.2765	0.5583
Syn1	_DAS[Phospho (STY)]PGRGSHSQSSSPGALTLGR_.3	#N/A	8.1115	9.7470	10.7247	10.6141	8.4662	8.9865	9.4266	9.5475	9.0271	8.3477	1.0390	0.0552	0.5712	0.2432
Syn1	_VKVDNQHDFQDIASVVALTK_.3	#N/A	7.5937	7.7571	8.3561	8.0138	7.2239	7.6126	7.7173	7.2812	7.6520	8.1168	1.0088	0.0126	0.7852	0.1050
Syn1	_QLIVELVVNK_.2	#N/A	4.8485	4.5056	3.8271	4.9096	3.8047	3.9626	3.4993	4.0820	4.4708	5.0823	1.0061	0.0088	0.9454	0.0244
Syn1	_TSVSGNWK_.2	#N/A	7.5598	7.8354	7.7356	7.7177	8.2819	7.8150	7.0312	7.7375	7.8996	7.8186	1.0266	0.0378	0.3435	0.4641
Syn1	_GSHSQSSS[Phospho (STY)]PGALTLGR_.2	#N/A	10.4830	10.4804	11.1335	11.0190	10.1750	10.4336	10.7186	10.1915	8.7567	10.5541	1.0562	0.0789	0.1961	0.7074
Syn1	_KIHGEIDIK_.2	#N/A	6.7608	3.5803	6.2750	3.9645	5.8864	5.9926	4.3600	4.7143	5.9749	4.9315	1.0830	0.1151	0.5817	0.2353
Syn1	_SQSLTNAFNLPEPAPPRPSLSQDEVKAETIR_.4	#N/A	3.8060	3.3903	4.6783	3.9895	3.6527	4.5341	3.5884	3.4106	3.9906	2.9856	1.0779	0.1082	0.4224	0.3743
Syn1	_QTTAAAAATFSEQVGGGSGGAGR_.2	#N/A	7.1742	6.5067	7.9636	6.7837	6.2158	6.7105	6.8059	5.8907	6.7962	6.4890	1.0611	0.0856	0.2970	0.5273
Syn1	_PQPPPPPPSAAS[Phospho (STY)]PGATPGSATASAER_.3	O88935_S39_M1_Syn1	9.7240	10.5340	10.2498	11.3573	8.4133	9.6248	9.8307	10.1433	9.6530	9.4359	1.0223	0.0319	0.6858	0.1638
Syn1	_QAS[Phospho (STY)]QAGPGPR_.2	#N/A	10.6155	9.5168	10.6132	10.6792	9.5147	10.8069	9.6428	10.0450	10.7929	10.4663	1.0053	0.0076	0.8858	0.0526
Syn1	_LPSPT[Phospho (STY)]AAPQQSASQATPVTQGQGR_.3	O88935_T512_M1_Syn1	3.7364	4.0169	5.9703	6.5929	3.8904	3.7335	5.8792	6.5459	4.6278	3.8907	0.8894	-0.1691	0.4930	0.3072
Syn1	_QHAFSMAR_.2	#N/A	3.7471	5.9781	3.5993	4.9500	4.6410	5.1077	4.5512	3.7491	4.3981	4.7689	1.0695	0.0970	0.5504	0.2593
Syn1	_SQSLTNAFNLPEPAPPRPSLSQDEVK_.3	#N/A	5.0215	5.0793	5.4322	8.7212	5.7481	5.6942	5.9374	6.9220	5.5483	5.5413	0.9937	-0.0091	0.9613	0.0172
Syn1	_RLS[Phospho (STY)]DSNFM[Oxidation (M)]ANLPNGYM[Oxidation (M)]TDLQR_.3	#N/A	3.6402	7.2144	3.8221	8.7728	5.8300	6.6778	5.6779	8.3525	7.1221	4.0678	0.9505	-0.0733	0.8098	0.0916
Syn1	_PVAGGPGAPPAARPPASPS[Phospho (STY)]PQR_.3	#N/A	3.6758	5.5736	3.9197	8.3179	3.8064	3.8967	4.0883	5.2236	4.6354	3.6174	1.1079	0.1478	0.6398	0.1939
Syn1	_QSRPVAGGPGAPPAARPPASPS[Phospho (STY)]PQR_.4	#N/A	3.4998	4.0242	6.3580	4.1900	3.6847	4.9857	3.7720	3.7582	3.1560	3.9680	1.2166	0.2829	0.1947	0.7106
Syn1	_DHIIEVVGSSM[Oxidation (M)]PLIGDHQDEDK_.3	#N/A	4.5515	4.6532	4.8443	5.0343	4.9303	5.1037	5.0748	5.1488	4.6250	5.0647	0.9748	-0.0368	0.4118	0.3853
Syn1	_VEQAEFSDLNLVAHANGGFSVDM[Oxidation (M)]EVLR_.3	#N/A	3.2932	4.0495	4.5278	5.0316	3.9378	4.1876	4.2198	4.4305	3.2287	3.7488	1.0677	0.0944	0.4902	0.3097
Syn1	_LWVDTC[Carbamidomethyl (C)]SEIFGGLDIC[Carbamidomethyl (C)]AVEALHGK_.3	#N/A	4.2094	3.5137	3.3954	4.6069	3.3351	4.3944	3.2544	1.9095	4.1941	3.8650	1.1825	0.2419	0.2534	0.5961
Zbtb7a	_HFKDEEEDEEEAS[Phospho (STY)]PDGSGR_.3	O88939_S537_M1_Zbtb7a	3.3496	3.6539	8.6492	8.0836	3.4753	7.8861	7.4989	8.2254	3.7289	7.6855	0.8867	-0.1734	0.6185	0.2087
Lin7b	_[Acetyl (Protein N-term)]AALVEPLGLER_.2	#N/A	5.8908	6.5464	6.1506	6.1131	6.6621	6.1888	6.2770	6.2049	6.0529	6.1208	1.0154	0.0220	0.5521	0.2580
Lin7b	_QQHHSYTS[Phospho (STY)]LESRG_.3	O88951_S202_M1_Lin7b	4.5197	3.9263	4.9438	3.9631	4.3591	3.7061	3.6778	4.1724	4.8958	3.9301	1.0161	0.0231	0.8348	0.0784
Lin7b	_QQHHSY[Phospho (STY)]TSLESR_.3	O88951_Y200_M1_Lin7b	3.3119	4.1171	3.9987	4.0036	3.9384	3.7911	4.1519	2.9976	3.2966	3.6809	1.0930	0.1283	0.2191	0.6593
Lin7c	_VLQSEFC[Carbamidomethyl (C)]NAVR_.2	#N/A	3.4518	5.2907	5.3797	5.4285	5.5877	4.6556	5.7910	4.8011	5.1593	5.2584	0.9454	-0.0810	0.5355	0.2712
Cpd	_KSLLSHEFQDET[Phospho (STY)]DT[Phospho (STY)]EEETLYSSKH_.4	#N/A	3.2337	3.9899	4.4682	4.4257	3.8782	4.2472	4.1404	2.9892	3.3414	3.6892	1.1414	0.1908	0.1388	0.8575
Cpd	_SLLSHEFQDET[Phospho (STY)]DT[Phospho (STY)]EEETLYSSKH_.3	#N/A	3.3570	3.9854	4.5915	4.1353	4.0016	4.1239	4.2835	4.3667	3.1650	3.8126	1.0321	0.0456	0.6846	0.1645
Cpd	_SLLSHEFQDET[Phospho (STY)]DT[Phospho (STY)]EEETLYSSK_.3	#N/A	4.1661	3.4215	3.8997	3.4435	3.3098	3.3148	3.2080	3.5577	3.8567	3.1208	1.0456	0.0644	0.5063	0.2956
Lgmn	_DYTGEDVTPENFLAVLR_.2	#N/A	2.8138	3.2731	3.8030	3.3468	3.1071	4.3752	3.4950	3.6544	3.5461	3.6952	0.9598	-0.0592	0.6269	0.2028
Sh3pxd2a	_AAS[Phospho (STY)]QGSESPLLPTQR_.2	O89032_S1029_M1_Sh3pxd2a	10.1914	10.0594	10.2819	3.4009	3.4022	10.0697	10.3254	10.1978	10.1359	10.1186	0.7750	-0.3677	0.2334	0.6318
Sh3pxd2a	_EAEENPVGAC[Carbamidomethyl (C)]ESQGS[Phospho (STY)]PLK_.2	O89032_S546_M1_Sh3pxd2a	7.9301	7.0265	7.7385	3.5873	6.6693	3.3747	7.8734	7.5016	3.7130	7.1591	0.9227	-0.1161	0.7047	0.1520
Sh3pxd2a	_GSSGDSDS[Phospho (STY)]PGSSSLSLAVK_.2	O89032_S643_M1_Sh3pxd2a	4.3529	4.3741	3.5388	6.0196	4.2624	4.2108	3.7562	4.6881	3.7256	5.0026	1.0388	0.0549	0.7441	0.1283
Sh3pxd2a	_SAS[Phospho (STY)]DAGIRDTPK_.2	O89032_S721_M1_Sh3pxd2a	4.3141	4.6485	3.5000	4.0027	3.4398	3.8242	2.6895	4.1158	3.7644	3.9697	1.0880	0.1217	0.3876	0.4116
Sh3pxd2a	_RIS[Phospho (STY)]PASSLQR_.2	O89032_S592_M1_Sh3pxd2a	5.0133	3.8434	4.0696	4.5723	3.7317	4.3938	4.2869	6.1017	3.1948	3.0384	1.0277	0.0395	0.8549	0.0681
Sh3pxd2a	_NSS[Phospho (STY)]FSTAR_.2	O89032_S1008_M1_Sh3pxd2a	4.1170	3.3725	3.8507	3.3945	3.2608	3.3639	3.2570	3.6067	3.9058	3.0718	1.0288	0.0409	0.6738	0.1715
Sh3pxd2a	_PEDVNPPK_.2	#N/A	3.4715	3.7546	4.1583	3.1601	3.6429	2.9817	4.3757	3.4575	3.1061	3.1271	1.0033	0.0048	0.9719	0.0124
Coro1a	_ATPEPSGTPSSDTVSR_.2	#N/A	8.8971	8.7027	8.8888	9.2351	9.1904	9.0496	8.7728	8.7724	8.7428	8.9386	1.0213	0.0304	0.1246	0.9045
Coro1a	_DGALIC[Carbamidomethyl (C)]TSC[Carbamidomethyl (C)]R_.2	#N/A	7.7411	7.3051	7.5575	7.8292	7.5637	7.4859	7.4658	7.3989	7.6409	7.7136	1.0034	0.0049	0.8239	0.0841
Coro1a	_ILTTGFSR_.2	#N/A	6.8208	7.1633	6.9764	6.3543	7.3698	7.0196	6.4716	7.4323	7.3730	7.1300	0.9787	-0.0310	0.5592	0.2524
Coro1a	_QVALWDTK_.2	#N/A	6.8026	6.3818	7.0879	7.0172	7.1268	6.7571	6.7128	6.7879	7.0547	6.8054	1.0032	0.0046	0.8899	0.0506
Coro1a	_DAGPLLISLK_.2	#N/A	5.2971	6.3212	5.7702	6.0906	5.9627	6.4049	6.0514	6.5563	6.1395	5.5874	0.9821	-0.0261	0.6852	0.1642
Coro1a	_HVFGQPAK_.2	#N/A	6.2813	6.3669	5.2107	6.0033	6.8577	5.3098	5.9155	6.6224	6.3982	6.0718	0.9605	-0.0582	0.5004	0.3007
Coro1a	_VSQTTWDSGFC[Carbamidomethyl (C)]AVNPK_.2	#N/A	4.0307	4.3067	3.2166	3.7193	5.2390	5.0202	4.8560	4.2402	4.9823	5.6407	0.8632	-0.2122	0.1759	0.7547
Coro1a	_LDRLEETVQAK_.2	#N/A	4.2694	3.7156	3.9350	4.0507	4.3443	4.3875	4.1008	4.1828	4.1491	4.1168	0.9951	-0.0071	0.8844	0.0534
Coro1a	_NLNAIVQK_.2	#N/A	3.2655	2.4331	4.3120	2.5512	3.8314	4.4034	4.0040	3.1454	4.3951	1.1844	1.0892	0.1233	0.7017	0.1539
Coro1a	_KSDLFQEDLYPPTAGPDPALTAEEWLGGR_.3	#N/A	3.3876	4.1438	3.1212	4.2718	4.0321	4.0933	3.9865	4.3362	3.4953	3.8431	0.9812	-0.0274	0.7966	0.0988

Cope	_APPVPGAVSGGS[Phospho (STY)]GEVDELFDVK_.2	#N/A	3.9906	6.2602	7.6062	6.8464	5.9832	6.4277	3.9185	9.0114	8.2454	3.6462	0.9968	-0.0046	0.9881	0.0052
Pde4a	_S[Phospho (STY)]PLGGPPSVC[Carbamidomethyl (C)]K_.2	O89084_S204_M1_Pde4a	8.5561	7.9433	8.0214	8.5172	7.6659	8.2014	8.3642	7.9711	8.3281	8.2261	0.9731	-0.0394	0.5347	0.2719
Pde4a	_GYS[Phospho (STY)]DSAER_.2	O89084_S49_M1_Pde4a	3.2725	4.0287	4.5070	4.0508	3.9170	4.2084	4.1016	4.4513	3.2495	3.7280	1.0296	0.0420	0.7044	0.1522
Pde4a	_SLS[Phospho (STY)]LSLPGPR_.2	O89084_S13_M1_Pde4a	3.4474	4.2037	3.1811	8.3462	4.0920	4.0335	3.9266	7.3687	3.0745	3.9030	0.9962	-0.0056	0.9889	0.0048
Pde4a	_QS[Phospho (STY)]PSPTLEEEPGVLSDPALPDK_.2	O89084_S672_M1_Pde4a	4.3407	4.3863	3.5267	4.0293	3.4664	4.2631	3.7440	4.0891	3.7377	3.9963	1.0283	0.0403	0.6315	0.1996
Pde4a	_S[Phospho (STY)]GNQVSEYISNTFLDK_.2	O89084_S274_M1_Pde4a	4.1337	3.4381	3.3197	3.1818	3.2594	2.9693	3.3300	3.8194	4.1184	3.7893	0.8989	-0.1538	0.1529	0.8156
Pde4a	_TLSPS[Phospho (STY)]EEAQGLLGLPSM[Oxidation (M)]AAEVEAPR_.3	O89084_S788_M1_Pde4a	4.1669	3.1755	3.9006	3.4444	3.3107	3.3139	3.5927	5.4496	3.8558	3.1217	0.8869	-0.1732	0.3401	0.4685
Pde4a	_SDS[Phospho (STY)]DYDMSPK_.2	O89084_S147_M1_Pde4a	4.0700	3.3254	3.8037	3.5893	3.2137	3.4109	3.3040	3.6537	4.1777	3.0247	1.0081	0.0117	0.9132	0.0394
Rbm3	_YDSRPGGYGYGYGR_.3	#N/A	5.5769	4.9645	6.0996	5.6209	6.5156	5.2613	4.0612	5.2967	5.4644	5.8811	1.0961	0.1324	0.2721	0.5653
Rbm3	_YSGGNYRDNYDN_.2	#N/A	5.6160	6.3666	7.1876	6.0396	6.1047	5.9310	6.0981	5.5717	6.1539	5.2562	1.0758	0.1055	0.2131	0.6714
Lancl1	_SLAENYFDSTGR_.2	#N/A	5.7173	5.4025	6.5752	5.2681	5.1287	4.9348	6.3317	5.7240	6.0501	3.9871	0.9966	-0.0049	0.9717	0.0125
Lancl1	_IPQSHIQQIC[Carbamidomethyl (C)]ENILTSGENLSR_.3	#N/A	4.5313	5.3061	3.3269	5.6166	3.9916	5.3737	3.7808	5.8709	4.4641	5.0954	0.9767	-0.0340	0.8520	0.0695
Mt-Cyb	_DVNYGWLIR_.2	#N/A	4.9496	2.8694	4.9123	5.0078	4.5117	4.8793	5.5296	4.7568	4.8794	4.9669	0.8984	-0.1546	0.2823	0.5492
Mtco2	_VVLPM[Oxidation (M)]ELPIR_.2	#N/A	9.2208	10.1823	9.2461	9.6686	9.3167	9.7339	10.0304	9.4624	9.0508	9.8710	0.9956	-0.0064	0.8741	0.0584
Mtco2	_LLEVDNR_.2	#N/A	10.5049	10.4991	10.7957	10.1836	10.0195	10.1505	10.2382	10.6884	10.5279	10.4030	0.9899	-0.0146	0.5433	0.2650
Mtco2	_ILYM[Oxidation (M)]M[Oxidation (M)]DEINNPVLTVK_.2	#N/A	6.3554	6.7637	7.0026	6.1194	6.1994	6.8619	7.0379	7.2116	6.7671	6.5821	0.9494	-0.0749	0.1509	0.8212
Mtco2	_M[Oxidation (M)]LISSEDVLHSWAVPSLGLK_.3	#N/A	6.2480	6.4299	6.6050	5.8113	5.2542	6.4508	6.0884	6.8346	6.5912	6.2659	0.9516	-0.0715	0.3163	0.4999
Hprt1	_VIGGDDLSTLTGK_.2	#N/A	6.9598	7.1403	7.1302	6.9503	7.0205	6.7231	6.5841	7.1540	6.8790	6.7463	1.0214	0.0306	0.2684	0.5713
Hprt1	_NVLIVEDIIDTGK_.2	#N/A	5.4819	6.2043	6.0199	6.1238	5.9276	6.4305	6.4063	5.7977	6.1759	6.1111	0.9851	-0.0217	0.6447	0.1906
Hprt1	_SIPM[Oxidation (M)]TVDFIR_.2	#N/A	5.6914	6.3835	4.2029	3.7466	5.1934	6.4752	6.7073	6.1849	5.4547	6.1899	0.8611	-0.2157	0.1996	0.6998
Hprt1	_EM[Oxidation (M)]GGHHIVALC[Carbamidomethyl (C)]VLK_.3	#N/A	2.7569	4.0475	4.4676	4.0732	4.1506	4.1860	4.2214	2.7552	3.2271	3.7505	1.1314	0.1781	0.2814	0.5507
Hprt1	_DLNHVC[Carbamidomethyl (C)]VISETGK_.2	#N/A	4.0383	3.0097	3.4032	3.9059	4.1034	4.9046	3.6206	4.2134	3.6686	3.8729	1.0131	0.0188	0.8886	0.0513
Abl1	_SC[Carbamidomethyl (C)]S[Phospho (STY)]ASC[Carbamidomethyl (C)]M[Oxidation (M)]PHGAR_.2	#N/A	3.7695	3.7859	3.6675	4.1702	3.6072	3.4720	3.7936	4.0101	3.6587	4.3046	0.9502	-0.0737	0.2674	0.5728
Ca2	_DFPIANGDR_.2	#N/A	9.7881	9.5867	9.7421	10.0332	10.1209	9.8200	9.5845	9.5127	9.6938	10.0616	1.0139	0.0200	0.3589	0.4450
Ca2	_GGPLSDSYR_.2	#N/A	8.0886	8.4144	8.6754	8.5724	9.0164	8.3951	8.0923	8.3634	8.4007	8.4998	1.0225	0.0322	0.3097	0.5090
Ca2	_VLEALHSIK_.2	#N/A	8.5144	9.1154	9.1083	8.9463	9.5430	8.8455	8.9180	8.8639	8.9449	8.8994	1.0119	0.0170	0.5618	0.2504
Ca2	_[Acetyl (Protein N-term)]S[Phospho (STY)]HHWGYSK_.2	P00920_S2_M1_Ca2	7.0505	7.4864	7.8747	7.5605	7.5297	7.2058	7.3304	7.0942	7.3012	7.3688	1.0244	0.0348	0.2866	0.5428
Ca2	_AVQQPDGLAVLGIFLK_.2	#N/A	7.2592	7.3876	7.0829	6.9279	7.7224	7.5581	7.0807	7.1756	7.3675	7.5194	1.0051	0.0073	0.8319	0.0799
Ca2	_IGPASQGLQK_.2	#N/A	5.7807	6.5741	6.7125	6.9172	6.9716	6.1055	6.2415	6.7129	6.5779	6.4353	1.0028	0.0041	0.9441	0.0250
Ca2	_SIVNNGHSFNVEFDDSQDNAVLK_.3	#N/A	6.2113	6.5003	6.7537	6.7984	6.4924	6.2845	6.7642	6.2935	6.3440	6.6521	0.9990	-0.0015	0.9658	0.0151
Ca2	_QSPVDIDTATAQHDPALQPLLISYDK_.3	#N/A	5.1086	5.5318	5.5005	6.3198	6.0771	6.0249	6.2502	5.6850	5.6582	5.6838	0.9899	-0.0147	0.8251	0.0835
Ca2	_YAAELHLVHWNTK_.3	#N/A	5.1278	5.1201	6.0839	5.1983	5.8077	5.5324	5.2651	3.9154	4.3953	6.1377	1.1116	0.1526	0.2452	0.6104
Ca2	_TLNFNEEGDAEEAM[Oxidation (M)]VDNWRPAQPLK_.3	#N/A	4.1537	4.5262	5.8817	4.4268	4.3209	4.7210	5.5040	3.7497	4.5222	4.8765	1.0019	0.0027	0.9845	0.0068
Ca2	_EPITVSSEQM[Oxidation (M)]SHFR_.2	#N/A	3.9987	3.8534	4.3578	4.7371	4.5866	4.4253	4.0021	4.7104	3.8048	4.2717	1.0308	0.0438	0.5937	0.2264
Ca2	_HNGPENWHKDFPIANGDR_.4	#N/A	4.1981	3.4535	3.9318	4.9932	7.1697	6.6264	3.6238	6.0919	5.8344	6.3744	0.9236	-0.1147	0.6609	0.1799
Thy1	_VTLSNQPYIK_.2	#N/A	8.9259	9.0160	9.1447	9.0105	8.6393	8.5672	8.9660	9.3846	8.8570	8.7639	0.9879	-0.0176	0.5138	0.2892
Thy1	_DNSIQHEFSLTR_.2	#N/A	6.2385	6.0304	5.9416	6.8609	5.8570	5.8631	6.3396	6.9658	6.0928	6.2093	0.9578	-0.0622	0.3101	0.5085
Thy1	_HVLSGTLGIPEHTYR_.3	#N/A	9.4622	9.3910	9.8939	9.7052	9.2266	9.3271	9.5508	9.7399	9.3466	9.3895	0.9994	-0.0009	0.9698	0.0133
Thy1	_VTSLTAC[Carbamidomethyl (C)]LVNQNLR_.2	#N/A	7.7270	7.5217	7.9826	7.9333	7.6094	7.5806	8.1417	7.9061	7.9033	7.8728	0.9711	-0.0424	0.0695	1.1579
Hba	_M[Oxidation (M)]FASFPTTK_.2	#N/A	6.7737	6.8578	7.5786	7.0508	8.4247	6.5012	7.2632	7.4026	6.4022	6.4834	1.0450	0.0635	0.4733	0.3248
Hba	_FLASVSTVLTSK_.2	#N/A	6.1574	5.4502	6.6470	5.9113	7.4055	5.8725	6.8221	6.1736	5.5019	5.7400	1.0299	0.0425	0.6783	0.1686
Hba	_VDPVNFK_.2	#N/A	5.4376	5.9428	6.6150	5.0817	7.8103	5.4669	5.6083	5.8296	5.7097	5.9250	1.0504	0.0710	0.5893	0.2296
Hba	_VLSGEDKSNIK_.2	#N/A	4.7044	5.5670	6.9190	4.9582	6.5120	5.2586	5.9389	5.4530	5.1427	4.4994	1.0751	0.1044	0.4605	0.3368
Hba	_IGGHGAEYGAEALER_.3	#N/A	4.8873	5.4868	6.3274	5.1670	6.3432	4.8488	5.3783	6.1288	4.6322	5.1397	1.0358	0.0507	0.6662	0.1764
Hba	_TYFPHFDVSHGSAQVK_.3	#N/A	6.6743	6.6447	7.5816	6.0027	7.9490	6.5109	7.1065	6.9069	6.2597	6.4456	1.0321	0.0455	0.6086	0.2157
Hbb-b1	_LLVVYPWTQR_.2	#N/A	9.4886	9.8270	10.7103	9.7108	10.6669	9.6862	10.4803	10.4161	9.4189	9.6151	1.0032	0.0047	0.9280	0.0324
Hbb-b1	_LHVDPENFR_.2	#N/A	8.9019	8.9976	9.4186	9.1270	10.4746	8.6842	9.8245	9.7165	8.7416	8.5516	1.0064	0.0092	0.8913	0.0500
Hbb-b1	_DFTPAAQAAFQK_.2	#N/A	7.8647	8.1478	8.7697	8.1792	9.5301	7.6800	9.0352	8.5609	7.9400	7.9189	0.9998	-0.0003	0.9965	0.0015
Hbb-b1	_VHLTDAEK_.2	#N/A	7.7766	7.9314	8.6705	7.5803	9.1999	7.8666	8.1006	8.5334	7.8141	7.6949	1.0168	0.0241	0.7111	0.1481
Hbb-b1	_VNSDEVGGEALGR_.2	#N/A	7.7565	7.8581	8.6997	8.0729	9.2275	7.9773	8.6984	8.1925	8.0001	7.7330	1.0134	0.0192	0.7527	0.1234
Hbb-b1	_VITAFNDGLNHLDSLK_.3	#N/A	5.5907	5.9318	6.4804	6.2727	7.1159	6.1177	6.6528	6.4956	5.1356	5.5267	1.0502	0.0707	0.4706	0.3273
Hbb-b1	_GTFASLSELHC[Carbamidomethyl (C)]DK_.2	#N/A	6.6042	6.7127	6.5788	6.4761	7.9218	5.6554	6.7041	6.7546	6.5373	6.3542	1.0107	0.0154	0.8566	0.0672
Hbb-b1	_YFDSFGDLSSASAIM[Oxidation (M)]GNAK_.2	#N/A	3.9129	4.3150	6.0363	4.7605	5.4324	4.2852	5.4600	4.6613	4.5040	4.6361	0.9948	-0.0075	0.9565	0.0193
Hbb-b2	_VNPDEVGGEALGR_.2	#N/A	7.8170	7.9471	8.8133	7.9331	9.0816	8.2488	8.5384	8.1497	8.1686	7.9157	1.0139	0.0199	0.7011	0.1542
H3-5;H3-3a	_YRPGTVALR_.2	#N/A	10.0443	10.4987	10.6065	10.4188	10.7767	10.2027	10.3955	10.7786	10.5108	10.1767	0.9961	-0.0056	0.8149	0.0889
H3-5;H3-3a	_RVTIM[Oxidation (M)]PK_.2	#N/A	7.9136	8.4028	8.7346	7.8146	9.4405	8.6249	8.6682	9.4885	8.8984	8.7695	0.9478	-0.0774	0.2028	0.6930
H3-5;H3-3a	_STELLIR_.2	#N/A	7.8542	6.9962	8.6620	7.5953	7.6974	8.1441	5.1356	8.4628	8.6133	8.1153	1.0321	0.0455	0.7407	0.1304
H3-5;H3-3a	_EIAQDFK_.2	#N/A	5.7021	5.9809	6.0679	5.2945	6.3837	6.1526	3.0290	6.8353	5.9775	6.5673	1.0586	0.0821	0.6607	0.1800
H3-5;H3-3a	_EIAQDFKTDLR_.2	#N/A	3.9332	5.4498	5.4743	4.2064	5.4600	5.7187	4.0814	5.2830	4.8514	4.3766	1.0844	0.1169	0.4008	0.3971
H3-5;H3-3a	_FQSAAIGALQEASEAYLVGLFEDTNLC[Carbamidomethyl (C)]AIHAK_.3	#N/A	3.4520	4.2572	4.1388	3.8635	4.0785	3.6510	4.0118	3.4770	3.1256	3.1076	1.1388	0.1876	0.0723	1.1407
Mt1	ethyl (C)]C[Carbamidomethyl (C)]SC[Carbamidomethyl (C)]C[Carbamidomethyl (C)]PVGC[Carbamidor	#N/A	2.9817	4.2445	3.6685	4.1712	6.3316	3.9927	3.8858	3.9473	4.5480	4.1382	1.0247	0.0352	0.8666	0.0622
Mtnd3	_ANPYEC[Carbamidomethyl (C)]GFDPTSSAR_.2	#N/A	7.4389	7.6567	4.5938	4.1376	4.0038	4.1216	8.4730	8.0026	7.7392	7.2130	0.6778	-0.5611	0.0238	1.6240
Mtnd4	_DNNVLQK_.2	#N/A	3.3175	4.0249	3.0380	4.4454	3.8463	3.8832	4.2440	4.4063	3.8658	3.7730	0.9231	-0.1154	0.3030	0.5186
Mtatp8	_IYLPHSLPQQ_.2	#N/A	7.8247	7.5787	8.0845	7.3630	7.3247	7.3133	7.7727	8.0899	7.9723	7.4806	0.9684	-0.0463	0.2345	0.6298
Gfap	_FADLTDAASR_.2	#N/A	7.6188	8.2652	8.3520	8.3669	8.5401	7.7091	8.0486	8.2285	8.0341	8.0334	1.0069	0.0099	0.7855	0.1049
Gfap	_LADVYQAELR_.2	#N/A	7.2023	7.9137	7.7553	7.9856	7.9801	7.2437	7.4871	8.0421	7.7629	7.8462	0.9866	-0.0195	0.6272	0.2026
Gfap	_ITIPVQTFSNLQIR_.2	#N/A	7.5023	7.6118	7.8400	7.5959	7.7880	7.2283	7.2823	7.5943	7.6701	7.6842	1.0049	0.0070	0.7918	0.1014
Gfap	_ESASYQEALAR_.2	#N/A	6.9037	6.9390	7.2818	7.5477	7.7115	7.3333	7.1876	7.2035	7.1092	7.6877	0.9985	-0.0021	0.9570	0.0191
Gfap	_FLEQQNK_.2	#N/A	6.7737	7.7832	7.6210	7.3427	7.8915	6.7212	6.8913	7.5853	7.4608	7.4741	1.0004	0.0005	0.9927	0.0032
Gfap	_KLLEGEENR_.2	#N/A	6.7357	6.9586	7.0063	7.1196	7.1771	7.2282	6.8676	7.1058	6.7728	7.2544	1.0053	0.0077	0.7745	0.1110
Gfap	_ALAAELNQLR_.2	#N/A	6.1827	6.6800	7.0096	6.1625	7.1177	6.4975	6.2703	6.9141	6.2090	6.4735	1.0219	0.0312	0.5744	0.2408
Gfap	_QEADEATLAR_.2	#N/A	5.8358	5.8391	5.1072	6.4154	7.0227	6.3182	6.0362	6.2937	6.1764	5.9775	0.9949	-0.0074	0.9284	0.0322
Gfap	_HLQEYQDLLNVK_.2	#N/A	4.7879	4.9449	5.5457	5.2130	4.9075	4.9629	4.0815	4.9146	5.5028	4.7425	1.0520	0.0731	0.3826	0.4172
Gfap	_KVESLEEEIQFLR_.3	#N/A	5.6597	5.8438	5.6955	6.1286	5.7471	5.2282	5.6466	5.4713	5.0820	4.8851	1.0846	0.1172	0.0598	1.2235
Gfap	_LEAENNLAAYR_.2	#N/A	6.3996	6.1722	3.2654	6.0605	7.0015	6.6584	6.4196	6.6500	6.3628	6.8201	0.9030	-0.1473	0.3847	0.4149
Gfap	_S[Phospho (STY)]YASETVVR_.2	P03995_S12_M1_Gfap	8.6111	5.5443	6.6722	5.6890	7.2348	7.0623	3.4667	6.1471	9.0510	3.7190	1.2156	0.2816	0.3360	0.4737
Gfap	_QLQALTC[Carbamidomethyl (C)]DLESLR_.2	#N/A	4.0520	4.4386	3.9397	5.5839	6.0246	4.8667	5.0303	5.2404	4.7297	4.9293	0.9669	-0.0485	0.7164	0.1449
Gfap	_DGEVIKDSK_.2	#N/A	3.9238	4.1940	3.6572	4.9772	5.0985	3.4078	5.3399	4.7516	4.6533	4.0623	0.8954	-0.1595	0.2646	0.5773
Gfap	_QKLQDETNLR_.2	#N/A	4.8408	4.6975	3.8503	4.5928	5.0221	4.1744	4.6542	3.9645	5.2404	4.6375	0.9796	-0.0298	0.7639	0.1170

Gfap	_MTPPLPAR_.2	#N/A	5.2048	4.7579	4.1099	7.3301	6.6150	3.7384	5.8097	5.0476	4.7993	4.3755	1.0568	0.0798	0.7178	0.1440
Gfap	_DNFAQDLGTLR_.2	#N/A	6.4732	6.4258	6.5330	7.3116	6.3656	6.5959	6.7003	6.8711	6.8296	6.4914	0.9843	-0.0228	0.5948	0.2256
Gfap	_LRLDQLTANSAR_.2	#N/A	3.2276	3.4323	4.4622	4.4317	4.6775	3.9875	4.4617	3.5381	2.9440	3.6832	1.1038	0.1425	0.3603	0.4433
Gfap	_LEVERDNFAQDLGTLR_.3	#N/A	3.3190	4.6492	4.8821	4.1765	5.0189	3.8321	4.3166	4.5825	4.7778	3.7746	0.9886	-0.0166	0.8983	0.0466
Gfap	_FASYIEK_.2	#N/A	3.9093	3.3520	3.6430	3.1868	4.0375	2.1080	3.3350	3.8144	3.6983	4.0225	0.9073	-0.1404	0.3836	0.4161
Gfap	_TQYEAVATSNM[Oxidation (M)]QETEEWYR_.2	#N/A	6.2338	6.8678	8.6993	8.3805	6.8729	7.2362	6.7708	6.6690	6.3188	6.4210	1.1279	0.1736	0.1302	0.8854
Gfap	_EQLAQQQVHVEM[Oxidation (M)]DVAKPDLTAALR_.3	#N/A	4.1111	4.3359	5.1837	3.5482	4.6503	3.8807	5.1003	3.6126	5.0176	3.0659	1.0205	0.0292	0.8677	0.0616
Gfap	_AEMMELNDR_.2	#N/A	5.3109	3.7254	3.3507	6.5182	4.4159	4.1828	4.5436	4.1067	4.4057	4.0726	1.0705	0.0983	0.6276	0.2023
Gfap	_LEEEGQSLKEEMAR_.2	#N/A	3.7609	3.5413	3.7189	3.7012	3.4748	4.0431	3.9363	4.0162	3.6648	3.4165	0.9862	-0.0200	0.7358	0.1332
Gfap	_LALDIEIATYR_.2	#N/A	3.7842	3.0396	3.5179	4.5625	4.3046	3.6967	3.2099	3.9395	4.2386	4.2398	0.9771	-0.0334	0.7989	0.0975
Gfap	_GTNES[Phospho (STY)]LER_.2	P03995_S302_M1_Gfap	3.6027	3.7397	3.3364	4.3810	3.5610	4.1684	4.5292	4.1210	4.4201	4.0583	0.8870	-0.1730	0.0606	1.2173
Mbp	_FFS[Phospho (STY)]GDRGAPK_.2	P04370_S178_M1_Mbp	7.0866	7.8027	7.9607	7.6802	6.8974	7.1145	7.9226	6.8182	7.1270	8.6046	0.9745	-0.0373	0.6321	0.1992
Mbp	_TQDENPVVHFFK_.2	#N/A	6.3003	12.5144	12.7552	12.7997	12.6866	12.5117	12.5913	12.2340	12.3141	12.4640	0.9350	-0.0970	0.5602	0.2517
Mbp	_LFS[Phospho (STY)]RDAPGR_.2	P04370_S96_M1_Mbp	3.4002	4.0406	5.2308	4.7779	4.9940	4.6375	4.7229	3.6314	4.5481	3.8973	1.0747	0.1039	0.4579	0.3393
Mbp	_NIVTPRT[Phospho (STY)]PPPSQGK_.2	#N/A	11.1941	12.7246	13.7178	12.7265	12.5430	12.4849	12.5160	12.3314	12.1015	12.0232	1.0263	0.0375	0.4671	0.3306
Mbp	_DTGILDS[Phospho (STY)]IGR_.2	#N/A	3.7308	11.2770	3.6448	9.2282	8.8675	11.4021	11.0723	11.1233	11.3413	8.7582	0.7590	-0.3979	0.2071	0.6838
Mbp	_TTHYGS[Phospho (STY)]LPQK_.2	P04370_S201_M1_Mbp	12.7307	12.9397	13.3709	13.3916	13.3217	13.0060	13.0727	12.7429	12.8678	12.9387	1.0171	0.0245	0.1781	0.7494
Mbp	_HRDTGILDSIGR_.3	#N/A	11.5327	12.0577	12.4725	12.0830	12.3550	12.0842	11.8222	11.7587	11.7674	11.8560	1.0251	0.0358	0.1135	0.9450
Mbp	_TTHYGSLPQKS[Phospho (STY)]QHGR_.3	#N/A	6.2032	5.8499	7.1975	7.0835	6.7841	6.2296	6.6303	5.6841	5.9085	6.7968	1.0485	0.0683	0.4117	0.3854
Mbp	_DSRSGS[Phospho (STY)]PMAR_.2	#N/A	3.8937	3.6007	3.9923	4.0584	3.7296	3.7601	3.9510	3.5607	3.5288	3.9236	1.0262	0.0373	0.4584	0.3387
Mbp	_S[Phospho (STY)]KYLATASTM[Oxidation (M)]DHAR_.2	#N/A	7.2896	7.3941	6.9204	7.9626	8.1376	7.1981	7.7868	6.6855	6.7515	6.7599	1.0697	0.0972	0.1633	0.7870
Mbp	_YLATASTM[Oxidation (M)]DHAR_.2	#N/A	10.3969	10.6773	10.2836	10.9663	11.3109	10.6791	10.3722	10.4803	10.6087	10.4710	1.0225	0.0321	0.2627	0.5806
Mbp	_T[Phospho (STY)]PPPSQGK_.2	#N/A	3.5125	4.2128	4.1993	3.2011	4.1390	3.5905	4.4166	3.4165	3.0651	3.1681	1.0832	0.1153	0.3994	0.3986
Mbp	_S[Phospho (STY)]QHGRTQDENPVVHFFK_.4	#N/A	3.4836	3.8588	3.2173	5.9152	3.6801	4.0493	4.4101	4.2401	3.0384	3.9392	1.0325	0.0462	0.8227	0.0848
Mbp	_SGS[Phospho (STY)]PM[Oxidation (M)]AR_.2	#N/A	3.4766	3.7496	4.1634	3.1652	3.6379	2.9867	4.3807	3.4524	3.1010	3.1322	1.0038	0.0054	0.9679	0.0142
Prnp	_YPGQGSPGGNR_.2	#N/A	7.2885	6.9330	4.0160	6.9808	6.8318	7.0689	6.8281	7.0803	7.2615	6.8930	0.9293	-0.1058	0.4577	0.3394
Prnp	_VVEQM[Oxidation (M)]C[Carbamidomethyl (C)]VTQYQK_.2	#N/A	5.9115	6.4452	6.4678	4.0918	6.2630	6.5428	6.7272	6.3315	6.1840	6.3552	0.9303	-0.1042	0.3879	0.4113
Prnp	_PM[Oxidation (M)]IHFGNDWEDR_.3	#N/A	4.9527	3.5292	4.0074	4.0090	4.4966	4.9380	4.2464	5.3565	5.1062	3.9853	0.9248	-0.1128	0.3956	0.4028
Prnp	_ESQAYYDGRR_.2	#N/A	3.8484	3.3778	4.5352	3.5370	3.2660	3.3586	3.2517	3.0806	4.2300	3.5040	1.0390	0.0552	0.6751	0.1706
Prnp	_HVAGAAAAGAVVGGLGGYM[Oxidation (M)]LGSAM[Oxidation (M)]SR_.3	#N/A	3.0374	3.7936	4.2719	4.6220	3.6819	2.9427	4.3367	3.4965	3.1451	3.4929	1.0296	0.0421	0.7921	0.1012
Aldoc	_DDNGVPFVR_.2	#N/A	10.1361	9.5478	9.6815	9.8202	9.9295	9.8309	9.4201	9.7002	9.9696	10.0869	1.0031	0.0044	0.8521	0.0695
Aldoc	_QVLFSADDR_.2	#N/A	9.1607	9.2417	9.3658	9.3467	9.3934	9.2740	9.2693	9.0409	9.2851	9.3949	1.0054	0.0077	0.5222	0.2822
Aldoc	_ELSDIALR_.2	#N/A	9.1939	9.2377	9.6941	9.1985	9.6661	9.4058	8.4869	9.4345	9.5115	9.4913	1.0182	0.0261	0.4837	0.3154
Aldoc	_ALQASALNAWR_.2	#N/A	8.5509	8.7195	8.4454	8.7849	9.0437	8.7225	8.5093	8.3492	8.5952	8.7085	1.0200	0.0285	0.1963	0.7070
Aldoc	_DNAGAATEEFIK_.2	#N/A	8.2959	8.1824	8.0267	8.3771	8.4310	8.4326	8.8194	7.9211	8.2003	8.6451	0.9874	-0.0182	0.5774	0.2386
Aldoc	_PHSYPALSAEQKK_.3	#N/A	8.0621	8.0910	8.2966	8.4945	8.9641	8.4071	8.4287	8.0507	8.2917	8.1546	1.0188	0.0268	0.4176	0.3793
Aldoc	_LSQIGVENTEENR_.2	#N/A	8.2190	8.0239	7.8752	8.3122	7.8318	7.9906	8.5157	7.7484	8.1787	8.2499	0.9840	-0.0233	0.4327	0.3639
Aldoc	_VDKGVVPLAGTDGETTTQGLDGLLER_.3	#N/A	6.3432	6.4341	6.7651	7.1773	6.7927	6.6869	6.3157	6.1295	6.6890	6.2871	1.0542	0.0762	0.0887	1.0522
Aldoc	_ISDRTPSALAILENANVLAR_.3	#N/A	6.1259	7.0728	6.7129	6.6954	6.4856	6.9978	6.4013	6.3474	6.2507	6.4770	1.0491	0.0691	0.1222	0.9128
Aldoc	_YEGSGDGGAAAQSLYIANHAY_.2	#N/A	5.9032	6.1829	6.3965	6.0599	6.0786	6.3429	6.2555	5.7455	6.0342	5.7887	1.0344	0.0487	0.1629	0.7882
Aldoc	_TPSALAILENANVLAR_.2	#N/A	5.7630	5.7896	4.9881	5.6013	5.1966	4.1486	4.9161	5.3519	5.3473	6.0263	0.9700	-0.0440	0.6700	0.1739
Aldoc	_PHSYPALSAEQK_.2	#N/A	7.4372	7.4128	6.6384	7.6686	7.7501	7.4326	7.1243	7.4927	7.2605	7.4795	1.0069	0.0099	0.8181	0.0872
Aldoc	_TVPPAVPGVTFLSGGQSEEEASLNLNAINR_.3	#N/A	5.7574	5.7784	6.1582	6.7972	5.4068	6.4146	6.8227	6.1324	5.9030	6.7111	0.9468	-0.0789	0.3074	0.5123
Aldoc	_GILAADESVGS[Phospho (STY)]M[Oxidation (M)]AK_.2	#N/A	9.1060	8.6707	8.9248	8.6144	9.3994	9.8470	7.6312	8.6904	8.9085	8.8791	1.0664	0.0928	0.1326	0.8775
Aldoc	_GVVPLAGTDGETTTQGLDGLLER_.2	#N/A	6.5763	6.9344	6.8620	7.2444	7.2569	7.1522	7.1820	6.7861	7.0471	7.1048	0.9964	-0.0053	0.8698	0.0606
Aldoc	_YSPEEIAM[Oxidation (M)]ATVTALR_.2	#N/A	8.5849	8.4409	8.5221	8.5776	8.7194	8.5390	8.7384	8.5569	8.6024	8.6078	0.9928	-0.0105	0.2991	0.5242
Aldoc	_AEM[Oxidation (M)]NGLAAQGR_.2	#N/A	6.6400	6.8825	6.6752	7.0187	7.5655	6.7745	6.6145	6.5942	6.8049	6.8068	1.0330	0.0468	0.2565	0.5909
Aldoc	_LSQIGVENTEENRR_.3	#N/A	5.2557	5.2362	6.1681	5.3357	6.0490	5.9675	4.5514	5.2290	5.8725	5.8912	1.0525	0.0738	0.4243	0.3723
Aldoc	_RLS[Phospho (STY)]QIGVENTEENRR_.3	P05063_S45_M1_Aldoc	5.3711	5.1530	5.8818	5.9310	5.3380	5.4009	4.5931	5.4444	5.6062	5.6854	1.0338	0.0480	0.5018	0.2995
Aldoc	_DNAGAATEEFIKR_.2	#N/A	8.6873	9.1312	9.2492	9.2863	9.1845	9.2338	8.8959	8.9048	9.0165	9.0663	1.0176	0.0252	0.2195	0.6586
Aldoc	_GILAADESVGS[Phospho (STY)]M[Oxidation (M)]AKR_.2	#N/A	4.7845	5.5981	6.4997	6.4252	5.5475	6.0877	5.6140	5.6138	5.3073	5.7584	1.0449	0.0634	0.4810	0.3179
Aldoa	_GILAADESTGS[Phospho (STY)]IAK_.2	#N/A	11.1150	11.1918	10.8321	11.8369	11.2833	11.4708	11.6568	11.2547	11.4044	11.5911	0.9836	-0.0239	0.3466	0.4601
Aldoa	_ELSDIAHR_.2	#N/A	11.3679	11.4695	11.3989	11.5649	11.7814	11.4611	11.2754	11.3808	11.3106	11.4717	1.0130	0.0186	0.1172	0.9312
Aldoa	_ALANSLAC[Carbamidomethyl (C)]QGK_.2	#N/A	10.7869	11.1911	11.1548	11.2139	11.6510	10.8242	10.8257	11.0334	11.0994	10.9367	1.0149	0.0213	0.3576	0.4467
Aldoa	_PHPYPALTPEQK_.2	#N/A	10.0519	10.1560	9.9574	10.2300	10.3704	9.9884	10.1021	10.1369	10.0424	10.1203	1.0025	0.0036	0.7664	0.1155
Aldoa	_DGADFAKWR_.2	#N/A	11.0569	10.9413	11.5774	11.3285	10.2548	10.3471	11.5955	10.8372	10.5860	11.0269	0.9915	-0.0123	0.7756	0.1104
Aldoa	_AAQEEYIKR_.2	#N/A	10.6264	10.7813	11.0773	10.9642	11.1136	10.9805	10.6609	10.7431	10.7127	10.8889	1.0160	0.0230	0.1311	0.8824
Aldoa	_LQSIGTENTEENR_.2	#N/A	8.6885	8.3272	8.3013	9.0779	8.2921	8.6264	8.8767	8.3507	8.4338	8.7811	0.9932	-0.0098	0.7647	0.1165
Aldoa	_GVVPLAGTNGETTTQGLDGLSER_.2	#N/A	8.6542	8.5092	8.2578	9.0538	9.0132	8.5502	8.7145	8.5429	8.4759	8.4250	1.0156	0.0224	0.4421	0.3545
Aldoa	_YASIC[Carbamidomethyl (C)]QQNGIVPIVEPEILPDGDHDLKR_.4	#N/A	8.9833	9.3399	9.1928	9.8114	9.6945	9.1569	9.0941	9.4591	9.4502	9.3122	1.0037	0.0053	0.8531	0.0690
Aldoa	_PHPYPALTPEQKK_.3	#N/A	8.7107	9.6243	9.7160	9.3322	9.6358	9.3045	9.2146	9.2223	9.2926	9.2502	1.0154	0.0220	0.4766	0.3219
Aldoa	_IGEHTPSALAIM[Oxidation (M)]ENANVLAR_.3	#N/A	8.4340	8.0691	8.3778	9.0669	8.0110	7.7040	8.3813	8.5017	7.9323	8.3018	0.9997	-0.0004	0.9936	0.0028
Aldoa	_KELSDIAHR_.2	#N/A	8.4094	8.1945	8.2758	8.2751	8.7469	8.3201	8.3487	8.1937	8.4677	8.3561	1.0034	0.0050	0.8005	0.0966
Aldoa	_VDKGVVPLAGTNGETTTQGLDGLSER_.3	#N/A	8.1297	8.6377	8.9460	8.3666	8.8222	8.9480	8.4163	8.2436	8.2102	8.2621	1.0433	0.0612	0.0733	1.1347
Aldoa	_KDGADFAK_.2	#N/A	7.6817	7.1413	7.2670	7.3685	7.6771	7.5172	7.4430	7.1965	7.4602	7.4602	1.0071	0.0102	0.6848	0.1644
Aldoa	_AAQEEYIK_.2	#N/A	7.4981	7.2272	6.6144	7.5781	8.0335	7.0838	6.7807	6.9095	6.9130	7.4295	1.0472	0.0666	0.2575	0.5892
Aldoa	_GGVVGIKVDK_.2	#N/A	6.4940	6.8923	7.5641	7.4409	6.7357	7.0422	6.8641	6.5537	7.0175	6.9625	1.0261	0.0372	0.4508	0.3461
Aldoa	_QLLLTADDR_.2	#N/A	10.9353	10.5742	10.7051	10.8956	10.7053	10.6525	10.8746	10.5437	10.6133	10.9195	1.0006	0.0009	0.9483	0.0231
Aldoa	_LQSIGTENTEENRR_.3	#N/A	7.9607	7.6345	7.9279	7.6732	8.0823	7.5795	7.1465	7.3838	7.7072	7.9495	1.0348	0.0494	0.1714	0.7660
Aldoa	_SKGGVVGIK_.2	#N/A	7.1874	7.2638	6.7880	7.5011	7.6359	7.2014	7.2517	7.3674	7.3976	7.2822	0.9916	-0.0122	0.6956	0.1577
Aldoa	_ALQASALK_.2	#N/A	6.1959	6.6099	6.7004	5.3256	6.9016	5.6761	3.8946	6.2107	6.6399	6.6354	1.0667	0.0931	0.5405	0.2672
Aldoa	_ADDGRPFPQVIK_.2	#N/A	8.9013	9.1584	9.0167	9.3194	9.3589	9.0872	9.2313	9.0132	8.9377	8.9766	1.0111	0.0160	0.3612	0.4422
Aldoa	_GILAADESTGS[Phospho (STY)]IAKR_.2	#N/A	10.3824	10.6463	11.2513	11.0366	10.4107	10.7544	10.8113	10.5757	10.6285	10.7282	1.0057	0.0082	0.7444	0.1282
Aldoa	_ALSDHHVYLEGTLLKPNM[Oxidation (M)]VTPGHAC[Carbamidomethyl (C)]TQK_.5	#N/A	7.7206	8.3882	8.5545	8.2666	8.3668	8.2995	8.3791	8.3437	7.5971	8.2615	1.0148	0.0212	0.5743	0.2409
Aldoa	_FSNEEIAM[Oxidation (M)]ATVTALR_.2	#N/A	7.3936	7.1481	7.4827	7.5151	7.0308	7.5218	7.4654	6.8842	7.6075	7.1704	1.0092	0.0132	0.6978	0.1563
Aldoa	_C[Carbamidomethyl (C)]QYVTEK_.2	#N/A	3.2677	4.0729	3.9545	4.4572	8.0644	7.8794	7.6391	7.9130	7.2291	8.1532	0.6831	-0.5499	0.0552	1.2584
Aldoa	_RLQSIGTENTEENRR_.3	#N/A	4.6303	4.6407	3.8924	5.2652	5.3061	5.5352	5.1415	4.5014	4.8282	5.0753	0.9983	-0.0025	0.9806	0.0085
Aldoa	_YTPSGQSGAAASESLFISNHAY_.2	#N/A	6.7953	6.9181	7.0493	7.9638	7.2790	7.3252	7.1502	7.2152	6.8204	7.2187	1.0170	0.0243	0.6070	0.2168
Aldoa	_VNPC[Carbamidomethyl (C)]IGGVILFHETLYQK_.2	#N/A	4.1853	4.0455	3.8638	4.1259	4.3201	3.8735	4.2922	4.2229	4.6667	4.7555	0.9074	-0.1402	0.0176	1.7550

Aldoa	_TVPPAVTGVTFLSGGQSEEEASINLNAINK_.3	#N/A	4.1503	5.2620	6.0964	6.2525	5.4905	3.8745	5.8753	5.5080	5.4681	4.3082	0.9807	-0.0281	0.8622	0.0644
Aldoa	_FSNEEIAMATVTALRR_.3	#N/A	3.7761	3.0315	3.3806	5.4363	2.9269	3.7048	3.5980	4.2352	3.8838	3.5575	0.9714	-0.0419	0.8264	0.0828
Prkaca	_FKGPGDTSNFDDYEEEEIRVS[Phospho (STY)]INEK_.4	P05132_S339_M1_Prkaca	6.9389	4.1473	4.0289	4.5316	4.6131	4.5373	6.8809	3.5869	6.4354	2.9977	0.9647	-0.0519	0.8580	0.0665
Prkaca	_WETPSQNTAQLDQFDR_.2	#N/A	5.1711	3.8362	3.2399	4.2845	3.8354	4.0720	4.4328	5.3158	3.0157	3.9618	0.9741	-0.0379	0.8341	0.0788
Got1	_HIYLLPSGR_.2	#N/A	9.6870	9.7671	10.0879	9.7923	9.6017	9.6805	9.7260	9.6900	9.7929	9.6296	1.0062	0.0089	0.5295	0.2761
Got1	_ITWSNPPAQGAR_.2	#N/A	9.3423	9.3391	9.5115	9.5576	9.3007	9.3012	9.5228	9.1733	9.3161	9.3631	1.0052	0.0074	0.5678	0.2458
Got1	_NFGLYNER_.2	#N/A	9.2912	9.6612	9.8156	9.3964	8.9769	9.2422	9.4813	9.5193	9.5426	9.4527	0.9893	-0.0155	0.5301	0.2756
Got1	_VGGVQSLGGTGALR_.2	#N/A	8.3345	8.7151	8.6207	9.0080	8.8365	8.7525	8.4864	8.9924	8.7409	8.7158	0.9974	-0.0038	0.8765	0.0572
Got1	_IVAATLSDPELFK_.2	#N/A	8.6344	8.5164	8.7173	8.7744	8.6745	8.7140	8.7724	8.7875	8.8349	8.5834	0.9917	-0.0121	0.2830	0.5483
Got1	_LVLGDNSLAIR_.2	#N/A	8.6527	9.0581	8.9531	8.8511	8.9001	8.7606	8.7969	8.9195	8.8729	8.8444	1.0005	0.0007	0.9572	0.0190
Got1	_DIRPYC[Carbamidomethyl (C)]YWDAEKR_.3	#N/A	7.9535	8.2261	8.5016	8.3480	8.0412	8.2807	8.2535	7.9945	8.1760	8.0376	1.0135	0.0194	0.3602	0.4434
Got1	_QVEYLVNEK_.2	#N/A	8.0123	8.0219	8.3972	8.1104	8.3555	8.2833	7.8818	7.8627	8.3206	8.2829	1.0136	0.0194	0.4270	0.3696
Got1	_APPSVFAQVPQAPPVLVFK_.2	#N/A	6.9271	6.8186	6.9634	7.3142	7.1399	6.7218	6.8426	7.1083	6.8692	7.1665	0.9977	-0.0033	0.9044	0.0437
Got1	_NTPIYVSSPTWENHNAVFSAAGFK_.3	#N/A	7.8374	7.6268	7.7782	7.6261	7.4914	7.2917	7.7526	7.5940	7.5539	7.6297	0.9969	-0.0045	0.8278	0.0821
Got1	_IGADFLGR_.2	#N/A	7.1615	7.2225	7.4997	6.8399	6.5498	6.8386	7.0246	7.2622	6.8439	7.1280	0.9935	-0.0094	0.8119	0.0905
Got1	_VNLGVGAYR_.2	#N/A	5.3569	6.3990	5.2104	5.7858	6.3785	5.9161	5.6078	6.0685	5.8636	5.5287	1.0128	0.0184	0.7928	0.1009
Got1	_VGNLTVVGK_.2	#N/A	5.9532	5.8693	6.3452	5.3232	5.5912	4.9705	3.9510	5.9828	5.5733	5.6766	1.0717	0.0998	0.4121	0.3850
Got1	_IANDNSLNHEYLPILGLAEFR_.3	#N/A	5.4528	5.0398	3.9021	5.4270	5.0331	5.3968	4.7481	4.5227	5.3312	4.8037	1.0393	0.0556	0.5799	0.2367
Got1	_IVAATLSDPELFKEWK_.3	#N/A	3.6157	4.4904	5.0245	4.1835	3.9489	4.7984	3.4288	5.0807	4.5540	4.7939	0.9729	-0.0396	0.7666	0.1154
Got1	_QIAAVM[Oxidation (M)]QR_.2	#N/A	6.9246	6.4718	6.5431	7.3945	7.2723	6.7553	5.9800	6.8353	7.0349	6.6184	1.0418	0.0590	0.3265	0.4861
Got1	_INM[Oxidation (M)]C[Carbamidomethyl (C)]GLTTK_.2	#N/A	5.8002	6.2055	6.2755	6.1376	6.9342	5.7090	5.8826	6.2463	6.3948	6.0848	1.0040	0.0058	0.9194	0.0365
Got1	_TPGTWSHITEQIGM[Oxidation (M)]FSFTGLNPK_.3	#N/A	6.6055	6.6674	7.7107	7.2696	6.0184	6.6950	6.9470	5.6857	6.3426	7.4139	1.0349	0.0495	0.5992	0.2224
Got1	_TDESQPWVLPVVR_.2	#N/A	8.6450	8.7444	8.9135	8.9159	8.9255	8.9384	8.8832	8.7893	8.7469	8.6101	1.0102	0.0147	0.2771	0.5574
Got1	_FLFPFFDSAYQGFASGDLEK_.2	#N/A	3.8199	4.2467	3.4405	4.6937	2.5053	4.1599	5.2523	3.8897	4.5223	4.7353	0.8285	-0.2714	0.1176	0.9295
Got1	_LTADFRDDPDPR_.2	#N/A	6.7110	6.8384	6.9783	6.7261	7.5333	7.9527	7.4357	6.9831	7.3907	7.4917	0.9724	-0.0403	0.4828	0.3162
Got1	_DIRPYC[Carbamidomethyl (C)]YWDAEK_.3	#N/A	3.6916	3.4073	4.3784	4.5521	4.4953	4.1102	2.6431	3.2374	4.4714	3.3472	1.1989	0.2617	0.1141	0.9426
Got1	_NLDYVATSIHEAVTK_.2	#N/A	7.2148	7.4099	8.1573	7.6715	6.9234	7.5634	7.5896	7.3691	7.5765	7.5150	0.9970	-0.0043	0.9206	0.0359
Got1	_WYNGTDNK_.2	#N/A	4.1712	4.9451	3.3572	4.5495	5.3727	4.4326	3.2926	4.6540	4.1559	3.8268	1.1228	0.1671	0.2759	0.5593
Got1	_VLSQMEK_.2	#N/A	4.2420	2.9841	3.4280	4.2210	3.3423	3.7522	3.6453	4.1878	3.8364	3.5500	0.9623	-0.0554	0.6234	0.2053
Got1	_YFVSEGFELFC[Carbamidomethyl (C)]AQSFSK_.2	#N/A	3.6053	4.3615	3.3389	4.3835	4.2498	3.8756	3.7688	4.1185	4.4175	4.0608	0.9701	-0.0438	0.6315	0.1996
Got2	_IAATILTSPDLR_.2	#N/A	10.5640	10.6937	10.8028	10.9839	10.6145	10.8795	10.7702	10.8573	10.9098	10.6111	0.9972	-0.0041	0.7600	0.1192
Got2	_ASAELALGENNEVLK_.2	#N/A	9.6642	9.4990	9.9820	10.0903	9.5174	9.7222	9.8426	9.7055	9.7447	9.6504	1.0010	0.0015	0.9392	0.0272
Got2	_TQLVSNLKK_.2	#N/A	8.5292	8.9458	8.9519	8.9401	8.9129	8.7511	8.4548	8.8957	8.9250	8.7200	1.0102	0.0147	0.4817	0.3172
Got2	_DDNGKPYVLPSVR_.2	#N/A	8.9919	8.5584	8.5883	8.8567	8.7533	8.6494	8.6642	8.4429	8.8179	8.6885	1.0092	0.0132	0.4724	0.3256
Got2	_PSWGNHTPIFR_.3	#N/A	8.4889	8.5962	8.6963	8.7831	8.6180	8.5684	8.3659	8.6728	8.5335	8.5399	1.0114	0.0163	0.2156	0.6664
Got2	_FVTVQTISGTGALR_.2	#N/A	9.2551	9.0368	9.7737	9.1894	9.2082	9.2923	8.9774	9.0740	9.4185	9.2520	1.0122	0.0175	0.4755	0.3229
Got2	_VGAFTVVC[Carbamidomethyl (C)]K_.2	#N/A	9.1105	9.3368	9.1889	9.3281	8.7809	8.8976	9.1073	9.2338	8.9969	8.9129	1.0049	0.0071	0.7383	0.1318
Got2	_VGASFLQR_.2	#N/A	8.8960	9.0353	9.3125	8.9682	9.0035	8.8444	9.0527	9.1567	9.1437	9.0082	0.9912	-0.0128	0.3898	0.4091
Got2	_NLDKEYLPIGGLAEFC[Carbamidomethyl (C)]K_.3	#N/A	7.4266	7.5221	7.9060	8.0269	7.7798	7.6769	7.7243	7.7321	7.6133	7.8637	0.9987	-0.0019	0.9354	0.0290
Got2	_KQWLQEVK_.2	#N/A	8.2462	8.4649	8.2039	8.6154	8.5067	8.3933	8.6971	8.5204	8.7998	8.5413	0.9728	-0.0397	0.0403	1.3949
Got2	_KAEAQIAAK_.2	#N/A	8.0622	7.5067	7.5862	7.5284	8.2076	8.0308	7.7966	7.8089	7.9194	7.5885	1.0054	0.0078	0.8101	0.0914
Got2	_QWLQEVK_.2	#N/A	7.0862	7.1102	7.1854	7.1285	7.1325	6.8960	7.0201	7.4413	7.2159	7.2893	0.9790	-0.0306	0.1158	0.9364
Got2	_TQLVSNLK_.2	#N/A	7.3140	6.8729	6.7641	7.0031	7.7287	7.0656	6.0815	6.9171	7.3850	7.1548	1.0349	0.0495	0.4271	0.3694
Got2	_TC[Carbamidomethyl (C)]GFDFSGALEDISK_.2	#N/A	6.3652	6.3427	6.3733	6.5353	6.7723	6.6250	6.9572	6.0385	6.3314	6.5841	1.0038	0.0054	0.8938	0.0488
Got2	_M[Oxidation (M)]NLGVGAYR_.2	#N/A	6.8072	6.7409	6.4649	6.4577	6.7604	7.2049	7.0019	6.9685	6.9040	6.6901	0.9780	-0.0321	0.3429	0.4648
Got2	_DAGM[Oxidation (M)]QLQGYR_.2	#N/A	9.2886	9.4817	9.5886	9.5546	9.5402	9.2426	9.3885	9.3465	9.3787	9.3177	1.0098	0.0140	0.2640	0.5783
Got2	_IAATILTSPDLRK_.2	#N/A	5.9938	7.0157	7.0733	7.1145	6.7716	6.9723	7.0627	7.1773	6.8208	7.1645	0.9670	-0.0484	0.3327	0.4779
Got2	_NM[Oxidation (M)]GLYGER_.2	#N/A	8.2657	9.2623	8.1610	8.5469	8.3558	8.1382	7.8173	8.3352	8.1965	8.1485	1.0407	0.0575	0.1927	0.7151
Got2	_EYLPIGGLAEFC[Carbamidomethyl (C)]K_.2	#N/A	5.2606	5.5299	3.3591	5.6007	5.2426	5.5160	5.9063	4.9955	5.5416	5.3632	0.9327	-0.1005	0.4510	0.3459
Got2	_EFSVYMTK_.2	#N/A	6.5489	4.6580	4.1009	7.0532	5.7293	3.1136	3.7929	4.8226	3.6555	5.1586	1.1935	0.2552	0.3365	0.4731
Got2	_IPEQSVLLLHAC[Carbamidomethyl (C)]AHNPTGVDPRPEQWK_.5	#N/A	6.4891	5.7454	5.8011	6.0360	6.1417	3.8889	3.7821	6.3081	6.3459	5.9261	1.0167	0.0238	0.8933	0.0490
Got2	_ISVAGVTSGNVGYLAHAIHQVTK_.3	#N/A	5.4223	5.3383	6.4278	5.7674	5.3454	5.3551	5.6583	5.2134	5.2765	5.9283	1.0164	0.0234	0.7353	0.1335
Tuba1b	_EIIDLVLDR_.2	#N/A	13.9241	13.8640	13.9029	14.0083	13.7429	13.9567	13.8003	13.8569	13.9395	13.8221	1.0033	0.0047	0.4144	0.3826
Tuba1b	_VGINYQPPTVVPGGDLAK_.2	#N/A	13.5861	13.5163	13.6964	13.6863	13.1149	13.3517	13.6239	13.4846	13.3111	13.3770	1.0032	0.0046	0.7437	0.1286
Tuba1b	_IHFPLATYAPVISAEK_.2	#N/A	11.9568	12.0711	12.1844	12.2778	11.6884	11.8745	12.0020	12.0983	11.9713	11.9605	1.0001	0.0001	0.9944	0.0024
Tuba1b	_LSVDYGKK_.2	#N/A	11.5471	11.7184	11.9436	11.7549	11.9001	11.7020	11.5898	11.7134	11.5513	11.6830	1.0109	0.0156	0.1498	0.8246
Tuba1b	_DVNAAIATIK_.2	#N/A	12.5266	12.4470	12.8931	12.4720	12.5266	12.6203	11.8031	12.5319	12.6654	12.5156	1.0163	0.0233	0.2828	0.5486
Tuba1b	_QLFHPEQLITGK_.2	#N/A	10.7561	10.6650	10.7759	10.9002	10.9108	10.5935	10.9027	10.8024	10.8400	10.8317	0.9929	-0.0103	0.2773	0.5570
Tuba1b	_SIQFVDWC[Carbamidomethyl (C)]PTGFK_.2	#N/A	8.6489	9.2905	9.7839	10.2213	9.9689	9.9600	9.7329	8.8838	9.5274	8.9812	1.0392	0.0555	0.3118	0.5061
Tuba1b	_QLFHPEQLITGKEDAANNYAR_.4	#N/A	9.4318	9.7509	10.3456	9.6351	9.6445	9.7881	9.6783	9.3982	9.6879	9.7617	1.0140	0.0200	0.4525	0.3444
Tuba1b	_EDAANNYAR_.2	#N/A	10.6657	10.3683	10.2908	10.3211	10.9003	10.3790	9.7644	10.4951	10.7295	10.5466	1.0100	0.0143	0.6329	0.1987
Tuba1b	_RNLDIERPTYTNLNR_.3	#N/A	8.0730	7.6746	8.5307	8.4452	7.8943	8.1064	7.9092	8.0100	8.0938	7.6284	1.0266	0.0379	0.2857	0.5440
Tuba1b	_DVNAAIATIKTK_.2	#N/A	6.5036	6.4080	6.3905	7.6009	5.6158	6.2460	6.8720	6.6123	6.6083	6.3940	0.9757	-0.0355	0.6462	0.1897
Tuba1b	_YM[Oxidation (M)]AC[Carbamidomethyl (C)]C[Carbamidomethyl (C)]LLYR_.2	#N/A	10.6421	11.1726	11.2900	10.6851	10.7665	10.7334	10.9347	11.1076	11.0423	10.8726	0.9902	-0.0142	0.4853	0.3140
Tuba1b	_AYHEQLSVAEITNAC[Carbamidomethyl (C)]FEPANQM[Oxidation (M)]VK_.3	#N/A	10.0237	9.9657	10.7497	10.7251	9.7898	10.1103	10.3089	9.8748	10.1867	10.5357	1.0001	0.0001	0.9972	0.0012
Tuba1b	_DYEEVGVDSVEGEGEEEGEEY_.2	#N/A	6.8470	7.9550	7.6305	9.4942	7.5446	8.5359	8.7958	7.3190	6.9574	7.3860	1.0508	0.0715	0.5141	0.2889
Tuba1b	_FDGALNVDLTEFQTNLVPYPR_.3	#N/A	7.3991	7.8127	7.8143	8.1985	7.4972	7.6723	7.8285	7.6865	7.6827	7.8535	0.9961	-0.0057	0.8429	0.0742
Tuba1b	_LDHKFDLM[Oxidation (M)]YAK_.2	#N/A	7.7467	8.7169	8.7381	8.8180	8.5882	8.5734	8.4104	8.7632	8.4613	8.3181	1.0049	0.0071	0.8503	0.0704
Tuba1b	_NLDIERPTYTNLNR_.3	#N/A	13.0461	12.9344	13.4751	13.3712	12.8373	13.0537	13.2236	12.7231	13.0048	13.1179	1.0079	0.0113	0.5241	0.2806
Tuba1b	_AVFVDLEPTVIDEVR_.2	#N/A	12.8912	12.7768	13.2443	12.9189	12.8820	13.0467	13.0298	12.7526	12.9805	12.8838	1.0037	0.0054	0.6306	0.2003
Tuba1b	_EDMAALEK_.2	#N/A	8.5983	7.0421	3.1165	8.7999	8.3358	3.4407	6.1976	6.7276	7.1454	7.5915	0.9479	-0.0771	0.7971	0.0985
Tuba1b	_TIGGGDDS[Phospho (STY)]FNTFFSETGAGK_.2	P05213_S48_M1_Tuba1b	6.0316	6.0405	5.5151	8.6767	5.7513	4.3078	6.2015	8.2860	6.1918	5.6714	0.9190	-0.1219	0.5539	0.2565
Tuba1b	_LISQIVSSITASLR_.2	#N/A	7.4931	7.3927	8.0923	6.3849	8.1027	8.3059	8.2997	7.1993	8.1937	7.6532	0.9735	-0.0388	0.6296	0.2009
Tuba1b	_FDLM[Oxidation (M)]YAK_.2	#N/A	8.8956	8.7533	9.0248	8.1650	8.7346	8.7468	7.7224	9.0856	9.0774	9.1417	0.9958	-0.0061	0.9089	0.0415
Tuba1b	_AFVHWYVGEGM[Oxidation (M)]EEGEFSEAR_.3	#N/A	10.5391	11.3740	12.0678	12.7908	10.9341	11.3039	11.3866	11.3480	11.3159	11.0602	1.0199	0.0284	0.6066	0.2171
Tuba1b	_AVC[Carbamidomethyl (C)]M[Oxidation (M)]LSNTTAIAEAWAR_.3	#N/A	9.3121	8.9468	9.8791	10.2719	8.9064	9.0853	9.1067	8.6348	8.9034	9.6068	1.0372	0.0527	0.3323	0.4784
Tuba1b	_GDVVPKDVNAAIATIK_.2	#N/A	4.8933	5.3289	5.9924	5.9561	5.0478	3.8286	5.4445	5.4462	5.6334	5.3204	0.9475	-0.0778	0.5065	0.2955
Tuba1b	_AFVHWYVGEGM[Oxidation (M)]EEGEFSEAREDM[Oxidation (M)]AALEK_.4	#N/A	5.8433	7.6838	8.1976	7.4611	5.6902	6.7582	6.0377	6.8179	6.8737	7.3347	1.0256	0.0364	0.7662	0.1157
Tuba1b	_LS[Phospho (STY)]VDYGK_.2	#N/A	5.5982	3.3724	3.7037	3.2475	3.1138	3.5108	3.3958	3.7537	4.0527	4.4256	0.9618	-0.0562	0.7741	0.1112

Tuba1b	_LADQC[Carbamidomethyl (C)]TGLQGFLVFHSFGGGTGSGFTSLLM[Oxidation (M)]ER_.3	#N/A	4.3443	3.6487	3.5303	4.4720	3.4700	4.2595	3.1195	4.0855	4.3290	3.9999	1.0182	0.0260	0.8261	0.0830
Tuba1b	_EDM[Oxidation (M)]AALEKDYEEVGVDSVEGEGEEEGEEY_.3	#N/A	3.9016	3.4408	3.6353	3.1791	3.0454	3.5793	3.3273	3.8221	4.1212	4.3572	0.8865	-0.1738	0.0989	1.0050
Tuba1b	_RAFVHWYVGEGMEEGEFSEAR_.3	#N/A	3.7331	3.0374	4.4199	3.5824	2.8587	3.3699	3.7307	3.4187	3.7178	3.3887	0.9821	-0.0260	0.8334	0.0791
Tuba3a	_TIQFVDWC[Carbamidomethyl (C)]PTGFK_.2	#N/A	10.4568	10.4638	10.7791	10.5237	10.5818	10.4366	10.5557	10.4198	10.6553	10.4305	1.0024	0.0034	0.7606	0.1189
Tuba3a	_LIGQIVSSITASLR_.2	#N/A	7.2962	7.4557	7.5628	7.3742	6.6672	6.8852	7.7809	8.3551	7.9436	7.3209	0.9181	-0.1233	0.0312	1.5055
Src	_S[Phospho (STY)]LEPSENVHGAGGAFPASQTPSKPASADGHR_.4	P05480_S17_M1_Src	4.3838	9.9849	9.8282	11.1876	4.2315	8.9958	9.4711	10.2948	9.8077	8.7374	0.8459	-0.2414	0.3729	0.4285
Src	_LFGGFNSSDTVTS[Phospho (STY)]PQR_.2	P05480_S74_M1_Src	3.8996	7.6755	8.1337	9.0277	5.6461	7.1897	7.0888	8.2399	7.4381	6.9571	0.9324	-0.1010	0.6208	0.2070
Kit	_VNS[Phospho (STY)]VGSSASSTQPLLVHEDA_.2	P05532_S962_M1_Kit	3.7851	3.0895	3.9866	3.5304	2.9108	3.3178	3.6786	3.4708	3.7699	4.1291	0.9135	-0.1305	0.2055	0.6872
Jun;Jund	_LAS[Phospho (STY)]PELER_.2	P05627_S73_M1_Jun;Jund	6.8580	5.9242	7.3552	6.5847	5.4639	6.7785	6.3471	6.3849	6.4881	6.5472	1.0081	0.0117	0.8855	0.0528
Ldha	_VTLTPEEEAR_.2	#N/A	9.6708	9.5499	9.9549	9.6747	9.5883	9.6590	9.6837	9.7855	9.6417	9.6386	0.9995	-0.0007	0.9558	0.0196
Ldha	_LVIITAGAR_.2	#N/A	8.7609	8.5740	8.9552	9.1847	8.7130	8.9339	8.4020	9.4509	9.0979	9.0707	0.9831	-0.0245	0.4807	0.3181
Ldha	_SADTLWGIQK_.2	#N/A	8.8196	8.6741	9.0059	8.7980	8.7206	8.8052	8.9677	8.8375	8.7476	8.5695	1.0027	0.0038	0.7979	0.0981
Ldha	_VIGSGC[Carbamidomethyl (C)]NLDSAR_.2	#N/A	8.4961	8.7013	8.7713	8.5809	9.1008	8.4153	8.6569	8.7916	8.6247	8.3585	1.0081	0.0116	0.6414	0.1928
Ldha	_QVVDSAYEVIK_.2	#N/A	7.8904	7.3557	7.2776	7.7392	7.6646	7.8153	7.6106	7.6380	7.8734	7.5544	0.9941	-0.0085	0.7538	0.1227
Ldha	_DQLIVNLLK_.2	#N/A	7.6880	6.9270	6.4301	7.1348	6.8454	7.0986	6.5865	7.3036	7.0996	7.3143	0.9922	-0.0113	0.8304	0.0807
Ldha	_DYC[Carbamidomethyl (C)]VTANSK_.2	#N/A	7.1932	6.5725	6.7556	7.0483	7.3558	6.4787	6.8030	6.9877	6.7343	7.0797	0.9999	-0.0001	0.9980	0.0009
Ldha	_DQLIVNLLKEEQAPQNK_.3	#N/A	6.9103	7.2048	7.7343	7.6386	6.3063	7.4335	7.1664	7.5587	7.0563	7.4882	0.9846	-0.0224	0.7057	0.1514
Ldha	_DLADELALVDVM[Oxidation (M)]EDK_.2	#N/A	7.3658	6.7056	6.9364	6.6445	6.6793	7.1643	7.1553	6.8162	7.1234	7.2952	0.9744	-0.0374	0.3190	0.4962
Ldha	_GEM[Oxidation (M)]M[Oxidation (M)]DLQHGSLFLK_.3	#N/A	3.3511	4.3775	3.3237	3.6425	4.2389	5.3137	3.0293	5.1115	4.4709	4.5024	0.9445	-0.0823	0.6624	0.1789
Ldha	_SLNPELGTDADKEQWK_.2	#N/A	7.4634	6.9377	7.1598	7.2303	7.1572	7.3928	7.6861	7.3532	7.4974	7.3612	0.9664	-0.0493	0.0588	1.2309
Ldha	_DLADELALVDVM[Oxidation (M)]EDKLK_.2	#N/A	4.8229	5.0160	4.5783	4.5239	4.3138	4.7453	5.0058	5.0762	5.4009	4.8812	0.9166	-0.1256	0.0246	1.6096
Ldha	_LLIVSNPVDILTYVAWK_.2	#N/A	3.7558	3.0112	3.4895	4.5341	2.1193	2.6130	3.1815	1.7428	2.2604	4.2114	1.1421	0.1917	0.5283	0.2771
Gpi	_SNT[Phospho (STY)]PIKVDGK_.2	#N/A	8.6860	8.4575	8.8147	8.9418	8.4512	8.6746	8.7856	8.7471	8.7317	8.6326	0.9939	-0.0088	0.6164	0.2101
Gpi	_ELFEADPER_.2	#N/A	9.6984	9.3549	9.4286	9.4271	9.0657	9.1287	9.5597	9.5506	9.1782	9.1911	0.9979	-0.0030	0.8969	0.0473
Gpi	_INYTENR_.2	#N/A	8.7771	8.6763	9.1462	8.5710	8.7422	8.6944	8.5727	9.0268	8.7399	8.7511	0.9995	-0.0008	0.9708	0.0129
Gpi	_AVLHVALR_.2	#N/A	8.1546	8.4930	8.2805	8.4382	8.2202	8.0202	8.3231	8.8618	8.3629	8.1995	0.9800	-0.0292	0.2815	0.5505
Gpi	_VFEGNRPTNSIVFTK_.3	#N/A	8.4284	7.7892	8.2651	7.9596	8.2216	8.5299	7.1399	8.0932	8.5329	8.3731	1.0204	0.0292	0.5804	0.2363
Gpi	_GKLPEEARK_.2	#N/A	8.3252	8.0302	8.2859	7.8746	7.5536	8.3292	8.2059	8.0855	7.9370	8.0256	1.0004	0.0005	0.9863	0.0060
Gpi	_HFVALSTNTAK_.2	#N/A	6.9607	7.3558	7.2017	7.4758	7.4938	7.7019	7.6472	7.4814	7.6186	7.3381	0.9792	-0.0303	0.3063	0.5138
Gpi	_TLASLSPETSLFIIASK_.2	#N/A	7.5964	7.4204	7.4330	6.9447	7.2090	7.2272	7.3578	7.7693	7.7750	7.5193	0.9605	-0.0581	0.0665	1.1772
Gpi	_EWFLEAAKDPSAVAK_.2	#N/A	6.4119	5.6205	6.4836	5.7811	5.8806	6.3768	6.5288	6.1763	5.6612	5.8929	1.0045	0.0065	0.9119	0.0401
Gpi	_NRS[Phospho (STY)]NTPIK_.2	#N/A	3.4275	3.7986	4.1143	4.6170	3.6869	2.9377	4.3317	3.5015	3.1501	3.0831	1.0703	0.0980	0.5240	0.2807
Gpi	_LRELFEADPER_.2	#N/A	6.4871	7.0633	7.2432	6.8279	6.8514	7.0580	6.6490	7.1238	6.6014	6.6741	1.0236	0.0337	0.3609	0.4426
Gpi	_NRS[Phospho (STY)]NTPIKVDGK_.3	#N/A	4.8410	4.5234	3.8413	5.0355	4.5969	4.7275	3.8752	5.3382	4.7164	4.5496	0.9945	-0.0080	0.9377	0.0280
Gpi	_EWFLEAAK_.2	#N/A	4.8823	5.2318	3.1082	4.5090	4.7342	4.7236	2.6865	4.9907	5.5274	5.5623	0.9659	-0.0501	0.8135	0.0897
Gpi	_DVM[Oxidation (M)]PEVNR_.2	#N/A	8.6173	8.2718	9.0858	8.7940	8.2541	8.7385	8.3062	8.4410	7.9963	8.2298	1.0465	0.0656	0.0654	1.1846
Gpi	_TFTTQETITNAETAK_.2	#N/A	5.8899	5.9218	5.9424	6.5974	5.8863	5.9423	6.1161	6.5199	6.0107	6.0915	0.9750	-0.0365	0.3859	0.4135
Gpi	_TFTTQETITNAETAKEWFLEAAK_.3	#N/A	4.7641	4.1211	5.2373	5.3285	4.2466	4.1477	4.5023	3.9046	5.0688	5.2108	0.9934	-0.0095	0.9352	0.0291
Gpi	_VWFVSNIDGTHIAK_.2	#N/A	7.1462	7.1983	7.3710	7.1546	6.4962	7.0577	7.4256	7.0624	7.1372	7.0435	0.9865	-0.0196	0.5821	0.2350
Gpi	_M[Oxidation (M)]KS[Phospho (STY)]FC[Carbamidomethyl (C)]QR_.2	#N/A	6.4187	5.8858	4.1359	6.5849	6.2231	5.7806	6.6293	6.4243	6.3636	6.2674	0.9092	-0.1373	0.2382	0.6230
Gpi	_KIEPELEGSSAVTSHDSSTNGLISFIK_.4	#N/A	5.5151	5.8911	6.2430	6.4410	5.8272	5.3788	5.2944	5.5623	5.8112	5.9118	1.0421	0.0595	0.3416	0.4664
Gpi	_EVM[Oxidation (M)]QM[Oxidation (M)]LVELAK_.2	#N/A	5.6574	5.0557	5.5972	4.9259	4.7423	4.9896	4.8697	5.4169	5.5062	5.3241	0.9777	-0.0326	0.6102	0.2145
Gpi	_ILLANFLAQTEALM[Oxidation (M)]K_.2	#N/A	4.8581	5.3245	4.9106	4.9850	5.1842	5.0519	5.7146	5.7579	5.6744	4.8081	0.9205	-0.1195	0.0616	1.2102
Gpi	_FAAYFQQGDMESNGK_.2	#N/A	5.6992	3.3712	4.5651	5.9920	4.8230	3.9226	3.4082	5.4047	4.0516	4.7105	1.0763	0.1060	0.6010	0.2211
Gpi	_SPEDLEK_.2	#N/A	3.3015	4.1067	3.9883	4.4909	3.5249	3.8015	4.2056	2.8859	4.3299	3.2542	1.0545	0.0766	0.5880	0.2307
Gpi	_DNM[Oxidation (M)]FSGSK_.2	#N/A	3.9196	4.2970	3.7030	3.7648	4.0225	3.7741	3.4795	4.2272	4.0770	3.6763	1.0126	0.0180	0.7916	0.1015
Gpi	_ELQAAGKS[Phospho (STY)]PEDLEK_.2	P06745_S455_M1_Gpi	6.6300	6.6449	4.3232	7.0446	3.7333	4.3922	4.2853	3.5478	3.1964	3.5443	1.4990	0.5840	0.0455	1.3421
Ptprc	_NRNS[Phospho (STY)]NVVPYDFNR_.2	#N/A	6.8750	6.1835	7.5089	5.4093	3.8738	5.2532	7.1623	7.3931	5.5334	6.6777	0.8743	-0.1938	0.2867	0.5425
Me1	_AIFASGSPFDPVTLPDGR_.2	#N/A	5.2670	5.1596	5.6941	5.8597	5.5640	4.9724	5.4888	5.2230	5.3152	4.8381	1.0390	0.0551	0.3509	0.4549
Gap43	_EGDGSATTDAAPATS[Phospho (STY)]PK_.2	#N/A	12.9492	12.9172	13.1247	13.4250	9.5195	12.7992	13.2752	12.8702	12.8363	13.0540	0.9575	-0.0627	0.4800	0.3188
Gap43	_EKDDAPVADGVEKK_.3	#N/A	6.9147	7.3164	7.5094	7.1577	7.7826	7.4536	6.7931	6.4476	7.2742	7.4416	1.0525	0.0738	0.1587	0.7993
Gap43	_AEDGPAKEEPK_.2	#N/A	7.0947	7.4522	7.4868	7.6799	8.1332	7.7602	7.3018	7.2875	7.1094	7.1857	1.0526	0.0740	0.0693	1.1590
Gap43	_KGDAPAAEAEAK_.2	#N/A	7.1059	7.3401	7.0312	6.9331	7.3530	7.0908	7.0592	7.2261	6.6635	7.0205	1.0215	0.0306	0.2728	0.5642
Gap43	_KGEGDAAPSEEK_.2	#N/A	5.8629	6.0106	5.1418	5.7469	7.4693	6.2076	5.4887	6.0936	5.3275	5.9832	1.0611	0.0856	0.4302	0.3664
Gap43	_EGDGSATTDAAPATS[Phospho (STY)]PKAEEPSK_.3	#N/A	11.8132	11.9759	12.4890	11.5583	11.6285	11.8906	12.0947	11.8989	11.6907	12.1962	0.9935	-0.0094	0.6953	0.1578
Gap43	_KGEGDAAPSEEKAGS[Phospho (STY)]AETESAAK_.2	#N/A	6.1321	6.2614	6.4802	6.8347	6.2878	6.3810	6.7196	6.3721	6.4514	6.1347	0.9964	-0.0052	0.8859	0.0526
Gap43	_QADVPAAVTDAAATT[Phospho (STY)]PAAEDAATK_.2	#N/A	13.1697	13.5047	14.1377	13.8668	13.0964	13.4472	13.7281	13.1803	13.2874	13.4661	1.0091	0.0130	0.6050	0.2183
Gap43	_NDEDQKIEQDGVKPEDK_.3	#N/A	6.6909	6.3099	6.5331	6.7349	7.0427	6.2943	6.0925	6.1512	6.4592	6.3792	1.0527	0.0741	0.0743	1.1292
Gap43	_KEGDGSATTDAAPATS[Phospho (STY)]PK_.2	#N/A	7.3043	3.7674	6.8052	7.6171	6.6866	7.0428	4.2034	6.7154	7.2770	7.2275	1.0285	0.0406	0.8481	0.0716
Gap43	_ATTDNS[Phospho (STY)]PSSKAEDGPAKEEPK_.3	#N/A	11.0301	11.6592	12.4748	12.0870	11.8027	11.6985	11.6819	11.5694	8.7549	11.4898	1.0844	0.1169	0.1713	0.7662
Gap43	_QDEGKEDPEADQEHA_.2	#N/A	4.7648	5.3331	4.4347	4.4592	5.9225	5.0650	4.1739	4.5251	4.7622	4.9202	1.0873	0.1208	0.2435	0.6135
Gap43	_AGS[Phospho (STY)]AETESAAK_.2	#N/A	8.6845	8.4732	8.3491	8.8623	8.3822	7.8486	8.5053	7.6148	3.5226	8.8552	1.1837	0.2433	0.2215	0.6545
Gap43	_AEDGPAKEEPKQADVPAAVTDAAATT[Phospho (STY)]PAAEDAATK_.4	#N/A	3.9151	3.2245	4.6019	3.4004	3.0408	3.1878	3.5486	3.6008	3.8999	3.0776	1.0085	0.0122	0.9300	0.0315
Gap43	_AAQPPTETAESSQAEEEKDAVDEAKPK_.4	#N/A	8.6826	10.4322	9.1230	10.2855	10.1456	8.7451	9.5094	10.3117	9.3297	10.1518	0.9739	-0.0382	0.5867	0.2316
Gap43	_EGDGSATTDAAPATS[Phospho (STY)]PKAEEPSKAGDAPSEEK_.4	#N/A	8.3083	8.2120	9.4071	4.1773	8.3367	4.3960	4.3326	8.4785	8.4876	4.8862	1.0906	0.1252	0.6938	0.1588
Gap43	_AGDAPS[Phospho (STY)]EEKKGEGDAAPSEEK_.3	#N/A	3.8957	2.9032	4.4694	3.5833	3.0550	2.7459	3.5360	3.2819	3.1912	3.4094	1.0261	0.0371	0.8058	0.0938
Gap43	_KEGDGSATTDAAPATS[Phospho (STY)]PKAEEPSK_.3	#N/A	3.7118	4.3172	5.9642	4.9018	6.3352	5.4638	5.3414	5.4479	3.9534	5.1279	1.0298	0.0424	0.8043	0.0946
Gap43	_ATTDNS[Phospho (STY)]PSSKAEDGPAK_.2	#N/A	7.9461	7.9769	7.6390	8.7822	8.2429	8.2739	8.3068	7.9894	7.9463	7.9743	1.0111	0.0159	0.6811	0.1668
Gap43	_AGSAETESAAKATTDNS[Phospho (STY)]PSSK_.2	#N/A	3.4230	4.0814	3.6850	4.9572	3.9710	4.1783	4.0715	3.9403	4.0889	3.5547	1.0346	0.0491	0.6471	0.1890
Gap43	_GEGDAAPS[Phospho (STY)]EEKAGSAETESAAK_.2	P06837_S122_M1_Gap43	4.2639	3.0785	3.9976	3.5414	3.4077	3.2169	3.6897	3.4598	3.7588	3.2187	1.0149	0.0213	0.8410	0.0752
Gap43	_AGDAPSEEK_.2	#N/A	3.3417	3.2046	3.6828	3.2266	3.0929	3.5317	3.3749	3.7746	4.0736	3.5579	0.9057	-0.1429	0.0665	1.1774
Gap43	_AEEPSKAGDAPSEEK_.3	#N/A	3.2527	4.0090	4.4872	4.4066	5.2598	4.2282	4.1213	3.8075	3.3604	4.3736	1.0915	0.1263	0.3693	0.4326
Gap43	_ESARQDEGKEDPEADQEHA_.3	#N/A	3.5186	3.7076	4.2054	3.2072	4.1451	3.0287	4.4227	3.4105	3.0590	3.1742	1.0338	0.0479	0.7410	0.1302
Alb	_LVQEVTDFAK_.2	#N/A	3.9310	4.8604	5.1683	5.4187	5.0419	3.7485	5.0764	3.8961	4.9435	4.1399	1.0401	0.0567	0.6786	0.1684
Alb	_APQVSTPTLVEAAR_.2	#N/A	2.9576	5.4823	6.5804	5.2417	6.0960	5.0343	6.0461	5.6868	5.8151	5.2015	0.9199	-0.1204	0.5062	0.2957
Alb	_QTALAELVK_.2	#N/A	4.3414	3.6447	3.5274	2.6208	3.1794	3.8515	4.1017	0.3383	3.7370	3.9970	1.1590	0.2129	0.5500	0.2596
Alb	_TC[Carbamidomethyl (C)]VADESAANC[Carbamidomethyl (C)]DK_.2	#N/A	4.2731	2.9531	3.4590	3.9617	4.4958	3.5446	5.2816	4.4957	3.8054	4.7274	0.8260	-0.2757	0.0686	1.1638
Hsp90aa1	_DQVANSAFVER_.2	#N/A	9.3415	9.2907	9.6330	9.2770	9.3241	9.5063	9.2491	9.3296	9.2329	9.2101	1.0151	0.0217	0.1013	0.9942
Hsp90aa1	_NPDDITNEEYGEFYK_.2	#N/A	8.6098	8.4455	9.3172	9.2088	8.5777	8.9721	9.0673	8.8363	8.5829	8.7532	1.0051	0.0074	0.8279	0.0820

Hsp90aa1	_LGIHEDSQNR_.2	#N/A	7.6342	7.8700	7.7962	7.9256	7.2811	7.7272	8.2038	7.8278	7.7077	7.6751	0.9812	-0.0274	0.3603	0.4433
Hsp90aa1	_ELISNSSDALDK_.2	#N/A	8.9322	8.6506	8.0760	8.9677	8.5969	8.6176	8.8184	8.6101	8.6096	8.6025	0.9977	-0.0033	0.9087	0.0416
Hsp90aa1	_DKEVS[Phospho (STY)]DDEAEEK_.2	#N/A	8.3881	7.9722	8.3184	8.8683	7.7740	8.1632	8.4849	8.4475	8.3260	7.9258	0.9941	-0.0085	0.8289	0.0815
Hsp90aa1	_HIYFITGETK_.2	#N/A	7.8711	7.8318	8.1074	8.1545	7.6797	8.0380	7.9020	7.9775	7.6821	7.7589	1.0149	0.0214	0.3076	0.5120
Hsp90aa1	_ELHINLIPSK_.2	#N/A	7.9986	7.3366	7.2358	8.0332	7.9040	7.9534	8.2456	7.9152	8.0983	7.6113	0.9719	-0.0411	0.3224	0.4916
Hsp90aa1	_ELHINLIPSKQDR_.3	#N/A	7.8759	7.7301	8.0068	7.9520	7.9135	8.3409	7.5125	7.9465	8.2170	8.2199	0.9995	-0.0007	0.9811	0.0083
Hsp90aa1	_APFDLFENR_.2	#N/A	6.2854	6.1381	6.2103	6.3911	6.2380	6.0643	5.9297	5.8911	6.4435	5.7893	1.0346	0.0490	0.1474	0.8316
Hsp90aa1	_RAPFDLFENR_.2	#N/A	7.9815	8.0919	7.4362	7.7735	7.3411	7.5752	7.7380	7.9251	7.9736	8.0490	0.9720	-0.0409	0.2091	0.6796
Hsp90aa1	_ESDDKPEIEDVGS[Phospho (STY)]DEEEEK_.2	#N/A	3.3368	4.9694	4.0236	6.6502	6.0296	5.2809	6.1393	5.4767	6.1238	5.2161	0.8797	-0.1850	0.3221	0.4921
Hsp90aa1	_ESDDKPEIEDVGS[Phospho (STY)]DEEEEKK_.3	#N/A	8.5287	8.1834	7.6206	9.1993	7.9260	8.2961	8.7298	8.4998	8.4248	8.5648	0.9693	-0.0450	0.3792	0.4211
Hsp90aa1	_ELISNSSDALDKIR_.2	#N/A	5.6403	5.6588	6.3056	5.8177	5.7897	5.5833	5.8121	5.7459	5.6038	5.4013	1.0281	0.0400	0.3297	0.4818
Hsp90aa1	_HGLEVIYM[Oxidation (M)]IEPIDEYC[Carbamidomethyl (C)]VQQLK_.3	#N/A	3.7015	3.6711	4.9228	4.7508	4.0530	4.3153	5.0342	4.5022	4.6832	4.4387	0.9081	-0.1391	0.1752	0.7565
Hsp90aa1	_DKEVS[Phospho (STY)]DDEAEEKEEK_.2	#N/A	5.4749	5.4378	4.9533	6.4960	4.6782	4.9395	5.6476	5.3938	5.6302	5.4941	0.9618	-0.0561	0.5455	0.2632
Hsp90aa1	_FYEQFSK_.2	#N/A	5.0247	4.2838	3.9380	3.8074	4.3820	4.3553	3.1697	4.2323	3.9799	4.2060	1.1030	0.1415	0.2083	0.6814
Hsp90aa1	_SLTNDWEEHLAVK_.2	#N/A	5.0591	5.6012	6.0529	5.1209	5.8339	5.6562	5.6432	5.6032	4.9134	4.8238	1.0587	0.0823	0.2781	0.5557
Hsp90aa1	_HLEINPDHSIIETLR_.3	#N/A	6.7200	6.8157	6.7562	6.6504	6.5085	7.2608	6.7913	6.2735	7.0020	6.6206	1.0170	0.0243	0.5428	0.2653
Hsp90aa1	_DNSTM[Oxidation (M)]GYMAAK_.2	#N/A	3.6596	3.5751	4.0103	5.6344	5.3625	3.6792	3.9155	5.4843	3.7664	3.7142	1.0237	0.0338	0.8673	0.0618
Hsp90aa1	_LVTSPC[Carbamidomethyl (C)]C[Carbamidomethyl (C)]IVTSTYGWTANM[Oxidation (M)]ER_.3	#N/A	3.8862	3.4562	3.6199	4.6596	3.0299	3.5947	3.3119	3.5323	4.1366	4.3418	0.9679	-0.0470	0.7256	0.1393
Hsp90aa1	_KHLEINPDHSIIETLR_.4	#N/A	3.4137	4.1700	3.1474	4.1920	4.1964	4.0671	4.3403	4.3100	3.1082	4.2664	0.9646	-0.0520	0.6828	0.1657
Hsp90aa1	_ERDKEVS[Phospho (STY)]DDEAEEK_.2	#N/A	3.9765	4.0133	4.1421	3.1377	3.8595	3.0284	3.5161	3.3982	3.0699	2.6607	1.1682	0.2243	0.1031	0.9869
Hsp90aa1	_LGLGIDEDDPTVDDTSAAVTEEMPPLEGDDDTSR_.3	#N/A	3.8376	3.0930	3.5713	4.6159	2.9813	3.6433	3.2633	3.8861	4.1852	4.2931	0.9275	-0.1086	0.4434	0.3532
Hsp90aa1	_LGIHEDSQNRK_.3	#N/A	2.9151	2.9265	4.1496	3.6934	4.2486	3.4808	3.8416	3.3078	3.6069	3.2426	1.0198	0.0283	0.8309	0.0804
Hsp90b1	_SILFVPTSAPR_.2	#N/A	8.0608	8.4472	8.6292	8.9055	8.3315	8.5527	8.5036	8.3236	8.1487	8.4745	1.0150	0.0214	0.4543	0.3427
Hsp90b1	_TFEINPR_.2	#N/A	6.8921	7.1858	6.5398	6.7884	7.2441	6.5820	6.7665	7.0777	6.5697	7.2421	0.9939	-0.0088	0.8331	0.0793
Hsp90b1	_FAFQAEVNR_.2	#N/A	6.4245	6.6526	6.2741	6.6079	6.4995	6.3105	6.7689	6.6706	6.8028	6.3857	0.9706	-0.0430	0.1094	0.9608
Hsp90b1	_GLFDEYGSK_.2	#N/A	6.9162	6.4670	6.5161	7.0811	6.6089	6.9986	6.4669	6.5523	6.4169	7.0128	1.0231	0.0329	0.4044	0.3932
Hsp90b1	_TDDEVVQREEEAIQLDGLNASQIR_.3	#N/A	5.4611	4.6480	5.8251	5.9381	5.1653	4.7775	6.0735	5.2644	4.3169	5.9331	0.9825	-0.0255	0.8269	0.0826
Hsp90b1	_GVVDSDDLPLNVSR_.2	#N/A	6.4444	7.6695	7.5712	8.2402	7.8863	7.5022	7.6989	7.7131	7.5825	7.6267	0.9865	-0.0195	0.7479	0.1261
Hsp90b1	_SGTSEFLNK_.2	#N/A	5.4145	4.4471	5.5303	5.2442	5.0065	4.4423	3.6806	5.4309	4.3982	5.5535	1.0521	0.0733	0.5770	0.2388
Hsp90b1	_DISTNYYASQK_.2	#N/A	5.8927	5.4018	6.3379	6.2323	5.5998	5.9044	4.5346	5.4318	5.1392	6.0021	1.1171	0.1598	0.0756	1.1216
Hsp90b1	_SGYLLPDTK_.2	#N/A	4.8657	4.9047	5.0634	5.2215	3.9188	4.2067	4.2007	4.9074	4.9466	4.5335	1.0107	0.0154	0.8713	0.0598
Hsp90b1	_NLLHVTDTGVGM[Oxidation (M)]TR_.2	#N/A	4.0773	4.7290	4.8484	4.4166	3.5804	4.0713	4.9799	4.6363	4.6140	4.1213	0.9345	-0.0978	0.3121	0.5057
Hsp90b1	_FQSSHHSTDITSLDQYVER_.3	#N/A	5.7052	5.6167	6.1846	5.4487	5.5328	5.7517	5.6066	4.0700	5.3457	5.2431	1.1264	0.1717	0.0654	1.1842
Hsp90b1	_LISLTDENALAGNEELTVK_.2	#N/A	4.9060	5.1286	5.6223	4.5342	3.9713	4.4854	4.2489	3.5842	5.1061	5.0840	1.0597	0.0836	0.5323	0.2739
Apoe	_ELEEQLGPVAEETR_.2	#N/A	7.4680	7.9833	7.9949	8.1821	7.6978	8.1664	7.6987	7.9174	7.9517	7.9538	1.0044	0.0064	0.8224	0.0849
Apoe	_LQAEIFQAR_.2	#N/A	7.0735	7.3783	7.5537	7.4076	7.3118	7.3127	7.5311	7.5273	7.2437	7.1066	0.9983	-0.0025	0.9164	0.0379
Apoe	_LGPLVEQGR_.2	#N/A	7.0740	7.4471	7.7479	7.1785	7.1300	6.8963	7.2008	7.4733	7.1934	7.2333	0.9959	-0.0059	0.8613	0.0649
Apoe	_DRLEEVR_.2	#N/A	6.4748	5.7252	5.5935	6.0799	6.5299	5.5086	5.6801	6.1405	6.4311	5.3917	1.0126	0.0181	0.8054	0.0940
Apoe	_IQASVATNPIITPVAQENQ_.2	#N/A	8.5541	8.9189	9.0573	9.9373	8.7218	8.4770	8.9867	9.0129	8.6827	8.5618	1.0151	0.0217	0.6534	0.1848
Apoe	_NEVHTM[Oxidation (M)]LGQS[Phospho (STY)]TEEIR_.2	#N/A	5.2103	5.1059	5.4630	5.8201	5.0782	4.3755	3.9183	4.4745	4.1783	4.5572	1.2087	0.2734	0.0109	1.9625
Apoe	_LGADMEDLR_.2	#N/A	3.7805	3.0282	3.5064	4.1628	4.5018	3.7081	3.6013	2.0440	3.8804	3.8536	1.1305	0.1769	0.3440	0.4634
Apoe	_DAEDLQKR_.2	#N/A	3.6473	4.4035	3.3809	4.0121	2.7910	3.8336	3.7268	4.1220	3.7550	3.9791	0.9441	-0.0829	0.4784	0.3202
Apoe	_SKMEEQTQQIR_.2	#N/A	3.6632	2.9675	4.1086	3.6523	2.7889	3.4398	3.8006	3.3488	3.6479	3.3296	0.9731	-0.0394	0.7292	0.1371
Sod1	_DGVANVSIEDR_.2	#N/A	9.2411	9.4640	9.5329	9.8061	9.3145	9.5326	9.6688	9.3068	9.2397	9.5711	1.0037	0.0054	0.7928	0.1008
Sod1	_HVGDLGNVTAGK_.2	#N/A	8.8497	9.4229	9.2848	9.3987	10.1541	9.4995	9.2240	9.0340	9.2570	9.2046	1.0278	0.0395	0.2770	0.5576
Sod1	_VISLSGEHSIIGR_.2	#N/A	6.7514	6.7285	7.2534	6.9652	8.7903	8.2496	7.5425	7.0691	7.8765	7.9288	0.9806	-0.0283	0.7597	0.1194
Sod1	_TM[Oxidation (M)]VVHEK_.2	#N/A	6.4017	4.2596	6.3294	6.4332	8.0852	6.8846	5.9264	6.0346	6.6187	6.2600	1.0304	0.0433	0.7763	0.1099
Sod1	_LAC[Carbamidomethyl (C)]GVIGIAQ_.2	#N/A	4.9524	4.8788	4.8309	6.4494	5.1847	5.3158	4.1582	5.3054	4.6281	5.0080	1.0406	0.0574	0.4279	0.3686
Sod1	_GDGPVQGTIHFEQK_.2	#N/A	7.3539	7.8390	7.7276	8.4569	8.5853	8.1577	7.8530	8.0012	8.0348	7.9616	1.0072	0.0104	0.8172	0.0877
Sod1	_KHGGPADEER_.2	#N/A	5.8279	2.9465	3.4656	3.9683	3.4053	5.4511	6.6245	4.8023	5.1877	3.9353	0.8131	-0.2984	0.2360	0.6271
Sod1	_QDDLGKGGNEESTK_.2	#N/A	4.5948	5.3498	6.3560	5.1896	6.2975	6.2823	5.2295	5.2513	5.5156	4.6511	1.1000	0.1376	0.2365	0.6262
Mdh2	_GC[Carbamidomethyl (C)]DVVVIPAGVPR_.2	#N/A	12.7725	12.7082	12.8435	13.1242	12.8884	12.8174	12.8239	12.4845	12.6431	12.7204	1.0151	0.0216	0.0731	1.1363
Mdh2	_VDFPQDQLATLTGR_.2	#N/A	12.2905	12.3528	12.7091	12.6342	12.7648	12.4153	12.4534	12.3091	12.3639	12.4911	1.0099	0.0143	0.2828	0.5485
Mdh2	_GYLGPEQLPDC[Carbamidomethyl (C)]LK_.2	#N/A	11.6265	11.7003	11.7871	11.9189	12.0062	11.6605	11.7449	11.6759	11.6162	11.6894	1.0087	0.0125	0.2399	0.6201
Mdh2	_IFGVTTLDIVR_.2	#N/A	11.5097	11.6704	11.7384	11.7288	11.6184	11.6785	11.4922	11.4833	11.5457	11.5915	1.0112	0.0161	0.0261	1.5829
Mdh2	_M[Oxidation (M)]IAEAIPELK_.2	#N/A	10.3397	10.6949	10.6551	10.5636	10.7132	10.4488	10.3647	10.4459	10.3722	10.4926	1.0144	0.0207	0.0964	1.0160
Mdh2	_TIIPLISQC[Carbamidomethyl (C)]TPK_.2	#N/A	10.7233	10.9988	11.0421	11.1531	11.1393	10.7706	10.9880	11.0490	10.8623	10.9819	1.0001	0.0001	0.9930	0.0030
Mdh2	_IQEAGTEVVK_.2	#N/A	9.8111	9.8452	9.9801	10.0270	10.2766	10.0502	9.7215	9.7714	9.8145	10.0195	1.0169	0.0242	0.1334	0.8749
Mdh2	_ETEC[Carbamidomethyl (C)]TYFSTPLLLGK_.2	#N/A	10.6093	10.5194	10.8223	10.6353	10.8613	10.6770	10.6198	10.2512	10.5198	10.6314	1.0173	0.0248	0.0967	1.0147
Mdh2	_VNVPVIGGHAGK_.2	#N/A	10.5532	10.7639	10.5679	9.8710	10.9432	10.5975	10.7651	9.9223	10.3576	10.6500	1.0121	0.0173	0.6116	0.2135
Mdh2	_EGVVEC[Carbamidomethyl (C)]SFVQSK_.2	#N/A	9.0909	9.2203	9.2226	9.7715	9.0362	9.0183	9.4481	9.2120	9.0949	9.5092	0.9904	-0.0139	0.5981	0.2232
Mdh2	_VAVLGASGGIGQPLSLLLK_.2	#N/A	8.9750	8.9138	9.0836	8.9894	9.1701	9.1516	8.8154	9.0376	9.0652	9.2065	1.0018	0.0026	0.8508	0.0702
Mdh2	_ANTFVAELK_.2	#N/A	8.7142	8.8129	8.5344	8.8997	8.9286	9.1286	7.0951	8.7385	8.9196	9.0836	1.0446	0.0629	0.3477	0.4588
Mdh2	_KGEDFVK_.2	#N/A	8.5562	8.1775	8.3062	8.4984	8.4918	8.1346	7.9747	8.1088	8.2208	8.5679	1.0174	0.0248	0.3249	0.4883
Mdh2	_LTLYDIAHTPGVAADLSHIETR_.3	#N/A	7.0194	7.3063	7.5652	7.7446	7.3869	7.2581	7.1070	7.2651	7.2103	7.5683	1.0127	0.0182	0.5565	0.2545
Mdh2	_HGVYNPNK_.2	#N/A	8.5639	7.9947	8.1377	8.0117	8.3439	7.8292	8.0573	8.2471	3.9196	8.3064	1.1422	0.1918	0.2714	0.5664
Mdh2	_NSPLVS[Phospho (STY)]R_.2	P08249_S51_M1_Mdh2	7.8439	7.5213	4.5709	8.1492	7.4620	7.8712	7.4951	7.5894	7.9551	7.9774	0.9332	-0.0997	0.4717	0.3263
Mdh2	_FVFSLVDAM[Oxidation (M)]NGK_.2	#N/A	8.6340	8.7776	8.4959	8.9362	8.8814	8.7439	8.7324	8.6946	8.7194	8.9449	0.9968	-0.0046	0.7741	0.1112
Mdh2	_AGAGSATLSM[Oxidation (M)]AYAGAR_.2	#N/A	7.4552	7.7324	7.1777	7.4687	7.2660	7.4018	7.6664	7.7397	7.4732	7.6963	0.9703	-0.0435	0.0696	1.1575
Mdh2	_ITPFEEK_.2	#N/A	3.6221	4.3783	3.3558	4.4004	4.2666	3.8588	10.2275	4.1017	4.4007	4.0776	0.6981	-0.5185	0.1941	0.7120
Camk4	_ASS[Phospho (STY)]DPPSTQDAK_.2	P08414_S353_M1_Camk4	3.9207	3.1761	3.6544	3.1981	3.0644	3.5602	3.3464	3.8030	4.1021	4.3763	0.8777	-0.1883	0.0858	1.0664
Camk4	_DSTDLLGKK_.2	#N/A	4.0853	4.1742	4.6523	4.0369	5.2580	4.4882	4.0927	4.2960	3.6131	4.1433	1.1023	0.1405	0.1562	0.8064
Camk4	_RDS[Phospho (STY)]EEKLK_.2	P08414_S433_M1_Camk4	4.2944	3.6852	4.7657	3.6621	3.1384	3.4884	4.7873	4.5193	4.2599	3.1396	0.9192	-0.1215	0.4397	0.3568
Camk4	_TEIGVLLR_.2	#N/A	3.8432	3.6205	3.8482	3.9597	3.5636	2.9548	3.3156	4.6407	4.1126	2.9318	0.9684	-0.0463	0.7484	0.1259
Camk4	_LGS[Phospho (STY)]ASSHTSIQENHK_.3	P08414_S337_M1_Camk4	3.2478	4.0041	4.4824	4.0261	3.8924	4.2330	4.1744	2.9751	3.2741	3.7034	1.1272	0.1727	0.1675	0.7760
Nefl	_AQLQDLNDR_.2	#N/A	12.1994	12.4200	12.7134	12.8676	12.4626	12.3133	12.4888	12.2060	12.3703	12.4788	1.0089	0.0128	0.4480	0.3487
Nefl	_EYQDLLNVK_.2	#N/A	10.6888	10.7469	10.8794	10.9509	10.8985	10.7723	10.7118	10.2982	10.6071	10.8500	1.0194	0.0277	0.0884	1.0536
Nefl	_KLLEGEETR_.2	#N/A	9.9388	10.2661	10.3767	10.3816	10.4703	10.1961	10.2277	9.8412	9.9914	10.2941	1.0181	0.0259	0.1887	0.7243
Nefl	_VHELEQQNK_.2	#N/A	9.5440	9.6937	9.7694	9.8621	9.9776	9.5766	9.6515	9.4134	9.5523	9.6963	1.0166	0.0237	0.1458	0.8363

Nefl	_YEEEVLSR_.2	#N/A	9.2285	9.3427	8.9982	9.6174	9.7150	9.2359	9.2253	9.1806	9.1704	9.4847	1.0098	0.0141	0.5564	0.2546
Nefl	_ALYEQEIR_.2	#N/A	9.5677	9.4933	9.7113	9.4585	9.5454	9.0721	8.7044	9.3137	9.0970	9.3637	1.0389	0.0551	0.0595	1.2253
Nefl	_FTVLTESAAK_.2	#N/A	8.5761	8.9539	9.0542	9.3713	9.1740	8.8883	8.5372	8.5747	8.9091	8.9369	1.0301	0.0429	0.1416	0.8490
Nefl	_TLEIEAC[Carbamidomethyl (C)]R_.2	#N/A	8.3985	8.5226	8.7132	8.9261	8.6695	8.4594	8.2232	8.1991	8.1903	8.6847	1.0349	0.0495	0.0675	1.1708
Nefl	_VLEAELLVLR_.2	#N/A	8.2594	8.7471	8.6101	8.5975	8.8292	8.5265	8.7033	8.4588	8.5355	8.6433	1.0011	0.0016	0.9314	0.0309
Nefl	_KGADEAALAR_.2	#N/A	8.1684	8.0573	7.9291	8.0920	7.8914	7.7847	8.1041	7.6070	7.5326	7.9232	1.0251	0.0357	0.1676	0.7757
Nefl	_[Acetyl (Protein N-term)]SSFGYDPYFSTSYK_.2	#N/A	7.4326	7.6188	7.6648	7.4005	7.4544	7.5024	7.6845	7.0934	7.3336	7.7030	1.0079	0.0113	0.6599	0.1805
Nefl	_FASFIER_.2	#N/A	7.5164	7.5419	7.1432	7.3063	7.9165	7.8370	5.6641	7.7184	7.7974	7.9446	1.0360	0.0511	0.5790	0.2373
Nefl	_AAKDEVSESR_.2	#N/A	7.3097	7.2628	7.3495	7.4296	8.3382	7.7468	6.8412	6.6440	7.4530	6.8241	1.0911	0.1258	0.0368	1.4340
Nefl	_EGLEETLR_.2	#N/A	7.5866	6.9544	6.4303	7.6586	7.2741	7.1996	6.8454	7.3819	7.2752	7.4955	0.9910	-0.0131	0.8043	0.0946
Nefl	_KEES[Phospho (STY)]AGEEQVAK_.2	#N/A	7.1509	6.9987	6.8392	7.7056	6.6778	6.7079	7.3079	6.6037	6.8199	7.0979	1.0080	0.0116	0.8143	0.0892
Nefl	_YLKEYQDLLNVK_.2	#N/A	6.5587	7.0070	7.6223	7.2983	7.1561	6.7197	6.4529	6.6529	6.5092	7.1306	1.0559	0.0785	0.1462	0.8351
Nefl	_AEEAKDEPPS[Phospho (STY)]EGEAEEEEK_.2	#N/A	7.9800	5.4318	6.8365	8.4562	6.8165	7.0331	7.8161	7.0891	7.6613	7.4091	0.9464	-0.0795	0.4878	0.3118
Nefl	_LLEGEETR_.2	#N/A	8.0417	8.2202	8.0662	8.5448	8.8031	8.3395	7.9726	7.9159	8.0950	8.2285	1.0351	0.0498	0.1153	0.9383
Nefl	_SFPAYYTSHVQEEQTEVEETIEATK_.3	#N/A	8.0572	8.7422	8.8278	10.6662	7.4884	7.8707	8.4350	8.5572	7.5967	8.2191	1.0496	0.0698	0.5180	0.2856
Nefl	_EES[Phospho (STY)]AGEEQVAK_.2	#N/A	6.0837	6.0345	3.2978	6.5801	5.5326	6.1693	6.3010	5.8995	6.2928	5.6071	0.9322	-0.1014	0.5273	0.2779
Nefl	_IDSLM[Oxidation (M)]DEIAFLKK_.3	#N/A	5.3551	5.4971	5.4391	4.3159	4.4663	4.4713	4.1185	4.4274	4.6917	5.1445	1.0715	0.0996	0.3528	0.4525
Nefl	_QALQGEREGLEETLR_.3	#N/A	5.9908	6.9211	7.7675	7.3472	7.5050	6.9895	6.6289	6.5646	6.9874	6.7862	1.0512	0.0720	0.3219	0.4923
Nefl	_RYVETPR_.2	#N/A	5.0014	5.0756	3.7052	3.6965	4.9658	4.6409	4.3418	3.8415	4.7917	4.7019	1.0215	0.0307	0.8048	0.0943
Nefl	_NM[Oxidation (M)]QNAEEWFK_.2	#N/A	6.9291	7.1392	7.1970	7.1197	6.9553	6.9301	7.2711	6.9327	6.8289	7.0686	1.0028	0.0040	0.8444	0.0735
Nefl	_ESEEEKKEES[Phospho (STY)]AGEEQVAK_.3	#N/A	4.4982	4.0019	3.2834	4.5443	3.6978	3.7458	4.0821	3.6628	4.9449	4.3328	0.9310	-0.1032	0.3969	0.4013
Nefl	_IDSLM[Oxidation (M)]DEIAFLK_.2	#N/A	4.8986	5.2443	4.9598	5.3983	5.0106	5.2032	4.7628	5.0796	4.9044	5.0068	1.0366	0.0519	0.1469	0.8331
Nefl	_SAYSGLQSSSYLM[Oxidation (M)]SAR_.2	#N/A	6.9303	6.9787	7.4033	7.7417	7.1299	7.2909	6.8711	7.1222	7.0950	7.2083	1.0243	0.0346	0.3271	0.4854
Nefl	_M[Oxidation (M)]ALDIEIAAYR_.2	#N/A	6.9229	7.2005	7.1621	7.2635	7.2810	7.2713	7.4795	7.1198	7.2486	7.3235	0.9850	-0.0218	0.2655	0.5759
Nefl	_AAKDEVSESRR_.3	#N/A	4.3808	3.2293	3.9288	5.5054	5.6981	5.1606	4.1461	3.7962	4.6436	4.3984	1.0952	0.1312	0.4551	0.3419
Nefl	_SAYSSYSAPVSSSLSVR_.2	#N/A	7.0689	7.6622	7.7226	7.9239	7.7116	7.4198	7.5656	7.1570	7.3712	7.3133	1.0317	0.0450	0.2002	0.6986
Nefl	_LSFTSVGSITSGYSQSSQVFGR_.2	#N/A	4.9479	5.1742	6.0750	6.5129	4.6439	5.4972	5.3175	4.7965	5.0096	4.9268	1.0923	0.1273	0.2478	0.6059
Nefl	_SYSSSSGSLM[Oxidation (M)]PSLENLDLSQVAAISNDLK_.3	#N/A	3.1126	4.2298	4.3471	3.8909	3.7571	4.3683	4.0391	3.1103	3.4094	3.5681	1.1187	0.1618	0.1850	0.7328
Nefl	_TQEKAQLQDLNDR_.3	#N/A	4.4473	4.2527	3.1696	3.6723	4.9492	4.9523	3.3870	4.4462	5.3198	4.9424	0.9374	-0.0933	0.5798	0.2367
Nefl	_YEEEVLSREDAEGR_.3	#N/A	3.8874	4.0859	3.6211	3.1649	4.4352	5.1208	3.3131	3.8363	5.1245	4.8078	0.9490	-0.0756	0.6615	0.1794
Nefl	_AEEAKDEPPS[Phospho (STY)]EGEAEEEEKEK_.3	#N/A	2.9998	3.7561	4.2343	8.6347	7.4005	2.9803	4.3743	3.4589	3.1075	7.9771	1.0574	0.0805	0.8623	0.0644
Nefm	_S[Phospho (STY)]PVEEVKPKPEAK_.3	#N/A	13.1248	12.7703	12.9273	13.5524	12.9537	13.0459	12.8866	12.7123	13.1432	13.1531	1.0068	0.0098	0.5967	0.2243
Nefm	_GVVTNGLDVS[Phospho (STY)]PAEEK_.2	#N/A	11.0344	10.9928	10.9433	11.8895	10.7555	11.1485	11.6718	10.7922	11.1198	11.3078	0.9915	-0.0123	0.7106	0.1484
Nefm	_AES[Phospho (STY)]PVKEK_.2	#N/A	10.2105	10.0298	10.3544	10.0542	9.6196	9.8053	10.2545	9.8464	9.9118	10.2946	0.9936	-0.0093	0.7038	0.1526
Nefm	_QLS[Phospho (STY)]DIEER_.2	P08553_S344_M1_Nefm	8.5865	8.7644	9.0893	9.1544	8.8248	8.7435	8.8473	8.4615	8.5993	8.9338	1.0172	0.0246	0.3180	0.4976
Nefm	_VHYLEQQNK_.2	#N/A	8.6784	8.8186	8.0713	9.4207	9.0033	8.8005	8.6416	8.6679	8.5726	8.8256	1.0140	0.0201	0.6090	0.2154
Nefm	_EIEAEIQALR_.2	#N/A	8.3431	8.0899	8.1999	8.7477	8.6017	8.1550	8.1658	8.2254	8.3957	8.5329	1.0032	0.0045	0.8656	0.0627
Nefm	_[Acetyl (Protein N-term)]SYTLDSLGNPSAYR_.2	#N/A	8.2697	8.3192	8.4047	8.4797	8.7548	8.3900	8.6386	7.8235	7.9781	8.1440	1.0356	0.0505	0.1166	0.9334
Nefm	_VQSLQDEVAFLR_.2	#N/A	7.4758	8.0569	8.1270	8.2207	8.4388	7.8791	8.1051	7.8027	7.9082	7.8308	1.0153	0.0220	0.5117	0.2910
Nefm	_FSTFSGSITGPLYTHR_.2	#N/A	6.8199	6.7480	6.8080	6.7136	7.0690	6.7991	6.6615	5.9723	6.6308	6.9561	1.0414	0.0585	0.1626	0.7889
Nefm	_LTEAAEQNK_.2	#N/A	7.4879	7.7625	7.6543	7.9313	7.9651	7.7590	7.7461	7.5469	7.4528	7.9232	1.0121	0.0173	0.4713	0.3267
Nefm	_SAKEEIAEYR_.2	#N/A	7.2784	6.9675	7.1022	7.4586	7.7299	7.5125	6.5237	6.8971	7.0987	7.2386	1.0579	0.0812	0.0659	1.1814
Nefm	_AVEEVITISK_.2	#N/A	5.8252	6.5438	6.2912	7.7100	7.2810	6.6696	6.7077	6.5955	6.8422	6.6938	1.0015	0.0022	0.9771	0.0101
Nefm	_SAKEEIAEYRR_.3	#N/A	7.0111	6.8090	6.3151	6.3018	7.5210	7.4302	6.7165	6.5620	7.0010	7.3171	0.9998	-0.0002	0.9971	0.0013
Nefm	_VTSHAIVK_.2	#N/A	5.6490	7.0674	5.9553	6.8115	7.0746	6.5917	6.3265	6.5757	6.5895	6.4543	1.0059	0.0085	0.9038	0.0439
Nefm	_LRDDTEAAIR_.2	#N/A	6.0244	6.2821	5.9610	6.2002	6.6393	6.7832	6.4064	5.8003	6.3901	6.1191	1.0220	0.0314	0.5217	0.2825
Nefm	_SNHEEEVADLLAQIQASHITVER_.3	#N/A	5.8829	5.8159	5.7187	6.4680	6.1678	5.9321	5.8372	4.6696	5.7854	6.3584	1.0591	0.0829	0.3174	0.4984
Nefm	_GS[Phospho (STY)]PSTVSSSYKR_.2	#N/A	8.2659	8.8110	9.0804	8.9411	8.5690	8.6463	8.6874	8.3726	8.3995	8.6983	1.0210	0.0300	0.3055	0.5150
Nefm	_KGEDS[Phospho (STY)]SDDKVVVTK_.2	#N/A	6.0414	6.8555	7.3516	7.4472	5.6347	6.8615	7.2031	6.9285	6.7135	7.0451	0.9607	-0.0578	0.4883	0.3113
Nefm	_KAES[Phospho (STY)]PVKEK_.2	#N/A	8.6049	3.8588	8.6329	8.3761	8.7898	8.5704	8.8896	8.2256	8.3795	8.9191	0.9072	-0.1404	0.4474	0.3493
Nefm	_EEEPEAEKS[Phospho (STY)]PVKS[Phospho (STY)]PEAK_.3	#N/A	6.1529	6.5198	5.5565	6.3613	5.1081	5.5376	5.6978	5.3100	5.6079	5.6427	1.0284	0.0404	0.5335	0.2729
Nefm	_HNHDLSSYQDTIQQLENELR_.3	#N/A	5.9856	6.0746	5.2480	6.6018	5.1568	5.4560	5.8753	5.5380	5.9585	6.0732	0.9817	-0.0267	0.7301	0.1366
Nefm	_VSGS[Phospho (STY)]PSSGFR_.2	P08553_S30_M1_Nefm	11.4175	11.5093	11.7964	11.8630	11.4327	11.5496	11.5596	11.2535	11.3636	11.7386	1.0101	0.0145	0.3939	0.4046
Nefm	_SIELESVR_.2	#N/A	6.5096	5.3477	6.5043	6.9368	7.0745	7.1159	5.5298	6.8752	7.4035	7.5906	0.9608	-0.0576	0.6056	0.2178
Nefm	_VKEKAEEEGGS[Phospho (STY)]EEEGSDR_.3	#N/A	4.7726	4.6916	5.7858	5.7693	5.8845	5.0377	4.6147	4.6536	5.1273	4.8924	1.1040	0.1428	0.1285	0.8910
Nefm	_ATLEMVNHEK_.2	#N/A	5.8607	5.1547	4.3552	7.4189	6.0341	3.1785	5.1156	4.3651	4.7987	5.3662	1.0860	0.1190	0.5972	0.2239
Nefm	_S[Phospho (STY)]PVEEVKPK_.2	#N/A	6.0692	5.5028	3.4175	6.2241	5.6858	5.5719	5.8648	5.5797	6.0651	5.8034	0.9286	-0.1069	0.4508	0.3460
Nefm	_SNEKEQLQGLNDR_.2	#N/A	8.2294	8.6903	9.0706	9.1633	8.6493	8.6243	8.5562	8.4090	8.6432	8.7349	1.0177	0.0253	0.4259	0.3707
Nefm	_EEEKGVVTNGLDVS[Phospho (STY)]PAEEK_.3	#N/A	3.3418	7.4354	3.5661	8.8470	7.9489	8.2969	6.7438	7.9524	8.2677	8.6004	0.8329	-0.2637	0.3387	0.4702
Nefm	_GS[Phospho (STY)]PSTVSSSYK_.2	#N/A	8.5281	8.4481	8.5874	8.9874	8.6228	8.6868	8.7164	8.2206	8.3631	8.7192	1.0163	0.0233	0.3452	0.4619
Nefm	_AEEEGGS[Phospho (STY)]EEEGSDRSPQESK_.3	#N/A	4.8311	5.6171	5.7850	7.0640	5.5788	5.5103	5.6152	5.7412	5.8915	5.6024	1.0032	0.0047	0.9621	0.0168
Nefm	_EEIAEYRR_.2	#N/A	5.0228	5.7319	5.3278	5.7260	5.5192	5.5476	3.0947	3.2376	5.3162	4.9073	1.3238	0.4047	0.0214	1.6690
Nefm	_GTKES[Phospho (STY)]LER_.2	P08553_S338_M1_Nefm	4.0217	6.0768	3.2077	3.7104	3.0927	3.5319	5.9685	4.4081	4.0567	3.6774	0.8702	-0.2005	0.4178	0.3790
Nefm	_ITS[Phospho (STY)]EGGDGATK_.2	P08553_S795_M1_Nefm	4.1152	3.3707	4.8392	3.3927	3.2590	3.3656	5.3242	3.6085	3.9075	3.0700	0.9362	-0.0951	0.6238	0.2050
Nefm	_QASHAQLGDAYDQEIR_.3	#N/A	5.1646	5.2162	4.9746	6.0447	5.9902	6.1447	4.7924	4.8348	5.6928	5.6388	1.0667	0.0931	0.3218	0.4924
Nefm	_EEKPQPQEK_.2	#N/A	4.4075	4.4550	3.5595	4.6226	4.8199	4.2303	4.9530	4.0564	3.7050	4.9443	0.9852	-0.0216	0.8496	0.0708
Nefm	_AQVQLDSDHLEEDIHR_.3	#N/A	8.1761	8.2956	8.4829	8.8461	8.2337	8.2526	8.3059	7.6943	7.8829	8.2840	1.0422	0.0596	0.0886	1.0528
Nefm	_FAGYIEK_.2	#N/A	4.1944	4.5923	4.9198	3.5773	4.9518	4.6201	3.2920	5.1370	4.5644	4.2845	1.0362	0.0513	0.7084	0.1497
Nefm	_LTEAAEQNKEAIR_.2	#N/A	3.0288	4.0982	3.9788	4.4664	3.9744	5.0364	3.9388	2.5811	3.5488	3.9676	1.1676	0.2235	0.2026	0.6934
Nefm	_LS[Phospho (STY)]RSNEKEQLQGLNDR_.3	P08553_S95_M1_Nefm	4.0955	3.2469	3.8292	3.3730	3.2393	3.3854	3.5212	3.6282	3.9273	3.0503	0.9990	-0.0014	0.9881	0.0052
Nefm	_TDISTALK_.2	#N/A	3.2948	4.1000	2.6958	4.4843	3.9818	2.4825	4.1989	3.6449	4.1166	2.7808	0.9515	-0.0717	0.7231	0.1408
Nefm	_EVTKES[Phospho (STY)]PKEEK_.2	#N/A	7.0654	6.9705	4.4116	7.9556	7.3816	6.4524	7.2756	6.6994	7.6676	7.2224	0.8744	-0.1937	0.3266	0.4860
Nefm	_AEEEGGS[Phospho (STY)]EEEGSDR_.2	#N/A	6.8173	6.0670	6.1453	7.3889	7.2941	6.3154	7.1795	6.3441	6.7390	6.8660	0.9837	-0.0238	0.7433	0.1288
Nefm	_VEEHEETFEEK_.2	#N/A	5.6744	6.1204	5.3275	5.8000	6.5772	6.4752	6.0800	5.8132	5.7336	5.9790	1.0160	0.0229	0.7207	0.1422
Nefm	_SPVKS[Phospho (STY)]PEAK_.2	P08553_S506_M1_Nefm	6.8422	6.8983	3.9248	7.5668	5.4714	3.2897	6.9550	3.1495	6.8017	3.4352	1.1141	0.1559	0.6446	0.1907
Nefm	_EKAEEEGGS[Phospho (STY)]EEEGSDR_.2	#N/A	3.2909	4.5561	3.9777	6.1818	5.9589	3.8121	5.3537	5.1560	3.2867	4.4474	1.0150	0.0215	0.9254	0.0337
Nefm	_QPSVTISSK_.2	#N/A	3.9022	4.9238	3.6359	3.1797	5.1078	4.1043	3.3279	3.8215	5.1998	5.2316	0.9425	-0.0855	0.6521	0.1857
Nefm	_AKS[Phospho (STY)]PMPK_.2	P08553_S605_M1_Nefm	5.7828	4.3184	3.2958	6.5980	4.2067	3.9188	4.4887	4.1616	2.9598	4.0177	1.1996	0.2625	0.2891	0.5389
Nefm	_EGSS[Phospho (STY)]EKDEGEQEEEGETEAEGEGEEAEAKEEK_.4	P08553_S551_M1_Nefm	4.2854	3.5674	4.1046	4.9271	3.4195	3.8301	3.5311	3.6522	4.0108	3.7834	1.0742	0.1033	0.3678	0.4344

Nefm	_SVTVTQKVEEHEETFEEK_.3	#N/A	7.5389	8.1631	9.8360	5.0141	8.0262	8.0111	7.8301	5.0761	7.1152	7.2465	1.1391	0.1878	0.3368	0.4726
Nefm	_AEEEGGS[Phospho (STY)]EEEGSDRSPQESKK_.3	#N/A	3.7590	3.7769	3.4926	3.4010	3.6736	3.7989	3.7569	3.2478	3.9974	3.4846	1.0079	0.0114	0.8572	0.0669
Nefm	_EKAEEEGGS[Phospho (STY)]EEEGSDRSPQESK_.3	#N/A	2.7194	3.9107	4.1697	4.1266	3.7320	4.0086	4.3693	3.5550	3.4209	3.9597	0.9873	-0.0184	0.8850	0.0530
Nefm	_SQLEC[Carbamidomethyl (C)]HSDQNMHQAEEWFK_.3	#N/A	3.6473	4.0025	4.2963	3.5832	3.4174	3.3853	3.5618	4.0584	4.0331	3.3915	0.9896	-0.0151	0.8665	0.0622
Nefm	_DTKEEKPQPQEK_.3	#N/A	3.6811	3.5451	4.3679	3.3697	2.8067	3.4219	3.0844	3.2480	4.3974	3.3367	1.0044	0.0063	0.9658	0.0151
Gnai2	_LFDSIC[Carbamidomethyl (C)]NNK_.2	#N/A	8.5569	8.8507	8.7793	8.3256	8.0986	8.0641	9.1734	8.5130	8.2047	8.4040	0.9851	-0.0217	0.6070	0.2168
Gnai2	_IAQSDYIPTQQDVLR_.2	#N/A	7.9961	8.1085	8.2059	7.8184	7.5033	7.9841	8.2412	7.8100	7.9208	8.0200	0.9923	-0.0112	0.6830	0.1656
Gnai2	_EYQLNDSAAYYLNDLER_.2	#N/A	5.5559	5.2679	5.8677	5.9383	4.6884	5.7269	5.9926	6.2393	5.6720	5.6634	0.9348	-0.0973	0.1817	0.7406
Gnai2	_AVVYSNTIQSIM[Oxidation (M)]AIVK_.2	#N/A	4.9363	3.3659	5.6087	4.6532	2.3908	4.0187	4.1699	4.3502	3.9321	4.2591	0.9963	-0.0054	0.9798	0.0088
Gnai2	_NLREDGEK_.2	#N/A	5.6363	5.6948	4.7171	5.7405	6.6264	5.5491	5.5342	5.9767	5.1598	5.0388	1.0430	0.0607	0.5261	0.2790
Gnai2	_NNLKDC[Carbamidomethyl (C)]GLF_.2	#N/A	5.6955	5.3222	5.6495	4.9865	4.5292	5.2052	5.3612	5.1109	4.9661	4.8500	1.0314	0.0446	0.5240	0.2806
Gnai2	_M[Oxidation (M)]FDVGGQR_.2	#N/A	6.5675	6.5110	5.8067	6.7208	5.7940	4.9320	6.1053	6.3307	6.2621	6.1912	0.9732	-0.0392	0.6447	0.1906
Gnai2	_YDEAASYIQSK_.2	#N/A	5.4520	3.5803	5.3818	3.3345	5.6118	5.6781	5.4241	5.3374	5.1997	5.3442	0.9086	-0.1382	0.4036	0.3941
Gnai2	_SREYQLNDSAAYYLNDLER_.3	#N/A	5.2541	5.3673	5.3295	3.2108	4.9308	3.5475	5.9105	5.5474	4.0894	4.6607	0.9118	-0.1331	0.4743	0.3239
Gnai2	_IIHEDGYSEEEC[Carbamidomethyl (C)]R_.3	#N/A	4.8253	3.4493	4.0080	3.5517	2.5456	3.3392	3.1100	4.3834	2.0347	3.9981	1.0705	0.0982	0.6846	0.1646
Gnai2	_TTGIVETHFTFK_.2	#N/A	7.2903	6.9176	7.2765	6.3717	6.7911	6.8595	6.8822	6.8707	6.9153	6.7463	1.0094	0.0134	0.7265	0.1388
Gnai2	_MFDVGGQRS[Phospho (STY)]ER_.2	P08752_S207_M1_Gnai2	5.0597	4.3711	3.3485	4.0445	4.2594	3.8660	3.7592	4.0740	3.7226	4.0115	1.0685	0.0955	0.3988	0.3992
Gnai2	_QLFALSC[Carbamidomethyl (C)]AAEEQGM[Oxidation (M)]LPEDLSGVIR_.3	#N/A	1.8542	3.0477	4.4536	3.8671	2.9360	3.6886	3.5818	4.2514	3.8999	3.8341	0.8500	-0.2345	0.2532	0.5965
Gnai2	_LWADHGVQAC[Carbamidomethyl (C)]FGR_.3	#N/A	3.6590	4.3480	4.3906	3.6292	4.0633	3.4325	3.5939	3.1868	3.9506	3.3595	1.1129	0.1543	0.1418	0.8485
Gnai2	_EIYTHFTC[Carbamidomethyl (C)]ATDTK_.3	#N/A	3.6225	3.6710	3.7412	3.8800	3.5593	3.9168	3.8099	3.8122	3.8196	4.3322	0.9463	-0.0796	0.1306	0.8841
Gnai2	_AMGNLQIDFADPQR_.2	#N/A	3.2372	3.9935	4.4718	4.4221	3.8818	4.2436	4.1368	3.6964	3.3450	3.6928	1.0871	0.1205	0.2584	0.5877
Gnai2	_NVQFVFDAVTDVIIK_.2	#N/A	3.3800	3.9624	3.1137	4.1583	4.0245	4.1009	4.3065	4.3437	3.1420	3.8355	0.9701	-0.0439	0.7185	0.1436
Polr2a	_YSPTS[Phospho (STY)]PTYSPTTPK_.2	P08775_S1878_M1_Polr2a	3.9561	3.2115	3.6898	3.2335	3.0998	3.5248	3.3818	3.7676	4.0667	4.4117	0.8837	-0.1784	0.0968	1.0139
Polr2a	_YSPTS[Phospho (STY)]PTYSPTSPK_.2	P08775_S1913_M1_Polr2a	4.0089	7.2905	7.7497	4.0435	6.2042	6.0931	4.1918	7.1713	4.1037	3.7208	1.2296	0.2982	0.3137	0.5035
Polr2a	_YTPTSPSYSPSS[Phospho (STY)]PEYTPASPK_.2	P08775_S1850_M1_Polr2a	3.1219	4.1043	3.8087	4.3113	3.9926	4.1328	4.0260	3.8072	3.4557	4.2783	1.0052	0.0074	0.9391	0.0273
P4hb	_EADDIVNWLK_.2	#N/A	5.9567	6.0496	5.4730	6.1611	6.3089	5.9648	5.8366	6.5354	6.1850	5.8507	0.9810	-0.0277	0.5680	0.2456
P4hb	_NNFEGEITK_.2	#N/A	6.1967	5.9193	6.4090	6.6276	6.2175	6.2303	6.1606	6.1608	6.5791	6.6856	0.9797	-0.0296	0.4479	0.3488
P4hb	_TGPAATTLSDTAAAESLVDSSEVTVIGFFK_.3	#N/A	3.2077	3.7220	4.7602	5.3370	2.4944	4.4230	3.2272	3.6667	3.3228	3.7645	1.1417	0.1912	0.3906	0.4083
P4hb	_TVIDYNGER_.2	#N/A	5.1993	6.6394	6.7176	7.0541	7.0370	4.9473	6.8474	6.1137	6.0556	6.5183	0.9815	-0.0269	0.8203	0.0860
P4hb	_ILFIFIDSDHTDNQR_.3	#N/A	3.1999	4.0263	3.8867	5.0898	3.9146	4.2109	4.1040	3.7291	3.3777	4.3563	1.0419	0.0591	0.6590	0.1811
Cck	_NLQSLDPSHR_.2	#N/A	6.4551	6.7597	6.8385	4.3493	7.3398	6.7674	6.0944	6.2322	6.5168	6.8776	0.9981	-0.0027	0.9833	0.0073
Alpl	_TYNTNAQVPDS[Phospho (STY)]AGTATAYLC[Carbamidomethyl (C)]GVK_.3	P09242_S110_M1_Alpl	3.3743	3.3864	4.2857	5.3181	3.2490	3.2310	3.7960	3.2572	4.0297	3.3751	1.0534	0.0750	0.6803	0.1673
Ncl	_NDLAVVDVR_.2	#N/A	5.4015	4.8067	6.1008	4.8128	5.2976	5.9973	5.1346	5.3894	5.8886	5.3759	0.9919	-0.0118	0.8902	0.0505
Ncl	_LELQGSNSR_.2	#N/A	6.2434	6.3218	5.8580	6.1841	6.3086	5.3382	6.0277	6.2933	6.3524	5.6624	0.9932	-0.0099	0.8624	0.0643
Ncl	_ETLEEVFEK_.2	#N/A	5.8970	5.1993	3.6314	5.7382	5.5650	5.5496	5.7831	5.7179	5.7674	5.0135	0.9449	-0.0818	0.5140	0.2890
Ncl	_VEGSEPTTPFNLFIGNLNPNK_.2	#N/A	3.4276	3.6137	4.9513	4.2643	3.3369	2.9857	4.1700	3.4526	2.2199	3.4499	1.1324	0.1794	0.3924	0.4063
Ncl	_NLS[Phospho (STY)]FNITEDELK_.2	P09405_S403_M1_Ncl	3.7386	3.0429	4.0332	3.5769	2.8643	3.3644	6.6033	5.6791	3.7233	3.2542	0.7138	-0.4865	0.0724	1.1401
Ncl	_GLS[Phospho (STY)]EDTTEETLK_.2	P09405_S577_M1_Ncl	3.9867	3.2421	3.7204	3.6726	3.1304	3.4942	7.1654	4.4458	6.1830	3.6396	0.6608	-0.5976	0.0241	1.6172
Pgk1	_NNQITNNQR_.2	#N/A	11.6793	12.1436	12.0743	12.9317	12.3335	12.1120	12.0703	12.0030	12.1152	12.0389	1.0129	0.0185	0.4826	0.3164
Pgk1	_LTLDKLDVK_.2	#N/A	8.2153	8.9488	8.8369	8.9785	8.8694	8.6581	8.6885	8.4873	8.4124	8.5978	1.0239	0.0341	0.2191	0.6594
Pgk1	_FHVEEEGKGK_.2	#N/A	8.5327	7.7703	7.9591	7.8817	8.4969	8.6446	8.3260	8.0283	8.3985	8.6496	0.9837	-0.0238	0.5538	0.2567
Pgk1	_ITLPVDFVTADKFDENAK_.2	#N/A	7.0801	7.4762	8.0472	7.9557	7.5254	7.5702	8.0512	7.3571	7.4728	7.2774	1.0092	0.0132	0.7669	0.1152
Pgk1	_YSLEPVAAELK_.2	#N/A	8.8124	8.3399	8.0247	8.6829	8.5536	8.3176	8.6410	8.4643	8.3552	8.6133	0.9926	-0.0108	0.6935	0.1589
Pgk1	_GC[Carbamidomethyl (C)]ITIIGGGDTATC[Carbamidomethyl (C)]C[Carbamidomethyl (C)]AK_.2	#N/A	7.7417	8.0930	8.1364	9.1533	7.9303	7.5253	7.7210	8.1138	7.8029	7.8129	1.0298	0.0423	0.4527	0.3442
Pgk1	_LGDVYVNDAFGTAHR_.3	#N/A	7.3991	6.8891	5.9677	6.8722	6.8953	6.7849	7.1640	6.7788	6.9617	6.9602	0.9763	-0.0345	0.5194	0.2845
Pgk1	_FC[Carbamidomethyl (C)]LDNGAK_.2	#N/A	7.8619	7.9142	7.9290	7.9679	8.3888	7.6904	7.2769	7.9180	7.7675	7.9674	1.0293	0.0416	0.2247	0.6485
Pgk1	_FHVEEEGK_.2	#N/A	7.8948	7.3870	6.9265	7.5573	7.9844	7.6277	7.6843	7.6906	7.7160	7.5911	0.9860	-0.0204	0.5980	0.2233
Pgk1	_KYAEAVGR_.2	#N/A	7.3218	6.8880	7.2443	6.9700	7.5019	7.1515	7.2482	6.7830	6.9486	7.1561	1.0207	0.0296	0.3364	0.4732
Pgk1	_IQLINNM[Oxidation (M)]LDK_.2	#N/A	6.4710	6.9750	6.3460	6.5993	6.8745	6.5510	6.9962	6.9714	7.1298	6.7770	0.9523	-0.0705	0.0403	1.3947
Pgk1	_ELNYFAK_.2	#N/A	5.9788	5.0983	7.1124	5.3085	5.9757	5.6162	4.1722	6.7151	6.4483	6.1088	0.9978	-0.0031	0.9831	0.0074
Pgk1	_VNEM[Oxidation (M)]IIGGGM[Oxidation (M)]AFTFLK_.2	#N/A	6.8294	6.0544	4.3338	5.9731	6.7597	6.7274	6.9837	6.4246	6.3238	7.0268	0.9138	-0.1301	0.2867	0.5426
Pgk1	_AEPAKIDAFR_.2	#N/A	5.7509	6.5450	7.5143	6.3272	6.1427	6.5943	6.8993	5.2975	6.2183	5.6914	1.0751	0.1044	0.2998	0.5231
Pgk1	_VLNNM[Oxidation (M)]EIGTSLYDEEGAK_.2	#N/A	6.4776	6.2137	6.2555	6.8039	6.4534	5.9004	6.5822	5.9330	6.0981	6.3042	1.0195	0.0278	0.5421	0.2659
Pgk1	_SLM[Oxidation (M)]DEVVK_.2	#N/A	7.0897	6.5120	6.6543	7.2426	7.1001	6.7982	5.7979	6.9138	6.8046	6.9239	1.0438	0.0618	0.2988	0.5246
Pgk1	_VDFNVPMK_.2	#N/A	6.6998	4.6632	3.2568	7.1962	6.4246	3.0321	3.4741	3.4901	4.3535	5.1854	1.2633	0.3372	0.3006	0.5220
Pgk1	_ALESPERPFLAILGGAK_.3	#N/A	6.6743	7.2605	7.5739	7.8124	7.2094	7.0404	7.1799	7.4948	7.3485	7.5731	0.9815	-0.0270	0.5411	0.2667
Pgk1	_AHSSM[Oxidation (M)]VGVNLPQK_.2	#N/A	6.8977	6.8886	6.9969	7.1284	7.2516	7.2679	6.7082	6.7539	7.0225	7.1760	1.0227	0.0323	0.2396	0.6206
Pgk1	_DC[Carbamidomethyl (C)]VGPEVENAC[Carbamidomethyl (C)]ANPAAGTVILLENLR_.3	#N/A	4.5052	3.3422	4.3768	4.6918	5.3956	3.4129	3.0933	3.2390	4.3884	3.3456	1.2192	0.2859	0.1359	0.8667
Pgk1	_VSHVSTGGGASLELLEGK_.3	#N/A	5.5655	4.6442	3.8783	5.3496	3.8180	5.1046	5.4603	3.7376	3.5787	5.5543	1.0314	0.0446	0.8067	0.0933
Pgk1	_WNTEDKVSHVSTGGGASLELLEGK_.3	#N/A	3.3330	4.0094	4.5675	4.1113	3.9776	4.1479	4.2596	4.3907	3.1889	3.2954	1.0636	0.0890	0.4754	0.3229
Pgk1	_QIVWNGPVGVFEWEAFAR_.2	#N/A	3.2645	3.9617	3.9513	4.4540	3.8910	4.2755	4.1686	3.6645	3.3131	4.4210	1.0191	0.0274	0.8013	0.0962
Fth1	_[Acetyl (Protein N-term)]TTAS[Phospho (STY)]PSQVR_.2	P09528_S5_M1_Fth1	11.1216	11.3438	11.3896	11.7922	11.2010	11.1980	11.4745	10.9850	11.1419	11.2678	1.0110	0.0158	0.4285	0.3681
Fth1	_QNYHQDAEAAINR_.3	#N/A	3.8989	3.1544	3.6326	3.1764	3.0427	3.5820	3.3246	3.8248	4.1239	4.3545	0.8739	-0.1944	0.0807	1.0930
Sod2	_GELLEAIKR_.2	#N/A	9.9577	10.3127	10.4428	10.4956	10.4657	10.3174	10.1358	10.1068	10.0112	10.1205	1.0236	0.0337	0.0508	1.2941
Sod2	_NVRPDYLK_.2	#N/A	9.2416	9.7094	9.5202	9.6979	10.2001	9.4444	9.3508	9.5124	9.4171	9.5440	1.0190	0.0271	0.3225	0.4915
Sod2	_GDVTTQVALQPALK_.2	#N/A	8.0693	8.6204	8.4770	9.3711	9.3486	9.0336	8.4148	8.7227	8.6834	8.7452	1.0207	0.0295	0.5301	0.2756
Sod2	_AIWNVINWENVTER_.2	#N/A	8.4623	8.3548	8.7106	8.6427	8.6431	8.6273	8.6238	8.1616	8.4744	8.5439	1.0145	0.0208	0.2796	0.5535
Sod2	_YHEALAK_.2	#N/A	5.7241	5.8444	4.6490	5.5343	6.2223	5.6230	5.0832	6.1629	5.6217	5.1981	1.0150	0.0216	0.8077	0.0928
Sod2	_GELLEAIK_.2	#N/A	5.8410	4.4247	4.1857	4.7797	4.5009	5.3965	3.0432	4.6670	4.4442	5.0425	1.1292	0.1753	0.2750	0.5606
Sod2	_HHAAYVNNLNATEEK_.3	#N/A	6.2198	5.9002	3.1207	4.1653	7.4774	6.4871	4.3136	5.7106	3.1349	6.7082	1.1198	0.1632	0.5792	0.2371
Sod2	_FNGGGHINHTIFWTNLSPK_.3	#N/A	3.0848	4.9232	4.3194	5.0267	4.8285	4.3960	4.0114	4.9920	3.4371	3.5404	1.1088	0.1490	0.3742	0.4269
Sod2	_DFGSFEK_.2	#N/A	4.2167	3.0140	4.0621	3.6058	3.4721	3.9793	3.7541	3.3953	3.6944	3.9148	1.0096	0.0138	0.8884	0.0514
Nudt10;Nudt11	_LGGS[Phospho (STY)]PTNGNSAAPSPPESEP_.2	P0C027_S148_M1_Nudt10;Nudt11	3.1848	11.0787	4.4193	11.3985	3.8294	10.9238	4.1114	10.8906	10.9243	3.6404	1.0109	0.0157	0.9761	0.0105
Nudt10;Nudt11	_LKLGGSPT[Phospho (STY)]NGNSAAPSPPESEP_.2	P0C027_T150_M1_Nudt10;Nudt11	3.4167	4.2219	4.1035	4.6061	9.9853	3.6863	4.3208	3.5123	7.5659	3.0723	1.0835	0.1157	0.8033	0.0951
Nudt10;Nudt11	_SEREDEVLLVSSSR_.3	#N/A	4.2126	3.1298	3.9463	3.4901	2.9511	3.2775	3.6383	3.5111	3.8102	3.1673	0.9914	-0.0125	0.9134	0.0394
Rc3h2	_T[Phospho (STY)]GDPANLNRLR_.2	P0C090_T341_M1_Rc3h2	3.9349	3.1903	3.6686	7.8488	3.0786	3.5460	3.4392	3.7889	4.0879	4.3904	1.0725	0.1010	0.7691	0.1140
Chmp6	_MHQVM[Oxidation (M)]S[Phospho (STY)]IEEVER_.2	#N/A	5.5687	3.7577	4.1175	5.9322	4.3661	4.5594	4.6038	4.6385	4.8996	5.0690	0.9821	-0.0260	0.8514	0.0699
H2az1;H2az2	_ATIAGGGVIPHIHK_.3	#N/A	6.8656	5.8267	7.0587	5.8125	6.9435	6.3678	5.2217	6.3163	6.4251	6.2659	1.0696	0.0971	0.2784	0.5553
H2az1;H2az2	_GDEELDSLIK_.2	#N/A	3.4639	4.2691	4.1507	3.8516	4.0904	3.6391	3.9999	3.1495	3.4486	3.5289	1.1073	0.1471	0.1124	0.9493

Lrrc4b	_SGS[Phospho (STY)]KENVQETQI_.2	P0C192_S700_M1_Lrrc4b	10.0747	10.0564	10.8888	10.5548	9.2502	3.8876	10.5768	10.2343	3.2605	10.1307	1.0664	0.0928	0.7764	0.1099
Gpr52	_RRANS[Phospho (STY)]C[Carbamidomethyl (C)]S[Phospho (STY)]I_.2	P0C5J4_S358_M2_Gpr52	5.9462	6.3168	7.1874	6.6446	5.4365	6.1783	6.9553	6.9605	6.5866	6.0640	0.9463	-0.0796	0.3356	0.4741
Igsf11	_GSS[Phospho (STY)]PQVLPR_.2	P0C673_S378_M1_Igsf11	9.9322	10.1707	10.6730	10.1147	9.1665	9.9254	10.5025	10.1218	9.8449	9.9502	0.9893	-0.0155	0.7052	0.1517
Wipf3	_AQLVS[Phospho (STY)]PPAPPTK_.2	P0C7L0_S211_M1_Wipf3	12.7930	13.0025	13.4765	13.3680	12.4844	12.8472	13.2971	12.7209	12.6768	12.9873	1.0058	0.0083	0.7443	0.1282
Wipf3	_TAVAPPPLPGANNSGSET[Phospho (STY)]PPPLPPK_.3	P0C7L0_T338_M1_Wipf3	3.2956	4.0519	4.5301	4.3637	3.9402	4.1853	4.0784	3.7547	3.4033	4.3307	1.0435	0.0614	0.5498	0.2598
Clec2l	_SGS[Phospho (STY)]GYEGSTSWK_.2	P0C7M9_S47_M1_Clec2l	7.9928	7.1109	8.4211	8.4096	6.1427	6.8259	8.8285	8.4251	7.0974	7.2082	0.9485	-0.0762	0.5091	0.2932
Clec2l	_S[Phospho (STY)]PAEAEAR_.2	#N/A	9.9249	9.3917	9.6155	9.4095	8.3844	8.8881	9.8975	9.8324	8.9917	9.5236	0.9694	-0.0448	0.3944	0.4041
Clec2l	_PRS[Phospho (STY)]PAEAEAR_.2	#N/A	7.6497	7.7705	7.8919	7.5748	7.0443	7.6509	8.4293	7.9369	8.0126	7.6729	0.9481	-0.0769	0.0645	1.1903
Clec2l	_PAPAAPRPRS[Phospho (STY)]PAEAEAR_.3	#N/A	8.0798	8.8299	10.2807	8.5283	7.9192	8.7743	8.8779	9.0778	8.4310	8.4824	1.0021	0.0030	0.9687	0.0138
	DTTEDS[Phospho (STY)]ITEDDKRR.3	#N/A	7.4008	5.1205	7.7567	7.3833	7.4353	5.1168	6.6812	5.3831	7.2894	7.0850	1.0140	0.0201	0.9001	0.0457
	DTTEDS[Phospho (STY)]ITEDDKR.2	#N/A	3.5883	3.7541	3.3220	4.3666	4.2329	4.1541	4.5149	4.1354	4.4344	4.0439	0.9115	-0.1338	0.1354	0.8683
Ubb;Ubc;Rps27a;Uba	_TLSDYNIQK_.2	#N/A	9.6378	9.6343	9.6990	9.9010	10.1502	9.8688	9.8763	9.7357	9.7807	9.7354	1.0034	0.0049	0.7613	0.1184
Ubb;Ubc;Rps27a;Uba	_ESTLHLVLR_.2	#N/A	9.6879	9.7428	10.1587	9.9675	10.3636	10.0543	10.1212	10.0176	10.3104	9.9340	0.9901	-0.0144	0.5101	0.2924
Ubb;Ubc;Rps27a;Uba	_TITLEVEPSDTIENVK_.2	#N/A	9.1743	9.1189	9.4874	9.5252	9.7161	9.4730	9.4424	9.2143	9.2861	9.2001	1.0140	0.0201	0.3238	0.4898
Ubb;Ubc;Rps27a;Uba	_QLEDGRTLS[Phospho (STY)]DYNIQK_.3	#N/A	3.6176	3.3747	4.1284	3.2938	3.6437	3.3143	4.1154	3.4220	4.3533	3.2547	0.9408	-0.0881	0.4213	0.3754
Nrxn1	_FNVGTDDIAIEESNAIINDGK_.2	#N/A	3.4599	5.8355	4.1467	3.1485	3.6546	2.9700	4.3640	4.2204	3.1177	5.1690	0.9174	-0.1244	0.5952	0.2253
Nrxn1	_DEGS[Phospho (STY)]YHVDESR_.2	P0DI97_S423_M1_Nrxn1	3.4742	4.2305	3.2079	4.2525	4.1188	4.0067	3.8998	4.2495	3.0477	3.9298	1.0265	0.0377	0.7485	0.1258
Nrxn1	_YPAGNNDNER_.2	#N/A	4.3187	4.4083	4.6190	4.0073	2.7958	3.8288	4.5986	4.4977	3.7597	4.8110	0.9048	-0.1443	0.2995	0.5236
Nrxn1	_NYIS[Phospho (STY)]NSAQSNGAVVK_.2	P0DI97_S434_M1_Nrxn1	4.0171	3.3508	3.2031	3.7058	3.1428	4.1937	4.2294	3.8133	4.0663	3.8065	0.9053	-0.1435	0.1532	0.8147
Ndufb1	_TLFQLLR_.2	#N/A	7.1186	7.4870	7.3052	6.8065	6.9284	7.3748	6.7969	7.4075	7.0057	7.1037	1.0129	0.0186	0.6020	0.2204
Ndufb1	_ELRPNEEVTWK_.2	#N/A	4.7999	4.8775	5.1726	5.2061	5.7084	5.1174	4.6271	5.0453	4.7301	5.2113	1.0497	0.0699	0.2483	0.6049
Calm1;Calm2;Calm3	_EAFSLFDK_.2	#N/A	6.7835	6.3606	4.3376	6.1725	7.1594	5.8276	5.7185	5.8336	6.5815	6.3149	0.9991	-0.0012	0.9923	0.0034
Calm1;Calm2;Calm3	_EAFSLFDKDGDGTITTK_.3	#N/A	10.5984	10.3415	10.3234	10.8804	11.6011	11.1057	10.5990	10.2062	10.9187	10.9660	1.0127	0.0183	0.6492	0.1876
Calm1;Calm2;Calm3	_ELGTVMR_.2	#N/A	8.4477	6.9704	3.5794	9.0751	8.8166	4.4115	5.3629	6.3599	6.9805	7.3561	1.0566	0.0794	0.7767	0.1098
Calm1;Calm2;Calm3	_VFDKDGNGYISAAELR_.3	#N/A	8.6940	8.5475	8.4445	9.0541	9.3916	8.8476	8.7981	8.6094	8.7004	8.7384	1.0136	0.0195	0.5325	0.2737
Calm1;Calm2;Calm3	_DGNGYISAAELR_.2	#N/A	6.6028	3.3860	3.8944	6.4409	4.9281	5.1376	6.5336	6.2487	6.5624	7.1808	0.7638	-0.3888	0.0508	1.2945
Calm1;Calm2;Calm3	_M[Oxidation (M)]KDTDSEEEIREAFR_.3	#N/A	8.2193	8.3552	8.2761	9.2797	9.1044	8.2993	8.1506	8.2362	8.4529	8.5326	1.0295	0.0419	0.3564	0.4480
Calm1;Calm2;Calm3	_DTDSEEEIREAFR_.2	#N/A	4.9684	5.6167	5.1424	6.1386	6.5625	6.1758	6.0563	6.0256	6.0931	6.4235	0.9378	-0.0926	0.2801	0.5526
Calm1;Calm2;Calm3	_M[Oxidation (M)]KDTDSEEEIR_.2	#N/A	5.6643	4.8585	4.8326	6.1705	5.8048	4.8358	4.6048	4.7289	4.7810	5.1122	1.1153	0.1575	0.1154	0.9377
Calm1;Calm2;Calm3	_[Acetyl (Protein N-term)]ADQLTEEQIAEFKEAFSLFDKDGDGTITTK_.3	#N/A	3.6036	3.2001	3.3373	5.2866	3.2347	1.6606	3.7704	2.7812	3.7113	2.2194	1.0854	0.1183	0.6974	0.1565
Calm1;Calm2;Calm3	_[Acetyl (Protein N-term)]ADQLTEEQIAEFK_.2	#N/A	4.6794	3.9272	3.9858	12.1123	3.9255	4.3099	4.2031	11.5594	3.2786	2.9546	0.9984	-0.0023	0.9970	0.0013
Calm1;Calm2;Calm3	_DTDSEEEIR_.2	#N/A	2.9810	3.7373	4.2155	3.1775	3.6256	2.9991	4.3930	3.4401	3.0887	5.2184	0.8565	-0.2235	0.2389	0.6219
Eef1a1	_IGGIGTVPVGR_.2	#N/A	10.5656	10.6423	10.8153	10.3027	10.5158	10.3522	10.5339	10.7363	10.6198	10.5965	0.9916	-0.0122	0.4072	0.3902
Eef1a1	_RYEEIVK_.2	#N/A	8.9030	8.8378	8.9087	8.8893	9.0096	8.9901	8.7249	8.9457	8.8505	8.8407	1.0094	0.0134	0.1281	0.8926
Eef1a1	_LPLQDVYK_.2	#N/A	8.9691	8.8623	8.5126	8.5220	8.9360	8.4828	8.5930	8.8235	8.7871	8.7429	0.9974	-0.0037	0.8613	0.0649
Eef1a1	_QLIVGVNK_.2	#N/A	8.5651	8.8980	8.8730	8.9639	8.4787	8.5671	8.5753	8.9376	8.8588	8.5362	0.9997	-0.0004	0.9846	0.0068
Eef1a1	_QTVAVGVIK_.2	#N/A	8.8199	8.5874	8.5361	8.6216	8.6967	8.8428	8.2497	8.6036	8.8257	8.7207	1.0098	0.0141	0.4958	0.3047
Eef1a1	_STTTGHLIYK_.2	#N/A	8.0521	8.6917	8.5239	8.6766	8.9531	8.5767	8.3941	8.7156	8.8644	8.3963	0.9984	-0.0023	0.9410	0.0264
Eef1a1	_TIEKFEK_.2	#N/A	7.4823	7.5650	7.6491	7.7081	7.8768	7.6905	7.2955	7.6850	7.6769	7.3855	1.0201	0.0288	0.1869	0.7283
Eef1a1	_EHALLAYTLGVK_.2	#N/A	6.7695	6.8598	7.4679	6.4868	6.5665	6.9716	6.3222	6.4518	6.9116	6.6464	1.0411	0.0581	0.2247	0.6483
Eef1a1	_M[Oxidation (M)]DSTEPPYSQK_.2	#N/A	7.6942	7.4667	7.3919	7.9123	7.6631	7.8581	7.3462	7.4495	7.1808	7.3883	1.0440	0.0622	0.0226	1.6452
Eef1a1	_VETGVLKPGM[Oxidation (M)]VVTFAPVNVTTEVK_.3	#N/A	6.1352	5.9038	6.5488	6.6927	6.3212	6.1862	5.9555	6.5206	6.4552	5.9019	1.0144	0.0207	0.6577	0.1820
Eef1a1	_YYVTIIDAPGHR_.2	#N/A	8.1327	8.5430	8.5809	8.6119	8.0370	8.6210	8.5337	8.4575	8.4710	8.2469	0.9993	-0.0011	0.9667	0.0147
Eef1a1	_DGSASGTTLLEALDC[Carbamidomethyl (C)]ILPPTRPTDKPLR_.3	#N/A	4.2663	3.7996	3.2765	4.1669	3.4257	2.9806	4.1667	3.7094	1.5996	3.9984	1.0843	0.1168	0.6128	0.2127
Eef1a1	_NMITGTSQADC[Carbamidomethyl (C)]AVLIVAAGVGEFEAGISK_.3	#N/A	3.9056	3.9888	4.1000	3.6146	3.8330	3.4179	3.5793	3.2014	3.9651	3.4326	1.0749	0.1042	0.1798	0.7452
Eef1a1	_THINIVVIGHVDSGK_.3	#N/A	3.7334	3.6090	3.4671	4.5117	2.8771	3.7475	3.1591	3.9903	4.2894	4.1889	0.9362	-0.0951	0.4808	0.3180
Alad	_DAAQSS[Phospho (STY)]PAFGDR_.2	#N/A	10.1417	10.1883	10.4653	10.8431	3.2613	10.2008	10.5584	10.0880	10.2316	10.2429	0.8933	-0.1628	0.4823	0.3167
Alad	_DAAQSS[Phospho (STY)]PAFGDRR_.2	#N/A	8.0288	8.3108	8.6484	8.6686	8.0075	8.3356	8.2882	8.1591	8.1305	8.0923	1.0203	0.0290	0.3017	0.5204
Ctsb	_EQWSNC[Carbamidomethyl (C)]PTIGQIR_.2	#N/A	7.6166	7.5913	7.1199	7.8964	7.8267	7.6429	7.3932	7.1045	7.1855	7.5996	1.0403	0.0570	0.1104	0.9571
Ctsb	_GENHC[Carbamidomethyl (C)]GIESEIVAGIPR_.3	#N/A	6.9498	6.3485	5.7073	7.0891	7.3149	7.0336	6.7793	7.0657	6.4437	7.6623	0.9646	-0.0520	0.5209	0.2833
Ctsb	_SC[Carbamidomethyl (C)]EAGYSPSYKEDK_.2	#N/A	4.0708	3.9100	3.1661	4.7550	4.9103	4.1658	3.7319	4.8578	4.0029	3.9878	1.0043	0.0062	0.9631	0.0163
Ctsb	_DQGSC[Carbamidomethyl (C)]GSC[Carbamidomethyl (C)]WAFGAVEAISDR_.2	#N/A	4.6537	5.4262	5.4412	6.1601	4.9322	5.1443	5.3519	4.8479	5.2364	4.8505	1.0436	0.0616	0.4595	0.3377
Ctsb	_NGPVEGAFTVFSDFLTYK_.2	#N/A	3.7466	3.0020	3.4803	2.8966	2.8903	3.7343	3.1723	3.9771	4.2762	5.2957	0.7874	-0.3448	0.0596	1.2250
Ctsb	_EIMAEIYK_.2	#N/A	3.1478	3.9530	3.8346	4.3373	3.7744	3.9551	4.0520	1.1239	3.4298	4.3043	1.1878	0.2483	0.3468	0.4600
Eif4a2	_LQAEAPHIVVGTPGR_.3	#N/A	6.2036	5.0800	6.4103	6.0463	6.1412	6.5072	5.1294	6.0849	6.4667	5.5730	1.0432	0.0610	0.4919	0.3082
Eif4a2	_ETQALVLAPTR_.2	#N/A	5.0338	5.5015	4.2347	6.1146	4.2121	4.8191	4.8806	5.7899	5.1485	5.5226	0.9345	-0.0977	0.4181	0.3788
MAPT	_SGYSSPGS[Phospho (STY)]PGTPGSR_.2	#N/A	14.5840	14.8898	15.5797	15.4325	14.6664	14.8157	15.3150	14.6830	14.5246	14.9746	1.0081	0.0116	0.6453	0.1902
MAPT	_SKIGS[Phospho (STY)]TENLK_.2	#N/A	9.5329	9.8645	9.9427	10.3526	9.1282	9.8960	9.4876	9.6684	9.6567	10.1364	1.0050	0.0072	0.8428	0.0743
MAPT	_TDHGAEIVYK_.2	#N/A	7.4549	7.4704	7.5006	7.5559	8.1970	7.7003	7.5166	7.3994	7.1673	7.6333	1.0293	0.0416	0.2239	0.6500
MAPT	_LDLSNVQSK_.2	#N/A	7.4460	7.5619	7.6820	7.9077	8.1559	7.7331	7.7101	7.2750	7.5860	7.9432	1.0156	0.0224	0.5031	0.2984
MAPT	_SEKLDFKDR_.2	#N/A	7.5489	7.7220	7.9684	7.8523	8.1390	8.1782	7.7622	7.7838	7.4122	7.5252	1.0368	0.0522	0.0863	1.0638
MAPT	_HVPGGGSVQIVYKPVDLSK_.3	#N/A	8.8430	9.1139	9.5888	9.4215	9.4234	8.9175	9.1151	8.9218	9.4478	9.2768	1.0030	0.0043	0.8809	0.0551
MAPT	_SEKLDFK_.2	#N/A	6.2690	5.9122	6.0812	6.3342	6.7408	6.4666	5.8871	6.0703	5.5049	6.4032	1.0560	0.0786	0.1494	0.8257
MAPT	_VQIINKK_.2	#N/A	4.9165	5.1082	4.2898	5.3804	5.5121	4.8544	4.0860	5.2794	4.4875	5.2612	1.0485	0.0683	0.4932	0.3070
MAPT	_LQTAPVPM[Oxidation (M)]PDLK_.2	#N/A	9.0415	9.0945	8.5902	9.2683	9.4398	8.6462	8.8108	8.9747	8.1988	8.3390	1.0504	0.0710	0.0919	1.0365
MAPT	_T[Phospho (STY)]PPKSPSSAK_.2	#N/A	10.0973	9.8324	10.7880	11.5780	9.3849	9.9071	10.0410	10.8485	10.3747	11.1053	0.9691	-0.0453	0.4821	0.3169
MAPT	_IGS[Phospho (STY)]LDNITHVPGGGNK_.2	#N/A	9.4625	9.5276	9.7899	9.8449	8.2841	9.2345	9.0630	8.9558	9.0633	9.9008	1.0121	0.0173	0.7509	0.1244
MAPT	_IGSTENLK_.2	#N/A	7.3513	7.4359	7.2937	7.2079	7.6561	7.1972	7.0818	7.2702	6.9919	7.8174	1.0091	0.0131	0.7059	0.1512
MAPT	_VAVVRT[Phospho (STY)]PPKS[Phospho (STY)]PSSAK_.2	#N/A	10.7106	10.7830	10.8977	11.4364	9.7050	10.9945	10.2280	10.3910	10.6013	10.7625	1.0247	0.0351	0.4240	0.3727
MAPT	_TPS[Phospho (STY)]LPTPPTR_.2	#N/A	10.9829	10.9920	10.6444	11.4914	10.2526	10.5289	10.7425	10.3595	10.6400	10.7085	1.0191	0.0273	0.4081	0.3893
MAPT	_IGS[Phospho (STY)]LDNITHVPGGGNKK_.3	#N/A	6.2395	6.6623	7.2228	7.2092	6.3050	5.6382	6.5419	6.9363	6.1622	7.0017	0.9828	-0.0250	0.7518	0.1239
MAPT	_SRT[Phospho (STY)]PS[Phospho (STY)]LPTPPTREPK_.3	#N/A	3.7019	8.3615	10.3275	9.7733	7.1417	9.8275	9.5358	9.6041	9.1108	9.9146	0.8583	-0.2205	0.3208	0.4937
MAPT	_AKTDHGAEIVYKS[Phospho (STY)]PVVS[Phospho (STY)]GDTSPR_.4	#N/A	7.5079	9.8005	8.3852	10.0005	8.9788	9.7745	6.9479	9.6565	8.7011	9.4091	1.0456	0.0644	0.5850	0.2328
MAPT	_IGS[Phospho (STY)]TENLKHQPGGGK_.3	#N/A	3.6526	3.8054	3.9408	4.1140	3.6809	3.7928	4.0926	3.7053	3.5328	4.0078	0.9991	-0.0013	0.9798	0.0088
MAPT	_SPVVS[Phospho (STY)]GDTS[Phospho (STY)]PR_.2	#N/A	13.9636	14.3335	14.9791	14.5773	13.9609	14.2428	14.5956	14.0425	14.1148	14.3742	1.0043	0.0062	0.7908	0.1020
MAPT	_TDHGAEIVYKS[Phospho (STY)]PVVSGDTS[Phospho (STY)]PR_.3	#N/A	10.0876	10.6189	9.9949	11.0432	9.9760	10.8364	10.6777	10.1721	10.5849	10.8721	0.9858	-0.0207	0.5860	0.2321
MAPT	_TPS[Phospho (STY)]LPTPPTREPK_.2	#N/A	10.2839	10.8451	11.1820	11.1215	10.2926	10.6026	10.9515	10.3275	10.3213	10.4464	1.0199	0.0285	0.3952	0.4032
MAPT	_AKTDHGAEIVYK_.2	#N/A	4.4478	3.7884	4.1958	4.5146	5.3572	4.9911	3.8878	4.4185	3.8667	4.8103	1.0714	0.0995	0.3947	0.4037
MAPT	_SRT[Phospho (STY)]PS[Phospho (STY)]LPTPPTR_.2	#N/A	10.2939	3.9202	4.0203	4.1507	7.6505	10.2456	8.4630	8.2255	3.4881	8.6933	0.9302	-0.1044	0.7933	0.1005

MAPT	_KLDLSNVQSK_.2	#N/A	6.4968	6.5098	6.9842	7.0204	7.1532	6.4240	6.8088	6.7029	6.4790	6.6137	1.0171	0.0244	0.5301	0.2756
MAPT	_IPAKT[Phospho (STY)]PPAPK_.2	#N/A	3.2345	1.6289	4.4690	4.4249	3.8791	4.2464	4.1395	3.6936	3.3422	5.5239	0.8736	-0.1950	0.4552	0.3418
MAPT	_T[Phospho (STY)]PPSSGEPPK_.2	#N/A	3.8656	3.4837	3.5993	4.0319	3.0613	3.4140	3.2913	3.8651	4.1641	4.3211	0.9145	-0.1290	0.2195	0.6585
MAPT	_TPPAPKT[Phospho (STY)]PPSSGEPPK_.2	#N/A	6.3606	5.1306	5.9670	5.9803	3.5424	3.0428	6.2365	5.6443	3.7737	7.3292	0.8709	-0.1995	0.4448	0.3518
MAPT	_SKIGS[Phospho (STY)]TENLKHQPGGGK_.3	#N/A	4.1160	3.2264	3.8497	6.5815	4.8535	3.3649	3.5417	3.6077	3.9068	3.0707	1.2266	0.2947	0.2537	0.5957
MAPT	_DQGGYTM[Oxidation (M)]HQDQEGDTDAGLK_.3	#N/A	4.2517	3.5072	3.9854	3.5292	3.3955	3.2291	3.6774	3.4720	3.7711	3.2065	1.0334	0.0474	0.6084	0.2158
MAPT	_QEFEVM[Oxidation (M)]EDHAGTYGLGDR_.3	#N/A	3.7739	3.5127	4.0363	4.1358	2.9091	3.5244	3.1890	3.5896	4.2827	3.9052	0.9752	-0.0363	0.7580	0.1203
MAPT	_VQS[Phospho (STY)]KIGS[Phospho (STY)]LDNITHVPGGGNK_.3	P10636_S669_M2_MAPT	3.7267	2.9821	3.4300	3.9326	2.8704	3.7542	3.6473	4.1858	3.8344	5.9045	0.7852	-0.3489	0.0770	1.1137
MAPT	_SGDRSGYSSPGS[Phospho (STY)]PGTPGSR_.3	#N/A	3.9203	3.6248	3.6961	3.9351	3.4648	3.9533	3.9442	4.0085	3.6185	3.7217	0.9850	-0.0219	0.6598	0.1806
MAPT	_IPAKT[Phospho (STY)]PPAPKT[Phospho (STY)]PPSSGEPPK_.3	#N/A	3.8621	3.1378	3.2855	3.7882	2.9961	3.6097	3.5028	4.3415	3.9901	4.7338	0.8321	-0.2652	0.0358	1.4465
MAPT	_TPPAPKT[Phospho (STY)]PPSSGEPPKSGDR_.3	#N/A	3.7384	3.5949	3.9930	3.8379	3.3718	3.6038	3.6652	3.8400	3.8906	4.3773	0.9358	-0.0958	0.1598	0.7965
MAPT	_C[Carbamidomethyl (C)]GSLGNIHHKPGGGQVEVK_.3	#N/A	3.4162	4.5457	4.4344	5.6182	5.3273	5.1432	5.1864	4.4392	3.6728	5.4057	1.0153	0.0219	0.8923	0.0495
MAPT	_T[Phospho (STY)]PPSSGEPPKSGDR_.2	#N/A	3.2702	4.0817	4.0872	4.2666	3.8967	4.0863	4.1100	4.4901	3.3742	3.6907	1.0081	0.0117	0.9066	0.0426
MAPT	_HLSNVSSTGS[Phospho (STY)]IDM[Oxidation (M)]VDSPQLATLADEVSASLAK_.4	#N/A	3.8578	3.5933	3.6901	6.6018	5.3322	4.1130	3.7815	5.7850	3.7645	4.2470	1.0311	0.0442	0.8534	0.0689
MAPT	_QEFEVMEDHAGTYGLGDRK_.3	#N/A	3.5206	3.5908	4.0690	4.2989	3.4791	4.0029	3.7610	3.5596	3.8586	3.9762	1.0100	0.0144	0.8445	0.0734
Mapt	_IPAKTT[Phospho (STY)]PS[Phospho (STY)]PK_.2	#N/A	3.9289	10.5655	10.8449	11.1036	10.6229	3.1741	10.6634	10.5216	10.5692	10.5647	0.7914	-0.3374	0.2819	0.5499
Mapt	_IPAKTT[Phospho (STY)]PS[Phospho (STY)]PKT[Phospho (STY)]PPGSGEPPK_.2	#N/A	5.5295	3.6084	6.4124	4.3894	3.5511	7.5250	3.6405	4.1312	6.5885	5.6795	1.0318	0.0452	0.8750	0.0580
Mapt	_T[Phospho (STY)]PPKSPSASK_.2	#N/A	11.1539	10.0484	10.8540	11.6892	9.5793	10.1484	10.2775	10.8994	10.5818	11.2161	0.9847	-0.0223	0.7132	0.1468
Mapt	_ESPPQPPADDGAEEPGS[Phospho (STY)]ETSDAK_.2	#N/A	8.6793	9.1257	9.5120	9.8614	8.8186	9.2618	9.4759	8.6955	8.7093	8.9417	1.0284	0.0404	0.3674	0.4349
Mapt	_VAVVRT[Phospho (STY)]PPKS[Phospho (STY)]PSASK_.2	#N/A	10.5527	10.6436	10.7121	11.1933	9.5494	10.8312	10.0755	10.1376	10.4115	10.6116	1.0263	0.0375	0.3898	0.4091
Mapt	_TTPS[Phospho (STY)]PKT[Phospho (STY)]PPGSGEPPKSGER_.3	#N/A	8.5750	6.8016	9.3333	9.1595	8.9001	9.3830	9.2519	8.8674	7.0785	8.3447	1.0365	0.0518	0.6355	0.1969
Mapt	_QEFDTM[Oxidation (M)]EDHAGDYTLLQDQEGDM[Oxidation (M)]DHGLK_.4	#N/A	4.3392	4.6815	4.1385	3.3003	3.8975	5.1006	3.3543	3.4553	4.4003	5.2726	1.0297	0.0422	0.8046	0.0944
Mapt	_TTPS[Phospho (STY)]PKT[Phospho (STY)]PPGSGEPPK_.2	#N/A	12.0422	12.2648	12.5913	12.8910	11.5574	12.2992	12.5364	12.0979	12.1453	12.3322	0.9997	-0.0004	0.9886	0.0050
Mapt	_ST[Phospho (STY)]PTAEDVTAPLVDER_.2	P10637_T58_M1_Mapt	9.5682	9.5354	3.8953	10.2993	9.4129	9.7500	10.1046	9.3457	9.7228	9.8995	0.8951	-0.1599	0.4287	0.3679
Mapt	_T[Phospho (STY)]PPGSGEPPK_.2	#N/A	11.5996	10.1460	9.4560	11.6561	11.3377	11.1740	11.3916	10.8738	11.2310	11.5160	0.9682	-0.0467	0.4665	0.3312
Mapt	_SGERS[Phospho (STY)]GYSS[Phospho (STY)]PGSPGTPGSR_.2	P10637_S487_M2_Mapt	3.4474	3.7605	5.0346	3.7259	3.6352	3.5055	4.1930	3.7138	5.3315	3.5651	0.9168	-0.1252	0.4476	0.3491
Mapt	_T[Phospho (STY)]PPGSGEPPKSGER_.2	#N/A	9.1498	8.9656	8.6433	10.8446	9.7190	10.4742	10.4367	3.3662	3.6653	10.3529	1.3850	0.4698	0.1398	0.8546
Txn	_EAFQEALAAAGDK_.2	#N/A	8.9934	8.8409	8.8975	9.2962	9.5258	8.9776	9.1372	8.9672	9.2038	9.1780	0.9964	-0.0052	0.8222	0.0850
Txn	_VGEFSGANK_.2	#N/A	7.8126	7.8892	7.8228	8.0525	8.5293	7.8185	7.6713	7.9905	7.6641	8.1127	1.0163	0.0233	0.4712	0.3268
Txn	_VKLIESK_.2	#N/A	6.5843	7.0926	7.8604	6.7941	6.8399	6.7049	7.0211	7.0235	6.6646	7.0968	1.0040	0.0058	0.9136	0.0392
Txn	_VGEFSGANKEK_.2	#N/A	5.0531	5.6101	5.6074	4.6745	5.6008	5.5250	5.4100	5.0127	4.9335	5.3746	1.0314	0.0445	0.4862	0.3132
Gstm1	_ITQSNAILR_.2	#N/A	9.2725	9.6575	9.8722	9.7082	9.8040	9.5276	9.5070	9.6461	9.6848	9.3977	1.0085	0.0122	0.5231	0.2814
Gstm1	_YIATPIFSK_.2	#N/A	8.7145	9.0763	8.8391	8.7516	9.0192	8.7871	8.6639	8.9538	9.0601	8.8709	0.9975	-0.0037	0.8287	0.0816
Gstm1	_PM[Oxidation (M)]ILGYWNVR_.2	#N/A	7.8625	8.1904	8.1323	7.8774	8.0524	8.1157	8.0807	8.2581	8.2975	8.2020	0.9792	-0.0304	0.0639	1.1947
Gstm1	_SQWLNEK_.2	#N/A	7.9421	7.9134	8.0561	7.9653	8.1722	7.8810	7.1482	8.0088	7.9936	8.1987	1.0193	0.0275	0.4572	0.3399
Gstm1	_C[Carbamidomethyl (C)]LDAFPNLR_.2	#N/A	7.4743	8.0040	7.7204	7.6280	8.0095	7.7815	7.7668	7.6330	7.8986	7.3755	1.0132	0.0189	0.4882	0.3114
Gstm1	_RPWFAGDK_.2	#N/A	7.3923	7.6066	6.9642	7.8575	7.8636	7.3061	7.5554	7.6825	7.0346	7.3540	1.0124	0.0178	0.6738	0.1715
Gstm1	_LYSEFLGK_.2	#N/A	6.7259	7.1569	7.1501	6.8554	7.2209	7.1797	5.4975	7.0716	7.6867	7.1984	1.0269	0.0383	0.6480	0.1884
Gstm1	_YTM[Oxidation (M)]GDAPDFDR_.2	#N/A	8.7407	8.6990	8.8030	8.7539	8.7293	8.7263	8.7227	8.6157	8.6195	8.7969	1.0061	0.0088	0.2081	0.6818
Gstm1	_ADIVENQVM[Oxidation (M)]DTR_.2	#N/A	8.1224	8.1912	8.2083	8.4750	8.4641	8.1041	8.1288	8.1568	8.1346	8.3418	1.0086	0.0123	0.4755	0.3229
Gstm1	_LGLDFPNLPYLIDGSHK_.3	#N/A	6.1123	6.1259	5.7842	5.9328	5.6697	5.8668	6.5045	6.1130	6.1145	6.0355	0.9553	-0.0659	0.0571	1.2437
Gstm1	_MLLEYTDSSYDEK_.2	#N/A	3.8484	3.1527	4.5352	5.7727	3.6441	3.2546	3.6154	3.0505	3.1414	3.5040	1.2124	0.2779	0.2063	0.6855
Gstm1	_M[Oxidation (M)]LLEYTDSSYDEKR_.2	#N/A	5.0530	5.7430	5.9695	5.9991	6.0955	5.9921	5.6879	5.5048	5.5702	5.8486	1.0276	0.0392	0.4730	0.3252
Gstm1	_IRADIVENQVM[Oxidation (M)]DTR_.3	#N/A	3.5247	5.1752	6.0058	4.7811	5.5528	5.5069	4.7659	5.3129	5.0093	5.0877	1.0093	0.0134	0.9196	0.0364
Gstm1	_VTYVDFLAYDILDQYR_.2	#N/A	3.9233	3.1787	3.6570	2.9982	1.8323	3.5576	3.4507	4.3824	2.2879	3.7030	0.9234	-0.1150	0.6197	0.2078
Tcea1	_EPAISSQNS[Phospho (STY)]PEAR_.2	#N/A	9.4308	9.1973	3.2815	9.9699	9.1109	9.4398	9.6399	8.8690	9.3586	9.5131	0.8994	-0.1530	0.4891	0.3106
Tcea1	_KKEPAISSQNS[Phospho (STY)]PEAR_.3	#N/A	9.6788	8.7104	10.2024	10.3830	10.1321	10.0628	10.0041	9.9610	9.4484	9.9722	1.0015	0.0022	0.9643	0.0158
Tcea1	_DTYVSSFPR_.2	#N/A	2.9505	4.2757	3.6373	4.1399	3.5770	4.1525	3.8546	3.9786	3.6271	4.1069	0.9735	-0.0387	0.7117	0.1477
Slc3a2	_LLLSTDSAR_.2	#N/A	7.5919	7.4582	7.6013	7.0895	7.1156	7.4304	7.1637	7.7806	7.7469	7.2021	0.9877	-0.0179	0.6149	0.2112
Slc3a2	_GQNEDPGSLLTQFR_.2	#N/A	7.2213	6.9169	6.9376	7.1645	6.8076	6.9710	7.4338	7.1002	6.9653	7.0884	0.9799	-0.0293	0.2393	0.6210
Slc3a2	_LGASNLPAGISLPASAK_.2	#N/A	7.0252	6.7252	6.2952	7.0954	6.3664	6.0861	6.6619	7.4207	5.9243	6.7864	0.9852	-0.0216	0.7650	0.1163
Slc3a2	_IGDLQAFVGR_.2	#N/A	6.7124	6.6978	6.3548	6.0182	6.0729	5.8753	6.5272	6.8946	6.2830	6.3011	0.9672	-0.0480	0.3503	0.4555
Slc3a2	_QINPTLGSQEDFKDLLQSAK_.3	#N/A	5.8971	5.3272	5.6915	5.6392	5.8211	5.3194	5.9061	6.1085	6.2350	5.4815	0.9466	-0.0792	0.1172	0.9312
Slc3a2	_EALSSWLQDGVDGFQFR_.2	#N/A	4.8489	4.9943	4.6117	4.7738	4.8245	4.7567	5.4896	4.9741	4.6465	4.0578	1.0020	0.0029	0.9696	0.0134
Slc3a2	_SHLEYLSTLK_.2	#N/A	4.8032	4.6864	5.5478	4.8116	5.0361	4.8478	4.3044	4.5668	4.6691	4.7140	1.0859	0.1189	0.0552	1.2583
Slc3a2	_HWDQNER_.2	#N/A	5.5156	4.8505	3.4415	4.4861	2.8515	5.0431	3.1335	4.0159	5.4291	5.1706	0.9836	-0.0238	0.9162	0.0380
Slc3a2	_DAGGIAGLK_.2	#N/A	3.9244	3.4180	3.6581	3.2019	2.5423	2.9894	3.3502	2.6380	4.0983	1.6716	1.1189	0.1621	0.4872	0.3123
Slc3a2	_NQKDEINETDLK_.3	#N/A	3.7964	3.0518	3.5301	3.8630	2.9401	3.6845	3.5776	4.2555	3.9041	3.8300	0.8936	-0.1623	0.1072	0.9697
H2bc7;H2bc14;H2bc3	_LLLPGELAK_.2	#N/A	11.8504	12.1697	11.9875	11.6559	12.2161	11.9933	11.7994	11.9512	12.1737	12.0438	0.9989	-0.0016	0.9167	0.0378
H2bc7;H2bc14;H2bc3	_ESYSVYVYK_.2	#N/A	8.0023	7.5065	8.0621	8.4650	8.1962	8.3475	6.0303	8.3866	8.2450	8.3331	1.0449	0.0634	0.4936	0.3066
H2bc7;H2bc14;H2bc3	_EIQTAVR_.2	#N/A	7.4531	7.2484	7.6902	6.8500	8.2459	7.4790	5.6903	7.5971	7.7633	7.6580	1.0442	0.0624	0.5089	0.2934
H2bc7;H2bc14;H2bc3	_KESYSVYVYK_.2	#N/A	6.9531	6.7446	7.7350	7.5635	7.2153	7.5010	7.1498	7.5052	7.2209	7.5335	0.9909	-0.0132	0.7584	0.1201
H2bc7;H2bc14;H2bc3	_AM[Oxidation (M)]GIM[Oxidation (M)]NSFVNDIFER_.2	#N/A	8.5308	8.6331	8.6956	8.0154	8.3265	8.5893	8.4665	8.4264	8.5418	8.3277	1.0029	0.0042	0.8596	0.0657
H2bc7;H2bc14;H2bc3	_QVHPDTGISSK_.2	#N/A	10.5309	10.8089	10.9623	10.7904	11.1962	10.9192	10.6355	10.7849	10.7246	10.8174	1.0119	0.0170	0.3069	0.5131
H1-0	_VGENADSQIK_.2	#N/A	6.0507	5.8880	3.9026	5.7604	6.5134	5.1980	6.2341	6.3469	5.7471	6.0746	0.9101	-0.1359	0.2840	0.5467
H1-0	_[Acetyl (Protein N-term)]TENS[Phospho (STY)]TSAPAAKPK_.2	#N/A	4.4933	5.3936	4.7669	3.5245	4.1389	3.8747	3.2900	4.7101	4.3708	4.2029	1.0535	0.0752	0.6092	0.2152
H1-0	_GVGASGSFR_.2	#N/A	4.8861	5.6692	4.3204	4.8299	6.3312	4.9840	3.8301	5.7755	5.4648	4.9843	1.0312	0.0443	0.7608	0.1187
Sub1	_EQISDIDDAVR_.2	#N/A	6.5434	6.5826	6.2078	6.5206	6.3357	6.0562	6.7361	6.2860	6.2562	6.2389	0.9992	-0.0011	0.9735	0.0117
Sub1	_QSS[Phospho (STY)]SSRDDNMFQIGK_.3	#N/A	3.6948	3.6375	3.5992	4.1018	3.4832	3.7543	3.8165	4.0837	3.7323	4.0688	0.9456	-0.0807	0.1364	0.8652
Gpx1	_TIDIEPDIETLLSQQSGNS_.2	#N/A	8.6880	9.7170	9.9897	10.2730	9.1530	8.9239	9.6069	9.3770	8.9784	8.8354	1.0280	0.0399	0.4832	0.3159
Fabp3	_SLGVGFATR_.2	#N/A	7.5389	8.1408	8.0167	8.2887	8.3722	7.8845	7.8307	8.3551	8.2444	8.0062	0.9915	-0.0123	0.7129	0.1470
Fabp3	_[Acetyl (Protein N-term)]ADAFVGTWK_.2	#N/A	7.5743	7.7488	7.3811	7.4897	7.9155	8.0734	7.7130	7.8217	7.9424	7.4878	0.9943	-0.0082	0.7834	0.1060
Fabp3	_SLVTLDGGK_.2	#N/A	6.8689	6.4736	7.2843	6.2093	7.6517	7.0119	5.6148	7.2044	7.3775	7.4625	1.0003	0.0004	0.9968	0.0014
Fabp3	_WNGQETTLTR_.2	#N/A	6.4525	6.2020	6.6983	6.1684	7.2523	6.4354	6.1140	6.5444	6.3126	5.6351	1.0623	0.0872	0.1717	0.7652
Fabp3	_LILTLTHGSVVSTR_.2	#N/A	2.4005	3.5001	3.7326	4.1773	3.3627	3.1172	3.8920	3.3075	3.7960	3.4889	0.9339	-0.0987	0.4819	0.3170
Fabp3	_NGDTITIK_.2	#N/A	2.8956	3.7008	3.5824	4.0851	3.5222	4.2073	3.7998	4.0334	3.6820	3.4463	0.9800	-0.0291	0.7787	0.1086
Hsp90ab1	_IEDVGS[Phospho (STY)]DEEDDSGK_.2	#N/A	12.2660	11.9205	11.6824	12.8398	11.4881	11.9515	12.3851	11.9922	12.0052	12.1609	0.9908	-0.0133	0.6739	0.1714
Hsp90ab1	_TKPIWTR_.2	#N/A	10.0879	10.6107	10.6214	10.4701	10.7734	10.1755	10.3011	10.6601	10.5280	10.2536	1.0020	0.0029	0.8981	0.0467
Hsp90ab1	_ADLINNLGTIAK_.2	#N/A	10.3684	10.4748	10.5663	10.7182	10.5910	10.3086	10.3889	10.6266	10.3555	10.3001	1.0083	0.0120	0.3926	0.4060

Hsp90ab1	_IDIIPNPQER_.2	#N/A	10.1778	10.2288	10.2592	10.2501	10.1491	10.1248	10.3599	10.2633	10.1924	10.0099	0.9992	-0.0011	0.9038	0.0439
Hsp90ab1	_YIDQEELNK_.2	#N/A	9.8129	9.9772	10.1884	10.1989	10.0403	9.9346	9.9750	9.9612	9.7806	9.8642	1.0132	0.0188	0.1626	0.7890
Hsp90ab1	_EDQTEYLEER_.2	#N/A	9.6293	9.2241	9.2803	9.7129	9.3155	9.2442	9.5345	9.2047	9.1296	9.4354	1.0080	0.0116	0.5863	0.2319
Hsp90ab1	_ELISNASDALDK_.2	#N/A	9.5094	9.1657	9.1798	9.5922	9.0887	9.0976	9.3425	9.2751	9.1463	9.1867	1.0037	0.0054	0.7764	0.1099
Hsp90ab1	_SIYYITGESK_.2	#N/A	8.8957	8.6079	9.0201	9.0896	8.3841	8.8642	8.6402	8.8031	8.7006	8.9179	1.0051	0.0074	0.7639	0.1169
Hsp90ab1	_EGLELPEDEEEKKK_.3	#N/A	8.6575	8.2200	8.3027	8.3900	8.3202	8.2410	8.0456	8.4642	8.1433	8.5910	1.0053	0.0077	0.7441	0.1284
Hsp90ab1	_NPDDITQEEYGEFYK_.2	#N/A	8.2916	8.1340	7.7506	8.2199	7.9826	8.4544	8.4458	8.2516	7.9321	8.2102	0.9913	-0.0125	0.6510	0.1864
Hsp90ab1	_ADHGEPIGR_.2	#N/A	8.5119	7.9670	8.3611	8.1210	8.3319	8.1565	7.8883	8.3580	8.1973	8.1707	1.0108	0.0155	0.5063	0.2956
Hsp90ab1	_EQVANSAFVER_.2	#N/A	7.3913	7.6210	8.1192	7.8537	7.7910	7.9244	7.6511	7.5937	7.9833	7.8956	1.0003	0.0005	0.9869	0.0057
Hsp90ab1	_ALLFIPR_.2	#N/A	7.1228	7.9299	7.4205	7.1158	7.3884	7.5990	6.4610	7.9156	7.7778	7.2141	1.0119	0.0170	0.7820	0.1068
Hsp90ab1	_EIS[Phospho (STY)]DDEAEEEK_.2	#N/A	7.3882	6.8959	7.0496	7.6969	6.5742	7.3135	7.6484	7.2028	6.8881	7.2391	0.9874	-0.0183	0.7096	0.1490
Hsp90ab1	_YESLTDPSK_.2	#N/A	7.5114	6.7849	6.6619	7.3590	7.4489	7.2368	6.2186	7.2913	7.3124	7.5321	1.0111	0.0159	0.7976	0.0982
Hsp90ab1	_EKEIS[Phospho (STY)]DDEAEEEK_.2	#N/A	6.9099	6.8497	6.2997	7.6299	6.5693	6.5789	7.1092	6.9609	6.4313	6.7464	0.9992	-0.0012	0.9831	0.0074
Hsp90ab1	_EKYIDQEELNK_.2	#N/A	6.8395	6.2918	6.5733	6.7058	6.1144	6.2811	6.7055	6.6545	6.4510	6.0933	0.9987	-0.0019	0.9640	0.0159
Hsp90ab1	_VFIM[Oxidation (M)]DSC[Carbamidomethyl (C)]DELIPEYLNFIR_.2	#N/A	5.7985	6.2929	6.6749	6.3579	5.0735	5.2360	4.9915	5.7674	5.7283	5.6806	1.0656	0.0917	0.3436	0.4639
Hsp90ab1	_GVVDSEDLPLNIS[Phospho (STY)]R_.2	P11499_S391_M1_Hsp90ab1	6.9487	6.5152	3.6982	7.7949	6.6599	6.8598	6.9008	7.3594	7.1200	6.7477	0.9119	-0.1330	0.4170	0.3799
Hsp90ab1	_APFDLFENK_.2	#N/A	5.0915	5.0008	4.1651	3.7262	4.2493	4.8340	4.8450	5.0446	5.0894	5.3992	0.8855	-0.1755	0.0812	1.0903
Hsp90ab1	_LGIHEDSTNR_.2	#N/A	7.0557	7.4160	6.7368	7.0256	6.2463	6.6464	7.1256	7.0350	6.9372	6.9144	0.9788	-0.0309	0.4977	0.3031
Hsp90ab1	_ELISNASDALDKIR_.2	#N/A	5.7992	6.2351	6.3742	6.1036	6.0665	6.3638	6.1237	5.8739	6.1509	5.9349	1.0226	0.0323	0.3011	0.5213
Hsp90ab1	_EKEIS[Phospho (STY)]DDEAEEEKGEK_.2	#N/A	5.4389	4.6627	5.4161	5.8775	4.3630	4.9734	4.9874	5.8110	5.4219	5.1171	0.9602	-0.0586	0.5259	0.2791
Hsp90ab1	_EIS[Phospho (STY)]DDEAEEEKGEK_.2	#N/A	5.1188	3.9645	3.5618	4.7426	3.8475	3.8452	4.8388	4.2872	4.1979	3.8583	0.9731	-0.0393	0.7496	0.1252
Hsp90ab1	_AKFENLC[Carbamidomethyl (C)]K_.2	#N/A	7.4486	7.4241	8.0412	7.5460	7.4960	7.3100	7.5575	7.5223	7.6175	7.6233	0.9953	-0.0068	0.7928	0.1008
Hsp90ab1	_IEDVGS[Phospho (STY)]DEEDDSGKDK_.2	#N/A	10.9431	11.0258	11.4497	11.6810	10.5962	10.9159	11.3081	11.0404	10.9206	10.9700	1.0038	0.0055	0.8475	0.0718
Hsp90ab1	_FYEAFSK_.2	#N/A	3.7473	3.0027	4.9217	2.2650	5.2405	5.0702	3.1730	5.6455	5.3822	4.4462	0.8669	-0.2061	0.4428	0.3538
Hsp90ab1	_IEDVGS[Phospho (STY)]DEEDDSGKDKK_.3	#N/A	12.9984	12.6342	13.2042	13.0155	12.8589	12.8652	12.8343	12.7928	13.0644	12.7513	1.0053	0.0077	0.5580	0.2533
Hsp90ab1	_EM[Oxidation (M)]LQQSK_.2	#N/A	5.3645	5.2304	5.0455	5.4015	6.9878	6.2308	5.1668	5.7526	5.8430	5.4515	1.0282	0.0401	0.7057	0.1514
Hsp90ab1	_YESLTDPSKLDSGK_.2	#N/A	7.6205	7.6300	7.8388	7.9735	7.5588	7.7773	7.6456	7.6478	7.3030	7.4044	1.0311	0.0441	0.0591	1.2281
Hsp90ab1	_SLTNDWEDHLAVK_.2	#N/A	5.9618	5.8932	5.7220	5.4530	5.9336	5.4214	6.3749	5.4491	6.1058	5.5790	0.9751	-0.0364	0.5102	0.2923
Hsp90ab1	_HLEINPDHPIVETLR_.3	#N/A	6.7619	7.2974	4.2356	6.6885	6.1328	6.6538	7.1988	6.7274	6.7524	6.7566	0.9178	-0.1237	0.3400	0.4685
Hsp90ab1	_YHTSQSGDEM[Oxidation (M)]TSLSEYVSR_.3	#N/A	6.4755	6.2555	6.1302	6.5145	5.3199	5.2439	5.9501	4.0682	5.6545	4.8330	1.1684	0.2246	0.0872	1.0596
Hsp90ab1	_HNDDEQYAWESSAGGSFTVR_.3	#N/A	5.5992	3.5056	3.9722	6.3350	4.8717	5.9227	6.4055	5.6894	5.5341	5.3712	0.8755	-0.1918	0.2672	0.5731
Hsp90ab1	_EGLELPEDEEEKK_.3	#N/A	3.5903	3.7521	3.3240	4.3686	4.2349	3.9611	4.5169	4.1334	3.2040	4.0459	0.9740	-0.0380	0.7376	0.1322
Hsp90ab1	_VILHLKEDQTEYLEER_.3	#N/A	5.1842	5.9485	6.4294	3.8638	3.3009	5.7972	5.2407	4.2547	3.9033	5.3531	1.0852	0.1180	0.5818	0.2353
Hsp90ab1	_KHLEINPDHPIVETLR_.4	#N/A	4.9416	3.2018	3.6800	3.2238	3.0901	3.5345	3.3721	3.7774	5.4960	4.4019	0.8475	-0.2387	0.2360	0.6271
Hsp90ab1	_LGIHEDSTNRR_.3	#N/A	3.3741	4.1303	3.1077	4.2853	4.0186	4.1068	4.0000	3.8332	3.4818	4.2523	0.9860	-0.0204	0.8474	0.0719
Hsp90ab1	_LGLGIDEDEVTAEEPSAAVPDEIPPLEGDEDASR_.3	#N/A	3.3988	4.1551	3.7579	4.2606	4.0434	4.0821	3.9752	3.8579	3.5065	4.2276	1.0149	0.0213	0.7808	0.1075
Dmd	_SDSS[Phospho (STY)]QPMLLR_.2	P11531_S3617_M1_Dmd	3.2966	3.9097	3.9834	4.4861	3.7630	4.1863	4.2923	3.6405	5.3002	3.8370	0.9227	-0.1161	0.3828	0.4170
Dmd	_NAPGKPMREDT[Phospho (STY)]M_.2	P11531_T3677_M1_Dmd	5.9910	4.4185	4.3001	3.3019	4.2398	3.4897	4.5174	3.3158	2.9643	3.2689	1.2200	0.2869	0.2017	0.6953
L1cam	_SLES[Phospho (STY)]DNEEK_.2	P11627_S1184_M1_L1cam	10.9620	10.6834	10.7251	11.0524	9.8418	10.3838	11.1120	11.0151	10.7263	10.6849	0.9746	-0.0371	0.2841	0.5466
L1cam	_EKEAAGGNDSSGAT[Phospho (STY)]SPINPAVALE_.2	P11627_T1250_M1_L1cam	3.1244	3.9649	4.3589	3.9027	5.4178	6.4656	6.7730	3.1047	3.4038	3.5799	1.0768	0.1067	0.7328	0.1350
L1cam	_TLQLLNVGEEDDGEYTC[Carbamidomethyl (C)]LAENSLGSAR_.3	#N/A	5.0994	5.1873	5.6325	6.4896	5.1466	5.7921	5.5933	5.0052	5.2164	5.5236	1.0419	0.0592	0.4708	0.3272
L1cam	_AQLLVVGSPGPVPHLELSDR_.3	#N/A	5.2399	4.7848	4.1237	5.4899	5.2509	3.0908	5.4625	5.5246	5.6222	5.0350	0.8618	-0.2146	0.1545	0.8112
L1cam	_EAAGGNDSSGATS[Phospho (STY)]PINPAVALE_.2	#N/A	8.0775	7.6923	8.2157	8.2066	8.1433	7.8783	8.5079	9.3903	8.4839	8.1691	0.9303	-0.1042	0.0325	1.4880
L1cam	_DETFGEYRS[Phospho (STY)]LES[Phospho (STY)]DNEEK_.2	#N/A	3.4404	3.9901	4.0656	4.1555	4.4092	3.8240	4.3038	5.3010	4.8544	5.3146	0.8053	-0.3125	0.0051	2.2920
L1cam	_PM[Oxidation (M)]KDETFGEY[Phospho (STY)]RSLES[Phospho (STY)]DNEEK_.3	#N/A	3.6551	5.0085	4.1242	4.1944	3.9375	4.6743	4.3858	5.1881	5.3823	5.0330	0.8536	-0.2284	0.0432	1.3648
L1cam	_DKEDTQVDS[Phospho (STY)]EARPMK_.3	P11627_S1166_M1_L1cam	3.9955	3.3469	3.7292	3.2729	3.1682	3.0604	3.4212	3.7282	4.0273	4.4511	0.8776	-0.1883	0.0950	1.0225
L1cam	_LVALQGQSLILEC[Carbamidomethyl (C)]IAEGFPTPTIK_.3	#N/A	3.2137	4.0189	4.5580	4.1018	3.8402	3.8893	4.2500	4.4002	3.1984	3.7791	1.0077	0.0110	0.9250	0.0339
Camk2a	_ITQYLDAGGIPR_.2	#N/A	11.3658	11.3895	11.7050	11.3266	11.1842	11.2413	11.6615	11.4671	11.3420	11.0616	0.9987	-0.0018	0.9189	0.0367
Camk2a	_HPWIS[Phospho (STY)]HR_.2	P11798_S272_M1_Camk2a	9.4177	9.5061	9.8567	9.5111	9.5300	9.3094	10.1192	10.0538	9.8790	9.4965	0.9631	-0.0543	0.0357	1.4470
Camk2a	_FTEEYQLFEELGK_.2	#N/A	9.6980	9.4251	9.9056	9.3540	9.0811	9.4800	9.7517	9.3526	9.6240	9.5852	0.9908	-0.0133	0.5976	0.2236
Camk2a	_VLAGQEYAAK_.2	#N/A	9.4268	9.6110	9.5658	9.2668	9.5292	9.2556	9.4423	9.9343	9.4859	9.3417	0.9886	-0.0165	0.4276	0.3690
Camk2a	_WQIVHFHR_.2	#N/A	8.1442	8.9350	9.0479	8.7906	8.0121	8.9507	9.1627	8.7151	8.6775	8.1720	0.9960	-0.0058	0.9034	0.0441
Camk2a	_FYFENLWSR_.2	#N/A	8.6949	9.2476	8.9415	9.1934	8.5280	8.6339	9.3121	9.2052	9.1246	8.7435	0.9755	-0.0358	0.2561	0.5915
Camk2a	_ITAAEALK_.2	#N/A	6.7985	6.8978	6.8775	5.8309	6.9010	6.9763	4.7187	7.0012	7.0061	7.1950	1.0360	0.0510	0.6631	0.1784
Camk2a	_ESSES[Phospho (STY)]TNT[Phospho (STY)]T[Phospho (STY)]IEDEDTKVR_.2	#N/A	10.7444	11.1939	4.0298	8.7838	7.8384	11.1742	11.3703	11.2557	10.8753	8.2886	0.8577	-0.2215	0.3600	0.4437
Camk2a	_M[Oxidation (M)]C[Carbamidomethyl (C)]DPGM[Oxidation (M)]TAFEPEALGNLVEGLDFHR_.3	#N/A	4.7136	4.0016	5.2332	4.0225	4.5766	4.3498	4.5108	4.3319	4.4467	4.3569	1.0162	0.0231	0.7740	0.1112
Camk2a	_ESS[Phospho (STY)]ESTNTTIEDEDTK_.2	#N/A	11.6467	11.3086	11.2422	11.6532	10.6939	10.9227	11.8362	11.1300	11.4098	11.5414	0.9795	-0.0298	0.3332	0.4773
Camk2a	_NFS[Phospho (STY)]GGKSGGNK_.2	P11798_S314_M1_Camk2a	4.0276	3.3319	6.1618	3.7162	3.1533	3.0754	5.8706	6.2578	6.1020	3.6832	0.7139	-0.4862	0.0732	1.1356
Camk2a	_[Acetyl (Protein N-term)]ATITC[Carbamidomethyl (C)]TR_.2	#N/A	4.7559	5.2506	3.1588	4.8998	4.8019	3.9909	3.9516	4.2986	4.9206	3.8807	1.0501	0.0705	0.6358	0.1966
Camk2a	_S[Phospho (STY)]TVASC[Carbamidomethyl (C)]M[Oxidation (M)]HR_.2	#N/A	9.4275	8.9638	9.0118	10.0124	10.0001	8.7256	9.7372	9.1557	9.0310	9.1095	1.0106	0.0153	0.7581	0.1203
Camk2a	_NDGVKES[Phospho (STY)]SESTNTTIEDEDTK_.2	#N/A	4.8755	4.9482	3.9482	4.2789	3.8808	5.4371	3.8263	4.4357	5.1514	5.0055	0.9906	-0.0136	0.9159	0.0381
Camk2a	_VTEQLIEAISNGDFESYTK_.2	#N/A	8.8556	8.8936	9.7279	9.7064	8.3062	8.9490	9.3203	8.8961	8.7650	9.1671	1.0040	0.0057	0.9068	0.0425
Camk2a	_QETVDC[Carbamidomethyl (C)]LKK_.2	#N/A	6.0396	4.7029	4.9497	5.5850	5.2164	5.3279	5.7427	5.2428	5.0493	4.7297	1.0217	0.0309	0.7117	0.1477
Camk2a	_QET[Phospho (STY)]VDC[Carbamidomethyl (C)]LK_.2	#N/A	5.7957	4.1774	3.1548	4.1994	4.2538	4.0597	4.3477	4.3026	3.1008	5.4506	0.9937	-0.0091	0.9639	0.0160
Camk2a	_SGAPSVLPH_.2	#N/A	7.9322	7.8814	7.7404	7.4907	7.6761	7.1834	7.6896	8.1479	7.3219	7.4230	1.0007	0.0010	0.9805	0.0086
Camk2a	_NDGVKESS[Phospho (STY)]ESTNTTIEDEDTKVR_.3	#N/A	3.8349	5.4491	4.1534	3.7867	4.9340	5.8699	5.9084	6.4684	5.9583	3.6687	0.8492	-0.2358	0.2485	0.6047
Camk2a	_KNDGVKESS[Phospho (STY)]ESTNTTIEDEDTK_.3	#N/A	3.0622	3.7152	3.8227	3.8556	3.5644	3.7888	4.0512	3.6168	3.7163	3.8930	0.9517	-0.0714	0.3106	0.5078
Gas2	_VDGKTS[Phospho (STY)]PVQSK_.2	P11862_S283_M1_Gas2	5.9206	6.5858	4.2279	5.9887	4.1703	3.5592	3.9200	3.2295	3.5285	6.1038	1.2097	0.2747	0.3119	0.5060
Tcp1	_SLLVIPNTLAVNAAQDSTDLVAK_.2	#N/A	6.8380	7.2895	7.6982	6.8704	6.9169	7.0460	7.3624	7.5760	7.0352	7.2750	0.9723	-0.0405	0.3208	0.4938
Tcp1	_SSFGPVGLDK_.2	#N/A	6.3435	6.6746	6.7373	6.2344	6.4437	6.1858	5.5396	6.7023	6.5137	5.9826	1.0407	0.0576	0.3217	0.4926
Tcp1	_LLEVEHPAAK_.2	#N/A	6.3482	6.7906	6.2102	5.7418	6.4907	5.9838	6.3860	6.4953	6.2554	6.0638	0.9938	-0.0090	0.8517	0.0697
Tcp1	_ILATGANVILTTGGIDDM[Oxidation (M)]YLK_.2	#N/A	5.1080	3.9598	5.3065	4.1793	4.3517	4.4927	3.2476	4.6783	3.9624	5.1205	1.0739	0.1028	0.4792	0.3195
Tcp1	_YINENLIINTDELGR_.2	#N/A	6.5748	6.1213	5.8271	6.4962	5.6285	6.0784	6.5614	5.9720	6.3042	6.4058	0.9699	-0.0441	0.3970	0.4012
Tcp1	_FATEAAITILR_.2	#N/A	5.5849	5.3025	5.3308	5.6606	5.2329	4.7072	5.8748	3.2712	5.5663	5.4675	1.0512	0.0720	0.6214	0.2066
Tcp1	_IC[Carbamidomethyl (C)]DDELILIK_.2	#N/A	4.9025	5.2268	3.8500	4.6902	3.1045	4.7906	4.6006	4.9477	3.9008	4.2439	1.0009	0.0014	0.9927	0.0032
Tcp1	_DDKHGS[Phospho (STY)]YENAVHSGALDD_.3	#N/A	6.6498	6.6482	7.0223	6.6983	6.1636	6.6753	6.2272	6.5932	6.8413	6.5311	1.0145	0.0207	0.5973	0.2238
Tcp1	_M[Oxidation (M)]LVDDIGDVTITNDGATILK_.2	#N/A	5.7456	5.9348	6.0346	6.1892	4.8331	5.3593	5.3880	5.2804	5.3058	5.7087	1.0483	0.0681	0.3588	0.4451
Tcp1	_HGSYENAVHS[Phospho (STY)]GALDD_.2	#N/A	5.5139	4.6929	4.6052	4.7913	4.0572	4.7954	5.1356	4.9606	4.3432	5.0187	0.9749	-0.0366	0.6712	0.1731
Tcp1	_DC[Carbamidomethyl (C)]LINAAK_.2	#N/A	4.2179	4.3879	3.5156	4.6497	5.1969	4.3477	3.2076	4.3847	0.8686	2.1606	1.6517	0.7240	0.0297	1.5274

Tcp1	_SLHDALC[Carbamidomethyl (C)]VVK_.2	#N/A	3.5544	5.3074	4.7349	5.4118	4.6042	4.4880	4.5019	4.9412	5.0591	4.5190	0.9849	-0.0220	0.8468	0.0722
Tcp1	_IAC[Carbamidomethyl (C)]LDFSLQK_.2	#N/A	3.2675	4.0727	3.9543	4.5771	3.8940	3.8355	4.1963	3.6546	4.0792	3.7253	1.0050	0.0072	0.9365	0.0285
Tcp1	_SQNVM[Oxidation (M)]AAASIANIVK_.2	#N/A	2.9584	3.0522	4.1929	3.7367	3.1736	3.0217	3.8849	3.6601	4.0016	3.4139	0.8973	-0.1564	0.1986	0.7021
App	_VES[Phospho (STY)]LEQEAANER_.2	P12023_S441_M1_App	8.1671	7.5514	7.6073	7.9822	7.3445	7.6179	8.0805	7.1502	7.4758	7.6948	1.0147	0.0210	0.6243	0.2046
App	_C[Carbamidomethyl (C)]LVGEFVSDALLVPDK_.2	#N/A	3.9730	4.2591	3.1299	4.4959	4.2453	3.4541	3.3472	2.9851	4.1345	4.6274	1.0405	0.0572	0.7121	0.1475
App	_QYTSIHHGVVEVDAAVT[Phospho (STY)]PEER_.3	P12023_T743_M1_App	3.5291	3.6356	5.4104	3.2177	3.5854	3.0392	4.4332	3.3999	3.0485	3.1847	1.0625	0.0874	0.6726	0.1722
Prkar2a	_TDEHVIDQGDDGDNFYVIER_.3	#N/A	8.0087	8.0293	8.4588	8.8261	7.3057	8.2420	8.1585	7.9835	8.1428	8.4022	0.9967	-0.0047	0.9237	0.0345
Prkar2a	_SVGQYDNR_.2	#N/A	6.1778	6.8166	6.4968	6.1467	6.8413	6.7032	6.2549	6.5133	6.3849	6.3260	1.0252	0.0359	0.3563	0.4482
Prkar2a	_AATIIATSEGSLWGLDR_.2	#N/A	6.0909	5.9245	6.9796	6.0598	5.1132	6.3471	6.2576	5.9430	5.3467	6.4489	1.0145	0.0207	0.8173	0.0876
Prkar2a	_GTYDILVTK_.2	#N/A	4.7858	5.1464	5.2874	5.5195	5.1815	4.9405	4.3325	4.3432	5.0981	5.4229	1.0718	0.1000	0.2119	0.6739
Prkar2a	_M[Oxidation (M)]FGS[Phospho (STY)]NLDLM[Oxidation (M)]DPGQ_.2	#N/A	7.4541	7.3745	7.9980	7.6911	7.3691	8.1457	7.8843	7.8012	8.0842	7.9360	0.9679	-0.0471	0.1886	0.7245
Prkar2a	_NISHYEEQLVK_.2	#N/A	4.2720	4.6961	5.2746	5.0868	4.6913	5.5566	5.5374	5.0944	5.3111	5.2581	0.9301	-0.1046	0.1734	0.7610
Prkar2a	_RVS[Phospho (STY)]VC[Carbamidomethyl (C)]AETFNPDEEEEDNDPR_.3	P12367_S96_M1_Prkar2a	3.9231	5.9967	4.0352	8.4155	5.7573	6.1041	5.6476	7.6691	6.4684	5.2434	0.9118	-0.1332	0.5737	0.2413
Prkar2a	_VSVC[Carbamidomethyl (C)]AETFNPDEEEEDNDPR_.2	#N/A	4.3030	3.5584	4.0366	3.3564	3.4467	3.1779	3.0711	3.2612	4.4107	3.3234	1.0369	0.0523	0.7003	0.1547
Pfkl	_RTLS[Phospho (STY)]IDKGF_.2	#N/A	12.1092	11.8140	12.6657	11.9813	10.3404	11.9490	12.4709	12.0317	11.9545	11.8927	0.9770	-0.0335	0.5181	0.2856
Pfkl	_RTLS[Phospho (STY)]IDK_.2	#N/A	9.8842	9.2319	9.5396	9.9207	8.0999	9.2557	9.8136	9.7032	9.5515	9.3290	0.9711	-0.0423	0.4518	0.3451
Pfkl	_GGTPSAFDR_.2	#N/A	8.7245	7.2927	8.9247	8.8329	8.2298	7.3165	8.8628	7.6984	8.7476	8.7030	0.9667	-0.0488	0.5364	0.2705
Pfkl	_VTVLGHVQR_.2	#N/A	7.0735	7.4872	7.4155	7.4877	7.2232	7.0933	7.0159	7.7744	7.6381	7.2914	0.9821	-0.0261	0.4483	0.3485
Pfkl	_TNVLGHLQQGGAPTPFDR_.3	#N/A	6.7097	6.0568	6.6097	6.7285	5.7787	6.2733	6.5357	6.4647	6.3266	6.4186	0.9880	-0.0173	0.7128	0.1470
Pfkl	_SFENNWK_.2	#N/A	6.6142	5.5802	5.4941	6.1951	4.3264	5.5412	6.1454	6.6430	5.5709	5.2647	0.9525	-0.0703	0.5622	0.2501
Pfkl	_VFANAPDSAC[Carbamidomethyl (C)]VIGLR_.2	#N/A	5.3862	4.8141	5.4163	5.2617	4.9962	4.1988	5.6023	4.7355	5.7863	5.2045	0.9400	-0.0893	0.3160	0.5003
Pfkl	_TLS[Phospho (STY)]IDKGF_.2	#N/A	2.9462	7.9091	8.6184	7.8812	3.5908	8.3022	3.8728	8.1877	8.8272	8.8323	0.8804	-0.1838	0.5964	0.2245
Pfkl	_TFVLEVM[Oxidation (M)]GR_.2	#N/A	5.6959	5.4244	5.9014	6.1805	5.2257	5.1691	4.0668	6.3233	5.6981	5.4214	1.0413	0.0584	0.6176	0.2093
Pfkl	_NEWGSLLEELVK_.2	#N/A	4.5939	3.9002	4.3624	3.5278	3.8391	2.5949	3.7968	3.9859	3.6517	3.3258	1.0306	0.0435	0.7716	0.1126
Pfkl	_AIGVLTSGGDAQGM[Oxidation (M)]NAAVR_.2	#N/A	5.0702	5.1433	5.5644	4.6650	5.2845	5.4174	5.4448	5.9534	5.0245	5.4598	0.9488	-0.0757	0.2383	0.6228
Pfkl	_IM[Oxidation (M)]EVIDAITTTAQSHQR_.2	#N/A	3.3717	4.7914	5.0904	4.0946	3.6501	4.3315	5.4716	4.7313	4.1791	4.3411	0.9019	-0.1489	0.2899	0.5377
Pfkl	_TGISEGHTVYIVHDGFEGLAK_.3	#N/A	4.2997	2.9265	3.4856	3.9883	2.8148	3.8098	4.4927	4.1302	3.7788	3.9553	0.8691	-0.2023	0.1387	0.8578
Chrm1	_SQPGAEGSPES[Phospho (STY)]PPGR_.2	P12657_S254_M1_Chrm1	10.4920	10.5310	11.3116	10.3285	9.1056	10.1605	11.0480	10.8792	10.5476	10.2301	0.9668	-0.0487	0.3922	0.4065
Chrm1	_RPGS[Phospho (STY)]VHRT[Phospho (STY)]PSR_.3	#N/A	3.4453	5.6383	5.2180	5.2769	4.0821	5.1741	3.9474	3.9123	6.1039	4.1997	1.0583	0.0818	0.6703	0.1738
Calb1	_TDLALILSAGDN_.2	#N/A	8.7981	8.9861	8.8586	9.6085	9.0622	8.9023	9.1372	9.1235	9.2474	8.7702	0.9963	-0.0054	0.8496	0.0708
Calb1	_SFVDQYGQR_.2	#N/A	7.0142	6.5376	6.1339	6.7859	7.5184	7.0172	7.1265	6.8886	7.0010	7.0970	0.9724	-0.0403	0.4512	0.3456
Calb1	_ELQNLIQELLQAR_.2	#N/A	4.9667	5.3232	4.0094	4.8389	4.6429	4.8099	5.4306	5.0361	5.3158	4.6610	0.9324	-0.1010	0.2203	0.6570
Calb1	_NIMALSDGGK_.2	#N/A	6.0340	3.2526	3.7308	5.9674	3.1409	3.4837	3.3769	3.7266	4.0256	2.9519	1.2125	0.2780	0.3270	0.4855
Cox5a	_LNDFASAVR_.2	#N/A	9.7093	10.0385	9.9978	10.0590	10.6479	9.8809	9.8108	9.9615	9.9334	9.9027	1.0155	0.0222	0.3774	0.4232
Cox5a	_ILEVVKDK_.2	#N/A	8.5839	9.4821	9.8337	9.5540	9.8052	9.3958	9.0807	9.0334	9.2879	9.0466	1.0362	0.0514	0.2013	0.6962
Cox5a	_GM[Oxidation (M)]NTLVGYDLVPEPK_.2	#N/A	8.2898	8.4947	9.3745	8.9717	8.7289	9.3975	8.3506	8.1753	8.1951	8.1563	1.0799	0.1109	0.0235	1.6282
Cox5a	_RLNDFASAVR_.2	#N/A	6.0497	7.0026	6.8493	6.8670	7.2277	7.1299	6.6652	6.3038	6.5603	6.2698	1.0627	0.0878	0.1138	0.9440
Nr4a1;Nr4a2;Nr4a3	_TDS[Phospho (STY)]LKGR_.2	P12813_S344_M1_Nr4a1;Nr4a2;Nr4a3	3.2823	3.9755	3.5383	4.1452	3.9185	3.9327	6.0235	4.3569	6.0871	3.8225	0.7489	-0.4172	0.0305	1.5158
Pdcd6	_LSDQFHDILIR_.2	#N/A	4.3260	4.5421	4.8325	3.4830	4.5925	4.0041	4.6049	3.9490	4.5551	3.5740	1.0302	0.0429	0.7012	0.1542
Pdcd6	_YITDWQNVFR_.2	#N/A	3.6039	5.1036	3.3376	6.0387	4.6912	4.1697	5.1225	4.1198	4.6947	4.0595	0.9981	-0.0027	0.9883	0.0051
Prkar1b	_NVLFSHLDDNER_.3	#N/A	5.6542	5.0649	4.1594	5.1826	5.0051	5.4071	5.3672	5.0985	4.8945	5.0648	0.9946	-0.0078	0.9221	0.0352
Prkar1b	_S[Phospho (STY)]PNEEYVEVGR_.2	P12849_S307_M1_Prkar1b	6.3205	3.1103	3.9658	6.7493	3.3759	3.2488	3.6578	6.6572	3.7907	3.1869	1.0321	0.0455	0.8975	0.0470
Prkar1b	_[Acetyl (Protein N-term)]ASPS[Phospho (STY)]C[Carbamidomethyl (C)]FHSEDEDSLK_.2	P12849_S5_M1_Prkar1b	3.6830	3.8140	3.4167	3.9875	3.7023	3.8090	4.6989	4.1422	3.7908	3.9545	0.9008	-0.1506	0.0558	1.2536
Prkar1b	_SNSQC[Carbamidomethyl (C)]DSHDEEIS[Phospho (STY)]PTPPNPVVK_.3	P12849_S83_M1_Prkar1b	6.1782	6.0453	7.0199	7.7367	6.5839	6.9565	6.7481	7.1789	7.2956	6.0643	0.9900	-0.0145	0.8642	0.0634
Prkar1b	_GGVSAEVYTEEDAVSYVR_.2	#N/A	5.6248	5.7812	6.6624	4.1144	3.8276	3.9019	4.2627	4.3876	3.1858	3.7917	1.2760	0.3517	0.1337	0.8738
Prkar1b	_IVVQGEPGDDFYIITEGTASVLQR_.3	#N/A	3.1218	3.9270	3.8087	4.1936	3.7484	3.9811	4.3419	4.3084	3.1066	3.8709	0.9718	-0.0413	0.7175	0.1442
Cntn1	_NDGGIYTC[Carbamidomethyl (C)]FAENNR_.2	#N/A	10.9907	10.9571	11.6426	12.1997	10.5209	11.0766	11.4403	11.5923	11.3209	11.1896	0.9864	-0.0197	0.6326	0.1989
Cntn1	_M[Oxidation (M)]NNGDVDLTNDR_.2	#N/A	9.4027	9.1579	9.5324	9.8017	8.6980	9.0583	9.6648	9.2917	9.2929	9.3142	0.9877	-0.0179	0.5981	0.2233
Cntn1	_GPPGPPGGLR_.2	#N/A	8.4274	8.8726	8.7530	8.4735	8.7523	8.3377	8.4852	8.9258	8.4710	8.5522	0.9993	-0.0010	0.9677	0.0143
Cntn1	_IKTDGAAPNVAPSDVGGGGGTNR_.3	#N/A	8.2104	8.3889	8.7696	8.4615	8.0797	8.3712	8.4457	8.5788	8.3550	8.3169	0.9948	-0.0075	0.7419	0.1297
Cntn1	_VTVTNPDTGR_.2	#N/A	7.8576	7.9016	8.2132	8.1388	8.1793	7.9283	7.9556	8.0781	8.0052	8.3109	0.9937	-0.0091	0.6293	0.2011
Cntn1	_FIPLIPIPER_.2	#N/A	8.0857	8.2184	8.1130	7.7033	7.8052	7.9485	8.2786	8.3930	7.7992	7.9973	0.9830	-0.0247	0.3735	0.4277
Cntn1	_IVESYQIR_.2	#N/A	8.0655	7.9931	8.3243	8.0784	7.8687	8.1256	7.7935	8.2681	8.0513	7.7395	1.0142	0.0203	0.3882	0.4109
Cntn1	_ELTITWAPLSR_.2	#N/A	7.8144	7.7188	7.7034	7.8421	7.4889	7.6880	8.0038	7.8831	8.0373	7.7346	0.9740	-0.0379	0.0395	1.4035
Cntn1	_VQVTSQEYSAR_.2	#N/A	7.1804	7.1429	7.6999	7.0355	7.3626	7.2845	7.4002	7.1168	7.4977	7.5692	0.9849	-0.0219	0.4558	0.3412
Cntn1	_TDGAAPNVAPSDVGGGGGTNR_.2	#N/A	7.1575	7.2039	7.2722	7.5854	6.7240	7.0598	7.9326	7.3278	7.4712	6.9325	0.9664	-0.0493	0.2852	0.5449
Cntn1	_DGEYVVEVR_.2	#N/A	7.4674	7.3714	7.1000	7.5735	6.1771	7.0405	7.0966	7.6324	7.0204	7.4158	0.9767	-0.0340	0.5643	0.2485
Cntn1	_GM[Oxidation (M)]VLLC[Carbamidomethyl (C)]DPPYHFPDDLSYR_.3	#N/A	6.3780	6.5704	6.6114	6.5613	6.5767	6.7481	6.8480	7.1270	6.7675	6.4542	0.9669	-0.0485	0.1098	0.9594
Cntn1	_HSIEVPIPR_.2	#N/A	6.3071	6.9496	6.4663	6.8467	6.4439	6.1775	6.6549	6.9767	6.3149	6.9008	0.9732	-0.0392	0.3825	0.4174
Cntn1	_ILYRPDGQHDGK_.3	#N/A	5.7812	5.2752	5.6455	5.5868	6.4695	5.7671	5.2328	6.0922	6.0031	5.9692	0.9880	-0.0175	0.7909	0.1019
Cntn1	_AHSDGGDGVVSQVK_.2	#N/A	5.9148	5.4870	5.9396	6.2697	5.1283	6.0515	5.9169	5.9062	5.7183	5.8481	0.9916	-0.0121	0.8262	0.0829
Cntn1	_VVATNTLGTGEPSIPSNR_.2	#N/A	8.2503	8.6859	4.2217	8.6353	8.8024	8.8375	9.1075	9.1306	8.2501	8.4013	0.9063	-0.1419	0.4125	0.3846
Cntn1	_ILALAPTFEM[Oxidation (M)]NPM[Oxidation (M)]K_.2	#N/A	6.7289	7.6195	7.2958	6.3580	6.7698	7.5693	7.3983	7.8216	7.5162	7.1297	0.9451	-0.0814	0.1888	0.7239
Cntn1	_GSDNHSPISK_.2	#N/A	5.0918	4.4350	5.5019	5.5019	5.6670	5.5610	5.6141	5.5766	5.5388	5.1027	0.9698	-0.0443	0.5355	0.2712
Cntn1	_STEATLSFGYLDPFPPEERPEVK_.3	#N/A	3.8056	5.3879	4.4598	5.4900	5.1110	5.3453	5.0417	5.1345	4.5881	5.2290	0.9870	-0.0189	0.8602	0.0654
Cntn1	_VLEPM[Oxidation (M)]PSTAEISTSGAVLK_.2	#N/A	5.8971	6.3941	6.3855	6.2112	5.6800	5.8353	5.9096	6.0600	6.0482	5.9520	1.0125	0.0179	0.6483	0.1882
Cntn1	_TDPPIIEGNMESAK_.2	#N/A	7.6624	7.3219	3.6814	7.9116	7.0769	3.5331	3.4263	5.7747	4.0750	6.4148	1.2590	0.3323	0.3090	0.5100
Cntn1	_KVTVTNPDTGR_.2	#N/A	4.7227	3.4904	4.7277	3.5887	4.3742	3.8280	3.3034	5.0502	3.8473	3.5557	1.0464	0.0654	0.6735	0.1717
Cntn1	_IFNIQLEDEGLYEC[Carbamidomethyl (C)]EAENIR_.3	#N/A	4.1904	5.3642	3.3763	3.8790	2.9241	4.4890	5.3741	5.3554	4.1124	5.0841	0.8104	-0.3032	0.0941	1.0262
Cntn1	_FVSQTNGNLYIANVESSDR_.2	#N/A	3.5238	3.9711	3.9418	4.4445	3.8816	3.8479	4.1592	3.6740	3.3226	4.4115	1.0111	0.0160	0.8641	0.0634
Cntn1	_VLSSSEISVHWK_.2	#N/A	3.0162	3.8214	3.7030	4.2056	3.6427	3.7769	3.9203	3.9128	3.5614	4.1726	0.9493	-0.0751	0.3972	0.4010
Cntn1	_DAGVYYC[Carbamidomethyl (C)]LASNNYGM[Oxidation (M)]VR_.2	#N/A	5.3571	3.8876	5.3131	5.0717	3.9651	3.7644	4.2427	3.5904	4.8098	5.2553	1.0191	0.0272	0.8640	0.0635
Scg5	_TADDGC[Carbamidomethyl (C)]LENAPDT[Phospho (STY)]AEFSR_.2	P12961_T137_M1_Scg5	5.0742	7.0694	6.3490	7.6540	7.2538	7.0167	7.6390	6.6429	7.2109	7.0952	0.9425	-0.0854	0.4312	0.3654
Scg5	_KSVPHFS[Phospho (STY)]EEEKEAE_.3	#N/A	3.4860	3.4960	4.1728	3.1746	3.6284	3.6477	4.3902	3.5446	4.4950	3.1416	0.9250	-0.1125	0.3722	0.4292
Scg5	_SVPHFS[Phospho (STY)]EEEKEAE_.2	#N/A	8.3113	8.7724	9.4317	9.2486	9.3781	9.1437	9.3402	8.5717	9.3290	8.9794	0.9992	-0.0012	0.9781	0.0096
Scg5	_DFSEDQGYPDPPNPC[Carbamidomethyl (C)]PLGK_.2	#N/A	5.4455	5.1264	4.4147	5.4658	5.0669	4.3007	4.1067	4.6597	4.5141	3.6358	1.1752	0.2329	0.0461	1.3363
Rpl7a	_QTATQLLK_.2	#N/A	6.2318	2.7530	3.9113	2.5856	4.0386	2.6569	4.1120	3.1803	3.1837	2.3109	1.1562	0.2094	0.5353	0.2714
Rpl7a	_NFGIGQDIQPK_.2	#N/A	3.0774	4.1488	6.2038	4.2668	6.0807	6.2131	6.4692	6.2782	5.8406	6.1397	0.8086	-0.3066	0.1267	0.8972
Gsn	_HVVPNEVVVQR_.2	#N/A	5.8598	5.0557	5.0453	5.7889	5.8257	4.8610	5.7157	5.3978	5.1700	5.6536	0.9857	-0.0207	0.7685	0.1143

Gsn	_DSQEEEKTEALTSAK_.2	#N/A	3.9016	3.7455	3.7619	4.2646	3.3653	4.0861	3.5465	3.6494	3.5025	3.5036	1.0855	0.1184	0.0961	1.0171
Ncam1	_NPPEAATAPAS[Phospho (STY)]PK_.2	#N/A	14.4856	14.6933	15.3196	14.9451	14.0003	14.5969	15.1388	14.8191	14.6494	14.5802	0.9917	-0.0121	0.6309	0.2000
Ncam1	_GVTASSSS[Phospho (STY)]PASAPK_.2	P13595_S887_M1_Ncam1	11.8316	11.8577	12.4327	11.8849	10.9058	11.7371	12.3270	12.1094	11.9112	11.7712	0.9788	-0.0309	0.3710	0.4306
Ncam1	_FFLC[Carbamidomethyl (C)]QVAGDAK_.2	#N/A	8.4185	8.2449	8.1503	8.3059	7.9956	7.8098	8.3611	8.3258	8.2193	8.1089	0.9879	-0.0175	0.4363	0.3603
Ncam1	_GLGEISAATEFK_.2	#N/A	7.3871	7.3956	7.6197	7.1378	7.5977	7.7153	7.6062	8.0896	7.7557	7.3480	0.9709	-0.0426	0.2057	0.6868
Ncam1	_ALASEWKPEIR_.2	#N/A	7.8384	7.8091	7.9640	7.7550	7.6818	7.5475	8.1597	7.9811	7.6680	7.6645	0.9870	-0.0189	0.4215	0.3752
Ncam1	_NAPTPQEFK_.2	#N/A	7.8070	7.1829	7.3102	7.2631	7.2524	6.8732	7.3917	7.1686	6.9972	7.4540	1.0039	0.0057	0.8741	0.0584
Ncam1	_AGEQDASIHLK_.2	#N/A	6.8263	6.3889	6.0079	6.7383	6.0736	6.0235	6.0159	6.8906	6.2283	6.0969	1.0056	0.0080	0.8896	0.0508
Ncam1	_FIVLSNNYLQIR_.2	#N/A	6.1703	6.1429	6.7270	6.2452	5.6065	5.7830	7.0509	6.4998	6.3783	6.3370	0.9309	-0.1034	0.0933	1.0299
Ncam1	_NPPEAATAPAS[Phospho (STY)]PKSK_.2	#N/A	7.8079	4.3833	8.7271	3.7373	6.5547	3.0210	3.8856	7.9428	8.0172	3.4146	0.9811	-0.0275	0.9451	0.0245
Ncam1	_VEPYSSTAQVQFDEPEATGGVPILK_.3	#N/A	5.6676	5.3221	5.3243	4.3857	5.6651	4.7059	5.5096	5.0012	5.0575	5.3027	0.9925	-0.0109	0.8928	0.0492
Ncam1	_NVDKNDEAEYVC[Carbamidomethyl (C)]IAENK_.3	#N/A	7.5720	7.1956	8.0855	7.9179	6.8768	7.4354	7.3873	7.7670	7.6028	7.5391	0.9921	-0.0115	0.8062	0.0936
Ncam1	_AAHFVFR_.2	#N/A	5.0513	5.1920	4.2850	4.7868	4.7465	4.8390	5.0264	5.5937	5.2603	3.5633	0.9909	-0.0132	0.9122	0.0399
Ncam1	_AAFSKDES[Phospho (STY)]KEPIVEVR_.3	#N/A	12.1176	12.3327	12.9295	12.4540	11.2528	12.2115	12.8983	12.6033	12.4715	12.1947	0.9740	-0.0380	0.3152	0.5015
Ncam1	_GPDPEPTQPGTVKNPPEAATAPAS[Phospho (STY)]PK_.3	#N/A	4.2825	6.9623	9.4810	6.5673	3.4150	5.5754	5.7514	7.2089	3.7960	5.2131	1.1010	0.1389	0.6643	0.1776
Ncam1	_DES[Phospho (STY)]KEPIVEVR_.2	#N/A	11.7195	11.3057	11.8491	11.8338	10.2905	11.1652	12.0884	11.7328	11.5049	11.3910	0.9727	-0.0399	0.3593	0.4445
Ncam1	_EANM[Oxidation (M)]EGIVTIM[Oxidation (M)]GLKPETR_.3	#N/A	5.5518	5.1626	5.6422	4.4485	4.2907	5.1169	4.9487	5.6123	5.2332	5.1979	0.9595	-0.0597	0.5051	0.2967
Ncam1	_C[Carbamidomethyl (C)]VVTAEDGTQSEATVNVK_.2	#N/A	5.6600	4.2421	6.4683	6.9576	4.0634	3.6661	6.0267	6.0291	3.1407	3.0925	1.1321	0.1790	0.5495	0.2600
Ncam1	_LEGQM[Oxidation (M)]GEDGNSIK_.2	#N/A	6.0115	6.1106	6.4485	5.4111	6.0868	6.0554	6.3116	6.3178	6.0928	6.1783	0.9671	-0.0482	0.2822	0.5494
Ncam1	_ASPAPT[Phospho (STY)]PTPAGAASPLAAVAAPATDAPQAK_.3	P13595_T950_M1_Ncam1	3.1977	6.6296	7.2182	7.5930	4.2717	4.1037	6.3012	6.3111	5.8843	4.1722	0.9709	-0.0426	0.8771	0.0570
Ncam1	_DGEPIENEEEDDEKHIFSDDSSELTIR_.4	#N/A	9.3489	9.2300	9.2270	9.6144	7.9560	8.7647	9.4104	9.0731	8.9060	9.5342	0.9775	-0.0328	0.5385	0.2688
Ncam1	_SLGEESWHFK_.2	#N/A	6.9039	7.0277	6.9811	7.0163	6.3912	6.9608	7.0907	6.8716	6.8121	6.6044	1.0052	0.0075	0.8158	0.0884
Ncam1	_IGQESLEFILVQADTPSSPSIDR_.3	#N/A	3.6133	3.2854	3.3470	4.3916	4.2579	3.8675	4.5399	4.0370	3.1611	3.2279	1.0140	0.0200	0.8858	0.0527
Ncam1	_LPSGSDHVMLK_.2	#N/A	5.3566	3.0776	3.9985	6.5768	3.4085	3.3297	3.6905	3.4589	3.7580	3.2195	1.2151	0.2810	0.3188	0.4965
Ncam1	_TEEERT[Phospho (STY)]PNHDGGK_.2	P13595_T788_M1_Ncam1	4.3738	5.4776	3.5598	4.0624	3.4995	4.2300	3.7771	6.0242	3.7046	4.0294	0.9582	-0.0616	0.7558	0.1216
Gpd1	_LISEVIGER_.2	#N/A	7.0529	6.8044	7.2352	6.9406	6.8541	6.8188	7.4654	6.7761	7.4856	6.8954	0.9714	-0.0419	0.2636	0.5790
Gpd1	_DLM[Oxidation (M)]QTPNFR_.2	#N/A	5.8488	6.3995	3.3768	3.8794	6.2833	6.0777	3.5941	6.3719	6.1301	5.8284	0.9689	-0.0455	0.8453	0.0730
Dnmt1	_EADDDEEADDDVSEM[Oxidation (M)]PS[Phospho (STY)]PK_.2	#N/A	4.9678	5.1324	5.2684	4.6860	3.5058	5.4862	5.4532	4.9368	3.8604	5.1437	0.9985	-0.0022	0.9874	0.0055
Dnmt1	_S[Phospho (STY)]KSDSDTLSVETSPSSVATR_.3	P13864_S138_M1_Dnmt1	3.4241	4.2293	4.1109	4.6135	4.0506	3.6789	4.0397	3.5050	3.1535	3.0797	1.1665	0.2221	0.0706	1.1513
Atp1b1	_YNPNVLPVQC[Carbamidomethyl (C)]TGK_.2	#N/A	11.2504	11.1104	11.2579	11.0985	11.2067	10.7637	11.5628	11.2791	11.0470	10.8127	0.9946	-0.0079	0.7105	0.1484
Atp1b1	_SYEAYVLNIIR_.2	#N/A	10.0109	9.9030	10.1551	9.7996	9.6938	9.9999	10.3178	10.2031	10.1879	9.8478	0.9791	-0.0305	0.1052	0.9780
Atp1b1	_AYGENIGYSEK_.2	#N/A	9.3714	9.0092	9.4338	9.2609	9.2985	9.0197	9.3622	9.2801	9.1414	9.2668	0.9967	-0.0047	0.7647	0.1165
Atp1b1	_AKEEGSWKK_.2	#N/A	8.0231	7.7163	8.3165	7.6439	7.6385	7.9456	8.3243	8.0324	7.8428	7.6803	0.9888	-0.0163	0.6230	0.2055
Atp1b1	_EGKPC[Carbamidomethyl (C)]IIIK_.2	#N/A	8.5623	8.7434	8.8234	8.5780	8.0099	7.9630	8.8956	8.6707	8.2872	8.3593	0.9875	-0.0181	0.6405	0.1935
Atp1b1	_FIWNSEK_.2	#N/A	9.0251	8.5301	8.8529	8.4320	8.5908	8.3061	7.3607	9.0795	8.9238	8.7165	1.0121	0.0173	0.7703	0.1133
Atp1b1	_AKEEGSWK_.2	#N/A	8.0222	7.3543	7.6309	7.3665	7.8534	7.6375	7.5621	7.8777	7.8178	7.5228	0.9934	-0.0096	0.7460	0.1273
Atp1b1	_VAPPGLTQIPQIQK_.2	#N/A	11.8837	11.8213	12.1844	11.7684	11.7713	11.6749	12.1323	12.0421	11.8739	11.6381	0.9940	-0.0086	0.5853	0.2327
Atp1b1	_AYGENIGYSEKDR_.2	#N/A	8.6160	8.5204	9.0655	8.3705	8.5165	8.7796	8.8129	8.5983	8.6699	8.5476	0.9986	-0.0021	0.9283	0.0323
Atp1b1	_KFIWNSEK_.2	#N/A	3.5771	6.1425	9.8317	7.5159	6.1318	5.3388	7.0356	7.7947	6.3945	4.0326	1.0172	0.0246	0.9331	0.0301
Atp1b1	_YKDSAQKDDM[Oxidation (M)]IFEDC[Carbamidomethyl (C)]GNVPSEPK_.4	#N/A	8.7145	8.0866	8.9816	8.4826	7.9918	8.2577	8.2068	8.3198	8.4803	8.5900	1.0024	0.0034	0.9255	0.0336
Atp1b1	_DDM[Oxidation (M)]IFEDC[Carbamidomethyl (C)]GNVPSEPK_.2	#N/A	8.0954	7.7778	8.0021	8.0112	7.6165	7.9045	8.5497	8.1956	8.2338	7.9019	0.9612	-0.0571	0.0499	1.3020
Atp1b1	_DSAQKDDM[Oxidation (M)]IFEDC[Carbamidomethyl (C)]GNVPSEPK_.3	#N/A	7.8326	7.4584	7.4493	7.8003	6.6270	7.2915	8.0468	7.5916	7.4146	7.2955	0.9766	-0.0341	0.5130	0.2899
Atp1b1	_TEISFRPNDPK_.2	#N/A	9.2515	9.3572	9.5918	9.3748	9.2327	9.0708	9.5615	9.5621	9.2179	9.1058	0.9948	-0.0075	0.7154	0.1454
Atp1b1	_GDINHER_.2	#N/A	3.8678	3.3583	4.5546	3.5564	3.2466	3.3780	3.2711	3.0612	4.2106	3.5234	1.0409	0.0578	0.6636	0.1781
Rpl27a	_TGVAPIIDVVR_.2	#N/A	6.8326	6.8337	6.9771	6.7230	6.1506	6.2698	6.8503	6.9434	6.3163	6.7807	0.9864	-0.0198	0.6667	0.1761
Rpl27a	_LWTLVSEQTR_.2	#N/A	7.1542	6.2059	3.9082	6.2845	6.1137	6.6386	3.6003	6.5893	3.8482	3.1293	1.4099	0.4956	0.0695	1.1579
Rps16	_LLEPVLLLGK_.2	#N/A	6.4660	5.7999	5.9058	5.7363	4.7765	4.9246	5.0489	5.8675	5.4516	5.5065	1.0243	0.0346	0.7153	0.1455
Rps16	_DILIQYDR_.2	#N/A	5.6037	4.7532	4.3068	3.8672	5.5383	5.2850	4.6102	6.0532	6.7180	4.5945	0.8905	-0.1673	0.3091	0.5099
Rps16	_TATAVAHC[Carbamidomethyl (C)]K_.2	#N/A	3.9048	3.1603	3.6385	3.7545	4.9666	3.5760	3.4692	3.3090	4.0125	4.3604	1.0121	0.0173	0.9036	0.0440
Rps16	_EIKDILIQYDR_.3	#N/A	3.1311	3.9363	3.8179	4.1844	1.6732	3.9718	4.3326	4.3176	1.7905	4.2876	0.9376	-0.0929	0.7488	0.1256
Mdh1	_FVEGLPINDFSR_.2	#N/A	11.3614	11.5961	11.6733	11.7941	11.8228	11.5545	11.6586	11.6738	11.5928	11.5240	1.0018	0.0027	0.8200	0.0862
Mdh1	_GEFITTVQQR_.2	#N/A	11.2742	11.2827	11.3117	11.6146	11.3860	11.3178	11.4242	11.2811	11.3964	11.2692	1.0019	0.0028	0.7723	0.1122
Mdh1	_VIVVGNPANTNC[Carbamidomethyl (C)]LTASK_.2	#N/A	10.5741	10.6159	11.0203	10.9761	10.7061	10.7024	10.7616	10.5875	10.7376	10.5130	1.0109	0.0156	0.3092	0.5097
Mdh1	_ENFSC[Carbamidomethyl (C)]LTR_.2	#N/A	10.1176	10.2734	10.2120	10.4240	10.6208	10.1525	10.0934	10.2083	10.2490	10.1685	1.0118	0.0169	0.2669	0.5737
Mdh1	_DLDVAVLVGSM[Oxidation (M)]PR_.2	#N/A	8.6677	8.7537	8.7129	8.9201	9.0669	8.8433	8.9942	8.8852	9.0445	9.0143	0.9825	-0.0255	0.0870	1.0606
Mdh1	_LGVTADDVK_.2	#N/A	9.3682	9.4662	9.3521	9.4655	9.3577	9.0232	9.1343	9.4816	9.2782	9.4657	0.9999	-0.0002	0.9917	0.0036
Mdh1	_EVGVYEALKDDSWLK_.2	#N/A	7.2638	7.3138	6.7650	7.5839	7.0961	7.4042	7.3444	6.7439	7.2427	7.2845	1.0117	0.0168	0.6546	0.1840
Mdh1	_SQGTALEK_.2	#N/A	6.5648	6.7848	6.4461	6.4826	7.0867	6.7595	5.8279	7.0810	6.9265	6.7712	1.0054	0.0077	0.8917	0.0498
Mdh1	_ELTEEKETAFEFLSSA_.2	#N/A	10.2733	10.8215	12.1322	11.8609	10.6042	11.2522	11.0501	10.4525	10.5985	10.7679	1.0411	0.0581	0.2865	0.5428
Mdh1	_NVIIWGNHSSTQYPDVNHAK_.3	#N/A	8.8658	8.9106	9.2852	9.2794	9.0136	9.0839	9.2047	9.0647	9.2190	9.1017	0.9919	-0.0118	0.4615	0.3358
Mdh1	_KLS[Phospho (STY)]SAM[Oxidation (M)]SAAK_.2	#N/A	8.8550	8.7339	9.0991	9.6639	8.6541	8.4952	9.0496	8.8218	8.7256	8.6310	1.0125	0.0179	0.6383	0.1950
Mdh1	_EKM[Oxidation (M)]DLTAK_.2	#N/A	7.2593	6.0500	6.2699	7.2437	7.4428	6.6935	5.9456	7.7430	6.7035	6.4410	1.0570	0.0800	0.2952	0.5299
Mdh1	_IFKS[Phospho (STY)]QGTALEK_.2	P14152_S111_M1_Mdh1	4.2018	5.0566	5.8503	4.6145	5.1400	5.4081	3.2619	5.2683	5.0767	3.8575	1.1555	0.2086	0.1975	0.7045
Mdh1	_LSSAMSAAK_.2	#N/A	3.2823	4.0385	4.5168	4.3771	3.9268	4.1986	4.0918	3.7414	3.3900	4.3441	1.0424	0.0599	0.5678	0.2458
Mdh1	_VLVTGAAGQIAYSLLYSIGNGSVFGK_.3	#N/A	3.9853	3.2896	3.1712	3.6739	3.1110	3.1177	3.4785	4.4446	4.0932	3.6409	0.8664	-0.2068	0.0720	1.1425
Rpsa	_YVDIAIPC[Carbamidomethyl (C)]NNK_.2	#N/A	8.0825	8.3400	8.2805	8.6449	8.3379	8.1009	8.3141	7.8746	8.1428	8.2907	1.0174	0.0249	0.3107	0.5077
Rpsa	_FAAATGATPIAGR_.2	#N/A	7.8147	7.6106	7.5441	7.8354	8.2406	7.8185	7.7864	7.5769	7.9314	7.7735	1.0056	0.0081	0.7583	0.1201
Rpsa	_AIVAIENPADVSVISSR_.2	#N/A	6.7760	6.5509	6.4403	7.2649	7.0018	7.2211	6.8443	6.4026	6.9537	7.1451	1.0058	0.0083	0.8593	0.0659
Rpsa	_LLVVTDPR_.2	#N/A	6.2506	6.4955	6.5543	6.6643	6.4244	6.7633	5.5522	6.4278	6.1139	5.9763	1.0844	0.1169	0.0177	1.7517
Rpsa	_[Acetyl (Protein N-term)]SGALDVLQM[Oxidation (M)]KEEDVLK_.2	#N/A	4.1457	4.5458	5.0930	4.9924	4.4359	4.5322	3.8990	5.6293	4.3508	4.6704	0.9971	-0.0041	0.9701	0.0132
Rpsa	_ADHQPLTEASYVNLPTIALC[Carbamidomethyl (C)]NTDSPLR_.3	#N/A	3.0394	4.3374	3.7262	4.2761	4.1333	3.5409	4.4244	4.2259	3.9940	3.9534	0.9260	-0.1110	0.2899	0.5377
Calr	_EQFLDGDAWTNR_.2	#N/A	8.9715	8.9909	9.1193	9.1341	9.0806	9.0433	8.9070	8.8683	8.7568	9.1772	1.0145	0.0207	0.1372	0.8626
Calr	_LFPSGLDQK_.2	#N/A	8.4803	8.7245	8.9201	8.7750	9.1767	8.2338	8.3358	8.8192	8.5536	8.7963	1.0107	0.0153	0.6425	0.1921
Calr	_FYGDLEKDK_.2	#N/A	8.0327	8.4078	8.3271	8.4418	8.7601	8.1801	8.0728	8.2906	7.9447	8.2365	1.0273	0.0389	0.1549	0.8099
Calr	_QIDNPDYK_.2	#N/A	5.6708	5.7976	6.0446	5.6960	6.9379	6.1482	5.5641	5.6574	5.5589	6.2049	1.0527	0.0741	0.2974	0.5267
Calr	_FEPFSNK_.2	#N/A	7.1437	8.0076	6.6865	7.4029	8.2110	7.7161	7.7077	8.1855	7.6742	8.2081	0.9476	-0.0776	0.2182	0.6611
Calr	_IDDPTDSKPEDWDKPEHIPDPDAK_.4	#N/A	6.7414	6.6011	6.6540	7.1832	7.4477	7.1458	6.5320	6.4020	6.7685	7.1356	1.0377	0.0533	0.2777	0.5565
Calr	_DKGLQTSQDAR_.2	#N/A	5.4237	4.4036	4.8290	5.5309	5.9315	5.5656	5.0724	5.5166	5.5800	4.9824	0.9986	-0.0020	0.9821	0.0078
Calr	_VHVIFNYK_.2	#N/A	5.0266	4.2842	5.0760	5.6136	5.4179	4.1610	6.2454	5.6635	5.3006	5.1745	0.8810	-0.1828	0.0984	1.0072

Calr	_HEQNIDC[Carbamidomethyl (C)]GGGYVK_.2	#N/A	4.8502	4.8783	3.7365	5.1481	3.6762	5.4461	3.9539	4.8503	3.0564	4.6939	1.1169	0.1595	0.3601	0.4435
Calr	_IKDPDAAKPEDWDER_.3	#N/A	6.9780	6.8405	7.3185	7.1149	7.3075	6.9067	6.5173	6.6595	6.7424	6.9575	1.0534	0.0750	0.0223	1.6513
Calr	_LKEEEEDKK_.2	#N/A	2.7176	3.7432	3.3329	4.3775	3.5645	4.1650	4.5258	4.1245	4.4236	3.2949	0.8920	-0.1649	0.2735	0.5630
Calr	_DM[Oxidation (M)]HGDSEYNIM[Oxidation (M)]FGPDIC[Carbamidomethyl (C)]GPGTK_.3	#N/A	4.0766	3.3321	3.8103	3.5827	3.2204	3.4042	3.2974	3.0349	4.1843	3.5497	1.0155	0.0222	0.8362	0.0777
Atp1b2	_FLNVTPNVEVNVEC[Carbamidomethyl (C)]R_.2	#N/A	6.5686	6.3160	7.0398	6.6886	6.2585	6.3086	6.9758	6.7629	6.5698	6.0998	0.9891	-0.0158	0.7442	0.1283
Atp1b2	_SC[Carbamidomethyl (C)]GQVVEEWK_.2	#N/A	6.7889	5.7073	6.8396	6.0421	6.3421	5.7544	6.1574	6.1622	5.5885	6.3874	1.0283	0.0403	0.5655	0.2476
Atp1b2	_NDVC[Carbamidomethyl (C)]RPGR_.2	#N/A	5.6326	5.0413	5.8535	5.8861	6.0661	5.3933	6.2046	6.3636	6.3053	5.7128	0.9185	-0.1227	0.0564	1.2486
Atp1b2	_INAANIATDDERDK_.2	#N/A	6.0868	5.6102	5.4125	5.8674	5.5693	5.3827	5.9178	5.8288	5.2675	5.6004	1.0002	0.0003	0.9948	0.0023
Atp1b2	_EFVWNPR_.2	#N/A	5.8380	5.6424	5.0633	6.0062	5.1962	5.2352	5.4593	5.7209	5.9126	5.5188	0.9724	-0.0404	0.4849	0.3144
Atp1b2	_YYEQPDNGVLNYPK_.2	#N/A	5.7809	5.5147	4.4767	5.3950	5.0798	4.2387	6.3089	5.7365	5.0275	5.2012	0.9124	-0.1322	0.2417	0.6168
Atp1b2	_SC[Carbamidomethyl (C)]GQVVEEWKEFVWNPR_.3	#N/A	4.7777	4.7622	4.9048	5.6754	4.7455	3.6054	4.3800	4.6057	5.0288	5.1233	0.9918	-0.0119	0.9170	0.0376
Atp1b2	_INAANIATDDERDKFAGR_.3	#N/A	7.4658	7.8618	8.3632	7.8807	7.7582	7.6414	8.0474	7.8392	7.7767	7.6354	1.0005	0.0007	0.9826	0.0076
Atp1b2	_TQLGDC[Carbamidomethyl (C)]SGIGDPTHYGYSTGQPC[Carbamidomethyl (C)]VFIK_.3	#N/A	2.1850	2.7903	4.1985	3.6979	3.6085	3.0161	1.9409	3.2589	3.5580	3.4195	1.0673	0.0940	0.6762	0.1699
Hspb1	_QLS[Phospho (STY)]SGVSEIR_.2	P14602_S86_M1_Hspb1	9.7332	9.7293	10.3468	10.3867	9.8962	9.7502	9.9416	9.3811	9.8530	10.1176	1.0153	0.0219	0.4770	0.3215
Hspb1	_SPS[Phospho (STY)]WEPFR_.2	P14602_S15_M1_Hspb1	3.2813	4.0865	3.9681	9.2076	8.1388	3.8217	8.1890	8.5040	3.2963	8.3360	0.7650	-0.3865	0.3411	0.4671
Psmd3	_SVFPEQANNNEWAR_.2	#N/A	7.6173	3.4790	8.1695	8.2238	3.3003	7.5222	8.2326	8.0060	7.7414	7.3987	0.8140	-0.2970	0.2592	0.5864
Psmd3	_LQLDS[Phospho (STY)]PEDAEFIVAK_.2	P14685_S426_M1_Psmd3	3.8749	3.1792	4.5617	3.5635	3.0006	3.2281	3.2782	3.0541	4.2036	3.5305	1.0146	0.0209	0.8883	0.0514
Lmnb1	_[Acetyl (Protein N-term)]ATAT[Phospho (STY)]PVQQQR_.2	P14733_T5_M1_Lmnb1	9.0744	9.1895	9.6450	9.5265	8.6229	9.2631	9.0316	9.1152	8.9753	9.0700	1.0190	0.0272	0.3820	0.4179
Lmnb1	_ASAPATPLS[Phospho (STY)]PTR_.2	P14733_S24_M1_Lmnb1	7.6731	3.8588	8.6159	4.5568	7.7605	4.3784	4.2715	3.5616	7.9837	7.6899	1.0449	0.0634	0.8551	0.0680
Lmnb1	_LKLS[Phospho (STY)]PSPSSR_.2	P14733_S392_M1_Lmnb1	5.8655	7.7939	6.3181	8.1386	7.3940	8.2350	6.3755	7.9806	7.7792	6.0764	1.0337	0.0479	0.7161	0.1450
Lmnb1	_LSPS[Phospho (STY)]PSSR_.2	P14733_S394_M1_Lmnb1	8.1670	8.4956	8.8958	8.6967	8.4043	8.6057	8.6130	8.5251	8.5236	8.4715	1.0013	0.0018	0.9353	0.0290
Lmnb1	_NQNSWGTGEDVK_.2	#N/A	5.2635	5.2373	4.5941	6.2122	5.1856	3.1957	5.1914	5.6239	5.6821	5.5843	0.8963	-0.1579	0.3027	0.5190
Lmnb1	_AGSRAS[Phospho (STY)]APAT[Phospho (STY)]PLSPTR_.2	P14733_S17_M2_Lmnb1	5.1679	3.5589	4.0543	6.1436	4.8893	5.7954	6.0823	5.4049	5.5864	5.6499	0.8687	-0.2031	0.1886	0.7244
Anxa6	_SEIDLLNIR_.2	#N/A	7.1535	6.8085	6.1767	7.0488	6.5810	6.6635	6.4176	6.7540	6.6667	6.6931	1.0160	0.0228	0.5906	0.2287
Anxa6	_DLESDIIGDTSGHFQK_.2	#N/A	6.9760	6.4567	6.9366	6.9476	6.4198	6.7111	6.4767	6.6887	6.6381	5.9410	1.0474	0.0668	0.1416	0.8489
Anxa6	_TLIEILATR_.2	#N/A	6.2290	5.9763	3.4584	5.3028	6.1274	6.3567	6.1548	6.2472	6.4048	6.5209	0.8805	-0.1837	0.2175	0.6625
Anxa6	_SLEDALSSDTSGHFR_.2	#N/A	5.5102	5.1752	5.5779	5.2865	4.5119	5.3384	5.3151	5.0819	5.0102	4.5889	1.0469	0.0661	0.3365	0.4731
Anxa6	_M[Oxidation (M)]LVVLLQGTR_.2	#N/A	4.4152	4.5057	3.3513	4.9262	4.5872	5.2755	5.0189	5.4482	4.9530	5.4555	0.8642	-0.2106	0.0766	1.1155
Anxa6	_ENDDVVSEDLVQQDVQDLYEAGELK_.3	#N/A	4.8836	5.0910	4.0096	5.1613	4.7842	4.3168	4.4849	4.8738	3.1376	5.1396	1.0678	0.0946	0.4980	0.3027
Anxa6	_GIGTDEATIIDIVTHR_.2	#N/A	4.2319	3.7881	3.2048	4.4098	4.1021	3.6300	4.5458	3.8116	4.8733	3.9803	0.9051	-0.1438	0.2084	0.6811
Anxa6	_WGTDEAQFIYILGNR_.2	#N/A	3.2097	4.1137	3.9470	4.4497	3.8542	3.9787	4.5145	3.6688	3.3174	4.4167	0.9865	-0.0196	0.8664	0.0623
Anxa6	_ILISLATGNR_.2	#N/A	7.8677	3.9061	3.1699	7.3047	4.0808	4.0446	4.3628	3.9636	3.0857	3.8918	1.3231	0.4040	0.2676	0.5725
Anxa6	_AINEAYKEDYHK_.3	#N/A	3.3015	4.0409	4.5361	1.3350	3.2979	4.1407	4.2281	1.4614	2.6281	3.7571	1.1402	0.1893	0.5931	0.2269
Anxa6	_IAQGAMYR_.2	#N/A	4.2918	3.5961	3.4777	4.5246	3.4175	4.3120	3.1720	4.1381	4.2765	3.9474	1.0137	0.0196	0.8714	0.0598
Rplp0	_IIQLLDDYPK_.2	#N/A	6.2679	6.3717	6.2759	6.4460	6.2399	6.1327	6.1811	6.5698	6.5191	5.6862	1.0080	0.0115	0.7765	0.1099
Rplp0	_GHLENNPALEK_.2	#N/A	6.1489	5.7354	5.1604	6.1070	6.0379	6.3175	5.8701	5.8696	5.9047	6.2877	0.9891	-0.0158	0.7819	0.1068
Rplp0	_TSFFQALGITTK_.2	#N/A	5.4448	5.3850	5.6650	5.9602	5.2496	5.4363	5.1673	5.1632	5.3496	5.6985	1.0335	0.0475	0.3037	0.5176
Rplp0	_C[Carbamidomethyl (C)]FIVGADNVGSK_.2	#N/A	6.3767	5.9868	6.1486	5.2578	6.2625	4.2793	5.6414	6.5369	6.2378	6.1081	0.9683	-0.0465	0.6750	0.1707
Rplp0	_AFLADPSAFAAAAPAAAATTAAPAAAAAPAK_.3	#N/A	4.4200	4.2100	3.2865	4.0000	4.2166	3.5310	3.9831	3.7739	3.5558	4.4054	1.0037	0.0053	0.9582	0.0185
Rplp0	_EDLTEIR_.2	#N/A	3.8660	3.4765	3.5996	3.1434	3.5465	3.6327	3.2917	4.7049	2.7664	3.7412	0.9767	-0.0340	0.8131	0.0899
Rplp0	_AEAKEES[Phospho (STY)]EESDEDM[Oxidation (M)]GFGLFD_.2	P14869_S304_M1_Rplp0	3.5896	3.8443	3.8057	4.6806	4.6013	3.6589	4.0197	5.0995	3.8457	4.1067	0.9443	-0.0827	0.4948	0.3055
Map1b	_DLTGQVPT[Phospho (STY)]PPVK_.2	P14873_T527_M1_Map1b	12.2757	11.9935	12.8313	12.5115	11.6024	12.2548	12.7521	12.1885	12.1947	12.1992	0.9928	-0.0104	0.7234	0.1406
Map1b	_VLS[Phospho (STY)]PLRS[Phospho (STY)]PPLLGSESPYEDFLSADSK_.3	P14873_S1391_M2_Map1b	8.8964	10.3081	10.7741	11.1085	9.6624	9.6634	9.6722	10.3270	10.1461	9.5664	1.0142	0.0203	0.7578	0.1205
Map1b	_SKPLAAS[Phospho (STY)]PKPGALK_.2	#N/A	8.6550	9.1784	8.7576	9.9463	7.9924	8.4857	9.0553	9.2334	8.9513	8.4484	0.9903	-0.0140	0.8194	0.0865
Map1b	_T[Phospho (STY)]PEEGGYSYEISEK_.2	#N/A	8.9986	9.4120	8.7735	10.4057	8.6874	9.6490	9.7536	9.6586	9.6760	9.9204	0.9558	-0.0652	0.2327	0.6332
Map1b	_QPGIQS[Phospho (STY)]PSR_.2	P14873_S1073_M1_Map1b	9.6679	9.3046	9.1560	4.3304	8.6178	4.1519	9.6925	3.7881	9.7022	9.6350	0.9188	-0.1222	0.7142	0.1462
Map1b	_C[Carbamidomethyl (C)]YTTEKKS[Phospho (STY)]PSEAR_.3	#N/A	7.8678	7.9339	8.3850	8.2739	7.8845	7.9362	8.2421	8.0516	8.3165	7.9365	0.9890	-0.0160	0.5188	0.2850
Map1b	_SSYYVVSGNDPAAEEPSR_.2	#N/A	8.0553	8.1203	8.2187	8.6274	8.2173	8.6092	8.5211	7.9359	8.2798	8.4419	1.0016	0.0023	0.9368	0.0283
Map1b	_AVLDALLEGK_.2	#N/A	7.6175	7.8957	7.7077	7.9894	8.1216	7.9551	7.3654	7.9684	7.8689	8.0540	1.0086	0.0123	0.6768	0.1695
Map1b	_VLFPGNSTQYNILEGLEK_.2	#N/A	7.6792	7.7536	7.9972	7.5800	7.6374	7.3997	7.4554	7.7402	7.1829	7.4990	1.0275	0.0391	0.1686	0.7730
Map1b	_FSPEVPGQK_.2	#N/A	7.0067	7.2112	7.0680	7.1142	7.4870	7.3742	7.1520	7.4180	7.2102	7.2676	0.9929	-0.0103	0.6371	0.1958
Map1b	_T[Phospho (STY)]PEDGGYTC[Carbamidomethyl (C)]EITEK_.2	#N/A	6.7012	6.4639	6.0927	7.6984	5.7476	6.1844	7.3620	5.9907	6.6917	6.8319	0.9646	-0.0520	0.5802	0.2364
Map1b	_AIGNIELGIR_.2	#N/A	6.2462	6.4671	7.0768	6.3434	6.4045	6.4941	6.4562	6.5430	6.4187	6.1702	1.0169	0.0242	0.5242	0.2805
Map1b	_SVGNTIEPVILFQK_.2	#N/A	6.3566	6.1778	7.0093	6.2802	5.9901	6.7180	6.4786	6.2262	6.7390	6.2721	0.9989	-0.0016	0.9747	0.0111
Map1b	_DVSDERLS[Phospho (STY)]PAK_.2	#N/A	6.6513	6.0420	5.6271	6.8921	5.1792	6.4515	6.6587	6.0774	6.0933	5.8646	0.9677	-0.0474	0.6075	0.2164
Map1b	_TPEVSGYTYEK_.2	#N/A	6.4933	6.5537	6.1888	7.0734	6.1754	6.5197	6.6698	6.4601	6.3538	6.5449	0.9990	-0.0014	0.9716	0.0125
Map1b	_QGFPDRES[Phospho (STY)]PVSDLTSTGLYQDK_.3	#N/A	7.2808	3.7538	3.6354	7.9369	5.9041	6.4861	7.1060	3.9804	6.7300	6.4790	0.9603	-0.0584	0.8281	0.0819
Map1b	_TTEAAATAVGTAATTAAVVAAAGIAASGPVK_.3	#N/A	6.4822	6.3210	6.9696	7.4474	5.5118	6.0307	6.7291	6.0265	6.2896	7.2886	0.9813	-0.0272	0.7727	0.1120
Map1b	_SWDTNLIEC[Carbamidomethyl (C)]NLDQELK_.2	#N/A	6.2291	5.9465	5.6835	6.0304	5.7920	6.0957	5.8320	5.4237	6.1324	5.7170	1.0323	0.0459	0.2616	0.5823
Map1b	_SVNFSLTPNEIK_.2	#N/A	5.3644	5.6801	6.1713	5.7478	5.9105	5.5874	5.9822	5.9724	5.8455	6.0395	0.9637	-0.0533	0.1738	0.7599
Map1b	_DAYC[Carbamidomethyl (C)]S[Phospho (STY)]EEKELK_.2	P14873_S1225_M1_Map1b	3.2315	4.0367	3.9183	7.5432	3.8580	3.8715	4.2323	3.6975	3.3461	4.3880	1.1261	0.1714	0.5631	0.2494
Map1b	_SPSLSPSPPS[Phospho (STY)]PIEK_.2	#N/A	12.9357	13.2123	13.6997	13.6530	12.4118	13.3033	13.7045	13.1547	13.2568	13.3362	0.9880	-0.0174	0.5590	0.2526
Map1b	_SDIS[Phospho (STY)]PLTPR_.2	P14873_S1781_M1_Map1b	10.4422	10.7624	11.0569	10.9045	9.8772	10.7170	11.0507	10.6695	10.7258	10.7534	0.9840	-0.0233	0.4637	0.3337
Map1b	_S[Phospho (STY)]PPLLGSESPYEDFLSADSK_.2	#N/A	9.9112	9.3837	9.2556	10.2122	9.3883	9.6394	10.2503	9.7533	9.8567	9.6414	0.9753	-0.0360	0.2900	0.5376
Map1b	_TIKS[Phospho (STY)]PC[Carbamidomethyl (C)]DSGYSYETIEK_.2	#N/A	7.2396	7.4298	7.0504	8.3111	5.7061	7.7859	7.2707	7.6387	6.9819	7.7718	0.9782	-0.0319	0.7397	0.1310
Map1b	_ESVVS[Phospho (STY)]GDDR_.2	#N/A	8.4100	7.9562	7.9480	3.8635	3.5183	7.9233	4.0117	8.0880	8.3233	8.3407	0.9183	-0.1230	0.6916	0.1602
Map1b	_ADS[Phospho (STY)]RESLKPATK_.3	#N/A	6.1806	6.1521	3.7734	6.3628	4.9368	5.8130	6.2506	6.0121	5.8731	5.5645	0.9344	-0.0979	0.4793	0.3194
Map1b	_TIKT[Phospho (STY)]PEDGGYTC[Carbamidomethyl (C)]EITEK_.2	#N/A	4.8787	5.6877	6.2011	6.0016	5.4274	5.7225	6.1355	6.2425	5.4387	6.1387	0.9439	-0.0832	0.2625	0.5808
Map1b	_ASLTLFC[Carbamidomethyl (C)]PEEGDWK_.2	#N/A	6.0861	5.9545	6.3030	6.3328	6.0395	5.8429	6.6064	5.6414	5.9181	5.5521	1.0276	0.0393	0.4642	0.3333
Map1b	_KLGGDVS[Phospho (STY)]PTQIDVSQFGSFK_.3	#N/A	3.2198	7.1389	3.9066	8.4828	5.1346	4.2308	4.1240	7.3859	6.2759	4.2741	0.9705	-0.0432	0.8970	0.0472
Map1b	_THDVGGYYYEK_.2	#N/A	5.5198	5.0236	5.8389	3.4862	5.5169	5.2547	5.7435	5.4184	4.7195	5.7204	0.9456	-0.0807	0.5481	0.2611
Map1b	_SVSPGVTQAVVEEHC[Carbamidomethyl (C)]AS[Phospho (STY)]PEEK_.3	#N/A	12.3565	11.4551	12.9182	11.9059	11.8039	11.3478	12.6154	11.6026	12.0008	11.1070	1.0113	0.0161	0.7430	0.1290
Map1b	_FEDEGAGFEESS[Phospho (STY)]ETGDYEEK_.2	P14873_S934_M1_Map1b	3.8466	3.8900	4.0370	3.9808	3.7114	5.2048	3.7320	5.3586	3.5722	5.7064	0.8954	-0.1595	0.3787	0.4217
Map1b	_SPC[Carbamidomethyl (C)]DSGYSYETIEK_.2	#N/A	4.9042	5.6228	5.1139	6.3153	5.6848	5.6732	6.0055	5.9532	5.7261	5.5539	0.9557	-0.0654	0.3624	0.4408
Map1b	_AAEAGVTEEQYGYLGTSAK_.2	#N/A	5.5417	5.2521	6.7323	6.7690	3.4680	5.4715	5.7025	4.7857	4.7625	4.8845	1.1004	0.1380	0.4540	0.3429
Map1b	_HNLQDFINIK_.2	#N/A	4.9754	5.0363	5.3040	5.2161	4.7799	5.1843	5.1642	4.9469	5.5708	6.0604	0.9351	-0.0969	0.1424	0.8465
Map1b	_LSPAKS[Phospho (STY)]PS[Phospho (STY)]LSPSPPSPIEK_.2	#N/A	5.3075	3.3690	3.7958	3.9106	3.2058	3.7388	3.3900	3.6978	6.0417	3.5878	0.9303	-0.1043	0.6525	0.1854
Map1b	_TTRT[Phospho (STY)]PEEGGYSYEISEK_.3	#N/A	5.9709	4.7782	5.0106	7.1077	3.5805	5.1633	5.5656	6.4563	6.1816	4.0798	0.9457	-0.0805	0.6924	0.1597
Map1b	_EVSSKEEQS[Phospho (STY)]PVK_.2	#N/A	2.9409	5.6404	5.4519	3.2176	4.9491	3.0392	4.4331	5.9715	6.0461	6.7117	0.7264	-0.4611	0.0686	1.1638

Map1b	_ESS[Phospho (STY)]PLYSPGFSDSTSAAK_.2	P14873_S1789_M1_Map1b	11.7394	11.7842	12.3663	12.1997	11.0408	11.9316	12.2573	11.7431	11.8912	11.8882	0.9915	-0.0123	0.6974	0.1565
Map1b	_ETAAAHQASS[Phospho (STY)]SPPIDAATAEPYGFR_.3	P14873_S1814_M1_Map1b	4.5542	6.1836	3.4810	6.8028	4.3301	4.6706	4.9678	6.1425	4.5048	4.3570	1.0021	0.0031	0.9883	0.0051
Map1b	_TTRS[Phospho (STY)]PDTSAYC[Carbamidomethyl (C)]YETM[Oxidation (M)]EK_.2	#N/A	5.6413	5.5721	6.0977	3.9884	5.1589	5.6965	5.6313	3.6074	3.9064	3.6657	1.2752	0.3507	0.0622	1.2059
Map1b	_RES[Phospho (STY)]VVS[Phospho (STY)]GDDR_.2	#N/A	5.4809	5.3325	4.9139	6.1976	4.9341	5.6214	6.0659	5.5278	5.9314	5.4911	0.9408	-0.0880	0.2423	0.6157
Map1b	_GEAEQS[Phospho (STY)]EEEGEEEDKAEDAR_.3	#N/A	5.8662	4.7577	3.8509	5.6498	4.8098	4.6700	4.1175	5.0101	5.0628	5.4422	1.0053	0.0076	0.9538	0.0205
Map1b	_VDSILLTHIGDDNLPGINSM[Oxidation (M)]LQR_.3	#N/A	4.3881	5.3757	4.0893	4.7387	4.1050	5.2354	5.6204	4.6458	5.7091	4.6790	0.9016	-0.1495	0.2020	0.6947
Map1b	_ASLSPM[Oxidation (M)]DEPVPDSES[Phospho (STY)]PVEK_.2	#N/A	11.2830	11.5367	12.5337	11.3262	9.9899	11.6979	12.0632	11.6055	11.6562	11.5942	0.9714	-0.0418	0.4581	0.3390
Map1b	_SQGSTSNS[Phospho (STY)]DWM[Oxidation (M)]K_.2	#N/A	6.7323	3.8819	3.8556	7.5120	5.4710	3.9105	5.9883	4.5045	4.6007	5.5775	1.0115	0.0165	0.9473	0.0235
Map1b	_DLTTSS[Phospho (STY)]VEKDSGGK_.2	P14873_S1851_M1_Map1b	5.6242	5.3153	4.0172	5.3750	4.5025	3.7725	5.3240	3.0161	5.5046	5.9156	0.9651	-0.0512	0.7981	0.0980
Map1b	_TPQASTYSYETSDR_.2	#N/A	5.5528	6.3527	3.9339	6.6763	6.0343	6.0349	4.2167	6.4019	6.0224	6.5655	0.9935	-0.0093	0.9558	0.0197
Map1b	_TTEAAATAVGTAATTAAVVAAAGIAASGPVKELEAER_.3	#N/A	3.9750	4.3296	3.9486	4.0459	3.9777	4.2795	4.3548	4.5153	3.8850	4.4155	0.9534	-0.0688	0.1909	0.7193
Map1b	_[Acetyl (Protein N-term)]ATVVVEATEPEPSGSIGNPAASTS[Phospho (STY)]PSLSHR_.3	#N/A	4.8342	9.6601	9.8737	11.2252	7.7821	5.9900	5.5312	10.1059	9.1367	4.6443	1.1187	0.1618	0.6098	0.2148
Map1b	_RSES[Phospho (STY)]PFEGK_.2	P14873_S1422_M1_Map1b	6.9032	7.5198	7.7220	7.5826	6.5592	7.4219	7.5518	7.0832	7.4633	7.1800	0.9952	-0.0069	0.8917	0.0498
Map1b	_TESKPS[Phospho (STY)]VTEK_.2	P14873_S601_M1_Map1b	5.6315	4.9993	3.4176	5.1667	3.3573	4.5718	3.2322	4.8275	4.2163	3.8872	1.1196	0.1630	0.4042	0.3935
Map1b	_SLM[Oxidation (M)]S[Phospho (STY)]S[Phospho (STY)]PEDLTK_.2	#N/A	3.5420	10.9762	3.3475	11.7912	10.5533	11.0164	12.2043	10.9481	11.1267	11.0821	0.7529	-0.4095	0.2061	0.6859
Map1b	_SPSLSPSPPS[Phospho (STY)]PIEKTPLGER_.3	#N/A	3.9830	6.3733	4.2209	6.0345	3.1187	3.3175	5.4359	4.8080	6.1831	4.6628	0.8550	-0.2260	0.3412	0.4670
Map1b	_LGGDVS[Phospho (STY)]PTQIDVSQFGSFK_.2	#N/A	4.0619	6.5807	3.2479	7.5275	3.1876	3.0410	3.4652	6.3611	4.0165	3.7175	1.0496	0.0698	0.8516	0.0698
Map1b	_KES[Phospho (STY)]KEETPEVTK_.2	#N/A	9.1044	9.3712	9.7411	9.4990	8.8689	9.1159	9.2880	9.2619	9.4231	9.1661	0.9999	-0.0002	0.9937	0.0027
Map1b	_MSISEGTVS[Phospho (STY)]DK_.2	P14873_S1523_M1_Map1b	7.3554	6.1629	3.6329	8.2780	6.3423	3.5956	6.7711	5.3395	5.7625	6.6447	0.9617	-0.0564	0.8234	0.0844
Map1b	_VQSLEGEKLS[Phospho (STY)]PK_.2	#N/A	11.4455	11.6399	12.2468	12.0579	11.0563	11.6175	12.0354	11.7173	11.5925	11.6403	0.9941	-0.0085	0.7734	0.1116
Map1b	_EKRES[Phospho (STY)]VVSGDDR_.2	#N/A	5.7784	4.1743	4.0744	5.9200	3.8068	4.8570	4.4985	4.5472	5.2444	4.9494	0.9914	-0.0125	0.9339	0.0297
Map1b	_HM[Oxidation (M)]DPPPAPMQDRS[Phospho (STY)]PS[Phospho (STY)]PR_.3	#N/A	6.0562	3.9989	4.0949	7.2214	4.6201	3.6884	5.4314	3.5443	4.4913	4.6025	1.0950	0.1310	0.5944	0.2259
Map1b	_SSHLPTEVTEKPQAVPVSFEFSEAKDENER_.4	#N/A	5.1353	5.6369	6.0028	5.9773	5.4419	5.5525	6.1307	5.6360	5.1174	5.4327	1.0081	0.0117	0.8539	0.0686
Map1b	_EVSSKEEQS[Phospho (STY)]PVKAEVAEK_.2	#N/A	8.8563	8.7572	9.2252	9.3277	8.6955	8.9467	9.1433	9.1885	9.1341	8.9348	0.9855	-0.0211	0.3662	0.4362
Map1b	_SRT[Phospho (STY)]PVQDHR_.2	#N/A	5.8097	6.1000	6.2094	6.1523	3.5523	6.1501	5.9204	6.2204	5.6218	5.8303	0.9600	-0.0589	0.6745	0.1710
Map1b	_LM[Oxidation (M)]ITDAAR_.2	#N/A	4.7690	5.6220	5.1369	4.5802	5.3913	4.6138	5.2907	5.0638	5.3348	5.5744	0.9441	-0.0830	0.2431	0.6143
Map1b	_LEMYVLNPVK_.2	#N/A	4.5639	3.2870	3.7891	4.6211	3.8453	3.1203	3.4811	3.6683	3.9674	3.0101	1.0961	0.1324	0.3688	0.4333
Map1b	_KLGGDVS[Phospho (STY)]PTQIDVSQFGSFKEDTK_.3	#N/A	3.7376	4.7898	5.7742	6.1250	2.8632	3.3654	3.7262	3.1915	4.1653	3.3932	1.2275	0.2958	0.2735	0.5630
Map1b	_T[Phospho (STY)]PVQDHR_.2	#N/A	4.8738	4.4544	3.7873	4.6419	3.7270	4.1114	4.8669	3.8286	3.6369	4.2475	1.0292	0.0415	0.7161	0.1450
Map1b	_NVDVEFFK_.2	#N/A	3.9182	3.3915	4.6050	2.5632	3.0439	3.4284	3.3215	1.3826	3.8711	4.0174	1.1091	0.1494	0.5830	0.2344
Map1b	_ITSFPESESYSYETSTK_.2	#N/A	7.5655	3.5516	3.7162	4.0327	7.6617	7.6152	7.6091	7.7789	7.5213	7.5948	0.7462	-0.4224	0.1106	0.9561
Map1b	_VQSLEGEKLS[Phospho (STY)]PKS[Phospho (STY)]DISPLTPR_.3	P14873_S1778_M2_Map1b	3.8205	3.6794	3.5835	9.8440	5.8137	4.0369	3.6063	9.1728	5.2063	4.2153	0.9242	-0.1137	0.7988	0.0975
Map1b	_EEQS[Phospho (STY)]PVKAEVAEK_.2	#N/A	10.1166	9.8764	9.6914	10.8811	9.7876	9.8121	10.6463	10.2194	10.2671	10.2746	0.9687	-0.0459	0.2113	0.6751
Map1b	_S[Phospho (STY)]PDTSAYC[Carbamidomethyl (C)]YETMEK_.2	#N/A	8.3001	6.5788	5.0397	9.2651	6.4853	4.5917	7.6510	6.0129	6.1534	6.9362	1.0033	0.0047	0.9827	0.0076
Map1b	_TSDVETM[Oxidation (M)]SSQSALALDER_.2	#N/A	4.9395	5.4541	5.6921	6.8489	4.9616	5.4708	5.5120	6.1144	5.7542	5.8441	0.9578	-0.0622	0.5263	0.2787
Map1b	_EWYQETHEK_.2	#N/A	5.8035	5.0019	5.2605	6.2181	6.5702	6.1236	5.4289	5.6344	6.6437	6.3973	0.9674	-0.0478	0.6225	0.2059
Map1b	_SDVLETVVLINPSDEAVSTEVR_.2	#N/A	4.7219	5.3149	5.4043	5.3848	4.3773	5.3402	5.0663	5.4548	4.8181	5.2014	0.9913	-0.0126	0.8603	0.0654
Map1b	_ESVVS[Phospho (STY)]GDDRAEEDM[Oxidation (M)]DDVLEK_.3	#N/A	3.1745	6.5691	4.4090	7.1426	6.4430	4.3064	4.1996	7.1152	7.0530	2.9511	1.0021	0.0030	0.9926	0.0032
Map1b	_AEEDM[Oxidation (M)]DDVLEKGEAEQS[Phospho (STY)]EEEGEEEDKAEDAR_.4	#N/A	3.5963	3.9695	3.9836	6.4653	3.2837	4.6332	3.9948	4.3291	4.5869	4.0283	1.0206	0.0294	0.8869	0.0521
Map1b	_PMSIS[Phospho (STY)]PPDFS[Phospho (STY)]PK_.2	P14873_S1616_M2_Map1b	3.6375	3.8746	4.3243	7.6616	5.1675	3.4930	3.8802	3.8783	3.8070	3.2931	1.2634	0.3373	0.2623	0.5812
Map1b	_QGFPDRES[Phospho (STY)]PVSDLTSTGLYQDKQEEK_.4	#N/A	3.5337	3.5223	3.6696	4.0580	3.3704	3.8592	4.0485	4.2859	3.6003	3.8580	0.9293	-0.1059	0.1436	0.8429
Map1b	_AETEEAEEPEEDGEDNASGSASK_.2	#N/A	4.2378	3.1046	3.7866	1.6016	2.9798	3.8681	4.3330	3.4859	3.8534	3.1925	0.8781	-0.1876	0.4085	0.3888
Map1b	_LGGDVS[Phospho (STY)]PTQIDVSQFGSFKEDTK_.3	#N/A	3.7160	2.9715	3.4497	5.6689	2.8598	3.7648	3.1418	4.0077	4.3067	4.1716	0.9569	-0.0636	0.7708	0.1131
Map1b	_DYNASASTIS[Phospho (STY)]PPSSMEEDKFSK_.3	#N/A	4.9559	3.7419	3.8170	4.1329	3.5879	4.2805	3.6727	3.8251	3.8987	3.9069	1.0680	0.0949	0.3415	0.4666
Map1b	_DVM[Oxidation (M)]SDETNNEETES[Phospho (STY)]PSQEFVNITK_.3	#N/A	8.4682	9.1451	9.7418	9.6239	8.6651	9.3041	9.2268	9.3801	9.3472	9.1058	0.9885	-0.0167	0.6968	0.1569
Map1b	_IAELEEERS[Phospho (STY)]QGSTSNSDWM[Oxidation (M)]K_.3	#N/A	7.3045	7.1683	7.7956	8.1541	7.1147	7.5413	7.5377	7.4742	7.4540	7.7518	0.9945	-0.0079	0.8509	0.0701
Map1b	_SSM[Oxidation (M)]LFDTM[Oxidation (M)]QHHLALNR_.3	#N/A	3.9605	4.2237	3.6942	3.2379	3.1042	3.5204	6.0865	4.9089	5.2959	4.4160	0.6999	-0.5147	0.0023	2.6370
Map1b	_ASAELDIKDVS[Phospho (STY)]DER_.2	#N/A	4.3605	5.5186	3.5465	6.3741	3.4862	6.0094	5.1145	6.6365	5.9938	5.9973	0.8226	-0.2817	0.1642	0.7845
Map1b	_GEAEQS[Phospho (STY)]EEEGEEEDK_.2	#N/A	4.0316	3.1946	3.2176	6.2681	3.0829	3.5417	3.4349	4.3983	4.0468	3.6872	0.9994	-0.0009	0.9971	0.0013
Map1b	_TAKS[Phospho (STY)]RT[Phospho (STY)]PVQDHR_.3	#N/A	3.5199	3.7063	4.2067	3.2085	3.5946	3.0300	4.4240	3.4092	3.0577	2.3035	1.0745	0.1037	0.5622	0.2501
Map1b	_ASAELDIKDVS[Phospho (STY)]DERLSPAK_.3	#N/A	4.0092	3.3612	4.2414	4.8879	3.1450	4.0309	3.4250	3.3450	4.0652	3.3154	1.1154	0.1576	0.2765	0.5583
Map1b	_DENERAS[Phospho (STY)]LS[Phospho (STY)]PM[Oxidation (M)]DEPVPDSESPVEK_.3	P14873_S1371_M2_Map1b	3.8945	3.1989	4.5814	3.5832	3.0202	3.2084	3.2979	3.0345	4.1839	3.5502	1.0183	0.0262	0.8605	0.0653
Map1b	_ELEAERS[Phospho (STY)]LM[Oxidation (M)]S[Phospho (STY)]S[Phospho (STY)]PEDLTK_.2	P14873_S825_M3_Map1b	3.4028	4.1591	3.1365	10.0890	4.0474	4.0781	3.9712	9.5610	3.5105	4.2235	0.9064	-0.1418	0.7827	0.1064
Map1b	_TDATDGKDYNASASTIS[Phospho (STY)]PPSSM[Oxidation (M)]EEDKFSK_.4	#N/A	3.7718	3.3354	4.2060	6.2884	2.9035	3.5034	3.1826	3.4764	4.2911	3.7450	1.0892	0.1232	0.6231	0.2055
Map1b	_QQDLNIM[Oxidation (M)]VLASSSTVVM[Oxidation (M)]QDESFPAC[Carbamidomethyl (C)]K_.3	#N/A	3.7932	3.0486	3.5269	4.5715	4.1095	5.0673	3.2189	4.2373	1.8440	4.2488	1.1867	0.2469	0.3096	0.5092
Map1b	_SEQSSM[Oxidation (M)]SIEFGQES[Phospho (STY)]PEHSLAMDFSR_.3	#N/A	3.9811	3.2706	3.7148	4.4475	3.1243	3.3204	3.4296	4.1034	4.0358	3.3635	0.9759	-0.0352	0.7725	0.1121
Map1b	_KIAELEEERS[Phospho (STY)]QGSTSNSDWM[Oxidation (M)]K_.3	#N/A	3.9460	3.8986	3.5461	4.9324	3.4189	3.6372	3.9317	4.4809	3.7413	3.7265	0.9815	-0.0270	0.8196	0.0864
Map1b	_RES[Phospho (STY)]VVS[Phospho (STY)]GDDRAEEDMDDVLEK_.3	#N/A	3.9615	2.9357	3.6952	3.8105	2.7207	3.5213	3.4442	3.0928	3.8685	3.8278	0.9670	-0.0485	0.6991	0.1555
Map1b	_DIKPQLELIEDEEK_.3	#N/A	3.7561	3.4701	4.4429	3.4447	2.8818	3.3469	3.1594	3.1729	3.2158	3.4117	1.0979	0.1347	0.2718	0.5658
Map1b	_NLISPDLGVVFLNVPENLKDPEPNIK_.3	#N/A	3.2239	4.0023	3.9107	4.4133	3.8504	3.8791	4.1280	3.7051	3.3537	4.3803	0.9970	-0.0044	0.9654	0.0153
Map1b	_AQWGSNM[Oxidation (M)]QVTLIPTHDSEVM[Oxidation (M)]R_.3	#N/A	3.4074	4.1637	3.1411	4.2520	7.0703	4.0735	6.8527	3.8665	7.2906	4.2190	0.7830	-0.3529	0.2623	0.5811
Map1b	_EEC[Carbamidomethyl (C)]PRPMSIS[Phospho (STY)]PPDFSPK_.3	P14873_S1616_M1_Map1b	3.6175	4.4227	4.3043	3.6980	4.2440	3.4855	3.8463	3.3031	3.6022	3.2731	1.1300	0.1763	0.0865	1.0632
Map1b	_TLEVVSPSQSVTGSAGHTPYYQSPTDEK_.3	#N/A	4.3060	4.4210	3.4919	3.9946	2.8085	3.8161	3.7093	4.1239	3.7725	3.9616	0.9780	-0.0320	0.7908	0.1019
Map1b	_SDISPLTPRES[Phospho (STY)]S[Phospho (STY)]PLYSPGFSDSTSAAK_.3	#N/A	3.1412	3.9464	3.8280	4.3307	3.7677	3.9618	4.3226	3.7878	3.4364	4.2977	0.9667	-0.0488	0.6273	0.2025
Map1b	_HSPTEDDES[Phospho (STY)]AKAEADVHLK_.3	P14873_S974_M1_Map1b	4.0225	3.2779	3.7561	3.6369	3.1662	3.4584	3.3516	2.9807	4.1302	2.9772	1.0575	0.0806	0.4929	0.3072
Glul	_DIVEAHYR_.2	#N/A	10.8939	10.8311	11.0313	11.0139	11.2157	11.0260	10.7159	10.8043	10.9560	10.9615	1.0131	0.0188	0.1232	0.9093
Glul	_[Acetyl (Protein N-term)]ATSASSHLNK_.2	#N/A	10.1399	10.5697	10.7066	10.6049	10.9750	10.3956	10.3813	10.4866	10.4591	10.4467	1.0117	0.0167	0.4259	0.3706
Glul	_KPAETNLR_.2	#N/A	10.4197	10.1607	10.1858	10.4204	10.7751	10.2682	10.1655	10.1720	10.2623	10.3469	1.0132	0.0189	0.2955	0.5294
Glul	_TC[Carbamidomethyl (C)]LLNETGDEPFQYKN_.2	#N/A	10.2913	10.1229	10.7064	10.5655	10.3814	10.3518	10.6629	10.0999	10.3484	10.3542	1.0036	0.0051	0.7979	0.0981
Glul	_LVLC[Carbamidomethyl (C)]EVFK_.2	#N/A	7.8978	7.8787	7.2052	7.8377	8.0248	7.8750	7.2157	7.8175	8.0442	8.0669	1.0001	0.0001	0.9983	0.0008
Glul	_TLDC[Carbamidomethyl (C)]EPK_.2	#N/A	7.0164	7.7403	7.6404	7.1959	8.2048	7.3710	6.9245	7.7522	7.6741	7.8349	0.9976	-0.0035	0.9484	0.0230
Glul	_TC[Carbamidomethyl (C)]LLNETGDEPFQYK_.2	#N/A	7.6633	7.1965	7.2526	7.9950	7.6699	7.4571	7.8519	7.2101	7.2499	7.7890	1.0018	0.0027	0.9475	0.0234
Glul	_KGYFEDR_.2	#N/A	7.5702	7.5769	7.7278	7.4863	7.5066	7.2990	7.6401	7.4861	7.0882	7.7245	1.0058	0.0083	0.7536	0.1229
Glul	_AC[Carbamidomethyl (C)]LYAGVK_.2	#N/A	6.9472	6.9819	6.7675	7.1420	7.4967	7.2133	5.5756	6.9584	7.3578	7.3083	1.0429	0.0605	0.4349	0.3616
Glul	_C[Carbamidomethyl (C)]IEEAIDK_.2	#N/A	6.0486	5.9335	6.1437	6.1904	7.3179	6.2457	5.3151	6.2850	6.5519	6.7348	1.0147	0.0211	0.8048	0.0943
Glul	_M[Oxidation (M)]GDHLWIAR_.2	#N/A	7.5371	8.0682	8.3501	7.7804	7.4189	7.5710	7.7333	7.7372	7.5766	7.8052	1.0097	0.0139	0.7001	0.1548
Glul	_VQAM[Oxidation (M)]YIWVDGTGEGLR_.2	#N/A	7.6976	7.5174	8.0874	7.7426	7.6932	7.8167	8.1841	7.4525	7.8200	8.0100	0.9863	-0.0199	0.5127	0.2902
Glul	_ITGTNAEVM[Oxidation (M)]PAQWEFQIGPC[Carbamidomethyl (C)]EGIR_.3	#N/A	7.5519	7.6297	7.8093	8.3130	7.7823	8.0477	7.9158	7.6799	8.5684	7.7218	0.9855	-0.0211	0.6079	0.2161
Glul	_QM[Oxidation (M)]YM[Oxidation (M)]SLPQGEK_.2	#N/A	7.3664	8.0349	8.9090	6.8199	8.0568	8.1402	8.4786	8.1065	7.8855	7.7291	0.9799	-0.0293	0.6869	0.1631

Glul	_Y[Phospho (STY)]NRKPAETNLR_.3	#N/A	3.7815	3.6512	4.9599	3.8583	4.4981	3.8084	3.9864	4.3134	4.5443	4.4554	0.9464	-0.0795	0.4341	0.3624
Glul	_RPSANC[Carbamidomethyl (C)]DPYAVTEAIVR_.3	#N/A	6.1854	5.9662	5.8830	7.0025	6.9502	6.4185	6.1890	6.8140	6.8782	6.9711	0.9535	-0.0687	0.3035	0.5179
Glul	_AMREENGLK_.2	#N/A	4.0082	4.2636	4.1452	3.8097	2.9930	3.6446	1.6947	3.4706	3.1192	3.1140	1.3373	0.4193	0.0389	1.4100
Glul	_C[Carbamidomethyl (C)]IEEAIDKLSK_.2	#N/A	5.1727	6.0421	6.6343	5.9292	6.4797	5.8025	5.7037	5.5877	6.1045	5.8376	1.0347	0.0493	0.4924	0.3077
Glul	_RLTGFHETSNINDFSAGVANR_.4	#N/A	6.7589	5.8123	6.5785	3.6575	5.1432	6.5884	6.6026	3.4180	5.4546	5.5520	1.0950	0.1310	0.5526	0.2576
Glul	_LTGFHETSNINDFSAGVANR_.3	#N/A	4.1622	3.0648	3.3482	3.8508	2.9144	4.0733	3.7428	4.2685	3.9170	3.9308	0.9002	-0.1517	0.2027	0.6932
Glul	_VC[Carbamidomethyl (C)]EDFGVIATFDPK_.2	#N/A	4.1251	3.3805	3.8588	3.4025	3.2688	3.3558	3.5508	3.9063	3.8977	3.0798	0.9880	-0.0175	0.8572	0.0669
Cdh2	_SAAPHPGDIGDFINEGLK_.3	#N/A	4.3603	4.2643	4.7388	3.9256	3.2739	3.9467	3.3543	4.2560	4.5818	4.0421	1.0065	0.0094	0.9375	0.0280
Ntrk2	_IWC[Carbamidomethyl (C)]TEPSPGIVAFPR_.2	#N/A	5.9733	5.4547	3.4807	5.5808	3.4167	5.1949	4.9822	3.9767	5.8594	5.3161	0.9636	-0.0535	0.7847	0.1053
Prph	_LLGSGS[Phospho (STY)]PSSSAR_.2	P15331_S57_M1_Prph	3.0790	4.1471	3.7659	4.2685	3.7056	4.0239	3.9832	3.8500	3.4986	4.2355	0.9845	-0.0225	0.8157	0.0885
Kcnc1	_LAWLAEPDAHS[Phospho (STY)]HFDYDPR_.3	P15388_S44_M1_Kcnc1	4.3548	3.6102	4.0885	6.5483	3.4985	3.1261	4.5201	6.1856	2.9617	3.2716	0.9928	-0.0104	0.9725	0.0121
Kcnc1	_HIPRPPQLGS[Phospho (STY)]PNYC[Carbamidomethyl (C)]K_.3	P15388_S468_M1_Kcnc1	4.3452	3.6496	3.5312	4.0338	3.4709	4.2586	3.1186	4.0847	3.7332	4.0008	1.0394	0.0558	0.5868	0.2315
Sptb	_ILS[Phospho (STY)]PVDSGNK_.2	P15508_S1218_M1_Sptb	5.9974	6.2004	3.3968	5.4179	2.8069	5.3828	6.6170	6.2612	3.7709	5.4280	0.8818	-0.1814	0.4795	0.3192
Sptb	_LLTS[Phospho (STY)]PDVSYDEAR_.2	P15508_S1289_M1_Sptb	5.6146	5.1358	6.0777	4.3862	4.8739	5.5643	6.5298	6.2011	3.3809	4.3532	1.0311	0.0442	0.8178	0.0874
Nme1	_NIIHGSDSVK_.2	#N/A	7.4491	7.5804	7.7898	7.8730	7.9329	7.5753	7.5126	7.5306	7.5736	7.6950	1.0161	0.0231	0.2714	0.5664
Nme1	_FLQASEDLLK_.2	#N/A	8.1378	7.9056	7.3935	8.1839	8.2690	7.9751	7.7524	8.0179	8.0698	8.3307	0.9919	-0.0117	0.7357	0.1333
Nme1	_YM[Oxidation (M)]HSGPVVAM[Oxidation (M)]VWEGLNVVK_.3	#N/A	7.9639	8.0273	7.9854	7.7205	8.0013	8.2716	8.1569	8.1853	8.5015	8.0036	0.9736	-0.0386	0.1130	0.9469
Nme1	_FLQASEDLLKEHYTDLK_.3	#N/A	6.9718	7.0137	7.3159	7.4559	7.6141	6.7987	7.1826	7.1481	7.7104	7.6076	0.9707	-0.0429	0.3031	0.5184
Nme1	_EISLWFQPEELVEYK_.2	#N/A	6.3004	6.1684	6.6073	6.9778	6.2378	6.6862	6.5993	5.9321	6.4803	6.7535	1.0085	0.0123	0.8033	0.0951
Nme1	_SC[Carbamidomethyl (C)]AQNWIYE_.2	#N/A	3.1372	4.5496	4.3717	4.5222	4.7703	4.3838	4.3195	4.1428	4.5818	3.5928	1.0312	0.0444	0.7125	0.1472
H1-2	_[Acetyl (Protein N-term)]S[Phospho (STY)]EAAPAAPAAAPPAEK_.2	#N/A	10.0865	9.8123	10.4588	10.3864	10.0604	10.0082	10.7468	10.0951	9.8396	10.2318	0.9909	-0.0132	0.6474	0.1888
H1-2	_ALAAAGYDVEK_.2	#N/A	7.5530	7.4612	7.9810	7.9663	8.0968	7.5667	7.6530	8.1206	7.3929	7.6276	1.0094	0.0135	0.7051	0.1518
H1-2	_GTGASGSFK_.2	#N/A	6.1542	5.7447	6.5404	6.7133	6.6560	6.5672	5.7530	6.0577	6.0924	6.2508	1.0592	0.0830	0.1240	0.9065
H1-2	_ASGPPVSELITK_.2	#N/A	6.0858	5.6697	6.5530	6.4734	5.5507	6.0359	5.7580	6.4755	5.4570	5.7180	1.0358	0.0507	0.4604	0.3369
H1-2	_[Acetyl (Protein N-term)]S[Phospho (STY)]EAAPAAPAAAPPAEKAPAK_.2	#N/A	4.4330	5.8812	7.2980	5.7131	5.6521	5.8731	6.1697	5.5923	5.6988	5.2061	1.0250	0.0356	0.7802	0.1078
H1-2	_ALAAAGYDVEKNNSR_.2	#N/A	3.6928	3.6905	3.2268	4.0125	3.7091	2.9235	2.9329	4.3116	3.5991	2.9431	1.0278	0.0396	0.7774	0.1094
Atp6v0a2	_KDS[Phospho (STY)]EEEVSLLGNQDIEEGNSR_.3	P15920_S695_M1_Atp6v0a2	2.9114	2.9302	4.1459	3.6897	4.2524	3.4771	3.8379	5.4755	3.6106	3.3670	0.8760	-0.1910	0.3222	0.4919
Chgb	_IYHS[Phospho (STY)]EER_.2	P16014_S155_M1_Chgb	11.1903	11.7133	12.1524	12.0183	12.6141	12.0794	11.6508	11.7149	11.9569	11.6995	1.0175	0.0250	0.4329	0.3636
Chgb	_WASS[Phospho (STY)]REDAGAPVEDSQGQTK_.3	#N/A	8.7334	8.5393	9.4108	9.0248	9.3806	9.1308	5.4907	8.5196	9.2704	3.6198	1.3437	0.4262	0.0603	1.2196
Chgb	_SSYEGHPLS[Phospho (STY)]EER_.2	#N/A	9.1286	9.0934	9.2588	9.8014	9.6052	9.4770	9.3748	9.0436	9.4596	9.5612	1.0037	0.0053	0.8443	0.0735
Chgb	_WWQQEEQLGPEES[Phospho (STY)]REEVRFPDR_.4	#N/A	6.2678	6.7617	6.4683	7.7402	7.7721	6.7521	6.0298	7.4497	7.3385	6.6716	1.0128	0.0183	0.8390	0.0762
Chgb	_ES[Phospho (STY)]KEADVATVR_.2	P16014_S318_M1_Chgb	7.3745	7.3811	3.8569	8.0361	7.6682	7.5620	7.5095	7.3807	7.5600	7.4805	0.9328	-0.1004	0.5427	0.2654
Chgb	_EGVDDQESLRPSNQQAS[Phospho (STY)]KEAK_.3	#N/A	4.9382	3.8253	5.5005	5.6355	5.4061	5.2550	5.2639	5.4965	5.2322	5.4540	0.9500	-0.0740	0.4576	0.3395
Chgb	_AS[Phospho (STY)]EEEPEYGEESR_.2	#N/A	10.0436	10.3335	10.0999	10.9140	10.4319	10.6860	10.3960	10.0217	9.9115	10.5266	1.0200	0.0286	0.3536	0.4515
Chgb	_WTEGGGHS[Phospho (STY)]R_.2	#N/A	10.0188	9.9244	9.8908	10.4748	10.4812	10.2524	10.1735	9.9126	10.3373	10.0785	1.0048	0.0069	0.7614	0.1184
Chgb	_EGVDDQESLRPSNQQAS[Phospho (STY)]K_.3	#N/A	4.9444	3.8582	4.3365	3.8803	5.1265	4.3709	4.0285	4.5740	3.4200	5.3941	1.0150	0.0215	0.8822	0.0544
Chgb	_KHIEDS[Phospho (STY)]GEKPNTFSNK_.3	#N/A	3.5311	4.3363	4.9170	5.3096	5.3389	4.0840	4.5803	3.3979	5.1730	4.5499	1.0364	0.0515	0.7421	0.1295
Chgb	_WWQQEEQLGPEES[Phospho (STY)]REEVR_.3	#N/A	4.0217	3.2772	3.7554	6.5411	5.5448	3.4591	3.4475	5.0677	5.9122	5.3325	0.8974	-0.1561	0.5384	0.2689
Chgb	_SGSNKSS[Phospho (STY)]YEGHPLSEER_.3	P16014_S301_M1_Chgb	3.3504	5.3824	3.7365	5.4523	3.7127	4.9292	4.3607	3.8730	3.2356	3.8609	1.1552	0.2081	0.2775	0.5568
Chgb	_S[Phospho (STY)]HHLAHYRASEEEPEYGEESR_.4	P16014_S333_M1_Chgb	7.9098	3.3873	8.6517	8.2516	3.0258	3.4614	3.3064	3.6036	4.1608	6.8238	1.2923	0.3699	0.4195	0.3773
Chgb	_GRGS[Phospho (STY)]EEDRAPR_.2	#N/A	4.7684	4.9821	5.0932	5.4890	6.1481	5.7081	5.2274	5.3505	5.7761	5.2991	0.9910	-0.0130	0.8669	0.0620
Chgb	_EDAGAPVEDSQGQTK_.2	#N/A	7.8624	4.0967	3.8162	4.3189	4.9268	5.2849	4.0336	3.7996	5.0821	4.2859	1.1746	0.2321	0.3678	0.4344
Chgb	_WTEGGGHS[Phospho (STY)]REGVDDQESLRPSNQQASK_.4	#N/A	7.3781	8.1988	8.3178	8.0841	3.3847	8.5859	3.6667	8.1421	8.0925	8.0104	1.0497	0.0700	0.8011	0.0963
Chgb	_SSYEGHPLS[Phospho (STY)]EERRPSPK_.3	#N/A	7.0438	4.0797	8.0862	7.9006	7.9637	7.9014	7.7975	7.7716	3.4311	4.3029	1.2295	0.2980	0.2991	0.5241
Chgb	_ADAHS[Phospho (STY)]M[Oxidation (M)]ELEEK_.2	#N/A	9.2454	9.4368	9.4499	9.9475	9.4013	8.3443	9.5816	9.2896	9.3017	9.2492	0.9945	-0.0079	0.8569	0.0671
Chgb	_SEES[Phospho (STY)]QEREYKR_.3	P16014_S378_M1_Chgb	3.2568	4.0130	4.4913	4.4026	3.9013	4.2241	4.1173	3.7159	3.3645	3.7123	1.0860	0.1191	0.2504	0.6013
Chgb	_ADAHS[Phospho (STY)]MELEEKTHS[Phospho (STY)]R_.2	P16014_S220_M2_Chgb	2.9076	2.9339	4.1422	3.6859	3.5522	3.0724	3.8342	3.3152	3.6143	3.3632	0.9577	-0.0623	0.5946	0.2258
Chgb	_HIEDS[Phospho (STY)]GEKPNTFSNK_.3	#N/A	3.3858	4.1421	3.1195	4.2735	4.0304	4.0951	3.9882	3.8450	3.4935	4.7626	0.9549	-0.0665	0.5866	0.2316
Chgb	_ADEFPDFY[Phospho (STY)]DS[Phospho (STY)]EEQM[Oxidation (M)]GPHQEANDEK_.3	P16014_S626_M2_Chgb	4.3680	3.6724	3.5540	4.0567	3.4937	4.2358	3.7713	4.0618	3.7104	4.0237	1.0013	0.0018	0.9810	0.0083
Prkce	_LAAGAESPQPASGNS[Phospho (STY)]PSEDDR_.2	#N/A	10.0585	9.8186	9.9611	10.4735	9.1091	9.7577	10.3384	10.0495	9.9690	9.8532	0.9812	-0.0274	0.4574	0.3397
Prkce	_SAPTS[Phospho (STY)]PC[Carbamidomethyl (C)]DQELK_.2	#N/A	9.9365	9.9882	3.6302	9.3625	9.0332	8.2325	11.0562	10.1669	9.5000	3.8433	0.9679	-0.0471	0.8799	0.0556
Prkce	_ALS[Phospho (STY)]FDNRGEEHR_.3	#N/A	8.8606	8.7414	9.5866	9.1974	8.2098	8.8935	8.8608	9.0032	9.0504	8.9424	0.9945	-0.0080	0.8406	0.0754
Prkce	_KALS[Phospho (STY)]FDNR_.2	#N/A	9.4413	9.0376	9.3961	9.8178	7.9325	9.0523	9.3126	9.3049	9.2678	8.8795	0.9915	-0.0123	0.8236	0.0843
Prkce	_ASSATDGQLAS[Phospho (STY)]PGENGEVRPGQAK_.3	#N/A	8.6067	8.9816	9.1798	9.7710	8.1898	8.9555	9.1479	9.2245	9.1000	8.4458	0.9964	-0.0052	0.9193	0.0366
Prkce	_KQET[Phospho (STY)]PDEVGSQR_.2	P16054_T228_M1_Prkce	7.5024	3.2981	8.3531	7.9768	7.1021	7.4520	7.7964	7.6841	7.6296	2.9991	1.0644	0.0900	0.7588	0.1199
Prkce	_SAPTS[Phospho (STY)]PC[Carbamidomethyl (C)]DQELKELENNIRK_.3	#N/A	3.4239	4.1801	4.0593	4.2393	6.9886	4.0608	3.9539	4.0330	7.0998	4.1239	0.9353	-0.0965	0.7339	0.1343
Prkce	_ALS[Phospho (STY)]FDNR_.2	#N/A	7.8868	4.0255	7.1980	4.0952	6.3847	7.6458	7.1772	4.4069	7.7222	7.6077	0.9223	-0.1166	0.6421	0.1924
Prkce	_SKS[Phospho (STY)]APTS[Phospho (STY)]PC[Carbamidomethyl (C)]DQELK_.2	#N/A	9.3278	7.0007	9.1425	9.8309	8.1108	8.8707	7.4300	9.3306	7.1560	9.1406	1.0544	0.0764	0.5289	0.2766
Prkce	_KLAAGAESPQPASGNS[Phospho (STY)]PSEDDR_.3	#N/A	6.4470	5.5377	5.7478	6.7504	5.6436	6.1648	6.3380	6.5709	6.6510	5.8208	0.9532	-0.0691	0.3341	0.4761
Prkce	_LAAGAESPQPASGNS[Phospho (STY)]PSEDDRSK_.3	#N/A	7.7810	7.9792	8.6897	8.4076	7.7151	8.0193	8.1393	8.2891	8.0330	8.0139	0.9975	-0.0036	0.9218	0.0353
Prkce	_KALS[Phospho (STY)]FDNRGEEHR_.3	#N/A	7.1678	6.7914	7.7572	7.3319	6.7024	7.2606	6.9979	7.2285	7.5108	7.1284	0.9934	-0.0096	0.8292	0.0814
Prkce	_SAPTS[Phospho (STY)]PC[Carbamidomethyl (C)]DQELKELENNIR_.3	#N/A	3.2963	3.4636	4.0090	3.9195	3.8128	3.7136	3.8737	3.6008	3.7295	3.7518	0.9902	-0.0142	0.8097	0.0917
Prkce	_ASSATDGQLAS[Phospho (STY)]PGENGEVRPGQAKR_.3	#N/A	3.2143	3.9706	4.4488	4.4450	3.8589	3.7851	4.1597	3.6734	3.3220	4.4120	1.0159	0.0228	0.8440	0.0736
Prkce	_DVILQDDDVDC[Carbamidomethyl (C)]TMTEK_.2	#N/A	4.3236	3.5791	4.0573	3.3357	3.4674	3.1572	4.5512	3.4001	4.4313	3.2784	0.9331	-0.0999	0.4748	0.3235
Prkce	_SKS[Phospho (STY)]APTS[Phospho (STY)]PC[Carbamidomethyl (C)]DQELKELENNIR_.3	#N/A	4.2312	2.9949	3.4172	3.9199	2.8832	3.7414	3.6345	3.2021	3.8472	3.8869	0.9694	-0.0448	0.7180	0.1439
Ldhb	_LIASVADDEAAVPNNK_.2	#N/A	11.6549	11.6844	12.0609	12.0778	11.4444	11.7916	12.1265	11.6070	11.7714	11.8474	0.9956	-0.0064	0.7398	0.1309
Ldhb	_FIIPQIVK_.2	#N/A	10.1153	10.3526	10.4415	10.1696	10.1488	10.3176	10.1224	10.4986	10.4320	10.3946	0.9899	-0.0146	0.2975	0.5265
Ldhb	_LKDDEVAQLR_.2	#N/A	9.8417	9.6432	9.8456	9.9256	9.9197	9.8078	9.9114	9.6262	9.7295	9.8277	1.0058	0.0084	0.4499	0.3469
Ldhb	_IVVVTAGVR_.2	#N/A	9.1668	9.4157	9.2439	9.7154	9.6772	9.7926	9.1564	9.6884	9.6295	9.5728	0.9990	-0.0015	0.9539	0.0205
Ldhb	_SADTLWDIQK_.2	#N/A	9.4789	9.4143	9.8059	9.4601	9.4449	9.2955	9.5646	9.3294	9.4730	9.4183	1.0039	0.0056	0.7092	0.1492
Ldhb	_GLTSVINQK_.2	#N/A	8.9071	8.7218	8.8515	8.7698	9.0649	8.7713	8.2999	8.7664	8.9107	8.8267	1.0169	0.0241	0.2765	0.5584
Ldhb	_LKDDEVAQLRK_.3	#N/A	7.0654	7.2381	7.6159	6.9935	7.4552	7.2153	7.1288	7.1693	7.3696	7.3431	1.0015	0.0022	0.9331	0.0301
Ldhb	_SLADELALVDVLEDK_.2	#N/A	8.0252	7.8202	7.7253	7.6870	7.7097	7.6955	8.0502	7.4500	7.5237	8.0040	1.0026	0.0038	0.8895	0.0509
Ldhb	_EKLIASVADDEAAVPNNK_.3	#N/A	6.5092	6.6011	7.0685	6.9905	6.6165	6.5465	6.9776	6.8871	6.9580	6.9036	0.9698	-0.0443	0.1320	0.8795
Ldhb	_KSADTLWDIQK_.2	#N/A	5.9204	6.5459	6.0972	6.8082	6.3301	6.4084	5.9083	6.2338	6.6196	6.4452	1.0079	0.0114	0.8106	0.0912
Ldhb	_M[Oxidation (M)]VVDSAYEVIK_.2	#N/A	8.6412	8.8730	10.1077	8.8209	8.6599	10.0435	8.9332	8.9133	8.9234	8.8296	1.0327	0.0464	0.4337	0.3628
Ldhb	_IHPVSTM[Oxidation (M)]VK_.2	#N/A	7.3675	8.1652	7.9488	7.4817	8.2501	7.8359	7.6479	8.2366	8.0818	7.6808	0.9911	-0.0129	0.7533	0.1230
Ldhb	_IVADKDYSVTANSK_.2	#N/A	7.4881	7.2286	7.5586	7.5009	7.7939	7.6601	7.3823	7.5206	7.7736	7.6935	0.9929	-0.0103	0.6617	0.1793
Ldhb	_SLADELALVDVLEDKLK_.2	#N/A	4.6620	4.6068	4.2385	4.9461	4.4900	4.4359	4.7059	4.6962	4.3362	4.4556	1.0032	0.0047	0.9197	0.0364

Ldhb	_ITVVGVGQVGM[Oxidation (M)]AC[Carbamidomethyl (C)]AISILGK_.2	#N/A	5.1644	4.9925	5.4336	5.8482	4.5993	5.1155	5.7895	5.5922	4.8832	5.1032	0.9720	-0.0410	0.5965	0.2244
Ldhb	_GEM[Oxidation (M)]M[Oxidation (M)]DLQHGSLFLQTPK_.3	#N/A	4.9651	4.8742	6.5192	3.3128	6.2521	6.1436	5.0372	5.8884	5.7555	5.9222	0.9458	-0.0804	0.6448	0.1906
Ldhb	_GM[Oxidation (M)]YGIENEVFLSLPC[Carbamidomethyl (C)]ILNAR_.3	#N/A	3.7369	2.9924	3.4706	4.5152	2.8807	3.7439	3.1627	3.9868	4.2858	4.1925	0.9103	-0.1355	0.3650	0.4377
Srp14	_KSS[Phospho (STY)]VEGLEPAENK_.2	P16254_S45_M1_Srp14	8.4747	3.0309	8.8898	3.8838	8.0809	8.3737	3.5985	3.9482	8.6148	8.5690	1.0981	0.1350	0.7346	0.1339
Slc4a3	_ALPSVGLQSDQS[Phospho (STY)]PQR_.2	P16283_S198_M1_Slc4a3	10.8730	10.8546	11.2934	11.0782	9.5284	10.7541	11.4804	11.2406	10.9515	10.8182	0.9647	-0.0518	0.2781	0.5558
Slc4a3	_SYS[Phospho (STY)]ERDFEFHR_.3	P16283_S67_M1_Slc4a3	2.9921	3.7973	3.6789	4.3234	3.6186	4.1109	4.4717	4.1786	2.9768	4.0007	0.9607	-0.0578	0.6709	0.1733
Slc4a3	_FSIGS[Phospho (STY)]DEDDSPGLPVK_.2	#N/A	9.1187	9.4549	4.2896	10.4874	8.9311	9.3737	10.0574	10.2903	9.6145	9.5081	0.8725	-0.1968	0.2954	0.5296
Slc4a3	_AKFSIGS[Phospho (STY)]DEDDSPGLPVK_.2	#N/A	3.7353	3.0144	4.0029	6.8508	4.7507	3.5635	3.6889	5.7276	4.1770	3.8623	0.9898	-0.0147	0.9565	0.0193
Slc4a3	_SRPWS[Phospho (STY)]PSASYDLR_.2	P16283_S224_M1_Slc4a3	3.5464	4.3516	4.2253	3.7691	4.1730	3.5565	3.9173	6.3450	3.5312	3.4464	0.9135	-0.1306	0.5327	0.2735
Slc4a3	_GS[Phospho (STY)]PSGLAPILR_.2	#N/A	3.9434	3.1988	3.6770	9.1353	3.0871	3.5375	3.3691	3.7804	4.0794	4.3989	1.1338	0.1812	0.6745	0.1710
Slc4a3	_IQGGRGS[Phospho (STY)]PSGLAPILR_.3	#N/A	3.4038	4.2090	4.0906	4.5932	4.0303	3.6992	4.0600	3.5252	3.1738	3.0594	1.1591	0.2130	0.0811	1.0911
Cnp	_NQWQLS[Phospho (STY)]ADDLKK_.2	#N/A	9.0163	9.1853	9.3219	9.6890	8.7133	9.0417	9.7915	9.3066	9.2047	9.1906	0.9774	-0.0330	0.3240	0.4895
Cnp	_HFISGDEPK_.2	#N/A	8.4939	8.1528	7.9361	8.2791	8.2224	7.9895	8.6459	8.3279	8.1378	8.1976	0.9822	-0.0259	0.3108	0.5075
Cnp	_KS[Phospho (STY)]HTFLPK_.2	#N/A	9.1855	9.5083	9.5626	9.5176	8.7519	9.0436	9.9260	9.2786	9.1852	9.1613	0.9866	-0.0195	0.5810	0.2358
Cnp	_LDEDLAGYC[Carbamidomethyl (C)]R_.2	#N/A	9.0771	9.0267	9.2080	9.0455	9.0076	8.7270	9.2218	8.9773	9.0751	8.9227	0.9962	-0.0054	0.7327	0.1350
Cnp	_ADFSEAYKR_.2	#N/A	8.9485	8.7993	8.9566	8.7069	8.7831	8.7763	8.7969	8.8791	8.7482	8.8106	1.0022	0.0032	0.7325	0.1352
Cnp	_LSISALFVTPK_.2	#N/A	8.2615	8.2355	8.3218	8.0804	7.9701	7.9983	8.2104	8.2812	8.2163	8.3138	0.9866	-0.0195	0.1934	0.7135
Cnp	_AAGAEEYAQQEVVK_.2	#N/A	8.2356	8.1690	8.1878	8.2273	7.7424	8.1154	8.4585	7.7584	7.6430	8.3633	1.0071	0.0102	0.7709	0.1130
Cnp	_AAGAEEYAQQEVVKR_.2	#N/A	6.6402	6.8762	7.2374	7.1175	6.3744	6.5641	6.8379	6.8860	6.7614	6.7436	0.9992	-0.0012	0.9749	0.0110
Cnp	_KAGQVFLEELGNHK_.3	#N/A	7.9036	7.5919	7.4681	7.2301	7.2191	7.6102	7.9736	7.7484	7.7933	8.0282	0.9516	-0.0716	0.0281	1.5508
Cnp	_AIFTGYYGK_.2	#N/A	7.9456	7.4254	7.9448	7.1580	7.0246	6.8605	6.2272	8.1414	7.4663	7.7640	0.9991	-0.0013	0.9874	0.0055
Cnp	_RPPGVLHC[Carbamidomethyl (C)]TTK_.3	#N/A	7.3888	7.3904	7.3240	5.7812	7.4817	7.2072	6.4022	7.6234	7.1491	6.9800	1.0081	0.0116	0.8868	0.0522
Cnp	_EKPELQFPFLQDEDTVATLHEC[Carbamidomethyl (C)]K_.4	#N/A	7.2182	6.9890	6.9392	7.2795	6.2924	6.4451	6.0243	6.8624	7.0216	6.9166	1.0230	0.0328	0.5904	0.2289
Cnp	_LDC[Carbamidomethyl (C)]AQLK_.2	#N/A	5.0571	4.8654	4.7947	3.6196	5.5194	5.2045	4.1816	5.1969	4.7258	4.9663	1.0159	0.0227	0.8450	0.0732
Cnp	_GKLY[Phospho (STY)]SLGK_.2	P16330_Y372_M1_Cnp	3.0601	6.4218	3.7469	4.2495	3.6866	4.0429	3.9642	6.1982	3.5175	4.2165	0.9390	-0.0908	0.7272	0.1383
Cnp	_GGSQGEAVGELPR_.2	#N/A	10.5242	10.5111	10.8755	10.3921	10.1880	10.3035	10.7143	10.4186	10.2833	10.3828	1.0015	0.0022	0.9130	0.0395
Cnp	_VLVLDDTNHER_.2	#N/A	10.1103	9.7397	9.9650	9.7660	9.6334	9.7841	10.1359	9.7891	9.7476	9.7840	0.9968	-0.0046	0.7920	0.1013
Cnp	_[Acetyl (Protein N-term)]M[Oxidation (M)]NTS[Phospho (STY)]FTRK_.2	#N/A	8.6500	9.6725	3.9980	4.0850	9.3698	8.8640	9.8356	9.2025	9.1690	9.2822	0.7938	-0.3331	0.1936	0.7131
Cnp	_NQWQLSADDLK_.2	#N/A	7.5614	7.9669	7.6485	8.2756	7.5150	7.7207	8.1973	8.1996	7.9775	8.0392	0.9603	-0.0585	0.0704	1.1525
Cnp	_EKNQWQLSADDLKK_.3	#N/A	6.2238	6.6680	6.5431	7.0717	6.5667	6.7342	6.6833	6.9354	6.9364	6.6883	0.9741	-0.0378	0.2811	0.5511
Cnp	_S[Phospho (STY)]HTFLPK_.2	#N/A	11.3666	11.4686	11.8290	11.5164	10.6583	10.7411	11.8192	11.2613	10.7779	3.6062	1.2026	0.2661	0.2545	0.5943
Cnp	_RLDEDLAGYC[Carbamidomethyl (C)]R_.3	#N/A	8.1619	7.5571	7.8144	7.7888	7.9641	7.4588	7.6682	7.6702	7.6633	7.3967	1.0252	0.0359	0.2155	0.6666
Cnp	_AGQVFLEELGNHK_.2	#N/A	6.6071	6.8375	7.1855	6.5744	6.2945	6.3628	7.0068	6.9709	7.0270	6.7399	0.9578	-0.0622	0.1340	0.8728
Cnp	_AHVTLGC[Carbamidomethyl (C)]AADVQPVQTGLDLLDILQQVK_.3	#N/A	4.4054	3.0396	3.8526	4.6578	2.8931	3.0104	4.7655	4.2606	3.9738	2.8811	0.9176	-0.1241	0.5348	0.2718
Cnp	_EKLELVSYFGK_.2	#N/A	5.9657	5.7573	6.0998	5.9976	6.0432	6.0836	6.3712	6.1828	6.2059	6.3293	0.9552	-0.0662	0.0051	2.2946
Cnp	_ADFSEAYK_.2	#N/A	6.0854	5.8746	5.9626	4.4496	6.0192	6.1234	3.9340	6.2018	5.4060	6.0278	1.0668	0.0932	0.5111	0.2915
Cnp	_SSETLRK_.2	#N/A	6.0914	4.6189	4.0048	4.2119	4.8478	4.1370	5.0323	4.9918	4.2545	5.6261	0.9349	-0.0972	0.4943	0.3060
Cnp	_VLVLDDTNHERER_.3	#N/A	6.9971	7.9047	8.4334	7.7716	7.5820	7.9712	7.7445	7.8124	7.8083	7.6988	1.0014	0.0020	0.9660	0.0150
Cnp	_HFISGDEPKEK_.2	#N/A	5.8230	5.8856	5.8971	5.6625	5.9274	6.3523	6.2985	5.8530	5.7595	5.5651	1.0095	0.0136	0.7516	0.1240
Cnp	_M[Oxidation (M)]VSADAYK_.2	#N/A	4.7239	4.6883	5.9235	4.9370	5.4396	5.4067	4.9936	5.5311	4.7900	4.3247	1.0563	0.0791	0.4083	0.3890
Cnp	_LELVSYFGK_.2	#N/A	8.1121	8.3884	8.0209	8.1208	7.8279	8.0801	7.7822	8.3792	8.3410	8.0529	0.9942	-0.0084	0.7517	0.1240
Cnp	_LDQLFEM[Oxidation (M)]ADQYQYQVVLVEPK_.3	#N/A	4.1212	2.9331	3.8549	3.3986	2.1293	3.1861	4.0007	2.9974	3.9016	3.4301	0.9129	-0.1314	0.4632	0.3343
Cnp	_[Acetyl (Protein N-term)]M[Oxidation (M)]NTS[Phospho (STY)]FTR_.2	#N/A	7.1417	7.8458	3.7097	4.0993	7.5952	3.9008	8.0910	3.8774	4.0649	3.5907	1.1650	0.2203	0.5580	0.2533
Cnp	_EKNQWQLSADDLK_.3	#N/A	3.6392	3.5680	3.4496	4.5527	3.1071	3.4302	3.2002	3.9493	4.2483	4.2300	0.9665	-0.0491	0.7129	0.1470
Cnp	_ERLDQLFEM[Oxidation (M)]ADQYQYQVVLVEPK_.3	#N/A	4.3563	3.6117	4.0900	3.6337	3.5000	3.1246	4.5186	3.3674	3.6665	3.3110	1.0009	0.0014	0.9914	0.0038
Ass1	_IDIVENR_.2	#N/A	6.3919	6.1839	6.0387	6.0572	6.1875	6.4971	5.3553	6.2482	5.9865	6.4057	1.0379	0.0536	0.3010	0.5215
Ass1	_RQVEIAQR_.2	#N/A	4.3193	5.7800	3.2751	7.7165	5.2993	4.3449	3.4925	7.3867	3.9893	3.7448	1.1008	0.1386	0.6723	0.1724
Ass1	_TTSLELFM[Oxidation (M)]YLNEVAGK_.2	#N/A	4.1338	3.4382	3.6379	3.1817	3.2595	2.9692	3.3300	3.8195	3.8967	3.3915	0.9522	-0.0707	0.4899	0.3099
Sptan1	_S[Phospho (STY)]LQQLAEER_.2	#N/A	11.6799	11.3927	11.7595	12.0256	11.1954	11.5070	11.8636	11.3096	11.5599	11.5119	1.0028	0.0040	0.8586	0.0662
Sptan1	_KLDPAQSAS[Phospho (STY)]R_.2	#N/A	9.6909	9.2808	9.0573	9.7455	9.0592	9.4778	9.4919	9.3454	9.4185	9.4774	0.9949	-0.0074	0.7666	0.1154
Sptan1	_EANQQQFNR_.2	#N/A	9.5109	9.6930	10.0886	10.0196	9.5081	9.6120	9.7166	9.6923	9.7740	9.7085	1.0016	0.0023	0.9066	0.0426
Sptan1	_EKEPIAASTNR_.2	#N/A	9.3759	9.4851	9.8389	9.7095	9.6824	9.6718	9.5301	9.6604	9.5454	9.6687	1.0027	0.0039	0.7797	0.1081
Sptan1	_DLTGVQNLR_.2	#N/A	9.2133	9.2706	9.2298	9.2743	9.5557	8.8908	9.1756	9.1522	9.2279	9.1301	1.0074	0.0106	0.5536	0.2568
Sptan1	_LSDDNTIGQEEIQQR_.2	#N/A	8.9003	9.1008	9.4054	9.3815	8.9321	9.1695	9.4339	9.0606	9.2599	9.1803	0.9908	-0.0134	0.5171	0.2864
Sptan1	_SSEEIESAFR_.2	#N/A	8.8031	8.6814	9.1782	9.0846	9.0036	8.5257	8.6614	8.9190	8.6787	8.8498	1.0116	0.0167	0.4800	0.3187
Sptan1	_ELPTAFDYVEFTR_.2	#N/A	8.6349	8.6551	9.0211	8.7717	8.9547	8.9232	8.9745	8.6276	8.8924	8.6757	1.0039	0.0056	0.7556	0.1217
Sptan1	_LFGAAEVQR_.2	#N/A	8.6310	8.9874	8.7643	8.8614	8.7999	8.5643	8.9413	8.9243	8.6207	8.7297	0.9959	-0.0059	0.7275	0.1382
Sptan1	_LLVSSEDYGR_.2	#N/A	8.8803	8.7753	8.9217	9.1117	8.7967	8.6738	8.7927	8.6382	8.7902	8.8743	1.0098	0.0141	0.3470	0.4597
Sptan1	_EELYQNLTR_.2	#N/A	9.0709	8.5935	8.4808	8.9864	8.8396	8.7130	8.6958	8.5932	8.8445	8.8427	1.0042	0.0060	0.7785	0.1087
Sptan1	_LIQEQHPEEELIK_.2	#N/A	7.7393	7.3840	7.9530	7.4598	7.4449	7.6673	7.4057	7.3577	7.5889	7.6366	1.0148	0.0212	0.3967	0.4015
Sptan1	_ADVVESWIGEK_.2	#N/A	8.7882	8.0845	8.4889	8.6256	8.4300	8.6336	8.8662	8.1816	8.2892	8.6102	1.0026	0.0037	0.9041	0.0438
Sptan1	_WTQLLANSATR_.2	#N/A	8.2770	8.4988	8.6296	8.4346	8.4045	8.7488	8.3884	8.4858	8.5692	8.4017	1.0044	0.0064	0.6936	0.1589
Sptan1	_EANELQQWITEK_.2	#N/A	8.4349	8.3130	8.5569	8.7241	8.2198	8.5343	8.6964	8.4516	8.5041	8.7210	0.9849	-0.0219	0.2605	0.5841
Sptan1	_DLAALEDKVK_.2	#N/A	8.0293	8.6909	8.9825	8.4268	8.6417	8.7515	8.3747	8.7107	8.5504	8.7214	0.9997	-0.0004	0.9904	0.0042
Sptan1	_DVEDEETWIR_.2	#N/A	8.7382	8.6404	8.8955	8.8503	8.0503	8.8660	8.6797	8.5435	8.6474	8.6937	1.0037	0.0054	0.8498	0.0707
Sptan1	_DLASVQALLR_.2	#N/A	8.6745	8.2255	7.7822	8.4582	8.2707	8.2175	8.3701	8.6032	8.5229	8.5136	0.9728	-0.0397	0.1780	0.7495
Sptan1	_DLSSVQTLLTK_.2	#N/A	8.5131	8.3223	8.4117	8.5075	8.4784	8.1178	8.5882	8.3158	8.4257	8.5053	0.9921	-0.0115	0.4794	0.3193
Sptan1	_ITALDEFATK_.2	#N/A	8.5063	8.7160	8.4620	8.5210	8.2992	8.5122	8.5024	8.7183	8.3507	8.5558	0.9966	-0.0049	0.7570	0.1209
Sptan1	_VLETAEDIQER_.2	#N/A	8.1154	8.1011	8.1570	8.3799	8.2069	8.4859	8.2259	8.1470	8.3387	8.4501	0.9940	-0.0086	0.6192	0.2082
Sptan1	_WRS[Phospho (STY)]LQQLAEER_.3	#N/A	7.2922	7.1174	8.0610	7.8886	6.3971	7.5836	7.1746	7.6183	7.4441	7.2859	1.0013	0.0018	0.9774	0.0099
Sptan1	_TATDEAYKDPSNLQGK_.2	#N/A	7.0142	7.1734	7.0788	7.2860	7.4923	7.3489	6.4500	7.1306	7.2039	7.1865	1.0343	0.0486	0.1963	0.7071
Sptan1	_GLVSSDELAK_.2	#N/A	8.2921	8.0269	7.6268	7.7675	8.1626	7.8784	7.7094	7.9887	7.5157	8.1998	1.0135	0.0193	0.5612	0.2509
Sptan1	_LLEAQSHFR_.2	#N/A	6.6571	6.8560	7.1258	7.3120	6.9718	6.6647	7.4126	7.1462	6.7032	7.0237	0.9802	-0.0289	0.4492	0.3476
Sptan1	_KFEEFQTDLAAHEER_.3	#N/A	7.7817	7.6123	8.2051	8.4679	7.7745	7.8348	7.8797	8.0534	7.9751	7.9183	0.9987	-0.0019	0.9512	0.0217
Sptan1	_IDGITIQAR_.2	#N/A	7.8726	7.6477	7.1320	8.3842	7.5639	7.6491	7.7707	7.6850	7.8237	7.9301	0.9879	-0.0175	0.6711	0.1732
Sptan1	_LIDVNHYAK_.2	#N/A	7.5593	8.0767	7.6678	7.7456	7.9057	7.5734	7.9150	7.8230	8.0453	7.6141	0.9880	-0.0175	0.4738	0.3244
Sptan1	_VNEVSQFAAK_.2	#N/A	7.5216	7.6562	7.7257	7.7495	7.7168	7.5676	7.5205	7.6402	7.5963	7.3916	1.0158	0.0226	0.1001	0.9997
Sptan1	_REELITNWEQIR_.3	#N/A	8.1369	7.7135	8.2451	7.7460	6.6780	7.6948	7.6072	7.1436	8.0498	7.9978	1.0004	0.0005	0.9935	0.0028
Sptan1	_VNSLGETAQR_.2	#N/A	7.7073	7.5847	7.4647	7.7661	7.6525	7.3028	7.7798	7.4926	7.7294	7.5601	0.9920	-0.0115	0.5696	0.2444

Sptan1	_QKLEDSYR_.2	#N/A	7.8519	7.4659	7.7277	7.6616	8.0254	7.5960	7.3407	7.6609	7.6180	7.3457	1.0307	0.0436	0.0946	1.0242
Sptan1	_LAQFVEHWK_.2	#N/A	7.5044	7.7165	7.8721	7.8537	7.6602	7.7185	8.0298	7.6091	7.6315	7.8873	0.9912	-0.0127	0.5362	0.2706
Sptan1	_DVDEIEAWISEK_.2	#N/A	7.5644	6.9342	7.8424	7.6179	7.6108	7.8635	7.9643	7.3357	7.7093	7.6301	0.9886	-0.0166	0.6732	0.1719
Sptan1	_LDPAQSAS[Phospho (STY)]RENLLEEQGSIALR_.3	#N/A	7.6768	8.0204	8.1911	8.4867	7.0802	7.4135	8.0127	7.9836	7.3737	7.4995	1.0122	0.0175	0.7585	0.1200
Sptan1	_DVDETIGWIK_.2	#N/A	7.7385	6.9379	7.1003	7.8277	7.3176	7.3517	6.9635	7.4247	7.3619	7.7552	1.0004	0.0005	0.9907	0.0041
Sptan1	_NTTGVTEEALK_.2	#N/A	7.7961	7.1122	7.0442	7.4447	7.5740	7.2934	7.1226	6.9556	7.3793	7.4257	1.0217	0.0310	0.3834	0.4163
Sptan1	_M[Oxidation (M)]NEVISLWK_.2	#N/A	6.9031	7.1781	7.4529	7.1092	7.3031	7.7417	7.2241	7.5254	7.4440	7.4588	0.9822	-0.0259	0.4273	0.3692
Sptan1	_LSILSEER_.2	#N/A	7.1628	7.3008	7.5271	7.2861	7.1102	7.6923	6.6146	7.4176	7.5344	7.5071	1.0107	0.0154	0.7162	0.1450
Sptan1	_LGESQTLQQFSR_.2	#N/A	7.1369	7.3091	7.0701	7.5896	6.9941	6.6158	7.1795	6.8981	7.0057	6.9867	1.0145	0.0208	0.5713	0.2431
Sptan1	_LDENSAFLQFNWK_.2	#N/A	6.6591	6.6953	7.2079	6.4673	6.7350	6.7253	6.4871	6.4039	7.0815	6.9052	1.0043	0.0062	0.8763	0.0573
Sptan1	_QDEVNAAWQR_.2	#N/A	6.5240	6.4668	6.1173	5.4060	6.5613	7.0991	6.8537	5.8797	6.6180	7.0626	0.9635	-0.0536	0.5139	0.2891
Sptan1	_DLTNVQNLQK_.2	#N/A	6.8726	6.4804	6.6430	6.9885	6.6852	6.5816	6.7255	6.6807	6.6337	6.6726	1.0046	0.0066	0.7630	0.1175
Sptan1	_KLLEATELK_.2	#N/A	6.7567	6.4842	6.3109	6.8065	6.7761	6.7973	6.6217	6.1648	6.3669	6.5011	1.0377	0.0534	0.1029	0.9877
Sptan1	_EELITNWEQIR_.2	#N/A	6.3306	6.2233	6.5186	6.6028	6.6404	6.9350	6.5831	6.3911	6.9104	6.4178	0.9949	-0.0074	0.8371	0.0772
Sptan1	_KVEDLFLTFAK_.2	#N/A	6.0027	6.4227	6.6929	6.8904	6.2016	6.4213	6.1784	6.5275	6.4388	6.3833	1.0089	0.0127	0.7533	0.1230
Sptan1	_LGDSHDLQR_.2	#N/A	6.9396	6.3565	6.1827	5.8746	6.5873	6.3922	6.4580	6.5958	6.5879	6.6090	0.9735	-0.0387	0.3778	0.4228
Sptan1	_KFDDFQK_.2	#N/A	6.4741	6.5117	5.7226	6.4966	7.0092	6.5036	6.2481	6.4572	6.4031	6.1489	1.0220	0.0313	0.5424	0.2657
Sptan1	_LLEATELK_.2	#N/A	6.3564	6.1561	7.1751	5.8490	5.5232	6.0039	4.8536	6.9208	6.1580	6.3507	1.0175	0.0251	0.8188	0.0868
Sptan1	_QWELLLEK_.2	#N/A	6.3159	5.9206	5.9866	6.7380	6.1933	6.2669	6.2003	6.4168	6.1681	6.3629	0.9920	-0.0115	0.7562	0.1214
Sptan1	_VNDVC[Carbamidomethyl (C)]TNGQDLIK_.2	#N/A	6.2530	5.9914	6.0290	5.9372	6.7222	6.3721	6.5262	6.1057	6.2352	6.1376	0.9946	-0.0078	0.8479	0.0717
Sptan1	_DLM[Oxidation (M)]SWINGIR_.2	#N/A	6.3375	6.4709	5.5485	4.8428	6.0300	6.0150	6.1571	5.9993	6.4912	6.2861	0.9424	-0.0857	0.2881	0.5404
Sptan1	_C[Carbamidomethyl (C)]TELNQAWTSLGK_.2	#N/A	5.3812	4.9620	6.2191	5.8437	6.1560	5.4451	5.5186	5.7455	5.4212	5.5600	1.0192	0.0274	0.6881	0.1623
Sptan1	_LAALADQWQFLVQK_.2	#N/A	6.2210	6.5303	6.5461	6.3067	5.9455	6.2091	6.4191	5.6339	6.0864	6.4251	1.0247	0.0353	0.4400	0.3565
Sptan1	_IAALQAFADQLIAVDHYAK_.3	#N/A	4.8113	5.0846	5.2292	5.9443	4.7361	5.2288	5.5332	5.0860	4.9366	4.8531	1.0137	0.0197	0.7867	0.1042
Sptan1	_KLSDDNTIGQEEIQQR_.3	#N/A	5.9620	5.8570	6.8039	6.3046	6.2373	5.9182	5.3790	5.8701	5.9174	6.2370	1.0563	0.0791	0.1876	0.7267
Sptan1	_QVEELYQSLLELGEK_.2	#N/A	5.7835	5.6426	6.0091	6.1228	5.7108	5.4795	5.6266	5.7136	6.0287	5.4791	1.0139	0.0199	0.6162	0.2103
Sptan1	_DVTGAEALLER_.2	#N/A	9.1210	8.6243	8.6969	8.9325	8.5376	8.3100	9.1570	8.8265	8.5118	8.7717	0.9872	-0.0186	0.5489	0.2605
Sptan1	_EAIVTSEELGQDLEHVEVLQK_.3	#N/A	7.3874	7.1150	7.2818	8.3147	6.3232	7.2123	7.5198	7.2344	7.0610	7.4532	0.9939	-0.0088	0.8973	0.0471
Sptan1	_HQALQAEIAGHEPR_.3	#N/A	6.7260	6.4199	3.3835	7.5147	7.5234	7.1665	5.7253	7.1717	7.2108	6.8069	0.9594	-0.0598	0.7550	0.1221
Sptan1	_LQQSHPLSASQIQVK_.2	#N/A	6.2140	5.8997	4.4652	6.1813	6.2281	6.0474	6.0215	5.9157	6.1770	6.0238	0.9677	-0.0474	0.5943	0.2260
Sptan1	_ENLLEEQGSIALR_.2	#N/A	6.3947	6.4356	3.1323	6.5094	6.7528	6.2348	6.0613	6.4497	7.1412	6.8159	0.8931	-0.1630	0.3571	0.4472
Sptan1	_EAHDAFR_.2	#N/A	6.4224	6.5393	3.5670	6.1876	6.6380	6.1394	6.1295	6.0005	6.2183	6.3065	0.9597	-0.0593	0.6890	0.1618
Sptan1	_TALLELWELR_.2	#N/A	6.4074	5.5048	3.3099	5.9572	5.6679	5.3759	6.3506	5.7437	6.0463	5.9786	0.8907	-0.1670	0.2706	0.5676
Sptan1	_RQQVLDR_.2	#N/A	6.1760	6.0986	6.5130	5.3026	6.0653	6.0621	5.9585	5.3929	5.8272	5.8087	1.0504	0.0709	0.2341	0.6307
Sptan1	_TALLELWELRR_.2	#N/A	4.4646	5.0023	4.1245	3.6788	4.9682	4.5948	4.4711	5.1972	5.4403	5.2639	0.8781	-0.1876	0.0798	1.0980
Sptan1	_LKDLNSQADSLM[Oxidation (M)]TSSAFDTSQVK_.3	#N/A	5.3000	6.3515	5.9064	5.2698	4.7048	5.0455	6.2949	5.2085	4.6711	5.7583	0.9902	-0.0142	0.8998	0.0458
Sptan1	_DAEELEKWIQEK_.2	#N/A	3.3282	6.2419	7.0379	5.4485	5.5092	5.6944	5.3546	5.7435	5.1346	5.2067	1.0342	0.0486	0.7818	0.1069
Sptan1	_C[Carbamidomethyl (C)]NSLEEIK_.2	#N/A	4.2622	4.7770	4.6590	4.2995	5.0138	4.7172	4.0671	4.6591	4.6666	4.1307	1.0549	0.0771	0.2558	0.5921
Sptan1	_KHEGFER_.2	#N/A	9.3667	7.6638	8.0072	8.2635	8.7874	8.7973	9.2601	7.8014	8.7400	8.5438	0.9877	-0.0178	0.7970	0.0985
Sptan1	_VAEDLESEGLM[Oxidation (M)]AEEVQAVQQQEVYGAM[Oxidation (M)]PR_.3	#N/A	6.4993	7.5277	7.9831	7.5536	6.0121	7.3389	7.6791	7.2956	7.2292	7.6052	0.9598	-0.0592	0.4628	0.3346
Sptan1	_ALINADELANDVAGAEALLDR_.2	#N/A	5.5498	5.9484	6.1048	6.1539	5.2462	5.1643	5.2724	5.9723	6.0461	5.7783	0.9874	-0.0183	0.7880	0.1035
Sptan1	_LNHQEFK_.2	#N/A	5.9679	5.5550	5.6238	5.7736	6.3793	5.6844	5.8507	6.0329	5.9988	5.6506	0.9911	-0.0129	0.7649	0.1164
Sptan1	_M[Oxidation (M)]M[Oxidation (M)]LDHC[Carbamidomethyl (C)]LELQLFHR_.3	#N/A	6.2559	5.2455	6.2634	5.5290	6.0133	6.1523	6.1773	6.6631	6.7059	6.0177	0.9247	-0.1129	0.0971	1.0129
Sptan1	_GEIDAHEDSFK_.2	#N/A	5.2773	4.8759	4.4219	4.9622	5.5742	5.2306	5.4367	5.4311	5.5004	5.3023	0.9334	-0.0994	0.1176	0.9296
Sptan1	_DTEQVDNWM[Oxidation (M)]SK_.2	#N/A	7.7991	7.8738	7.9848	8.0794	7.6796	7.6789	7.9948	7.5606	7.7840	7.9449	1.0036	0.0052	0.8099	0.0915
Sptan1	_GVIDM[Oxidation (M)]GNSLIER_.2	#N/A	6.9844	6.8520	6.8644	7.4430	7.0483	6.7435	6.9816	6.8158	6.5370	6.7525	1.0321	0.0456	0.1728	0.7624
Sptan1	_DLAALGDKVNSLGETAQR_.3	#N/A	5.9501	6.1923	6.4053	6.0401	6.2246	6.3382	5.9210	6.3455	6.1169	6.5941	0.9916	-0.0122	0.7260	0.1391
Sptan1	_AQLADSFHLQQFFR_.3	#N/A	5.2212	4.9371	5.5968	5.1377	4.9304	5.1564	5.0523	4.9239	4.8741	5.4873	1.0155	0.0222	0.6480	0.1884
Sptan1	_QQVAPM[Oxidation (M)]DDETGKELVLALYDYQEKS[Phospho (STY)]PR_.3	#N/A	3.2948	4.3022	3.9816	5.4859	5.3774	4.5935	4.1989	5.7393	3.3389	4.6516	1.0053	0.0076	0.9685	0.0139
Sptan1	_SADESGQALLAASHYASDEVR_.3	#N/A	5.6661	5.5016	5.7360	6.4166	4.8829	5.8618	5.8546	5.4621	5.3855	5.9975	1.0005	0.0007	0.9929	0.0031
Sptan1	_DLIGVQNLLK_.2	#N/A	5.2183	5.0459	3.2472	5.1096	5.7055	5.0402	5.0477	4.8575	5.5974	5.8049	0.9188	-0.1221	0.3804	0.4197
Sptan1	_DC[Carbamidomethyl (C)]EQAENWM[Oxidation (M)]AAR_.2	#N/A	7.2370	6.7070	5.8495	7.1019	7.1149	6.5603	7.1984	6.8799	6.8343	7.2060	0.9619	-0.0561	0.3601	0.4435
Sptan1	_DSDELKSWVNEK_.2	#N/A	5.3971	6.1127	6.1003	5.6910	5.8055	6.3963	5.7036	5.7767	6.0162	6.2898	0.9951	-0.0072	0.8920	0.0497
Sptan1	_AINVQEEK_.2	#N/A	7.0750	6.1864	5.6927	6.2288	6.8766	6.8068	4.5020	5.8738	6.6011	6.6750	1.0955	0.1316	0.2737	0.5627
Sptan1	_SSLSSAQADFNQLAELDR_.2	#N/A	7.2506	7.1899	7.3643	7.2650	7.0338	7.1590	7.0603	6.9846	7.0886	6.8978	1.0289	0.0411	0.0157	1.8037
Sptan1	_GLVSSDELAKDVTGAEALLER_.3	#N/A	3.1820	3.6739	4.6774	2.6875	3.3251	3.9563	4.1920	3.0408	4.1722	4.3963	0.9072	-0.1405	0.4160	0.3809
Sptan1	_ASAFNSWFENAEEDLTDPVR_.2	#N/A	3.8858	3.1114	3.1654	4.1569	3.3958	1.2319	4.4030	3.8810	3.2730	2.9813	0.8688	-0.2028	0.4365	0.3600
Sptan1	_LIQSHPESAEDLKEK_.3	#N/A	5.9549	6.0409	6.3029	6.3142	6.6343	6.3307	6.0609	6.1931	6.1248	6.0841	1.0241	0.0343	0.2733	0.5633
Sptan1	_DM[Oxidation (M)]DDEESWIK_.2	#N/A	5.6881	6.5467	5.9276	6.6525	6.2081	6.4787	6.5269	6.4935	6.4592	6.8186	0.9507	-0.0730	0.1520	0.8180
Sptan1	_EKLDILDQERTDLEK_.3	#N/A	6.0687	5.6828	7.1650	6.4622	6.3912	5.7359	6.3418	5.8107	5.8256	3.8531	1.1453	0.1958	0.1637	0.7859
Sptan1	_M[Oxidation (M)]QHNLEQQIQAR_.3	#N/A	5.2072	5.3739	6.0611	5.1445	5.6494	5.9972	4.5300	5.3911	5.7936	5.4725	1.0520	0.0731	0.3757	0.4251
Sptan1	_DLNSQADSLM[Oxidation (M)]TSSAFDTSQVK_.2	#N/A	4.1867	4.8868	5.0050	5.9502	4.5776	4.9075	4.9033	4.7185	4.2213	4.7007	1.0610	0.0855	0.4038	0.3939
Sptan1	_DLTSWVTEMK_.2	#N/A	5.6778	4.0785	4.2902	6.2586	4.3218	4.4252	4.0321	3.5148	3.1633	3.5911	1.3543	0.4375	0.0297	1.5272
Sptan1	_M[Oxidation (M)]TLVASEDYGDTLAAIQGLLK_.2	#N/A	4.6907	5.4519	5.9053	5.6784	5.1888	5.5780	5.4635	5.4991	5.2834	5.3446	1.0033	0.0048	0.9378	0.0279
Sptan1	_ADVVESWIGEKENSLK_.2	#N/A	4.2919	3.6605	4.7178	4.3896	4.1923	4.6169	4.4148	4.4103	4.4608	4.7176	0.9579	-0.0620	0.3711	0.4305
Sptan1	_QQNFNTGIK_.2	#N/A	3.8803	3.9911	3.9232	4.7848	6.3154	4.1263	3.1845	4.7526	5.3602	3.4368	1.0765	0.1063	0.6282	0.2019
Sptan1	_YTEHSTVGLAQQWDQLDQLGM[Oxidation (M)]R_.3	#N/A	5.6843	5.2255	5.7298	5.2149	4.1249	4.1325	5.6768	5.4070	4.9164	4.7513	0.9674	-0.0478	0.6878	0.1625
Sptan1	_LDETGNLM[Oxidation (M)]SEGHFASETIR_.3	#N/A	6.0972	5.9197	6.6977	7.0420	5.1046	5.7293	5.7952	5.5570	5.9067	5.8993	1.0533	0.0750	0.4156	0.3813
Sptan1	_RNEVLDR_.2	#N/A	5.2069	4.4080	3.4126	4.6704	5.9085	5.1391	5.7696	4.0283	4.7601	4.1345	1.0252	0.0359	0.8316	0.0801
Sptan1	_KLLVSSEDYGR_.2	#N/A	6.1540	6.2089	6.1801	6.3737	6.3395	6.4370	6.5457	6.0342	6.3839	5.9262	1.0096	0.0138	0.6568	0.1825
Sptan1	_EC[Carbamidomethyl (C)]EDVM[Oxidation (M)]DWINDK_.2	#N/A	6.0604	5.4345	5.6489	6.4372	4.2057	5.4069	6.0369	5.4493	5.7197	5.9886	0.9541	-0.0679	0.5271	0.2781
Sptan1	_EAFLNTEDKGDSLDSVEALIK_.3	#N/A	5.5305	5.6059	5.8310	6.0233	4.5742	5.6080	5.5184	5.4545	5.3707	5.6669	1.0048	0.0068	0.9225	0.0350
Sptan1	_KLDPAQSAS[Phospho (STY)]RENLLEEQGSIALR_.3	#N/A	3.7328	7.2269	3.9206	3.8657	4.6293	4.0833	3.8133	3.7997	3.6868	3.6951	1.2208	0.2878	0.2595	0.5858
Sptan1	_ELVLALYDYQEKS[Phospho (STY)]PR_.2	#N/A	3.6005	3.5734	3.7195	3.9052	3.4107	3.8210	4.8605	3.9547	3.5719	4.1568	0.8877	-0.1718	0.0810	1.0918
Sptan1	_SLGYDLPM[Oxidation (M)]VEEGEPDPEFEAILDTVDPNR_.3	#N/A	4.0065	3.2197	3.1924	3.6951	3.1322	3.0965	1.5873	4.4234	1.2600	3.6621	1.2405	0.3109	0.3355	0.4744
Sptan1	_DEADS[Phospho (STY)]KTAS[Phospho (STY)]PWK_.2	P16546_S1186_M2_Sptan1	8.6646	8.9331	9.6279	9.2544	7.6716	8.9198	9.0323	8.2690	8.6196	8.8499	1.0176	0.0251	0.6852	0.1642
Sptan1	_DLAALEDK_.2	#N/A	5.5804	3.0077	4.0684	3.6122	5.1001	4.9326	3.7604	4.3740	4.8293	4.9245	0.9802	-0.0288	0.8748	0.0581
Sptan1	_LQVASDENYKDPTNLQGK_.3	#N/A	5.8474	6.0172	6.4405	6.7442	6.3584	6.4359	6.0141	6.2697	6.1041	6.3772	1.0187	0.0268	0.5314	0.2746
Sptan1	_YEALKEPM[Oxidation (M)]VAR_.2	#N/A	6.2845	5.8228	5.6773	6.4478	6.2439	6.2682	6.3722	5.7172	5.9013	6.0915	1.0172	0.0246	0.6001	0.2218

Sptan1	_EAALTNEEVGADLEQVEVLQK_.2	#N/A	3.5495	3.8008	3.2832	8.9449	3.3343	3.9190	3.8606	8.2231	3.8657	4.0051	0.8964	-0.1577	0.7239	0.1403
Sptan1	_SQLLGSAHEVQR_.2	#N/A	7.3007	7.2547	7.2402	7.3496	7.8816	7.4594	7.6555	7.5393	7.5016	7.4849	0.9826	-0.0253	0.3335	0.4768
Sptan1	_EKEPIVGSTDYGKDEDSAEALLK_.3	#N/A	6.6741	6.4490	6.7244	7.2947	6.4350	6.5079	6.9281	6.5940	6.0354	6.6520	1.0196	0.0280	0.5781	0.2380
Sptan1	_QQVAPM[Oxidation (M)]DDETGKELVLALYDYQEK_.3	#N/A	3.7967	5.2232	5.7113	5.5032	4.6100	4.3503	5.6236	4.7035	4.5204	4.5246	1.0047	0.0068	0.9591	0.0181
Sptan1	_TYLLDGSC[Carbamidomethyl (C)]MVEESGTLESQLEATK_.3	#N/A	4.3402	4.3868	3.5262	5.7011	3.4659	4.2636	3.7435	3.2423	3.7382	3.9958	1.1632	0.2181	0.2008	0.6972
Sptan1	_NQALNTDNYGHDLASVQALQR_.3	#N/A	6.8298	6.6043	6.9437	7.0400	6.7122	6.3826	7.0667	6.5615	6.8009	6.7932	0.9921	-0.0114	0.7253	0.1395
Sptan1	_NNHHEENISSK_.2	#N/A	4.7577	4.2487	4.3591	3.9337	4.1146	4.1361	4.9682	4.0431	4.3099	4.3417	0.9644	-0.0524	0.4787	0.3200
Sptan1	_EKLDILDQER_.2	#N/A	7.6286	7.3388	7.4841	7.1760	7.3947	7.1184	7.7167	6.8892	7.2603	7.4413	1.0041	0.0059	0.8633	0.0638
Sptan1	_DADETKEWIEEK_.2	#N/A	6.0465	5.9106	5.7235	6.2469	6.0534	6.1043	6.4149	5.9289	6.2299	5.9518	0.9809	-0.0278	0.3930	0.4057
Sptan1	_HQEHKGEIDAHEDSFK_.4	#N/A	5.5904	4.2327	3.6835	4.1862	5.3259	4.0312	3.5831	4.0955	5.7297	4.1532	1.0269	0.0382	0.8313	0.0802
Sptan1	_QFQDAGHFDAENIKK_.3	#N/A	5.1040	5.9248	5.0811	5.5058	4.9671	5.3578	5.3227	6.1847	5.4499	4.8174	0.9779	-0.0322	0.6867	0.1632
Sptan1	_KIEDLGAAM[Oxidation (M)]EEALILDNK_.3	#N/A	4.4312	4.9824	5.0936	4.4901	4.2723	4.1161	5.2745	4.0397	5.4024	4.8874	0.9313	-0.1027	0.3154	0.5012
Sptan1	_TKQDEVNAAWQR_.3	#N/A	4.0299	4.3574	3.1168	3.6195	4.7767	3.1721	3.3341	4.4555	4.1476	5.0413	0.9059	-0.1425	0.3906	0.4082
Sptan1	_QEFAQHANAFHQWIQETR_.3	#N/A	6.4510	5.7096	6.5923	6.8756	6.2796	5.8147	6.8496	6.3300	6.3448	6.5884	0.9631	-0.0543	0.3637	0.4393
Sptan1	_EKLSILSEER_.2	#N/A	4.0198	3.2752	3.7535	5.8097	5.1953	3.4611	3.3542	5.4760	5.9071	2.9745	0.9604	-0.0584	0.8281	0.0819
Sptan1	_SADESGQALLAASHYASDEVREK_.3	#N/A	4.8040	6.0052	7.1456	5.8980	5.7576	6.7849	6.0057	5.2833	5.8464	6.1820	1.0406	0.0574	0.6120	0.2133
Sptan1	_GNAM[Oxidation (M)]VEEGHFAAEDVK_.2	#N/A	3.3962	4.2902	3.8128	4.6418	4.1853	4.5202	4.4473	4.4854	3.8381	4.5009	0.9590	-0.0603	0.5294	0.2762
Sptan1	_EQLMASDDFGR_.2	#N/A	5.3065	4.4011	4.2827	3.2845	4.2224	3.5071	4.5000	3.3331	2.9817	3.2515	1.1851	0.2450	0.1888	0.7240
Sptan1	_QETFDAGLQAFQQEGIANITALK_.3	#N/A	3.4801	3.0499	3.3623	4.6967	2.9382	3.6865	3.5796	4.2535	3.2759	4.0583	0.9324	-0.1009	0.5053	0.2964
Sptan1	_HALLEADVAAHQDR_.3	#N/A	4.5976	4.0234	4.5016	4.3922	3.9117	4.2138	4.1069	3.7262	3.3748	3.7227	1.1449	0.1952	0.0179	1.7477
Sptan1	_HQLLEADISAHEDR_.3	#N/A	5.1143	3.6626	4.1408	3.6846	4.5107	3.0737	4.6375	3.3166	4.7455	4.5058	0.9372	-0.0936	0.5659	0.2473
Sptan1	_LIQNNHYAM[Oxidation (M)]EDVATR_.3	#N/A	3.7439	3.7441	3.3790	4.0611	4.0823	3.7721	3.9534	4.0058	3.5953	3.9266	0.9811	-0.0275	0.6404	0.1936
Sptan1	_KHEAFETDFTVHK_.3	#N/A	4.9715	3.3419	3.1317	3.6343	3.0714	3.1572	3.5180	2.9833	4.1327	3.6013	0.9979	-0.0030	0.9860	0.0061
Sptan1	_AKLGDSHDLQR_.3	#N/A	4.3873	3.4018	4.1210	3.6648	4.2772	3.4522	3.8130	3.3364	3.4487	3.3421	1.0469	0.0661	0.5663	0.2470
Sptan1	_DMDDEESWIKEK_.2	#N/A	3.6965	3.5901	3.9466	4.8950	3.5889	3.9933	3.6856	4.0351	3.6837	3.3966	1.0680	0.0949	0.3828	0.4171
Sptan1	_LM[Oxidation (M)]VHTVATFNSIK_.2	#N/A	4.0047	1.3753	3.7383	3.2821	3.1484	3.5847	3.3694	3.7191	4.0181	2.9594	0.9068	-0.1411	0.5408	0.2670
Sptan1	_AGTFQAFEQFGQQLLAHGHYASPEIK_.3	#N/A	4.1785	3.1904	3.3644	3.8671	3.3041	4.4254	1.4793	3.7415	0.9639	1.7129	1.8849	0.9145	0.0128	1.8915
Sptan1	_HQAFEAELHANADR_.3	#N/A	5.2847	3.0437	4.4261	3.5762	2.8650	3.3636	3.7244	3.1897	3.7241	3.3950	1.0717	0.0999	0.6166	0.2100
Sptan1	_LIQNNHYAMEDVATRR_.3	#N/A	3.6154	4.4206	4.3022	5.4094	4.2419	3.4876	3.8484	3.3136	3.6001	3.2710	1.2103	0.2754	0.0788	1.1033
Sptan1	_HEAFETDFTVHK_.3	#N/A	3.4578	3.2755	3.8005	3.3443	3.0969	3.1318	3.4926	3.6569	3.9559	3.0216	0.9489	-0.0757	0.3991	0.3990
Sptan1	_QETFDAGLQAFQQEGIANITALKDQLLAAK_.3	#N/A	3.1104	3.9156	3.1604	4.2051	3.7370	3.9925	4.3533	4.2970	3.0952	3.8823	0.9437	-0.0837	0.5183	0.2854
Sptan1	_EKEQLMASDDFGR_.3	#N/A	4.3874	3.6428	4.1211	3.6648	3.5311	3.0935	3.8131	3.3363	3.6354	3.3421	1.0590	0.0827	0.4297	0.3669
Sptan1	_IEDLGAAMEEALILDNK_.2	#N/A	3.9909	3.2353	3.1769	3.6795	3.1166	3.5011	3.3942	4.4389	4.0875	3.6465	0.8865	-0.1738	0.1184	0.9265
Sptan1	_RQDLEDSLQAQQYFADANEAESWM[Oxidation (M)]R_.3	#N/A	3.4215	4.2267	4.1083	4.6109	4.0480	3.6815	4.3256	3.5076	3.1561	3.0771	1.1421	0.1916	0.1463	0.8347
Sptan1	_KHEAFETDFTVHKDR_.4	#N/A	3.0551	4.2873	4.2896	3.8334	4.1086	3.6209	3.9817	3.1678	3.4668	3.5107	1.0946	0.1304	0.2624	0.5811
Gapdh	_GAAQNIIPASTGAAK_.2	#N/A	12.9012	13.0132	13.4463	13.0267	13.2460	12.9031	13.1720	12.8720	13.1715	12.8452	1.0057	0.0082	0.5869	0.2314
Gapdh	_VPTPNVSVVDLTC[Carbamidomethyl (C)]R_.2	#N/A	10.6705	10.6767	10.8709	11.0485	10.8051	10.7300	10.8554	10.4803	10.7612	10.6194	1.0114	0.0163	0.2514	0.5996
Gapdh	_VIPELNGK_.2	#N/A	10.7416	10.8409	10.7273	10.5126	11.2720	10.5609	9.5376	10.8172	10.8136	10.8462	1.0259	0.0369	0.3753	0.4256
Gapdh	_LTGM[Oxidation (M)]AFR_.2	#N/A	10.1535	10.9001	10.7639	9.8632	11.0173	10.6627	10.2452	11.0055	10.9352	10.7112	0.9847	-0.0223	0.5575	0.2538
Gapdh	_VGVNGFGR_.2	#N/A	9.5758	9.8844	9.6053	9.5879	10.1851	9.3633	9.5766	9.9563	9.6717	9.7558	0.9959	-0.0059	0.8110	0.0910
Gapdh	_LVINGKPITIFQER_.3	#N/A	8.9613	8.6733	8.5089	9.0940	8.6162	8.5854	8.7837	8.8040	8.6956	9.0183	0.9903	-0.0141	0.5308	0.2750
Gapdh	_PITIFQER_.2	#N/A	9.0213	8.4576	7.8963	8.9145	8.4980	8.7432	8.6768	8.5138	8.8151	8.6906	0.9901	-0.0143	0.6979	0.1562
Gapdh	_VVKQASEGPLK_.2	#N/A	7.5238	7.1538	7.3163	7.2588	7.5856	7.0445	7.5902	7.2481	6.9609	7.2811	1.0060	0.0087	0.7744	0.1110
Gapdh	_VIISAPSADAPM[Oxidation (M)]FVM[Oxidation (M)]GVNHEKYDNSLK_.4	#N/A	6.9600	7.7759	8.8448	6.2256	7.0853	8.4788	7.1744	7.6570	7.9407	7.6569	0.9940	-0.0087	0.9325	0.0303
Gapdh	_PITIFQERDPTNIK_.3	#N/A	7.7029	7.5178	7.5852	8.0460	7.5191	7.7162	7.5415	7.5039	7.5835	7.6242	1.0156	0.0223	0.2868	0.5424
Gapdh	_WGEAGAEYVVESTGVFTTM[Oxidation (M)]EK_.2	#N/A	8.5813	8.9440	10.0467	10.0754	8.6166	9.4332	9.1004	8.9494	8.6465	8.9020	1.0431	0.0608	0.3091	0.5099
Gapdh	_VIISAPSADAPM[Oxidation (M)]FVM[Oxidation (M)]GVNHEK_.3	#N/A	8.3489	7.8000	7.4498	8.5987	7.8208	7.7860	7.4430	7.7575	7.2114	7.9054	1.0512	0.0720	0.1572	0.8036
Gapdh	_RVIISAPSADAPM[Oxidation (M)]FVM[Oxidation (M)]GVNHEK_.3	#N/A	7.9324	8.5523	9.3912	8.2290	8.6215	8.9633	8.4260	8.7460	8.8250	9.0815	0.9824	-0.0257	0.6024	0.2201
Gapdh	_LISWYDNEYGYSNR_.2	#N/A	10.5877	10.4349	11.1230	10.6050	10.2385	10.6417	10.7735	10.1679	10.5669	10.5771	1.0080	0.0114	0.6553	0.1836
Gapdh	_DPTNIKWGEAGAEYVVESTGVFTTM[Oxidation (M)]EK_.3	#N/A	5.1439	4.8676	6.8277	5.2174	4.3515	5.7609	5.3699	4.5578	5.3844	5.3772	1.0366	0.0518	0.6945	0.1583
Gapdh	_VVDLM[Oxidation (M)]AYM[Oxidation (M)]ASK_.2	#N/A	7.5324	7.8607	7.9362	7.1822	7.7849	8.0027	7.6975	7.6133	8.0153	8.1582	0.9825	-0.0255	0.4898	0.3100
Gapdh	_VVDLM[Oxidation (M)]AYM[Oxidation (M)]ASKE_.2	#N/A	7.1458	8.5067	8.4190	8.3508	8.4740	8.4272	8.3111	8.8379	8.5343	8.4303	0.9639	-0.0530	0.3106	0.5078
Gapdh	_QASEGPLK_.2	#N/A	10.2872	10.5981	10.4048	10.4406	3.5808	4.1487	3.8584	3.9747	3.6233	10.5641	1.4974	0.5825	0.2461	0.6089
Gapdh	_LEKPAKYDDIK_.3	#N/A	4.5608	3.6186	4.2944	4.2203	5.2808	3.4954	2.6841	4.9234	5.1546	4.8733	0.9628	-0.0546	0.7795	0.1082
Gapdh	_IVSNASC[Carbamidomethyl (C)]TTNC[Carbamidomethyl (C)]LAPLAK_.2	#N/A	10.8743	10.9210	11.1796	11.3508	11.3452	10.9329	11.0092	10.7727	10.9797	10.9523	1.0157	0.0225	0.1878	0.7263
Gapdh	_TVDGPSGKLWR_.2	#N/A	7.0793	6.2737	6.5861	7.1069	6.5057	6.2692	7.2516	6.2820	6.3051	6.7747	0.9975	-0.0036	0.9516	0.0216
Gapdh	_VIHDNFGIVEGLM[Oxidation (M)]TTVHAITATQK_.4	#N/A	8.1204	8.2568	8.3734	8.2682	7.9686	8.5799	7.0222	8.1281	8.5642	8.3852	1.0294	0.0419	0.4444	0.3522
Gapdh	_FNGTVKAENGK_.2	#N/A	3.8499	3.4925	3.5836	4.6282	2.4954	4.4156	3.2756	3.8738	4.1729	3.1549	1.0345	0.0489	0.7803	0.1078
Gapdh	_VEIVAINDPFIDLNYMVYMFQYDSTHGK_.3	#N/A	3.2132	3.8318	3.9000	4.4026	3.6872	4.2492	4.3699	3.7392	3.3878	3.7160	1.0204	0.0291	0.7816	0.1070
Atf2	_NDSVIVADQTPT[Phospho (STY)]PTR_.2	P16951_T53_M1_Atf2	6.4573	6.9404	7.0893	7.6292	3.3533	7.0595	7.4267	7.0400	6.5198	6.8039	0.9243	-0.1136	0.5316	0.2744
Atf2	_SKIEEPSVVETTHQDS[Phospho (STY)]PLPHPESTTSDEK_.4	P16951_S118_M1_Atf2	3.8733	3.3529	4.5601	3.5619	2.9990	3.2297	3.2766	3.0557	4.2052	3.5289	1.0226	0.0323	0.8241	0.0840
Hmga1	_[Acetyl (Protein N-term)]SESGS[Phospho (STY)]KSSQPLASK_.2	P17095_S6_M1_Hmga1	4.0174	3.2842	3.7919	3.3356	3.1056	4.5517	4.2724	3.6655	3.9903	3.0129	0.9855	-0.0211	0.8812	0.0549
Hmga1	_KLEKEEEEGISQES[Phospho (STY)]S[Phospho (STY)]EEEQ_.2	#N/A	7.2594	7.4898	7.9114	7.7922	7.2920	7.2346	8.0881	7.7683	7.2688	7.2885	0.9859	-0.0204	0.6347	0.1974
Zfp37	_IHSEEQS[Phospho (STY)]EEED_.2	P17141_S590_M1_Zfp37	7.7748	3.4226	8.9535	8.3937	8.3568	7.7260	3.3456	8.2880	8.0197	8.4063	1.0603	0.0845	0.7725	0.1121
Hspa2	_DAGTITGLNVLR_.2	#N/A	6.4518	6.5792	6.5194	6.9474	6.5673	6.8018	6.5082	6.1952	6.5612	6.5945	1.0278	0.0396	0.1751	0.7567
Hspa2	_LYQGGPGGGGSSGGPTIEEVD_.2	#N/A	4.6292	4.7073	4.0176	4.4546	4.9470	3.6110	4.4451	4.5628	5.3388	4.2765	0.9439	-0.0833	0.4280	0.3686
Hspa2	_QTQTFTTYSDNQSSVLVQVYEGER_.3	#N/A	3.8677	3.1721	4.5545	3.4478	2.9934	6.8030	3.5960	3.5534	3.8525	3.5233	1.1400	0.1891	0.5039	0.2977
Eno1	_YITPDQLADLYK_.2	#N/A	11.4682	11.6888	11.6456	11.6546	11.7386	11.6752	11.6548	11.5568	11.4737	11.7265	1.0036	0.0052	0.5307	0.2752
Eno1	_GVSQAVEHINK_.2	#N/A	10.8197	11.2462	11.1267	11.4002	11.6749	11.1256	11.0200	11.0919	11.1061	11.0277	1.0154	0.0221	0.2830	0.5482
Eno1	_GNPTVEVDLYTAK_.2	#N/A	10.7930	10.9452	11.3753	11.3961	10.9850	11.0188	10.7083	10.9706	10.6923	11.1505	1.0189	0.0269	0.2154	0.6667
Eno1	_TIAPALVSK_.2	#N/A	8.4075	8.4584	8.6260	8.6633	8.9280	8.6843	7.8508	8.4204	8.7868	8.8635	1.0174	0.0249	0.4937	0.3066
Eno1	_SC[Carbamidomethyl (C)]NC[Carbamidomethyl (C)]LLLK_.2	#N/A	9.3170	9.5040	9.4748	9.5102	9.6187	9.0799	9.2458	9.3384	9.2895	9.3057	1.0132	0.0189	0.2511	0.6002
Eno1	_IGAEVYHNLK_.2	#N/A	8.7935	9.2843	8.5705	9.3561	8.8716	8.7352	9.0356	9.1950	8.7755	8.9724	0.9934	-0.0096	0.7426	0.1292
Eno1	_KVNVVEQEK_.2	#N/A	8.8880	8.8814	8.6568	9.1261	9.5088	9.0144	8.6383	8.6376	8.8924	8.9927	1.0253	0.0360	0.2122	0.6734
Eno1	_IEEELGSK_.2	#N/A	8.1065	7.5967	8.1491	7.2993	8.6773	7.9843	5.9570	7.8568	8.2553	8.3544	1.0477	0.0673	0.4930	0.3071
Eno1	_DATNVGDEGGFAPNILENKEALELLK_.3	#N/A	6.5619	7.7660	8.5464	7.4542	7.8286	7.8299	7.5338	7.3604	7.3666	7.5130	1.0297	0.0422	0.5254	0.2795
Eno1	_VNVVEQEK_.2	#N/A	6.8474	6.3569	5.6601	5.9863	6.8375	6.6268	5.8757	5.9142	6.5982	6.7686	1.0154	0.0220	0.7603	0.1190
Eno1	_HIADLAGNPEVILPVPAFNVINGGSHAGNK_.4	#N/A	6.9495	6.5690	7.6950	7.9131	8.1679	7.7340	7.3041	6.9864	7.6450	8.0177	1.0022	0.0032	0.9645	0.0157
Eno1	_AAVPSGASTGIYEALELRDNDK_.3	#N/A	6.9769	7.9745	8.5686	8.2740	7.7376	7.2221	7.3120	7.6988	7.5763	7.2119	1.0460	0.0649	0.3212	0.4932

Eno1	_SGKYDLDFK_.2	#N/A	8.0237	8.0968	8.1103	8.6625	8.1877	7.9166	8.2658	8.0523	7.5850	8.6045	1.0048	0.0070	0.8590	0.0660
Eno1	_KVNVVEQEKIDK_.2	#N/A	4.7984	4.9323	5.4926	4.2887	5.8886	5.7822	5.4585	4.9919	5.4415	5.0114	0.9945	-0.0079	0.9340	0.0297
Eno1	_IEEELGSKAK_.2	#N/A	5.3669	8.3438	7.3658	8.6504	7.3867	8.1757	8.3002	7.7760	5.1511	7.7120	1.0433	0.0612	0.7138	0.1464
Eno1	_DATNVGDEGGFAPNILENK_.2	#N/A	11.6144	11.4751	11.6544	11.9924	11.3813	11.5769	11.8794	11.2388	11.3649	11.6577	1.0070	0.0100	0.6206	0.2072
Eno1	_LAM[Oxidation (M)]QEFM[Oxidation (M)]ILPVGASSFR_.2	#N/A	9.2426	9.5277	9.5821	8.9842	9.5832	9.7395	9.3149	9.4887	9.5263	9.6354	0.9949	-0.0073	0.7584	0.1201
Eno1	_YGKDATNVGDEGGFAPNILENK_.3	#N/A	9.1272	8.6960	9.2555	9.3441	8.5521	8.6732	9.4205	8.2203	8.5780	8.9334	1.0174	0.0249	0.5814	0.2355
Eno1	_FGANAILGVSLAVC[Carbamidomethyl (C)]K_.2	#N/A	6.4138	6.0907	3.4232	4.4678	5.6008	5.7656	4.9615	4.1037	5.4626	6.8055	0.9926	-0.0108	0.9580	0.0186
Eno1	_SGKYDLDFKS[Phospho (STY)]PDDPSR_.3	#N/A	10.1418	10.8005	11.1808	11.3537	9.9520	10.7623	10.9230	10.8950	10.7143	10.9439	0.9843	-0.0228	0.5682	0.2455
Eno1	_LM[Oxidation (M)]IEM[Oxidation (M)]DGTENK_.2	#N/A	7.4642	7.6052	7.8662	7.5161	7.5627	7.8842	7.6317	7.7669	7.8719	8.5251	0.9624	-0.0553	0.1387	0.8579
Eno1	_AAVPSGASTGIYEALELR_.2	#N/A	10.4446	10.7040	10.9852	10.7008	10.6481	10.5053	10.3562	10.1907	10.5493	10.5058	1.0254	0.0362	0.0525	1.2798
Eno1	_YDLDFKS[Phospho (STY)]PDDPSR_.2	#N/A	8.7148	10.0418	9.2282	11.0621	10.1196	10.1712	10.3322	10.2336	10.3871	10.5342	0.9535	-0.0687	0.2847	0.5456
Eno1	_SGETEDTFIADLVVGLC[Carbamidomethyl (C)]TGQIK_.2	#N/A	3.9530	4.5022	4.9846	5.4800	3.5821	4.2927	4.9499	3.8574	3.8802	4.0348	1.0682	0.0952	0.5033	0.2981
Eno1	_FTASAGIQVVGDDLTVTNPK_.2	#N/A	7.5043	7.7994	7.8697	8.8849	7.1938	7.5660	7.6918	7.8277	7.6995	8.0675	0.9976	-0.0034	0.9529	0.0209
Eno1	_SFVQNYPVVSIEDPFDQDDWGAWQK_.3	#N/A	3.9068	2.6888	4.5936	3.1807	3.0325	3.1961	3.3101	3.5128	2.5237	3.5625	1.0638	0.0892	0.6224	0.2060
Eno1	_VNVVEQEKIDK_.2	#N/A	5.4526	6.1188	3.2453	6.3837	6.1527	5.9723	5.3184	5.9572	5.3920	5.8023	0.9887	-0.0163	0.9201	0.0362
Eno1	_LAQSNGWGVM[Oxidation (M)]VSHR_.2	#N/A	5.0838	5.0707	4.4541	7.5072	4.6960	4.2335	5.9402	5.6228	5.4085	4.7111	0.9545	-0.0671	0.7110	0.1481
Eno1	_FTASAGIQVVGDDLTVTNPKR_.3	#N/A	3.0286	4.1958	4.7281	3.8069	3.6731	3.3573	3.3917	5.6549	5.7185	5.5668	0.7473	-0.4203	0.0454	1.3431
Eno1	_AGYTDQVVIGM[Oxidation (M)]DVAASEFYR_.2	#N/A	6.3687	7.0552	7.6526	8.4543	6.2422	7.1454	7.7778	7.2229	6.5112	7.0819	1.0006	0.0009	0.9924	0.0033
Eno1	_IDKLMIEM[Oxidation (M)]DGTENK_.3	#N/A	3.9664	3.2630	3.5055	3.5239	3.1101	3.5155	3.4379	4.0917	4.0273	3.3755	0.9324	-0.1010	0.2659	0.5753
Eno1	_EALELLK_.2	#N/A	3.8944	3.4480	3.6281	3.1718	3.2694	2.9593	3.3201	3.8293	1.6235	4.3499	1.0349	0.0495	0.8239	0.0841
Eno2	_IEEELGDEAR_.2	#N/A	11.6865	11.9035	11.9066	12.0979	12.3624	12.1045	11.6609	11.6072	11.6721	11.9642	1.0242	0.0345	0.0670	1.1736
Eno2	_VNQIGSVTEAIQAC[Carbamidomethyl (C)]K_.2	#N/A	10.5748	10.6496	11.1243	11.1196	11.0550	10.7679	10.7260	10.5691	10.6058	10.7927	1.0195	0.0279	0.1553	0.8089
Eno2	_AVDHINSR_.2	#N/A	10.1881	10.2941	10.3164	10.3840	11.0368	10.3387	9.9659	10.0018	10.1849	10.2685	1.0318	0.0451	0.0898	1.0467
Eno2	_YITGDQLGALYQDFVR_.2	#N/A	9.3773	9.6565	9.9313	9.8993	9.6568	9.6533	9.4974	9.3760	9.4207	9.5704	1.0243	0.0346	0.0668	1.1751
Eno2	_DGKYDLDFK_.2	#N/A	9.0389	9.2322	9.2430	9.2923	8.9085	9.0376	9.3806	8.8337	8.8379	9.2151	1.0065	0.0093	0.6724	0.1724
Eno2	_AC[Carbamidomethyl (C)]NC[Carbamidomethyl (C)]LLLK_.2	#N/A	8.6709	9.1538	9.3659	9.0742	9.4180	8.7772	8.7514	8.7504	8.6578	8.7534	1.0399	0.0565	0.0560	1.2520
Eno2	_LGAEVYHTLK_.2	#N/A	8.1976	8.1872	8.4354	7.4783	8.5692	8.2126	8.3221	7.3502	8.3320	8.6875	1.0009	0.0013	0.9816	0.0081
Eno2	_DATNVGDEGGFAPNILENSEALELVK_.3	#N/A	7.7272	7.9754	8.3143	8.2196	8.3913	7.9463	8.2496	7.7564	7.8076	8.3142	1.0079	0.0114	0.7225	0.1412
Eno2	_FAGHNFR_.2	#N/A	6.9695	8.1339	7.3956	7.5271	8.5056	7.9657	7.4743	7.5900	7.6478	7.4904	1.0264	0.0375	0.5062	0.2957
Eno2	_LDNLM[Oxidation (M)]LELDGTENK_.2	#N/A	6.9426	6.9751	7.1181	7.3271	7.3802	7.4270	7.4221	7.1682	7.3245	7.1844	0.9890	-0.0159	0.5179	0.2857
Eno2	_DKYGKDATNVGDEGGFAPNILENSEALELVK_.4	#N/A	6.3257	5.8680	6.4377	6.5327	7.1192	6.6907	6.9049	5.3211	6.5087	6.5007	1.0296	0.0421	0.6010	0.2211
Eno2	_[Acetyl (Protein N-term)]S[Phospho (STY)]IEKIWAR_.2	P17183_S2_M1_Eno2	5.1848	6.8049	6.7250	6.0352	6.4355	6.1794	5.7865	6.2503	6.0912	6.3036	1.0196	0.0280	0.7148	0.1458
Eno2	_DGKYDLDFKS[Phospho (STY)]PADPSR_.3	#N/A	7.7346	8.7836	8.8316	9.5575	8.1689	8.2835	8.9681	8.6171	8.4599	8.7928	0.9828	-0.0250	0.6686	0.1748
Eno2	_IAPALISSGISVVEQEK_.2	#N/A	6.9621	6.3395	5.9356	7.2526	6.9683	6.8361	6.7595	5.7243	6.3633	7.0294	1.0381	0.0540	0.4814	0.3175
Eno2	_HIAQLAGNSDLILPVPAFNVINGGSHAGNK_.3	#N/A	4.9736	5.5392	6.3806	6.6063	7.6394	6.1187	5.2945	6.2910	4.8653	7.2001	1.0502	0.0707	0.6474	0.1889
Eno2	_M[Oxidation (M)]VIGM[Oxidation (M)]DVAASEFYR_.2	#N/A	7.6142	7.8986	8.6622	7.5402	7.9321	8.1655	7.9162	7.8441	7.8157	7.8262	1.0151	0.0216	0.5877	0.2309
Eno2	_LAM[Oxidation (M)]QEFM[Oxidation (M)]ILPVGAESFR_.3	#N/A	7.3060	7.4027	7.4990	6.6588	7.5002	7.7939	6.7846	7.3683	7.4106	7.3569	1.0180	0.0257	0.5827	0.2346
Eno2	_YDLDFKS[Phospho (STY)]PADPSR_.2	#N/A	6.0415	6.7215	6.3314	7.5962	6.4052	6.5028	6.7201	6.4871	6.6261	6.8959	0.9877	-0.0179	0.7773	0.1094
Eno2	_IAPALISSGISVVEQEKLDNLM[Oxidation (M)]LELDGTENK_.4	#N/A	5.2273	6.0189	6.3725	6.5731	5.5030	5.9071	5.8761	5.8318	5.8391	5.5257	1.0287	0.0408	0.5533	0.2570
Eno2	_FTANVGIQIVGDDLTVTNPK_.2	#N/A	6.2902	6.7818	6.9810	8.5618	5.7586	6.4781	6.9485	7.1776	6.5895	6.7888	0.9902	-0.0142	0.8956	0.0479
Eno2	_LAQENGWGVM[Oxidation (M)]VSHR_.3	#N/A	5.5251	5.0409	4.2720	3.3554	5.2707	4.8464	3.3620	3.5539	6.1255	6.1726	0.9823	-0.0258	0.9108	0.0406
Eno2	_NYPVVSIEDPFDQDDWAAWSK_.3	#N/A	3.6631	4.4193	3.3967	3.9963	2.8068	3.8178	3.7110	4.0607	3.7708	4.1186	0.9408	-0.0881	0.4495	0.3473
Ap2a1	_NADVELQQR_.2	#N/A	8.4571	8.7245	8.7472	8.1078	7.9017	8.4494	8.7128	8.7446	8.4394	8.3490	0.9809	-0.0278	0.4108	0.3863
Ap2a1	_DFLTPPLLSVR_.2	#N/A	8.4257	8.7066	8.3411	8.0706	7.8141	7.7855	8.8908	8.7247	8.6123	8.3613	0.9472	-0.0783	0.0572	1.2426
Ap2a1	_LLQC[Carbamidomethyl (C)]YPPPEDAAVK_.2	#N/A	8.2273	8.2354	7.9957	8.0925	8.0346	7.3475	8.4587	8.4006	8.1825	7.8901	0.9703	-0.0434	0.2491	0.6036
Ap2a1	_GLAVFISDIR_.2	#N/A	7.9604	7.6405	8.0254	7.1989	6.6470	7.5548	7.7545	7.9259	7.7819	7.4143	0.9722	-0.0407	0.4602	0.3371
Ap2a1	_QLSLPLQEAQK_.2	#N/A	7.3747	7.4499	6.2893	7.1788	7.2928	6.9651	7.7794	7.5251	7.4705	7.2047	0.9462	-0.0798	0.1280	0.8927
Ap2a1	_HLC[Carbamidomethyl (C)]ELLAQQF_.2	#N/A	6.6752	7.0472	7.0015	6.6808	6.3312	6.7207	7.1229	6.8788	6.7446	6.7209	0.9819	-0.0263	0.4363	0.3602
Ap2a1	_QSAALC[Carbamidomethyl (C)]LLR_.2	#N/A	6.9187	6.8906	6.9842	5.7982	6.7021	6.3962	7.3509	7.2221	6.8061	6.6024	0.9456	-0.0807	0.1959	0.7080
Ap2a1	_ATIQGVLR_.2	#N/A	6.9321	6.8994	6.7776	6.6062	6.3437	5.9308	6.8486	7.3684	6.7310	6.6537	0.9538	-0.0682	0.2108	0.6762
Ap2a1	_LVEC[Carbamidomethyl (C)]LETVLNK_.2	#N/A	6.3191	6.3885	5.9193	6.2079	5.9667	6.3350	6.6037	6.6251	6.3156	6.0679	0.9666	-0.0490	0.1819	0.7403
Ap2a1	_NSGVLFENQLLQIGVK_.2	#N/A	6.1902	6.2235	6.3079	5.7508	5.6707	6.2432	6.3853	6.5153	6.0553	6.4236	0.9558	-0.0652	0.1225	0.9120
Ap2a1	_THIDTVINALK_.2	#N/A	6.5237	6.0121	6.5381	6.5215	4.5391	5.7431	6.7915	6.4646	5.7840	6.6040	0.9327	-0.1005	0.3490	0.4572
Ap2a1	_YGGTAQSLTLK_.2	#N/A	5.1116	4.9708	5.4687	5.6669	5.5183	4.8140	5.2709	6.0547	5.4404	5.4164	0.9482	-0.0767	0.2311	0.6362
Ap2a1	_IAGDYVSEEVWYR_.2	#N/A	6.0779	5.5018	7.0285	4.0012	4.3075	5.3982	6.4412	5.1540	5.7517	5.6969	0.9349	-0.0971	0.5546	0.2560
Ap2a1	_TSKEPVSR_.2	#N/A	5.3884	4.3711	5.1961	3.8438	5.0979	5.1555	4.1166	4.0939	4.3669	5.4966	1.0716	0.0998	0.4458	0.3508
Ap2a1	_LLGFGSALLDNVDPNPENFVGAGIIQTK_.3	#N/A	4.3561	4.5966	4.9177	4.2968	4.1869	4.7143	4.7173	4.9058	5.4178	3.6294	0.9665	-0.0491	0.6485	0.1881
Ap2a1	_AC[Carbamidomethyl (C)]NQLGQFLQHR_.3	#N/A	6.9126	6.6293	6.4942	6.3043	6.0771	5.9476	6.3870	6.5501	6.0878	6.4298	1.0048	0.0069	0.8819	0.0546
Ap2a1	_VGGYILGEFGNLIAGDPR_.2	#N/A	5.2480	4.3364	3.9578	4.4970	4.0049	4.2361	4.9133	5.1291	4.6819	3.6747	0.9522	-0.0706	0.5474	0.2617
Ap2a1	_AADLLYAM[Oxidation (M)]C[Carbamidomethyl (C)]DR_.2	#N/A	6.2028	6.1908	6.2996	6.0377	5.6740	5.8973	6.7846	6.7352	6.3638	6.3914	0.9211	-0.1186	0.0079	2.1008
Ap2a1	_FFQPTEM[Oxidation (M)]AAQDFFQR_.2	#N/A	6.8889	6.4759	6.9072	6.0595	6.0675	6.3360	7.0986	6.8557	6.4225	6.4395	0.9630	-0.0544	0.3176	0.4981
Ap2a1	_ILVAGDSMDSVK_.2	#N/A	5.4562	3.4844	3.9627	5.6463	2.9750	3.2519	3.6547	3.4947	3.7938	3.1837	1.1692	0.2256	0.3451	0.4620
Ap2a1	_ANHPMDAEVTK_.2	#N/A	5.9548	3.3612	3.7149	6.3436	5.1609	3.4997	3.4069	3.7425	4.0416	4.4368	1.1959	0.2582	0.3024	0.5194
Ap2a1	_YLALESM[Oxidation (M)]C[Carbamidomethyl (C)]TLASSEFSHEAVK_.3	#N/A	4.8495	4.5163	6.2576	5.1528	3.5335	4.3976	4.7951	4.4171	4.5656	5.3636	0.9998	-0.0002	0.9987	0.0006
Ap2a1	_TSVQFQNFLPTVVHPGDLQTQLAVQTK_.3	#N/A	3.4570	3.4038	3.6384	3.1821	3.0484	3.5762	3.4581	2.1989	3.5842	4.3603	0.9953	-0.0068	0.9667	0.0147
Ap2a1	_ASPDLVPMGEWTAR_.2	#N/A	5.5845	3.5888	3.4873	6.2707	3.4101	4.3194	3.1793	3.9701	4.2692	4.2092	1.1373	0.1856	0.4334	0.3631
Ap2a1	_VAAQVDGGAQVQQVLNIEC[Carbamidomethyl (C)]LR_.2	#N/A	3.6766	3.9276	3.5077	4.2683	3.5883	3.6868	3.9756	4.3089	2.9319	3.9420	0.9964	-0.0052	0.9614	0.0171
Ap2a1	_LEPNAQAQMYR_.2	#N/A	5.6733	4.1741	3.7389	4.2415	3.6786	4.0509	3.9562	3.8770	3.5255	4.2085	1.0945	0.1303	0.3715	0.4300
Ap2a1	_AVEYLTLSSVASTDVLATVLEEM[Oxidation (M)]PPFPER_.3	#N/A	3.0014	2.3392	4.2359	3.7797	3.6460	2.9786	3.9280	3.2215	3.5205	3.4570	0.9429	-0.0848	0.5984	0.2230
Ap2a2	_TTQIGC[Carbamidomethyl (C)]LLR_.2	#N/A	7.2975	7.3030	7.0209	6.5180	7.2081	6.9379	7.3960	7.5923	7.3476	6.9010	0.9642	-0.0526	0.2085	0.6809
Ap2a2	_LLQC[Carbamidomethyl (C)]YPPPDPAVR_.2	#N/A	7.4854	7.0987	6.5049	6.9379	6.4897	6.4573	7.2109	7.3412	6.9630	6.4852	0.9756	-0.0357	0.5295	0.2762
Ap2a2	_ATIQDVLR_.2	#N/A	7.1178	6.7231	7.0873	6.3278	6.5110	6.7723	6.6131	7.3904	6.4831	6.4263	1.0042	0.0061	0.9082	0.0418
Ap2a2	_QLSNPQQEVQNIFK_.2	#N/A	6.8155	6.7043	6.8750	5.4077	5.9170	6.1054	6.5321	6.5690	6.3582	6.1273	0.9855	-0.0210	0.7741	0.1112
Ap2a2	_SSPLIQFNLLHSK_.2	#N/A	6.1353	6.1222	6.5087	5.5863	5.6486	5.7108	6.4002	6.4726	5.5231	6.5402	0.9548	-0.0668	0.3169	0.4991
Ap2a2	_KGPSTVTDLEETKR_.3	#N/A	6.4353	6.2631	6.3271	5.8940	6.2647	5.4372	6.3101	5.9446	6.0625	5.9167	1.0074	0.0107	0.8302	0.0808
Ap2a2	_LTEC[Carbamidomethyl (C)]LETILNK_.2	#N/A	5.3286	4.9513	6.3311	4.3293	4.3677	5.1651	5.5755	5.1733	5.1574	4.8395	0.9793	-0.0302	0.7922	0.1012
Ap2a2	_FFQPTEMASQDFFQR_.2	#N/A	7.0051	6.9858	6.1662	6.5953	6.3853	6.5977	7.3424	7.2595	6.9482	6.5489	0.9427	-0.0851	0.1054	0.9771
Ap2a2	_THIETVINALK_.2	#N/A	5.8843	5.2427	4.3735	3.9519	4.3593	5.2087	5.0780	6.1441	3.8152	3.9678	1.0180	0.0257	0.8838	0.0536
Ap2a2	_YGGTFQNVSVK_.2	#N/A	4.8923	3.0310	4.8455	3.5888	3.9517	4.8490	5.7657	5.4104	5.6468	4.9612	0.7699	-0.3772	0.0188	1.7251

Ap2a2	_LSTVASTDILATVLEEM[Oxidation (M)]PPFPER_.3	#N/A	3.0716	4.2172	5.2192	4.5611	3.7427	4.7836	5.5353	4.6553	5.0092	4.2940	0.8753	-0.1921	0.2095	0.6787
Ap2a2	_TVFEALQAPAC[Carbamidomethyl (C)]HENLVK_.3	#N/A	4.3340	4.0707	4.3196	3.3214	3.4284	3.1429	4.5369	4.0907	5.1791	3.2884	0.8820	-0.1811	0.2595	0.5859
Ap2a2	_VIQIVINR_.2	#N/A	3.2397	4.1027	4.4742	3.7242	3.9241	3.8054	4.1662	4.6422	3.2823	4.4026	0.9406	-0.0884	0.4587	0.3385
Ap2a2	_AVDLLYAMC[Carbamidomethyl (C)]DR_.2	#N/A	3.8755	3.1799	3.8962	5.3290	3.0012	3.2275	3.5883	3.5612	3.4784	2.6706	1.1284	0.1743	0.3915	0.4072
Ap2a2	_TSPDLVPMGDWTSR_.2	#N/A	6.3665	3.3989	3.6772	3.2210	3.2202	3.0085	3.3693	3.7802	4.0792	4.3991	0.9766	-0.0342	0.8948	0.0483
Ap2a2	_ILVAGDTMDSVK_.2	#N/A	3.5521	3.7903	3.2858	4.3304	1.9559	3.9288	4.4787	4.1716	2.9698	4.0077	0.8892	-0.1695	0.4056	0.3919
Cox7c	_SHYEEGPGK_.2	#N/A	8.0206	7.4161	7.7748	7.5989	8.5953	8.0510	7.8718	7.7207	7.8893	7.5626	1.0191	0.0273	0.5194	0.2845
Cox7c	_NLPFSVENK_.2	#N/A	6.4445	6.7366	7.1477	6.0742	7.1159	5.9225	6.5494	7.0534	6.8124	6.7542	0.9678	-0.0472	0.4518	0.3451
Hk1	_FLSQIESDR_.2	#N/A	9.1418	9.0393	9.1273	8.9219	8.2436	8.5558	9.3657	8.9938	9.0359	8.7558	0.9779	-0.0322	0.3696	0.4323
Hk1	_ITPELLTR_.2	#N/A	8.9107	8.9834	8.9978	8.1266	8.4112	8.4732	8.6120	9.0841	8.9167	8.7814	0.9776	-0.0327	0.3554	0.4493
Hk1	_ESLLFEGR_.2	#N/A	8.9962	8.8071	8.9307	8.4586	8.0164	8.3766	8.8268	8.8827	8.7111	8.6725	0.9800	-0.0292	0.3996	0.3984
Hk1	_HIDLVEGDEGR_.2	#N/A	8.7818	8.4101	8.9128	8.4239	8.1742	8.6255	8.9689	8.6279	8.6176	8.3522	0.9899	-0.0146	0.6242	0.2047
Hk1	_ASGVEGADVVK_.2	#N/A	8.8411	8.2249	8.6886	8.3822	8.4546	8.2056	8.5218	8.5568	8.6120	8.5289	0.9896	-0.0150	0.5167	0.2868
Hk1	_LVPDSDVR_.2	#N/A	8.5175	8.4011	8.2616	8.2494	8.3894	8.3187	7.9023	8.6234	8.2849	8.2324	1.0116	0.0166	0.4754	0.3230
Hk1	_GAALITAVGVR_.2	#N/A	7.6467	7.8987	8.3348	8.2105	7.6529	7.9216	7.7395	8.5383	8.4595	8.4031	0.9589	-0.0606	0.1346	0.8709
Hk1	_SIPDGTEHGDFLALDLGGTNFR_.3	#N/A	8.1806	8.2164	8.1850	8.3256	7.2194	8.0232	8.3298	8.3301	7.8906	8.1289	0.9823	-0.0258	0.5346	0.2719
Hk1	_ATDC[Carbamidomethyl (C)]VGHDVATLLR_.3	#N/A	6.5882	6.8948	6.6347	7.1863	6.3397	6.7959	6.9567	6.8815	6.8400	6.1384	1.0053	0.0077	0.8694	0.0608
Hk1	_TTVGVDGSLYK_.2	#N/A	7.2947	7.6435	7.6999	7.5948	7.6608	7.6048	7.4563	8.1801	7.5836	7.3784	0.9913	-0.0126	0.6921	0.1598
Hk1	_GDFIALDLGGSSFR_.2	#N/A	7.5468	7.2783	7.9557	7.3727	7.4588	7.6919	7.9576	7.3371	7.7162	7.4043	0.9930	-0.0101	0.7611	0.1186
Hk1	_LSDEILIDILTR_.2	#N/A	7.5148	7.7013	7.6067	7.7357	6.8598	7.3594	7.9502	7.7931	7.5767	7.5205	0.9679	-0.0470	0.2151	0.6673
Hk1	_VVDEYSLNSGK_.2	#N/A	7.2807	6.9777	7.1455	6.9568	7.0255	6.8735	7.1415	7.2174	6.8830	7.1429	0.9925	-0.0108	0.5918	0.2278
Hk1	_RLVPDSDVR_.2	#N/A	7.2823	7.0069	7.2360	7.1860	6.9588	7.1548	7.3483	7.1822	7.1347	7.1293	0.9915	-0.0123	0.4500	0.3468
Hk1	_IDEAVLITWTK_.2	#N/A	6.8614	6.6758	6.3402	6.6846	6.4829	6.0954	6.9394	7.1966	7.1694	6.6708	0.9327	-0.1005	0.0249	1.6031
Hk1	_GQISEPLK_.2	#N/A	6.7098	6.9802	6.1422	5.7454	6.3417	6.3808	5.7295	6.7866	6.7022	7.0659	0.9714	-0.0418	0.5717	0.2428
Hk1	_AILQQLGLNSTC[Carbamidomethyl (C)]DDSILVK_.2	#N/A	6.7472	6.7897	7.1659	7.2750	5.3770	6.5116	6.4608	6.4875	6.4266	6.3995	1.0312	0.0443	0.5802	0.2365
Hk1	_NILIDFTK_.2	#N/A	6.4820	6.5913	6.2186	6.2062	5.7700	5.9037	5.5671	6.8009	6.6600	6.4323	0.9733	-0.0390	0.5504	0.2594
Hk1	_M[Oxidation (M)]HPQYSR_.2	#N/A	6.9847	3.2720	7.4260	6.7071	8.3348	7.5302	6.8609	7.4532	7.4385	7.3836	0.9211	-0.1186	0.5459	0.2629
Hk1	_M[Oxidation (M)]LPTFVR_.2	#N/A	7.3442	8.0224	7.9940	6.3997	7.4137	7.6022	7.3453	8.2439	8.8424	7.6656	0.9300	-0.1047	0.1977	0.7039
Hk1	_TDFDKVVDEYSLNSGK_.3	#N/A	5.7974	6.1129	6.6204	6.2451	6.3935	6.8917	6.8142	6.1525	6.5650	6.4775	0.9756	-0.0357	0.4989	0.3020
Hk1	_M[Oxidation (M)]ISGM[Oxidation (M)]YLGEIVR_.2	#N/A	5.5429	5.4799	6.1170	5.3016	5.1717	5.3857	5.7816	5.9555	5.8799	5.8529	0.9373	-0.0934	0.0632	1.1992
Hk1	_IM[Oxidation (M)]HQTVK_.2	#N/A	4.4516	2.9854	5.9578	5.0181	7.0214	6.0148	5.8093	6.4297	6.4327	5.9633	0.8511	-0.2327	0.2469	0.6075
Hk1	_GAAM[Oxidation (M)]VTAVAYR_.2	#N/A	5.6269	6.5154	6.9416	6.3997	5.6474	6.1982	6.2500	6.7379	6.2012	6.0032	0.9878	-0.0176	0.7983	0.0978
Hk1	_LPVGFTFSFPC[Carbamidomethyl (C)]R_.2	#N/A	3.6180	4.3742	4.8454	5.3716	4.2625	3.8629	3.7561	5.7099	4.8124	4.9452	0.9133	-0.1309	0.3879	0.4113
Hk1	_DYNPTASVK_.2	#N/A	6.0485	5.8082	3.4149	5.2663	5.3425	4.9772	3.2348	5.6803	3.8495	3.8846	1.2356	0.3052	0.1594	0.7976
Hk1	_C[Carbamidomethyl (C)]TVSFLLSEDGSGK_.2	#N/A	5.8421	4.8329	3.4589	3.9341	2.8690	3.7556	5.1431	3.9985	5.4676	5.1908	0.8314	-0.2664	0.2017	0.6953
Hk1	_QIEETLSHFR_.2	#N/A	6.9349	7.1183	7.0134	6.8851	6.4918	6.6115	7.4178	7.1439	6.6585	6.7511	0.9785	-0.0314	0.4428	0.3538
Hk1	_FTTSDVAAIETDKEGVQNAK_.3	#N/A	5.0741	5.1749	5.5486	5.8973	5.6180	5.3844	5.4668	5.4337	6.0273	5.2105	0.9846	-0.0223	0.6919	0.1599
Hk1	_SANLVAATLGAILNR_.2	#N/A	4.3431	3.8070	4.0503	3.2454	3.4604	3.0514	3.8511	2.6138	3.7251	4.0626	1.0271	0.0385	0.7953	0.0995
Hk1	_M[Oxidation (M)]C[Carbamidomethyl (C)]INTEWGAFGDDGSLEDIR_.2	#N/A	3.3346	4.1741	4.1090	4.6174	3.2767	3.8170	3.6480	3.5860	3.0617	4.3337	1.0631	0.0883	0.5114	0.2913
Hk1	_FLLSESGSGK_.2	#N/A	7.3986	7.4229	7.7599	7.5782	7.0611	7.6333	7.1969	7.8961	7.8714	7.6868	0.9756	-0.0357	0.3253	0.4878
Hk1	_LGVEPSHDDC[Carbamidomethyl (C)]VSVQHVC[Carbamidomethyl (C)]TIVSFR_.4	#N/A	3.1374	5.2133	4.3804	4.3269	3.7640	3.9655	4.5243	3.7916	4.3982	4.2939	0.9716	-0.0416	0.7571	0.1209
Hk1	_QALMEVK_.2	#N/A	3.4819	4.2382	3.2156	4.2602	1.3968	3.9990	3.8921	4.2418	3.0400	2.2640	1.0216	0.0308	0.9145	0.0388
Hk1	_AAQLC[Carbamidomethyl (C)]GAGMAAVVEK_.2	#N/A	3.1290	3.9342	3.8158	5.5029	3.1390	3.9740	4.3348	4.3155	3.1137	3.8638	1.0023	0.0033	0.9862	0.0060
Hk1	_TVC[Carbamidomethyl (C)]GVVSK_.2	#N/A	3.1966	3.9529	4.4311	4.4628	4.4848	4.2843	4.1774	3.6557	4.8580	3.6522	1.0121	0.0174	0.8880	0.0516
Hk1	_SEMEMGLR_.2	#N/A	3.7668	3.4594	4.4536	3.4554	2.8924	3.2769	3.1701	3.1623	4.3117	3.4224	1.0097	0.0139	0.9234	0.0346
Ppia	_SIYGEKFEDENFILK_.3	#N/A	9.9535	10.3205	10.6756	10.6555	10.1776	10.4084	9.8571	10.0222	10.1281	10.4066	1.0259	0.0369	0.1603	0.7951
Ppia	_VSFELFADK_.2	#N/A	8.4734	8.4775	8.1672	8.6674	8.7934	8.3307	7.8695	8.3754	8.4278	8.5097	1.0228	0.0326	0.2765	0.5584
Ppia	_KITISDC[Carbamidomethyl (C)]GQL_.2	#N/A	7.8762	8.0504	7.5985	8.6903	8.4128	7.6585	8.0964	8.2372	7.6422	7.9465	1.0084	0.0121	0.7882	0.1034
Ppia	_VNPTVFFDITADDEPLGR_.2	#N/A	9.0207	9.3621	9.5822	9.5437	9.5497	9.4970	9.4202	9.1868	9.4554	9.4565	1.0049	0.0071	0.7110	0.1481
Ppia	_VSFELFADKVPK_.2	#N/A	9.2231	9.4606	10.0390	9.6044	9.7706	9.6546	9.5327	9.4237	9.7173	9.6998	1.0033	0.0048	0.8381	0.0767
Ppia	_IIPGFMC[Carbamidomethyl (C)]QGGDFTR_.2	#N/A	9.4655	8.1714	3.5208	10.4148	8.9765	3.6938	8.5271	7.5036	7.7830	8.5618	0.9110	-0.1344	0.6544	0.1841
Ppia	_HTGPGILSM[Oxidation (M)]ANAGPNTNGSQFFIC[Carbamidomethyl (C)]TAK_.3	#N/A	6.4337	6.8582	7.3094	7.4245	7.2833	7.0097	7.0130	7.0114	6.9730	7.2203	0.9998	-0.0003	0.9947	0.0023
Ppia	_FEDENFILK_.2	#N/A	4.3447	9.0223	9.3392	9.7358	9.6889	9.2063	9.4149	9.3307	9.1663	9.2277	0.9215	-0.1179	0.5124	0.2904
Ppia	_EGM[Oxidation (M)]NIVEAMER_.2	#N/A	5.6338	3.3870	3.9030	8.1654	5.4154	3.7124	6.5157	3.7948	3.9052	5.4808	1.0228	0.0325	0.9177	0.0373
Ppia	_VKEGM[Oxidation (M)]NIVEAM[Oxidation (M)]ER_.3	#N/A	6.7624	7.0050	7.6163	7.1699	7.7782	7.7545	6.3230	7.2024	7.4744	7.6983	1.0241	0.0344	0.6066	0.2171
Ppia	_TEWLDGK_.2	#N/A	4.2118	3.4672	3.9455	4.4705	3.3555	5.8824	3.6767	3.5119	3.8110	6.0510	0.9905	-0.0138	0.9531	0.0209
Ppia	_[Acetyl (Protein N-term)]M[Oxidation (M)]VNPTVFFDITADDEPLGR_.2	#N/A	3.5165	4.2727	3.2501	4.2947	1.4367	3.9644	3.8576	4.2073	3.0055	3.9720	0.9190	-0.1219	0.6167	0.2099
Tpi1	_VVLAYEPVWAIGTGK_.2	#N/A	10.7375	10.6616	11.0479	10.8481	11.0383	10.9173	10.8617	10.6153	10.8381	10.9083	1.0064	0.0092	0.4882	0.3114
Tpi1	_IIYGGSVTGATC[Carbamidomethyl (C)]K_.2	#N/A	8.7437	8.9825	9.0469	9.4451	9.1777	8.9612	9.1511	8.9453	7.7176	9.1511	1.0364	0.0516	0.3166	0.4995
Tpi1	_SNVNDGVAQSTR_.2	#N/A	9.9145	10.1177	10.2179	10.2936	10.5856	10.2457	9.8068	10.0180	9.9935	10.2672	1.0207	0.0296	0.1625	0.7891
Tpi1	_FFVGGNWK_.2	#N/A	9.4486	9.6963	9.4755	9.3716	9.3914	9.3452	9.1513	9.5969	9.5274	9.6818	0.9964	-0.0053	0.7678	0.1148
Tpi1	_IAVAAQNC[Carbamidomethyl (C)]YK_.2	#N/A	8.0930	8.5221	8.0523	8.7552	9.2807	8.5314	8.5045	8.5519	8.4476	8.7593	0.9969	-0.0045	0.9134	0.0394
Tpi1	_VSHALAEGLGVIAC[Carbamidomethyl (C)]IGEK_.3	#N/A	6.7681	6.7796	7.2994	6.8947	6.7210	6.5865	7.0758	6.3454	6.7791	7.1203	1.0017	0.0024	0.9533	0.0208
Tpi1	_VIADNVKDWSK_.2	#N/A	7.2748	8.3974	8.8785	7.8904	8.3878	8.0949	8.1853	8.1272	7.9511	8.0245	1.0101	0.0146	0.7781	0.1089
Tpi1	_DLGATWVVLGHSER_.2	#N/A	8.6054	8.3191	7.1460	8.3108	8.6432	8.5428	7.9657	8.0256	8.2373	8.7590	1.0017	0.0025	0.9655	0.0152
Tpi1	_VVFEQTK_.2	#N/A	7.1575	7.0231	7.3286	6.5770	7.5040	7.5490	5.4518	7.4080	7.4072	7.3727	1.0405	0.0573	0.5297	0.2760
Tpi1	_VTNGAFTGEISPGM[Oxidation (M)]IK_.2	#N/A	7.9590	8.0523	7.1495	8.1567	8.7205	8.3825	8.2231	8.1386	8.2082	7.8419	0.9959	-0.0059	0.9088	0.0415
Tpi1	_TATPQQAQEVHEK_.2	#N/A	9.9240	10.2062	10.1725	10.2016	10.5873	10.2882	9.9970	10.1010	9.8928	10.2203	1.0176	0.0252	0.1863	0.7297
Tpi1	_HVFGESDELIGQK_.2	#N/A	6.5903	6.9520	7.0839	7.5938	7.1367	7.3479	7.0849	7.0052	6.9981	7.3645	1.0006	0.0009	0.9826	0.0076
Tpi1	_ELASQPDVDGFLVGGASLKPEFVDIINAK_.3	#N/A	3.0672	4.9522	5.4282	6.3235	5.3770	3.6758	5.4829	5.0766	5.0045	4.4248	0.9613	-0.0569	0.7710	0.1129
Tpi1	bamidomethyl (C)]LGELIC[Carbamidomethyl (C)]TLNAANVPAGTEVVC[Carbamidomethyl (C)]APPTAYID	#N/A	3.0940	3.5642	4.4299	4.4639	3.7071	4.2854	4.3484	3.3880	3.3487	3.6510	1.0652	0.0911	0.4970	0.3036
Tpi1	_RHVFGESDELIGQK_.3	#N/A	5.5681	5.4618	3.7816	6.1000	6.0001	5.0116	4.0759	5.7916	5.4382	5.7022	1.0131	0.0187	0.9014	0.0451
Slc2a1	_TFDEIASGFR_.2	#N/A	6.4763	6.7161	6.6976	5.9099	5.8573	5.8312	6.3359	7.0047	6.1288	6.1567	0.9753	-0.0361	0.5757	0.2398
Slc2a1	_AKS[Phospho (STY)]VLKK_.2	P17809_S226_M1_Slc2a1	5.1131	4.6448	6.1404	4.4108	1.8550	4.2361	6.2464	4.6839	4.5322	5.1101	0.8555	-0.2251	0.3729	0.4284
Slc2a1	_VTILELFR_.2	#N/A	4.9972	4.9393	5.3398	4.3397	3.6161	3.7071	4.4880	4.6859	4.5384	4.0170	1.0202	0.0288	0.8225	0.0849
Slc2a1	_QGGASQSDKT[Phospho (STY)]PEELFHPLGADSQV_.3	P17809_T478_M1_Slc2a1	4.1024	3.2400	3.8361	3.3799	3.0613	3.1674	3.5282	9.5676	3.9203	8.3764	0.5458	-0.8737	0.0475	1.3235
Hspa1b;Hspa1a	_IINEPTAAAIAYGLDR_.2	#N/A	4.9487	5.0629	4.9355	5.4264	4.5063	4.7760	5.4524	4.9555	5.4980	4.4094	0.9732	-0.0392	0.6073	0.2166
Hspa1b;Hspa1a	_NQVALNPQNTVFDAK_.2	#N/A	6.2682	6.3693	6.1770	6.4890	6.3084	6.2846	6.6359	6.3242	6.3176	5.9939	0.9997	-0.0004	0.9880	0.0052
Hspa1b;Hspa1a	_ATAGDTHLGGEDFDNR_.3	#N/A	3.7644	3.6930	4.1713	4.0837	3.5813	9.0542	3.7984	8.7211	3.8721	10.9008	0.6924	-0.5302	0.2723	0.5649

Ptpra	_QAGSHSNS[Phospho (STY)]FR_.2	P18052_S180_M1_Ptpra	4.9812	5.2408	3.6079	5.1624	5.2148	5.5949	5.8431	6.5021	3.4957	5.7186	0.9215	-0.1179	0.5192	0.2846
Ptpra	_VVQEYIDAFSDY[Phospho (STY)]ANFK_.2	P18052_Y825_M1_Ptpra	6.4517	7.4912	7.6110	5.5723	4.7982	6.3307	6.5928	7.3328	6.5364	6.5043	0.9952	-0.0069	0.9570	0.0191
Ctsd	_VSSLPTVYLK_.2	#N/A	7.3028	7.5868	7.5722	7.5596	7.5623	7.5344	7.4431	7.4853	7.0745	7.5129	1.0191	0.0273	0.1885	0.7247
Ctsd	_TTEPVSELLK_.2	#N/A	6.9073	6.7789	6.4694	6.9469	6.9264	7.0275	6.8118	6.3354	6.8712	7.3585	0.9998	-0.0003	0.9940	0.0026
Ctsd	_QPGIVFVAAK_.2	#N/A	6.0391	5.8053	5.6259	5.7147	6.5463	5.7344	5.8802	5.6894	5.7534	6.1865	1.0057	0.0082	0.8673	0.0618
Ctsd	_QIFGEATK_.2	#N/A	4.9290	4.9335	4.7278	5.5607	5.9934	5.7016	4.2220	4.8869	4.9301	5.3032	1.0976	0.1344	0.1734	0.7611
Ctsd	_ILDIAC[Carbamidomethyl (C)]WVHHK_.3	#N/A	3.6437	3.8639	3.9354	3.4080	3.3173	3.1891	4.2152	3.1835	3.3290	4.4695	0.9369	-0.0940	0.4414	0.3552
Ctsd	_AIGAVPLIQGEYMIPC[Carbamidomethyl (C)]EK_.2	#N/A	4.1532	3.4030	3.6602	6.1833	4.4654	3.9844	3.8776	3.9556	3.6042	3.9837	1.1175	0.1603	0.4006	0.3973
Bsg	_YIVDADDR_.2	#N/A	6.9524	7.1887	6.8756	7.1843	6.7277	6.5250	6.7541	6.9396	6.8208	7.0225	1.0036	0.0052	0.8650	0.0630
Bsg	_SEINVEGPPR_.2	#N/A	6.7389	6.9375	6.9784	6.9247	6.7691	6.8700	6.4268	6.7741	6.5431	6.5420	1.0454	0.0640	0.0043	2.3639
Bsg	_SGEYSC[Carbamidomethyl (C)]IFLPEPVGR_.2	#N/A	6.7204	6.9311	6.5823	6.8013	6.4181	6.4777	6.9147	6.8075	6.4018	6.5889	0.9965	-0.0050	0.8687	0.0611
Bsg	_YVVVSTPEK_.2	#N/A	6.8450	6.2966	6.9892	6.9738	6.8572	6.4144	6.3611	6.7780	6.6253	6.1437	1.0390	0.0551	0.2169	0.6637
Bsg	_VLQEDTLPDLHTK_.3	#N/A	5.0314	3.3002	3.1818	3.6844	4.4668	3.1072	3.3991	4.4340	4.0826	4.3288	0.9345	-0.0977	0.5634	0.2492
Slc7a2	_NLS[Phospho (STY)]LPFILHEK_.2	P18581_S645_M1_Slc7a2	5.1456	7.2343	7.1907	7.7068	4.5565	5.1459	6.5996	7.7613	6.4333	5.3419	0.9433	-0.0843	0.6540	0.1844
Rps6ka3	_NQS[Phospho (STY)]PVLEPVGR_.2	P18654_S715_M1_Rps6ka3	8.6646	8.5138	8.9965	8.6958	7.6932	8.5504	8.7773	8.5876	8.6146	8.5919	0.9857	-0.0208	0.6001	0.2218
Rps6ka3	_DLKPENILLDEEGHIK_.3	#N/A	4.0550	4.8788	3.2410	4.8723	3.9993	4.5039	4.7635	4.3193	4.2516	4.0339	0.9807	-0.0281	0.8129	0.0900
Cfl1	_[Acetyl (Protein N-term)]AS[Phospho (STY)]GVAVSDGVIK_.2	P18760_S3_M1_Cfl1	14.3716	14.2551	15.1361	14.7036	14.1076	14.6555	14.8877	14.2171	14.4002	14.4701	1.0031	0.0044	0.8452	0.0730
Cfl1	_YALYDATYETK_.2	#N/A	10.5639	10.5577	10.7977	10.9035	10.7943	10.9315	10.6523	10.4491	10.5974	10.7497	1.0138	0.0197	0.1694	0.7711
Cfl1	_KSSTPEEVK_.2	#N/A	7.3495	7.2695	7.1244	7.6547	7.4026	6.8114	6.5993	7.4719	7.5797	7.4886	0.9978	-0.0032	0.9463	0.0240
Cfl1	_LGGSAVISLEGKPL_.2	#N/A	8.0513	7.9180	7.6063	7.8950	7.8095	7.7964	8.2947	7.9483	7.9251	8.3900	0.9640	-0.0530	0.0413	1.3840
Cfl1	_SSTPEEVKK_.2	#N/A	6.2473	6.4777	6.1769	6.8286	7.1918	7.0881	6.6052	6.1905	6.7512	6.5830	1.0208	0.0297	0.5863	0.2319
Cfl1	_EILVGDVGQTVDDPYTTFVK_.2	#N/A	6.7146	6.1841	6.1675	6.7239	7.1861	6.5387	6.6426	6.9017	6.3238	6.6422	0.9937	-0.0091	0.8521	0.0695
Cfl1	_M[Oxidation (M)]LPDKDC[Carbamidomethyl (C)]R_.2	#N/A	8.6417	8.7125	7.9560	9.2031	9.4803	8.4133	8.4843	8.6802	8.5991	8.5943	1.0169	0.0242	0.6195	0.2079
Cfl1	_NIILEEGKEILVGDVGQTVDDPYTTFVK_.3	#N/A	2.7506	5.6947	6.3628	7.4964	3.2118	4.0435	4.8847	5.1835	4.4526	1.7331	1.2124	0.2779	0.4730	0.3252
Cfl1	_NIILEEGK_.2	#N/A	6.3045	5.8944	5.3998	5.4423	6.3477	5.2885	3.2187	5.8017	5.7954	6.1212	1.1042	0.1430	0.3795	0.4208
Cfl1	_AVLFC[Carbamidomethyl (C)]LSEDKK_.2	#N/A	6.4891	6.9262	6.9182	7.7076	7.1205	7.0239	7.0159	7.2443	7.0043	7.2000	0.9880	-0.0174	0.6926	0.1595
Cfl1	_[Acetyl (Protein N-term)]ASGVAVSDGVIKVFNDM[Oxidation (M)]K_.2	#N/A	4.0031	3.5521	5.0528	4.0840	3.9914	4.4733	3.6338	3.3082	2.1914	2.5163	1.4396	0.5257	0.0089	2.0498
Cfl1	_KEDLVFIFWAPENAPLK_.3	#N/A	5.5002	5.4228	5.3388	5.4619	5.2347	4.8879	5.5062	5.4874	5.7123	4.9405	0.9808	-0.0280	0.5678	0.2458
Cfl1	_HELQANC[Carbamidomethyl (C)]YEEVKDR_.3	#N/A	6.0286	5.3782	4.3402	6.4852	7.0430	6.4033	5.0086	6.1739	6.6660	5.9913	0.9977	-0.0033	0.9814	0.0082
Cfl1	_SST[Phospho (STY)]PEEVKKR_.2	#N/A	3.7856	3.5568	3.5193	4.5639	3.3781	4.3514	3.2113	3.9381	4.2372	4.2412	0.9878	-0.0177	0.8827	0.0542
Cfl1	_KSST[Phospho (STY)]PEEVK_.2	#N/A	6.2366	4.9436	5.4906	6.0466	4.8280	4.8552	6.3250	4.2237	6.1403	6.0520	0.9498	-0.0742	0.5859	0.2322
Cfl1	_LTGIKHELQANC[Carbamidomethyl (C)]YEEVKDR_.4	#N/A	5.7175	4.3380	3.5750	4.0777	6.2192	5.0062	3.7923	3.9131	3.6894	4.0447	1.2493	0.3212	0.1016	0.9929
Cfl1	_MIYASSK_.2	#N/A	4.0679	3.2320	3.8016	3.3453	3.2785	3.3469	3.4936	3.6589	3.9042	3.0226	0.9978	-0.0032	0.9735	0.0117
Gnao1	_IGAGDYQPTEQDILR_.2	#N/A	11.0458	10.8904	11.0852	11.0718	10.3190	10.6802	11.2169	10.8570	10.6838	10.7214	0.9981	-0.0028	0.9105	0.0407
Gnao1	_LFDVGGQR_.2	#N/A	10.3389	10.6818	10.7110	10.1539	10.1191	9.9414	10.4731	10.5949	10.4037	10.1502	0.9922	-0.0113	0.6588	0.1813
Gnao1	_NLKEDGISAAK_.2	#N/A	8.3609	8.4069	8.3544	8.5828	8.3008	8.5879	8.5982	8.5157	8.4665	8.5091	0.9894	-0.0153	0.2129	0.6719
Gnao1	_LWGDSGIQEC[Carbamidomethyl (C)]FNR_.2	#N/A	8.6053	8.4925	9.1123	8.7655	8.5682	8.3151	8.5029	8.4701	8.6039	8.3806	1.0181	0.0259	0.3166	0.4995
Gnao1	_EYQLNDSAK_.2	#N/A	8.7920	8.4304	8.8444	8.4935	8.7546	8.5614	8.5150	8.8733	8.6670	8.8431	0.9910	-0.0131	0.4958	0.3047
Gnao1	_KDLFGEK_.2	#N/A	6.0244	5.3449	5.2109	5.0994	5.9712	5.7123	5.1011	5.6089	5.5555	4.9699	1.0474	0.0668	0.3232	0.4905
Gnao1	_YYLDSLDR_.2	#N/A	9.8620	9.6768	10.1157	9.5613	9.4252	9.5321	9.3948	9.8670	9.5978	9.7172	1.0053	0.0077	0.7437	0.1286
Gnao1	_M[Oxidation (M)]VC[Carbamidomethyl (C)]DVVSR_.2	#N/A	8.4297	8.5696	8.3980	8.4922	8.8811	9.2171	8.3420	8.6263	8.5701	8.3734	1.0220	0.0314	0.3130	0.5045
Gnao1	_IIHEDGFSGEDVK_.3	#N/A	8.8254	8.4566	8.7715	8.6048	8.7447	8.6906	7.9802	8.6785	8.8383	8.7495	1.0141	0.0202	0.4973	0.3034
Gnao1	_QYKPVVYSNTIQSLAAIVR_.3	#N/A	7.4119	7.2203	7.7485	7.3942	6.7248	7.1872	7.2520	7.4199	7.3110	7.6651	0.9823	-0.0257	0.5035	0.2980
Gnao1	_LFDVGGQRS[Phospho (STY)]ER_.2	#N/A	8.8942	8.8473	9.7064	9.3713	8.3453	8.9954	9.4023	9.1231	8.9149	9.0765	0.9888	-0.0163	0.6947	0.1582
Gnao1	_M[Oxidation (M)]HESLM[Oxidation (M)]LFDSIC[Carbamidomethyl (C)]NNK_.3	#N/A	6.1830	6.1681	6.6788	6.3140	5.1967	6.1556	6.4073	6.0512	6.1388	6.5425	0.9731	-0.0393	0.5444	0.2641
Gnao1	_M[Oxidation (M)]EDTEPFSAELLSAM[Oxidation (M)]MR_.2	#N/A	5.8440	4.6758	3.9044	6.7962	3.2910	3.9018	3.6738	3.6467	3.8562	4.1139	1.2388	0.3090	0.2212	0.6552
Gnao1	_AM[Oxidation (M)]DTLGVEYGDKER_.2	#N/A	6.7764	6.9288	6.5492	6.7914	6.8591	7.2003	6.6948	7.1108	6.8613	7.1660	0.9846	-0.0224	0.4637	0.3338
Gnao1	_NLKEDGIS[Phospho (STY)]AAKDVK_.3	#N/A	4.7185	4.7977	5.4934	5.0017	2.9058	4.2403	4.2575	4.5149	4.7219	3.8467	1.0440	0.0622	0.7006	0.1545
Gnao1	_SREYQLNDSAK_.2	#N/A	5.6727	6.1071	6.0467	5.8282	5.4198	6.1560	6.2140	5.4074	5.6583	6.1920	1.0006	0.0009	0.9863	0.0060
Gnao1	_SPLTIC[Carbamidomethyl (C)]FPEYPGSNTYEDAAAYIQTQFESK_.3	#N/A	3.7641	3.5783	3.4978	4.1811	3.3996	4.3299	3.1898	3.9596	4.2586	4.2197	0.9705	-0.0432	0.6879	0.1625
Gnao1	_PVVYSNTIQSLAAIVR_.2	#N/A	3.7035	2.9590	3.4372	3.9558	2.8473	3.7773	3.6705	4.1627	3.8112	3.9228	0.8856	-0.1752	0.1079	0.9668
Fasn	_SRS[Phospho (STY)]DTSLK_.2	P19096_S2201_M1_Fasn	6.7480	6.4924	4.0778	6.1675	3.7455	5.3800	7.9203	6.7179	6.4958	6.3338	0.7915	-0.3373	0.0779	1.1085
Fasn	_VQEVQQVSTNK_.2	#N/A	6.1724	5.4791	5.8351	5.4322	5.4032	5.9878	6.2967	5.8954	5.7025	5.5957	0.9737	-0.0384	0.4758	0.3226
Fasn	_VTAIYIDPATHR_.2	#N/A	5.3968	6.0788	4.7229	5.0889	4.6924	5.2307	6.1240	6.1435	5.6790	4.7406	0.9171	-0.1248	0.2376	0.6241
Fasn	_TLEAVQDLLEQGR_.2	#N/A	4.0741	5.0778	4.5035	4.3011	5.2719	5.2935	5.7146	5.8695	5.2456	5.5610	0.8492	-0.2358	0.0194	1.7129
Fasn	_GYTVLGVEGR_.2	#N/A	5.4719	5.2794	3.8437	3.3875	5.5447	5.6209	5.7467	5.9934	5.4088	5.6438	0.8526	-0.2301	0.1375	0.8618
Fasn	_LFDHPEVPTPPESASVSR_.3	#N/A	4.1734	3.4778	3.3594	5.1571	5.9389	5.7060	3.6717	5.9485	5.7960	3.8291	0.8449	-0.2431	0.2753	0.5602
Gstp1	_PPYTIVYFPVR_.2	#N/A	6.1034	6.8170	6.8625	6.7905	7.1979	6.9685	6.7033	6.9733	6.5969	6.7409	1.0054	0.0078	0.8586	0.0662
Gstp1	_YVTLIYTNYENGK_.2	#N/A	6.7296	6.5317	6.2486	6.8693	6.8052	6.3343	6.7801	6.3428	6.1698	6.7728	1.0108	0.0154	0.7055	0.1515
Gstp1	_MLLADQGQSWK_.2	#N/A	5.5886	3.6545	4.1328	6.7818	6.4579	3.5696	5.4346	4.9304	5.0961	5.0815	0.9796	-0.0297	0.8903	0.0505
Gstp1	_EAAQM[Oxidation (M)]DM[Oxidation (M)]VNDGVEDLR_.2	#N/A	5.8462	6.3529	6.8154	5.7288	6.5442	6.5314	6.2556	6.1245	6.3855	6.2467	1.0080	0.0115	0.8272	0.0824
Gstp1	_ALPGHLKPFETLLSQNQGGK_.3	#N/A	8.0635	8.2431	8.4422	8.7679	8.2909	8.3498	8.3691	8.2201	8.1286	8.5832	1.0041	0.0059	0.8176	0.0875
Gstp1	_FEDGDLTLYQSNAILR_.2	#N/A	8.0961	8.3779	8.7379	8.6315	8.5298	8.3704	8.3957	8.0868	8.3237	8.3550	1.0201	0.0288	0.2296	0.6390
Gstp1	_EAAQMDMVNDGVEDLRGK_.3	#N/A	4.0053	3.2208	3.1913	4.5153	3.1091	3.5155	3.4086	4.4245	4.0731	3.6610	0.9232	-0.1153	0.3993	0.3987
Nefh	_AKPLDVKS[Phospho (STY)]PEAQTPVQEEAK_.3	#N/A	11.0947	10.9506	11.2466	11.6719	10.9374	10.8925	11.3096	10.6846	10.9106	11.0159	1.0139	0.0199	0.4276	0.3690
Nefh	_S[Phospho (STY)]PGEAKS[Phospho (STY)]PAEAK_.2	#N/A	10.2749	10.0736	9.8707	10.7054	9.4061	10.0263	10.4937	9.8084	10.1212	10.4172	0.9852	-0.0214	0.5667	0.2467
Nefh	_SPAEAKS[Phospho (STY)]PIEVK_.2	#N/A	10.0628	10.2186	10.1305	10.8260	10.0239	10.1335	10.5945	9.9325	10.0239	10.1850	1.0048	0.0069	0.8060	0.0937
Nefh	_S[Phospho (STY)]PAEAKS[Phospho (STY)]PAAVK_.2	#N/A	9.4164	9.4649	9.4086	9.9644	9.4208	9.5189	9.8134	9.2727	9.5864	9.6697	0.9944	-0.0080	0.7182	0.1437
Nefh	_S[Phospho (STY)]PGEAKS[Phospho (STY)]PAAVK_.2	#N/A	9.5132	9.1852	9.2807	9.8847	9.1953	9.3648	9.5511	9.1217	9.3684	9.5884	0.9996	-0.0005	0.9833	0.0073
Nefh	_TSVSSVSAS[Phospho (STY)]PSR_.2	P19246_S61_M1_Nefh	8.5758	8.8354	9.5553	9.3152	8.7039	8.8083	9.7090	8.5142	8.6540	8.8373	1.0041	0.0060	0.9010	0.0453
Nefh	_S[Phospho (STY)]PAEAKS[Phospho (STY)]PGEAK_.2	#N/A	9.0136	8.9296	8.6963	9.6067	8.1124	8.7487	9.3042	8.6636	8.9164	9.1845	0.9816	-0.0268	0.5599	0.2519
Nefh	_SPIEVKS[Phospho (STY)]PEK_.2	#N/A	8.8851	8.7515	8.6714	9.4626	8.7101	8.6894	9.3901	8.4629	8.8400	8.9282	0.9951	-0.0071	0.8452	0.0730
Nefh	_SEKEQLQALNDR_.2	#N/A	8.4351	8.8478	8.9933	9.2116	8.7012	8.8043	9.0518	8.4330	8.6488	8.8788	1.0090	0.0130	0.6564	0.1828
Nefh	_S[Phospho (STY)]PGEAKS[Phospho (STY)]PAEPK_.2	#N/A	8.6213	8.3202	8.0995	8.8091	8.2893	8.3104	8.7895	8.0779	8.4966	8.4374	0.9950	-0.0072	0.8162	0.0882
Nefh	_S[Phospho (STY)]PSEAKS[Phospho (STY)]PAEAK_.2	#N/A	7.8244	7.7466	7.7855	8.4918	7.2642	7.8644	8.0734	7.4536	7.6686	7.8812	1.0078	0.0112	0.7966	0.0988
Nefh	_S[Phospho (STY)]PVKEDIKPPAEAK_.3	P19246_S795_M1_Nefh	8.7072	8.3557	8.3503	8.8836	8.7134	8.6418	8.1382	8.0337	8.6916	8.7098	1.0257	0.0365	0.2621	0.5816
Nefh	_S[Phospho (STY)]PAEAKS[Phospho (STY)]PAEPK_.2	#N/A	7.5528	7.4853	7.2302	8.5155	7.9716	7.5872	7.9448	7.0017	7.3470	7.7506	1.0283	0.0403	0.4780	0.3206
Nefh	_EQLQALNDR_.2	#N/A	7.4124	7.2911	7.2952	8.0826	7.2602	6.9560	7.6143	6.9405	7.4489	7.0950	1.0149	0.0213	0.6469	0.1891
Nefh	_AVAS[Phospho (STY)]EEETPAK_.2	P19246_S965_M1_Nefh	6.9456	7.6475	8.1556	8.0256	7.4075	7.1906	3.4781	4.3550	7.2368	3.7304	1.6089	0.6861	0.0043	2.3702

Nefh	_S[Phospho (STY)]PVKEEVK_.2	#N/A	6.7191	5.6500	5.6084	6.6598	6.0832	5.7517	5.9492	6.1994	6.5780	6.5045	0.9637	-0.0534	0.4379	0.3586
Nefh	_IPSISTHIK_.2	#N/A	5.9277	6.1251	5.1048	5.4894	6.0532	5.9255	5.2429	5.3331	6.1442	5.8024	1.0249	0.0355	0.6057	0.2177
Nefh	_HQADIASYQDAIQQLDSELR_.3	#N/A	5.5010	6.2241	6.3331	6.3402	6.1296	5.2126	5.4453	5.3853	5.8201	5.7998	1.0613	0.0859	0.2240	0.6497
Nefh	_S[Phospho (STY)]PEAQTPVQEEAK_.2	#N/A	8.7454	8.1155	6.4501	9.1144	7.8956	8.1942	8.7120	8.0251	8.1478	8.5681	0.9668	-0.0487	0.5841	0.2335
Nefh	_SPIEVKS[Phospho (STY)]PEKAK_.2	#N/A	6.5611	7.3419	8.1886	7.7718	7.2839	7.2794	7.5061	7.2380	7.1467	7.1158	1.0211	0.0301	0.6097	0.2149
Nefh	_S[Phospho (STY)]PAEAKS[Phospho (STY)]PEK_.2	#N/A	7.7849	7.3541	7.1446	8.0900	6.5183	6.8481	7.8874	7.3953	7.1254	7.3274	0.9806	-0.0282	0.6687	0.1748
Nefh	_C[Carbamidomethyl (C)]DVTSALR_.2	#N/A	5.3351	5.3738	4.7488	3.6787	5.5231	5.2736	4.8896	4.4536	3.2590	4.4748	1.1686	0.2247	0.1486	0.8281
Nefh	_SPAEAKS[Phospho (STY)]PAEAKS[Phospho (STY)]PIEVK_.3	#N/A	9.0090	9.1233	9.1739	9.6737	9.1894	9.2295	9.4271	8.8539	9.1902	9.2886	1.0047	0.0068	0.7829	0.1063
Nefh	_S[Phospho (STY)]LEGEAAALR_.2	P19246_S122_M1_Nefh	6.3788	5.7298	6.3497	6.4162	5.7341	6.4035	6.6104	5.7704	6.3626	6.4413	0.9797	-0.0295	0.5870	0.2313
Nefh	_S[Phospho (STY)]PEKAKS[Phospho (STY)]PVK_.2	P19246_S795_M2_Nefh	5.0370	3.7257	4.3062	3.6394	4.2459	3.4836	4.8603	3.2791	3.6041	3.5912	1.0624	0.0874	0.5705	0.2437
Nefh	_VKEEAKS[Phospho (STY)]PGEAK_.2	#N/A	8.3371	8.7879	8.7506	9.1851	8.3322	8.9968	8.7222	8.4664	8.7155	8.7469	1.0080	0.0114	0.7172	0.1444
Nefh	_SPAAVKS[Phospho (STY)]PAEAKS[Phospho (STY)]PAAVK_.2	#N/A	8.4421	8.5249	8.5870	9.2355	8.1916	8.6645	8.8334	8.3128	8.3983	8.3661	1.0153	0.0219	0.5366	0.2704
Nefh	_SPAEPKS[Phospho (STY)]PAEAKS[Phospho (STY)]PAEVK_.2	#N/A	7.6970	7.8281	7.9125	8.3985	7.9626	7.8973	8.0666	7.4681	7.7879	7.7261	1.0241	0.0344	0.2637	0.5789
Nefh	_SPAEAKS[Phospho (STY)]PAEVKS[Phospho (STY)]PAEAK_.2	#N/A	6.9208	7.0501	7.0405	7.5466	6.9078	7.1930	7.3570	6.7944	6.8955	6.9041	1.0175	0.0250	0.4586	0.3386
Nefh	_ETVIVEGQTEEIR_.2	#N/A	5.3463	5.5280	5.9348	6.8980	3.2549	4.8492	6.0182	2.4822	4.5961	6.0248	1.1091	0.1494	0.5811	0.2358
Nefh	_SPAEAKS[Phospho (STY)]PIEVKS[Phospho (STY)]PEK_.2	#N/A	8.2697	7.9618	7.4790	8.6564	7.8672	8.0347	8.5429	7.8555	7.9721	8.2159	0.9875	-0.0181	0.6763	0.1699
Nefh	_SPAEPKS[Phospho (STY)]PAEAKS[Phospho (STY)]PAEPK_.2	#N/A	7.3343	7.8296	7.9663	8.0634	8.0687	7.5114	8.2084	7.5253	7.7111	7.4295	1.0100	0.0143	0.7207	0.1422
Nefh	_SPAEAKS[Phospho (STY)]PAEAKS[Phospho (STY)]PAEVK_.2	#N/A	7.1455	7.0897	7.1537	7.7466	7.1506	7.1107	7.4466	6.8344	6.8735	7.1310	1.0228	0.0326	0.3721	0.4294
Nefh	_SPAEAKS[Phospho (STY)]PATVKS[Phospho (STY)]PGEAK_.2	#N/A	4.7560	5.6053	5.6542	5.9181	5.0948	5.8332	6.0795	3.9312	4.7901	5.3089	1.0894	0.1236	0.3218	0.4924
Nefh	_AQALQEEC[Carbamidomethyl (C)]GYLR_.2	#N/A	4.1784	4.6710	3.8003	3.8885	5.4687	5.0523	4.0176	4.7037	4.4792	5.1933	0.9807	-0.0281	0.8270	0.0825
Nefh	_SPGEAKS[Phospho (STY)]PAAVKS[Phospho (STY)]PAEAK_.2	#N/A	8.7791	8.5972	8.5854	9.1328	8.2371	8.7613	8.9987	8.3506	8.6170	8.9185	0.9955	-0.0065	0.8427	0.0743
Nefh	_SPGEAKS[Phospho (STY)]PAEPKS[Phospho (STY)]PAEAK_.2	#N/A	7.9753	8.4002	8.5983	9.0611	8.4187	8.1610	8.6556	8.2434	8.1923	8.2381	1.0124	0.0178	0.6358	0.1967
Nefh	_SPSEAKS[Phospho (STY)]PAEAKS[Phospho (STY)]PAEAK_.2	#N/A	7.3096	7.6304	7.7691	8.1839	7.4027	7.2375	7.9292	7.4765	7.7200	7.7758	0.9823	-0.0257	0.5038	0.2977
Nefh	_SPAEVKS[Phospho (STY)]PAEAKS[Phospho (STY)]PAEVK_.2	#N/A	7.5148	7.6073	7.6780	8.6289	7.7648	7.8203	8.1322	7.6078	7.8834	7.8121	0.9971	-0.0043	0.9198	0.0363
Nefh	_S[Phospho (STY)]PGEAKS[Phospho (STY)]PAEAKS[Phospho (STY)]PAEAK_.2	#N/A	9.9297	9.9643	9.8178	10.5285	9.9522	10.0003	10.3405	9.6691	9.9518	10.2069	0.9990	-0.0014	0.9559	0.0196
Nefh	_SPAEAKS[Phospho (STY)]PAEAKS[Phospho (STY)]PAEAK_.2	#N/A	9.1709	9.5346	9.6787	9.9039	9.4201	9.5469	9.6667	9.3296	9.3781	9.4922	1.0080	0.0115	0.5995	0.2222
Nefh	_S[Phospho (STY)]PAEAKS[Phospho (STY)]PGEAKS[Phospho (STY)]PGEAK_.2	#N/A	9.2821	9.2787	8.9740	10.0318	9.2108	9.1774	9.6574	8.9147	9.3421	9.4099	0.9994	-0.0008	0.9818	0.0080
Nefh	_SPAEAKS[Phospho (STY)]PAEVKS[Phospho (STY)]PGEAK_.2	#N/A	8.5259	9.0241	8.8617	9.3823	8.8127	8.7326	9.0565	8.6044	8.5141	8.6986	1.0197	0.0281	0.3582	0.4459
Nefh	_S[Phospho (STY)]PGEAKS[Phospho (STY)]PAEPKS[Phospho (STY)]PAEPK_.2	#N/A	7.6806	7.4889	7.5784	8.3084	7.7582	7.6953	7.9816	7.3892	7.8443	7.4683	1.0105	0.0151	0.6756	0.1703
Nefh	_SPAEAKS[Phospho (STY)]PAEAK_.2	#N/A	10.5553	10.9273	11.1400	11.3536	10.3923	10.9078	11.0979	10.7074	10.8094	10.8673	1.0008	0.0012	0.9646	0.0156
Nefh	_S[Phospho (STY)]PGEAKS[Phospho (STY)]PGEAKS[Phospho (STY)]PAEAK_.2	#N/A	9.6696	9.7228	9.6345	10.3435	9.7811	9.5718	10.0170	9.3780	9.7653	9.7174	1.0070	0.0100	0.7123	0.1474
Nefh	_S[Phospho (STY)]PGEAKS[Phospho (STY)]PAEAKS[Phospho (STY)]PGEAK_.2	#N/A	9.3438	9.4285	9.2102	10.0000	9.4673	9.2859	9.7017	9.0181	9.4722	9.4380	1.0052	0.0074	0.7976	0.0982
Nefh	_SPATVKS[Phospho (STY)]PGEAK_.2	P19246_S589_M1_Nefh	8.7149	9.2133	9.3997	9.4611	9.1493	8.5666	9.3614	8.5048	9.0479	9.0812	1.0095	0.0136	0.7249	0.1397
Nefh	_VKS[Phospho (STY)]EEMIK_.2	#N/A	6.2737	5.3642	4.2674	7.3671	5.2042	2.9471	5.6034	4.7449	4.8588	5.3202	1.0206	0.0293	0.8984	0.0465
Nefh	_S[Phospho (STY)]PAEAKS[Phospho (STY)]PATVK_.2	#N/A	7.6601	8.1491	7.8181	8.7534	8.0759	8.2037	8.5212	7.9470	8.0903	8.3318	0.9863	-0.0199	0.6188	0.2084
Nefh	_SPGEAKS[Phospho (STY)]PSEAK_.2	#N/A	9.6727	10.0877	10.1713	10.5619	9.3966	9.9226	10.1960	9.7919	9.7930	10.1096	0.9996	-0.0005	0.9869	0.0057
Nefh	_SPAEPKS[Phospho (STY)]PAEPKS[Phospho (STY)]PAEAK_.2	#N/A	7.8951	8.2201	8.4616	8.8277	8.3624	8.0380	7.0696	7.8099	8.0364	7.8643	1.0787	0.1093	0.0350	1.4556
Nefh	_SPAEAKS[Phospho (STY)]PAAVKS[Phospho (STY)]PGEAK_.2	#N/A	7.5098	8.1724	8.0273	8.4251	7.4021	8.2044	8.2709	7.8122	7.9897	7.9516	0.9938	-0.0089	0.8304	0.0807
Nefh	_SPAEVKS[Phospho (STY)]PGEAKS[Phospho (STY)]PAEPK_.2	#N/A	7.2919	7.2045	7.8330	7.6116	7.8024	7.5442	7.3429	7.2656	7.3233	7.5010	1.0258	0.0367	0.2055	0.6871
Nefh	_TPVKEGAKS[Phospho (STY)]PAEAK_.2	#N/A	4.6502	4.4381	4.3926	4.6441	4.7103	3.6171	4.5422	4.1415	3.7254	4.4532	1.0458	0.0646	0.4692	0.3286
Nefh	_SPAEVKS[Phospho (STY)]PGEAK_.2	P19246_S679_M1_Nefh	11.0511	11.4561	11.7857	11.7969	11.3431	11.4056	11.6353	11.1771	11.2732	11.3620	1.0098	0.0140	0.5180	0.2856
Nefh	_S[Phospho (STY)]PGEAKS[Phospho (STY)]PGEAK_.2	#N/A	10.0316	10.3746	10.4069	10.8480	9.7500	10.1852	10.5180	10.0518	10.0255	10.3140	1.0038	0.0055	0.8596	0.0657
Nefh	_S[Phospho (STY)]PAEAKS[Phospho (STY)]PAEVK_.2	#N/A	10.0852	10.1493	9.8675	10.6891	9.9912	10.1169	10.4816	9.8602	10.0037	10.3047	0.9988	-0.0018	0.9463	0.0240
Nefh	_SPAAVKS[Phospho (STY)]PAEAK_.2	P19246_S727_M1_Nefh	9.8903	9.9456	10.1125	10.4852	10.0123	9.9575	10.1754	9.8552	9.9617	10.0945	1.0045	0.0065	0.7243	0.1401
Nefh	_S[Phospho (STY)]PAEVKS[Phospho (STY)]PAEAK_.2	#N/A	9.8273	9.9083	9.8596	10.4612	9.7510	9.8923	10.2778	9.6126	9.7729	10.0618	1.0019	0.0027	0.9179	0.0372
Nefh	_S[Phospho (STY)]PAEPKS[Phospho (STY)]PAEAK_.2	#N/A	9.8258	9.9548	10.0860	10.3689	9.8476	9.9011	10.1987	9.6713	9.8416	10.0159	1.0066	0.0095	0.6465	0.1894
Nefh	_SPAAVKS[Phospho (STY)]PGEAK_.2	P19246_S739_M1_Nefh	9.5657	9.7752	9.9565	10.1750	9.8327	9.7573	10.0759	9.5684	9.7032	9.8473	1.0046	0.0066	0.7484	0.1258
Nefh	_S[Phospho (STY)]PAEPKS[Phospho (STY)]PATVK_.2	#N/A	9.8737	9.5736	9.2790	10.2768	9.3386	9.5957	10.0530	9.2607	9.6816	9.8817	0.9935	-0.0094	0.7931	0.1007
Nefh	_SPATVKS[Phospho (STY)]PGEAKS[Phospho (STY)]PSEAK_.2	#N/A	7.4327	5.7004	8.2326	8.3146	8.2212	8.1155	8.3065	7.8314	7.9420	7.7568	0.9636	-0.0535	0.5981	0.2233
Nefh	_SPAEVKS[Phospho (STY)]PGEAKS[Phospho (STY)]PAAVK_.2	#N/A	6.7685	7.3924	7.5178	8.2721	7.5688	7.5422	7.6078	7.1221	7.1472	7.5428	1.0211	0.0301	0.5736	0.2414
Nefh	_SPAEAKS[Phospho (STY)]PAEAKS[Phospho (STY)]PATVK_.2	#N/A	7.6282	7.8711	7.6594	8.4552	7.5874	7.7430	8.1730	7.4797	7.7838	7.9219	0.9980	-0.0029	0.9402	0.0268
Nefh	_EEVKS[Phospho (STY)]PVKEEVK_.2	#N/A	5.1170	4.8067	5.4585	5.3554	4.7291	5.3690	4.8988	4.7594	4.4795	5.2453	1.0606	0.0848	0.1846	0.7338
Nefh	_AKT[Phospho (STY)]PVKEGAK_.2	P19246_T775_M1_Nefh	3.1542	3.4972	3.4021	4.2679	3.3418	4.3877	4.2038	2.9257	4.2009	4.2466	0.9437	-0.0835	0.5680	0.2456
Nefh	_HPTDIRPPEQVKS[Phospho (STY)]PAK_.3	#N/A	8.0579	3.6262	4.0005	8.5367	7.4507	7.2867	8.0221	7.6270	7.2979	7.3734	0.8566	-0.2233	0.3490	0.4572
Nefh	_SPAEAKS[Phospho (STY)]PAEPKS[Phospho (STY)]PATVK_.2	#N/A	7.4573	7.7664	7.6441	8.4749	7.4043	7.5395	8.0123	7.3095	6.6762	7.6978	1.0391	0.0554	0.3670	0.4353
Nefh	_S[Phospho (STY)]PGEAKS[Phospho (STY)]PSEAKS[Phospho (STY)]PAEAK_.2	#N/A	8.3827	8.6637	8.6524	8.2727	7.2576	4.5491	7.7502	6.9659	7.7897	7.6689	1.0114	0.0164	0.9199	0.0363
Nefh	_S[Phospho (STY)]PGEAKS[Phospho (STY)]PGEAKS[Phospho (STY)]PGEAK_.2	#N/A	6.3700	6.3188	6.5766	7.0389	6.6444	5.7287	7.0281	6.1587	6.1499	6.6517	0.9922	-0.0113	0.8595	0.0658
Nefh	_SPAEPKS[Phospho (STY)]PAEPK_.2	P19246_S565_M1_Nefh	7.1124	7.3221	7.6920	7.9109	7.8845	8.0246	7.7437	7.4986	7.7344	8.0949	0.9858	-0.0206	0.6132	0.2124
Nefh	_S[Phospho (STY)]PAEPKS[Phospho (STY)]PATVKS[Phospho (STY)]PGEAK_.2	#N/A	5.1740	4.1399	5.1990	5.9109	5.2509	4.6945	4.2044	4.7930	5.5453	4.3322	1.0726	0.1012	0.4016	0.3962
Nefh	_QREEAEAAAR_.2	#N/A	3.5229	4.3281	4.2488	3.7926	3.1473	2.3240	3.9408	3.2086	1.7595	0.6108	1.4961	0.5812	0.1310	0.8828
Nefh	_EGAKS[Phospho (STY)]PAEAKS[Phospho (STY)]PEK_.2	#N/A	7.3931	7.6253	6.8254	8.2999	6.8897	7.4400	7.9694	7.3735	7.4834	7.7003	0.9712	-0.0421	0.4768	0.3217
Nefh	_S[Phospho (STY)]PVKEGAKPPEK_.2	#N/A	6.9454	7.0597	6.4289	7.7577	6.9505	7.2214	7.4833	6.7524	6.9504	7.2043	0.9948	-0.0075	0.8877	0.0517
Nefh	_S[Phospho (STY)]PGEAKS[Phospho (STY)]PGEAKS[Phospho (STY)]PAEPK_.2	#N/A	5.7373	6.5166	6.1288	7.0486	6.2794	5.9757	6.8155	6.1102	6.1674	5.9113	1.0048	0.0069	0.9178	0.0373
Nefh	_S[Phospho (STY)]PAEAKS[Phospho (STY)]PEKAK_.2	#N/A	5.5519	5.3976	5.8387	6.1272	4.6666	5.3459	5.5349	5.5156	5.4491	4.5800	1.0414	0.0585	0.5053	0.2965
Nefh	_LEQEHLLEDIAHVR_.3	#N/A	6.8369	6.8489	6.9620	7.4023	7.1493	6.8650	6.8647	6.5449	6.7076	6.9040	1.0378	0.0535	0.0885	1.0531
Nefh	_SPAAVKS[Phospho (STY)]PGEAKS[Phospho (STY)]PGEAK_.3	#N/A	6.0432	6.4820	6.8003	6.8642	6.7181	6.4945	6.3223	5.9421	6.5165	6.6917	1.0312	0.0444	0.3475	0.4591
Nefh	_EEAKS[Phospho (STY)]PGEAKS[Phospho (STY)]PGEAK_.2	#N/A	3.5862	2.6390	3.6262	2.6160	4.0880	4.4146	4.1897	4.3516	1.7788	3.5398	1.0087	0.0125	0.9612	0.0172
Nefh	_SAAGSSSGFHSWAR_.2	#N/A	5.3498	5.3282	4.5690	5.9537	5.6717	5.4569	5.8995	5.6542	4.6941	3.7900	1.0756	0.1052	0.4237	0.3729
Nefh	_VKS[Phospho (STY)]EEMIKVVEK_.3	#N/A	3.5487	4.3539	4.2355	3.7668	4.1752	3.5543	3.9151	3.3803	3.5334	3.2043	1.1228	0.1671	0.0847	1.0720
Nefh	_EGAKS[Phospho (STY)]PAEAK_.2	#N/A	3.7927	4.8070	4.4795	3.5227	2.9184	3.3102	4.8754	3.7592	4.2857	4.9451	0.8520	-0.2312	0.1600	0.7959
Nefh	_AKS[Phospho (STY)]PEKEEAK_.2	P19246_S867_M1_Nefh	4.3131	4.4139	3.4991	4.0018	3.4388	4.2907	3.7165	4.1167	3.7653	3.9688	1.0260	0.0370	0.6729	0.1720
Nefh	_VTEGVTEEEDKEAQGQEGEEAEEGEEKEEEEGAAATS[Phospho (STY)]PPAEEAASPEK_.5	P19246_S500_M1_Nefh	3.3782	4.1834	4.0650	3.9373	4.0047	3.7248	4.0856	3.5508	3.1994	3.0338	1.1196	0.1630	0.1169	0.9320
Nefh	_EDIKPPAEAKS[Phospho (STY)]PEK_.3	#N/A	5.9873	3.1498	3.6280	3.1718	3.0381	3.5865	3.4797	3.8294	4.1284	4.3499	0.9527	-0.0699	0.7597	0.1193
Nefh	_SEKETVIVEGQTEEIR_.3	#N/A	5.5172	4.1347	4.0163	4.5190	4.3543	3.7734	4.1342	3.5995	3.2481	2.9852	1.2561	0.3289	0.0416	1.3806
Nefh	_AQLEGHAVQSTLQSEEWFR_.3	#N/A	3.2748	3.9514	3.9616	4.4643	3.9013	3.8282	4.1790	3.6542	3.3028	4.4313	1.0013	0.0019	0.9858	0.0062
Nefh	_PPAEAKS[Phospho (STY)]PEK_.2	#N/A	4.0441	3.2995	3.7778	3.3215	3.1878	3.4368	3.4698	3.6796	3.9787	2.9988	0.9942	-0.0084	0.9325	0.0304
Nefh	_SAQEEITEYRR_.3	#N/A	4.2743	2.9518	3.4603	3.9630	2.8401	3.7845	3.6776	4.1555	3.8041	3.9300	0.9111	-0.1344	0.2840	0.5467
Rpl13a	_YQAVTATLEEK_.2	#N/A	3.3086	4.1343	3.4524	4.6172	3.9734	3.7622	4.9339	5.0785	3.5764	4.3912	0.8620	-0.2142	0.1257	0.9007
Nelfe	_SLS[Phospho (STY)]EQPVVDTATATEQAK_.2	P19426_S51_M1_Nelfe	8.5664	8.8435	9.3980	3.3312	8.3230	9.0848	9.3668	8.6033	8.6878	8.9686	0.8897	-0.1686	0.4265	0.3701

Nelfe	_SM[Oxidation (M)]S[Phospho (STY)]ADEDLQEPSR_.2	#N/A	8.9830	9.0006	9.3666	9.7837	8.7315	9.1313	9.2081	8.7755	8.9691	9.1185	1.0164	0.0235	0.4829	0.3161
Cox5b	_EDPNLVPSISNK_.2	#N/A	9.8962	9.5487	9.3282	9.8496	10.1034	9.8531	9.7526	9.4052	9.5844	9.7917	1.0135	0.0193	0.4347	0.3618
Cox5b	_AASGTKEDPNLVPSISNK_.3	#N/A	7.5417	7.6641	8.3008	8.1632	8.5872	8.0723	7.4344	7.5121	7.9641	7.9277	1.0448	0.0632	0.1686	0.7732
Cox5b	_EIM[Oxidation (M)]IAAQK_.2	#N/A	7.3103	7.2140	7.3462	7.4708	7.9772	8.1164	6.3042	7.0860	7.4058	7.5066	1.0702	0.0979	0.1244	0.9052
Cox5b	_LVPHQMAH_.2	#N/A	6.0482	6.4620	3.5232	7.4257	6.9442	4.3553	5.0812	4.7531	4.5478	5.4832	1.1665	0.2222	0.3304	0.4809
Cox5b	_AASGTKEDPNLVPSISNKR_.3	#N/A	9.4556	10.0604	10.5635	10.0842	10.6138	10.1712	9.7158	9.7673	9.8693	9.7149	1.0401	0.0567	0.1078	0.9675
Cox5b	_EDPNLVPSISNKR_.2	#N/A	9.7346	9.7708	9.8144	9.9971	10.4483	9.8500	9.8965	9.5150	9.7131	9.5344	1.0281	0.0399	0.1151	0.9389
Cox5b	_C[Carbamidomethyl (C)]PNC[Carbamidomethyl (C)]GTHYK_.2	#N/A	5.2079	4.4188	3.3962	3.3968	6.2722	3.8183	3.7115	5.2381	4.3602	4.1181	1.0370	0.0525	0.7936	0.1004
Cox4i1	_SEDYAFPTYADR_.2	#N/A	10.3766	9.7713	9.9332	10.1107	9.7036	9.9073	10.3774	9.9559	10.0207	9.9622	0.9889	-0.0161	0.4718	0.3262
Cox4i1	_ANPIQGFSAK_.2	#N/A	9.4049	9.5368	9.8673	9.1759	9.5031	9.1055	9.3444	9.8621	9.2282	9.2576	1.0010	0.0014	0.9612	0.0172
Cox4i1	_ADWSSLSR_.2	#N/A	9.1215	9.0611	9.5229	8.9138	8.9627	8.6556	8.7008	9.1239	8.9301	8.9050	1.0140	0.0200	0.4621	0.3353
Cox4i1	_EKADWSSLSR_.2	#N/A	7.9849	8.2745	8.5260	8.1453	8.0295	8.0263	8.5029	8.2126	8.1363	8.0415	0.9928	-0.0104	0.6660	0.1765
Cox4i1	_SEDYAFPTYADRR_.2	#N/A	7.1456	7.1294	7.4206	7.0795	7.0372	7.3834	6.8718	7.2250	7.1144	7.1109	1.0168	0.0240	0.2756	0.5597
Cox4i1	_IQFNESFAEM[Oxidation (M)]NR_.2	#N/A	6.3062	6.1542	6.6554	6.2894	6.0547	6.1855	6.8124	6.3203	6.7607	6.4169	0.9539	-0.0681	0.0681	1.1667
Cox4i1	_DWVAM[Oxidation (M)]QTK_.2	#N/A	6.8206	6.6690	6.6736	6.4914	6.4496	6.3292	6.6937	7.0184	6.6830	6.7775	0.9675	-0.0477	0.0809	1.0921
Cox4i1	_DYPLPDVAHVTM[Oxidation (M)]LSASQK_.3	#N/A	7.7820	8.1065	8.3908	7.4072	7.3460	7.9719	7.9238	7.9957	8.2999	7.6967	0.9818	-0.0265	0.5455	0.2632
Lsp1	_QPS[Phospho (STY)]IELPSMAVASTK_.2	P19973_S243_M1_Lsp1	4.1654	3.4698	3.3514	3.1501	3.2911	2.9375	3.2983	3.8511	4.1501	3.8210	0.8979	-0.1554	0.1699	0.7699
Hspa5	_ITITNDQNR_.2	#N/A	9.1633	9.3745	9.5828	9.6071	9.4837	9.1209	9.0929	9.1786	9.1824	9.4753	1.0169	0.0242	0.2469	0.6075
Hspa5	_ITPSYVAFTPEGER_.2	#N/A	8.1862	8.7201	8.4768	8.6012	8.6708	8.5720	8.7906	8.2720	8.1684	8.4043	1.0153	0.0220	0.4006	0.3972
Hspa5	_LTPEEIER_.2	#N/A	8.1787	8.4382	8.8154	8.5508	8.7651	8.1266	8.1766	8.5565	8.1546	8.5308	1.0149	0.0213	0.4867	0.3127
Hspa5	_NQLTSNPENTVFDAK_.2	#N/A	7.8166	8.0345	8.1772	8.2805	8.2217	8.0024	8.0161	7.6806	7.7608	8.3246	1.0180	0.0258	0.3498	0.4562
Hspa5	_SQIFSTASDNQPTVTIK_.2	#N/A	7.5478	7.2188	7.7317	7.5179	7.9195	7.7441	7.0718	6.9246	7.3528	7.3172	1.0623	0.0872	0.0165	1.7837
Hspa5	_TWNDPSVQQDIK_.2	#N/A	7.5155	7.4006	7.8556	7.8575	7.7970	7.2326	7.1849	7.4177	7.5351	7.6430	1.0221	0.0316	0.3222	0.4919
Hspa5	_VYEGERPLTK_.2	#N/A	7.0238	7.6573	7.2760	7.7570	7.8514	7.2945	7.6343	7.2917	6.9973	7.5298	1.0154	0.0221	0.5868	0.2315
Hspa5	_NELESYAYSLK_.2	#N/A	7.3660	7.2163	7.5112	7.7679	7.3340	7.2184	6.8960	7.0662	6.7560	7.4573	1.0509	0.0716	0.0563	1.2494
Hspa5	_ELEEIVQPIISK_.2	#N/A	6.2700	6.2648	6.3008	6.7476	6.3736	6.7330	6.3069	5.7950	6.5064	6.2823	1.0363	0.0514	0.2144	0.6687
Hspa5	_NTVVPTKK_.2	#N/A	6.2158	6.4149	6.5802	6.1490	7.0610	6.2879	5.7911	6.5430	6.2020	6.6562	1.0244	0.0347	0.5240	0.2807
Hspa5	_IEIESFFEGEDFSETLTR_.2	#N/A	4.8845	5.9035	6.7995	7.4019	5.5513	6.3571	5.3562	5.6297	5.4417	4.9841	1.1488	0.2002	0.1297	0.8870
Hspa5	_VTHAVVTVPAYFNDAQR_.3	#N/A	6.4795	6.3816	6.2632	7.1486	6.3770	6.5945	6.2592	6.4115	6.4848	6.5900	1.0162	0.0232	0.5592	0.2524
Hspa5	_NQLTSNPENTVFDAKR_.3	#N/A	5.4087	5.9694	4.2089	4.8008	5.3114	5.8256	5.2485	4.7905	5.3952	6.0092	0.9801	-0.0290	0.7914	0.1016
Hspa5	_IINEPTAAAIAYGLDKR_.3	#N/A	5.2219	5.1274	5.2822	5.4541	5.2101	4.9638	3.0305	4.8889	5.3359	5.5180	1.1101	0.1506	0.2921	0.5344
Hspa5	_TFAPEEISAM[Oxidation (M)]VLTK_.2	#N/A	5.6235	6.0385	5.5507	6.5794	6.1055	5.6153	6.1101	5.5839	5.7594	5.8547	1.0157	0.0225	0.6900	0.1612
Hspa5	_KSDIDEIVLVGGSTR_.3	#N/A	6.2642	4.9054	5.5068	5.9380	5.9035	5.5842	5.4098	4.5477	4.9100	5.5004	1.1162	0.1586	0.0815	1.0887
Hspa5	_ALSSQHQAR_.2	#N/A	3.7705	4.0936	2.8636	3.7250	5.0142	3.8146	3.8511	3.1424	4.1085	4.7469	0.9793	-0.0302	0.8570	0.0670
Hspa5	_IEWLESHQDADIEDFK_.3	#N/A	4.8852	5.3424	5.4687	6.1560	4.3771	5.1025	5.1018	4.8956	5.1233	5.8575	0.9957	-0.0062	0.9498	0.0224
Hspa5	_VLEDSDLK_.2	#N/A	4.6338	4.1646	3.6755	3.2193	5.3243	3.5390	1.7737	4.1475	4.4884	4.6782	1.0851	0.1178	0.6431	0.1917
Hspa5	_DAGTIAGLNVMR_.2	#N/A	3.4906	4.2958	4.1774	6.8660	5.6999	3.6124	3.9732	3.4384	3.0870	3.6162	1.3292	0.4106	0.1315	0.8811
Hspa5	_LYGSGGPPPT[Phospho (STY)]GEEDTSEKDEL_.2	P20029_T644_M1_Hspa5	3.3000	4.1052	3.9868	4.4894	3.9265	3.8156	4.2041	5.3288	3.2902	3.8272	0.9459	-0.0803	0.5838	0.2338
Hspa5	_AKFEELNM[Oxidation (M)]DLFR_.3	#N/A	3.7290	4.0092	4.0269	3.3661	3.4370	4.3776	3.0808	3.4761	3.7295	3.8752	1.0802	0.1113	0.2723	0.5649
Tmsb4x	_NPLPSKETIEQEK_.3	#N/A	6.9510	7.1853	7.2728	7.5985	8.5880	7.6911	7.6820	7.1987	8.0749	7.3721	0.9955	-0.0065	0.9206	0.0359
Tmsb4x	_ETIEQEKQAGES[Phospho (STY)]_.2	P20065_S50_M1_Tmsb4x	6.8796	7.3984	7.8103	7.4311	7.9914	7.6272	7.2413	7.1602	7.7003	7.0977	1.0306	0.0434	0.3510	0.4547
Tmsb4x	_TETQEKNPLPSKETIEQEK_.3	#N/A	5.8799	5.9653	6.7173	6.4446	7.3688	6.5253	6.6116	6.1696	7.3256	6.1708	0.9869	-0.0190	0.8130	0.0899
Tmsb4x	_KTETQEKNPLPSK_.3	#N/A	6.3708	5.3907	5.6044	6.9134	7.0988	6.9302	5.7985	6.2521	7.0604	5.6908	1.0297	0.0422	0.6915	0.1602
Tmsb4x	_TET[Phospho (STY)]QEKNPLPSK_.2	#N/A	7.9351	7.7411	7.6874	8.2484	8.6030	8.3493	7.8622	7.6465	8.2724	8.0743	1.0163	0.0234	0.5603	0.2516
Prdx3	_GLFIIDPNGVVK_.2	#N/A	8.4808	8.6774	8.5317	8.7330	8.6399	8.6445	8.4330	8.5017	8.5073	8.6407	1.0114	0.0164	0.1381	0.8598
Prdx3	_HLSVNDLPVGR_.2	#N/A	8.0017	8.3642	8.1172	8.2672	8.7105	8.3385	8.2225	7.9695	8.2237	7.9926	1.0244	0.0348	0.1847	0.7336
Prdx3	_DYGVLLESAGIALR_.2	#N/A	6.3443	6.4581	7.1656	6.7323	6.9545	7.0028	6.8520	6.0344	6.2690	7.1547	1.0302	0.0429	0.4707	0.3273
Prdx3	_GTAVVNGEFK_.2	#N/A	6.6196	6.4092	5.7433	6.5752	6.8069	6.6443	6.1418	6.4507	6.3121	6.3481	1.0243	0.0346	0.4629	0.3345
Prdx3	_ELSLDDFK_.2	#N/A	6.7634	6.6969	5.9002	5.5091	6.4612	6.8322	5.5464	6.3153	6.7515	7.0762	0.9865	-0.0196	0.8190	0.0867
Vim	_VELQELNDR_.2	#N/A	7.6976	7.3649	7.1809	7.7969	7.1224	7.1192	7.4985	6.7911	7.5565	7.5296	1.0050	0.0071	0.8677	0.0616
Vim	_FADLSEAANR_.2	#N/A	6.7902	7.3240	7.1104	7.1623	7.1731	6.5946	6.8328	6.5695	6.8734	6.9061	1.0339	0.0481	0.1703	0.7689
Vim	_NLQEAEEWYK_.2	#N/A	5.2110	5.7395	6.3786	5.9366	6.2976	5.5029	6.1115	5.5305	6.2901	6.0069	0.9765	-0.0342	0.6101	0.2146
Vim	_QDVDNAS[Phospho (STY)]LAR_.2	P20152_S214_M1_Vim	4.7884	5.3754	4.8985	4.2691	4.6057	5.1363	5.1666	4.8001	4.9259	4.9769	0.9755	-0.0358	0.5743	0.2409
Vim	_TNEKVELQELNDR_.3	#N/A	6.0900	6.3111	6.5886	7.1216	5.5757	6.0498	5.3996	6.3786	5.7406	6.3042	1.0560	0.0786	0.3365	0.4730
Vim	_ISLPLPTFSSLNLR_.2	#N/A	4.2796	5.7899	6.2116	5.2418	5.6197	5.1219	5.5826	5.8042	5.9467	5.8914	0.9261	-0.1107	0.2496	0.6027
Vim	_ILLAELEQLK_.2	#N/A	5.3913	5.2207	4.5878	5.1212	5.4762	4.7093	5.2924	5.4175	5.0733	5.5079	0.9552	-0.0661	0.2656	0.5758
Vim	_SLYSSS[Phospho (STY)]PGGAYVTR_.2	P20152_S56_M1_Vim	3.9080	7.0199	7.4575	3.4626	7.1291	5.3608	3.5345	3.5485	3.8475	4.8961	1.4464	0.5325	0.0927	1.0329
Vim	_QQYESVAAK_.2	#N/A	4.3312	3.5638	4.0420	4.5842	5.4502	4.7335	3.0657	4.0032	4.7496	3.8368	1.1372	0.1855	0.2438	0.6130
Vim	_EM[Oxidation (M)]EENFALEAANYQDTIGR_.2	#N/A	3.9845	4.5266	5.2102	5.1453	4.0872	4.5967	4.6743	3.9118	4.0869	4.2781	1.0835	0.1157	0.2601	0.5849
Vim	_FANYIDK_.2	#N/A	3.1072	3.9124	3.7940	3.9823	4.5912	3.9958	4.0114	3.8218	4.0091	4.2637	0.9679	-0.0471	0.6235	0.2052
Vim	_SYVTTSTR_.2	#N/A	4.2740	3.6814	4.0077	3.5515	4.5742	3.3389	3.6997	3.7751	3.7488	3.2288	1.0807	0.1120	0.2933	0.5327
Vim	_DNLAEDIMR_.2	#N/A	4.6291	4.2728	4.1545	3.8478	4.0942	3.6353	3.9961	3.1533	3.4524	3.5251	1.1625	0.2172	0.0331	1.4798
Vim	_ETNLESLPLVDTHSK_.3	#N/A	3.8706	3.3556	4.5574	3.5592	3.2439	3.3807	3.2739	3.0585	4.2079	3.5262	1.0411	0.0581	0.6621	0.1791
Vim	_SVSSSSYR_.2	#N/A	3.7495	3.0538	4.4363	3.4381	2.8752	3.3535	3.1528	3.1795	4.3290	3.4051	0.9908	-0.0133	0.9307	0.0312
Map2	_ARVDHGAEIITQS[Phospho (STY)]PSR_.3	#N/A	11.7462	12.3955	13.1650	13.0039	11.9688	12.3245	12.3331	12.4822	12.3212	12.3855	1.0043	0.0062	0.8567	0.0672
Map2	_IST[Phospho (STY)]PERK_.2	#N/A	13.3357	13.5393	14.0177	14.0571	13.1066	13.7620	13.6370	13.5983	13.6849	13.7617	0.9975	-0.0036	0.8659	0.0625
Map2	_KSEVQAHS[Phospho (STY)]PSRK_.3	#N/A	9.3715	9.7111	9.1343	9.9351	8.4942	8.0151	8.6640	9.4765	9.7656	9.4264	0.9761	-0.0349	0.6083	0.2159
Map2	_GLSS[Phospho (STY)]VPEVAEVEPTTK_.2	P20357_S1023_M1_Map2	9.3916	9.7426	8.8983	10.2678	9.6149	9.9715	10.0623	9.6112	9.5676	9.8430	0.9874	-0.0183	0.6464	0.1895
Map2	_SSVAS[Phospho (STY)]PRR_.2	P20357_S1791_M1_Map2	9.4117	9.9929	10.6461	10.3287	9.8206	9.9945	9.9618	10.0495	9.9409	10.0023	1.0044	0.0063	0.8452	0.0731
Map2	_DGS[Phospho (STY)]PDAPATPEK_.2	#N/A	9.6939	9.4513	9.5401	10.3518	9.5305	9.5846	10.0633	9.5129	9.4813	9.7229	0.9997	-0.0005	0.9882	0.0051
Map2	_GRIST[Phospho (STY)]PER_.2	#N/A	8.8808	3.7265	3.3496	4.3942	3.5478	4.1817	4.5424	4.1078	9.4092	4.0715	0.8459	-0.2415	0.5806	0.2361
Map2	_FAAPAQPEEER_.2	#N/A	9.1275	8.8312	8.8786	9.3023	9.2514	9.0733	9.0588	8.8384	9.0561	9.1884	1.0046	0.0067	0.7209	0.1421
Map2	_LINQPLPDLK_.2	#N/A	8.9217	9.2944	9.2143	9.1393	9.3605	8.9203	9.1765	9.3227	9.1275	9.0905	0.9959	-0.0059	0.7256	0.1393
Map2	_NKDDLTLSR_.2	#N/A	8.6505	8.8611	8.8927	9.1977	8.9518	8.9112	8.5122	8.7845	8.5692	8.9268	1.0244	0.0348	0.1082	0.9659
Map2	_AEPSQLDIK_.2	#N/A	8.3869	8.3205	8.3791	8.3795	8.5094	8.4338	7.9881	8.3263	8.1086	8.2137	1.0297	0.0422	0.0062	2.2071
Map2	_ADQGLDFAATK_.2	#N/A	8.0088	8.0402	7.8852	8.1829	8.3134	8.3193	8.1044	8.1497	7.8662	8.2376	1.0044	0.0063	0.7543	0.1225
Map2	_ELITTKDTS[Phospho (STY)]PEK_.2	#N/A	7.7986	8.2849	8.6710	8.4612	7.9277	8.1323	8.2701	3.0205	7.7026	8.1375	1.2108	0.2760	0.1946	0.7110
Map2	_VKDEFTAEK_.2	#N/A	7.9262	7.5822	7.7053	7.9460	8.0067	7.9723	7.8592	7.6381	7.6274	7.9990	1.0097	0.0139	0.5224	0.2820
Map2	_EIQGLFEEK_.2	#N/A	7.8216	7.9178	7.8359	8.1524	7.5174	8.0810	7.7992	7.7234	7.9111	8.0927	1.0008	0.0011	0.9642	0.0159

Map2	_VKIESVK_.2	#N/A	7.4035	7.5468	7.5816	7.6452	7.5615	7.4721	7.0903	7.4009	7.2173	7.5683	1.0295	0.0420	0.0497	1.3033
Map2	_ETSAPSVQEPTLTETEPQTK_.2	#N/A	7.2891	7.2112	7.2992	7.9189	7.6296	7.6418	7.6440	7.1851	7.0062	7.3569	1.0274	0.0390	0.2908	0.5364
Map2	_SDTLQISDLLVSESR_.2	#N/A	7.0478	6.8875	7.4507	7.1987	6.9853	7.4479	7.2038	6.4629	6.9785	7.1996	1.0299	0.0426	0.2901	0.5375
Map2	_GGQVQIVTK_.2	#N/A	6.2521	6.2785	6.9130	7.1675	6.8209	6.7959	6.1526	6.2953	6.7528	6.9018	1.0274	0.0390	0.4662	0.3314
Map2	_SKIGS[Phospho (STY)]TDNIK_.2	P20357_S1680_M1_Map2	7.0187	6.8220	6.5557	7.4626	3.0074	6.9801	5.5071	7.0968	7.3147	7.1805	0.9311	-0.1030	0.6192	0.2081
Map2	_VTDGISKS[Phospho (STY)]PEK_.2	#N/A	6.7973	6.5620	6.4538	7.1969	3.6467	6.3774	7.1836	6.8072	6.9657	6.5290	0.8983	-0.1548	0.3193	0.4958
Map2	_GSAQESLDTIS[Phospho (STY)]PK_.2	P20357_S608_M1_Map2	12.5142	12.4371	13.1592	12.8675	12.2506	12.6195	13.0519	12.2329	12.6066	12.6361	1.0008	0.0011	0.9655	0.0153
Map2	_TPGTPGT[Phospho (STY)]PSYPR_.2	P20357_T1623_M1_Map2	11.2716	11.5677	11.9970	11.7887	7.8696	11.6818	11.0327	11.1190	10.9251	11.4076	0.9918	-0.0119	0.9120	0.0400
Map2	_VAIIRT[Phospho (STY)]PPKS[Phospho (STY)]PATPK_.2	#N/A	9.1293	8.4154	10.3843	10.7257	6.1405	9.7297	8.8315	9.7330	8.6760	9.0402	1.0019	0.0027	0.9847	0.0067
Map2	_LAS[Phospho (STY)]VSADAEVAR_.2	P20357_S823_M1_Map2	5.7012	5.3553	10.0061	10.7141	9.6634	9.9777	9.7724	10.3269	10.2353	10.2892	0.8438	-0.2450	0.2299	0.6385
Map2	_T[Phospho (STY)]PPKSPATPK_.2	P20357_T1650_M1_Map2	9.7559	8.9895	9.0636	10.3913	9.4619	9.5543	9.6259	9.2695	9.4229	9.3324	1.0131	0.0188	0.6576	0.1820
Map2	_KETS[Phospho (STY)]PETSLIQDEVALK_.2	#N/A	6.3861	6.2339	6.8115	7.2931	5.9853	6.6667	7.1975	3.7376	6.5589	6.7110	1.0845	0.1171	0.4625	0.3349
Map2	_TTAASGDLAQAPGAFK_.2	#N/A	6.8363	6.8792	7.3237	7.6397	7.3804	7.0145	7.0263	6.8741	7.1535	6.8151	1.0304	0.0432	0.2572	0.5897
Map2	_STVS[Phospho (STY)]IEEAVAK_.2	P20357_S447_M1_Map2	5.2304	6.9442	5.7542	7.9592	5.7364	5.7597	6.8708	5.8278	5.3335	7.4654	0.9775	-0.0329	0.8295	0.0812
Map2	_SILTEQLETIPKEER_.2	#N/A	5.7877	6.3806	6.0932	6.6275	6.0521	6.5850	6.0340	6.5606	6.1648	6.2496	1.0003	0.0005	0.9916	0.0037
Map2	_SILTEQLETIPK_.2	#N/A	3.4816	6.1163	5.5486	6.3447	5.8958	5.7533	6.0656	5.9552	6.3583	6.3909	0.8919	-0.1650	0.2476	0.6062
Map2	_SGILVPSEK_.2	#N/A	7.9652	7.5999	7.8793	7.8823	7.2900	7.7592	7.0714	7.7094	7.1348	7.6601	1.0454	0.0640	0.1066	0.9721
Map2	_VELFGLGITYDQASTK_.2	#N/A	5.2168	5.2922	3.1734	6.1869	5.5083	5.6326	5.8839	5.2137	5.3029	5.8793	0.9279	-0.1080	0.4843	0.3149
Map2	_IDLSHVTSK_.2	#N/A	5.4073	5.3939	5.0862	5.7647	5.6489	5.4488	5.1190	5.1872	5.1085	4.4241	1.1005	0.1382	0.0277	1.5577
Map2	_KSEVQAHS[Phospho (STY)]PSR_.2	#N/A	10.7596	11.3867	11.7420	11.7355	11.2812	11.6416	11.1848	11.1899	11.4145	11.2650	1.0143	0.0205	0.4372	0.3593
Map2	_STELGSDYYELSDSR_.2	#N/A	7.2378	6.7451	6.9479	7.2533	6.5738	7.0277	6.7746	6.5211	6.6550	8.8622	0.9668	-0.0487	0.6176	0.2093
Map2	_DLATDLSLIEVK_.2	#N/A	6.5017	6.6916	6.7125	6.3431	6.7619	6.6671	6.4697	6.3299	6.3834	6.8028	1.0179	0.0257	0.3472	0.4594
Map2	_VTS[Phospho (STY)]EPEAVSERR_.2	#N/A	3.9526	4.1700	4.5765	6.3203	4.1083	4.5844	5.9415	5.6246	5.6865	5.6406	0.8070	-0.3094	0.0393	1.4058
Map2	_SEVQAHS[Phospho (STY)]PSR_.2	#N/A	11.7737	12.0535	12.3496	12.4889	11.9457	12.2730	11.9704	11.9070	12.0869	12.0796	1.0114	0.0163	0.3639	0.4391
Map2	_ELITTKDTS[Phospho (STY)]PEKTEK_.3	#N/A	9.1649	10.2276	11.4056	10.4370	10.0660	9.9639	9.6160	10.1442	10.9415	10.0570	1.0021	0.0030	0.9621	0.0168
Map2	_DGS[Phospho (STY)]PDAPATPEKEEVAFSEYK_.3	#N/A	11.2432	10.9781	11.1578	11.7806	11.2473	11.4707	11.4327	11.2838	11.4863	11.6871	0.9861	-0.0202	0.3388	0.4701
Map2	_VTS[Phospho (STY)]EPEAVSER_.2	#N/A	9.3955	9.3044	9.3947	9.9635	9.2850	9.3502	9.6338	9.3499	9.4837	9.6584	0.9913	-0.0126	0.5782	0.2379
Map2	_DWFIEM[Oxidation (M)]PTESK_.2	#N/A	5.7253	5.5891	5.5428	6.4896	5.7049	5.8355	6.2614	5.2451	5.5686	6.1235	1.0026	0.0037	0.9555	0.0198
Map2	_VDHGAEIITQS[Phospho (STY)]PSR_.2	#N/A	14.1043	14.0381	14.8426	14.5128	13.7691	14.1310	14.2598	13.9603	14.2059	14.2655	1.0042	0.0061	0.7749	0.1107
Map2	_VTDGISKS[Phospho (STY)]PEKR_.2	#N/A	9.7008	10.3504	10.7049	10.7503	10.1198	10.3813	10.3800	10.4115	10.4032	10.4119	0.9936	-0.0093	0.7449	0.1279
Map2	_VDHGAEIITQSPSRSS[Phospho (STY)]VAS[Phospho (STY)]PR_.3	P20357_S1788_M2_Map2	3.5899	5.9641	3.7449	6.2692	4.7598	6.3651	5.2422	3.7972	5.9331	6.0276	0.9744	-0.0375	0.8641	0.0634
Map2	_S[Phospho (STY)]GILVPSEKK_.2	#N/A	3.5580	6.2676	4.2448	5.8436	4.1845	6.1439	4.4621	5.6284	5.6663	3.2136	1.0628	0.0879	0.7041	0.1524
Map2	_ETS[Phospho (STY)]PETSLIQDEVALK_.2	#N/A	9.3712	3.8708	9.6964	12.8506	8.6538	4.1663	9.4116	12.0905	3.4978	9.1177	0.9498	-0.0743	0.8556	0.0677
Map2	_EKDVLEDIPR_.2	#N/A	7.7536	7.4085	7.4569	7.8426	7.6873	7.7295	7.9239	7.6088	7.6663	7.7955	0.9868	-0.0192	0.3573	0.4469
Map2	_VSDFGQM[Oxidation (M)]ASGM[Oxidation (M)]NVDAGK_.2	#N/A	5.7595	6.4805	6.3620	5.6854	6.4821	6.7095	6.1039	6.2380	6.3962	5.9776	1.0109	0.0157	0.7732	0.1117
Map2	_M[Oxidation (M)]PSKPGEDFEHAALVPDTSK_.4	#N/A	6.1105	5.5960	6.8322	6.2215	6.4410	6.0865	4.8165	6.1721	6.3464	6.4214	1.0464	0.0654	0.4718	0.3263
Map2	_YFETSALKEDM[Oxidation (M)]TR_.2	#N/A	5.0759	4.8730	4.4531	6.0536	5.2786	5.2534	4.8713	5.1324	5.1557	5.1750	1.0159	0.0228	0.7772	0.1094
Map2	_ESS[Phospho (STY)]KDEEPLKDK_.2	#N/A	7.1751	5.0143	7.1288	7.8572	6.5788	7.2363	7.2764	7.3940	7.2757	7.3469	0.9329	-0.1002	0.3543	0.4506
Map2	_TGVIQTS[Phospho (STY)]TEQSFSK_.2	#N/A	4.6862	3.6140	4.0821	5.6539	3.4553	3.8376	3.6939	4.8409	4.4562	3.7635	1.0079	0.0113	0.9463	0.0240
Map2	_VGS[Phospho (STY)]LDNAHHVPGGGNVK_.3	#N/A	8.4347	8.1486	8.2938	8.7546	6.9796	8.2310	7.5746	8.3562	8.4298	8.6425	0.9866	-0.0194	0.7673	0.1150
Map2	_SEVQAHS[Phospho (STY)]PSRK_.2	#N/A	8.5384	9.0267	9.2661	9.3445	8.4548	9.0615	8.7150	3.8625	8.7600	8.9810	1.1806	0.2396	0.2077	0.6826
Map2	_NQHDEKELQAK_.2	#N/A	4.8034	4.4707	4.0088	5.0462	5.4409	5.3894	5.3845	4.6858	4.3638	4.9950	1.0005	0.0008	0.9939	0.0027
Map2	_GVSGDREENS[Phospho (STY)]FSLNSSISSAR_.3	P20357_S1567_M1_Map2	5.0783	4.2920	5.6338	4.0929	3.7599	4.0447	4.0400	5.4659	4.7396	5.5211	0.9073	-0.1403	0.3476	0.4589
Map2	_SGTSTPTTPGSTAITPGT[Phospho (STY)]PPSYSSR_.3	P20357_T1609_M1_Map2	3.7749	8.9776	9.5061	10.6216	3.3629	7.5069	8.6778	8.7956	3.7789	3.7512	1.1665	0.2222	0.6041	0.2189
Map2	_GRIST[Phospho (STY)]PERK_.2	#N/A	3.0748	4.0447	3.7616	9.6199	9.2381	9.5867	9.2341	9.4831	9.2297	4.2313	0.8148	-0.2956	0.4620	0.3354
Map2	_AGKS[Phospho (STY)]GTSTPTTPGSTAITPGTPPSY[Phospho (STY)]SSR_.3	P20357_S1592_M2_Map2	3.6110	4.3673	3.3447	4.3893	4.2556	3.8698	4.5376	4.1127	4.4117	4.0666	0.9278	-0.1081	0.2330	0.6327
Map2	_VTGGQTIQVETSSESPFPAK_.2	#N/A	4.3117	4.4623	4.4602	5.0976	3.5548	4.0921	4.0045	3.9070	4.0979	4.6723	1.0382	0.0541	0.6000	0.2218
Map2	_MFTIDPK_.2	#N/A	3.0971	3.6905	3.7839	5.4014	3.9733	4.0058	3.0131	4.0939	3.4805	3.9546	1.0981	0.1350	0.4369	0.3596
Map2	_EAS[Phospho (STY)]PPTSADK_.2	#N/A	14.0685	13.9246	14.0690	14.7881	13.6937	13.9784	14.1537	14.0447	14.0202	14.1900	0.9989	-0.0015	0.9391	0.0273
Map2	_KANDKLDTVLEK_.3	#N/A	2.9008	4.1608	3.5876	4.6481	5.7461	4.4280	3.0621	4.7049	4.3864	4.4950	1.0200	0.0285	0.8887	0.0512
Map2	_VKDEFTAEKEAS[Phospho (STY)]PPTSADK_.3	#N/A	11.5521	12.0378	12.8769	12.8805	11.8921	12.1941	12.1737	12.1740	11.9211	12.0285	1.0136	0.0195	0.5720	0.2426
Map2	_TGVIQTSTEQS[Phospho (STY)]FSKEDQK_.3	#N/A	5.1925	5.2281	4.5735	3.7133	3.6806	4.8888	4.9505	5.1161	4.2374	5.0647	0.9389	-0.0910	0.4715	0.3266
Map2	_DKVTDGISKS[Phospho (STY)]PEK_.2	#N/A	7.3534	7.4442	7.5876	8.0968	7.3149	7.3878	7.7109	7.3296	7.3513	7.3058	1.0143	0.0205	0.5436	0.2647
Map2	_IVQVVTAEAVAVLKGEQEK_.2	#N/A	4.7143	4.1806	5.5057	5.4028	3.7606	4.0686	5.6259	4.2439	4.3227	4.5588	0.9824	-0.0256	0.8589	0.0661
Map2	_TPGT[Phospho (STY)]PKS[Phospho (STY)]GILVPSEK_.2	#N/A	3.8235	7.5490	3.8805	7.8900	3.7535	8.0726	5.3591	6.9564	7.1951	7.2200	0.8721	-0.1974	0.4897	0.3101
Map2	_APHWTSASLTEAAAHPHS[Phospho (STY)]PEM[Oxidation (M)]K_.3	#N/A	7.0857	8.7223	7.7938	10.7063	7.1838	8.0813	7.7895	9.1326	8.6091	8.1742	0.9805	-0.0284	0.8256	0.0832
Map2	_EEFVETC[Carbamidomethyl (C)]PGELK_.2	#N/A	3.9907	3.3517	3.7244	7.7461	7.5022	7.3318	3.4165	3.7330	6.9384	4.4463	1.2103	0.2753	0.4583	0.3388
Map2	_LEGAGSATIAEVEM[Oxidation (M)]PFYEDK_.2	#N/A	5.0453	4.8541	3.4858	5.6453	4.8987	3.0089	5.0205	4.3893	4.0390	4.8949	0.9790	-0.0306	0.8650	0.0630
Map2	_DQPAALPLAAEETANLPPS[Phospho (STY)]PPPS[Phospho (STY)]PASEQTATVEEDLLTASK_.4	#N/A	3.3588	4.0203	3.7878	3.9803	3.9914	3.8635	4.1708	3.7231	3.9066	3.9473	0.9738	-0.0383	0.5004	0.3007
Map2	_TPGTPGTPSYPRT[Phospho (STY)]PGT[Phospho (STY)]PK_.2	#N/A	3.6364	5.5839	3.9096	6.1245	4.3929	6.4810	5.3684	5.6982	3.6664	4.7913	1.0287	0.0409	0.8477	0.0718
Map2	_ARVDHGAEIITQS[Phospho (STY)]PSRSS[Phospho (STY)]VAS[Phospho (STY)]PR_.3	#N/A	3.7595	3.6109	4.0388	3.7510	3.4488	3.6454	3.6529	4.0153	3.8482	6.3213	0.8317	-0.2658	0.1751	0.7568
Map2	_IGSTDNIK_.2	#N/A	4.3585	3.4041	3.1657	4.4354	4.8280	3.5652	3.6902	3.7415	4.2118	3.9333	1.0168	0.0240	0.8582	0.0664
Map2	_YFETSALK_.2	#N/A	4.1281	3.1856	3.7218	4.3519	4.3771	3.7917	4.4288	3.8941	4.4241	4.1914	0.9271	-0.1092	0.2566	0.5907
Map2	_GHDLSPLASDILTNTSGSM[Oxidation (M)]DEGDDY[Phospho (STY)]LPPTTPAVEK_.4	#N/A	3.8423	3.5679	3.4961	5.3355	3.4060	4.0060	3.7901	4.0915	3.9791	4.0517	0.9910	-0.0130	0.9254	0.0337
Map2	_SM[Oxidation (M)]SINLPM[Oxidation (M)]SC[Carbamidomethyl (C)]LDSIALGFNFGR_.3	#N/A	3.0629	3.8681	3.7497	4.2526	3.6894	4.0401	4.4009	4.2494	3.3227	3.9299	0.9500	-0.0739	0.4984	0.3024
Map2	_LSNVSS[Phospho (STY)]SGSINLLESPQLATLAEDVTAALAK_.3	P20357_S1800_M1_Map2	4.0413	5.9783	3.2273	3.2741	3.1670	3.0616	3.4224	3.7270	4.0261	2.9514	1.0736	0.1024	0.6779	0.1688
Map2	_APHWTSASLTEAAAHPHS[Phospho (STY)]PEM[Oxidation (M)]KDQGGAGEGLSR_.5	#N/A	3.7152	3.6955	6.5111	3.4450	3.9287	4.8654	4.0079	3.4694	5.6165	3.7877	1.0331	0.0470	0.8475	0.0719
Map2	_ASQPSPPAQEAGYSTLAQSYTPDHPSELPEEPSS[Phospho (STY)]PQER_.4	P20357_S655_M1_Map2	3.9925	3.6616	3.9257	3.9469	3.5146	3.7457	3.9655	5.9258	4.4248	3.7266	0.8420	-0.2482	0.1140	0.9432
Map2	_KDEWGLAAPIS[Phospho (STY)]PGPLTPM[Oxidation (M)]R_.3	P20357_S283_M1_Map2	3.7366	4.9677	3.9357	5.6985	3.5728	3.9072	3.9243	4.3645	4.0373	3.0312	1.1208	0.1645	0.3667	0.4357
Map2	_DTS[Phospho (STY)]PEKTEK_.2	#N/A	3.2777	4.0647	4.5122	4.0560	3.9223	3.8435	4.2043	4.4460	3.2442	3.7333	1.0100	0.0144	0.8972	0.0471
Map2	_ANDKLDTVLEKS[Phospho (STY)]EEHIDSK_.3	P20357_S969_M1_Map2	3.3326	3.8936	4.0194	4.5220	3.7819	4.3436	4.2367	3.5964	3.2450	4.8315	1.0012	0.0017	0.9895	0.0046
Map2	_ESEEM[Oxidation (M)]GGKVELFGLGITYDQASTK_.3	#N/A	3.9484	3.2039	3.6821	3.7109	3.0922	5.2989	3.4256	4.4076	4.6795	4.4040	0.9039	-0.1458	0.4023	0.3955
Map2	_YTVPLPSPVQDSENLSGESGSFYEGTDDKVR_.3	#N/A	3.0112	3.7675	4.2457	3.7895	3.6558	2.9688	4.3628	3.2117	3.5107	3.4668	0.9822	-0.0260	0.8447	0.0733
Map2	_IVQVVTAEAVAVLK_.2	#N/A	3.0393	4.3031	4.2738	3.8176	4.1244	3.6050	3.9658	3.1836	3.4826	3.4949	1.0931	0.1284	0.2720	0.5655
Prkca	_VIS[Phospho (STY)]PSEDR_.2	#N/A	12.0181	11.6648	12.0850	11.9779	10.9896	12.1824	11.8219	11.9756	12.1258	11.9261	0.9881	-0.0173	0.5544	0.2562
Prkca	_[Acetyl (Protein N-term)]ADVYPANDSTASQDVANR_.2	#N/A	6.8528	8.1896	8.3850	8.0485	7.5502	8.0712	8.3783	8.1624	7.3367	6.9604	1.0182	0.0260	0.7290	0.1373
Prkca	_VIS[Phospho (STY)]PSEDRKQPSNNLDR_.3	#N/A	6.8716	8.0449	9.1569	7.8116	7.2884	7.8894	7.7402	8.0329	7.5792	7.5403	1.0156	0.0224	0.7743	0.1111
Prkca	_VIS[Phospho (STY)]PSEDRK_.2	#N/A	7.9081	7.8362	3.8111	3.5819	7.5908	3.4035	3.2966	3.0357	7.7738	7.7692	1.0402	0.0568	0.8924	0.0494
Prkca	_LTDFNFLM[Oxidation (M)]VLGK_.2	#N/A	5.7105	5.6616	5.6899	4.6520	5.1454	6.0679	6.0917	5.9721	5.7029	5.3694	0.9488	-0.0758	0.3323	0.4785

Prkca	_DVVIQDDDVEC[Carbamidomethyl (C)]TM[Oxidation (M)]VEK_.2	#N/A	6.4595	6.6264	7.0454	8.6444	6.1949	6.7217	6.8449	7.1700	6.3553	6.7062	1.0265	0.0378	0.7106	0.1484
Prkca	_T[Phospho (STY)]FC[Carbamidomethyl (C)]GTPDYIAPEIIAYQPYGK_.3	P20444_T497_M1_Prkca	6.0966	4.1541	4.7974	3.9034	4.1591	6.0697	4.4898	5.7737	4.3349	4.5266	1.0172	0.0246	0.8890	0.0511
Prkca	_LKPS[Phospho (STY)]DKDR_.2	P20444_S234_M1_Prkca	3.5771	3.7653	5.8818	4.3553	4.2216	4.5719	5.4538	5.7635	4.7898	4.0326	0.8774	-0.1888	0.2677	0.5724
Prkca	_LSVEIWDWDR_.2	#N/A	4.0765	3.9240	5.3415	4.2186	3.8123	4.3132	4.2063	3.0552	3.2754	3.6233	1.2093	0.2742	0.0635	1.1972
Prkca	_NLIPM[Oxidation (M)]DPNGLSDPYVK_.2	#N/A	6.9769	7.1652	7.1350	6.1586	6.6782	7.0422	6.8040	7.1843	7.2072	6.9721	0.9741	-0.0379	0.4108	0.3864
Prkca	_DVVIQDDDVEC[Carbamidomethyl (C)]TM[Oxidation (M)]VEKR_.3	#N/A	4.6235	5.5523	6.3334	6.7962	4.5988	5.4918	5.5534	5.8436	5.0615	5.6591	1.0066	0.0095	0.9400	0.0269
Prkca	_KDVVIQDDDVEC[Carbamidomethyl (C)]TMVEK_.3	#N/A	4.3501	3.6055	4.0838	3.3092	3.4938	3.1308	4.5248	3.3084	2.9570	3.3048	1.0393	0.0556	0.7116	0.1478
Mag	_LLGDLGLR_.2	#N/A	8.1318	8.2712	8.1692	7.8268	7.4643	7.6331	8.2367	8.0111	7.8581	8.1894	0.9805	-0.0285	0.4053	0.3922
Mag	_NLYGTQSLELPFQGAHR_.2	#N/A	5.2050	5.1740	4.7761	5.2713	5.6364	4.8362	4.4478	5.2612	5.6963	5.2808	0.9958	-0.0061	0.9359	0.0287
Mag	_TQVVHESFQGR_.2	#N/A	5.3466	5.2666	5.2846	4.8268	5.2670	5.4350	5.5094	5.4266	5.2940	5.5015	0.9641	-0.0528	0.1279	0.8933
Mag	_SGLLLTSILTIR_.2	#N/A	4.0983	3.5235	4.1486	3.9230	3.4118	3.6930	5.1767	4.4705	5.0751	3.9714	0.8130	-0.2986	0.0122	1.9129
Mag	_GESPELDLSYSHSDLGK_.2	#N/A	4.0470	4.8764	4.2694	6.2730	4.6312	4.0901	5.6429	5.7015	5.0026	3.8948	0.9283	-0.1073	0.5211	0.2831
Mag	_NVTESSSFS[Phospho (STY)]GGDNPHVLYSPEFR_.3	P20917_S549_M1_Mag	6.5982	7.2618	7.6093	7.9254	5.4107	6.4588	6.9210	7.2772	6.9095	6.2268	1.0064	0.0092	0.9322	0.0305
Mag	_LREDEGTWVQVSLLHFVPTR_.3	#N/A	2.8970	3.6533	4.1315	3.2615	3.5416	3.0830	4.4770	3.3259	2.5639	3.3526	0.9995	-0.0008	0.9963	0.0016
Tpm3	_IQLVEEELDR_.2	#N/A	9.6437	9.3730	9.3629	9.8409	9.3893	9.4966	9.4756	9.1986	9.2267	9.6892	1.0128	0.0183	0.3941	0.4043
Tpm3	_AADAEAEVASLNR_.2	#N/A	8.4449	8.2656	8.4063	8.5889	8.6117	8.4180	8.2146	7.9768	8.2846	8.4732	1.0265	0.0378	0.0692	1.1600
Tpm3	_KYEEVAR_.2	#N/A	7.6837	7.3077	7.8711	7.5065	8.5786	7.4798	7.1799	7.3117	7.4098	7.6419	1.0477	0.0672	0.1876	0.7267
Tpm3	_HIAEEADR_.2	#N/A	7.5106	6.9473	7.0773	7.3845	8.3701	7.3011	6.7318	7.0930	7.4727	7.3064	1.0393	0.0556	0.3553	0.4494
Tpm3	_YSQKEDKYEEEIK_.3	#N/A	6.3426	6.7240	6.7854	6.6890	7.1444	6.5096	6.1360	6.4380	6.5994	6.9671	1.0251	0.0358	0.4243	0.3724
Tpm3	_KLVIIEGDLER_.2	#N/A	5.8720	5.7984	5.9282	6.1827	6.4488	5.9904	6.3502	5.5865	6.1713	6.2379	0.9918	-0.0118	0.7920	0.1013
Tpm3	_IQLVEEELDRAQER_.3	#N/A	7.0630	6.6386	7.6727	6.7352	6.4503	7.0985	6.4519	6.4802	6.4524	7.2494	1.0427	0.0604	0.3253	0.4877
Tpm3	_LEEAEKAADESER_.2	#N/A	4.7970	5.7994	5.4666	5.6940	5.3655	5.4860	5.6878	5.4743	5.1899	4.7506	1.0302	0.0429	0.5263	0.2788
Tpm3	_KLVIIEGDLERTEER_.3	#N/A	6.2317	6.7090	7.7523	7.0850	6.7094	6.3034	6.3099	6.6090	6.2020	7.2656	1.0306	0.0435	0.5725	0.2422
Tpm3	_C[Carbamidomethyl (C)]SELEEELKNVTNNLK_.2	#N/A	2.9793	4.2094	3.8416	4.2240	3.6249	4.3098	3.8512	3.1223	4.0458	3.4166	1.0709	0.0988	0.4290	0.3675
Tpm3	_ALKDEEKM[Oxidation (M)]ELQEIQLK_.3	#N/A	5.0441	5.2866	5.9199	3.7434	4.6259	5.6331	4.7055	4.6859	4.7440	5.2623	1.0397	0.0562	0.6528	0.1852
Tpm3	_AADAEAEVASLNRR_.2	#N/A	3.7744	2.9661	3.8050	3.4229	3.2655	3.6592	3.4901	3.9590	4.0567	3.8878	0.9048	-0.1443	0.0959	1.0182
Tpm3	_TEERAELAESK_.2	#N/A	2.4390	3.5699	3.2077	4.2523	3.3421	3.7414	4.4006	4.2497	3.0479	3.9296	0.8767	-0.1898	0.2528	0.5973
Tpm3	_LVIIEGDLERTEER_.3	#N/A	4.7668	5.0278	3.1168	3.6195	5.0465	3.4410	3.3341	2.9982	4.1476	3.5865	1.1857	0.2458	0.2147	0.6682
Tpm3	_RIQLVEEELDR_.2	#N/A	3.1899	4.0363	3.8767	10.1036	3.8164	9.3743	4.0940	3.7391	3.3877	4.3463	1.4731	0.5588	0.2840	0.5467
Tpm3	_HIAEEADRKYEEVAR_.4	#N/A	4.0782	3.3826	3.2642	3.7668	3.2039	3.0247	3.3855	4.3516	3.3428	3.7338	0.9325	-0.1008	0.3860	0.4135
Tpm3	_AISDELDHALNDMTS[Phospho (STY)]I_.2	P21107_S284_M1_Tpm3	4.0093	3.3136	3.1952	3.3062	3.1350	3.0937	3.4545	3.6950	3.9940	2.9835	0.9463	-0.0796	0.4552	0.3418
Gna11	_LLLLGTGESGK_.2	#N/A	7.6828	7.6307	7.4247	7.3153	6.7637	7.0720	7.4911	7.6363	7.4957	7.5994	0.9681	-0.0467	0.2192	0.6592
Gna11	_IATVGYLPTQQDVLR_.2	#N/A	7.0101	6.3105	6.9618	6.9111	6.3272	6.8238	6.7693	6.7150	6.8749	6.4595	1.0029	0.0042	0.9157	0.0383
Gna11	_DTILQLNLK_.2	#N/A	5.0901	3.3108	3.4729	4.7379	4.6767	4.4762	4.6139	5.2298	4.7855	4.6981	0.8887	-0.1702	0.2023	0.6940
Gna11	_MVDVGGQR_.2	#N/A	6.0132	5.1541	3.5174	5.5570	6.7142	4.3495	4.7339	4.3628	4.8603	4.2393	1.1470	0.1978	0.2971	0.5271
Gna11	_TLWSDPGVQEC[Carbamidomethyl (C)]YDR_.2	#N/A	3.9898	3.3526	3.7235	3.2672	4.7648	3.4911	3.4155	3.7339	4.0330	5.0195	0.9295	-0.1055	0.4896	0.3102
Gnaq	_VADPSYLPTQQDVLR_.2	#N/A	8.4366	8.4326	8.4532	8.6964	8.3689	8.6274	8.6840	8.5966	8.6208	8.1154	0.9998	-0.0003	0.9893	0.0047
Gnaq	_YYLNDLDR_.2	#N/A	7.0538	6.6819	7.3875	6.2897	6.5595	7.0656	6.5448	7.2483	6.9756	6.8201	0.9917	-0.0121	0.8130	0.0899
Gnaq	_VSAFENPYVDAIK_.2	#N/A	6.6606	6.0981	6.6572	6.3663	6.5688	6.4290	6.3595	6.4642	6.3796	5.6801	1.0390	0.0552	0.2167	0.6642
Gnaq	_[Acetyl (Protein N-term)]TLESIMAC[Carbamidomethyl (C)]C[Carbamidomethyl (C)]LSEEAK_.2	#N/A	2.4097	3.1549	4.5373	3.4395	2.9762	3.2524	3.6132	3.0785	4.2279	3.5061	0.9136	-0.1303	0.4638	0.3337
Gnaq	_EVDVEKVS[Phospho (STY)]AFENPYVDAIK_.2	#N/A	3.6576	3.5802	3.7756	3.8618	3.4660	3.6781	3.7455	3.7673	3.7493	3.7326	0.9790	-0.0307	0.3030	0.5185
Gnaq	_VPTTGIIEYPFDLQSVIFR_.2	#N/A	3.8875	3.1918	4.5743	3.5761	3.0132	3.2155	3.1249	2.9220	4.1910	3.5431	1.0381	0.0539	0.7322	0.1353
Abcb1a	_S[Phospho (STY)]TRKS[Phospho (STY)]IC[Carbamidomethyl (C)]GPHDQDR_.3	P21447_S663_M2_Abcb1a	3.0722	3.8774	3.7590	4.2433	3.6987	4.0308	4.3916	4.2587	3.0569	3.9206	0.9676	-0.0476	0.6958	0.1575
Abcb1a	_S[Phospho (STY)]IC[Carbamidomethyl (C)]GPHDQDR_.2	P21447_S667_M1_Abcb1a	8.8263	8.6043	8.4723	8.8063	7.7369	8.3044	8.9731	9.0723	8.7819	8.4960	0.9578	-0.0622	0.1440	0.8417
Abcb1a	_DSGSS[Phospho (STY)]LIR_.2	P21447_S657_M1_Abcb1a	3.1889	3.9451	4.4234	4.4705	3.8334	4.2920	4.1852	3.6480	3.2966	4.4375	1.0344	0.0487	0.6871	0.1630
Cst3	_ALDFAVSEYNK_.2	#N/A	6.2994	6.7211	6.5026	7.1202	7.0346	6.9480	7.2803	6.7581	6.7597	7.0089	0.9740	-0.0380	0.3732	0.4281
Lmnb2	_SQT[Phospho (STY)]NWGPGESFR_.2	P21619_T528_M1_Lmnb2	3.4175	5.6948	3.9233	6.4464	3.5765	4.3581	3.6153	5.6301	6.2091	3.8731	0.9457	-0.0806	0.7533	0.1231
Lmnb2	_RRLETEDTSGS[Phospho (STY)]PSR_.3	#N/A	3.9316	3.7262	5.7661	5.2532	5.5716	5.2012	4.8109	5.3339	5.7011	5.4413	0.9223	-0.1167	0.3999	0.3981
Lmnb2	_RLETEDTSGS[Phospho (STY)]PSR_.2	#N/A	9.2918	9.5860	9.9633	9.9586	9.2929	9.5694	9.6494	9.4812	9.5659	9.4841	1.0068	0.0098	0.6877	0.1626
Lmnb2	_LETEDTSGS[Phospho (STY)]PSR_.2	#N/A	8.3823	8.4509	8.7290	4.4232	8.4191	8.7258	8.6455	8.4806	8.5014	8.5969	0.9181	-0.1233	0.4396	0.3569
Lmnb2	_ATSSSSSSS[Phospho (STY)]GVGM[Oxidation (M)]SVGQGR_.2	#N/A	3.7188	4.1877	4.0103	4.0179	3.8668	3.8440	3.8441	3.9920	3.5915	4.8534	0.9682	-0.0466	0.5928	0.2271
Pou3f1	_WLEETDSSGS[Phospho (STY)]PTNLDK_.2	P21952_S324_M1_Pou3f1	4.7454	3.7127	3.6055	5.5278	4.2947	4.2058	4.6568	4.6933	3.7700	4.1971	1.0045	0.0064	0.9628	0.0165
Emb	_SDDS[Phospho (STY)]NGIENNVPR_.2	P21995_S312_M1_Emb	3.9802	3.4061	7.0317	3.3701	5.9471	3.1765	3.2938	3.4906	6.3546	6.4489	0.9159	-0.1267	0.7110	0.1481
Penk	_FAESLPSDEEGENYS[Phospho (STY)]K_.2	#N/A	8.7179	7.9170	7.3165	8.4482	8.4345	9.3758	8.4302	7.9179	9.3888	8.3964	0.9807	-0.0282	0.7130	0.1469
Penk	_ELLGTGDNR_.2	#N/A	8.3989	8.4978	8.6441	8.5978	9.2893	8.6172	8.4893	8.6890	8.6602	8.7359	1.0035	0.0051	0.8586	0.0662
Penk	_FAESLPSDEEGENY[Phospho (STY)]SKEVPEIEK_.3	#N/A	6.3333	3.9048	6.3902	7.3985	6.7600	6.9614	4.1596	6.8295	3.3087	6.9042	1.1869	0.2472	0.3333	0.4772
Penk	_FAESLPSDEEGENYS[Phospho (STY)]KEVPEIEKR_.3	#N/A	3.6639	3.6640	3.9339	4.0049	3.5261	6.7625	3.8659	4.0704	3.7190	3.6582	1.1125	0.1539	0.5177	0.2859
Penk	_LVRPGDINFLAC[Carbamidomethyl (C)]TLEC[Carbamidomethyl (C)]EGQLPSFK_.3	#N/A	3.6572	3.8880	3.1881	4.3057	3.6692	4.5663	4.3810	4.4437	3.9791	3.5441	0.9491	-0.0753	0.5100	0.2924
Penk	_DADEGDTLANS[Phospho (STY)]SDLLK_.2	P22005_S153_M1_Penk	3.6708	5.2385	4.1949	5.2041	4.4086	3.8002	5.4256	4.8651	6.4033	3.4160	0.8791	-0.1859	0.3420	0.4660
Gabrg2	_VPEGDVTVILNNLLEGYDNK_.2	#N/A	3.4561	4.9701	5.4731	5.4770	3.1237	3.4572	4.1762	5.0176	4.0720	3.1370	1.0550	0.0772	0.7325	0.1352
Ap1g1	_NLIM[Oxidation (M)]SGYS[Phospho (STY)]PEHDVSGISDPFLQVR_.3	P22892_S232_M1_Ap1g1	4.2094	3.0168	3.3954	3.8980	3.3351	3.7195	3.6127	4.8687	3.8690	3.8650	0.8870	-0.1730	0.1787	0.7479
Cbx3	_LTWHSC[Carbamidomethyl (C)]PEDEAQ_.2	#N/A	5.2039	5.0628	5.7703	6.0616	5.0642	4.9390	5.7191	4.5876	4.7404	5.8011	1.0265	0.0378	0.6972	0.1567
Cbx3	_KVEEAEPEEFVVEK_.2	#N/A	5.3920	5.7423	5.9797	6.4979	6.1172	5.9499	5.8751	5.2283	4.3014	5.5782	1.1336	0.1809	0.0660	1.1804
Cbx3	_SLS[Phospho (STY)]DSESDDSKSK_.2	#N/A	6.7913	7.6877	8.4731	7.9677	8.4708	7.8982	7.6038	7.7340	7.9773	7.8331	1.0121	0.0174	0.7776	0.1093
Cbx3	_KSLS[Phospho (STY)]DSESDDSK_.2	#N/A	5.7052	5.7161	5.7774	5.9816	6.4611	7.9947	6.0787	5.9592	8.3681	5.7377	0.9597	-0.0593	0.7027	0.1532
Cbx3	_SLS[Phospho (STY)]DSESDDSK_.2	#N/A	6.0810	5.4316	5.4262	7.5026	3.1556	4.0752	3.1184	5.7515	5.5143	6.9946	0.9876	-0.0179	0.9493	0.0226
Cbx3	_RKS[Phospho (STY)]LS[Phospho (STY)]DSESDDSK_.2	P23198_S93_M2_Cbx3	5.3224	5.7116	6.2217	6.5223	6.8198	6.0415	5.9245	5.8639	5.5012	6.3281	1.0342	0.0486	0.5293	0.2763
Cbx3	_KSLS[Phospho (STY)]DSESDDSKSK_.2	#N/A	3.6508	4.4071	3.5059	4.0085	7.0109	3.8300	3.7232	4.1100	3.7585	3.9755	1.1311	0.1778	0.4714	0.3266
Cbx3	_C[Carbamidomethyl (C)]PQIVIAFYEER_.2	#N/A	3.6987	3.5275	4.3855	3.3873	3.4158	3.2089	3.1020	3.2303	4.3797	3.3543	1.0248	0.0354	0.7877	0.1036
Gja1	_ASS[Phospho (STY)]RPRPDDLEI_.2	#N/A	10.2996	10.1185	11.0191	10.1135	7.8590	10.0298	10.8829	10.3725	10.0049	10.0010	0.9604	-0.0583	0.4927	0.3074
Gja1	_PRPDDLEI_.2	#N/A	2.8984	5.1648	5.1492	3.9898	5.2966	3.7773	5.9402	5.8761	5.7429	5.5587	0.7577	-0.4002	0.0236	1.6263
Gja1	_LVTGDRNNS[Phospho (STY)]S[Phospho (STY)]C[Carbamidomethyl (C)]RNYNK_.3	P23242_S296_M2_Gja1	3.2051	3.9614	4.0182	4.4542	3.8497	4.2758	4.1689	3.6642	3.3128	4.4212	1.0177	0.0253	0.8212	0.0855
Gja1	_QASEQNWANYSAEQNR_.2	#N/A	6.0196	5.6471	6.8764	6.5215	4.8855	6.5626	6.4838	6.5171	6.6184	5.8575	0.9554	-0.0658	0.4959	0.3046
Gja1	_SDPY[Phospho (STY)]HATTGPLSPSK_.2	P23242_Y247_M1_Gja1	3.9462	5.9229	3.6799	3.2237	3.0900	3.5346	3.3720	5.6031	4.0765	4.4018	0.8937	-0.1621	0.4927	0.3074
Gja1	_LVTGDRNNS[Phospho (STY)]SC[Carbamidomethyl (C)]R_.2	P23242_S296_M1_Gja1	4.1946	4.3803	5.8943	4.9713	3.3384	5.2337	5.4534	5.2072	4.7976	4.8949	0.9176	-0.1241	0.3991	0.3989
Gja1	_VAAGHELQPLAIVDQRPSS[Phospho (STY)]RAS[Phospho (STY)]S[Phospho (STY)]R_.4	P23242_S365_M3_Gja1	3.6602	3.5659	4.3470	7.5483	2.7859	3.4427	3.0635	3.2688	4.4182	3.3159	1.2015	0.2648	0.4548	0.3422
Gja1	_C[Carbamidomethyl (C)]NTQQPGC[Carbamidomethyl (C)]ENVC[Carbamidomethyl (C)]YDK_.2	#N/A	3.4953	4.3005	4.1821	3.8202	4.1218	3.6077	3.9685	3.4337	3.4800	3.1509	1.1177	0.1606	0.0911	1.0407
Xrcc6	_VELS[Phospho (STY)]EEELK_.2	P23475_S558_M1_Xrcc6	3.1437	3.9489	3.8305	6.3398	5.8651	5.7726	4.3201	5.5047	6.1540	6.2145	0.8682	-0.2040	0.3658	0.4368
Pcmt1	_ELVDDSITNVK_.2	#N/A	7.3964	6.9546	7.1641	6.9102	7.7326	7.1598	6.8133	6.8601	7.0907	7.7330	1.0134	0.0192	0.6880	0.1624

Pcmt1	_ALDVGSGSGILTAC[Carbamidomethyl (C)]FAR_.2	#N/A	5.1191	6.0124	6.9264	6.0507	6.0543	5.9461	5.9000	5.3343	6.2029	6.1925	1.0187	0.0268	0.7485	0.1258
Pcmt1	_LILPVGPAGGNQM[Oxidation (M)]LEQYDKLQDGSVK_.3	#N/A	5.5912	6.5905	6.7785	6.6207	6.8056	5.7087	6.5802	6.4791	6.6097	6.5985	0.9669	-0.0486	0.4614	0.3359
Pcmt1	_VFEVM[Oxidation (M)]LATDR_.2	#N/A	3.8414	5.1722	4.6021	5.2323	4.7699	3.4614	3.3546	5.2530	5.5847	5.2038	0.9307	-0.1035	0.5530	0.2573
Pcmt1	_SGGASHSELIHNLR_.3	#N/A	4.9250	5.0179	5.4277	4.9580	5.1353	5.1111	5.3053	4.1443	5.0070	5.6238	1.0151	0.0216	0.7847	0.1053
Pcmt1	_TDKVFEVM[Oxidation (M)]LATDR_.3	#N/A	4.2602	3.6433	4.6977	4.0491	3.6803	2.9444	4.1728	4.1400	3.1434	3.0898	1.0667	0.0932	0.5495	0.2600
Pcmt1	_ELVDDSITNVKK_.3	#N/A	3.8625	2.3380	3.5962	3.1400	3.1996	3.9461	3.2882	3.8612	2.6822	1.7354	1.1575	0.2110	0.3614	0.4420
Gria2	_ADIAIAPLTITLVR_.2	#N/A	4.5802	5.0752	4.0346	4.6791	4.8549	3.7457	4.8834	5.0436	3.9999	4.6851	0.9660	-0.0499	0.6302	0.2005
Gria2	_NAQNINPSSSQNS[Phospho (STY)]QNFATYK_.2	P23819_S863_M1_Gria2	3.3739	4.1791	4.3978	3.9416	4.0004	3.7291	7.2002	3.0596	3.3586	3.6189	0.9136	-0.1304	0.6494	0.1875
Gria2	_YAYLLESTM[Oxidation (M)]NEYIEQR_.2	#N/A	3.2846	4.0578	5.6852	4.0629	3.8792	3.8503	4.2111	3.4999	3.5093	3.7401	1.1060	0.1454	0.3876	0.4116
Gria2	_YTSALTYDAVQVM[Oxidation (M)]TEAFR_.2	#N/A	3.6818	2.9372	3.4155	3.9776	7.9033	4.2304	4.2709	4.0570	3.7895	4.1374	1.0723	0.1008	0.7573	0.1207
Cryab	_VLGDVIEVHGK_.2	#N/A	5.6728	6.0425	5.1450	6.2106	6.4236	5.7723	6.7080	5.3142	5.6182	6.3881	0.9785	-0.0314	0.7180	0.1439
Cryab	_[Acetyl (Protein N-term)]M[Oxidation (M)]DIAIHHPWIR_.3	#N/A	5.1129	6.3012	6.0466	5.1942	6.3052	5.6021	5.5173	5.2049	6.2090	6.1551	0.9981	-0.0028	0.9742	0.0114
Cryab	_QDEHGFISR_.2	#N/A	3.4903	5.2370	4.1771	5.7033	6.1285	4.4729	4.4354	5.8450	5.1193	3.1459	1.0500	0.0704	0.7424	0.1293
Cryab	_FSVNLDVK_.2	#N/A	3.1499	4.1925	4.3845	3.9282	4.2662	3.7157	3.3012	3.8681	2.3386	3.5168	1.2099	0.2748	0.0854	1.0686
Cryab	_APSWIDTGLSEMR_.2	#N/A	4.2687	3.9784	3.9345	5.4327	4.3128	4.2587	4.1519	3.6813	3.3299	4.1666	1.1388	0.1875	0.1369	0.8638
Cryab	_HEERQDEHGFISR_.4	#N/A	4.3110	4.4160	3.4970	3.9997	3.4367	4.2928	3.7143	4.1188	3.7674	3.9667	1.0258	0.0367	0.6761	0.1700
Bcat1	_LTDIQYGR_.2	#N/A	5.5651	5.7045	3.2067	5.1599	5.8120	5.3655	4.8147	5.5484	5.3227	5.0715	0.9897	-0.0150	0.9191	0.0366
Bcat1	_HLTM[Oxidation (M)]DDLATALEGNR_.3	#N/A	4.4904	4.8130	4.1229	5.1470	4.2221	4.5506	4.1511	5.3037	5.0316	4.3610	0.9673	-0.0480	0.6088	0.2155
Bcat1	_QSILELAQQWGEFK_.2	#N/A	3.1624	3.9187	4.3969	3.9407	3.8070	4.3185	4.0889	3.3405	3.3596	3.6180	1.0895	0.1236	0.2569	0.5902
Ppib	_TVDNFVALATGEK_.2	#N/A	6.8759	7.1472	7.1662	7.5721	7.1132	7.1885	7.0676	7.1621	7.0441	7.0324	1.0142	0.0204	0.4143	0.3827
Ppib	_VLEGMDVVR_.2	#N/A	5.3936	3.1560	4.5384	6.0763	4.6159	3.2514	3.2549	3.0774	4.2268	3.5072	1.2811	0.3574	0.1506	0.8221
Ppib	_DTNGSQFFITTVK_.2	#N/A	4.2307	3.4862	3.9644	3.4286	4.7087	4.0564	3.1433	3.1890	4.3384	3.3956	1.1315	0.1783	0.1980	0.7034
Ppib	_VYFDLQIGDESVGR_.2	#N/A	5.2251	4.1377	3.7753	4.2779	3.7150	4.0995	3.9926	3.8405	3.4891	4.4585	1.0659	0.0920	0.4408	0.3558
Gsta4	_FLQPGSQR_.2	#N/A	5.8749	7.0599	6.7862	6.4289	7.2138	6.7829	6.4789	6.6615	6.8560	6.7380	1.0011	0.0016	0.9772	0.0100
Gsta4	_EKEESYDLILSR_.3	#N/A	4.1085	3.3639	3.8421	3.3859	3.2522	3.3724	3.5342	3.6153	3.9143	3.0632	1.0064	0.0091	0.9222	0.0352
Th	_PTPSASS[Phospho (STY)]PQPK_.2	P24529_S8_M1_Th	10.8268	10.9693	11.2740	11.0751	10.6601	11.0752	10.8713	3.4047	10.9186	10.9356	1.2156	0.2817	0.2267	0.6446
Th	_RQS[Phospho (STY)]LIEDARK_.3	#N/A	7.6319	8.0404	8.0555	7.7581	7.5512	7.7749	7.9317	7.5740	7.8396	7.6863	1.0057	0.0082	0.7297	0.1368
Th	_RQS[Phospho (STY)]LIEDAR_.2	#N/A	5.9100	6.7838	6.2494	6.2917	5.4443	6.3619	6.4663	5.7750	6.1787	5.5960	1.0282	0.0402	0.5600	0.2518
Th	_QAEAVTS[Phospho (STY)]PR_.2	P24529_S31_M1_Th	10.0109	10.1156	10.2087	10.4230	9.8484	10.0581	10.1264	10.1044	10.0649	10.2247	0.9981	-0.0028	0.8556	0.0677
Th	_RAVS[Phospho (STY)]EQDTK_.2	P24529_S19_M1_Th	5.3806	3.3523	4.5607	5.5832	3.2406	3.3840	5.8194	5.5480	4.7297	3.5295	0.8662	-0.2072	0.3632	0.4399
Th	_FEVPSGDLAALLSSVR_.2	#N/A	5.0155	4.7375	3.2055	4.8296	4.8322	4.0090	4.8223	4.2844	5.2770	5.6189	0.8875	-0.1721	0.2205	0.6566
Th	_FDPYTLAIDVLDS[Phospho (STY)]PHTIR_.2	#N/A	3.3572	4.1331	3.5460	4.3518	3.9947	3.9710	4.5000	4.0750	4.1344	3.7689	0.9448	-0.0819	0.3397	0.4690
Th	_SLEGVQDELHTLTQALSAIS_.2	#N/A	3.9138	3.4286	3.6475	3.9065	4.2680	2.9787	3.3395	3.1049	2.5860	4.3694	1.1017	0.1397	0.3895	0.4095
Th	_VFETFEAK_.2	#N/A	3.7465	3.0020	3.4802	4.5248	2.8903	3.7344	3.1722	3.9772	4.2763	4.2021	0.9120	-0.1329	0.3712	0.4304
Impdh2	_GMGS[Phospho (STY)]LDAMDK_.2	P24547_S416_M1_Impdh2	4.2988	3.6031	3.4847	4.4526	3.4245	4.3050	3.1650	4.1311	3.7797	3.9544	1.0454	0.0641	0.5755	0.2399
Aldh1a1	_[Acetyl (Protein N-term)]SS[Phospho (STY)]PAQPAVPAPLADLK_.2	P24549_S3_M1_Aldh1a1	9.2159	9.4270	9.7027	10.0281	8.9942	9.3008	3.9130	9.2993	9.1546	9.0429	1.2028	0.2664	0.1700	0.7696
Aldh1a1	_GFFVQPTVFSNVTDEM[Oxidation (M)]R_.2	#N/A	4.8950	6.1675	6.1222	5.3950	6.0009	6.1161	5.9475	5.7429	5.7128	5.7874	0.9974	-0.0037	0.9574	0.0189
Aldh1a1	_IHGQTIPSDGDIFTYTR_.3	#N/A	6.1519	5.6994	3.6183	5.2290	6.1088	6.0852	5.9898	5.6516	5.3431	5.9079	0.9579	-0.0621	0.6508	0.1865
Aldh1a1	_VFANAYLSDLGGC[Carbamidomethyl (C)]IK_.2	#N/A	5.1615	4.9118	4.9561	4.9053	3.1493	3.0793	5.2617	5.1510	3.5672	4.8961	0.9240	-0.1140	0.5563	0.2547
Aldh1a1	_VTLELGGK_.2	#N/A	3.7190	4.3998	4.3984	3.8868	4.3429	5.1883	3.4318	5.0235	4.6288	4.9287	0.9599	-0.0591	0.6561	0.1830
Aldh1a1	_VAFTGSTQVGK_.2	#N/A	4.1130	3.2295	3.8466	5.0811	3.2567	3.3679	3.5387	3.6108	3.9098	3.0677	1.0804	0.1116	0.4866	0.3129
M6pr	_SFESTVGQGSDTYSYIFR_.2	#N/A	4.4677	3.3672	4.1922	4.5902	3.9862	4.4084	4.4904	4.3023	2.4307	4.5037	1.0602	0.0844	0.6191	0.2082
M6pr	_GVGDDQLGEES[Phospho (STY)]EERDDHLLPM[Oxidation (M)]_.3	P24668_S268_M1_M6pr	3.0636	1.9783	2.3385	2.9055	1.1304	4.0394	4.4002	4.2501	3.0483	3.9292	0.6593	-0.6009	0.0457	1.3399
Cdk11b	_S[Phospho (STY)]PLRPPR_.2	P24788_S234_M1_Cdk11b	9.4551	9.2801	7.7280	9.4299	8.0117	8.7543	9.7398	9.5177	9.1379	8.9131	0.9410	-0.0878	0.2169	0.6637
Cdk11b	_DLLSDLQDIS[Phospho (STY)]DSER_.2	P24788_S270_M1_Cdk11b	3.8086	3.0641	3.5423	3.8507	6.7572	3.6722	3.5654	3.9151	3.9164	4.2642	1.0512	0.0721	0.7772	0.1095
Rps2	_TYSYLTPDLWK_.2	#N/A	5.6372	6.0152	6.6159	6.1213	5.8261	6.1037	5.7483	5.7060	5.9941	5.4399	1.0579	0.0812	0.1221	0.9133
Rps2	_AEDKEWIPVTK_.3	#N/A	3.4881	3.4746	4.7108	3.0990	3.9646	2.8171	3.8860	3.9839	3.6253	3.2057	0.9775	-0.0329	0.8282	0.0819
Tln1	_VLVQNAAGS[Phospho (STY)]QEK_.2	P26039_S2040_M1_Tln1	6.5048	6.3817	6.7999	6.5872	5.7722	6.1743	6.9651	6.9916	6.4345	6.4361	0.9498	-0.0743	0.1667	0.7780
Ezr	_APDFVFYAPR_.2	#N/A	6.6123	6.9245	6.9860	7.4162	6.9060	6.9723	6.6044	7.1858	7.0921	7.3764	0.9865	-0.0196	0.6212	0.2067
Ezr	_IGFPWSEIR_.2	#N/A	6.5604	6.8141	5.6427	6.2706	5.8162	5.9896	6.5030	6.5511	5.6081	6.3683	0.9880	-0.0175	0.8003	0.0968
Ezr	_NISFNDKK_.2	#N/A	5.9639	5.5395	6.2026	6.5412	6.6722	6.1321	3.4258	5.6764	6.0689	6.2713	1.1520	0.2041	0.1834	0.7367
Ezr	_QLLTLSNELSQAR_.2	#N/A	4.2547	4.4224	3.4406	3.5100	3.3803	4.3491	3.2091	4.2783	4.1334	3.6500	1.0197	0.0281	0.8192	0.0866
Ezr	_IAQDLEM[Oxidation (M)]YGINYFEIK_.2	#N/A	3.7475	3.4656	4.5399	4.0268	3.8541	3.7851	3.3842	3.2770	4.3246	3.1240	1.1065	0.1460	0.2205	0.6566
Ezr	_RKEDEVEEWQHR_.3	#N/A	5.5048	3.5625	4.3523	3.3541	4.5250	5.1030	5.1919	3.2654	3.8266	4.8360	1.0281	0.0400	0.8336	0.0790
Ezr	_GTDLWLGVDALGLNIYEK_.2	#N/A	4.1753	3.0509	3.3612	3.8639	3.3010	4.4285	3.5786	4.2546	3.9032	3.8309	0.9499	-0.0742	0.5321	0.2740
Gabra3	_LLDGYDNR_.2	#N/A	6.5521	6.8256	6.9206	6.5619	5.8529	5.9348	6.7888	6.9293	6.5766	6.7429	0.9529	-0.0695	0.2148	0.6679
Gabra3	_SAAAPSASSTPTAIAS[Phospho (STY)]PK_.2	P26049_S433_M1_Gabra3	8.6247	8.7496	9.0921	8.3579	6.9771	8.6878	9.3463	9.1385	8.4495	8.6147	0.9469	-0.0788	0.2875	0.5414
Ctnna1	_SRTS[Phospho (STY)]VQTEDDQLIAGQSAR_.3	#N/A	3.7867	8.8088	3.9644	9.1518	8.6930	6.9594	8.6820	6.9428	7.0125	9.0014	0.8716	-0.1983	0.4658	0.3318
Ctnna1	_QDLLAYLQR_.2	#N/A	5.4931	5.6581	3.1568	5.1294	4.7462	3.1525	3.9509	5.4585	3.0988	5.4738	1.0135	0.0193	0.9368	0.0283
Ctnna1	_EYAQVFR_.2	#N/A	5.2685	4.5928	5.0380	3.2049	4.9873	4.9450	4.8789	4.8366	5.4692	3.1719	1.0182	0.0260	0.8824	0.0543
Ctnna1	_TS[Phospho (STY)]VQTEDDQLIAGQSAR_.2	#N/A	7.5561	7.1721	3.9512	7.9708	6.8711	7.6759	8.3170	7.4752	7.8012	7.3084	0.8888	-0.1701	0.2998	0.5231
Ctnna1	_TPEELDDSDFET[Phospho (STY)]EDFDVR_.2	P26231_T645_M1_Ctnna1	3.8113	3.6715	3.8837	6.4258	3.8745	3.9607	3.6821	6.4464	3.8866	3.9398	0.9515	-0.0717	0.7788	0.1086
Ctnna1	_LESIISGAALM[Oxidation (M)]ADSSC[Carbamidomethyl (C)]TR_.2	#N/A	4.3463	3.6017	4.0800	3.1584	3.4900	3.1346	3.7720	3.3774	3.6765	3.3010	1.0293	0.0416	0.7076	0.1502
Chga	_GWRPSS[Phospho (STY)]REDS[Phospho (STY)]VEAR_.2	#N/A	7.1788	7.7213	7.5527	8.2025	8.0078	7.7776	5.4821	7.4055	7.7742	7.7408	1.0901	0.1244	0.2094	0.6791
Chga	_DDGQSDS[Phospho (STY)]QAVDGDGK_.2	P26339_S282_M1_Chga	4.3089	3.0335	4.0425	3.5863	3.4526	3.1720	3.7346	3.4149	3.7139	3.2636	1.0191	0.0273	0.8076	0.0928
Ptma	_[Acetyl (Protein N-term)]SDAAVDTSSEITTK_.2	#N/A	6.7075	9.1008	7.8231	9.5089	9.3194	9.0575	8.9057	8.3466	8.9058	8.9788	0.9775	-0.0329	0.7375	0.1323
Ptma	_VAEDDEDDDVDTKK_.2	#N/A	3.8261	4.7924	4.1883	3.8846	5.3928	4.6914	4.6281	3.9533	4.8287	3.5592	1.0519	0.0730	0.5853	0.2326
Ptma	_RVAEDDEDDDVDTKK_.3	#N/A	3.4950	5.2382	5.7882	5.8006	6.3987	5.3961	5.2981	5.2987	5.1921	5.0024	1.0298	0.0424	0.7693	0.1139
U2af2	_GAKEEHGGLIRS[Phospho (STY)]PR_.3	#N/A	9.3528	9.6819	10.6698	9.7137	9.3190	9.5643	9.7687	9.7611	9.7919	9.4897	1.0015	0.0021	0.9579	0.0187
U2af2	_EEHGGLIRS[Phospho (STY)]PR_.3	#N/A	9.1593	8.8518	9.3169	9.4851	8.6164	8.9342	9.3351	9.0877	9.0809	9.1487	0.9888	-0.0162	0.5633	0.2492
U2af2	_[Acetyl (Protein N-term)]S[Phospho (STY)]DFDEFER_.2	P26369_S2_M1_U2af2	4.2757	3.5311	4.0093	7.8985	6.5757	5.3969	3.0984	6.6871	7.2702	3.2304	1.0413	0.0584	0.8687	0.0611
Glud1	_NYTDNELEK_.2	#N/A	8.6501	8.7907	8.7218	9.0214	8.8650	8.7731	8.9402	8.7448	8.7113	8.7985	1.0006	0.0008	0.9497	0.0224
Glud1	_TAAYVNAIEK_.2	#N/A	8.1291	8.3362	9.0359	8.4521	8.1003	8.1670	7.4174	8.4851	7.6176	7.8659	1.0667	0.0932	0.0760	1.1194
Glud1	_YSTDVSVDEVK_.2	#N/A	8.1551	8.2879	8.3554	8.4974	8.0788	8.6630	7.9494	8.1000	8.2570	8.2821	1.0236	0.0337	0.1659	0.7800
Glud1	_C[Carbamidomethyl (C)]AVVDVPFGGAK_.2	#N/A	7.6871	7.9015	8.0028	7.8319	8.2908	8.0229	7.7317	7.9791	8.0966	7.8797	1.0043	0.0063	0.7834	0.1060
Glud1	_YNLGLDLR_.2	#N/A	7.9992	8.1782	8.1712	7.8076	8.0277	7.9432	7.4535	8.1909	7.8091	7.9432	1.0219	0.0313	0.2581	0.5882
Glud1	_HGGTIPVVPTAEFQDR_.2	#N/A	7.2989	7.3824	7.4657	7.6892	6.6603	7.0919	7.4130	6.9293	6.9813	6.9697	1.0271	0.0385	0.3719	0.4296
Glud1	_NLNHVSYGR_.2	#N/A	7.2325	7.5966	7.5399	7.7719	7.9615	7.1610	7.6192	7.5918	8.0590	7.3732	0.9847	-0.0222	0.5629	0.2496
Glud1	_C[Carbamidomethyl (C)]VGVGESDGSIWNPDGIDPK_.2	#N/A	6.2773	6.1599	6.3761	6.9413	6.7726	6.5377	6.6340	5.9412	6.2763	5.7721	1.0577	0.0809	0.1373	0.8625

Glud1	_RDDGSWEVIEGYR_.3	#N/A	4.6856	4.7264	5.1635	3.9150	5.3152	5.7880	5.5563	5.2204	5.9632	5.8871	0.8719	-0.1977	0.0755	1.1220
Glud1	_M[Oxidation (M)]VEGFFDR_.2	#N/A	7.3167	7.8282	7.6740	7.6419	7.4970	7.5760	7.2895	7.8507	7.8577	7.4075	0.9984	-0.0024	0.9348	0.0293
Glud1	_GFIGPGIDVPAPDM[Oxidation (M)]STGER_.2	#N/A	7.5056	7.4557	7.4138	8.0154	7.2450	7.3443	7.6725	7.4601	7.2063	7.4529	1.0065	0.0094	0.7644	0.1167
Glud1	_DDGSWEVIEGYR_.2	#N/A	8.4990	8.3937	8.7897	9.2871	8.5254	8.6023	8.6738	8.7699	8.8255	8.8959	0.9877	-0.0179	0.5403	0.2673
Glud1	_IIAEGANGPTTPEADKIFLER_.3	#N/A	7.4003	7.0827	7.3096	7.4474	6.8657	6.9843	7.1134	7.2779	6.9645	7.0423	1.0116	0.0166	0.5524	0.2577
Glud1	_TFVVQGFGNVGLHSM[Oxidation (M)]R_.2	#N/A	4.0415	4.5766	5.0623	2.5466	4.8963	5.0096	3.3835	3.4846	5.5963	5.4152	0.9744	-0.0374	0.8711	0.0599
Glud1	_IIAEGANGPTTPEADK_.2	#N/A	5.7191	3.1667	3.6449	5.1654	5.3241	5.1495	3.4628	4.3704	5.0377	4.9881	1.0516	0.0725	0.7115	0.1478
Glud1	_GASIVEDKLVEDLK_.2	#N/A	5.5909	6.0872	6.2585	5.8418	5.0680	5.7125	6.0184	5.7886	5.6249	5.1852	1.0187	0.0267	0.6893	0.1616
Glud1	_ALASLMTYK_.2	#N/A	3.4235	3.9600	3.1161	5.0745	3.1261	3.9482	4.3090	4.3413	3.5483	2.5560	1.0233	0.0333	0.8682	0.0614
Glud1	_DSNYHLLM[Oxidation (M)]SVQESLER_.3	#N/A	4.9029	5.4313	5.7192	5.4889	4.7986	5.7791	4.7952	5.1977	6.0778	5.1542	1.0089	0.0127	0.8796	0.0557
Glud1	_VYEGSILEADC[Carbamidomethyl (C)]DILIPAASEK_.2	#N/A	7.7458	8.0495	8.4082	8.3789	7.6050	7.9877	7.8778	7.5483	7.7493	8.0358	1.0290	0.0413	0.2555	0.5926
Glud1	_DIVHSGLAYTM[Oxidation (M)]ER_.3	#N/A	5.2611	5.0209	4.5693	5.1967	5.0131	5.2882	5.6654	5.3494	5.1240	4.8704	0.9630	-0.0543	0.3385	0.4705
Glud1	_FTMELAK_.2	#N/A	3.3800	3.8461	4.0668	2.7469	1.7826	3.7229	4.2842	3.5490	3.1976	3.0357	0.9263	-0.1104	0.6108	0.2141
Psmd7	_SVVALHNLINNK_.2	#N/A	3.4156	4.7477	5.9270	4.1275	3.5183	2.6637	4.0481	4.8051	5.0466	5.4030	0.8427	-0.2469	0.2623	0.5812
Sars1	_LLIDEAIQK_.2	#N/A	5.8352	5.9299	5.3131	5.9932	6.4137	5.6578	3.1754	6.3183	6.5199	6.0774	1.0605	0.0848	0.6209	0.2070
Sars1	_VLDLDLFR_.2	#N/A	5.8201	5.8387	5.6696	5.2477	5.4358	5.5927	3.1896	5.5717	5.8034	4.5766	1.1704	0.2270	0.1308	0.8833
Sars1	_YLIATSEQPIAALHR_.3	#N/A	5.1042	5.1730	4.7288	4.3694	5.1225	5.5340	3.4950	5.2079	5.7508	5.5880	0.9990	-0.0015	0.9914	0.0038
Sars1	_TIC[Carbamidomethyl (C)]AILENYQAEK_.2	#N/A	4.9411	5.2264	4.2623	4.4145	5.0601	4.5647	5.2998	4.0168	4.9075	4.9288	0.9909	-0.0131	0.8855	0.0528
Sars1	_YAGLSTC[Carbamidomethyl (C)]FR_.2	#N/A	4.8131	4.5108	4.1282	5.1854	5.7739	4.6320	4.1623	5.0757	5.3654	5.0519	0.9851	-0.0217	0.8431	0.0741
Sars1	_IEQFVYSSPHDNK_.2	#N/A	5.5690	5.7661	5.6579	6.1829	6.4327	6.0143	6.1855	5.6795	6.1265	6.1390	0.9842	-0.0230	0.6359	0.1966
Sars1	_EVPLENQLQS[Phospho (STY)]M[Oxidation (M)]EVTEA_.2	#N/A	3.6307	6.6906	3.3644	7.1171	4.0878	6.5926	4.5572	7.1321	6.1895	4.0863	0.9555	-0.0656	0.8210	0.0857
Sars1	_ELVSC[Carbamidomethyl (C)]SNC[Carbamidomethyl (C)]TDYQAR_.2	#N/A	3.5230	3.8194	3.2566	4.1900	4.5707	3.0610	4.1714	4.2008	4.2423	3.4961	0.9278	-0.1081	0.3960	0.4023
Sars1	_AKEVPLENQLQS[Phospho (STY)]MEVTEA_.2	#N/A	3.6696	3.5566	4.3564	3.3582	3.4449	3.1797	3.0729	3.2594	4.4089	3.3252	1.0221	0.0315	0.8129	0.0900
Sars1	_EIGNLLHPSVPISNDEDADNKVER_.4	#N/A	4.3462	3.6506	3.5322	4.0835	3.4719	4.2576	3.7495	4.0836	3.7322	4.0018	0.9996	-0.0005	0.9945	0.0024
Marcks	_AEDGAAPS[Phospho (STY)]PSSETPK_.2	#N/A	14.0336	14.2945	14.7087	13.9131	14.0504	14.3007	14.4121	14.0676	13.8637	14.3347	1.0033	0.0048	0.7956	0.0993
Marcks	_LSGFS[Phospho (STY)]FKK_.2	#N/A	14.1200	14.1506	14.8471	14.7197	13.5893	14.0890	14.3619	14.0733	13.9115	14.2690	1.0070	0.0100	0.7020	0.1537
Marcks	_GEATAERPGEAAVASS[Phospho (STY)]PSK_.2	#N/A	10.2629	9.7286	10.0601	10.4658	10.3971	10.1491	9.9082	9.7035	10.2261	9.4048	1.0374	0.0529	0.0936	1.0288
Marcks	_VNGDAS[Phospho (STY)]PAAAEPGAK_.2	P26645_S46_M1_Marcks	10.7822	10.7075	10.4350	11.4679	10.5578	10.7362	10.8834	10.5700	10.6019	10.9357	1.0031	0.0045	0.8706	0.0602
Marcks	_FS[Phospho (STY)]FKKS[Phospho (STY)]FK_.2	P26645_S152_M2_Marcks	9.6443	10.0720	10.6935	10.3471	9.8239	10.2818	10.1044	9.7229	10.0314	10.2491	1.0116	0.0167	0.5977	0.2235
Marcks	_LSGFS[Phospho (STY)]FKKS[Phospho (STY)]K_.2	P26645_S163_M2_Marcks	7.9589	7.5291	8.0248	8.4985	7.4064	8.2204	8.0880	7.5308	8.1647	8.3574	0.9881	-0.0173	0.7155	0.1454
Marcks	_DEAAAATEPGAGAADKEAAEAEPAEPSS[Phospho (STY)]PAAEAEGASASSTSSPK_.4	#N/A	6.2755	3.8963	4.3745	3.6505	6.6630	3.7309	4.2674	3.2487	3.2022	3.4361	1.3466	0.4293	0.1250	0.9029
Marcks	_ESGEGAEAEGATAEGAK_.2	#N/A	5.6342	5.8542	4.4573	5.3604	5.8618	5.3687	4.3401	4.9396	5.4659	6.2347	1.0339	0.0481	0.6797	0.1677
Marcks	_PGEAAVASS[Phospho (STY)]PSK_.2	#N/A	2.8960	5.8890	4.1305	6.7662	4.2677	3.4618	6.4491	3.3269	5.8832	3.3516	0.9613	-0.0570	0.8578	0.0666
Marcks	_SKKES[Phospho (STY)]GEGAEAEGATAEGAK_.3	#N/A	6.3582	6.0621	6.5828	6.7543	6.5301	6.1774	6.1606	6.2593	6.6307	4.1051	1.1074	0.1472	0.2225	0.6528
Marcks	_AEDGAAPS[Phospho (STY)]PSSET[Phospho (STY)]PKKK_.2	#N/A	8.4834	9.2770	10.1222	8.4979	8.4102	8.2479	7.9871	9.3372	7.1223	9.4230	1.0440	0.0621	0.5347	0.2719
Marcks	_EAAEAEPAEPSS[Phospho (STY)]PAAEAEGASASSTSSPK_.3	#N/A	12.1611	11.8835	12.0959	12.6404	11.9993	12.1424	12.4967	11.7483	11.9478	12.2270	1.0040	0.0058	0.7986	0.0977
Marcks	_S[Phospho (STY)]FKLS[Phospho (STY)]GFSFK_.2	P26645_S160_M2_Marcks	3.8721	3.1764	4.5589	6.6676	5.6743	3.2309	3.2754	5.8239	4.2064	3.5277	1.0764	0.1063	0.7140	0.1463
Marcks	_AEDGAAPSPSSET[Phospho (STY)]PKK_.2	#N/A	10.5390	10.8773	11.3060	11.3972	10.4512	10.9803	10.8468	10.2922	10.7140	10.8664	1.0230	0.0328	0.3048	0.5160
Marcks	_GEATAERPGEAAVASS[Phospho (STY)]PSKANGQENGHVK_.4	#N/A	8.4770	3.5784	8.6366	3.6462	8.8140	3.4336	3.7944	7.7906	8.8253	3.8985	1.0034	0.0048	0.9910	0.0039
Marcks	_LSGFS[Phospho (STY)]FK_.2	#N/A	3.9336	3.2926	3.1195	3.6222	3.0593	3.1694	3.3369	9.1570	4.1449	3.5892	0.6656	-0.5872	0.1637	0.7860
Marcks	_TAAKGEATAERPGEAAVASS[Phospho (STY)]PSK_.3	#N/A	6.0360	6.4348	7.1586	6.8037	6.4943	6.8109	3.9858	6.6810	3.7670	6.5625	1.2618	0.3354	0.0703	1.1530
Marcks	_KES[Phospho (STY)]GEGAEAEGATAEGAKDEAAAAAGGEGAAAPGEQAGGAGAEGAAGGEPR_.4	#N/A	3.8087	3.8634	4.2157	3.7174	3.6258	3.8607	3.7049	5.1763	3.9296	4.4703	0.8908	-0.1668	0.1289	0.8896
Marcks	_ESGEGAEAEGATAEGAKDEAAAAAGGEGAAAPGEQAGGAGAEGAAGGEPR_.4	#N/A	3.4925	4.2488	3.2262	4.1669	4.1371	3.9884	3.8815	3.9516	3.6002	4.1339	0.9961	-0.0056	0.9491	0.0227
Marcks	_KES[Phospho (STY)]GEGAEAEGATAEGAK_.2	#N/A	3.8856	3.4568	3.6192	5.4411	3.0293	2.9505	3.3113	3.8382	4.1372	4.3411	0.9548	-0.0667	0.7315	0.1358
Fkbp1a	_GVQVETISPGDGR_.2	#N/A	10.8937	11.1971	11.6066	11.4672	11.4195	11.3470	11.3802	10.9148	11.1054	11.1846	1.0158	0.0226	0.2705	0.5678
Fkbp1a	_GWEEGVAQM[Oxidation (M)]SVGQR_.2	#N/A	8.2405	8.6108	8.8447	8.6063	8.9376	8.7395	8.4831	8.2461	8.3939	8.4961	1.0307	0.0437	0.0875	1.0580
Map4	_SKVGS[Phospho (STY)]TENIK_.2	#N/A	9.7403	9.7859	10.2805	10.3939	3.9032	9.8857	10.0814	9.9665	9.8754	9.8878	0.9041	-0.1455	0.4777	0.3208
Map4	_SAS[Phospho (STY)]ADLSR_.2	P27546_S828_M1_Map4	7.5878	7.9200	7.8342	7.7967	6.3403	7.7732	7.0666	7.4958	7.8015	7.9560	0.9950	-0.0072	0.9146	0.0388
Map4	_LATTVS[Phospho (STY)]APDLK_.2	P27546_S901_M1_Map4	4.0398	7.5041	3.7734	8.3145	7.6165	8.2971	3.3343	7.7121	4.1475	8.0522	1.1341	0.1816	0.6028	0.2198
Map4	_AAVGVTGNDITTPPNKEPPPS[Phospho (STY)]PEK_.3	#N/A	8.2432	8.2854	8.4742	4.0493	3.9156	7.9668	8.4500	4.4527	3.2509	8.0320	1.1283	0.1742	0.6236	0.2051
Map4	_DM[Oxidation (M)]S[Phospho (STY)]PSAETEAPLAK_.2	#N/A	10.8180	10.6776	10.9530	11.5014	10.4441	10.6050	11.2079	10.3348	10.7673	10.8445	1.0041	0.0059	0.8552	0.0679
Map4	_TEFIPLLDGDEK_.2	#N/A	7.6884	7.3194	3.6815	7.6133	8.1296	7.8154	7.2109	6.7249	7.4843	7.7028	0.9671	-0.0482	0.7894	0.1027
Map4	_NTT[Phospho (STY)]PTGAAPPAGM[Oxidation (M)]TSTR_.2	#N/A	11.0182	11.3427	11.8405	11.9557	10.8851	11.3271	11.5911	11.2125	11.0484	11.1854	1.0120	0.0173	0.5842	0.2335
Map4	_ATS[Phospho (STY)]PSTLVSTGPSSR_.2	P27546_S785_M1_Map4	8.7598	9.4980	9.9409	3.9421	8.3908	9.1691	5.6525	4.9557	9.0815	8.9940	1.1551	0.2081	0.4535	0.3435
Map4	_DM[Oxidation (M)]S[Phospho (STY)]PLPESEVTLGK_.2	#N/A	7.7421	8.2588	7.9623	9.6755	8.2148	8.1994	8.6837	8.1233	8.3268	8.4870	0.9925	-0.0109	0.8655	0.0627
Map4	_GQSTVPPC[Carbamidomethyl (C)]TAS[Phospho (STY)]PEPVK_.2	P27546_S598_M1_Map4	8.5795	8.6058	8.9263	10.3152	3.9311	3.7984	4.2087	9.4206	7.8901	2.9602	1.2025	0.2661	0.5236	0.2810
Map4	_SKVGST[Phospho (STY)]ENIKHQPGGGR_.3	P27546_T915_M1_Map4	4.1206	3.2218	3.8542	5.8702	5.1670	3.3603	3.5463	3.6032	3.9022	3.0753	1.2078	0.2724	0.2198	0.6579
Map4	_TTSASS[Phospho (STY)]VKR_.2	P27546_S841_M1_Map4	2.9085	3.6648	4.1430	3.2500	3.5531	3.0716	4.4656	3.3676	3.0162	3.3641	0.9658	-0.0502	0.7290	0.1373
Map4	_VGS[Phospho (STY)]TENIKHQPGGGR_.3	#N/A	4.1351	3.3905	3.8688	3.5242	3.2788	3.3458	3.2389	3.5886	4.2428	3.0898	1.0143	0.0204	0.8545	0.0683
Map4	_DM[Oxidation (M)]S[Phospho (STY)]PLPESEVTLGKDVVILPETK_.3	#N/A	2.8994	5.4934	4.9300	6.3006	5.0882	3.4652	3.8260	3.3234	3.6225	3.3550	1.3297	0.4111	0.1151	0.9390
Map4	_VGS[Phospho (STY)]LDNVGHLPAGGAVK_.2	#N/A	4.9869	5.9855	6.1470	7.0151	4.6351	5.5312	5.9870	6.2604	5.8166	5.8939	0.9545	-0.0672	0.5566	0.2545
Map4	_VAEFNNVT[Phospho (STY)]PLS[Phospho (STY)]EEEVTSVK_.2	P27546_S506_M2_Map4	4.1461	3.0801	3.3321	3.8347	3.6607	3.6563	3.5494	5.1822	3.9323	3.8017	0.8790	-0.1861	0.1873	0.7276
Map4	_AAVGVTGNDITT[Phospho (STY)]PPNKEPPPS[Phospho (STY)]PEKK_.3	P27546_T658_M2_Map4	9.8640	10.5283	10.8751	11.3775	9.8565	10.4297	10.6884	10.4709	10.2376	10.3285	1.0055	0.0079	0.8585	0.0663
Map4	_DFMAALEAEPYDDIVGETVEK_.2	#N/A	4.2012	3.1412	3.9349	3.2795	4.1371	3.2661	3.6269	3.5225	3.8216	3.1559	1.0363	0.0515	0.6475	0.1888
Map4	_S[Phospho (STY)]PATTLPK_.2	P27546_S798_M1_Map4	3.4729	3.8695	3.2066	4.2512	3.6908	3.7010	4.3995	4.2508	3.0490	3.9285	0.9467	-0.0790	0.5068	0.2951
Map4	_VGS[Phospho (STY)]TENIK_.2	#N/A	2.8958	3.6521	4.1303	3.6741	3.5404	3.0842	3.8224	3.3271	3.6261	3.3514	0.9899	-0.0146	0.8884	0.0514
Map4	_AAEQM[Oxidation (M)]STLPIDAPS[Phospho (STY)]PLENLEQK_.3	P27546_S617_M1_Map4	3.0767	4.1495	3.7635	4.2662	4.0378	4.0877	3.9808	3.8128	3.5009	4.2332	1.0039	0.0055	0.9543	0.0203
Gna13	_LTESLNIFETIVNNR_.2	#N/A	4.1938	3.4631	4.8913	4.0319	4.1786	3.9146	4.3059	4.3524	3.4098	3.5616	1.0524	0.0737	0.5234	0.2811
Gna13	_ALWEDSGIQNAYDR_.2	#N/A	3.9831	5.5383	3.1691	5.1482	5.8392	5.1682	6.1403	5.5303	5.8984	5.3529	0.8390	-0.2533	0.1255	0.9013
Rpl3	_NNASTDYDLSDK_.2	#N/A	7.0187	6.4577	6.6567	7.2866	6.3410	6.9519	7.2518	6.8915	6.4217	6.9743	0.9856	-0.0210	0.6765	0.1697
H2ax	_LLGGVTIAQGGVLPNIQAVLLPK_.2	#N/A	2.0914	3.3611	3.9248	4.5011	3.9007	4.2471	4.9433	4.5344	4.5600	2.6774	0.8785	-0.1869	0.4197	0.3771
Rasgrf1	_LLYGDAPKS[Phospho (STY)]PR_.2	P27671_S718_M1_Rasgrf1	3.8382	3.1425	3.9336	3.4773	2.9638	3.2648	3.6256	6.5064	3.8229	3.1546	0.8035	-0.3157	0.2185	0.6605
Rasgrf1	_AGS[Phospho (STY)]ADDEDSDIKK_.2	P27671_S199_M1_Rasgrf1	3.5759	4.3322	3.5808	4.0834	4.2205	3.9049	3.7981	4.0351	3.6836	4.0504	1.0149	0.0213	0.7546	0.1223
Pdia3	_SEPIPESNEGPVK_.2	#N/A	8.5994	8.8064	8.6027	9.0782	8.8632	8.8300	8.5276	8.6596	8.5681	9.0449	1.0111	0.0159	0.4819	0.3171
Pdia3	_LAPEYEAAATR_.2	#N/A	8.2171	8.7919	8.5345	8.5537	8.7953	8.5037	8.2467	8.5601	8.4954	8.6630	1.0088	0.0126	0.5810	0.2358
Pdia3	_EATNPPIIQEEKPK_.2	#N/A	7.0140	7.7049	7.3930	7.8052	7.6766	7.6460	7.3691	7.2787	7.0145	7.6771	1.0280	0.0398	0.2973	0.5268
Pdia3	_ELNDFISYLQR_.2	#N/A	7.0098	7.4170	7.3179	7.3125	7.3990	7.2039	7.2152	7.0661	6.7118	7.2569	1.0303	0.0431	0.1240	0.9065
Pdia3	_DLFSDGHSEFLK_.2	#N/A	6.8542	6.9325	7.5421	7.3936	7.1167	7.0373	7.2723	6.8933	6.8245	6.9455	1.0232	0.0331	0.3350	0.4749
Pdia3	_TADGIVSHLK_.2	#N/A	5.8318	5.4658	5.6317	5.7080	6.3318	5.6776	5.5283	5.6004	5.4228	5.5149	1.0467	0.0659	0.1341	0.8726

Pdia3	_VDC[Carbamidomethyl (C)]TANTNTC[Carbamidomethyl (C)]NK_.2	#N/A	4.3019	3.8141	4.9947	3.6288	4.7177	4.8187	4.2351	3.7650	4.6309	3.7008	1.0726	0.1011	0.4012	0.3967
Pdia3	_FISDKDASVVGFFR_.3	#N/A	6.6573	6.5376	7.0458	7.0029	7.0591	6.5190	6.7859	6.5452	6.8257	7.0476	1.0004	0.0005	0.9875	0.0055
Pdia3	_DLLTAYYDVDYEK_.2	#N/A	6.0560	6.8349	6.5614	6.1573	6.3318	6.7835	6.0330	6.5399	6.0429	6.6616	1.0317	0.0450	0.4496	0.3472
Pdia3	_TFSHELSDFGLESTTGEVPVVAIR_.3	#N/A	5.3132	5.7955	3.1914	6.3983	5.1421	5.5893	5.9096	5.9217	5.8359	5.9246	0.8882	-0.1711	0.2715	0.5663
Pdia3	_QAGPASVPLR_.2	#N/A	4.4994	3.5923	3.2612	4.0015	5.4993	4.3488	4.4541	4.7738	4.5488	4.5518	0.9167	-0.1255	0.3732	0.4280
Pdia3	_FVM[Oxidation (M)]QEEFSR_.2	#N/A	5.6135	5.7971	6.2126	6.6583	6.3088	5.7171	5.7140	6.1127	5.9193	5.8980	1.0237	0.0338	0.5372	0.2698
Pdia3	_FAHTNIESLVK_.2	#N/A	5.5196	5.6629	5.9213	5.7976	5.7734	5.3416	5.7025	5.4221	5.1933	5.3659	1.0458	0.0646	0.1050	0.9787
Pdia3	_DGEEAGAYDGPR_.2	#N/A	7.6740	7.5729	3.1101	3.3914	7.1761	7.3844	7.2371	7.3609	7.2408	7.8871	0.8143	-0.2964	0.2521	0.5984
Pdia3	_MDATANDVPSPYEVK_.2	#N/A	3.8704	5.4054	4.5572	7.6280	6.7070	3.3805	3.2737	3.0587	4.2081	3.5260	1.4952	0.5804	0.0794	1.1002
Braf	_SAS[Phospho (STY)]EPSLNR_.2	P28028_S714_M1_Braf	12.4928	12.3782	13.1082	12.9632	12.1582	12.8333	12.9489	12.7549	12.8778	12.6122	0.9888	-0.0162	0.4898	0.3099
Braf	_NNPKS[Phospho (STY)]PQKPIVR_.3	#N/A	9.0006	9.3081	9.4253	9.8572	7.3747	8.9883	8.6761	9.2298	9.1350	9.0289	0.9972	-0.0040	0.9565	0.0193
Braf	_S[Phospho (STY)]PQKPIVR_.2	#N/A	8.1323	7.6498	6.7797	7.9354	6.1526	7.4985	7.5265	7.0771	7.6919	7.4253	0.9903	-0.0141	0.8603	0.0653
Braf	_SSS[Phospho (STY)]APNVHINTIEPVNIDDLIR_.3	P28028_S348_M1_Braf	2.9721	8.5430	4.2066	9.0173	6.5250	6.9246	7.5265	8.3511	3.5499	3.4277	1.1139	0.1557	0.6927	0.1595
Braf	_RDS[Phospho (STY)]SDDWEIPDGQITVGQR_.3	#N/A	3.4561	6.7552	9.2153	9.1544	8.1721	7.7280	4.3743	9.3976	8.8375	3.6588	1.1289	0.1749	0.6123	0.2130
Braf	_AGFQTEDFSLYAC[Carbamidomethyl (C)]AS[Phospho (STY)]PK_.2	#N/A	7.2230	2.9765	3.4209	7.1143	2.8648	6.4243	7.2886	7.0851	3.8288	6.2245	0.8194	-0.2873	0.4045	0.3931
Braf	_DS[Phospho (STY)]SDDWEIPDGQITVGQR_.2	#N/A	3.0881	4.2543	4.3226	3.8664	4.0756	3.6538	4.0146	3.1348	3.4339	3.5437	1.0977	0.1345	0.2451	0.6106
Mt3	nethyl (C)]C[Carbamidomethyl (C)]SC[Carbamidomethyl (C)]C[Carbamidomethyl (C)]PAGC[Carbamidor	#N/A	6.2676	6.5508	6.9858	7.1264	7.8409	6.1746	6.2215	6.5882	7.0604	6.3190	1.0423	0.0598	0.4540	0.3429
Mt3	_GEEGAKAEAEK_.2	#N/A	3.9509	2.9418	4.1441	4.3013	4.8346	3.4899	4.0446	3.1149	4.0649	3.3553	1.0820	0.1137	0.4620	0.3354
Mt3	_DC[Carbamidomethyl (C)]VC[Carbamidomethyl (C)]KGEEGAK_.2	#N/A	4.6153	5.5626	5.3406	5.2645	6.2383	5.6525	5.6867	5.5849	4.9928	5.0108	1.0239	0.0340	0.6916	0.1602
Mt3	Dxidation (M)]DPETC[Carbamidomethyl (C)]PC[Carbamidomethyl (C)]PTGGSC[Carbamidomethyl (C)]TC	#N/A	6.4538	6.6882	6.8096	7.1530	7.3354	6.7219	6.8008	6.5126	6.7048	6.7399	1.0255	0.0364	0.3523	0.4530
Mt3	_DC[Carbamidomethyl (C)]VC[Carbamidomethyl (C)]KGEEGAKAEAEK_.3	#N/A	4.6998	5.3226	5.9455	5.4363	6.0847	5.5945	5.2760	4.9347	5.4492	5.4480	1.0449	0.0634	0.4059	0.3916
Gjb1	_KGS[Phospho (STY)]GFGHR_.2	P28230_S233_M1_Gjb1	5.6370	5.1354	5.8245	4.0962	3.5333	4.3216	6.0060	5.7129	5.3742	5.1933	0.8540	-0.2277	0.1340	0.8729
Aco1	_VILQDFTGVPAVVDFAAM[Oxidation (M)]R_.2	#N/A	3.5932	2.8752	3.3269	4.3715	3.5705	4.1590	4.5198	4.1305	1.6061	4.0488	1.0204	0.0292	0.9052	0.0432
Apex1	_AAADDGEEPKS[Phospho (STY)]EPETKK_.3	P28352_S18_M1_Apex1	3.0010	3.8062	3.6878	5.2659	3.6275	4.1020	3.9051	3.9281	3.5766	4.1574	1.0060	0.0086	0.9547	0.0201
Adh5	_IDPSAPLDK_.2	#N/A	6.4418	5.9910	5.4268	5.8284	6.6592	5.9096	6.4641	6.5754	6.3391	6.1897	0.9454	-0.0811	0.1768	0.7524
Adh5	_AAVAWEAGKPLSIEEIEVAPPK_.3	#N/A	4.2088	4.4044	4.3009	4.0671	4.9006	4.4678	3.4989	4.3985	4.4981	4.6174	1.0325	0.0462	0.5942	0.2261
Adh5	_EFGASEC[Carbamidomethyl (C)]ISPQDFS[Phospho (STY)]K_.2	#N/A	7.6722	6.3050	3.6839	7.6965	6.1997	6.8944	3.9012	7.0679	6.8265	7.0554	1.0315	0.0448	0.8453	0.0730
Adh5	_AKEFGASEC[Carbamidomethyl (C)]ISPQDFS[Phospho (STY)]K_.3	#N/A	3.3001	4.0564	4.5346	5.6226	3.9447	4.1808	4.0739	5.1819	3.4078	3.7557	1.0410	0.0580	0.7442	0.1283
Adh5	_LVSEYMSK_.2	#N/A	3.6546	3.5715	4.3414	3.3433	3.4598	3.1648	4.5588	3.2744	4.4238	3.3103	0.9223	-0.1168	0.4051	0.3925
Adh5	_SIQEVLVEM[Oxidation (M)]TDGGVDYSFEC[Carbamidomethyl (C)]IGNVK_.3	#N/A	3.8828	3.1382	3.6164	3.7766	3.0265	3.5981	3.4913	4.3419	3.9905	3.7436	0.9010	-0.1504	0.1291	0.8892
Slc6a9	_YAPTTTPS[Phospho (STY)]PEDGFEVQPLHPDK_.3	P28571_S659_M1_Slc6a9	3.2621	4.3248	4.2527	3.7965	3.8962	3.3342	3.9447	3.2053	3.5043	3.4737	1.0790	0.1097	0.3106	0.5078
Adss1	_VVDLLATDADIVSR_.2	#N/A	3.9150	3.3134	3.7916	5.1940	5.3079	4.4026	3.3161	5.0510	5.1998	4.3060	0.9670	-0.0484	0.7886	0.1031
Adss1	_IC[Carbamidomethyl (C)]DLLSDFDEFSAR_.2	#N/A	3.7537	3.0581	4.0180	3.5618	4.0300	3.1357	3.7100	3.4394	3.7384	3.2391	1.0173	0.0248	0.8015	0.0961
Camk2b	_ESSDS[Phospho (STY)]TNTTIEDEDAK_.2	#N/A	10.7724	11.1918	12.3941	12.4352	12.2410	11.7681	12.3065	12.4795	11.9114	11.8572	0.9721	-0.0408	0.3895	0.4095
Camk2b	_FTDEYQLYEDIGK_.2	#N/A	7.7236	7.3735	8.1209	7.0499	6.9743	7.9891	7.6135	7.1886	7.2154	7.7495	1.0130	0.0186	0.7292	0.1371
Camk2b	_HPWVC[Carbamidomethyl (C)]QR_.2	#N/A	7.4712	7.3854	6.6121	7.5234	7.8167	7.1190	7.1416	7.7220	7.5259	7.0416	0.9950	-0.0072	0.8860	0.0525
Camk2b	_ITAHEALK_.2	#N/A	6.5097	5.9554	5.9875	6.1526	6.5596	6.3030	5.9943	6.7154	6.9359	6.1730	0.9675	-0.0477	0.3660	0.4365
Camk2b	_LC[Carbamidomethyl (C)]TGHEYAAK_.2	#N/A	5.9595	5.8010	5.4679	5.7100	6.2732	5.5951	5.3437	6.3484	5.4883	5.4015	1.0276	0.0392	0.5300	0.2758
Camk2b	_ADGVKPQTNS[Phospho (STY)]TK_.2	#N/A	3.6348	3.7076	3.3685	4.4131	3.5289	4.2006	3.0605	4.0889	5.0745	4.0904	0.9339	-0.0987	0.5045	0.2971
Camk2b	_FYFENLLAK_.2	#N/A	5.5036	4.4027	3.9301	3.7994	5.3443	4.9103	4.5452	2.6224	4.6147	6.3549	1.0252	0.0359	0.8755	0.0577
Camk2b	_AGAYDFPSPEWDTVTPEAK_.2	#N/A	9.6665	9.6935	9.6323	11.1396	9.0496	9.3373	11.2711	11.1676	9.2629	9.3662	0.9500	-0.0741	0.3943	0.4042
Camk2b	_NLINQM[Oxidation (M)]LTINPAK_.2	#N/A	5.8732	5.7433	6.0355	5.6920	5.9675	4.7755	5.7345	6.3274	5.6983	6.0505	0.9544	-0.0674	0.3326	0.4781
Camk2b	_QET[Phospho (STY)]VEC[Carbamidomethyl (C)]LKK_.2	#N/A	5.9068	3.5963	3.4798	5.5170	4.4429	5.5758	5.7341	4.9423	4.2767	5.5878	0.9256	-0.1116	0.5441	0.2644
Camk2b	_GAILTTM[Oxidation (M)]LATR_.2	#N/A	6.2775	6.2276	5.4391	6.7631	6.3495	5.9552	6.2442	6.8052	6.4204	6.3514	0.9556	-0.0655	0.2768	0.5578
Camk2b	_WQNVHFHC[Carbamidomethyl (C)]SGAPVAPLQ_.3	#N/A	5.3331	4.8150	5.0843	5.0318	5.4622	5.8482	5.5303	5.3598	5.0620	5.3192	0.9896	-0.0151	0.7911	0.1018
Camk2b	_LTQYIDGQGR_.2	#N/A	6.4286	5.5833	3.3807	4.4253	5.9589	5.7974	6.0199	6.0720	5.7473	5.2030	0.9135	-0.1305	0.4317	0.3648
Camk2b	_QET[Phospho (STY)]VEC[Carbamidomethyl (C)]LK_.2	#N/A	5.5878	3.8676	4.3458	3.8896	3.7559	5.5235	4.4313	3.1116	4.6739	3.5669	1.0713	0.0993	0.6314	0.1997
Camk2b	_ESSDST[Phospho (STY)]NT[Phospho (STY)]T[Phospho (STY)]IEDEDAKAR_.2	#N/A	3.2570	3.9871	7.8669	7.5132	6.5181	6.1910	7.5193	3.6493	3.3167	3.6705	1.2974	0.3756	0.3079	0.5116
Camk2b	_DLKPENLLLASK_.2	#N/A	9.9736	10.1006	10.3976	10.0473	9.9273	9.9209	10.2015	10.2142	10.0788	9.8946	0.9964	-0.0052	0.7481	0.1260
Camk2b	_NSSAITS[Phospho (STY)]PK_.2	P28652_S367_M1_Camk2b	12.9656	12.9743	13.2773	13.1576	12.3790	12.8694	13.0425	13.1558	13.0466	12.9942	0.9906	-0.0136	0.4678	0.3299
Camk2b	_LTQYIDGQGRPR_.2	#N/A	5.9078	6.7673	5.6115	6.7428	6.5737	6.1052	6.4184	6.3948	6.4634	6.0260	0.9935	-0.0094	0.8778	0.0566
Camk2b	_S[Phospho (STY)]TVASMMHR_.2	P28652_S276_M1_Camk2b	4.4439	3.4519	3.3942	6.2818	4.8958	3.7421	3.8344	4.3248	3.7399	4.0389	1.0963	0.1327	0.5226	0.2818
Camk2b	_IC[Carbamidomethyl (C)]DPGLTSFEPEALGNLVEGM[Oxidation (M)]DFHR_.3	#N/A	4.3622	4.7884	5.4547	4.1260	4.8295	4.3132	4.5272	3.9925	4.4465	3.4142	1.1344	0.1820	0.1227	0.9113
Camk2b	_TTEQLIEAVNNGDFEAYAK_.2	#N/A	6.7841	6.8080	7.2634	6.8337	5.9831	6.8866	7.0141	6.8937	6.6459	6.7287	0.9911	-0.0129	0.7928	0.1008
Camk2b	_KADGVKPQTNS[Phospho (STY)]TK_.2	#N/A	3.6724	3.7981	3.9018	4.4865	3.4037	3.9378	3.7664	4.8134	3.8048	3.6065	0.9672	-0.0481	0.6575	0.1821
Camk2b	_QTTAPATMSTAASGTTM[Oxidation (M)]GLVEQAK_.2	#N/A	3.4846	3.9658	4.1338	3.6362	3.6779	4.9859	3.9549	4.5476	4.3211	4.0479	0.9438	-0.0835	0.4494	0.3474
Nap1l1	_LDGLVDTPTGYIESLPK_.2	#N/A	5.6835	5.3768	6.0297	5.7527	6.1676	5.9368	5.5905	5.3142	5.7350	5.9007	1.0336	0.0477	0.3089	0.5103
Nap1l1	_NVDLLSDMVQEHDEPILK_.3	#N/A	3.5175	3.7086	4.2043	3.2061	3.5969	3.0277	4.4217	3.4115	3.0601	3.1731	1.0077	0.0110	0.9356	0.0289
Celf1	_TWS[Phospho (STY)]EKDLR_.2	P28659_S28_M1_Celf1	7.0856	7.1076	4.5837	7.7237	3.2176	6.9580	3.3002	7.4676	4.1816	7.7524	1.0770	0.1071	0.7411	0.1301
Nckap1	_NNNQQLAQLQK_.2	#N/A	5.0173	5.4129	3.9856	5.9017	3.5945	5.6082	5.8129	5.0472	5.3545	5.4399	0.9088	-0.1379	0.3436	0.4639
Nckap1	_NLSADQWR_.2	#N/A	5.3463	5.6708	3.8165	4.7831	5.9243	5.3452	5.0415	5.4629	5.1467	4.7362	1.0100	0.0143	0.9029	0.0444
Nckap1	_AAEDLFVNIR_.2	#N/A	4.2718	4.5957	4.2024	4.5901	3.9837	3.5293	3.9105	4.4893	4.8151	5.1159	0.9155	-0.1274	0.2179	0.6618
Nckap1	_SVLQPSQQK_.2	#N/A	6.3954	6.1991	5.7992	5.9582	5.6670	5.7191	5.8624	6.4997	3.8990	3.0785	1.2319	0.3009	0.1246	0.9045
Nckap1	_NAYHAVYK_.2	#N/A	4.4799	4.1547	4.3874	4.2431	3.7974	3.7186	4.7743	4.6336	3.3691	3.6084	1.0083	0.0119	0.9191	0.0367
Nckap1	_LVVENVDVLTQM[Oxidation (M)]R_.2	#N/A	3.3506	4.1558	4.0374	3.9649	3.9771	3.7530	4.1132	3.9946	4.2685	3.9186	0.9508	-0.0728	0.2417	0.6166
Nckap1	_ASLSLADHR_.2	#N/A	4.2171	3.0091	3.4031	3.9057	3.3428	4.3867	3.6204	4.2128	3.4473	3.8727	0.9795	-0.0298	0.8067	0.0933
Septin4	_PQS[Phospho (STY)]PDLC[Carbamidomethyl (C)]DDDVEFR_.2	#N/A	8.7135	8.9451	8.7376	9.9811	8.6534	9.1467	9.5969	9.0510	9.2332	9.3801	0.9693	-0.0449	0.3230	0.4908
Septin4	_PRS[Phospho (STY)]PWGK_.2	P28661_S107_M1_Septin4	6.6002	7.3137	7.1469	7.4991	6.3680	7.0185	7.7293	6.8554	7.2183	7.1251	0.9667	-0.0489	0.3865	0.4128
Septin4	_ESIPFAVIGSNTVVEAR_.2	#N/A	5.3104	5.1551	6.1341	6.0102	5.6537	5.0338	5.3614	5.4695	5.6204	6.3298	0.9744	-0.0374	0.6288	0.2015
Septin4	_VARPQILEPRPQS[Phospho (STY)]PDLC[Carbamidomethyl (C)]DDDVEFR_.4	#N/A	6.0690	7.4120	7.5706	9.3883	6.2983	6.4453	6.6324	8.1520	7.4160	6.6888	0.9965	-0.0050	0.9720	0.0123
Septin4	_PQILEPRPQS[Phospho (STY)]PDLC[Carbamidomethyl (C)]DDDVEFR_.3	#N/A	7.1010	7.2360	6.6862	9.2370	7.2074	6.9956	7.5973	7.9348	7.5546	7.4189	0.9717	-0.0414	0.6620	0.1791
Septin4	_IYQFPDC[Carbamidomethyl (C)]DS[Phospho (STY)]DEDEDFKLQDQALK_.3	#N/A	4.0089	3.2644	3.7426	3.2864	3.1527	3.4719	3.4347	8.6118	4.0138	2.9637	0.7334	-0.4474	0.2590	0.5867
Septin4	_IYQFPDC[Carbamidomethyl (C)]DS[Phospho (STY)]DEDEDFK_.2	#N/A	3.4771	4.2823	4.1640	9.9887	4.1037	3.6258	3.9866	8.6233	3.4619	3.1328	1.0290	0.0412	0.9340	0.0296
Septin4	_STLVNSLFLTDLYR_.2	#N/A	2.6703	3.6570	3.6522	4.2102	3.8513	3.7984	3.8695	3.8184	4.0622	4.0518	0.9214	-0.1181	0.2804	0.5522
Septin4	_HFLEDS[Phospho (STY)]SDAELSK_.2	P28661_S28_M1_Septin4	3.8056	5.7005	3.8377	6.2883	5.3031	5.3490	3.7037	5.3054	5.7885	5.4581	0.9967	-0.0047	0.9798	0.0089
Septin4	_LDPY[Phospho (STY)]DSSEDDKEYVGFATLPNQVHR_.3	#N/A	3.8496	3.4747	7.6230	10.3410	8.9294	8.4905	9.8317	9.6812	9.4580	9.7354	0.7356	-0.4430	0.1136	0.9446
Napb	_VAAYAAQLEQYQK_.2	#N/A	7.2574	7.5450	8.0120	8.1676	7.5405	7.5963	7.3947	8.1149	7.6394	7.4620	1.0044	0.0063	0.8794	0.0558
Napb	_NWSAAGNAFC[Carbamidomethyl (C)]QAAK_.2	#N/A	6.0442	6.1985	5.5517	6.1810	6.0943	6.1676	5.7219	5.7445	6.0762	6.0819	1.0226	0.0322	0.3946	0.4038
Napb	_KADPQEAINC[Carbamidomethyl (C)]LNAAIDIYTDM[Oxidation (M)]GR_.3	#N/A	5.7172	5.3558	5.9499	5.4479	5.1773	6.0471	5.8330	6.1747	6.1387	6.0443	0.9286	-0.1069	0.0491	1.3089

Napb	_YEEM[Oxidation (M)]FPAFTDSR_.2	#N/A	8.3779	7.9765	8.0466	8.3772	8.0036	8.1214	8.0721	7.8574	8.0212	8.1480	1.0157	0.0224	0.2655	0.5760
Napb	_AALC[Carbamidomethyl (C)]HFIVDELNAK_.3	#N/A	5.3432	6.1415	6.2034	5.9934	5.9947	5.8685	5.9005	6.1600	5.7745	6.3582	0.9795	-0.0299	0.5281	0.2773
Napb	_SIQGDGEGDGDLK_.2	#N/A	7.4411	5.0067	7.8498	7.1566	7.7407	7.6536	7.0731	4.7315	7.5768	7.4567	1.0644	0.0900	0.5860	0.2321
Napb	_HDSATSFVDAGNAYK_.2	#N/A	6.1352	5.3036	5.8472	5.8239	5.7649	5.0017	5.1071	5.2698	5.7632	5.0631	1.0651	0.0910	0.1989	0.7014
Napb	_LDQWLTTM[Oxidation (M)]LLR_.2	#N/A	7.9506	8.1023	7.7655	7.6430	7.8839	7.7696	7.7653	7.9271	8.2078	7.6884	0.9943	-0.0082	0.7256	0.1393
Napb	_LLEAHEEQNSEAYTEAVK_.3	#N/A	5.8294	5.9049	6.1911	6.6653	4.3716	5.4774	5.5544	5.7374	3.9664	5.3258	1.1154	0.1576	0.2770	0.5576
Napb	_ASHS[Phospho (STY)]FLR_.2	P28663_S26_M1_Napb	3.7029	3.0073	4.3897	3.6126	2.8286	3.4000	4.6423	3.3886	3.6877	3.2898	0.9302	-0.1044	0.5040	0.2976
Napb	_AIEIYEQVGANTM[Oxidation (M)]DNPLLK_.2	#N/A	4.5053	4.8829	5.1970	5.3681	4.1249	4.9314	5.2673	5.3143	4.6419	5.0128	0.9557	-0.0654	0.4182	0.3786
Napb	_LHMQLQSK_.2	#N/A	5.3405	3.0700	3.3422	6.5227	5.0229	3.6663	3.5595	4.2737	3.9222	3.8118	1.1548	0.2076	0.4148	0.3822
Napb	_IEEAC[Carbamidomethyl (C)]EMYTR_.2	#N/A	4.7724	4.3075	3.6055	5.9389	4.8502	3.9296	3.8228	3.1415	3.6589	4.0751	1.2430	0.3138	0.0819	1.0865
Napb	_LLEAHEEQNSEAYTEAVKEFDSISR_.4	#N/A	4.1224	4.5281	4.3922	5.0990	4.1088	3.9497	3.7451	4.1793	4.6294	4.1976	1.0427	0.0603	0.5039	0.2977
Napb	_AIAHYEQSADYYKGEESNSSANK_.3	#N/A	6.7567	7.1111	7.3377	8.1660	6.3436	6.7884	6.6860	7.2942	7.0858	7.0932	1.0063	0.0090	0.8990	0.0462
Napb	_EAVQLMAEAEKR_.2	#N/A	4.2325	2.9702	3.4419	4.9732	3.2707	3.7661	3.6593	4.1739	3.8225	3.9116	0.9702	-0.0437	0.7689	0.1141
Napb	_KLLEAHEEQNSEAYTEAVK_.3	#N/A	3.3714	2.0764	4.0582	1.2518	3.9979	3.7316	4.0924	3.0571	3.3561	3.6214	0.8724	-0.1969	0.4840	0.3152
Napb	_AIAHYEQSADYYK_.3	#N/A	2.8960	3.7012	3.5828	4.0854	3.5225	4.2070	3.8001	4.0331	3.8387	4.0524	0.9325	-0.1008	0.3082	0.5111
Marcksl1	_AAAT[Phospho (STY)]PESQEPQAK_.2	P28667_T148_M1_Marcksl1	10.7438	11.0448	11.7055	11.5419	11.5143	11.2521	11.2851	11.1138	11.1428	11.2120	1.0100	0.0144	0.5636	0.2491
Marcksl1	_GDVTAEEAAGAS[Phospho (STY)]PAK_.2	P28667_S22_M1_Marcksl1	11.4061	11.2238	11.5305	11.9932	11.6198	11.7441	11.6224	11.3573	11.7578	11.7265	0.9974	-0.0037	0.8520	0.0696
Marcksl1	_LSGLS[Phospho (STY)]FKR_.2	#N/A	10.6817	10.9526	11.7747	11.8605	10.7705	10.6697	11.0616	11.0249	11.0408	10.7318	1.0140	0.0201	0.6087	0.2156
Marcksl1	_GEVAPKET[Phospho (STY)]PK_.2	#N/A	10.6390	10.8067	11.0756	11.2205	11.3148	10.8882	10.9975	10.8660	11.0198	10.9606	1.0027	0.0039	0.8299	0.0810
Marcksl1	_LSGLS[Phospho (STY)]FK_.2	#N/A	3.2582	4.1714	3.9450	4.4743	3.8562	4.2692	4.1624	3.6708	3.3194	5.9153	0.9364	-0.0947	0.6053	0.2180
Marcksl1	_GEVAPKET[Phospho (STY)]PKK_.2	#N/A	5.9562	6.0903	7.0134	6.9430	7.3579	6.6991	5.8982	6.1880	6.2841	6.3221	1.0816	0.1131	0.1215	0.9156
Kif5c	_SVELDS[Phospho (STY)]DDGGGSAAQK_.2	P28738_S820_M1_Kif5c	6.3240	6.5096	7.1933	7.4049	6.1626	6.4816	7.2175	6.7136	6.5974	6.6579	0.9827	-0.0251	0.6854	0.1641
Kif5c	_LYLVDLAGSEK_.2	#N/A	5.9323	6.0196	6.1917	6.7367	5.6297	6.1195	6.6757	6.2416	6.2917	6.1272	0.9638	-0.0532	0.3051	0.5156
Kif5c	_ILQDSLGGNC[Carbamidomethyl (C)]R_.2	#N/A	6.5321	6.2802	5.2000	3.1753	6.3473	5.8708	6.2991	5.8995	5.3650	6.1396	0.9396	-0.0899	0.6049	0.2183
Kif5c	_SLEPC[Carbamidomethyl (C)]DNT[Phospho (STY)]PIIDNITPVVDGISAEK_.3	P28738_T396_M1_Kif5c	4.1631	5.3589	5.0930	6.6431	3.3548	4.2329	3.5888	5.6037	3.8597	4.5579	1.0920	0.1270	0.5711	0.2433
Kif5c	_SLSALGNVISALAEGTK_.2	#N/A	4.9836	4.7986	4.6358	4.5652	4.3000	4.7312	3.4638	4.4381	4.4790	4.9167	1.0797	0.1106	0.2370	0.6252
Kif5c	_ISFLENNLEQLTK_.2	#N/A	4.5142	4.1046	3.9862	4.4889	3.2210	4.2037	4.2036	3.6001	3.2782	4.1007	1.0766	0.1065	0.3548	0.4500
Kif5c	_TTIVIC[Carbamidomethyl (C)]C[Carbamidomethyl (C)]SPSVFNEAETK_.2	#N/A	3.7162	3.0205	4.4030	3.5993	3.1681	3.3868	3.7476	3.2128	3.7009	3.3718	1.0116	0.0166	0.8844	0.0534
Kif5c	_HVAVTNMNEHSSR_.3	#N/A	4.1590	3.4144	4.4985	4.1736	3.3027	3.3219	3.2150	3.1173	4.2667	3.4673	1.0839	0.1163	0.4083	0.3891
Kif2a	_GSLDYRPLTTADPIDEHR_.3	#N/A	6.2098	6.0866	6.7985	6.1127	6.3840	6.0526	6.4968	6.1810	5.9591	6.1523	1.0124	0.0178	0.6624	0.1789
Kif2a	_PSQLPEQSSSAQQNGSVSDIS[Phospho (STY)]PVQAAK_.3	P28740_S139_M1_Kif2a	4.2362	5.8200	6.1201	7.2939	3.3619	5.4181	5.8013	6.1348	5.9754	5.7814	0.9075	-0.1401	0.4665	0.3312
Kif2a	_YLENQTFR_.2	#N/A	5.6034	5.8768	3.7570	5.9324	6.1020	5.6065	5.6277	5.5215	4.8801	5.4807	1.0190	0.0272	0.8303	0.0807
Kif2a	_IDILTELR_.2	#N/A	4.5633	4.5149	3.4412	2.9253	4.2053	3.4184	3.3672	4.7737	4.6934	4.7164	0.8763	-0.1906	0.2499	0.6022
Kif2a	_LIDIGNSC[Carbamidomethyl (C)]R_.2	#N/A	4.7798	4.4970	4.3039	3.3057	4.2584	4.0816	4.9660	4.2863	4.1293	4.4651	0.9423	-0.0857	0.4055	0.3920
Kif2a	_DFRGS[Phospho (STY)]LDYRPLTTADPIDEHR_.4	P28740_S206_M1_Kif2a	3.6718	3.6706	3.4055	4.4501	2.8155	4.4038	3.0975	4.0519	4.3510	4.1274	0.9563	-0.0645	0.6690	0.1746
Kif2a	_AVFQESIR_.2	#N/A	3.5503	3.6759	4.2371	6.1133	3.5642	3.3549	4.4545	3.3787	2.9308	3.2059	1.1690	0.2252	0.3481	0.4583
Kif3a	_S[Phospho (STY)]AKPETVIDSLLQ_.2	P28741_S689_M1_Kif3a	6.4572	3.5018	6.5764	6.8790	6.1630	4.4064	3.6007	6.5121	6.0142	6.4675	1.0027	0.0039	0.9867	0.0058
Kif3a	_KQT[Phospho (STY)]PVPDKK_.2	P28741_T635_M1_Kif3a	3.0350	3.6789	1.9246	3.0408	3.5672	3.0574	4.4514	2.7482	3.0304	2.6581	0.9468	-0.0788	0.7178	0.1440
Prkcd	_LLAEALNQVTQR_.2	#N/A	4.3934	3.5823	3.4408	4.5988	4.5198	3.7650	4.2130	4.3861	4.7021	4.2256	0.9243	-0.1136	0.2644	0.5777
Prkcd	_SPSDYSNFDPEFLNEKPQLS[Phospho (STY)]FSDK_.3	P28867_S643_M1_Prkcd	3.8929	3.3333	4.5797	3.5815	3.2216	3.4030	3.2962	3.0362	4.1856	3.5485	1.0432	0.0611	0.6500	0.1871
Pabpc1	_FSPAGPILSIR_.2	#N/A	7.4136	7.2218	7.5561	7.7611	7.3049	7.0740	7.4701	7.3258	7.1517	7.2227	1.0132	0.0189	0.5024	0.2990
Pabpc1	_SLGYAYVNFQQPADAER_.2	#N/A	5.7459	6.2396	6.4122	5.9810	3.4282	5.7667	6.0534	5.6042	5.8686	5.6323	0.9665	-0.0492	0.7399	0.1308
Pabpc1	_ALYDTFSAFGNILSC[Carbamidomethyl (C)]K_.2	#N/A	2.9122	4.2747	3.5990	4.1016	4.5412	3.9577	4.2989	4.3050	3.6654	4.0686	0.9543	-0.0675	0.5722	0.2424
Pabpc1	_SKVDEAVAVLQAHQAK_.3	#N/A	4.9921	4.5285	3.2808	4.4548	4.8278	5.1207	3.4981	4.9183	3.9836	5.1810	1.0316	0.0449	0.7710	0.1130
Pabpc1	_NLDDGIDDERLR_.3	#N/A	3.2185	4.1239	4.4530	3.9968	3.8630	4.2624	4.1450	3.0044	3.3035	3.6740	1.1287	0.1747	0.1590	0.7986
Gnb4	_SGVLAGHDNR_.2	#N/A	10.6379	3.1265	3.2856	10.4611	5.5971	10.2075	10.5212	10.6128	10.3377	10.4335	0.6891	-0.5372	0.1171	0.9315
Vcam1	_QEFSS[Phospho (STY)]EEM[Oxidation (M)]TK_.2	#N/A	8.2946	8.2440	8.8065	9.0136	8.1357	8.2022	8.7472	8.3043	8.2483	3.1796	1.1867	0.2470	0.2446	0.6115
Nedd8	_EIEIDIEPTDKVER_.2	#N/A	5.8102	6.1530	6.5885	6.5798	6.7601	6.6629	6.5330	6.4456	6.5728	6.7762	0.9763	-0.0346	0.4464	0.3503
Ahsg	_VM[Oxidation (M)]HTQC[Carbamidomethyl (C)]HSTPDS[Phospho (STY)]AEDVR_.3	#N/A	3.1465	3.8509	4.7193	3.5989	3.9951	3.5629	4.5381	3.6704	4.0748	4.0324	0.9346	-0.0975	0.4078	0.3896
Ahsg	_VM[Oxidation (M)]HTQC[Carbamidomethyl (C)]HSTPDS[Phospho (STY)]AEDVRK_.4	#N/A	3.4536	3.5936	4.1115	4.4296	3.4858	3.7095	4.0403	3.5835	3.5515	3.9681	1.0030	0.0043	0.9604	0.0175
Oat	_LPSDVVTSVR_.2	#N/A	6.4717	6.2237	6.2130	6.2549	6.8378	6.5429	6.4033	6.4403	6.2613	6.2765	1.0124	0.0178	0.5622	0.2501
Oat	_IAIAALEVLEEENLAENADK_.2	#N/A	5.3390	5.1886	5.5384	6.1105	5.2148	4.5458	5.3240	5.4146	4.3858	5.2152	1.0468	0.0660	0.4791	0.3196
Oat	_IVFADGNFWGR_.2	#N/A	4.7726	5.0680	3.1588	5.3946	4.5200	5.3338	5.5473	5.2443	5.1292	4.6709	0.9145	-0.1289	0.3534	0.4518
Oat	_AFYNNVLGEYEEYITK_.2	#N/A	4.1301	4.6221	4.7185	5.0036	5.1132	4.7400	4.7768	4.3069	3.2209	4.9207	1.0964	0.1327	0.2715	0.5662
Oat	_KTEQGPPSSEYIFER_.3	#N/A	4.1782	5.4285	3.9119	5.2146	5.6522	5.2524	3.6039	5.5221	6.2123	3.1329	1.0697	0.0972	0.6535	0.1847
Ckmt1	_LYPPSAEYPDLR_.2	#N/A	10.6666	10.6123	10.5288	10.6592	10.6365	10.5448	10.5733	10.3859	10.3398	10.5769	1.0133	0.0190	0.0417	1.3800
Ckmt1	_VVVDALSGLK_.2	#N/A	10.0545	10.3955	10.2646	10.3068	10.2668	10.1219	10.0845	10.1641	10.0890	10.1613	1.0109	0.0156	0.1339	0.8731
Ckmt1	_TTPTGWTLDQC[Carbamidomethyl (C)]IQTGVDNPGHPFIK_.3	#N/A	8.6029	8.7243	9.0827	8.8021	8.8937	8.7362	8.5852	8.3554	8.5644	8.6007	1.0329	0.0467	0.0192	1.7176
Ckmt1	_GWEFM[Oxidation (M)]WNER_.2	#N/A	8.4989	8.4269	8.8984	8.3425	8.4425	8.6204	8.6148	8.2804	8.4002	8.7612	1.0028	0.0041	0.8601	0.0654
Ckmt1	_LYPPSAEYPDLRK_.3	#N/A	8.1536	8.0430	8.3886	8.0612	8.5924	8.5642	7.3623	8.0425	8.2884	8.5741	1.0290	0.0412	0.3598	0.4439
Ckmt1	_TVGM[Oxidation (M)]VAGDEETYEVFAELFDPVIQER_.3	#N/A	7.4065	7.6630	8.3459	9.2808	7.9995	8.2275	8.1766	7.4458	7.3921	7.7491	1.0602	0.0843	0.2374	0.6245
Ckmt1	_HTTDLDASK_.2	#N/A	7.9700	7.6747	7.4483	7.8858	8.4858	8.0044	7.5100	7.3199	7.6435	7.9491	1.0402	0.0569	0.1780	0.7497
Ckmt1	_GIWHNNEK_.2	#N/A	7.8268	7.8139	7.6601	7.9609	7.8980	7.7341	7.5587	7.7309	7.5856	7.6831	1.0230	0.0329	0.0249	1.6030
Ckmt1	_HNGYDPR_.2	#N/A	6.3640	5.3247	4.9922	6.0918	6.6053	5.8063	5.6367	6.1573	5.4584	6.0789	1.0053	0.0077	0.9296	0.0317
Ckmt1	_HTTDLDAS[Phospho (STY)]KIR_.2	P30275_S148_M1_Ckmt1	4.9626	5.3957	6.1281	6.0811	5.1453	5.4942	5.8984	5.2442	5.3677	5.7645	0.9939	-0.0089	0.9037	0.0440
Ckmt1	_GLSLPPAC[Carbamidomethyl (C)]TR_.2	#N/A	8.6233	8.6622	4.3056	9.6810	8.5510	7.8460	8.3820	9.2221	8.4746	9.2179	0.9004	-0.1514	0.3933	0.4052
Ckmt1	_GLKEVEK_.2	#N/A	6.8947	7.2038	7.3515	4.3033	7.2960	7.1366	4.4516	6.8542	6.9549	7.1428	1.0546	0.0767	0.6707	0.1735
Ckmt1	_HNNC[Carbamidomethyl (C)]M[Oxidation (M)]ASHLTPAVYAR_.3	#N/A	7.9102	8.0628	8.5685	8.3377	8.6555	8.0952	7.8863	7.8346	7.9440	8.1609	1.0396	0.0561	0.0884	1.0536
Ckmt1	_GTGGVDTAATGSVFDISNLDR_.2	#N/A	7.3179	7.3477	7.8536	8.3947	7.4212	7.2379	7.2207	7.5549	7.1273	7.2600	1.0418	0.0591	0.2395	0.6206
Ckmt1	_GWEFMWNERLGYILTC[Carbamidomethyl (C)]PSNLGTGLR_.3	#N/A	4.4262	4.6154	3.2153	5.6489	5.0222	4.6823	5.3871	5.3359	4.8971	4.1630	0.9304	-0.1040	0.4827	0.3163
Ckmt1	_SFLIWVNEEDHTR_.2	#N/A	7.0385	6.9923	7.1391	7.2494	7.1191	6.9433	7.0245	6.2641	6.9324	7.0502	1.0385	0.0545	0.1349	0.8700
Ckmt1	_LGYILTC[Carbamidomethyl (C)]PSNLGTGLR_.2	#N/A	6.8456	7.2936	7.1149	7.4052	6.8181	6.9076	6.9662	6.4971	6.5987	7.0591	1.0419	0.0592	0.1258	0.9002
Ckmt1	_VVVDALSGLKGDLAGR_.2	#N/A	4.1399	2.9264	4.8156	3.8818	3.3884	4.7882	4.5776	3.7041	4.4225	3.8874	0.9619	-0.0560	0.7162	0.1450
Ckmt1	_SGYFDER_.2	#N/A	5.6356	5.1222	5.5586	5.3759	5.4580	5.7087	4.0289	5.3980	5.3639	5.9557	1.0559	0.0785	0.4197	0.3771
Ckmt1	_IRS[Phospho (STY)]GYFDER_.2	P30275_S152_M1_Ckmt1	7.9371	7.9994	8.4623	8.1825	7.8918	8.1201	8.2901	7.6600	8.0295	8.0856	1.0103	0.0148	0.5944	0.2259
Ckmt1	_RGTGGVDTAATGSVFDISNLDR_.3	#N/A	6.6632	6.8279	6.4463	7.1040	6.1251	6.1314	7.0495	6.3948	6.1974	6.6555	0.9963	-0.0054	0.9229	0.0348
Nktr	_TC[Carbamidomethyl (C)]DEGSS[Phospho (STY)]PSKPK_.2	P30415_S1060_M1_Nktr	3.0438	4.2986	4.2784	3.8221	3.6884	2.9362	3.9704	3.1790	3.4781	3.4994	1.0414	0.0585	0.6657	0.1767
Fkbp4	_TQLAVC[Carbamidomethyl (C)]QQR_.2	#N/A	3.4519	5.0171	3.1856	4.2302	5.8173	4.0176	4.3784	4.2719	5.4150	4.8730	0.9054	-0.1434	0.4342	0.3623
Sstr1	_AYS[Phospho (STY)]VEDFQPENLESGGVFR_.2	P30873_S364_M1_Sstr1	3.7217	3.5044	4.4085	3.4104	3.3927	3.2319	3.1250	4.9646	5.0659	3.3774	0.8738	-0.1946	0.2884	0.5400

Ctnnd1	_VGGSS[Phospho (STY)]VDLHR_.2	P30999_S269_M1_Ctnnd1	5.7032	5.3643	4.7903	4.9212	3.9427	5.0388	5.3596	5.6016	6.2379	4.9749	0.8948	-0.1604	0.1536	0.8136
Ctnnd1	_HYEDGYPGGSDNYGS[Phospho (STY)]LSR_.3	#N/A	6.2279	6.3220	6.6841	3.6737	3.5400	5.7496	5.2397	6.5109	3.7109	5.3472	1.0315	0.0448	0.8509	0.0701
Ctnnd1	_GSLAS[Phospho (STY)]LDSLRK_.2	P30999_S349_M1_Ctnnd1	7.8294	7.6079	7.9333	8.8628	7.3891	7.8492	8.1321	7.9652	8.2573	7.9563	0.9795	-0.0299	0.5484	0.2609
Ctnnd1	_SQSSHSY[Phospho (STY)]DDSTLPLIDR_.3	P30999_Y865_M1_Ctnnd1	3.1656	6.3288	6.0496	7.7310	5.9461	3.9420	4.3028	7.0776	7.2426	4.1880	0.9692	-0.0451	0.8753	0.0578
Ctnnd1	_GS[Phospho (STY)]LASLDSLR_.2	#N/A	4.5698	4.0087	3.8425	4.7493	3.4785	3.8361	3.5533	5.4990	3.7127	3.6732	0.9930	-0.0101	0.9498	0.0224
Ctnnd1	_S[Phospho (STY)]M[Oxidation (M)]GYDDLDYGM[Oxidation (M)]M[Oxidation (M)]SDYGTAR_.2	P30999_S288_M1_Ctnnd1	3.1364	3.9416	3.1345	4.1791	3.7630	3.9665	4.9624	5.4788	5.1338	5.4451	0.7016	-0.5113	0.0002	3.6377
Ctnnd1	_TLDRS[Phospho (STY)]GDLGDMEPLK_.3	P30999_S920_M1_Ctnnd1	4.2657	3.5211	3.9993	3.3937	3.4094	3.2152	3.1084	3.2239	4.3734	3.2204	1.0438	0.0619	0.6405	0.1934
Prkar2b	_AATITATSPGALWGLDR_.2	#N/A	7.1107	6.7841	6.9645	7.1222	7.2675	7.4610	7.0927	7.0731	7.1394	7.1146	1.0019	0.0027	0.9142	0.0390
Prkar2b	_TWGDAGAAAGGGIPSK_.2	#N/A	6.6593	6.2618	6.9600	6.9645	6.7479	6.8743	6.5692	6.1794	6.6394	6.4169	1.0455	0.0642	0.0995	1.0022
Prkar2b	_C[Carbamidomethyl (C)]VGNYDNR_.2	#N/A	5.6031	5.1243	4.8337	5.9334	5.6778	5.7381	5.4422	5.3852	5.8867	5.6616	0.9805	-0.0283	0.6505	0.1867
Prkar2b	_ASVC[Carbamidomethyl (C)]AEAYNPDEEEDDAESR_.2	#N/A	4.5232	5.1299	3.9520	6.0780	5.1254	5.5783	5.8218	4.6682	5.6710	5.0502	0.9551	-0.0663	0.6020	0.2204
Prkar2b	_AASAHAIGTVK_.2	#N/A	4.6624	5.3504	4.2552	4.8752	5.1727	5.1233	4.9705	4.7824	4.8274	5.1145	0.9965	-0.0050	0.9376	0.0280
Prkar2b	_VYNDGEQIIAQGDLADSFFIVESGEVK_.3	#N/A	4.1352	5.4330	6.0632	6.5889	4.8559	5.6736	4.4952	4.7372	4.1645	5.3608	1.1640	0.2190	0.1537	0.8133
Prkar2b	_LQQENER_.2	#N/A	5.3852	5.2128	4.3886	5.8463	3.7986	5.1135	5.6364	3.0688	4.7882	6.1349	1.0103	0.0147	0.9403	0.0268
Prkar2b	_GSFGELALM[Oxidation (M)]YNTPR_.2	#N/A	5.5090	5.1140	6.0252	5.8502	5.9327	5.7898	5.3531	5.4853	5.4611	5.2109	1.0606	0.0849	0.1067	0.9719
Prkar2b	_VVDVIGTK_.2	#N/A	3.7985	3.7900	3.5171	3.8759	5.4589	4.9458	3.5906	4.8610	5.1299	4.6521	0.9282	-0.1075	0.5134	0.2896
Prkar2b	_EGEHVIDQGDDGDNFYVIDR_.3	#N/A	7.8031	7.4381	7.9106	8.0272	7.0203	7.5428	7.7377	7.6508	7.3375	7.7306	1.0013	0.0018	0.9635	0.0161
Prkar2b	_GTFDIYVK_.2	#N/A	3.0718	3.8280	4.3063	2.3239	3.5124	4.4091	3.9983	3.6645	3.1355	3.5273	0.9983	-0.0025	0.9888	0.0049
Prkar2b	_GVNFAEEPM[Oxidation (M)]RS[Phospho (STY)]DSENGEEEEAAEAGAFNAPVINR_.4	P31324_S83_M1_Prkar2b	3.8843	3.6127	3.4945	5.2461	4.4748	3.9809	3.7920	7.3276	3.8104	3.6548	0.8858	-0.1750	0.5161	0.2873
Prkar2b	_HQPADLLEFALQHFTR_.3	#N/A	3.7688	3.4235	4.4894	3.4913	3.3118	3.0923	3.2059	3.9532	3.8925	3.4583	0.9914	-0.0125	0.9160	0.0381
Prkar2b	_NLDPEQM[Oxidation (M)]SQVLDAMFEK_.2	#N/A	3.9141	3.1696	3.6478	3.1916	3.0578	3.5668	3.3398	3.8096	4.1087	4.3697	0.8765	-0.1901	0.0842	1.0749
Prkar2b	_RAS[Phospho (STY)]VC[Carbamidomethyl (C)]AEAYNPDEEEDDAESR_.3	P31324_S112_M1_Prkar2b	4.1298	3.4341	3.3158	6.0791	3.2555	2.9732	3.5331	4.6286	5.4323	3.7854	0.8895	-0.1690	0.4997	0.3013
Prkar2b	_C[Carbamidomethyl (C)]LAMDVQAFER_.2	#N/A	3.0969	3.9021	3.7837	4.2186	3.7235	4.0060	4.3668	4.2835	3.0817	4.2534	0.9480	-0.0771	0.5220	0.2824
Pou3f2	_WLEEADSSSGS[Phospho (STY)]PTSIDK_.2	P31360_S343_M1_Pou3f2	5.3964	5.5276	4.2306	6.4278	3.8577	5.8637	6.3398	4.1362	3.3414	4.0401	1.1686	0.2248	0.3250	0.4881
Pou3f3;Pou3f4	_WLEEADSSTGS[Phospho (STY)]PTSIDK_.2	P31361_S390_M1_Pou3f3;Pou3f4	6.2149	6.2694	6.3399	6.8940	6.0512	6.9062	3.2254	3.9353	6.8925	6.6162	1.2474	0.3190	0.1301	0.8858
Slc6a1	_QC[Carbamidomethyl (C)]DNPWNTDR_.2	#N/A	7.4841	7.1637	7.6249	7.0205	6.9277	6.7181	7.7914	7.2893	7.4259	7.1007	0.9669	-0.0486	0.2760	0.5590
Slc6a1	_AGDLPDRDTWK_.2	#N/A	5.2695	5.7583	5.2091	5.2699	2.9445	3.6801	5.5466	5.1989	4.7858	5.1654	0.9062	-0.1422	0.4261	0.3705
Slc6a1	_VADGQIS[Phospho (STY)]TEVSEAPVASDKPK_.3	#N/A	6.5584	6.6787	7.1389	5.2109	5.6171	5.2875	6.9860	7.3932	7.0895	5.6151	0.8982	-0.1548	0.2211	0.6553
Slc6a11	_GS[Phospho (STY)]ETLGGGGGGAAGTR_.2	#N/A	5.9048	6.9672	7.1350	7.0256	5.6731	7.1182	7.5205	5.8638	7.1471	7.2404	0.9560	-0.0650	0.5132	0.2897
Slc6a11	_LTVPSADLK_.2	#N/A	7.2728	7.3034	7.6676	6.4481	7.1280	7.1163	7.1134	7.4801	7.2817	7.2365	0.9833	-0.0244	0.5828	0.2345
Slc6a11	_AAEEARGS[Phospho (STY)]ETLGGGGGGAAGTR_.3	#N/A	4.2009	3.4063	3.7144	3.6772	3.2946	3.3599	3.6166	3.9699	3.8685	3.5121	0.9645	-0.0522	0.5075	0.2946
Slc6a11	_VKGDGTISAITEK_.3	#N/A	4.1461	3.1963	3.8798	3.4236	3.2899	3.3347	3.5719	3.5776	3.8766	3.1009	1.0038	0.0054	0.9555	0.0198
Akt1	_SGS[Phospho (STY)]PSDNSGAEEM[Oxidation (M)]EVSLAKPK_.3	#N/A	3.5838	7.4410	4.0346	7.0740	3.7731	3.8526	3.8463	7.6320	3.6855	4.3295	1.0178	0.0254	0.9430	0.0255
Dbi	_AKWDSWNK_.2	#N/A	7.6353	7.9438	8.4174	8.2033	8.6857	8.0086	7.7344	7.4922	7.9568	8.0174	1.0447	0.0631	0.1379	0.8605
Dbi	_TYVEKVDELKK_.3	#N/A	5.4309	6.0812	6.8263	5.7377	7.1385	6.2501	6.1507	5.7452	6.5882	6.6616	0.9933	-0.0097	0.9117	0.0401
Dbi	_[Acetyl (Protein N-term)]SQAEFDKAAEEVKR_.3	#N/A	7.2971	7.7120	8.2923	7.9688	8.0109	7.8753	7.5014	7.4961	7.6903	7.5918	1.0382	0.0541	0.1360	0.8664
Dbi	_TQPTDEEM[Oxidation (M)]LFIYSHFK_.3	#N/A	7.1669	7.1106	7.0001	6.9263	7.7634	7.5031	7.0930	6.7682	7.7547	7.4932	0.9956	-0.0064	0.8958	0.0478
Dbi	_QATVGDVNTDRPGLLDLK_.3	#N/A	7.9616	8.1406	8.4973	8.3418	8.9131	8.6764	8.0869	8.0529	8.4223	8.0354	1.0334	0.0474	0.1943	0.7116
Dbi	_[Acetyl (Protein N-term)]SQAEFDKAAEEVK_.2	#N/A	2.8142	2.9880	3.5448	4.5952	4.0780	4.6347	3.7621	3.3399	4.0047	4.1067	0.9928	-0.0105	0.9498	0.0223
Dbi	_TYVEKVDELK_.2	#N/A	7.8857	8.3376	8.7841	8.3149	8.8230	8.4703	8.0002	8.2238	8.2136	8.1690	1.0349	0.0494	0.1557	0.8077
Map2k1	_DVKPSNILVNSR_.2	#N/A	8.1456	8.4185	8.3111	8.5955	8.7067	8.1854	8.4092	7.8568	8.1446	8.0343	1.0348	0.0494	0.0897	1.0471
Map2k1	_KLEELELDEQQR_.3	#N/A	6.6177	6.8354	6.6288	6.8842	6.8486	7.3219	6.2960	7.0181	7.2574	6.9380	0.9969	-0.0045	0.9212	0.0357
Map2k1	_IPEQILGK_.2	#N/A	5.1307	4.8581	2.2775	4.6825	4.6645	5.3378	4.2936	4.1444	4.3421	4.2707	1.0538	0.0755	0.6984	0.1559
Map2k1	_LEAFLTQK_.2	#N/A	5.7836	3.9011	3.8425	4.5530	4.0139	4.0287	4.0598	4.9939	4.1634	4.3121	0.9935	-0.0094	0.9469	0.0237
Map2k1	_ISELGAGNGGVVFK_.2	#N/A	5.1662	5.8449	4.0208	5.5838	4.3020	3.3520	3.7128	4.9192	3.7356	5.2189	1.0716	0.0998	0.6057	0.2178
Map2k1	_LPSGVFSLEFQDFVNK_.2	#N/A	5.0689	4.0069	3.9342	3.4069	2.8531	3.3847	3.7454	3.8297	4.8085	3.3739	0.9585	-0.0612	0.7298	0.1368
Map2k1	_LC[Carbamidomethyl (C)]DFGVSGQLIDSM[Oxidation (M)]ANSFVGTR_.2	#N/A	3.8516	4.1958	4.5084	3.8839	4.3226	4.6781	4.7184	4.8563	4.7432	4.8342	0.8856	-0.1753	0.0127	1.8971
Map2k1	_KLEELELDEQQRK_.3	#N/A	3.6666	4.2871	3.2645	4.1285	2.5609	3.9501	3.8432	4.1929	2.9911	2.3835	1.0866	0.1198	0.5463	0.2626
Map2k1	_VGELKDDDFEK_.3	#N/A	4.1288	3.4331	2.7244	3.1867	2.5273	2.9742	3.3350	3.8145	4.1135	3.2410	0.8722	-0.1973	0.2028	0.6929
Map2k1	_LEELELDEQQR_.2	#N/A	3.7632	3.5792	3.4969	4.5415	3.4005	4.3289	3.1889	3.9605	4.2596	4.2188	0.9859	-0.0205	0.8634	0.0638
Map2k1	_LQGTHYSVQSDIWSM[Oxidation (M)]GLSLVEM[Oxidation (M)]AVGR_.3	#N/A	3.4497	3.7765	4.1365	3.1383	3.6648	2.9599	4.3539	3.4793	3.1279	3.1053	1.0012	0.0018	0.9895	0.0046
Scp2	_KLEEEGEQFVKK_.3	#N/A	4.2957	3.6000	3.4816	3.9843	4.9281	3.3487	3.6990	4.1342	3.7828	3.4810	1.0439	0.0619	0.6221	0.2062
Scp2	_GSVLPNSDKK_.2	#N/A	4.1499	3.4053	3.8836	3.5095	5.5458	4.9533	3.2241	3.7936	4.9693	3.1046	1.1241	0.1688	0.4174	0.3795
Scp2	_EATWVVDVK_.2	#N/A	3.6188	4.4240	4.1529	2.7816	4.2453	3.4842	3.8449	3.4902	3.6035	3.3740	1.0577	0.0809	0.5407	0.2670
Slc2a3	_EEDQATEILQR_.2	#N/A	7.2260	6.3835	6.9407	6.3134	6.4416	6.4725	7.0842	7.0747	6.6382	6.7545	0.9625	-0.0551	0.2485	0.6047
Slc2a3	_QVTVLELFR_.2	#N/A	6.2975	6.2671	6.3808	5.9160	5.6256	6.0354	6.7678	6.6315	6.0733	6.2642	0.9461	-0.0800	0.1102	0.9577
Slc2a3	_LWGTSDVVQEIQEM[Oxidation (M)]KDESVR_.3	#N/A	6.4751	6.5211	7.0103	6.8743	6.2569	4.1535	7.3271	6.6904	6.7583	7.0908	0.8921	-0.1647	0.2068	0.6845
Slc2a3	_TFEDIAR_.2	#N/A	4.8241	4.7298	3.9282	4.4309	3.8731	4.7461	4.1456	4.3518	4.5567	4.3979	1.0135	0.0194	0.8016	0.0960
Ssb	_S[Phospho (STY)]PSRPLPEVTDEYKNDVK_.3	P32067_S92_M1_Ssb	3.8744	3.1788	4.5612	3.4411	5.8726	3.2286	3.5894	3.5601	5.7810	6.5318	0.8275	-0.2732	0.3261	0.4867
Chrm4	_KFAS[Phospho (STY)]IAR_.2	P32211_S379_M1_Chrm4	10.6613	10.5632	11.0903	10.4251	9.3070	10.2169	10.9918	10.7451	10.5028	10.3505	0.9746	-0.0371	0.4305	0.3660
Kras	_LVVVGAGGVGK_.2	#N/A	6.9658	7.3945	6.9820	7.3743	7.3743	7.0750	6.9357	7.5699	7.3984	7.2468	0.9872	-0.0186	0.5516	0.2583
Kras	_SYGIPFIETSAK_.2	#N/A	7.4023	7.5521	7.4392	7.6894	7.5411	7.2623	7.5550	7.3665	7.4617	6.9457	1.0203	0.0290	0.2856	0.5443
Kras	_TGEGFLC[Carbamidomethyl (C)]VFAINNTK_.2	#N/A	5.2444	4.8588	5.8653	5.6413	4.9782	4.9903	5.7158	4.6982	5.2666	6.2123	0.9616	-0.0565	0.5403	0.2673
Kras	_QVVIDGETC[Carbamidomethyl (C)]LLDILDTAGQEEYSAM[Oxidation (M)]R_.3	#N/A	3.1614	3.7684	5.1002	5.1520	3.1866	3.8356	3.6570	3.5129	2.4807	3.7089	1.2078	0.2724	0.2100	0.6777
Kras	_SFEDIHHYR_.3	#N/A	5.1808	4.5724	4.2380	3.2398	4.7069	5.2930	3.3162	5.2579	4.1664	4.7533	1.0377	0.0534	0.7517	0.1240
Wars1	_ASEDFVDPWTVR_.2	#N/A	7.0549	6.6829	6.3820	7.0274	6.8519	6.6690	6.9627	6.9520	6.4380	6.0064	1.0286	0.0406	0.4234	0.3733
Wars1	_TLIDVLQPLIAEHQAR_.2	#N/A	4.2637	4.0300	3.9974	4.0852	3.8211	3.7500	4.2973	3.7030	2.8710	3.3342	1.1239	0.1685	0.1245	0.9049
Wars1	_DLTLEQAYSYTVENAK_.2	#N/A	3.7166	3.6258	3.4503	4.4949	2.8604	5.5933	3.1424	4.0071	3.4061	4.1722	1.0128	0.0183	0.9274	0.0327
Kif1a	_C[Carbamidomethyl (C)]NAIINEDPNNK_.2	#N/A	6.8793	6.1924	6.8844	6.3331	5.5120	6.6092	6.9072	6.8017	6.7391	6.3610	0.9552	-0.0662	0.3157	0.5007
Kif1a	_AASVSS[Phospho (STY)]LHER_.2	P33173_S419_M1_Kif1a	2.9632	3.7195	4.1977	3.7415	3.6078	3.0168	3.8898	6.7255	3.5587	5.9607	0.7035	-0.5074	0.0531	1.2747
Kif1a	_NLFGSGS[Phospho (STY)]LR_.2	P33173_S1317_M1_Kif1a	3.6434	3.5828	4.3302	3.3320	4.2699	3.4596	4.5475	3.2856	5.4659	3.2990	0.9085	-0.1385	0.4435	0.3531
Kif1a	_DVLS[Phospho (STY)]PLRPSR_.2	P33173_S1093_M1_Kif1a	8.4550	8.3347	8.4372	8.4924	5.5337	7.9969	8.3708	8.0490	7.5877	7.8516	0.9887	-0.0164	0.8860	0.0526
Kif1a	_SGTS[Phospho (STY)]QEELR_.2	P33173_S937_M1_Kif1a	9.4709	9.1021	9.5236	9.0712	7.8175	8.9350	8.9695	9.7040	9.1815	9.1357	0.9718	-0.0413	0.4644	0.3331
Kif1a	_KTPS[Phospho (STY)]PVR_.2	P33173_S1553_M1_Kif1a	8.7316	8.7053	8.9784	4.6359	7.5387	8.3297	9.0177	8.9198	8.6880	4.5083	1.0047	0.0067	0.9765	0.0103
Kif1a	_ISLVDLAGSER_.2	#N/A	4.9722	8.0426	4.2426	7.1677	7.2846	3.5472	5.2493	4.6062	5.0074	7.7299	1.0403	0.0571	0.8411	0.0752
Kif1a	_FQSESC[Carbamidomethyl (C)]PVVGMS[Phospho (STY)]R_.2	P33173_S932_M1_Kif1a	3.4050	3.8403	4.3186	4.9614	3.7286	4.3968	4.2900	3.1388	3.4379	3.5396	1.2333	0.3025	0.0591	1.2283
Kif1a	_IS[Phospho (STY)]FDDQHFEK_.2	P33173_S912_M1_Kif1a	3.6708	3.6922	4.3576	5.1447	3.3312	3.4321	3.8905	5.2333	4.1468	3.3264	0.9491	-0.0753	0.6676	0.1755
Kif1a	_S[Phospho (STY)]DSLILDHQWELEK_.2	P33173_S1373_M1_Kif1a	3.8076	3.6447	4.2610	3.8048	3.4880	3.5923	3.9531	4.0480	3.5532	3.4821	1.0019	0.0028	0.9683	0.0140
Kif1a	_VTGVYELSLC[Carbamidomethyl (C)]HVADAGS[Phospho (STY)]PGM[Oxidation (M)]QR_.3	P33173_S1342_M1_Kif1a	3.2694	4.0746	3.9562	4.4588	3.8959	3.8336	4.1735	3.6596	3.3082	4.4258	1.0059	0.0085	0.9367	0.0284

Kif1a	_TPQPC[Carbamidomethyl (C)]S[Phospho (STY)]RPAS[Phospho (STY)]PEPELLPELDSK_.3	P33173_S1533_M2_Kif1a	3.9204	3.4220	3.6541	6.4947	3.0641	3.5605	3.3461	6.3152	4.1024	4.3759	0.8863	-0.1741	0.5414	0.2665
Kif1a	_PASPEPELLPELDSKKT[Phospho (STY)]PS[Phospho (STY)]PVR_.4	#N/A	3.9185	3.1943	4.1236	3.5935	3.8622	3.3971	3.5534	3.2004	3.9767	3.3898	1.0429	0.0606	0.5126	0.2902
Kif1a	_PAS[Phospho (STY)]PEPELLPELDSK_.2	P33173_S1537_M1_Kif1a	4.1170	3.7457	3.8507	3.3944	3.0467	3.1819	3.5427	3.6067	3.9058	3.0717	1.0069	0.0099	0.9254	0.0336
Ranbp1	_FLNAENAQK_.2	#N/A	6.4732	6.7209	7.3117	7.0267	7.3619	6.7201	6.6174	6.9917	6.9524	6.5450	1.0235	0.0335	0.4564	0.3406
Ranbp1	_FASENDLPEWK_.2	#N/A	6.4387	7.5985	6.5927	8.7243	7.6297	8.0210	7.8566	8.0220	8.3339	8.3625	0.9211	-0.1186	0.1930	0.7145
Ranbp1	_TLEEDEEELFK_.2	#N/A	3.1672	3.9235	5.8587	5.8438	5.8806	4.3137	4.9011	5.5091	3.3547	3.6228	1.1114	0.1524	0.5253	0.2796
Ranbp1	_VAEKLEALSVR_.3	#N/A	3.9064	3.2943	5.0606	3.6673	4.1584	4.7001	3.4738	4.1311	3.9072	4.2183	1.0505	0.0711	0.5961	0.2247
Ptk2	_YMEDSTY[Phospho (STY)]YK_.2	P34152_Y576_M1_Ptk2	5.8749	4.1962	3.1736	5.9628	4.0845	4.0410	3.9341	3.8990	3.5476	4.1865	1.1705	0.2272	0.2854	0.5445
Ptk2	_LQPQEIS[Phospho (STY)]PPPTANLDR_.2	P34152_S910_M1_Ptk2	12.8118	12.6636	13.3519	13.1023	11.5355	12.4682	13.3482	12.9132	12.7205	12.5078	0.9832	-0.0245	0.5552	0.2555
Mif	_LHISPDR_.2	#N/A	9.7990	9.9594	10.0990	9.9564	10.5548	9.8889	9.6004	9.8541	9.9882	9.9335	1.0202	0.0289	0.2307	0.6370
Mif	_LLC[Carbamidomethyl (C)]GLLSDR_.2	#N/A	9.9109	10.3060	10.3378	10.2502	10.0727	10.0701	9.9412	10.0983	9.9760	10.1180	1.0124	0.0178	0.2120	0.6737
Mif	_ASVPEGFLSELTQQLAQATGK_.2	#N/A	8.0293	7.9690	7.6448	8.8223	8.2643	8.2793	7.9529	7.9010	8.1461	8.0520	1.0194	0.0277	0.4737	0.3245
Mif	_PM[Oxidation (M)]FIVNTNVPR_.2	#N/A	9.2074	9.5424	9.8974	10.0777	9.7190	9.9229	9.4206	9.4807	9.6089	9.6837	1.0188	0.0268	0.3150	0.5017
Mif	QYIAVHVVPDQLM[Oxidation (M)]TFSGTNDPC[Carbamidomethyl (C)]ALC[Carbamidomethyl (C)]SLHSIG	#N/A	3.0162	3.0227	3.9303	3.7206	3.7498	3.0377	3.8689	3.1856	3.5796	3.4729	0.9677	-0.0473	0.6563	0.1829
Mif	_VYINYYDM[Oxidation (M)]NAANVGWNGSTFA_.2	#N/A	4.0845	3.3622	3.5969	4.6804	3.2202	3.2153	3.3540	3.0911	4.2405	5.6315	0.9054	-0.1434	0.4964	0.3041
Ptpn11	_IQNTGDY[Phospho (STY)]YDLYGGEK_.2	P35235_Y62_M1_Ptpn11	4.4810	7.1611	7.5064	7.4720	3.3988	7.3660	4.9318	7.3983	7.3237	6.8203	0.9414	-0.0871	0.7166	0.1447
Ptpn11	_ESQSHPGDFVLSVR_.3	#N/A	3.5975	3.6287	4.2843	3.2861	4.2240	3.5055	4.5016	3.5317	2.9801	3.2531	1.0526	0.0740	0.5894	0.2296
Rab5c	_FEIWDTAGQER_.2	#N/A	7.2129	6.6887	6.5367	7.0118	6.6378	6.3443	7.1357	6.8828	6.7577	7.1478	0.9653	-0.0510	0.2143	0.6689
Rab5c	_GVDLQESNPASR_.2	#N/A	7.0848	7.3209	6.9859	7.2512	6.9762	7.2047	7.6499	7.6794	7.2853	7.0833	0.9613	-0.0569	0.0675	1.1707
Rab5c	_LVLLGESAVGK_.2	#N/A	6.4659	7.0032	6.9522	7.3433	6.0708	6.4245	7.0231	7.6492	6.8835	6.9485	0.9416	-0.0868	0.1711	0.7669
Rab5c	_QASPNIVIALAGNK_.2	#N/A	5.0816	4.6163	5.9221	4.9980	4.0998	5.0894	5.0729	5.2000	5.1515	5.7487	0.9385	-0.0915	0.3540	0.4509
Rab5c	_YHSLAPM[Oxidation (M)]YYR_.3	#N/A	4.8144	5.0737	4.9590	4.3435	4.0707	3.6588	4.3483	5.8706	3.1334	3.0998	1.0908	0.1254	0.5442	0.2642
Rab5c	_GAQAAIVVYDITNTDTFAR_.2	#N/A	6.0996	6.2420	6.9705	6.6655	4.6837	6.0669	6.6478	6.1180	6.0847	6.3941	0.9699	-0.0440	0.6598	0.1806
Rab5c	_AVEFQEAQAYADDNSLLFM[Oxidation (M)]ETSAK_.3	#N/A	3.5002	4.3054	4.1870	3.8153	4.1267	3.6028	3.9636	3.1859	3.4849	3.4926	1.1108	0.1515	0.1018	0.9924
Rab6a	_[Acetyl (Protein N-term)]S[Phospho (STY)]AGGDFGNPLRK_.2	P35279_S2_M1_Rab6a	6.9324	6.6820	7.1929	6.7480	4.6160	6.9147	7.1061	6.9099	6.4880	6.2917	0.9724	-0.0403	0.7244	0.1400
Rab6a	_TDLADKR_.2	#N/A	5.9972	4.7933	4.9202	4.8213	5.1754	6.3375	5.0925	5.5974	5.4910	5.4343	0.9883	-0.0169	0.8613	0.0648
Rab6a	_LVFLGEQSVGK_.2	#N/A	6.2548	6.1507	3.9681	6.2052	6.9321	5.8840	5.2060	6.5077	5.1792	5.1164	1.0721	0.1005	0.5123	0.2905
Rab6a	_SREDMIDIK_.2	#N/A	3.3047	1.7651	4.2891	5.0346	4.2288	3.1124	4.5064	3.3267	2.9753	3.2579	1.0301	0.0428	0.8737	0.0586
Rab6a	_VAAALPGMESTQDR_.2	#N/A	4.0988	3.2436	3.8325	3.3763	3.2426	3.3820	3.5245	3.6249	3.9240	3.0536	0.9993	-0.0010	0.9918	0.0036
Rab21	_DSNGAILVYDVTDEDSFQK_.2	#N/A	5.1017	5.5907	6.5040	6.1003	4.2168	5.2101	5.9793	5.6028	5.4912	5.1556	0.9814	-0.0271	0.8175	0.0875
Rab21	_VNLAIWDTAGQER_.2	#N/A	3.8059	3.0613	4.7080	4.0812	4.2733	3.6750	4.3325	3.6083	3.7072	4.1742	0.9946	-0.0078	0.9481	0.0232
Rab12	_FNS[Phospho (STY)]ITSAYYR_.2	P35283_S105_M1_Rab12	8.0562	7.8656	8.4712	4.0927	3.9590	7.5622	8.4735	8.2209	8.1458	7.5865	0.8225	-0.2819	0.2139	0.6698
Rab18	_IIQTPGLWESENQNK_.2	#N/A	6.7096	6.7449	7.0864	7.4330	5.7909	6.5279	7.2238	6.9654	6.8720	6.9314	0.9596	-0.0595	0.3583	0.4457
Rab18	_LAIWDTAGQER_.2	#N/A	6.6693	6.5138	4.0478	6.4473	6.4365	6.3467	7.0827	6.5791	6.2857	6.8739	0.9063	-0.1420	0.2684	0.5712
Rab18	_TC[Carbamidomethyl (C)]DGVQC[Carbamidomethyl (C)]AFEELVEK_.2	#N/A	4.3358	4.4174	3.1744	4.2190	4.0853	4.5775	4.3672	4.7453	4.8115	4.6114	0.8923	-0.1644	0.0981	1.0084
Crhr1;Crhr2	_AMSIPTS[Phospho (STY)]PTR_.2	P35347_S400_M1_Crhr1;Crhr2	3.3411	4.0013	4.5756	4.1194	3.9857	4.1398	4.2676	4.3826	3.1809	3.7967	1.0308	0.0437	0.6961	0.1573
Htr2a	_S[Phospho (STY)]IHREPGSYAGR_.3	P35363_S298_M1_Htr2a	6.3561	4.0441	4.5328	4.0766	4.8640	3.8641	4.2249	6.1036	3.2236	5.8265	0.9542	-0.0676	0.7647	0.1165
Grin2a	_QHS[Phospho (STY)]YDNILDKPR_.3	#N/A	5.6969	3.8759	3.8491	4.1219	3.6721	4.9201	3.8365	6.3892	3.7468	4.0889	0.9647	-0.0518	0.8097	0.0917
Grin2a	_YLPEEVAHS[Phospho (STY)]DIS[Phospho (STY)]ETSSR_.2	#N/A	3.7959	3.4940	3.7684	6.8425	4.9181	5.2804	6.3236	7.5816	7.5370	7.3175	0.6514	-0.6185	0.0067	2.1752
Grin2a	_KMPS[Phospho (STY)]IESDV_.2	P35436_S1459_M1_Grin2a	6.6098	4.2312	3.2086	7.1580	4.6464	4.0060	3.8991	4.2488	3.0470	3.9305	1.3161	0.3963	0.1837	0.7359
Grin2a	_SM[Oxidation (M)]ESLRQDS[Phospho (STY)]LNQNPVSQR_.3	#N/A	6.4056	5.8336	6.6277	6.3624	4.5248	6.0997	5.9915	6.3613	5.9901	5.9830	0.9826	-0.0253	0.7958	0.0992
Grin2a	_SIS[Phospho (STY)]LKDR_.2	P35436_S1312_M1_Grin2a	3.3507	4.1070	4.5852	4.3087	3.9953	4.1302	4.0234	3.8098	3.4584	4.2757	1.0482	0.0680	0.4759	0.3225
Grin2a	_DKGS[Phospho (STY)]PHSEGSDRYR_.3	P35436_S1198_M1_Grin2a	4.6892	4.6478	5.5991	4.1647	4.7279	3.8372	4.1980	5.4252	5.2693	4.1367	0.9692	-0.0451	0.7294	0.1371
Grin2a	_SPDFNLTGS[Phospho (STY)]QSNM[Oxidation (M)]LK_.2	#N/A	3.6708	6.6984	7.1726	5.8526	3.5102	3.9613	7.0453	3.8724	3.7336	3.6669	1.1233	0.1678	0.6072	0.2167
Grin2a	_QDS[Phospho (STY)]LNQNPVSQR_.2	#N/A	3.5227	4.3279	4.2490	6.0018	4.1492	3.5803	6.5320	3.2084	3.5074	3.4701	1.0301	0.0427	0.8749	0.0580
Grin2a	_KKS[Phospho (STY)]PDFNLTGS[Phospho (STY)]QSNMLK_.3	P35436_S882_M2_Grin2a	3.3249	4.0175	4.5594	5.1563	3.8388	3.8907	4.2514	4.3988	3.1970	3.7805	1.0574	0.0805	0.5810	0.2358
Grin2a	_STASYC[Carbamidomethyl (C)]SRDS[Phospho (STY)]R_.2	P35436_S1416_M1_Grin2a	3.5895	4.1148	3.7981	4.3008	3.7379	3.9916	4.0155	4.2180	3.4663	4.2678	0.9825	-0.0254	0.7331	0.1348
Pdha1	_LEEGPPVTTVLTR_.2	#N/A	10.3383	10.3795	10.4365	10.6009	10.5288	10.4595	10.3483	10.2098	10.3582	10.3746	1.0130	0.0187	0.0478	1.3210
Pdha1	_AILAELTGR_.2	#N/A	8.4592	8.3426	7.9536	8.3674	8.2914	8.4770	7.6186	8.6193	8.5972	8.8424	0.9876	-0.0180	0.6712	0.1731
Pdha1	_FAAAYC[Carbamidomethyl (C)]R_.2	#N/A	7.5729	7.5420	7.8048	7.5726	8.0520	7.2057	7.4606	7.8726	8.0862	7.8074	0.9767	-0.0340	0.3360	0.4737
Pdha1	_TREEIQEVR_.2	#N/A	7.1637	7.0858	6.5492	7.3971	7.1473	6.9289	6.8601	6.8323	6.5900	7.1064	1.0289	0.0412	0.2727	0.5644
Pdha1	_AAASTDYYKR_.2	#N/A	6.3268	5.8752	6.2877	6.1208	6.6836	6.1780	5.7308	5.8050	6.2077	6.0735	1.0489	0.0689	0.1114	0.9531
Pdha1	_VDGM[Oxidation (M)]DILC[Carbamidomethyl (C)]VR_.2	#N/A	6.7874	6.6765	6.3578	7.5389	7.0074	6.4892	7.2309	6.4843	6.8973	6.9066	0.9898	-0.0148	0.7839	0.1057
Pdha1	_YGM[Oxidation (M)]GTSVER_.2	#N/A	7.0916	6.9205	6.5767	7.2489	6.6657	6.7254	6.6260	6.7982	6.8592	6.9500	1.0093	0.0133	0.6733	0.1718
Pdha1	_NFYGGNGIVGAQVPLGAGIALAC[Carbamidomethyl (C)]K_.3	#N/A	4.2418	3.9183	3.5962	4.3160	3.8084	4.4282	3.1249	3.3589	5.0366	4.1015	1.0374	0.0530	0.7101	0.1487
Pdha1	_GPILM[Oxidation (M)]ELQTYR_.2	#N/A	7.0373	7.0046	6.3831	7.2499	7.0926	6.7492	6.8626	6.4640	6.7628	6.8390	1.0278	0.0396	0.3117	0.5063
Pdha1	_M[Oxidation (M)]VNSNLASVEELKEIDVEVR_.3	#N/A	6.2522	6.4625	7.2599	7.4442	6.2341	7.2127	7.1434	7.2878	6.6184	6.9072	0.9745	-0.0373	0.5752	0.2402
Pdha1	_YHGHS[Phospho (STY)]M[Oxidation (M)]SDPGVSYR_.3	#N/A	10.0654	9.5238	10.4800	10.4200	10.1920	10.2563	10.2294	9.6136	10.2939	9.8967	1.0148	0.0212	0.5124	0.2904
Pdha1	_LPC[Carbamidomethyl (C)]IFIC[Carbamidomethyl (C)]ENNR_.2	#N/A	3.1198	4.1064	3.8066	10.6823	3.7463	3.9832	4.0240	3.8092	3.4578	4.2763	1.2610	0.3345	0.5065	0.2954
Pdha1	_YHGHSMSDPGVS[Phospho (STY)]YRT[Phospho (STY)]REEIQEVR_.4	#N/A	3.8365	3.9290	3.9129	5.6820	3.5787	3.6758	4.2609	3.6586	3.2804	3.8136	1.0930	0.1283	0.4423	0.3543
Pdha1	_M[Oxidation (M)]VNSNLASVEELKEIDVEVRK_.4	#N/A	3.7249	3.3472	3.4585	3.9345	3.7784	3.0004	3.6492	3.9989	3.8326	4.1804	0.9043	-0.1451	0.0922	1.0355
Fah	_ASLQNLLSASQAR_.2	#N/A	4.8361	5.5278	6.0258	6.0266	6.2923	5.4726	5.5627	6.0412	5.9701	5.7535	0.9769	-0.0338	0.6456	0.1900
Fah	_ASSIVVSGTPIR_.2	#N/A	3.3380	5.5544	5.7695	5.9631	5.9742	5.5648	5.9244	6.0036	5.4376	5.5895	0.9341	-0.0983	0.4920	0.3080
Canx	_APVPTGEVYFADSFDR_.2	#N/A	7.3011	6.9931	7.1981	7.1456	7.0745	7.1276	6.8592	7.0761	7.4505	7.2730	0.9966	-0.0050	0.8341	0.0788
Canx	_TYFTDKK_.2	#N/A	6.4229	6.2125	6.2167	6.0483	5.9483	5.4387	5.4456	6.2350	5.6791	5.9533	1.0377	0.0534	0.3468	0.4599
Canx	_AEEDEILNRS[Phospho (STY)]PR_.2	#N/A	11.0358	10.8861	11.3588	11.0973	10.2080	10.8412	11.3658	10.9275	10.9833	11.0102	0.9849	-0.0220	0.4546	0.3424
Canx	_QKS[Phospho (STY)]DAEEDGVTGS[Phospho (STY)]QDEEDSKPK_.3	P35564_S563_M2_Canx	6.7950	6.5906	5.6807	7.0223	5.3809	6.5629	6.6112	6.9216	6.9516	6.5869	0.9366	-0.0945	0.2458	0.6094
Canx	_AEEDEILNR_.2	#N/A	6.7832	6.4202	3.4653	6.4546	6.1843	5.8750	6.2379	6.4984	6.1529	5.9478	0.9444	-0.0826	0.5959	0.2248
Canx	_SDAEEDGVTGS[Phospho (STY)]QDEEDSKPK_.3	#N/A	5.1383	4.6791	4.4152	5.3496	4.7090	4.2310	4.5776	5.0299	5.4238	3.5636	1.0226	0.0322	0.7913	0.1017
Canx	_VVDDWANDGWGLK_.2	#N/A	4.1889	4.6156	5.2363	3.4664	4.4301	4.7860	5.8039	3.5348	3.8338	4.3683	1.0157	0.0224	0.8946	0.0484
Irs1	_[Acetyl (Protein N-term)]AS[Phospho (STY)]PPDTDGFSDVRK_.2	#N/A	8.0832	8.9518	9.1853	4.2465	8.1585	8.2693	8.9706	8.7714	8.8664	8.3238	0.8950	-0.1601	0.3520	0.4535
Irs1	_[Acetyl (Protein N-term)]AS[Phospho (STY)]PPDTDGFSDVR_.2	#N/A	4.1635	6.8276	3.3494	7.5384	6.5578	6.5674	3.5668	7.4060	7.2288	7.6662	0.9021	-0.1486	0.5959	0.2248
Ap1m1	_VLFDNTGR_.2	#N/A	4.9456	4.7517	4.4757	4.4153	4.3960	4.9560	3.6748	5.0930	3.7736	4.3989	1.0996	0.1369	0.1860	0.7305
Prdx1	_IGYPAPNFK_.2	#N/A	8.1762	8.7556	8.2322	8.1752	8.5580	8.3373	8.7838	8.5266	8.5553	8.5177	0.9740	-0.0380	0.1254	0.9017
Prdx1	_TIAQDYGVLK_.2	#N/A	8.0780	8.2826	8.1751	8.2471	8.7457	8.2514	7.4973	8.3418	8.3485	8.4783	1.0159	0.0228	0.5595	0.2522
Prdx1	_LVQAFQFTDK_.2	#N/A	6.6565	7.1201	7.1253	7.7833	7.4381	7.5729	7.3323	7.1969	7.4399	7.2443	0.9972	-0.0041	0.9237	0.0345
Prdx1	_ADEGISFR_.2	#N/A	7.2307	7.1373	6.7802	6.9526	7.9186	7.4418	6.2611	6.7836	7.3790	7.5816	1.0346	0.0491	0.4612	0.3361
Prdx1	_SKEYFSK_.2	#N/A	5.5877	6.2473	6.5041	6.1238	6.8237	6.5338	5.7453	5.7769	6.2483	6.0806	1.0571	0.0802	0.1905	0.7202
Prdx1	_GLFIIDDK_.2	#N/A	6.6488	6.8215	5.9314	6.4581	6.9364	7.0507	5.2666	6.0749	7.0841	6.8615	1.0505	0.0711	0.4328	0.3637

Prdx1	_ATAVM[Oxidation (M)]PDGQFK_.2	#N/A	8.0536	8.5577	7.9825	8.3169	8.9391	8.1274	8.2278	8.2562	8.2390	8.3694	1.0068	0.0098	0.7709	0.1130
Prdx1	_DISLSEYK_.2	#N/A	5.6581	3.4442	4.4688	2.0233	5.2809	6.2357	3.1853	5.4190	6.6068	5.8255	0.8592	-0.2190	0.4742	0.3241
Prdx1	_SVDEIIR_.2	#N/A	5.6093	5.5338	4.3729	5.4683	5.3483	4.9609	3.6955	5.5213	5.7765	5.5411	1.0160	0.0229	0.8603	0.0653
Prdx1	_LNC[Carbamidomethyl (C)]QVIGASVDSHFC[Carbamidomethyl (C)]HLAWINTPK_.3	#N/A	3.9396	4.2280	3.7909	5.4085	3.7306	4.0285	3.9892	3.6107	3.8959	3.3184	1.1307	0.1773	0.1924	0.7158
Cd81	_QFYDQALQQAVM[Oxidation (M)]DDDANNAK_.3	#N/A	8.5698	8.9165	10.1544	9.1407	7.5706	8.5359	9.3494	9.2990	8.7405	8.5765	0.9803	-0.0286	0.7106	0.1483
Cd81	_NSLC[Carbamidomethyl (C)]PSGGNILTPLLQQDC[Carbamidomethyl (C)]HQK_.3	#N/A	6.5909	6.2939	7.0113	6.4779	6.5683	3.6656	7.2860	6.9590	6.7460	6.7117	0.8810	-0.1828	0.2266	0.6447
Cd81	_TFHETLNC[Carbamidomethyl (C)]C[Carbamidomethyl (C)]GSNALTTLTTTILR_.3	#N/A	3.8858	4.5786	5.1791	4.8496	3.6765	3.9205	5.2947	5.2691	5.0873	4.7802	0.8513	-0.2322	0.0469	1.3284
Gpm6a	_QFGIVTIGEEK_.2	#N/A	10.0663	9.6952	10.0326	9.9537	9.1628	9.7393	10.1028	9.9983	9.9762	9.6626	0.9839	-0.0234	0.4178	0.3790
Gpm6a	_IC[Carbamidomethyl (C)]TASENFLR_.2	#N/A	9.9544	9.8293	10.0666	9.9402	9.6430	9.4152	10.1274	10.0541	9.8589	9.6473	0.9801	-0.0290	0.2579	0.5885
Gpm6a	_KIC[Carbamidomethyl (C)]TASENFLR_.2	#N/A	6.1164	6.3866	6.7064	5.9085	5.7450	5.7679	6.4086	6.4272	6.2312	5.9747	0.9752	-0.0362	0.4829	0.3161
Gpm6a	_SKEEQELHDIHSTR_.3	#N/A	9.2008	8.1188	8.6400	8.0421	8.6661	8.6730	9.2358	8.4753	8.9247	7.9705	0.9890	-0.0159	0.7658	0.1159
Gpm6a	_M[Oxidation (M)]QKYEDIK_.2	#N/A	5.2196	6.2180	6.3196	5.4407	5.6227	5.9352	6.2492	6.5365	6.4983	5.5464	0.9331	-0.0998	0.1875	0.7271
Gpm6a	_QFGIVTIGEEKK_.2	#N/A	5.5326	5.6527	6.0175	5.0422	4.8820	5.4316	5.8201	5.5950	5.3918	5.5709	0.9700	-0.0440	0.4713	0.3267
Gpm6a	_DLYGDFK_.2	#N/A	5.3863	3.8247	3.5783	4.3825	4.5420	4.4007	3.0714	5.3001	5.9802	4.9910	0.9001	-0.1519	0.4353	0.3612
Gpm6a	_EEQELHDIHSTRS[Phospho (STY)]K_.3	#N/A	4.0715	5.5044	7.3131	5.4557	4.7989	5.5410	5.5363	6.0619	3.9292	5.0909	1.0568	0.0797	0.6675	0.1756
Gpm6a	_S[Phospho (STY)]KERLNAYT_.2	#N/A	5.7080	6.2700	6.9458	6.5073	5.7411	6.3351	6.4765	6.7798	6.1452	5.9812	0.9851	-0.0216	0.7433	0.1289
Gpm6a	_SKEEQELHDIHSTRS[Phospho (STY)]K_.3	#N/A	7.0962	8.7862	9.2870	8.5504	7.5179	8.4370	9.0165	9.4827	9.0924	7.7475	0.9371	-0.0937	0.3102	0.5083
Gpm6a	_EEQELHDIHSTRS[Phospho (STY)]KER_.3	#N/A	3.4886	6.1642	7.7727	5.9478	4.6218	5.3899	3.6738	6.1458	3.9384	4.9785	1.1879	0.2484	0.3395	0.4692
Gpm6a	_YEDIKS[Phospho (STY)]KEEQELHDIHSTR_.4	#N/A	9.1584	9.1191	9.7358	9.2329	7.9873	8.8952	9.4788	9.5894	9.2907	9.2081	0.9606	-0.0580	0.2560	0.5917
Gpm6a	_EEQELHDIHSTR_.3	#N/A	5.2519	4.8113	3.9626	3.6998	4.6245	5.0116	4.0712	5.1293	5.0809	4.6692	0.9626	-0.0550	0.6406	0.1934
Gpm6a	_[Acetyl (Protein N-term)]MEENMEEGQTQK_.2	#N/A	5.3210	3.6887	4.2242	6.0230	3.5770	3.0476	4.4416	3.3916	3.0402	3.1930	1.2266	0.2947	0.2432	0.6140
Gpm6b	_AKEEQELQDIQS[Phospho (STY)]R_.2	#N/A	7.8525	7.4708	7.9522	7.8086	6.7250	7.5309	8.0705	7.6984	7.9867	7.3895	0.9705	-0.0432	0.4021	0.3956
Gpm6b	_EEQELQDIQS[Phospho (STY)]R_.2	#N/A	8.7442	8.0157	8.3041	8.2434	6.9150	8.3745	8.8466	8.2120	8.4457	8.3565	0.9568	-0.0637	0.3100	0.5087
Gpm6b	_QYGIIPWNAFPGK_.2	#N/A	7.7753	7.8837	10.9784	6.9878	7.5562	7.2385	7.9827	8.0277	7.5177	7.5172	1.0398	0.0563	0.6935	0.1590
Gpm6b	_ELHGEFK_.2	#N/A	6.9766	6.3673	6.3792	6.2194	6.3711	6.5884	6.8179	6.9937	6.7338	6.6626	0.9532	-0.0692	0.0641	1.1935
Gpm6b	_S[Phospho (STY)]KEQLNSYT_.2	#N/A	11.6855	11.6241	12.3681	11.9047	10.8770	11.4392	11.9578	11.8602	11.7748	11.5890	0.9876	-0.0179	0.5906	0.2287
Gpm6b	_MQAYQDIK_.2	#N/A	5.1213	3.2250	3.8510	3.5684	3.9761	3.1823	4.7509	3.6064	3.9054	3.0721	0.9966	-0.0049	0.9780	0.0096
Gpm6b	_[Acetyl (Protein N-term)]M[Oxidation (M)]KPAM[Oxidation (M)]ETAAEENTEQS[Phospho (STY)]QER_.	#N/A	3.7286	2.8408	3.4622	5.9757	2.8107	3.7777	3.9977	3.8444	3.6622	4.1841	0.9602	-0.0586	0.8010	0.0964
Ptpn12	_DADVSEES[Phospho (STY)]PPPLPER_.2	P35831_S673_M1_Ptpn12	3.4922	3.8502	3.2258	4.2705	3.6716	4.0579	4.4187	7.5112	3.0298	3.9477	0.7957	-0.3296	0.2578	0.5887
Ptpn12	_NLS[Phospho (STY)]FEIKK_.2	#N/A	4.0266	4.1616	4.2340	3.8800	3.2141	4.3223	4.6164	4.9911	3.0670	4.8871	0.9050	-0.1441	0.3382	0.4708
Ptpn12	_S[Phospho (STY)]PAEVTDIGFGNR_.2	#N/A	3.1826	4.1598	4.4171	3.9609	3.8272	4.2983	5.1482	3.0403	3.3393	3.6382	1.0482	0.0679	0.6847	0.1645
Ptpn12	_DQITKS[Phospho (STY)]PAEVTDIGFGNR_.3	#N/A	4.3463	3.6018	4.0800	3.3130	3.4901	3.1346	4.5286	3.3046	2.9532	3.3011	1.0395	0.0559	0.7104	0.1485
Ptpn12	_NLS[Phospho (STY)]FEIK_.2	#N/A	3.2441	3.7110	3.9309	4.0714	3.8706	3.8589	4.2197	0.7267	3.2288	2.8855	1.3674	0.4515	0.1289	0.8897
Flt1	_DGFADPHFHSGS[Phospho (STY)]SDDVR_.3	P35969_S1207_M1_Flt1	3.6235	4.3798	3.3572	4.4018	4.4558	3.8573	3.7505	4.1002	4.3992	4.0791	0.9829	-0.0248	0.7956	0.0993
Rpl12	_EILGTAQSVGC[Carbamidomethyl (C)]NVDGR_.2	#N/A	6.3132	6.1493	5.4216	7.1685	6.8265	6.1383	6.5959	6.7082	6.5429	6.4762	0.9628	-0.0546	0.4562	0.3408
Rpl12	_QAQIEVVPSASALIIK_.2	#N/A	6.0896	5.5248	5.9842	5.3361	6.2849	5.8857	5.7952	5.2849	6.4427	6.4691	0.9755	-0.0358	0.6246	0.2044
Rpl12	_HSGNITFDEIVNIAR_.3	#N/A	4.3196	3.0228	4.0532	3.5970	3.9543	3.1613	3.7453	4.0651	4.0383	3.2743	0.9746	-0.0371	0.7577	0.1205
Rpl18	_ILTFDQLALESPK_.2	#N/A	3.1569	4.6855	4.3914	4.5203	3.6710	5.0388	5.9882	5.4923	5.0827	4.4435	0.8081	-0.3074	0.0512	1.2903
Rpl18	_SQDIYLR_.2	#N/A	4.2054	4.2935	4.1751	3.1770	4.1149	3.6363	4.3925	4.4741	4.4989	4.6761	0.8721	-0.1974	0.0353	1.4522
Sar1a;Sar1b	_LVFLGLDNAGK_.2	#N/A	5.5221	5.6593	4.0833	5.8406	5.0827	6.0020	4.1151	6.0296	5.7262	5.5909	0.9999	-0.0001	0.9993	0.0003
Cpox	_SPS[Phospho (STY)]PGRREEDGDELAR_.3	P36552_S101_M1_Cpox	3.4288	4.2340	4.3429	3.8867	4.0553	3.6742	4.0350	3.1145	3.4135	3.5640	1.1147	0.1567	0.1197	0.9219
Gnl1	_GLQDGLRS[Phospho (STY)]SS[Phospho (STY)]NSR_.2	P36916_S32_M2_Gnl1	5.3087	3.7351	4.5555	3.5574	2.9944	3.2342	3.5950	3.0603	5.5748	3.5244	0.9896	-0.0151	0.9495	0.0225
Gnl1	_REEQTDTS[Phospho (STY)]DGESVTHHIR_.4	#N/A	8.9147	8.8802	9.6091	9.1932	7.8760	9.1711	9.1876	9.2658	9.1355	9.0573	0.9759	-0.0352	0.4822	0.3167
Gnl1	_EEQTDTS[Phospho (STY)]DGESVTHHIR_.3	#N/A	8.2567	7.7728	7.9765	8.2187	7.1604	7.8880	8.2325	8.1904	7.9661	7.9120	0.9757	-0.0355	0.3836	0.4162
Gnl1	_ERREEQTDT[Phospho (STY)]S[Phospho (STY)]DGESVTHHIR_.4	#N/A	3.3830	4.2122	7.9003	7.2752	4.0335	7.3552	6.7818	7.2433	4.1963	3.3954	1.0535	0.0752	0.8263	0.0829
Gnl1	_EVYQPGSVLDFPR_.2	#N/A	5.1641	3.1414	3.9347	3.4785	4.4283	3.2799	3.6267	3.5227	4.9117	3.1557	1.0264	0.0375	0.8457	0.0728
Hmgcl	_LLEAGDFIC[Carbamidomethyl (C)]QALNR_.2	#N/A	3.6918	3.6506	5.1663	5.0418	4.9467	5.1046	5.6881	4.8808	5.5768	5.6800	0.8431	-0.2462	0.0645	1.1901
Hmgcl	_EVSVFGAVSELFTR_.2	#N/A	3.9228	3.2272	3.6600	3.6115	3.0485	3.1801	3.5409	3.2402	4.1556	3.5785	0.9484	-0.0764	0.4395	0.3570
Hsf1	_VKQEPPS[Phospho (STY)]PPHS[Phospho (STY)]PR_.2	#N/A	6.9226	7.5014	6.8238	8.0629	6.9186	7.3361	7.6609	7.2364	7.4428	7.3597	0.9779	-0.0322	0.5342	0.2723
Hspa9	_DAGQISGLNVLR_.2	#N/A	8.5249	8.6111	8.7163	8.9396	8.9006	8.5809	8.4268	8.6205	8.7299	8.5956	1.0138	0.0198	0.2738	0.5625
Hspa9	_NAVITVPAYFNDSQR_.2	#N/A	8.0384	7.8231	8.3633	8.4891	7.6695	7.9745	8.3508	7.8712	7.5334	7.9747	1.0160	0.0229	0.5584	0.2531
Hspa9	_TTPSVVAFTADGER_.2	#N/A	7.9097	7.7580	7.8713	7.7561	7.8025	7.9471	8.0021	7.6786	7.4171	8.0467	1.0070	0.0101	0.6707	0.1735
Hspa9	_VQQTVQDLFGR_.2	#N/A	7.9868	8.0412	8.1167	8.1198	8.0795	8.1107	7.9392	7.5131	7.8209	7.7178	1.0423	0.0598	0.0026	2.5799
Hspa9	_AQFEGIVTDLIK_.2	#N/A	6.8525	6.3861	6.6989	7.1285	6.9678	6.9888	6.1069	6.6397	6.1909	6.8566	1.0603	0.0844	0.0821	1.0857
Hspa9	_NTTIPTKK_.2	#N/A	6.2405	6.6264	7.2285	7.2330	7.3090	6.6017	6.5534	6.7609	6.5365	6.7082	1.0352	0.0498	0.3404	0.4680
Hspa9	_VINEPTAAALAYGLDK_.2	#N/A	6.4074	6.0058	4.8761	6.7669	6.6847	6.2837	6.3280	5.5108	5.9668	6.1400	1.0308	0.0438	0.6398	0.1940
Hspa9	_QAVTNPNNTFYATK_.2	#N/A	5.8960	6.0398	5.9955	6.3855	6.4508	5.8983	6.1287	5.9286	5.7470	5.9398	1.0295	0.0419	0.2452	0.6104
Hspa9	_QAASSLQQASLK_.2	#N/A	6.4321	6.2016	5.6916	6.0138	6.3580	6.4995	5.8764	5.9435	6.2852	5.9166	1.0323	0.0459	0.2922	0.5343
Hspa9	_STNGDTFLGGEDFDQALLR_.2	#N/A	4.9919	5.4375	5.0824	4.9928	5.5685	5.5578	5.6264	4.9521	4.8086	4.7444	1.0475	0.0669	0.2972	0.5269
Hspa9	_EQQIVIQSSGGLSKDDIENM[Oxidation (M)]VK_.3	#N/A	5.6364	5.9845	6.6437	6.2781	4.8235	5.4717	5.4711	5.7042	5.4682	5.9368	1.0286	0.0406	0.6474	0.1888
Hspa9	_LLGQFTLIGIPPAPR_.2	#N/A	5.9732	6.2209	6.1532	6.6570	7.3955	6.8923	6.5827	7.2868	6.6707	7.8225	0.9236	-0.1147	0.1677	0.7755
Hspa9	_ETAENYLGHTAK_.2	#N/A	4.7098	4.9573	4.2514	5.4079	5.9705	4.9753	4.7764	3.2060	5.3501	5.5494	1.0688	0.0960	0.5469	0.2621
Hspa9	_ALLAGKDSETGENIR_.2	#N/A	4.0190	4.9446	4.6813	4.0599	3.4945	3.8336	4.0759	3.3209	4.2266	3.5059	1.1031	0.1415	0.2658	0.5754
Hspa9	_ETGVDLTKDNM[Oxidation (M)]ALQR_.3	#N/A	5.2608	5.9156	6.2299	3.4965	5.4226	5.9947	3.2112	5.3284	5.4570	5.9872	1.0782	0.1086	0.5927	0.2272
Hspa9	_S[Phospho (STY)]DIGEVILVGGMT[Phospho (STY)]R_.2	P38647_S378_M2_Hspa9	10.6535	10.8489	11.0389	11.4149	10.4242	11.0057	8.9535	10.8635	10.8899	10.9046	1.0476	0.0670	0.2726	0.5644
Hspa9	_AM[Oxidation (M)]QDAEVSK_.2	#N/A	4.1063	4.5303	4.4751	4.0189	6.4098	4.5972	4.1671	4.7456	3.6726	4.2516	1.1141	0.1559	0.3454	0.4616
Hspa9	_RYDDPEVQKDTK_.3	#N/A	5.3830	3.2388	5.0900	4.9653	6.0756	5.7338	4.8883	5.2423	5.0021	5.0064	1.0092	0.0132	0.9298	0.0316
Hspa9	_M[Oxidation (M)]EEFKDQLPADEC[Carbamidomethyl (C)]NK_.3	#N/A	5.0739	4.3111	3.5485	4.1932	5.2851	4.7838	3.2405	4.5647	4.5530	4.9125	1.0498	0.0701	0.6370	0.1959
Hspa9	_AM[Oxidation (M)]QDAEVSKSDIGEVILVGGMTR_.3	#N/A	4.0418	4.3356	3.7755	6.4855	3.1855	3.4391	3.4675	3.6819	3.9810	2.9965	1.1922	0.2536	0.3112	0.5069
Hspa9	_ASNGDAWVEAHGK_.2	#N/A	5.1610	5.1285	4.5500	5.3049	6.2279	5.5369	5.4810	4.6341	5.6205	5.8486	0.9856	-0.0210	0.8301	0.0809
Hspa9	_VEAVNM[Oxidation (M)]AEGIIHDTETK_.3	#N/A	3.7971	3.8405	5.0424	5.1724	4.0581	3.4952	4.3466	3.0148	3.9556	4.2536	1.0878	0.1214	0.4509	0.3459
Hspa9	_VINEPTAAALAYGLDKSEDK_.3	#N/A	3.0306	3.7868	4.2651	3.8088	3.6751	2.9495	3.9571	3.1923	3.4914	3.6682	1.0024	0.0035	0.9765	0.0103
Hspa9	_TIAPC[Carbamidomethyl (C)]QK_.2	#N/A	3.1642	3.9694	3.1067	3.8896	4.3051	3.9387	4.2995	4.3507	3.1490	4.7038	0.9038	-0.1459	0.3066	0.5134
Hspa9	_MKETAENYLGHTAK_.3	#N/A	3.2843	4.0895	3.9711	5.2325	4.3952	3.8187	4.1795	2.9699	3.2690	4.4407	1.1123	0.1535	0.3650	0.4377
Hspa9	_GAVVGIDLGTTNSC[Carbamidomethyl (C)]VAVM[Oxidation (M)]EGK_.2	#N/A	3.3790	3.9336	4.3207	4.4752	3.7907	4.1437	4.3056	4.3544	3.1526	3.8346	1.0244	0.0348	0.7578	0.1204
Hspa9	_SQVFSTAADGQTQVEIK_.2	#N/A	3.6988	3.5273	4.3856	3.3874	6.8209	3.4041	3.1021	3.2302	4.3796	3.3544	1.1955	0.2576	0.3670	0.4353
Dnm1	_S[Phospho (STY)]PTSSPTPQR_.2	#N/A	9.4702	9.6096	8.1080	9.8184	8.4900	9.3810	9.5852	8.9978	9.3811	9.1029	0.9870	-0.0189	0.7492	0.1254
Dnm1	_GISPVPINLR_.2	#N/A	10.4254	10.6599	10.6994	10.3904	10.5832	10.4811	10.5085	10.6409	10.5992	10.4775	0.9984	-0.0023	0.8220	0.0851
Dnm1	_SSVLENFVGR_.2	#N/A	10.1011	10.3269	10.6037	10.3925	10.1075	10.1216	10.2779	10.3142	10.1068	9.9897	1.0102	0.0146	0.4129	0.3841

Dnm1	_VPVGDQPPDIEFQIR_.2	#N/A	9.3405	9.4000	9.7692	9.7207	9.2311	9.4738	9.5197	9.3825	9.4934	9.3035	1.0068	0.0098	0.5942	0.2261
Dnm1	_DITAALAAER_.2	#N/A	9.0997	9.1256	9.0106	9.5174	9.1661	9.0713	9.1448	9.2111	9.0692	9.0771	1.0043	0.0062	0.6900	0.1612
Dnm1	_VLNQQLTNHIR_.2	#N/A	7.7097	7.9643	7.9545	8.0506	8.1357	7.6934	7.7289	8.0931	7.8960	7.8766	1.0025	0.0035	0.8636	0.0637
Dnm1	_VYSPHVLNLTLVDLPGM[Oxidation (M)]TK_.3	#N/A	8.8428	8.8957	9.2225	8.9055	8.6996	9.0644	8.9958	9.1856	9.0345	8.9927	0.9874	-0.0182	0.2865	0.5429
Dnm1	_IVTTHIR_.2	#N/A	8.2910	8.8853	8.7288	8.8136	8.9723	8.4750	8.5865	8.7129	8.9179	8.7719	0.9939	-0.0088	0.7221	0.1414
Dnm1	_FFLSHPSYR_.2	#N/A	8.6304	8.8376	8.9409	8.6761	8.1417	8.3723	8.6874	8.8035	8.7360	8.7139	0.9845	-0.0225	0.4017	0.3961
Dnm1	_NFRPDDPAR_.2	#N/A	8.6676	8.1238	7.7043	8.7076	8.6549	8.1403	8.5329	8.4560	8.2945	8.4480	0.9882	-0.0172	0.6503	0.1869
Dnm1	_GYIGVVNR_.2	#N/A	8.4529	7.9896	8.7717	8.2273	8.3659	8.4471	6.5964	8.5198	8.4991	8.2778	1.0505	0.0710	0.3309	0.4803
Dnm1	_LQDAFSAIGQNADLDLPQIAVVGGQSAGK_.3	#N/A	7.4678	7.5070	7.8415	8.0631	7.6962	7.8023	7.8281	7.7157	7.6505	7.6857	1.0012	0.0018	0.9367	0.0284
Dnm1	_NVYKDYR_.2	#N/A	7.4262	7.2525	7.6148	7.3102	7.3369	7.5231	6.9231	7.3892	7.1276	7.3354	1.0301	0.0428	0.0835	1.0783
Dnm1	_C[Carbamidomethyl (C)]VDM[Oxidation (M)]VISELISTVR_.2	#N/A	5.2881	5.1438	5.2813	4.6964	5.7625	5.9824	5.3480	4.9082	5.8197	5.3009	1.0028	0.0040	0.9585	0.0184
Dnm1	_GM[Oxidation (M)]EDLIPLVNR_.2	#N/A	9.8879	9.8267	9.8616	9.9359	9.6208	9.5772	9.9319	10.0173	9.8884	9.9001	0.9850	-0.0219	0.0969	1.0136
Dnm1	_KKTS[Phospho (STY)]GNQDEILVIR_.3	#N/A	3.6522	3.4686	3.4832	8.2987	3.4229	7.5477	7.3470	7.9852	4.2183	7.6569	0.7320	-0.4501	0.2164	0.6647
Dnm1	_EVDEYKNFRPDDPAR_.3	#N/A	6.6355	6.7127	4.4797	7.1971	7.4740	7.4295	6.6461	6.8228	7.1193	6.8299	0.9709	-0.0427	0.7385	0.1316
Dnm1	_M[Oxidation (M)]GTPYLQK_.2	#N/A	6.3210	7.0518	6.9136	6.2611	6.6768	6.6139	6.4071	7.1249	6.2391	7.0068	0.9918	-0.0118	0.8220	0.0851
Dnm1	_FTDFEEVR_.2	#N/A	6.8343	6.0655	4.0172	6.3139	6.3593	5.9866	6.2157	6.3954	6.4763	5.9605	0.9469	-0.0787	0.5324	0.2738
Dnm1	_ALLQM[Oxidation (M)]VQQFAVDFEKR_.3	#N/A	4.5382	5.0857	4.7236	3.8745	4.3400	4.4855	5.0846	5.1857	3.7997	4.1537	0.9895	-0.0153	0.8912	0.0500
Dnm1	_LDLM[Oxidation (M)]DEGTDAR_.2	#N/A	8.3294	7.8733	7.8411	8.6554	8.1385	7.6158	8.3544	8.1149	7.8590	7.8866	1.0027	0.0039	0.9208	0.0359
Dnm1	_NLVDSYM[Oxidation (M)]AIVNK_.2	#N/A	6.8957	6.6596	6.8315	6.8804	6.0828	6.2014	6.4946	6.4507	6.4012	6.8855	1.0052	0.0074	0.8718	0.0596
Dnm1	_LREEM[Oxidation (M)]ER_.2	#N/A	5.8024	4.9484	4.6723	5.8744	5.9044	5.4865	4.5602	4.6519	4.2002	4.6102	1.2092	0.2740	0.0097	2.0127
Dnm1	_EISYAIK_.2	#N/A	6.5211	6.2169	6.6306	5.7852	6.8545	6.3978	4.0636	6.6470	6.4320	7.0912	1.0565	0.0794	0.5639	0.2488
Dnm1	_KDITAALAAER_.2	#N/A	5.5540	3.8327	4.3110	5.0341	5.3120	5.2412	5.8768	4.8362	5.4025	8.1925	0.8032	-0.3162	0.1139	0.9434
Dnm1	_TSGNQDEILVIR_.2	#N/A	6.7015	6.9458	6.7897	7.9608	6.8309	7.3380	7.4290	7.5721	7.0185	7.6341	0.9570	-0.0635	0.2680	0.5719
Dnm1	_ENC[Carbamidomethyl (C)]LILAVSPANSDLANSDALK_.2	#N/A	7.1022	7.2704	7.2804	7.5966	6.8702	7.2158	7.2654	7.1939	7.1691	7.2436	1.0006	0.0009	0.9712	0.0127
Dnm1	_RSPTSS[Phospho (STY)]PTPQR_.2	#N/A	6.7402	3.9514	7.3399	7.1110	7.4900	6.5703	7.1169	6.7995	3.8084	9.1138	0.9738	-0.0383	0.8763	0.0573
Dnm1	_LEIEAETDRVTGTNK_.2	#N/A	4.7535	3.8346	4.2252	4.9107	3.7198	4.6979	3.6988	4.4246	4.4894	4.7741	1.0023	0.0034	0.9749	0.0110
Dnm1	_S[Phospho (STY)]PTSS[Phospho (STY)]PTPQRR_.2	#N/A	6.9993	7.8255	7.8275	3.8039	4.8693	3.4305	7.5393	7.1149	7.1546	7.3195	0.7955	-0.3301	0.1848	0.7333
Dnm1	_IEGSGDQIDTYELSGGAR_.2	#N/A	5.8859	6.0593	6.2519	7.3846	6.0165	5.9458	5.9162	6.6923	5.8158	6.4806	1.0050	0.0072	0.9282	0.0324
Dnm1	_KTSGNQDEILVIR_.3	#N/A	4.6611	4.5115	3.7489	3.9781	4.0383	4.4229	4.7212	4.1811	5.2680	4.8919	0.8870	-0.1731	0.0674	1.1716
Dnm1	_RS[Phospho (STY)]PT[Phospho (STY)]SSPTPQRR_.2	#N/A	3.4221	3.9834	3.9085	3.6385	4.0119	3.6784	4.1995	4.5135	4.0930	3.5316	0.9240	-0.1141	0.1602	0.7953
Dnm1	_YM[Oxidation (M)]LSVDNLK_.2	#N/A	6.7933	6.8058	6.7635	7.0484	6.4554	6.4300	6.4827	7.2745	6.6902	6.5611	0.9947	-0.0077	0.8509	0.0701
Dnm1	_TIM[Oxidation (M)]HLM[Oxidation (M)]INNTK_.2	#N/A	4.1716	5.5895	5.4546	3.8649	4.8925	5.6930	4.9796	5.2605	5.2093	5.0812	0.9633	-0.0539	0.6492	0.1876
Dnm1	_LQSQLLSIEKEVDEYK_.3	#N/A	3.9156	3.8998	4.4842	4.1734	3.7657	3.3778	4.2438	4.4433	3.6277	4.3063	0.9473	-0.0782	0.3847	0.4148
Dnm1	_LDLM[Oxidation (M)]DEGTDARDVLENK_.3	#N/A	5.0221	5.4194	6.0067	5.6680	5.0030	5.5909	4.6166	5.1209	5.5356	5.0705	1.0719	0.1002	0.1792	0.7468
Dnm1	_ALLQM[Oxidation (M)]VQQFAVDFEK_.2	#N/A	5.2672	4.7778	4.9374	4.8722	4.7313	4.9000	5.4632	5.3704	4.0742	4.2044	1.0285	0.0406	0.6701	0.1738
Dnm1	_HIFALFNTEQR_.2	#N/A	7.1125	6.6670	7.4962	7.3698	6.5474	6.8073	7.0522	6.9231	7.1414	6.6968	1.0067	0.0096	0.8311	0.0804
Dnm1	_RIEGSGDQIDTYELSGGAR_.3	#N/A	4.6502	4.9979	5.1381	5.4180	4.5732	4.4006	4.7597	5.0529	4.3341	4.7691	1.0283	0.0403	0.5747	0.2406
Dnm1	_QLELAC[Carbamidomethyl (C)]ETQEEVDSWK_.2	#N/A	3.2824	4.5679	4.5169	8.5493	3.9269	4.1985	4.2089	7.4491	3.2396	3.7379	1.0389	0.0551	0.8852	0.0529
Dnm1	_RPLVLQLVNSTTEYAEFLHC[Carbamidomethyl (C)]K_.3	#N/A	4.0445	3.2979	3.7782	3.6432	3.1192	3.1094	3.4702	3.6792	3.9783	2.9992	0.9907	-0.0135	0.8999	0.0458
Dnm1	_EALSIIGDINTTTVSTPM[Oxidation (M)]PPVDDSWLQVQSVPAGR_.4	#N/A	4.0518	3.3562	3.2378	3.2636	3.1775	3.0511	3.4119	4.3780	2.7729	3.7075	0.9408	-0.0880	0.5275	0.2778
Dnm1	_EFIFSELLANLYSC[Carbamidomethyl (C)]GDQNTLM[Oxidation (M)]EESAEQAQR_.3	#N/A	4.1780	3.1644	3.9117	3.4555	2.9857	3.2430	3.6038	3.5457	3.8447	3.1328	0.9881	-0.0173	0.8774	0.0568
Dnm1	_ASETEENGSDSFMHSMDPQLER_.3	#N/A	3.7449	3.5975	3.4786	4.5232	2.8886	4.3107	3.1706	3.9788	4.2779	4.2005	0.9617	-0.0564	0.6904	0.1609
Tjp1	_ERS[Phospho (STY)]LS[Phospho (STY)]PR_.2	P39447_S166_M2_Tjp1	9.6412	9.6951	10.0439	10.1150	9.4127	9.7213	9.9611	9.6007	9.7158	9.7837	1.0006	0.0009	0.9673	0.0144
Tjp1	_AVPVS[Phospho (STY)]PSAVEEDEDEDGHTVVATAR_.3	P39447_S1614_M1_Tjp1	9.0154	9.2447	3.6298	10.0036	3.5695	9.3899	4.5208	4.1295	9.2152	9.0883	1.1094	0.1498	0.7075	0.1503
Tjp1	_EIS[Phospho (STY)]QDSLAAR_.2	P39447_S212_M1_Tjp1	3.0267	4.1994	3.7135	4.2162	6.1263	4.0377	3.9309	3.9023	3.5509	4.1832	1.0843	0.1168	0.5586	0.2529
Tjp1	_S[Phospho (STY)]VAS[Phospho (STY)]SQPAKPTK_.2	P39447_S175_M2_Tjp1	8.1916	8.3883	8.4531	8.9352	7.6394	8.3234	8.6468	7.7773	8.3801	8.3644	1.0036	0.0052	0.9115	0.0402
Tjp1	_ADGAT[Phospho (STY)]SDDLDLHDDR_.2	P39447_T809_M1_Tjp1	3.6118	3.7418	4.4569	5.4898	3.5632	3.5267	3.8875	3.2974	3.5965	3.4165	1.1452	0.1957	0.2444	0.6119
Tjp1	_KS[Phospho (STY)]REDLSAQPVQTK_.3	#N/A	4.0165	4.2512	6.7609	3.6429	3.1602	5.5155	3.3576	5.5342	5.6068	5.2559	0.9229	-0.1157	0.6477	0.1886
Tjp1	_LSYLSAPGSEYSM[Oxidation (M)]YSTDS[Phospho (STY)]R_.2	P39447_S837_M1_Tjp1	4.1061	3.3616	3.8398	3.5532	4.6792	3.3747	3.2679	3.6176	4.2139	3.0609	1.0788	0.1095	0.4194	0.3774
Tjp1	_DDISEIQSLAS[Phospho (STY)]DHS[Phospho (STY)]GR_.2	P39447_S297_M2_Tjp1	3.5518	4.3570	4.2386	6.0216	4.6019	3.5512	3.9120	3.9015	3.5365	3.2074	1.2054	0.2696	0.1601	0.7955
Tjp1	_SAS[Phospho (STY)]LENKK_.2	P39447_S1277_M1_Tjp1	3.6172	3.3235	4.5894	3.5912	3.2118	3.4128	3.3059	3.0264	4.1758	3.5582	1.0306	0.0435	0.7446	0.1281
Tjp1	_S[Phospho (STY)]REDLSAQPVQTK_.2	#N/A	8.3719	8.5474	8.7753	8.9764	8.0734	8.4462	8.7748	8.3803	8.3475	8.6347	0.9997	-0.0004	0.9890	0.0048
Tjp1	_IDS[Phospho (STY)]PGLKPASQQK_.2	#N/A	5.0943	5.8475	3.5239	5.6649	5.3347	5.5652	6.1692	5.3006	5.4624	4.9448	0.9655	-0.0507	0.7269	0.1385
Tjp1	_ATLLNVPDLSDSIHS[Phospho (STY)]ANAS[Phospho (STY)]ER_.3	P39447_S284_M2_Tjp1	3.3086	4.1138	3.9954	3.8045	3.9351	3.8250	4.3946	3.9080	3.0705	3.7980	1.0099	0.0142	0.8882	0.0515
Tjp1	_VQIPVSHPDPEPVS[Phospho (STY)]DNEDDSYDEEVHDPR_.4	P39447_S125_M1_Tjp1	3.2160	3.8837	4.0404	9.2403	6.6550	4.2808	4.2946	8.8192	4.8486	3.5532	0.9703	-0.0434	0.9176	0.0373
Tjp1	_KVQIPVSHPDPEPVS[Phospho (STY)]DNEDDSY[Phospho (STY)]DEEVHDPR_.4	#N/A	3.3392	4.0955	4.5737	4.3202	3.9838	4.1417	4.0348	4.3845	3.4469	3.7948	1.0410	0.0579	0.5594	0.2523
Tjp1	_QAS[Phospho (STY)]RDLEQPSYR_.2	P39447_S1051_M1_Tjp1	3.1961	4.1463	4.4306	3.9744	3.9677	6.3150	4.1226	3.0268	3.3259	5.7664	1.0684	0.0955	0.7109	0.1482
Fyn	_DGS[Phospho (STY)]LNQSSGYR_.2	#N/A	10.1431	9.7928	10.1043	10.4043	9.0640	9.7958	10.0167	10.0722	9.9415	9.9970	0.9877	-0.0178	0.6192	0.2082
Fyn	_GAY[Phospho (STY)]SLSIR_.2	P39688_Y185_M1_Fyn	5.4231	5.2505	4.1302	5.2767	4.7317	5.7758	4.0204	3.1290	3.4281	5.5723	1.2627	0.3365	0.0769	1.1138
Fyn	_WTAPEAALYGR_.2	#N/A	5.2654	5.8636	4.3614	3.6475	5.4193	5.3069	5.6494	5.9030	5.4430	5.2151	0.8964	-0.1578	0.2194	0.6588
Fyn	_LT[Phospho (STY)]EERDGSLNQSSGYR_.3	P39688_T15_M1_Fyn	4.0113	3.7237	5.1750	3.5719	4.7538	4.0669	4.5011	3.6610	5.0398	4.4441	0.9559	-0.0650	0.6302	0.2005
Cap1	_ALLATASQC[Carbamidomethyl (C)]QQPAGNK_.2	#N/A	7.2367	7.8820	7.2202	8.0089	7.7457	7.3921	7.6906	7.4566	7.6042	7.7823	0.9931	-0.0100	0.7815	0.1071
Cap1	_NSLDC[Carbamidomethyl (C)]EIVSAK_.2	#N/A	7.2180	6.8692	7.2997	7.6872	7.2209	7.1426	7.2558	7.0245	7.1892	7.4010	1.0030	0.0044	0.8861	0.0525
Cap1	_VPTISINK_.2	#N/A	7.1498	7.0466	7.7841	7.3283	7.5684	7.5134	6.8118	7.5083	7.6086	7.5406	1.0042	0.0061	0.8825	0.0543
Cap1	_KEPALLELEGKK_.3	#N/A	5.7464	5.2158	5.6413	5.7865	6.1528	6.4270	6.3213	5.9604	5.9304	5.9855	0.9635	-0.0537	0.3570	0.4473
Cap1	_TDGC[Carbamidomethyl (C)]HAYLSK_.2	#N/A	5.3372	5.0547	5.8582	5.7535	5.7410	5.6002	5.5767	5.1574	5.6603	4.9350	1.0422	0.0597	0.3081	0.5113
Cap1	_INSITVDNC[Carbamidomethyl (C)]KK_.2	#N/A	5.6894	5.0253	3.4947	5.6549	6.0651	4.8595	5.0275	5.5642	4.9169	5.2754	0.9876	-0.0180	0.8969	0.0472
Cap1	_SALFAQINQGESITHALK_.2	#N/A	5.1716	5.3829	5.7750	5.2414	4.1224	3.9603	4.8027	4.8397	4.4999	5.1112	1.0268	0.0381	0.7467	0.1268
Cap1	_LSDLLAPISEQIQEVITFR_.2	#N/A	4.6103	4.7647	4.7804	5.4936	3.5701	4.5039	5.6237	5.3804	4.9483	4.0549	0.9238	-0.1144	0.3884	0.4108
Cap1	_TLWNGQK_.2	#N/A	3.6541	4.1229	3.9673	2.5699	4.5689	3.2472	3.1723	4.5888	4.1472	3.8993	0.9333	-0.0995	0.5567	0.2544
Cap1	_[Acetyl (Protein N-term)]ADMQNLVER_.2	#N/A	6.0884	4.7200	3.8871	6.8841	5.5667	3.3274	3.2206	3.1117	4.2612	4.9776	1.3047	0.3837	0.1640	0.7851
Cap1	_LEAVSHTSDM[Oxidation (M)]HC[Carbamidomethyl (C)]GYGDS[Phospho (STY)]PSK_.3	#N/A	6.2405	6.0235	5.8955	5.2605	5.9021	5.8688	6.1411	5.8303	5.9255	6.0185	0.9810	-0.0277	0.5336	0.2728
Cap1	_C[Carbamidomethyl (C)]VNTTLQIK_.2	#N/A	3.1514	3.9731	4.3860	4.7776	4.2892	4.3294	3.8205	3.6106	3.2592	3.6070	1.1614	0.2158	0.0879	1.0559
Cap1	_VENQENVSNLVIDDTELK_.2	#N/A	4.1466	3.4020	7.3516	7.3725	3.2903	7.0387	6.8909	6.8542	6.6299	3.1607	0.9235	-0.1149	0.7294	0.1371
Cap1	_EM[Oxidation (M)]NDAAM[Oxidation (M)]FYTNR_.2	#N/A	5.3564	5.8291	5.4664	5.0340	5.7872	6.0525	5.9181	5.9278	5.7655	5.8887	0.9511	-0.0724	0.1722	0.7638
Cap1	_PQTS[Phospho (STY)]PSPKPATK_.2	P40124_S307_M1_Cap1	3.0671	7.8960	8.1195	8.7870	7.5731	8.4062	8.4560	8.2820	3.4548	8.3961	1.0225	0.0321	0.9147	0.0387
Cap1	_EFHTTGLAWSK_.3	#N/A	2.0219	3.9555	4.1956	4.4601	3.8972	4.2816	4.1748	3.6584	1.7412	4.4271	1.0862	0.1192	0.6615	0.1795
Tkt	_VLDPFTIKPLDR_.2	#N/A	6.7568	6.8703	7.0803	7.2346	7.1311	6.8313	7.2050	6.9173	6.8044	7.0992	0.9968	-0.0046	0.8569	0.0671
Tkt	_LAVSQVPR_.2	#N/A	7.4489	7.8878	6.8975	7.5692	8.1465	7.7171	6.3446	7.9365	7.9860	7.9893	1.0062	0.0090	0.9065	0.0426

Tkt	_LDNLVAIFDINR_.2	#N/A	7.2699	6.9448	7.6946	7.5036	7.8628	7.9321	8.0040	7.7378	7.6457	7.7852	0.9668	-0.0487	0.2353	0.6283
Tkt	_LGQSDPAPLQHQVDIYQK_.3	#N/A	6.8206	6.6261	7.6164	7.4699	7.0853	6.8096	7.0320	6.9671	6.7784	7.1527	1.0127	0.0182	0.6858	0.1638
Tkt	_HQPTAIIAK_.2	#N/A	6.6872	7.2212	6.6439	6.5602	7.3002	6.8819	6.7145	6.5880	7.0352	7.1323	1.0022	0.0031	0.9392	0.0272
Tkt	_IIALDGDTK_.2	#N/A	6.6317	6.7666	6.2612	6.4620	6.9212	6.7302	5.5532	6.2330	6.8564	6.4690	1.0559	0.0785	0.1940	0.7123
Tkt	_NSTFSELFK_.2	#N/A	6.5485	6.2586	6.2541	6.7656	6.2455	6.2463	5.5546	6.2019	6.0110	6.3992	1.0571	0.0801	0.0946	1.0241
Tkt	_NM[Oxidation (M)]AEQIIQEIYSQVQSK_.2	#N/A	5.8653	5.2791	4.4817	6.1746	5.6787	6.0396	5.5240	5.8400	5.7493	5.7682	0.9766	-0.0342	0.6898	0.1613
Tkt	_ILATPPQEDAPSVDIANIR_.2	#N/A	7.3606	7.6322	7.8290	8.1646	7.7278	8.0536	7.7530	7.6102	7.6057	7.5281	1.0224	0.0319	0.2984	0.5253
Tkt	_TVPFC[Carbamidomethyl (C)]STFAAFFTR_.2	#N/A	3.6244	5.9792	7.2217	6.6060	6.1361	5.7773	6.4419	5.4120	6.1562	5.9544	0.9833	-0.0244	0.8808	0.0551
Tkt	_SVPM[Oxidation (M)]STVFYPSDGVATEK_.2	#N/A	8.2156	8.3738	8.2032	8.4931	8.1275	8.4147	8.2217	7.7724	7.7726	8.0683	1.0435	0.0614	0.0167	1.7783
Tkt	_M[Oxidation (M)]PTPPSYK_.2	#N/A	4.7136	5.6817	3.8175	5.0908	5.8402	5.0787	5.0381	5.3958	4.4893	5.7191	0.9761	-0.0349	0.7794	0.1082
Tkt	_TSRPENAIIYSNNEDFQVGQAK_.3	#N/A	5.2019	4.7870	5.2658	6.1282	4.9251	4.8764	4.8134	5.1373	5.7208	4.8435	1.0134	0.0192	0.8258	0.0831
Tkt	_C[Carbamidomethyl (C)]EAFGWHTIIVDGHSVEELC[Carbamidomethyl (C)]K_.3	#N/A	3.5333	3.8091	4.2538	4.4698	4.1778	3.9476	4.4598	3.6248	2.9887	2.8861	1.1553	0.2083	0.1433	0.8438
Tkt	_ISSDLDGHPVPK_.3	#N/A	2.9357	1.0214	3.6225	4.1252	2.0148	4.1672	3.8399	3.9933	3.6419	4.0922	0.7660	-0.3846	0.1965	0.7067
Tkt	_ILTVEDHYYEGGIGEAVSAAVVGEPGVTVTR_.3	#N/A	3.0009	1.6861	4.2354	3.4813	3.6455	2.9791	4.3731	3.2220	3.5210	3.4565	0.8705	-0.2001	0.3576	0.4466
Tkt	_VYC[Carbamidomethyl (C)]M[Oxidation (M)]LGDGEVSEGSVWEAM[Oxidation (M)]AFAGIYK_.3	#N/A	2.9119	3.6681	4.1464	3.6902	3.5564	3.0682	3.8384	3.3110	3.6101	3.3674	0.9930	-0.0102	0.9230	0.0348
Vps26a	_FES[Phospho (STY)]PDSQASAEQPEM_.2	P40336_S315_M1_Vps26a	3.4510	4.2073	3.1847	9.5565	4.0956	4.0298	3.9230	6.4823	3.0709	3.9066	1.0940	0.1296	0.7701	0.1135
Syt4	_LFTETEKEANS[Phospho (STY)]PESLK_.2	P40749_S135_M1_Syt4	3.8416	3.1459	6.5699	6.2916	4.8289	5.6015	7.0325	6.3799	6.0901	5.6621	0.8022	-0.3180	0.1274	0.8947
Rpl28	_QTYSTEPNNLK_.2	#N/A	5.4545	5.8995	4.7847	5.3467	5.7934	5.3725	5.2104	5.5854	4.9030	5.1315	1.0450	0.0635	0.3392	0.4696
Rpl28	_[Acetyl (Protein N-term)]SAHLQWMVVR_.2	#N/A	4.1305	3.5565	4.1873	4.0283	3.2742	3.8560	4.4030	3.6385	3.8950	4.5794	0.9297	-0.1051	0.2819	0.5499
Abca2	_VSEEDQS[Phospho (STY)]LENS[Phospho (STY)]EADVK_.2	#N/A	6.2352	5.7659	6.4390	5.7957	5.1757	5.8258	6.8174	6.4750	6.0744	5.8164	0.9328	-0.1003	0.1738	0.7600
Abca2	_QPAEPGTSQEPGLASS[Phospho (STY)]PSGC[Carbamidomethyl (C)]PR_.2	P41234_S1238_M1_Abca2	3.5094	5.0896	3.2431	4.2877	3.3519	4.0752	4.4360	5.4409	3.0125	3.9650	0.9318	-0.1019	0.6070	0.2168
Abca2	_VSEEDQS[Phospho (STY)]LENS[Phospho (STY)]EADVKESR_.2	#N/A	4.2308	3.6650	4.2583	4.5258	3.6568	4.1578	5.2210	5.0257	4.1561	3.8543	0.8944	-0.1610	0.1666	0.7783
Tac1	_DADSS[Phospho (STY)]VEKQVALLK_.2	P41539_S76_M1_Tac1	4.1095	6.0697	4.5480	6.8088	6.8973	6.6445	6.4176	3.0678	4.2172	6.3055	1.1688	0.2250	0.3746	0.4264
Eci1	_EADIQNFTSFISK_.2	#N/A	3.7494	3.0049	3.4073	3.9099	3.6369	3.7320	4.2809	3.5163	3.7975	3.4868	0.9478	-0.0774	0.3971	0.4011
Foxk1	_QS[Phospho (STY)]PGPALAR_.2	P42128_S87_M1_Foxk1	8.1871	8.0479	8.2226	8.8029	7.9613	8.1759	8.5391	8.0255	8.2195	8.4185	0.9918	-0.0118	0.7098	0.1489
Foxk1	_EGS[Phospho (STY)]PIPHDPDLGSK_.2	#N/A	8.2976	8.2019	8.7701	8.8691	7.6815	8.2484	8.6519	8.3196	8.2988	8.3559	0.9926	-0.0106	0.7944	0.1000
Foxk1	_S[Phospho (STY)]APASPT[Phospho (STY)]HPGLM[Oxidation (M)]S[Phospho (STY)]PR_.2	P42128_T408_M3_Foxk1	3.3371	3.8213	3.9271	8.0800	4.0546	3.7200	4.0808	3.7120	3.4128	3.5360	1.2183	0.2849	0.4040	0.3936
Septin2	_LTVVDTPGYGDAINC[Carbamidomethyl (C)]R_.2	#N/A	5.4233	5.3469	3.1857	6.1407	5.3787	5.1121	6.0591	5.2538	5.5500	4.3837	0.9598	-0.0593	0.7223	0.1413
Septin2	_TVQIEASTVEIEER_.2	#N/A	7.0543	7.6812	8.6094	8.3164	6.7956	7.2982	7.4292	6.6923	6.7598	6.9882	1.0945	0.1303	0.1288	0.8901
Septin2	_TIISYIDEQFER_.2	#N/A	4.7062	4.7003	5.1036	4.1058	4.9801	3.5274	4.3530	5.1742	4.9104	4.4961	0.9550	-0.0664	0.5472	0.2618
Septin2	_IYHLPDAES[Phospho (STY)]DEDEDFKEQTR_.3	#N/A	8.1596	9.6779	8.6920	11.8079	10.3699	9.8183	8.9796	10.8948	10.7984	10.5734	0.9460	-0.0802	0.4767	0.3218
Septin2	_STLINSLFLTDLYPER_.2	#N/A	3.7074	4.3151	4.3482	3.5983	4.4733	4.1862	3.3130	2.7501	3.5426	4.7587	1.1430	0.1929	0.2163	0.6649
Septin2	_IYHLPDAES[Phospho (STY)]DEDEDFK_.2	#N/A	4.1251	3.9454	3.3110	3.7240	2.9337	3.5321	3.6271	3.8898	3.8419	3.5826	0.9625	-0.0552	0.5586	0.2529
Septin2	_QQPTQFINPETPGYVGFANLPNQVHR_.3	#N/A	3.1456	4.9895	4.3801	3.9239	3.7902	4.3353	5.3005	3.8407	4.1153	3.6012	0.9714	-0.0418	0.7901	0.1023
Eps15	_AEITSQESQISSYEEELLK_.2	#N/A	5.0618	5.5773	5.9443	6.7129	4.7664	4.9471	6.0399	5.0360	4.1305	5.3181	1.0722	0.1006	0.4699	0.3280
Eps15	_NITGSS[Phospho (STY)]PVADFSAIK_.2	P42567_S324_M1_Eps15	7.3650	3.0752	7.4619	8.2303	6.6061	3.6611	7.7638	7.1851	7.1941	7.2166	0.8265	-0.2749	0.2838	0.5470
Pura	_VSEVKPTYR_.2	#N/A	8.7068	8.9475	8.8804	9.2759	9.2795	8.8825	8.6436	9.0401	9.0560	8.8932	1.0098	0.0141	0.5527	0.2575
Pura	_IAEVGAGGNK_.2	#N/A	8.0550	8.5163	8.8309	8.2889	8.8378	8.5533	7.9897	8.4796	8.6136	8.2475	1.0217	0.0310	0.3686	0.4335
Pura	_FFFDVGSNK_.2	#N/A	7.8839	8.0362	7.9523	8.0477	7.9192	7.8912	8.1251	7.9463	7.8911	7.8503	1.0002	0.0003	0.9759	0.0106
Pura	_LIDDYGVEEEPAELPEGTSLTVDNKR_.3	#N/A	7.1435	7.0550	6.7796	7.9603	7.3800	7.5876	7.0544	6.9684	7.1952	6.9206	1.0402	0.0569	0.2326	0.6334
Pura	_GPGLGSTQGQTIALPAQGLIEFR_.3	#N/A	5.0786	5.1927	5.5878	5.7084	5.5579	5.8919	5.1680	5.3009	5.4340	5.3720	1.0346	0.0491	0.2953	0.5298
Pura	_VDIQNKR_.2	#N/A	6.0697	5.5561	5.6547	5.4083	6.4190	5.7266	5.1646	5.9254	6.3964	5.2741	1.0203	0.0290	0.7077	0.1502
Pura	_NSITVPYK_.2	#N/A	4.7263	5.2152	4.8567	4.7356	4.8951	5.1174	4.2872	5.3830	5.4459	5.3697	0.9615	-0.0566	0.4408	0.3557
Pura	_YSEEMKK_.2	#N/A	3.2754	4.0806	3.9622	5.9084	4.1011	3.8276	4.1795	3.6536	3.4160	3.5692	1.1317	0.1785	0.3339	0.4764
Htt	_RHS[Phospho (STY)]LSC[Carbamidomethyl (C)]TK_.2	P42859_S1841_M1_Htt	4.1365	4.8129	3.6352	3.1790	3.2622	1.5103	4.8149	5.0701	4.1213	4.0989	0.7562	-0.4032	0.1041	0.9827
Htt	_S[Phospho (STY)]GEEEDSGSAAQLGMC[Carbamidomethyl (C)]NR_.2	#N/A	3.6081	4.4133	4.2949	3.2967	4.2346	3.4949	3.8557	3.3210	2.9695	3.2637	1.1605	0.2147	0.0952	1.0214
Htt	_DEVAEAS[Phospho (STY)]DPESKPC[Carbamidomethyl (C)]R_.2	P42859_S637_M1_Htt	3.9207	3.4217	3.6544	3.1982	3.0644	3.5602	3.3464	3.8030	4.1021	4.3763	0.8881	-0.1711	0.1012	0.9949
Htt	_SGS[Phospho (STY)]IVELLAGGGSS[Phospho (STY)]C[Carbamidomethyl (C)]SPVLSR_.2	P42859_S409_M2_Htt	3.7502	3.5890	4.1811	3.9444	3.4287	3.5110	3.7379	4.8460	3.8038	3.4876	0.9408	-0.0880	0.4238	0.3729
Htt	_SLNPQKS[Phospho (STY)]GEEEDSGSAAQLGM[Oxidation (M)]C[Carbamidomethyl (C)]NR_.3	#N/A	7.2634	6.8405	7.8858	7.0446	5.3542	3.9598	7.1806	7.7315	7.5817	7.1670	0.8619	-0.2144	0.2104	0.6769
Oprm1	_EFC[Carbamidomethyl (C)]IPTSSTIEQQNS[Phospho (STY)]AR_.2	P42866_S363_M1_Oprm1	8.3693	7.7680	8.0461	8.2156	7.0970	7.9724	8.1611	8.5400	8.1390	8.1224	0.9600	-0.0588	0.2114	0.6748
Cct8	_KDWDDDQND_.2	#N/A	6.9889	7.2539	6.6572	7.0077	6.6300	4.9160	7.2998	7.0701	7.3516	7.0470	0.9143	-0.1293	0.1953	0.7092
Cct8	_DVDEVSSLLR_.2	#N/A	6.9883	6.8440	7.0551	7.0401	6.4356	6.6235	6.9136	6.5048	6.8037	6.5475	1.0207	0.0296	0.3842	0.4155
Cct8	_FAEAFEAIPR_.2	#N/A	6.0056	6.4499	5.7694	6.2174	6.0033	6.1323	6.9610	6.2793	6.4006	6.1791	0.9444	-0.0825	0.0832	1.0800
Cct8	_LATNAAVTVLR_.2	#N/A	5.2409	5.8256	5.6906	6.1153	5.7443	5.5966	6.1782	6.0969	5.9369	5.8748	0.9469	-0.0786	0.0756	1.1214
Cct8	_AVDDGVNTFK_.2	#N/A	5.6421	6.3046	6.3764	6.0755	5.4216	6.1101	5.0768	6.1307	6.0162	5.6660	1.0465	0.0655	0.3514	0.4541
Cct8	_LFVTNDAATILR_.2	#N/A	5.8896	6.3104	6.4205	5.7977	5.6161	5.8651	5.7738	5.8958	6.4837	5.5975	1.0077	0.0110	0.8415	0.0749
Cct8	_LYSVHQEGNK_.2	#N/A	5.0821	5.8081	5.8616	6.0340	5.6590	5.6717	5.7186	5.9531	5.7031	5.7790	0.9823	-0.0257	0.5695	0.2445
Cct8	_EDGAISTIVLR_.2	#N/A	4.9194	4.7526	5.5106	5.9565	5.1469	5.5830	5.8372	5.5277	5.0520	3.0612	1.0908	0.1253	0.4406	0.3560
Cct8	_AIAGTGANVIVTGGK_.2	#N/A	3.3816	3.4599	3.1152	4.3378	4.2006	3.9905	3.9925	4.5197	4.0763	3.0821	0.9566	-0.0640	0.6378	0.1953
Cct8	_HEKEDGAISTIVLR_.3	#N/A	3.9519	3.3822	3.2638	3.2377	4.6879	2.9124	3.3859	4.6634	3.9133	3.7334	0.9105	-0.1353	0.3954	0.4029
Cct8	_GSTDNLM[Oxidation (M)]DDIER_.2	#N/A	4.9123	5.3403	5.4126	5.5702	5.3496	5.0272	5.4749	5.3218	5.1242	4.0496	1.0553	0.0777	0.3605	0.4431
Cct8	_QYGSETFLAK_.2	#N/A	4.2907	5.8652	3.4766	3.9793	5.6157	4.3131	3.6940	4.1392	5.5882	5.0823	0.9923	-0.0112	0.9531	0.0209
Cct8	_HFSGLEEAVYR_.2	#N/A	4.0178	3.2084	3.2037	3.7064	3.0967	3.5279	3.4211	4.3905	4.0499	4.8263	0.8294	-0.2699	0.0433	1.3632
Slc1a2	_SADC[Carbamidomethyl (C)]S[Phospho (STY)]VEEEPWK_.2	#N/A	6.3161	6.8016	3.7722	7.1423	5.8007	6.8399	8.0520	7.3620	7.3955	3.7391	0.9209	-0.1189	0.6136	0.2121
Slc1a2	_NLFPENLVQAC[Carbamidomethyl (C)]FQQIQTVTK_.2	#N/A	6.3666	5.9701	5.9030	6.5343	5.3196	5.5470	6.2490	6.4686	6.2314	6.5126	0.9332	-0.0998	0.1191	0.9240
Slc1a2	_KNDEVSSLDAFLDLIR_.3	#N/A	5.9357	5.6362	5.7870	5.8394	4.9568	5.5234	5.8131	6.1391	6.0105	5.8868	0.9414	-0.0871	0.1019	0.9919
Slc1a2	_[Acetyl (Protein N-term)]ASTEGANNM[Oxidation (M)]PK_.2	#N/A	8.5670	7.4631	7.6117	8.2957	6.9376	7.0014	8.0096	8.0322	7.4882	7.5208	0.9850	-0.0218	0.7544	0.1224
Slc1a2	_TSVNVVGDSFGAGIVYHLSK_.2	#N/A	6.0960	6.0107	6.8310	6.9596	4.8804	5.7528	7.0565	7.1584	6.6586	6.5364	0.8885	-0.1706	0.0965	1.0156
Slc1a2	_SELDTIDSQHR_.2	#N/A	9.6716	9.5896	9.7483	9.4552	9.3711	9.3961	9.8075	9.8558	9.5881	9.4760	0.9852	-0.0215	0.2148	0.6680
Slc1a2	_C[Carbamidomethyl (C)]LEDNLGIDKR_.2	#N/A	7.6353	7.3156	7.4369	7.1993	7.4774	7.2745	7.7886	7.6497	7.6555	7.4099	0.9690	-0.0454	0.0493	1.3067
Slc1a2	_SADC[Carbamidomethyl (C)]S[Phospho (STY)]VEEEPWKR_.2	#N/A	8.8700	8.8782	9.1614	9.1525	8.1137	8.6590	9.4071	9.2450	8.9674	8.5816	0.9730	-0.0395	0.3467	0.4600
Slc1a2	_M[Oxidation (M)]QEDIEMTK_.2	#N/A	6.9894	5.6122	3.6669	7.8166	6.4055	3.4696	5.8281	5.5076	5.3626	6.0172	0.9967	-0.0048	0.9841	0.0070
Slc1a2	_TQSIYDDKNHR_.2	#N/A	5.2045	4.4468	5.1261	4.5824	5.1919	4.8325	5.0088	5.4831	5.0495	4.4478	0.9800	-0.0291	0.6846	0.1645
Slc1a2	_M[Oxidation (M)]HDSHLS[Phospho (STY)]SDEPK_.2	#N/A	6.2029	7.5143	8.0436	7.0444	6.4254	7.6112	7.9832	8.2393	6.7533	6.6883	0.9628	-0.0547	0.5865	0.2317
Slc1a2	_ESNS[Phospho (STY)]NQC[Carbamidomethyl (C)]VYAAHNSVVIDEC[Carbamidomethyl (C)]K_.3	#N/A	7.0874	6.6460	7.1910	8.0667	4.1518	6.0559	7.1042	7.7649	6.9019	6.6909	0.9182	-0.1232	0.4352	0.3613
Slc1a2	_NDEVSSLDAFLDLIR_.2	#N/A	7.4549	7.3698	8.0171	7.3690	6.9681	7.3856	7.8176	7.6295	7.7663	7.1772	0.9776	-0.0327	0.4347	0.3618
Slc1a2	_TQSIYDDK_.2	#N/A	7.3635	6.6426	7.0540	6.7502	6.9533	6.7866	6.6279	7.5719	7.1899	6.5306	0.9921	-0.0114	0.8206	0.0859
Slc1a2	_M[Oxidation (M)]HDSHLS[Phospho (STY)]S[Phospho (STY)]DEPKHR_.3	#N/A	5.0085	5.7185	6.8855	5.0685	4.3318	5.5804	5.9617	6.1723	6.4507	5.1346	0.9161	-0.1264	0.3441	0.4633
Slc1a2	_DLEVVAR_.2	#N/A	3.9663	3.9309	4.4091	3.5036	3.8192	4.3063	4.1012	4.1105	3.3473	4.4137	0.9990	-0.0014	0.9875	0.0055

Slc1a2	_NHRESNS[Phospho (STY)]NQC[Carbamidomethyl (C)]VYAAHNSVVIDEC[Carbamidomethyl (C)]K_.4	#N/A	3.8264	4.3577	3.9311	3.9158	3.5652	3.4794	3.8452	3.7141	3.6273	3.7574	1.0294	0.0418	0.5198	0.2842
Slc1a2	…SIY[Phospho (STY)]DDKNHRESNSNQC[Carbamidomethyl (C)]VYAAHNSVVIDEC[Carbamidomethyl (C)]K	P43006_Y522_M1_Slc1a2	8.9370	4.3925	10.3827	3.7282	3.5944	3.5156	3.8764	3.2730	3.5721	3.4054	1.6305	0.7053	0.1941	0.7119
Cox6a1	_HEEHERPPFVAYPHLR_.4	#N/A	7.9443	8.7959	8.1828	7.7290	8.0476	8.8752	8.8878	7.6412	8.2594	8.9070	0.9808	-0.0279	0.6453	0.1903
Cox6a1	_ALTYFVALPGVGVSM[Oxidation (M)]LNVFLK_.2	#N/A	4.0617	3.3171	3.7953	3.5977	3.2054	3.4192	3.3124	3.0199	4.1694	3.5647	1.0141	0.0202	0.8499	0.0706
H1-4	_[Acetyl (Protein N-term)]S[Phospho (STY)]ETAPAAPAAPAPAEK_.2	#N/A	13.1401	13.3665	14.3193	13.6442	13.2897	13.2790	14.0105	13.4226	11.4903	13.3832	1.0329	0.0467	0.4023	0.3955
H1-4	_TSGPPVSELITK_.2	#N/A	8.7289	8.8263	9.3149	9.8822	9.1124	8.7221	9.1656	8.9328	8.6147	8.5964	1.0136	0.0195	0.4772	0.3213
H1-4	_[Acetyl (Protein N-term)]S[Phospho (STY)]ETAPAAPAAPAPAEKTPVK_.2	#N/A	6.5423	7.4861	8.8952	7.4891	7.2109	5.5900	7.8903	7.5611	7.0274	6.8734	0.9815	-0.0269	0.8244	0.0839
H1-5	_[Acetyl (Protein N-term)]S[Phospho (STY)]ETAPAETAAPAPVEK_.2	#N/A	6.0631	11.2620	11.5447	11.5570	11.1706	11.2203	12.0314	11.5326	11.2090	11.2737	0.9095	-0.1369	0.3772	0.4234
H1-5	_[Acetyl (Protein N-term)]SETAPAETAAPAPVEKS[Phospho (STY)]PAKK_.3	#N/A	7.1822	7.2807	8.4873	7.9271	7.3976	6.9729	8.0639	7.6761	7.4682	7.1185	0.9947	-0.0077	0.9048	0.0434
H1-5	_ALAAGGYDVEK_.2	#N/A	4.2010	4.9849	5.0075	4.4700	4.9487	4.2958	4.7724	3.8323	4.0525	4.5654	1.0803	0.1114	0.2145	0.6685
H1-5	_[Acetyl (Protein N-term)]SETAPAETAAPAPVEKS[Phospho (STY)]PAK_.2	#N/A	7.9948	8.2776	9.5822	8.7910	8.1187	8.2045	9.0071	8.5193	8.2418	8.0506	1.0047	0.0068	0.9108	0.0406
H1-3	_[Acetyl (Protein N-term)]S[Phospho (STY)]ETAPAAPAAPAPVEK_.2	P43277_S2_M1_H1-3	12.1714	12.1815	13.0636	12.6091	12.2262	12.2714	12.9852	12.5697	12.2634	12.3272	0.9908	-0.0134	0.6165	0.2101
H1-3	_[Acetyl (Protein N-term)]SETAPAAPAAPAPVEKT[Phospho (STY)]PVK_.2	P43277_T18_M1_H1-3	4.4030	4.5684	6.5417	4.9061	4.6582	4.5610	5.3643	5.1030	4.0506	4.4294	1.0428	0.0605	0.6797	0.1677
Dck	_FC[Carbamidomethyl (C)]PS[Phospho (STY)]PSTSSEGTR_.2	P43346_S11_M1_Dck	8.7865	3.8266	3.2495	9.1754	8.6751	9.0774	8.8058	8.5926	8.7196	3.9714	0.9481	-0.0769	0.8247	0.0837
Itgav	_EQLQPHENGEGNS[Phospho (STY)]ET_.2	P43406_S1042_M1_Itgav	3.4332	4.2384	4.1200	4.6226	4.0597	3.6698	5.4283	3.4958	3.1444	3.0888	1.0619	0.0867	0.6434	0.1915
Itgav	_YNNQLATR_.2	#N/A	3.7731	3.0774	4.4599	3.5424	2.8988	3.3299	3.6907	3.1559	3.7578	3.4287	1.0015	0.0022	0.9866	0.0059
Akr1b1	_TIGVSNFNPLQIER_.2	#N/A	6.5914	6.2490	6.2498	7.0350	6.5029	6.7911	6.7656	6.5223	6.5001	6.5130	0.9992	-0.0012	0.9748	0.0111
Akr1b1	_[Acetyl (Protein N-term)]ASHLELNNGTK_.2	#N/A	6.5434	6.6667	6.5415	6.4175	6.7845	6.2485	6.1272	6.2422	6.3222	6.4752	1.0385	0.0544	0.0623	1.2058
Akr1b1	_TTAQVLIR_.2	#N/A	6.0813	5.9245	5.4960	5.3133	6.0704	6.5046	5.4748	6.2299	6.5425	6.0444	0.9713	-0.0421	0.5547	0.2559
Akr1b1	_VAIDLGYR_.2	#N/A	6.1098	6.4398	4.2814	6.0623	6.2961	6.4762	4.9578	6.5107	6.3907	6.3465	0.9823	-0.0258	0.8400	0.0757
Akr1b1	_SPPGQVTEAVK_.2	#N/A	6.8890	6.9146	6.8757	7.4050	7.4453	7.3100	6.5493	7.0078	7.2597	7.5153	1.0080	0.0115	0.7975	0.0982
Akr1b1	_LIEYC[Carbamidomethyl (C)]HSK_.2	#N/A	5.3352	5.1510	5.1622	5.5024	6.0190	5.1996	5.2945	4.7821	4.9437	4.6214	1.0987	0.1357	0.0455	1.3416
Akr1b1	_HIDC[Carbamidomethyl (C)]AQVYQNEK_.2	#N/A	5.6291	2.9303	3.4819	3.9845	3.4216	4.3079	3.6992	5.9302	3.7825	3.9515	0.9121	-0.1328	0.5680	0.2457
Cfl2	_[Acetyl (Protein N-term)]AS[Phospho (STY)]GVTVNDEVIK_.2	P45591_S3_M1_Cfl2	9.6724	9.2934	10.0520	9.9833	9.1139	9.9874	10.0698	9.9730	9.6096	9.8557	0.9955	-0.0064	0.8589	0.0661
Cfl2	_QIIVEEAK_.2	#N/A	6.0884	6.1877	6.8177	5.5283	6.4241	6.2739	5.3476	5.8401	6.2736	5.3540	1.0905	0.1250	0.1011	0.9951
Cfl2	_QILVGDIGDTVEDPYTSFVK_.2	#N/A	4.7322	4.9085	5.2692	5.0790	4.3521	3.5024	4.3492	4.4476	4.7764	4.7151	1.0150	0.0215	0.8438	0.0738
Cfl2	_LLPLNDC[Carbamidomethyl (C)]R_.2	#N/A	7.0234	7.4293	6.9771	6.4712	7.2994	6.7577	7.1519	7.3050	6.9402	6.9411	0.9871	-0.0188	0.6462	0.1896
Cfl2	_HEWQVNGLDDIKDR_.3	#N/A	5.4454	5.6752	5.5710	5.7714	6.6711	5.8637	4.3467	5.6171	5.7570	5.4589	1.1016	0.1396	0.1501	0.8235
Fkbp2	_LVIPSELGYGER_.2	#N/A	6.7505	6.9830	6.9963	7.0673	7.3546	6.6294	6.4582	6.8920	6.6432	6.5531	1.0493	0.0694	0.0596	1.2251
Fkbp2	_IPGGATLVFEVELLK_.2	#N/A	3.0761	3.8324	4.0806	4.5832	3.7207	4.4048	4.2979	3.5352	3.1838	3.0494	1.1231	0.1675	0.2550	0.5934
Rangap1	_GSGEEPAT[Phospho (STY)]PSRK_.2	P46061_T411_M1_Rangap1	5.8958	4.9659	3.5471	3.8459	2.9572	3.6674	3.5606	3.9103	3.9212	4.2690	1.0591	0.0828	0.6918	0.1600
Syt1	_VFVGYNSTGAELR_.2	#N/A	8.9151	8.9511	9.4306	8.4743	8.0436	8.5078	9.1934	9.1976	8.8851	8.5487	0.9737	-0.0385	0.4151	0.3819
Syt1	_HDIIGEFK_.2	#N/A	8.3533	8.0684	8.3083	7.7582	7.7466	7.6922	8.4532	8.1835	7.9942	7.8232	0.9845	-0.0225	0.5169	0.2866
Syt1	_TLNPVFNEQFTFK_.2	#N/A	7.8560	7.8632	7.8116	7.0618	7.1222	7.3480	7.7839	8.1474	7.9732	7.2803	0.9634	-0.0539	0.2736	0.5630
Syt1	_VPYSELGGK_.2	#N/A	8.0557	7.7005	8.0327	7.3113	7.2742	7.7421	7.1139	8.1622	7.8790	7.9373	0.9888	-0.0162	0.7361	0.1331
Syt1	_LTVVILEAK_.2	#N/A	6.9044	6.6421	6.2362	5.7356	5.2682	6.5847	6.1410	6.5080	6.8700	6.5986	0.9539	-0.0681	0.3993	0.3987
Syt1	_M[Oxidation (M)]DVGGLSDPYVK_.2	#N/A	7.5537	7.2659	7.3398	7.8679	6.9112	6.6185	7.6162	7.2803	7.1502	6.8300	1.0056	0.0080	0.8814	0.0549
Syt1	_DDDAETGLT[Phospho (STY)]DGEEKEEPKEEEK_.3	P46096_T128_M1_Syt1	3.4609	4.2172	3.1946	4.2392	4.1055	4.0200	3.9131	4.2628	3.0610	3.9165	1.0223	0.0318	0.7862	0.1044
Syt1	_HWSDM[Oxidation (M)]LANPR_.2	#N/A	6.7025	6.3304	5.2023	6.7946	5.6855	5.5347	6.6107	6.1627	5.5773	5.2837	1.0225	0.0321	0.7541	0.1226
Syt1	_DLQSAEKEEQEK_.2	#N/A	6.8919	6.7047	6.6689	6.5378	5.5058	6.1559	6.9098	6.8688	6.7704	6.8164	0.9371	-0.0938	0.1361	0.8662
Syt1	_VFLLPDK_.2	#N/A	6.1385	6.2275	6.2673	5.7157	4.8679	4.8459	3.6074	6.1934	6.0272	5.7028	1.0547	0.0768	0.6275	0.2024
Syt1	_VQVVVTVLDYDK_.2	#N/A	6.6572	6.1615	6.3810	6.4089	5.6322	5.9525	7.1541	6.4978	3.0276	6.6012	1.0651	0.0909	0.6351	0.1972
Syt1	_TLVM[Oxidation (M)]AVYDFDR_.2	#N/A	7.9093	7.6919	8.0209	7.6014	6.8887	7.6481	8.4124	8.1842	7.8703	7.6362	0.9503	-0.0736	0.1395	0.8554
Syt1	_VPM[Oxidation (M)]NTVDFGHVTEEWR_.3	#N/A	7.8179	7.5412	7.9881	7.8887	7.0943	7.1409	8.0488	7.8359	7.5337	7.2696	0.9878	-0.0177	0.7061	0.1511
Syt1	_NTLNPYYNESFS[Phospho (STY)]FEVPFEQIQK_.3	P46096_S344_M1_Syt1	3.8341	3.1385	4.5209	3.5227	2.9598	3.2689	3.2374	3.0949	4.2443	3.4897	1.0069	0.0099	0.9472	0.0236
Cdkn1b	_VSNGS[Phospho (STY)]PSLER_.2	P46414_S10_M1_Cdkn1b	8.7280	8.8369	4.0148	9.9616	8.9832	9.0771	9.0514	8.7558	8.7510	8.0076	0.9567	-0.0639	0.7424	0.1294
Cdkn1b	_TEENVSDGS[Phospho (STY)]PNAGTVEQTPK_.2	P46414_S178_M1_Cdkn1b	6.0102	3.5286	6.5725	6.6560	5.8034	6.3652	6.6879	6.2448	6.2052	3.8602	1.0127	0.0182	0.9278	0.0325
Cdkn1b	_VLAQES[Phospho (STY)]QDVSGSR_.2	P46414_S106_M1_Cdkn1b	4.1477	3.4521	3.3337	3.8364	3.2734	6.7700	3.3160	6.5257	3.9307	3.8034	0.9412	-0.0874	0.7786	0.1087
Nsf	_GILLYGPPGC[Carbamidomethyl (C)]GK_.2	#N/A	9.8163	9.7557	10.0503	9.7654	9.6297	9.5546	9.7128	9.8532	9.7725	9.8333	0.9968	-0.0046	0.7423	0.1294
Nsf	_LLDYVPIGPR_.2	#N/A	9.6921	10.0806	9.9487	9.5752	9.4397	9.7404	9.8644	10.0919	9.9788	9.6197	0.9856	-0.0210	0.3530	0.4523
Nsf	_YVGESEANIR_.2	#N/A	9.3970	9.3720	9.4678	9.4729	9.4773	9.5059	9.3873	9.2730	9.5431	9.3103	1.0075	0.0108	0.2306	0.6371
Nsf	_VLDDGELLVQQTK_.2	#N/A	9.4470	9.3634	9.6460	9.5204	9.1307	9.3383	9.5332	9.2925	9.4734	9.2868	1.0012	0.0017	0.9161	0.0381
Nsf	_SQLSC[Carbamidomethyl (C)]VVVDDIER_.2	#N/A	9.2258	8.9965	9.3584	9.1394	8.6201	8.8809	9.4030	8.9574	9.0339	8.9464	0.9947	-0.0077	0.7697	0.1137
Nsf	_NFSGAELEGLVR_.2	#N/A	8.8628	9.0839	9.1759	8.8312	8.7767	8.7933	8.9375	8.7902	8.9227	8.8575	1.0049	0.0071	0.6389	0.1945
Nsf	_LLIGTTSR_.2	#N/A	8.6488	8.3140	8.6126	8.6708	7.4709	8.6949	8.5367	8.6003	8.4810	8.6602	0.9804	-0.0285	0.5140	0.2890
Nsf	_NIDSNPYDTDK_.2	#N/A	8.4651	8.2988	8.2381	8.6698	8.4518	8.5764	8.7445	8.3912	8.4981	8.4812	0.9908	-0.0134	0.4634	0.3340
Nsf	_KLLIGTTSR_.2	#N/A	7.5654	8.2409	8.4333	8.1293	7.9169	8.1927	8.5002	8.2283	8.3220	8.2600	0.9702	-0.0436	0.1636	0.7862
Nsf	_IAEESNFPFIK_.2	#N/A	8.4436	8.4252	8.4483	8.2854	8.2386	8.1835	8.3795	8.5222	8.3428	8.0876	1.0005	0.0008	0.9632	0.0163
Nsf	_WGDPVTR_.2	#N/A	8.0215	8.3816	7.9804	8.2286	8.0292	7.7856	7.4774	8.4029	8.0671	7.7746	1.0177	0.0254	0.4784	0.3202
Nsf	_VVNGPEILNK_.2	#N/A	7.9832	8.1588	8.1545	8.0228	8.5559	7.7730	8.2050	8.1904	8.0452	7.7650	1.0070	0.0101	0.7255	0.1393
Nsf	_AENSSLNLIGK_.2	#N/A	7.6525	7.6639	7.7935	7.3579	7.7429	7.3084	7.6766	7.5193	7.7947	7.5446	0.9938	-0.0090	0.6931	0.1592
Nsf	_AESLQVTR_.2	#N/A	7.8419	7.2711	7.1334	7.0295	7.4415	7.2295	6.2101	7.3169	7.6146	7.7928	1.0126	0.0180	0.7816	0.1070
Nsf	_TTIAQQVK_.2	#N/A	7.1598	7.3263	7.0733	7.1942	7.5028	7.1447	6.4537	7.5195	7.2548	7.0653	1.0226	0.0323	0.4374	0.3591
Nsf	_TKENRQS[Phospho (STY)]IINPDWNFEK_.3	#N/A	7.1309	7.2375	7.7082	8.1894	3.7219	7.3791	7.1615	7.5294	7.3679	7.2455	0.9411	-0.0876	0.6127	0.2128
Nsf	_YVGESEANIRK_.2	#N/A	5.4001	5.8374	6.1665	5.6601	6.2679	5.9189	5.7001	6.0478	5.7103	6.1596	0.9950	-0.0072	0.8801	0.0554
Nsf	_M[Oxidation (M)]GIGGLDKEFSDIFR_.3	#N/A	5.7822	5.9488	4.5183	5.6314	4.5570	5.2368	5.8486	5.7475	5.5280	5.6704	0.9264	-0.1103	0.2280	0.6421
Nsf	_LQILHIHTAR_.3	#N/A	7.4781	7.5647	7.6735	7.6759	6.8660	7.4236	7.6552	7.5010	7.2516	7.1035	1.0094	0.0135	0.7148	0.1458
Nsf	_KIFDDAYK_.2	#N/A	6.7914	6.8255	5.7485	6.3814	6.2885	6.5332	6.6555	6.6712	6.5743	6.4110	0.9772	-0.0333	0.4905	0.3093
Nsf	_IEVGLVVGNSQVAFEK_.2	#N/A	6.0089	4.6953	4.9322	5.2952	4.7384	5.2903	5.4679	5.5754	5.6256	5.8986	0.9146	-0.1288	0.1015	0.9936
Nsf	_QC[Carbamidomethyl (C)]IGTM[Oxidation (M)]TIEIDFLQK_.2	#N/A	5.0856	2.9547	4.4677	3.5122	4.0051	4.5414	4.9617	4.2225	4.7286	3.8548	0.9218	-0.1175	0.4522	0.3446
Nsf	_M[Oxidation (M)]EIGLPDEK_.2	#N/A	7.3387	7.5103	6.4591	7.3320	7.0623	7.1650	6.8310	7.3174	7.2584	7.3671	0.9932	-0.0098	0.8235	0.0844
Nsf	_DIEAM[Oxidation (M)]DPSILK_.2	#N/A	7.3602	6.8236	6.7766	7.1340	7.0186	6.8898	7.0858	7.1219	6.9056	7.0912	0.9928	-0.0104	0.6806	0.1671
Nsf	_TPLVSVLLEGPPHSGK_.2	#N/A	6.7752	6.7632	6.6331	6.8189	6.1811	6.7735	7.0012	6.8598	6.8236	7.0312	0.9608	-0.0577	0.0699	1.1553
Nsf	_IDGVEQLNNILVIGM[Oxidation (M)]TNRPDLIDEALLRPGR_.4	#N/A	4.9012	4.4967	5.4591	4.7947	4.3244	3.5190	5.7834	5.0130	4.7676	4.0654	0.9338	-0.0988	0.4762	0.3222
Nsf	_KLFADAEEEQR_.3	#N/A	5.2296	4.9704	3.6923	4.4909	5.3831	4.6825	3.6747	4.2972	5.2816	5.0571	1.0358	0.0507	0.7108	0.1483
Nsf	_M[Oxidation (M)]IGFSETAK_.2	#N/A	4.7904	4.8383	3.9234	5.0537	4.9552	4.5629	4.0815	4.7975	4.6292	4.3090	1.0523	0.0736	0.3682	0.4339
Nsf	_AAQSTAMNR_.2	#N/A	5.6721	3.1700	4.5524	6.9255	5.6506	3.2373	4.9783	3.0634	4.3763	4.8367	1.1285	0.1744	0.5259	0.2791
Nsf	_EFSDIFR_.2	#N/A	5.5443	4.3121	3.5746	5.3183	3.8196	3.8988	3.3163	4.3545	4.7644	5.2686	0.9967	-0.0048	0.9788	0.0093
Nsf	_GHQLLSADVDIK_.2	#N/A	5.5891	5.3179	4.2537	3.7975	5.5411	4.8395	5.3122	5.0942	4.5413	5.5043	0.9563	-0.0644	0.6004	0.2216

Nsf	_ENRQS[Phospho (STY)]IINPDWNFEK_.3	#N/A	6.0715	5.6974	5.6495	6.0651	5.1909	6.1919	5.4783	5.7744	6.4659	6.1969	0.9719	-0.0411	0.5337	0.2727
Nsf	_KLFADAEEEQRR_.3	#N/A	6.1080	5.7320	5.6841	5.7118	6.2290	6.0084	6.1879	6.3196	6.5164	6.1857	0.9381	-0.0922	0.0196	1.7071
Nsf	_QSIINPDWNFEK_.2	#N/A	7.0342	6.9761	7.3424	6.9910	6.9307	7.0584	7.1060	7.0517	6.9861	6.9027	1.0063	0.0090	0.6110	0.2139
Nsf	_GSM[Oxidation (M)]AGSTGVHDTVVNQLLSK_.3	#N/A	7.9818	8.0094	8.8747	7.7603	7.6072	7.8707	7.8845	7.9659	7.9949	8.0377	1.0058	0.0084	0.8437	0.0738
Nsf	_VEVDM[Oxidation (M)]EKAESLQVTR_.3	#N/A	4.1419	3.3477	3.8756	3.3559	3.2856	3.6158	3.2321	3.8260	4.2496	3.0966	1.0007	0.0011	0.9926	0.0032
Nsf	_C[Carbamidomethyl (C)]PTDELSLSNC[Carbamidomethyl (C)]AVVNEK_.2	#N/A	5.0066	4.2338	4.3431	3.8869	3.7532	4.3722	4.1432	4.2579	3.4133	3.5642	1.1096	0.1500	0.1699	0.7699
Nsf	_KNIDSNPYDTDK_.2	#N/A	3.0650	3.8212	4.2995	3.8432	3.7095	5.6133	5.9429	5.5386	6.0292	3.5205	0.7719	-0.3735	0.0976	1.0107
Nsf	_LFADAEEEQRR_.2	#N/A	3.8935	3.4565	3.7039	3.4272	3.3159	4.8653	4.2988	3.4401	3.4721	3.6239	0.9602	-0.0586	0.6633	0.1783
Nsf	_FLALM[Oxidation (M)]REEGAS[Phospho (STY)]PLDFD_.2	#N/A	3.9398	3.4026	3.6735	3.2172	3.0835	3.1339	3.3655	3.7839	4.0830	4.3953	0.8724	-0.1969	0.0758	1.1203
Nsf	_EEGAS[Phospho (STY)]PLDFD_.1	#N/A	3.3320	4.1372	4.0188	3.3205	3.9586	3.7709	4.1317	3.0177	3.3168	3.6607	1.0636	0.0890	0.4152	0.3817
Nsf	_DFQSGQHVM[Oxidation (M)]VR_.2	#N/A	3.7298	3.8541	3.8536	5.3393	6.2608	3.6348	3.7627	3.8072	4.8862	3.6679	1.1028	0.1412	0.5104	0.2921
Nsf	_VEVDMEK_.2	#N/A	3.6764	2.9318	3.4101	3.9829	2.8201	3.8045	3.6976	4.0473	3.7841	4.1320	0.8780	-0.1877	0.0988	1.0053
Vps4b	_GNDS[Phospho (STY)]DGEAESDDPEK_.2	#N/A	7.1729	4.1278	7.1980	9.0155	6.4208	7.2917	8.1002	7.7018	3.4792	8.2387	0.9987	-0.0019	0.9943	0.0025
Vps4b	_GNDS[Phospho (STY)]DGEAESDDPEKKK_.2	#N/A	4.8932	5.0443	4.6733	5.8726	5.3829	5.1393	5.1215	4.9492	4.7790	5.3896	1.0213	0.0304	0.6620	0.1792
Psmc2	_TM[Oxidation (M)]LELINQLDGFDPR_.2	#N/A	4.5026	4.7765	4.4252	4.0094	4.4780	5.4612	5.2786	4.9868	5.2017	5.0061	0.9005	-0.1513	0.0798	1.0980
Psmc2	_FVVDLSDQVAPTDIEEGM[Oxidation (M)]R_.2	#N/A	3.9028	4.5738	3.6365	3.1803	3.9686	4.2060	3.3285	4.8761	4.2155	4.3179	0.9347	-0.0974	0.4591	0.3381
Rab11b	_AQIWDTAGQER_.2	#N/A	7.7280	7.7830	8.0136	7.3269	7.1934	7.3779	7.7997	7.7440	7.7175	7.6145	0.9808	-0.0280	0.3947	0.4038
Rab11b	_VVLIGDSGVGK_.2	#N/A	7.2065	7.5713	6.7871	7.4486	7.0381	7.2415	7.3787	7.5759	7.4177	7.3634	0.9706	-0.0430	0.1804	0.7439
Rab11b	_[Acetyl (Protein N-term)]GTRDDEYDYLFK_.2	#N/A	5.4048	5.4659	6.3100	5.8511	4.9628	6.0518	6.0735	5.7738	5.9563	6.1177	0.9488	-0.0758	0.2685	0.5710
Rab11b	_NNLSFIETSALDSTNVEEAFK_.2	#N/A	4.3470	5.1401	5.4537	6.2330	5.2177	5.1456	5.0100	5.3666	4.9927	5.8175	0.9924	-0.0111	0.9097	0.0411
Rab11b	_HLTYENVER_.2	#N/A	6.9787	6.5502	7.2556	6.4869	6.7349	6.7748	7.0050	6.7395	6.9694	6.5918	0.9957	-0.0063	0.8619	0.0646
Rab11b	_NILTEIYR_.2	#N/A	5.5259	5.5643	5.8494	5.6626	5.1477	4.9826	4.4914	5.5238	5.8137	5.3731	1.0292	0.0416	0.5953	0.2252
Rab11b	_STIGVEFATR_.2	#N/A	6.1430	3.9954	4.4736	5.5029	3.8837	5.6177	4.1349	6.3698	6.0959	3.6947	0.9728	-0.0397	0.8531	0.0690
Rab11b	_AITSAYYR_.2	#N/A	4.2124	3.6713	3.7879	4.2906	4.6497	4.1121	4.0052	3.9316	3.1492	4.2575	1.0742	0.1033	0.3086	0.5106
Rab11b	_NEFNLESK_.2	#N/A	4.6092	4.1258	4.4511	3.9948	3.8611	3.7823	4.1431	5.1181	4.8840	5.0641	0.8615	-0.2150	0.0265	1.5762
Rab11b	_AAHDES[Phospho (STY)]PGNNVVDISVPPTTDGQR_.3	P46638_S190_M1_Rab11b	3.2762	4.0814	3.9630	4.4656	3.9027	3.8268	4.1876	3.6529	3.3014	4.4326	1.0066	0.0095	0.9300	0.0315
Ina	_TNEKEQLQGLNDR_.3	#N/A	8.7319	9.0423	9.3208	9.2754	9.0342	9.0796	8.9965	8.7995	8.9676	9.3161	1.0067	0.0097	0.6690	0.1746
Ina	_DGLAEEVQR_.2	#N/A	9.4122	9.4953	9.5856	9.6898	9.2926	9.2865	9.3478	9.1696	9.3383	9.7054	1.0075	0.0107	0.5797	0.2368
Ina	_VHQLETQNR_.2	#N/A	9.0561	9.4194	8.8475	9.2600	9.7556	9.1146	9.4794	9.0391	9.3055	9.2043	0.9984	-0.0023	0.9351	0.0291
Ina	_ALEAELAALR_.2	#N/A	9.0759	9.4773	9.2567	9.4057	9.0949	9.3143	9.1715	9.3848	9.3270	9.3289	0.9965	-0.0050	0.7308	0.1362
Ina	_QILELEER_.2	#N/A	9.2292	9.2922	9.0029	9.1283	9.2090	9.3741	9.0330	8.6424	9.0453	9.4111	1.0192	0.0274	0.2533	0.5963
Ina	_AQLEEASSAR_.2	#N/A	8.9220	9.0041	9.0220	8.9066	9.4014	9.2217	8.7963	8.7604	9.0220	9.0155	1.0203	0.0291	0.1483	0.8289
Ina	_TIEIEGLR_.2	#N/A	8.8878	8.7137	8.7255	8.7549	8.9024	9.0955	8.1755	8.6267	8.6435	9.1616	1.0225	0.0321	0.3006	0.5221
Ina	_FANLNEQAAR_.2	#N/A	8.3059	8.5640	8.5556	8.9878	9.1021	8.5248	8.7190	8.2479	8.3668	8.6428	1.0211	0.0301	0.3459	0.4610
Ina	_NLQSAEEWYK_.2	#N/A	8.6380	8.7433	8.8023	8.7557	8.6811	8.7037	8.4479	8.3322	8.4349	8.6200	1.0310	0.0440	0.0016	2.8087
Ina	_KVESLLDELAFVR_.3	#N/A	6.9270	7.1380	7.1875	7.4213	7.3962	7.1903	7.2922	7.0830	7.2641	7.2849	0.9971	-0.0042	0.8401	0.0757
Ina	_RLPASDGLDLSQAAAR_.3	#N/A	7.2565	7.4400	7.5934	7.5225	7.7871	7.4187	6.6259	7.2341	7.4840	7.5507	1.0387	0.0547	0.1797	0.7454
Ina	_DVDGATLAR_.2	#N/A	7.2140	7.4777	7.6019	7.2441	7.8599	7.3891	5.6505	7.4442	7.5460	7.7588	1.0514	0.0722	0.3940	0.4045
Ina	_LPASDGLDLSQAAAR_.2	#N/A	7.4557	7.0212	6.7979	7.6451	7.7361	7.2896	7.2635	6.8223	7.1155	6.7930	1.0465	0.0656	0.1531	0.8152
Ina	_AQALLER_.2	#N/A	6.3703	7.0688	6.8263	6.4747	7.0360	6.7787	5.3680	6.6564	6.7525	6.9130	1.0524	0.0737	0.3188	0.4965
Ina	_FAVFIEK_.2	#N/A	6.5406	6.2559	6.2592	6.6068	6.6247	6.6918	5.8306	6.4815	6.7686	7.1099	0.9922	-0.0113	0.8336	0.0790
Ina	_AQYESLAAK_.2	#N/A	6.5287	6.7034	6.1873	6.3327	6.9625	6.3503	5.2408	5.9364	6.9248	6.5447	1.0567	0.0795	0.3144	0.5025
Ina	_VGELFQR_.2	#N/A	6.6654	6.7596	6.2352	6.5535	6.4791	5.9827	6.3294	6.5661	5.9695	6.2707	1.0258	0.0367	0.3859	0.4135
Ina	_KVFGDSSR_.2	#N/A	4.6620	5.3082	5.2343	5.3753	5.5553	4.8286	4.8592	5.1985	5.7050	5.5721	0.9675	-0.0476	0.4754	0.3229
Ina	_LSGPGGSGSFR_.2	#N/A	8.1278	8.4012	8.0282	8.7816	8.2147	7.6269	8.2728	7.8984	8.1599	8.1662	1.0089	0.0128	0.7348	0.1338
Ina	_VESLLDELAFVR_.2	#N/A	5.5825	5.0688	3.3541	5.1852	5.1269	5.1811	3.7489	4.7282	4.4001	4.8092	1.1119	0.1530	0.2978	0.5261
Ina	_SNVASTAAC[Carbamidomethyl (C)]SSASSLGLGLAYR_.2	#N/A	5.6705	5.0478	5.9329	5.4753	4.7099	5.3210	6.0242	4.4824	5.1488	5.8065	0.9989	-0.0016	0.9871	0.0056
Ina	_GANES[Phospho (STY)]LER_.2	P46660_S335_M1_Ina	6.7894	7.0362	3.2237	6.7791	7.3379	7.0129	5.9702	6.4985	6.7020	7.4820	0.9550	-0.0665	0.7270	0.1385
Ina	_[Acetyl (Protein N-term)]SFGSEHYLC[Carbamidomethyl (C)]SASSYR_.2	#N/A	5.1237	4.5893	4.9142	6.0162	5.1300	5.0834	5.0132	4.9314	5.0637	4.6148	1.0483	0.0681	0.3796	0.4207
Ina	_[Acetyl (Protein N-term)]SFGSEHYLC[Carbamidomethyl (C)]SAS[Phospho (STY)]SYRK_.3	#N/A	3.6791	5.0832	6.2273	5.9511	5.3810	3.7480	3.9480	5.4040	3.7534	3.7407	1.1900	0.2509	0.2447	0.6114
Ina	_EEIHEYRR_.2	#N/A	5.7096	4.9841	3.4000	5.7124	5.3520	4.9504	5.5212	5.6873	5.5301	4.5726	0.9419	-0.0864	0.5390	0.2684
Ina	_VSSAGLSLK_.2	#N/A	4.0950	3.7746	4.4089	4.4857	5.2032	4.6805	3.1254	2.7781	4.3109	4.0289	1.2473	0.3188	0.0497	1.3039
Ina	_VGEGFEETLGEAVISTK_.2	#N/A	8.1632	8.0643	8.4650	8.4948	7.2400	8.2747	8.3040	7.4024	7.8045	7.9627	1.0316	0.0449	0.3971	0.4011
Ina	_KEEEEEEEASKEVSK_.3	#N/A	5.0024	4.9357	5.1256	5.7717	5.0522	5.1541	5.3922	4.4223	5.0959	4.9567	1.0364	0.0516	0.4136	0.3834
Ina	_SATEESTSS[Phospho (STY)]SQKM_.2	P46660_S497_M1_Ina	4.0059	3.7238	4.2020	5.8632	3.8691	4.1017	3.9949	3.5022	3.7047	3.7821	1.1464	0.1971	0.2174	0.6627
Ina	_HSAEVAGYQDSIGQLESDLR_.3	#N/A	7.2781	7.6818	7.3134	7.6647	7.3259	7.3244	6.9203	6.4095	6.9917	7.5533	1.0664	0.0927	0.0572	1.2423
Ina	_ILSSTASK_.2	#N/A	4.2890	3.5933	3.4828	4.5274	3.4677	4.3149	3.1748	3.9746	4.2737	4.2778	1.0052	0.0075	0.9503	0.0221
Adss2	_VVDLLAQDADIVC[Carbamidomethyl (C)]R_.2	#N/A	5.3171	5.1038	4.2645	4.8038	5.2104	5.0982	4.5927	4.7770	5.4785	5.7095	0.9663	-0.0494	0.5662	0.2470
Adss2	_ELPVNAQNYVR_.2	#N/A	3.5424	5.8663	4.2292	5.8686	6.1844	6.0386	5.7363	6.1238	6.2727	5.1570	0.9083	-0.1388	0.4005	0.3974
Nedd4	_RQIS[Phospho (STY)]EDVDGPDNR_.2	#N/A	7.5291	7.5370	8.0426	8.1266	6.6466	7.5453	7.9733	7.8261	7.6403	7.5832	0.9762	-0.0347	0.5256	0.2794
Nedd4	_QIS[Phospho (STY)]EDVDGPDNR_.2	#N/A	8.2638	8.0996	8.6288	8.4185	7.3351	8.1468	8.3208	8.2291	8.1058	8.1894	0.9924	-0.0110	0.7916	0.1015
Nedd4	_RPSPDDDLT[Phospho (STY)]DEDNDDM[Oxidation (M)]QLQAQR_.3	P46935_T287_M1_Nedd4	3.5521	3.7903	3.2857	4.3304	4.1966	4.1178	2.6806	4.1635	2.9699	4.0076	1.1225	0.1668	0.2718	0.5658
Yap1	_QAS[Phospho (STY)]TDAGTAGALTPQHVR_.2	#N/A	3.4375	6.5198	6.8776	7.0502	6.3174	6.5930	7.0006	6.2149	6.4782	6.5521	0.9346	-0.0975	0.5565	0.2546
Yap1	_GDS[Phospho (STY)]ETDLEALFNAVM[Oxidation (M)]NPK_.2	P46938_S46_M1_Yap1	4.0746	6.0024	3.2606	6.5958	6.5157	4.9915	3.4779	4.3552	5.6611	3.7302	1.2169	0.2832	0.2758	0.5594
Gal	_EAGALDSLPGIPLATS[Phospho (STY)]SEDLEKS_.2	P47212_S117_M1_Gal	3.7114	3.5147	4.3982	3.4000	3.4030	8.5194	3.1147	8.9065	4.3670	3.3670	0.9094	-0.1371	0.7700	0.1135
Rph3a	_LHLLPGASK_.2	#N/A	6.6876	7.0237	6.7775	7.1442	7.3300	6.7826	6.5218	6.9186	5.7848	6.7890	1.0698	0.0974	0.0925	1.0340
Rph3a	_KQEELTDEEKEINR_.3	#N/A	6.0313	6.0822	4.3623	6.5974	5.6163	6.4365	6.6060	6.1570	6.1869	6.4137	0.9233	-0.1152	0.2803	0.5524
Rph3a	_TKPQQPAGEPATQEQPTPESR_.3	#N/A	5.9265	5.6482	5.5126	6.3363	6.3779	5.9982	5.1321	5.8803	5.7415	6.4115	1.0303	0.0430	0.5408	0.2669
Rph3a	_TGPAGGFQAAPHTAAPYSQAAPAR_.3	#N/A	4.7398	4.6155	4.6164	4.5282	4.1217	3.8588	4.6237	3.7432	4.6486	3.7609	1.0523	0.0735	0.4365	0.3600
Rph3a	_DSEGWDHAHGGGTGDTSR_.3	#N/A	4.8498	4.2000	4.3200	3.6000	5.6505	3.8439	4.0778	4.7086	4.5205	4.5284	0.9892	-0.0157	0.9057	0.0430
Rph3a	_WHQLQNENHVSS[Phospho (STY)]D_.2	#N/A	10.2105	10.7670	11.5459	11.3749	10.9064	11.1291	10.9192	10.9098	10.8304	10.9642	1.0076	0.0109	0.7431	0.1289
Rph3a	_RANS[Phospho (STY)]VQAAR_.2	#N/A	3.9848	3.6654	3.5470	4.0497	3.4867	2.1942	2.6500	3.6054	2.9870	4.4526	1.0188	0.0268	0.8936	0.0489
Rph3a	_DSEGWDHAHGGGTGDTSRS[Phospho (STY)]PAGLR_.4	P47708_S262_M1_Rph3a	3.7844	6.3129	7.4466	6.6927	3.3794	6.7283	3.2101	5.8664	3.3848	4.2399	1.1622	0.2168	0.4664	0.3312
Rph3a	_ANS[Phospho (STY)]VQAAR_.2	#N/A	4.3525	3.6569	3.5385	4.0411	3.4782	4.2513	3.1112	4.0773	3.7259	4.0081	1.0418	0.0590	0.5653	0.2477
Rph3a	_ISVC[Carbamidomethyl (C)]DEDKFGHNEFIGETR_.3	#N/A	3.2053	4.1371	4.4398	3.9836	3.8499	3.9265	4.1319	3.0176	3.3166	3.6609	1.1110	0.1518	0.2017	0.6953
Aldh2	_TIPIDGDFFSYTR_.2	#N/A	7.4806	6.9024	7.6357	7.5219	7.3392	7.6333	7.3810	7.2464	7.5654	7.3991	1.0028	0.0041	0.8927	0.0493
Aldh2	_AAFQLGSPWR_.2	#N/A	7.0448	7.3795	6.6291	7.3644	6.6567	7.0029	6.9413	7.0606	7.2134	7.3385	0.9824	-0.0256	0.5057	0.2961
Aldh2	_VVGNPFDSR_.2	#N/A	6.5211	6.9006	7.3778	6.8194	7.1041	6.4975	6.3839	6.9192	6.7202	6.7156	1.0277	0.0395	0.3679	0.4343
Aldh2	_TFVQENVYDEFVER_.2	#N/A	6.7104	6.1776	7.6872	6.9041	5.7792	6.7814	6.7546	5.8119	5.7805	6.5083	1.0739	0.1029	0.2686	0.5710

Aldh2	_VAEQTPLTALYVANLIK_.2	#N/A	4.9715	5.1429	5.8537	5.3815	5.8220	5.0876	4.3398	5.5251	4.9601	5.6543	1.0501	0.0706	0.4277	0.3689
Aldh2	_TEQGPQVDETQFK_.2	#N/A	6.4238	6.1830	3.3720	5.6130	6.3675	5.9773	3.0641	5.5658	5.6965	6.0821	1.1086	0.1487	0.5097	0.2927
Aldh2	_TIEEVVGR_.2	#N/A	5.6139	6.1262	4.4577	3.8905	6.7658	6.2527	4.1497	5.9153	6.3664	6.1500	0.9774	-0.0330	0.8594	0.0658
Aldh2	_LLC[Carbamidomethyl (C)]GGGAAADR_.2	#N/A	5.7579	6.5353	4.3584	6.6223	6.8777	6.2069	6.2970	6.5332	6.2685	6.5266	0.9459	-0.0802	0.4839	0.3152
Aldh2	_ELGEYGLQAYTEVK_.2	#N/A	5.0040	4.6688	4.3861	3.9298	5.0582	4.3293	4.0781	3.0713	4.9423	4.8006	1.0804	0.1116	0.4252	0.3715
Aldh2	_LADLIER_.2	#N/A	4.8681	4.0787	4.5569	3.2748	3.4638	3.9640	4.2490	4.3450	4.9439	4.2359	0.9079	-0.1393	0.2632	0.5797
Capza2	_EATDPRPYEAENAIESWR_.3	#N/A	7.9992	7.9326	8.5001	8.3879	7.5525	8.2792	8.0288	7.8070	8.0470	8.2394	1.0097	0.0140	0.6948	0.1581
Capza2	_LLLNNDNLLR_.2	#N/A	7.7371	7.6345	7.7252	7.6219	7.7189	7.7012	7.7155	7.9892	7.6682	7.3946	0.9997	-0.0004	0.9838	0.0071
Capza2	_FTVTPSTTQVVGILK_.2	#N/A	7.1183	6.7360	7.4769	7.4043	7.0304	7.3590	7.3229	6.9321	7.4580	6.5948	1.0156	0.0224	0.6141	0.2118
Capza2	_EHYPNGVC[Carbamidomethyl (C)]TVYGK_.2	#N/A	6.0785	5.4953	6.4799	6.0502	6.1855	5.8624	3.5665	6.5858	3.8820	5.8547	1.2118	0.2771	0.1214	0.9159
Capza2	_TSVETALR_.2	#N/A	5.3908	5.1762	4.6605	4.7218	5.2287	5.4399	4.2822	5.8835	5.9186	5.4903	0.9461	-0.0799	0.4272	0.3693
Capza2	_EGAAHAFAQYNLDQFTPVK_.3	#N/A	4.0274	3.2387	3.8374	4.5264	3.8688	4.5614	3.4857	3.6305	4.4936	3.5533	1.0578	0.0811	0.5039	0.2976
Capza2	_[Acetyl (Protein N-term)]ADLEEQLSDEEKVR_.2	#N/A	5.8641	5.7107	5.6758	6.7812	6.6179	5.7259	5.3625	6.3384	5.5162	5.7413	1.0563	0.0790	0.3225	0.4915
Capza2	_[Acetyl (Protein N-term)]ADLEEQLSDEEK_.2	#N/A	5.5013	5.0321	3.7828	6.4059	5.4869	4.7059	4.8951	6.0804	5.7981	5.6151	0.9205	-0.1194	0.3932	0.4054
Capza2	_FIIHAPPGEFNEVFNDVR_.3	#N/A	6.4422	6.5843	6.4118	7.0947	6.7572	6.5432	6.6570	6.7018	6.7472	6.4427	1.0003	0.0004	0.9905	0.0041
Capza2	_DIQDSLTVSNEVQTAK_.2	#N/A	6.0308	5.6718	4.2564	5.9023	4.1419	5.3729	5.4518	3.2010	3.5001	5.4644	1.1873	0.2477	0.2351	0.6288
Capza2	_IVEAAENEYQTAISENYQTMSDTTFK_.3	#N/A	3.5812	4.3375	3.3149	2.6277	4.2258	3.8997	3.7928	4.1425	4.4416	4.0368	0.8930	-0.1632	0.2351	0.6288
Capzb	_DYLLC[Carbamidomethyl (C)]DYNR_.2	#N/A	9.1510	8.8786	9.1067	9.1475	8.5551	8.7713	9.2020	8.7157	9.0642	9.0885	0.9908	-0.0133	0.5952	0.2254
Capzb	_STLNEIYFGK_.2	#N/A	6.6915	6.3230	6.4949	6.6654	6.4563	6.6022	6.6850	6.7600	6.8972	6.6597	0.9687	-0.0459	0.0345	1.4623
Capzb	_ELS[Phospho (STY)]QVLTQR_.2	P47757_S263_M1_Capzb	10.6586	10.4619	10.9971	10.8526	9.7272	10.7412	10.9551	10.4468	10.5332	10.6597	0.9929	-0.0103	0.7671	0.1151
Capzb	_GC[Carbamidomethyl (C)]WDSIHVVEVQEK_.2	#N/A	4.6944	3.2031	4.5292	4.3663	3.3409	4.2087	4.3570	2.4723	4.1744	4.3860	1.0588	0.0825	0.6491	0.1877
Capzb	_KLEVEANNAFDQYR_.3	#N/A	6.2242	6.1175	6.6200	6.3716	6.0808	6.2210	6.0374	5.9849	6.3142	5.9447	1.0333	0.0473	0.1325	0.8776
Capzb	_LEVEANNAFDQYR_.2	#N/A	2.9763	3.7815	3.2945	6.1451	3.6029	4.1266	4.4874	5.5040	2.9611	5.1828	0.8796	-0.1851	0.4754	0.3230
Capzb	_SGSGTM[Oxidation (M)]NLGGSLTR_.2	#N/A	6.0999	6.6402	6.9290	3.9474	6.1881	6.8350	6.5516	6.5926	6.4729	6.2665	0.9437	-0.0836	0.5401	0.2675
Capzb	_[Acetyl (Protein N-term)]SDQQLDC[Carbamidomethyl (C)]ALDLMR_.2	#N/A	3.9726	3.3645	4.5487	4.3043	3.5218	3.3260	3.2652	3.0673	4.2168	3.0318	1.1309	0.1775	0.2312	0.6360
Gsr	_ALLTPVAIAAGR_.2	#N/A	6.0543	6.5349	6.9814	6.1631	6.3041	6.2166	6.4493	6.4063	6.2449	6.7244	0.9875	-0.0181	0.6823	0.1660
Map2k4	_LC[Carbamidomethyl (C)]DFGISGQLVDSIAK_.2	#N/A	4.0488	4.9299	4.0440	4.2604	4.3105	4.8540	4.5764	3.5718	4.3267	4.4215	1.0435	0.0614	0.5098	0.2926
Map2k4	_LRTHS[Phospho (STY)]IESSGK_.3	#N/A	4.0600	4.0463	4.1119	4.3826	3.6820	4.6849	3.4633	3.7498	5.1393	3.7156	1.0359	0.0509	0.6882	0.1623
Map2k4	_YVYSVLDDVIPEEILGK_.2	#N/A	4.3280	4.5678	4.5214	1.2777	3.5918	3.9204	3.8724	4.2638	3.8193	3.4902	0.9585	-0.0612	0.8108	0.0911
Map2k4	_FTLNPNTTGVQNPHIER_.3	#N/A	6.1748	5.6064	3.4405	6.3261	5.8565	6.4124	5.4971	5.4859	5.5516	5.9454	1.0029	0.0041	0.9784	0.0095
Map2k4	_THS[Phospho (STY)]IESSGK_.2	#N/A	5.0938	4.0306	4.5463	4.0901	3.9564	4.1691	4.2384	4.4119	3.2101	3.7674	1.1043	0.1431	0.2213	0.6550
Map2k4	_LKIS[Phospho (STY)]PEQHWDFTAEDLKDLGEIGR_.4	P47809_S88_M1_Map2k4	3.4140	4.1702	3.7427	4.2454	4.0585	4.0669	3.9601	3.8731	3.5217	4.3257	1.0075	0.0108	0.8899	0.0506
Map2k4	_ILDQM[Oxidation (M)]PATPSS[Phospho (STY)]PM[Oxidation (M)]YVD_.2	#N/A	3.7213	7.8222	12.6936	11.3120	5.7094	3.3439	12.1989	3.4603	7.3061	3.7932	1.1112	0.1522	0.7787	0.1086
Mapk14	_HTDDEM[Oxidation (M)]TGY[Phospho (STY)]VATR_.2	#N/A	3.6961	3.6983	3.7405	4.1408	3.8974	3.8132	4.2474	4.0133	4.5507	4.0479	0.9090	-0.1377	0.0187	1.7280
Gfpt1	_VDS[Phospho (STY)]TTC[Carbamidomethyl (C)]LFPVEEK_.2	P47856_S259_M1_Gfpt1	3.7681	3.0724	4.4549	10.0362	2.8938	3.3349	3.1714	9.9818	4.3104	3.4237	0.8797	-0.1850	0.7471	0.1266
Pfkm	_KRS[Phospho (STY)]GEAAV_.2	P47857_S775_M1_Pfkm	9.2888	9.0341	9.6200	9.1475	8.8877	9.3208	9.3908	9.3761	9.5121	9.5286	0.9751	-0.0364	0.1167	0.9331
Pfkm	_ALVFQPVTELKDQTDFEHR_.3	#N/A	6.9044	7.3410	8.0679	7.4603	7.1763	7.1559	7.8262	7.4592	7.5909	7.3342	0.9733	-0.0390	0.3855	0.4140
Pfkm	_RFDEAIK_.2	#N/A	7.6011	7.5645	7.3255	7.1763	7.3644	7.0983	7.4614	7.5246	7.5098	7.4352	0.9829	-0.0248	0.2552	0.5931
Pfkm	_DLQVNVEHLVQK_.2	#N/A	6.8378	7.1523	7.3002	7.0026	6.2298	6.8428	7.0645	6.9467	7.0388	7.2549	0.9743	-0.0376	0.3813	0.4187
Pfkm	_GQIEEAGWSYVGGWTGQGGSK_.2	#N/A	6.7395	6.7837	7.5797	7.6470	6.2645	7.2180	7.3560	4.4698	6.6344	6.7074	1.0363	0.0515	0.4555	0.3415
Pfkm	_ALVFQPVTELK_.2	#N/A	6.1687	5.5468	5.7430	6.2076	6.1641	6.1319	6.2898	6.4172	6.3859	5.9431	0.9576	-0.0625	0.1486	0.8279
Pfkm	_NLEQISANITK_.2	#N/A	5.8586	6.3681	5.7948	5.8466	5.5479	5.6522	5.2736	6.4749	6.4552	5.8943	0.9701	-0.0437	0.5199	0.2841
Pfkm	_IVEIVDAITTTAQSHQR_.2	#N/A	6.2147	5.8834	6.8073	5.8067	4.7414	5.5193	6.6419	5.8013	5.9970	6.2092	0.9459	-0.0803	0.4061	0.3914
Pfkm	_SEWSDLLNDLQK_.2	#N/A	6.2327	5.3700	3.2507	5.9727	5.4062	5.5611	6.2397	6.0387	5.6411	5.8360	0.8922	-0.1645	0.2780	0.5559
Pfkm	_GITNLC[Carbamidomethyl (C)]VIGGDGSLTGADTFR_.2	#N/A	4.9126	4.5022	5.7800	4.9706	4.1224	4.5067	4.0596	4.5026	4.9995	5.0020	1.0341	0.0483	0.6565	0.1827
Pfkm	_IFANTPDSGC[Carbamidomethyl (C)]VLGM[Oxidation (M)]R_.2	#N/A	5.5043	6.1228	5.9336	6.3880	5.6908	5.1628	5.7754	5.9117	5.6770	5.0152	1.0367	0.0521	0.4757	0.3226
Pfkm	_VLVVHDGFEGLAK_.2	#N/A	3.4088	4.6356	4.9318	4.7316	4.4671	3.7868	4.0065	4.6731	4.3151	4.3053	1.0004	0.0006	0.9954	0.0020
Pfkm	_AIAVLTSGGDAQGM[Oxidation (M)]NAAVR_.2	#N/A	4.8105	5.1276	5.4852	4.9591	4.6155	5.6285	5.4528	5.6751	5.2672	5.5221	0.9316	-0.1022	0.1131	0.9464
Pfkm	_NVLGHM[Oxidation (M)]QQGGSPTPFDR_.3	#N/A	5.7376	6.0245	6.5137	5.6859	5.9770	5.6700	6.0691	6.0879	6.1007	6.2013	0.9706	-0.0431	0.3087	0.5104
Pfkm	_LNIIIVAEGAIDK_.2	#N/A	4.1667	3.4222	3.9004	4.0649	3.3105	3.3142	3.2073	4.4047	4.2744	3.4596	0.9635	-0.0536	0.6614	0.1795
Pfkm	_SSYLNIVGLVGSIDNDFC[Carbamidomethyl (C)]GTDM[Oxidation (M)]TIGTDSALHR_.3	#N/A	4.2012	3.5056	3.3872	3.8899	3.3269	4.4026	3.6045	4.2286	3.8772	3.8569	0.9727	-0.0399	0.6832	0.1655
Pfkm	_VFFVHEGYQGLVDGGEHIR_.3	#N/A	6.6878	5.7790	4.3416	5.4291	3.7516	4.3738	6.1845	5.6237	3.2147	3.5626	1.0891	0.1232	0.6223	0.2060
Pfkm	_EATWESVSM[Oxidation (M)]M[Oxidation (M)]LQLGGTVIGSAR_.3	#N/A	3.4683	3.7579	4.1551	3.1569	3.6462	2.9784	4.3724	3.4608	3.1093	3.1239	1.0030	0.0043	0.9745	0.0112
Scg3	_ELS[Phospho (STY)]AERPLNEQIAEAEADK_.3	P47867_S40_M1_Scg3	5.3120	5.5667	3.4512	7.3051	5.0132	5.5819	4.8645	5.7345	5.8924	5.9835	0.9560	-0.0649	0.7190	0.1433
Rpl6	_AVPQLQGYLR_.2	#N/A	6.4173	6.5385	6.2682	5.8195	6.9444	6.6588	6.8857	6.5629	6.5090	6.5648	0.9714	-0.0418	0.3849	0.4147
Rpl6	_QLDSGLLLVTGPLVINR_.2	#N/A	3.9310	4.2393	5.0900	4.4768	4.7421	4.1097	3.2492	4.3509	4.3251	4.2496	1.0959	0.1321	0.2374	0.6245
Rpl6	_SSITPGTVLIILTGR_.2	#N/A	4.9498	3.8880	3.1880	4.7522	5.1396	5.2391	5.0924	4.8861	3.8065	5.4224	0.9426	-0.0853	0.5957	0.2250
Rpl6	_KAGS[Phospho (STY)]DAAASRPR_.2	#N/A	4.3445	4.3222	5.2179	4.0722	4.1436	3.5859	4.5292	5.4358	4.2228	3.4757	0.9695	-0.0447	0.7570	0.1209
Rpl6	_HQEGEIFDTEKEKYEITEQR_.4	#N/A	5.3103	4.1539	6.7616	4.5451	3.9822	5.5298	5.2487	3.5733	5.1843	5.9832	1.0100	0.0143	0.9423	0.0258
Rpl6	_AGS[Phospho (STY)]DAAASRPR_.2	#N/A	3.8618	3.1172	3.5954	3.7976	2.3890	3.6191	3.5123	4.3209	3.9695	4.3173	0.8429	-0.2466	0.0853	1.0693
Fosl2	_S[Phospho (STY)]PPTSGLQSLR_.2	P47930_S200_M1_Fosl2	7.3926	3.7386	3.3375	7.9951	3.5599	7.2767	7.6579	4.1199	6.7858	7.1424	0.8636	-0.2115	0.5162	0.2872
Crkl	_Y[Phospho (STY)]PSPPVGSVSAPNLPTAEENLEYVR_.3	P47941_Y105_M1_Crkl	7.2922	8.0995	8.5040	9.2107	7.9010	8.0574	8.2306	7.8465	8.1083	7.4197	1.0350	0.0496	0.4609	0.3364
Crkl	_IFDPQNPDDNE_.2	#N/A	8.7360	8.8064	9.3966	9.1467	8.7976	9.0073	9.1798	8.6012	8.8181	8.9846	1.0096	0.0139	0.6115	0.2136
Crkl	_DSS[Phospho (STY)]TC[Carbamidomethyl (C)]PGDYVLSVSENSR_.2	P47941_S42_M1_Crkl	3.5998	3.9220	3.6108	6.7055	3.7743	3.7172	3.9233	3.9170	4.0563	4.2465	1.0461	0.0650	0.7744	0.1110
Rplp1	_AAGVSVEPFWPGLFAK_.2	#N/A	7.5260	7.4898	7.6743	7.6318	7.8133	7.3736	7.1483	7.2849	7.5748	7.7262	1.0203	0.0291	0.2809	0.5514
Rplp1	_ALANVNIGSLIC[Carbamidomethyl (C)]NVGAGGPAPAAGAAPAGGAAPSTAAAPAEEK_.4	#N/A	2.0765	0.8870	4.4392	3.5631	2.6274	3.3506	3.7114	2.6800	3.7371	2.4717	0.8965	-0.1577	0.6488	0.1879
Rplp1	_KEES[Phospho (STY)]EES[Phospho (STY)]EDDM[Oxidation (M)]GFGLFD_.2	#N/A	5.6247	5.0708	6.3137	11.6026	9.8685	5.9416	4.9715	12.0212	10.3051	7.4451	0.8524	-0.2304	0.5048	0.2969
Rpl5	_EFNAEVHRK_.2	#N/A	4.1610	3.8014	3.2747	3.2417	3.6227	4.8824	3.9959	3.0778	2.9809	3.9966	1.0905	0.1250	0.4337	0.3628
Rpl5	_VGLTNYAAAYC[Carbamidomethyl (C)]TGLLLAR_.2	#N/A	4.1214	3.2210	3.8551	3.3989	3.2652	3.3594	3.5472	2.7147	3.9013	3.0762	1.0686	0.0957	0.4376	0.3589
Rpl5	_YLMEEDEDAYKK_.3	#N/A	3.0299	3.7861	4.2644	3.8082	3.6744	2.9502	4.3442	3.1930	3.4921	3.4854	0.9881	-0.0173	0.8973	0.0471
Rpl13	_VDTWFNQPAR_.2	#N/A	7.1744	7.0841	7.5405	7.1351	7.2802	7.1806	7.5981	7.0755	7.3462	7.1826	0.9907	-0.0135	0.5947	0.2257
Rpl13	_LATQLTGPVM[Oxidation (M)]PIR_.2	#N/A	6.1346	6.3980	7.0791	5.6280	6.4839	6.4739	6.2948	6.8908	6.5208	6.4578	0.9733	-0.0391	0.5243	0.2804
Rpl13	_GFSLEELR_.2	#N/A	6.2588	6.5276	6.3455	6.4184	5.2214	6.3338	5.4241	6.3204	5.7994	6.3788	1.0340	0.0483	0.5215	0.2827
Rpl13	_KGDSS[Phospho (STY)]AEELK_.2	P47963_S140_M1_Rpl13	3.3322	3.8939	5.6311	4.5217	3.9588	5.7600	4.2364	3.5968	5.5558	2.9878	1.1031	0.1416	0.5440	0.2644
Rpl13	_TIGISVDPR_.2	#N/A	5.4481	5.8428	5.8908	5.8110	5.8824	5.9014	5.8180	6.2524	5.6467	5.6767	0.9910	-0.0130	0.7213	0.1419
Rpl13	_STESLQANVQR_.2	#N/A	4.8720	5.6054	3.3507	5.8515	5.9286	2.9382	3.5681	5.8089	5.5583	3.8204	1.0147	0.0210	0.9344	0.0294
Rpl36	_EVC[Carbamidomethyl (C)]GFAPYER_.2	#N/A	7.4085	7.1528	7.8001	7.4632	7.4269	7.2675	7.6288	7.2467	7.2848	7.5791	0.9980	-0.0029	0.9150	0.0386
Anxa5	_GTVTDFPGFDGR_.2	#N/A	8.8785	8.8389	9.2520	9.0435	8.8250	9.1342	9.0235	8.5861	8.6964	8.7501	1.0264	0.0376	0.0813	1.0899
Anxa5	_VLTEIIASR_.2	#N/A	8.0537	7.9129	8.1881	8.4556	7.7976	8.3500	8.1252	8.0800	8.3475	8.0987	0.9955	-0.0065	0.7984	0.0978

Anxa5	_SEIDLFNIR_.2	#N/A	7.7698	7.7619	7.3264	7.7368	7.3677	7.6175	7.7296	7.4708	7.5328	7.7165	0.9979	-0.0030	0.8946	0.0484
Anxa5	_GLGTDEDSILNLLTSR_.2	#N/A	7.2447	7.4237	7.2907	6.9580	7.2700	7.2997	7.3939	6.6544	6.9964	7.1175	1.0294	0.0419	0.1898	0.7216
Anxa5	_GAGTDDHTLIR_.2	#N/A	6.8873	6.8312	7.3515	6.8101	7.1751	6.2442	6.2184	7.1675	7.1728	6.7231	1.0092	0.0132	0.8179	0.0873
Anxa5	_QEIAQEFK_.2	#N/A	6.1778	6.3387	5.8878	5.9475	6.4949	6.2598	6.7162	6.3252	6.2048	5.9967	0.9800	-0.0292	0.4747	0.3236
Anxa5	_ADAEVLRK_.2	#N/A	5.6693	6.1639	6.1911	6.0577	6.3743	6.0225	5.9274	6.0717	6.5070	6.2951	0.9806	-0.0283	0.4645	0.3330
Anxa5	_DLVDDLKSELTGK_.2	#N/A	5.2578	5.1803	4.2875	5.5970	5.2570	5.4679	5.4593	4.8333	5.4522	4.9796	0.9987	-0.0018	0.9812	0.0082
Anxa5	_ETSGNLEQLLLAVVK_.2	#N/A	5.3488	4.0927	4.2613	3.2512	4.6494	4.5676	3.8545	4.0461	4.5359	4.5717	1.0258	0.0368	0.7810	0.1073
Anxa5	_LYDAYELK_.2	#N/A	5.7276	5.4650	6.4027	5.4268	5.5484	5.6877	3.5249	6.2609	6.2190	5.6433	1.0550	0.0772	0.5976	0.2236
Anxa5	_TPEELSAIK_.2	#N/A	5.7942	5.3250	5.9879	4.2356	5.5436	5.1489	4.0231	5.5719	5.5837	5.7175	1.0220	0.0315	0.8038	0.0949
Epb41	_S[Phospho (STY)]LDGAAAAESTDR_.2	P48193_S543_M1_Epb41	8.6689	8.5249	8.8077	8.9214	8.0725	8.6160	8.8648	8.4600	8.7397	8.7066	0.9895	-0.0152	0.5963	0.2245
Epb41	_LSTHS[Phospho (STY)]PFR_.2	P48193_S706_M1_Epb41	3.5223	4.2786	3.6344	4.1371	4.1669	5.0660	3.8517	3.9814	3.6300	4.1041	1.0623	0.0872	0.4306	0.3659
Epb41	_RAS[Phospho (STY)]RS[Phospho (STY)]LDGAAAAESTDR_.3	P48193_S541_M2_Epb41	3.4851	4.2414	3.2188	4.2634	4.1297	3.9958	4.4117	4.2386	2.2306	3.4266	1.0873	0.1207	0.5090	0.2933
Gad1	_GVPDS[Phospho (STY)]PERR_.2	P48318_S527_M1_Gad1	7.7014	7.8155	7.9754	7.5263	7.4149	7.7257	7.7854	8.2573	7.8449	7.5470	0.9789	-0.0307	0.3176	0.4981
Gad1	_ANSVTWNPHK_.2	#N/A	6.8745	6.8342	7.0135	6.9754	7.4096	7.1002	6.7633	7.1279	6.6823	6.9112	1.0238	0.0339	0.2476	0.6063
Gad1	_GTVGFENQINK_.2	#N/A	5.8621	5.8094	6.0054	4.7668	5.0789	5.6437	6.2754	5.9148	5.5365	5.8525	0.9377	-0.0928	0.2239	0.6500
Gad1	_NLLS[Phospho (STY)]C[Carbamidomethyl (C)]ENSDQGAR_.2	P48318_S77_M1_Gad1	7.9780	7.7024	8.3007	8.1083	4.9062	7.6776	7.7613	7.4737	7.7303	7.8981	0.9650	-0.0515	0.6885	0.1621
Gad1	_TNS[Phospho (STY)]LEEKSR_.2	P48318_S55_M1_Gad1	4.8295	3.7056	6.5705	4.4151	4.2813	3.8441	5.1352	4.0869	5.0121	5.4157	0.9380	-0.0924	0.6128	0.2126
Gad1	_ERQS[Phospho (STY)]SKNLLSC[Carbamidomethyl (C)]ENSDQGAR_.3	#N/A	3.9395	3.4559	3.3550	3.6606	3.5231	3.9548	3.6546	4.1851	3.4646	3.9477	0.9568	-0.0638	0.3860	0.4134
Gad1	_QSS[Phospho (STY)]KNLLSC[Carbamidomethyl (C)]ENSDQGAR_.3	P48318_S72_M1_Gad1	4.1783	3.4570	3.6797	3.3894	3.3136	3.1199	3.5377	3.6288	4.5618	3.9274	0.9001	-0.1518	0.1751	0.7568
Gad2	_M[Oxidation (M)]M[Oxidation (M)]GVPLQC[Carbamidomethyl (C)]SALLVR_.2	#N/A	6.4942	7.0234	7.3626	5.2232	6.0424	7.1630	6.7163	7.4900	6.7251	6.9323	0.9405	-0.0885	0.3714	0.4302
Gad2	_HYDLSYDTGDK_.2	#N/A	5.4882	5.9364	5.3473	5.9043	4.7857	6.2336	6.1696	5.6352	5.8150	6.0141	0.9505	-0.0733	0.3271	0.4854
Gad2	_M[Oxidation (M)]VISNPAATHQDIDFLIEEIER_.3	#N/A	4.4636	3.9053	3.5042	4.6935	4.1701	5.6083	5.1934	4.5856	4.4315	4.1837	0.9548	-0.0667	0.6248	0.2042
Gad2	_[Acetyl (Protein N-term)]ASPGSGFWSFGS[Phospho (STY)]EDGSADPENPGTAR_.3	P48320_S13_M1_Gad2	3.2128	5.0696	5.8414	4.0936	3.8574	4.2751	4.1878	5.7495	3.3798	3.8936	1.0207	0.0295	0.8901	0.0506
Prox1	_DRAS[Phospho (STY)]PESLDLTR_.2	P48437_S511_M1_Prox1	3.2380	3.9882	3.9248	4.4275	3.8645	3.8650	4.1422	3.6910	3.3396	4.3945	0.9982	-0.0027	0.9794	0.0090
Prox1	_GMSHS[Phospho (STY)]PSVALR_.2	P48437_S179_M1_Prox1	3.5490	3.7934	3.2827	4.3273	4.1936	3.9319	4.4755	4.1747	2.9730	4.0046	0.9845	-0.0226	0.8572	0.0669
Ppp3cb	_GFS[Phospho (STY)]PPHR_.2	P48453_S472_M1_Ppp3cb	10.7047	10.5454	10.8988	10.7346	9.9849	10.3699	10.8733	10.5935	10.6131	10.2348	0.9963	-0.0053	0.8474	0.0719
Ppp3cb	_LFEVGGSPANTR_.2	#N/A	9.1172	8.9684	9.3097	9.0853	8.7813	8.8723	9.3849	9.1493	9.0399	8.9825	0.9872	-0.0186	0.3569	0.4474
Ppp3cb	_IC[Carbamidomethyl (C)]S[Phospho (STY)]FEEAK_.2	#N/A	8.7050	8.0548	8.1782	8.2853	7.0514	7.9680	7.1799	8.1607	8.4368	8.2765	1.0034	0.0048	0.9419	0.0260
Ppp3cb	_YLFLGDYVDR_.2	#N/A	9.1204	9.1930	9.1057	9.1070	8.0851	8.6736	9.3097	8.9322	8.9280	8.7724	0.9883	-0.0169	0.6717	0.1728
Ppp3cb	_EESESVLTLK_.2	#N/A	8.5872	8.0571	8.1657	8.4669	8.0230	8.5905	7.8151	7.8964	8.4264	8.5633	1.0171	0.0245	0.5046	0.2971
Ppp3cb	_AHEAQDAGYR_.2	#N/A	7.7678	7.5529	7.7331	7.7488	8.2364	7.6581	7.4367	7.7539	7.7047	7.3589	1.0290	0.0412	0.1635	0.7866
Ppp3cb	_IINEGAAILR_.2	#N/A	6.8341	6.8277	6.9530	6.2436	6.3529	6.6094	7.1082	6.8880	6.7736	6.4254	0.9762	-0.0348	0.4060	0.3915
Ppp3cb	_YENNVM[Oxidation (M)]NIR_.2	#N/A	8.0676	7.9997	7.7012	8.1599	8.3054	7.5936	7.8829	7.8845	7.9687	7.8682	1.0089	0.0128	0.6311	0.1999
Ppp3cb	_HLTEYFTFK_.2	#N/A	6.7013	6.5523	6.9427	6.6168	6.0450	6.2948	6.7381	6.4304	7.0487	6.4563	0.9786	-0.0312	0.4901	0.3097
Ppp3cb	_IC[Carbamidomethyl (C)]S[Phospho (STY)]FEEAKGLDR_.2	#N/A	6.8585	7.3729	8.2336	7.1409	6.2751	7.2626	6.9641	7.1682	7.2767	7.3479	1.0002	0.0003	0.9969	0.0014
Ppp3cb	_VDEEIALR_.2	#N/A	4.2234	3.0028	3.4093	3.9120	2.8911	3.7335	3.6267	4.2065	2.8626	4.3228	0.9398	-0.0895	0.5640	0.2487
Ppp3cc	_IRS[Phospho (STY)]FEEAR_.2	P48455_S462_M1_Ppp3cc	5.6280	4.6951	3.4762	5.5207	2.8863	5.4764	5.6808	5.9376	5.4770	5.3154	0.8235	-0.2802	0.1425	0.8463
Kcnj9	_[Acetyl (Protein N-term)]AQENAAFS[Phospho (STY)]PGSEEPPR_.2	P48543_S9_M1_Kcnj9	2.8997	2.9419	4.1342	8.5540	3.5442	3.0804	3.8262	3.3232	3.6223	3.3552	1.1871	0.2474	0.5716	0.2429
Lmna	_KLES[Phospho (STY)]SESR_.2	P48678_S423_M1_Lmna	8.9142	8.9972	9.5216	4.6243	9.6753	9.3208	9.2048	3.8777	9.5152	9.1297	1.0728	0.1013	0.7016	0.1539
Lmna	_LADALQELR_.2	#N/A	5.6768	6.5406	6.7462	5.8929	6.1365	5.8376	5.9141	5.9172	6.0739	5.9998	1.0271	0.0386	0.4781	0.3205
Lmna	_LQEKEDLQELNDR_.3	#N/A	5.8010	5.9157	6.4174	5.9858	6.0058	5.8810	5.6597	5.9136	6.4042	6.3360	0.9873	-0.0185	0.6761	0.1700
Lmna	_SGAQASSTPLS[Phospho (STY)]PTR_.2	P48678_S22_M1_Lmna	7.9152	8.1352	8.2161	8.5536	7.4994	7.7873	8.2387	4.6370	8.0844	8.0559	1.1053	0.1444	0.3168	0.4993
Lmna	_LSPS[Phospho (STY)]PTSQR_.2	P48678_S392_M1_Lmna	10.8002	11.0191	11.4749	11.4832	10.6650	11.0589	11.1697	10.8773	10.9646	11.0910	1.0053	0.0076	0.7563	0.1213
Lmna	_SS[Phospho (STY)]FSQHAR_.2	P48678_S429_M1_Lmna	3.3712	3.9712	4.6057	6.7986	7.7516	4.1097	4.2977	4.3526	3.1508	3.8267	1.3057	0.3848	0.2315	0.6355
Lmna	_GSHC[Carbamidomethyl (C)]S[Phospho (STY)]GSGDPAEYNLR_.2	P48678_S573_M1_Lmna	4.0330	3.3094	3.7667	3.3104	3.1767	3.4479	3.4587	3.6907	3.9898	2.9877	0.9931	-0.0100	0.9203	0.0361
Lmna	_NSNLVGAAHEELQQSR_.2	#N/A	2.5396	3.5331	3.1425	4.1733	3.1930	3.4361	4.3354	4.2164	3.5843	2.7742	0.8950	-0.1600	0.3497	0.4563
Lmna	_SVGGSGGGS[Phospho (STY)]FGDNLVTR_.2	P48678_S637_M1_Lmna	3.0017	3.8069	3.6885	4.3138	6.0841	4.1013	6.9149	6.5122	2.9864	4.1582	0.8101	-0.3039	0.3165	0.4996
Lmna	_LRLS[Phospho (STY)]PS[Phospho (STY)]PTSQR_.2	P48678_S390_M2_Lmna	9.3653	9.5386	9.9240	10.1401	9.3134	9.6479	9.9447	9.5726	9.6508	9.6912	0.9938	-0.0089	0.7432	0.1289
Lmna	_MQQQLDEYQELLDIK_.2	#N/A	7.0102	3.3231	3.2047	7.0664	3.1444	3.0842	7.4928	4.4111	4.0597	3.6744	0.9109	-0.1346	0.7310	0.1361
Lmna	_NKS[Phospho (STY)]NEDQSM[Oxidation (M)]GNWQIR_.3	#N/A	4.5072	4.4757	5.0899	5.3478	4.4530	4.5772	4.0545	4.0273	4.8226	4.2359	1.1066	0.1461	0.0972	1.0122
Lmna	_ASS[Phospho (STY)]HSSQSQGGGSVTK_.2	P48678_S404_M1_Lmna	5.3223	5.7706	6.7076	6.4287	3.1693	5.3333	6.1188	5.6970	4.2876	5.8615	0.9935	-0.0095	0.9613	0.0171
Lmna	_AQHEDQVEQYKK_.3	#N/A	3.8375	3.2395	3.8366	3.3804	5.0432	3.9371	3.5286	3.6208	3.9199	3.0577	1.0983	0.1353	0.3550	0.4498
Lmna	_LEAALGEAK_.2	#N/A	4.2245	3.5288	3.4104	4.5919	3.3502	4.3793	3.2393	4.2054	3.8540	3.8801	1.0315	0.0447	0.7195	0.1430
Hspa4l	_S[Phospho (STY)]FDDPIVQTER_.2	#N/A	13.3737	13.1778	13.3309	13.7670	12.7094	13.3323	13.8470	13.2640	13.4631	13.4523	0.9834	-0.0242	0.2934	0.5325
Hspa4l	_LHGRS[Phospho (STY)]FDDPIVQTER_.3	#N/A	8.6098	10.0753	10.3945	9.7346	9.5034	9.8925	10.1519	9.7766	9.8861	9.6692	0.9829	-0.0250	0.6157	0.2106
Hspa4l	_QLT[Phospho (STY)]QDLLNSYIENEGK_.2	P48722_T593_M1_Hspa4l	6.3123	6.1671	6.4155	6.3818	5.8316	6.4186	6.4635	6.2673	6.2985	6.4076	0.9835	-0.0240	0.4144	0.3826
Hspa4l	_SGGIETIANEYSDR_.2	#N/A	7.2736	7.0122	7.0269	7.2252	6.5675	7.2111	7.4172	6.7684	6.8688	7.0537	1.0037	0.0053	0.8866	0.0523
Hspa4l	_VLATTFDPYLGGR_.2	#N/A	6.0435	6.4639	6.8216	6.8584	6.1609	6.3779	6.8840	6.8572	6.4877	6.5479	0.9642	-0.0526	0.2401	0.6196
Hspa4l	_SIDLPIQSSLYR_.2	#N/A	6.3854	6.5022	6.9964	6.9954	6.7130	6.8031	6.7063	6.3910	6.8478	6.7153	1.0101	0.0145	0.6641	0.1777
Hspa4l	_SQIVTNVR_.2	#N/A	6.2939	6.2315	6.4494	6.5689	6.8734	6.3793	6.2378	6.3218	6.8344	6.3797	1.0035	0.0051	0.8901	0.0506
Hspa4l	_LSLTQDPVVK_.2	#N/A	5.7208	6.2001	6.0653	5.8181	5.7332	5.2649	5.9650	6.2641	6.0141	5.8848	0.9616	-0.0565	0.2293	0.6395
Hspa4l	_DKLGTVYEK_.2	#N/A	5.9062	5.4912	6.2390	5.6383	5.7926	5.7850	5.6731	5.5919	5.1858	4.8217	1.0923	0.1273	0.0420	1.3770
Hspa4l	_QDLPSLDEKPR_.3	#N/A	5.3678	4.8802	3.9394	3.4831	5.3307	5.1578	4.9651	5.3366	5.1571	4.9011	0.9220	-0.1171	0.3638	0.4391
Hspa4l	_AQFEQLC[Carbamidomethyl (C)]ASLLAR_.2	#N/A	3.2867	4.0417	4.1108	3.6546	3.3480	3.6616	4.1681	3.8852	4.3248	3.3319	0.9380	-0.0924	0.3496	0.4564
Hspa4l	_C[Carbamidomethyl (C)]HAEHT[Phospho (STY)]PEEEIDHTGAK_.3	#N/A	5.8987	5.2311	6.0219	6.0641	5.6379	5.3490	6.2036	5.6629	5.9500	5.9716	0.9585	-0.0611	0.2530	0.5968
Hspa4l	_GSSQHTDS[Phospho (STY)]GEMEVD_.2	P48722_S832_M1_Hspa4l	4.0426	3.3003	3.7763	7.2565	3.1863	3.4388	5.6658	3.6817	3.9807	3.8643	0.9695	-0.0448	0.8836	0.0537
Hspa4l	_FITPEDMNK_.2	#N/A	5.5632	3.0144	3.3978	5.0076	2.9027	3.7219	3.6151	4.2181	3.8666	3.4642	1.0379	0.0536	0.8095	0.0918
Hspa4l	_TSFEEGTGEC[Carbamidomethyl (C)]EVFSK_.2	#N/A	3.8956	4.8554	3.8761	5.9289	3.0213	3.2074	3.5682	3.5813	3.8803	3.0972	1.1696	0.2260	0.3254	0.4876
Hspa4l	_SVM[Oxidation (M)]AAAQVAGLNC[Carbamidomethyl (C)]LR_.2	#N/A	3.5390	3.8687	5.1742	3.9074	3.3444	3.5981	3.6793	4.0301	3.8981	4.2235	0.9867	-0.0192	0.8836	0.0538
Hspa4l	_NHPAPFSK_.2	#N/A	3.1039	2.5343	3.7907	4.2934	2.8897	3.9990	4.0081	3.8251	3.4737	4.2604	0.8827	-0.1800	0.2603	0.5845
Hspa4l	_TGSEHNGPMDGQSGSET[Phospho (STY)]SPDPPK_.3	P48722_T818_M1_Hspa4l	3.1362	3.8925	4.0205	4.5232	3.7808	4.3447	4.2378	3.5953	3.2439	2.9893	1.1231	0.1675	0.2229	0.6518
Hspa4l	_[Acetyl (Protein N-term)]SVVGIDLGFLNC[Carbamidomethyl (C)]YIAVAR_.2	#N/A	3.1202	4.2222	4.3547	3.8985	3.7648	4.3607	4.0468	3.1027	3.4017	3.5758	1.1194	0.1628	0.1822	0.7396
Pcnt	_RNS[Phospho (STY)]EIDELK_.2	P48725_S1437_M1_Pcnt	5.3227	5.4940	5.1805	5.4050	4.1682	5.0297	4.9968	5.2975	5.3290	4.4830	1.0146	0.0209	0.8079	0.0927
Cbr1	_LQAEGLSPR_.2	#N/A	7.5731	7.8587	8.0568	7.9550	8.3017	7.8058	7.7175	8.0422	8.0120	7.9324	0.9999	-0.0002	0.9952	0.0021
Cbr1	_ILLNAC[Carbamidomethyl (C)]C[Carbamidomethyl (C)]PGWVR_.2	#N/A	5.7552	6.1788	6.7456	6.6576	6.0877	5.9952	6.6023	6.5019	6.1426	6.0813	0.9849	-0.0219	0.6798	0.1676
Cbr1	_FVEDTKK_.2	#N/A	5.5656	5.6029	5.3268	5.4320	6.3073	5.3424	5.1223	5.8000	5.5561	5.2603	1.0297	0.0422	0.4878	0.3117
Cbr1	_FHQLDIDNPQSIR_.3	#N/A	7.0290	7.0824	7.2765	7.3030	7.3143	7.3326	7.2074	7.0471	6.9998	7.1302	1.0179	0.0256	0.1357	0.8674
Cbr1	_GVHAEEGWPNSAYGVTK_.2	#N/A	5.7060	3.9905	4.4688	5.0702	4.5425	4.2466	4.1608	2.9886	3.2877	3.6898	1.3225	0.4033	0.0162	1.7901
Cbr1	_[Acetyl (Protein N-term)]SS[Phospho (STY)]SRPVALVTGANK_.2	P48758_S3_M1_Cbr1	3.7576	3.5848	3.4913	5.6765	3.4061	4.3234	3.1834	3.9661	4.2651	4.2132	1.0340	0.0483	0.7904	0.1021

Cbr1	_FSGDVVLAAR_.2	#N/A	3.3766	4.1818	4.0634	4.5868	4.0031	2.9623	3.5049	3.4702	3.3068	3.6162	1.1116	0.1527	0.2388	0.6220
Cox7a2	_GGASDALLYR_.2	#N/A	9.0880	9.1752	9.2576	9.2284	9.3567	8.9795	8.7258	9.1179	9.2653	8.9008	1.0198	0.0283	0.1620	0.7906
Cox7a2	_LFQEDNGM[Oxidation (M)]PVHLK_.2	#N/A	6.9902	6.8849	6.9363	7.1963	7.1916	6.7829	7.1448	6.9693	6.8112	6.8616	1.0072	0.0104	0.6397	0.1940
Gstm5	_IAAFLQSDR_.2	#N/A	7.6072	7.6378	7.1211	7.3914	6.5275	6.9921	7.4384	7.9451	7.2184	6.8321	0.9802	-0.0288	0.6212	0.2067
Gstm5	_YIC[Carbamidomethyl (C)]GEAPDYDR_.2	#N/A	7.4154	7.1926	7.0484	6.5478	6.9090	7.1324	7.4824	7.1032	7.2692	7.1316	0.9716	-0.0415	0.2477	0.6061
Gstm5	_SM[Oxidation (M)]VLGYWDIR_.2	#N/A	6.3345	6.4758	5.7156	6.3982	5.9842	6.4330	6.9451	6.9687	6.4761	6.4682	0.9269	-0.1095	0.0331	1.4796
Gstm5	_SQWLDVK_.2	#N/A	2.3020	3.1248	3.2873	2.5170	3.7344	3.7371	3.5046	4.0009	3.8989	1.9265	0.9353	-0.0965	0.6709	0.1733
Gstm5	_LTFVDFLTYDVLDQNR_.2	#N/A	3.0513	4.1749	3.7381	0.5121	4.0632	4.0622	3.9554	3.8778	3.5263	4.2077	0.8394	-0.2525	0.4159	0.3810
Gstm5	_M[Oxidation (M)]LLEFTDTSYEEKR_.3	#N/A	3.8256	3.5168	3.5593	4.6039	2.9693	3.6553	3.2513	3.8981	4.1972	4.2812	0.9441	-0.0831	0.5249	0.2800
Slc25a4	_QIFLGGVDR_.2	#N/A	11.0726	11.1794	11.2336	10.7180	10.4698	10.7162	11.3496	11.3128	11.0317	10.7748	0.9803	-0.0287	0.2808	0.5516
Slc25a4	_EQGFLSFWR_.2	#N/A	10.6911	10.7856	10.9224	10.2707	9.9852	10.2858	10.8583	10.7836	10.5900	10.2972	0.9866	-0.0194	0.5178	0.2858
Slc25a4	_EFNGLGDC[Carbamidomethyl (C)]LTK_.2	#N/A	10.1506	9.9560	10.0634	9.7363	9.6789	9.5827	10.1971	10.1562	9.9296	9.6395	0.9881	-0.0173	0.4616	0.3358
Slc25a4	_[Acetyl (Protein N-term)]GDQALSFLK_.2	#N/A	8.6640	8.7445	8.5136	8.2063	8.3707	8.4158	8.4096	9.1177	8.4911	8.6465	0.9788	-0.0309	0.2831	0.5480
Slc25a4	_DFLAGGIAAAVSK_.2	#N/A	8.2351	7.7886	7.3523	6.9135	7.2813	7.5386	7.9876	7.8746	7.6903	7.6898	0.9626	-0.0550	0.2570	0.5901
Slc25a4	_AAYFGVYDTAK_.2	#N/A	6.9786	7.0636	6.6237	7.0874	6.2503	6.8146	6.9132	7.4155	7.3057	6.9025	0.9536	-0.0686	0.1275	0.8945
Slc25a4	_LAADVGKGS[Phospho (STY)]SQR_.2	P48962_S149_M1_Slc25a4	5.3069	5.1006	5.2481	7.1205	6.3752	4.3781	7.8419	7.5347	4.8642	7.1111	0.8172	-0.2912	0.1268	0.8969
Slc25a4	_IAKDEGANAFFK_.3	#N/A	4.9012	5.1081	4.5899	4.2264	3.4202	4.5179	3.3889	5.2260	4.1284	4.5028	1.0346	0.0490	0.7370	0.1325
Slc25a4	_GADIM[Oxidation (M)]YTGTLDC[Carbamidomethyl (C)]WR_.2	#N/A	6.1824	6.0080	6.4494	6.9513	5.8362	6.5998	6.5894	6.4619	6.7798	6.0288	0.9803	-0.0286	0.6171	0.2097
Slc25a4	_DEGANAFFK_.2	#N/A	3.3870	3.9554	7.3375	5.8604	6.0274	3.9527	4.3135	4.3367	6.4051	6.3832	0.9491	-0.0754	0.7745	0.1110
Slc25a4	_GSS[Phospho (STY)]QREFNGLGDC[Carbamidomethyl (C)]LTK_.3	P48962_S150_M1_Slc25a4	3.6653	3.6771	3.3990	4.4436	3.4984	4.2310	3.0910	4.0584	4.3575	4.1209	0.9775	-0.0328	0.7826	0.1065
Cit	_RVS[Phospho (STY)]EVEAVLSQK_.2	#N/A	6.0613	6.0492	6.5214	7.0513	4.5554	6.0033	5.6454	6.4870	6.6033	5.5065	0.9967	-0.0048	0.9674	0.0144
Cit	_MNS[Phospho (STY)]PGLQSK_.2	#N/A	5.1503	3.9087	3.4168	5.5380	3.6317	4.2488	4.5004	4.0407	4.3397	4.1386	1.0143	0.0205	0.8941	0.0486
Cit	_DKS[Phospho (STY)]PGRPLER_.2	#N/A	7.0866	6.9008	7.2369	7.4249	6.3801	6.6890	6.8235	7.1375	6.9889	6.9415	0.9972	-0.0041	0.9239	0.0344
Cit	_DKMNS[Phospho (STY)]PGLQSK_.2	#N/A	4.1000	3.3756	4.3017	3.5601	3.2363	3.2180	3.5165	3.2566	5.3607	3.3836	0.9362	-0.0951	0.6037	0.2192
Cit	_ERS[Phospho (STY)]PGRLFEDSSR_.3	P49025_S2021_M1_Cit	3.4130	5.0840	4.0998	4.6025	4.0396	3.6899	3.9692	3.5160	4.4780	3.0686	1.1056	0.1448	0.3405	0.4679
Cit	_S[Phospho (STY)]PEHQPSAMSLLAPPSSR_.3	P49025_S1348_M1_Cit	4.3404	4.3866	3.5264	6.8276	3.4661	4.2634	3.7437	4.0894	3.7380	3.9961	1.1482	0.1993	0.3867	0.4126
Camlg	_VVLGDS[Phospho (STY)]VDGGGADQLGGVAEVR_.2	P49070_S88_M1_Camlg	4.0363	3.3406	3.2222	3.2792	3.1619	3.0667	3.4275	4.5585	4.8918	3.6919	0.7673	-0.3821	0.0425	1.3717
Camlg	_IMGFHRPGS[Phospho (STY)]GS[Phospho (STY)]EEENQTK_.3	P49070_S53_M2_Camlg	1.3765	2.9624	4.1137	3.6575	2.7837	3.4449	3.8057	3.3437	3.6428	3.3347	0.8654	-0.2085	0.3647	0.4380
Hnrnpa1	_GFAFVTFDDHDSVDKIVIQK_.3	#N/A	6.6462	6.5449	8.7273	8.6397	7.4163	7.5291	6.9751	6.6799	7.5176	7.9465	1.0418	0.0591	0.5809	0.2359
Hnrnpa1	_SES[Phospho (STY)]PKEPEQLRK_.2	#N/A	11.2521	11.5581	12.1545	12.1427	11.5594	11.5154	11.5923	11.5829	11.5327	11.5817	1.0108	0.0155	0.5262	0.2788
Hnrnpa1	_LFIGGLSFETTDESLR_.2	#N/A	5.3469	4.7643	4.2644	5.0282	5.2934	5.0349	4.9078	4.4089	4.7442	5.4250	1.0172	0.0246	0.7582	0.1202
Hnrnpa1	_EDSQRPGAHLTVK_.3	#N/A	5.5386	5.6434	5.5033	5.6479	5.8411	6.0316	5.1670	5.4795	5.6955	5.6771	1.0356	0.0505	0.2014	0.6960
Hnrnpa1	_IEVIEIM[Oxidation (M)]TDR_.2	#N/A	6.2313	6.6086	6.7737	7.2087	6.5118	6.4078	6.5312	6.5496	6.7151	6.9586	0.9903	-0.0141	0.7416	0.1298
Hnrnpa1	_[Acetyl (Protein N-term)]S[Phospho (STY)]KSES[Phospho (STY)]PKEPEQLR_.2	P49312_S2_M2_Hnrnpa1	3.8300	6.2345	6.5977	9.4245	3.7321	3.5541	9.0214	8.6399	3.6255	8.6869	0.7423	-0.4300	0.2514	0.5996
Hnrnpa1	_SES[Phospho (STY)]PKEPEQLR_.2	#N/A	11.2431	11.4126	11.7922	12.0902	11.3604	11.3914	11.6919	11.4123	11.4223	11.4424	1.0049	0.0070	0.7548	0.1222
Hnrnpa1	_NQGGYGGSSSSSSYGSGR_.2	#N/A	4.4734	5.4768	4.5889	5.0353	5.5822	5.3935	5.3733	4.8823	5.1067	5.2082	0.9901	-0.0144	0.8464	0.0724
Hnrnpa1	_GFAFVTFDDHDSVDK_.2	#N/A	3.6978	4.0060	3.5189	4.2758	2.7823	4.0799	4.2304	4.0425	3.7369	4.0795	0.9265	-0.1101	0.3314	0.4796
Hnrnpa1	_GFGFVTYATVEEVDAAM[Oxidation (M)]NAR_.2	#N/A	3.8162	4.3502	3.5498	4.5945	2.9599	3.6647	4.8324	3.5400	4.2066	4.2717	0.9074	-0.1402	0.3163	0.4999
Hnrnpa1	_SHFEQWGTLTDC[Carbamidomethyl (C)]VVM[Oxidation (M)]R_.3	#N/A	5.0916	3.6852	5.1756	3.5434	3.4904	3.2149	5.4665	3.4578	3.7568	5.6226	0.8815	-0.1820	0.4126	0.3845
Hnrnpa1	_YHTVNGHNC[Carbamidomethyl (C)]EVR_.3	#N/A	4.0182	3.2737	3.7519	3.2957	3.1620	3.4626	3.4439	3.7055	4.0046	2.9730	0.9893	-0.0155	0.8794	0.0558
Hnrnpa1	_DYFEQYGK_.2	#N/A	3.2135	4.1289	4.4480	3.9917	3.8580	4.2674	4.1400	3.0094	3.3085	3.6690	1.1282	0.1741	0.1596	0.7970
Inpp1	_TLADVLVQEVIK_.2	#N/A	2.8399	4.7390	4.2944	4.3888	4.6915	4.5051	4.8322	3.7995	4.5617	5.4002	0.9128	-0.1316	0.3910	0.4078
Ppm1a	_NVIEAVYNR_.2	#N/A	5.8855	6.4910	6.6106	6.5406	6.5344	6.3951	6.5594	6.7383	6.4785	6.1034	0.9907	-0.0135	0.7349	0.1338
Ppm1a	_ALGDFDYK_.2	#N/A	4.2715	4.9608	5.4778	4.9526	4.9713	5.4364	4.8890	5.2411	4.9404	5.1399	0.9919	-0.0117	0.8647	0.0632
Ppm1a	_GPTEQLVSPEPEVHDIER_.3	#N/A	4.8663	4.9439	5.0237	5.3044	4.6530	5.3429	5.0449	4.8242	4.6455	4.7318	1.0438	0.0618	0.2003	0.6983
Ppm1a	_LNPYKNDDTDSAS[Phospho (STY)]TDDM[Oxidation (M)]W_.2	P49443_S377_M1_Ppm1a	3.5313	4.2875	3.5667	5.9445	3.5591	3.9580	3.8511	3.9904	5.4457	5.1478	0.8985	-0.1543	0.4378	0.3587
Pcyt1a	_TS[Phospho (STY)]PSSSPASLSR_.2	P49586_S343_M1_Pcyt1a	7.7934	7.8958	8.3791	9.2662	7.5558	7.9116	8.9072	7.8681	7.9493	8.0176	0.9937	-0.0092	0.8914	0.0499
Pcyt1a	_AVTC[Carbamidomethyl (C)]DIS[Phospho (STY)]EDEED_.2	P49586_S362_M1_Pcyt1a	9.0333	9.2644	9.3413	9.8592	9.1404	9.2534	9.6619	8.9332	9.3715	9.4611	0.9956	-0.0064	0.8326	0.0795
Pcyt1a	_QS[Phospho (STY)]PSSS[Phospho (STY)]PTHER_.2	P49586_S323_M2_Pcyt1a	3.9780	4.9370	3.5979	4.5813	4.3401	4.2548	3.5157	4.8200	3.8979	4.3608	1.0320	0.0455	0.6943	0.1585
Pcyt1a	_SPS[Phospho (STY)]PSFR_.2	P49586_S331_M1_Pcyt1a	8.1746	3.7260	8.2739	8.7499	8.2189	8.4434	8.5269	3.3196	8.3525	8.7273	1.0506	0.0713	0.8029	0.0953
Pcyt1a	_MLQAIS[Phospho (STY)]PK_.2	P49586_S315_M1_Pcyt1a	4.0672	3.3227	3.8009	3.3447	3.2110	3.4136	3.3068	3.6565	3.9555	3.0220	1.0119	0.0171	0.8637	0.0636
Psma2	_HIGLVYSGM[Oxidation (M)]GPDYR_.3	#N/A	3.2453	5.8361	4.4798	6.2652	5.9986	5.7976	4.1718	6.0147	5.9995	6.0309	0.9489	-0.0757	0.6955	0.1577
Psma2	_LAQQYYLVYQEPIPTAQLVQR_.3	#N/A	4.2300	3.5343	3.4159	4.1268	2.5927	3.5191	3.2338	3.3677	3.0193	4.5094	1.0105	0.0151	0.9280	0.0325
Psma2	_LVQIEYALAAVAGGAPSVGIK_.3	#N/A	0.9105	3.7368	3.7077	4.3098	3.9941	4.1314	4.0245	3.8086	3.4572	2.3098	1.0191	0.0273	0.9299	0.0316
Psma2	_YNEDLELEDAIHTAILTLK_.3	#N/A	4.2141	3.0670	3.9478	3.4916	3.1043	3.2667	3.6399	3.5096	3.8086	3.1810	0.9945	-0.0080	0.9425	0.0257
Psen1	_AAVQELSGSILT[Phospho (STY)]SEDPEER_.2	P49769_T370_M1_Psen1	3.0452	4.2247	3.9352	6.3613	4.0278	3.8556	3.9568	5.8557	5.5600	5.1933	0.8250	-0.2776	0.2085	0.6810
Tmod1	_NNDPELEEVNLNNIR_.2	#N/A	7.1719	7.1493	7.0308	7.7963	7.0595	7.3815	7.6242	7.0496	7.3454	7.3964	0.9879	-0.0176	0.6231	0.2054
S100b	_AM[Oxidation (M)]VALIDVFHQYSGR_.2	#N/A	4.5508	4.6766	5.1285	4.6645	4.4391	4.5581	4.6039	4.5585	4.8426	4.8050	0.9930	-0.0101	0.8140	0.0894
Bckdha	_S[Phospho (STY)]VDEVNYWDK_.2	#N/A	9.1590	8.1872	7.7800	9.3579	8.1735	8.6084	9.3325	8.1406	8.7396	8.9885	0.9709	-0.0426	0.5104	0.2921
Bckdha	_IGHHS[Phospho (STY)]TSDDSSAYR_.2	#N/A	4.8880	4.5419	3.3501	5.8990	4.2486	3.7257	5.4611	5.7202	4.2798	5.5317	0.8464	-0.2405	0.1657	0.7807
Bckdha	_S[Phospho (STY)]VDEVNYWDKQDHPISR_.3	#N/A	7.7193	8.2145	7.3611	8.9434	7.7064	8.3090	8.4050	8.0576	8.1816	8.4890	0.9709	-0.0426	0.4429	0.3537
Bckdha	_IGHHS[Phospho (STY)]TSDDSSAY[Phospho (STY)]RSVDEVNYWDKQDHPISR_.5	P50136_S334_M2_Bckdha	3.5403	4.2965	3.2739	4.1191	4.1848	3.9406	3.8338	3.9994	3.6480	4.0861	1.0002	0.0003	0.9974	0.0011
Bckdha	_NNGYAISTPTSEQYR_.2	#N/A	4.2699	3.2996	3.7779	5.5330	3.1879	3.4367	4.8845	3.0025	4.1519	4.9249	0.9237	-0.1145	0.5884	0.2303
Gng4	_EDPLIIPVPASENPFR_.2	#N/A	7.4474	7.3322	7.6465	7.0148	7.4080	7.4178	7.8741	7.6277	7.7367	7.4826	0.9606	-0.0580	0.0408	1.3896
Gng4	_VSQAASDLLAYC[Carbamidomethyl (C)]EAHVR_.2	#N/A	3.7222	4.2960	3.8509	3.6026	3.9661	3.5931	4.4264	4.0980	4.2535	3.2966	0.9552	-0.0662	0.4740	0.3242
Gng4	_EGMSNNSTT[Phospho (STY)]S[Phospho (STY)]ISQAR_.2	P50153_S12_M2_Gng4	3.6433	3.5829	4.3301	3.3319	3.4712	3.1534	4.5474	3.2857	4.4352	3.2989	0.9213	-0.1183	0.3985	0.3995
Ahcy	_VAVVAGYGDVGK_.2	#N/A	5.8831	5.6745	6.5480	6.7610	5.7231	6.1125	6.1673	5.9575	5.7182	6.0123	1.0257	0.0366	0.5418	0.2662
Ahcy	_VADIGLAAWGR_.2	#N/A	3.4415	6.0018	5.3493	5.5050	5.4716	5.3151	5.2180	5.4100	5.8006	5.3265	0.9526	-0.0701	0.5931	0.2269
Ahcy	_VPAINVNDSVTK_.2	#N/A	6.4965	5.7694	3.3067	3.1948	3.2464	2.9822	3.3430	6.2725	5.8907	6.0526	0.7730	-0.3715	0.2369	0.6254
Ahcy	_KLDEAVAEAHLGK_.3	#N/A	2.6102	4.2405	4.1221	4.6247	4.0618	3.6677	4.3394	3.5642	4.0338	3.0909	1.0348	0.0494	0.7616	0.1183
Ahcy	_GISEETTTGVHNLYK_.2	#N/A	3.9981	3.2535	3.1586	3.5369	3.0278	3.4828	3.3760	4.4572	4.1058	3.6283	0.8761	-0.1908	0.1012	0.9948
Gdi1	_KQNDVFGEADQ_.2	#N/A	10.4968	10.5736	11.0182	10.6500	10.3042	10.5352	10.9772	10.5191	10.5980	10.5621	0.9936	-0.0092	0.6567	0.1826
Gdi1	_TDDYLDQPC[Carbamidomethyl (C)]LETINR_.2	#N/A	9.8248	9.5715	10.0194	9.6876	9.4870	9.6642	9.9186	9.7017	9.7574	9.5950	0.9965	-0.0051	0.7659	0.1158
Gdi1	_QLIC[Carbamidomethyl (C)]DPSYIPDR_.2	#N/A	9.2641	9.2891	9.2744	9.2264	9.2030	9.1828	9.3983	9.0639	9.1617	9.0834	1.0069	0.0099	0.3560	0.4485
Gdi1	_IIC[Carbamidomethyl (C)]ILSHPIK_.2	#N/A	7.9176	8.2608	7.6584	7.9968	7.6666	7.4833	8.1851	8.0770	8.0226	7.7935	0.9764	-0.0344	0.2659	0.5752
Gdi1	_FLVFVANFDENDPK_.2	#N/A	6.1024	5.8633	6.0139	5.5742	5.9517	6.3878	6.3840	5.8987	6.2499	6.5115	0.9555	-0.0657	0.1445	0.8401
Gdi1	_VVEGSFVYK_.2	#N/A	6.3661	5.7680	6.7025	6.4331	6.0614	6.2780	5.1611	7.0045	6.8818	6.3679	0.9865	-0.0196	0.8233	0.0845
Gdi1	_NPYYGGESSSITPLEELYKR_.3	#N/A	6.2640	6.3369	6.8634	6.4031	6.2697	6.3047	6.1236	6.3460	6.3296	6.2974	1.0212	0.0302	0.3148	0.5020
Gdi1	_NPYYGGESSSITPLEELYK_.2	#N/A	5.0355	5.5084	3.6521	5.4511	5.2165	5.1811	6.1975	5.3493	5.4522	5.0091	0.9101	-0.1359	0.2542	0.5948

Gdi1	_M[Oxidation (M)]LLYTEVTR_.2	#N/A	8.2627	8.4271	8.8026	8.5889	7.8309	8.5841	8.4875	8.4533	8.4068	8.2675	1.0015	0.0021	0.9463	0.0240
Gdi1	_TFEGVDPQNTSM[Oxidation (M)]R_.2	#N/A	7.9160	7.7583	7.6455	8.0968	7.9189	7.1966	7.7100	7.5767	7.4713	7.3975	1.0287	0.0408	0.2362	0.6266
Gdi1	_QNDVFGEADQ_.2	#N/A	5.0768	4.5334	5.1561	4.6703	4.1451	5.0357	4.1956	4.6387	4.8613	4.6596	1.0394	0.0557	0.4526	0.3443
Gdi1	_YIAIASTTVETAEPEKEVEPALELLEPIDQK_.3	#N/A	3.8867	3.2538	4.2354	3.6205	2.6108	3.2174	3.2900	3.8125	4.1929	3.0306	0.9691	-0.0453	0.7644	0.1167
Gdi1	_VPSTETEALASNLM[Oxidation (M)]GM[Oxidation (M)]FEK_.3	#N/A	3.4232	4.2284	4.1100	3.8923	3.0491	3.6798	4.0406	3.5059	3.1544	3.0788	1.0829	0.1149	0.3453	0.4618
Gdi1	_KSDIYVC[Carbamidomethyl (C)]M[Oxidation (M)]ISYAHNVAAQGK_.3	#N/A	3.2622	4.5104	4.5096	4.0533	3.8887	3.8408	4.2016	4.4487	3.2469	3.7306	1.0266	0.0379	0.7529	0.1233
Gdi1	_FDLGQDVIDFTGHALALYR_.3	#N/A	3.8630	3.9359	3.5967	3.1761	3.8242	3.6587	4.0361	3.9015	4.2006	3.6352	0.9321	-0.1014	0.1533	0.8144
Atp6v1a	_LPANHPLLTGQR_.2	#N/A	7.9220	8.2884	7.8944	8.1046	8.5726	8.0313	7.6870	8.3004	8.1697	8.1114	1.0085	0.0122	0.6943	0.1584
Atp6v1a	_TVISQSLSK_.2	#N/A	8.6410	8.8593	8.9439	8.7141	9.1690	8.8666	8.4314	8.9551	8.9262	8.6854	1.0133	0.0190	0.4150	0.3820
Atp6v1a	_VGHSELVGEIIR_.2	#N/A	7.9471	7.9134	8.1758	7.9563	7.3334	7.6645	7.5650	7.7925	7.5720	7.7766	1.0202	0.0289	0.3535	0.4516
Atp6v1a	_LIKDDFLQQNGYTPYDR_.3	#N/A	8.0595	8.0064	8.7561	8.3167	8.6520	8.4501	8.0292	8.0543	8.4871	8.4548	1.0142	0.0203	0.5427	0.2654
Atp6v1a	_YSNSDVIIYVGC[Carbamidomethyl (C)]GER_.2	#N/A	7.7947	7.7705	8.5679	8.5776	7.9991	7.7424	8.0769	7.8967	8.1994	8.1148	1.0004	0.0006	0.9873	0.0056
Atp6v1a	_DIKWEFIPSK_.2	#N/A	7.6264	8.2387	8.5828	8.6098	7.9795	8.3762	8.3696	8.3044	8.0650	8.0713	1.0040	0.0058	0.8750	0.0580
Atp6v1a	_VGSHITGGDIYGIVNENSLIK_.3	#N/A	8.4344	8.4305	8.9441	8.4304	7.7964	8.1510	8.3369	7.9926	8.2477	8.5690	1.0094	0.0135	0.7265	0.1388
Atp6v1a	_GVNVSALSR_.2	#N/A	8.3184	8.2391	8.4521	8.3069	8.5273	8.2708	7.5530	8.4503	8.4413	8.4094	1.0169	0.0242	0.4705	0.3274
Atp6v1a	_AVETTAQSDNK_.2	#N/A	7.9730	8.1467	7.8081	8.1145	7.9803	7.6696	7.9451	8.1604	8.2511	7.9084	0.9854	-0.0212	0.3316	0.4793
Atp6v1a	_ALDEYYDKHFTEFVPLR_.3	#N/A	7.9135	8.0830	8.2984	8.1284	7.7078	7.7396	8.0161	8.0071	8.0673	8.0739	0.9922	-0.0113	0.6141	0.2117
Atp6v1a	_EILQEEEDLAEIVQLVGK_.2	#N/A	6.1666	6.2709	7.2836	7.8274	6.3899	7.0571	6.5104	6.5007	6.7153	6.8386	1.0288	0.0410	0.5939	0.2263
Atp6v1a	_HFTEFVPLR_.2	#N/A	7.1620	7.0522	6.8378	7.3225	6.8285	6.8473	6.7261	6.9198	6.8858	7.4719	1.0011	0.0015	0.9652	0.0154
Atp6v1a	_AKEILQEEEDLAEIVQLVGK_.3	#N/A	6.1423	7.0774	7.5939	7.7674	6.5215	6.1356	6.8104	6.6668	6.2348	6.8011	1.0369	0.0523	0.5382	0.2690
Atp6v1a	_IKADYAQLLEDM[Oxidation (M)]QNAFR_.3	#N/A	6.0497	6.6647	6.5880	6.0843	5.6264	6.1964	6.0295	6.5687	6.1244	6.3221	0.9905	-0.0138	0.7903	0.1022
Atp6v1a	_QVRPVTEK_.2	#N/A	5.3501	5.4290	4.8115	5.4473	5.8456	5.4313	4.8800	4.1028	5.5128	4.8666	1.1126	0.1540	0.0911	1.0403
Atp6v1a	_LAEM[Oxidation (M)]PADSGYPAYLGAR_.2	#N/A	8.9542	8.8247	7.8228	9.4510	8.8473	8.5155	9.2143	8.5218	8.2737	8.7905	1.0041	0.0059	0.9133	0.0394
Atp6v1a	_TALVANTSNM[Oxidation (M)]PVAAR_.2	#N/A	7.4218	7.6128	7.5445	7.6940	7.4878	7.3760	7.2656	7.5735	7.4362	7.2367	1.0196	0.0280	0.1343	0.8719
Atp6v1a	_LASFYER_.2	#N/A	6.5192	6.8118	6.9842	6.2878	6.4189	6.9076	4.2274	6.6170	6.7527	6.2240	1.1175	0.1603	0.1880	0.7257
Atp6v1a	_ITWSIIR_.2	#N/A	6.5745	7.2170	5.8045	6.3559	6.1281	6.8555	6.0121	6.9267	6.9730	7.0009	0.9645	-0.0522	0.4776	0.3209
Atp6v1a	_RAVETTAQSDNK_.2	#N/A	4.9197	4.1159	4.6892	5.2664	5.3474	5.6659	5.0846	4.4826	5.2559	5.2824	0.9949	-0.0074	0.9377	0.0280
Atp6v1a	_GNEM[Oxidation (M)]SEVLR_.2	#N/A	7.9333	8.0798	7.6482	8.2902	8.1735	7.7614	7.7530	7.8943	7.6673	8.1498	1.0146	0.0209	0.4680	0.3297
Atp6v1a	_ALDEYYDK_.2	#N/A	4.9331	4.4389	3.8205	5.0362	4.5432	4.8549	3.4062	4.6619	5.4207	5.0851	0.9916	-0.0122	0.9277	0.0326
Atp6v1a	_EHM[Oxidation (M)]GEILYK_.2	#N/A	7.2306	6.8508	7.0078	7.4708	7.2785	7.1310	6.6622	6.8447	6.8247	6.9924	1.0484	0.0682	0.0275	1.5607
Atp6v1a	_LEGDM[Oxidation (M)]ATIQVYEETSGVSVGDPVLR_.3	#N/A	7.8159	7.9276	8.6372	9.0220	7.8418	8.4514	8.2814	8.6048	7.9640	8.2077	1.0022	0.0032	0.9488	0.0228
Atp6v1a	_DFPELTM[Oxidation (M)]EVDGK_.2	#N/A	8.0242	7.8743	7.4692	8.0174	7.5448	7.9590	7.9808	7.8237	7.9430	8.0474	0.9831	-0.0245	0.3354	0.4744
Atp6v1a	_FSMVQVWPVR_.2	#N/A	5.2552	4.4284	4.2737	6.7636	4.6454	3.6049	5.1273	3.1837	3.9353	3.9527	1.1923	0.2537	0.2582	0.5880
Atp6v1a	_FKDPVKDGEAK_.3	#N/A	6.5745	5.8988	6.2998	5.1250	6.5332	5.9640	3.8106	5.8136	5.4182	6.0772	1.1489	0.2002	0.1452	0.8380
Atp6v1a	_DDFLQQNGYTPYDR_.2	#N/A	7.4450	7.1784	4.4656	4.0094	7.1063	7.5115	7.6301	7.0896	7.3975	3.6867	0.9744	-0.0374	0.8843	0.0534
Atp6v1a	_ADYAQLLEDM[Oxidation (M)]QNAFR_.2	#N/A	6.1292	5.9954	5.8994	6.2520	5.8768	5.8994	6.2019	6.0751	6.0443	5.9713	0.9894	-0.0154	0.4765	0.3219
Atp6v1a	_ADYAQLLEDM[Oxidation (M)]QNAFRS[Phospho (STY)]LED_.2	P50516_S614_M1_Atp6v1a	3.6737	4.4703	5.1262	3.6417	2.2666	3.4292	3.5173	4.7368	3.6585	3.3190	0.9895	-0.0152	0.9446	0.0248
Atp6v1a	_EASIYTGITLSEYFR_.2	#N/A	3.5419	4.3471	4.2287	1.0850	4.1684	4.1960	4.4460	3.3872	3.6593	3.1975	0.9788	-0.0310	0.9114	0.0403
Atp6v1a	_TVGM[Oxidation (M)]LSNM[Oxidation (M)]ISFYDMAR_.2	#N/A	3.4141	3.9284	3.1477	4.1923	3.7497	3.9798	4.3406	4.3097	3.1079	3.8696	0.9561	-0.0648	0.5848	0.2330
Atp6v1a	_ASLAETDKITLEVAK_.3	#N/A	3.6277	3.5985	4.3145	3.3163	4.2542	3.4753	4.5318	3.3013	2.9499	3.2833	1.0705	0.0982	0.4967	0.3039
Atp6v1a	_GSVTYIAPPGNYDASDVVLELEFEGVK_.3	#N/A	3.9580	3.2624	3.8137	3.3575	3.0837	3.1449	3.5057	3.2272	3.9428	3.0348	1.0027	0.0038	0.9709	0.0128
Atp6v1e1	_GALFGANANR_.2	#N/A	8.8967	9.0581	9.1496	9.1096	9.3656	8.7437	8.8091	8.9595	9.0675	8.9100	1.0131	0.0188	0.3470	0.4597
Atp6v1e1	_KQDFPLVK_.2	#N/A	7.9427	8.2976	7.8957	8.1835	8.5132	7.9515	7.8348	7.9709	8.2754	8.1276	1.0098	0.0140	0.6053	0.2180
Atp6v1e1	_[Acetyl (Protein N-term)]ALSDADVQK_.2	#N/A	5.6010	6.3043	7.4641	6.1311	7.1964	6.1077	5.6898	6.3623	6.2808	6.6697	1.0347	0.0492	0.6012	0.2210
Atp6v1e1	_VSNTLESR_.2	#N/A	7.1031	6.9209	6.9131	6.8990	7.8176	6.9807	6.2659	7.1580	7.2471	7.4228	1.0117	0.0168	0.7713	0.1128
Atp6v1e1	_AEEEFNIEK_.2	#N/A	7.1253	6.3863	7.0495	6.9714	6.9736	6.9928	6.0779	6.7971	6.7808	6.9627	1.0394	0.0557	0.2403	0.6192
Atp6v1e1	_DDLITDLLNEAK_.2	#N/A	5.8177	5.3567	3.7922	5.1202	5.4116	5.5526	5.5159	5.7060	5.2038	5.7538	0.9333	-0.0996	0.3569	0.4475
Atp6v1e1	_LDLIAQQM[Oxidation (M)]M[Oxidation (M)]PEVR_.2	#N/A	7.3279	8.0627	8.3205	7.5972	7.6714	8.0843	7.8384	8.0514	7.9667	7.9128	0.9876	-0.0181	0.6222	0.2061
Atp6v1e1	_ARDDLITDLLNEAK_.2	#N/A	6.5127	6.7790	6.6308	6.9804	6.7094	6.5266	6.6141	6.2505	6.2717	6.5979	1.0398	0.0564	0.0641	1.1934
Atp6v1e1	_IQM[Oxidation (M)]SNLM[Oxidation (M)]NQAR_.2	#N/A	4.8179	5.2959	5.8009	4.1042	5.4986	5.4556	3.7899	5.7377	5.7251	5.4119	0.9992	-0.0011	0.9936	0.0028
Atp6v1e1	_IMEYYEK_.2	#N/A	3.9910	3.8793	4.3575	4.8498	4.5815	4.3579	4.2510	2.3917	3.3989	3.5786	1.2734	0.3487	0.0307	1.5134
Atp6v1e1	_IKVSNT[Phospho (STY)]LESR_.2	P50518_T195_M1_Atp6v1e1	3.7646	3.0201	3.4983	4.5429	2.9084	3.7162	3.1904	3.9591	4.2581	4.2202	0.9151	-0.1281	0.3836	0.4161
Acadvl	_SGELAVQALDQFATVVEAK_.2	#N/A	3.2678	4.0730	3.9546	3.5787	3.8943	3.8351	3.9169	3.6612	3.6352	4.4243	0.9637	-0.0534	0.5152	0.2880
Pa2g4	_[Acetyl (Protein N-term)]S[Phospho (STY)]GEDEQQEQTIAEDLVVTK_.2	P50580_S2_M1_Pa2g4	4.0011	7.6950	7.7961	8.2673	6.8328	8.6089	8.6197	8.4437	7.6426	7.9387	0.8823	-0.1807	0.3041	0.5170
Cast	_EQKPFTPAS[Phospho (STY)]PVQSTPSKPSDK_.3	P51125_S219_M1_Cast	6.2432	5.8207	6.7554	7.5025	5.9193	6.4287	6.5722	6.7234	5.8961	6.7926	0.9921	-0.0114	0.8893	0.0510
Cast	_SNDTSQT[Phospho (STY)]PPGETVPR_.2	P51125_T479_M1_Cast	7.9508	4.0983	9.0619	9.1577	3.9866	8.5239	8.8453	8.1791	3.1799	8.7141	0.9862	-0.0200	0.9530	0.0209
Cast	_S[Phospho (STY)]NEQM[Oxidation (M)]VSEKPSESK_.2	P51125_S165_M1_Cast	3.3016	4.1068	4.6062	4.4911	3.9282	3.8013	4.2058	3.2209	3.2760	2.9573	1.1828	0.2422	0.0915	1.0387
Cast	_SLSES[Phospho (STY)]ELIGELSADFDR_.2	P51125_S446_M1_Cast	3.8212	3.5213	3.5548	4.5994	2.9649	3.6597	3.2469	3.9026	4.2016	4.2767	0.9437	-0.0836	0.5223	0.2821
Rab7a	_NNIPYFETSAK_.2	#N/A	7.9322	7.9131	8.2598	7.5640	7.3796	7.8217	8.4545	7.7393	7.8793	7.6690	0.9844	-0.0227	0.5738	0.2413
Rab7a	_VIILGDSGVGK_.2	#N/A	7.6327	7.6126	7.3225	7.2156	6.8386	7.2830	7.4429	7.5064	7.8003	7.2016	0.9773	-0.0332	0.3671	0.4352
Rab7a	_FQSLGVAFYR_.2	#N/A	5.6902	5.9556	6.5597	6.1190	4.9403	5.8280	6.0242	5.8498	6.2499	6.2447	0.9601	-0.0588	0.4170	0.3799
Rab7a	_EAINVEQAFQTIAR_.2	#N/A	5.4803	5.3856	6.6047	5.0158	5.0280	5.6998	5.8824	5.3563	5.8417	5.6140	0.9757	-0.0355	0.6726	0.1722
Rab7a	_ATIGADFLTK_.2	#N/A	5.6596	6.0295	5.6931	6.2531	6.1027	6.2197	6.7124	6.4275	6.3718	6.0227	0.9388	-0.0911	0.0538	1.2694
Rab7a	_TLDSWRDEFLIQASPR_.3	#N/A	3.1268	5.9689	6.3420	5.8987	4.9296	3.9762	4.0309	6.2715	5.0010	4.2496	1.0311	0.0442	0.8469	0.0722
Rab7a	_LVTM[Oxidation (M)]QIWDTAGQER_.2	#N/A	6.2438	5.8150	6.7378	5.9362	5.4965	5.8542	6.6022	6.4971	6.3715	6.0169	0.9438	-0.0834	0.1749	0.7572
Acadl	_LPANALLGEENK_.2	#N/A	6.3678	5.3492	5.6011	6.0331	6.3901	5.5275	6.7043	5.6183	6.4013	5.5121	0.9701	-0.0437	0.5935	0.2266
Acadl	_AQDTAELFFEDVR_.2	#N/A	5.3435	4.5834	5.0269	4.6214	4.7344	4.6388	5.3502	4.3598	4.2888	4.4866	1.0440	0.0621	0.4359	0.3606
Acadl	_THIC[Carbamidomethyl (C)]VTR_.2	#N/A	4.0979	5.6766	3.8316	3.3754	6.0569	5.0692	5.3527	5.7295	3.9248	3.0527	1.0376	0.0532	0.8240	0.0841
Acadl	_IFSS[Phospho (STY)]EHDIFR_.2	P51174_S55_M1_Acadl	3.2409	3.9971	3.7134	4.4231	3.8854	2.7238	4.1578	3.9584	4.7472	3.5324	0.8939	-0.1618	0.2682	0.5715
Rpl9	_TILSNQTVDIPENVEITLK_.2	#N/A	4.6157	5.2528	5.0186	5.4466	4.5323	5.0270	5.2458	5.2678	4.6980	4.6665	1.0026	0.0037	0.9562	0.0195
Rpl9	_FLDGIYVSEK_.2	#N/A	5.0236	3.1380	5.4356	3.7768	4.9987	5.2057	5.1478	5.0491	3.9902	5.1462	0.9510	-0.0725	0.6602	0.1803
Plcb3	_RNNS[Phospho (STY)]ISEAK_.2	P51432_S1105_M1_Plcb3	3.5547	4.3110	3.2884	3.8267	4.1993	3.9262	3.8193	4.0138	3.6624	4.0716	0.9895	-0.0152	0.8511	0.0700
Hsd17b4	_VDSEGIS[Phospho (STY)]PNR_.2	P51660_S308_M1_Hsd17b4	6.4510	6.0058	3.2174	3.7201	3.1572	3.0715	3.4348	4.3984	5.9886	3.6871	0.9756	-0.0356	0.9093	0.0413
Hsd17b4	_VVLVTGAGGGLGR_.2	#N/A	3.4761	4.4107	4.1010	4.4031	4.4682	3.6888	4.3184	4.8352	4.8217	3.0698	0.9601	-0.0587	0.6760	0.1701
Hsd17b4	_IDVVVNNAGILR_.2	#N/A	4.2201	3.5244	4.4096	4.5963	3.3457	4.3837	4.0819	3.9057	3.6744	4.2736	1.0241	0.0344	0.7424	0.1294
Hsd17b4	_ISDEDWDIIHR_.3	#N/A	3.1220	4.1042	3.8088	4.3115	3.7485	4.1330	4.0262	3.8070	3.4556	4.2785	0.9947	-0.0076	0.9383	0.0277
Adcy9	_ASLGS[Phospho (STY)]DDSTQAK_.2	P51830_S1295_M1_Adcy9	8.2211	8.4032	8.7241	8.2449	7.5502	8.1300	8.6785	8.4396	8.0683	8.0614	0.9880	-0.0174	0.6763	0.1698
Adcy9	_GQGTAS[Phospho (STY)]PGSVSDLAQTVK_.2	P51830_S610_M1_Adcy9	8.3405	8.7771	9.4140	9.2940	7.1457	8.2242	9.2049	9.4242	9.1065	8.3370	0.9462	-0.0799	0.3264	0.4862
Adcy9	_AIEKDS[Phospho (STY)]C[Carbamidomethyl (C)]EDIGVEEASELSK_.3	P51830_S1332_M1_Adcy9	4.0895	3.3449	3.8232	5.6559	3.2332	3.3914	3.5152	5.6697	3.8816	4.1876	0.9095	-0.1369	0.5305	0.2754

Adcy9	_QGDEES[Phospho (STY)]ENSVKR_.2	P51830_S354_M1_Adcy9	4.1225	3.3928	3.7122	3.5795	3.2609	3.2401	3.2522	3.7887	4.0462	3.4590	0.9766	-0.0342	0.7087	0.1495
Adcy9	_IIADDLMKQGDEES[Phospho (STY)]ENS[Phospho (STY)]VKR_.3	#N/A	3.7953	4.9727	3.7323	6.8604	3.8038	3.4727	4.3502	5.8627	4.9080	3.4951	0.9539	-0.0681	0.7875	0.1038
Gss	_QIEINTISASFGGLASR_.2	#N/A	3.6485	3.5776	4.3353	4.1511	4.2750	3.4544	4.5527	3.2805	4.4299	3.3041	1.0039	0.0056	0.9653	0.0153
Hdgf	_AGDVLEDS[Phospho (STY)]PK_.2	#N/A	10.8949	10.4582	10.3060	11.1575	10.2517	10.3094	10.8953	10.3599	10.6324	10.8012	0.9898	-0.0148	0.6211	0.2068
Hdgf	_RAGDVLEDS[Phospho (STY)]PK_.2	#N/A	8.4278	8.2650	7.9118	8.9324	8.0880	8.3138	8.5330	8.1964	8.3159	8.4744	0.9932	-0.0098	0.7705	0.1132
Hdgf	_AGDVLEDS[Phospho (STY)]PKRPK_.2	#N/A	9.1105	9.5369	9.5899	9.9323	9.1686	9.2563	9.5754	9.3316	9.3858	9.4907	0.9986	-0.0021	0.9371	0.0282
Hdgf	_GSAEGSS[Phospho (STY)]DEEGKLVIDEPAKEK_.3	#N/A	3.7491	7.6737	3.6798	8.6698	7.6614	7.8077	6.9054	6.9244	7.6959	7.5298	0.9004	-0.1514	0.5449	0.2637
Hdgf	_GSAEGSS[Phospho (STY)]DEEGKLVIDEPAK_.3	#N/A	3.7844	4.0216	4.2697	9.2107	8.6676	6.8459	4.4192	8.1602	8.5108	8.9236	0.8174	-0.2909	0.3858	0.4137
Hdgf	_GFSEGLWEIENNPTVK_.2	#N/A	4.9813	5.0783	5.3391	5.5887	5.6431	5.4703	4.3676	4.0223	4.6218	3.9972	1.2582	0.3314	0.0003	3.5081
Hdgf	_KGSAEGS[Phospho (STY)]S[Phospho (STY)]DEEGKLVIDEPAK_.3	#N/A	3.7980	3.3632	3.7249	7.3759	6.2058	4.7685	3.9638	4.7822	4.6030	6.5072	0.9816	-0.0268	0.9236	0.0345
Hdgf	_SC[Carbamidomethyl (C)]AAEPEVEPEAHEGDGDKKGSAEGS[Phospho (STY)]S[Phospho (STY)]DEEGK_.4	#N/A	4.1163	3.4206	3.3022	3.1992	3.2419	2.9867	3.3475	3.8019	4.1010	3.7719	0.8994	-0.1529	0.1440	0.8417
Atp6v0d1	_LYPEGLAQLAR_.2	#N/A	9.7141	9.6865	9.6084	9.4273	9.4805	9.3901	9.6989	9.9155	9.8252	9.4623	0.9821	-0.0261	0.1345	0.8713
Atp6v0d1	_NVADYYPEYK_.2	#N/A	9.2469	8.7297	9.2674	8.6768	8.6236	8.6777	8.9584	9.0067	8.7403	8.7280	1.0014	0.0020	0.9435	0.0253
Atp6v0d1	_FFEHEVK_.2	#N/A	6.8214	7.2936	6.7273	6.9065	6.7927	6.3940	6.7462	7.1568	6.3438	6.8944	1.0055	0.0079	0.8569	0.0670
Atp6v0d1	_NIVWIAEC[Carbamidomethyl (C)]IAQR_.2	#N/A	7.0312	6.7310	7.0856	6.5648	5.9033	6.3993	6.8143	5.8868	6.7164	6.4187	1.0248	0.0353	0.5806	0.2361
Atp6v0d1	_ADDYEQVK_.2	#N/A	5.8314	5.8802	5.6107	5.9471	5.8448	5.3989	5.0917	6.0267	6.3934	5.3202	1.0077	0.0111	0.8701	0.0604
Atp6v0d1	_AYLESFYK_.2	#N/A	6.3929	6.1435	5.7678	4.9969	5.8644	6.5315	3.4602	5.6276	6.5483	7.0266	1.0501	0.0705	0.6896	0.1614
Atp6v0d1	_LKEQEC[Carbamidomethyl (C)]R_.2	#N/A	5.8357	5.6349	5.6195	5.6275	5.4714	3.7962	4.1570	6.2714	4.9442	5.6105	1.0162	0.0232	0.8760	0.0575
Atp6v0d1	_LLFEGAGSNPGDK_.2	#N/A	8.3046	8.3553	8.3075	8.4238	7.9540	8.2111	8.6886	8.3966	8.3973	8.2369	0.9798	-0.0295	0.1678	0.7751
Atp6v0d1	_AFIITINSFGTELSKEDR_.2	#N/A	4.0774	5.3914	5.5905	6.0343	3.9717	5.5776	4.9322	5.2199	5.0516	5.5247	0.9855	-0.0210	0.8727	0.0591
Atp6v0d1	_LHLQSTDYGNFLANEASPLTVSVIDDK_.3	#N/A	4.0458	3.6203	4.3333	4.6211	4.2324	3.4971	4.5100	3.8488	4.3131	3.7972	0.9857	-0.0208	0.8263	0.0829
Atp6v0d1	_LLFEGAGSNPGDKTLEDR_.3	#N/A	5.7836	6.0833	6.2042	5.9259	5.1830	5.8915	6.0164	5.4673	6.2692	5.4996	1.0055	0.0080	0.8967	0.0474
Atp6v0d1	_FC[Carbamidomethyl (C)]TLLGGTTADAM[Oxidation (M)]C[Carbamidomethyl (C)]PILEFEADRR_.3	#N/A	3.4814	4.0432	3.2151	4.2378	4.1260	4.0419	4.8582	4.2426	4.2668	3.9370	0.8917	-0.1654	0.1076	0.9682
Atp6v0d1	_AGVLSQADYLNLVQC[Carbamidomethyl (C)]ETLEDLK_.3	#N/A	3.0239	3.7802	4.2584	3.8022	3.6685	2.9561	4.3501	3.1990	3.4980	3.4795	0.9862	-0.0200	0.8805	0.0552
Fabp7	_NTEINFQLGEEFEETSIDDR_.2	#N/A	4.0156	3.3268	6.0882	4.4196	3.3206	4.7078	3.4413	5.2837	5.2260	2.9703	1.0196	0.0280	0.9100	0.0409
Fabp7	_LTDSQNFDEYMK_.2	#N/A	3.1796	4.0466	3.8664	4.3691	3.9349	4.1906	4.0838	3.7494	3.3980	4.3361	1.0101	0.0145	0.8852	0.0530
Slc25a5	_GNLANVIR_.2	#N/A	11.1910	11.2424	11.4927	10.5601	11.1148	10.7224	11.0891	11.5424	11.3033	11.0726	0.9824	-0.0256	0.3459	0.4610
Slc25a5	_TAVAPIER_.2	#N/A	10.8174	10.6108	10.7460	10.2159	10.4623	10.1799	10.1327	11.1791	10.8276	10.6696	0.9816	-0.0268	0.3962	0.4020
Slc25a5	_YFPTQALNFAFK_.2	#N/A	10.3335	10.2416	10.7411	9.7835	9.7597	9.8290	10.5683	10.7048	10.3793	10.2823	0.9648	-0.0517	0.1233	0.9089
Slc25a5	_EQGVLSFWR_.2	#N/A	10.3392	10.3428	10.2328	9.9681	9.5519	10.0103	10.4024	10.4439	10.1546	9.9699	0.9835	-0.0239	0.3698	0.4321
Slc25a5	_LLLQVQHASK_.2	#N/A	9.6359	9.3496	9.7106	8.7578	8.9817	9.1985	9.8711	9.7184	9.3141	9.4378	0.9673	-0.0479	0.1818	0.7405
Slc25a5	_QIFLGGVDKR_.2	#N/A	9.5538	9.7377	9.6576	9.2059	9.2668	9.2520	9.8376	9.6883	9.4612	9.3715	0.9850	-0.0218	0.3499	0.4560
Slc25a5	_DFLAGGVAAAISK_.2	#N/A	8.7093	8.4297	8.8239	8.0059	8.0949	8.4308	9.0087	8.7581	8.7873	8.1361	0.9704	-0.0434	0.2805	0.5520
Slc25a5	_GAWSNVLR_.2	#N/A	8.6606	8.3382	8.4448	7.7950	8.2861	8.4637	7.0587	9.1223	8.7968	8.6926	0.9898	-0.0149	0.8326	0.0796
Slc25a5	_GLGDC[Carbamidomethyl (C)]LVK_.2	#N/A	7.7054	7.4432	7.7973	6.6835	7.2936	7.1800	6.6200	8.1134	7.8307	7.5112	0.9776	-0.0327	0.6221	0.2062
Slc25a5	_AAYFGIYDTAK_.2	#N/A	6.5859	6.2105	6.6780	6.2715	5.2946	6.1759	6.5601	7.0354	6.5901	6.5824	0.9269	-0.1095	0.1034	0.9856
Slc25a5	_QIFLGGVDK_.2	#N/A	6.1140	6.4216	6.1183	5.7848	6.3800	6.2258	6.3835	6.8107	6.7023	6.0314	0.9525	-0.0702	0.1286	0.8907
Slc25a5	_[Acetyl (Protein N-term)]TDAAVSFAK_.2	#N/A	6.7778	6.0067	6.9207	3.1335	6.3899	6.1312	4.2185	7.2015	6.9956	6.6261	0.9414	-0.0872	0.6936	0.1589
Slc25a5	_GM[Oxidation (M)]GGAFVLVLYDEIKK_.2	#N/A	3.9931	3.8122	3.9150	3.8396	2.8469	4.0216	3.6618	4.1938	4.0391	3.9431	0.9441	-0.0830	0.3896	0.4094
Slc25a5	_MM[Oxidation (M)]MQSGR_.2	#N/A	4.2924	4.0649	4.1106	4.7523	3.9433	4.1891	4.1197	4.0309	3.3613	4.1555	1.0788	0.1094	0.1741	0.7592
Slc25a5	_GTDIMYTGTLDC[Carbamidomethyl (C)]WR_.2	#N/A	4.8844	3.2183	3.8578	3.4015	3.2678	3.3568	3.5498	3.5996	3.8987	3.0788	1.0376	0.0532	0.7166	0.1447
Slc25a5	_GM[Oxidation (M)]GGAFVLVLYDEIK_.2	#N/A	3.3714	4.1276	4.6059	4.2880	4.0159	4.1095	4.0027	4.3524	3.1506	3.8269	1.0661	0.0923	0.4050	0.3926
Nek1	_TC[Carbamidomethyl (C)]S[Phospho (STY)]LPDLSK_.2	P51954_S997_M1_Nek1	8.5716	8.3066	8.6333	8.7142	7.3178	8.1104	8.8580	8.4583	8.6025	8.4156	0.9641	-0.0527	0.2986	0.5249
Nek1	_SSDVPLPLELLET[Phospho (STY)]GGSPSK_.2	P51954_T615_M1_Nek1	3.7667	3.8411	3.5004	3.8631	4.5586	5.3656	3.6471	5.7659	3.6139	4.2223	0.9622	-0.0556	0.7672	0.1151
Nek1	_EQPGDEYS[Phospho (STY)]EEEESVLK_.2	P51954_S1071_M1_Nek1	3.4254	4.1817	3.1591	4.2339	4.0700	4.0555	3.9486	4.2983	3.0965	3.8810	1.0127	0.0181	0.8782	0.0564
Tst	_VLDASWYSPGTR_.2	#N/A	6.6630	6.3262	6.4268	6.5970	6.6406	6.3725	6.8237	6.5189	6.6435	6.3674	0.9872	-0.0185	0.4545	0.3425
Usp10	_LLS[Phospho (STY)]PTHEK_.2	P52479_S541_M1_Usp10	4.1440	7.5668	6.9521	3.5154	6.3381	3.3369	7.6508	7.5368	7.0771	3.0987	0.8373	-0.2563	0.4439	0.3528
Usp10	_C[Carbamidomethyl (C)]S[Phospho (STY)]PPVPSPLASEK_.2	P52479_S359_M1_Usp10	3.1682	4.0580	3.8550	4.3576	3.7947	4.1792	7.7338	3.7608	3.4094	7.1837	0.7067	-0.5009	0.1147	0.9405
Usp10	_TC[Carbamidomethyl (C)]DS[Phospho (STY)]PQNPVDFISGPVPDSPFPR_.3	P52479_S208_M1_Usp10	3.2621	4.0673	3.9489	5.5831	3.8886	3.8409	4.1662	3.6669	3.3155	4.4185	1.0531	0.0746	0.6535	0.1848
Pkm	_LDIDSAPITAR_.2	#N/A	12.1478	12.3357	12.4573	12.2692	12.3343	12.2102	12.3397	12.1610	12.2486	12.0747	1.0071	0.0102	0.2609	0.5835
Pkm	_NTGIIC[Carbamidomethyl (C)]TIGPASR_.2	#N/A	11.5919	11.5975	11.8819	11.6893	11.5292	11.5752	11.7472	11.4297	11.5928	11.4571	1.0076	0.0109	0.3437	0.4638
Pkm	_GDYPLEAVR_.2	#N/A	11.2850	10.9565	11.3038	11.0773	11.0984	11.0080	10.5697	11.1205	11.0136	11.2014	1.0132	0.0190	0.3075	0.5121
Pkm	_APIIAVTR_.2	#N/A	10.8273	10.9209	10.9318	10.8008	10.7962	10.7072	10.4057	11.0626	10.9985	10.7034	1.0035	0.0051	0.7712	0.1128
Pkm	_DAVLNAWAEDVDLR_.2	#N/A	10.6907	10.4474	11.0699	10.5894	10.5121	10.7678	10.7160	10.3403	10.6429	10.7288	1.0068	0.0098	0.6056	0.2178
Pkm	_IENHEGVR_.2	#N/A	9.1133	9.0973	9.2248	9.1094	9.8315	9.2500	8.8978	9.2110	9.2275	9.1524	1.0163	0.0234	0.3682	0.4339
Pkm	_IYVDDGLISLQVK_.2	#N/A	8.8093	8.5461	9.0794	9.0444	8.6333	8.9328	8.9370	8.7137	8.9481	8.7389	1.0007	0.0011	0.9587	0.0183
Pkm	_C[Carbamidomethyl (C)]DENILWLDYK_.2	#N/A	8.2985	7.8180	8.3910	8.0368	7.9994	8.4311	8.3541	7.9737	8.2425	8.3516	0.9917	-0.0120	0.6501	0.1870
Pkm	_GVNLPGAAVDLPAVSEK_.2	#N/A	8.5978	7.9200	8.1071	8.7869	8.1272	8.0781	8.2614	8.1998	8.4262	8.3789	0.9943	-0.0082	0.7990	0.0975
Pkm	_KAADVHEVR_.2	#N/A	9.2470	7.9530	7.8898	8.0599	8.3186	8.5139	8.5989	7.8513	8.4328	8.4019	1.0011	0.0016	0.9756	0.0107
Pkm	_EKGADFLVTEVENGGSLGSK_.3	#N/A	7.5187	7.0598	7.2078	7.3710	7.4379	7.5723	7.6613	7.0381	7.4502	7.5504	0.9914	-0.0124	0.6747	0.1709
Pkm	_AADVHEVRK_.2	#N/A	7.1622	6.4757	6.4267	7.0224	6.8303	7.3682	6.8557	6.3269	6.9020	6.6475	1.0296	0.0421	0.3913	0.4075
Pkm	_AADVHEVR_.2	#N/A	6.6095	6.2944	5.7526	6.4516	6.9856	6.4960	6.1261	6.3140	6.4296	6.4727	1.0152	0.0217	0.6684	0.1750
Pkm	_GDLGIEIPAEK_.2	#N/A	11.9571	11.8686	12.0697	11.9100	11.7195	11.9625	11.8883	11.7730	11.7379	11.8323	1.0090	0.0130	0.1402	0.8533
Pkm	_M[Oxidation (M)]QHLIAR_.2	#N/A	8.5748	9.0440	8.9502	9.0776	9.3282	8.8339	8.7879	9.1738	9.0401	8.9563	0.9976	-0.0034	0.8855	0.0528
Pkm	_ITLDNAYM[Oxidation (M)]EK_.2	#N/A	8.8351	8.7505	9.1645	8.5638	8.8636	8.9652	8.6993	8.8569	8.8833	8.9030	1.0024	0.0035	0.8491	0.0710
Pkm	_GVNLPGAAVDLPAVSEKDIQDLK_.3	#N/A	6.3559	6.4822	7.2888	6.9895	6.0762	6.1718	6.8482	6.4930	6.1604	6.5537	1.0072	0.0104	0.8657	0.0626
Pkm	_GADFLVTEVENGGSLGSKK_.3	#N/A	6.6732	6.9919	7.2237	6.9711	6.1737	6.6826	7.0309	6.5097	6.0893	6.7581	1.0287	0.0408	0.4614	0.3359
Pkm	_VNLAM[Oxidation (M)]DVGK_.2	#N/A	8.2290	8.6785	8.6476	8.0508	8.5981	8.6545	8.4981	8.8238	8.6504	8.5110	0.9832	-0.0244	0.3618	0.4415
Pkm	_AEGSDVANAVLDGADC[Carbamidomethyl (C)]IM[Oxidation (M)]LSGETAK_.3	#N/A	6.1676	5.9263	5.6605	7.1915	5.3015	5.5861	6.2532	6.0144	6.2339	6.5484	0.9537	-0.0685	0.4325	0.3641
Pkm	_EATESFASDPILYR_.2	#N/A	5.4493	4.7992	4.8674	5.4478	5.4313	5.7588	5.6967	4.6336	4.7889	3.9239	1.1116	0.1527	0.1634	0.7867
Pkm	_AEGSDVANAVLDGADC[Carbamidomethyl (C)]IM[Oxidation (M)]LSGETAKGDYPLEAVR_.3	#N/A	2.8227	4.9207	5.6537	5.1748	4.0168	4.1697	4.3105	4.4810	4.0694	4.1443	1.0490	0.0690	0.6996	0.1552
Pkm	_FGVEQDVDM[Oxidation (M)]VFASFIR_.2	#N/A	6.4885	6.7790	7.1875	6.9542	6.7150	7.5003	7.4985	6.7795	7.4269	6.9311	0.9690	-0.0454	0.3686	0.4334
Pkm	_EATESFASDPILYRPVAVALDTK_.3	#N/A	9.0971	8.9883	9.1605	9.4986	9.0609	9.1466	9.1422	9.2770	9.2107	9.0375	0.9991	-0.0013	0.9364	0.0286
Pkm	_LAPITSDPTEAAAVGAVEASFK_.2	#N/A	5.1180	5.4636	5.8280	5.8224	4.8980	5.0598	5.2727	5.5795	5.1885	4.5993	1.0397	0.0562	0.4552	0.3418
Pkm	_GSGTAEVELKK_.2	#N/A	8.2554	8.1778	8.3183	8.5406	8.7684	8.5556	8.1383	8.5983	8.5174	8.6942	0.9940	-0.0087	0.7408	0.1303
Pkm	_EATESFASDPILYRPVAVALDTKGPEIR_.4	#N/A	3.3958	4.7461	5.6678	4.3044	3.6306	5.1348	3.9782	5.3470	4.8015	4.6602	0.9538	-0.0682	0.6757	0.1702
Pkm	_RFDEILEASDGIM[Oxidation (M)]VAR_.3	#N/A	7.3321	7.1839	7.7273	7.7433	7.1445	7.5083	7.4708	7.4083	7.5816	7.9754	0.9778	-0.0324	0.3420	0.4660
Pkm	_GADFLVTEVENGGSLGSK_.2	#N/A	7.9641	7.6683	8.3095	8.0021	7.7736	7.8519	7.8835	7.5587	7.8505	7.9091	1.0164	0.0234	0.3575	0.4467
Pkm	_LNFSHGTHEYHAETIK_.3	#N/A	4.6194	4.4966	4.0252	4.8236	5.2426	3.3279	4.5010	4.5199	4.1200	3.9656	1.0341	0.0484	0.6945	0.1583

Pkm	_GSGTAEVELK_.2	#N/A	4.0879	3.1382	3.2739	6.2499	3.2136	3.5981	3.4912	4.3419	3.9905	3.7436	1.0090	0.0130	0.9565	0.0193
Pkm	_FDEILEASDGIMVAR_.2	#N/A	3.3762	4.1325	3.1099	4.2831	4.0208	4.1046	3.9978	3.8354	3.4839	4.2501	0.9861	-0.0201	0.8489	0.0712
Ndufs6	_TGTC[Carbamidomethyl (C)]GYC[Carbamidomethyl (C)]GLQFK_.2	#N/A	5.4200	5.3594	4.0981	5.7014	4.0378	5.3617	4.9384	4.7721	5.1323	5.4561	0.9846	-0.0224	0.8460	0.0726
Ndufs6	_EVNENFAIDLIAQQPVNEVEHR_.3	#N/A	3.8833	3.5627	3.6170	3.1608	4.2146	3.7560	4.6363	3.0856	4.1394	3.3822	0.9707	-0.0430	0.7449	0.1279
Ndufs6	_ITHTGQVYDEK_.2	#N/A	5.8505	5.6484	5.0371	5.7206	6.3800	5.4707	5.5796	5.3740	5.2132	5.6443	1.0425	0.0601	0.3602	0.4435
Ndufs6	_VYINLDKETK_.2	#N/A	6.4165	6.3449	6.7965	6.7238	7.1267	6.8371	6.4569	6.5856	6.5747	6.5633	1.0248	0.0354	0.3079	0.5115
Ndufs6	_IIAC[Carbamidomethyl (C)]DGGGGALGHPK_.2	#N/A	5.3612	5.4358	5.2839	5.8487	6.4886	5.3743	5.2639	5.5782	5.5909	5.3260	1.0354	0.0501	0.4577	0.3394
Ndufs6	_QKEVNENFAIDLIAQQPVNEVEHR_.4	#N/A	3.7627	3.0181	3.4963	3.8967	4.7176	4.4667	3.6114	3.9611	4.2601	4.3485	0.9624	-0.0553	0.6705	0.1736
Fgd1	_ALRFSY[Phospho (STY)]HLEGS[Phospho (STY)]QPR_.2	P52734_S98_M2_Fgd1	8.8079	8.7414	7.8797	9.1130	8.2800	8.1561	9.6518	8.0519	8.3849	8.8763	0.9720	-0.0410	0.5189	0.2849
Fgd1	_SLS[Phospho (STY)]LDPGQSLEPHPEGPQR_.3	#N/A	3.9887	2.9531	6.3109	3.9622	5.9207	3.7837	5.6146	6.0547	3.8053	7.5283	0.7802	-0.3582	0.2010	0.6967
Fgd1	_LLNSTNRDDEDT[Phospho (STY)]PPNSPNVDLGK_.3	P52734_T710_M1_Fgd1	3.5904	3.6357	4.2772	3.2791	3.5240	3.1006	4.4946	3.3386	5.3312	3.2461	0.8697	-0.2015	0.2633	0.5796
Fgd1	_QES[Phospho (STY)]VELTVQQK_.2	P52734_S364_M1_Fgd1	4.0627	3.3181	3.7963	3.3401	3.2064	3.4182	3.4884	3.6611	3.9601	3.0174	0.9977	-0.0033	0.9728	0.0120
Rida	_AAYQVAALPR_.2	#N/A	7.4322	7.9731	7.8490	7.6239	8.2739	7.8522	7.6817	8.2071	7.8792	7.9104	0.9892	-0.0157	0.6299	0.2008
Rida	_APAAIGPYSQAVQVDR_.2	#N/A	6.9158	6.8174	7.2820	7.8517	7.6709	7.3711	7.5497	7.4660	7.1274	6.9533	1.0061	0.0087	0.8562	0.0674
Rida	_AAGC[Carbamidomethyl (C)]DFNNVVK_.2	#N/A	5.9206	5.7772	6.1404	5.6496	5.9706	5.1303	5.3967	5.9796	5.0867	5.2824	1.0604	0.0846	0.2009	0.6970
Rida	_TYFQGSLPAR_.2	#N/A	6.6104	7.4891	7.7253	6.8734	7.6096	7.4222	7.4496	7.2870	7.1797	6.7260	1.0178	0.0255	0.6332	0.1985
Rida	_TTVLLADM[Oxidation (M)]NDFGTVNEIYK_.2	#N/A	4.5825	5.1319	5.3762	5.3009	5.0893	5.3676	3.8239	4.9688	4.4305	5.0692	1.1243	0.1690	0.0709	1.1491
Rida	_TIYISGQVGLDPSSGQLVPGGVVEEAK_.3	#N/A	3.5572	4.3135	3.2909	4.3355	4.1214	3.9236	3.8168	4.1665	2.9647	4.0128	1.0491	0.0691	0.5611	0.2510
Hmga2	_KPAQETEETSSQES[Phospho (STY)]AEED_.2	P52927_S104_M1_Hmga2	3.0073	3.8125	3.4692	4.1967	3.3050	4.0957	3.9114	3.9217	3.5703	4.1637	0.9373	-0.0934	0.3699	0.4319
Dbt	_LSDIGEGIR_.2	#N/A	4.2826	5.2384	4.6599	4.8468	5.0470	3.9099	5.2827	5.0791	4.7426	5.1080	0.9230	-0.1156	0.1844	0.7342
Cux1	_AAS[Phospho (STY)]REEPIEWEF_.2	P53564_S1506_M1_Cux1	6.1440	6.0309	6.3175	6.5114	6.2890	6.4974	6.3892	6.7157	6.6772	6.3495	0.9641	-0.0527	0.0925	1.0340
Limk1	_SC[Carbamidomethyl (C)]S[Phospho (STY)]IDTSPGTSSLASPASQR_.2	P53668_S298_M1_Limk1	3.7936	3.6937	6.5044	6.1602	4.5439	5.6489	3.7452	3.4483	5.5810	5.2836	1.1203	0.1638	0.4905	0.3094
Limk1	_VQGVDPGC[Carbamidomethyl (C)]M[Oxidation (M)]S[Phospho (STY)]PDVK_.2	#N/A	6.9744	6.5477	6.4231	7.0728	5.7035	5.7594	6.8179	5.6933	5.8522	6.0724	1.0499	0.0702	0.4180	0.3788
Sox1	_SEPSGS[Phospho (STY)]PPAPAHSR_.2	P53783_S325_M1_Sox1	8.6372	9.3446	3.4393	9.9810	9.0757	7.9637	9.5146	8.9658	8.2933	4.1612	1.0439	0.0620	0.8314	0.0802
Pitpna	_LFTNFHR_.2	#N/A	6.6372	7.0023	6.3600	6.8172	6.8711	6.7257	6.6685	6.5760	6.5458	6.3977	1.0288	0.0410	0.1603	0.7952
Pitpna	_LEPEAWK_.2	#N/A	5.9297	3.2978	3.9474	5.6231	5.8749	5.3185	4.9102	5.8229	5.6078	5.5836	0.9120	-0.1330	0.4341	0.3624
Pitpna	_VENFIHK_.2	#N/A	6.0433	5.3611	3.8049	5.1574	5.6123	5.3512	5.2145	5.7746	5.2905	5.1670	0.9739	-0.0381	0.7371	0.1325
Pitpna	_VILPVSVDEYQVGQLYSVAEASK_.3	#N/A	3.4172	4.9398	6.1354	5.6549	4.0618	5.6188	5.0235	4.9220	4.9551	5.4672	0.9763	-0.0346	0.8300	0.0809
Pitpna	_NETGGGEGVEVLVNEPYEKDDGEKGQYTHK_.4	#N/A	4.9813	5.2935	5.1750	5.1509	5.3046	4.7597	5.1266	5.2751	4.4407	4.9739	1.0316	0.0449	0.4061	0.3914
Pitpna	_HVEAIYIDIADR_.2	#N/A	3.1820	4.0442	3.8688	4.2914	3.9325	4.1929	4.0861	3.7471	4.5140	4.3384	0.9394	-0.0902	0.3234	0.4902
Pitpna	_MLAPEGALNIHEK_.3	#N/A	4.0527	3.1735	3.2386	3.7413	3.0618	3.5628	3.4560	4.3772	4.0258	3.7083	0.8921	-0.1648	0.1324	0.8781
Slc16a1	_SDANTDLIGGS[Phospho (STY)]PK_.2	#N/A	9.6453	9.4567	9.6548	9.5048	8.5570	9.4126	10.3054	9.6558	9.5646	9.4046	0.9629	-0.0545	0.2055	0.6873
Slc16a1	_SKES[Phospho (STY)]LQEAGK_.2	#N/A	8.9447	8.4616	8.9026	8.9393	7.8832	8.7420	9.2462	9.2182	8.7737	8.5271	0.9669	-0.0485	0.2773	0.5571
Slc16a1	_EGKEDEAS[Phospho (STY)]T[Phospho (STY)]DVDEKPK_.2	#N/A	6.1740	5.4328	5.2774	5.3262	4.5550	5.4419	5.9037	5.3316	5.7936	4.9689	0.9761	-0.0349	0.6859	0.1637
Slc16a1	_LKS[Phospho (STY)]KES[Phospho (STY)]LQEAGK_.2	#N/A	8.6840	8.4608	8.8567	8.8426	7.6567	8.4647	9.1342	8.9289	8.5615	8.4567	0.9685	-0.0461	0.3183	0.4972
Slc16a1	_AAQSPQQHSSGDPTEEES[Phospho (STY)]PV_.2	#N/A	8.2545	8.3114	8.7775	8.4917	7.0253	8.0088	8.9271	8.6064	8.2611	8.1520	0.9597	-0.0593	0.3426	0.4653
Slc16a1	_SDANTDLIGGS[Phospho (STY)]PKGEK_.2	#N/A	6.0224	7.0755	7.8728	7.2541	5.3380	6.5074	7.0902	7.0826	6.6535	6.2720	0.9858	-0.0206	0.8496	0.0708
Slc16a1	_S[Phospho (STY)]KESLQEAGKSDANTDLIGGSPK_.3	#N/A	4.1312	3.2576	3.6299	4.1341	3.2664	3.1582	3.5463	4.0085	3.9244	3.4618	0.9628	-0.0547	0.5954	0.2252
Rab2a	_YIIIGDTGVGK_.2	#N/A	6.9163	6.5425	6.8742	6.2355	5.8248	6.5050	7.0307	6.9475	6.5639	6.2462	0.9680	-0.0469	0.4231	0.3735
Rab2a	_TASNVEEAFINTAK_.2	#N/A	6.9319	6.3216	6.7528	6.5990	6.4180	6.3725	6.9916	6.8715	6.6016	6.4538	0.9757	-0.0355	0.3263	0.4864
Rab2a	_IQEGVFDINNEANGIK_.2	#N/A	6.5274	6.4351	6.8645	6.5714	5.9663	6.0935	6.8601	6.8307	6.6974	5.9835	0.9722	-0.0407	0.4569	0.3402
Rab2a	_LQIWDTAGQESFR_.2	#N/A	6.0105	5.8264	6.3193	5.6165	4.7252	5.9621	6.0927	6.1844	5.8465	5.7030	0.9642	-0.0526	0.4885	0.3112
Rab2a	_DTFNHLTTWLEDAR_.3	#N/A	6.3702	5.9911	5.8859	6.2804	5.1363	6.0880	6.1726	6.4297	5.6417	5.1803	1.0175	0.0250	0.7530	0.1232
Anapc1	_SFDFEGSLS[Phospho (STY)]PVIAPK_.2	P53995_S688_M1_Anapc1	6.1855	6.6875	7.2038	3.6255	5.7046	6.4552	7.1148	7.1481	6.9690	6.1272	0.8739	-0.1945	0.2329	0.6328
Idh2	_ATDFVVDR_.2	#N/A	6.4080	6.5307	6.7336	6.3961	6.5132	6.8959	5.9051	6.9540	6.1461	6.3518	1.0379	0.0537	0.2722	0.5651
Idh2	_GKLDGNQDLIR_.2	#N/A	6.4366	6.2187	6.3460	6.2470	6.2380	5.9152	6.6026	6.1705	6.5203	6.4503	0.9686	-0.0461	0.1210	0.9171
Idh2	_LNEHFLNTTDFLDTIK_.3	#N/A	6.0178	6.1564	6.3096	6.3200	5.7664	5.0932	5.8578	5.7518	5.6015	6.1269	1.0188	0.0268	0.6767	0.1696
Idh2	_YFDLGLPNR_.2	#N/A	5.6608	6.2227	3.2377	6.0277	5.5098	5.8213	4.4306	5.7465	5.7629	4.9013	1.0390	0.0552	0.7504	0.1247
Idh2	_DQTNDQVTIDSALATQK_.2	#N/A	3.0928	3.8980	3.7796	6.1635	5.5866	5.0240	3.9970	3.8362	5.0901	5.9440	0.9733	-0.0391	0.8655	0.0627
Idh2	_TIEAEAAHGTVTR_.2	#N/A	4.2900	4.8775	3.4760	4.8585	3.2948	3.7449	3.8016	3.9959	4.5122	4.0892	0.9977	-0.0033	0.9804	0.0086
Idh2	_FAQTLEK_.2	#N/A	3.8759	3.4665	3.6095	3.1533	3.0196	2.9408	3.3016	3.8479	4.1469	4.3314	0.8560	-0.2244	0.0610	1.2149
Stmn1	_DKHVEEVRK_.2	#N/A	9.4692	7.7074	7.5396	8.1014	7.5280	9.1616	9.7719	7.6205	8.4593	9.1752	0.9423	-0.0858	0.4013	0.3966
Stmn1	_EHEKEVLQK_.2	#N/A	6.3501	6.9221	6.9461	6.3658	7.4357	7.1709	5.6053	6.2784	6.5277	6.9239	1.0839	0.1162	0.1265	0.8980
Stmn1	_SKESVPDFPLS[Phospho (STY)]PPKK_.3	#N/A	5.4701	7.8253	7.9931	9.0837	6.8824	6.2201	7.4050	7.7764	6.7694	6.5615	1.0165	0.0236	0.8716	0.0597
Stmn1	_SHEAEVLK_.2	#N/A	8.6415	8.0949	8.3079	8.8076	8.0535	8.5137	8.2514	7.1103	8.5733	8.7664	1.0279	0.0397	0.5110	0.2916
Stmn1	_ESVPDFPLS[Phospho (STY)]PPKKK_.3	#N/A	8.9863	9.7467	10.2478	10.4448	9.5253	9.3453	9.6190	9.6608	8.9745	9.1271	1.0397	0.0561	0.2703	0.5682
Stmn1	_ASGQAFELILS[Phospho (STY)]PR_.2	#N/A	10.6309	11.6153	12.1251	12.6777	11.3529	11.1369	11.3730	11.6347	11.6447	10.9526	1.0165	0.0237	0.6443	0.1909
Stmn1	_SKESVPDFPLS[Phospho (STY)]PPK_.2	#N/A	5.8179	8.1626	13.7271	13.9568	12.9690	13.1101	13.4681	13.1151	13.3203	13.2491	0.8497	-0.2350	0.2873	0.5416
Stmn1	_RKS[Phospho (STY)]HEAEVLK_.2	#N/A	10.1311	10.0643	10.9225	11.1175	10.3222	10.7732	9.9709	10.3372	10.6321	10.7519	1.0127	0.0182	0.6301	0.2006
Stmn1	_KSHEAEVLK_.2	#N/A	5.1173	4.9734	3.1648	4.2094	5.1295	4.7350	5.4936	4.6317	4.9443	4.5512	0.9286	-0.1069	0.4328	0.3637
Stmn1	_NKES[Phospho (STY)]KDPADETEAD_.2	P54227_S139_M1_Stmn1	5.8568	7.1428	7.5233	7.7033	7.3115	7.0869	6.1641	6.9544	6.3907	6.9282	1.0749	0.1041	0.2158	0.6659
Stmn1	_REHEKEVLQK_.3	#N/A	3.4616	4.2668	4.1484	3.1503	6.3289	7.3775	6.3193	3.4674	6.7304	5.4856	0.8706	-0.1999	0.5106	0.2919
Stmn1	_[Acetyl (Protein N-term)]ASSDIQVKELEKR_.3	#N/A	4.2608	4.2775	3.9462	5.6056	4.8731	4.0863	4.7217	3.9804	3.6289	4.7709	1.0544	0.0765	0.5655	0.2476
Stmn1	_RAS[Phospho (STY)]GQAFELILS[Phospho (STY)]PR_.2	#N/A	5.3280	6.2425	6.5998	8.0128	6.9319	6.8146	5.6238	7.3601	6.8797	5.7419	1.0396	0.0560	0.6640	0.1779
Stmn1	_[Acetyl (Protein N-term)]ASSDIQVK_.2	#N/A	2.3673	4.3044	4.1860	3.1878	2.4534	3.8503	3.9646	3.2738	2.2470	3.5978	1.0369	0.0523	0.8237	0.0842
Stmn1	_ESVPDFPLS[Phospho (STY)]PPK_.2	#N/A	3.1652	2.9306	4.3997	3.9435	3.8098	4.3157	4.0918	3.0577	3.3567	3.6208	1.0648	0.0906	0.5330	0.2732
Atxn1	_EAS[Phospho (STY)]PSTLNDK_.2	P54254_S382_M1_Atxn1	8.3114	8.6808	4.1648	3.8375	4.1046	8.8990	3.9857	8.5324	8.5413	3.1336	1.0471	0.0664	0.8729	0.0590
Atxn1	_RWS[Phospho (STY)]APETR_.2	#N/A	8.0603	8.0632	8.5795	8.5228	7.7296	8.0134	8.3599	7.9932	7.6317	7.9533	1.0222	0.0316	0.4114	0.3858
Atxn1	_WS[Phospho (STY)]APETR_.2	#N/A	7.6034	7.8474	7.7171	7.8848	7.8313	7.7537	7.4011	7.5323	7.8182	7.7858	1.0182	0.0260	0.1849	0.7330
Atxn1	_ALS[Phospho (STY)]AGLDYSPPSAPR_.2	P54254_S81_M1_Atxn1	3.5079	6.9845	6.4028	7.0977	5.8798	6.7572	6.6717	7.2839	6.7949	7.3137	0.8701	-0.2007	0.2290	0.6401
Atxn1	_HGS[Phospho (STY)]AGTSGEHGLQGM[Oxidation (M)]GLHK_.3	P54254_S62_M1_Atxn1	3.0215	3.8267	3.7083	4.4365	3.6480	4.0815	3.9256	3.9075	3.5561	4.1779	0.9731	-0.0394	0.6998	0.1551
Atxn1	_RRWS[Phospho (STY)]APETR_.3	#N/A	4.2508	3.5552	3.4368	3.9394	3.3765	4.3530	3.2130	4.1790	3.8276	3.9064	1.0098	0.0141	0.8940	0.0487
Msh6	_SAAVSASS[Phospho (STY)]PEAK_.2	P54276_S63_M1_Msh6	4.3082	6.9400	3.4941	3.9968	2.8063	6.5054	3.7115	6.7830	3.7703	6.2524	0.9115	-0.1337	0.6813	0.1667
Cacnb3	_VEEHS[Phospho (STY)]PLER_.2	P54285_S393_M1_Cacnb3	10.4626	10.5056	10.6314	11.0181	9.9784	10.4818	10.7628	10.4738	10.5174	10.4814	0.9957	-0.0063	0.8043	0.0946
Cacnb3	_RS[Phospho (STY)]PPPSLAK_.2	P54285_S152_M1_Cacnb3	6.9006	3.4656	7.0265	6.9036	7.3916	7.0922	7.2769	6.0683	3.7778	6.6311	1.0884	0.1222	0.6016	0.2207
Cacnb3	_LLAQDS[Phospho (STY)]EHDHNDR_.3	P54285_S464_M1_Cacnb3	5.4211	3.0895	3.9866	4.4252	2.9108	4.2213	3.6786	4.6896	4.9694	3.8602	0.9325	-0.1009	0.6003	0.2216
Cacnb3	_QAWTGSS[Phospho (STY)]QR_.2	P54285_S418_M1_Cacnb3	4.1940	3.4494	3.9277	3.4714	3.3377	3.2869	3.1800	3.5297	3.8288	3.1487	1.0553	0.0777	0.4253	0.3713
Cacnb3	_DSLMPS[Phospho (STY)]DEASESSR_.2	P54285_S403_M1_Cacnb3	6.7408	3.5864	3.5217	9.3451	6.4924	3.6844	7.3447	5.2857	3.5974	6.9150	0.9613	-0.0569	0.8757	0.0576
Lipe	_SVS[Phospho (STY)]EAALAQPEGLLGTDTLK_.2	P54310_S559_M1_Lipe	5.3747	3.0111	4.0650	6.5479	2.8324	3.3963	3.7571	3.3924	6.1441	5.4185	0.8988	-0.1539	0.6185	0.2087

Lipe	_TTS[Phospho (STY)]PTAESVRPTESMR_.3	P54310_S542_M1_Lipe	4.2443	3.5486	3.4302	4.5721	3.3700	4.3595	3.2195	4.1856	4.2290	3.8999	1.0096	0.0138	0.9121	0.0400
Rad23a	_NENLAANFLLSQNFDDE_.2	#N/A	2.9242	4.9563	4.1587	5.7145	4.1571	3.0559	4.0561	4.3771	4.9150	5.1507	0.8998	-0.1524	0.4500	0.3468
Rad23b	_IDIDPEETVK_.2	#N/A	7.5073	7.1036	7.6470	7.6386	7.6634	7.2700	6.7565	6.9627	7.0687	7.0127	1.0750	0.1044	0.0040	2.3958
Rad23b	_ASFNNPDR_.2	#N/A	6.7933	6.4647	5.5881	6.3631	7.0063	6.2723	6.0004	6.0781	6.4639	6.6806	1.0173	0.0247	0.7082	0.1498
Rad23b	_TLQQTFK_.2	#N/A	4.7801	4.9876	4.6807	4.5681	5.5298	5.0230	4.6240	4.8234	5.3255	4.9874	0.9976	-0.0035	0.9564	0.0193
Rad23b	_NENLAANFLLQQNFDED_.2	#N/A	5.8212	3.4537	3.3353	5.6032	6.0356	5.2458	3.5527	3.1505	3.9291	5.8897	1.1901	0.2511	0.3443	0.4631
Rad23b	_QIIQQNPSLLPALLQQIGR_.3	#N/A	4.0102	3.9053	3.3828	4.0928	3.7817	3.8291	3.5817	3.9528	3.5398	3.8715	1.0260	0.0371	0.5374	0.2697
Faf1	_RTS[Phospho (STY)]PVQTR_.2	P54731_S269_M1_Faf1	9.2024	9.3555	9.7685	9.3460	8.3872	9.0126	9.5579	9.4188	9.2609	9.2267	0.9800	-0.0292	0.4624	0.3350
Faf1	_TPS[Phospho (STY)]GEFLER_.2	P54731_S581_M1_Faf1	3.5555	4.3607	4.2423	3.2441	4.1820	3.5475	3.9083	6.2137	3.0221	3.2111	0.9429	-0.0848	0.7189	0.1433
Ephb3	_FLEDDPSDPTYTSS[Phospho (STY)]LGGK_.2	P54754_S790_M1_Ephb3	4.0262	3.3305	6.2003	3.7148	3.1518	3.0768	3.4295	4.4037	6.5645	3.6818	0.8666	-0.2066	0.4843	0.3149
Ephb2	_FLEDDTS[Phospho (STY)]DPTYTSALGGK_.2	P54763_S776_M1_Ephb2	3.6606	2.9401	3.9370	4.0070	3.7119	3.6400	3.9907	3.7114	3.7982	3.7802	0.9553	-0.0660	0.4161	0.3808
Psmc4	_ENAPAIIFIDEIDAIATK_.2	#N/A	3.7178	3.2752	3.4515	4.4961	3.9349	3.3384	3.1435	2.6744	4.3050	3.6258	1.0771	0.1072	0.4850	0.3142
Psmc4	_LQQELEFLEVQEEYIKDEQK_.3	#N/A	3.8764	3.1808	6.6112	6.2264	3.0021	3.2265	3.5873	5.0597	3.8612	5.3723	0.9740	-0.0380	0.9006	0.0454
Slc12a2	_GEGTPAAGDGLGRPLGPTPS[Phospho (STY)]QSR_.3	#N/A	3.4175	8.4060	3.7239	4.3635	7.3132	3.8650	8.5684	3.8448	3.7344	7.8815	0.8625	-0.2133	0.5940	0.2262
Slc12a2	_PLGPTPS[Phospho (STY)]QSR_.2	#N/A	3.1644	4.0617	3.8512	4.3539	3.7910	4.1754	4.0686	7.7906	3.4132	7.5592	0.6832	-0.5496	0.0876	1.0577
Slc12a2	_KES[Phospho (STY)]KGPIVPLNVADQK_.3	P55012_S987_M1_Slc12a2	5.7134	5.1838	5.8696	5.6571	2.9527	4.3748	6.1145	6.1997	5.2958	5.2903	0.8661	-0.2074	0.2416	0.6169
Slc12a2	_S[Phospho (STY)]PGTKDVVVNVDYSK_.2	P55012_S937_M1_Slc12a2	4.1157	3.1104	3.3017	3.8043	5.2377	6.2498	3.5190	6.5157	6.0540	6.1307	0.7747	-0.3683	0.1670	0.7772
Ncan	_ELGGEVFYVGPAR_.2	#N/A	6.6621	6.9402	7.0342	7.2729	6.8909	7.3245	6.8241	6.8359	7.0524	6.7248	1.0235	0.0336	0.2753	0.5602
Ncan	_LSSATIAAPR_.2	#N/A	6.7031	7.1507	6.5835	7.0161	7.5634	6.5795	6.6297	6.6540	6.9473	6.6309	1.0323	0.0459	0.3249	0.4882
Ncan	_FDAYC[Carbamidomethyl (C)]FR_.2	#N/A	6.5413	6.1850	6.5247	6.1608	6.7310	6.0252	5.3130	6.2634	6.4829	6.7210	1.0268	0.0382	0.5712	0.2432
Ncan	_VSLPAYPR_.2	#N/A	7.4067	7.5442	7.7851	7.6105	7.8640	7.2552	7.1110	7.6640	7.4756	7.4072	1.0220	0.0314	0.3013	0.5210
Ncan	_YPIQTPR_.2	#N/A	6.7973	7.2861	7.1203	7.3287	7.3012	6.9750	6.8328	7.1131	7.0107	7.1139	1.0167	0.0239	0.3609	0.4427
Ncan	_AALAELVALPC[Carbamidomethyl (C)]FFTLQPR_.2	#N/A	3.5790	4.2775	3.0532	4.8168	3.7451	3.8137	4.0944	4.2662	3.9980	3.2168	0.9967	-0.0048	0.9721	0.0123
Ncan	_YPITQSR_.2	#N/A	3.0680	5.7833	4.3025	4.9785	6.3834	5.7349	5.4677	5.5323	5.7375	5.5176	0.9062	-0.1421	0.4224	0.3743
Ncan	_YQC[Carbamidomethyl (C)]DEGFSQHR_.2	#N/A	4.6751	4.6598	4.9993	5.3238	5.5429	5.7539	4.1422	4.6915	5.1636	5.8153	1.0416	0.0588	0.5875	0.2310
Ncan	_ASDSGLYR_.2	#N/A	5.4410	5.2191	4.0334	3.5772	5.7235	3.1811	3.0743	5.1987	5.5327	5.0511	0.9608	-0.0577	0.7983	0.0978
Ncan	_YNVHATVR_.2	#N/A	3.6833	6.3376	5.3848	5.7151	6.1442	6.2135	5.0333	5.3396	5.4258	5.8188	1.0325	0.0461	0.7462	0.1271
Ncan	_DFQWTDNTGLQYENWR_.2	#N/A	3.5692	3.7732	5.1890	4.3475	3.9832	4.1350	4.4958	4.6152	4.2957	3.2778	0.9988	-0.0017	0.9899	0.0044
Ncan	_C[Carbamidomethyl (C)]GGPAPGVR_.2	#N/A	3.0066	4.4244	3.6934	4.1960	5.4992	4.0964	3.9107	5.0722	5.2879	5.2508	0.8509	-0.2330	0.1794	0.7462
Ncan	_HLQAAFEDGFDNC[Carbamidomethyl (C)]DAGWLSDR_.3	#N/A	4.4934	5.3015	4.4886	5.5317	4.9627	5.3688	5.8182	2.8175	4.7089	4.5483	1.1232	0.1676	0.3388	0.4700
Ncan	_FINSFGHENSWIGLNDR_.3	#N/A	6.1669	5.9594	6.5037	6.0230	6.2750	5.9556	6.1600	5.0076	6.0530	6.4221	1.0400	0.0566	0.4050	0.3926
Ncan	_QDLPILVAK_.2	#N/A	4.0066	3.2620	3.7402	3.2840	3.1503	3.4743	3.4323	3.7172	4.0162	2.9613	0.9871	-0.0187	0.8567	0.0672
Ncan	_AQGM[Oxidation (M)]PTLTSTS[Phospho (STY)]SEGHPEPK_.3	P55066_S869_M1_Ncan	3.3646	4.1209	4.5991	4.2947	4.0092	4.1163	4.0094	3.8237	3.4723	4.2617	1.0494	0.0696	0.4577	0.3394
Aqp4	_GSYM[Oxidation (M)]EVEDNR_.2	#N/A	8.6147	8.6905	9.2380	8.0086	7.7629	8.3184	8.4213	8.2636	7.9246	8.7186	1.0128	0.0184	0.7301	0.1366
Tyro3	_AEQPTESGS[Phospho (STY)]PEVHC[Carbamidomethyl (C)]GER_.2	P55144_S808_M1_Tyro3	4.0578	3.3132	3.7914	3.6016	3.2015	3.4231	3.3163	3.0160	4.1655	3.5686	1.0137	0.0196	0.8535	0.0688
Sh3bp1	_ERTEADLPKPTS[Phospho (STY)]PK_.3	P55194_S535_M1_Sh3bp1	3.3979	7.3580	8.8548	8.8272	4.0244	3.7051	8.0847	3.5311	8.6971	8.6905	0.8313	-0.2665	0.4812	0.3177
Sh3bp1	_DNHSQADHS[Phospho (STY)]PLTTAAPFSR_.3	P55194_S262_M1_Sh3bp1	6.8745	7.3071	7.8568	7.7637	6.3254	7.1471	7.3691	7.1242	6.9596	6.9491	1.0158	0.0226	0.7204	0.1424
Rab8a	_KLEGNS[Phospho (STY)]PQGS[Phospho (STY)]SHGVK_.2	#N/A	5.7542	5.3093	6.6035	5.2751	4.5452	5.7462	6.0447	5.9086	6.3673	5.9123	0.9143	-0.1293	0.1858	0.7310
Rab8a	_LEGNS[Phospho (STY)]PQGS[Phospho (STY)]SHGVK_.2	#N/A	7.6784	7.9263	8.2434	7.8586	6.8976	7.8592	8.2359	8.0511	7.8252	7.8068	0.9704	-0.0433	0.3643	0.4385
Adk	_VAQWLIQEPHK_.2	#N/A	3.6911	4.4087	4.0874	3.5644	3.7025	3.6477	4.3767	4.0631	4.3650	3.7519	0.9302	-0.1044	0.1951	0.7098
Adk	_YAAENNR_.2	#N/A	3.2376	4.1048	4.4721	4.0158	3.9262	3.8033	4.1641	2.9853	3.2844	3.6931	1.1118	0.1529	0.2102	0.6774
Cdh11	_LADLYGS[Phospho (STY)]KDTFDDDS_.2	P55288_S788_M1_Cdh11	4.6007	7.9078	7.5090	8.4479	6.9260	7.5850	8.4178	8.2346	7.7865	7.7005	0.8915	-0.1658	0.2498	0.6024
Cdh11	_PGLRPAPNS[Phospho (STY)]VDVDDFINTR_.3	P55288_S714_M1_Cdh11	3.3618	3.8644	4.0486	5.5435	3.7527	4.3728	4.2659	4.4312	3.2158	3.0174	1.1138	0.1555	0.4016	0.3962
Lrpap1	_IQEYNVLLDTLSR_.2	#N/A	4.5896	4.1481	4.1988	4.5641	4.8223	4.4388	4.4098	4.2220	4.4479	4.6143	1.0083	0.0120	0.8058	0.0938
Lrpap1	_RES[Phospho (STY)]GEEFR_.2	P55302_S53_M1_Lrpap1	4.6996	3.1562	4.0642	5.0555	5.0114	4.9863	4.4501	4.0779	4.6954	4.5553	1.0114	0.0164	0.9017	0.0450
Stmn2	_DLS[Phospho (STY)]LEEIQK_.2	#N/A	14.0876	13.9763	14.7653	14.4232	13.7861	14.2212	14.2867	13.8102	14.0970	14.1445	1.0089	0.0128	0.5365	0.2705
Stmn2	_DLS[Phospho (STY)]LEEIQKK_.2	#N/A	13.0628	13.2959	14.1675	13.8552	13.1571	13.4250	13.6132	13.1458	13.2895	13.3660	1.0105	0.0151	0.5638	0.2489
Stmn2	_KLEAAEER_.2	#N/A	8.2308	8.0236	8.4417	8.3677	9.1397	8.4750	8.1079	7.8169	8.5294	8.4294	1.0274	0.0390	0.3582	0.4459
Stmn2	_KDLS[Phospho (STY)]LEEIQK_.2	#N/A	6.3973	6.4473	6.8227	7.1636	7.1025	6.8117	6.9977	6.2644	6.6899	6.7883	1.0158	0.0226	0.6175	0.2094
Stmn2	_KKDLS[Phospho (STY)]LEEIQK_.2	#N/A	6.4670	7.0264	7.5151	7.4067	7.2474	7.2096	7.4401	6.9155	7.1378	6.9100	1.0063	0.0090	0.8405	0.0754
Stmn2	_RKS[Phospho (STY)]QEAQVLK_.2	P55821_S97_M1_Stmn2	4.9769	4.4937	5.8063	5.4547	3.7568	5.2778	5.7817	5.7251	5.3487	4.4583	0.9310	-0.1031	0.4355	0.3610
Stmn2	_RAS[Phospho (STY)]GQAFELILKPPS[Phospho (STY)]PISEAPR_.3	#N/A	3.4335	4.1897	3.1672	5.2582	4.0780	4.0474	4.3600	5.1022	3.0885	3.8890	0.9803	-0.0287	0.8752	0.0579
Stmn2	_AS[Phospho (STY)]GQAFELILKPPS[Phospho (STY)]PISEAPR_.3	#N/A	4.0687	3.3647	3.2547	4.6121	3.1944	3.0406	3.4720	4.6706	4.0160	3.7244	0.9039	-0.1457	0.3384	0.4705
Stmn2	_NKELQVELS[Phospho (STY)]G_.2	P55821_S178_M1_Stmn2	3.8696	3.1250	3.6033	3.1470	3.0133	3.6113	3.5045	3.8541	4.1219	3.7013	0.8945	-0.1609	0.0852	1.0696
Stmn2	_KDLS[Phospho (STY)]LEEIQKK_.3	#N/A	1.4107	1.8773	3.5155	4.5601	2.9255	2.7148	3.2075	3.9419	4.2410	1.4248	0.8846	-0.1770	0.6411	0.1931
Golga3	_GFKEEGS[Phospho (STY)]PDR_.2	P55937_S60_M1_Golga3	9.8055	9.7897	10.0624	10.2755	9.5888	9.6763	10.0057	9.7226	9.8216	9.8295	1.0022	0.0032	0.8806	0.0552
Golga3	_LHNQNS[Phospho (STY)]VPR_.2	#N/A	7.4565	7.1858	4.4436	7.3825	6.3941	6.9797	6.2391	7.0633	7.1438	6.8086	0.9746	-0.0372	0.7816	0.1070
Golga3	_LHNQNS[Phospho (STY)]VPRDGLGQ_.2	#N/A	5.5604	5.7684	6.0389	6.3531	3.2977	5.8008	5.6664	5.5932	5.8635	5.3075	0.9754	-0.0359	0.8143	0.0892
Atp5mf	_DFTPSGIAGAFR_.2	#N/A	9.3302	9.3580	9.0991	8.9773	8.9921	9.0576	9.4812	9.5412	9.1748	9.0941	0.9799	-0.0292	0.1647	0.7832
Rrp1	_EAGS[Phospho (STY)]EAESSSADPGPGR_.2	P56183_S434_M1_Rrp1	8.2594	8.4735	9.0392	8.6321	7.7948	8.3225	8.7365	8.7918	8.5415	8.2194	0.9823	-0.0258	0.5353	0.2714
Arpp19	_VTS[Phospho (STY)]PEKAEEAK_.2	P56212_S23_M1_Arpp19	7.8067	7.1919	7.3550	7.7983	3.3838	7.4999	7.4482	7.4471	7.9667	3.9138	1.0217	0.0310	0.9020	0.0448
Arpp19	_YFDS[Phospho (STY)]GDYNM[Oxidation (M)]AK_.2	#N/A	10.8875	11.4349	11.7191	11.4847	11.0412	11.3332	11.0516	11.0905	11.2435	11.2569	1.0140	0.0200	0.3604	0.4432
Arpp19	_KPS[Phospho (STY)]LVASK_.2	P56212_S104_M1_Arpp19	3.5621	3.6641	4.2489	5.6384	4.1886	3.0722	4.4662	5.7544	3.0155	4.8651	0.8977	-0.1557	0.4892	0.3105
Arpp19	_GQKYFDS[Phospho (STY)]GDYNM[Oxidation (M)]AK_.2	#N/A	3.9002	6.6143	7.3940	3.4153	3.0259	3.2027	3.5635	3.5859	5.8522	3.0926	1.1413	0.1907	0.6160	0.2104
Acyp2	_SVDYEVFGTVQGVC[Carbamidomethyl (C)]FR_.2	#N/A	4.5621	4.9763	6.1034	5.0522	5.2303	4.9255	3.6243	4.8027	5.7037	5.4235	1.0518	0.0728	0.5901	0.2290
Acyp2	_NTS[Phospho (STY)]KGTVTGQVQGPEEK_.2	P56375_S51_M1_Acyp2	4.0727	3.3282	3.8064	3.5866	3.2165	3.4081	3.3013	3.0310	4.1804	3.5536	1.0151	0.0216	0.8397	0.0759
Acyp1	_VQGVFFR_.2	#N/A	6.0600	6.7799	6.0130	6.5021	6.3907	6.3881	6.5288	6.1138	6.3841	6.2784	1.0046	0.0067	0.8602	0.0654
Acyp1	_[Acetyl (Protein N-term)]AEGDTLVSVDYEIFGK_.2	#N/A	5.1413	5.8997	7.1082	6.7341	6.4489	6.4927	6.3199	5.7367	5.6243	6.6382	1.0369	0.0523	0.5917	0.2279
Acyp1	_VIANLDYSDFQIVK_.2	#N/A	5.2168	5.1670	5.8815	4.4536	5.4311	5.6717	5.3216	4.6128	5.1577	6.1205	1.0001	0.0001	0.9990	0.0004
Acyp1	_FM[Oxidation (M)]QQWLETR_.2	#N/A	4.1696	6.0852	5.0598	3.8582	6.2881	6.4116	6.3110	6.2319	5.8812	6.0073	0.8697	-0.2014	0.2042	0.6900
Acyp1	_LGLVGWVQNTDR_.2	#N/A	5.3170	4.7344	3.6323	5.3309	4.1690	4.9119	4.5029	5.1250	3.6321	5.0521	1.0228	0.0326	0.8170	0.0878
Acyp1	_GTVQGQLQGPVSK_.2	#N/A	2.9921	4.2340	3.6790	5.4698	5.3555	5.0601	3.8963	3.9369	5.1773	4.1486	1.0409	0.0578	0.7634	0.1172
Atp5mpl	_MFQTLIQK_.2	#N/A	6.0018	4.8923	4.4927	6.5394	4.6327	3.2970	5.1968	4.3307	4.1980	4.9575	1.0654	0.0913	0.6355	0.1969
Nudt2	_EETGIEASQLTIIEGFR_.2	#N/A	4.2040	3.0221	3.3900	5.1108	4.2969	4.7201	3.6073	4.2258	4.4054	3.8597	1.0247	0.0352	0.8218	0.0852
Nudt2	_ATLQEGHQFLC[Carbamidomethyl (C)]ST[Phospho (STY)]PA_.2	P56380_T145_M1_Nudt2	3.8120	3.8849	3.7665	3.5827	3.7063	3.3701	3.7309	3.6548	3.6979	3.8753	0.9859	-0.0204	0.6202	0.2075
Atp5f1e	_QAGLSYIR_.2	#N/A	8.5672	8.8756	8.8358	8.5642	8.9434	8.6303	8.1283	8.8672	8.8206	8.7309	1.0115	0.0165	0.5537	0.2567
Atp5f1e	_DALKTEFK_.2	#N/A	8.2967	8.4512	8.8207	8.5487	8.6259	8.6581	8.5146	8.5277	8.5540	8.4584	1.0063	0.0090	0.5847	0.2330
Atp5f1e	_FSQIC[Carbamidomethyl (C)]AK_.2	#N/A	5.9228	6.2472	5.5138	6.0757	7.0349	6.0456	5.2122	6.0325	6.5994	6.0144	1.0294	0.0418	0.6215	0.2065
Cartpt	_ALDIYSAVDDAS[Phospho (STY)]HEK_.2	#N/A	3.9755	5.6198	4.8653	6.5302	5.3974	6.6604	6.3349	5.9874	5.8284	5.8879	0.9165	-0.1257	0.3683	0.4338

Cartpt	_ALDIYSAVDDAS[Phospho (STY)]HEKELPR_.3	#N/A	3.4997	4.2560	3.2334	4.1597	4.1443	6.2511	6.3792	3.9588	6.4519	4.1267	0.8142	-0.2966	0.2402	0.6193
Cox6b1	_SLC[Carbamidomethyl (C)]PVSWVSAWDDR_.2	#N/A	9.8791	10.0127	10.3449	10.2501	10.5363	10.0552	10.1837	9.7397	9.9082	9.9546	1.0234	0.0334	0.1421	0.8474
Cox6b1	_IAEGTFPGKI_.2	#N/A	9.3354	9.6121	9.6484	9.5215	10.2348	9.6166	9.9231	9.4891	9.4886	9.2578	1.0128	0.0183	0.5388	0.2686
Cox6b1	_NC[Carbamidomethyl (C)]WQNYLDFHR_.3	#N/A	8.0441	8.3350	8.3862	8.4066	8.6265	8.0953	8.0474	8.2174	8.0661	8.2782	1.0200	0.0286	0.2065	0.6851
Cox6b1	_IAEGTFPGK_.2	#N/A	7.8111	7.9400	7.6846	7.6073	8.8807	7.6793	7.4303	7.7816	8.0895	8.5041	0.9978	-0.0032	0.9553	0.0199
Cox6b1	_[Acetyl (Protein N-term)]AEDIKTK_.2	#N/A	4.0583	5.4607	5.3049	4.0247	5.8150	5.1401	3.9857	4.5991	4.8942	4.8099	1.0864	0.1196	0.3703	0.4314
Cox6b1	_FPNQNQTK_.2	#N/A	9.1783	2.9833	3.4615	9.6344	10.5583	9.0852	3.6462	9.3117	7.4376	8.8964	1.0219	0.0313	0.9376	0.0280
Cox6b1	_GGDVSVC[Carbamidomethyl (C)]EWYR_.2	#N/A	7.8655	7.7052	8.0206	8.2347	8.4080	7.9674	7.8825	7.6948	7.7679	8.0322	1.0241	0.0344	0.2190	0.6596
Cox7b	_APSFHDK_.2	#N/A	5.9016	6.0808	4.8543	6.0689	6.0990	6.0340	6.1118	6.5137	6.7989	5.3777	0.9418	-0.0865	0.3309	0.4803
Cyb5a	_FLEEHPGGEEVLR_.3	#N/A	6.5142	5.7273	6.5778	6.1802	6.6143	6.2584	6.0023	6.0803	5.9144	6.1582	1.0452	0.0638	0.1601	0.7956
Cyb5a	_YYTLEEIQK_.2	#N/A	5.9829	5.7867	5.9641	6.3752	6.4694	6.5516	5.7759	5.9598	6.2610	6.1804	1.0238	0.0340	0.4545	0.3425
Cyb5a	_EQAGGDATENFEDVGHSTDAR_.3	#N/A	5.9308	6.6482	5.7689	6.7179	6.1208	6.8916	5.9735	6.1632	6.6466	6.6472	0.9982	-0.0026	0.9681	0.0141
Cyb5a	_[Acetyl (Protein N-term)]AGQS[Phospho (STY)]DKDVK_.2	P56395_S5_M1_Cyb5a	3.0618	4.8834	3.9633	4.5555	1.9835	4.2798	3.6548	3.0218	3.3209	4.4226	1.0507	0.0714	0.7687	0.1142
Cyb5a	_TYIIGELHPDDR_.2	#N/A	5.1897	4.8564	5.2928	5.2816	5.4354	5.6998	5.3046	5.5722	5.0111	5.3721	0.9958	-0.0061	0.8979	0.0467
Usp5	_GTGLQPGEEELPDIAPPLVT[Phospho (STY)]PDEPK_.3	P56399_T623_M1_Usp5	10.3687	10.8225	11.0623	11.4353	10.2169	10.7873	10.7377	11.0929	10.7705	10.3840	1.0033	0.0048	0.8916	0.0498
Usp5	_ATNNS[Phospho (STY)]LER_.2	P56399_S751_M1_Usp5	10.6035	10.6258	11.1188	11.2358	10.4667	10.9129	10.8587	10.8579	10.8764	10.7573	0.9991	-0.0014	0.9502	0.0222
Usp5	_DGLGGLPDIVR_.2	#N/A	7.5836	7.6397	6.7128	7.4483	7.0648	6.9436	7.6922	7.4593	7.4662	7.5853	0.9578	-0.0622	0.1465	0.8340
Usp5	_IFQNAPTDPTQDFSTQVAK_.2	#N/A	6.7787	6.6777	6.7685	7.5579	6.9467	7.1519	6.8748	6.8848	6.5355	6.6262	1.0371	0.0526	0.2066	0.6849
Usp5	_IVILPDYLEIAR_.2	#N/A	6.2076	5.0389	5.6295	4.9834	6.3195	5.5703	5.6876	5.3881	5.7507	6.6521	0.9583	-0.0614	0.5144	0.2887
Usp5	_WVIYNDQK_.2	#N/A	6.1530	6.2392	6.6389	6.3424	6.0738	6.0685	6.6042	6.3633	6.2660	5.6159	1.0065	0.0093	0.8454	0.0729
Usp5	_[Acetyl (Protein N-term)]AELSEEALLSVLPTIR_.2	#N/A	6.5156	5.8102	4.7116	4.7808	6.1738	5.9527	5.6972	5.2838	6.1543	6.1933	0.9700	-0.0439	0.6859	0.1637
Usp5	_DLGYIYFYQR_.2	#N/A	6.1347	6.0957	4.9214	6.2082	5.9047	5.7792	6.3544	5.5006	5.5125	5.9673	1.0012	0.0017	0.9817	0.0080
Usp5	_ENLWLNLTDGSILC[Carbamidomethyl (C)]GR_.2	#N/A	5.7764	5.4059	6.1249	5.4380	5.2239	5.5142	5.7178	5.4138	4.8946	5.4921	1.0374	0.0529	0.3755	0.4254
Usp5	_SSENPNEVFR_.2	#N/A	7.5122	7.5016	3.3082	7.4923	7.5702	7.4035	7.4248	7.4560	7.4008	7.6790	0.9076	-0.1399	0.4514	0.3454
Usp5	_ETGYPLAVK_.2	#N/A	6.0980	6.3486	3.5037	5.9368	6.3927	6.1901	5.2512	6.1143	6.1751	6.0172	0.9755	-0.0358	0.8133	0.0898
Usp5	_AVDWIFSHIDDLDAEAAM[Oxidation (M)]DIS[Phospho (STY)]EGR_.3	P56399_S775_M1_Usp5	4.2294	5.6654	6.4199	6.4789	4.9798	5.3037	3.6327	5.7357	4.7497	3.5905	1.2452	0.3164	0.1077	0.9676
Usp5	_QAEEEKVPLPELVR_.3	#N/A	7.3520	6.9930	7.4704	6.8506	7.0752	6.9081	7.1377	6.6649	6.8709	6.9876	1.0279	0.0397	0.2337	0.6313
Usp5	_GHPEFSTNR_.2	#N/A	6.7436	6.6388	6.6664	7.0458	7.0114	6.6775	6.6320	6.9438	6.8989	6.6936	1.0008	0.0011	0.9640	0.0159
Usp5	_YFDGSGGNNHAVEHYR_.3	#N/A	6.0654	6.2564	6.6418	6.6383	6.5500	6.3915	6.2769	5.8382	6.1717	6.7043	1.0282	0.0401	0.3664	0.4360
Usp5	_FASFPDYLVIQIK_.2	#N/A	3.6932	4.1102	3.5850	4.0607	3.8348	3.4926	4.1555	3.6314	3.9238	3.6355	0.9895	-0.0153	0.8101	0.0915
Usp5	_KQEVQAWDGEVR_.3	#N/A	5.7971	5.3327	3.5978	4.9032	5.5080	5.6530	5.4945	4.7839	5.8542	5.8194	0.9351	-0.0968	0.4601	0.3372
Usp5	_SAAES[Phospho (STY)]ISESVPVGPK_.2	P56399_S783_M1_Usp5	10.3332	10.3377	10.5742	11.0338	9.9822	10.6457	10.7999	10.3395	10.6081	10.7187	0.9876	-0.0181	0.5237	0.2809
Usp5	_VTSAVEALLSADSASR_.2	#N/A	5.4501	5.4790	5.8625	5.4450	5.3735	5.4918	5.7691	5.6528	5.6030	5.4290	0.9828	-0.0250	0.3850	0.4145
Atp5f1b	_VLDSGAPIKIPVGPETLGR_.3	#N/A	10.4134	10.7440	10.9561	10.9598	10.7925	10.7300	10.5984	10.5078	10.5677	10.6562	1.0173	0.0248	0.1190	0.9246
Atp5f1b	_AIAELGIYPAVDPLDSTSR_.2	#N/A	10.2547	10.2519	10.5215	10.5086	10.7194	10.2878	10.4735	10.1193	10.3920	10.3022	1.0099	0.0142	0.3966	0.4016
Atp5f1b	_IPVGPETLGR_.2	#N/A	11.0421	11.1138	10.7340	11.0140	11.4265	10.7126	10.8922	11.0797	11.0353	10.9114	1.0025	0.0036	0.8489	0.0712
Atp5f1b	_LVPLKETIK_.2	#N/A	10.4594	11.2649	11.4654	10.9919	11.2633	10.8209	10.8663	11.0578	10.8990	10.9204	1.0099	0.0142	0.5831	0.2342
Atp5f1b	_VVDLLAPYAK_.2	#N/A	10.5872	11.3794	10.9061	10.7232	11.0433	10.6647	10.0527	11.1237	11.0659	10.8850	1.0095	0.0136	0.6901	0.1611
Atp5f1b	_VALTGLTVAEYFR_.2	#N/A	10.4765	10.4912	10.7769	10.4288	10.8316	10.6375	10.6496	10.3102	10.7480	10.7710	0.9988	-0.0017	0.9192	0.0366
Atp5f1b	_IGLFGGAGVGK_.2	#N/A	9.0252	9.6929	9.6792	9.7834	9.5709	9.0781	9.3056	9.9324	9.5984	9.8738	0.9787	-0.0310	0.3430	0.4648
Atp5f1b	_ADKLAEEHGS_.2	#N/A	8.1741	8.6304	9.0277	8.8082	9.1896	8.8233	8.4400	8.4403	8.5986	8.5596	1.0312	0.0444	0.1837	0.7358
Atp5f1b	_VLDSGAPIK_.2	#N/A	7.2990	7.2015	7.5943	7.5613	7.8334	7.3028	5.7843	7.3811	7.6425	7.4040	1.0585	0.0820	0.2837	0.5472
Atp5f1b	_IM[Oxidation (M)]NVIGEPIDER_.2	#N/A	11.7185	11.9168	11.8298	11.9431	11.9822	11.7668	11.8787	11.7220	11.7704	11.8699	1.0042	0.0060	0.4452	0.3514
Atp5f1b	_TIAM[Oxidation (M)]DGTEGLVR_.2	#N/A	9.9344	10.3298	10.4815	10.4248	10.5403	10.0332	10.2323	10.0195	10.1463	10.1041	1.0163	0.0233	0.2466	0.6080
Atp5f1b	_TVLIM[Oxidation (M)]ELINNVAK_.2	#N/A	8.9766	8.7354	8.9442	8.8927	8.9288	9.1061	9.0630	8.8642	9.1735	8.9556	0.9907	-0.0134	0.3338	0.4766
Atp5f1b	_IM[Oxidation (M)]DPNIVGNEHYDVAR_.3	#N/A	8.2687	8.5028	8.5789	9.0995	8.9849	8.5512	8.4664	8.6014	8.4033	8.5331	1.0192	0.0274	0.3487	0.4575
Atp5f1b	_VALVYGQM[Oxidation (M)]NEPPGAR_.2	#N/A	10.7472	11.0394	11.0436	11.3052	11.0774	10.9877	10.7919	10.9562	10.9756	10.8092	1.0138	0.0198	0.1672	0.7767
Atp5f1b	_AHGGYSVFAGVGER_.3	#N/A	6.9459	6.7764	6.8283	5.7062	7.1051	7.2662	6.7534	5.7461	6.5697	6.6400	1.0535	0.0752	0.3351	0.4749
Atp5f1b	_FLSQPFQVAEVFTGHM[Oxidation (M)]GK_.3	#N/A	8.5685	8.8989	8.9508	8.8613	8.6562	8.9054	8.5461	8.9137	9.0237	8.8755	0.9963	-0.0054	0.7795	0.1082
Atp5f1b	_KGSITSVQAIYVPADDLTDPAPATTFAHLDATTVLSR_.4	#N/A	4.2337	4.2295	5.0169	5.1826	3.5650	4.6177	6.0252	4.5426	4.0836	4.5010	0.9344	-0.0978	0.5075	0.2945
Atp5f1b	_IPSAVGYQPTLATDM[Oxidation (M)]GTM[Oxidation (M)]QER_.3	#N/A	6.6117	7.1891	7.6129	6.4030	7.2273	7.4099	7.1989	7.1767	7.0461	6.8672	1.0005	0.0007	0.9893	0.0047
Atp5f1b	_FTQAGSEVSALLGR_.2	#N/A	4.9006	4.9608	6.2565	6.4564	4.4447	5.4160	6.0548	5.9475	5.4038	6.0667	0.9212	-0.1184	0.3104	0.5081
Atp5f1b	_TREGNDLYHEM[Oxidation (M)]IESGVINLK_.4	#N/A	5.8740	4.7496	6.6455	5.8268	5.9655	5.6515	4.7455	5.4996	5.3739	6.0140	1.0697	0.0973	0.3429	0.4648
Atp5f1b	_EGNDLYHEM[Oxidation (M)]IESGVINLK_.3	#N/A	6.4684	5.3568	5.5807	6.6833	5.4710	5.2566	5.9187	5.8400	4.5088	5.5182	1.0654	0.0914	0.4037	0.3940
Atp5f1b	_DQEGQDVLLFIDNIFR_.2	#N/A	5.7832	6.3888	6.2489	7.0453	5.5361	5.8662	6.4761	6.5360	6.4927	6.1998	0.9562	-0.0646	0.3481	0.4583
Atp5f1b	_IM[Oxidation (M)]NVIGEPIDERGPIK_.3	#N/A	3.8919	5.1778	5.5848	5.2708	5.2787	4.6380	4.5739	4.9606	4.2937	4.6705	1.0755	0.1050	0.3228	0.4910
Atp5f1b	_LVLEVAQHLGESTVR_.3	#N/A	8.0290	7.7710	8.0425	7.8659	8.0197	7.9042	7.6694	7.5878	8.0421	8.4677	0.9996	-0.0005	0.9862	0.0061
Atp5f1b	_QFAPIHAEAPEFIEM[Oxidation (M)]SVEQEILVTGIK_.3	#N/A	4.1862	4.3832	4.3329	6.1346	6.1282	3.0399	5.1891	5.4595	3.6815	4.2768	1.0106	0.0152	0.9457	0.0243
Atp5f1b	_SLQDIIAILGM[Oxidation (M)]DELSEEDKLTVSR_.3	#N/A	2.9765	4.4085	5.0640	5.1942	3.9057	4.9398	5.0645	4.8789	4.2348	4.4533	0.9478	-0.0773	0.6118	0.2134
Atp5f1b	_GFQQILAGEYDHLPEQAFYM[Oxidation (M)]VGPIEEAVAK_.4	#N/A	3.5241	3.8285	3.5971	3.7586	3.8019	4.1824	3.3132	2.7944	2.9267	3.9441	1.1657	0.2212	0.0516	1.2872
Atp5f1b	_IVAVIGAVVDVQFDEGLPPILNALEVQGR_.3	#N/A	3.5308	3.8116	3.2645	4.3091	1.4830	4.0966	4.4574	4.1929	2.9911	3.9864	0.8743	-0.1938	0.4205	0.3763
Slc1a3	_TTTNVLGDSLGAGIVEHLSR_.2	#N/A	4.6296	4.4131	5.3324	5.4626	4.7756	5.0263	4.9044	6.2576	4.8679	5.1325	0.9337	-0.0989	0.3225	0.4915
Slc1a3	_IVQVTAADAFLDLIR_.2	#N/A	5.0064	4.4859	4.5337	4.9089	3.8243	4.5604	5.3638	4.6931	4.1726	4.4118	0.9770	-0.0335	0.7255	0.1393
Slc1a3	_DVEM[Oxidation (M)]GNS[Phospho (STY)]VIEENEMK_.2	#N/A	5.2110	3.6900	3.7887	6.8463	3.2660	3.5611	6.2065	4.0898	5.1480	3.6418	0.9209	-0.1190	0.6638	0.1779
Slc1a3	_NRDVEMGNS[Phospho (STY)]VIEENEM[Oxidation (M)]K_.3	#N/A	3.8859	3.1761	3.8325	4.2495	3.0169	3.3983	3.4786	3.9468	4.0380	3.7309	0.9459	-0.0802	0.4534	0.3435
S100a1	_DLLQTELSGFLDVQK_.2	#N/A	4.1469	3.1010	4.4834	4.1100	4.7287	3.1274	3.6672	3.1324	3.6969	3.9604	1.0928	0.1280	0.3985	0.3996
Wfs1	_LNATAS[Phospho (STY)]LEQDKIEPPR_.3	#N/A	8.6592	3.8404	7.0757	8.0549	7.1145	3.8808	4.3601	3.9272	8.8723	3.5645	1.2425	0.3133	0.4104	0.3868
Wfs1	_GITS[Phospho (STY)]ENEAEVK_.2	P56695_S158_M1_Wfs1	3.2632	4.0684	3.9500	4.4526	3.8897	7.5445	4.1673	3.6658	3.3144	4.4196	1.1635	0.2185	0.4513	0.3455
Wfs1	_LNATAS[Phospho (STY)]LEQDK_.2	#N/A	4.0917	3.3961	3.2777	3.7803	3.2174	3.0112	3.4950	4.3381	3.9867	3.7473	0.8897	-0.1687	0.1223	0.9127
Wfs1	_[Acetyl (Protein N-term)]MNSGT[Phospho (STY)]PPPSPSGPPPPPAPQPQAR_.3	P56695_T5_M1_Wfs1	4.2695	3.0729	4.0032	5.6536	3.4132	3.2114	3.6952	3.4542	3.7533	3.2242	1.1148	0.1568	0.4401	0.3565
Pdcd5	_KVM[Oxidation (M)]DS[Phospho (STY)]DEDDADY_.2	#N/A	9.1846	9.0582	9.4699	9.8808	8.9342	9.2981	9.3123	8.7384	8.9536	9.4656	1.0205	0.0293	0.4137	0.3834
Pdcd5	_[Acetyl (Protein N-term)]ADEELEALRK_.2	#N/A	5.8917	6.6064	6.3054	6.1999	6.7074	6.4166	6.4766	6.3048	6.3194	5.8126	1.0203	0.0289	0.5216	0.2827
Pdcd5	_RKVM[Oxidation (M)]DS[Phospho (STY)]DEDDADY_.2	#N/A	7.0049	7.8053	8.8272	8.2530	7.9577	7.9404	7.4493	7.7102	7.6009	7.4967	1.0529	0.0744	0.2290	0.6402
Pdcd5	_VSEQGLIEILEK_.2	#N/A	4.3179	4.5653	3.9309	4.2575	5.0336	4.4813	3.7680	4.8219	4.1772	3.9582	1.0597	0.0837	0.3665	0.4359
Pdcd5	_VMDS[Phospho (STY)]DEDDADY_.2	#N/A	6.3404	6.3008	3.6047	8.6424	6.3947	3.9289	6.3907	4.2088	5.2356	5.9423	1.0779	0.1083	0.6884	0.1621
Fus	_AAIDWFDGK_.2	#N/A	6.6431	5.9437	5.9592	6.8479	6.5800	6.4292	6.4174	5.8188	6.0993	6.7517	1.0205	0.0293	0.6179	0.2091
Fus	_GEATVS[Phospho (STY)]FDDPPSAK_.2	P56959_S333_M1_Fus	3.8211	7.2429	7.5165	7.5722	6.9755	7.0445	7.6127	3.1079	7.4292	7.8243	1.0311	0.0442	0.8654	0.0628
Fus	_HDSEQDNSDNNTIFVQGLGENVTIESVADYFK_.3	#N/A	3.8473	3.1027	3.5810	4.6256	2.9910	3.6336	3.5268	3.8764	4.1755	4.3028	0.9143	-0.1292	0.3321	0.4787
Nrg2	_SDS[Phospho (STY)]PPLC[Carbamidomethyl (C)]PAADSR_.2	P56974_S715_M1_Nrg2	3.5477	9.1281	9.5705	9.2474	4.1922	3.9332	3.8264	4.0068	3.6554	9.1442	1.2801	0.3563	0.4572	0.3399

Samsn1	_SSS[Phospho (STY)]FGNFDR_.2	P57725_S23_M1_Samsn1	4.1411	3.0851	3.3270	3.8297	2.9734	3.6512	3.5444	4.2888	3.9374	3.7967	0.8996	-0.1526	0.1734	0.7609
Atp6v1d	_FTAGDFSTTVIQNVNK_.2	#N/A	8.2025	7.6379	8.7918	8.2944	7.3887	8.2455	8.1861	7.0722	7.9111	8.2580	1.0301	0.0428	0.5001	0.3010
Atp6v1d	_IEIFPSR_.2	#N/A	7.7487	7.8192	7.4486	7.1112	7.3085	7.5836	7.3561	7.5848	7.7940	7.4086	0.9957	-0.0062	0.8417	0.0748
Atp6v1d	_VNAIEHVIIPR_.2	#N/A	5.1858	5.6526	6.1127	5.6775	5.4114	5.0934	5.8314	5.5081	5.3509	5.7659	0.9836	-0.0238	0.6740	0.1714
Atp6v1d	_EAAFSLAEAK_.2	#N/A	6.6363	6.0548	6.8673	6.2552	5.8664	6.1518	5.3753	6.5758	6.4239	6.3200	1.0213	0.0304	0.6597	0.1807
Atp6v1d	_TLAYIITELDER_.2	#N/A	6.1412	5.7257	5.8171	6.1291	5.9765	5.6559	5.3365	6.1780	5.9132	6.1434	1.0025	0.0036	0.9387	0.0275
Npy	_SSPETLISDLLM[Oxidation (M)]KES[Phospho (STY)]TENAPR_.3	#N/A	3.7796	3.6168	3.8996	3.8513	3.5912	3.6387	6.4470	6.8686	6.7125	3.5285	0.6333	-0.6591	0.0089	2.0486
Eef1d	_ITSLEVENQNLR_.2	#N/A	6.0128	5.8327	3.4929	6.4912	6.4897	6.0725	5.7440	5.7473	6.2019	6.3385	0.9541	-0.0678	0.6517	0.1860
Eef1d	_[Acetyl (Protein N-term)]ATNFLAHEK_.2	#N/A	5.6993	6.4393	6.3134	6.2736	6.2567	5.7095	6.3147	6.0719	5.5966	6.4430	1.0014	0.0021	0.9695	0.0135
Eef1d	_ATAPQTQHVS[Phospho (STY)]PM[Oxidation (M)]R_.2	#N/A	11.1500	11.3141	11.7189	12.0441	11.2054	11.2535	11.5357	11.1976	11.1299	11.3718	1.0123	0.0176	0.4970	0.3036
Eef1d	_LSSLEKSS[Phospho (STY)]PTPR_.2	P57776_S119_M1_Eef1d	3.5170	5.6536	6.7033	6.0802	3.6467	5.8037	4.4436	4.2067	3.0049	5.1821	1.2435	0.3144	0.2183	0.6610
Eef1d	_GATPAEDDEDKDIDLFGSDEEEEDKEAAR_.4	#N/A	3.6391	4.5402	3.5847	10.4756	8.9575	4.7382	3.9238	9.8183	8.0714	5.0348	0.8923	-0.1644	0.7068	0.1507
Eef1d	_DIDLFGS[Phospho (STY)]DEEEEDKEAAR_.3	#N/A	3.6878	3.6145	3.9145	4.5171	3.3503	3.3403	3.9433	3.6659	3.7214	3.8479	0.9849	-0.0219	0.8095	0.0918
Actn4	_EAILAIHK_.2	#N/A	5.2238	4.5394	4.4532	4.7992	3.4001	0.9514	5.5341	4.4400	4.5805	4.2428	0.8287	-0.2710	0.3602	0.4434
Actn4	_DGLAFNALIHR_.2	#N/A	4.1610	4.3214	4.5184	5.0173	3.7818	3.5750	5.0412	4.8511	4.9896	4.8280	0.8583	-0.2205	0.0313	1.5042
Actn4	_M[Oxidation (M)]APYQGPDAAPGALDYK_.2	#N/A	6.6053	6.3958	6.7894	6.9414	6.6748	7.2398	6.9378	7.2600	6.4650	7.0403	0.9781	-0.0319	0.4697	0.3281
Actn4	_AGTQIENIDEDFRDGLK_.3	#N/A	4.4379	3.7984	3.2777	4.3223	3.6197	4.4253	4.4706	4.1797	4.5230	5.0647	0.8729	-0.1960	0.0789	1.1029
Actn4	_IC[Carbamidomethyl (C)]DQWDNLGSLTHSR_.3	#N/A	5.6100	6.0502	6.2331	6.4431	6.2708	6.2156	4.1605	6.4759	6.5960	6.1411	1.0503	0.0708	0.5520	0.2580
Actn4	_VLAVNQENEHLMEDYER_.3	#N/A	5.2587	4.0962	4.4807	4.9511	3.8907	4.2347	4.1727	2.9767	3.2758	3.7017	1.2700	0.3448	0.0225	1.6477
Snrpa1	_LTAELIEQAAQYTNAVR_.2	#N/A	3.4704	3.5787	4.1572	3.1590	4.0969	4.1524	4.3746	3.4586	4.1483	4.0765	0.9389	-0.0910	0.3840	0.4157
Nadk	_S[Phospho (STY)]LSASPALGSTK_.2	P58058_S46_M1_Nadk	3.6458	4.4021	8.8195	4.4241	2.7896	3.8350	3.0715	8.0671	4.3769	4.1014	0.9487	-0.0760	0.8606	0.0652
Eef2	_VFS[Phospho (STY)]GVVS[Phospho (STY)]T[Phospho (STY)]GLK_.2	P58252_S418_M3_Eef2	10.3654	10.4525	10.6259	11.1832	8.4433	10.5771	9.9660	10.2084	10.3214	10.6063	0.9999	-0.0001	0.9985	0.0007
Eef2	_GEGQLSAAER_.2	#N/A	7.5643	7.8486	7.5655	7.8946	7.8664	7.6202	7.6158	7.8328	7.6514	7.7597	1.0015	0.0022	0.9001	0.0457
Eef2	_VNFTVDQIR_.2	#N/A	7.8539	7.6538	8.0519	7.8701	7.6310	7.4084	7.2841	7.7988	7.7337	7.4840	1.0224	0.0320	0.2867	0.5425
Eef2	_FSVSPVVR_.2	#N/A	7.2487	7.7178	7.0065	7.0535	7.6192	7.2476	7.3542	7.5438	7.6586	7.4159	0.9763	-0.0346	0.2951	0.5300
Eef2	_YEWDVAEAR_.2	#N/A	7.4776	7.0568	6.9691	7.1932	6.5526	7.3436	7.3195	7.2211	6.8983	7.3330	0.9869	-0.0190	0.6232	0.2054
Eef2	_STLTDSLVC[Carbamidomethyl (C)]K_.2	#N/A	6.9529	6.7198	7.4411	6.8073	7.2481	6.9762	7.1608	6.7083	6.7967	6.6289	1.0294	0.0418	0.2646	0.5774
Eef2	_DLEEDHAC[Carbamidomethyl (C)]IPIKK_.3	#N/A	7.1181	6.7469	6.7645	6.9697	7.0515	7.1835	6.4149	6.7399	7.1410	6.9564	1.0234	0.0334	0.3324	0.4783
Eef2	_SDPVVSYR_.2	#N/A	6.3634	6.4841	6.6436	6.7963	6.5260	6.5743	5.4379	6.5334	6.6256	6.5151	1.0457	0.0644	0.2565	0.5909
Eef2	_TFC[Carbamidomethyl (C)]QLILDPIFK_.2	#N/A	4.7757	5.9256	5.9682	5.3966	5.8618	5.8166	6.3276	5.8307	5.7906	5.6699	0.9525	-0.0703	0.3169	0.4991
Eef2	_ETVSEESNVLC[Carbamidomethyl (C)]LSK_.2	#N/A	5.7706	5.2477	5.1301	6.4516	5.0595	5.1634	5.8658	5.3118	5.5950	6.0446	0.9590	-0.0604	0.4657	0.3319
Eef2	_AYLPVNESFGFTADLR_.2	#N/A	5.7384	5.6714	6.0103	5.5647	2.9592	3.2695	3.6303	6.2236	6.3965	3.1593	1.0034	0.0049	0.9868	0.0058
Eef2	_C[Carbamidomethyl (C)]ELLYEGPPDDEAAM[Oxidation (M)]GIK_.2	#N/A	5.6281	5.3687	5.8588	5.2592	4.8720	5.9832	3.5512	5.6727	5.0823	5.4731	1.1113	0.1522	0.2407	0.6186
Eef2	_EGIPALDNFLDKL_.2	#N/A	4.0830	5.4493	3.8167	3.5763	4.3900	3.3979	3.2910	5.6607	4.6407	3.5433	0.9615	-0.0567	0.7807	0.1075
Eef2	_FT[Phospho (STY)]DTRKDEQER_.2	#N/A	3.6682	4.4245	3.4019	4.4465	4.5577	3.8127	5.2556	4.0555	4.3546	4.1238	0.9111	-0.1344	0.2657	0.5755
Eef2	_VFDAIMNFR_.2	#N/A	4.1037	3.7611	3.8373	5.2450	4.6016	3.1686	3.5294	3.6201	4.6825	3.4723	1.0767	0.1066	0.5171	0.2865
Eef2	_AGETRFT[Phospho (STY)]DTR_.2	#N/A	2.9318	5.8713	3.6186	4.3837	3.5583	4.1712	4.5320	6.0096	3.6458	4.0882	0.8950	-0.1601	0.4857	0.3136
Eef2	_ALLELQLEPEELYQTFQR_.2	#N/A	4.3010	4.3558	4.0320	5.0832	3.4304	4.1453	4.5188	4.4438	4.2442	3.7954	0.9939	-0.0088	0.9336	0.0298
Eef2	_YFDPANGK_.2	#N/A	5.8677	4.0363	4.1403	3.1421	3.6610	4.7074	4.3576	5.3511	5.1893	5.1963	0.8478	-0.2382	0.1750	0.7570
Eef2	_EDLYLKPIQR_.3	#N/A	3.5274	4.3326	4.2142	3.2160	4.1539	3.5756	4.4315	3.4016	3.0502	3.1830	1.0910	0.1257	0.3739	0.4272
Eef2	_STAISLFYELSENDLNFIK_.2	#N/A	4.2921	3.0503	4.0258	3.5696	2.8716	3.3570	3.7178	3.4316	3.7307	3.2468	0.9989	-0.0016	0.9896	0.0046
Opa1	_VVVVGDQSAGK_.2	#N/A	6.6744	6.7976	6.7590	6.3293	6.8595	6.8048	6.1934	6.6320	6.5931	6.5896	1.0311	0.0442	0.1535	0.8138
Opa1	_EVLEDFAEDGEK_.2	#N/A	6.4976	6.4506	6.6682	6.5017	6.5213	6.4510	6.7455	6.0379	6.2442	6.5048	1.0207	0.0295	0.3344	0.4758
Opa1	_ESVEQQADSFK_.2	#N/A	6.3665	6.4264	6.9119	5.8789	6.2228	6.2274	6.5722	6.6287	6.6688	6.2942	0.9691	-0.0453	0.3076	0.5119
Opa1	_AVEVAWETLQEEFSR_.2	#N/A	6.1768	4.8953	6.2780	5.1512	5.3162	5.9385	5.9787	4.9126	4.9064	5.9535	1.0346	0.0491	0.6353	0.1971
Opa1	_EFDLTKEEDLAALR_.3	#N/A	5.8596	5.0144	5.3470	5.0537	5.5163	5.7774	5.3711	5.3873	4.9475	5.6507	1.0167	0.0238	0.6900	0.1611
Opa1	_FNLETEWK_.2	#N/A	4.2707	4.6609	4.6292	4.8565	4.5185	5.0356	4.2231	5.5882	4.7974	4.7179	0.9649	-0.0516	0.5339	0.2726
Opa1	_TSVLEM[Oxidation (M)]IAQAR_.2	#N/A	5.6984	5.8029	6.0989	5.7825	5.3234	5.8598	5.5536	5.7168	6.3570	6.0227	0.9744	-0.0375	0.4498	0.3470
Opa1	_DFFTAGSPGETAFR_.2	#N/A	5.4878	3.8357	3.2404	4.2850	5.9627	5.8765	6.2479	4.2170	6.4651	6.1784	0.8276	-0.2729	0.2029	0.6927
Opa1	_VNDEHPAYLASDEITTVR_.3	#N/A	5.4434	5.1752	5.3245	5.5695	4.9370	5.0819	5.4900	5.4571	4.9271	5.1520	0.9998	-0.0004	0.9937	0.0027
Opa1	_IQQIIEGK_.2	#N/A	4.6163	2.3271	3.5912	3.1349	3.0012	2.9610	3.2832	3.8898	3.1737	4.0726	0.9077	-0.1398	0.4629	0.3345
Opa1	_IDQLQEELLHTQLK_.3	#N/A	4.0078	3.5735	3.1938	3.6965	2.4855	3.5180	3.4111	3.1221	3.9008	3.6635	0.9683	-0.0465	0.7179	0.1439
Opa1	_LDAFIEALHQEK_.2	#N/A	5.2260	4.5403	5.6125	4.4283	5.4070	5.0881	5.4772	5.0435	5.3222	4.8174	0.9778	-0.0324	0.6801	0.1674
Opa1	_SIVTDLVSQM[Oxidation (M)]DPHGR_.3	#N/A	3.1879	4.1545	4.4224	3.9662	1.3534	3.7537	4.1145	3.0350	3.3340	3.5816	0.9877	-0.0178	0.9442	0.0249
Ptpa	_LVALLDTLDR_.2	#N/A	6.8292	7.1365	6.2722	6.9250	6.5400	6.3410	6.2300	6.9865	7.1709	6.8334	0.9807	-0.0281	0.5969	0.2241
Ptpa	_FGSLLPIHPVTSG_.2	#N/A	5.3865	6.2155	6.2938	5.8925	4.6611	5.6694	6.2969	6.2038	5.3668	5.8543	0.9589	-0.0606	0.5066	0.2953
Ptpa	_LDQEAENLVATVVPTHLAAAVPEVAVYLK_.3	#N/A	3.9481	3.2035	3.2086	3.7113	3.0918	3.5328	3.4260	4.4072	4.0558	3.6783	0.8863	-0.1741	0.1051	0.9786
Strn4	_S[Phospho (STY)]LELNGAGEPVEGAPR_.2	P58404_S206_M1_Strn4	11.1427	11.1450	11.2719	11.9480	10.5332	11.4174	11.4349	11.2591	11.5403	11.5978	0.9812	-0.0273	0.3990	0.3991
Strn4	_AS[Phospho (STY)]PGPGGLSGGESLLVK_.2	P58404_S223_M1_Strn4	2.9485	9.2128	9.7954	10.3982	9.0040	9.4772	9.7138	9.6182	3.0562	9.5589	1.0608	0.0852	0.8059	0.0937
Hrh3	_GSKPSASSAS[Phospho (STY)]LEKR_.3	P58406_S332_M1_Hrh3	4.4976	3.1573	4.5397	3.4626	2.9786	3.2501	3.6109	4.9760	3.8376	4.0962	0.8832	-0.1792	0.2892	0.5387
Hrh3	_VQPHGS[Phospho (STY)]LEQC[Carbamidomethyl (C)]WK_.2	#N/A	8.2630	7.9640	8.4287	8.2142	7.0281	8.0921	8.8216	8.4177	8.2704	8.2082	0.9489	-0.0757	0.1592	0.7980
Foxp2	_RDS[Phospho (STY)]SSHEETGASHTLYGHGVC[Carbamidomethyl (C)]K_.4	P58463_S329_M1_Foxp2	3.3488	4.1540	4.4230	3.9667	3.9753	3.7542	4.1150	3.0345	3.3335	3.6440	1.1147	0.1567	0.1586	0.7997
Fam207a	_AVEPALPQS[Phospho (STY)]PAGGAGAK_.2	P58468_S38_M1_Fam207a	8.8463	8.6946	9.4799	9.4209	8.5537	9.0111	9.1639	8.7184	8.6625	8.7198	1.0210	0.0299	0.4146	0.3824
	LEFQQQLGEAPGDAS[Phospho (STY)]P.2	P58686_S82_M1_	4.0013	7.9037	8.2808	9.5413	9.0717	8.4423	7.5511	8.8028	9.0635	8.5184	0.9281	-0.1077	0.5756	0.2399
Aaas	_FS[Phospho (STY)]PVLGR_.2	P58742_S495_M1_Aaas	9.6375	9.3349	9.9919	9.5395	8.6168	9.3939	9.8894	9.5840	9.3221	9.4002	0.9864	-0.0198	0.6221	0.2062
Tpm1	_KLVIIESDLERAEER_.3	#N/A	6.3927	6.5165	7.7054	6.9950	6.9233	6.2424	6.6257	6.6341	6.1146	6.4748	1.0516	0.0726	0.2822	0.5495
Tpm1	_KLVIIESDLER_.3	#N/A	4.5312	4.3660	3.3831	4.4277	4.5360	3.8314	3.0751	3.3518	4.3733	4.1050	1.1215	0.1655	0.2204	0.6568
Tpm1	_TVTNNLKS[Phospho (STY)]LEAQAEK_.2	P58771_S206_M1_Tpm1	3.8521	3.5784	3.5858	4.6598	3.7276	4.4179	3.2778	3.8716	4.1706	5.0525	0.9700	-0.0440	0.7512	0.1243
Tpm1	_C[Carbamidomethyl (C)]AELEEELKTVTNNLK_.3	#N/A	3.6838	4.0184	3.4175	4.4621	2.9791	3.3301	3.1095	3.6564	4.3390	4.1394	0.9573	-0.0629	0.6515	0.1861
Tpm1	_AQKDEEKMEIQEIQLK_.3	#N/A	5.2263	5.3708	4.4431	6.6352	4.7473	4.8827	4.8078	2.8604	5.0175	3.4119	1.2965	0.3746	0.0707	1.1504
Tpm1	_LVIIESDLERAEER_.3	#N/A	3.1100	4.5698	3.7968	4.2994	3.7365	5.1086	4.0141	3.8191	4.1880	4.2664	1.0078	0.0112	0.9336	0.0298
Tbc1d10a	_EPAAGGS[Phospho (STY)]LSGTR_.2	P58802_S18_M1_Tbc1d10a	3.6294	2.9337	4.3162	3.3180	4.2559	3.4736	4.5335	3.2997	2.9483	3.2850	1.0392	0.0555	0.7330	0.1349
Tnks1bp1	_ENYEDQEPLVGHES[Phospho (STY)]PITLAAR_.3	P58871_S602_M1_Tnks1bp1	8.6711	8.4006	3.5244	9.5226	8.1115	8.7442	9.0020	8.9062	8.8843	8.5476	0.8861	-0.1744	0.3895	0.4095
Tnks1bp1	_LANISVPASES[Phospho (STY)]PR_.2	P58871_S300_M1_Tnks1bp1	3.5100	4.2663	8.4619	8.0456	3.5090	3.9903	7.8542	3.9691	3.6177	8.0587	0.9017	-0.1494	0.7131	0.1469
Tnks1bp1	_VSGAGLS[Phospho (STY)]PSRK_.2	#N/A	6.8530	7.1723	3.8389	7.4339	3.8669	7.0763	7.0271	6.9339	7.0282	3.7262	0.9776	-0.0327	0.9014	0.0451
Tnks1bp1	_DS[Phospho (STY)]LGSFSTR_.2	#N/A	7.6749	6.9905	7.7187	8.0123	6.5820	7.7070	7.4258	4.3643	7.4764	7.3912	1.1175	0.1603	0.2737	0.5627
Tnks1bp1	_VPSS[Phospho (STY)]DEEVVEEPQSR_.2	P58871_S1612_M1_Tnks1bp1	3.8404	10.0508	7.7706	10.6010	9.8916	10.1421	10.6966	10.0741	10.2573	10.4375	0.8408	-0.2502	0.2477	0.6061
Tnks1bp1	_VSGAGLSPS[Phospho (STY)]R_.2	#N/A	3.9945	3.2317	5.5846	5.5936	3.1202	5.6350	3.3978	5.3498	5.7199	6.0881	0.8809	-0.1830	0.4562	0.3409
Tnks1bp1	_SLSSGFS[Phospho (STY)]PEEAQQQDEEFEK_.2	P58871_S980_M1_Tnks1bp1	4.9823	4.8841	3.9580	3.9478	4.8017	3.6987	4.8663	4.7884	3.6629	3.6356	1.0331	0.0470	0.7322	0.1354

Tnks1bp1	_WLDDLLAS[Phospho (STY)]PPPNSGSAR_.2	P58871_S692_M1_Tnks1bp1	3.7223	3.5038	4.4091	9.8764	3.3921	3.2325	3.1256	8.7043	8.4070	7.2683	0.6820	-0.5522	0.2247	0.6484
Tnks1bp1	_M[Oxidation (M)]QAESQS[Phospho (STY)]PTNVDLEDKER_.2	#N/A	5.8737	6.4374	5.9425	6.4634	4.9395	6.5285	5.9240	6.4275	6.2113	5.4280	1.0055	0.0079	0.9274	0.0327
Tnks1bp1	_SSGSLS[Phospho (STY)]PGLETEDPLEAR_.2	P58871_S1375_M1_Tnks1bp1	7.5272	7.5761	7.7465	9.7447	7.6214	7.7808	7.9868	9.0254	7.8284	7.8025	0.9802	-0.0288	0.7532	0.1231
Tnks1bp1	_GEGVSQVGPGT[Phospho (STY)]PPAPESPR_.2	P58871_T533_M1_Tnks1bp1	7.1816	7.5426	8.2582	8.5853	7.7255	3.5683	8.6607	7.8253	7.9129	8.0753	0.8799	-0.1846	0.3308	0.4804
Tnks1bp1	_RFS[Phospho (STY)]EGVLQPPSQDQEK_.2	#N/A	3.6732	7.4852	8.1059	8.5471	6.9631	7.7776	7.9934	8.0670	7.8781	8.1801	0.8832	-0.1791	0.3276	0.4847
Tnks1bp1	_NM[Oxidation (M)]APGAGC[Carbamidomethyl (C)]S[Phospho (STY)]PGEPR_.2	#N/A	7.1769	6.6290	6.8596	7.7289	5.4625	7.0240	6.9158	6.6611	7.0909	7.1677	0.9791	-0.0305	0.7237	0.1404
Tnks1bp1	_DVGHLEEGASGGLLS[Phospho (STY)]PSTPHSR_.3	P58871_S1022_M1_Tnks1bp1	5.9217	5.8825	6.4273	7.2138	3.2581	5.9675	6.0749	6.5978	6.1348	6.2057	0.9241	-0.1139	0.5091	0.2932
Tnks1bp1	_GYSS[Phospho (STY)]QDAEEQDREFEKR_.3	P58871_S912_M1_Tnks1bp1	6.6754	6.4914	3.7928	3.8177	5.8345	6.6732	3.7287	6.8555	6.7980	7.0071	0.9098	-0.1363	0.5761	0.2395
Tnks1bp1	_ASRVPS[Phospho (STY)]S[Phospho (STY)]DEEVVEEPQSR_.2	#N/A	3.4878	4.8579	3.7728	6.0815	5.1181	5.6136	6.0061	5.9637	4.9702	3.6003	0.9390	-0.0908	0.6598	0.1806
Tnks1bp1	_RDS[Phospho (STY)]LGSFSTR_.2	#N/A	5.8932	3.8493	4.0637	5.9160	4.0034	3.7261	4.2811	3.5521	3.2007	5.1138	1.1334	0.1806	0.4148	0.3821
Tnks1bp1	_LDS[Phospho (STY)]PPPSPITEASEAAEAAEADSWAVSGR_.3	P58871_S496_M1_Tnks1bp1	5.6059	6.5853	6.6312	7.1687	5.5735	5.2432	6.3258	6.5723	3.5680	5.9115	1.0966	0.1330	0.4430	0.3536
Tnks1bp1	_HS[Phospho (STY)]LGQEVIGIGGSQDESEVPVR_.3	P58871_S796_M1_Tnks1bp1	6.5661	6.2368	3.9783	7.3288	4.7677	5.7116	4.0519	4.7812	6.5999	6.4261	1.0549	0.0771	0.7161	0.1450
Tnks1bp1	_NRS[Phospho (STY)]AEEGEVTESK_.2	P58871_S1657_M1_Tnks1bp1	3.0900	4.0777	4.2144	4.4240	3.7166	4.4830	4.3737	3.3112	4.0469	3.9027	1.0236	0.0337	0.7803	0.1078
Tnks1bp1	_S[Phospho (STY)]PPSGSQSLLEGIM[Oxidation (M)]TASSSK_.2	#N/A	3.3294	5.5412	5.3871	7.2978	4.3937	4.6231	4.6091	5.2661	4.5394	4.5982	1.0720	0.1003	0.6366	0.1962
Tnks1bp1	_DRQS[Phospho (STY)]PSTC[Carbamidomethyl (C)]SEGLLGWAQK_.3	P58871_S713_M1_Tnks1bp1	3.2473	4.0951	4.4818	4.0256	3.8919	4.2336	4.1739	2.9756	3.2746	3.7029	1.1314	0.1781	0.1565	0.8054
Bcl2l13	_S[Phospho (STY)]HTGEAAAVR_.2	#N/A	6.4087	5.8336	4.0138	3.8854	3.5040	3.5717	3.8677	3.8597	6.4646	3.6912	1.0146	0.0209	0.9391	0.0273
Bcl2l13	_ALQTILSQPVTYEAYR_.2	#N/A	4.9787	4.4337	3.4978	4.7642	4.4078	4.6475	4.7892	4.9167	3.7666	5.2522	0.9517	-0.0715	0.5525	0.2577
Bcl2l13	_KKSHT[Phospho (STY)]GEAAAVR_.3	#N/A	3.7907	6.3809	4.4787	6.3333	3.6625	3.5702	3.7489	3.0590	3.8546	3.5751	1.3212	0.4018	0.1353	0.8688
Pcif1	_[Acetyl (Protein N-term)]ANENHGS[Phospho (STY)]PR_.2	P59114_S8_M1_Pcif1	7.8055	7.5758	7.7059	7.9876	6.8644	6.5389	8.2611	7.8216	7.3369	7.6353	0.9548	-0.0667	0.3211	0.4934
Pcif1	_QLS[Phospho (STY)]EEQPSGNGVK_.2	P59114_S116_M1_Pcif1	4.0840	3.3884	3.6877	3.2315	3.2097	3.0189	3.3797	3.7697	4.0688	4.4096	0.8796	-0.1850	0.1116	0.9524
Arhgap39	_AFS[Phospho (STY)]EDEALAQQDSK_.2	P59281_S597_M1_Arhgap39	11.0103	11.0048	11.3935	11.1583	9.7485	10.9808	11.4673	10.9743	11.0872	10.9706	0.9782	-0.0317	0.4553	0.3417
Arhgap39	_KPSS[Phospho (STY)]ETDIENWASK_.2	P59281_S683_M1_Arhgap39	3.8667	3.1418	6.0394	3.6759	3.0105	5.5397	3.5738	5.5243	4.1758	3.6761	0.9941	-0.0086	0.9737	0.0116
Arhgap39	_DGST[Phospho (STY)]GSSLEPELEER_.2	P59281_T133_M1_Arhgap39	3.4237	4.2289	4.1105	4.6131	4.0502	3.6793	4.3278	3.5053	3.1539	3.0793	1.1425	0.1922	0.1459	0.8361
Arhgap39	_QLVYVEQAGS[Phospho (STY)]SPK_.2	P59281_S402_M1_Arhgap39	3.6765	3.5497	4.3633	3.3651	2.8022	3.4265	3.0798	3.2525	5.3403	5.5763	0.8187	-0.2885	0.2185	0.6605
Arhgap39	_EDTSSC[Carbamidomethyl (C)]S[Phospho (STY)]PPGVLLEK_.2	P59281_S169_M1_Arhgap39	3.1732	4.0530	3.8600	4.3627	3.9413	4.1842	4.0773	3.7558	3.4044	4.3297	1.0096	0.0137	0.8908	0.0502
Eif5	_SVS[Phospho (STY)]DQFYR_.2	P59325_S10_M1_Eif5	7.2684	7.6678	7.2653	3.6406	7.2554	7.7286	7.2604	7.3176	7.3238	7.7082	0.9192	-0.1215	0.4774	0.3211
Eif5	_AM[Oxidation (M)]GPLVLTEVLFDEK_.2	#N/A	3.7595	4.2215	3.6267	3.1705	3.8454	3.8652	3.3187	2.5975	4.1617	4.3486	1.0392	0.0555	0.7082	0.1498
Eif5	_GLTLS[Phospho (STY)]DDLER_.2	P59325_S227_M1_Eif5	4.3492	3.6047	4.0829	3.6267	3.4930	3.1317	3.7749	3.3745	3.6736	3.3040	1.0518	0.0729	0.4672	0.3305
Eif5	_VETVKS[Phospho (STY)]DNKDDDIDIDAI_.2	P59325_S417_M1_Eif5	3.9117	3.5888	3.8756	3.6003	3.9489	4.0401	4.0929	3.8662	3.5148	3.5673	1.0179	0.0256	0.6525	0.1854
Eif5	_EEGIIDSSDKEIVAEAER_.3	#N/A	2.9710	4.2551	3.6578	4.1605	3.5975	3.9820	3.8752	3.9580	3.6066	4.1732	0.9660	-0.0499	0.6213	0.2067
Inpp5j	_AAGPATS[Phospho (STY)]PPR_.2	P59644_S266_M1_Inpp5j	4.0048	3.2603	3.1519	7.9103	5.7837	3.4760	3.3692	4.4640	4.1126	8.0678	0.9189	-0.1219	0.7578	0.1204
Inpp5j	_QLS[Phospho (STY)]PTSVGPK_.2	P59644_S171_M1_Inpp5j	3.2214	4.0266	4.6562	6.0804	3.8479	3.8816	4.2424	4.4079	3.2061	3.7714	1.0969	0.1335	0.5098	0.2926
Inpp5j	_SRS[Phospho (STY)]PGLAR_.2	P59644_S885_M1_Inpp5j	4.0845	6.5313	3.2705	3.7732	3.2102	6.2559	6.6347	4.3453	3.9939	3.7402	0.9663	-0.0494	0.8687	0.0611
Inpp5j	_VKAPSGGPS[Phospho (STY)]PSGR_.2	P59644_S691_M1_Inpp5j	3.3822	3.8439	4.0690	4.5717	4.0088	4.3932	4.2864	3.5468	6.3242	3.0378	0.9409	-0.0879	0.6879	0.1624
Inpp5j	_HRS[Phospho (STY)]RS[Phospho (STY)]PGLAR_.3	P59644_S883_M2_Inpp5j	4.3512	4.7868	3.5372	4.2684	3.4769	3.8614	3.7545	4.0786	6.6989	4.1922	0.8645	-0.2100	0.3191	0.4961
Inpp5j	_GGSRS[Phospho (STY)]PS[Phospho (STY)]PQSR_.2	P59644_S909_M2_Inpp5j	3.2915	4.6567	4.5260	4.0698	3.9361	3.8573	4.2181	4.4322	3.2304	3.7471	1.0382	0.0541	0.6619	0.1792
Fxyd7	_S[Phospho (STY)]C[Carbamidomethyl (C)]KSELPSSAPGGGGV_.2	P59648_S65_M1_Fxyd7	10.5573	10.0790	4.0169	10.5126	3.8117	10.1426	10.6340	10.1140	10.2243	3.8254	0.9411	-0.0876	0.8155	0.0886
Fxyd7	_SELPSS[Phospho (STY)]APGGGGV_.2	P59648_S73_M1_Fxyd7	6.7070	5.2933	6.3965	5.5088	5.0760	5.1109	4.9279	5.6217	5.3897	6.4365	1.0158	0.0225	0.8445	0.0734
Fxyd7	_ADSRS[Phospho (STY)]ES[Phospho (STY)]PTC[Carbamidomethyl (C)]K_.2	#N/A	5.0157	6.4418	6.4947	6.5236	3.2915	3.5758	5.1753	7.2501	4.0895	5.7861	0.9370	-0.0939	0.7145	0.1460
Fxyd7	_SESPTC[Carbamidomethyl (C)]KSC[Carbamidomethyl (C)]KS[Phospho (STY)]ELPSSAPGGGGV_.3	#N/A	7.3537	7.2973	4.1534	7.5139	4.2448	3.4847	3.8455	3.3040	3.6030	7.2623	1.2600	0.3334	0.3629	0.4402
Fxyd7	_KADS[Phospho (STY)]RSES[Phospho (STY)]PTC[Carbamidomethyl (C)]K_.2	#N/A	3.3159	7.7648	3.8434	4.1911	3.9556	6.5132	4.2125	7.9125	3.2890	8.1932	0.8354	-0.2594	0.4912	0.3087
Anks1a	_SES[Phospho (STY)]LSNC[Carbamidomethyl (C)]SIGK_.2	#N/A	7.7187	7.8255	3.5096	8.3818	7.5644	7.9208	8.3811	3.9478	4.2469	7.8876	1.1697	0.2261	0.4500	0.3468
Anks1a	_SES[Phospho (STY)]LSNC[Carbamidomethyl (C)]SIGKK_.2	#N/A	6.1216	5.9624	3.2641	6.4954	5.8357	6.2742	3.3856	6.3682	6.0751	5.9909	1.0374	0.0530	0.8101	0.0915
Anks1a	_GTESTQDAC[Carbamidomethyl (C)]AK_.2	#N/A	3.7291	3.6133	3.4628	4.5074	3.4346	4.2949	3.1548	3.9946	4.2937	4.1847	0.9830	-0.0248	0.8343	0.0787
Dock4	_S[Phospho (STY)]DPNLSAPEK_.2	P59764_S1759_M1_Dock4	7.2023	4.0414	3.9230	4.4257	6.2028	7.2120	8.0136	3.6928	6.7385	7.4116	0.8510	-0.2327	0.4060	0.3914
Dock4	_NSAPASM[Oxidation (M)]S[Phospho (STY)]PDGTR_.2	#N/A	8.6662	9.0123	8.9475	8.1993	7.2711	8.3499	3.8395	5.9650	8.2690	8.3448	1.2730	0.3482	0.0830	1.0810
Dock4	_AVNPTPSSWS[Phospho (STY)]LDSGK_.2	P59764_S1778_M1_Dock4	5.5845	4.0910	4.2357	5.7272	4.1754	3.2917	4.1728	3.3810	3.0296	5.7791	1.1044	0.1432	0.5496	0.2600
Dock4	_AS[Phospho (STY)]PLLSDK_.2	P59764_S1709_M1_Dock4	3.1281	3.8844	4.3626	3.9064	3.7727	4.3527	4.0547	3.0948	3.3938	3.5837	1.1046	0.1435	0.2252	0.6474
Sash1	_S[Phospho (STY)]LPVSIC[Carbamidomethyl (C)]R_.2	P59808_S805_M1_Sash1	8.7718	8.5980	9.3247	9.2903	8.0346	8.8080	8.5363	8.7848	8.6434	8.6802	1.0166	0.0237	0.5781	0.2380
Sash1	_EQS[Phospho (STY)]DDETEDSVK_.2	P59808_S241_M1_Sash1	7.3883	8.9537	9.2133	8.5259	7.9187	7.3346	9.2235	3.3497	8.4878	8.7707	1.1025	0.1408	0.5294	0.2762
Sash1	_AGGS[Phospho (STY)]VESLR_.2	#N/A	6.8372	3.3241	7.2113	6.9748	3.1454	6.8580	5.9077	4.4101	4.0587	7.0787	1.0674	0.0940	0.7576	0.1206
Sash1	_S[Phospho (STY)]C[Carbamidomethyl (C)]ETLEGPEPVESWPR_.2	P59808_S813_M1_Sash1	5.9971	7.0300	3.5078	8.9696	7.6302	7.1169	3.7252	8.1170	8.1495	7.7339	0.9679	-0.0471	0.8647	0.0631
Sash1	_DSGC[Carbamidomethyl (C)]YES[Phospho (STY)]SENLENAK_.2	P59808_S723_M1_Sash1	3.8000	4.2401	5.4643	4.0032	3.4403	4.2892	5.4106	4.1153	3.7638	3.9702	0.9748	-0.0368	0.8186	0.0869
Sash1	_LKAGGS[Phospho (STY)]VESLR_.2	#N/A	4.2736	2.9526	3.4595	3.9622	2.8409	3.7837	3.6769	4.7577	3.8049	5.0561	0.8200	-0.2864	0.0856	1.0674
Sash1	_SHS[Phospho (STY)]LDDLQGDADVGK_.2	P59808_S831_M1_Sash1	6.3304	7.6673	7.3523	8.4206	7.0408	7.6666	8.0760	7.8027	3.2201	7.8574	1.1000	0.1375	0.5173	0.2862
Sash1	_AVLLTAVELLQEYDS[Phospho (STY)]NSDQSGSQEK_.3	P59808_S692_M1_Sash1	3.4878	3.9249	3.6255	3.7316	3.3272	3.9024	3.6066	3.9127	3.9085	4.2255	0.9370	-0.0940	0.1523	0.8173
Arpc4	_ELLLQPVTISR_.2	#N/A	8.9248	8.6544	8.6894	8.7982	8.0594	8.1647	8.9130	8.9566	8.4948	8.4895	0.9811	-0.0276	0.4473	0.3494
Arpc4	_VLIEGSINSVR_.2	#N/A	6.5082	6.5613	6.6603	6.4151	5.0898	5.9388	6.7141	6.8580	6.5099	6.1907	0.9433	-0.0843	0.2855	0.5444
Arpc4	_AENFFILR_.2	#N/A	5.2571	6.2365	5.8299	5.0366	4.5407	5.0273	5.5738	6.4705	5.5518	6.3435	0.8891	-0.1695	0.1089	0.9629
Arpc4	_IVAEEFLK_.2	#N/A	3.1131	3.3652	4.1264	3.8096	4.3923	4.0362	3.3328	4.1204	4.9099	4.1574	0.9218	-0.1175	0.3894	0.4096
Arpc4	_ATLQAALC[Carbamidomethyl (C)]LENFSSQVVER_.2	#N/A	3.8100	3.7562	4.7308	4.8956	3.3253	3.2930	4.4730	3.1190	3.8258	4.8051	0.9785	-0.0314	0.8540	0.0685
Rims4	_QASQLS[Phospho (STY)]LESTVGPC[Carbamidomethyl (C)]GERS_.2	#N/A	4.3176	4.8710	3.8604	5.5039	4.1842	5.0053	3.8720	3.8555	3.3551	3.6759	1.2532	0.3256	0.0206	1.6860
Rims4	_QASQLS[Phospho (STY)]LESTVGPC[Carbamidomethyl (C)]GER_.2	#N/A	3.7159	3.6089	3.2610	5.3022	3.2661	3.6835	3.7252	4.8825	4.8998	3.9112	0.8741	-0.1942	0.2671	0.5734
Plp1	_TSASIGSLC[Carbamidomethyl (C)]ADAR_.2	#N/A	12.4387	12.7882	12.8341	12.5876	12.5713	12.3270	13.0402	12.9068	12.7216	12.4494	0.9853	-0.0214	0.2217	0.6543
Plp1	_M[Oxidation (M)]YGVLPWNAFPGK_.2	#N/A	11.3385	11.7034	11.6869	10.9424	10.8772	11.0001	11.6395	11.7232	11.5867	11.3478	0.9727	-0.0400	0.1548	0.8101
Plp1	_LIETYFSK_.2	#N/A	10.8616	10.7933	11.1955	10.3744	10.2825	10.5379	10.1927	11.0669	11.0489	10.6907	0.9930	-0.0102	0.7588	0.1199
Plp1	_VC[Carbamidomethyl (C)]GSNLLSIC[Carbamidomethyl (C)]K_.2	#N/A	10.3697	10.3550	10.1086	10.3154	9.9758	9.9552	10.6411	10.5986	10.3276	10.0979	0.9773	-0.0331	0.1294	0.8881
Plp1	_GLLEC[Carbamidomethyl (C)]C[Carbamidomethyl (C)]AR_.2	#N/A	10.0202	10.2897	10.2410	9.8083	9.7675	9.4493	10.3261	10.2146	9.9847	9.9029	0.9824	-0.0256	0.3526	0.4527
Plp1	_QIFGDYK_.2	#N/A	9.9659	9.8955	10.2998	9.2812	9.5337	9.5872	9.2004	10.1743	9.8976	9.7997	0.9992	-0.0011	0.9766	0.0103
Plp1	_VC[Carbamidomethyl (C)]HC[Carbamidomethyl (C)]LGK_.2	#N/A	8.1201	8.4156	8.2160	7.6743	8.4842	7.5953	8.2492	8.3786	8.3387	7.9165	0.9834	-0.0242	0.5294	0.2762
Plp1	_WLGHPDK_.2	#N/A	7.5518	7.8211	7.3307	7.1167	7.2179	7.2019	7.5747	7.9242	7.7396	7.1421	0.9708	-0.0428	0.2751	0.5604
Plp1	_GQHQAHSLER_.2	#N/A	11.0131	10.1493	10.2649	9.8074	9.7159	10.3681	11.0384	10.1378	10.3864	10.2003	0.9788	-0.0309	0.4650	0.3326
Plp1	_GLSATVTGGQK_.2	#N/A	10.2094	10.5019	10.2507	10.0870	10.1338	10.0542	10.5433	10.5736	10.4198	10.1276	0.9798	-0.0294	0.1068	0.9715
Pcbp1	_LVVPATQC[Carbamidomethyl (C)]GSLIGK_.2	#N/A	7.3902	7.9435	8.2126	7.9467	8.1322	7.6651	7.6299	7.7080	7.8091	7.7006	1.0220	0.0314	0.3165	0.4996
Pcbp1	_AITIAGVPQSVTEC[Carbamidomethyl (C)]VK_.2	#N/A	7.3084	6.9906	7.3490	6.9976	7.0018	7.3620	7.6893	7.0602	6.9930	7.3758	0.9847	-0.0222	0.5038	0.2977
Pcbp1	_IITLTGPTNAIFK_.2	#N/A	6.5672	6.8537	6.9367	6.3611	6.6871	6.7486	6.4674	6.9253	7.0566	6.5202	0.9926	-0.0107	0.7579	0.1204
Pcbp1	_IANPVEGSSGR_.2	#N/A	6.8875	6.8810	3.2986	6.6789	7.2314	6.9475	6.7618	6.9040	7.0157	6.6909	0.9237	-0.1145	0.5131	0.2898
Pcbp1	_QGANINEIR_.2	#N/A	5.0506	4.7798	4.9750	5.4277	5.0130	4.9214	4.9166	4.9638	4.9627	5.0666	1.0101	0.0146	0.6687	0.1748
Pcbp1	_QVTITGSAASISLAQYLINAR_.2	#N/A	2.8450	4.0960	3.4447	4.1771	3.9538	3.9821	3.5480	4.1511	3.3708	3.7148	1.0145	0.0208	0.8592	0.0659

Pcbp1	_QIC[Carbamidomethyl (C)]LVM[Oxidation (M)]LETLSQS[Phospho (STY)]PQGR_.2	#N/A	3.6753	6.9075	7.0478	9.1232	3.7465	3.8469	4.1402	6.7854	6.1141	3.1236	1.1356	0.1835	0.6263	0.2032
Ppfia3	_GEGPAVPGDT[Phospho (STY)]PPPTPR_.2	P60469_T714_M1_Ppfia3	13.3072	13.2515	13.8712	13.5811	12.5439	13.2951	13.7672	13.1686	13.2385	13.2137	0.9971	-0.0042	0.8817	0.0547
Ppfia3	_LAPPS[Phospho (STY)]PAR_.2	P60469_S683_M1_Ppfia3	10.3321	10.1063	10.1798	10.3394	9.0164	10.2625	9.9990	10.2132	10.2544	10.1654	0.9883	-0.0169	0.6648	0.1773
Ppfia3	_QAQS[Phospho (STY)]PGGVSSEVEVLK_.2	#N/A	9.3813	9.4288	10.1155	9.9343	8.9692	9.5177	9.7845	9.3748	9.4792	9.3942	1.0052	0.0075	0.8298	0.0810
Ppfia3	_RGS[Phospho (STY)]ALGPDEAGGELER_.3	#N/A	8.0918	7.7441	7.9384	8.3468	6.5491	7.8851	7.3704	8.0873	7.9971	8.1629	0.9816	-0.0267	0.6898	0.1613
Ppfia3	_YSQAPTLPSGAPLDPYGAGS[Phospho (STY)]GR_.2	P60469_S537_M1_Ppfia3	6.5932	3.6333	7.1812	8.0223	3.5216	3.1030	4.4970	3.3458	3.6449	3.3326	1.4419	0.5280	0.1835	0.7365
Ppfia3	_VSSSGLDS[Phospho (STY)]LGR_.2	P60469_S645_M1_Ppfia3	7.8975	7.8548	8.3897	7.9093	6.8406	7.8004	7.9572	7.7493	7.5602	7.5052	1.0116	0.0166	0.7514	0.1241
Ppfia3	_ESVSLAGTPSDETLATDPLGLAK_.2	#N/A	4.3268	4.5784	4.8076	4.5996	4.3283	3.7640	4.8596	4.6575	5.1262	4.4132	0.9237	-0.1145	0.1369	0.8635
Ppfia3	_GS[Phospho (STY)]ALGPDEAGGELER_.2	#N/A	5.9801	5.4187	6.6492	4.2079	4.0961	6.4660	3.9225	5.8852	4.9122	6.4050	1.0357	0.0506	0.7980	0.0980
Ppfia3	_TAELEEALER_.2	#N/A	5.0514	5.6113	3.1282	4.9304	5.3012	5.5186	4.3210	5.6472	5.4767	5.8693	0.9240	-0.1141	0.4763	0.3222
Ppfia3	_S[Phospho (STY)]LPGSALELR_.2	P60469_S508_M1_Ppfia3	3.2097	11.3940	11.9953	11.9212	10.4539	11.9456	11.7270	11.4925	11.6883	11.6751	0.8718	-0.1979	0.4216	0.3751
Ppfia3	_M[Oxidation (M)]AQALALQAGS[Phospho (STY)]PEDGAPPR_.2	#N/A	8.4787	9.3448	9.0813	10.4771	8.3411	9.2179	9.6338	9.6866	9.4655	9.2987	0.9617	-0.0563	0.3841	0.4156
Ppfia3	_RQAQS[Phospho (STY)]PGGVSSEVEVLK_.3	#N/A	3.8777	3.1331	3.6114	3.7817	3.2483	3.6032	3.4963	3.8460	3.9854	5.1473	0.8601	-0.2174	0.1097	0.9598
Ppfia3	_QM[Oxidation (M)]S[Phospho (STY)]QLEEELGTAHR_.3	#N/A	5.5408	4.6980	4.1089	5.6523	5.0260	6.0180	4.6072	5.7663	6.1066	6.0400	0.9190	-0.1219	0.3424	0.4655
Ppfia3	_WSGAKDESS[Phospho (STY)]KDWDR_.3	P60469_S554_M1_Ppfia3	3.6871	2.9915	4.3739	3.6284	2.8128	3.4159	3.7766	3.3728	3.6718	3.3057	0.9867	-0.0193	0.8792	0.0559
Pdxp	_IVPGAPELLQR_.2	#N/A	8.1483	8.6040	8.4022	7.9440	8.5533	8.1555	8.1202	8.3143	7.9712	8.0277	1.0238	0.0339	0.2210	0.6555
Pdxp	_NTLFVSNNSR_.2	#N/A	7.8403	7.5856	7.9282	8.1355	7.6980	7.7207	7.7590	7.6356	7.7612	7.7498	1.0119	0.0170	0.3982	0.3999
Pdxp	_LAGDPGEDPR_.2	#N/A	6.6678	6.7140	6.9919	6.8051	7.2804	6.8661	6.6818	7.0726	6.6949	6.2424	1.0322	0.0457	0.2580	0.5883
Pdxp	_AEQLFSSALC[Carbamidomethyl (C)]AAR_.2	#N/A	5.9364	6.0116	6.2067	6.2029	6.5705	6.5985	5.8531	6.3766	6.5897	6.1353	1.0025	0.0036	0.9354	0.0290
Pdxp	_LSGPPDASGAVFVLGGEGLR_.2	#N/A	5.1993	5.6381	6.0177	5.2665	4.7899	5.4320	5.6090	4.4988	5.9134	5.8440	0.9862	-0.0201	0.8274	0.0823
Pdxp	_TPGTGSLAAAVETASGR_.2	#N/A	6.1744	6.2985	3.1861	6.2711	6.7027	6.4106	6.5072	6.3661	5.8282	6.3575	0.9323	-0.1011	0.5504	0.2593
Pdxp	_C[Carbamidomethyl (C)]GM[Oxidation (M)]TTVLTLTGVSSLEEAQAYLTAGQR_.3	#N/A	1.6575	3.6930	3.3831	3.9800	3.5143	4.2152	3.6723	4.0743	4.3734	4.1050	0.8400	-0.2516	0.2116	0.6746
Pdxp	_AVLVGYDEQFSFSR_.2	#N/A	3.8981	3.2025	4.5849	3.5867	3.0238	3.2049	3.5657	3.0309	4.1803	3.5537	1.0002	0.0003	0.9982	0.0008
Gabarapl2	_EDHS[Phospho (STY)]LEHR_.2	#N/A	6.0507	5.8379	5.4602	6.3324	6.0736	6.3058	6.1405	5.9416	6.1898	6.1088	0.9860	-0.0203	0.6329	0.1987
Gabarapl2	_WM[Oxidation (M)]FKEDHS[Phospho (STY)]LEHR_.3	#N/A	3.2593	4.0832	4.4938	4.0375	3.9038	3.8250	4.1858	4.4645	3.2627	3.7148	1.0069	0.0099	0.9295	0.0318
Actb	_VAPEEHPVLLTEAPLNPK_.3	#N/A	13.1250	13.0598	13.5636	13.3605	13.1999	13.2840	13.2088	13.0046	13.1791	13.0418	1.0120	0.0172	0.1579	0.8016
Actb	_AGFAGDDAPR_.2	#N/A	13.4706	13.5793	13.4314	13.5474	13.4536	13.2256	13.3666	13.5366	13.2492	13.4539	1.0037	0.0053	0.5514	0.2585
Actb	_DSYVGDEAQSKR_.2	#N/A	11.8883	12.2861	12.4790	12.2355	12.2643	12.2692	12.1400	12.2214	12.1916	12.0961	1.0062	0.0088	0.4775	0.3211
Actb	_IIAPPERK_.2	#N/A	11.6736	12.5049	12.3502	12.1754	12.6823	12.0592	12.0134	12.4405	12.2803	12.0797	1.0031	0.0044	0.8544	0.0683
Actb	_AVFPSIVGRPR_.2	#N/A	11.1358	11.1993	11.4019	10.7458	11.1082	11.1503	11.1177	11.0217	11.1143	11.1660	1.0017	0.0024	0.8712	0.0599
Actb	_DSYVGDEAQSK_.2	#N/A	11.9535	11.9548	12.0398	12.2185	12.1532	12.0379	12.0251	12.0372	12.1622	12.0701	0.9988	-0.0017	0.8205	0.0859
Actb	_AVFPSIVGR_.2	#N/A	11.3852	11.2781	11.2389	11.0270	11.2501	11.0605	11.0316	11.2822	11.3772	11.3290	0.9957	-0.0062	0.6158	0.2105
Actb	_C[Carbamidomethyl (C)]DVDIRK_.2	#N/A	10.7105	10.7166	10.9718	10.5456	11.3420	10.9110	10.3464	10.6393	10.7905	10.6689	1.0240	0.0343	0.1511	0.8209
Actb	_IKIIAPPER_.2	#N/A	8.6120	9.3189	9.9309	8.9987	8.6239	9.1724	9.2658	9.0120	9.1538	8.8439	1.0045	0.0064	0.8808	0.0551
Actb	_GYSFTTTAER_.2	#N/A	12.2070	12.2179	12.4274	12.6040	12.4380	12.4615	12.1398	12.4225	12.2500	12.3229	1.0089	0.0127	0.2678	0.5722
Actb	_TTGIVM[Oxidation (M)]DSGDGVTHTVPIYEGYALPHAILR_.3	#N/A	7.6425	8.6579	9.0760	8.2305	8.3425	8.7543	7.9657	8.8081	8.7336	8.5966	0.9912	-0.0128	0.8053	0.0940
Actb	_KDLYANTVLSGGTTM[Oxidation (M)]YPGIADR_.3	#N/A	7.7970	7.4324	7.6310	8.3097	7.6198	8.6814	7.4781	7.2843	7.4798	7.3898	1.0680	0.0949	0.0769	1.1140
Actb	_[Acetyl (Protein N-term)]DDDIAALVVDNGSGMC[Carbamidomethyl (C)]K_.2	#N/A	10.5113	10.6242	11.0179	11.8073	8.7865	9.4033	11.2382	11.4897	9.2926	10.8011	0.9676	-0.0475	0.6244	0.2046
Actb	Acetyl (Protein N-term)]DDDIAALVVDNGSGM[Oxidation (M)]C[Carbamidomethyl (C)]KAGFAGDDAPR_	#N/A	8.1378	8.2950	8.6400	8.7659	7.6981	7.8678	8.8866	7.8759	8.0210	7.8535	1.0092	0.0132	0.8022	0.0957
Actb	_LC[Carbamidomethyl (C)]YVALDFEQEM[Oxidation (M)]ATAASSSSLEK_.3	#N/A	8.8961	8.6230	9.5120	9.8445	8.6474	9.2215	9.2502	8.5656	8.9573	9.2325	1.0136	0.0195	0.6740	0.1713
Actb	_EKLC[Carbamidomethyl (C)]YVALDFEQEM[Oxidation (M)]ATAASSSSLEK_.3	#N/A	6.1453	6.4233	8.2186	7.9029	6.0439	6.5253	7.2990	6.5118	7.0209	7.2501	0.9795	-0.0299	0.7808	0.1074
Actb	_EITALAPSTM[Oxidation (M)]K_.2	#N/A	12.3587	12.5682	12.7835	12.4544	12.7472	12.3299	12.4237	12.5731	12.4839	12.3085	1.0075	0.0107	0.4145	0.3825
Actb	·bamidomethyl (C)]PEALFQPSFLGM[Oxidation (M)]ESC[Carbamidomethyl (C)]GIHETTFNSIM[Oxidation	#N/A	4.6751	5.7256	7.5630	6.6020	4.4997	5.5638	6.0625	5.8647	5.6290	6.2079	0.9715	-0.0418	0.7854	0.1049
Actb	_HQGVM[Oxidation (M)]VGM[Oxidation (M)]GQK_.2	#N/A	8.2731	8.4724	8.2948	8.4859	8.8786	8.4014	8.1902	8.3531	8.4941	8.4753	1.0107	0.0153	0.4944	0.3059
Actb	_DLYANTVLSGGTTM[Oxidation (M)]YPGIADR_.2	#N/A	10.5195	10.6561	11.3569	10.7965	10.6987	10.8223	10.8419	10.6590	10.7311	10.6593	1.0080	0.0115	0.5885	0.2302
Actb	_IWHHTFYNELR_.3	#N/A	9.9643	9.8949	9.5152	6.7291	9.1249	10.3810	10.3775	9.2251	10.0105	10.0931	0.9337	-0.0990	0.3737	0.4275
Actb	_QEYDESGPSIVHR_.2	#N/A	10.6803	10.6907	10.9754	10.8314	10.8936	10.8350	10.5940	10.5825	10.7425	10.7073	1.0151	0.0217	0.0421	1.3752
Actb	_QEYDESGPSIVHRK_.3	#N/A	4.6696	5.5290	6.1530	5.3563	5.2866	5.5043	4.4591	5.0409	5.3532	5.1276	1.0843	0.1168	0.1797	0.7455
Actb	_DLTDYLM[Oxidation (M)]K_.2	#N/A	10.6519	10.7977	11.3648	10.2515	10.2720	10.9831	10.4522	10.9992	10.9202	10.9838	0.9891	-0.0159	0.6361	0.1964
Actb	ıamidomethyl (C)]PEALFQPSFLGM[Oxidation (M)]ESC[Carbamidomethyl (C)]GIHETTFNSIM[Oxidation (	#N/A	5.2131	5.5780	6.4752	6.4552	5.2578	5.5402	5.7219	5.9794	6.0711	5.9413	0.9705	-0.0433	0.5715	0.2430
Actb	_DLTDYLM[Oxidation (M)]KILTER_.2	#N/A	3.7621	3.7096	3.3946	3.7519	2.9059	3.0140	4.1663	4.2213	3.1131	3.2660	0.9272	-0.1090	0.4011	0.3968
Actb	_S[Phospho (STY)]YELPDGQVITIGNER_.2	P60710_S239_M1_Actb	8.7082	8.2140	8.3442	12.3103	8.1401	8.5256	8.9677	11.9406	8.5894	8.4684	0.9525	-0.0703	0.6788	0.1682
Nrgn	_KGPGPGGPGGAGGAR_.2	#N/A	7.9926	8.4679	8.2625	8.7163	9.1300	8.5968	8.3427	8.1782	8.1966	7.9923	1.0428	0.0605	0.1296	0.8874
Nrgn	/I[Oxidation (M)]DC[Carbamidomethyl (C)]C[Carbamidomethyl (C)]TESAC[Carbamidomethyl (C)]SKPDI	#N/A	4.8632	5.4531	4.0634	4.8814	5.6073	5.1256	5.4150	4.6614	5.1035	5.1346	0.9843	-0.0228	0.8009	0.0964
Nrgn	_GPGPGGPGGAGGAR_.2	#N/A	4.0252	3.3296	3.2112	3.7138	3.1509	3.0778	3.4386	4.4047	4.0532	3.6808	0.8777	-0.1882	0.0970	1.0132
Nrgn	_IKS[Phospho (STY)]GEC[Carbamidomethyl (C)]GR_.2	P60761_S48_M1_Nrgn	3.0570	4.1692	3.7438	4.2465	3.6835	4.0460	3.9611	3.8720	3.5206	4.2135	0.9827	-0.0252	0.7947	0.0998
Cdc42	_QKPITPETAEK_.2	#N/A	7.7464	7.9084	7.9061	7.4936	7.5865	7.6133	7.7021	7.5936	7.5755	7.6120	1.0116	0.0166	0.3620	0.4413
Cdc42	_WVPEITHHC[Carbamidomethyl (C)]PK_.3	#N/A	7.0452	4.9202	6.5779	6.0689	7.1115	6.6157	6.0379	6.3011	6.0901	6.3430	1.0318	0.0452	0.6508	0.1865
Cdc42	_TPFLLVGTQIDLR_.2	#N/A	6.3234	4.9200	3.8948	5.6436	5.4175	5.0609	6.0042	5.3268	5.7605	5.3354	0.9292	-0.1059	0.3889	0.4101
Cdc42	_TC[Carbamidomethyl (C)]LLISYTTNK_.2	#N/A	5.2382	4.8295	5.3869	5.6170	5.4648	5.2268	5.6915	5.5766	5.5677	5.2083	0.9606	-0.0580	0.2141	0.6693
Cdc42	_TPFLLVGTQIDLRDDPSTIEK_.3	#N/A	4.0711	3.3266	3.8048	3.3486	3.2149	4.7623	3.2262	4.3722	4.8057	4.4143	0.8930	-0.1633	0.2988	0.5246
Cdc42	_YVEC[Carbamidomethyl (C)]SALTQK_.2	#N/A	3.6932	5.8736	3.4269	3.9662	5.5374	3.7877	3.6808	4.0305	3.8009	4.1488	1.1189	0.1621	0.4141	0.3829
Cdc42	_LRPLSYPQTDVFLVC[Carbamidomethyl (C)]FSVVSPSSFENVK_.3	#N/A	3.0869	4.1393	3.7737	4.2764	4.0276	4.0979	3.9910	3.8421	3.4907	4.2434	1.0022	0.0031	0.9741	0.0114
Cirbp	_S[Phospho (STY)]QGGSYGYR_.2	P60824_S146_M1_Cirbp	3.3754	4.1806	4.0622	5.3625	4.0019	3.7276	4.0884	5.0806	3.2022	6.2800	0.8832	-0.1791	0.4099	0.3874
Ensa	_KSS[Phospho (STY)]LVTSK_.2	P60840_S109_M1_Ensa	7.2496	6.3910	7.3127	6.7433	7.0078	6.9767	6.8077	6.8562	7.4974	6.9966	0.9868	-0.0191	0.6767	0.1696
Ensa	_[Acetyl (Protein N-term)]S[Phospho (STY)]QKQEEENPAEETGEEK_.2	P60840_S2_M1_Ensa	3.6097	4.4149	3.2276	3.2984	4.2363	3.4932	4.5139	3.3193	2.9679	3.2654	1.0560	0.0785	0.6095	0.2150
Eif4a1	_ATQALVLAPTR_.2	#N/A	5.6910	5.9592	5.5490	6.5626	5.8182	5.8004	6.4496	6.3228	5.8292	5.1371	0.9936	-0.0092	0.9014	0.0451
Eif4a1	_GYDVIAQAQSGTGK_.2	#N/A	4.3867	6.2981	6.4028	6.6521	6.3633	6.2535	6.6091	6.6190	6.6380	6.5981	0.9159	-0.1268	0.2258	0.6462
Eif4a1	_GIDVQQVSLVINYDLPTNR_.2	#N/A	3.4101	3.6580	3.1438	4.1884	3.7537	3.9758	4.3366	3.5891	3.2079	1.5459	1.1635	0.2185	0.3343	0.4759
Lzts1	_FGFSQDS[Phospho (STY)]GRGK_.2	P60853_S50_M1_Lzts1	6.3818	4.0494	4.5276	7.0290	5.0217	4.1878	7.0850	4.4306	7.3364	6.6002	0.8172	-0.2913	0.1925	0.7155
Lzts1	_ALSFS[Phospho (STY)]DGGSK_.2	P60853_S233_M1_Lzts1	2.9551	4.2711	3.6419	4.1445	4.1594	3.9661	3.8592	3.9739	3.6225	4.1115	0.9909	-0.0132	0.8967	0.0473
Lzts1	_S[Phospho (STY)]FDEKEFASGQTFEERPR_.3	P60853_S283_M1_Lzts1	2.9779	4.9301	4.2124	3.1806	3.6224	3.0022	4.3962	3.4370	3.0856	3.1476	1.0391	0.0554	0.7747	0.1109
Rps20	_DTGKTPVEPEVAIHR_.3	#N/A	6.3437	6.2897	6.4331	6.7097	6.2264	6.1824	6.3376	5.9303	5.9160	5.7440	1.0639	0.0893	0.0253	1.5972
Rps20	_LIDLHSPSEIVK_.2	#N/A	5.4283	5.9151	4.5843	6.0681	6.0201	4.9570	5.9212	5.7928	5.3579	5.2092	0.9866	-0.0195	0.8326	0.0796
Rps20	_VC[Carbamidomethyl (C)]ADLIR_.2	#N/A	4.1547	3.4591	3.3407	3.8014	4.7667	3.5289	3.5580	4.5913	4.0959	3.8104	0.9571	-0.0632	0.6120	0.2132
Rps20	_TPVEPEVAIHR_.3	#N/A	2.8314	4.1693	4.4076	3.9514	4.6092	3.2144	4.0997	1.8110	3.3488	4.1751	1.1504	0.2022	0.3944	0.4040
Snap25	_RADQLADESLESTRR_.3	#N/A	5.6807	5.8985	6.3958	5.9869	5.8859	5.8879	5.6579	5.6837	6.0265	5.7688	1.0297	0.0422	0.2500	0.6020
Snap25	[Carbamidomethyl (C)]GLC[Carbamidomethyl (C)]VC[Carbamidomethyl (C)]PC[Carbamidomethyl (C)]N	#N/A	6.4861	6.4872	5.9133	6.5612	6.9426	6.0058	6.6411	6.6609	6.1597	6.3968	0.9899	-0.0146	0.7701	0.1134
Snap25	_ADQLADESLESTR_.2	#N/A	6.1976	5.8446	4.0873	5.5014	5.9110	5.3892	6.5408	6.1425	5.7318	5.9517	0.9010	-0.1504	0.1738	0.7598

Snap25	_IEEGM[Oxidation (M)]DQINK_.2	#N/A	7.5443	7.3456	6.9311	7.7596	7.3324	6.9468	7.3597	7.4048	7.0364	7.5277	0.9970	-0.0044	0.9081	0.0419
Snap25	_TLVM[Oxidation (M)]LDEQGEQLER_.2	#N/A	7.5916	7.3609	7.5546	8.3072	7.3093	7.3811	7.5184	7.4562	7.2488	7.5303	1.0196	0.0280	0.4796	0.3191
Snap25	_IDEANQR_.2	#N/A	5.2399	6.0324	6.2879	5.7661	6.6144	4.2882	5.9220	6.2561	5.8814	6.3672	0.9342	-0.0982	0.3853	0.4142
Snap25	_EQM[Oxidation (M)]AISGGFIR_.2	#N/A	9.2210	9.1582	9.3054	8.9117	8.5099	9.1645	9.3933	9.3517	9.1063	9.1757	0.9771	-0.0334	0.2204	0.6567
Snap25	_MLQLVEESK_.2	#N/A	6.9235	2.9584	3.4537	6.7823	5.9365	4.3361	5.3551	4.1621	5.3264	6.2555	0.9602	-0.0585	0.8289	0.0815
Snap25	_AWGNNQDGVVASQPAR_.2	#N/A	8.7045	8.9754	9.2589	9.1035	8.8134	8.7267	9.2908	8.9876	8.9683	8.8848	0.9887	-0.0165	0.4640	0.3335
Snap25	_RADQLADESLESTR_.3	#N/A	5.5287	4.6766	3.9917	5.5598	5.3595	5.2045	4.2090	3.6242	5.6071	5.7142	1.0553	0.0777	0.6208	0.2071
Snap25	_ENEM[Oxidation (M)]DENLEQVSGIIGNLR_.2	#N/A	7.6048	7.6892	7.7770	8.1591	7.3120	7.5601	7.8842	7.7215	7.7265	7.8857	0.9845	-0.0225	0.4380	0.3586
Snap25	_NELEEMQR_.2	#N/A	4.2148	4.3007	3.2781	8.5868	4.1890	3.9365	7.8151	6.8418	2.9775	1.2251	1.0077	0.0110	0.9823	0.0078
Snap25	_ADQLADESLESTRR_.2	#N/A	4.7689	4.7256	5.3765	4.8643	4.9085	4.8073	4.5774	3.9989	4.7809	4.4763	1.1010	0.1388	0.0359	1.4444
Snap25	_HM[Oxidation (M)]ALDM[Oxidation (M)]GNEIDTQNR_.3	#N/A	6.3619	6.6827	7.2455	5.5551	6.8583	6.7357	5.9071	6.8151	6.7629	6.8418	0.9987	-0.0019	0.9808	0.0084
Snap25	_M[Oxidation (M)]LQLVEESKDAGIR_.2	#N/A	5.2238	5.8368	6.7343	4.7594	5.7470	5.8449	5.5567	5.8453	5.2804	5.6829	1.0178	0.0255	0.7848	0.1053
Snap25	_[Acetyl (Protein N-term)]AEDADM[Oxidation (M)]RNELEEMQR_.2	#N/A	4.3201	3.9426	4.0377	4.9712	3.4477	3.2538	3.8932	3.4967	3.7957	3.7737	1.0684	0.0954	0.4509	0.3459
Snap25	_VVDEREQM[Oxidation (M)]AISGGFIR_.3	#N/A	3.9423	3.2467	7.0229	3.3732	3.0680	3.1606	3.5214	3.6280	3.9271	3.0504	1.1238	0.1684	0.5963	0.2245
Dnajc5	_EINNAHAILTDATKR_.3	#N/A	7.9118	7.7370	7.6006	7.4335	7.6861	7.6675	7.5883	8.1931	7.7287	7.4343	0.9918	-0.0119	0.6885	0.1621
Dnajc5	_SLS[Phospho (STY)]TSGESLYHVLGLDKNATSDDIKK_.4	#N/A	6.5836	8.0604	4.2358	7.6706	3.1352	3.3015	7.7662	8.4070	8.1025	7.2438	0.6977	-0.5193	0.0713	1.1472
Dnajc5	_NATSDDIKK_.2	#N/A	5.5047	5.1885	5.9624	4.9077	5.7707	5.8434	5.4573	5.7356	5.3217	6.1431	0.9762	-0.0348	0.6103	0.2144
Dnajc5	_SLS[Phospho (STY)]TSGESLYHVLGLDK_.2	#N/A	11.8230	12.0435	12.6195	8.3275	10.9506	11.7679	12.2277	11.8230	11.8217	11.6020	0.9483	-0.0765	0.4591	0.3381
Dnajc5	_EINNAHAILTDATK_.2	#N/A	4.1080	3.7947	3.5082	3.5205	3.6764	2.5817	4.3129	4.6062	4.3070	4.2860	0.8067	-0.3100	0.0138	1.8600
Dnajc5	_APEGEETEFYVS[Phospho (STY)]PEDLEAQLQSDER_.3	P60904_S151_M1_Dnajc5	5.2211	5.5712	5.6773	5.8814	5.2940	3.6754	5.6432	6.3116	5.8991	5.0804	0.9104	-0.1354	0.2908	0.5364
Dnajc5	_QRS[Phospho (STY)]LS[Phospho (STY)]TSGESLYHVLGLDK_.3	P60904_S10_M2_Dnajc5	3.6220	3.6042	4.3088	3.3106	4.2485	3.1321	4.5261	3.3070	2.9556	3.2776	1.0534	0.0750	0.6234	0.2053
Dnajc5	_YHPDKNPDNPEAADKFK_.4	#N/A	4.1269	3.0993	3.3128	3.8155	3.2526	2.9761	3.5302	4.3030	5.1575	3.7825	0.8181	-0.2897	0.0695	1.1582
Rab5b	_GVDLHEQSQQNK_.2	#N/A	4.0921	4.4276	5.0686	3.3943	3.5517	4.7242	5.2282	4.3952	4.4236	3.6970	0.9490	-0.0755	0.6021	0.2204
Rab5b	_GAQAAIVVYDITNQETFAR_.2	#N/A	6.2322	6.2067	7.6528	7.0709	5.3126	6.5796	7.0170	6.6835	5.9199	6.3786	1.0014	0.0021	0.9839	0.0071
Chp1	_DELLQVLR_.2	#N/A	4.1316	4.1615	4.4306	3.6278	3.5424	3.2322	3.7200	4.0971	4.1198	4.2236	0.9540	-0.0679	0.4759	0.3225
Chp1	_IINAFFSEGEDQVNFR_.2	#N/A	3.5367	3.8453	3.2704	4.3150	4.1813	3.9442	4.4633	4.1870	2.6169	5.1286	0.9390	-0.0908	0.6059	0.2176
Chp1	_ETGFSHSQITR_.3	#N/A	3.7457	3.0501	4.4325	3.5698	2.8714	3.3572	3.7180	3.1833	3.7305	3.4013	0.9989	-0.0016	0.9902	0.0043
Rab10	_AFLTLAEDILR_.2	#N/A	7.3548	7.0928	7.0660	6.7916	7.0484	6.6390	7.1145	7.3523	7.6752	6.6093	0.9737	-0.0385	0.4128	0.3842
Rab10	_GKGEQIAR_.2	#N/A	6.6454	5.3330	5.4690	5.5357	6.5502	6.0182	5.4556	6.4653	6.4172	5.7152	0.9853	-0.0213	0.8093	0.0919
Rab10	_EPNSENVDISS[Phospho (STY)]GGGVTGWK_.2	#N/A	3.9757	5.9677	3.8955	3.4393	5.3643	3.2267	7.0563	6.9052	6.5978	3.1165	0.7284	-0.4571	0.1226	0.9113
Rab10	_KTPVKEPNSENVDISS[Phospho (STY)]GGGVTGWK_.3	#N/A	3.2714	3.9548	3.9582	4.4608	3.8979	4.2824	4.1755	5.4729	3.3062	4.4278	0.9138	-0.1301	0.3869	0.4124
Rab10	_ANINIEK_.2	#N/A	3.2895	3.3868	3.2402	3.5623	2.3124	3.7896	4.4331	3.8642	3.8221	3.5792	0.8315	-0.2662	0.0558	1.2534
Rab10	_NIDEHANEDVER_.2	#N/A	5.5327	3.0415	3.3706	3.8732	5.1116	3.6948	3.5879	4.9692	5.0152	3.8402	0.9428	-0.0850	0.6824	0.1660
Rab10	_TPVKEPNSENVDISS[Phospho (STY)]GGGVTGWK_.3	#N/A	3.5702	4.0777	3.8490	3.9140	4.2060	3.8930	4.4858	4.7863	3.8916	3.9381	0.9165	-0.1258	0.1185	0.9263
Rab8b	_SSTNVEEAFFTLAR_.2	#N/A	4.1469	4.1844	3.8806	3.4243	3.2906	3.3340	3.9977	3.5768	3.8759	3.6311	0.9840	-0.0232	0.7948	0.0997
Ube2d3	_SQWSPALTISK_.2	#N/A	7.7411	7.8287	7.9616	7.7614	7.8589	7.5342	7.7050	7.6837	7.3785	7.5751	1.0258	0.0367	0.0721	1.1423
Ube2d3	_ELS[Phospho (STY)]DLAR_.2	P61079_S11_M1_Ube2d3	3.8248	3.1291	3.9469	3.4907	2.9505	3.2782	3.6390	5.6756	3.8095	3.1680	0.8438	-0.2451	0.2212	0.6553
Ube2d1	_ELS[Phospho (STY)]DLQR_.2	P61080_S11_M1_Ube2d1	5.6994	5.3621	3.6416	6.0706	5.2093	5.9265	5.8270	5.8712	5.6741	5.7999	0.9180	-0.1234	0.3245	0.4888
Ube2m	_EAAEVLQNNR_.2	#N/A	9.7399	9.4938	9.7495	10.0903	9.5615	9.8265	9.9663	9.7457	9.8725	9.8714	0.9878	-0.0177	0.3198	0.4951
Ube2m	_GGYIGSTYFER_.2	#N/A	5.7021	5.2086	4.5348	5.4628	5.6548	4.6933	5.6196	5.1118	5.2404	5.4932	0.9708	-0.0428	0.5756	0.2398
Ube2m	_DINELNLPK_.2	#N/A	4.7180	3.8730	4.9544	4.5427	4.9084	3.7003	4.2573	3.7886	3.0410	3.0088	1.2626	0.3364	0.0346	1.4604
Ube2m	_TC[Carbamidomethyl (C)]DISFSDPDDLLNFK_.2	#N/A	3.8226	3.1269	4.5094	3.5112	4.1917	3.2804	3.6412	3.1065	4.2559	3.4782	1.0331	0.0470	0.7282	0.1378
Ube2n	_LLAEPVPGIK_.2	#N/A	9.2599	9.6904	9.3424	9.2054	9.8301	9.3857	9.4973	9.4792	9.4481	9.3552	1.0008	0.0011	0.9563	0.0194
Ube2n	_TNEAQAIETAR_.2	#N/A	8.8212	9.1319	9.2095	9.2209	9.4951	9.1394	8.9872	9.0895	8.9534	9.1290	1.0144	0.0206	0.2920	0.5346
Ube2n	_IYHPNVDK_.2	#N/A	8.0271	7.9362	7.8246	8.2701	8.6858	8.1521	7.8101	8.0665	7.9749	8.1960	1.0172	0.0245	0.4390	0.3575
Ube2n	_WSPALQIR_.2	#N/A	7.4259	8.0293	7.9185	7.8903	7.9559	7.8630	7.7288	7.7375	7.7415	7.8128	1.0119	0.0170	0.4291	0.3674
Ube2n	_YFHVVIAGPQDSPFEGGTFK_.3	#N/A	5.7789	5.6589	5.7747	6.2340	5.9617	5.6544	5.9498	5.7691	5.5206	6.1129	1.0010	0.0014	0.9710	0.0128
Ube2n	_LLAEPVPGIKAEPDESNAR_.3	#N/A	5.1749	5.6663	7.0440	6.0829	4.9751	5.6500	5.3005	5.1580	5.2347	5.0048	1.1142	0.1560	0.1590	0.7986
Ube2n	_LELFLPEEYPM[Oxidation (M)]AAPK_.2	#N/A	7.7651	8.0153	7.9694	7.6050	8.0294	8.0783	7.9412	8.0311	7.9332	8.0291	0.9908	-0.0133	0.4710	0.3270
Ube2n	_AEPDESNAR_.2	#N/A	6.2978	3.1791	3.6574	3.2011	4.6674	6.3504	3.3494	3.8001	5.5631	5.8969	0.9799	-0.0293	0.9199	0.0362
Ube2n	_TVLLSIQALLSAPNPDDPLANDVAEQWK_.3	#N/A	4.3453	3.6007	4.0790	3.3141	3.4890	1.5972	4.5296	3.3036	2.9522	3.2810	0.9680	-0.0469	0.8470	0.0721
Actr2	_ILLTEPPM[Oxidation (M)]NPTK_.2	#N/A	8.0183	8.2612	8.6457	7.6646	7.9802	8.1313	8.1891	8.6592	8.3501	7.9821	0.9785	-0.0313	0.4019	0.3959
Actr2	_HLWDYTFGPEK_.2	#N/A	5.7150	5.9294	6.3560	5.9399	4.3625	6.0232	5.0145	6.2910	5.3621	5.6000	1.0277	0.0394	0.7199	0.1427
Actr2	_DLM[Oxidation (M)]VGDEASELR_.2	#N/A	7.4452	6.9173	7.2978	7.0578	6.6821	7.1172	6.9737	7.2647	6.9512	7.0074	1.0052	0.0075	0.8112	0.0909
Actr2	_LC[Carbamidomethyl (C)]YVGYNIEQEQK_.2	#N/A	4.0259	5.4556	3.7459	3.2896	5.1655	5.7316	6.1181	6.4099	4.0106	6.2059	0.8035	-0.3156	0.1393	0.8560
Actr2	_GYAFNHSADFETVR_.3	#N/A	4.5191	3.9292	3.9837	4.4864	4.5395	3.8060	4.2011	3.9899	3.2807	2.9526	1.1677	0.2236	0.0701	1.1542
Actr2	_HIVLSGGSTM[Oxidation (M)]YPGLPSR_.2	#N/A	3.8214	3.6555	3.4206	2.8468	3.4768	3.5262	4.5172	4.0368	4.3358	4.2466	0.8071	-0.3091	0.0022	2.6529
Actr2	_LALETTVLVESYTLPDGR_.2	#N/A	3.8916	3.4508	3.6253	3.1690	3.0353	2.9565	3.3173	3.6897	4.1312	4.3472	0.8666	-0.2066	0.0823	1.0844
Actr1a	_AQYYLPDGSTIEIGPSR_.2	#N/A	7.4257	7.1222	7.8110	7.7658	7.2272	7.4205	7.2545	7.2907	7.3674	7.0834	1.0294	0.0418	0.1928	0.7150
Actr1a	_DQLQTFSEEHPVLLTEAPLNPR_.3	#N/A	5.0415	5.5457	5.7529	6.1332	5.2203	5.9612	5.1310	5.4356	5.3574	5.4547	1.0495	0.0697	0.2715	0.5662
Actr1a	_AC[Carbamidomethyl (C)]YLSINPQKDETLETEK_.3	#N/A	3.4354	4.3508	4.1222	4.6249	5.7560	5.3375	4.3396	5.2448	4.2806	4.9744	0.9776	-0.0326	0.8275	0.0822
Actr1a	_KEYEEDGAR_.2	#N/A	4.1153	3.2271	3.8490	3.3928	3.0484	3.1803	3.5410	3.6084	3.9074	3.0701	0.9822	-0.0259	0.8112	0.0909
Arf3;Arf1	_NISFTVWDVGGQDK_.2	#N/A	7.9012	7.6664	8.3726	7.7020	7.1537	7.4766	8.1604	8.0259	7.8332	7.8232	0.9688	-0.0458	0.2891	0.5390
Arf3;Arf1	_DAVLLVFANK_.2	#N/A	7.7313	6.9418	6.6039	7.6704	6.6676	7.2285	7.3159	7.6256	7.5264	7.4065	0.9561	-0.0648	0.2335	0.6317
Arf3;Arf1	_LGEIVTTIPTIGFNVETVEYK_.2	#N/A	6.2543	6.2873	6.6078	7.1726	5.2462	5.9024	6.6958	6.5169	6.4928	6.3596	0.9584	-0.0613	0.4428	0.3538
Arf3;Arf1	_M[Oxidation (M)]LAEDELRDAVLLVFANK_.3	#N/A	6.1655	5.9137	6.1556	5.1358	5.7566	6.1494	6.0803	6.4241	6.5068	5.8808	0.9448	-0.0819	0.1816	0.7410
Arf3;Arf1	_M[Oxidation (M)]LAEDELR_.2	#N/A	7.5834	6.5126	6.2768	7.3812	6.7201	6.2185	6.4802	6.8110	6.8706	6.8805	1.0032	0.0046	0.9451	0.0245
Arf3;Arf1	_QDLPNAM[Oxidation (M)]NAAEITDK_.2	#N/A	8.4453	7.7340	7.9688	8.2914	7.6920	7.3902	8.2275	7.5933	7.6119	7.8880	1.0115	0.0165	0.7098	0.1488
Arf3;Arf1	_ILM[Oxidation (M)]VGLDAAGK_.2	#N/A	7.1470	7.5810	7.1992	7.4476	6.9742	7.0244	7.4915	7.9069	7.2279	7.4011	0.9630	-0.0544	0.1345	0.8712
Arf3;Arf1	_HYFQNTQGLIFVVDSNDRER_.3	#N/A	6.9334	5.2141	7.0542	5.7211	4.9035	6.0666	6.2851	6.0412	6.0064	6.4196	0.9667	-0.0488	0.6639	0.1779
Arf3;Arf1	_HYFQNTQGLIFVVDSNDR_.3	#N/A	4.0826	3.3870	3.2686	3.7712	4.8046	3.0204	5.2980	5.0769	3.9958	3.7382	0.8222	-0.2824	0.1126	0.9485
Rap2b	_YDPTIEDFYR_.2	#N/A	8.0512	7.4891	7.4784	7.6475	7.5560	7.2492	7.4675	7.5578	7.2881	7.6458	1.0118	0.0170	0.5675	0.2460
Rap2b	_VVVLGSGGVGK_.2	#N/A	6.2736	6.6191	6.4455	6.3182	6.8219	6.8098	6.2278	6.9902	6.6795	6.6510	0.9866	-0.0195	0.6223	0.2060
Rap2b	_ALAEEWSC[Carbamidomethyl (C)]PFM[Oxidation (M)]ETSAK_.2	#N/A	5.3694	4.5658	5.5491	4.9040	4.9549	5.6882	5.9258	5.8122	6.0441	5.0654	0.9055	-0.1433	0.0911	1.0407
Rap2b	_ASVDELFAEIVR_.2	#N/A	4.4307	3.7252	4.9304	5.5153	4.7834	4.9621	5.3110	4.7148	4.9992	5.2226	0.9334	-0.0995	0.3293	0.4824
Rap2b	_SALTVQFVTGSFIEK_.2	#N/A	4.4590	3.8137	4.2920	3.8357	4.7030	4.4311	4.3930	3.1654	5.0866	4.0115	1.0220	0.0314	0.8085	0.0923
Rap2b	_EIEVDSSPSVLEILDTAGTEQFASM[Oxidation (M)]R_.3	#N/A	3.2507	2.8713	4.5736	4.5060	3.1311	3.3085	4.4064	4.2438	3.1499	2.2949	1.0236	0.0336	0.8823	0.0544
Rpl26	_FNPFVTSDR_.2	#N/A	6.6757	6.9461	7.2278	6.8516	6.8218	7.0088	6.9812	7.0468	6.7963	6.7057	1.0057	0.0082	0.7398	0.1309
Rpl26	_YVIYIER_.2	#N/A	5.3217	3.8159	5.1677	4.8034	5.2473	5.7114	4.2809	5.3353	5.2188	5.1907	1.0010	0.0014	0.9903	0.0042

Stx1b	_TKQELEDLTADIKK_.3	#N/A	8.9216	9.1350	9.5974	8.6367	8.5354	8.8450	9.3480	9.2098	8.7911	8.9251	0.9864	-0.0198	0.5907	0.2286
Stx1b	_LAIFTDDIK_.2	#N/A	8.1854	7.8243	8.1188	7.6001	7.2778	7.6379	7.7935	8.3316	8.0437	7.5619	0.9800	-0.0291	0.4878	0.3117
Stx1b	_QLEITGR_.2	#N/A	7.8002	7.7111	7.6813	6.8795	7.3615	7.4617	7.1337	7.9882	7.9532	7.4597	0.9802	-0.0289	0.5415	0.2664
Stx1b	_LSEDVEQVKK_.2	#N/A	7.4233	7.3876	7.8749	7.2795	7.4532	7.6108	7.7592	7.9515	7.4248	7.6206	0.9761	-0.0350	0.2215	0.6546
Stx1b	_QALNEIETR_.2	#N/A	7.8769	7.2431	7.4446	7.3513	7.5322	7.1651	7.8410	7.5954	7.4737	7.3963	0.9814	-0.0271	0.3760	0.4248
Stx1b	_LSEDVEQVK_.2	#N/A	5.6383	5.3162	5.2322	5.2713	5.3779	4.4980	5.4450	6.1018	5.1758	3.8119	1.0173	0.0247	0.8410	0.0752
Stx1b	_TQHSTLSR_.2	#N/A	6.8621	3.3739	6.3561	5.9054	7.1564	6.7227	6.5653	6.7133	6.9797	6.7554	0.8977	-0.1556	0.3596	0.4441
Stx1b	_DHFM[Oxidation (M)]DEFFEQVEEIR_.3	#N/A	5.9283	5.9257	6.7229	6.5088	5.1425	6.1628	6.2357	6.1204	6.1133	6.0602	0.9890	-0.0159	0.8186	0.0869
Stx1b	_TTTNEELEDM[Oxidation (M)]LESGK_.2	#N/A	5.8518	5.9829	5.5610	5.8146	5.2227	5.3247	6.5982	5.8779	5.4766	5.7958	0.9476	-0.0776	0.2387	0.6222
Stx1b	_IEYNVEHSVDYVER_.3	#N/A	6.0974	5.0892	5.4997	5.1466	4.2840	5.2876	3.9267	5.2187	5.3936	5.1225	1.0648	0.0906	0.4499	0.3469
Stx1b	_SAKDS[Phospho (STY)]DDEEEVVHVDR_.3	#N/A	13.9647	13.7587	14.2319	13.9901	12.7479	13.5658	14.1970	13.9666	13.9442	13.7301	0.9821	-0.0260	0.3939	0.4046
Stx1b	_AIEQSIEQEEGLNRS[Phospho (STY)]SADLR_.3	P61264_S108_M1_Stx1b	4.1411	4.1928	3.6634	4.9348	3.2770	3.3031	4.0644	3.2126	3.9411	3.4404	1.0693	0.0967	0.5016	0.2996
Stx1b	_KQHS[Phospho (STY)]AILAAPNPDEK_.3	#N/A	7.8725	7.6054	8.6414	7.7102	6.4673	7.6051	8.2545	8.1535	7.8149	7.5727	0.9624	-0.0552	0.4516	0.3453
Stx1b	_AIEQSIEQEEGLNR_.2	#N/A	9.1320	9.0305	9.8456	9.3426	8.5534	8.9670	9.7112	9.5905	9.3819	9.2067	0.9654	-0.0507	0.2038	0.6909
Stx1b	_LKAIEQSIEQEEGLNR_.3	#N/A	5.8018	5.7813	4.4424	4.7865	4.8462	5.2737	5.7911	5.8446	5.5101	4.3427	0.9596	-0.0594	0.6008	0.2213
Stx1b	_QHSAILAAPNPDEK_.2	#N/A	6.2254	6.0982	5.2594	5.6985	6.1270	6.0347	5.7319	5.9547	6.0959	5.7409	1.0045	0.0065	0.8978	0.0468
Stx1b	_DS[Phospho (STY)]DDEEEVVHVDR_.2	#N/A	7.5065	8.2630	4.1932	8.7100	6.9216	8.1155	9.1215	8.3493	8.5972	8.3507	0.8466	-0.2402	0.1584	0.8004
Stx1b	_TQELRS[Phospho (STY)]AKDS[Phospho (STY)]DDEEEVVHVDR_.4	#N/A	3.8035	3.5936	3.9500	4.0650	3.4573	3.8755	3.9361	3.5877	3.5897	6.0452	0.8837	-0.1784	0.3303	0.4811
Stx1b	_FVEVM[Oxidation (M)]TEYNATQSK_.2	#N/A	4.0125	4.0180	3.4206	4.1935	3.8803	4.0958	4.1606	5.2579	4.2711	4.0097	0.8897	-0.1686	0.1011	0.9952
Stx1b	_KFVEVM[Oxidation (M)]TEYNATQSK_.3	#N/A	4.0179	3.2083	3.2039	3.7065	3.1436	3.0851	3.4212	6.5002	4.0605	3.6735	0.7690	-0.3790	0.1232	0.9092
Fgf12	_EPS[Phospho (STY)]LHEIGEK_.2	P61329_S212_M1_Fgf12	14.3055	13.9989	14.6553	14.5998	13.8489	14.2915	14.5711	14.0902	14.2653	14.1993	1.0001	0.0002	0.9920	0.0035
Fgf12	_VVAIQGVK_.2	#N/A	3.7330	4.7600	4.0038	3.6923	4.3503	4.2197	3.8406	4.8475	4.6327	4.1969	0.9423	-0.0858	0.3805	0.4197
Fgf12	_KS[Phospho (STY)]SGTPTMNGGK_.2	P61329_S226_M1_Fgf12	3.3707	3.9012	3.7691	6.2843	4.0040	3.8668	4.8800	4.1259	3.1395	4.3031	1.0212	0.0303	0.8889	0.0511
Fgf12	_SRKS[Phospho (STY)]S[Phospho (STY)]GTPTMNGGK_.2	#N/A	4.2892	3.8413	3.8939	4.0799	3.4368	3.8355	4.4330	3.7634	3.4120	3.9990	1.0374	0.0530	0.4977	0.3030
Rpl27	_YSVDIPLDK_.2	#N/A	6.4140	5.4255	6.2330	6.5869	6.8131	6.4452	6.3084	5.9074	6.3581	6.3418	1.0146	0.0208	0.7351	0.1336
Rpl27	_NIDDGTSDRPYSHALVAGIDR_.3	#N/A	3.7458	3.6575	3.9565	4.0227	3.5218	4.2830	4.4623	4.0043	3.8556	3.3050	0.9892	-0.0157	0.8622	0.0644
Smg6	_NWS[Phospho (STY)]GC[Carbamidomethyl (C)]GEGEQK_.2	P61406_S331_M1_Smg6	6.2115	6.2724	4.2510	3.7513	4.1908	5.7257	6.1225	5.6192	6.1977	6.4746	0.8302	-0.2685	0.1184	0.9265
Snn	_LM[Oxidation (M)]TANS[Phospho (STY)]PEVHG_.2	#N/A	7.6043	7.0184	7.9454	6.4810	6.1820	7.0605	7.4554	7.3457	7.2578	6.9473	0.9720	-0.0409	0.5763	0.2394
Abat	_GNYLVDVDGNR_.2	#N/A	12.0318	11.9108	12.4792	12.2621	11.4903	12.2322	12.3422	12.0395	12.1253	12.1066	0.9930	-0.0102	0.6527	0.1853
Abat	_GTFC[Carbamidomethyl (C)]SFDTPDEAIR_.2	#N/A	8.4567	8.5808	8.5754	8.4352	8.5278	8.6293	8.7973	8.5122	8.6422	8.1407	1.0013	0.0019	0.9271	0.0329
Abat	_GVVLGGC[Carbamidomethyl (C)]GDK_.2	#N/A	8.2342	8.1637	8.0073	8.1423	8.3283	8.0916	7.9720	8.2715	8.2766	8.0603	1.0020	0.0029	0.8511	0.0700
Abat	_TVAGIIVEPIQSEGGDNHASDDFFR_.3	#N/A	7.9082	7.9857	8.2760	8.3762	7.5888	7.8507	7.7709	8.1029	8.1682	8.0075	0.9982	-0.0027	0.9299	0.0315
Abat	_EDLLNNVAR_.2	#N/A	8.1017	7.8283	7.7229	8.0060	7.7492	7.8812	7.7356	8.0383	8.1306	7.9006	0.9912	-0.0127	0.5121	0.2906
Abat	_IFNTWLGDPSK_.2	#N/A	7.7746	7.9101	7.4520	7.9437	7.5722	7.6949	7.6268	7.9183	7.7479	7.6084	0.9999	-0.0001	0.9946	0.0024
Abat	_IDIPSFDWPIAPFPR_.2	#N/A	7.9673	7.9174	7.8853	7.6764	7.4200	7.6574	7.6219	7.6508	7.7949	7.4462	1.0165	0.0235	0.3273	0.4850
Abat	_LKYPLEEFTTDNQQEEAR_.3	#N/A	7.7661	7.5934	6.6500	7.2600	7.7784	8.0931	8.2261	7.7086	7.7817	7.4710	0.9649	-0.0515	0.3691	0.4328
Abat	_M[Oxidation (M)]LDLYSQISSVPIGYNHPALAK_.3	#N/A	6.1782	5.9671	6.2671	5.3709	5.7182	6.5997	5.7611	5.8337	5.7726	5.8904	1.0348	0.0494	0.3890	0.4100
Abat	_NLLLAEVINIIK_.2	#N/A	5.7939	5.6419	3.4435	5.9273	5.2369	5.6994	6.0392	6.2405	5.9572	5.5526	0.8895	-0.1689	0.2171	0.6634
Abat	_C[Carbamidomethyl (C)]LEEVEDLIVK_.2	#N/A	4.4977	5.0506	5.0151	4.9912	5.1090	5.3939	5.3154	5.0619	4.6882	5.0474	0.9963	-0.0054	0.9200	0.0362
Abat	_TLLTGLLDLQAQYPQFISR_.2	#N/A	3.1907	5.9027	5.4274	5.9750	5.4221	5.4667	5.3201	5.9970	5.6160	5.0404	0.9522	-0.0707	0.6456	0.1900
Abat	_EEFRPSAPYR_.3	#N/A	5.9673	4.7613	4.3910	5.5324	5.3504	5.8347	4.0831	5.5759	6.0260	5.9918	0.9791	-0.0304	0.8195	0.0865
Abat	_REDLLNNVAR_.2	#N/A	6.0277	5.4729	4.1812	6.3088	4.6037	5.4358	5.6423	5.3196	5.0316	4.3842	1.0479	0.0675	0.6155	0.2108
Abat	_QLNTIQNAEAVHFFC[Carbamidomethyl (C)]NYEESR_.3	#N/A	5.2642	5.3747	5.2697	5.2034	4.6760	4.4315	5.2994	4.0378	4.8075	5.4357	1.0289	0.0411	0.6682	0.1751
Abat	_TM[Oxidation (M)]GC[Carbamidomethyl (C)]LATTHSK_.2	#N/A	5.3990	5.8840	5.3467	6.0929	6.0857	5.4582	5.2918	5.5136	5.5723	5.3205	1.0528	0.0743	0.1632	0.7874
Abat	_VDIEFDYDGPLM[Oxidation (M)]K_.2	#N/A	6.0248	5.2663	5.8451	5.7731	5.6021	5.9096	5.7752	5.9539	5.8005	5.5947	0.9923	-0.0111	0.7752	0.1106
Abat	_YPLEEFTTDNQQEEAR_.2	#N/A	4.4241	3.2055	3.6838	3.7092	4.2181	3.5308	5.6702	4.4092	4.0578	4.6171	0.8095	-0.3050	0.0371	1.4308
Abat	_GM[Oxidation (M)]SQLITMAC[Carbamidomethyl (C)]GSC[Carbamidomethyl (C)]SNENAFK_.2	#N/A	3.9040	3.1594	3.6377	3.7554	3.0477	3.5769	3.4701	4.3631	4.0117	3.7224	0.9028	-0.1475	0.1387	0.8580
Ufm1	_VLSVPESTPFTAVLK_.2	#N/A	5.3437	5.6800	5.8496	5.1038	6.2112	5.9769	5.9188	6.0025	5.4784	5.5561	0.9922	-0.0113	0.8527	0.0692
Ufm1	_ITLTSDPR_.2	#N/A	5.0827	5.0142	3.9975	5.7638	5.7528	4.9227	4.8876	4.7320	5.0231	5.1605	1.0279	0.0397	0.6953	0.1578
Dcaf7	_LEC[Carbamidomethyl (C)]LLNNNK_.2	#N/A	6.5419	5.8018	6.3392	5.9983	5.3646	5.6402	5.5808	5.6692	6.0795	5.9544	1.0218	0.0311	0.6157	0.2106
Dcaf7	_LALGSFVEEYNNK_.2	#N/A	5.8513	6.1368	6.6971	7.3648	5.7489	6.2426	6.5024	6.2089	5.6837	5.9686	1.0409	0.0579	0.4805	0.3183
Ap1s1	_AIEQADLLQEEDES[Phospho (STY)]PR_.2	P61967_S147_M1_Ap1s1	8.2103	8.8993	8.1793	9.5531	8.0996	8.6993	9.6897	9.3144	9.3431	9.1932	0.9171	-0.1249	0.0317	1.4986
Hnrnpk	_TDYNASVSVPDSSGPER_.2	#N/A	8.3756	8.8346	8.6838	9.0641	8.8790	8.7570	8.6373	8.6045	8.6888	8.8688	1.0076	0.0109	0.6169	0.2098
Hnrnpk	_IILDLISESPIK_.2	#N/A	7.4891	7.8662	7.8393	7.7718	7.7447	7.8617	7.6904	7.4980	7.7724	7.4683	1.0204	0.0291	0.1355	0.8681
Hnrnpk	_GSDFDC[Carbamidomethyl (C)]ELR_.2	#N/A	6.5748	6.0157	6.7748	6.8866	6.5761	6.5707	6.2020	6.1648	6.3428	6.4788	1.0428	0.0604	0.1374	0.8621
Hnrnpk	_IITITGTQDQIQNAQYLLQNSVK_.3	#N/A	5.9015	5.4042	5.7969	5.7848	5.1141	6.1882	4.2216	5.1882	5.4603	5.7451	1.0853	0.1181	0.3916	0.4071
Hnrnpk	_DYDDM[Oxidation (M)]S[Phospho (STY)]PR_.2	#N/A	11.7898	11.8953	12.3156	12.2544	11.5694	11.9685	11.8151	11.8180	11.8358	11.9605	1.0091	0.0131	0.4820	0.3169
Hnrnpk	_LFQEC[Carbamidomethyl (C)]C[Carbamidomethyl (C)]PHSTDR_.3	#N/A	6.1222	6.3230	5.6172	6.8642	6.9264	6.2697	6.2022	5.8688	6.3345	6.4473	1.0226	0.0323	0.6144	0.2116
Hnrnpk	_DLAGSIIGK_.2	#N/A	6.0331	5.8925	5.8062	4.9330	5.5878	5.5753	4.4016	6.1337	5.9128	6.2442	0.9938	-0.0089	0.9311	0.0310
Hnrnpk	_RDYDDM[Oxidation (M)]S[Phospho (STY)]PR_.2	#N/A	9.6921	8.3441	8.9291	10.6054	9.1030	8.6384	9.2802	8.6486	8.3587	9.1604	1.0403	0.0569	0.4520	0.3449
Hnrnpk	_RPAEDM[Oxidation (M)]EEEQAFKR_.3	#N/A	6.0151	6.2216	5.4164	5.6805	7.0511	6.1340	6.7827	5.8848	5.9148	6.2123	0.9819	-0.0263	0.7426	0.1292
Hnrnpk	_GSYGDLGGPIITTQVTIPK_.2	#N/A	6.0671	5.8039	5.7920	6.1395	5.9031	5.9925	5.9579	5.6028	5.9993	6.1229	1.0049	0.0070	0.8057	0.0938
Hnrnpk	_LLIHQSLAGGIIGVK_.3	#N/A	4.7904	5.0987	4.8055	4.5638	5.0346	4.3023	4.8474	4.3958	5.1993	5.2129	0.9699	-0.0441	0.5101	0.2923
Hnrnpk	_IIPTLEEGLQLPS[Phospho (STY)]PTATSQLPLESDAVEC[Carbamidomethyl (C)]LNYQHYK_.4	P61979_S116_M1_Hnrnpk	8.5182	9.5817	9.7381	10.5760	9.2153	9.1556	9.0194	9.1841	9.5081	9.0874	1.0287	0.0409	0.4862	0.3132
Hnrnpk	_NTDEMVELR_.2	#N/A	6.3869	3.8598	3.7414	6.9661	3.6811	4.0483	4.4091	3.8744	3.5230	5.8709	1.0817	0.1134	0.6859	0.1638
Hnrnpk	_GGDLM[Oxidation (M)]AYDRR_.2	#N/A	3.9333	3.4049	4.1683	4.1442	4.9625	4.5255	4.2874	5.5000	4.2571	3.4795	0.9564	-0.0644	0.6654	0.1769
Hnrnpk	_ILSISADIETIGEILKK_.2	#N/A	3.1981	3.9544	4.4327	4.4612	3.8427	4.2827	4.1759	0.6385	3.3058	4.4282	1.2842	0.3609	0.2553	0.5930
Hnrnpk	_ILSISADIETIGEILK_.2	#N/A	3.3484	3.9940	4.5829	4.1267	3.8153	3.9142	4.2750	4.3753	3.1735	3.8040	1.0145	0.0208	0.8545	0.0683
Ywhag	_[Acetyl (Protein N-term)]VDREQLVQK_.2	#N/A	10.0845	10.7804	10.9347	10.5450	10.6785	10.5502	10.4832	10.5552	10.5089	10.3272	1.0121	0.0174	0.4316	0.3649
Ywhag	_MKGDYYR_.2	#N/A	8.8336	8.2348	7.4023	9.2189	9.2303	7.6402	8.0269	8.1325	8.1613	8.2665	1.0344	0.0487	0.5106	0.2920
Ywhag	_AYSEAHEISK_.2	#N/A	9.3938	9.5479	9.1886	9.3995	9.8904	9.5070	9.1513	9.4646	9.4497	9.3714	1.0137	0.0197	0.3586	0.4454
Ywhag	_ATVVESSEKAYSEAHEISK_.3	#N/A	8.0357	8.0895	9.1665	7.4170	7.2765	7.6656	7.5591	7.1457	7.8147	7.3770	1.0626	0.0876	0.2366	0.6260
Ywhag	_RATVVESSEK_.2	#N/A	4.7068	5.1091	5.2065	4.0033	5.4570	5.6749	5.0521	4.5965	4.5493	5.3120	1.0305	0.0434	0.6719	0.1727
Ywhag	_NC[Carbamidomethyl (C)]SETQYESK_.2	#N/A	6.9919	7.1897	6.9000	7.4167	7.3024	6.9744	6.7042	6.9631	6.7628	6.6094	1.0546	0.0767	0.0155	1.8110
Ywhag	_EHM[Oxidation (M)]QPTHPIR_.2	#N/A	8.2485	8.4840	8.5775	8.4794	9.1361	8.7923	8.4356	8.5711	8.8531	8.4882	1.0038	0.0055	0.8556	0.0678
Ywhag	_NVTELNEPLSNEER_.2	#N/A	10.6958	10.8380	11.2511	10.8950	10.7882	10.8828	10.9238	10.6978	10.8431	10.7450	1.0083	0.0119	0.4194	0.3774
Ywhag	_YLAEVATGEK_.2	#N/A	9.0358	8.8838	9.0113	9.2757	9.0483	8.9912	8.8287	8.9876	9.0576	9.0097	1.0078	0.0112	0.3869	0.4124
Ywhag	_NC[Carbamidomethyl (C)]SETQYES[Phospho (STY)]KVFYLK_.2	P61982_S119_M1_Ywhag	3.2784	4.0346	4.5129	4.3810	3.9229	4.2025	4.0957	3.7375	3.3861	4.3480	1.0420	0.0594	0.5730	0.2418
Ywhag	_DNLTLWT[Phospho (STY)]SDQQDDDGGEGNN_.2	P61982_T234_M1_Ywhag	14.3699	14.3320	4.1470	9.3722	13.7712	14.5334	14.4045	14.4504	14.3700	13.8463	0.8238	-0.2796	0.2768	0.5578

Ywhag	_TAFDDAIAELDTLNEDSYK_.2	#N/A	8.0283	7.6777	7.5185	8.0730	7.4323	7.7667	7.7887	7.4974	7.6698	7.7830	1.0084	0.0121	0.6649	0.1772
Ywhag	_YLAEVATGEKR_.2	#N/A	8.0670	8.5720	8.6675	8.6304	8.5793	8.5904	8.1822	8.3679	8.4544	8.2371	1.0249	0.0356	0.1336	0.8742
Ywhag	_ELEAVC[Carbamidomethyl (C)]QDVLSLLDNYLIK_.2	#N/A	4.3615	3.0121	3.5474	5.2352	3.0294	3.6665	3.3763	3.3846	3.4440	4.0171	1.0008	0.0012	0.9948	0.0022
Ywhag	_VISSIEQKTS[Phospho (STY)]ADGNEKK_.2	#N/A	3.2170	2.9941	4.3766	3.6257	2.8155	3.4132	3.7740	3.3754	3.6745	3.3030	0.9647	-0.0519	0.6862	0.1636
Ywhag	_VISSIEQKTS[Phospho (STY)]ADGNEK_.2	#N/A	7.2634	7.6284	7.7646	7.9546	7.6206	7.9606	7.8901	7.7766	7.2267	7.5211	1.0125	0.0179	0.6051	0.2181
Rras2	_FQEQEC[Carbamidomethyl (C)]PPS[Phospho (STY)]PEPTR_.2	#N/A	10.6290	10.4616	10.8615	10.9141	10.1446	10.4423	11.0326	10.5141	10.6675	10.6094	0.9878	-0.0177	0.4709	0.3270
Rras2	_KFQEQEC[Carbamidomethyl (C)]PPS[Phospho (STY)]PEPTRK_.3	#N/A	8.5848	8.3631	9.2368	8.9734	8.2988	8.5133	8.8660	8.9430	8.7470	8.6074	0.9853	-0.0213	0.5294	0.2762
Rras2	_FQEQEC[Carbamidomethyl (C)]PPS[Phospho (STY)]PEPTRK_.2	#N/A	8.2496	8.1547	7.5503	8.7989	6.9407	7.1868	8.0062	8.6088	8.3724	8.2795	0.9395	-0.0900	0.2155	0.6665
Rras2	_KFQEQEC[Carbamidomethyl (C)]PPS[Phospho (STY)]PEPTR_.2	#N/A	7.1466	4.7890	5.9819	7.2470	6.9370	7.1320	4.3671	7.1502	7.1860	6.9797	1.0184	0.0263	0.8765	0.0573
Rras2	_[Acetyl (Protein N-term)]AAAGWRDGS[Phospho (STY)]GQEK_.2	P62071_S10_M1_Rras2	2.9941	4.6950	4.4369	5.9439	3.6207	4.8030	4.2138	4.1816	3.5835	5.2972	1.0224	0.0319	0.8746	0.0582
Timm10	_YLDIHER_.2	#N/A	6.2846	7.0565	6.7728	6.7298	7.2280	6.8962	6.6635	6.7444	6.4161	7.1289	1.0133	0.0191	0.6693	0.1744
Timm10	_GESVC[Carbamidomethyl (C)]LDR_.2	#N/A	6.4085	5.2239	3.3975	6.5531	7.1321	6.2233	5.8604	5.8926	6.4348	6.3515	0.9492	-0.0752	0.6654	0.1769
Timm10	_EAELSKGESVC[Carbamidomethyl (C)]LDR_.2	#N/A	2.9957	3.7663	3.1996	4.5727	4.2557	3.3804	3.6440	3.7786	3.3311	3.7950	1.0159	0.0228	0.8641	0.0634
Timm13	_VQIAVANAQELLQR_.2	#N/A	5.2891	5.3079	5.9447	5.1296	5.9524	6.2043	6.0957	5.3539	5.4329	5.6056	1.0028	0.0041	0.9531	0.0208
Timm13	_YM[Oxidation (M)]DAWNTVSR_.2	#N/A	6.6616	6.4718	6.7945	4.9164	6.9328	7.1537	6.6486	6.4135	6.7663	6.6069	0.9818	-0.0265	0.7789	0.1085
Timm13	_LDPGAIMEQVK_.2	#N/A	3.9801	3.2844	3.1660	4.7837	5.3920	3.1229	3.4837	3.6658	3.9648	3.0127	1.1198	0.1632	0.4308	0.3657
Timm8b	_FIDTTLAITGR_.2	#N/A	2.8892	3.2705	3.7487	3.2925	5.1028	4.4089	3.4407	4.0781	4.0077	2.9698	1.0445	0.0628	0.7405	0.1305
Rps7	_HVVFIAQR_.2	#N/A	5.8245	6.7024	6.4639	6.7866	6.2771	6.6309	5.9150	6.4499	6.5598	6.4073	1.0181	0.0259	0.6064	0.2173
Rps7	_AIIIFVPVPQLK_.2	#N/A	6.0719	5.4158	6.1581	5.5154	5.9780	5.8838	6.1613	5.7882	5.4398	5.5543	1.0177	0.0252	0.6257	0.2036
Ppp1ca	_LLEVQGS[Phospho (STY)]RPGK_.2	P62137_S22_M1_Ppp1ca	5.1349	4.5914	5.0753	5.1916	4.7977	5.3851	4.9434	4.7451	5.1230	5.5285	0.9891	-0.0159	0.7848	0.1052
Ppp1ca	_[Acetyl (Protein N-term)]SDSEKLNLDSIIGR_.2	#N/A	4.2451	3.5162	3.4664	3.7471	3.5536	4.1195	3.4185	4.3256	4.2036	4.0804	1.0169	0.0242	0.8672	0.0619
Ppp1cb	_[Acetyl (Protein N-term)]ADGELNVDSLITR_.2	#N/A	5.6113	5.7723	5.5234	5.3595	6.0827	6.1252	5.5541	5.3138	5.5836	4.7944	1.0818	0.1134	0.0764	1.1170
Psmc1	_VAEEHAPSIVFIDEIDAIGTK_.3	#N/A	3.0372	4.0318	3.7240	4.4947	3.8510	4.0657	3.9414	3.5197	3.5037	3.3678	1.0793	0.1101	0.3158	0.5006
Psmc1	_AVANQTSATFLR_.2	#N/A	3.1023	4.1239	5.0997	4.1943	5.1642	4.1133	4.0064	4.8890	4.4878	5.1446	0.9282	-0.1074	0.4671	0.3306
Psmc1	_APQETYADIGGLDNQIQEIK_.2	#N/A	3.5136	6.5157	3.2473	4.2919	4.1582	3.9673	4.4402	4.2101	3.0083	3.9692	1.0961	0.1324	0.5758	0.2397
Psmc5	_NDSYTLHK_.2	#N/A	4.2773	3.3511	4.5619	3.5637	4.0574	3.3852	3.2784	4.9139	3.9228	3.5307	0.9884	-0.0168	0.9089	0.0415
Rps8	_LTPEEEEILNK_.2	#N/A	8.1922	8.3440	8.4081	8.2633	8.3285	8.1133	8.4122	8.4455	8.0396	8.4154	0.9936	-0.0093	0.5868	0.2315
Rps8	_IIDVVYNASNNELVR_.2	#N/A	6.3152	5.5101	7.0950	5.9993	5.3740	6.0509	6.5652	5.4678	5.0900	6.1510	1.0411	0.0581	0.5765	0.2392
Rps8	_NC[Carbamidomethyl (C)]IVLIDSTPYR_.2	#N/A	5.6628	5.6765	5.9771	6.0349	4.8947	5.2713	6.0296	5.0415	4.9239	4.9667	1.0660	0.0922	0.2893	0.5386
Rps8	_ISSLLEEQFQQGK_.2	#N/A	4.7787	5.0760	5.4530	5.4811	4.7348	5.0499	5.2769	5.7081	4.8631	5.5436	0.9528	-0.0697	0.2817	0.5502
Rps8	_LDVGNFSWGSEC[Carbamidomethyl (C)]C[Carbamidomethyl (C)]TR_.2	#N/A	3.0863	4.6695	3.7731	4.2292	4.3060	4.1336	4.3775	4.2728	4.8183	4.6404	0.8908	-0.1668	0.1330	0.8763
Rps15a	_IVVNLTGR_.2	#N/A	5.7292	5.9005	4.3499	5.9460	5.1073	5.6612	4.4048	6.3702	6.2736	5.9559	0.9475	-0.0779	0.5460	0.2628
Rps15a	_M[Oxidation (M)]NVLADALK_.2	#N/A	5.6828	5.8652	5.4202	3.3666	5.6746	4.9058	4.9495	5.5697	5.0046	5.4196	0.9841	-0.0231	0.8700	0.0605
Rps15a	_HGYIGEFEIIDDHR_.3	#N/A	5.4325	4.6919	4.3649	3.3157	2.8038	3.1883	5.2926	3.5299	3.9190	5.5551	0.8671	-0.2058	0.3798	0.4205
Ywhae	_EALQDVEDENQ_.2	#N/A	11.7530	11.4763	11.9316	11.9567	11.2047	11.4122	11.9161	11.3688	11.6001	11.6730	0.9985	-0.0021	0.9264	0.0332
Ywhae	_YLAEFATGNDR_.2	#N/A	9.8926	9.6518	10.0243	10.2635	9.5723	9.9322	10.1131	9.7774	9.9432	9.9911	0.9933	-0.0097	0.6458	0.1899
Ywhae	_LIC[Carbamidomethyl (C)]C[Carbamidomethyl (C)]DILDVLDK_.2	#N/A	9.7015	9.5464	9.1385	9.8653	10.1302	9.4298	9.8131	9.4742	9.6725	9.6065	0.9993	-0.0009	0.9736	0.0116
Ywhae	_VAGM[Oxidation (M)]DVELTVEER_.2	#N/A	9.1595	8.0686	10.0457	9.6844	9.5157	9.7286	9.5718	9.8407	9.8659	9.8784	0.9569	-0.0636	0.2764	0.5585
Ywhae	_EAAENSLVAYK_.2	#N/A	9.5688	9.4670	9.9324	9.6522	9.7981	9.7949	9.4600	9.5360	9.5621	9.6052	1.0169	0.0242	0.1127	0.9479
Ywhae	_IISSIEQK_.2	#N/A	8.1420	7.9047	7.8502	7.8931	8.6162	7.8867	7.0752	8.0805	8.2813	7.9630	1.0253	0.0361	0.4657	0.3319
Ywhae	_LIC[Carbamidomethyl (C)]C[Carbamidomethyl (C)]DILDVLDKHLIPAANTGESK_.4	#N/A	5.4395	5.6481	5.3057	5.9226	5.3501	5.6157	5.1327	5.5307	5.8317	5.4029	1.0132	0.0190	0.6705	0.1736
Ywhae	_HLIPAANTGESK_.2	#N/A	9.2439	9.2122	8.8510	9.6020	9.5783	9.2674	9.0013	9.2344	9.3112	9.1802	1.0121	0.0173	0.4825	0.3165
Ywhae	_QM[Oxidation (M)]VETELK_.2	#N/A	7.0355	6.7846	6.2370	7.3764	7.3652	7.0653	6.3958	6.7866	6.7801	7.0858	1.0318	0.0452	0.4040	0.3936
Ywhae	_YLAEFATGNDRK_.2	#N/A	7.9693	8.3398	8.5738	8.6232	8.2702	8.2399	8.1905	8.1375	8.1707	8.0545	1.0243	0.0346	0.1511	0.8209
Ywhae	_AAFDDAIAELDTLSEESYK_.2	#N/A	8.9213	8.8429	9.0135	9.8531	8.8472	9.1686	9.1161	8.8951	8.7441	9.2792	1.0043	0.0063	0.8797	0.0556
Ywhae	_[Acetyl (Protein N-term)]M[Oxidation (M)]DDREDLVYQAK_.2	#N/A	7.4050	7.5286	7.8473	7.9035	7.6737	7.8867	7.3806	7.5092	7.3438	7.7555	1.0280	0.0399	0.1414	0.8495
Ywhae	_M[Oxidation (M)]KGDYHR_.2	#N/A	3.9504	3.2547	3.1363	3.3651	3.0761	3.1526	6.8188	5.2287	5.5314	5.6729	0.5716	-0.8070	0.0001	4.2655
Ywhae	_KEAAENSLVAYK_.2	#N/A	4.7744	2.8273	5.2756	4.9532	3.1050	4.8860	3.4127	4.6326	4.0690	5.4648	0.9792	-0.0303	0.8899	0.0507
Ywhae	_AASDIAM[Oxidation (M)]TELPPTHPIR_.2	#N/A	7.9475	7.2444	6.6652	7.4808	8.3077	7.0975	7.6982	7.2403	8.5225	7.4247	0.9658	-0.0502	0.5032	0.2983
Ywhae	_YDEM[Oxidation (M)]VESM[Oxidation (M)]KK_.2	#N/A	5.4932	5.0048	4.8674	5.9191	5.8633	4.8798	5.1609	5.2210	4.8124	5.2798	1.0429	0.0606	0.4257	0.3709
Ywhae	_EENKGGEDKLK_.2	#N/A	4.7680	4.8588	4.2754	4.3815	5.3599	4.6258	4.6022	4.4731	4.4387	4.8697	1.0252	0.0358	0.6011	0.2211
Ywhae	_IISSIEQKEENK_.2	#N/A	5.8382	5.8332	5.6853	6.4661	5.9062	6.0767	6.0252	5.6221	6.0772	5.7659	1.0162	0.0232	0.5788	0.2375
Ywhae	_KVAGM[Oxidation (M)]DVELTVEER_.3	#N/A	5.1782	5.2408	6.0262	5.0130	5.3879	5.7392	4.7264	5.4258	5.4067	5.4199	1.0355	0.0503	0.4559	0.3411
Ywhae	_YDEM[Oxidation (M)]VESMK_.2	#N/A	3.1180	3.8743	4.3525	4.5414	3.7626	4.3629	4.2560	3.5771	3.2257	3.0075	1.1380	0.1865	0.1978	0.7037
Ywhae	_LAEQAERYDEM[Oxidation (M)]VESM[Oxidation (M)]KK_.3	#N/A	6.1158	7.0359	7.7702	6.7290	6.8937	7.2887	6.1870	6.6347	6.7422	6.5513	1.0679	0.0948	0.1761	0.7543
Ywhae	_IISSIEQKEENKGGEDK_.3	#N/A	6.2500	6.1775	6.5613	6.5856	6.7704	6.3462	6.1338	6.0364	6.5175	6.1441	1.0387	0.0548	0.1313	0.8816
Ywhae	_DNLTLWTSDMQGDGEEQNK_.2	#N/A	3.7783	12.3316	3.8736	12.2042	11.1901	3.7247	4.0855	11.6557	11.4825	11.8343	0.8040	-0.3148	0.5025	0.2989
Ywhae	_AAFDDAIAELDTLSEESYKDSTLIMQLLR_.3	#N/A	3.5004	3.8055	4.1508	2.7831	3.4739	3.8601	3.6809	3.5084	3.9706	3.9332	0.9529	-0.0696	0.5059	0.2959
Ywhae	_LAEQAERYDEMVESMK_.3	#N/A	4.0931	3.2788	3.8268	3.3706	2.9487	3.1581	3.5188	3.6601	3.9592	3.8836	0.9176	-0.1241	0.2225	0.6527
Rps14	_IEDVTPIPSDSTR_.2	#N/A	6.8328	7.3466	7.2165	6.9511	7.0973	6.6905	6.8058	7.2281	6.5182	6.9118	1.0228	0.0325	0.3852	0.4143
Rps14	_TPGPGAQSALR_.2	#N/A	4.2486	6.0970	5.4023	5.6831	6.3156	5.5529	5.6888	6.0285	3.7742	6.4157	1.0133	0.0191	0.9049	0.0434
Rps23	_ANPFGGASHAK_.2	#N/A	6.1439	6.5739	5.3411	6.3450	6.7568	6.4069	5.2577	6.5339	5.8412	6.0114	1.0592	0.0830	0.3164	0.4998
Rps18	_VLNTNIDGR_.2	#N/A	7.9986	7.8423	7.7645	7.7927	7.9588	8.0238	7.7587	7.6158	8.0660	8.1836	0.9988	-0.0017	0.9399	0.0269
Rps18	_IPDWFLNR_.2	#N/A	6.9096	6.9141	6.7983	6.7285	6.8161	6.6889	6.8379	7.0431	7.1851	6.8522	0.9756	-0.0356	0.0676	1.1701
Rps18	_YSQVLANGLDNK_.2	#N/A	7.2564	6.6269	7.0525	7.3512	6.8437	6.6573	6.6622	6.7643	6.5353	6.5977	1.0489	0.0689	0.0775	1.1106
Rps18	_LREDLER_.2	#N/A	5.8378	6.3026	5.8806	5.8229	6.0737	6.2541	4.8535	6.4934	5.9349	6.1786	1.0279	0.0397	0.6028	0.2198
Rps29	_GHQQLYWSHPR_.3	#N/A	5.9971	6.1578	5.6308	6.6507	7.0638	6.8625	5.7179	6.4506	6.3578	6.1230	1.0376	0.0532	0.4772	0.3213
Rps29	_DIGFIKLD_.2	#N/A	2.2340	3.2329	3.3552	3.8579	3.4035	4.2816	3.5726	2.8648	2.8925	3.8249	1.0321	0.0456	0.7989	0.0975
Rps11	_DYLHYIR_.2	#N/A	6.3609	6.2644	6.9394	6.2693	5.6239	5.7200	6.7376	6.5280	6.3741	5.4933	0.9862	-0.0201	0.7966	0.0988
Rps11	_EAIEGTYIDKK_.2	#N/A	6.1128	5.4276	6.3009	5.4773	5.3499	5.8118	5.3305	5.3419	5.7750	5.9877	1.0246	0.0351	0.5797	0.2368
Rps13	_DSHGVAQVR_.2	#N/A	7.0066	6.4523	6.6930	6.3602	6.9648	6.7393	6.2427	6.8767	6.2316	6.5068	1.0369	0.0522	0.2207	0.6562
Rps13	_LILIESR_.2	#N/A	4.9983	3.0255	3.5592	4.4442	3.9868	3.5801	3.2512	4.7489	4.7239	4.6739	0.9041	-0.1454	0.3930	0.4056
Snrpd2	_NNTQVLINC[Carbamidomethyl (C)]R_.2	#N/A	5.4098	5.4995	3.7325	3.2762	5.2140	5.1383	5.8300	5.4098	5.2877	5.3776	0.8604	-0.2169	0.1623	0.7897
Snrpd3	_VAQLEQVYIR_.2	#N/A	5.4406	5.5964	5.1878	5.5971	5.2255	5.7096	5.4227	5.6685	5.7810	5.9251	0.9579	-0.0620	0.1199	0.9212
Snrpd3	_FLILPDM[Oxidation (M)]LK_.2	#N/A	3.1650	5.3939	4.3995	4.2202	5.6486	5.9068	5.5671	6.1777	5.6702	3.6205	0.9106	-0.1350	0.5177	0.2859
Lsm5	_[Acetyl (Protein N-term)]AANATTNPSQLLPLELVDK_.2	#N/A	5.8528	5.9528	6.4987	5.7288	6.5430	5.8973	6.0057	5.1149	5.7203	5.9260	1.0680	0.0949	0.1446	0.8398
Arf6	_FNVWDVGGQDK_.2	#N/A	6.8855	6.5324	7.0757	6.9243	6.0824	6.3433	7.0073	6.9961	6.7444	6.3292	0.9810	-0.0277	0.5962	0.2246
Psmc6	_EVIELPLTNPELFQR_.2	#N/A	4.0015	5.0420	4.4369	5.2067	4.1735	4.5367	4.1982	4.5006	3.4654	4.3935	1.1031	0.1416	0.1986	0.7020
Psmc6	_ALQSVGQIVGEVLK_.2	#N/A	4.3335	4.8490	4.1632	3.7070	4.5065	4.7840	3.8552	4.5064	3.7893	4.8139	1.0352	0.0499	0.6245	0.2045

Psmc6	_ADHDFVVQEDFM[Oxidation (M)]K_.3	#N/A	3.2191	3.9754	4.4536	3.9974	5.1843	4.2618	4.1457	5.5130	3.3028	3.6747	1.0055	0.0079	0.9649	0.0155
Kcnab2	_RSS[Phospho (STY)]LVITTK_.2	#N/A	8.5553	3.9439	8.8372	3.9659	7.3715	4.2932	4.1142	3.0352	8.6043	3.6432	1.2705	0.3454	0.4259	0.3707
Kcnab2	_QTGS[Phospho (STY)]PGM[Oxidation (M)]IYSTR_.2	#N/A	8.1562	8.1687	8.1573	8.6695	3.1053	8.1209	8.5536	7.5436	7.3802	8.2131	0.9336	-0.0992	0.6451	0.1904
Kcnab2	_AEVVLGNIIK_.2	#N/A	4.4772	4.9095	3.6034	5.3810	3.3439	2.9346	3.7873	5.0302	4.2220	4.6677	0.9280	-0.1077	0.5677	0.2459
Kcnab2	_SS[Phospho (STY)]LVITTK_.2	#N/A	3.7686	3.7615	6.0830	3.8196	3.6090	4.5581	3.8061	5.0529	4.7534	5.2057	0.9069	-0.1410	0.4448	0.3518
Kcnab2	_YPESTTGS[Phospho (STY)]PAR_.2	#N/A	3.3076	4.0639	4.5422	4.0859	3.9522	4.1732	5.6293	4.4161	3.2143	3.7632	0.9448	-0.0819	0.6230	0.2055
Kcnab2	_[Acetyl (Protein N-term)]M[Oxidation (M)]YPESTTGS[Phospho (STY)]PAR_.2	#N/A	11.2618	10.4805	11.1126	10.5752	9.4758	10.3659	10.7112	10.5819	10.5037	10.8130	0.9899	-0.0146	0.7528	0.1233
Kcnab2	_[Acetyl (Protein N-term)]MYPESTTGS[Phospho (STY)]PARLS[Phospho (STY)]LR_.2	P62482_S14_M2_Kcnab2	4.0394	3.2095	4.0739	6.4882	3.1651	3.9669	3.4427	3.7608	4.0617	3.6951	1.1115	0.1525	0.5251	0.2797
Kcnab2	_NEGVSSVLLGASNAEQLM[Oxidation (M)]ENIGAIQVLPK_.3	#N/A	3.6706	3.6718	3.4042	4.4489	2.8143	3.8103	3.0963	4.0532	4.3522	4.1261	0.9308	-0.1034	0.4618	0.3356
Kcnab2	_LQLEYVDVVFANRPDPNTPM[Oxidation (M)]EETVR_.3	#N/A	3.8486	3.3776	4.5354	3.5372	2.9743	3.2544	3.2519	3.0804	4.2298	3.5042	1.0203	0.0290	0.8409	0.0753
Abi2	_EIGILTTNK_.2	#N/A	6.3306	5.9360	5.8085	6.1016	5.9863	5.7303	5.8077	6.1157	6.3131	6.0405	0.9857	-0.0208	0.5439	0.2645
Abi2	_ALFDSYTNLER_.2	#N/A	6.0977	5.9611	6.4337	6.0123	6.0887	5.5455	5.6680	5.7941	5.8843	3.6380	1.1481	0.1993	0.1222	0.9128
Abi2	_VADYC[Carbamidomethyl (C)]ENNYIQSPDK_.2	#N/A	6.7141	6.5172	4.2305	7.2220	6.6752	6.7637	6.8415	3.2269	6.9792	6.7167	1.0695	0.0969	0.6587	0.1813
Abi2	_VADYC[Carbamidomethyl (C)]ENNYIQS[Phospho (STY)]PDKQR_.3	P62484_S40_M1_Abi2	4.9028	4.4052	4.1718	3.4916	4.2265	3.5030	3.8638	3.2856	3.5847	3.3928	1.1657	0.2211	0.0831	1.0806
Abi2	_NMAPSQQS[Phospho (STY)]PVR_.2	#N/A	8.7479	7.7826	3.3133	9.7990	7.1320	5.3765	8.0234	6.7283	6.9400	8.0110	0.9461	-0.0800	0.7535	0.1229
Abi2	_HTPPTIGGS[Phospho (STY)]LPYR_.2	P62484_S301_M1_Abi2	3.7817	10.2098	10.7809	10.4306	3.7808	10.1089	8.7172	10.3932	7.5483	9.1506	0.9140	-0.1298	0.6807	0.1670
Abi2	_[Acetyl (Protein N-term)]AELQM[Oxidation (M)]LLEEEIPGGR_.2	#N/A	4.0979	3.3534	3.8316	3.5614	4.1328	3.3829	3.2761	3.0562	4.0729	5.0267	0.9660	-0.0500	0.7480	0.1261
Abi2	_TTPPTQKPPS[Phospho (STY)]PPM[Oxidation (M)]SGK_.2	#N/A	10.9351	11.3274	9.6742	12.5969	10.7192	10.7785	11.4188	11.1982	10.7105	10.9010	0.9953	-0.0068	0.9202	0.0361
Rab11a	_ENDM[Oxidation (M)]S[Phospho (STY)]PSNNVVPIHVPPTTENKPK_.3	#N/A	3.6285	6.3308	9.7099	7.1652	5.6450	6.7175	3.8168	7.9178	4.0204	3.8452	1.3332	0.4149	0.2413	0.6175
Tsc22d1	_NSQLEQENNLLK_.2	#N/A	7.0811	7.3416	7.2180	7.7284	7.5452	7.1631	7.6775	7.0189	6.9581	7.0765	1.0228	0.0325	0.3965	0.4018
Tsc22d1	_EEVEVLK_.2	#N/A	3.1588	0.4168	3.8456	4.3482	3.9557	4.1697	4.0629	3.7703	3.4188	4.3152	0.8520	-0.2311	0.4754	0.3229
Dynlrb1	_[Acetyl (Protein N-term)]AEVEETLKR_.2	#N/A	7.4563	7.8815	7.8471	8.2325	8.4961	7.9088	7.5722	7.8331	7.9451	7.8036	1.0234	0.0333	0.3718	0.4296
Dynlrb1	_EIDPQNDLTFLR_.2	#N/A	7.5534	7.6461	7.9198	7.9615	8.4800	7.9442	7.3484	7.6553	7.8124	7.3198	1.0509	0.0716	0.0791	1.1019
Dynlrb1	_GVVQGIIVVNTEGIPIK_.2	#N/A	4.5213	3.4875	4.1610	4.3468	4.8371	4.1692	4.4952	4.5805	3.8970	4.5470	0.9712	-0.0421	0.6456	0.1900
Eef1a2	_RYDEIVK_.2	#N/A	9.3626	9.2090	9.4532	9.2731	9.4156	9.2639	9.0213	9.3199	9.2892	9.2055	1.0131	0.0188	0.1329	0.8764
Eef1a2	_KLEDNPK_.2	#N/A	5.1354	5.1688	4.0598	4.6669	5.4974	3.1698	5.3310	4.4345	5.2527	4.6864	0.9371	-0.0937	0.5323	0.2739
Eef1a2	_VETGILRPGM[Oxidation (M)]VVTFAPVNITTEVK_.3	#N/A	6.5979	6.4343	6.8027	6.8880	6.7178	6.5021	6.8254	7.6096	6.6911	6.9545	0.9483	-0.0766	0.0846	1.0726
Eef1a2	_YYITIIDAPGHR_.2	#N/A	7.9133	7.8538	8.3179	7.9973	7.6629	8.1031	8.2851	8.0828	8.1862	7.9132	0.9825	-0.0255	0.3074	0.5123
Eef1a2	_EVSAYIKK_.2	#N/A	4.4926	4.2081	4.0897	3.5252	4.3164	5.3213	4.3391	4.4535	3.1747	4.7844	1.0329	0.0467	0.7449	0.1279
Eef1a2	_SGDAAIVEM[Oxidation (M)]VPGKPM[Oxidation (M)]C[Carbamidomethyl (C)]VESFSQYPPLGR_.3	#N/A	3.3845	3.6400	4.1182	3.2748	3.5283	3.0963	4.4903	3.3392	2.9914	3.3393	1.0378	0.0535	0.7208	0.1422
Rps4x	_LSNIFVIGK_.2	#N/A	4.2894	4.8003	4.4354	4.5328	3.9096	4.3106	4.1274	5.1197	5.0298	4.3521	0.9404	-0.0887	0.2922	0.5343
Rps4x	_EC[Carbamidomethyl (C)]LPLIIFLR_.2	#N/A	4.3622	4.7860	4.0958	3.6101	4.6026	3.1187	3.7879	4.2771	3.6606	3.5091	1.0754	0.1049	0.4332	0.3633
Rps4x	_TDITYPAGFM[Oxidation (M)]DVISIDK_.2	#N/A	4.5004	4.8632	5.7966	4.8802	4.5369	4.5287	5.3581	4.9125	3.7288	4.4685	1.0507	0.0713	0.5476	0.2615
Rps4x	_FDTGNLC[Carbamidomethyl (C)]M[Oxidation (M)]VTGGANLGR_.2	#N/A	4.6012	3.9655	3.8471	3.5642	4.3098	4.4807	3.9853	3.9034	3.6488	3.5312	1.0958	0.1320	0.1416	0.8490
Ppp2cb	_YSFLQFDPAPR_.2	#N/A	8.7677	8.6904	8.6178	8.8186	8.2972	8.6531	8.7093	8.8691	8.5649	8.6035	0.9947	-0.0076	0.6824	0.1660
Ppp2cb	_NVVTIFSAPNYC[Carbamidomethyl (C)]YR_.2	#N/A	6.3900	6.9744	7.3266	6.6149	6.4274	6.6863	6.9386	6.7547	7.1129	6.7965	0.9762	-0.0347	0.4203	0.3764
Ppp2cb	_ELDQWVEQLNEC[Carbamidomethyl (C)]K_.2	#N/A	6.2057	5.7564	6.9578	6.3120	5.9232	5.9294	6.0417	5.9263	5.8868	6.2671	1.0249	0.0355	0.5325	0.2737
Ppp2cb	_GAGYTFGQDISETFNHANGLTLVSR_.3	#N/A	5.2783	4.9896	5.7293	5.2691	3.8991	4.8465	5.0352	4.6883	4.9680	5.5759	0.9872	-0.0186	0.8569	0.0671
Ppp2cb	_QITQVYGFYDEC[Carbamidomethyl (C)]LR_.2	#N/A	4.9343	4.3600	5.4450	5.2884	5.1701	5.0590	5.4249	4.7729	4.8964	4.7906	1.0144	0.0206	0.7617	0.1182
Ppp2cb	_SPDTNYLFM[Oxidation (M)]GDYVDR_.2	#N/A	5.9642	5.7980	5.9287	6.2414	5.1243	5.8742	5.4712	5.6677	5.5683	6.1347	1.0195	0.0279	0.6313	0.1997
Ppp2cb	_LQEVPHEGPM[Oxidation (M)]C[Carbamidomethyl (C)]DLLWSDPDDR_.3	#N/A	4.0575	3.1903	3.5334	5.9734	5.5629	3.5495	3.4426	5.9368	5.5136	5.3025	0.8539	-0.2279	0.3474	0.4592
Ppp2cb	_AHQLVM[Oxidation (M)]EGYNWC[Carbamidomethyl (C)]HDR_.3	#N/A	4.9732	5.1021	4.1873	4.5138	4.7193	3.0107	5.7726	5.5610	6.2310	4.7016	0.7936	-0.3335	0.0385	1.4145
Rpl18a	_SSGEIVYC[Carbamidomethyl (C)]GQVFEKS[Phospho (STY)]PLR_.3	P62717_S71_M1_Rpl18a	3.3808	3.8713	3.8404	3.8226	3.7110	3.6285	4.9036	3.8090	3.7205	3.6634	0.9217	-0.1176	0.2454	0.6101
Rpl18a	_DLTTAGAVTQC[Carbamidomethyl (C)]YR_.2	#N/A	3.6728	3.6696	3.4065	4.4511	3.4909	4.2386	3.0985	4.0509	4.3499	4.1284	0.9782	-0.0319	0.7884	0.1032
Acta2;Actg2	_YPIEHGIITNWDDM[Oxidation (M)]EK_.3	#N/A	11.0749	10.9509	11.0843	11.1401	11.1010	11.1420	11.1669	11.0245	11.2226	11.1323	0.9951	-0.0071	0.2964	0.5282
Acta2;Actg2	_VAPEEHPTLLTEAPLNPK_.2	#N/A	8.6061	8.8871	8.8245	9.0857	6.0453	8.6094	8.9338	8.8143	8.7465	8.4940	0.9538	-0.0682	0.5102	0.2923
Acta2;Actg2	_DLYANNVLSGGTTM[Oxidation (M)]YPGIADR_.2	#N/A	3.2161	4.0100	3.9029	4.4056	6.0245	6.0547	4.1203	6.4162	5.8160	5.7370	0.8334	-0.2629	0.2344	0.6300
Ap2s1	_FILIQNR_.2	#N/A	9.2679	9.1216	9.4976	8.9446	8.4120	8.8450	9.4105	9.3877	9.2546	8.9823	0.9736	-0.0385	0.2721	0.5652
Ap2s1	_HTNFVEFR_.2	#N/A	5.9460	6.3183	5.6081	5.6243	5.7871	5.5030	5.9552	6.2328	5.8371	6.0485	0.9633	-0.0539	0.2207	0.6561
Ap2s1	_VYTVVDEM[Oxidation (M)]FLAGEIR_.2	#N/A	4.6961	5.0318	6.0991	3.2125	4.3076	5.0113	5.9835	5.2628	5.5736	4.5447	0.8849	-0.1764	0.2893	0.5387
Rhob	_LVVVGDGAC[Carbamidomethyl (C)]GK_.2	#N/A	7.6355	7.7266	7.6275	7.3904	7.6357	7.1348	7.7657	7.2164	7.6158	7.3437	1.0053	0.0076	0.7985	0.0977
Rhob	_EVFETATR_.2	#N/A	7.5391	7.0653	6.9940	7.2179	7.6104	7.0334	6.8172	7.4589	7.2154	7.2164	1.0092	0.0133	0.7107	0.1483
Rhob	_IQAYDYLEC[Carbamidomethyl (C)]SAK_.2	#N/A	4.3205	5.0575	5.4692	5.0708	5.1685	3.9644	4.4431	5.3245	6.4493	4.8433	1.0056	0.0080	0.9369	0.0283
Rhob	_DLRS[Phospho (STY)]DEHVR_.2	P62746_S123_M1_Rhob	5.5151	4.4860	4.8713	6.3666	4.3014	4.1758	5.6862	5.1221	4.8495	4.8802	0.9646	-0.0520	0.7014	0.1540
Hpcal1	_EHTEFTDHELQEWYK_.3	#N/A	5.1318	4.0965	4.4010	3.4028	3.9618	4.5424	3.7495	3.2148	4.3642	3.3698	1.1582	0.2119	0.1454	0.8373
Rpl23a	_LAPDYDALDVANK_.2	#N/A	8.6874	8.6620	8.9657	8.9006	8.6285	8.8145	8.7839	8.6066	8.3979	8.6868	1.0183	0.0261	0.1388	0.8577
Rpl23a	_IRTS[Phospho (STY)]PTFR_.2	P62751_S43_M1_Rpl23a	8.7983	4.1237	3.7893	9.3955	8.6713	4.0005	9.2933	8.8883	8.7595	4.2589	0.8286	-0.2712	0.4503	0.3465
Rpl23a	_KLYDIDVAK_.2	#N/A	5.3058	5.0515	5.7116	5.3108	5.7015	5.6198	5.6644	5.3543	4.9844	5.9511	0.9930	-0.0101	0.8619	0.0645
Rps6	_DIPGLTDTTVPR_.2	#N/A	8.0074	7.7925	8.0974	7.9716	7.7353	7.7099	7.7012	7.9568	7.5569	7.5378	1.0257	0.0366	0.1162	0.9350
Rps6	_LIEVDDER_.2	#N/A	6.7407	6.2569	6.4672	6.6620	6.0116	6.4899	5.9361	6.5607	6.1215	6.7117	1.0167	0.0238	0.6097	0.2149
Vsnl1	_FFPYGDASK_.2	#N/A	9.2999	10.0007	9.4856	8.7911	8.2764	9.1716	8.8810	9.0673	8.5616	8.7488	1.0404	0.0572	0.2885	0.5398
Vsnl1	_FAQHAFR_.2	#N/A	8.3017	8.9841	8.5524	8.7362	9.1344	8.5119	8.5397	8.6723	8.7333	8.4174	1.0131	0.0188	0.5226	0.2818
Vsnl1	_STEFNEHELK_.2	#N/A	8.0456	7.7947	7.7289	7.9877	8.3214	7.8669	8.0588	7.9316	8.1362	8.0543	0.9891	-0.0158	0.4639	0.3336
Vsnl1	_EFIC[Carbamidomethyl (C)]ALSITSR_.2	#N/A	7.0776	7.3066	7.2059	7.5012	7.3743	7.4704	7.4159	7.0082	7.2614	7.4030	1.0069	0.0100	0.6619	0.1792
Vsnl1	_NKDDQITLDEFKEAAK_.3	#N/A	6.8739	7.2526	6.9956	7.1971	6.9930	7.2090	7.3948	6.9816	6.9628	6.9946	1.0005	0.0007	0.9767	0.0103
Vsnl1	_LNLEEFQQLYVK_.2	#N/A	6.5516	6.4457	6.8761	6.1873	6.8302	6.4953	6.6195	6.1193	6.9021	6.8699	0.9904	-0.0139	0.7523	0.1236
Vsnl1	_NGDGTIDFR_.2	#N/A	6.6310	6.1888	4.1842	6.5487	5.5391	5.9122	5.8623	6.0816	6.1010	5.9148	0.9740	-0.0381	0.7453	0.1276
Vsnl1	_LAPEVM[Oxidation (M)]EDLVK_.2	#N/A	8.6959	8.7916	8.8258	8.7850	8.6159	8.5024	8.8262	8.7189	8.5530	8.5853	1.0037	0.0053	0.7028	0.1531
Vsnl1	_MNEDGLTPEQR_.2	#N/A	8.4498	7.5831	3.9834	8.9865	8.0691	3.8064	7.8077	7.0181	7.2426	7.7726	0.9132	-0.1309	0.6006	0.2214
Vsnl1	_LNWAFNM[Oxidation (M)]YDLDGDGK_.2	#N/A	6.4980	6.5832	6.2755	6.8185	6.5773	6.3174	6.4867	6.5482	6.5274	6.4685	1.0006	0.0009	0.9701	0.0132
Vsnl1	_M[Oxidation (M)]DKNKDDQITLDEFK_.3	#N/A	6.4316	6.3244	5.9832	6.5769	6.1831	6.7283	7.2014	5.8405	6.3502	6.3349	0.9906	-0.0136	0.8232	0.0845
Vsnl1	_VEM[Oxidation (M)]LEIIEAIYK_.2	#N/A	6.2743	6.5415	5.5514	6.0489	6.4722	5.8784	6.5504	6.4758	7.6293	6.5339	0.9015	-0.1496	0.0511	1.2919
Vsnl1	_TFDKNGDGTIDFR_.3	#N/A	3.4050	3.8654	4.2631	5.1954	3.5382	5.3150	4.4804	4.5079	5.4023	4.5802	0.9306	-0.1037	0.4373	0.3592
Vsnl1	_NKDDQITLDEFK_.2	#N/A	5.6336	5.2247	5.1767	5.8809	5.2882	5.3397	5.8490	5.2318	5.0380	5.2249	1.0165	0.0236	0.6686	0.1748
Vsnl1	_SDPSIVLLLQC[Carbamidomethyl (C)]DIQK_.2	#N/A	6.4370	6.1033	6.1382	6.1124	6.3208	6.1608	6.4340	5.8183	6.5969	6.4396	0.9826	-0.0253	0.4915	0.3085
Mtpn	_GPDGLTALEATDNQAIK_.2	#N/A	8.2543	8.3856	8.8552	8.6332	8.7812	8.6749	8.3239	8.2985	8.4747	8.5260	1.0228	0.0325	0.1685	0.7735
Mtpn	_GEDVNRT[Phospho (STY)]LEGGR_.2	#N/A	6.9694	6.8868	6.9278	7.3128	7.4065	7.2197	6.9273	7.1921	6.7483	7.1586	1.0163	0.0233	0.4378	0.3587
Mtpn	_NGDLDEVKDYVAK_.2	#N/A	4.8085	4.9920	4.5670	5.1440	4.9557	5.0979	5.1973	4.5001	4.3142	4.8261	1.0463	0.0653	0.2788	0.5547
H4c1	_VFLENVIR_.2	#N/A	10.5543	10.9248	10.9561	10.8441	10.4536	10.6151	10.4205	11.0411	10.7838	10.6220	1.0007	0.0011	0.9596	0.0179

H4c1	_DAVTYTEHAK_.2	#N/A	10.2956	10.2951	10.6932	10.5419	10.4598	10.3201	10.2602	10.4784	10.4841	10.2024	1.0075	0.0108	0.4611	0.3362
H4c1	_ISGLIYEETR_.2	#N/A	9.5035	9.6483	10.0437	10.3831	9.5544	9.9300	9.8994	10.1630	10.0451	9.8189	0.9862	-0.0201	0.4742	0.3241
H4c1	_TVTAM[Oxidation (M)]DVVYALK_.2	#N/A	6.6258	6.5448	6.4180	6.5193	6.5743	6.5074	6.4292	6.6901	6.5652	6.3314	1.0042	0.0061	0.7095	0.1490
H4c1	_DNIQGITKPAIR_.2	#N/A	10.1047	10.4026	10.8957	10.5318	10.4611	10.1771	10.6208	10.6445	10.3430	10.0726	1.0008	0.0012	0.9630	0.0164
H4c1	_TVTAM[Oxidation (M)]DVVYALKR_.2	#N/A	5.5094	5.6300	6.6814	5.4691	5.4194	5.9332	6.0457	6.1582	6.0948	6.0927	0.9469	-0.0788	0.2247	0.6485
H4c1	_RIS[Phospho (STY)]GLIYEETR_.2	P62806_S48_M1_H4c1	3.1848	3.9900	4.5869	5.3203	4.4347	3.9181	4.2789	4.3714	3.1696	3.8079	1.0850	0.1177	0.4597	0.3375
H4c1	_DAVTYTEHAKR_.3	#N/A	2.9970	4.2291	3.6838	4.1865	3.6236	4.0063	3.9012	3.9320	4.5179	4.9842	0.8740	-0.1943	0.1183	0.9271
H4c1	_DNIQGITK_.2	#N/A	4.0303	3.3346	3.2163	3.2852	3.1560	3.0727	3.4335	4.2048	4.3174	3.6859	0.8565	-0.2235	0.0494	1.3061
Gabra1	_NNTYAPTATSY[Phospho (STY)]TPNLAR_.2	P62812_Y374_M1_Gabra1	3.3919	6.5136	7.3684	7.1262	4.7073	7.1727	6.8793	6.8829	6.7379	6.7328	0.8881	-0.1711	0.3863	0.4131
Atp6v1b2	_NFITQGPYENR_.2	#N/A	10.6376	10.7008	10.9644	10.7385	10.2378	10.5525	10.7904	10.5877	10.6265	10.6431	0.9978	-0.0032	0.8593	0.0659
Atp6v1b2	_NYLSQPR_.2	#N/A	7.1527	7.1126	7.2018	7.3709	8.6042	8.9087	8.5161	8.5008	7.6133	8.7584	0.9255	-0.1117	0.2119	0.6738
Atp6v1b2	_QIYPPINVLPSLSR_.2	#N/A	8.7821	8.7759	9.0168	8.8812	9.0252	8.9791	8.7712	8.6633	9.0113	8.8879	1.0087	0.0125	0.3823	0.4176
Atp6v1b2	_EQALAVSR_.2	#N/A	9.0935	9.0099	9.1922	9.0620	9.3848	9.1570	8.2354	9.0687	9.1992	9.0913	1.0282	0.0402	0.2198	0.6580
Atp6v1b2	_IPQSTLSEFYPR_.2	#N/A	8.8498	9.0316	8.8861	8.9607	9.0833	8.8683	8.7759	8.9103	8.8858	8.6612	1.0157	0.0225	0.0704	1.1527
Atp6v1b2	_AVVQVFEGTSGIDAK_.2	#N/A	8.7232	8.6319	8.8705	9.1499	8.6571	8.5908	8.9767	8.7196	8.7328	8.7645	0.9968	-0.0046	0.8183	0.0871
Atp6v1b2	_GFPGYM[Oxidation (M)]YTDLATIYER_.2	#N/A	8.5489	8.9580	9.1420	8.0349	8.2954	8.8841	9.1509	8.6194	8.9861	8.3385	0.9852	-0.0215	0.6320	0.1993
Atp6v1b2	_SGQVLEVSGSK_.2	#N/A	8.3932	8.3710	8.7286	8.5612	8.1967	8.3094	8.1702	8.3525	8.4347	8.4672	1.0084	0.0121	0.5400	0.2676
Atp6v1b2	_AVVGEEALTSDDLLYLEFLQK_.2	#N/A	5.7788	6.0624	6.7396	7.1515	5.8157	6.2658	6.4695	5.9044	6.0175	6.3374	1.0194	0.0278	0.6965	0.1571
Atp6v1b2	_EEVPGRR_.2	#N/A	6.7013	6.5353	7.1836	6.1782	7.1616	7.1022	6.2778	6.4201	6.6077	6.5562	1.0534	0.0750	0.1499	0.8241
Atp6v1b2	_IPIFSAAGLPHNEIAAQIC[Carbamidomethyl (C)]R_.3	#N/A	6.2071	6.1472	6.3443	6.7740	6.1437	6.0712	6.4225	6.2850	6.5814	6.4798	0.9750	-0.0365	0.2851	0.5450
Atp6v1b2	_GPVVLAEDFLDIM[Oxidation (M)]GQPINPQC[Carbamidomethyl (C)]R_.3	#N/A	6.4879	5.6042	5.9296	6.0493	5.9545	6.3094	6.1380	6.2718	6.5079	5.9314	0.9748	-0.0368	0.4221	0.3746
Atp6v1b2	_TSC[Carbamidomethyl (C)]EFTGDILR_.2	#N/A	5.7209	6.0728	3.8162	6.1283	6.1219	5.6476	6.1383	6.1671	6.0302	6.0953	0.9144	-0.1292	0.2839	0.5468
Atp6v1b2	_TPVSEDMLGR_.2	#N/A	7.9612	6.8974	4.5813	8.8392	7.6563	3.2085	6.6903	6.3449	6.3867	6.5018	1.0066	0.0095	0.9700	0.0132
Atp6v1b2	_KTSC[Carbamidomethyl (C)]EFTGDILR_.3	#N/A	6.5724	6.6873	7.0918	7.0465	7.3141	7.0645	6.6942	6.5209	6.9191	6.9341	1.0289	0.0411	0.2610	0.5834
Atp6v1b2	_IYPEEM[Oxidation (M)]IQTGISAIDGM[Oxidation (M)]NSIAR_.3	#N/A	5.3479	6.4483	5.8646	5.2279	5.5474	6.5232	6.0426	5.4842	6.5699	5.6735	0.9805	-0.0284	0.7421	0.1295
Atp6v1b2	_SAIGEGMTR_.2	#N/A	7.6290	6.4390	3.5640	8.5951	7.4778	3.8881	5.9789	4.0519	5.7757	5.9963	1.1495	0.2010	0.4902	0.3096
Atp6v1b2	_YAEIVHLTLPDGTKR_.3	#N/A	7.1641	7.3993	7.9182	7.4088	7.4037	7.4473	7.2535	7.3609	7.5554	7.3913	1.0090	0.0130	0.6367	0.1961
Atp6v1b2	_LALTTAEFLAYQC[Carbamidomethyl (C)]EK_.2	#N/A	6.4849	6.3074	6.9593	6.2191	5.9959	6.2950	6.4202	5.6860	6.1608	6.3045	1.0381	0.0539	0.2968	0.5276
Atp6v1b2	_YAEIVHLTLPDGTK_.2	#N/A	5.1004	4.8449	5.1194	5.5796	4.6831	5.0741	4.8741	5.1343	4.7316	5.1385	1.0196	0.0280	0.5924	0.2274
Atp6v1b2	_TVSGVNGPLVILDHVK_.3	#N/A	3.0280	3.6838	4.2291	3.2310	3.7095	3.5606	4.4465	3.3262	3.0353	3.5543	0.9953	-0.0068	0.9594	0.0180
Atp6v1b2	_TVYETLDIGWQLLR_.2	#N/A	3.1470	3.9032	4.3815	4.6462	3.7915	4.3339	4.2271	4.8974	3.3750	3.6025	1.0021	0.0030	0.9831	0.0074
Atp6v1b2	_HVLVILTDM[Oxidation (M)]SSYAEALR_.3	#N/A	5.1951	3.8414	5.7753	5.1269	4.3475	5.4998	4.6622	4.8716	4.9000	4.9753	1.0231	0.0329	0.7735	0.1115
Atp6v1b2	_SDFEENGSM[Oxidation (M)]DNVC[Carbamidomethyl (C)]LFLNLANDPTIER_.3	#N/A	3.9262	3.3000	3.1121	3.6148	3.1883	3.4363	3.3295	3.0028	4.1523	3.5818	0.9753	-0.0361	0.7330	0.1349
Rab1A	_QWLQEIDR_.2	#N/A	8.5791	8.3693	8.7199	7.9510	7.4646	7.7933	8.6727	8.5077	8.0139	8.1539	0.9771	-0.0334	0.5104	0.2921
Rab1A	_EFADSLGIPFLETSAK_.2	#N/A	8.2695	8.0001	8.1285	7.2876	7.3880	7.8547	8.0370	7.7472	7.8923	7.8486	0.9924	-0.0110	0.7826	0.1065
Rab1A	_IQST[Phospho (STY)]PVK_.2	P62821_T195_M1_Rab1A	3.1875	7.4471	8.5694	7.1330	3.8320	7.4408	4.1140	3.0354	7.9810	7.4471	1.1105	0.1513	0.6846	0.1646
Rab1A	_KVVDYTTAK_.2	#N/A	6.2446	6.5261	5.6028	5.9086	5.9813	6.0477	6.3826	6.6100	6.0202	6.0073	0.9675	-0.0476	0.3334	0.4770
Rab1A	_TITS[Phospho (STY)]SYYR_.2	P62821_S78_M1_Rab1A	3.7005	4.6678	5.0791	4.2790	4.4604	4.4175	3.7203	6.2607	5.0017	4.2631	0.9216	-0.1178	0.4658	0.3318
Rab1A	_GAHGIIVVYDVTDQESFNNVK_.3	#N/A	5.9942	6.0208	6.6095	6.8826	3.4173	4.7278	6.2686	6.0513	5.3355	5.2774	0.9783	-0.0317	0.8622	0.0644
Rab1A	_YASENVNK_.2	#N/A	7.3422	7.0898	7.2517	7.2884	6.9525	6.9478	7.4994	7.5775	7.3330	7.2371	0.9641	-0.0528	0.0382	1.4184
Rab1A	_FADDTYTESYISTIGVDFK_.2	#N/A	5.2631	5.7703	6.6193	6.1613	3.9674	5.4553	6.0760	5.6601	5.3229	5.2523	0.9931	-0.0100	0.9393	0.0272
Rab1A	_QWLQEIDRYAS[Phospho (STY)]ENVNK_.3	P62821_S114_M1_Rab1A	3.8511	3.1065	3.5848	4.6294	2.9948	3.6298	3.5229	4.8373	4.1717	4.3067	0.8630	-0.2126	0.1558	0.8073
Rab1A	_MGPGATAGGAEK_.2	#N/A	4.2114	3.1310	3.9450	3.4888	2.9524	3.2763	3.6371	3.5124	3.8114	3.1661	0.9912	-0.0127	0.9121	0.0400
Rab3c	_TITTAYYR_.2	#N/A	8.3268	8.1903	8.5301	7.8701	7.6780	7.9895	7.2633	8.3949	8.0808	8.1018	1.0172	0.0247	0.5975	0.2237
Rab3c	_TYSWDNAQVILAGNK_.2	#N/A	6.5847	6.0405	6.8348	6.4334	5.4723	6.1793	6.6245	6.5457	6.1014	5.8303	0.9971	-0.0041	0.9513	0.0217
Rab3c	_LVDIIC[Carbamidomethyl (C)]DK_.2	#N/A	6.8193	6.4999	4.5105	4.3834	6.3772	6.6275	7.3588	6.7923	6.8165	8.0580	0.8089	-0.3060	0.0540	1.2678
Rab3c	_MSESLETDPAITAAK_.2	#N/A	5.2706	6.4459	6.5333	6.3264	5.6514	6.3412	6.6161	6.5601	6.1682	5.9266	0.9647	-0.0518	0.4660	0.3316
Rab3c	_LGEQLGFEFFETSAK_.2	#N/A	5.4260	4.8903	4.5546	4.2430	4.1092	4.8090	4.9303	5.2210	4.6012	5.2120	0.9361	-0.0953	0.2723	0.5650
Rab3c	_DNINVKQTFER_.3	#N/A	3.4352	4.1915	3.1689	4.2135	4.0798	4.0457	4.3617	4.2885	3.0868	3.8908	0.9869	-0.0190	0.8779	0.0566
Ran	_VC[Carbamidomethyl (C)]ENIPIVLC[Carbamidomethyl (C)]GNK_.2	#N/A	8.6973	8.2455	8.0808	8.5781	8.7586	8.3854	8.7808	8.2679	8.4474	8.1498	1.0055	0.0079	0.7976	0.0982
Ran	_LVLVGDGGTGK_.2	#N/A	8.5845	8.0628	8.8679	8.3921	8.4749	8.4536	7.9173	8.3222	8.5421	8.2567	1.0258	0.0367	0.2415	0.6172
Ran	_[Acetyl (Protein N-term)]AAQGEPQVQFK_.2	#N/A	8.3603	8.4473	8.6788	8.3377	8.3215	8.3868	8.2797	8.4027	8.2030	8.5075	1.0088	0.0127	0.4169	0.3799
Ran	_NLQYYDISAK_.2	#N/A	8.0441	7.7997	7.7449	7.8492	8.1833	8.2278	7.5814	7.9143	7.8228	8.2630	1.0101	0.0144	0.6181	0.2089
Ran	_FNVWDTAGQEK_.2	#N/A	8.1651	8.1565	8.3646	8.2736	8.2421	8.0056	8.3654	8.2244	8.1210	8.1091	0.9995	-0.0007	0.9631	0.0163
Ran	_HLTGEFEK_.2	#N/A	5.9772	5.6983	5.6422	5.4210	6.4954	5.3826	5.9677	5.0173	6.0859	6.0921	0.9963	-0.0053	0.9441	0.0250
Ran	_YVATLGVEVHPLVFHTNR_.3	#N/A	4.7848	3.3597	4.5532	5.4296	2.9921	4.8109	5.7729	5.4259	4.8216	5.5052	0.8031	-0.3164	0.0695	1.1582
Rpl23	_ISLGLPVGAVINC[Carbamidomethyl (C)]ADNTGAK_.2	#N/A	6.2827	6.3972	6.7079	5.7045	6.1217	6.4112	6.6428	6.5247	5.9629	6.3357	0.9850	-0.0218	0.6586	0.1813
Rpl23	_GSAITGPVAK_.2	#N/A	5.8757	5.2837	5.7830	6.2208	6.3415	5.6152	5.8921	6.3172	5.7953	6.0288	0.9742	-0.0377	0.4977	0.3031
Rap1a	_YDPTIEDSYR_.2	#N/A	8.2526	8.1545	8.6133	8.2273	7.9159	8.3073	8.3557	8.1723	8.1562	8.2439	1.0016	0.0023	0.9161	0.0381
Rap1a	_YDPTIEDSYRK_.2	#N/A	7.6806	7.5359	8.1540	7.8093	7.9283	7.8844	7.8059	7.9244	7.5961	7.9107	1.0029	0.0042	0.8586	0.0662
Rap1a	_LVVLGSGGVGK_.2	#N/A	6.9153	7.5584	7.1628	7.3147	6.8203	6.7462	6.5687	7.7133	7.0217	6.6713	1.0132	0.0190	0.7313	0.1359
Rap1a	_EQGQNLAR_.2	#N/A	6.9441	6.9195	7.0086	6.8281	7.3578	6.7583	6.9857	7.0533	7.1940	6.7868	0.9949	-0.0073	0.7853	0.1049
Rap1a	_INVNEIFYDLVR_.2	#N/A	4.5857	4.6058	5.1493	4.5383	5.1763	5.2069	5.3265	5.1905	5.0765	4.7493	0.9590	-0.0604	0.3151	0.5016
Rap1a	)C[Carbamidomethyl (C)]QQC[Carbamidomethyl (C)]M[Oxidation (M)]LEILDTAGTEQFTAM[Oxidation (	#N/A	3.8872	3.7862	3.4870	4.3018	4.0804	4.5028	4.3825	3.6370	3.8133	4.2518	0.9966	-0.0049	0.9551	0.0199
Rap1a	_QWC[Carbamidomethyl (C)]NC[Carbamidomethyl (C)]AFLESSAK_.2	#N/A	4.3115	3.5669	4.0452	3.3479	4.0302	3.1694	3.0626	3.2698	3.7534	3.3149	1.1179	0.1608	0.1625	0.7891
Rps24	_TTPDVIFVFGFR_.2	#N/A	3.1569	3.9621	3.8437	4.3464	3.7835	3.7440	4.3068	3.4312	4.0659	4.3134	0.9446	-0.0822	0.4090	0.3883
Rps25	_LITPAVVSER_.2	#N/A	7.8566	8.0918	7.7868	7.7696	8.4363	7.9950	7.8897	8.0543	7.7095	7.9875	1.0100	0.0144	0.5916	0.2279
Rps25	_AALQELLSK_.2	#N/A	5.9778	6.5302	6.5134	6.3913	6.8546	6.6222	6.2551	6.5062	6.1488	5.9942	1.0410	0.0580	0.1741	0.7591
Rps25	_AQVIYTR_.2	#N/A	5.4605	5.7269	5.7712	4.7428	5.8853	5.6432	4.4746	5.6583	5.2661	5.5503	1.0575	0.0806	0.3437	0.4638
Rps26	_DISEASVFDAYVLPK_.2	#N/A	3.9849	4.6738	4.5474	4.0911	4.8334	4.4722	3.3538	4.4109	3.2091	3.7793	1.2021	0.2656	0.0255	1.5937
Rps28	_EGDVLTLLESER_.2	#N/A	7.9636	7.3365	7.5001	7.7361	7.1144	7.4439	7.5686	7.3735	7.3341	7.3960	1.0132	0.0189	0.5537	0.2567
Rps28	_TGS[Phospho (STY)]QGQC[Carbamidomethyl (C)]TQVR_.2	P62858_S23_M1_Rps28	7.1184	7.3444	7.4716	7.7370	6.9402	6.9945	7.1762	7.0355	7.2878	7.1694	1.0140	0.0201	0.5524	0.2578
Elob	_ADDTFEALR_.2	#N/A	5.8154	5.6117	6.6187	6.7131	6.7227	6.0785	6.0225	5.9968	6.4418	6.4780	1.0040	0.0058	0.9279	0.0325
Elob	_ESSTVFELK_.2	#N/A	4.3781	4.1397	4.4372	4.4997	4.6640	4.6764	4.4891	4.8479	4.8727	4.3985	0.9600	-0.0589	0.2209	0.6559
Elob	_TTIFTDAK_.2	#N/A	3.1164	3.4482	3.8032	4.1991	4.4141	3.3986	4.3474	3.5374	3.1011	3.8764	1.0039	0.0056	0.9664	0.0148
Gnb1	_VHAIPLR_.2	#N/A	10.6861	11.3262	11.1806	11.0832	11.4299	10.8495	10.8438	11.3143	11.2483	10.9367	1.0006	0.0009	0.9693	0.0135
Gnb1	_LLVSASQDGK_.2	#N/A	10.5948	10.6798	10.7089	10.7305	10.7459	10.8746	10.5621	10.5589	10.8302	10.7156	1.0052	0.0075	0.4483	0.3484
Gnb1	_AGVLAGHDNR_.2	#N/A	10.6310	10.6326	10.4169	10.4574	10.2687	10.1898	10.5178	10.5626	10.3301	10.4239	0.9975	-0.0036	0.8053	0.0941
Gnb1	_LIIWDSYTTNK_.2	#N/A	10.1717	10.0878	10.5682	10.6170	9.9967	10.2751	10.3059	10.2746	10.3121	10.1982	1.0013	0.0019	0.9216	0.0355

Gnb1	_ELAGHTGYLSC[Carbamidomethyl (C)]C[Carbamidomethyl (C)]R_.3	#N/A	7.7712	8.0461	7.8947	7.9770	8.4361	7.9055	7.8874	7.7961	8.1933	7.8781	1.0084	0.0120	0.6391	0.1944
Gnb1	_LFVSGAC[Carbamidomethyl (C)]DASAK_.2	#N/A	8.2913	8.6747	8.5274	8.9844	8.8307	8.5561	8.5587	9.0723	8.8577	8.4912	0.9885	-0.0167	0.5555	0.2553
Gnb1	_KAC[Carbamidomethyl (C)]ADATLSQITNNIDPVGR_.3	#N/A	8.5061	8.2646	8.6532	8.3870	8.3875	8.4969	8.8306	8.2957	8.4192	8.3372	0.9975	-0.0037	0.8606	0.0652
Gnb1	_VSC[Carbamidomethyl (C)]LGVTDDGM[Oxidation (M)]AVATGSWDSFLK_.3	#N/A	6.9479	7.1714	8.1006	7.0927	6.6802	7.5142	7.9126	6.7929	7.1711	7.3156	0.9936	-0.0093	0.8850	0.0531
Gnb1	_LLLAGYDDFNC[Carbamidomethyl (C)]NVWDALK_.2	#N/A	6.4332	6.5840	7.0593	7.2854	6.2720	6.7795	6.9571	6.7949	6.7426	6.2168	1.0086	0.0124	0.8110	0.0910
Gnb1	_ADQELM[Oxidation (M)]TYSHDNIIC[Carbamidomethyl (C)]GITSVSFSK_.3	#N/A	7.5435	7.9916	8.7919	9.4364	6.9347	8.2031	8.4544	9.4565	7.9637	9.4128	0.9239	-0.1142	0.2474	0.6066
Gnb1	_[Acetyl (Protein N-term)]SELDQLRQEAEQLK_.2	#N/A	6.5515	6.8939	6.9065	6.9777	6.6943	6.6907	6.9309	6.7858	6.7876	6.6139	1.0009	0.0013	0.9511	0.0218
Gnb1	_[Acetyl (Protein N-term)]SELDQLRQEAEQLKNQIR_.3	#N/A	4.3126	5.1221	5.6814	5.6333	4.6462	4.7263	5.0555	4.6788	3.9673	5.1653	1.0644	0.0900	0.4182	0.3786
Gnb1	_IYAM[Oxidation (M)]HWGTDSR_.2	#N/A	7.7480	8.2491	8.4041	8.4272	8.3674	8.4181	8.4189	8.3713	8.3417	8.2184	0.9918	-0.0119	0.6341	0.1979
Gnb1	_AC[Carbamidomethyl (C)]ADATLSQITNNIDPVGR_.2	#N/A	8.3053	8.0059	7.8553	8.5890	8.4456	8.1932	8.4822	8.0154	8.2074	8.2182	1.0002	0.0003	0.9923	0.0034
Gnb1	_[Acetyl (Protein N-term)]SELDQLR_.2	#N/A	4.6008	3.5656	4.0438	3.5876	3.4539	3.1707	3.7359	3.4136	3.2589	4.0099	1.0368	0.0521	0.6614	0.1795
Gnb1	dation (M)]TC[Carbamidomethyl (C)]AYAPSGNYVAC[Carbamidomethyl (C)]GGLDNIC[Carbamidomethyl	#N/A	4.0253	3.2807	3.7590	3.6340	3.1690	3.4556	3.3487	2.9836	4.1330	3.6010	1.0106	0.0152	0.8850	0.0530
Gnb1	_QTFTGHESDINAIC[Carbamidomethyl (C)]FFPNGNAFATGSDDATC[Carbamidomethyl (C)]R_.3	#N/A	4.2643	3.0781	3.9980	3.5418	3.4081	3.2165	3.6900	3.4594	3.7584	3.2191	1.0149	0.0214	0.8406	0.0754
Rbx1	_QVC[Carbamidomethyl (C)]PLDNR_.2	#N/A	3.7036	6.7803	6.4549	6.6230	7.0542	3.7772	3.1294	6.4458	4.3191	4.1592	1.2700	0.3449	0.2425	0.6152
Rbx1	_[Acetyl (Protein N-term)]AAAMDVDTPSGTNSGAGK_.2	#N/A	5.4929	3.1469	3.6252	3.7678	4.9917	3.5894	3.4825	4.3506	3.9992	3.7348	1.0541	0.0760	0.6802	0.1673
Gnb2	_TFVSGAC[Carbamidomethyl (C)]DASIK_.2	#N/A	7.8742	8.0991	8.2889	8.4871	8.6788	8.2731	8.1155	8.5571	8.3942	8.2159	0.9955	-0.0064	0.8262	0.0829
Gnb2	_KAC[Carbamidomethyl (C)]GDSTLTQITAGLDPVGR_.3	#N/A	8.0533	8.4461	8.6873	8.7948	8.3032	8.4523	8.4148	8.3939	8.2196	8.2705	1.0158	0.0226	0.3779	0.4227
Gnb2	_AC[Carbamidomethyl (C)]GDSTLTQITAGLDPVGR_.2	#N/A	7.2341	6.0332	6.1134	6.8815	6.4865	6.4567	6.6223	6.2468	6.4334	6.5864	1.0096	0.0138	0.8051	0.0941
Gnb2	_[Acetyl (Protein N-term)]S[Phospho (STY)]ELEQLRQEAEQLR_.2	#N/A	5.4278	5.6813	6.1929	5.9034	5.5535	5.5714	6.0438	5.9164	5.9271	5.5605	0.9761	-0.0349	0.4212	0.3755
Gnb2	_LLLAGYDDFNC[Carbamidomethyl (C)]NIWDAM[Oxidation (M)]K_.2	#N/A	6.7876	6.8559	7.1447	6.9932	6.7557	6.3747	7.1076	6.5899	6.9951	6.9020	0.9884	-0.0168	0.6294	0.2011
Gnb2	_ELPGHTGYLSC[Carbamidomethyl (C)]C[Carbamidomethyl (C)]R_.3	#N/A	8.3417	8.1726	7.4080	8.4687	8.7815	8.1476	8.2269	8.3065	8.4235	8.1262	0.9939	-0.0089	0.8377	0.0769
Gnb2	_[Acetyl (Protein N-term)]SELEQLR_.2	#N/A	5.2610	3.5624	3.8072	4.2420	4.8627	4.1721	3.9853	4.7590	4.6777	4.5416	0.9615	-0.0567	0.6384	0.1949
Gnb2	_ADQELLM[Oxidation (M)]YSHDNIIC[Carbamidomethyl (C)]GITSVAFSR_.3	#N/A	3.0455	3.8018	4.2800	4.4138	4.1613	6.4571	4.4988	3.1821	3.1532	3.5011	1.2166	0.2828	0.2546	0.5942
Gnb2	_QTFIGHESDINAVAFFPNGYAFTTGSDDATC[Carbamidomethyl (C)]R_.3	#N/A	3.8762	3.3499	4.5630	3.5649	3.2382	3.3864	3.2795	3.0528	4.2022	3.5319	1.0417	0.0589	0.6589	0.1812
Gnb5	_SEAES[Phospho (STY)]LKGK_.2	#N/A	8.0930	7.9074	3.4208	8.5595	7.7357	7.7507	8.0258	7.9056	7.9662	8.0205	0.9079	-0.1394	0.4698	0.3281
Gnb5	_VIVWDSFTTNK_.2	#N/A	6.1129	5.7733	5.7630	6.1240	5.2648	5.6399	6.0995	5.8149	6.1251	5.9623	0.9632	-0.0541	0.2381	0.6232
Gnb5	_SEAES[Phospho (STY)]LKGKLEEER_.2	#N/A	3.0330	4.3561	3.7198	4.2976	4.7817	4.0714	4.2499	4.0219	3.2604	4.4468	1.0121	0.0174	0.8992	0.0461
Gnb5	_SGQC[Carbamidomethyl (C)]VQAFETHESDVNSVR_.3	#N/A	4.2698	4.0176	5.0025	5.2772	3.5870	4.4225	3.8749	4.7387	4.5436	4.7575	0.9890	-0.0160	0.8941	0.0486
Gnb5	_LHDVELHQVAER_.3	#N/A	3.3372	4.1424	4.0240	4.1400	3.9637	4.8869	4.2413	3.5918	3.2404	4.6397	1.0392	0.0555	0.6757	0.1703
Gnb5	_VSPDGTAFC[Carbamidomethyl (C)]SGSWDHTLR_.2	#N/A	3.3188	4.0750	4.5533	4.3406	3.9633	4.1621	4.0553	4.4050	4.4703	3.7743	0.9743	-0.0376	0.6797	0.1677
Rpl30	_VC[Carbamidomethyl (C)]TLAIIDPGDSDIIR_.2	#N/A	6.7960	6.7671	7.3593	7.3580	6.7925	7.0123	6.9328	6.8862	6.8531	6.6300	1.0276	0.0393	0.2524	0.5980
Rpl30	_LVILANNC[Carbamidomethyl (C)]PALR_.2	#N/A	6.2695	6.3127	5.6580	6.1057	5.7883	6.1985	6.0368	5.9405	6.2041	5.9999	1.0017	0.0024	0.9459	0.0242
Cycs	_TGPNLHGLFGR_.2	#N/A	9.0918	9.5941	9.9071	9.9460	9.5459	9.2829	9.6558	9.4748	9.3645	9.4170	1.0088	0.0126	0.6542	0.1843
Cycs	_TGQAAGFSYTDANK_.2	#N/A	6.3754	6.3025	6.5575	7.4592	6.2446	6.4209	6.9521	6.5946	6.6922	6.8055	0.9703	-0.0436	0.4250	0.3716
Cycs	_GITWGEDTLM[Oxidation (M)]EYLENPKK_.3	#N/A	6.2081	6.7655	7.2530	6.8358	6.5219	6.5203	6.9381	6.9783	6.8992	6.7699	0.9692	-0.0451	0.2845	0.5459
Cycs	_ADLIAYLK_.2	#N/A	4.8633	6.2573	3.9941	5.0681	5.9307	5.1693	3.2676	5.4689	5.8839	4.4254	1.0950	0.1309	0.4853	0.3140
Cycs	_KTGQAAGFSYTDANK_.3	#N/A	5.7243	5.2185	4.9897	5.5381	5.0715	5.9674	4.8546	5.1537	5.4653	5.4426	1.0362	0.0513	0.4312	0.3653
Rpl31	_SAINEVVTR_.2	#N/A	6.9464	6.7681	7.4443	6.6977	7.5687	7.2675	7.1712	7.2775	6.6273	6.8040	1.0209	0.0298	0.5295	0.2761
Rpl31	_EYTINIHK_.2	#N/A	5.3709	4.6658	5.1920	5.8838	5.7866	5.7692	5.4829	5.3639	5.7184	5.6585	0.9800	-0.0292	0.6643	0.1776
Rpl31	_LYTLVTYVPVTTFK_.2	#N/A	3.5413	4.4151	5.1011	4.6988	4.3196	4.1183	4.6073	3.3028	3.8628	4.7039	1.0598	0.0838	0.5302	0.2756
Rpl31	_NLQTVNVDEN_.2	#N/A	4.0687	3.2767	5.7810	3.3432	4.2784	4.5054	4.8734	3.6580	4.1499	5.7102	0.9154	-0.1275	0.5257	0.2793
Rps3	_DEILPTT[Phospho (STY)]PISEQK_.2	P62908_T221_M1_Rps3	3.6377	10.1239	10.6952	10.7363	9.6644	3.7309	11.0513	4.1480	10.6196	10.5563	0.8905	-0.1673	0.6613	0.1796
Rps3	_AELNEFLTR_.2	#N/A	6.7524	7.0297	7.2668	6.4705	6.5129	6.4537	6.6484	6.8391	6.7271	6.4709	1.0114	0.0164	0.6870	0.1631
Rps3	_GLC[Carbamidomethyl (C)]AIAQAESLR_.2	#N/A	6.1786	6.3390	4.3497	6.1207	5.7142	6.0825	6.6731	6.0749	6.4132	6.0177	0.9210	-0.1187	0.2445	0.6116
Rpl32	_SYC[Carbamidomethyl (C)]AEIAHNVSSK_.2	#N/A	4.7492	5.2944	3.2700	6.0241	5.8689	4.7578	5.6756	5.3716	4.9764	5.0869	0.9463	-0.0797	0.6041	0.2189
Rpl8	_DIIHDPGR_.2	#N/A	6.6352	5.6518	6.5687	6.2408	6.4364	6.1945	6.1511	6.5729	6.0162	6.5530	0.9944	-0.0081	0.8724	0.0593
Rpl8	_AVVGVVAGGGR_.2	#N/A	5.1332	5.4199	4.5823	4.9748	5.2976	4.5785	5.2501	4.8460	5.1965	5.4370	0.9644	-0.0523	0.3971	0.4011
Cacng7	_YPDHLHISTS[Phospho (STY)]PC[Carbamidomethyl (C)]_.2	P62956_S273_M1_Cacng7	7.9895	8.3161	4.4188	8.5472	7.3800	8.5312	9.2367	8.5561	8.7394	8.3410	0.8638	-0.2113	0.1870	0.7283
Cacng7	_LS[Phospho (STY)]DC[Carbamidomethyl (C)]SDYSGQFLQPEAWR_.2	P62956_S222_M1_Cacng7	3.6782	3.6642	3.4119	4.4565	3.4855	4.2440	3.1039	4.0455	4.3445	4.1338	0.9786	-0.0312	0.7927	0.1009
Ybx1	_AADPPAENSS[Phospho (STY)]APEAEQGGAE_.2	P62960_S312_M1_Ybx1	6.4007	6.6748	4.0315	4.3055	3.6397	4.3082	3.7495	3.5480	3.8470	6.8294	1.0890	0.1230	0.6707	0.1735
Pfn1	_DSPSVWAAVPGK_.2	#N/A	9.2764	9.3607	9.4976	9.5482	9.4953	9.4577	9.5275	9.2369	9.2761	9.3195	1.0106	0.0153	0.2096	0.6785
Pfn1	_TFVSITPAEVGVLVGK_.2	#N/A	8.2030	8.1099	8.2071	7.8022	8.1137	8.0225	8.0479	7.8934	8.2431	8.0766	1.0014	0.0020	0.9100	0.0409
Pfn1	_STGGAPTFNVTVTM[Oxidation (M)]TAK_.2	#N/A	5.9014	6.0920	6.6020	5.1439	5.8724	6.6455	6.3119	5.9689	5.9415	6.6243	0.9728	-0.0397	0.6012	0.2210
Pfn1	_DSLLQDGEFTM[Oxidation (M)]DLR_.2	#N/A	7.4585	7.5479	8.2238	7.4653	7.5211	7.6452	7.8630	7.2650	7.7341	7.4943	1.0072	0.0103	0.7721	0.1123
Pfn1	_[Acetyl (Protein N-term)]AGWNAYIDSLM[Oxidation (M)]ADGTC[Carbamidomethyl (C)]QDAAIVGYK_.3	#N/A	4.6619	3.8973	4.8859	3.7264	4.7275	4.8780	4.6023	4.5357	4.3370	5.2925	0.9512	-0.0722	0.4808	0.3181
Pfn1	_C[Carbamidomethyl (C)]YEM[Oxidation (M)]ASHLR_.2	#N/A	3.6526	4.5173	4.2872	3.9267	5.8826	3.9219	4.5187	4.5350	3.9128	4.0683	1.0249	0.0355	0.8109	0.0911
Tra2b	_[Acetyl (Protein N-term)]S[Phospho (STY)]DSGEQNYGER_.2	P62996_S2_M1_Tra2b	10.4215	10.5976	10.9292	10.9134	9.9048	10.4932	10.9596	10.8395	10.6621	10.3659	0.9847	-0.0222	0.4750	0.3233
Tra2b	_[Acetyl (Protein N-term)]SDSGEQNYGERES[Phospho (STY)]R_.2	P62996_S14_M1_Tra2b	6.5705	5.6492	3.8948	6.2200	3.3048	4.9897	6.4913	6.6913	6.4678	5.9532	0.7975	-0.3264	0.0904	1.0438
Tra2b	_S[Phospho (STY)]RS[Phospho (STY)]YS[Phospho (STY)]RDYR_.2	P62996_S87_M3_Tra2b	3.9788	6.5078	6.9864	6.8932	2.9512	6.7070	3.5668	7.9860	6.5957	4.0405	1.0223	0.0318	0.9218	0.0354
Tra2b	_RPHT[Phospho (STY)]PTPGIYMGR_.3	P62996_T201_M1_Tra2b	3.9905	4.0095	4.5674	5.9140	3.8309	3.8986	4.2594	4.3909	3.1891	3.7884	1.1181	0.1611	0.3470	0.4597
Tra2b	_RHS[Phospho (STY)]HS[Phospho (STY)]HS[Phospho (STY)]PMSTR_.3	#N/A	5.7280	3.7450	3.9587	5.3754	3.8961	3.9816	3.6485	4.1883	4.8088	4.1592	1.0586	0.0822	0.6220	0.2062
Tra2b	_RRHS[Phospho (STY)]HS[Phospho (STY)]HS[Phospho (STY)]PM[Oxidation (M)]STR_.3	#N/A	3.5500	3.8888	4.1618	3.9227	3.6779	5.5800	4.3077	4.0877	3.7515	3.6895	1.0432	0.0611	0.6764	0.1698
Tra2b	_HSHS[Phospho (STY)]HS[Phospho (STY)]PMSTR_.2	#N/A	3.7959	3.0514	3.5296	4.5742	2.9397	3.6850	3.5781	3.9278	4.2269	4.2515	0.8999	-0.1522	0.2539	0.5953
Rac1	_C[Carbamidomethyl (C)]VVVGDGAVGK_.2	#N/A	8.4192	8.4028	8.2844	8.1803	8.7452	8.1323	8.4017	8.4819	8.4359	8.4307	0.9909	-0.0132	0.5170	0.2865
Rac1	_YLEC[Carbamidomethyl (C)]SALTQR_.2	#N/A	8.5295	8.6655	8.6955	8.3983	8.5033	8.2954	8.7370	8.7614	8.3570	8.3663	0.9952	-0.0069	0.7385	0.1316
Rac1	_AVLC[Carbamidomethyl (C)]PPPVK_.2	#N/A	8.3931	8.6391	7.8243	7.8681	8.3279	6.7036	8.5749	9.1282	8.1485	8.4762	0.9275	-0.1087	0.1470	0.8326
Rac1	_HHC[Carbamidomethyl (C)]PNTPIILVGTK_.3	#N/A	8.3106	8.2731	8.8022	8.0645	7.6967	7.7312	8.2426	8.2761	8.3282	7.8236	0.9974	-0.0038	0.9287	0.0321
Rac1	_TVFDEAIR_.2	#N/A	8.4074	7.8383	8.4466	7.4839	8.0627	7.8188	6.6983	8.4870	8.1031	8.0477	1.0224	0.0320	0.6415	0.1928
Rac1	_LDLRDDKDTIEK_.3	#N/A	7.1032	7.1348	7.7508	6.8172	7.3530	7.1135	7.1467	7.0440	7.2870	7.2020	1.0059	0.0085	0.8051	0.0941
Rac1	_LTPITYPQGLAM[Oxidation (M)]AK_.2	#N/A	7.6497	7.6759	8.1645	7.0417	7.2547	7.2077	8.2080	8.0062	7.7762	7.4246	0.9548	-0.0667	0.1920	0.7168
Rac1	_DDKDTIEK_.2	#N/A	4.4058	3.6925	4.2402	3.8000	4.9253	3.5495	3.8730	3.3756	3.9562	4.8294	1.0234	0.0333	0.7997	0.0971
Rac1	_LRPLSYPQTDVFLIC[Carbamidomethyl (C)]FSLVSPASFENVR_.3	#N/A	3.3797	3.8464	4.0665	4.5692	3.7347	4.3907	4.2839	3.5493	3.1979	3.0354	1.1369	0.1851	0.1628	0.7882
Pafah1b1	_TAPYVVTGSVDQTVK_.2	#N/A	6.0404	6.5345	6.2450	6.9259	6.7409	6.5896	6.2610	6.9799	6.6066	6.3291	0.9952	-0.0069	0.8846	0.0533
Pafah1b1	_VWDYETGDFER_.2	#N/A	6.9527	6.8091	7.0373	6.5975	6.4457	6.6463	6.7674	6.5314	6.6452	6.4755	1.0217	0.0310	0.2878	0.5408
Pafah1b1	_KWTS[Phospho (STY)]VIR_.2	P63005_S57_M1_Pafah1b1	3.4021	6.7735	3.1357	7.0306	3.7617	6.0456	6.9664	4.3217	3.1199	5.7236	0.9984	-0.0023	0.9945	0.0024
Pafah1b1	_FILSC[Carbamidomethyl (C)]ADDK_.2	#N/A	6.9466	6.4019	3.6228	6.6446	6.5608	6.3179	4.5278	6.8127	6.9241	6.7570	0.9723	-0.0405	0.8287	0.0816
Pafah1b1	_YALSGHR_.2	#N/A	6.3578	5.8590	4.1815	6.4975	6.8463	6.2325	6.2105	6.8574	6.7859	6.4189	0.9129	-0.1315	0.2832	0.5479
Pafah1b1	_LWDFQGFEC[Carbamidomethyl (C)]IR_.2	#N/A	4.0777	5.6523	6.0255	3.3551	4.8626	5.7829	3.5034	5.4422	5.7565	5.8384	0.9658	-0.0503	0.8069	0.0932
Pafah1b1	_QRDELNR_.2	#N/A	4.5830	4.4901	4.1072	5.0076	5.0919	3.0169	4.5014	4.0835	4.8130	3.2528	1.0529	0.0743	0.6527	0.1853

Pafah1b1	_GHTDSVQDISFDHSGK_.3	#N/A	5.5630	5.2731	5.8266	5.9825	5.7292	5.1431	5.4090	3.7881	6.1456	6.1362	1.0403	0.0570	0.6566	0.1827
Rab3a	_M[Oxidation (M)]S[Phospho (STY)]ESLDTADPAVTGAK_.2	#N/A	7.9017	10.1884	10.8051	10.2061	9.4885	10.1806	10.6341	10.4024	10.3901	10.1829	0.9416	-0.0868	0.2800	0.5529
Rab3a	_LVDVIC[Carbamidomethyl (C)]EK_.2	#N/A	8.7398	8.4308	8.7937	8.0453	8.4194	8.3813	7.8019	8.9474	8.5793	8.4608	1.0025	0.0036	0.9307	0.0312
Rab3a	_YADDSFTPAFVSTVGIDFK_.2	#N/A	6.4123	6.6590	6.6654	6.2585	5.6162	6.0960	6.8987	7.0722	6.6480	6.4105	0.9300	-0.1046	0.0762	1.1180
Rab3a	_QLADHLGFEFFEASAK_.3	#N/A	6.5744	6.2479	6.8349	6.4603	5.1813	6.1313	6.3050	6.6407	6.2886	6.3569	0.9751	-0.0364	0.6096	0.2150
Rab3a	_ESSDQNFDYMFK_.2	#N/A	5.6460	4.3847	3.9334	5.9543	4.3717	3.8598	4.6816	3.7758	4.2803	3.7435	1.1387	0.1873	0.2753	0.5602
Rab3a	_LVDVIC[Carbamidomethyl (C)]EKMS[Phospho (STY)]ESLDTADPAVTGAK_.3	#N/A	3.4006	4.1568	3.1342	5.4357	4.0451	4.0803	3.9735	3.8597	3.5083	4.2258	1.0386	0.0547	0.7324	0.1353
Hspa8	_VEIIANDQGNR_.2	#N/A	14.7636	14.7623	15.3673	15.3383	14.8090	15.0220	15.1412	14.6623	14.9028	15.1144	1.0037	0.0053	0.7517	0.1239
Hspa8	_TTPSYVAFTDTER_.2	#N/A	12.1222	12.1811	12.5160	12.3824	12.1029	12.3732	12.3449	11.8808	12.0315	12.3299	1.0109	0.0157	0.3171	0.4988
Hspa8	_TVTNAVVTVPAYFNDSQR_.2	#N/A	10.1488	10.2377	10.3089	10.6318	10.2696	10.3581	10.3756	10.1737	10.2121	10.2807	1.0064	0.0092	0.4958	0.3047
Hspa8	_LSKEDIER_.2	#N/A	10.7805	11.0460	11.1617	11.2220	11.3279	11.0951	10.9736	10.7925	10.8661	11.1062	1.0156	0.0224	0.1580	0.8012
Hspa8	_DAGTIAGLNVLR_.2	#N/A	10.7168	10.9402	11.0178	11.2063	10.9742	10.8966	10.8719	10.9795	10.8753	10.8947	1.0049	0.0070	0.5434	0.2648
Hspa8	_IINEPTAAAIAYGLDKK_.2	#N/A	8.9230	8.9222	9.0990	8.9115	9.3086	9.1199	9.0421	8.9674	9.1572	9.2477	0.9938	-0.0089	0.5688	0.2451
Hspa8	_GTLDPVEK_.2	#N/A	9.8691	10.0867	10.1484	10.0377	10.5030	9.9794	9.2382	9.9999	9.9852	10.2220	1.0246	0.0351	0.2656	0.5758
Hspa8	_ITITNDKGR_.2	#N/A	8.9474	9.7544	10.0637	9.8338	9.9617	9.6183	9.2957	9.4872	9.5489	9.4540	1.0265	0.0377	0.2633	0.5795
Hspa8	_SQIHDIVLVGGSTR_.3	#N/A	8.0254	7.8125	8.0013	8.6327	8.3106	8.1140	7.3195	8.1030	7.9154	8.2743	1.0312	0.0443	0.2955	0.5295
Hspa8	_LLQDFFNGK_.2	#N/A	9.2751	9.6314	9.5362	9.4837	9.4243	9.3242	9.5124	9.2966	9.2116	9.5536	1.0056	0.0080	0.5947	0.2257
Hspa8	_VC[Carbamidomethyl (C)]NPIITK_.2	#N/A	8.7399	9.2164	8.7891	8.9928	9.6015	8.8019	8.8595	9.0719	9.0208	8.9298	1.0059	0.0085	0.7684	0.1144
Hspa8	_FEELNADLFR_.2	#N/A	9.0252	8.9862	8.8703	9.2180	9.0460	8.7363	8.8703	8.8802	8.8149	8.7950	1.0159	0.0227	0.1387	0.8580
Hspa8	_VQVEYKGETK_.2	#N/A	7.0743	7.3816	7.8184	7.5347	7.7205	7.7224	7.2982	7.2762	7.5367	7.5526	1.0170	0.0243	0.4351	0.3614
Hspa8	_EIAEAYLGK_.2	#N/A	8.1956	7.7637	7.6192	8.3020	7.9827	7.8612	7.6150	7.8469	7.8663	8.1719	1.0100	0.0144	0.6353	0.1970
Hspa8	_C[Carbamidomethyl (C)]NEIISWLDK_.2	#N/A	7.5794	7.2064	6.9282	7.6911	7.6930	7.2357	7.0928	7.1556	7.3334	7.4208	1.0191	0.0273	0.4410	0.3556
Hspa8	_YKAEDEKQR_.2	#N/A	7.0929	7.2246	7.2279	6.8716	7.7945	7.0823	7.2376	6.9099	6.4887	6.2754	1.0725	0.1010	0.0695	1.1577
Hspa8	_SFYPEEVSSM[Oxidation (M)]VLTK_.2	#N/A	10.0220	10.3319	11.1132	10.5634	10.9306	10.8628	10.9950	10.6439	10.4901	10.8731	0.9895	-0.0153	0.6323	0.1991
Hspa8	_DIS[Phospho (STY)]ENKR_.2	P63017_S254_M1_Hspa8	8.9280	8.5453	9.3050	9.1273	8.7815	8.5646	9.1349	8.9069	8.7366	9.0516	0.9908	-0.0133	0.6422	0.1923
Hspa8	_ARFEELNADLFR_.2	#N/A	7.3713	7.5417	7.5278	7.8355	7.3904	7.4971	7.4119	7.4079	7.3755	7.6435	1.0091	0.0130	0.5105	0.2920
Hspa8	_NQTAEKEEFEHQQK_.3	#N/A	6.5402	6.1105	5.5510	6.5315	6.8229	6.5534	6.7870	6.7371	6.5322	6.6932	0.9498	-0.0743	0.1920	0.7166
Hspa8	_ITITNDK_.2	#N/A	7.8886	7.2067	7.5765	7.6599	8.1733	7.7509	6.5024	7.5881	8.0046	7.9754	1.0255	0.0363	0.5693	0.2446
Hspa8	_NSLESYAFNM[Oxidation (M)]K_.2	#N/A	8.8949	9.1327	9.1755	9.2057	9.1645	8.9576	9.0939	8.9463	9.0333	9.1316	1.0041	0.0059	0.6255	0.2038
Hspa8	_GPAVGIDLGTTYSC[Carbamidomethyl (C)]VGVFQHGK_.3	#N/A	4.6671	4.7169	6.0524	6.2253	5.1829	4.9192	6.3959	5.9095	5.4264	5.7851	0.9005	-0.1513	0.1641	0.7848
Hspa8	_NQTAEKEEFEHQQKELEK_.4	#N/A	6.8441	6.0411	6.8750	6.1835	6.5345	6.9291	6.4352	7.0941	5.7333	6.6094	1.0154	0.0221	0.7443	0.1283
Hspa8	_HWPFM[Oxidation (M)]VVNDAGRPK_.3	#N/A	9.8675	10.1755	10.5030	10.0273	9.8356	9.9569	10.1073	9.8892	10.0576	10.1810	1.0002	0.0003	0.9876	0.0054
Hspa8	_NQVAM[Oxidation (M)]NPTNTVFDAK_.2	#N/A	9.7861	9.8124	9.9719	10.2515	10.0363	9.6911	9.9532	9.4833	9.6688	9.7227	1.0224	0.0320	0.1301	0.8858
Hspa8	_SINPDEAVAYGAAVQAAILSGDK_.2	#N/A	5.8899	5.7160	5.1513	6.3288	5.6047	5.4412	5.9960	5.5927	5.6203	5.7147	0.9926	-0.0107	0.8508	0.0702
Hspa8	_FDDAVVQSDM[Oxidation (M)]K_.2	#N/A	7.8851	7.9152	7.3596	8.2283	8.1744	7.7808	7.5361	7.7143	7.5028	7.8889	1.0300	0.0427	0.2239	0.6499
Hspa8	_QTQTFTTYSDNQPGVLIQVYEGER_.3	#N/A	4.3615	3.9054	6.0341	5.7742	4.6653	3.7822	4.2249	4.9075	5.4520	5.0396	0.9690	-0.0455	0.7791	0.1084
Hspa8	_NQVAM[Oxidation (M)]NPTNTVFDAKR_.3	#N/A	5.9361	6.9228	7.5097	7.0183	7.0133	6.8891	6.4428	6.7604	6.6000	6.1278	1.0615	0.0861	0.1973	0.7048
Hspa8	_GRLS[Phospho (STY)]KEDIER_.3	P63017_S511_M1_Hspa8	4.1871	3.3788	3.8571	4.5854	4.8880	3.3575	3.5491	4.0759	3.5624	5.1290	0.9910	-0.0131	0.9346	0.0294
Hspa8	_STAGDTHLGGEDFDNR_.3	#N/A	11.3100	11.3711	11.8379	11.7405	11.4794	11.4901	11.2559	11.1761	11.4186	11.5478	1.0166	0.0238	0.1695	0.7708
Hspa8	_RFDDAVVQSDM[Oxidation (M)]K_.2	#N/A	6.9867	6.8759	6.9322	7.2190	7.2225	6.9506	7.0268	6.7879	6.7861	7.1165	1.0147	0.0210	0.3469	0.4598
Hspa8	_M[Oxidation (M)]KEIAEAYLGK_.2	#N/A	6.5745	7.2210	7.6698	7.8318	7.7951	7.6806	7.4084	7.3587	7.3818	8.2310	0.9825	-0.0255	0.6694	0.1743
Hspa8	_MVNHFIAEFK_.2	#N/A	5.1397	5.0259	3.5651	5.6744	6.0860	4.0965	5.3837	5.4535	6.0573	4.8495	0.9071	-0.1406	0.3620	0.4413
Hspa8	_IINEPTAAAIAYGLDK_.2	#N/A	8.1222	8.0580	8.1405	8.3014	8.1038	7.9322	8.0890	7.9177	7.9224	8.1764	1.0104	0.0149	0.3248	0.4884
Hspa8	_MVQEAEKYK_.2	#N/A	4.5577	3.2100	3.2021	5.7856	4.2684	3.5263	3.4194	4.4137	4.0623	3.8575	1.0390	0.0551	0.7821	0.1067
Hspa8	_LDKSQIHDIVLVGGSTR_.3	#N/A	6.0185	6.4678	7.6333	6.2179	6.0949	6.6939	6.1042	5.5129	6.2777	6.1747	1.0837	0.1160	0.1708	0.7676
Hspa8	_LYQSAGGM[Oxidation (M)]PGGM[Oxidation (M)]PGGFPGGGAPPSGGASSGPTIEEVD_.3	#N/A	3.1024	3.8587	4.3369	3.8807	3.7470	4.3785	4.0289	3.1205	3.4196	3.5580	1.0997	0.1372	0.2444	0.6118
Hspa8	_YKAEDEK_.2	#N/A	3.5256	4.3308	4.2124	3.2142	4.1521	3.5774	4.4297	3.4035	3.0520	3.1812	1.0907	0.1252	0.3750	0.4260
Tpt1	_DLISHDELFSDIYK_.2	#N/A	5.0458	5.0790	4.9639	5.0421	5.2849	5.3730	4.9993	4.8014	5.1159	5.7036	0.9954	-0.0066	0.8953	0.0480
Tpt1	_LEEQKPER_.2	#N/A	5.0763	5.1401	3.9942	5.3134	5.9847	5.5887	3.6450	4.0733	4.8032	4.9695	1.1853	0.2452	0.0908	1.0417
Tpt1	_GKLEEQKPER_.3	#N/A	6.4429	4.1470	5.3695	4.5692	6.5636	6.1352	3.6311	5.1513	5.3790	6.1718	1.0894	0.1236	0.5141	0.2890
Tpt1	_EIADGLC[Carbamidomethyl (C)]LEVEGK_.2	#N/A	5.1080	3.3943	3.2759	3.7786	5.0335	5.3769	3.4932	4.3399	3.9885	5.0761	1.0245	0.0349	0.8557	0.0677
Mpc1	_KAADYVR_.2	#N/A	6.1872	5.8404	6.1207	5.5841	6.2768	5.1256	5.7521	6.3385	6.0947	6.0780	0.9654	-0.0508	0.4134	0.3836
Dnaja1	_TIVITSHPGQIVK_.2	#N/A	5.3569	6.0198	6.2521	6.4607	6.4374	6.1159	5.7396	5.9270	5.5350	5.4643	1.0778	0.1080	0.0839	1.0765
Dnaja1	_QISQAYEVLADSK_.2	#N/A	4.2809	5.0521	3.2086	5.0602	4.9708	3.4043	4.4392	4.3593	4.0782	4.5998	0.9909	-0.0131	0.9305	0.0313
Dnaja1	_DHAVFTR_.2	#N/A	3.7435	5.4215	3.8582	4.3608	5.1730	4.9287	3.8673	3.7576	4.9044	3.8253	1.1204	0.1640	0.2720	0.5654
Dnaja1	_ITFHGEGDQEPGLEPGDIIIVLDQK_.3	#N/A	3.6342	4.1085	3.7174	5.1197	4.4093	4.0415	3.9347	4.1450	4.5086	4.1870	0.9947	-0.0076	0.9417	0.0261
Dnaja1	_NVVHQLS[Phospho (STY)]VTLEDLYNGATR_.3	P63037_S112_M1_Dnaja1	3.3964	3.8298	4.0832	5.1064	4.0229	3.7066	4.3005	3.5326	3.1812	3.0520	1.1443	0.1945	0.2090	0.6798
Hspd1	_VTDALNATR_.2	#N/A	8.5128	8.9055	8.7943	8.6584	9.3664	8.4726	8.3829	8.8186	8.8205	8.6949	1.0122	0.0175	0.5863	0.2319
Hspd1	_APGFGDNRK_.2	#N/A	8.0717	8.2976	8.2407	8.3322	8.8498	8.4790	8.1310	8.4666	8.2638	8.3078	1.0104	0.0149	0.5721	0.2425
Hspd1	_APGFGDNR_.2	#N/A	8.2983	8.4030	8.4816	8.4944	8.6543	8.3699	8.3078	8.3300	8.2604	8.5337	1.0110	0.0158	0.2769	0.5577
Hspd1	_AAVEEGIVLGGGC[Carbamidomethyl (C)]ALLR_.2	#N/A	8.2344	7.7988	8.1498	8.2239	7.7608	8.2891	8.1246	7.6940	8.0017	8.3652	1.0037	0.0053	0.8595	0.0658
Hspd1	_GANPVEIR_.2	#N/A	7.8337	8.0625	8.1010	8.1163	8.7550	8.2211	7.6193	8.0476	8.0511	8.3524	1.0205	0.0292	0.4305	0.3661
Hspd1	_LVQDVANNTNEEAGDGTTTATVLAR_.3	#N/A	7.9897	7.6406	8.3584	8.6240	8.0508	7.9188	8.1019	7.5721	7.8596	8.0992	1.0239	0.0341	0.3787	0.4216
Hspd1	_GYISPYFINTSK_.2	#N/A	7.9132	7.9669	8.1863	7.8873	8.3062	8.1118	8.2499	7.9766	7.9864	7.9700	1.0020	0.0029	0.8761	0.0574
Hspd1	_ISSVQSIVPALEIANAHR_.3	#N/A	7.1448	6.8728	6.7991	7.2754	7.2347	7.1340	6.6705	6.9437	7.1856	7.5834	0.9973	-0.0039	0.9197	0.0363
Hspd1	_LSDGVAVLK_.2	#N/A	7.7811	7.8921	7.7561	7.8470	7.8236	7.5456	7.5090	8.0123	7.7680	7.9113	0.9967	-0.0048	0.8138	0.0895
Hspd1	_VGLQVVAVK_.2	#N/A	7.2163	7.3486	6.8805	7.6470	7.1968	7.4806	7.1406	7.3935	6.9427	7.3862	1.0110	0.0158	0.6331	0.1985
Hspd1	_IQEITEQLDITTSEYEK_.2	#N/A	6.3410	6.9134	7.8518	8.4808	7.1609	7.2006	7.2434	7.2790	5.7531	7.1573	1.0680	0.0949	0.3599	0.4439
Hspd1	_C[Carbamidomethyl (C)]EFQDAYVLLSEK_.2	#N/A	6.1397	6.2967	6.0358	5.5113	6.0902	5.8893	5.2011	5.7320	5.5587	6.0322	1.0644	0.0901	0.0996	1.0017
Hspd1	_TVIIEQSWGSPK_.2	#N/A	6.8511	7.1674	7.1231	8.0392	7.5728	7.4089	7.2344	7.7927	7.6671	7.5914	0.9721	-0.0408	0.3897	0.4093
Hspd1	_NAGVEGSLIVEK_.2	#N/A	6.9976	6.3871	3.5085	7.3411	7.0671	6.3131	6.6556	6.4707	7.0216	6.5819	0.9381	-0.0921	0.5848	0.2330
Hspd1	_KPLVIIAEDVDGEALSTLVLNR_.3	#N/A	6.5787	6.7213	6.7207	8.2616	5.7791	6.0901	7.0496	7.0393	6.6327	6.0649	0.9993	-0.0010	0.9923	0.0034
Hspd1	_SIDLKDK_.2	#N/A	5.9435	5.9021	6.6122	6.3291	6.1016	6.1928	5.9772	5.8252	5.5331	6.3435	1.0440	0.0621	0.2066	0.6849
Hspd1	_VGEVIVTK_.2	#N/A	3.7697	4.6560	3.2446	4.5196	4.7159	4.7137	4.2261	4.7469	4.5156	4.7832	0.9348	-0.0973	0.3946	0.4039
Hspd1	_IQEITEQLDITTSEYEKEK_.3	#N/A	6.7197	7.4363	8.4926	8.2959	7.0669	8.0138	7.1808	6.6662	7.1573	7.7205	1.0682	0.0952	0.2552	0.5931
Hspd1	_C[Carbamidomethyl (C)]IPALDSLKPANEDQK_.2	#N/A	3.5577	3.3503	3.2914	6.0850	5.4828	3.9232	3.8163	4.0168	5.9440	5.7186	0.8785	-0.1869	0.4547	0.3423
Hspd1	_TLNDELEIIEGM[Oxidation (M)]K_.2	#N/A	6.5943	6.5021	6.6410	7.0629	6.6640	6.9612	6.8422	6.4999	6.6861	6.8137	1.0040	0.0058	0.8387	0.0764
Hspd1	_VGGTSDVEVNEKK_.2	#N/A	4.0754	3.3308	3.1493	3.5005	3.2419	4.5103	4.3218	2.8151	3.6116	3.8936	0.9929	-0.0102	0.9464	0.0239
Hspd1	_ALMLQGVDLLADAVAVTM[Oxidation (M)]GPK_.3	#N/A	3.8070	3.7193	3.8072	4.5542	3.5773	4.3591	3.8409	3.7727	3.8300	3.7390	1.0461	0.0650	0.4076	0.3898

Hspd1	AVVTEIPKEEKDPGM[Oxidation (M)]GAM[Oxidation (M)]GGM[Oxidation (M)]GGGM[Oxidation (M)]GG	#N/A	3.1056	4.2368	4.3401	3.0802	3.7502	4.3753	4.0322	3.1173	3.4163	3.5612	1.0801	0.1112	0.4326	0.3639
Hspd1	_ILQSSSEVGYDAM[Oxidation (M)]LGDFVNM[Oxidation (M)]VEK_.3	#N/A	3.6910	3.5352	4.3778	3.3796	4.2413	3.2011	3.0943	3.2381	4.3875	3.3466	1.0629	0.0880	0.5289	0.2766
Hspd1	_DM[Oxidation (M)]AIATGGAVFGEEGLNLNLEDVQAHDLGK_.3	#N/A	4.3674	4.3597	3.5533	4.0560	3.4930	4.2365	3.7707	2.2205	3.7111	4.0230	1.1689	0.2252	0.1668	0.7779
Cplx1	_KAIPPGC[Carbamidomethyl (C)]GDEPEEEDESILDTVIK_.3	#N/A	6.7692	7.3712	6.7542	7.6288	7.7087	7.6215	7.2616	7.4025	7.5995	7.4153	0.9851	-0.0217	0.6437	0.1913
Cplx1	_EAEAQAAM[Oxidation (M)]EANSEGS[Phospho (STY)]LTRPK_.2	#N/A	5.4041	6.9687	6.4941	3.7084	6.9931	7.0996	7.0512	6.7346	6.1252	6.0821	0.9405	-0.0886	0.5989	0.2227
Stmn4	_RRDPS[Phospho (STY)]LEEIQK_.3	#N/A	7.9054	7.8909	8.3380	8.0381	7.6022	7.9592	8.0652	8.0083	8.2304	8.0148	0.9846	-0.0223	0.3625	0.4407
Vamp2	_DQKLS[Phospho (STY)]ELDDR_.2	P63044_S61_M1_Vamp2	7.7009	7.3651	7.7720	7.8538	5.3456	7.6395	7.9153	7.7337	7.4451	6.9057	0.9706	-0.0430	0.6833	0.1654
Vamp2	_LSELDDR_.2	#N/A	5.6245	5.3234	5.3494	4.2051	4.2584	4.8594	4.1716	5.6371	5.2104	4.4158	1.0160	0.0230	0.8531	0.0690
Vamp2	_ADALQAGASQFETSAAK_.2	#N/A	8.8879	8.6501	9.1683	8.7428	8.1147	8.6791	9.2775	8.7913	8.8527	8.7361	0.9767	-0.0339	0.3351	0.4748
Vamp2	_[Acetyl (Protein N-term)]SATAATVPPAAPAGEGGPPAPPPNLTSNR_.3	#N/A	3.7213	9.3581	4.4081	3.4100	10.4896	9.5383	3.1246	8.7792	8.4864	3.3769	1.1480	0.1991	0.6842	0.1648
Vamp2	_LQQTQAQVDEVVDIM[Oxidation (M)]R_.2	#N/A	6.7276	6.4441	7.0626	6.5729	5.4426	6.3122	6.6472	6.4572	6.6269	6.5849	0.9769	-0.0337	0.6037	0.2192
Vamp2	_LSELDDRADALQAGASQFETSAAK_.3	#N/A	6.3072	7.3299	7.4324	6.9681	6.4165	6.7367	7.3850	7.0048	7.0033	6.8303	0.9730	-0.0395	0.4749	0.3234
Sult4a1	_[Acetyl (Protein N-term)]AESEAETPGT[Phospho (STY)]PGEFESK_.2	P63046_T11_M1_Sult4a1	9.1312	9.6081	9.4188	9.7610	7.9104	9.5261	9.5541	9.5022	9.3795	9.5667	0.9711	-0.0423	0.4523	0.3446
Pcp4	_AAVAIQSQFR_.2	#N/A	9.4538	9.8883	10.1057	10.3115	10.9749	10.3796	9.7712	10.2252	10.3512	10.2250	1.0042	0.0060	0.8830	0.0540
Pcp4	_VQEEFDIDM[Oxidation (M)]DAPETER_.2	#N/A	8.0891	8.5380	8.9204	9.0199	9.2152	9.0629	8.4751	8.4750	8.6194	8.7413	1.0268	0.0382	0.3262	0.4865
Pcp4	_KVQEEFDIDM[Oxidation (M)]DAPETER_.3	#N/A	7.4461	7.1319	8.1979	7.9258	8.4680	7.8145	7.0691	7.9039	7.3428	7.5294	1.0495	0.0697	0.2305	0.6374
Thra	_VEC[Carbamidomethyl (C)]GS[Phospho (STY)]DPEENSAR_.2	P63058_S12_M1_Thra	6.1963	3.0956	4.4780	6.6715	2.9169	3.3118	6.3668	5.7941	5.7628	3.4468	0.8320	-0.2654	0.3871	0.4122
Eif4e	_DAVTHIGR_.2	#N/A	5.9295	5.4304	5.2204	5.6852	6.1215	5.4749	6.0375	5.5607	5.5453	5.5671	0.9940	-0.0087	0.8666	0.0622
Eif4e	_IAIWTTEC[Carbamidomethyl (C)]ENR_.2	#N/A	3.2565	3.9697	3.9433	4.4459	4.1402	4.1792	4.1606	3.6725	3.3211	5.1465	0.9789	-0.0308	0.8240	0.0841
Gabrb3	_NSAIS[Phospho (STY)]FDNSGIQYR_.2	P63080_S394_M1_Gabrb3	3.8956	3.1511	3.2611	6.0174	3.0394	5.1742	4.0987	6.1348	5.7973	4.6613	0.7906	-0.3390	0.1780	0.7496
Gabrb3	_KQS[Phospho (STY)]MPKEGHGR_.3	P63080_S406_M1_Gabrb3	3.7077	3.5184	4.3945	3.3963	3.4067	3.2179	3.1110	3.2213	4.3707	3.3633	1.0257	0.0366	0.7801	0.1079
Mapk1	_[Acetyl (Protein N-term)]AAAAAAGPEM[Oxidation (M)]VR_.2	#N/A	8.2397	8.9348	9.1368	7.7776	8.5834	8.8633	8.6784	9.0293	8.8080	8.6208	0.9778	-0.0324	0.4872	0.3123
Mapk1	_GQVFDVGPR_.2	#N/A	8.4223	8.3253	8.5971	8.0828	8.2781	8.1714	8.5294	8.5031	8.5002	7.9972	0.9917	-0.0120	0.6273	0.2025
Mapk1	_NYLLSLPHK_.2	#N/A	7.6869	8.1165	7.9531	7.8152	7.8317	7.6219	8.0039	8.1476	8.2708	8.1139	0.9635	-0.0536	0.0191	1.7188
Mapk1	_ELIFEETAR_.2	#N/A	5.7141	5.2714	5.7414	5.3189	5.1214	5.6625	3.6204	5.6798	6.0987	5.6230	1.0411	0.0582	0.6506	0.1867
Mapk1	_LKELIFEETAR_.3	#N/A	4.6583	3.2623	3.1439	3.3576	4.1938	3.7546	3.5058	3.8785	5.1072	4.9540	0.8549	-0.2262	0.1847	0.7336
Mapk1	_DVYIVQDLM[Oxidation (M)]ETDLYK_.2	#N/A	5.7149	5.8737	6.9113	6.5059	6.0187	6.0746	6.2749	5.8736	6.0682	6.2315	1.0116	0.0167	0.7726	0.1121
Mapk1	_VADPDHDHTGFLTEYVATR_.3	#N/A	6.1501	7.2324	7.3255	8.3620	5.8292	7.1797	7.1531	7.8959	7.5232	7.6294	0.9288	-0.1065	0.2955	0.5295
Mapk1	_ISPFEHQTYC[Carbamidomethyl (C)]QR_.3	#N/A	5.5736	4.9418	5.2496	5.0214	5.0560	4.6017	4.9043	5.1813	4.7907	5.2624	1.0078	0.0112	0.8389	0.0763
Mapk1	_YTNLSYIGEGAYGM[Oxidation (M)]VC[Carbamidomethyl (C)]SAYDNLNK_.3	#N/A	4.1172	4.5713	3.3032	3.8058	3.2429	2.9857	3.3678	3.7407	3.9612	3.7685	0.9896	-0.0151	0.9079	0.0420
Mapk1	_APEIMLNSK_.2	#N/A	3.5850	4.3902	4.2718	5.2879	4.5341	3.5180	3.8788	3.6256	3.5697	3.4078	1.1779	0.2362	0.0979	1.0091
Mapk1	_YIHSANVLHR_.3	#N/A	3.2511	4.1037	4.5819	4.1257	3.9920	4.1335	1.7566	4.3763	3.9853	3.8030	1.1583	0.2120	0.3149	0.5018
Ppp1cc	_GNHEC[Carbamidomethyl (C)]AS[Phospho (STY)]INR_.2	P63087_S129_M1_Ppp1cc	6.9840	6.9407	7.1904	7.2681	7.2829	7.0031	7.2108	6.8563	6.7077	6.8500	1.0297	0.0423	0.1127	0.9480
Ppp1cc	_HDLDLIC[Carbamidomethyl (C)]R_.2	#N/A	7.6957	7.4170	7.2503	7.5155	7.7220	7.2809	7.2531	6.5347	7.4099	7.2623	1.0513	0.0722	0.0858	1.0667
Ppp1cc	_NVQLQENEIR_.2	#N/A	7.2881	7.1838	7.4851	7.2001	7.5452	7.8973	7.5625	6.9669	7.6193	7.6854	0.9966	-0.0049	0.8978	0.0468
Ppp1cc	_IYGFYDEC[Carbamidomethyl (C)]K_.2	#N/A	7.3614	7.1010	7.4201	7.5192	7.3783	7.1899	7.5225	6.9886	6.7052	6.7383	1.0486	0.0685	0.0790	1.1022
Ppp1cc	_TFTDC[Carbamidomethyl (C)]FNC[Carbamidomethyl (C)]LPIAAIVDEK_.2	#N/A	7.0109	7.1516	7.1392	7.6966	7.3616	7.0049	7.1431	6.9964	6.9328	6.9020	1.0334	0.0475	0.1361	0.8662
Ppp1cc	_EIFLSQPILLELEAPLK_.2	#N/A	5.4946	5.7237	6.2285	5.5677	5.7164	5.5080	5.6131	5.9766	5.5969	5.7457	0.9954	-0.0067	0.8690	0.0610
Ppp1cc	_YPENFFLLR_.2	#N/A	5.5300	5.2986	4.0431	5.4464	3.7750	3.7467	4.6031	4.1697	4.3806	5.2500	1.0085	0.0122	0.9371	0.0282
Ppp1cc	_[Acetyl (Protein N-term)]ADIDKLNIDSIIQR_.2	#N/A	5.3827	4.9896	5.8110	5.3469	5.7015	5.6170	5.7624	5.4875	5.4890	5.3924	0.9895	-0.0152	0.7338	0.1344
Ppp1cc	_IFC[Carbamidomethyl (C)]C[Carbamidomethyl (C)]HGGLSPDLQSM[Oxidation (M)]EQIR_.3	#N/A	4.9707	5.1902	5.3059	5.3529	5.4496	5.0682	5.0543	4.8309	5.2914	5.4098	1.0148	0.0212	0.5939	0.2263
Ppp1cc	_AHQVVEDGYEFFAK_.2	#N/A	6.4217	5.8049	6.2273	5.8709	5.7413	6.0801	6.2809	5.2099	5.9491	6.1284	1.0224	0.0320	0.5836	0.2339
Ppp1cc	_QSLETIC[Carbamidomethyl (C)]LLLAYK_.2	#N/A	3.0723	3.0114	3.7591	4.2432	3.6989	4.0306	4.3914	3.8567	3.5053	3.6325	0.9453	-0.0812	0.5006	0.3005
Ppp1cc	_IC[Carbamidomethyl (C)]GDIHGQYYDLLR_.3	#N/A	3.4494	4.0727	3.6093	4.3120	3.6419	4.1698	4.2407	4.0387	3.2312	3.4377	1.0371	0.0526	0.6100	0.2147
Ywhaz	_EKIETELR_.2	#N/A	11.1018	10.7407	10.8378	11.0418	11.2191	11.0294	10.8354	10.8060	11.0086	11.0466	1.0065	0.0093	0.5049	0.2968
Ywhaz	_GIVDQSQQAYQEAFEISKK_.3	#N/A	9.3580	9.8653	10.3760	10.1721	9.2875	9.5993	9.7457	9.4191	9.6099	9.7210	1.0158	0.0227	0.5300	0.2757
Ywhaz	_VVSSIEQK_.2	#N/A	8.4056	8.4159	8.5894	8.4202	8.7549	8.5281	7.9329	7.8388	8.0404	8.6527	1.0496	0.0699	0.0369	1.4332
Ywhaz	_YLAEVAAGDDK_.2	#N/A	7.5799	6.8866	6.9951	7.0966	7.0366	7.3388	6.8721	7.2407	7.3686	7.3105	0.9941	-0.0085	0.7954	0.0994
Ywhaz	_FLIPNASQPESK_.2	#N/A	11.2674	11.5145	11.6738	11.5527	11.7299	11.4314	11.4806	11.3905	11.4481	11.3819	1.0090	0.0129	0.2726	0.5645
Ywhaz	_[Acetyl (Protein N-term)]M[Oxidation (M)]DKNELVQK_.2	#N/A	8.7508	9.3729	9.0806	9.4078	9.2506	9.2357	9.0869	9.2224	8.6408	9.3049	1.0132	0.0189	0.5023	0.2990
Ywhaz	_SVTEQGAELSNEER_.2	#N/A	10.3018	10.4991	10.6741	10.7970	10.7449	10.6387	10.4509	10.4306	10.3957	10.5885	1.0136	0.0196	0.1856	0.7314
Ywhaz	_KGIVDQSQQAYQEAFEISK_.3	#N/A	5.7431	4.9050	5.7703	6.0146	4.6539	4.2892	5.7181	3.9125	4.7911	5.1223	1.0703	0.0980	0.4838	0.3154
Ywhaz	_YDDM[Oxidation (M)]AAC[Carbamidomethyl (C)]M[Oxidation (M)]K_.2	#N/A	5.7754	5.4424	5.4786	6.4200	6.3145	5.4406	4.6743	5.7034	5.3454	5.4945	1.0957	0.1318	0.1171	0.9314
Ywhaz	_DIC[Carbamidomethyl (C)]NDVLSLLEK_.2	#N/A	9.4832	9.4416	8.9788	9.5170	9.6494	9.5046	9.6004	9.2208	9.5472	9.4036	0.9985	-0.0021	0.9211	0.0357
Ywhaz	_TAFDEAIAELDTLSEESYK_.2	#N/A	9.3378	9.3145	9.7847	10.3463	9.6027	9.5060	9.8555	9.5014	9.5000	9.9479	0.9946	-0.0078	0.8145	0.0891
Ywhaz	_YLAEVAAGDDKK_.2	#N/A	10.0122	10.2067	10.2159	10.4516	10.5294	10.4387	10.0265	10.2154	10.2615	10.3748	1.0088	0.0126	0.4607	0.3366
Ywhaz	_VVSS[Phospho (STY)]IEQKTEGAEKK_.3	#N/A	3.9922	3.2339	3.1782	3.6809	6.5318	3.1107	3.3955	4.4376	4.0862	3.6478	1.0161	0.0231	0.9299	0.0316
Ywhaz	_GIVDQSQQAYQEAFEISK_.2	#N/A	3.0054	3.8106	3.6922	10.8006	3.6319	4.0975	4.4583	9.8875	2.9902	4.1619	0.9005	-0.1512	0.7889	0.1030
Ywhaz	_VVSS[Phospho (STY)]IEQKTEGAEK_.2	#N/A	3.4202	3.9663	3.8479	4.1544	3.7876	3.9419	4.3027	3.7679	3.3166	4.3175	0.9814	-0.0271	0.7562	0.1214
Ywhaz	_TAFDEAIAELDTLSEESYKDSTLIMQLLR_.3	#N/A	3.5625	3.6637	4.2493	3.2511	4.1890	3.5405	4.4666	3.3665	3.0151	3.2181	1.0643	0.0899	0.5079	0.2943
Kcna2	_IPS[Phospho (STY)]S[Phospho (STY)]PDLKK_.2	#N/A	11.6577	11.2583	11.0150	11.1148	10.4284	10.8071	11.9738	11.4493	11.4607	11.3414	0.9559	-0.0650	0.0664	1.1777
Kcna2	_ETEGEEQAQYLQVTS[Phospho (STY)]C[Carbamidomethyl (C)]PK_.2	#N/A	7.5741	7.7442	8.1982	8.1133	6.9794	3.9835	8.5696	8.3186	8.3830	8.0498	0.8522	-0.2308	0.1690	0.7721
Kcna2	_IPS[Phospho (STY)]S[Phospho (STY)]PDLKKS[Phospho (STY)]R_.2	#N/A	10.3834	10.8559	10.6402	10.9627	9.9429	10.6559	11.2858	10.9790	10.7596	10.6503	0.9684	-0.0463	0.1519	0.8185
Kcna2	_SDYMEIQEGVNNS[Phospho (STY)]NEDFREENLK_.3	#N/A	4.4041	3.6468	3.9910	3.8915	3.5351	4.2199	4.7162	3.4627	3.9946	3.7068	0.9945	-0.0080	0.9378	0.0279
Kcna2	_IPS[Phospho (STY)]S[Phospho (STY)]PDLK_.2	#N/A	4.3679	3.6233	4.1016	3.6453	3.5116	3.1130	4.5070	3.3558	3.6549	3.3226	1.0046	0.0066	0.9582	0.0186
Ube2b	_VSAIVEQSWNDS_.2	#N/A	3.7408	2.9962	3.4744	3.9186	5.7718	3.7401	3.6333	4.1999	3.8485	3.8856	1.0125	0.0179	0.9244	0.0342
Hmgb1	_GKFEDMAK_.2	#N/A	2.9571	3.7134	4.1916	3.6983	3.8385	3.0229	3.8837	3.2658	3.5648	3.4127	1.0109	0.0157	0.8892	0.0510
Hmgb1	_M[Oxidation (M)]SSYAFFVQTC[Carbamidomethyl (C)]R_.2	#N/A	4.3718	3.6762	3.5578	4.0605	3.4975	4.2320	3.7751	4.0580	5.4484	4.0275	0.9011	-0.1502	0.2617	0.5821
Sumo1	_[Acetyl (Protein N-term)]S[Phospho (STY)]DQEAKPSTEDLGDK_.2	#N/A	3.8721	3.3540	6.6041	6.6313	3.2423	6.4425	3.2754	6.5561	6.3813	3.5277	1.0181	0.0259	0.9381	0.0277
Sumo1	_[Acetyl (Protein N-term)]S[Phospho (STY)]DQEAKPSTEDLGDKK_.2	#N/A	8.4488	8.5118	9.0531	9.1711	8.5264	8.7550	8.9014	8.5350	8.6722	8.6449	1.0064	0.0093	0.7464	0.1270
Dynll1	_NADM[Oxidation (M)]SEEM[Oxidation (M)]QQDSVEC[Carbamidomethyl (C)]ATQALEK_.3	#N/A	5.7174	5.9793	6.6474	5.0652	4.6733	5.5956	6.3454	6.1947	5.8152	6.1239	0.9172	-0.1247	0.2016	0.6955
Gng2	_[Acetyl (Protein N-term)]ASNNTAS[Phospho (STY)]IAQAR_.2	P63213_S8_M1_Gng2	8.8118	8.6937	9.4473	9.0253	8.3364	8.5941	8.8972	8.8459	8.9611	8.3276	1.0069	0.0099	0.7978	0.0981
Gng2	_EDPLLTPVPASENPFR_.2	#N/A	7.8191	7.6825	7.7619	7.7440	7.6017	7.4580	8.1979	7.7245	7.9041	7.5832	0.9778	-0.0324	0.1977	0.7041
Gng2	_KLVEQLK_.2	#N/A	7.5170	7.5942	7.4161	7.5310	8.0030	7.5619	7.8712	7.8208	7.9671	7.5397	0.9749	-0.0367	0.1622	0.7901
Gng3	_IEASLC[Carbamidomethyl (C)]R_.2	#N/A	5.0477	5.2827	5.0620	4.3726	5.2320	4.7557	5.1907	5.9722	5.5730	5.6528	0.8859	-0.1747	0.0181	1.7419
Gng3	_GETPVNSTM[Oxidation (M)]S[Phospho (STY)]IGQAR_.2	#N/A	9.9307	9.6416	10.2659	9.9963	9.3062	9.7965	10.5080	9.9460	9.9351	9.7449	0.9790	-0.0306	0.3500	0.4560
Gng3	_MKGET[Phospho (STY)]PVNSTM[Oxidation (M)]SIGQAR_.3	#N/A	4.1581	3.6829	4.2730	6.2570	3.4636	3.4177	3.6415	3.3097	3.8995	3.3962	1.1817	0.2408	0.2757	0.5596
Eif5a	_VHLVGIDIFTGK_.3	#N/A	5.6749	5.6757	5.6917	6.4002	6.0675	5.9562	4.6494	5.9470	5.8052	6.4422	1.0350	0.0497	0.5665	0.2468
Eif5a	_EDLRLPEGDLGK_.3	#N/A	3.3760	2.7209	4.3957	3.9592	4.7160	4.7319	4.0878	4.2437	3.4501	3.9986	1.0097	0.0139	0.9317	0.0307

Eif5a	_EDLRLPEGDLGKEIEQK_.3	#N/A	5.6211	5.9724	6.0699	5.6783	5.7969	6.4011	6.4361	5.4996	5.5235	6.5840	0.9854	-0.0212	0.7564	0.1212
Eif5a	_RNDFQLIGIQDGYLSLLQDSGEVR_.3	#N/A	2.3261	3.5283	4.3847	3.7975	3.4166	3.2080	3.1012	3.2312	4.3806	2.3767	1.0523	0.0735	0.7295	0.1370
Eif5a	_IVEMSTSK_.2	#N/A	4.0832	3.1430	3.2691	3.7718	3.0313	3.5933	3.4865	3.4367	3.9953	3.7388	0.8947	-0.1605	0.1435	0.8430
Pkia	_[Acetyl (Protein N-term)]TDVETTYADFIASGR_.2	#N/A	4.4249	4.6794	4.9328	4.5083	5.6581	5.0132	5.2525	4.0587	4.0651	4.7682	1.0735	0.1023	0.3350	0.4749
Pkia	_SSTEQSGEAQGEAAK_.2	#N/A	4.1448	3.7132	3.9498	3.0313	4.6955	3.8744	4.3261	3.6031	3.9430	3.6382	1.0062	0.0089	0.9401	0.0268
Pkia	_SSTEQSGEAQGEAAKS[Phospho (STY)]ES_.2	P63248_S74_M1_Pkia	3.4946	4.1800	3.7506	3.9899	3.8479	3.6502	4.1382	3.8657	3.3595	3.6672	1.0163	0.0233	0.7426	0.1293
Kcnj3	_ERHNS[Phospho (STY)]VEC[Carbamidomethyl (C)]LDGLDDISTK_.3	#N/A	4.1961	5.7862	3.9298	5.6327	3.7879	5.2988	5.7759	5.7389	6.4422	5.9217	0.7994	-0.3231	0.0364	1.4391
Kcnj3	_HNS[Phospho (STY)]VEC[Carbamidomethyl (C)]LDGLDDISTK_.2	#N/A	3.9322	4.2194	4.0898	3.7032	3.8028	3.4297	3.8514	4.5470	3.6109	3.1836	1.0170	0.0243	0.8154	0.0886
Actg1	_[Acetyl (Protein N-term)]EEEIAALVIDNGSGM[Oxidation (M)]C[Carbamidomethyl (C)]K_.2	#N/A	11.7492	11.9004	12.5158	12.0530	11.5173	11.5506	11.9746	11.6206	12.2148	11.5658	1.0031	0.0045	0.8733	0.0589
Actg1	[Acetyl (Protein N-term)]EEEIAALVIDNGSGM[Oxidation (M)]C[Carbamidomethyl (C)]KAGFAGDDAPR_.	#N/A	8.5415	9.1764	8.8418	9.2416	8.4534	8.1317	9.1769	8.7913	8.6069	8.4265	0.9978	-0.0032	0.9415	0.0262
Prkcg	_AAPALT[Phospho (STY)]PPDR_.2	P63318_T655_M1_Prkcg	16.7896	16.6543	17.3737	16.9527	16.0607	16.6525	17.0796	16.7850	16.7606	16.6768	0.9954	-0.0067	0.7428	0.1291
Prkcg	_ENVFPGSTTR_.2	#N/A	8.0781	8.4624	8.6175	8.2997	8.4582	7.8822	7.9760	8.4182	8.1760	8.1153	1.0157	0.0225	0.4397	0.3568
Prkcg	_DVIVQDDDVDC[Carbamidomethyl (C)]TLVEK_.2	#N/A	7.7100	7.9583	8.6718	9.1743	6.9339	7.8561	8.4384	7.8733	7.5435	8.0072	1.0107	0.0153	0.8463	0.0725
Prkcg	_RGS[Phospho (STY)]DELYAIK_.2	P63318_S373_M1_Prkcg	7.9296	8.1672	4.4437	8.2898	7.3609	7.7879	8.8677	8.3972	8.1636	7.9165	0.8793	-0.1856	0.2206	0.6564
Prkcg	_FEAC[Carbamidomethyl (C)]NYPLELYER_.2	#N/A	7.4706	7.4895	7.4064	7.5485	6.9501	7.6291	7.4265	7.3466	7.5793	7.0967	1.0073	0.0104	0.7243	0.1401
Prkcg	_C[Carbamidomethyl (C)]FFGAS[Phospho (STY)]PGR_.2	P63318_S342_M1_Prkcg	7.1239	6.9269	6.4801	7.0021	6.2939	6.5150	7.0802	6.7725	6.7887	6.7807	0.9808	-0.0280	0.4886	0.3110
Prkcg	_APVDGWYK_.2	#N/A	6.3744	5.9297	6.3828	5.7797	6.0807	6.1725	5.5495	6.4835	6.1341	5.6208	1.0291	0.0414	0.4418	0.3548
Prkcg	_LSVEVWDWDR_.2	#N/A	5.8412	5.2636	3.8679	5.4904	5.0784	5.2112	6.0708	5.5155	5.8562	6.0068	0.8743	-0.1938	0.0726	1.1392
Prkcg	_S[Phospho (STY)]GENFDKFFTR_.2	P63318_S639_M1_Prkcg	2.9651	7.0359	7.2211	7.5740	6.1398	7.1794	7.4133	7.4513	6.8413	6.6012	0.8977	-0.1558	0.4428	0.3538
Prkcg	_DVIVQDDDVDC[Carbamidomethyl (C)]TLVEKR_.3	#N/A	4.9875	5.7021	7.0081	6.5450	4.4056	5.6706	5.2767	6.2215	5.2724	5.8213	1.0127	0.0182	0.8941	0.0486
Prkcg	_KDVIVQDDDVDC[Carbamidomethyl (C)]TLVEK_.3	#N/A	4.6755	4.1905	3.7728	4.8875	3.1828	3.5992	4.1994	3.8859	3.8760	4.6404	0.9761	-0.0349	0.7919	0.1013
Prkcg	_S[Phospho (STY)]PTSPVPVPVM_.2	P63318_S687_M1_Prkcg	8.8692	7.1282	4.8304	9.6815	4.8926	4.9792	7.8322	5.1122	6.9436	5.4277	1.0634	0.0887	0.7500	0.1249
Prkcg	_SVPSLC[Carbamidomethyl (C)]GVDHTER_.2	#N/A	6.1576	6.6166	5.9317	6.3396	6.7296	6.3068	7.0718	6.7472	6.7266	6.7648	0.9296	-0.1053	0.0184	1.7351
Prkcg	_M[Oxidation (M)]GPSSSPIPSPS[Phospho (STY)]PS[Phospho (STY)]PTDSKR_.3	#N/A	5.8130	7.9780	3.9378	6.7233	7.2182	7.9022	8.2412	8.1868	8.1624	7.9680	0.8103	-0.3035	0.0837	1.0771
Prkcg	_NDFM[Oxidation (M)]GAM[Oxidation (M)]SFGVSELLK_.2	#N/A	5.1775	4.8037	4.3235	4.3616	4.2950	5.4985	3.8025	4.7315	5.1605	3.3315	1.1144	0.1562	0.2801	0.5527
Prkcg	_APTS[Phospho (STY)]DEIHITVGEAR_.2	#N/A	3.2919	4.1782	4.1693	5.1383	3.9175	4.2071	4.1543	4.5359	4.6171	3.5952	0.9822	-0.0259	0.8376	0.0770
Prkcg	_DLKLDNVMLDAEGHIK_.3	#N/A	5.6102	3.2673	3.1489	6.1398	3.0886	3.1400	3.5008	3.6486	3.9477	3.2131	1.1365	0.1846	0.5229	0.2816
Prkcg	_S[Phospho (STY)]VPSLC[Carbamidomethyl (C)]GVDHTERR_.3	P63318_S145_M1_Prkcg	3.0447	3.8009	4.2792	4.6147	3.6892	3.4164	4.3294	3.5038	3.1524	3.0808	1.0827	0.1147	0.4525	0.3444
Prkcg	_LLNQEEGEYYNVPVADADNC[Carbamidomethyl (C)]SLLQK_.3	#N/A	4.3293	3.6350	3.5193	4.7366	3.6120	3.1523	3.7542	4.8762	4.1423	3.7762	0.9259	-0.1110	0.4235	0.3731
Prkcg	_GSDELYAIK_.2	#N/A	3.8021	3.0576	3.1848	3.8572	2.9459	2.9562	3.5719	4.2613	3.9099	4.2577	0.8251	-0.2773	0.0234	1.6300
Prkcg	_LGSGPDGEPTIR_.2	#N/A	5.2823	5.9419	3.6354	3.1792	3.0454	3.5792	3.3274	3.8220	4.1211	4.3573	1.0521	0.0733	0.7580	0.1203
Prkcg	_MGPSSSPIPSPSPS[Phospho (STY)]PTDSK_.2	#N/A	3.1657	4.0854	4.0442	4.9470	3.7978	4.1040	4.1128	3.5415	3.3770	3.6833	1.0939	0.1295	0.3099	0.5088
Rala	_VIM[Oxidation (M)]VGSGGVGK_.2	#N/A	7.4713	7.6879	7.7346	6.8505	7.5552	7.6062	7.1312	7.7479	7.6629	7.5257	0.9957	-0.0063	0.8728	0.0591
Rala	_ADQWNVNYVETSAK_.2	#N/A	5.6054	6.1848	6.4390	6.7485	5.9820	6.1982	6.2386	5.7932	5.7824	6.7533	1.0083	0.0120	0.8542	0.0684
Rala	_SDLEDKR_.2	#N/A	5.7582	5.8469	5.6642	5.7750	6.7588	6.3470	5.4338	6.4022	5.7279	4.9219	1.0718	0.1000	0.2553	0.5929
Rala	_VKEDENVPFLLVGNK_.3	#N/A	5.8113	5.8872	4.7741	5.3869	5.6038	5.8443	4.8872	5.2424	5.5989	6.0758	1.0184	0.0263	0.7430	0.1290
Rala	_GQNSLALHK_.2	#N/A	3.9730	4.7414	4.7317	4.6767	5.0149	4.6095	4.9557	5.3051	4.5632	5.0869	0.9290	-0.1062	0.1408	0.8515
Rala	_VVLDGEEVQIDILDTAGQEDYAAIR_.3	#N/A	4.2393	4.9735	5.0415	5.0715	3.3831	3.2415	3.6650	3.4844	3.7834	3.1941	1.2246	0.2923	0.1107	0.9560
Rps12	_TALIHDGLAR_.2	#N/A	7.2607	7.4092	6.7620	7.5915	7.8383	7.5310	7.0896	7.7199	7.5666	7.3053	0.9971	-0.0042	0.9233	0.0346
Rps12	_[Acetyl (Protein N-term)]AEEGIAAGGVM[Oxidation (M)]DVNTALQEVLK_.2	#N/A	5.7566	4.7774	4.9176	6.2537	5.2848	5.7695	4.8614	4.4401	3.6144	6.2159	1.1415	0.1910	0.2274	0.6433
Rps12	_DVIEEYFK_.2	#N/A	3.1800	3.1223	4.4145	3.9583	2.5978	3.8312	4.1066	3.2809	3.3419	3.8216	0.9669	-0.0486	0.7556	0.1217
Rps10	_AEAGAGS[Phospho (STY)]ATEFQFR_.2	P63325_S146_M1_Rps10	4.2094	3.1330	3.9430	3.4868	6.0748	6.9829	3.6351	6.3669	3.8134	6.9082	0.8953	-0.1596	0.6135	0.2122
Rps10	_IAIYELLFK_.2	#N/A	4.5801	4.9324	3.8344	4.3356	4.2291	4.3324	4.0847	5.0296	4.4595	5.2035	0.9318	-0.1020	0.2801	0.5526
Ppp3ca	_IITEGASILR_.2	#N/A	9.6598	9.8234	9.9212	9.7050	9.6004	9.7092	9.8806	10.1205	9.8344	9.6502	0.9863	-0.0199	0.2012	0.6965
Ppp3ca	_EVFDNDGKPR_.2	#N/A	8.6515	8.3316	8.4619	8.5254	8.9287	8.6805	8.2448	8.6105	8.6491	8.6981	1.0054	0.0077	0.7394	0.1311
Ppp3ca	_QTLQSATVEAIEADEAIK_.2	#N/A	7.1413	6.6125	6.9385	6.6681	6.3832	6.9722	6.9473	6.4773	6.6793	7.4096	0.9866	-0.0195	0.6771	0.1694
Ppp3ca	_GFS[Phospho (STY)]PQHK_.2	#N/A	8.8208	8.6905	8.6038	8.7095	8.0011	8.6764	8.3831	8.3954	8.4998	8.3575	1.0208	0.0297	0.2835	0.5474
Ppp3ca	_ITSFEEAK_.2	#N/A	7.5875	6.8240	7.5276	6.7602	7.0751	7.5339	5.8137	7.3873	7.7659	6.9116	1.0356	0.0505	0.5382	0.2691
Ppp3ca	_TQEHFTHNTVR_.2	#N/A	7.0278	7.1593	7.0563	7.1334	7.4352	7.2702	7.2654	7.2336	6.9193	6.8701	1.0153	0.0219	0.3630	0.4400
Ppp3ca	_GLTPTGMLPSGVLSGGK_.2	#N/A	7.8276	6.8124	5.3560	8.9810	7.2010	3.5706	6.9611	6.2214	6.2709	6.5712	1.0182	0.0261	0.9072	0.0423
Ppp3ca	_DAM[Oxidation (M)]PSDANLNSINK_.2	#N/A	9.2694	8.8726	3.3487	9.3501	7.1271	8.6162	9.2747	8.7324	8.7958	9.0271	0.8668	-0.2063	0.3421	0.4658
Ppp3ca	_ALASETNGTDSNGSNSSNIQ_.2	#N/A	4.6359	4.7705	7.0676	5.6851	4.4398	5.6324	8.0160	5.3229	4.9998	5.6886	0.8943	-0.1612	0.4141	0.3829
Ppp3ca	_LEESVALR_.2	#N/A	5.8374	5.7714	5.3828	5.8810	5.9566	6.0814	3.7679	6.0869	6.2039	6.0847	1.0510	0.0718	0.5739	0.2412
Ppp3ca	_ITS[Phospho (STY)]FEEAKGLDR_.2	#N/A	4.2547	9.7362	10.7223	9.4852	2.8598	9.6139	8.8981	9.6091	9.5982	9.1640	0.8349	-0.2604	0.3929	0.4057
Ppp3ca	_VTEMLVNVLNIC[Carbamidomethyl (C)]SDDELGSEEDGFDGATAAAR_.3	#N/A	3.2912	4.0512	4.5258	4.0695	3.9358	4.1896	4.2178	4.4325	3.2307	3.7468	1.0265	0.0377	0.7354	0.1335
Ppp3ca	_QTLQSATVEAIEADEAIKGFS[Phospho (STY)]PQHK_.3	#N/A	3.6568	3.6235	3.9028	4.1421	3.5118	3.9636	3.8567	7.3847	3.7645	2.9924	0.8445	-0.2438	0.3990	0.3990
Ppp2ca	_ELDQWIEQLNEC[Carbamidomethyl (C)]K_.2	#N/A	6.9794	6.9332	7.0933	7.3476	6.7919	6.8282	6.9764	7.1255	6.9554	6.9033	1.0008	0.0011	0.9617	0.0170
Phb	_VLPSITTEILK_.2	#N/A	8.0581	8.2168	8.2153	7.9394	7.9689	8.0428	8.0170	8.3114	8.4222	8.0497	0.9846	-0.0224	0.2375	0.6243
Phb	_DLQNVNITLR_.2	#N/A	6.6919	7.3752	7.3751	7.6345	7.7949	7.3514	7.2253	7.4016	7.5006	7.4581	0.9965	-0.0051	0.8993	0.0461
Phb	_FDAGELITQR_.2	#N/A	7.8930	7.3286	7.4839	7.4868	7.5241	7.5636	7.3348	7.2693	7.3924	6.7717	1.0261	0.0372	0.3031	0.5185
Phb	_IYTSIGEDYDER_.2	#N/A	5.6002	5.9137	5.5719	6.4961	6.2985	6.5041	6.2033	6.0437	5.8258	6.0889	1.0039	0.0056	0.9196	0.0364
Phb	_NVPVITGSK_.2	#N/A	6.3537	6.5617	5.8937	6.0635	6.7955	6.3576	4.9774	6.1610	6.8644	6.7404	1.0245	0.0350	0.6994	0.1553
Phb	_NITYLPAGQSVLLQLPQ_.2	#N/A	6.1892	5.6233	6.9426	5.7135	6.0587	6.1726	6.3053	6.0234	6.0045	6.9883	0.9662	-0.0496	0.4971	0.3035
Phb	_QVSDDLTER_.2	#N/A	4.1377	7.4276	7.3994	7.4790	7.4710	7.2731	6.9155	7.2614	7.2392	7.3039	0.9561	-0.0648	0.6582	0.1817
Phb	_AAIISAEGDSK_.2	#N/A	4.3365	3.6409	8.0209	7.7591	8.2042	7.9360	7.4939	7.8216	7.6292	7.6472	0.8695	-0.2018	0.3745	0.4265
Phb	_EFTEAVEAK_.2	#N/A	4.0450	5.6486	3.2310	5.7762	6.1350	6.1448	5.6170	5.7252	6.1603	5.8896	0.8829	-0.1796	0.3103	0.5082
Phb	_AAELIANSLATAGDGLIELR_.3	#N/A	3.2388	4.0138	3.6950	4.4016	3.5521	4.2301	3.9203	3.8982	4.0795	3.9468	0.9732	-0.0391	0.6518	0.1859
Csnk2b	_YQQGDFGYC[Carbamidomethyl (C)]PR_.2	#N/A	6.3933	6.2529	6.7874	6.5308	6.6460	6.2839	6.7333	6.6298	6.4464	6.4953	0.9857	-0.0207	0.4542	0.3427
Csnk2b	_GNEFFC[Carbamidomethyl (C)]EVDEDYIQDK_.2	#N/A	4.8135	4.4878	5.6260	5.4894	5.0588	3.9102	5.1286	4.4770	5.0392	5.5722	0.9690	-0.0454	0.6852	0.1642
Rpl22	_ITVTSEVPFSK_.2	#N/A	6.7070	6.1965	6.5111	6.6823	6.8071	6.6617	6.3127	6.1888	6.3992	6.7015	1.0303	0.0430	0.2050	0.6883
Rpl22	_AGNLGGGVVTIER_.2	#N/A	5.7563	5.4094	5.4750	5.7992	5.7716	5.7115	5.9925	5.5422	4.8146	5.3724	1.0411	0.0582	0.3198	0.4952
Rpl22	_YFQINQDEEEEEDED_.2	#N/A	4.2148	3.5192	3.4008	7.3412	5.5879	6.0669	3.2490	7.2592	6.6301	5.9683	0.8693	-0.2020	0.4977	0.3031
Ube2l3	_TDQVIQSLIALVNDPQPEHPLR_.3	#N/A	3.2746	4.2397	3.9614	4.2291	4.3240	3.8284	4.1179	3.6544	3.4469	4.1053	1.0379	0.0536	0.5621	0.2502
Ube2l3	_NIQVDEANLLTWQGLIVPDNPPYDK_.3	#N/A	3.5302	4.2864	3.2638	4.3084	4.1747	3.9507	3.8439	4.1936	2.9918	3.9857	1.0440	0.0622	0.6009	0.2212
Rack1	_FSPNSSNPIIVSC[Carbamidomethyl (C)]GWDK_.2	#N/A	7.0986	7.2109	7.1789	7.4794	7.0286	7.2103	6.7866	7.1079	7.0419	6.4187	1.0530	0.0745	0.0384	1.4160
Rack1	_IIVDELKQEVISTSSK_.3	#N/A	5.2592	5.7243	5.6305	5.6621	4.6926	5.3150	4.8981	5.2857	5.1446	5.8066	1.0183	0.0262	0.7078	0.1501
Rack1	_DVLSVAFSSDNR_.2	#N/A	6.4599	6.4724	6.8217	6.8606	5.9593	6.5761	6.6395	6.4678	6.6690	6.2507	1.0028	0.0040	0.9229	0.0348
Rack1	_VWNLANC[Carbamidomethyl (C)]K_.2	#N/A	4.1674	5.6492	5.6486	5.4915	5.9602	5.1414	4.8710	5.3615	5.5983	5.4188	1.0058	0.0083	0.9317	0.0307
Rack1	_YWLC[Carbamidomethyl (C)]AATGPSIK_.2	#N/A	5.8933	6.1410	6.0618	5.3135	6.2906	5.7919	6.1616	5.9870	6.1386	5.8734	0.9793	-0.0301	0.5156	0.2877

Rack1	_AEPPQC[Carbamidomethyl (C)]TSLAWSADGQTLFAGYTDNLVR_.3	#N/A	3.3185	2.2194	4.0053	4.5079	3.7960	2.1328	4.2226	3.6105	3.2591	2.9741	0.9469	-0.0787	0.7385	0.1316
Rack1	_YTVQDESHSEWVSC[Carbamidomethyl (C)]VR_.3	#N/A	3.5649	3.2389	3.1205	3.6232	3.0602	3.1684	3.3378	2.9945	4.1439	3.5901	0.9373	-0.0935	0.3595	0.4443
Prkacb	_AKEDFLR_.2	#N/A	7.2015	6.7500	7.4652	6.7473	7.0335	6.5527	6.9100	7.3018	7.1403	6.6351	0.9945	-0.0080	0.8575	0.0668
Prkacb	_TLGTGSFGR_.2	#N/A	6.1962	6.2035	5.9531	5.2287	5.8092	5.4498	4.7671	6.4828	6.4344	5.3453	1.0086	0.0123	0.9025	0.0446
Prkacb	_ILQAVEFPFLVR_.2	#N/A	4.8737	4.7183	4.2272	3.8017	4.1570	4.8546	5.5668	5.6782	5.3924	4.5084	0.8396	-0.2522	0.0249	1.6038
Prkacb	_TWTLC[Carbamidomethyl (C)]GTPEYLAPEIILSK_.2	#N/A	5.0119	5.2784	5.3479	5.5945	4.6434	4.8984	4.8840	5.3362	4.9772	4.9113	1.0203	0.0290	0.6133	0.2123
Prkacb	_FRGS[Phospho (STY)]GDTSNFDDYEEEEIR_.3	#N/A	3.3619	4.1182	4.5964	6.8093	4.0065	4.1190	5.7303	6.3873	3.1600	6.2154	0.8378	-0.2553	0.3363	0.4732
Prkacb	_GS[Phospho (STY)]GDTSNFDDYEEEEIR_.2	#N/A	4.1954	3.4509	3.9291	3.4639	3.3392	3.2854	3.1786	3.5283	4.3032	3.1502	1.0199	0.0285	0.8091	0.0920
Prkacb	_ATEQYYAMK_.2	#N/A	3.8641	3.3621	4.5509	6.9977	2.9897	3.2389	3.2674	3.0650	4.2144	3.5197	1.1850	0.2449	0.4325	0.3640
Ywhaq	_AVTEQGAELSNEER_.2	#N/A	9.7597	9.8715	9.9741	9.8770	9.9411	9.9051	9.7693	9.7835	9.9270	9.9941	1.0020	0.0029	0.7422	0.1295
Ywhaq	_EKVESELR_.2	#N/A	9.3581	9.4302	9.0481	9.4742	9.6171	9.5097	9.4070	9.4022	9.4360	9.5280	0.9961	-0.0057	0.7267	0.1387
Ywhaq	_YLAEVAC[Carbamidomethyl (C)]GDDR_.2	#N/A	9.3465	9.2680	9.4353	9.4079	9.1637	9.3502	9.4045	8.9767	9.4173	9.3791	1.0037	0.0053	0.7365	0.1328
Ywhaq	_YLIANATNPESK_.2	#N/A	8.8445	8.5724	8.5234	8.5798	8.7524	8.3320	8.5785	8.6332	8.5937	8.3790	1.0064	0.0092	0.6075	0.2165
Ywhaq	_SIC[Carbamidomethyl (C)]TTVLELLDK_.2	#N/A	7.3228	7.1971	6.4554	7.2976	7.0442	6.9144	7.3391	7.0931	7.3892	7.1836	0.9707	-0.0429	0.2578	0.5887
Ywhaq	_YLAEVAC[Carbamidomethyl (C)]GDDRK_.2	#N/A	6.6319	6.5539	5.9401	7.0537	7.3640	6.7057	6.3774	6.8385	6.4886	6.8657	1.0099	0.0142	0.8107	0.0912
Ywhaq	_KQTIENSQGAYQEAFDISK_.3	#N/A	5.6428	4.8921	5.1744	5.2835	3.7287	4.8285	5.6454	4.4179	4.8646	4.8990	0.9936	-0.0093	0.9372	0.0282
Ywhaq	_TAFDEAIAELDTLNEDSYK_.2	#N/A	6.6687	5.8550	6.2149	6.8701	6.0946	6.2706	6.6214	6.3343	6.1114	6.4034	0.9939	-0.0088	0.8577	0.0667
Ywhaq	_KQTIENSQGAYQEAFDISKK_.3	#N/A	4.2494	4.5897	5.5469	4.0438	4.3860	4.2968	3.7585	4.4043	5.3420	4.7093	0.9924	-0.0111	0.9288	0.0321
Ywhaq	_YDDMATC[Carbamidomethyl (C)]MK_.2	#N/A	3.1603	0.7649	4.3948	4.9165	3.8048	4.3206	4.0868	3.0626	3.3617	1.1514	1.2211	0.2882	0.4982	0.3026
Ywhaq	_QTIENSQGAYQEAFDISK_.2	#N/A	2.5262	4.2922	3.6208	4.1234	4.1805	3.9450	3.8381	3.9951	3.6436	4.0904	0.9716	-0.0415	0.7567	0.1211
Tuba4a	_DVNAAIAAIK_.2	#N/A	9.8570	9.9124	9.6765	9.7843	9.8536	9.9724	9.2727	9.8778	9.9393	9.8703	1.0105	0.0151	0.4671	0.3306
Tuba4a	_EIIDPVLDR_.2	#N/A	13.3168	13.3417	13.3024	13.0258	13.2256	13.2324	13.2961	13.1871	13.1479	13.3342	1.0000	-0.0001	0.9938	0.0027
Tuba4a	_AVFVDLEPTVIDEIR_.2	#N/A	10.7816	10.8127	11.0247	10.9791	10.8403	10.8568	10.9370	10.6128	10.8016	10.7422	1.0101	0.0145	0.1715	0.7657
Tuba4a	_TIGGGDDSFTTFFC[Carbamidomethyl (C)]ETGAGK_.2	#N/A	3.9953	7.5976	7.8773	7.9140	7.2967	7.3894	7.5479	7.0296	7.3741	7.5569	0.9505	-0.0733	0.6482	0.1883
Tubb4b	_FPGQLNADLR_.2	#N/A	13.7754	13.9702	13.8036	13.9095	14.0604	13.7479	13.9380	13.9868	13.9018	13.8255	0.9975	-0.0037	0.6204	0.2073
Tubb4b	_EVDEQM[Oxidation (M)]LNVQNK_.2	#N/A	11.7876	11.9646	13.2834	12.8051	11.9783	12.0063	12.0646	12.7195	11.9677	12.0212	1.0091	0.0131	0.7493	0.1253
Tubb4b	_AVLVDLEPGTM[Oxidation (M)]DSVR_.2	#N/A	12.1250	12.2718	12.6606	12.3764	12.2062	12.4738	12.4379	12.1658	12.2862	12.3213	1.0040	0.0058	0.6613	0.1796
Tubb4b	_LAVNM[Oxidation (M)]VPFPR_.2	#N/A	12.1193	12.2742	12.3330	12.3854	11.9825	11.8462	12.0763	12.1914	12.0727	12.1648	1.0025	0.0036	0.7907	0.1020
Tubb4b	_LHFFM[Oxidation (M)]PGFAPLTSR_.3	#N/A	11.4821	11.6544	11.6634	11.3732	11.2465	11.5639	11.4452	11.6753	11.5689	11.4885	0.9959	-0.0059	0.6253	0.2039
Tubb4b	_TAVC[Carbamidomethyl (C)]DIPPR_.2	#N/A	12.2207	12.6864	11.1601	12.8916	13.6221	12.9618	12.4852	11.7534	13.2163	12.2432	1.0134	0.0191	0.7433	0.1288
Tubb4b	_FPGQLNADLRK_.2	#N/A	10.8692	11.4532	11.1949	11.3762	11.6512	11.2643	10.8943	11.4751	11.2718	11.2601	1.0068	0.0098	0.6575	0.1821
Tubb4b	_IREEYPDR_.2	#N/A	12.1021	12.1881	12.3770	12.3424	12.4200	12.2493	12.1615	12.0880	12.1075	12.1265	1.0131	0.0188	0.0363	1.4405
Tubb4b	_YLTVAAVFR_.2	#N/A	11.4278	11.4198	11.4607	11.4283	11.3405	11.4686	11.3357	11.5675	11.6172	11.5795	0.9913	-0.0127	0.1080	0.9668
Tubb4b	_GHYTEGAELVDSVLDVVR_.2	#N/A	9.5473	9.8872	10.1860	10.7695	9.4162	9.9783	9.7972	10.0285	9.6771	10.2013	1.0038	0.0055	0.8890	0.0511
Tubb4b	_INVYYNEATGGK_.2	#N/A	9.7497	9.9573	10.1858	10.5438	10.2646	10.1892	9.8733	10.1535	10.0302	10.2231	1.0078	0.0112	0.6183	0.2088
Tubb4b	_IM[Oxidation (M)]NTFSVVPSPK_.2	#N/A	11.4818	11.8640	12.0215	11.7665	11.7890	11.8248	11.6766	11.8003	11.7712	11.7408	1.0038	0.0054	0.6466	0.1894
Tubb4b	_ALTVPELTQQM[Oxidation (M)]FDAK_.2	#N/A	11.5448	11.8091	11.9976	11.6522	11.5261	11.6618	11.7503	11.7568	11.6047	11.7672	0.9982	-0.0026	0.8308	0.0805
Tubb4b	_M[Oxidation (M)]SATFIGNSTAIQELFKR_.3	#N/A	6.1611	7.3531	8.4427	7.1601	6.7705	7.2224	6.5424	6.9136	7.1184	7.3741	1.0283	0.0403	0.6410	0.1931
Tubb4b	_KLAVNM[Oxidation (M)]VPFPR_.3	#N/A	10.4544	10.9199	11.3250	10.4662	10.4132	10.7515	10.6770	10.8064	10.6460	10.8325	0.9983	-0.0025	0.9220	0.0353
Tubb4b	_NM[Oxidation (M)]M[Oxidation (M)]AAC[Carbamidomethyl (C)]DPR_.2	#N/A	8.7071	7.9513	7.8921	9.2203	9.3212	8.0083	7.3656	8.0533	8.3449	8.5866	1.0531	0.0746	0.3081	0.5113
Tubb4b	_SGPFGQIFRPDNFVFGQSGAGNNWAK_.3	#N/A	7.3719	7.8903	8.7712	9.2780	7.1553	6.9244	8.0332	7.9503	7.5814	7.9322	1.0031	0.0044	0.9615	0.0170
Tubb4b	_NSSYFVEWIPNNVK_.2	#N/A	11.4965	11.6429	12.0483	12.0269	11.4514	11.6454	11.8214	11.4982	11.6168	11.6903	1.0053	0.0076	0.6759	0.1701
Tubb4b	_GHYTEGAELVDSVLDVVRK_.3	#N/A	9.1662	9.7390	10.7023	10.3817	9.6242	9.9791	9.6022	9.8781	9.8925	10.1238	1.0059	0.0084	0.8484	0.0714
Tubb4b	_LTTPTYGDLNHLVSATMSGVTTC[Carbamidomethyl (C)]LR_.3	#N/A	6.1170	5.1548	4.8890	7.8625	5.9318	5.0041	5.9508	4.4948	5.4880	6.0559	1.0599	0.0839	0.6189	0.2084
Tubb4b	AESC[Carbamidomethyl (C)]DC[Carbamidomethyl (C)]LQGFQLTHSLGGGTGSGM[Oxidation (M)]GTLLISI	#N/A	5.6728	5.6011	5.6465	6.1512	4.6260	4.7661	4.8273	4.7577	5.2710	5.3797	1.0695	0.0970	0.3112	0.5070
Tubb4b	AESC[Carbamidomethyl (C)]DC[Carbamidomethyl (C)]LQGFQLTHSLGGGTGSGM[Oxidation (M)]GTLLISK	#N/A	4.2791	3.3386	3.5018	3.4715	3.3277	3.7586	3.6232	3.2169	3.7174	4.6121	0.9527	-0.0700	0.5620	0.2502
Tubb4b	_FWEVISDEHGIDPTGTYHGDSDLQLER_.4	#N/A	5.4043	5.6830	6.5826	6.5698	5.9549	5.6148	5.5322	5.6576	6.2329	6.1686	1.0119	0.0171	0.8157	0.0885
Tubb4b	_ISEQFTAM[Oxidation (M)]FR_.2	#N/A	10.1722	10.5174	10.7767	10.0711	10.2420	10.4764	10.4430	10.4719	10.5532	10.4711	0.9896	-0.0151	0.4434	0.3532
Tubb4b	_TLKLTTPTYGDLNHLVSATM[Oxidation (M)]SGVTTC[Carbamidomethyl (C)]LR_.3	#N/A	3.3476	4.1528	3.7734	4.0321	4.2104	4.1340	4.1804	4.6711	5.0953	3.5122	0.9031	-0.1471	0.2190	0.6595
Tubb4b	_MSATFIGNSTAIQELFK_.2	#N/A	3.2889	4.0535	4.5235	3.5846	2.1460	3.8547	4.2155	2.0529	3.2330	3.7445	1.0796	0.1106	0.6477	0.1886
Prkcb	_[Acetyl (Protein N-term)]ADPAAGPPPS[Phospho (STY)]EGEESTVR_.2	P68404_S11_M1_Prkcb	9.9614	10.4840	10.1448	10.9969	10.1219	7.8876	11.5678	10.9173	10.4793	10.6428	0.9111	-0.1343	0.1315	0.8810
Prkcb	_LIPDPKS[Phospho (STY)]ESK_.2	P68404_S206_M1_Prkcb	9.1699	9.0808	9.4870	9.3926	8.5615	8.7576	9.4278	9.3137	8.9990	9.0311	0.9872	-0.0186	0.5737	0.2413
Prkcb	_ENIWDGVTTK_.2	#N/A	8.4159	8.1242	8.1600	8.0684	8.1719	8.0411	8.5141	8.1872	8.0993	8.2003	0.9895	-0.0152	0.4070	0.3904
Prkcb	_QPVELT[Phospho (STY)]PTDK_.2	P68404_T642_M1_Prkcb	3.6313	3.7111	3.3649	4.4095	7.8127	8.4858	4.5578	4.0925	4.3915	8.5027	0.9721	-0.0408	0.9190	0.0367
Prkcb	_KGTDELYAVK_.2	#N/A	4.9368	5.4269	4.3674	5.5418	5.0369	3.8320	5.5330	5.3219	3.5384	5.1398	0.9946	-0.0078	0.9585	0.0184
Prkcb	_C[Carbamidomethyl (C)]SLNPEWNETFR_.2	#N/A	6.2144	5.5544	3.2831	5.8438	5.6939	3.9438	5.5687	4.3468	5.4473	5.4025	0.9803	-0.0287	0.8776	0.0567
Prkcb	_LSVEIWDWDLTSR_.2	#N/A	5.6751	4.0705	6.2295	5.4757	5.0851	5.8922	6.1147	5.1565	6.0453	5.8550	0.9330	-0.1001	0.3869	0.4124
Prkcb	_DLKLDNVM[Oxidation (M)]LDSEGHIK_.3	#N/A	6.4212	5.7675	6.1547	6.3875	5.7399	5.7629	5.8034	6.2634	6.2484	6.1331	0.9880	-0.0174	0.7031	0.1530
Prkcb	_AGVDGWFK_.2	#N/A	4.7397	3.8515	4.1579	4.1511	3.8346	2.9022	2.9110	5.0000	4.4406	4.6290	0.9280	-0.1078	0.5394	0.2681
Prkcb	_NLVPM[Oxidation (M)]DPNGLSDPY[Phospho (STY)]VK_.2	#N/A	7.0944	7.3352	7.6376	7.2913	6.8286	7.3789	7.8050	7.7623	7.4226	7.3658	0.9568	-0.0637	0.0841	1.0750
Prkcb	_C[Carbamidomethyl (C)]VM[Oxidation (M)]NVPSLC[Carbamidomethyl (C)]GTDHTER_.3	#N/A	6.1150	4.2098	3.7031	4.2058	4.0981	4.0273	3.9205	3.9127	4.7892	4.1728	1.0463	0.0653	0.6899	0.1612
Prkcb	_FQLKES[Phospho (STY)]DKDR_.3	P68404_S234_M1_Prkcb	4.1079	3.2345	3.8416	3.3854	3.0558	3.1728	3.5336	3.4728	3.9149	3.0626	0.9915	-0.0123	0.9098	0.0411
Ywhah	_AVTELNEPLSNEDR_.2	#N/A	10.7202	10.4487	10.8365	10.8099	10.5814	10.5634	10.6551	10.5409	10.7156	10.6183	1.0026	0.0037	0.7496	0.1252
Ywhah	_NSVVEASEAAYK_.2	#N/A	8.5369	8.4725	8.9346	8.8505	8.7913	8.7811	8.8185	8.4725	8.6973	8.8182	1.0030	0.0043	0.8227	0.0847
Ywhah	_YLAEVASGEK_.2	#N/A	8.4113	8.4236	8.4500	8.5314	8.7150	8.6798	8.0766	8.2547	8.5874	8.6042	1.0184	0.0264	0.2437	0.6131
Ywhah	_YLAEVASGEKK_.2	#N/A	7.9829	8.2423	8.1918	8.2656	8.4311	8.3358	7.9308	7.9955	8.1997	8.1764	1.0206	0.0293	0.1137	0.9443
Ywhah	_NC[Carbamidomethyl (C)]NDFQYESK_.2	#N/A	7.8131	7.8707	7.7809	8.2576	8.0309	7.7559	7.7555	7.9283	7.9597	7.9133	1.0037	0.0053	0.7892	0.1028
Ywhah	_[Acetyl (Protein N-term)]GDREQLLQR_.2	#N/A	7.2809	7.7246	7.6039	7.6973	7.6866	7.3955	7.5978	7.2372	7.7192	7.1339	1.0192	0.0275	0.3545	0.4504
Ywhah	_ELETVC[Carbamidomethyl (C)]NDVLALLDK_.2	#N/A	6.9775	6.7832	5.7849	7.0368	6.9014	6.6256	6.8374	6.7166	6.7667	6.9479	0.9806	-0.0283	0.5973	0.2238
Ywhah	_QAFDDAIAELDTLNEDSYK_.2	#N/A	6.7572	6.5727	6.2739	6.8970	6.7580	6.6095	7.2453	6.2052	6.6126	6.5409	0.9991	-0.0014	0.9762	0.0105
Ywhah	_AVTELNEPLSNEDRNLLSVAYK_.3	#N/A	6.3260	7.1636	7.1699	6.7380	6.7756	6.8050	6.8963	6.8242	6.2969	6.4130	1.0336	0.0477	0.2960	0.5288
Ywhah	_NSVVEASEAAYKEAFEISK_.3	#N/A	6.1254	7.2679	8.4484	7.2307	5.9690	5.8859	5.9172	6.5597	6.2416	7.0132	1.0604	0.0846	0.4983	0.3025
Ywhah	_EAFEISK_.2	#N/A	4.6245	4.4065	5.1664	4.6502	5.3029	4.2012	3.3060	5.0699	4.7590	5.3689	1.0215	0.0306	0.8201	0.0861
Ywhah	_DNLTLWTSDQQDEEAGEGN_.2	#N/A	11.0439	10.5131	3.7163	12.6721	11.7243	12.7746	12.3456	12.1274	12.6083	12.1135	0.8462	-0.2409	0.3075	0.5122
Ywhah	_YDDMASAMK_.2	#N/A	3.1215	3.8778	4.3561	5.4412	3.7661	4.3593	4.2525	3.5807	3.2293	3.0040	1.1812	0.2402	0.1964	0.7068
Kpnb1	_AAVENLPTFLVELSR_.2	#N/A	6.3458	6.4837	6.3086	5.9469	5.8798	6.3010	5.9108	6.3171	6.4159	5.7741	1.0174	0.0249	0.5566	0.2544
Kpnb1	_LAATNALLNSLEFTK_.2	#N/A	6.1322	5.7445	5.8802	5.3969	5.3759	5.5895	6.3767	5.9890	5.4849	5.4529	0.9761	-0.0349	0.5611	0.2510
Kpnb1	_VLANPGNSQVAR_.2	#N/A	6.3907	6.2527	6.6930	5.7720	6.4604	6.1316	3.5538	6.4767	6.9141	6.1470	1.0884	0.1222	0.4335	0.3630
Kpnb1	_WLAIDANAR_.2	#N/A	2.9310	3.7362	3.6178	4.1204	4.7142	4.7341	4.0046	5.2629	4.7910	4.2406	0.8690	-0.2025	0.1909	0.7193

Kpnb1	_SNEILTAIIQGM[Oxidation (M)]R_.2	#N/A	5.2374	4.4430	3.3370	3.8397	3.9276	2.9519	4.7188	5.0013	4.1803	4.7205	0.8498	-0.2348	0.1469	0.8329
Kpnb1	_[Acetyl (Protein N-term)]M[Oxidation (M)]ELITILEK_.2	#N/A	4.3773	2.9651	4.1110	3.6547	3.5210	3.1036	3.8030	2.4442	3.0627	3.3320	1.1461	0.1967	0.2354	0.6282
Doc2b	_WHTLTNELPGAVLS[Phospho (STY)]D_.2	P70169_S411_M1_Doc2b	4.3382	3.0042	4.0718	8.8269	6.7856	3.1427	3.7639	8.0515	6.3632	3.2929	0.9367	-0.0943	0.8234	0.0844
Hrh1	_SRS[Phospho (STY)]NS[Phospho (STY)]GLDYIK_.2	P70174_S381_M2_Hrh1	3.7305	3.0348	4.4173	3.4191	2.8562	3.3725	3.1338	3.1985	4.3480	3.3861	0.9872	-0.0185	0.9041	0.0438
Hrh1	_SNS[Phospho (STY)]GLDYIK_.2	P70174_S383_M1_Hrh1	3.5152	3.8272	3.2489	4.2935	3.6485	4.0810	4.4418	4.2085	3.0067	3.9708	0.9647	-0.0518	0.6740	0.1713
Hrh1	_RIS[Phospho (STY)]ETSEDQTLVDR_.2	P70174_S344_M1_Hrh1	4.3264	4.6116	3.5123	4.0150	3.4521	4.2774	3.8520	4.1035	3.6787	3.9820	1.0329	0.0467	0.6197	0.2078
Dlg3	_GVTSNTS[Phospho (STY)]DSESSSK_.2	P70175_S632_M1_Dlg3	7.1702	7.2950	7.5689	7.7691	7.0726	3.3361	7.5527	4.0014	7.4530	7.1902	1.0233	0.0332	0.8917	0.0498
Dlg3	_LLAVNNTNLQDVR_.2	#N/A	3.6837	4.6758	4.5528	4.0965	4.3542	4.8891	4.2448	5.2118	4.4125	3.6440	0.9993	-0.0010	0.9932	0.0029
Dlg3	_EQMMNSSMS[Phospho (STY)]SGS[Phospho (STY)]GSLR_.2	P70175_S508_M2_Dlg3	6.7046	3.8580	5.0227	4.0454	3.8063	3.7604	4.3378	3.8498	3.1483	3.9176	1.1887	0.2494	0.2830	0.5482
Dlg3	_FIEAGQFNDNLYGTSIQSVR_.2	#N/A	3.9521	4.1536	4.3473	4.1778	3.6607	3.8297	4.0393	3.1102	2.5446	3.5683	1.2125	0.2780	0.0378	1.4221
Dlg3	_VNDDLISEFPHK_.3	#N/A	3.9338	3.0136	3.6675	3.2113	3.0775	2.2924	3.3595	1.6236	4.0890	1.7473	1.1828	0.2422	0.4045	0.3931
Dlg3	_ALFDYDR_.2	#N/A	3.9665	3.2597	3.1524	3.6551	3.0922	3.1365	3.3698	4.4634	4.1120	3.6221	0.8677	-0.2047	0.0880	1.0554
Dlg3	_NATHEQAAAALKR_.3	#N/A	3.1826	3.9878	3.8694	4.1329	3.8091	3.9204	4.2812	3.7464	3.3950	4.3390	0.9687	-0.0459	0.6284	0.2017
Dlg3	_VNEVDVSEVVHSR_.3	#N/A	2.9267	3.6830	4.1612	3.7050	3.5713	3.0533	3.8533	3.2962	3.5952	3.3823	0.9958	-0.0061	0.9543	0.0203
Pip5k1b	_IQALKASPS[Phospho (STY)]K_.2	P70181_S407_M1_Pip5k1b	6.3636	4.2472	3.6658	4.1685	4.1355	3.9900	3.8832	3.9500	3.5986	4.1355	1.1379	0.1863	0.3170	0.4989
Pip5k1a	_IPLKPS[Phospho (STY)]PTK_.2	P70182_S443_M1_Pip5k1a	3.6567	3.5695	6.0806	3.3453	4.9667	5.6458	6.7705	3.2723	4.4217	3.3123	1.0225	0.0321	0.9128	0.0396
Pip5k1a	_S[Phospho (STY)]GPSGNSC[Carbamidomethyl (C)]TSQLMASGEHR_.3	P70182_S458_M1_Pip5k1a	2.5188	4.1377	3.1151	4.2780	4.0260	4.0995	3.9926	3.8405	3.4891	4.2450	0.9496	-0.0745	0.6234	0.2052
Kifap3	_RDS[Phospho (STY)]LPGK_.2	#N/A	8.8453	8.4122	8.7702	8.8316	7.4289	8.2433	8.7872	8.9153	8.8788	8.3584	0.9642	-0.0526	0.3215	0.4929
Kifap3	_RDS[Phospho (STY)]LPGKEK_.2	#N/A	6.0500	6.8814	6.9447	6.9169	5.5872	6.2812	6.6727	6.6805	6.7390	6.2859	0.9771	-0.0334	0.6267	0.2030
Psmb7	_DGIVLGADTR_.2	#N/A	6.6364	5.7879	4.4324	5.9911	5.7379	5.9161	5.7267	4.9774	5.5389	5.6089	1.0526	0.0739	0.4839	0.3152
Psmb7	_VTPLEIEVLEETVQTM[Oxidation (M)]DTS_.2	#N/A	4.8033	5.3076	6.0052	5.5737	5.9101	5.6302	5.3211	5.2529	5.6162	5.2153	1.0349	0.0496	0.4488	0.3479
Psmb7	_ATEGMVVADKNC[Carbamidomethyl (C)]SK_.2	#N/A	3.0882	4.2542	4.3227	3.8665	2.6789	3.6540	4.0148	3.1347	3.4337	3.5438	1.0318	0.0452	0.7646	0.1166
Lxn	_LFLVQTVQQASK_.2	#N/A	5.5692	5.5558	5.3777	6.1436	5.9198	6.3225	5.7997	5.9709	6.0243	5.6527	0.9920	-0.0117	0.8211	0.0856
Lxn	_FSVEEIIQK_.2	#N/A	3.9197	4.0609	3.6906	4.7166	4.4985	3.5023	3.5440	3.8801	2.6540	3.6756	1.1337	0.1810	0.1856	0.7315
Lxn	_AASVAENC[Carbamidomethyl (C)]INYQQGTPHK_.2	#N/A	3.5893	3.5954	3.9503	3.5736	3.0936	4.3876	3.7218	3.5300	3.8290	3.8394	0.9915	-0.0123	0.8930	0.0491
Plxna1	_TASS[Phospho (STY)]PDSLR_.2	P70206_S1630_M1_Plxna1	7.5652	7.5017	4.3779	6.9222	2.8167	3.4119	7.5000	3.2380	7.7027	7.5905	0.8348	-0.2605	0.4630	0.3344
Plxna1	_QTSAYNISNS[Phospho (STY)]STFTK_.2	P70206_S1611_M1_Plxna1	3.3930	4.1982	4.0798	3.9225	4.0195	3.7100	4.0708	3.0787	3.3777	3.5998	1.1006	0.1383	0.1492	0.8263
Plxna1	_LGKDS[Phospho (STY)]PSNK_.2	P70206_S1793_M1_Plxna1	3.0386	3.7948	4.2731	4.6208	3.6831	2.9415	4.3355	3.1843	3.4834	3.4941	1.0279	0.0397	0.8025	0.0955
Plxna2;Plxna4	_YTGS[Phospho (STY)]PDSLR_.2	P70207_S1630_M1_Plxna2;Plxna4	8.4185	8.0793	8.6250	7.8441	3.7721	3.9574	4.3182	8.4614	8.3458	8.3472	1.0864	0.1196	0.7328	0.1350
Plxna2;Plxna4	_YTGS[Phospho (STY)]PDS[Phospho (STY)]LRSR_.2	P70207_S1630_M2_Plxna2;Plxna4	5.1543	5.6858	4.0368	4.5394	3.9765	3.7530	5.4903	3.5790	3.2276	4.8009	1.0585	0.0820	0.6721	0.1725
Fv1	_S[Phospho (STY)]LSDLTSQK_.2	P70213_S244_M1_Fv1	6.8283	6.9585	3.2708	7.1707	3.2105	5.8436	3.3789	6.6019	6.0104	3.7405	1.1245	0.1693	0.6035	0.2193
Chl1	_DETFGEYS[Phospho (STY)]DSDEKPLKGS[Phospho (STY)]LR_.3	P70232_S1148_M2_Chl1	3.5486	3.4855	3.8089	4.3116	3.3415	4.1331	4.0262	4.1949	3.5201	3.5631	0.9857	-0.0207	0.8238	0.0842
Nfic	_SPFNSPS[Phospho (STY)]PQDSPR_.2	P70255_S339_M1_Nfic	3.8934	3.4490	3.6271	3.1708	3.0371	3.5875	6.9256	3.8303	4.1294	4.3490	0.7197	-0.4745	0.0507	1.2954
Nfic	_NWTEDIEGGIS[Phospho (STY)]PVKK_.2	#N/A	4.6050	3.2773	4.3994	3.2139	3.5514	3.3240	5.3285	4.4662	4.4411	4.2959	0.8048	-0.3133	0.0378	1.4222
Nfix	_S[Phospho (STY)]IDDSEMESPVDDVFYPGTGR_.2	P70257_S280_M1_Nfix	3.7008	3.8566	3.8064	3.9053	3.4896	3.7268	3.6200	3.5595	3.9500	3.7471	1.0077	0.0110	0.7868	0.1041
Nfix	_S[Phospho (STY)]PAAGSSQSSGWPNDVDAGPASLKK_.3	#N/A	4.2401	3.7012	3.4261	4.4818	3.3658	3.8785	4.4692	4.1898	3.8384	3.8957	0.9392	-0.0906	0.3558	0.4488
Nfix	_S[Phospho (STY)]PAAGSSQSSGWPNDVDAGPASLK_.2	#N/A	3.3079	4.0345	4.5424	4.0862	3.9525	3.8737	4.2345	4.4158	3.2140	3.7635	1.0152	0.0217	0.8453	0.0730
Pfkfb2	_NS[Phospho (STY)]FTPLSSSNTIR_.2	#N/A	3.9946	6.3265	6.5539	6.9101	3.1383	3.4863	3.4203	5.9491	4.0282	6.0000	1.0451	0.0637	0.8348	0.0784
Pfkfb2	_RNS[Phospho (STY)]FTPLSSSNTIR_.3	#N/A	3.5398	3.8026	3.2735	4.3181	3.6239	4.1056	4.4664	4.1839	2.9821	3.9954	0.9668	-0.0487	0.6974	0.1565
Pkn1	_TDVSNFDEEFTGEAPTLS[Phospho (STY)]PPR_.2	P70268_S920_M1_Pkn1	3.0809	3.8861	3.7677	4.2703	3.7074	4.0221	3.9850	3.8481	3.4967	4.2373	0.9736	-0.0386	0.6778	0.1689
Pdlim4	_RSS[Phospho (STY)]VSGISLEDNR_.2	#N/A	6.5972	6.5058	7.2335	7.4456	4.0214	6.9785	7.4735	6.7804	6.0294	7.0373	0.9463	-0.0796	0.6052	0.2181
Pdlim4	_S[Phospho (STY)]SVSGISLEDNR_.2	P70271_S118_M1_Pdlim4	5.9727	4.2117	4.3977	3.9415	3.8078	3.8396	4.0898	5.6159	5.7722	3.6188	0.9136	-0.1303	0.5105	0.2920
Pdlim4	_IHIDPESQDC[Carbamidomethyl (C)]S[Phospho (STY)]PATSR_.2	#N/A	4.1194	4.7274	5.2186	4.4102	4.4211	4.1690	4.0622	3.7404	3.1329	5.1767	1.1199	0.1633	0.2596	0.5857
Hdac2	_IAC[Carbamidomethyl (C)]DEEFS[Phospho (STY)]DS[Phospho (STY)]EDEGEGGRR_.2	#N/A	5.6795	6.2488	3.7991	6.9542	5.5859	6.5615	6.4936	6.1290	6.2795	6.0869	0.9292	-0.1060	0.4614	0.3359
Hdac2	_M[Oxidation (M)]LPHAPGVQM[Oxidation (M)]QAIPEDAVHEDS[Phospho (STY)]GDEDGEDPDKR_.4	#N/A	2.9635	8.5633	7.2080	10.2241	9.2038	9.1910	7.4513	10.2215	10.1628	9.7849	0.8391	-0.2530	0.3210	0.4936
Pebp1	_LYTLVLTDPDAPSR_.2	#N/A	10.8205	10.8957	11.1029	11.4299	11.1924	11.0432	11.0492	10.7541	10.9495	10.9892	1.0133	0.0190	0.2689	0.5704
Pebp1	_VDYAGVTVDELGK_.2	#N/A	10.6323	10.8463	11.1888	11.1875	11.1189	11.1371	10.7946	10.7048	10.6637	11.0440	1.0201	0.0287	0.1460	0.8356
Pebp1	_FKVETFR_.2	#N/A	10.2664	10.5310	10.8320	10.7320	10.9154	10.4764	10.4606	10.4146	10.3321	10.4262	1.0209	0.0298	0.1242	0.9057
Pebp1	_VLTPTQVM[Oxidation (M)]NRPSSISWDGLDPGK_.3	#N/A	9.6322	10.1328	10.2286	10.0491	10.3155	10.1662	10.1469	10.1086	10.1045	10.0126	0.9994	-0.0008	0.9643	0.0158
Pebp1	_LYEQLSGK_.2	#N/A	7.4383	7.1353	6.8527	7.7523	8.1088	7.6899	6.7163	7.5505	6.9135	7.9037	1.0310	0.0440	0.4987	0.3021
Pebp1	_[Acetyl (Protein N-term)]AADISQWAGPLC[Carbamidomethyl (C)]LQEVDEPPQHALR_.3	#N/A	7.6841	7.9148	8.0679	8.5506	8.5362	8.1972	7.9969	8.0028	8.1847	7.7697	1.0213	0.0304	0.3920	0.4068
Pebp1	_GNDISSGTVLSDYVGSGPPSGTGLHR_.3	#N/A	8.2733	8.0612	7.8591	7.6538	8.0741	8.1642	8.1006	7.1017	7.1781	7.8656	1.0599	0.0839	0.0814	1.0895
Pebp1	_YVWLVYEQEQPLSC[Carbamidomethyl (C)]DEPILSNK_.3	#N/A	5.1612	5.1786	4.9590	6.3047	6.2196	5.4331	5.2769	5.0731	5.3940	5.5841	1.0395	0.0559	0.5117	0.2910
Pebp1	_YNLGAPVAGTC[Carbamidomethyl (C)]YQAEWDDYVPK_.3	#N/A	3.1625	4.0636	3.8493	4.3520	3.7890	4.1735	4.0667	3.7665	4.3151	4.3190	1.0017	0.0024	0.9807	0.0084
Pebp1	_EWHHFLVNM[Oxidation (M)]K_.2	#N/A	4.6663	3.5293	5.5663	3.3855	3.4176	3.2070	3.1002	3.2322	4.3816	3.3525	1.1267	0.1720	0.4289	0.3677
Stam	_AS[Phospho (STY)]PALVAK_.2	P70297_S156_M1_Stam	6.8940	5.8346	6.6497	6.7699	3.2952	6.5266	3.2942	4.2603	6.6892	6.7776	1.1407	0.1900	0.4744	0.3239
Stim1	_NTGASSGATS[Phospho (STY)]EESTEAEFC[Carbamidomethyl (C)]R_.2	P70302_S40_M1_Stim1	3.5624	3.6014	4.2492	3.9131	3.3203	3.6535	4.0143	3.7183	3.7026	3.7849	0.9768	-0.0339	0.6257	0.2036
Stim1	_DLTHS[Phospho (STY)]DSESSLHMSDR_.3	P70302_S519_M1_Stim1	3.9124	3.9230	3.6461	3.9450	3.8113	3.6603	4.0933	3.9031	3.4565	3.6223	1.0126	0.0181	0.7274	0.1382
Hnrnph2	_IQNGTSGVR_.2	#N/A	5.0707	4.1803	3.3018	4.7603	5.5115	4.1339	4.4947	4.1556	2.9538	4.3367	1.1274	0.1731	0.3280	0.4841
Rock2	_NDQWNWDNIR_.2	#N/A	6.7815	6.3043	7.0090	7.0479	6.0398	6.5214	7.2756	6.8022	6.7005	6.9334	0.9552	-0.0662	0.2096	0.6787
Rock2	_TLTSDVANLANEKEELNNK_.3	#N/A	8.0117	8.1780	9.0980	9.1320	6.8577	7.8891	8.1659	8.2808	8.0828	7.8939	1.0109	0.0157	0.8449	0.0732
Rock2	_QIQQLESNNR_.2	#N/A	4.3523	5.8010	7.7281	8.0063	6.3677	7.3546	7.3967	6.0911	7.2807	5.5574	1.0031	0.0044	0.9803	0.0087
Rock2	_ENLLLSDS[Phospho (STY)]PPC[Carbamidomethyl (C)]RENDAIQTR_.3	P70336_S425_M1_Rock2	4.1134	3.3689	3.8471	8.0599	6.9778	7.0394	7.6155	3.6103	7.1319	7.2113	0.8710	-0.1992	0.5328	0.2734
Rock2	_NLLLANSTEEQQK_.2	#N/A	4.2034	3.0202	3.3894	3.8975	4.0668	4.9152	4.3366	4.3639	4.4733	4.9631	0.8635	-0.2117	0.1207	0.9181
Rock2	_KS[Phospho (STY)]EESQEIQKK_.2	P70336_S439_M1_Rock2	2.3037	3.8856	4.3638	3.9076	3.7739	4.3516	4.2447	3.0936	3.3926	3.5849	1.0518	0.0729	0.6792	0.1680
Rock2	_DSDIEQLR_.2	#N/A	3.9638	3.3786	3.6975	3.2413	3.1075	3.5171	3.3895	3.7599	6.2137	4.4194	0.7838	-0.3515	0.1019	0.9917
Frat1	_AAC[Carbamidomethyl (C)]S[Phospho (STY)]DPGAFMGR_.2	P70339_S252_M1_Frat1	2.9981	4.3443	4.2326	7.0420	3.6426	2.9820	6.0787	3.2248	5.3239	5.5484	0.9158	-0.1270	0.6956	0.1576
Hint1	_AQVAQPGGDTIFGK_.2	#N/A	9.2533	9.4971	9.6287	9.5845	9.9070	9.3687	9.2244	8.9598	9.4139	9.2056	1.0368	0.0522	0.0388	1.4114
Hint1	_IIFEDDR_.2	#N/A	8.1819	7.4621	8.4758	7.9738	8.2703	8.0246	7.3341	8.4915	8.3429	8.4436	0.9892	-0.0157	0.7604	0.1189
Hint1	_M[Oxidation (M)]VVNEGADGGQSVYHIHLHVLGGR_.4	#N/A	4.4326	4.1181	3.9997	4.5023	3.9394	3.7901	4.2170	3.7212	5.5134	6.2071	0.8404	-0.2508	0.1376	0.8614
Hint1	_C[Carbamidomethyl (C)]LAFHDIS[Phospho (STY)]PQAPTHFLVIPK_.3	P70349_S45_M1_Hint1	4.2730	3.5767	4.1175	3.8227	3.4070	3.8406	3.5373	3.6475	3.9994	3.7897	1.0257	0.0366	0.6132	0.2124
Hint1	_C[Carbamidomethyl (C)]AADLGLK_.2	#N/A	3.0139	3.7702	4.2484	3.7922	3.6585	2.9661	3.9405	3.2090	3.5080	3.4695	1.0122	0.0175	0.8814	0.0548
Ufd1	_FIAFSGEGQS[Phospho (STY)]LR_.2	P70362_S299_M1_Ufd1	4.3129	7.2667	7.5438	8.6658	7.2953	7.5293	7.7453	8.1899	7.7729	8.0027	0.8959	-0.1586	0.3031	0.5185
Ncoa1	_LLQEGS[Phospho (STY)]PSDITTLSVEPEKK_.3	P70365_S702_M1_Ncoa1	3.1458	3.9021	4.0109	4.5136	3.7904	4.3351	4.2282	3.6049	3.2535	2.9797	1.1231	0.1675	0.2182	0.6612
Ncoa1	_KGS[Phospho (STY)]PC[Carbamidomethyl (C)]DTLASSTEKR_.3	#N/A	3.8394	3.5030	3.5730	4.6176	3.7081	3.6415	3.2651	4.3571	4.1834	4.2949	0.9475	-0.0778	0.4887	0.3110
Ncoa1	_EHS[Phospho (STY)]GLSPQDDSNSGMSIPR_.3	P70365_S369_M1_Ncoa1	3.3089	3.8723	3.8933	5.5020	3.7277	4.2091	4.2355	3.7863	3.2347	3.7645	1.0880	0.1216	0.4511	0.3457
Ncoa1	_QMSSQNS[Phospho (STY)]PSR_.2	P70365_S570_M1_Ncoa1	4.0631	8.0100	3.2491	3.7518	3.1888	3.0398	3.4664	4.3667	4.0153	3.7188	1.0836	0.1158	0.7486	0.1258
Ncoa1	_KGS[Phospho (STY)]PC[Carbamidomethyl (C)]DTLASSTEK_.2	#N/A	2.9099	3.6662	4.1444	3.2486	3.5545	3.0701	4.4641	3.3690	3.0176	3.2156	0.9760	-0.0350	0.8126	0.0901
Elavl1	_VLVDQTTGLSR_.2	#N/A	5.5007	5.5866	5.4946	5.6944	5.5864	5.3936	5.2352	4.9915	4.9406	5.3168	1.0823	0.1142	0.0015	2.8177

Elavl1	_FAANPNQNK_.2	#N/A	3.8558	5.2472	3.5895	6.0414	5.4526	4.4215	5.2639	5.1396	6.1770	4.3113	0.9129	-0.1315	0.4538	0.3432
Elavl1	_TNLIVNYLPQNM[Oxidation (M)]TQEELR_.2	#N/A	3.4600	4.9404	4.3612	3.3096	4.6377	5.0476	5.3986	4.3770	4.3598	4.2366	0.9346	-0.0975	0.5105	0.2920
Rasgrf2	_LGS[Phospho (STY)]LSLKK_.2	P70392_S484_M1_Rasgrf2	7.3040	6.3101	7.1907	6.4705	2.7951	5.6695	6.4460	7.2471	5.8615	5.7446	0.9418	-0.0865	0.6901	0.1611
Rasgrf2	_KFS[Phospho (STY)]S[Phospho (STY)]PPPLAVSR_.2	P70392_S725_M2_Rasgrf2	4.0235	6.3836	3.7483	3.2920	3.1492	3.0795	3.4403	8.8929	8.2923	2.9693	0.6690	-0.5800	0.1978	0.7037
Rasgrf2	_TLNS[Phospho (STY)]C[Carbamidomethyl (C)]KVPQIR_.2	P70392_S633_M1_Rasgrf2	3.0677	4.2747	4.3022	3.8460	3.7123	4.4131	3.9943	3.1552	6.9813	5.3233	0.8918	-0.1652	0.5353	0.2714
Rasgrf2	_EHLVDGKS[Phospho (STY)]PR_.2	#N/A	5.5322	5.5234	4.0258	3.6028	3.0399	3.1892	3.3175	5.5782	5.1397	3.5698	0.9434	-0.0840	0.7397	0.1310
Rasgrf2	_KLS[Phospho (STY)]LTSSLNSR_.2	P70392_S745_M1_Rasgrf2	3.9128	5.3745	4.5996	3.6014	3.0384	3.1902	3.3161	5.7994	4.1657	3.5684	0.9384	-0.0918	0.6930	0.1593
Dlx5	_VPS[Phospho (STY)]AT[Phospho (STY)]S[Phospho (STY)]QPEK_.2	P70396_S113_M3_Dlx5	9.2530	8.4807	8.5452	9.0781	7.4475	8.3575	9.3891	9.1616	8.8769	8.6438	0.9456	-0.0807	0.1978	0.7038
Usp9x	_AQENYEGGEEVS[Phospho (STY)]PPQTK_.2	P70398_S2547_M1_Usp9x	8.5902	8.3834	8.3803	8.4000	7.3417	4.1645	8.6360	8.6835	8.4041	8.2697	0.7986	-0.3245	0.1673	0.7765
Tp53bp1	_STPFIVPS[Phospho (STY)]SPTEQGGR_.2	P70399_S381_M1_Tp53bp1	3.3738	10.2460	10.3962	3.6183	9.6719	4.0021	10.7619	10.3402	3.2122	3.4176	0.9930	-0.0101	0.9848	0.0067
Tp53bp1	_SEDRPSS[Phospho (STY)]PQVSVAAVETK_.3	#N/A	7.9268	7.1236	8.5038	8.6639	7.5043	8.3498	7.7279	8.2788	8.3642	6.0364	1.0540	0.0758	0.4591	0.3381
Tp53bp1	_MESLGS[Phospho (STY)]PR_.2	#N/A	9.3203	8.5980	8.7250	9.8610	8.4936	8.5749	8.9661	8.7274	8.8943	9.0079	1.0034	0.0048	0.9184	0.0369
Tp53bp1	_AETTEKDAVTEDS[Phospho (STY)]PQPPLPSVR_.3	#N/A	6.9379	7.1850	7.6673	9.5819	3.3773	7.6222	7.2402	3.9373	4.2363	7.6530	1.2246	0.2923	0.3455	0.4615
Tp53bp1	_DAVTEDS[Phospho (STY)]PQPPLPSVR_.2	#N/A	8.0117	8.1723	7.5142	9.1032	7.7650	8.2724	8.9119	8.5131	8.5957	8.4040	0.9458	-0.0804	0.1490	0.8268
Tp53bp1	_GGPGKLS[Phospho (STY)]PR_.2	P70399_S1359_M1_Tp53bp1	7.0886	7.2763	7.3029	7.6328	6.9167	6.6321	7.3666	7.1550	7.0856	7.4687	0.9825	-0.0255	0.5218	0.2825
Tp53bp1	_ENTQIDDTEPLS[Phospho (STY)]PVSNSK_.2	#N/A	5.3300	5.1846	3.1375	3.6401	4.0528	5.4189	4.9734	4.9090	5.5284	5.3914	0.8577	-0.2214	0.1879	0.7262
Tp53bp1	_KLPTSEEERS[Phospho (STY)]PAK_.3	P70399_S1675_M1_Tp53bp1	5.1573	3.0473	4.4297	3.5726	5.2711	3.3600	3.7208	3.1861	3.7277	5.2299	1.0438	0.0618	0.7787	0.1086
Tp53bp1	_ASQEPFS[Phospho (STY)]PAEDVM[Oxidation (M)]ETDLLEGLAANQDRPSK_.3	#N/A	3.3715	5.7206	3.5400	6.0670	3.6411	3.8826	3.5382	6.0009	5.2302	4.8533	0.8909	-0.1666	0.4866	0.3129
Tp53bp1	_EAPKEEC[Carbamidomethyl (C)]PEAM[Oxidation (M)]EVETSVISIDS[Phospho (STY)]PQK_.3	P70399_S719_M1_Tp53bp1	6.6110	8.5089	8.3212	8.3822	7.2250	7.3719	8.8801	7.6604	7.1897	7.5073	0.9907	-0.0135	0.8864	0.0524
Tp53bp1	_EHHPEEGFS[Phospho (STY)]GSEVEEVPETPC[Carbamidomethyl (C)]GSHR_.4	P70399_S652_M1_Tp53bp1	3.6089	3.8663	3.7158	6.5263	5.2693	3.7118	3.6794	5.9098	5.4998	4.9181	0.8896	-0.1687	0.4650	0.3325
Tp53bp1	_S[Phospho (STY)]NISSPVTPTAASSSSTTPTR_.2	P70399_S1628_M1_Tp53bp1	3.7548	5.0527	3.4885	4.5331	2.8986	4.3206	3.1806	3.9689	4.2679	4.2104	1.0259	0.0369	0.8255	0.0833
Tp53bp1	_GSGEKPASAPVDDTES[Phospho (STY)]LHS[Phospho (STY)]QGEEEFEM[Oxidation (M)]PQPPHGHVLHR_.5	#N/A	3.5999	3.8285	3.6762	7.4835	5.3157	3.4885	4.0400	7.9854	4.0414	3.7571	0.9212	-0.1184	0.7399	0.1308
Tp53bp1	_TEEDRENTQIDDTEPLS[Phospho (STY)]PVSNSK_.3	#N/A	3.9669	3.2593	3.1528	4.3272	0.5428	3.4770	3.3702	4.4630	4.1116	3.6225	0.8019	-0.3184	0.3094	0.5095
Tp53bp1	_SEALS[Phospho (STY)]S[Phospho (STY)]VLDQEEAM[Oxidation (M)]DTK_.2	#N/A	4.1762	3.4970	3.6305	3.6971	3.3099	3.9292	3.5895	4.8011	3.8906	3.5624	0.9358	-0.0957	0.3873	0.4119
Idh3g	_ENTEGEYSSLEHESVAGVVESLK_.3	#N/A	9.2198	9.6885	10.2864	10.5666	8.3468	9.3986	9.8109	9.6273	9.2354	9.6174	1.0012	0.0018	0.9783	0.0095
Idh3g	_LHSYATSIR_.2	#N/A	7.0230	7.4702	7.0834	7.3978	7.3251	7.5965	3.7102	7.0703	7.4737	7.1413	1.1523	0.2046	0.2091	0.6796
Idh3g	_LGDGLFLQC[Carbamidomethyl (C)]C[Carbamidomethyl (C)]R_.2	#N/A	6.8315	6.9924	6.5387	7.0826	6.1138	6.4354	6.7599	6.9647	6.6766	6.7408	0.9824	-0.0257	0.5557	0.2551
Idh3g	_HKDIDILIVR_.3	#N/A	6.3563	5.0290	7.0975	6.3140	6.2365	6.8559	6.2850	5.9035	2.9512	6.2527	1.1808	0.2397	0.2237	0.6503
Idh3g	_HAC[Carbamidomethyl (C)]VPVDFEEVHVSSNADEEDIR_.4	#N/A	6.1948	5.3241	6.2585	6.4295	4.9228	6.3038	5.9120	5.4113	6.2312	6.2753	0.9913	-0.0126	0.8875	0.0518
Idh3g	_TSLDLYANVIHC[Carbamidomethyl (C)]K_.2	#N/A	5.2237	5.4439	5.3490	4.9369	5.8147	5.9043	4.6351	5.4448	6.1397	5.9653	0.9818	-0.0265	0.7646	0.1166
Idh3g	_DIDILIVR_.2	#N/A	4.3745	3.6299	4.1082	3.2849	3.5182	3.1064	4.5004	3.3328	3.1212	3.2519	1.0334	0.0475	0.7456	0.1275
Idh3g	_AVLASM[Oxidation (M)]DNENM[Oxidation (M)]HTPDIGGQGTTSQAIQDIIR_.3	#N/A	2.0634	3.9862	3.7623	3.4500	3.1206	2.9573	2.8867	3.4864	3.2699	3.5007	0.9809	-0.0278	0.8690	0.0610
Cdh10	_LAEMYGGGES[Phospho (STY)]DKDA_.2	#N/A	8.4708	7.4828	3.6514	9.3678	6.8801	3.9232	8.1384	6.8457	6.9441	7.5310	0.9001	-0.1518	0.5657	0.2474
Evl	_TPSVAKS[Phospho (STY)]PEAK_.2	P70429_S347_M1_Evl	4.6933	5.0209	5.0098	4.7497	5.0303	5.2426	3.0950	5.4768	5.0667	4.6106	1.0867	0.1199	0.3783	0.4222
Evl	_SNS[Phospho (STY)]VEKPVSSLLSR_.2	#N/A	7.6595	8.4597	8.7794	9.5311	7.0116	8.4136	8.3559	8.6310	8.3544	8.0726	0.9947	-0.0077	0.9251	0.0338
Evl	_QVQNGPS[Phospho (STY)]PEEM[Oxidation (M)]DIQR_.2	#N/A	4.7538	5.7717	5.9348	5.8622	5.2605	5.9677	5.8250	5.5783	5.2448	3.8977	1.0886	0.1225	0.3103	0.5083
Evl	_KEDESQTEDPSTS[Phospho (STY)]PSPGTR_.2	P70429_S302_M1_Evl	3.9132	3.3130	4.6000	3.6018	3.0389	3.4234	3.3165	3.0158	4.2995	3.5688	1.0277	0.0394	0.7888	0.1030
Evl	_VQRPEDAS[Phospho (STY)]GGSSPSGTSK_.2	P70429_S240_M1_Evl	3.4112	4.2164	4.0980	4.6007	4.0377	3.6917	4.3154	3.5178	3.1664	3.0668	1.1401	0.1892	0.1485	0.8284
Evl	_VKPAGS[Phospho (STY)]VNDVGLDALDLDR_.2	#N/A	3.9137	3.4077	3.9681	3.3636	3.2642	3.1693	3.2973	3.4768	4.1798	3.5693	0.9679	-0.0470	0.6274	0.2025
Slc9a3r1	_SAS[Phospho (STY)]SDTSEELNSQDSPK_.2	#N/A	9.8759	10.0938	10.3437	10.8236	10.0375	10.3305	10.4935	9.9446	10.0453	10.3585	1.0040	0.0057	0.8437	0.0738
Slc9a3r1	_[Acetyl (Protein N-term)]S[Phospho (STY)]ADAAAGEPLPR_.2	P70441_S2_M1_Slc9a3r1	10.1117	10.0339	10.8107	10.6255	10.0995	10.1665	10.4394	9.9695	10.0627	10.2972	1.0114	0.0163	0.5526	0.2576
Slc9a3r1	_LLVVDPETDER_.2	#N/A	8.3594	8.1668	8.2645	8.6675	8.2846	8.3668	8.3497	8.1164	8.1680	8.4787	1.0089	0.0127	0.5216	0.2827
Slc9a3r1	_AVDPDSPAEASGLR_.2	#N/A	7.5195	7.7289	8.1827	7.6198	8.1272	8.0883	8.1670	7.9250	7.6259	7.6525	1.0045	0.0064	0.8489	0.0711
Slc9a3r1	_EALVEPASES[Phospho (STY)]PRPALAR_.3	#N/A	12.0482	12.0475	13.0200	12.4626	11.7192	12.1329	12.2652	12.0622	11.9907	12.1641	1.0097	0.0140	0.6292	0.2012
Slc9a3r1	_SEQAEPPAAADTHEAGDQNEAEK_.3	#N/A	5.9449	5.8893	6.2030	6.4860	6.3153	6.0737	5.6234	5.5430	6.0360	6.4108	1.0421	0.0595	0.2415	0.6172
Slc9a3r1	_LVEVNGENVEKETHQQVVSR_.3	#N/A	5.7765	5.9802	5.1711	3.3885	5.5056	3.1760	5.4829	5.2109	5.5777	5.1465	0.9026	-0.1478	0.4351	0.3614
Slc9a3r1	_SASSDTSEELNSQDS[Phospho (STY)]PKR_.2	#N/A	7.6167	8.3474	8.5421	8.5902	8.3999	8.0971	8.4277	7.7601	8.0131	8.3187	1.0167	0.0239	0.5549	0.2558
Stx4	_QGDNIS[Phospho (STY)]DDEDEVR_.2	P70452_S15_M1_Stx4	9.0196	8.9927	9.5958	9.1438	7.6167	8.4767	9.6554	9.1383	8.8967	8.9251	0.9622	-0.0556	0.3837	0.4160
Pde7a	_RGS[Phospho (STY)]HPYIDFR_.3	#N/A	3.8986	3.1119	6.0605	3.5872	3.0243	3.4647	4.6609	5.7764	4.1965	3.5542	0.8484	-0.2371	0.3427	0.4650
Vasp	_KVS[Phospho (STY)]KQEEASGGPLAPK_.3	P70460_S235_M1_Vasp	4.9302	2.9774	5.3994	5.6853	2.7987	3.4299	3.7907	3.2560	4.4054	3.3286	1.1376	0.1859	0.4807	0.3181
Sparcl1	KAESSPNAEPSDEGNS[Phospho (STY)]REHSAGSC[Carbamidomethyl (C)]TNFQC[Carbamidomethyl (C)]K_	#N/A	3.6711	3.8464	4.3854	4.0527	4.5089	3.9248	3.9125	4.3409	4.8725	3.7350	0.9643	-0.0524	0.5782	0.2379
Sparcl1	_HGAGDDY[Phospho (STY)]FIPS[Phospho (STY)]QEFLEAER_.2	P70663_S353_M2_Sparcl1	3.8787	3.5136	4.2488	6.0101	3.4733	3.4461	3.4104	5.4340	3.8268	3.1364	1.0362	0.0514	0.8305	0.0806
Sparcl1	_AAGS[Phospho (STY)]KEHIPHTEQQDQEGK_.3	#N/A	5.9939	5.6344	5.8243	6.8176	6.3947	5.9977	6.5062	5.7551	5.7409	6.2232	1.0089	0.0128	0.8427	0.0743
Sparcl1	_KAESSPNAEPSDEGNS[Phospho (STY)]R_.2	#N/A	3.8076	3.6805	3.6563	4.6927	4.1220	4.3620	4.1777	4.2432	4.1285	4.4180	0.9556	-0.0655	0.4132	0.3839
Sparcl1	_HQSEQGNQGQESDSEAEGEDKAAGSKEHIPHT[Phospho (STY)]EQQDQEGK_.5	P70663_T290_M1_Sparcl1	7.3202	3.8636	4.3418	3.8856	3.7519	4.3736	4.0338	3.1156	3.4147	3.5629	1.2995	0.3779	0.1756	0.7554
Sparcl1	_AEKPSALNS[Phospho (STY)]EEETHEQSTEQDK_.3	P70663_S70_M1_Sparcl1	4.8668	3.7364	3.6965	6.4758	5.0476	3.2903	4.3299	3.5332	3.8322	3.1453	1.2180	0.2845	0.2395	0.6206
Naca	_SPASDTYIVFGEAK_.2	#N/A	5.5569	6.1620	6.2596	6.6529	5.7163	5.8961	5.8399	5.2075	4.8677	4.8117	1.1658	0.2213	0.0145	1.8385
Naca	_NNSNDIVNAIM[Oxidation (M)]ELTM[Oxidation (M)]_.2	#N/A	3.3730	4.1293	3.1067	4.2864	5.8252	5.6405	4.0010	4.6594	5.9253	3.8286	0.9544	-0.0674	0.7680	0.1146
Naca	_DIELVMSQANVSR_.2	#N/A	3.6929	2.9483	3.4266	8.2389	2.8366	3.7880	3.6812	4.0309	3.8006	4.1484	1.0613	0.0858	0.8238	0.0842
Irf3	_SSLPHS[Phospho (STY)]QENLPK_.2	P70671_S135_M1_Irf3	7.9013	8.5121	8.8932	8.8428	8.0264	8.3719	8.8076	8.3716	8.2426	3.3538	1.1711	0.2278	0.2696	0.5692
Casp3	_S[Phospho (STY)]VDSGIYLDSSYK_.2	P70677_S26_M1_Casp3	3.7443	6.5788	4.4312	8.1246	6.8690	6.9154	3.1477	7.7970	4.3341	7.5615	1.0701	0.0978	0.7575	0.1206
Ctps1	_SGSSS[Phospho (STY)]PDSEITELKFPSISQD_.2	#N/A	3.5386	3.8038	3.2723	4.3169	4.1832	6.4940	4.4652	4.1851	2.9833	3.9942	1.0925	0.1276	0.5900	0.2291
Ctps1	_SGSSS[Phospho (STY)]PDSEITELK_.2	#N/A	4.2249	3.5293	3.4109	4.5914	3.3506	4.3789	3.2389	3.9106	4.2096	4.2687	1.0019	0.0027	0.9830	0.0074
Ctps1	_GC[Carbamidomethyl (C)]RLS[Phospho (STY)]PR_.2	P70698_S562_M1_Ctps1	9.1210	9.0609	9.6283	9.2557	8.5874	8.7479	9.1799	9.1327	8.9421	8.9972	1.0004	0.0006	0.9844	0.0068
Atp8a1	_RTVS[Phospho (STY)]EIR_.2	P70704_S9_M1_Atp8a1	4.1875	3.4919	9.4404	8.6849	7.1331	8.6638	3.2762	3.8732	8.8298	8.5301	1.1316	0.1783	0.6552	0.1836
Atp8a1	_IIYQAASPDEGALVR_.2	#N/A	6.6035	5.8647	6.1786	5.2716	5.7211	5.8076	6.7186	5.6748	6.2149	5.4439	0.9825	-0.0255	0.7519	0.1238
Atp8a1	_LDGHGTVPLGADQILLR_.3	#N/A	5.4185	5.9771	6.0920	5.8634	5.0488	5.7156	6.1622	6.5503	5.6863	4.6668	0.9860	-0.0203	0.8368	0.0774
Atp8a1	_T[Phospho (STY)]SLADQEEVR_.2	#N/A	3.9158	3.2202	4.6026	3.6045	3.0415	3.1871	3.3192	3.0132	4.1626	3.5715	1.0224	0.0319	0.8309	0.0804
Atp8a1	_TDDVS[Phospho (STY)]EKT[Phospho (STY)]S[Phospho (STY)]LADQEEVR_.2	#N/A	4.5014	8.4747	3.8404	8.8416	7.3598	8.2030	9.0497	8.7465	8.5749	7.9024	0.8018	-0.3187	0.1671	0.7771
Atp8a1	_SESLQQNLLHGYAFSQDENGIVSQSEVIR_.3	#N/A	3.7887	3.0931	4.4755	3.4773	2.9144	3.3143	3.1920	3.1403	4.2897	3.4443	0.9983	-0.0025	0.9868	0.0058
Cct7	_TC[Carbamidomethyl (C)]TIILR_.2	#N/A	8.4207	9.0181	8.4472	8.3949	8.9715	9.2788	8.8434	5.4778	8.8477	9.2712	1.0796	0.1104	0.3994	0.3986
Cct7	_LLDVVHPAAK_.2	#N/A	5.8246	6.1497	5.4807	5.9907	6.0650	5.3517	6.6028	5.9752	5.8274	5.9267	0.9552	-0.0662	0.2442	0.6122
Cct7	_ATISNDGATILK_.2	#N/A	4.8626	5.1276	4.4066	5.2635	5.6877	5.2906	5.8788	4.9875	5.0053	5.5158	0.9550	-0.0664	0.4153	0.3817
Cct7	_LPIGDVATQYFADR_.2	#N/A	5.5308	5.4850	3.7804	5.7549	5.2745	5.4429	6.1428	5.5498	5.2841	5.3583	0.9333	-0.0995	0.3762	0.4245
Cct7	_SLHDAIM[Oxidation (M)]IVR_.2	#N/A	4.6937	5.6272	5.0984	3.4893	4.6813	4.8268	5.1469	3.5118	5.0875	4.6814	1.0280	0.0399	0.7898	0.1025
Cct7	_SQDAEVGDGTTSVTLLAAEFLK_.2	#N/A	4.7821	4.9913	5.6733	5.6157	4.0315	4.4392	5.0920	5.3484	4.3881	4.9137	0.9973	-0.0039	0.9718	0.0124
Cct7	_QLC[Carbamidomethyl (C)]DNAGFDATNILNK_.2	#N/A	4.2425	3.5407	4.4754	4.0192	3.8854	3.5175	4.1332	2.9820	2.3127	3.6964	1.2029	0.2665	0.1110	0.9548
Cct7	_TATQLAVNK_.2	#N/A	1.9234	3.6327	4.4759	3.4778	3.1797	3.3138	3.1924	2.5473	4.2893	3.4448	0.9897	-0.0149	0.9476	0.0234
Cct7	_ALEIIPR_.2	#N/A	4.2917	3.5620	4.0254	3.3676	4.1368	3.1892	3.0823	5.0267	3.9136	3.1362	0.9927	-0.0105	0.9500	0.0223

Cct7	_INALTAASEAAC[Carbamidomethyl (C)]LIVSVDETIKNPR_.3	#N/A	2.9683	3.7735	3.6551	4.1578	3.5949	4.1346	3.8725	3.9607	3.6093	4.1248	0.9543	-0.0674	0.4771	0.3214
Cct7	_INALTAASEAAC[Carbamidomethyl (C)]LIVSVDETIK_.3	#N/A	3.7472	3.0026	3.4808	3.9122	2.8909	3.7337	3.6269	4.2063	3.8549	3.8792	0.8894	-0.1692	0.1051	0.9785
Cct2	_GATQQILDEAER_.2	#N/A	8.3864	8.3162	8.5423	8.3208	8.1302	8.7331	8.3664	8.3091	8.2435	8.4634	1.0071	0.0102	0.6130	0.2125
Cct2	_[Acetyl (Protein N-term)]ASLSLAPVNIFK_.2	#N/A	6.2708	6.9775	6.6779	6.2861	6.7484	6.6836	6.6854	6.2670	6.0529	6.8678	1.0215	0.0307	0.5158	0.2875
Cct2	_LSSFIGAIAIGDLVK_.2	#N/A	5.1296	4.4632	5.3077	4.7501	4.8714	4.4557	5.2186	5.1871	4.7364	5.4785	0.9369	-0.0941	0.1680	0.7746
Cct2	_EALLSSAVDHGSDEAR_.2	#N/A	6.3298	5.9194	6.0535	5.8434	6.0615	5.5905	5.4530	5.4985	5.5652	5.9538	1.0621	0.0869	0.0552	1.2579
Cct2	_VQDDEVGDGTTSVTVLAAELLR_.2	#N/A	4.8943	4.9330	6.2707	6.3104	4.3596	5.1307	5.4896	5.3267	4.9996	5.2444	1.0098	0.0140	0.9044	0.0436
Cct2	_LAVEAVLR_.2	#N/A	6.6760	4.7806	5.1089	5.2188	4.5484	4.2504	3.7567	5.7254	4.8073	5.2303	1.0445	0.0628	0.7013	0.1541
Cct2	_FWQDLM[Oxidation (M)]NIAGTTLSSK_.2	#N/A	4.9488	3.7100	5.8209	5.4225	4.8621	4.9012	5.0810	5.1862	3.0614	3.1718	1.1986	0.2613	0.2002	0.6986
Cct2	_NIGVDNPAAK_.2	#N/A	2.9768	4.7501	4.2113	3.7551	5.1447	3.0033	4.0330	5.3439	3.5452	4.7296	0.9004	-0.1513	0.4501	0.3467
Cct2	_M[Oxidation (M)]LPTIIADNAGYDSADLVAQLR_.2	#N/A	3.6265	4.1051	4.5680	4.2382	3.7686	3.9246	4.2436	4.4263	3.8395	4.0656	0.9746	-0.0371	0.6131	0.2124
Cct2	_LALVTGGEIASTFDHPELVK_.3	#N/A	3.1363	3.8926	4.0204	4.5230	3.7809	4.3446	4.2377	3.5954	3.2440	2.9892	1.1231	0.1675	0.2229	0.6519
Cct2	_VAEIEHAEKEK_.3	#N/A	3.1927	4.0335	3.8795	4.3822	3.9218	4.2037	4.0969	3.7363	3.3849	4.3492	1.0112	0.0161	0.8739	0.0585
Cct4	_IDDVVNTR_.2	#N/A	6.2568	6.0186	5.6973	5.3544	6.7237	6.5559	5.7028	6.3568	6.3264	6.3337	0.9872	-0.0185	0.7948	0.0997
Cct4	_VIDPATATSVDLR_.2	#N/A	6.3063	6.3801	5.9669	5.9177	5.8713	5.8605	5.8357	5.4687	5.9213	5.5648	1.0619	0.0867	0.0415	1.3824
Cct4	_LVIEEAER_.2	#N/A	5.9602	5.9383	6.3474	5.1549	5.5370	5.4711	5.0470	6.3912	6.3533	5.8643	0.9697	-0.0444	0.6017	0.2206
Cct4	_DALSDLALHFLNK_.2	#N/A	4.2032	4.7733	3.1752	3.8038	3.8186	3.8042	4.2713	4.5687	4.0892	4.1222	0.9219	-0.1174	0.2738	0.5626
Cct4	_ALIAGGGAPEIELALR_.2	#N/A	2.1659	3.7197	3.6202	3.9050	4.2464	3.6977	3.3122	4.3242	4.4830	4.3421	0.8648	-0.2095	0.2263	0.6453
Cct4	_AQDIEAGDGTTSVVIIAGSLLDSC[Carbamidomethyl (C)]TK_.3	#N/A	3.7388	2.7304	3.8607	4.0272	2.8462	3.7410	3.2532	4.0412	1.7353	4.1381	1.0604	0.0846	0.7136	0.1465
Cct4	_ITGC[Carbamidomethyl (C)]TSPGK_.2	#N/A	4.8057	3.9745	4.6024	4.1462	4.9343	3.0258	4.2945	4.1136	4.6686	3.8235	1.0055	0.0079	0.9537	0.0206
Cct4	_VANSGITR_.2	#N/A	3.6194	3.0044	3.3531	4.3977	5.1042	3.8615	4.5460	4.1043	4.0221	4.3136	0.9161	-0.1265	0.3967	0.4015
Cct4	_AFADAM[Oxidation (M)]EVIPSTLAENAGLNPISTVTELR_.3	#N/A	3.9854	3.2898	3.1714	3.6740	3.1111	3.1176	3.4784	4.4444	4.0930	3.6410	0.8665	-0.2068	0.0721	1.1421
Cct4	_DVEREDIEFIC[Carbamidomethyl (C)]K_.3	#N/A	3.0168	4.3256	4.2513	3.7951	4.1469	3.5826	3.9434	1.2534	3.5051	3.4724	1.2660	0.3402	0.1742	0.7590
Cct5	_VLVDINNPEPLIQTAK_.2	#N/A	6.8510	6.6880	6.6671	7.0389	6.5816	6.8518	6.4328	6.6259	6.4340	6.7840	1.0321	0.0455	0.0871	1.0600
Cct5	_LGFAGVVQEISFGTTK_.2	#N/A	5.4757	4.8155	5.8376	5.1991	4.0181	3.8457	4.5490	4.9983	5.4702	5.5769	0.9450	-0.0817	0.5450	0.2636
Cct5	_[Acetyl (Protein N-term)]ASVGTLAFDEYGRPFLIIK_.2	#N/A	2.9445	2.0264	2.5624	2.9734	4.0876	3.6397	1.5144	3.8115	2.7524	2.9336	1.1039	0.1426	0.6047	0.2184
Cct5	_TSLGPNGLDK_.2	#N/A	5.8123	6.1464	3.2629	6.3227	6.2467	5.7076	3.8448	5.7612	6.4905	5.7031	1.0244	0.0348	0.8620	0.0645
Cct5	_WVGGPEIELIAIATGGR_.2	#N/A	3.3151	3.6211	4.2919	4.5334	4.9352	4.7800	4.5092	4.4640	4.9857	4.9198	0.8997	-0.1525	0.2104	0.6769
Cct5	_ESNPALGIDC[Carbamidomethyl (C)]LHK_.2	#N/A	5.7708	5.7815	3.6625	5.6743	5.6148	4.9503	5.6246	5.7513	4.7678	5.1699	0.9839	-0.0235	0.8567	0.0672
Cct5	_DVDFELIK_.2	#N/A	0.9759	3.2002	3.8759	3.4197	3.2860	3.3387	3.5679	3.5815	3.8806	0.7316	1.0257	0.0366	0.9256	0.0336
Cct5	_DVDFELIKVEGK_.3	#N/A	4.2273	3.1151	3.9610	3.5047	3.3710	3.2536	3.6530	3.4964	3.7955	3.1820	1.0114	0.0164	0.8727	0.0591
Cct6a	_GLVLDHGAR_.2	#N/A	6.2102	6.6146	6.2353	6.7792	6.7924	6.4931	6.0524	6.7983	6.5112	6.4324	1.0112	0.0161	0.6963	0.1572
Cct6a	_GIDPFSLDALAK_.2	#N/A	5.5796	6.3612	6.3769	5.8419	5.7997	5.4994	5.5264	6.1511	5.8139	5.4065	1.0324	0.0460	0.4503	0.3465
Cct6a	_IITEGFEAAK_.2	#N/A	6.1865	6.3194	5.9986	6.4036	5.9587	5.9595	6.2845	6.1857	5.4967	5.8848	1.0293	0.0417	0.3383	0.4707
Cct6a	_VLAQNSGFDLQETLVK_.2	#N/A	3.8883	3.7823	4.1680	4.0294	3.7964	4.1085	4.2416	3.7162	3.9781	4.4869	0.9650	-0.0513	0.3835	0.4163
Cct6a	_GLQDVLR_.2	#N/A	3.8832	3.2879	6.1605	4.0358	4.6463	4.1889	3.5913	4.3998	3.6148	4.6657	1.0735	0.1024	0.5990	0.2226
Cct6a	_VATAQDDITGDGTTSNVLIIGELLK_.3	#N/A	3.7629	4.3266	4.8044	4.6999	3.0569	3.1995	3.8037	2.4852	3.9241	3.6506	1.1469	0.1977	0.3051	0.5155
Cct6a	_EMDRETLIDVAR_.3	#N/A	3.3262	4.0163	4.5607	3.9241	3.9707	4.1547	4.2527	4.3976	3.1958	3.7817	1.0218	0.0311	0.7808	0.1075
Cct6a	_QADLYISEGLHPR_.2	#N/A	3.4415	3.4184	3.8227	3.5660	3.0788	3.2649	4.0601	3.9007	4.0339	3.5330	0.8841	-0.1777	0.0237	1.6255
Cct3	_ISTPVDVNNR_.2	#N/A	8.0550	8.4803	9.0688	8.7001	7.6955	7.6835	8.3791	8.0363	7.9929	8.2269	1.0149	0.0214	0.6917	0.1601
Cct3	_AVAQALEVIPR_.2	#N/A	6.9209	7.5283	7.6446	7.3241	6.9706	7.3195	7.0110	7.5055	7.2100	7.1021	1.0108	0.0154	0.6623	0.1790
Cct3	_IPGGIIEDSC[Carbamidomethyl (C)]VLR_.2	#N/A	6.5003	6.3694	5.9030	6.8130	6.5667	6.4727	6.6641	6.7162	6.9283	6.3321	0.9666	-0.0491	0.2559	0.5919
Cct3	_ELGIWEPLAVK_.2	#N/A	6.3819	6.8613	6.6324	6.1539	6.3153	6.2293	6.6071	6.8222	6.3272	6.2893	0.9873	-0.0184	0.6387	0.1947
Cct3	_EIQVQHPAAK_.2	#N/A	5.9830	5.9460	5.6760	5.5215	6.0483	5.8672	5.6673	5.9725	5.7664	5.6835	1.0118	0.0169	0.5778	0.2382
Cct3	_EILSEVER_.2	#N/A	6.9365	6.6048	7.4026	6.4847	6.7520	7.1759	5.9409	6.8215	6.8824	7.0262	1.0337	0.0479	0.4188	0.3780
Cct3	_TAVETAVLLLR_.2	#N/A	4.5545	3.4698	4.0647	3.4230	5.2732	4.0863	4.5209	3.9896	5.6032	5.9762	0.8253	-0.2769	0.1237	0.9076
Cct3	_IVLLDSSLEYK_.2	#N/A	5.4663	5.3793	5.0150	5.0242	4.7367	5.1066	5.5749	5.3400	5.5148	4.9747	0.9571	-0.0633	0.2203	0.6569
Cct3	_NVLLDPQLVPGGGASEM[Oxidation (M)]AVAHALTEK_.3	#N/A	4.9188	5.3090	5.1927	5.8458	5.1505	4.7713	5.1573	5.3219	5.4492	5.1953	0.9843	-0.0228	0.6852	0.1642
Cct3	_TLIQNC[Carbamidomethyl (C)]GASTIR_.2	#N/A	5.6760	4.7767	5.4885	5.8523	5.5805	3.7001	5.5273	5.5275	5.1172	3.8456	1.0349	0.0495	0.7461	0.1272
Cct3	_WSSLAC[Carbamidomethyl (C)]NIALDAVK_.2	#N/A	3.5678	4.0547	4.2546	3.2564	4.1943	3.2757	3.0288	3.8928	3.0098	2.6998	1.1930	0.2546	0.0840	1.0755
Cct3	_NLQDAM[Oxidation (M)]QVC[Carbamidomethyl (C)]R_.2	#N/A	3.3382	5.5857	5.5674	4.7251	5.8933	4.9820	4.0359	5.1818	3.4459	5.6627	1.0947	0.1305	0.5050	0.2967
Cct3	_QTGAPDAGQE_.1	#N/A	3.7625	3.5799	3.4962	4.5408	2.9062	4.3283	3.1882	3.9612	4.2603	4.2181	0.9647	-0.0519	0.7124	0.1473
Cct3	_KGESQTDIEITREEDFTR_.4	#N/A	3.9203	3.3652	3.4054	3.4184	3.2190	3.2255	3.3349	3.4610	4.1305	3.9540	0.9209	-0.1190	0.1798	0.7453
Ptprn2	_ADSVAGAIQSDPAEGS[Phospho (STY)]QESHGR_.3	#N/A	9.9445	9.8387	10.4535	10.2557	10.0903	10.2424	10.0302	9.6943	9.9366	10.0498	1.0211	0.0302	0.1501	0.8237
Ptprn2	_SEQPEEVLSS[Phospho (STY)]EEETAGVEHVR_.3	#N/A	4.0053	8.6269	9.2702	9.0103	8.5749	9.0224	8.8019	8.7556	9.0398	8.9138	0.9107	-0.1350	0.4636	0.3339
Ptprn2	_INSVSSQFS[Phospho (STY)]DGPM[Oxidation (M)]PS[Phospho (STY)]PSAR_.2	#N/A	3.9500	5.5397	3.8661	3.9193	3.8058	5.9398	3.8554	3.7214	3.6248	5.8107	1.0589	0.0825	0.7071	0.1505
Ptprn2	_SLQQNADNEKWFS[Phospho (STY)]LER_.3	P80560_S139_M1_Ptprn2	4.2496	3.0928	3.9833	3.5271	3.3933	3.2313	3.6753	3.4741	3.7732	3.2043	1.0135	0.0194	0.8529	0.0691
Ptprn2	_KSEQPEEVLS[Phospho (STY)]S[Phospho (STY)]EEETAGVEHVR_.3	#N/A	3.7630	3.5707	3.9546	4.1128	3.6861	4.8735	3.9652	4.3061	3.6296	3.0392	1.0692	0.0965	0.4455	0.3512
Nucb2	_SGRLS[Phospho (STY)]QELDLVSHK_.3	P81117_S89_M1_Nucb2	4.1401	3.3955	3.8737	3.4175	3.2838	3.3408	3.5658	4.9173	3.8827	3.0948	0.9250	-0.1125	0.4356	0.3610
Irs2	_VAS[Phospho (STY)]PTSGLKR_.2	P81122_S1089_M1_Irs2	4.2319	8.7934	3.4179	3.9205	7.8172	8.7726	8.7611	8.7693	8.8013	3.8875	0.8152	-0.2947	0.4153	0.3817
Irs2	_RVS[Phospho (STY)]GDGAQDLDR_.2	P81122_S556_M1_Irs2	7.1350	7.4617	7.7576	8.0249	6.4514	7.3068	7.4591	7.3356	7.5519	7.3374	0.9913	-0.0127	0.8238	0.0842
Irs2	_HNSAS[Phospho (STY)]VENVSLRK_.3	P81122_S1165_M1_Irs2	3.3998	4.1561	3.1335	4.1781	4.0444	5.8911	4.3264	4.3239	3.1221	3.8554	1.0581	0.0814	0.6856	0.1639
Irs2	_TYS[Phospho (STY)]LTTPAR_.2	P81122_S573_M1_Irs2	4.3579	7.4191	4.2996	3.3014	6.7638	7.6901	7.8137	8.0598	2.9648	7.7354	0.8488	-0.2366	0.4819	0.3170
Irs2	_SNT[Phospho (STY)]PESIAETPPAR_.2	P81122_T517_M1_Irs2	6.3808	6.5514	6.8159	7.4320	5.5647	6.4437	6.8274	6.4033	6.9148	7.3726	0.9494	-0.0749	0.3470	0.4596
Irs2	_ASSPAESS[Phospho (STY)]PEDSGYMR_.2	P81122_S728_M1_Irs2	5.6617	3.2420	3.8735	4.8780	3.3970	3.0365	3.5655	3.6233	3.9224	3.9140	1.0688	0.0960	0.6427	0.1920
Irs2	_QVPQPSSAS[Phospho (STY)]LDEYTLMR_.2	P81122_S590_M1_Irs2	3.0985	3.9037	3.7853	4.2879	3.7250	4.0045	4.0026	3.8306	3.4791	4.2549	0.9766	-0.0341	0.7145	0.1460
Irs2	_GPGTGGDEASAAGGS[Phospho (STY)]PPQPPR_.2	P81122_S66_M1_Irs2	3.6642	2.9686	4.3510	3.3528	2.7899	3.4388	3.0675	3.2648	4.4142	3.3198	0.9747	-0.0370	0.8163	0.0881
Stim2	_SIVPSS[Phospho (STY)]PQSQR_.2	P83093_S523_M1_Stim2	3.6918	2.9961	4.3786	3.6237	7.9291	3.4112	3.7720	3.2372	3.6765	3.3474	1.2366	0.3064	0.3991	0.3989
Stim2	_GS[Phospho (STY)]PEC[Carbamidomethyl (C)]VGLTETK_.2	#N/A	7.8061	7.3749	7.3319	7.8375	6.4458	7.3199	8.1608	3.0809	7.5785	7.2041	1.1301	0.1765	0.3994	0.3986
Stim2	_GESPVTADVSRGS[Phospho (STY)]PEC[Carbamidomethyl (C)]VGLTETK_.3	#N/A	3.3643	7.1327	4.0511	8.1711	6.6544	3.7387	7.5862	7.8395	7.5182	7.3547	0.7286	-0.4568	0.0850	1.0707
Stim2	_DELS[Phospho (STY)]LEDSSR_.2	P83093_S631_M1_Stim2	4.0489	6.3749	3.1078	6.7730	3.1926	3.4320	5.8537	6.6588	6.4831	6.8561	0.6945	-0.5260	0.0510	1.2924
Stim2	_DELSLEDSSRGES[Phospho (STY)]PVTADVSR_.3	P83093_S640_M1_Stim2	4.1249	3.3804	3.8586	3.4024	3.2687	3.3559	3.5507	3.5988	3.8978	3.0797	1.0095	0.0136	0.8834	0.0538
Stim2	_RAS[Phospho (STY)]GSAGAAASPSAAAAGER_.2	P83093_S28_M1_Stim2	3.1267	3.9319	3.8135	4.3162	3.7533	3.9762	4.3370	3.8023	3.4509	4.2832	0.9625	-0.0551	0.5832	0.2342
Stim2	_S[Phospho (STY)]MIFSPASR_.2	P83093_S661_M1_Stim2	3.0841	3.8404	4.3186	3.8624	3.7287	4.3968	4.2899	3.1388	3.4378	3.5397	1.0750	0.1044	0.4075	0.3898
Tnik	_QNS[Phospho (STY)]DPTSENPPLPTR_.2	P83510_S611_M1_Tnik	12.2532	12.0527	12.8571	12.2162	11.1939	12.0408	12.7318	12.2084	12.2549	11.9496	0.9850	-0.0218	0.5601	0.2518
Tnik	_TTSIS[Phospho (STY)]PALAR_.2	P83510_S651_M1_Tnik	7.7346	7.6575	8.2845	3.8177	3.8605	6.1159	4.4144	3.8939	5.7065	5.8890	1.2551	0.3278	0.2779	0.5561
Tnik	_ANS[Phospho (STY)]KSEGS[Phospho (STY)]PVLPHEPSK_.2	#N/A	3.9267	7.7954	3.6865	8.6152	5.1043	3.9532	6.6123	6.7067	6.9123	3.9649	0.9115	-0.1337	0.6752	0.1706
Tnik	_VRANS[Phospho (STY)]KSEGS[Phospho (STY)]PVLPHEPSK_.3	#N/A	8.4104	9.3558	3.9441	3.9891	8.1312	8.3136	8.6148	8.7499	8.5765	8.1059	0.8252	-0.2772	0.2620	0.5817
Tnik	_SES[Phospho (STY)]FSISGVQPAR_.2	P83510_S541_M1_Tnik	3.2889	3.9503	3.9757	5.7610	4.0087	3.9385	4.1931	3.6532	5.9433	5.2695	0.8718	-0.1979	0.3306	0.4807

Tnik	_KNS[Phospho (STY)]PGNGSALGPR_.2	#N/A	7.0729	7.2285	7.5730	7.5062	5.7535	7.1495	7.6000	7.3093	7.0946	7.1257	0.9677	-0.0473	0.5219	0.2824
Tnik	_SEGS[Phospho (STY)]PVLPHEPSK_.2	P83510_S740_M1_Tnik	12.9432	12.9559	13.7559	13.2201	12.0491	12.7837	13.6639	13.2442	13.0028	12.7930	0.9830	-0.0248	0.5042	0.2974
Tnik	_VYQTSPT[Phospho (STY)]DEDEEDDESSAAALFTSELLR_.3	P83510_T950_M1_Tnik	3.5247	4.2810	3.2584	4.3030	4.1693	3.9561	4.4513	4.1990	2.9972	3.9803	1.0022	0.0031	0.9804	0.0086
Tnik	_AS[Phospho (STY)]NPDLR_.2	#N/A	3.5641	3.7783	3.2977	4.3424	4.2086	5.4712	3.2641	4.1597	2.9579	4.0196	1.1417	0.1912	0.2961	0.5285
Tnik	_LGSQPIRAS[Phospho (STY)]NPDLR_.3	#N/A	4.2732	3.5776	3.4985	4.5431	3.3989	4.4306	3.1905	3.9589	4.2580	4.2204	1.0077	0.0110	0.9278	0.0325
Wnk1	_GTEDGSGSPHS[Phospho (STY)]PPHLC[Carbamidomethyl (C)]SK_.3	P83741_S2027_M1_Wnk1	5.2449	4.9010	3.9862	6.1177	5.5728	4.2585	5.4634	5.7079	6.0174	5.5198	0.8831	-0.1793	0.1540	0.8124
Rpl36a	_LEC[Carbamidomethyl (C)]VEPNC[Carbamidomethyl (C)]R_.2	#N/A	6.8809	6.8350	7.1102	6.9681	7.3124	6.3488	7.3645	7.0944	6.7697	7.2193	0.9715	-0.0417	0.3255	0.4874
Cbx1	_KKVEEVLEEEEEEYVVEK_.3	#N/A	4.8237	5.5225	6.5164	6.3457	4.7183	5.2497	5.9656	3.8428	4.4351	5.8590	1.1002	0.1378	0.4012	0.3966
Cbx1	_VEEVLEEEEEEYVVEK_.2	#N/A	3.0549	4.1745	6.1544	4.6585	3.6814	5.6611	3.9590	3.8741	3.5227	4.2114	1.1728	0.2299	0.3040	0.5172
Cbx1	_ADS[Phospho (STY)]DSEDKGEESKPK_.2	P83917_S89_M1_Cbx1	3.9107	3.1661	3.6444	1.9641	3.0544	3.5702	3.4633	4.3698	4.0184	4.4663	0.7938	-0.3332	0.0649	1.1877
Eloc	_EIPSHVLSK_.2	#N/A	6.4095	6.6583	6.5242	6.4516	7.1974	6.0404	6.2214	6.4980	6.9160	6.5032	1.0019	0.0027	0.9579	0.0187
Eloc	_LISSDGHEFIVK_.2	#N/A	4.1777	4.6086	4.0805	4.8069	5.0626	3.8384	4.7051	4.4473	4.6622	4.6957	0.9571	-0.0632	0.4416	0.3549
Eloc	_EHALTSGTIK_.2	#N/A	5.6629	6.0483	5.7259	6.1460	6.3046	6.2668	5.7563	6.0028	5.8473	5.9874	1.0216	0.0308	0.4112	0.3859
Hpca	_GAKS[Phospho (STY)]DPSIVR_.2	P84075_S175_M1_Hpca	9.9714	10.2499	10.7078	10.5556	9.9565	10.2211	10.5124	10.2717	10.3078	10.1618	0.9965	-0.0051	0.8318	0.0800
Hpca	_LSLEEFIR_.2	#N/A	8.7457	8.9836	8.5000	8.7268	8.5961	8.6735	8.5271	9.0749	8.5974	8.5048	1.0033	0.0047	0.8400	0.0757
Hpca	_FAEHVFR_.2	#N/A	8.5793	8.9092	8.8182	8.6007	8.9894	8.4703	8.9058	8.8148	8.6764	8.6760	0.9954	-0.0067	0.7335	0.1346
Hpca	_IYANFFPYGDASK_.2	#N/A	8.4124	8.3462	8.6748	8.5437	8.3460	8.5184	8.5440	8.3380	8.4712	8.4285	1.0033	0.0048	0.7147	0.1458
Hpca	_ENTEFSELELQEWYK_.2	#N/A	7.3230	6.4959	7.5176	7.6479	7.1468	7.2904	7.3884	6.4675	6.3303	7.1658	1.0583	0.0818	0.2069	0.6841
Hpca	_LLQC[Carbamidomethyl (C)]DPSSASQF_.2	#N/A	6.6715	6.3849	6.6732	6.4302	6.5211	6.3111	6.6877	6.3177	6.5515	6.1618	1.0107	0.0154	0.5830	0.2344
Hpca	_TFDTNSDGTIDFR_.2	#N/A	6.0767	6.2216	4.5455	6.2263	6.3170	3.3688	5.7185	6.9008	5.6880	5.6514	0.9115	-0.1338	0.4510	0.3459
Hpca	_EEM[Oxidation (M)]LEIVQAIYK_.2	#N/A	5.1628	3.2468	4.5972	5.0202	4.6945	4.5847	5.2708	4.4250	4.4184	4.8622	0.9593	-0.0599	0.6279	0.2021
Hpca	_LRPEMLQDLR_.2	#N/A	5.3490	5.0739	3.6937	6.1024	4.7833	3.4458	4.7544	3.9140	5.4002	3.6245	1.0719	0.1002	0.6144	0.2116
Hpca	_DC[Carbamidomethyl (C)]PTGILNVDEFKK_.3	#N/A	4.1347	3.4391	3.3207	3.8234	0.1816	2.9682	3.3290	4.2951	3.9437	2.8415	0.8267	-0.2746	0.4422	0.3544
Hpca	_MPEDESTPEKR_.2	#N/A	4.9429	4.3689	4.2860	5.3052	5.0885	3.7403	5.0155	3.8684	3.1650	3.3608	1.1998	0.2627	0.1223	0.9126
Arf5	_VQESADELQK_.2	#N/A	5.5588	6.1543	6.3110	6.2854	5.3320	5.3735	6.1903	5.7678	5.2660	5.4628	1.0289	0.0412	0.5806	0.2361
Cplx2	_AALEQPC[Carbamidomethyl (C)]EGS[Phospho (STY)]LTRPK_.2	#N/A	9.3011	9.5841	9.7925	9.9111	9.4773	9.5369	9.6471	9.2002	7.3058	9.4041	1.0800	0.1110	0.1432	0.8440
Cplx2	_KAIPAGC[Carbamidomethyl (C)]GDEEEEEEESILDTVLK_.3	#N/A	8.3603	8.7845	9.5555	9.6982	9.1962	9.1149	8.9441	8.8247	8.4762	8.9584	1.0361	0.0511	0.2690	0.5703
Cplx2	_AIPAGC[Carbamidomethyl (C)]GDEEEEEEESILDTVLK_.3	#N/A	6.5575	6.5388	7.1750	7.3151	7.1279	7.0149	6.5045	6.4271	6.0445	7.0335	1.0696	0.0971	0.0878	1.0565
Cplx2	_YLPGPLQDMFK_.2	#N/A	7.9700	6.7620	3.4646	8.7055	7.6943	4.3252	6.7531	6.0109	6.0355	6.8734	1.0107	0.0154	0.9516	0.0215
Erh	_[Acetyl (Protein N-term)]SHTILLVQPTK_.2	#N/A	6.4609	6.7663	6.9732	7.2107	7.1849	7.1280	6.5925	6.4126	6.8223	6.5692	1.0538	0.0756	0.0619	1.2084
Erh	_TYADYESVNEC[Carbamidomethyl (C)]M[Oxidation (M)]EGVC[Carbamidomethyl (C)]K_.2	#N/A	4.8280	5.0165	6.0939	6.0474	4.7015	5.4104	5.6121	4.8544	5.2479	5.5186	1.0078	0.0112	0.9057	0.0430
Erh	_MYEEHLK_.2	#N/A	4.1316	3.2108	3.8653	2.0425	3.0114	3.3493	3.5573	1.6224	3.8912	1.9985	1.1811	0.2401	0.4131	0.3839
Erh	_ADTQTYQPYNK_.2	#N/A	2.9504	4.2758	3.6372	4.1398	3.5769	4.1526	3.8545	3.9786	3.6272	4.1068	0.9735	-0.0387	0.7116	0.1477
Ap2m1	_TFITQQGIK_.2	#N/A	8.3957	8.4699	8.5220	7.7689	8.3483	8.0730	8.4923	8.6689	8.6898	8.1261	0.9728	-0.0398	0.2338	0.6311
Ap2m1	_ESQISAEIELLPTNDK_.2	#N/A	7.7178	7.4537	7.4065	7.1528	6.6008	7.3259	7.5074	7.3214	7.6022	7.8565	0.9610	-0.0575	0.2015	0.6958
Ap2m1	_VNVIHAR_.2	#N/A	7.1071	7.5914	7.3323	6.7240	7.3875	6.9341	7.4890	7.4923	7.5030	7.1320	0.9697	-0.0445	0.2424	0.6155
Ap2m1	_DIILPFR_.2	#N/A	6.5005	6.7344	6.2128	5.9004	6.1416	6.5475	5.4111	7.0718	6.8024	6.8375	0.9707	-0.0429	0.5866	0.2316
Ap2m1	_VYRDDIGR_.2	#N/A	5.6183	6.3812	6.3459	5.4532	5.7783	5.3488	5.4338	5.9408	5.8208	6.0037	1.0036	0.0053	0.9343	0.0295
Ap2m1	_TSFFHVK_.2	#N/A	5.6224	5.9091	6.1572	5.5644	5.5746	5.1047	5.8405	6.2493	5.9202	5.3393	0.9688	-0.0457	0.4610	0.3363
Ap2m1	_M[Oxidation (M)]IGGLFIYNHK_.2	#N/A	5.6185	5.9645	6.5914	5.3240	5.1332	5.5145	5.4923	6.6554	5.8657	3.4376	1.0612	0.0857	0.6019	0.2205
Ap2m1	_ASENAIVWK_.2	#N/A	6.2875	5.4010	6.0287	5.6853	4.6190	5.8773	4.9458	5.9702	6.0785	5.2546	1.0157	0.0225	0.8192	0.0866
Ap2m1	_SNIWLAAVTK_.2	#N/A	3.0177	5.7448	5.7346	5.7495	4.4207	4.8873	5.4904	6.3404	5.7748	5.7965	0.8419	-0.2482	0.1445	0.8401
Ap2m1	_S[Phospho (STY)]PVTNIAR_.2	P84091_S45_M1_Ap2m1	7.4240	6.9340	5.1986	6.9981	5.6812	6.8861	7.2890	7.3278	7.3097	7.2044	0.8953	-0.1595	0.1253	0.9019
Ap2m1	_SGIYETR_.2	#N/A	5.2114	4.2784	3.5669	4.6115	4.8004	3.6477	3.2589	5.5140	4.3538	4.7501	0.9739	-0.0381	0.8211	0.0856
Ap2m1	_QSIAIDDC[Carbamidomethyl (C)]TFHQC[Carbamidomethyl (C)]VR_.3	#N/A	5.5293	5.0378	5.7178	5.3734	5.2398	4.9191	5.5106	5.4140	5.0896	4.9947	1.0096	0.0138	0.7878	0.1036
Ap2m1	_LNYSDHDVIK_.2	#N/A	6.4330	6.0905	6.3572	6.4001	6.0641	5.9602	6.7421	6.3498	6.2511	6.2328	0.9724	-0.0404	0.2421	0.6159
Ap2m1	_LS[Phospho (STY)]KFDSER_.2	P84091_S255_M1_Ap2m1	3.7938	3.0492	3.5275	3.8656	2.9375	3.6871	3.5802	3.9299	4.2290	4.2494	0.8698	-0.2012	0.0584	1.2336
Ap2m1	_EEQSQITSQVTGQIGWR_.2	#N/A	3.1119	3.9171	3.7987	4.2036	3.7384	3.9911	4.3519	3.8172	3.4657	4.2683	0.9541	-0.0677	0.4870	0.3124
Rhog	_EYIPTVFDNYSAQSAVDGR_.2	#N/A	4.9575	4.1735	3.9170	3.4608	3.3271	3.2975	3.6091	5.3063	5.2333	4.7352	0.8167	-0.2921	0.0913	1.0393
Rhog	_YLEC[Carbamidomethyl (C)]SALQQDGVK_.2	#N/A	4.0475	4.6589	3.2335	3.7361	3.1732	3.0555	3.4508	4.4239	4.0309	3.7031	0.9356	-0.0961	0.5031	0.2983
Rpl19	_VWLDPNETNEIANANSR_.2	#N/A	8.3392	8.4149	9.0553	8.9169	7.8401	8.2685	8.7383	8.3946	8.3049	8.1892	1.0078	0.0112	0.7966	0.0988
Rpl19	_LLADQAEAR_.2	#N/A	6.7388	6.7088	6.9042	6.6363	7.1688	6.7047	6.8136	7.3431	7.1431	6.8875	0.9664	-0.0493	0.1272	0.8954
Rpl19	_TLS[Phospho (STY)]KEETKK_.2	P84099_S189_M1_Rpl19	4.5862	5.2723	4.8916	5.9064	3.4923	4.4765	5.0120	5.8815	3.6742	3.3033	1.0678	0.0947	0.6427	0.1920
Srsf3	_S[Phospho (STY)]RS[Phospho (STY)]LS[Phospho (STY)]RDR_.2	P84104_S126_M3_Srsf3	6.6095	6.4426	6.2585	6.8681	6.4553	6.4511	6.8447	6.5000	7.2367	5.7566	0.9893	-0.0155	0.8010	0.0964
Srsf3	_RRS[Phospho (STY)]PPPR_.2	P84104_S108_M1_Srsf3	8.4872	4.3034	8.2216	7.9564	5.9688	5.2343	8.5541	8.2836	6.4240	6.5731	0.8976	-0.1558	0.4677	0.3300
Srsf3	_NPPGFAFVEFEDPRDAADAVR_.3	#N/A	4.8848	5.3047	3.7387	3.6809	4.3254	3.4684	3.8292	5.3257	4.7624	3.3981	0.9765	-0.0343	0.8464	0.0724
Srsf3	_NPPGFAFVEFEDPR_.2	#N/A	5.4558	4.1181	4.5963	4.1401	3.6520	4.1191	5.8538	4.6027	6.0424	3.8174	0.8558	-0.2246	0.1997	0.6995
Srsf3	_ERS[Phospho (STY)]LS[Phospho (STY)]RER_.2	#N/A	8.6393	8.5292	9.0176	8.6267	8.8406	8.6730	8.9069	8.6433	8.8904	8.2540	1.0055	0.0079	0.7609	0.1187
Srsf3	_VYVGNLGNNGNK_.2	#N/A	6.6399	6.9559	3.7018	7.5844	6.7096	3.5127	5.1517	7.5668	6.8850	4.4237	0.9740	-0.0380	0.8879	0.0516
Srsf3	_NHKPS[Phospho (STY)]RS[Phospho (STY)]FSR_.3	P84104_S148_M2_Srsf3	3.2882	4.0445	4.5227	4.0665	3.9328	4.1927	4.2147	2.5614	3.2338	3.7437	1.1656	0.2211	0.1411	0.8505
Srsf3	Acetyl (Protein N-term)]MHRDS[Phospho (STY)]C[Carbamidomethyl (C)]PLDC[Carbamidomethyl (C)]K_	P84104_S5_M1_Srsf3	3.5471	4.3034	3.2808	4.1122	4.1917	3.9337	3.8269	4.0063	3.6548	4.0792	1.0008	0.0011	0.9892	0.0047
Adcy5	_SVS[Phospho (STY)]PPGYAAQTAASPAPR_.2	P84309_S8_M1_Adcy5	10.3167	6.4352	11.1911	10.6721	8.9865	10.0591	11.0561	11.0475	10.4680	10.0916	0.8541	-0.2274	0.3237	0.4899
Adcy5	_SGGAVT[Phospho (STY)]PQQQQR_.2	P84309_T62_M1_Adcy5	5.9291	6.3853	4.3901	5.9118	3.8002	3.7214	6.5707	6.5263	5.9019	5.3011	0.8268	-0.2743	0.1447	0.8394
Adcy5	_S[Phospho (STY)]VELGLEER_.2	P84309_S156_M1_Adcy5	3.7036	7.7428	3.4373	8.5989	6.8043	8.1171	7.6985	8.8422	8.3518	8.7103	0.7619	-0.3923	0.1276	0.8941
Adcy5	_DVAAHFLAR_.2	#N/A	4.3943	4.3416	4.2017	4.3093	4.1414	3.6288	4.4190	4.6229	4.2500	3.7240	0.9801	-0.0289	0.6966	0.1570
Adcy5	_ILGDC[Carbamidomethyl (C)]YYC[Carbamidomethyl (C)]VSGLPEAR_.2	#N/A	3.9219	4.3111	3.1079	3.6105	3.1926	3.4321	3.3252	5.3684	4.1565	3.5775	0.8756	-0.1917	0.2676	0.5725
Isoc2a	_DASHPQFK_.2	#N/A	4.6951	3.9416	3.1345	4.1791	4.0454	4.4723	4.2145	4.5674	4.1208	4.3711	0.9559	-0.0651	0.5600	0.2518
Isoc2a	_QSGAFLATSESLILQLVR_.2	#N/A	2.9387	3.6949	4.1732	3.7170	3.5832	3.0414	3.8652	3.2842	3.5833	3.3942	0.9980	-0.0029	0.9789	0.0093
Rpl10l;Rpl10	_LIPDGC[Carbamidomethyl (C)]GVK_.2	#N/A	4.6779	3.1774	4.5599	5.5602	6.1062	3.2299	3.2764	5.7681	4.2054	3.5287	1.0852	0.1179	0.6473	0.1889
Cdh8	_LGELYSVGES[Phospho (STY)]DKET_.2	P97291_S795_M1_Cdh8	3.3960	6.2685	4.0828	7.4580	4.0225	3.7070	6.7562	3.5331	6.7954	6.7564	0.8091	-0.3056	0.3121	0.5056
Hrh2	_LNNS[Phospho (STY)]LLSR_.2	P97292_S321_M1_Hrh2	4.2307	3.4861	3.9644	5.0603	3.3744	3.2502	5.9956	3.1890	4.6233	3.3957	0.9055	-0.1433	0.5299	0.2758
Nptn	_QNPSITWIR_.2	#N/A	8.0061	8.3274	7.6724	8.2007	7.4190	8.0289	7.9671	8.2880	7.8873	8.0059	0.9882	-0.0171	0.6246	0.2044
Nptn	_IVTSEEVIIR_.2	#N/A	7.2292	7.1180	7.2979	6.7271	7.8772	7.8127	7.6734	6.6220	7.3303	7.4593	1.0100	0.0143	0.8066	0.0934
Nptn	_AAPDITGHK_.2	#N/A	7.4241	6.9081	7.1484	7.3790	7.7712	6.9102	7.0313	7.4658	7.0886	7.5505	0.9963	-0.0054	0.8952	0.0481
Nptn	_SVGYPHPEWIWR_.2	#N/A	7.1288	7.2729	7.1809	7.7948	6.5821	7.2534	7.7539	7.5469	7.1452	6.9447	0.9802	-0.0289	0.5698	0.2443
Nptn	_VTVNTAYGSNGVSVLR_.2	#N/A	6.1041	5.4777	5.7552	5.2231	5.6996	5.5670	6.2011	5.4698	6.1036	6.0344	0.9472	-0.0783	0.1531	0.8150
Nptn	_QLWDGAR_.2	#N/A	5.9895	6.2305	5.6691	5.5189	5.7986	5.7713	6.4085	6.3360	5.8302	5.7468	0.9588	-0.0608	0.2132	0.6713
Nptn	_LTLEDSGTYEC[Carbamidomethyl (C)]R_.2	#N/A	5.5007	5.2130	4.5175	6.0093	5.1462	3.8487	4.2095	5.6383	4.9110	5.0240	1.0189	0.0270	0.8411	0.0752
Nptn	_NGVELTATR_.2	#N/A	6.1016	7.8213	3.6830	3.2268	3.2144	7.8823	3.3751	6.1232	8.0437	6.2547	0.8945	-0.1608	0.6592	0.1810

Nptn	_AEDSGEYHC[Carbamidomethyl (C)]VYHFVSAPK_.3	#N/A	5.9644	5.4345	6.0454	6.0513	5.0301	5.6688	5.8621	5.1214	4.9355	5.6842	1.0552	0.0776	0.3047	0.5161
Nptn	_SENKNEGQDAMMYC[Carbamidomethyl (C)]K_.3	#N/A	3.6567	3.7218	4.1473	3.6911	3.5842	3.7552	3.8393	3.4132	3.7123	4.1123	0.9974	-0.0038	0.9501	0.0222
Csrp1	_C[Carbamidomethyl (C)]SQAVYAAEK_.2	#N/A	5.3390	5.7785	5.7647	6.2446	6.3787	6.1567	5.3999	5.4657	6.3882	5.7649	1.0328	0.0466	0.4979	0.3029
Csrp1	_GFGFGQGAGALVHSE_.2	#N/A	5.6531	7.6343	8.3799	8.9900	8.1916	7.8389	8.0093	8.2256	7.9159	7.6454	0.9789	-0.0308	0.7839	0.1058
Csrp1	_GYGYGQGAGTLSTDKGESLGIK_.3	#N/A	6.2955	5.7805	4.3742	6.6688	6.7377	5.7939	5.4191	5.4924	5.3531	5.9255	1.0711	0.0990	0.4124	0.3846
Csrp1	_TVYFAEEVQC[Carbamidomethyl (C)]EGNSFHK_.2	#N/A	4.5844	5.0899	6.1948	6.0023	4.4915	5.5529	4.4546	3.3794	4.8128	5.0179	1.2045	0.2684	0.0883	1.0538
Csrp1	_HEEAPGHRPTTNPNASK_.4	#N/A	3.5713	4.1249	3.8651	4.0588	9.4329	3.7358	3.9525	3.8147	3.7728	3.6763	1.2613	0.3349	0.4180	0.3788
Cdh6	_LADMYGGM[Oxidation (M)]DS[Phospho (STY)]DKDS_.2	#N/A	3.6434	3.9002	3.9225	7.4131	3.3325	3.8893	4.7431	3.6660	3.9651	4.3980	1.0375	0.0531	0.8486	0.0713
Rps3a	_LITEDVQGK_.2	#N/A	6.8254	6.5966	7.2708	6.5292	7.0940	6.8560	6.3066	6.8414	6.8056	6.9517	1.0202	0.0288	0.4819	0.3170
Rps3a	_TTDGYLLR_.2	#N/A	6.8193	6.6185	6.5966	6.4348	6.8753	6.3128	6.1297	6.9562	6.6270	6.2218	1.0194	0.0277	0.5197	0.2843
Rps3a	_ADGYEPPVQES[Phospho (STY)]V_.2	P97351_S263_M1_Rps3a	8.8876	8.9429	9.3235	9.2188	8.4831	9.0681	9.3791	9.0281	9.0557	9.0162	0.9855	-0.0211	0.4483	0.3485
Rps3a	_VFEVSLADLQNDEVAFR_.2	#N/A	4.9033	4.2748	5.7075	3.5474	4.0055	5.4586	5.6467	4.3409	4.3298	4.5990	0.9832	-0.0245	0.8772	0.0569
Rps3a	_KDWYDVK_.2	#N/A	4.0606	3.4643	3.3459	3.1556	4.1550	4.6459	2.3100	4.2699	4.1447	4.1983	1.0198	0.0283	0.8805	0.0553
Rps3a	_EVQTNDLKEVVNK_.3	#N/A	3.6453	3.6971	3.3790	4.4236	3.5184	4.2111	3.0710	4.0784	4.3775	4.1009	0.9758	-0.0353	0.7676	0.1149
Rps3a	_LIPDSIGKDIEK_.3	#N/A	4.1371	3.4414	3.6346	3.1784	3.2628	2.9659	3.3267	3.8228	4.1218	4.3565	0.8796	-0.1850	0.1243	0.9054
S100a13	_KGS[Phospho (STY)]LNINEFK_.2	P97352_S32_M1_S100a13	4.9357	4.5363	4.3864	4.8352	4.5552	4.2928	5.1479	5.4125	5.1614	5.2923	0.8737	-0.1947	0.0013	2.8925
Evi5	_SDS[Phospho (STY)]NQYIR_.2	P97366_S689_M1_Evi5	3.9598	3.2641	3.1457	5.1558	3.0855	3.1432	3.5040	2.9692	4.1187	3.6154	1.0208	0.0297	0.8755	0.0577
Atp1b3	_LFIYNPSSGEFLGR_.2	#N/A	6.4226	6.1588	6.2511	5.9860	6.4983	5.6751	6.7331	6.1479	6.0010	5.9058	0.9949	-0.0074	0.8854	0.0528
Atp1b3	_FDSGLNKK_.2	#N/A	5.3913	4.6363	5.2652	3.8965	4.9268	4.3627	4.0447	3.1047	3.4038	5.1097	1.2122	0.2776	0.1048	0.9795
G3bp2	_SAT[Phospho (STY)]PPPAEPASLPQEPPK_.2	P97379_T227_M1_G3bp2	14.8380	14.8317	15.4274	15.3906	14.5841	14.8766	15.3707	14.6729	14.8250	15.0148	1.0014	0.0020	0.9246	0.0340
G3bp2	_LPNFGFVVFDDSEPVQR_.2	#N/A	3.0562	3.6431	4.4085	4.2551	5.0201	4.3201	4.4056	3.5883	3.4484	3.9453	1.0703	0.0980	0.5034	0.2980
G3bp2	_VDAKPEVQSQPPR_.3	#N/A	4.7601	4.1426	3.9245	3.7827	5.3516	3.8652	4.1419	3.6913	3.9325	5.3604	1.0054	0.0077	0.9590	0.0182
G3bp2	_HLEELEEKS[Phospho (STY)]AT[Phospho (STY)]PPPAEPASLPQEPPK_.3	P97379_S225_M2_G3bp2	3.9345	3.1899	3.6682	6.8077	3.0782	3.5464	3.3602	4.7874	4.0883	4.3900	0.9714	-0.0419	0.8778	0.0566
G3bp2	_GDMEQNDSDNRR_.2	#N/A	3.1933	3.9496	4.4278	4.4660	3.8379	4.2876	4.1807	3.0296	3.3010	3.6489	1.1376	0.1859	0.1600	0.7958
Anxa11	_DVQELYAAGENR_.2	#N/A	7.5435	7.4900	8.3730	7.7436	7.2092	7.2014	8.2198	7.7658	7.3206	7.8304	0.9755	-0.0358	0.4927	0.3074
Anxa11	_SDTSGHFQR_.2	#N/A	5.1052	4.3769	4.2585	3.2603	4.1982	3.5313	4.4758	3.3573	4.5835	3.2273	1.0539	0.0757	0.6437	0.1913
Arhgap5	_GSEEDPLLS[Phospho (STY)]PVETWK_.2	#N/A	4.2078	3.1346	3.9415	7.0316	3.3515	6.1910	7.3110	7.1896	6.6769	3.1625	0.7630	-0.3902	0.2362	0.6268
Arhgap5	_GGIDNPAITS[Phospho (STY)]DQEVDDKK_.2	#N/A	3.8343	3.9181	3.7997	3.5230	4.9239	3.3382	3.9575	3.9437	3.5923	3.4900	1.0384	0.0543	0.6414	0.1928
Arhgap5	_HRGSEEDPLLS[Phospho (STY)]PVETWK_.3	#N/A	3.9653	4.0904	3.5705	6.3653	4.4724	4.4180	4.5646	6.6674	4.3105	4.5064	0.8939	-0.1619	0.4469	0.3498
Lyst	_QPEPFS[Phospho (STY)]PR_.2	P97412_S375_M1_Lyst	7.2544	7.0248	6.9011	6.1864	4.7317	6.5257	7.8254	7.8448	7.2057	6.8299	0.8668	-0.2062	0.0872	1.0597
Lyst	_RM[Oxidation (M)]S[Phospho (STY)]QEHPSQASEAELAQR_.3	P97412_S2614_M1_Lyst	3.9309	3.2952	4.6709	3.6196	3.1835	3.4411	4.3173	4.2625	4.6356	3.0485	0.9076	-0.1399	0.3666	0.4358
Lyst	_STANHDSES[Phospho (STY)]PVHS[Phospho (STY)]PSAHR_.3	P97412_S2560_M2_Lyst	5.0107	4.4077	5.6413	4.5011	3.9382	4.5698	5.1304	5.5657	3.2660	4.6776	1.0039	0.0056	0.9715	0.0125
Lyst	_MFLAYQDQS[Phospho (STY)]FDIPDR_.2	P97412_S3251_M1_Lyst	2.9531	3.7583	3.6399	4.3624	3.5796	4.1499	4.5107	4.1396	4.4386	4.1095	0.8700	-0.2010	0.0669	1.1745
Crmp1	_KS[Phospho (STY)]IPHITSDR_.2	#N/A	8.6119	8.8417	9.3180	9.4027	8.5119	8.9697	9.0498	8.7448	8.6242	9.0249	1.0092	0.0132	0.6977	0.1563
Crmp1	_DNFTLIPEGVNGIEER_.2	#N/A	10.0419	9.9002	10.1663	10.3808	10.0443	9.9851	10.1105	9.7609	9.8883	9.8815	1.0178	0.0254	0.1270	0.8961
Crmp1	_VFGLHSVSR_.2	#N/A	7.8248	8.3976	8.2212	8.9511	7.7667	7.7258	8.4327	8.2196	7.9487	7.8657	1.0038	0.0055	0.9091	0.0414
Crmp1	_SAADIIALAR_.2	#N/A	8.9830	9.3385	9.3868	9.0428	8.9738	9.1572	9.1324	9.1599	9.0680	9.2636	0.9990	-0.0014	0.9288	0.0321
Crmp1	_IFNLYPR_.2	#N/A	8.9095	8.5427	8.9871	8.6553	8.9967	8.9311	8.7333	8.6026	8.7038	8.9358	1.0107	0.0153	0.4291	0.3674
Crmp1	_INC[Carbamidomethyl (C)]PVYITK_.2	#N/A	8.2983	8.2963	8.2704	8.5713	8.8304	8.1394	8.4496	8.2884	8.3890	8.3330	1.0043	0.0062	0.7920	0.1013
Crmp1	_IVFEDGNISVSK_.2	#N/A	7.4210	7.3470	7.6143	7.9310	7.7468	7.5616	7.5495	7.4022	7.6793	7.7790	1.0001	0.0002	0.9934	0.0029
Crmp1	_KPFPEHLYQR_.3	#N/A	7.5081	7.7294	7.4081	7.6836	8.0915	7.5148	6.5489	7.3398	6.9225	7.5119	1.0812	0.1127	0.0263	1.5793
Crmp1	_AALAGGTTM[Oxidation (M)]IIDHVVPEPGSSLLTSFEK_.3	#N/A	6.6830	6.1168	6.6548	6.5302	6.3306	6.2829	7.0555	6.0698	6.1295	6.7197	0.9907	-0.0135	0.7905	0.1021
Crmp1	_WHEAADTK_.2	#N/A	5.8814	6.0330	5.9332	6.1030	6.6748	5.7928	6.1668	6.1883	5.0385	5.6245	1.0548	0.0769	0.2747	0.5611
Crmp1	_DLYQM[Oxidation (M)]SDSQLYEAFTFLK_.2	#N/A	5.1643	5.5835	7.0603	7.4175	6.5258	6.4438	6.3787	5.4434	5.7549	6.0614	1.0772	0.1073	0.3557	0.4489
Crmp1	_IINDDQSFYADVYLEDGLIK_.2	#N/A	5.3262	5.5569	6.6141	6.3273	5.4317	5.8325	5.9894	5.7590	5.2264	5.9808	1.0190	0.0272	0.7255	0.1394
Crmp1	_STVEYNIFEGM[Oxidation (M)]EC[Carbamidomethyl (C)]HGSPLVVISQGK_.3	#N/A	5.3831	5.4664	6.3984	5.8020	5.4519	5.0151	5.9022	4.9585	5.3064	4.9213	1.0596	0.0835	0.3254	0.4875
Crmp1	_HAAPAPS[Phospho (STY)]AK_.2	#N/A	10.0159	9.7346	10.0818	10.4110	8.2766	9.4259	9.9768	9.8784	9.5853	10.0219	0.9789	-0.0307	0.6106	0.2142
Crmp1	_S[Phospho (STY)]IPHITSDR_.2	#N/A	14.2992	14.3009	15.0906	14.8861	14.0473	14.3946	14.7031	14.1203	14.3331	14.5331	1.0056	0.0081	0.7307	0.1363
Crmp1	_GM[Oxidation (M)]YDGPVYEVPAT[Phospho (STY)]PK_.2	#N/A	12.7114	12.6104	13.3981	12.9585	12.2048	12.7247	13.0658	12.4666	12.7455	12.8066	0.9998	-0.0004	0.9891	0.0047
Crmp1	_HAAPAPS[Phospho (STY)]AKSS[Phospho (STY)]PSK_.2	#N/A	6.7999	9.0051	9.2933	9.4507	8.1788	8.7376	9.2542	8.9372	9.0651	8.8554	0.9501	-0.0738	0.3983	0.3998
Crmp1	_HAAPAPSAKS[Phospho (STY)]S[Phospho (STY)]PSKHQPPPIR_.4	#N/A	11.5768	11.8135	12.6491	12.2871	11.9154	12.0077	11.7981	11.9232	12.0297	11.9117	1.0106	0.0152	0.5395	0.2680
Crmp1	_M[Oxidation (M)]TVVWDK_.2	#N/A	4.3034	5.2446	4.5332	4.7928	6.2301	4.1106	4.9244	4.2098	5.3015	6.2221	0.9428	-0.0850	0.5828	0.2345
Crmp1	_IVAPPGGRS[Phospho (STY)]NITS[Phospho (STY)]LG_.2	P97427_S570_M2_Crmp1	3.7689	3.6857	3.8614	8.7685	3.5370	3.7058	3.5913	3.5888	4.0057	8.1265	0.9433	-0.0841	0.8466	0.0723
Crmp1	_SS[Phospho (STY)]PSKHQPPPIR_.2	#N/A	9.6916	12.4700	13.1203	3.4233	12.0779	12.4437	12.8469	12.3251	3.9098	12.2596	1.0196	0.0280	0.9382	0.0277
Crmp1	_EELEVLVQDK_.2	#N/A	3.2452	3.9809	3.9320	4.4347	4.8784	4.3850	4.1494	3.6838	5.0467	5.5175	0.9007	-0.1508	0.3253	0.4878
Crmp1	_GLGAVILVHAENGDLIAQEQK_.3	#N/A	4.2261	4.3242	4.2527	3.7965	3.7126	4.5617	3.9448	4.4404	4.1834	4.7355	0.9583	-0.0614	0.4236	0.3730
Crmp1	_NLHQSNFSLS[Phospho (STY)]GAQIDDNNPR_.3	#N/A	7.8955	7.4499	8.0016	8.9044	6.7004	7.3928	7.4868	7.8614	7.3966	7.6027	1.0181	0.0259	0.7311	0.1360
Crmp1	_NLHQSNFSLSGAQIDDNNPRR_.4	#N/A	5.2969	5.1494	6.2132	5.7342	5.7286	5.3832	4.6841	4.9164	4.9713	5.2460	1.1271	0.1726	0.0201	1.6958
Crmp1	_HQPPPIR_.2	#N/A	4.1033	6.7338	3.2892	6.4093	3.2290	3.8359	3.5066	6.0422	3.5469	3.7589	1.0917	0.1266	0.6899	0.1612
Crmp1	_IAVGSDADVVIWDPDKM[Oxidation (M)]K_.2	#N/A	4.8236	4.7917	4.3819	4.7862	4.5268	4.2653	4.7379	4.9943	5.0102	5.0734	0.9277	-0.1082	0.0295	1.5301
Crmp1	_ILEM[Oxidation (M)]GITGPEGHALSRPEELEAEAVFR_.4	#N/A	3.8950	4.0766	3.9659	3.6925	4.6036	4.7018	4.1033	3.9538	4.4719	4.8149	0.9585	-0.0612	0.5040	0.2976
Crmp1	_[Acetyl (Protein N-term)]S[Phospho (STY)]HQGKKS[Phospho (STY)]IPHITSDR_.3	P97427_S2_M2_Crmp1	3.4622	4.6786	5.2336	3.7836	4.0976	3.8126	4.3773	4.0752	3.0880	4.0522	1.0718	0.1000	0.5068	0.2951
Mprip	_SKS[Phospho (STY)]NPDFLK_.2	#N/A	4.0339	3.2894	3.7676	5.8282	3.1777	5.1418	6.1987	3.6898	5.9672	2.9887	0.8929	-0.1635	0.5629	0.2496
Mprip	_SKS[Phospho (STY)]NPDFLKK_.2	#N/A	4.1593	6.2023	4.1343	6.7005	5.3500	6.2338	6.4138	6.2716	5.9675	6.5007	0.8688	-0.2029	0.1882	0.7254
Mprip	_AEEQLPPLLS[Phospho (STY)]PPS[Phospho (STY)]PSTPHSR_.3	P97434_S289_M2_Mprip	2.9445	3.7497	3.6313	4.3710	3.5710	4.1585	4.5193	9.7467	4.4301	7.5129	0.5704	-0.8098	0.0275	1.5611
Mprip	_DFASEAPTAPLSDAC[Carbamidomethyl (C)]PLS[Phospho (STY)]PHRR_.3	P97434_S364_M1_Mprip	4.0129	3.2683	3.7465	3.2903	3.1566	3.4680	3.3612	3.7109	4.0099	2.9676	0.9937	-0.0090	0.9306	0.0312
Mprip	_AATEALGEKS[Phospho (STY)]PEGTTVSGYDIM[Oxidation (M)]K_.3	P97434_S976_M1_Mprip	4.1112	7.5057	3.8449	9.5727	3.2549	3.3697	3.2628	7.6257	3.9116	7.3121	0.9545	-0.0672	0.8799	0.0556
Mprip	_DFASEAPTAPLS[Phospho (STY)]DAC[Carbamidomethyl (C)]PLSPHR_.3	P97434_S358_M1_Mprip	2.9568	4.2694	3.6436	4.1462	3.5833	3.9678	3.8609	1.6651	3.6208	4.1132	1.1346	0.1822	0.4026	0.3952
Kcnk2	_RLS[Phospho (STY)]VEIYDK_.2	P97438_S348_M1_Kcnk2	4.9377	4.3577	3.7997	3.5934	3.2097	3.4149	3.3080	3.6577	4.7482	5.7761	0.8886	-0.1704	0.4036	0.3941
Slc30a3	_DPVPQSGLS[Phospho (STY)]PER_.2	P97441_S66_M1_Slc30a3	3.5885	3.7539	9.5428	4.3667	4.2330	4.1542	9.7644	9.1180	9.1675	4.0440	0.6157	-0.6998	0.0849	1.0712
Slc30a3	_DRSS[Phospho (STY)]AGGGLR_.2	P97441_S23_M1_Slc30a3	3.3465	4.1517	4.4252	3.9690	3.9730	3.7564	4.1172	3.0322	3.3313	3.6463	1.1147	0.1567	0.1599	0.7961
Slc30a3	_SLFTEPS[Phospho (STY)]EPLPEEPK_.2	P97441_S38_M1_Slc30a3	3.7416	3.6008	3.4753	4.5199	2.8854	4.3074	3.1674	3.9821	4.2811	4.1972	0.9611	-0.0572	0.6864	0.1634
Cacna1a	_ESPS[Phospho (STY)]WVTQR_.2	P97445_S1981_M1_Cacna1a	10.0669	9.4588	4.0430	4.5457	3.9828	3.7467	9.8752	3.5728	3.2214	9.3994	0.9166	-0.1256	0.8001	0.0968
Cacna1a	_RAWPGS[Phospho (STY)]PER_.2	#N/A	8.7785	8.5363	8.6225	8.5133	7.0769	8.0431	8.9789	8.9157	8.4165	8.2761	0.9555	-0.0657	0.3041	0.5169
Cacna1a	_WSRS[Phospho (STY)]PSEGR_.2	P97445_S2200_M1_Cacna1a	3.1978	7.0795	7.7441	7.2428	3.8243	3.9052	8.0017	3.7312	3.3798	4.3543	1.1299	0.1762	0.6512	0.1863
Cacna1a	_M[Oxidation (M)]AGPAAPPGGS[Phospho (STY)]PR_.2	#N/A	10.7088	10.6643	11.1302	10.7574	9.5057	10.3418	11.1567	10.9243	10.5058	10.5067	0.9763	-0.0346	0.4356	0.3609
Cacna1a	_KQNLLAS[Phospho (STY)]R_.2	#N/A	11.1843	10.6949	11.2456	11.0268	9.6346	10.6361	11.4443	11.2676	11.1005	10.7528	0.9637	-0.0533	0.2490	0.6038
Cacna1a	_PLVSYS[Phospho (STY)]PAPR_.2	P97445_S2252_M1_Cacna1a	6.8808	4.4098	4.1671	3.7109	4.2311	6.1428	3.8591	3.2903	3.5893	3.3882	1.3941	0.4794	0.0670	1.1738
Cacna1a	_AWPGS[Phospho (STY)]PER_.2	#N/A	6.9782	6.1033	3.7849	4.2875	3.7246	5.8340	6.8376	6.9538	6.8256	7.1097	0.7385	-0.4374	0.0321	1.4936

Cacna1a	_KQNLLAS[Phospho (STY)]REALYGDAAER_.3	#N/A	3.7753	3.3583	5.4904	3.4639	2.9010	3.3276	3.6884	3.8674	4.3105	3.4309	0.9726	-0.0401	0.8352	0.0782
Cacna1a	_GADYTEPDS[Phospho (STY)]PREPPGGAHDPAPR_.3	P97445_S2303_M1_Cacna1a	3.5553	3.7871	6.2297	4.3336	4.1999	3.9256	4.4819	4.1684	2.9666	4.0109	1.1105	0.1512	0.4615	0.3358
Cacna1a	_GADYTEPDSPREPPGGAHDPAPRS[Phospho (STY)]PR_.4	P97445_S2318_M1_Cacna1a	5.7259	6.0129	6.7287	4.2499	3.6870	4.0425	6.1385	5.7887	3.5171	4.2169	1.0324	0.0460	0.8480	0.0716
Cacna1a	_EEQNRT[Phospho (STY)]PLM[Oxidation (M)]FQR_.3	#N/A	4.9846	3.5668	4.1870	3.9007	3.8887	3.8625	4.0828	4.4862	3.7242	3.7287	1.0149	0.0213	0.8416	0.0749
Cacna1a	_GNDLSTISDTS[Phospho (STY)]PM[Oxidation (M)]KR_.2	#N/A	3.1500	4.1390	4.1562	6.1362	3.7943	5.6168	4.0662	3.5017	3.4099	5.7664	1.0747	0.1039	0.6774	0.1692
Cacna1a	_QGSSSVSGS[Phospho (STY)]PAPSTSGTSTPR_.2	P97445_S2220_M1_Cacna1a	3.6902	3.5436	4.3770	3.5781	3.8321	3.4159	3.9775	3.2464	3.7813	3.3458	1.0423	0.0598	0.5147	0.2885
Cacna1a	_AS[Phospho (STY)]IKS[Phospho (STY)]AKLENSTFFHK_.3	P97445_S459_M2_Cacna1a	3.3063	3.9199	3.9931	3.8381	3.8082	4.3172	4.2104	2.5221	3.2713	2.9619	1.1920	0.2534	0.0950	1.0223
Cacna1a	_EVAEVS[Phospho (STY)]PLS[Phospho (STY)]AANM[Oxidation (M)]SIAVK_.2	#N/A	3.2675	4.2497	3.7561	4.4007	3.9334	6.3792	4.1000	7.0506	3.2221	3.8666	0.9498	-0.0742	0.7991	0.0974
Cacna1a	_RMAGPAAPPGGS[Phospho (STY)]PR_.2	#N/A	3.8519	3.1563	4.5387	3.4636	2.9776	4.9634	3.5971	3.5376	3.8366	3.1409	1.0842	0.1167	0.4971	0.3035
Cacna1a	_AKEVAEVS[Phospho (STY)]PLS[Phospho (STY)]AANM[Oxidation (M)]SIAVK_.3	#N/A	3.6259	3.6647	3.9330	3.9374	3.8698	3.6019	3.9530	4.3668	3.8467	3.7194	0.9498	-0.0743	0.1837	0.7360
Cacna1a	_WSRS[Phospho (STY)]PS[Phospho (STY)]EGREHTTHR_.4	P97445_S2200_M2_Cacna1a	2.9968	4.2294	3.6836	4.1863	4.1177	4.0078	3.9010	3.9322	3.5808	4.1533	0.9945	-0.0080	0.9354	0.0290
Cacna1a	_EM[Oxidation (M)]GTDGYSDSEHY[Phospho (STY)]LPMEGQTR_.3	P97445_Y2033_M1_Cacna1a	3.7055	3.5207	4.3923	3.3941	2.8312	3.2157	3.1088	3.2235	4.3729	3.3611	0.9981	-0.0027	0.9853	0.0064
Atp5pf	_FEVIDKPQS_.2	#N/A	6.1936	6.0072	6.6729	6.7163	7.7624	6.1921	6.3930	6.1423	6.4912	6.2403	1.0434	0.0613	0.4339	0.3626
Atp5pf	_QASGGPVDIGPEYQQDLDRELYK_.3	#N/A	6.0705	6.8553	7.2927	7.4237	7.9512	6.8592	6.5075	6.3926	6.9844	6.5748	1.0696	0.0971	0.2144	0.6688
Atp5pf	_QASGGPVDIGPEYQQDLDR_.2	#N/A	5.0203	4.9888	4.5141	5.7958	6.0435	4.6408	4.8999	5.0015	4.6560	5.3224	1.0397	0.0562	0.5717	0.2428
Atp5pf	_GEM[Oxidation (M)]DTFPTFK_.2	#N/A	7.4315	7.9780	8.1582	8.2560	8.7849	8.1845	7.9675	8.0191	8.1709	7.9371	1.0135	0.0194	0.6458	0.1899
Atp5pf	_FDDPKFEVIDKPQS_.2	#N/A	7.9029	7.9254	8.4286	8.0642	8.6228	8.4740	8.2561	7.7654	8.2228	8.0866	1.0190	0.0272	0.4215	0.3752
Rps5	_VNQAIWLLC[Carbamidomethyl (C)]TGAR_.2	#N/A	5.3864	4.7983	4.9606	5.4712	4.5706	4.7963	5.9503	5.1994	5.4712	4.7201	0.9366	-0.0944	0.2511	0.6002
Rps5	_GSSNSYAIK_.2	#N/A	4.8613	4.2097	3.9882	4.0117	5.0618	3.6467	4.2477	4.3784	4.4943	4.2234	0.9909	-0.0132	0.8935	0.0489
Rps5	_WSTDDVQINDISLQDYIAVK_.2	#N/A	0.1554	2.5670	3.0085	3.0074	3.2917	3.8322	3.1128	2.2188	3.3841	2.4721	0.9452	-0.0813	0.8302	0.0808
Rgs14	_PLREPGSSHLGS[Phospho (STY)]PDTAR_.3	#N/A	8.8084	8.8254	8.8120	9.4253	8.0331	8.7291	8.8924	8.8669	8.7772	8.7301	0.9950	-0.0073	0.8505	0.0703
Rgs14	_EPGSSHLGS[Phospho (STY)]PDTAR_.2	#N/A	9.0755	8.4235	4.0491	9.3059	7.9754	8.8346	8.9990	8.8139	8.8783	8.6755	0.8985	-0.1545	0.3977	0.4004
Rgs14	_S[Phospho (STY)]LGSGESESESRPGK_.2	#N/A	8.5244	8.8587	8.8862	9.2925	8.4331	8.9270	8.9733	8.8250	8.7418	8.9705	0.9935	-0.0094	0.7356	0.1334
Rgs14	_S[Phospho (STY)]LPLGVEELGQLPLAEGPC[Carbamidomethyl (C)]GR_.2	#N/A	3.9897	5.8513	5.3905	7.2271	3.7852	3.9197	4.6194	5.6942	5.3317	4.4768	0.9993	-0.0009	0.9966	0.0015
Rgs14	_SPLYQEC[Carbamidomethyl (C)]LLAEAEGRPLREPGSSHLGS[Phospho (STY)]PDTAR_.4	#N/A	3.7574	3.0618	4.4442	3.5581	2.8831	3.3455	3.7063	3.1716	3.7422	3.4131	1.0000	0.0000	0.9998	0.0001
Rgs14	_GLS[Phospho (STY)]LPDIK_.2	P97492_S339_M1_Rgs14	3.5717	3.0149	4.1224	3.2707	2.3612	1.8027	4.4862	3.3470	3.2678	3.7109	0.8166	-0.2923	0.1951	0.7098
Rgs14	_S[Phospho (STY)]LGSGESESESR_.2	#N/A	5.9181	5.4897	5.5576	6.8144	5.7326	5.8235	5.7888	5.3794	5.9152	5.7664	1.0310	0.0440	0.5184	0.2853
Gclc	_GLLSQGS[Phospho (STY)]PLSWEETQR_.2	P97494_S8_M1_Gclc	6.0143	6.0788	6.4694	6.4479	5.0487	4.3155	3.1754	6.1569	6.0392	3.9439	1.1864	0.2466	0.2576	0.5890
Smarcc1	_RKPS[Phospho (STY)]PS[Phospho (STY)]PPPPTATESR_.3	#N/A	7.4468	7.2225	7.4477	7.8685	7.3010	7.3467	7.5328	7.4219	7.2419	7.7388	0.9940	-0.0087	0.7600	0.1192
Smarcc1	_NEEPVRS[Phospho (STY)]PERR_.2	#N/A	4.1469	3.5952	4.1745	3.4771	3.3231	3.7138	3.9347	3.7726	4.2718	4.0232	0.9345	-0.0978	0.2212	0.6553
Myt1l	_LEEQLS[Phospho (STY)]PR_.2	P97500_S391_M1_Myt1l	8.1676	8.2189	8.4195	8.5354	7.7626	8.0218	8.6754	8.1994	8.4718	8.1315	0.9783	-0.0317	0.3235	0.4902
Myt1l	_KLS[Phospho (STY)]ETNPQDR_.2	P97500_S343_M1_Myt1l	3.5063	3.8361	3.2400	4.2846	4.1508	4.0720	4.9809	4.2175	3.0157	3.9618	0.9516	-0.0715	0.6217	0.2064
Myt1l	M[Oxidation (M)]VEES[Phospho (STY)]DEEVC[Carbamidomethyl (C)]LSSLEC[Carbamidomethyl (C)]LR_.	P97500_S319_M1_Myt1l	3.3875	4.1926	4.0743	4.5769	4.0140	3.7155	4.2916	3.5416	3.1902	3.0431	1.1356	0.1835	0.1543	0.8117
Grm1	_SAFTTS[Phospho (STY)]DVVR_.2	P97772_S853_M1_Grm1	7.0892	7.4162	7.4082	3.4277	5.9818	7.0120	7.1058	6.9809	7.5574	7.0912	0.8894	-0.1691	0.3462	0.4607
Mllt11	_APIASIHS[Phospho (STY)]VDLDLL_.2	P97783_S84_M1_Mllt11	3.7107	3.7389	5.9601	3.9199	3.6471	3.8582	3.7907	3.7335	3.7361	3.7988	1.0994	0.1368	0.4376	0.3589
Cxadr	_APQS[Phospho (STY)]PTLAPAK_.2	#N/A	7.1780	9.7065	9.9951	9.9510	8.8089	9.5039	10.1260	10.0116	7.9240	9.7069	0.9734	-0.0390	0.7221	0.1414
Cxadr	_TQYNQVPSEDFERAPQS[Phospho (STY)]PTLAPAK_.3	#N/A	5.5837	3.8796	7.5412	7.0155	3.3325	5.8935	6.2758	6.4245	6.0885	5.0612	0.9293	-0.1058	0.6470	0.1891
Sirpa	_HDQQPAITR_.2	#N/A	8.1015	7.4811	7.2810	7.4365	7.9494	7.7932	7.4261	7.8926	7.6302	7.8059	0.9981	-0.0028	0.9373	0.0281
Sirpa	_LLIYSFAGEYVPR_.2	#N/A	6.0071	5.5216	5.8974	5.6295	5.2460	5.6036	5.5288	4.9704	5.3420	6.0228	1.0338	0.0480	0.4291	0.3675
Sirpa	_PSPPEVSGPADR_.2	#N/A	9.2522	8.9691	8.5954	9.1867	8.9974	8.9692	9.2208	9.0630	8.9284	9.2284	0.9874	-0.0184	0.4022	0.3956
Sirpa	_NNM[Oxidation (M)]DFSIR_.2	#N/A	6.8115	6.8394	7.0675	6.8074	6.8961	6.7928	7.0848	7.1949	6.7954	6.9644	0.9799	-0.0293	0.1402	0.8533
Sirpa	_GSTSS[Phospho (STY)]TRLHEPEK_.3	P97797_S409_M1_Sirpa	3.4189	5.6500	3.5652	4.3564	6.8473	4.1583	3.5511	3.9725	3.9743	5.9004	1.0727	0.1013	0.7015	0.1540
Sirpa	_DGQELHPLETTVNPSGK_.3	#N/A	4.7331	3.9020	3.8234	3.7523	3.2749	4.3789	3.8897	4.0748	3.7022	3.8178	1.0275	0.0391	0.7020	0.1537
Sirpa	_VIC[Carbamidomethyl (C)]EVAHITLDR_.2	#N/A	5.0645	4.2052	4.9213	3.8428	4.2122	4.0493	4.7226	3.1851	4.0283	4.4571	1.0694	0.0968	0.4600	0.3373
Sirpa	_VPRPEDTLTYADLDMVHLSR_.4	#N/A	4.0741	3.6037	4.0819	3.3111	3.4920	3.1326	4.5266	3.3755	3.6745	3.3030	0.9720	-0.0409	0.7368	0.1326
Neo1	_NS[Phospho (STY)]QDITPVDNSM[Oxidation (M)]DSNIHQR_.3	#N/A	6.0072	5.4902	5.8131	6.4263	4.7617	5.8751	6.3179	6.1155	5.9197	6.0773	0.9380	-0.0923	0.2345	0.6299
Smn1	_GTGQSDDS[Phospho (STY)]DIWDDTALIK_.2	P97801_S28_M1_Smn1	3.5834	3.9046	3.9647	4.2627	4.8894	3.9473	4.4497	4.8359	4.7884	3.6091	0.9256	-0.1115	0.3340	0.4763
Smn1	_[Acetyl (Protein N-term)]AM[Oxidation (M)]GSGGAGS[Phospho (STY)]EQEDTVLFR_.2	#N/A	8.6201	8.4599	8.8152	9.2013	8.2569	9.0246	9.0799	8.6392	8.7939	8.7907	0.9891	-0.0158	0.6340	0.1979
Smn1	_[Acetyl (Protein N-term)]M[Oxidation (M)]AM[Oxidation (M)]GSGGAGSEQEDTVLFR_.2	#N/A	3.5350	4.3402	4.6135	6.4252	4.1615	3.5680	3.9288	3.2207	3.5197	3.7686	1.2303	0.2990	0.1730	0.7620
Smn1	_RGT[Phospho (STY)]GQSDDSDIWDDTALIK_.3	P97801_T22_M1_Smn1	3.6132	3.5653	3.3469	4.3915	3.3744	3.8796	3.6563	4.1225	4.0177	4.0688	0.9316	-0.1022	0.2458	0.6093
Fh	_SGLGELILPENEPGSSIM[Oxidation (M)]PGK_.2	#N/A	7.5246	7.8377	7.5359	7.6439	7.3229	7.6008	7.6184	7.6870	7.4948	7.5593	0.9984	-0.0023	0.8973	0.0471
Fh	_IYELAAGGTAVGTGLNTR_.2	#N/A	7.4954	6.9945	7.5807	6.9216	7.5524	7.5294	7.5915	7.0835	7.2831	7.5165	0.9969	-0.0045	0.9011	0.0452
Fh	_LHDALSAK_.2	#N/A	7.0449	7.2802	7.2866	7.5215	7.7973	6.3853	6.9547	7.1098	7.2054	7.6254	0.9994	-0.0009	0.9871	0.0056
Fh	_AAAEVNQEYGLDPK_.2	#N/A	6.3550	6.3052	3.5325	7.1387	6.6331	6.0624	6.4208	6.0829	6.0468	6.2273	0.9693	-0.0449	0.7773	0.1094
Fh	_YYGAQTVR_.2	#N/A	7.4167	7.3382	7.1114	7.4121	7.9841	7.1631	7.1929	7.4278	7.3185	7.7751	0.9967	-0.0047	0.8999	0.0458
Fh	_VEFDTFGELK_.2	#N/A	5.3052	4.8659	3.4506	5.0037	5.4862	4.3663	3.1426	4.5907	4.7538	4.5987	1.1112	0.1521	0.3544	0.4506
Fh	_AIEM[Oxidation (M)]LGGELGSK_.2	#N/A	6.4659	6.9106	3.8423	7.1905	7.1539	6.8757	6.5707	6.8493	4.3793	6.8483	1.0397	0.0562	0.7697	0.1137
Fh	_MPIPVIQAFGILK_.2	#N/A	4.8573	4.4109	4.5750	5.0138	4.4817	5.9573	3.2915	3.6950	4.3960	5.5424	1.1540	0.2066	0.2184	0.6607
Fh	_ETAIELGYLTAEQFDEWVKPK_.3	#N/A	3.0312	2.4186	3.7180	3.6087	3.2698	4.0717	3.9354	3.8978	3.2822	2.1511	1.0110	0.0157	0.9366	0.0284
Fh	_LNDHFPLVVWQTGSGTQTNM[Oxidation (M)]NVNEVISNR_.3	#N/A	3.4252	3.6916	3.6053	4.1021	3.3306	3.9236	3.8168	4.0325	3.6811	4.1938	0.9361	-0.0953	0.1879	0.7260
Fh	_SQSSNDTFPTAM[Oxidation (M)]HIAAAVEVHK_.3	#N/A	3.6656	4.4218	3.3992	4.4438	5.9757	5.8743	3.0913	4.0582	4.3572	6.7144	1.0164	0.0235	0.9297	0.0317
Fh	_THTQDAVPLTLGQEFSGYVQQVQYAMVR_.3	#N/A	3.8708	3.7664	3.8071	3.5594	3.7468	4.0791	4.0244	3.9051	3.5537	3.5264	1.0140	0.0200	0.7004	0.1547
Map4k4	_AASS[Phospho (STY)]PNLSNGETESVK_.2	P97820_S795_M1_Map4k4	6.9326	3.6475	7.3923	7.5279	4.2052	3.5243	7.5500	7.2747	6.6348	6.7289	0.7859	-0.3476	0.1710	0.7670
Map4k4	_TTSRS[Phospho (STY)]PVLSR_.2	P97820_S621_M1_Map4k4	7.0017	7.0515	3.9053	7.7221	5.8892	7.5043	7.8711	7.5144	3.5086	7.4698	0.9881	-0.0173	0.9444	0.0248
Map4k4	_RDS[Phospho (STY)]PLQGGGQQNSQAGQR_.3	#N/A	4.8566	3.8070	3.8837	4.2758	3.1146	4.2630	3.3727	4.3785	3.9779	4.5366	0.9919	-0.0117	0.9296	0.0317
Map4k4	_SSS[Phospho (STY)]KSEGSPSPR_.2	P97820_S701_M1_Map4k4	5.2455	4.4592	5.2002	5.3531	3.3013	3.3473	4.0865	5.5677	4.2653	4.9516	0.9505	-0.0732	0.6854	0.1641
Map4k4	_DSPLQGGGQQNSQAGQR_.2	#N/A	4.3139	4.3671	4.2099	3.7536	5.3303	3.5411	3.9019	3.2475	3.5466	3.4309	1.2041	0.2680	0.0640	1.1937
Map4k4	_S[Phospho (STY)]EGSPSPR_.2	P97820_S703_M1_Map4k4	3.5685	4.3247	3.3022	4.3468	4.2130	3.9124	4.4950	4.1552	2.9535	4.0240	1.0096	0.0138	0.9154	0.0384
Jpt1	_SSGGREDSES[Phospho (STY)]PGTQR_.2	#N/A	6.1660	6.4979	7.0123	6.9047	7.2945	7.2768	6.4408	6.3148	6.8234	6.4435	1.0543	0.0763	0.1864	0.7296
Jpt1	_SNS[Phospho (STY)]SEASSGDFLDLK_.2	P97825_S87_M1_Jpt1	3.9299	4.2939	3.6300	9.8627	3.7772	3.6518	4.3648	8.9723	3.6453	4.0997	0.9217	-0.1177	0.8023	0.0956
G3bp1	_STS[Phospho (STY)]PAPADVAPAQEDLR_.2	P97855_S231_M1_G3bp1	12.4235	12.5946	13.3793	13.0242	12.2925	12.5984	12.9161	12.3012	12.5081	12.6240	1.0104	0.0150	0.5853	0.2326
G3bp1	_YQDEVFGGFVTEPQEES[Phospho (STY)]EEEVEEPEER_.3	P97855_S149_M1_G3bp1	3.3586	3.9838	4.5931	4.1369	3.8051	3.9243	4.2851	4.3651	3.1634	3.8142	1.0154	0.0220	0.8467	0.0723
Nfib	_KPEKPLFSSTS[Phospho (STY)]PQDSSPR_.3	P97863_S328_M1_Nfib	3.9285	6.9950	7.7705	7.7351	6.1883	3.3697	7.2388	7.1794	3.9179	6.7004	0.9583	-0.0615	0.8275	0.0822
Rbbp6	_VEGTEIVKPS[Phospho (STY)]PK_.2	P97868_S1179_M1_Rbbp6	7.0292	6.5314	7.5077	6.9802	6.1031	6.9036	7.8442	7.5018	6.6700	7.0433	0.9419	-0.0864	0.2205	0.6566
Rbbp6	_SRS[Phospho (STY)]PQAFR_.2	P97868_S773_M1_Rbbp6	6.1973	6.0464	6.6553	6.2300	3.1866	4.0497	6.4043	6.2736	3.9083	6.0355	0.9538	-0.0682	0.7689	0.1141
Rbbp6	_YHS[Phospho (STY)]RS[Phospho (STY)]RS[Phospho (STY)]PQAFR_.3	P97868_S769_M3_Rbbp6	3.2566	3.9696	3.9434	4.4460	3.8831	5.3529	5.1649	5.2524	5.5611	5.4945	0.7716	-0.3741	0.0102	1.9907
Rbbp6	_WDKDDFES[Phospho (STY)]EEEDVK_.2	P97868_S1329_M1_Rbbp6	3.0842	4.1420	3.7710	5.1249	3.7107	4.0188	3.9883	4.5098	4.8377	4.9685	0.8687	-0.2031	0.1562	0.8064
Rbbp6	_PSANREDFS[Phospho (STY)]PER_.2	#N/A	3.5701	6.7322	7.1384	7.3482	3.5681	6.5540	7.0318	3.5719	7.0281	6.5228	0.9635	-0.0536	0.8484	0.0714
Rbbp6	_VDGDREKS[Phospho (STY)]PR_.2	P97868_S1053_M1_Rbbp6	3.4186	3.8243	4.1375	3.9460	3.9960	3.7335	4.0943	3.0552	3.3542	4.1372	1.0498	0.0702	0.4844	0.3148

Rab33a	_S[Phospho (STY)]MVEHYYR_.2	P97950_S101_M1_Rab33a	3.6533	4.4095	3.3869	4.0061	2.7970	3.8276	3.7208	4.1124	3.7610	3.9731	0.9456	-0.0807	0.4879	0.3117
Apba2	_SAS[Phospho (STY)]QDC[Carbamidomethyl (C)]IETTPGAQEGK_.2	#N/A	9.5167	9.5472	9.4885	9.8774	8.1166	9.3707	9.5862	9.2349	9.2704	9.3821	0.9948	-0.0076	0.8820	0.0545
Apba2	_SAS[Phospho (STY)]QDC[Carbamidomethyl (C)]IETTPGAQEGKK_.2	#N/A	3.5106	6.3973	6.2577	6.6923	3.3625	6.1924	6.2652	5.7453	3.7917	6.1663	0.9836	-0.0238	0.9233	0.0347
C1qa	_DSLSFSNTNNK_.2	#N/A	8.6357	8.4892	9.0069	9.2683	8.4830	8.9588	9.0021	8.5977	8.9382	8.8198	0.9963	-0.0053	0.8595	0.0657
Cds1	_GDS[Phospho (STY)]DVPEVPPSSDR_.2	P98191_S56_M1_Cds1	8.6781	9.5186	8.3466	4.1479	3.5850	8.8797	10.4525	9.6137	8.7186	7.8140	0.7861	-0.3472	0.2015	0.6958
Atp8a2	_ATS[Phospho (STY)]VGDQLEAPAR_.2	P98200_S6_M1_Atp8a2	10.5174	10.4309	10.9711	10.4853	9.1287	10.1901	11.1434	10.8128	10.5175	10.3054	0.9619	-0.0560	0.2758	0.5594
Atp8a2	_EQS[Phospho (STY)]SDDFC[Carbamidomethyl (C)]R_.2	P98200_S421_M1_Atp8a2	6.4698	6.7852	7.1006	4.1144	3.8294	3.9019	4.2627	7.2753	6.9231	3.7917	0.9647	-0.0518	0.8588	0.0661
Atp8a2	_[Acetyl (Protein N-term)]S[Phospho (STY)]RATS[Phospho (STY)]VGDQLEAPAR_.2	P98200_S2_M2_Atp8a2	4.0231	3.3193	3.7567	3.3005	3.1668	3.4578	3.4488	3.7007	3.9997	2.9778	0.9922	-0.0114	0.9102	0.0409
Atp8a2	_M[Oxidation (M)]TSC[Carbamidomethyl (C)]TNDS[Phospho (STY)]C[Carbamidomethyl (C)]DFNDPR_.2	#N/A	4.1543	4.8288	4.9990	4.2025	4.2487	4.5258	5.2468	4.3683	4.2225	3.8443	1.0164	0.0235	0.8123	0.0903
Arvcf	_DIPSYGS[Phospho (STY)]LSR_.2	P98203_S205_M1_Arvcf	7.6061	4.0166	3.8982	8.2541	3.8379	3.8916	4.2524	4.3979	7.9740	3.7814	1.0293	0.0416	0.9121	0.0400
Arvcf	_GSLGS[Phospho (STY)]LDR_.2	P98203_S337_M1_Arvcf	3.6485	2.9529	4.1232	7.0425	4.2750	3.4545	3.8153	3.3342	3.6332	3.3443	1.2032	0.2669	0.3636	0.4394
Arvcf	_S[Phospho (STY)]LAADDEGGPDLEPDYSTATR_.2	P98203_S269_M1_Arvcf	5.4248	6.6556	6.1137	7.9445	6.1949	7.2185	5.8252	7.3540	7.8710	7.4516	0.9251	-0.1123	0.3818	0.4181
Arvcf	_RSPS[Phospho (STY)]VDSTR_.2	P98203_S347_M1_Arvcf	5.1150	4.5577	5.4176	5.0992	3.2254	3.2423	3.7583	3.8242	3.6911	4.9539	1.0952	0.1311	0.5042	0.2974
Arvcf	_S[Phospho (STY)]LPEHFQAEPYGLEDDTR_.3	P98203_S251_M1_Arvcf	3.5155	3.8269	3.2492	5.7060	3.6482	4.0813	4.4421	5.8457	5.5109	5.0569	0.7680	-0.3807	0.0449	1.3480
Arvcf	_S[Phospho (STY)]PSVDSTR_.2	#N/A	6.6869	3.1587	3.6369	6.5412	3.0470	3.5776	3.4708	6.3818	4.1195	4.3588	0.9692	-0.0452	0.8910	0.0501
Arvcf	_NFDT[Phospho (STY)]LDLPK_.2	P98203_T643_M1_Arvcf	3.6829	3.6595	3.4166	4.4612	2.8266	4.2486	3.1086	4.0409	4.3399	4.1384	0.9511	-0.0723	0.6190	0.2083
Tubb5	_ISVYYNEATGGK_.2	#N/A	9.5551	9.3770	9.8107	10.1733	9.9002	9.5235	9.4391	9.7369	9.6906	9.5921	1.0113	0.0162	0.5122	0.2906
Tubb5	_M[Oxidation (M)]AVTFIGNSTAIQELFKR_.3	#N/A	5.4867	5.3839	6.9177	4.4447	5.3898	5.8879	5.3080	5.8324	5.3394	6.2020	0.9850	-0.0219	0.8528	0.0692
Tubb5	_MAVTFIGNSTAIQELFK_.2	#N/A	5.7559	4.4177	5.4829	7.2169	4.9561	4.5029	5.9369	4.6553	4.9312	5.2128	1.0395	0.0559	0.7303	0.1365
Tubb5	_ALTVPELTQQVFDAK_.2	#N/A	10.4185	10.3929	10.5895	10.2355	10.5165	10.4295	10.3429	10.1868	10.4192	10.4912	1.0068	0.0098	0.4056	0.3919
Tubb5	_FWEVISDEHGIDPTGTYHGDSDLQLDR_.4	#N/A	6.7946	6.3186	7.2634	6.8342	7.1272	7.2562	6.6904	6.8041	6.9955	7.3272	0.9968	-0.0046	0.9214	0.0355
Psmb4	_QPVLSQTEAR_.2	#N/A	6.5068	6.6585	6.8826	6.9002	7.0777	6.7465	6.2374	6.5063	6.3296	7.0167	1.0418	0.0591	0.1506	0.8223
Psmb4	_FQIATVTEK_.2	#N/A	6.2629	6.2088	6.6535	6.6262	6.6920	6.3060	6.0155	6.2671	6.5275	6.2890	1.0292	0.0416	0.2262	0.6456
Psmb4	_AIHSWLTR_.2	#N/A	6.3168	6.9148	6.2337	6.6605	6.6575	6.0568	5.0999	6.4519	6.4707	6.2863	1.0652	0.0911	0.2334	0.6318
Psmb4	_TQNPM[Oxidation (M)]VTGTSVLGVK_.2	#N/A	4.0446	5.6686	3.2306	5.2332	3.1703	3.5548	3.4479	5.2316	5.2064	3.7003	0.9440	-0.0831	0.7192	0.1432
Psmb4	_FDGGVVIAADM[Oxidation (M)]LGSYGSLAR_.2	#N/A	3.9217	4.1421	4.2470	4.1355	4.2412	4.5884	4.4985	3.8251	4.1572	4.2312	1.0083	0.0119	0.8302	0.0808
Psmb4	_VNDSTM[Oxidation (M)]LGASGDYADFQYLK_.2	#N/A	4.0190	4.0611	4.4835	3.9100	4.1589	3.9537	3.8351	3.7146	3.4963	3.7544	1.1075	0.1473	0.0109	1.9631
Rplp2	_LASVPAGGAVAVSAAPGSAAPAAGSAPAAAEEK_.3	#N/A	5.1228	6.3443	5.9197	6.1934	7.0270	6.0929	5.6767	6.1422	5.0415	5.9578	1.0722	0.1006	0.2961	0.5286
Rplp2	_NIEDVIAQGVGK_.2	#N/A	9.9863	10.3459	10.7043	10.6090	10.4095	10.2129	10.3763	9.9878	10.0860	10.2879	1.0190	0.0272	0.2362	0.6266
Rplp2	_YVASYLLAALGGNSSPSAK_.2	#N/A	5.8909	5.8822	6.4032	6.2933	6.4846	6.1563	5.9385	5.5185	6.0828	6.2869	1.0383	0.0543	0.2481	0.6053
Rplp2	_ILDSVGIEADDDRLNK_.2	#N/A	4.1581	4.5299	4.4844	4.7140	4.9449	4.8374	4.7743	4.3542	4.1819	4.8336	1.0166	0.0238	0.7040	0.1524
Rplp2	_KEESEES[Phospho (STY)]DDDM[Oxidation (M)]GFGLFD_.2	#N/A	3.9573	3.3499	4.0619	10.2997	10.4059	5.8717	4.7264	12.8251	8.2840	5.8698	0.7979	-0.3257	0.4825	0.3165
Rplp2	_LASVPAGGAVAVSAAPGSAAPAAGSAPAAAEEKKDEK_.4	#N/A	4.1382	5.0929	3.8718	5.4406	5.0599	3.2031	3.5639	3.5856	3.8846	4.4682	1.1528	0.2051	0.2453	0.6102
Uqcrh	_LELC[Carbamidomethyl (C)]DNR_.2	#N/A	8.7003	9.0680	9.0760	9.0827	9.3545	9.0836	8.8361	8.7907	9.0083	9.0092	1.0168	0.0240	0.2314	0.6356
Uqcrh	_EHC[Carbamidomethyl (C)]EQLEK_.2	#N/A	7.2624	7.7999	7.7381	7.7556	8.7885	7.7196	7.2659	7.6478	7.8508	7.6488	1.0317	0.0450	0.4060	0.3915
Uqcrh	_ERLELC[Carbamidomethyl (C)]DNR_.2	#N/A	7.5113	7.3718	7.9646	7.7848	7.9998	7.6947	7.5752	7.4918	7.8459	7.5486	1.0139	0.0199	0.4749	0.3234
Uqcrh	_SQTEEDC[Carbamidomethyl (C)]TEELFDFLHAR_.3	#N/A	6.8680	6.7539	7.3584	7.1556	7.5418	7.2995	6.9988	6.4801	6.9456	7.4931	1.0263	0.0374	0.4378	0.3587
Prdx5	_LLADPTGAFGK_.2	#N/A	10.2801	10.6605	10.6516	10.4399	10.6142	10.4715	10.3240	10.5290	10.4899	10.5048	1.0055	0.0079	0.5159	0.2874
Prdx5	_THLPGFVEQAGALK_.2	#N/A	8.5370	8.9945	8.8644	8.8772	9.1546	8.8675	8.7360	8.6955	8.6645	8.8489	1.0167	0.0240	0.2144	0.6687
Prdx5	_VGDAIPSVEVFEGEPGK_.2	#N/A	9.0406	8.8618	9.3021	9.2127	8.8166	8.9964	9.1001	8.7749	8.6502	9.1008	1.0148	0.0212	0.3509	0.4548
Prdx5	_VGDAIPSVEVFEGEPGKK_.2	#N/A	7.1080	7.2319	7.2014	7.8717	7.3297	7.4177	7.6120	7.2552	6.8556	7.4056	1.0107	0.0154	0.6890	0.1618
Prdx5	_KVNLAELFK_.2	#N/A	6.9209	6.9388	7.0329	7.4981	7.4055	7.0227	6.9912	6.8459	7.0926	7.1973	1.0149	0.0213	0.4777	0.3208
Prdx5	_ALNVEPDGTGLTC[Carbamidomethyl (C)]SLAPNILSQL_.2	#N/A	5.6476	6.4102	6.2498	7.0308	6.6998	5.9261	6.4371	6.5049	6.5805	5.9932	0.9919	-0.0117	0.8572	0.0669
Prdx5	_VNLAELFK_.2	#N/A	5.8512	6.9354	5.7583	6.1349	6.6388	6.5312	4.9084	6.4646	6.8501	6.6574	1.0142	0.0203	0.8407	0.0754
Prdx5	_ATDLLLDDSLVSLFGNR_.2	#N/A	6.8936	6.6367	5.9116	5.6399	6.6880	6.3393	6.8629	5.8745	6.2539	6.5130	0.9961	-0.0056	0.9364	0.0286
Prdx5	_ATDLLLDDSLVSLFGNRR_.3	#N/A	5.5163	6.3098	6.2182	6.4802	6.1068	6.1209	5.9405	5.8622	6.2987	6.0405	1.0149	0.0213	0.6378	0.1953
Prdx5	_FSM[Oxidation (M)]VIDNGIVK_.2	#N/A	7.3828	7.3657	7.3616	7.5439	7.3940	7.2954	7.2357	7.1928	7.2508	7.0296	1.0297	0.0423	0.0063	2.1984
Cpe	_SNAQGIDLNR_.2	#N/A	6.9672	6.9097	6.8785	7.6078	7.1286	7.1505	7.2516	6.8077	7.1522	7.0494	1.0059	0.0085	0.7966	0.0988
Cpe	_LTASAPGYLAITK_.2	#N/A	5.3285	5.8672	5.5908	5.7147	5.4143	5.2952	5.6016	4.7066	5.0851	5.4725	1.0611	0.0855	0.1459	0.8358
Cpe	_IVYVNEK_.2	#N/A	3.9346	3.1425	4.1044	4.0250	4.4894	3.5938	3.3128	4.4787	4.1357	4.6535	0.9364	-0.0948	0.4520	0.3448
Cpe	_YIGNM[Oxidation (M)]HGNEAVGR_.3	#N/A	3.9466	4.5479	4.1131	4.6158	5.1617	3.6767	4.3305	4.6550	5.1577	3.0819	1.0087	0.0125	0.9351	0.0292
Cpe	_EALVSVWLQC[Carbamidomethyl (C)]TAISR_.2	#N/A	3.2378	4.0430	3.9246	4.4272	3.8643	3.8652	4.1419	2.9765	3.3398	4.3942	1.0486	0.0685	0.5974	0.2237
Il6st	_SHIAQWS[Phospho (STY)]PHT[Phospho (STY)]PPR_.3	Q00560_S665_M2_Il6st	2.9076	4.3185	3.5944	5.5432	3.5341	5.1002	4.1314	5.7688	5.3397	5.4801	0.8043	-0.3142	0.1236	0.9079
G6pdx	_DVAGDIFHQQC[Carbamidomethyl (C)]K_.2	#N/A	4.2874	4.1646	5.9164	8.7039	4.1623	3.5672	3.9280	3.2215	4.5432	3.7159	1.3327	0.4143	0.2372	0.6250
Hnrnpul2	_S[Phospho (STY)]GDETPGSEAPGDK_.2	#N/A	12.0838	11.9423	11.6011	12.7379	11.2570	11.9741	12.4759	11.8978	11.9103	12.2417	0.9836	-0.0238	0.4936	0.3067
Hnrnpul2	_DLLVQQASQC[Carbamidomethyl (C)]LSK_.2	#N/A	5.3126	5.2907	4.7213	5.1930	5.6594	5.3076	5.4832	5.0964	5.2535	5.3858	0.9892	-0.0157	0.7423	0.1294
Hnrnpul2	_YNVLGAETVLTQM[Oxidation (M)]R_.2	#N/A	5.1853	6.0190	6.7128	5.1480	6.0570	5.8799	6.2191	5.9203	6.0907	6.1489	0.9572	-0.0632	0.4184	0.3784
Hnrnpul2	_NFILDQC[Carbamidomethyl (C)]NVYNSGQR_.2	#N/A	3.4378	5.3945	3.1714	5.3865	5.1345	6.0398	4.3643	4.4958	5.4321	5.4488	0.9646	-0.0519	0.7921	0.1012
Hnrnpul2	_AVEEQGDDQDS[Phospho (STY)]EK_.2	Q00PI9_S183_M1_Hnrnpul2	5.5117	4.6666	5.5753	6.9363	5.1861	4.4124	5.8510	6.0677	4.8363	6.0485	0.9440	-0.0832	0.5482	0.2611
Hnrnpul2	_S[Phospho (STY)]KSPPPPEEEAKDEEEDQTLVNLDTYTSDLHFQISK_.5	Q00PI9_S224_M1_Hnrnpul2	6.2030	3.0933	3.3188	3.8215	3.2586	2.9701	3.5362	4.2970	3.9456	3.7885	0.9706	-0.0430	0.8621	0.0645
Hnrnpul2	_S[Phospho (STY)]GDETPGSEAPGDKAVEEQGDDQDSEK_.3	#N/A	2.9839	4.3585	4.2185	3.7622	1.6685	3.5497	3.9105	3.2390	3.5669	3.4395	0.9674	-0.0478	0.8292	0.0813
Hnrnpul2	_AVEEQGDDQDS[Phospho (STY)]EKS[Phospho (STY)]KPAGS[Phospho (STY)]DGER_.3	Q00PI9_S183_M3_Hnrnpul2	3.3078	4.1130	3.9946	4.0077	3.9343	3.7952	4.1560	3.6213	3.2698	2.9634	1.1017	0.1397	0.1929	0.7147
Pde4d	_QHEVEIPSPT[Phospho (STY)]QKEK_.3	Q01063_T289_M1_Pde4d	4.3585	2.9839	4.0922	3.6359	3.5022	3.1224	3.7842	3.3652	3.6643	3.3132	1.0238	0.0340	0.7776	0.1092
Pde4d	_NNFAALTNLQDR_.2	#N/A	5.2599	4.2081	4.0897	4.8978	4.5845	5.0625	4.8642	4.5483	4.0573	5.1331	1.0071	0.0102	0.9156	0.0383
Pde4d	_SDS[Phospho (STY)]DYDLSPK_.2	Q01063_S136_M1_Pde4d	3.9792	6.5202	7.0177	3.2669	5.4921	6.8823	5.8501	6.6381	6.8672	7.0605	0.8368	-0.2570	0.2331	0.6324
Pde4d	_HSVSEMASNK_.2	#N/A	5.8323	5.1340	5.8380	5.6641	3.4925	5.4952	3.7744	5.8885	5.6297	3.3035	1.1277	0.1734	0.4134	0.3836
Pde4d	_QHEVEIPS[Phospho (STY)]PTQK_.2	Q01063_S287_M1_Pde4d	3.3530	4.1582	4.0398	4.5424	7.9362	3.7500	4.1108	3.5761	3.2246	3.0086	1.3304	0.4119	0.2239	0.6499
Pde1b	_TALQQLER_.2	#N/A	6.8886	6.7807	6.1269	5.4939	6.6191	6.3361	5.8466	6.5994	6.0973	6.8616	1.0036	0.0052	0.9445	0.0248
Pde1b	_TIVFELLTR_.2	#N/A	5.3655	5.6849	5.0161	4.4760	5.3719	5.0606	5.7856	5.6935	5.5973	5.8025	0.9026	-0.1479	0.0320	1.4942
Pde1b	_QILDTEDELR_.2	#N/A	6.2007	6.1755	3.8878	5.4483	5.0060	5.4488	5.6719	6.3312	5.8647	5.3283	0.9245	-0.1133	0.3769	0.4238
Pde1b	_DWLASTFTQQTR_.2	#N/A	4.9496	4.6507	5.4528	3.2186	4.7037	4.8884	4.4341	5.2932	4.9743	5.2387	0.9316	-0.1023	0.4349	0.3617
Pde1b	_S[Phospho (STY)]PPEM[Oxidation (M)]LESDC[Carbamidomethyl (C)]PSPLELK_.2	#N/A	8.6961	8.2139	8.1440	9.0304	7.6324	7.9958	8.9382	8.7054	8.5674	8.4131	0.9572	-0.0631	0.2084	0.6812
Pde1b	_QILDTEDELRELR_.3	#N/A	3.5166	4.2729	3.2503	4.2949	4.1612	5.0018	3.6659	3.7302	4.5304	4.0594	1.0216	0.0309	0.8135	0.0896
Pde1b	_NLEYTASLLEAVYIDETR_.2	#N/A	3.5502	4.3554	4.2370	3.7653	3.6351	3.2013	3.9136	3.3788	3.5349	3.2058	1.0805	0.1117	0.2953	0.5298
Grin2b	_S[Phospho (STY)]FTHSDC[Carbamidomethyl (C)]K_.2	Q01097_S940_M1_Grin2b	7.9132	7.4204	7.8984	7.4700	6.5781	7.3882	7.8818	7.7243	7.5967	7.3775	0.9738	-0.0383	0.4662	0.3314
Grin2b	_YPQS[Phospho (STY)]PTNSK_.2	Q01097_S1284_M1_Grin2b	8.6652	8.3718	8.8407	8.2923	7.3098	8.2580	8.7926	8.8073	8.3918	8.3157	0.9665	-0.0491	0.3509	0.4548
Grin2b	_HSQLSDLY[Phospho (STY)]GK_.2	Q01097_Y1039_M1_Grin2b	8.3019	7.4958	8.3832	8.0068	5.2446	7.5307	8.3678	8.0251	7.7666	7.8802	0.9356	-0.0961	0.4166	0.3802
Grin2b	_SDRYS[Phospho (STY)]GHDDLIR_.3	Q01097_S1050_M1_Grin2b	2.9858	3.7909	3.6726	4.1752	3.6123	4.1172	4.4780	5.9935	5.8184	4.1422	0.7294	-0.4553	0.0123	1.9115
Grin2b	_SVS[Phospho (STY)]LKDK_.2	Q01097_S1323_M1_Grin2b	7.3284	4.3116	7.8520	7.2403	5.4394	7.3947	6.9590	7.5832	7.1715	7.1923	0.9125	-0.1321	0.4050	0.3926

Grin2b	_RQHS[Phospho (STY)]YDTFVDLQKEEAALAPR_.4	#N/A	3.8276	3.3985	7.9154	7.9770	3.2868	3.3378	3.2309	8.2144	4.2508	6.9920	0.8740	-0.1943	0.6463	0.1895
Grin2b	_ENS[Phospho (STY)]PHWEHVDLTDIYK_.3	Q01097_S1143_M1_Grin2b	4.0969	3.4013	3.2829	5.4667	3.2226	3.0061	3.3669	5.5785	4.9901	4.4774	0.8138	-0.2973	0.1902	0.7208
Grin2b	_RES[Phospho (STY)]S[Phospho (STY)]VYDISEHRR_.3	Q01097_S929_M2_Grin2b	3.9523	3.3902	5.6373	3.2297	3.2115	3.0172	4.9866	5.5324	4.0705	4.8190	0.7707	-0.3757	0.0801	1.0961
Grin2b	_QHSY[Phospho (STY)]DTFVDLQKEEAALAPR_.3	#N/A	2.9343	3.1153	3.5935	3.1373	3.0036	3.6210	3.2856	3.8639	4.1629	4.3154	0.8278	-0.2726	0.0213	1.6717
Grin2b	_RQHS[Phospho (STY)]YDTFVDLQK_.3	#N/A	3.3087	4.0650	4.5432	6.4406	4.1520	6.0309	6.0276	6.5072	5.8847	6.1702	0.7738	-0.3700	0.0588	1.2304
Grin2b	_QHSY[Phospho (STY)]DTFVDLQK_.2	#N/A	3.4038	4.1601	3.9665	4.2890	4.0484	4.1106	4.0037	3.8630	3.5115	3.5841	1.0684	0.0954	0.1992	0.7008
Grin2b	_S[Phospho (STY)]DVSDISTHTVTYGNIEGNAAK_.3	Q01097_S1058_M1_Grin2b	3.2775	3.8336	3.7304	4.4460	3.6881	3.5929	4.3669	3.9225	3.0997	3.5688	1.0059	0.0084	0.9414	0.0262
Grin2b	_NM[Oxidation (M)]ANLSGVNGS[Phospho (STY)]PQSALDFIR_.2	Q01097_S917_M1_Grin2b	3.3554	3.9870	4.5899	5.8185	3.8083	3.9212	4.2820	4.3683	3.1665	3.8110	1.0870	0.1203	0.5099	0.2925
Grin2b	_AGNLYDIS[Phospho (STY)]EDNSLQELDQPAAPVAVSSNASTTK_.3	Q01097_S1255_M1_Grin2b	3.7906	3.0949	3.9811	3.5249	2.9163	3.3124	3.6732	3.7976	5.0224	3.2022	0.8759	-0.1912	0.2253	0.6473
Grin2b	_ESSVY[Phospho (STY)]DISEHRR_.3	Q01097_Y932_M1_Grin2b	4.0747	3.3790	3.6970	3.2408	3.2004	3.0283	3.3891	3.7604	2.9968	4.4189	0.9438	-0.0834	0.5269	0.2783
Creb1	_DLYC[Carbamidomethyl (C)]HKS[Phospho (STY)]D_.2	Q01147_S326_M1_Creb1	4.1564	4.6886	3.5114	5.9201	4.7452	4.2784	3.1383	4.0111	4.3102	4.5167	1.1392	0.1880	0.2763	0.5586
Creb1	_ILNDLS[Phospho (STY)]SDAPGVPR_.2	Q01147_S128_M1_Creb1	6.9198	6.7717	6.9073	7.4068	4.0832	6.8382	7.0193	7.2311	7.0961	7.4921	0.8999	-0.1522	0.2761	0.5589
Adra2c	_TEVEPDES[Phospho (STY)]SAAER_.2	Q01337_S286_M1_Adra2c	3.8251	3.0805	3.5587	3.8343	2.9688	3.6558	3.5490	4.2842	3.9328	3.8013	0.8960	-0.1584	0.1117	0.9521
Adra2a	_RGPGAAGPGASGS[Phospho (STY)]GHGEER_.3	Q01338_S362_M1_Adra2a	3.7624	3.0178	6.6890	3.8970	2.9061	3.7185	6.7786	7.1644	3.8701	3.8640	0.7378	-0.4387	0.1937	0.7129
Adra2a	_TRAS[Phospho (STY)]QVKPGDS[Phospho (STY)]LPR_.3	Q01338_S339_M2_Adra2a	4.5636	4.1091	3.9907	4.4933	3.9304	3.7991	4.2080	3.6251	3.2737	2.9595	1.1795	0.2381	0.0443	1.3533
Adra2a	_ASQVKPGDS[Phospho (STY)]LPR_.2	#N/A	7.9478	7.6794	8.1103	7.6683	6.4701	7.4164	8.0494	8.1543	7.6276	7.4804	0.9643	-0.0524	0.4126	0.3845
Adcy6	_NAEPPS[Phospho (STY)]PTPAAHTR_.2	#N/A	8.6994	8.5551	9.1689	3.5413	3.2221	3.1784	9.2523	8.8622	8.6648	8.4467	0.6882	-0.5390	0.1137	0.9442
Nme2	_GLVGEIIKR_.2	#N/A	9.6460	9.9576	9.8347	9.9205	9.9932	9.8107	9.8305	9.6974	9.6724	9.8717	1.0095	0.0136	0.2539	0.5954
Nme2	_GDFC[Carbamidomethyl (C)]IQVGR_.2	#N/A	8.5405	8.6341	8.9856	9.0259	9.2062	8.8238	8.5064	8.9063	9.0990	8.8078	1.0045	0.0064	0.8130	0.0899
Nme2	_DRPFFPGLVK_.2	#N/A	7.9820	7.9459	8.1735	8.0016	8.2527	7.8423	7.6895	7.9704	7.9156	7.8994	1.0209	0.0298	0.1107	0.9559
Nme2	_SC[Carbamidomethyl (C)]AHDWVYE_.2	#N/A	7.5413	7.0844	7.8674	7.6476	7.7507	7.0294	6.9491	7.2224	7.2387	7.0567	1.0520	0.0731	0.0833	1.0791
Nme2	_GLVGEIIK_.2	#N/A	7.3919	6.8567	7.1792	6.1409	7.5890	7.7433	6.8469	6.9515	7.7327	7.6374	0.9805	-0.0284	0.6947	0.1582
Nme2	_TFIAIKPDGVQR_.2	#N/A	9.3151	9.5373	9.6809	9.5942	9.5307	9.6015	9.5981	9.2162	9.4251	9.4788	1.0121	0.0173	0.2391	0.6214
Nme2	_NIIHGSDSVESAEK_.2	#N/A	5.3204	5.3142	3.6334	5.4618	5.7839	5.7288	5.0271	5.7937	5.2375	4.6688	1.0049	0.0070	0.9562	0.0194
Nme2	_VM[Oxidation (M)]LGETNPADSKPGTIR_.3	#N/A	8.0410	8.1698	8.6278	7.8730	8.5471	8.2857	7.6652	8.3469	8.4424	8.1510	1.0130	0.0186	0.6140	0.2118
Cacna1c	_SITADGES[Phospho (STY)]PPTTK_.2	Q01815_S815_M1_Cacna1c	4.3397	3.6440	3.5256	10.2038	9.4141	9.7203	10.8324	10.6672	10.6561	9.7153	0.6504	-0.6207	0.0615	1.2110
Cacna1c	_EAVSAAS[Phospho (STY)]EDDIFR_.2	#N/A	3.1624	4.0638	3.8492	4.3518	3.9521	4.1734	4.0665	3.7666	3.4152	4.3188	1.0086	0.0124	0.9006	0.0455
Cacna1c	_TGNNQADTES[Phospho (STY)]PSHEK_.2	Q01815_S1742_M1_Cacna1c	4.7016	4.0761	5.3779	4.2421	3.9270	4.4217	5.0826	5.1035	4.8288	3.3013	0.9735	-0.0387	0.7866	0.1043
Cacna1c	_EAVSAAS[Phospho (STY)]EDDIFRR_.2	#N/A	3.5092	3.7757	3.7910	3.8497	3.8969	3.6480	4.4248	3.6671	3.0414	3.6245	1.0151	0.0216	0.8193	0.0866
Vcp	_EVDIGIPDATGR_.2	#N/A	9.1115	9.3257	9.0562	9.4402	8.9660	9.2058	9.2178	8.7745	9.0541	9.3412	1.0096	0.0138	0.5273	0.2780
Vcp	_SVS[Phospho (STY)]DNDIR_.2	#N/A	8.9767	9.1806	9.4835	9.6804	8.8651	9.0576	9.2746	9.0931	8.8581	9.2556	1.0095	0.0137	0.6372	0.1957
Vcp	_LDQLIYIPLPDEK_.2	#N/A	8.3967	8.7031	8.9787	8.8854	8.9872	8.7040	8.7354	8.3534	8.4990	8.8101	1.0205	0.0293	0.2485	0.6047
Vcp	_GVLFYGPPGC[Carbamidomethyl (C)]GK_.2	#N/A	8.6260	8.5840	8.5288	8.7750	8.6354	8.5312	8.5690	8.3458	8.7097	8.7319	1.0028	0.0041	0.7800	0.1079
Vcp	_RSVS[Phospho (STY)]DNDIR_.2	#N/A	7.4241	7.5421	7.9265	8.2800	7.4199	7.5494	7.7205	7.6887	7.2852	7.6706	1.0131	0.0187	0.6215	0.2065
Vcp	_GGNIGDGGGAADR_.2	#N/A	8.1983	8.2473	8.0352	8.5466	8.3912	8.1273	8.2162	7.9244	8.0876	8.1320	1.0207	0.0296	0.1532	0.8147
Vcp	_WALSQSNPSALR_.2	#N/A	7.3956	7.6099	7.7160	8.1675	8.0059	7.8034	7.7750	7.7693	7.7798	7.8947	0.9972	-0.0040	0.8836	0.0538
Vcp	_M[Oxidation (M)]DELQLFR_.2	#N/A	7.1634	7.4775	7.2890	7.1822	7.4354	7.6053	7.3589	7.4719	7.6292	7.5029	0.9824	-0.0256	0.2235	0.6507
Vcp	_ETVVEVPQVTWEDIGGLEDVKR_.3	#N/A	6.8735	7.0249	7.7371	7.4751	7.5425	7.0562	7.4231	7.0897	7.2438	7.1795	1.0070	0.0101	0.7903	0.1022
Vcp	_LGDVISIQPC[Carbamidomethyl (C)]PDVK_.2	#N/A	6.6412	6.7798	5.9149	7.5428	6.5752	6.3055	6.9594	7.1187	6.5148	6.5454	0.9767	-0.0340	0.6147	0.2113
Vcp	_LEILQIHTK_.2	#N/A	6.2449	6.6156	6.3150	6.8473	6.8612	6.8649	7.1162	6.4197	7.0720	6.9117	0.9629	-0.0545	0.2210	0.6557
Vcp	_ESIESEIRR_.2	#N/A	6.2735	6.4160	6.0953	6.2356	6.4036	6.4060	5.8956	5.9128	6.3768	5.3863	1.0699	0.0975	0.0442	1.3545
Vcp	_AIANEC[Carbamidomethyl (C)]QANFISIK_.2	#N/A	5.3335	5.3419	4.9530	5.6177	5.3958	4.4238	4.9053	5.2471	5.2205	4.5425	0.9945	-0.0080	0.9059	0.0429
Vcp	_SVS[Phospho (STY)]DNDIRK_.2	#N/A	4.2244	5.5547	6.1337	3.9131	3.3501	5.7857	3.2393	5.6738	3.8540	5.9590	1.0311	0.0441	0.8582	0.0664
Vcp	_LAGESESNLR_.2	#N/A	7.5122	7.6781	7.8758	8.0779	8.0483	7.5787	7.7553	7.5736	7.5978	7.8799	1.0121	0.0174	0.5099	0.2925
Vcp	_QAAPC[Carbamidomethyl (C)]VLFFDELDSIAK_.2	#N/A	3.3062	5.6598	4.7711	4.8419	4.9291	4.3596	4.3629	5.0927	4.0317	4.7913	1.0164	0.0235	0.8685	0.0612
Vcp	_LIVDEAINEDNSVVSLSQPK_.2	#N/A	7.1720	7.5488	8.0565	8.3653	7.2672	7.8137	7.9538	7.2384	7.2010	7.8418	1.0192	0.0274	0.6220	0.2062
Vcp	_ELQELVQYPVEHPDK_.2	#N/A	6.6744	6.1537	6.4438	6.5494	6.3698	6.5198	6.2577	6.0602	6.1142	6.6620	1.0284	0.0404	0.2415	0.6170
Vcp	_NAPAIIFIDELDAIAPK_.2	#N/A	6.1565	5.9124	3.2558	5.5564	6.4257	5.3372	4.4487	5.3964	5.7377	6.5472	0.9834	-0.0241	0.8953	0.0480
Vcp	_EM[Oxidation (M)]VELPLR_.2	#N/A	7.5189	7.4446	6.8041	7.5643	7.6076	7.3773	7.4063	7.3360	7.4446	7.5439	0.9937	-0.0091	0.7720	0.1124
Vcp	_DHFEEAM[Oxidation (M)]R_.2	#N/A	5.9971	5.2427	4.0239	6.6709	5.8329	5.2190	4.8548	4.9952	5.1920	5.0917	1.0923	0.1273	0.3452	0.4619
Vcp	_IVSQLLTLM[Oxidation (M)]DGLK_.2	#N/A	4.9749	4.6107	4.3722	4.1766	4.6750	4.9059	4.6519	4.9510	4.6766	5.0958	0.9536	-0.0685	0.2427	0.6149
Vcp	_ESIESEIR_.2	#N/A	6.0636	5.6452	3.2923	5.8537	6.5001	5.2921	4.7489	4.1651	5.8997	6.0065	1.0454	0.0640	0.7359	0.1332
Vcp	_EAVC[Carbamidomethyl (C)]IVLSDDTC[Carbamidomethyl (C)]SDEK_.2	#N/A	5.4176	3.4721	4.8314	6.7519	3.3604	5.8133	5.9610	2.7590	3.6168	5.4474	1.1114	0.1523	0.5998	0.2220
Vcp	_[Acetyl (Protein N-term)]ASGADSKGDDLSTAILK_.2	#N/A	4.6105	4.8739	4.3889	5.2489	4.9109	4.7193	5.2237	4.8660	4.6860	4.8226	0.9781	-0.0320	0.5561	0.2548
Vcp	_M[Oxidation (M)]TNGFSGADLTEIC[Carbamidomethyl (C)]QR_.2	#N/A	3.1663	4.0567	4.1567	6.0881	5.8302	5.1747	4.7865	3.4577	5.0962	5.1211	1.0282	0.0401	0.8484	0.0714
Vcp	_VINQILTEM[Oxidation (M)]DGM[Oxidation (M)]STK_.2	#N/A	6.8444	7.5213	7.6540	6.9094	7.2653	7.8063	7.6490	7.4109	7.3174	7.4169	0.9845	-0.0225	0.5981	0.2232
Vcp	_EDEEES[Phospho (STY)]LNEVGYDDIGGC[Carbamidomethyl (C)]R_.2	Q01853_S197_M1_Vcp	4.0464	3.6979	3.8546	4.7462	3.3481	3.7275	3.8118	3.7002	3.6663	4.1922	1.0158	0.0227	0.8203	0.0860
Vcp	_LADDVDLEQVANETHGHVGADLAALC[Carbamidomethyl (C)]SEAALQAIR_.4	#N/A	4.3600	3.3316	3.5460	3.7226	3.1331	3.8702	3.7633	4.0698	3.1538	3.3246	1.0231	0.0330	0.7717	0.1125
Vcp	_DVDLEFLAK_.2	#N/A	3.9646	4.6498	3.1506	2.3159	3.5406	4.3222	3.4991	4.6550	4.1138	3.7761	0.9118	-0.1332	0.4778	0.3207
Vcp	_EAVC[Carbamidomethyl (C)]IVLSDDTC[Carbamidomethyl (C)]SDEKIR_.3	#N/A	3.8332	3.0886	3.5669	3.8262	2.9769	3.6477	3.5408	4.2923	3.9409	4.2582	0.8707	-0.1997	0.0578	1.2383
Vcp	_LAGESESNLRK_.3	#N/A	3.3920	3.9504	3.1257	4.1703	3.7717	3.9578	4.3186	4.3317	3.1299	3.8476	0.9542	-0.0676	0.5678	0.2458
Uba1	_AENYDIS[Phospho (STY)]PADR_.2	Q02053_S876_M1_Uba1	8.9664	8.9787	9.2771	9.3059	8.5789	8.9814	9.2539	8.9191	8.8480	9.0453	0.9998	-0.0003	0.9906	0.0041
Uba1	_DNPGVVTC[Carbamidomethyl (C)]LDEAR_.2	#N/A	8.5409	8.7182	8.8277	8.6491	8.6454	8.7793	8.9258	8.5645	8.7011	8.4771	1.0030	0.0044	0.7857	0.1047
Uba1	_RVS[Phospho (STY)]GPDPK_.2	Q02053_S13_M1_Uba1	8.2902	7.6818	7.9366	8.4846	7.3805	7.9374	8.6299	8.0749	8.3484	8.3356	0.9526	-0.0700	0.1138	0.9438
Uba1	_YDGQVAVFGSDFQEK_.2	#N/A	8.0504	7.6124	8.2932	8.4035	7.7679	7.9683	8.3121	7.9234	7.7614	7.7630	1.0096	0.0137	0.6921	0.1598
Uba1	_[Acetyl (Protein N-term)]SSS[Phospho (STY)]PLSKK_.2	Q02053_S4_M1_Uba1	7.0183	6.9793	7.9675	6.9671	6.7635	7.1747	7.1529	3.5229	7.2292	6.9427	1.1502	0.2019	0.2443	0.6121
Uba1	_LKSDTAAAAVR_.2	#N/A	7.8884	6.7891	7.9361	7.0800	8.1653	6.8947	7.0553	7.2730	8.2405	6.9574	1.0105	0.0150	0.8460	0.0726
Uba1	_QNSLDEDLIR_.2	#N/A	7.3265	6.6924	7.3750	7.1966	7.0716	6.8210	7.2043	7.0317	6.9958	7.3390	0.9913	-0.0126	0.6968	0.1569
Uba1	_QPAENVNQYLTDSK_.2	#N/A	7.2324	7.5041	7.1979	7.3251	7.0648	7.2262	7.6322	7.0703	7.1322	6.9552	1.0085	0.0122	0.6746	0.1710
Uba1	_SLPASLVEPDFVM[Oxidation (M)]TDFAK_.2	#N/A	6.8074	7.1144	6.7757	6.6279	6.8320	6.5746	7.2965	7.4284	7.1206	7.0962	0.9383	-0.0919	0.0046	2.3343
Uba1	_AAVASLLQSVQVPEFTPK_.2	#N/A	6.4351	6.5197	6.7896	5.9885	6.3167	6.3914	6.4493	6.2143	6.5440	6.3254	1.0037	0.0053	0.8751	0.0580
Uba1	_SIPIC[Carbamidomethyl (C)]TLK_.2	#N/A	5.7185	6.4666	6.0396	5.8365	6.7942	5.7950	5.3063	6.4103	6.1392	5.5612	1.0434	0.0613	0.4184	0.3784
Uba1	_YFLVGAGAIGC[Carbamidomethyl (C)]ELLK_.2	#N/A	5.5323	5.0736	6.0961	4.5146	5.1131	5.5692	5.4870	4.6312	5.6112	5.1750	1.0173	0.0247	0.7883	0.1033
Uba1	_NEEDATELVGLAQAVNAR_.2	#N/A	7.1460	7.2829	7.5764	7.1752	7.0616	7.0354	7.2336	7.1504	7.4085	6.8137	1.0086	0.0123	0.6759	0.1701
Uba1	_ATLPS[Phospho (STY)]PDKLPGFK_.2	Q02053_S835_M1_Uba1	8.0163	9.3453	4.4212	10.7791	2.8601	8.1716	3.7293	3.4201	3.7192	3.2583	2.0572	1.0407	0.0419	1.3782
Uba1	_VGEFC[Carbamidomethyl (C)]HSR_.2	#N/A	6.1461	6.9198	6.5102	6.8303	6.1567	6.9533	7.1523	6.5185	6.4155	6.6245	0.9863	-0.0199	0.7002	0.1548
Uba1	_LAGTQPLEVLEAVQR_.2	#N/A	7.3064	6.7135	6.6369	6.8855	6.6957	6.8302	6.8469	6.9138	7.1253	6.8022	0.9888	-0.0162	0.5873	0.2311
Uba1	_NGS[Phospho (STY)]EADIDESLYSR_.2	Q02053_S46_M1_Uba1	3.2399	6.4212	4.4744	6.9338	5.2121	6.2922	6.7941	6.2055	5.9966	6.1372	0.8640	-0.2109	0.2730	0.5639
Uba1	_VVQGHQQLDSYK_.2	#N/A	4.6078	4.4503	3.9874	3.6531	4.6020	3.9688	3.2058	3.7928	4.7213	3.8139	1.0845	0.1170	0.3347	0.4753

Uba1	_QFLDYFK_.2	#N/A	3.4627	4.3387	3.1963	3.5618	3.4180	4.2857	4.3922	4.5314	3.9717	4.0889	0.8739	-0.1945	0.0792	1.1015
Uba1	_LDQPM[Oxidation (M)]TEIVSR_.2	#N/A	7.2134	7.1926	7.3148	7.1859	7.4443	7.2903	7.0855	7.3320	7.0917	7.1800	1.0141	0.0202	0.1746	0.7580
Uba1	_M[Oxidation (M)]LQTSSVLVSGLR_.2	#N/A	5.2968	5.3059	5.7188	4.8996	5.0336	5.9602	4.9976	5.9710	5.3324	5.3353	0.9926	-0.0107	0.8822	0.0544
Uba1	_NFPNAIEHTLQWAR_.2	#N/A	5.6238	5.4138	4.5350	5.3404	3.4224	5.2803	5.4895	5.7011	5.3393	5.4697	0.8975	-0.1561	0.2232	0.6513
Uba1	_LAYVAAGDLAPINAFIGGLAAQEVM[Oxidation (M)]K_.3	#N/A	3.9705	3.2259	2.9658	2.7908	3.1142	2.2088	2.8989	1.8077	2.7559	2.5143	1.2212	0.2883	0.1548	0.8103
Uba1	_NFAM[Oxidation (M)]IGLGC[Carbamidomethyl (C)]GEGGEVVVTDM[Oxidation (M)]DTIEK_.3	#N/A	3.9293	5.2163	5.3868	3.7301	3.0730	6.4352	3.4448	3.7944	4.9387	5.3017	1.0592	0.0829	0.7330	0.1349
Uba1	_DEFEGLFK_.2	#N/A	4.0079	5.0989	4.1128	4.6155	4.3562	4.7863	3.5448	3.9395	4.5045	5.2104	1.0457	0.0645	0.5964	0.2245
Uba1	_QM[Oxidation (M)]NPYIQVTSHQNR_.3	#N/A	3.4411	4.2621	4.1387	4.1093	4.7737	4.8919	4.0010	4.8311	4.0931	4.9376	0.9561	-0.0648	0.5677	0.2459
Uba1	_IHVS[Phospho (STY)]DQELQSANASVDDSRLEELK_.3	Q02053_S810_M1_Uba1	4.1740	4.4490	3.9848	3.3757	3.3763	3.2644	4.0620	3.3911	3.7888	3.1108	1.0509	0.0716	0.5641	0.2486
Uba1	_IYDDDFFQNLDGVANALDNIDAR_.3	#N/A	3.5598	4.3650	4.2466	3.7557	4.1864	3.5431	3.9039	3.2455	3.5446	3.4329	1.1164	0.1588	0.0944	1.0248
Uba1	_VLGPYTFSIC[Carbamidomethyl (C)]DTSNFSDYIR_.2	#N/A	3.7316	3.6108	3.4653	4.5099	3.4322	4.2973	3.1573	3.9921	4.2912	4.1871	0.9832	-0.0245	0.8364	0.0776
Uba1	_QNSLDEDLIRK_.3	#N/A	0.6489	3.2248	3.1064	3.6090	3.0461	3.1825	3.5433	3.0086	4.1580	3.5760	0.7848	-0.3496	0.2206	0.6563
Ctnnb1	_TS[Phospho (STY)]M[Oxidation (M)]GGTQQQFVEGVR_.2	#N/A	6.7459	8.5494	9.5750	7.9746	4.9003	8.7201	9.2745	9.0505	9.0062	8.5999	0.8621	-0.2140	0.1895	0.7223
Ctnnb1	_HAVVNLINYQDDAELATR_.3	#N/A	5.4638	5.0850	4.2022	4.6734	3.4451	3.7653	4.3971	3.0492	4.8173	4.9626	1.0308	0.0437	0.8071	0.0930
Ctnnb1	_RTS[Phospho (STY)]M[Oxidation (M)]GGTQQQFVEGVR_.3	#N/A	3.9274	3.2415	3.8871	5.3559	4.9306	3.3916	5.0608	5.7534	4.1496	3.3580	0.9000	-0.1520	0.4656	0.3320
Ctnnb1	_TMQNTNDVETAR_.2	#N/A	5.2022	4.0106	4.5664	4.2422	3.9764	3.8976	4.2584	4.3919	3.1901	3.7874	1.1047	0.1436	0.2538	0.5955
Ank1	_GPPETGS[Phospho (STY)]PK_.2	Q02357_S1759_M1_Ank1	8.6057	8.3846	4.1215	8.4529	3.2748	3.8040	4.0269	3.6300	4.3378	3.8048	1.5462	0.6287	0.1461	0.8354
Ank1	_LDQVVES[Phospho (STY)]PAIPR_.2	Q02357_S852_M1_Ank1	6.5965	6.7898	7.1906	7.0328	4.2719	6.9370	7.0051	6.5699	6.1564	6.7243	0.9782	-0.0318	0.8088	0.0921
Ank1	_AEDS[Phospho (STY)]DAIPEWK_.2	Q02357_S1589_M1_Ank1	3.5599	3.6662	4.2467	8.7298	6.9910	3.5430	5.8122	7.9034	7.9791	8.2451	0.6844	-0.5471	0.0868	1.0617
Nfia	_SVEDEM[Oxidation (M)]DS[Phospho (STY)]PGEEPFYTGQGR_.2	#N/A	3.5491	6.1239	3.5767	7.1037	6.0295	4.0263	6.4118	6.7863	6.9339	6.5302	0.7604	-0.3952	0.0777	1.1097
Nfia	_LKS[Phospho (STY)]VEDEM[Oxidation (M)]DS[Phospho (STY)]PGEEPFYTGQGR_.3	Q02780_S303_M2_Nfia	3.3098	4.1150	3.9966	4.0057	3.9364	3.7931	4.1539	3.6192	3.2678	2.9654	1.1022	0.1404	0.1901	0.7210
Nfia	_S[Phospho (STY)]PGSGSQSSGWHEVEPGLPSPSTLK_.3	Q02780_S323_M1_Nfia	3.7831	3.0875	4.4699	6.2797	2.9088	3.3199	3.6807	3.1459	4.2953	3.4387	1.0919	0.1269	0.6318	0.1995
Nucb1	_KVPEQPPELPQLDS[Phospho (STY)]QHL_.3	Q02819_S456_M1_Nucb1	6.0885	6.7847	5.5866	7.4687	6.1246	6.3461	5.7620	6.9386	6.7315	6.3451	0.9931	-0.0100	0.9122	0.0399
Nucb1	_SGKLS[Phospho (STY)]QELDFVSHNVR_.3	Q02819_S85_M1_Nucb1	3.9878	5.9196	3.6091	6.9006	3.2883	4.5048	5.0055	4.8734	5.4057	4.3310	0.9588	-0.0607	0.7929	0.1008
Nucb1	_AQRLS[Phospho (STY)]QETEALGR_.2	#N/A	3.8296	4.9820	4.0316	4.2320	3.9046	4.2912	3.7238	3.4571	3.7562	4.3794	1.1000	0.1374	0.1841	0.7350
Nucb1	_LSQELDFVSHNVR_.3	#N/A	4.0292	3.3132	3.7629	3.3067	3.1729	3.4517	3.4549	3.6945	3.9936	2.9839	0.9927	-0.0105	0.9164	0.0379
Epha4	_NPNS[Phospho (STY)]LKR_.2	Q03137_S887_M1_Epha4	7.7005	7.4896	7.4194	7.8799	6.1935	6.8888	7.3910	7.7942	7.2036	7.3893	0.9755	-0.0358	0.5975	0.2237
Epha4	_IDTIAADESFTQVDIGDR_.2	#N/A	5.1667	4.8160	5.2330	6.1061	3.0771	5.5390	5.4348	4.5964	5.4602	5.0279	0.9727	-0.0400	0.8055	0.0939
Epha4	_TYVDPFTYEDPNQAVR_.2	#N/A	4.6438	4.7568	4.0141	4.5167	3.3968	4.5912	5.7879	4.7230	4.7007	4.6455	0.8702	-0.2006	0.0973	1.0120
Mark3	_RYS[Phospho (STY)]DHAGPAIPSVVAYPK_.3	Q03141_S419_M1_Mark3	3.7795	8.5118	8.6329	4.5578	7.1192	8.0552	3.5946	8.5706	8.4450	7.9629	0.9486	-0.0761	0.8035	0.0950
Mark3	_SRGS[Phospho (STY)]TNLFSK_.2	Q03141_S601_M1_Mark3	3.4878	4.2441	3.2215	4.1716	4.1324	3.9931	3.8863	3.9469	5.9588	4.1386	0.8645	-0.2101	0.2103	0.6771
Mark3	_SAELDASDSSS[Phospho (STY)]SSNLSLAK_.2	Q03141_S378_M1_Mark3	3.3174	3.5831	4.0042	5.2176	3.9003	4.1227	4.7942	5.0613	4.2197	3.5215	0.9148	-0.1285	0.4080	0.3893
Mark3	_IDIMVGM[Oxidation (M)]GYS[Phospho (STY)]QEEIQESLSK_.2	Q03141_S341_M1_Mark3	2.9862	3.7914	3.2847	4.3293	3.6127	4.1168	4.4776	4.1727	2.9709	4.0066	0.9437	-0.0837	0.5625	0.2499
Mark3	_VRPNSDLSNSTGQS[Phospho (STY)]PHHK_.3	#N/A	5.0502	5.1656	5.4305	5.5513	4.4907	5.2363	5.3137	5.5528	5.1924	5.1372	0.9727	-0.0400	0.4963	0.3042
Mark3	_TPVAS[Phospho (STY)]THS[Phospho (STY)]ISSATTPDR_.2	Q03141_S540_M2_Mark3	3.4367	4.2419	4.1235	3.8788	4.0632	3.6663	4.0271	3.1224	3.4214	3.5561	1.1048	0.1437	0.1238	0.9072
Cdk7	_SFGS[Phospho (STY)]PNR_.2	Q03147_S164_M1_Cdk7	4.1864	3.0398	3.3724	3.8750	3.3121	4.4174	3.5897	4.2435	7.9543	7.5921	0.6331	-0.6595	0.0501	1.2998
Enah	_PSS[Phospho (STY)]PVNTPSSQPPAAK_.2	#N/A	3.7179	2.9733	3.4388	3.9415	2.8616	7.5239	3.6562	4.1770	7.5515	3.9085	0.8451	-0.2428	0.5325	0.2737
Enah	_S[Phospho (STY)]PVISRPK_.2	Q03173_S740_M1_Enah	7.3249	4.2437	3.6693	4.1720	5.7009	3.9935	3.8866	3.9465	7.2541	4.1390	1.0092	0.0132	0.9646	0.0157
Enah	_NSRPSS[Phospho (STY)]PVNTPSSQPPAAK_.3	#N/A	8.5630	8.3268	8.7777	9.5808	8.3267	8.8605	8.7616	8.1985	8.5157	8.4586	1.0301	0.0428	0.3468	0.4599
Atp5f1a	_AVDSLVPIGR_.2	#N/A	12.2132	12.4356	12.5501	12.2423	12.1868	12.1060	12.1276	12.2752	12.0167	12.0453	1.0143	0.0204	0.1137	0.9442
Atp5f1a	_VVDALGNAIDGK_.2	#N/A	11.9426	11.8715	12.0462	12.1298	12.1483	11.9032	12.0000	11.8507	11.9087	11.8819	1.0081	0.0117	0.1782	0.7490
Atp5f1a	_VLSIGDGIAR_.2	#N/A	11.8300	11.9892	11.9680	11.8968	11.9254	11.9104	11.8450	11.8274	11.9233	11.8124	1.0057	0.0082	0.0861	1.0648
Atp5f1a	_TGAIVDVPVGEELLGR_.2	#N/A	11.4067	11.2124	11.6040	11.3943	10.9705	11.2597	11.3264	11.1543	11.1576	11.2472	1.0077	0.0111	0.4703	0.3276
Atp5f1a	_ELIIGDR_.2	#N/A	11.4260	11.3846	11.6489	11.3416	11.3410	11.3793	10.9119	11.2624	11.3340	11.3740	1.0178	0.0254	0.0869	1.0610
Atp5f1a	_LELAQYR_.2	#N/A	10.9552	11.1023	11.1957	10.9355	10.9915	10.8474	10.6250	11.0234	10.8551	11.0109	1.0116	0.0166	0.2311	0.6362
Atp5f1a	_TSIAIDTIINQK_.2	#N/A	9.6111	9.4416	9.9003	9.9588	9.6888	9.9091	9.8446	9.6587	9.8714	9.5907	1.0011	0.0015	0.9327	0.0303
Atp5f1a	_GYLDKLEPSK_.2	#N/A	9.8907	9.8348	10.4225	10.1214	9.7672	9.8702	10.0058	9.9416	9.5748	9.7142	1.0179	0.0256	0.2709	0.5671
Atp5f1a	_HALIIYDDLSK_.2	#N/A	9.3064	9.4738	9.9594	9.9713	9.5122	9.5719	9.4551	9.5526	9.5277	9.5012	1.0130	0.0186	0.4041	0.3936
Atp5f1a	_ILGADTSVDLEETGR_.2	#N/A	9.2664	8.8508	9.3581	9.1805	9.0576	9.1383	9.2830	9.3626	9.0568	9.1487	0.9923	-0.0111	0.5189	0.2849
Atp5f1a	_VVDALGNAIDGKGPIGSK_.2	#N/A	6.8478	7.4811	7.7221	7.0543	7.1393	7.4776	6.5620	6.9026	7.0839	7.2458	1.0487	0.0686	0.1338	0.8735
Atp5f1a	_STVAQLVK_.2	#N/A	8.1359	8.4599	7.6541	8.1557	8.2814	7.5285	6.7325	8.1902	8.1606	8.0018	1.0341	0.0483	0.4495	0.3473
Atp5f1a	_RTGAIVDVPVGEELLGR_.3	#N/A	7.6446	7.6841	7.7685	7.7556	7.8440	8.0195	7.8612	7.7039	7.9003	8.0163	0.9893	-0.0156	0.3512	0.4545
Atp5f1a	_EGDVVKR_.2	#N/A	7.9022	7.3884	7.6539	7.5302	7.9938	7.3335	7.2374	7.5553	6.8360	7.6309	1.0436	0.0615	0.1469	0.8331
Atp5f1a	_LKEIVTNFLAGFEP_.2	#N/A	6.6250	6.8547	7.4887	7.0489	6.6967	7.0128	6.7109	6.4712	6.9071	6.8559	1.0324	0.0460	0.2510	0.6003
Atp5f1a	_RFNDGTDEK_.2	#N/A	6.8900	6.9589	6.9359	7.1606	7.6730	7.1258	6.9743	6.5289	7.1064	6.4497	1.0531	0.0746	0.1038	0.9836
Atp5f1a	_EIVTNFLAGFEP_.2	#N/A	7.0797	6.8273	6.9101	6.8494	6.6114	6.6483	6.4956	6.3334	6.5876	6.8896	1.0372	0.0527	0.0919	1.0365
Atp5f1a	_EAYPGDVFYLHSR_.2	#N/A	10.6677	10.8258	11.1264	10.9876	10.5026	10.7908	10.9083	10.7716	10.6942	10.7557	1.0032	0.0046	0.7800	0.1079
Atp5f1a	_FNDGTDEK_.2	#N/A	7.8408	6.6348	7.0987	6.3813	7.6591	6.9406	5.5739	7.3089	7.4547	7.4452	1.0211	0.0302	0.7599	0.1192
Atp5f1a	_ISEQSDAK_.2	#N/A	7.2138	6.8689	7.4090	7.0762	8.2836	4.5010	6.6228	6.6081	6.7782	6.8906	1.0249	0.0354	0.8035	0.0950
Atp5f1a	_QM[Oxidation (M)]SLLLR_.2	#N/A	9.3033	9.7044	9.8705	9.3776	9.5143	9.6191	9.3946	9.6748	9.6713	9.6794	0.9958	-0.0060	0.7484	0.1258
Atp5f1a	_RLTDADAM[Oxidation (M)]K_.2	#N/A	8.1385	8.1254	7.8822	8.4432	8.4226	7.8029	7.7306	7.8101	7.8004	8.0439	1.0369	0.0523	0.0822	1.0852
Atp5f1a	_FNDGTDEKK_.2	#N/A	5.7315	5.4129	4.9874	4.5156	5.9191	5.7286	5.4694	4.3813	5.7391	5.3292	1.0292	0.0415	0.6823	0.1660
Atp5f1a	_LYC[Carbamidomethyl (C)]IYVAIGQK_.2	#N/A	4.9357	5.5519	3.7900	4.2926	4.6177	5.7461	5.8199	4.9171	5.9848	5.5767	0.8651	-0.2091	0.1138	0.9440
Atp5f1a	_GM[Oxidation (M)]SLNLEPDNVGVVVFGNDK_.2	#N/A	8.6327	8.7630	8.7008	9.0292	8.7144	8.8857	8.9380	8.7998	8.7703	8.8171	0.9951	-0.0071	0.5986	0.2228
Atp5f1a	_QGQYSPM[Oxidation (M)]AIEEQVAVIYAGVR_.2	#N/A	5.6400	4.6949	5.3187	5.1078	4.6580	4.8829	5.2946	5.6481	5.2487	5.4280	0.9344	-0.0979	0.1261	0.8993
Atp5f1a	_EPMQTGIK_.2	#N/A	7.3461	5.3974	3.3521	8.0774	7.5048	3.6763	3.5695	5.4654	5.5822	6.2674	1.1286	0.1745	0.5734	0.2415
Atp5f1a	_TGTAEM[Oxidation (M)]SSILEER_.2	#N/A	9.0216	8.9907	8.9815	9.4511	8.6094	8.8350	8.9160	8.9025	8.7903	9.0001	1.0089	0.0128	0.5997	0.2220
Atp5f1a	_FENAFLSHVISQHQSLLGNIR_.3	#N/A	5.8199	5.7904	6.2527	6.3575	5.5503	5.8227	6.1672	6.0335	6.2824	6.0941	0.9655	-0.0507	0.2289	0.6404
Atp5f1a	_EVAAFAQFGSDLDAATQQLLSR_.3	#N/A	7.1677	6.9079	7.2985	7.6900	6.5622	7.1277	7.2645	6.9297	7.2605	7.3663	0.9890	-0.0160	0.7108	0.1483
Atp5f1a	_LTDADAMK_.2	#N/A	4.8830	4.0473	4.5296	4.6056	4.5798	4.1858	4.2216	4.4287	3.2269	3.4640	1.1660	0.2215	0.0506	1.2956
Atp5f1a	_YTIVVSATASDAAPLQYLAPYSGC[Carbamidomethyl (C)]SM[Oxidation (M)]GEYFR_.3	#N/A	3.1309	4.2115	4.3654	3.9092	4.0328	3.6967	4.0575	3.0920	3.3910	3.5865	1.1017	0.1398	0.2282	0.6416
Atp5f1a	_NVQAEEMVEFSSGLK_.2	#N/A	3.8449	3.3813	4.5317	3.5335	3.2696	3.3550	3.2482	3.0841	4.2336	2.8260	1.0910	0.1257	0.4013	0.3965
Grin2d	_S[Phospho (STY)]LEDLSSC[Carbamidomethyl (C)]PR_.2	Q03391_S1258_M1_Grin2d	7.3874	6.7330	7.2294	7.2126	5.8083	7.0685	8.1013	7.6260	7.1204	7.3769	0.9140	-0.1297	0.0922	1.0352
Grin2d	_LAFEDES[Phospho (STY)]PPAPSR_.2	Q03391_S1049_M1_Grin2d	4.0289	3.3333	3.2149	3.7176	3.1546	7.9227	8.9229	8.6949	7.9941	3.6846	0.5774	-0.7924	0.0512	1.2906
Grin2d	_RGS[Phospho (STY)]AHFSSLESEV_.2	Q03391_S1313_M1_Grin2d	4.1120	3.2304	3.8456	3.3894	3.0518	3.1769	5.1463	3.6118	3.9108	3.0667	0.8815	-0.1820	0.2868	0.5424
Scg2	_VAWIPDVESR_.2	#N/A	7.4627	7.9853	7.8067	7.6796	8.0069	7.6035	7.5163	7.3831	7.2695	7.0701	1.0612	0.0858	0.0099	2.0048
Scg2	_IETQTQEEVRDS[Phospho (STY)]KENTEK_.3	#N/A	5.5691	4.1828	5.1784	4.0949	6.2574	6.2018	5.9042	5.8333	6.1867	4.2106	0.9483	-0.0766	0.6459	0.1899
Scg2	_IETQTQEEVRDS[Phospho (STY)]K_.2	#N/A	5.6146	5.3430	4.2559	4.8608	5.2023	4.6814	5.1599	3.2015	5.7163	5.7771	1.0059	0.0085	0.9580	0.0186
Scg2	_QAPYENLNDQELGEYLAR_.2	#N/A	4.2374	5.3389	4.1599	3.8433	4.9822	4.9491	5.0737	3.0728	4.5014	4.2546	1.0851	0.1178	0.4444	0.3522

Scg2	_EHLGPGSS[Phospho (STY)]QEM[Oxidation (M)]ER_.2	#N/A	3.8685	4.1435	4.0421	10.6828	7.9215	9.9556	9.7305	3.6171	9.8810	9.9093	0.8171	-0.2915	0.4753	0.3230
Scg2	_VPSPVS[Phospho (STY)]SEDDLQEEEQLEQAIK_.3	Q03517_S532_M1_Scg2	3.9358	3.4066	3.6695	3.2133	3.2279	3.0008	3.3616	3.7879	4.0869	4.3914	0.8725	-0.1967	0.0775	1.1104
Scg2	_SPYFPNQYSQDK_.2	#N/A	6.4723	6.4789	4.2039	6.3182	6.6587	6.6098	4.2057	5.9858	6.1525	6.1931	1.0869	0.1202	0.4485	0.3483
Scg2	_S[Phospho (STY)]GQLGLPDEENRR_.2	#N/A	5.8167	6.5895	6.6747	6.4807	5.9868	6.2130	6.1176	5.7503	6.1721	6.0629	1.0445	0.0628	0.1991	0.7009
Scg2	_ESKDQLSEDASK_.2	#N/A	3.7363	3.0919	3.9842	3.5280	4.9881	3.2303	3.6763	3.4732	4.5310	3.2053	1.0103	0.0148	0.9288	0.0321
Scg2	_VPSPVSSEDDLQEEEQLEQAIKEHLGPGS[Phospho (STY)]SQEM[Oxidation (M)]ER_.4	#N/A	2.9203	3.7255	3.6071	4.3952	3.5469	4.1826	4.5434	4.1068	4.4059	4.0768	0.8707	-0.1997	0.0836	1.0776
Scg2	_FPLMYEENS[Phospho (STY)]RENPFKR_.3	Q03517_S176_M1_Scg2	4.2291	3.4846	3.9628	5.2067	3.3729	3.2518	3.6548	3.4946	3.7937	3.1839	1.1094	0.1497	0.3490	0.4572
Kcnb1	_NHFESSPLPTS[Phospho (STY)]PK_.2	#N/A	8.5381	8.5657	3.1905	8.4670	6.6946	8.5004	8.7090	8.6064	8.4023	3.6602	0.9975	-0.0036	0.9903	0.0042
Kcnb1	_SLHGSTS[Phospho (STY)]PK_.2	Q03717_S782_M1_Kcnb1	6.7389	6.4648	3.8473	4.1550	3.7870	3.9425	4.3033	6.8911	3.1452	3.8323	1.0615	0.0862	0.7780	0.1090
Kcnb1	_VQDNHLS[Phospho (STY)]PNK_.2	#N/A	5.5011	5.1786	3.9809	5.8681	3.6885	5.3326	5.9305	5.5835	5.3478	5.3677	0.8862	-0.1743	0.2079	0.6821
Kcnb1	_T[Phospho (STY)]PPRSPEK_.2	Q03717_T732_M1_Kcnb1	8.0268	7.4827	5.8092	6.8264	3.2335	5.9050	7.2628	8.0885	6.4356	6.9287	0.8656	-0.2083	0.3194	0.4957
Kcnb1	_TQS[Phospho (STY)]QPILNTK_.2	Q03717_S541_M1_Kcnb1	8.2174	8.0401	7.6427	7.9628	5.1536	7.4600	7.7386	7.7603	7.6939	7.4059	0.9690	-0.0454	0.6964	0.1572
Kcnb1	_TEKNHFESSPLPTS[Phospho (STY)]PK_.3	#N/A	6.7455	6.1766	8.2306	6.6678	5.0882	5.5877	4.3196	6.3508	3.1289	3.8487	1.4542	0.5403	0.0333	1.4778
Kcnb1	_VQDNHLS[Phospho (STY)]PNKWK_.3	#N/A	6.2728	5.7734	6.2181	6.0080	4.8356	5.9645	6.0666	6.5089	6.2723	6.0297	0.9399	-0.0895	0.2222	0.6532
Kcnb1	_DKVQDNHLS[Phospho (STY)]PNKWK_.3	#N/A	5.6575	4.7803	4.9619	5.1865	4.4519	5.0438	5.5524	5.4286	5.3274	4.7063	0.9543	-0.0675	0.3731	0.4282
Kcnb1	_SSSS[Phospho (STY)]PQHLNVQQLQDMYSK_.3	Q03717_S520_M1_Kcnb1	3.2887	4.2152	4.1104	4.0254	4.0799	3.9362	4.0293	3.9399	3.4477	3.8158	1.0353	0.0501	0.5161	0.2873
Kcnb1	_S[Phospho (STY)]IEM[Oxidation (M)]M[Oxidation (M)]DIVVEK_.2	#N/A	6.2766	6.6867	6.9398	5.7943	5.1549	6.7077	7.2288	7.1404	6.8733	6.6464	0.8978	-0.1555	0.0835	1.0783
Kcnb1	_STSS[Phospho (STY)]LPPEPMEIVR_.2	Q03717_S15_M1_Kcnb1	3.8959	3.6371	3.8942	4.0550	3.4949	3.8518	4.9243	3.6928	3.7522	3.8167	0.9403	-0.0889	0.3695	0.4324
Kcnb1	_NGS[Phospho (STY)]IVSMNMK_.2	Q03717_S444_M1_Kcnb1	4.1307	3.3861	3.8644	3.5287	3.2744	3.3502	3.2434	3.0890	4.2384	3.4957	1.0206	0.0294	0.7914	0.1016
Kcnb1	_EQGS[Phospho (STY)]PEKAR_.2	Q03717_S511_M1_Kcnb1	3.1652	3.9215	4.3997	4.4942	3.8098	4.3157	4.2088	3.6243	3.2729	2.9603	1.1425	0.1922	0.1683	0.7740
Kcnd1	_QNGGLEDSGS[Phospho (STY)]GDGQM[Oxidation (M)]LC[Carbamidomethyl (C)]VR_.2	#N/A	3.7904	4.0400	4.1889	4.9189	3.4774	3.7637	6.3411	4.2237	3.8933	3.7220	0.8867	-0.1735	0.3713	0.4302
Pfdn6	_ETLAQLQQEFQR_.2	#N/A	4.6562	5.8813	5.3835	6.0264	6.0011	5.6827	5.4704	3.2150	5.2144	5.4013	1.1616	0.2162	0.1596	0.7969
Ckb	_VLTPELYAELR_.2	#N/A	11.4805	11.8402	11.8972	11.9634	11.9004	11.7278	11.8682	11.8364	11.8437	11.8972	0.9950	-0.0073	0.5275	0.2778
Ckb	_FSEVLKR_.2	#N/A	10.5668	11.0721	11.0046	11.1090	11.4880	10.8506	10.8112	10.8626	10.9214	10.9064	1.0129	0.0184	0.3983	0.3998
Ckb	_DLFDPIIEER_.2	#N/A	11.9541	11.4902	11.1824	11.9567	11.6264	11.5539	11.6878	11.3571	11.3899	11.7500	1.0070	0.0101	0.6474	0.1888
Ckb	_GIWHNDNK_.2	#N/A	10.8977	11.1667	11.0568	11.3761	11.3423	11.0743	11.0906	10.9541	11.0102	11.1697	1.0087	0.0125	0.3654	0.4372
Ckb	_LLIEMEQR_.2	#N/A	9.9368	9.6867	9.6060	10.8469	9.7429	9.1482	9.7065	9.4419	9.3415	9.6861	1.0297	0.0423	0.3662	0.4363
Ckb	_HGGYQPSDEHKTDLNPDNLQGGDDLDPNYVLSSR_.5	#N/A	9.3445	9.5790	9.7266	9.8154	8.6933	9.3942	9.2326	8.9356	9.1113	9.4291	1.0271	0.0385	0.2940	0.5316
Ckb	_FC[Carbamidomethyl (C)]TGLTQIETLFK_.2	#N/A	9.8089	10.0619	10.1628	10.0858	10.2365	9.9397	9.9826	9.9264	9.7395	10.1791	1.0093	0.0133	0.4106	0.3866
Ckb	_TFLVWINEEDHLR_.2	#N/A	6.6997	7.1050	7.0863	7.9578	6.5784	6.8612	7.2148	6.8143	7.7136	7.4730	0.9650	-0.0514	0.4087	0.3886
Ckb	_HEKFS[Phospho (STY)]EVLKR_.3	#N/A	7.5654	6.9128	7.5594	7.8817	7.4501	7.6282	7.3762	7.3660	7.1988	7.7431	1.0106	0.0152	0.6862	0.1635
Ckb	_NYEFM[Oxidation (M)]WNPHLGYILTC[Carbamidomethyl (C)]PSNLGTGLR_.3	#N/A	5.0313	5.0236	5.0441	6.0724	5.5223	5.0615	5.3960	5.5607	4.7069	4.2449	1.0634	0.0886	0.3618	0.4415
Ckb	_LEQGQAIDDLM[Oxidation (M)]PAQK_.2	#N/A	11.0802	11.1629	12.2098	11.7237	12.1703	11.8152	12.0291	11.6385	10.9009	11.0340	1.0257	0.0366	0.3907	0.4081
Ckb	_LAVEALSSLDGDLSGR_.2	#N/A	9.6183	9.6573	10.1303	9.9567	9.8711	9.9158	9.8288	9.3473	9.7472	9.8612	1.0167	0.0239	0.2669	0.5737
Ckb	_HEKFS[Phospho (STY)]EVLK_.2	#N/A	6.0189	5.7420	5.6438	5.3863	4.9277	5.1027	5.8353	5.6905	5.7710	6.0695	0.9364	-0.0948	0.1285	0.8911
Ckb	_TDLNPDNLQGGDDLDPNYVLSSR_.3	#N/A	4.8361	4.5748	4.2716	5.4698	5.2078	4.8368	4.9268	4.3200	5.3553	4.6754	1.0097	0.0139	0.8708	0.0601
Ckb	_FPAEDEFPDLSSHNNHM[Oxidation (M)]AK_.3	#N/A	8.4342	7.8941	7.8587	8.5944	8.5300	8.1333	7.7545	7.6819	7.9701	8.1218	1.0455	0.0642	0.0861	1.0648
Ckb	_LGFSEVELVQM[Oxidation (M)]VVDGVK_.2	#N/A	7.7760	7.9893	8.1750	8.0439	7.1596	7.7800	7.9283	7.6498	7.6732	8.0794	0.9985	-0.0022	0.9537	0.0206
Ckb	_PFSNSHNTQK_.2	#N/A	7.2311	7.6366	7.5014	7.8234	8.6937	7.5977	7.5197	7.2627	7.6713	7.5753	1.0320	0.0454	0.3925	0.4062
Ckb	_LRFPAEDEFPDLSSHNNHM[Oxidation (M)]AK_.4	#N/A	10.0960	10.4997	11.4321	10.7827	10.6741	10.5104	10.4763	10.0607	10.2791	10.5136	1.0323	0.0458	0.2025	0.6936
Ckb	_SMTEAEQQQLIDDHFLFDKPVSPLLLASGM[Oxidation (M)]AR_.4	#N/A	5.2798	4.7723	3.2942	5.8770	4.8346	4.2554	4.0581	4.7022	4.3213	4.6454	1.0648	0.0906	0.5557	0.2551
Ckb	_GTGGVDTAAVGGVFDVSNADR_.2	#N/A	8.5785	8.6700	9.0188	9.2068	8.8879	8.6877	8.8393	8.4602	8.7259	8.5639	1.0225	0.0321	0.2015	0.6957
Ckb	_HGGYQPSDEHK_.3	#N/A	5.0524	3.1653	3.9108	3.4545	3.2320	3.2420	3.6028	4.1565	4.3282	3.1318	0.9662	-0.0496	0.7719	0.1124
Ckb	_RGTGGVDTAAVGGVFDVSNADR_.3	#N/A	4.7372	4.7433	3.1110	3.6136	4.1810	3.1779	3.3283	3.0040	4.9526	3.5806	1.0568	0.0797	0.6878	0.1626
Npy1r	_QAS[Phospho (STY)]PVAFKK_.2	#N/A	7.1028	7.4295	8.3507	7.6828	6.2575	7.2571	7.7036	7.8769	7.5337	7.4993	0.9599	-0.0590	0.4177	0.3792
Npy1r	_QAS[Phospho (STY)]PVAFK_.2	#N/A	6.8587	5.6705	6.8482	6.1050	4.1869	6.0114	6.0232	6.5937	6.3204	6.4874	0.9356	-0.0960	0.4470	0.3497
Npy1r	_TS[Phospho (STY)]LKQAS[Phospho (STY)]PVAFK_.2	#N/A	4.3816	4.3454	3.5676	4.0702	4.2337	3.8917	3.7849	5.0168	5.2878	4.0372	0.9007	-0.1509	0.2100	0.6778
Npy1r	_TS[Phospho (STY)]LKQAS[Phospho (STY)]PVAFKK_.3	#N/A	4.0382	3.2937	3.7719	3.3157	3.1820	3.4427	3.4639	3.6855	3.9846	2.9930	0.9931	-0.0100	0.9202	0.0361
Nf1	_S[Phospho (STY)]FDHLISDTK_.2	#N/A	10.8070	10.6461	11.0387	10.7769	9.1806	10.4884	11.2204	10.9203	10.6994	10.4343	0.9696	-0.0446	0.3936	0.4049
Nf1	_KVS[Phospho (STY)]VSESNVLLDEEVLTDPK_.3	Q04690_S2599_M1_Nf1	6.2325	5.5627	3.3861	4.4307	3.5113	4.2182	6.4361	6.0190	6.0484	5.3450	0.7643	-0.3878	0.0488	1.3120
Nf1	_SFS[Phospho (STY)]PGSYER_.2	Q04690_S2190_M1_Nf1	7.6122	7.1065	4.5124	6.4973	3.8858	6.5531	7.1144	7.4838	3.2440	6.7302	0.9812	-0.0273	0.9179	0.0372
Nf1	_SSSGLATYS[Phospho (STY)]PPM[Oxidation (M)]GAVSER_.2	#N/A	7.2185	7.6554	8.7156	7.7788	3.8791	7.5979	7.5622	3.3944	8.0530	7.8087	1.0651	0.0910	0.7306	0.1363
Nf1	_KS[Phospho (STY)]FDHLISDTK_.2	#N/A	6.9414	6.7616	7.1266	6.6580	4.9079	6.6168	7.1613	7.2579	7.0224	6.4406	0.9328	-0.1004	0.3128	0.5047
Nf1	_SM[Oxidation (M)]S[Phospho (STY)]LDM[Oxidation (M)]GQPSQANTK_.2	#N/A	6.8706	7.4451	8.1585	3.8265	6.0539	7.8482	5.8526	8.4031	7.8809	7.7816	0.8958	-0.1587	0.4245	0.3721
Nf1	_SAGS[Phospho (STY)]FKR_.2	Q04690_S2831_M1_Nf1	6.9539	6.9512	7.3733	6.9170	6.0008	4.0137	7.3331	7.3842	6.9894	7.0716	0.8852	-0.1760	0.2306	0.6371
Nf1	_GSEGYLAATYPAVGQTS[Phospho (STY)]PR_.2	Q04690_S2517_M1_Nf1	2.9296	9.0755	3.6164	9.1674	7.2934	9.0955	3.8338	3.9994	9.0430	9.0672	1.0581	0.0815	0.8459	0.0727
Nf1	_KS[Phospho (STY)]M[Oxidation (M)]SLDMGQPSQANTK_.2	#N/A	3.9901	3.6437	3.9289	3.8249	3.5152	3.8217	3.7956	3.9134	3.7690	3.6387	1.0022	0.0032	0.9369	0.0283
Nf1	_SMS[Phospho (STY)]LDMGQPSQANTKK_.2	#N/A	3.4137	4.1699	3.1473	4.3070	4.0582	4.0672	4.3402	4.3101	3.1083	3.8692	0.9881	-0.0172	0.8913	0.0500
Cdk16	_KIS[Phospho (STY)]TEDINK_.2	#N/A	5.5170	5.6740	6.1480	4.0133	2.7898	5.5418	3.7280	5.6149	5.5986	3.9803	1.0458	0.0647	0.7844	0.1055
Cdk16	_KIS[Phospho (STY)]TEDINKR_.2	#N/A	3.7160	4.3876	3.8499	4.0602	3.7793	3.7865	4.2983	4.5280	3.8553	4.4623	0.9169	-0.1251	0.0782	1.1068
Cdk16	_QLS[Phospho (STY)]MTLR_.2	Q04735_S12_M1_Cdk16	3.0353	4.3071	4.2698	6.3240	3.6799	2.9447	3.9619	3.1876	3.4866	3.4909	1.1591	0.2129	0.4087	0.3886
Sox10	_AS[Phospho (STY)]PGPGELGK_.2	Q04888_S45_M1_Sox10	7.2811	7.0091	6.6943	7.9680	6.9625	7.0291	7.4213	6.6949	6.9823	7.5476	0.9994	-0.0008	0.9881	0.0052
Sox10	_[Acetyl (Protein N-term)]AEEQDLS[Phospho (STY)]EVELSPVGSEEPR_.2	Q04888_S8_M1_Sox10	3.9859	3.2902	3.1718	5.4598	4.8329	3.1171	3.4779	4.4440	4.0926	5.0568	0.9317	-0.1021	0.6190	0.2083
Cdk18	_RAS[Phospho (STY)]LSDIGFGK_.2	#N/A	4.1976	8.0710	8.4264	8.9667	7.1542	7.9803	8.1411	8.3593	7.9424	8.1481	0.9163	-0.1260	0.4580	0.3392
Cdk18	_RFS[Phospho (STY)]M[Oxidation (M)]EDLNKR_.3	#N/A	6.8970	6.3064	6.5121	6.5992	5.6325	6.2964	5.9677	6.1117	6.1999	6.6115	1.0243	0.0346	0.5508	0.2590
Cdk18	_RFS[Phospho (STY)]MEDLNK_.2	#N/A	6.0414	5.0154	4.0902	7.7243	5.4383	3.1244	5.8780	4.4343	4.3609	5.4628	1.0407	0.0576	0.8189	0.0868
Cdk18	_AS[Phospho (STY)]LSDIGFGK_.2	#N/A	3.0841	3.8893	3.7709	4.1759	3.7106	4.0229	4.3837	3.8490	4.2675	4.2406	0.9021	-0.1486	0.0920	1.0364
Cdk18	_FS[Phospho (STY)]MEDLNK_.2	#N/A	3.7383	2.9937	3.4720	3.9210	2.8820	3.7426	3.6357	4.1974	3.8460	3.8880	0.8886	-0.1704	0.1053	0.9775
Mark2	_SS[Phospho (STY)]DQAVPAIPTSNSYSK_.2	#N/A	3.4385	8.5173	4.2969	8.1548	6.7081	3.2850	8.1434	3.3226	5.9136	8.1681	0.8977	-0.1557	0.6766	0.1697
Mark2	_SRNS[Phospho (STY)]PLLDR_.2	#N/A	8.6870	8.9580	8.9257	8.9640	3.7229	8.7159	8.6890	8.8001	8.5344	8.7125	0.9207	-0.1192	0.5383	0.2690
Mark2	_RSS[Phospho (STY)]DQAVPAIPTSNSYSK_.3	#N/A	7.5249	7.2834	3.9158	3.7982	5.3634	5.7199	7.7002	6.5206	3.6720	3.6404	1.0404	0.0572	0.8544	0.0683
Mark2	_S[Phospho (STY)]SELEGDTITLKPR_.2	Q05512_S365_M1_Mark2	6.7131	4.1909	4.3860	3.9298	3.7961	6.5355	7.1455	3.0714	6.6522	3.6071	0.9621	-0.0557	0.8603	0.0654
Mark2	_DQQNLPYGVTPAS[Phospho (STY)]PSGHSQGR_.3	Q05512_S616_M1_Mark2	4.3904	3.6458	4.1241	3.6678	3.5341	6.4782	6.9958	7.3065	3.6324	3.3451	0.8095	-0.3048	0.3465	0.4603
Mark2	_NS[Phospho (STY)]PLLDR_.2	#N/A	3.7616	5.2768	4.0030	4.5056	3.9427	2.6166	4.1476	4.8204	3.2614	3.5751	1.0169	0.0241	0.9020	0.0448
Mark2	_PSADLTNSSAPS[Phospho (STY)]PSHK_.2	#N/A	5.0183	3.8539	3.9887	5.2974	3.7007	5.1494	4.8004	4.7507	4.7098	3.0829	1.0382	0.0540	0.7477	0.1263
Mark2	_GRNS[Phospho (STY)]ATSADEQPHIGNYR_.3	Q05512_S40_M1_Mark2	3.9370	3.1924	3.6707	3.2144	3.0807	3.5439	3.3627	3.7867	4.0858	4.3926	0.8804	-0.1837	0.0904	1.0436
Mark2	_VPVAS[Phospho (STY)]PSAHNISSSSGAPDR_.3	Q05512_S566_M1_Mark2	4.1186	2.9990	4.0771	3.6209	3.4871	3.4083	3.7691	3.3803	3.6794	4.8943	0.9206	-0.1194	0.3859	0.4135
Mark2	_NSATS[Phospho (STY)]ADEQPHIGNYR_.2	Q05512_S43_M1_Mark2	4.3325	3.5879	4.0662	3.3269	3.4762	3.1484	4.5424	3.2908	4.4402	3.2939	0.9395	-0.0900	0.5301	0.2757
Fabp5	_ELGVGLALR_.2	#N/A	7.3008	7.2561	7.1061	7.0007	7.7334	7.1993	6.7754	7.6065	7.4675	7.2765	0.9979	-0.0031	0.9382	0.0277

Fabp5	_[Acetyl (Protein N-term)]ASLKDLEGK_.2	#N/A	5.8774	6.7821	6.4875	6.9498	6.7938	6.8070	6.5179	6.7395	6.5658	6.7511	0.9959	-0.0059	0.8977	0.0469
Fabp5	[Oxidation (M)]AAM[Oxidation (M)]AKPDC[Carbamidomethyl (C)]IITC[Carbamidomethyl (C)]DGNNITV	#N/A	5.3056	5.0582	5.9062	3.6716	5.3869	5.6002	3.0692	4.0218	4.9059	5.2173	1.1978	0.2604	0.1616	0.7916
Fabp5	_M[Oxidation (M)]IVEC[Carbamidomethyl (C)]VM[Oxidation (M)]NNATC[Carbamidomethyl (C)]TR_.2	#N/A	5.2693	5.3582	5.5075	6.1684	5.6849	5.4451	5.3149	5.7783	5.4824	5.1426	1.0263	0.0374	0.4897	0.3100
Fabp5	_TTVFSC[Carbamidomethyl (C)]NLGEKFDETTADGR_.3	#N/A	5.0355	4.2429	5.1238	3.8778	4.0642	3.6653	4.0261	3.1234	3.4224	3.5551	1.2274	0.2956	0.0479	1.3192
Fabp5	_TETVC[Carbamidomethyl (C)]TFQDGALVQHQQWDGK_.3	#N/A	3.2659	4.0765	4.5004	4.0442	3.8978	3.8317	4.1925	4.4578	3.2560	3.7215	1.0075	0.0107	0.9234	0.0346
Fabp5	_KTETVC[Carbamidomethyl (C)]TFQDGALVQHQQWDGK_.3	#N/A	3.9427	3.2835	3.1286	3.6313	3.1718	3.4528	3.3460	2.9864	4.1358	5.3498	0.8687	-0.2031	0.2749	0.5608
Fabp5	_LMESHGFEEYMK_.2	#N/A	3.9398	3.2442	3.1258	3.3757	3.0655	3.1632	3.5240	2.9892	3.9245	3.5954	0.9461	-0.0800	0.4243	0.3723
Gch1	_HRS[Phospho (STY)]EEENQVNLPK_.3	#N/A	4.3048	4.1631	4.4820	5.8719	4.2448	5.2474	3.9670	5.3378	5.1540	4.7943	0.9804	-0.0285	0.8303	0.0808
Pc	_ALAVSDLNR_.2	#N/A	7.0499	7.0773	6.9938	6.9846	7.0414	7.0580	6.6747	6.7394	6.8410	7.0040	1.0322	0.0457	0.0063	2.1999
Pc	_DFTATFGPLDSLNTR_.2	#N/A	6.6266	6.2208	6.0687	6.5863	6.0869	6.5860	6.1982	6.4482	6.3784	6.1944	1.0092	0.0132	0.7001	0.1549
Pc	_GANAVGYTNYPDNVVFK_.2	#N/A	5.8670	5.6025	3.2320	5.1777	5.8175	5.3229	5.6555	5.3242	5.0340	5.6697	0.9537	-0.0684	0.6410	0.1932
Pc	_FIGPSPEVVR_.2	#N/A	7.2618	6.5425	3.4303	7.0318	7.0829	6.6338	6.5842	6.9852	6.6353	6.9030	0.9341	-0.0983	0.5644	0.2484
Pc	_AC[Carbamidomethyl (C)]TELGIR_.2	#N/A	5.3882	5.2250	5.1945	4.2753	5.2711	4.6520	4.3269	5.0454	5.1277	5.2331	1.0137	0.0197	0.8125	0.0902
Pc	_DM[Oxidation (M)]TLEGDDLILEIE_.2	#N/A	4.4821	4.8457	5.9903	5.2625	4.8401	5.6909	5.2800	3.6122	5.2787	4.8901	1.0881	0.1219	0.3548	0.4500
Pc	_VVEIAPATHLDPQLR_.2	#N/A	5.6870	5.7994	4.9683	5.8006	5.8065	4.3840	4.9782	5.7798	5.7820	5.6606	0.9743	-0.0375	0.6870	0.1631
Pc	_DAHQSLLATR_.2	#N/A	5.4089	5.4165	6.1319	6.2281	5.9633	6.5212	6.0341	6.3282	6.0646	5.7660	0.9829	-0.0248	0.6871	0.1630
Pc	_VFDYSEYWEGAR_.2	#N/A	5.3043	5.0074	3.9413	5.7910	4.8288	4.7954	5.7342	4.6392	5.2482	4.6670	0.9749	-0.0367	0.7433	0.1289
Pc	_AEAEAQAEELSFPR_.2	#N/A	4.5622	3.5652	5.4866	4.7214	4.3957	4.4268	5.2544	4.4826	3.2272	5.0789	1.0034	0.0050	0.9750	0.0110
Pc	_EGPEGFAR_.2	#N/A	5.5888	5.3660	3.8325	3.3763	5.9192	5.5789	3.5246	5.4494	4.9844	5.6426	1.0088	0.0127	0.9493	0.0226
Pc	_VVHSYEELEENYTR_.3	#N/A	4.5301	4.6091	5.7247	5.3957	4.8135	5.2530	4.1149	4.9865	5.3651	5.5881	1.0081	0.0117	0.9111	0.0404
Pc	_ADFAQAC[Carbamidomethyl (C)]QDAGVR_.2	#N/A	5.7160	3.2340	3.8421	3.3859	5.1565	3.3724	5.2366	3.6153	5.2899	4.9601	0.8623	-0.2138	0.3216	0.4927
Dnal1	_LSLS[Phospho (STY)]TNC[Carbamidomethyl (C)]IEK_.2	Q05A62_S56_M1_Dnal1	9.9125	9.8390	10.4716	10.2072	9.7251	9.9823	10.2523	9.8359	10.0985	9.9540	0.9988	-0.0018	0.9394	0.0271
Drp2	_GQTT[Phospho (STY)]PDTEVADDVGSK_.2	#N/A	5.9408	5.7686	3.5944	6.0706	5.2637	5.4158	6.3744	5.8865	4.1620	5.8590	0.9590	-0.0603	0.7144	0.1461
Drp2	_EKGQTT[Phospho (STY)]PDTEVADDVGSK_.3	#N/A	3.0038	4.2224	3.6906	4.1932	3.6303	4.0992	3.9079	3.9253	3.5739	6.0207	0.8737	-0.1948	0.3061	0.5141
Eml1	_TSSS[Phospho (STY)]ERVS[Phospho (STY)]PGGR_.2	Q05BC3_S135_M2_Eml1	4.2966	4.6584	4.9485	4.3999	5.1336	5.7864	5.8181	5.8300	3.2235	4.2374	1.0195	0.0279	0.8757	0.0577
Larp7	_TAS[Phospho (STY)]EGS[Phospho (STY)]EAETPEAPK_.2	#N/A	8.6320	8.3263	8.4548	9.1448	8.2361	8.4669	8.9059	8.3638	8.2824	8.5703	1.0015	0.0022	0.9498	0.0224
Larp7	_TAS[Phospho (STY)]EGS[Phospho (STY)]EAETPEAPKQPAK_.2	#N/A	6.2059	6.7221	7.2912	7.2254	6.2757	6.7866	7.0645	6.8997	6.7080	6.5834	0.9908	-0.0134	0.8062	0.0936
Larp7	_DLEFC[Carbamidomethyl (C)]S[Phospho (STY)]T[Phospho (STY)]EEEKETDR_.2	Q05CL8_S334_M2_Larp7	3.1745	3.9308	4.4090	3.9528	3.8191	2.9097	4.1995	3.0484	3.3474	3.6301	1.0402	0.0569	0.6876	0.1627
Eif5b	_IEPIPGES[Phospho (STY)]PK_.2	Q05D44_S1164_M1_Eif5b	7.9922	7.8696	8.0567	8.2388	7.2840	7.8979	8.6830	8.2692	8.1482	8.2188	0.9472	-0.0783	0.0503	1.2982
Eif5b	_SVPTVDS[Phospho (STY)]GNEDDDSSFK_.2	Q05D44_S215_M1_Eif5b	12.4144	13.0963	13.1769	13.3900	12.3704	12.9807	13.6457	13.1686	13.0299	12.8720	0.9792	-0.0303	0.3069	0.5130
Eif5b	_TARPNSEAPLS[Phospho (STY)]GSEDADDSNK_.3	#N/A	6.1800	4.0924	4.3049	6.3398	5.6131	5.8296	5.7040	3.9993	6.2999	3.4456	1.1092	0.1496	0.4861	0.3133
Eif5b	_KTARPNSEAPLS[Phospho (STY)]GSEDADDSNK_.3	#N/A	3.7608	3.8524	4.4832	3.9652	3.9768	3.7527	4.8071	3.0360	3.3350	4.4333	1.0160	0.0229	0.8682	0.0614
Eif5b	_PNSEAPLS[Phospho (STY)]GSEDADDSNK_.2	#N/A	4.1923	3.1217	3.9544	4.9995	3.3644	4.3369	3.6464	3.1435	4.3719	3.1754	1.1146	0.1565	0.3519	0.4536
Eif5b	_TSFDENDS[Phospho (STY)]EELEDKDSK_.2	#N/A	4.2534	4.0125	4.2606	4.3383	3.2068	3.9645	3.4764	3.6475	3.6234	3.3428	1.1373	0.1856	0.0601	1.2214
Eif5b	_KTS[Phospho (STY)]FDENDS[Phospho (STY)]EELEDKDSK_.3	#N/A	3.9048	3.7042	4.0786	3.7515	3.5793	3.5589	3.6953	3.6390	4.0681	3.5834	1.0044	0.0063	0.9054	0.0432
Cab39	_DVAQIFNNILR_.2	#N/A	5.1310	4.7539	5.7639	4.6472	4.9769	3.3106	5.0914	4.9760	5.5779	5.3406	0.9080	-0.1392	0.2926	0.5338
Atp5me	_VPPVQVSPLIK_.2	#N/A	9.7018	10.2336	9.9693	9.7999	10.3992	10.1337	9.9087	10.2169	10.4068	9.9688	0.9915	-0.0123	0.6136	0.2121
Atp5me	_ELAEAQDDSILK_.2	#N/A	9.4811	9.2476	9.6833	9.5452	9.8495	9.5818	9.6206	9.4769	9.6677	9.5838	0.9977	-0.0034	0.8399	0.0758
Atp5me	_YSYLKPR_.2	#N/A	8.6752	9.1281	8.9754	9.1715	9.5329	8.8990	8.7882	9.1505	9.0927	8.8736	1.0097	0.0140	0.6068	0.2169
Atp5me	_YSALIIGM[Oxidation (M)]AYGAK_.2	#N/A	6.7319	6.7984	7.1301	6.3772	7.0743	6.8826	6.7250	6.9936	6.9480	6.5722	1.0033	0.0048	0.8899	0.0507
Sh3bp2	_S[Phospho (STY)]PPDGQSFR_.2	Q06649_S414_M1_Sh3bp2	6.5085	6.7192	6.5568	6.8397	3.4227	4.3068	3.1668	6.5191	6.9253	6.6318	0.9854	-0.0213	0.9360	0.0287
Clu	_ASGIIDTLFQDR_.2	#N/A	5.7151	5.7031	5.1059	5.8265	6.1003	5.7044	3.4759	5.8549	5.8179	5.5723	1.0989	0.1361	0.3183	0.4972
Clu	_VSTVTTHSSDSEVPSR_.3	#N/A	4.1616	3.1808	3.8953	3.4390	5.7924	3.3193	3.5873	3.5621	3.8612	3.1163	1.1226	0.1668	0.4198	0.3770
Clu	_QQSQVLDAMQDSFAR_.2	#N/A	3.7527	3.0081	3.4863	6.3368	2.8964	3.7282	3.1784	5.4533	4.2701	7.7413	0.7495	-0.4160	0.2347	0.6295
Anxa7	_SDTSGHFER_.2	#N/A	5.5092	5.3078	4.6990	5.1838	5.5285	5.4413	4.8278	3.8440	5.2527	4.8728	1.1232	0.1676	0.0777	1.1097
Anxa7	_GFGTDEQAIVDVVSNR_.2	#N/A	5.8867	5.7718	6.4193	6.3590	5.4868	6.0883	6.4551	5.6864	6.1576	5.7592	0.9979	-0.0030	0.9579	0.0187
Anxa7	_LYQAGEGR_.2	#N/A	3.2339	3.7592	4.4684	4.0121	3.7993	3.1527	4.1604	3.6827	3.2881	3.6894	1.0088	0.0126	0.9131	0.0395
Igf2r	_AEALSSLHGDDQDS[Phospho (STY)]EDEVLTVPEVK_.3	Q07113_S2401_M1_Igf2r	3.5117	4.3169	4.1985	3.2003	5.8707	3.5913	4.4158	7.8821	3.0659	3.1673	0.8882	-0.1710	0.6239	0.2049
Igf2r	_LVSFHDDS[Phospho (STY)]DEDLLHI_.2	#N/A	3.7757	3.3948	4.1017	3.6455	3.4259	3.2216	4.1342	3.3582	3.6572	3.3228	0.9934	-0.0096	0.9159	0.0381
Mtf1	_[Acetyl (Protein N-term)]GEHS[Phospho (STY)]PDDNIIFFK_.2	Q07243_S5_M1_Mtf1	3.4650	4.2212	3.1987	4.9481	4.1846	4.0159	4.3915	4.6720	4.4208	3.9205	0.9206	-0.1194	0.3376	0.4716
Tle3	_ESSTNNSVS[Phospho (STY)]PSESLR_.2	Q08122_S203_M1_Tle3	9.1069	9.3890	9.8453	10.0930	8.9924	8.7556	9.6005	9.0933	3.9327	9.4617	1.1672	0.2231	0.2624	0.5811
Tle3	_DAPTS[Phospho (STY)]PASVASSSSTPSSK_.2	Q08122_S286_M1_Tle3	3.3500	4.1063	4.5845	10.0412	3.9946	4.1309	9.6485	4.3737	9.4848	9.3422	0.6131	-0.7059	0.0859	1.0661
Tle3	_VS[Phospho (STY)]PAHS[Phospho (STY)]PPENGLDK_.2	Q08122_S263_M2_Tle3	8.0241	4.1408	3.1182	8.9794	4.0291	4.0964	3.9895	3.8436	3.4922	4.2419	1.3870	0.4720	0.2653	0.5762
Dmwd	_SVVEGISSQPGSS[Phospho (STY)]PSGTVV_.2	Q08274_S659_M1_Dmwd	3.4891	9.7387	10.2878	10.0535	9.0696	10.0137	10.3262	3.9482	9.9466	4.1372	1.2378	0.3078	0.4082	0.3891
Dmwd	_YHS[Phospho (STY)]LGNISR_.2	#N/A	9.1518	9.2014	9.7016	9.6173	8.1439	9.3072	9.1647	9.1368	9.1604	9.1245	1.0044	0.0064	0.8900	0.0506
Dmwd	_TLPGT[Phospho (STY)]PGAT[Phospho (STY)]PPASGSSR_.2	Q08274_T457_M2_Dmwd	8.5604	8.7160	9.6861	4.5707	6.8252	9.6334	3.2181	9.0507	8.3131	6.2001	1.1946	0.2566	0.3929	0.4058
Dmwd	_RYHS[Phospho (STY)]LGNISR_.3	#N/A	5.2089	4.1902	5.4524	5.4364	3.9190	4.6904	4.0993	4.8056	4.8668	4.5521	1.0514	0.0723	0.5344	0.2722
Dmwd	_AFTDEETEAQAGQASWPRS[Phospho (STY)]PSK_.3	Q08274_S643_M1_Dmwd	3.6849	2.9893	4.3717	3.6306	3.7731	3.4180	3.7788	3.3706	3.6697	3.3078	1.0320	0.0454	0.6615	0.1795
Dmwd	_LGDPDGAGEPPST[Phospho (STY)]PSGLGAGGDR_.2	Q08274_T112_M1_Dmwd	3.4767	3.7495	4.1635	3.1653	3.6378	2.9868	4.3808	3.4523	3.1009	3.1323	1.0038	0.0055	0.9678	0.0142
Dmwd	_GGSGGNSS[Phospho (STY)]NDKLSGPAPR_.2	#N/A	4.3248	3.9833	4.2223	4.2591	3.8418	4.0965	3.9738	3.7664	3.5090	4.0134	1.0801	0.1112	0.0478	1.3206
Dmwd	_SNS[Phospho (STY)]LPHPAGGGK_.2	Q08274_S487_M1_Dmwd	11.1841	11.1895	11.6740	11.7550	10.7107	11.4364	11.4951	11.3446	11.3197	11.2932	0.9966	-0.0049	0.8525	0.0693
Dmwd	_S[Phospho (STY)]IDLNKPIDKR_.3	Q08274_S145_M1_Dmwd	5.3338	4.3431	3.4077	4.0655	3.8982	3.7319	3.6251	4.5588	4.6983	4.9322	0.9274	-0.1088	0.4522	0.3447
Calb2	_LLPVQENFLLK_.2	#N/A	8.7618	9.1383	9.2134	8.8098	9.4703	9.0865	8.9577	9.1113	9.3626	9.0805	0.9947	-0.0076	0.7577	0.1205
Calb2	_SGYIEANELK_.2	#N/A	6.9670	7.4951	7.2491	7.5437	7.3687	7.7073	7.2680	7.1479	7.4687	7.5482	1.0041	0.0059	0.8457	0.0728
Calb2	_EM[Oxidation (M)]NIQQLTTYR_.2	#N/A	6.7537	7.2245	7.6823	6.5764	7.4102	7.3928	6.8266	6.9960	7.3846	6.9299	1.0198	0.0282	0.5733	0.2416
Calb2	_ELENFFQELEK_.2	#N/A	6.4745	6.5132	6.6558	6.6681	7.0112	7.2696	6.5111	6.3000	7.2818	7.0155	0.9983	-0.0025	0.9622	0.0168
Calb2	_SVMSLAEAGK_.2	#N/A	3.3143	4.1194	4.0011	4.5037	3.9408	3.7887	4.1495	3.1062	3.2634	2.9699	1.1697	0.2262	0.0840	1.0758
Kcnma1	_GGYST[Phospho (STY)]PQTLANR_.2	Q08460_T1061_M1_Kcnma1	10.5820	10.4690	11.0408	10.7734	9.1077	10.0098	11.2735	10.9288	10.4314	10.3726	0.9608	-0.0576	0.3125	0.5051
Kcnma1	_LEDEQPPTLS[Phospho (STY)]PK_.2	#N/A	2.9832	9.1077	9.1926	7.6777	6.1862	8.9280	8.3030	9.5277	7.7022	8.9992	0.8509	-0.2329	0.3438	0.4637
Kcnma1	_RLEDEQPPTLS[Phospho (STY)]PK_.2	#N/A	8.7778	9.5442	9.9390	9.5869	8.1120	9.3202	10.2829	10.0158	9.6767	9.4583	0.9346	-0.0976	0.1169	0.9320
Kcnma1	_SS[Phospho (STY)]PDNSPVHGMLR_.2	Q08460_S924_M1_Kcnma1	3.5548	5.7162	3.8101	3.9245	3.5745	3.7433	5.9679	3.8054	3.8791	3.6920	0.9349	-0.0971	0.6523	0.1855
Eps8	_DSVSS[Phospho (STY)]VSDVSQYR_.2	Q08509_S58_M1_Eps8	6.6192	3.8940	3.6295	6.8242	5.4672	3.6893	6.6216	6.0306	6.1914	3.5933	0.8951	-0.1600	0.5442	0.2642
Eps8	_QNSSSS[Phospho (STY)]DSGGSIVR_.2	Q08509_S661_M1_Eps8	2.9396	3.7448	3.3313	4.3759	3.5661	4.1634	4.5242	4.1261	4.4251	3.6816	0.8801	-0.1843	0.1427	0.8455
Eps8	_RKS[Phospho (STY)]QMEEVQDELFQR_.3	Q08509_S684_M1_Eps8	3.0212	3.7775	4.1355	2.7669	3.6658	2.9588	4.3528	3.4804	3.1289	3.1043	0.9633	-0.0539	0.7307	0.1363
Ssrp1	_EGINPGYDDY[Phospho (STY)]ADSDEDQHDAYLER_.3	Q08943_Y441_M1_Ssrp1	3.6283	3.8631	4.3079	8.0228	4.8113	3.7974	3.9596	7.5065	4.8251	3.9274	0.9374	-0.0932	0.7772	0.1095
Agbl4	_SQTAPEAGNDTGNEDAIGGNVNK_.2	#N/A	2.8192	2.6106	3.7536	3.6743	3.6934	4.0240	4.3567	3.0384	3.5108	3.1083	0.9788	-0.0309	0.8487	0.0713
Chd8	_TAS[Phospho (STY)]PSPLRPDAPVEK_.2	Q09XV5_S1997_M1_Chd8	3.7040	3.6328	3.8038	3.8856	4.7276	3.7551	6.1878	3.5792	3.9678	5.7354	0.8050	-0.3130	0.1221	0.9134
Cnnm1	_GT[Phospho (STY)]PQT[Phospho (STY)]PKDDPVLTLLSNR_.3	Q0GA42_T821_M2_Cnnm1	4.1687	6.2086	7.3124	8.1399	5.8318	6.7532	6.5688	8.3377	7.6794	6.5287	0.8796	-0.1851	0.2937	0.5322
Cnnm1	_SDGLRS[Phospho (STY)]PGEVVYLR_.3	Q0GA42_S850_M1_Cnnm1	3.9855	4.5667	3.8867	3.6742	4.1785	3.7561	3.7312	3.7935	3.4421	3.6412	1.0975	0.1342	0.0792	1.1012

Inf2	_SFSEDAVTDSS[Phospho (STY)]GSGTLPR_.2	Q0GNC1_S1226_M1_Inf2	3.6320	5.6362	4.1826	6.2894	4.5278	4.5485	4.0991	4.5782	5.8789	4.7759	0.9937	-0.0091	0.9598	0.0178
Inf2	_AVLLASDAQAC[Carbamidomethyl (C)]TLEEVVER_.2	#N/A	3.9322	2.6858	3.6659	3.8250	3.0759	3.5487	3.1400	4.3913	2.3328	3.6941	1.0195	0.0278	0.8797	0.0557
Unc79	_ALS[Phospho (STY)]LPETLTSK_.2	Q0KK59_S1242_M1_Unc79	7.5396	7.2634	7.6368	7.4515	3.0327	7.4868	7.9663	7.6294	7.1415	7.1624	0.9010	-0.1503	0.4544	0.3425
Unc79	_VDS[Phospho (STY)]PGKPAPR_.2	Q0KK59_S1596_M1_Unc79	3.1872	5.0498	5.0794	5.3122	3.5573	4.3609	5.8474	5.8956	4.9249	5.3489	0.8038	-0.3150	0.0568	1.2454
Ubxn2b	_FKAFS[Phospho (STY)]GEGQK_.2	#N/A	8.4805	9.0432	9.5375	9.1823	7.4300	8.9676	8.6036	8.6822	8.7630	8.9575	1.0025	0.0036	0.9557	0.0197
Ubxn2b	_STPATC[Carbamidomethyl (C)]RS[Phospho (STY)]PRT[Phospho (STY)]PPHR_.3	Q0KL01_S56_M2_Ubxn2b	6.6467	7.0823	7.3651	7.3146	5.7817	7.1396	3.9526	7.2018	6.6799	7.2217	1.0997	0.1371	0.3911	0.4077
Ubxn2b	_AFS[Phospho (STY)]GEGQK_.2	#N/A	3.2620	4.0804	4.4966	4.0403	3.9066	4.2188	4.1886	4.4617	3.2599	3.7176	1.0240	0.0342	0.7604	0.1190
Trio	_DSDDSAAT[Phospho (STY)]PQDETIEER_.2	Q0KL02_T1824_M1_Trio	6.6716	6.6261	4.2921	3.8359	5.0825	3.6234	3.9842	6.9285	6.4631	6.5446	0.8398	-0.2519	0.3043	0.5167
Trio	_NFLNALTS[Phospho (STY)]PIEYQR_.2	Q0KL02_S2282_M1_Trio	3.0350	4.3074	4.2695	6.1528	3.6795	3.6007	3.9615	3.1879	3.4870	3.4905	1.1819	0.2411	0.2881	0.5405
Trio	_SADAGSQKDS[Phospho (STY)]DDSAATPQDETIEER_.3	Q0KL02_S1818_M1_Trio	3.0266	4.1996	3.7134	4.2160	3.6531	4.0375	3.9307	3.9025	3.5511	4.1830	0.9784	-0.0315	0.7471	0.1266
Spata18	_SARS[Phospho (STY)]PS[Phospho (STY)]PSTGTR_.2	Q0P557_S244_M2_Spata18	3.2559	4.0611	3.9427	4.0596	3.8824	3.8471	4.2079	3.6731	3.2406	4.4123	0.9892	-0.0157	0.8753	0.0578
Spata18	_SNS[Phospho (STY)]PC[Carbamidomethyl (C)]TTVAK_.2	Q0P557_S273_M1_Spata18	4.3705	3.6259	4.1042	3.6480	3.5142	3.1104	4.5044	3.3532	3.6523	3.3252	1.0054	0.0078	0.9507	0.0219
Sobp	_SADS[Phospho (STY)]PPGSSGQALSLAPAER_.2	Q0P5V2_S597_M1_Sobp	7.5813	3.3417	3.8200	3.3638	3.2300	3.3946	8.6597	7.8918	3.9365	3.0410	0.7007	-0.5131	0.2472	0.6070
Slc45a4	_GS[Phospho (STY)]PPINSLSR_.2	Q0P5V9_S408_M1_Slc45a4	4.3664	3.6218	4.1001	7.1388	3.5101	3.1145	4.5085	7.2935	6.5961	7.3602	0.6691	-0.5797	0.0474	1.3241
Slc45a4	_S[Phospho (STY)]M[Oxidation (M)]SDLYDLQQR_.2	#N/A	3.9594	6.4554	6.8971	5.5827	3.0851	6.7796	7.1306	6.7578	6.8656	6.9233	0.7891	-0.3418	0.1114	0.9530
Zc3h18	_LGVSVS[Phospho (STY)]PSR_.2	Q0P678_S530_M1_Zc3h18	3.3976	8.8046	9.4466	3.9179	4.0241	3.7054	9.0225	3.0833	3.3823	3.5952	1.1632	0.2181	0.6788	0.1683
Zc3h18	_KRPLS[Phospho (STY)]PQSK_.2	Q0P678_S888_M1_Zc3h18	3.6395	7.8219	8.1136	4.4178	7.1819	3.8413	8.2724	4.0842	4.3832	4.0951	1.1204	0.1641	0.6515	0.1861
Zc3h18	_KANLS[Phospho (STY)]PDR_.2	#N/A	6.3585	6.6656	6.7699	3.7122	6.1888	5.8745	6.5526	6.9082	6.5621	6.7156	0.8868	-0.1732	0.2299	0.6385
Zc3h18	_ANLS[Phospho (STY)]PDRGSR_.2	#N/A	4.5920	3.6030	6.3154	5.4733	5.2848	4.3051	3.7020	5.6077	3.7798	5.0254	1.0884	0.1222	0.5334	0.2730
Zc3h18	_M[Oxidation (M)]GS[Phospho (STY)]PKPER_.2	#N/A	6.2444	6.2916	6.0815	7.1713	5.9318	5.8344	6.4862	6.6178	5.9596	6.2724	0.9882	-0.0172	0.7886	0.1031
Zc3h18	_ANLS[Phospho (STY)]PDR_.2	#N/A	5.9369	5.5221	5.8017	5.3818	4.9182	5.8256	5.6705	3.7749	5.4272	6.0264	1.0650	0.0909	0.4614	0.3359
Zc3h18	_S[Phospho (STY)]PQPPSR_.2	Q0P678_S483_M1_Zc3h18	5.6879	3.3981	3.6780	3.2218	3.2194	3.3644	3.3700	5.6116	5.3712	4.6528	0.7917	-0.3370	0.1551	0.8093
Zc3h18	_SQDQDS[Phospho (STY)]EAHELSR_.2	Q0P678_S80_M1_Zc3h18	3.0517	4.1745	3.7385	4.2411	3.6782	4.0513	3.9558	3.8773	3.5259	4.2081	0.9822	-0.0259	0.7898	0.1025
Zc3h18	_VQSQEETRS[Phospho (STY)]DEEDRASEPK_.3	Q0P678_S64_M1_Zc3h18	3.8768	4.2238	4.0732	4.0142	3.6836	4.2619	3.9638	3.6559	3.5403	3.8424	1.0724	0.1009	0.0768	1.1149
Dact3	_S[Phospho (STY)]LGDASPSAPESVGAR_.2	Q0PHV7_S165_M1_Dact3	10.3894	10.3238	10.2822	10.6068	9.3575	10.3495	10.9036	10.5384	10.5081	10.3940	0.9653	-0.0510	0.1629	0.7881
Dact3	_GRS[Phospho (STY)]VEQS[Phospho (STY)]PPR_.2	#N/A	8.7079	7.7284	8.1784	8.5426	7.2224	8.0723	8.4693	8.5077	8.6538	8.1122	0.9573	-0.0630	0.2519	0.5987
Dact3	_SQS[Phospho (STY)]ETSLLGR_.2	Q0PHV7_S409_M1_Dact3	8.1147	7.5765	8.6949	7.8205	3.4818	8.0998	8.1787	8.3452	7.9261	8.1150	0.8964	-0.1577	0.4125	0.3846
Dact3	_AFSFPVS[Phospho (STY)]PER_.2	Q0PHV7_S10_M1_Dact3	4.2136	7.9382	8.1820	8.6090	7.7062	7.5707	8.2975	8.6372	8.5360	8.3734	0.8710	-0.1992	0.2167	0.6642
Dact3	_PRPPEAS[Phospho (STY)]PPPGGAR_.2	#N/A	7.0277	3.9371	6.9432	5.4306	3.7768	5.3152	7.4599	7.3607	5.5362	6.7815	0.7967	-0.3279	0.1213	0.9163
Dact3	_SFS[Phospho (STY)]APYPTAAAGAETC[Carbamidomethyl (C)]SSAER_.2	Q0PHV7_S188_M1_Dact3	3.5558	3.5681	3.8116	3.7251	3.4564	3.6840	3.6875	3.0907	3.8501	3.5445	1.0255	0.0363	0.5510	0.2588
Dact3	_GRS[Phospho (STY)]VEQS[Phospho (STY)]PPRER_.3	#N/A	3.6773	3.5489	4.3641	3.3659	3.4372	3.1874	3.0806	3.2517	4.4012	3.3329	1.0228	0.0325	0.8062	0.0935
Stum	_GASSSS[Phospho (STY)]GVVVQVR_.2	Q0VBF8_S29_M1_Stum	8.9744	8.6400	9.1030	8.5915	7.4820	8.6615	9.4244	8.8322	8.4233	8.8290	0.9660	-0.0499	0.3939	0.4046
Stum	_DAETAAAAAAVAAADPR_.2	#N/A	4.1202	3.2222	3.8539	3.3977	3.0435	3.1851	3.5459	3.6035	3.9026	3.0750	0.9826	-0.0253	0.8163	0.0882
Rbm15	_SLS[Phospho (STY)]PGGAALGYR_.2	Q0VBL3_S293_M1_Rbm15	12.1165	12.0289	12.6669	12.4752	11.4549	12.1223	12.5506	11.9493	12.0420	12.1915	0.9968	-0.0047	0.8728	0.0591
Rbm15	_LHSYSS[Phospho (STY)]PSTK_.2	Q0VBL3_S127_M1_Rbm15	3.0156	3.8208	3.7024	4.2998	3.6422	4.0873	4.4481	7.0119	7.1891	3.9771	0.6650	-0.5887	0.0270	1.5693
Rbm15	_HLDRS[Phospho (STY)]PESERPR_.3	Q0VBL3_S655_M1_Rbm15	3.9870	3.3554	3.7207	3.2644	3.1307	3.0519	3.4127	3.7367	4.0358	4.4426	0.8749	-0.1927	0.0903	1.0441
Rbm15	_S[Phospho (STY)]RS[Phospho (STY)]PLDKDAYAPSSSVVGTSVGSHR_.3	#N/A	3.8925	4.1879	3.7203	9.8697	7.6623	7.6428	4.0462	8.9282	7.2456	8.4858	0.8587	-0.2197	0.5384	0.2689
Rbm15	_HC[Carbamidomethyl (C)]T[Phospho (STY)]PS[Phospho (STY)]PDRS[Phospho (STY)]PELSSNR_.3	Q0VBL3_S670_M3_Rbm15	4.6659	3.8285	4.0844	3.9523	3.7168	3.7772	3.9988	3.4812	3.8760	3.4824	1.0794	0.1102	0.1935	0.7132
Rbm15	_DRT[Phospho (STY)]PPLLYR_.2	#N/A	9.2233	9.2923	9.8955	9.9774	8.8598	9.4162	9.8608	9.1410	9.2742	9.4819	1.0005	0.0007	0.9857	0.0063
Fam83b	_KSEPKVDSS[Phospho (STY)]PR_.2	Q0VBM2_S827_M1_Fam83b	3.5862	4.8070	3.7598	4.8442	3.6964	3.5766	3.9771	3.9657	3.6143	3.7220	1.0590	0.0826	0.4998	0.3012
Ppp4r2	_TS[Phospho (STY)]APEPR_.2	Q0VGB7_S63_M1_Ppp4r2	3.8021	3.4241	4.4889	3.4907	3.3124	3.3122	3.2054	3.1269	4.2764	3.4577	1.0346	0.0491	0.7058	0.1513
Vgf	_LLQQGLAQVEAGR_.2	#N/A	4.5624	2.8357	4.1658	3.7096	4.8212	3.0300	4.1769	4.0184	3.5906	3.3869	1.0161	0.0230	0.8927	0.0493
Vgf	_S[Phospho (STY)]QEEAPGHR_.2	#N/A	11.6846	11.9743	12.2667	12.0386	11.8061	11.8561	11.9268	11.8050	11.6998	11.9648	1.0075	0.0108	0.4607	0.3366
Vgf	_QNALLFAEEEDGEAGAEDKRS[Phospho (STY)]QEEAPGHR_.4	#N/A	5.2177	5.1714	4.2164	6.0928	5.6672	4.9353	3.9084	4.8786	5.3880	4.6254	1.1099	0.1505	0.2406	0.6188
Tanc1	_SPC[Carbamidomethyl (C)]ETISS[Phospho (STY)]PSSTLESK_.2	Q0VGY8_S211_M1_Tanc1	3.9142	3.4282	6.2512	3.1917	3.0580	3.5666	3.3400	5.9255	5.8278	4.3698	0.8019	-0.3186	0.2518	0.5990
Tanc1	_QQGPPPAPANDS[Phospho (STY)]DNEEDAPASSLK_.3	Q0VGY8_S1436_M1_Tanc1	3.3964	3.8298	4.0832	4.5859	3.7181	4.4074	4.3005	3.5326	3.1812	3.0520	1.1384	0.1871	0.1634	0.7868
Npepps	_VLGATLSPELIQK_.2	#N/A	5.9444	5.9857	5.2458	5.4028	6.1412	6.0884	6.9481	6.4475	6.3808	5.7938	0.9075	-0.1400	0.0593	1.2272
Npepps	_DNWEELHNR_.2	#N/A	5.9524	5.9824	6.2839	5.9530	5.8093	5.4257	6.5226	5.9645	5.8797	5.9926	0.9690	-0.0454	0.3349	0.4751
Npepps	_LSVEGFAVDK_.2	#N/A	6.0706	6.3926	5.6034	6.0204	5.4048	5.7380	6.5837	6.2687	5.8722	5.9044	0.9536	-0.0685	0.2418	0.6166
Npepps	_LGLQNDLFSLAR_.2	#N/A	5.0528	5.0613	4.7374	4.7953	3.1368	4.3258	4.1272	5.3309	5.1785	4.1889	0.9600	-0.0589	0.6861	0.1636
Npepps	_AGIISTVEVLK_.2	#N/A	5.0187	7.5562	7.8237	7.9810	7.8220	8.0974	7.4640	5.5680	7.8273	7.6375	1.0363	0.0515	0.7312	0.1360
Npepps	_QILSADLR_.2	#N/A	6.2542	5.9840	6.5862	5.8097	5.7761	6.2676	4.4195	5.8554	5.8058	5.8475	1.1151	0.1572	0.0861	1.0649
Npepps	_IDFVGELNDK_.2	#N/A	5.6695	5.6700	4.3035	6.0501	5.7186	5.0014	5.8385	6.1476	5.8702	5.6075	0.9209	-0.1188	0.2058	0.6866
Npepps	_YQGGFLISR_.2	#N/A	5.4985	6.0952	6.0751	3.6166	3.4829	5.8794	5.5885	5.9105	5.8250	5.9381	0.8783	-0.1872	0.2933	0.5326
Npepps	_YAAVTQFEATDAR_.2	#N/A	3.7358	4.1383	3.1302	4.6512	4.2148	4.7398	4.6160	5.3106	4.1342	4.5246	0.8828	-0.1799	0.1711	0.7667
Npepps	_DVFSPIGER_.2	#N/A	6.0336	5.4463	5.6190	6.3111	5.1537	5.6726	5.8566	5.8010	5.4667	5.6155	1.0037	0.0053	0.9268	0.0330
Npepps	_ETALLIDPK_.2	#N/A	3.4694	3.5556	3.3116	4.3563	3.3022	4.3788	4.5045	4.0123	3.5651	3.9090	0.9328	-0.1004	0.3957	0.4027
Npepps	_DADSIHQYLLQR_.2	#N/A	5.3673	4.9646	4.2444	4.0775	4.7422	4.7016	4.8685	5.1940	4.2436	4.6557	0.9879	-0.0176	0.8463	0.0725
Npepps	_AFFESHPAPSAER_.2	#N/A	5.7050	6.0879	5.9918	3.6834	5.8566	6.1742	3.4427	5.9284	5.6793	5.6417	1.0793	0.1101	0.5550	0.2557
Npepps	_S[Phospho (STY)]PVYLTVLK_.2	Q11011_S746_M1_Npepps	3.5976	3.7448	3.3313	4.3759	4.2422	4.1633	4.5241	4.0075	4.4252	4.0532	0.9193	-0.1214	0.1811	0.7420
Npepps	_VALSNMNVIDR_.2	#N/A	3.8303	3.1081	4.0740	7.5419	3.5724	3.4690	5.4183	3.5261	6.7198	3.9768	0.8688	-0.2029	0.5428	0.2653
Npepps	_QM[Oxidation (M)]GFPLIYVEAEQVEDDR_.2	#N/A	3.7735	3.5004	4.2618	3.6502	3.3477	3.4463	3.8695	4.4466	3.8691	3.4054	0.9399	-0.0895	0.3540	0.4510
Npepps	_ATFDISLVVPK_.2	#N/A	3.2491	3.1802	3.9359	4.4385	2.7470	4.2601	4.1532	3.6799	3.4715	0.6714	1.2141	0.2799	0.3936	0.4049
Pepd	_EAS[Phospho (STY)]FEGISK_.2	Q11136_S162_M1_Pepd	4.1582	3.1842	3.8919	6.0528	4.7590	3.3227	5.6151	3.5656	3.8646	4.9542	0.9396	-0.0899	0.6914	0.1603
Pepd	_YAVDDVQYTDEIASVLTSR_.2	#N/A	3.5312	3.1817	4.5471	4.0425	3.4132	4.3388	4.1309	3.4799	3.3428	4.4693	0.9965	-0.0050	0.9706	0.0130
Relch	_NYKSS[Phospho (STY)]PEIQEPIKPLEK_.3	#N/A	9.5715	9.5292	4.5408	9.9824	8.7233	9.5807	9.9197	9.9119	9.3749	9.4577	0.8954	-0.1594	0.3662	0.4363
Relch	_YS[Phospho (STY)]DDGNRETDER_.2	Q148V7_S193_M1_Relch	2.9297	4.4127	4.1642	6.1059	4.2340	3.4955	6.2908	6.0433	3.5922	3.3853	0.8748	-0.1929	0.4839	0.3152
Relch	_SS[Phospho (STY)]PEIQEPIKPLEK_.2	#N/A	9.6645	4.1766	9.9019	9.9813	8.4816	9.5282	10.3662	9.6042	9.6984	9.4265	0.8822	-0.1808	0.3486	0.4577
Relch	_AGS[Phospho (STY)]ISTLDSLDFAR_.2	Q148V7_S180_M1_Relch	7.5202	6.9743	7.2679	7.4695	5.8624	3.3160	7.4512	7.5275	7.4427	6.9805	0.8709	-0.1994	0.2901	0.5374
Relch	_VVPALITLSSDPEISVR_.2	#N/A	2.5075	3.3134	3.7627	3.3065	1.9660	3.0939	3.0795	3.8391	2.4849	2.9837	0.9660	-0.0498	0.7980	0.0980
Relch	_AGSISTLDSLDFARY[Phospho (STY)]SDDGNRETDER_.3	Q148V7_Y192_M1_Relch	2.9199	3.6761	4.1544	3.6981	3.5644	3.0602	3.8464	5.1784	3.6021	3.3754	0.8779	-0.1878	0.2511	0.6001
Relch	_[Acetyl (Protein N-term)]AAMAPGGGGSGSGVNPFLS[Phospho (STY)]DSDEDDDEVAATEDRR_.3	Q148V7_S20_M1_Relch	2.9283	3.7335	3.3426	4.3872	3.5548	4.1747	4.5355	4.1148	4.4138	4.0645	0.8610	-0.2160	0.0730	1.1365
Traf3ip1	_QES[Phospho (STY)]TETLVVDR_.2	#N/A	4.1924	3.1500	3.9261	3.4698	2.9713	3.2573	3.6181	3.5313	3.8304	3.1471	0.9895	-0.0153	0.8925	0.0494
Traf3ip1	_RQES[Phospho (STY)]TETLVVDR_.2	#N/A	3.0653	4.1609	3.7521	4.2547	4.0492	4.0762	3.9694	3.8638	3.5123	4.2217	1.0003	0.0005	0.9961	0.0017
Cdk12	_NNS[Phospho (STY)]PAPPQPAPVK_.2	#N/A	7.0702	6.6680	6.4176	7.2298	6.2048	7.4323	7.3216	6.5956	6.7046	6.7244	1.0001	0.0001	0.9984	0.0007
Cdk12	_ETTSGTTAEPVKNNS[Phospho (STY)]PAPPQPAPVK_.3	#N/A	4.9464	4.7900	5.5447	3.4882	2.9253	4.7338	5.6593	5.5743	3.4873	3.4552	0.9693	-0.0449	0.8478	0.0717
Cdk12	_RRS[Phospho (STY)]S[Phospho (STY)]SPFLSK_.2	Q14AX6_S332_M2_Cdk12	6.4393	6.4974	3.9682	7.0118	4.0285	6.2583	4.3350	6.6703	3.1473	6.5206	1.1030	0.1414	0.5962	0.2246
Cdk12	_RQS[Phospho (STY)]IS[Phospho (STY)]PPYKEPSAYQSSTR_.3	Q14AX6_S273_M2_Cdk12	4.2855	5.6021	3.4714	6.2346	3.4112	4.7258	3.1783	4.1444	5.6569	5.7085	0.9892	-0.0156	0.9488	0.0228
Cdk12	_S[Phospho (STY)]RS[Phospho (STY)]PAYSR_.2	Q14AX6_S356_M2_Cdk12	4.9657	5.4454	3.9125	3.4805	3.3226	3.3020	3.1952	3.1371	5.3336	4.9518	0.9800	-0.0291	0.9021	0.0447

Kcnc2	_SVLSGDDSTGSEPPLS[Phospho (STY)]PPER_.2	Q14B80_S557_M1_Kcnc2	8.5645	7.5706	4.3648	8.9680	7.0660	7.5307	8.8657	3.2511	7.8935	8.1224	1.0442	0.0624	0.8178	0.0873
Kcnc2	TELNM[Oxidation (M)]AC[Carbamidomethyl (C)]NSTQS[Phospho (STY)]DTC[Carbamidomethyl (C)]LGK_	#N/A	3.7277	3.8587	3.8738	3.6175	3.2299	3.3555	3.9023	3.4099	3.8298	3.1127	1.0131	0.0188	0.8207	0.0858
Map6d1	_AGS[Phospho (STY)]RDAAQERPSGPGGR_.3	Q14BB9_S82_M1_Map6d1	6.5734	6.3563	7.5087	6.8021	6.5713	6.7059	6.7673	6.6685	7.0240	6.9113	0.9869	-0.0191	0.6845	0.1646
Map6d1	_TVYVLPVGDADAAVVATTSYR_.2	#N/A	5.1746	5.5621	5.6717	5.8394	4.8353	5.0900	3.8201	4.9414	3.6284	5.0966	1.2266	0.2946	0.0243	1.6136
Map6d1	_VVTTHS[Phospho (STY)]SGWDPS[Phospho (STY)]PGASFQVPEVR_.3	Q14BB9_S154_M2_Map6d1	4.3391	5.0646	3.9229	6.9036	4.2607	4.7746	4.2729	5.2085	4.1206	4.5879	1.0726	0.1011	0.5840	0.2336
Map6d1	_SDVAVPLTLHGYS[Phospho (STY)]DPGSEESGADC[Carbamidomethyl (C)]SVSR_.3	Q14BB9_S34_M1_Map6d1	3.2783	4.0633	3.6407	6.3153	3.9124	3.9864	4.2178	4.9334	4.9949	4.8484	0.8843	-0.1773	0.3610	0.4425
Fam171b	_STVEDFEANTS[Phospho (STY)]PTK_.2	#N/A	10.4626	10.1156	11.0932	10.2044	3.3616	10.3013	11.1084	10.2504	10.4889	3.1726	1.0573	0.0803	0.8169	0.0879
Fam171b	_RDS[Phospho (STY)]KTNIWK_.2	#N/A	5.4042	5.7200	4.8306	5.7238	3.6519	5.9572	6.1392	5.9275	6.0909	5.6760	0.8752	-0.1924	0.1341	0.8726
Fam171b	_DQTTS[Phospho (STY)]TTHINHISSVK_.3	Q14CH0_S405_M1_Fam171b	5.0236	3.3943	3.6818	4.9911	3.2156	3.0130	3.3738	3.7756	4.0747	4.4037	0.9948	-0.0075	0.9678	0.0142
Fam171b	_HILEGGSGVIIEHPGEES[Phospho (STY)]PGRK_.3	#N/A	3.8996	3.6687	3.3024	4.5848	3.5570	3.9465	3.8397	5.8543	3.5061	3.8889	0.8957	-0.1590	0.3786	0.4219
Fam171b	_KSTVEDFEANTS[Phospho (STY)]PTK_.2	#N/A	3.7615	6.8760	3.4952	4.5398	6.1772	6.4636	7.5097	7.1808	7.0225	6.5428	0.7388	-0.4367	0.0425	1.3714
Fam171b	_STVEDFEANTSPT[Phospho (STY)]KK_.2	Q14CH0_T794_M1_Fam171b	3.3521	3.9662	3.9824	5.3450	3.8227	4.1116	5.7970	5.4433	3.1747	3.8077	0.8992	-0.1532	0.4693	0.3285
Nsun2	_FQQPPQPEGEEDAS[Phospho (STY)]DGGR_.2	Q1HFZ0_S23_M1_Nsun2	8.1631	8.4948	8.2625	9.1640	7.7304	8.4657	9.0392	8.5412	7.9044	7.8399	1.0059	0.0084	0.8858	0.0527
Nsun2	_EGVILTNENAAS[Phospho (STY)]PEQPGDEDAK_.2	#N/A	4.2659	3.5702	3.4518	4.5505	3.3916	4.3379	3.1979	7.4408	7.2954	3.9215	0.7189	-0.4761	0.1319	0.8798
Nsun2	_KKEGVILTNENAAS[Phospho (STY)]PEQPGDEDAK_.3	#N/A	3.6616	3.6808	3.3953	4.4399	4.3766	4.2274	3.0873	4.0621	4.3612	4.1172	1.0145	0.0208	0.8619	0.0646
Specc1l	_RSS[Phospho (STY)]TSSEPTPTVK_.2	Q2KN98_S833_M1_Specc1l	7.1403	3.4231	7.5527	7.6025	2.9288	7.4138	3.2064	7.2384	7.3756	3.4587	1.1298	0.1761	0.6460	0.1898
Specc1l	_SKS[Phospho (STY)]DNQISDK_.2	Q2KN98_S171_M1_Specc1l	6.1769	3.4018	6.7796	6.7704	5.7886	5.4132	6.8243	6.6550	5.0472	4.1873	1.0076	0.0110	0.9590	0.0182
Specc1l	_KGS[Phospho (STY)]SGNASEVSVAC[Carbamidomethyl (C)]LTER_.3	Q2KN98_S385_M1_Specc1l	3.3024	4.0712	3.3417	4.2489	3.9425	4.0808	3.8371	4.3241	4.8945	4.0215	0.8974	-0.1562	0.1518	0.8187
Phactr1	_S[Phospho (STY)]KFANLGR_.2	Q2M3X8_S111_M1_Phactr1	8.2380	7.5074	8.6688	8.7021	8.1124	8.1791	8.3009	8.4942	8.6043	8.2518	0.9788	-0.0309	0.4651	0.3324
Phactr1	_KLS[Phospho (STY)]QRPTVEELR_.3	Q2M3X8_S505_M1_Phactr1	7.0742	7.1035	7.7316	8.0380	7.3050	3.2604	7.2975	7.1476	7.6740	7.1652	0.9223	-0.1167	0.5445	0.2640
Phactr1	_SDSLVPGTHT[Phospho (STY)]PPIR_.3	Q2M3X8_T104_M1_Phactr1	3.8665	4.8308	4.5533	3.5551	2.9922	3.2365	3.2698	4.6838	4.2120	3.5221	0.9789	-0.0308	0.8588	0.0661
Phactr1	_RLS[Phospho (STY)]QRPTAEELEQR_.3	Q2M3X8_S467_M1_Phactr1	4.2451	3.5495	3.4311	4.5712	2.1034	4.3587	3.7940	4.1847	2.2280	3.4177	1.0892	0.1232	0.6097	0.2149
Phactr1	_SKS[Phospho (STY)]DTPYLAEAR_.2	Q2M3X8_S67_M1_Phactr1	10.0403	9.9271	10.4319	10.5723	9.2255	10.1379	10.1699	10.0663	9.9421	10.0786	0.9992	-0.0012	0.9734	0.0117
Phactr1	_SDT[Phospho (STY)]PYLAEAR_.2	Q2M3X8_T69_M1_Phactr1	4.0047	3.2601	3.7383	3.2821	3.1484	3.4762	3.4304	3.7191	4.0181	2.9594	0.9868	-0.0192	0.8531	0.0690
Phactr1	_NEQEEQEEKR_.2	#N/A	3.9404	3.2448	3.1264	3.6290	3.0661	3.1625	3.5233	2.9886	4.8813	3.5960	0.8971	-0.1567	0.3196	0.4954
Gsk3a	_GEPNVSY[Phospho (STY)]IC[Carbamidomethyl (C)]SR_.2	Q2NL51_Y279_M1_Gsk3a	16.0182	15.7447	16.5782	15.9302	14.9507	15.6144	16.3860	15.8097	15.9043	15.6517	0.9917	-0.0120	0.6720	0.1726
Gsk3a	_[Acetyl (Protein N-term)]S[Phospho (STY)]GGGPSGGGPGGSGR_.2	Q2NL51_S2_M1_Gsk3a	10.3761	10.4359	10.8638	10.5993	9.3998	10.4516	10.7437	10.5531	10.4896	10.3384	0.9832	-0.0244	0.5210	0.2832
Gsk3a	_T[Phospho (STY)]SSFAEPGGGGGGGGGPGGSASGPGGTGGGK_.3	Q2NL51_T19_M1_Gsk3a	3.4717	5.9668	4.1585	3.1603	4.0982	3.6313	3.9921	3.4573	3.1059	3.1273	1.1931	0.2547	0.2521	0.5985
Psd3	_KSHS[Phospho (STY)]SPSLNPDASPVTAK_.3	#N/A	10.7962	11.0887	11.3879	11.4221	10.9311	11.3582	10.9269	11.1094	11.3842	10.7002	1.0121	0.0174	0.4709	0.3270
Psd3	_VLLFQTQSPEEM[Oxidation (M)]QGWINK_.2	#N/A	5.3792	5.5705	6.0092	5.2951	4.8530	5.6237	5.5915	5.7407	5.5208	5.4834	0.9769	-0.0337	0.5404	0.2673
Psd3	_IGS[Phospho (STY)]TTNPFLDIPHDPNAAVYK_.3	#N/A	7.1882	8.3631	8.0091	9.0687	6.0478	7.2782	6.9551	8.2454	7.6994	7.1791	1.0185	0.0265	0.8175	0.0875
Psd3	_DHYLEFEK_.2	#N/A	5.2895	5.1083	5.3908	5.3597	5.2395	5.9620	4.3196	5.8112	5.2323	5.5491	1.0313	0.0444	0.5984	0.2230
Psd3	_SHS[Phospho (STY)]SPSLNPDASPVTAK_.2	#N/A	12.2211	12.4634	13.1157	12.8452	11.1578	12.4323	12.6777	12.2318	11.8346	12.4586	1.0058	0.0084	0.8521	0.0695
Psd3	_ALYNS[Phospho (STY)]IKNEK_.2	Q2PFD7_S719_M1_Psd3	4.1298	3.3853	4.5277	6.0372	3.2736	3.3510	3.2442	3.0881	4.7422	5.3801	1.0009	0.0013	0.9959	0.0018
Psd3	_INC[Carbamidomethyl (C)]VAAVFSAPPFPAAIGSQK_.2	#N/A	4.0100	5.1443	3.9243	4.8266	3.6284	4.6091	3.9399	4.0812	4.6516	4.1784	1.0343	0.0486	0.6687	0.1748
Psd3	_KDHRPET[Phospho (STY)]PGIK_.3	#N/A	6.7462	6.8973	3.5346	7.1721	5.6109	6.3262	6.6699	6.6367	5.9447	5.6171	0.9728	-0.0398	0.8194	0.0865
Psd3	_LEWAVDDEEKKK_.3	#N/A	3.0388	4.3036	4.2734	3.8171	2.6604	2.9376	3.9654	3.1840	3.4831	3.4944	0.9925	-0.0109	0.9471	0.0236
Psd3	_DEYKPEKS[Phospho (STY)]LSDEDLK_.2	#N/A	3.5973	3.8453	3.4651	3.9677	3.7107	3.6196	3.6917	4.1905	3.8391	3.9347	0.9456	-0.0807	0.1251	0.9028
Pi4k2a	_VAAAAGSGPS[Phospho (STY)]PPC[Carbamidomethyl (C)]S[Phospho (STY)]PGHDRER_.3	#N/A	9.6445	10.1301	4.1726	3.7164	9.3430	3.0419	10.3045	9.9107	9.7040	9.8858	0.6707	-0.5762	0.0920	1.0362
Pi4k2a	_SAS[Phospho (STY)]ESYTQSFQSR_.2	Q2TBE6_S462_M1_Pi4k2a	8.3405	8.6574	8.6183	9.2077	4.0361	8.6102	8.9967	8.7957	8.7730	8.4824	0.9030	-0.1473	0.4124	0.3847
Pi4k2a	_[Acetyl (Protein N-term)]M[Oxidation (M)]DETS[Phospho (STY)]PLVS[Phospho (STY)]PER_.2	Q2TBE6_S5_M2_Pi4k2a	6.7372	9.7629	3.9970	9.7077	8.9171	9.9873	10.4841	9.8070	9.8218	9.8119	0.8200	-0.2863	0.1790	0.7470
Pi4k2a	_VAAAAGSGPS[Phospho (STY)]PPC[Carbamidomethyl (C)]S[Phospho (STY)]PGHDR_.2	#N/A	6.8304	6.7258	7.2228	7.8204	6.3846	6.5897	7.0619	7.1310	7.3449	3.8409	1.0921	0.1271	0.4359	0.3606
Msto1	_M[Oxidation (M)]AEDEES[Phospho (STY)]PGELC[Carbamidomethyl (C)]PDVLYR_.2	Q2YDW2_S41_M1_Msto1	4.3149	3.6193	3.4568	6.9698	3.4406	4.2889	3.1488	6.7784	4.2997	4.1787	0.9450	-0.0816	0.7896	0.1026
Acot10;Acot9	_DSYIEVLLPLGTDPELR_.2	#N/A	4.5577	4.2804	3.3550	4.8476	4.4245	5.2891	4.0715	4.7885	4.5181	4.4105	1.0027	0.0039	0.9738	0.0115
Cnnm3	_IPVYEEER_.2	#N/A	3.3624	3.7363	5.6065	3.6272	3.6672	4.4028	3.5043	5.0214	4.7019	3.8928	0.9502	-0.0737	0.6850	0.1643
Osbp	_SLS[Phospho (STY)]ELESLKLPAESNEK_.2	#N/A	6.6557	6.5899	7.3185	7.3517	6.1749	3.5708	7.2468	7.4802	6.9247	6.6796	0.8862	-0.1743	0.3007	0.5219
Osbp	_M[Oxidation (M)]LAES[Phospho (STY)]DDS[Phospho (STY)]GDEESVSQTDK_.2	#N/A	5.5806	6.4336	6.6953	6.9380	5.7016	6.1969	7.0750	6.6494	6.3525	5.8697	0.9647	-0.0518	0.5204	0.2836
Osbp	_TGS[Phospho (STY)]NIS[Phospho (STY)]GASSDVSLDEQYK_.2	Q3B7Z2_S377_M2_Osbp	4.3143	3.5945	3.7989	3.8128	3.4490	3.8325	4.0223	3.8039	3.7365	3.6630	0.9984	-0.0023	0.9710	0.0128
Osbp	_SLS[Phospho (STY)]ELESLK_.2	#N/A	4.1620	3.1804	3.8957	3.4395	3.3057	3.3189	3.5877	3.5617	3.8608	3.1167	1.0053	0.0076	0.9382	0.0277
Osbp	/LPANPPGS[Phospho (STY)]AGS[Phospho (STY)]GKDQC[Carbamidomethyl (C)]C[Carbamidomethyl (C)]S	Q3B7Z2_S333_M2_Osbp	3.3671	4.1234	4.6017	4.1454	4.0117	4.1137	4.2937	4.3566	4.7664	3.8227	0.9422	-0.0860	0.3550	0.4498
Nhej1	_DSGETQASSSTS[Phospho (STY)]PR_.2	Q3KNJ2_S245_M1_Nhej1	3.8913	5.8610	3.8786	3.7781	3.8183	3.2638	3.7180	3.8700	6.0944	6.1006	0.8253	-0.2770	0.2518	0.5989
Hddc2	_ELYELWEEYETQSSEEAK_.2	#N/A	4.9860	6.0492	7.1732	7.2077	6.0734	6.6434	6.1236	5.6382	5.0877	5.1570	1.1552	0.2081	0.1061	0.9742
Arhgap45	_YDFEPYVSNNSWS[Phospho (STY)]PIM[Oxidation (M)]R_.2	Q3TBD2_S568_M1_Arhgap45	3.8427	3.1471	3.9290	5.6557	2.9684	3.2603	3.6211	3.5284	5.1429	3.1501	0.9844	-0.0226	0.9242	0.0342
Ppp1r21	_T[Phospho (STY)]PDSEAPDVESR_.2	#N/A	7.6072	7.2670	7.5007	8.0003	6.2033	7.6328	7.4937	7.0429	7.3300	7.9061	0.9900	-0.0145	0.8347	0.0785
Ppp1r21	_SGESSSQLS[Phospho (STY)]QEQK_.2	Q3TDD9_S101_M1_Ppp1r21	6.2891	5.9177	5.7889	6.2135	4.2173	6.0777	5.5548	6.2139	5.8308	5.6903	0.9877	-0.0179	0.8655	0.0627
Ppp1r21	_T[Phospho (STY)]PDSEAPDVESREDLIK_.2	#N/A	3.3841	4.1893	4.0709	5.2808	4.0106	3.7189	4.2882	3.5449	3.1935	3.0397	1.1685	0.2246	0.1721	0.7643
Rogdi	_LTTPATLTLPEIAASGLTR_.2	#N/A	4.2369	3.9144	4.7010	4.0460	4.1249	3.6590	3.4154	5.0906	5.0889	4.0049	0.9349	-0.0970	0.4663	0.3314
Stt3b	_ENPPVEDS[Phospho (STY)]S[Phospho (STY)]DEDDKR_.2	#N/A	7.3442	6.6921	7.3887	6.4013	5.6210	6.5262	7.2897	7.2934	7.0235	6.7056	0.9413	-0.0873	0.2732	0.5636
Stt3b	_ENPPVEDS[Phospho (STY)]S[Phospho (STY)]DEDDKRNPGNLYDK_.3	#N/A	3.5905	6.7480	3.8916	7.1564	4.2258	3.7205	7.2213	7.3678	6.7331	6.3218	0.7074	-0.4994	0.0440	1.3567
Hp1bp3	_EASYSLIR_.2	#N/A	3.1895	2.5708	4.1298	3.2533	3.5398	1.0025	4.4788	2.7126	2.7767	3.3182	0.8874	-0.1723	0.5744	0.2408
Rnf157	_TLS[Phospho (STY)]PLDR_.2	Q3TEL6_S414_M1_Rnf157	4.1077	3.4121	3.2937	3.7963	3.2334	2.9952	3.3560	4.3221	3.9707	3.7633	0.9014	-0.1498	0.1850	0.7328
Iqsec3	_AQAPELQQEEERPGAVGS[Phospho (STY)]PR_.3	Q3TES0_S256_M1_Iqsec3	10.9905	10.9654	11.7505	10.9934	10.0054	10.9590	11.2487	11.0316	10.7726	10.7301	0.9998	-0.0002	0.9956	0.0019
Iqsec3	_LQTSQHS[Phospho (STY)]PK_.2	Q3TES0_S1055_M1_Iqsec3	7.8091	7.8570	8.1096	8.1424	3.7974	7.5488	8.0464	8.1841	7.7274	7.7330	0.9101	-0.1358	0.4344	0.3621
Iqsec3	_VQTPQS[Phospho (STY)]PHQHPVAPGAIADKEK_.4	#N/A	3.7435	3.7183	6.6087	7.0411	3.6065	3.6248	3.8978	5.8559	6.7814	3.4268	0.9466	-0.0792	0.8050	0.0942
Iqsec3	_VQTPQS[Phospho (STY)]PHQHPVAPGAIADK_.3	#N/A	3.0701	5.5114	4.3046	3.8484	3.7147	4.4108	4.3040	3.1528	4.9862	3.5257	1.0379	0.0536	0.7828	0.1063
Iqsec3	_S[Phospho (STY)]PTLSTDTLR_.2	Q3TES0_S646_M1_Iqsec3	6.7235	3.8679	3.2419	4.0500	3.7562	6.5624	6.6523	4.3012	3.6227	3.8475	1.0205	0.0293	0.9237	0.0345
Eepd1	_GNSAQHS[Phospho (STY)]PSSLR_.2	#N/A	4.6004	3.7845	3.6986	5.3606	4.8678	5.1223	5.4358	4.1759	3.8245	4.2984	1.0313	0.0445	0.7648	0.1165
Eepd1	_GNSAQHS[Phospho (STY)]PSSLRR_.3	#N/A	3.7540	5.2674	6.5560	5.8261	5.5052	5.8200	5.8100	6.0233	4.3245	3.4096	1.1151	0.1571	0.4361	0.3604
Eepd1	_EHGPFRS[Phospho (STY)]VEDLVR_.3	#N/A	3.8569	3.3693	4.5437	7.2290	3.2575	3.3671	3.2602	6.7445	5.7834	3.5125	0.8851	-0.1761	0.6054	0.2179
Eepd1	_S[Phospho (STY)]VEDLVR_.2	#N/A	3.0731	3.8783	3.7599	5.8329	2.0795	5.6519	3.9772	6.5656	3.7974	6.5273	0.7755	-0.3667	0.2584	0.5877
Myl12b	_LNGTDPEDVIR_.2	#N/A	7.5665	7.4641	7.2719	7.6601	7.7658	7.3735	7.5667	7.2295	7.4246	7.6333	1.0072	0.0103	0.6605	0.1801
Myl12b	_GNFNYIEFTR_.2	#N/A	7.0632	7.3183	7.5684	7.8482	7.1588	7.5838	7.5224	7.2412	7.1754	7.0665	1.0237	0.0338	0.3396	0.4690
Myl12b	_FTDEEVDELYR_.2	#N/A	7.0764	6.9471	7.1623	7.4406	7.3501	7.4062	7.1488	7.0450	6.9374	7.3043	1.0171	0.0245	0.3363	0.4732
Myl12b	_ATSNVFAM[Oxidation (M)]FDQSQIQEFK_.2	#N/A	4.7122	5.0618	5.2377	6.0967	4.7615	5.2218	4.9145	4.7296	4.6926	5.0142	1.0712	0.0992	0.2261	0.6457
Myl12b	_EAFNMIDQNR_.2	#N/A	7.5861	5.8448	4.4933	7.7854	6.7631	4.2221	6.3934	5.5124	5.9866	6.3613	1.0086	0.0124	0.9491	0.0227
Aarsd1	_RAEAQALLQDYVSTQS[Phospho (STY)]AEE_.2	#N/A	4.1245	3.4289	3.3105	3.8131	3.2502	2.9785	8.0028	4.3053	3.9539	3.7801	0.6954	-0.5241	0.1001	0.9996
Aarsd1	_ELS[Phospho (STY)]LDDPEVEQVR_.2	Q3THG9_S174_M1_Aarsd1	3.3815	8.8003	3.1151	9.6187	8.8692	8.6066	8.4239	9.0948	9.1741	8.9357	0.7932	-0.3342	0.2617	0.5821
Aarsd1	_GAQADHFTESPLS[Phospho (STY)]PGSQVQVR_.3	#N/A	6.4578	7.7367	7.9934	8.6177	5.7940	6.8844	7.4750	7.6448	6.2574	6.2015	1.0512	0.0720	0.5837	0.2338
Aarsd1	_AEAQALLQDYVSTQS[Phospho (STY)]AEE_.2	#N/A	3.0341	3.8393	3.7209	4.2814	3.6606	4.0689	4.4297	3.8949	4.6480	4.1905	0.8781	-0.1876	0.0721	1.1422

Gmps	_TLNM[Oxidation (M)]TTS[Phospho (STY)]PEEK_.2	#N/A	10.4966	10.2560	10.8088	10.9924	9.9565	10.2358	10.6247	10.2484	10.4293	10.4780	1.0012	0.0017	0.9530	0.0209
Gmps	_TLNMTTS[Phospho (STY)]PEEKR_.2	#N/A	3.2478	4.0041	3.9089	6.0788	3.8924	4.2331	4.1262	3.7069	3.8864	4.3785	1.0504	0.0710	0.6986	0.1558
Gmps	_NFLYDIAGC[Carbamidomethyl (C)]SGNFTVQNR_.2	#N/A	4.2285	0.8756	3.9622	3.7930	3.3723	3.2523	4.2960	3.6385	3.7942	3.1833	0.8711	-0.1992	0.4790	0.3197
Gmps	_VIC[Carbamidomethyl (C)]AEEPYIC[Carbamidomethyl (C)]K_.2	#N/A	3.0365	4.1896	3.7233	4.2260	4.1019	4.0475	3.9407	3.8925	4.7239	5.1295	0.8792	-0.1857	0.1476	0.8308
Gmps	_QADFEAHNILR_.3	#N/A	3.6914	3.5348	4.3782	3.3800	2.8171	3.2015	3.0947	3.2376	4.3871	3.3470	0.9954	-0.0066	0.9650	0.0155
Mat2a	_FVIGGPQGDAGLTGR_.2	#N/A	4.7889	5.7315	5.6791	5.3465	5.0477	4.7755	3.5755	5.6654	5.2102	4.8536	1.0833	0.1154	0.3618	0.4415
Mat2a	_TQVTVQYMQDR_.2	#N/A	4.1393	3.4437	3.3253	3.8280	3.2650	2.9636	3.3244	4.2905	3.9391	3.7950	0.9106	-0.1351	0.2355	0.6281
Usp39	_EADEDS[Phospho (STY)]EPEREVR_.2	#N/A	5.1579	5.1261	6.4588	6.0135	5.3048	5.3068	4.5197	5.5548	5.5629	5.3672	1.0591	0.0828	0.3894	0.4096
Usp39	_EREADEDS[Phospho (STY)]EPEREVR_.3	#N/A	4.0462	4.2492	4.6793	4.7770	4.0738	4.3842	4.0577	4.7459	4.4105	4.1011	1.0091	0.0131	0.8491	0.0711
Usp39	_EADEDS[Phospho (STY)]EPER_.2	#N/A	3.9941	3.2985	3.1801	4.9555	3.1198	3.5413	3.4696	5.6249	6.4794	2.9986	0.7929	-0.3348	0.2371	0.6251
Usp39	_EREADEDS[Phospho (STY)]EPER_.2	#N/A	3.4364	3.8499	3.7316	4.2342	3.6713	4.0582	4.5672	3.8843	3.5329	4.2012	0.9466	-0.0792	0.3693	0.4326
Smarca4	_DSEAGSST[Phospho (STY)]PTTSTR_.2	Q3TKT4_T1390_M1_Smarca4	3.7000	3.5261	4.3868	3.3886	2.8257	3.2102	3.1033	3.2290	4.3784	3.3556	0.9971	-0.0042	0.9774	0.0099
Cwc27	_NFSLLSFGEEAEEEEEEVNR_.2	#N/A	3.8751	2.7118	3.6087	3.1525	3.1605	5.1093	3.3008	3.8487	3.9136	4.3306	0.9362	-0.0951	0.6086	0.2157
Prrc2c	_S[Phospho (STY)]LEDVKPR_.2	Q3TLH4_S853_M1_Prrc2c	8.8325	9.0343	9.5221	9.7234	8.3572	8.8528	8.9691	9.0664	8.8880	8.9790	1.0087	0.0125	0.7682	0.1145
Prrc2c	_SFS[Phospho (STY)]SQRPGVDR_.2	Q3TLH4_S1516_M1_Prrc2c	3.5892	4.3455	6.8832	4.3675	5.7139	3.8916	3.7848	4.1345	4.4335	4.0448	1.1705	0.2272	0.3124	0.5052
Prrc2c	_SESS[Phospho (STY)]DFEVVPK_.2	Q3TLH4_S1222_M1_Prrc2c	4.2942	3.5496	4.0279	5.3315	3.4379	3.1867	3.0799	3.2525	4.4019	3.2489	1.1360	0.1840	0.3363	0.4733
Prrc2c	_S[Phospho (STY)]VSHGSNHAQNAEEQR_.3	#N/A	3.8786	3.1106	3.6123	3.9769	3.4976	3.6837	3.7758	4.0446	4.0533	3.6811	0.9326	-0.1007	0.1700	0.7695
Trappc10	_SPS[Phospho (STY)]DNSLNTTGIIC[Carbamidomethyl (C)]R_.2	Q3TLI0_S684_M1_Trappc10	4.3461	8.4688	8.8980	8.9828	6.9542	8.2002	9.1946	9.0006	8.3298	3.3009	1.0248	0.0354	0.9000	0.0458
Tmem38a	_AKEELGEGS[Phospho (STY)]R_.2	#N/A	5.9854	6.2870	6.9707	5.8120	6.4844	6.1348	6.9838	6.4858	6.3338	6.6918	0.9480	-0.0771	0.1847	0.7336
Tmem38a	_AKEELGEGS[Phospho (STY)]RK_.3	#N/A	4.2419	2.9843	3.4278	3.9305	3.3676	4.3619	3.6452	4.1880	1.6048	3.8975	1.1155	0.1577	0.4965	0.3040
Exoc5	_VC[Carbamidomethyl (C)]HLGDQLEGVNT[Phospho (STY)]PR_.3	Q3TPX4_T122_M1_Exoc5	5.1555	4.1159	3.1402	4.1848	4.0511	4.0743	4.1833	5.0387	4.6659	3.8621	0.9285	-0.1070	0.4344	0.3621
	ADS[Phospho (STY)]ES[Phospho (STY)]EEDEQESEEVR.2	#N/A	5.8618	6.6432	6.8768	6.9083	6.2108	5.6644	6.6967	6.2697	6.5315	6.0110	0.9974	-0.0037	0.9573	0.0190
	VGDTEKPEPERS[Phospho (STY)]PPNR.3	#N/A	8.2541	8.1327	8.8392	8.9045	7.9755	7.9798	8.3974	8.0504	8.0906	8.1190	1.0224	0.0320	0.4351	0.3614
	RRADS[Phospho (STY)]ES[Phospho (STY)]EEDEQESEEVR.2	#N/A	3.8452	3.5268	4.0270	3.9557	4.0890	3.7982	4.4614	4.2669	4.2952	3.4065	0.9431	-0.0846	0.3067	0.5132
	RADS[Phospho (STY)]ES[Phospho (STY)]EEDEQESEEVR.2	#N/A	4.0727	3.8693	3.8290	4.2150	3.0076	3.4656	3.9580	3.8752	3.6333	3.2982	1.0141	0.0202	0.8425	0.0744
Fndc4	_GKGPEQS[Phospho (STY)]PQGRPVGTTR_.3	Q3TR08_S209_M1_Fndc4	3.3676	5.0695	5.2128	5.2923	5.4685	5.1348	4.1204	5.4631	5.1210	3.8231	1.0631	0.0883	0.5768	0.2390
Pnpla6	_DEGGS[Phospho (STY)]PEGASPSTASEVEEEK_.2	Q3TRM4_S1319_M1_Pnpla6	3.3779	4.1831	4.0647	4.5673	4.0044	3.7251	4.2820	3.5511	3.1997	3.0335	1.1338	0.1812	0.1569	0.8043
Map9	_ESPGGC[Carbamidomethyl (C)]TS[Phospho (STY)]PGSQEK_.2	Q3TRR0_S257_M1_Map9	9.3640	9.4421	9.5542	10.2377	9.4084	9.4307	9.5652	9.4690	9.5084	9.5795	1.0044	0.0064	0.8102	0.0914
Map9	_S[Phospho (STY)]PSAATSSHYLGSLK_.2	Q3TRR0_S389_M1_Map9	6.1992	4.1818	6.7205	4.2339	5.6432	6.4508	5.7363	3.8846	3.5332	4.2009	1.2841	0.3608	0.1101	0.9581
Map9	_KQS[Phospho (STY)]LEPDKADHIR_.3	#N/A	8.1362	7.9024	8.1647	8.1781	7.1998	8.0733	7.3917	7.7884	8.1869	8.2366	1.0053	0.0076	0.8711	0.0599
Map9	_STS[Phospho (STY)]SGETSSGLDADGHFKPSPQPR_.3	Q3TRR0_S160_M1_Map9	4.0963	3.3517	4.5612	3.5630	5.7744	3.3846	3.2777	3.0546	4.2040	3.5300	1.1721	0.2291	0.2747	0.5611
Map9	_RTS[Phospho (STY)]FQDELIR_.2	Q3TRR0_S23_M1_Map9	3.9479	3.2522	3.1338	6.3470	4.6446	5.4831	3.5159	5.2039	4.1306	5.0314	0.9995	-0.0008	0.9975	0.0011
Map9	_M[Oxidation (M)]NDFHIS[Phospho (STY)]DDEEKNSPR_.3	#N/A	5.4837	3.8681	4.0592	7.0921	4.6045	6.3960	6.0120	6.4136	6.1681	6.3399	0.8423	-0.2475	0.1802	0.7442
Cblb	_QNSPVTS[Phospho (STY)]PGSS[Phospho (STY)]PLAQR_.2	Q3TTA7_S480_M2_Cblb	3.9053	3.5416	3.9935	4.0485	4.0462	3.7240	3.9460	3.5673	3.5277	3.9106	1.0371	0.0525	0.3376	0.4716
Ccdc136	_GLS[Phospho (STY)]LTETELEELR_.2	Q3TVA9_S50_M1_Ccdc136	11.0863	10.7565	11.2686	10.8705	10.1396	10.9367	11.0574	10.6499	10.9337	10.9211	0.9956	-0.0063	0.8262	0.0829
Hexim2	_TQS[Phospho (STY)]PGGC[Carbamidomethyl (C)]SVEAVLAR_.2	Q3TVI4_S75_M1_Hexim2	3.1491	4.1933	4.3837	7.4366	5.8417	3.7149	6.2341	3.0738	6.0626	6.1176	0.8910	-0.1665	0.5776	0.2383
Pbxip1	_GREPS[Phospho (STY)]SSQPVVPVDVEDQAK_.3	Q3TVI8_S164_M1_Pbxip1	3.6220	3.8724	4.1161	3.7738	5.1910	3.4696	3.6395	3.5578	3.8710	3.6558	1.0887	0.1226	0.3387	0.4702
Tmem63b	_LTSVSS[Phospho (STY)]S[Phospho (STY)]VDFDQR_.2	Q3TWI9_S115_M2_Tmem63b	3.5503	3.5358	3.2840	5.7344	3.3798	3.8742	4.0336	4.1893	5.4697	4.0059	0.8799	-0.1846	0.3597	0.4441
Srsf6	_SHS[Phospho (STY)]PLPAPPSK_.2	Q3TWW8_S303_M1_Srsf6	9.3068	9.1173	9.7378	9.5531	8.1344	9.0443	9.5832	9.5966	9.2083	9.2430	0.9725	-0.0402	0.4110	0.3861
Srsf6	_LIVENLSSR_.2	#N/A	6.1113	5.7885	6.1700	5.7596	5.8472	6.6598	5.9507	6.0987	6.9666	6.3354	0.9555	-0.0656	0.2897	0.5381
Srsf6	_ARS[Phospho (STY)]MS[Phospho (STY)]PPPK_.2	#N/A	7.5132	4.1402	3.1176	8.3365	4.0285	4.0970	3.9901	5.4822	3.4916	3.8395	1.2391	0.3093	0.4093	0.3880
Srsf6	_S[Phospho (STY)]M[Oxidation (M)]S[Phospho (STY)]PPPK_.2	#N/A	5.6726	3.0335	5.8876	5.9686	2.8548	7.4264	5.5318	8.7116	7.5782	6.8238	0.7178	-0.4783	0.0942	1.0257
Srsf6	_SRS[Phospho (STY)]QS[Phospho (STY)]RS[Phospho (STY)]HS[Phospho (STY)]PLPAPPSK_.3	Q3TWW8_S297_M3_Srsf6	4.0519	3.2905	3.7856	3.3294	3.1956	3.1168	3.4776	3.6718	3.9709	3.0066	0.9802	-0.0289	0.7846	0.1053
Srsf6	_ARS[Phospho (STY)]MS[Phospho (STY)]PPPKR_.3	#N/A	3.6486	2.9530	4.3354	3.3373	4.2752	3.4543	4.5528	3.2804	4.4298	3.3043	0.9423	-0.0857	0.5810	0.2358
Psmd1	_TVGT[Phospho (STY)]PIASVPGSTNTGTVPGSEK_.2	Q3TXS7_T273_M1_Psmd1	6.1164	5.8065	3.7318	6.6549	4.0328	3.4828	6.9206	6.2257	5.6822	5.9003	0.8041	-0.3146	0.1376	0.8613
Psmd1	_TPDAS[Phospho (STY)]PEPK_.2	Q3TXS7_S315_M1_Psmd1	6.8380	4.0906	7.5994	4.1126	6.5456	4.1465	7.2709	7.3010	6.8911	6.7980	0.7863	-0.3468	0.1063	0.9735
Psmd1	_TASAVAGKT[Phospho (STY)]PDAS[Phospho (STY)]PEPKDQTLK_.3	#N/A	6.3760	7.2222	8.1282	6.6679	6.1079	6.8730	7.2748	6.9665	7.0692	6.7865	0.9817	-0.0266	0.7406	0.1304
Psmd1	_IEEEEQEPEPPEPFEYIDD_.2	#N/A	3.9907	5.0311	3.7810	5.1777	4.4651	4.7454	5.1980	5.3294	4.8705	5.4722	0.8686	-0.2033	0.0539	1.2682
Psmd1	_TASAVAGKT[Phospho (STY)]PDAS[Phospho (STY)]PEPK_.2	#N/A	9.8227	9.8627	10.3172	10.1944	9.0345	9.7014	10.3842	10.0777	9.9415	9.8483	0.9761	-0.0349	0.3608	0.4427
Psmd1	_AAVESLGFILFR_.2	#N/A	4.0660	3.3717	4.2492	3.2510	3.5521	3.3874	3.2824	3.3667	3.8916	3.2180	1.0601	0.0841	0.4194	0.3774
Psmd1	_TPEQC[Carbamidomethyl (C)]PSVVSLLSESYNPHVR_.3	#N/A	3.9574	3.2618	3.1434	3.6461	2.1823	3.1455	3.5063	2.9716	4.1210	3.6131	0.9071	-0.1407	0.3852	0.4143
Slc17a7	_WAPPLER_.2	#N/A	8.5447	8.5856	8.9426	7.7886	8.3627	7.9131	8.6564	9.0681	8.7474	8.0184	0.9691	-0.0453	0.3731	0.4281
Slc17a7	_DPPVVDC[Carbamidomethyl (C)]TC[Carbamidomethyl (C)]FGLPR_.2	#N/A	6.9434	4.6615	6.4313	5.8747	6.3155	6.3804	7.0258	6.1693	6.5252	6.2605	0.9855	-0.0211	0.6948	0.1581
Slc17a7	_YIEDAIGESAK_.2	#N/A	7.2458	6.6231	7.4244	6.7003	6.9397	6.7426	7.0157	7.3360	6.7626	6.8393	0.9939	-0.0088	0.8321	0.0798
Slc17a7	_GGHVVVQK_.2	#N/A	5.3014	4.5934	4.5857	4.7349	5.3600	5.1090	4.4232	5.4485	4.9266	5.2222	0.9885	-0.0167	0.8241	0.0840
Slc17a7	_KYIEDAIGESAK_.2	#N/A	5.0020	5.0356	4.3157	5.1877	4.5168	4.7675	4.5004	5.0004	4.1391	5.0834	1.0264	0.0376	0.6279	0.2021
Slc17a7	_HIM[Oxidation (M)]STTNVR_.2	#N/A	5.8572	5.5208	5.4925	5.6475	5.4817	4.5026	5.6196	5.5076	5.6866	4.8857	0.9986	-0.0021	0.9784	0.0095
Slc17a7	_QPWAEPEEM[Oxidation (M)]S[Phospho (STY)]EEK_.2	#N/A	6.7586	6.8641	6.8482	7.1688	5.9603	6.6602	7.4544	7.3284	6.6565	6.4280	0.9631	-0.0542	0.3962	0.4021
Slc17a7	omethyl (C)]GFVGHDQLAGS[Phospho (STY)]DES[Phospho (STY)]EMEDEAEPPGAPPAPPPSYGATHSTVQP	Q3TXX4_S519_M2_Slc17a7	3.0592	4.2832	4.2937	3.8375	3.7038	3.6250	3.9858	3.1637	3.4627	3.5148	1.0761	0.1058	0.3511	0.4545
Slc17a7	_RQEGAETLELS[Phospho (STY)]ADGRPVTTHTR_.4	Q3TXX4_S36_M1_Slc17a7	4.0931	3.3974	3.2790	3.7817	3.2187	3.0099	3.4964	4.3368	3.9854	3.7487	0.8899	-0.1683	0.1229	0.9105
Fam131b	_DTDAYSDLS[Phospho (STY)]DGEK_.2	#N/A	9.5286	9.2145	9.4014	10.0802	9.2353	9.1531	9.8422	9.2321	9.4076	9.5818	0.9916	-0.0122	0.7035	0.1528
Fam131b	_NS[Phospho (STY)]NAYGIGALAK_.2	#N/A	9.6526	3.3770	9.7672	9.7332	8.7128	9.6378	9.9787	3.7583	4.0573	9.6468	1.2361	0.3058	0.4111	0.3860
Fam131b	_RNS[Phospho (STY)]NAYGIGALAK_.2	#N/A	6.7197	3.0519	6.7592	6.8316	2.8732	3.3554	3.1508	5.9071	4.3309	3.4032	1.1748	0.2324	0.5387	0.2687
Fam131b	_VSDVTSSGVQSFDEEEGDANN_.2	#N/A	6.1366	8.8881	7.9500	9.5313	8.7190	9.1027	8.9001	8.8394	8.8678	9.0263	0.9416	-0.0868	0.4279	0.3686
Fam131b	_DLESLS[Phospho (STY)]PR_.2	#N/A	4.3307	6.6424	3.5166	4.8857	3.4563	4.2731	6.9037	4.0992	4.3154	3.9863	0.9222	-0.1168	0.6545	0.1841
Fam131b	_DTDAYSDLS[Phospho (STY)]DGEKEAR_.2	#N/A	7.8253	9.1041	9.0875	9.6840	9.0785	9.1424	9.5600	9.0983	9.0155	9.1285	0.9768	-0.0339	0.5336	0.2728
Fam131b	_RDTDAYSDLS[Phospho (STY)]DGEKEAR_.3	#N/A	6.1367	6.1504	6.5541	7.0420	6.4823	6.2591	6.8925	6.7271	6.2720	6.6343	0.9707	-0.0428	0.3675	0.4348
Fam131b	_KVSDVTSSGVQS[Phospho (STY)]FDEEEGDANN_.2	#N/A	8.7629	9.6185	9.9787	10.3131	9.5584	9.9155	10.0799	9.6614	9.6945	9.8385	0.9870	-0.0188	0.6621	0.1791
Fam131b	_APTIQPQHS[Phospho (STY)]HEAVR_.3	Q3TY60_S102_M1_Fam131b	3.1505	4.0757	3.8373	5.3002	3.7770	4.7709	4.0546	3.7786	3.4271	4.3069	1.0668	0.0933	0.5524	0.2577
Fam131b	_RDTDAYSDLS[Phospho (STY)]DGEK_.2	#N/A	4.1963	3.6086	3.5923	4.6654	3.4969	2.9526	3.2844	3.8651	4.1641	3.4142	0.9603	-0.0584	0.6731	0.1719
Fam131b	_DLESLS[Phospho (STY)]PREDPEM[Oxidation (M)]STALSR_.3	#N/A	6.0536	5.6670	6.0528	6.5980	5.5651	6.0484	3.5838	6.1116	6.1571	6.3838	1.0789	0.1095	0.4522	0.3447
Ica1l	_VALS[Phospho (STY)]EEEEEERFER_.2	#N/A	3.3563	3.9974	4.3119	3.7607	4.0608	3.4753	3.8191	3.1845	3.3736	3.6111	1.0944	0.1301	0.1656	0.7809
Ica1l	_ALPRDS[Phospho (STY)]LEGDDFEK_.3	#N/A	3.2988	3.8443	4.2574	4.3180	3.9335	4.0397	4.2130	3.2993	3.2635	3.3687	1.1166	0.1592	0.1502	0.8233
Aifm3	_GKEELSASGKGS[Phospho (STY)]PR_.2	#N/A	5.3417	5.5458	5.9657	5.5381	4.7687	5.6641	5.8883	5.8188	5.6249	5.2981	0.9670	-0.0484	0.4392	0.3573
Aifm3	_LPTPQPYPS[Phospho (STY)]PQDC[Carbamidomethyl (C)]VEATVC[Carbamidomethyl (C)]HVK_.3	Q3TY86_S63_M1_Aifm3	3.8214	3.0769	3.5551	6.9147	2.9652	3.6595	3.2471	3.9023	4.2014	4.2770	1.0235	0.0335	0.9082	0.0418
Aifm3	_EELSASGKGS[Phospho (STY)]PR_.2	#N/A	3.4589	3.8835	3.1926	2.6179	4.1035	4.0220	4.3854	4.2648	3.0631	3.9145	0.9077	-0.1397	0.3657	0.4369
Mphosph8	_GTVAVGDS[Phospho (STY)]EEDGEDVFEVER_.2	Q3TYA6_S51_M1_Mphosph8	3.8111	3.1155	4.4979	3.4998	2.9368	3.2918	3.2144	3.1179	4.2673	3.4668	1.0025	0.0036	0.9806	0.0085
Lmtk2	_GQS[Phospho (STY)]PPESVVPEESSDVR_.2	Q3TYD6_S1104_M1_Lmtk2	10.3679	10.1886	11.0421	10.4371	9.7916	10.0934	11.0651	10.3816	10.4218	10.1953	0.9814	-0.0271	0.4759	0.3225
Lmtk2	_YFS[Phospho (STY)]PPPPAR_.2	Q3TYD6_S1418_M1_Lmtk2	7.9949	8.6950	8.6328	3.8326	3.2697	3.6541	3.5473	4.2859	7.2780	3.7996	1.2719	0.3470	0.4244	0.3722

Lmtk2	_HSGTSPQASPALLTEEGS[Phospho (STY)]PTAPTDPILKPEETK_.4	Q3TYD6_S797_M1_Lmtk2	3.1550	3.9602	3.8418	4.1605	4.4367	3.9479	5.6348	6.1167	4.9408	5.3082	0.7122	-0.4897	0.0007	3.1761
Lmtk2	_NAGFTSALLES[Phospho (STY)]PQR_.2	Q3TYD6_S742_M1_Lmtk2	6.9502	6.3406	6.6488	7.8663	5.4184	6.5242	6.7167	7.3558	7.0132	6.1956	0.9713	-0.0420	0.6766	0.1697
Esyt2	_SSS[Phospho (STY)]SLLASPSHIAAK_.2	Q3TZZ7_S662_M1_Esyt2	4.2262	3.1162	3.9599	6.8973	2.9376	3.2911	3.6519	7.6654	3.7966	3.1809	0.9362	-0.0951	0.7865	0.1043
Tbc1d2b	_DTSPDKGELVS[Phospho (STY)]DEEEDT_.2	Q3U0J8_S959_M1_Tbc1d2b	4.3665	6.1698	4.1001	3.6439	4.1634	5.3522	6.0474	5.5979	4.8751	3.3212	0.9339	-0.0986	0.6397	0.1940
Trappc9	_FNFESVPES[Phospho (STY)]PGEK_.2	Q3U0M1_S953_M1_Trappc9	2.9135	9.1468	7.9127	9.9749	8.2457	3.0665	10.2295	10.1558	9.8196	9.7774	0.6880	-0.5396	0.0842	1.0745
Trappc9	_VESRPTNPS[Phospho (STY)]EGS[Phospho (STY)]KTGDLGHVK_.3	Q3U0M1_S840_M2_Trappc9	6.8924	4.2336	4.3371	4.0398	4.0915	4.1115	6.7708	6.5711	6.8656	6.5329	0.6907	-0.5338	0.0070	2.1540
Rasip1	_AAS[Phospho (STY)]GGAALASPGPGSGSGTPTGSGGK_.2	Q3U0S6_S286_M1_Rasip1	4.3709	3.6753	3.4008	5.6225	3.4966	3.8367	3.0928	4.0842	4.1640	4.2806	1.0414	0.0585	0.7439	0.1285
Rasip1	_SVS[Phospho (STY)]ELSLQGR_.2	Q3U0S6_S322_M1_Rasip1	7.2151	7.8948	7.5783	7.9739	6.8088	7.8641	8.4746	8.0589	8.0323	7.8186	0.9333	-0.0996	0.0702	1.1535
Khsrp	_GS[Phospho (STY)]PQQIDHAK_.2	Q3U0V1_S481_M1_Khsrp	6.4909	6.4508	6.7928	6.9153	6.8895	7.1029	7.2503	6.6090	7.1081	6.5369	0.9851	-0.0216	0.6081	0.2160
Khsrp	_IINDLLQSLR_.2	#N/A	5.4964	5.7531	5.6987	5.5227	6.0414	5.4373	6.1226	5.7985	5.6085	5.6138	0.9779	-0.0322	0.4162	0.3807
Khsrp	_SVSLTGAPESVQK_.2	#N/A	5.9571	5.9899	6.5566	6.2879	6.3784	6.0295	6.3131	6.0025	6.1055	5.8004	1.0239	0.0340	0.3655	0.4371
Khsrp	_VQIS[Phospho (STY)]PDSGGLPER_.2	Q3U0V1_S182_M1_Khsrp	10.0876	10.3813	10.9241	10.9413	10.2907	10.5189	10.8383	10.3105	10.3684	10.6468	0.9984	-0.0023	0.9348	0.0293
Khsrp	_IGGDAATTVNNNTPDFGFGGQK_.2	#N/A	3.2223	3.5230	5.0511	4.5977	3.2347	3.4912	3.2451	3.9043	4.2034	3.6007	1.0307	0.0437	0.7950	0.0996
Pag1	_SSSS[Phospho (STY)]C[Carbamidomethyl (C)]NDLYATVK_.2	Q3U1F9_S351_M1_Pag1	8.9687	9.0028	9.5223	9.3040	8.3707	3.5686	9.5127	8.7062	8.9079	9.1170	0.8965	-0.1577	0.4435	0.3531
Pag1	_RPS[Phospho (STY)]EEPEPDYEAIQTLNR_.3	Q3U1F9_S379_M1_Pag1	4.7503	3.9384	3.6024	5.5813	3.8267	3.6551	4.0316	4.6375	5.2931	5.6299	0.8627	-0.2130	0.2059	0.6863
Pag1	_HSANAESILGT[Phospho (STY)]C[Carbamidomethyl (C)]SDLDEESPPPVPVK_.3	Q3U1F9_T245_M1_Pag1	4.0617	3.2569	3.5111	6.4834	4.8707	4.1256	3.4748	6.0714	5.1751	4.1195	0.9309	-0.1032	0.6758	0.1702
Tfpt	_TTATLDPTS[Phospho (STY)]PAPGEGPSGR_.2	Q3U1J1_S180_M1_Tfpt	7.0891	7.1267	7.5859	7.9274	6.7712	7.4316	7.5017	7.2430	7.1930	3.5943	1.1471	0.1980	0.2552	0.5932
Tfpt	_EGLS[Phospho (STY)]PSQR_.2	Q3U1J1_S167_M1_Tfpt	3.6987	3.0030	4.3855	3.6168	2.8244	3.4043	5.7886	3.2304	3.6834	5.6573	0.7601	-0.3957	0.1013	0.9942
Tfpt	_LLPYPTLAS[Phospho (STY)]PPFD_.2	Q3U1J1_S255_M1_Tfpt	3.9049	3.1604	3.6386	3.7544	3.0487	3.5760	3.4691	3.8188	4.0126	4.3605	0.8975	-0.1561	0.1147	0.9405
Dennd1b	_ASKIS[Phospho (STY)]S[Phospho (STY)]EDGEETR_.2	Q3U1T9_S516_M2_Dennd1b	3.0049	4.8829	5.8494	4.9664	4.9707	5.4639	6.0244	5.4588	5.5930	4.7876	0.8885	-0.1706	0.2916	0.5352
Uevld	_DGVLS[Phospho (STY)]PSSQAQLSSR_.2	Q3U1V6_S365_M1_Uevld	8.7571	8.6508	9.2849	9.1770	7.5092	8.6655	9.2114	8.7001	8.6644	8.4897	0.9895	-0.0153	0.7956	0.0993
Map3k9	_SS[Phospho (STY)]PPASPTIIPR_.2	Q3U1V8_S541_M1_Map3k9	3.4729	7.1526	7.5995	4.2512	3.4344	7.1082	3.7483	4.2729	3.9208	3.9285	1.3870	0.4720	0.1690	0.7720
Dennd4b	_S[Phospho (STY)]APSS[Phospho (STY)]PAPR_.2	Q3U1Y4_S732_M2_Dennd4b	7.9783	7.8212	7.4036	5.9236	3.8537	3.3854	7.4525	8.0877	3.1769	7.8900	0.9112	-0.1342	0.6814	0.1666
Dennd4b	_SGS[Phospho (STY)]LGSAR_.2	Q3U1Y4_S961_M1_Dennd4b	3.7821	3.0375	3.5157	3.8773	2.9258	3.6988	3.5920	4.2412	3.8898	3.8443	0.8924	-0.1643	0.1059	0.9750
Gpsm3	_SAPPS[Phospho (STY)]PPPPGTR_.2	Q3U1Z5_S38_M1_Gpsm3	9.0157	9.9798	3.9759	10.4644	6.6300	9.4252	9.7189	9.8293	7.9442	3.1969	1.0751	0.1045	0.7523	0.1236
Mast3	_RLS[Phospho (STY)]ADLR_.2	Q3U214_S769_M1_Mast3	8.6466	7.8799	8.8196	8.0918	7.0772	8.5361	8.3822	8.9517	8.6354	8.4421	0.9503	-0.0736	0.2483	0.6050
Mast3	_RGQS[Phospho (STY)]ADKLGLGTSER_.3	#N/A	8.3314	8.0111	8.3934	7.9864	6.8521	7.8052	8.1728	8.1728	7.8509	7.9937	0.9812	-0.0273	0.6183	0.2088
Mast3	_DPLEEIQPLNDLDESQPPAPGS[Phospho (STY)]PESR_.3	Q3U214_S370_M1_Mast3	6.3486	6.6385	7.1280	7.5232	6.0765	6.6071	4.3269	7.4107	6.9444	6.3559	1.0736	0.1025	0.4648	0.3327
Mast3	_KQEAVQEVS[Phospho (STY)]FDEEPGPPR_.2	#N/A	4.5035	6.2066	6.4933	6.9821	5.5561	6.5660	5.7922	7.1099	6.6157	6.9282	0.9153	-0.1277	0.3047	0.5161
Mast3	_GQS[Phospho (STY)]ADKLGLGTSER_.2	#N/A	8.7607	8.5757	9.1027	8.9674	7.1368	8.5948	8.9145	8.9221	8.4739	8.6508	0.9751	-0.0363	0.5756	0.2399
Mast3	_QEAVQEVS[Phospho (STY)]FDEEPGPPR_.2	#N/A	5.6620	6.3009	6.8732	7.8010	6.2544	6.2784	7.3313	7.6044	7.1589	6.9681	0.8985	-0.1544	0.0942	1.0258
Mast3	_SFS[Phospho (STY)]EDREDGWGQSLGDR_.3	#N/A	3.2412	3.6939	4.1023	3.7925	3.7926	3.5272	3.9363	4.9153	5.0498	3.6714	0.8403	-0.2510	0.0538	1.2691
Mast3	_S[Phospho (STY)]SESVVDEDGGR_.2	Q3U214_S156_M1_Mast3	3.0976	3.9028	3.7844	4.2871	3.7242	8.4832	4.0018	3.8314	8.6775	4.2541	0.8758	-0.1913	0.6487	0.1879
Mast3	_HLGS[Phospho (STY)]EDDETNDEESSTEIPQFSSC[Carbamidomethyl (C)]SHR_.4	Q3U214_S702_M1_Mast3	4.0051	3.3095	3.1911	3.8083	3.1308	3.0978	3.4084	4.4247	4.0733	3.6607	0.8797	-0.1849	0.1154	0.9376
Mast3	_VY[Phospho (STY)]SSSEFLAVQPTPTFAER_.2	Q3U214_Y730_M1_Mast3	3.4618	3.7644	4.1486	3.1504	4.0883	2.9719	4.3659	6.4083	3.1159	3.1174	0.8461	-0.2411	0.3521	0.4533
Fam160a2	_QQS[Phospho (STY)]LGGSESPGPVPR_.2	Q3U2I3_S510_M1_Fam160a2	7.2231	4.3337	4.2153	3.2171	4.1550	6.6413	4.4326	3.4005	6.5970	3.1841	1.1273	0.1729	0.5967	0.2242
Gse1	_ERS[Phospho (STY)]PS[Phospho (STY)]PPAVQC[Carbamidomethyl (C)]K_.2	Q3U3C9_S826_M2_Gse1	8.1993	8.3247	9.3116	9.0993	8.3645	8.7890	9.0552	7.9673	8.4117	8.6737	1.0181	0.0259	0.6147	0.2114
Gse1	_YSPDEM[Oxidation (M)]NNS[Phospho (STY)]PNFEEK_.2	Q3U3C9_S857_M1_Gse1	4.2189	3.0073	6.1495	3.9075	2.8956	5.8652	3.6222	4.2110	6.3107	3.8745	0.9636	-0.0535	0.8535	0.0688
Gse1	_RQT[Phospho (STY)]PS[Phospho (STY)]PR_.2	Q3U3C9_S840_M2_Gse1	4.1749	3.4303	3.8578	3.4523	3.3186	3.3060	3.1992	3.5489	3.8479	3.1296	1.0462	0.0652	0.4950	0.3054
Fam117b	_SSS[Phospho (STY)]WGSTEQLK_.2	#N/A	8.8917	8.4226	3.5388	8.8523	8.2082	8.6163	9.0773	4.0770	8.5596	8.6334	1.0222	0.0316	0.9081	0.0419
Fam117b	_TS[Phospho (STY)]PTVATQTGASVTSTR_.2	Q3U3E2_S102_M1_Fam117b	3.4442	6.7809	3.1779	4.2225	4.0888	4.0367	6.2020	4.2795	3.0777	6.3840	0.8608	-0.2162	0.4667	0.3309
Fam117b	_SPS[Phospho (STY)]APVC[Carbamidomethyl (C)]K_.2	#N/A	3.4623	3.7639	4.1491	3.1509	7.3420	2.9724	4.3664	6.5021	7.6413	3.1179	0.7657	-0.3851	0.3055	0.5150
Fam117b	_SSS[Phospho (STY)]WGSTEQLKEIAK_.2	#N/A	3.6702	3.5833	4.0616	3.2816	3.4716	3.1785	4.0155	5.1739	3.7204	3.2826	0.8748	-0.1930	0.1940	0.7122
Fam117b	_RSPS[Phospho (STY)]APVC[Carbamidomethyl (C)]K_.2	#N/A	3.4574	4.2626	4.1442	3.1461	4.0840	3.6455	4.3616	3.4716	3.1202	3.1130	1.0777	0.1080	0.4235	0.3731
Fam117b	_S[Phospho (STY)]PEQGRPSAEKR_.3	Q3U3E2_S176_M1_Fam117b	3.8458	3.1012	3.5795	3.8135	2.9895	3.6351	3.5282	4.3049	3.9535	3.7805	0.8978	-0.1555	0.1165	0.9335
Fam117b	_SIDTQTPGGADKGS[Phospho (STY)]NNSSR_.3	Q3U3E2_S399_M1_Fam117b	3.0571	4.2853	4.2916	3.8354	3.7017	4.4238	3.9836	3.1658	3.4649	3.5126	1.1135	0.1551	0.2109	0.6759
Tepsin	_QQLQELGAGS[Phospho (STY)]PGPVTNK_.2	Q3U3N6_S400_M1_Tepsin	6.3894	4.0451	7.1872	5.9539	3.9334	4.1920	6.8192	3.7480	6.0800	6.3944	0.9172	-0.1247	0.6102	0.2145
Prickle1	_S[Phospho (STY)]AESLQSLNSGLC[Carbamidomethyl (C)]PEK_.2	Q3U5C7_S592_M1_Prickle1	6.0224	6.4341	6.5097	6.9691	4.0102	4.0002	6.9139	6.8090	4.3323	6.2609	0.9307	-0.1036	0.6218	0.2063
Prickle1	_NLFQQQS[Phospho (STY)]SEVDPR_.2	Q3U5C7_S433_M1_Prickle1	3.0824	4.2600	4.3169	3.8607	3.7270	3.6482	4.0090	3.1405	3.4395	3.5380	1.0804	0.1116	0.3257	0.4872
Prickle1	_KS[Phospho (STY)]RSDNALNLVTER_.3	#N/A	3.7338	2.9892	3.4675	3.9256	2.8775	3.7471	3.6403	4.5186	3.8415	3.8926	0.8700	-0.2009	0.0890	1.0507
Prickle1	_SKS[Phospho (STY)]QSRPQQVK_.2	#N/A	4.1077	3.5051	4.8229	3.2286	3.0949	3.5297	3.3768	3.8501	4.0717	4.0782	0.9663	-0.0494	0.7242	0.1401
Prickle1	_S[Phospho (STY)]QSRPQQVK_.2	#N/A	3.0824	3.8387	4.3169	3.8607	3.7270	4.3985	4.2916	3.1405	3.4395	3.5380	1.0745	0.1036	0.4114	0.3858
Prickle1	_S[Phospho (STY)]RSDNALNLVTER_.2	#N/A	3.7766	3.6829	4.0101	3.9338	3.6580	4.1420	3.8914	3.7591	3.6324	3.7565	1.0286	0.0406	0.3451	0.4621
Foxk2	_EGS[Phospho (STY)]PAPLEPEPGASQPK_.2	Q3UCQ1_S389_M1_Foxk2	9.7138	9.7613	9.9801	10.3929	9.0209	9.6078	10.1781	9.6633	9.8537	9.7135	0.9892	-0.0156	0.6801	0.1674
Ttll12	_[Acetyl (Protein N-term)]M[Oxidation (M)]EIQSGPQPGS[Phospho (STY)]PGR_.2	#N/A	12.2710	12.1933	12.5056	12.5856	11.6790	12.1017	12.7168	12.0373	12.1824	12.3305	0.9924	-0.0111	0.6536	0.1847
Ttll12	_[Acetyl (Protein N-term)]M[Oxidation (M)]EIQSGPQPGS[Phospho (STY)]PGRAER_.2	#N/A	5.6737	6.3383	7.0287	6.5859	5.5061	5.9143	6.2923	6.1246	5.9576	5.7856	1.0223	0.0318	0.6749	0.1708
Wdr41	_LEENGDLYPES[Phospho (STY)]P_.2	Q3UDP0_S459_M1_Wdr41	3.4360	7.9114	8.8923	10.3815	9.5475	4.0449	8.9207	10.4906	10.4116	10.4916	0.7311	-0.4518	0.1138	0.9438
Hgsnat	_LINSELGS[Phospho (STY)]PSR_.2	Q3UDW8_S238_M1_Hgsnat	7.6983	7.2579	7.6066	7.3205	4.1929	3.7566	8.0748	7.4743	7.3319	7.4765	0.8308	-0.2674	0.2078	0.6823
	VTSFS[Phospho (STY)]TPPT[Phospho (STY)]PER.2	Q3UE31_S289_M2_	8.3606	9.2377	4.1389	3.6827	8.2319	9.0710	3.8309	8.7370	8.5847	8.8787	0.9484	-0.0764	0.8160	0.0883
	SRS[Phospho (STY)]LS[Phospho (STY)]GTGR.2	Q3UE31_S351_M2_	3.6557	5.5299	6.7603	6.2477	5.3282	6.4747	6.2716	6.5884	6.5362	5.6762	0.8561	-0.2242	0.2636	0.5791
	SLVGS[Phospho (STY)]WLK.2	Q3UE31_S362_M1_	6.1531	5.6457	4.3934	3.9372	4.0048	6.3310	4.0855	3.0640	6.1319	3.6145	1.2021	0.2655	0.2991	0.5242
	SHS[Phospho (STY)]ANDSEEFFREDDSGADLHNATNLR.4	Q3UE31_S324_M1_	3.8286	3.1330	4.5154	3.5172	2.9543	3.2743	3.6351	3.1004	4.2498	3.4842	0.9778	-0.0324	0.8225	0.0849
Frmd8	_SS[Phospho (STY)]VSS[Phospho (STY)]VGAR_.2	#N/A	5.7498	3.8966	4.3148	3.5852	3.7179	3.4146	3.7754	3.2310	3.9972	3.4023	1.1421	0.1917	0.2977	0.5262
Frmd8	_SHRSS[Phospho (STY)]VSS[Phospho (STY)]VGAR_.2	#N/A	5.2767	5.3635	3.9466	5.7676	3.3567	3.2790	5.2548	3.2253	3.9604	3.4226	1.1343	0.1818	0.4483	0.3485
Garem1	_TDWNEDC[Carbamidomethyl (C)]KS[Phospho (STY)]PK_.2	Q3UFT3_S356_M1_Garem1	3.5869	4.8268	4.2737	5.3787	4.9211	3.5161	4.7818	4.4636	4.1334	5.3382	0.9440	-0.0831	0.5649	0.2481
Garem1	_STC[Carbamidomethyl (C)]APSS[Phospho (STY)]PPTPATLGATIK_.2	Q3UFT3_S493_M1_Garem1	4.2293	3.1131	3.9630	7.2374	2.9344	3.2942	3.6550	3.4944	3.7935	3.1840	1.1690	0.2253	0.4899	0.3099
Eif3j1	_[Acetyl (Protein N-term)]AAAAAAAAAGDS[Phospho (STY)]DSWDADTFSM[Oxidation (M)]EDPVRK_.3	Q3UGC7_S14_M1_Eif3j1	4.0085	3.3339	6.6012	7.5805	7.7923	5.8949	3.4342	3.7152	6.9222	2.9632	1.3780	0.4626	0.2101	0.6775
Hdhd2	_TFFLEALR_.2	#N/A	5.5410	5.1727	5.4810	3.2494	4.9921	5.2954	4.3422	4.9757	4.8205	4.6920	1.0526	0.0740	0.5982	0.2232
Hdhd2	_FVTNTTK_.2	#N/A	2.9271	4.2991	3.6139	5.5252	4.1874	3.9381	3.8312	4.0019	3.6505	4.0836	1.0488	0.0688	0.6816	0.1665
Mcrip1	_S[Phospho (STY)]PTNSSEIFTPAHEENVR_.2	#N/A	4.0205	3.5387	3.8760	6.3014	7.8649	6.5077	8.2789	6.1816	8.2425	7.8345	0.7010	-0.5126	0.0496	1.3048
Arfgef3	_EHLS[Phospho (STY)]EPQSLR_.2	Q3UGY8_S1966_M1_Arfgef3	6.9700	4.2563	3.2337	4.2783	4.1446	3.9809	7.3037	6.8962	6.7374	3.9556	0.7195	-0.4750	0.0856	1.0673
Arfgef3	_VAAPSSS[Phospho (STY)]PSAEAEYWR_.2	Q3UGY8_S1646_M1_Arfgef3	10.3949	10.3077	10.9598	10.0931	3.5262	5.2596	11.2815	3.4932	3.7922	10.2213	1.1704	0.2270	0.6087	0.2156
Arfgef3	_SGS[Phospho (STY)]TGSSLSVSVR_.2	Q3UGY8_S2090_M1_Arfgef3	3.7622	3.4639	4.4490	3.4508	2.8879	3.2724	3.1655	8.2004	4.3162	3.4178	0.7430	-0.4286	0.2378	0.6239
Arfgef3	_YSESNFS[Phospho (STY)]VDDQDLSR_.2	Q3UGY8_S593_M1_Arfgef3	4.0421	3.3003	3.7758	3.3196	3.1859	3.1071	3.4678	3.6816	3.9806	2.9969	0.9783	-0.0316	0.7668	0.1153
Arfgef3	_DGAESSRDS[Phospho (STY)]M[Oxidation (M)]EINEADFR_.3	#N/A	3.5974	3.6688	3.7845	4.9462	3.5136	3.7323	5.5401	3.7645	3.7941	5.6106	0.8282	-0.2719	0.1413	0.8498
Arfgef3	_SDVSDIGS[Phospho (STY)]DNC[Carbamidomethyl (C)]SLADEEQTPR_.2	Q3UGY8_S628_M1_Arfgef3	3.2518	3.9744	3.9386	4.4412	3.8783	3.8512	4.1559	4.2760	3.3259	4.4082	0.9623	-0.0554	0.5931	0.2269
Arfgef3	_HSFS[Phospho (STY)]AGPELLR_.2	Q3UGY8_S2074_M1_Arfgef3	3.3054	3.9208	3.9922	4.4948	3.9319	3.7976	4.2095	3.6237	3.2722	2.9610	1.1110	0.1519	0.2115	0.6747
Dip2b	_LLSPYSPQT[Phospho (STY)]QETDSIGQK_.2	Q3UH60_T56_M1_Dip2b	3.7778	3.7338	3.4640	3.9666	3.6027	5.3395	5.2321	4.1635	5.4547	4.9869	0.8027	-0.3171	0.0462	1.3349
Wnk2	_AGS[Phospho (STY)]LGPETPSR_.2	Q3UH66_S1797_M1_Wnk2	8.6569	7.8727	4.1455	8.6338	7.0203	8.1614	8.5047	8.2829	8.5344	8.6274	0.8737	-0.1948	0.2553	0.5929

Wnk2	_QSS[Phospho (STY)]LPGTGGVASDFVK_.2	Q3UH66_S1770_M1_Wnk2	9.6696	9.2599	9.8590	9.7751	8.1352	9.5355	9.6488	9.1063	9.3921	9.5404	0.9947	-0.0076	0.8879	0.0516
Wnk2	_YS[Phospho (STY)]APPDVYLDEIPSSPEVK_.2	Q3UH66_S1713_M1_Wnk2	3.4019	3.8252	4.0887	4.5913	3.7135	3.8318	4.3060	3.5281	3.1767	3.0575	1.1114	0.1523	0.2314	0.6357
Limch1	_TSVPESIASAGTGS[Phospho (STY)]PSK_.2	Q3UH68_S523_M1_Limch1	9.2403	3.8086	4.2868	9.5402	3.6969	4.4286	9.4046	8.9237	3.4696	3.5079	0.9221	-0.1170	0.8038	0.0949
Limch1	_S[Phospho (STY)]PEPEATLTFPFLDK_.2	Q3UH68_S719_M1_Limch1	7.7371	7.4519	3.3611	8.7798	7.4704	7.8608	8.0674	7.6162	8.0245	7.3964	0.9144	-0.1292	0.5151	0.2881
Limch1	_QTPS[Phospho (STY)]PDVVLR_.2	Q3UH68_S217_M1_Limch1	3.4076	3.8185	4.0944	4.5971	8.9640	10.0632	4.3118	3.5214	3.1700	3.0632	1.6562	0.7279	0.1623	0.7897
Limch1	_S[Phospho (STY)]ASQDLIKK_.2	#N/A	5.9374	3.4743	4.4387	3.4405	2.8776	5.9765	3.1552	5.5457	4.3266	3.4075	1.0605	0.0848	0.7653	0.1162
Limch1	_SRQT[Phospho (STY)]PS[Phospho (STY)]PDVVLR_.2	Q3UH68_S217_M2_Limch1	4.0134	3.3178	3.7583	8.8218	7.4439	3.0896	3.4504	8.0711	8.2152	8.5876	0.7166	-0.4808	0.2367	0.6259
Limch1	_DDS[Phospho (STY)]FDSLDSFGSR_.2	#N/A	3.3512	4.1074	4.5857	7.2614	5.5378	4.1297	4.0229	6.6058	6.1279	7.3589	0.8010	-0.3202	0.2226	0.6526
Limch1	_SAS[Phospho (STY)]QDLIKKEEER_.3	Q3UH68_S379_M1_Limch1	3.6451	3.7824	3.9778	3.9494	3.6504	3.6922	4.0976	3.8988	3.4444	4.3248	0.9598	-0.0592	0.3683	0.4338
Limch1	_SDSLS[Phospho (STY)]PPR_.2	Q3UH68_S192_M1_Limch1	4.2826	3.5381	4.0163	8.0559	3.4264	7.4674	6.9298	3.4411	3.7401	7.2922	0.9589	-0.0605	0.8728	0.0591
Limch1	_KETDDIDS[Phospho (STY)]PKR_.3	Q3UH68_S169_M1_Limch1	4.0809	3.3852	3.6199	4.8143	4.7710	3.0221	3.3829	4.3490	4.6947	3.7365	0.9773	-0.0332	0.8410	0.0752
Limch1	_GSSDGRGS[Phospho (STY)]DSESDLPHR_.3	#N/A	8.4457	8.4439	9.1099	8.9241	8.4797	8.6454	8.5552	8.5094	8.9321	8.8092	0.9969	-0.0044	0.8752	0.0579
Limch1	_TINHQM[Oxidation (M)]ES[Phospho (STY)]PGER_.2	#N/A	7.3380	7.4226	7.8819	8.5285	7.4154	7.1187	7.7447	7.0361	7.1878	7.6318	1.0294	0.0418	0.4802	0.3186
Limch1	_HGRDDS[Phospho (STY)]FDSLDSFGSR_.3	#N/A	4.2819	4.7741	3.4678	5.9180	4.2365	4.9065	3.1819	5.7840	4.2666	6.0843	0.9520	-0.0709	0.7423	0.1294
Shisa6	_LHSQDPLLS[Phospho (STY)]PER_.2	#N/A	6.9643	4.9344	6.7282	6.7427	5.0104	6.0655	7.0665	6.6840	6.5799	6.6766	0.8997	-0.1526	0.1872	0.7277
Shisa6	_ETISAIDTS[Phospho (STY)]PKENTPVR_.2	Q3UH99_S247_M1_Shisa6	4.4379	5.7437	3.5602	6.5729	4.8110	5.9271	5.8178	5.6757	4.7218	6.1220	0.9268	-0.1097	0.5238	0.2809
Shisa6	_ILSDEQLLS[Phospho (STY)]TER_.2	Q3UH99_S422_M1_Shisa6	5.4171	3.2752	5.8300	6.7739	2.8659	5.2606	5.4733	5.7380	5.5304	5.5400	0.8803	-0.1839	0.4159	0.3810
Ccdc177	_YVLTS[Phospho (STY)]PR_.2	Q3UHB8_S90_M1_Ccdc177	11.0815	2.9568	11.4894	10.5771	9.7018	10.8673	9.9050	10.9341	10.8198	10.8920	0.8879	-0.1715	0.4937	0.3066
Ccdc177	_SHS[Phospho (STY)]LDSLSR_.2	#N/A	8.0070	7.1172	8.6838	8.1402	6.7458	7.8464	8.0543	7.9913	7.7753	7.8159	0.9807	-0.0281	0.6874	0.1628
Ccdc177	_RTS[Phospho (STY)]PS[Phospho (STY)]PPAR_.2	Q3UHB8_S209_M2_Ccdc177	10.2426	9.9557	10.5677	3.8801	3.5796	5.0893	10.3982	10.3030	10.0609	10.0984	0.7067	-0.5008	0.1199	0.9213
Ccdc177	_NSC[Carbamidomethyl (C)]PAGSASS[Phospho (STY)]APNPLGR_.2	Q3UHB8_S281_M1_Ccdc177	4.6691	3.0517	4.0809	3.9969	3.1490	3.3847	3.4297	4.5967	4.0813	3.7457	0.9391	-0.0906	0.5408	0.2670
Ccdc177	_KSHS[Phospho (STY)]LDS[Phospho (STY)]LSR_.2	#N/A	8.0046	8.0970	8.6422	8.0097	6.9003	8.0197	8.0698	8.0646	8.0171	7.8539	0.9930	-0.0101	0.8536	0.0687
Ccdc177	_SFSLGDLSHS[Phospho (STY)]PQTAQHVER_.3	Q3UHB8_S310_M1_Ccdc177	6.1781	7.1449	4.2198	7.9530	6.0022	6.2799	6.6472	7.7588	6.6570	6.7716	0.9048	-0.1443	0.3550	0.4497
Ccdc177	_DGALSSESGASSSSY[Phospho (STY)]SGESLR_.2	Q3UHB8_Y254_M1_Ccdc177	3.3893	3.9531	3.1230	4.1676	4.0339	4.0915	3.7683	4.3344	3.1326	3.8449	1.0061	0.0088	0.9394	0.0271
Tnrc6c	_DSSEATGWEEPS[Phospho (STY)]PPSIR_.2	#N/A	7.7875	8.2882	7.0782	3.8965	7.8776	8.4926	8.6710	3.1046	8.2243	8.6131	1.0117	0.0167	0.9532	0.0208
Tnrc6c	_AET[Phospho (STY)]PPSHQAGTQLNR_.3	Q3UHC0_T776_M1_Tnrc6c	2.9140	3.7192	3.6008	4.1034	3.1079	4.1890	3.8181	4.0150	4.5845	4.0704	0.8747	-0.1931	0.1160	0.9356
Tnrc6c	_QKDSSEATGWEEPS[Phospho (STY)]PPSIR_.3	#N/A	4.1369	3.3923	3.8706	3.5224	3.5391	3.3440	3.2371	3.0952	4.2446	3.4894	1.0335	0.0475	0.6582	0.1816
Dab2ip	_GDS[Phospho (STY)]PELKPR_.2	#N/A	9.2913	8.8571	9.1043	9.3665	8.4516	8.8027	9.4607	9.2411	9.1876	9.0331	0.9727	-0.0399	0.2182	0.6611
Dab2ip	_LPS[Phospho (STY)]PTPENK_.2	Q3UHC7_S702_M1_Dab2ip	8.4252	7.6550	7.4328	4.4185	2.7840	8.0156	3.7338	7.7165	7.5912	4.0958	1.1160	0.1583	0.6560	0.1831
Dab2ip	_RQMS[Phospho (STY)]LTEK_.2	Q3UHC7_S895_M1_Dab2ip	2.9687	4.3737	4.2032	3.7470	4.1950	3.5345	3.8953	3.2542	3.5532	3.4243	1.0864	0.1196	0.3247	0.4886
Dab2ip	_QQSSSSKGDS[Phospho (STY)]PELKPR_.3	#N/A	5.7322	5.5695	6.7711	5.9421	5.5738	5.9129	5.8980	6.0888	6.3602	5.7007	0.9842	-0.0230	0.7183	0.1437
Adgrb1	_LHLHGS[Phospho (STY)]PR_.2	Q3UHD1_S1289_M1_Adgrb1	6.4637	7.1062	7.2124	6.2308	4.6178	6.2360	7.0265	6.7754	6.1624	6.1285	0.9675	-0.0477	0.6872	0.1629
Adgrb1	_NENVATLSVSS[Phospho (STY)]LERR_.2	#N/A	3.1917	4.6290	4.4262	5.3635	3.8363	4.0161	4.1183	5.2888	4.4981	4.7405	0.9104	-0.1354	0.3555	0.4492
Adgrb1	_NENVATLSVSS[Phospho (STY)]LER_.2	#N/A	9.0905	8.7179	9.1597	4.0029	7.4204	9.2157	9.4439	9.5310	9.2334	9.2951	0.8463	-0.2408	0.2042	0.6899
Adgrb1	_RPS[Phospho (STY)]LPEEEKMK_.3	Q3UHD1_S1257_M1_Adgrb1	3.4638	3.8786	3.1975	4.2421	4.1084	4.0295	4.3903	4.2599	3.0582	3.9194	0.9777	-0.0325	0.7893	0.1028
Mtus2	_QLS[Phospho (STY)]EENANLQEYVEK_.2	Q3UHD3_S1286_M1_Mtus2	4.1712	3.4267	3.9049	6.0727	3.3150	5.3962	3.2028	5.6778	3.8515	3.1260	1.1051	0.1442	0.5876	0.2309
Mtus2	_QLS[Phospho (STY)]EEHAVKR_.3	Q3UHD3_S109_M1_Mtus2	4.5373	3.2348	3.8625	3.6800	3.1231	3.5015	3.3947	3.9722	3.7022	5.2441	0.8966	-0.1575	0.3377	0.4715
Mtus2	_SM[Oxidation (M)]S[Phospho (STY)]PVER_.2	#N/A	7.1463	7.3878	7.7535	7.2703	7.4892	3.2837	7.5524	7.1588	7.3714	3.4547	1.0529	0.0743	0.7789	0.1085
Snx27	_SES[Phospho (STY)]GYGFNVR_.2	#N/A	11.4897	11.3338	11.9810	11.6342	10.7263	11.5151	11.6829	11.4287	11.5846	11.4839	0.9915	-0.0123	0.6611	0.1797
Snx27	_IVKSES[Phospho (STY)]GYGFNVR_.2	#N/A	2.9033	2.9383	4.1378	3.6816	3.5479	6.0285	3.8298	3.3196	3.6187	3.3589	1.0966	0.1330	0.5834	0.2340
Agap2	_ALS[Phospho (STY)]TDC[Carbamidomethyl (C)]TPSGDLSPLSR_.2	Q3UHD9_S802_M1_Agap2	11.7929	11.2992	12.1821	12.1591	3.7249	11.6004	11.9241	11.6988	11.5382	11.4184	0.8982	-0.1548	0.5042	0.2974
Agap2	_GSEPRDPGS[Phospho (STY)]PR_.2	Q3UHD9_S54_M1_Agap2	8.5108	9.4707	10.1635	8.9272	8.0311	9.1122	9.2159	9.0353	8.5820	8.8333	1.0134	0.0192	0.7700	0.1135
Agap2	_GAGSRLS[Phospho (STY)]WPESEGKPR_.3	#N/A	7.1607	7.4809	4.1019	7.8529	3.4878	3.8557	8.2191	7.8309	7.7279	7.4536	0.7245	-0.4650	0.0741	1.1299
Agap2	_RSS[Phospho (STY)]AASLGR_.2	#N/A	6.4943	6.3966	5.9719	6.3723	5.9368	6.6315	6.8626	6.8666	6.3697	6.6865	0.9409	-0.0879	0.0499	1.3017
Agap2	_ELLGAELRTS[Phospho (STY)]PK_.2	Q3UHD9_S382_M1_Agap2	6.0137	6.8731	3.6698	3.2136	3.2276	7.0044	6.6591	3.7876	7.0784	6.8851	0.8194	-0.2874	0.3521	0.4534
Agap2	_LRTDS[Phospho (STY)]QSEAVAIQAIR_.3	#N/A	5.9295	7.3476	3.5081	7.8768	3.8991	4.1009	3.4943	7.3824	7.0829	6.4287	0.8928	-0.1635	0.5982	0.2232
Agap2	_FLTGS[Phospho (STY)]YQVLEK_.2	Q3UHD9_S424_M1_Agap2	5.7742	3.7802	4.1327	3.1346	4.0725	5.8523	4.3501	6.4392	5.7488	3.1015	0.9079	-0.1394	0.5944	0.2259
Agap2	_LKS[Phospho (STY)]FGS[Phospho (STY)]LR_.2	Q3UHD9_S856_M2_Agap2	10.5612	11.8293	9.6117	12.4284	11.1293	12.2319	12.3180	12.5288	12.5276	12.4001	0.9080	-0.1393	0.0723	1.1407
Agap2	_SGNS[Phospho (STY)]LNKEWK_.2	Q3UHD9_S684_M1_Agap2	5.9351	5.5716	3.3158	3.8184	3.2555	5.6923	5.6879	5.7889	5.9338	5.8791	0.7897	-0.3406	0.0948	1.0231
Agap2	_TDS[Phospho (STY)]QSEAVAIQAIR_.2	#N/A	8.5631	3.4265	8.5590	9.4512	7.6099	8.7499	8.6873	9.2904	9.1277	9.0256	0.8554	-0.2253	0.2774	0.5569
Agap2	_RTS[Phospho (STY)]LFANR_.2	Q3UHD9_S642_M1_Agap2	3.1149	4.8366	3.8017	6.2630	3.7414	3.9881	4.3489	4.3014	3.0996	3.8779	1.0983	0.1353	0.5483	0.2610
Agap2	_RRS[Phospho (STY)]S[Phospho (STY)]AASLGR_.2	Q3UHD9_S1171_M2_Agap2	8.4877	7.8944	8.5991	8.2143	7.5318	8.4009	8.2954	8.3771	8.4648	8.4504	0.9751	-0.0363	0.3471	0.4595
Agap2	_ELTLVLTAIGNDTANR_.2	#N/A	3.4635	3.8789	3.1972	4.2418	5.2009	4.0293	4.3901	4.2602	3.0584	5.3119	0.9405	-0.0885	0.6336	0.1982
Agap2	_LS[Phospho (STY)]WPESEGKPR_.2	#N/A	4.1695	3.4738	3.3554	7.5940	3.2951	2.9335	3.2943	3.8551	4.1542	4.3242	1.0589	0.0825	0.8061	0.0936
Agap2	_SS[Phospho (STY)]AASLGR_.2	#N/A	3.5306	4.3358	4.2174	3.2193	4.1572	3.5723	3.9331	3.3984	3.0470	3.1863	1.1320	0.1789	0.1475	0.8313
Agap2	_AEAAAVAGLSTPGS[Phospho (STY)]LHR_.2	Q3UHD9_S632_M1_Agap2	3.9306	3.3443	3.9225	3.6193	3.7514	3.3918	4.1398	3.8530	4.1966	3.5863	0.9280	-0.1078	0.1356	0.8677
Agap2	_AVVNSQEWTLSR_.2	#N/A	4.2642	3.0782	3.9979	3.5416	2.6929	3.3291	3.6899	3.4595	3.7586	3.2189	0.9865	-0.0196	0.8822	0.0545
Pitpnm3	_AQS[Phospho (STY)]QPESDKDHERPLPALSWAR_.4	Q3UHE1_S946_M1_Pitpnm3	7.2261	7.9281	3.3842	9.1465	2.9162	7.6453	3.6015	8.8515	8.1884	7.9525	0.8917	-0.1653	0.6461	0.1897
Pitpnm3	_NVLSDS[Phospho (STY)]VESSDDEFFDAR_.2	Q3UHE1_S27_M1_Pitpnm3	3.2444	4.0496	3.9312	4.0711	3.8709	3.8586	4.2194	4.4309	3.2291	3.7484	0.9823	-0.0258	0.7980	0.0980
Pitpnm3	_GS[Phospho (STY)]PPLLDAPAS[Phospho (STY)]PPQAPR_.2	#N/A	8.9917	10.4878	9.6019	10.9515	9.6077	10.1916	11.1040	10.7884	10.6748	10.4766	0.9267	-0.1098	0.0690	1.1612
Pitpnm3	_KGS[Phospho (STY)]ISSTQDTPVVVEEDC[Carbamidomethyl (C)]SLASSK_.3	Q3UHE1_S295_M1_Pitpnm3	3.6696	3.6728	3.4033	4.4479	2.8134	3.8113	3.0953	4.0541	4.3532	4.1780	0.9276	-0.1084	0.4431	0.3535
Hivep2	_GSHS[Phospho (STY)]FDER_.2	Q3UHF7_S565_M1_Hivep2	3.0161	3.8212	3.7029	4.2994	3.6426	4.0869	4.4477	3.9130	3.5616	4.1725	0.9348	-0.0972	0.3600	0.4437
Hivep2	_QASFSGS[Phospho (STY)]PEIAQGEAGVDPSVK_.2	Q3UHF7_S1081_M1_Hivep2	3.4336	3.8024	3.9803	4.0796	3.6604	4.2758	4.7555	3.6461	3.9452	3.8891	0.9539	-0.0680	0.4682	0.3296
Hivep2	_ELSLS[Phospho (STY)]TEEGNEPSPEK_.2	Q3UHF7_S2415_M1_Hivep2	3.5501	5.7832	4.0113	7.1444	6.0903	6.2153	6.1853	6.8760	6.0074	6.3481	0.8602	-0.2173	0.2549	0.5935
Hivep2	_SPS[Phospho (STY)]LGSEDLPLAADPDK_.2	Q3UHF7_S764_M1_Hivep2	3.0480	3.8532	3.7348	4.2375	3.6746	4.0549	4.4157	3.8810	3.5296	4.2045	0.9400	-0.0893	0.3803	0.4199
Hivep2	_EM[Oxidation (M)]S[Phospho (STY)]QGDASPR_.2	Q3UHF7_S2085_M1_Hivep2	4.2402	4.3619	4.9403	3.5176	3.3839	3.7218	3.6659	2.8772	3.7826	3.5951	1.1573	0.2108	0.1493	0.8259
Hivep2	_LADLEHSSGESS[Phospho (STY)]FESTGTGLSR_.3	Q3UHF7_S947_M1_Hivep2	3.5158	4.3210	4.2026	3.7997	4.1423	3.5872	3.9480	3.2014	3.5005	3.4770	1.1122	0.1534	0.0987	1.0055
Hivep2	_SES[Phospho (STY)]TEMVAC[Carbamidomethyl (C)]PQDK_.2	Q3UHF7_S811_M1_Hivep2	3.0515	3.8567	3.7383	4.2410	3.6780	4.0515	4.4123	3.8775	3.5261	4.2080	0.9410	-0.0878	0.3877	0.4115
Hivep2	_S[Phospho (STY)]IEQAEDAHKK_.2	#N/A	4.4740	4.5309	4.5623	4.7337	3.9752	4.8038	4.7242	3.7568	4.7994	5.0183	0.9866	-0.0195	0.8238	0.0842
Hivep2	_MSTFSS[Phospho (STY)]QEGPR_.2	Q3UHF7_S41_M1_Hivep2	3.4587	4.2639	4.1455	3.1474	4.0853	3.6442	4.3629	3.4703	3.1189	3.1143	1.0780	0.1083	0.4225	0.3742
Hivep2	_QSTS[Phospho (STY)]EDSLNSTER_.2	Q3UHF7_S2295_M1_Hivep2	4.1636	3.4190	3.8973	3.4957	3.3073	3.3173	3.2104	3.1219	4.2713	3.4627	1.0237	0.0338	0.7681	0.1146
Zswim8	_GWGS[Phospho (STY)]PGRPK_.2	Q3UHH1_S1132_M1_Zswim8	6.6240	6.2405	3.3312	5.9965	5.5003	6.1126	6.1903	6.3130	5.7002	6.0873	0.9278	-0.1081	0.4955	0.3050
Zswim8	_QSAGPNS[Phospho (STY)]PTGGGGGGGSGGTR_.2	#N/A	5.4552	3.1834	3.2287	4.9885	3.1685	3.0602	3.4461	6.0399	6.0609	3.6984	0.7997	-0.3225	0.2568	0.5904
Zswim8	_RLS[Phospho (STY)]AEGGDK_.2	Q3UHH1_S564_M1_Zswim8	5.5120	4.3602	3.5527	5.5729	4.2485	3.8769	5.8990	5.3940	5.8983	5.4571	0.7984	-0.3248	0.0328	1.4847
Zswim8	_KQS[Phospho (STY)]AGPNS[Phospho (STY)]PTGGGGGGGSGGTR_.2	#N/A	3.6447	3.8783	3.9763	3.2505	3.9957	2.9314	4.3653	3.1704	3.1546	3.3539	1.0290	0.0412	0.7572	0.1208
Ccser2	_SSS[Phospho (STY)]GESLAQSPDNAK_.2	Q3UHI0_S198_M1_Ccser2	6.2888	5.4854	7.0178	6.0580	6.1472	6.3554	6.1702	6.8110	5.6424	6.5781	0.9881	-0.0173	0.8229	0.0847
Ccser2	_HTSGNNLIS[Phospho (STY)]PDTDYR_.2	#N/A	4.0881	4.8105	4.3043	5.2418	4.0541	6.0314	4.6936	5.9805	5.1610	3.1949	0.9995	-0.0007	0.9969	0.0014
Tmed8	_RGS[Phospho (STY)]AGGVGDRR_.2	#N/A	4.8652	5.1988	6.1287	5.9326	4.4941	4.9011	5.4141	5.4429	4.9712	5.0479	1.0066	0.0095	0.9226	0.0350
Tmed8	_RGS[Phospho (STY)]AGGVGDR_.2	#N/A	6.1012	5.2909	5.6620	6.2736	4.2370	3.4925	5.7211	6.0112	5.7263	6.1879	0.8756	-0.1917	0.2311	0.6361

Aak1	_VGSLT[Phospho (STY)]PPSS[Phospho (STY)]PK_.2	#N/A	13.5096	13.4706	13.5492	13.8520	12.2085	13.3263	13.7702	13.2786	13.2444	13.2904	0.9943	-0.0083	0.8098	0.0916
Aak1	_VQTT[Phospho (STY)]PPPTIQGQK_.2	Q3UHJ0_T604_M1_Aak1	13.2894	13.4158	14.1121	13.7642	13.0356	13.3964	13.7612	13.3301	13.3745	13.4233	1.0022	0.0032	0.8895	0.0508
Aak1	_EQGSSGLGSGSS[Phospho (STY)]GGGGSSSGLGSGYIGR_.3	#N/A	2.8978	2.9438	4.1323	7.7815	6.4723	3.4636	3.8243	3.3251	3.6241	7.9693	0.9850	-0.0219	0.9598	0.0178
Aak1	_LTDPIPTTETSIAPR_.2	#N/A	8.0978	7.8751	8.0854	7.8316	8.0381	7.9119	7.8480	7.9449	7.8795	7.4646	1.0243	0.0346	0.1060	0.9746
Aak1	_VAEDEFDPIPVLITK_.2	#N/A	6.8462	6.9445	6.7442	7.0377	6.9895	7.1434	6.9194	6.6496	7.1447	6.5593	1.0195	0.0278	0.3285	0.4835
Aak1	_AAEDSNLISGFGVAEGSEK_.2	#N/A	6.2300	6.0102	6.5812	7.0400	5.6445	5.7526	6.3071	6.4021	6.0629	6.6509	0.9770	-0.0335	0.6238	0.2049
Aak1	_DLKVENILLHDR_.3	#N/A	5.8160	5.9404	5.5576	6.5535	6.0910	5.9346	6.6610	5.1081	6.0894	6.0782	0.9997	-0.0005	0.9950	0.0022
Aak1	_FQNPQAEGVNAVEDEIKK_.3	#N/A	6.1323	5.0028	5.8014	6.3442	6.0287	6.0540	6.1110	6.3406	5.8196	6.3160	0.9589	-0.0606	0.3562	0.4483
Aak1	_SKS[Phospho (STY)]ATTT[Phospho (STY)]PSGS[Phospho (STY)]PR_.2	Q3UHJ0_S676_M3_Aak1	4.9167	5.2431	7.6126	6.7594	4.5987	5.2044	6.5294	4.9615	3.8514	4.9096	1.1303	0.1767	0.4030	0.3947
Aak1	_LPSQTESVTSNR_.2	#N/A	6.5040	6.5247	6.5042	6.6208	6.5418	6.6540	6.4922	6.4197	6.7421	6.0833	1.0193	0.0275	0.3017	0.5204
Aak1	_NSS[Phospho (STY)]GSSESSLPNLAR_.2	Q3UHJ0_S936_M1_Aak1	9.7147	9.6981	10.1128	10.1130	9.0125	9.7381	9.9276	9.5145	6.3911	9.7758	1.0932	0.1285	0.2677	0.5724
Aak1	_AQATPSQPLQSSQPK_.2	#N/A	5.1695	4.4921	4.0127	4.5154	4.6516	4.1745	4.6006	4.9363	4.6361	4.1754	0.9816	-0.0268	0.7343	0.1341
Aak1	_SATTTPSGS[Phospho (STY)]PR_.2	#N/A	11.5263	11.7036	12.0797	11.7516	10.8987	11.6362	11.5675	11.5841	11.5576	11.5076	1.0039	0.0056	0.8269	0.0826
Aak1	_VGSLT[Phospho (STY)]PPSS[Phospho (STY)]PKTQR_.2	#N/A	10.3491	11.6956	12.5980	11.4757	10.4946	3.5858	11.3791	4.1313	10.4781	9.2733	1.1381	0.1867	0.5782	0.2379
Aak1	_REQGSSGLGSGSS[Phospho (STY)]GGGGSSSGLGSGYIGR_.3	#N/A	3.8534	3.1088	3.5870	3.1308	2.9971	3.6275	3.2791	3.6338	4.1694	4.3089	0.8795	-0.1853	0.1121	0.9504
Aak1	_ILSDVTHSAVFGVPASK_.2	#N/A	4.2124	4.0409	4.7618	6.0819	3.8016	3.2990	3.6924	4.3832	4.1270	3.3946	1.1198	0.1632	0.3988	0.3992
Aak1	_STQLLQAAAAEASLNK_.2	#N/A	5.8790	3.0328	5.9453	3.9435	5.7289	3.7035	5.5873	4.8894	5.4677	5.3178	0.8852	-0.1759	0.3893	0.4098
Aak1	_YTTLSYR_.2	#N/A	3.3588	4.1640	4.0456	4.5483	3.9853	3.7442	4.2629	3.5702	4.0677	3.0144	1.0658	0.0920	0.4381	0.3584
Aak1	_GHYVLC[Carbamidomethyl (C)]DFGSATNK_.3	#N/A	3.7729	3.0284	3.5066	4.5512	2.9167	3.7079	3.6011	3.9508	4.2498	4.2285	0.8935	-0.1625	0.2248	0.6482
Aak1	_LQTGFTENEVLQIFC[Carbamidomethyl (C)]DTC[Carbamidomethyl (C)]EAVAR_.3	#N/A	3.8679	3.1233	3.6016	3.7915	3.9377	3.6130	3.5061	3.8558	3.9756	4.3235	0.9337	-0.0989	0.2319	0.6347
Slc2a13	_GSNYHLSDNDAS[Phospho (STY)]DVE_.2	#N/A	9.0544	10.5901	8.5149	10.4398	9.3040	10.3456	11.2127	10.7444	10.4996	10.3374	0.9074	-0.1401	0.0663	1.1785
Slc2a13	_KAS[Phospho (STY)]EDVEYTLR_.2	Q3UHK1_S6_M1_Slc2a13	6.5670	3.7017	4.2113	3.2131	4.1510	6.7814	4.4287	6.9474	6.7667	6.7290	0.7673	-0.3822	0.1497	0.8248
Slc2a13	_VKGSNYHLS[Phospho (STY)]DNDASDVE_.2	#N/A	4.6431	5.4043	4.5271	5.6639	3.7793	6.1804	6.0040	6.9248	5.1867	3.5032	0.9312	-0.1028	0.6233	0.2053
Slc2a13	_SLSS[Phospho (STY)]LMGER_.2	Q3UHK1_S18_M1_Slc2a13	4.0542	3.2882	3.7879	6.1773	3.1096	3.1191	3.4799	3.6696	3.9686	3.0089	1.1107	0.1515	0.5441	0.2643
Slc2a13	_SLLAAESAAS[Phospho (STY)]LQGAELER_.2	Q3UHK1_S47_M1_Slc2a13	3.2895	4.0589	3.8573	4.0678	4.1143	3.8249	3.9411	3.7980	3.7656	3.7451	1.0148	0.0212	0.7352	0.1336
Camkv	_AAAT[Phospho (STY)]PEPAVAQPDSTALEGATGQAPPSSK_.3	Q3UHL1_T470_M1_Camkv	8.5095	8.3383	8.2933	8.4892	7.0446	8.2474	8.2042	8.2079	8.3135	8.1994	0.9906	-0.0137	0.7911	0.1017
Camkv	_TEEFC[Carbamidomethyl (C)]EIFR_.2	#N/A	7.1498	6.6540	6.5676	7.0078	6.6185	6.4533	7.0365	7.0629	7.0706	6.7335	0.9664	-0.0492	0.1659	0.7800
Camkv	_EPC[Carbamidomethyl (C)]GTPEYLAPEVVGR_.2	#N/A	6.7649	6.1189	5.9228	7.1263	6.6673	6.4998	7.0176	6.5100	6.4851	6.6953	0.9760	-0.0351	0.5305	0.2753
Camkv	_LENLVYYNR_.2	#N/A	7.0536	6.6973	6.4716	6.8711	5.7617	6.0188	6.6388	7.0043	6.6265	5.9784	0.9874	-0.0184	0.7935	0.1004
Camkv	_IVISDFHLAK_.2	#N/A	6.0927	6.2022	6.3429	6.6252	5.6491	6.0449	6.7172	6.8838	5.1731	5.9552	0.9963	-0.0053	0.9499	0.0223
Camkv	_AT[Phospho (STY)]PATEESTVPATQSSALPAAK_.2	#N/A	7.9665	7.5847	7.7648	8.0905	4.7948	7.6460	8.3091	7.7601	7.7783	7.7516	0.9251	-0.1124	0.3855	0.4139
Camkv	_EVFDWILDQGYYSER_.2	#N/A	4.8044	5.4728	6.3738	5.3967	4.9747	5.7470	5.8225	5.0001	4.6352	5.1779	1.0587	0.0822	0.4100	0.3872
Camkv	_SDDIAS[Phospho (STY)]ADR_.1	#N/A	6.7385	6.2269	5.9630	5.9498	4.9772	6.3232	6.6669	6.0856	7.0141	6.1450	0.9308	-0.1034	0.2346	0.6296
Camkv	_ITAEEAISHEWISGNAASDK_.3	#N/A	5.6416	6.0633	6.5292	5.5597	5.2865	5.5363	5.9624	5.1853	5.2581	5.9363	1.0329	0.0467	0.5348	0.2718
Camkv	_LMEVEQDQR_.2	#N/A	5.4416	4.4082	4.2487	6.2846	4.8374	3.5411	4.4660	3.0583	4.2433	3.2175	1.2796	0.3557	0.1020	0.9913
Camkv	_GEEATGC[Carbamidomethyl (C)]AQESQR_.2	#N/A	6.9981	3.2672	3.1449	3.6476	3.1555	7.2950	3.3622	2.9701	6.9211	3.6146	1.0872	0.1206	0.7756	0.1104
Camkv	_SAT[Phospho (STY)]PATDGR_.2	Q3UHL1_T438_M1_Camkv	3.4316	3.6452	4.3514	4.1483	3.5335	3.1075	3.8630	3.1874	3.8590	3.4448	1.0319	0.0453	0.6841	0.1649
Camkv	_NYNQPSEVTDR_.2	#N/A	6.0285	3.9615	3.9515	4.4541	3.8498	4.2756	4.1688	3.6644	3.3129	4.4211	1.1358	0.1837	0.2864	0.5431
Dop1b	_S[Phospho (STY)]DLSEEDLPYYVDLPDR_.2	Q3UHQ6_S1080_M1_Dop1b	3.5981	4.4033	4.2849	3.7174	4.2246	3.5049	3.8657	3.3309	2.9795	3.2537	1.1781	0.2365	0.0424	1.3721
Dop1b	_AALLAAFQPES[Phospho (STY)]PR_.2	Q3UHQ6_S1167_M1_Dop1b	3.2868	3.8406	4.5213	0.8480	1.9197	3.8525	4.0942	5.0024	3.2352	3.7423	0.7577	-0.4003	0.2396	0.6204
Dop1b	_SEDSGIGLSASS[Phospho (STY)]PELSEHLR_.3	Q3UHQ6_S597_M1_Dop1b	4.2715	3.0709	4.0052	3.5490	2.8922	3.3364	3.6972	3.4522	3.7513	3.2262	0.9969	-0.0044	0.9709	0.0128
Mtcl1	_QELGPGQETGTSS[Phospho (STY)]R_.2	Q3UHU5_S1795_M1_Mtcl1	7.7155	7.5079	4.1757	7.7193	3.5858	3.5070	3.8678	3.2817	3.5807	7.5307	1.2490	0.3208	0.4237	0.3729
Mtcl1	_EDVTPPLS[Phospho (STY)]PDDLK_.2	Q3UHU5_S1407_M1_Mtcl1	3.6272	3.5401	3.2695	6.7123	3.6207	3.8758	4.0242	6.7774	6.0244	3.8625	0.7942	-0.3325	0.2579	0.5885
Mtcl1	_GLPSAGSKEDVT[Phospho (STY)]PPLS[Phospho (STY)]PDDLK_.3	#N/A	3.7099	3.8619	4.0600	7.2201	3.6862	3.7261	3.6192	6.6304	3.9202	3.8716	0.9705	-0.0432	0.8872	0.0520
Mtcl1	_GRS[Phospho (STY)]PS[Phospho (STY)]PLGVGSETFR_.3	Q3UHU5_S1799_M2_Mtcl1	3.8787	3.1342	3.6124	3.7806	3.0225	3.6022	3.4953	4.0069	3.9864	4.3343	0.8861	-0.1745	0.0794	1.1003
Pdap1	_SLDS[Phospho (STY)]DES[Phospho (STY)]EDEDDDYQQK_.2	#N/A	7.4890	8.2646	8.5892	9.1983	8.6420	8.4050	8.5390	8.4324	8.0732	7.8147	1.0264	0.0375	0.5114	0.2912
Pdap1	_GVEGLIDIENPNR_.2	#N/A	6.3965	6.7101	6.5719	6.6844	6.8901	6.5269	6.7031	6.4578	6.7218	5.6786	1.0375	0.0531	0.2914	0.5355
Pdap1	_M[Oxidation (M)]QSLS[Phospho (STY)]LNK_.2	#N/A	5.8547	6.1743	6.9329	6.0598	5.3860	6.4052	5.6725	6.0142	6.2605	6.1678	1.0177	0.0253	0.7175	0.1442
Pdap1	_QYTS[Phospho (STY)]PEEIDAQLQAEK_.2	Q3UHX2_S19_M1_Pdap1	3.5009	4.6522	4.1247	4.8883	4.5566	4.2671	3.9914	4.0769	4.4850	4.0607	1.0429	0.0606	0.5209	0.2833
Pdap1	_SLDS[Phospho (STY)]DESEDEDDDYQQKR_.2	#N/A	3.7358	4.6451	4.9609	5.7627	4.9014	4.6562	5.0876	5.0171	4.7545	4.3441	0.9950	-0.0072	0.9487	0.0228
Pdap1	_KSLDS[Phospho (STY)]DES[Phospho (STY)]EDEDDDYQQK_.2	#N/A	5.9276	6.9292	7.3263	7.6474	7.2215	6.8563	7.2780	6.9184	4.7612	6.7034	1.0888	0.1227	0.3211	0.4934
Pdap1	_KSLDS[Phospho (STY)]DESEDEDDDYQQKR_.2	#N/A	3.5813	3.8787	3.8191	3.8401	3.5533	3.6362	3.9884	3.7871	3.5665	4.1755	0.9584	-0.0612	0.2398	0.6202
Arhgap17	_LGEQGPEPGPTPPQTPT[Phospho (STY)]PPST[Phospho (STY)]PPLAK_.3	#N/A	3.5759	3.6297	3.9907	3.5119	3.7291	3.6556	6.6601	3.4072	3.5213	3.4677	0.8635	-0.2117	0.3880	0.4112
Plekha7	_SVDISLGGS[Phospho (STY)]PR_.2	Q3UIL6_S609_M1_Plekha7	5.9921	7.1006	6.8684	7.4108	6.4234	7.0098	7.2606	6.8802	6.9853	7.3235	0.9562	-0.0646	0.2887	0.5395
Plekha7	_KVMS[Phospho (STY)]PLQS[Phospho (STY)]PTK_.2	Q3UIL6_S900_M2_Plekha7	3.5433	5.1046	4.2301	6.0383	3.4719	3.6106	3.9714	3.4366	3.8547	4.7165	1.0847	0.1173	0.5697	0.2444
Plekha7	_HLSGSSS[Phospho (STY)]PPPR_.2	Q3UIL6_S482_M1_Plekha7	10.8422	3.4917	3.5844	4.6290	2.9944	3.6302	3.2764	9.7572	4.1721	4.3063	0.9041	-0.1455	0.7946	0.0999
Plekha7	_IEDVM[Oxidation (M)]AGLS[Phospho (STY)]ANKENY[Phospho (STY)]R_.3	Q3UIL6_S820_M2_Plekha7	3.5872	3.6389	4.2740	3.2759	4.2138	3.0974	4.4914	3.3418	2.9904	3.2429	1.0468	0.0660	0.6593	0.1809
Edc4	_DSQDTSAEQS[Phospho (STY)]DHDDEVASLASASGGFGSK_.3	Q3UJB9_S884_M1_Edc4	3.4905	4.2957	4.1773	8.2499	4.1170	3.6125	4.3946	7.8051	3.0871	3.1461	1.0106	0.0152	0.9701	0.0132
Edc4	_[Acetyl (Protein N-term)]ASC[Carbamidomethyl (C)]AS[Phospho (STY)]IDIEDATQHLR_.2	Q3UJB9_S6_M1_Edc4	3.5689	3.7700	3.3026	4.3472	3.5948	4.1347	4.4955	4.1548	2.9530	4.0245	0.9691	-0.0452	0.7240	0.1403
Usp19	_RNS[Phospho (STY)]PVER_.2	Q3UJD6_S1258_M1_Usp19	4.3765	2.9659	4.1102	3.6540	2.7872	3.4415	3.8023	8.8899	3.6462	3.9004	0.7028	-0.5088	0.1934	0.7135
Rmdn3	_SHS[Phospho (STY)]LPNSLDYAQASER_.2	#N/A	7.4598	7.5762	8.9615	8.0883	6.6664	7.7526	8.3798	7.7296	7.6490	8.1937	0.9703	-0.0435	0.5799	0.2366
Rmdn3	_SLQGLAGEIVGEVR_.2	#N/A	3.9617	3.8301	3.1477	3.2755	4.4228	4.3718	4.3475	4.0070	4.9599	4.2693	0.8724	-0.1970	0.1142	0.9422
Rmdn3	_AIPGEAGDAAILPS[Phospho (STY)]LSQEGQEK_.2	Q3UJU9_S81_M1_Rmdn3	3.8591	3.7720	3.9743	8.0315	3.6300	6.9324	3.7880	3.6323	3.9313	3.8987	1.3202	0.4007	0.2513	0.5997
Rmdn3	_DSDKES[Phospho (STY)]GDAEDEVSC[Carbamidomethyl (C)]ETVR_.2	Q3UJU9_S193_M1_Rmdn3	3.2347	4.0399	3.9215	4.0808	3.8613	3.8682	4.2290	4.4212	3.2195	3.7580	0.9814	-0.0270	0.7902	0.1023
Rmdn3	_KDS[Phospho (STY)]LDLDVEAASSPAAAALEEDDSSGR_.3	Q3UJU9_S212_M1_Rmdn3	4.1856	3.4410	3.9193	3.4738	3.3293	3.2953	3.1884	3.5381	4.2933	3.1403	1.0190	0.0272	0.8159	0.0884
Tax1bp1	_KLEGQS[Phospho (STY)]PQQVSR_.2	Q3UKC1_S632_M1_Tax1bp1	7.2332	7.4419	8.1658	4.5704	3.7335	7.9670	4.2851	3.5481	7.5346	7.7208	1.1293	0.1755	0.5778	0.2382
Tax1bp1	_VPS[Phospho (STY)]WEDNVVC[Carbamidomethyl (C)]SQPAR_.2	Q3UKC1_S693_M1_Tax1bp1	7.6374	8.0061	3.6414	8.6178	5.9434	4.1484	4.5092	7.9790	7.7995	7.7386	0.9038	-0.1460	0.6057	0.2177
Tax1bp1	_AAS[Phospho (STY)]PVEELLTM[Oxidation (M)]EDEGNSDM[Oxidation (M)]LVVTTK_.3	Q3UKC1_S124_M1_Tax1bp1	4.1847	3.1577	6.7255	5.1120	5.2859	4.8262	5.9832	6.3104	5.3189	5.6515	0.8394	-0.2525	0.1766	0.7531
Ell2	_LSNAS[Phospho (STY)]PNPNEGVK_.2	Q3UKU1_S501_M1_Ell2	3.5790	3.6472	6.3360	3.2676	6.5783	3.5240	4.4831	3.3500	2.9986	3.2346	1.2764	0.3521	0.2722	0.5652
Arglu1	_ASS[Phospho (STY)]PPDRIDIFGR_.2	#N/A	3.5886	3.5285	9.4634	9.4821	7.8338	8.6005	9.3352	9.0957	8.8232	3.8499	0.9109	-0.1347	0.7048	0.1519
Arglu1	_ERASS[Phospho (STY)]PPDR_.2	#N/A	3.9687	3.3737	3.7024	3.2462	3.1125	3.5121	3.3945	3.7550	4.0540	4.4243	0.8922	-0.1645	0.1117	0.9520
Gpd1l	_ELLQTPNFR_.2	#N/A	7.0645	7.2284	7.2464	6.9653	7.0552	7.2331	7.6517	7.3530	7.2073	6.7336	0.9856	-0.0209	0.5404	0.2673
Gpd1l	_IC[Carbamidomethyl (C)]DEITGR_.2	#N/A	6.3211	5.8610	3.5239	6.0791	6.2243	5.8698	5.6874	6.1746	6.0322	6.2631	0.9350	-0.0970	0.4941	0.3062
Gpd1l	_GIDEGPDGLK_.2	#N/A	6.7917	6.6840	4.4440	6.4615	6.5158	6.8908	6.6917	6.5205	6.6378	6.5056	0.9558	-0.0651	0.5549	0.2558
Gpd1l	_KLTDIINNDHENVK_.3	#N/A	4.7826	4.4084	4.7851	3.5772	4.6744	4.9603	3.7079	3.9932	3.7758	3.9722	1.1732	0.2305	0.0340	1.4687
Gpd1l	_FC[Carbamidomethyl (C)]ETTIGSK_.2	#N/A	5.2208	5.4447	5.7322	5.1286	5.6259	4.5882	3.3007	5.0355	2.9950	4.6977	1.3201	0.4007	0.0218	1.6623
Gpd1l	_LISDIIR_.2	#N/A	3.8691	4.3430	5.1434	4.4669	4.4898	4.3401	3.6670	5.4233	3.8147	4.6381	1.0128	0.0184	0.8870	0.0521
Gpd1l	_LQGPQTSAEVYR_.2	#N/A	5.3654	4.8076	4.5324	4.3615	3.9424	5.4621	4.0762	4.6773	4.7155	5.3845	1.0068	0.0097	0.9331	0.0301
Gpd1l	_VC[Carbamidomethyl (C)]IVGSGNWGSAVAK_.2	#N/A	3.7475	3.0030	3.4812	3.9118	2.8913	3.7333	3.6265	4.2067	3.8552	4.2031	0.8712	-0.1988	0.0686	1.1637

Gpd1l	_TIEELEKELLNGQK_.3	#N/A	3.5718	4.3281	3.3055	4.3501	4.2164	3.9091	4.4984	4.1519	2.9501	4.0274	1.0102	0.0146	0.9106	0.0407
Ppp1r7	_ELYLSNNGIEVIEGLENNNK_.2	#N/A	8.8836	9.7589	9.9841	10.8481	8.9333	9.0134	9.3708	9.7547	9.1165	9.0596	1.0263	0.0374	0.5727	0.2421
Ppp1r7	_ELDLYDNQIK_.2	#N/A	7.3719	7.2786	7.4488	7.7111	7.3639	7.2908	7.2523	7.1806	7.2363	7.4351	1.0185	0.0265	0.1826	0.7385
Ppp1r7	_IEGLQSLVNLR_.2	#N/A	6.8771	7.4769	6.9031	7.4860	7.0681	7.2839	7.0286	7.2763	7.0083	7.2339	1.0064	0.0092	0.7677	0.1148
Ppp1r7	_QIDATYVR_.2	#N/A	6.1373	6.3963	5.7602	6.2167	6.7433	6.6123	6.0089	6.1838	6.4219	6.3815	1.0099	0.0142	0.7597	0.1193
Ppp1r7	_SLETVYLER_.2	#N/A	7.8546	8.0851	8.4823	8.4949	7.4911	8.2428	8.3254	8.1398	7.9630	8.3568	0.9893	-0.0155	0.6880	0.1624
Ppp1r7	_C[Carbamidomethyl (C)]IENLEELQSLR_.2	#N/A	5.1121	5.5058	4.9616	5.5855	5.7865	5.3861	3.7682	5.1952	5.1404	5.2680	1.1129	0.1543	0.1303	0.8852
Ppp1r7	_HGGGGIVANLS[Phospho (STY)]EQSLKDGVDR_.3	#N/A	4.0816	3.7756	3.4603	7.7346	6.0440	4.9828	4.5224	7.0923	5.9003	6.2172	0.8450	-0.2431	0.3528	0.4525
Ppp1r7	_LQNLDALTNLTVLSVQSNR_.2	#N/A	5.4517	5.9366	6.1248	6.4244	5.7764	5.3260	6.4064	5.6572	5.8725	6.2350	0.9664	-0.0492	0.4404	0.3561
Ppp1r7	_RVES[Phospho (STY)]EES[Phospho (STY)]GDEEGKK_.2	#N/A	7.6075	7.5832	7.8754	8.1538	8.8195	8.3142	7.7485	7.5616	8.1892	7.7690	1.0309	0.0440	0.3845	0.4151
Ppp1r7	_LTMLDIASNR_.2	#N/A	5.7483	5.0967	3.5953	6.4731	4.1822	4.4274	3.2873	3.8621	4.1612	4.3818	1.2542	0.3268	0.1210	0.9172
Ppp1r7	_IENISNLHQLQM[Oxidation (M)]LELGSNR_.3	#N/A	4.7980	4.9277	5.0736	5.4450	4.5514	5.2054	4.8867	4.6764	4.6609	5.1735	1.0311	0.0442	0.4412	0.3554
Ppp1r7	_KHGGGGIVANLS[Phospho (STY)]EQS[Phospho (STY)]LKDGVDR_.3	#N/A	3.3140	3.8914	4.0008	5.4221	3.5445	3.9405	4.3118	4.6816	4.5718	4.1923	0.9053	-0.1436	0.3082	0.5111
Ppp1r7	_VES[Phospho (STY)]EES[Phospho (STY)]GDEEGKK_.2	#N/A	6.2810	7.5626	7.1624	7.8273	7.5253	6.6279	7.5373	6.3409	6.9872	7.7621	1.0011	0.0015	0.9853	0.0064
Ppp1r7	_VESEES[Phospho (STY)]GDEEGKKHGGGGIVANLSEQSLK_.4	#N/A	3.8327	5.5872	3.5664	7.4250	5.7107	3.6482	3.2584	7.1916	5.3472	4.2883	0.9881	-0.0173	0.9553	0.0199
Ppp1r7	_HGGGGIVANLS[Phospho (STY)]EQSLK_.2	#N/A	4.0877	3.1384	3.2737	3.7763	3.0267	3.5979	3.4910	4.3421	3.9907	3.7433	0.8951	-0.1599	0.1455	0.8372
Ppp1r7	_RVES[Phospho (STY)]EES[Phospho (STY)]GDEEGK_.2	#N/A	5.2817	5.2117	5.0384	5.7819	5.2457	3.9060	5.4217	4.9705	4.9119	5.3241	0.9846	-0.0224	0.8182	0.0871
Ppp1r7	_GAGQQQS[Phospho (STY)]QEMM[Oxidation (M)]EVDR_.2	#N/A	6.1384	4.7520	4.1376	7.3913	3.5806	3.5757	3.4688	3.6714	3.8612	4.2685	1.2912	0.3687	0.2003	0.6983
Ppp1r7	_GAGQQQS[Phospho (STY)]QEM[Oxidation (M)]M[Oxidation (M)]EVDRR_.3	#N/A	3.6707	3.7659	3.9716	4.0692	3.6743	4.0210	3.9536	3.7347	3.2994	4.1626	1.0197	0.0281	0.6768	0.1695
Ppp1r7	_DAEDVDLTHYR_.3	#N/A	2.9467	4.2794	3.6336	5.8523	3.5733	3.9577	3.8509	4.7598	3.6309	4.1032	0.9888	-0.0162	0.9349	0.0292
Ppp1r7	_ELDLYDNQIKK_.3	#N/A	3.3720	4.1283	3.7847	4.2873	4.0166	4.1089	4.0020	0.8120	3.4797	4.2543	1.2590	0.3323	0.2451	0.6107
Ppp1r7	_GAEDPEEEHELAVDMETINLDR_.3	#N/A	4.1168	3.2256	3.8505	3.4075	3.2606	3.1818	3.5426	3.6069	3.9059	3.0716	0.9930	-0.0101	0.9207	0.0359
Smcr8	_VAS[Phospho (STY)]PANVGTLHTLSR_.2	#N/A	4.7185	7.0275	6.5263	8.1913	4.4623	4.2837	6.2374	6.9778	5.8277	3.8113	1.0271	0.0385	0.8786	0.0562
Smcr8	_SS[Phospho (STY)]VESVLIK_.2	Q3UMB5_S420_M1_Smcr8	3.1249	3.9301	3.1460	4.1906	3.7514	3.9781	4.3389	4.3114	3.1096	4.5493	0.9042	-0.1452	0.2912	0.5358
Smcr8	_SDS[Phospho (STY)]QASLTVPLSPHVVR_.2	Q3UMB5_S488_M1_Smcr8	4.1160	6.1885	3.5921	7.3867	3.2504	3.1967	3.3038	6.1321	5.7838	5.1768	0.9064	-0.1418	0.6532	0.1850
Washc4	_[Acetyl (Protein N-term)]AVDTLS[Phospho (STY)]PDWDFDRVDDGSQK_.3	#N/A	3.7960	3.6416	4.0497	3.8812	3.4827	3.4794	3.8320	5.2184	3.8991	3.3610	0.9127	-0.1317	0.3205	0.4941
Cobll1	_RQSS[Phospho (STY)]LTFQSSDPEHVR_.3	#N/A	4.0022	3.3402	3.7358	3.2796	3.7568	3.0671	3.4279	3.7216	4.0206	2.9569	1.1695	0.2259	0.4984	0.3025
Cobll1	_TLS[Phospho (STY)]SPTGTETNPPK_.2	Q3UMF0_S896_M1_Cobll1	7.4962	4.1186	4.0002	8.1085	3.9399	3.7896	7.2517	3.6157	7.1848	2.9690	0.9975	-0.0036	0.9925	0.0033
Cobll1	_QDS[Phospho (STY)]NPKPKPSNEITR_.3	Q3UMF0_S794_M1_Cobll1	3.0692	3.8744	3.2017	4.2463	3.6957	4.0338	2.7856	2.7543	3.0539	3.9236	1.1782	0.2365	0.1208	0.9178
Cobll1	_ITVEKDPDSALGIS[Phospho (STY)]DGETSPSSK_.3	Q3UMF0_S546_M1_Cobll1	3.6067	3.7357	3.3403	4.3849	3.5571	4.1724	4.5332	4.1171	4.4161	4.0622	0.8873	-0.1725	0.0618	1.2093
Cobll1	_QSS[Phospho (STY)]LTFQSSDPEHVR_.2	#N/A	3.5675	4.3727	4.2543	3.2561	4.1940	3.5355	3.8963	3.3616	3.0101	6.6570	0.9130	-0.1312	0.6119	0.2133
Faxc	_TETFEDEGAENS[Phospho (STY)]FSR_.2	Q3UMF9_S378_M1_Faxc	4.2091	4.9263	3.4261	3.9288	2.8743	3.7503	3.6435	4.1897	4.6893	4.6089	0.8995	-0.1528	0.3172	0.4987
Ankrd27	_PSEVPAQS[Phospho (STY)]PTR_.2	Q3UMR0_S634_M1_Ankrd27	6.8643	6.2864	7.4041	7.1377	5.9692	3.3819	6.8202	6.7127	6.7227	6.8660	0.9106	-0.1352	0.4418	0.3548
Mcu	_VAIYSPDGVR_.2	#N/A	6.3790	6.0846	5.9854	5.3385	5.9384	6.0929	6.5435	6.3794	6.4595	5.7231	0.9512	-0.0722	0.2196	0.6584
Mcu	_DLLSHEDAATLNDVK_.3	#N/A	4.1613	3.1811	3.8950	2.7697	3.4647	3.3195	3.5871	4.2316	3.1938	3.1161	0.9811	-0.0276	0.8423	0.0745
Ppp1r12c	_TGS[Phospho (STY)]TGALGPSER_.2	Q3UMT1_S431_M1_Ppp1r12c	9.4802	9.8510	10.0910	10.2086	8.9019	9.6035	9.9764	9.7917	9.3807	9.7603	0.9961	-0.0056	0.8885	0.0513
Ppp1r12c	_ALNGVSS[Phospho (STY)]PVSSNPK_.2	Q3UMT1_S403_M1_Ppp1r12c	3.5935	7.2066	7.5031	7.5304	4.2200	7.1310	6.7733	3.2792	6.6090	6.8288	1.0553	0.0777	0.7833	0.1061
Hdgfl2	_GGSSEELHDS[Phospho (STY)]PR_.2	Q3UMU9_S635_M1_Hdgfl2	6.4517	5.6873	5.8433	6.0749	5.1308	5.6717	6.6674	6.3981	5.9903	5.5288	0.9453	-0.0812	0.2938	0.5319
Hdgfl2	_GGS[Phospho (STY)]S[Phospho (STY)]GEELEDEEPVKK_.2	#N/A	9.3204	9.7805	4.4407	10.3644	8.4620	9.3866	10.3909	9.9096	9.8560	9.2891	0.8747	-0.1931	0.2971	0.5270
Hdgfl2	_TRLAS[Phospho (STY)]ESANDDNEDS_.2	#N/A	5.2062	5.5521	6.8013	6.0746	5.5528	5.7145	6.2122	6.1259	5.2537	5.1693	1.0222	0.0317	0.7339	0.1344
Hdgfl2	_AERGGS[Phospho (STY)]S[Phospho (STY)]GEELEDEEPVK_.2	#N/A	3.1119	5.7660	4.3464	5.6107	4.7945	5.9350	5.7636	5.6142	5.4017	5.4702	0.8858	-0.1749	0.2856	0.5442
Hdgfl2	_AQEDGQDS[Phospho (STY)]EDGPR_.2	Q3UMU9_S620_M1_Hdgfl2	4.0803	5.9191	6.0121	4.4160	4.4029	3.4006	3.2937	5.0824	5.3862	5.8039	0.9619	-0.0561	0.7936	0.1004
Hdgfl2	_LAS[Phospho (STY)]ESANDDNEDS_.2	#N/A	6.9354	6.8755	7.0697	7.1450	6.3067	6.1167	7.1016	7.0609	7.0889	6.5713	0.9692	-0.0451	0.3977	0.4004
Hdgfl2	_GGS[Phospho (STY)]S[Phospho (STY)]GEELEDEEPVKKR_.3	#N/A	4.8084	5.4082	4.9458	5.7810	5.1214	5.7486	5.4402	6.0439	5.8933	4.9717	0.9490	-0.0755	0.3446	0.4627
Hdgfl2	_GGS[Phospho (STY)]SGEELEDEEPVK_.2	Q3UMU9_S366_M1_Hdgfl2	3.8338	9.5277	3.7833	9.8983	8.8926	9.5303	10.2318	9.4851	9.6686	9.6875	0.7757	-0.3664	0.1835	0.7365
Hdgfl2	_KRS[Phospho (STY)]EGLSLER_.3	Q3UMU9_S450_M1_Hdgfl2	3.6204	3.6058	4.3072	3.3090	3.4941	3.1305	4.5245	3.3087	2.9573	3.2760	1.0174	0.0249	0.8623	0.0643
Hdgfl2	_AERGGS[Phospho (STY)]S[Phospho (STY)]GEELEDEEPVKK_.3	#N/A	4.2561	4.3753	4.2440	3.3010	3.5210	4.2266	3.5370	4.1789	4.4330	3.0640	1.0484	0.0682	0.6004	0.2215
Gpr162	_RLS[Phospho (STY)]HDETNIFSTPR_.3	Q3UN16_S413_M1_Gpr162	4.1679	7.2286	7.1505	7.1613	5.4455	6.6779	7.6632	7.8140	7.4550	7.0605	0.8409	-0.2500	0.1030	0.9872
Gpr162	_RSS[Phospho (STY)]LDGSESAK_.2	Q3UN16_S256_M1_Gpr162	6.6403	3.5952	4.3178	3.3196	3.4835	3.1412	4.5351	7.0059	7.0134	6.3919	0.6547	-0.6112	0.0297	1.5271
Gpr162	_C[Carbamidomethyl (C)]SLTGS[Phospho (STY)]EGSSR_.2	Q3UN16_S565_M1_Gpr162	3.4842	3.7420	4.1710	3.1728	4.1107	3.6188	4.3883	3.4449	3.0934	3.1398	1.0569	0.0798	0.5329	0.2734
Gpr162	_RLS[Phospho (STY)]LGSPDSR_.2	Q3UN16_S539_M1_Gpr162	7.3064	7.3296	6.1720	6.5322	4.4190	7.3057	7.7428	7.3066	6.6454	7.7008	0.8860	-0.1747	0.2084	0.6812
Gpr162	_RC[Carbamidomethyl (C)]S[Phospho (STY)]LT[Phospho (STY)]GSEGSSR_.2	Q3UN16_T563_M2_Gpr162	4.3178	4.2300	3.7254	4.2280	3.7778	3.8107	3.9427	4.2181	3.6011	3.7311	1.0366	0.0518	0.4397	0.3568
Gprin1	_DLAAAAAQKS[Phospho (STY)]PSAEAAAPPPGPR_.3	#N/A	12.1018	12.4526	13.3002	3.8923	11.2382	12.0911	12.6402	3.7339	12.2481	12.2487	1.0615	0.0861	0.8050	0.0942
Gprin1	_KAS[Phospho (STY)]LPASEK_.2	#N/A	7.0992	7.0796	6.9203	7.7632	6.1430	7.2883	6.3324	6.8800	7.1055	7.0829	1.1415	0.1909	0.2619	0.5819
Gprin1	_SLGTWGS[Phospho (STY)]LSAAK_.2	Q3UNH4_S446_M1_Gprin1	3.7406	6.7792	3.4743	7.3145	3.4232	6.9953	3.1663	6.6992	4.2822	4.1961	1.1530	0.2055	0.5566	0.2545
Gprin1	_RERPGS[Phospho (STY)]TGEGDLVSLR_.3	#N/A	7.2781	6.8905	3.7177	7.7030	3.6574	6.9735	3.9350	3.8982	6.7035	7.0276	1.1198	0.1632	0.5922	0.2275
Gprin1	_VITQIPGATSPGKVETPS[Phospho (STY)]LQK_.3	Q3UNH4_S584_M1_Gprin1	4.2390	2.9872	3.4249	3.9276	2.8755	3.7491	3.6423	4.1909	3.8395	3.8946	0.9080	-0.1392	0.2498	0.6024
Gprin1	_VDIVS[Phospho (STY)]PGGDNAGSLR_.2	#N/A	9.2331	9.5302	9.6821	9.9976	8.3973	9.6576	9.2392	9.1443	9.2497	9.5146	1.0139	0.0200	0.6685	0.1749
Gprin1	_ERPGS[Phospho (STY)]TGEGDLVSLR_.2	#N/A	8.4217	8.1756	7.4304	7.9534	8.0772	8.6970	7.7866	8.2544	7.2198	7.5720	1.0542	0.0761	0.1726	0.7630
Gprin1	_AETVS[Phospho (STY)]PGEVDAM[Oxidation (M)]TLGK_.2	#N/A	8.9478	9.3879	10.1862	9.9411	9.1565	9.5457	9.8471	9.3651	9.4753	9.6226	0.9948	-0.0076	0.8482	0.0715
Gprin1	_KAS[Phospho (STY)]LPASEKTDPLASSK_.3	#N/A	5.3092	5.3954	6.3222	5.9705	3.6382	5.2236	4.8233	4.3034	5.2960	3.0092	1.2184	0.2850	0.1584	0.8002
Gprin1	_VDIVSPGGDNAGS[Phospho (STY)]LRK_.2	#N/A	5.2301	5.6976	5.1662	6.4126	4.1649	6.3240	4.9255	5.3898	4.9892	6.1562	1.0250	0.0356	0.7884	0.1032
Gprin1	_NSELVS[Phospho (STY)]PVKPER_.2	#N/A	10.1420	10.3556	10.6580	10.9225	9.2188	10.4144	9.9690	10.0579	9.9516	10.1554	1.0251	0.0357	0.4288	0.3678
Gprin1	_KTDPEFSGKLT[Phospho (STY)]PGSSGK_.3	Q3UNH4_T256_M1_Gprin1	6.7247	6.8086	4.4232	7.1610	3.8333	6.6933	4.1153	6.4490	3.3332	3.6443	1.3546	0.4379	0.1284	0.8914
Gprin1	_ADSASPS[Phospho (STY)]PR_.2	Q3UNH4_S622_M1_Gprin1	7.8700	8.2138	8.2386	8.0516	3.3821	7.5373	7.5484	7.7660	7.4118	8.3535	0.9287	-0.1068	0.5876	0.2309
Gprin1	_APS[Phospho (STY)]PAPRPPSR_.2	#N/A	9.8757	10.1264	10.0021	10.5221	9.0412	10.0314	9.7436	9.7804	9.6796	10.0152	1.0131	0.0188	0.6298	0.2008
Gprin1	_SVATGPM[Oxidation (M)]T[Phospho (STY)]PQAAAPPAVPPVFPEVR_.3	#N/A	6.3202	8.8961	8.5066	9.9905	3.3708	3.6073	3.8158	9.7078	3.7235	6.6414	1.1356	0.1834	0.6686	0.1748
Gprin1	_APS[Phospho (STY)]WDAGAPPPR_.2	Q3UNH4_S714_M1_Gprin1	14.8927	14.4293	14.8168	15.2894	14.1196	14.6028	15.0044	14.5153	14.5850	14.6830	0.9996	-0.0005	0.9822	0.0078
Gprin1	_S[Phospho (STY)]PSAEAAAPPPGPR_.2	#N/A	3.8021	3.9232	3.5358	12.4215	11.2858	11.7130	11.8365	11.4217	4.2356	11.8426	0.7911	-0.3380	0.4686	0.3292
Gprin1	_MDGNS[Phospho (STY)]LKQADSTSTR_.2	Q3UNH4_S110_M1_Gprin1	3.0253	3.7816	4.2598	3.1332	3.6699	2.9547	3.4387	3.1976	3.1330	3.4809	0.9804	-0.0285	0.8456	0.0728
Arhgef40	_IQQQLGEEAS[Phospho (STY)]PR_.2	Q3UPH7_S959_M1_Arhgef40	6.4420	6.3949	6.9939	6.2476	5.6901	5.9870	6.6963	6.4245	5.8720	6.3164	0.9945	-0.0079	0.8985	0.0465
Sec31a	_LVTFESVAVPLQQGAEQQR_.2	#N/A	4.6421	4.5362	5.2175	4.5617	3.9361	4.5068	4.6493	4.5917	4.5155	3.9413	1.0322	0.0457	0.5772	0.2387
Sec31a	_DSDQVAQS[Phospho (STY)]DGEESPAAEEQLLGER_.3	#N/A	3.9519	5.9325	3.8534	7.8322	7.2232	6.0932	3.8963	8.2894	7.8590	7.1285	0.8559	-0.2245	0.4190	0.3778
Sec31a	_AQDGSS[Phospho (STY)]PLSLQDLIEK_.2	Q3UPL0_S712_M1_Sec31a	3.7547	3.5877	3.4884	4.5330	2.8985	4.3205	3.1804	3.9690	4.2681	4.2103	0.9634	-0.0539	0.7026	0.1533
Sec31a	_VDGPDVALKDSDQVAQS[Phospho (STY)]DGEESPAAEEQLLGER_.4	#N/A	3.3686	3.9738	4.6031	4.1469	4.0132	3.9343	4.2951	4.3551	4.8603	3.8242	0.9245	-0.1132	0.2488	0.6041
Ag2	_S[Phospho (STY)]TQSLSLQR_.2	Q3UPL5_S217_M1_Ag2	6.7921	3.7598	4.0120	4.2939	3.4221	4.0498	3.7041	3.5546	3.8536	7.0475	0.9666	-0.0490	0.8714	0.0598
Ag2	_TQPVTFDEIQEVEEEGVS[Phospho (STY)]PM[Oxidation (M)]EEEK_.3	#N/A	3.6362	3.6095	3.4912	3.8739	4.6815	3.6614	4.0222	3.9909	4.5159	4.2202	0.9136	-0.1303	0.1745	0.7581
Fcho2	_NS[Phospho (STY)]SNEELTK_.2	Q3UQN2_S402_M1_Fcho2	3.3311	3.8950	4.0179	4.5206	3.9576	3.7718	4.2353	3.5979	3.2465	2.9867	1.1135	0.1551	0.2064	0.6852
Fcho2	_HS[Phospho (STY)]PVQMNR_.2	Q3UQN2_S394_M1_Fcho2	3.6446	3.6978	3.3783	4.4714	3.5192	4.2103	3.0703	4.0792	4.3782	4.1001	0.9778	-0.0324	0.7897	0.1025

Rbm20	_SGS[Phospho (STY)]PGPLHSVSGYK_.2	Q3UQS8_S728_M1_Rbm20	4.0712	3.3755	3.2571	5.9196	3.1969	3.0318	3.4745	4.3587	4.0073	3.7268	0.9786	-0.0311	0.8893	0.0509
Brd9	_EKEHC[Carbamidomethyl (C)]DS[Phospho (STY)]EGEADAFDPGKK_.3	#N/A	4.8149	5.2583	3.3740	6.4459	2.7841	5.3855	5.8149	6.0787	5.6900	5.3728	0.8150	-0.2952	0.1701	0.7694
Brd9	_EKEHC[Carbamidomethyl (C)]DS[Phospho (STY)]EGEADAFDPGK_.3	#N/A	3.1909	3.7114	3.9129	4.5725	3.4407	3.7108	4.4891	4.4494	3.3548	3.6126	0.9447	-0.0821	0.5266	0.2785
Tgfbrap1	_HTAPS[Phospho (STY)]SPSPGTR_.2	#N/A	3.5048	5.4474	4.4248	3.7081	3.6029	5.6330	6.0548	5.6596	3.9000	3.5183	0.9171	-0.1248	0.5846	0.2331
Tgfbrap1	_HTAPSS[Phospho (STY)]PSPGTRT_.2	#N/A	4.3072	5.8498	3.4932	3.9958	3.2373	3.9931	3.7105	5.7486	5.5279	5.4526	0.8114	-0.3016	0.1462	0.8351
Slmap	_ESDLSDTLS[Phospho (STY)]PSKEK_.2	Q3URD3_S469_M1_Slmap	3.3645	3.6847	3.5501	4.1037	4.3384	3.9012	4.1380	4.0792	4.7540	4.1714	0.8922	-0.1645	0.0716	1.1449
Slmap	_SDVIHAPLPS[Phospho (STY)]PVDK_.2	#N/A	6.6397	5.9933	4.0958	6.6924	5.0319	6.3338	6.7527	6.3830	6.0282	6.3982	0.9073	-0.1404	0.3036	0.5177
Slmap	_AKESDLSDTLSPS[Phospho (STY)]KEK_.2	Q3URD3_S471_M1_Slmap	4.2617	3.5172	3.9954	3.5392	3.4055	3.2191	3.6875	3.4620	3.7610	3.2165	1.0353	0.0500	0.5906	0.2287
Lingo2	_HKNS[Phospho (STY)]IDLEYVPR_.3	Q3URE9_S576_M1_Lingo2	5.6188	5.4816	5.5740	6.8594	3.6174	5.6721	4.4729	6.3595	2.9756	4.0019	1.2287	0.2971	0.2240	0.6497
Plcl1	_RSS[Phospho (STY)]IIKDPSNQK_.2	#N/A	7.8738	7.3797	3.6291	8.1127	6.9581	7.9851	7.8934	7.8137	3.6005	7.5879	1.0395	0.0559	0.8299	0.0810
Plcl1	_TVS[Phospho (STY)]FSSM[Oxidation (M)]PSEK_.2	#N/A	8.5578	3.8066	8.3646	7.3902	7.4276	7.7063	8.5680	7.8081	7.8287	7.7635	0.9020	-0.1488	0.4088	0.3885
Plcl1	_S[Phospho (STY)]GVALPGAAGVPADSEAGLLEAAR_.2	Q3USB7_S48_M1_Plcl1	3.2687	4.0250	6.0068	4.3906	3.9133	4.2122	4.1053	5.3080	3.3764	4.3576	1.0037	0.0054	0.9782	0.0096
Plcl1	_RM[Oxidation (M)]S[Phospho (STY)]GDYNGEQK_.2	#N/A	3.7093	5.2699	5.4933	5.3109	3.2198	4.2247	4.1510	3.8345	5.2712	3.6033	1.0766	0.1065	0.5856	0.2324
Plcl1	_KKT[Phospho (STY)]VS[Phospho (STY)]FSSMPSEK_.2	#N/A	3.8751	3.1542	3.6087	5.7799	3.0176	3.4948	4.8023	3.6059	3.7443	3.7281	0.9626	-0.0550	0.7976	0.0982
Plcl1	_KLPSESDLLEGEVT[Phospho (STY)]DEDEEAEM[Oxidation (M)]SR_.3	Q3USB7_T557_M1_Plcl1	2.9739	4.2523	3.6607	4.2127	4.1406	3.9849	3.8780	3.9551	3.6037	4.1303	0.9946	-0.0078	0.9387	0.0275
Insyn2a	_STGVQTS[Phospho (STY)]PDLRK_.2	#N/A	2.9442	8.1199	8.6022	3.7224	7.6724	8.0122	8.3468	7.9028	3.5778	8.2687	0.9271	-0.1091	0.7520	0.1238
Insyn2a	_STGVQTS[Phospho (STY)]PDLR_.2	#N/A	7.1054	7.1042	4.3893	3.9331	6.8231	7.4808	7.5391	6.5876	3.3672	7.4952	0.9827	-0.0252	0.9250	0.0339
Sfswap	_GVS[Phospho (STY)]QEKDGQISSAIVSSVQSK_.3	Q3USH5_S903_M1_Sfswap	3.6395	2.9439	4.3263	6.3428	4.2660	3.4635	4.5437	5.5386	4.4389	3.2951	0.9348	-0.0973	0.6916	0.1602
Sfswap	_VKLDDDS[Phospho (STY)]EEDEESR_.2	Q3USH5_S601_M1_Sfswap	3.1344	4.0918	3.8212	4.3238	3.9801	4.1454	4.0385	3.7946	3.4432	4.2908	1.0062	0.0090	0.9269	0.0330
Sorbs2	_RKS[Phospho (STY)]EPAVGPLR_.3	#N/A	10.7775	10.3249	10.9976	10.9658	10.1643	10.3512	10.3978	10.5822	10.7339	10.7462	0.9983	-0.0025	0.9282	0.0324
Sorbs2	_RGS[Phospho (STY)]LPDNSILHR_.3	#N/A	9.2493	9.1998	9.8366	10.2424	7.6944	8.8536	8.7545	9.7097	9.1144	9.0423	1.0026	0.0038	0.9609	0.0173
Sorbs2	_ESQS[Phospho (STY)]PDSAWR_.2	Q3UTJ2_S40_M1_Sorbs2	9.6674	9.6957	10.2672	10.0009	9.2990	9.6951	10.0862	9.5394	9.7793	9.8624	0.9953	-0.0068	0.8154	0.0886
Sorbs2	_S[Phospho (STY)]EPAVGPLR_.2	#N/A	10.0477	8.9181	9.3811	10.0971	8.9328	9.2468	9.6945	9.2088	9.4343	9.7142	0.9920	-0.0115	0.7963	0.0989
Sorbs2	_GAEDYPDPPLPHSYS[Phospho (STY)]SDR_.2	#N/A	3.5768	7.9416	3.6467	9.5260	3.7138	3.7561	8.2645	3.7602	7.8318	4.1164	0.8944	-0.1611	0.7117	0.1477
Sorbs2	_VQS[Phospho (STY)]SPNLLAAGR_.2	Q3UTJ2_S27_M1_Sorbs2	3.7474	3.5950	3.4811	4.5257	2.8912	3.7334	3.1732	3.9763	4.2753	4.2030	0.9374	-0.0933	0.4862	0.3132
Sorbs2	_SFISSSPSS[Phospho (STY)]PSR_.2	Q3UTJ2_S382_M1_Sorbs2	10.0050	9.6742	10.1211	10.3969	9.7658	9.4422	10.2729	3.5499	9.9340	9.9337	1.1755	0.2333	0.2875	0.5413
Sorbs2	_TS[Phospho (STY)]PGRADLPGSSSTFTK_.3	#N/A	3.3307	6.3675	6.9649	6.4890	5.8040	3.2457	5.6454	3.4885	5.1014	5.2735	1.0071	0.0102	0.9716	0.0125
Sorbs2	_APHYPGIGPVDESGIPTAIR_.3	#N/A	5.8394	6.1398	4.5052	5.8112	5.7749	5.7093	5.8119	5.6960	5.5615	5.8748	0.9815	-0.0269	0.7296	0.1369
Sorbs2	_SC[Carbamidomethyl (C)]DDLLNDDC[Carbamidomethyl (C)]DS[Phospho (STY)]FPDPK_.2	Q3UTJ2_S474_M1_Sorbs2	3.7367	3.8626	3.7416	8.7196	6.4837	4.4907	3.9589	7.7494	6.3387	5.4847	0.8792	-0.1857	0.5735	0.2415
Sorbs2	_GLLHGWSQSS[Phospho (STY)]TEEVPR_.2	#N/A	3.7608	6.6971	6.8497	7.6001	6.1036	3.8024	8.1407	3.8377	6.7962	3.9054	1.0233	0.0333	0.9143	0.0389
Sorbs2	_QNAEIWSSTEETVS[Phospho (STY)]PK_.2	Q3UTJ2_S456_M1_Sorbs2	3.8653	3.4199	4.1372	6.7397	3.0003	3.4343	3.2802	6.3683	4.1882	3.9887	0.9199	-0.1204	0.6918	0.1600
Sorbs2	_SRS[Phospho (STY)]C[Carbamidomethyl (C)]DDLLNDDC[Carbamidomethyl (C)]DSFPDPK_.3	#N/A	2.9737	4.1240	4.2082	5.9472	4.3764	3.2578	3.9003	3.2502	3.5493	4.6635	1.0799	0.1110	0.6141	0.2117
Sorbs2	_GS[Phospho (STY)]LPDNSILHR_.2	#N/A	7.6656	6.9916	7.5977	7.8576	6.5721	3.1775	7.5665	3.2244	7.4881	7.6275	1.0258	0.0367	0.8964	0.0475
Sorbs2	_RFS[Phospho (STY)]IESLLEEETQVR_.3	Q3UTJ2_S630_M1_Sorbs2	4.7152	4.1104	4.4665	4.0102	3.8765	3.7977	4.1585	2.9909	3.2900	3.6875	1.1787	0.2372	0.0481	1.3182
Sorbs2	_RRGS[Phospho (STY)]LPDNSILHR_.4	#N/A	3.2861	4.0913	3.9729	4.0294	3.9126	3.8169	4.1777	4.5107	3.2708	4.4426	0.9393	-0.0903	0.3849	0.4146
Cdkl5	_LLDRS[Phospho (STY)]PSR_.2	Q3UTQ8_S306_M1_Cdkl5	8.8786	8.9763	4.2043	9.0050	4.1440	9.0439	8.9072	9.0721	3.0601	8.9438	0.9839	-0.0234	0.9460	0.0241
Cdkl5	_SNS[Phospho (STY)]KDIQNLSVGLPR_.3	#N/A	4.4627	5.6252	3.8035	7.0704	3.7921	3.2933	3.6541	6.3639	3.8383	3.9959	1.0474	0.0668	0.8170	0.0878
Cdkl5	_NLSEGNNANYTEY[Phospho (STY)]VATR_.2	Q3UTQ8_Y171_M1_Cdkl5	3.9019	3.6547	4.0422	5.4809	3.6323	3.7815	4.1644	3.5683	3.6404	4.3405	1.0392	0.0554	0.7027	0.1532
Cdkl5	_VSS[Phospho (STY)]LPSDSSSGTNHSK_.2	Q3UTQ8_S738_M1_Cdkl5	3.7537	3.5887	3.4874	4.5320	2.8974	4.3194	3.1794	4.2437	4.2691	4.2093	0.9466	-0.0792	0.5802	0.2364
Cdkl5	_DLTNNNIPHLLS[Phospho (STY)]PK_.2	Q3UTQ8_S407_M1_Cdkl5	3.0825	3.8388	4.3170	8.8595	3.7271	4.3984	4.2915	7.3995	3.1902	3.0430	1.0497	0.0700	0.8714	0.0598
Trappc14	_KSS[Phospho (STY)]PSSPAVR_.2	Q3UTZ3_S495_M1_Trappc14	7.9192	6.7758	4.0363	6.5397	3.7284	3.6140	8.0489	8.0933	7.3803	7.7938	0.6943	-0.5264	0.0373	1.4279
Trappc14	_SQS[Phospho (STY)]FSHQQPSR_.2	Q3UTZ3_S517_M1_Trappc14	5.9882	4.0128	5.9086	6.4913	3.4316	6.0945	5.9295	6.1121	5.7967	5.9351	0.8953	-0.1595	0.3659	0.4367
Ankrd34b	_VAS[Phospho (STY)]LQEELQDITPEEEIAYK_.2	Q3UUF8_S260_M1_Ankrd34b	5.6305	5.6921	5.9038	6.4743	5.1194	5.8050	5.9009	5.7288	5.7550	5.7784	0.9966	-0.0050	0.9320	0.0306
Ankrd34b	_NSGANHY[Phospho (STY)]SSDSQLAEGVTPPTVEDGK_.3	Q3UUF8_Y365_M1_Ankrd34b	4.3721	4.3549	3.3580	4.0607	4.2432	3.8822	3.7754	4.0578	3.7064	4.0277	1.0480	0.0676	0.3192	0.4959
Ankrd34b	_HQS[Phospho (STY)]IDVK_.2	Q3UUF8_S293_M1_Ankrd34b	5.1398	3.2764	3.7997	3.3435	4.8393	3.1310	3.4918	3.6577	3.9337	3.0208	1.1122	0.1534	0.4199	0.3769
Ankrd34b	_RGS[Phospho (STY)]GAFPLDHSLAQSR_.3	Q3UUF8_S426_M1_Ankrd34b	3.9118	3.2162	4.5986	3.4037	3.0375	3.1911	3.5519	0.7807	3.8966	3.5674	1.2071	0.2715	0.3746	0.4264
Tbc1d24	_SVS[Phospho (STY)]LSKR_.2	Q3UUG6_S324_M1_Tbc1d24	8.5322	8.8680	9.0266	8.6304	3.5020	3.1226	8.8656	8.9600	8.4997	8.4017	0.8002	-0.3216	0.2642	0.5781
Tbc1d24	_QKS[Phospho (STY)]VSLSK_.2	Q3UUG6_S322_M1_Tbc1d24	4.1286	3.0976	5.9124	6.1832	3.2543	3.6387	5.5708	5.6839	6.0949	5.1802	0.7757	-0.3664	0.1110	0.9547
Ksr2	_S[Phospho (STY)]KSHEFQLGNR_.3	Q3UVC0_S312_M1_Ksr2	3.3137	4.0699	4.5482	4.0919	3.9582	4.1672	4.0604	4.4101	3.2083	3.7692	1.0422	0.0596	0.5865	0.2317
Ksr2	_TES[Phospho (STY)]VPC[Carbamidomethyl (C)]DINNPVR_.2	Q3UVC0_S484_M1_Ksr2	4.0134	3.2688	3.7471	3.2908	3.1571	7.8420	3.4391	3.7103	7.4975	2.9681	0.9582	-0.0615	0.8854	0.0529
Ksr2	_TPNIVTTVT[Phospho (STY)]PPGT[Phospho (STY)]PPMR_.2	Q3UVC0_T273_M2_Ksr2	3.9722	3.2276	3.7059	3.2496	3.1159	3.5087	3.4019	3.7515	4.0506	4.4277	0.8862	-0.1743	0.1020	0.9915
Fam91a1	_KLS[Phospho (STY)]EASDER_.2	Q3UVG3_S671_M1_Fam91a1	6.8032	5.8236	6.0287	6.6946	6.1018	6.1950	5.9689	6.2792	5.8702	6.0688	1.0377	0.0533	0.3100	0.5086
Vps51	_[Acetyl (Protein N-term)]AAAAAVGPGLGSGPGDS[Phospho (STY)]PEGPEADAPER_.3	#N/A	3.6876	10.4333	9.3988	11.7072	10.1852	10.2266	7.6293	11.8482	11.3608	10.7872	0.8911	-0.1663	0.5068	0.2951
Vps51	_[Acetyl (Protein N-term)]AAAAAVGPGLGSGPGDS[Phospho (STY)]PEGPEADAPERR_.3	#N/A	3.0573	10.8637	10.4908	11.5648	3.6924	10.4887	11.0374	11.7043	11.3392	10.9004	0.7434	-0.4278	0.1847	0.7335
Vps51	_IDVFS[Phospho (STY)]PVEFNK_.2	Q3UVL4_S690_M1_Vps51	4.0415	3.3009	3.7752	3.3190	3.7874	3.1064	3.4672	5.4439	4.7512	2.9963	0.8536	-0.2283	0.2431	0.6142
Ttbk2	_SIHS[Phospho (STY)]FELEKR_.3	Q3UVR3_S448_M1_Ttbk2	4.1326	3.8996	4.3428	3.8865	3.7528	3.7479	4.0348	3.9907	3.4804	4.6228	0.9851	-0.0216	0.7986	0.0977
Grm5	_RGS[Phospho (STY)]SGETLR_.2	#N/A	5.7885	3.9411	6.1024	5.3744	3.7625	3.9670	4.3278	5.8882	5.3096	3.8568	0.9953	-0.0068	0.9726	0.0121
Grm5	_TDDDAPS[Phospho (STY)]LHS[Phospho (STY)]ETAAR_.2	Q3UVX5_S1037_M2_Grm5	5.7784	2.9632	3.4415	6.3624	2.8515	3.7731	3.6663	6.0372	5.7499	3.8291	0.8702	-0.2005	0.5116	0.2910
Grm5	_SVTWAQNEKST[Phospho (STY)]R_.2	Q3UVX5_T923_M1_Grm5	3.3009	4.1061	3.9877	4.4903	3.9274	3.8021	4.1629	3.6281	3.2767	2.9565	1.1226	0.1668	0.1703	0.7689
Grm5	_DSVDSGSTTPNS[Phospho (STY)]PVSESALC[Carbamidomethyl (C)]IPSSPK_.3	Q3UVX5_S1173_M1_Grm5	4.0385	3.3429	3.7332	3.2770	3.1642	3.0645	6.1444	6.0256	4.0232	2.9543	0.7179	-0.4781	0.0701	1.1545
Grm5	_SSS[Phospho (STY)]LVNLWK_.2	Q3UVX5_S860_M1_Grm5	3.4899	4.2462	3.2236	7.0710	4.1345	3.9909	3.8841	7.5692	3.0320	3.9455	0.9461	-0.0799	0.8216	0.0853
Grm5	_S[Phospho (STY)]PS[Phospho (STY)]PISTLSHLAGSAGR_.2	Q3UVX5_S1014_M2_Grm5	3.3763	3.8498	4.0631	6.1389	3.7381	4.3873	4.2805	7.0521	3.2013	3.0319	0.9698	-0.0442	0.8852	0.0530
Grm5	_GS[Phospho (STY)]SGETLR_.2	#N/A	3.7524	3.4737	4.4392	3.4410	2.8781	3.3505	3.1557	3.1766	4.3260	3.4080	1.0112	0.0160	0.9116	0.0402
Tubal3	_SFGGGTGSGFTSLLM[Oxidation (M)]ER_.2	#N/A	6.1145	5.7005	5.4650	5.7242	5.2470	5.8339	5.9958	5.3771	5.8467	5.7554	0.9891	-0.0159	0.7427	0.1292
Tubal3	_AVC[Carbamidomethyl (C)]M[Oxidation (M)]LSNTTAIVEAWAR_.2	#N/A	3.4301	7.6047	3.7266	4.2292	4.0747	4.0508	3.9439	5.1232	6.6160	6.5692	0.8124	-0.2998	0.2965	0.5280
Fam81a	_IKDDIVSS[Phospho (STY)]LQK_.2	Q3UXZ6_S58_M1_Fam81a	3.6078	4.0580	5.3269	3.7037	3.9463	5.6429	6.5323	3.7609	5.0596	3.7233	0.9186	-0.1225	0.5892	0.2297
Ppm1h	_GGVGAPGS[Phospho (STY)]PSTPPTR_.2	Q3UYC0_S220_M1_Ppm1h	10.0139	9.9918	10.0328	4.4109	3.3396	9.7154	3.9666	9.7559	9.6007	9.7806	0.9567	-0.0639	0.8600	0.0655
Ppm1h	_SS[Phospho (STY)]LPNGEGLQLK_.2	#N/A	8.7843	4.0692	8.0920	8.6414	7.1208	4.0810	3.4626	8.0909	8.2110	4.1575	1.1367	0.1849	0.5999	0.2219
Ppm1h	_AGTITST[Phospho (STY)]PNR_.2	#N/A	8.0109	8.0463	3.1495	8.6704	3.7479	7.9018	8.1354	8.1653	3.1061	8.0827	0.9586	-0.0610	0.8631	0.0639
Ppm1h	_KAGTITST[Phospho (STY)]PNR_.2	#N/A	3.7326	3.0370	4.4194	3.4213	2.8583	3.3703	3.1359	3.1964	4.3458	3.3883	0.9877	-0.0179	0.9071	0.0423
Ppm1h	_AGTITSTPNRNS[Phospho (STY)]K_.2	Q3UYC0_S118_M1_Ppm1h	5.6674	4.4119	3.3893	4.4339	2.7993	5.0023	3.7185	4.0681	4.3672	4.1111	1.0536	0.0753	0.6995	0.1552
Ppm1h	_RSS[Phospho (STY)]LPNGEGLQLK_.2	#N/A	3.8022	7.4943	8.2730	8.2313	6.7871	7.6115	3.6616	7.6091	7.7084	7.8114	1.0501	0.0705	0.7817	0.1070
Macrod2	_GLS[Phospho (STY)]PPHKK_.2	#N/A	10.4256	10.5087	10.9900	10.8943	10.5342	10.7241	10.7757	10.6873	10.5617	10.5953	1.0023	0.0033	0.8465	0.0724
Macrod2	_GLS[Phospho (STY)]PPHK_.2	#N/A	8.8950	8.2984	8.2718	7.4790	8.4859	8.5486	8.0007	8.5022	8.5654	8.9292	0.9800	-0.0291	0.5680	0.2456
Rgl3	_VIEPPAASC[Carbamidomethyl (C)]PSS[Phospho (STY)]PR_.2	Q3UYI5_S510_M1_Rgl3	3.4149	4.1711	3.9731	4.2807	4.0594	4.1022	7.2993	3.6688	3.9426	3.8704	0.8520	-0.2311	0.3531	0.4520
Lrrfip1	_RGS[Phospho (STY)]GDTSISM[Oxidation (M)]DTEASIR_.3	Q3UZ39_S88_M1_Lrrfip1	4.0154	3.3198	3.2014	3.3001	3.1411	4.4024	3.4483	3.7011	4.0002	3.6711	0.9617	-0.0563	0.6257	0.2037
Rcsd1	_S[Phospho (STY)]PDANMPEEEGVVR_.2	#N/A	5.8303	4.0364	3.8766	6.7784	4.6849	4.2008	4.0939	3.7392	3.3878	4.9110	1.2153	0.2813	0.2155	0.6666
Rcsd1	_LGQKS[Phospho (STY)]PDANMPEEEGVVR_.3	#N/A	3.1954	4.2160	3.4104	5.0617	3.8323	4.1143	3.9165	4.1587	3.3935	3.8978	1.0339	0.0480	0.7287	0.1375
Rcsd1	_S[Phospho (STY)]KDPGSPQLNQEAMADGVEGTPWSAEKPR_.4	Q3UZA1_S211_M1_Rcsd1	3.4568	3.7254	4.1436	3.9310	3.5756	3.6601	3.6456	3.5367	3.9708	3.8980	0.9963	-0.0054	0.9283	0.0323

Garnl3	_SFSDVLPES[Phospho (STY)]PK_.2	Q3V0G7_S455_M1_Garnl3	7.5755	8.0628	7.7559	8.8206	7.6908	8.1543	8.7278	8.4897	8.3357	8.1703	0.9501	-0.0739	0.1314	0.8814
Garnl3	_IC[Carbamidomethyl (C)]LPYS[Phospho (STY)]PTK_.2	Q3V0G7_S179_M1_Garnl3	3.6360	2.9403	4.3228	3.6795	4.2625	3.4670	3.8278	5.4934	5.5453	3.2916	0.8190	-0.2880	0.1574	0.8031
Garnl3	_GASAHTS[Phospho (STY)]PQT[Phospho (STY)]PPAR_.2	Q3V0G7_S848_M2_Garnl3	3.2969	7.2894	4.5314	8.0319	3.9414	4.1840	7.9038	7.5773	3.2251	3.7524	0.9284	-0.1072	0.7804	0.1077
Prr7	_ALELEPLELEGSLAGS[Phospho (STY)]PPGLAPPPPPHR_.3	Q3V0I2_S68_M1_Prr7	3.5356	4.5978	3.8119	5.8092	3.4424	4.0295	3.8288	3.7478	3.7883	3.7862	1.1100	0.1505	0.3850	0.4146
Nt5c2	_NRTS[Phospho (STY)]VDFK_.2	Q3V1L4_S511_M1_Nt5c2	5.1052	3.4191	3.8974	3.5162	3.3074	3.3377	4.0839	3.9750	4.2714	3.9715	0.9235	-0.1148	0.4081	0.3892
Pacs2	_STS[Phospho (STY)]LKER_.2	Q3V3Q7_S408_M1_Pacs2	3.4799	9.1506	9.6263	9.2895	4.1244	8.9732	9.1801	9.2846	9.1665	3.9354	0.9428	-0.0849	0.8067	0.0933
Pacs2	_TES[Phospho (STY)]LVIPSTR_.2	Q3V3Q7_S387_M1_Pacs2	6.3206	3.9544	3.1217	4.1663	4.9192	6.2931	6.1756	4.3357	3.1339	6.1871	0.9673	-0.0480	0.8599	0.0656
Pacs2	_ANS[Phospho (STY)]LDNER_.2	#N/A	6.4901	6.1805	3.3073	7.4827	6.2033	5.9191	7.1987	6.8114	7.0206	7.0636	0.8444	-0.2441	0.1649	0.7828
Pacs2	_ANS[Phospho (STY)]LDNERC[Carbamidomethyl (C)]PDTR_.2	#N/A	4.4839	4.7177	4.9790	5.4377	4.1857	3.8012	5.8679	5.0637	4.9836	3.8333	0.9319	-0.1018	0.4706	0.3274
Pacs2	_EPSGQPEDS[Phospho (STY)]PEAETSTLDVFTEK_.3	Q3V3Q7_S361_M1_Pacs2	2.9279	4.4145	4.1624	3.7062	4.2359	3.4936	3.8544	6.3644	3.5941	3.3834	0.8894	-0.1692	0.4643	0.3332
Carmil2	_LQRS[Phospho (STY)]PVLK_.2	Q3V3V9_S1242_M1_Carmil2	5.2760	6.1945	4.9570	6.0607	4.9603	5.4657	5.3411	5.2804	5.2302	5.3702	1.0340	0.0482	0.5296	0.2760
Carmil2	_GPRPDLETS[Phospho (STY)]PGAAAR_.2	#N/A	3.0992	3.5799	7.2951	3.8775	4.0946	4.6751	3.9764	3.9952	6.3160	3.5548	0.9947	-0.0077	0.9799	0.0088
Carmil2	_AGS[Phospho (STY)]DGDIM[Oxidation (M)]DSSTETPPISIK_.2	#N/A	6.4029	8.9258	8.4574	9.8231	8.7244	9.1031	9.1635	9.1715	8.8995	9.2217	0.9406	-0.0883	0.3889	0.4102
Carmil2	_KAGS[Phospho (STY)]DGDIM[Oxidation (M)]DSSTETPPISIK_.2	#N/A	6.1932	6.8538	6.4720	7.5554	6.5374	7.3158	5.9379	6.8508	6.9573	7.1222	1.0155	0.0222	0.7675	0.1149
Carmil2	_PLPPYPTEPSSPERSPPS[Phospho (STY)]PAT[Phospho (STY)]DQR_.3	Q3V3V9_S1284_M2_Carmil2	3.5723	3.7701	3.3060	4.3506	4.2169	4.1381	4.4989	4.1514	2.9496	4.0279	0.9963	-0.0054	0.9664	0.0148
Vezt	_TEYVC[Carbamidomethyl (C)]DS[Phospho (STY)]PTEGPSK_.2	Q3ZK22_S667_M1_Vezt	5.7812	5.3881	3.5996	3.6233	3.8095	4.8923	6.1737	3.9912	3.9304	3.4764	1.0279	0.0398	0.8624	0.0643
Bahd1	_DLS[Phospho (STY)]PELAPDEGAR_.2	Q497V6_S182_M1_Bahd1	3.6760	6.3319	4.3628	7.4732	3.6652	6.4062	3.7877	7.1260	6.7531	3.3316	1.0990	0.1362	0.6399	0.1939
Shank3	_AHS[Phospho (STY)]PQGEGEIPLHR_.3	#N/A	10.5556	10.3958	11.1095	11.0313	10.0593	10.5172	10.8409	10.4824	10.4761	10.5845	1.0015	0.0021	0.9440	0.0250
Shank3	_S[Phospho (STY)]LGEEPVGGLGSLLDPAK_.2	#N/A	10.0030	9.9509	10.5444	10.4347	9.0975	9.8738	9.6595	10.0443	9.8699	9.7120	1.0166	0.0237	0.5647	0.2482
Shank3	_S[Phospho (STY)]KLWGDPVESR_.2	Q4ACU6_S1471_M1_Shank3	10.0562	9.2367	10.8000	10.3405	9.5090	10.1738	10.0778	10.1193	10.3136	10.0741	0.9875	-0.0181	0.6766	0.1696
Shank3	_STS[Phospho (STY)]M[Oxidation (M)]QDTVR_.2	#N/A	7.7749	7.2808	8.3514	7.8592	3.0817	3.4453	8.7633	3.8475	3.9096	9.2886	0.9762	-0.0347	0.9300	0.0315
Shank3	_S[Phospho (STY)]M[Oxidation (M)]TAELEELASIR_.2	#N/A	5.7539	5.1774	5.6524	5.6318	5.1782	5.6776	4.2814	3.6085	5.9287	5.9033	1.1179	0.1608	0.2619	0.5818
Shank3	_SAS[Phospho (STY)]DINLKGDQPAASPGPTLR_.3	#N/A	3.2060	8.2898	8.5433	9.5587	7.8234	8.4175	8.0956	9.0141	8.8333	8.6655	0.8830	-0.1795	0.4070	0.3904
Shank3	_RAPSPVKPAS[Phospho (STY)]LER_.3	Q4ACU6_S1583_M1_Shank3	3.5989	4.4041	4.2857	3.2875	4.2255	3.5040	3.8648	6.5928	2.9787	3.2545	0.9309	-0.1033	0.6903	0.1610
Shank3	_S[Phospho (STY)]KSM[Oxidation (M)]TAELEELASIR_.3	#N/A	3.8421	5.6510	4.0439	5.7185	4.7634	5.8280	4.0830	6.0488	3.6898	3.7725	1.1309	0.1775	0.3882	0.4110
Shank3	_TKS[Phospho (STY)]VGEDEKLASLLEGR_.3	#N/A	3.2281	9.5667	9.8983	10.4129	8.8689	9.7114	9.6341	9.7305	9.7397	9.8519	0.8845	-0.1770	0.4358	0.3607
Shank3	_FM[Oxidation (M)]DY[Phospho (STY)]VQLHSTDK_.2	#N/A	6.0520	5.9455	6.6283	7.1096	5.4873	6.3527	6.7058	6.5246	6.2264	6.5312	0.9639	-0.0530	0.4565	0.3405
Shank3	_LFSSLGELSTISAQR_.2	#N/A	5.0026	4.9442	3.5285	4.9818	5.2753	5.5154	3.7459	3.8816	5.4199	4.4767	1.1127	0.1540	0.3197	0.4952
Shank3	_S[Phospho (STY)]LGEEPVGGLGSLLDPAKK_.2	#N/A	4.0792	3.3835	3.2651	3.7678	3.2049	3.0238	3.3846	4.3507	5.8224	3.7348	0.7990	-0.3238	0.1020	0.9914
Shank3	_APS[Phospho (STY)]TTLTLR_.2	Q4ACU6_S685_M1_Shank3	3.3135	4.0289	4.5480	4.0918	3.9581	4.1674	4.2401	4.4102	3.2084	3.7691	1.0284	0.0404	0.7172	0.1444
Shank3	_HYTVGSY[Phospho (STY)]DSLTSHSDYVIDDK_.3	Q4ACU6_Y555_M1_Shank3	3.6487	3.5775	4.3355	2.0186	4.2752	3.4543	4.5528	1.5535	4.4297	3.3043	1.0265	0.0377	0.8986	0.0464
Shank3	_SAS[Phospho (STY)]DINLK_.2	#N/A	4.2121	3.0140	3.3981	3.9007	3.3378	3.7223	3.6154	4.2177	3.8663	3.8677	0.9244	-0.1134	0.2571	0.5899
Shank3	_LDEILAVAAEPTLRPDIADADSR_.3	#N/A	3.2740	4.0303	4.5085	4.3853	4.2934	4.2069	4.1000	3.8117	3.3817	4.3523	1.0524	0.0737	0.4848	0.3145
Shank3	_GPRPGSLDY[Phospho (STY)]SSGEGLGLTFGGPSPGPVK_.3	Q4ACU6_Y998_M1_Shank3	4.3792	3.6347	4.2783	5.4524	3.5230	3.1017	4.4957	3.3375	2.9861	3.2471	1.1550	0.2078	0.3085	0.5108
Shank3	_SPTPVHS[Phospho (STY)]PDADRPGPLFVDVQTR_.3	Q4ACU6_S1134_M1_Shank3	3.6980	4.6055	4.3848	5.3615	3.4164	3.2082	3.1013	3.2310	4.3804	3.3537	1.1694	0.2258	0.2474	0.6066
Prpf38a	_VPS[Phospho (STY)]PDHR_.2	Q4FK66_S209_M1_Prpf38a	8.6500	8.3218	8.8631	8.2993	7.0321	8.2445	8.9060	8.6168	8.5580	8.3187	0.9576	-0.0625	0.3127	0.5049
Fam189a2	_SKS[Phospho (STY)]DPVLHHSEER_.3	Q4FZH1_S114_M1_Fam189a2	5.2277	5.2259	4.2422	3.7601	4.1819	3.5476	5.5974	5.5967	3.5401	3.2110	0.9728	-0.0398	0.8506	0.0703
Abl2	_DKS[Phospho (STY)]PSSLLEDAK_.2	Q4JIM5_S632_M1_Abl2	3.4776	4.2828	4.1644	3.1662	4.1041	3.6254	4.3817	7.6211	7.1118	7.4001	0.5738	-0.8014	0.0022	2.6504
Abl2	_STQASSGS[Phospho (STY)]PALPR_.2	Q4JIM5_S621_M1_Abl2	8.0352	8.0425	4.0160	7.7277	3.9557	7.9052	8.3244	8.0015	7.9774	8.2283	0.8132	-0.2983	0.1832	0.7371
Abl2	_S[Phospho (STY)]NSTSSM[Oxidation (M)]SSGLPEQDR_.2	Q4JIM5_S783_M1_Abl2	6.1709	4.0579	4.2070	4.3146	3.9195	4.1284	4.1414	3.3213	3.3422	3.3652	1.2608	0.3343	0.0792	1.1010
Abl2	_TVS[Phospho (STY)]TSSQPEENVDR_.2	Q4JIM5_S819_M1_Abl2	3.6251	4.3813	3.3587	7.2977	4.2696	3.8558	3.7490	7.0056	3.7328	6.9183	0.8343	-0.2614	0.4185	0.3783
Abracl	_[Acetyl (Protein N-term)]M[Oxidation (M)]NVEHEVNLLVEEIHR_.3	#N/A	5.5603	5.8121	5.4480	4.9608	5.1858	5.7517	5.5625	5.2909	5.7318	5.4372	0.9905	-0.0138	0.7823	0.1066
Oxr1	_SQS[Phospho (STY)]VDITAPGFNPLGGAGK_.2	Q4KMM3_S15_M1_Oxr1	6.6849	7.2509	7.9524	9.0732	6.1889	6.8813	6.7808	7.8657	7.4311	6.2377	1.0367	0.0520	0.6760	0.1700
Oxr1	_ESLPIELDQLS[Phospho (STY)]GR_.2	#N/A	7.8398	7.4994	7.8522	8.3053	7.4954	7.6873	8.2323	7.9233	8.1777	7.7969	0.9685	-0.0461	0.1846	0.7337
Oxr1	_DTIQQVSQR_.2	#N/A	7.2174	7.4314	7.5132	7.7898	7.5399	6.7930	7.0709	7.0598	6.9757	7.2660	1.0406	0.0574	0.1510	0.8211
Oxr1	_GTIEYTVES[Phospho (STY)]R_.2	Q4KMM3_S104_M1_Oxr1	8.2756	7.9500	8.2047	8.1800	7.5134	8.0181	8.6743	8.0882	8.3307	8.1830	0.9645	-0.0522	0.1289	0.8897
Oxr1	_TSSNPGALSHETGLSGLETATK_.3	#N/A	5.5740	5.4807	6.0315	5.6363	5.5070	5.3751	5.7714	5.1505	5.0034	4.9190	1.0748	0.1040	0.0769	1.1142
Oxr1	_DSETEVEELRK_.2	#N/A	5.3811	5.5702	5.4216	6.2332	5.6783	5.8721	6.2970	5.4821	5.6801	5.8757	0.9758	-0.0353	0.5273	0.2780
Oxr1	_DQGNDS[Phospho (STY)]ASTAPR_.2	#N/A	5.3476	5.5680	5.5286	4.6764	3.7851	4.1944	4.8523	4.0221	5.4314	4.2578	1.0451	0.0636	0.6597	0.1807
Oxr1	_DQQGQELKR_.2	#N/A	5.1319	4.5815	4.8908	4.6099	5.4647	4.5420	4.4464	4.0213	5.2444	5.1165	1.0346	0.0491	0.5963	0.2246
Oxr1	_DQQGQELKRDS[Phospho (STY)]ETEVEELR_.3	#N/A	4.3237	5.7827	5.9098	4.0123	2.7908	3.8338	5.7508	4.1062	3.7548	3.9793	1.0101	0.0145	0.9519	0.0214
Oxr1	_RM[Oxidation (M)]S[Phospho (STY)]FQKPK_.2	#N/A	7.0293	6.0433	6.1498	7.6260	5.9085	6.5151	6.6628	6.2027	6.2306	6.3305	1.0061	0.0088	0.9254	0.0337
Oxr1	_VVSSTS[Phospho (STY)]EEEEAFTEK_.2	Q4KMM3_S204_M1_Oxr1	10.7136	12.0031	12.1623	12.3612	11.6456	12.0267	12.3487	11.8945	12.1303	12.1091	0.9751	-0.0364	0.3585	0.4455
Oxr1	_DSDGQVFGALASEPFK_.2	#N/A	6.7101	6.2709	7.1124	6.6148	5.7738	4.2647	6.7195	6.5433	6.0523	6.2555	0.9580	-0.0618	0.6276	0.2023
Oxr1	_TPIIEEEQNNSANTQK_.2	#N/A	6.1284	5.8809	6.1689	6.8627	6.2759	6.2866	6.5817	6.3059	5.9344	5.9830	1.0106	0.0153	0.7559	0.1215
Oxr1	_FDTTPNELVQLNK_.2	#N/A	6.1966	6.3305	6.2055	6.6653	5.8906	6.2403	6.4547	5.8739	6.1185	6.3148	1.0104	0.0149	0.7015	0.1539
Oxr1	_KATGVS[Phospho (STY)]AEDADPR_.2	Q4KMM3_S308_M1_Oxr1	6.1316	4.0301	3.9117	4.4144	3.8515	3.8780	4.1291	3.7041	3.3527	4.3814	1.1228	0.1670	0.3558	0.4488
Oxr1	_RDS[Phospho (STY)]ETEVEELRK_.3	#N/A	6.5781	3.8417	3.8332	6.6388	3.9218	3.5498	6.4142	3.7683	3.7015	3.6987	1.0754	0.1049	0.7269	0.1385
Oxr1	_ADLEPESFRPNLSDPSELLLPDQIEK_.3	#N/A	4.5091	4.9503	3.1673	4.3802	4.8262	4.4478	4.7874	4.5878	4.6833	3.6370	1.0081	0.0116	0.9288	0.0321
Oxr1	_M[Oxidation (M)]S[Phospho (STY)]FQKPK_.2	#N/A	3.3586	3.3492	3.5098	3.8511	3.4719	4.0709	3.3425	3.9346	3.7402	3.7346	0.9767	-0.0341	0.6437	0.1913
Oxr1	_IKESLPIELDQLSGR_.3	#N/A	5.3346	5.2290	5.2849	5.8391	5.1712	5.4981	5.0849	5.6741	5.0508	5.0002	1.0366	0.0518	0.3136	0.5036
Oxr1	_DSLNSIALK_.2	#N/A	3.8794	4.0389	3.8613	4.5663	4.1411	4.5998	3.5534	3.5961	3.8951	4.3473	1.0866	0.1198	0.1707	0.7678
Oxr1	_KS[Phospho (STY)]QSVDITAPGFNPLGGAGK_.3	#N/A	3.2278	4.0330	3.9146	4.4172	3.8543	3.8752	4.1319	3.7012	3.3498	4.3842	0.9988	-0.0018	0.9861	0.0061
Oxr1	_TFVSQASATM[Oxidation (M)]QQYAQR_.2	#N/A	4.0400	3.3309	4.3308	3.4810	4.2082	3.7424	4.1984	3.7909	4.6328	4.3594	0.9082	-0.1390	0.1552	0.8091
Oxr1	_SSDASSESVQTVSQMEVQSLTATSEAANVPDR_.3	#N/A	5.1172	4.7569	4.0292	7.8640	3.4026	3.7606	5.6138	3.5866	3.8261	2.9980	1.2036	0.2673	0.4103	0.3869
Oxr1	_ARDQGNDS[Phospho (STY)]ASTAPR_.2	#N/A	4.0033	3.2588	3.7370	3.2808	3.1471	3.4776	3.4290	3.7204	4.0195	2.9581	0.9865	-0.0196	0.8506	0.0703
Oxr1	_HEYWFAVPQER_.2	#N/A	3.3427	4.1479	4.0295	3.9728	3.9692	3.7603	4.6059	4.5421	3.2349	2.9983	1.0065	0.0094	0.9470	0.0236
Unc13a	_ALS[Phospho (STY)]PTGSSR_.2	Q4KUS2_S255_M1_Unc13a	12.0203	12.0856	12.6045	12.0626	11.1935	11.8851	12.4109	12.4179	12.0271	12.0419	0.9796	-0.0297	0.3445	0.4628
Unc13a	_ESYSDSM[Oxidation (M)]HS[Phospho (STY)]YEEFSEPR_.3	#N/A	3.6433	3.5217	4.1190	3.7675	3.3781	3.6378	3.4822	3.4422	4.1021	3.7345	0.9967	-0.0048	0.9459	0.0242
Trp53i11	_HS[Phospho (STY)]QTDLVSR_.2	#N/A	7.4012	7.2934	7.4947	6.8717	6.2445	6.9454	7.4193	7.2438	6.8452	6.7120	0.9981	-0.0027	0.9621	0.0168
Trp53i11	_KHS[Phospho (STY)]QTDLVSR_.2	#N/A	9.8596	9.9393	10.5125	10.0724	8.7415	9.6720	10.2746	10.4827	9.8147	9.7512	0.9721	-0.0408	0.4219	0.3747
Pds5b	_APS[Phospho (STY)]PSQPPKK_.2	Q4VA53_S1381_M1_Pds5b	9.4400	9.2408	9.8056	4.4969	7.8597	3.7955	9.7737	9.6720	9.2866	9.3121	0.7822	-0.3544	0.1643	0.7844
Pds5b	_TAS[Phospho (STY)]DSDEQQWPEEK_.2	Q4VA53_S1255_M1_Pds5b	9.5419	9.3674	3.2652	3.7678	3.0352	3.5894	10.1808	9.7392	9.3228	3.7348	0.6584	-0.6030	0.1958	0.7081
Pds5b	_M[Oxidation (M)]ETVSNASSSSNPSS[Phospho (STY)]PGR_.2	#N/A	5.6967	6.4694	4.1479	5.7780	5.3193	6.1637	6.7499	7.1164	6.3696	6.0230	0.8524	-0.2304	0.0658	1.1820
Pds5b	_AES[Phospho (STY)]PETSAVESTQSTPQK_.2	Q4VA53_S1356_M1_Pds5b	9.7836	9.9595	10.4775	10.1521	8.8256	9.4988	10.5007	10.2976	10.0426	9.6175	0.9672	-0.0481	0.3432	0.4644
Pds5b	_HKEELLENEDEQNS[Phospho (STY)]PPKK_.4	Q4VA53_S1281_M1_Pds5b	5.4368	3.1141	5.7415	3.5057	2.9355	5.0030	3.6540	4.0746	3.7945	5.2631	1.0221	0.0316	0.8975	0.0470
Phactr4	_SSS[Phospho (STY)]PVLVEEEPER_.2	Q501J7_S118_M1_Phactr4	9.6341	9.9905	10.5622	10.4245	9.7709	10.0051	10.4029	9.7163	9.9462	9.9642	1.0057	0.0082	0.7990	0.0975
Lrrc47	_QHRES[Phospho (STY)]GEGEEEVADSAR_.3	#N/A	5.7545	5.0724	5.6845	3.3787	4.7026	5.2755	5.2824	4.4737	5.3620	5.2846	0.9760	-0.0351	0.8035	0.0950
Lrrc47	_KRQS[Phospho (STY)]VSGLHR_.3	#N/A	3.2052	4.7297	5.2177	3.9835	3.8498	4.2756	4.1318	3.0176	3.3167	3.6608	1.1921	0.2535	0.1334	0.8750

Lrrc47	_RQS[Phospho (STY)]VSGLHR_.2	#N/A	3.4310	4.1873	3.1647	4.2093	3.6429	4.0499	4.3576	4.2347	3.7626	3.8866	0.9312	-0.1029	0.2922	0.5343
Lrrc47	_KQHRES[Phospho (STY)]GEGEEEVADSAR_.3	#N/A	3.8320	4.0564	3.8005	3.3443	3.5175	3.8302	3.3321	3.6604	4.2712	3.9248	0.9824	-0.0257	0.7515	0.1240
Kctd8	_IVS[Phospho (STY)]PKQEHEDR_.2	#N/A	5.1231	5.8660	6.1942	5.7096	5.4992	5.8103	5.5255	5.7578	5.2468	5.7750	1.0223	0.0318	0.5694	0.2446
Kctd8	_RNS[Phospho (STY)]ELFQSLISK_.2	Q50H33_S413_M1_Kctd8	3.5439	3.6823	4.2307	6.0850	4.1704	3.0541	4.4481	4.8585	3.0337	3.1995	1.0625	0.0874	0.7165	0.1448
C2cd3	_SDVGTSELPS[Phospho (STY)]EDDGVEPSR_.2	Q52K86_S453_M1_C2cd3	4.0457	3.3501	3.2317	3.7344	4.4186	3.0572	3.4490	5.5480	5.4186	4.6928	0.7619	-0.3924	0.0401	1.3970
Ccnl1	_EVKAEEKS[Phospho (STY)]PVSINVK_.3	Q52KE7_S358_M1_Ccnl1	4.0543	4.3749	5.8780	3.3318	2.9536	3.1192	5.5694	3.6694	4.0680	4.1343	0.9064	-0.1419	0.5459	0.2629
Ccnl1	_QQAS[Phospho (STY)]KSPYNGVR_.3	Q52KE7_S378_M1_Ccnl1	4.3230	3.5784	4.3346	3.3364	3.4667	3.1579	4.5519	3.2812	4.4307	3.3034	0.9506	-0.0731	0.6239	0.2049
Spire1	_LRPVS[Phospho (STY)]PEEIRR_.3	Q52KF3_S229_M1_Spire1	7.4014	7.6752	7.6734	7.8533	5.3721	7.3060	7.4920	7.4432	7.4146	6.9114	0.9861	-0.0202	0.8386	0.0765
Spire1	_S[Phospho (STY)]VDKSDEELQFPK_.2	#N/A	6.4050	6.2584	3.9976	7.0972	5.4915	6.4499	6.6211	6.6091	6.7164	6.8707	0.8875	-0.1722	0.2118	0.6740
Spire1	_RHS[Phospho (STY)]IEKETPTNVR_.3	#N/A	4.6385	5.9833	6.8341	6.3730	5.3158	3.3315	5.8240	6.1389	6.0732	6.2156	0.8928	-0.1637	0.3515	0.4540
Spire1	_SRS[Phospho (STY)]VDKSDEELQFPK_.3	#N/A	5.4943	5.7867	5.9028	4.0390	4.2664	5.6548	4.1872	6.2973	5.6703	5.8706	0.9427	-0.0852	0.5839	0.2337
Spire1	_APTLAELDSS[Phospho (STY)]DS[Phospho (STY)]EEEKSLHK_.3	Q52KF3_S306_M2_Spire1	3.9152	3.5703	3.7628	6.9615	3.4818	3.5741	3.6632	3.8869	3.9454	3.8700	1.0962	0.1325	0.6088	0.2155
Spire1	_HS[Phospho (STY)]IEKETPTNVR_.3	#N/A	3.5432	3.7992	3.2769	4.3215	3.6205	4.1090	4.4698	4.1805	2.9787	3.9988	0.9671	-0.0483	0.7007	0.1545
Srrm1	_RLS[Phospho (STY)]PS[Phospho (STY)]AS[Phospho (STY)]PPR_.2	Q52KI8_S387_M3_Srrm1	11.5479	11.7288	12.1060	11.9636	11.0720	11.5976	12.2089	11.8911	11.8214	11.7003	0.9802	-0.0289	0.2805	0.5520
Srrm1	_TAS[Phospho (STY)]PPPPPKR_.2	#N/A	11.1228	11.2746	11.7292	11.3754	10.6880	11.0708	11.6065	11.4242	11.2898	11.0807	0.9876	-0.0179	0.4977	0.3030
Srrm1	_RRT[Phospho (STY)]PS[Phospho (STY)]PPPR_.2	#N/A	11.5099	11.6193	11.9279	11.8216	11.1270	11.5000	11.9027	11.6645	11.5784	11.4097	0.9953	-0.0068	0.7497	0.1251
Srrm1	_APQTSS[Phospho (STY)]PPPVR_.2	#N/A	11.5510	11.6499	12.2535	11.9964	10.9974	11.5804	12.0426	11.8448	11.5129	11.6253	0.9928	-0.0105	0.7303	0.1365
Srrm1	_SRVS[Phospho (STY)]VS[Phospho (STY)]PGR_.2	#N/A	11.1763	11.1682	11.5559	11.3475	10.5991	11.1070	11.5607	11.2858	11.2589	11.2309	0.9846	-0.0225	0.3432	0.4644
Srrm1	_AAS[Phospho (STY)]PSPQSVR_.2	Q52KI8_S779_M1_Srrm1	10.9804	11.0529	11.5786	11.3015	10.5373	10.9501	11.5331	11.3405	11.0601	11.1054	0.9829	-0.0249	0.3616	0.4418
Srrm1	_RVS[Phospho (STY)]HS[Phospho (STY)]PPPK_.2	#N/A	11.5227	11.2524	11.1718	11.6487	10.3444	11.1659	11.8167	11.5498	11.3414	11.4609	0.9690	-0.0454	0.1840	0.7351
Srrm1	_RRS[Phospho (STY)]PT[Phospho (STY)]PPPR_.2	#N/A	10.1800	10.3727	10.6356	10.6713	10.5847	10.3062	10.7028	10.1435	10.2953	11.0872	0.9906	-0.0136	0.6290	0.2013
Srrm1	_S[Phospho (STY)]PS[Phospho (STY)]PPPAR_.2	#N/A	11.1430	10.5641	10.7587	8.7962	9.5400	10.6420	11.0148	10.0212	10.8844	10.8838	0.9351	-0.0968	0.1556	0.8079
Srrm1	_RRS[Phospho (STY)]PS[Phospho (STY)]PPPAR_.2	#N/A	9.6687	9.7074	10.2225	9.9385	9.1388	9.6144	10.1801	9.8619	9.7511	9.6383	0.9855	-0.0210	0.5080	0.2942
Srrm1	_RYS[Phospho (STY)]PPIQR_.2	Q52KI8_S616_M1_Srrm1	10.8878	10.7303	11.3664	11.2660	3.1045	10.5307	11.1282	11.1652	10.8327	10.6826	0.8809	-0.1830	0.4510	0.3459
Srrm1	_WQS[Phospho (STY)]PVTK_.2	#N/A	10.0516	10.0019	10.4497	10.2874	9.4753	10.1738	10.3876	10.2161	10.3287	10.0371	0.9835	-0.0240	0.3787	0.4217
Srrm1	_S[Phospho (STY)]PT[Phospho (STY)]PPPR_.2	#N/A	10.5500	10.0405	8.6993	10.3662	7.2418	10.1887	8.7138	9.1918	7.8455	8.7784	1.1022	0.1404	0.2411	0.6178
Srrm1	_RRS[Phospho (STY)]PS[Phospho (STY)]LSSK_.2	Q52KI8_S674_M2_Srrm1	7.2179	7.3116	7.8020	7.6581	7.2160	5.1778	8.0065	7.6425	7.6563	7.4670	0.9182	-0.1231	0.2400	0.6199
Srrm1	_RAS[Phospho (STY)]PS[Phospho (STY)]PPPK_.2	#N/A	8.9301	8.9171	8.6028	8.1515	6.5675	8.7828	9.2497	8.9937	8.6050	8.6389	0.9384	-0.0917	0.2869	0.5423
Srrm1	_S[Phospho (STY)]PELPEPSVR_.2	#N/A	9.2831	8.6569	4.3470	8.0661	3.7570	8.8910	9.2816	8.7042	8.8737	8.4438	0.8120	-0.3004	0.2241	0.6496
Srrm1	_S[Phospho (STY)]RS[Phospho (STY)]YS[Phospho (STY)]PR_.2	Q52KI8_S304_M3_Srrm1	8.2710	3.4884	6.5039	6.9234	6.0144	3.9766	6.8999	7.3808	7.6752	3.1696	0.9334	-0.0995	0.7455	0.1276
Srrm1	_AAS[Phospho (STY)]PS[Phospho (STY)]PQSVRR_.2	#N/A	4.0584	8.2692	8.9995	8.7619	3.0560	8.5973	9.0482	8.8504	8.7167	8.3450	0.7960	-0.3292	0.2275	0.6430
Srrm1	_KAQVS[Phospho (STY)]PQS_.2	Q52KI8_S943_M1_Srrm1	7.5633	7.8277	7.9604	7.7504	6.1479	3.5364	8.0792	7.8990	7.4991	7.6820	0.8726	-0.1965	0.2966	0.5279
Srrm1	_AKS[Phospho (STY)]PT[Phospho (STY)]PSLS[Phospho (STY)]PAR_.2	Q52KI8_S832_M3_Srrm1	7.0801	6.8784	3.8852	7.6368	6.3847	7.0933	7.4969	7.6420	7.3999	6.3648	0.8986	-0.1543	0.3394	0.4692
Srrm1	_RWQS[Phospho (STY)]PVTK_.2	#N/A	6.3429	3.1503	3.2618	3.7645	3.0386	3.5860	3.4792	4.3540	4.0026	3.7315	0.9911	-0.0128	0.9593	0.0181
Srrm1	_QRS[Phospho (STY)]PTVTK_.2	Q52KI8_S664_M1_Srrm1	5.7601	3.8427	6.0393	5.8799	5.2959	5.4788	6.1371	6.0456	3.0222	3.9553	1.1237	0.1683	0.4444	0.3523
Srrm1	_S[Phospho (STY)]PS[Phospho (STY)]PPPARR_.2	#N/A	8.0246	7.4428	7.8115	8.7597	6.6206	8.0010	8.9137	8.2755	8.5008	7.7433	0.9304	-0.1041	0.1946	0.7108
Srrm1	_GAS[Phospho (STY)]AS[Phospho (STY)]PQGRQS[Phospho (STY)]PSPSTRPIR_.3	#N/A	6.0037	5.6533	5.6572	5.5087	4.9808	5.9262	6.4731	5.8142	5.2120	5.8537	0.9629	-0.0545	0.4549	0.3421
Srrm1	_RS[Phospho (STY)]PT[Phospho (STY)]PPPR_.2	#N/A	8.5925	8.6750	3.1302	8.6116	8.2261	9.0995	8.2805	9.3914	7.5849	8.5823	0.9128	-0.1316	0.5541	0.2564
Srrm1	_RS[Phospho (STY)]PS[Phospho (STY)]PPPAR_.2	#N/A	7.1301	7.0823	7.6714	7.1150	6.5068	7.4231	7.6585	6.6774	7.3366	6.3731	1.0204	0.0292	0.6530	0.1851
Srrm1	_APKPEPVPEPKEPS[Phospho (STY)]PEK_.3	#N/A	7.6754	7.5376	8.0785	7.5947	7.0758	7.4756	8.4454	7.5713	7.4968	7.3212	0.9824	-0.0256	0.6136	0.2121
Srrm1	_RVS[Phospho (STY)]HS[Phospho (STY)]PPPKQR_.3	#N/A	6.2656	7.3533	7.1987	7.6672	4.2720	7.0516	5.6005	6.6741	7.7386	7.1811	0.9759	-0.0352	0.8284	0.0818
Srrm1	_SVS[Phospho (STY)]GS[Phospho (STY)]PEPAAK_.2	Q52KI8_S795_M2_Srrm1	3.6503	3.9722	3.8205	3.5015	4.1712	5.1453	3.6498	5.0742	3.7749	3.6058	1.0043	0.0062	0.9673	0.0145
Srrm1	_TAS[Phospho (STY)]PPPPPK_.2	#N/A	9.7550	9.7652	10.2960	10.0111	9.0240	9.7765	10.2400	9.9715	9.6434	9.8206	0.9851	-0.0216	0.5517	0.2583
Srrm1	_AS[Phospho (STY)]PS[Phospho (STY)]PPPKR_.2	#N/A	7.3162	8.0193	4.4113	6.5753	2.8502	6.8939	8.9894	8.3829	7.7067	8.1685	0.7232	-0.4676	0.0553	1.2571
Srrm1	_RRS[Phospho (STY)]PS[Phospho (STY)]PAPPPPPPPPPR_.3	#N/A	9.5320	10.7255	8.5926	11.5983	8.1131	9.7212	11.1158	11.0482	9.5679	8.7250	0.9604	-0.0583	0.6335	0.1983
Srrm1	_VSHS[Phospho (STY)]PPPK_.2	Q52KI8_S657_M1_Srrm1	6.5454	3.5575	3.4391	6.5244	3.3789	6.3986	6.7579	4.1767	6.1769	6.4971	0.8427	-0.2468	0.3652	0.4375
Srrm1	_KETES[Phospho (STY)]EAEDDNLDDLER_.2	#N/A	3.9117	7.0347	3.6734	7.7667	6.3198	6.8284	6.3406	7.5390	7.3115	7.3667	0.8295	-0.2696	0.2139	0.6698
Srrm1	_S[Phospho (STY)]PSAPPPPPPPPPR_.2	Q52KI8_S572_M1_Srrm1	4.0671	9.7942	3.8008	10.6982	8.3022	10.3972	10.6111	10.8437	10.4895	10.2984	0.7427	-0.4292	0.1289	0.8899
Srrm1	_RES[Phospho (STY)]PS[Phospho (STY)]PAPKPR_.2	#N/A	8.4983	8.4929	8.7192	7.8715	7.8237	8.2259	8.8741	8.7735	8.6067	8.3192	0.9570	-0.0634	0.1137	0.9444
Srrm1	_EKS[Phospho (STY)]PELPEPSVR_.2	#N/A	10.5625	10.8615	11.4347	11.0854	10.0632	10.7037	11.4758	10.8973	10.7825	10.6777	0.9842	-0.0230	0.5497	0.2599
Srrm1	_RHS[Phospho (STY)]PS[Phospho (STY)]PRPR_.2	#N/A	7.0328	6.7662	7.1572	6.8239	5.1843	6.0337	7.3652	7.2656	6.5191	6.6132	0.9364	-0.0947	0.3259	0.4869
Srrm1	_QS[Phospho (STY)]PSPSTRPIR_.2	Q52KI8_S731_M1_Srrm1	3.6183	6.4949	6.7512	7.2955	5.9337	3.6431	6.8052	6.9782	7.0559	6.8788	0.8114	-0.3015	0.1496	0.8251
Srrm1	_RYS[Phospho (STY)]PS[Phospho (STY)]PPPK_.2	#N/A	12.7636	12.8433	13.1373	13.1192	12.2190	12.7988	13.3578	12.9629	12.7889	12.7569	0.9882	-0.0171	0.4704	0.3275
Srrm1	_Y[Phospho (STY)]SPPIQR_.2	Q52KI8_Y615_M1_Srrm1	4.2429	3.5472	3.5288	4.5735	3.3686	4.3609	3.2209	3.9286	4.2276	4.2507	1.0077	0.0110	0.9284	0.0323
Srrm1	_S[Phospho (STY)]RS[Phospho (STY)]PS[Phospho (STY)]PAPEKK_.2	Q52KI8_S207_M3_Srrm1	5.6822	3.3506	4.9034	5.6439	3.0013	3.3857	5.1245	5.5150	4.2028	5.5403	0.8493	-0.2356	0.2863	0.5432
Srrm1	_RVS[Phospho (STY)]RT[Phospho (STY)]PEPK_.2	#N/A	9.0382	9.0662	9.5513	9.3695	8.2355	9.1807	9.4429	9.2302	9.2615	9.0620	0.9810	-0.0276	0.4850	0.3143
Srrm1	_TRHS[Phospho (STY)]PTPQQSNR_.3	#N/A	6.4567	6.8163	6.5789	7.2695	3.3374	5.7545	7.0795	7.1236	3.8417	6.3453	0.9898	-0.0147	0.9493	0.0226
Srrm1	_RES[Phospho (STY)]PSPAPK_.2	#N/A	3.3980	3.8282	4.0848	4.5874	4.0245	3.7050	4.3021	3.5310	3.1796	3.0536	1.1198	0.1633	0.2008	0.6973
Srrm1	_RAS[Phospho (STY)]PS[Phospho (STY)]PPPKR_.2	#N/A	11.3125	11.7348	11.7675	11.8512	10.2390	11.6207	12.0054	11.7618	11.6368	11.0846	0.9827	-0.0252	0.5777	0.2383
Srrm1	_KSRVS[Phospho (STY)]VS[Phospho (STY)]PGR_.2	#N/A	8.2097	8.3462	4.1114	8.3889	7.8261	8.3193	8.7106	8.4476	3.7259	8.1971	1.0362	0.0513	0.8414	0.0750
Srrm1	_RRT[Phospho (STY)]AS[Phospho (STY)]PPPPPK_.2	#N/A	7.7376	7.7144	8.2267	7.9324	7.4761	7.3525	8.3203	5.7527	7.3444	7.6397	1.0655	0.0915	0.3302	0.4812
Srrm1	_RYS[Phospho (STY)]PS[Phospho (STY)]PPPKR_.2	#N/A	8.3828	3.5707	9.2698	9.6292	9.0738	4.3374	3.6697	4.1635	9.3297	8.7689	1.1380	0.1864	0.6345	0.1976
Srrm1	_S[Phospho (STY)]PS[Phospho (STY)]PAPPPPPPPPPR_.3	#N/A	3.9676	6.6212	3.7012	6.7995	3.1113	3.5133	6.9876	6.3337	5.0976	6.3147	0.7470	-0.4208	0.1187	0.9255
Srrm1	_RRAS[Phospho (STY)]PS[Phospho (STY)]PPPK_.2	#N/A	5.3480	6.9189	7.0583	6.8859	5.1652	5.1670	6.6475	7.1765	6.6897	6.5857	0.8990	-0.1536	0.2056	0.6870
Srrm1	_KVELS[Phospho (STY)]ES[Phospho (STY)]EEDKGSK_.2	Q52KI8_S461_M2_Srrm1	6.0405	6.1436	6.1943	6.4537	4.2774	6.3543	6.7379	6.5634	6.1678	5.9622	0.9297	-0.1052	0.3376	0.4717
Srrm1	_HRPSS[Phospho (STY)]PAT[Phospho (STY)]PPPK_.2	Q52KI8_S401_M2_Srrm1	10.0487	4.1494	11.4164	4.1454	4.0104	9.5487	10.6569	4.1714	10.1043	10.6536	0.8115	-0.3013	0.4614	0.3359
Srrm1	_KPPAPPS[Phospho (STY)]PVQSQS[Phospho (STY)]PSTNWSPAVPAK_.3	Q52KI8_S810_M2_Srrm1	4.2612	3.5655	3.4471	4.5552	3.3869	4.3426	3.2026	3.9469	4.7566	4.2324	0.9732	-0.0392	0.7759	0.1102
Srrm1	_KPPAPPS[Phospho (STY)]PVQS[Phospho (STY)]QSPSTNWS[Phospho (STY)]PAVPAKK_.4	Q52KI8_S814_M3_Srrm1	2.9690	3.7504	3.9503	3.8709	3.6044	3.6693	4.4502	3.8440	3.0154	3.7275	0.9671	-0.0482	0.6848	0.1644
Srrm1	_APQTSS[Phospho (STY)]PPPVRR_.2	#N/A	3.5661	3.7763	3.2997	4.3443	3.5977	4.1318	4.4926	4.1577	2.9559	10.6923	0.6791	-0.5582	0.2335	0.6318
Srrm1	_RS[Phospho (STY)]PS[Phospho (STY)]PAPPPPPPPPPR_.3	#N/A	3.7513	3.5911	3.4850	5.7203	4.6950	5.3442	6.1617	3.9724	5.7348	6.0916	0.8071	-0.3092	0.1353	0.8688
Srrm1	_LS[Phospho (STY)]PSAS[Phospho (STY)]PPR_.2	#N/A	3.6908	2.9462	3.4245	3.9686	4.8539	3.7901	3.6832	4.1499	3.7985	5.8005	0.8671	-0.2057	0.2853	0.5446
Srrm1	_VS[Phospho (STY)]VS[Phospho (STY)]PGR_.2	#N/A	3.1619	3.5994	3.8487	4.3513	3.7884	3.9411	4.0660	3.7672	5.3500	4.3183	0.8643	-0.2103	0.1169	0.9321
Srrm1	_RGAS[Phospho (STY)]AS[Phospho (STY)]PQGR_.2	Q52KI8_S723_M2_Srrm1	3.8969	4.4916	3.6306	4.4850	3.0407	3.5839	3.4771	4.3561	4.0047	4.3525	0.9524	-0.0704	0.5765	0.2392
Srrm1	_EARS[Phospho (STY)]PQPNK_.2	Q52KI8_S694_M1_Srrm1	4.2056	5.3046	5.9242	3.4830	3.3493	3.2753	5.6147	3.5181	3.8172	3.1603	1.0570	0.0799	0.7568	0.1210
Srrm1	_HS[Phospho (STY)]PS[Phospho (STY)]PRPR_.2	#N/A	3.7455	4.0232	3.8898	4.3924	3.8295	4.2139	4.5152	3.7261	3.3746	4.3594	1.0055	0.0079	0.9314	0.0309
Srrm1	_RRVS[Phospho (STY)]HS[Phospho (STY)]PPPK_.3	#N/A	3.1782	3.9834	3.8650	4.3677	3.5868	3.9247	4.2855	3.7508	3.3994	4.3347	0.9683	-0.0465	0.6570	0.1825
Srrm1	_RRS[Phospho (STY)]PS[Phospho (STY)]PR_.2	Q52KI8_S609_M2_Srrm1	2.9739	3.7791	3.6607	4.3416	3.6004	4.1291	4.4899	4.1604	2.9586	4.1303	0.9524	-0.0704	0.6161	0.2103
Prune2	_SSGQESESIPEYTAEEEREDNR_.3	#N/A	7.6984	7.6907	8.0704	6.9162	5.7985	7.8676	8.1650	8.0018	7.0317	6.7958	0.9789	-0.0308	0.7650	0.1163
Prune2	_SSGQESES[Phospho (STY)]IPEYTAEEER_.2	#N/A	3.4677	5.4245	4.1545	5.4478	4.5058	3.7576	3.9874	3.5836	5.3373	3.8494	1.0645	0.0902	0.6216	0.2065

Prune2	_NLSLTC[Carbamidomethyl (C)]FAGEEPSS[Phospho (STY)]PER_.2	Q52KR3_S594_M1_Prune2	4.0714	3.3757	3.2573	3.7600	3.1971	3.0316	3.4747	4.3585	4.0071	3.7270	0.8862	-0.1743	0.1139	0.9436
Prune2	_DTSLTS[Phospho (STY)]GSPTSDLGQTWNNSK_.2	Q52KR3_S876_M1_Prune2	3.3024	4.1043	3.8114	4.3141	3.9926	4.1356	4.0288	3.8072	3.4558	4.2811	1.0129	0.0185	0.8312	0.0803
Thrap3	_ASVSDLS[Phospho (STY)]PR_.2	Q569Z6_S243_M1_Thrap3	12.0729	12.1199	12.7605	12.2545	11.4287	11.9147	12.4661	12.2878	12.1136	11.9965	0.9898	-0.0147	0.6137	0.2120
Thrap3	_ERS[Phospho (STY)]PALK_.2	Q569Z6_S248_M1_Thrap3	10.1884	10.1604	10.6043	10.3451	9.1750	9.7866	10.5869	10.4223	10.3518	10.0395	0.9704	-0.0434	0.2916	0.5351
Thrap3	_S[Phospho (STY)]REEEWDPEYTPK_.2	Q569Z6_S860_M1_Thrap3	9.0006	9.2613	9.6000	9.8604	8.0200	9.2085	9.5813	9.5316	9.3635	9.0018	0.9775	-0.0329	0.5527	0.2575
Thrap3	_NKKS[Phospho (STY)]PEIHR_.2	Q569Z6_S669_M1_Thrap3	8.9326	9.1329	9.8024	9.3894	7.0828	8.6577	9.6540	9.4189	8.8556	8.9790	0.9573	-0.0629	0.4563	0.3407
Thrap3	_IDIS[Phospho (STY)]PSTFR_.2	#N/A	11.1902	10.8797	11.4517	11.3036	10.1150	3.0004	11.6336	11.1950	11.0612	11.0302	0.8599	-0.2177	0.3779	0.4227
Thrap3	_ERS[Phospho (STY)]PALKS[Phospho (STY)]PLQSVVVR_.3	Q569Z6_S248_M2_Thrap3	5.6680	7.9816	6.4077	9.3912	7.3076	7.3350	7.7574	8.4973	8.4018	7.2797	0.9204	-0.1197	0.3871	0.4121
Thrap3	_S[Phospho (STY)]YS[Phospho (STY)]PAHNR_.2	Q569Z6_S55_M2_Thrap3	3.1565	3.8654	4.1188	7.1725	3.7912	6.3353	7.5609	7.1156	6.2925	4.0348	0.7583	-0.3992	0.1808	0.7428
Thrap3	_M[Oxidation (M)]DS[Phospho (STY)]FDEDLAR_.2	#N/A	5.4259	7.4003	6.8230	7.9484	6.3356	7.2915	8.0136	7.6919	7.5387	7.4647	0.8950	-0.1601	0.1219	0.9139
Thrap3	_FSGEEGEIEDDESGT[Phospho (STY)]ENREEK_.3	Q569Z6_T937_M1_Thrap3	3.2269	4.0207	3.8010	4.5489	3.7074	3.5575	4.3478	3.8634	3.5805	3.6825	0.9850	-0.0219	0.8320	0.0799
Thrap3	_IDIS[Phospho (STY)]PSTFRK_.2	#N/A	4.3588	3.6142	4.0925	3.3006	3.5025	6.5184	4.5161	7.2281	6.4136	3.3135	0.7882	-0.3433	0.2556	0.5924
Thrap3	_S[Phospho (STY)]PRPSPVPK_.2	Q569Z6_S264_M1_Thrap3	4.0356	3.2991	3.5258	3.5298	3.1204	4.0136	3.4503	4.0902	4.0162	3.3771	0.9609	-0.0576	0.5595	0.2522
Thrap3	_M[Oxidation (M)]DS[Phospho (STY)]FDEDLARPSGLLAQER_.3	#N/A	3.6849	4.9376	5.3798	5.3094	4.2933	5.1823	3.7851	6.7107	5.5571	4.7497	0.9226	-0.1163	0.5199	0.2841
Thrap3	_S[Phospho (STY)]RS[Phospho (STY)]YS[Phospho (STY)]PAHNR_.2	Q569Z6_S51_M3_Thrap3	3.8651	5.2649	4.1317	3.3534	3.0002	3.4336	4.7038	3.5007	5.6954	5.4802	0.7929	-0.3348	0.1148	0.9400
Thrap3	_WAHDKFS[Phospho (STY)]GEEGEIEDDESGTENR_.3	#N/A	3.1024	4.1238	3.7892	4.2918	3.7289	4.1133	4.0065	3.8267	3.4752	4.2588	0.9914	-0.0125	0.8982	0.0466
Thrap3	_SS[Phospho (STY)]FSITR_.2	Q569Z6_S557_M1_Thrap3	3.4960	4.2522	3.2296	4.2742	4.1405	4.1642	3.8781	4.2278	3.0260	3.9515	1.0412	0.0582	0.6265	0.2031
Thrap3	_WAHDKFSGEEGEIEDDES[Phospho (STY)]GTENREEK_.4	#N/A	3.1244	4.0584	4.2151	3.9553	3.9467	3.7769	4.0998	3.7612	3.4098	3.8283	1.0189	0.0270	0.7591	0.1197
Thrap3	_FSGEEGEIEDDES[Phospho (STY)]GTENR_.2	#N/A	3.9554	3.2707	3.1414	3.6441	3.0811	3.1475	3.3587	2.9736	4.1230	3.6111	0.9593	-0.0600	0.5984	0.2230
Scn2b	_EQKLS[Phospho (STY)]TDDLKTEEEGK_.3	#N/A	5.4725	5.4581	3.4561	5.6836	4.4546	5.6444	4.9406	5.6870	5.5828	5.5858	0.9228	-0.1160	0.4021	0.3957
Scn2b	_EQKLS[Phospho (STY)]TDDLK_.2	#N/A	6.9846	6.8493	6.8571	6.9133	2.9764	6.7340	7.4380	7.1673	4.2277	6.3643	0.9873	-0.0185	0.9377	0.0279
Scn2b	_LS[Phospho (STY)]TDDLKTEEEGK_.2	#N/A	4.4346	4.2304	3.9380	4.1193	3.9856	4.1803	4.2676	4.4416	3.1968	3.7966	1.0567	0.0795	0.3784	0.4221
Scn2b	_YDVSVTLK_.2	#N/A	3.5441	3.6821	4.2309	3.2327	3.5704	3.0542	4.4482	3.3849	3.0335	1.8892	1.1140	0.1557	0.4577	0.3394
Gpkow	_KHS[Phospho (STY)]PDRQDGPVPK_.3	#N/A	5.9352	6.3395	6.7356	6.6624	5.9524	6.3197	6.1945	6.4281	6.1681	5.9752	1.0214	0.0306	0.4999	0.3011
Znf608	_DRHS[Phospho (STY)]PFSQR_.3	Q56A10_S1452_M1_Znf608	3.8575	3.1619	4.5443	3.4580	2.9832	3.2454	3.6062	3.5432	4.0786	3.5131	0.9610	-0.0573	0.6575	0.1821
Znf608	_APGS[Phospho (STY)]PGAGNPPGTPK_.2	Q56A10_S626_M1_Znf608	3.1841	3.9893	3.8709	4.3736	3.8107	3.9188	4.2796	3.7449	3.3935	4.3406	0.9793	-0.0302	0.7663	0.1156
Deptor	_STSFMSVS[Phospho (STY)]PSK_.2	Q570Y9_S265_M1_Deptor	4.1314	3.2110	3.8651	3.4089	3.0323	3.1964	3.5571	3.5923	3.8913	3.0862	0.9837	-0.0237	0.8280	0.0819
Prag1	_ANS[Phospho (STY)]LPAGTR_.2	Q571I4_S40_M1_Prag1	8.0993	7.9154	8.4144	8.5805	6.5658	8.0820	7.9744	7.9419	8.0165	8.1899	0.9891	-0.0159	0.8177	0.0874
Tab3	_TLVHSSS[Phospho (STY)]DGHIDPQHTAGK_.3	#N/A	4.3393	3.5549	3.8349	3.6598	5.5146	3.6897	3.7455	3.8547	4.2710	4.7738	0.9850	-0.0218	0.8865	0.0523
Tab3	_YLYM[Oxidation (M)]EYHS[Phospho (STY)]PEDNR_.2	#N/A	5.0378	3.9985	3.5288	4.3000	3.5676	5.9342	5.7816	4.0171	4.0425	4.1171	0.9788	-0.0309	0.8753	0.0579
Tab3	_SSSSGSDDY[Phospho (STY)]AYTQALLLHQR_.2	Q571K4_Y516_M1_Tab3	3.4367	3.9057	3.1704	4.2150	4.0813	4.0442	4.3633	4.2870	3.0852	3.8923	0.9749	-0.0367	0.7619	0.1181
Spag9	_ETDYPAGEELSES[Phospho (STY)]GQVDK_.2	Q58A65_S815_M1_Spag9	3.1488	4.9698	4.0986	4.1744	3.7832	4.6142	5.2327	4.8335	4.6396	5.0374	0.8370	-0.2566	0.0457	1.3404
Spag9	_SASQSS[Phospho (STY)]LDKLDQELKEQQK_.3	#N/A	6.9773	7.0405	4.3576	8.1201	6.6631	7.4924	7.6720	6.3855	7.8448	7.5130	0.9213	-0.1182	0.4368	0.3597
Spag9	_SASQSS[Phospho (STY)]LDKLDQELK_.2	#N/A	3.3351	5.2793	3.7124	7.4736	3.9689	5.6914	6.0983	5.6576	3.8259	6.8167	0.8769	-0.1896	0.4846	0.3146
Pex1	_SQS[Phospho (STY)]GEDESLNQPGPIK_.2	Q5BL07_S1212_M1_Pex1	8.3281	8.3156	8.4822	8.8089	7.4709	8.3799	8.5837	8.3251	8.3594	8.5464	0.9815	-0.0269	0.5209	0.2832
Pex1	_TPS[Phospho (STY)]QEGC[Carbamidomethyl (C)]QDLTQEQR_.2	Q5BL07_S1182_M1_Pex1	5.6350	5.6149	6.4042	4.1999	3.7421	6.0889	6.3143	4.3021	5.7751	3.8772	1.0422	0.0596	0.7719	0.1124
Tut7	_NTTEEVGS[Phospho (STY)]PAKEK_.2	Q5BLK4_S705_M1_Tut7	5.7917	6.0235	6.6792	5.6661	3.8049	5.3585	6.0292	6.4090	5.3337	5.8038	0.9423	-0.0857	0.5342	0.2723
Stxbp5l	_SSS[Phospho (STY)]ISS[Phospho (STY)]IDKDSK_.2	Q5DQR4_S819_M2_Stxbp5l	10.3510	10.3017	10.7223	10.3848	9.2631	10.0662	10.5692	10.5140	10.2645	10.1879	0.9805	-0.0284	0.4653	0.3323
Stxbp5l	_RLS[Phospho (STY)]SADVSK_.2	Q5DQR4_S770_M1_Stxbp5l	7.5525	3.7155	8.1461	7.1115	4.1372	7.4237	8.2057	8.0061	7.9484	3.1663	0.9292	-0.1060	0.7336	0.1345
Stxbp5l	_SLSGSTNTVSS[Phospho (STY)]EGVTK_.2	Q5DQR4_S598_M1_Stxbp5l	7.9334	7.3801	7.6599	7.7954	6.5125	7.7665	8.0884	7.3990	7.9165	7.1558	0.9827	-0.0251	0.6882	0.1623
Stxbp5l	_TSYQS[Phospho (STY)]LTELNDSPVPLELER_.2	Q5DQR4_S727_M1_Stxbp5l	4.1344	3.3898	4.5232	3.5250	3.2781	3.3465	3.2397	3.0927	4.2421	3.4920	1.0520	0.0731	0.5936	0.2265
Stxbp5l	_EEIQESLTSDYFQIC[Carbamidomethyl (C)]K_.2	#N/A	3.3238	4.2913	4.3991	4.9407	3.9503	3.0031	4.4692	3.6052	3.2538	3.8721	1.0486	0.0685	0.6711	0.1732
Stxbp5l	_SRSS[Phospho (STY)]S[Phospho (STY)]ISS[Phospho (STY)]IDKDSK_.2	#N/A	3.4375	6.2169	6.2876	4.3893	3.3432	3.8987	6.5053	6.4976	6.0794	4.2063	0.7893	-0.3413	0.1674	0.7763
Slc4a10	_FPSKSSPS[Phospho (STY)]_.2	Q5DTL9_S1118_M1_Slc4a10	3.1028	10.2068	10.7233	4.1188	9.1975	10.7804	4.0172	10.6388	11.5192	3.9804	1.0640	0.0895	0.8459	0.0727
Slc4a10	_S[Phospho (STY)]FADIGKK_.2	#N/A	6.6739	6.3766	7.5392	6.2770	5.1360	5.9955	6.6757	6.5669	6.7436	6.3306	0.9626	-0.0550	0.5645	0.2483
Slc4a10	_ENS[Phospho (STY)]TVDFSK_.2	Q5DTL9_S280_M1_Slc4a10	3.5806	6.0803	3.9027	3.8411	3.8096	3.6626	3.5558	3.6242	6.7935	6.5528	0.8080	-0.3076	0.2834	0.5477
Slc4a10	_NAGQVVS[Phospho (STY)]PQSAPAC[Carbamidomethyl (C)]AENK_.2	Q5DTL9_S261_M1_Slc4a10	6.7871	4.1592	4.0408	6.0066	3.9806	3.7489	4.1097	3.5750	3.2236	3.0097	1.3758	0.4603	0.0904	1.0439
Slc4a10	_GHTS[Phospho (STY)]PC[Carbamidomethyl (C)]GMK_.2	Q5DTL9_S297_M1_Slc4a10	3.4216	5.8148	4.1084	3.8939	4.0482	3.6813	4.0421	3.1073	3.4064	3.5711	1.1783	0.2367	0.2080	0.6820
Slc4a10	_ERDSGLEDGRES[Phospho (STY)]PSFDTPSQR_.4	#N/A	5.7158	5.3374	6.6183	4.7594	5.0521	5.5658	5.9885	6.2183	5.7959	5.5030	0.9373	-0.0934	0.3234	0.4902
Slc4a10	_DSGLEDGRES[Phospho (STY)]PSFDTPSQR_.3	#N/A	11.4462	10.9416	12.0516	11.2888	10.0259	11.0450	11.3791	11.2232	11.4708	11.2168	0.9833	-0.0243	0.5980	0.2233
Slc4a10	_DDPSVINIS[Phospho (STY)]DEM[Oxidation (M)]SK_.2	#N/A	3.7829	7.0222	4.0911	7.8429	5.8087	7.0473	7.5752	7.4126	7.6268	7.1736	0.7966	-0.3280	0.1166	0.9333
Slc4a10	_ESRFPSKS[Phospho (STY)]SPS_.2	Q5DTL9_S1115_M1_Slc4a10	3.8086	3.0640	3.3481	3.8508	2.9523	3.6723	3.5655	4.2677	3.9163	3.8178	0.8863	-0.1741	0.0885	1.0533
Slc4a10	_KQS[Phospho (STY)]EPNSMDK_.2	Q5DTL9_S247_M1_Slc4a10	5.5994	3.2139	3.1982	3.7009	3.1022	3.5224	4.9894	4.4176	4.4189	3.6679	0.8512	-0.2324	0.2533	0.5964
Slc4a10	_NDVS[Phospho (STY)]RENSTVDFSK_.2	Q5DTL9_S276_M1_Slc4a10	3.0290	3.7852	4.1321	4.0328	3.6735	3.8543	4.6006	3.4881	3.1367	3.1009	1.0473	0.0667	0.6329	0.1987
Slc4a10	_S[Phospho (STY)]FADIGK_.2	#N/A	3.7447	3.4814	4.4315	3.4333	3.3697	3.2549	3.1480	3.1843	4.3337	2.5768	1.0932	0.1286	0.4219	0.3748
Rnf20	_ALVVPEPEPDSDS[Phospho (STY)]NQER_.2	#N/A	4.1216	3.5264	3.5694	9.9583	3.3477	8.9617	3.2472	9.1044	4.2068	4.1771	1.0766	0.1065	0.8371	0.0772
Rnf20	_KALVVPEPEPDSDS[Phospho (STY)]NQER_.2	#N/A	4.1222	3.4079	3.8559	3.3997	3.2660	3.1686	3.5480	3.6094	3.9085	3.0770	1.0003	0.0004	0.9966	0.0015
Jakmip3	_S[Phospho (STY)]PAINFHHTPFVDGK_.3	Q5DTN8_S622_M1_Jakmip3	3.6624	4.4187	3.3961	5.9599	2.8061	3.8185	3.0881	4.4999	4.3604	4.1180	0.9984	-0.0023	0.9920	0.0035
Jakmip3	_RPS[Phospho (STY)]SLNDLDQSQDER_.2	#N/A	3.7962	4.2090	3.8119	3.9199	3.4204	4.4249	3.7440	3.9436	3.8592	4.2606	0.9946	-0.0079	0.9169	0.0377
Psd	_S[Phospho (STY)]LSELADPNPK_.2	Q5DTT2_S720_M1_Psd	2.9743	11.1832	11.5974	4.1638	4.1402	11.2981	11.1261	11.2309	11.1935	4.1308	0.8025	-0.3175	0.4866	0.3128
Psd	_FC[Carbamidomethyl (C)]SEGDC[Carbamidomethyl (C)]AIS[Phospho (STY)]PPR_.2	Q5DTT2_S17_M1_Psd	10.1030	8.8098	9.5759	10.0844	8.9900	9.5159	10.1548	9.3205	9.4647	9.6606	0.9858	-0.0206	0.6702	0.1738
Psd	_LEASTS[Phospho (STY)]DPLPAGGGSVLPGSR_.2	Q5DTT2_S156_M1_Psd	3.4031	4.1594	7.1786	4.1814	4.0477	4.0778	4.3296	8.0213	3.1189	3.8586	0.9329	-0.1002	0.7760	0.1101
Psd	_LALGS[Phospho (STY)]TDTLSNGQK_.2	Q5DTT2_S543_M1_Psd	3.6655	5.6956	5.0608	4.1232	3.5820	6.0172	3.8379	5.4305	3.7599	4.0902	1.0961	0.1323	0.5247	0.2801
Psd	_AQRPGS[Phospho (STY)]ETR_.2	Q5DTT2_S1011_M1_Psd	3.5379	4.2942	3.2716	4.3162	4.1825	3.9430	4.4645	4.1858	2.9840	3.9935	1.0044	0.0064	0.9604	0.0176
Zc3h12c	_RQS[Phospho (STY)]DPSIR_.2	Q5DTV4_S492_M1_Zc3h12c	8.1954	3.7159	4.1970	8.6988	4.1367	3.5927	8.2976	3.4188	3.0674	3.1658	1.2084	0.2732	0.5675	0.2460
Jcad	_DFS[Phospho (STY)]PRPVSR_.2	Q5DTX6_S954_M1_Jcad	7.4254	7.9815	8.3549	8.1907	3.7728	3.9566	7.9937	7.5421	7.4715	7.6049	0.8642	-0.2106	0.3735	0.4278
Jcad	_AAPLYC[Carbamidomethyl (C)]LSEPRGS[Phospho (STY)]QELTK_.3	Q5DTX6_S980_M1_Jcad	3.3031	4.1083	3.9899	4.4925	3.9296	3.7999	4.2072	3.6259	3.2745	2.9587	1.1196	0.1630	0.1857	0.7313
Jcad	_SADS[Phospho (STY)]VEEPDPLK_.2	Q5DTX6_S1248_M1_Jcad	3.8082	8.5594	3.9496	8.5897	3.6002	3.8821	3.7752	8.0865	8.2383	8.7532	0.7484	-0.4182	0.2763	0.5586
Jcad	_S[Phospho (STY)]PPQHIPNPYLEDPVPR_.3	Q5DTX6_S351_M1_Jcad	6.5851	6.2271	3.8292	4.3319	3.9720	6.0524	4.0466	6.3318	5.8690	6.2955	0.9167	-0.1255	0.5581	0.2533
Jcad	_GPT[Phospho (STY)]PS[Phospho (STY)]PVNEQSSAFVHSSPR_.3	#N/A	3.5488	3.8103	3.7436	5.2400	5.3819	5.2749	3.8146	6.2592	5.8025	6.1942	0.8156	-0.2941	0.1505	0.8224
Jcad	_DQQTQTS[Phospho (STY)]FHEDSK_.2	Q5DTX6_S693_M1_Jcad	5.8011	5.8646	6.1168	5.9284	6.0516	5.7889	6.0646	4.0089	3.6575	5.4108	1.2382	0.3082	0.0370	1.4320
Jcad	_TAQQPAGLALENVAS[Phospho (STY)]PDRR_.3	Q5DTX6_S888_M1_Jcad	3.6140	6.0989	4.3008	6.8370	4.2405	3.4889	5.5791	5.5535	2.9636	5.2007	0.9874	-0.0183	0.9454	0.0244
Jcad	_SESLVLATDTNNNDFK_.2	#N/A	4.0699	3.3742	3.2558	5.3969	3.8748	4.2970	3.3939	4.9555	4.5827	5.1433	0.8951	-0.1599	0.3729	0.4285
Jcad	_AEGHASS[Phospho (STY)]PQPQSEGTC[Carbamidomethyl (C)]K_.2	Q5DTX6_S543_M1_Jcad	4.0901	3.1361	4.0935	3.7787	4.9633	3.6002	3.4934	4.1602	3.9884	3.7457	1.0251	0.0358	0.7791	0.1084
Jcad	_APQASIC[Carbamidomethyl (C)]SDVDGFPTSQAT[Phospho (STY)]SPEPGKKDEEAK_.4	Q5DTX6_T1164_M1_Jcad	3.4983	4.3035	4.2734	3.8172	4.1248	3.6046	3.9654	3.1840	3.4831	3.4945	1.1147	0.1567	0.1002	0.9993
Kctd16	_RKS[Phospho (STY)]DLLR_.2	Q5DTY9_S362_M1_Kctd16	9.4319	9.3353	10.1831	9.7356	8.5888	9.4109	9.7660	9.6391	9.4964	9.3497	0.9880	-0.0175	0.6883	0.1622
Kctd16	_TLTSGS[Phospho (STY)]RESNISSKK_.3	#N/A	3.4461	3.7800	4.1329	3.1347	4.0727	3.6568	6.0413	3.4829	3.1315	3.1017	0.9402	-0.0889	0.7006	0.1545
Kctd16	pho (STY)]HC[Carbamidomethyl (C)]DC[Carbamidomethyl (C)]C[Carbamidomethyl (C)]C[Carbamidome	Q5DTY9_S286_M1_Kctd16	4.5145	3.0188	3.4971	3.7799	2.9071	3.7175	3.1891	5.1201	4.2594	4.2189	0.8512	-0.2324	0.1861	0.7302
Kctd16	_TLTSGS[Phospho (STY)]RESNISSK_.2	#N/A	8.0527	7.8474	8.5643	8.1976	6.9921	7.9143	8.1178	8.1319	8.1248	7.7539	0.9870	-0.0188	0.7176	0.1441

Kctd16	_QSPDEFC[Carbamidomethyl (C)]HS[Phospho (STY)]DFEDASQGSDTR_.3	Q5DTY9_S137_M1_Kctd16	3.2341	4.8822	3.9209	5.7971	3.9693	4.9655	4.6333	5.3212	5.7413	4.1574	0.8989	-0.1538	0.3859	0.4135
Kctd16	_EKLS[Phospho (STY)]IEEELEK_.2	Q5DTY9_S389_M1_Kctd16	3.8873	4.5425	4.5741	3.5759	3.9978	3.2157	3.2906	4.1052	3.6303	4.8495	0.9991	-0.0012	0.9931	0.0030
Kctd16	_QSNIQT[Phospho (STY)]LDRPIKK_.3	#N/A	3.3721	4.1773	4.0589	4.5616	3.9986	3.7309	4.2762	3.5569	3.2055	3.0277	1.1327	0.1798	0.1586	0.7996
Kctd16	_QSNIQT[Phospho (STY)]LDRPIK_.2	#N/A	3.5135	4.3187	4.2003	3.8020	4.1400	2.6707	3.9502	3.4155	3.4982	3.1691	1.0758	0.1054	0.4564	0.3406
Kctd16	_LLAPEDVKQS[Phospho (STY)]PDEFC[Carbamidomethyl (C)]HSDFEDASQGSDTR_.4	Q5DTY9_S130_M1_Kctd16	4.0658	3.3689	3.7995	4.0979	3.2096	3.5074	3.4916	3.7503	4.0493	3.7752	0.9757	-0.0356	0.6729	0.1721
Usp22	_S[Phospho (STY)]PHIPYK_.2	Q5DU02_S237_M1_Usp22	6.6110	3.6130	6.6601	7.0949	5.3280	6.5266	7.1844	6.4622	6.6591	6.6002	0.8879	-0.1716	0.2958	0.5290
Iqsec2	_DPLGARDS[Phospho (STY)]PGR_.2	#N/A	9.0814	8.3293	8.3833	9.0279	7.8276	8.2261	9.2213	8.8604	8.7059	8.5642	0.9594	-0.0598	0.2243	0.6492
Iqsec2	_HSWDS[Phospho (STY)]PAFNNDVVQR_.2	#N/A	5.8635	5.3358	7.2776	6.4028	3.3916	6.0676	3.5373	7.4862	7.2626	6.3545	0.9291	-0.1062	0.6679	0.1753
Iqsec2	_SSS[Phospho (STY)]PGAGGGHSTSTSTSPATTLQR_.3	#N/A	4.0325	3.6313	3.8602	6.1020	7.3860	7.7211	7.8627	8.1939	8.1794	7.5422	0.6867	-0.5423	0.0311	1.5068
Iqsec2	_SS[Phospho (STY)]LEDTYGAGDGLK_.2	#N/A	4.3231	7.2681	3.5091	4.0117	3.4488	4.2807	3.7264	4.1067	3.7553	3.9787	1.1495	0.2010	0.4470	0.3497
Iqsec2	_LIEAFSQR_.2	#N/A	5.8729	6.5789	4.0862	6.4028	5.5569	5.0873	6.4208	6.5353	6.3270	5.8256	0.8917	-0.1653	0.1991	0.7008
Iqsec2	_M[Oxidation (M)]QFS[Phospho (STY)]FEEYEK_.2	#N/A	8.2398	8.3508	8.6912	8.2337	7.2457	8.6701	8.4818	9.0669	8.6881	8.7480	0.9420	-0.0863	0.1134	0.9456
Iqsec2	_GALSSS[Phospho (STY)]LR_.2	#N/A	6.4069	3.3245	3.8027	3.5903	3.2128	3.4118	3.3050	4.6099	4.1767	3.0238	1.0475	0.0669	0.8006	0.0966
Iqsec2	_QLVYEADGC[Carbamidomethyl (C)]S[Phospho (STY)]PHGTLK_.2	#N/A	5.3235	5.6548	4.5447	5.5845	3.0780	5.3992	5.8986	3.6809	5.2777	3.8134	1.0564	0.0791	0.7028	0.1532
Iqsec2	_ESQY[Phospho (STY)]QNLR_.2	Q5DU25_Y89_M1_Iqsec2	7.4362	7.6286	7.6349	7.5645	6.4815	7.0809	7.6191	7.6509	7.5986	7.5055	0.9619	-0.0560	0.2490	0.6038
Iqsec2	_DPLGARDS[Phospho (STY)]PGRESQYQNLR_.3	#N/A	3.3522	4.1085	4.5867	4.1305	3.9968	4.1287	4.0218	6.7773	3.1697	3.8078	0.9114	-0.1338	0.5693	0.2447
Iqsec2	_ETRHS[Phospho (STY)]WDSPAFNNDVVQR_.3	#N/A	3.8598	3.5122	3.6951	7.2141	3.4786	5.1005	3.7787	6.8944	5.5404	4.2539	0.8749	-0.1928	0.5116	0.2911
Iqsec2	_[Acetyl (Protein N-term)]M[Oxidation (M)]EAGSGPPGGPGSES[Phospho (STY)]PNR_.2	#N/A	9.4602	8.9586	8.5010	10.0551	8.5150	8.7472	9.1420	9.0512	8.8764	9.1021	0.9996	-0.0005	0.9917	0.0036
Iqsec2	_GELHRDPLGARDS[Phospho (STY)]PGR_.3	#N/A	7.4851	7.1577	7.8372	7.4519	6.7209	6.9742	7.9544	7.6171	7.4788	7.4074	0.9549	-0.0666	0.1671	0.7771
Iqsec2	_SS[Phospho (STY)]LEDTYGAGDGLKR_.2	#N/A	3.3940	7.0134	7.3438	7.5685	5.8351	7.0455	7.4832	7.3092	7.3905	3.8496	0.9783	-0.0317	0.8978	0.0468
Iqsec2	_AAHLPLLTIEPPSDSS[Phospho (STY)]VDLSDR_.3	Q5DU25_S593_M1_Iqsec2	3.5457	4.3509	4.2325	3.7698	4.1723	3.5572	3.9180	3.2314	3.5305	3.4470	1.1151	0.1571	0.0952	1.0215
Iqsec2	_DS[Phospho (STY)]PGRESQYQNLR_.2	#N/A	3.6584	3.5685	4.3452	3.3471	5.7789	6.5900	3.9830	3.2714	6.7061	3.3141	1.0531	0.0747	0.8127	0.0901
Iqsec2	_LRSS[Phospho (STY)]ASESR_.2	Q5DU25_S363_M1_Iqsec2	4.4131	3.4391	3.8288	3.9418	3.0682	3.6710	4.4037	3.4502	4.2357	3.7773	0.9396	-0.0899	0.4322	0.3643
Iqsec2	_ALSDSYELS[Phospho (STY)]TDLQDK_.2	Q5DU25_S316_M1_Iqsec2	4.0788	5.8398	3.2648	3.7674	3.2045	3.0242	3.3850	4.3511	3.9997	3.7344	0.9989	-0.0016	0.9940	0.0026
Iqsec2	_EAS[Phospho (STY)]YQLQDTTGYTAR_.2	Q5DU25_S138_M1_Iqsec2	3.0314	3.8365	3.7182	4.2208	3.6579	5.0874	5.5444	3.8977	4.8747	4.1878	0.8485	-0.2370	0.1627	0.7887
Iqsec2	_AQNPAYFEGKPAS[Phospho (STY)]LDEGAM[Oxidation (M)]AGAR_.3	#N/A	7.1128	7.4128	7.9635	7.7679	6.2316	7.6399	7.4776	8.4268	7.7583	7.5970	0.9411	-0.0876	0.2367	0.6258
Iqsec2	_GALSSS[Phospho (STY)]LRDLSDAGK_.2	#N/A	3.2749	3.9512	3.9325	4.4644	3.8395	4.2859	4.1791	3.6541	3.3027	4.4314	1.0170	0.0244	0.8254	0.0833
Iqsec2	_RNS[Phospho (STY)]VGS[Phospho (STY)]LDSTIEGSVISSPRPHQR_.4	Q5DU25_S1148_M2_Iqsec2	2.9693	4.2569	3.6561	4.1588	3.5958	3.9803	3.8735	7.5829	3.6083	4.1258	0.7857	-0.3479	0.2224	0.6528
Zfyve26	_GPLGLRS[Phospho (STY)]PSES[Phospho (STY)]PQHIAATER_.3	Q5DU37_S605_M2_Zfyve26	3.7311	3.0355	4.4179	3.5844	2.8568	3.3719	3.7327	3.4168	3.7158	3.2617	0.9909	-0.0132	0.9168	0.0377
Lrrc8b	_S[Phospho (STY)]KTLLSTSGGSADIDASK_.2	Q5DU41_S186_M1_Lrrc8b	3.0123	3.7686	5.7037	3.1462	3.6569	2.9677	4.3617	3.2106	3.1200	3.4679	1.0478	0.0673	0.7753	0.1105
Rassf5	_S[Phospho (STY)]PESTLTPTLNQNVC[Carbamidomethyl (C)]K_.2	Q5EBH1_S177_M1_Rassf5	3.6677	6.4454	3.9450	7.1578	6.0003	3.9211	3.5195	6.3016	6.1637	3.7868	1.0499	0.0702	0.8068	0.0932
Ermn	_HREGS[Phospho (STY)]PLKEESLAR_.3	#N/A	8.9676	9.3050	9.5921	9.8305	9.2577	9.1855	9.5024	9.0099	9.1335	9.2586	1.0141	0.0202	0.4842	0.3150
Ermn	_ILNENPEENLFVVHQAIK_.3	#N/A	3.7861	5.0986	4.5666	4.1104	4.9552	4.9310	4.3518	3.8601	4.0623	4.9607	1.0617	0.0864	0.4417	0.3549
Ermn	_YNTISYR_.2	#N/A	3.9503	3.6889	3.1362	4.5248	4.8150	3.1527	3.5135	3.8562	4.2718	3.6059	1.0174	0.0248	0.8658	0.0626
Ermn	_EDSPLGS[Phospho (STY)]PGSQPGTPDEQPVFGKK_.3	#N/A	3.4483	4.0333	4.1352	3.5497	4.2499	3.6546	4.1957	3.8609	3.2872	3.3429	1.0472	0.0666	0.4950	0.3054
Ermn	_EGS[Phospho (STY)]PLKEESLAR_.2	#N/A	10.2853	10.3277	10.7367	10.9803	10.0428	10.2172	10.7302	10.0647	10.2240	10.4278	1.0068	0.0097	0.7510	0.1244
Ermn	_EDSPLGS[Phospho (STY)]PGSQPGTPDEQPVFGK_.3	#N/A	2.9594	4.0998	4.1939	3.7377	3.6040	3.0207	3.8860	3.2636	3.5626	3.4150	1.0201	0.0286	0.8112	0.0909
Ermn	_SRPVDILHS[Phospho (STY)]K_.3	Q5EBJ4_S166_M1_Ermn	3.8358	3.1401	4.5226	3.5244	2.9614	3.2672	3.2391	3.0933	4.2427	3.4914	1.0072	0.0103	0.9448	0.0247
Prkaa1	_SGS[Phospho (STY)]ISNYR_.2	Q5EG47_S496_M1_Prkaa1	8.2495	8.3108	8.7694	8.5223	3.1358	8.0497	7.5571	8.2137	8.3633	8.4132	0.9225	-0.1164	0.5858	0.2322
Prkaa1	_SIDDEITEAKS[Phospho (STY)]GTATPQR_.2	#N/A	3.8584	3.6973	5.1219	4.6838	4.3928	3.8547	4.4907	5.6903	3.7844	5.2440	0.8888	-0.1701	0.2582	0.5880
Nufip2	_GADNDGS[Phospho (STY)]GSESGYTTPK_.2	Q5F2E7_S213_M1_Nufip2	7.4446	7.7644	8.0070	8.3473	7.6837	7.5576	7.9823	7.6842	7.6174	7.8319	1.0028	0.0040	0.9065	0.0426
Nufip2	_SLS[Phospho (STY)]SDEATNPISR_.2	Q5F2E7_S113_M1_Nufip2	6.8954	6.9513	3.8499	7.2273	6.4988	7.3637	7.5925	7.0006	7.0543	7.3865	0.8906	-0.1672	0.2767	0.5579
Nufip2	_KSS[Phospho (STY)]DIKPGLSSK_.2	Q5F2E7_S304_M1_Nufip2	3.9578	3.3846	3.6915	3.2352	3.9809	3.0227	3.3835	3.7659	4.0650	4.4134	0.9075	-0.1401	0.2102	0.6774
Nufip2	_DYEIENQNPLAS[Phospho (STY)]PTNTLLGSAK_.3	Q5F2E7_S626_M1_Nufip2	3.1183	4.2241	4.3528	3.8966	3.7629	4.3626	4.0449	3.1046	3.4036	3.5739	1.1192	0.1625	0.1828	0.7379
Nufip2	_NDS[Phospho (STY)]WGSFDLR_.2	Q5F2E7_S649_M1_Nufip2	3.5060	4.3112	4.1928	3.1946	4.1325	3.5970	4.4101	3.4231	3.0716	3.1616	1.0869	0.1203	0.3875	0.4118
Taok1	_NS[Phospho (STY)]PQALR_.2	#N/A	9.0948	8.9067	8.8563	9.3229	8.1029	8.9761	8.7482	8.9103	8.9216	9.0498	0.9965	-0.0050	0.8904	0.0504
Taok1	_NS[Phospho (STY)]PQALRR_.2	#N/A	7.4500	7.6380	7.6796	7.6111	6.8637	7.6010	7.6232	7.4763	7.1220	7.6654	1.0003	0.0004	0.9908	0.0040
Taok1	_ASDPQS[Phospho (STY)]PPQVSR_.2	Q5F2E8_S421_M1_Taok1	11.9068	12.1471	12.4646	12.2708	11.7583	12.2188	12.3924	12.0410	11.9890	12.1449	0.9988	-0.0017	0.9270	0.0329
Taok1	_AGS[Phospho (STY)]LKDPEIAELFFKEDPEK_.3	Q5F2E8_S9_M1_Taok1	4.3933	4.3356	3.5793	5.7783	3.2142	3.1747	3.7966	4.3074	3.4195	3.5463	1.0828	0.1147	0.5704	0.2438
Mlip	_AEYVFIVDS[Phospho (STY)]DGEDEATC[Carbamidomethyl (C)]R_.2	Q5FW52_S85_M1_Mlip	3.4835	3.5759	4.4253	7.9981	3.4420	3.8896	3.9674	7.6758	3.5033	3.5290	0.9572	-0.0631	0.8723	0.0593
Arhgap1	_SSS[Phospho (STY)]PEPVTHLK_.2	#N/A	12.6177	12.6074	3.1662	13.0378	12.4272	12.5741	12.9796	12.7858	12.7272	12.6380	0.8662	-0.2073	0.4127	0.3844
Arhgap1	_STQKS[Phospho (STY)]PATAPK_.2	Q5FWK3_S223_M1_Arhgap1	7.3500	7.4280	7.6739	7.5075	6.5022	6.7883	7.6950	7.4309	7.0107	7.1054	0.9860	-0.0203	0.7093	0.1492
Arhgap1	_FLLDHQGELFPSTDAQGV_.2	#N/A	6.0787	6.2584	6.1151	5.9173	6.0799	6.6427	6.2390	6.1983	6.3915	5.9793	0.9968	-0.0047	0.8932	0.0490
Arhgap1	_SDDSKSS[Phospho (STY)]S[Phospho (STY)]PEPVTHLK_.2	#N/A	6.7104	5.6033	7.3399	8.2844	3.4229	4.1498	7.8705	3.6235	7.4487	7.0048	0.9124	-0.1323	0.6561	0.1831
Arhgap1	_LAS[Phospho (STY)]IDEK_.2	Q5FWK3_S27_M1_Arhgap1	4.8638	3.1968	3.6751	3.7180	3.0851	3.5395	3.4327	4.6254	5.1458	4.1888	0.8463	-0.2408	0.1606	0.7944
Xkr7	_TKEGS[Phospho (STY)]AELVPR_.2	#N/A	4.3175	3.6219	3.4542	4.4988	3.4432	4.2863	3.1463	4.0032	4.3022	4.1761	1.0077	0.0110	0.9277	0.0326
Xkr7	_EGS[Phospho (STY)]AELVPR_.2	#N/A	7.0687	7.0539	4.2024	6.7788	4.1421	3.5873	6.9692	6.9545	6.5354	6.9434	0.7988	-0.3241	0.1429	0.8449
Xkr4	_GS[Phospho (STY)]GAGSAALC[Carbamidomethyl (C)]LR_.2	Q5GH67_S87_M1_Xkr4	4.2283	6.4594	3.4142	4.5881	3.3539	4.3755	6.7790	3.9139	6.6019	3.8839	0.8316	-0.2660	0.3300	0.4815
Xkr4	_TVVSSGSAAGEGEVRPST[Phospho (STY)]PQR_.3	Q5GH67_T208_M1_Xkr4	3.7029	3.6395	3.4366	4.4812	2.8466	3.7780	3.1286	6.4027	5.6152	4.1585	0.7558	-0.4040	0.1022	0.9904
Vps13a	_EYTEAS[Phospho (STY)]PLEDKVVELDNIPVR_.3	Q5H8C4_S2581_M1_Vps13a	3.3473	4.1036	4.5818	4.1256	3.9919	4.1336	4.2739	4.3764	3.1746	3.8029	1.0359	0.0509	0.6495	0.1874
C2cd4c	_HGS[Phospho (STY)]LSADDSTPDTSPGVR_.2	#N/A	5.8916	5.5342	5.8541	6.2815	5.7766	5.7104	5.6959	5.8173	6.0235	5.2500	1.0254	0.0362	0.4484	0.3483
C2cd4c	_AQTSYGFATLAES[Phospho (STY)]PHTR_.2	#N/A	4.0749	4.6697	5.0086	5.4453	4.2965	3.6858	4.9996	5.0708	3.5595	5.0487	0.9701	-0.0438	0.7590	0.1198
C2cd4c	_ESLFHSEHGALAQVGS[Phospho (STY)]PGAGR_.3	Q5HZI2_S175_M1_C2cd4c	4.5270	5.0805	5.7789	4.2325	4.0714	4.0540	3.9472	5.0092	3.5346	4.1995	1.1082	0.1482	0.3208	0.4937
C2cd4c	_RAT[Phospho (STY)]PEPGPESGQAPR_.3	Q5HZI2_T282_M1_C2cd4c	3.6470	4.4033	3.3807	4.4253	2.7907	3.8339	3.0727	4.0767	4.3758	4.1026	0.9590	-0.0604	0.6931	0.1592
Cnrip1	_IQPNDGPVFFK_.2	#N/A	8.7134	8.5796	9.0738	9.0567	8.8698	8.8636	8.6696	8.8432	8.7917	8.8825	1.0071	0.0102	0.5656	0.2475
Cnrip1	_VVYTGIYDTEGVAPTK_.2	#N/A	7.5636	7.7378	7.7234	7.9703	7.9566	7.7994	7.7123	7.5129	7.4267	7.8175	1.0229	0.0327	0.1378	0.8608
Cnrip1	_IKPTTLQVENISIGGVLVPLELK_.3	#N/A	4.3551	4.6112	3.3348	5.2846	4.8112	5.4473	5.3230	5.0377	5.3096	4.9838	0.8987	-0.1540	0.2209	0.6558
Cnrip1	_[Acetyl (Protein N-term)]GDLPGLVR_.2	#N/A	4.8712	5.3412	4.0044	3.3886	5.9382	5.3180	5.1226	5.3289	5.2940	5.5605	0.9031	-0.1471	0.3216	0.4927
Cnrip1	_DHC[Carbamidomethyl (C)]QWGSPFSVIEYEC[Carbamidomethyl (C)]KPNETR_.4	#N/A	4.0499	4.3807	3.5500	4.5909	4.6415	4.6584	3.7673	4.5346	4.6474	4.2199	1.0046	0.0066	0.9445	0.0248
Cnrip1	_LLTGSSYK_.2	#N/A	4.4439	4.8703	4.6805	4.0331	4.9279	4.6779	3.8964	4.9994	4.4638	4.3236	1.0418	0.0591	0.4736	0.3246
Kctd1	_S[Phospho (STY)]PAS[Phospho (STY)]PLNNQGIPTPAQLTK_.2	Q5M956_S12_M2_Kctd1	4.2482	3.0942	3.9819	6.9761	3.3919	3.2327	3.6739	5.6997	3.7746	3.2029	1.0162	0.0232	0.9404	0.0267
Cobl	_TIS[Phospho (STY)]PPSAVVETDTPPIGK_.2	Q5NBX1_S826_M1_Cobl	8.7094	4.2923	4.2846	3.8284	8.1112	8.7840	9.0246	3.1728	8.5973	8.8626	0.8544	-0.2270	0.5361	0.2707
Cobl	_AEHLGLSGADS[Phospho (STY)]DEDPAK_.2	Q5NBX1_S272_M1_Cobl	4.3792	4.3478	3.5651	4.0678	3.5049	7.3272	3.7825	4.0507	3.6993	4.0348	1.1645	0.2197	0.4047	0.3928
Cobl	_KEPDPS[Phospho (STY)]PPSQDNR_.2	Q5NBX1_S676_M1_Cobl	4.0546	7.3556	3.2405	3.7432	3.1803	3.0484	3.4579	4.3753	4.0239	3.7102	1.0545	0.0765	0.8095	0.0918
Cobl	_S[Phospho (STY)]SLGNDETDKEK_.2	#N/A	3.1773	3.9825	3.8641	4.3668	3.8039	3.9256	4.0815	3.7517	3.4003	3.7773	1.0268	0.0382	0.6673	0.1757
Cobl	_KSS[Phospho (STY)]LGNDETDKEK_.2	Q5NBX1_S235_M1_Cobl	4.4161	4.0304	5.0935	4.5862	4.0102	4.0780	4.3009	4.3913	4.3503	4.2617	1.0099	0.0143	0.8484	0.0714
Cobl	_S[Phospho (STY)]PPTRKDDAAPNLHSEAR_.4	Q5NBX1_S982_M1_Cobl	4.2905	3.5460	4.3670	3.3688	3.4343	3.1903	3.0835	3.2488	4.3982	3.3358	1.0520	0.0731	0.6136	0.2121
Dph1	[Phospho (STY)]PAPAC[Carbamidomethyl (C)]ESC[Carbamidomethyl (C)]NC[Carbamidomethyl (C)]AD	Q5NCQ5_S418_M1_Dph1	4.2468	3.1342	3.9419	2.9362	2.9555	3.2732	3.6340	3.0082	3.8145	3.1630	1.0028	0.0041	0.9764	0.0104
Neurl4	_ALS[Phospho (STY)]PEGALRR_.2	#N/A	5.0271	3.4738	4.4392	5.6051	3.3621	5.5844	5.9033	5.6055	5.5271	3.4080	0.8965	-0.1576	0.4603	0.3370

Neurl4	_ALS[Phospho (STY)]PEGALR_.2	#N/A	2.9594	4.8724	4.1939	4.8711	4.7283	4.5297	4.4147	4.8773	3.5626	4.7791	0.9888	-0.0162	0.9140	0.0391
Neurl4	_S[Phospho (STY)]FPLHS[Phospho (STY)]PVAGVAHR_.3	Q5NCX5_S904_M2_Neurl4	4.1680	3.1744	3.9017	3.4454	3.3117	3.3129	3.5937	3.5557	3.8548	3.1227	1.0058	0.0084	0.9318	0.0307
Crad	_ESSDNQPPS[Phospho (STY)]PSLPEELK_.2	#N/A	8.6821	8.1555	4.0074	9.1658	3.4174	8.4520	9.0435	8.1219	8.2785	8.5990	0.8201	-0.2861	0.2783	0.5555
Crad	_ETAQTDS[Phospho (STY)]PQPQER_.2	Q5PR69_S487_M1_Crad	4.2285	7.6037	3.4144	8.4132	7.1552	7.7609	8.1386	7.6453	7.7456	7.7794	0.8214	-0.2838	0.2249	0.6481
Crad	_VNLS[Phospho (STY)]PVTPAK_.2	Q5PR69_S552_M1_Crad	3.8201	3.1244	4.5069	5.5390	2.9458	3.2829	3.2234	3.1089	4.2584	3.4757	1.1005	0.1381	0.5368	0.2702
Crad	_ESSDNQPPS[Phospho (STY)]PSLPEELKGQK_.3	#N/A	3.6221	8.1385	8.3559	4.4414	3.4637	8.1689	5.1187	3.4546	7.8583	8.2075	0.9792	-0.0303	0.9353	0.0291
Crad	_DLPSFLVPGLPS[Phospho (STY)]PQK_.2	Q5PR69_S783_M1_Crad	3.5885	7.1148	6.6188	8.2075	4.2330	3.8924	3.7856	6.6725	5.9333	4.0379	1.0983	0.1352	0.6716	0.1729
Crad	_PASQT[Phospho (STY)]PPSS[Phospho (STY)]PLSK_.2	Q5PR69_S953_M2_Crad	3.1237	4.1024	3.8105	4.3132	3.7502	3.9792	4.0279	3.8053	3.4539	4.2802	0.9884	-0.0169	0.8610	0.0650
Diras2	_C[Carbamidomethyl (C)]DES[Phospho (STY)]PNREVQSSEAEALAR_.3	Q5PR73_S126_M1_Diras2	3.9571	6.3227	6.7925	7.1105	6.1235	6.9050	6.7215	6.6835	7.1267	6.6097	0.9140	-0.1297	0.3586	0.4454
Diras2	_VAVFGAGGVGK_.2	#N/A	5.6790	5.8928	5.5445	5.4591	3.9814	5.2482	4.5912	6.2874	5.2472	5.5819	0.9768	-0.0339	0.7845	0.1054
Diras2	_ELFQELLNLEK_.2	#N/A	5.7853	5.7267	6.2412	5.8362	5.5464	5.4607	5.4839	5.9111	5.0604	5.5227	1.0494	0.0696	0.2031	0.6922
Diras2	_PEQSNDYR_.2	#N/A	4.1648	3.2095	4.7311	4.4573	4.9338	3.1978	3.6092	5.1333	4.1702	2.8022	1.0476	0.0671	0.7405	0.1305
Diras2	_GTFRES[Phospho (STY)]YIPTVEDTYR_.3	Q5PR73_S35_M1_Diras2	3.9513	3.3911	3.6850	3.2287	3.0950	3.5296	3.3770	3.7725	4.0715	4.4068	0.8908	-0.1669	0.1071	0.9704
Mdc1	_VLLAADS[Phospho (STY)]EEEGDFPSGR_.2	Q5PSV9_S168_M1_Mdc1	4.3120	3.5675	4.0457	9.7866	8.3407	3.1688	3.0620	9.0841	8.4494	7.8613	0.7783	-0.3616	0.4048	0.3927
Osbp2	_IVHSS[Phospho (STY)]PSSDGR_.2	Q5QNQ6_S760_M1_Osbp2	3.4471	8.4011	8.8202	8.8683	4.0917	8.5138	8.8037	8.8453	3.0748	8.2571	0.9694	-0.0448	0.8996	0.0460
Brsk1	_STPLPGPPGS[Phospho (STY)]PR_.2	Q5RJI5_S508_M1_Brsk1	9.9647	10.4525	10.9116	10.3668	8.7943	9.9311	10.3338	10.1711	9.7358	10.3380	0.9927	-0.0106	0.8512	0.0699
Brsk1	_SRLNS[Phospho (STY)]IR_.2	Q5RJI5_S555_M1_Brsk1	5.4573	6.2392	6.4951	5.7658	5.9079	6.8275	5.8932	5.8551	6.4552	5.8018	1.0190	0.0272	0.6977	0.1563
Brsk1	_NSFLGS[Phospho (STY)]PR_.2	Q5RJI5_S563_M1_Brsk1	3.7569	12.1974	13.0899	12.8229	2.8826	12.6537	12.4607	12.5594	3.7416	3.4125	1.1894	0.2503	0.6476	0.1887
Brsk1	_FQVDISSSEGPEPS[Phospho (STY)]PR_.2	Q5RJI5_S676_M1_Brsk1	2.9528	3.7091	4.1873	3.2057	3.5974	3.0272	4.4212	3.4119	3.0605	3.4084	0.9639	-0.0530	0.7103	0.1486
Brsk1	_SSGGT[Phospho (STY)]PLHS[Phospho (STY)]PLHTPR_.2	#N/A	4.7969	5.0328	3.9236	5.6131	3.4645	4.5578	4.2923	4.7318	4.9914	4.5727	0.9823	-0.0258	0.8465	0.0724
Brsk1	_S[Phospho (STY)]PVFSFSPEPGAGDEAR_.2	Q5RJI5_S450_M1_Brsk1	4.0698	3.3252	3.8035	3.3473	3.2135	3.4111	3.4955	3.6539	3.9530	3.0245	0.9991	-0.0014	0.9887	0.0049
Brsk1	_SVSGASTGLSSS[Phospho (STY)]PLSS[Phospho (STY)]PR_.2	Q5RJI5_S443_M2_Brsk1	3.7830	4.1986	3.4840	5.1194	3.8898	4.8506	3.9165	4.6242	3.9083	4.1103	1.0196	0.0280	0.8239	0.0841
Brsk1	_S[Phospho (STY)]M[Oxidation (M)]EVLSITDAGSGGSPVPTR_.2	Q5RJI5_S399_M1_Brsk1	3.5508	6.3779	3.2845	4.3291	4.9817	5.9772	5.7897	4.1729	6.7054	5.6132	0.8528	-0.2298	0.3156	0.5009
Brsk1	_VDS[Phospho (STY)]PMLSR_.2	Q5RJI5_S384_M1_Brsk1	4.3504	3.6547	3.5363	4.1262	3.4760	4.2535	3.7537	4.0795	3.7281	4.0060	1.0020	0.0029	0.9716	0.0125
Brsk1	_SLPS[Phospho (STY)]NGELDPDVLESM[Oxidation (M)]ASLGC[Carbamidomethyl (C)]FR_.3	Q5RJI5_S311_M1_Brsk1	3.3608	4.1660	4.0476	4.4058	3.9874	3.7630	4.0120	3.5891	3.4559	3.8688	1.0599	0.0840	0.3163	0.4998
Islr2	_EESLAGC[Carbamidomethyl (C)]S[Phospho (STY)]LVESQSK_.2	Q5RKR3_S700_M1_Islr2	4.9895	4.9257	3.8830	5.7772	3.3554	3.7273	6.7770	5.5763	5.2537	4.8107	0.7928	-0.3350	0.0800	1.0968
Islr2	_ASYLES[Phospho (STY)]EKS[Phospho (STY)]YPAR_.2	Q5RKR3_S654_M2_Islr2	3.5215	4.3267	4.2502	3.7940	4.1480	3.5815	5.8550	5.1166	3.5062	3.4713	0.8774	-0.1887	0.3066	0.5134
Lrrk2	_SNS[Phospho (STY)]ISVGEVYR_.2	Q5S006_S910_M1_Lrrk2	5.8813	6.1783	3.2049	5.7021	3.1446	3.0840	3.4448	4.4109	4.0595	6.4867	0.9852	-0.0215	0.9441	0.0250
Ccdc88a	_AGS[Phospho (STY)]PGSEVVTLQQFLEESNK_.2	Q5SNZ0_S1677_M1_Ccdc88a	3.5535	4.0377	4.2403	3.6358	3.2951	3.5578	3.6219	4.3142	4.0060	4.1277	0.9260	-0.1110	0.1999	0.6992
Ccdc88a	_FYDPS[Phospho (STY)]PPR_.2	Q5SNZ0_S1387_M1_Ccdc88a	4.1360	3.0902	3.3220	3.8246	3.2617	2.9670	3.5393	8.5242	3.9424	3.7916	0.6937	-0.5275	0.1576	0.8025
Ccdc88a	_RT[Phospho (STY)]SIHDFLSK_.3	Q5SNZ0_T1821_M1_Ccdc88a	4.0679	3.3722	6.6598	3.7565	3.1935	3.0351	3.4712	4.3620	4.0106	3.7235	1.0314	0.0447	0.8666	0.0622
Ccdc88a	_QKS[Phospho (STY)]LTLTPTR_.2	Q5SNZ0_S1417_M1_Ccdc88a	6.1917	3.6103	6.6029	6.8840	3.4986	6.1325	3.7805	6.5524	6.3899	5.8052	0.9742	-0.0377	0.8791	0.0559
Ccdc88a	_DT[Phospho (STY)]TSFEDISPQGISDDSSTGSR_.2	Q5SNZ0_T1560_M1_Ccdc88a	4.1496	5.6815	3.8832	6.5681	3.3883	3.2145	3.5753	3.6105	3.9096	4.1926	1.1724	0.2295	0.3718	0.4297
Ccdc88a	_SVS[Phospho (STY)]GKTPGDFYDRR_.3	Q5SNZ0_S1738_M1_Ccdc88a	3.8144	3.4117	4.5012	3.5031	3.1063	3.3246	3.2177	3.1146	4.2640	3.4701	1.0266	0.0379	0.7809	0.1074
Ccdc88a	_VHASRPAS[Phospho (STY)]LDSGR_.3	#N/A	3.5874	3.8929	5.1944	3.7435	3.7142	4.1686	3.1744	3.9480	4.7386	3.5665	1.0501	0.0705	0.6436	0.1914
Ccdc88a	_SSS[Phospho (STY)]QENLLDEVMK_.2	Q5SNZ0_S1704_M1_Ccdc88a	3.0445	3.8496	3.7313	4.2339	3.6710	4.0585	4.4193	3.8846	3.5332	4.2009	0.9390	-0.0908	0.3727	0.4286
Ccdc88a	_S[Phospho (STY)]SPMLQHK_.2	Q5SNZ0_S1653_M1_Ccdc88a	3.4410	4.1973	3.1747	4.2193	4.0856	4.0398	4.3676	4.2827	3.0809	3.8966	0.9879	-0.0176	0.8873	0.0519
Ccdc85a	_STS[Phospho (STY)]PEHLHKPR_.2	#N/A	9.2471	9.2505	9.7791	3.4150	8.9184	9.1772	9.3501	9.1824	8.7799	9.1583	0.9101	-0.1359	0.5247	0.2801
Ccdc85a	_GTS[Phospho (STY)]PEHLK_.2	#N/A	6.7559	7.0463	4.1259	7.2013	6.8914	6.8736	4.3432	3.4900	7.2397	6.6205	1.1953	0.2573	0.2852	0.5448
Ccdc85a	_HALGGS[Phospho (STY)]LEHLPR_.3	#N/A	6.1664	5.6828	6.3499	5.9913	3.6878	5.7350	5.4432	5.8364	5.3577	6.0627	0.9872	-0.0186	0.8906	0.0503
Ccdc85a	_ARGTS[Phospho (STY)]PEHLK_.2	#N/A	2.9430	3.6993	4.1775	3.7213	3.5876	4.3443	4.4310	3.2799	3.5789	3.3986	1.0200	0.0286	0.8271	0.0824
Ccdc85a	_HRPGGS[Phospho (STY)]PEHTR_.3	Q5SP85_S320_M1_Ccdc85a	4.2708	3.0716	4.0045	3.5483	2.8929	3.3358	3.6966	3.4529	3.7519	5.7896	0.8437	-0.2452	0.2356	0.6279
Ccdc85a	_AAGGSAPAAESC[Carbamidomethyl (C)]PSAPAGAST[Phospho (STY)]PTGVDDLSK_.3	Q5SP85_T24_M1_Ccdc85a	3.1884	3.9936	3.8752	4.3779	3.8150	3.9145	4.2753	3.7406	3.3892	4.3449	0.9805	-0.0284	0.7806	0.1076
Ccdc85a	_GS[Phospho (STY)]PEQQRHPHPGSS[Phospho (STY)]PEVLPK_.3	Q5SP85_S284_M2_Ccdc85a	3.7022	3.6929	5.5485	3.8116	3.5410	3.6887	4.7061	4.7605	4.5912	4.9329	0.8420	-0.2481	0.0936	1.0289
Ccdc85a	_HASGSGGSGGGS[Phospho (STY)]REGTLR_.3	Q5SP85_S380_M1_Ccdc85a	3.9989	3.2543	3.7326	3.6605	3.1426	3.4820	3.3751	4.4580	4.1066	3.6275	0.9109	-0.1346	0.2044	0.6895
Ccdc85a	_HVLSGS[Phospho (STY)]PEHFQK_.2	#N/A	5.9228	3.4431	6.4060	6.4852	2.9205	6.3725	5.9210	3.8244	6.1110	5.8933	0.9671	-0.0483	0.8528	0.0692
Ccdc85a	_HHASGGS[Phospho (STY)]PEHLQKPR_.3	#N/A	3.4085	3.9500	4.0953	3.9963	3.8006	3.4376	4.2685	4.4136	4.7425	3.0641	0.9173	-0.1245	0.3265	0.4861
Ccdc85a	_HSGGS[Phospho (STY)]PEHLQK_.2	Q5SP85_S330_M1_Ccdc85a	7.8031	8.0369	8.5718	8.2450	8.1513	8.0331	8.0498	7.9771	7.9749	7.8666	1.0217	0.0310	0.2359	0.6273
Ccdc85a	_HPHPGS[Phospho (STY)]SPEVLPK_.3	Q5SP85_S295_M1_Ccdc85a	3.5055	3.8369	3.2391	4.2837	4.1500	3.9754	4.4320	4.2183	3.0165	3.9610	0.9808	-0.0280	0.8196	0.0864
Ccdc85a	_GPS[Phospho (STY)]PEHHKPLC[Carbamidomethyl (C)]K_.3	Q5SP85_S273_M1_Ccdc85a	4.0062	3.2200	3.1922	3.6948	3.1319	3.0968	3.4095	4.4236	4.0722	3.6618	0.8711	-0.1990	0.0895	1.0482
Cyfip2	_SYLQDPIWR_.2	#N/A	6.3989	6.3896	6.1993	5.2630	5.3569	5.9291	6.2503	6.2582	5.9193	6.3506	0.9561	-0.0647	0.3410	0.4673
Cyfip2	_YAPLHLVPLIER_.2	#N/A	5.9310	4.5618	5.9470	5.2382	3.4283	4.8397	6.1342	6.1412	5.7414	5.3723	0.8536	-0.2284	0.1290	0.8895
Cyfip2	_FISELAR_.2	#N/A	5.7658	5.9182	3.5077	4.9568	4.8883	3.8608	5.5979	5.8781	5.6434	4.9274	0.8738	-0.1946	0.2209	0.6559
Cyfip2	_QLQVVPLFGDM[Oxidation (M)]QIELAR_.2	#N/A	5.2591	5.6109	5.1011	3.2390	3.9829	3.8108	5.2877	5.4620	5.2603	4.5515	0.8755	-0.1917	0.2449	0.6109
Cyfip2	_SLDQAISR_.2	#N/A	5.3316	4.4584	5.8590	6.8785	4.5701	3.8067	4.6705	5.0486	4.6240	3.4383	1.1587	0.2125	0.2948	0.5304
Cyfip2	_SVETDSSTVEHVR_.2	#N/A	5.0695	4.8616	4.7328	3.1952	4.5489	4.0014	4.7053	4.6220	4.6844	4.1032	0.9719	-0.0411	0.7410	0.1302
Cyfip2	_LGT[Phospho (STY)]PQQIAIAR_.2	Q5SQX6_T1067_M1_Cyfip2	5.2462	3.7059	4.4617	4.9152	3.5942	5.1854	5.0689	5.4344	5.0109	3.9572	0.9282	-0.1076	0.4579	0.3393
Cyfip2	_NAFVTGIAR_.2	#N/A	3.9143	3.7290	3.5270	4.5716	3.6968	4.3591	3.2191	4.6624	4.7074	4.1329	0.9488	-0.0759	0.5518	0.2582
Cyfip2	_YSNSEVVTGSGLDSQK_.2	#N/A	4.0264	3.3160	3.7601	3.3038	5.6415	4.8639	5.8771	5.9010	5.4075	2.9811	0.8235	-0.2801	0.2554	0.5927
Cyfip2	_M[Oxidation (M)]ESVFNQAIR_.2	#N/A	6.0258	5.5409	3.1453	5.5359	3.0850	5.5932	6.4927	6.3236	4.1191	5.6204	0.8550	-0.2261	0.3387	0.4702
Cyfip2	_EANHNVSAPYGR_.2	#N/A	4.1588	3.4632	3.3448	3.1567	3.2845	2.9442	3.3049	3.8445	4.1435	3.8144	0.8981	-0.1550	0.1663	0.7792
Tom1l2	_NNPPTIVQDK_.2	#N/A	8.2053	8.2608	8.4023	8.4083	8.2196	8.0347	8.2369	7.9614	7.7890	8.2372	1.0247	0.0352	0.1150	0.9394
Tom1l2	_FHLLVANR_.2	#N/A	6.5360	6.4872	6.5262	6.4917	6.3092	6.4373	6.2658	6.3267	6.5966	6.8300	0.9938	-0.0089	0.7276	0.1381
Tom1l2	_TVTYEDPQAVGGLASALDNR_.2	#N/A	2.4870	4.7293	5.3907	5.4347	4.1096	4.1800	4.9044	4.7388	4.8560	4.0385	0.9469	-0.0786	0.6824	0.1660
Tom1l2	_GNS[Phospho (STY)]LAEQR_.2	Q5SRX1_S394_M1_Tom1l2	7.5292	7.1961	7.3957	7.8868	6.5523	7.3246	6.7166	7.3194	7.4892	7.4422	1.0100	0.0143	0.7927	0.1009
Tom1l2	_GIEFPM[Oxidation (M)]ADLDALS[Phospho (STY)]PIHTPQR_.3	#N/A	6.3442	8.6054	8.3960	9.4677	7.0844	6.9950	6.8870	8.7177	7.8260	5.4705	1.0817	0.1133	0.4911	0.3088
Tom1l2	_IVELISR_.2	#N/A	4.7858	3.9141	3.9988	4.7510	4.5060	4.8510	4.2162	5.2449	3.2656	4.7087	1.0250	0.0356	0.7891	0.1029
Tom1l2	_AAETVPDLPS[Phospho (STY)]PPTEAPAPASNTSTR_.3	Q5SRX1_S479_M1_Tom1l2	11.4489	12.6859	13.1368	13.5384	11.9744	11.8843	12.1702	12.4447	12.1161	11.3226	1.0359	0.0509	0.3699	0.4319
Tom1l2	_DFIDSVLVK_.2	#N/A	3.9449	3.7437	4.0953	3.5704	2.9210	4.3311	3.3482	2.9841	4.1882	3.6006	1.0673	0.0939	0.4809	0.3179
Tom1l2	_EVM[Oxidation (M)]LALTVLETC[Carbamidomethyl (C)]VK_.2	#N/A	3.8395	3.0949	3.5732	4.4349	3.8739	3.9231	4.0891	3.8843	4.1833	4.2950	0.9215	-0.1180	0.2073	0.6835
Tom1l2	_VSNEEVTEELLHVNDDLNNVFLR_.3	#N/A	3.0593	2.8477	3.7461	4.2461	3.6858	3.4558	4.4045	2.7105	3.5183	3.3711	1.0016	0.0023	0.9884	0.0051
Zzef1	_GDQEEELDRPVSS[Phospho (STY)]PGEAEQK_.3	Q5SSH7_S2407_M1_Zzef1	3.1594	6.7167	4.1199	3.6506	3.7941	6.0898	7.2412	3.2467	3.2010	6.2462	0.9207	-0.1192	0.7297	0.1368
Zzef1	_S[Phospho (STY)]M[Oxidation (M)]EETRPVPTVK_.2	#N/A	7.1102	3.7858	3.4751	5.7723	3.6741	6.5125	7.0015	6.9576	3.8602	6.4000	0.8349	-0.2604	0.3505	0.4553
Zzef1	_S[Phospho (STY)]PEMDKLK_.2	Q5SSH7_S240_M1_Zzef1	6.0805	4.2092	3.5553	6.4031	3.3422	4.3873	5.6242	3.9021	4.2012	4.2772	1.0359	0.0509	0.8287	0.0816
Zzef1	_LLPSSGPC[Carbamidomethyl (C)]VAEVSTAEEPS[Phospho (STY)]PPST[Phospho (STY)]PTR_.3	Q5SSH7_S1515_M2_Zzef1	3.1968	5.3744	4.1794	7.0715	3.8322	3.8155	4.1120	6.4259	3.3531	4.0499	1.0207	0.0296	0.9199	0.0363
Zzef1	_TSSVVEEHFQGSAS[Phospho (STY)]PTEAATPAAGDR_.3	Q5SSH7_S1475_M1_Zzef1	3.0258	4.3166	4.2604	3.8041	4.1379	4.7531	3.9524	3.1970	3.4961	3.4814	1.1466	0.1974	0.1490	0.8270
Utp18	_GQLHGS[Phospho (STY)]S[Phospho (STY)]DES[Phospho (STY)]EVENEAK_.2	Q5SSI6_S114_M3_Utp18	3.0576	4.5827	3.7444	5.5570	1.5128	3.1350	4.4062	4.2455	4.9467	4.2140	0.8080	-0.3075	0.2709	0.5673
Abr	_SQGGGDSVS[Phospho (STY)]PTPPEGLAPGVEAGK_.2	#N/A	3.6555	7.3841	7.8765	7.6088	5.8148	7.3147	7.6991	3.8159	7.8321	7.1829	0.9965	-0.0051	0.9837	0.0071
Abr	_GIEEVGIYR_.2	#N/A	4.6817	2.8176	3.9704	3.3092	2.9270	3.3017	3.6625	4.3502	4.1829	4.5108	0.8383	-0.2545	0.1201	0.9204

Abr	_ISQNFLSSINEDIDPR_.2	#N/A	4.0719	3.3273	3.8055	3.3493	3.2156	3.4090	3.3022	3.5048	3.9509	3.0266	1.0243	0.0346	0.7250	0.1396
Abr	_GPPEGSETMPYIDES[Phospho (STY)]PTMSPQLSAR_.3	Q5SSL4_S53_M1_Abr	3.6185	3.7239	3.3522	7.1671	4.2630	4.1842	4.5450	4.1052	4.4043	4.0740	1.0240	0.0342	0.8906	0.0503
Abr	_C[Carbamidomethyl (C)]SQSNNQFQK_.2	#N/A	3.0858	4.2566	4.3203	3.8640	3.7303	4.3951	4.0123	3.1371	3.4362	3.5413	1.1162	0.1586	0.1964	0.7068
Arhgap44	_HSATDKRDS[Phospho (STY)]EEESESTAL_.3	#N/A	6.9840	6.6902	7.0872	5.6037	5.9209	7.0384	5.5239	7.0625	7.2917	5.1383	1.0480	0.0676	0.5917	0.2279
Arhgap44	_SKELS[Phospho (STY)]PGSGQK_.2	Q5SSM3_S598_M1_Arhgap44	7.2125	7.1766	7.7743	7.5495	6.7638	7.4382	7.2832	7.2872	4.3431	6.7662	1.1401	0.1891	0.1621	0.7902
Tns3	_RES[Phospho (STY)]PPSAER_.2	Q5SSZ5_S934_M1_Tns3	9.1914	4.0054	3.9076	4.4103	3.8473	3.8822	4.1249	3.7082	3.3568	9.2652	0.9531	-0.0693	0.8805	0.0552
Coil	_VTGS[Phospho (STY)]PAEEDEEETK_.2	#N/A	7.8580	7.9757	7.2410	8.4866	7.1805	8.0030	3.4623	4.3708	7.3196	7.9359	1.3497	0.4327	0.0556	1.2551
Coil	_VTGS[Phospho (STY)]PAEEDEEETKKK_.3	#N/A	4.2533	3.9854	6.3824	4.7695	4.1219	3.9546	5.5277	5.3056	4.7789	2.1225	1.0325	0.0462	0.8585	0.0663
Coil	_RQEDSGQNEKAS[Phospho (STY)]DLETK_.3	#N/A	4.0492	2.4653	4.2956	3.6329	2.3775	3.6623	3.8506	4.4196	4.2766	3.8078	0.8349	-0.2603	0.1549	0.8099
Coil	_QEDSGQNEKAS[Phospho (STY)]DLETK_.2	#N/A	3.9859	3.2902	3.3589	3.3296	2.9546	3.1171	3.4779	3.6715	3.9706	3.0069	0.9455	-0.0808	0.4466	0.3501
Luc7l3	_NEVNGTSEDIKSEGDTQS[Phospho (STY)]N_.2	Q5SUF2_S431_M1_Luc7l3	3.7600	3.4857	4.0453	3.8839	3.3117	4.2532	4.0770	3.9397	3.6001	3.9688	0.9727	-0.0400	0.6021	0.2203
Synrg	_SLS[Phospho (STY)]LGDKEISR_.2	Q5SV85_S1067_M1_Synrg	9.0548	9.2101	9.5404	9.6665	3.2299	9.2285	9.2156	9.0988	9.1719	9.3272	0.9042	-0.1453	0.5100	0.2924
Synrg	_S[Phospho (STY)]GSIDDSFTDFQEM[Oxidation (M)]PASSK_.2	#N/A	3.6612	6.1136	5.7402	7.0466	3.8804	3.7158	3.7641	3.9217	3.7319	6.9069	1.0972	0.1338	0.6571	0.1824
Synrg	_TADSVS[Phospho (STY)]PLEPPTK_.2	Q5SV85_S576_M1_Synrg	3.8152	3.1196	4.5020	7.1013	2.9409	8.5037	8.7135	3.1138	7.9559	3.4708	0.8596	-0.2183	0.6329	0.1987
Synrg	_QLS[Phospho (STY)]LEGAGLAMEEFKENTSSTK_.3	#N/A	3.7884	3.4378	4.4752	5.1919	2.9141	3.2985	3.1917	3.1406	4.2900	3.4440	1.0951	0.1310	0.5045	0.2972
Synrg	_QLS[Phospho (STY)]LEGAGLAMEEFK_.2	#N/A	4.2785	3.0639	4.0122	3.5559	3.4222	3.2024	3.7042	3.4452	3.7443	3.2332	1.0163	0.0233	0.8295	0.0812
Rap1gap2	_SETSS[Phospho (STY)]NPSS[Phospho (STY)]PEIC[Carbamidomethyl (C)]PNK_.2	Q5SVL6_S590_M2_Rap1gap2	11.2431	10.9582	11.2073	11.9068	10.1499	11.2380	10.9041	10.4312	11.2844	11.3172	1.0121	0.0174	0.7006	0.1546
Rap1gap2	_NQSRS[Phospho (STY)]PIK_.2	#N/A	6.6567	7.1384	6.6170	6.7171	4.7828	6.3542	6.6851	6.6682	5.9947	6.7424	0.9778	-0.0324	0.7514	0.1241
Rap1gap2	_TTFS[Phospho (STY)]PPVAAATAK_.2	Q5SVL6_S525_M1_Rap1gap2	3.3066	6.4015	3.9934	4.4961	3.9332	3.7963	4.1571	7.6043	3.2710	7.1441	0.7794	-0.3595	0.2631	0.5799
Rap1gap2	_NQSRS[Phospho (STY)]PIKR_.2	#N/A	4.1520	3.4564	3.3380	3.8407	3.2777	2.9509	3.5554	5.8997	3.9264	5.7830	0.7311	-0.4519	0.0413	1.3836
Rap1gap2	_TPDGGHSS[Phospho (STY)]QEIK_.2	Q5SVL6_S581_M1_Rap1gap2	3.3733	4.1296	6.1269	4.2860	4.6268	5.3952	4.0007	5.8364	3.4810	4.2530	1.0600	0.0840	0.6913	0.1604
Rap1gap2	_SHS[Phospho (STY)]M[Oxidation (M)]ETM[Oxidation (M)]VGSQR_.2	#N/A	3.9411	4.3823	4.3970	5.2158	3.8071	3.9152	4.0271	4.6339	4.0699	4.5101	0.9921	-0.0114	0.9110	0.0405
Rap1gap2	_LHS[Phospho (STY)]GSEGQGDSR_.2	Q5SVL6_S553_M1_Rap1gap2	3.0108	4.3633	3.6976	4.3054	3.6373	4.1094	4.2610	4.0125	3.2575	4.1295	0.9844	-0.0227	0.8514	0.0699
Kat7	_LSQSSQDSS[Phospho (STY)]PVR_.2	Q5SVQ0_S59_M1_Kat7	7.5433	7.6477	8.3152	7.8842	7.2640	7.7350	7.8273	7.8122	7.6663	7.5132	1.0035	0.0050	0.8910	0.0501
Kat7	_S[Phospho (STY)]SGSETEQVVDFSDR_.2	Q5SVQ0_S101_M1_Kat7	4.2275	3.5318	3.4134	4.5889	3.3532	4.3763	3.2363	3.9131	4.2122	4.2661	1.0021	0.0030	0.9810	0.0083
Tbc1d9b	_GTS[Phospho (STY)]PPDYR_.2	Q5SVR0_S997_M1_Tbc1d9b	9.4976	9.2882	9.3221	4.3694	8.4307	9.3393	9.7512	9.7249	9.3058	9.3887	0.8776	-0.1884	0.2869	0.5423
Tbc1d9b	_VFQKNS[Phospho (STY)]PM[Oxidation (M)]EDLGAK_.2	#N/A	3.0743	5.7085	3.7611	4.2638	4.0401	4.6747	5.6327	5.7237	5.8543	4.7654	0.7743	-0.3691	0.0366	1.4360
Tbc1d9b	_NS[Phospho (STY)]PMEDLGAK_.2	#N/A	4.6121	3.7291	3.6107	4.1134	3.5504	4.1791	4.5399	4.0051	3.6537	4.0804	0.9745	-0.0373	0.6930	0.1593
Cluh	_AVEDM[Oxidation (M)]GS[Phospho (STY)]PQTAK_.2	#N/A	7.0691	3.9979	4.2093	4.0763	3.8532	4.0837	4.2246	3.4087	6.7202	4.0588	0.9881	-0.0173	0.9505	0.0221
Ssh2	_IPHSSS[Phospho (STY)]SENIR_.2	Q5SW75_S1217_M1_Ssh2	7.9536	7.9341	4.0791	8.1790	2.9283	7.6706	3.4685	7.6578	4.0150	3.8239	1.3620	0.4457	0.2599	0.5852
Ssh2	_EM[Oxidation (M)]TTS[Phospho (STY)]ADQIAEVK_.2	Q5SW75_S487_M1_Ssh2	8.6014	8.7781	9.4961	8.4872	7.9915	9.0480	9.2056	8.8920	8.8940	8.8342	0.9751	-0.0363	0.4328	0.3637
Nacad	_DEDS[Phospho (STY)]LEEDAQR_.2	#N/A	6.9901	6.0594	6.4540	6.9503	5.7102	6.3412	6.9506	6.3692	6.1378	6.8121	0.9772	-0.0333	0.6259	0.2035
Nacad	_VSLS[Phospho (STY)]PHSTLNPK_.2	Q5SWP3_S1216_M1_Nacad	5.5920	6.3319	3.2219	4.2665	4.1328	5.6304	6.7031	5.9079	5.9658	3.9438	0.8637	-0.2114	0.3412	0.4670
Nacad	_EFPALEQKDEDS[Phospho (STY)]LEEDAQR_.3	#N/A	3.6026	3.5361	4.2894	8.3980	6.6851	5.9023	3.9935	7.8053	7.2925	6.9097	0.8311	-0.2669	0.3867	0.4127
Acaca	_SSM[Oxidation (M)]S[Phospho (STY)]GLHLVK_.2	#N/A	4.5959	4.4150	5.7305	3.7027	3.0790	3.0957	5.3962	3.8109	4.9420	5.5310	0.8340	-0.2619	0.2144	0.6688
Acaca	_FIIGSVSEDNS[Phospho (STY)]EDEISNLVK_.2	Q5SWU9_S29_M1_Acaca	3.3101	5.3418	6.3969	3.9734	5.2992	3.8877	3.8770	6.2423	5.7164	3.9562	0.9502	-0.0737	0.7549	0.1221
Wwc1	_LNRSDS[Phospho (STY)]DSSTLSK_.2	Q5SXA9_S939_M1_Wwc1	3.3602	4.1164	4.5947	4.1384	4.0047	2.9422	4.2867	4.3636	3.1618	3.8157	0.9878	-0.0177	0.9025	0.0445
Specc1	_LGSS[Phospho (STY)]PTSSC[Carbamidomethyl (C)]NPTPTK_.2	#N/A	6.2843	5.6055	3.6380	3.7550	3.0481	5.3652	6.3600	6.0645	5.3178	5.5527	0.7926	-0.3353	0.1184	0.9266
Specc1	_ASS[Phospho (STY)]EDTLNKPGSASSGVAR_.2	#N/A	6.4019	6.2760	4.7719	6.9005	5.8235	6.2248	3.8471	7.0122	6.5684	6.4048	1.0182	0.0260	0.8766	0.0572
Specc1	_SSKGS[Phospho (STY)]PTGSSPNNASELSLASLTEK_.3	Q5SXY1_S355_M1_Specc1	5.6910	3.6340	7.1061	7.6615	3.4165	6.1971	3.6985	6.4268	3.8558	3.7643	1.2663	0.3406	0.2893	0.5386
Specc1	_S[Phospho (STY)]STSLAFESR_.2	Q5SXY1_S37_M1_Specc1	3.7645	5.3938	4.0072	3.5510	2.8902	3.3384	3.6992	3.4502	6.1888	3.2283	0.9234	-0.1150	0.6618	0.1793
Specc1	_T[Phospho (STY)]SPAPSDSATTVK_.2	Q5SXY1_T809_M1_Specc1	3.3988	3.9437	3.1324	4.1770	3.7650	3.9645	4.3253	4.3250	3.1232	3.8543	0.9548	-0.0668	0.5729	0.2419
Specc1	_KLGSS[Phospho (STY)]PTSSC[Carbamidomethyl (C)]NPTPTK_.2	#N/A	4.5849	3.7812	4.1434	3.6872	5.4623	5.0683	5.5252	4.9126	5.1376	4.8649	0.8717	-0.1981	0.1256	0.9011
Specc1	_S[Phospho (STY)]PLSGIPVR_.2	Q5SXY1_S847_M1_Specc1	3.1190	3.8752	4.3535	4.9769	3.7635	4.3619	4.2551	4.0787	3.2267	3.0065	1.1190	0.1622	0.3172	0.4987
Specc1	_GSPT[Phospho (STY)]GSS[Phospho (STY)]PNNASELSLASLTEK_.2	Q5SXY1_T357_M2_Specc1	3.3842	4.1894	4.0710	4.9826	4.0108	3.7187	4.0795	3.5448	3.1934	3.0398	1.1718	0.2287	0.1084	0.9650
Nkapl;Nkap	_IGELGAPEVWGLS[Phospho (STY)]PK_.2	Q5SZT7_S141_M1_Nkapl;Nkap	4.1404	6.3053	3.3264	7.0102	3.2661	5.9852	3.3234	6.8459	6.1043	3.7960	0.9976	-0.0034	0.9915	0.0037
Rabl6	_ESS[Phospho (STY)]DEDRDSKLPSK_.2	#N/A	7.0418	6.7347	4.0372	7.3332	7.5452	6.5305	7.0345	7.4942	6.5306	3.5764	1.0614	0.0860	0.7028	0.1532
Rabl6	_VAPQQC[Carbamidomethyl (C)]S[Phospho (STY)]EPETK_.2	Q5U3K5_S502_M1_Rabl6	9.3058	3.2342	3.8419	9.8538	3.0555	3.1731	3.5339	3.6155	9.3224	3.0629	1.1079	0.1479	0.8017	0.0960
Rabl6	_EDLS[Phospho (STY)]DVTDEDTGPAQPPPPSK_.2	#N/A	3.6236	3.4893	4.2003	3.7705	3.2825	3.8350	7.1677	7.4693	4.0024	3.8214	0.6590	-0.6017	0.0428	1.3687
Rabl6	_NISLSS[Phospho (STY)]EEEAEGLAGHPR_.3	#N/A	7.5067	7.5538	8.1520	8.0930	7.8057	7.6089	7.9639	9.1108	7.6808	7.7040	0.9596	-0.0596	0.3105	0.5080
Rabl6	_FPVREDLS[Phospho (STY)]DVTDEDTGPAQPPPPSK_.3	#N/A	3.7709	3.2933	4.4577	8.6118	4.8504	3.3534	3.4270	7.6884	5.5121	3.4265	0.9420	-0.0861	0.8288	0.0816
Tmprss13	_NKPGVYTK_.2	#N/A	6.7617	6.7391	6.9677	6.6102	7.6288	6.8689	7.0722	6.8620	6.6558	6.7697	1.0131	0.0188	0.6635	0.1782
Dnajc11	_[Acetyl (Protein N-term)]ATALS[Phospho (STY)]EEELDNEDYYSLLNVR_.3	Q5U458_S6_M1_Dnajc11	4.2607	3.0817	3.9944	5.5580	2.9030	3.3256	3.6864	3.4630	3.7621	4.4884	1.0010	0.0015	0.9943	0.0025
Dnajc11	_RTPAEIR_.2	#N/A	3.4446	4.2009	3.1783	4.2229	4.0892	4.0362	4.3712	1.4941	3.0773	1.2361	1.5177	0.6019	0.0667	1.1760
Gprasp1	_VESTS[Phospho (STY)]GSEC[Carbamidomethyl (C)]ENPTK_.2	Q5U4C1_S295_M1_Gprasp1	3.8356	3.9299	3.4930	5.4263	4.9077	3.9117	3.9350	4.0331	4.9699	4.5584	0.9718	-0.0413	0.7792	0.1083
Scaf1	_APS[Phospho (STY)]PAPAVS[Phospho (STY)]PK_.2	#N/A	10.4458	10.5317	10.5351	10.9320	9.8994	10.4846	11.0837	10.6537	10.7076	10.4728	0.9760	-0.0351	0.2267	0.6445
Scaf1	_S[Phospho (STY)]AS[Phospho (STY)]PPPAASSSSSSR_.2	#N/A	9.9420	10.1799	10.2727	10.5046	9.1063	9.8718	10.6060	10.3107	10.0097	9.6340	0.9842	-0.0230	0.6039	0.2191
Scaf1	_S[Phospho (STY)]AS[Phospho (STY)]PGPPPAR_.2	#N/A	9.3967	9.0101	8.9648	7.8854	7.0947	9.0249	9.4738	9.4810	8.8593	9.4586	0.9189	-0.1220	0.1435	0.8431
Scaf1	_S[Phospho (STY)]PFLKPDER_.2	Q5U4C3_S821_M1_Scaf1	8.7208	8.0823	8.5706	8.6217	7.1892	7.8256	8.9973	8.4519	8.4745	8.1761	0.9582	-0.0616	0.3133	0.5040
Scaf1	_FDIYDPFHPTDEAYS[Phospho (STY)]PPPAPEQK_.3	Q5U4C3_S240_M1_Scaf1	3.8710	3.3552	4.5578	7.1941	5.5356	6.4773	6.8452	7.0369	6.8486	5.1758	0.7975	-0.3264	0.1575	0.8028
Scaf1	_GTEETSWS[Phospho (STY)]GEER_.2	Q5U4C3_S912_M1_Scaf1	6.9387	7.3423	7.6772	7.5331	6.3760	7.5138	3.2263	7.4240	7.3091	6.9026	1.1632	0.2182	0.2569	0.5902
Scaf1	_APS[Phospho (STY)]PAPAVS[Phospho (STY)]PKR_.2	#N/A	7.4063	5.9632	6.9992	7.8798	6.7546	7.5594	7.9385	7.5456	7.6572	7.6728	0.9208	-0.1190	0.1241	0.9063
Scaf1	_SGDPAPPDS[Phospho (STY)]PSWEAK_.2	Q5U4C3_S518_M1_Scaf1	4.0499	6.5065	7.3764	7.0616	3.9392	6.7261	7.1537	6.8546	4.0979	8.0606	0.9085	-0.1384	0.5793	0.2371
Scaf1	_QRS[Phospho (STY)]AS[Phospho (STY)]PGPPPAR_.2	#N/A	5.6255	7.4511	3.6692	7.4898	4.0200	7.2061	3.8866	7.4043	3.5952	4.1389	1.2426	0.3134	0.3389	0.4699
Scaf1	_QRS[Phospho (STY)]GDPAPPDS[Phospho (STY)]PSWEAK_.2	#N/A	3.6489	6.3515	3.3826	6.6215	2.7926	4.2147	6.7283	5.7318	4.3739	5.7972	0.7957	-0.3297	0.2373	0.6248
Scaf1	_RRS[Phospho (STY)]AS[Phospho (STY)]PPPAASSSSSSR_.3	#N/A	3.4649	4.2212	3.1986	4.1944	4.1095	4.0159	3.9091	4.6361	4.2613	3.9205	0.9248	-0.1127	0.2577	0.5888
Scaf1	_REVLY[Phospho (STY)]DSEGLS[Phospho (STY)]ADER_.2	Q5U4C3_S695_M2_Scaf1	3.4912	4.2964	4.1780	3.1798	4.1177	3.6118	3.9726	3.4379	3.0864	3.1468	1.1177	0.1606	0.1882	0.7253
Dyrk2;Dyrk4	_VYTY[Phospho (STY)]IQSR_.2	Q5U4C9_Y380_M1_Dyrk2;Dyrk4	8.0317	7.9056	8.4171	8.0253	6.4790	7.7609	8.2795	8.4010	3.0954	7.6839	1.1318	0.1787	0.4171	0.3798
Dync2i2	_VGS[Phospho (STY)]AGAAALAAGGAGGAER_.2	Q5U4F6_S15_M1_Dync2i2	5.4413	6.0660	3.3712	4.4158	5.1432	5.7565	6.0585	6.2011	6.2058	6.3189	0.8122	-0.3001	0.0510	1.2921
Hykk	_YMGALS[Phospho (STY)]QSR_.2	Q5U5V2_S195_M1_Hykk	4.3506	2.9918	4.0842	3.6280	2.8132	3.4155	3.7763	3.3732	3.6722	3.3053	1.0044	0.0063	0.9626	0.0166
Fam169a	_TS[Phospho (STY)]PTASVNK_.2	Q5XG69_S359_M1_Fam169a	5.3479	3.7940	4.0939	3.8316	5.3343	3.6854	3.7821	3.6334	5.8672	3.7986	1.0182	0.0260	0.8970	0.0472
Fam169a	_HFQDSEFSSS[Phospho (STY)]QGEDENVAK_.3	Q5XG69_S348_M1_Fam169a	5.6367	5.4581	3.1776	4.2222	4.0885	4.0370	3.9301	6.1823	3.0780	3.8995	1.0384	0.0544	0.8234	0.0844
Fam169a	_TLLGS[Phospho (STY)]SDNVATVSNIEK_.2	Q5XG69_S523_M1_Fam169a	7.1694	4.2289	3.2063	4.2509	6.6284	4.0083	4.3992	4.2511	3.0493	3.9282	1.2581	0.3313	0.2679	0.5721
Fam169a	_TSPDTDSEM[Oxidation (M)]LIDQSPSDDKGHTEENLS[Phospho (STY)]PVSK_.4	#N/A	3.9958	6.3246	7.3139	3.3878	5.9917	7.0064	5.9395	7.0773	4.1578	3.6514	1.0890	0.1230	0.6663	0.1763
Fam169a	_AVDS[Phospho (STY)]S[Phospho (STY)]SEEIEVEVPVVDRR_.2	#N/A	3.3241	4.1293	4.2844	4.3145	3.9506	3.7799	4.2233	3.2993	3.2428	3.4037	1.1190	0.1622	0.1476	0.8308
Fam169a	_SQSEEQSEASS[Phospho (STY)]EHLEQFTQSAEK_.3	Q5XG69_S614_M1_Fam169a	3.6401	3.3741	3.6985	3.8300	3.2418	3.3089	3.8928	3.9919	3.6550	3.7544	0.9195	-0.1212	0.0525	1.2799
Leo1	_KYVIS[Phospho (STY)]DEEEEEDD_.2	#N/A	2.9578	7.5806	3.6446	7.8327	6.5502	7.3308	8.1653	7.5986	3.6198	7.2024	0.9001	-0.1518	0.6382	0.1951
Leo1	_YVIS[Phospho (STY)]DEEEEEDD_.2	#N/A	4.1535	3.4089	3.8871	6.5852	3.2972	3.3274	3.2206	3.5703	3.8693	3.1082	1.1940	0.2558	0.3396	0.4691
Leo1	_LNS[Phospho (STY)]DEEGESSGKR_.2	#N/A	4.3049	3.5603	4.0385	4.2388	3.4486	3.1760	3.0692	3.2632	4.4126	3.3215	1.0790	0.1097	0.4334	0.3632

Leo1	_KLNS[Phospho (STY)]DEEGESSGKR_.3	#N/A	3.8016	3.0972	4.0317	3.6800	3.8963	3.6692	3.6026	3.6743	3.6746	3.5260	1.0212	0.0302	0.6594	0.1809
Leo1	_[Acetyl (Protein N-term)]ADM[Oxidation (M)]EDLFGS[Phospho (STY)]EAESEAER_.2	#N/A	3.7330	3.3282	3.4667	4.5113	2.8767	3.7479	3.1587	3.9907	4.2898	4.1886	0.9242	-0.1138	0.4146	0.3824
Leo1	_KLNS[Phospho (STY)]DEEGESSGK_.2	#N/A	3.4481	3.8943	3.1818	4.3993	3.7847	4.0139	4.3747	3.8720	4.1088	3.9037	0.9317	-0.1021	0.2752	0.5603
Leo1	_MQNT[Phospho (STY)]DDEDR_.2	Q5XJE5_T189_M1_Leo1	4.1272	3.4316	3.3132	3.1883	3.2529	2.9758	3.3365	3.8129	4.1119	4.3664	0.8655	-0.2084	0.0846	1.0725
Lmtk3	_S[Phospho (STY)]PSVSSEYYIR_.2	Q5XJV6_S531_M1_Lmtk3	7.4358	3.4334	3.9117	7.8478	6.6555	6.3586	3.6037	8.0468	7.7035	7.9086	0.8716	-0.1983	0.5090	0.2933
Lmtk3	_ALVNGEPM[Oxidation (M)]S[Phospho (STY)]PEAGEK_.2	#N/A	8.6652	8.5965	8.9207	8.8083	7.6266	8.5277	8.9980	8.9626	8.2661	8.4840	0.9823	-0.0258	0.5929	0.2270
Lmtk3	_AQRPPEGM[Oxidation (M)]S[Phospho (STY)]PELPPR_.3	#N/A	7.2027	6.6939	7.5077	6.3764	5.3374	5.8513	7.4684	7.7784	5.6323	5.3363	0.9910	-0.0130	0.9295	0.0317
Lmtk3	_VAENGLES[Phospho (STY)]PEKEER_.2	Q5XJV6_S947_M1_Lmtk3	6.4959	6.5200	6.8477	6.7759	5.8670	3.1169	6.8819	7.1226	6.4318	6.6475	0.8769	-0.1896	0.2903	0.5371
Zdhhc8	_QGLPS[Phospho (STY)]PPGTPR_.2	Q5Y5T5_S672_M1_Zdhhc8	4.0920	3.2504	3.8257	3.3695	3.0717	3.1569	8.4356	3.6317	3.9308	3.0467	0.7269	-0.4601	0.2301	0.6380
Zdhhc8	_TPRPGS[Phospho (STY)]AESALSVQR_.2	#N/A	3.8719	3.4902	3.9492	3.9812	4.0192	3.7686	4.1294	3.8715	3.3774	3.6585	1.0233	0.0332	0.6016	0.2207
Zdhhc8	_TS[Phospho (STY)]PPTPAMYK_.2	Q5Y5T5_S346_M1_Zdhhc8	3.2430	3.9993	4.4775	4.4164	3.8876	4.2379	4.1311	3.7021	3.3507	3.6986	1.0868	0.1201	0.2560	0.5917
Kif1b	_MADTGS[Phospho (STY)]PGM[Oxidation (M)]QR_.2	#N/A	3.9175	3.6813	3.3211	4.0894	7.8051	3.9397	3.8328	4.2273	3.4880	3.9081	1.1540	0.2066	0.5068	0.2952
Kif1b	_IVEGQGQSSEVIS[Phospho (STY)]PPEEVNR_.2	Q60575_S1075_M1_Kif1b	4.3584	4.3686	3.5443	4.0470	4.2569	3.8685	3.7617	4.0715	3.7201	4.0140	1.0468	0.0660	0.3398	0.4688
Kif1b	_SGLS[Phospho (STY)]LEELR_.2	Q60575_S1057_M1_Kif1b	3.8545	7.7407	4.5413	7.6850	5.6017	7.5652	7.7435	7.9797	7.6726	7.6717	0.7937	-0.3333	0.1086	0.9644
Kif1b	_EFSQVHGSIS[Phospho (STY)]DC[Carbamidomethyl (C)]K_.2	Q60575_S1605_M1_Kif1b	3.8767	3.1322	3.6104	3.7826	3.0205	3.6041	3.4973	4.3359	3.7038	3.7496	0.9170	-0.1250	0.2017	0.6952
Kif1b	_GDS[Phospho (STY)]LILEHQWELEK_.2	Q60575_S1487_M1_Kif1b	4.1082	3.4126	3.6635	3.2073	3.2339	2.9947	3.3555	3.7939	4.0930	4.3854	0.8796	-0.1850	0.1166	0.9331
Mast2	_KLS[Phospho (STY)]NPDIFAPTGK_.2	Q60592_S14_M1_Mast2	3.9449	3.2493	3.8268	5.9971	3.0706	3.1580	3.5188	5.3271	3.9297	4.7556	0.8840	-0.1779	0.4549	0.3421
Mast2	_SLS[Phospho (STY)]EEKEDHSDGLAGLK_.3	Q60592_S817_M1_Mast2	4.3163	4.5597	3.5023	5.1587	3.4420	4.2875	3.1474	4.9604	3.8695	3.9719	1.0561	0.0788	0.6284	0.2018
Xrcc1	_TQAAGPS[Phospho (STY)]SPPRPPT[Phospho (STY)]PK_.2	Q60596_S445_M2_Xrcc1	7.0328	7.0089	4.3142	6.5286	6.3547	4.1021	6.2908	6.9689	7.0411	6.9240	0.8654	-0.2085	0.2228	0.6520
Ogdh	_SSPYPTDVAR_.2	#N/A	8.0887	8.2431	8.3112	8.3535	8.6140	8.2160	8.1226	8.4364	8.2097	8.3060	1.0043	0.0062	0.7418	0.1297
Ogdh	_LNVLANVIR_.2	#N/A	8.1998	8.2540	8.1369	8.0892	7.7912	7.9932	7.9532	8.2668	7.9351	8.2085	0.9983	-0.0024	0.9042	0.0437
Ogdh	_FEEFLQR_.2	#N/A	7.6374	7.6037	7.2099	7.5514	7.6338	7.4660	7.4933	7.6574	7.7099	7.3667	0.9947	-0.0076	0.7118	0.1477
Ogdh	_DVVVDLVC[Carbamidomethyl (C)]YR_.2	#N/A	6.6867	6.9673	6.3726	7.3336	6.4344	6.9194	7.2164	6.6947	7.4040	7.1290	0.9542	-0.0676	0.1766	0.7530
Ogdh	_QILLPFR_.2	#N/A	6.5438	7.2858	6.9611	6.3109	6.7912	6.8476	6.7606	7.4820	6.8736	6.6414	0.9785	-0.0314	0.5289	0.2766
Ogdh	_IC[Carbamidomethyl (C)]EEAFTR_.2	#N/A	6.6059	6.4369	6.6960	6.7729	6.6686	5.9241	7.0722	6.9232	6.0842	6.4855	0.9813	-0.0272	0.6165	0.2101
Ogdh	_THLTELQR_.2	#N/A	6.0360	5.7610	6.5596	5.7035	6.1250	4.1141	5.4271	6.1929	6.0512	5.6905	0.9788	-0.0309	0.7907	0.1020
Ogdh	_ELEQIFC[Carbamidomethyl (C)]QFDSK_.2	#N/A	4.9027	4.4622	3.4706	5.4560	4.1650	5.0702	5.5679	4.5364	4.2859	4.8943	0.9516	-0.0716	0.5975	0.2237
Ogdh	_TSFDEM[Oxidation (M)]LPGTHFQR_.3	#N/A	6.3993	6.6522	6.6545	5.1927	6.0075	5.7416	6.3302	6.5466	6.4945	6.6090	0.9404	-0.0887	0.2301	0.6381
Ogdh	_VASSVPVENFTIHGGLSR_.3	#N/A	5.6653	3.7482	4.0213	5.5606	5.4132	5.8562	6.1197	6.6590	5.5290	6.3066	0.8197	-0.2868	0.0582	1.2351
Ogdh	_SWDIFFR_.2	#N/A	4.7177	3.0856	4.7407	3.5280	4.7602	4.9889	3.6825	4.5563	5.6800	5.5222	0.8854	-0.1755	0.3374	0.4719
Ogdh	_LVEDHLAVQSLIR_.3	#N/A	4.2150	3.9625	4.8124	3.5251	4.6373	3.9988	3.5123	4.2060	4.6624	4.5650	0.9895	-0.0153	0.8918	0.0498
Ogdh	_VVNAPIFHVNSDDPEAVMYVC[Carbamidomethyl (C)]K_.3	#N/A	3.7770	3.5654	3.5107	4.0533	2.9207	3.7039	3.2027	3.9467	4.2458	4.2325	0.9185	-0.1227	0.2778	0.5563
Ogdh	_TKAEQFYC[Carbamidomethyl (C)]GDTEGKK_.3	#N/A	5.1153	4.5433	4.0223	4.8468	5.5245	3.1922	3.7144	3.4351	4.4729	3.2434	1.2218	0.2890	0.1217	0.9147
Ogdh	_VFHLPTTTFIGGQEPALPLR_.3	#N/A	4.1555	3.4599	3.3415	3.8441	3.2812	2.9475	3.5588	4.2744	3.9229	3.8111	0.9006	-0.1511	0.1592	0.7981
Ogdh	_HHVLHDQNVDKR_.3	#N/A	2.9442	4.2820	3.6310	4.1337	4.1703	3.9552	3.8483	3.9848	3.6334	4.1007	0.9900	-0.0146	0.8873	0.0519
Cttn	_TVPIEAVTSK_.2	#N/A	6.5764	7.0676	7.0463	6.9137	7.6870	6.9768	6.3879	6.6445	6.8354	6.7320	1.0594	0.0832	0.0832	1.0797
Cttn	_NAST[Phospho (STY)]FEEVVQVPSAYQK_.2	Q60598_T323_M1_Cttn	4.2462	6.3546	3.9799	3.5236	3.3899	3.2347	3.6719	6.9585	3.7766	3.2009	0.9363	-0.0950	0.7636	0.1171
Cttn	_SAVGFDYQGK_.2	#N/A	5.7250	5.2453	4.1338	6.7248	6.4254	6.0648	3.8258	5.6862	5.6591	6.3640	1.0624	0.0874	0.6151	0.2111
Cttn	_HC[Carbamidomethyl (C)]S[Phospho (STY)]QVDSVR_.2	Q60598_S113_M1_Cttn	4.9354	4.6055	3.6228	4.3598	5.0432	4.4185	4.4905	4.7161	4.7219	4.7097	0.9652	-0.0511	0.5552	0.2555
Cttn	_YGVQADRVDK_.2	#N/A	4.9980	5.0780	4.7321	5.4174	4.4618	4.8339	4.5964	4.5814	4.2713	4.6612	1.0867	0.1200	0.0608	1.2162
Cttn	_SAVGFEYQGK_.2	#N/A	5.7956	5.5403	4.7840	4.1981	5.8639	5.6737	5.2918	5.3788	5.2681	3.8754	1.0718	0.1001	0.4469	0.3498
Cttn	_TVQGSGHQEHINIHK_.3	#N/A	5.3453	5.6328	5.8957	5.4342	7.0579	5.8325	4.0537	5.3383	5.5917	5.7696	1.1307	0.1772	0.1632	0.7871
Cttn	_KQT[Phospho (STY)]PPASPSPQPIEDRPPSSPIYEDAAPFK_.4	Q60598_T401_M1_Cttn	7.1716	7.9006	7.8711	9.1246	7.8917	8.3608	7.6336	8.3757	7.8594	7.6199	1.0230	0.0328	0.6274	0.2025
Cttn	_VDQSAVGFEYQGK_.2	#N/A	3.6229	2.9273	4.3097	7.0479	5.9699	3.4801	3.8408	3.3061	5.7731	3.2785	1.1259	0.1711	0.6038	0.2191
Cttn	_SAVGHEYQSK_.2	#N/A	3.2810	4.0862	4.1558	4.4704	5.6485	3.8220	4.1828	4.9752	4.0392	5.3247	0.9165	-0.1258	0.4384	0.3582
Cttn	_YGIDKDKVDK_.2	#N/A	3.0566	4.3884	4.7590	4.1428	3.5799	5.5650	4.6940	4.9322	3.8894	5.0593	0.9149	-0.1283	0.4485	0.3482
Cttn	_DYSSGFGGK_.2	#N/A	3.0318	3.7881	4.4075	4.7189	3.6764	2.9482	4.3422	3.4909	2.9700	3.5278	1.0500	0.0704	0.6859	0.1637
Kcnh1	_S[Phospho (STY)]PQDRS[Phospho (STY)]PILAEVK_.2	Q60603_S899_M2_Kcnh1	2.9681	4.3743	4.2026	3.7464	3.6126	3.0120	3.8946	3.2548	3.5539	3.4236	1.0342	0.0486	0.7131	0.1469
Myl6	_ALGQNPTNAEVLK_.2	#N/A	7.3967	7.7071	8.0644	7.3451	8.3347	7.7745	7.6953	7.4324	7.5556	7.5381	1.0285	0.0405	0.3126	0.5050
Myl6	_NKDQGTYEDYVEGLR_.3	#N/A	7.4583	7.6355	7.4255	7.5054	7.9735	7.8866	7.7663	7.2328	7.6514	7.6850	1.0084	0.0120	0.6856	0.1639
Myl6	_EAFQLFDR_.2	#N/A	7.6500	7.5357	7.4728	7.3302	7.6342	7.7525	7.1410	7.2987	7.5037	7.5191	1.0267	0.0381	0.0959	1.0180
Myl6	_HVLVTLGEK_.2	#N/A	4.7683	4.9836	4.5322	5.6318	5.0478	4.9884	5.4942	4.7926	4.8785	5.2148	0.9798	-0.0295	0.6615	0.1794
Myl6	_ILYSQC[Carbamidomethyl (C)]GDVMR_.2	#N/A	4.1450	3.4005	3.8787	5.0714	4.5728	3.3358	3.2290	3.5787	3.8777	3.0998	1.1802	0.2390	0.1335	0.8745
Tiam1	_ANS[Phospho (STY)]LGDLYAQK_.2	Q60610_S231_M1_Tiam1	9.1370	9.4593	9.7962	9.6787	8.3679	9.4892	9.6117	9.6980	9.4989	9.5557	0.9719	-0.0411	0.3415	0.4666
Tiam1	_TES[Phospho (STY)]LPSAQQYVPFGGK_.2	Q60610_S1407_M1_Tiam1	6.7244	6.1276	4.3059	3.6946	3.5950	6.7734	3.6724	6.9013	6.9475	6.3455	0.8721	-0.1975	0.4597	0.3375
Tiam1	_SNATNSSYS[Phospho (STY)]PPTGR_.2	#N/A	8.8208	3.7376	9.1154	3.9347	3.5993	8.8951	9.3198	9.3554	8.5300	3.7358	0.8210	-0.2846	0.4639	0.3336
Tiam1	_SKS[Phospho (STY)]ADIWR_.2	Q60610_S172_M1_Tiam1	2.8995	2.9421	4.1340	3.6778	4.2642	5.7575	3.8260	3.3234	3.6225	6.5924	0.9090	-0.1377	0.6371	0.1958
Tiam1	_RSNATNSSYS[Phospho (STY)]PPTGR_.2	#N/A	4.3281	3.0143	4.0618	3.6055	2.8356	3.3930	8.4973	3.3956	3.6947	3.2828	0.7503	-0.4144	0.2909	0.5363
Tiam1	_S[Phospho (STY)]LEGIFDDTVPDGK_.2	#N/A	3.2090	4.0142	3.8958	4.1065	3.8355	3.8940	4.2548	4.3955	3.1937	3.7838	0.9792	-0.0303	0.7705	0.1132
Tiam1	_FTIDSDAISASS[Phospho (STY)]PEKEPQQPAGGGDTDR_.3	Q60610_S1466_M1_Tiam1	3.3197	3.9064	4.0065	4.5092	3.7947	4.3307	4.2239	3.6093	3.2579	2.9754	1.1312	0.1778	0.1648	0.7829
Tiam1	_S[Phospho (STY)]LEGIFDDTVPDGKR_.2	#N/A	4.1662	3.3293	3.8999	6.6111	3.3099	3.3151	3.3874	3.5580	3.8570	3.1209	1.1794	0.2380	0.3747	0.4264
Satb1	_LEQNGS[Phospho (STY)]PLGR_.2	Q60611_S38_M1_Satb1	6.1601	6.3276	3.1106	7.0752	6.1110	6.1122	6.6950	5.8429	5.7820	6.0457	0.9548	-0.0667	0.7126	0.1472
Icam5	_LDDLDC[Carbamidomethyl (C)]PR_.2	#N/A	5.8724	5.9357	5.9323	6.1191	6.2632	5.5859	5.4748	6.3152	5.5647	5.8073	1.0278	0.0395	0.4213	0.3754
Icam5	_SFVGEPPR_.2	#N/A	5.4990	6.5346	6.7578	5.5745	6.6784	5.7073	6.1895	6.5950	6.4893	6.1661	0.9631	-0.0542	0.4750	0.3233
Icam5	_ASLTLTLLR_.2	#N/A	4.3490	4.8700	3.3641	5.5551	5.5144	3.8987	3.2857	4.6084	5.0631	4.8986	1.0287	0.0408	0.8227	0.0848
Icam5	_QLM[Oxidation (M)]C[Carbamidomethyl (C)]VVTLGGESR_.2	#N/A	3.0902	4.1359	3.7770	5.7911	4.4877	4.1012	3.9944	5.6443	3.6196	4.2467	0.9667	-0.0488	0.8068	0.0932
Icam5	_C[Carbamidomethyl (C)]TAVNGQGQAVK_.2	#N/A	3.4586	4.2149	3.1923	2.6097	4.1032	2.9716	3.9154	4.2651	3.0633	3.9142	0.9038	-0.1459	0.3674	0.4348
Icam5	_SGKEEVMEGPLR_.3	#N/A	3.0628	4.1634	3.7496	4.2522	3.6893	4.0738	3.9669	3.8662	3.5148	4.2192	0.9846	-0.0224	0.8179	0.0873
Grb2	_NYVTPVNR_.2	#N/A	7.0802	7.2272	7.2750	7.0673	7.6381	6.8317	6.6761	7.2288	6.8741	7.3859	1.0206	0.0295	0.4621	0.3353
Grb2	_NQQIFLR_.2	#N/A	6.4256	6.7768	6.7306	6.2753	7.0665	6.2329	6.8087	6.4911	6.6982	6.6529	0.9883	-0.0170	0.6670	0.1759
Grb2	_FNSLNELVDYHR_.2	#N/A	4.6130	5.3709	3.3455	5.1551	5.2336	5.3878	5.4590	5.2639	5.2817	4.9367	0.9266	-0.1100	0.3784	0.4221
Grb2	_ATADDELSFK_.2	#N/A	3.1004	5.5227	3.1705	4.2151	3.7269	3.3864	4.3634	4.2869	3.0851	4.3227	0.9599	-0.0590	0.7681	0.1146
Flot2	_NVVLQTLEGHLR_.2	#N/A	3.2546	4.1325	3.9829	4.9560	3.9470	2.7071	3.9552	4.5607	3.9237	3.8288	0.9417	-0.0866	0.5843	0.2333
Hnrnpd	_IFVGGLSPDTPEEK_.2	#N/A	7.9210	8.3929	8.5931	8.5768	8.3006	8.2429	8.5237	8.1454	8.1431	8.2229	1.0096	0.0138	0.6018	0.2205
Hnrnpd	_YHNVGLSK_.2	#N/A	5.4506	5.3873	5.6158	6.0595	6.0116	5.4297	4.7531	5.2595	5.3054	5.2243	1.1019	0.1400	0.0220	1.6578
Hnrnpd	_IDASKNEEDEGHSNSS[Phospho (STY)]PR_.3	#N/A	8.5907	8.8317	9.5776	8.8895	8.8467	9.0206	8.9274	8.8476	9.0632	8.9730	1.0007	0.0011	0.9705	0.0130
Hnrnpd	_IREYFGGFGEVESIELPM[Oxidation (M)]DNK_.3	#N/A	5.2397	5.8852	6.6752	6.3098	4.8930	6.1089	5.8117	5.7285	5.8896	6.0804	0.9956	-0.0063	0.9431	0.0254
Hnrnpd	_EYFGGFGEVESIELPM[Oxidation (M)]DNK_.2	#N/A	5.8444	6.4019	6.7165	6.6443	6.3759	6.5266	6.6033	6.1474	6.2220	6.3776	1.0127	0.0182	0.6616	0.1794
Hnrnpd	_NEEDEGHSNS[Phospho (STY)]SPR_.2	Q60668_S82_M1_Hnrnpd	6.5009	7.1161	7.3713	7.9984	3.0871	3.1430	3.3647	4.1018	4.1185	5.9664	1.3377	0.4197	0.2517	0.5992

Hnrnpd	_IDASKNEEDEGHSNS[Phospho (STY)]S[Phospho (STY)]PRHTEAAAAQR_.4	#N/A	3.2363	4.0415	3.9231	4.4257	3.8628	3.8667	4.1404	3.6928	3.3413	4.3927	1.0002	0.0003	0.9975	0.0011
Ptprn	_AEDS[Phospho (STY)]SEGHEEEVLGGRGEK_.3	#N/A	4.0481	3.3524	8.9884	3.7367	3.1737	8.4245	3.4514	4.3818	4.0304	3.7037	1.3586	0.4421	0.3391	0.4697
Ptprn	_AEDS[Phospho (STY)]SEGHEEEVLGGR_.3	#N/A	7.5815	6.9285	7.0094	7.0229	5.8949	3.8238	7.3578	6.2741	4.3877	3.9104	1.1631	0.2180	0.3715	0.4300
Ppp5c	_SVVDSLDIESM[Oxidation (M)]TIEDEYSGPK_.2	#N/A	5.8678	6.8193	6.9077	7.7125	6.4438	7.4483	6.4701	6.8066	6.5930	5.7661	1.0714	0.0995	0.2685	0.5711
Ppp5c	_AEGYEVAHGGR_.2	#N/A	5.3322	4.7751	4.4735	4.8714	5.5217	5.1394	4.8142	5.1095	5.3548	5.2141	0.9796	-0.0297	0.6433	0.1916
Ppp5c	_AFLEENQLDYIIR_.2	#N/A	4.5148	5.0440	5.7576	4.6829	3.3560	4.7128	4.9798	3.8624	4.8267	4.3486	1.0385	0.0546	0.7080	0.1500
Ppp5c	_FYSQAIELNPGNAIYYGNR_.2	#N/A	5.5839	5.4714	5.0171	5.2960	5.1456	5.4166	5.9153	5.7769	5.3915	4.6347	0.9801	-0.0290	0.6793	0.1679
Ppp5c	_TQANDYFK_.2	#N/A	4.2422	3.3562	4.5567	4.6791	4.6738	4.1561	3.2732	4.3547	4.2085	3.5749	1.1102	0.1508	0.2303	0.6377
Ppp5c	_TEC[Carbamidomethyl (C)]YGYALGDATR_.2	#N/A	6.0796	3.1216	3.9545	5.6843	5.0392	6.0901	3.6465	3.5029	6.0567	3.1755	1.2196	0.2864	0.3007	0.5218
Ppp5c	_GNHETDNMNQIYGFEGEVK_.3	#N/A	4.3528	3.6571	3.5387	4.0414	3.4784	4.2511	3.7561	4.0771	3.7257	4.0084	0.9987	-0.0019	0.9803	0.0086
Psmb6	_LAAIQESGVER_.2	#N/A	6.0465	6.4824	6.6894	7.0830	7.0469	6.8272	6.7963	6.9405	7.0408	6.9126	0.9673	-0.0480	0.2957	0.5291
Psmb6	_QVLLGDQIPK_.2	#N/A	6.4742	6.7046	6.3975	5.6554	6.8447	5.9022	5.3855	6.4371	6.1109	6.2551	1.0467	0.0659	0.3709	0.4308
Psmb6	_TTTGSYIANR_.2	#N/A	5.3977	6.2661	5.5299	5.7852	5.9921	5.7476	5.7142	5.8554	5.7078	5.1937	1.0300	0.0427	0.4166	0.3803
Tcf4	_SSSGSWGTGGHPS[Phospho (STY)]PSR_.2	Q60722_S66_M1_Tcf4	3.0659	4.9301	4.3004	5.4897	3.7105	4.4150	5.8180	3.1570	5.1190	3.5215	0.9806	-0.0282	0.9014	0.0451
Tcf4	_NGGQASSS[Phospho (STY)]PNYEGPLHSLQSR_.3	Q60722_S371_M1_Tcf4	3.9382	3.8639	4.1142	3.3874	3.6853	3.4320	4.1165	3.4296	3.4518	3.2030	1.0526	0.0739	0.4094	0.3879
Tcf4	_DLGSHDNLS[Phospho (STY)]PPFVNSR_.3	Q60722_S92_M1_Tcf4	4.1154	3.4198	3.3014	3.8040	3.2411	2.9875	3.5187	4.3144	3.9630	3.7710	0.8937	-0.1621	0.1340	0.8729
Csnk2a1	_QLYQTLTDYDIR_.2	#N/A	3.0909	5.0215	4.6161	5.2901	4.4291	4.5974	4.4791	4.8785	4.7869	4.5961	0.9621	-0.0557	0.6655	0.1768
Csnk2a1	_FNDILGR_.2	#N/A	5.0512	4.3238	4.7165	4.9025	5.0869	5.0346	4.0104	5.1921	4.4821	4.4290	1.0716	0.0997	0.2217	0.6542
Csnk2a1	_FVHSENQHLVSPEALDFLDK_.4	#N/A	4.0154	4.0845	3.2013	4.1948	4.8869	3.9327	3.4187	4.6750	4.0632	5.0684	0.9411	-0.0876	0.5403	0.2673
Csnk2a1	_YSEVFEAINITNNEK_.2	#N/A	12.6436	5.0414	3.7007	12.8938	4.1006	7.3929	12.5239	12.4878	3.5637	12.5390	0.7422	-0.4301	0.3672	0.4351
Csnk2a1	_VLGTEDLYDYIDKYNIELDPR_.3	#N/A	3.4530	4.2582	3.7417	3.7447	3.4202	3.7321	4.0108	1.1825	3.1246	3.1086	1.3040	0.3829	0.1181	0.9279
Csnk2a1	_TPALVFEHVNNTDFK_.2	#N/A	3.8580	4.1730	4.1226	4.0320	3.9192	4.2540	3.8336	4.2848	3.7040	3.4387	1.0641	0.0896	0.1647	0.7834
Csnk2a1	_EYWDYESHVVEWGNQDDYQLVR_.3	#N/A	3.8896	3.3366	4.5764	3.5102	1.5307	3.3997	3.2929	3.0395	4.1889	3.5452	0.9594	-0.0598	0.8026	0.0955
Khdrbs1	_KDDEENYLDLFSHK_.3	#N/A	7.2104	7.0501	7.4491	7.3198	7.3791	6.8745	7.2934	6.6000	6.9678	6.9850	1.0362	0.0514	0.1482	0.8291
Khdrbs1	_DPSGAHPSVR_.2	#N/A	5.3680	6.4052	5.2787	6.3308	6.5116	6.0613	6.0334	6.0287	5.5636	5.9683	1.0160	0.0228	0.7535	0.1229
Khdrbs1	_S[Phospho (STY)]C[Carbamidomethyl (C)]SKDPSGAHPSVR_.2	#N/A	3.5200	4.0318	3.9186	3.7827	4.0252	4.8076	4.2408	3.7223	3.2602	3.5285	1.0885	0.1223	0.2694	0.5696
Cldn11	_MGHEPGVAK_.2	#N/A	5.5923	5.5085	4.2140	6.1185	5.1383	3.0006	5.6575	5.1932	3.9529	4.9482	0.9981	-0.0027	0.9889	0.0048
Cldn11	_KM[Oxidation (M)]DELGSK_.2	#N/A	6.6263	5.9580	6.0246	6.3385	6.6200	6.2327	6.2149	6.1507	6.2899	6.0309	1.0208	0.0297	0.4228	0.3739
Cldn11	_FYYSSGSSS[Phospho (STY)]PTHAK_.2	#N/A	12.8530	13.1154	13.7596	13.3250	12.1826	12.8083	13.6202	12.8903	13.0708	12.9757	0.9900	-0.0146	0.6734	0.1717
Cldn11	_FYYSSGSSS[Phospho (STY)]PTHAKSAHV_.3	#N/A	7.6243	3.5980	5.5142	3.8077	5.0932	6.3948	5.3141	6.1662	6.0566	5.5991	0.9230	-0.1156	0.5929	0.2270
Gas7	_KSTGDSQNLGSSS[Phospho (STY)]PGRK_.2	#N/A	5.5908	5.0005	6.2271	6.1363	6.4850	6.4487	3.6354	5.8208	5.5068	5.7649	1.1543	0.2070	0.1528	0.8159
Gas7	_HETDM[Oxidation (M)]FNQSTVEPVDQLLR_.3	#N/A	4.0354	5.4793	5.3038	4.0470	5.2836	5.5436	5.1963	5.1899	4.9983	5.6355	0.9417	-0.0866	0.4410	0.3556
Gas7	_STGDSQNLGSSS[Phospho (STY)]PGR_.2	#N/A	7.9570	8.5363	8.6963	9.2830	8.3829	8.2343	8.0730	8.6078	8.3999	7.9929	1.0298	0.0424	0.3671	0.4352
Gas7	_STGDSQNLGSSS[Phospho (STY)]PGRK_.2	#N/A	7.8753	8.1228	8.7887	8.6561	7.9797	8.2118	8.3835	4.3435	8.1843	8.1174	1.1399	0.1889	0.2357	0.6276
Gas7	_KSTGDSQNLGS[Phospho (STY)]SSPGR_.2	Q60780_S106_M1_Gas7	3.2870	3.9391	3.9738	4.4765	3.9136	4.2980	4.1912	3.6420	3.2906	4.4435	1.0230	0.0328	0.7679	0.1147
Gas7	_C[Carbamidomethyl (C)]VDLYNQAQSK_.2	#N/A	3.1930	3.9982	3.8798	4.3825	3.8195	3.9100	4.0971	3.7360	3.3846	4.9211	0.9576	-0.0624	0.6145	0.2114
Rasa3	_YGS[Phospho (STY)]QEHPIGDK_.2	#N/A	9.3518	9.1124	9.9195	9.6643	8.4720	9.0731	9.6065	9.6937	9.5244	9.3622	0.9705	-0.0431	0.3190	0.4962
Rasa3	_T[Phospho (STY)]RYGSQEHPIGDK_.3	Q60790_T805_M1_Rasa3	3.9422	4.1351	4.0168	4.3845	3.8416	3.8013	4.8966	4.7096	4.6240	4.9892	0.8367	-0.2572	0.0003	3.5461
Ppp1r1b	_DGNSEDQVEGR_.2	#N/A	7.6875	7.4702	7.5170	8.0395	7.8730	7.4167	7.3327	7.6606	7.4825	7.6782	1.0171	0.0244	0.3898	0.4091
Ppp1r1b	_ATLSEPGEEPQHPS[Phospho (STY)]PP_.2	Q60829_S192_M1_Ppp1r1b	15.1542	15.2688	16.0313	15.6211	15.3712	15.3473	15.5584	15.1207	15.0955	15.5092	1.0094	0.0136	0.4658	0.3318
Ppp1r1b	_GLEGPWERPPPLDEPQR_.3	#N/A	7.8558	7.7440	7.6441	7.8667	8.4262	8.2291	7.5616	6.4375	7.6334	8.2345	1.0662	0.0925	0.1772	0.7517
Ppp1r1b	_GTVGQKPTC[Carbamidomethyl (C)]GR_.2	#N/A	6.8445	7.0899	6.6280	6.8522	8.1215	6.9729	6.3639	6.6212	6.8306	7.2354	1.0476	0.0671	0.3255	0.4875
Ppp1r1b	_VSEHSS[Phospho (STY)]PEEEASPHQR_.3	#N/A	11.2630	11.0158	11.9411	11.6028	11.8027	11.4781	11.2150	11.3611	11.4298	11.6765	1.0085	0.0122	0.6257	0.2036
Ppp1r1b	_IQFSVPAPPSQLDPR_.2	#N/A	5.5500	6.3051	7.1942	4.0227	6.7085	6.2790	5.7181	6.2181	5.6628	6.4588	0.9993	-0.0011	0.9941	0.0026
Ppp1r1b	_GLEGPWERPPPLDEPQRDGNSEDQVEGR_.4	#N/A	6.4035	6.6212	6.7681	7.1792	6.6943	6.6689	6.3656	6.4006	5.7342	5.4644	1.1221	0.1662	0.0119	1.9259
Ppp1r1b	_ELGYPQEDDEEDEDEEEDEEEDSQAEVLK_.3	#N/A	4.3391	3.0033	4.0728	3.6166	3.4829	3.1418	3.7648	3.3846	3.6837	3.2938	1.0220	0.0314	0.7885	0.1032
Serpinb6	_FYEAELEELDFQGATEESR_.2	#N/A	6.7463	7.1019	7.9328	7.7241	6.8201	7.6966	6.9511	6.7750	6.8179	6.8093	1.0729	0.1015	0.0941	1.0265
Stip1	_LAYINPDLALEEK_.2	#N/A	7.6502	8.1119	8.2359	8.3042	8.0204	7.9864	8.1549	8.2220	7.6295	8.2064	0.9998	-0.0003	0.9920	0.0035
Stip1	_LDPQNHVLYSNR_.3	#N/A	7.1387	6.8088	6.5372	6.9941	7.4445	7.0086	6.5974	6.2633	6.9682	7.2030	1.0341	0.0484	0.3370	0.4724
Stip1	_HDS[Phospho (STY)]PEDVKR_.2	#N/A	7.0737	7.2374	7.4857	7.8199	7.4025	7.5056	7.3438	7.1616	7.0694	7.3955	1.0246	0.0351	0.2493	0.6032
Stip1	_ALDLDSSC[Carbamidomethyl (C)]K_.2	#N/A	6.7327	6.5006	6.3724	6.9672	7.1749	6.7201	5.8964	6.2486	6.3690	6.2843	1.0879	0.1216	0.0130	1.8868
Stip1	_DPQALSEHLK_.2	#N/A	5.9872	5.9377	4.7128	6.0543	6.5299	5.4639	5.9998	5.9675	5.9241	6.1014	0.9638	-0.0532	0.5159	0.2875
Stip1	_ALSAGNIDDALQC[Carbamidomethyl (C)]YSEAIK_.2	#N/A	5.7949	5.9728	6.2515	6.8998	6.0128	5.9446	6.4986	6.0314	5.0856	6.1211	1.0357	0.0506	0.5167	0.2867
Stip1	_DPQALSEHLKNPVIAQK_.3	#N/A	5.4594	6.0788	6.5657	6.1070	6.9748	5.1596	6.1151	6.2146	6.0746	6.0093	0.9925	-0.0109	0.8976	0.0469
Stip1	_TPDVLKK_.2	#N/A	5.6153	5.9656	5.6184	6.0695	5.8898	5.0878	5.1743	5.7631	5.5500	5.5816	1.0345	0.0490	0.3821	0.4178
Stip1	_HDS[Phospho (STY)]PEDVK_.2	#N/A	7.7099	7.7168	8.0944	8.3852	7.7821	7.6564	7.9736	7.7090	7.7951	8.1389	0.9983	-0.0024	0.9376	0.0280
Stip1	_ELIEQLQNKPSDLGTK_.3	#N/A	6.2566	6.1316	5.9340	6.6161	6.2102	6.4550	5.9850	5.9426	6.1542	6.2949	1.0284	0.0404	0.2469	0.6074
Stip1	_LM[Oxidation (M)]DVGLIAIR_.2	#N/A	7.0026	6.9447	6.4151	6.8644	6.8536	6.8453	6.8374	6.7712	6.6304	6.9581	1.0032	0.0046	0.8603	0.0654
Stip1	_HYTEAIKR_.2	#N/A	6.9518	6.3570	3.1879	6.3046	6.0995	6.7401	6.7524	6.2451	6.5598	6.5643	0.9096	-0.1367	0.4304	0.3661
Stip1	_HDS[Phospho (STY)]PEDVKRR_.3	#N/A	4.5127	5.4008	7.2359	5.7020	5.8939	5.9443	5.5934	5.6857	5.8288	5.4074	1.0271	0.0386	0.7467	0.1268
Stip1	_HYTEAIK_.2	#N/A	2.8334	3.2999	3.4029	3.9056	3.1111	3.2980	3.6203	2.5222	3.3751	3.7327	0.9988	-0.0018	0.9888	0.0049
Stip1	_S[Phospho (STY)]LLSDPTYR_.2	Q60864_S145_M1_Stip1	5.0859	3.4901	4.4228	3.4246	2.8617	6.0529	5.8499	5.7127	4.3424	5.8634	0.7760	-0.3659	0.1092	0.9617
Stip1	_LILEQMQK_.2	#N/A	4.3709	4.0151	4.1431	5.5475	4.3186	3.6467	4.3604	3.4727	3.1213	3.1119	1.2342	0.3036	0.0752	1.1237
Stip1	_DAIHFYNK_.2	#N/A	4.5328	3.3923	4.3310	4.5419	4.3660	3.1543	4.1786	4.3317	4.4343	3.2998	0.9980	-0.0029	0.9834	0.0073
Stip1	_TYEEGLKHEANNLQLK_.3	#N/A	6.0531	6.6451	6.9684	6.9353	6.8339	6.3969	6.5462	6.1412	6.5091	6.6472	1.0275	0.0392	0.4040	0.3936
Stip1	_IGNSYFKEEK_.2	#N/A	4.6780	4.9421	4.4516	5.4263	5.2321	4.9768	4.0321	4.5403	4.5984	4.9160	1.0950	0.1309	0.1010	0.9959
Stip1	_NKGNEC[Carbamidomethyl (C)]FQK_.2	#N/A	4.3572	3.9717	3.5951	4.2019	4.9017	4.0729	3.8124	3.5359	3.6693	4.0647	1.1095	0.1499	0.1233	0.9089
Stip1	_AMDVYQK_.2	#N/A	2.9805	3.7368	4.2150	3.7588	3.6251	2.9995	4.3935	3.2424	3.5414	3.4361	0.9724	-0.0403	0.7589	0.1198
Stip1	_AMADPEVQQIMSDPAMR_.2	#N/A	3.1283	3.8846	4.0284	4.5311	3.7728	4.3526	4.2458	3.5874	3.2360	2.9972	1.1231	0.1675	0.2270	0.6441
Caprin1	_YQEVTNNLEFAK_.2	#N/A	3.0452	3.7442	3.7320	5.6569	4.6675	4.0577	3.9494	3.8838	3.5324	4.2017	1.0665	0.0929	0.6009	0.2212
Reep5	_HESQVDSVVK_.2	#N/A	5.6799	5.6008	5.9266	5.6389	5.5606	5.9781	5.6960	6.0681	6.1568	5.7077	0.9701	-0.0437	0.2149	0.6679
Arhgef2	_EAQELGS[Phospho (STY)]PEDR_.2	#N/A	12.5955	12.5479	12.8530	13.0606	11.6068	12.5406	13.0021	12.7721	12.5686	12.6464	0.9833	-0.0243	0.4446	0.3520
Arhgef2	_LS[Phospho (STY)]PPHSPR_.2	#N/A	9.6914	9.3384	8.9778	9.8315	8.2836	9.0113	3.3645	9.0287	9.4501	9.3814	1.1771	0.2353	0.2852	0.5448
Arhgef2	_LQDSS[Phospho (STY)]DPDTGSEEEVSSR_.2	Q60875_S940_M1_Arhgef2	3.2451	4.0279	4.2104	5.4658	5.2209	5.7752	6.1266	5.4542	5.5186	3.3248	0.9122	-0.1326	0.5377	0.2695
Arhgef2	_LES[Phospho (STY)]FESLR_.2	#N/A	7.5977	6.8374	3.2309	7.8377	3.1707	7.1172	7.1525	7.5566	7.4242	3.7006	0.9236	-0.1146	0.7196	0.1429
Arhgef2	_SLHRPFDDREAQELGS[Phospho (STY)]PEDR_.3	#N/A	3.5644	8.2299	4.0646	9.4178	7.9648	9.1422	7.5307	9.3247	9.0016	8.8273	0.8147	-0.2957	0.2689	0.5704
Arhgef2	_LQEIYNR_.2	#N/A	4.0844	4.5839	3.8621	2.9569	4.8103	3.5476	4.7721	4.6538	4.5235	4.9805	0.8398	-0.2519	0.0654	1.1842
Arhgef2	_TFNGS[Phospho (STY)]IELC[Carbamidomethyl (C)]R_.2	Q60875_S710_M1_Arhgef2	3.3362	3.8900	4.0230	4.5256	3.7783	4.3471	4.2403	3.5929	3.2414	2.9918	1.1327	0.1798	0.1636	0.7861
Arhgef2	_QILSQS[Phospho (STY)]TDSLNMR_.2	Q60875_S174_M1_Arhgef2	5.4415	3.9197	3.2604	4.0769	3.8080	3.6863	5.2473	4.2899	4.2611	3.8699	0.9128	-0.1316	0.4118	0.3853

Arhgef2	_S[Phospho (STY)]LPAGDALYLSFNPPQPSR_.2	Q60875_S885_M1_Arhgef2	3.7012	7.9159	8.2499	3.3898	5.9384	3.4018	3.1045	3.2278	7.5616	3.3568	1.2597	0.3331	0.4587	0.3384
Arhgef2	_LES[Phospho (STY)]FESLRGER_.2	#N/A	3.3200	4.0762	4.5545	4.0982	3.9645	7.7577	7.8583	4.4038	7.5057	3.7755	0.7864	-0.3467	0.3088	0.5103
Arhgef2	_SVSTTNIAGHFNDES[Phospho (STY)]PLGLR_.3	Q60875_S163_M1_Arhgef2	3.9733	3.2776	3.1592	6.1652	3.0990	3.1297	3.3766	5.7240	4.1052	3.6289	0.9031	-0.1471	0.5976	0.2236
Arhgef2	_LQDSSDPDTGSEEEVSSRLS[Phospho (STY)]PPHSPR_.3	#N/A	5.2182	5.7143	3.9991	6.2567	4.1603	5.8279	3.3765	3.5446	4.1091	5.6255	1.2479	0.3195	0.1359	0.8667
Elavl2	_VSYARPSS[Phospho (STY)]ASIR_.2	Q60899_S119_M1_Elavl2	6.6328	7.0013	3.3446	4.0484	4.2555	6.6417	3.7631	6.9104	3.7186	4.0154	1.1562	0.2094	0.5023	0.2990
Elavl2	_ELEQLFSQYGR_.2	#N/A	3.7327	6.0778	5.4668	6.3204	5.7338	5.8323	6.1004	5.9453	5.8594	5.7473	0.9348	-0.0973	0.4415	0.3551
Elavl2	_IEAEEAIK_.2	#N/A	4.2411	3.5455	3.4271	6.0944	3.3668	4.8007	3.2226	4.1887	4.8869	3.8968	1.0487	0.0686	0.7538	0.1227
Elavl2	_TNQAILSQLYQS[Phospho (STY)]PNR_.2	Q60899_S221_M1_Elavl2	3.1254	6.8641	6.8552	7.9693	3.7614	4.1731	6.5654	3.7679	3.4165	5.3574	1.1426	0.1924	0.5787	0.2376
Elavl2	_FANNPSQK_.2	#N/A	3.7403	3.5494	3.4310	3.9337	3.3707	4.3588	3.6483	4.1848	3.8334	3.1580	1.0066	0.0095	0.9254	0.0337
Eps15l1	_LQQEETQLEQSIQAGR_.2	#N/A	4.8645	5.5710	6.6976	3.1563	5.3150	4.7855	5.6322	5.1729	5.2325	4.4585	0.9846	-0.0223	0.9016	0.0450
Eps15l1	_STPSHGSVSSLNSTGS[Phospho (STY)]LS[Phospho (STY)]PK_.2	#N/A	3.8506	4.2575	4.1131	4.8957	4.8208	4.4268	5.1051	4.2997	5.2224	4.7896	0.9052	-0.1437	0.1195	0.9227
Eps15l1	_ESDPFHS[Phospho (STY)]SSSDDFFKK_.3	Q60902_S669_M1_Eps15l1	3.6860	3.4406	3.4196	4.4642	3.2876	3.9438	4.1250	4.0443	4.3433	4.1415	0.8903	-0.1676	0.0814	1.0893
Eps15l1	_GIDPPQVLS[Phospho (STY)]PDM[Oxidation (M)]VPPSER_.2	Q60902_S353_M1_Eps15l1	7.8652	9.5640	8.7797	10.0000	8.5524	9.2394	8.3496	10.2919	10.1134	7.0663	1.0050	0.0072	0.9520	0.0214
Eps15l1	_TVFAGAVPVLPAS[Phospho (STY)]PPPK_.2	#N/A	3.2132	4.0184	3.9000	8.0991	3.8398	3.8897	4.2505	4.3998	3.1980	3.7795	1.1501	0.2018	0.5492	0.2603
Eps15l1	_TVFAGAVPVLPAS[Phospho (STY)]PPPKDSLR_.3	#N/A	4.1503	3.4547	4.1444	7.9775	3.2760	3.8079	3.1751	5.9379	4.2955	3.8423	1.0361	0.0512	0.8812	0.0549
Mef2a	_SEPIS[Phospho (STY)]PPRDR_.2	Q60929_S406_M1_Mef2a	3.7192	7.0796	3.4529	9.1450	2.8630	3.7616	8.7229	8.7542	4.3036	4.1748	0.7711	-0.3750	0.3927	0.4060
Vdac2	_YQLDPTASISAK_.2	#N/A	9.2116	9.2762	9.4141	9.6534	9.6328	9.4722	9.1766	9.3116	9.5245	9.2292	1.0143	0.0205	0.2627	0.5806
Vdac2	etyl (Protein N-term)]AEC[Carbamidomethyl (C)]C[Carbamidomethyl (C)]VPVC[Carbamidomethyl (C)]F	#N/A	8.9381	8.9636	8.4680	9.1515	9.1610	8.5226	8.8214	8.8456	9.1192	8.6905	0.9998	-0.0003	0.9922	0.0034
Vdac2	_SNFAVGYR_.2	#N/A	8.2774	8.3879	8.5617	8.2979	8.4292	8.3642	6.2996	8.4118	8.5754	8.3985	1.0587	0.0823	0.3129	0.5047
Vdac2	_LTLSALVDGK_.2	#N/A	7.2845	8.1905	7.6198	7.5611	7.7651	7.8398	6.3376	8.0041	7.7769	7.6298	1.0367	0.0520	0.4377	0.3588
Vdac2	_WC[Carbamidomethyl (C)]EYGLTFTEK_.2	#N/A	6.7572	6.7990	6.7193	7.2320	6.9266	6.9807	6.9679	6.8806	7.2861	6.7404	0.9905	-0.0138	0.6324	0.1990
Vdac2	_GFGFGLVK_.2	#N/A	6.0705	6.8523	6.3271	6.2545	6.4739	6.3229	5.4875	6.2384	6.7477	6.6843	1.0150	0.0214	0.7330	0.1349
Vdac2	_LTFDTTFSPNTGK_.2	#N/A	6.9239	6.4451	6.2913	6.7222	7.0369	6.7354	6.7430	6.8153	6.8384	6.4244	0.9981	-0.0028	0.9390	0.0273
Vdac2	_SFNAGGHK_.2	#N/A	7.8151	3.9780	7.3683	7.5528	8.3709	7.1711	7.0794	7.5867	7.6599	7.2502	0.9525	-0.0702	0.6731	0.1719
Vdac2	_VNNSSLIGVGYTQTLRPGVK_.2	#N/A	6.0490	5.3700	5.3727	5.8340	5.7127	5.5540	5.0138	5.5400	6.1822	5.8534	1.0002	0.0004	0.9955	0.0020
Vdac2	_LTFDTTFSPNTGKK_.2	#N/A	4.3493	6.6272	6.6285	7.1494	6.7814	6.7225	6.6981	6.5935	6.5882	6.4154	0.9700	-0.0440	0.7132	0.1468
Vdac2	_TGDFQLHTNVNNGTEFGGSIYQK_.3	#N/A	5.8012	4.3997	3.3772	4.4218	2.7872	4.9817	5.5421	4.0803	4.3793	4.7363	0.9168	-0.1253	0.5390	0.2684
Vdac2	_WNTDNTLGTEIAIEDQIC[Carbamidomethyl (C)]QGLK_.3	#N/A	3.7259	3.2814	4.7008	4.5042	2.8697	3.7549	3.1516	3.9978	3.6378	4.1815	1.0037	0.0054	0.9745	0.0112
Vdac2	_SC[Carbamidomethyl (C)]SGVEFSTSGSSNTDTGK_.2	#N/A	3.4725	4.2288	3.2062	4.2508	4.1171	4.0083	4.3991	4.2512	3.0494	3.9281	0.9933	-0.0097	0.9378	0.0279
Vdac3	[Acetyl (Protein N-term)]C[Carbamidomethyl (C)]NT[Phospho (STY)]PTYC[Carbamidomethyl (C)]DLGK_	Q60931_T4_M1_Vdac3	8.7291	8.4486	8.7894	8.8843	8.2383	8.5238	8.9984	8.5034	8.5582	8.5824	0.9933	-0.0097	0.7127	0.1471
Vdac3	_LTLDTIFVPNTGK_.2	#N/A	8.6357	8.2708	8.6869	8.6409	8.4551	8.6609	8.8790	8.6396	8.7719	8.4738	0.9847	-0.0222	0.2551	0.5932
Vdac3	_LSQNNFALGYK_.2	#N/A	7.7529	7.6757	7.4900	8.2839	7.7517	7.7115	7.6085	7.9078	7.8998	8.0324	0.9893	-0.0156	0.5968	0.2242
Vdac3	_LTLDTIFVPNTGKK_.2	#N/A	7.8621	7.7494	8.5724	7.9690	7.4144	7.9639	7.8271	7.8219	8.0167	7.9006	1.0038	0.0055	0.8813	0.0549
Vdac3	_VC[Carbamidomethyl (C)]NYGLTFTQK_.2	#N/A	7.4887	6.7715	7.0120	7.5776	7.0437	6.8240	7.0832	6.9033	6.8875	6.9405	1.0239	0.0340	0.3737	0.4275
Vdac3	_NFNAGGHK_.2	#N/A	7.6386	7.2028	7.0433	7.2970	8.3005	6.9968	7.0551	7.2850	7.4158	7.1572	1.0256	0.0364	0.4942	0.3061
Vdac3	_VNNASLIGLGYTQTLRPGVK_.2	#N/A	5.6510	5.5933	6.2887	6.0884	5.2599	5.6276	5.8129	5.7617	5.9143	5.4450	1.0031	0.0045	0.9326	0.0303
Vdac3	_IETSINLAWTAGSNNTR_.2	#N/A	6.5272	4.0058	6.7812	6.1154	5.3486	4.9579	6.9866	5.0951	6.2179	6.4789	0.9077	-0.1398	0.3854	0.4140
Vdac3	_WNTDNTLGTEISWENK_.2	#N/A	5.5766	5.4944	3.7391	4.2418	3.6789	4.0506	6.4064	5.4509	5.3122	5.9016	0.7739	-0.3698	0.0260	1.5848
Vdac1	_YQVDPDAC[Carbamidomethyl (C)]FSAK_.2	#N/A	9.5666	9.8272	9.8456	10.1466	10.1394	9.8216	9.6449	9.9129	9.7122	9.8735	1.0108	0.0154	0.4183	0.3785
Vdac1	_LTFDSSFS[Phospho (STY)]PNTGK_.2	#N/A	8.8883	8.6988	8.6388	9.4261	8.6812	8.9757	9.0300	8.8985	8.9828	8.9962	0.9897	-0.0149	0.5630	0.2495
Vdac1	_VTQSNFAVGYK_.2	#N/A	9.3590	9.3955	9.5179	9.7515	9.7142	9.5224	9.2466	9.3417	9.4779	9.5863	1.0138	0.0198	0.2337	0.6314
Vdac1	_WTEYGLTFTEK_.2	#N/A	9.3803	9.2282	9.4017	9.4947	9.4377	9.4763	9.2758	9.2121	9.4958	9.3817	1.0066	0.0095	0.3992	0.3988
Vdac1	_TDEFQLHTNVNDGTEFGGSIYQK_.3	#N/A	8.9739	9.1015	9.1514	9.5435	8.5706	8.7088	9.2950	8.5819	8.9993	8.8594	1.0083	0.0120	0.7347	0.1339
Vdac1	_GYGFGLIK_.2	#N/A	8.3536	8.4579	8.4890	8.2468	8.1460	8.4714	8.0939	8.3601	8.7156	8.8457	0.9832	-0.0245	0.3763	0.4244
Vdac1	_SENGLEFTSSGSANTETTK_.2	#N/A	5.9116	6.1043	6.2453	8.3190	5.6391	6.2007	3.9053	6.6950	5.9915	2.7708	1.3228	0.4036	0.1106	0.9561
Vdac1	_TKSENGLEFTSSGSANTETTK_.3	#N/A	5.1596	5.2951	3.1180	5.9444	5.7488	5.7941	4.5723	5.4006	5.1297	5.1705	1.0214	0.0305	0.8501	0.0705
Vdac1	_LTFDSSFS[Phospho (STY)]PNTGKK_.2	#N/A	9.4088	9.7284	10.1787	10.5591	9.5432	9.7752	10.0143	9.8550	9.8426	9.7956	0.9989	-0.0017	0.9605	0.0175
Vdac1	_LETAVNLAWTAGNSNTR_.2	#N/A	6.0155	6.0027	6.0312	3.2682	5.4530	5.7422	7.0625	3.7330	5.6113	6.3930	0.9507	-0.0730	0.7315	0.1358
Vdac1	_VNNSSLIGLGYTQTLKPGIK_.2	#N/A	8.0422	8.2286	8.4989	8.3743	8.1562	8.2153	8.2475	7.9683	8.0827	8.3671	1.0106	0.0151	0.4481	0.3487
Vdac1	_EHINLGC[Carbamidomethyl (C)]DVDFDIAGPSIR_.3	#N/A	8.0778	7.4897	6.9542	8.0475	7.8155	8.0813	7.9843	7.5080	7.6498	8.3842	0.9826	-0.0253	0.6329	0.1986
Vdac1	_WNTDNTLGTEITVEDQLAR_.2	#N/A	6.3734	5.8232	6.5745	6.8508	6.2032	6.1878	6.3541	6.1347	6.4977	6.3554	1.0000	0.0000	1.0000	0.0000
Vdac1	_KLETAVNLAWTAGNSNTR_.3	#N/A	5.5373	5.4222	6.2765	3.4826	4.1110	5.9676	5.7510	3.4458	5.4328	5.8397	1.0030	0.0044	0.9832	0.0074
Tbc1d1	_KSFS[Phospho (STY)]QPGLR_.2	#N/A	3.7175	2.9729	3.4511	4.4957	2.8612	3.7634	3.1432	4.0063	4.3053	4.1730	0.9070	-0.1408	0.3531	0.4521
Tbc1d1	_QSARPST[Phospho (STY)]PEPDC[Carbamidomethyl (C)]TQLEPTGD_.2	Q60949_T1242_M1_Tbc1d1	5.5666	4.2894	4.1710	3.8313	4.7993	3.6188	7.1087	3.1699	5.3978	5.7670	0.8169	-0.2917	0.2218	0.6540
Tbc1d1	_LNPSAS[Phospho (STY)]SPNFFK_.2	Q60949_S660_M1_Tbc1d1	4.1797	3.4840	3.3656	3.8683	3.3054	4.4241	7.1140	4.2502	3.8988	3.8353	0.7898	-0.3404	0.1692	0.7717
Tbc1d1	_SFS[Phospho (STY)]QPGLR_.2	#N/A	6.0209	3.2782	7.0487	3.6366	3.1665	3.4581	3.3513	3.7010	6.6843	6.5107	0.8761	-0.1908	0.5856	0.2324
Pml	_VPAPPGS[Phospho (STY)]PC[Carbamidomethyl (C)]R_.2	Q60953_S17_M1_Pml	9.5918	9.1560	9.6216	10.0256	9.0354	9.2554	9.8019	9.5533	9.5656	9.4400	0.9851	-0.0216	0.4902	0.3096
Pml	_LATSS[Phospho (STY)]PEQSWPSTFK_.2	Q60953_S515_M1_Pml	6.1142	3.4160	3.4157	3.4575	3.2373	5.8844	3.3609	4.1563	5.8772	4.0327	0.9764	-0.0344	0.9025	0.0446
Rbbp4	_[Acetyl (Protein N-term)]ADKEAAFDDAVEER_.2	#N/A	5.8848	5.3277	5.3994	5.9955	5.8426	5.1667	5.3400	5.4884	5.4164	5.5689	1.0274	0.0390	0.4325	0.3640
Ncor1	_AQLS[Phospho (STY)]PGLYDDSSAR_.2	Q60974_S1481_M1_Ncor1	6.2071	6.5674	6.9707	7.1257	6.1205	6.8526	7.1839	6.6851	6.6640	7.0094	0.9644	-0.0523	0.3248	0.4884
Ncor1	_VS[Phospho (STY)]PENLVDK_.2	Q60974_S2134_M1_Ncor1	6.5270	5.9179	6.1557	6.4565	6.0775	6.0194	6.5068	6.5158	6.1251	6.6188	0.9613	-0.0569	0.1393	0.8559
Ncor1	_SHIPSEPYEPIS[Phospho (STY)]PPQGPAVHEK_.3	Q60974_S2165_M1_Ncor1	3.6936	2.9491	3.4273	6.2077	2.8374	3.7872	3.6804	5.9633	4.3292	5.8506	0.7702	-0.3767	0.1782	0.7491
Ncor1	_EPAPLLSAQYETLS[Phospho (STY)]DSDD_.2	Q60974_S2449_M1_Ncor1	4.2869	3.5424	4.0206	3.5644	3.4307	3.1940	3.7126	5.8253	3.7359	3.2417	0.8896	-0.1687	0.3891	0.4100
Ncor1	_YS[Phospho (STY)]PESQSQTVLHPR_.2	Q60974_S2116_M1_Ncor1	3.6773	3.6651	3.4110	4.4556	3.4864	4.2431	3.1030	4.0464	4.3455	4.1329	0.9785	-0.0313	0.7919	0.1013
Klf3	_GS[Phospho (STY)]PPAAGGSPSSLK_.2	#N/A	3.2458	3.9803	3.9326	4.4353	3.8686	4.2568	4.1500	3.6832	3.3318	4.4023	1.0158	0.0226	0.8325	0.0796
Ppp2r5c	_RKS[Phospho (STY)]ELPQDPHTEK_.3	#N/A	5.0487	3.6653	4.9800	4.9392	3.4005	4.4792	3.9670	5.3420	4.9029	4.1638	0.9619	-0.0561	0.7051	0.1518
Gng7	_[Acetyl (Protein N-term)]S[Phospho (STY)]GTNNVAQAR_.2	Q61016_S2_M1_Gng7	11.6329	11.7795	12.1988	12.1572	11.6155	11.8504	11.9691	11.8553	11.6812	11.9094	1.0016	0.0023	0.8960	0.0477
Gng7	_KLVEQLR_.2	#N/A	8.2799	8.3485	8.1880	8.4299	9.0852	8.3641	8.3587	8.7711	8.7233	8.4217	0.9861	-0.0203	0.5343	0.2722
Gng7	_NDPLLVGVPASENPFK_.2	#N/A	7.6572	7.5058	7.4764	7.4899	7.7451	7.3940	7.7000	7.6945	7.4406	7.3979	0.9982	-0.0026	0.8868	0.0522
Gng7	_NDPLLVGVPASENPFKDK_.3	#N/A	3.8482	3.5337	3.3085	3.8111	4.4255	4.8670	3.5258	4.3397	3.9559	5.0591	0.9397	-0.0897	0.5344	0.2721
Gng7	_ASSDLMGYC[Carbamidomethyl (C)]EQHAR_.2	#N/A	4.2902	3.7736	3.5172	4.5389	4.0358	3.9889	3.8848	4.0181	3.8891	4.1040	1.0126	0.0181	0.7983	0.0978
Ncoa2	_LLQDSSS[Phospho (STY)]PVDLAK_.2	Q61026_S699_M1_Ncoa2	3.2923	3.9339	3.9791	4.4818	7.4026	3.8107	8.1251	3.6367	3.2853	4.4488	0.9199	-0.1205	0.7446	0.1281
Tmpo	_SST[Phospho (STY)]PLPTVSSSAENTR_.2	Q61029_T159_M1_Tmpo	10.1180	10.1096	11.0504	10.4760	9.3202	10.1333	10.7281	10.2295	10.1678	10.0938	0.9899	-0.0146	0.7466	0.1269
Tmpo	_EQGTESRSS[Phospho (STY)]T[Phospho (STY)]PLPTVSSSAENTR_.3	#N/A	4.0150	3.2705	3.7487	7.8695	7.1875	3.4659	7.6260	7.8465	7.4129	7.2257	0.6544	-0.6118	0.0380	1.4208
Tmpo	_QNGSNDSDRYS[Phospho (STY)]DNDEDSKIELK_.3	Q61029_S183_M1_Tmpo	2.9625	4.2637	3.6493	4.1186	3.5890	4.1405	3.8666	4.4361	4.3012	4.1190	0.9059	-0.1426	0.1807	0.7430
Hars1	_DQGGELLSLR_.2	#N/A	6.3048	5.7485	5.8616	6.1663	5.7172	6.0889	5.7376	5.6795	5.6445	5.5647	1.0574	0.0805	0.0327	1.4852
Hars1	_IFSIVEQR_.2	#N/A	4.7932	5.2974	6.3365	4.9444	4.8612	5.2153	5.3555	5.2748	5.2579	4.7416	1.0163	0.0233	0.7950	0.0996
Hars1	_AALEELVR_.2	#N/A	4.4693	4.9169	4.9058	4.9117	5.2789	4.5453	4.0856	4.8839	5.4824	5.7725	0.9569	-0.0636	0.5279	0.2774
Hars1	_YDLTVPFAR_.2	#N/A	5.5545	5.4810	3.7892	5.1270	5.5541	3.4253	4.9400	5.5953	5.9336	5.2234	0.8891	-0.1695	0.2805	0.5521

Hars1	_ASAEQIEEEVTK_.2	#N/A	3.7852	6.0033	4.4720	6.2245	6.1998	6.2184	6.4937	6.3260	5.9795	6.7825	0.8575	-0.2218	0.1450	0.8387
Hars1	_TIC[Carbamidomethyl (C)]SSVDKLDK_.3	#N/A	4.2396	1.1777	3.9733	3.4197	2.5293	3.1416	3.1344	3.4841	4.3473	3.1943	0.8701	-0.2007	0.4719	0.3261
Pak3	_[Acetyl (Protein N-term)]SDS[Phospho (STY)]LDNEEKPPAPPLR_.2	Q61036_S4_M1_Pak3	5.1211	8.8836	9.0677	10.0174	7.8319	9.0154	5.2636	8.8293	8.8527	8.9637	1.0433	0.0612	0.7677	0.1148
Tsc2	_STS[Phospho (STY)]LNERPK_.2	Q61037_S939_M1_Tsc2	8.6770	8.5373	9.0412	4.4924	7.3422	8.3717	8.7244	8.7402	8.6582	8.4572	0.8957	-0.1589	0.3278	0.4844
Tsc2	_IDIGRLS[Phospho (STY)]PEAK_.2	Q61037_S1412_M1_Tsc2	7.5981	6.5011	6.9914	6.5187	5.5184	6.3302	7.8927	7.6273	6.8532	7.1065	0.8923	-0.1644	0.0829	1.0814
Tsc2	_S[Phospho (STY)]PSGLRPR_.2	Q61037_S1455_M1_Tsc2	7.7093	7.6459	7.7006	3.6537	3.0908	7.3753	7.5476	7.7012	7.4654	7.4034	0.8229	-0.2812	0.2690	0.5702
Tsc2	_SSS[Phospho (STY)]SPELQTLQDILGDLGDK_.2	Q61037_S1388_M1_Tsc2	3.3940	4.6592	4.0808	4.5835	4.0206	3.7089	4.2982	3.5350	3.1836	3.0497	1.1587	0.2124	0.1333	0.8752
Tsc2	_SQS[Phospho (STY)]GILDGEAATWSATGEESR_.2	Q61037_S1421_M1_Tsc2	3.5304	4.3356	4.2172	5.3475	4.1569	3.5726	3.9334	4.5913	3.8593	3.1478	1.0799	0.1110	0.4705	0.3275
Tsc2	_SIS[Phospho (STY)]VSEHVVR_.2	Q61037_S981_M1_Tsc2	3.2501	3.9761	3.9369	4.4396	3.8766	3.8529	4.1542	3.6789	3.3275	5.7911	0.9176	-0.1241	0.4808	0.3181
Tsc2	_SM[Oxidation (M)]S[Phospho (STY)]GGHGLR_.2	#N/A	4.7260	4.7288	4.8604	3.7296	3.3606	3.9705	3.8778	4.8405	3.5847	4.3156	1.0180	0.0257	0.8515	0.0698
Tsc2	_LISS[Phospho (STY)]VDDFTEFV_.2	Q61037_S1806_M1_Tsc2	3.4569	3.8856	3.1905	4.2351	4.1014	4.0226	4.3834	4.2669	3.0651	3.9124	0.9766	-0.0342	0.7778	0.1092
Wbp4	_S[Phospho (STY)]LDKAKEEEK_.2	Q61048_S51_M1_Wbp4	3.6200	3.2441	3.0057	2.8616	2.9363	2.7048	2.3100	1.8586	1.2587	4.0064	1.2984	0.3767	0.1931	0.7142
Usf1;Usf2	_THPYS[Phospho (STY)]PK_.2	Q61069_S186_M1_Usf1;Usf2	7.0000	7.3385	7.2421	7.1241	3.9438	4.1816	4.0748	6.9970	7.0225	4.3271	1.0951	0.1311	0.6235	0.2051
Ei24	_RPQS[Phospho (STY)]VER_.2	Q61070_S56_M1_Ei24	5.5252	5.9563	6.6831	5.1857	4.2137	4.9486	5.4986	6.0404	5.4858	5.5476	0.9602	-0.0585	0.6294	0.2011
Ei24	_FPS[Phospho (STY)]PHPS[Phospho (STY)]PAK_.2	Q61070_S326_M2_Ei24	8.3986	7.9933	3.8526	7.7460	3.2626	3.3620	7.6184	8.4778	6.3986	7.3460	0.7733	-0.3708	0.2377	0.6239
Ppm1g	_KLEEALST[Phospho (STY)]EGAEDTGNSDK_.2	Q61074_T525_M1_Ppm1g	3.9376	4.0044	4.3341	4.0752	4.0259	3.8263	4.3849	4.0545	3.1248	3.9076	1.0429	0.0606	0.4880	0.3115
Map3k2	_AQS[Phospho (STY)]YPDNHQEFTDYDNPIFEK_.3	Q61083_S239_M1_Map3k2	3.8235	4.4946	3.5572	6.0890	2.9672	3.6574	3.2492	3.9002	4.7036	4.4088	1.0080	0.0115	0.9587	0.0183
Map3k3	_AIDILDRS[Phospho (STY)]S[Phospho (STY)]SM[Oxidation (M)]K_.2	Q61084_S111_M2_Map3k3	3.4616	4.2396	3.6927	4.2329	4.0983	3.8409	4.3812	4.2921	3.9382	4.2678	0.9308	-0.1035	0.1405	0.8523
Ksr1	_TES[Phospho (STY)]VPSDINNPVDR_.2	Q61097_S392_M1_Ksr1	7.6976	3.0860	3.5642	4.6088	2.9743	3.6503	3.5435	7.2928	4.1922	7.7059	0.7502	-0.4147	0.2836	0.5473
Ksr1	_SKS[Phospho (STY)]HESQLGNR_.2	Q61097_S297_M1_Ksr1	7.6517	7.5633	4.4077	8.2764	7.4555	7.9048	3.7428	7.7538	8.0358	7.6765	1.0599	0.0840	0.7157	0.1453
Dpf2	_VDDDS[Phospho (STY)]LGEFPVSNSR_.2	Q61103_S142_M1_Dpf2	4.2920	7.7589	4.0256	9.2569	8.3500	3.1889	3.0821	9.2141	8.9441	8.4457	0.8281	-0.2722	0.4882	0.3114
Shc3	_QGS[Phospho (STY)]LDIYSTAEGK_.2	Q61120_S282_M1_Shc3	3.3389	4.0035	7.7144	4.1172	3.9835	7.5099	4.2655	4.3848	7.7393	7.5102	0.8554	-0.2253	0.5097	0.2927
Shc3	_AAS[Phospho (STY)]VEC[Carbamidomethyl (C)]ISPVTPR_.2	Q61120_S354_M1_Shc3	9.1946	8.8548	8.9308	9.1438	7.5079	8.8857	9.1124	8.9025	8.9720	9.0397	0.9718	-0.0412	0.4525	0.3444
Nab2	_SFS[Phospho (STY)]PKS[Phospho (STY)]PLELGEK_.2	Q61127_S159_M2_Nab2	3.9542	3.2096	3.6878	8.0824	7.1118	3.5267	3.4199	7.9427	6.0772	4.4097	0.9023	-0.1483	0.6986	0.1558
Prpf4b	_KS[Phospho (STY)]KS[Phospho (STY)]PPLR_.2	#N/A	10.6666	10.4827	10.7976	10.4867	9.4930	10.3857	10.9343	10.7068	10.6963	10.4513	0.9709	-0.0427	0.2443	0.6121
Prpf4b	_KKS[Phospho (STY)]PIVNER_.2	Q61136_S278_M1_Prpf4b	9.0123	8.9064	9.5949	9.3248	8.2782	8.9918	9.5005	9.3585	9.0565	8.9404	0.9787	-0.0310	0.4541	0.3429
Prpf4b	_SKDAS[Phospho (STY)]PINRWS[Phospho (STY)]PTR_.2	#N/A	8.1686	8.0137	8.8933	8.2670	7.0872	8.1682	8.1248	8.3649	8.1723	6.9304	1.0255	0.0364	0.6227	0.2057
Prpf4b	_S[Phospho (STY)]RS[Phospho (STY)]PLLNDR_.2	#N/A	8.8713	8.6479	9.2689	9.0596	8.1549	8.7342	9.2911	8.8802	8.8406	8.6526	0.9858	-0.0206	0.5851	0.2327
Prpf4b	_RRS[Phospho (STY)]LS[Phospho (STY)]PK_.2	Q61136_S354_M2_Prpf4b	8.2017	7.9996	8.9621	8.1659	7.4204	8.3193	8.4315	8.5913	8.4908	7.9154	0.9786	-0.0312	0.5416	0.2664
Prpf4b	_S[Phospho (STY)]RS[Phospho (STY)]PLRR_.2	Q61136_S451_M2_Prpf4b	7.5403	7.8403	7.6543	7.8950	7.1464	7.9085	8.4135	8.0061	8.1875	7.7163	0.9484	-0.0764	0.0589	1.2301
Prpf4b	_AKS[Phospho (STY)]RS[Phospho (STY)]LER_.2	Q61136_S394_M2_Prpf4b	6.6350	6.6427	6.6738	7.4019	5.8888	7.2666	7.2356	7.3081	7.4484	6.6940	0.9414	-0.0871	0.2064	0.6854
Prpf4b	_TLS[Phospho (STY)]PGRR_.2	Q61136_S387_M1_Prpf4b	7.3490	7.0386	7.7770	4.0798	5.9472	6.8491	7.1749	4.0386	6.9966	6.8152	1.0400	0.0566	0.7878	0.1036
Prpf4b	_SKDAS[Phospho (STY)]PINR_.2	Q61136_S431_M1_Prpf4b	7.2530	6.6311	6.9497	7.2473	3.5413	6.4479	7.1370	7.2601	3.0044	7.0970	1.0360	0.0510	0.8452	0.0730
Prpf4b	_S[Phospho (STY)]KS[Phospho (STY)]PVDLR_.2	#N/A	10.2244	9.6411	9.3528	10.2060	8.6764	9.7126	10.4044	9.7777	10.0399	9.9838	0.9586	-0.0610	0.2207	0.6562
Prpf4b	_S[Phospho (STY)]RS[Phospho (STY)]PLLNDRR_.2	#N/A	7.5720	7.3158	7.8904	7.5096	6.7196	7.5629	7.8839	7.6722	7.7682	7.4415	0.9658	-0.0502	0.2540	0.5952
Prpf4b	_DAS[Phospho (STY)]PINRWS[Phospho (STY)]PTR_.2	#N/A	6.4624	3.8716	3.7532	8.3316	3.6929	8.0372	8.7722	8.0084	7.9524	4.2228	0.7862	-0.3470	0.2954	0.5296
Prpf4b	_EVIEASDKEGLS[Phospho (STY)]PAKR_.3	#N/A	6.8406	6.8349	7.2861	7.6188	6.5149	7.1637	7.3057	7.4690	7.6247	7.4451	0.9440	-0.0832	0.0777	1.1096
Prpf4b	_LC[Carbamidomethyl (C)]DFGSASHVADNDITPY[Phospho (STY)]LVSR_.3	Q61136_Y849_M1_Prpf4b	4.3512	8.4123	8.1729	9.7993	6.1640	6.1786	7.1161	8.6591	7.2832	5.7443	0.9971	-0.0042	0.9854	0.0064
Prpf4b	_S[Phospho (STY)]KS[Phospho (STY)]PVDLRDK_.2	#N/A	8.0296	7.6441	8.1491	8.0365	7.3476	7.6104	8.3900	7.9522	8.0674	8.0751	0.9608	-0.0577	0.1098	0.9594
Prpf4b	_RS[Phospho (STY)]RS[Phospho (STY)]PLLNDR_.3	#N/A	3.9570	4.2918	3.6212	4.1239	1.1101	3.9454	3.8386	2.5143	3.6432	4.0909	0.9962	-0.0055	0.9844	0.0068
Prpf4b	_S[Phospho (STY)]KS[Phospho (STY)]PPLR_.2	#N/A	2.9403	3.7455	3.6271	3.2198	3.5669	4.1626	4.5234	3.9887	4.4259	4.0968	0.8321	-0.2652	0.0173	1.7615
Astn1	_VGS[Phospho (STY)]PRS[Phospho (STY)]PVNK_.2	Q61137_S376_M2_Astn1	6.7547	6.7752	7.2185	7.1028	6.1500	6.5132	7.2037	7.0253	6.6977	6.6414	0.9797	-0.0295	0.5529	0.2573
Astn1	_SAS[Phospho (STY)]AEAANEIHYIPSVLIGGHGR_.3	Q61137_S194_M1_Astn1	6.4795	7.1134	7.3352	7.8941	3.0717	5.7317	6.6668	6.9673	6.8186	5.6283	0.9618	-0.0562	0.7927	0.1009
Astn1	_GNSPSDES[Phospho (STY)]EERERDPK_.3	#N/A	5.3424	3.6335	5.7207	4.1878	3.4819	3.9898	5.3193	3.7816	5.3848	3.4703	0.9786	-0.0313	0.8798	0.0556
Astn1	_VQGHNS[Phospho (STY)]SGTLSIR_.2	Q61137_S227_M1_Astn1	6.3735	6.2700	3.4482	6.6442	3.1547	4.0755	7.0163	4.0895	4.0912	6.6113	0.9160	-0.1265	0.6685	0.1749
Astn1	_GNSPSDES[Phospho (STY)]EERER_.2	#N/A	3.9498	3.2052	3.6834	3.7096	3.0935	3.5311	3.4243	4.4089	4.0575	4.4053	0.8662	-0.2073	0.0584	1.2336
Bcar1	_GS[Phospho (STY)]PGFTPEDLDR_.2	Q61140_S572_M1_Bcar1	2.9912	4.3512	4.2257	3.7695	4.1725	7.2545	7.6104	7.0393	3.5307	3.4468	0.8250	-0.2775	0.4353	0.3612
Psen2	_TQES[Phospho (STY)]EEDC[Carbamidomethyl (C)]EEDPDR_.2	Q61144_S52_M1_Psen2	3.7346	5.0766	3.4683	4.5129	2.8783	3.7463	5.3985	3.9891	4.2882	4.1902	0.8738	-0.1946	0.2650	0.5768
Psen2	_TSLMSAES[Phospho (STY)]PTSR_.2	Q61144_S25_M1_Psen2	3.5337	4.3389	4.2206	3.2224	4.1603	3.5692	4.4379	3.3953	3.0439	3.1894	1.0922	0.1272	0.3701	0.4316
Ocln	_YSS[Phospho (STY)]NGNLETPSKR_.2	#N/A	3.0647	3.8210	6.0498	3.8430	3.7093	4.4161	3.9913	6.2683	3.4572	3.5203	0.9632	-0.0541	0.8359	0.0779
Ocln	_YSS[Phospho (STY)]NGNLETPSK_.2	#N/A	3.8963	3.2007	4.5831	3.4192	3.0220	3.2066	3.5674	7.3317	4.1821	3.5520	0.7631	-0.3901	0.1919	0.7170
Ocln	_NVS[Phospho (STY)]AGTQDM[Oxidation (M)]PPPPSDYAER_.2	#N/A	3.9035	5.1983	5.9903	5.2738	4.2852	5.1325	6.1798	5.4580	5.7365	5.2711	0.8768	-0.1897	0.1303	0.8850
Ppp2r5e	_SQS[Phospho (STY)]SSQFR_.2	Q61151_S32_M1_Ppp2r5e	4.0891	6.4017	7.0335	3.4382	3.2125	6.4717	9.4839	4.1754	4.1217	4.0231	0.9370	-0.0938	0.8093	0.0919
Map4k2	_TPS[Phospho (STY)]EIQFHQVK_.2	Q61161_S328_M1_Map4k2	3.9395	8.4996	3.8322	8.8869	3.0652	8.3332	8.7409	8.5784	8.4792	8.5003	0.7106	-0.4930	0.1140	0.9432
Map4k2	_ASDPHLGTLS[Phospho (STY)]PEDSELETHDMFPDTIHSR_.4	Q61161_S298_M1_Map4k2	4.3016	3.5570	4.0352	3.3578	3.4453	3.1793	3.0725	3.2598	4.4093	3.2563	1.0419	0.0592	0.6662	0.1764
Ctcf	_[Acetyl (Protein N-term)]M[Oxidation (M)]EGEAVEAIVEESETFIK_.2	#N/A	4.3998	5.4364	7.0969	6.6367	5.6665	6.3300	6.1862	5.3108	4.4528	5.0038	1.1316	0.1784	0.2615	0.5825
Slc9a1	_LDS[Phospho (STY)]PTLSR_.2	#N/A	11.1806	10.5555	11.0499	10.7271	9.6133	10.4577	11.0956	11.1424	10.8114	10.7640	0.9675	-0.0477	0.2610	0.5834
Slc9a1	_IGS[Phospho (STY)]DPLAYEPK_.2	Q61165_S707_M1_Slc9a1	9.6808	9.4822	10.0092	9.5035	7.6739	9.4188	10.3293	9.8716	9.6264	9.4547	0.9465	-0.0794	0.2720	0.5654
Slc9a1	_ADLPVITIDPAS[Phospho (STY)]PQSPESVDLVNEELK_.3	Q61165_S727_M1_Slc9a1	3.6833	2.9387	3.4170	5.4138	2.8270	3.7976	3.1090	4.0405	4.3395	4.1388	0.9418	-0.0865	0.6755	0.1704
Slc9a1	_ITNYLTVPAHKLDS[Phospho (STY)]PTLSR_.3	#N/A	3.9987	3.3031	3.1847	7.9624	4.0075	3.1043	3.4020	4.4311	5.3609	3.6543	1.0114	0.0163	0.9633	0.0163
Slc9a1	_C[Carbamidomethyl (C)]LS[Phospho (STY)]DPGPHPEPGEGEPFIPK_.3	Q61165_S801_M1_Slc9a1	3.5786	3.7638	3.3123	4.3569	4.2231	3.9023	4.5051	4.1452	2.9434	4.0341	0.9870	-0.0189	0.8825	0.0543
Mapre1	_[Acetyl (Protein N-term)]AVNVYSTSVTSDNLSR_.2	#N/A	6.0653	5.5266	6.5320	6.1348	6.1817	5.9094	6.1587	5.9125	6.0243	5.8749	1.0110	0.0157	0.7196	0.1429
Mapre1	_NIELIC[Carbamidomethyl (C)]QENEGENDPVLQR_.2	#N/A	4.5096	4.9450	4.8400	5.8460	5.2962	4.9312	4.7548	4.5125	5.1618	4.6839	1.0593	0.0830	0.3051	0.5155
Mapre1	_LEHEYIQNFK_.3	#N/A	4.9597	4.6730	4.5629	4.1807	5.0280	4.7545	3.0665	4.8869	5.4158	5.1182	1.0154	0.0221	0.8772	0.0569
Mapre1	_LTVEDLEKER_.2	#N/A	5.3421	5.3561	5.8668	5.6696	5.3519	5.6283	5.2555	5.2308	5.2549	5.0132	1.0669	0.0934	0.0208	1.6809
Prdx2	_QITVNDLPVGR_.2	#N/A	9.9227	10.1659	10.2612	10.1820	10.3603	10.3232	10.0735	9.9032	10.1884	9.9703	1.0168	0.0241	0.1104	0.9572
Prdx2	_SLSQNYGVLK_.2	#N/A	8.8083	9.1528	9.3886	9.1226	9.5672	9.0664	9.1148	9.2102	9.0576	8.9891	1.0100	0.0144	0.5314	0.2746
Prdx2	_NDEGIAYR_.2	#N/A	8.5886	8.7936	8.9797	8.8688	9.3988	8.9197	7.9359	8.7847	8.7991	8.9114	1.0368	0.0522	0.1966	0.7065
Prdx2	_EGGLGPLNIPLLADVTK_.2	#N/A	6.9947	6.7953	6.7152	6.6238	6.5908	6.8127	6.7702	6.3293	6.4106	6.9889	1.0197	0.0282	0.3890	0.4101
Prdx2	_GLFIIDAK_.2	#N/A	6.1072	7.0420	5.2645	6.0569	6.6519	6.7714	4.6754	6.4151	6.9974	6.8599	1.0126	0.0181	0.8867	0.0522
Prdx2	_KEGGLGPLNIPLLADVTK_.3	#N/A	8.1979	8.2370	8.3610	8.3566	8.5478	8.1759	8.0782	8.1800	7.8940	8.1127	1.0306	0.0434	0.0210	1.6786
Prdx2	_SAPDFTATAVVDGAFK_.2	#N/A	6.3680	6.3975	6.8374	6.6886	7.0317	6.9230	6.6069	6.6694	7.0312	6.8120	0.9894	-0.0154	0.6624	0.1789
Prdx2	_LGC[Carbamidomethyl (C)]EVLGVSVDSQFTHLAWINTPR_.3	#N/A	3.3426	3.8836	4.0294	4.5321	3.9691	3.7604	4.2467	3.5864	3.2350	2.9982	1.1146	0.1565	0.2047	0.6888
Rgl2	_VSC[Carbamidomethyl (C)]EVEPPGTSDS[Phospho (STY)]PAAR_.2	Q61193_S518_M1_Rgl2	3.8244	3.1288	4.5112	3.4087	4.4782	3.2846	3.3552	3.4145	4.1425	3.2389	1.0664	0.0927	0.5196	0.2843
Rgl2	_SASC[Carbamidomethyl (C)]GS[Phospho (STY)]PLSGNTGEGTSR_.2	Q61193_S622_M1_Rgl2	4.6432	3.1884	3.2237	3.7264	3.0767	3.5479	3.4410	4.3921	4.0407	3.6934	0.9167	-0.1254	0.3664	0.4360
Pafah1b3	_LLAQDQGQGIPLPETAS_.2	#N/A	6.1024	6.2318	6.8773	5.9407	6.1355	6.7138	6.4972	5.8661	6.1475	6.2707	1.0223	0.0318	0.5422	0.2659
Pafah1b2	_IIVLGLLPR_.2	#N/A	8.3965	8.7181	8.7203	8.5576	8.7375	8.5736	8.3178	8.5391	8.5107	8.4671	1.0188	0.0268	0.0782	1.1066
Pafah1b2	_GEKPNPLR_.2	#N/A	8.0430	8.4818	8.3767	8.5172	9.0409	8.2455	8.1203	8.1271	8.3363	8.2585	1.0293	0.0416	0.2114	0.6750
Pafah1b2	_[Acetyl (Protein N-term)]SQGDSNPAAIPHAAEDIQGDDR_.3	#N/A	4.8462	4.2897	4.0908	5.2423	4.4419	4.4068	4.7725	4.8810	4.8251	5.1890	0.9260	-0.1110	0.1463	0.8348

Pafah1b2	_ELFSPLHALNFGIGGDTTR_.3	#N/A	5.6606	5.5850	5.4293	6.3234	6.2075	5.9130	5.7002	5.7176	5.5845	6.0422	1.0160	0.0229	0.6549	0.1838
Pafah1b2	_[Acetyl (Protein N-term)]SQGDSNPAAIPHAAEDIQGDDRWM[Oxidation (M)]SQHNR_.4	#N/A	5.9252	6.5981	7.6157	5.9583	6.5476	6.3638	6.3901	6.3172	6.1065	6.2454	1.0378	0.0535	0.4782	0.3204
Psap	_GC[Carbamidomethyl (C)]SFLPDPYQK_.2	#N/A	9.6001	10.0329	10.1293	10.0810	10.4847	10.1073	10.0147	9.8131	9.9792	9.8605	1.0157	0.0225	0.3269	0.4855
Psap	_SLPC[Carbamidomethyl (C)]DIC[Carbamidomethyl (C)]K_.2	#N/A	7.8054	8.2714	8.5531	8.1939	8.8647	7.8352	7.8145	8.0352	8.2437	8.2483	1.0208	0.0298	0.4750	0.3233
Psap	_TVVTEAGNLLK_.2	#N/A	6.5672	7.0318	6.3363	6.8557	7.3210	6.2133	6.4094	6.5318	6.7249	6.3032	1.0352	0.0499	0.3465	0.4603
Psap	_LVSDVQTAVK_.2	#N/A	5.7504	5.9868	5.5705	5.2401	6.5194	5.7760	5.5949	5.9021	5.4207	5.7644	1.0241	0.0344	0.5753	0.2401
Psap	_LVLYLEHNLEK_.2	#N/A	5.0812	3.9156	5.1287	5.7821	5.4773	5.1660	5.4505	4.9828	5.2530	4.6406	1.0020	0.0029	0.9779	0.0097
Psap	_LGPGVSDIC[Carbamidomethyl (C)]K_.2	#N/A	5.2508	4.8662	5.3882	5.8895	5.2705	4.3714	4.7735	5.3447	5.1271	5.6427	0.9906	-0.0137	0.8732	0.0589
Psap	_EVVDSYLPVILDM[Oxidation (M)]IK_.2	#N/A	5.7889	6.1905	5.8427	5.8766	6.4557	6.3858	6.0943	5.9207	6.0070	5.9130	1.0178	0.0254	0.5073	0.2948
Psap	_TC[Carbamidomethyl (C)]EWIHDSSLSASC[Carbamidomethyl (C)]K_.3	#N/A	3.8078	3.5169	4.2203	4.1900	4.9913	4.7861	3.9390	4.8717	3.9152	4.0224	1.0155	0.0222	0.8528	0.0692
Dyrk1a	_IYQY[Phospho (STY)]IQSR_.2	Q61214_Y321_M1_Dyrk1a	12.0795	11.9337	12.5613	12.0456	11.1462	11.7923	12.3213	12.0309	12.0545	11.8292	0.9890	-0.0159	0.6089	0.2154
Lcp1	_FSLVGIAGQDLNEGNR_.2	#N/A	5.5084	4.8540	5.0603	5.2384	4.2771	5.1923	5.6129	5.2723	4.7649	5.2224	0.9624	-0.0553	0.4655	0.3320
Lcp1	_GS[Phospho (STY)]VSDEEMMELR_.2	Q61233_S5_M1_Lcp1	4.2782	2.9480	3.4641	3.9668	3.4039	4.3256	3.6815	4.1517	3.8003	3.9338	0.9587	-0.0608	0.5954	0.2252
Lcp1	_EGES[Phospho (STY)]LEDLMK_.2	Q61233_S257_M1_Lcp1	3.7974	3.1018	4.4842	3.5180	2.9231	3.3055	3.6663	3.1316	4.2810	3.4531	0.9694	-0.0449	0.7555	0.1218
Snta1	_QPSS[Phospho (STY)]PGPQPR_.2	Q61234_S195_M1_Snta1	3.9298	3.2529	8.8339	8.7966	5.0227	3.4360	5.8268	6.0404	8.5030	8.7433	0.7619	-0.3923	0.2711	0.5670
Snta1	_NSAGGTSVGWDS[Phospho (STY)]PPASPLQR_.2	Q61234_S183_M1_Snta1	3.9738	4.8658	6.5095	9.1230	6.0092	6.1030	6.0311	8.0506	6.5859	7.0147	0.8811	-0.1827	0.4143	0.3827
Snta1	_YLEIC[Carbamidomethyl (C)]AADGQDAVFLR_.2	#N/A	2.9097	3.6660	4.1442	3.6880	3.5543	3.0703	3.8363	3.3132	3.6122	3.3653	0.9925	-0.0108	0.9183	0.0370
Sntb2	_GPAGEASAS[Phospho (STY)]PPVR_.2	#N/A	8.7078	3.5792	3.4608	3.9634	3.4005	4.3290	3.1889	4.1550	7.4858	3.9304	0.9751	-0.0363	0.9303	0.0314
Sntb2	_GLGPPS[Phospho (STY)]PPAPPR_.2	Q61235_S75_M1_Sntb2	9.4542	9.3607	3.6855	9.7418	7.3927	8.5945	9.4965	9.1910	9.2332	9.2372	0.8653	-0.2087	0.3170	0.4990
Sntb2	_GPAGEASAS[Phospho (STY)]PPVRR_.3	#N/A	4.0382	3.2936	3.7719	3.3156	3.1819	3.4427	3.4639	3.6855	3.9846	2.9929	0.9931	-0.0100	0.9201	0.0362
Igbp1	_[Acetyl (Protein N-term)]AAS[Phospho (STY)]EDELLLPR_.2	Q61249_S4_M1_Igbp1	5.5581	6.5970	6.8573	7.1047	6.2481	7.2744	6.9299	6.2975	7.1574	6.5536	0.9810	-0.0277	0.7281	0.1378
Abcd2	_AEAYS[Phospho (STY)]PAENR_.2	Q61285_S58_M1_Abcd2	6.9097	3.2237	7.3574	7.1061	3.0451	3.1836	3.5444	3.0096	4.1591	3.5750	1.4383	0.5244	0.2029	0.6928
Tcf12	_TSS[Phospho (STY)]TNEDEDLNPEQK_.2	Q61286_S583_M1_Tcf12	3.9839	3.2422	3.1699	3.6725	3.1305	3.4941	3.3872	4.4459	4.0945	3.6395	0.8862	-0.1743	0.1178	0.9289
Cacna1e	_GTQADWES[Phospho (STY)]PER_.2	#N/A	8.4652	8.1154	8.8105	8.5065	6.4225	7.9318	8.4155	8.3973	8.1519	8.0368	0.9747	-0.0369	0.6490	0.1878
Cacna1e	_QGT[Phospho (STY)]PVPASGPAAAYK_.2	Q61290_T29_M1_Cacna1e	8.1405	7.5500	3.6066	7.6331	3.5463	7.2443	8.2524	8.1363	7.9489	7.7890	0.7828	-0.3534	0.1463	0.8348
Cacna1e	_GTQADWES[Phospho (STY)]PERR_.2	#N/A	7.4538	7.2938	7.8795	7.4830	6.1130	6.9673	7.5499	7.6481	7.0504	7.0317	0.9834	-0.0242	0.7267	0.1386
Cacna1e	_S[Phospho (STY)]YHSSLR_.2	Q61290_S2013_M1_Cacna1e	7.6708	6.6483	4.1640	7.0752	4.8337	7.1105	7.1273	7.4979	6.9535	6.9714	0.8757	-0.1915	0.2571	0.5899
Cacna1e	_HLLS[Phospho (STY)]PDVSR_.2	#N/A	3.5970	7.1684	7.5151	6.9681	4.2415	7.0751	7.5954	7.5038	7.2963	6.8269	0.8342	-0.2616	0.2066	0.6848
Cacna1e	_KHLLS[Phospho (STY)]PDVSR_.2	#N/A	6.9276	3.9009	4.1602	4.0250	4.0999	6.0219	4.0104	7.1212	6.2654	6.4115	0.8158	-0.2937	0.2314	0.6356
Cacna1e	_AS[Phospho (STY)]IKS[Phospho (STY)]TKVDGASYFR_.3	Q61290_S447_M2_Cacna1e	3.1517	4.8396	4.1295	5.0511	3.7870	4.9194	4.0667	3.4574	5.0002	4.8744	0.9916	-0.0122	0.9410	0.0264
Cacna1e	_FGEAVVVGRPGS[Phospho (STY)]GDGDSDQSR_.3	#N/A	3.3908	6.8812	4.0226	7.9260	5.8601	7.1044	7.3661	8.4009	7.0700	7.3367	0.7774	-0.3633	0.1155	0.9376
Cacna1e	_DSSDEHC[Carbamidomethyl (C)]VDISSVGT[Phospho (STY)]PLAR_.3	Q61290_T441_M1_Cacna1e	4.9460	4.8151	4.7004	4.2895	4.2358	4.8383	5.4074	5.4869	4.9121	4.9478	0.8938	-0.1620	0.0219	1.6588
Cacna1e	_GISQS[Phospho (STY)]EPDLSC[Carbamidomethyl (C)]M[Oxidation (M)]TANM[Oxidation (M)]DK_.2	#N/A	6.6495	6.5871	7.0661	7.1798	3.8470	6.0651	7.0182	7.2069	6.3260	3.9733	1.0165	0.0236	0.9093	0.0413
Cacna1e	_SAS[Phospho (STY)]QERS[Phospho (STY)]LDEGVSVEGEKEHEPHSSHR_.4	Q61290_S948_M2_Cacna1e	4.7338	5.0663	3.4779	6.0073	3.4176	4.8627	5.0309	6.0620	5.7666	5.2962	0.8294	-0.2698	0.1179	0.9283
Cacna1e	_TDGEAS[Phospho (STY)]PLKEAETKEEEEEVEK_.3	Q61290_S1099_M1_Cacna1e	3.9248	3.8986	4.3768	3.9206	3.7868	1.5585	3.4367	3.0806	2.2059	3.5978	1.1615	0.2160	0.4090	0.3882
Cacna1e	_S[Phospho (STY)]LDEGVSVEGEKEHEPHSSHR_.4	#N/A	5.0752	3.6074	3.9022	4.8427	4.1598	3.8526	3.9756	5.5775	4.5402	4.7559	0.8998	-0.1524	0.2703	0.5681
Cacna1e	_EVS[Phospho (STY)]PM[Oxidation (M)]SAPNM[Oxidation (M)]PSIER_.2	#N/A	7.5416	7.0704	7.8448	7.2912	5.6245	6.8177	7.6015	7.5553	7.2005	6.9980	0.9581	-0.0617	0.4778	0.3207
Cacna1e	_GGS[Phospho (STY)]LKGDIGGLTSALDNQR_.3	#N/A	3.8996	4.7191	3.9084	4.8867	3.7515	3.7246	3.6177	4.7092	4.0073	3.8366	1.0261	0.0372	0.7517	0.1239
Cacna1e	_HMQMSS[Phospho (STY)]QEALNK_.2	Q61290_S794_M1_Cacna1e	3.6809	3.5452	4.3677	3.3695	2.8066	3.1911	3.0842	3.2481	4.3975	3.3365	0.9934	-0.0095	0.9501	0.0222
Cacna1e	_AKEVS[Phospho (STY)]PMSAPNMPSIER_.3	#N/A	3.3579	3.9845	4.5924	4.1361	4.0024	4.1230	4.2844	4.3659	4.1944	3.8134	1.0322	0.0457	0.6840	0.1649
Ctnna2	_VYGTAAVNS[Phospho (STY)]PVVSWK_.2	Q61301_S901_M1_Ctnna2	6.8745	7.7520	8.2618	4.4681	6.0144	7.2172	3.1155	7.9270	7.3879	7.0174	1.0633	0.0886	0.7266	0.1387
Ctnna2	_NATNEQDLANR_.2	#N/A	5.9762	5.5182	5.9511	5.7012	5.4585	5.6934	6.4276	5.9122	5.5882	5.9165	0.9590	-0.0605	0.2003	0.6982
Ctnna2	_GHTGIGELAAALNEFDNK_.3	#N/A	5.9149	6.0547	6.5305	6.5729	5.2569	4.8658	5.9585	6.3297	6.2129	5.1784	0.9909	-0.0132	0.8975	0.0470
Ctnna2	_QVQEAIAGISSAAQATS[Phospho (STY)]PTDEAK_.2	#N/A	8.8411	9.6148	10.3198	10.6828	8.6906	9.0694	9.5169	10.2901	9.6566	8.9787	0.9923	-0.0112	0.8783	0.0564
Ctnna2	_[Acetyl (Protein N-term)]TSATS[Phospho (STY)]PIILK_.2	Q61301_S6_M1_Ctnna2	2.9594	9.6799	10.2527	9.8712	8.9524	9.9279	10.2945	9.7225	9.9350	9.5991	0.8705	-0.2001	0.3980	0.4001
Ctnna2	_LVYDGVR_.2	#N/A	4.6792	4.9346	5.4153	4.8025	3.6614	5.1052	4.4414	5.3360	5.2371	3.5030	1.0296	0.0421	0.7708	0.1131
Ctnna2	_IVEEALEAVK_.2	#N/A	4.4750	4.5118	3.4938	4.1626	3.9068	4.7347	4.1130	3.8140	5.1146	4.2243	0.9763	-0.0346	0.7583	0.1202
Ctnna2	_LLEPLVTQVTTLVNTSNK_.2	#N/A	4.6807	4.7862	3.6384	4.5321	3.7293	4.1514	4.8262	4.4004	4.3154	4.1080	0.9639	-0.0531	0.5815	0.2354
Ctnna2	_TPEELEDDSDFEQEDYDVR_.2	#N/A	3.5991	4.0448	3.8519	7.6038	5.2235	4.1004	4.3820	4.0094	4.3154	4.0832	1.1286	0.1745	0.5056	0.2962
Ctnna2	_SKLDAEVAK_.2	#N/A	5.0960	4.7619	4.1240	4.4362	4.2820	3.0905	4.8003	3.3334	3.1663	5.0827	1.0495	0.0697	0.7089	0.1494
Ctnna2	_NATM[Oxidation (M)]LYTASQAFLR_.2	#N/A	4.4508	4.1095	4.1482	3.6920	3.5583	4.0569	3.8402	4.0294	3.6082	4.1228	1.0263	0.0374	0.6025	0.2201
Ctnna2	_LVEVANLAC[Carbamidomethyl (C)]SISNNEEGVK_.2	#N/A	2.7448	3.0981	2.4030	3.8167	2.9864	3.1301	1.6962	3.4018	3.4492	3.7837	0.9160	-0.1266	0.5988	0.2227
Ctnna2	_VLTEAVDDITSVDDFLSVSENHILEDVNK_.3	#N/A	3.3740	4.1792	4.0608	3.8749	4.0005	3.7290	4.2781	3.5550	3.2036	3.0296	1.1004	0.1381	0.2168	0.6640
Ctnna2	_HIS[Phospho (STY)]PVQALSEFK_.2	Q61301_S939_M1_Ctnna2	4.2007	3.4562	4.4568	6.3775	3.3445	3.2802	3.1733	3.1590	4.3084	3.4256	1.1903	0.2514	0.3249	0.4883
Apc	_QSVGSGS[Phospho (STY)]PVQTVGLETR_.2	Q61315_S2713_M1_Apc	9.9144	9.8417	10.3707	9.9757	9.1281	9.8464	10.4313	9.9390	9.7706	9.7007	0.9885	-0.0166	0.6513	0.1862
Apc	_SELS[Phospho (STY)]PITR_.2	Q61315_S2283_M1_Apc	6.9586	6.5270	7.2265	6.7646	6.0753	7.0790	7.6049	6.7177	6.6684	4.2458	1.0649	0.0907	0.5219	0.2824
Apc	_SGRS[Phospho (STY)]PTGNTPPVIDSVSEK_.3	#N/A	3.4394	5.7014	3.8994	4.2176	3.7103	3.6030	3.5914	3.6319	6.2148	3.8949	0.9451	-0.0815	0.7255	0.1394
Apc	_AVEFSSGAKS[Phospho (STY)]PSK_.2	Q61315_S1359_M1_Apc	4.9888	3.1689	4.5514	5.4494	4.0885	4.9465	3.2679	5.4807	5.8814	5.3792	0.9060	-0.1424	0.4715	0.3265
Apc	_HSS[Phospho (STY)]PSGTVAAR_.2	Q61315_S2774_M1_Apc	3.6476	3.5785	4.3344	5.4287	4.2742	3.4553	4.5518	3.2814	4.4308	3.3032	1.0586	0.0821	0.6384	0.1949
Apc	_SPT[Phospho (STY)]GNTPPVIDSVSEK_.2	Q61315_T2676_M1_Apc	6.2360	5.2166	3.6028	6.1766	4.5938	5.9065	3.8201	3.6689	3.6783	5.7553	1.0781	0.1085	0.6256	0.2037
Apc	_YSDEQLNSGRQS[Phospho (STY)]PSQNER_.3	#N/A	4.8096	4.3492	3.5734	5.2523	3.5132	3.5630	5.1190	5.5003	5.2508	5.0511	0.7986	-0.3245	0.0264	1.5791
Apc	_ALEAELDAQHLSETFDNIDNLS[Phospho (STY)]PK_.3	Q61315_S778_M1_Apc	4.0235	5.3990	6.4781	3.3009	5.1591	3.4574	3.4492	6.2177	5.6663	2.9782	1.0128	0.0183	0.9497	0.0224
Apc	_T[Phospho (STY)]GSSSSILSASSESSEK_.2	Q61315_T2567_M1_Apc	3.7152	3.6272	3.4488	4.4934	3.4486	4.2809	3.1409	4.0086	4.3076	4.1707	0.9818	-0.0266	0.8226	0.0848
Apc	_QAS[Phospho (STY)]SDSDSILSLK_.2	Q61315_S2129_M1_Apc	3.6818	2.9372	3.4155	4.4601	2.8255	3.7991	3.6922	4.0419	6.2100	4.1374	0.7787	-0.3609	0.1046	0.9806
Apc	_LKSESEKQS[Phospho (STY)]PR_.2	#N/A	4.0639	3.9995	4.7535	4.3984	3.2295	3.6407	4.8181	4.8226	4.1233	3.6394	0.9226	-0.1162	0.3737	0.4275
Apc	_SHS[Phospho (STY)]ES[Phospho (STY)]PSRLPINR_.2	#N/A	3.0755	3.8558	4.0689	4.0966	3.7113	4.2249	4.2448	4.2276	4.1804	4.1016	0.9165	-0.1258	0.1409	0.8510
Apc	_C[Carbamidomethyl (C)]TSVSS[Phospho (STY)]LDSFESR_.2	Q61315_S1391_M1_Apc	3.8106	3.0888	4.0613	3.4265	3.4664	4.9427	3.5747	3.6060	5.2235	3.9364	0.9668	-0.0488	0.7741	0.1112
Apc	_S[Phospho (STY)]PPEHYVQETPLVFSR_.2	#N/A	3.6855	4.7886	3.8824	3.8071	3.7211	3.7646	3.7427	4.9775	4.8595	3.8736	0.9033	-0.1467	0.2413	0.6174
Apc	_NDS[Phospho (STY)]LSSLDFDDDDVDLSR_.2	Q61315_S1859_M1_Apc	3.7839	3.0883	3.9878	3.5316	2.9096	3.3190	3.6798	6.6895	3.7687	3.2088	0.7925	-0.3356	0.2109	0.6759
Apc	_SKT[Phospho (STY)]PPPPPQTVQAK_.2	Q61315_T1437_M1_Apc	8.6184	9.1204	9.3036	9.0473	8.0078	9.2423	9.4252	9.3168	8.9096	8.8573	0.9740	-0.0380	0.4161	0.3808
Apc	_TPASKS[Phospho (STY)]PSEGPGATTSPR_.2	#N/A	4.1820	3.8872	4.1530	4.6455	3.7505	4.2428	4.8666	3.6345	3.7366	4.0181	1.0196	0.0280	0.7774	0.1094
Apc	_KPTS[Phospho (STY)]PVKPMPQNTEYR_.3	Q61315_S1772_M1_Apc	3.8885	3.1929	4.5753	3.5771	3.0142	3.2145	3.5753	3.0405	4.1899	3.5441	0.9971	-0.0042	0.9771	0.0101
Apc	_SFHVEDTPVC[Carbamidomethyl (C)]FS[Phospho (STY)]R_.3	Q61315_S2021_M1_Apc	3.5167	4.3219	4.2035	3.7988	4.1432	3.5863	3.9471	3.2024	3.5014	3.4761	1.1123	0.1536	0.0986	1.0061
Hspa4	_GC[Carbamidomethyl (C)]ALQC[Carbamidomethyl (C)]AILSPAFK_.2	#N/A	6.6878	7.0409	7.3506	7.2148	7.4169	6.8068	6.9727	7.2124	7.0120	7.0799	1.0024	0.0035	0.9157	0.0383
Hspa4	_EFSITDVVPYPISLR_.2	#N/A	6.9150	7.0795	7.0268	6.8909	6.9637	7.2472	7.1862	6.8758	6.9744	6.9413	1.0037	0.0054	0.7676	0.1148
Hspa4	_AGGIETIANEYSDR_.2	#N/A	7.3417	7.1037	7.1484	7.4110	6.5859	6.9001	7.4188	7.0419	6.7651	7.0128	1.0031	0.0045	0.9090	0.0414
Hspa4	_VLATAFDTTLGGR_.2	#N/A	6.4682	6.8966	5.8463	6.9244	6.6608	6.7556	6.9941	6.8333	6.9455	6.6800	0.9605	-0.0582	0.2370	0.6252
Hspa4	_QSLTVDPVVK_.2	#N/A	6.2981	6.6276	6.3600	6.6415	7.2927	6.9036	6.7820	6.7286	6.6819	6.8878	0.9878	-0.0178	0.6763	0.1699
Hspa4	_NHAAPFS[Phospho (STY)]K_.2	Q61316_S429_M1_Hspa4	5.5642	5.7433	5.8640	6.1041	6.0472	5.9416	5.7672	6.1901	5.7842	6.1143	0.9855	-0.0211	0.5369	0.2701

Hspa4	_SNLAYDIVQLPTGLTGIK_.2	#N/A	6.1277	5.8510	6.2263	6.3407	5.6829	6.4216	6.8229	6.4372	6.2139	6.4684	0.9418	-0.0865	0.0661	1.1798
Hspa4	_LEDTENWLYEDGEDQPK_.2	#N/A	6.4100	6.2725	6.3513	6.4663	5.3608	6.5146	6.0740	5.9937	5.9093	5.5151	1.0607	0.0850	0.1795	0.7460
Hspa4	_KPVVDC[Carbamidomethyl (C)]VVSVPSFYTDAER_.3	#N/A	5.6363	5.2330	4.8223	6.0936	5.8662	5.4742	5.6880	4.7793	5.6962	5.7214	1.0091	0.0131	0.8704	0.0603
Hspa4	_LFEELGK_.2	#N/A	5.4239	5.4814	5.3287	5.6907	5.2263	5.3999	4.2560	5.1840	5.7629	4.9921	1.0746	0.1037	0.1835	0.7364
Hspa4	_DKLSGEYEK_.2	#N/A	6.5985	6.3934	6.7683	5.7717	7.1963	6.7187	6.4644	3.0962	6.5530	6.8101	1.1472	0.1981	0.2858	0.5440
Hspa4	_FQESEERPK_.2	#N/A	5.4888	5.9604	5.9745	5.5054	6.2434	5.7670	4.6126	5.7386	5.7094	6.0213	1.0548	0.0770	0.3238	0.4897
Hspa4	_LM[Oxidation (M)]NETTAVALAYGIYK_.2	#N/A	4.6283	5.0886	6.0819	4.3736	4.9595	4.9167	5.3010	5.3302	5.7129	5.6490	0.9108	-0.1347	0.1533	0.8146
Hspa4	_LKETAESVLK_.2	#N/A	4.8644	3.8142	5.5004	4.1984	6.0042	4.0940	5.4045	5.2153	5.7649	4.6868	0.9009	-0.1505	0.3055	0.5149
Hspa4	_QDLPALEEKPR_.3	#N/A	8.3847	7.1967	7.5599	8.0492	8.8227	7.9583	7.5458	8.2564	8.5352	8.5632	0.9721	-0.0409	0.5288	0.2767
Hspa4	_SVM[Oxidation (M)]DATQIAGLNC[Carbamidomethyl (C)]LR_.2	#N/A	5.6733	5.6339	6.0646	6.0058	5.8705	5.7261	5.9434	5.9009	5.8768	5.6563	0.9974	-0.0038	0.8872	0.0520
Hspa4	_AFSDPFVEAEK_.2	#N/A	7.0492	6.7491	6.9860	7.8609	6.5953	6.6471	6.9259	6.2524	6.3427	6.9963	1.0531	0.0746	0.2491	0.6036
Hspa4	_NKEDQYEHLDAADVTK_.3	#N/A	4.8109	4.6753	4.8461	4.6946	4.9755	4.5740	4.4084	4.9376	5.2647	5.0315	0.9699	-0.0441	0.3844	0.4153
Hspa4	_FLEM[Oxidation (M)]C[Carbamidomethyl (C)]DDLLAR_.2	#N/A	3.8297	4.1676	4.7002	4.5844	4.5360	3.9230	4.0332	4.5306	4.3934	4.1039	1.0058	0.0084	0.9083	0.0418
Hspa4	_NAVEEYVYEMR_.2	#N/A	4.9025	3.8597	3.2163	6.2338	3.8012	4.0484	4.4092	4.2411	3.0393	3.9382	1.1118	0.1529	0.4868	0.3127
Hspa4	_WNSPAEEGLS[Phospho (STY)]DC[Carbamidomethyl (C)]EVFPK_.2	Q61316_S415_M1_Hspa4	3.9654	3.2697	3.1514	3.3501	3.0911	3.1376	3.4984	3.6511	3.9501	3.0274	0.9422	-0.0859	0.3921	0.4066
Hspa4	_SQVISNAK_.2	#N/A	4.0833	3.2591	3.8170	3.3608	3.2271	3.3975	3.5091	3.7238	4.9879	3.0381	0.9238	-0.1143	0.4592	0.3380
Hspa4	_ELSTTLNADEAVTR_.2	#N/A	3.5621	3.7803	3.2958	4.3404	3.6016	4.1278	4.4886	5.6663	2.9599	4.0177	0.8836	-0.1785	0.3346	0.4754
Cntn2	_TTGPGGDGIPAEVHIVR_.3	#N/A	5.0250	4.8792	3.1819	5.1162	4.6643	4.9776	3.3992	3.1793	4.0825	2.9711	1.3617	0.4454	0.0187	1.7291
Cntn2	_FAQLNLAAEDPR_.2	#N/A	4.2646	3.5200	3.9983	3.5420	8.2421	3.2163	3.6903	3.4591	3.7582	3.2193	1.2639	0.3379	0.3640	0.4389
Bad	_S[Phospho (STY)]APPNLWAAQR_.2	Q61337_S136_M1_Bad	10.2728	10.2787	10.5044	11.0185	10.6133	10.3088	9.5824	9.4981	10.8879	10.7329	1.0319	0.0452	0.3500	0.4559
Bad	_S[Phospho (STY)]AGTATQMR_.2	Q61337_S170_M1_Bad	8.8933	7.4743	3.3355	9.9257	8.0823	3.8791	7.7244	4.1219	6.9450	7.7636	1.0441	0.0623	0.8539	0.0686
Bad	_RM[Oxidation (M)]S[Phospho (STY)]DEFEGSFK_.2	#N/A	6.1981	6.9031	6.9800	6.4474	6.5826	7.0638	6.8190	6.2997	6.6140	6.8020	1.0094	0.0134	0.7622	0.1179
Bcan	_ELETPS[Phospho (STY)]EEK_.2	#N/A	10.3106	10.0262	10.2216	10.8468	9.9614	10.1665	10.4673	10.1754	10.1685	10.5283	0.9923	-0.0111	0.6677	0.1754
Bcan	_EYQWIGLNDR_.2	#N/A	9.6256	9.5834	9.6886	9.8522	9.4347	9.7839	9.8264	9.4226	9.5920	9.7144	1.0023	0.0034	0.8313	0.0802
Bcan	_EVGGETGS[Phospho (STY)]PELSGVPR_.2	Q61361_S546_M1_Bcan	8.7251	8.9537	9.2971	9.4618	8.3260	8.9778	9.4149	8.9861	8.9681	9.0611	0.9835	-0.0241	0.5181	0.2856
Bcan	_SWEEAESQC[Carbamidomethyl (C)]R_.2	#N/A	7.5646	7.7725	8.0496	8.2377	8.1572	8.0926	8.1312	7.7681	8.1352	8.0289	0.9954	-0.0066	0.8095	0.0918
Bcan	_YPIITPSQR_.2	#N/A	7.5036	7.9517	7.6078	7.9933	8.1728	7.8371	7.6610	7.8941	8.0954	7.7793	0.9983	-0.0024	0.9315	0.0308
Bcan	_FNVYC[Carbamidomethyl (C)]FR_.2	#N/A	6.5541	6.2743	6.2779	6.7269	5.9433	6.0502	4.9181	6.6209	6.4595	6.6311	1.0239	0.0340	0.6946	0.1583
Bcan	_C[Carbamidomethyl (C)]QENGLWEAPQISC[Carbamidomethyl (C)]VPR_.2	#N/A	4.5265	6.1303	3.6239	7.0777	5.8898	5.6766	5.6048	5.9836	5.7171	5.1809	0.9761	-0.0348	0.8393	0.0761
Bcan	_LTWEEAR_.2	#N/A	5.3387	5.3674	5.5802	5.1154	5.8223	5.3246	3.3525	5.5418	5.4887	5.8198	1.0741	0.1031	0.4473	0.3494
Bcan	_GAIYSIPIS[Phospho (STY)]EDGGGGSSTPEDPAEAPR_.3	Q61361_S406_M1_Bcan	3.7527	3.4735	4.4395	6.1037	3.3618	3.2628	5.1157	3.1763	4.3258	3.4083	1.0148	0.0211	0.9304	0.0313
Bcan	_ALGAHLTSIC[Carbamidomethyl (C)]TPEEQDFVNDR_.3	#N/A	8.1041	8.1549	8.3416	9.0316	7.7962	7.5532	8.1242	7.8137	7.7165	7.9267	1.0340	0.0482	0.3476	0.4590
Bcan	_C[Carbamidomethyl (C)]SPGWLADGSVR_.2	#N/A	6.0047	5.5454	3.3244	6.0739	6.5811	6.1520	4.5173	6.1370	6.0644	6.2751	0.9765	-0.0342	0.8478	0.0717
Bcan	_ELETPS[Phospho (STY)]EEKSGR_.2	#N/A	10.5534	10.9261	11.5819	11.5654	10.8216	10.9801	11.1505	10.9975	10.8185	11.0075	1.0071	0.0102	0.7311	0.1360
Bcan	_DSAHPSAS[Phospho (STY)]SEASSPASDGLEAIVTVTEK_.3	Q61361_S361_M1_Bcan	3.6435	2.9479	4.3303	3.6720	4.2700	3.4595	3.8203	3.3292	3.6282	3.3493	1.0535	0.0751	0.5204	0.2837
Bcan	_ALGAHLTSIC[Carbamidomethyl (C)]TPEEQDFVNDRYR_.4	#N/A	5.4867	6.3316	5.5001	5.5438	5.5605	5.6021	3.4236	5.4223	5.2959	5.2656	1.1688	0.2250	0.0823	1.0844
Bcan	_EVEVLVAR_.2	#N/A	3.3489	4.1541	4.0357	4.2122	3.3702	5.2506	4.1148	2.1360	4.5419	5.1634	1.0183	0.0261	0.9104	0.0408
Bcan	_C[Carbamidomethyl (C)]EVQHGIDDSSDAVEVK_.2	#N/A	3.8299	2.0381	3.6299	3.3661	2.4248	4.0582	3.5944	3.9172	3.8925	3.9600	0.8395	-0.2524	0.1797	0.7455
Bcan	_EAC[Carbamidomethyl (C)]S[Phospho (STY)]GDM[Oxidation (M)]DGYPGVR_.2	#N/A	3.8524	3.5552	3.7169	6.6166	3.6566	4.1105	3.9342	3.9366	3.5852	4.1865	1.0871	0.1205	0.5901	0.2291
Cox7a2l	_NKVPELQK_.2	#N/A	6.3114	5.2587	6.5252	5.9158	6.2878	5.7322	5.7644	6.0573	6.1327	6.3858	0.9869	-0.0191	0.7650	0.1163
Cox7a2l	_LTSSVTAYDYSGK_.2	#N/A	4.2295	6.3497	3.4154	4.5868	5.6030	4.3743	3.2343	3.9152	4.2142	4.2641	1.2183	0.2849	0.1712	0.7665
Prkg2	_LQDVIHVQGGS[Phospho (STY)]PLQAS[Phospho (STY)]PDKVPLDVHR_.4	Q61410_S92_M2_Prkg2	4.2243	3.4797	3.9579	3.5017	1.8496	3.2566	3.6500	4.1288	3.7985	3.1790	0.9158	-0.1270	0.5098	0.2926
Prkg2	_ALS[Phospho (STY)]LEMIQLK_.2	Q61410_S431_M1_Prkg2	3.9728	3.2533	3.1588	4.7020	3.1416	3.4830	3.3761	4.4570	4.1056	3.6285	0.9298	-0.1050	0.4783	0.3203
Hras	_SFEDIHQYR_.2	#N/A	6.8009	6.7518	7.0484	6.8916	7.0725	6.9019	7.0286	6.6304	6.1775	6.8784	1.0348	0.0494	0.1872	0.7277
Hras	_QGVEDAFYTLVR_.2	#N/A	4.3415	4.1954	5.0123	4.8650	4.2939	5.1529	4.6331	4.6249	5.0593	4.7360	0.9748	-0.0368	0.6108	0.2141
Kcna4	_GAYSSHDPQGS[Phospho (STY)]RGSR_.3	Q61423_S79_M1_Kcna4	3.7443	3.6737	3.5553	4.4580	3.4951	4.2455	3.9812	4.0551	3.7291	4.1353	0.9715	-0.0417	0.6100	0.2147
Kcna4	_QSSFPHC[Carbamidomethyl (C)]SDLMPS[Phospho (STY)]GSEEK_.2	Q61423_S111_M1_Kcna4	3.8556	3.1111	3.3010	3.8037	2.9994	3.6252	3.5184	1.5732	3.9634	3.7707	1.0758	0.1054	0.6222	0.2060
Hadh	_DTPGFIVNR_.2	#N/A	6.0522	5.7966	6.4688	6.6433	6.5762	5.8796	6.3427	6.1725	6.0532	5.9772	1.0162	0.0232	0.6311	0.1999
Hadh	_TFESLVDFC[Carbamidomethyl (C)]K_.2	#N/A	4.1472	3.8591	4.2739	3.8177	4.1603	4.6852	3.9659	4.1994	3.4510	4.5654	1.0276	0.0393	0.6600	0.1805
Hadh	_AGDEFVEK_.2	#N/A	3.5305	3.6957	4.2173	3.2191	2.6523	3.0407	4.4347	2.8063	3.0471	3.1861	1.0071	0.0103	0.9536	0.0206
Hadh	_FAAEHTIFASNTSSLQITNIANATTR_.3	#N/A	3.2465	3.5232	3.5408	4.5854	3.3566	4.3729	3.2328	3.9166	2.9881	4.2627	1.0475	0.0669	0.6570	0.1824
Znf638	_SYRS[Phospho (STY)]AS[Phospho (STY)]PER_.2	Q61464_S560_M2_Znf638	8.1231	8.6960	9.5428	8.7717	8.0320	8.6108	8.7202	8.6449	8.4489	8.3250	1.0111	0.0159	0.7493	0.1254
Znf638	_GLS[Phospho (STY)]PAQKPK_.2	#N/A	8.2295	7.8075	7.6901	8.6407	7.5386	8.1583	8.3340	8.0056	8.1758	7.9791	0.9861	-0.0202	0.6193	0.2081
Znf638	_QSSVTQVTEQS[Phospho (STY)]PK_.2	Q61464_S128_M1_Znf638	7.3295	7.1589	7.5920	7.5608	6.7636	7.2005	7.6482	7.1284	7.0123	6.9822	1.0104	0.0149	0.7157	0.1452
Znf638	_HTETVDKGLS[Phospho (STY)]PAQKPK_.3	#N/A	4.0441	5.0161	3.5069	3.7327	3.1698	3.0589	4.1341	4.3858	4.2927	3.6997	0.9096	-0.1368	0.3601	0.4435
Znf638	_GSTFS[Phospho (STY)]PDLK_.2	Q61464_S1099_M1_Znf638	3.7939	3.5485	3.5275	4.5722	2.9376	3.6870	3.2196	3.9299	4.2289	4.2494	0.9413	-0.0872	0.5074	0.2946
Msi1	etyl (Protein N-term)]M[Oxidation (M)]ETDAPQPGLAS[Phospho (STY)]PDSPHDPC[Carbamidomethyl (C	#N/A	3.9497	3.9569	4.0181	3.7467	3.4432	4.1543	4.1890	3.8665	3.7650	3.9295	0.9849	-0.0219	0.6959	0.1575
Pde1a	_NNLVDIIQQNK_.2	#N/A	5.8339	5.5459	6.5696	5.8728	4.7493	4.2819	5.9525	5.3749	4.8724	5.5766	1.0058	0.0083	0.9471	0.0236
Pde1a	_NSLQQPEGIDR_.2	#N/A	7.4316	7.2598	7.8862	7.6126	6.8375	7.4116	7.8002	7.3155	7.4390	7.2934	0.9926	-0.0108	0.7905	0.1021
Pde1a	_[Acetyl (Protein N-term)]GS[Phospho (STY)]TDTDIEELENATYK_.2	Q61481_S3_M1_Pde1a	3.4988	7.3168	8.0924	8.0978	3.8722	7.7393	8.1943	8.1487	3.6537	3.9544	1.0749	0.1042	0.7702	0.1134
Alcam	_APFLETDQLK_.2	#N/A	6.5553	6.0598	6.5842	6.2771	5.8279	5.9584	6.0916	6.2509	6.3594	6.3038	0.9934	-0.0095	0.8124	0.0902
Alcam	_SSNTYTLTDVR_.2	#N/A	5.6728	5.6997	6.1104	5.5302	5.5710	5.4154	5.8267	5.1753	5.9313	5.2129	1.0235	0.0335	0.5328	0.2735
Alcam	_EIDPGTQLYTVTSSLEYK_.2	#N/A	5.4368	5.4592	6.0505	6.2857	5.0980	6.0828	5.6052	5.4646	5.6334	5.7320	1.0226	0.0322	0.6158	0.2106
Alcam	_VLQPVEGEVAILFK_.2	#N/A	4.0385	3.8755	4.2846	4.5019	2.6807	4.1876	4.3240	4.7766	4.4688	5.0193	0.8453	-0.2425	0.0757	1.1208
Alcam	_NM[Oxidation (M)]AASTTITVHYLDLSLNPSGEVTK_.3	#N/A	1.5790	4.0606	4.5163	5.4930	4.3177	3.9770	5.0774	4.2281	4.9690	4.8940	0.8327	-0.2641	0.2730	0.5639
Alcam	_KTDPSGLSK_.2	#N/A	5.1826	3.5839	4.3291	4.3011	4.4416	3.9257	4.5464	3.2867	5.4092	4.9376	0.9448	-0.0820	0.5945	0.2258
Alcam	_SVQYDDVPEYKDR_.3	#N/A	3.5702	4.3264	3.3038	4.3484	0.9690	3.9107	3.8039	4.1536	1.2947	4.0257	1.0257	0.0366	0.9215	0.0355
Snap91	_DALEIYKR_.2	#N/A	9.0739	9.0089	9.1435	9.2044	8.9655	9.1937	9.3534	9.0657	9.2037	9.2184	0.9878	-0.0176	0.1408	0.8514
Snap91	_NTLFNLSNFLDK_.2	#N/A	7.5038	7.2892	7.9772	6.9451	6.6222	7.3299	7.4909	7.5263	7.5527	7.2207	0.9772	-0.0333	0.5083	0.2938
Snap91	_GLGSDLDSSLASLVGNLGISGTTSK_.2	#N/A	5.5916	5.6913	6.0089	5.8475	4.3860	5.8709	5.4075	5.4112	5.2565	5.6528	1.0247	0.0352	0.6779	0.1688
Snap91	_DPLADLNIKDFL_.2	#N/A	5.1909	5.2883	4.8367	5.2195	5.1745	5.5898	5.2360	5.5115	4.5977	5.2356	1.0139	0.0199	0.7257	0.1393
Snap91	_SSPATTVTS[Phospho (STY)]PNSTPAK_.2	Q61548_S313_M1_Snap91	12.2077	11.7981	12.5540	12.5915	11.4832	12.0474	12.5491	12.2725	12.3610	11.7571	0.9901	-0.0144	0.6507	0.1866
Snap91	_QM[Oxidation (M)]AFDFAR_.2	#N/A	7.2066	7.4397	7.0084	6.8584	6.5781	6.9783	7.0901	7.4081	7.2084	6.8114	0.9835	-0.0241	0.5303	0.2755
Snap91	_IAAAQYSVTGSAVAR_.2	#N/A	6.1666	6.4870	3.6398	5.8064	6.3875	6.5243	6.2427	6.2416	6.6752	6.8593	0.8971	-0.1567	0.2807	0.5518
Snap91	_ATTHEVM[Oxidation (M)]GPK_.2	#N/A	6.3881	6.0613	5.7090	5.9696	6.0544	5.5343	5.4801	5.9076	5.8057	5.0020	1.0728	0.1014	0.1061	0.9742
Snap91	_LTGGANWQPK_.2	#N/A	5.4930	4.3874	5.1290	5.3231	5.9605	4.6050	5.7292	5.6840	5.5445	5.6784	0.9100	-0.1361	0.1255	0.9012
Snap91	_KPGNNEGSGAPS[Phospho (STY)]PLSK_.2	#N/A	11.5005	11.6584	12.2066	12.1950	10.8901	11.6083	11.9955	11.8554	11.6799	11.5986	0.9910	-0.0130	0.6947	0.1582
Snap91	_GASPVPESSLTADLLSVDAFAAPSPASTASPAK_.3	#N/A	5.6588	6.1126	6.6547	6.6537	5.3751	3.9686	5.5796	5.7665	5.3038	5.2752	1.0467	0.0659	0.6377	0.1954
Snap91	_KGDLQWNAGEK_.2	#N/A	4.3956	2.9468	4.0972	3.6731	6.0542	3.4606	5.9416	4.8313	5.1832	5.5281	0.7642	-0.3880	0.0612	1.2129

Snap91	_TIDTS[Phospho (STY)]PPVDIFATASAAAPVSSAK_.2	#N/A	3.9243	7.3902	3.2854	8.7120	3.5639	5.7526	3.5740	7.5992	6.8357	3.9334	0.9913	-0.0125	0.9737	0.0116
Snap91	_PDAAPSIDLFGTDAFSS[Phospho (STY)]PPR_.2	Q61548_S594_M1_Snap91	3.4849	4.2412	3.2186	7.2128	5.0595	3.9960	3.8891	4.2388	3.0371	3.9405	1.2010	0.2643	0.3546	0.4503
Snap91	_SGSHGYDM[Oxidation (M)]STFIR_.3	#N/A	4.6133	5.2276	4.9256	4.8332	5.0217	4.8860	3.8379	5.5636	5.2611	4.8915	1.0060	0.0086	0.9280	0.0324
Snap91	_DALEIYK_.2	#N/A	3.0136	3.7699	4.2482	3.7919	3.6582	2.9664	3.9402	3.2092	1.8147	3.4692	1.1500	0.2017	0.3213	0.4931
Fscn1	_VNASASS[Phospho (STY)]LKK_.2	#N/A	8.9452	8.9588	9.2716	9.2664	8.8429	8.9860	9.2014	9.0878	8.9223	9.0733	0.9971	-0.0042	0.8056	0.0939
Fscn1	_FLVVAHDDGR_.2	#N/A	8.5111	8.6274	8.4531	8.6988	8.4320	8.4385	8.6064	8.2906	8.5509	8.5323	1.0037	0.0054	0.6995	0.1552
Fscn1	_YLAPSGPSGTLK_.2	#N/A	8.3565	8.5155	8.9244	8.4750	8.7971	8.2430	8.2928	8.5884	8.2640	8.4604	1.0179	0.0256	0.3325	0.4782
Fscn1	_GEHGFIGC[Carbamidomethyl (C)]R_.2	#N/A	7.8050	7.4895	7.2618	7.9343	8.1920	7.5228	7.4400	7.8492	7.5358	7.7365	1.0079	0.0114	0.7560	0.1215
Fscn1	_LSC[Carbamidomethyl (C)]FAQSVSPAEK_.2	#N/A	7.3705	7.7310	7.7686	7.8205	8.1051	7.3212	7.3824	7.4608	7.6650	7.4384	1.0266	0.0379	0.2429	0.6145
Fscn1	_KVTGTLDANR_.2	#N/A	6.5261	6.4629	5.9459	6.6183	7.2338	6.7895	6.9385	6.9654	6.6208	6.7493	0.9674	-0.0478	0.3513	0.4543
Fscn1	_VNASASS[Phospho (STY)]LKKK_.2	#N/A	6.4178	6.6714	7.2847	6.5388	6.5872	6.6981	6.9575	4.3725	6.6986	3.7130	1.2326	0.3017	0.0935	1.0293
Fscn1	_YSVQTSDHR_.2	#N/A	7.2920	6.9345	6.9822	7.0475	7.7046	7.0572	7.2271	7.0878	7.1507	7.4025	0.9934	-0.0095	0.7709	0.1130
Fscn1	_LVARPEPATGFTLEFR_.3	#N/A	7.3446	7.1241	7.2174	7.4241	7.0968	6.9898	7.2121	7.3818	7.5709	7.1701	0.9817	-0.0267	0.2568	0.5903
Fscn1	_WSLQSEAHR_.2	#N/A	6.5721	6.9203	7.1071	7.0141	7.2255	7.0144	7.2532	7.3535	7.2929	7.0560	0.9636	-0.0535	0.0676	1.1700
Fscn1	_YFGGTEDR_.2	#N/A	6.6538	6.7606	6.6761	6.4203	6.8945	6.8484	6.3823	6.7107	6.9737	6.9747	0.9924	-0.0110	0.7246	0.1399
Fscn1	_YLTAEAFGFK_.2	#N/A	6.4757	6.6576	6.7909	6.2110	6.2830	6.1631	6.3956	6.6744	6.7179	5.6935	1.0094	0.0135	0.7995	0.0972
Fscn1	_AC[Carbamidomethyl (C)]AETIDPASLWEY_.2	#N/A	5.7827	6.5159	6.5112	5.8163	6.4833	6.1008	5.9912	5.7242	6.0471	5.9246	1.0473	0.0666	0.1717	0.7652
Fscn1	_SSYDVFQLEFNDGAYNIK_.2	#N/A	6.0504	6.5168	6.5095	6.7246	6.0743	6.1951	6.2913	6.1129	5.6800	6.2601	1.0426	0.0601	0.1879	0.7262
Fscn1	_VGKDELFALEQSC[Carbamidomethyl (C)]AQVVLQAANER_.3	#N/A	6.1924	6.0543	6.5324	6.4279	4.6970	5.6528	6.6162	5.6228	6.2110	6.0508	0.9675	-0.0477	0.6162	0.2103
Fscn1	_VTGTLDANR_.2	#N/A	4.9524	5.1936	4.9156	4.9956	5.6100	5.7703	5.7145	5.7594	5.6704	5.0419	0.9447	-0.0821	0.2178	0.6619
Fscn1	_YLAADKDGNVTC[Carbamidomethyl (C)]ER_.2	#N/A	6.4609	5.8688	5.8292	6.4989	6.5342	6.2713	6.0623	6.0411	6.2192	6.1385	1.0210	0.0300	0.4610	0.3363
Fscn1	_YAHLSAR_.2	#N/A	6.1693	5.7451	5.2450	5.1625	6.5079	5.8274	5.2211	6.0558	6.3589	5.3582	1.0048	0.0069	0.9375	0.0280
Fscn1	_QIWTLEQPPDEAGSAAVC[Carbamidomethyl (C)]LR_.2	#N/A	3.6157	4.6902	4.3025	3.3043	5.0830	4.5845	5.8488	3.3133	2.9619	4.9006	1.0017	0.0024	0.9913	0.0038
Fscn1	_DVPWGVDSLITLAFQDQR_.2	#N/A	4.0798	3.2626	3.8135	4.7070	3.4182	3.8776	3.5055	3.9043	4.1720	3.5986	1.0170	0.0244	0.8282	0.0819
Fscn1	_VNASASS[Phospho (STY)]LK_.2	#N/A	6.7817	6.3774	6.1946	6.7005	6.1688	6.7047	6.7528	6.5221	6.4974	6.7086	0.9800	-0.0291	0.4018	0.3959
Fscn1	_QGM[Oxidation (M)]DLSANQDEETDQETFQLEIDR_.3	#N/A	3.7917	3.1612	3.5253	4.5699	3.3721	3.4644	3.4853	2.7770	4.2311	4.2472	0.9898	-0.0148	0.9225	0.0350
Ktn1	_EIQNGTLRES[Phospho (STY)]DSEHVPR_.3	Q61595_S75_M1_Ktn1	5.4985	6.1092	5.8417	4.0951	5.4364	5.8973	3.9926	5.1851	3.4813	3.9573	1.2442	0.3153	0.1130	0.9469
Ktn1	_DLLT[Phospho (STY)]ELQK_.2	Q61595_T1211_M1_Ktn1	6.0220	5.3465	6.6958	6.6836	6.8240	6.4198	5.3453	5.6803	5.8978	5.8932	1.1101	0.1506	0.0729	1.1375
Gdi2	_NTNDANSC[Carbamidomethyl (C)]QIIIPQNQVNR_.2	#N/A	9.9247	10.0197	10.2258	10.6958	9.5932	9.6723	10.1975	9.7156	9.9222	9.9855	1.0067	0.0096	0.7695	0.1138
Gdi2	_LYSESLAR_.2	#N/A	8.8535	8.9115	9.0244	8.7489	9.1576	8.7458	7.9570	9.2462	8.9174	9.0536	1.0129	0.0185	0.6509	0.1865
Gdi2	_TDDYLDQPC[Carbamidomethyl (C)]C[Carbamidomethyl (C)]ETINR_.2	#N/A	7.5589	7.6741	7.7318	7.7266	7.1323	6.9814	7.8340	7.1363	7.5614	7.4917	0.9949	-0.0074	0.8543	0.0684
Gdi2	_VIC[Carbamidomethyl (C)]ILSHPIK_.2	#N/A	6.2963	6.8746	6.9478	6.7217	6.5518	6.3480	6.9314	6.7630	7.1009	6.5739	0.9680	-0.0469	0.2197	0.6581
Gdi2	_FVSISDLFVPK_.2	#N/A	6.2869	6.4161	6.8502	6.4435	6.2050	5.8754	6.6705	6.5922	6.5067	6.1515	0.9793	-0.0302	0.4938	0.3065
Gdi2	_DLGTDSQIFISR_.2	#N/A	5.1663	5.7104	5.6649	6.2449	6.1333	6.2651	5.2326	6.2910	6.1674	5.7627	1.0001	0.0002	0.9980	0.0009
Gdi2	_M[Oxidation (M)]LLFTEVTR_.2	#N/A	6.4823	7.0478	6.1557	7.0071	5.4406	6.8189	6.3184	6.8284	5.7637	6.8628	1.0076	0.0109	0.8998	0.0459
Gdi2	_FLM[Oxidation (M)]ANGQLVK_.2	#N/A	6.7069	6.2852	6.3921	6.2858	6.3736	6.2460	6.2160	6.5434	6.3329	6.3344	1.0039	0.0056	0.8124	0.0902
Gdi2	_SPYLYPLYGLGELPQGFAR_.2	#N/A	6.5987	6.9720	7.2532	7.5707	6.5572	6.8588	6.9909	7.2022	6.9394	6.4475	1.0106	0.0153	0.7634	0.1172
Gdi2	_AYDATTHFETTC[Carbamidomethyl (C)]DDIKDIYKR_.4	#N/A	4.7270	5.4645	5.3445	4.7458	2.8797	5.0429	4.1457	2.9710	4.1204	4.6157	1.1861	0.2462	0.2201	0.6574
Gdi2	_VLHM[Oxidation (M)]DQNPYYGGESASITPLEDLYK_.3	#N/A	3.6762	3.4989	3.2349	3.5655	2.9217	3.3751	3.9612	3.1882	3.8807	3.6342	0.9216	-0.1178	0.1787	0.7478
Gdi2	_FLVYVANFDEKDPR_.2	#N/A	4.2945	4.4371	4.1841	4.4482	3.8410	4.1668	4.5997	3.9632	3.9952	4.0474	1.0186	0.0266	0.6525	0.1854
Gdi2	_VIEGSFVYK_.2	#N/A	6.1325	3.7186	3.6002	4.4021	4.0327	2.8127	4.5504	4.4991	4.3658	3.3905	0.9798	-0.0295	0.8929	0.0492
Gdi2	_FDLGQDVIDFTGHSLALYR_.3	#N/A	3.8850	4.0314	3.8903	3.8229	3.5961	3.6231	3.8245	3.7866	3.4969	3.6969	1.0289	0.0411	0.3325	0.4782
Gdi2	_MTGSEFDFEEMKR_.3	#N/A	3.3691	4.1743	4.4027	3.9464	3.9956	3.7339	4.0947	3.0547	3.3538	3.6237	1.1147	0.1567	0.1473	0.8318
Slc20a1	_EVKSS[Phospho (STY)]PSES[Phospho (STY)]PLMEKK_.2	#N/A	3.5933	3.6433	4.0467	4.1805	3.6222	3.9577	4.1546	3.8014	3.3680	4.0179	1.0013	0.0019	0.9787	0.0093
Slc20a1	_EVKSS[Phospho (STY)]PSES[Phospho (STY)]PLM[Oxidation (M)]EK_.2	#N/A	4.7254	7.1950	5.2428	3.6123	6.0524	6.4120	7.5553	3.1549	5.9253	3.4664	1.1024	0.1406	0.6389	0.1946
Slc20a1	_SS[Phospho (STY)]PSES[Phospho (STY)]PLMEKK_.2	#N/A	3.4615	4.2667	4.1483	3.1501	4.0880	3.6415	4.3656	3.4675	3.1161	3.1171	1.0785	0.1090	0.4202	0.3765
Slc20a1	_SS[Phospho (STY)]PSESPLMEK_.2	#N/A	3.2401	4.1672	4.1085	3.8104	3.8719	3.5942	5.5885	3.5191	3.3696	3.6865	0.9401	-0.0892	0.6026	0.2200
Drd1	_IQPVTHSGQHS[Phospho (STY)]T_.2	Q61616_S445_M1_Drd1	11.4874	11.4734	11.9073	11.4289	10.5176	11.3453	11.7705	11.7111	2.9740	3.3592	1.5292	0.6128	0.0827	1.0827
Znf148	_GGLLTSEEDS[Phospho (STY)]GFSTSPKDNSLPK_.3	Q61624_S301_M1_Znf148	4.3670	4.3600	3.5530	4.0556	4.2483	3.8772	3.7703	4.0628	3.7114	4.0226	1.0475	0.0670	0.3266	0.4860
Znf148	_GGLLTSEEDSGFSTS[Phospho (STY)]PK_.2	Q61624_S306_M1_Znf148	3.8481	3.4943	3.5817	4.6263	3.1037	3.6328	3.2738	3.8757	4.1747	4.3036	0.9507	-0.0729	0.5603	0.2516
Znf148	_AGMTSS[Phospho (STY)]PDATTGQTFG_.2	Q61624_S784_M1_Znf148	3.8501	3.7601	3.6576	4.3872	3.5814	4.1746	3.6197	4.1414	3.8132	4.0644	0.9980	-0.0029	0.9674	0.0144
Grid2	_VPS[Phospho (STY)]KEDDKEIDLEHLHR_.4	Q61625_S865_M1_Grid2	6.2719	6.2637	3.3088	6.5912	4.2196	3.9058	5.0168	6.4256	5.6310	5.6773	0.8955	-0.1592	0.4620	0.3353
Pacsin1	_TEQSVT[Phospho (STY)]PEQQK_.2	#N/A	10.7862	11.0244	11.3080	11.4890	10.9834	11.0587	11.1481	10.9322	10.9985	11.1269	1.0051	0.0074	0.6828	0.1657
Pacsin1	_GPQYGSLER_.2	#N/A	9.7944	9.6888	10.2163	9.9531	10.0165	9.8099	9.7643	9.4118	9.5615	9.8050	1.0288	0.0410	0.0507	1.2952
Pacsin1	_GRLDS[Phospho (STY)]GQLGLYPANYVEAI_.2	#N/A	8.6645	8.7675	9.3026	9.3683	8.8908	8.8477	8.9312	8.7721	9.0343	8.8952	1.0073	0.0106	0.6838	0.1651
Pacsin1	_EVLLDIKR_.2	#N/A	9.1597	9.7489	10.0075	9.8683	9.7902	9.6879	9.5667	9.4270	9.5121	9.7189	1.0161	0.0231	0.3531	0.4521
Pacsin1	_NSLLNEDLEK_.2	#N/A	9.7376	9.4854	9.6309	9.8168	9.8252	9.6919	9.8596	9.4722	9.5739	9.8821	1.0001	0.0001	0.9925	0.0032
Pacsin1	_AYAQQLTDWAK_.2	#N/A	8.4482	8.8935	8.6540	9.1020	9.1200	8.5563	8.7091	8.5465	8.6645	8.7558	1.0146	0.0209	0.4222	0.3745
Pacsin1	_LGEEDEQGWC[Carbamidomethyl (C)]R_.2	#N/A	8.4370	8.2794	8.3579	8.3202	8.2828	8.1038	8.4014	7.9110	8.1034	8.3244	1.0137	0.0196	0.3161	0.5002
Pacsin1	_GADAQEDLR_.2	#N/A	8.0703	8.3330	8.4633	7.9974	8.8516	8.2785	7.8324	8.1146	8.0914	8.2322	1.0328	0.0466	0.1584	0.8004
Pacsin1	_ALYDYDGQEQDELSFK_.2	#N/A	8.0722	7.9493	8.6843	8.3690	8.0939	8.2862	8.1419	7.6564	7.5318	8.1756	1.0465	0.0655	0.0871	1.0598
Pacsin1	_EAEDGFRK_.2	#N/A	7.8184	7.6758	7.4149	7.6801	7.9360	7.6350	7.3492	7.5201	7.5329	7.9659	1.0133	0.0191	0.4824	0.3166
Pacsin1	_LDS[Phospho (STY)]GQLGLYPANYVEAI_.2	#N/A	7.0006	6.3867	5.4353	5.7123	6.8521	7.2504	6.7781	5.7012	6.4759	6.6722	1.0051	0.0073	0.9399	0.0269
Pacsin1	_NSLLNEDLEKVK_.2	#N/A	5.6329	6.5350	6.3592	6.0237	5.4210	5.7898	6.2155	5.3740	5.3494	5.3003	1.0720	0.1004	0.1894	0.7227
Pacsin1	_KAEGATLSNATGAVESTSQAGDR_.3	#N/A	7.2842	7.6659	7.8080	8.0535	7.4526	7.1171	7.4911	7.2511	7.3659	7.5611	1.0197	0.0282	0.4518	0.3451
Pacsin1	_AYHLAC[Carbamidomethyl (C)]K_.2	#N/A	4.2045	5.0920	3.3905	5.7493	6.1811	5.3514	4.9997	5.3490	5.6530	5.7184	0.9198	-0.1205	0.4450	0.3516
Pacsin1	_LC[Carbamidomethyl (C)]NDLM[Oxidation (M)]SC[Carbamidomethyl (C)]VQER_.2	#N/A	6.1576	5.9141	5.3873	7.4261	6.8762	5.5676	5.3176	5.6225	6.0794	6.3960	1.0628	0.0879	0.4319	0.3647
Pacsin1	_AYHLAC[Carbamidomethyl (C)]KEER_.2	#N/A	6.2000	6.0753	6.1756	6.3224	6.2107	6.7877	6.3066	6.3190	5.9899	6.1504	1.0168	0.0240	0.4894	0.3104
Pacsin1	_GSVSS[Phospho (STY)]YDR_.2	Q61644_S346_M1_Pacsin1	8.7688	8.4342	8.8115	3.6028	8.3475	8.6829	3.5957	4.1296	8.9472	9.0786	1.2077	0.2722	0.4212	0.3756
Pacsin1	_TEQSVT[Phospho (STY)]PEQQKK_.2	#N/A	8.0311	8.4339	8.9372	9.0333	8.5849	8.5022	8.6339	8.5379	8.2703	8.5203	1.0114	0.0163	0.6348	0.1974
Pacsin1	_HLNLAENS[Phospho (STY)]SYM[Oxidation (M)]HVYR_.3	#N/A	6.2254	6.6031	7.3247	3.5045	6.7765	6.8280	7.0681	6.8670	7.0120	6.8898	0.8924	-0.1643	0.3169	0.4991
Pacsin1	_[Acetyl (Protein N-term)]SGSYDEASEEITDSFWEVGNYKR_.3	#N/A	5.5765	6.6174	7.7541	7.6908	6.4905	6.7722	5.9482	6.0065	5.5472	6.3162	1.1448	0.1951	0.0826	1.0832
Pacsin1	_AWGAM[Oxidation (M)]M[Oxidation (M)]TEADKVSELHQEVK_.3	#N/A	5.9602	6.7680	7.8326	6.9628	6.6798	6.9100	6.9887	6.9550	6.8647	7.1842	0.9791	-0.0304	0.6519	0.1858
Pacsin1	_KAEGATLSNATGAVESTS[Phospho (STY)]QAGDRGSVSSYDR_.4	#N/A	6.5839	6.8047	3.8476	3.7175	3.4944	3.5186	3.7006	8.6487	3.8123	6.5878	0.8196	-0.2871	0.4321	0.3644
Pacsin1	_AEGATLSNATGAVESTSQAGDR_.2	#N/A	4.1860	3.4414	4.4716	4.4074	3.3297	3.2949	3.1881	3.1443	4.2937	3.4404	1.0963	0.1326	0.3674	0.4348
Pacsin1	_TTPQYM[Oxidation (M)]EGM[Oxidation (M)]EQVFEQC[Carbamidomethyl (C)]QQFEEK_.3	#N/A	3.8270	3.4958	4.1990	4.4454	3.6269	3.8671	3.7603	3.4414	3.9347	3.1679	1.0934	0.1289	0.1770	0.7520
Pacsin1	_STSGPGM[Oxidation (M)]PM[Oxidation (M)]NWPQFEEWNPDLPHTTAK_.3	#N/A	4.0001	3.2260	3.1861	3.6887	3.1258	3.1028	3.4034	4.4297	4.0783	3.6557	0.8706	-0.1999	0.0891	1.0502
Ddx5	_APILIATDVASR_.2	#N/A	7.6338	7.9531	7.8631	7.9431	7.6305	7.8688	7.6249	7.8793	7.9377	7.7637	1.0018	0.0026	0.8837	0.0537
Ddx5	_QVSDLISVLR_.2	#N/A	5.6152	5.9184	5.6359	4.9049	5.1536	5.5742	4.6543	5.0116	5.6991	5.7459	1.0359	0.0508	0.5222	0.2821

Ddx5	_LIDFLEC[Carbamidomethyl (C)]GK_.2	#N/A	4.1617	4.5891	4.8563	4.8089	3.9980	4.0243	3.8553	3.9743	4.2728	5.1487	1.0217	0.0310	0.7673	0.1150
Ddx5	_GVEIC[Carbamidomethyl (C)]IATPGR_.2	#N/A	5.0261	5.6768	5.6375	5.4041	5.2020	4.3718	6.2904	5.4206	5.2983	3.8876	0.9991	-0.0012	0.9925	0.0033
Ddx5	_ELAQQVQQVAAEYC[Carbamidomethyl (C)]R_.2	#N/A	6.2402	3.8959	6.5771	6.3006	5.8961	6.3446	6.5172	5.7166	6.5456	6.0610	0.9462	-0.0798	0.5466	0.2623
Ddx5	_LLQLVEDR_.2	#N/A	5.5169	4.8710	5.7143	5.7677	5.6026	5.4182	3.5262	5.2459	6.1381	5.3527	1.0821	0.1139	0.3977	0.4004
Ddx5	_STC[Carbamidomethyl (C)]IYGGAPK_.2	#N/A	4.6882	5.4918	3.3508	5.9318	5.3121	5.2385	3.7569	5.4831	5.4887	5.5082	0.9887	-0.0164	0.9234	0.0346
Ddx5	_NFYQEHPDLAR_.2	#N/A	5.6640	5.4820	5.4900	6.0255	5.6480	5.4635	5.4425	5.4745	5.0248	5.2824	1.0608	0.0852	0.0447	1.3499
Ddx5	_FVINYDYPNSSEDYIHR_.3	#N/A	3.9935	3.3489	4.9619	3.2709	4.7793	5.1729	4.9173	3.7302	6.2574	4.9741	0.8561	-0.2242	0.2596	0.5856
Ddx5	_DWVLNEFK_.2	#N/A	3.3012	4.4904	4.5358	4.3581	4.6192	4.1796	4.0728	4.0211	3.4090	4.3251	1.0734	0.1022	0.3503	0.4555
Cbx5	_KSS[Phospho (STY)]FSNSADDIK_.2	#N/A	8.1579	8.3793	8.8830	8.7952	8.2655	3.5422	8.9535	8.3635	8.2738	8.3904	0.9029	-0.1473	0.4547	0.3423
Cbx5	_SS[Phospho (STY)]FSNSADDIK_.2	#N/A	6.5984	6.4956	3.5881	7.2968	6.3373	6.6637	7.3718	4.0374	6.6389	4.0577	1.1152	0.1573	0.5237	0.2809
Cbx5	_TADSSSS[Phospho (STY)]EDEEEYVVEK_.2	Q61686_S14_M1_Cbx5	3.9322	3.9319	3.6659	3.2096	4.0134	5.1995	4.5725	3.7957	4.0948	4.4592	0.9436	-0.0837	0.5321	0.2740
Atrx	_EENVNS[Phospho (STY)]PEDKR_.2	Q61687_S912_M1_Atrx	4.8992	5.5143	5.8803	5.9171	4.9015	5.0594	6.0516	5.6827	4.9303	4.9212	0.9936	-0.0093	0.9189	0.0367
Atrx	_LTLS[Phospho (STY)]DGES[Phospho (STY)]GEEKPTKPK_.2	#N/A	5.9671	5.8085	5.5582	5.9531	3.7500	5.4218	6.5105	5.6729	5.4026	4.2362	0.9916	-0.0121	0.9378	0.0279
Atrx	_VC[Carbamidomethyl (C)]DQTSKFS[Phospho (STY)]PK_.2	Q61687_S315_M1_Atrx	6.8295	6.9842	6.9372	7.3383	6.4396	7.0521	7.7587	3.6429	7.1826	6.7942	1.0923	0.1274	0.4541	0.3428
Atrx	_QNYSESSNYDS[Phospho (STY)]ELER_.2	Q61687_S801_M1_Atrx	3.4749	4.2311	3.2085	4.2531	4.1194	4.0060	3.8992	4.2489	3.0471	4.6110	0.9825	-0.0255	0.8460	0.0726
Atrx	_YVES[Phospho (STY)]DDEKPTDENVNEK_.2	#N/A	5.0145	5.2804	6.2255	6.0209	5.0962	5.7346	6.0232	6.1166	5.2173	5.2027	0.9862	-0.0201	0.8162	0.0882
Atrx	_TTSTSNPS[Phospho (STY)]S[Phospho (STY)]PAPDWYK_.2	Q61687_S1978_M2_Atrx	3.8543	3.1097	3.5879	3.1317	2.9980	3.6266	3.2800	3.8695	4.1685	4.3098	0.8663	-0.2070	0.0734	1.1341
Atrx	_EEIS[Phospho (STY)]DHENNVTILLEDSDLR_.3	Q61687_S626_M1_Atrx	3.9244	3.2287	3.1103	3.3911	3.0501	3.1786	3.5394	3.6101	3.9091	3.0684	0.9383	-0.0919	0.3382	0.4708
Atrx	_TTQEGS[Phospho (STY)]S[Phospho (STY)]ADDTGDTEGR_.2	Q61687_S854_M2_Atrx	3.7380	3.6331	3.4717	3.8064	3.4806	3.7494	3.9547	4.0458	3.6349	3.4837	0.9648	-0.0518	0.3271	0.4853
Atrx	_AKQPVIGDQNS[Phospho (STY)]DS[Phospho (STY)]DEM[Oxidation (M)]LAVLK_.3	#N/A	3.9238	3.4222	4.1532	7.4691	3.0596	3.3177	3.3397	7.8011	4.1528	3.2279	0.9123	-0.1324	0.7425	0.1293
Atrx	_IGKQS[Phospho (STY)]EES[Phospho (STY)]PADDGELRR_.3	Q61687_S1287_M2_Atrx	3.0815	4.2609	4.3161	3.8598	4.0822	3.6473	4.0081	3.1413	3.4080	3.5371	1.0996	0.1370	0.2392	0.6213
Hsph1	_IES[Phospho (STY)]PKLER_.2	#N/A	13.2827	13.5092	14.0838	13.9112	13.0778	13.4159	13.8047	13.5770	13.4057	13.4612	0.9989	-0.0016	0.9426	0.0257
Hsph1	_PKIES[Phospho (STY)]PKLER_.2	#N/A	6.7419	6.5979	6.9244	6.9847	5.4452	6.8098	7.1211	6.6826	7.0104	6.4798	0.9649	-0.0515	0.4695	0.3283
Hsph1	_SVLDAAQIVGLNC[Carbamidomethyl (C)]LR_.2	#N/A	6.7878	6.9761	6.4503	6.4997	6.8537	6.6092	7.2419	6.7815	6.7915	6.8386	0.9686	-0.0461	0.1533	0.8146
Hsph1	_FVVQNVSAQK_.2	#N/A	6.9231	6.5164	6.1840	6.6092	6.7555	6.1198	6.5317	7.0189	6.5428	6.1401	0.9938	-0.0089	0.8558	0.0676
Hsph1	_AGGIETIANEFSDR_.2	#N/A	6.7098	6.8277	6.8713	6.8748	5.8113	6.4925	6.9796	6.5533	6.5533	6.4436	0.9948	-0.0075	0.8843	0.0534
Hsph1	_[Acetyl (Protein N-term)]SVVGLDVGSQSC[Carbamidomethyl (C)]YIAVAR_.2	#N/A	6.7937	6.0086	7.1243	7.0157	5.2054	6.2593	6.5708	5.9833	6.2336	6.8940	0.9970	-0.0043	0.9632	0.0163
Hsph1	_FIC[Carbamidomethyl (C)]EQEHEK_.2	#N/A	6.0231	5.9257	3.7269	5.6703	5.7728	5.9945	5.4407	6.0011	5.9635	6.1731	0.9363	-0.0950	0.4473	0.3494
Hsph1	_LLTETEDWLYEEGEDQAK_.2	#N/A	6.4112	6.1887	6.8853	7.3824	5.4344	6.3804	6.6063	6.4203	5.7123	6.3125	1.0294	0.0418	0.6309	0.2000
Hsph1	_NAVEEC[Carbamidomethyl (C)]VYEFR_.2	#N/A	5.7257	5.7475	6.2118	6.4623	5.1644	5.9387	5.4844	6.0563	5.4813	5.2098	1.0571	0.0801	0.2719	0.5656
Hsph1	_SQFEELC[Carbamidomethyl (C)]AELLQK_.2	#N/A	4.5761	4.7461	5.2817	5.2585	3.6516	4.3346	4.6668	3.9943	4.2539	4.1039	1.0909	0.1255	0.2802	0.5526
Hsph1	_S[Phospho (STY)]LDQDPVVR_.2	Q61699_S774_M1_Hsph1	3.0623	6.2868	4.2969	6.8262	5.9435	6.6378	6.4206	6.3397	5.8037	6.3043	0.8861	-0.1745	0.3868	0.4125
Hsph1	_VVNVELPVEANLVWQLGR_.2	#N/A	4.0633	3.6249	3.8576	4.8108	6.4460	4.8557	5.0959	4.3245	4.4433	3.9627	1.0344	0.0487	0.7907	0.1020
Hsph1	_VLEELGQR_.2	#N/A	6.3110	3.4319	4.4811	6.4463	6.9383	5.8327	3.1976	6.5598	6.2088	6.4885	0.9929	-0.0103	0.9669	0.0146
Hsph1	_QDLPNAEEKPR_.3	#N/A	4.0433	3.6264	3.2749	3.7776	3.7292	3.1928	3.4923	3.0448	3.8795	3.7446	1.0190	0.0271	0.7672	0.1151
Hsph1	_NQQITHANNTVSSFK_.2	#N/A	4.2152	4.0216	3.9489	3.4927	4.4786	4.6105	3.6409	3.8214	3.8076	3.1699	1.1435	0.1934	0.0613	1.2123
Hsph1	_T[Phospho (STY)]PNGPNIDKKEDLEGK_.3	Q61699_T816_M1_Hsph1	4.8475	3.8884	3.1877	4.2323	3.7097	4.0198	5.3958	7.3582	3.0679	3.9096	0.8070	-0.3093	0.2656	0.5757
Elavl4	_SSQALLSQLYQS[Phospho (STY)]PNR_.2	Q61701_S233_M1_Elavl4	3.4195	7.1037	4.1063	8.7188	4.0460	3.6834	6.8451	6.8124	3.1581	3.0751	1.0416	0.0588	0.8864	0.0524
Elavl4	_ILVDQVTGVSR_.2	#N/A	3.9532	5.2067	3.6869	5.1002	5.1031	3.5277	4.8752	5.3708	4.7998	4.4088	0.9108	-0.1348	0.3435	0.4641
Elavl4	_NC[Carbamidomethyl (C)]PS[Phospho (STY)]PMQTGAATDDSK_.2	#N/A	9.9453	8.4144	3.3698	11.2130	8.2291	3.8495	8.9762	4.0093	7.6682	8.4358	1.0318	0.0451	0.9043	0.0437
Cd47	_ISVSDLINGIASLK_.2	#N/A	5.6788	5.4401	5.3488	5.6374	5.4630	5.4419	5.8581	6.0066	5.4193	5.9575	0.9469	-0.0788	0.0381	1.4194
Cd47	_FVASNQR_.2	#N/A	4.1038	4.9491	3.8375	3.5555	5.2922	4.7242	3.2702	5.4185	4.7250	3.0586	1.0710	0.0989	0.6214	0.2066
Cd47	_NVEAQSTEEMFVK_.2	#N/A	4.1850	3.0390	3.9187	8.8850	6.7725	3.2959	8.1065	8.0056	7.5491	3.1397	0.7486	-0.4177	0.2977	0.5262
Phgdh	_GTIQVVTQGTSLK_.2	#N/A	5.7190	6.8021	7.2838	6.7168	6.6320	6.8547	6.7592	6.9812	6.6235	6.8473	0.9802	-0.0289	0.6317	0.1995
Phgdh	_GGIVDEGALLR_.2	#N/A	7.0492	6.8394	6.6846	6.6411	6.9938	6.5170	6.9616	6.8487	6.8573	6.9252	0.9840	-0.0233	0.3394	0.4693
Phgdh	_VTADVINAAEK_.2	#N/A	6.6920	6.6545	6.7713	6.5300	6.7409	6.8362	6.3619	6.6351	6.4274	6.5457	1.0326	0.0463	0.0192	1.7160
Phgdh	_QADVNLVNAK_.2	#N/A	4.9421	4.6150	4.9532	5.8776	5.2875	5.8585	5.2688	5.5234	5.0061	5.6564	0.9799	-0.0294	0.7179	0.1439
Phgdh	_NAGTC[Carbamidomethyl (C)]LSPAVIVGLLR_.2	#N/A	4.9811	5.4079	3.8936	5.2920	5.5274	4.7712	3.9511	4.8883	4.9960	5.2491	1.0435	0.0615	0.5993	0.2224
Phgdh	_VLISDSLDPC[Carbamidomethyl (C)]C[Carbamidomethyl (C)]R_.2	#N/A	2.9137	5.8618	5.9666	6.4318	6.3752	5.3189	5.6476	5.5764	6.0558	5.4130	0.9656	-0.0505	0.7819	0.1069
Phgdh	_ILQDGGLQVVEK_.2	#N/A	3.6838	3.6705	4.3706	3.8885	4.2379	4.1910	3.9753	4.3820	3.0628	3.3394	1.0859	0.1190	0.2935	0.5325
Phgdh	_AGTGVDNVDLEAATR_.2	#N/A	4.3452	4.9519	3.6541	4.9801	4.5468	5.2932	5.0342	5.3505	3.6103	5.3492	0.9571	-0.0633	0.6523	0.1856
Phgdh	_ALVDHENVISC[Carbamidomethyl (C)]PHLGASTK_.3	#N/A	3.6532	4.4622	4.1704	4.9422	4.0681	4.0817	4.2385	3.7301	4.0321	3.4732	1.0934	0.1288	0.2002	0.6986
Kcna5	_RES[Phospho (STY)]GLDTGGQR_.2	Q61762_S535_M1_Kcna5	6.0257	3.4169	6.6453	3.4390	3.3052	3.3194	3.5872	6.1902	6.4700	5.8983	0.7873	-0.3451	0.2481	0.6055
Kif3b	_DYEGPAIS[Phospho (STY)]PK_.2	Q61771_S681_M1_Kif3b	9.8322	9.9455	10.1193	10.3046	9.3131	9.9175	10.1981	9.8265	9.8186	9.8727	0.9976	-0.0034	0.9021	0.0448
Kif3b	_SQNQQYLVR_.2	#N/A	3.6667	3.6248	4.3535	3.6488	3.8176	3.4362	3.7970	3.2623	3.6514	3.8510	1.0323	0.0458	0.5586	0.2529
Lasp1	_GFSVVADTPELQR_.2	#N/A	7.5750	7.6133	8.0325	8.1257	8.2475	7.9603	7.7068	7.5547	7.6129	7.9710	1.0278	0.0396	0.2115	0.6746
Lasp1	_YHEEFEK_.2	#N/A	7.1339	7.2752	7.2989	7.5924	7.9026	7.3944	6.9189	7.4206	7.5029	7.3034	1.0201	0.0287	0.4239	0.3728
Lasp1	_YKEEFEK_.2	#N/A	7.1549	7.2549	7.3533	7.2445	7.7168	7.2828	6.8207	7.2457	7.3890	7.4117	1.0163	0.0234	0.4495	0.3473
Lasp1	_QSFTM[Oxidation (M)]VADTPENLR_.2	#N/A	6.6347	6.9054	7.5777	6.7318	7.0353	7.4378	6.8818	6.4766	6.9469	7.4013	1.0184	0.0262	0.6181	0.2089
Lasp1	_TQDQISNIK_.2	#N/A	5.8442	6.4774	5.7553	6.9727	6.8343	6.3061	5.9937	6.2594	6.0911	6.7369	1.0151	0.0216	0.7493	0.1253
Lasp1	_IVYPTEK_.2	#N/A	5.1634	5.3348	3.4340	4.5683	5.8744	5.3112	3.2157	5.6989	5.0655	5.3058	1.0262	0.0373	0.8426	0.0744
Lasp1	_EAQDSSSYR_.2	#N/A	4.3113	4.4152	3.6215	3.8308	7.0645	3.2779	3.6287	4.5803	4.4792	4.4096	1.0341	0.0484	0.8440	0.0737
Lasp1	_TGDTGMLPANYVEAI_.2	#N/A	4.5037	3.9908	3.8724	4.6175	2.9037	3.9173	4.0898	3.7434	3.3920	4.3421	1.0195	0.0278	0.8345	0.0785
Lasp1	_MGPSGGEGVEPERR_.3	#N/A	3.8941	3.1984	4.5809	4.4180	3.0198	3.2089	3.5697	3.0349	4.1844	3.5497	1.0378	0.0535	0.7386	0.1316
Lasp1	_LKQQSELQSQVR_.3	#N/A	3.6584	2.9628	4.3452	3.3470	2.7841	3.4445	3.0617	4.8098	4.4200	3.3140	0.8776	-0.1884	0.3067	0.5132
Rai1	_S[Phospho (STY)]PALPER_.2	Q61818_S1332_M1_Rai1	3.4367	4.4189	3.1704	4.2226	4.2393	4.0442	3.9373	3.8958	5.1597	4.1896	0.9130	-0.1313	0.3118	0.5061
Pdcd4	_SGVAVPTS[Phospho (STY)]PK_.2	#N/A	10.2895	10.1033	10.4217	10.4048	9.0820	9.8081	10.2783	10.1860	3.2521	10.1118	1.1846	0.2444	0.2961	0.5286
Pdcd4	_SGVAVPTS[Phospho (STY)]PKGR_.2	#N/A	3.1064	7.9022	8.7545	3.8847	6.6356	7.4193	7.3936	7.6113	7.1567	6.9646	0.8630	-0.2126	0.4181	0.3787
Myh10	_TSDVNDTQPPQS[Phospho (STY)]E_.2	Q61879_S1975_M1_Myh10	11.2476	11.1903	11.3849	11.1662	10.2552	10.9987	11.6696	11.3063	10.9750	11.0893	0.9805	-0.0284	0.3855	0.4140
Myh10	_SDLLLEGFNNYR_.2	#N/A	5.7529	5.8777	6.6692	5.6979	4.6077	4.9482	5.9288	6.4287	6.2389	4.9049	0.9518	-0.0712	0.5546	0.2560
Myh10	_IVFQEFR_.2	#N/A	5.5540	5.8038	5.6554	5.5641	4.5567	5.8834	5.1980	5.8986	5.4889	5.7405	0.9859	-0.0205	0.7815	0.1071
Myh10	_TQLEELEDELQATEDAK_.2	#N/A	5.2466	4.8390	5.0376	4.3995	4.2258	4.6417	5.6446	5.4513	5.2034	4.5263	0.9088	-0.1379	0.1234	0.9088
Myh10	_GGPISFS[Phospho (STY)]SSR_.2	Q61879_S1937_M1_Myh10	3.1324	7.9100	3.8192	4.4567	3.7589	3.9991	4.3599	3.8756	3.9390	3.6299	1.1421	0.1917	0.5434	0.2649
Myh10	_EQEVAELKK_.2	#N/A	5.3423	5.0807	5.8345	5.1256	5.3163	5.8036	6.1603	5.3823	4.8803	4.6910	1.0263	0.0374	0.6651	0.1771
Myh10	_LQQELDDLTVDLDHQR_.3	#N/A	5.6210	5.3743	5.1883	4.6898	4.1530	4.3772	4.8149	5.0489	4.8020	4.8245	1.0058	0.0083	0.9282	0.0324
Myh10	_LDPHLVLDQLR_.2	#N/A	6.1489	6.1442	3.7726	5.7465	5.4850	5.2898	6.3058	6.6151	6.0944	6.2589	0.8596	-0.2183	0.0890	1.0504
Myh10	_DAAGLESQLQDTQELLQEETR_.2	#N/A	5.1191	4.5075	4.9867	4.9759	4.0594	4.3225	5.3555	5.4025	4.6660	4.2885	0.9460	-0.0801	0.4099	0.3873
Myh10	_QLLQANPILESFGNAK_.2	#N/A	4.2126	3.1561	4.2298	2.1799	3.3902	4.1458	3.3156	3.2947	3.6676	3.4779	1.0330	0.0468	0.7944	0.1000
Myh10	_LVQEQGSHSK_.2	#N/A	3.0417	4.0736	4.3503	4.0471	4.2672	3.8346	3.6753	2.4137	2.3846	2.8690	1.3880	0.4730	0.0118	1.9268

Myh10	_DLEAQIEAANK_.2	#N/A	4.7801	3.4789	3.3605	3.1409	5.0175	4.4293	5.0091	4.9531	5.8001	5.4690	0.7601	-0.3957	0.0201	1.6958
Myh10	_QLHIEGAS[Phospho (STY)]LELSDDDTESK_.3	#N/A	3.4557	3.9090	4.1018	7.1681	5.9386	3.7492	4.0928	7.3194	6.5715	4.5676	0.8373	-0.2562	0.3748	0.4262
Myh10	_ELQAQIAELQEDFESEK_.2	#N/A	5.4742	5.5973	5.6608	6.1596	4.6430	4.8521	5.7356	5.6981	5.2016	5.2760	0.9854	-0.0212	0.8002	0.0968
Myh10	_IAQLEEELEEEQSNM[Oxidation (M)]ELLNDR_.3	#N/A	5.9635	5.9928	7.3207	6.0266	5.2071	6.1887	6.9419	6.4559	5.8699	6.2863	0.9574	-0.0628	0.5053	0.2964
Myh10	_SLEAEILQLQEELASSER_.2	#N/A	3.8140	3.1183	4.5008	4.4991	2.9397	3.5506	3.6498	2.1139	3.7987	3.1788	1.1732	0.2305	0.2592	0.5864
Myh10	_RQLHIEGASLELS[Phospho (STY)]DDDTESK_.3	#N/A	4.1208	3.5784	4.0509	6.3805	3.2645	3.4154	3.3936	6.2545	5.4410	3.6816	0.8812	-0.1825	0.5065	0.2954
Myh10	_VIQYLAHVASSHK_.3	#N/A	5.1379	3.9620	4.4403	3.5174	3.8503	4.2751	4.3849	4.6896	3.3135	3.6613	1.0461	0.0650	0.6411	0.1931
Myh10	_IAEC[Carbamidomethyl (C)]SSQLAEEEEKAK_.2	#N/A	3.2436	4.0988	4.4781	4.0219	3.8882	4.2373	3.1123	2.9793	3.2784	3.6992	1.2226	0.2900	0.0186	1.7296
Myh10	_AVIYNPATQADWTAK_.2	#N/A	3.8768	3.3494	4.5636	3.5654	3.0025	3.3869	3.2801	3.0522	4.2016	3.5324	1.0306	0.0434	0.7594	0.1195
Myh10	_NKQEVMISDLEER_.2	#N/A	3.8002	3.5422	3.5338	4.5785	3.3636	4.3659	3.2259	3.9236	4.2226	4.2557	0.9890	-0.0159	0.8953	0.0480
Myh10	_LQQLFNHTM[Oxidation (M)]FILEQEEYQR_.3	#N/A	3.8516	1.9136	3.5853	3.1290	2.9953	3.6293	3.2773	3.8721	4.1712	4.3072	0.8150	-0.2952	0.1090	0.9627
Mog	_VIGPGYPIR_.2	#N/A	9.0913	9.3137	9.2574	8.5548	8.8566	8.5824	9.3321	9.4755	9.0451	8.7594	0.9770	-0.0335	0.3466	0.4602
Mog	_ALVGDEAELPC[Carbamidomethyl (C)]R_.2	#N/A	8.6991	8.7341	8.8427	8.3232	8.3868	8.5865	8.9910	8.7109	8.5698	8.3899	0.9919	-0.0117	0.6414	0.1929
Mog	_AEVENLHR_.2	#N/A	7.9496	8.0545	7.6859	7.8697	7.8266	7.7586	8.0572	7.8282	7.7908	7.7397	1.0004	0.0006	0.9692	0.0136
Mog	_TFDPHFLR_.2	#N/A	7.4906	7.6198	7.5605	7.0716	6.9156	6.5186	7.9991	7.6441	7.3333	7.1653	0.9550	-0.0665	0.2376	0.6241
Mog	_FSDEGGYTC[Carbamidomethyl (C)]FFR_.2	#N/A	6.7488	6.1792	6.2918	6.2285	5.7511	5.6778	6.6901	6.4788	5.2664	6.1904	0.9983	-0.0024	0.9752	0.0109
Mog	_LAGQFLEELR_.2	#N/A	6.2469	6.4759	6.1620	5.9797	5.7258	5.7782	6.6492	6.5143	6.3676	5.9288	0.9523	-0.0705	0.1529	0.8156
Mog	_LAGQFLEELRNPF_.2	#N/A	5.2954	4.5769	3.5684	4.7276	4.6330	4.6822	5.5571	5.9376	4.9383	4.3565	0.8813	-0.1823	0.1586	0.7997
Mog	_DQDAEQAPEYR_.2	#N/A	7.4064	6.7920	6.2226	7.2634	6.7298	6.5731	7.2627	7.0533	6.8000	6.8246	0.9780	-0.0322	0.5396	0.2680
Mog	_LRAEVENLHR_.3	#N/A	4.8123	5.2261	5.0787	4.6769	4.8011	3.8294	4.5929	5.1068	5.4922	4.4850	0.9630	-0.0543	0.5744	0.2408
Mog	_DHSYQEEAAM[Oxidation (M)]ELK_.2	#N/A	4.7930	4.0300	4.4387	3.6647	3.7349	3.6764	4.9454	4.3601	4.6507	4.1387	0.8967	-0.1574	0.1284	0.8914
Mog	_NGKDQDAEQAPEYR_.2	#N/A	4.0666	3.2758	3.8003	3.3440	3.0971	3.1315	3.4923	5.4184	3.9562	3.0213	0.8692	-0.2022	0.2878	0.5409
Runx1t1	_STT[Phospho (STY)]PGTPSTIETTPR_.2	Q61909_T565_M1_Runx1t1	3.3876	7.5350	7.9318	4.1659	7.3081	7.1409	7.6439	7.4467	7.5674	7.4165	0.8306	-0.2678	0.2358	0.6274
Runx1t1	_QQS[Phospho (STY)]PVNPDPVALDAHR_.3	#N/A	6.0682	5.5534	6.2120	6.0917	5.4062	5.9451	4.7701	5.7168	5.5408	5.7281	1.0810	0.1123	0.1088	0.9632
Kcna6	_S[Phospho (STY)]LTLAAPGEVR_.2	#N/A	8.1138	4.2036	3.1810	4.2256	6.0973	4.0336	8.4345	4.2764	3.0746	7.3492	0.8603	-0.2171	0.5686	0.2452
Kcna6	_SEKS[Phospho (STY)]LTLAAPGEVR_.2	#N/A	6.3596	4.4101	7.0390	6.1963	2.7975	3.8271	6.5843	4.0699	4.3690	4.1094	1.0673	0.0939	0.7503	0.1248
Kcna6	_MRS[Phospho (STY)]EKS[Phospho (STY)]LTLAAPGEVR_.3	Q61923_S3_M2_Kcna6	3.9623	3.2666	3.1482	3.3532	4.1251	3.1407	3.5015	3.6480	3.9470	3.0305	0.9908	-0.0133	0.9065	0.0426
Npm1	_M[Oxidation (M)]TDQEAIQDLWQWR_.2	#N/A	6.3709	6.2909	6.3958	5.9874	6.2740	6.5390	5.6258	6.3009	6.5166	6.4874	1.0124	0.0177	0.6948	0.1582
Npm1	_GPSSVEDIK_.2	#N/A	5.9308	5.4282	6.3935	5.4737	6.3093	5.8428	5.3697	5.4197	5.5625	6.2022	1.0457	0.0645	0.3442	0.4631
Npm1	_VDNDENEHQLSLR_.2	#N/A	4.2930	4.7596	4.2454	5.1888	5.5016	4.7908	5.1032	5.2017	4.3670	4.4386	1.0040	0.0057	0.9520	0.0213
Npm1	_DELHIVEAEAM[Oxidation (M)]NYEGSPIK_.3	#N/A	3.4986	3.6190	3.2323	3.8319	5.3319	4.6549	5.3175	4.0960	4.1337	3.9541	0.9206	-0.1193	0.4896	0.3102
Npm1	_MSVQPTVSLGGFEITPPVVLR_.2	#N/A	3.7473	3.0517	4.4341	5.6891	4.1062	3.3556	3.7164	3.4330	3.7321	3.4030	1.1380	0.1865	0.3385	0.4705
Npm1	Carbamidomethyl (C)]GSGPVHISGQHLVAVEEDAES[Phospho (STY)]EDEDEEDVKLLGM[Oxidation (M)]SGI	Q61937_S125_M1_Npm1	4.2404	2.9857	3.4264	3.9291	3.3661	4.3634	3.6437	4.1894	3.8380	3.8961	0.9555	-0.0657	0.5687	0.2451
Pcbp2	_LVVPASQC[Carbamidomethyl (C)]GSLIGK_.2	#N/A	7.8150	8.2289	8.0973	8.2224	8.1780	7.9546	8.0660	8.2518	8.0971	7.9324	0.9995	-0.0007	0.9681	0.0141
Pcbp2	_INISEGNC[Carbamidomethyl (C)]PER_.2	#N/A	7.9238	8.0680	7.8765	7.9351	7.9745	7.8247	7.7188	7.8690	7.5712	7.7259	1.0275	0.0392	0.0108	1.9685
Pcbp2	_AITIAGIPQSIIEC[Carbamidomethyl (C)]VK_.2	#N/A	7.0762	6.8244	7.4406	7.2926	7.5298	7.6027	7.1486	6.8406	7.0011	7.3966	1.0279	0.0396	0.2988	0.5246
Pcbp2	_IITLAGPTNAIFK_.2	#N/A	6.8520	6.8915	6.8351	7.0835	7.0297	7.2711	6.9335	7.2088	7.5821	7.0215	0.9732	-0.0392	0.2135	0.6706
Pcbp2	_PSSS[Phospho (STY)]PVIFAGGQDR_.2	Q61990_S185_M1_Pcbp2	3.5754	3.6957	3.7385	3.8696	3.5840	3.8922	3.7337	3.8013	3.8147	4.3316	0.9504	-0.0734	0.1714	0.7660
Pcbp2	_ESTGAQVQVAGDM[Oxidation (M)]LPNSTER_.2	#N/A	4.2778	5.1878	3.8525	4.8052	5.0497	4.8658	4.6650	5.4330	4.7688	4.9356	0.9440	-0.0832	0.3700	0.4318
Pcbp2	_QVTITGSAASISLAQYLINVR_.2	#N/A	3.0351	4.3074	4.2696	3.8133	3.6796	3.6008	3.9616	3.1878	3.4869	3.4906	1.0715	0.0996	0.3807	0.4195
Ctr9	_KGGEFDEFVNDDT[Phospho (STY)]DDDLPVSK_.3	#N/A	3.4026	4.2078	4.0894	3.9129	4.0291	3.7004	4.0612	7.2378	3.3873	3.5902	0.8514	-0.2320	0.3783	0.4222
Ctr9	_GGEFDEFVNDDT[Phospho (STY)]DDDLPVSK_.2	#N/A	3.3172	3.9089	4.0040	4.5067	3.7972	4.3282	4.2214	3.6118	3.2604	2.9728	1.1309	0.1775	0.1651	0.7823
Ctr9	_NSNS[Phospho (STY)]DS[Phospho (STY)]DDDERPNR_.2	Q62018_S1039_M2_Ctr9	3.4894	4.2946	4.1762	3.8261	4.1159	3.6136	3.9744	3.1751	3.4741	3.1450	1.1386	0.1873	0.0673	1.1723
Ctr9	_KGS[Phospho (STY)]GS[Phospho (STY)]EQEGEEEEGGER_.2	#N/A	3.2803	3.9459	3.9671	4.4697	3.9068	3.8227	4.1844	3.6487	3.2973	4.4367	1.0018	0.0026	0.9809	0.0084
Ctr9	_RKGS[Phospho (STY)]GS[Phospho (STY)]EQEGEEEEGGER_.2	#N/A	4.0895	3.1366	3.2755	3.7781	3.2152	3.5997	1.4723	4.3403	3.9889	3.7451	1.0381	0.0540	0.8196	0.0864
Cep131	_SNS[Phospho (STY)]TTQVNQSWTGSPR_.2	Q62036_S78_M1_Cep131	3.4988	4.0129	3.7610	4.1437	4.1349	3.7995	4.2919	3.8646	3.1925	4.6685	0.9719	-0.0411	0.6995	0.1552
Pea15	_QPS[Phospho (STY)]EEEIIK_.2	#N/A	14.1747	13.9692	14.5227	14.4419	14.0192	14.2173	13.8661	13.7808	14.1842	14.2517	1.0145	0.0208	0.2001	0.6988
Pea15	_SAC[Carbamidomethyl (C)]KEDIPSEK_.2	#N/A	6.3086	6.6406	6.5305	6.6404	7.0919	6.3156	6.2556	6.7596	6.3885	6.6555	1.0112	0.0161	0.6845	0.1646
Pea15	_YKDIIRQPS[Phospho (STY)]EEEIIK_.3	#N/A	10.7542	11.6445	12.6696	11.8826	11.4818	11.8847	11.3931	11.5746	11.6001	11.6474	1.0143	0.0206	0.6204	0.2074
Pea15	_LTRIPS[Phospho (STY)]AK_.2	Q62048_S104_M1_Pea15	4.9543	6.8857	8.4950	6.2055	6.6779	6.9612	3.1744	6.2277	6.1427	5.8284	1.2533	0.3257	0.1384	0.8590
Pea15	_DIIRQPS[Phospho (STY)]EEEIIK_.2	#N/A	10.8686	11.5295	12.4484	11.8238	11.1628	11.7935	11.6646	11.3767	11.5176	11.5756	1.0061	0.0088	0.8114	0.0908
Pea15	_RPDLLTM[Oxidation (M)]VVDYR_.2	#N/A	4.7044	5.2255	5.0402	4.7631	5.3575	5.2916	4.9391	5.0577	5.5783	5.4571	0.9630	-0.0543	0.3281	0.4841
Pea15	_[Acetyl (Protein N-term)]AEYGTLLQDLTNNITLEDLEQLK_.3	#N/A	3.3764	4.6085	3.1100	4.6986	4.4878	4.1751	4.3029	3.8587	4.2273	4.3860	0.9719	-0.0411	0.7496	0.1252
Pea15	_QPS[Phospho (STY)]EEEIIKLAPPPK_.2	#N/A	7.6523	6.4834	7.0364	7.8656	6.9613	8.0757	7.8972	7.5179	7.2723	7.7766	0.9645	-0.0521	0.4393	0.3572
Pea15	_SEEITTGSAWFSFLESHNK_.2	#N/A	3.1479	4.0422	4.3468	4.7508	3.1228	4.4940	4.6291	3.7613	3.7037	4.3245	0.9706	-0.0430	0.7691	0.1140
Pea15	_DNLSYIEHIFEISR_.2	#N/A	3.4704	3.4368	4.1496	3.5711	3.5828	3.3100	4.3381	3.3405	2.7119	2.7437	1.0924	0.1274	0.3927	0.4060
Vcan	_YEINSLIR_.2	#N/A	6.5756	6.7570	6.7938	6.8968	6.8265	6.8418	6.5189	6.6972	6.7397	6.6750	1.0187	0.0267	0.1072	0.9700
Vcan	_AQC[Carbamidomethyl (C)]GGGLLGVR_.2	#N/A	6.1535	6.7330	6.0099	6.7933	6.9494	6.2756	6.0815	6.2291	6.8967	6.2296	1.0199	0.0284	0.6193	0.2081
Vcan	_GTVAC[Carbamidomethyl (C)]GQPPVVENAK_.2	#N/A	5.8597	5.7276	6.5387	5.7885	6.2785	5.8356	6.2759	5.6179	6.0122	6.3391	0.9907	-0.0135	0.7958	0.0992
Vcan	_LATVGELQAAWR_.2	#N/A	5.5855	5.5174	5.8825	5.2989	5.9165	5.9620	5.6606	4.9740	5.4935	5.9616	1.0310	0.0441	0.4441	0.3526
Vcan	_FTFEEAEAEC[Carbamidomethyl (C)]TSR_.2	#N/A	3.2502	5.4398	4.4847	6.0944	5.2137	4.2306	4.1238	2.9727	3.3579	4.5432	1.2033	0.2671	0.2633	0.5796
Vcan	_NGFDQC[Carbamidomethyl (C)]DYGWLSDASVR_.2	#N/A	3.0709	3.8761	3.7577	4.2446	6.2260	4.0321	6.6344	3.8581	3.0556	6.5477	0.8362	-0.2580	0.3918	0.4070
Vcan	_VGHDYQWIGLNDK_.3	#N/A	3.2172	4.0090	3.9040	4.4067	4.5765	4.2282	4.1213	3.7118	4.7250	4.3737	0.9584	-0.0613	0.5703	0.2439
Map3k7	_S[Phospho (STY)]IQDLTVTGTEPGQVSSR_.2	Q62073_S412_M1_Map3k7	3.5396	8.8263	7.3152	9.4786	8.3132	3.0497	9.0676	9.0633	9.3347	9.3259	0.7343	-0.4456	0.1230	0.9101
Plcg1	_AREGS[Phospho (STY)]FEAR_.2	Q62077_S1248_M1_Plcg1	6.1189	5.9559	6.9216	4.2208	5.3578	6.3392	6.1358	6.4574	6.4503	6.4703	0.9123	-0.1324	0.2783	0.5555
Srsf2	_S[Phospho (STY)]RS[Phospho (STY)]PPPVSK_.2	Q62093_S189_M2_Srsf2	12.1422	11.6264	11.5450	12.0655	10.8701	11.4806	12.2676	11.9805	12.0101	11.6910	0.9695	-0.0447	0.1858	0.7309
Srsf2	_TS[Phospho (STY)]PDTLRR_.2	#N/A	7.9865	7.9613	8.4623	8.1158	7.3866	7.9999	8.3852	8.4412	8.0861	7.6265	0.9816	-0.0267	0.5353	0.2714
Srsf2	_VGDVYIPR_.2	#N/A	5.8620	4.8773	5.9145	5.0081	5.8635	5.6364	5.7394	5.9487	5.3478	5.8310	0.9668	-0.0487	0.4837	0.3154
Srsf2	_S[Phospho (STY)]KS[Phospho (STY)]PPKS[Phospho (STY)]PEEEGAVSS_.2	Q62093_S206_M3_Srsf2	8.2320	7.4584	7.1776	8.1554	6.7378	7.5586	8.4169	7.9070	7.7922	7.7395	0.9484	-0.0764	0.2311	0.6363
Srsf2	_VDNLTYRTS[Phospho (STY)]PDTLR_.2	#N/A	7.3782	7.3251	8.4506	3.9468	3.9001	7.4577	8.1379	3.0544	7.7218	7.3944	0.9746	-0.0372	0.9060	0.0429
Srsf2	_TS[Phospho (STY)]PDTLR_.2	#N/A	6.2048	4.3444	3.5686	4.0712	4.2327	3.8927	3.7859	4.0473	3.6959	4.0382	1.1269	0.1724	0.3344	0.4758
Srsf2	_S[Phospho (STY)]RS[Phospho (STY)]RS[Phospho (STY)]PPPVSK_.2	#N/A	9.6452	8.9329	8.8798	9.3468	8.0742	8.9124	9.4844	9.2979	8.8252	9.0368	0.9786	-0.0312	0.5246	0.2802
Srsf2	_VDNLTYR_.2	#N/A	3.6266	2.9310	4.3134	3.6889	4.2531	3.4764	3.8372	3.3123	3.6113	3.3662	1.0519	0.0729	0.5311	0.2748
Ddx3y	_YLVLDEADR_.2	#N/A	6.5044	6.3978	7.1012	6.4019	6.0557	6.2500	6.7523	6.1223	6.1410	5.9049	1.0356	0.0504	0.3655	0.4371
Ddx3y	_GKPNYFSDRGS[Phospho (STY)]GSR_.3	Q62095_S90_M1_Ddx3y	4.0627	3.2226	3.2487	3.5696	3.0754	3.3771	3.4801	4.0446	6.2800	3.4234	0.7954	-0.3302	0.1552	0.8091
Ddx3y	_FS[Phospho (STY)]GGFGAR_.2	Q62095_S592_M1_Ddx3y	4.9593	5.0676	5.3590	5.6068	4.4976	5.0703	5.4151	6.0296	4.8865	4.9854	0.9558	-0.0653	0.4265	0.3701
Ddx3y	_HVINFDLPSDIEEYVHR_.3	#N/A	3.8419	4.8700	3.5755	6.1348	5.2033	5.3184	5.3115	5.6018	4.1809	5.6645	0.9295	-0.1054	0.5333	0.2730
Ddx3y	_DLLDLLVEAK_.2	#N/A	4.7035	3.6240	4.1022	4.4587	4.4039	4.0794	4.5063	4.7718	4.8198	4.9742	0.8869	-0.1732	0.0316	1.4997
Ddx3y	_VGNLGLATSFFNER_.2	#N/A	3.9868	4.0203	3.7204	3.2642	3.1305	3.9098	3.4125	3.6808	3.4237	4.4423	0.9819	-0.0264	0.8108	0.0911

Dlg4	_RYS[Phospho (STY)]PVAK_.2	Q62108_S295_M1_Dlg4	12.5815	12.6108	13.0954	12.5583	11.8629	12.3780	12.8331	12.7883	12.5946	12.4733	0.9875	-0.0181	0.4824	0.3166
Dlg4	_IIPGGAAAQDGR_.2	#N/A	8.1690	8.3275	8.0628	8.2062	8.5818	7.9097	8.2800	8.3959	8.3041	8.0662	0.9937	-0.0091	0.6990	0.1555
Dlg4	_DYHFVSSR_.2	#N/A	6.7404	6.3829	6.4862	6.6874	6.4564	6.6812	6.7122	6.8029	6.5886	6.1886	0.9999	-0.0001	0.9962	0.0017
Dlg4	_VIEDLSGPYIWVPAR_.2	#N/A	7.5488	7.5357	7.3752	7.0906	7.5387	7.4550	7.6932	7.5344	7.4207	7.4065	0.9881	-0.0173	0.4144	0.3826
Dlg4	_PIIILGPTK_.2	#N/A	7.2483	6.8659	7.1928	7.1890	7.0297	7.2576	7.3994	7.4634	7.2005	7.2516	0.9730	-0.0395	0.0637	1.1958
Dlg4	_EVTHSAAVEALK_.2	#N/A	4.4663	4.5644	5.0177	4.6923	5.3764	4.8637	4.7284	5.3316	5.0232	5.2508	0.9502	-0.0738	0.2430	0.6143
Dlg4	_VHSDS[Phospho (STY)]ETDDIGFIPSKR_.3	#N/A	4.2706	8.8997	3.4566	3.9592	8.1121	4.3332	3.6739	4.1592	3.8078	3.9262	1.4146	0.5004	0.2178	0.6620
Dlg4	_REYEIDGR_.2	#N/A	5.6880	4.9710	4.2395	5.2577	5.7137	4.9798	4.3690	5.0066	5.5098	5.0957	1.0293	0.0417	0.6753	0.1705
Dlg4	_VNDSILFVNEVDVR_.2	#N/A	6.6796	6.1142	3.1753	5.7947	5.4174	5.7574	6.5008	6.0053	6.4126	5.8098	0.8880	-0.1714	0.3048	0.5159
Dlg4	_NTYDVVY[Phospho (STY)]LK_.2	Q62108_Y240_M1_Dlg4	8.6224	8.2587	8.8284	8.7111	7.5560	8.5249	8.7678	8.6999	8.6125	8.5615	0.9719	-0.0411	0.3390	0.4698
Dlg4	_AKDWGS[Phospho (STY)]SSGSQGR_.2	Q62108_S509_M1_Dlg4	3.2299	3.3874	4.1593	3.4520	4.1997	3.5332	4.1430	3.3785	3.3775	3.2572	1.0343	0.0486	0.6603	0.1802
Dlg4	_EQLM[Oxidation (M)]NSSLGS[Phospho (STY)]GTASLR_.2	#N/A	8.4980	8.7056	9.3999	8.5218	4.3857	5.3990	7.8360	5.6074	8.7856	6.9045	1.0277	0.0394	0.8685	0.0612
Dlg4	_RVHSDS[Phospho (STY)]ETDDIGFIPSK_.3	#N/A	3.3534	4.3193	4.2605	4.9780	4.1406	3.5917	3.9525	5.0867	6.6223	6.3831	0.7453	-0.4242	0.0395	1.4037
Dlg4	_VHSDSETDDIGFIPSK_.3	#N/A	5.0982	4.5489	4.3407	7.1955	5.5807	4.3821	6.2613	7.0877	5.0463	6.2600	0.8553	-0.2255	0.1976	0.7042
Dlg4	_HC[Carbamidomethyl (C)]ILDVSANAVR_.2	#N/A	4.1997	4.0442	3.3856	3.8883	3.7056	4.4041	4.0795	3.8064	4.1869	3.8553	0.9889	-0.0161	0.8298	0.0810
Dlg4	_RVHS[Phospho (STY)]DS[Phospho (STY)]ETDDIGFIPSKR_.3	#N/A	3.3059	3.6699	3.9927	3.7594	5.4847	4.9607	4.2100	6.2115	6.2842	6.3447	0.7281	-0.4579	0.0298	1.5265
Dlg4	_EVTHSAAVEALKEAGS[Phospho (STY)]IVR_.3	Q62108_S142_M1_Dlg4	3.5190	5.5048	5.8318	7.1965	4.1635	5.4698	3.8551	6.4970	5.3291	4.3397	1.0551	0.0773	0.7415	0.1299
Dlg4	_DWGSSSGS[Phospho (STY)]QGR_.2	#N/A	4.6910	4.0820	3.9101	3.9812	5.1287	3.6663	4.7174	4.7771	6.7872	4.9416	0.7997	-0.3224	0.0589	1.2297
Dlg4	_GNS[Phospho (STY)]GLGFSIAGGTDNPHIGDDPSIFITK_.3	Q62108_S73_M1_Dlg4	3.7414	2.8512	3.4750	5.0497	2.8851	3.7395	3.1671	4.0345	4.2814	4.1969	0.9244	-0.1134	0.5350	0.2716
Dlg4	_FIEAGQYNSHLYGTSVQSVR_.3	#N/A	5.5927	5.7773	5.9888	3.8108	3.2479	2.9808	3.5255	4.3077	5.1902	5.6894	0.9761	-0.0349	0.8915	0.0499
Dlg4	_DLLGEEDIPREPR_.3	#N/A	2.8302	3.3871	3.8653	3.4091	0.9142	3.2034	3.5573	3.5921	3.8911	3.0864	0.8310	-0.2671	0.3089	0.5102
Dlg4	_EQLMNSSLGSGT[Phospho (STY)]ASLRS[Phospho (STY)]NPKR_.3	Q62108_S425_M2_Dlg4	4.0080	4.3857	3.4267	4.4781	3.9053	4.0924	3.7450	4.0885	3.7371	3.9973	1.0404	0.0572	0.4640	0.3335
Dlg4	_S[Phospho (STY)]LENVLEINKR_.2	Q62108_S654_M1_Dlg4	3.8971	3.2014	3.8747	3.2029	3.0227	3.2059	3.5667	3.5827	3.8818	3.0957	0.9629	-0.0545	0.5903	0.2289
Dlg4	_ILAVNSVGLEDVMHEDAVAALK_.3	#N/A	3.3398	4.0961	4.5743	4.1181	3.9844	4.1411	4.0342	4.3839	3.1821	3.7954	1.0502	0.0707	0.5174	0.2861
Dlg4	_IIEGGAAHKDGR_.3	#N/A	3.0715	3.8767	3.7583	4.2610	3.6981	4.0314	3.9757	3.8575	3.5061	4.2280	0.9720	-0.0410	0.6586	0.1814
Jak2	_TNGISDVQIS[Phospho (STY)]PTLQR_.2	Q62120_S523_M1_Jak2	7.3474	3.1451	3.2670	3.7697	5.8166	7.1291	3.4843	7.6709	7.1133	6.8914	0.8075	-0.3085	0.3568	0.4475
Ptprr	_NQEIHLS[Phospho (STY)]PIAR_.2	#N/A	6.6293	6.2459	7.1637	6.3074	4.9452	6.1652	7.2895	6.6854	6.2636	5.8537	0.9570	-0.0634	0.5473	0.2618
Ralbp1	_TPS[Phospho (STY)]SEEISPTK_.2	Q62172_S29_M1_Ralbp1	3.7122	6.8201	3.9892	7.2001	6.0067	3.5885	7.2987	3.6601	4.0033	3.8314	1.1109	0.1517	0.6440	0.1911
Ralbp1	_[Acetyl (Protein N-term)]TEC[Carbamidomethyl (C)]FLPPSS[Phospho (STY)]SPSEHR_.2	Q62172_S10_M1_Ralbp1	7.4197	3.7894	3.6623	7.5328	3.7373	6.9356	4.2009	6.9146	3.5492	6.4700	1.0434	0.0613	0.8530	0.0691
Ralbp1	_[Acetyl (Protein N-term)]TEC[Carbamidomethyl (C)]FLPPS[Phospho (STY)]SSPSEHRR_.3	#N/A	3.6842	3.6261	4.1261	3.6699	3.4886	7.1763	3.8181	3.3900	3.6891	3.3472	1.2061	0.2704	0.3466	0.4601
Ralbp1	_TEGYAAFQEDS[Phospho (STY)]S[Phospho (STY)]GDEAESPSK_.2	#N/A	4.2171	3.4726	4.4404	8.1118	3.3609	7.0178	3.1569	7.9426	7.3424	7.6936	0.7811	-0.3565	0.3205	0.4942
Ralbp1	_TPS[Phospho (STY)]SEEIS[Phospho (STY)]PTKFPGLYR_.2	#N/A	4.0118	3.7099	3.5818	7.5176	3.7038	3.4126	3.9812	4.7925	6.4881	3.7141	0.9112	-0.1341	0.6676	0.1755
Ralbp1	_TGEPS[Phospho (STY)]PPHDVLHEPPDT[Phospho (STY)]VSDDDKDHGK_.4	Q62172_T60_M2_Ralbp1	3.4774	3.8758	3.6966	4.4460	8.1425	3.8300	3.7606	9.7225	3.9270	4.2748	0.8445	-0.2439	0.5778	0.2382
Ralbp1	_RTEGYAAFQEDS[Phospho (STY)]S[Phospho (STY)]GDEAESPSK_.3	#N/A	3.3230	4.0194	4.5576	4.9512	3.9676	3.8888	4.2496	4.4007	3.1989	3.7786	1.0540	0.0759	0.5736	0.2414
Sema4a	_TSAS[Phospho (STY)]DVDADNNHLGAEVA_.2	Q62178_S746_M1_Sema4a	3.4536	3.6231	4.4048	3.7189	4.0881	3.6576	4.0184	3.1439	3.1170	3.5128	1.1091	0.1494	0.1635	0.7866
Dpysl3	_DNFTAIPEGTNGVEER_.2	#N/A	8.2543	8.2079	8.0352	8.7366	8.1284	8.1261	8.5836	7.9455	8.1433	8.2922	1.0008	0.0012	0.9679	0.0142
Dpysl3	_GAPLVVIC[Carbamidomethyl (C)]QGK_.2	#N/A	6.6204	7.4467	6.9167	6.8352	6.7744	7.0361	7.5249	6.4909	6.7013	6.9403	1.0035	0.0050	0.9194	0.0365
Dpysl3	_QIGDNLIVPGGVK_.2	#N/A	7.1330	6.9004	7.1965	7.0056	7.4214	6.9689	7.3034	7.2859	7.0155	6.9405	0.9955	-0.0065	0.7986	0.0977
Dpysl3	_IAVGSDSDLVIWDPDALK_.2	#N/A	6.8860	6.9905	7.1850	7.1808	7.4274	7.3022	6.9967	7.0214	6.8900	7.2085	1.0189	0.0270	0.2772	0.5571
Dpysl3	_AITVASQTNC[Carbamidomethyl (C)]PLYVTK_.2	#N/A	6.2553	6.6427	7.0827	6.9623	7.0500	6.8473	7.0368	6.4038	6.4635	6.2815	1.0398	0.0563	0.2459	0.6092
Dpysl3	_GM[Oxidation (M)]YDGPVFDLTTT[Phospho (STY)]PK_.2	#N/A	11.6396	11.4363	11.8095	11.9699	11.3646	11.6248	11.9940	11.4129	11.6111	11.7008	0.9967	-0.0048	0.8015	0.0961
Dpysl3	_SAADLISQAR_.2	#N/A	7.4214	7.6235	7.7645	7.9763	8.3066	7.5783	7.9733	7.6634	7.6478	7.4915	1.0110	0.0157	0.6540	0.1844
Dpysl3	_GS[Phospho (STY)]PTRPNPPVR_.2	#N/A	12.8340	12.8016	13.3659	13.4075	12.8555	12.9033	13.1070	12.8008	12.9100	13.0261	1.0052	0.0074	0.6720	0.1726
Dpysl3	_GGT[Phospho (STY)]PAGSTRGS[Phospho (STY)]PTRPNPPVR_.3	#N/A	10.9459	11.4690	11.8659	12.1303	10.7776	11.6245	11.4141	11.5084	11.7924	11.7947	0.9864	-0.0198	0.5844	0.2333
Dpysl3	_IVAPPGGRS[Phospho (STY)]NITS[Phospho (STY)]LS_.2	Q62188_S564_M2_Dpysl3	3.3009	4.1061	3.9877	11.8891	10.4173	10.0304	4.1629	11.1791	11.1899	3.6919	0.9646	-0.0520	0.9201	0.0362
Dpysl3	_NLHQSGFS[Phospho (STY)]LSGTQVDEGVR_.3	#N/A	7.5110	6.7680	7.8036	7.9799	5.2770	7.0710	7.2637	7.4354	7.3364	7.3710	0.9615	-0.0567	0.5898	0.2293
Dpysl3	_GM[Oxidation (M)]TTVDDFFQGTK_.2	#N/A	5.6841	6.0354	6.8774	6.1051	6.4244	6.4907	6.0688	6.1044	6.1383	5.9567	1.0334	0.0474	0.3734	0.4278
Dpysl3	_NHQSVAEYNIFEGM[Oxidation (M)]ELR_.3	#N/A	5.4733	5.2136	6.1802	5.7035	5.5513	5.8973	5.8716	5.3141	6.0370	6.3340	0.9628	-0.0548	0.3914	0.4074
Dpysl3	_GM[Oxidation (M)]YDGPVFDLTTT[Phospho (STY)]PKGGT[Phospho (STY)]PAGSTR_.3	#N/A	5.1857	8.3189	8.7428	9.2375	8.2260	3.6901	5.2053	8.6976	9.4271	8.2133	0.9173	-0.1246	0.6444	0.1908
Dpysl3	_AALAGGTTM[Oxidation (M)]IIDHVVPEPESSLTEAYEK_.3	#N/A	4.7341	4.1923	5.4873	5.7636	5.1325	5.3972	5.5945	5.5551	4.9694	5.3454	0.9537	-0.0683	0.4506	0.3462
Dpysl3	_MADLHAVPR_.2	#N/A	5.2160	3.6692	3.5508	6.7709	5.8559	4.2389	5.3850	4.0650	4.4619	4.0205	1.0893	0.1235	0.5845	0.2332
Dpysl3	_IM[Oxidation (M)]LEDGNLHVTQGAGR_.3	#N/A	4.2968	5.5892	5.3033	5.1802	6.1646	5.9073	4.8278	4.7716	5.1509	4.8850	1.1015	0.1394	0.1826	0.7385
Dpysl3	_SC[Carbamidomethyl (C)]C[Carbamidomethyl (C)]DYALHVDITHWNDSVK_.3	#N/A	3.6947	3.9324	4.7871	4.9000	3.3569	3.1711	4.0774	3.8718	4.2018	3.9166	0.9892	-0.0156	0.9110	0.0405
Dpysl3	_NLHQSGFS[Phospho (STY)]LSGTQVDEGVRS[Phospho (STY)]ASKR_.4	Q62188_S551_M2_Dpysl3	3.5361	3.8063	3.2698	4.3144	4.1807	3.9448	4.4627	4.1876	2.9858	3.9917	0.9834	-0.0242	0.8461	0.0726
Scn9a	_S[Phospho (STY)]GS[Phospho (STY)]EES[Phospho (STY)]IRKK_.2	Q62205_S502_M3_Scn9a	4.4557	4.3899	4.0325	4.2875	4.0682	4.3812	4.3724	5.0483	4.6455	4.4092	0.9243	-0.1136	0.0514	1.2888
Tgfb1i1	_EGC[Carbamidomethyl (C)]PS[Phospho (STY)]PPGQTSK_.2	Q62219_S194_M1_Tgfb1i1	9.6197	9.3191	9.8427	10.2912	9.2073	8.7628	9.9508	9.4730	9.2575	9.7884	0.9885	-0.0166	0.7212	0.1419
Sos1	_IPESETESTASAPNS[Phospho (STY)]PR_.2	Q62245_S1082_M1_Sos1	6.8843	3.7079	6.5674	6.8140	3.5292	5.8841	7.2094	6.8992	4.3882	6.3665	0.8952	-0.1597	0.5062	0.2957
Sos1	_RLS[Phospho (STY)]ESAC[Carbamidomethyl (C)]R_.2	Q62245_S401_M1_Sos1	4.0677	3.2747	3.8014	3.3451	3.2114	3.1326	3.5002	3.6560	3.9551	3.0224	0.9827	-0.0252	0.8091	0.0920
Sos1	_SAS[Phospho (STY)]VSSISLSK_.2	Q62245_S1120_M1_Sos1	3.9885	3.2376	5.8353	3.6771	3.1142	3.1144	3.3918	4.4413	4.0899	3.6441	0.9836	-0.0239	0.9125	0.0398
Sptbn1	_ETWLSENQR_.2	#N/A	9.3729	9.2655	9.6875	9.2812	8.7944	9.1605	9.4208	9.4011	9.1993	9.1726	0.9959	-0.0059	0.8145	0.0891
Sptbn1	_DVEDEILWVGER_.2	#N/A	9.0876	8.5999	8.9730	8.7609	8.1180	8.7669	9.1831	8.7525	8.8615	8.8312	0.9787	-0.0310	0.3464	0.4604
Sptbn1	_DQNTVETLQR_.2	#N/A	8.7160	8.4756	8.4681	8.9045	8.3412	8.5828	8.7005	8.7211	8.5624	8.7441	0.9884	-0.0168	0.3789	0.4215
Sptbn1	_SALPAQSAATLPAR_.2	#N/A	8.5224	8.5569	8.6017	8.2232	8.4172	8.2534	8.3458	8.5283	8.5041	8.3260	1.0004	0.0005	0.9737	0.0116
Sptbn1	_EIGQSVDEVEK_.2	#N/A	7.7455	7.3706	7.7329	7.4379	7.1343	7.2662	7.6822	7.4845	7.2782	7.3980	0.9983	-0.0025	0.9308	0.0312
Sptbn1	_VAVVNQIAR_.2	#N/A	8.2981	7.9642	8.4827	8.1005	7.9129	7.7398	8.1276	8.1199	8.1020	7.8633	1.0037	0.0053	0.8444	0.0734
Sptbn1	_FM[Oxidation (M)]ELLEPLSER_.2	#N/A	7.9335	8.3530	8.2344	7.9306	7.4856	8.0096	8.4251	8.6707	8.4298	8.0626	0.9517	-0.0715	0.0571	1.2431
Sptbn1	_LLEVLSGER_.2	#N/A	8.5206	8.2567	8.1130	8.1795	7.3266	7.9492	8.3225	8.6167	8.1071	8.2336	0.9685	-0.0462	0.2742	0.5620
Sptbn1	_IHC[Carbamidomethyl (C)]LENVDK_.2	#N/A	8.0205	7.8351	8.0032	7.9093	7.8648	7.7274	8.0032	8.0408	7.9103	8.1748	0.9827	-0.0252	0.0856	1.0678
Sptbn1	_VLLLSQDYGK_.2	#N/A	8.1280	8.0511	8.2421	7.8626	7.3230	7.7821	8.3570	8.2045	7.6664	7.5601	0.9939	-0.0089	0.8362	0.0777
Sptbn1	_EVDDLEQWIAER_.2	#N/A	7.8839	7.5845	8.2032	7.4441	7.4671	7.6075	7.8804	7.6253	7.5684	8.0273	0.9901	-0.0144	0.6665	0.1762
Sptbn1	_EIEELQSQAQALSQEGK_.2	#N/A	7.9874	7.1906	7.2521	7.5854	7.0537	7.6148	8.1433	7.3988	7.4079	7.5190	0.9777	-0.0326	0.4725	0.3256
Sptbn1	_ENEVLEAWK_.2	#N/A	7.9013	7.4212	8.3062	7.0032	7.4778	7.8886	7.5460	7.8426	8.0777	7.9242	0.9769	-0.0337	0.4890	0.3107
Sptbn1	_SQNIITDSSSLNAEAIR_.2	#N/A	7.5994	7.3663	7.4442	7.4248	7.3136	7.5474	7.5552	7.4058	7.6457	7.3950	0.9932	-0.0099	0.5034	0.2981
Sptbn1	_LVSDGNINSDR_.2	#N/A	7.8180	7.6220	7.3680	7.5879	7.4792	7.3956	7.8939	7.6664	7.6610	7.6013	0.9792	-0.0304	0.1454	0.8374
Sptbn1	_KLPEELGR_.2	#N/A	7.7405	7.6776	7.1277	7.6059	7.3450	6.4456	7.3900	7.7192	7.7377	7.6163	0.9616	-0.0564	0.2881	0.5404
Sptbn1	_LVDTGDKFR_.2	#N/A	7.1975	7.7348	6.9858	7.2588	7.4510	6.9898	7.7974	7.6221	7.4218	7.1481	0.9696	-0.0445	0.2499	0.6023
Sptbn1	_LTTLELLEVR_.2	#N/A	7.3486	7.5560	7.3368	7.2962	6.9848	7.3809	7.3758	7.4275	7.2969	7.2903	0.9959	-0.0060	0.7657	0.1159
Sptbn1	_ITDLYTDLR_.2	#N/A	7.4658	7.2193	7.6438	6.9722	6.5738	7.1430	7.3952	7.6851	7.6242	7.0060	0.9653	-0.0510	0.2896	0.5382
Sptbn1	_VLDNAIETEK_.2	#N/A	6.9994	7.3371	7.3711	7.0634	6.9686	6.6605	7.6911	7.3029	7.1825	6.9073	0.9719	-0.0411	0.3035	0.5179

Sptbn1	_SLLDAC[Carbamidomethyl (C)]EGR_.2	#N/A	7.4558	6.5642	6.7230	7.1126	6.8816	6.6336	6.5692	6.9137	7.0086	6.7477	1.0125	0.0180	0.6627	0.1787
Sptbn1	_VQAVVAVAR_.2	#N/A	6.8290	7.1773	7.3028	6.5565	6.6760	6.3660	6.4621	7.0965	6.8459	6.1278	1.0279	0.0397	0.4810	0.3179
Sptbn1	_IDDIFER_.2	#N/A	7.0738	7.0992	6.6567	6.4349	6.5199	6.6558	5.5540	7.4376	7.6123	7.5344	0.9581	-0.0617	0.4998	0.3012
Sptbn1	_LVSQDNFGFDLPAVEAATK_.2	#N/A	6.9948	7.1815	7.0811	7.0872	6.4466	6.8724	6.9432	7.0825	7.0875	6.5913	1.0026	0.0037	0.9161	0.0381
Sptbn1	_LQALDTGWNELHK_.2	#N/A	6.4382	6.0570	6.1088	5.8652	6.0752	6.0335	5.9771	6.2272	5.7227	5.7193	1.0313	0.0444	0.2101	0.6776
Sptbn1	_TQTAIASEDM[Oxidation (M)]PNTLTEAEK_.2	#N/A	6.9157	6.9610	7.3832	5.9893	6.7279	6.9049	7.1866	6.9631	6.9410	6.9477	0.9720	-0.0409	0.4355	0.3610
Sptbn1	_LQAAYAGDKADDIQKR_.3	#N/A	6.3079	6.9140	6.9904	6.1405	6.6565	6.9892	6.6149	7.0393	6.7351	6.7962	0.9809	-0.0279	0.5348	0.2718
Sptbn1	_TAGYPNVNIHNFTTSWR_.3	#N/A	6.8305	6.5947	6.2011	6.7486	6.2285	6.4661	6.0958	6.9941	6.7541	6.5004	0.9887	-0.0164	0.7222	0.1413
Sptbn1	_HLLGVEDLLQK_.2	#N/A	7.0803	6.6710	6.8626	6.9258	6.1518	6.3299	6.8645	7.2716	6.7823	6.5819	0.9702	-0.0436	0.3736	0.4276
Sptbn1	_LSDGNEYLFQAK_.2	#N/A	6.5483	6.3827	6.4791	6.5322	6.3661	6.4373	6.8131	6.7761	6.1704	6.4391	0.9859	-0.0204	0.4860	0.3133
Sptbn1	_EQWANLEQLSAIR_.2	#N/A	6.6958	6.4613	6.5006	6.1059	6.2929	6.5668	7.1704	6.3391	6.5485	6.3773	0.9740	-0.0380	0.3832	0.4165
Sptbn1	_IVSSNDVGHDEYSTQSLVK_.3	#N/A	5.6743	4.9520	6.4077	5.5146	5.7228	6.1950	6.5115	6.0110	5.9190	6.4109	0.9246	-0.1132	0.1414	0.8495
Sptbn1	_EAEKLESEHPDQAQAILSR_.3	#N/A	5.4436	5.4374	6.1119	5.9078	5.4935	5.6326	5.9709	5.8405	5.1265	5.3952	1.0157	0.0225	0.6872	0.1629
Sptbn1	_LQAAYAGDK_.2	#N/A	5.3659	5.1765	4.9761	4.6929	5.4811	4.5331	5.6579	5.1976	5.6710	5.6227	0.9098	-0.1364	0.0455	1.3421
Sptbn1	_QLWGLLIEETEKR_.2	#N/A	4.9931	4.0955	4.7271	4.6222	3.9376	4.3325	5.2074	4.6370	4.3055	4.0110	0.9804	-0.0285	0.7655	0.1160
Sptbn1	_RPPS[Phospho (STY)]PDPNTK_.2	#N/A	14.4084	14.4992	14.9359	14.7733	13.6966	14.2947	14.7729	14.7172	14.5026	14.3601	0.9895	-0.0153	0.5291	0.2764
Sptbn1	_TSS[Phospho (STY)]KESS[Phospho (STY)]PVPS[Phospho (STY)]PTLDRK_.3	#N/A	11.2599	10.5649	10.0185	10.4196	9.1991	10.6373	11.4468	11.6735	11.7505	9.8402	0.9259	-0.1110	0.1375	0.8618
Sptbn1	_[Acetyl (Protein N-term)]TTTVATDYDNIEIQQQYSDVNNR_.3	#N/A	11.8186	11.1059	12.1593	11.9128	10.6244	11.7251	12.1779	11.7774	11.7705	11.5273	0.9784	-0.0316	0.4373	0.3592
Sptbn1	_ESS[Phospho (STY)]PVPSPTLDR_.2	#N/A	9.1755	10.9619	4.1015	11.3778	10.7673	8.6721	12.0006	10.7636	9.1255	10.5634	0.8646	-0.2099	0.3527	0.4526
Sptbn1	_FQIQDISVETEDNK_.2	#N/A	7.9472	7.6560	8.2774	7.8512	6.4940	7.1731	8.3478	7.9684	7.7077	7.5128	0.9597	-0.0593	0.3985	0.3996
Sptbn1	_EIQGHQPR_.2	#N/A	6.1938	6.2260	5.7143	5.7065	6.4599	6.1598	5.9670	5.8088	6.1252	6.0294	1.0157	0.0225	0.5798	0.2367
Sptbn1	_QIEAQEKPR_.2	#N/A	5.2254	4.0232	4.3821	3.9545	5.4814	5.0837	4.6502	5.2869	5.2273	4.7868	0.9406	-0.0883	0.4312	0.3653
Sptbn1	_QALQDTLALYK_.2	#N/A	4.4844	4.6995	4.0487	4.6806	3.7048	5.3704	5.4715	5.2905	3.4640	4.3382	0.9692	-0.0452	0.7690	0.1141
Sptbn1	_DLDDFQSWLSR_.2	#N/A	3.0286	6.1485	5.9906	6.6596	6.6120	6.2476	6.9062	6.8093	6.8690	6.4912	0.8541	-0.2276	0.1988	0.7017
Sptbn1	_HQILEQAVEDYAETVHQLSK_.3	#N/A	6.3564	5.5255	4.3442	5.0623	6.1374	5.2562	6.6476	6.1537	6.0303	6.5900	0.8571	-0.2225	0.0505	1.2964
Sptbn1	_SAATWDER_.2	#N/A	5.7650	5.8132	5.8455	5.0552	6.0395	5.8447	4.4090	5.8747	5.5694	5.6153	1.0671	0.0937	0.2812	0.5510
Sptbn1	_LPEELGR_.2	#N/A	5.0894	5.5240	3.3409	4.3855	5.5867	5.4141	5.5551	6.0197	5.6360	5.4281	0.8640	-0.2109	0.1324	0.8783
Sptbn1	_AQTLPTSVVTITSESS[Phospho (STY)]PGKR_.3	#N/A	6.7847	8.2394	8.4631	9.8375	5.6952	6.9296	7.7013	9.1010	7.7562	5.7700	1.0100	0.0144	0.9366	0.0284
Sptbn1	_FATDGEGYKPC[Carbamidomethyl (C)]DPQVIR_.3	#N/A	7.0903	5.8649	3.2364	6.7587	6.1160	5.8510	5.9480	6.8333	6.2260	5.3972	0.9538	-0.0682	0.7111	0.1481
Sptbn1	_EAIC[Carbamidomethyl (C)]EVALDYK_.2	#N/A	6.6688	5.9633	3.6891	6.0100	5.3338	5.3639	5.7740	5.9849	5.3278	6.2923	0.9418	-0.0864	0.5487	0.2607
Sptbn1	_ILSSDDYGK_.2	#N/A	3.7658	3.3082	3.3412	3.4387	4.4297	3.7734	3.3086	3.8784	4.3663	3.9755	0.9469	-0.0787	0.4766	0.3218
Sptbn1	_HRPDLIDFDK_.3	#N/A	7.2424	5.9657	6.8934	6.1219	6.0299	6.1279	6.0199	6.5805	6.6141	6.2125	1.0063	0.0091	0.8955	0.0479
Sptbn1	_SAASGIPYHSEVPVSLK_.3	#N/A	6.9378	6.5455	5.9765	6.2560	5.7904	6.8528	6.3043	3.6357	6.1717	6.1091	1.1508	0.2027	0.1729	0.7623
Sptbn1	_FANSLVGVQQQLQAFNTYR_.2	#N/A	4.5540	4.2708	5.3447	5.2293	3.9004	4.5345	4.6832	5.3023	4.6088	4.6948	0.9620	-0.0559	0.5718	0.2427
Sptbn1	_VIESTQDLGNDLAGVM[Oxidation (M)]ALQR_.3	#N/A	5.9022	5.7088	6.5314	5.9992	5.7978	6.0367	6.2534	6.3565	6.2440	6.0978	0.9612	-0.0571	0.1539	0.8128
Sptbn1	_LQAAYAGDKADDIQK_.2	#N/A	4.4451	4.3408	3.5722	4.2254	4.0768	3.9093	4.3941	3.1823	3.7337	2.5517	1.1816	0.2408	0.1101	0.9580
Sptbn1	_ESS[Phospho (STY)]PVPS[Phospho (STY)]PTLDRK_.2	#N/A	9.8312	9.9767	9.8782	10.2907	9.4249	9.6540	10.5970	9.9706	10.0266	9.7754	0.9753	-0.0362	0.2572	0.5898
Sptbn1	_FESLEPEM[Oxidation (M)]NNQASR_.2	#N/A	7.7726	7.5198	7.9227	7.8537	7.2873	7.3551	7.9722	7.7671	7.4717	7.3861	0.9960	-0.0058	0.8639	0.0636
Sptbn1	_DDEEMNTWIQAISSAISSDKHDTSASTQS[Phospho (STY)]TPASSR_.4	#N/A	3.5265	3.8159	3.2602	7.1579	4.1711	3.9543	6.7184	4.1972	2.9954	3.9821	0.9645	-0.0522	0.8728	0.0591
Sptbn1	_INAVVETGR_.2	#N/A	5.6580	5.0575	3.3462	4.3908	5.4412	5.2270	5.2422	4.9388	4.7567	4.0681	1.0215	0.0306	0.8366	0.0775
Sptbn1	_AQTLPTSVVTITSESSPGK_.2	#N/A	4.0887	3.5371	4.3078	3.5851	4.0445	3.2817	4.6047	4.3698	3.9210	3.9376	0.9048	-0.1444	0.1361	0.8660
Sptbn1	_STDEVDSKR_.2	#N/A	3.4197	4.3000	4.5379	4.0789	2.9768	3.3612	4.2484	3.9987	2.9791	4.1287	0.9845	-0.0226	0.8818	0.0546
Sptbn1	_TSS[Phospho (STY)]KESS[Phospho (STY)]PVPSPTLDR_.2	#N/A	3.8833	8.0161	8.1616	8.9074	3.7083	8.4523	8.8177	8.1275	8.3018	3.9330	0.9397	-0.0898	0.7783	0.1088
Sptbn1	_GDQVSQNGLPAEQGS[Phospho (STY)]PR_.2	#N/A	11.4437	8.3785	10.7183	11.4467	10.0880	11.1547	11.4804	10.7239	11.3368	11.3035	0.9400	-0.0893	0.3057	0.5147
Sptbn1	_EGM[Oxidation (M)]QLISEKPETEAVVK_.3	#N/A	6.1397	5.7387	6.5892	6.0401	5.0842	6.2933	5.9650	5.9875	6.0244	6.1772	0.9905	-0.0138	0.8355	0.0781
Sptbn1	_DLVAIEAK_.2	#N/A	4.3384	3.5466	3.8960	4.0603	3.4289	4.8365	3.5880	4.2641	4.3318	4.5593	0.9599	-0.0591	0.6061	0.2174
Sptbn1	_ELEAENYHDIKR_.3	#N/A	5.5808	5.2620	5.0270	5.2346	5.2748	5.6136	5.2381	5.5323	5.5769	5.5792	0.9727	-0.0399	0.2888	0.5395
Sptbn1	_LILEVHQFSR_.2	#N/A	5.3335	5.1784	5.5256	4.3013	4.7841	4.6754	5.4808	5.5206	5.1074	5.0631	0.9383	-0.0919	0.2325	0.6337
Sptbn1	_LISDINK_.2	#N/A	4.6096	3.4658	3.5665	3.4809	4.5751	3.2503	2.5214	5.1390	5.0815	4.0976	0.9085	-0.1384	0.5205	0.2835
Sptbn1	_HDTSASTQS[Phospho (STY)]T[Phospho (STY)]PASSR_.2	Q62261_T2319_M2_Sptbn1	5.8687	5.3675	5.0789	5.7718	3.8525	5.5076	5.5476	5.0140	5.2486	5.4057	0.9882	-0.0172	0.8751	0.0579
Sptbn1	_QNLLSQSHAYQQFLR_.3	#N/A	5.0432	5.0013	5.3888	3.2626	4.5961	4.0587	4.7520	4.8516	4.8535	5.2314	0.9261	-0.1107	0.3980	0.4001
Sptbn1	_DVSSVELLM[Oxidation (M)]NNHQGIK_.2	#N/A	4.3611	3.8731	4.1530	4.2887	3.4174	4.5342	4.3349	4.8090	4.5987	4.2436	0.9128	-0.1316	0.1259	0.8998
Sptbn1	_VDTVNNM[Oxidation (M)]ADELINSGHSDAATIAEWK_.3	#N/A	4.0284	4.7050	4.7478	5.7093	3.8562	4.4126	5.1142	4.9148	4.5245	4.3427	0.9688	-0.0458	0.6962	0.1572
Sptbn1	_EAASELLMR_.2	#N/A	5.2220	3.8885	3.1876	5.4419	3.6434	4.0196	4.3804	4.2698	3.0681	2.2279	1.2143	0.2802	0.2576	0.5891
Sptbn1	_DASVAEAWLLGQEPYLSSR_.2	#N/A	5.6966	5.5986	3.3474	5.5664	5.1368	5.3585	6.0974	5.6903	5.1516	5.3343	0.9190	-0.1219	0.3780	0.4226
Sptbn1	_MWEVLESTTQTK_.2	#N/A	5.2522	2.9683	4.1078	5.6721	4.9969	3.4390	4.9325	3.3496	3.6487	3.3288	1.1549	0.2078	0.3716	0.4299
Sptbn1	_DALLSALSIQNYHLEC[Carbamidomethyl (C)]NETK_.3	#N/A	3.7579	2.7132	3.4916	2.8384	3.6705	3.7685	3.6617	3.6989	4.0405	3.9843	0.8770	-0.1893	0.0998	1.0007
Sptbn1	_LAEISDVWEEMK_.2	#N/A	4.4972	3.5769	3.4585	5.2890	3.3982	4.3313	4.4606	4.1573	3.8059	3.9281	1.0009	0.0014	0.9926	0.0032
Sptbn1	_WDVDDWDNENS[Phospho (STY)]SAR_.2	Q62261_S35_M1_Sptbn1	3.7080	2.9635	3.4417	4.4863	2.8518	3.7728	3.1338	4.0157	4.3147	4.1636	0.9054	-0.1434	0.3477	0.4588
Sptbn1	_GNLEVLLFTIQSK_.2	#N/A	3.4465	4.2436	3.6694	4.1721	3.6091	4.3006	3.8868	2.9962	3.7594	4.0342	1.0648	0.0906	0.3940	0.4045
Sptbn1	_RRPPS[Phospho (STY)]PDPNTK_.2	#N/A	3.9561	3.2115	9.1050	8.8642	3.0998	3.5248	3.3818	3.7676	8.3960	4.4117	1.0610	0.0854	0.8646	0.0632
Sptbn1	_VAHM[Oxidation (M)]EFC[Carbamidomethyl (C)]YQELC[Carbamidomethyl (C)]QLAAER_.3	#N/A	5.8594	5.7519	6.1121	5.7511	5.0020	5.5416	5.9356	5.4947	5.4717	6.2685	0.9788	-0.0309	0.6284	0.2018
Sptbn1	_TQILAASYELHK_.2	#N/A	6.2443	5.0381	6.7136	6.0223	5.7450	5.7246	6.0184	6.7895	6.5350	5.5745	0.9495	-0.0748	0.4060	0.3914
Sptbn1	_FQIQDISVETEDNKEK_.3	#N/A	4.8225	3.7486	3.8475	4.5890	3.5915	4.1376	3.8285	4.6960	4.6630	4.2709	0.9446	-0.0822	0.4399	0.3566
Sptbn1	_RPPS[Phospho (STY)]PDPNTKVSEEAESQQWDTSK_.3	#N/A	3.6188	4.3532	6.2133	5.7540	4.6473	4.8262	5.1897	6.0680	3.9771	4.5533	0.9909	-0.0131	0.9421	0.0259
Sptbn1	_TLETPAAQM[Oxidation (M)]EGFLNR_.2	#N/A	7.3107	7.8470	8.0413	8.0327	6.7533	7.1354	7.7799	8.0462	7.4462	7.4008	0.9807	-0.0282	0.6315	0.1996
Sptbn1	_LLDPEDISVDHPDEK_.2	#N/A	6.9471	6.4442	6.6893	6.3440	5.7976	6.3852	6.8095	6.9205	6.3582	6.3288	0.9743	-0.0376	0.4832	0.3159
Sptbn1	_EVVAGSHELGQDYEHVTM[Oxidation (M)]LQER_.3	#N/A	4.8468	4.8308	5.0676	5.7812	3.6840	5.1070	5.5507	5.3051	4.5751	4.8800	0.9623	-0.0555	0.6355	0.1969
Sptbn1	_KHEAIETDIAAYEER_.3	#N/A	7.0873	6.5096	7.0121	5.5539	5.8083	6.9623	7.7665	5.8156	7.5154	6.9407	0.9257	-0.1113	0.3112	0.5070
Sptbn1	_ITDLYTDLRDGR_.3	#N/A	3.8209	3.1329	3.6111	3.7819	4.1420	3.6034	3.4966	4.3366	3.8411	3.5355	0.9683	-0.0464	0.6130	0.2125
Sptbn1	_LEMNLGLQK_.2	#N/A	2.5696	3.7812	4.1318	3.1336	3.6695	2.9551	4.3491	3.4841	3.1327	3.1006	0.9593	-0.0600	0.7137	0.1465
Sptbn1	_M[Oxidation (M)]HTTFEHDIQALGTQVR_.3	#N/A	4.7164	5.2260	5.6731	4.4671	4.8839	5.2325	5.5806	4.9106	5.4863	4.9839	0.9605	-0.0582	0.4455	0.3512
Sptbn1	_VSEEAESQQWDTSKGDQVSQNGLPAEQGS[Phospho (STY)]PR_.4	#N/A	3.7050	6.4433	4.3918	7.1213	6.5929	7.6520	3.1083	8.6834	7.8910	7.0325	0.8960	-0.1584	0.5982	0.2232
Sptbn1	_IQEKVDSIDDR_.3	#N/A	3.6748	2.9791	4.3616	3.6407	2.8004	4.5289	3.7890	3.2543	3.6595	3.3304	1.0445	0.0628	0.6855	0.1640
Sptbn1	_IFQEM[Oxidation (M)]LYIM[Oxidation (M)]DWM[Oxidation (M)]DEM[Oxidation (M)]K_.2	#N/A	3.3803	4.1855	4.3914	3.9352	4.0068	3.7227	4.0835	3.0660	3.3650	3.6125	1.1147	0.1567	0.1414	0.8494
Sptbn1	_QAEAFLNNQEYVLAHTEM[Oxidation (M)]PTTLEGAEAAIK_.3	#N/A	3.8216	3.2259	3.5396	3.6063	2.9653	3.4152	3.4987	4.0130	3.9815	4.1502	0.8768	-0.1897	0.0353	1.4522
Sptbn1	_HYASEEIKEK_.3	#N/A	2.2309	4.3043	4.1859	3.1877	4.1256	3.6039	4.4032	3.4300	3.0785	3.1547	1.0255	0.0364	0.8539	0.0686
Sptbn1	_SNAHYNLQNAFNLAEQHLGLTK_.3	#N/A	3.1205	3.8768	4.3550	3.2808	1.9365	4.3603	4.0471	3.1024	3.4014	3.5761	0.9877	-0.0178	0.9324	0.0304
Sptbn1	_MLTAQDMSYDEAR_.2	#N/A	3.4641	4.2693	4.1509	5.0964	4.0906	3.6389	3.9997	3.1498	3.4488	3.5287	1.1661	0.2217	0.1075	0.9688

Sptbn1	_FFWEM[Oxidation (M)]AEEEGWIR_.2	#N/A	3.2746	4.0678	4.5091	4.0529	3.9192	3.8404	4.2012	4.4491	3.2473	3.7302	1.0095	0.0136	0.9026	0.0445
Sptbn1	_EAIC[Carbamidomethyl (C)]EVALDYKK_.3	#N/A	4.3660	4.3611	3.5519	4.0546	3.4916	4.2379	3.7693	4.0639	3.7125	4.0216	1.0305	0.0433	0.5936	0.2265
Sptbn1	_AQQYYFDAAEAEAWM[Oxidation (M)]SEQELYM[Oxidation (M)]M[Oxidation (M)]SEEK_.3	#N/A	3.9187	3.2231	4.6055	3.6073	3.0444	3.1843	3.3220	3.0103	4.1597	3.5743	1.0229	0.0327	0.8270	0.0825
Syp	_M[Oxidation (M)]ATDPENIIK_.2	#N/A	9.1621	8.7704	8.5019	8.7845	8.5618	8.1096	8.9263	8.9846	8.6051	8.6526	0.9836	-0.0238	0.4808	0.3181
Syp	_LHQVY[Phospho (STY)]FDAPSC[Carbamidomethyl (C)]VK_.2	#N/A	8.9709	9.1958	9.5647	9.3080	8.2766	8.6581	9.4006	9.3469	9.0029	8.7362	0.9862	-0.0201	0.6515	0.1861
Syp	_ETGWAAPFM[Oxidation (M)]R_.2	#N/A	8.0035	8.1861	8.2608	7.7387	7.5094	7.7103	8.2173	8.2800	7.8159	7.7394	0.9861	-0.0202	0.5648	0.2481
Syp	_M[Oxidation (M)]LLLADM[Oxidation (M)]DVVNQLVAGGQFR_.2	#N/A	4.2975	4.2499	4.1315	3.1333	4.0712	3.6583	4.3488	4.0790	3.1329	3.1003	1.0705	0.0983	0.4718	0.3262
Tspan7	_AVDHVQR_.2	#N/A	6.8380	6.2115	6.2099	5.1182	6.4758	5.6257	6.1435	6.1463	6.1810	5.6686	1.0075	0.0107	0.8944	0.0484
Tspan7	_TYTDAM[Oxidation (M)]QNYNGNDER_.2	#N/A	6.5698	5.7563	5.9851	6.7535	5.0858	5.7265	7.3439	6.1456	6.1692	5.9975	0.9323	-0.1012	0.3059	0.5145
Tgoln1;Tgoln2	_DADS[Phospho (STY)]GDSQNPPNQPSK_.2	Q62313_S28_M1_Tgoln1;Tgoln2	6.4968	5.9089	4.4091	6.6532	5.8856	4.3063	4.1011	3.0483	3.3474	3.6302	1.5884	0.6676	0.0053	2.2727
Tgoln1;Tgoln2	_TES[Phospho (STY)]GETLAGDSDFSLKPEKGDK_.3	#N/A	2.9882	4.2380	3.6750	4.1776	3.6147	4.3275	3.8923	3.9408	3.5894	4.1446	0.9859	-0.0205	0.8475	0.0718
Tgoln1;Tgoln2	_TES[Phospho (STY)]GETLAGDSDFSLKPEK_.2	#N/A	3.5541	3.8168	3.4412	3.8392	3.6827	3.6485	3.7253	3.7867	3.7648	3.7085	0.9780	-0.0322	0.3272	0.4852
Trim28	_S[Phospho (STY)]GEGEVSGLLR_.2	#N/A	10.0464	9.8151	9.9809	10.2262	9.4596	9.8422	10.2364	9.6859	10.2295	10.1625	0.9818	-0.0265	0.3089	0.5102
Trim28	_SRS[Phospho (STY)]GEGEVSGLLR_.2	#N/A	7.7513	7.6032	7.4118	7.3628	6.5316	7.5651	7.9627	8.2880	6.9208	6.8389	0.9825	-0.0255	0.7273	0.1383
Trim28	_[Acetyl (Protein N-term)]AASAAATAAASAATAASAASGS[Phospho (STY)]PGSGEGSAGGEK_.3	Q62318_S23_M1_Trim28	4.1234	9.3904	3.8448	10.8346	7.7137	7.9379	8.8784	9.5210	8.9959	7.6002	0.8352	-0.2597	0.3554	0.4493
Trim28	_LSPPYSSPQEFAQDVGR_.2	#N/A	7.7806	4.0209	7.4336	8.1343	8.0585	4.2163	4.1094	6.8885	7.5373	5.8613	1.0833	0.1155	0.6716	0.1729
Trim28	_VS[Phospho (STY)]LERLDLDLTSDSQPPVFK_.3	Q62318_S489_M1_Trim28	3.0220	4.9572	4.1347	3.1366	3.6665	2.9581	4.3521	3.4811	3.1297	3.4046	1.0150	0.0215	0.9078	0.0420
Trim28	ASSAAAASAAASS[Phospho (STY)]PAGGGGEAQELLEHC[Carbamidomethyl (C)]GVC[Carbamidomethyl (C	Q62318_S51_M1_Trim28	3.7191	3.5071	4.4059	8.8069	3.3954	3.2292	3.1224	3.2099	4.3593	3.3747	1.2827	0.3591	0.3996	0.3984
Trim28	_[Acetyl (Protein N-term)]AASAAATAAASAATAASAASGSPGSGEGS[Phospho (STY)]AGGEKR_.3	Q62318_S30_M1_Trim28	3.3649	3.9775	4.5994	4.1432	4.0095	3.9307	4.2915	5.1443	3.1570	3.8205	0.9758	-0.0353	0.8046	0.0944
Snrnp70	_YDERPGPS[Phospho (STY)]PLPHR_.3	#N/A	8.7868	8.5930	8.9923	9.0652	7.6487	8.0467	8.6069	8.6430	8.1214	8.2938	1.0126	0.0180	0.7363	0.1329
Snrnp70	_GGGGS[Phospho (STY)]GQDNGLEGLGSDGR_.2	Q62376_S408_M1_Snrnp70	7.1004	6.9411	6.8526	7.8609	6.2718	6.9011	7.4871	7.0660	6.7071	7.0403	0.9877	-0.0179	0.7720	0.1124
Tpd52	_NSPTFKS[Phospho (STY)]FEEK_.2	#N/A	10.6938	10.5309	10.8455	11.2339	9.9226	10.5996	10.7113	10.3405	10.5315	10.8055	1.0038	0.0055	0.8668	0.0621
Tpd52	_S[Phospho (STY)]FEEKVENLK_.2	#N/A	10.4681	10.2933	10.0901	11.1408	9.8169	10.4240	10.6565	10.0952	10.5109	10.5691	0.9918	-0.0119	0.7387	0.1316
Tpd52	_NSPTFKS[Phospho (STY)]FEEKVENLK_.3	#N/A	7.9451	8.6808	9.3434	10.0157	7.8728	8.4338	8.5656	8.8058	8.5436	8.2811	1.0194	0.0278	0.7114	0.1479
Tpd52	_ASAAFSSVGSVITK_.2	#N/A	3.9161	3.1715	3.6498	3.7432	3.6803	3.1253	3.4579	3.3958	4.1067	4.3717	0.9256	-0.1116	0.2903	0.5372
Tpd52	_PAGGDFGEVLNSTANATSTM[Oxidation (M)]TTEPPPEQMTES[Phospho (STY)]P_.4	Q62393_S223_M1_Tpd52	3.1335	4.2089	4.3680	6.6330	3.7780	4.3474	4.0600	3.0894	3.3885	3.5890	1.2491	0.3209	0.1973	0.7048
Speg	_SSS[Phospho (STY)]FSQGEAEPR_.2	Q62407_S2135_M1_Speg	9.4708	3.2201	10.0222	9.8595	8.4909	9.4438	9.6003	9.5686	9.4695	9.5127	0.8826	-0.1802	0.4235	0.3732
Speg	_AAS[Phospho (STY)]VELPQR_.2	#N/A	9.3002	8.7638	9.0089	8.9345	7.6042	8.8002	9.1289	9.0800	8.8727	9.1338	0.9648	-0.0517	0.3240	0.4895
Speg	_ARS[Phospho (STY)]LEQPK_.2	Q62407_S439_M1_Speg	8.6063	8.6943	9.2653	8.9673	8.3590	8.5935	8.9102	8.8396	8.5238	8.5470	1.0049	0.0070	0.8208	0.0858
Speg	_AAS[Phospho (STY)]VELPQRR_.2	#N/A	7.2490	6.9202	7.3235	7.1904	5.6102	6.9180	7.2882	6.9482	6.9625	6.7276	0.9838	-0.0236	0.7472	0.1266
Speg	_AS[Phospho (STY)]PVLAVR_.2	Q62407_S2451_M1_Speg	5.3263	5.7744	4.2993	5.0461	4.4699	5.2664	5.3786	4.8103	4.4551	4.7189	1.0392	0.0555	0.5729	0.2419
Speg	_SRS[Phospho (STY)]VQDLR_.2	Q62407_S2396_M1_Speg	3.6948	6.5525	3.4284	3.9646	5.3799	6.3792	3.6793	7.1075	6.7544	6.7862	0.8057	-0.3118	0.2498	0.6025
Speg	_SATS[Phospho (STY)]DLELR_.2	Q62407_S493_M1_Speg	2.9938	3.7990	7.2538	4.3217	3.6203	4.1091	4.4699	7.7573	2.9786	7.5725	0.7638	-0.3887	0.2963	0.5282
Speg	_LSPS[Phospho (STY)]QDHDSSDSSSK_.2	Q62407_S860_M1_Speg	4.0716	5.2079	3.2576	5.0686	4.3257	5.5338	5.2051	5.6704	5.7870	3.7272	0.8980	-0.1552	0.3909	0.4080
Speg	_ATS[Phospho (STY)]EGESLRR_.2	Q62407_S2499_M1_Speg	3.4938	4.2990	6.1134	3.8216	4.1204	3.6091	5.1946	5.7212	3.4786	3.1495	0.9674	-0.0478	0.8435	0.0739
Speg	_SEGGAAWGT[Phospho (STY)]PEASQEELR_.2	Q62407_T453_M1_Speg	5.8539	5.3029	5.9184	6.6174	5.7789	5.9345	6.2749	6.3849	6.5330	6.6543	0.9132	-0.1310	0.0374	1.4269
Speg	_SEGGAAWGT[Phospho (STY)]PEAS[Phospho (STY)]QEELRSPR_.3	#N/A	3.0872	3.8435	4.3218	4.5721	3.7318	4.3936	4.2868	3.5464	3.1950	3.0383	1.1351	0.1828	0.2195	0.6587
Speg	_S[Phospho (STY)]TS[Phospho (STY)]REELVR_.2	Q62407_S509_M2_Speg	4.1668	5.4786	5.8452	3.4064	4.5419	3.3976	3.3222	3.4747	4.1431	4.0160	1.1962	0.2585	0.2176	0.6624
Speg	_AAS[Phospho (STY)]SEAAPHHQPPPESR_.3	Q62407_S2114_M1_Speg	3.7421	3.4019	4.1230	3.3847	3.2622	3.1884	4.1359	3.4313	3.4668	5.0732	0.8734	-0.1953	0.1871	0.7280
Speg	_RGSS[Phospho (STY)]AESALPR_.2	Q62407_S2020_M1_Speg	4.3373	3.5928	4.3202	3.3220	3.4811	3.1435	4.5375	7.1458	2.9442	3.2890	0.8259	-0.2759	0.3565	0.4479
Speg	_RLS[Phospho (STY)]STLER_.2	Q62407_S2461_M1_Speg	3.9422	3.2840	3.1281	3.6308	3.0678	3.1608	3.3455	2.9869	4.1363	3.5978	0.9580	-0.0619	0.5856	0.2324
Speg	_MPS[Phospho (STY)]IPEEPEHGDLER_.3	#N/A	3.7805	3.7060	3.8782	6.1765	3.9692	3.6829	4.0765	3.8017	3.6428	3.8084	1.0956	0.1318	0.4865	0.3129
Speg	_QRS[Phospho (STY)]ATS[Phospho (STY)]DLELR_.2	Q62407_S490_M2_Speg	4.3074	3.5628	4.0410	3.5848	3.4511	3.1735	3.7331	3.4164	3.7154	3.2621	1.0439	0.0620	0.5193	0.2846
Ppp1r13b	_SS[Phospho (STY)]ITEPEGPGGPNIQK_.2	Q62415_S708_M1_Ppp1r13b	3.1672	4.1752	4.4017	3.9455	3.8117	3.7329	4.0937	3.0557	3.3548	3.6227	1.0964	0.1328	0.2547	0.5940
Ppp1r13b	_VNGTSSPQS[Phospho (STY)]PLSTSGR_.2	Q62415_S335_M1_Ppp1r13b	3.8416	3.0971	3.3151	3.8177	2.9854	3.6392	3.5324	4.3008	3.9493	3.7847	0.8863	-0.1741	0.0878	1.0567
Sorbs1	_AGEQDPVPTPAELTS[Phospho (STY)]PGR_.2	#N/A	9.2256	3.3357	3.2173	3.7200	3.1571	3.0716	9.5694	4.3985	9.0515	9.3618	0.5297	-0.9168	0.0424	1.3724
Sorbs1	_SATVS[Phospho (STY)]PQQPQAQQR_.2	Q62417_S1209_M1_Sorbs1	8.6448	3.9511	9.5676	9.3798	8.5406	8.9783	9.1003	3.7831	8.7065	8.9504	1.0710	0.0989	0.7238	0.1404
Sorbs1	_NNNPQSELAAAHGDSES[Phospho (STY)]PR_.3	#N/A	7.0495	7.1758	6.8014	7.7777	6.9119	6.9750	7.4544	3.8636	7.0954	7.2159	1.1105	0.1512	0.3389	0.4699
Sorbs1	_S[Phospho (STY)]AESLLESTK_.2	Q62417_S722_M1_Sorbs1	7.8805	7.4948	8.2039	8.3973	7.1299	7.9900	7.7095	7.8850	7.9924	8.3442	0.9833	-0.0243	0.6228	0.2057
Sorbs1	_EGS[Phospho (STY)]GGSVHGDFPK_.2	Q62417_S791_M1_Sorbs1	7.6629	5.6500	5.8656	8.3116	2.8195	7.7345	6.4165	4.9778	7.5341	5.7792	1.0265	0.0378	0.8875	0.0518
Sorbs1	_RAGEQDPVPTPAELTS[Phospho (STY)]PGR_.3	#N/A	9.1020	9.0170	3.9386	3.4824	3.3487	3.2759	3.6307	9.1045	3.8178	3.1597	1.0878	0.1214	0.8197	0.0863
Sorbs1	_SAT[Phospho (STY)]LPLPAR_.2	Q62417_T286_M1_Sorbs1	7.6610	3.5053	4.4077	3.4095	2.8466	7.5767	3.1242	7.1128	7.6667	7.7032	0.7656	-0.3853	0.3170	0.4990
Sorbs1	_S[Phospho (STY)]M[Oxidation (M)]PSLDFSGR_.2	#N/A	8.2526	7.4041	8.1588	8.5703	6.7766	7.7491	8.1521	7.7961	7.8530	7.9152	0.9861	-0.0203	0.7526	0.1234
Sorbs1	_GTPSSSPVSPQES[Phospho (STY)]PKHESK_.3	#N/A	10.3026	10.4630	11.0736	10.8922	10.3257	10.6240	10.6139	10.3845	10.4279	10.5568	1.0112	0.0161	0.4972	0.3035
Sorbs1	_SKS[Phospho (STY)]EM[Oxidation (M)]NYIEGEK_.2	#N/A	6.1661	3.1706	3.6488	4.0621	6.8963	6.9429	6.8939	3.8220	4.1211	4.6962	1.0542	0.0761	0.8041	0.0947
Sorbs1	_GTPSSSPVS[Phospho (STY)]PQESPK_.2	Q62417_S58_M1_Sorbs1	8.2950	7.5842	4.2594	8.3869	3.9484	7.7323	7.8352	7.7614	7.7560	8.0500	0.8536	-0.2284	0.3017	0.5204
Sorbs1	_DIS[Phospho (STY)]PEEIDLK_.2	#N/A	4.2294	6.9751	4.4282	9.0645	3.3731	7.3458	7.7927	8.0755	7.8533	8.0539	0.7431	-0.4285	0.1094	0.9610
Sorbs1	_SVLEGGDIPLQGLSGLK_.2	#N/A	4.1680	4.0886	3.3540	3.8567	3.2937	4.0840	3.2957	4.2618	3.9104	4.6318	0.9460	-0.0801	0.4891	0.3106
Sorbs1	_S[Phospho (STY)]PTPVLSR_.2	Q62417_S707_M1_Sorbs1	4.8925	3.6404	4.2833	4.0156	3.6933	3.8249	3.9016	5.0182	3.5886	3.8849	0.9902	-0.0142	0.9109	0.0405
Sorbs1	_SSS[Phospho (STY)]LKSSPER_.2	Q62417_S295_M1_Sorbs1	3.1246	4.1016	3.8114	4.9268	3.9899	4.1355	4.0287	4.7820	3.4530	3.2288	1.0366	0.0519	0.7345	0.1340
Sorbs1	_RES[Phospho (STY)]DGTPGGLASLENER_.2	Q62417_S432_M1_Sorbs1	3.2080	4.0182	3.8948	4.3974	3.9065	4.2190	4.1121	3.7210	3.3696	4.3644	1.0125	0.0180	0.8613	0.0649
Sorbs1	_YS[Phospho (STY)]FSDDTKSPLSVPR_.3	#N/A	2.8980	3.6543	4.1325	3.6763	3.5426	3.0820	3.8245	3.3249	3.6239	3.3536	0.9903	-0.0140	0.8931	0.0491
Sorbs1	_AQS[Phospho (STY)]C[Carbamidomethyl (C)]ENLC[Carbamidomethyl (C)]VSLNDSK_.2	#N/A	3.0758	3.8321	4.3104	3.8541	3.7204	4.4050	4.0024	3.1470	3.4461	3.5314	1.0947	0.1306	0.2673	0.5730
Sorbs1	_SAQDLSDVST[Phospho (STY)]DEVGIPLR_.2	Q62417_T213_M1_Sorbs1	3.7760	3.6042	3.7394	4.2421	3.4645	3.8958	3.9341	4.0017	3.6503	3.4317	1.0087	0.0125	0.8545	0.0683
Sorbs1	_DDDSDVHS[Phospho (STY)]PR_.2	Q62417_S250_M1_Sorbs1	4.0702	3.1559	3.2562	3.7589	3.1959	3.0327	3.4736	4.3596	4.0082	3.7259	0.8766	-0.1900	0.0987	1.0055
Sorbs1	_YS[Phospho (STY)]FSDDTK_.2	#N/A	3.9348	4.2411	4.1227	3.8796	4.0625	3.6670	4.0278	3.1216	3.4207	2.3477	1.2338	0.3032	0.0336	1.4743
Sorbs1	_AQS[Phospho (STY)]C[Carbamidomethyl (C)]ENLC[Carbamidomethyl (C)]VSLNDSKR_.3	#N/A	3.2823	4.0601	4.5168	5.8515	3.9269	4.1986	4.2088	4.4414	3.2396	3.7379	1.1021	0.1403	0.4360	0.3606
Sorbs1	_TPVDYIDLPYSSS[Phospho (STY)]PSR_.2	Q62417_S1201_M1_Sorbs1	2.9129	3.7181	3.3580	9.1917	3.5394	4.1901	4.5509	8.2644	4.3984	4.0799	0.8425	-0.2472	0.5731	0.2417
Sorbs1	_DDDSDVHSPRYS[Phospho (STY)]FSDDTK_.3	#N/A	3.8262	3.3999	4.5130	3.5149	2.9519	3.3364	3.2295	3.1028	4.2522	3.4818	1.0210	0.0300	0.8335	0.0791
Sorbs1	_VGGS[Phospho (STY)]IENLLMR_.2	Q62417_S576_M1_Sorbs1	3.1821	3.9384	3.9746	5.9154	3.8267	4.2988	4.1919	3.6412	3.2898	4.4443	1.0764	0.1063	0.5784	0.2377
Sorbs1	_DIS[Phospho (STY)]PEEIDLKNEPWYK_.3	#N/A	3.7887	4.0942	3.5567	4.2269	3.4964	4.0292	4.2030	4.0963	4.1061	4.0369	0.9403	-0.0887	0.1523	0.8172
Sorbs1	_GELGAS[Phospho (STY)]QEGSEHIPK_.2	Q62417_S666_M1_Sorbs1	3.1471	3.9034	4.3816	4.5122	3.7917	4.3338	4.2269	3.0758	3.2548	3.6027	1.1332	0.1804	0.1890	0.7235
Dbnl	_NGPALQEAYVR_.2	#N/A	6.3881	6.3271	6.2984	6.8899	6.5870	6.1096	6.0699	6.2995	5.9198	6.7186	1.0290	0.0413	0.3800	0.4202
Dbnl	_VAGTGEGGLEELVEELNSGK_.2	#N/A	5.1828	4.8704	4.7198	5.4649	4.4733	5.8239	5.6272	5.3416	5.4176	5.0440	0.9499	-0.0742	0.3538	0.4512
Dbnl	_ESTSFQDVGPQAPVGSVYQK_.2	#N/A	4.3217	5.1011	5.6422	5.0999	5.2470	4.9605	5.3322	4.6765	4.6541	4.5794	1.0523	0.0735	0.3620	0.4412
Dbnl	_SPTDWALFTYEGNSNDIR_.2	#N/A	5.0440	5.0449	4.4480	4.7983	4.6460	5.1465	5.6534	4.1245	4.7825	4.6891	1.0088	0.0126	0.8861	0.0525
Dbnl	_TRQEWESAGQQAPHPR_.3	#N/A	5.7907	7.1473	7.1494	7.5055	6.8934	6.9162	3.9556	7.2865	6.3762	6.3073	1.1536	0.2062	0.1871	0.7280
Dbnl	_VVTEKS[Phospho (STY)]PTDWALFTYEGNSNDIR_.3	Q62418_S24_M1_Dbnl	3.8281	3.3612	4.9583	5.9948	4.7232	4.0641	3.9267	5.4441	4.6814	3.9818	0.9955	-0.0065	0.9719	0.0124
Dbnl	_VKDPNSGLPK_.2	#N/A	3.5483	5.9764	4.2351	3.2369	6.3872	6.2170	5.7013	6.3630	5.6668	5.9349	0.8339	-0.2621	0.2193	0.6590

Dbnl	_FVLINWTGEGVNDVR_.2	#N/A	4.7613	4.9069	3.1205	5.0004	4.5608	4.6914	3.3378	4.6440	4.6952	4.5675	1.0454	0.0641	0.6674	0.1756
Dbnl	_AMS[Phospho (STY)]TTSVTSSQPGK_.2	Q62418_S277_M1_Dbnl	5.1539	3.7261	3.7724	5.9232	4.2515	3.7755	4.3942	3.9960	3.8175	3.7868	1.1088	0.1490	0.3887	0.4104
Dbnl	_QLT[Phospho (STY)]QPETSYGR_.2	Q62418_T299_M1_Dbnl	4.3324	4.3946	3.5184	4.0210	3.4581	4.2714	3.7357	4.0975	5.4622	3.9880	0.9256	-0.1116	0.4138	0.3832
Dbnl	_AEEDVEPEC[Carbamidomethyl (C)]IMEK_.2	#N/A	4.2567	3.0857	3.9904	5.5533	2.9070	3.3217	3.6825	3.4670	3.7660	3.2115	1.0908	0.1254	0.5481	0.2611
Dbnl	_ASGANYSFHK_.2	#N/A	3.2848	3.9414	3.9716	4.8069	3.8297	5.0619	4.1889	3.6442	3.2928	4.4413	1.0662	0.0925	0.5329	0.2733
Sh3gl1	_ITAS[Phospho (STY)]SSFR_.2	Q62419_S286_M1_Sh3gl1	3.9343	4.3274	4.2090	3.7933	4.1488	3.5807	3.9415	3.2079	3.5070	3.1779	1.1562	0.2094	0.0274	1.5626
Sh3gl2	_TIEYLQPNPASR_.2	#N/A	10.4737	10.6679	10.9584	10.9522	11.0029	10.7295	10.7498	10.4722	10.7483	10.6239	1.0140	0.0200	0.2448	0.6112
Sh3gl2	_IPDEELR_.2	#N/A	9.9162	9.9709	9.9522	9.7854	10.3668	10.0616	9.3841	10.0474	10.0057	10.0833	1.0130	0.0187	0.4587	0.3384
Sh3gl2	_ALYDFEPENEGELGFK_.2	#N/A	9.7759	9.6178	10.0118	10.0786	9.8643	9.8757	9.8681	9.4708	9.6368	9.6665	1.0217	0.0310	0.0829	1.0817
Sh3gl2	_RLDFDYK_.2	#N/A	9.1232	9.0166	8.9927	9.2799	9.0359	9.0969	9.0133	8.8848	9.0321	9.2138	1.0061	0.0087	0.4893	0.3105
Sh3gl2	_KVDVTSR_.2	#N/A	8.7330	8.6793	8.6591	8.6116	9.2099	8.6827	8.4303	8.5962	8.7052	8.6082	1.0207	0.0295	0.1842	0.7346
Sh3gl2	_[Acetyl (Protein N-term)]S[Phospho (STY)]VAGLKK_.2	Q62420_S2_M1_Sh3gl2	8.2883	7.6527	8.2697	7.9403	6.6855	8.0437	7.5404	7.9361	8.3012	8.1962	0.9775	-0.0329	0.6047	0.2185
Sh3gl2	_RLDFDYKK_.2	#N/A	7.8798	7.8533	7.6276	8.2742	8.0544	7.9697	8.3054	7.7163	7.8685	7.6710	1.0067	0.0096	0.7482	0.1260
Sh3gl2	_QAVQILQQVTVR_.2	#N/A	7.3300	7.0772	7.6050	7.0625	7.8222	7.0992	7.3926	6.9770	7.5462	7.4196	0.9998	-0.0002	0.9953	0.0020
Sh3gl2	_EIQHHLK_.2	#N/A	8.0790	6.7249	6.2002	7.1274	6.9660	7.4135	7.1624	6.5218	7.6276	7.2537	0.9921	-0.0114	0.8837	0.0537
Sh3gl2	_EYQPKPR_.2	#N/A	6.4097	6.3805	6.6399	6.7992	7.0996	6.8060	6.1525	6.6956	6.3476	6.6156	1.0366	0.0519	0.2026	0.6934
Sh3gl2	_QNFIDPLQNLHDKDLR_.3	#N/A	7.9651	8.1748	7.9347	8.5319	8.2539	8.4430	8.2881	7.9400	8.0419	7.7664	1.0260	0.0370	0.2061	0.6860
Sh3gl2	_ELGDDC[Carbamidomethyl (C)]NFGPALGEVGEAM[Oxidation (M)]R_.2	#N/A	6.2382	6.5080	6.6190	7.3445	6.7871	6.8947	6.5486	6.3287	6.5318	6.2907	1.0478	0.0673	0.1627	0.7885
Sh3gl2	_GPGYPQAEALLAEAM[Oxidation (M)]LK_.2	#N/A	5.0266	5.4556	5.7227	5.9993	5.7254	5.7115	5.6124	5.3767	5.6078	5.6442	1.0084	0.0120	0.7984	0.0978
Sh3gl2	_VGGAEGTKLDDDFKEM[Oxidation (M)]ER_.3	#N/A	7.3252	6.7489	6.6927	7.6245	6.9764	6.6596	6.8781	6.5538	6.4795	6.8803	1.0458	0.0646	0.1953	0.7093
Sh3gl2	_ELSEVKDSLDM[Oxidation (M)]EVK_.2	#N/A	6.0599	6.5278	7.6873	7.4691	7.3199	6.9811	6.9513	7.3545	6.8549	6.2324	1.0233	0.0332	0.6735	0.1717
Sh3gl2	_AVMEIMTK_.2	#N/A	3.8705	3.1209	4.4340	5.7272	3.9643	3.7652	4.1260	3.0234	1.6610	3.6550	1.3307	0.4122	0.1337	0.8738
Sh3gl2	_LDDDFKEMER_.3	#N/A	2.6101	4.2134	4.3635	3.9073	4.0347	3.6947	4.0555	3.0939	3.3930	3.5846	1.0771	0.1071	0.4697	0.3282
Ostf1	_TLSNAEDYLDDEDS[Phospho (STY)]D_.2	Q62422_S214_M1_Ostf1	4.3797	3.6841	3.5657	11.7429	9.4166	4.2241	4.0175	10.9489	8.0850	4.0353	0.9110	-0.1345	0.7941	0.1001
Ndufa4	_LALFNPDVSWDR_.2	#N/A	9.8466	9.7742	9.9576	10.0753	9.9817	9.8182	9.8158	9.7513	10.0375	9.6866	1.0088	0.0126	0.3351	0.4749
Ndufa4	_FYSVNVDYSK_.2	#N/A	9.4274	9.1497	9.4010	9.5546	9.3308	9.6022	9.4089	9.3338	9.3593	9.4210	1.0030	0.0044	0.7417	0.1298
Ndufa4	_KNNPEPWNK_.2	#N/A	8.4474	8.1156	8.0923	8.0360	8.4840	8.4120	8.1543	8.3113	8.4570	8.0501	1.0026	0.0037	0.8692	0.0609
Ndufa4	_LGPNEQYK_.2	#N/A	7.0540	6.7407	6.6022	6.8007	7.0677	6.7648	6.8198	6.8611	6.3807	6.9129	1.0140	0.0201	0.5037	0.2979
Ndufa4	_LALFNPDVSWDRK_.3	#N/A	4.5673	5.2603	5.8009	5.8329	5.2290	5.6887	4.9173	5.3418	5.4325	5.4793	1.0196	0.0280	0.7078	0.1501
Ndufa4	_NNPEPWNK_.2	#N/A	7.2794	6.8538	3.3203	7.5243	7.5000	7.2133	3.5376	7.0151	7.4834	7.7275	1.0271	0.0385	0.8820	0.0545
Ndrg1	_TAS[Phospho (STY)]GSSVTSLEGTR_.2	Q62433_S330_M1_Ndrg1	9.2796	9.1991	9.6303	9.4707	9.7445	9.2416	9.6899	8.8387	9.4703	9.6939	1.0005	0.0007	0.9826	0.0076
Ndrg1	_LNITPNSGATGNNAGPK_.2	#N/A	7.0294	7.1441	6.8466	7.4925	7.1688	6.9322	7.2559	7.0693	6.7434	7.2224	1.0042	0.0060	0.8471	0.0721
Ndrg1	_[Acetyl (Protein N-term)]S[Phospho (STY)]RELHDVDLAEVKPLVEK_.3	Q62433_S2_M1_Ndrg1	8.8626	9.3868	10.1264	10.2563	9.1200	9.3285	9.7611	9.6738	9.3650	9.2643	0.9997	-0.0004	0.9932	0.0030
Ndrg1	_SRT[Phospho (STY)]AS[Phospho (STY)]GSSVTSLEGTR_.2	#N/A	3.4448	3.9994	3.1785	4.2766	4.0893	4.5367	4.6648	4.0459	3.2763	4.9888	0.9239	-0.1142	0.4393	0.3572
Tle1	_DASGS[Phospho (STY)]PASTASSGSSSSLK_.2	Q62440_S284_M1_Tle1	3.3327	4.0890	5.8362	4.3266	3.9773	4.1482	5.1644	3.7918	3.4404	5.2727	0.9700	-0.0439	0.8203	0.0860
Tle4	_DAPIS[Phospho (STY)]PASVASSSSTPSSK_.2	Q62441_S292_M1_Tle4	9.3798	9.6001	10.1585	10.0835	8.6106	9.4587	9.9824	9.5579	9.2625	9.2818	1.0029	0.0041	0.9330	0.0301
Tle4	_SSSVS[Phospho (STY)]PSASFR_.2	Q62441_S208_M1_Tle4	9.0887	9.2980	9.0183	9.6505	8.6714	9.3710	9.7015	8.2625	9.2748	9.2537	1.0066	0.0094	0.8446	0.0733
Nptx1	_FIC[Carbamidomethyl (C)]TSVPVDADM[Oxidation (M)]C[Carbamidomethyl (C)]AASVAAGGAEELR_.3	#N/A	4.4270	4.4980	4.3880	4.9795	4.5539	4.5140	3.3009	4.9073	4.6716	5.0633	1.0166	0.0237	0.8310	0.0804
Fkbp3	_FLQDHGSDS[Phospho (STY)]FLAEHK_.3	Q62446_S36_M1_Fkbp3	4.9927	3.2495	5.0614	5.7596	5.1082	5.2271	4.3498	5.3381	5.1417	3.6167	1.0625	0.0874	0.6054	0.2179
Eif4g2	_TQT[Phospho (STY)]PPLGQTPQLGLK_.2	Q62448_T507_M1_Eif4g2	7.3706	2.9503	8.0057	9.4231	5.3559	3.4571	8.2584	8.4409	3.6306	6.4941	0.8635	-0.2118	0.6004	0.2216
Eif4g2	_LTEAVVTDYLNSGNANDAVSGVR_.2	#N/A	2.4410	4.1525	3.4247	4.7897	3.3180	3.3384	4.3754	4.5909	3.5922	3.1034	0.9137	-0.1303	0.5124	0.2904
Eif4g2	_FS[Phospho (STY)]PTMGR_.2	Q62448_S394_M1_Eif4g2	3.1055	4.1206	3.7923	4.2950	3.7320	3.9974	4.0097	3.8235	3.4721	4.2620	0.9868	-0.0191	0.8422	0.0746
Vat1	_VLLVPGPEKET_.2	#N/A	6.8509	8.6850	6.7208	6.5626	6.4321	6.5593	8.8019	8.4742	8.4330	8.4828	0.8152	-0.2947	0.0071	2.1460
Spen	_SQS[Phospho (STY)]PVHLR_.2	Q62504_S749_M1_Spen	6.3346	7.5510	8.0851	7.7982	6.8222	7.3463	8.0858	7.7554	7.4605	7.5607	0.9491	-0.0754	0.2908	0.5364
Spen	_DLEPGEVPS[Phospho (STY)]DS[Phospho (STY)]DEDAEHR_.2	Q62504_S1395_M2_Spen	4.3161	5.7337	4.0497	6.2856	3.4598	5.4667	6.5359	6.7599	6.2696	5.2572	0.7872	-0.3451	0.0660	1.1804
Spen	_S[Phospho (STY)]RSPNRFDAHDPR_.4	Q62504_S188_M1_Spen	4.3579	3.7491	4.2435	4.0134	3.4504	3.6259	3.7280	3.4520	4.0146	3.9804	1.0298	0.0423	0.6049	0.2183
Spen	_EAGPAAAS[Phospho (STY)]PQESESPQK_.2	Q62504_S2128_M1_Spen	4.0492	3.3535	3.7225	3.2663	3.1749	3.0538	3.4146	1.5080	4.0339	4.4444	1.0258	0.0368	0.8786	0.0562
Spen	_RPQS[Phospho (STY)]PGVS[Phospho (STY)]PAHSER_.3	Q62504_S758_M2_Spen	3.7955	3.0509	3.5292	4.5738	2.9392	3.6854	3.2212	3.9282	4.2273	4.2510	0.9203	-0.1198	0.4069	0.3905
Zyx	_S[Phospho (STY)]PGGPGPLTLK_.2	Q62523_S336_M1_Zyx	10.1984	10.0622	10.4204	10.6358	9.5657	10.2131	10.2406	9.6870	9.7983	10.2042	1.0200	0.0286	0.3812	0.4189
Ppp3r1	_DTQLQQIVDK_.2	#N/A	7.6802	7.0655	7.0012	7.2821	8.2856	7.5063	7.6580	7.0660	7.3484	7.5218	1.0097	0.0139	0.7922	0.1012
Ppp3r1	_VIDIFDTDGNGEVDFK_.2	#N/A	6.8441	5.4641	5.5000	6.4405	6.6619	6.4328	5.9347	5.7368	5.7665	6.7429	1.0296	0.0420	0.6297	0.2009
Ppp3r1	_IYDM[Oxidation (M)]DKDGYISNGELFQVLK_.3	#N/A	5.6190	4.6747	4.8561	5.6743	6.2900	5.6771	5.4377	4.7241	5.4087	5.7071	1.0274	0.0390	0.6856	0.1639
Ppp3r1	_EFIEGVSQFSVK_.2	#N/A	6.3350	5.7275	6.0907	6.2806	6.7562	6.1079	5.9697	5.6053	6.5162	6.3508	1.0173	0.0248	0.6665	0.1762
Ppp3r1	_ISFEEFC[Carbamidomethyl (C)]AVVGGLDIHK_.3	#N/A	3.3850	3.4125	4.0718	4.3136	4.0116	3.7179	4.0787	3.5440	3.1926	4.4670	0.9995	-0.0007	0.9953	0.0021
Ppp3r1	_KLDLDNSGSLSVEEFM[Oxidation (M)]SLPELQQNPLVQR_.3	#N/A	3.7173	3.0216	4.4041	3.5982	2.8430	3.3857	3.7465	3.4030	3.7020	3.3729	0.9828	-0.0250	0.8407	0.0754
Ppp3r1	_LDLDNSGSLSVEEFM[Oxidation (M)]SLPELQQNPLVQR_.3	#N/A	3.0379	4.3045	4.2724	3.8162	3.6825	3.6037	3.9645	3.1850	3.4840	3.4935	1.0721	0.1004	0.3770	0.4237
Ppp3r1	_TIINADKDGDGR_.3	#N/A	4.0483	3.1778	3.2343	3.7369	3.1740	3.0546	3.4516	4.3815	4.0301	3.7039	0.8747	-0.1931	0.0945	1.0245
Mapk3	_[Acetyl (Protein N-term)]AAAAAAPGGGGGEPR_.2	#N/A	7.6240	7.8302	7.9691	7.6210	7.8490	7.6609	8.1251	8.2543	7.8627	7.7811	0.9692	-0.0452	0.0630	1.2003
Mapk3	_IADPEHDHTGFLTEY[Phospho (STY)]VATR_.3	Q63844_Y205_M1_Mapk3	5.5799	6.0934	6.7456	7.4543	4.7897	6.3320	6.3278	6.8345	7.0536	6.4254	0.9258	-0.1113	0.3428	0.4650
Nup62	_ELEDLLS[Phospho (STY)]PLEESVK_.2	Q63850_S412_M1_Nup62	2.9267	3.6830	4.1612	3.7050	3.5713	3.0533	3.8533	3.2962	3.5952	3.3823	0.9958	-0.0061	0.9542	0.0203
Omg	_TLDISNNR_.2	#N/A	10.0746	9.9419	9.9947	10.4042	9.6756	9.8951	10.1595	10.0402	9.9224	10.1670	0.9926	-0.0107	0.5842	0.2335
Omg	_LESLPAQLPR_.2	#N/A	8.7676	8.8246	8.6157	8.7124	8.8226	8.7176	8.9621	8.7471	8.6144	8.7460	0.9973	-0.0040	0.7399	0.1308
Omg	_LWTVPTNM[Oxidation (M)]PSK_.2	#N/A	7.7304	8.2043	8.2715	7.7731	7.7894	8.0443	7.9701	8.1083	7.9936	8.1167	0.9903	-0.0141	0.5468	0.2622
Omg	_AHVIGTPC[Carbamidomethyl (C)]SK_.2	#N/A	6.4416	6.3056	6.6303	6.7331	7.3186	6.3938	6.1729	7.0801	6.5143	6.4556	1.0124	0.0178	0.7441	0.1284
Omg	_SDTAYQWNLK_.2	#N/A	6.6361	4.1554	6.2471	6.8972	6.7230	6.7707	3.9749	6.8513	6.5664	7.3037	1.0104	0.0149	0.9378	0.0279
Omg	_FTFIPEQSFDQLLQLQEITLHNNR_.3	#N/A	3.0431	4.9589	4.4088	5.4254	4.1372	4.3971	5.0491	4.9721	5.2027	4.8713	0.8749	-0.1929	0.1697	0.7703
Cavin2	_S[Phospho (STY)]SPFKVS[Phospho (STY)]PLSFGR_.2	Q63918_S287_M2_Cavin2	3.2909	3.6869	4.2200	5.0889	3.9287	3.0498	4.4438	3.3178	4.1308	3.3178	1.0197	0.0282	0.8675	0.0617
Map2k2	_LKQPST[Phospho (STY)]PTR_.2	Q63932_T395_M1_Map2k2	11.9508	12.0490	12.4670	12.3516	4.2061	12.0802	12.1554	12.0457	12.0760	12.0929	0.8973	-0.1563	0.4770	0.3215
Mef2d	_SEPVS[Phospho (STY)]PSRER_.2	Q63943_S437_M1_Mef2d	7.9768	8.8236	4.0411	3.3520	8.5650	8.6330	3.0667	8.9309	8.4133	8.5812	0.9518	-0.0713	0.8414	0.0750
Kcnc3	_LAPLATPPGS[Phospho (STY)]PR_.2	Q63959_S754_M1_Kcnc3	3.2837	8.6275	3.8698	8.6965	3.9282	7.5654	8.1497	8.1321	7.8600	8.7253	0.7296	-0.4548	0.1298	0.8869
Rab34	_INS[Phospho (STY)]DDKNLYLTASK_.2	Q64008_S241_M1_Rab34	3.6423	4.3985	3.3760	4.4206	2.7860	3.8386	4.9786	4.0815	4.2258	4.0978	0.8614	-0.2152	0.1340	0.8729
Crk	_QEAVALLQGQR_.2	#N/A	6.5782	6.1886	6.1357	6.8549	6.8954	5.9155	6.3096	5.5547	6.3547	6.6701	1.0331	0.0469	0.4827	0.3164
Crk	_QGS[Phospho (STY)]GVILR_.2	Q64010_S125_M1_Crk	5.2403	4.8758	3.4028	5.6973	3.3425	5.2388	5.1002	4.2131	3.8616	5.8496	0.9741	-0.0379	0.8488	0.0712
Crk	_ALFDFNGNDEEDLPFK_.2	#N/A	5.1394	5.2303	4.4950	4.0388	5.4582	4.8718	5.1166	4.7368	5.6906	5.4422	0.9287	-0.1068	0.2673	0.5730
Crk	_DSS[Phospho (STY)]TSPGDYVLSVSENSR_.2	Q64010_S41_M1_Crk	5.2909	11.3834	10.5084	11.4307	11.0825	10.7645	11.0746	10.7528	10.9242	10.7523	0.9265	-0.1101	0.5280	0.2773
Crk	_LLDQQNPDEDFS_.2	#N/A	3.8936	5.0694	5.7882	3.5822	5.1910	5.1453	3.2969	3.0354	4.9117	4.8902	1.1846	0.2444	0.2419	0.6164
Crk	_IHYLDTTTLIEPVAR_.3	#N/A	5.2456	5.3443	5.0546	5.8542	5.5709	6.0363	5.6620	6.1498	6.0042	5.7396	0.9370	-0.0939	0.1172	0.9309
Raly	_GRLS[Phospho (STY)]PVPVPR_.2	#N/A	7.6651	9.3770	9.5175	10.2027	7.5916	7.9244	8.9560	9.1401	8.4105	8.0336	1.0090	0.0130	0.9008	0.0454
Raly	_LPAPQEDTAS[Phospho (STY)]EAGTPQGEVQTR_.3	#N/A	8.6385	8.9455	9.1372	9.3405	8.3175	8.9428	9.0870	8.8085	8.6182	8.8014	1.0607	0.0850	0.2956	0.5293

Raly	_LS[Phospho (STY)]PVPVPR_.2	#N/A	4.3530	3.6084	4.3046	4.8960	4.0875	3.1279	4.5219	3.3113	4.0113	3.2734	1.0750	0.1043	0.4936	0.3066
Tfe3	_RS[Phospho (STY)]SFSMEEES_.2	Q64092_S564_M1_Tfe3	4.3453	3.6496	3.5312	6.1251	3.4710	4.2589	3.1188	4.0309	4.3300	4.0980	1.0862	0.1193	0.5602	0.2517
Mcf2l	_TSS[Phospho (STY)]TGEEEESLAILR_.2	Q64096_S621_M1_Mcf2l	4.3015	3.6059	3.4875	7.8946	3.4272	4.3023	3.1622	7.0253	3.7769	3.9571	1.0051	0.0073	0.9841	0.0070
Mcf2l	_QLC[Carbamidomethyl (C)]PEPAEILS[Phospho (STY)]PEPV_.2	Q64096_S1145_M1_Mcf2l	3.6157	4.4209	4.3025	5.5634	4.6588	3.4873	3.8481	5.4824	2.9619	3.2713	1.1158	0.1581	0.4656	0.3320
Mcf2l	_SSS[Phospho (STY)]AQC[Carbamidomethyl (C)]LSSSGK_.2	Q64096_S1120_M1_Mcf2l	3.1296	4.0965	3.8164	7.7026	3.9848	4.1406	4.0338	3.7994	7.0300	4.2861	0.9355	-0.0962	0.7698	0.1136
Arl13b	_EALSQQLDS[Phospho (STY)]EDEQDQR_.2	Q640N2_S328_M1_Arl13b	3.3862	7.2326	3.1199	7.6131	6.4589	7.5707	7.8171	7.4448	7.5864	3.8418	0.8838	-0.1783	0.5682	0.2455
Hepacam	_THTS[Phospho (STY)]PPR_.2	#N/A	9.6779	3.5542	9.4864	9.3627	7.8457	3.1821	9.6651	9.5909	3.7240	9.3613	0.8886	-0.1703	0.6528	0.1852
Hepacam	_QNS[Phospho (STY)]LEYM[Oxidation (M)]DQNDDR_.2	#N/A	9.3281	9.0881	9.4370	8.6300	8.1921	8.8252	9.7135	9.2426	9.1961	9.1258	0.9568	-0.0637	0.1601	0.7956
Hepacam	_STTEPGPPGYSVS[Phospho (STY)]PPVPGR_.2	Q640R3_S345_M1_Hepacam	6.0735	7.4841	7.7532	9.0599	3.6637	7.5202	6.8958	8.0675	7.5589	3.3052	1.0726	0.1011	0.7225	0.1411
Hepacam	_SPARS[Phospho (STY)]PATGR_.2	Q640R3_S370_M1_Hepacam	6.3508	3.4106	6.3506	4.0867	3.3134	3.9447	6.5866	3.7919	4.0909	6.2255	0.8845	-0.1771	0.5320	0.2741
Hepacam	_THTS[Phospho (STY)]PPRAPSS[Phospho (STY)]PGR_.2	#N/A	11.6233	11.8106	4.1643	11.9622	11.2433	11.8541	12.3517	12.1825	11.9815	11.9307	0.8622	-0.2139	0.3209	0.4937
Hepacam	_QNS[Phospho (STY)]LEYM[Oxidation (M)]DQNDDRLK_.3	#N/A	8.8299	9.2727	10.0302	7.6163	8.0586	7.5004	9.6505	9.4152	9.3390	7.4571	0.9538	-0.0682	0.5414	0.2665
Hepacam	_DKDSS[Phospho (STY)]EPDENPATEPR_.2	Q640R3_S321_M1_Hepacam	3.4987	6.6957	7.2215	6.8033	6.1294	6.5160	4.0382	7.1015	3.4797	6.3689	1.1709	0.2277	0.3853	0.4142
Hepacam	_SEADTLPRS[Phospho (STY)]GEQER_.2	#N/A	3.3772	4.2267	5.7494	4.5779	4.1600	4.4870	5.1432	3.7082	3.3568	3.9180	1.0988	0.1359	0.4487	0.3481
Hepacam	_S[Phospho (STY)]PGLPIR_.2	Q640R3_S352_M1_Hepacam	3.5685	3.7739	11.4545	4.3468	4.2131	3.9124	4.4951	4.1552	2.9534	4.0241	1.3339	0.4157	0.4355	0.3610
Spr	_ETSKDPELR_.2	#N/A	5.9631	5.5013	3.1247	5.9989	5.9884	6.4067	5.5898	5.6169	5.3810	5.6729	0.9878	-0.0177	0.9144	0.0388
Spr	_ALAPQLAR_.2	#N/A	5.4261	5.1198	5.2969	4.0712	6.5136	3.8936	4.6262	5.4531	5.8886	5.4554	0.9436	-0.0838	0.5870	0.2313
Spr	_MEADGLGC[Carbamidomethyl (C)]AVC[Carbamidomethyl (C)]VLTGASR_.2	#N/A	5.0665	3.9626	5.6621	5.5031	4.9539	5.7399	4.8318	5.5934	5.4325	6.1333	0.9364	-0.0948	0.4058	0.3916
Spr	_LLLINNAATLGDVSK_.2	#N/A	3.6045	4.3534	4.7944	4.7711	4.9152	4.8885	3.5918	3.2482	4.6402	4.3211	1.1529	0.2053	0.1342	0.8723
Spr	_DM[Oxidation (M)]LYQVLAAEEPSVR_.2	#N/A	6.2432	6.5265	6.6195	6.2241	6.3020	5.9998	6.9438	6.4782	6.2882	6.5339	0.9631	-0.0542	0.1642	0.7846
Spr	_LKS[Phospho (STY)]DGALVDC[Carbamidomethyl (C)]GTSAQK_.2	Q64105_S228_M1_Spr	5.3309	5.6308	4.5954	3.5972	4.9274	5.3023	5.8103	5.0277	5.1341	3.5642	1.0027	0.0039	0.9806	0.0085
Trim24	_SEWSDASQKS[Phospho (STY)]PVHVGETR_.3	Q64127_S812_M1_Trim24	4.1644	4.2303	3.3504	3.8530	3.2901	2.9385	3.5677	5.1350	3.9140	4.7513	0.8378	-0.2553	0.1099	0.9589
Trim24	_HEAEDC[Carbamidomethyl (C)]KFS[Phospho (STY)]DDS[Phospho (STY)]DDDFVQPR_.3	Q64127_S1026_M2_Trim24	2.9841	4.3583	4.2186	3.7624	3.6286	2.9960	3.9106	3.2388	3.5379	3.4396	1.0357	0.0507	0.7021	0.1536
Maoa	_EIPVDAPWQAR_.2	#N/A	6.6101	6.4977	5.8664	6.4966	6.1553	6.3048	6.5609	6.7425	5.6684	6.5547	0.9906	-0.0136	0.8074	0.0929
Maoa	_DVPALEITHTFLER_.2	#N/A	5.8303	4.9201	5.5166	5.5648	4.2004	3.6129	5.6487	5.8493	5.5326	4.8627	0.9027	-0.1477	0.2969	0.5274
Maoa	_VLGSQEALS[Phospho (STY)]PVHYEEK_.2	#N/A	4.4340	3.8467	4.0638	3.9603	3.2426	4.1433	4.2811	3.8551	3.2060	4.0359	1.0270	0.0385	0.7134	0.1467
Maoa	_IYFAGTETATQWSGYM[Oxidation (M)]EGAVEAGER_.3	#N/A	3.2986	3.9275	3.9854	4.4881	3.9252	3.8043	4.2028	3.6304	3.2790	2.9543	1.1104	0.1511	0.2131	0.6714
Agtpbp1	_VTS[Phospho (STY)]PTTYVLDEDEPR_.2	Q641K1_S1160_M1_Agtpbp1	4.0259	3.3165	3.7596	6.7945	3.1696	3.4550	3.4516	3.6979	6.7870	2.9806	0.9663	-0.0494	0.8875	0.0518
Nuak1	_ENDFPQSGQDSVPES[Phospho (STY)]PSK_.2	Q641K5_S389_M1_Nuak1	7.3476	7.0057	3.2433	7.9705	4.1542	3.9712	7.7662	7.4903	7.5635	7.7511	0.7347	-0.4447	0.0886	1.0528
Nuak1	_SSVC[Carbamidomethyl (C)]DC[Carbamidomethyl (C)]DALPDSES[Phospho (STY)]PLLAR_.2	Q641K5_S326_M1_Nuak1	4.0373	4.1612	3.2233	5.5372	4.9396	3.0657	5.4235	5.1005	5.8901	5.6000	0.7560	-0.4035	0.0292	1.5352
Nuak1	_TAIPLPSS[Phospho (STY)]PEADM[Oxidation (M)]SGK_.2	#N/A	3.7919	3.4128	4.1243	5.9734	3.5343	3.2113	4.1161	3.3368	3.4228	3.3453	1.1273	0.1729	0.4246	0.3720
Actr3b	_LSQELSGGR_.2	#N/A	3.6330	4.0364	3.9181	4.0842	3.8372	3.8717	4.2325	4.9130	3.2160	3.5875	0.9773	-0.0331	0.7752	0.1106
Actr3b	_TLTGIVIDSGDGVTHVIPVAEGYVIGSC[Carbamidomethyl (C)]IK_.3	#N/A	2.9019	3.6582	4.1365	3.6802	3.5465	3.0781	4.4721	3.3209	3.6200	1.9083	1.0510	0.0718	0.7321	0.1354
Sf1	_TGDLGIPPNPEDRSPSPEPIY[Phospho (STY)]NS[Phospho (STY)]EGKR_.4	#N/A	3.6354	3.8623	4.0899	7.9435	6.3555	3.6239	4.1533	8.8843	7.0416	5.5613	0.7673	-0.3822	0.2570	0.5901
Sf1	_TGDLGIPPNPEDRSPSPEPIY[Phospho (STY)]NS[Phospho (STY)]EGK_.3	#N/A	3.3190	4.0234	4.5535	4.0973	3.9636	4.1619	4.2456	5.9608	3.2029	3.7746	0.9357	-0.0959	0.6047	0.2184
Zeb1	_S[Phospho (STY)]C[Carbamidomethyl (C)]TSS[Phospho (STY)]PSPLNLC[Carbamidomethyl (C)]SAR_.2	Q64318_S682_M2_Zeb1	3.1550	3.8758	4.1789	4.1245	3.7918	4.1513	4.0719	4.1987	4.9333	4.6638	0.8685	-0.2034	0.0496	1.3043
Npdc1	_GPTS[Phospho (STY)]PSTPR_.2	Q64322_S236_M1_Npdc1	9.9832	10.0073	10.3528	9.8826	3.3897	9.8595	10.3525	10.3452	9.9799	9.9928	0.8766	-0.1901	0.3917	0.4071
Npdc1	_IS[Phospho (STY)]PGDQR_.2	Q64322_S243_M1_Npdc1	8.3397	8.3017	7.9204	8.2482	7.1744	7.9751	8.0316	8.1143	8.2071	8.0295	0.9874	-0.0184	0.6621	0.1791
Npdc1	_GPTS[Phospho (STY)]PST[Phospho (STY)]PRIS[Phospho (STY)]PGDQR_.2	Q64322_S236_M3_Npdc1	5.8648	3.1788	3.9058	3.6904	3.0002	6.7221	3.6308	3.9585	3.8558	3.5247	1.1740	0.2315	0.4313	0.3653
Syn2	_VLLVVDEPHTDWAK_.2	#N/A	8.1415	7.8928	8.4620	8.1143	8.0803	8.3326	8.2920	8.0276	8.0697	8.2470	1.0014	0.0020	0.9224	0.0351
Syn2	_FPLIEQTYYPNHR_.3	#N/A	8.3970	7.7362	7.5120	8.0648	7.8876	8.1298	7.5829	7.6724	8.0924	8.4481	1.0007	0.0010	0.9805	0.0085
Syn2	_TLGGEKFPLIEQTYYPNHR_.3	#N/A	8.6688	8.9161	8.9986	9.0699	8.8004	9.0924	8.9473	8.8714	8.8257	8.9463	1.0030	0.0043	0.7676	0.1149
Syn2	_QLITDLVISK_.2	#N/A	7.9680	7.8877	8.0304	8.2904	8.0543	8.4468	7.7178	8.1873	8.2254	7.9978	1.0101	0.0145	0.5843	0.2334
Syn2	_ILGDYDIK_.2	#N/A	8.1743	7.7191	7.8364	7.6644	7.8866	7.3843	7.4559	8.1267	7.8129	8.1832	0.9852	-0.0216	0.5508	0.2590
Syn2	_SFRPDFVLIR_.2	#N/A	7.3528	7.6116	7.3303	7.6834	7.2147	7.6333	7.3006	7.5427	7.5821	7.3385	1.0040	0.0058	0.7995	0.0972
Syn2	_EM[Oxidation (M)]LTLPTFPVVVK_.2	#N/A	9.0817	9.2283	9.4185	8.9818	9.1717	9.2518	9.1952	9.1960	9.1314	9.1916	1.0011	0.0016	0.8968	0.0473
Syn2	_M[Oxidation (M)]NQLLSR_.2	#N/A	6.9533	7.6676	7.3897	7.5903	8.1320	7.5706	7.0474	7.8059	7.7007	7.4024	1.0082	0.0118	0.8023	0.0957
Syn2	_QTAASAGLVDAPAPSAASR_.2	#N/A	7.4950	7.8026	7.9231	7.7571	7.7462	8.0518	7.8846	7.5399	7.9202	7.6192	1.0071	0.0102	0.6631	0.1784
Syn2	_KSFASLFSD_.2	#N/A	5.1464	4.9367	5.2934	6.4133	5.3920	5.5749	4.9967	6.3813	6.2488	6.3512	0.9107	-0.1349	0.1888	0.7240
Syn2	_TNTGSAM[Oxidation (M)]LEQIAM[Oxidation (M)]SDR_.2	#N/A	7.6798	8.8251	8.2379	8.4595	7.8170	7.9355	7.9841	7.8883	8.1100	7.8352	1.0257	0.0367	0.3914	0.4074
Syn2	_T[Phospho (STY)]PALS[Phospho (STY)]PQRPLTTQQPQSGTLK_.3	#N/A	5.3196	6.3320	3.3724	7.2655	4.8786	5.5310	5.5772	5.8690	6.0824	5.2174	0.9584	-0.0613	0.7407	0.1303
Syn2	_VEQAEFSELNLVAHADGTYAVDM[Oxidation (M)]QVLR_.3	#N/A	4.3203	5.0638	5.3676	6.0446	4.4633	4.4567	3.2358	5.1948	4.9227	5.4620	1.0529	0.0744	0.6477	0.1886
Syn2	_TPALS[Phospho (STY)]PQRPLTTQQPQSGTLKEPDSSK_.4	#N/A	6.5798	7.1911	5.6803	8.3824	6.2154	6.4149	6.4878	7.1850	6.7501	6.5189	1.0013	0.0018	0.9868	0.0058
Syn2	_SS[Phospho (STY)]ANEDEAKAETIR_.2	#N/A	6.1296	6.1299	6.0575	6.5843	5.6008	6.3682	5.9525	6.0955	5.1771	6.2361	1.0477	0.0672	0.2991	0.5242
Syn2	_LWVDAC[Carbamidomethyl (C)]SEM[Oxidation (M)]FGGLDIC[Carbamidomethyl (C)]AVK_.2	#N/A	4.8575	4.0439	3.1863	6.0114	4.3735	4.4302	4.5146	4.5237	4.4145	3.9082	1.0331	0.0469	0.7773	0.1094
Syn2	_QHAFGM[Oxidation (M)]AENEDFR_.3	#N/A	5.6121	5.2965	5.3533	5.8052	5.6346	5.8946	5.2960	5.2655	5.5105	5.7136	1.0281	0.0400	0.3280	0.4842
Syn2	_TSISGNWK_.2	#N/A	7.2979	7.6253	7.4564	7.2663	8.0339	7.7354	6.9909	7.5246	7.6898	7.4124	1.0223	0.0318	0.4106	0.3866
Syn2	_DGKDYIFEVM[Oxidation (M)]DC[Carbamidomethyl (C)]SM[Oxidation (M)]PLIGEHQVEDR_.4	#N/A	5.8924	6.5610	4.0478	6.5311	5.2272	6.1940	6.1141	6.3269	6.5241	6.3489	0.9074	-0.1402	0.2720	0.5655
Syn2	_SQS[Phospho (STY)]LTNAFSFSESSFFR_.2	Q64332_S546_M1_Syn2	4.3569	4.1984	4.3551	6.5677	3.7651	3.9259	4.6441	3.1155	3.4146	4.2573	1.1737	0.2311	0.2926	0.5337
Syn2	_VENHYDFQDIASVVALTQTYATAEPFIDAK_.3	#N/A	3.2474	0.5506	3.9342	4.4368	3.8671	4.2584	4.1515	3.6816	3.3302	4.4038	0.8691	-0.2024	0.5226	0.2819
Tbr1	_S[Phospho (STY)]PPQYC[Carbamidomethyl (C)]GAK_.2	Q64336_S549_M1_Tbr1	8.4329	7.7083	7.8684	8.9659	8.1898	8.3275	8.6698	7.9684	8.3111	8.3758	0.9901	-0.0144	0.7541	0.1225
Tbr1	_LTPS[Phospho (STY)]PNDSPR_.2	Q64336_S410_M1_Tbr1	4.3460	8.3769	9.0072	9.3112	3.4717	8.9331	3.7493	8.4353	3.7325	4.0016	1.4541	0.5401	0.1992	0.7008
Tbr1	_SIDS[Phospho (STY)]SDSGIYEQAK_.2	Q64336_S625_M1_Tbr1	3.4857	4.2909	4.1725	3.1744	4.1123	3.6172	4.3899	3.4433	3.0919	3.1414	1.0831	0.1152	0.4015	0.3963
Tbr1	_RIS[Phospho (STY)]PADTPVSESSSPLK_.3	Q64336_S640_M1_Tbr1	3.6989	4.0647	3.4325	4.4772	2.8426	3.7820	3.1246	4.0249	4.3239	4.1544	0.9512	-0.0722	0.6052	0.2181
Tbr1	_SKLS[Phospho (STY)]PVLDGVS[Phospho (STY)]ELRHSFDGSAADR_.4	Q64336_S84_M2_Tbr1	3.5901	4.3953	4.2769	3.2787	4.2167	3.5128	4.4943	3.3389	2.9875	3.2457	1.1029	0.1413	0.3414	0.4667
Pde1c	_RSS[Phospho (STY)]LNSINSSDAKR_.3	Q64338_S469_M1_Pde1c	6.3617	6.7377	6.8638	6.3477	6.2025	3.7386	6.0347	3.5647	6.7094	3.0207	1.2503	0.3223	0.2288	0.6406
Pde1c	_[Acetyl (Protein N-term)]MES[Phospho (STY)]PTKEIEEFESNSLK_.3	Q64338_S3_M1_Pde1c	3.8614	3.1657	4.5482	3.4541	2.9870	3.2416	3.6024	3.5470	3.8461	3.1314	1.0032	0.0046	0.9725	0.0121
Aktip	_TLAGDVKTS[Phospho (STY)]PPR_.2	Q64362_S30_M1_Aktip	8.3951	8.5306	9.2064	8.7570	3.3493	8.7411	9.3033	4.3001	8.4983	8.3358	1.0290	0.0412	0.8818	0.0546
Atp7a	_VSIASEVESTASS[Phospho (STY)]PSSSSLQK_.2	Q64430_S357_M1_Atp7a	3.7826	3.0381	3.5163	4.6242	2.9264	3.6982	3.5914	4.2418	3.8903	3.8437	0.9244	-0.1134	0.3979	0.4002
Hspe1	_VLLPEYGGTK_.2	#N/A	10.4269	10.7317	10.9818	10.7316	11.1102	10.7024	10.3876	10.6159	10.5292	10.5953	1.0236	0.0337	0.0889	1.0509
Hspe1	_FLPLFDR_.2	#N/A	9.0383	9.4296	9.1633	8.9615	9.2967	9.4533	9.0912	9.1945	9.4015	9.0530	1.0042	0.0061	0.7568	0.1210
Hspe1	_KFLPLFDR_.2	#N/A	9.0016	9.4890	9.2807	9.3669	9.2543	9.2897	9.0780	9.1712	9.2308	9.0702	1.0156	0.0224	0.1417	0.8486
Hspe1	_SGEIEPVSVK_.2	#N/A	8.8755	8.8349	9.0744	9.1564	9.4512	9.0487	8.5551	8.8547	8.7041	8.9682	1.0345	0.0490	0.0532	1.2739
Hspe1	_VVLDDKDYFLFR_.2	#N/A	7.2533	7.5291	7.3310	7.4493	7.7673	7.4005	6.9447	7.2463	7.1381	7.5181	1.0337	0.0479	0.1023	0.9901
Hspe1	_VLQATVVAVGSGGK_.2	#N/A	7.4930	7.8199	7.9067	8.2162	8.0144	7.9478	7.4599	7.6895	8.0229	7.8881	1.0173	0.0248	0.4139	0.3831
Hspe1	_GKSGEIEPVSVK_.2	#N/A	5.1606	5.2038	4.3668	4.5959	5.5307	5.3263	5.1066	4.3565	5.4233	5.7477	0.9752	-0.0362	0.7077	0.1501
Hspe1	_GGIM[Oxidation (M)]LPEK_.2	#N/A	6.8592	7.3895	7.4662	7.0056	7.7132	7.0361	6.3752	7.3976	6.9675	7.0484	1.0429	0.0605	0.2446	0.6115
Ptprd	_KSS[Phospho (STY)]LPNSK_.2	Q64487_S1303_M1_Ptprd	8.5501	4.2401	4.1217	8.5987	4.0614	8.2790	8.8931	9.0151	8.6676	8.4405	0.7206	-0.4727	0.0794	1.1001
Ptprd	_DFLPVDTSNNNGR_.2	#N/A	7.6269	7.3286	7.7879	8.6267	7.0156	7.5023	7.6885	7.4893	7.2038	7.3048	1.0305	0.0434	0.4605	0.3367

Ptprd	_YANVIAYDHSR_.2	#N/A	5.2464	4.9091	4.6959	4.9744	4.4951	4.7079	5.3598	5.1701	5.1661	4.4068	0.9627	-0.0549	0.4066	0.3908
Ptprd	_GALQIEQSEESDQGKYEC[Carbamidomethyl (C)]VATNSAGTR_.3	#N/A	4.3612	3.2172	3.4240	2.5088	3.4734	4.2561	4.1006	4.0334	4.3324	4.1459	0.8524	-0.2304	0.1227	0.9113
Top2b	_ASGS[Phospho (STY)]ENEGDYNPGR_.2	Q64511_S1539_M1_Top2b	6.2054	3.4614	4.0735	3.3554	6.5393	7.5539	7.3558	3.4657	3.4775	6.5874	0.9955	-0.0065	0.9851	0.0065
Top2b	_KAS[Phospho (STY)]GS[Phospho (STY)]ENEGDYNPGR_.2	Q64511_S1537_M2_Top2b	6.2319	6.5863	6.7047	6.3026	5.3992	4.9062	6.6241	6.5445	6.3907	6.2773	0.9323	-0.1011	0.2698	0.5690
Top2b	_YFAES[Phospho (STY)]DEEEDVDFAM[Oxidation (M)]FN_.2	Q64511_S1600_M1_Top2b	4.3069	3.6113	3.4929	4.5094	7.2890	4.2969	3.1568	3.9926	6.8002	3.9625	1.0237	0.0339	0.9135	0.0393
Top2b	_IVETINS[Phospho (STY)]DS[Phospho (STY)]DSEFGIPKK_.2	#N/A	3.7039	3.6884	3.6744	4.1079	3.5306	3.9021	3.7033	6.0655	4.0616	3.8366	0.8531	-0.2292	0.1879	0.7261
Top2b	_IVETINSDS[Phospho (STY)]DSEFGIPK_.2	#N/A	4.0100	3.5461	3.5548	6.5255	3.6533	3.9552	3.8360	7.2134	3.7708	3.6973	0.9089	-0.1379	0.6518	0.1859
Top2b	_KIVETINS[Phospho (STY)]DS[Phospho (STY)]DSEFGIPKK_.3	#N/A	2.9505	4.2757	3.6373	4.1399	4.1640	3.9615	3.8546	3.9785	3.6271	4.1069	0.9905	-0.0138	0.8927	0.0493
Top2b	_KTSFDQDS[Phospho (STY)]DVDIFPSDFTSEPPALPR_.3	Q64511_S1568_M1_Top2b	3.0441	3.8003	4.2786	3.8223	3.6886	2.9360	4.3300	3.1788	3.4779	3.4996	0.9927	-0.0106	0.9370	0.0283
Top2b	_KIVETINS[Phospho (STY)]DS[Phospho (STY)]DSEFGIPK_.2	#N/A	3.5205	3.9787	4.0418	3.8394	3.6841	3.9321	3.8575	3.6380	3.6345	3.6196	1.0394	0.0558	0.2238	0.6502
Top2b	_AS[Phospho (STY)]PITNDGEDEFVPSDGLDKDEYAFSSGK_.3	#N/A	4.1988	4.6419	5.8140	5.7531	4.3076	4.4114	5.6412	5.1119	4.7157	3.8545	1.0049	0.0070	0.9618	0.0169
Top2b	_VKAS[Phospho (STY)]PITNDGEDEFVPS[Phospho (STY)]DGLDKDEYAFSSGK_.4	Q64511_S1387_M2_Top2b	3.5349	3.5960	4.0743	4.3132	3.4843	3.9949	3.7663	3.5426	3.8417	3.9904	1.0126	0.0181	0.8073	0.0930
Sdc3	_KKDEGS[Phospho (STY)]YTLEEPK_.3	Q64519_S418_M1_Sdc3	4.0495	3.1767	3.2355	3.7381	3.0650	3.5596	3.4528	4.3804	4.0289	3.7051	0.8918	-0.1652	0.1314	0.8813
Sdc3	_QAS[Phospho (STY)]VTYQKPDKQEEFYA_.3	Q64519_S428_M1_Sdc3	2.9238	4.3024	3.6106	4.1132	4.1907	3.9347	3.8279	4.0053	3.6538	4.0802	0.9882	-0.0171	0.8702	0.0604
Guk1	_IC[Carbamidomethyl (C)]VLDVDLQGVR_.2	#N/A	4.3118	3.9039	4.3316	4.9916	4.7859	4.3451	5.2665	4.3522	4.5128	4.4054	0.9592	-0.0602	0.4869	0.3125
Gpd2	_NYLSSDVEVR_.2	#N/A	6.0447	6.1747	6.3587	6.5601	6.1773	5.9816	5.9894	6.4785	6.4405	6.0783	0.9951	-0.0071	0.8402	0.0756
Gpd2	_ELNWSELR_.2	#N/A	6.4818	6.6007	6.6573	6.2382	5.9953	6.3250	6.6847	6.4454	6.3125	6.9086	0.9689	-0.0456	0.2467	0.6078
Gpd2	_TVGLFLQGGK_.2	#N/A	6.0014	6.2317	4.4571	6.0219	5.7918	5.7426	5.3580	6.3107	6.2057	6.2941	0.9447	-0.0821	0.3955	0.4029
Gpd2	_LAFLNVQAAEEALPR_.2	#N/A	5.8327	5.7485	5.4207	5.1818	5.6888	5.9077	5.0439	5.7612	5.8510	5.8842	0.9991	-0.0013	0.9815	0.0081
Gpd2	_KQEELETATR_.2	#N/A	5.3373	4.3467	5.1704	5.5322	5.7890	5.5709	5.4802	5.7928	5.1563	5.0888	0.9836	-0.0239	0.7678	0.1148
Gpd2	_FHNLNAGPSR_.2	#N/A	6.3795	6.9878	6.6777	6.8093	7.3225	7.0145	6.6932	6.8549	7.0977	6.8993	0.9969	-0.0044	0.9083	0.0418
Gpd2	_DWSPTLYIR_.2	#N/A	5.7757	5.5715	5.9317	5.8558	5.2043	5.5578	5.3518	6.5330	4.9497	5.8674	0.9954	-0.0066	0.9336	0.0298
Gpd2	_TEQLTDSTEISLLPSDIDR_.2	#N/A	6.4589	6.7451	6.1794	7.5364	6.4099	6.5107	6.5350	6.7949	6.9317	6.4861	0.9930	-0.0101	0.8596	0.0657
Gpd2	_LVQDYGLESEVAQHLAK_.2	#N/A	4.4284	4.8021	5.2430	5.3174	4.9405	5.0851	4.7155	5.2461	4.3002	5.0027	1.0318	0.0452	0.5260	0.2790
Gpd2	_YGAATANYM[Oxidation (M)]EVVSLLK_.2	#N/A	2.5811	3.4217	3.5462	3.7071	4.2550	3.6173	3.7636	4.1096	3.7182	3.6702	0.9230	-0.1157	0.3378	0.4713
Gpd2	_C[Carbamidomethyl (C)]VINASGPFTDSVR_.2	#N/A	3.4149	3.9275	3.1486	4.0066	4.9104	5.1638	5.9493	5.2096	5.0498	4.7754	0.7806	-0.3573	0.0350	1.4560
Gpd2	_M[Oxidation (M)]NLAIALTAAR_.2	#N/A	4.2162	4.7082	4.3022	3.9048	4.5212	4.5954	4.4796	6.0222	5.4415	5.2410	0.7977	-0.3261	0.0172	1.7648
Gpd2	_NTPEFDILVIGGGATGC[Carbamidomethyl (C)]GC[Carbamidomethyl (C)]ALDAVTR_.3	#N/A	4.2466	3.3292	3.2108	4.5220	3.1505	3.0781	4.7402	4.2576	4.3616	4.7179	0.7943	-0.3323	0.0239	1.6210
Gpd2	_AIM[Oxidation (M)]NLDVEQYR_.2	#N/A	4.0168	6.7676	4.0090	6.7977	6.6573	6.8777	3.7836	6.9525	6.6979	6.4969	0.9785	-0.0313	0.8941	0.0486
Gpd2	_TALVERDDFSSGTSSR_.3	#N/A	3.7822	3.0865	4.4690	3.5333	3.6604	3.3208	3.6816	3.4679	3.7669	3.2106	1.0312	0.0444	0.6842	0.1648
Gpd2	_LVGAIVYYDGQHNDAR_.2	#N/A	3.7425	3.6646	3.8833	2.5096	3.4991	3.5771	3.8662	3.6546	3.6526	3.8697	0.9252	-0.1122	0.3045	0.5164
Gpd2	_EYAC[Carbamidomethyl (C)]TAVDMISR_.2	#N/A	4.3893	3.6448	4.1230	3.6668	3.5331	4.7806	3.8151	3.3344	3.5374	3.3441	1.1391	0.1879	0.1057	0.9761
Gpd2	_M[Oxidation (M)]TIAGTTDTPTDVTHHPIPSEEDINFILNEVR_.4	#N/A	3.3320	4.0883	4.5666	4.3273	3.9766	4.1488	4.0420	4.3917	3.1899	3.7876	1.0572	0.0803	0.4725	0.3256
H2ac20;Hist2h2aa1	_AGLQFPVGR_.2	#N/A	11.1373	11.3524	11.4263	11.0541	10.9542	10.8909	11.2323	11.3304	11.0286	11.1674	0.9952	-0.0070	0.6671	0.1758
H2ac20;Hist2h2aa1	_HLQLAIR_.2	#N/A	10.3306	10.8746	10.8654	11.1579	11.0416	10.8968	10.8260	11.1619	10.8602	10.9636	0.9916	-0.0121	0.5745	0.2407
H2ac20;Hist2h2aa1	_VTIAQGGVLPNIQAVLLPK_.2	#N/A	8.7202	8.9513	9.3441	9.1517	9.0645	8.8255	8.9280	9.0231	8.8153	9.1526	1.0033	0.0048	0.8225	0.0848
H2ac20;Hist2h2aa1	_NDEELNKLLGK_.2	#N/A	7.2454	7.7295	8.3904	7.5485	7.6057	7.7203	7.6305	7.5986	7.6014	7.5228	1.0156	0.0223	0.5590	0.2526
H2ac20;Hist2h2aa1	_NDEELNK_.2	#N/A	3.1246	4.2924	3.2698	4.1232	4.1807	3.9447	3.8379	3.9953	3.6438	4.0902	0.9822	-0.0259	0.8015	0.0961
H2ac20;Hist2h2aa1	_KGNYAER_.2	#N/A	3.0818	3.8870	3.1891	4.2337	3.7083	4.0212	4.3820	2.9016	3.0665	3.9110	1.0341	0.0484	0.7468	0.1268
Srm	_YQDILVFR_.2	#N/A	4.6393	5.4496	4.4015	6.1382	5.0384	5.1980	5.6932	5.1665	5.2194	5.1110	0.9711	-0.0424	0.6565	0.1828
Srm	_AAFVLPEFTR_.2	#N/A	6.7922	7.2235	6.8847	7.2493	6.5972	7.1622	7.1189	7.2735	6.6834	6.7484	1.0041	0.0060	0.8747	0.0581
Srm	_VLIIGGGDGGVLR_.2	#N/A	5.5577	3.3134	4.5996	6.0098	5.2717	3.7502	5.6448	5.0810	5.1607	5.3058	0.8966	-0.1574	0.3476	0.4590
Rbl2	_AQPAGS[Phospho (STY)]PSHQIQQR_.2	Q64700_S37_M1_Rbl2	3.0375	4.3049	4.2720	3.8158	4.1263	3.6032	3.9640	3.1854	3.4845	3.4930	1.0929	0.1282	0.2731	0.5636
Zrsr1	_GREEDSS[Phospho (STY)]PGPQSQSHR_.3	#N/A	3.6300	3.6036	3.6556	4.1583	4.1427	3.9798	3.8730	4.0500	4.6263	4.4106	0.9108	-0.1348	0.0816	1.0885
Zrsr1	_ALEAPPEEDDDVS[Phospho (STY)]ANEELAER_.2	Q64707_S50_M1_Zrsr1	3.1185	3.9237	3.8053	7.2821	7.2152	3.9844	4.3452	6.7473	7.3146	6.2393	0.7933	-0.3340	0.2701	0.5685
Zrsr1	_GREEDSS[Phospho (STY)]PGPQSQSHRT_.3	#N/A	3.7646	3.5778	3.4983	4.5429	3.3991	4.3303	3.1903	3.9591	4.2582	4.2202	0.9860	-0.0204	0.8646	0.0632
Vcl	_DPNAS[Phospho (STY)]PGDAGEQAIR_.2	#N/A	8.9898	8.6028	8.6837	9.4344	8.3327	8.6141	9.2306	8.7535	8.7927	8.9511	0.9826	-0.0254	0.4880	0.3115
Vcl	_GWLRDPNAS[Phospho (STY)]PGDAGEQAIR_.3	#N/A	3.9388	6.0902	3.1247	8.0010	6.3524	5.8329	6.0491	7.4794	6.6392	6.7765	0.8249	-0.2777	0.2395	0.6207
Vcl	_GQGAS[Phospho (STY)]PVAMQK_.2	Q64727_S346_M1_Vcl	5.6887	3.4803	4.4327	7.2534	4.8829	3.3571	3.1492	3.1831	4.3325	3.4015	1.3789	0.4636	0.1260	0.8997
Sp9	_KGS[Phospho (STY)]DS[Phospho (STY)]DTDASNLETPR_.2	Q64HY3_S424_M2_Sp9	4.2788	3.8468	3.8640	5.9763	4.6047	3.3505	3.5561	4.6886	4.9273	4.9414	0.9540	-0.0679	0.7075	0.1503
Sp9	_SES[Phospho (STY)]PDLILHDSGVSAAR_.3	Q64HY3_S440_M1_Sp9	4.0944	3.3988	3.2804	3.7831	4.7874	3.0085	3.3693	4.3354	3.9840	4.4902	0.9211	-0.1186	0.4296	0.3669
Ctnna3	_TPEELEDVS[Phospho (STY)]DLEDDHEVR_.2	#N/A	4.1524	3.2027	3.6291	4.3043	3.2869	3.9803	3.5666	3.9572	3.8991	3.5110	1.0069	0.0099	0.9206	0.0359
Wapl	_TES[Phospho (STY)]PSESC[Carbamidomethyl (C)]PVK_.2	#N/A	7.3313	7.2435	7.9570	7.5176	3.1566	6.7810	7.7910	7.5011	7.4470	7.2400	0.8892	-0.1694	0.3847	0.4149
Wapl	_RTES[Phospho (STY)]PSESC[Carbamidomethyl (C)]PVK_.2	#N/A	3.6433	2.9477	4.3301	3.3319	4.2698	3.4597	3.8205	3.2857	4.4351	3.2989	0.9875	-0.0181	0.8983	0.0466
Eif3j2	Acetyl (Protein N-term)]AAAAAAAAAAAAGDS[Phospho (STY)]DSWDADTFSM[Oxidation (M)]EDPVRK_	Q66JS6_S16_M1_Eif3j2	3.5923	3.7429	6.5058	7.0799	3.6114	3.7365	3.8921	3.8285	3.8593	4.1105	1.2011	0.2644	0.3698	0.4321
Rbm12b2;Rbm12b1	_RVS[Phospho (STY)]VEDLR_.2	Q66JV4_S681_M1_Rbm12b2;Rbm12b1	6.7754	6.6828	7.7683	7.5402	5.9505	6.9751	7.5101	7.1802	7.0144	6.8595	0.9731	-0.0394	0.5977	0.2235
Rbm12b2;Rbm12b1	_S[Phospho (STY)]RS[Phospho (STY)]PLGFYVHLK_.3	Q66JV4_S277_M2_Rbm12b2;Rbm12b1	3.0951	3.9003	3.7819	4.9856	3.7217	4.0078	3.9993	4.7972	3.4825	4.2516	0.9474	-0.0779	0.5837	0.2338
Rbm12b2;Rbm12b1	_MRS[Phospho (STY)]EEHS[Phospho (STY)]PSR_.2	Q66JV4_S250_M2_Rbm12b2;Rbm12b1	4.1267	3.3821	3.8603	3.4041	3.2704	3.3542	3.2474	3.5971	3.8961	3.0814	1.0321	0.0455	0.6387	0.1947
Map3k10	_GSDGAS[Phospho (STY)]PPASPSIIPR_.2	#N/A	3.8777	7.7940	8.7497	8.4331	6.9852	8.1496	8.6284	8.2843	8.1033	7.9122	0.8906	-0.1671	0.3589	0.4450
Map3k10	_QWSSS[Phospho (STY)]APNLGK_.2	Q66L42_S583_M1_Map3k10	3.3768	4.1820	6.6200	4.5663	4.0034	3.7261	4.2810	3.5522	3.2008	3.0325	1.2547	0.3274	0.1918	0.7172
Map3k10	_KGSDGAS[Phospho (STY)]PPASPSIIPR_.2	#N/A	3.9703	7.1767	6.7083	3.4970	3.2829	3.3482	6.0813	6.7215	5.9659	6.3282	0.7433	-0.4279	0.1187	0.9254
Cbarp	_HAS[Phospho (STY)]LDGASPYFK_.2	#N/A	9.0882	9.0376	9.4908	9.5829	7.7289	9.1641	9.3785	9.4934	9.0557	9.3225	0.9681	-0.0468	0.4189	0.3779
Cbarp	_ASS[Phospho (STY)]LDTRGS[Phospho (STY)]PK_.2	#N/A	7.3698	6.7729	7.1708	2.8536	5.7579	7.0923	7.1814	4.2564	7.2249	7.2981	0.9506	-0.0731	0.7695	0.1138
Cbarp	_RDYS[Phospho (STY)]IDEK_.2	Q66L44_S537_M1_Cbarp	7.6308	3.6833	7.2798	7.3757	3.5047	6.8946	3.0848	4.0509	3.6995	7.6199	1.3138	0.3937	0.2882	0.5404
Cbarp	_GAGDEVSELPAPARS[Phospho (STY)]PPR_.3	#N/A	8.6697	7.9091	8.8297	8.2319	7.4370	8.0274	8.5155	8.4991	8.1589	8.4118	0.9747	-0.0369	0.4538	0.3432
Cbarp	_LAEPVASSQVLIVAAAAPTS[Phospho (STY)]PDHSPA_.3	Q66L44_S692_M1_Cbarp	3.6853	6.7770	4.2360	7.4152	3.5127	3.7172	3.9094	3.8101	3.7526	3.9526	1.2682	0.3428	0.2773	0.5571
Cbarp	_RHAS[Phospho (STY)]LDGASPYFK_.3	#N/A	5.3624	4.9160	6.3943	5.4067	3.2853	3.2675	4.6960	5.8480	5.3662	5.1029	0.9084	-0.1386	0.4920	0.3080
Cbarp	_GAGDEVSELPAPARS[Phospho (STY)]PPRS[Phospho (STY)]PR_.3	Q66L44_S520_M2_Cbarp	7.0584	8.2774	4.3091	7.9145	6.5719	8.1483	8.0977	7.8916	7.8552	8.3988	0.8742	-0.1940	0.2243	0.6491
Cbarp	_ASS[Phospho (STY)]LDTRGS[Phospho (STY)]PKR_.2	#N/A	6.6669	6.6994	7.0930	3.2267	5.9883	6.6618	6.6259	6.8190	6.5120	6.4604	0.9170	-0.1250	0.4759	0.3225
Cbarp	_LLQMDSGYAS[Phospho (STY)]IEGR_.2	#N/A	3.8083	3.9412	3.8228	5.3258	3.7625	3.3284	3.6743	3.9505	3.8330	3.5505	1.0656	0.0916	0.5086	0.2936
Cbarp	_RGDS[Phospho (STY)]VDC[Carbamidomethyl (C)]PPEGR_.2	Q66L44_S610_M1_Cbarp	6.9825	3.5536	3.5225	4.5671	2.9325	3.6921	3.2145	3.9349	4.2340	4.2444	1.0772	0.1072	0.7049	0.1519
Cbarp	GGAS[Phospho (STY)]PET[Phospho (STY)]PPEHGISLGPEHAQQQDPQQEQDAEHAQC[Carbamidomethyl	Q66L44_S389_M2_Cbarp	3.4207	4.1770	3.1544	4.2386	4.0653	4.0601	3.9533	3.8799	3.5284	4.2056	0.9900	-0.0146	0.8820	0.0545
Cbarp	_RVS[Phospho (STY)]FNEAALFEQSR_.3	Q66L44_S129_M1_Cbarp	3.5117	3.2448	4.1985	3.8038	4.1382	3.5913	3.9521	3.4173	3.0659	3.1673	1.1022	0.1403	0.1957	0.7083
Tusc1	_GHGREEPGS[Phospho (STY)]PR_.3	#N/A	3.4317	3.8111	4.9290	4.8816	6.8758	4.0020	5.6823	5.6519	4.5959	6.3192	0.8369	-0.2568	0.2269	0.6441
Bcl9l	_EAPGS[Phospho (STY)]PPLSPR_.2	Q67FY2_S21_M1_Bcl9l	6.9137	7.5840	3.5986	9.2524	8.5012	7.7908	8.9706	7.6385	7.6526	8.7535	0.8812	-0.1824	0.3752	0.4258
Bcl9l	_TAMPS[Phospho (STY)]PGVSQNK_.2	Q67FY2_S1014_M1_Bcl9l	3.1279	4.2145	4.3624	3.9062	4.0359	3.6936	4.0544	3.0950	3.3941	3.5834	1.1015	0.1394	0.2292	0.6397
Grm7	_TELC[Carbamidomethyl (C)]ENVDPNS[Phospho (STY)]PAAK_.2	Q68ED2_S900_M1_Grm7	10.2690	10.2338	10.9839	10.4093	9.2215	9.9836	10.8433	10.5087	10.3606	10.0342	0.9757	-0.0354	0.4559	0.3411
Crtc1	_RPLS[Phospho (STY)]VDKHGR_.3	#N/A	5.8570	4.8847	5.3194	5.3090	3.3318	6.5468	4.3793	2.9910	6.5066	3.1428	1.2240	0.2916	0.2932	0.5329
Crtc1	_RPLS[Phospho (STY)]VDK_.2	#N/A	4.6696	7.9942	4.2962	5.8560	4.9561	5.2231	4.1090	5.0441	5.5494	5.8753	1.0690	0.0962	0.6464	0.1895

Rab3ip	_TLVLSSSPTS[Phospho (STY)]PTQEPLAAAK_.2	Q68EF0_S218_M1_Rab3ip	6.0143	6.5854	7.2230	7.4931	5.3145	4.0741	6.6737	6.2422	7.2003	6.0274	0.9360	-0.0955	0.5574	0.2539
Begain	_AS[Phospho (STY)]PLYATYK_.2	Q68EF6_S473_M1_Begain	8.9186	8.5418	9.0046	8.9652	8.1282	8.6569	8.9836	8.8812	8.6331	8.6094	0.9915	-0.0123	0.7007	0.1544
Begain	_AGGDLSLS[Phospho (STY)]PSR_.2	Q68EF6_S510_M1_Begain	8.6976	8.5843	4.4001	3.4019	7.6625	8.2549	3.1166	8.6507	4.3651	8.5725	1.1064	0.1459	0.6985	0.1558
Begain	_VLEKPDPGS[Phospho (STY)]LSSR_.2	Q68EF6_S194_M1_Begain	3.3582	4.1634	4.0450	4.5477	7.0889	3.7447	4.2624	3.5708	3.2194	3.0139	1.2772	0.3530	0.2096	0.6787
Begain	_GDIYC[Carbamidomethyl (C)]S[Phospho (STY)]DPALYC[Carbamidomethyl (C)]PDER_.2	Q68EF6_S229_M1_Begain	8.7842	9.6423	8.5420	10.6116	9.2764	9.4606	9.9484	10.0032	9.9572	10.0622	0.9393	-0.0903	0.1423	0.8467
Begain	_YYGGGGGGGAAGGGS[Phospho (STY)]PGDKAEGR_.3	#N/A	5.4158	4.0709	3.9496	4.4523	4.1047	4.4163	4.8214	4.9538	5.2015	5.6854	0.8521	-0.2309	0.0400	1.3980
Begain	_KPSATYGS[Phospho (STY)]GPR_.2	Q68EF6_S344_M1_Begain	5.4158	3.0162	4.3987	5.3298	2.8375	3.3911	3.7519	6.3034	3.6966	3.3675	0.9498	-0.0744	0.7937	0.1003
Begain	_AQNST[Phospho (STY)]DSAAEEEEEAEAAAFPEAYR_.3	Q68EF6_T263_M1_Begain	4.0122	5.6842	5.3888	6.5146	5.5237	6.0053	5.7889	6.3294	5.3937	5.4160	0.9633	-0.0540	0.6614	0.1795
Begain	_SADALAGYAAS[Phospho (STY)]DGDGDRLR_.2	#N/A	3.4591	3.4227	3.9009	4.2374	3.8669	3.0834	3.9745	5.1365	4.7007	5.2659	0.7678	-0.3813	0.0076	2.1172
Begain	_LS[Phospho (STY)]PQQAFPR_.2	Q68EF6_S570_M1_Begain	8.5759	2.7088	4.4124	6.2637	3.8224	6.0145	4.1962	3.0450	5.2927	5.9228	1.1486	0.1998	0.5768	0.2390
Begain	_VQLC[Carbamidomethyl (C)]GAGS[Phospho (STY)]S[Phospho (STY)]PEPEHGSR_.2	#N/A	4.5939	5.7253	5.7506	5.7752	5.4607	5.4422	5.8268	5.5263	5.3761	5.3401	0.9892	-0.0156	0.8147	0.0890
Begain	_ADS[Phospho (STY)]FSEGDDLSQGHLAEPC[Carbamidomethyl (C)]FLR_.3	Q68EF6_S483_M1_Begain	3.0966	4.1296	3.7834	4.2860	4.0179	4.1075	4.0007	7.0691	3.4810	6.2085	0.7521	-0.4109	0.1098	0.9594
Begain	_YYGGGGGGGAAGGGS[Phospho (STY)]PGDK_.2	#N/A	3.9705	3.2259	3.1862	3.6889	3.1142	5.3815	3.4036	4.4296	4.0782	3.6559	0.9664	-0.0492	0.7902	0.1022
Begain	_GDIY[Phospho (STY)]C[Carbamidomethyl (C)]SDPALYC[Carbamidomethyl (C)]PDEREHAR_.4	#N/A	3.9276	3.4148	3.6613	3.2051	3.0714	2.9926	3.3533	3.7961	4.0951	4.7937	0.8427	-0.2469	0.0704	1.1525
Begain	_AASTLGS[Phospho (STY)]PLEAQVAPGFAR_.2	Q68EF6_S357_M1_Begain	3.3772	3.9652	3.1109	4.1555	4.0218	3.9430	4.3038	4.3465	3.1447	3.8328	0.9630	-0.0544	0.6497	0.1873
Begain	_RPS[Phospho (STY)]VDT[Phospho (STY)]PVTDVGFLR_.2	Q68EF6_S246_M2_Begain	3.2497	4.0060	4.4842	4.0280	3.8942	4.2312	4.1762	8.1813	3.2723	3.7052	0.8238	-0.2796	0.3812	0.4189
Begain	_ESLEPS[Phospho (STY)]SMEAS[Phospho (STY)]PEMHPPTR_.2	Q68EF6_S555_M2_Begain	3.8009	3.5415	3.5346	4.5792	2.9447	4.3667	3.2267	3.9228	4.2218	4.2565	0.9712	-0.0421	0.7627	0.1177
Cltc	_IYIDSNNNPER_.2	#N/A	12.9315	12.8998	13.3825	13.1201	11.8375	12.7022	13.3149	13.1909	13.0106	12.7659	0.9802	-0.0288	0.3934	0.4051
Cltc	_LLLPWLEAR_.2	#N/A	9.9946	10.1043	10.0008	9.2684	9.0837	9.6215	10.0507	10.1595	9.7942	9.5528	0.9787	-0.0310	0.4114	0.3858
Cltc	_NNLAGAEELFAR_.2	#N/A	9.5829	9.3987	9.6941	9.2215	8.8878	9.1374	9.5726	9.6005	9.3084	9.3511	0.9854	-0.0212	0.4237	0.3729
Cltc	_DPHLAC[Carbamidomethyl (C)]VAYER_.2	#N/A	8.3778	7.9336	8.1324	7.8081	7.4860	8.2130	8.2135	8.1793	8.0400	8.1262	0.9818	-0.0265	0.3983	0.3997
Cltc	_LLYNNVSNFGR_.2	#N/A	8.9727	9.0636	9.1514	8.4479	8.5280	8.6941	9.3604	9.2320	8.9676	8.6412	0.9734	-0.0389	0.2535	0.5960
Cltc	_ESNC[Carbamidomethyl (C)]YDPER_.2	#N/A	8.8112	8.6648	8.5482	8.3488	8.5430	8.2394	8.7173	8.9179	8.5931	8.6598	0.9775	-0.0328	0.1391	0.8565
Cltc	_LHIIEVGTPPTGNQPFPK_.3	#N/A	7.8337	7.5715	7.9812	7.4579	6.7146	7.4957	7.7365	7.7614	7.4355	7.5195	0.9863	-0.0199	0.6669	0.1759
Cltc	_EVC[Carbamidomethyl (C)]FAC[Carbamidomethyl (C)]VDGK_.2	#N/A	8.8902	8.6994	8.6083	8.4971	7.9767	8.2878	8.5808	8.5795	8.3751	8.5695	0.9961	-0.0056	0.8508	0.0702
Cltc	_LAELEEFINGPNNAHIQQVGDR_.3	#N/A	8.8470	8.6118	8.7823	8.8071	7.8033	8.0072	9.0735	9.0656	8.5729	8.3136	0.9680	-0.0469	0.3394	0.4693
Cltc	_AHIAQLC[Carbamidomethyl (C)]EK_.2	#N/A	8.3525	8.3915	8.2718	8.2068	8.2850	7.9092	8.5094	8.5438	8.2693	8.2688	0.9807	-0.0280	0.1659	0.7801
Cltc	_GQFSTDELVAEVEKR_.2	#N/A	7.0770	7.1332	7.3729	7.2251	5.9545	6.5637	7.1189	7.1123	7.0498	6.6536	0.9863	-0.0200	0.7458	0.1274
Cltc	_ENPYYDSR_.2	#N/A	7.6521	7.5261	7.9288	7.1857	7.3877	7.6211	7.2756	8.1289	7.7951	7.8548	0.9725	-0.0402	0.2961	0.5286
Cltc	_LTDQLPLIIVC[Carbamidomethyl (C)]DR_.2	#N/A	7.8847	7.5461	7.3474	7.3412	6.9076	7.1161	8.0658	7.7046	7.3360	7.4555	0.9629	-0.0545	0.2238	0.6501
Cltc	_C[Carbamidomethyl (C)]NEPAVWSQLAK_.2	#N/A	7.4691	7.8752	7.7094	7.3155	7.5972	7.3269	7.8739	7.9782	7.5797	7.0205	0.9916	-0.0122	0.7609	0.1187
Cltc	_SVDPTLALSVYLR_.2	#N/A	7.5979	7.6871	7.5109	7.1337	7.0694	7.0854	7.7366	7.9904	7.8533	7.5721	0.9434	-0.0840	0.0248	1.6050
Cltc	_NLQNLLILTAIK_.2	#N/A	7.8547	7.8641	8.0416	6.8742	6.9559	6.8318	7.9972	8.0151	7.8644	7.3834	0.9474	-0.0780	0.2265	0.6450
Cltc	_EHLELFWSR_.2	#N/A	7.9849	7.3950	7.8030	7.2497	6.6889	7.2745	7.7642	7.8588	7.5305	7.3079	0.9716	-0.0415	0.4185	0.3783
Cltc	_HELIEFR_.2	#N/A	7.9841	7.7946	7.6912	7.3703	6.7033	7.1343	7.7863	7.5160	7.1127	7.4269	0.9981	-0.0027	0.9587	0.0183
Cltc	_WLLLTGISAQQNR_.2	#N/A	7.5295	6.9221	8.0395	7.1309	5.6997	6.8794	7.6526	7.3983	7.2340	7.5631	0.9426	-0.0853	0.3236	0.4900
Cltc	_HDVVFLITK_.2	#N/A	7.3890	6.9517	7.2249	7.2187	5.2328	6.6510	7.3787	7.7508	6.5188	6.9185	0.9491	-0.0754	0.4522	0.3447
Cltc	_LASTLVHLGEYQAAVDGAR_.3	#N/A	6.4219	6.2639	6.0819	6.1435	5.1320	5.9042	6.3659	6.1653	5.9751	6.2737	0.9671	-0.0482	0.4237	0.3729
Cltc	_AFM[Oxidation (M)]TADLPNELIELLEK_.2	#N/A	6.8484	7.0396	6.7064	6.7683	6.3048	6.4529	7.0067	7.1869	6.9861	6.6996	0.9594	-0.0598	0.1112	0.9539
Cltc	_VIQC[Carbamidomethyl (C)]FAETGQVQK_.2	#N/A	6.1256	6.0191	6.0981	6.4546	6.4053	5.3467	6.8897	6.8146	6.3872	6.2235	0.9234	-0.1150	0.0689	1.1617
Cltc	_LEKHELIEFR_.3	#N/A	6.7217	7.1978	7.2922	6.7982	5.6417	6.7038	6.8311	7.5745	7.1034	6.5450	0.9590	-0.0604	0.4301	0.3664
Cltc	_IAAYLFK_.2	#N/A	7.1453	7.0127	7.6248	5.7947	5.6580	5.8988	6.9741	7.1344	6.8945	6.9291	0.9340	-0.0985	0.3148	0.5019
Cltc	_YIEIYVQK_.2	#N/A	6.4343	6.4492	6.9627	6.0133	5.5654	6.3228	5.6833	7.2215	6.8480	6.8171	0.9471	-0.0784	0.3524	0.4529
Cltc	_SVNESLNNLFITEEDYQALR_.2	#N/A	5.5369	5.6600	6.4980	6.1109	4.4897	5.2562	6.1040	6.0156	5.8937	5.8110	0.9389	-0.0910	0.3403	0.4681
Cltc	_ISGETIFVTAPHEATAGIIGVNR_.3	#N/A	6.4790	6.8407	7.2874	6.5331	5.4069	5.7368	6.3672	6.9953	5.6057	5.5097	1.0427	0.0603	0.5780	0.2381
Cltc	_YESLELC[Carbamidomethyl (C)]RPVLQQGR_.3	#N/A	6.3448	5.0688	6.1378	6.1078	4.7565	5.5992	6.3611	6.1944	6.3487	5.6840	0.9223	-0.1168	0.2108	0.6761
Cltc	_QSVELC[Carbamidomethyl (C)]KK_.2	#N/A	5.2636	6.2672	4.5774	6.5121	5.0702	6.7658	6.5688	6.3990	5.4263	5.9929	0.9419	-0.0863	0.4959	0.3046
Cltc	_RPLIDQVVQTALSETQDPEEVSVTVK_.3	#N/A	5.5282	5.2795	6.2585	5.9358	4.6384	5.5154	6.8782	6.5570	6.1719	5.8591	0.8680	-0.2043	0.0361	1.4419
Cltc	_EAIDSYIK_.2	#N/A	6.1330	4.8435	5.5939	4.6139	5.1312	6.0905	4.5190	5.9641	5.8291	6.1741	0.9608	-0.0577	0.6304	0.2004
Cltc	_GQFSTDELVAEVEK_.2	#N/A	5.7118	5.3066	5.8921	5.5161	4.2174	5.1257	5.9968	4.9275	4.8683	5.4087	0.9990	-0.0015	0.9887	0.0049
Cltc	_IVLDNSVFSEHR_.2	#N/A	7.5487	7.3737	7.8984	7.4930	6.8763	7.4263	8.1014	7.8823	7.5230	7.3729	0.9632	-0.0540	0.2211	0.6555
Cltc	_KFNALFAQGNYSEAAK_.3	#N/A	6.5014	6.1521	6.4736	5.7166	5.9275	6.5670	6.2085	6.4810	6.4324	5.9956	0.9910	-0.0130	0.7837	0.1058
Cltc	_AYEFAER_.2	#N/A	6.4495	6.8098	6.7967	5.3475	6.4084	6.0134	3.0132	6.9285	6.9249	5.9971	1.1029	0.1413	0.4753	0.3230
Cltc	_RDPHLAC[Carbamidomethyl (C)]VAYER_.3	#N/A	7.0710	6.8887	7.1724	3.6259	6.7509	6.4103	6.6943	7.0887	6.8218	6.8379	0.9212	-0.1185	0.4559	0.3412
Cltc	_VGYTPDWIFLLR_.2	#N/A	4.7044	4.9655	4.1965	4.5230	3.5780	3.5651	5.5096	5.9868	4.9180	4.8963	0.7987	-0.3242	0.0184	1.7341
Cltc	_QSVELC[Carbamidomethyl (C)]K_.2	#N/A	4.7611	5.1555	4.2675	3.8112	5.5141	2.9471	4.5436	5.0114	5.1403	4.6205	0.9131	-0.1311	0.4198	0.3770
Cltc	_VM[Oxidation (M)]EYINR_.2	#N/A	7.1013	7.5497	6.0330	6.4127	5.5058	5.3926	5.9636	6.2660	5.6956	5.6683	1.0227	0.0324	0.7077	0.1502
Cltc	_VANVELYYK_.2	#N/A	3.9441	3.8838	2.9339	4.6236	3.7113	4.4111	3.9017	4.9250	4.5611	4.3009	0.8860	-0.1747	0.1843	0.7346
Cltc	_VVGAM[Oxidation (M)]QLYSVDR_.2	#N/A	5.9579	5.5911	5.6968	5.7432	4.4533	4.9768	5.8734	5.7173	5.2878	5.0578	0.9852	-0.0214	0.8109	0.0911
Cltc	_M[Oxidation (M)]EGNAEESTLFC[Carbamidomethyl (C)]FAVR_.2	#N/A	6.0476	6.9150	7.3706	6.6425	3.3414	6.2756	6.8327	6.4894	6.4796	5.8465	0.9511	-0.0723	0.6861	0.1636
Cltc	_LLEM[Oxidation (M)]NLM[Oxidation (M)]HAPQVADAILGNQM[Oxidation (M)]FTHYDR_.4	#N/A	5.2269	5.5930	3.8479	3.3916	4.6553	5.0616	5.5944	5.8695	5.3921	5.6509	0.8227	-0.2815	0.0535	1.2714
Cltc	_FNALFAQGNYSEAAK_.2	#N/A	4.5978	4.4821	3.2376	5.1178	4.0128	4.1485	5.1056	4.4803	5.4083	3.7073	0.9481	-0.0769	0.6466	0.1894
Cltc	_TLQIFNIEMK_.2	#N/A	5.1315	3.8555	4.3337	5.8003	2.4290	4.3817	4.6741	2.4817	3.2069	4.0391	1.2004	0.2635	0.3326	0.4781
Cltc	_VDKLDASESLR_.2	#N/A	6.5160	6.3228	6.4585	6.2367	6.7245	6.7358	6.7754	6.6893	6.8757	6.5083	0.9683	-0.0465	0.1165	0.9338
Cltc	_VSQPIEGHAASFAQFK_.3	#N/A	6.1968	6.3910	6.8936	5.8206	5.8639	6.1457	6.2027	6.1221	5.2362	5.4680	1.0801	0.1112	0.1331	0.8759
Cltc	_GQC[Carbamidomethyl (C)]DLELINVC[Carbamidomethyl (C)]NENSLFK_.2	#N/A	6.2285	5.9727	6.3491	6.2509	5.5443	5.6953	6.4600	6.2026	6.0870	6.0307	0.9696	-0.0445	0.3350	0.4749
Cltc	_AM[Oxidation (M)]LSANIR_.2	#N/A	5.4910	5.5027	5.3987	5.5837	5.2614	4.8061	5.6294	5.9595	6.4805	5.3549	0.9120	-0.1329	0.0640	1.1936
Cltc	_ALEHFTDLYDIK_.2	#N/A	6.2064	5.4210	5.3899	5.4256	5.0343	5.3436	5.8806	6.1290	5.8532	5.7498	0.9266	-0.1099	0.0717	1.1445
Cltc	_ESYVETELIFALAK_.2	#N/A	4.7516	3.7480	4.4911	4.2335	3.1430	3.7812	4.2259	5.3936	5.1532	4.3164	0.8434	-0.2458	0.0832	1.0798
Cltc	_HSSLAGC[Carbamidomethyl (C)]QIINYR_.3	#N/A	5.6606	4.7344	5.2828	4.8880	4.7979	4.5501	5.3182	5.6768	5.6716	5.2009	0.9938	-0.0090	0.9470	0.0237
Cltc	_DAMQYASESK_.2	#N/A	6.2232	4.6802	4.3299	6.6900	5.4471	3.4598	4.5473	3.2859	4.4353	3.2988	1.3203	0.4009	0.1029	0.9875
Cltc	_IHEGC[Carbamidomethyl (C)]EEPATHNALAK_.3	#N/A	6.1609	5.7712	5.9856	5.5095	5.8042	5.5995	5.9721	6.1633	5.9419	5.6949	0.9768	-0.0339	0.3680	0.4342
Cltc	_ALEHFTDLYDIKR_.3	#N/A	7.4717	7.5061	8.0305	6.8710	6.7907	7.3605	7.7496	7.4455	7.4508	7.2813	0.9808	-0.0279	0.5754	0.2400
Cltc	_LPVVIGGLLDVDC[Carbamidomethyl (C)]SEDVIK_.2	#N/A	6.2391	6.2057	6.7774	6.4900	5.4616	6.0689	6.7443	6.6989	5.9770	6.2954	0.9655	-0.0506	0.4314	0.3651
Cltc	_ADDPSSYM[Oxidation (M)]EVVQAANASGNWEELVK_.3	#N/A	5.1245	4.8576	5.7706	4.8613	4.1204	5.1480	5.2885	4.7500	4.9126	4.8036	1.0085	0.0121	0.8893	0.0510
Cltc	_FQSVPAQPGQTSPLLQYFGILLDQGQLNK_.3	#N/A	3.7009	4.0548	2.3803	5.5818	3.3548	2.7630	4.9599	4.3519	3.5986	3.4153	0.8917	-0.1654	0.5103	0.2922
Cltc	_GILRT[Phospho (STY)]PDTIR_.2	Q68FD5_T394_M1_Cltc	3.8744	3.1298	3.6080	7.8555	3.0181	3.6065	3.3001	8.1212	4.1484	4.3299	0.8406	-0.2505	0.5469	0.2621
Cltc	_KFDVNTSAVQVLIEHIGNLDR_.3	#N/A	4.6593	3.5918	4.7115	5.0766	3.4801	3.1445	3.7621	4.7964	4.7246	3.2911	0.9920	-0.0115	0.9492	0.0226
Cltc	_TSIDAYDNFDNISLAQR_.2	#N/A	6.9964	6.8694	7.2434	6.6310	5.9018	6.7278	7.3010	7.0030	6.6166	6.6943	0.9746	-0.0371	0.5259	0.2791

Cltc	_AVNYFSK_.2	#N/A	3.1291	2.3264	3.0859	3.0473	3.2719	3.5512	3.3868	2.9314	3.0486	3.1606	0.9798	-0.0294	0.7827	0.1064
Cltc	_YIQAAC[Carbamidomethyl (C)]K_.2	#N/A	4.9495	3.7907	5.0394	3.8127	4.8451	3.3759	4.3396	5.0657	4.8698	4.0877	0.9372	-0.0936	0.5022	0.2991
Cltc	_NNRPSEGPLQTR_.2	#N/A	5.9134	4.6738	5.5148	5.2790	5.0929	4.6555	5.3825	5.6073	5.5548	5.4162	0.9450	-0.0816	0.2676	0.5724
Cltc	_VGEQAQVVIIDM[Oxidation (M)]NDPSNPIR_.2	#N/A	4.2009	3.9699	4.4831	4.0705	3.0974	3.4922	4.1588	4.2540	4.1896	4.5271	0.9074	-0.1402	0.1742	0.7590
Cltc	_AHM[Oxidation (M)]GM[Oxidation (M)]FTELAILYSK_.2	#N/A	4.0775	3.5496	4.7599	4.5711	3.3709	4.3586	4.2630	4.3017	4.1227	4.2484	0.9718	-0.0413	0.6879	0.1625
Cltc	_AHTM[Oxidation (M)]TDDVTFWK_.2	#N/A	3.5733	4.8154	4.2601	3.2619	3.5412	3.0834	5.1114	5.7326	3.4352	4.7451	0.7897	-0.3406	0.0851	1.0702
Cltc	_RPIS[Phospho (STY)]ADSAIMNPASK_.2	Q68FD5_S67_M1_Cltc	3.4287	4.1849	3.1623	4.2307	4.0732	4.0522	3.9454	4.2951	3.5364	3.8842	0.9847	-0.0222	0.8229	0.0847
Cltc	_FDVNTSAVQVLIEHIGNLDR_.3	#N/A	4.1681	3.4725	3.3541	3.8568	3.2938	2.9348	3.5714	4.2617	3.9103	3.8238	0.9028	-0.1476	0.1687	0.7729
Cltc	_YGYIHLYDLETGTC[Carbamidomethyl (C)]IYM[Oxidation (M)]NR_.3	#N/A	3.1967	4.0295	3.8835	4.3862	3.9178	4.2077	4.1008	3.7323	3.3809	4.3531	1.0116	0.0166	0.8706	0.0602
Cltc	_EDKLEC[Carbamidomethyl (C)]SEELGDLVK_.3	#N/A	4.0120	3.3163	3.1979	3.7006	3.1376	3.0910	3.4153	4.4179	4.0665	3.6676	0.8760	-0.1910	0.0965	1.0156
Fnip1	_NESSDSALGDS[Phospho (STY)]ESEDTGPDIR_.2	#N/A	2.9615	4.2646	3.6483	6.0411	4.4276	5.0126	6.2310	5.6389	5.2316	5.3552	0.7824	-0.3540	0.0658	1.1817
Fnip1	_NESSDSALGDS[Phospho (STY)]ESEDTGPDIRR_.3	#N/A	4.0188	3.3232	3.7529	3.2967	3.1445	3.0842	3.4450	3.7045	4.0035	2.9740	0.9731	-0.0394	0.7195	0.1430
Kiaa1549	_DHTTPSENGDVPS[Phospho (STY)]PK_.2	Q68FD9_S1384_M1_Kiaa1549	7.6894	6.7650	7.9167	8.1094	7.0185	7.2430	8.3595	7.9028	7.7138	7.1547	0.9582	-0.0617	0.3594	0.4445
Kiaa1549	_LITTDS[Phospho (STY)]DGTYK_.2	Q68FD9_S1613_M1_Kiaa1549	6.7148	7.8607	4.4931	8.3917	7.3895	8.2359	9.4045	8.4371	8.4026	8.6256	0.8237	-0.2797	0.0789	1.1030
Kiaa1549	_GHYEFPVVDDLS[Phospho (STY)]SGDTKER_.3	#N/A	3.8996	4.9514	3.9910	8.4221	5.3516	5.5043	4.5544	8.2429	6.0607	3.6248	0.9524	-0.0703	0.8233	0.0845
Kiaa1549	_GLQGPGLGYPTSS[Phospho (STY)]TEDLQPGHSSASLIK_.3	Q68FD9_S1904_M1_Kiaa1549	3.5743	3.7552	4.5098	4.0089	3.6435	3.8304	3.7236	3.4580	3.8881	3.6106	1.0591	0.0829	0.2821	0.5497
Kiaa1549	_GHYEFPVVDDLS[Phospho (STY)]SGDTK_.2	#N/A	3.7576	3.8753	3.4913	6.5170	3.7435	3.8512	3.9554	3.9919	5.6674	3.7828	0.9670	-0.0484	0.8380	0.0768
Kiaa1549	_RGHYEFPVVDDLS[Phospho (STY)]S[Phospho (STY)]GDTKER_.4	#N/A	3.9575	3.2619	3.1435	3.3580	3.0832	3.1454	3.5062	3.6432	3.9423	3.6131	0.9044	-0.1449	0.0892	1.0497
Atg9a	_LEASYSDS[Phospho (STY)]PPGEEDLLVHVAEGSK_.3	Q68FE2_S18_M1_Atg9a	7.0576	7.2661	7.6905	7.4828	6.7701	5.6817	7.2492	7.9224	7.1204	6.7785	0.9620	-0.0559	0.5219	0.2824
Ripor1	_FSTYSQS[Phospho (STY)]PPDTPSLR_.2	Q68FE6_S347_M1_Ripor1	10.3837	10.5126	11.1264	11.0628	9.0886	10.4562	10.9272	10.6617	10.3930	10.2702	0.9882	-0.0171	0.7587	0.1199
Ripor1	_PLTSPAPDS[Phospho (STY)]PEQIPK_.2	Q68FE6_S748_M1_Ripor1	7.7804	7.7255	3.9167	8.5287	7.5235	7.8900	8.7644	7.9764	8.0997	7.9152	0.8826	-0.1802	0.2959	0.5288
Ripor1	_HTSS[Phospho (STY)]PEVVAEDR_.2	Q68FE6_S875_M1_Ripor1	6.8044	6.4212	3.9096	3.7587	6.1148	6.5486	4.1899	3.6881	3.6234	3.7789	1.4641	0.5500	0.0375	1.4257
Tmem151b	_SGS[Phospho (STY)]VNEASC[Carbamidomethyl (C)]PTEQTR_.2	Q68FE7_S488_M1_Tmem151b	4.1143	3.9057	3.8480	3.5840	3.2580	3.3755	3.5196	4.6579	3.9599	3.9249	0.9167	-0.1255	0.2205	0.6566
Znf280d	_SVNS[Phospho (STY)]DAS[Phospho (STY)]DPGSVR_.2	Q68FE8_S904_M2_Znf280d	3.7371	5.0829	5.6362	3.4257	2.8628	3.3659	6.6283	3.1920	4.3414	3.3927	0.9157	-0.1271	0.6693	0.1744
Git1	_LQAENLQLR_.2	#N/A	5.5359	5.4910	5.5601	6.0670	5.7143	5.2093	5.6276	5.7396	5.9351	5.7090	0.9728	-0.0398	0.3376	0.4716
Git1	_SAVPFLPVNPEYSATR_.2	#N/A	5.7513	5.7218	5.9983	5.6116	5.6042	5.3888	3.5397	5.5333	5.2134	5.4099	1.1534	0.2059	0.0838	1.0766
Git1	_S[Phospho (STY)]M[Oxidation (M)]DSSDLSDGAVTLQEYLELKK_.3	#N/A	3.5266	4.3318	4.2134	5.4491	5.2495	5.3609	3.9372	3.2122	6.4517	5.5233	0.9806	-0.0282	0.8989	0.0463
Git1	_SLSS[Phospho (STY)]PTDNLELSAR_.2	Q68FF6_S371_M1_Git1	11.2398	11.5678	11.9037	12.2228	11.0943	11.8161	12.0058	11.6084	11.6214	11.7121	0.9918	-0.0119	0.6855	0.1640
Git1	_ARS[Phospho (STY)]M[Oxidation (M)]DSSDLSDGAVTLQEYLELKK_.3	#N/A	4.0856	3.3411	3.8193	3.7430	3.2294	3.4450	3.6712	3.6879	5.0778	3.8449	0.8870	-0.1730	0.1823	0.7393
Git1	_S[Phospho (STY)]M[Oxidation (M)]DSSDLSDGAVTLQEYLELK_.2	#N/A	4.3960	3.7004	3.5820	4.4203	3.5217	4.2078	3.0678	4.0339	4.3807	4.0516	1.0226	0.0323	0.7831	0.1062
Git1	_HGS[Phospho (STY)]GADSDYENTQSGDPLLGLEGK_.3	#N/A	3.8018	6.4015	3.9962	7.2048	3.4442	6.5287	3.5827	7.2339	3.8130	6.7770	0.9772	-0.0333	0.9170	0.0376
Git1	_HGS[Phospho (STY)]GADSDYENTQSGDPLLGLEGKR_.4	#N/A	3.5349	4.3160	3.8250	3.8659	4.1704	3.7705	3.8845	3.8477	3.0150	3.6430	1.0879	0.1215	0.1797	0.7454
Git1	_AEVC[Carbamidomethyl (C)]ADC[Carbamidomethyl (C)]SAPDPGWASISR_.2	#N/A	2.5278	1.9968	3.3751	4.4198	2.7852	3.7517	3.0672	4.0823	4.3813	4.0970	0.8044	-0.3140	0.1677	0.7754
Git1	_SQSELDDQHDYDSVASDEDT[Phospho (STY)]DQEPLPSAGATR_.4	Q68FF6_T401_M1_Git1	3.3660	3.9201	4.3381	3.6721	4.0011	3.7022	4.2808	6.8899	6.0318	3.4555	0.7422	-0.4301	0.0728	1.1376
Git1	_ENDAVWLATQNHSTLVTER_.3	#N/A	3.8164	4.2240	4.5032	3.5051	1.7923	3.3266	3.2197	3.1126	2.8203	3.4721	1.1178	0.1607	0.4780	0.3206
Slain1	_S[Phospho (STY)]MPNLAR_.2	Q68FF7_S401_M1_Slain1	6.1696	4.6155	3.8804	7.1971	3.2904	3.3342	4.7330	3.9361	3.8761	4.4061	1.1204	0.1640	0.5586	0.2529
Slain1	_SSQS[Phospho (STY)]FDSSLHGAGSGVSR_.2	#N/A	3.9233	3.4351	3.3886	4.2178	3.2101	3.4030	3.3399	4.1782	4.1336	4.0042	0.9188	-0.1221	0.2404	0.6191
Pkp4	_VGS[Phospho (STY)]PLTLTDAQTR_.2	Q68FH0_S313_M1_Pkp4	12.4915	12.3010	13.0477	12.6133	11.2275	12.2182	12.8504	12.3703	12.2686	12.2714	0.9901	-0.0144	0.7170	0.1445
Pkp4	_AQS[Phospho (STY)]PSYVTSTGVSPSR_.2	Q68FH0_S220_M1_Pkp4	9.8204	9.7507	10.7138	10.1530	9.7693	10.3034	10.7467	10.2315	9.8512	9.7099	0.9951	-0.0071	0.8571	0.0670
Pkp4	_LYLQS[Phospho (STY)]PR_.2	Q68FH0_S1133_M1_Pkp4	10.9707	10.8434	11.3163	4.2878	9.7507	10.7581	11.0574	10.7913	10.7786	10.6860	0.8916	-0.1655	0.4163	0.3806
Pkp4	_TGS[Phospho (STY)]VGIGNLQR_.2	Q68FH0_S446_M1_Pkp4	9.6826	9.6635	9.9337	9.6862	8.1100	9.1910	9.8239	9.4712	9.4037	9.4022	0.9845	-0.0225	0.6841	0.1649
Pkp4	_VAS[Phospho (STY)]PSQGQVGSSSPK_.2	Q68FH0_S326_M1_Pkp4	8.9391	7.7863	8.5642	9.2176	7.9076	3.8057	8.5877	4.0101	8.9250	3.7603	1.2188	0.2854	0.3858	0.4137
Pkp4	_PSPIYISSYSS[Phospho (STY)]PAR_.2	#N/A	8.8253	4.2244	3.6886	4.1912	4.1127	8.5099	8.6788	8.2200	8.6461	8.3761	0.6594	-0.6007	0.0460	1.3376
Pkp4	_TYYS[Phospho (STY)]PVYR_.2	Q68FH0_S421_M1_Pkp4	3.3871	7.3776	7.9140	7.5183	4.0137	3.7158	7.3120	3.5419	3.1905	7.4813	1.0507	0.0714	0.8537	0.0687
Pkp4	_S[Phospho (STY)]YEDPYC[Carbamidomethyl (C)]DDR_.2	Q68FH0_S1136_M1_Pkp4	6.0746	5.4375	6.1986	3.9444	5.3157	3.7660	6.4432	4.1740	6.3038	5.6370	0.9084	-0.1386	0.4639	0.3336
Pkp4	_PAS[Phospho (STY)]PTAVR_.2	#N/A	3.5196	4.2759	3.2533	4.1397	4.1642	6.6603	3.8544	4.2041	3.0023	7.1227	0.9537	-0.0684	0.8284	0.0818
Pkp4	_AAS[Phospho (STY)]PYSQRPAS[Phospho (STY)]PTAVRR_.3	#N/A	6.5778	6.3089	3.8384	3.3822	3.2485	3.3761	3.2693	3.6190	3.9181	3.0595	1.2853	0.3621	0.2557	0.5923
Pkp4	_TSS[Phospho (STY)]QRS[Phospho (STY)]TLTYQR_.2	Q68FH0_S457_M2_Pkp4	3.0651	3.8213	4.2996	4.5943	3.7096	4.4158	4.3090	3.1578	4.8667	3.5206	1.0052	0.0075	0.9618	0.0169
Pkp4	_AASPYSQRPAS[Phospho (STY)]PTAVR_.3	#N/A	8.0014	3.5096	8.2909	7.1869	7.0070	7.5885	8.5291	7.7610	7.8070	7.5255	0.8767	-0.1899	0.3134	0.5040
Pkp4	_GLYPGSSKPSPIY[Phospho (STY)]ISSYS[Phospho (STY)]SPAR_.3	Q68FH0_S1098_M2_Pkp4	4.1943	3.4498	3.9280	3.4771	3.3381	3.2987	3.1918	6.1670	4.3020	3.3775	0.8485	-0.2370	0.2896	0.5382
Pkp4	_VASPSQGQVGSSS[Phospho (STY)]PKR_.2	#N/A	8.3562	9.2451	9.6340	9.6293	8.6966	8.0169	9.5512	9.4411	9.2853	4.8812	1.0772	0.1073	0.5252	0.2796
Pkp4	_LQHTGPADDGATRS[Phospho (STY)]PS[Phospho (STY)]IDSIQKDPR_.4	#N/A	4.0875	7.3194	3.6637	6.5738	7.0552	7.3508	6.9577	3.8331	7.5029	7.5750	0.9291	-0.1062	0.6899	0.1612
Pkp4	_SAVS[Phospho (STY)]PDLHITPIYEGR_.2	#N/A	3.8436	7.5327	7.8869	8.1941	6.7896	7.6495	7.7589	8.2740	7.5430	7.4308	0.9008	-0.1507	0.3833	0.4165
Pkp4	_LQHTGPADDGATRS[Phospho (STY)]PS[Phospho (STY)]IDSIQK_.3	#N/A	3.7580	5.2512	3.7114	5.7814	5.3301	5.0830	6.3227	5.5870	5.0810	5.6004	0.8533	-0.2289	0.1282	0.8921
Pkp4	_S[Phospho (STY)]PNHGTVELQGSQTALYR_.3	Q68FH0_S426_M1_Pkp4	3.3800	3.7015	3.8776	4.9207	3.2877	3.4474	3.8843	3.7726	3.8457	3.7252	0.9901	-0.0144	0.9060	0.0429
Pkp4	_AEQYPGS[Phospho (STY)]PDSWV_.2	Q68FH0_S1185_M1_Pkp4	3.5634	4.3197	3.2971	4.3417	4.2080	3.9175	3.8106	4.1603	2.9585	4.0190	1.0546	0.0767	0.5241	0.2806
Pkp4	_SSYAS[Phospho (STY)]QHS[Phospho (STY)]QLGQELR_.2	Q68FH0_S391_M2_Pkp4	2.9275	3.7327	3.6143	4.1169	3.5540	4.1755	4.5363	4.0016	3.6501	4.0839	0.9063	-0.1419	0.1992	0.7008
Ahcyl2	_DGGEALVS[Phospho (STY)]PDGTVTEAPR_.2	#N/A	9.5646	9.6312	9.8411	10.2578	8.4875	9.6320	10.4310	9.7192	9.9679	9.8650	0.9573	-0.0629	0.2237	0.6504
Ahcyl2	_IVLLAEGR_.2	#N/A	8.1949	8.0441	7.4771	7.6857	8.3309	7.9274	6.9674	8.1142	8.2656	8.0716	1.0113	0.0162	0.7651	0.1163
Ahcyl2	_GESEDDFWWC[Carbamidomethyl (C)]IDR_.2	#N/A	7.1810	6.8514	7.6186	7.1337	6.1846	6.8256	6.8225	6.7647	7.2104	7.0029	1.0023	0.0032	0.9528	0.0210
Ahcyl2	_AQGEKPLAGAK_.2	#N/A	6.3802	6.5379	5.4089	6.0858	6.4002	6.3495	6.0884	6.3319	6.1888	6.2641	0.9961	-0.0057	0.9116	0.0402
Ahcyl2	_TPELTWER_.2	#N/A	7.0586	6.7736	7.0329	6.6608	6.7079	7.0511	5.6001	6.4585	6.5500	7.0227	1.0738	0.1027	0.0982	1.0078
Ahcyl2	_HRDGGEALVS[Phospho (STY)]PDGTVTEAPR_.3	#N/A	7.8275	8.0631	8.5851	8.3607	7.2869	8.1952	8.4804	8.1088	8.1650	8.0284	0.9826	-0.0253	0.5767	0.2390
Ahcyl2	_LC[Carbamidomethyl (C)]VPAM[Oxidation (M)]NVNDSVTK_.2	#N/A	6.4493	6.8422	7.0103	6.2574	6.7910	6.8834	6.9556	7.0182	6.8871	6.6113	0.9763	-0.0345	0.3496	0.4564
Ahcyl2	_GIVEESVTGVHR_.2	#N/A	6.4027	6.0892	6.2723	6.4961	6.4269	6.2906	6.5551	6.5077	6.7007	6.2729	0.9724	-0.0403	0.1166	0.9334
Ahcyl2	_FDNLYC[Carbamidomethyl (C)]C[Carbamidomethyl (C)]R_.2	#N/A	4.6213	3.7643	5.0326	4.3564	0.6979	4.1439	4.5047	4.1456	5.1106	4.0337	0.8473	-0.2390	0.4321	0.3644
Ahcyl2	_QVVVC[Carbamidomethyl (C)]GYGEVGK_.2	#N/A	3.7059	3.0102	4.3927	3.6096	3.6182	3.3971	3.7579	4.4606	3.6906	3.2869	0.9535	-0.0687	0.5733	0.2416
Mars1	_NNSELLNNLGNFINR_.2	#N/A	5.5569	5.6859	5.8928	5.6349	4.9069	3.9468	5.5528	5.4818	5.6578	5.6618	0.9431	-0.0845	0.4197	0.3770
Elfn2	_DSTYSQLS[Phospho (STY)]PR_.2	Q68FM6_S750_M1_Elfn2	10.2826	10.3530	11.0585	10.4236	9.4978	10.1052	10.8042	10.4501	10.0942	10.2450	0.9893	-0.0156	0.7054	0.1516
Elfn2	_QLS[Phospho (STY)]ADAAVSRK_.2	#N/A	5.7367	5.9535	6.3779	5.6697	4.3780	5.8120	5.3192	5.7547	5.1557	5.1460	1.0581	0.0815	0.4158	0.3811
Elfn2	_QLS[Phospho (STY)]ADAAVSR_.2	#N/A	8.3127	7.6578	8.4507	8.2740	3.5131	8.3138	8.1754	8.3173	7.8642	8.4482	0.9048	-0.1444	0.4539	0.3431
Elfn2	_YIEKGS[Phospho (STY)]PLNSPLDR_.2	#N/A	6.7697	6.4566	3.8341	3.3778	3.2441	5.8520	7.0124	4.8806	6.0957	5.0761	0.8537	-0.2283	0.3815	0.4185
Elfn2	_GS[Phospho (STY)]PLNSPLDR_.2	#N/A	4.3189	3.5743	4.0526	3.5964	3.4626	3.1620	6.3567	3.4048	5.2713	3.2736	0.8072	-0.3089	0.1985	0.7022
Elfn2	_HYYSGYSS[Phospho (STY)]S[Phospho (STY)]PEYSSESTHK_.3	Q68FM6_S760_M2_Elfn2	4.6515	4.1587	3.6282	4.2722	4.0470	5.1745	3.9868	3.9624	3.5102	5.3008	1.0315	0.0447	0.7552	0.1219
Ttc41	_SISELVS[Phospho (STY)]EK_.2	Q692V3_S1279_M1_Ttc41	3.1790	4.1634	4.4135	3.9573	3.8236	4.3019	4.1056	3.0439	4.3548	3.6346	1.0498	0.0701	0.5746	0.2407
Peak1	_ANTLS[Phospho (STY)]PVR_.2	Q69Z38_S282_M1_Peak1	7.2637	4.1014	4.4756	4.0193	6.0327	4.2398	4.1676	2.9818	7.2912	3.6966	1.1076	0.1474	0.6427	0.1920
Peak1	_DPSTKPVT[Phospho (STY)]SPPSK_.2	Q69Z38_T851_M1_Peak1	4.1859	3.4902	6.2018	4.0106	3.3116	3.7980	6.4313	6.8490	4.1833	6.7521	0.6882	-0.5391	0.0325	1.4887
Peak1	_SYSSTHSS[Phospho (STY)]PAK_.2	Q69Z38_S727_M1_Peak1	3.4931	4.0689	3.6144	2.7787	3.8902	3.8393	3.6645	4.4502	3.2484	4.4201	0.9159	-0.1267	0.3443	0.4631
Peak1	_SLFTSQSSGEGEAHQTTES[Phospho (STY)]PT[Phospho (STY)]AK_.3	Q69Z38_S836_M2_Peak1	3.8716	3.1759	4.5584	3.5602	2.9973	3.2314	3.2749	3.0574	4.2069	3.5272	1.0140	0.0200	0.8930	0.0491

Radil	_GTPALTSEAAQSS[Phospho (STY)]PPTR_.2	Q69Z89_S235_M1_Radil	8.4418	8.4982	9.1448	3.6314	7.5168	8.3702	8.9934	8.6992	3.5455	8.1620	1.0341	0.0484	0.8664	0.0623
Radil	_S[Phospho (STY)]LLLEFEPDVEDTLLQR_.2	Q69Z89_S432_M1_Radil	3.5215	3.8209	3.2552	4.2998	4.1661	3.9594	4.4481	4.2570	2.1203	3.9771	1.0369	0.0523	0.7782	0.1089
Brsk2	_TRLNS[Phospho (STY)]IK_.2	Q69Z98_S482_M1_Brsk2	7.5005	6.8646	7.4104	3.1944	6.9139	6.9200	7.5626	7.7486	3.0718	7.1250	1.0142	0.0203	0.9422	0.0258
Brsk2	_VTPHPS[Phospho (STY)]PR_.2	Q69Z98_S436_M1_Brsk2	7.1615	3.7322	7.6757	3.1826	6.0837	7.1103	7.1683	7.4818	7.1885	7.2496	0.8009	-0.3203	0.1770	0.7521
Brsk2	_S[Phospho (STY)]M[Oxidation (M)]EVLSVTDGGSPVPAR_.2	#N/A	7.3397	8.1079	7.7474	8.6843	6.5800	8.2751	7.8910	8.7906	8.5088	8.4693	0.9256	-0.1115	0.1657	0.7808
Brsk2	_VDS[Phospho (STY)]PMLNR_.2	#N/A	5.7644	4.6849	4.3651	7.4988	4.0331	3.6964	4.5736	3.0923	3.6559	4.5827	1.2593	0.3326	0.2208	0.6559
Brsk2	_S[Phospho (STY)]ISGASSGLSTS[Phospho (STY)]PLSSPR_.2	Q69Z98_S413_M2_Brsk2	3.6534	3.9914	3.7318	6.8943	3.8534	3.7274	3.7863	6.5414	6.7054	4.0500	0.8175	-0.2908	0.3149	0.5018
Brsk2	_VTPHPS[Phospho (STY)]PRGS[Phospho (STY)]PLPTPK_.3	Q69Z98_S436_M2_Brsk2	3.2644	4.0207	4.4990	4.3949	3.9090	4.2164	4.1096	3.7236	3.3721	4.3619	1.0408	0.0577	0.5915	0.2280
Brsk2	_GS[Phospho (STY)]PLPTPK_.2	Q69Z98_S440_M1_Brsk2	3.0229	4.3195	4.2574	3.5769	4.1408	3.5887	3.9495	3.2000	3.4990	3.4785	1.0810	0.1123	0.3480	0.4584
Brsk2	_KLQVPTPEEM[Oxidation (M)]SNLT[Phospho (STY)]PESS[Phospho (STY)]PELAK_.3	#N/A	3.4462	5.3084	3.6121	7.4569	3.6195	3.8811	3.7742	4.0774	7.3608	6.3884	0.8433	-0.2459	0.4485	0.3482
Brsk2	_LQVPTPEEM[Oxidation (M)]SNLT[Phospho (STY)]PESS[Phospho (STY)]PELAK_.3	#N/A	3.8142	3.8544	3.8020	3.5029	3.6968	3.5806	3.7277	3.9683	4.9805	3.4699	0.9187	-0.1223	0.2609	0.5836
Brsk2	_LQVPTPEEMSNLT[Phospho (STY)]PESS[Phospho (STY)]PELAKK_.3	#N/A	3.0823	4.1439	3.7691	4.2717	4.0322	4.0933	3.9864	3.8467	3.4953	4.2387	1.0018	0.0026	0.9788	0.0093
Brsk2	_RVDS[Phospho (STY)]PMLNR_.2	#N/A	3.1395	3.8958	4.3740	4.5199	3.7841	4.3414	4.2345	3.0796	3.2472	3.5951	1.1328	0.1799	0.1938	0.7128
Cdk13	_GGDVS[Phospho (STY)]PSPYSSSSWR_.2	Q69ZA1_S384_M1_Cdk13	7.2864	3.4748	8.0184	7.8225	6.4582	7.2236	7.9859	7.2232	7.4955	7.2913	0.8953	-0.1595	0.3917	0.4071
Cdk13	_QTDPST[Phospho (STY)]PQQESSK_.2	Q69ZA1_T1147_M1_Cdk13	5.1802	5.5301	6.5804	6.0798	3.7993	5.7982	6.2121	3.4420	3.3875	4.0275	1.2876	0.3647	0.1255	0.9014
Cdk13	_TEHAPS[Phospho (STY)]PSSGGTVK_.2	Q69ZA1_S526_M1_Cdk13	6.7906	2.9801	7.8591	4.5029	6.3796	3.7562	3.1504	3.9991	4.2981	4.1802	1.3765	0.4611	0.1785	0.7483
Cdk13	_HSS[Phospho (STY)]IS[Phospho (STY)]PSTLTLK_.2	Q69ZA1_S438_M2_Cdk13	2.9918	3.7970	3.6786	4.1813	3.6183	4.1112	3.8960	7.5824	3.5858	7.1834	0.6706	-0.5765	0.0666	1.1768
Cdk13	_S[Phospho (STY)]LS[Phospho (STY)]PLGGRDES[Phospho (STY)]PVSHR_.3	#N/A	3.5869	4.4423	3.2397	5.4858	4.5203	4.9427	3.9175	5.4328	5.1650	5.4280	0.8764	-0.1903	0.2644	0.5778
Cdk13	_S[Phospho (STY)]RS[Phospho (STY)]PY[Phospho (STY)]SPVLR_.2	Q69ZA1_Y400_M3_Cdk13	3.7707	3.7026	3.8785	6.8435	4.6280	3.7634	3.8425	6.2803	3.6437	7.2514	0.8433	-0.2459	0.4097	0.3875
Cdk13	_S[Phospho (STY)]RS[Phospho (STY)]PYS[Phospho (STY)]PVLRR_.3	#N/A	3.9195	3.4229	3.6532	3.1970	3.0633	3.5613	3.3452	3.8042	4.1032	4.3751	0.8880	-0.1713	0.1010	0.9956
Tspyl5	_AAAEDAWHDEKPPQS[Phospho (STY)]PR_.3	Q69ZB3_S41_M1_Tspyl5	5.5585	6.0651	5.4784	3.9260	4.0160	5.1427	5.3758	3.9448	5.3670	5.4006	1.0018	0.0026	0.9867	0.0058
Cnnm4	_NSLLHRAS[Phospho (STY)]EEETI_.2	Q69ZF7_S766_M1_Cnnm4	5.8804	5.6631	3.9531	6.0866	3.3631	5.8323	3.1546	6.7670	5.9760	5.7342	0.9486	-0.0762	0.7539	0.1227
Arhgap23	_SYS[Phospho (STY)]PSFQR_.2	Q69ZH9_S421_M1_Arhgap23	3.1074	3.8636	8.9261	4.5520	8.1172	8.4265	8.4162	8.4255	8.3912	8.3326	0.7347	-0.4447	0.1319	0.8796
Arhgap23	_RHS[Phospho (STY)]TSDLSDATFSDIR_.3	#N/A	3.5590	7.3712	3.3780	7.7356	6.5126	3.9258	7.3872	4.0181	7.4661	7.7872	0.8123	-0.2999	0.3436	0.4639
Arhgap23	_S[Phospho (STY)]AEALGPGALVSPR_.2	Q69ZH9_S361_M1_Arhgap23	11.0978	11.1243	11.5066	12.2204	10.5985	11.2429	11.4044	11.6182	11.5462	11.4538	0.9820	-0.0262	0.4773	0.3212
Arhgap23	_SKS[Phospho (STY)]C[Carbamidomethyl (C)]DDGLNTFRDEGR_.3	#N/A	8.4103	8.1774	8.9364	8.9513	7.6033	8.2984	8.2073	8.5410	3.4163	8.2930	1.1802	0.2390	0.2400	0.6197
Arhgap23	_HYS[Phospho (STY)]QDC[Carbamidomethyl (C)]SSIK_.2	Q69ZH9_S594_M1_Arhgap23	5.8751	6.4853	6.1037	6.5574	5.6314	6.2177	6.1436	6.2418	6.4003	6.3960	0.9761	-0.0349	0.4463	0.3504
Arhgap23	_HS[Phospho (STY)]TSDLSDATFSDIRR_.3	#N/A	3.7158	6.3603	4.4026	6.8570	5.4127	6.6336	3.7480	6.5378	6.2775	6.6923	0.9570	-0.0635	0.7771	0.1095
Arhgap23	_QMNLGFGDES[Phospho (STY)]PEPEAR_.2	Q69ZH9_S515_M1_Arhgap23	3.2706	4.0758	3.9574	5.8992	3.8971	3.8324	4.1932	3.6660	3.3070	4.4270	1.0659	0.0921	0.6214	0.2066
Arhgap23	_ARS[Phospho (STY)]DDYLSR_.2	Q69ZH9_S351_M1_Arhgap23	8.4264	8.6025	9.0865	8.9797	7.9194	8.4779	8.4928	8.3828	8.3442	8.5182	1.0175	0.0250	0.5167	0.2868
Arhgap23	_SKS[Phospho (STY)]C[Carbamidomethyl (C)]DDGLNTFR_.2	#N/A	8.1981	7.4190	7.4315	8.1084	6.8954	7.6784	8.0176	7.3521	7.5065	7.8217	0.9931	-0.0099	0.8527	0.0692
Arhgap23	_HS[Phospho (STY)]TSDLSDATFSDIR_.2	#N/A	3.0854	3.8906	3.1854	6.1010	3.7120	5.3172	4.3783	4.2720	3.0702	5.4778	0.9804	-0.0286	0.9115	0.0402
Arhgap23	_RS[Phospho (STY)]S[Phospho (STY)]YLLAITTER_.2	Q69ZH9_S606_M2_Arhgap23	3.0814	4.2610	4.3159	3.8597	4.0823	3.6472	4.0079	3.1415	3.4405	3.5370	1.0971	0.1337	0.2483	0.6050
Sh3rf1	_TSS[Phospho (STY)]LDSAVPIAPPPR_.2	Q69ZI1_S805_M1_Sh3rf1	3.1282	4.2142	4.3627	3.9065	3.7728	3.6940	4.0548	8.9290	8.6198	3.5838	0.6108	-0.7111	0.0669	1.1748
Prex1	_DSVLS[Phospho (STY)]YTSVR_.2	Q69ZK0_S1173_M1_Prex1	3.2600	3.9662	3.9468	4.4494	3.8865	4.2710	5.1790	3.6691	3.3176	4.4164	0.9560	-0.0649	0.6509	0.1865
Jmjd1c	_LVSRT[Phospho (STY)]PT[Phospho (STY)]PK_.2	Q69ZK6_T322_M2_Jmjd1c	5.1464	3.6748	3.4013	5.2943	5.2004	4.2334	3.0933	5.5804	4.3552	5.7433	0.9571	-0.0632	0.7640	0.1169
Nlgn2	_RLS[Phospho (STY)]PPGGSGSGVPGGGPLLPTAGR_.3	Q69ZK9_S714_M1_Nlgn2	3.8234	8.9131	8.7999	10.3924	6.7564	3.2796	3.6404	8.6358	3.8081	3.1694	1.4530	0.5391	0.2599	0.5852
Fgd6	_AAS[Phospho (STY)]EELVEK_.2	Q69ZL1_S494_M1_Fgd6	5.6415	3.0143	5.6189	4.7295	3.3819	4.3926	5.5016	4.1608	4.2226	3.6453	1.0184	0.0263	0.9034	0.0441
Fgd6	_S[Phospho (STY)]LDEDSERKEEVSPLGAK_.3	Q69ZL1_S1167_M1_Fgd6	3.8313	3.1356	4.5181	3.4842	2.9570	3.2717	3.6325	3.0977	3.4476	3.4869	1.0342	0.0485	0.7108	0.1483
Hectd1	_SSS[Phospho (STY)]DNNTNTLGR_.2	Q69ZR2_S1576_M1_Hectd1	7.8809	8.0953	8.3180	7.7025	4.2017	7.7457	3.8885	7.9257	3.5599	7.8074	1.2638	0.3377	0.2501	0.6018
Hectd1	_RLDS[Phospho (STY)]SGER_.2	Q69ZR2_S357_M1_Hectd1	3.7041	4.9734	6.1811	5.7709	2.8298	6.3795	6.1065	3.2249	4.3744	3.3597	1.1657	0.2211	0.4553	0.3417
Hectd1	_AS[Phospho (STY)]LALIR_.2	Q69ZR2_S545_M1_Hectd1	3.2798	4.0626	4.5143	4.0581	3.8840	3.8455	4.2063	4.4440	3.2422	3.7353	1.0086	0.0124	0.9113	0.0403
Tasor	_NNS[Phospho (STY)]RDETTEPEQQK_.2	Q69ZR9_S653_M1_Tasor	4.1636	3.4190	3.8973	3.4410	3.3073	3.3173	3.2104	3.5601	3.8592	3.5559	1.0125	0.0180	0.8369	0.0774
Hbs1l	_SSQS[Phospho (STY)]ESEIVPK_.2	Q69ZS7_S150_M1_Hbs1l	6.3166	3.9944	6.4098	4.3787	3.8157	6.5288	4.2745	3.7398	3.3884	4.3457	1.3311	0.4126	0.0954	1.0205
Kazn	_SSTPSDINS[Phospho (STY)]PR_.2	Q69ZS8_S343_M1_Kazn	9.3978	9.6433	9.6395	9.8142	8.9127	9.4770	9.3655	9.2383	9.1062	9.8287	1.0102	0.0147	0.6482	0.1883
Kazn	_VS[Phospho (STY)]PC[Carbamidomethyl (C)]HSR_.2	#N/A	5.7723	5.8585	5.3285	5.8144	3.2068	3.4178	5.5609	5.0110	4.6205	5.2570	0.9584	-0.0613	0.7538	0.1227
Kazn	_THSLC[Carbamidomethyl (C)]NGDS[Phospho (STY)]PGPVQK_.2	Q69ZS8_S356_M1_Kazn	6.2813	6.6354	5.4702	7.0850	6.2072	6.8639	3.2006	6.5420	6.0957	6.2257	1.1646	0.2198	0.2193	0.6590
Kazn	_QVRVS[Phospho (STY)]PC[Carbamidomethyl (C)]HSR_.3	#N/A	3.2066	3.9629	4.4411	4.1238	3.8512	4.2743	4.1332	3.0163	4.4200	3.6622	1.0443	0.0625	0.6207	0.2071
Kazn	_S[Phospho (STY)]LDPGLFDDSDSQC[Carbamidomethyl (C)]SPTR_.2	Q69ZS8_S404_M1_Kazn	3.0914	3.8477	4.3260	4.5679	3.7360	4.3894	4.2826	3.5506	3.1992	3.0341	1.1355	0.1833	0.2164	0.6648
Ehbp1	_S[Phospho (STY)]IQEDTKR_.2	#N/A	9.5121	9.5186	9.7639	9.7557	9.2236	9.7204	9.3761	9.4194	9.5061	9.7247	1.0080	0.0115	0.5558	0.2551
Ehbp1	_QRS[Phospho (STY)]IQEDTKR_.3	#N/A	4.3867	5.0811	4.9513	4.4712	5.0838	4.4398	3.8997	4.3915	5.1626	4.8978	1.0322	0.0457	0.6120	0.2133
Ehbp1	_SRS[Phospho (STY)]LEC[Carbamidomethyl (C)]R_.2	Q69ZW3_S747_M1_Ehbp1	3.6974	4.7085	3.8703	4.8129	3.5191	4.6322	4.4938	4.9836	5.0351	4.9075	0.8665	-0.2068	0.0688	1.1623
Ehbp1	_T[Phospho (STY)]SGSDDPGLSSSTDSAQALASLGK_.2	Q69ZW3_T673_M1_Ehbp1	3.7240	3.6336	3.4577	3.8664	3.7327	3.7721	4.0147	4.0149	3.6781	4.1796	0.9310	-0.1032	0.0369	1.4324
Ehbp1	_QRS[Phospho (STY)]IQEDTK_.2	#N/A	3.8752	4.2094	4.5620	3.4403	3.0009	3.4960	4.3203	3.5608	3.8599	3.1176	1.0133	0.0190	0.8920	0.0496
Ehbp1	_LDGELAITKPNVSS[Phospho (STY)]PSK_.2	Q69ZW3_S765_M1_Ehbp1	3.2568	3.9693	3.9436	4.4463	4.3615	4.2678	4.1610	3.6722	5.7589	4.4133	0.8977	-0.1557	0.3017	0.5204
Ehbp1	_VGNYETDTNSS[Phospho (STY)]VDQEK_.2	Q69ZW3_S588_M1_Ehbp1	3.0570	4.1692	3.7438	4.2464	3.6835	4.0460	3.9611	3.8721	3.5206	4.2134	0.9827	-0.0252	0.7947	0.0998
Ehbp1	_ITEIVNQLNALSS[Phospho (STY)]LDEDQDDC[Carbamidomethyl (C)]IK_.3	Q69ZW3_S222_M1_Ehbp1	4.0419	3.1842	3.2279	3.7305	3.0725	3.5521	3.4452	4.3879	4.0365	3.6975	0.8912	-0.1663	0.1292	0.8887
Morc2a	_S[Phospho (STY)]PPLPAVIK_.2	Q69ZX6_S614_M1_Morc2a	3.6276	8.0014	8.5448	8.5789	7.5874	8.2223	8.4017	8.2092	8.5501	8.4476	0.8840	-0.1780	0.3442	0.4632
Ablim3	_ASS[Phospho (STY)]PGYIDSPTYSR_.2	Q69ZX8_S373_M1_Ablim3	3.4419	6.9108	6.6216	3.1305	4.0684	3.6611	4.3460	6.6555	3.1357	3.0975	1.0767	0.1066	0.7677	0.1148
Ablim3	_SS[Phospho (STY)]PYHSQLDVR_.2	#N/A	4.2057	6.7171	5.6867	4.6106	6.0783	6.6771	3.2581	3.8914	4.1904	3.8613	1.4900	0.5753	0.0104	1.9841
Ablim3	_SGPESGRSS[Phospho (STY)]PYHSQLDVR_.3	#N/A	3.5162	4.2725	5.9269	4.1431	4.1608	3.9646	3.8578	4.2075	3.6240	3.9718	1.1061	0.1455	0.3648	0.4380
Ablim3	_RFS[Phospho (STY)]SGGEEEDFDR_.2	Q69ZX8_S502_M1_Ablim3	5.2585	4.6628	5.9921	5.7267	5.5063	5.1133	5.7947	4.9447	6.2229	5.4044	0.9615	-0.0566	0.5244	0.2804
Ablim3	_YTS[Phospho (STY)]DEMLER_.2	Q69ZX8_S337_M1_Ablim3	4.2457	3.0967	3.9794	3.5231	2.9181	3.3106	3.6714	3.4781	3.7771	3.2004	0.9945	-0.0080	0.9464	0.0239
Ablim3	_TSETSIS[Phospho (STY)]PPGSSIGSPNR_.2	Q69ZX8_S282_M1_Ablim3	8.3898	8.2963	9.0505	8.9657	8.1896	8.5956	8.9133	8.3486	8.5582	8.5217	0.9995	-0.0007	0.9842	0.0069
Ablim3	_SAS[Phospho (STY)]LPAYR_.2	Q69ZX8_S584_M1_Ablim3	3.1818	4.0444	3.8686	5.9667	3.9327	6.3806	4.0859	3.7473	5.4328	4.3382	1.0367	0.0520	0.8280	0.0820
Ablim3	_SHYLADS[Phospho (STY)]DPLISK_.2	#N/A	3.9197	4.1745	3.7646	6.1499	5.3320	6.1428	5.6888	5.7849	6.2690	5.9984	0.8279	-0.2724	0.1116	0.9522
Ablim3	_RAS[Phospho (STY)]SPGYIDSPTYSR_.2	Q69ZX8_S372_M1_Ablim3	5.4472	2.9967	3.4154	3.9181	2.8850	3.7396	3.6327	5.5950	4.8178	3.8851	0.8329	-0.2638	0.2425	0.6152
Ablim3	_RTS[Phospho (STY)]ETSIS[Phospho (STY)]PPGSSIGSPNR_.2	Q69ZX8_S277_M2_Ablim3	3.1515	3.9567	3.8383	4.3410	3.7780	3.9515	4.3123	3.7775	3.4261	4.3080	0.9697	-0.0444	0.6597	0.1807
Tmcc1	_NKFGS[Phospho (STY)]ADNIPNLK_.2	#N/A	5.6774	5.6401	4.2521	5.8919	4.2912	5.2739	6.0577	5.7139	4.5252	5.3256	0.9566	-0.0640	0.6181	0.2089
Tmcc1	_FGS[Phospho (STY)]ADNIPNLK_.2	#N/A	5.5592	5.5959	3.4792	5.7988	2.8893	3.7353	6.3032	5.7265	4.0582	4.2011	0.8891	-0.1696	0.4962	0.3043
Kiaa0754	_GS[Phospho (STY)]PPDC[Carbamidomethyl (C)]TC[Carbamidomethyl (C)]VPAGDTVISEVK_.2	Q69ZZ9_S276_M1_Kiaa0754	3.0583	4.1679	3.7451	4.2478	4.0562	4.0693	3.9625	3.8707	3.5193	4.2148	0.9997	-0.0004	0.9968	0.0014
N4bp1	_KQFS[Phospho (STY)]LENVPEGELLPDGK_.2	#N/A	3.9399	3.5523	4.1269	3.3937	3.8558	3.3900	3.9809	3.4221	3.4516	3.2127	1.0549	0.0770	0.3712	0.4303
Cep170	_ALLHSGS[Phospho (STY)]SSSK_.2	#N/A	3.8258	7.7464	7.4913	7.7523	5.9049	3.2772	7.1439	7.2648	7.1173	7.6886	0.8215	-0.2837	0.2447	0.6115
Cep170	_SKTS[Phospho (STY)]PVASGSTSK_.2	Q6A065_S1069_M1_Cep170	6.5707	7.1682	7.5489	7.3763	6.3668	3.2192	7.2182	7.1230	7.1829	7.2323	0.8868	-0.1734	0.3517	0.4538
Cep170	_SRS[Phospho (STY)]ADIGAR_.2	Q6A065_S985_M1_Cep170	5.6264	4.3378	6.4917	3.7829	4.1591	3.5704	5.2761	5.8925	5.8300	3.4602	0.9114	-0.1339	0.5565	0.2545
Cep170	_LGEAS[Phospho (STY)]DSELADADK_.2	Q6A065_S1102_M1_Cep170	3.4233	6.7641	4.1102	7.2006	4.0499	3.6796	4.3275	3.5057	3.1543	3.0790	1.3852	0.4701	0.1609	0.7934
Cep170	_LRTS[Phospho (STY)]PALK_.2	Q6A065_S1250_M1_Cep170	3.7961	3.1004	4.4829	3.4847	2.9217	3.3069	6.3365	3.1330	6.4253	3.4517	0.7268	-0.4603	0.1216	0.9151
Cep170	_SIKS[Phospho (STY)]DVPVYLKR_.3	#N/A	3.8583	6.2377	6.3852	7.2650	5.5081	5.9965	6.0695	6.4048	6.8685	4.6924	0.9778	-0.0325	0.8515	0.0698
Cep170	_S[Phospho (STY)]VDSRPQPAEHPDHLTITR_.4	Q6A065_S1384_M1_Cep170	4.0399	5.7103	3.7319	3.2756	3.1655	3.0631	3.4239	3.7255	4.0246	2.9529	1.0847	0.1174	0.5931	0.2269

Cep170	_SIKS[Phospho (STY)]DVPVYLK_.2	#N/A	6.1310	6.7594	6.5306	8.4387	6.8824	6.9979	7.2666	6.8989	6.4966	6.7408	1.0155	0.0221	0.8081	0.0925
Cep170	_TEEDSKS[Phospho (STY)]IKS[Phospho (STY)]DVPVYLK_.2	#N/A	3.6522	6.4458	3.9872	8.2885	7.3672	3.7311	3.9345	7.5069	7.9056	7.1495	0.8422	-0.2478	0.4336	0.3629
Cep170	_QGS[Phospho (STY)]FTIDKPSSNIPIELIPHINK_.3	Q6A065_S829_M1_Cep170	3.2051	4.0103	3.8919	4.1104	3.8316	3.8979	4.2587	4.3916	3.1898	3.7877	0.9789	-0.0308	0.7678	0.1148
Cep170	_C[Carbamidomethyl (C)]STSS[Phospho (STY)]PSKDVTK_.2	Q6A065_S963_M1_Cep170	4.2719	3.0741	4.0056	3.5493	3.1049	3.3368	4.6976	3.4555	3.7545	3.2266	1.0067	0.0096	0.9320	0.0306
Cep170	_ALLHSGS[Phospho (STY)]SSSKEK_.2	#N/A	2.7154	3.5811	3.4627	4.5396	3.4025	4.3270	3.1870	3.4737	4.2615	4.2168	0.9700	-0.0439	0.7846	0.1053
Cep170	_SDS[Phospho (STY)]EATISR_.2	Q6A065_S1157_M1_Cep170	3.3864	4.1426	6.4010	4.3101	4.0309	4.1316	4.0248	3.8455	3.4941	3.5994	1.1763	0.2342	0.2523	0.5981
Cep170	_SDS[Phospho (STY)]LDTDSSM[Oxidation (M)]DTTLILK_.2	#N/A	6.2208	5.4746	3.8523	4.3033	3.5171	6.1657	6.3542	6.1502	3.6323	6.0410	0.8878	-0.1717	0.4533	0.3436
Cep170	_GNKHDDGTQS[Phospho (STY)]DSENAGAHR_.3	#N/A	3.5783	3.7787	3.3309	4.7041	3.3398	4.4710	4.1364	4.2135	3.4817	3.9201	0.9820	-0.0262	0.8337	0.0790
Cep170	_HDDGTQS[Phospho (STY)]DSENAGAHRR_.3	#N/A	3.1541	4.1308	4.1477	4.1472	3.8616	3.8770	4.0698	3.4747	3.3977	3.9932	1.0409	0.0578	0.5404	0.2672
Cep170	_RFPTDYAS[Phospho (STY)]TS[Phospho (STY)]EDEFGSNR_.2	Q6A065_S1229_M2_Cep170	3.1085	4.0533	4.1056	6.5311	3.7445	4.1906	4.0883	3.7561	3.4047	3.7151	1.1465	0.1972	0.3923	0.4063
Cep170	_NQATSASSEKDNDDDQSDKGTY[Phospho (STY)]TIELENPNSEEVEAR_.4	Q6A065_Y499_M1_Cep170	1.9302	4.2675	3.2449	4.1482	4.1558	3.9697	3.8629	4.2125	3.6189	3.9668	0.9244	-0.1134	0.5485	0.2608
Cep170	_GNKHDDGTQS[Phospho (STY)]DSENAGAHR_.4	#N/A	3.2106	4.0158	3.8974	4.1049	3.8372	3.8923	4.2531	4.3971	3.1954	3.7821	0.9794	-0.0301	0.7718	0.1125
Larp4b	_SPS[Phospho (STY)]PVHLPEDPKVAEK_.3	#N/A	8.3194	3.0934	9.4864	8.7284	8.3298	8.7970	3.6747	8.3746	3.7738	3.2037	1.6382	0.7121	0.0828	1.0820
Larp4b	_EQNTPPKS[Phospho (STY)]PQ_.2	Q6A0A2_S739_M1_Larp4b	8.0117	8.4900	7.5751	9.1504	7.3308	7.2717	7.2269	8.5065	7.4536	7.7077	1.0321	0.0456	0.5852	0.2327
Larp4b	_RAS[Phospho (STY)]PPAAGKR_.2	#N/A	7.4066	8.1136	8.1851	8.5314	7.2534	8.4534	8.1217	6.5181	7.8046	7.9050	1.0531	0.0747	0.3391	0.4697
Larp4b	_KNS[Phospho (STY)]FGYR_.2	Q6A0A2_S500_M1_Larp4b	7.7204	8.0271	3.3111	8.1826	7.2753	7.6321	7.8565	4.3047	7.7928	8.0695	1.0027	0.0039	0.9877	0.0054
Larp4b	_RAS[Phospho (STY)]PPAAGK_.2	#N/A	6.7186	4.3057	7.0036	7.2017	3.3759	5.7325	6.1120	6.6598	6.7619	7.0410	0.8614	-0.2152	0.2920	0.5346
Larp4b	_SPS[Phospho (STY)]PVHLPEDPK_.2	#N/A	9.9357	9.6438	4.3343	10.4221	8.8440	3.4555	3.8163	3.3331	9.4638	10.0168	1.1675	0.2234	0.6107	0.2142
Rftn1	_DSVALRHS[Phospho (STY)]NPR_.2	#N/A	4.3961	4.2563	4.2880	4.0740	3.9639	4.5530	4.5753	4.0780	4.6124	4.6733	0.9488	-0.0758	0.1733	0.7612
Cgnl1	_SYGSQPNS[Phospho (STY)]PTSEDLAK_.2	Q6AW69_S203_M1_Cgnl1	5.1407	3.8186	3.7002	4.4088	4.6628	4.1062	3.6658	3.9088	3.9482	6.4759	0.9570	-0.0634	0.7523	0.1236
Cgnl1	_RQDS[Phospho (STY)]AGPILDGAR_.2	Q6AW69_S284_M1_Cgnl1	5.8960	4.2318	4.3451	6.3131	5.2257	5.3999	5.7544	5.3396	5.9021	5.7993	0.9187	-0.1224	0.3161	0.5002
Nlrp14	_KVM[Oxidation (M)]LPESYLLVATR_.2	#N/A	12.1281	11.1308	12.3829	11.2980	11.8729	12.2051	12.0210	10.9877	11.6867	12.0445	1.0129	0.0186	0.6547	0.1840
Arhgap21	_DQGEPVS[Phospho (STY)]PSEDEPFSWPGPK_.2	Q6DFV3_S41_M1_Arhgap21	10.5554	10.3617	10.5822	11.3319	9.7243	10.5016	11.0523	10.9951	3.6766	10.4197	1.1631	0.2180	0.3364	0.4732
Arhgap21	_NRS[Phospho (STY)]PTLSC[Carbamidomethyl (C)]R_.2	Q6DFV3_S1501_M1_Arhgap21	7.2076	7.5803	7.7768	7.8513	7.4579	7.1081	7.7886	7.5485	7.4883	7.2951	0.9956	-0.0064	0.8526	0.0693
Arhgap21	_QLS[Phospho (STY)]HDQESVGPPSLDGQHSSK_.3	#N/A	8.4188	8.0115	8.7203	8.6478	6.8301	8.3930	4.3617	4.2886	3.0868	8.4609	1.6180	0.6942	0.0139	1.8578
Arhgap21	_VGGS[Phospho (STY)]IRPWK_.2	Q6DFV3_S947_M1_Arhgap21	7.5759	7.6512	7.5653	8.0459	4.0747	7.0708	7.1795	7.5874	7.2362	7.4299	0.9509	-0.0726	0.6437	0.1913
Arhgap21	_LLSKDDTS[Phospho (STY)]PPKDK_.3	Q6DFV3_S1108_M1_Arhgap21	3.7387	3.4875	4.4255	3.4273	2.8644	3.2488	3.1420	6.4058	4.4397	3.3943	0.8175	-0.2907	0.2560	0.5917
Arhgap21	_KGS[Phospho (STY)]SSEVFSDAAR_.2	Q6DFV3_S917_M1_Arhgap21	3.5379	6.0619	6.0762	7.0841	5.9801	6.5055	7.1338	6.7193	6.9137	6.0451	0.8764	-0.1904	0.2370	0.6252
Arhgap21	_SKS[Phospho (STY)]YDEGLDDYREDAK_.3	#N/A	7.5429	7.9801	7.8507	8.5291	6.6383	7.9642	7.7327	8.2172	7.9224	7.9263	0.9750	-0.0365	0.5657	0.2474
Arhgap21	_VRTS[Phospho (STY)]ASDLSR_.2	Q6DFV3_S1847_M1_Arhgap21	4.3497	3.6541	3.5357	4.4666	3.4754	4.2541	3.1140	4.0354	4.1214	4.1439	1.0265	0.0378	0.7428	0.1291
Arhgap21	_EKPQS[Phospho (STY)]GRQTPQPLR_.3	Q6DFV3_S736_M1_Arhgap21	2.9161	4.4263	6.2160	3.6944	4.2476	3.4819	3.8427	6.1881	3.6058	5.6437	0.8638	-0.2112	0.4212	0.3755
Arhgap21	_T[Phospho (STY)]SQGFGFTLR_.2	Q6DFV3_T61_M1_Arhgap21	3.6572	3.6852	3.3909	4.4355	3.5065	4.2229	3.0829	6.0252	4.3656	4.1128	0.8680	-0.2042	0.3027	0.5190
Arhgap21	_S[Phospho (STY)]TS[Phospho (STY)]HDRVPQSVQIR_.2	#N/A	3.5665	3.7935	4.1940	4.1149	3.5096	3.9712	3.4759	3.8582	4.1313	3.7812	1.0122	0.0175	0.8018	0.0959
Arhgap21	_RQLS[Phospho (STY)]HDQESVGPPSLDGQHSSK_.4	#N/A	7.5039	7.6510	7.8677	7.6324	6.0690	5.1228	7.3274	7.5555	7.2128	7.5407	0.9413	-0.0872	0.4698	0.3281
Arhgap21	_TQSPHS[Phospho (STY)]PKEESER_.3	Q6DFV3_S1092_M1_Arhgap21	4.1790	4.2134	5.0216	3.9157	3.9378	4.5481	4.0640	4.4626	3.3847	5.3930	1.1101	0.1506	0.1758	0.7549
Arhgap21	_GIPS[Phospho (STY)]IVR_.2	Q6DFV3_S1122_M1_Arhgap21	5.5471	4.5624	3.8776	5.0135	3.2877	5.2178	3.2301	5.7364	3.8788	5.6991	0.9888	-0.0162	0.9402	0.0268
Arhgap21	_SVS[Phospho (STY)]QERLEDSVLMK_.2	Q6DFV3_S453_M1_Arhgap21	3.8205	3.5219	3.5542	4.5988	3.3432	4.3863	3.2462	3.9032	4.2023	4.2761	0.9908	-0.0134	0.9127	0.0397
Ncoa7	_EVLDSLGSS[Phospho (STY)]LDK_.2	Q6DFV7_S612_M1_Ncoa7	3.9292	3.4132	3.6629	3.2067	3.2345	5.9540	3.3549	3.7945	4.0936	4.3848	0.9982	-0.0025	0.9905	0.0041
Ncoa7	_HSGS[Phospho (STY)]PENLGESTLNIHEDLDKIK_.4	Q6DFV7_S500_M1_Ncoa7	3.5073	4.2636	3.2410	4.2856	4.1519	3.9735	3.8667	5.5866	3.0146	3.9629	0.9504	-0.0734	0.6814	0.1666
Ncoa7	_VLS[Phospho (STY)]STSEEDEPGVVK_.2	Q6DFV7_S211_M1_Ncoa7	3.5740	3.9561	5.0466	6.8582	3.7774	6.3753	5.3688	3.8098	5.5939	3.8844	1.0573	0.0803	0.7505	0.1247
Ncoa7	_YYS[Phospho (STY)]VDDNQNK_.2	Q6DFV7_S92_M1_Ncoa7	6.5623	4.1661	6.3720	7.0691	6.1996	6.4794	6.7318	4.0075	4.1110	3.2076	1.3604	0.4440	0.0751	1.1243
Ncoa7	_SVRS[Phospho (STY)]PEESTER_.2	Q6DFV7_S353_M1_Ncoa7	7.0504	3.9051	7.5852	3.9271	6.4082	6.8626	4.0754	3.0741	6.5296	3.6044	1.3785	0.4631	0.1495	0.8252
	[Acetyl (Protein N-term)]STIC[Carbamidomethyl (C)]PPPS[Phospho (STY)]PAVAK.2	Q6DFW0_S9_M1_	7.2764	4.3212	4.2557	3.7995	3.6658	2.9589	7.9059	3.2017	3.5008	7.4177	0.7953	-0.3304	0.3938	0.4048
Nop58	_EEPLS[Phospho (STY)]EEEPC[Carbamidomethyl (C)]TSTAVPS[Phospho (STY)]PEK_.2	#N/A	3.7179	3.2948	3.4516	5.7837	3.1425	5.8421	5.0237	6.8691	4.3060	4.1735	0.8257	-0.2763	0.3043	0.5166
Nop58	_HIKEEPLS[Phospho (STY)]EEEPC[Carbamidomethyl (C)]TSTAVPS[Phospho (STY)]PEK_.3	#N/A	3.4327	4.7837	3.7378	3.7466	8.8375	3.6547	4.3477	3.9646	3.1184	3.5280	1.0336	0.0477	0.7038	0.1525
Nol4l	_NTPES[Phospho (STY)]PPYSSGSYDSIK_.2	Q6DIB4_S189_M1_Nol4l	3.9680	7.3205	3.1539	3.3475	7.0521	3.1350	3.4958	3.6536	3.9527	7.7320	0.9903	-0.0141	0.9726	0.0121
Nol4l	_LEIYQSS[Phospho (STY)]QEEPIALDK_.2	Q6DIB4_S354_M1_Nol4l	3.8216	3.4045	4.5085	8.4236	3.2928	3.3318	3.2250	7.6061	4.2568	7.7305	0.7825	-0.3539	0.3903	0.4086
Scaf8	_ASEPVKEPVQTAQS[Phospho (STY)]PAPVEK_.3	#N/A	7.6104	7.5627	7.7882	7.9052	7.0306	7.6848	8.0270	7.6405	7.5188	7.4592	0.9916	-0.0122	0.7366	0.1328
Lemd2	_WTKPSSFS[Phospho (STY)]DSER_.2	Q6DVA0_S507_M1_Lemd2	3.9548	3.6209	3.9212	6.9857	3.4772	6.9865	7.5451	3.6911	7.1487	3.8073	0.8696	-0.2016	0.5601	0.2517
Lemd2	_AS[Phospho (STY)]VRGS[Phospho (STY)]SEDDEDTRTPDR_.3	Q6DVA0_S145_M2_Lemd2	5.9576	3.5481	3.9572	4.0980	3.4037	3.8957	3.1532	3.6264	4.3179	3.8679	1.1075	0.1473	0.4534	0.3435
Utp14b	_S[Phospho (STY)]KAIMAKY[Phospho (STY)]DPEAR_.2	Q6EJB6_S307_M2_Utp14b	8.2784	7.1486	7.2752	7.0796	6.8944	8.3068	8.8271	8.7137	7.9900	7.5733	0.9059	-0.1426	0.0862	1.0643
Tmem151a	_SLS[Phospho (STY)]GGPLGR_.2	Q6GQT5_S423_M1_Tmem151a	7.2019	4.4872	3.8886	3.9058	3.5709	4.5722	4.0540	4.7601	5.0757	5.0991	0.9699	-0.0441	0.8445	0.0734
Ralgapa1	_SQT[Phospho (STY)]PS[Phospho (STY)]PSTLSR_.2	Q6GYP7_S1003_M2_Ralgapa1	8.9419	8.3975	8.9535	8.2148	6.6832	8.3853	8.8842	8.5492	8.6924	8.7787	0.9469	-0.0787	0.3113	0.5069
Ralgapa1	_SSS[Phospho (STY)]TSDILEPFTVER_.2	Q6GYP7_S796_M1_Ralgapa1	7.6329	3.7447	3.9946	4.2641	3.4047	3.6967	3.6867	3.5855	7.1271	3.9414	0.9719	-0.0411	0.9054	0.0431
Ralgapa1	_SATTGS[Phospho (STY)]PGTEK_.2	Q6GYP7_S739_M1_Ralgapa1	9.0241	9.2865	9.6061	9.0056	3.8212	3.9083	9.4862	9.5740	8.9688	8.9306	0.8054	-0.3122	0.2425	0.6153
Ralgapa1	_RGS[Phospho (STY)]SPGSLEIPK_.2	Q6GYP7_S859_M1_Ralgapa1	7.3240	7.0105	3.8686	3.3939	3.2347	3.1992	8.4761	8.0081	6.9531	6.9204	0.6156	-0.7000	0.0232	1.6353
Ralgapa1	_GVGHEFQKVS[Phospho (STY)]VDK_.3	Q6GYP7_S710_M1_Ralgapa1	3.3693	2.7293	3.2739	3.7765	3.2136	3.5167	3.4912	2.7007	3.9905	3.6492	0.9582	-0.0617	0.6194	0.2080
Ralgapa1	_GWS[Phospho (STY)]RDQPGQAPMR_.2	#N/A	4.0918	4.0171	3.9104	3.8886	3.5410	3.9836	3.8210	3.7458	3.1502	3.7282	1.0815	0.1130	0.0983	1.0074
Hecw2	_ANS[Phospho (STY)]DTDLVTSESR_.2	Q6I6G8_S48_M1_Hecw2	11.2731	11.2694	11.7684	11.3061	10.2137	11.0661	11.6424	11.2232	11.1645	11.2526	0.9849	-0.0220	0.5522	0.2579
Hecw2	_SHS[Phospho (STY)]AGEVGEDSR_.2	#N/A	5.7825	6.6442	6.8669	6.5201	5.3676	5.1343	6.4022	6.1409	5.6416	6.0644	0.9984	-0.0023	0.9809	0.0084
Hecw2	_TSSTLEIDTEDLISTSSRNS[Phospho (STY)]PPR_.3	Q6I6G8_S406_M1_Hecw2	7.4030	3.4760	3.6000	3.1438	3.2974	4.4321	3.2921	3.8574	4.1564	4.3219	1.0815	0.1130	0.7164	0.1449
Hecw2	_SNS[Phospho (STY)]IQQMEQLNR_.2	#N/A	6.9129	5.3724	4.0743	6.8377	4.7789	3.8358	6.1288	4.2527	3.8962	5.5844	1.0678	0.0946	0.6850	0.1643
Hecw2	_QRSHS[Phospho (STY)]AGEVGEDSR_.3	#N/A	3.6731	3.7780	4.2433	3.6972	3.6687	3.9382	4.0489	4.3589	3.4693	3.7938	0.9784	-0.0315	0.6652	0.1771
Tmem132e	_DQTEDPASS[Phospho (STY)]PTSK_.2	#N/A	4.3583	3.6627	3.4134	6.9172	3.4840	4.2455	3.1055	7.5924	4.4330	4.1353	0.9067	-0.1413	0.6730	0.1720
Tmem132e	_DQTEDPASS[Phospho (STY)]PTSKR_.2	#N/A	4.0135	5.5280	6.0996	4.4462	3.9130	4.2337	4.0079	4.0574	4.3564	4.1235	1.1376	0.1861	0.2523	0.5982
Arhgap20	_KSS[Phospho (STY)]C[Carbamidomethyl (C)]DAVLSR_.2	Q6IFT4_S730_M1_Arhgap20	5.9783	3.3912	6.2743	6.2579	5.1973	5.9246	6.8064	6.2811	5.9810	6.0729	0.8757	-0.1915	0.2171	0.6633
Arhgap20	_S[Phospho (STY)]APSLILDK_.2	Q6IFT4_S46_M1_Arhgap20	2.9794	3.7357	4.1773	3.1791	3.6240	5.3410	4.3946	6.4916	6.9165	3.1461	0.7331	-0.4479	0.1288	0.8901
Arhgap20	_SS[Phospho (STY)]EPSIDYLDTK_.2	#N/A	2.9804	3.7367	4.2150	3.1781	3.6250	2.9996	4.3936	4.9386	3.0881	3.4360	0.8718	-0.1980	0.2600	0.5850
Arhgap20	_RC[Carbamidomethyl (C)]S[Phospho (STY)]EPSIDDQNYK_.2	Q6IFT4_S847_M1_Arhgap20	3.0586	3.8638	3.7454	4.2481	3.6851	4.0444	3.9627	3.8704	3.5190	4.2151	0.9698	-0.0443	0.6327	0.1988
Arhgap20	_RSS[Phospho (STY)]EPSIDYLDTK_.2	#N/A	3.9583	3.2627	3.1443	3.3572	3.0840	3.1447	3.5055	3.6440	3.9430	3.6139	0.9044	-0.1449	0.0893	1.0492
Gpsm1	_AAQSSVTAS[Phospho (STY)]PQTEEFFDLIASSQSR_.3	Q6IR34_S543_M1_Gpsm1	3.8897	3.1451	3.6234	3.1671	3.0334	3.5912	3.3154	3.8340	4.1331	4.3453	0.8724	-0.1970	0.0789	1.1030
Gpsm1	_APS[Phospho (STY)]SDEEC[Carbamidomethyl (C)]FFDLLSK_.2	Q6IR34_S490_M1_Gpsm1	3.4683	7.3460	4.1551	8.2168	7.2942	7.6832	7.4798	7.6558	7.8077	7.3954	0.8386	-0.2539	0.2687	0.5707
Gpsm1	_AS[Phospho (STY)]VGSLPGLR_.2	Q6IR34_S567_M1_Gpsm1	3.3899	4.1462	3.1236	7.4708	4.0345	4.0910	3.9841	6.7155	7.9007	5.9495	0.7130	-0.4880	0.1265	0.8978
Gpsm1	_EGSHS[Phospho (STY)]PLDSADVR_.2	Q6IR34_S469_M1_Gpsm1	3.4726	4.2778	4.1594	6.1456	4.0992	3.6303	3.9911	3.1583	3.4574	3.5201	1.2168	0.2831	0.1702	0.7691
Gpsm1	_VDLAGS[Phospho (STY)]PEQEASGLPDPQQQC[Carbamidomethyl (C)]PPGAS_.3	Q6IR34_S653_M1_Gpsm1	6.1872	3.6818	3.5634	4.4389	3.5032	4.2263	3.0863	4.0632	4.3622	4.0331	1.0979	0.1348	0.5172	0.2864
Cltb	_DLEEWNQR_.2	#N/A	9.4538	9.4271	9.9674	9.6759	9.7464	9.1854	9.0816	9.0697	9.0279	9.2485	1.0515	0.0725	0.0124	1.9052
Cltb	_LTQEPESIRK_.2	#N/A	8.4649	8.7525	8.7249	8.7246	9.3195	8.9269	8.4276	8.6755	8.6102	8.7008	1.0250	0.0357	0.1995	0.7001
Cltb	_VAQLC[Carbamidomethyl (C)]DFNPK_.2	#N/A	8.4616	8.7472	8.8364	8.6323	9.1006	8.3311	8.4904	8.4034	8.0372	8.7024	1.0329	0.0467	0.1589	0.7988
Cltb	_LTQEPESIR_.2	#N/A	8.4342	8.5612	8.5249	8.4301	9.1116	8.5794	7.9782	8.3621	8.1796	8.4318	1.0448	0.0632	0.0424	1.3728

Cltb	_LQELDAASK_.2	#N/A	8.0679	8.1340	8.5373	8.3895	8.7484	8.4057	8.0757	7.8533	8.1832	8.4778	1.0286	0.0407	0.1950	0.7099
Cltb	_VTEQEWR_.2	#N/A	7.7708	7.1318	7.3392	7.7590	7.4195	7.5721	7.3194	6.8456	7.4711	7.3496	1.0348	0.0494	0.1711	0.7668
Cltb	_KDLEEWNQR_.2	#N/A	4.0667	3.3711	3.2527	3.7553	5.5882	4.5393	3.4700	4.0000	4.1367	4.5648	1.0130	0.0187	0.9150	0.0386
Cltb	_ESKEETPGTEWEK_.2	#N/A	5.3394	5.5058	5.8681	5.7328	6.6591	6.0683	5.6095	5.4809	5.8622	5.9380	1.0244	0.0348	0.5965	0.2244
Cltb	_AFYQQPDADTIGYVASEEAFVK_.3	#N/A	4.0326	4.1836	3.7663	3.3100	4.4848	5.3689	3.4583	3.6911	3.9902	4.2028	1.0927	0.1279	0.3774	0.4232
Nipbl	_AITSLLGGGS[Phospho (STY)]PK_.2	Q6KCD5_S2652_M1_Nipbl	4.0798	7.2575	7.5302	7.8869	5.4885	6.5250	7.6048	7.7726	3.9986	3.7354	1.1183	0.1613	0.5664	0.2469
Dgkb	_FPHSS[Phospho (STY)]PNVK_.2	Q6NS52_S95_M1_Dgkb	4.2642	8.7015	8.9554	8.9993	7.7302	3.2166	8.9443	3.4595	4.3720	3.2190	1.3959	0.4812	0.2740	0.5622
Dgkb	_VLAC[Carbamidomethyl (C)]GGDGTVGWILDC[Carbamidomethyl (C)]IEK_.2	#N/A	3.3264	4.0827	4.8973	4.1047	3.9710	4.1544	4.2530	4.3973	3.0656	3.7618	1.0569	0.0798	0.5458	0.2629
Dgkb	_QDILNQTIDFEGFK_.2	#N/A	3.3176	4.0739	4.5521	4.0959	3.1476	3.5546	4.2441	4.4061	3.2043	3.7732	0.9701	-0.0437	0.7458	0.1274
Fbxo41	_GGGGS[Phospho (STY)]ASGPGVR_.2	Q6NS60_S363_M1_Fbxo41	6.7766	3.2240	3.7023	3.2461	4.8853	6.7334	7.0685	3.7551	4.0542	4.4242	0.9867	-0.0193	0.9522	0.0213
Fbxo41	_EHHAGSAVPSTYAVS[Phospho (STY)]R_.3	Q6NS60_S391_M1_Fbxo41	4.4623	4.0004	4.2758	3.5837	3.8470	4.5237	4.6661	3.2437	4.0395	4.6505	0.9917	-0.0120	0.9182	0.0370
Fbxo41	_RHS[Phospho (STY)]TEGEEGDVSDVGSR_.3	#N/A	8.4912	8.1952	9.2624	5.6912	7.7734	8.1153	8.5844	8.6780	8.6273	8.3031	0.9267	-0.1099	0.3397	0.4690
Fbxo41	_LSARPEGGS[Phospho (STY)]GR_.2	Q6NS60_S526_M1_Fbxo41	3.1146	4.1116	3.8014	4.3041	8.9363	4.1256	4.0187	3.8144	8.8755	4.2711	0.9023	-0.1484	0.7309	0.1361
Fbxo41	_[Acetyl (Protein N-term)]AS[Phospho (STY)]LDLPYR_.2	Q6NS60_S3_M1_Fbxo41	2.8986	6.3505	3.5854	4.0881	3.5252	4.2043	3.8028	6.8504	3.6790	5.9642	0.8097	-0.3045	0.3008	0.5217
Fbxo41	_HGSS[Phospho (STY)]PSTGASSR_.2	Q6NS60_S396_M1_Fbxo41	3.8672	3.3590	4.5540	3.5558	3.2473	3.3773	3.2705	3.0618	4.2113	3.5228	1.0408	0.0577	0.6640	0.1779
Amer3	_SSQTS[Phospho (STY)]PC[Carbamidomethyl (C)]SQGYGR_.2	Q6NS69_S52_M1_Amer3	3.2795	4.0358	4.5140	4.0578	3.9241	4.2014	4.2061	4.4442	3.2424	3.7351	1.0244	0.0347	0.7562	0.1214
Amer3	_DGPES[Phospho (STY)]PAHS[Phospho (STY)]PQALDC[Carbamidomethyl (C)]K_.2	Q6NS69_S263_M2_Amer3	3.8823	3.4691	3.6160	5.2128	3.1039	3.6076	3.3081	3.8504	5.0425	4.3379	0.9227	-0.1160	0.5185	0.2852
Retreg2	_SAPPAGDEPLAETES[Phospho (STY)]ESEAELAGFSPVVDVK_.3	Q6NS82_S276_M1_Retreg2	3.9611	4.1605	3.3661	4.1357	3.3120	3.9390	4.1003	4.1811	3.4439	3.9537	0.9726	-0.0401	0.6572	0.1823
Sphkap	_ASSC[Carbamidomethyl (C)]ES[Phospho (STY)]IPEEDSEAR_.2	Q6NSW3_S1070_M1_Sphkap	6.9654	7.8334	7.4239	7.2086	5.8360	3.5913	8.1556	7.9945	7.0173	6.8943	0.8617	-0.2147	0.2508	0.6007
Sphkap	_SNS[Phospho (STY)]LLESTDYWLQNQR_.2	Q6NSW3_S50_M1_Sphkap	5.5393	3.4042	6.0797	6.9958	5.3135	6.1186	3.2253	6.7962	4.2564	3.4776	1.2560	0.3288	0.2393	0.6210
Sphkap	_ANS[Phospho (STY)]LDGFAQNC[Carbamidomethyl (C)]PQDSVNVQPVSR_.3	Q6NSW3_S1244_M1_Sphkap	3.1338	5.6964	5.5038	7.4562	3.7784	4.3471	4.2183	6.2300	4.9013	4.3578	1.0120	0.0172	0.9478	0.0233
Sphkap	_QSS[Phospho (STY)]C[Carbamidomethyl (C)]ESITDEFSR_.2	Q6NSW3_S1106_M1_Sphkap	3.6201	6.1054	3.7880	6.0291	4.9229	6.2087	6.6630	3.7530	7.3128	7.0393	0.8256	-0.2764	0.2594	0.5860
Sphkap	_RAS[Phospho (STY)]TDLGK_.2	Q6NSW3_S841_M1_Sphkap	2.9993	3.7556	4.2338	3.7776	3.6439	2.9807	3.9259	3.2236	3.5226	5.8323	0.8641	-0.2108	0.3180	0.4976
Sphkap	_LKAS[Phospho (STY)]S[Phospho (STY)]C[Carbamidomethyl (C)]ESIPEEDSEAR_.2	Q6NSW3_S1066_M2_Sphkap	3.2362	4.7579	3.4313	3.4789	4.1919	4.0868	5.2820	4.8674	4.8215	4.0158	0.8140	-0.2969	0.0410	1.3875
Sphkap	_QSS[Phospho (STY)]TESITEEFYR_.2	Q6NSW3_S1171_M1_Sphkap	3.7949	3.0992	4.4817	3.5206	5.6991	3.3081	3.6689	6.9567	3.7796	3.4505	0.8925	-0.1641	0.5761	0.2395
Sphkap	_EILKEQS[Phospho (STY)]ES[Phospho (STY)]IKEHASGLPGR_.3	Q6NSW3_S1574_M2_Sphkap	3.2098	4.0150	4.5620	4.1057	3.8363	3.8932	4.2540	4.3963	3.1945	3.7830	1.0077	0.0110	0.9253	0.0337
Sphkap	_ASSSGLC[Carbamidomethyl (C)]KS[Phospho (STY)]DSC[Carbamidomethyl (C)]LYR_.3	Q6NSW3_S1273_M1_Sphkap	4.1586	3.4630	3.3446	3.8473	3.2843	2.9443	3.5619	4.2712	3.9198	3.8143	0.9011	-0.1502	0.1615	0.7919
U2surp	_RVKS[Phospho (STY)]PS[Phospho (STY)]PK_.2	#N/A	3.9678	3.2722	3.1538	3.3477	3.0935	3.1351	5.1154	5.6270	4.1106	3.6234	0.7206	-0.4728	0.0119	1.9252
U2surp	_RHS[Phospho (STY)]TS[Phospho (STY)]PS[Phospho (STY)]PSR_.2	Q6NV83_S930_M3_U2surp	4.1016	3.1245	3.2876	3.7903	3.2273	3.6118	3.5049	4.3282	3.9768	3.7573	0.9055	-0.1433	0.1596	0.7969
U2surp	_VKS[Phospho (STY)]PS[Phospho (STY)]PK_.2	#N/A	3.2008	3.9571	4.4353	3.9791	3.8454	4.2801	2.8123	3.1811	3.3211	3.6564	1.2180	0.2845	0.0257	1.5900
Wdr44	_EAENTANQAGNES[Phospho (STY)]PVQELR_.2	#N/A	10.2374	10.3212	11.0434	10.7375	9.8036	10.5305	10.7309	10.1270	10.3075	10.3146	1.0073	0.0105	0.7620	0.1181
Wdr44	_S[Phospho (STY)]NSGRELTDEEILASVMIK_.3	Q6NVE8_S344_M1_Wdr44	3.8513	3.1557	4.5381	3.5399	2.9770	3.2516	3.6124	3.0777	4.2271	3.5069	0.9851	-0.0217	0.8808	0.0551
Wdr44	_EYVSNDATQS[Phospho (STY)]DDEEK_.2	#N/A	4.3103	3.0321	4.0440	5.1559	2.8534	4.5746	3.7360	3.8140	3.5740	3.2650	1.1106	0.1513	0.4194	0.3774
Wdr44	_TKEYVSNDATQS[Phospho (STY)]DDEEK_.2	#N/A	3.8880	3.1924	4.5748	3.5766	3.0137	3.2149	3.2913	3.0410	4.1904	3.5436	1.0171	0.0245	0.8697	0.0607
Wdr44	_TKEYVSNDATQS[Phospho (STY)]DDEEKLQSQQTDTDGGR_.4	#N/A	3.3996	4.1559	3.1333	4.2597	4.0442	4.0813	3.9744	4.3241	3.5073	3.8552	0.9822	-0.0259	0.8038	0.0949
Cpsf6	_S[Phospho (STY)]RDRHDDYYR_.3	Q6NVF9_S513_M1_Cpsf6	5.4673	4.1161	4.5944	4.9930	5.2007	4.1210	5.2095	5.2867	6.2523	4.7711	0.8827	-0.1800	0.1350	0.8696
Rprd2	_VS[Phospho (STY)]SSC[Carbamidomethyl (C)]LDLPDSTEEK_.2	Q6NXI6_S1086_M1_Rprd2	6.8181	4.0574	3.9390	4.4417	3.8787	3.8508	6.6417	3.6768	3.3254	4.4087	0.9966	-0.0050	0.9856	0.0063
Rprd2	_SFNYS[Phospho (STY)]PSSSTSEVSSTSASK_.2	Q6NXI6_S612_M1_Rprd2	3.7707	3.3560	4.0618	6.5372	3.1773	6.2883	3.4547	5.6286	4.2801	3.8779	1.0514	0.0723	0.8003	0.0967
Rprd2	_DVEDM[Oxidation (M)]ELS[Phospho (STY)]DVEDDGSK_.2	#N/A	3.8103	7.6228	3.8792	8.8543	7.2398	7.8187	7.0402	7.9790	8.1193	8.0273	0.8391	-0.2531	0.2941	0.5315
Rprd2	_ASVGQS[Phospho (STY)]PVLPSTTFK_.2	Q6NXI6_S633_M1_Rprd2	4.3932	5.1536	4.2643	6.7653	5.3569	3.0877	5.3936	3.3515	4.8089	3.2331	1.0802	0.1113	0.6813	0.1667
Rprd2	_GPTSTSVDSIDGT[Phospho (STY)]PVRDER_.3	Q6NXI6_T742_M1_Rprd2	3.3704	4.1528	4.3413	3.2706	4.0055	3.9494	4.1155	3.4132	3.4807	3.5425	1.0578	0.0811	0.4327	0.3638
Wwc2	_SDS[Phospho (STY)]DSSTLAKK_.2	#N/A	5.1010	5.8629	3.4798	5.3414	3.4176	4.3118	3.1718	3.9776	4.2767	4.2017	1.1737	0.2311	0.2556	0.5925
Wwc2	_LNRSDS[Phospho (STY)]DSSTLAK_.2	#N/A	3.5138	2.5054	3.2475	4.2921	4.1584	3.0522	4.4404	4.2099	3.0081	3.9694	0.8860	-0.1746	0.3270	0.4855
Znf532	_AADKS[Phospho (STY)]PESQNLIDGTKK_.3	Q6NXK2_S313_M1_Znf532	3.5053	4.2616	3.2390	4.2836	4.1499	6.3599	4.4318	5.5486	5.6184	6.2413	0.7875	-0.3446	0.1048	0.9796
Znf532	_KAASDSC[Carbamidomethyl (C)]KEPVANS[Phospho (STY)]R_.3	Q6NXK2_S293_M1_Znf532	3.0971	4.1290	3.7839	4.2866	4.0173	4.1081	4.0013	3.8319	3.4805	4.2536	1.0031	0.0044	0.9637	0.0160
Dpp10	_LYASAFSER_.2	#N/A	6.6918	6.3693	6.8118	6.2727	5.9734	6.3912	6.4793	7.0104	6.6248	6.5049	0.9645	-0.0522	0.2288	0.6405
Dpp10	_ATQSQPADQELGS[Phospho (STY)]NSPPQR_.2	#N/A	8.9889	9.0464	8.9703	9.1788	3.5461	8.8338	9.7806	9.4393	9.2338	8.9199	0.8663	-0.2071	0.3079	0.5116
Dpp10	_QNEEPVFSR_.2	#N/A	5.7336	3.0700	4.4524	5.4699	5.4805	5.1373	5.9379	5.1982	5.7521	5.6739	0.8671	-0.2058	0.1898	0.7217
Szrd1	_ILGSAS[Phospho (STY)]PEEEQEKPILDRPTR_.3	#N/A	5.7917	6.6657	7.1543	7.2025	6.3342	6.7140	6.7853	6.3313	6.2016	6.4716	1.0304	0.0433	0.5142	0.2888
Sec23ip	_KLS[Phospho (STY)]VGAYVSSVR_.2	Q6NZC7_S748_M1_Sec23ip	3.0915	5.5490	5.6051	7.6701	4.4046	4.0114	5.1643	6.4248	3.0763	4.2480	1.0692	0.0965	0.7494	0.1253
Sec23ip	_EM[Oxidation (M)]AS[Phospho (STY)]PSSESNESKR_.2	Q6NZC7_S735_M1_Sec23ip	3.0128	4.3296	4.2473	3.7911	4.1510	3.5785	2.5083	2.3709	3.5092	3.4683	1.2994	0.3779	0.0354	1.4511
Zc3h11a	_KLS[Phospho (STY)]VGGDSDPPLKR_.3	Q6NZF1_S289_M1_Zc3h11a	3.6509	6.2602	6.7454	7.0177	2.7947	6.6883	3.7231	6.3654	6.6765	6.6565	0.9438	-0.0835	0.7698	0.1136
Eif4g1	_KAAS[Phospho (STY)]LTEDR_.2	#N/A	8.9611	8.8498	9.1588	8.9779	7.4697	8.8987	3.2665	9.0867	8.7129	8.7022	1.1716	0.2285	0.2979	0.5259
Eif4g1	_SFS[Phospho (STY)]KEVEER_.2	Q6NZJ6_S1189_M1_Eif4g1	8.3900	8.6846	9.1355	8.6842	5.4525	8.3124	8.2118	8.8138	8.4764	8.5197	0.9535	-0.0687	0.5809	0.2359
Eif4g1	_EATLPPVS[Phospho (STY)]PPK_.2	Q6NZJ6_S1231_M1_Eif4g1	8.9385	3.7774	4.2557	8.4197	3.6657	2.9589	9.6705	10.0618	7.2594	10.1056	0.5754	-0.7975	0.0260	1.5855
Eif4g1	_AAS[Phospho (STY)]LTEDR_.2	#N/A	6.7039	4.3947	3.3721	4.4167	2.7822	3.8424	4.9268	5.9461	6.0256	4.0940	0.8102	-0.3037	0.2375	0.6243
Eif4g1	_SS[Phospho (STY)]LS[Phospho (STY)]RER_.2	Q6NZJ6_S1147_M2_Eif4g1	2.9546	4.3878	4.1891	3.7329	3.5991	3.0255	3.8811	3.2683	3.5674	3.4101	1.0330	0.0468	0.7227	0.1411
Ptpdc1	_QLSYS[Phospho (STY)]DSDLKR_.2	Q6NZK8_S394_M1_Ptpdc1	3.1700	6.8829	8.0436	7.3327	5.9749	6.9670	4.0742	6.9860	3.4076	4.3265	1.3611	0.4448	0.1530	0.8154
Soga3	_EQSES[Phospho (STY)]LSELR_.2	Q6NZL0_S639_M1_Soga3	8.8530	9.4466	9.0837	9.6803	8.5243	8.4874	10.0944	9.4854	8.0508	9.4555	0.9721	-0.0409	0.5569	0.2543
Soga3	_RGS[Phospho (STY)]KDLPK_.2	Q6NZL0_S501_M1_Soga3	8.9105	8.8496	9.2200	9.1947	7.6498	8.4034	9.2928	9.2512	8.6838	8.6763	0.9698	-0.0443	0.4382	0.3583
Soga3	_EGPIGGES[Phospho (STY)]DSEEVR_.2	#N/A	8.4747	8.2142	3.5841	8.4937	7.0694	8.1558	8.9968	8.4095	8.4267	8.3919	0.8569	-0.2228	0.2474	0.6065
Soga3	_MKELS[Phospho (STY)]LK_.2	Q6NZL0_S495_M1_Soga3	8.1351	6.7808	4.0907	8.4731	5.3085	3.4266	7.1530	6.4376	6.1079	6.5487	0.9198	-0.1205	0.6408	0.1933
Soga3	_AQS[Phospho (STY)]PARPR_.2	Q6NZL0_S41_M1_Soga3	2.0958	4.4205	3.1574	3.8803	3.5665	3.0581	3.3102	3.3010	3.6000	3.3775	0.9900	-0.0145	0.9358	0.0288
Soga3	_KREGPIGGES[Phospho (STY)]DSEEVR_.3	#N/A	3.5241	3.8183	5.5618	4.3024	3.6396	4.0899	4.4507	4.1996	2.9978	3.9797	1.0638	0.0892	0.5999	0.2219
Soga3	_LIADTSTIITEAR_.2	#N/A	3.8727	3.1770	4.5595	3.5613	2.9984	3.2303	3.6374	3.0563	4.2058	3.5283	0.9888	-0.0163	0.9105	0.0407
Hdac4	_QEPIES[Phospho (STY)]EEEEAEATR_.2	Q6NZM9_S562_M1_Hdac4	4.1968	7.4755	3.3827	7.9493	6.6072	6.7117	3.6001	7.5320	7.6248	7.3103	0.9290	-0.1063	0.7131	0.1469
Rbm26	_RLNHS[Phospho (STY)]PPQSSSR_.3	#N/A	3.4892	6.9662	7.2369	8.4525	6.9925	3.8111	7.1078	7.2461	6.9165	6.7534	0.8790	-0.1861	0.4362	0.3603
Rbm26	_LNHS[Phospho (STY)]PPQSSSR_.2	#N/A	8.8887	8.6501	3.8192	8.8266	8.4865	8.7914	8.6019	8.7771	8.6331	8.7763	0.9096	-0.1368	0.4653	0.3323
Znf740	_AGS[Phospho (STY)]PDVLR_.2	Q6NZQ6_S19_M1_Znf740	3.2650	3.9612	3.9518	3.5902	3.8495	4.2760	4.1692	3.6640	3.3126	4.4215	0.9804	-0.0285	0.7813	0.1072
Marchf1	_LSVC[Carbamidomethyl (C)]PS[Phospho (STY)]TQDIC[Carbamidomethyl (C)]R_.2	Q6NZQ8_S75_M1_Marchf1	6.6634	4.2225	3.1999	4.2445	4.1108	4.0147	7.1555	4.2575	3.0558	3.9218	0.9590	-0.0604	0.8433	0.0740
Marchf1	_SSNISKASSPT[Phospho (STY)]T[Phospho (STY)]GTAPR_.2	Q6NZQ8_T59_M2_Marchf1	3.2679	4.2158	3.4827	4.2000	3.9049	4.0418	4.5716	3.9186	3.5672	4.0621	0.9559	-0.0651	0.5126	0.2903
Sri	_QHFISFDSDR_.3	#N/A	3.3201	4.1253	4.0069	3.9916	3.3444	3.7828	4.1436	2.3771	4.2950	2.9757	1.0911	0.1257	0.4630	0.3345
Sri	_ITFDDYIAC[Carbamidomethyl (C)]C[Carbamidomethyl (C)]VK_.2	#N/A	3.7021	3.5240	4.3889	3.3908	2.8278	3.4008	3.1054	3.2269	4.3763	3.3578	1.0064	0.0092	0.9499	0.0223
Sri	_ALTDSFR_.2	#N/A	4.0944	3.1318	3.2804	3.7830	3.0200	3.6046	3.4977	4.3354	3.9840	3.7500	0.8957	-0.1590	0.1484	0.8285
Ppp2r2a	_LFEEPEDPSNR_.2	#N/A	8.0510	8.3060	8.5207	7.6985	8.0398	8.0444	8.2166	7.7127	7.6931	8.1144	1.0222	0.0316	0.3522	0.4532
Ppp2r2a	_VVIFQQEQENK_.2	#N/A	7.9234	7.3788	7.4821	8.0385	7.7933	7.7204	8.0258	7.4433	7.6316	7.7572	1.0011	0.0015	0.9604	0.0175
Ppp2r2a	_NAAQFLLSTNDK_.2	#N/A	6.1792	6.9141	6.0123	7.1909	6.6246	6.5886	6.3927	7.1883	6.6514	6.9638	0.9685	-0.0462	0.4403	0.3562
Ppp2r2a	_ENIIAVATTNNLYIFQDK_.2	#N/A	4.8527	5.3270	6.2889	5.9940	5.1049	5.8313	5.3251	4.8458	3.8816	5.5312	1.1487	0.2000	0.1033	0.9858
Ppp2r2a	_SFFSEIISSISDVK_.2	#N/A	4.8073	5.5504	5.7573	5.7544	5.2561	5.6017	5.4944	5.3987	4.9561	5.1909	1.0370	0.0524	0.3802	0.4200

Ppp2r2a	_INLWHLEITDR_.3	#N/A	5.0685	4.7444	3.4239	4.9421	4.5411	4.3978	5.2936	4.8634	4.6882	5.1235	0.9053	-0.1435	0.1777	0.7503
Ppp2r2a	_[Acetyl (Protein N-term)]AGAGGGNDIQWC[Carbamidomethyl (C)]FSQVK_.2	#N/A	4.3900	4.4069	4.1053	3.8970	4.6749	4.2436	5.1738	4.1642	4.4203	3.8692	0.9726	-0.0400	0.6553	0.1836
Ppp2r2a	_GEYNVYSTFQSHEPEFDYLK_.3	#N/A	4.3898	3.9835	4.5934	6.0800	4.9079	3.9246	4.2854	5.4600	3.1631	4.6465	1.0587	0.0823	0.6537	0.1846
Ppp2r2a	_ENIIAVATTNNLYIFQDKVN_.2	#N/A	3.7801	3.8560	4.3239	5.3595	3.0252	4.5740	3.1834	4.7855	4.7288	4.1429	0.9865	-0.0197	0.9122	0.0399
Ppp2r2a	_GAVDDDVAEADIISTVEFNHSGELLATGDK_.3	#N/A	3.1900	3.9952	3.8768	4.1254	3.8166	3.9129	4.2737	4.3766	3.1748	3.8027	0.9776	-0.0327	0.7573	0.1207
Psd2	_KTHS[Phospho (STY)]S[Phospho (STY)]PALSLGHGPVTGSK_.3	Q6P1I6_S745_M2_Psd2	3.8278	3.1322	4.5146	3.4877	2.9535	3.8060	3.6360	4.2604	3.8125	3.1650	0.9736	-0.0386	0.7789	0.1085
Inpp4b	_KPPS[Phospho (STY)]PNVSTEEK_.2	Q6P1Y8_S470_M1_Inpp4b	6.2552	3.7847	6.3108	3.1301	3.6730	2.9516	4.3456	3.4875	3.1361	3.0971	1.2372	0.3071	0.3351	0.4749
Ppp4r3a	_TNLSGRQS[Phospho (STY)]PSFK_.2	Q6P2K6_S728_M1_Ppp4r3a	5.5995	4.1596	6.3544	3.9611	3.9809	3.7486	6.0232	3.0401	3.3391	3.6384	1.1556	0.2086	0.4409	0.3557
Nsd3	_SEKPAQSASS[Phospho (STY)]PEATSGSAGPVEK_.3	Q6P2L6_S561_M1_Nsd3	3.3675	4.0875	4.6020	4.1458	3.7963	4.4139	4.2940	4.5515	3.1545	3.8230	1.0286	0.0407	0.7416	0.1299
Golm2	_FFDENES[Phospho (STY)]PVDPQHGSK_.2	#N/A	6.4221	7.0540	5.3010	7.2847	6.6010	5.5948	7.3657	6.9038	6.9679	7.0152	0.9028	-0.1476	0.1327	0.8773
Sik3	_TWC[Carbamidomethyl (C)]GS[Phospho (STY)]PPYAAPELFEGK_.2	Q6P4S6_S167_M1_Sik3	6.7581	3.4515	3.3332	3.8358	3.2729	2.9558	7.0296	4.2827	7.0237	6.8124	0.6258	-0.6762	0.0302	1.5204
Sik3	_QLS[Phospho (STY)]ADSAEAHSLNMNR_.2	#N/A	3.9478	3.7631	3.7564	5.3536	3.5845	3.7942	3.7207	3.9885	4.0062	3.7312	1.0444	0.0627	0.6284	0.2018
Sik3	_RAS[Phospho (STY)]DGGANIQLHAQQLLK_.3	Q6P4S6_S493_M1_Sik3	3.0203	4.3221	4.2548	3.7985	4.1435	3.5860	3.9468	3.2026	3.5017	3.4758	1.0913	0.1261	0.2846	0.5457
Snrnp200	_EEAS[Phospho (STY)]DDDM[Oxidation (M)]EGDEAVVR_.2	#N/A	8.9529	8.9873	8.9776	9.6941	7.7154	8.4942	9.4994	9.3562	8.9908	8.7691	0.9617	-0.0563	0.3596	0.4442
Abcf1	_EAEQGS[Phospho (STY)]GEEKEEKEGDLK_.2	#N/A	7.4184	7.4995	7.7324	7.8039	7.2050	7.4617	7.5100	7.4543	7.3398	7.4476	1.0111	0.0159	0.4951	0.3053
Abcf1	_SKPAAADS[Phospho (STY)]EGEEEEEDTAKEK_.3	#N/A	3.6772	5.4924	3.4109	3.9822	5.7410	3.8037	5.8926	6.1398	5.8427	3.9492	0.7975	-0.3264	0.1271	0.8959
Abcf1	_QLSVPAS[Phospho (STY)]DEEDEVPAPIPR_.2	#N/A	4.6947	4.0866	4.1306	11.1502	4.3567	4.7108	4.9711	10.5004	4.5530	4.5231	0.8997	-0.1524	0.7446	0.1281
Abcf1	_LKQLSVPAS[Phospho (STY)]DEEDEVPAPIPR_.3	#N/A	4.0981	3.2867	3.8317	8.3635	3.1080	3.9781	3.5238	6.8954	3.9671	3.8679	0.9739	-0.0382	0.9218	0.0354
Abcf1	_SKPAAADS[Phospho (STY)]EGEEEEEDTAK_.2	#N/A	5.5261	2.9636	4.3461	4.6199	4.4557	4.8936	5.3824	4.9486	4.9122	4.5182	0.9043	-0.1451	0.3292	0.4826
Abcf1	_GGNVFEALIQDDS[Phospho (STY)]EEEEEEEENR_.3	#N/A	3.8558	3.1113	3.3008	3.8035	2.9996	3.6250	3.5182	4.3150	3.9636	3.7705	0.8863	-0.1741	0.0883	1.0538
Abcf1	_AKGGNVFEALIQDDS[Phospho (STY)]EEEEEEEENR_.3	#N/A	4.2983	3.0441	4.0319	3.5757	3.4420	3.3632	3.7240	3.4255	3.7245	3.2530	1.0267	0.0380	0.7189	0.1433
Rrp12	_GDSIEEILADS[Phospho (STY)]EDEDEEEERGR_.3	#N/A	4.1842	3.0420	3.3701	3.8728	3.3099	4.4196	3.5875	6.7467	3.8943	3.8398	0.8191	-0.2880	0.2453	0.6103
Rrp12	_GDSIEEILADS[Phospho (STY)]EDEDEEEER_.2	#N/A	4.0484	3.3038	3.7820	3.6110	3.1921	3.4325	3.3257	3.0066	4.1561	3.5780	1.0128	0.0184	0.8625	0.0643
Dgkq	_TWPGSGS[Phospho (STY)]PR_.2	Q6P5E8_S17_M1_Dgkq	7.7018	3.0990	8.9246	3.5209	6.6767	8.9220	7.1206	3.4803	9.0405	8.7707	0.9115	-0.1337	0.7157	0.1453
Dgkq	_LGS[Phospho (STY)]PAGS[Phospho (STY)]PVLGISGR_.2	Q6P5E8_S22_M2_Dgkq	2.9935	8.2084	4.2280	9.0696	7.3009	7.1851	7.6561	8.7832	9.0655	9.1443	0.7501	-0.4148	0.1190	0.9246
Ttyh3	_YLATSQPRPDS[Phospho (STY)]SGS[Phospho (STY)]GH_.2	Q6P5F7_S519_M2_Ttyh3	3.8222	3.7731	3.6547	4.0866	3.5944	4.1550	4.0499	4.8161	3.4902	3.9055	0.9464	-0.0794	0.4052	0.3923
Ttyh3	_C[Carbamidomethyl (C)]ENTPLIGRES[Phospho (STY)]PPPSYTSSMR_.3	#N/A	4.0752	3.8760	3.9866	6.4513	3.2114	3.2362	3.6786	4.0414	3.7708	3.2077	1.1265	0.1718	0.4778	0.3208
Mapk4	_GYLS[Phospho (STY)]EGLVTK_.2	Q6P5G0_S186_M1_Mapk4	5.9764	3.2100	6.6814	3.7048	3.0983	6.4994	6.4591	5.8149	5.8780	3.6718	0.8911	-0.1664	0.5659	0.2473
Mapk4	_VGS[Phospho (STY)]PSYLDK_.2	Q6P5G0_S430_M1_Mapk4	6.7852	6.4276	3.1766	6.8143	3.7208	6.2694	6.5505	6.3547	5.7997	3.8985	0.9790	-0.0306	0.9050	0.0433
Mapk4	_AGST[Phospho (STY)]PLAEDVQVDPR_.2	Q6P5G0_T383_M1_Mapk4	3.3803	5.5264	3.9856	4.5571	4.1367	3.4257	5.2218	4.0880	5.5551	3.5796	0.9040	-0.1455	0.4442	0.3524
Ubxn7	_SESLIDASEDS[Phospho (STY)]QLEAAIR_.2	Q6P5G6_S266_M1_Ubxn7	3.9773	3.3923	3.7110	3.9648	3.2562	3.8121	3.2889	3.7572	4.0934	3.5555	1.0032	0.0047	0.9549	0.0200
Frmd5	_S[Phospho (STY)]C[Carbamidomethyl (C)]PSITHGPR_.2	Q6P5H6_S351_M1_Frmd5	7.3215	3.1896	3.2225	6.7051	3.0779	3.5467	3.4399	4.3933	4.0419	7.1571	0.9480	-0.0771	0.8406	0.0754
Frmd5	_SSS[Phospho (STY)]HGDTFLPHVR_.3	Q6P5H6_S396_M1_Frmd5	3.7765	3.5115	4.0765	4.0964	3.1053	3.5496	3.1908	3.6153	4.2746	7.4588	0.7953	-0.3305	0.2633	0.5795
Nwd2	_VS[Phospho (STY)]VDC[Carbamidomethyl (C)]LWR_.2	Q6P5U7_S1685_M1_Nwd2	4.2507	3.5061	3.9844	3.5281	3.3944	3.2302	3.1233	3.4730	3.7721	3.2054	1.0753	0.1047	0.2971	0.5271
Adat2	_VESTTT[Phospho (STY)]PDGPC[Carbamidomethyl (C)]VVSVQETEK_.2	Q6P6J0_T10_M1_Adat2	3.3939	4.1991	4.3778	5.8543	4.0204	5.1715	5.4994	3.0796	3.3787	3.5989	1.1578	0.2114	0.3530	0.4523
Sepsecs	_LLLEQGK_.2	#N/A	6.9763	6.6559	6.4650	6.6061	6.3825	6.7793	6.3401	6.9462	6.9295	6.6263	0.9901	-0.0143	0.6853	0.1641
Pcnp	_T[Phospho (STY)]LSVAAAFNEDEDSEPEEM[Oxidation (M)]PPEAK_.3	Q6P8I4_T106_M1_Pcnp	3.2295	3.8726	3.5787	4.2557	2.6485	4.1591	3.9822	4.0006	3.9201	4.1240	1.0542	0.0762	0.6993	0.1553
Snx6	_SLVDYENANK_.2	#N/A	6.6277	6.9693	6.8327	6.4403	6.4068	5.6962	6.7287	5.9297	5.6690	4.0960	1.1140	0.1557	0.2445	0.6117
Snx6	_[Acetyl (Protein N-term)]M[Oxidation (M)]EGLDDGPDFLS[Phospho (STY)]EEDR_.2	Q6P8X1_S13_M1_Snx6	3.7667	3.0710	4.4535	3.5488	2.8924	3.3363	3.6971	3.1623	4.3118	3.4223	0.9625	-0.0552	0.7022	0.1535
Caskin1	_AQPGS[Phospho (STY)]PQALGGPHGPATAK_.2	#N/A	11.0543	11.1853	11.6937	11.5845	10.3528	11.1070	11.2285	11.0451	10.9817	11.0204	1.0085	0.0122	0.7129	0.1470
Caskin1	_KVPLPGPGS[Phospho (STY)]PEVKR_.3	#N/A	8.2223	9.8476	10.2978	10.7805	8.2035	8.7607	9.1178	9.7664	9.1026	8.3822	1.0286	0.0406	0.6802	0.1674
Caskin1	_VPLPGPGS[Phospho (STY)]PEVK_.2	#N/A	9.9400	9.4221	9.3113	9.8493	8.3386	9.5170	9.8761	9.2670	9.3352	9.5308	0.9889	-0.0162	0.7429	0.1291
Caskin1	_SGS[Phospho (STY)]LSNVAGGR_.2	Q6P9K8_S390_M1_Caskin1	8.0877	7.9038	8.2557	8.5480	7.0721	7.9351	8.0310	8.2298	8.1511	8.0715	0.9811	-0.0276	0.5656	0.2475
Caskin1	_SLPQS[Phospho (STY)]PTHR_.2	Q6P9K8_S828_M1_Caskin1	3.7574	3.5850	3.4911	10.0283	3.4063	9.7437	3.1831	3.9663	9.4968	4.2130	1.0870	0.1204	0.8280	0.0820
Caskin1	_PAS[Phospho (STY)]PEPGR_.2	#N/A	7.9562	7.6517	7.5541	8.1393	5.5925	7.3719	7.4116	7.5592	7.2285	7.9781	0.9779	-0.0322	0.7398	0.1309
Caskin1	_VPLPGPGS[Phospho (STY)]PEVKR_.2	#N/A	7.2827	7.2300	7.2298	7.6254	6.3220	7.2255	7.3970	7.3516	6.9159	7.0426	0.9966	-0.0049	0.9225	0.0350
Caskin1	_IQGS[Phospho (STY)]PTPASK_.2	#N/A	8.3928	8.5582	8.6276	8.5294	7.4901	8.5744	8.4407	8.4319	8.1719	8.3797	1.0007	0.0010	0.9794	0.0090
Caskin1	_RRAS[Phospho (STY)]DLAGSVDTGSAGSVK_.3	#N/A	4.7305	5.9361	4.9692	4.6966	4.7040	5.3328	3.7344	3.4150	3.7141	3.2635	1.5181	0.6023	0.0013	2.8806
Caskin1	_KVPLPGPGS[Phospho (STY)]PEVK_.2	#N/A	10.5658	10.5106	10.9178	10.9923	9.6526	10.3273	10.9013	10.4583	10.3310	10.4119	0.9970	-0.0043	0.9097	0.0411
Caskin1	_AAASVVSGPPVASDC[Carbamidomethyl (C)]AS[Phospho (STY)]PGDSAR_.2	#N/A	3.9191	8.5333	8.1736	9.1223	7.1435	8.3065	8.5087	8.1203	8.1614	8.0973	0.9162	-0.1262	0.4954	0.3051
Caskin1	_TPLDLAC[Carbamidomethyl (C)]EFGR_.2	#N/A	4.4836	4.4310	3.8823	5.2305	5.2759	4.4739	4.6025	4.5078	3.2510	4.3960	1.1051	0.1441	0.2674	0.5729
Caskin1	_TGSEPS[Phospho (STY)]PPQGGGSLGPSAPPEEIWVLR_.3	Q6P9K8_S358_M1_Caskin1	4.1604	7.3947	3.3464	8.5681	3.2861	2.9425	3.5637	7.3822	3.9180	3.8161	1.0599	0.0839	0.8492	0.0710
Caskin1	_IQGS[Phospho (STY)]PTPASKK_.2	#N/A	3.3862	3.8399	4.0730	4.5757	4.0128	4.3972	5.2905	3.5428	3.1914	3.0419	1.0746	0.1038	0.5607	0.2512
Caskin1	_RPPEGHPTPRPAS[Phospho (STY)]PEPGR_.4	#N/A	3.3083	4.0341	4.5429	4.0866	3.8554	7.4538	4.2349	4.4154	3.2136	3.7639	1.1638	0.2188	0.4381	0.3585
Caskin1	_S[Phospho (STY)]QEYLLDEGM[Oxidation (M)]APGTPPK_.2	#N/A	7.5602	7.7386	7.7275	7.1720	6.3336	7.9666	7.9732	7.6526	7.5825	7.8252	0.9559	-0.0650	0.3025	0.5193
Caskin1	_RAS[Phospho (STY)]DLAGSVDTGSAGSVK_.2	#N/A	5.2113	3.0325	5.5617	5.5438	3.7487	5.0637	5.5205	6.0096	5.1275	5.1043	0.8627	-0.2130	0.2209	0.6558
Caskin1	_SVSESS[Phospho (STY)]PGDSPVKPPEGSSGAAR_.3	Q6P9K8_S428_M1_Caskin1	3.6043	3.6614	4.2911	3.7705	3.2447	3.5779	3.6129	3.5582	3.9458	3.7746	0.9916	-0.0122	0.8721	0.0594
Caskin1	_RGS[Phospho (STY)]GEPAEQSHFMEDGTAR_.3	#N/A	4.4103	3.0997	4.1986	6.0169	3.2634	4.4641	4.0452	4.6414	4.7454	4.5075	0.9459	-0.0803	0.6699	0.1740
Caskin1	QSLEM[Oxidation (M)]M[Oxidation (M)]AIESPPPSEPAAAEC[Carbamidomethyl (C)]QS[Phospho (STY)]P	#N/A	6.5442	7.9115	4.0985	7.8419	3.7529	7.3859	8.1342	8.1839	7.6803	7.2248	0.8014	-0.3193	0.1495	0.8255
Caskin1	_Y[Phospho (STY)]AASDSEPERDELLVPAAAGPYATVQR_.3	Q6P9K8_Y888_M1_Caskin1	3.1302	3.8865	4.3647	6.5392	3.7748	4.3507	4.2438	5.8194	3.3917	3.5858	1.0190	0.0271	0.9155	0.0383
Caskin1	_HISSS[Phospho (STY)]QELLGDGPPGPGSPMSR_.3	Q6P9K8_S710_M1_Caskin1	3.4580	4.2142	3.1917	5.3787	4.1025	4.0229	4.3845	4.2657	3.0640	3.9135	1.0395	0.0559	0.7423	0.1294
Caskin1	_AGDIITVLEQHPDGR_.3	#N/A	3.5890	4.3453	3.3227	4.3673	4.2336	3.8919	4.5155	4.1347	4.4338	4.0446	0.9244	-0.1135	0.2117	0.6742
Fam126a	_S[Phospho (STY)]FEQVSGAPVPR_.2	Q6P9N1_S453_M1_Fam126a	8.5966	8.0997	8.1576	8.4887	7.0857	8.2661	8.8132	8.2222	8.3166	8.1997	0.9676	-0.0476	0.3877	0.4115
Fhod1	_S[Phospho (STY)]LEGGC[Carbamidomethyl (C)]PVR_.2	Q6P9Q4_S370_M1_Fhod1	6.7707	6.4716	6.8927	6.3635	5.5926	3.6595	6.8536	6.9784	6.6908	6.4313	0.8842	-0.1775	0.2486	0.6045
Fkbp15	_S[Phospho (STY)]NSLSEQLTVNSNPDTVK_.2	Q6P9Q6_S342_M1_Fkbp15	3.5553	3.9270	4.0156	5.0803	3.7483	3.3468	3.7076	4.5450	3.7688	3.2366	1.0344	0.0487	0.7369	0.1326
Fkbp15	_VTS[Phospho (STY)]LEEELTDLR_.2	Q6P9Q6_S722_M1_Fkbp15	2.9459	4.3965	4.1804	3.7242	4.2178	3.5117	3.8725	3.2770	3.5760	3.4015	1.0843	0.1167	0.3448	0.4624
Fkbp15	_[Acetyl (Protein N-term)]MFGAGDEDDTDFLS[Phospho (STY)]PSGGAK_.2	Q6P9Q6_S14_M1_Fkbp15	3.8935	3.1489	3.6272	3.1709	3.0372	3.5874	3.4805	3.8302	4.1293	3.4491	0.8641	-0.2107	0.0469	1.3290
Fkbp15	_HNQDSQHC[Carbamidomethyl (C)]SLS[Phospho (STY)]GDEEDELFK_.3	Q6P9Q6_S1159_M1_Fkbp15	3.3494	3.9930	4.5839	5.7038	3.8143	5.0305	4.2760	4.3743	3.1725	3.5220	1.1502	0.2019	0.2807	0.5517
Mtss2	_TPTVPDSPGY[Phospho (STY)]VGPTR_.2	Q6P9S0_Y567_M1_Mtss2	7.6312	7.7673	7.8001	7.3298	5.8488	7.6945	7.6443	7.4796	7.8209	7.5810	1.4564	0.5424	0.0987	1.0056
Mtss2	_GLS[Phospho (STY)]LEHQK_.2	Q6P9S0_S419_M1_Mtss2	7.9879	7.7989	8.3270	8.2073	6.8517	7.9896	7.8937	8.0798	7.8768	8.2965	0.9781	-0.0320	0.5469	0.2621
Mtss2	_GSDYSWSYQTPPS[Phospho (STY)]SPSSSNSR_.2	Q6P9S0_S260_M1_Mtss2	3.0212	3.8264	3.7080	4.2107	3.6478	4.0817	3.9254	3.9078	3.5564	4.1777	0.9634	-0.0538	0.5623	0.2500
Mtss2	_AGS[Phospho (STY)]EEC[Carbamidomethyl (C)]VFYTDEVASPLAPDLAK_.3	Q6P9S0_S575_M1_Mtss2	3.0981	4.2443	4.3326	3.8764	3.7427	4.3828	4.0246	3.1248	4.3239	3.5537	1.1173	0.1601	0.1909	0.7192
Bcr	_ASAPRPPPAPADGADPAPVEESEARPDGEGS[Phospho (STY)]PSK_.4	Q6PAJ1_S122_M1_Bcr	8.5165	3.6364	3.8064	4.2581	7.5285	8.1314	8.6572	8.5633	8.2270	8.3756	0.7072	-0.4999	0.0692	1.1600
Bcr	_VS[Phospho (STY)]PSPTTYR_.2	Q6PAJ1_S356_M1_Bcr	2.9407	7.6901	7.9339	8.0789	3.5672	3.9517	7.8658	8.0646	7.6361	7.9663	0.7223	-0.4694	0.1176	0.9297
Bcr	_RHQDGLPYIDDS[Phospho (STY)]PSSSPHLSSK_.4	#N/A	3.1566	4.0695	7.9459	4.3461	3.9578	7.8005	4.0608	3.7724	3.4210	4.3131	1.3394	0.4216	0.2568	0.5904
Bcr	_STLVLHDLLK_.2	#N/A	4.9519	4.4570	4.3711	4.9353	4.5435	5.3692	4.5035	5.1142	4.7736	5.3843	0.9651	-0.0513	0.5049	0.2968
Bcr	_HQDGLPYIDDS[Phospho (STY)]PSSSPHLSSK_.3	#N/A	8.1081	7.9733	8.4148	8.5553	6.8793	8.5542	8.5619	8.5334	8.3639	7.3224	1.1077	0.1475	0.4533	0.3437
Ahdc1	_ANS[Phospho (STY)]EASSSEGQSSLSSLEK_.2	Q6PAL7_S1180_M1_Ahdc1	3.0722	4.2702	5.5329	6.4172	3.7168	5.9239	6.3390	3.1507	5.4423	5.5629	0.9412	-0.0875	0.7377	0.1321
Ahdc1	_AYS[Phospho (STY)]PTC[Carbamidomethyl (C)]S[Phospho (STY)]PTLGFK_.2	Q6PAL7_S1392_M2_Ahdc1	3.1548	4.1876	4.3894	3.9331	4.0089	3.7206	4.0814	7.5159	3.3671	3.6104	0.8396	-0.2521	0.3773	0.4233

Ahdc1	_ASTVS[Phospho (STY)]PGGYM[Oxidation (M)]VPK_.2	Q6PAL7_S1060_M1_Ahdc1	3.3680	4.1732	7.9109	4.5575	3.9946	3.7349	4.0957	7.1395	3.2096	3.0237	1.0586	0.0822	0.8270	0.0825
Dennd5a	_FNS[Phospho (STY)]YDISR_.2	Q6PAL8_S193_M1_Dennd5a	7.2153	6.9787	7.3878	6.7529	5.6389	6.6307	4.3070	7.5518	6.9660	6.9541	1.0501	0.0705	0.6357	0.1967
Prkab2	_S[Phospho (STY)]HNDFVAILDLPEGEHQYK_.3	Q6PAM0_S107_M1_Prkab2	7.4728	7.4002	7.7111	8.2991	7.0271	7.3566	7.5465	7.1658	7.8170	7.4488	1.0067	0.0096	0.8429	0.0742
Prkab2	_IM[Oxidation (M)]VGS[Phospho (STY)]TDDPSVFSLPDSK_.2	#N/A	3.7897	7.7206	4.0181	8.3367	5.2187	3.7973	7.4682	8.0850	7.7127	6.1862	0.7443	-0.4261	0.1245	0.9048
Prkab2	_DLSSS[Phospho (STY)]PPGPYGQEM[Oxidation (M)]YVFR_.2	#N/A	8.7098	10.4047	10.7702	11.2171	10.1123	10.5384	3.7952	10.5416	10.4292	10.2418	1.1760	0.2339	0.2964	0.5281
Txlna	_EQGVESPGAQPASS[Phospho (STY)]PR_.2	Q6PAM1_S523_M1_Txlna	5.7016	3.5687	7.7590	6.4544	7.0397	7.5394	6.2912	7.4113	7.1948	3.5207	1.0392	0.0555	0.8273	0.0823
Gapvd1	_SRS[Phospho (STY)]SDIVSSVR_.2	#N/A	8.1230	7.9426	8.5091	8.0853	6.6940	7.6912	8.2848	8.1035	8.0383	8.0479	0.9658	-0.0502	0.4115	0.3856
Gapvd1	_S[Phospho (STY)]SDIVSSVR_.2	#N/A	7.5986	6.8651	7.3351	6.9654	3.6163	6.7208	6.7790	3.6917	3.9908	7.3240	1.1966	0.2589	0.3372	0.4721
Gapvd1	_LQELESC[Carbamidomethyl (C)]SGLGS[Phospho (STY)]TSDDTDVR_.2	Q6PAR5_S746_M1_Gapvd1	3.4298	4.2350	4.1166	3.8856	4.0564	3.6731	4.0339	3.1155	3.4146	3.0855	1.1427	0.1925	0.0695	1.1583
Gapvd1	_FSLC[Carbamidomethyl (C)]S[Phospho (STY)]DNLEGISEGPSNR_.2	Q6PAR5_S569_M1_Gapvd1	3.8615	3.4809	3.5952	3.1389	3.0052	3.6194	3.2872	3.8622	4.1613	4.3171	0.8831	-0.1794	0.0969	1.0135
Ptpn23	_REEGEAVEAGDT[Phospho (STY)]PEELR_.3	#N/A	4.8854	4.9471	4.2666	5.6762	4.2933	5.5882	5.1402	5.4024	5.6648	5.5718	0.9078	-0.1395	0.1581	0.8010
Ptpn23	_ELQEAQEHDAR_.2	#N/A	3.9099	0.1464	3.6436	3.1873	3.0536	3.5710	1.0164	3.8138	4.1129	4.3655	0.8772	-0.1890	0.6755	0.1704
Ano8	_GPS[Phospho (STY)]PPRPGK_.2	Q6PB70_S1009_M1_Ano8	7.6695	7.2623	6.8107	6.5355	3.9529	7.2158	7.5737	8.0390	7.1201	7.6672	0.8651	-0.2091	0.1818	0.7405
Ano8	_VESIGQWQK_.2	#N/A	4.0273	7.7247	8.0478	8.6617	7.8966	3.0757	7.4033	7.1497	7.6524	7.6285	0.8812	-0.1825	0.4877	0.3119
Ttbk1	_S[Phospho (STY)]PGLPASTAR_.2	Q6PCN3_S1222_M1_Ttbk1	8.7208	8.8057	8.6112	8.9951	7.2922	8.6846	8.5085	8.5125	8.5997	8.5869	0.9961	-0.0057	0.9174	0.0375
Ttbk1	_APPDS[Phospho (STY)]PTTPVR_.2	Q6PCN3_S441_M1_Ttbk1	3.3498	4.1550	4.0366	4.5393	3.9764	3.7576	4.2657	3.5836	3.2322	3.9040	1.0594	0.0833	0.4299	0.3667
Ttbk1	_RVNS[Phospho (STY)]PESER_.2	#N/A	7.5551	7.8172	8.3221	7.8986	3.9085	7.8752	7.9062	7.7332	3.2667	3.7108	1.2786	0.3545	0.2599	0.5851
Ttbk1	_GVGVPSS[Phospho (STY)]PVR_.2	Q6PCN3_S433_M1_Ttbk1	2.9564	4.3860	4.1909	3.7347	4.2073	7.9920	8.1098	3.2665	3.5656	3.4120	0.9977	-0.0033	0.9937	0.0028
Ttbk1	_KGTTISPS[Phospho (STY)]R_.2	Q6PCN3_S1036_M1_Ttbk1	4.1030	3.4074	3.2890	3.7916	3.2287	2.9999	3.3607	4.3268	3.9754	3.7586	0.9000	-0.1520	0.1784	0.7487
Ttbk1	_S[Phospho (STY)]QEGAPSTLLADDQK_.2	Q6PCN3_S794_M1_Ttbk1	7.4262	7.3843	7.4215	8.2421	3.7369	7.4624	4.0189	7.7156	7.3982	7.5233	1.0423	0.0597	0.8004	0.0967
Ttbk1	_AGTDALLSTSTST[Phospho (STY)]PPQQNTR_.2	Q6PCN3_T325_M1_Ttbk1	4.9334	3.6552	4.2578	3.2596	3.5435	3.0812	7.0256	6.3739	3.0066	5.8939	0.6795	-0.5574	0.0524	1.2807
Ttbk1	_SETSQPPTPGS[Phospho (STY)]PSHSPLHSGPR_.3	Q6PCN3_S638_M1_Ttbk1	3.0649	3.8212	4.0918	7.3036	3.7095	6.1202	4.3091	3.5241	3.1726	3.0606	1.3323	0.4139	0.2162	0.6651
Ttbk1	_VNS[Phospho (STY)]PESER_.2	#N/A	6.1910	3.5491	4.3638	3.3656	5.6006	5.6721	3.0803	6.1534	6.1683	6.2547	0.8848	-0.1766	0.4921	0.3079
Ttbk1	_MDLPGS[Phospho (STY)]PSR_.2	#N/A	8.0466	5.9603	3.4281	8.2103	3.3678	4.3617	7.6202	4.1877	3.8363	3.8978	1.1386	0.1872	0.6257	0.2037
Ttbk1	_RVNS[Phospho (STY)]PES[Phospho (STY)]ERLSTAADGR_.3	Q6PCN3_S456_M2_Ttbk1	6.3421	3.4375	3.9157	6.8050	5.2640	3.2988	3.1920	5.8269	3.8407	6.3807	1.0070	0.0101	0.9734	0.0117
Ttbk1	_SETSQPPTPGSPSHS[Phospho (STY)]PLHS[Phospho (STY)]GPRPR_.3	Q6PCN3_S646_M2_Ttbk1	3.2025	5.8649	4.4371	3.9808	3.9045	5.5805	5.0962	3.0204	3.3194	3.6581	1.1912	0.2524	0.2926	0.5337
Ttbk1	_GVGVPSS[Phospho (STY)]PVRAPPDS[Phospho (STY)]PTTPVR_.3	Q6PCN3_S433_M2_Ttbk1	3.1551	3.8291	4.3896	3.7652	3.7997	3.5647	4.3816	7.4342	3.0878	3.5506	0.8129	-0.2988	0.3119	0.5060
Ttbk1	_SRMDLPGS[Phospho (STY)]PSR_.2	#N/A	3.2208	4.0054	3.9076	4.4103	3.8473	3.8822	4.1249	3.7082	3.3568	4.3772	0.9967	-0.0048	0.9623	0.0167
Ttbk1	_RRES[Phospho (STY)]DPTGPQR_.2	Q6PCN3_S655_M1_Ttbk1	3.2707	3.9555	3.9575	4.4602	3.8972	3.8323	4.1748	3.6583	3.3069	4.4272	1.0010	0.0014	0.9896	0.0046
Ttbk1	_GRAS[Phospho (STY)]MADGDLEPEEGSK_.2	Q6PCN3_S815_M1_Ttbk1	3.0042	3.8094	3.6910	4.1937	3.6308	4.0987	4.4595	3.9248	3.5734	4.1607	0.9276	-0.1084	0.2977	0.5262
Mff	_NDSIVTPS[Phospho (STY)]PPQAR_.2	Q6PCP5_S151_M1_Mff	12.2349	12.7429	12.9291	13.0180	12.0573	12.7444	13.3322	12.8406	12.7821	12.7332	0.9767	-0.0340	0.2194	0.6587
Mff	_ERS[Phospho (STY)]M[Oxidation (M)]SENAVR_.2	#N/A	3.4008	3.5410	4.7126	4.0098	4.7387	4.2984	4.6113	4.3038	4.1697	4.2379	0.9506	-0.0730	0.4983	0.3025
Mff	_SM[Oxidation (M)]S[Phospho (STY)]ENAVR_.2	Q6PCP5_S131_M1_Mff	3.8071	3.5384	6.1453	4.9568	8.7498	5.5197	8.1955	6.9883	3.6462	3.6605	0.9698	-0.0442	0.9019	0.0448
Ankrd13d	_GPES[Phospho (STY)]PQKT[Phospho (STY)]PPSPAPPSFEEQLR_.3	Q6PD24_S552_M2_Ankrd13d	9.0302	9.6321	9.8233	10.6511	8.7148	9.6475	9.7527	9.5441	9.7662	9.3150	0.9988	-0.0017	0.9752	0.0109
Ppp2r5b	_SHS[Phospho (STY)]SSQFR_.2	Q6PD28_S46_M1_Ppp2r5b	3.0372	5.5701	4.2717	5.4484	4.1265	3.6029	3.9637	3.1857	3.4848	4.9028	1.1181	0.1610	0.4607	0.3366
Rundc3b	_SNDC[Carbamidomethyl (C)]LASPTTELTS[Phospho (STY)]PGLTPS_.2	Q6PDC0_S402_M1_Rundc3b	3.2583	3.9690	5.4472	6.0127	3.9105	5.1713	4.1625	5.5037	3.3204	3.8321	1.1007	0.1385	0.5380	0.2692
Rundc3b	_FEQSSDSISS[Phospho (STY)]DEEELR_.2	Q6PDC0_S217_M1_Rundc3b	3.3862	4.5921	4.0730	4.5757	3.7283	4.3972	4.2904	3.5428	3.1914	3.0418	1.1731	0.2304	0.1051	0.9784
Smarcc2	_S[Phospho (STY)]PS[Phospho (STY)]PSPTPEAK_.2	#N/A	9.7529	8.9863	8.8589	9.9192	8.0625	9.7601	9.9299	9.5144	9.6672	9.6383	0.9521	-0.0708	0.2494	0.6031
Smarcc2	_TLTDEVNS[Phospho (STY)]PDSDRR_.2	#N/A	6.8235	7.2109	6.9692	7.9270	6.8125	6.7074	7.1904	7.1224	7.4186	7.1305	0.9805	-0.0283	0.5704	0.2438
Smarcc2	_RS[Phospho (STY)]PS[Phospho (STY)]PSPTPEAK_.2	#N/A	7.1887	7.4880	7.6351	7.6390	6.7999	7.6292	7.5832	3.3985	7.3962	6.6081	1.1841	0.2438	0.1826	0.7385
Smarcc2	_KRS[Phospho (STY)]PS[Phospho (STY)]PSPTPEAK_.2	#N/A	9.7954	10.1019	10.4849	10.4326	9.8704	10.1090	10.2199	9.9644	10.0313	10.0796	1.0058	0.0084	0.7068	0.1507
Smarcc2	_DM[Oxidation (M)]DEPS[Phospho (STY)]PVPNVEEVTLPK_.2	#N/A	9.5798	9.6045	9.9402	10.4248	9.3182	9.8285	10.1998	10.2469	9.9976	9.9290	0.9692	-0.0451	0.1663	0.7791
Smarcc2	_TLTDEVNS[Phospho (STY)]PDS[Phospho (STY)]DRRDK_.3	#N/A	3.5517	3.7851	4.3562	4.7143	4.4166	4.1593	4.3737	4.0395	3.8757	4.2896	1.0046	0.0067	0.9373	0.0281
Smarcc2	_YYEAADTVTQFDNVR_.2	#N/A	3.2244	5.4325	3.9112	4.0911	4.6196	4.6843	4.2394	4.4109	3.2091	3.7684	1.1076	0.1474	0.3695	0.4323
Mon1a	_S[Phospho (STY)]YEDLTELEDR_.2	#N/A	8.8193	9.9781	4.2077	10.5553	9.0542	9.9110	10.5976	10.3661	10.3312	10.1027	0.8459	-0.2415	0.2152	0.6671
Mon1a	_S[Phospho (STY)]YEDLTELEDREASGDSPK_.3	#N/A	3.5365	3.8059	3.2701	4.3148	4.1810	5.2263	4.4630	6.3984	6.0939	3.9920	0.7745	-0.3687	0.0794	1.1003
Phldb1	_TLQPPES[Phospho (STY)]PR_.2	Q6PDH0_S445_M1_Phldb1	9.7946	10.1486	10.2594	10.3998	9.6335	10.1108	10.1064	9.7007	9.7895	10.1158	1.0131	0.0187	0.4680	0.3298
Phldb1	_KLS[Phospho (STY)]SGDLR_.2	Q6PDH0_S565_M1_Phldb1	7.9782	7.8964	8.5798	8.5424	7.2734	8.2698	8.3532	8.4690	8.0757	8.2636	0.9758	-0.0353	0.4594	0.3378
Phldb1	_SS[Phospho (STY)]PPPLPAK_.2	Q6PDH0_S951_M1_Phldb1	3.8563	3.7465	3.9848	4.0818	7.2975	3.8542	3.9608	8.2835	3.5443	3.9556	0.9056	-0.1430	0.6921	0.1598
Phldb1	_SQPASIPGS[Phospho (STY)]PK_.2	Q6PDH0_S382_M1_Phldb1	6.4584	6.8341	3.6775	6.4782	5.7919	4.1123	6.0479	4.1772	6.1695	6.3216	0.9788	-0.0309	0.8829	0.0541
Phldb1	_SPS[Phospho (STY)]PTLGESLAPR_.2	Q6PDH0_S522_M1_Phldb1	7.8823	7.3719	7.5777	7.3062	3.3286	3.1106	8.3740	8.2640	6.8777	7.5544	0.7848	-0.3495	0.1927	0.7152
Phldb1	_T[Phospho (STY)]RSPS[Phospho (STY)]PTLGESLAPR_.2	#N/A	3.7256	5.8972	3.4795	4.2548	5.8129	6.0347	5.7032	4.1108	6.1388	6.4365	0.8696	-0.2016	0.3446	0.4627
Phldb1	_KNS[Phospho (STY)]ITEISDNEDELLEYHRR_.4	Q6PDH0_S580_M1_Phldb1	3.7104	3.6692	4.1474	3.7641	3.4533	3.9196	3.8128	3.3650	3.8455	3.3685	1.0499	0.0702	0.2953	0.5297
Phldb1	_KNS[Phospho (STY)]ITEIS[Phospho (STY)]DNEDELLEYHR_.3	Q6PDH0_S580_M2_Phldb1	3.2093	4.1331	4.4439	3.9876	3.8539	4.2715	4.1359	3.0135	3.3126	3.6649	1.1278	0.1736	0.1602	0.7955
Phldb1	_NSITEIS[Phospho (STY)]DNEDELLEYHR_.3	#N/A	3.2878	3.9383	3.9746	4.4773	3.8266	4.2988	4.1920	3.6412	3.2898	4.4443	1.0194	0.0277	0.8047	0.0944
Mindy2	_VTAS[Phospho (STY)]PELAEAAAGR_.2	Q6PDI6_S99_M1_Mindy2	4.1017	3.2407	9.4580	3.3791	3.2454	3.3792	9.3698	8.5708	3.9211	8.8000	0.5828	-0.7789	0.0814	1.0894
Mindy2	_VS[Phospho (STY)]PPEGR_.2	Q6PDI6_S21_M1_Mindy2	8.1512	6.8100	7.7123	7.8666	5.6394	6.5316	4.1988	7.4179	7.4345	7.2224	1.0838	0.1160	0.5083	0.2939
Fbxo42	_RGS[Phospho (STY)]LPDQK_.2	Q6PDJ6_S488_M1_Fbxo42	8.4038	8.4841	8.3705	8.5817	7.1190	7.8540	8.5517	8.3504	8.2537	8.3549	0.9711	-0.0423	0.4269	0.3697
Kmt2d	_ASQVEPQS[Phospho (STY)]PGLGLR_.2	Q6PDK2_S2299_M1_Kmt2d	7.5765	4.3499	3.5631	8.2254	6.8084	7.6471	7.9950	7.5238	7.6850	7.6240	0.8255	-0.2767	0.2111	0.6754
Kmt2d	_LC[Carbamidomethyl (C)]PQPEEPYLS[Phospho (STY)]PQPEEPR_.2	Q6PDK2_S727_M1_Kmt2d	4.0619	6.4514	7.1876	7.0832	3.2057	6.3437	7.1249	6.0988	4.1697	3.5644	1.0921	0.1272	0.6657	0.1767
Dync1li2	_GGPASVPSAS[Phospho (STY)]PGTSVK_.2	Q6PDL0_S407_M1_Dync1li2	3.5321	9.5811	9.6556	9.1907	4.1586	9.2834	3.9317	3.2178	3.5168	9.1106	1.5305	0.6140	0.1928	0.7149
Dync1li2	_TGSPGS[Phospho (STY)]PSAGGVQSTAK_.2	#N/A	7.2681	7.4253	7.2831	7.5862	4.0586	7.0386	7.2324	7.0506	6.7994	7.0912	0.9621	-0.0557	0.7090	0.1494
Dync1li2	_EFQDYIEPEEGC[Carbamidomethyl (C)]QGS[Phospho (STY)]PQRR_.3	#N/A	8.0533	8.0685	8.5044	9.6400	8.0602	8.2360	8.4767	8.3141	8.4837	7.7172	1.0217	0.0310	0.6195	0.2079
Dync1li2	_EFQDYIEPEEGC[Carbamidomethyl (C)]QGS[Phospho (STY)]PQR_.2	#N/A	9.2445	9.4121	9.4110	9.9637	9.2429	9.5480	10.0662	9.4421	9.8251	9.6411	0.9720	-0.0410	0.1518	0.8187
Dync1li2	_FAVSAESLR_.2	#N/A	5.4361	5.7187	3.9053	5.4192	3.8960	4.2295	4.6942	5.1615	4.1652	4.7238	1.0173	0.0248	0.8642	0.0634
Dync1li2	_KPDSM[Oxidation (M)]VTNSS[Phospho (STY)]TENEA_.2	#N/A	3.9415	3.7471	4.1049	3.6487	5.1336	7.3617	4.0893	3.3864	6.5419	3.3259	1.0739	0.1028	0.7427	0.1292
Dync1li2	_KTGSPGS[Phospho (STY)]PSAGGVQSTAK_.2	#N/A	5.4390	5.3317	5.7820	5.0734	4.4032	3.2946	5.7242	3.1445	5.1864	4.7529	1.0394	0.0558	0.7789	0.1085
Dync1li2	_TSES[Phospho (STY)]PARGPSGSPR_.2	Q6PDL0_S383_M1_Dync1li2	3.1251	4.1010	3.8119	5.3692	3.9893	4.1361	4.0293	3.8039	3.4525	4.2816	1.0506	0.0712	0.6332	0.1984
Srsf1	_VKVDGPRS[Phospho (STY)]PS[Phospho (STY)]YGR_.3	#N/A	10.6448	10.2122	10.9816	10.5691	9.9203	10.5473	10.5258	10.6550	10.9049	9.4515	1.0091	0.0131	0.7712	0.1128
Srsf1	_YS[Phospho (STY)]PRHS[Phospho (STY)]R_.2	Q6PDM2_S242_M2_Srsf1	8.7490	3.6020	3.4837	8.5535	7.2027	4.3061	3.1661	8.4523	4.2824	8.4097	0.9844	-0.0227	0.9560	0.0195
Srsf1	_DGTGVVEFVR_.2	#N/A	6.2320	5.7728	6.3403	6.1214	5.3644	5.4115	5.7784	5.8564	5.6228	5.8115	1.0185	0.0264	0.6400	0.1938
Srsf1	_VDGPRS[Phospho (STY)]PSYGRS[Phospho (STY)]R_.2	#N/A	4.2502	3.7324	7.3383	4.1396	5.9821	6.6046	3.5681	4.1842	3.8328	5.9128	1.2210	0.2881	0.2993	0.5240
Srsf1	_VDGPRS[Phospho (STY)]PSYGR_.2	#N/A	10.7944	8.9796	10.4574	10.2758	3.5640	10.4876	10.2586	10.7382	9.3593	10.5015	0.8902	-0.1678	0.4584	0.3388
Srsf1	_GS[Phospho (STY)]PRYS[Phospho (STY)]PR_.2	#N/A	11.1683	10.8102	11.2396	3.5587	10.4376	3.1996	11.2717	11.0539	3.7415	10.8846	0.9095	-0.1368	0.7432	0.1289
Srsf1	_SRGS[Phospho (STY)]PRYS[Phospho (STY)]PR_.2	#N/A	9.5696	9.4280	4.5896	9.7442	3.8086	3.9209	4.2817	9.7664	3.1668	9.5233	1.0238	0.0339	0.9402	0.0268
Mylk	_KSS[Phospho (STY)]TGSPTSPINAEK_.2	Q6PDN3_S1795_M1_Mylk	3.0521	4.1741	3.7389	4.2415	4.7782	4.0509	3.9562	3.8769	3.5255	5.7349	0.9374	-0.0932	0.6003	0.2216
Chd4	_MSQPGS[Phospho (STY)]PSPK_.2	Q6PDQ2_S1528_M1_Chd4	7.3765	3.3246	3.8029	3.3466	3.2129	3.4117	6.6820	3.6545	3.9536	3.0239	0.9424	-0.0856	0.8179	0.0873
Chd4	_KMS[Phospho (STY)]QPGS[Phospho (STY)]PSPK_.2	Q6PDQ2_S1528_M2_Chd4	3.5927	3.3854	3.8509	6.9255	3.6168	3.4580	4.0912	3.8284	3.4769	3.6035	1.1035	0.1421	0.5993	0.2224
Sarm1	_EM[Oxidation (M)]LHS[Phospho (STY)]PLPC[Carbamidomethyl (C)]TGGK_.2	#N/A	6.2096	6.2957	6.7268	6.1098	5.3875	6.1802	6.6986	6.5448	6.0266	5.8379	0.9800	-0.0291	0.6599	0.1805

Prrt3	_S[Phospho (STY)]LTEVC[Carbamidomethyl (C)]LR_.2	#N/A	8.9580	8.0178	3.9975	8.3697	6.2593	8.2688	8.3280	8.4492	8.7933	8.4631	0.8594	-0.2187	0.2449	0.6110
Prrt3	_SSESHSAS[Phospho (STY)]SDTIEL_.2	Q6PE13_S965_M1_Prrt3	3.8884	3.9140	3.7956	4.2761	3.7353	4.0636	3.7032	3.7266	6.9244	3.7653	0.8710	-0.1993	0.3872	0.4120
Prrt3	_RGS[Phospho (STY)]QPDAELDGAGTSLLR_.3	#N/A	3.6899	3.6363	4.0442	6.8213	3.4852	6.7529	6.7732	9.6836	8.4156	3.7411	0.6624	-0.5943	0.1007	0.9969
Prrt3	_C[Carbamidomethyl (C)]RS[Phospho (STY)]LTEVC[Carbamidomethyl (C)]LR_.2	#N/A	3.6265	4.3827	3.5302	4.0329	4.2710	3.8544	3.7476	4.0856	3.7342	3.9999	1.0149	0.0213	0.7668	0.1153
Prrt3	_GS[Phospho (STY)]QPDAELDGAGTSLLR_.2	#N/A	3.7441	3.4821	4.4309	3.4327	2.8697	3.3589	3.1474	3.1850	4.3344	3.3997	1.0104	0.0149	0.9180	0.0372
Prrt3	_GLASS[Phospho (STY)]PTGGALRPR_.2	Q6PE13_S833_M1_Prrt3	3.8071	8.0842	3.5408	4.5854	2.9509	3.6738	3.2328	8.2004	4.2157	4.2627	0.8920	-0.1649	0.6873	0.1628
Prrt3	_TSLPQHVM[Oxidation (M)]EPPVGAAAAGTSGS[Phospho (STY)]SLDSFSK_.3	Q6PE13_S894_M1_Prrt3	3.4600	4.2162	3.1936	4.2382	4.1045	4.0209	3.9141	4.2638	3.0620	3.9155	1.0220	0.0314	0.7890	0.1029
Prrt3	_GSVGPAPSLSELDLRPPS[Phospho (STY)]PINLSR_.3	Q6PE13_S799_M1_Prrt3	3.7721	3.5703	3.5058	7.0009	3.3917	4.3378	3.1978	5.0321	4.2507	4.2276	1.0206	0.0294	0.9133	0.0394
Prrt3	_S[Phospho (STY)]IDAALFR_.2	Q6PE13_S806_M1_Prrt3	3.0132	3.7694	4.2477	7.6488	3.6577	2.9669	3.9397	3.2097	3.5088	3.4687	1.1941	0.2559	0.4684	0.3293
Gpatch2l	_EVAPLTNFSDS[Phospho (STY)]DDTVVAK_.2	Q6PE65_S88_M1_Gpatch2l	3.9678	3.2722	4.0013	3.6564	3.0935	3.1584	3.2522	3.8320	4.1339	3.6234	0.9500	-0.0740	0.4858	0.3136
Mob4	_DNLIVPILEEEVQNSVSGESEA_.2	#N/A	7.8355	8.0599	8.6316	8.8152	7.0749	7.6453	7.9776	7.9948	7.2963	7.7862	1.0318	0.0451	0.5045	0.2971
Mob4	_HTLDGAAC[Carbamidomethyl (C)]LLNSNK_.3	#N/A	3.2585	4.0637	3.9453	4.4479	3.8850	3.8445	4.1626	3.6705	3.3191	4.4149	1.0040	0.0058	0.9562	0.0195
Ctif	_S[Phospho (STY)]QEIEELER_.2	Q6PEE2_S18_M1_Ctif	9.7802	9.3889	9.3271	9.6519	9.1154	9.4493	9.9995	9.1039	9.6063	9.6345	0.9860	-0.0203	0.4990	0.3019
Slc46a1	_VNPHPEFQQFPQS[Phospho (STY)]P_.2	Q6PEM8_S458_M1_Slc46a1	8.2913	8.4742	8.7758	8.7637	6.9349	8.2640	9.1563	8.9869	8.5912	8.0999	0.9474	-0.0779	0.2789	0.5545
Mapre3	_LSNVAPPC[Carbamidomethyl (C)]ILR_.2	#N/A	8.0959	8.2293	8.0284	8.3657	8.4392	7.9515	8.0115	8.2000	8.0907	8.1360	1.0093	0.0134	0.4860	0.3134
Mapre3	_LEHEYIHNFK_.2	#N/A	6.0004	5.9546	5.7559	6.3405	5.6314	5.6174	5.7366	5.5921	5.9432	5.2298	1.0459	0.0647	0.1983	0.7028
Mapre3	_FFDANYDGKDYNPLLAR_.3	#N/A	6.3812	6.8558	6.7206	6.9151	6.7480	6.8359	6.6567	6.6002	6.7175	6.2326	1.0292	0.0415	0.1803	0.7440
Mapre3	_QGQDVAPPPNPGDQIFNK_.2	#N/A	5.9373	6.4871	6.4427	6.1990	5.8632	5.9592	6.9058	6.8873	6.2612	6.3392	0.9318	-0.1020	0.0491	1.3092
Mapre3	_FFDANYDGK_.2	#N/A	5.9239	5.9021	5.9416	6.3746	6.4153	6.1845	6.2446	6.2739	6.3958	5.9443	0.9854	-0.0213	0.5380	0.2692
Mapre3	_KLIGTAVPQRT[Phospho (STY)]SPTGPK_.3	#N/A	5.5442	3.4001	3.2817	3.2198	3.2214	3.0072	3.3680	3.7814	4.0805	3.7513	0.9645	-0.0521	0.7974	0.0983
Mapre3	_LTVDGLEK_.2	#N/A	6.5566	6.1089	4.0260	6.1299	6.2182	6.7569	4.8424	6.2645	6.6062	6.3359	0.9923	-0.0111	0.9398	0.0270
Mapre3	_DYNPLLAR_.2	#N/A	5.5322	5.2587	5.4982	5.4083	6.1539	5.6918	4.3052	6.2601	6.3236	6.1792	0.9694	-0.0448	0.6834	0.1653
Mapre3	_[Acetyl (Protein N-term)]AVNVYSTSVTSENLSR_.2	#N/A	7.2657	6.6416	7.4594	7.2914	6.7615	7.0491	7.2088	6.6161	6.6596	7.0978	1.0265	0.0377	0.3946	0.4038
Mapre3	_LIGTAVPQRTS[Phospho (STY)]PTGPK_.2	Q6PER3_S162_M1_Mapre3	3.7820	3.7610	3.9672	3.7873	3.4319	3.9242	3.6865	6.2230	5.3349	3.6483	0.7994	-0.3231	0.1013	0.9944
Wipf2	_HSSS[Phospho (STY)]APPPPPPGR_.2	Q6PEV3_S175_M1_Wipf2	8.2014	3.1693	9.4342	9.6038	3.3464	9.2702	9.4905	3.8914	4.0216	3.5805	1.3669	0.4509	0.3470	0.4596
Pik3c3	_DGDESS[Phospho (STY)]PILTSFELVK_.2	Q6PF93_S244_M1_Pik3c3	3.1906	6.1962	6.1244	4.3801	5.2285	3.9123	4.0948	3.7384	5.9606	6.2531	0.9655	-0.0507	0.8345	0.0786
Dlgap3	_SHS[Phospho (STY)]LEAPGKR_.2	#N/A	10.2182	10.3986	10.8452	10.5365	9.3879	10.2765	10.0770	10.3803	10.1421	10.4277	1.0020	0.0029	0.9393	0.0272
Dlgap3	_S[Phospho (STY)]LDSVDR_.2	#N/A	9.2205	8.7529	9.0537	9.0989	7.9422	8.9209	8.7542	8.8443	8.8060	9.1825	0.9927	-0.0106	0.8000	0.0969
Dlgap3	_SHS[Phospho (STY)]LEAPGK_.2	#N/A	6.8302	3.1505	7.2433	7.4442	3.0388	6.7536	3.4789	6.5167	6.7741	7.2447	0.9567	-0.0639	0.8407	0.0753
Dlgap3	_S[Phospho (STY)]LPDS[Phospho (STY)]GRTSPC[Carbamidomethyl (C)]PR_.2	Q6PFD5_S783_M2_Dlgap3	6.7972	6.9992	6.8972	7.2392	6.3559	6.8584	6.8279	6.4684	6.4863	7.1546	1.0183	0.0262	0.5469	0.2621
Dlgap3	_LFAKS[Phospho (STY)]HS[Phospho (STY)]LEAPGKR_.3	Q6PFD5_S183_M2_Dlgap3	5.2971	5.5564	3.6282	5.9065	3.5679	5.6968	5.2262	5.5665	6.2207	5.2798	0.8868	-0.1734	0.3005	0.5221
Dlgap3	_RDS[Phospho (STY)]WMER_.2	Q6PFD5_S771_M1_Dlgap3	6.0930	4.2723	4.1341	6.6599	4.4169	3.6557	4.5737	4.7807	4.3236	4.3460	1.0812	0.1127	0.5722	0.2424
Dlgap3	_ERS[Phospho (STY)]LDSVDR_.2	#N/A	8.4819	8.4834	8.9900	8.9207	7.9383	8.6900	8.2224	8.6051	8.5850	8.4826	1.0130	0.0187	0.6080	0.2161
Dlgap3	_HAS[Phospho (STY)]EPQPGPR_.2	Q6PFD5_S712_M1_Dlgap3	6.2043	5.8435	6.4608	7.5502	8.1794	6.4378	6.2310	6.4712	6.4559	6.4453	1.0591	0.0829	0.4320	0.3645
Dlgap3	_WRPSIGVQVETIS[Phospho (STY)]DSDTENR_.3	Q6PFD5_S641_M1_Dlgap3	3.5871	4.3923	4.2739	5.5278	4.1281	3.5158	3.8766	5.3939	3.5719	3.2428	1.0538	0.0756	0.6931	0.1592
Dlgap3	_S[Phospho (STY)]NSVTAGVQADLELEGLAGLATVATEDK_.3	Q6PFD5_S672_M1_Dlgap3	3.2174	3.9737	4.4519	3.9957	3.8620	4.2635	4.1440	3.0055	3.3045	3.6730	1.1215	0.1654	0.1780	0.7496
Dlgap3	_HSSATES[Phospho (STY)]ADSIEIYIPEAQTR_.3	#N/A	6.1595	5.9121	6.6415	7.8691	6.3909	5.5569	6.0015	6.7693	6.7671	6.5856	0.9833	-0.0243	0.8077	0.0927
Dlgap3	_AMGDEESGDS[Phospho (STY)]DGS[Phospho (STY)]PKTS[Phospho (STY)]PK_.2	Q6PFD5_S407_M3_Dlgap3	3.9720	3.3378	4.0570	4.7693	3.6650	3.6141	3.2417	4.0920	4.2006	3.8840	1.0124	0.0178	0.8791	0.0560
Dlgap3	_AIQAGC[Carbamidomethyl (C)]S[Phospho (STY)]QDDDC[Carbamidomethyl (C)]LPLLAAPASVSGR_.3	Q6PFD5_S510_M1_Dlgap3	3.4430	3.7832	4.1298	3.1316	3.6714	2.9532	4.3472	3.4860	6.3622	3.0986	0.8139	-0.2972	0.2281	0.6418
Dlgap3	_RSSS[Phospho (STY)]VDTAR_.2	Q6PFD5_S429_M1_Dlgap3	4.0800	3.2450	3.2660	3.7686	3.2057	3.0234	3.4833	4.3503	3.9989	3.7356	0.8817	-0.1817	0.1059	0.9750
Dlgap3	_S[Phospho (STY)]LPDSGR_.2	Q6PFD5_S779_M1_Dlgap3	2.9735	5.2579	3.6603	4.3420	3.6000	4.1294	4.1714	4.1600	6.0443	4.0193	0.8685	-0.2035	0.3048	0.5159
Dlgap3	_AMGDEESGDSDGS[Phospho (STY)]PK_.2	Q6PFD5_S410_M1_Dlgap3	4.8241	4.1237	3.8737	5.6812	4.1002	3.6991	4.0911	3.8266	3.4752	3.8445	1.1508	0.2026	0.1808	0.7428
Dlgap3	_EAEDYELPEEILEK_.2	#N/A	3.0594	3.8646	3.7462	4.2488	3.6859	4.0436	3.9635	6.7838	3.5182	4.2158	0.8170	-0.2916	0.2079	0.6821
Dlgap3	_EGYPLPYEPPATDGS[Phospho (STY)]PGPTPVPAPGPGSGR_.3	Q6PFD5_S753_M1_Dlgap3	3.6084	3.7340	3.3421	5.6304	4.2530	3.8725	4.5350	4.8991	4.4144	4.0640	0.9096	-0.1367	0.3847	0.4149
Dlgap3	_C[Carbamidomethyl (C)]S[Phospho (STY)]SADGLDGPTMGAR_.2	Q6PFD5_S578_M1_Dlgap3	3.9139	3.6011	3.3483	3.9044	3.8545	3.6918	4.0526	4.1906	4.2814	3.9497	0.9030	-0.1472	0.0134	1.8721
Dlgap3	_DSWMERGS[Phospho (STY)]RS[Phospho (STY)]LPDSGR_.3	Q6PFD5_S777_M2_Dlgap3	2.9912	3.7964	3.6780	4.3243	3.6178	4.1117	4.4725	3.9378	2.9760	4.1477	0.9665	-0.0492	0.7167	0.1447
Nup98	_YGLQDS[Phospho (STY)]DEEEEEHPPK_.2	#N/A	4.0585	4.9426	3.5356	3.5625	3.0830	3.3700	5.7220	4.0516	3.9929	3.4166	0.8750	-0.1927	0.3299	0.4816
Nup98	_GETVQEIC[Carbamidomethyl (C)]S[Phospho (STY)]PR_.2	Q6PFD9_S1027_M1_Nup98	3.0866	3.8918	3.7734	4.2761	3.7132	4.0163	3.9908	3.8424	3.4910	4.2431	0.9746	-0.0371	0.6897	0.1613
Tra2a	_[Acetyl (Protein N-term)]S[Phospho (STY)]DVEENNFEGR_.2	Q6PFR5_S2_M1_Tra2a	8.7694	9.3547	9.0792	9.7698	8.7545	9.2905	10.0415	9.5257	9.6420	9.5775	0.9457	-0.0806	0.0423	1.3741
Tra2a	_S[Phospho (STY)]RS[Phospho (STY)]YT[Phospho (STY)]PEYR_.2	Q6PFR5_S82_M3_Tra2a	8.8185	4.1734	3.1508	4.1954	4.0617	4.0637	3.9569	4.3066	3.1048	3.8727	1.2450	0.3162	0.4030	0.3947
Tra2a	_RRS[Phospho (STY)]PS[Phospho (STY)]PYYSR_.2	#N/A	10.7591	10.4959	11.0211	10.6836	10.2385	10.5957	10.8778	10.6379	10.9403	10.5902	0.9880	-0.0174	0.4156	0.3814
Tra2a	_S[Phospho (STY)]PSPYYSR_.2	#N/A	8.7851	8.7120	8.9328	8.8328	8.1231	8.4766	8.6243	8.7723	8.6623	8.9468	0.9877	-0.0179	0.5256	0.2793
Tra2a	_RS[Phospho (STY)]PS[Phospho (STY)]PYYSR_.2	#N/A	9.3038	9.2186	9.7943	9.5421	8.8203	9.2376	9.6570	9.5053	9.4742	9.1675	0.9861	-0.0202	0.5006	0.3005
Tra2a	_[Acetyl (Protein N-term)]S[Phospho (STY)]DVEENNFEGRES[Phospho (STY)]R_.2	Q6PFR5_S14_M2_Tra2a	3.4777	5.1758	3.7443	5.9201	4.0556	5.5066	5.9606	5.3379	5.2416	5.2252	0.8540	-0.2277	0.1773	0.7514
Tra2a	_S[Phospho (STY)]RS[Phospho (STY)]HS[Phospho (STY)]PMSNR_.2	Q6PFR5_S94_M3_Tra2a	3.1602	5.1596	3.8470	7.6381	5.4865	3.9428	5.7432	3.7688	3.4174	5.4875	1.0582	0.0817	0.7838	0.1058
Tra2a	_S[Phospho (STY)]HS[Phospho (STY)]PMSNR_.2	Q6PFR5_S96_M2_Tra2a	3.0739	4.2685	4.3084	3.8521	4.0899	3.6396	4.0004	3.1490	3.4481	3.5294	1.0964	0.1327	0.2521	0.5984
Tra2a	_AHT[Phospho (STY)]PTPGIYMGR_.2	#N/A	3.9071	3.2180	3.6408	5.8560	3.0509	3.5791	3.4892	3.8219	4.1210	4.3627	0.9814	-0.0271	0.8954	0.0480
Nyap1	_RPPAKPR_.2	#N/A	4.3924	3.6152	3.3749	4.3643	4.1692	3.9889	3.7385	4.2410	3.8895	4.3206	0.9844	-0.0227	0.7969	0.0986
Slc6a7	_KYGGITSFENT[Phospho (STY)]AIEVDR_.3	Q6PGE7_T620_M1_Slc6a7	3.7606	3.8921	4.0367	5.2050	3.8398	3.3679	3.7287	3.4752	3.7742	4.0559	1.0688	0.0960	0.4585	0.3386
Morn4	_NEGLFENNK_.2	#N/A	5.4470	5.6548	3.1800	6.2591	6.1197	4.0121	4.3729	5.6538	5.9055	5.2880	0.9636	-0.0534	0.7856	0.1048
Gmip	_DLS[Phospho (STY)]PEGPAPLTEEELDLR_.2	Q6PGG2_S75_M1_Gmip	2.9857	4.2404	3.6725	6.3456	5.4575	3.9967	3.8899	3.9433	5.9738	4.1422	0.9916	-0.0121	0.9609	0.0173
Gmip	_S[Phospho (STY)]QGSPEDPPSQASPGSNK_.2	Q6PGG2_S235_M1_Gmip	3.9881	3.0560	4.1885	3.4477	4.0400	3.3308	3.7142	3.3688	4.1175	3.2708	1.0158	0.0227	0.8420	0.0747
Washc2	_VS[Phospho (STY)]PEVGSADVASIAQK_.2	#N/A	8.5475	8.2981	8.4716	9.0463	7.7844	8.4311	9.0953	8.0456	8.2036	8.4687	0.9972	-0.0040	0.9345	0.0294
Washc2	_VPLLFS[Phospho (STY)]DEEDSEVPSGVKPEDLK_.3	Q6PGL7_S723_M1_Washc2	2.9131	3.6694	4.2436	3.2454	7.5515	3.0669	4.4609	8.1770	3.0209	7.4972	0.7108	-0.4924	0.2399	0.6201
Washc2	_VDNARVS[Phospho (STY)]PEVGSADVASIAQK_.3	#N/A	6.4392	7.0071	7.3371	7.7088	6.2222	6.6381	7.1173	6.3255	6.6918	6.1658	1.0482	0.0679	0.3696	0.4323
Washc2	_LAAQESS[Phospho (STY)]EAEDVTVDR_.2	Q6PGL7_S1049_M1_Washc2	3.9558	4.3750	3.7915	6.2650	3.5506	3.3843	4.8446	4.4991	3.6895	4.7826	0.9476	-0.0777	0.6976	0.1564
Washc2	_EGLLPASDQEAGGPSDIFSSSS[Phospho (STY)]PLDK_.3	Q6PGL7_S783_M1_Washc2	4.3937	4.6322	3.5797	4.0823	3.5194	4.2101	3.7970	4.0361	3.6847	4.0493	1.0457	0.0644	0.4753	0.3230
Washc2	_GLFS[Phospho (STY)]DEEDSEDLFSSQSSSKPK_.3	#N/A	3.2540	3.9721	3.9408	8.3619	3.8806	3.8489	4.1582	8.1132	3.3236	4.4105	0.9084	-0.1387	0.7294	0.1370
Washc2	_TSALLFSS[Phospho (STY)]DEEDQWNIADSHTK_.3	Q6PGL7_S614_M1_Washc2	3.8602	5.2643	3.5939	3.1377	5.6586	3.6207	3.2859	6.4756	4.1626	5.4854	0.8633	-0.2120	0.4100	0.3873
Washc2	_VASIFDDPLNAFGS[Phospho (STY)]Q_.2	Q6PGL7_S1333_M1_Washc2	3.1294	4.2130	4.3640	4.6057	3.9129	3.6952	4.0560	4.2165	3.3925	3.5850	1.0457	0.0645	0.5903	0.2289
Washc2	_GLFS[Phospho (STY)]DEEDSEDLFSSQSSSK_.2	#N/A	3.1660	3.9712	4.6058	4.1495	3.7925	3.9370	4.2978	4.3525	3.1507	3.8268	1.0077	0.0110	0.9288	0.0321
Washc2	_AGNS[Phospho (STY)]DS[Phospho (STY)]EEDDANER_.2	#N/A	3.9039	3.2082	4.5907	3.5925	3.0296	3.1991	3.3072	3.0251	4.1746	3.5595	1.0201	0.0287	0.8474	0.0719
Washc2	_AGNS[Phospho (STY)]DS[Phospho (STY)]EEDDANERVDLILEPK_.3	#N/A	3.1709	3.9761	3.8577	4.3604	3.7975	3.9320	4.0751	3.7581	3.4067	4.3274	0.9890	-0.0159	0.8699	0.0605
Dclk2	_SSSSS[Phospho (STY)]PTSPGSFR_.2	Q6PGN3_S340_M1_Dclk2	7.6265	8.4621	9.2618	8.1007	7.2134	8.5466	8.1273	3.8064	7.8011	7.3525	1.2112	0.2764	0.1403	0.8529
Dclk2	_C[Carbamidomethyl (C)]LS[Phospho (STY)]PEGVNGNR_.2	Q6PGN3_S374_M1_Dclk2	9.0979	8.9609	9.3015	9.5858	8.6259	9.2496	9.3677	9.0645	9.0451	8.9934	1.0021	0.0030	0.9174	0.0374
Dclk2	_SSS[Phospho (STY)]NVNGGPELDR_.2	Q6PGN3_S361_M1_Dclk2	7.4068	3.1539	3.9222	3.4660	2.9752	3.2534	3.6142	3.5352	3.8343	7.5763	0.8684	-0.2035	0.6125	0.2129
Dclk2	_S[Phospho (STY)]IELEHFEER_.2	Q6PGN3_S6_M1_Dclk2	5.8684	6.1465	6.3575	7.2883	4.3670	6.3785	5.7684	6.4847	6.8547	6.7037	0.9403	-0.0888	0.4845	0.3147
Dclk2	_YSGSRS[Phospho (STY)]PGFSR_.2	Q6PGN3_S307_M1_Dclk2	3.8534	3.3727	8.4208	3.5420	3.2610	3.3636	3.2567	3.0756	4.2250	3.5090	1.2234	0.2909	0.4774	0.3211
Erc2	_TISNLEGS[Phospho (STY)]PSR_.2	Q6PH08_S14_M1_Erc2	10.0262	3.9335	10.6090	10.6908	9.9799	10.1695	4.1968	9.9951	3.2849	10.2905	1.3303	0.4118	0.2812	0.5509

Erc2	_S[Phospho (STY)]QLQPEPAK_.2	Q6PH08_S364_M1_Erc2	6.4556	6.4763	6.5350	6.9951	6.3297	6.7769	6.9811	6.8132	6.5395	7.0292	0.9640	-0.0528	0.1457	0.8364
Erc2	_AAILQTEVDALR_.2	#N/A	4.8522	4.9035	6.0873	5.8514	5.9779	4.7155	5.8338	5.6294	5.6530	5.6940	0.9466	-0.0792	0.3785	0.4219
Erc2	_DLNHLLQQESGNR_.3	#N/A	7.3371	7.1914	7.5740	7.9148	6.6864	7.1519	7.3838	7.0491	6.9823	7.1339	1.0241	0.0344	0.4631	0.3343
Erc2	_ESELLALQTK_.2	#N/A	5.9642	5.6558	5.6940	6.0105	5.2812	5.4712	5.4654	5.8995	5.3936	3.5935	1.1163	0.1587	0.2046	0.6892
Erc2	_SQHSNHRPS[Phospho (STY)]PDQDDEEGIWA_.3	#N/A	3.7757	10.2267	3.9090	11.2752	9.8666	10.2674	10.4811	10.3737	10.1008	9.9910	0.8030	-0.3165	0.2823	0.5493
Erc2	_IAELESLTLR_.2	#N/A	4.5671	4.2499	3.7174	3.2612	4.5346	3.4972	3.5047	3.2588	3.9332	3.9742	1.0827	0.1147	0.3631	0.4400
Erc2	_TISNLEGS[Phospho (STY)]PSRS[Phospho (STY)]PR_.2	Q6PH08_S14_M2_Erc2	3.3764	3.9679	3.9656	4.3111	4.3273	3.3283	4.2952	3.5380	4.1795	3.7847	0.9823	-0.0258	0.7970	0.0985
Rab35	_DYDHLFK_.2	#N/A	4.5239	4.1558	4.4111	3.8533	3.4114	4.0002	4.5673	4.8050	3.5477	4.4052	0.9372	-0.0936	0.3892	0.4098
Rab35	_QQQQQQNDVVK_.2	#N/A	3.4069	4.1416	1.8623	2.4205	3.3487	2.9523	4.6112	3.3720	1.9342	3.0305	0.9336	-0.0991	0.7286	0.1375
Rab35	_FAGQM[Oxidation (M)]GIQLFETSAK_.2	#N/A	4.7379	3.8617	4.4621	4.4147	4.1824	4.0465	5.4301	4.1240	4.2512	4.4466	0.9389	-0.0909	0.3561	0.4485
Rab35	_WLHEINQNC[Carbamidomethyl (C)]DDVC[Carbamidomethyl (C)]R_.3	#N/A	4.2942	3.3993	3.5524	4.8831	3.2454	3.9901	3.7698	4.0768	3.7162	4.0221	0.9995	-0.0008	0.9949	0.0022
Sort1	_SGYHDDS[Phospho (STY)]DEDLLE_.2	Q6PHU5_S819_M1_Sort1	4.2079	9.9085	5.3699	3.8965	8.7668	9.9060	9.9943	10.4133	10.1820	10.2964	0.6857	-0.5443	0.0572	1.2426
Camk2d	_FTDEYQLFEELGK_.2	#N/A	7.4285	6.9346	7.0634	8.9254	6.7583	7.2718	7.3736	6.7632	9.2360	7.1211	0.9703	-0.0435	0.7123	0.1473
Camk2d	_FYFENALSK_.2	#N/A	5.5805	6.1627	5.7847	5.1182	5.9606	5.7828	5.3273	6.2532	6.2561	5.8642	0.9673	-0.0479	0.4647	0.3328
Camk2d	_IPTGQEYAAK_.2	#N/A	6.2071	6.5688	6.9458	6.7226	6.9809	6.7850	3.1212	7.0966	6.5565	6.3266	1.1604	0.2147	0.2375	0.6243
Camk2d	_ESTESSNTT[Phospho (STY)]IEDEDVK_.2	Q6PHZ2_T337_M1_Camk2d	3.2477	4.0040	4.4822	4.4116	3.8923	4.2332	4.1263	3.7068	3.3554	4.3786	1.0394	0.0558	0.6135	0.2122
Camk2d	_HPWIC[Carbamidomethyl (C)]QR_.2	#N/A	4.4483	3.5358	3.4174	4.5849	4.9421	4.3724	4.4842	5.2562	4.9880	5.1422	0.8489	-0.2364	0.0569	1.2448
Camk2d	_IC[Carbamidomethyl (C)]DPGLTAFEPEALGNLVEGM[Oxidation (M)]DFHR_.3	#N/A	6.1359	4.4210	5.9497	6.8388	5.7394	4.3439	3.2039	4.7779	4.2446	4.2338	1.3539	0.4371	0.0338	1.4713
Camk2d	_VTEQLIEAINNGDFEAYTK_.2	#N/A	5.2830	5.5187	5.2396	5.6863	4.8034	5.1475	5.6613	5.1901	4.5831	5.5267	1.0075	0.0108	0.8766	0.0572
Camk2d	_ITASEALK_.2	#N/A	2.9547	2.9518	3.5896	3.3361	3.4441	3.4555	1.7905	3.2816	3.4111	3.3031	1.1161	0.1585	0.3378	0.4714
Rbm15b	_ASGDPGAGGAS[Phospho (STY)]PR_.2	Q6PHZ5_S107_M1_Rbm15b	8.2358	8.2704	8.6013	8.8347	7.8091	8.1882	8.3303	8.4902	8.3095	7.9266	1.0072	0.0103	0.7800	0.1079
Rbm15b	_TTHS[Phospho (STY)]PYEER_.2	Q6PHZ5_S606_M1_Rbm15b	5.8294	6.0471	6.2281	3.5450	3.4113	3.2133	3.6933	3.4562	5.5797	3.2223	1.1817	0.2408	0.4227	0.3739
Rbm15b	_QRS[Phospho (STY)]LS[Phospho (STY)]PVAAPPLREPR_.3	Q6PHZ5_S261_M2_Rbm15b	3.3863	4.1119	3.1200	5.5215	3.4664	5.4174	4.3128	4.7928	5.1417	4.4805	0.8908	-0.1669	0.3870	0.4123
Rbm15b	_RNS[Phospho (STY)]LEGYSR_.2	Q6PHZ5_S559_M1_Rbm15b	4.0248	3.2014	3.2107	3.7134	3.0897	3.5349	3.4281	4.8559	4.2289	4.7062	0.8043	-0.3141	0.0279	1.5551
Kcnip4	_TSS[Phospho (STY)]PAIQNS[Phospho (STY)]VEDELEM[Oxidation (M)]ATVR_.2	Q6PHZ8_S50_M2_Kcnip4	3.7505	4.0386	4.0757	4.0065	4.9216	4.8531	10.4487	4.9299	4.4540	3.2193	0.7417	-0.4311	0.2824	0.5492
Atp1a3	_YQLSIHETEDPNDNR_.3	#N/A	10.7506	10.5463	10.8012	11.0104	10.1456	10.7723	11.2712	11.2023	11.1533	10.9748	0.9570	-0.0634	0.0169	1.7712
Atp1a3	_C[Carbamidomethyl (C)]IELSSGS[Phospho (STY)]VK_.2	Q6PIC6_S456_M1_Atp1a3	8.7294	8.3100	9.0044	8.2621	8.2120	8.2621	8.5735	8.6921	8.7962	8.4588	0.9807	-0.0282	0.3711	0.4305
Atp1a3	_TDKLVNER_.2	#N/A	10.1443	10.1157	10.2794	9.4425	9.8563	9.8523	10.0910	10.4318	9.8955	9.7471	0.9907	-0.0134	0.6424	0.1922
Atp1a3	_GGQDNIPVLK_.2	#N/A	10.1563	9.8578	9.9187	9.6139	9.5498	9.3915	10.2311	10.1572	9.7581	9.6498	0.9798	-0.0295	0.3032	0.5182
Atp1a3	_IATLASGLEVGK_.2	#N/A	9.2181	9.3596	9.3051	9.3912	9.1058	9.0077	9.3946	9.9213	9.3221	9.0338	0.9802	-0.0289	0.2911	0.5359
Atp1a3	_GGQDNIPVLKR_.2	#N/A	8.7217	9.1485	9.6314	8.8366	9.0069	9.0473	9.4951	9.4366	9.2295	8.9607	0.9768	-0.0338	0.2851	0.5450
Atp1a3	_IISAHGC[Carbamidomethyl (C)]K_.2	#N/A	7.6449	8.0254	8.0505	7.5551	8.0548	7.3890	7.9813	8.3162	7.9752	7.7862	0.9715	-0.0417	0.2243	0.6491
Atp1a3	_VLGFC[Carbamidomethyl (C)]HYYLPEEQFPK_.3	#N/A	7.9300	8.3266	8.3421	8.3725	7.0314	7.4356	8.3435	8.6985	7.4334	7.4507	0.9906	-0.0136	0.8489	0.0712
Atp1a3	_GVVVATGDR_.2	#N/A	9.0720	8.9021	9.3890	8.3449	8.6419	8.8166	8.1097	9.1841	8.8766	9.0966	1.0050	0.0072	0.8717	0.0596
Atp1a3	_AC[Carbamidomethyl (C)]VIHGTDLK_.2	#N/A	8.8111	8.2857	8.0379	8.2455	8.7419	8.1836	8.6431	8.7190	8.5853	8.1589	0.9833	-0.0243	0.4740	0.3242
Atp1a3	_C[Carbamidomethyl (C)]ATILLQGK_.2	#N/A	7.8941	7.9567	7.7918	7.5083	7.4727	7.8468	8.2096	8.1136	8.1763	7.9515	0.9547	-0.0669	0.0121	1.9172
Atp1a3	_RDLDDLKK_.2	#N/A	8.4826	8.1596	8.0592	7.8462	7.2393	8.1720	8.7571	8.3183	8.0766	7.9447	0.9660	-0.0498	0.3075	0.5122
Atp1a3	_RNPGGWVEK_.2	#N/A	7.9477	7.7793	7.9806	7.8150	8.1501	7.7548	8.2705	8.3722	8.0274	8.0317	0.9669	-0.0486	0.0304	1.5172
Atp1a3	_EAFQNAYLELGGLGER_.2	#N/A	7.1630	6.4267	6.8461	6.6810	6.5333	6.6485	7.5486	6.8360	6.6913	6.7184	0.9666	-0.0490	0.2970	0.5273
Atp1a3	_AQEILAR_.2	#N/A	7.2065	6.7381	7.4210	6.3157	6.8341	6.6526	4.4643	7.3225	7.4561	7.5299	1.0251	0.0358	0.7939	0.1002
Atp1a3	_NITFFSTNC[Carbamidomethyl (C)]VEGTAR_.2	#N/A	6.7509	6.0014	7.3202	6.1446	6.0105	5.4441	6.3552	6.2060	6.8462	6.4228	0.9723	-0.0405	0.6263	0.2032
Atp1a3	_NPGGWVEKETYY_.2	#N/A	6.1300	6.1883	7.5524	5.4939	5.3125	6.2130	6.6622	6.5363	6.2986	5.9662	0.9658	-0.0501	0.6185	0.2086
Atp1a3	_EQPLDEEM[Oxidation (M)]KEAFQNAYLELGGLGER_.3	#N/A	5.9635	6.9913	7.0346	6.9403	5.4952	5.8362	7.0595	7.6112	6.7982	6.0283	0.9276	-0.1084	0.2879	0.5407
Atp1a3	_NPGGWVEK_.2	#N/A	5.8263	5.6284	6.1000	4.7703	5.4422	5.5528	5.1412	6.2279	6.2917	6.0826	0.9356	-0.0961	0.2551	0.5933
Atp1a3	_M[Oxidation (M)]QVNAEEVVVGDLVEIK_.2	#N/A	9.3678	9.5713	10.5123	9.7583	8.6695	9.8458	9.9021	9.7685	9.3527	9.5326	0.9981	-0.0027	0.9567	0.0192
Atp1a3	_KYNTDC[Carbamidomethyl (C)]VQGLTHSK_.3	#N/A	8.3748	8.3953	8.6165	7.8690	8.3415	8.0218	8.1602	8.2435	8.1848	7.8914	1.0185	0.0264	0.3552	0.4496
Atp1a3	_DFTSEQIDEILQNHTEIVFAR_.3	#N/A	3.0963	5.6220	5.5329	6.0165	3.9967	4.6588	6.1530	5.6762	5.5800	4.1357	0.8950	-0.1601	0.4207	0.3760
Atp1a3	_S[Phospho (STY)]PDC[Carbamidomethyl (C)]THDNPLETR_.2	#N/A	9.9490	9.7696	10.0848	10.0211	9.2195	9.5479	10.1998	10.0481	9.8105	9.6293	0.9842	-0.0230	0.4474	0.3493
Atp1a3	_MSVEEVC[Carbamidomethyl (C)]R_.2	#N/A	8.3424	7.9273	7.8759	8.2150	7.7735	7.2433	7.9078	7.9291	7.8192	7.8278	1.0032	0.0046	0.9016	0.0450
Atp1a3	_EVAMTEHK_.2	#N/A	7.1882	4.3525	3.1998	6.8212	6.0508	4.0148	5.7880	5.0608	4.7361	5.5160	0.9992	-0.0011	0.9964	0.0016
Atp1a3	[Carbamidomethyl (C)]DDVNFTTDNLC[Carbamidomethyl (C)]FVGLM[Oxidation (M)]SM[Oxidation (M)	#N/A	4.9364	5.5915	4.6220	6.5014	3.6961	5.1308	6.5018	5.6055	5.3326	4.9828	0.9062	-0.1421	0.3625	0.4407
Atp1a3	_SSHTWVALSHIAGLC[Carbamidomethyl (C)]NR_.3	#N/A	8.2125	8.1207	8.7178	8.2382	7.4001	7.7149	8.3221	8.2340	7.8662	8.3069	0.9860	-0.0204	0.6557	0.1833
Atp1a3	_DVAGDASESALLK_.2	#N/A	7.7677	7.5780	7.3231	7.9228	7.6736	7.7059	7.7069	8.8353	7.8923	7.8529	0.9492	-0.0752	0.1101	0.9584
Atp1a3	_ADIGVAM[Oxidation (M)]GIAGSDVSK_.2	#N/A	6.8026	7.2910	6.8693	6.8763	6.6535	7.1526	7.2831	7.8291	7.1765	7.2156	0.9410	-0.0877	0.0340	1.4683
Atp1a3	_KADIGVAM[Oxidation (M)]GIAGSDVSK_.2	#N/A	5.3299	5.6182	5.5174	5.3106	5.6118	5.7939	4.2938	6.3254	6.2388	6.0896	0.9640	-0.0529	0.6141	0.2118
Atp1a3	_MSVEEVC[Carbamidomethyl (C)]RK_.2	#N/A	3.3973	4.2609	3.6521	6.2655	5.7411	3.9763	3.8694	3.9637	4.6713	4.1218	1.0944	0.1301	0.5423	0.2658
Atp1a3	_TVNDLEDSYGQQWTYEQR_.2	#N/A	8.8455	8.7158	9.6365	8.8273	7.3120	8.4891	9.3634	8.5429	8.1946	8.4472	1.0001	0.0001	0.9988	0.0005
Atp1a3	_YNTDC[Carbamidomethyl (C)]VQGLTHSK_.2	#N/A	6.8173	6.7611	6.9296	6.8814	6.6091	6.2532	7.2973	7.0246	6.5585	6.6398	0.9751	-0.0364	0.3844	0.4152
Atp1a3	_M[Oxidation (M)]TVAHM[Oxidation (M)]WFDNQIHEADTTEDQSGTSFDK_.4	#N/A	7.1059	7.3829	8.0592	6.5857	5.9850	7.4517	8.0297	7.5589	7.3557	7.8738	0.9209	-0.1189	0.1560	0.8068
Atp1a3	_VDNSSLTGESEPQTRS[Phospho (STY)]PDC[Carbamidomethyl (C)]THDNPLETR_.4	#N/A	2.9559	3.7611	3.6427	4.3595	3.5825	4.1470	4.5078	3.9731	3.6217	4.1124	0.9230	-0.1156	0.3112	0.5070
Atp1a3	_AC[Carbamidomethyl (C)]VIHGTDLKDFTSEQIDEILQNHTEIVFAR_.4	#N/A	3.8452	3.1007	4.6773	4.6235	2.9890	3.6356	4.0282	3.8785	4.1775	4.3008	0.9306	-0.1038	0.4719	0.3262
Atp1a2	_VDNSS[Phospho (STY)]LTGESEPQTR_.2	Q6PIE5_S215_M1_Atp1a2	11.9973	11.9928	12.4327	12.1112	11.1717	11.7378	12.4403	12.2973	11.9927	11.8021	0.9814	-0.0271	0.3838	0.4159
Atp1a2	_NM[Oxidation (M)]VPQQALVIR_.2	#N/A	10.9961	11.0807	11.3123	10.5836	10.3842	10.6034	11.1387	11.2370	10.9687	10.6785	0.9837	-0.0237	0.4098	0.3874
Atp1a2	_LIIVEGC[Carbamidomethyl (C)]QR_.2	#N/A	11.6136	11.5523	11.6514	11.3417	10.9595	11.2730	11.8922	11.6963	11.6420	11.3449	0.9789	-0.0307	0.1668	0.7778
Atp1a2	_LIFDNLKK_.2	#N/A	10.4896	10.7576	10.9417	10.2241	10.0961	10.1002	10.9183	10.8310	10.4921	10.3898	0.9791	-0.0305	0.3155	0.5010
Atp1a2	_AAVPDAVGK_.2	#N/A	9.6177	9.7403	9.7711	9.1606	9.6270	9.2576	9.1022	10.0711	9.5148	9.5669	0.9964	-0.0052	0.8695	0.0607
Atp1a2	_C[Carbamidomethyl (C)]IELSC[Carbamidomethyl (C)]GS[Phospho (STY)]VR_.2	#N/A	8.4835	8.1505	8.4745	8.1642	7.2711	7.9790	8.9324	8.3864	8.2758	8.1951	0.9573	-0.0629	0.2077	0.6825
Atp1a2	_TGTLTQNR_.2	#N/A	9.2190	9.2240	9.4145	8.8740	9.1551	8.5449	9.1515	9.5595	9.1567	9.0484	0.9830	-0.0248	0.4141	0.3829
Atp1a2	_IAGLC[Carbamidomethyl (C)]NR_.2	#N/A	8.2451	8.7149	8.6375	8.4213	8.6342	8.0199	8.5935	8.9082	8.8031	8.2058	0.9789	-0.0308	0.3532	0.4520
Atp1a2	_DHTEIVFAR_.2	#N/A	8.2837	8.1244	7.3200	8.1428	7.5752	7.8274	8.6925	8.4468	8.2894	7.9053	0.9455	-0.0809	0.0851	1.0703
Atp1a2	_LSLDELGR_.2	#N/A	8.1945	8.1265	8.6259	8.0130	8.1377	7.8225	8.3140	8.6139	8.4508	7.8350	0.9819	-0.0263	0.4522	0.3446
Atp1a2	_FDTDELNFPTEK_.2	#N/A	8.0749	7.3486	7.7969	7.3670	7.2467	7.3991	8.0977	7.7726	7.5665	7.5617	0.9728	-0.0398	0.3066	0.5134
Atp1a2	_VLGFC[Carbamidomethyl (C)]QLNLPSGK_.2	#N/A	7.5821	7.6136	7.8253	7.2418	7.5888	6.9407	7.8886	7.9301	7.5242	7.4116	0.9710	-0.0425	0.2784	0.5554
Atp1a2	_KYQVDLSK_.2	#N/A	6.9672	6.6621	6.5281	6.7198	6.0470	6.3188	7.3972	7.0590	6.6551	6.7469	0.9391	-0.0906	0.0802	1.0957
Atp1a2	_YQLSIHER_.2	#N/A	7.0584	7.1343	7.2079	7.0960	6.8835	7.0503	7.8938	7.7405	7.4611	7.2307	0.9328	-0.1004	0.0042	2.3744
Atp1a2	_TTNDLEDSYGQEWTYEQR_.2	#N/A	6.1365	6.1383	6.8230	6.6561	5.6666	5.6641	7.1975	6.5450	5.6971	5.6734	0.9845	-0.0226	0.8047	0.0944
Atp1a2	_NLEAVETLGSTSTIC[Carbamidomethyl (C)]SDKTGTLTQNR_.3	#N/A	6.3351	7.1655	7.9670	6.5288	5.9131	6.7103	7.5441	6.6962	6.7603	6.6447	0.9796	-0.0298	0.7348	0.1338
Atp1a2	_LIFDNLK_.2	#N/A	6.3390	6.5247	5.8010	6.0289	6.1139	6.6453	5.5633	7.2876	7.0988	6.6587	0.9384	-0.0918	0.2700	0.5687
Atp1a2	_YPGGWVEK_.2	#N/A	6.8227	6.5291	7.2901	5.7409	5.7080	5.6851	5.9379	6.6694	5.9253	6.4040	1.0099	0.0142	0.8742	0.0584

Atp1a2	_ELDELKK_.2	#N/A	6.2639	6.0190	5.8876	5.5284	6.0793	5.4908	5.9942	6.4055	5.9724	5.0809	1.0025	0.0037	0.9576	0.0188
Atp1a2	_C[Carbamidomethyl (C)]STILVQGK_.2	#N/A	6.0847	5.5101	5.4301	5.0340	5.7542	5.8380	5.7097	6.1075	5.5690	5.7636	0.9691	-0.0453	0.4143	0.3827
Atp1a2	_VIM[Oxidation (M)]VTGDHPITAK_.2	#N/A	8.6768	8.7631	9.1502	8.2903	8.3430	8.6152	9.0862	8.5937	8.7011	8.3170	0.9960	-0.0058	0.8684	0.0613
Atp1a2	_NLEAVETLGSTSTIC[Carbamidomethyl (C)]SDK_.2	#N/A	8.6876	8.4585	8.9651	8.6585	7.7629	8.3189	8.8543	8.3506	8.3540	8.0474	1.0088	0.0126	0.7744	0.1110
Atp1a2	_AC[Carbamidomethyl (C)]VVHGSDLK_.2	#N/A	7.3725	7.1233	7.1922	6.9802	7.4126	6.8556	7.3217	7.5612	7.4735	7.0251	0.9742	-0.0377	0.2273	0.6433
Atp1a2	_DTAGDASESALLK_.2	#N/A	7.3402	7.4004	7.7991	6.7520	6.6349	7.2570	7.3791	7.1253	7.5024	7.0984	0.9891	-0.0158	0.7450	0.1278
Atp1a2	_LC[Carbamidomethyl (C)]FVGLM[Oxidation (M)]SM[Oxidation (M)]IDPPR_.2	#N/A	7.7438	8.0544	7.5703	4.0427	7.1154	8.0825	8.0075	8.7296	8.2814	7.7190	0.8677	-0.2047	0.2150	0.6676
Atp1a2	_AGQENISVSK_.2	#N/A	6.5387	6.3673	4.4123	6.1499	6.3414	6.2937	6.9510	6.5588	6.2599	6.0494	0.9322	-0.1013	0.3437	0.4639
Atp1a2	_NSVFQQGM[Oxidation (M)]K_.2	#N/A	7.9239	7.9501	7.4184	7.6689	7.7333	7.1745	7.7991	8.1959	7.6887	7.3905	0.9841	-0.0232	0.5577	0.2536
Atp1a2	_GFKFDTDELNFPTEK_.3	#N/A	5.7174	6.1115	5.6811	5.4233	5.7090	5.9785	4.8179	6.3998	6.1198	6.0742	0.9859	-0.0206	0.7929	0.1008
Atp1a2	_QGAIVAVTGDGVNDSPALK_.2	#N/A	7.7118	8.0117	7.8426	8.0125	6.8675	7.8519	8.4321	8.3749	8.0024	7.9755	0.9414	-0.0870	0.0803	1.0952
Atp1a2	_DM[Oxidation (M)]TSEQLDEILR_.2	#N/A	6.9995	6.4309	6.3151	6.4450	6.2840	6.3194	7.0579	6.7619	6.5730	6.6089	0.9578	-0.0622	0.1187	0.9257
Atp1a2	_QAADM[Oxidation (M)]ILLDDNFASIVTGVEEGR_.3	#N/A	6.9112	6.0609	7.1456	6.5169	6.1497	7.0506	6.6608	6.3613	6.4775	6.7895	1.0102	0.0146	0.7957	0.0992
Atp1a2	_DGPNALTPPPTTPEWVK_.2	#N/A	10.0832	9.6794	10.0046	9.7658	9.4615	9.5464	10.2598	9.8965	9.7116	9.5950	0.9890	-0.0160	0.5410	0.2668
Atp1a2	_AIAKGVGIISEGNETVEDIAAR_.3	#N/A	4.9489	4.7389	4.5691	5.7931	3.6170	4.1439	5.6884	5.0861	4.5497	5.3611	0.8963	-0.1579	0.2402	0.6194
Atp1a2	_QGAIVAVTGDGVNDSPALKK_.2	#N/A	5.1739	4.7452	3.7794	5.7862	4.3139	4.8480	5.2716	5.6452	5.2653	4.9448	0.9040	-0.1457	0.2083	0.6813
Atp1a2	_NSQTDKLVNER_.2	#N/A	4.9839	3.9467	5.1296	5.2113	5.2418	4.4524	5.2988	5.4228	5.1875	4.6488	0.9393	-0.0903	0.3252	0.4878
Atp1a2	_IISSHGC[Carbamidomethyl (C)]K_.2	#N/A	4.7738	5.5550	5.5089	4.9689	5.9952	4.6983	5.6000	5.9489	5.5138	4.7449	0.9630	-0.0544	0.5583	0.2532
Atp1a2	_EIPLDKEM[Oxidation (M)]QDAFQNAYM[Oxidation (M)]ELGGLGER_.3	#N/A	4.3196	6.3690	6.4619	6.6447	5.1462	3.9819	6.3488	7.0375	6.6875	3.9348	0.9142	-0.1294	0.5451	0.2636
Atp1a2	_YQVDLSK_.2	#N/A	3.5648	4.4771	3.8953	4.3980	4.5900	4.0636	3.1493	4.2489	4.8326	4.2972	1.0079	0.0114	0.9264	0.0332
Atp1a2	_M[Oxidation (M)]QINAEEVVVGDLVEVK_.2	#N/A	6.8907	7.0871	8.1535	7.2300	6.0082	7.3912	7.4428	7.4237	6.9495	7.0347	0.9881	-0.0173	0.8227	0.0848
Atp1a2	_LNIPVSQVNPR_.2	#N/A	11.1508	11.2090	11.4628	10.8952	10.7820	10.8300	11.3503	11.3609	11.1445	10.8204	0.9898	-0.0148	0.5163	0.2871
Atp1a2	_ADIGIAM[Oxidation (M)]GISGSDVSK_.2	#N/A	5.6360	5.2040	6.0532	5.1417	3.9369	3.7926	6.0897	6.0347	5.8237	3.6824	0.9174	-0.1244	0.5125	0.2903
Atp1a2	_VAEIPFNSTNK_.2	#N/A	10.8092	10.7899	11.2237	10.5520	10.5400	10.4416	11.0564	10.9302	10.6823	10.7273	0.9887	-0.0164	0.4664	0.3312
Atp1a2	_SPEFTHENPLETR_.2	#N/A	6.8579	6.2698	6.5648	5.8549	6.0312	6.2265	6.5931	6.8643	6.2774	6.0844	0.9762	-0.0348	0.5217	0.2826
Atp1a2	_AGQENIS[Phospho (STY)]VSKR_.2	Q6PIE5_S439_M1_Atp1a2	5.4479	6.5551	7.4254	7.0228	4.9381	4.9762	5.1608	7.4388	7.2768	4.9037	0.9784	-0.0316	0.8656	0.0627
Atp1a2	_GVGIISEGNETVEDIAAR_.2	#N/A	9.4061	9.4861	10.1440	9.7309	8.2887	9.3221	9.9707	9.4972	9.3170	9.2811	0.9874	-0.0183	0.7325	0.1352
Atp1a2	_SPTWTALSR_.2	#N/A	7.5529	7.5820	8.1062	7.2193	7.0740	7.2060	7.3356	7.9081	7.4594	7.1711	0.9984	-0.0023	0.9601	0.0177
Atp1a2	_C[Carbamidomethyl (C)]IELSC[Carbamidomethyl (C)]GS[Phospho (STY)]VRK_.2	#N/A	7.5042	7.4027	8.2218	7.8318	6.6351	7.5227	8.5177	7.8437	7.5488	7.4728	0.9584	-0.0612	0.3494	0.4567
Atp1a2	_EDSPQSHVLVM[Oxidation (M)]K_.2	#N/A	6.5265	6.2789	5.3478	6.0097	5.7698	5.9544	6.6733	6.4166	5.9754	5.8949	0.9585	-0.0611	0.3384	0.4705
Atp1a2	_NIC[Carbamidomethyl (C)]FFSTNC[Carbamidomethyl (C)]VEGTAR_.2	#N/A	5.5087	5.5302	3.8436	3.3874	6.0626	4.5038	3.5357	3.7729	3.9128	6.0382	1.1138	0.1555	0.5083	0.2939
Atp1a2	_YQVDLS[Phospho (STY)]KGLTNQR_.3	#N/A	4.2495	3.0929	3.9832	3.5270	3.3933	3.2314	3.6752	3.4742	3.1302	3.2043	1.0619	0.0866	0.4277	0.3689
Atp1a2	_M[Oxidation (M)]TVAHM[Oxidation (M)]WFDNQIHEADTTEDQSGATFDKR_.4	#N/A	4.2970	3.5524	4.0307	3.5744	3.4407	3.1839	3.7227	3.4267	3.7258	3.2517	1.0419	0.0593	0.5341	0.2724
Nfrkb	_SPS[Phospho (STY)]PAVPLR_.2	Q6PIJ4_S228_M1_Nfrkb	3.5915	4.3967	4.2783	3.7240	4.2180	3.5115	3.8722	3.2772	3.5762	3.4013	1.1194	0.1627	0.0948	1.0234
Rhbdf1	_QTS[Phospho (STY)]ITQTIR_.2	Q6PIX5_S76_M1_Rhbdf1	7.3760	3.5946	3.4815	4.5261	5.6804	6.8299	3.1735	3.9759	4.2750	6.7359	1.1559	0.2091	0.5143	0.2888
Dmxl1	_DSS[Phospho (STY)]LKLDVR_.2	Q6PNC0_S1904_M1_Dmxl1	6.3901	7.0593	7.3302	7.4236	6.0373	6.8269	7.5603	7.8029	4.1747	7.2516	1.0220	0.0314	0.8444	0.0735
Dmxl1	_TGS[Phospho (STY)]ESS[Phospho (STY)]LKK_.2	Q6PNC0_S1426_M2_Dmxl1	3.8765	3.4430	3.6481	4.0575	3.2643	3.8450	3.3401	4.7886	4.1233	3.7348	0.9230	-0.1156	0.3149	0.5019
Dmxl1	_WDS[Phospho (STY)]DNDEENEDPPISMK_.2	Q6PNC0_S1965_M1_Dmxl1	4.1541	3.4585	3.3401	8.5392	4.9584	2.9489	3.3097	5.1933	2.0177	3.8097	1.2746	0.3501	0.4277	0.3689
Dmxl1	_ISEASWLPEEHY[Phospho (STY)]SSSPEK_.2	#N/A	3.8503	6.2570	6.7295	3.9890	3.5098	4.0760	3.7630	4.2641	4.0297	3.8553	1.1904	0.2514	0.3182	0.4974
Dmxl1	_GSSFLTSKDS[Phospho (STY)]S[Phospho (STY)]LKLDVR_.3	#N/A	3.6702	3.4840	4.2665	4.2667	3.3917	3.7662	3.7294	4.8193	4.3782	3.6944	0.9163	-0.1261	0.2633	0.5796
Atp2b4	_TQDGVALEIQPLNS[Phospho (STY)]QEGLDSEDKEK_.3	#N/A	7.2262	6.5101	7.4118	8.2699	6.9593	7.0227	7.6971	8.4984	7.4949	7.1245	0.9389	-0.0909	0.2498	0.6023
Atp2b4	_GIIDSTAGEQR_.2	#N/A	5.9680	5.7510	5.0629	5.4145	5.7272	6.2786	5.8453	6.4392	5.7331	5.8558	0.9551	-0.0663	0.3154	0.5011
Atp2b4	_EAGHGS[Phospho (STY)]DKEDISR_.3	Q6Q477_S1065_M1_Atp2b4	5.6939	4.4217	3.7890	4.2917	4.0122	4.1132	4.0064	5.7289	3.4754	4.2587	1.0045	0.0065	0.9705	0.0130
Atp2b4	_TQDGVALEIQPLNS[Phospho (STY)]QEGLDSEDKEKK_.3	#N/A	5.5899	5.3700	6.2836	6.4566	5.3640	5.4809	5.9183	6.5995	6.6253	5.7799	0.9240	-0.1140	0.1574	0.8029
Atp2b4	_DDFLC[Carbamidomethyl (C)]LEGK_.2	#N/A	3.7037	3.5225	4.3905	3.3923	2.8294	3.3993	3.1070	1.2950	4.3748	3.3593	1.1666	0.2224	0.4007	0.3972
Atp2b4	_NEKGEVEQEKLDK_.3	#N/A	3.0091	4.3333	4.2436	3.7874	3.6537	2.9709	3.9357	3.2138	3.3836	3.4647	1.0477	0.0672	0.6186	0.2086
Rictor	_NDS[Phospho (STY)]GEENVPLDLTR_.2	#N/A	3.1840	8.0712	3.8708	8.7353	7.0653	8.0340	8.9300	8.9239	8.7801	8.3968	0.7415	-0.4316	0.0988	1.0051
Rictor	_GRNDS[Phospho (STY)]GEENVPLDLTR_.3	#N/A	4.2000	3.0262	3.3860	3.8886	3.3257	5.0006	3.6033	4.2299	3.8784	3.8556	0.9776	-0.0327	0.8250	0.0835
Camk2n1	_[Acetyl (Protein N-term)]S[Phospho (STY)]EVLPYGDEK_.2	Q6QWF9_S2_M1_Camk2n1	3.9120	3.3142	4.5988	7.8435	3.2025	7.0409	3.3153	7.3526	7.5634	7.9595	0.7614	-0.3933	0.2727	0.5643
Camk2n1	_LSPYGDGGDVGQIFSC[Carbamidomethyl (C)]R_.2	#N/A	4.5902	3.8694	3.4050	4.5972	3.9037	4.3847	3.2447	4.2108	4.2165	4.2044	1.0393	0.0556	0.6280	0.2021
Camk2n1	_LQDTNNFFGAGQSK_.2	#N/A	5.8760	5.8235	4.2566	3.2584	5.7112	3.0799	4.4739	3.3592	5.8653	3.2254	1.1032	0.1417	0.6109	0.2140
Gnas	_ISTAS[Phospho (STY)]GDGR_.2	Q6R0H7_S1091_M1_Gnas	4.1048	3.3602	3.8385	3.3822	3.2485	3.4077	3.3009	3.6506	3.9496	3.8917	0.9618	-0.0562	0.5155	0.2878
Gnas	_ALWEDEGVR_.2	#N/A	6.3240	6.4468	6.5018	3.9737	5.9768	6.3875	6.0892	6.1028	6.3837	6.1206	0.9613	-0.0570	0.6470	0.1891
Gnas	_VLTSGIFETK_.2	#N/A	4.9255	5.3837	4.2717	5.2584	3.6817	4.8586	5.1884	5.1883	4.4870	4.7778	0.9633	-0.0540	0.6251	0.2040
Gnas	_EAAEM[Oxidation (M)]EGS[Phospho (STY)]PTTATAVEGK_.2	Q6R0H7_S367_M1_Gnas	3.2784	3.9478	3.9652	4.4679	3.9049	3.8246	4.1825	3.6506	3.2992	4.4349	1.0016	0.0023	0.9826	0.0076
Ppp1r9b	_AQLEQSVEENKER_.2	#N/A	3.7624	4.0369	3.2776	3.8915	4.7488	3.3029	3.8967	3.6891	4.2989	4.1544	0.9568	-0.0637	0.5766	0.2391
Ppp1r9b	_ERPGEQSEVAQLIQQTLEQER_.3	#N/A	3.5773	4.3336	5.6218	5.7085	3.8133	5.1020	5.4450	5.1671	4.9771	4.9137	0.9155	-0.1273	0.3911	0.4077
Ppp1r9b	_ASS[Phospho (STY)]LNENVDHSALLK_.2	#N/A	8.7814	8.8479	9.0956	9.5940	7.3687	9.1113	8.5000	8.7498	8.9569	9.1407	0.9958	-0.0061	0.9287	0.0321
Ppp1r9b	_TVTEGGAAHR_.2	#N/A	3.9469	3.3963	3.3518	3.2315	4.5391	3.2731	3.2332	2.7596	4.2486	2.0497	1.1791	0.2377	0.2567	0.5906
Ppp1r9b	_ETQAQYQALER_.2	#N/A	3.7949	3.0503	6.6842	6.8134	6.7868	3.6860	6.4337	4.2540	3.9026	6.4050	0.9785	-0.0314	0.9181	0.0371
Magi1	_KDS[Phospho (STY)]QNSSQHSVSSHR_.3	Q6RHR9_S733_M1_Magi1	6.0036	3.8095	6.0361	6.3959	3.6308	5.3116	5.5534	5.8144	5.1149	5.7476	0.9353	-0.0965	0.5804	0.2363
Magi1	_SLHTASPSHGIQVLPEY[Phospho (STY)]LPADAPAPDQTDSSGQK_.4	Q6RHR9_Y762_M1_Magi1	4.0576	3.1686	3.2435	3.7462	3.1833	3.0454	3.4609	4.3723	4.0209	3.7132	0.8755	-0.1918	0.0962	1.0170
Sdk2	_S[Phospho (STY)]PPRPSPGSLHYS[Phospho (STY)]DEDVTK_.3	#N/A	3.7581	3.3375	3.3990	3.7893	3.3554	3.4404	3.8135	4.1686	3.7865	3.5627	0.9166	-0.1256	0.0574	1.2414
Ranbp10	_SQDSYPGSPSLS[Phospho (STY)]PR_.2	Q6VN19_S369_M1_Ranbp10	7.0629	7.1729	4.1246	7.5738	3.9613	3.7846	7.7804	8.0955	7.1973	7.2769	0.7398	-0.4348	0.0705	1.1515
Wdfy3	_LKISS[Phospho (STY)]PVR_.2	Q6VNB8_S3483_M1_Wdfy3	6.7508	7.2423	4.3911	3.3929	6.1253	6.5408	3.1076	7.3408	6.9870	3.3599	1.1042	0.1430	0.6596	0.1807
Wdfy3	_SQS[Phospho (STY)]EYC[Carbamidomethyl (C)]NVGTK_.2	Q6VNB8_S1942_M1_Wdfy3	4.8622	3.4452	4.4678	4.4454	3.3335	3.2911	5.2577	5.2935	5.3269	4.6589	0.7741	-0.3695	0.0150	1.8239
Ntrk3	_VFLAEC[Carbamidomethyl (C)]YNLS[Phospho (STY)]PTKDK_.2	#N/A	3.4346	4.1491	6.4001	4.1257	5.3083	4.0523	4.0721	6.2720	3.4254	5.8258	0.9346	-0.0976	0.6887	0.1619
Ntrk3	_VFLAEC[Carbamidomethyl (C)]YNLS[Phospho (STY)]PTK_.2	#N/A	3.2818	4.0870	3.9686	4.0337	3.9084	3.8211	4.1819	3.6472	3.2958	4.4383	0.9895	-0.0152	0.8762	0.0574
Npr2	_GSS[Phospho (STY)]YGSLM[Oxidation (M)]TAHGK_.2	Q6VVW5_S523_M1_Npr2	4.2962	2.9300	6.2102	3.9848	3.4219	6.2733	3.6995	4.1337	3.7822	3.9518	1.1613	0.2157	0.4123	0.3847
Pcp4l1	_TPPAANQAS[Phospho (STY)]DPEEK_.2	#N/A	9.2047	9.1515	9.5803	9.7054	9.7470	9.6956	9.2828	9.2028	9.5076	9.5595	1.0134	0.0192	0.4321	0.3644
Pcp4l1	_[Acetyl (Protein N-term)]SELNTKT[Phospho (STY)]PPAANQASDPEEK_.2	#N/A	5.7363	6.4248	3.7248	7.0305	6.5895	6.6001	3.4169	3.7326	4.0316	5.8303	1.4150	0.5008	0.0457	1.3404
Pcp4l1	_TPPAANQAS[Phospho (STY)]DPEEKGKPGSIK_.3	#N/A	4.3814	6.6650	3.5673	6.3851	7.2276	6.5854	3.7847	6.3345	6.6453	6.2157	1.0099	0.0142	0.9518	0.0215
Pcp4l1	_KAEEEEEIDIDLTAPETEK_.3	#N/A	4.1090	3.2334	3.8427	3.3864	3.2527	3.3719	3.5347	3.6147	4.8624	3.0637	0.9373	-0.0934	0.5241	0.2806
Dagla	_TSTPTGHGAS[Phospho (STY)]PTKQDDLVISAR_.3	Q6WQJ1_S1032_M1_Dagla	3.4752	4.0334	4.1620	3.1638	3.8825	5.5269	4.7871	3.4538	3.1024	3.1308	1.1166	0.1592	0.4422	0.3544
Dagla	_GS[Phospho (STY)]PSLHAVLER_.2	#N/A	3.7993	3.6701	3.5330	3.8434	3.7097	3.7113	3.9041	4.0053	3.5702	3.5207	0.9896	-0.0151	0.7324	0.1352
Dagla	_HSQDTQPLEAALGSGGVTPERPPS[Phospho (STY)]AT[Phospho (STY)]IEEEEAAGGSEGGGVAPR_.4	Q6WQJ1_S874_M2_Dagla	2.9800	4.3624	4.2145	3.7583	3.4525	3.5458	3.9066	3.2429	3.7976	3.4356	1.0343	0.0486	0.6803	0.1673
Kctd12	_DLQLVLPDYFPER_.2	#N/A	6.0197	6.3070	6.6612	6.6357	6.4863	6.5891	6.3361	6.3913	6.2813	6.1772	1.0244	0.0347	0.2759	0.5592
Kctd12	_EAEYFELPELVR_.2	#N/A	5.9946	5.6752	5.9361	5.4573	6.0562	6.4971	5.4930	5.8739	6.0249	6.0592	1.0125	0.0179	0.7337	0.1345
Kctd12	_YILDYLR_.2	#N/A	5.3483	5.1338	3.3395	4.4877	4.5474	5.1251	4.3274	5.3830	5.5349	5.3251	0.9069	-0.1411	0.3014	0.5208

Kctd12	_S[Phospho (STY)]PSGGAAGPLLTPSQSLDGSR_.2	#N/A	3.8098	8.1512	9.0219	9.0124	6.7985	7.7522	7.6855	3.5473	7.9292	7.2573	1.1241	0.1687	0.5424	0.2657
Kctd12	_SPSGGAAGPLLT[Phospho (STY)]PS[Phospho (STY)]QSLDGSRR_.3	#N/A	3.2496	4.0548	3.9364	6.3556	5.0888	6.7516	5.7083	6.1287	6.7568	6.2710	0.7968	-0.3278	0.1395	0.8554
Kctd12	_MFTQQQPQELAR_.2	#N/A	3.6151	4.4203	4.3019	3.7004	4.2416	3.4878	3.8486	3.3008	3.5999	3.3777	1.1216	0.1656	0.0966	1.0152
Tacc1	_EADEQLVAEVIEKC[Carbamidomethyl (C)]S[Phospho (STY)]PDTC[Carbamidomethyl (C)]SR_.3	Q6Y685_S111_M1_Tacc1	5.4558	4.4177	6.7040	7.0799	6.1029	3.4905	3.8513	6.2538	3.5972	5.5057	1.1541	0.2067	0.4189	0.3779
Tacc1	_KYEAQPLDLDAC[Carbamidomethyl (C)]S[Phospho (STY)]QDEGAVISK_.3	Q6Y685_S480_M1_Tacc1	3.7142	3.6282	3.4479	6.1193	4.1290	4.8244	3.1399	5.0427	4.3850	4.1698	1.0301	0.0429	0.8398	0.0758
Tacc1	_ESC[Carbamidomethyl (C)]DSS[Phospho (STY)]PGLAEPEAKPQESR_.3	Q6Y685_S82_M1_Tacc1	3.0191	3.7754	4.2536	3.7974	3.6637	2.9609	3.9457	3.2038	3.5028	3.4747	1.0132	0.0189	0.8728	0.0591
Gigyf2	_SQS[Phospho (STY)]WEER_.2	#N/A	8.7042	8.7391	3.7855	9.3388	3.7252	4.0043	8.9119	8.7209	8.6421	4.2551	0.8363	-0.2580	0.4790	0.3196
Gigyf2	_WRPHS[Phospho (STY)]PDGPR_.3	#N/A	8.2965	8.2280	7.6749	7.7267	6.9891	8.3240	8.1356	8.2616	7.5007	8.2434	0.9798	-0.0294	0.6050	0.2183
Gigyf2	_S[Phospho (STY)]FDEVEGVFGR_.2	Q6Y7W8_S140_M1_Gigyf2	3.4867	4.2430	3.6700	6.5486	4.6795	3.9942	3.8873	5.8681	5.0786	4.1397	0.9354	-0.0964	0.6613	0.1796
Gigyf2	_SQS[Phospho (STY)]WEERGDR_.2	#N/A	6.9585	3.9212	3.1549	4.1995	3.7425	3.9870	3.4478	6.1026	3.1007	6.9525	0.8442	-0.2444	0.4326	0.3640
Gigyf2	_ALSSGGSITS[Phospho (STY)]PPLSPALPK_.2	Q6Y7W8_S26_M1_Gigyf2	2.9472	4.3952	4.1817	8.8410	3.5918	3.0328	3.8738	6.6488	3.5747	3.4028	1.0282	0.0401	0.9255	0.0336
Gigyf2	_QQQQQQQQQQQHQQSNR_.3	#N/A	3.8726	3.4698	3.6063	3.1500	3.0163	3.6083	3.2983	3.8512	4.1502	4.3281	0.8840	-0.1778	0.0971	1.0128
Sbf1	_GS[Phospho (STY)]GEGQGQILQR_.2	Q6ZPE2_S20_M1_Sbf1	8.2806	8.4399	9.0630	8.3373	7.3613	7.9801	8.8742	8.5121	8.3562	8.2357	0.9705	-0.0432	0.4361	0.3604
Sbf1	_DKGPS[Phospho (STY)]FR_.2	Q6ZPE2_S1047_M1_Sbf1	7.7917	7.7162	8.3170	7.7015	5.4627	7.2059	7.6409	8.1575	7.4810	7.3215	0.9628	-0.0547	0.6051	0.2182
Sbf1	_ALYLEPSDGVS[Phospho (STY)]PTQETGEAQSQDDER_.3	Q6ZPE2_S706_M1_Sbf1	4.0796	3.2628	3.8133	6.5826	3.2233	3.1445	3.5053	3.6441	3.9432	3.0343	1.1376	0.1860	0.4984	0.3024
Sbf1	_C[Carbamidomethyl (C)]SGPHINS[Phospho (STY)]AR_.2	Q6ZPE2_S559_M1_Sbf1	4.0939	3.8267	3.2799	3.7825	3.1529	3.9302	3.4972	5.1483	3.9845	4.6232	0.8526	-0.2300	0.1018	0.9924
Tiam2	_TSSS[Phospho (STY)]SEWPSEPSK_.2	Q6ZPF3_S1524_M1_Tiam2	3.8239	4.0256	3.8750	3.8891	3.5252	3.5582	3.8050	3.7275	3.6603	3.7394	1.0133	0.0191	0.6450	0.1904
Tiam2	_QGS[Phospho (STY)]PTKDIEIQFQR_.2	#N/A	3.5801	3.7060	8.2161	8.5633	7.1806	3.7371	3.8929	8.0866	8.0305	8.6837	0.8128	-0.2990	0.3986	0.3995
Tiam2	_HLS[Phospho (STY)]DADR_.2	Q6ZPF3_S1116_M1_Tiam2	4.5725	4.5467	4.6547	4.8202	3.4164	4.2807	4.4614	4.9467	4.1450	4.7951	0.9553	-0.0660	0.5047	0.2970
Tiam2	_ESDILSDEDEDFHHPLK_.3	#N/A	5.3016	4.5996	4.1740	10.4834	8.4710	4.9645	4.9905	10.0266	9.7026	5.1708	0.8474	-0.2389	0.5208	0.2833
Tiam2	_HQS[Phospho (STY)]LDSHPETASIDLNLVLER_.3	Q6ZPF3_S1672_M1_Tiam2	4.1116	3.2309	3.8452	3.3890	3.2553	3.3693	3.5373	3.6122	3.9112	3.0663	1.0005	0.0007	0.9939	0.0026
Tex2	_TAPSS[Phospho (STY)]PLT[Phospho (STY)]SPSDTR_.2	Q6ZPJ0_T268_M2_Tex2	7.8369	3.7983	7.4702	6.4080	5.9256	6.4822	7.2293	4.1554	7.5856	3.8244	1.1091	0.1493	0.5772	0.2386
Tex2	_SLS[Phospho (STY)]TEVEPK_.2	Q6ZPJ0_S195_M1_Tex2	4.2035	3.4590	3.9372	3.4810	3.3472	3.5786	3.1705	3.5202	4.9747	3.1582	0.9897	-0.0149	0.9226	0.0350
Ube2o	_KEEKPEVQS[Phospho (STY)]PVK_.2	Q6ZPJ3_S893_M1_Ube2o	6.4361	6.1005	5.3025	6.7873	4.9042	5.8706	7.0502	6.7737	6.2552	6.3935	0.8915	-0.1657	0.0998	1.0009
Ube2o	_LIHGEDS[Phospho (STY)]DS[Phospho (STY)]EGDDDGR_.2	#N/A	6.4438	5.6410	3.6873	5.8529	4.8162	5.7061	6.3715	5.8083	5.6933	5.7067	0.9089	-0.1378	0.3243	0.4891
Ube2o	_DSSSLEPMAAAELS[Phospho (STY)]DSGR_.2	Q6ZPJ3_S1175_M1_Ube2o	4.3711	2.9713	4.1047	6.1502	2.7927	3.4360	3.7968	3.3527	3.6517	3.3258	1.1244	0.1691	0.5091	0.2932
Ube2o	_DHS[Phospho (STY)]M[Oxidation (M)]EDPDKKGEAR_.2	Q6ZPJ3_S406_M1_Ube2o	4.4078	2.9373	3.4749	5.1086	2.8256	3.7990	3.6922	4.1410	3.7896	3.6612	0.9837	-0.0236	0.8953	0.0480
Ube2o	_S[Phospho (STY)]IPLSIK_.2	Q6ZPJ3_S510_M1_Ube2o	3.7322	3.0366	4.0395	3.5833	2.8579	3.3707	3.7315	3.4179	3.7170	3.2605	0.9731	-0.0394	0.7058	0.1513
Ube2o	_NM[Oxidation (M)]TVEQLLTGSPTS[Phospho (STY)]PTVEPEKPTR_.3	Q6ZPJ3_S836_M1_Ube2o	3.3061	4.8625	4.9071	5.4859	3.8274	4.0523	4.2439	3.7325	3.2211	3.5631	1.1942	0.2561	0.1477	0.8307
Ube2o	_NC[Carbamidomethyl (C)]AQGEGS[Phospho (STY)]M[Oxidation (M)]AKK_.2	Q6ZPJ3_S376_M1_Ube2o	4.3339	3.6383	3.4378	4.4824	3.4596	4.2699	3.1298	4.0196	4.3187	4.1597	1.0077	0.0110	0.9278	0.0325
Zfyve28	_LKSGS[Phospho (STY)]DEEER_.2	Q6ZPK7_S394_M1_Zfyve28	3.9943	3.6833	3.1803	3.6251	3.0734	3.3667	3.4807	3.8511	4.0856	3.6499	0.9258	-0.1113	0.2076	0.6828
Ddx55	_TVQLTDLGVS[Phospho (STY)]DLEEDS_.2	Q6ZPL9_S594_M1_Ddx55	3.8107	3.5317	3.5444	4.5890	2.9544	4.3765	3.2364	3.9130	4.2121	4.2663	0.9729	-0.0396	0.7758	0.1102
Pitpnm2	_KQWS[Phospho (STY)]TSSK_.2	Q6ZPQ6_S302_M1_Pitpnm2	7.9696	7.8114	8.1346	4.5592	3.3828	7.8316	8.0818	3.9428	4.2419	7.4205	1.1170	0.1597	0.6234	0.2052
Pitpnm2	_GAS[Phospho (STY)]PSRHS[Phospho (STY)]ISEWR_.3	#N/A	7.6548	6.5764	8.1065	7.8975	5.9640	7.3694	7.6388	7.4268	7.5413	7.2671	0.9723	-0.0406	0.6414	0.1929
Pitpnm2	_HS[Phospho (STY)]ISEWR_.2	Q6ZPQ6_S321_M1_Pitpnm2	3.6346	3.9304	4.3214	4.4231	4.2611	3.4684	4.5387	3.2944	4.4265	3.9349	0.9896	-0.0151	0.8920	0.0496
Pitpnm2	_TDTGENNNVFNLS[Phospho (STY)]PVEK_.2	Q6ZPQ6_S129_M1_Pitpnm2	6.7236	6.3886	3.5079	7.4443	6.2446	6.7830	7.4276	6.7768	6.6870	6.7985	0.8930	-0.1632	0.3301	0.4813
Pitpnm2	_WNS[Phospho (STY)]NDLMDK_.2	Q6ZPQ6_S365_M1_Pitpnm2	6.4324	3.4129	3.8911	6.3548	3.3012	3.3234	3.2166	3.5663	3.7183	3.1122	1.3083	0.3877	0.2172	0.6632
Pitpnm2	_RGAS[Phospho (STY)]PSRHS[Phospho (STY)]ISEWR_.3	#N/A	4.0599	3.1663	3.2459	7.3618	3.1856	7.3662	3.4632	7.6130	7.3852	7.3550	0.7330	-0.4481	0.2272	0.6435
Pitpnm2	_S[Phospho (STY)]NIDIPR_.2	Q6ZPQ6_S630_M1_Pitpnm2	2.9133	3.2339	3.6001	4.1028	3.5399	4.1896	3.8175	4.2762	4.6689	4.5621	0.8304	-0.2681	0.0363	1.4395
Pitpnm2	_QWSTSSKSS[Phospho (STY)]R_.2	Q6ZPQ6_S308_M1_Pitpnm2	3.6324	2.9368	4.3192	3.3210	4.2590	3.4705	3.8313	3.2966	2.9452	3.2880	1.0947	0.1305	0.3406	0.4678
Kcnt1	_GGGSVGS[Phospho (STY)]DVGQR_.2	Q6ZPR4_S47_M1_Kcnt1	6.9864	4.1729	4.0545	6.5892	5.3507	3.7357	6.9930	3.5618	3.3082	6.6017	1.0063	0.0090	0.9763	0.0104
Ankib1	_GVAPADS[Phospho (STY)]PDAPR_.2	#N/A	9.6948	9.7106	10.2954	10.1617	9.4462	9.9352	9.9066	9.7427	9.7524	9.9633	1.0033	0.0048	0.8505	0.0703
Ankib1	_GVAPADS[Phospho (STY)]PDAPRR_.2	#N/A	8.5955	8.6453	9.1432	8.9996	8.1706	8.7523	8.8193	8.7625	8.7100	8.7993	0.9937	-0.0091	0.7613	0.1184
Klhl9	_AC[Carbamidomethyl (C)]TLTVFPPEENPGS[Phospho (STY)]PSR_.2	Q6ZPT1_S603_M1_Klhl9	8.3455	8.0826	4.5703	8.4781	6.9030	7.9033	8.1610	3.0455	7.6185	7.6083	1.1168	0.1594	0.5415	0.2664
Zc3h4	_TGTGS[Phospho (STY)]PFAGNS[Phospho (STY)]PAR_.2	Q6ZPZ3_S1270_M2_Zc3h4	7.8657	7.7417	7.3989	8.2898	4.9465	7.2274	8.1763	7.5946	7.5334	7.7940	0.9319	-0.1018	0.4149	0.3821
Efr3b	_S[Phospho (STY)]PSPLQAPEK_.2	Q6ZQ18_S214_M1_Efr3b	8.3972	3.4452	3.6707	4.0334	3.2665	3.8209	3.3627	3.8265	4.1255	3.7107	1.1817	0.2409	0.5169	0.2866
Cand1	_SVILEAFSSPSEEVK_.2	#N/A	6.5157	6.1742	6.5000	6.3690	5.4834	5.7165	5.8968	6.4548	5.8453	5.9825	1.0135	0.0193	0.7493	0.1254
Cand1	_LTLIDPETLLPR_.2	#N/A	6.3047	5.8139	6.5043	5.4881	4.7475	5.0782	6.2829	6.3554	5.7404	5.8817	0.9326	-0.1007	0.3007	0.5219
Cand1	_AVAALLTIPEAEK_.2	#N/A	5.6156	5.6907	5.3734	5.0796	5.2646	5.7557	6.4189	6.0986	5.7859	5.7358	0.9091	-0.1375	0.0182	1.7393
Cand1	_EGPAVVGQFIQDVK_.2	#N/A	6.1971	5.6048	5.6256	5.7911	4.5222	4.9083	6.3438	5.9200	5.6085	5.8829	0.9163	-0.1262	0.1765	0.7534
Cand1	_ITSEALLVTQQLVK_.2	#N/A	4.9666	5.4534	5.7501	3.3372	3.3514	5.1199	5.3167	5.4061	4.2520	4.7561	0.9453	-0.0811	0.6542	0.1843
Cand1	_[Acetyl (Protein N-term)]ASASYHISNLLEK_.2	#N/A	3.9719	3.9444	5.3790	4.0533	3.8895	2.9311	5.1575	4.6563	4.3145	3.9213	0.8927	-0.1638	0.3130	0.5044
Cand1	_ISGSILNELIGLVR_.2	#N/A	3.6324	2.9926	4.1884	1.3991	4.1281	1.7796	4.0716	4.6375	3.8984	3.2155	0.7634	-0.3894	0.1892	0.7231
Cand1	_DS[Phospho (STY)]SSTNLESMDTS_.2	Q6ZQ38_S1219_M1_Cand1	2.9951	3.7514	4.2297	3.1634	3.6397	2.9849	4.3789	3.4543	3.1028	3.1304	0.9841	-0.0231	0.8759	0.0575
Cand1	_NVVAEC[Carbamidomethyl (C)]LGK_.2	#N/A	4.3718	4.3552	3.5578	4.0604	4.3064	3.7744	3.7751	4.8111	5.5702	4.8350	0.8574	-0.2219	0.0813	1.0901
Cand1	_QEFEKQDELKR_.3	#N/A	3.5740	3.3759	3.7002	3.2440	4.1048	4.4672	3.3922	3.9000	4.2294	2.8789	1.0401	0.0567	0.6757	0.1702
Cand1	_HTVDDGLDIRK_.3	#N/A	3.7397	3.6027	3.4734	4.5180	3.4240	4.3055	3.1655	3.9840	3.3856	2.5019	1.1794	0.2380	0.1205	0.9191
Cand1	_DLLDSVLPHLYNETK_.3	#N/A	3.9178	3.2222	3.8539	1.8525	3.0435	3.1851	3.5459	3.6035	2.2313	3.0749	1.0210	0.0299	0.8897	0.0508
Cand1	_QSYYSIAK_.2	#N/A	3.0838	3.8890	3.7706	4.2733	3.7104	4.0191	3.9880	3.8616	3.4938	4.2403	0.9731	-0.0394	0.6717	0.1728
Cand1	_HC[Carbamidomethyl (C)]EC[Carbamidomethyl (C)]AEEGTR_.2	#N/A	3.2062	4.1362	4.4407	3.9845	3.9576	3.7719	4.1327	3.0167	3.3158	3.6617	1.1089	0.1491	0.2123	0.6730
Larp1	_GLSAS[Phospho (STY)]LPDLDSESWIEVK_.2	Q6ZQ58_S525_M1_Larp1	6.3319	3.8285	7.6394	3.7971	3.6712	6.8641	6.8742	7.5304	6.8101	6.9871	0.7596	-0.3967	0.1037	0.9843
Larp1	_S[Phospho (STY)]LPTTVPESPNYR_.2	Q6ZQ58_S743_M1_Larp1	10.9815	10.9631	11.4197	11.2397	9.6485	11.0943	11.1226	10.8140	10.8526	10.8896	0.9974	-0.0038	0.9326	0.0303
Larp1	_ETESAPGS[Phospho (STY)]PR_.2	Q6ZQ58_S498_M1_Larp1	9.4110	9.4240	9.8219	9.7912	8.9370	9.6201	9.4991	8.9753	9.2758	9.4150	1.0226	0.0322	0.3018	0.5203
Larp1	_ESPRPPAAAEAPAGS[Phospho (STY)]DGEDGGRR_.3	#N/A	6.8314	6.8686	6.9104	7.2453	6.5894	6.8528	7.0876	6.9587	6.7076	6.8122	0.9988	-0.0018	0.9475	0.0234
Larp1	_NTFTAWSEEDS[Phospho (STY)]DYEIDDRDVNK_.3	Q6ZQ58_S608_M1_Larp1	3.2579	4.1418	3.8967	4.1658	4.0301	4.1017	4.1781	3.5007	3.3069	3.9502	1.0531	0.0747	0.4259	0.3707
Larp1	_TASISSS[Phospho (STY)]PSEGTPAVGSYGC[Carbamidomethyl (C)]TPQSLPK_.3	Q6ZQ58_S828_M1_Larp1	3.7893	3.0447	3.5230	3.8701	2.9330	3.6916	3.5848	3.9344	4.5570	4.2448	0.8517	-0.2315	0.0488	1.3114
Larp1	_QHYQKET[Phospho (STY)]ESAPGS[Phospho (STY)]PR_.2	Q6ZQ58_T492_M2_Larp1	4.0811	3.3365	4.5764	3.5783	3.2248	3.3998	3.2929	3.0394	4.1888	3.5453	1.0520	0.0731	0.5959	0.2248
Larp1	_KNTFTAWS[Phospho (STY)]EEDSDY[Phospho (STY)]EIDDRDVNK_.3	#N/A	3.4001	4.1090	4.3811	4.0236	4.0355	3.9086	4.1718	3.7234	3.2886	3.7008	1.0686	0.0957	0.2760	0.5591
Arhgap26	_DSQSEGTAQLDSIGFSIIR_.2	#N/A	3.3189	2.0043	4.2941	4.3205	3.9635	2.2462	4.2455	3.0118	3.2030	3.8367	0.9395	-0.0901	0.7136	0.1465
Kdm1a	_NGY[Phospho (STY)]S[Phospho (STY)]C[Carbamidomethyl (C)]VPVALAEGLDIK_.3	Q6ZQ88_S573_M2_Kdm1a	5.6330	3.2015	3.8746	6.3082	3.2847	3.3399	3.5667	5.4900	6.3389	3.0957	0.9245	-0.1133	0.7145	0.1460
Kdm1a	_QATPGVPAQQS[Phospho (STY)]PSM_.2	Q6ZQ88_S850_M1_Kdm1a	4.2024	3.1400	3.9361	3.4798	2.9613	3.2673	3.6281	3.5213	3.8204	3.1571	0.9904	-0.0139	0.9029	0.0444
Usp34	_DSSIIDPGTEQDLPS[Phospho (STY)]PENSSVK_.2	Q6ZQ93_S3443_M1_Usp34	3.7397	5.4821	5.8879	6.1627	2.8834	5.6146	6.1274	3.9841	4.2831	4.1952	1.0676	0.0944	0.6987	0.1557
Usp34	_RVS[Phospho (STY)]S[Phospho (STY)]DEEHTVDSC[Carbamidomethyl (C)]IGDIK_.3	Q6ZQ93_S3395_M2_Usp34	3.7781	3.4480	4.4649	3.4667	2.9038	3.3248	3.1814	3.1509	3.4003	3.4337	1.0136	0.0195	0.8921	0.0496
Acap2	_FVDKYSALLS[Phospho (STY)]PSEQEKR_.3	#N/A	5.8444	6.7462	7.6391	7.2452	5.5616	6.2040	6.5917	6.9638	6.1759	6.3502	1.0030	0.0044	0.9650	0.0155
Acap2	_KS[Phospho (STY)]SPSTGSLDSGNESK_.2	Q6ZQK5_S378_M1_Acap2	8.5560	8.6910	4.0537	4.0763	4.7234	8.9690	5.3545	3.6247	3.1271	8.8094	1.2453	0.3165	0.4495	0.3473
Acap2	_YSALLS[Phospho (STY)]PSEQEK_.2	#N/A	7.6751	7.1795	3.5871	4.0897	3.5268	7.2377	7.9629	7.4295	7.3192	7.7186	0.7294	-0.4551	0.0806	1.0935
Acap2	_SSPSTGS[Phospho (STY)]LDSGNESK_.2	#N/A	9.0821	8.8750	9.0024	7.8751	8.5667	8.5537	9.5193	9.3851	8.7035	8.6690	0.9548	-0.0668	0.1900	0.7213
Acap2	_SSPSTGS[Phospho (STY)]LDSGNESKEK_.2	#N/A	3.7355	3.6854	4.1636	4.0842	3.5737	3.9057	5.7545	3.0762	3.8432	5.5823	0.8453	-0.2425	0.2221	0.6535
Acap2	_FVDKYSALLS[Phospho (STY)]PSEQEK_.3	#N/A	4.3682	4.7192	3.5542	4.0568	3.4939	3.8783	3.7715	4.5959	3.7102	4.5693	0.9640	-0.0529	0.6407	0.1934

Acap2	_ESLPSTVS[Phospho (STY)]ANSLYEPEGER_.2	Q6ZQK5_S573_M1_Acap2	5.3205	3.5110	3.7724	4.4477	3.5200	3.7559	3.9689	3.8524	3.5752	3.7135	1.0734	0.1021	0.4726	0.3255
Acap2	_YSALLS[Phospho (STY)]PSEQEKR_.2	#N/A	7.6249	7.2723	7.8303	7.7293	3.9998	7.0618	7.9864	7.5162	7.5038	3.4654	1.0456	0.0643	0.7943	0.1000
Rps9	_LIGEYGLR_.2	#N/A	6.3449	6.3981	5.0945	6.2663	5.4976	6.3824	6.9143	6.6734	6.4899	5.9234	0.9226	-0.1162	0.1669	0.7775
Rps9	_LFEGNALLR_.2	#N/A	6.3980	5.4910	6.4816	5.6360	5.4399	5.3686	6.0664	6.2850	5.7701	5.7198	0.9735	-0.0387	0.5843	0.2334
Rps9	_IEDFLER_.2	#N/A	5.3192	4.2933	4.2028	3.9232	4.7510	3.9612	4.4058	4.9764	4.0975	4.6339	0.9735	-0.0387	0.7093	0.1492
Rps9	_ELLTLDEKDPR_.3	#N/A	4.1877	3.1547	3.9214	3.4651	1.4653	3.2526	3.6134	3.5360	2.4750	3.1424	1.0155	0.0222	0.9279	0.0325
Syne2	_SISEGHPWHVPDS[Phospho (STY)]PSHSK_.3	Q6ZWQ0_S6448_M1_Syne2	3.3573	3.9851	6.9567	6.0423	4.8434	6.0565	6.4593	4.3664	6.1373	5.3875	0.9319	-0.1018	0.6446	0.1907
Syne2	_LTSHTPGLDDEKEAS[Phospho (STY)]ENETDIEDPR_.3	Q6ZWQ0_S6348_M1_Syne2	3.6693	2.9737	4.3561	3.6462	2.7950	5.6328	3.7945	3.3550	3.6540	3.3235	1.0888	0.1228	0.5755	0.2399
Syne1	_NPET[Phospho (STY)]PTELQFIEADLR_.2	Q6ZWR6_T2268_M1_Syne1	4.0503	3.2921	3.7839	3.3277	4.7666	3.4306	3.4760	3.6735	3.9725	3.0050	1.0689	0.0962	0.4832	0.3158
Syne1	_YLFQTGSS[Phospho (STY)]HER_.2	Q6ZWR6_S1360_M1_Syne1	7.5705	7.3532	7.3646	6.9841	5.3661	6.9974	7.4711	7.3194	7.0763	6.9336	0.9638	-0.0532	0.5530	0.2573
Syne1	_TLPS[Phospho (STY)]EDEEGEEDKEFYLR_.3	Q6ZWR6_S8308_M1_Syne1	3.7837	3.0881	4.4705	7.2430	2.9094	3.3192	3.1870	3.1453	4.2947	3.4393	1.1760	0.2339	0.4894	0.3104
Rps27	_LTEGC[Carbamidomethyl (C)]SFR_.2	#N/A	6.0737	5.7903	4.0482	5.6332	6.0095	5.9401	5.3764	6.4352	5.9938	5.8741	0.9430	-0.0847	0.4534	0.3435
Rps27	_DLLHPS[Phospho (STY)]PEEEKRK_.3	Q6ZWU9_S11_M1_Rps27	6.5758	6.2600	6.4999	6.8359	6.1409	6.3572	6.8957	6.5687	6.4554	6.3221	0.9824	-0.0256	0.4892	0.3105
Rpl35	_VLTVINQTQK_.2	#N/A	5.5850	4.7916	4.8375	6.0080	5.9618	5.9592	6.0703	6.2623	6.0593	5.3682	0.9299	-0.1048	0.2426	0.6151
Rpl35	_QLDDLKVELSQLR_.3	#N/A	4.1155	3.0109	4.3933	4.0828	2.8322	3.3965	3.1098	4.0325	3.4529	4.4036	0.9704	-0.0434	0.7891	0.1029
Eif2s1	_ADIEVAC[Carbamidomethyl (C)]YGYEGIDAVK_.2	#N/A	3.5263	3.8161	5.6271	4.3045	3.6375	4.9347	4.4528	4.1975	2.9957	4.2050	1.0871	0.1204	0.5065	0.2954
Tmsb10	_ETIEQEKR_.2	#N/A	7.9915	7.8298	7.9615	7.7749	9.4378	8.3248	7.6570	7.6083	8.2910	7.8079	1.0483	0.0681	0.3009	0.5216
Tmsb10	_TET[Phospho (STY)]QEKNTLPTK_.2	Q6ZWY8_T23_M1_Tmsb10	6.5719	6.1823	6.3966	4.3233	3.7603	3.9691	6.2627	3.7952	6.5630	5.8971	0.9238	-0.1143	0.6208	0.2070
Tmsb10	_NTLPTKETIEQEKR_.3	#N/A	4.7637	5.5954	7.2240	5.3263	6.8113	5.9706	4.1218	5.5045	6.0209	5.7601	1.1115	0.1525	0.3329	0.4777
Tmsb10	_[Acetyl (Protein N-term)]ADKPDM[Oxidation (M)]GEIASFDK_.2	#N/A	6.3333	6.4193	6.1478	7.0011	7.2317	6.8243	5.8028	5.8982	6.4286	6.1883	1.0954	0.1315	0.0445	1.3514
Tmsb10	_[Acetyl (Protein N-term)]ADKPDMGEIAS[Phospho (STY)]FDKAK_.3	Q6ZWY8_S12_M1_Tmsb10	3.2413	3.9849	3.9281	4.4307	3.8678	3.8617	4.1454	3.6878	3.3363	4.3977	0.9984	-0.0023	0.9826	0.0076
Synm	_IDFSTPFQVEEVDDVSPS[Phospho (STY)]PK_.2	Q70IV5_S780_M1_Synm	4.1153	3.9851	3.5706	3.6354	4.6664	3.4442	3.5286	3.9832	4.7899	4.1817	0.9471	-0.0784	0.5022	0.2992
Synm	_QFTQS[Phospho (STY)]PGAEEEATSFPDTK_.2	Q70IV5_S655_M1_Synm	3.9257	3.3005	3.1116	3.6143	3.0514	3.1773	3.3290	3.0033	4.1528	3.5813	0.9565	-0.0642	0.5704	0.2438
Znrf2	_S[Phospho (STY)]LGGAVGSASGGR_.2	Q71FD5_S75_M1_Znrf2	8.4855	8.2033	8.6368	8.9963	8.1573	8.6095	8.5585	7.8050	8.3893	8.5519	1.0226	0.0323	0.3994	0.3986
Znrf2	_AYS[Phospho (STY)]GSDLPSGTGSGGGADGAR_.2	Q71FD5_S18_M1_Znrf2	4.1006	8.6373	9.3021	3.3780	8.5993	8.8026	3.2735	3.6231	3.9222	3.0553	2.0575	1.0409	0.0274	1.5623
Tln2	_TSS[Phospho (STY)]PEESIR_.2	Q71LX4_S2083_M1_Tln2	3.1617	5.3335	4.3962	3.9400	3.8063	4.3191	4.0883	5.5972	3.3602	6.2658	0.8616	-0.2150	0.3304	0.4809
Tln2	_DRFGLEGDEESTM[Oxidation (M)]LEESVS[Phospho (STY)]PK_.3	Q71LX4_S428_M1_Tln2	4.9702	3.5207	3.3085	3.8111	3.2482	5.8799	3.3412	5.6507	6.0411	4.8787	0.8283	-0.2718	0.2694	0.5696
Cspg5	_VSS[Phospho (STY)]PHAR_.2	Q71M36_S498_M1_Cspg5	6.7825	3.0901	6.9446	3.8246	2.9784	3.6462	3.5393	4.2938	3.9424	6.3039	1.0054	0.0078	0.9819	0.0079
Cspg5	_GDQDDLGVNC[Carbamidomethyl (C)]LQNNLT_.2	#N/A	5.9657	5.2982	3.9802	5.3860	4.8122	4.7838	6.0851	5.1148	5.9199	5.3343	0.8974	-0.1561	0.1785	0.7484
Cspg5	_LEGGKGDQDDLGVNC[Carbamidomethyl (C)]LQNNLT_.2	#N/A	5.3442	5.7987	6.2386	6.5587	4.4821	5.2303	5.4042	5.7131	5.2619	5.1872	1.0403	0.0570	0.5965	0.2244
Cspg5	_KFC[Carbamidomethyl (C)]DTPRVS[Phospho (STY)]S[Phospho (STY)]PHAR_.3	Q71M36_S497_M2_Cspg5	3.7678	3.5746	6.0012	4.5461	3.3960	5.9500	3.1935	3.9559	5.7766	4.2233	1.0588	0.0824	0.7420	0.1296
Cspg5	_LKEEESFNIQNSMS[Phospho (STY)]PK_.2	#N/A	3.5846	4.1319	3.8797	4.0573	4.2200	3.7232	4.2056	3.8026	2.9654	3.6885	1.0729	0.1015	0.2980	0.5259
Ssh1	_SSS[Phospho (STY)]SDSIHSVR_.2	Q76I79_S929_M1_Ssh1	7.6357	7.9563	8.3569	8.0424	7.3269	7.9236	8.2658	8.1298	8.0801	7.7547	0.9772	-0.0333	0.3845	0.4151
Ssh1	_SDS[Phospho (STY)]LPQVEELEK_.2	Q76I79_S585_M1_Ssh1	3.7987	3.5437	3.5324	4.5770	2.9424	6.7890	6.7917	3.9250	4.2241	4.2543	0.8746	-0.1932	0.5130	0.2899
Mindy1	_TRAPS[Phospho (STY)]PQGR_.2	Q76LS9_S440_M1_Mindy1	9.6845	9.9551	10.3267	10.3665	9.7248	10.1205	10.0966	9.8630	10.0821	9.9676	1.0027	0.0039	0.8646	0.0632
Mindy1	_VETAEVC[Carbamidomethyl (C)]SRPQELPQS[Phospho (STY)]PR_.3	#N/A	7.0267	7.1688	7.3468	7.9797	6.6504	6.9055	7.3332	7.4525	7.1398	6.9416	0.9949	-0.0074	0.8855	0.0528
Mindy1	_PQELPQS[Phospho (STY)]PR_.2	#N/A	5.9826	5.7375	5.4457	6.0701	5.1236	5.7173	6.5860	6.0231	5.7302	5.8046	0.9409	-0.0878	0.1681	0.7744
Ppp2r1a	_LAGGDWFTSR_.2	#N/A	8.2365	7.9551	8.2465	7.8831	7.9090	7.9779	8.1868	7.9925	7.8907	7.8024	1.0084	0.0120	0.5475	0.2616
Ppp2r1a	_DKAVESLR_.2	#N/A	7.8396	8.0018	7.6754	7.8959	7.9021	7.9284	7.7685	7.6284	7.8925	7.9720	1.0075	0.0108	0.4955	0.3050
Ppp2r1a	_IGPILDNSTLQSEVKPILEK_.3	#N/A	7.7051	7.2371	7.8994	7.4720	7.3237	7.4967	7.6696	7.8377	7.2511	7.9011	0.9814	-0.0271	0.4268	0.3698
Ppp2r1a	_VLELDNVK_.2	#N/A	7.9302	7.8009	8.0498	7.6070	7.5364	7.6817	7.3845	8.0648	7.6109	8.0086	1.0001	0.0001	0.9978	0.0010
Ppp2r1a	_NEDVQLR_.2	#N/A	7.4980	7.0978	6.5076	6.8637	7.2478	6.8846	6.5496	7.0391	7.2177	7.2619	0.9999	-0.0001	0.9983	0.0007
Ppp2r1a	_AVGPEITK_.2	#N/A	7.0618	6.6431	6.8015	5.5054	7.1015	6.7079	6.7208	7.3136	6.5825	6.9576	0.9628	-0.0548	0.4510	0.3458
Ppp2r1a	_LSTIALALGVER_.2	#N/A	6.6832	6.6489	6.7582	6.3370	6.8392	6.2550	6.6978	6.7751	6.9940	6.7412	0.9684	-0.0464	0.1399	0.8542
Ppp2r1a	_EWAHATIIPK_.2	#N/A	6.3821	6.4491	7.2940	6.6785	6.0872	6.6574	6.3115	6.5997	6.8159	6.3179	1.0123	0.0176	0.7348	0.1338
Ppp2r1a	_SALASVIM[Oxidation (M)]GLSPILGK_.2	#N/A	6.0636	6.4881	5.6584	5.6386	6.4838	6.3772	6.1745	6.3347	6.1888	5.5425	1.0096	0.0138	0.8185	0.0870
Ppp2r1a	_LTQDQDVDVK_.2	#N/A	7.3104	7.2468	7.0804	7.8844	7.4523	7.4062	7.2764	7.3200	7.2682	7.2978	1.0146	0.0209	0.4679	0.3298
Ppp2r1a	_TSAC[Carbamidomethyl (C)]GLFSVC[Carbamidomethyl (C)]YPR_.2	#N/A	6.7549	6.6602	6.6102	6.1638	6.7042	6.0732	6.5069	6.1285	6.2026	6.4671	1.0266	0.0378	0.3480	0.4584
Ppp2r1a	_AISHEHSPSDLEAHFVPLVK_.3	#N/A	5.3139	4.9732	3.7669	3.6261	5.2913	6.7277	6.6253	5.8427	5.5200	6.6257	0.8044	-0.3140	0.0904	1.0437
Ppp2r1a	_QLSQSLLPAIVELAEDAK_.2	#N/A	5.3375	5.2117	4.7936	5.2003	4.5570	5.0819	5.0458	5.4568	4.6645	4.8178	1.0068	0.0098	0.8709	0.0600
Ppp2r1a	_SEIIPM[Oxidation (M)]FSNLASDEQDSVR_.2	#N/A	6.8268	7.9210	6.7920	7.2288	7.4386	6.5757	7.8535	7.7505	6.5419	6.2930	1.0395	0.0559	0.4882	0.3114
Ppp2r1a	_NLC[Carbamidomethyl (C)]SDDTPM[Oxidation (M)]VR_.2	#N/A	7.0543	6.5419	6.5725	6.0488	6.6400	6.4134	6.5837	6.5980	6.5478	6.6105	0.9939	-0.0088	0.8173	0.0876
Ppp2r1a	_MAGDPVANVR_.2	#N/A	7.3469	6.9184	3.2651	8.0742	7.0520	7.7467	7.1059	6.6035	6.4383	6.2223	1.0214	0.0306	0.8800	0.0555
Ppp2r1a	_YFAQEALTVLSLA_.2	#N/A	4.5157	4.9603	4.0181	5.1624	4.3280	4.6995	4.8365	4.0668	4.2253	3.3419	1.1205	0.1642	0.1633	0.7871
Ppp2r1a	_LNIISNLDC[Carbamidomethyl (C)]VNEVIGIR_.2	#N/A	5.2919	4.9431	5.8604	5.9007	5.1290	5.5219	5.4960	5.3550	5.3826	5.2183	1.0146	0.0209	0.7117	0.1477
Ppp2r1a	_HM[Oxidation (M)]LPTVLR_.2	#N/A	6.9048	7.2460	7.4826	6.4240	6.9307	6.9291	6.8665	7.3950	6.9414	7.2638	0.9817	-0.0267	0.5490	0.2604
Ppp2r1a	_ENVIM[Oxidation (M)]TQILPC[Carbamidomethyl (C)]IK_.2	#N/A	6.2460	6.2583	6.4827	5.9309	6.0305	6.1706	6.2873	5.7837	6.7358	6.2813	0.9864	-0.0198	0.6519	0.1858
Ppp2r1a	_VLAM[Oxidation (M)]SGDPNYLHR_.2	#N/A	4.9543	6.2448	6.0350	5.9431	6.2073	6.1808	6.2146	6.1303	6.1054	6.0320	0.9685	-0.0462	0.4657	0.3319
Ppp2r1a	_ELVSDANQHVK_.2	#N/A	7.0321	7.5387	7.4270	7.7841	7.7326	7.8787	7.4206	7.7614	7.0578	7.4784	1.0183	0.0262	0.5050	0.2967
Ppp2r1a	_LNSLC[Carbamidomethyl (C)]M[Oxidation (M)]AWLVDHVYAIR_.3	#N/A	3.2761	4.0663	4.5106	4.0543	3.8877	3.8418	4.2026	3.7027	3.2459	3.7316	1.0588	0.0824	0.4190	0.3778
Ppp2r1a	_VKEFC[Carbamidomethyl (C)]ENLSADC[Carbamidomethyl (C)]R_.2	#N/A	4.2722	3.0702	4.0059	3.5497	3.4159	3.2087	3.6979	3.4515	3.7506	3.2269	1.0157	0.0224	0.8344	0.0786
Stambpl1	_SDGSNFANYS[Phospho (STY)]PPVNR_.2	Q76N33_S242_M1_Stambpl1	10.4695	10.4617	10.9097	11.0399	9.3566	10.6688	10.7113	10.3935	10.3658	10.5291	0.9985	-0.0021	0.9615	0.0171
Mvb12a	_GLS[Phospho (STY)]LDPPKEPSK_.2	Q78HU3_S168_M1_Mvb12a	9.1564	8.3274	8.9789	9.6279	8.1306	9.2751	8.9300	8.8261	8.9575	9.0967	0.9959	-0.0059	0.9053	0.0432
Mvb12a	_TLS[Phospho (STY)]QDMR_.2	Q78HU3_S161_M1_Mvb12a	4.0210	3.3254	3.2070	3.7097	3.1467	3.0819	3.4427	4.4088	4.0574	3.6766	0.8765	-0.1901	0.0939	1.0271
Atp5md	_YFNSYTLTGR_.2	#N/A	7.3485	7.3182	6.9361	7.0972	6.9485	7.1072	7.2466	7.4329	7.1578	7.2193	0.9810	-0.0277	0.2099	0.6779
Atp5md	_AGAESDGQFQFTGIK_.2	#N/A	3.7925	3.7060	3.5262	3.8669	2.9362	3.6884	4.2639	3.9312	3.9002	4.2481	0.8777	-0.1883	0.0297	1.5266
Ccdc25	_VENM[Oxidation (M)]SSNQDGNDS[Phospho (STY)]DEFM[Oxidation (M)]_.2	#N/A	7.1723	8.3777	8.5750	8.8201	8.0456	8.3797	8.7546	8.2881	8.2270	8.3002	0.9805	-0.0285	0.6107	0.2141
Fam102a	_SSS[Phospho (STY)]LSDLTHR_.2	Q78T81_S252_M1_Fam102a	6.7159	4.1615	4.4154	3.9592	3.9828	3.7467	4.1075	6.5499	3.3410	4.4662	0.8790	-0.1861	0.4921	0.3080
Nap1l4	_FYEEVHDLER_.2	#N/A	6.6850	6.9897	6.7210	7.0097	7.1172	7.0665	6.7657	6.8174	7.3929	7.1607	0.9854	-0.0212	0.5127	0.2901
Nap1l4	_LTDQVM[Oxidation (M)]QNPQVLAALQER_.2	#N/A	4.1283	4.9487	4.9592	5.5776	4.9803	5.1645	4.6771	5.0146	4.7077	4.5537	1.0467	0.0659	0.4071	0.3903
Nap1l4	_[Acetyl (Protein N-term)]AENSLSDGGPADSVEAAK_.2	#N/A	5.7998	8.2121	5.8448	8.9622	8.1138	8.5169	8.2491	8.2156	8.1931	8.5128	0.9135	-0.1306	0.3426	0.4652
Nap1l4	_FY[Phospho (STY)]EEVHDLERK_.3	Q78ZA7_Y85_M1_Nap1l4	7.3758	7.2955	4.5152	8.0363	6.7747	7.6736	7.3880	3.5661	7.4257	7.2068	1.0858	0.1187	0.5933	0.2267
Nap1l4	_EFITGDVEPTDAES[Phospho (STY)]AWHSENEEEDK_.3	#N/A	3.1324	3.9376	3.6800	4.8311	3.7589	3.9891	4.3560	3.9161	3.1280	4.2527	0.9936	-0.0093	0.9460	0.0241
Nap1l4	_EFITGDVEPTDAESAWHS[Phospho (STY)]ENEEEDKLAGDM[Oxidation (M)]K_.4	#N/A	5.6343	5.7400	6.5503	3.7894	7.1595	3.5440	3.8270	6.4993	3.8082	6.4870	1.0480	0.0677	0.8035	0.0950
Nap1l4	_LDNVSHTPSSYIETLPK_.3	#N/A	4.2610	5.0296	4.9794	4.5800	4.6326	4.5245	4.3681	4.2418	4.8928	4.1464	1.0579	0.0812	0.2336	0.6315
Nap1l4	_REFITGDVEPTDAESAWHS[Phospho (STY)]ENEEEDKLAGDM[Oxidation (M)]K_.4	#N/A	3.6214	3.7210	3.3550	4.3996	4.2659	3.8595	4.5479	4.1024	4.4014	4.0769	0.9038	-0.1458	0.1007	0.9970
Mtch1	_RVS[Phospho (STY)]SGSC[Carbamidomethyl (C)]FALE_.2	Q791T5_S381_M1_Mtch1	3.1486	7.5926	7.4263	8.1548	6.3306	7.8023	8.4018	8.1581	3.3733	8.0709	0.9631	-0.0543	0.8529	0.0691
Mtch2	_VLQYYQESEKPEELGSVTVQK_.3	#N/A	5.1626	5.9109	5.2809	5.2078	4.1098	4.5965	5.6992	5.5859	5.3246	5.0174	0.9330	-0.1000	0.3158	0.5006
Mtch2	_TYC[Carbamidomethyl (C)]YDLR_.2	#N/A	4.9708	3.1820	3.6602	3.1298	5.3459	4.1426	3.3577	4.9349	6.2305	5.2906	0.8220	-0.2827	0.2237	0.6504

Mtch2	_EYSSSFDR_.2	#N/A	4.0255	3.3169	3.7592	3.3030	5.1138	3.4554	3.4512	5.7761	3.9973	5.7561	0.8069	-0.3095	0.1593	0.7979
Picalm	_ATTLSNAVSSLASTGLSLTK_.2	#N/A	3.6738	3.5725	3.5085	5.1268	3.6643	2.4972	4.7400	3.9052	2.5179	4.0861	0.9637	-0.0534	0.8130	0.0899
Scn4b	_EC[Carbamidomethyl (C)]LVSSSGNDNTENGLPGSK_.2	#N/A	4.0227	4.7035	5.1841	3.8944	3.3924	3.5312	5.5880	5.3883	4.8573	3.9724	0.8324	-0.2647	0.1054	0.9770
Opalin	_IS[Phospho (STY)]ENPRRSPTHEM[Oxidation (M)]NPR_.4	#N/A	7.7246	8.4277	9.3891	8.1258	8.7293	8.4436	8.3069	8.3500	8.4827	8.2794	1.0142	0.0203	0.6931	0.1592
Opalin	_RS[Phospho (STY)]PTHEM[Oxidation (M)]NPR_.3	#N/A	6.8176	4.8469	5.0621	8.0006	6.6878	4.9688	4.3189	5.0365	5.4543	4.6503	1.2464	0.3178	0.1200	0.9208
Opalin	_GS[Phospho (STY)]QEGHIYVK_.2	Q7M750_S100_M1_Opalin	8.1974	8.4963	8.3849	8.8042	8.4924	8.6112	8.6622	8.1313	8.2431	8.4983	1.0136	0.0195	0.4435	0.3531
Opalin	_TVSGSEEPLPDTYRPPEELER_.3	#N/A	5.7958	5.9911	5.8550	6.2775	6.0017	6.4426	6.2878	5.4431	6.1014	5.2199	1.0516	0.0726	0.2491	0.6035
Opalin	_S[Phospho (STY)]PTHEMNPR_.2	#N/A	7.3709	5.6383	3.7422	8.1668	6.5120	3.7672	5.8956	5.1384	3.8745	6.5369	1.0942	0.1298	0.6412	0.1930
Asap2	_LLHEDLDES[Phospho (STY)]DDDVDEK_.2	#N/A	3.5812	8.6850	3.8513	9.2910	7.9462	8.7806	9.0457	8.7876	8.9124	8.9228	0.7875	-0.3446	0.1911	0.7188
Asap2	_LLHEDLDES[Phospho (STY)]DDDVDEKLQPSPNRR_.4	#N/A	4.1266	3.4310	3.3126	8.8635	6.9161	2.9764	3.3371	8.3632	4.1113	4.3670	0.9788	-0.0309	0.9454	0.0244
Asap2	_LLHEDLDES[Phospho (STY)]DDDVDEKLQPSPNR_.4	#N/A	3.2504	3.9758	3.9372	7.4587	3.8641	4.2613	4.1545	6.8927	3.3272	4.4068	0.9494	-0.0749	0.8147	0.0890
Cyfip1	_SGDGEST[Phospho (STY)]PVEHVR_.2	Q7TMB8_T1234_M1_Cyfip1	6.8098	6.8753	3.4368	6.4025	5.5512	6.6610	3.1289	4.0206	6.5857	6.6803	1.1670	0.2228	0.4110	0.3862
Plppr4	_PGSSQLVHIPEETQENISTS[Phospho (STY)]PK_.3	Q7TME0_S549_M1_Plppr4	9.8447	10.0900	10.5275	10.8188	8.2333	4.2981	3.1580	10.6810	4.2904	3.9613	1.6240	0.6995	0.1010	0.9958
Plppr4	_DASS[Phospho (STY)]LTNLKR_.2	Q7TME0_S386_M1_Plppr4	8.4179	8.2952	8.8447	8.5965	6.4279	8.2357	8.3875	8.7444	8.2234	7.8292	0.9807	-0.0281	0.7408	0.1303
Plppr4	_QQGVLQSS[Phospho (STY)]PK_.2	Q7TME0_S594_M1_Plppr4	9.2092	9.4917	9.6156	9.4147	8.6491	9.1517	3.5074	9.4638	9.4496	9.1363	1.1732	0.2304	0.2764	0.5585
Plppr4	_DALRS[Phospho (STY)]LTDLNQDPSR_.2	#N/A	5.5616	5.7363	6.4991	7.0323	5.6171	5.9980	4.5829	7.0155	6.4404	5.7222	1.0225	0.0321	0.7995	0.0972
Plppr4	_M[Oxidation (M)]SLQVM[Oxidation (M)]DTEPEGQS[Phospho (STY)]PPR_.2	#N/A	6.5720	6.5974	7.3972	6.9447	4.9253	7.3577	7.3674	7.1012	6.9906	7.0597	0.9302	-0.1043	0.3191	0.4960
Plppr4	_TVHTS[Phospho (STY)]PGGGLGAR_.2	#N/A	3.7326	3.5620	4.0724	3.7676	3.3355	3.4901	3.7708	3.4208	4.1016	4.1215	0.9498	-0.0744	0.3262	0.4866
Plppr4	_S[Phospho (STY)]LTDLNQDPSR_.2	#N/A	7.5890	6.7823	7.2167	8.1667	6.0974	7.4844	7.7236	7.4365	7.2903	3.2770	1.1230	0.1673	0.4104	0.3868
Plppr4	_NASIHAS[Phospho (STY)]M[Oxidation (M)]DSAR_.2	Q7TME0_S439_M1_Plppr4	3.0249	5.0816	3.7117	4.2144	3.6514	4.0780	4.4388	3.9041	3.5527	4.1814	0.9853	-0.0213	0.8813	0.0549
Plppr4	_KM[Oxidation (M)]SLQVMDTEPEGQS[Phospho (STY)]PPR_.3	#N/A	3.7906	3.0950	4.4774	3.5037	2.9163	3.3124	3.4548	3.3158	4.0528	3.3308	0.9936	-0.0093	0.9450	0.0246
Plppr4	_DSESC[Carbamidomethyl (C)]ESLKDSFGS[Phospho (STY)]GDR_.2	#N/A	4.0115	4.0788	3.9604	3.7001	3.9001	3.8899	4.1777	3.7649	3.4135	3.6671	1.0446	0.0630	0.2721	0.5653
Plppr4	_SNS[Phospho (STY)]PENTR_.2	Q7TME0_S746_M1_Plppr4	4.2278	3.1146	3.9615	3.5053	3.3715	3.2531	3.6535	3.4959	3.7950	3.1825	1.0115	0.0165	0.8722	0.0594
Ndufa12	_FNVSATPEQYVPYSTTR_.2	#N/A	8.1751	8.1353	7.9745	8.3746	8.0366	8.2553	8.3341	7.9521	8.2974	7.7046	1.0107	0.0154	0.5528	0.2574
Ndufa12	_IGTLVGEDK_.2	#N/A	5.2504	5.9265	4.2365	6.1965	5.7015	5.6936	5.4276	6.2111	5.2047	6.1751	0.9559	-0.0651	0.5514	0.2585
Ndufa12	_[Acetyl (Protein N-term)]M[Oxidation (M)]ELVEVLKR_.2	#N/A	7.6460	7.8793	7.5414	7.8327	7.5148	7.6007	7.5618	7.7462	7.5847	7.7418	1.0014	0.0020	0.9069	0.0424
Ndufa12	_GVQQVTGHGGLR_.2	#N/A	7.0567	7.3709	7.3642	7.4891	8.0294	7.1818	6.7375	7.0840	7.7444	7.4850	1.0210	0.0300	0.5518	0.2582
Ndufa12	_WLHC[Carbamidomethyl (C)]M[Oxidation (M)]TDDPPTTNPPTAR_.3	#N/A	3.3275	3.8987	4.0143	4.5169	5.6468	4.3385	4.2316	6.4068	6.5196	5.8268	0.7467	-0.4215	0.0356	1.4490
Ndufa12	_[Acetyl (Protein N-term)]M[Oxidation (M)]ELVEVLK_.2	#N/A	3.9443	3.3981	3.6779	3.2217	2.7670	3.0092	3.3700	3.7795	3.5813	4.3998	0.8820	-0.1811	0.1521	0.8178
Uhrf2	_RPAS[Phospho (STY)]DDEC[Carbamidomethyl (C)]PGDSK_.2	Q7TMI3_S668_M1_Uhrf2	4.2933	3.5977	3.4793	3.9820	3.4190	3.8757	3.1704	4.1365	3.7851	3.9490	1.0038	0.0055	0.9538	0.0205
Syncrip	_NLANTVTEEILEK_.2	#N/A	4.3470	3.7477	4.4581	5.2507	4.6387	5.3941	5.0067	5.0152	4.7666	4.5657	0.9588	-0.0606	0.5540	0.2565
Syncrip	_DLFEDELVPLFEK_.2	#N/A	4.7604	5.5612	3.5416	5.0503	5.2037	5.7726	5.0726	5.0520	5.0163	5.9367	0.9454	-0.0810	0.5323	0.2738
Syncrip	_DSDLSHVQNK_.2	#N/A	4.3066	4.1708	4.8076	5.3090	4.6495	4.9547	3.7529	4.0188	3.9700	5.1872	1.1105	0.1512	0.1991	0.7010
Syncrip	_LYNNHEIR_.2	#N/A	3.7740	5.1758	3.5076	4.5522	4.3761	5.2896	3.1997	3.9498	4.2488	5.2924	1.0655	0.0915	0.6015	0.2208
Syncrip	_TGYTLDVTTGQR_.2	#N/A	5.2168	3.1214	4.5038	6.0246	5.0158	5.7868	3.2203	5.8832	4.2614	3.4727	1.1747	0.2323	0.3333	0.4771
Syncrip	_GFC[Carbamidomethyl (C)]FLEYEDHK_.3	#N/A	3.8521	3.3741	4.5389	3.5407	3.2624	3.3622	3.2554	3.0770	4.2264	3.5077	1.0394	0.0557	0.6729	0.1721
Syncrip	_VTEGLTDVILYHQPDDK_.3	#N/A	4.2729	3.5772	3.4588	4.5434	3.3986	4.3309	3.1909	4.1570	3.8056	3.9285	1.0424	0.0599	0.6153	0.2109
Tubb2a	_AILVDLEPGTM[Oxidation (M)]DSVR_.2	#N/A	12.8603	12.8284	13.2180	12.9376	12.9932	13.1711	13.0129	12.7440	13.0274	13.0271	1.0037	0.0054	0.6367	0.1960
Tubb2a	_ALTVPELTQQM[Oxidation (M)]FDSK_.2	#N/A	11.5334	11.7243	11.8047	11.6333	11.5706	11.5277	11.6731	11.6109	11.5197	11.6646	1.0013	0.0019	0.8162	0.0882
Tubb2a	_YLTVAAIFR_.2	#N/A	11.3253	11.3809	11.3973	11.3958	11.2455	11.3753	11.1873	11.4581	11.5686	11.4396	0.9947	-0.0076	0.4190	0.3778
Tubb2a	_INVYYNEAAGNK_.2	#N/A	11.4335	11.5329	12.1236	12.2234	11.6793	11.8998	11.7464	12.0333	11.8159	12.0386	0.9922	-0.0113	0.6067	0.2170
Tubb2a	ESESC[Carbamidomethyl (C)]DC[Carbamidomethyl (C)]LQGFQLTHSLGGGTGSGM[Oxidation (M)]GTLLISK	#N/A	6.2317	5.6659	5.5815	6.4858	4.9746	5.2729	5.5211	5.1334	4.8602	5.9987	1.0602	0.0843	0.3826	0.4172
Tubb2a	_IM[Oxidation (M)]NTFSVM[Oxidation (M)]PSPK_.2	#N/A	8.9408	9.2310	9.2102	9.5541	9.4818	9.2127	9.0979	9.3558	9.2675	9.2999	1.0018	0.0026	0.8946	0.0484
Tubb2a	_FWEVISDEHGIDPTGSYHGDSDLQLER_.4	#N/A	6.6771	6.4782	6.9223	6.6945	7.0422	6.9681	5.9817	6.5929	6.6775	7.2613	1.0255	0.0363	0.4920	0.3080
Tubb2a	_INVYYNEAAGNKYVPR_.3	#N/A	4.5337	4.7772	5.2499	4.1676	4.7330	4.5926	3.7755	4.1518	4.2351	4.4497	1.1258	0.1710	0.0392	1.4062
Tubb2a	SESC[Carbamidomethyl (C)]DC[Carbamidomethyl (C)]LQGFQLTHSLGGGTGSGM[Oxidation (M)]GTLLISK	#N/A	3.0180	3.7742	3.9590	4.6668	3.6625	2.9621	3.9445	2.8305	3.5040	3.4735	1.0685	0.0956	0.5433	0.2650
Huwe1	_AES[Phospho (STY)]PEEVAC[Carbamidomethyl (C)]R_.2	#N/A	10.7333	10.4261	10.5895	10.5997	9.3468	10.0663	11.0495	10.6764	10.5124	10.4971	0.9635	-0.0537	0.2053	0.6876
Huwe1	_RAQC[Carbamidomethyl (C)]ETLS[Phospho (STY)]PDGLPEEQPQTTK_.3	#N/A	6.6635	7.0141	7.7750	7.0222	3.0036	6.4781	7.5878	7.4717	4.1629	6.3836	0.9882	-0.0171	0.9453	0.0244
Huwe1	_ESNQPPEDSS[Phospho (STY)]PPASSESSSTR_.2	Q7TMY8_S2918_M1_Huwe1	7.9882	4.2759	8.5706	4.1397	3.5767	4.1527	8.7889	3.9788	3.6274	4.1067	1.0634	0.0888	0.8324	0.0797
Huwe1	_GSGTAS[Phospho (STY)]DDEFENLR_.2	Q7TMY8_S1907_M1_Huwe1	3.4056	9.4641	9.6999	9.7146	8.2557	9.4099	10.2119	9.9352	9.6821	9.5168	0.8463	-0.2407	0.2667	0.5739
Huwe1	_AQC[Carbamidomethyl (C)]ETLS[Phospho (STY)]PDGLPEEQPQTTK_.2	#N/A	7.3117	6.8441	7.0545	7.4063	5.9779	5.2766	7.9043	7.5528	6.9412	7.2332	0.8970	-0.1568	0.1360	0.8665
Huwe1	_DGGS[Phospho (STY)]GNSTIIVSR_.2	Q7TMY8_S2362_M1_Huwe1	2.8988	4.3273	3.5856	4.0883	3.5253	4.2041	3.8030	4.0302	3.6788	6.6478	0.8308	-0.2675	0.2521	0.5984
Huwe1	_AES[Phospho (STY)]PEEVAC[Carbamidomethyl (C)]RK_.2	#N/A	7.7378	6.8457	7.3604	7.3237	5.8359	6.3177	7.8640	7.5040	7.1996	7.2936	0.9248	-0.1129	0.1814	0.7413
Huwe1	_DLS[Phospho (STY)]MSEEDQMMR_.2	Q7TMY8_S1368_M1_Huwe1	3.9084	3.4340	3.6421	3.1859	3.2553	2.9733	3.3341	3.8153	4.1144	4.3640	0.8702	-0.2006	0.0726	1.1394
Smap2	_DLDLLASVPS[Phospho (STY)]PSSVSR_.2	Q7TN29_S219_M1_Smap2	3.8788	7.9832	8.1493	9.2452	6.9262	6.9581	3.9961	8.0191	7.4315	3.5252	1.2520	0.3242	0.3008	0.5217
Smap2	_S[Phospho (STY)]LDINVLR_.2	Q7TN29_S127_M1_Smap2	3.1256	1.6383	3.8124	4.3150	3.9889	4.1366	4.0297	3.8034	3.4520	4.2820	0.9000	-0.1519	0.4836	0.3155
Smap2	_KAVGSMPTAGSAGS[Phospho (STY)]VPENLNLFPEPGSK_.3	Q7TN29_S239_M1_Smap2	3.1219	4.1042	3.8087	9.4540	3.9925	4.1329	4.0261	3.8071	3.4557	4.2784	1.2254	0.2933	0.4837	0.3154
Cpeb4	_QQLSPS[Phospho (STY)]PGQEAGILPETEK_.2	Q7TN98_S99_M1_Cpeb4	6.9640	7.1499	6.1796	7.6769	6.2916	7.2676	6.1956	3.8589	3.6982	3.9212	1.5665	0.6476	0.0020	2.7011
Cpeb4	_S[Phospho (STY)]PASPHPPPFTHR_.3	Q7TN98_S252_M1_Cpeb4	3.9089	3.4335	3.6425	3.1863	3.2549	2.9738	3.3346	3.8149	4.1139	3.7545	0.9056	-0.1431	0.1357	0.8675
Cpeb3	_SPAS[Phospho (STY)]PSQAPYAQR_.2	Q7TN99_S197_M1_Cpeb3	8.3549	8.4903	8.6539	9.0432	8.0691	8.5052	8.7120	8.3039	8.2911	7.9896	1.0235	0.0335	0.3631	0.4400
Cpeb3	_RS[Phospho (STY)]PAS[Phospho (STY)]PSQAPYAQR_.2	#N/A	3.8525	4.3444	4.2325	3.7762	3.6425	2.9821	3.9245	3.2249	3.5240	3.4535	1.0774	0.1075	0.3467	0.4600
Spag7	_EFAPS[Phospho (STY)]DEELDSYR_.2	Q7TNE3_S114_M1_Spag7	3.3892	4.1944	4.0760	7.1516	5.4203	3.7138	4.2933	6.1682	3.1884	6.1140	0.9426	-0.0852	0.7657	0.1159
Tspoap1	_GGGS[Phospho (STY)]PEKLPNR_.2	Q7TNF8_S1402_M1_Tspoap1	4.7129	4.4503	6.0087	5.4171	3.4941	4.0139	5.2266	5.0687	5.3069	5.2357	0.8989	-0.1537	0.2951	0.5300
Trim46	_LQESFQGAPDVIS[Phospho (STY)]PR_.2	Q7TNM2_S627_M1_Trim46	9.6392	9.5700	10.1076	10.3919	9.0930	9.5393	10.0457	9.8738	9.7211	9.6684	0.9894	-0.0153	0.6819	0.1663
Plppr3	_SVS[Phospho (STY)]TDELGPPGR_.2	Q7TPB0_S320_M1_Plppr3	9.8319	9.7234	10.3264	9.6546	7.9002	9.5242	10.1602	9.7984	9.7154	9.5135	0.9690	-0.0454	0.5059	0.2959
Plppr3	_GLGLPDEAS[Phospho (STY)]PVHLR_.2	#N/A	6.7794	6.6304	6.4431	6.8084	5.0232	6.6459	6.7475	7.2311	7.1044	6.6784	0.9205	-0.1195	0.1675	0.7760
Plppr3	_QKS[Phospho (STY)]LEGR_.2	Q7TPB0_S413_M1_Plppr3	4.0837	4.5201	3.8174	3.8222	4.3997	3.3971	4.4002	3.4014	4.6831	4.2970	0.9550	-0.0664	0.5532	0.2571
Plppr3	_EKTS[Phospho (STY)]LGS[Phospho (STY)]LKR_.2	Q7TPB0_S343_M2_Plppr3	6.4889	6.6413	3.7273	6.6179	3.6670	6.6479	7.0864	3.8886	3.5372	6.1790	1.0887	0.1226	0.6668	0.1760
Plppr3	_RAS[Phospho (STY)]VDVDLLAPR_.2	Q7TPB0_S351_M1_Plppr3	3.0116	4.4689	3.2593	3.9214	2.9044	4.0913	4.2714	4.1981	4.1070	4.7566	0.8330	-0.2637	0.0698	1.1563
Plppr3	_QPGM[Oxidation (M)]GPGS[Phospho (STY)]PVSDVDQEEPR_.2	#N/A	4.6435	5.0462	3.5943	4.2866	3.8642	5.0093	4.0155	4.4841	4.6788	4.9930	0.9702	-0.0437	0.7056	0.1514
Slc7a6os	_EFDYDSPHGLDS[Phospho (STY)]D_.2	Q7TPE5_S305_M1_Slc7a6os	3.1007	3.8570	4.3352	7.5984	7.1735	4.3802	4.2734	6.9509	6.8770	7.2303	0.8012	-0.3197	0.2824	0.5491
Mycbp2	_SKS[Phospho (STY)]DSYTLDPDTLR_.2	Q7TPH6_S2943_M1_Mycbp2	3.8752	8.0029	4.5620	7.9533	3.0009	7.8986	3.5886	8.4543	8.1419	3.5308	0.9921	-0.0114	0.9775	0.0099
Mycbp2	_LISGDAEPT[Phospho (STY)]PEQEEK_.2	Q7TPH6_T3992_M1_Mycbp2	4.2176	3.4731	8.6426	8.1723	3.3614	3.2632	8.4208	8.5272	8.0791	7.5335	0.6374	-0.6498	0.0526	1.2790
Mycbp2	_ALLSS[Phospho (STY)]PEGEEK_.2	Q7TPH6_S4003_M1_Mycbp2	3.5261	5.5261	3.7470	6.3223	5.4289	6.3185	6.2448	6.7692	7.0683	6.6151	0.7708	-0.3755	0.0444	1.3526
Mycbp2	_SSS[Phospho (STY)]PQDKNLPQK_.2	Q7TPH6_S2916_M1_Mycbp2	7.6922	8.0441	8.8725	7.6452	2.9278	7.8902	8.3752	8.3014	7.8844	7.4503	0.8970	-0.1568	0.4751	0.3232
Mycbp2	_STS[Phospho (STY)]PKPKPVPK_.2	Q7TPH6_S2970_M1_Mycbp2	4.5142	4.4039	4.1731	4.8857	4.2252	3.5043	3.8651	3.2844	4.1651	3.3941	1.1651	0.2205	0.0658	1.1816
Mycbp2	_DPKDSPGS[Phospho (STY)]ENR_.2	Q7TPH6_S2986_M1_Mycbp2	3.9802	5.5883	3.1662	3.6688	3.1059	3.1228	3.3835	4.4497	4.0982	3.6358	0.9692	-0.0451	0.8248	0.0837
Mycbp2	_SLS[Phospho (STY)]PNHNTLQTLK_.2	#N/A	3.8148	3.6591	4.2562	4.1538	3.5244	3.8058	3.8776	4.4513	3.5587	3.4772	1.0072	0.0104	0.9053	0.0432
Mycbp2	_QVS[Phospho (STY)]TENDSTLVHR_.2	Q7TPH6_S1621_M1_Mycbp2	6.7190	7.1918	7.5699	6.6000	5.5571	6.7299	7.2449	7.2655	6.9738	6.7759	0.9523	-0.0705	0.3753	0.4256

Prrc2b	_ESGS[Phospho (STY)]PAQEFSK_.2	Q7TPM1_S621_M1_Prrc2b	8.1560	7.5002	7.2002	8.0163	6.8734	7.5078	8.1546	7.5615	7.5985	7.8585	0.9678	-0.0472	0.3792	0.4212
Prrc2b	_ALS[Phospho (STY)]LSSADSTDAKR_.2	Q7TPM1_S415_M1_Prrc2b	3.2537	4.0100	4.4883	6.1324	3.8983	4.2271	4.1203	5.2543	3.3615	6.3510	0.9085	-0.1385	0.5593	0.2523
Prrc2b	_LKFS[Phospho (STY)]DDEDEEDVVKDGR_.3	#N/A	3.4246	5.5501	4.1115	7.2371	5.8715	6.1157	6.1540	6.6051	5.8477	5.7949	0.8827	-0.1799	0.3530	0.4522
Prrc2b	_LKFS[Phospho (STY)]DDEDEEDVVK_.2	#N/A	3.4531	4.2583	4.1399	4.6037	4.0796	3.6499	4.2351	3.8354	4.1116	4.1773	0.9855	-0.0210	0.7989	0.0975
Prrc2b	_S[Phospho (STY)]PDEALPGGLGSHSPYALER_.3	Q7TPM1_S793_M1_Prrc2b	4.1196	3.2228	3.8533	3.3970	3.2633	3.3613	3.5453	3.6041	3.9032	3.0743	1.0013	0.0018	0.9849	0.0066
Prrc2b	_QQQQQQQQQQQQQQQEQLYK_.3	#N/A	4.1028	3.2396	3.8365	3.3803	3.0609	3.1677	3.5285	3.6209	3.9200	3.0575	0.9810	-0.0277	0.7983	0.0979
Fsd1	_TAS[Phospho (STY)]PVNSPAR_.2	Q7TPM6_S313_M1_Fsd1	9.2061	9.2525	7.6701	9.3646	7.4693	9.2607	9.0332	8.9603	8.9099	9.1144	0.9666	-0.0490	0.5245	0.2803
Fsd1	_RGS[Phospho (STY)]ATSSSNTSLT_.2	Q7TPM6_S486_M1_Fsd1	8.9467	8.7783	3.9913	9.1838	3.2523	8.5964	8.7713	8.6661	8.7721	8.7442	0.8153	-0.2945	0.2803	0.5524
Fsd1	_LDGSTSHQNLR_.2	#N/A	4.3107	3.5669	4.0444	3.5882	4.3447	3.1701	3.7365	3.4130	3.7120	3.2655	1.0866	0.1198	0.2676	0.5726
Fsd1	_ALESSEELLETANQTLQASDSEDFSQAAK_.3	#N/A	3.2731	4.0294	3.8836	4.3863	3.9177	4.2078	4.1010	3.7322	3.3808	4.3533	1.0149	0.0213	0.8278	0.0821
Mtmr10	_IWLS[Phospho (STY)]TETLANED_.2	Q7TPM9_S763_M1_Mtmr10	3.8890	3.3372	4.5758	3.5776	3.2255	3.3991	3.2923	3.0401	4.1895	3.5446	1.0429	0.0606	0.6520	0.1857
Mtmr10	_RNS[Phospho (STY)]LVLK_.2	Q7TPM9_S603_M1_Mtmr10	3.5353	3.6908	4.2221	3.2239	3.5791	3.0455	4.4395	3.3937	3.0423	3.1909	1.0093	0.0134	0.9220	0.0353
Actn1	_ATLPDADKER_.2	#N/A	8.2451	8.2048	8.3562	8.2720	8.0501	8.2341	8.1789	8.4366	8.0921	7.9174	1.0087	0.0125	0.4982	0.3026
Actn1	_TINEVENQILTR_.2	#N/A	8.0678	7.6343	8.1333	7.6574	7.8851	7.7404	8.0459	8.0057	7.8992	7.2063	1.0082	0.0118	0.7450	0.1279
Actn1	_LASDLLEWIR_.2	#N/A	7.6801	7.2008	7.3905	7.6732	6.9043	7.5382	7.9801	7.6788	7.6065	7.5670	0.9597	-0.0593	0.1075	0.9686
Actn1	_ILAGDKNYITEDELR_.3	#N/A	7.4744	7.0885	7.4742	7.7982	6.9540	7.3798	7.5327	7.6492	7.2400	7.5707	0.9818	-0.0265	0.4441	0.3525
Actn1	_EGLLLWC[Carbamidomethyl (C)]QR_.2	#N/A	7.6067	7.2357	7.3493	7.3178	6.7302	6.9068	7.4543	7.6546	6.7586	6.9655	0.9976	-0.0034	0.9428	0.0256
Actn1	_GYEEWLLNEIR_.2	#N/A	7.2695	6.9367	7.0325	7.0421	6.7032	6.7380	7.1808	6.9648	7.0334	7.0316	0.9860	-0.0204	0.4094	0.3879
Actn1	_VGWEQLLTTIAR_.2	#N/A	7.2101	7.4409	7.0591	7.3134	6.3694	7.1725	7.3998	7.3124	7.3016	7.0251	0.9772	-0.0333	0.4392	0.3574
Actn1	_TIPWLENR_.2	#N/A	7.1028	7.4164	6.9054	6.6252	7.2290	7.0616	6.9948	7.3710	6.7753	7.1295	0.9985	-0.0022	0.9505	0.0221
Actn1	_IC[Carbamidomethyl (C)]DQWDNLGALTQK_.2	#N/A	6.4728	6.8392	7.0886	7.1135	6.5878	6.7661	7.1743	6.5638	6.7526	6.4463	1.0114	0.0164	0.6844	0.1647
Actn1	_FAIQDISVEETSAK_.2	#N/A	6.5537	6.4386	7.0205	6.9039	5.6972	6.4118	6.6944	6.7627	6.3866	6.7483	0.9784	-0.0315	0.5791	0.2372
Actn1	_LAILGIHNEVSK_.2	#N/A	5.8002	6.4279	6.2719	6.5181	5.5435	6.1407	6.6898	6.5791	6.3167	6.3292	0.9442	-0.0829	0.1172	0.9310
Actn1	_ASFNHFDR_.2	#N/A	6.0065	6.2087	6.1077	6.0892	6.2406	5.9932	5.8662	6.3016	6.0383	5.6833	1.0227	0.0323	0.2777	0.5563
Actn1	_DLLLDPAWEK_.2	#N/A	6.5570	6.0883	5.0800	5.9049	5.8202	5.8156	6.0131	6.0735	5.9810	5.9230	0.9800	-0.0291	0.6389	0.1946
Actn1	_KDDPLTNLNTAFDVAER_.3	#N/A	5.6661	5.7622	6.1732	5.3798	6.0875	5.3729	5.5334	5.6536	5.8947	5.6534	1.0099	0.0143	0.7661	0.1157
Actn1	_LLETIDQLYLEYAK_.2	#N/A	5.2459	4.8616	6.1646	4.9836	5.2392	5.3069	5.1484	5.4041	5.0191	5.2147	1.0200	0.0285	0.6794	0.1679
Actn1	_RDQALTEEHAR_.3	#N/A	7.0102	6.0654	6.3818	6.1881	6.5580	6.6329	6.6148	6.0493	6.7581	6.3510	1.0046	0.0066	0.8934	0.0490
Actn1	_DDPLTNLNTAFDVAER_.2	#N/A	6.1835	5.9016	3.1949	5.9654	6.0304	5.7010	6.0930	6.3039	5.6634	6.1508	0.9080	-0.1392	0.3738	0.4273
Actn1	_DGLGFC[Carbamidomethyl (C)]ALIHR_.2	#N/A	5.1506	3.8031	4.2819	4.4752	3.4534	3.5407	5.1955	5.3314	5.1759	4.9428	0.7977	-0.3260	0.0146	1.8358
Actn1	_IDQLEC[Carbamidomethyl (C)]DHQLIQEALIFDNK_.3	#N/A	4.2668	5.5759	4.5875	5.5906	2.8445	4.6672	5.3582	5.0420	4.7950	4.4676	0.9335	-0.0993	0.5602	0.2516
Actn1	_QFGAQANVIGPWIQTK_.2	#N/A	5.1622	4.9140	5.5537	5.1948	4.1172	5.1355	5.4306	5.3294	5.5238	5.1276	0.9365	-0.0947	0.2214	0.6549
Actn1	_HRPELIDYGK_.3	#N/A	5.8304	4.6979	5.9571	5.5285	5.3317	5.7503	5.2822	6.2192	5.8620	4.9064	0.9908	-0.0134	0.8797	0.0557
Actn1	_VLAVNQENEQLM[Oxidation (M)]EDYEK_.2	#N/A	4.3936	4.5642	5.7501	5.8046	3.9926	4.8225	5.3120	4.6076	4.3497	5.4238	0.9928	-0.0104	0.9367	0.0284
Actn1	_GISQEQM[Oxidation (M)]NEFR_.2	#N/A	5.9448	6.4797	7.1940	5.6717	6.3711	6.0563	5.8791	6.8820	6.1719	6.2462	0.9986	-0.0020	0.9793	0.0091
Actn1	_ALDFIASK_.2	#N/A	5.2220	3.6898	3.7031	5.7751	5.5852	5.0531	3.3952	4.9525	4.6004	5.1118	1.0716	0.0997	0.5806	0.2362
Actn1	_C[Carbamidomethyl (C)]QLEINFNTLQTK_.2	#N/A	5.7718	4.4744	4.1898	4.1898	5.6499	5.6410	3.9045	4.8337	5.4813	5.6223	0.9883	-0.0170	0.9189	0.0367
Actn1	_IMSIVDPNR_.2	#N/A	5.6595	4.3916	3.5213	6.4303	5.2885	4.2684	3.7387	3.8472	2.6878	3.9910	1.3815	0.4662	0.0500	1.3008
Actn1	_NVNIQNFHISWK_.2	#N/A	5.1661	4.5064	3.4768	4.6091	2.8868	4.7610	3.6310	4.2022	3.5168	3.8833	1.1119	0.1530	0.3794	0.4209
Actn1	_HTNYNM[Oxidation (M)]EHIR_.3	#N/A	6.0064	3.2794	3.4249	3.9666	4.3880	7.6129	6.3143	4.5246	3.8007	4.0245	1.0244	0.0347	0.9102	0.0408
Actn1	_MVSDINNAWGC[Carbamidomethyl (C)]LEQAEK_.2	#N/A	5.7326	4.0161	4.5608	6.0307	4.9820	4.1546	4.2528	4.3975	3.1957	3.7818	1.2575	0.3305	0.0665	1.1769
Actn1	_AIM[Oxidation (M)]TYVSSFYHAFSGAQK_.2	#N/A	3.3980	3.5253	4.6051	4.3182	3.2958	3.5862	4.2822	4.4349	3.2607	4.3852	0.9260	-0.1109	0.4153	0.3816
Actn1	_ASIHEAWTDGK_.2	#N/A	5.2778	5.1317	4.3681	5.8152	4.8767	5.1881	5.7408	5.0429	5.1945	5.3010	0.9605	-0.0582	0.4598	0.3375
Actn1	_KHEAFESDLAAHQDR_.3	#N/A	5.2626	3.4556	3.6526	3.7281	4.6033	3.9598	3.2447	5.0037	3.8765	3.5321	1.0501	0.0705	0.6847	0.1645
Actn1	_VEQIAAIAQELNELDYYDSPSVNAR_.3	#N/A	3.9986	3.2276	3.1846	3.6872	3.9115	3.5088	3.3586	4.4312	4.0798	4.1887	0.8933	-0.1627	0.1279	0.8933
Actn1	_QKDYETATLSEIK_.2	#N/A	3.8153	3.6460	3.9127	4.0030	3.4935	3.8904	3.9218	3.7080	4.1652	3.1380	1.0161	0.0231	0.7693	0.1139
Actn1	_ISIEM[Oxidation (M)]HGTLEDQLSHLR_.3	#N/A	5.7224	5.2594	6.0809	4.3169	4.9297	5.5695	6.1070	3.8016	3.4502	5.3366	1.1368	0.1850	0.3113	0.5069
Actn1	_MLDAEDIVGTARPDEK_.3	#N/A	3.0624	4.2800	4.2969	3.8407	3.7070	3.6281	3.9889	3.1605	3.4596	3.5180	1.0767	0.1066	0.3475	0.4590
C2cd5	_NQTYSFS[Phospho (STY)]PSK_.2	Q7TPS5_S295_M1_C2cd5	2.9799	4.3625	4.2144	3.7582	3.6245	3.5456	7.1625	3.2430	3.5421	6.7838	0.7231	-0.4678	0.1334	0.8748
C2cd5	_SQS[Phospho (STY)]ESSDEVTELDLSHGKK_.3	#N/A	5.3065	6.4341	5.4580	6.1397	5.2124	5.4299	6.1510	6.6793	6.6303	6.5713	0.8702	-0.2005	0.0146	1.8343
C2cd5	_SQSES[Phospho (STY)]DEVTELDLSHGK_.3	#N/A	3.8313	3.3916	3.5463	3.9054	3.2315	3.7036	3.9316	4.7523	4.2367	3.6796	0.8678	-0.2045	0.0413	1.3844
C2cd5	_LSSPAAFLPAC[Carbamidomethyl (C)]S[Phospho (STY)]SPSR_.2	Q7TPS5_S259_M1_C2cd5	3.9391	6.9275	4.1677	8.4615	2.9655	3.2690	3.3550	7.1919	4.1382	3.2426	1.1056	0.1448	0.7348	0.1339
Mybbp1a	_SPAPSNPTLS[Phospho (STY)]PSTPAK_.2	Q7TPV4_S1253_M1_Mybbp1a	2.9065	3.7830	3.8910	3.9064	4.2331	5.8747	3.8218	3.7099	3.6435	3.7570	1.0981	0.1350	0.4846	0.3147
Nexn	_TVS[Phospho (STY)]QESLTPGK_.2	Q7TPW1_S296_M1_Nexn	8.9741	8.6117	8.6411	9.5898	8.5263	8.7516	9.2300	8.6803	8.3826	8.8942	1.0059	0.0086	0.8368	0.0774
Nexn	_EM[Oxidation (M)]LAS[Phospho (STY)]DDEEESSPK_.2	#N/A	9.2687	9.3941	9.5535	10.2839	9.1882	9.2977	9.5260	9.2299	8.7154	9.4276	1.0296	0.0421	0.3088	0.5103
Nexn	_EMLAS[Phospho (STY)]DDEEESSPKIEK_.2	#N/A	3.3499	4.1061	3.8068	4.3095	3.9944	4.1310	4.0242	3.8090	3.4576	4.2765	1.0149	0.0213	0.7994	0.0972
Lnpk	_ADS[Phospho (STY)]VPNLEPSEESLVTK_.2	Q7TQ95_S411_M1_Lnpk	12.0341	11.8667	12.4546	12.2777	10.8961	11.9866	12.5077	12.3345	12.1921	12.0414	0.9715	-0.0417	0.2605	0.5841
Lnpk	_IVEYLVGDGPQNR_.2	#N/A	5.9565	5.9824	3.2967	3.7994	4.7518	5.9930	6.1142	5.6035	5.9414	4.6700	0.8891	-0.1695	0.3796	0.4206
Tppp	_AANKT[Phospho (STY)]PPKS[Phospho (STY)]PGDPAR_.2	#N/A	12.5651	12.8802	13.1412	13.2234	12.4772	12.9235	12.6083	12.5778	12.6624	12.8200	1.0159	0.0227	0.2403	0.6193
Tppp	_AVSS[Phospho (STY)]PTVSR_.2	Q7TQD2_S159_M1_Tppp	10.2580	10.4380	10.6071	10.4622	10.7046	10.4527	10.1427	10.0021	10.2355	10.4919	1.0263	0.0375	0.0448	1.3491
Tppp	_VDLVDESGYVPGYK_.2	#N/A	10.7696	10.5808	10.9170	10.8628	10.9370	10.9406	10.7080	10.4320	10.5674	10.8134	1.0192	0.0275	0.0682	1.1663
Tppp	_APVISGVTK_.2	#N/A	8.8663	9.1820	9.3042	9.1025	9.5645	9.1160	8.3452	9.1765	9.1034	9.1098	1.0286	0.0407	0.2282	0.6416
Tppp	_FAVHGDTR_.2	#N/A	8.8133	8.9987	8.7476	9.0565	9.4332	8.9691	8.7507	8.7012	8.7638	8.8977	1.0256	0.0365	0.1157	0.9365
Tppp	_NVTVTDVDIVFSK_.2	#N/A	7.1862	7.1215	8.0798	7.6267	7.7596	7.6153	7.5047	6.9441	7.5546	7.6852	1.0192	0.0275	0.5435	0.2648
Tppp	_TITFEQFQEALEELAK_.2	#N/A	5.4474	5.7958	5.9872	6.4918	6.0332	5.7274	5.8505	5.1294	5.2317	5.7357	1.0778	0.1081	0.0993	1.0032
Tppp	_RLS[Phospho (STY)]LESEGANEGATAAPELSALEEAFR_.3	#N/A	9.9242	11.0271	11.3780	11.9927	10.4902	10.2789	10.7358	11.0896	10.8291	10.3019	1.0102	0.0146	0.7970	0.0985
Tppp	_LSLESEGANEGATAAPELSALEEAFR_.3	#N/A	4.9578	4.6585	4.5240	4.6712	5.2946	4.5429	5.2017	4.3153	4.5425	5.0072	1.0017	0.0025	0.9716	0.0125
Tppp	_DC[Carbamidomethyl (C)]HVIDGK_.2	#N/A	3.9502	6.8595	3.1362	3.6388	3.1642	3.4604	6.3507	6.3438	6.8836	7.0112	0.6070	-0.7202	0.0075	2.1234
Tppp	_RLS[Phospho (STY)]LESEGANEGATAAPELSALEEAFRR_.4	#N/A	5.4787	8.0150	9.0347	8.6033	7.7902	7.2943	6.6793	7.4589	7.8418	7.4609	1.0465	0.0656	0.6210	0.2069
Tppp	_LSLESEGANEGATAAPELSALEEAFRR_.3	#N/A	3.0461	5.7965	5.3232	3.8244	5.1531	5.5270	3.9727	3.1767	5.2675	5.6822	1.0561	0.0787	0.7338	0.1344
Tppp	_TPPKS[Phospho (STY)]PGDPAR_.2	#N/A	10.8949	11.1083	3.9585	11.5671	10.6859	11.1535	10.8929	10.7528	10.7163	10.9095	0.9147	-0.1287	0.5533	0.2570
Tppp	_DKSSEEAVR_.2	#N/A	4.0451	3.7177	4.5243	3.5261	3.2770	3.3476	5.4804	3.0916	4.0188	4.1409	0.8940	-0.1616	0.3614	0.4420
Tppp	_[Acetyl (Protein N-term)]ADS[Phospho (STY)]KAKPAK_.2	Q7TQD2_S4_M1_Tppp	4.1879	3.4433	3.5618	3.4714	3.3316	3.2930	3.1861	3.1462	4.2956	3.5114	1.0038	0.0054	0.9617	0.0170
Maco1	_NASGVVNSS[Phospho (STY)]PR_.2	Q7TQE6_S332_M1_Maco1	7.6283	7.5910	8.1724	7.5911	6.7278	7.4318	8.2931	7.7808	7.6892	7.5035	0.9625	-0.0551	0.3137	0.5035
Fbxo10	_EAEVGS[Phospho (STY)]DGER_.2	#N/A	6.1666	6.0270	7.3126	7.1427	5.6711	5.7938	7.4176	7.6065	6.7220	6.8673	0.8880	-0.1713	0.0775	1.1105
Amph	_AALPAGEGGS[Phospho (STY)]PEGAK_.2	Q7TQF7_S500_M1_Amph	14.5006	14.5885	14.8998	15.2525	14.5372	14.6451	14.9724	14.5034	14.5898	14.7443	1.0024	0.0034	0.8415	0.0749
Amph	_GLFPENFTR_.2	#N/A	8.8153	8.9946	8.8825	8.9881	9.0514	9.0100	8.9956	8.9945	8.9548	8.9035	0.9994	-0.0008	0.9179	0.0372
Amph	_KLVDYDSAR_.2	#N/A	8.6357	8.9724	8.9242	8.8755	9.1929	9.0481	8.7971	8.7852	8.7716	8.9466	1.0132	0.0189	0.2801	0.5527
Amph	_ESDWLQYR_.2	#N/A	8.3620	8.1451	8.3660	8.2614	8.3211	8.2873	8.3155	8.0472	8.0587	8.2652	1.0145	0.0208	0.1234	0.9085

Amph	_EIAVLC[Carbamidomethyl (C)]HK_.2	#N/A	7.2513	7.6119	7.0767	7.8193	7.8629	7.2867	7.6058	6.9377	7.6535	7.3960	1.0117	0.0168	0.6919	0.1600
Amph	_VGFYVNTFK_.2	#N/A	6.4162	6.8861	6.6782	6.3450	5.4754	6.2510	6.5633	6.4665	6.1389	6.6302	0.9833	-0.0243	0.6914	0.1603
Amph	_LVDYDSAR_.2	#N/A	6.4050	6.5506	6.6225	6.5775	7.0249	6.6757	4.9065	6.4234	6.5065	7.0662	1.0670	0.0935	0.3051	0.5156
Amph	_ADETKDEQFEEYVQNFKR_.3	#N/A	5.4142	6.1149	6.3083	6.4084	5.7229	6.2802	5.4966	5.5688	5.6013	6.0740	1.0627	0.0877	0.1516	0.8192
Amph	_EAAEDVAAQGSAGEK_.2	#N/A	6.3565	5.3633	4.1149	6.0739	6.1118	6.2463	6.0551	5.6269	5.6533	6.1069	0.9745	-0.0373	0.7476	0.1264
Amph	_GDVVLVVPSDSEADQDAGWLVGVK_.3	#N/A	5.0244	3.0448	5.4406	6.0233	5.6308	5.5722	3.2151	5.4104	5.1451	5.0883	1.0865	0.1197	0.5625	0.2499
Amph	_ADETKDEQFEEYVQNFK_.3	#N/A	6.2247	6.3766	6.4637	7.4431	6.1906	6.4726	6.5270	6.3428	6.0962	7.0843	1.0025	0.0035	0.9572	0.0190
Amph	_LVDGSLLTLDTYLGQFPDIK_.2	#N/A	5.5104	5.7357	6.0361	6.0803	5.5901	5.3874	5.7269	6.0355	5.0395	5.7754	1.0140	0.0201	0.7308	0.1362
Amph	_AFSIQGAPSDS[Phospho (STY)]GPLR_.2	Q7TQF7_S252_M1_Amph	9.0457	9.8295	7.3561	11.0647	9.3757	9.9094	10.0120	7.2216	9.9237	9.9486	1.0166	0.0237	0.8572	0.0669
Amph	_TPS[Phospho (STY)]PPEEPSPLPSPTASPNHTLAPASPAPVRPR_.4	Q7TQF7_S262_M1_Amph	3.6409	3.6996	3.6681	10.3433	3.8005	3.7721	4.0810	9.3402	4.0021	3.8926	0.9046	-0.1446	0.7776	0.1093
Amph	_VETLHDFEAANSDELNLQR_.3	#N/A	5.8900	5.4750	6.5007	6.7519	6.5113	6.3901	5.7631	6.2150	5.9768	6.0992	1.0398	0.0564	0.3742	0.4269
Amph	_LTESLHEVYEPDWYGREDVK_.3	#N/A	3.3010	4.4719	3.9831	4.4006	4.5653	4.2656	4.1153	3.7601	4.1591	4.5764	1.0029	0.0041	0.9665	0.0148
Amph	_LYEVM[Oxidation (M)]TK_.2	#N/A	3.9272	0.9743	3.3437	3.7470	3.6170	4.0597	4.0988	4.1939	3.4770	3.9368	0.8349	-0.2604	0.3136	0.5037
Amph	_C[Carbamidomethyl (C)]DVLWEDFHQK_.3	#N/A	4.1436	3.3991	3.8773	3.4211	3.2874	3.3372	3.5694	3.5801	3.8791	3.0984	1.0130	0.0186	0.8393	0.0761
Plekha6	_TKS[Phospho (STY)]PAEEELTPSAVVR_.2	#N/A	7.1217	8.3188	8.9306	8.7971	7.8026	8.2381	7.8660	8.2885	8.3136	7.1264	1.0383	0.0543	0.4759	0.3225
Plekha6	_DPSQTS[Phospho (STY)]PLGTPR_.2	#N/A	8.1169	7.9612	8.2310	8.6469	7.4916	8.1360	8.6292	7.8750	7.9856	8.4062	0.9846	-0.0224	0.6082	0.2160
Plekha6	_SHSVPRS[Phospho (STY)]PSQGSYSR_.3	#N/A	3.2436	4.0248	6.3963	4.2445	4.9245	4.0631	4.1566	3.3711	4.9111	4.4786	1.0599	0.0840	0.6879	0.1625
Plekha6	_S[Phospho (STY)]PSQGSYSR_.2	#N/A	3.7552	3.8557	3.9605	3.7566	3.6992	3.5603	5.2563	3.9431	3.8134	3.5779	0.9076	-0.1398	0.2463	0.6086
Plekha6	_ANGLPSGPETAS[Phospho (STY)]EPGSPYPDGPR_.2	Q7TQG1_S247_M1_Plekha6	3.9904	5.9378	3.1763	3.6790	3.1161	3.1126	3.4734	5.5109	4.0881	3.6460	0.9176	-0.1240	0.6190	0.2083
Plekha6	_QPVFYDELDAASGS[Phospho (STY)]LRR_.3	Q7TQG1_S443_M1_Plekha6	3.4784	6.8480	4.1652	6.9314	4.1049	3.6246	4.3825	3.4506	6.1239	3.1340	1.1371	0.1854	0.5633	0.2492
Plekha6	_MVENSS[Phospho (STY)]PR_.2	Q7TQG1_S574_M1_Plekha6	5.7640	4.1042	4.5824	6.3483	4.3292	4.1330	4.2744	3.8978	3.1740	3.8035	1.2876	0.3647	0.0678	1.1687
Plekha6	_KDPSQTS[Phospho (STY)]PLGTPR_.2	#N/A	8.0956	8.3049	8.6268	8.8979	7.9816	3.6772	8.5447	8.1703	8.1922	8.1782	0.9185	-0.1226	0.5184	0.2853
Plekha6	_SISS[Phospho (STY)]PKYDYLGDR_.2	Q7TQG1_S506_M1_Plekha6	3.7848	3.0892	4.4716	3.4735	2.9105	3.3181	6.5074	3.1442	4.2936	6.0707	0.7010	-0.5124	0.0597	1.2243
Plekha6	_QDGTVWIPS[Phospho (STY)]PSR_.2	Q7TQG1_S426_M1_Plekha6	7.2194	3.7622	3.6438	4.1464	3.5835	8.5908	3.8119	4.0941	8.4548	8.4277	0.8323	-0.2649	0.5115	0.2911
Plekha6	_IYS[Phospho (STY)]PVRS[Phospho (STY)]PSAR_.2	Q7TQG1_S472_M2_Plekha6	9.2685	9.3023	3.1757	9.6660	8.5563	9.4149	9.4761	8.9484	8.9397	9.2040	0.9003	-0.1515	0.4972	0.3035
Plekha6	_AYVPLES[Phospho (STY)]PPTVPPLPNESR_.2	#N/A	3.8759	3.8164	8.8294	9.6709	3.6378	7.7529	3.6591	8.9335	7.4273	4.2261	1.0334	0.0474	0.9104	0.0408
Plekha6	_GVPPPEDLRS[Phospho (STY)]PSR_.2	Q7TQG1_S336_M1_Plekha6	3.7977	3.1021	3.9740	3.5177	2.9234	3.3052	3.6660	3.4834	3.7825	3.1950	0.9731	-0.0394	0.6902	0.1610
Plekha6	_S[Phospho (STY)]LQLPASPAPEPSTRPAYK_.3	Q7TQG1_S967_M1_Plekha6	3.9508	3.4286	3.6844	6.5938	3.0945	3.5412	3.3765	3.7840	4.0831	3.7813	1.0779	0.1082	0.6703	0.1737
Atxn2l	_LQPSSSPET[Phospho (STY)]GLDPFPSR_.2	Q7TQH0_T565_M1_Atxn2l	3.6259	4.0727	3.9041	8.3754	3.6798	7.5605	7.7075	3.7472	3.8351	3.7442	1.0934	0.1289	0.7504	0.1247
Atxn2l	_EVDGLLTSDPM[Oxidation (M)]GS[Phospho (STY)]PVSSK_.2	#N/A	9.0221	8.8391	9.4142	9.3295	8.3463	8.9919	9.3174	8.7714	8.8752	8.9143	1.0023	0.0034	0.9255	0.0336
Atxn2l	_EKEVDGLLTSDPM[Oxidation (M)]GS[Phospho (STY)]PVSSK_.3	#N/A	3.4980	4.5377	5.4553	5.9589	4.3540	5.1671	4.4625	4.9226	5.0972	5.0221	0.9902	-0.0141	0.9206	0.0359
Atxn2l	_GPPQS[Phospho (STY)]PVFEGVYNNSR_.2	#N/A	9.3574	9.7983	9.9484	10.6947	8.9706	9.8187	9.9102	9.7297	9.6197	9.5960	1.0052	0.0075	0.8709	0.0600
Atxn2l	_QGSGRES[Phospho (STY)]PSLVSR_.2	#N/A	3.9962	3.2875	3.3674	6.0817	3.2461	5.4260	3.4538	3.9518	5.2422	4.7574	0.9731	-0.0394	0.8714	0.0598
Atxn2l	_ES[Phospho (STY)]PSLVSR_.2	#N/A	3.0221	4.2040	3.7089	4.2116	4.0923	4.0331	3.9263	3.9069	3.5555	4.1786	0.9966	-0.0049	0.9602	0.0176
Otub1	_IQQEIAVQNPLVSER_.2	#N/A	7.9084	7.9532	8.0000	8.3725	8.1971	8.0918	7.8074	7.8808	8.2101	7.8959	1.0174	0.0249	0.2562	0.5914
Otub1	_LLTSGYLQR_.2	#N/A	7.6999	8.2321	8.0342	7.8826	7.8307	7.4345	7.7605	7.8979	7.8907	7.4422	1.0135	0.0193	0.5405	0.2672
Otub1	_EYAEDDNIYQQK_.2	#N/A	7.6329	7.7391	7.7112	7.7000	7.3770	7.5990	7.3511	7.3302	7.5428	7.2632	1.0346	0.0490	0.0152	1.8193
Otub1	_FFEHFIEGGR_.3	#N/A	6.8552	7.0158	7.4108	6.6945	6.5383	6.9462	7.0918	6.9496	6.8201	6.8516	0.9974	-0.0038	0.9128	0.0396
Otub1	_LELSVLYK_.2	#N/A	5.1455	5.5401	4.7370	5.2986	5.1926	5.0931	3.9247	5.1758	5.3766	5.4801	1.0358	0.0507	0.5867	0.2316
Otub1	_QEPLGSDS[Phospho (STY)]EGVNC[Carbamidomethyl (C)]LAYDEAIM[Oxidation (M)]AQQDR_.3	#N/A	3.8997	5.4346	3.4233	4.2505	3.5950	4.0142	3.6059	5.7834	3.9073	5.2727	0.8838	-0.1782	0.3582	0.4459
Otub1	_EFC[Carbamidomethyl (C)]QQEVEPMC[Carbamidomethyl (C)]K_.2	#N/A	5.8826	4.3711	3.3485	6.8658	4.2594	3.8660	3.7592	4.0740	3.7226	4.7969	1.1657	0.2212	0.3669	0.4355
Otub1	_AFGFSHLEALLDDSKELQR_.3	#N/A	3.8714	3.1289	3.6051	3.7880	3.0151	3.6095	3.5026	3.9279	3.4204	3.9718	0.9453	-0.0811	0.3661	0.4364
Otub1	_GEGGTTNPHVFPEGSEPK_.3	#N/A	2.9282	7.3782	3.6150	8.6753	3.5547	4.1748	8.1719	7.8514	7.9891	4.0846	0.7196	-0.4748	0.2079	0.6821
Abtb2	_SPTVDS[Phospho (STY)]GDIR_.2	Q7TQI7_S444_M1_Abtb2	2.9473	4.3951	4.1818	3.7256	4.2165	3.5130	3.8738	6.0688	3.5747	3.4028	0.9054	-0.1434	0.4981	0.3027
Ccdc93	_QSELSAEES[Phospho (STY)]PEKLGTSQLHQR_.3	#N/A	7.2679	7.1857	7.3056	8.1195	6.8553	7.8263	7.7679	7.7889	7.6084	7.4233	0.9712	-0.0422	0.3941	0.4044
Ccdc93	_QSELSAEES[Phospho (STY)]PEK_.2	#N/A	6.0702	3.0726	4.4550	6.0957	2.8939	5.8815	3.6956	3.4539	5.3530	5.6191	1.0473	0.0667	0.8138	0.0895
Prrc2a	_KGTS[Phospho (STY)]PGSELPPPK_.2	#N/A	4.1184	3.2761	3.6095	3.6509	3.1644	3.4724	3.3656	8.3959	3.9337	3.4587	0.7411	-0.4323	0.2375	0.6244
Prrc2a	_SDS[Phospho (STY)]GGSSSEPFER_.2	#N/A	3.8467	9.0752	9.4620	9.8279	8.3040	9.1877	9.2958	8.8835	8.7190	9.2355	0.9170	-0.1250	0.5308	0.2751
Prrc2a	_GTS[Phospho (STY)]PGSELPPPK_.2	#N/A	3.4559	9.4251	3.1896	4.2342	4.1005	4.0249	4.3825	4.2678	3.0660	3.9115	1.2128	0.2784	0.5124	0.2904
Prrc2a	_EGPEPPEEVPPPTT[Phospho (STY)]PPAPK_.2	Q7TSC1_T609_M1_Prrc2a	3.8702	3.1257	3.6039	3.1477	3.0140	3.6106	3.5038	9.3075	4.1525	4.3258	0.6379	-0.6485	0.1105	0.9566
Prrc2a	_S[Phospho (STY)]EGSEYEEIPKR_.2	#N/A	3.5612	3.7812	3.2949	4.3395	4.2057	3.9197	4.4877	6.9019	2.9608	4.0167	0.8385	-0.2541	0.3130	0.5044
Prrc2a	_ETPPGGNLS[Phospho (STY)]PAPR_.2	Q7TSC1_S1002_M1_Prrc2a	11.2496	11.5404	11.7724	11.8111	9.9302	11.3936	10.9184	10.8339	10.7529	11.4718	1.0263	0.0374	0.4672	0.3305
Prrc2a	_KQS[Phospho (STY)]SSEISLAVER_.2	Q7TSC1_S454_M1_Prrc2a	3.2160	3.9722	4.4505	4.4434	3.8605	4.2649	4.1581	3.6751	3.3237	6.1809	0.9308	-0.1034	0.6061	0.2174
Prrc2a	_TASETRS[Phospho (STY)]EGSEYEEIPKR_.3	#N/A	5.9386	3.5404	3.8358	3.8477	6.5365	5.1488	3.7790	4.0401	6.1369	3.6881	1.0900	0.1243	0.6276	0.2023
Prrc2a	_ERS[Phospho (STY)]DS[Phospho (STY)]GGSSSEPFER_.2	Q7TSC1_S759_M2_Prrc2a	4.6214	7.8260	5.3816	5.0613	6.5381	4.8679	5.0163	7.3247	4.4760	3.6809	1.1155	0.1576	0.5213	0.2829
Prrc2a	_TAS[Phospho (STY)]ETRS[Phospho (STY)]EGSEYEEIPK_.2	#N/A	3.8486	3.4906	4.1872	3.8934	3.4551	4.2010	3.7342	3.4361	3.8126	3.1081	1.0918	0.1267	0.1590	0.7985
Prrc2a	_FS[Phospho (STY)]REEFPTLQAAGDQDK_.3	Q7TSC1_S166_M1_Prrc2a	3.0645	3.8697	3.7513	4.2540	3.6911	4.0384	3.9687	4.7357	3.5131	4.2210	0.9194	-0.1213	0.2841	0.5465
Prrc2a	_S[Phospho (STY)]EGSEYEEIPK_.2	#N/A	3.4567	4.2130	3.1904	4.2026	4.1013	4.0241	3.9173	4.2670	3.5645	5.0469	0.9204	-0.1197	0.3465	0.4603
Prrc2a	_GSETGSETHES[Phospho (STY)]DLAPSDK_.2	Q7TSC1_S1113_M1_Prrc2a	4.0321	3.1941	3.2180	3.7207	3.6201	3.5422	3.4354	4.3978	4.0464	3.6877	0.9133	-0.1308	0.1841	0.7351
Prrc2a	_LKFS[Phospho (STY)]DEEDGRDS[Phospho (STY)]DEEGAEGHK_.4	#N/A	3.1872	4.1729	3.8740	4.1002	3.8137	3.9328	4.0306	3.7588	3.4428	3.7035	1.0302	0.0430	0.5930	0.2269
Lrrc75a	_AS[Phospho (STY)]PGAAPGPR_.2	#N/A	9.3989	8.9517	9.3058	9.6444	8.6067	9.3641	9.4874	9.2656	9.4447	9.4766	0.9781	-0.0320	0.3171	0.4988
Lrrc75a	_EGTDQQDPIGS[Phospho (STY)]PVTPAR_.2	Q7TSF4_S322_M1_Lrrc75a	8.1080	8.0652	8.0542	8.6608	7.0247	8.0669	8.5523	8.1477	8.3639	8.1807	0.9622	-0.0557	0.2955	0.5295
Lrrc75a	_GSLAERAS[Phospho (STY)]PGAAPGPR_.3	#N/A	3.4148	3.8114	4.1016	4.6043	4.0413	4.7284	4.3189	3.5142	3.1628	3.0704	1.1707	0.2274	0.1121	0.9505
Ctdp1	_DNS[Phospho (STY)]PAVFPDR_.2	Q7TSG2_S730_M1_Ctdp1	6.9723	6.5483	6.2225	6.9268	3.7411	6.8726	6.8519	6.1088	6.6984	6.8026	0.9393	-0.0903	0.5536	0.2568
Phkb	_[Acetyl (Protein N-term)]ANS[Phospho (STY)]PDAAFSSPALLR_.2	Q7TSH2_S4_M1_Phkb	6.7279	6.9048	3.6602	7.3063	3.0702	3.5544	7.6647	4.3856	7.1952	7.0876	0.7905	-0.3392	0.2692	0.5699
Phkb	_QSS[Phospho (STY)]TADAPEAQHEPGITITEWK_.3	Q7TSH2_S693_M1_Phkb	4.0518	3.3562	3.2378	3.7405	3.1775	3.0511	3.4119	4.3780	4.0266	3.7075	0.8853	-0.1758	0.1193	0.9234
Kcnf1	_LSHS[Phospho (STY)]DTFIPLLTEEK_.2	#N/A	3.9803	3.1867	4.0472	3.4528	3.1716	3.2157	3.6011	6.0450	3.8671	3.7534	0.8129	-0.2988	0.1463	0.8348
Tmem94	_LPS[Phospho (STY)]AETLK_.2	Q7TSH8_S664_M1_Tmem94	6.6626	4.0256	3.9072	4.4098	3.8469	3.8826	4.2434	5.8916	3.3572	3.4232	1.0537	0.0754	0.7638	0.1170
Tmem94	_VEPPHSS[Phospho (STY)]HEDLTDDLSTR_.3	#N/A	8.3723	7.2322	9.0540	7.5950	6.1182	6.8259	6.8243	9.4128	7.5726	7.5094	0.9621	-0.0558	0.6810	0.1669
Ppp6r1	_NTVDLVNTHHLHSSS[Phospho (STY)]DDEDDRLK_.4	Q7TSI3_S531_M1_Ppp6r1	3.4708	4.6160	3.9536	3.6069	3.5227	3.2092	3.9798	3.7499	3.5765	3.4714	1.0096	0.0138	0.8981	0.0467
Ppp6r1	_EQM[Oxidation (M)]IQGQDS[Phospho (STY)]PEPDQLLATLEK_.3	#N/A	3.9543	3.9404	3.6880	3.8223	3.7786	3.6438	3.5370	3.6433	3.6134	3.6601	1.0529	0.0744	0.0236	1.6267
Ppp6r1	_EADMSSIQIPSS[Phospho (STY)]PPAHGSPQLR_.3	Q7TSI3_S739_M1_Ppp6r1	4.0987	3.1274	3.2847	3.7874	3.2244	3.0042	3.5020	4.3311	3.9797	3.7544	0.8791	-0.1860	0.1060	0.9745
Map6	_ATGPAPGPS[Phospho (STY)]VDRETVAAPGR_.3	#N/A	8.0125	8.0200	8.5544	8.5812	7.7293	7.9323	7.8742	8.0812	7.9588	8.0895	1.0172	0.0246	0.4737	0.3245
Map6	_SEYQPSDAPFER_.2	#N/A	9.0920	8.9131	9.6344	9.2570	9.0026	9.0443	9.0356	8.6593	8.7247	8.8855	1.0375	0.0531	0.0565	1.2483
Map6	_DQSFPTPAPR_.2	#N/A	9.0697	8.9721	9.2150	9.1135	9.3987	8.9267	9.0414	8.7139	8.9131	9.0969	1.0195	0.0279	0.1532	0.8148
Map6	_DQVPVVPGSLK_.2	#N/A	8.4675	8.4867	8.3771	8.7434	8.7297	8.5200	8.6230	8.1814	8.3203	8.6060	1.0144	0.0206	0.3215	0.4928
Map6	_KDPGPVIPEPEKDR_.3	#N/A	8.4153	8.3508	8.2336	8.4361	8.4417	8.4043	8.3116	7.9887	8.1891	8.5225	1.0154	0.0221	0.2262	0.6454
Map6	_AVADALNR_.2	#N/A	7.5597	7.9398	6.9517	7.8953	8.3619	7.6148	7.3423	7.8325	7.6053	7.7523	1.0115	0.0164	0.7414	0.1299
Map6	_ADIAVPLVFTK_.2	#N/A	7.4145	7.8685	7.6932	7.9106	7.8147	7.6858	7.5786	7.7254	7.6083	7.8701	1.0046	0.0067	0.7447	0.1280

Map6	_NQGPVIPEPVK_.2	#N/A	7.8135	7.9248	7.4157	7.8180	8.1573	7.5436	8.0198	7.5687	7.4244	7.9028	1.0065	0.0093	0.7825	0.1065
Map6	_DQGPTAPDPLK_.2	#N/A	7.6792	7.4458	7.4039	8.0501	7.7798	7.5715	7.5560	7.2357	7.3033	7.7535	1.0259	0.0368	0.2460	0.6090
Map6	_NQGLAGPELVK_.2	#N/A	7.5822	7.7160	6.9927	7.5798	7.9031	7.3899	7.4956	7.3460	7.3585	7.5501	1.0121	0.0173	0.5997	0.2221
Map6	_NKDSVPLAPAK_.2	#N/A	6.5892	7.1419	7.0270	7.1091	7.4855	7.1127	6.7324	6.9238	6.8598	6.7766	1.0373	0.0528	0.1297	0.8870
Map6	_DQGAVLLGPVK_.2	#N/A	6.9950	6.5387	6.4543	7.1783	7.2107	6.9257	6.7090	6.5235	6.6578	6.5859	1.0400	0.0566	0.1501	0.8237
Map6	_NQSPVVPASTK_.2	#N/A	6.9971	6.5021	6.2906	6.9055	6.7896	6.6609	6.6834	6.3025	6.3032	6.6871	1.0303	0.0431	0.2539	0.5953
Map6	_GHDSVFVAPVK_.2	#N/A	6.4538	6.5879	5.9874	6.8613	6.7977	6.2857	6.4413	6.2499	6.1099	6.5398	1.0253	0.0361	0.4082	0.3891
Map6	_DTGTDTTAPR_.2	#N/A	6.4583	6.1604	6.1112	6.6967	6.7890	6.3756	6.0853	6.2932	6.2370	6.3058	1.0323	0.0459	0.2046	0.6891
Map6	_DKQVVSGQAAK_.2	#N/A	7.0590	5.9413	6.8051	6.3399	6.3035	6.7550	7.1153	6.3558	6.2800	7.0786	0.9741	-0.0378	0.5455	0.2632
Map6	_FWNQLDK_.2	#N/A	5.8965	5.9817	5.5584	5.5781	5.8403	6.0845	5.4052	6.1580	5.8303	6.3241	0.9821	-0.0261	0.5990	0.2226
Map6	_DPEGAGGAGVLAAGK_.2	#N/A	3.3218	3.7667	4.4118	4.2441	3.6117	3.6165	3.2156	2.8341	3.7707	4.9631	1.0360	0.0510	0.7619	0.1181
Map6	_AVAIETQPAQGESDAVAR_.2	#N/A	8.1969	8.4308	8.8193	8.6151	8.4171	8.5380	8.6208	8.2284	8.3026	8.5789	1.0083	0.0120	0.6100	0.2147
Map6	_ATGPAPGPS[Phospho (STY)]VDR_.2	#N/A	7.8183	7.5103	7.5553	8.3540	7.8224	7.7534	7.6341	7.4321	7.6628	7.8213	1.0216	0.0308	0.3507	0.4551
Map6	_GQS[Phospho (STY)]PTAPGPTKDQGAVLLGPVK_.3	#N/A	2.9076	9.5169	3.5944	10.3673	3.5341	4.1954	3.8117	7.0493	3.6700	7.0851	1.0522	0.0734	0.8834	0.0538
Map6	_NQS[Phospho (STY)]PVVLGPIK_.2	Q7TSJ2_S832_M1_Map6	7.4620	6.9036	6.4039	7.7930	5.3254	7.0197	6.9213	6.7613	6.5859	7.2355	0.9916	-0.0122	0.9024	0.0446
Map6	_VQDHIASELLK_.2	#N/A	5.3603	5.5175	5.8573	5.6604	5.6660	5.0659	5.1955	5.0087	5.4981	5.6392	1.0348	0.0494	0.3354	0.4744
Map6	_AGPAWM[Oxidation (M)]VTR_.2	#N/A	8.0314	8.2886	8.3181	8.3580	8.0664	7.6627	7.7683	8.0443	7.8075	8.0024	1.0272	0.0388	0.1748	0.7574
Map6	_AQS[Phospho (STY)]PLLPEPLKNQSPVVPASTK_.3	#N/A	4.6977	8.0236	7.2094	9.3418	5.6562	6.5517	6.5435	7.3712	6.5952	5.6851	1.0557	0.0782	0.6933	0.1591
Map6	_RPQS[Phospho (STY)]QERGPM[Oxidation (M)]QLSADAR_.3	#N/A	5.8560	3.9259	3.6843	3.8029	4.9045	5.5589	5.3460	5.6734	5.3067	4.2809	0.8972	-0.1565	0.3557	0.4489
Map6	_GPM[Oxidation (M)]QLS[Phospho (STY)]ADAR_.2	#N/A	6.7032	6.6678	6.3649	6.7874	6.6214	6.8061	6.7305	6.6047	6.6570	6.8419	0.9925	-0.0108	0.5982	0.2232
Map6	_S[Phospho (STY)]LYSEPFKEC[Carbamidomethyl (C)]PK_.2	Q7TSJ2_S491_M1_Map6	4.6190	3.9018	4.3801	3.9239	3.7901	4.3353	4.0721	3.0773	3.3764	3.6011	1.1774	0.2356	0.0294	1.5314
Map6	_DQVPVVPGSLKGQS[Phospho (STY)]PTAPGPTK_.3	#N/A	3.7056	9.7465	10.2279	10.3690	7.9383	8.4924	8.4885	9.1941	8.6431	3.4419	1.1305	0.1770	0.5745	0.2407
Map6	_EPHKDQGSVAPGLPK_.3	#N/A	5.7301	3.7614	3.6430	5.4604	5.5304	5.7175	4.5076	4.4565	5.7549	5.2852	0.9946	-0.0079	0.9627	0.0165
Map6	_RSEGHEQT[Phospho (STY)]PAAHAQGTGPEGGK_.3	Q7TSJ2_T392_M1_Map6	4.5445	4.1982	4.8630	5.1725	4.0726	4.3350	4.0075	4.5981	4.9607	4.0635	1.0280	0.0399	0.6704	0.1737
Map6	_AKPQYKPPDDK_.3	#N/A	3.5167	3.8257	4.8489	3.9807	5.9862	5.4848	4.7986	4.4465	5.1747	4.9642	0.9507	-0.0729	0.6587	0.1813
Map6	_EEVASTVSSSYR_.2	#N/A	6.3271	5.8121	4.1542	3.4309	6.2587	6.2647	5.8313	5.5982	5.6069	6.2904	0.9216	-0.1178	0.5055	0.2963
Map6	_GQS[Phospho (STY)]PTAPGPTK_.2	#N/A	13.8412	14.0523	14.2605	14.4114	13.6436	14.0573	14.0636	13.8193	13.7839	14.0530	1.0082	0.0118	0.4763	0.3221
Map6	_TEGHEETPLPPAQSQTQEGGPAAGK_.3	#N/A	5.7572	6.3700	3.6893	6.2680	6.3252	3.0206	3.3814	6.0945	4.0671	5.8332	1.0814	0.1129	0.6808	0.1670
Map6	_TTEGPSATKPDDKEQSK_.3	#N/A	3.8753	3.8313	3.6939	2.9597	4.0653	4.4961	4.0479	3.7611	4.6892	4.2747	0.9111	-0.1344	0.2501	0.6019
Map6	_YSEATEHPGAPPQPPAPLQPALAPPSR_.3	#N/A	3.1813	3.9865	3.8681	5.8913	5.4307	5.4348	5.1361	4.6424	4.0915	4.3377	1.0176	0.0252	0.8945	0.0484
Map6	_S[Phospho (STY)]EGHEQTPAAHAQGTGPEGGK_.3	#N/A	3.7749	2.8823	3.6708	3.1390	2.9455	3.3130	3.6530	3.2060	3.3518	3.4459	0.9629	-0.0545	0.5508	0.2590
Map6	_GTQLPTVSPSPPVM[Oxidation (M)]IPTVPHTEYIEGS[Phospho (STY)]P_.3	#N/A	3.2639	8.3787	7.4040	10.2442	5.4198	3.3342	5.9984	8.5136	6.4932	3.5028	1.0349	0.0495	0.9004	0.0456
Map6	_GPM[Oxidation (M)]QLS[Phospho (STY)]ADARDPEGAGGAGVLAAGK_.3	#N/A	3.3767	4.1819	4.0635	4.8158	3.0694	3.3287	3.3457	3.4064	2.7151	3.9774	1.1323	0.1793	0.2917	0.5351
Map6	_M[Oxidation (M)]VHETSYSAQFK_.3	#N/A	3.2283	3.4701	3.3409	3.2405	3.9050	3.7833	3.3124	4.4950	4.2802	4.4739	0.8440	-0.2446	0.0411	1.3865
Map6	_GEANKPSAADNK_.2	#N/A	5.1311	4.1014	5.4693	5.3905	4.0100	4.3944	5.4709	5.0744	3.1942	3.0391	1.1323	0.1792	0.3820	0.4179
Map6	_SEGHEEKPLPPAQSQTQEGGPAAGK_.4	#N/A	4.1029	3.4072	3.6688	3.2126	4.4365	3.0001	3.3609	5.0081	4.0876	4.3907	0.8638	-0.2113	0.1782	0.7492
Map6	_SGLGLGAASASTSGSGPADSVM[Oxidation (M)]R_.2	#N/A	3.6846	4.2369	3.9292	4.3031	3.5746	4.2106	3.6213	3.8603	3.7299	3.8084	1.0625	0.0875	0.1874	0.7272
Map6	_RRPQS[Phospho (STY)]QER_.2	#N/A	4.2216	3.0046	3.4075	3.9102	3.3473	4.3822	3.6249	4.2083	3.8569	3.8772	0.9539	-0.0682	0.5568	0.2543
Map6	_AQS[Phospho (STY)]PLLPEPLK_.2	#N/A	3.3060	4.0364	4.5405	12.5943	3.8577	3.8718	4.2326	4.4177	3.2159	4.7714	1.2905	0.3680	0.5291	0.2765
Map6	_NEGHEEKPLPPAQSQTQEGGPAAGK_.4	#N/A	3.7169	2.9724	3.4506	4.4952	2.8607	3.7639	3.1427	4.0068	4.3058	4.1725	0.9069	-0.1409	0.3528	0.4525
Sez6	_S[Phospho (STY)]PLQLPR_.2	Q7TSK2_S952_M1_Sez6	7.9411	6.8378	6.9596	7.5195	6.9303	7.7193	8.0087	7.6554	7.4310	7.7117	0.9502	-0.0737	0.1739	0.7597
Pcdh8	_EERPGAAGGGAS[Phospho (STY)]APGS[Phospho (STY)]PDETAR_.2	Q7TSK3_S798_M2_Pcdh8	3.6773	3.7089	4.0525	4.2954	3.5302	4.1796	3.9075	4.0902	3.6620	3.4365	1.0353	0.0501	0.5121	0.2907
Pcdh8	_DSGKGDSDFNDS[Phospho (STY)]DSDISGDALKK_.3	Q7TSK3_S926_M1_Pcdh8	3.3427	4.1479	4.0295	4.5322	3.9693	3.7602	4.1210	3.5863	3.2349	2.9984	1.1373	0.1856	0.1255	0.9012
Pgm2	_[Acetyl (Protein N-term)]AAAT[Phospho (STY)]PTETPAPEGSGLGMDAR_.2	Q7TSV4_T5_M1_Pgm2	3.7343	3.0387	4.4211	3.5812	2.8600	3.3686	3.7294	3.1947	4.3441	3.3900	0.9553	-0.0660	0.6493	0.1875
Atf7ip	_NKQEDLNSEALS[Phospho (STY)]PSITC[Carbamidomethyl (C)]DLSSR_.3	Q7TT18_S112_M1_Atf7ip	3.9767	3.2811	6.3413	6.9144	3.1024	3.1263	3.3800	5.3494	5.8832	3.6323	0.9772	-0.0333	0.9201	0.0362
Atf7ip	_SKS[Phospho (STY)]EDMDSVESKR_.2	#N/A	4.3728	2.9697	4.1064	3.6502	3.5165	3.4377	3.7985	3.3510	3.6500	3.3275	1.0407	0.0576	0.6104	0.2144
Atf7ip	_SKS[Phospho (STY)]EDMDSVESK_.2	#N/A	3.9757	3.2311	3.1810	3.6836	3.1194	3.5052	3.3983	4.4348	4.0834	3.6506	0.8863	-0.1741	0.1143	0.9419
Atf7ip	_EAFLVLS[Phospho (STY)]DEEDLSC[Carbamidomethyl (C)]GKDESEAVAQSK_.3	Q7TT18_S533_M1_Atf7ip	4.0672	3.2752	3.8008	3.3446	3.0966	3.1321	3.4929	3.6566	3.9556	3.0219	0.9776	-0.0326	0.7628	0.1176
Rexo1	_S[Phospho (STY)]LDEGAPQDTPK_.2	Q7TT28_S487_M1_Rexo1	7.7128	3.5708	7.9536	7.6171	6.8942	7.4578	8.0299	8.0294	7.8723	7.4707	0.8748	-0.1930	0.2811	0.5511
Rexo1	_ALS[Phospho (STY)]HAELFGDES[Phospho (STY)]EEEDSSLGAGAPR_.3	Q7TT28_S505_M2_Rexo1	4.0314	3.2868	3.7651	3.6280	3.1751	3.4495	3.3427	2.9897	4.1391	3.5950	1.0112	0.0161	0.8790	0.0560
Elp1	_SSGNSANDLANVAVIDGNR_.2	#N/A	4.8548	5.9661	5.9962	5.5182	3.6329	5.5699	5.9199	5.6789	5.1721	5.5729	0.9410	-0.0877	0.5062	0.2957
Cdc42bpb	_ALGNS[Phospho (STY)]NRDKEIK_.3	#N/A	5.3222	5.0472	5.3852	5.2335	4.0813	5.2034	4.9641	5.2774	5.4770	4.8447	0.9815	-0.0270	0.7360	0.1331
Cdc42bpb	_HSTPSNSSNPSGPPS[Phospho (STY)]PNS[Phospho (STY)]PHR_.3	Q7TT50_S1692_M2_Cdc42bpb	6.8548	6.5669	6.7144	7.0350	6.1203	6.6457	7.3257	6.9959	6.9355	6.6316	0.9547	-0.0669	0.1424	0.8464
Cdc42bpb	_SM[Oxidation (M)]S[Phospho (STY)]DPDQDFDKEPDSDSTK_.2	Q7TT50_S1661_M1_Cdc42bpb	3.9880	3.3544	3.7217	3.2655	3.1757	3.0529	4.4678	3.7357	4.0348	4.4436	0.8216	-0.2835	0.0117	1.9317
Ahcyl1	_VVLLAEGR_.2	#N/A	6.3232	7.5366	6.5479	6.2097	5.9647	6.1883	5.2101	7.0008	6.5177	6.2037	1.0367	0.0520	0.5955	0.2251
Ahcyl1	_[Acetyl (Protein N-term)]S[Phospho (STY)]M[Oxidation (M)]PDAM[Oxidation (M)]PLPGVGEELK_.2	#N/A	7.2602	7.6037	7.8725	7.5534	6.5238	7.3388	7.8960	7.5518	7.5181	7.2595	0.9738	-0.0382	0.4650	0.3325
Ahcyl1	_EIEIAEQDM[Oxidation (M)]SALISLR_.2	#N/A	5.4123	5.6024	6.7721	6.1434	5.4036	6.5738	6.4820	6.1316	6.0666	6.2436	0.9605	-0.0582	0.4565	0.3406
Ahcyl1	_QVDVVITC[Carbamidomethyl (C)]TGNK_.2	#N/A	3.4178	3.8083	4.1046	4.6073	3.6966	4.4288	4.3220	3.5112	3.1598	5.7823	0.9563	-0.0644	0.7315	0.1358
Ahcyl1	_GSSNFC[Carbamidomethyl (C)]VK_.2	#N/A	3.9857	3.2900	3.7477	3.3298	2.4403	3.1173	3.4781	3.6713	3.9704	2.7541	0.9567	-0.0638	0.6725	0.1723
Ahcyl1	_TTDVMFGGK_.2	#N/A	4.7183	3.0254	3.5036	3.8104	2.9137	3.7109	3.1957	3.9538	4.2528	4.2255	0.9249	-0.1126	0.4681	0.3296
Ahcyl1	_NSC[Carbamidomethyl (C)]IVC[Carbamidomethyl (C)]NM[Oxidation (M)]GHSNTEIDVTSLR_.3	#N/A	3.9104	3.0662	4.1125	3.5658	3.7603	3.3533	3.7141	3.4475	3.7466	3.2431	1.0255	0.0363	0.6871	0.1629
Mib1	_VAPAGSAISNAS[Phospho (STY)]GER_.2	Q80SY4_S411_M1_Mib1	4.1208	3.3762	8.7406	3.3982	3.2645	8.5189	3.2532	3.6029	3.9020	3.0755	1.5141	0.5985	0.2269	0.6441
Sytl5	_SYS[Phospho (STY)]LDLDSQNLQSFK_.2	Q80T23_S211_M1_Sytl5	4.9807	4.1963	3.7719	6.0436	3.1819	3.4427	3.4639	5.7841	3.9846	5.7223	0.9010	-0.1504	0.5343	0.2722
Sytl5	_NTSGTPSIAVSGTSLS[Phospho (STY)]SER_.2	Q80T23_S319_M1_Sytl5	5.2415	3.3890	3.2706	3.7733	3.2104	3.0183	3.4880	4.3452	3.9938	3.7403	0.9380	-0.0923	0.6002	0.2217
Sytl5	_DLNS[Phospho (STY)]LEDTEDGVDLVSSR_.2	Q80T23_S358_M1_Sytl5	4.2598	3.5153	3.9935	3.5373	3.4036	3.2210	3.6855	3.4639	3.7629	3.2146	1.0349	0.0495	0.5939	0.2263
Gabbr2	_TSTS[Phospho (STY)]VTSVNQASTSR_.2	Q80T41_S775_M1_Gabbr2	6.7172	6.3781	4.3762	6.5677	5.3487	6.7593	7.1664	4.7838	6.9559	3.7964	1.0615	0.0861	0.6804	0.1672
Gabbr2	_KEDS[Phospho (STY)]KTSTSVTSVNQASTSR_.3	Q80T41_S770_M1_Gabbr2	3.4844	3.8580	3.2181	4.2627	3.6793	4.0502	4.4110	4.2393	3.0375	3.9400	0.9621	-0.0558	0.6454	0.1902
Gabbr2	_ITELDKDLEEVTM[Oxidation (M)]QLQDT[Phospho (STY)]PEK_.3	#N/A	4.5011	4.4412	4.9161	4.8544	4.6641	3.1659	3.9742	6.0859	5.7100	3.3114	0.9273	-0.1088	0.5931	0.2268
Gabbr2	_TC[Carbamidomethyl (C)]KDPIEDINS[Phospho (STY)]PEHIQR_.3	#N/A	8.5063	8.0671	8.8709	8.3912	6.8828	7.9899	8.9245	8.7247	8.1828	8.0706	0.9578	-0.0622	0.3805	0.4197
Gabbr2	_FTQFQDSR_.2	#N/A	3.2400	3.8440	3.9268	4.0755	3.8665	3.8629	5.0686	5.3238	3.2248	4.3965	0.8444	-0.2440	0.1170	0.9318
Gabbr2	_DPIEDINS[Phospho (STY)]PEHIQR_.3	#N/A	3.7946	4.2508	3.4871	4.1132	4.1391	5.2327	4.2011	4.0724	4.1208	5.2662	0.9444	-0.0826	0.5325	0.2737
Gabbr2	_DLEEVTMQLQDT[Phospho (STY)]PEK_.2	#N/A	2.9255	3.7307	3.3454	4.3900	3.5520	4.1775	4.5383	4.1120	4.4111	4.0673	0.8610	-0.2160	0.0738	1.1322
Mtmr12	_QLS[Phospho (STY)]LPLTQSK_.2	Q80TA6_S564_M1_Mtmr12	7.2151	7.3580	7.7355	7.5477	5.3220	6.1517	7.9547	7.5400	7.0270	7.2541	0.8291	-0.2704	0.2200	0.6576
Vat1l	_ISAEGVDIVLDC[Carbamidomethyl (C)]LC[Carbamidomethyl (C)]GDNTGK_.2	#N/A	5.4226	4.9472	5.4934	5.4692	5.0699	4.3508	5.4374	5.1871	4.5870	3.9871	1.0679	0.0948	0.3679	0.4343
Vat1l	_DSVTHLFDR_.2	#N/A	5.7365	6.3336	3.9145	5.6686	5.4784	5.4714	5.2859	5.7036	5.5930	5.5763	0.9809	-0.0278	0.8067	0.0933
Vat1l	_TPLVPGFEC[Carbamidomethyl (C)]SGIVEALGDSVK_.2	#N/A	5.0975	5.2178	4.5634	5.0936	4.4077	5.0520	4.1548	4.5460	4.5830	4.5605	1.0996	0.1370	0.0461	1.3359
Vat1l	_AC[Carbamidomethyl (C)]GLNFIDLM[Oxidation (M)]VR_.2	#N/A	3.0125	5.0364	3.6993	4.5402	4.5934	4.0905	5.3423	5.0108	4.9727	5.1192	0.8143	-0.2964	0.0356	1.4482
Vat1l	_TPTPLM[Oxidation (M)]ANDS[Phospho (STY)]TETSEAGEEEEDHEGDSENKER_.4	#N/A	5.7319	7.2071	3.9876	3.8751	3.7040	7.6163	4.0126	7.3519	3.6479	3.6862	1.1453	0.1957	0.5691	0.2448
Vat1l	_LILDVEK_.2	#N/A	3.5134	4.3186	4.2002	4.3150	4.1400	3.5895	3.9503	4.7442	3.0642	1.9119	1.1741	0.2316	0.2815	0.5505

Vat1l	_ETSKEPAEGGDGSHR_.3	#N/A	2.9798	3.7850	3.6666	4.1693	3.6064	4.1231	4.4839	3.9492	3.5978	4.1363	0.9208	-0.1190	0.2606	0.5840
Sipa1l2	_EYGS[Phospho (STY)]TSSIDR_.2	Q80TE4_S195_M1_Sipa1l2	3.8401	8.1982	8.4302	4.6184	3.3236	8.0711	3.2658	3.8836	4.1827	7.5903	1.2853	0.3621	0.3775	0.4231
Sipa1l2	_TLS[Phospho (STY)]DESVC[Carbamidomethyl (C)]SNR_.2	Q80TE4_S1488_M1_Sipa1l2	8.0064	7.4873	4.0652	7.9758	6.8773	7.8251	8.1103	7.8056	7.7026	7.8807	0.8939	-0.1618	0.3137	0.5035
Sipa1l2	_YTIGDIFVHS[Phospho (STY)]PQR_.2	Q80TE4_S149_M1_Sipa1l2	5.6514	5.5260	3.7721	5.0327	3.7118	4.0177	5.6778	5.6238	4.6745	4.8272	0.8880	-0.1713	0.2771	0.5574
Sipa1l2	_SEGS[Phospho (STY)]PPPEEPEVTEC[Carbamidomethyl (C)]PR_.2	Q80TE4_S1406_M1_Sipa1l2	5.7262	3.6668	6.9178	6.5833	3.5328	6.8440	5.7487	7.2053	7.1798	4.0628	0.9167	-0.1255	0.6253	0.2039
Sipa1l2	_NEFWFS[Phospho (STY)]DGSLSDK_.2	Q80TE4_S1549_M1_Sipa1l2	3.1353	4.2071	4.3698	3.9136	6.2894	3.7010	4.0618	3.0876	3.3867	3.5909	1.2089	0.2736	0.2350	0.6290
Lrrc7	_TPS[Phospho (STY)]PFEDR_.2	Q80TE7_S850_M1_Lrrc7	9.1825	8.5670	9.6347	4.5247	3.7793	9.2011	9.3903	9.1076	3.3872	9.1428	0.9645	-0.0522	0.8797	0.0557
Lrrc7	_GVIAISKS[Phospho (STY)]TER_.2	Q80TE7_S928_M1_Lrrc7	2.9695	8.3674	4.2040	3.1891	7.4495	3.0106	8.5903	8.9321	8.7179	8.5264	0.5597	-0.8372	0.0147	1.8315
Lrrc7	_S[Phospho (STY)]M[Oxidation (M)]DGYPEQFC[Carbamidomethyl (C)]VR_.2	#N/A	13.1192	12.8609	13.2264	13.4588	12.4199	13.0914	13.3961	12.9430	13.1804	13.2621	0.9874	-0.0183	0.4235	0.3732
Lrrc7	_LETTPTTS[Phospho (STY)]PLPER_.2	#N/A	6.7760	6.0281	3.8186	4.0025	3.0757	4.9071	6.7650	3.8721	4.2005	3.8402	1.0211	0.0301	0.9165	0.0379
Lrrc7	_VYNIPLENYASGSDHLGS[Phospho (STY)]HERPDK_.3	#N/A	4.1460	3.1607	3.8797	3.6107	3.0181	3.4159	3.5507	3.5915	3.9353	3.1008	0.9983	-0.0025	0.9819	0.0079
Lrrc7	_TAFPSKLETT[Phospho (STY)]PTTS[Phospho (STY)]PLPERK_.3	#N/A	3.3023	4.0401	4.5368	4.0806	3.8615	3.8680	4.2288	7.2832	3.2197	3.7578	0.8542	-0.2274	0.3940	0.4045
Lrrc7	_SQS[Phospho (STY)]IDEIDVGTYK_.2	#N/A	10.1253	9.8675	10.3704	11.0556	9.8205	10.2087	10.3272	10.3581	10.2190	10.3019	0.9942	-0.0085	0.8007	0.0965
Lrrc7	_KSQS[Phospho (STY)]IDEIDVGTYK_.2	#N/A	5.7615	6.4628	3.7245	7.6497	6.1502	6.3730	6.6492	6.9329	6.6220	5.3346	0.9429	-0.0848	0.6248	0.2042
Lrrc7	_S[Phospho (STY)]VPAHGR_.2	Q80TE7_S801_M1_Lrrc7	6.8191	3.1716	5.6902	6.0356	2.9929	5.5253	6.0273	6.7410	5.9663	6.2818	0.8057	-0.3116	0.1761	0.7543
Lrrc7	_FKKS[Phospho (STY)]QS[Phospho (STY)]IDEIDVGTYK_.3	#N/A	3.8983	7.5747	3.9739	8.8177	8.1837	7.7466	5.7068	8.7215	8.7096	8.9609	0.8348	-0.2605	0.3276	0.4847
Lrrc7	_SYS[Phospho (STY)]TESYGASQTR_.2	#N/A	7.9381	3.3172	7.8557	8.5819	7.2282	7.6898	7.9993	7.6372	7.4775	7.8484	0.9175	-0.1243	0.5313	0.2747
Lrrc7	_STERLS[Phospho (STY)]PLM[Oxidation (M)]K_.2	Q80TE7_S933_M1_Lrrc7	6.6333	6.8820	7.4137	3.7888	3.4213	6.7690	3.7011	3.8260	6.7711	6.8017	1.1030	0.1414	0.6419	0.1925
Lrrc7	_LETT[Phospho (STY)]PTTS[Phospho (STY)]PLPERK_.2	#N/A	4.0188	3.4933	4.2288	6.6597	3.3570	3.7095	6.1642	3.4870	4.2840	4.6066	0.9157	-0.1271	0.6242	0.2047
Lrrc7	_SYS[Phospho (STY)]TESYGASQTRPVSAR_.2	#N/A	5.7220	4.7521	4.4687	5.6071	3.4998	3.8359	6.3979	3.5276	5.0393	3.9110	0.9849	-0.0220	0.9199	0.0362
Lrrc7	_TLAVDENFLPELPR_.2	#N/A	4.0003	5.0936	5.8357	5.5501	5.7179	3.0652	3.4260	5.5384	4.0225	5.3464	1.0641	0.0896	0.6844	0.1647
Lrrc7	_S[Phospho (STY)]REQQPYEGNINK_.2	#N/A	6.6323	6.7163	6.8014	7.0526	5.4073	6.4719	5.9380	6.5275	6.5316	6.5561	1.0196	0.0280	0.7027	0.1532
Lrrc7	_IPS[Phospho (STY)]DYNLGNYGDK_.2	Q80TE7_S1233_M1_Lrrc7	4.3067	3.5622	4.0404	8.8522	6.6867	8.1350	3.7324	8.5151	3.7161	8.4199	0.9729	-0.0397	0.9195	0.0364
Lrrc7	_S[Phospho (STY)]QSVPMLDDEMLM[Oxidation (M)]YGSSK_.2	Q80TE7_S997_M1_Lrrc7	3.7770	3.9810	4.2336	3.7646	3.6838	3.8517	3.9129	3.2839	3.5416	3.3745	1.1003	0.1378	0.0457	1.3404
Lrrc7	_RVS[Phospho (STY)]EPHELPPGDR_.3	Q80TE7_S1132_M1_Lrrc7	4.1327	3.4371	3.3187	3.8213	3.2584	2.9702	3.5360	4.2971	3.9457	3.7883	0.8967	-0.1573	0.1440	0.8417
Pcdh19	_DKES[Phospho (STY)]PSVKR_.2	Q80TF3_S1134_M1_Pcdh19	3.2953	4.1005	3.9821	4.0201	3.9219	3.8076	4.1684	3.6337	3.2801	4.4518	0.9926	-0.0108	0.9128	0.0396
Pcdh19	_SKS[Phospho (STY)]PEHVR_.2	Q80TF3_S983_M1_Pcdh19	3.1500	4.1924	4.3845	3.9283	4.0137	3.7158	4.0766	5.9338	3.3719	3.6056	0.9177	-0.1239	0.5120	0.2907
Pcdh19	_EAGNGC[Carbamidomethyl (C)]EAIS[Phospho (STY)]PVTS[Phospho (STY)]PLHLK_.2	Q80TF3_S1063_M2_Pcdh19	4.1462	3.4017	3.8799	3.4237	3.2900	3.3347	3.5719	3.5775	3.8766	3.1009	1.0135	0.0193	0.8332	0.0792
Erbin	_SES[Phospho (STY)]AENHSYAK_.2	Q80TH2_S1013_M1_Erbin	5.0572	4.7120	5.8189	5.3401	3.9467	3.7827	4.2244	5.7325	5.2193	5.4985	0.9241	-0.1139	0.4403	0.3563
Erbin	_VRQEDENFNSLLQNGVNLNNS[Phospho (STY)]PEEK_.3	Q80TH2_S712_M1_Erbin	3.7294	3.0338	4.4162	3.4180	2.8551	3.3735	3.7343	3.1996	4.3490	4.2532	0.8937	-0.1622	0.2751	0.5605
Erbin	_STEDLS[Phospho (STY)]PQR_.2	Q80TH2_S854_M1_Erbin	7.0525	7.3486	7.8347	7.8837	6.9723	7.3011	7.6177	7.5179	7.0489	7.6557	0.9918	-0.0119	0.7926	0.1009
Gramd1b	_STPAC[Carbamidomethyl (C)]S[Phospho (STY)]PILR_.2	Q80TI0_S21_M1_Gramd1b	3.2113	8.5266	8.9636	8.4005	7.1473	4.2223	8.8884	8.6732	8.7174	8.3829	0.7784	-0.3614	0.1629	0.7880
Gramd1b	_TESTYLAEIHRQS[Phospho (STY)]PK_.3	#N/A	7.3566	7.0823	7.4279	6.8730	5.8938	6.8130	6.7384	7.7024	7.4283	7.0965	0.9539	-0.0680	0.3385	0.4705
Gramd1b	_SSIETKPDAS[Phospho (STY)]PQLPKK_.3	#N/A	5.6379	5.3213	4.1948	3.1982	3.7188	3.0197	4.2741	5.9132	3.0680	5.5464	0.8897	-0.1687	0.5123	0.2905
Gramd1b	_S[Phospho (STY)]RSPTPQNQDGDTMVEK_.3	Q80TI0_S28_M1_Gramd1b	5.0477	3.5993	3.5411	5.2495	3.7209	3.8821	4.1122	3.9889	4.1243	4.1675	1.0184	0.0262	0.8528	0.0691
Gramd1b	_SSIETKPDAS[Phospho (STY)]PQLPK_.2	#N/A	8.7768	8.7469	9.5722	8.9737	7.3584	8.5122	9.2524	8.9468	8.6317	8.5659	0.9782	-0.0317	0.6375	0.1955
Gramd1b	_S[Phospho (STY)]PTPQNQDGDTM[Oxidation (M)]VEK_.2	#N/A	5.5744	3.8648	3.2113	4.2559	4.1222	5.5051	4.4042	4.2461	5.2666	4.9753	0.9363	-0.0949	0.5750	0.2404
Plekhh1	_SC[Carbamidomethyl (C)]DS[Phospho (STY)]DEDYEAGGTGR_.2	Q80TI1_S739_M1_Plekhh1	4.3283	3.5837	4.0620	4.6482	3.4720	3.1526	4.5466	4.1003	4.4360	3.2981	0.9461	-0.0799	0.5624	0.2500
Cadps	_VILHPTPNS[Phospho (STY)]PK_.2	Q80TJ1_S486_M1_Cadps	8.5114	3.2273	8.7839	8.7051	3.0486	8.0754	9.1447	8.7721	8.4865	8.1239	0.7791	-0.3601	0.2204	0.6568
Cadps	_DIVTPVPQEEVK_.2	#N/A	8.0941	7.5314	8.0464	7.2147	7.2906	7.5524	7.9666	7.8025	7.9085	7.6913	0.9719	-0.0412	0.2925	0.5339
Cadps	_LDALQTFIR_.2	#N/A	6.2498	6.1700	6.0991	4.6085	5.5131	5.7206	6.1134	6.5117	6.3445	6.0835	0.9144	-0.1292	0.1375	0.8618
Cadps	_EGDTVIFASDDEQDR_.2	#N/A	6.0541	6.0830	5.9310	6.4492	4.6040	5.5705	6.6528	6.0776	6.1395	5.8498	0.9356	-0.0960	0.2938	0.5320
Cadps	_S[Phospho (STY)]LPEIDGLSK_.2	Q80TJ1_S219_M1_Cadps	9.1023	8.3980	8.5439	8.7564	7.3865	8.5894	9.4375	8.9525	8.9123	8.5460	0.9443	-0.0827	0.1679	0.7749
Cadps	_SLTSNLPNVNLPNVNLPK_.2	#N/A	5.0841	5.5387	4.2891	5.4646	4.6917	5.1720	5.4440	6.0105	5.5234	4.9507	0.9194	-0.1213	0.1754	0.7559
Cadps	_LQGVLDSTLNSK_.2	#N/A	5.0166	5.2623	4.4954	4.8219	4.6142	4.7641	4.8234	5.2078	4.9918	3.4642	1.0448	0.0633	0.5612	0.2508
Cadps	_HSQDVLR_.2	#N/A	5.7936	5.6681	5.7540	7.8824	5.8785	5.3960	6.4410	5.7214	5.6650	5.0934	1.0579	0.0812	0.5348	0.2718
Cadps	_ATLSLLER_.2	#N/A	5.2911	4.1766	4.5222	4.9502	3.5396	4.2410	3.1021	5.0934	5.2244	4.5687	0.9903	-0.0141	0.9321	0.0305
Cadps	_LQKEEEER_.2	#N/A	4.7653	4.8685	5.0747	4.7356	4.9529	4.7076	3.2425	4.5882	4.4492	3.8769	1.2009	0.2641	0.0126	1.9012
Cadps	_EQLYEM[Oxidation (M)]FQNILGIK_.2	#N/A	3.6001	3.9429	3.1565	3.3450	3.0962	3.3623	3.5092	3.6562	3.5496	3.2936	0.9757	-0.0354	0.6316	0.1995
Cadps	_VLLENQITHFR_.2	#N/A	4.6816	5.6262	5.5878	4.6216	4.1125	4.4801	5.2695	5.3803	4.6113	4.2829	0.9930	-0.0102	0.9297	0.0316
Cadps	_LFTESTGVLALEDKELGR_.3	#N/A	3.6187	4.3750	3.3524	4.7088	4.2633	3.8622	5.8177	4.8783	3.7264	4.0076	0.8747	-0.1932	0.2395	0.6206
Cadps	_YVDLM[Oxidation (M)]ESSIAQSIHR_.2	#N/A	4.0039	3.0682	4.0745	3.4045	3.1295	3.2124	3.3254	3.4291	4.0827	3.2850	0.9863	-0.0199	0.8630	0.0640
Cadps	_M[Oxidation (M)]VQSGGC[Carbamidomethyl (C)]SANDSR_.2	#N/A	2.7742	4.1857	4.3677	3.6964	4.0071	3.7339	4.3158	4.1444	3.1546	3.4826	1.0053	0.0076	0.9574	0.0189
Phf8	_DAEYIYPSLES[Phospho (STY)]DDDDPALK_.2	Q80TJ7_S820_M1_Phf8	3.1103	4.1159	3.7971	4.2997	3.7368	4.1213	4.0144	3.8187	3.4673	4.2667	0.9927	-0.0105	0.9144	0.0389
Phf8	_SSGSSSSGLGTVSSS[Phospho (STY)]PASQR_.2	Q80TJ7_S768_M1_Phf8	3.0157	4.3267	4.2503	3.7940	4.1480	3.5815	3.9423	3.2072	3.5062	3.4713	1.0909	0.1255	0.2878	0.5409
Kiaa1107	_S[Phospho (STY)]LDSC[Carbamidomethyl (C)]PSQGLPQEGQVK_.2	Q80TK0_S1051_M1_Kiaa1107	3.2309	7.7574	3.9177	4.4204	3.8575	4.2419	8.0390	3.6981	3.3467	4.3874	0.9390	-0.0908	0.8086	0.0923
Kiaa1107	_SSS[Phospho (STY)]DTSTPEELK_.2	#N/A	7.1004	4.0776	4.2099	3.5044	5.8568	6.8160	7.0327	6.6304	3.3250	7.0075	0.8770	-0.1894	0.5042	0.2974
Kiaa1107	_SAPPTLAQAQGSQGESPHS[Phospho (STY)]VK_.2	Q80TK0_S343_M1_Kiaa1107	3.6028	5.0580	3.6372	4.1398	3.5769	4.9062	4.0263	4.8719	3.6323	4.7523	0.9613	-0.0569	0.6989	0.1556
Kiaa1107	_KQGGGDHQIHS[Phospho (STY)]AS[Phospho (STY)]DDEIPR_.3	#N/A	5.3871	5.6026	3.9571	5.9977	4.7564	5.8927	6.1904	6.0758	5.1448	5.6345	0.9140	-0.1298	0.2920	0.5346
Kiaa1107	_SEDYDAGGS[Phospho (STY)]QDDEGSHDR_.2	#N/A	4.2425	4.1506	3.9226	4.7950	4.0930	4.2988	4.4153	4.0671	4.5580	3.7652	1.0117	0.0167	0.8187	0.0869
Kiaa1107	_QGGGDHQIHS[Phospho (STY)]AS[Phospho (STY)]DDEIPR_.2	#N/A	7.5907	7.3411	4.2916	5.0002	4.2145	4.8348	8.1705	7.5756	4.4062	4.1119	0.9142	-0.1294	0.6593	0.1809
Kiaa1107	_SSS[Phospho (STY)]DTSTPEELKVHEGDLR_.3	#N/A	3.9297	3.3174	4.1930	7.3510	3.0649	3.4212	3.3448	3.4343	4.1266	7.0710	0.9374	-0.0933	0.7989	0.0975
Kiaa1107	_SSVSSRQS[Phospho (STY)]DENVTR_.2	Q80TK0_S353_M1_Kiaa1107	2.9606	3.7169	4.1951	3.7389	3.6052	3.0194	3.8872	3.2623	3.5613	3.4162	1.0022	0.0031	0.9776	0.0099
Ppm1e	_YNC[Carbamidomethyl (C)]PSFLAAALAR_.2	#N/A	5.4356	5.5838	4.4774	5.4730	5.5390	5.0502	5.2161	5.4915	5.6894	5.1390	0.9769	-0.0337	0.6194	0.2081
Ppm1e	_RPWPQHQC[Carbamidomethyl (C)]S[Phospho (STY)]APADLGYEGR_.3	#N/A	8.5628	8.6431	9.3985	10.2320	7.2853	7.7923	8.4656	8.8411	8.8642	7.6075	1.0246	0.0351	0.7343	0.1341
Ppm1e	_VDS[Phospho (STY)]FTDR_.2	Q80TL0_S545_M1_Ppm1e	3.3640	3.8622	4.0508	4.5535	3.9905	3.7390	4.2682	3.5650	3.2136	3.0196	1.1166	0.1591	0.2024	0.6938
Ppm1e	_AVNVS[Phospho (STY)]EESEWTENSFQGGQEDGGDDKETHGEC[Carbamidomethyl (C)]K_.4	Q80TL0_S495_M1_Ppm1e	4.0797	3.3840	3.2656	3.7683	3.2053	3.7258	3.4830	4.3502	3.9988	3.7353	0.9177	-0.1239	0.1974	0.7047
Adcy2	_S[Phospho (STY)]PQHLFR_.2	#N/A	9.2955	8.4442	8.7447	8.8237	7.3033	7.8273	9.3826	9.0308	8.8002	8.5616	0.9399	-0.0894	0.2111	0.6756
Adcy2	_S[Phospho (STY)]PQHLFRPR_.3	#N/A	5.0704	6.8958	6.2888	7.5431	3.2437	4.4179	6.6684	7.1097	5.8585	5.8345	0.8758	-0.1914	0.3867	0.4126
Phf24	_VIPDC[Carbamidomethyl (C)]SLTLEDFVR_.2	#N/A	8.7839	8.5996	8.9350	8.5886	8.7849	8.6831	8.7817	8.4034	8.8400	8.6043	1.0083	0.0119	0.5048	0.2969
Phf24	_ALLPTEQESR_.2	#N/A	8.3974	8.3285	8.3578	8.3129	8.2309	8.3675	8.3951	8.3358	8.4217	8.3009	0.9963	-0.0053	0.4250	0.3716
Phf24	_GVEPEEFDR_.2	#N/A	8.1886	8.0854	8.1065	8.0759	8.0613	7.7603	7.6127	8.0139	8.0485	8.1524	1.0112	0.0161	0.4775	0.3210
Phf24	_QFAALDPEQR_.2	#N/A	7.8252	7.8007	7.5655	7.8981	7.9773	7.6793	7.6209	7.7871	7.6594	7.7509	1.0112	0.0161	0.3219	0.4922
Phf24	_ALTDEQEEQAAR_.2	#N/A	7.3046	7.3548	7.5855	7.3118	7.5432	7.7478	7.4443	7.5108	7.2396	7.6359	1.0023	0.0033	0.8840	0.0535
Phf24	_YVDWPTFLR_.2	#N/A	6.7976	7.0480	6.4859	6.2104	6.5221	7.0582	6.4774	6.8472	6.4473	6.5030	1.0180	0.0258	0.5470	0.2620
Phf24	_ENVIYILAAR_.2	#N/A	6.2375	5.2963	4.3076	5.7925	5.7467	5.2278	5.8808	5.2749	5.7497	5.4746	0.9714	-0.0419	0.6640	0.1778
Phf24	_KLDDDKPPDIC[Carbamidomethyl (C)]LEPR_.3	#N/A	6.8953	6.3973	6.8937	6.8985	6.7959	6.7878	6.2396	6.3452	6.5083	6.5910	1.0556	0.0781	0.0157	1.8044
Phf24	_VFHDGC[Carbamidomethyl (C)]LR_.2	#N/A	6.6458	6.9716	3.7934	6.6250	7.3744	6.0305	6.7728	6.8077	6.5223	6.8656	0.9255	-0.1116	0.4654	0.3322
Phf24	_GSTISEAEC[Carbamidomethyl (C)]HHAR_.3	#N/A	6.8328	6.1957	6.2325	5.9027	7.2077	6.1380	5.9908	5.8430	6.6521	6.2985	1.0358	0.0508	0.4649	0.3326

Phf24	_EAEASAPVVQEESS[Phospho (STY)]INR_.2	Q80TL4_S73_M1_Phf24	6.6100	4.7534	3.7486	7.4984	5.3644	6.2332	6.9859	5.8858	5.9694	6.9441	0.8844	-0.1772	0.3376	0.4716
Phf24	_LTEAPSC[Carbamidomethyl (C)]SVSISHVGPIADSS[Phospho (STY)]PAASSSK_.3	Q80TL4_S348_M1_Phf24	3.7927	3.3752	6.9042	9.0726	3.0147	5.7692	3.3334	3.7305	6.4670	4.0125	1.2133	0.2790	0.5034	0.2981
Phf24	_RGT[Phospho (STY)]VEGSVQEVQEEKEAEASAPVVQEESSINR_.4	#N/A	4.1829	3.1595	3.9166	3.4603	2.9808	3.2478	3.6086	3.5408	6.1093	6.1690	0.7188	-0.4763	0.0627	1.2025
Phf24	_RGT[Phospho (STY)]VEGSVQEVQEEK_.2	#N/A	3.7504	3.5920	3.4841	4.5287	2.8941	3.7305	3.1761	3.4672	4.2724	4.2060	0.9690	-0.0454	0.7425	0.1293
Mon2	_DEQAAS[Phospho (STY)]DPM[Oxidation (M)]DQETVSR_.2	#N/A	3.7597	4.0057	4.5089	4.2052	3.4312	4.1177	4.1627	4.2803	3.5553	3.4548	1.0366	0.0519	0.5902	0.2290
R3hdm2	_PWSS[Phospho (STY)]TDSDGSVR_.2	#N/A	6.0807	2.9750	4.1010	3.6448	2.7964	6.1015	3.7931	3.3564	6.6363	3.3221	1.0015	0.0021	0.9950	0.0022
R3hdm2	_DSS[Phospho (STY)]QEYTDSTGIDLHEFLVNTLK_.3	Q80TM6_S143_M1_R3hdm2	3.2791	4.0354	4.5136	4.0574	3.9237	6.6377	4.2057	4.4446	3.2428	3.7347	1.1282	0.1740	0.4490	0.3477
R3hdm2	_SKS[Phospho (STY)]IEEREEEYQR_.3	#N/A	6.7349	4.1929	7.2237	5.4405	6.6564	7.4465	6.5934	4.8742	6.8254	6.9015	0.9974	-0.0037	0.9831	0.0074
R3hdm2	_ASS[Phospho (STY)]FSGISILTR_.2	Q80TM6_S381_M1_R3hdm2	3.9289	8.5993	3.8428	10.4778	3.0546	6.2094	7.6409	8.4028	7.3760	3.0638	0.9091	-0.1375	0.7460	0.1272
R3hdm2	_SLEPRPWSS[Phospho (STY)]TDSDGSVR_.2	#N/A	3.9778	6.5355	3.6397	7.0788	5.7707	6.7826	7.1399	7.3949	3.6841	7.7809	0.8663	-0.2071	0.4377	0.3588
R3hdm2	_S[Phospho (STY)]IEEREEEYQR_.2	#N/A	3.3491	7.0353	4.5836	8.1256	3.9937	7.5009	7.4148	7.1842	7.4334	3.8047	0.8925	-0.1641	0.5937	0.2265
R3hdm2	_SAS[Phospho (STY)]TDLGTADVVLGR_.2	Q80TM6_S923_M1_R3hdm2	3.7736	3.4526	4.4604	11.3457	3.3409	3.2837	3.1769	3.1554	4.3049	3.4292	1.4056	0.4912	0.4071	0.3903
R3hdm2	_TPS[Phospho (STY)]KEDVEKEGEENGLR_.3	Q80TM6_S37_M1_R3hdm2	3.7662	3.5762	3.4999	4.5445	2.9099	3.7147	3.1919	3.9575	4.2566	4.2217	0.9390	-0.0908	0.4942	0.3061
R3hdm2	_SKS[Phospho (STY)]IEER_.2	#N/A	4.2022	3.0240	3.3882	4.9497	2.9123	3.7123	3.6055	4.8740	3.8762	5.6602	0.8211	-0.2844	0.1762	0.7541
R3hdm2	_LEQEILDFINDNNNQFK_.2	#N/A	4.3032	4.5635	3.6036	6.0407	4.1977	3.9278	5.0075	4.0122	4.5299	4.0732	1.0076	0.0110	0.9448	0.0247
Nisch	_TPS[Phospho (STY)]PEPVDKDFYSEFGDK_.3	#N/A	5.3639	3.6946	3.6384	3.9826	4.4397	3.4900	4.5960	5.0813	4.6985	4.6802	0.8609	-0.2160	0.1105	0.9568
Nisch	_AC[Carbamidomethyl (C)]SDS[Phospho (STY)]LESIPAGQVASDDLR_.2	Q80TM9_S545_M1_Nisch	3.7186	5.0984	3.5356	6.4778	3.8806	4.1636	5.4043	6.2245	3.8093	5.5695	0.8529	-0.2296	0.3028	0.5188
Nav3	_SQS[Phospho (STY)]FNSIDK_.2	Q80TN7_S275_M1_Nav3	4.2042	3.4597	3.9379	5.8282	3.3480	5.3977	3.6299	3.5195	3.8186	3.1589	1.2353	0.3048	0.1592	0.7981
Nav3	_S[Phospho (STY)]LPRPSK_.2	Q80TN7_S1140_M1_Nav3	4.0194	8.2659	9.2099	8.4896	8.4442	8.2718	8.0308	8.1619	9.5866	2.9742	1.0828	0.1148	0.7005	0.1546
Nav3	_KTS[Phospho (STY)]LDGSQNQDDVVLHVSSK_.3	Q80TN7_S1116_M1_Nav3	3.7856	3.0411	3.5193	3.8737	2.9294	3.6952	3.5884	3.9381	4.2371	4.2412	0.8683	-0.2038	0.0569	1.2448
Ubr5	_WLDGAS[Phospho (STY)]FDNER_.2	Q80TP3_S321_M1_Ubr5	4.4425	6.2654	3.7840	6.3851	4.5306	6.0286	6.8640	6.9028	6.8100	6.8483	0.7642	-0.3880	0.0220	1.6575
Cyld	_SELFYTLNGSS[Phospho (STY)]VDSQQSK_.2	Q80TQ2_S359_M1_Cyld	4.0929	5.0730	3.5886	6.4095	3.2280	5.8033	3.5079	6.4310	6.2962	5.4864	0.8654	-0.2086	0.4086	0.3887
Cyld	_VTGSTS[Phospho (STY)]DPGSR_.2	Q80TQ2_S341_M1_Cyld	5.3020	3.5752	4.2144	5.8675	3.4044	5.8640	3.7353	3.2751	3.1789	5.8085	1.1763	0.2343	0.3746	0.4264
Adgrl1	_YYTGTQS[Phospho (STY)]R_.2	Q80TR1_S1141_M1_Adgrl1	7.5157	7.0815	7.7135	7.2710	5.8319	7.2788	7.6847	8.0242	7.4808	7.3851	0.9309	-0.1033	0.1780	0.7497
Adgrl1	_SPPGGTHGS[Phospho (STY)]LK_.2	Q80TR1_S1123_M1_Adgrl1	8.0816	7.6463	7.4127	7.5240	6.2979	7.6927	8.5446	8.2882	7.9118	7.6225	0.9198	-0.1207	0.0991	1.0038
Adgrl1	_PLSS[Phospho (STY)]PPGR_.2	Q80TR1_S1369_M1_Adgrl1	4.1985	3.5028	3.3844	4.6179	3.3242	4.4053	3.2653	3.8842	4.1832	4.2951	0.9996	-0.0005	0.9968	0.0014
Adgrl1	_QTESSFM[Oxidation (M)]AGDINST[Phospho (STY)]PTLNR_.2	#N/A	3.7066	3.7983	3.4403	3.7588	3.6866	3.8035	3.6330	4.0464	3.8838	4.1621	0.9409	-0.0879	0.0758	1.1205
Adgrl1	_RPS[Phospho (STY)]HEGYLAAPSLEGPGPDGDGQM[Oxidation (M)]QLVTSL_.3	Q80TR1_S1439_M1_Adgrl1	3.0725	3.8777	3.7593	4.2619	3.6990	4.0305	4.3913	3.8565	3.5051	4.2289	0.9469	-0.0787	0.4359	0.3606
Adgrl1	_TDTLTEYASWEDYVAAR_.2	#N/A	3.7628	3.0672	4.4496	3.4515	2.8885	3.3401	3.1661	3.1662	4.3156	3.4185	0.9934	-0.0096	0.9496	0.0224
Adgrl1	_SVYVDDDSEAAGNRVDYAFNTNANR_.3	#N/A	3.7381	3.6043	3.4718	4.5164	2.8818	4.3038	3.1638	3.9856	4.2847	4.1936	0.9605	-0.0581	0.6821	0.1662
Adgrl3	_GPAHLVTS[Phospho (STY)]L_.2	Q80TS3_S1536_M1_Adgrl3	3.3462	4.2429	3.9226	4.1245	4.0991	5.4538	4.2728	3.7451	3.2015	3.8018	1.1179	0.1608	0.2929	0.5332
Adgrl3	_S[Phospho (STY)]MPNLGSR_.2	Q80TS3_S1485_M1_Adgrl3	6.5371	3.2037	3.8724	6.1620	3.2824	3.2036	4.1275	3.5850	3.8841	3.0934	1.1918	0.2531	0.4109	0.3863
Adgrl3	_GSSDGFIVPPNKDGAS[Phospho (STY)]PEGTSK_.3	#N/A	5.6635	3.4783	3.7211	3.9817	5.4132	4.4013	6.1702	6.5777	6.3369	5.8248	0.7135	-0.4870	0.0058	2.2364
Adgrl3	_STESSIGS[Phospho (STY)]GK_.2	Q80TS3_S1222_M1_Adgrl3	3.3344	4.1396	4.0212	4.5239	3.9610	3.7685	4.1293	3.5946	3.2432	2.9900	1.1344	0.1819	0.1334	0.8749
Ddn	_S[Phospho (STY)]PLGQPRPEPR_.2	#N/A	8.0869	7.6348	8.3577	8.0980	7.2176	7.9097	8.4328	8.1864	7.7108	8.1959	0.9696	-0.0446	0.3302	0.4812
Ddn	_LRPGS[Phospho (STY)]PEPPPR_.2	Q80TS7_S77_M1_Ddn	8.9992	9.0995	9.7290	9.0195	8.4424	9.1177	9.4404	9.4070	9.0739	9.0441	0.9812	-0.0273	0.4631	0.3343
Ddn	_RS[Phospho (STY)]PLGQPRPEPR_.3	#N/A	3.0202	4.2060	3.7070	4.2096	4.0943	4.0312	3.0950	3.8990	3.2899	4.1766	1.0727	0.1013	0.4186	0.3782
Baiap3	_SGS[Phospho (STY)]PAPAEPVDPNR_.2	Q80TT2_S78_M1_Baiap3	11.9519	12.1811	12.4037	11.9665	10.1151	11.7077	12.4319	12.0936	11.4902	11.5234	0.9862	-0.0200	0.7290	0.1373
Cul9	_VGLQS[Phospho (STY)]PSVETR_.2	Q80TT8_S1775_M1_Cul9	8.7653	8.2501	8.7412	3.5705	6.7212	7.8575	8.9101	8.5124	8.1668	8.3395	0.8627	-0.2131	0.2867	0.5426
Cul9	_AVLETPSTQGQDGS[Phospho (STY)]PESLIR_.2	Q80TT8_S284_M1_Cul9	4.2768	3.5323	4.3807	3.3825	3.4205	3.2041	3.0972	3.2351	4.3845	5.4306	0.9164	-0.1259	0.5205	0.2836
Clasp1	_NSSNTGVGS[Phospho (STY)]PSNTIGR_.2	Q80TV8_S1088_M1_Clasp1	10.5553	10.6244	10.9136	10.6848	4.1015	10.3897	11.0536	10.9594	10.5415	10.2620	0.8917	-0.1654	0.4225	0.3741
Clasp1	_S[Phospho (STY)]QEDLNEPIKR_.2	#N/A	8.1806	7.5424	7.7449	7.6274	5.6370	7.5159	7.9108	7.5494	7.4234	7.7356	0.9634	-0.0538	0.5590	0.2526
Clasp1	_FSFRS[Phospho (STY)]QEDLNEPIKR_.3	#N/A	3.5074	4.3126	7.9221	7.3179	4.1339	3.5955	3.9563	7.5167	3.4921	6.7545	0.9451	-0.0815	0.8210	0.0856
Clasp1	_DGGAAS[Phospho (STY)]PATEGR_.2	Q80TV8_S1220_M1_Clasp1	8.4368	8.1901	8.6257	7.7537	3.2063	7.7960	8.4716	8.4007	7.7577	8.1157	0.8960	-0.1585	0.4428	0.3538
Clasp1	_SRS[Phospho (STY)]ANPAGAGSR_.2	Q80TV8_S676_M1_Clasp1	4.3767	3.7313	5.4707	4.1371	2.8976	4.5785	3.4745	3.7202	4.0192	3.8144	1.1175	0.1603	0.3537	0.4514
Clasp1	_TS[Phospho (STY)]PLTS[Phospho (STY)]PTNC[Carbamidomethyl (C)]SHGGLSPSR_.2	Q80TV8_S1106_M2_Clasp1	3.9161	3.8109	4.2892	5.6418	4.6555	3.8035	4.3166	5.5800	3.1683	3.4614	1.0536	0.0753	0.7038	0.1525
Clasp1	_SRS[Phospho (STY)]DIDVNAAASAK_.2	Q80TV8_S600_M1_Clasp1	5.7907	5.9415	5.5066	5.4943	4.7387	6.1005	6.0182	6.1278	3.6191	6.1764	1.0201	0.0286	0.8466	0.0723
Clasp1	_RS[Phospho (STY)]PTGSTASR_.2	Q80TV8_S572_M1_Clasp1	3.4867	4.2919	4.1735	3.8288	4.1132	3.6163	3.9771	3.4423	3.4714	3.1423	1.1169	0.1595	0.0929	1.0320
Clasp1	_NFDDEDS[Phospho (STY)]VDGNRPSSASSSSSK_.3	Q80TV8_S246_M1_Clasp1	4.1754	3.4308	3.9091	3.4528	3.3191	3.3055	3.1987	3.5483	3.8474	3.1301	1.0489	0.0688	0.4780	0.3206
Fnbp1	_TVS[Phospho (STY)]DNSLSSSK_.2	Q80TY0_S296_M1_Fnbp1	10.6899	10.9956	11.3854	11.4846	10.5745	10.9350	11.1500	10.8613	10.7163	10.9797	1.0077	0.0111	0.6849	0.1644
Fnbp1	_TVS[Phospho (STY)]DNS[Phospho (STY)]LSSSKEGKPELR_.3	#N/A	3.1788	4.4590	3.8657	4.3683	3.8054	3.9241	4.2849	3.7502	3.3988	4.3353	0.9978	-0.0032	0.9770	0.0101
Vps13b	[Phospho (STY)]EM[Oxidation (M)]PVPES[Phospho (STY)]APQM[Oxidation (M)]PS[Phospho (STY)]PVEK	#N/A	3.7114	3.8387	3.8624	9.1962	4.8278	3.3876	3.7645	5.2339	4.5581	4.3965	1.0704	0.0981	0.7906	0.1020
Vps13b	_RKEDELS[Phospho (STY)]IGS[Phospho (STY)]APLGK_.3	Q80TY5_S1001_M2_Vps13b	3.1855	4.0407	3.8723	4.3749	3.8120	3.9175	4.0896	3.7436	3.3921	4.3419	0.9937	-0.0092	0.9260	0.0334
Dnajc6	_RTATSASAS[Phospho (STY)]PTLR_.2	#N/A	7.0705	3.9104	7.1240	6.8797	5.9084	3.8689	6.6866	7.2076	6.7157	6.7809	0.8461	-0.2411	0.2195	0.6585
Dnajc6	_SAATS[Phospho (STY)]PTGSSHGTPTHQSK_.3	Q80TZ3_S683_M1_Dnajc6	5.6097	5.1770	4.5254	5.6595	2.9643	5.5017	3.6252	5.5663	5.6490	5.0092	0.9887	-0.0164	0.9330	0.0301
Dnajc6	_VSEC[Carbamidomethyl (C)]SWPIR_.2	#N/A	5.9076	6.2099	6.2293	5.1816	5.7626	5.7851	6.2092	6.0177	5.9309	6.0243	0.9670	-0.0484	0.3502	0.4556
Dnajc6	_IYSTC[Carbamidomethyl (C)]TDFER_.2	#N/A	5.6356	5.1650	3.3107	5.0743	5.3672	5.3757	5.0397	5.3338	5.0350	5.3170	0.9627	-0.0549	0.6692	0.1744
Dnajc6	_HLDHYTVYNLS[Phospho (STY)]PK_.3	Q80TZ3_S137_M1_Dnajc6	6.6052	6.4450	3.8238	6.2058	3.7636	4.1480	4.0412	7.1216	6.8178	6.5053	0.8438	-0.2450	0.3191	0.4961
Dnajc6	_TATSASAS[Phospho (STY)]PTLR_.2	#N/A	9.6003	9.5915	9.7980	9.8559	8.3795	3.2528	9.7787	9.6364	9.4904	9.5087	0.8760	-0.1909	0.3936	0.4049
Dnajc6	_GASS[Phospho (STY)]PDM[Oxidation (M)]EPSYGGGLFDMVK_.2	#N/A	5.2678	3.6501	3.9673	7.3881	4.6599	3.2996	3.7380	4.4227	3.6903	3.7107	1.2095	0.2744	0.3236	0.4900
Dnajc6	_DSENKGASS[Phospho (STY)]PDM[Oxidation (M)]EPSYGGGLFDM[Oxidation (M)]VK_.3	#N/A	3.6763	7.6446	3.4100	4.4546	6.5270	7.6840	3.9851	7.7416	7.4361	6.8475	0.8560	-0.2243	0.4607	0.3365
Dnajc6	_AADFEDLLSSQGFNAHK_.2	#N/A	4.9193	3.8450	4.1699	5.0990	4.3961	4.0478	4.5268	4.3152	4.2901	4.1829	1.0194	0.0278	0.7552	0.1219
Dnajc6	_ATGQPYEQYAK_.2	#N/A	3.2937	6.2455	3.9805	4.4831	5.7014	4.3046	4.1978	6.1881	6.4388	6.3552	0.8056	-0.3120	0.1482	0.8292
Dnajc6	Carbamidomethyl (C)]EEDHAALVNQES[Phospho (STY)]EQS[Phospho (STY)]DDELLTLSSPHGNAEGDKPHG	#N/A	3.5784	4.6314	3.9974	8.8969	7.6593	3.6106	4.1233	9.0725	8.1658	3.6503	0.8629	-0.2127	0.6073	0.2166
Dnajc6	arbamidomethyl (C)]EEDHAALVNQES[Phospho (STY)]EQS[Phospho (STY)]DDELLTLSSPHGNAEGDKPHG/	#N/A	4.3482	3.6525	3.5341	4.4682	3.4739	4.2556	3.1156	4.0339	4.3329	4.1454	1.0124	0.0178	0.8810	0.0550
Rere	_EKVAS[Phospho (STY)]DTEDTDR_.2	#N/A	4.8607	4.3201	4.2328	4.9328	3.8486	2.9817	4.9526	4.3336	4.7767	5.0056	0.8802	-0.1841	0.1783	0.7488
Rere	_PHVM[Oxidation (M)]S[Phospho (STY)]PTNR_.2	Q80TZ9_S1258_M1_Rere	4.2624	3.0800	3.9961	3.5399	3.4061	3.3273	3.6881	3.4613	3.7604	3.2171	1.0199	0.0284	0.7825	0.1065
Rere	_KQPTS[Phospho (STY)]PDGRAS[Phospho (STY)]PINEDIR_.3	#N/A	5.1061	4.2530	3.2304	4.8298	4.1413	3.9841	3.8773	4.2270	3.6045	5.1472	1.0103	0.0148	0.9219	0.0353
Rere	_AS[Phospho (STY)]PINEDIR_.2	Q80TZ9_S600_M1_Rere	6.3671	6.0435	4.0189	6.1234	3.7823	6.4373	6.5754	6.0560	6.0096	6.1774	0.8803	-0.1839	0.2732	0.5635
Rere	_VAS[Phospho (STY)]DTEDTDR_.2	#N/A	3.9690	3.2244	4.8988	3.6904	3.1127	3.5119	3.4051	4.4281	4.0767	4.6134	0.9041	-0.1455	0.3424	0.4655
Rere	_EKVAS[Phospho (STY)]DTEDTDRITSK_.3	#N/A	3.6738	3.8153	4.2936	4.6003	3.7036	4.4218	4.3150	3.5182	3.1668	3.0665	1.1616	0.2161	0.0984	1.0070
Rere	_QPTS[Phospho (STY)]PDGRAS[Phospho (STY)]PINEDIR_.2	#N/A	3.7197	3.5065	4.4065	3.4083	3.3948	3.2298	3.1230	3.2093	4.3587	3.3753	1.0268	0.0382	0.7700	0.1135
Pja2	_ANHHGS[Phospho (STY)]SPEQVVRPK_.4	Q80U04_S305_M1_Pja2	3.2048	4.0100	3.8916	4.1107	3.8313	3.8982	4.2590	4.3913	3.1895	4.3613	0.9442	-0.0828	0.4436	0.3530
Sgsm2	_S[Phospho (STY)]IDVEELGVGR_.2	Q80U12_S402_M1_Sgsm2	6.3319	6.9331	3.1378	7.4232	6.0158	7.1479	7.4100	7.1913	7.5102	7.1500	0.8427	-0.2468	0.1908	0.7193
Sgsm2	_FSSGSS[Phospho (STY)]IDSHVQR_.2	Q80U12_S637_M1_Sgsm2	4.0835	3.3389	3.8171	3.5759	3.2272	3.3974	3.2906	3.0417	4.1912	3.5429	1.0161	0.0231	0.8301	0.0809
Ripor2	_SQS[Phospho (STY)]FAGFSGLQER_.2	Q80U16_S46_M1_Ripor2	6.6186	4.3158	3.2932	6.1265	4.2041	3.9214	6.5343	5.9934	6.2163	5.8756	0.7712	-0.3748	0.0728	1.1376
Daam2	_ELGS[Phospho (STY)]TEDIYITSR_.2	Q80U19_S704_M1_Daam2	3.6366	9.2933	8.7816	9.7813	8.0684	9.0696	9.7493	9.8226	9.4435	9.3223	0.8457	-0.2419	0.2373	0.6247
Rusc2	_LSEGPAALAGPAS[Phospho (STY)]PPR_.2	Q80U22_S534_M1_Rusc2	6.8635	7.3309	7.8843	6.7346	5.7816	6.6392	7.9392	7.3712	7.1279	7.4964	0.9183	-0.1229	0.1509	0.8214
Rusc2	_NAGPEPETSRPSPLGSYS[Phospho (STY)]PVR_.3	Q80U22_S776_M1_Rusc2	3.7880	3.0924	4.4748	3.5274	2.9137	3.3149	3.6757	3.4737	3.7728	3.2047	0.9963	-0.0054	0.9664	0.0148

Rusc2	_ASVGDSSQEFS[Phospho (STY)]PIQEAQQDR_.2	Q80U22_S572_M1_Rusc2	4.0925	6.0096	3.2784	6.8100	5.0475	6.3289	3.4958	4.3374	6.4755	5.7908	1.0470	0.0663	0.7956	0.0993
Rusc2	_AAGSGS[Phospho (STY)]PPLR_.2	Q80U22_S557_M1_Rusc2	4.0955	3.2469	3.8291	3.3729	3.2392	7.2478	3.5212	3.6283	7.3458	7.1336	0.7715	-0.3742	0.3142	0.5028
Snph	_RTS[Phospho (STY)]PPVSVR_.2	#N/A	3.6244	10.8511	11.3776	4.1991	10.4255	10.7744	11.1835	4.0104	10.7625	10.8628	0.9280	-0.1078	0.7800	0.1079
Snph	_EEGTGESAGGS[Phospho (STY)]PAR_.2	Q80U23_S204_M1_Snph	9.0034	8.7564	8.9633	9.3512	8.5723	8.8446	9.0988	8.8584	9.0645	8.9213	0.9921	-0.0114	0.6327	0.1988
Snph	_TS[Phospho (STY)]PPVSVR_.2	#N/A	3.8052	3.8803	3.3995	3.9022	3.7686	3.7237	3.6169	7.1969	3.8914	7.5814	0.6724	-0.5725	0.0611	1.2137
Snph	_AIQTDFVQYQPDLNTILEK_.2	#N/A	4.7421	4.8983	3.5523	5.0915	4.2207	4.1009	3.5147	4.3146	3.5277	3.8451	1.1668	0.2225	0.0916	1.0382
Snph	_QPGDPSNTS[Phospho (STY)]AEDGADSGYVAADDTLSR_.3	Q80U23_S236_M1_Snph	4.3043	3.6086	3.4902	3.9929	3.4300	4.2995	3.7076	6.4052	3.7742	3.9599	0.8639	-0.2111	0.3036	0.5176
Snph	_SSTYTKLS[Phospho (STY)]DPAVC[Carbamidomethyl (C)]GDR_.2	#N/A	4.8100	3.8511	5.3454	5.0800	3.8682	4.6802	6.1807	3.4123	3.4851	3.8306	1.0896	0.1238	0.5514	0.2585
Snph	_EEGTGESAGGS[Phospho (STY)]PARS[Phospho (STY)]LTR_.2	Q80U23_S208_M2_Snph	5.7707	2.9853	3.4635	3.9295	2.8736	5.7446	6.3731	5.8024	5.5098	5.3784	0.7159	-0.4822	0.0460	1.3369
Madd	_EKPAGS[Phospho (STY)]PIR_.2	#N/A	11.3577	11.4395	11.9340	11.3819	10.6186	11.2053	11.6537	11.5677	11.3107	11.1928	0.9905	-0.0137	0.6539	0.1845
Madd	_S[Phospho (STY)]PTENVNTPVGK_.2	#N/A	10.0347	9.5689	10.0774	10.2618	9.6498	9.8616	10.3426	9.8119	10.0569	9.8692	0.9889	-0.0161	0.5206	0.2835
Madd	_KRS[Phospho (STY)]PTENVNTPVGK_.3	#N/A	9.0332	8.8661	9.5072	9.2723	8.5123	8.7341	9.4528	9.3197	9.2122	8.9740	0.9727	-0.0399	0.2467	0.6079
Madd	_REPSS[Phospho (STY)]PQGR_.2	Q80U28_S929_M1_Madd	3.4701	3.9009	3.6330	4.4146	3.7635	8.9364	9.6367	3.9067	3.9200	4.2262	0.8643	-0.2104	0.6469	0.1891
Madd	_LAS[Phospho (STY)]DSDAESDSR_.2	Q80U28_S817_M1_Madd	7.8404	7.6166	8.1301	7.7163	7.0731	7.6184	8.2800	7.7575	7.6600	7.5661	0.9808	-0.0280	0.5101	0.2923
Madd	_S[Phospho (STY)]LKEENFVASVELWNK_.3	#N/A	6.5263	6.3674	7.2551	6.1622	5.6516	6.4286	6.3354	6.7685	6.8590	6.4471	0.9691	-0.0453	0.4939	0.3063
Madd	_LLRPNS[Phospho (STY)]LK_.2	Q80U28_S812_M1_Madd	7.1677	7.1444	7.2729	7.4679	5.4540	6.3091	7.1132	7.4531	6.8468	6.5218	0.9741	-0.0379	0.6804	0.1672
Madd	_S[Phospho (STY)]PTENVNTPVGKDPGLAGR_.2	#N/A	3.4989	3.5901	4.0684	4.2772	3.4784	5.5950	3.6882	6.5898	3.8883	3.9545	0.9017	-0.1494	0.5328	0.2735
Madd	_SSVIKHS[Phospho (STY)]PTVK_.2	#N/A	6.4225	6.9456	7.1519	6.9406	5.8330	6.7043	6.9298	3.8218	6.4014	6.7298	1.1165	0.1590	0.2963	0.5282
Madd	_EKPAGS[Phospho (STY)]PIRSSEDVSQR_.3	#N/A	3.6478	6.2122	3.7924	3.7580	5.4835	5.4694	3.6961	3.8865	5.6192	3.5937	1.1258	0.1710	0.4635	0.3340
Madd	_RNGS[Phospho (STY)]ETQLNK_.2	Q80U28_S1499_M1_Madd	2.9329	3.7381	3.3380	4.3826	3.5595	4.1700	4.5308	3.4003	4.4185	4.0598	0.8987	-0.1541	0.2550	0.5935
Madd	_AT[Phospho (STY)]LS[Phospho (STY)]DSEIETNSATSAIFGK_.2	Q80U28_S1237_M2_Madd	3.1037	3.9089	3.7905	4.4299	3.7302	4.0210	4.3732	3.9645	3.9557	4.2623	0.9255	-0.1116	0.2291	0.6399
Clec16a	_RGS[Phospho (STY)]SDPTVQR_.2	Q80U30_S848_M1_Clec16a	4.1509	3.4063	3.8845	3.5085	3.2946	3.3300	5.8168	3.5729	3.8719	3.1056	0.8788	-0.1864	0.3532	0.4520
Arhgef17	_LPSLDHRDS[Phospho (STY)]PR_.3	Q80U35_S2005_M1_Arhgef17	5.0828	4.8922	5.7270	4.6972	3.6059	3.6908	4.1306	3.7510	5.4503	5.4624	0.9824	-0.0256	0.8841	0.0535
Arhgef17	_SLS[Phospho (STY)]DPIPQR_.2	Q80U35_S728_M1_Arhgef17	4.1013	3.1249	3.2872	3.7899	3.0132	3.6114	3.5046	4.3286	6.4141	3.7569	0.7749	-0.3679	0.1104	0.9570
Rimbp2	_RGTS[Phospho (STY)]VDDFLK_.2	Q80U40_S737_M1_Rimbp2	3.3726	4.1288	3.1062	4.1509	4.0171	4.1083	4.0015	4.3512	3.1494	3.8281	0.9952	-0.0070	0.9532	0.0208
Rimbp2	_DHLS[Phospho (STY)]PDFYDESETDPGAEELPAR_.3	Q80U40_S852_M1_Rimbp2	3.2150	4.9186	5.7906	7.5990	3.8596	4.2717	4.1649	3.6741	3.3227	3.8093	1.3205	0.4011	0.1792	0.7468
Cep170b	_GS[Phospho (STY)]LDWPEEER_.2	#N/A	8.7730	9.2971	9.0527	10.1609	8.5349	9.6938	9.6882	9.3768	9.5873	10.1186	0.9545	-0.0671	0.2193	0.6589
Cep170b	_SQS[Phospho (STY)]FTHTPPADPK_.2	Q80U49_S421_M1_Cep170b	3.7200	8.0146	8.0143	3.9393	2.8638	3.7608	3.6540	7.9018	3.8278	8.1221	0.8597	-0.2180	0.6061	0.2174
Cep170b	_HEDGTQS[Phospho (STY)]DSEDPLAK_.2	#N/A	8.4609	3.0685	3.5468	9.3320	2.9568	8.2390	3.2388	3.9107	8.2280	8.4474	0.9963	-0.0054	0.9909	0.0040
Cep170b	_VVQTS[Phospho (STY)]PSAR_.2	Q80U49_S819_M1_Cep170b	9.2592	9.5722	3.9929	4.4955	3.9326	9.5465	4.1577	3.6229	3.2715	2.9617	1.9409	0.9567	0.0599	1.2228
Cep170b	_AGS[Phospho (STY)]FTGPSDSETAPAR_.2	Q80U49_S1166_M1_Cep170b	7.8323	7.7550	7.8170	8.4404	7.0116	7.8856	8.1930	8.0106	7.9373	7.9086	0.9723	-0.0405	0.3788	0.4216
Cep170b	_ESPLS[Phospho (STY)]PPTVPDPGGATPGSAR_.2	Q80U49_S981_M1_Cep170b	4.3071	8.5495	8.6762	9.5991	3.4509	7.8931	8.5128	3.4166	3.7156	7.5489	1.2209	0.2879	0.4630	0.3344
Cep170b	_LGDAS[Phospho (STY)]DTEAVDGER_.2	Q80U49_S1122_M1_Cep170b	7.7392	7.8100	4.1361	3.6799	3.5462	7.4253	3.8282	7.6911	7.5050	7.5803	0.8604	-0.2169	0.5014	0.2998
Cep170b	_GASPVTPSTT[Phospho (STY)]PPPPTDPQLTK_.2	Q80U49_T541_M1_Cep170b	6.9709	4.3287	7.2817	7.5806	3.2799	7.5291	3.9687	7.0963	6.8432	7.5704	0.9674	-0.0479	0.8612	0.0649
Cep170b	_DGPGDDRHS[Phospho (STY)]TKS[Phospho (STY)]DLPVHTR_.3	#N/A	5.9393	7.2246	3.8750	3.9337	5.5234	6.7866	4.0923	5.9383	7.1437	7.3024	0.9065	-0.1416	0.5542	0.2563
Cep170b	_QES[Phospho (STY)]FTKEPTSGPPAPGK_.3	#N/A	4.2191	5.2768	3.6708	3.8708	4.6002	3.8843	3.5855	3.6089	3.9305	3.6084	1.1549	0.2077	0.1052	0.9779
Cep170b	_EIHDVAGDGDSLGS[Phospho (STY)]PGPTR_.2	#N/A	7.5296	7.8121	7.8007	8.1158	7.2628	8.0003	7.6198	7.2770	7.5312	7.9578	1.0207	0.0295	0.4425	0.3540
Cep170b	_GHGSRGS[Phospho (STY)]LDWPEEER_.3	#N/A	5.5717	6.0957	3.9357	6.6404	4.2676	6.3405	5.9107	6.0067	6.6147	6.6637	0.8692	-0.2022	0.2037	0.6911
Cep170b	_GHKHEDGTQS[Phospho (STY)]DSEDPLAK_.3	#N/A	7.3627	7.2487	7.9270	7.8198	7.1772	7.5243	7.5094	7.3487	7.4258	7.4607	1.0099	0.0142	0.6546	0.1840
Cep170b	_NGPS[Phospho (STY)]PTTPQTPGPQK_.2	Q80U49_S965_M1_Cep170b	9.5955	9.7205	9.8832	10.3603	9.0970	9.5235	10.0828	9.6610	9.5932	3.0324	1.1982	0.2609	0.2698	0.5690
Cep170b	_SNS[Phospho (STY)]LSTPRPTR_.2	#N/A	7.7175	4.3565	7.7051	8.1562	4.1778	7.4740	3.9124	7.3218	6.8435	7.9179	1.0152	0.0218	0.9344	0.0295
Cep170b	_RGHGS[Phospho (STY)]RGS[Phospho (STY)]LDWPEEER_.3	#N/A	3.8695	5.7722	4.0502	5.9937	4.3825	5.5837	5.6115	5.5790	5.5528	5.4007	0.8927	-0.1638	0.2543	0.5947
Cep170b	_S[Phospho (STY)]PSLGNVPNTPASTISAR_.2	Q80U49_S1347_M1_Cep170b	3.6329	3.8082	4.3681	3.6954	3.6295	3.4829	3.8437	3.1709	3.6177	3.4612	1.0699	0.0974	0.2410	0.6179
Cep170b	_SNS[Phospho (STY)]LSTPR_.2	#N/A	4.1940	3.4984	3.3800	3.8826	3.3197	4.4098	3.2697	4.2358	3.8844	3.8496	0.9923	-0.0111	0.9198	0.0363
Avl9	_DSLPSDS[Phospho (STY)]PPITVQPQANNR_.2	Q80U56_S368_M1_Avl9	8.5706	9.0562	9.2349	9.3896	7.9939	9.0691	9.6927	9.5069	9.1539	8.8517	0.9553	-0.0659	0.2062	0.6856
Avl9	_TEKPYFQVEGNNNK_.2	#N/A	4.2256	4.2025	3.9906	5.2708	3.6924	4.4735	4.8915	4.9628	4.4640	3.9514	0.9435	-0.0839	0.4578	0.3393
Rims3	_RLS[Phospho (STY)]QSS[Phospho (STY)]LESATSPSC[Carbamidomethyl (C)]S_.2	#N/A	10.2765	9.1597	9.6742	11.5405	10.5827	11.0404	11.6257	10.8882	11.1141	10.9933	0.9304	-0.1041	0.1332	0.8756
Rims3	_LSQSS[Phospho (STY)]LESATSPSC[Carbamidomethyl (C)]S_.2	#N/A	3.6054	4.3617	3.5513	8.6159	4.2500	3.8755	3.7686	7.8785	3.7131	4.0209	0.9721	-0.0409	0.9180	0.0372
Rims3	_SSS[Phospho (STY)]ISGEIC[Carbamidomethyl (C)]GSQQAGGGAGTTTAK_.2	Q80U57_S22_M1_Rims3	3.4544	4.2596	4.1412	3.1430	4.0810	3.6485	4.3586	3.4746	3.1232	3.1100	1.0772	0.1072	0.4260	0.3706
Rims3	_QGSRESTDGSTNS[Phospho (STY)]NSSEGTFIFPTR_.3	Q80U57_S114_M1_Rims3	3.8514	3.1558	4.5382	3.4641	2.9771	3.2515	3.6123	3.0776	4.2270	3.5070	0.9816	-0.0268	0.8535	0.0688
Pum2	_QAS[Phospho (STY)]PTEVVER_.2	Q80U58_S181_M1_Pum2	9.4572	9.6475	10.0738	9.9860	2.8345	9.5471	9.8081	4.1499	3.7985	9.6026	1.2560	0.3289	0.3953	0.4030
Pum2	_GKAS[Phospho (STY)]PFEEDQNR_.2	Q80U58_S136_M1_Pum2	7.4216	7.8569	8.2160	8.7615	7.6666	7.8062	8.1944	7.9628	7.6293	7.8880	1.0046	0.0066	0.8921	0.0496
Kiaa0232	_SAQTS[Phospho (STY)]LDS[Phospho (STY)]QEEATGILPK_.2	Q80U59_S1194_M2_Kiaa0232	3.8200	3.8171	3.7892	5.8104	3.6595	3.6173	4.0065	3.9586	3.6072	3.4756	1.0860	0.1190	0.4883	0.3113
Rubcn	_KQES[Phospho (STY)]PLLVTK_.2	Q80U62_S198_M1_Rubcn	4.3118	3.5672	4.0454	3.5892	3.4555	3.1691	3.7375	3.4120	3.7110	3.5882	1.0215	0.0306	0.7338	0.1344
Suz12	_ASM[Oxidation (M)]SEFLES[Phospho (STY)]EDGEVEQQR_.2	Q80U70_S548_M1_Suz12	3.0778	3.4849	4.3124	3.8561	3.7224	4.5524	4.0044	3.1450	4.6988	3.5334	0.9971	-0.0042	0.9775	0.0099
Scrib	_NS[Phospho (STY)]LESISSIDR_.2	Q80U72_S1206_M1_Scrib	8.6031	3.0341	7.7133	8.6121	7.0759	8.0048	8.7870	7.8752	8.1036	8.4053	0.8651	-0.2091	0.3336	0.4767
Scrib	_M[Oxidation (M)]QS[Phospho (STY)]PELPAPER_.2	#N/A	8.9009	8.5815	9.1170	9.0767	7.4574	8.7956	9.0053	8.6033	8.6132	8.8083	0.9883	-0.0170	0.7597	0.1194
Scrib	_VS[Phospho (STY)]PTGAAGR_.2	Q80U72_S1126_M1_Scrib	3.6920	2.9964	4.3788	3.3806	2.8177	3.4110	3.0953	3.2370	5.5692	3.3476	0.9039	-0.1457	0.5198	0.2842
Scrib	_M[Oxidation (M)]KS[Phospho (STY)]LEQDALR_.2	#N/A	5.8820	5.4898	6.2861	6.1366	4.7911	5.8307	5.9690	7.6533	6.8020	5.9740	0.8692	-0.2023	0.0738	1.1318
Scrib	_EDGES[Phospho (STY)]EEGQPEAAWPLPSGR_.2	Q80U72_S583_M1_Scrib	3.9281	3.1835	3.6618	7.3657	6.1838	3.5528	3.4459	6.4971	4.0358	4.3837	1.0120	0.0173	0.9580	0.0186
Pum1	_RDS[Phospho (STY)]LTGSSDLYKR_.3	#N/A	6.2933	5.9926	7.0095	6.4647	5.7880	6.2350	5.8650	6.4544	6.3470	6.3946	1.0051	0.0073	0.8977	0.0469
Pum1	_RDS[Phospho (STY)]LTGSSDLYK_.2	#N/A	6.1381	3.9082	3.7898	4.2925	3.7295	3.9999	4.0072	5.1907	3.4746	4.2595	1.0181	0.0259	0.8920	0.0496
Pum1	_S[Phospho (STY)]MDELNHDFQALALEGR_.3	#N/A	3.5244	4.0977	3.2581	4.2490	4.1690	3.9571	4.1514	4.1254	3.3793	4.0123	0.9895	-0.0153	0.8722	0.0594
Usp8	_SYS[Phospho (STY)]SPDITQALQEEEK_.2	#N/A	8.4470	8.4903	8.9545	9.0133	7.7417	8.4169	8.9031	8.6184	8.6857	8.0427	0.9939	-0.0088	0.8555	0.0678
Usp8	_SYS[Phospho (STY)]SPDITQALQEEEKR_.2	#N/A	3.8285	6.5782	6.0670	4.0214	5.1072	6.0083	5.1197	7.0863	7.2787	6.6029	0.8078	-0.3079	0.1113	0.9535
Usp8	_RSY[Phospho (STY)]SSPDITQALQEEEKR_.3	Q80U87_Y679_M1_Usp8	3.3241	4.0804	3.9230	4.0764	3.9687	4.8487	4.0056	3.5532	3.4667	3.9182	1.0806	0.1118	0.2974	0.5267
Usp8	_S[Phospho (STY)]VENLLDSK_.2	Q80U87_S153_M1_Usp8	3.6952	3.6472	3.4288	4.4734	3.4686	4.2609	3.1209	3.6503	4.1356	3.6555	1.0518	0.0728	0.5134	0.2895
Septin9	_S[Phospho (STY)]FEVEEIEPPNSTPPR_.2	#N/A	10.7933	10.6416	10.6339	12.0496	9.4970	11.0247	10.5199	10.9784	11.2665	11.2129	0.9799	-0.0293	0.6284	0.2018
Septin9	_S[Phospho (STY)]PKPSLR_.2	Q80UG5_S85_M1_Septin9	8.9257	8.4167	4.3409	9.1042	7.9335	8.8019	8.8080	8.7452	8.9159	3.3386	1.0629	0.0880	0.7502	0.1248
Septin9	_YLQEEVNINR_.2	#N/A	6.9352	6.9467	6.8180	8.2188	6.7135	6.7820	7.6699	7.2408	6.8789	7.3446	0.9705	-0.0431	0.5193	0.2846
Septin9	_SVQPT[Phospho (STY)]SEERIPK_.2	Q80UG5_T329_M1_Septin9	3.4493	3.8931	3.1830	4.2276	3.7144	4.0151	4.3759	7.9749	3.0726	3.9049	0.7755	-0.3669	0.2512	0.6000
Septin9	_KSVQPTS[Phospho (STY)]EERIPK_.3	Q80UG5_S330_M1_Septin9	4.2651	3.5205	5.5360	3.3943	3.4088	3.2158	3.1090	3.2234	4.3728	3.3613	1.1062	0.1456	0.4820	0.3169
Septin9	_S[Phospho (STY)]FEVEEIEPPNSTPPRR_.3	#N/A	6.1721	3.8221	4.0909	7.3770	4.0306	6.4289	4.3082	5.5381	6.2594	6.3987	0.9456	-0.0806	0.7326	0.1351
Rab3gap1	_KTS[Phospho (STY)]LSDSTTSAYPGDAGK_.2	Q80UJ7_S536_M1_Rab3gap1	3.5855	4.3907	4.1862	3.7300	4.2120	5.8442	3.8782	6.1888	6.0267	3.4073	0.8871	-0.1729	0.4552	0.3418
Rab3gap1	_FSVTHHYLVQES[Phospho (STY)]PDKER_.3	#N/A	3.0242	4.2607	4.0149	4.0195	3.8936	3.8772	3.9398	4.5757	3.5266	3.8671	0.9676	-0.0475	0.6551	0.1837
Naa15	_ITVNGDS[Phospho (STY)]SAETEELANEI_.2	Q80UM3_S854_M1_Naa15	3.9069	4.2285	4.1157	4.0509	3.4204	3.8551	4.0031	4.2687	3.5071	3.8772	1.0040	0.0057	0.9373	0.0281
Dgkz	_RPQNTLKAS[Phospho (STY)]K_.2	#N/A	4.4997	3.7843	4.8331	4.5064	3.4723	4.7441	5.3375	4.8297	3.8425	4.8930	0.9113	-0.1340	0.2969	0.5274
Slc20a2	_ESALSRAS[Phospho (STY)]DESLR_.2	#N/A	8.3656	4.2910	3.6219	4.1246	3.5617	8.5884	9.1535	8.5435	3.6425	4.0916	0.8534	-0.2288	0.5917	0.2279
Slc20a2	_ESALSRAS[Phospho (STY)]DESLRK_.3	#N/A	4.8492	3.4502	5.0385	3.7922	5.1239	4.3909	3.8795	5.9277	4.9401	4.1896	0.9380	-0.0923	0.5769	0.2389

Slc20a2	_AS[Phospho (STY)]DESLRK_.2	#N/A	5.3470	5.6018	5.8217	3.5245	4.6180	5.4024	3.2392	3.0931	4.2426	3.4915	1.4368	0.5228	0.0125	1.9024
Slc20a2	_ALS[Phospho (STY)]MTHGSAK_.2	Q80UP8_S316_M1_Slc20a2	2.9882	4.2380	3.6750	4.1777	3.6147	4.1148	3.8923	3.9408	3.5894	4.1447	0.9768	-0.0339	0.7372	0.1324
Slc20a2	_AS[Phospho (STY)]DES[Phospho (STY)]LRKVQEAESPGFK_.3	#N/A	3.8724	3.1278	3.6061	3.1498	3.0161	3.6085	3.2981	3.8513	4.1504	4.3280	0.8694	-0.2019	0.0759	1.1196
Pgrmc2	_GLC[Carbamidomethyl (C)]SGPGAGEES[Phospho (STY)]PAATLPR_.2	Q80UU9_S84_M1_Pgrmc2	7.3717	7.3684	7.6978	7.7085	6.6717	7.1666	8.2715	7.6347	7.2947	7.3886	0.9586	-0.0610	0.2622	0.5813
Pgrmc2	_DFS[Phospho (STY)]LEQLR_.2	Q80UU9_S98_M1_Pgrmc2	3.4460	5.0978	4.1328	3.1346	4.3444	3.6570	4.0178	5.6475	5.2980	3.1016	0.8788	-0.1864	0.3794	0.4208
Pgrmc2	_LLKPGEEPSEYT[Phospho (STY)]DEEDTKDHSK_.4	#N/A	3.1899	3.9951	3.8767	4.1256	3.8164	3.9130	4.2738	4.3764	3.1747	3.8029	0.9776	-0.0327	0.7572	0.1208
Pgrmc2	_LLKPGEEPSEYT[Phospho (STY)]DEEDTK_.3	#N/A	4.2131	3.0130	3.3991	3.9018	2.9013	3.7233	3.6164	3.9883	3.8653	3.8687	0.9193	-0.1214	0.2841	0.5465
Fbxo2	_VLAELPATELVQAC[Carbamidomethyl (C)]R_.2	#N/A	7.5971	7.4851	7.9638	7.9535	7.8190	7.7152	8.0435	7.5411	7.7695	7.8163	0.9953	-0.0069	0.7798	0.1080
Fbxo2	_TDAGSLYELTVR_.2	#N/A	5.9604	6.4991	3.2378	6.9413	6.3559	6.2482	6.4418	6.3914	6.3569	6.0714	0.9301	-0.1046	0.5357	0.2711
Fbxo2	_NPC[Carbamidomethyl (C)]GEEDLEGWS[Phospho (STY)]DVEHGGDGWR_.3	Q80UW2_S141_M1_Fbxo2	3.3487	4.1050	4.5832	5.3098	3.8382	4.1322	4.0253	4.4504	4.5010	3.8043	1.0058	0.0083	0.9489	0.0228
Fbxo2	_C[Carbamidomethyl (C)]QQEGLVPEGS[Phospho (STY)]ADEERDHWQQFYFLSK_.4	Q80UW2_S106_M1_Fbxo2	4.2058	3.4612	3.9395	3.4535	3.3495	3.2751	3.1682	3.1641	4.3135	3.4205	1.0277	0.0395	0.7427	0.1292
Fbxo2	_EAEEEEEAEAVEYLAELPEPLLLR_.3	#N/A	3.8470	3.4955	3.5806	3.9796	2.9907	4.4127	3.2727	3.8768	2.4286	2.4717	1.2341	0.3035	0.0923	1.0348
Fbxo2	_FEHGGQDSVYWK_.3	#N/A	2.4585	3.8120	4.2903	3.8340	3.7003	4.4251	3.9823	3.1671	3.4662	3.5113	1.0628	0.0878	0.5756	0.2399
Kcmf1	.cetyl (Protein N-term)]S[Phospho (STY)]RHEGVSC[Carbamidomethyl (C)]DAC[Carbamidomethyl (C)]LK	Q80UY2_S2_M1_Kcmf1	4.2358	4.9930	4.3314	5.5252	3.7415	4.3840	5.3470	4.8276	4.4250	3.5525	1.0576	0.0808	0.6299	0.2007
Ap4e1	_IIEQPEC[Carbamidomethyl (C)]SS[Phospho (STY)]PVIETER_.2	Q80V94_S962_M1_Ap4e1	7.1978	7.1808	3.5610	7.8486	6.0122	7.3713	3.2531	7.9014	7.3772	7.1110	1.0184	0.0263	0.9218	0.0354
Camsap3	_DLPDGHAVS[Phospho (STY)]PR_.2	Q80VC9_S334_M1_Camsap3	8.9166	8.7480	9.6367	9.0294	7.6564	8.9521	9.3060	8.9881	8.7636	9.0358	0.9778	-0.0324	0.5744	0.2408
Camsap3	_AVASS[Phospho (STY)]PAANNSEVK_.2	Q80VC9_S555_M1_Camsap3	7.2942	7.7082	8.0861	7.5076	6.0926	7.5767	7.8725	7.3497	7.4127	7.0880	0.9928	-0.0104	0.8897	0.0508
Camsap3	_RAS[Phospho (STY)]LLER_.2	Q80VC9_S903_M1_Camsap3	5.6736	5.2762	5.2710	5.3161	4.4233	5.4693	5.6368	5.4453	5.6973	4.6449	0.9780	-0.0321	0.6960	0.1574
Camsap3	_NTETVPS[Phospho (STY)]QNNSGSSSPVFNFR_.2	Q80VC9_S343_M1_Camsap3	4.0457	4.0791	3.6403	4.1429	3.5800	4.0209	3.6796	5.5132	3.6384	4.1099	0.9251	-0.1123	0.4146	0.3823
Camsap3	_KFS[Phospho (STY)]PSQVPVQTR_.2	Q80VC9_S812_M1_Camsap3	4.2704	5.8773	6.1227	6.5145	3.8418	4.1226	5.5333	5.6913	4.8133	4.0266	1.0217	0.0309	0.8751	0.0579
Camsap3	_APSPSGLM[Oxidation (M)]S[Phospho (STY)]PSR_.2	Q80VC9_S1083_M1_Camsap3	3.8861	3.1905	4.5729	3.4294	3.0118	3.2168	7.6881	3.5718	3.8709	3.5418	0.7607	-0.3945	0.2262	0.6456
Camsap3	_RS[Phospho (STY)]PGPGPSPTPR_.2	Q80VC9_S767_M1_Camsap3	3.7701	3.0255	3.5038	4.5484	2.9138	3.7108	5.2821	3.9536	4.2527	4.2257	0.8081	-0.3074	0.0558	1.2533
Camsap3	_SVS[Phospho (STY)]SDSLGPPRPVSTSSR_.3	Q80VC9_S431_M1_Camsap3	3.2032	3.9595	4.4377	5.0962	3.8478	4.2777	4.1708	3.6623	3.3109	3.6588	1.1179	0.1608	0.2516	0.5992
Znf579	_[Acetyl (Protein N-term)]M[Oxidation (M)]DPQPPPPAQGS[Phospho (STY)]PPHR_.2	Q80VM4_S12_M1_Znf579	3.5597	8.1340	9.1891	4.3380	6.6589	8.0443	8.3314	8.3092	2.9622	7.3287	0.9883	-0.0170	0.9602	0.0176
Tecpr1	_GSGTESAPSDTDAS[Phospho (STY)]LEVER_.2	Q80VP0_S418_M1_Tecpr1	3.7181	5.3519	3.9979	4.9027	3.6319	5.1774	5.2151	4.6773	5.0307	4.5543	0.9166	-0.1256	0.3517	0.4539
Epn1	_TALPTSGS[Phospho (STY)]STGELELLAGEVPAR_.2	#N/A	7.3851	6.0609	9.9026	5.2432	5.4052	8.1238	8.4172	4.8341	8.6380	7.9140	0.9422	-0.0859	0.7196	0.1429
Epn1	_[Acetyl (Protein N-term)]STSS[Phospho (STY)]LRR_.2	Q80VP1_S5_M1_Epn1	9.2532	9.5717	9.4241	9.7250	8.9755	9.5802	9.0653	9.2796	9.4518	9.5215	1.0099	0.0142	0.5809	0.2359
Epn1	_NIVHNYSEAEIK_.2	#N/A	4.1070	4.3013	3.9174	5.0854	4.5912	4.9241	3.2650	4.4425	4.2301	4.7134	1.0781	0.1084	0.3708	0.4309
Epn1	_T[Phospho (STY)]PESFLGPNAALVDLDSLVSR_.2	Q80VP1_T469_M1_Epn1	3.2247	3.9810	4.4592	4.0030	3.8693	4.2562	4.1493	2.9982	3.2972	3.6803	1.1230	0.1673	0.1745	0.7581
Epn1	_ENMYAVQTLK_.2	#N/A	3.5443	4.3495	4.2311	4.7554	4.1708	3.5587	4.4484	3.3848	3.0333	3.1999	1.1664	0.2220	0.1320	0.8795
Inka2	_HIETPATSS[Phospho (STY)]PK_.2	Q80VY2_S281_M1_Inka2	3.3469	3.8793	4.0337	5.4096	3.9734	3.7561	4.2510	4.5116	3.2307	4.9238	0.9615	-0.0566	0.7313	0.1359
Tmcc2	_GS[Phospho (STY)]PHLLR_.2	Q80W04_S189_M1_Tmcc2	5.8958	5.6158	5.3252	3.8319	4.1404	5.4973	5.6908	5.4989	5.6947	5.9024	0.8867	-0.1735	0.1798	0.7453
Tmcc2	_NKFGS[Phospho (STY)]ADNIAHLK_.3	#N/A	4.9237	4.5077	6.1149	5.4671	3.8794	4.0309	4.9568	5.6941	3.6435	4.6174	1.0196	0.0280	0.8713	0.0598
Tmcc2	_ALSGSATLVSS[Phospho (STY)]PK_.2	Q80W04_S467_M1_Tmcc2	3.5953	4.9350	4.2821	3.7202	4.2219	3.5076	3.8684	3.2810	5.7254	3.2510	1.0030	0.0044	0.9824	0.0077
Tmcc2	_NKFGS[Phospho (STY)]ADNIAHLKDPMEDGPPEEAAR_.4	#N/A	3.8953	3.4336	4.0913	3.8363	3.4158	3.2184	4.1042	3.4630	3.4985	3.1748	1.0248	0.0354	0.7136	0.1466
Tmcc2	Carbamidomethyl (C)]KS[Phospho (STY)]DELQQQQGEEDGAGMEDAAC[Carbamidomethyl (C)]LLPGADLI	Q80W04_S6_M1_Tmcc2	3.6867	2.9911	4.3735	3.6288	2.8124	3.4163	3.7771	3.3724	3.6714	3.3423	0.9842	-0.0230	0.8551	0.0680
Gstm7	_IRVDILENQLM[Oxidation (M)]DNR_.3	#N/A	4.0843	5.2570	6.0038	5.3978	4.5411	5.2901	3.2897	4.6149	4.1920	5.6208	1.1504	0.2022	0.2329	0.6328
Wipi2	_GAYVPSS[Phospho (STY)]PTR_.2	Q80W47_S395_M1_Wipi2	12.4377	12.5630	13.0854	12.6998	11.8435	12.5406	7.0341	6.2351	12.6457	12.5156	1.3040	0.3829	0.0670	1.1739
Agfg2	_ELGGC[Carbamidomethyl (C)]SQAGNR_.2	#N/A	4.1273	3.9974	3.8790	4.1233	3.9204	3.4184	4.2201	4.3787	4.2709	4.3316	0.9095	-0.1369	0.0214	1.6702
Mtdh	_SETNWES[Phospho (STY)]PK_.2	#N/A	9.6479	9.2687	9.8798	9.6965	8.8593	9.2143	10.0246	9.6801	9.5369	9.4905	0.9736	-0.0386	0.2707	0.5675
Mtdh	_SQEPISNDQKVS[Phospho (STY)]DDDKEK_.3	Q80WJ7_S423_M1_Mtdh	6.3693	5.9283	6.1262	6.0872	5.4686	5.5644	5.9986	5.9527	5.9843	5.3903	1.0159	0.0227	0.6742	0.1712
Mtdh	_SETNWES[Phospho (STY)]PKQIK_.2	#N/A	7.0688	7.8713	8.6024	7.6205	3.3257	7.7290	7.7276	7.7483	7.1715	7.4195	0.9361	-0.0953	0.6328	0.1987
Mtdh	_SETNWES[Phospho (STY)]PKQIKK_.2	#N/A	3.1944	4.0318	3.8812	4.3838	3.9201	4.2053	5.7327	3.7347	3.3832	4.3508	0.9153	-0.1277	0.4506	0.3462
Trmt9b	_S[Phospho (STY)]LDESTLRK_.2	#N/A	6.4836	4.2227	7.3068	7.8136	6.8109	7.3756	7.4338	7.4101	3.0555	7.1826	1.0635	0.0889	0.7199	0.1427
Trmt9b	_S[Phospho (STY)]LDESTLR_.2	#N/A	4.1199	5.3724	3.3059	3.8086	3.2456	2.9830	3.5232	5.7944	3.9585	6.4945	0.7700	-0.3770	0.1523	0.8174
Trmt9b	_S[Phospho (STY)]HSM[Oxidation (M)]DYGSAVAR_.2	Q80WQ4_S210_M1_Trmt9b	3.1868	3.9920	3.8736	4.1287	3.8133	3.9162	4.2769	3.7422	3.1715	4.3433	0.9832	-0.0244	0.8176	0.0874
Aftph	_SFS[Phospho (STY)]PGDFR_.2	Q80WT5_S151_M1_Aftph	2.9932	6.4582	4.2277	3.7715	6.1624	2.9868	4.3808	3.2297	3.5287	3.4488	1.2156	0.2817	0.3584	0.4456
Aftph	_HLLETHSS[Phospho (STY)]PAK_.2	#N/A	5.3605	5.5572	6.1287	5.8366	5.4652	5.8482	5.5095	5.4147	5.4497	5.6221	0.9758	-0.0353	0.7522	0.1236
Aftph	_S[Phospho (STY)]PDPDPTGQNALDDSAASMK_.2	Q80WT5_S395_M1_Aftph	4.3063	3.5617	4.0399	3.5837	3.4500	3.1746	3.0678	3.4175	3.7165	3.2610	1.0952	0.1312	0.2135	0.6706
Srrm3	_ARPAS[Phospho (STY)]TS[Phospho (STY)]PSPGTR_.2	Q80WV7_S464_M2_Srrm3	3.6995	3.6894	3.8543	3.9775	3.5657	3.6661	3.7877	5.4568	3.6917	3.8583	0.8913	-0.1661	0.2176	0.6624
Srrm3	_RRDS[Phospho (STY)]PSFMEPR_.3	#N/A	3.5495	3.8533	3.6692	3.7572	3.7416	3.6869	3.6401	4.3722	4.7097	4.2741	0.8730	-0.1959	0.0190	1.7216
Ubap2l	_RYPSSISSS[Phospho (STY)]PQKDLTQAK_.3	#N/A	8.0790	8.4557	9.2880	9.3109	7.9455	3.6424	8.2964	8.3613	8.1005	8.1353	0.9469	-0.0787	0.6965	0.1571
Ubap2l	_RYPSSISSS[Phospho (STY)]PQK_.2	#N/A	8.3957	8.1971	8.4987	9.1427	4.1450	8.4211	8.6315	8.1669	8.4232	8.4537	0.9265	-0.1101	0.5252	0.2797
Ubap2l	_YPSSISSS[Phospho (STY)]PQK_.2	#N/A	4.3187	4.4083	3.5046	7.4993	3.4444	7.2073	3.7220	4.1112	6.6367	7.2247	0.9336	-0.0991	0.7646	0.1166
Ubap2l	_TATEEWGTEDWNEDLSETK_.2	#N/A	4.6292	5.1979	6.5599	6.7807	4.3956	5.7784	6.0868	5.4621	4.4141	5.6922	1.0264	0.0376	0.8108	0.0911
Ubap2l	_YPSSISSS[Phospho (STY)]PQKDLTQAK_.2	#N/A	5.3052	3.8461	4.2827	3.4389	4.5338	3.2962	6.0072	4.9640	5.3133	5.1025	0.7700	-0.3770	0.0200	1.6981
Ubap2l	_NQDEC[Carbamidomethyl (C)]VIALHDC[Carbamidomethyl (C)]NGDVNR_.3	#N/A	5.1774	4.9889	4.0998	5.8811	3.7979	5.1661	5.3072	4.6416	4.4608	5.4081	0.9793	-0.0302	0.8195	0.0865
Ubap2l	_STSAPQMS[Phospho (STY)]PGSSDNQSSSPQPAQQK_.3	#N/A	6.8260	6.1083	4.1147	9.1031	3.4391	3.4970	6.3562	5.2070	5.3653	4.1648	1.0458	0.0646	0.8457	0.0728
Btbd10	_[Acetyl (Protein N-term)]AGRPHPYDSNSS[Phospho (STY)]DPENWDR_.3	Q80X66_S13_M1_Btbd10	3.1028	4.2396	4.3373	3.8811	3.7474	4.3781	4.0294	3.1201	3.4191	3.5584	1.1178	0.1606	0.1889	0.7237
C2cd2l	_NLGT[Phospho (STY)]PTSSTPR_.2	#N/A	10.2341	9.8160	10.0310	10.3400	9.0334	9.8396	10.3888	10.1738	9.7692	10.0721	0.9784	-0.0316	0.4220	0.3747
C2cd2l	_VDGKLDS[Phospho (STY)]PSR_.2	#N/A	4.3667	10.1111	10.6415	10.1255	9.6846	10.0302	10.5923	10.4831	10.1293	10.0698	0.8877	-0.1718	0.3674	0.4349
C2cd2l	_LDS[Phospho (STY)]PSRS[Phospho (STY)]PSKVEVTEK_.3	#N/A	8.1664	8.9734	3.6882	8.5390	7.1666	6.2179	8.0053	6.6517	3.8511	8.2849	1.0637	0.0892	0.7473	0.1265
C2cd2l	_NLGT[Phospho (STY)]PTS[Phospho (STY)]STPRPSITPTKK_.3	Q80X80_S420_M2_C2cd2l	3.7271	3.6705	7.7616	3.8018	3.4918	3.5893	3.9501	4.0518	3.6473	4.1827	1.0966	0.1331	0.6690	0.1746
C2cd2l	_LDS[Phospho (STY)]PSRSPSK_.2	#N/A	7.2695	7.9654	8.1875	7.9849	6.7576	7.6972	8.2857	8.0365	7.6525	7.8044	0.9621	-0.0557	0.3376	0.4716
C2cd2l	_KAGS[Phospho (STY)]FSR_.2	#N/A	8.0119	7.6157	8.1582	3.9138	2.8893	7.8297	3.6285	4.2047	3.8533	3.8808	1.6453	0.7183	0.0707	1.1506
C2cd2l	_EAGLSQS[Phospho (STY)]HDDLSNTTATPSVR_.3	#N/A	7.7050	6.9909	7.1961	7.7708	5.0992	7.6636	7.3408	7.4716	7.1918	7.6953	0.9523	-0.0705	0.5191	0.2847
C2cd2l	_S[Phospho (STY)]PSKVEVTEK_.2	#N/A	6.1825	3.4160	4.4970	6.0132	2.9358	5.4704	6.1959	3.1189	4.2683	3.4658	1.1150	0.1571	0.5941	0.2262
C2cd2l	_TST[Phospho (STY)]PEEAGVR_.2	Q80X80_T79_M1_C2cd2l	3.4949	3.6503	4.1285	6.0896	3.5386	5.7242	3.8206	6.6492	5.9459	3.3496	0.9284	-0.1071	0.6842	0.1648
C2cd2l	_KKAGS[Phospho (STY)]FSR_.2	#N/A	4.2927	3.6421	4.2138	4.8033	3.5304	3.0944	4.8806	3.3449	2.9935	3.2399	1.0871	0.1204	0.5159	0.2874
C2cd2l	_NLGT[Phospho (STY)]PTSSTPRPSIT[Phospho (STY)]PTK_.2	#N/A	3.3275	4.2915	3.5179	4.1268	3.9649	3.9994	6.7970	5.4591	3.6429	4.0938	0.7745	-0.3686	0.0947	1.0236
C2cd2l	_ESHLS[Phospho (STY)]NGLDPVAETAIR_.3	#N/A	4.8047	5.4529	4.2671	3.8109	4.6569	6.1889	6.3289	6.0346	5.6688	3.4881	0.9040	-0.1456	0.4625	0.3349
C2cd2l	_VDGKLDS[Phospho (STY)]PSRS[Phospho (STY)]PSK_.2	#N/A	3.3253	3.9388	4.3015	4.3316	3.8271	4.3039	4.1970	4.0719	3.2257	3.4207	1.0740	0.1030	0.3475	0.4591
C2cd2l	_SLSPAATVTAELHYEQGS[Phospho (STY)]PR_.3	#N/A	9.3901	9.4076	10.1438	9.6671	8.7611	9.0960	9.7772	9.9362	9.8120	9.3421	0.9685	-0.0461	0.2779	0.5560
C2cd2l	_QKEAGLSQS[Phospho (STY)]HDDLSNTTATPSVR_.3	#N/A	4.1855	3.4395	3.3715	3.8741	3.3112	4.4183	3.5888	4.2443	3.8929	3.8411	0.9679	-0.0471	0.6436	0.1914
C2cd2l	_SDISERPSVDDVES[Phospho (STY)]ETGSTGALETR_.3	Q80X80_S619_M1_C2cd2l	3.4188	3.6670	4.1452	5.4524	3.5553	3.4475	4.0503	5.2918	3.0756	3.7957	0.9739	-0.0381	0.8501	0.0705
Sympk	_TSS[Phospho (STY)]PETREPESK_.2	Q80X82_S1276_M1_Sympk	5.8745	5.3837	6.2838	5.2458	3.1657	4.8693	3.4238	6.4350	5.8522	2.9528	1.1010	0.1388	0.6073	0.2166
Reps2	_APS[Phospho (STY)]QAAESSPTK_.2	Q80XA6_S405_M1_Reps2	6.4488	6.3219	7.5398	7.0145	3.7262	7.7399	6.2513	7.5385	6.3024	6.4270	0.9752	-0.0363	0.8384	0.0766
Reps2	_ASS[Phospho (STY)]LDLNK_.2	Q80XA6_S354_M1_Reps2	4.7836	5.2536	3.8179	4.1844	3.7576	3.9719	4.3327	3.7980	3.1158	5.4544	1.0286	0.0407	0.8154	0.0886
Reps2	_LNS[Phospho (STY)]ELQQQLK_.2	Q80XA6_S492_M1_Reps2	5.9812	5.5637	6.2447	6.1295	5.4389	5.5289	6.1059	5.7157	5.9679	5.9862	0.9782	-0.0318	0.5105	0.2920

Reps2	_VS[Phospho (STY)]LSHGYSK_.2	Q80XA6_S67_M1_Reps2	3.5622	3.7802	3.2958	4.3404	4.7730	3.9187	4.4887	4.1616	4.3732	4.0177	0.9260	-0.1109	0.3025	0.5192
Reps2	_SYS[Phospho (STY)]STSIEEAMK_.2	Q80XA6_S324_M1_Reps2	4.1485	3.1939	3.8822	3.4260	3.0152	3.2134	3.5742	3.5752	3.8743	3.1032	0.9853	-0.0213	0.8461	0.0726
Reps2	_SFS[Phospho (STY)]VEREPQENNSNYPDEPWR_.3	Q80XA6_S119_M1_Reps2	4.0434	3.3477	3.2294	3.7320	3.1691	3.0596	3.4467	4.3865	4.0351	3.6990	0.8814	-0.1821	0.1045	0.9810
Reps2	_APSQAAESSPT[Phospho (STY)]KMDAPHAQPPSKPIR_.4	Q80XA6_T413_M1_Reps2	3.3354	4.1406	4.0222	4.5249	3.9620	3.7675	4.1283	3.5936	3.2422	2.9911	1.1347	0.1823	0.1324	0.8781
Reps2	_RGEDPPT[Phospho (STY)]PPPRPQK_.3	Q80XA6_T340_M1_Reps2	3.7961	3.5463	3.5297	4.5744	3.3677	4.3618	3.2218	3.9277	4.2267	4.2516	0.9887	-0.0164	0.8917	0.0498
Usp6nl	_SVGRPS[Phospho (STY)]PK_.2	Q80XC3_S394_M1_Usp6nl	6.3670	6.2897	6.4218	3.2366	5.3763	3.0241	6.7694	6.6979	6.7454	6.0877	0.7786	-0.3611	0.1141	0.9426
Ric8b	_GDNWYS[Phospho (STY)]EDEDTDTEEYK_.2	Q80XE1_S468_M1_Ric8b	3.9583	4.1384	3.5153	4.8461	3.9849	4.3284	5.0857	4.0244	4.5914	4.1703	0.9240	-0.1140	0.2821	0.5497
Lrrtm4	_AYEAVSIEQDDS[Phospho (STY)]PSLELGR_.2	Q80XG9_S560_M1_Lrrtm4	4.1831	3.4632	3.6692	3.3143	3.3179	3.9666	3.2366	3.9370	6.1090	5.1895	0.7909	-0.3384	0.1106	0.9562
Eif4g3	_TSS[Phospho (STY)]PTSLPPLAR_.2	Q80XI3_S267_M1_Eif4g3	14.1174	13.8247	14.4837	14.4326	12.7302	13.7664	14.4430	13.9334	13.8167	13.7166	0.9939	-0.0088	0.8153	0.0887
Eif4g3	_NPT[Phospho (STY)]PPIGR_.2	Q80XI3_T168_M1_Eif4g3	10.0991	8.7268	8.3774	10.6764	9.0362	8.9952	10.2659	10.0259	9.6492	9.7900	0.9382	-0.0921	0.2201	0.6574
Eif4g3	_GSS[Phospho (STY)]KDLLDNQSQEEQRR_.3	#N/A	7.2252	6.7841	7.2490	7.6792	3.2115	6.6861	3.3779	7.0656	3.9926	6.5625	1.2329	0.3021	0.3012	0.5211
Eif4g3	_GSS[Phospho (STY)]KDLLDNQSQEEQR_.2	#N/A	3.3816	4.1379	3.1153	4.4412	4.0262	4.0993	3.9925	2.9424	3.1404	3.8372	1.1118	0.1529	0.2712	0.5668
Eif4g3	_T[Phospho (STY)]PDEVLEAEAEPK_.2	Q80XI3_T513_M1_Eif4g3	3.9681	3.2236	3.7018	6.6485	3.1119	3.5127	6.3923	6.0658	5.9378	4.4237	0.7060	-0.5022	0.0578	1.2380
Eif4g3	_AESES[Phospho (STY)]DGQAEETADPQSLHSGR_.3	Q80XI3_S472_M1_Eif4g3	4.1462	3.1599	3.8798	3.4236	3.0173	3.4167	3.5719	3.5916	3.8906	3.1009	0.9911	-0.0129	0.9046	0.0436
Pip4k2b	_FGIDDQDYQNSVTR_.2	#N/A	5.2735	5.5518	3.4243	6.0666	4.7789	5.2706	3.2254	5.6144	5.3787	4.9843	1.0542	0.0762	0.6902	0.1610
Atg2b	_DFATAPPTS[Phospho (STY)]PAK_.2	Q80XK6_S1571_M1_Atg2b	9.2577	8.9107	9.5259	9.0685	3.3961	8.9328	9.3213	9.0165	8.8615	3.8507	1.0540	0.0759	0.7980	0.0980
Atg2b	_LS[Phospho (STY)]PSWNPK_.2	Q80XK6_S255_M1_Atg2b	3.6154	6.2245	5.7600	6.7026	4.2419	3.4876	4.5195	3.3136	6.0704	6.2698	0.9925	-0.0109	0.9674	0.0144
Atg2b	_S[Phospho (STY)]SPVC[Carbamidomethyl (C)]STAPVETEPK_.2	Q80XK6_S239_M1_Atg2b	4.3440	4.3830	7.5944	4.0326	4.2713	3.8541	3.7473	4.0859	3.7344	3.9996	1.2196	0.2864	0.2709	0.5671
Atg2b	_S[Phospho (STY)]PGADVTC[Carbamidomethyl (C)]SLPR_.2	Q80XK6_S1741_M1_Atg2b	6.5849	5.7238	4.3005	6.5317	4.0977	6.0555	6.7360	5.2750	5.8671	6.1037	0.9255	-0.1116	0.4834	0.3157
Atg2b	_SAS[Phospho (STY)]VDESRPEFIC[Carbamidomethyl (C)]R_.2	#N/A	4.6295	4.6592	4.8310	4.2038	3.8112	4.2975	3.9185	5.1160	4.5754	4.6226	0.9665	-0.0492	0.5904	0.2289
Atg2b	_AFTEVFLDDSHS[Phospho (STY)]PANR_.3	Q80XK6_S734_M1_Atg2b	3.0440	4.2984	4.2785	3.8223	4.1197	3.6098	3.9706	3.1789	3.4779	3.4996	1.0935	0.1290	0.2690	0.5702
Bdh1	_VSVVEPGNFIAATSLYSPER_.2	#N/A	6.6214	6.5741	6.5411	6.5764	6.4163	6.5472	6.1986	6.3270	6.4998	6.3573	1.0316	0.0449	0.0106	1.9750
Bdh1	_FGIEAFSDC[Carbamidomethyl (C)]LR_.2	#N/A	5.5345	6.3636	6.1921	5.7163	5.2452	5.7947	5.3844	5.9568	5.9128	6.2067	0.9902	-0.0142	0.8251	0.0835
Bdh1	_AILITGC[Carbamidomethyl (C)]DSGFGFSLAK_.2	#N/A	3.9613	3.3328	4.8851	3.5129	2.6823	4.3354	4.8926	3.0774	4.6974	3.6010	0.9306	-0.1037	0.6063	0.2173
Bdh1	_VVNISSMLGR_.2	#N/A	6.1797	4.3938	3.7099	6.6487	5.4986	3.5047	5.3264	4.6759	4.1329	4.4317	1.0749	0.1042	0.6328	0.1988
Bdh1	_SPYC[Carbamidomethyl (C)]ITK_.2	#N/A	4.3734	4.1349	4.1071	3.2860	5.2112	3.1075	4.5015	4.3764	2.9803	3.2530	1.0685	0.0956	0.6160	0.2104
Fam76b	_VSSLS[Phospho (STY)]PEQEQGLWK_.2	Q80XP8_S193_M1_Fam76b	8.0015	7.9993	3.1267	8.5595	7.8034	3.1622	8.4398	7.8061	7.9490	8.1820	0.7959	-0.3294	0.2443	0.6122
Wnk3	_EGETTVNTETSS[Phospho (STY)]PK_.2	Q80XP9_S1039_M1_Wnk3	3.6720	8.1668	8.1722	8.5865	2.8157	8.0796	3.0977	4.0517	8.0011	8.0575	1.1345	0.1820	0.6549	0.1838
Wnk3	_S[Phospho (STY)]VEM[Oxidation (M)]TEDDKVAESSR_.2	Q80XP9_S62_M1_Wnk3	4.1408	3.0854	3.3267	3.8294	2.9737	3.6509	3.5441	4.2891	3.9377	3.7964	0.8996	-0.1526	0.1732	0.7614
Tbc1d5	_TISSSPS[Phospho (STY)]IESLPGGR_.2	Q80XQ2_S565_M1_Tbc1d5	8.4880	8.0955	3.8329	8.5855	5.3261	7.9476	8.7557	8.4504	8.2882	8.4852	0.8294	-0.2698	0.1911	0.7188
Tbc1d5	_EFTGS[Phospho (STY)]PPPSATKK_.2	Q80XQ2_S578_M1_Tbc1d5	8.2748	4.2160	4.0976	4.6003	7.8097	3.6921	4.3150	3.5182	8.7202	3.0664	1.1108	0.1516	0.7194	0.1431
Tbc1d5	_SES[Phospho (STY)]MPVQLNK_.2	Q80XQ2_S546_M1_Tbc1d5	5.6762	4.3144	3.5986	4.1012	3.5383	3.9228	3.8159	4.0172	3.6658	4.0682	1.0771	0.1072	0.4820	0.3169
Fam168b	_[Acetyl (Protein N-term)]M[Oxidation (M)]NPVYS[Phospho (STY)]PGSSGVPYANAK_.2	#N/A	3.1915	4.1659	7.7487	6.9556	3.9821	4.0515	4.1180	3.6575	3.3454	7.1021	1.1010	0.1388	0.7041	0.1524
Nucks1	_ATVTPS[Phospho (STY)]PVKGK_.2	#N/A	10.3752	10.1287	10.9640	10.5823	9.4584	9.9517	10.7983	10.7114	10.3585	10.0784	0.9768	-0.0339	0.4369	0.3597
Nucks1	_ATVTPS[Phospho (STY)]PVK_.2	#N/A	7.6462	6.1852	3.8446	6.4558	3.2546	3.3700	6.2066	6.9640	3.9119	6.8810	0.8556	-0.2249	0.4576	0.3395
Nucks1	_LKATVTPS[Phospho (STY)]PVK_.2	#N/A	7.2412	6.3091	7.5718	3.5745	5.0536	3.3620	3.7228	3.4266	3.7257	3.2518	1.5626	0.6440	0.0652	1.1857
Nucks1	_TPSPKEEDEEAES[Phospho (STY)]PPEKK_.3	#N/A	7.6646	3.7160	7.2314	7.0844	3.5373	6.7998	7.7417	7.1733	7.5936	7.1492	0.8100	-0.3040	0.1799	0.7449
Nucks1	_EEDEEAES[Phospho (STY)]PPEKK_.2	#N/A	7.1127	3.8535	4.2135	6.2437	3.5878	3.0369	6.0570	4.9471	5.5314	4.2985	0.8975	-0.1560	0.5610	0.2510
Nucks1	_KVVDYSQFQES[Phospho (STY)]DDADEDYGRDSGPPAK_.4	#N/A	3.7070	3.0114	4.3938	3.3956	2.8327	3.3960	3.1103	3.2220	4.3714	3.3626	0.9828	-0.0250	0.8720	0.0595
Nucks1	_KVVDYSQFQES[Phospho (STY)]DDADEDYGR_.3	#N/A	3.4513	6.5982	3.7374	8.8641	7.1554	6.8427	3.9684	8.7073	8.0465	7.4900	0.8660	-0.2075	0.5073	0.2948
Nucks1	_VVDYSQFQESDDADEDY[Phospho (STY)]GRDSGPPAK_.3	Q80XU3_Y26_M1_Nucks1	3.8657	3.2396	4.5525	3.5318	2.9913	3.2379	3.4717	3.2990	4.4650	3.5213	0.9676	-0.0475	0.7460	0.1272
Nucks1	_LKATVT[Phospho (STY)]PS[Phospho (STY)]PVKGK_.2	Q80XU3_S181_M2_Nucks1	3.5170	4.2733	3.2507	4.1423	4.1616	3.9639	6.7440	3.9761	3.6247	4.1093	0.8420	-0.2480	0.2624	0.5811
Nucks1	_NSQEDS[Phospho (STY)]EDS[Phospho (STY)]EEKDVK_.2	#N/A	8.0959	7.8869	8.4013	8.0598	7.1641	7.1583	8.4679	8.3671	8.1638	7.5805	0.9570	-0.0635	0.2871	0.5420
Nucks1	_VVDYSQFQES[Phospho (STY)]DDADEDYGR_.2	#N/A	3.8958	6.6036	3.8331	9.4238	7.8979	6.5999	3.3068	9.1664	8.5528	8.4081	0.8664	-0.2068	0.5455	0.2632
Nucks1	_VVDYSQFQES[Phospho (STY)]DDADEDYGRDSGPPAKK_.4	#N/A	3.8724	3.7976	3.8229	4.9534	3.6392	3.6545	3.7948	3.9040	3.7001	3.5281	1.0603	0.0844	0.4145	0.3824
Nucks1	_SGKNS[Phospho (STY)]QEDS[Phospho (STY)]EDS[Phospho (STY)]EEKDVK_.2	Q80XU3_S54_M3_Nucks1	3.0966	3.2359	3.7383	4.0561	3.4932	3.7096	3.9703	3.4130	3.8022	3.2416	0.9856	-0.0209	0.8230	0.0846
Nucks1	_DDSHS[Phospho (STY)]AEDS[Phospho (STY)]EDEKDDHKNVR_.4	Q80XU3_S75_M2_Nucks1	3.9811	3.5353	3.7216	4.2242	3.5122	3.9864	3.9389	3.9600	3.6086	3.7114	1.0058	0.0084	0.8936	0.0489
Nucks1	_EM[Oxidation (M)]LLEDVGS[Phospho (STY)]EEEPEEDDEAPFQEK_.3	Q80XU3_S113_M1_Nucks1	3.2608	4.0660	3.9476	6.1791	3.8873	3.8422	4.2030	6.4792	3.3168	1.9880	1.0501	0.0706	0.8308	0.0805
Nucks1	_KDDS[Phospho (STY)]HS[Phospho (STY)]AEDS[Phospho (STY)]EDEKDDHK_.4	#N/A	3.6162	4.3725	3.3499	4.3945	4.2608	3.8646	3.7578	4.1075	4.4065	4.0718	0.9732	-0.0392	0.6677	0.1754
Lrfn4	_SC[Carbamidomethyl (C)]S[Phospho (STY)]LDLGDTGGC[Carbamidomethyl (C)]YGYAR_.2	Q80XU8_S585_M1_Lrfn4	3.4615	4.2667	8.9178	9.2738	7.1457	8.1672	8.8316	8.7994	8.7126	8.6450	0.7856	-0.3481	0.1729	0.7622
Lrfn4	_GVGGS[Phospho (STY)]AERLEESVV_.2	Q80XU8_S627_M1_Lrfn4	3.6709	9.7798	4.1008	10.0244	8.8941	10.0009	10.5382	9.9209	9.9337	9.9726	0.7675	-0.3818	0.1676	0.7757
Glrx5	_SALVDEKDQDS[Phospho (STY)]K_.2	Q80Y14_S151_M1_Glrx5	5.5119	5.4320	5.9045	6.2735	5.9604	5.8362	5.4428	5.7157	5.7783	5.7924	1.0242	0.0345	0.4435	0.3531
Llgl1	_S[Phospho (STY)]LRQS[Phospho (STY)]FRR_.2	#N/A	7.1245	6.7711	7.1045	7.3185	6.3132	7.1138	6.8931	6.4637	6.8898	7.0186	1.0207	0.0296	0.5161	0.2873
Llgl1	_S[Phospho (STY)]LRQS[Phospho (STY)]FR_.2	#N/A	6.1106	5.7482	5.6417	5.6928	4.5652	5.5728	5.2151	5.4374	5.8015	5.1460	1.0287	0.0409	0.6069	0.2169
Llgl1	_DIILAPES[Phospho (STY)]C[Carbamidomethyl (C)]EGS[Phospho (STY)]PSSAHSKR_.3	#N/A	3.4431	6.2766	4.1299	3.8723	4.0697	3.6598	7.0986	3.1288	3.4279	6.2990	0.8503	-0.2339	0.4549	0.3420
Llgl1	_DIILAPESC[Carbamidomethyl (C)]EGS[Phospho (STY)]PSSAHSK_.2	#N/A	3.3713	7.7138	7.6232	7.3855	7.1836	3.8566	8.0218	7.0908	6.8790	6.4322	0.8710	-0.1993	0.4117	0.3854
Prickle2	_S[Phospho (STY)]FQESLGQGSR_.2	#N/A	9.9879	9.3908	9.8880	9.8719	8.7407	9.6728	9.9009	9.3835	9.5698	9.8109	0.9923	-0.0111	0.7792	0.1083
Prickle2	_YVTS[Phospho (STY)]DELLHK_.2	Q80Y24_S807_M1_Prickle2	9.1438	9.0245	3.5712	9.4518	8.1347	9.3501	9.6796	9.2999	9.2126	9.1821	0.8683	-0.2038	0.3211	0.4934
Prickle2	_AC[Carbamidomethyl (C)]SAGEDPNGS[Phospho (STY)]DSSDSAFQNAR_.2	Q80Y24_S319_M1_Prickle2	3.8583	3.0953	3.3154	7.8809	2.9921	7.0598	8.0052	4.2404	6.8353	6.8048	0.7263	-0.4613	0.2033	0.6918
Prickle2	_S[Phospho (STY)]DNALHLASER_.2	#N/A	8.5037	8.0937	8.4706	8.6566	7.3620	8.5307	8.9505	8.1800	8.1762	8.6780	0.9733	-0.0390	0.4563	0.3407
Prickle2	_GHGGS[Phospho (STY)]FIQEC[Carbamidomethyl (C)]R_.2	Q80Y24_S463_M1_Prickle2	7.1174	6.5706	6.8121	7.1274	6.3955	6.7397	7.1552	6.6795	6.9234	6.7781	0.9869	-0.0190	0.6102	0.2145
Prickle2	_S[Phospho (STY)]REEPFHYGNK_.3	#N/A	7.7573	6.9268	7.8888	7.7894	6.8786	7.3294	7.6602	7.9733	7.7422	7.7241	0.9554	-0.0658	0.1803	0.7439
Prickle2	_SRS[Phospho (STY)]DNALHLASER_.3	#N/A	8.2096	8.0274	8.9577	8.7310	3.8732	8.1836	7.7692	8.2561	8.1726	8.4214	0.9398	-0.0896	0.6268	0.2029
Prickle2	_QRS[Phospho (STY)]FQESLGQGSR_.2	#N/A	5.1058	3.9289	5.9154	5.2097	4.7653	3.9023	5.0481	3.9612	3.2804	5.2122	1.0981	0.1350	0.4496	0.3472
Prickle2	_GS[Phospho (STY)]M[Oxidation (M)]ESLALSNATGLSAEGGAK_.2	#N/A	3.4911	3.5666	4.1779	3.9012	3.3880	3.6887	4.0495	3.9480	3.4759	3.5785	0.9839	-0.0235	0.7519	0.1238
Prickle2	_YTEDM[Oxidation (M)]T[Phospho (STY)]PTEQTPR_.2	#N/A	3.3803	3.8725	5.2164	3.6417	3.7608	4.7356	4.3015	3.7514	3.0504	3.4723	1.1255	0.1706	0.3069	0.5130
Prickle2	_TVS[Phospho (STY)]DLALQNAFGER_.2	Q80Y24_S754_M1_Prickle2	3.3219	3.9043	4.0087	4.5113	3.7926	4.3329	4.2260	7.9636	3.2557	2.9775	0.8638	-0.2112	0.5212	0.2830
Prickle2	_M[Oxidation (M)]HQS[Phospho (STY)]FDFDGGIASSK_.2	Q80Y24_S643_M1_Prickle2	3.3150	4.0713	4.5495	4.0933	3.9596	4.1659	4.0590	4.4087	3.2069	3.9836	1.0284	0.0404	0.7085	0.1497
Prickle2	_LMS[Phospho (STY)]QESYSDMSSQSFNETR_.2	Q80Y24_S479_M1_Prickle2	4.1733	3.4777	3.3593	3.8620	3.2990	4.4305	3.2904	4.2565	3.9051	3.8290	0.9861	-0.0203	0.8564	0.0673
Prickle2	_LPGQEGVHIQPMS[Phospho (STY)]ER_.3	Q80Y24_S667_M1_Prickle2	3.7682	3.0236	3.5019	4.5465	2.9119	3.7127	3.1939	3.9555	4.2546	4.2238	0.9157	-0.1271	0.3862	0.4132
Bsdc1	_ADQSISEEPGVWEEEEEELEGIVPS[Phospho (STY)]PK_.3	Q80Y55_S234_M1_Bsdc1	3.9262	5.8660	5.6408	5.9454	6.9582	5.9448	3.4879	6.1715	6.4706	6.2614	1.0207	0.0295	0.8816	0.0547
Rbsn	_DSLSTHTS[Phospho (STY)]PSQS[Phospho (STY)]PNSVHGSR_.3	#N/A	6.0356	6.5315	3.7900	7.3835	6.4512	7.3728	6.1154	7.0272	6.6983	6.8039	0.9399	-0.0894	0.5792	0.2372
Rbsn	_[Acetyl (Protein N-term)]AS[Phospho (STY)]LDDPGEVR_.2	Q80Y56_S3_M1_Rbsn	3.7098	5.3755	3.4435	6.4332	3.4539	6.0431	3.1356	6.0475	5.1677	5.9378	0.9351	-0.0967	0.7179	0.1440
Elac2	_TS[Phospho (STY)]PNRLS[Phospho (STY)]PK_.2	Q80Y81_S195_M2_Elac2	8.0562	7.7456	7.5892	8.1619	3.5829	7.8427	3.8605	3.9726	7.6859	8.0511	1.2156	0.2817	0.3487	0.4575
Elac2	_AHTDEPHS[Phospho (STY)]PQSK_.2	Q80Y81_S815_M1_Elac2	6.1172	6.1650	6.7192	3.5382	2.9030	3.3257	3.6865	6.5306	5.8597	3.2155	0.9941	-0.0085	0.9797	0.0089
Elac2	_QQPS[Phospho (STY)]QSPRTS[Phospho (STY)]PNRLS[Phospho (STY)]PK_.3	Q80Y81_S189_M3_Elac2	3.6297	4.3860	3.3634	4.4080	4.2743	3.8511	4.5563	4.0940	4.3930	4.0853	0.9307	-0.1036	0.2533	0.5964
Elac2	_QQPSQS[Phospho (STY)]PR_.2	Q80Y81_S191_M1_Elac2	3.8447	3.4977	3.5784	4.6230	2.9884	3.6362	3.2704	3.8790	4.1781	4.3003	0.9457	-0.0806	0.5365	0.2705
Dixdc1	_TDEPDS[Phospho (STY)]PSSR_.2	Q80Y83_S287_M1_Dixdc1	3.4109	4.1671	9.2676	9.2996	8.9875	4.0700	9.1952	3.8700	8.8698	3.8664	1.0129	0.0185	0.9660	0.0150
Cnksr2	_GSES[Phospho (STY)]PNSFLDQEYR_.2	#N/A	11.2190	11.2467	11.8307	11.6352	10.6153	11.3451	11.9351	11.2166	11.3320	11.1836	0.9911	-0.0129	0.7005	0.1546

Cnksr2	_RENS[Phospho (STY)]LLR_.2	Q80YA9_S467_M1_Cnksr2	5.6243	5.5484	5.9392	6.1837	4.8636	5.2178	6.0198	5.9808	5.9132	5.8419	0.9367	-0.0944	0.1641	0.7848
Cnksr2	_LGDS[Phospho (STY)]LQDLYR_.2	#N/A	4.1907	10.8993	3.9244	11.8425	10.0476	10.6660	10.5101	11.4755	11.0130	11.1569	0.7786	-0.3610	0.2174	0.6628
Cnksr2	_GSES[Phospho (STY)]PNSFLDQEYRK_.2	#N/A	6.0512	6.1523	3.7209	4.3101	3.6607	6.0634	6.7338	6.4387	6.1762	5.9918	0.7882	-0.3434	0.0681	1.1669
Cnksr2	_STYDGLHVITGTTENS[Phospho (STY)]PADR_.3	Q80YA9_S248_M1_Cnksr2	3.7795	3.4467	4.4663	5.3146	5.5658	3.2896	3.1828	5.1496	4.2990	3.4351	1.0731	0.1018	0.6414	0.1929
Cnksr2	_QEVTGSSAVS[Phospho (STY)]PIRK_.2	#N/A	5.8599	5.1240	3.7446	6.2061	4.5340	5.5759	5.9318	5.4862	5.7392	5.5867	0.9100	-0.1361	0.3091	0.5099
Cnksr2	_QEVTGSSAVS[Phospho (STY)]PIR_.2	#N/A	5.6953	3.1087	4.4911	5.5320	2.9300	3.2987	5.7615	4.6126	4.6607	3.4599	0.9032	-0.1469	0.5590	0.2526
Cnksr2	_IKQEQDYWS[Phospho (STY)]ES[Phospho (STY)]DKEEADTPSTPK_.3	Q80YA9_S685_M2_Cnksr2	4.2496	3.5288	3.7455	3.7314	3.3847	3.9215	3.1737	4.1067	4.5120	4.4107	0.9283	-0.1074	0.3402	0.4683
Cnksr2	_EEKLGDS[Phospho (STY)]LQDLYR_.2	#N/A	3.7797	3.9010	3.8034	3.7240	3.7437	3.5281	3.6778	3.9577	3.8363	3.4446	1.0047	0.0068	0.8749	0.0580
Cnksr2	_SPTSSVATPSSTIS[Phospho (STY)]T[Phospho (STY)]PTKR_.2	Q80YA9_S338_M2_Cnksr2	3.3108	4.1160	3.9976	4.5003	3.9374	3.7921	7.8058	3.6182	3.2668	5.3006	0.7888	-0.3422	0.2459	0.6093
Aatk	_EQDLRS[Phospho (STY)]PPS[Phospho (STY)]PGHQSVQAFPR_.3	Q80YE4_S1032_M2_Aatk	3.5610	4.3173	3.2947	4.3393	4.2056	3.9199	7.0039	4.1627	2.9609	4.0166	0.8685	-0.2034	0.4306	0.3659
Aatk	_AVDSGYDTENYES[Phospho (STY)]PEFVLK_.2	Q80YE4_S950_M1_Aatk	3.0392	4.1869	3.7260	4.2287	4.4264	4.0502	3.9434	5.0053	3.5384	4.1957	0.9454	-0.0810	0.5374	0.2697
Aatk	_SPPS[Phospho (STY)]PGHQSVQAFPR_.3	Q80YE4_S1035_M1_Aatk	3.5347	3.8077	3.2684	4.3130	3.6290	4.1005	4.4613	5.8285	2.9872	3.9903	0.8746	-0.1933	0.3158	0.5006
Aatk	_AS[Phospho (STY)]PEVGHLLSQEDPR_.2	#N/A	5.9781	5.0693	5.5181	5.7202	3.1267	4.9416	5.8047	5.8508	5.7819	5.5004	0.8822	-0.1808	0.2352	0.6286
Arhgap33	_QQS[Phospho (STY)]DGSLVR_.2	Q80YF9_S995_M1_Arhgap33	6.1943	3.2128	6.3476	3.2348	3.1011	3.5235	3.3831	3.7664	4.0654	4.4129	1.0927	0.1279	0.6681	0.1752
Arhgap33	_TPAS[Phospho (STY)]PVER_.2	Q80YF9_S594_M1_Arhgap33	8.7390	9.0548	9.3806	8.7210	7.9877	8.7578	9.3177	9.2484	8.4584	8.7706	0.9804	-0.0285	0.5566	0.2545
Arhgap33	_TSAWLDDGDDLDFS[Phospho (STY)]PPR_.2	Q80YF9_S749_M1_Arhgap33	3.3168	7.0937	7.8368	7.7967	7.6701	7.5147	7.9300	8.6196	7.9694	5.8125	0.9062	-0.1421	0.5056	0.2962
Arhgap33	_S[Phospho (STY)]AKSEESLSSQASGAGLQR_.3	Q80YF9_S657_M1_Arhgap33	3.6115	3.5339	3.6217	3.6698	3.1354	3.6536	3.8081	3.9866	3.6505	3.2998	0.9597	-0.0594	0.3656	0.4370
Arhgap33	_AQQVAEQQSQQEQGGTPPAPHS[Phospho (STY)]PFR_.3	Q80YF9_S949_M1_Arhgap33	3.5465	3.7720	3.7530	3.8290	3.4923	4.0251	3.7714	3.8089	3.8513	4.3345	0.9479	-0.0771	0.1920	0.7167
Bcas1	_RQS[Phospho (STY)]LGGFLK_.2	#N/A	9.2444	9.6788	10.5442	11.1433	8.9068	9.3512	10.1035	9.8136	9.6139	9.4002	1.0081	0.0116	0.8666	0.0622
Bcas1	_LGLS[Phospho (STY)]FRK_.2	Q80YN3_S347_M1_Bcas1	8.5634	9.2289	9.4983	9.8996	8.3039	8.2219	9.4297	8.8632	8.4358	8.6028	1.0136	0.0194	0.7675	0.1149
Bcas1	_ANFTPQETR_.2	#N/A	7.3409	7.0619	7.7880	7.6580	8.3491	7.2055	7.1445	7.5935	7.0964	7.8429	1.0199	0.0285	0.6105	0.2143
Bcas1	_AGPTS[Phospho (STY)]LPLGK_.2	Q80YN3_S414_M1_Bcas1	4.1225	3.2199	8.3506	8.3971	7.6054	7.5833	8.4369	3.6012	3.9002	7.9630	1.0956	0.1317	0.7210	0.1420
Bcas1	_TLVSPNKTETK_.2	#N/A	5.5043	5.0859	5.5389	5.2970	5.7746	5.0727	5.7439	4.9087	5.1832	5.0763	1.0289	0.0410	0.4749	0.3234
Bcas1	_ADS[Phospho (STY)]VC[Carbamidomethyl (C)]DGHAAGQK_.2	Q80YN3_S316_M1_Bcas1	5.1013	5.1859	4.5123	5.7377	5.9800	5.1875	5.3524	4.8389	4.8038	5.3902	1.0368	0.0522	0.5388	0.2686
Bcas1	_M[Oxidation (M)]SDAQVQTDPVSIGPVGK_.2	#N/A	4.7031	5.6530	6.1971	5.8330	6.2524	6.1245	6.1294	5.5884	6.0631	5.8186	0.9820	-0.0262	0.7432	0.1289
Bcas1	_TPS[Phospho (STY)]PPEPEPAGTAQK_.2	Q80YN3_S525_M1_Bcas1	11.8037	11.9332	12.0084	12.6630	12.1091	11.8196	12.2820	11.7096	11.7823	12.0377	1.0086	0.0124	0.6064	0.2172
Bcas1	_EPAPC[Carbamidomethyl (C)]VQPPTVEANAM[Oxidation (M)]QTGDKT[Phospho (STY)]PK_.3	#N/A	5.1456	5.9602	5.8032	7.2592	5.9595	6.3274	3.3892	3.8948	5.9079	3.5750	1.4495	0.5355	0.0119	1.9261
Bcas1	_SC[Carbamidomethyl (C)]S[Phospho (STY)]PPPPPPEPTSEGR_.2	Q80YN3_S390_M1_Bcas1	8.2364	8.7935	4.3257	4.3645	9.0809	8.6550	8.1396	8.4053	8.4251	9.0099	0.8526	-0.2301	0.3129	0.5046
Bcas1	_RM[Oxidation (M)]S[Phospho (STY)]DAQVQTDPVSIGPVGK_.3	#N/A	4.7684	5.6496	5.7437	6.5717	5.2652	5.5811	4.9669	5.5759	5.5848	5.4873	1.0215	0.0307	0.7371	0.1325
Bcas1	_S[Phospho (STY)]VKEDTLSTGAEENAVC[Carbamidomethyl (C)]ESPVETVR_.3	#N/A	3.9664	3.2707	4.0170	3.3603	3.0921	3.1519	3.2863	3.9357	4.1833	3.6220	0.9254	-0.1119	0.3117	0.5063
Bcas1	_KS[Phospho (STY)]VKEDTLSTGAEENAVC[Carbamidomethyl (C)]ESPVETVR_.3	#N/A	3.6153	4.3716	3.5414	4.0440	4.2599	3.8655	3.7587	4.0745	3.7231	4.0110	1.0149	0.0213	0.7635	0.1172
Bcas1	_EDTLSTGAEENAVC[Carbamidomethyl (C)]ES[Phospho (STY)]PVETVR_.3	Q80YN3_S443_M1_Bcas1	3.1354	3.9406	3.8222	4.3248	3.7619	3.9676	4.0395	3.7936	3.4422	4.2918	0.9829	-0.0248	0.7936	0.1004
Bcas1	_QS[Phospho (STY)]LGGFLK_.2	#N/A	2.9050	4.3212	3.5918	6.0856	4.2095	3.9160	3.8091	4.0240	3.6726	4.0615	1.0719	0.1001	0.6244	0.2045
Safb2	_MEEEGS[Phospho (STY)]EDNGLEEDSR_.2	Q80YR5_S95_M1_Safb2	3.7497	3.4764	4.4365	3.4383	2.8754	3.2599	3.1530	3.1793	4.3287	3.4053	1.0065	0.0093	0.9491	0.0227
Afap1	_SGTSSPQS[Phospho (STY)]PVFR_.2	Q80YS6_S669_M1_Afap1	8.4357	8.5961	9.1055	8.8732	7.0213	7.9251	8.8062	8.4125	8.0511	8.1283	0.9972	-0.0040	0.9559	0.0196
Zc3hc1	_LC[Carbamidomethyl (C)]SSSS[Phospho (STY)]SDTSPR_.2	Q80YV2_S409_M1_Zc3hc1	4.9108	6.1732	5.6088	6.6775	4.7067	5.6518	4.6848	6.5745	4.7446	6.1419	1.0154	0.0220	0.8784	0.0563
Zc3hc1	_SQDATVSPGSEQS[Phospho (STY)]EKSPGPIVSR_.3	Q80YV2_S340_M1_Zc3hc1	4.3434	3.4552	3.4500	6.9413	4.9309	3.9563	4.6927	4.0077	5.9966	4.1719	0.9567	-0.0639	0.7949	0.0997
Zc3hc1	_SQDATVS[Phospho (STY)]PGSEQSEK_.2	Q80YV2_S334_M1_Zc3hc1	5.8201	5.9628	5.9781	3.3721	5.0396	5.6489	6.0530	3.2456	4.3950	3.3391	1.2455	0.3167	0.1891	0.7233
Zc3hc1	_DTAATFQSVDGS[Phospho (STY)]PQAEQSPLESTSK_.3	Q80YV2_S62_M1_Zc3hc1	5.7407	5.8208	5.4190	6.8465	5.4814	6.4318	6.3876	5.9029	5.9837	5.8981	0.9857	-0.0208	0.7827	0.1064
Zc3hc1	_SWESSSPVDRPELEAAS[Phospho (STY)]PTTR_.3	Q80YV2_S369_M1_Zc3hc1	3.4713	3.7548	4.1581	3.1600	3.6431	5.9717	4.3755	2.6666	3.1063	3.1270	0.9544	-0.0673	0.8118	0.0905
Pank4	_VRS[Phospho (STY)]FDHPGK_.2	Q80YV4_S63_M1_Pank4	7.2412	6.7010	7.2360	7.3404	6.5209	7.1895	7.4783	7.3295	7.1827	7.5261	0.9538	-0.0683	0.1006	0.9974
Negr1	_VIVNFAPTIQEIK_.2	#N/A	7.1711	7.1356	7.5526	7.3183	7.2057	7.0293	6.9568	7.1962	7.0196	7.2403	1.0186	0.0266	0.2526	0.5976
Negr1	_DQAGEYEC[Carbamidomethyl (C)]SAENDVSFPDVK_.2	#N/A	6.9299	6.6103	6.6634	7.3041	5.4233	6.1132	7.2105	6.4769	6.1487	6.8435	0.9756	-0.0356	0.6822	0.1661
Negr1	_LFNGQQGIIIQNFSTR_.2	#N/A	5.0656	4.8062	3.1667	4.2113	4.0776	3.9993	5.4220	4.2059	6.8750	5.0636	0.7829	-0.3531	0.0687	1.1631
Topors	_LQQTVPADAS[Phospho (STY)]PDSK_.2	Q80Z37_S99_M1_Topors	7.1411	4.0838	4.4932	4.0369	3.9051	3.8244	4.1852	4.4651	3.2633	6.8540	0.9763	-0.0346	0.9035	0.0441
Shank2	_APSPVVS[Phospho (STY)]PTELSK_.2	#N/A	7.7704	4.1263	7.5139	4.2894	5.9327	7.1255	7.7408	3.8291	7.1454	7.7304	0.9266	-0.1099	0.6733	0.1718
Shank2	_NSPAFLS[Phospho (STY)]TDLGDEDVGLGPPAPR_.3	#N/A	4.0757	3.2667	3.8094	8.7518	3.2195	3.1407	3.5014	3.6480	3.9470	7.2599	0.9538	-0.0682	0.8761	0.0574
Shank2	_RNSPAFLS[Phospho (STY)]TDLGDEDVGLGPPAPR_.3	#N/A	3.2080	4.1344	4.4425	9.3381	3.9558	3.7737	4.1345	3.0149	3.3140	3.6635	1.3616	0.4453	0.3058	0.5146
Shank2	_QSNVEDS[Phospho (STY)]PEK_.2	#N/A	4.2081	3.4635	6.8135	7.6856	3.3518	5.6492	3.1660	3.1664	7.4170	3.4183	1.2105	0.2756	0.4866	0.3128
Shank2	_TIVAAGS[Phospho (STY)]VEEAVILPFR_.2	Q80Z38_S1005_M1_Shank2	3.7554	7.7548	3.4890	4.5336	3.4084	4.3211	3.1811	3.9684	4.2674	4.2109	1.1630	0.2178	0.4792	0.3194
Shank2	_S[Phospho (STY)]KSM[Oxidation (M)]TAELEELGLSLVDK_.3	Q80Z38_S370_M1_Shank2	4.8101	3.4267	3.9049	3.4487	3.3150	3.3096	3.2028	3.5525	3.8515	3.1260	1.0784	0.1090	0.4336	0.3629
Shank2	_ELDRFS[Phospho (STY)]LDSEDVYSR_.2	#N/A	4.1372	3.9560	3.3232	6.7367	5.2098	6.5125	3.5406	6.4823	5.6242	3.7929	1.0245	0.0350	0.8995	0.0460
Shank2	_S[Phospho (STY)]NSDNNLNAGAPEWAVC[Carbamidomethyl (C)]SAATSHR_.3	Q80Z38_S41_M1_Shank2	3.7874	3.5443	3.9805	3.2374	4.2194	3.0414	3.9979	5.4201	4.9369	3.0617	0.8348	-0.2604	0.1661	0.7797
Shank2	_ANVISELNSILQQM[Oxidation (M)]NR_.2	#N/A	5.0778	3.7824	4.2606	3.8044	3.6707	2.9539	5.5281	3.1968	4.8620	4.1888	0.8832	-0.1792	0.3588	0.4452
Shank2	_QSNVEDSPEKTC[Carbamidomethyl (C)]S[Phospho (STY)]IPIPTIIVK_.3	Q80Z38_S654_M1_Shank2	3.2317	4.1170	4.1662	3.4640	4.4600	3.0211	4.1450	5.5963	4.8652	3.2665	0.8378	-0.2554	0.1799	0.7450
Shank2	_DNEGFGFVLR_.2	#N/A	4.6722	3.4447	4.4597	5.2824	4.3004	4.4149	3.3234	4.4007	4.6016	5.2443	1.0083	0.0120	0.9356	0.0289
Shank2	_RAPSPVVS[Phospho (STY)]PTELSK_.2	#N/A	7.1283	7.7269	8.4434	9.2526	6.6631	8.3837	8.4196	8.2391	8.3777	7.9899	0.9608	-0.0577	0.5281	0.2773
Shank2	_RELDRFS[Phospho (STY)]LDSEDVYSR_.3	#N/A	3.2145	3.9708	4.4490	3.9928	3.8591	4.2664	4.1411	3.0084	3.3074	3.6701	1.1209	0.1647	0.1791	0.7469
Shank2	_IFLSGITEEER_.2	#N/A	4.9010	3.9244	4.4437	4.8688	3.7932	4.2828	3.7069	3.1721	4.2080	5.2186	1.0718	0.1000	0.5036	0.2979
Shank2	_SM[Oxidation (M)]T[Phospho (STY)]AELEELGLSLVDK_.2	Q80Z38_T374_M1_Shank2	3.0852	3.8677	4.0547	3.9686	3.7202	3.5347	4.1169	3.9026	3.3556	4.2667	0.9475	-0.0778	0.4180	0.3788
Amigo1	_RM[Oxidation (M)]S[Phospho (STY)]DPESVSSVFSDTPIVV_.2	#N/A	3.7102	3.8199	3.9213	3.7978	3.6631	3.6016	3.7549	3.5061	3.7676	4.0207	0.9973	-0.0038	0.9242	0.0342
Adgrb3	_SETGSTISM[Oxidation (M)]SS[Phospho (STY)]LER_.2	#N/A	8.6363	8.1416	9.0101	8.6992	7.4942	7.7486	9.0076	8.3618	8.4606	8.3437	0.9701	-0.0437	0.4574	0.3397
Wdtc1	_QSPS[Phospho (STY)]AGVHTFC[Carbamidomethyl (C)]DR_.2	Q80ZK9_S229_M1_Wdtc1	3.3211	3.9454	4.0079	3.8278	3.9476	3.8222	4.2252	3.6483	4.8180	4.1267	0.9066	-0.1414	0.1250	0.9030
Wdtc1	_KDS[Phospho (STY)]ISEDEM[Oxidation (M)]VLR_.2	Q80ZK9_S511_M1_Wdtc1	4.1584	6.3868	6.4428	5.3101	5.1432	6.3924	6.3149	5.5343	4.1431	5.5539	1.0469	0.0661	0.6821	0.1661
Wdtc1	_GC[Carbamidomethyl (C)]IS[Phospho (STY)]PQVELPPYLER_.2	Q80ZK9_S352_M1_Wdtc1	3.7357	2.9911	3.4694	3.9236	2.8794	3.7452	3.6383	4.1948	3.8434	3.8906	0.8884	-0.1707	0.1054	0.9771
Spag1	_ESGVDPSQVLLS[Phospho (STY)]PDSSEAAR_.2	Q80ZX8_S703_M1_Spag1	3.9764	6.9974	7.0391	7.2244	5.9955	7.1277	7.0008	6.6891	6.6609	6.6176	0.9483	-0.0766	0.6061	0.2175
Ddx42	_YM[Oxidation (M)]AENPTAGVVQEEEEDNLEY[Phospho (STY)]DSDGNPIAPSKK_.4	Q810A7_Y183_M1_Ddx42	3.5054	4.2617	3.6513	4.1540	5.1282	3.9755	3.8687	5.8109	3.6131	4.1210	0.9447	-0.0821	0.6367	0.1961
Ankfy1	_ESGAAEQVDNK_.2	#N/A	4.5051	4.0830	4.0300	4.8891	4.0606	3.6042	4.0915	4.6698	3.6336	3.8583	1.0325	0.0461	0.6576	0.1820
Slitrk1	_VYDC[Carbamidomethyl (C)]GSHSLS[Phospho (STY)]D_.2	Q810C1_S695_M1_Slitrk1	7.1377	6.9053	3.7558	4.2465	6.1621	4.0340	7.4208	6.8544	6.6723	6.9939	0.7693	-0.3785	0.0765	1.1164
Nfasc	_NLILAPGEDGR_.2	#N/A	8.2487	8.1755	8.0536	7.8202	7.7180	8.0422	8.4131	8.1819	7.9555	7.9090	0.9870	-0.0188	0.4704	0.3276
Nfasc	_DLELTDLAER_.2	#N/A	8.1262	7.6395	7.3622	7.3734	7.4075	7.6256	8.0855	7.8549	7.8262	7.8022	0.9616	-0.0565	0.0900	1.0458
Nfasc	_DNILIEC[Carbamidomethyl (C)]EAK_.2	#N/A	6.9384	6.8088	6.6085	6.7938	6.0072	6.6890	6.7461	6.6205	6.9315	6.8049	0.9801	-0.0290	0.4660	0.3316
Nfasc	_YVVGQTPVYVPYEIR_.2	#N/A	6.7654	6.8203	6.7065	6.3944	6.3617	6.3312	6.4605	6.7915	6.4904	6.0534	1.0177	0.0253	0.5094	0.2929
Nfasc	_EVAGDTIIFR_.2	#N/A	6.1525	6.4199	6.1816	6.8668	5.6806	6.8453	6.4083	6.6998	6.5111	6.3843	0.9780	-0.0321	0.5655	0.2476
Nfasc	_EDDSLTIFGVAER_.2	#N/A	6.4958	5.6131	6.6205	6.3417	5.5701	6.1354	6.9400	6.3813	5.9750	5.8235	0.9760	-0.0350	0.6313	0.1998
Nfasc	_AYLTVLADQATPTNR_.2	#N/A	5.6842	5.0383	4.2333	5.3692	5.2716	4.1671	5.5115	5.5315	5.4849	4.8692	0.9273	-0.1088	0.2901	0.5375
Nfasc	_LDC[Carbamidomethyl (C)]PFFGSPIPTLR_.2	#N/A	5.5599	5.3003	3.3243	4.9805	4.7964	4.3457	5.5796	5.3053	5.1156	5.3360	0.8845	-0.1771	0.1762	0.7540
Nfasc	_ITNVSEEDSGEYFC[Carbamidomethyl (C)]LASNK_.2	#N/A	5.3812	5.4585	6.6405	7.0088	3.4357	5.0202	6.2875	5.1747	4.7710	4.8989	1.0393	0.0556	0.7754	0.1105
Nfasc	_VIAVNEVGSSHPSLPSER_.2	#N/A	5.9678	5.7178	3.9834	3.7959	5.2343	3.2445	4.4558	4.3435	5.7347	5.5347	0.9283	-0.1074	0.5905	0.2287

Nfasc	_GTTVQLEC[Carbamidomethyl (C)]R_.2	#N/A	6.0262	4.5009	5.9419	5.8448	5.1838	4.1312	5.2643	4.3740	3.1722	5.3158	1.1633	0.2182	0.2317	0.6351
Nfasc	_SGTLVIDFR_.2	#N/A	3.8316	3.3945	4.5948	4.4269	3.2635	7.4358	4.3168	3.7555	5.0999	3.4873	1.0784	0.1088	0.7058	0.1513
Nfasc	_DQGSYTC[Carbamidomethyl (C)]MASTELDQDLAK_.2	#N/A	2.2488	4.4234	4.1536	4.6877	4.2447	3.4848	3.8456	3.3038	3.6029	3.3746	1.0969	0.1334	0.4830	0.3161
Nfasc	_DKEETEGNESSEATS[Phospho (STY)]PVNAIYSLA_.3	Q810U3_S1231_M1_Nfasc	4.0668	3.3222	4.5907	3.5926	3.2105	3.4141	3.3072	3.0251	2.1292	3.5596	1.2310	0.2998	0.0943	1.0254
Nrcam	_SVQLTWTPGDDNNSPITK_.2	#N/A	7.5764	7.5547	7.9571	8.3085	7.6958	7.7856	8.0940	7.3779	7.6907	7.8287	1.0084	0.0121	0.7361	0.1331
Nrcam	_GFHTPEGVPSAPSSLK_.3	#N/A	6.7881	7.2171	7.4205	7.3548	7.6183	7.1879	7.0436	7.3924	7.4631	6.8187	1.0118	0.0170	0.6606	0.1801
Nrcam	_DNGELPNDER_.2	#N/A	8.7110	8.9226	8.9247	9.5354	8.7673	8.9063	9.1498	8.6846	8.9022	9.2653	0.9956	-0.0063	0.8348	0.0784
Nrcam	_ENIVIQC[Carbamidomethyl (C)]EAK_.2	#N/A	6.6061	6.1354	5.8324	6.6016	6.8336	6.7796	7.0782	5.9678	6.8095	6.8283	0.9691	-0.0453	0.4798	0.3189
Nrcam	_DSTGTYTC[Carbamidomethyl (C)]VAR_.2	#N/A	2.9860	5.6975	3.6728	5.5840	6.4587	5.9443	5.6477	5.7502	5.9818	6.0007	0.8652	-0.2089	0.3007	0.5219
Nrcam	_EDDGTFGEYS[Phospho (STY)]DAEDHKPLK_.3	Q810U4_S1178_M1_Nrcam	3.4191	3.9233	3.1527	4.1974	4.0636	4.0618	4.3456	4.3047	5.7315	3.8746	0.8332	-0.2632	0.0827	1.0827
Nrcam	_NEVHLEIKDPTR_.3	#N/A	5.2485	4.8545	5.2106	5.5447	5.3486	5.7652	5.1951	5.4280	4.7817	5.4090	1.0241	0.0343	0.5449	0.2637
Nrcam	_ISWLTNGVPIEIALDDPSR_.2	#N/A	2.9999	4.0896	4.2344	3.7094	2.9436	2.9802	4.4158	3.6990	3.5221	3.5919	0.9174	-0.1243	0.3873	0.4120
Nrcam	_TLQITHVSEADSGNYQC[Carbamidomethyl (C)]IAK_.3	#N/A	4.1110	3.1316	3.2970	4.5546	3.2367	2.9919	3.3527	4.3188	3.9674	3.7667	0.9227	-0.1160	0.4299	0.3666
Nrcam	_HQAEVSGTQTTAQLK_.3	#N/A	3.6708	3.0863	4.0655	3.9663	3.3957	4.2854	4.1579	3.7571	3.7120	4.8600	0.9086	-0.1383	0.2597	0.5855
Nrcam	_EPAEGNESSEAPS[Phospho (STY)]PVNAMNSFV_.2	#N/A	3.4323	3.7939	4.1191	4.1100	3.6822	2.9424	4.3364	3.4967	3.1453	3.0879	1.0465	0.0655	0.6255	0.2038
Nrcam	_EKEPAEGNESSEAPS[Phospho (STY)]PVNAM[Oxidation (M)]NSFV_.3	#N/A	5.1910	5.3173	4.2332	3.7770	3.6432	2.9814	4.3754	5.9913	3.5233	3.4542	0.9664	-0.0493	0.8312	0.0803
Nrcam	_AETYEGVYQC[Carbamidomethyl (C)]TAR_.2	#N/A	4.0265	3.3159	3.7602	3.3040	3.1703	3.4543	3.4523	3.6972	3.9962	2.9813	0.9925	-0.0109	0.9137	0.0392
Atg4c	_RFST[Phospho (STY)]EEFVLL_.2	Q811C2_T452_M1_Atg4c	3.6133	4.3696	3.3470	4.0460	4.2579	4.9465	3.7607	4.5294	3.7211	4.0130	1.0226	0.0323	0.7877	0.1037
Atg4c	_DFDFTSTAAS[Phospho (STY)]EEDLFSEDERK_.3	Q811C2_S434_M1_Atg4c	2.8972	3.7024	3.5840	4.0867	3.5237	4.2057	3.8014	4.0318	3.6804	4.0537	0.9421	-0.0860	0.3915	0.4073
Dlg1	_DYHFVTSR_.2	#N/A	6.7661	6.4719	7.3031	7.0799	6.9600	6.6168	6.8727	6.3713	6.8761	6.8966	1.0166	0.0237	0.5647	0.2482
Dlg1	_AVEALKEAGS[Phospho (STY)]IVR_.2	#N/A	4.9895	6.6408	7.3173	7.4100	6.2450	6.7012	6.7114	7.2363	6.4579	5.9360	0.9947	-0.0076	0.9460	0.0241
Dlg1	_QVTPDGESDEVGVIPSKR_.3	#N/A	6.3094	5.9397	6.1751	6.5667	6.4286	6.3914	6.0341	6.1269	6.2334	6.7107	1.0041	0.0059	0.8798	0.0556
Dlg1	_QIIEEQSGPYIWVPAK_.2	#N/A	5.6081	5.3881	5.8088	5.9534	5.3324	5.4232	5.7688	5.4840	5.2788	5.3332	1.0219	0.0312	0.4623	0.3351
Dlg1	_VNDC[Carbamidomethyl (C)]ILR_.2	#N/A	5.9626	5.4136	4.5174	5.2811	5.5545	5.2702	4.9335	5.7214	5.2686	5.4577	0.9977	-0.0033	0.9661	0.0150
Dlg1	_IISVNSVDLR_.2	#N/A	6.0329	6.4489	5.9342	6.7078	5.4835	5.9384	6.9115	6.4747	6.0298	6.4622	0.9415	-0.0870	0.1864	0.7295
Dlg1	_IIEGGAAHK_.2	#N/A	5.0274	4.9606	3.3030	5.0007	4.2875	4.0895	3.2501	5.3117	4.4859	5.2485	0.9717	-0.0414	0.8088	0.0922
Dlg1	_IITGGAAAQDGR_.2	#N/A	6.5545	5.7953	3.3941	4.4387	3.5033	6.1824	5.5735	5.5007	4.3624	4.1160	1.0184	0.0263	0.9096	0.0412
Dlg1	_EQM[Oxidation (M)]M[Oxidation (M)]NSSVSSGSGS[Phospho (STY)]LR_.2	#N/A	9.0787	9.2593	10.0690	9.1010	8.5393	9.9841	9.6280	9.5509	9.4659	9.6577	0.9752	-0.0362	0.4538	0.3431
Dlg1	_ALFDYDK_.2	#N/A	2.5476	4.2382	3.6748	4.1775	2.6265	2.4424	3.8921	3.4935	3.5896	4.1444	0.8689	-0.2027	0.2982	0.5254
Dlg1	_NLSEIENVHGFVSHSHIS[Phospho (STY)]PIK_.3	Q811D0_S158_M1_Dlg1	3.0852	3.8414	4.0715	4.5742	3.7297	4.3957	4.2889	5.5105	3.1929	3.0403	0.9854	-0.0212	0.9144	0.0388
Dlg1	_T[Phospho (STY)]KDSGLPSQGLNFR_.3	Q811D0_T595_M1_Dlg1	3.3846	4.1409	3.7721	4.2747	4.0292	4.0963	3.9894	3.8437	3.4923	4.2417	1.0149	0.0213	0.7861	0.1045
Dlg1	_HC[Carbamidomethyl (C)]ILDVSGNAIK_.2	#N/A	3.6194	4.3757	3.3531	4.3977	2.9372	3.5662	4.5882	4.1043	4.4034	4.0750	0.8638	-0.2112	0.0966	1.0151
Dlg1	_HVTSNAS[Phospho (STY)]DSESSYR_.2	Q811D0_S688_M1_Dlg1	4.0250	3.2804	4.9733	3.6344	3.1687	3.4559	3.3490	2.9833	4.1327	3.6014	1.0682	0.0951	0.5565	0.2545
Dlg1	_AASHEQAAAALK_.2	#N/A	3.3315	3.0774	3.3348	3.8374	2.9657	3.6590	3.5521	4.2810	3.1409	3.8044	0.9115	-0.1337	0.2336	0.6316
Dlg1	_TRGDKGEIPDDM[Oxidation (M)]GS[Phospho (STY)]K_.3	#N/A	3.3823	4.1055	4.3637	4.4046	4.0177	3.9260	4.1724	3.6076	4.0566	3.7014	1.0383	0.0542	0.5137	0.2893
Dlg1	_FIEAGQYNNHLYGTSVQSVR_.3	#N/A	5.8312	4.0850	4.5632	4.1070	3.9733	4.1521	4.2553	4.3950	4.1052	3.7843	1.0767	0.1066	0.4219	0.3748
Dlg1	_NAGQAVTIVAQYRPEEYSR_.3	#N/A	3.1759	4.1665	4.4105	3.9542	3.9878	3.7417	4.1025	3.0469	4.7684	3.6315	1.0048	0.0069	0.9597	0.0179
Dlg1	_EAGS[Phospho (STY)]IVR_.2	#N/A	3.2798	4.0626	4.5143	4.0581	3.9244	4.2011	4.2064	4.4439	3.2421	3.7354	1.0255	0.0364	0.7450	0.1278
Atpaf1	_RGS[Phospho (STY)]GLPPEGADGSGVSELEANPFYDR_.3	Q811I0_S52_M1_Atpaf1	7.3307	2.9563	7.3721	8.4197	6.2618	6.9328	3.6732	8.0049	6.8942	3.9255	1.1638	0.2188	0.4951	0.3053
Mast4	_TPSPTPQPTS[Phospho (STY)]PQR_.2	Q811L6_S1389_M1_Mast4	3.8835	6.9159	7.4154	3.1609	3.0272	6.8984	7.2907	6.9786	6.9841	4.3390	0.8154	-0.2945	0.3489	0.4573
Mast4	_SDS[Phospho (STY)]LPSFR_.2	Q811L6_S2517_M1_Mast4	3.0377	6.4287	3.7245	4.2272	3.6643	4.0652	3.9419	3.8913	6.7874	4.1942	0.8911	-0.1664	0.5461	0.2627
Mast4	_KHS[Phospho (STY)]LEVTQEEVQR_.3	Q811L6_S1465_M1_Mast4	4.1745	2.4349	3.9082	4.6969	3.3182	3.8596	3.6002	3.5492	3.8483	3.1292	1.0567	0.0796	0.6417	0.1927
Mast4	_SLSYSAS[Phospho (STY)]FPEAQPGVR_.2	Q811L6_S2461_M1_Mast4	4.1213	3.5230	3.8550	3.3987	3.2650	5.8474	3.5470	3.6024	3.9015	3.0760	1.1188	0.1620	0.4472	0.3495
Arhgap32	_AVS[Phospho (STY)]PEGDER_.2	Q811P8_S1823_M1_Arhgap32	8.3223	8.0920	8.7886	8.5940	7.8719	8.3772	8.2667	8.3405	8.4065	8.3301	1.0006	0.0009	0.9772	0.0100
Arhgap32	_DVINRS[Phospho (STY)]PTQLQLGK_.2	#N/A	6.4943	7.4146	7.7284	7.9193	6.3953	6.5056	4.7705	3.6941	7.6165	7.5962	1.1954	0.2575	0.2175	0.6625
Arhgap32	_S[Phospho (STY)]PTQLQLGK_.2	#N/A	6.5089	5.1913	3.8333	3.7205	3.4826	5.9579	3.9881	3.7713	5.9065	3.6955	1.1018	0.1399	0.5846	0.2331
Arhgap32	_TQAQVSS[Phospho (STY)]PIVTENK_.2	Q811P8_S613_M1_Arhgap32	6.6086	6.4414	3.4724	6.0727	2.8825	3.7421	6.1781	4.1979	5.7993	3.8876	0.9709	-0.0425	0.8841	0.0535
Arhgap32	_NES[Phospho (STY)]EPSEM[Oxidation (M)]K_.2	#N/A	7.2349	6.1061	5.4012	7.6396	6.2612	5.4440	5.2912	5.5866	6.5358	6.5786	1.0583	0.0818	0.5328	0.2734
Arhgap32	_KLHDM[Oxidation (M)]GC[Carbamidomethyl (C)]S[Phospho (STY)]LPEHR_.3	Q811P8_S1876_M1_Arhgap32	5.6379	5.5579	6.8267	6.5441	5.2667	5.8356	6.2318	6.0270	6.6122	5.6860	0.9683	-0.0464	0.5900	0.2292
Arhgap32	_LSPFFTLDLS[Phospho (STY)]PTDDK_.2	Q811P8_S871_M1_Arhgap32	3.2571	4.0134	4.4917	6.7630	3.9017	4.2237	4.1169	4.4666	3.2648	3.7127	1.1418	0.1913	0.4211	0.3756
Arhgap32	_DAEAGGSQSQTPGSTASSEPVS[Phospho (STY)]PVQEK_.3	Q811P8_S856_M1_Arhgap32	8.7613	8.8808	9.4606	9.4985	8.0551	8.7687	9.2431	9.0780	8.9475	8.7075	0.9900	-0.0145	0.7619	0.1181
Arhgap32	_S[Phospho (STY)]SSDALSASFNGDVLGNR_.2	Q811P8_S730_M1_Arhgap32	3.5897	3.7195	4.4569	3.7495	3.5408	3.5489	3.7325	3.0808	3.8810	3.5548	1.0576	0.0808	0.3881	0.4111
Arhgap32	_SAKS[Phospho (STY)]EESLTSLHAVDGDSK_.3	#N/A	7.1998	7.4943	8.0925	7.5891	6.6436	7.2074	7.8848	7.5603	7.5548	7.1428	0.9782	-0.0319	0.5654	0.2477
Arhgap32	_SLLVSS[Phospho (STY)]PSTK_.2	Q811P8_S592_M1_Arhgap32	3.1842	4.2479	4.4187	4.4752	3.8287	4.2967	4.1899	3.6433	3.2919	3.7382	1.0967	0.1332	0.2513	0.5998
Arhgap32	_S[Phospho (STY)]EESLTSLHAVDGDSK_.2	#N/A	4.1244	3.1017	3.3104	7.2219	2.9900	3.6346	3.5277	3.4054	3.9540	3.7801	1.0442	0.0624	0.8404	0.0755
Arhgap32	_LSS[Phospho (STY)]RDFAFYNPR_.2	Q811P8_S1689_M1_Arhgap32	4.1701	3.4744	3.3560	3.1454	3.2957	4.4337	3.2937	3.8557	4.1548	4.3236	0.9332	-0.0998	0.4415	0.3550
Arhgap32	_S[Phospho (STY)]FFNLGK_.2	Q811P8_S662_M1_Arhgap32	3.8869	3.1912	3.8849	3.4286	3.0126	3.2161	3.5769	3.5726	3.8716	3.1059	0.9731	-0.0394	0.6870	0.1630
Arhgap32	_AES[Phospho (STY)]FPGHSC[Carbamidomethyl (C)]GFAAPVPPTR_.3	Q811P8_S1405_M1_Arhgap32	3.6264	2.9308	4.3132	3.6891	4.2529	3.4766	3.8374	3.3026	2.9512	3.2820	1.1111	0.1520	0.2510	0.6003
Pcyt1b	_QS[Phospho (STY)]PVSSPTR_.2	Q811Q9_S319_M1_Pcyt1b	3.3240	4.1292	4.0108	3.9915	3.9505	3.7790	4.1398	6.7994	6.8403	3.6688	0.7207	-0.4726	0.0604	1.2187
Pcyt1b	_M[Oxidation (M)]LQALS[Phospho (STY)]PK_.2	#N/A	4.9250	5.3212	4.9799	5.1959	3.7665	5.1771	4.7006	6.2926	5.0391	5.9384	0.8911	-0.1664	0.1872	0.7277
Pcyt1b	_AASASISS[Phospho (STY)]MS[Phospho (STY)]EGDEDEK_.2	Q811Q9_S360_M2_Pcyt1b	3.5612	3.6052	4.0035	5.6539	3.4649	2.9803	3.9372	3.5130	3.8121	4.0168	1.0153	0.0219	0.9066	0.0426
Pcyt1b	_MLQALS[Phospho (STY)]PKQS[Phospho (STY)]PVSSPTR_.2	Q811Q9_S315_M2_Pcyt1b	3.8684	3.1727	4.5552	5.4929	2.9941	3.2346	4.4246	3.5541	3.8531	3.5240	1.0123	0.0177	0.9303	0.0314
Srgap3	_GRIES[Phospho (STY)]PEK_.2	#N/A	7.7506	7.6862	7.8992	7.8208	6.6332	7.3362	7.7156	7.8203	7.6453	7.6084	0.9771	-0.0334	0.4937	0.3066
Srgap3	_GRIES[Phospho (STY)]PEKR_.2	#N/A	6.7310	6.9244	7.3361	7.2443	5.4789	6.7322	7.1266	7.5016	6.7435	6.6996	0.9606	-0.0580	0.4783	0.3203
Srgap3	_SGGDTHS[Phospho (STY)]PPR_.2	#N/A	8.0025	8.0724	7.8133	7.9942	3.6581	6.9137	8.3282	8.1978	7.9189	7.8569	0.8762	-0.1907	0.2922	0.5342
Srgap3	_ADSEASS[Phospho (STY)]GPLLDDK_.2	Q812A2_S821_M1_Srgap3	3.5451	3.5272	4.0304	4.1283	4.8021	3.9382	6.9802	3.5091	3.4991	3.1318	0.9335	-0.0994	0.7153	0.1455
Srgap3	_SSS[Phospho (STY)]SSSSGVGSPAVTPTEK_.2	Q812A2_S1070_M1_Srgap3	3.7363	9.5985	3.8056	9.8844	3.6319	3.7680	3.5494	3.6769	8.4554	4.0840	1.1611	0.2155	0.6762	0.1699
Srgap3	_RRS[Phospho (STY)]GGDTHS[Phospho (STY)]PPR_.2	#N/A	3.9750	3.5549	5.3030	3.9164	3.5642	4.1526	3.7627	5.3517	5.4104	3.8101	0.8896	-0.1688	0.3328	0.4779
Srgap3	_IES[Phospho (STY)]PEKR_.2	#N/A	11.9894	3.3706	12.2753	3.5442	3.2589	6.0433	12.2902	12.1918	12.3465	3.5112	0.6690	-0.5799	0.2666	0.5742
Srgap3	_NDLQS[Phospho (STY)]PTEHISDYGFGGVM[Oxidation (M)]GR_.3	Q812A2_S837_M1_Srgap3	3.7289	7.5923	3.9360	8.7447	5.7989	6.9170	3.9547	8.4234	7.6790	3.9041	1.0216	0.0308	0.9286	0.0322
Srgap3	_SGGDTHS[Phospho (STY)]PPRGLGPSIDTPPR_.3	#N/A	5.9743	4.2471	3.2245	6.8636	4.1354	6.2909	3.8832	3.9500	3.5986	4.1355	1.3163	0.3965	0.1364	0.8651
Srgap3	_MATFGS[Phospho (STY)]AGSINYPDKK_.2	#N/A	3.6947	3.3353	3.7668	3.7391	3.2236	3.4850	3.7098	3.7937	3.9458	3.9654	0.9188	-0.1222	0.0380	1.4200
Srgap3	_LRS[Phospho (STY)]DGAAIPR_.2	Q812A2_S858_M1_Srgap3	4.2817	6.1633	3.4677	5.4796	3.4074	6.1329	5.4019	6.1183	3.7967	5.6800	0.9186	-0.1224	0.5905	0.2288
Srgap3	_AAAC[Carbamidomethyl (C)]PSS[Phospho (STY)]PHKIPLSR_.3	Q812A2_S895_M1_Srgap3	3.4568	3.8856	3.1905	6.4408	3.7069	4.0226	4.3834	4.2669	3.0651	3.9124	1.0538	0.0756	0.7528	0.1233
Srgap3	_HSS[Phospho (STY)]LGDHKS[Phospho (STY)]LEAEALAEDIEK_.3	Q812A2_S948_M2_Srgap3	3.1301	4.0968	3.8169	4.3195	3.7566	3.9736	4.0342	4.4576	3.4482	4.2865	0.9488	-0.0758	0.4652	0.3324
Srgap3	_SLEAEALAEDIEK_.2	#N/A	3.1542	4.2194	4.3887	3.9324	4.1344	3.7199	4.0807	3.9328	3.3990	3.6097	1.0451	0.0636	0.5284	0.2770
Prr5	_S[Phospho (STY)]SPTPHSGPC[Carbamidomethyl (C)]PSR_.2	Q812A5_S310_M1_Prr5	4.1406	3.0855	3.3266	3.8293	3.2663	2.9623	3.5439	4.2892	3.9378	3.7963	0.8827	-0.1801	0.1208	0.9178
Tufm	_LLDAVDTYIPVPTR_.2	#N/A	7.8737	8.0681	8.5825	8.0540	8.0071	8.0828	8.2681	8.0073	7.9271	7.8760	1.0114	0.0164	0.5359	0.2709
Tufm	_GTVVTGTLER_.2	#N/A	7.4093	7.6474	7.7480	7.7729	7.8274	7.7673	7.4562	7.9438	7.5590	8.0412	0.9929	-0.0102	0.7005	0.1546

Tufm	_AEAGDNLGALVR_.2	#N/A	7.8151	7.9736	8.0733	8.1248	7.7757	7.8173	7.4640	7.4875	7.7815	7.7592	1.0403	0.0569	0.0164	1.7857
Tufm	_TVVTGIEM[Oxidation (M)]FHK_.2	#N/A	4.4202	5.8890	5.9565	6.1090	6.1249	6.3876	6.4193	6.4063	6.3534	6.1665	0.9176	-0.1240	0.1879	0.7261
Tufm	_TIGTGLVTDVPAM[Oxidation (M)]TEEDKNIK_.3	#N/A	4.5610	5.1384	5.5408	4.9378	5.3105	5.2904	5.4460	4.8846	4.5742	5.0967	1.0259	0.0369	0.5844	0.2333
Tufm	_KYEEIDNAPEER_.3	#N/A	6.5995	5.7055	3.9246	6.3375	6.8936	6.4728	6.4656	6.2297	6.1905	6.5772	0.9408	-0.0880	0.5190	0.2848
Tufm	_KGDEC[Carbamidomethyl (C)]ELLGHNK_.3	#N/A	5.9847	4.9431	5.7174	5.3864	6.1696	6.3825	6.3012	5.0113	5.6995	6.4464	0.9828	-0.0250	0.7952	0.0995
Tufm	_TTLTAAITK_.2	#N/A	5.5609	5.4060	5.5065	5.6017	5.6897	5.8481	4.5326	5.5702	5.3174	6.2009	1.0364	0.0516	0.5076	0.2945
Tufm	_YEEIDNAPEER_.2	#N/A	7.1115	7.1245	7.0873	3.1866	7.8141	7.3398	7.2800	7.2164	7.4999	7.4033	0.8994	-0.1529	0.4196	0.3772
Tufm	_ILAEGGGAK_.2	#N/A	5.0823	5.1117	4.4709	5.0585	5.9728	5.2143	4.3591	5.6858	5.1522	5.1571	1.0124	0.0178	0.8517	0.0697
Tufm	_ELAM[Oxidation (M)]PGEDLK_.2	#N/A	6.3162	7.1218	7.3705	7.3955	7.5834	7.1001	7.2549	7.2184	7.1587	7.4876	0.9819	-0.0264	0.5893	0.2297
Tufm	_ELLTEFGYKGEETPVIVGSALC[Carbamidomethyl (C)]ALEQR_.3	#N/A	4.0787	0.7906	3.2647	3.9537	3.2044	1.7668	2.1459	3.0097	3.4352	1.5694	1.1193	0.1626	0.6935	0.1590
Tufm	_GEETPVIVGSALC[Carbamidomethyl (C)]ALEQR_.2	#N/A	3.7334	4.2760	4.1577	4.5059	3.3113	4.5065	3.8016	3.4582	4.3633	3.8857	1.0528	0.0742	0.4901	0.3097
Tufm	_GDEC[Carbamidomethyl (C)]ELLGHNK_.2	#N/A	6.6486	2.9545	4.3369	7.1417	4.8667	3.4528	6.4185	5.2390	4.4283	3.4417	1.0037	0.0054	0.9858	0.0062
Tufm	_NM[Oxidation (M)]ITGTAPLDGC[Carbamidomethyl (C)]ILVVAANDGPM[Oxidation (M)]PQTR_.3	#N/A	3.9145	3.7615	4.2397	3.1533	3.3594	5.2753	4.3688	4.3577	2.6605	4.8230	0.9749	-0.0367	0.8549	0.0681
Tufm	_GITINAAHVEYSTAAR_.2	#N/A	4.5923	4.3928	3.5467	3.2142	3.7091	4.1358	4.1026	4.3515	4.6324	4.7423	0.8821	-0.1809	0.1111	0.9544
Tufm	_ADAVQDSEM[Oxidation (M)]VELVELEIR_.2	#N/A	5.7809	6.1959	7.6468	7.1123	6.5357	6.5770	6.3946	5.8471	5.9197	6.0564	1.0970	0.1335	0.1329	0.8766
Tufm	_DKPHVNVGTIGHVDHGK_.4	#N/A	3.5532	4.3584	4.2400	4.1832	4.1797	4.1779	3.9106	4.0982	3.6487	3.4396	1.0904	0.1248	0.1019	0.9920
Tufm	_HYAHTDC[Carbamidomethyl (C)]PGHADYVK_.3	#N/A	3.2530	4.0894	4.4875	4.0313	4.6685	3.8187	4.1795	2.9699	3.2690	3.7085	1.1490	0.2004	0.1502	0.8235
Tufm	_QIGVEHVVVYVNK_.2	#N/A	3.3227	3.9034	4.0095	4.5122	3.7917	4.3337	4.2269	3.6063	3.2549	2.9784	1.1315	0.1782	0.1646	0.7836
Svop	_SEDDAAS[Phospho (STY)]GEHDIQIEGVR_.3	#N/A	3.8284	3.4642	4.0909	6.7264	5.5099	6.0571	4.3287	6.9127	6.5873	6.6453	0.8084	-0.3069	0.1955	0.7087
Svop	_RTGESARS[Phospho (STY)]EDDAAS[Phospho (STY)]GEHDIQIEGVR_.4	#N/A	3.1969	3.9769	3.8675	4.2963	3.8329	4.1023	4.2264	4.0825	3.2468	4.3372	0.9762	-0.0347	0.7389	0.1314
Rnf214	_SDESLPDGLS[Phospho (STY)]PK_.2	Q8BFU3_S36_M1_Rnf214	8.9807	9.5214	9.3253	9.8535	9.1725	9.7884	9.9950	9.4131	9.5981	9.8093	0.9728	-0.0397	0.2290	0.6402
Rnf214	_VDQDDDQDS[Phospho (STY)]SSLK_.2	Q8BFU3_S194_M1_Rnf214	3.6845	2.9889	4.3713	6.0492	2.8102	3.4184	3.0878	3.2445	4.3939	3.3401	1.1054	0.1445	0.5862	0.2320
Rnf214	/l (Protein N-term)]AASEVAGLGAGT[Phospho (STY)]PS[Phospho (STY)]PSESSALC[Carbamidomethyl (C)]	Q8BFU3_S15_M2_Rnf214	3.2760	3.9502	3.9628	4.4654	3.4800	3.8270	4.1801	5.6902	3.3017	4.4324	0.8695	-0.2017	0.2321	0.6344
Rnf214	_NLS[Phospho (STY)]PPSVSSQMITK_.2	Q8BFU3_S48_M1_Rnf214	3.4252	4.1814	3.1588	4.2035	4.0697	4.0557	3.9489	4.2986	3.0968	3.8807	1.0113	0.0161	0.8909	0.0502
Pef1	_VC[Carbamidomethyl (C)]TQLQVLTEAFR_.2	#N/A	3.2458	4.0021	4.7769	4.4135	3.7042	4.2351	4.1306	3.9705	4.5751	4.2126	0.9623	-0.0555	0.6028	0.2199
Actbl2	_VAPDEHPILLTEAPLNPK_.3	#N/A	13.8258	13.7291	14.2603	14.0375	13.8851	13.9751	13.8623	13.6629	13.8638	13.7403	1.0123	0.0177	0.1364	0.8652
Hnrnpa3	_IFVGGIKEDTEEYNLR_.3	#N/A	7.2980	7.2461	7.9207	7.9584	7.3751	7.3364	7.2857	6.5795	7.3767	7.6659	1.0409	0.0578	0.2654	0.5761
Hnrnpa3	_GFAFVTFDDHDTVDKIVVQK_.3	#N/A	7.1983	8.1817	9.2118	8.8999	7.6941	7.8567	8.1418	8.1294	7.8988	8.1875	1.0104	0.0150	0.8345	0.0786
Hnrnpa3	_GFAFVTFDDHDTVDK_.2	#N/A	5.7438	5.5697	6.1830	5.9688	5.7314	6.5158	6.0401	5.8271	5.9799	5.8267	1.0057	0.0082	0.8590	0.0660
Hnrnpa3	_EDTEEYNLR_.2	#N/A	7.0286	6.3544	4.0107	6.7477	7.2788	6.6659	6.6844	6.7205	6.4460	6.6399	0.9585	-0.0612	0.6631	0.1784
Hnrnpa3	_IETIEVM[Oxidation (M)]EDR_.2	#N/A	7.9283	7.8524	7.6656	8.6470	8.0167	8.1021	7.9947	7.6870	7.8533	8.2412	1.0115	0.0165	0.6511	0.1863
Hnrnpa3	_EDSVKPGAHLTVK_.2	#N/A	5.6384	6.1633	6.2831	6.6141	6.8070	6.3766	6.2355	5.7809	6.2645	6.1584	1.0334	0.0474	0.3902	0.4087
Hnrnpa3	_SSGS[Phospho (STY)]PYGGGYGSGGGSGGYGSR_.2	Q8BG05_S359_M1_Hnrnpa3	7.7165	7.3819	7.7215	8.1849	6.8619	7.7976	7.5771	7.3581	10.4640	7.5237	0.9247	-0.1130	0.3556	0.4491
Hnrnpa3	_LFIGGLSFETTDDSLREHFEK_.3	#N/A	5.5466	6.3553	3.8266	7.0062	5.8116	3.3880	6.1419	5.8136	6.3421	6.4572	0.8600	-0.2176	0.2732	0.5635
Hnrnpa3	_LFIGGLSFETTDDSLR_.2	#N/A	4.4825	3.7814	3.6630	3.9356	3.9162	3.4747	3.9014	5.1114	5.3191	3.7479	0.8574	-0.2219	0.1153	0.9381
Hnrnpa3	_GEEGHDPKEPEQLRK_.4	#N/A	3.4208	4.2260	4.1076	3.8947	4.0473	3.6822	4.0430	3.1065	3.1606	3.5720	1.1227	0.1670	0.1010	0.9955
Hnrnpa3	_GFGFVTYSC[Carbamidomethyl (C)]VEEVDAAM[Oxidation (M)]C[Carbamidomethyl (C)]AR_.2	#N/A	3.6513	3.6214	3.9648	3.4171	2.7420	2.9587	3.6569	3.5605	3.8596	3.5629	0.9269	-0.1095	0.3000	0.5229
Hnrnpa3	_WGTLTDC[Carbamidomethyl (C)]VVMR_.2	#N/A	3.0264	4.1998	3.7132	4.2158	4.0881	4.0373	4.3042	3.9027	3.5512	4.1828	0.9736	-0.0386	0.7048	0.1519
Hnrnpa3	_YHTINGHNC[Carbamidomethyl (C)]EVK_.3	#N/A	3.0028	3.2553	3.8208	3.3646	3.0766	3.1521	3.5129	3.6366	3.9356	3.0419	0.9284	-0.1073	0.2633	0.5795
Hnrnpa3	_YGKIETIEVMEDR_.3	#N/A	3.3975	3.9449	3.1312	4.1758	3.7663	3.9632	4.3240	4.3263	3.1245	3.8530	0.9547	-0.0669	0.5719	0.2427
Slc6a15	_QSGS[Phospho (STY)]PTLDTAPNGR_.2	#N/A	3.0162	7.6417	7.5990	4.2993	7.0422	7.2601	7.8623	4.2027	7.6535	7.2189	0.9122	-0.1326	0.6396	0.1941
Slc6a15	_IPSEMSS[Phospho (STY)]PNFGK_.2	Q8BG16_S687_M1_Slc6a15	2.9933	3.7496	4.2279	3.7716	3.6379	2.9867	4.3807	3.2295	3.5286	6.7403	0.7967	-0.3278	0.2157	0.6662
Slc6a15	_KQS[Phospho (STY)]GS[Phospho (STY)]PTLDTAPNGR_.2	Q8BG16_S699_M2_Slc6a15	6.4948	6.3048	6.6907	6.5781	3.7454	6.3479	6.6121	7.0068	6.4318	6.2639	0.9161	-0.1264	0.3761	0.4247
Slc6a15	_DTDAEDGS[Phospho (STY)]EADDERPAWNSK_.2	Q8BG16_S55_M1_Slc6a15	3.2683	3.9579	3.9551	4.4578	3.8462	4.2793	4.1724	3.6607	3.3093	1.6065	1.2427	0.3135	0.1507	0.8218
Necab1	_ETSPSSNNS[Phospho (STY)]SEELSSTLQLSK_.2	Q8BG18_S14_M1_Necab1	3.9017	3.4533	3.8665	4.2241	3.2955	3.6398	3.6862	5.2124	3.8370	4.0097	0.8910	-0.1665	0.1974	0.7046
Psmd11	_AQS[Phospho (STY)]LLSTDR_.2	Q8BG32_S14_M1_Psmd11	7.6164	6.8652	6.9951	7.5811	5.1268	7.3231	6.8943	7.4008	7.4060	7.3971	0.9510	-0.0725	0.4825	0.3165
Psmd11	_LYDNLLEQNLIR_.2	#N/A	4.5474	5.3204	4.4353	4.8981	4.8090	3.2586	5.7511	5.4724	5.3415	4.5661	0.8603	-0.2171	0.1099	0.9590
Psmd11	_YQEALHLGSQLLR_.2	#N/A	3.3311	4.0113	3.8620	4.1094	2.7416	4.1498	4.2576	4.3927	3.1909	3.7866	0.9472	-0.0782	0.5786	0.2376
Psmd11	_TAYSYFYEAFEGYDSIDSPK_.2	#N/A	3.5315	3.8109	3.2652	3.8229	4.1761	3.9493	4.4581	4.1922	2.9904	3.9871	0.9622	-0.0556	0.6374	0.1956
Sv2b	_DNYEGYAPSDGYYR_.2	#N/A	8.0385	7.7108	7.5794	7.4508	7.2290	7.4978	8.1266	7.9043	7.7271	7.5018	0.9705	-0.0432	0.2230	0.6517
Sv2b	_YFQDEEYKS[Phospho (STY)]K_.2	Q8BG39_S425_M1_Sv2b	6.3104	6.1447	6.6820	6.3316	5.1486	6.2357	6.5720	6.9469	6.3183	6.6612	0.9272	-0.1091	0.1283	0.8918
Sv2b	_YFQDEEYK_.2	#N/A	6.7836	6.2827	6.0307	5.8875	5.9857	5.7857	6.1786	6.6459	6.2064	6.0561	0.9768	-0.0339	0.5100	0.2925
Sv2b	_HVLFEDTFFDK_.2	#N/A	4.9977	5.2680	5.0131	4.5129	4.4992	5.0228	4.5035	5.3191	5.3396	4.8786	0.9751	-0.0363	0.5927	0.2272
Sv2b	_QVHDTNMR_.2	#N/A	4.8416	3.5205	3.9987	4.5782	4.9463	3.2158	3.1090	3.0535	3.7577	0.9390	1.5410	0.6239	0.0421	1.3760
Sv2b	_DMC[Carbamidomethyl (C)]LSS[Phospho (STY)]SKK_.2	Q8BG39_S144_M1_Sv2b	2.9565	3.7128	4.1910	3.7348	3.6011	3.0235	4.4175	3.2664	3.5654	3.4121	0.9649	-0.0516	0.6932	0.1591
Sv2b	_ADGLGGQADLMAER_.2	#N/A	3.5132	3.8292	3.2469	4.2915	3.6505	4.0790	4.4398	4.2105	3.0087	3.9688	0.9645	-0.0521	0.6722	0.1725
Sv2b	_SNEQNQEEDAQS[Phospho (STY)]DVT[Phospho (STY)]EGHDEEDEIYEGEYQGIPHPDDVK_.5	Q8BG39_S33_M2_Sv2b	3.2344	4.1080	4.4689	4.0127	3.8790	4.2465	4.1609	9.0191	3.2875	3.6900	0.7921	-0.3363	0.3603	0.4433
Tmem169	\cetyl (Protein N-term)]M[Oxidation (M)]EESAPVESQGQLPS[Phospho (STY)]PHHGS[Phospho (STY)]LRR	#N/A	5.9077	6.3469	6.2115	7.0898	4.4286	5.8992	6.2220	6.6286	5.8534	5.5160	0.9877	-0.0178	0.8820	0.0545
Tmem169	_KDS[Phospho (STY)]RPESIIIYR_.3	Q8BG50_S47_M1_Tmem169	3.1903	4.8134	3.8771	4.3797	3.8168	5.1521	4.0944	5.7088	5.5729	4.3467	0.8528	-0.2297	0.1782	0.7490
Tmem169	_[Acetyl (Protein N-term)]MEESAPVESQGQLPS[Phospho (STY)]PHHGS[Phospho (STY)]LR_.3	#N/A	2.9991	3.7554	4.1576	3.1594	3.6437	2.9810	4.3749	3.4582	3.1068	3.1264	0.9809	-0.0279	0.8475	0.0719
Efr3a	_NLYFLT[Phospho (STY)]NK_.2	Q8BG67_T660_M1_Efr3a	7.4350	6.7605	7.6971	13.8158	6.7258	7.1356	12.9758	12.7633	7.3142	7.7169	0.8106	-0.3030	0.3298	0.4817
Efr3a	_LGPPSS[Phospho (STY)]PSAADKEENPAVLAESC[Carbamidomethyl (C)]FR_.3	Q8BG67_S220_M1_Efr3a	3.6626	3.1940	4.0203	3.8173	3.4304	3.2049	3.5567	3.4478	3.7469	3.2414	1.0162	0.0232	0.7738	0.1114
Sh3bgrl2	_FLEANKIEFEEVDITM[Oxidation (M)]SEEQR_.3	#N/A	6.3064	6.4492	7.6480	7.6261	7.4307	7.3698	7.4686	6.9088	6.6213	7.1505	1.0144	0.0206	0.7725	0.1121
Znf365	_AIHEPAES[Phospho (STY)]PR_.2	Q8BG89_S370_M1_Znf365	8.4397	8.3018	8.0556	8.8574	7.7014	7.9937	8.6312	8.3734	8.3479	8.4140	0.9743	-0.0375	0.3354	0.4744
Znf365	_ADSLDAPC[Carbamidomethyl (C)]AS[Phospho (STY)]PGLPTQDTK_.2	Q8BG89_S146_M1_Znf365	8.2440	8.3712	8.8048	9.3945	8.6477	8.2557	9.1816	8.8043	8.9782	8.9521	0.9600	-0.0589	0.1617	0.7914
Znf365	_MVEAVDRT[Phospho (STY)]IEKR_.3	#N/A	5.5726	3.3562	3.2378	5.8094	4.2576	3.0511	3.4552	4.3780	4.9636	3.7075	1.0213	0.0305	0.8999	0.0458
Znf365	_MVEAVDRT[Phospho (STY)]IEK_.2	#N/A	3.2887	3.0887	3.3234	4.0907	2.9770	3.6476	3.5407	4.2314	3.9410	3.7930	0.8778	-0.1881	0.0810	1.0914
Clvs2	_DVDKELS[Phospho (STY)]PK_.2	Q8BG92_S293_M1_Clvs2	8.3687	8.0890	8.2831	8.5266	7.2992	7.9881	8.8757	8.4488	8.2236	7.9381	0.9667	-0.0489	0.3334	0.4771
Clvs2	_SQS[Phospho (STY)]VVDPTALK_.2	#N/A	7.7361	3.2798	7.2588	7.7347	3.1681	6.9674	3.3497	7.5858	4.1321	7.7478	1.0562	0.0788	0.8293	0.0813
Clvs2	_SQS[Phospho (STY)]VVDPTALKR_.2	#N/A	8.1516	7.9213	8.2860	8.3301	6.1085	7.7918	7.6188	8.0565	8.4148	7.8762	0.9716	-0.0415	0.6261	0.2034
Limd2	_SKS[Phospho (STY)]FSLR_.2	Q8BGB5_S30_M1_Limd2	10.8703	10.5077	11.4587	11.3209	11.0141	11.2228	10.9142	10.8202	10.9990	11.0671	1.0106	0.0152	0.5413	0.2665
Limd2	_[Acetyl (Protein N-term)]MFQAAGAAQATPSHEAK_.2	#N/A	4.0297	3.2851	3.7634	3.4935	3.1734	3.4512	3.3443	3.6940	3.9931	2.9844	1.0082	0.0118	0.9061	0.0428
Htatsf1	_VLDEEGS[Phospho (STY)]ER_.2	Q8BGC0_S613_M1_Htatsf1	9.3610	8.4512	8.6111	9.1779	7.9100	8.4284	9.0481	8.9164	9.2063	8.8742	0.9606	-0.0579	0.2386	0.6223
Htatsf1	_[Acetyl (Protein N-term)]SGNNLSGNDEFDEQLR_.2	#N/A	5.7415	6.0543	6.8876	7.2654	3.7751	6.3599	4.0527	6.7676	6.4552	5.6955	1.0472	0.0666	0.7399	0.1308
Htatsf1	_LFDDS[Phospho (STY)]DER_.2	#N/A	7.7346	6.8377	7.1738	7.1975	6.3502	6.9414	7.2753	7.4324	4.3007	7.4937	1.0624	0.0874	0.5464	0.2625
Htatsf1	_AEEGGES[Phospho (STY)]EGDAS[Phospho (STY)]EKDAK_.2	Q8BGC0_S441_M2_Htatsf1	4.2672	4.3067	4.9737	5.1059	4.2822	3.7507	5.0385	4.4419	5.1298	4.7945	0.9168	-0.1253	0.1945	0.7110
Htatsf1	_LFDDS[Phospho (STY)]DERGTVGNVK_.2	#N/A	3.5572	3.6813	3.6583	5.1680	3.6835	4.0406	4.0862	4.6366	4.0486	3.8241	0.9556	-0.0655	0.6039	0.2190
Htatsf1	_VFDDDS[Phospho (STY)]DDIEEEEEADEK_.2	Q8BGC0_S645_M1_Htatsf1	3.4429	4.2481	4.1297	3.1315	4.0694	3.6601	4.0209	3.4862	3.1347	3.0985	1.1005	0.1381	0.2552	0.5931
Htatsf1	_QITAQAWDGTTDYQVEETSR_.2	#N/A	3.8037	3.5387	3.5373	4.5819	2.9474	3.6772	3.2294	3.9201	4.2191	4.2592	0.9422	-0.0859	0.5126	0.2903

Creg2	_RES[Phospho (STY)]GS[Phospho (STY)]SEASPGPR_.2	Q8BGC9_S109_M2_Creg2	3.6885	2.9439	3.4682	3.9709	2.8322	3.7924	3.6855	3.2814	3.5274	3.9379	0.9560	-0.0649	0.5605	0.2514
Coa6	_FEAGGFQSSQSTENS_.2	#N/A	6.7217	6.5770	7.0892	7.5222	7.0953	6.9799	7.2700	7.0898	7.0078	6.9710	0.9877	-0.0178	0.6353	0.1970
Coa6	_EKFEAGGFQSSQSTENS_.2	#N/A	7.0230	4.1431	4.2168	5.8853	5.4349	2.9977	5.9816	3.4414	3.0900	3.1432	1.2647	0.3388	0.2917	0.5350
Eif4b	_SQSS[Phospho (STY)]DTEQPS[Phospho (STY)]PTSGGGK_.2	#N/A	12.0517	12.3982	12.6545	12.8502	12.3877	12.4698	12.4593	12.1410	12.1588	12.4519	1.0135	0.0193	0.3145	0.5023
Eif4b	_AFGS[Phospho (STY)]GYRR_.2	#N/A	3.8254	3.5170	7.5649	7.4589	2.9691	7.0907	7.0906	6.8234	6.7426	4.2809	0.8669	-0.2061	0.5176	0.2860
Eif4b	_SWSS[Phospho (STY)]RDDYSR_.2	Q8BGD9_S312_M1_Eif4b	4.2401	3.4955	3.9738	3.4193	3.3838	3.2408	3.1339	3.1984	4.3478	6.1863	0.8598	-0.2179	0.3521	0.4534
Eif4b	_TGS[Phospho (STY)]ESSQTGASATSGR_.2	#N/A	7.6999	7.4099	7.6092	8.2739	6.9605	7.5257	7.5375	7.4857	7.3789	7.7030	1.0071	0.0102	0.8174	0.0876
Eif4b	_ERHPS[Phospho (STY)]WR_.2	#N/A	6.5289	6.0365	6.7184	7.0036	3.9115	6.9329	6.6740	6.3718	6.2082	3.7078	1.0781	0.1084	0.5930	0.2270
Eif4b	_RAFGS[Phospho (STY)]GYR_.2	#N/A	4.2931	3.5485	4.0267	3.5705	3.4368	3.1878	3.7188	3.4307	3.7297	3.2478	1.0412	0.0582	0.5399	0.2677
Eif4b	_YAALS[Phospho (STY)]VDGEDEDEGDDC[Carbamidomethyl (C)]TE_.2	Q8BGD9_S597_M1_Eif4b	3.5372	4.2076	3.9534	10.0324	8.4061	8.8845	5.9941	8.8916	8.4949	8.9727	0.8041	-0.3146	0.3469	0.4598
Eif4b	_ARPTT[Phospho (STY)]DSFDDYPPR_.2	Q8BGD9_T205_M1_Eif4b	7.6790	6.9658	3.7814	4.2271	3.9173	8.6275	4.1959	7.9291	8.0414	7.4984	0.8482	-0.2375	0.4462	0.3504
Eif4b	_SRTGS[Phospho (STY)]ESSQTGASATSGR_.2	#N/A	3.8406	3.1449	4.5274	3.5292	2.9663	1.8426	3.2439	3.0884	4.2379	3.4962	0.9408	-0.0880	0.6909	0.1606
Eif4b	_RGDDSFGDKYR_.3	#N/A	4.6130	4.6694	4.0259	4.9001	5.0547	3.9149	3.0818	3.4315	3.7306	4.6374	1.2175	0.2840	0.0515	1.2885
Eif4b	_ERHPS[Phospho (STY)]WRS[Phospho (STY)]EETQER_.3	Q8BGD9_S406_M2_Eif4b	4.2821	3.5533	3.9992	4.0712	3.5949	4.3103	3.6912	4.0637	3.9202	4.1495	1.0031	0.0045	0.9485	0.0230
Eif4b	_SAPKEDDASASTSQSSR_.2	#N/A	2.4725	4.8191	4.7778	4.3273	3.2630	2.9657	3.3265	3.8230	4.1220	4.3563	0.9652	-0.0511	0.8075	0.0928
Atg4b	_FFDS[Phospho (STY)]EDEDFEILSL_.2	Q8BGE6_S383_M1_Atg4b	4.1538	3.4582	6.1271	4.9494	6.9025	6.6315	4.9733	6.5163	6.8491	6.1950	0.8756	-0.1917	0.3596	0.4442
Pan2	_VPEPESQTS[Phospho (STY)]PK_.2	Q8BGF7_S1188_M1_Pan2	6.8410	3.9490	7.3061	6.9629	3.8115	4.1668	7.3325	7.0944	3.6894	4.0518	0.9935	-0.0094	0.9760	0.0106
Slc35d3	_DYLM[Oxidation (M)]ENEALPS[Phospho (STY)]P_.2	#N/A	8.2955	8.8739	9.3995	8.5398	8.3871	9.3257	9.1133	9.9716	9.4872	9.4978	0.9250	-0.1125	0.0342	1.4664
Slc35d3	_KDYLMENEALPS[Phospho (STY)]P_.2	#N/A	3.7387	3.6037	3.4724	8.9618	6.4949	3.7422	3.1644	6.1021	4.2841	6.2608	1.0100	0.0143	0.9704	0.0130
Samm50	_FYLGGPTSVR_.2	#N/A	7.0508	6.9798	6.9714	6.7584	6.7519	6.6365	6.9432	7.1401	6.4378	6.8763	1.0013	0.0018	0.9529	0.0209
Samm50	_LELNYC[Carbamidomethyl (C)]IPM[Oxidation (M)]GVQGGDR_.2	#N/A	5.3484	5.2050	3.4443	4.5579	4.8931	4.3454	3.2054	3.9441	5.7122	5.2386	1.0237	0.0338	0.8572	0.0669
Samm50	_THFFLNAGNLC[Carbamidomethyl (C)]NLNYGEGPK_.3	#N/A	3.2929	4.0495	4.5275	4.0712	3.8708	3.8587	4.2195	4.6945	4.9896	3.8370	0.8895	-0.1689	0.1263	0.8986
Samm50	_DVVVQHVHFDGLGR_.3	#N/A	4.2732	3.5775	3.4591	4.5432	3.3988	4.3306	3.1906	3.9588	4.2579	4.2205	1.0060	0.0086	0.9441	0.0250
Samm50	_SSLSHAM[Oxidation (M)]VIDSR_.2	#N/A	5.2592	3.5629	3.4445	3.9472	3.3842	4.3453	3.6619	4.1713	5.2192	3.9142	0.9408	-0.0880	0.5968	0.2242
Reep1	_SFS[Phospho (STY)]MQDLTTIR_.2	Q8BGH4_S152_M1_Reep1	4.3750	3.6304	4.1087	5.2415	3.5187	3.1059	4.4999	3.3487	2.9819	3.3297	1.1290	0.1750	0.3561	0.4484
Slc7a6	_[Acetyl (Protein N-term)]M[Oxidation (M)]EAQELGS[Phospho (STY)]PTPTYHLLPK_.2	#N/A	3.6579	3.5931	4.0714	4.1134	3.4814	4.0026	3.9708	3.5784	3.6027	3.0547	1.0756	0.1051	0.2253	0.6473
Slc7a6	_EDAGS[Phospho (STY)]PSQGSPETMQLK_.2	Q8BGK6_S30_M1_Slc7a6	4.2892	3.5446	4.0229	3.5667	3.4329	3.1917	3.0848	3.4345	3.7336	3.2439	1.0890	0.1231	0.2354	0.6283
Enpp6	_VDYLTPDFPSLS[Phospho (STY)]YPNYYTLM[Oxidation (M)]TGR_.3	Q8BGN3_S71_M1_Enpp6	4.1582	3.1842	3.8918	3.4356	3.0056	3.2231	3.5839	3.5656	3.8646	3.1129	0.9862	-0.0200	0.8563	0.0674
Vipas39	_AFT[Phospho (STY)]FDDEDDELSQLK_.2	Q8BGQ1_T21_M1_Vipas39	3.6486	3.5776	4.3354	3.3372	4.2751	3.1587	4.5527	3.2805	4.4299	3.3042	0.9564	-0.0643	0.6581	0.1817
Vipas39	_ETAGS[Phospho (STY)]SGSTPEGR_.2	Q8BGQ1_S77_M1_Vipas39	3.6258	4.3821	3.3595	4.0336	4.2704	3.8551	3.7482	4.0849	3.7335	4.0006	1.0075	0.0108	0.8928	0.0493
Vipas39	_TRPGS[Phospho (STY)]FQSLSDALSDTPAK_.3	Q8BGQ1_S119_M1_Vipas39	3.7230	3.7198	3.8670	3.9796	3.5724	3.6576	3.7580	3.6707	3.6710	3.8576	1.0037	0.0054	0.8707	0.0601
Aars1	_IVAVTGAEAQK_.2	#N/A	3.4101	5.0106	5.3577	4.3618	5.3220	4.3562	4.3142	5.7279	5.3351	5.1406	0.9039	-0.1458	0.3031	0.5184
Aars1	_ITC[Carbamidomethyl (C)]LC[Carbamidomethyl (C)]QVPQNAANR_.2	#N/A	6.4138	3.4228	4.4901	3.4919	6.9432	6.3113	6.7520	6.5205	4.2751	6.8199	0.8501	-0.2343	0.3573	0.4470
Aars1	_QIWQNLGLDEAR_.2	#N/A	5.9978	5.8824	5.9123	6.2591	5.1952	5.6980	4.4796	5.7613	5.4895	6.2831	1.0583	0.0817	0.3875	0.4118
Aars1	_M[Oxidation (M)]ALELLTQEFGIPVER_.2	#N/A	4.2875	4.5052	4.0212	3.3718	3.4312	6.0791	3.0865	5.9788	6.1043	3.2422	0.9304	-0.1041	0.7093	0.1492
Aars1	_LYVTYFGGDEAAGLEPDLEC[Carbamidomethyl (C)]R_.2	#N/A	3.6079	2.7236	4.2947	3.2965	3.4730	4.3471	3.8559	3.3212	2.9697	3.3110	1.0771	0.1071	0.4766	0.3219
Aars1	_MFVDEVVTGQEC[Carbamidomethyl (C)]GVVLDK_.2	#N/A	3.9783	3.2479	3.1642	4.3414	3.1040	3.4884	3.3816	4.4516	4.1002	3.6339	0.9132	-0.1310	0.3185	0.4969
Fam53b	_SSS[Phospho (STY)]FSLPAR_.2	Q8BGR5_S168_M1_Fam53b	8.2077	3.4971	3.9753	8.6106	7.8026	8.4128	3.6674	7.8082	3.7811	3.1964	1.4634	0.5493	0.1842	0.7347
Fam53b	_S[Phospho (STY)]RSQPC[Carbamidomethyl (C)]VLNDKK_.3	Q8BGR5_S267_M1_Fam53b	5.4761	6.2778	3.8866	6.3830	3.2966	3.3280	3.2212	5.4139	4.2606	3.4735	1.1668	0.2225	0.4364	0.3602
Zkscan1	_ETTGLS[Phospho (STY)]PQAAQEK_.2	Q8BGS3_S13_M1_Zkscan1	3.4553	4.2116	3.1890	4.2336	4.0999	4.0256	3.9187	4.2684	3.0666	3.9109	1.0206	0.0294	0.8024	0.0956
Micall1	_KPS[Phospho (STY)]PSASPTVR_.2	Q8BGT6_S647_M1_Micall1	6.1450	7.6868	3.4489	3.9515	6.8795	7.8852	5.5878	6.3821	6.7133	7.7602	0.9075	-0.1400	0.5688	0.2450
Micall1	_SLHPWYGITPT[Phospho (STY)]SSPK_.2	Q8BGT6_T479_M1_Micall1	3.8763	3.3929	4.1415	3.3784	3.0019	3.2375	5.0567	3.4536	4.1671	3.2135	0.8822	-0.1808	0.2671	0.5734
Micall1	_APS[Phospho (STY)]AS[Phospho (STY)]PLAIHASR_.2	Q8BGT6_S494_M2_Micall1	4.2044	3.4598	3.9381	3.4818	3.3481	3.2765	3.6301	3.5193	3.8184	3.1591	1.0245	0.0349	0.7019	0.1537
Micall1	_DLS[Phospho (STY)]SS[Phospho (STY)]FR_.2	Q8BGT6_S28_M2_Micall1	3.3621	4.1184	4.5966	4.2973	4.0067	4.1188	4.0120	3.8212	3.4698	4.2643	1.0492	0.0693	0.4610	0.3363
Phyhipl	_VHLTQLLEK_.2	#N/A	5.9929	6.9255	5.4198	6.5214	5.3179	6.6331	5.8390	7.0969	6.3566	6.7853	0.9410	-0.0877	0.3679	0.4342
Phyhipl	_TC[Carbamidomethyl (C)]NIS[Phospho (STY)]VGR_.2	Q8BGT8_S372_M1_Phyhipl	6.8928	6.8975	6.9866	5.9677	7.1442	6.7309	6.1263	7.0187	6.2101	6.9997	1.0275	0.0392	0.5444	0.2641
Phyhipl	_SQDSGIAEM[Oxidation (M)]EELPVPHNIK_.3	#N/A	5.8795	6.2958	6.0982	4.9383	6.1935	6.4605	6.5169	5.9280	6.0154	6.0427	0.9758	-0.0353	0.6317	0.1995
Phyhipl	_NLSLEAIQLC[Carbamidomethyl (C)]DR_.2	#N/A	4.3031	4.3422	5.9984	7.7301	6.3819	4.1864	7.2787	7.1954	7.2509	6.9801	0.7651	-0.3863	0.0526	1.2794
Phyhipl	_LDHALSSPSS[Phospho (STY)]PC[Carbamidomethyl (C)]EEIK_.2	#N/A	7.3540	7.2894	4.3104	7.8062	4.5256	7.1224	7.6835	7.1927	3.7210	7.4436	0.9833	-0.0243	0.9225	0.0350
Phyhipl	_GHWFLSPR_.2	#N/A	6.7117	6.9893	7.0405	8.2978	6.5914	6.6623	7.1208	7.1390	6.9862	7.0085	0.9979	-0.0030	0.9650	0.0155
Phyhipl	_TEYTVAVQTASK_.2	#N/A	3.0988	4.1274	3.7856	4.2883	4.0157	5.5316	4.0029	5.0598	3.6327	4.2552	0.9772	-0.0332	0.8432	0.0740
Phyhipl	_ISWDMDSK_.2	#N/A	3.6706	3.6718	3.4042	2.9777	2.1214	3.8103	3.0963	4.0532	4.3522	4.1261	0.8385	-0.2541	0.1468	0.8332
Phyhipl	_DNSGSHGSPISGK_.2	#N/A	3.7078	3.5183	4.3946	3.3964	3.8964	3.2180	3.1111	3.2212	4.3706	3.3634	1.0489	0.0689	0.5978	0.2235
Phyhipl	_FEIAAEK_.2	#N/A	3.2298	3.9861	3.9269	4.4295	3.8744	4.2511	4.1442	3.6889	4.0563	4.1859	0.9828	-0.0251	0.7695	0.1138
Phyhipl	_NLS[Phospho (STY)]LEAIQLC[Carbamidomethyl (C)]DRDGNK_.3	#N/A	3.8605	3.1159	3.5941	3.7989	3.0042	3.6204	3.5136	4.3196	3.9682	3.7659	0.8991	-0.1535	0.1209	0.9176
Ccny	_SAS[Phospho (STY)]ADNLILPR_.2	#N/A	14.8977	14.6204	15.4578	14.8661	13.5612	14.5353	15.3134	14.9370	14.7735	14.6165	0.9830	-0.0248	0.4777	0.3208
Ccny	_ASTIFLSKSQT[Phospho (STY)]DVR_.2	Q8BGU5_T75_M1_Ccny	3.0494	4.2930	4.2839	3.8277	3.6940	3.6152	3.9760	3.1735	3.4725	3.5050	1.0742	0.1033	0.3628	0.4404
Ccny	_LESYRPDTDLS[Phospho (STY)]R_.2	#N/A	5.8902	3.9965	3.7604	3.9332	3.8848	4.9805	6.0059	5.0475	5.4500	5.3166	0.8080	-0.3076	0.0532	1.2741
Ccny	_RSAS[Phospho (STY)]ADNLILPR_.2	#N/A	7.6103	3.2629	8.6533	3.6471	3.0842	3.1445	8.4244	8.0938	7.6688	7.3468	0.6216	-0.6859	0.0517	1.2866
Ccny	_LESYRPDTDLS[Phospho (STY)]REDTGC[Carbamidomethyl (C)]NLQHISDR_.4	#N/A	3.5459	4.3022	3.2796	4.3242	4.1905	3.9350	4.4724	4.1778	2.9761	4.0014	1.0058	0.0083	0.9484	0.0230
Mief1	_AISAPTS[Phospho (STY)]PTR_.2	Q8BGV8_S59_M1_Mief1	10.9654	11.0333	11.4885	3.9511	10.2596	8.3232	8.5924	8.2891	11.0929	10.9660	0.9591	-0.0603	0.8071	0.0931
Wbp1l	_GGVS[Phospho (STY)]PDSEDKTPGR_.2	Q8BGW2_S238_M1_Wbp1l	3.0588	3.8640	3.7456	4.2482	3.6853	4.0442	3.9629	3.8703	3.5188	4.2152	0.9698	-0.0442	0.6330	0.1986
Wbp1l	_SSTRPPSVADPQS[Phospho (STY)]PEVPTDR_.3	Q8BGW2_S183_M1_Wbp1l	4.6754	3.8596	4.3378	5.3166	4.5470	4.3776	4.0299	5.0098	3.4186	3.5589	1.1285	0.1745	0.2073	0.6834
Klhl26	_[Acetyl (Protein N-term)]AESGGSSGSSQS[Phospho (STY)]PERPSSLADR_.3	Q8BGY4_S13_M1_Klhl26	6.2938	5.5536	6.7873	6.4582	5.4180	5.9311	6.4794	6.4254	5.9227	5.5734	0.9956	-0.0063	0.9361	0.0287
Slc5a7	_QS[Phospho (STY)]LTLSSTFTNK_.2	Q8BGY9_S550_M1_Slc5a7	4.0386	3.2940	3.7723	7.7457	5.4089	3.4423	6.9008	7.6069	6.6757	6.3403	0.6710	-0.5757	0.0356	1.4483
Hpcal4	_EFIC[Carbamidomethyl (C)]ALSVTSR_.2	#N/A	6.2570	5.9809	6.3533	6.5689	6.2783	6.0579	6.3495	5.2651	6.5895	5.5464	1.0525	0.0738	0.2852	0.5449
Hpcal4	_SDPSIVLLLQC[Carbamidomethyl (C)]DM[Oxidation (M)]QK_.2	#N/A	5.3253	5.8165	6.3188	5.6701	5.3858	6.1876	5.4955	6.0083	6.0088	5.4856	1.0060	0.0086	0.8890	0.0511
Hpcal4	_LNWAFEM[Oxidation (M)]YDLDGDGR_.2	#N/A	5.9615	6.2264	5.8824	5.3318	5.2614	5.4866	5.7162	6.0868	6.0505	5.5291	0.9737	-0.0385	0.5124	0.2904
Hpcal4	_M[Oxidation (M)]NQDGLTPQQR_.2	#N/A	7.4780	7.5249	7.3961	7.4014	7.6598	7.3004	7.3912	7.4470	7.2824	7.4789	1.0081	0.0117	0.4278	0.3687
Hpcal4	_LAPEVLEDLVQNTEFSEQELK_.3	#N/A	3.7127	3.4963	4.4757	3.6028	4.1679	4.9196	3.7063	3.6317	3.6974	4.5453	1.0430	0.0607	0.6296	0.2010
Hpcal4	_LEM[Oxidation (M)]LEIIEAIYK_.2	#N/A	4.8174	3.8655	4.0475	4.7078	4.7734	4.3717	4.2648	3.5683	6.8845	3.0163	0.9993	-0.0010	0.9968	0.0014
Hpcal4	_MDQDKDDQITLEEFK_.3	#N/A	3.6608	2.9652	4.3476	3.3494	2.7865	3.4421	3.0641	3.2682	4.4176	3.3164	0.9740	-0.0380	0.8121	0.0904
Hpcal4	_M[Oxidation (M)]DQDKDDQITLEEFKEAAK_.4	#N/A	3.3210	3.9052	4.0078	4.5104	3.9475	3.7820	4.2251	3.6080	3.2566	2.9766	1.1125	0.1538	0.2082	0.6815
Fam168a	_[Acetyl (Protein N-term)]M[Oxidation (M)]NPVYS[Phospho (STY)]PVQPGAPYGNPK_.2	#N/A	4.4054	6.6249	3.5982	5.6470	6.3572	3.7816	4.0984	6.5727	3.4697	3.9148	1.1230	0.1673	0.5413	0.2666
Pck2	_YVAAAFPS[Phospho (STY)]AC[Carbamidomethyl (C)]GK_.2	Q8BH04_S304_M1_Pck2	9.1080	8.8663	9.4001	9.3442	8.4848	9.0852	9.4213	8.7432	9.1149	9.0823	0.9953	-0.0067	0.8408	0.0753
Mfsd8	_[Acetyl (Protein N-term)]ANLGSEAEREPLLGPGSPGS[Phospho (STY)]R_.3	Q8BH31_S21_M1_Mfsd8	3.5002	4.2565	3.2339	4.1592	4.1448	3.9807	3.8738	3.9593	3.6079	8.6641	0.7718	-0.3737	0.2771	0.5574
Ubap1	_VLS[Phospho (STY)]PPHTK_.2	Q8BH48_S146_M1_Ubap1	7.9471	8.0175	7.7168	7.8803	7.7832	7.5607	3.8489	8.2388	7.7866	4.1012	1.3043	0.3832	0.0853	1.0692
	RDS[Phospho (STY)]SESQLASTESDKPTTGR.3	Q8BH50_S66_M1_	4.0639	4.0902	4.0360	3.8326	3.8224	4.1941	4.0958	3.6546	3.4998	3.9399	1.0550	0.0773	0.1489	0.8270
	DSSESQLASTES[Phospho (STY)]DKPTTGR.2	Q8BH50_S76_M1_	4.0809	3.3363	3.8146	3.3583	3.2246	3.4000	3.2931	3.6428	3.9419	3.0356	1.0165	0.0236	0.8108	0.0911

	S[Phospho (STY)]RSESETSTMAAK.2	Q8BH50_S143_M1_	4.0076	3.2445	3.3343	4.2349	3.1384	3.8370	3.4514	4.9508	4.0311	3.9797	0.8853	-0.1757	0.2013	0.6961
Wdr48	_DTNNNVAYWDVLK_.2	#N/A	6.0109	6.0770	6.2164	6.5897	5.6356	6.1307	6.1808	5.8679	6.2407	5.7464	1.0168	0.0241	0.5977	0.2235
Tiprl	_IDPNPVDSQST[Phospho (STY)]PSE_.2	Q8BH58_T268_M1_Tiprl	7.9955	7.6086	3.8070	7.8328	3.5293	8.0084	8.2596	7.3824	7.8016	8.0187	0.8218	-0.2832	0.2453	0.6103
Slc25a12	_LTVNDFVR_.2	#N/A	8.3470	8.4431	8.3813	8.1096	7.8756	7.9687	8.2013	8.4871	8.3703	8.4207	0.9782	-0.0318	0.1984	0.7026
Slc25a12	_LLLADENGR_.2	#N/A	8.4113	8.0469	8.3195	7.7360	7.7740	7.9045	7.8086	8.2628	8.2682	8.0726	0.9912	-0.0127	0.6824	0.1659
Slc25a12	_YLGLYNDPNSNPK_.2	#N/A	7.9080	7.5991	7.8543	7.2641	7.3543	7.7013	7.6584	7.9100	7.8099	7.5160	0.9858	-0.0207	0.4838	0.3153
Slc25a12	_LTLADIER_.2	#N/A	8.1025	7.8658	7.6803	7.1300	6.9849	7.8626	7.4672	7.9314	8.1366	7.7301	0.9729	-0.0397	0.4290	0.3676
Slc25a12	_IAPLAEGALPYNLAELQR_.2	#N/A	6.5528	6.4525	6.3630	6.3105	6.0750	6.1808	6.5836	6.7955	6.7121	6.3808	0.9553	-0.0659	0.0323	1.4904
Slc25a12	_IVQLLAGVADQTK_.2	#N/A	7.2406	6.9888	7.1230	7.1103	6.4357	7.1959	7.2943	7.5014	7.0596	7.2727	0.9634	-0.0538	0.1504	0.8227
Slc25a12	_LATATFAGIENK_.2	#N/A	6.9193	6.8372	6.6598	7.4072	6.6983	6.7971	7.0524	7.1281	6.7351	6.6570	0.9990	-0.0014	0.9689	0.0137
Slc25a12	_YEGFFGLYR_.2	#N/A	7.1195	7.2340	6.6008	6.8660	6.1547	6.7788	7.0614	7.0229	6.9848	7.0222	0.9672	-0.0482	0.2782	0.5557
Slc25a12	_SGNGEVTFENVK_.2	#N/A	7.2451	6.5879	7.4753	7.1730	6.4624	6.5615	6.5933	7.0771	6.4368	6.4329	1.0426	0.0602	0.2919	0.5348
Slc25a12	_IYSTLAGTR_.2	#N/A	5.7926	5.4272	5.5767	5.6698	6.0732	5.0349	5.3201	5.9502	5.9287	5.3963	0.9906	-0.0136	0.8180	0.0872
Slc25a12	_VGGINLLTAGALAGVPAASLVTPADVIK_.3	#N/A	4.7002	4.4447	3.8125	3.5682	3.7044	3.3595	3.0124	4.2739	5.0378	4.2618	0.9482	-0.0768	0.6277	0.2022
Slc25a12	_DIEVTKEEFAQSAIR_.3	#N/A	5.2725	5.0572	6.0425	5.5745	5.2969	5.2518	4.8898	5.1357	5.5634	5.9845	1.0042	0.0060	0.9333	0.0300
Slc25a12	_LQVAGEITTGPR_.2	#N/A	6.5805	5.3744	4.5992	5.8182	5.4491	4.2358	6.1740	5.9906	5.9378	5.6394	0.9002	-0.1517	0.2138	0.6700
Slc25a12	_EEGPSAFWK_.2	#N/A	5.2892	5.6677	5.8662	6.1382	5.8444	5.2551	5.4480	5.9389	5.8258	5.9529	1.0728	0.1014	0.3715	0.4300
Slc25a12	_AGQTTYSGVVDC[Carbamidomethyl (C)]FR_.2	#N/A	6.0728	5.5674	3.4747	6.0670	5.3540	5.3404	5.8140	5.4344	5.3733	5.5209	0.9597	-0.0593	0.6645	0.1775
Slc25a12	_FTLGSVAGAVGATAVYPIDLVK_.2	#N/A	3.8502	4.4805	4.4955	4.3140	3.6065	4.1064	3.7949	4.0681	3.7156	3.6136	1.0906	0.1251	0.1211	0.9168
Slc25a12	_SSPQFGVTLVTYELLQR_.2	#N/A	4.0781	3.3336	3.8968	3.5812	2.9094	3.4028	3.2959	3.9793	4.4320	3.0329	0.9589	-0.0605	0.6593	0.1809
Slc25a12	_NSFDC[Carbamidomethyl (C)]FK_.2	#N/A	3.5288	3.7297	3.6113	4.3910	3.5510	2.4692	4.5393	4.1110	4.4100	3.4230	0.8607	-0.2164	0.1615	0.7920
Slc25a12	_NIFLQYASTEVDGEHYM[Oxidation (M)]TPEDFVQR_.3	#N/A	3.9071	3.1625	3.6408	3.1846	3.0508	3.5738	3.3328	3.8166	4.1157	4.3627	0.8753	-0.1921	0.0825	1.0835
Atl1	_ILIPWLLSPER_.2	#N/A	5.2139	5.5450	3.4645	4.9276	4.8062	5.3384	5.2223	5.3604	5.0792	5.0248	0.9441	-0.0830	0.4741	0.3241
Atl1	_SSDWSS[Phospho (STY)]EEEEPVR_.2	#N/A	6.6326	8.6673	3.7367	8.7311	7.4267	8.7905	9.1304	9.1077	8.7556	8.5477	0.8250	-0.2775	0.1618	0.7910
Atl1	_SSDWSS[Phospho (STY)]EEEEPVRK_.2	#N/A	7.4617	7.5059	8.2757	7.7126	6.7637	7.3081	7.4194	7.7956	7.6544	7.2741	0.9893	-0.0155	0.7804	0.1077
Atl1	_DDHSFELDEAALNR_.3	#N/A	3.9254	3.2298	3.1114	3.3901	3.0511	3.1776	3.5383	2.7248	1.8144	3.5810	1.1371	0.1853	0.3088	0.5104
Atl1	_DRNS[Phospho (STY)]WGGFSEK_.2	#N/A	3.6271	3.7791	8.0940	3.8205	3.5064	6.9361	3.7875	3.9040	6.9152	3.6368	1.0876	0.1212	0.7480	0.1261
Atl1	_VAVLLM[Oxidation (M)]DTQGTFDSQSTLR_.2	#N/A	2.4747	4.4174	3.4955	3.9982	2.8049	3.8197	2.3009	4.1203	3.7689	3.9652	0.9895	-0.0152	0.9429	0.0255
Fbxl4	_TS[Phospho (STY)]PLNAEVVQYAK_.2	Q8BH70_S45_M1_Fbxl4	3.6875	3.8152	3.5578	4.0604	3.6785	3.6557	3.7140	4.0995	4.9256	4.0274	0.8929	-0.1635	0.0791	1.1016
Nup107	_HPDISYILGTEGRS[Phospho (STY)]PR_.3	Q8BH74_S87_M1_Nup107	3.7349	3.0393	4.4218	6.0248	3.6718	3.3680	5.1010	6.3390	5.0107	3.3906	0.8152	-0.2949	0.2429	0.6146
Napepld	_S[Phospho (STY)]FKLDYR_.2	Q8BH82_S43_M1_Napepld	5.0971	5.3229	4.4098	5.6367	5.7697	4.3056	5.6319	5.8379	6.1129	5.0455	0.8998	-0.1523	0.1555	0.8083
Napepld	_QNS[Phospho (STY)]VQNSGGSVSSR_.2	Q8BH82_S27_M1_Napepld	3.5085	4.2648	3.2422	4.1508	4.1531	3.4745	3.8655	4.2152	3.6163	3.9641	0.9703	-0.0435	0.6475	0.1888
Echs1	_AQFGQPEILLGTIPGAGGTQR_.2	#N/A	6.6549	6.5491	6.0748	6.8536	6.0047	6.8205	6.2605	6.4118	6.6203	6.6647	1.0006	0.0008	0.9862	0.0060
Echs1	_LVEEAIQC[Carbamidomethyl (C)]AEK_.2	#N/A	6.7955	3.6327	3.4433	4.4879	6.5232	6.1759	6.6787	6.2003	4.3131	6.6424	0.8687	-0.2030	0.4027	0.3950
Echs1	_FLSHWDHITR_.3	#N/A	2.9807	3.7369	5.6796	5.8763	4.9390	5.9933	5.3812	6.0516	3.0884	4.9974	0.9975	-0.0036	0.9885	0.0050
Echs1	_AFAAGADIK_.2	#N/A	2.9836	3.9141	4.2181	3.7619	4.2236	3.5494	3.9102	4.2762	4.0210	4.4608	0.9059	-0.1425	0.1675	0.7760
Echs1	_LFYSTFATDDRR_.2	#N/A	3.4365	4.0857	3.4421	3.4312	4.0811	4.1623	4.2677	3.6408	3.3622	4.0194	0.9871	-0.0188	0.8461	0.0726
Echs1	_EGMTAFVEK_.2	#N/A	4.3636	3.6190	4.0973	3.6410	3.5073	3.1173	4.8638	3.3601	3.6592	3.3183	0.9800	-0.0292	0.8401	0.0757
Rab39b	_SGFVPNVVHSS[Phospho (STY)]EEVIK_.2	#N/A	6.6192	6.3322	7.0787	7.1584	3.4267	3.6723	7.1735	6.9983	6.9728	3.8955	0.9129	-0.1315	0.6243	0.2046
Gemin8	_S[Phospho (STY)]LEPPSEKPWER_.2	Q8BHE1_S181_M1_Gemin8	3.9504	3.3920	3.6841	3.2279	3.0941	3.5305	3.3761	3.7733	4.0724	4.4060	0.8907	-0.1670	0.1068	0.9712
Atcay	_LGGAVEDSSS[Phospho (STY)]PPSTLNLSGAHR_.3	Q8BHE3_S46_M1_Atcay	9.4537	9.7064	3.2858	11.0655	7.6868	9.1033	9.7537	4.3300	9.6194	9.4170	1.0125	0.0179	0.9539	0.0205
Atcay	_IQYVHSLEELER_.3	#N/A	3.6312	3.5143	4.3436	3.8873	3.3759	4.3718	4.2650	3.1138	3.4129	3.7694	1.0587	0.0823	0.4852	0.3141
Atcay	_AAEATEDQETS[Phospho (STY)]MS_.2	Q8BHE3_S370_M1_Atcay	4.3189	3.0235	4.0525	3.5963	2.8449	3.3838	3.7446	3.4049	3.7039	3.2736	1.0014	0.0020	0.9876	0.0054
Tmem108	_EIQSLETSEDQLS[Phospho (STY)]EPR_.2	Q8BHE4_S538_M1_Tmem108	4.0338	6.9414	3.7674	7.7574	6.2692	7.0044	7.7202	7.7456	7.1924	7.2389	0.7977	-0.3261	0.1164	0.9340
Nrdc	_LGADES[Phospho (STY)]EEEGR_.2	Q8BHG1_S85_M1_Nrdc	7.6493	7.7174	7.6866	7.8576	6.1260	7.3275	7.8503	7.7329	7.5843	7.2305	0.9730	-0.0395	0.5692	0.2448
Serinc5	_SSS[Phospho (STY)]DALQSR_.2	Q8BHJ6_S339_M1_Serinc5	3.0131	7.1836	7.3983	7.5573	6.3529	4.0899	3.9173	3.9159	7.4794	6.9733	1.0648	0.0906	0.7784	0.1088
Slu7	_LVEQANS[Phospho (STY)]PK_.2	Q8BHJ9_S215_M1_Slu7	8.5279	8.5825	8.6607	9.1497	8.6262	8.5675	8.8319	8.4830	8.6236	8.6430	1.0047	0.0067	0.7662	0.1156
Scn3b	_AEEAAQENASDY[Phospho (STY)]LAIPSENK_.2	Q8BHK2_Y198_M1_Scn3b	3.0662	3.8225	2.5325	3.8445	3.7108	4.4147	3.9927	6.2413	6.1599	5.3823	0.6549	-0.6107	0.0078	2.1073
Tbc1d10b	_ASPVPGPGT[Phospho (STY)]PTR_.2	Q8BHL3_T136_M1_Tbc1d10b	9.0728	9.3057	4.1677	3.7115	3.5778	8.8296	9.3065	9.1670	3.5888	3.3888	1.0128	0.0183	0.9681	0.0141
Tbc1d10b	_HGAPAAPS[Phospho (STY)]PPPR_.2	#N/A	7.5501	7.7245	7.7413	6.5223	5.2957	7.2655	8.5839	8.3897	6.2493	6.9092	0.9314	-0.1025	0.4588	0.3384
Tbc1d10b	_RHGAPAAPS[Phospho (STY)]PPPR_.3	#N/A	6.4117	6.8504	7.4090	7.8436	3.3024	4.4271	7.6878	7.6852	6.7605	5.5137	0.8740	-0.1943	0.4090	0.3883
Tbc1d10b	_ELLEQNPGKFEELER_.3	#N/A	4.2074	3.3247	3.3933	2.4314	2.7910	4.2513	3.8116	3.4900	3.8711	3.8630	0.9045	-0.1448	0.3738	0.4274
Txlng	_KHS[Phospho (STY)]LEGDEGSDFITK_.3	Q8BHN1_S79_M1_Txlng	3.0694	3.5830	3.7562	4.9135	3.7573	4.0336	3.9736	4.3607	4.1944	4.2259	0.9197	-0.1208	0.3207	0.4939
Ganab	_NPEPELLVR_.2	#N/A	6.0059	6.0877	5.7523	5.6664	6.2171	5.7463	6.0731	5.5218	5.4801	5.5933	1.0433	0.0612	0.1562	0.8064
Ganab	_LDLLEDR_.2	#N/A	6.2077	5.7760	3.2373	5.8512	5.9018	5.9212	5.2190	5.7459	6.1295	6.1003	0.9455	-0.0809	0.6070	0.2168
Ganab	_IILTAQPFR_.2	#N/A	5.7101	5.9241	8.1140	5.6847	5.9191	5.6664	5.8802	5.7817	6.0931	5.7479	1.0500	0.0704	0.5678	0.2458
Ganab	_M[Oxidation (M)]LDYLQGSGETPQTDIR_.2	#N/A	4.2464	4.5281	2.9475	3.3297	4.4449	5.0226	4.3518	4.4993	3.2666	5.0287	0.9533	-0.0690	0.6976	0.1564
Ganab	_DAVHYGGWEHR_.3	#N/A	3.6998	2.9553	3.4569	3.9595	1.8440	2.5290	3.6742	4.1590	3.8075	3.9265	0.7899	-0.3403	0.0830	1.0809
	KLDAES[Phospho (STY)]DGSTEETDESET.2	Q8BHR8_S319_M1_	4.0123	4.8172	4.7058	5.7923	2.6714	4.4883	5.4599	4.7454	3.6379	4.3134	0.9725	-0.0402	0.8428	0.0743
Armcx3	_YNDWS[Phospho (STY)]DDDDDSNESK_.2	Q8BHS6_S61_M1_Armcx3	8.9219	9.1582	9.5270	9.3744	8.3577	9.0113	9.5266	9.3647	8.8301	9.0167	0.9863	-0.0199	0.6195	0.2080
Armcx3	_ASPNSDDTVLS[Phospho (STY)]PQELQK_.2	Q8BHS6_S119_M1_Armcx3	6.6196	4.4120	6.6868	6.9724	5.7448	6.9602	6.8205	6.7717	7.1640	6.4134	0.9176	-0.1241	0.3164	0.4997
Armcx3	_RAS[Phospho (STY)]PNSDDTVLSPQELQK_.3	Q8BHS6_S110_M1_Armcx3	4.2023	3.5066	3.3882	3.8909	3.3279	6.0858	3.6056	4.2276	3.8762	3.8579	1.0450	0.0635	0.7549	0.1221
Sybu	_TPSDTGHSPIGFC[Carbamidomethyl (C)]PGS[Phospho (STY)]DEDFTR_.3	Q8BHS8_S111_M1_Sybu	3.6361	4.3924	3.3698	6.8816	4.5580	3.8448	3.7379	6.4264	3.7438	4.3109	0.9764	-0.0345	0.8992	0.0461
Sybu	_SGSS[Phospho (STY)]PPS[Phospho (STY)]PR_.2	Q8BHS8_S199_M2_Sybu	2.9944	3.7506	4.2289	3.7727	3.6389	2.9857	3.9209	3.2285	3.5276	3.4499	1.0085	0.0123	0.9150	0.0386
Cyria	_DAIQNPNDIQLQEK_.2	#N/A	6.4771	6.9418	7.1166	6.3175	6.0162	6.3116	6.5779	6.6813	6.5902	6.2901	0.9993	-0.0011	0.9835	0.0072
Cyria	_EQAPDSVEGLLNALR_.2	#N/A	5.1770	4.8110	3.5435	2.6012	4.0834	4.8853	4.9479	4.7759	5.4608	4.9678	0.8304	-0.2681	0.1345	0.8713
Cyria	_EIENYPHFFLDFENAQPTEGER_.3	#N/A	3.8284	5.0599	4.7346	3.1461	3.8649	2.9676	5.1636	4.9504	3.1201	3.1131	0.9625	-0.0551	0.8096	0.0917
Mal2	_VTLPAGPDILR_.2	#N/A	8.0511	8.0019	8.1824	7.8864	7.8393	7.4945	8.3592	8.5680	8.0522	7.7827	0.9657	-0.0504	0.1612	0.7927
Mal2	_[Acetyl (Protein N-term)]SAGGAVPPPPNPAVSFPAPR_.2	#N/A	8.0823	8.0149	8.1706	9.2082	7.7329	7.9208	8.3752	8.0948	7.9742	7.6748	1.0197	0.0282	0.5994	0.2223
Cdkn2aip	_SAAQQENSS[Phospho (STY)]PSR_.2	Q8BI72_S169_M1_Cdkn2aip	3.9898	6.9025	7.3035	7.4774	3.1335	3.4911	7.0762	7.5230	4.0330	6.3292	0.8626	-0.2132	0.4993	0.3016
Mia3	_EEAKPAS[Phospho (STY)]PSSVQDR_.2	#N/A	4.3176	4.4094	3.5036	4.0062	3.4433	3.8277	3.7209	4.1123	3.7608	3.9732	1.0067	0.0097	0.9076	0.0421
Mia3	_DSLPSGPREEAKPAS[Phospho (STY)]PSSVQDR_.3	#N/A	6.1329	6.2967	7.6713	4.4504	2.8158	6.1680	6.6295	6.2725	4.3507	6.1653	0.9547	-0.0669	0.7887	0.1031
Mia3	_DETAAQTPGS[Phospho (STY)]GEAVLSK_.2	Q8BI84_S820_M1_Mia3	6.1902	3.6188	4.0971	3.2960	3.5071	6.2387	6.4978	6.8628	6.2594	5.9961	0.7013	-0.5118	0.0272	1.5651
Mia3	_SEFGS[Phospho (STY)]LDR_.2	Q8BI84_S1733_M1_Mia3	4.2760	3.0664	4.0034	3.5535	3.4197	3.3409	3.7017	5.0497	3.7468	3.2307	0.9181	-0.1234	0.4260	0.3706
Mia3	_HSAS[Phospho (STY)]DPGPAPVVNSSS[Phospho (STY)]R_.2	Q8BI84_S1754_M2_Mia3	3.6490	3.6934	3.3827	4.4273	3.5147	4.2148	3.0747	4.0747	3.6738	4.1046	0.9761	-0.0349	0.7703	0.1133
Frmd4a	_LLGSENDTGS[Phospho (STY)]PDFYTPR_.2	Q8BIE6_S712_M1_Frmd4a	4.3895	4.3375	3.5755	4.0781	4.2258	3.8996	3.7928	6.1350	3.6889	4.0451	0.9250	-0.1125	0.5124	0.2904
Frmd4a	_MWSESS[Phospho (STY)]LDEPYEK_.2	Q8BIE6_S601_M1_Frmd4a	4.1055	3.4098	4.0694	6.0268	3.2311	3.8463	4.4094	3.6724	4.2599	3.7611	1.0221	0.0316	0.8707	0.0601
Frmd4a	_SSS[Phospho (STY)]LESQGK_.2	Q8BIE6_S696_M1_Frmd4a	7.1217	2.9419	4.3243	3.3262	4.2641	3.4654	3.8262	3.2915	4.4409	3.2932	1.1421	0.1917	0.5287	0.2768
Frmd4a	_TSNS[Phospho (STY)]YTAGGLFK_.2	Q8BIE6_S854_M1_Frmd4a	4.1246	3.4290	3.3106	3.1909	3.2503	2.9783	3.3391	3.8103	4.1093	3.7802	0.8992	-0.1534	0.1482	0.8292

Frmd4a	_SVDIS[Phospho (STY)]PTR_.2	Q8BIE6_S679_M1_Frmd4a	3.8161	3.1205	4.5029	3.5047	2.9418	3.2869	3.2194	7.2262	7.3931	3.4717	0.6624	-0.5943	0.0952	1.0214
Frmd4a	_IISGSSGSLLS[Phospho (STY)]SGSQESDSSQSAK_.2	Q8BIE6_S356_M1_Frmd4a	3.2329	4.1095	4.4674	4.0112	3.8775	4.2480	4.1594	2.9900	3.2890	3.6885	1.1301	0.1764	0.1575	0.8026
Cd99l2	_QETQS[Phospho (STY)]AEPPPPEPPRI_.2	Q8BIF0_S226_M1_Cd99l2	3.5470	7.5806	4.2338	3.2356	4.1735	3.5560	3.9168	3.3820	3.0306	3.2026	1.2970	0.3752	0.2640	0.5784
Cd99l2	_GENLEAVVC[Carbamidomethyl (C)]EEPQVTYSK_.2	#N/A	4.4021	4.1690	4.0506	4.5533	3.9903	4.7881	4.2680	3.5652	5.6300	3.0194	1.0497	0.0700	0.6766	0.1697
Rbfox3;Rbfox2;Rbfox	_KIEVNNATAR_.2	#N/A	4.9472	4.7973	4.2230	5.3876	5.3419	5.0064	5.2314	5.1525	4.4437	5.3452	0.9816	-0.0267	0.7402	0.1306
Iars2	_VLSELGVDAQSLSAM[Oxidation (M)]EIR_.2	#N/A	4.3565	5.2701	5.1670	4.4557	3.5159	5.0350	5.1135	4.1269	4.6660	4.6521	0.9987	-0.0019	0.9871	0.0056
Rufy1	_SQLPS[Phospho (STY)]PGELR_.2	Q8BIJ7_S52_M1_Rufy1	3.6966	8.0810	3.4303	8.2345	3.4671	7.1594	3.1223	4.0271	4.3262	4.1522	1.4533	0.5394	0.1891	0.7233
Dock9	_GSLISTDS[Phospho (STY)]GNSLPDRNPEK_.3	Q8BIK4_S1270_M1_Dock9	3.7497	3.3555	3.4834	4.0225	3.2143	3.8300	3.9340	4.2484	4.6192	4.0935	0.8545	-0.2269	0.0137	1.8629
Dock9	_LDKLPVHVYEVDEEADKDEDAAS[Phospho (STY)]LGSQK_.4	Q8BIK4_S178_M1_Dock9	3.6324	3.5938	4.3192	3.3210	4.2589	3.4706	4.5365	3.5831	2.9452	3.2880	1.0495	0.0698	0.6203	0.2074
Cstf2	_SLGTGAPVIESPYGESIS[Phospho (STY)]PEDAPESISK_.3	Q8BIQ5_S120_M1_Cstf2	3.6719	2.9273	3.4056	3.9874	2.8156	3.8090	3.7021	4.1310	3.7796	3.9544	0.8829	-0.1796	0.1123	0.9495
Anks1b	_NVSKS[Phospho (STY)]DSDLIAYPSKDK_.3	#N/A	3.3274	8.3213	3.8293	8.7295	7.2625	8.4745	8.8849	8.7100	3.4351	4.2989	1.0514	0.0723	0.8518	0.0697
Anks1b	_TLANLPWIVEPGQEAK_.2	#N/A	7.4265	6.6992	6.9911	6.7056	6.9873	6.7871	7.2431	6.8320	7.0228	6.6345	1.0000	-0.0001	0.9986	0.0006
Anks1b	_NIIAEHEIR_.2	#N/A	6.6770	6.7028	6.4073	6.8808	5.9612	6.5421	6.6283	6.8250	6.6169	6.2468	0.9923	-0.0112	0.7954	0.0994
Anks1b	_LIFQSC[Carbamidomethyl (C)]DYK_.2	#N/A	5.7800	5.9802	4.5336	5.5131	5.2905	5.7292	5.5775	6.0046	5.9210	4.6051	0.9899	-0.0147	0.8823	0.0544
Anks1b	_NVSKS[Phospho (STY)]DSDLIAYPSK_.2	#N/A	4.0688	3.3087	3.2548	7.5749	3.1945	3.0352	7.4904	7.0844	4.0107	3.7244	0.7302	-0.4536	0.2416	0.6169
Anks1b	_NSSTGPTPDC[Carbamidomethyl (C)]S[Phospho (STY)]PPS[Phospho (STY)]PDTALK_.2	Q8BIZ1_S507_M2_Anks1b	3.6539	4.4136	3.9943	4.0402	6.0739	3.7613	4.7817	7.2076	7.1760	6.6272	0.6704	-0.5769	0.0108	1.9673
Snip1	_LS[Phospho (STY)]PEPVAHR_.2	#N/A	7.2316	7.1504	7.4616	7.8143	7.0744	7.3284	7.6302	7.0652	7.1132	7.3704	1.0067	0.0096	0.7831	0.1062
Snip1	_QERLS[Phospho (STY)]PEPVAHR_.3	#N/A	6.5548	7.0019	7.0038	7.0730	6.8590	7.1134	7.3226	6.9299	6.9722	6.8002	0.9897	-0.0149	0.6137	0.2120
Snip1	_S[Phospho (STY)]GDALTTVVVK_.2	#N/A	3.2645	7.0100	6.8668	4.0428	5.8067	3.8302	4.1910	7.0240	6.6441	6.5940	0.8403	-0.2511	0.3477	0.4588
Snip1	_RPDAPAAS[Phospho (STY)]LS[Phospho (STY)]PPAAEPGHSGHR_.3	Q8BIZ6_S48_M2_Snip1	3.7224	3.6200	3.4561	4.5007	8.2761	8.1723	3.1481	8.0942	8.0223	4.1780	0.9028	-0.1474	0.7234	0.1406
Snip1	_HRS[Phospho (STY)]GDALTTVVVK_.3	#N/A	3.3446	3.9978	4.5792	4.1229	3.4469	3.9104	4.2712	4.3791	3.1773	5.0425	0.9248	-0.1128	0.4323	0.3642
Zc3h14	_DLVQPDKPAS[Phospho (STY)]PK_.2	Q8BJ05_S515_M1_Zc3h14	8.9865	9.2614	9.6985	9.8082	8.9975	9.3060	9.3454	9.1934	9.2173	9.2485	1.0099	0.0143	0.6204	0.2073
Zc3h14	_WIRPQS[Phospho (STY)]SE_.2	Q8BJ05_S733_M1_Zc3h14	6.3778	6.4606	6.9364	4.0633	3.9296	4.1959	4.0890	6.2736	3.2369	3.7406	1.2289	0.2974	0.2970	0.5273
Zc3h14	_IS[Phospho (STY)]PPVKEEEAKGDNTGK_.3	Q8BJ05_S409_M1_Zc3h14	3.1697	4.0564	5.7158	6.3418	3.7963	3.9332	4.0739	4.3333	4.4153	5.3027	0.9936	-0.0093	0.9662	0.0149
Zc3h14	_RFS[Phospho (STY)]HDGEEEEEDEDYGTR_.3	Q8BJ05_S309_M1_Zc3h14	2.6607	2.9885	3.1477	3.6504	3.1390	3.4719	3.3651	4.4681	3.6248	2.5863	0.9047	-0.1445	0.3735	0.4277
Dlgap2	_EKS[Phospho (STY)]LDLPDR_.2	#N/A	13.3018	13.3957	13.6670	13.9915	12.7315	13.5671	13.6185	13.4270	13.4594	13.5467	0.9948	-0.0075	0.7554	0.1218
Dlgap2	_SDVETATDS[Phospho (STY)]DTESR_.2	Q8BJ42_S745_M1_Dlgap2	11.5359	11.5721	12.0838	11.9247	11.2271	11.7338	11.8672	11.5360	11.5572	11.7038	1.0012	0.0017	0.9374	0.0281
Dlgap2	_IIES[Phospho (STY)]PERKEER_.3	#N/A	9.2802	9.5170	10.2200	9.6317	9.5077	9.5231	9.4461	9.5524	9.6814	9.4325	1.0089	0.0128	0.6277	0.2023
Dlgap2	_IIES[Phospho (STY)]PERK_.2	#N/A	10.4428	10.2706	10.4571	10.6585	9.9019	10.2013	10.6279	10.3934	10.3559	10.4760	0.9865	-0.0196	0.3467	0.4600
Dlgap2	_S[Phospho (STY)]IDKAVLASK_.2	Q8BJ42_S700_M1_Dlgap2	8.9642	8.7945	9.4082	9.2014	7.4761	8.8864	8.2367	9.1342	9.1300	8.7713	0.9966	-0.0048	0.9407	0.0265
Dlgap2	_EKS[Phospho (STY)]LDLPDRQR_.3	#N/A	5.8616	7.2991	8.8745	6.9349	6.9996	7.7555	6.1280	7.1997	6.8649	7.1473	1.0662	0.0925	0.4307	0.3659
Dlgap2	_GLQFGSS[Phospho (STY)]FQR_.2	Q8BJ42_S806_M1_Dlgap2	2.8989	3.6551	6.0020	3.2597	3.5434	3.0812	4.4752	3.3240	3.0066	3.3544	1.0565	0.0793	0.7614	0.1184
Dlgap2	_SHS[Phospho (STY)]LEGSSK_.2	Q8BJ42_S248_M1_Dlgap2	6.6963	7.0005	7.2738	6.9580	2.9034	3.7212	3.1854	6.8768	4.2631	6.8513	1.0878	0.1214	0.7147	0.1459
Dlgap2	_C[Carbamidomethyl (C)]SS[Phospho (STY)]IGVQDSEFPDHQPYPR_.3	Q8BJ42_S720_M1_Dlgap2	3.3813	4.1375	3.1149	4.1595	4.0258	4.0996	3.9928	5.5872	5.7398	5.5780	0.7311	-0.4518	0.0079	2.1024
Dlgap2	_GGLYNS[Phospho (STY)]M[Oxidation (M)]DSLDSNK_.2	#N/A	7.6925	7.8610	7.9521	7.9675	6.3316	8.4266	8.0994	7.6687	8.4156	8.4401	0.9447	-0.0820	0.2834	0.5475
Dlgap2	_T[Phospho (STY)]PPPVPPR_.2	Q8BJ42_T616_M1_Dlgap2	9.2910	8.5509	4.5968	9.0773	9.3120	9.3337	8.6844	8.1985	8.6704	9.5369	0.9530	-0.0694	0.6845	0.1646
Dlgap2	_ADS[Phospho (STY)]IEIYIPEAQTRL_.2	#N/A	3.8028	3.1071	4.4896	3.4914	2.9285	3.3002	3.2061	3.1262	5.6201	3.4584	0.9136	-0.1303	0.5621	0.2502
Dlgap2	_EAEENDLLEDILGK_.2	#N/A	4.7939	4.3375	4.5995	5.4434	4.1513	5.4269	3.3160	3.0163	4.1537	3.7607	1.3455	0.4281	0.0069	2.1629
Dlgap2	_TTSKPLISVTAQS[Phospho (STY)]STESTQDAYQDSR_.3	Q8BJ42_S636_M1_Dlgap2	3.9031	3.8694	3.9391	6.7774	3.7085	3.8128	3.4568	3.4864	4.0752	3.7631	1.1731	0.2303	0.3342	0.4760
Dlgap2	_IIES[Phospho (STY)]PER_.2	#N/A	6.5897	6.1814	6.3315	6.7732	6.1907	5.8350	6.3358	6.6018	6.0752	6.6292	0.9854	-0.0212	0.6489	0.1878
Dlgap2	_QNSATERADS[Phospho (STY)]IEIYIPEAQTR_.3	#N/A	3.4773	3.9954	3.7821	7.4551	4.2047	3.7451	3.7942	7.5161	3.7006	6.0700	0.8431	-0.2462	0.4572	0.3399
Dlgap2	_HS[Phospho (STY)]EPSTPTQYGALR_.2	Q8BJ42_S811_M1_Dlgap2	2.8973	3.6536	7.1019	8.0695	3.5419	7.3484	7.4143	3.3564	7.4491	6.9152	0.8650	-0.2092	0.5641	0.2486
Dlgap2	_SGM[Oxidation (M)]SSWWS[Phospho (STY)]SDDNLDSDSTYR_.2	#N/A	3.9215	3.5757	3.8904	3.5257	3.8764	3.6893	3.7385	3.6897	4.2242	3.5406	0.9864	-0.0198	0.7332	0.1348
Dlgap2	_MRS[Phospho (STY)]GSYIK_.2	Q8BJ42_S438_M1_Dlgap2	3.1828	3.9880	3.8696	4.3722	3.8093	3.9202	4.2810	3.7463	3.3948	4.3392	0.9789	-0.0308	0.7617	0.1182
Dlgap2	_AMGDEESGESDSS[Phospho (STY)]PK_.2	Q8BJ42_S456_M1_Dlgap2	4.5787	3.8443	4.1007	4.4146	3.6485	3.2538	4.0215	3.4600	3.9917	3.7955	1.0409	0.0579	0.5811	0.2358
Dlgap2	_ADSIEIYIPEAQTR_.2	#N/A	6.1076	3.6440	9.7198	3.6573	9.5545	6.5693	6.4123	5.3445	6.0534	9.4699	0.9592	-0.0600	0.8620	0.0645
Dlgap2	_S[Phospho (STY)]LDLPDR_.2	#N/A	3.5160	4.2723	3.2497	4.2943	4.1606	3.9649	3.8580	4.2077	4.9843	4.7134	0.8804	-0.1838	0.1120	0.9508
Dlgap2	_DGS[Phospho (STY)]WFLK_.2	Q8BJ42_S873_M1_Dlgap2	4.3450	3.6005	4.0787	3.6225	3.4888	3.1358	3.7708	3.3787	3.6777	3.2998	1.0510	0.0718	0.4719	0.3262
Chmp2b	_ATIS[Phospho (STY)]DEEIER_.2	Q8BJF9_S199_M1_Chmp2b	9.3886	9.6417	10.1669	9.9768	9.3296	9.8503	9.8796	9.7558	9.7788	9.6860	0.9949	-0.0073	0.7816	0.1070
Zc2hc1a	_NTT[Phospho (STY)]PPSLAR_.2	Q8BJH1_T243_M1_Zc2hc1a	11.9759	11.8193	12.4821	12.4913	11.6689	12.1227	11.9510	11.7842	4.2934	11.9064	1.2113	0.2766	0.1999	0.6992
Slc6a17	_YGSGYLLAST[Phospho (STY)]PESEL_.2	Q8BJI1_T722_M1_Slc6a17	8.6656	7.9808	8.7956	8.1252	6.4982	8.0725	8.5914	8.2500	7.9335	8.0573	0.9774	-0.0329	0.6802	0.1674
Slc6a17	_DIS[Phospho (STY)]NLEENDETR_.2	Q8BJI1_S665_M1_Slc6a17	10.1968	9.3955	9.9270	10.0220	7.9335	9.8495	9.9346	9.7863	9.7766	9.6580	0.9760	-0.0350	0.5998	0.2220
Slc6a17	_EHS[Phospho (STY)]NEHVTESVADLLALEEPVDYK_.3	#N/A	8.9182	9.2718	9.8126	9.7547	8.2568	8.9862	9.4886	10.0079	9.6782	8.9828	0.9609	-0.0575	0.3078	0.5118
Slc6a17	_SYLGPGSTS[Phospho (STY)]PLDNSNNPNGR_.2	#N/A	8.2845	8.4493	8.7286	8.5863	3.2923	3.2265	8.8834	8.6336	8.3247	8.1666	0.7952	-0.3305	0.2464	0.6083
Slc6a17	_ILGYLNSNVLSR_.2	#N/A	3.7501	4.5039	4.4199	3.9065	3.8300	3.7062	3.9783	4.4740	0.9771	3.0796	1.2853	0.3621	0.1986	0.7021
Slc6a17	_VPS[Phospho (STY)]EAPSPM[Oxidation (M)]PTHR_.2	Q8BJI1_S682_M1_Slc6a17	5.8194	3.6294	5.7980	6.4090	3.4803	5.4229	5.8964	5.9703	5.5017	5.0339	0.9094	-0.1370	0.4588	0.3384
Sgta	_ALELDPDNDTYK_.2	#N/A	6.1666	6.8792	3.3525	6.9618	6.9173	6.9404	6.6464	6.2210	6.4097	6.6029	0.9587	-0.0608	0.7252	0.1395
Sgta	_TPS[Phospho (STY)]ASHEEQQE_.2	#N/A	6.3160	5.7894	6.0914	6.7913	5.7984	6.3848	5.7888	5.9031	6.3558	3.1820	1.1673	0.2231	0.1781	0.7495
Sgta	_AIELNPANAVYFC[Carbamidomethyl (C)]NR_.2	#N/A	4.4583	4.2985	4.3201	5.5143	4.6983	4.7358	4.3976	4.6055	4.7128	4.7337	1.0127	0.0182	0.8127	0.0901
Sgta	_SRTPS[Phospho (STY)]ASHEEQQE_.2	#N/A	4.8556	5.9745	5.4417	6.3209	6.1733	6.2005	6.2604	5.7022	4.1120	5.5838	1.0763	0.1061	0.3996	0.3984
Sgta	_APDRT[Phospho (STY)]PPSEEDSAEAER_.3	#N/A	6.6854	8.0252	7.0783	6.8967	8.1819	8.0263	8.1190	6.6953	8.0106	7.1470	0.9986	-0.0021	0.9810	0.0083
Sgta	_HAEAVAYYK_.2	#N/A	3.3659	4.1711	4.0527	4.5553	3.9924	3.7371	4.0979	3.5631	3.2117	3.0215	1.1455	0.1960	0.1061	0.9743
Psmd5	_VFTAIADQPWAQR_.2	#N/A	3.6402	3.7022	3.3739	6.8613	3.5235	4.1215	3.0659	5.6606	4.3826	4.0958	0.9773	-0.0331	0.9058	0.0430
Psmd5	_IVENSEAVTEILNNAELLK_.2	#N/A	3.2779	3.7426	4.1369	3.6756	2.5563	4.2889	4.1820	3.6511	3.4577	3.3189	0.9892	-0.0157	0.9142	0.0390
Fbxo9	_S[Phospho (STY)]PDGDGVGSGYIEENEDASK_.2	Q8BK06_S126_M1_Fbxo9	3.4609	3.7652	4.1477	3.1496	3.6535	4.4677	4.3651	3.4681	3.1167	3.1165	1.0732	0.1020	0.4652	0.3323
Ndufv3	_TQSVLKEPEPTDTTTYK_.3	#N/A	6.9488	6.9602	7.4886	7.2752	7.6722	7.0577	5.8980	6.8369	7.0268	7.3031	1.0691	0.0964	0.1397	0.8549
Ndufv3	_LPQPSSGRES[Phospho (STY)]PRH_.3	#N/A	7.6647	4.3718	8.1318	3.7489	7.6279	7.7352	7.3914	3.2523	3.5514	3.2222	1.5035	0.5883	0.1241	0.9062
Ndufv3	_LPQPSSGR_.2	#N/A	6.2440	6.2499	5.7721	6.7760	7.5987	6.7218	6.0303	6.4617	6.5560	6.1640	1.0408	0.0577	0.4640	0.3335
Ndufv3	_LPQPSSGRES[Phospho (STY)]PR_.2	#N/A	3.0855	3.8418	4.3201	4.7590	3.7301	4.3953	4.0121	3.1373	3.4364	3.5411	1.1388	0.1875	0.1821	0.7397
Nop53	_EM[Oxidation (M)]C[Carbamidomethyl (C)]EGLLEES[Phospho (STY)]DGEDEHEAGR_.3	#N/A	3.5625	4.6609	3.9537	4.8159	4.0436	3.9448	4.3995	4.8297	4.3872	3.5265	0.9715	-0.0417	0.7173	0.1443
Csnk1a1	_AAQQAASSSGQGQQAQT[Phospho (STY)]PTGK_.2	Q8BK63_T321_M1_Csnk1a1	5.1126	4.5066	4.1778	5.5901	4.1175	4.2611	4.3951	4.6432	4.6319	4.3340	1.0281	0.0400	0.6942	0.1585
Ahsa1	_ETFLTSPEELYR_.2	#N/A	6.2874	6.8599	6.2919	6.7009	6.7760	6.6262	6.5764	6.3475	6.8319	6.6036	1.0001	0.0001	0.9971	0.0013
Ahsa1	_VFTTQELVQAFTHAPAALEADR_.3	#N/A	3.5132	3.3100	3.2468	4.2914	2.1574	3.4766	4.4397	4.2106	3.0088	3.4374	0.8830	-0.1795	0.3436	0.4639
Ahsa1	_QTFGYGAR_.2	#N/A	4.1106	3.0592	3.2965	3.7992	3.0839	2.9924	3.5139	4.4855	3.9679	3.9415	0.8524	-0.2303	0.0713	1.1469
Ahsa1	_ADATNVNNWHWTER_.2	#N/A	5.8848	4.2269	3.6861	4.1887	3.6258	4.0102	3.9034	3.9298	3.5784	4.1557	1.0973	0.1339	0.4080	0.3894
Srrm4	_DPT[Phospho (STY)]PPPSSRGK_.2	Q8BKA3_T101_M1_Srrm4	8.3359	4.2435	3.2209	9.1875	8.7438	3.9937	8.8116	4.2365	3.0347	3.9428	1.2559	0.3287	0.4805	0.3183
Srrm4	_SAS[Phospho (STY)]PLAATR_.2	Q8BKA3_S271_M1_Srrm4	6.2718	3.1500	4.5325	3.5343	2.9713	6.4976	3.2490	3.0834	6.3980	6.3480	0.9420	-0.0862	0.8048	0.0943
Srrm4	_GQEKGS[Phospho (STY)]PGGDLSK_.2	#N/A	4.8562	3.7167	3.5983	4.1010	3.5380	4.1915	4.5523	4.0175	3.6661	4.0680	0.9814	-0.0270	0.8018	0.0959
Srrm4	_SRGQEKGS[Phospho (STY)]PGGDLSK_.3	#N/A	3.7061	3.6363	3.4398	4.4844	2.8499	3.7747	3.1318	4.0176	4.3166	4.1617	0.9339	-0.0987	0.4715	0.3265

Ipo5	_NLIDEDGNNQWPEGLK_.2	#N/A	8.7116	7.8874	8.8597	8.5410	7.2760	8.4024	8.9278	8.4273	8.3045	8.2034	0.9780	-0.0321	0.5872	0.2312
Ipo5	_LVLEQVVTSIASVADTAEEK_.3	#N/A	4.7346	4.4744	4.1246	5.4667	3.4518	4.4248	4.7315	4.8974	4.8172	4.3493	0.9462	-0.0797	0.4947	0.3057
Ipo5	_HIVENAVQK_.2	#N/A	3.6871	2.9425	3.4208	3.9723	2.8308	4.0325	3.6869	4.1462	3.7948	4.1427	0.8829	-0.1797	0.1341	0.8725
Pi4kb	_S[Phospho (STY)]VENLPEC[Carbamidomethyl (C)]GITHEQR_.2	#N/A	7.0975	6.9935	7.1024	7.0683	3.6055	7.1073	7.2903	7.2385	7.0979	6.9264	0.9100	-0.1361	0.4018	0.3960
Mapkap1	_RTS[Phospho (STY)]FSFQK_.2	#N/A	6.4629	6.0047	6.8431	6.3206	3.5215	3.1183	6.2701	3.1926	3.9750	3.4394	1.2747	0.3502	0.2785	0.5552
Mapkap1	_YSS[Phospho (STY)]PGLTSK_.2	Q8BKH7_S270_M1_Mapkap1	5.0988	4.3396	4.2212	3.7811	4.3928	3.5685	3.9293	3.2201	3.5192	3.1900	1.2220	0.2892	0.0357	1.4467
Mapkap1	_TS[Phospho (STY)]FSFQK_.2	#N/A	4.1660	3.1764	3.8997	3.4435	2.9977	3.2309	3.5917	3.5577	3.8568	3.1207	0.9870	-0.0189	0.8647	0.0632
Tnrc6b	_DDEPSGWEEPS[Phospho (STY)]PQSISR_.2	Q8BKI2_S913_M1_Tnrc6b	6.4583	3.1329	6.4160	7.3781	3.2489	3.1918	6.9759	6.3034	6.6541	6.7421	0.7454	-0.4239	0.1334	0.8749
Tnrc6b	_M[Oxidation (M)]GS[Phospho (STY)]PAPLLPGDLLGGGSDSI_.2	Q8BKI2_S1793_M1_Tnrc6b	3.4320	5.4869	5.2631	5.6340	4.0585	4.3453	4.9913	4.6658	3.8539	4.4518	1.0473	0.0667	0.6769	0.1695
Tnrc6b	_SSSS[Phospho (STY)]AGSEVGGQSTGSNHK_.2	Q8BKI2_S601_M1_Tnrc6b	3.7318	3.0362	4.4186	3.4204	2.8575	3.3712	3.7320	3.1972	4.3466	3.3874	0.9473	-0.0781	0.5927	0.2272
Baiap2	_EGDLITLLVPEAR_.2	#N/A	5.9695	6.1159	5.7196	5.2309	5.7508	6.4122	5.6753	5.5950	5.8201	5.9676	1.0177	0.0253	0.6484	0.1881
Baiap2	_QIQNQLEETLK_.2	#N/A	5.7156	4.9944	5.3675	5.6468	5.5941	5.1949	5.3861	5.3448	5.8852	4.6514	1.0192	0.0274	0.6909	0.1606
Baiap2	_YSDKELQYIDAISNK_.2	#N/A	6.0925	6.1307	5.8447	6.4669	5.9622	6.3663	5.7284	5.7323	6.2773	6.2446	1.0247	0.0352	0.4112	0.3859
Baiap2	_LSDSYSNTLPVR_.2	#N/A	5.5325	5.0156	4.8201	5.6364	5.8665	5.2835	5.3634	5.7426	5.2626	5.5574	0.9688	-0.0457	0.4661	0.3315
Baiap2	_SLS[Phospho (STY)]PPQSQSK_.2	Q8BKX1_S326_M1_Baiap2	14.5249	14.7686	15.2324	15.2850	14.5829	14.8396	14.9759	14.6794	14.6261	14.8595	1.0059	0.0085	0.6343	0.1977
Baiap2	_LPLWQQAC[Carbamidomethyl (C)]ADPNKIPDR_.3	#N/A	5.6366	5.5326	5.9806	6.2640	5.6226	5.4694	5.6790	5.2595	5.7380	5.7847	1.0242	0.0344	0.4810	0.3179
Baiap2	_SFHNELLTQLEQK_.3	#N/A	4.8614	4.0863	4.8542	5.1103	5.1828	5.5839	4.5151	5.2919	5.6845	5.8320	0.9279	-0.1080	0.2977	0.5262
Baiap2	_SSS[Phospho (STY)]M[Oxidation (M)]AAGLER_.2	#N/A	3.8625	4.9923	5.7169	4.2561	3.7637	5.9425	3.8472	5.4665	6.0694	3.7650	0.9935	-0.0095	0.9635	0.0162
Baiap2	_SNLVISDPIPGAKPLPVPPELAPFVGR_.3	#N/A	4.4083	4.1362	3.4698	3.9724	3.3960	4.3200	3.6871	3.2035	3.7946	3.9314	1.0811	0.1125	0.2737	0.5627
Baiap2	_TIM[Oxidation (M)]EQFNPSLR_.2	#N/A	4.4214	5.8690	6.0654	5.8325	5.8993	5.8582	5.9516	5.4035	5.6814	6.5659	0.9588	-0.0607	0.5283	0.2771
Baiap2	_AIFSHAAGDNSTLLSFK_.2	#N/A	4.4430	3.7480	3.9937	4.0086	3.8119	4.4572	4.9125	2.9926	4.2640	4.2791	0.9915	-0.0123	0.9238	0.0344
Baiap2	_LTENVYK_.2	#N/A	3.3086	4.0648	4.5431	4.0869	3.9531	4.1723	4.0655	4.4152	3.2134	3.7641	1.0406	0.0574	0.6006	0.2214
Baiap2	_ELGDVLFQM[Oxidation (M)]AEVHR_.3	#N/A	3.5118	3.6123	3.9605	3.5042	3.3705	3.3183	3.6525	3.5612	3.8602	3.9674	0.9431	-0.0845	0.1596	0.7969
Pdhx	_VVDDELATR_.2	#N/A	7.2759	7.5528	7.6715	7.5559	7.9716	7.1240	6.8832	7.3742	7.2288	7.3061	1.0455	0.0641	0.0985	1.0067
Pdhx	_VSVNDFIIR_.2	#N/A	6.6626	7.0116	7.4076	6.9708	6.7660	6.5135	6.7861	6.8227	6.6075	6.9009	1.0161	0.0231	0.5342	0.2723
Pdhx	_DLVKDDIK_.2	#N/A	4.8681	5.2695	4.8636	5.1018	5.6273	5.4820	4.7986	5.0604	5.2041	5.2550	1.0241	0.0344	0.5164	0.2870
Pdhx	_GIQEIADSVK_.2	#N/A	5.9419	5.3532	4.1391	5.4107	6.0042	5.6420	4.3564	5.3387	5.6153	4.8754	1.0731	0.1017	0.3944	0.4041
Pdhx	_HSLDASQGTATGPR_.2	#N/A	2.9577	3.7629	3.6445	4.1471	3.5842	4.1453	4.5061	3.9713	3.6199	3.0420	0.9794	-0.0300	0.8199	0.0863
Pdhx	_LTEDEEGNPQLQQHQLITVTM[Oxidation (M)]SSDSR_.3	#N/A	3.0364	3.7927	4.2709	3.8147	3.6810	2.9437	3.9629	3.1865	3.4856	3.4920	1.0165	0.0236	0.8453	0.0730
Usp54	_NSSS[Phospho (STY)]PVSLDAAPPDSVNVYR_.2	Q8BL06_S613_M1_Usp54	2.9558	3.7121	4.1904	8.2478	3.6004	3.0242	8.4922	8.1579	3.5661	7.8524	0.6111	-0.7104	0.0813	1.0902
Ablim2	_S[Phospho (STY)]PQHYSR_.2	#N/A	10.3798	9.9148	9.7184	10.4352	9.1914	10.1837	10.1469	9.9104	9.9836	10.3924	0.9864	-0.0198	0.6025	0.2201
Ablim2	_IYPYDSLIVTNR_.2	#N/A	5.4546	6.4193	5.9019	7.0186	6.5761	6.2131	5.4433	6.2134	6.1177	6.3428	1.0389	0.0551	0.4841	0.3151
Ablim2	_TSSESIVSVPASSTSGS[Phospho (STY)]PSR_.2	#N/A	12.5509	12.5976	13.3100	13.1014	12.5345	12.6849	13.1282	12.4265	12.5670	12.7314	1.0065	0.0094	0.6965	0.1571
Ablim2	_TSS[Phospho (STY)]PSSAGSVSLGHYTPTSR_.3	#N/A	3.6397	4.9349	7.2114	8.4099	7.2791	3.7291	4.0748	6.6784	7.5626	6.6524	0.9400	-0.0893	0.7621	0.1180
Ablim2	_S[Phospho (STY)]PQHYSRPGSESGR_.3	#N/A	6.3129	6.3823	3.7882	3.3320	5.9824	5.6703	6.2583	5.4608	6.2042	6.3453	0.8644	-0.2102	0.2746	0.5614
Ablim2	_RLDVEDSS[Phospho (STY)]FDQDSR_.2	Q8BL65_S453_M1_Ablim2	4.0880	5.3258	3.6192	4.4083	4.3450	4.1374	3.9944	4.1336	3.9553	4.6008	0.9639	-0.0530	0.5166	0.2868
Ablim2	_T[Phospho (STY)]NSPDLDSQSLSLSSGTDQEPLQR_.3	Q8BL65_T475_M1_Ablim2	3.6702	3.9089	3.8756	8.1523	5.2388	3.3481	3.7639	7.1534	3.7446	3.6392	1.0270	0.0385	0.9168	0.0377
Eea1	_SLGS[Phospho (STY)]ADELFK_.2	Q8BL66_S52_M1_Eea1	3.4371	4.2423	4.1239	5.4007	4.0636	3.6659	4.0267	5.2349	4.8468	3.0927	0.9663	-0.0494	0.7845	0.1054
Eea1	_LQQSSQAAQELAAEK_.2	#N/A	2.0685	3.0936	3.9261	4.4288	3.7069	3.0858	3.3790	2.6441	3.3383	2.9000	1.1043	0.1431	0.4914	0.3086
Gtf3c2	_VSS[Phospho (STY)]PTKPK_.2	Q8BL74_S219_M1_Gtf3c2	4.6538	3.9967	5.9579	5.8441	3.8850	4.2405	4.1336	6.3844	3.3481	4.3860	1.0438	0.0619	0.7806	0.1076
Arhgap22	_LEEELDQER_.2	#N/A	11.6381	11.8383	11.8598	12.0373	12.2965	12.0412	11.5818	11.5582	11.5998	11.9147	1.0247	0.0352	0.0618	1.2088
Srsf7	_S[Phospho (STY)]RS[Phospho (STY)]AS[Phospho (STY)]LRR_.2	Q8BL97_S200_M3_Srsf7	6.9125	6.9709	5.1482	6.8623	6.9484	6.7978	7.0810	6.8938	7.1812	6.6990	0.9487	-0.0759	0.3696	0.4323
Srsf7	_S[Phospho (STY)]RS[Phospho (STY)]VSLR_.2	Q8BL97_S192_M2_Srsf7	3.1033	3.5643	5.1733	4.2122	3.7298	3.9997	5.6411	3.8649	3.4743	4.2597	0.9197	-0.1208	0.5227	0.2818
Srsf7	_S[Phospho (STY)]RS[Phospho (STY)]ASLR_.2	#N/A	5.4544	4.1958	5.3980	5.9654	4.0841	4.0414	5.8917	3.8986	3.5472	5.2809	1.0434	0.0613	0.7517	0.1239
Srsf7	_S[Phospho (STY)]RS[Phospho (STY)]GS[Phospho (STY)]IIGSR_.2	Q8BL97_S208_M3_Srsf7	8.9042	8.9908	9.3930	9.4189	8.3414	8.8881	9.4505	9.1139	9.1375	8.8931	0.9826	-0.0253	0.4925	0.3076
Srsf7	_SGS[Phospho (STY)]IIGSR_.2	Q8BL97_S212_M1_Srsf7	9.0161	4.3409	9.4468	3.5561	3.7561	4.2069	8.3510	3.3892	5.3103	4.4097	1.0663	0.0926	0.8332	0.0793
Ankrd34c	_VLNEPGS[Phospho (STY)]PTR_.2	Q8BLB8_S231_M1_Ankrd34c	3.6848	3.6576	3.4185	4.4631	6.8822	3.7961	3.1105	4.0389	4.3380	4.1403	1.1050	0.1440	0.5741	0.2410
Ankrd34c	_T[Phospho (STY)]NSIDSKDPTLFPTVQEQVLK_.3	Q8BLB8_T299_M1_Ankrd34c	4.5960	3.3819	3.9326	3.5375	2.9746	3.2587	3.2522	5.4258	4.2342	3.5045	0.8805	-0.1837	0.3410	0.4672
Ankrd34c	_RGS[Phospho (STY)]GTLLLDR_.2	Q8BLB8_S446_M1_Ankrd34c	3.2024	4.5196	3.8893	4.3919	3.8217	3.9005	4.1066	3.7266	3.3752	4.3858	1.0143	0.0205	0.8555	0.0678
Hirip3	_AVES[Phospho (STY)]TDEDHQTDLDAK_.2	Q8BLH7_S134_M1_Hirip3	5.8689	4.3132	5.8521	4.5098	3.6212	4.3148	3.7988	4.5275	3.8768	3.5843	1.2026	0.2662	0.1437	0.8426
Plcxd3	_LNYVFNEGEANT_.2	#N/A	3.4371	6.5701	7.4068	7.1112	6.2584	7.0904	7.1899	6.4552	6.2872	7.2213	0.9299	-0.1049	0.5554	0.2554
Lsamp	_AANEVSSADVK_.2	#N/A	7.6399	7.7317	7.5930	7.9398	7.5917	7.4673	7.2180	7.9468	7.5726	7.8338	1.0023	0.0034	0.9098	0.0410
Lsamp	_HALEYSLR_.2	#N/A	7.3714	7.2416	7.3034	7.6403	7.0212	7.1414	6.8377	7.3282	6.7433	7.3123	1.0328	0.0465	0.1943	0.7114
Lsamp	_QGDTAILR_.2	#N/A	6.1129	5.2559	6.2780	5.5476	5.7186	5.4524	4.7701	6.0703	5.2776	5.5122	1.0592	0.0829	0.3075	0.5121
Lsamp	_C[Carbamidomethyl (C)]EASAVPAPDFEWYR_.2	#N/A	5.2880	4.8737	3.5786	4.4303	5.2890	3.6360	4.5294	4.5083	4.6054	4.3809	1.0022	0.0032	0.9806	0.0085
Lsamp	_EFEGEEEYLEILGITR_.2	#N/A	6.0498	6.9306	7.4062	7.7460	6.3689	6.7662	6.7337	6.5344	6.3664	6.5686	1.0499	0.0703	0.3473	0.4593
Lsamp	_C[Carbamidomethyl (C)]EASAVPAPDFEWYRDDTR_.3	#N/A	4.2888	3.5931	3.4747	5.6730	5.3586	5.4304	3.1750	5.4163	5.2003	5.6179	0.9555	-0.0657	0.7556	0.1217
Lsamp	_SGIIFAGHDK_.2	#N/A	4.2771	3.0653	6.2807	3.5545	3.4208	3.2038	3.7028	6.1082	5.8088	5.9197	0.7367	-0.4408	0.0996	1.0017
Lsamp	_VDVYDEGSYTC[Carbamidomethyl (C)]SVQTQHEPK_.3	#N/A	4.2812	1.1371	3.8850	4.8843	3.2699	2.9588	3.3196	3.8298	4.1289	4.3494	0.8709	-0.1994	0.4858	0.3135
Lsamp	_IQKVDVYDEGSYTC[Carbamidomethyl (C)]SVQTQHEPK_.3	#N/A	3.4165	5.0843	4.1034	4.4520	4.0431	3.6864	4.3207	3.5125	3.7206	3.0722	1.1298	0.1760	0.2280	0.6421
Rps6kc1	_VC[Carbamidomethyl (C)]LQQPSAS[Phospho (STY)]PQGGSSFESR_.2	Q8BLK9_S448_M1_Rps6kc1	6.9931	7.0328	4.3616	7.5699	5.7118	6.6580	3.9857	6.6515	6.5397	4.8177	1.1617	0.2162	0.2930	0.5332
Rps6kc1	_SS[Phospho (STY)]PDQFLFSSLR_.2	Q8BLK9_S779_M1_Rps6kc1	5.2623	3.7191	3.6007	4.0280	5.4071	5.4821	3.8977	6.4830	3.6361	3.9081	1.0228	0.0325	0.8875	0.0518
Rps6kc1	_S[Phospho (STY)]SQESLDIK_.2	Q8BLK9_S422_M1_Rps6kc1	3.2253	4.0008	3.9121	4.4148	3.8891	4.2363	4.1295	3.7037	3.3523	4.3818	1.0140	0.0201	0.8476	0.0718
Rps6kc1	_VFSGGEDLEAVSS[Phospho (STY)]PPTSESLSR_.2	Q8BLK9_S577_M1_Rps6kc1	3.6479	3.9476	3.9031	4.1179	3.7126	3.8599	3.9463	5.1434	3.5362	4.0221	0.9286	-0.1069	0.3281	0.4840
Rps6kc1	_GVDLLLEGVQGESS[Phospho (STY)]PTR_.2	Q8BLK9_S281_M1_Rps6kc1	3.4980	3.5084	4.1848	4.1472	3.4793	5.0793	4.0319	3.4806	3.4610	3.1683	1.1265	0.1719	0.2390	0.6217
Rps6kc1	_FLNRSS[Phospho (STY)]QES[Phospho (STY)]LDIK_.2	Q8BLK9_S423_M2_Rps6kc1	3.8481	3.6141	3.7189	5.4258	3.6586	3.8113	3.9362	3.9387	5.5300	4.1886	0.9123	-0.1324	0.4328	0.3637
Rps6kc1	_ESEAQDSVSRGS[Phospho (STY)]DDSVPVISFK_.3	Q8BLK9_S658_M1_Rps6kc1	3.1995	4.0047	4.0379	7.1467	5.6276	3.9420	3.9510	6.6230	5.3222	3.8619	1.0379	0.0537	0.8600	0.0655
Rps6kc1	_ALFTLEDGDS[Phospho (STY)]PSQSLDPGESK_.2	#N/A	3.6053	4.4105	4.2921	3.2939	4.2318	3.4977	3.8585	3.3237	2.9723	3.2609	1.1594	0.2134	0.0966	1.0151
Rps6kc1	_ALFTLEDGDS[Phospho (STY)]PSQSLDPGESKR_.3	#N/A	4.0640	3.3683	3.2499	3.7526	3.1897	3.0390	3.3998	4.3659	4.0145	3.7196	0.8888	-0.1701	0.1312	0.8819
Unc80	_GRS[Phospho (STY)]PTVGNKR_.2	Q8BLN6_S1261_M1_Unc80	3.9293	3.2336	3.1152	3.6179	3.0549	3.1737	3.5345	2.9998	4.1492	3.5849	0.9403	-0.0888	0.4292	0.3674
Unc80	_VAS[Phospho (STY)]IQS[Phospho (STY)]EPGQQNVLLQQPLGR_.3	Q8BLN6_S3045_M2_Unc80	3.7349	2.9903	3.4686	3.9245	2.8786	3.7460	3.6391	3.9888	3.8426	4.1905	0.8830	-0.1795	0.0913	1.0397
Slc7a4	_ASPPSS[Phospho (STY)]PC[Carbamidomethyl (C)]LAS[Phospho (STY)]PGPTAK_.2	Q8BLQ7_S427_M2_Slc7a4	7.5058	7.5491	3.8868	7.6249	6.2596	3.5642	7.8357	3.7688	6.7028	3.4384	1.1156	0.1579	0.6387	0.1947
Slc7a4	_FQKAS[Phospho (STY)]PPSS[Phospho (STY)]PC[Carbamidomethyl (C)]LAS[Phospho (STY)]PGPTAK_.3	#N/A	3.8531	3.6194	4.0737	4.0034	3.6780	4.1094	3.9582	4.3148	3.5583	3.8280	0.9935	-0.0094	0.8804	0.0553
Cadm2	_DGAELPDPDR_.2	#N/A	10.1076	9.8129	9.8579	10.0688	9.4151	9.5952	10.2733	9.7817	9.7170	9.9584	0.9876	-0.0180	0.4865	0.3129
Cadm2	_GAEDAPDADTAIINAEGS[Phospho (STY)]QVNAEEKK_.3	#N/A	6.4262	7.9861	8.0064	6.9815	5.8188	7.8563	8.1180	6.0298	5.8670	8.0458	0.9997	-0.0005	0.9973	0.0012
Cadm2	_SDDGVAVIC[Carbamidomethyl (C)]R_.2	#N/A	8.8199	8.4036	8.8049	8.6332	8.6284	8.4527	8.3910	8.6128	8.4304	8.6295	1.0127	0.0182	0.3150	0.5017
Cadm2	_TSGSKPAADIR_.2	#N/A	7.6831	7.8008	7.8600	7.9317	8.1942	7.5696	7.4242	7.8054	7.3803	7.5447	1.0400	0.0565	0.0542	1.2658
Cadm2	_TFTVSSTLDFR_.2	#N/A	6.1010	4.2206	4.3563	6.3308	6.4062	6.0485	6.0359	5.7186	6.3300	6.3990	0.9112	-0.1342	0.3342	0.4760
Cadm2	_IIPSTPFPQEGQALTLTC[Carbamidomethyl (C)]ESK_.2	#N/A	5.8391	5.8660	5.6001	6.1657	5.4963	5.0020	6.0608	5.7010	5.5072	5.3194	1.0026	0.0037	0.9532	0.0208

Cadm2	_YLKEEDANRK_.3	#N/A	3.3729	2.6419	3.9589	3.5026	4.1712	2.3399	2.6280	3.4985	4.1550	3.1799	0.9898	-0.0147	0.9407	0.0265
Cadm2	_GAEDAPDADTAIINAEGS[Phospho (STY)]QVNAEEK_.3	#N/A	3.3473	3.9951	4.5818	4.1256	3.8164	3.9131	4.2739	4.3764	3.1746	4.3665	0.9791	-0.0305	0.7913	0.1017
Cadm2	_GAEDAPDADTAIINAEGS[Phospho (STY)]QVNAEEKKEYFI_.3	#N/A	3.3750	3.4236	4.3626	4.6791	3.5279	4.1760	3.8064	3.8046	4.1154	4.2666	0.9814	-0.0270	0.8090	0.0921
Sowaha	_QRS[Phospho (STY)]EEPSPR_.2	Q8BLS7_S242_M1_Sowaha	3.5572	3.5798	3.4614	3.7565	3.4011	4.3284	3.1698	1.8550	3.6946	3.9310	1.1638	0.2189	0.2374	0.6245
Slc9a7	_TKS[Phospho (STY)]SSEEVLER_.2	Q8BLV3_S694_M1_Slc9a7	4.8164	4.3360	4.2409	3.7847	4.1573	3.5722	4.9802	3.2165	3.5155	3.4620	1.0943	0.1300	0.3829	0.4169
Slc9a7	_VGVEELS[Phospho (STY)]EEDQNENR_.2	Q8BLV3_S546_M1_Slc9a7	3.9616	3.2660	3.1476	3.6502	3.0873	3.1414	3.3649	5.0184	4.1168	3.6172	0.8378	-0.2554	0.0907	1.0426
Plekhm3	_S[Phospho (STY)]RSDVTHVDWR_.3	Q8BM47_S150_M1_Plekhm3	3.3961	3.9463	3.1298	4.1744	3.7677	3.9618	4.3226	4.3276	3.1259	3.8516	0.9545	-0.0671	0.5708	0.2435
Nyap2	_LSTS[Phospho (STY)]SETVNSTAASK_.2	Q8BM65_S154_M1_Nyap2	7.7711	7.6411	7.7222	8.1604	6.2865	7.8430	7.7678	7.3387	7.4908	7.7966	0.9963	-0.0053	0.9379	0.0279
Nyap2	_RNPNTQLSTS[Phospho (STY)]FDETYIKK_.3	Q8BM65_S238_M1_Nyap2	3.7059	3.5203	4.3927	3.3945	2.8316	3.2160	3.1092	3.2231	4.3726	3.3615	0.9982	-0.0026	0.9859	0.0062
Nyap2	_FTVPASHS[Phospho (STY)]PPR_.2	Q8BM65_S184_M1_Nyap2	8.5672	8.4692	9.0789	9.4078	3.4504	3.8348	9.3246	4.1052	8.2526	3.9803	1.1121	0.1533	0.6957	0.1576
Nyap2	_KEEDDQSEAVY[Phospho (STY)]EEM[Oxidation (M)]KYPIFDDLGHDSK_.4	#N/A	3.8253	3.9751	3.9175	3.9376	3.5928	3.3725	4.0859	4.1566	4.1386	3.6149	0.9428	-0.0850	0.1873	0.7274
Nyap2	_DQTGALGPAPGASILSSS[Phospho (STY)]PPPPSTLYR_.3	Q8BM65_S442_M1_Nyap2	3.9264	3.2998	6.4069	3.6150	3.0521	3.1766	3.3297	3.0026	4.1520	3.5820	1.1127	0.1540	0.5719	0.2427
Emsy	_TNS[Phospho (STY)]SSSSPVVLK_.2	Q8BMB0_S173_M1_Emsy	2.9320	3.7372	3.6188	6.9534	6.3940	6.9236	6.9460	6.5557	3.6456	6.9673	0.8448	-0.2433	0.4349	0.3616
Me3	_VRGEEYDDLLDEFM[Oxidation (M)]QAVTDK_.3	#N/A	3.7768	4.4010	4.4742	4.5425	4.6918	5.6485	4.6929	5.1246	5.6170	4.5950	0.9165	-0.1258	0.2797	0.5533
Me3	_TFTPGQGNNAYVFPGVALGVIAGGIR_.3	#N/A	3.7084	2.1025	3.4182	3.9266	3.4577	4.3775	3.2315	4.3391	4.2377	4.6229	0.8517	-0.2316	0.2218	0.6541
Me3	_EMFAQDHPEVNSLEEVVR_.3	#N/A	3.6153	4.0270	3.9086	4.0937	3.8483	3.8812	4.2420	4.4083	3.2065	4.3783	0.9598	-0.0592	0.5189	0.2849
Dlat	_ILVPEGTR_.2	#N/A	9.9112	10.1968	10.1866	10.1804	10.5591	10.0302	9.8564	10.2096	9.9361	10.0552	1.0163	0.0233	0.2351	0.6287
Dlat	_DVPLGAPLC[Carbamidomethyl (C)]IIVEK_.2	#N/A	7.8826	7.8288	7.6386	8.1364	8.0695	8.1255	8.1792	7.2099	7.6197	8.0568	1.0232	0.0331	0.3972	0.4010
Dlat	_GLETIASDVVSLASK_.2	#N/A	7.5633	7.6955	7.7645	8.0821	7.5971	7.7426	7.3094	7.5402	7.6873	8.1370	1.0094	0.0136	0.6764	0.1698
Dlat	_VPLPSLSPTM[Oxidation (M)]QAGTIAR_.2	#N/A	7.3505	7.7830	8.4546	7.6868	7.7719	7.8958	7.9369	8.0610	7.8565	7.8646	0.9866	-0.0194	0.5879	0.2307
Dlat	_DIDSFVPSK_.2	#N/A	7.4514	7.5156	7.7765	7.2445	8.0595	7.7782	6.5721	7.5703	7.6227	7.9062	1.0296	0.0421	0.4454	0.3513
Dlat	_VFVSPLAK_.2	#N/A	7.4724	7.2403	7.5725	7.2326	7.6967	6.9423	5.7034	7.5212	7.5142	7.8127	1.0310	0.0441	0.6009	0.2212
Dlat	_YLEKPITM[Oxidation (M)]LL_.2	#N/A	5.1523	6.2078	5.7512	5.4858	5.2517	5.2878	5.5951	5.7442	5.3013	5.9149	0.9794	-0.0300	0.6232	0.2054
Dlat	_ISVNDFIIK_.2	#N/A	4.7717	4.9929	5.9171	6.0811	4.7582	5.5313	4.8725	5.6634	4.6139	4.4585	1.0898	0.1240	0.2628	0.5804
Dlat	_VAPAPAGVFTDIPISNIR_.2	#N/A	8.1703	8.3389	8.4805	8.5741	8.4992	8.2670	7.9601	8.4009	8.2162	8.3568	1.0188	0.0269	0.2021	0.6945
Dlat	_DVPVGSIIC[Carbamidomethyl (C)]ITVEKPQDIEAFK_.3	#N/A	6.5357	6.8174	3.4241	7.3455	6.9181	6.5503	6.7590	6.6127	7.1073	6.8060	0.9185	-0.1227	0.4686	0.3292
Dlat	_LQPHEFQGGTFTISNLGM[Oxidation (M)]FGIK_.3	#N/A	5.1364	4.8399	5.3627	5.5675	4.4042	5.4497	5.7128	4.7333	5.4962	6.0182	0.9338	-0.0988	0.2766	0.5581
Dlat	_VPEANSSWM[Oxidation (M)]DTVIR_.2	#N/A	6.7240	6.5762	7.1269	7.0470	6.8871	6.6260	6.3459	6.2903	6.4488	6.4275	1.0710	0.0990	0.0052	2.2855
Dlat	_AAPAAAAAM[Oxidation (M)]APPGPR_.2	#N/A	5.6031	6.2468	5.9233	5.7806	5.6002	5.8989	5.7226	6.2240	5.6789	6.3465	0.9748	-0.0368	0.4337	0.3628
Dlat	_ASALAC[Carbamidomethyl (C)]LK_.2	#N/A	2.9518	3.8732	4.1863	3.7301	4.5648	3.5175	3.8783	4.1338	3.4321	3.4074	1.0245	0.0350	0.7807	0.1075
Dlat	_VVDGAVGAQWLAEFK_.2	#N/A	4.0643	3.1618	3.2503	3.7530	3.1900	3.0386	3.4676	4.3655	4.0141	3.7200	0.8761	-0.1908	0.0975	1.0110
Rab3gap2	_GGFS[Phospho (STY)]PFGNTQGPSR_.2	Q8BMG7_S448_M1_Rab3gap2	9.9258	9.8537	10.2790	10.2852	7.2600	9.7082	9.9650	9.9439	9.5551	9.6021	0.9780	-0.0320	0.7262	0.1389
Rab3gap2	_AKS[Phospho (STY)]PRPDSFETEIK_.3	Q8BMG7_S566_M1_Rab3gap2	4.3972	2.9390	3.4173	3.9757	2.8273	3.7973	3.6904	4.1427	3.7913	4.1392	0.9031	-0.1471	0.2748	0.5610
Rab3gap2	_TSS[Phospho (STY)]WEDDSWGAWEETEPR_.2	Q8BMG7_S42_M1_Rab3gap2	3.2719	4.0705	4.5064	4.0502	3.8918	3.8377	4.1985	4.4518	3.2500	3.7275	1.0080	0.0115	0.9181	0.0371
Gga3	_LAS[Phospho (STY)]ETEDNDNSLGDILQASDNLSR_.3	Q8BMI3_S267_M1_Gga3	4.0531	3.1731	3.2390	3.7417	3.1788	3.0499	3.4564	4.3768	4.0254	3.7087	0.8752	-0.1924	0.0953	1.0207
Ckap4	_VGAHGS[Phospho (STY)]EEGAVFK_.2	Q8BMK4_S367_M1_Ckap4	5.6669	5.5640	4.5070	6.7034	4.4274	5.3515	5.8148	6.3020	5.5140	5.9051	0.9127	-0.1319	0.2845	0.5459
Hadha	_FVDLYGAQK_.2	#N/A	5.8190	6.3157	5.9364	5.3339	6.2464	5.6326	6.1039	5.6556	5.5697	5.5011	1.0303	0.0431	0.4499	0.3469
Hadha	_DGPGFYTTR_.2	#N/A	6.3774	6.1084	4.1262	5.9409	5.6862	6.1388	5.9327	6.5778	6.0120	6.0596	0.9323	-0.1011	0.3653	0.4373
Hadha	_AGLEQGSDAGYLAESQK_.2	#N/A	3.1975	3.0292	3.6818	3.6487	3.2156	4.4568	4.5275	3.8092	4.1707	4.8144	0.8171	-0.2915	0.0369	1.4333
Hadha	_SLNSEM[Oxidation (M)]DNILANLR_.2	#N/A	5.1380	5.1364	3.5257	4.5703	5.4037	5.7295	5.8388	5.6909	4.2308	5.8255	0.9112	-0.1342	0.3694	0.4325
Hadha	_DTTASAVAVGLR_.2	#N/A	3.5215	4.1457	4.2083	4.7894	4.5673	3.5815	5.0743	3.8335	4.3396	4.2385	0.9460	-0.0800	0.4965	0.3041
Hadha	_TVLGVPEVLLGILPGAGGTQR_.2	#N/A	3.6576	3.5910	4.0490	3.5436	3.2486	3.4495	3.8184	3.7981	3.2734	3.1500	1.0228	0.0325	0.6902	0.1610
Hadha	_LPAKPEVSSDEDVQYR_.3	#N/A	3.6666	3.4547	3.6214	4.2036	4.4330	3.5932	3.3134	3.8446	4.1351	4.3433	0.9794	-0.0300	0.7712	0.1128
Larp6	_SPGAS[Phospho (STY)]PLLSR_.2	Q8BN59_S451_M1_Larp6	3.5942	4.3505	3.3279	6.7005	4.2388	3.8867	6.9870	6.3878	5.6890	4.0498	0.7528	-0.4097	0.1108	0.9554
Larp6	_YLSPGWGSAS[Phospho (STY)]EEEPSR_.2	Q8BN59_S58_M1_Larp6	3.9149	3.2147	4.0135	3.5569	3.0406	7.0159	3.4895	5.9116	3.9864	3.5705	0.9732	-0.0391	0.8997	0.0459
Spata2l	_SGDLAPPESPSS[Phospho (STY)]PGQASPR_.2	Q8BNN1_S329_M1_Spata2l	3.6378	8.4198	4.0387	8.0960	7.7853	7.8248	9.1656	3.7133	7.7390	7.8527	0.9320	-0.1016	0.7475	0.1264
Kbtbd11	_ASAAEGSEAS[Phospho (STY)]PPSLR_.2	Q8BNW9_S70_M1_Kbtbd11	12.0569	11.9958	12.6454	12.4283	11.9645	12.3556	12.5928	12.2492	12.3980	12.5149	0.9841	-0.0231	0.2324	0.6337
Kbtbd11	_LLLADAYSGR_.2	#N/A	4.8744	5.6180	4.7237	4.1964	5.0548	5.6968	4.6772	5.6248	4.5750	5.4397	0.9898	-0.0148	0.8881	0.0515
Kbtbd11	_AC[Carbamidomethyl (C)]LLAAALGTTGER_.2	#N/A	4.8179	3.7833	4.1297	5.5259	4.9032	5.5824	5.2229	4.8815	5.1017	5.2042	0.9388	-0.0911	0.4298	0.3667
Kbtbd11	_SGS[Phospho (STY)]RPQS[Phospho (STY)]PSGDAESR_.2	Q8BNW9_S316_M2_Kbtbd11	8.5576	8.4276	8.3539	7.7295	7.8700	7.2414	8.7415	7.6081	8.3967	8.1207	0.9773	-0.0332	0.5756	0.2399
Kbtbd11	_GDAAVYC[Carbamidomethyl (C)]YQAEAGEWR_.2	#N/A	4.3315	4.6657	5.2765	5.6262	3.4752	3.1494	4.8585	3.3922	3.5953	3.2862	1.1686	0.2247	0.2999	0.5230
Kbtbd11	_PQS[Phospho (STY)]PSGDAESR_.2	#N/A	3.5865	3.7559	3.3202	4.2985	3.5772	4.1523	4.7083	4.1372	4.4363	4.0421	0.8732	-0.1956	0.0405	1.3921
Fam92a	_ANSKS[Phospho (STY)]PLQR_.2	Q8BP22_S312_M1_Fam92a	4.1298	3.3853	3.8635	3.4073	3.2736	4.5319	3.5555	3.5939	4.9628	3.0845	0.9911	-0.0130	0.9358	0.0288
Sfr1	_S[Phospho (STY)]SSHSFC[Carbamidomethyl (C)]SVVK_.2	Q8BP27_S139_M1_Sfr1	4.0667	3.5229	6.4159	6.5778	3.3686	6.3358	6.8614	6.3563	3.9992	3.5587	0.9719	-0.0411	0.8904	0.0504
Sfr1	_ENPPS[Phospho (STY)]PHSNSSGKQPLSGTPK_.3	#N/A	3.5858	3.6404	4.2726	3.2744	3.5287	3.0960	9.1866	3.3432	2.9918	9.0991	0.5794	-0.7874	0.0976	1.0107
Sfr1	_ENPPS[Phospho (STY)]PPTS[Phospho (STY)]PAAPQPR_.2	#N/A	14.1096	14.4073	14.9534	12.7420	14.3735	14.5017	14.7469	14.1102	14.4050	14.3854	0.9840	-0.0233	0.5798	0.2367
Sfr1	_ENPPS[Phospho (STY)]PHSNSSGK_.2	#N/A	10.3959	10.4798	10.8983	11.3429	10.6391	10.7208	10.5992	10.6588	10.6671	10.6474	1.0097	0.0139	0.5722	0.2424
NARS1	_ISALEGYR_.2	#N/A	5.5462	5.7625	5.6640	5.7854	4.5445	3.7705	5.8968	6.0301	5.2550	5.1555	0.9274	-0.1088	0.4032	0.3944
NARS1	_DVC[Carbamidomethyl (C)]LYPR_.2	#N/A	4.8206	5.2427	5.6133	5.1382	5.6213	4.4860	5.0052	5.5781	3.7623	5.2087	1.0542	0.0762	0.5112	0.2914
NARS1	_SWDSEEILEGYKR_.3	#N/A	3.5364	3.8060	3.2701	4.3147	4.1810	3.9445	4.4630	4.1873	2.9855	3.9920	0.9834	-0.0241	0.8463	0.0725
Fra10ac1	_TTPEC[Carbamidomethyl (C)]DES[Phospho (STY)]PRKK_.2	Q8BP78_S243_M1_Fra10ac1	4.3227	6.2835	6.2910	3.6001	6.1727	5.3590	5.6966	5.5826	5.9016	3.2774	1.0437	0.0617	0.7762	0.1100
Fra10ac1	_S[Phospho (STY)]RSPPSEEASK_.2	Q8BP78_S248_M1_Fra10ac1	4.4237	3.6451	4.0501	3.9564	3.6419	4.9956	3.9222	5.0494	4.8628	3.7736	0.9357	-0.0959	0.4638	0.3337
Fra10ac1	_NAEEEDSAS[Phospho (STY)]DSELWK_.2	Q8BP78_S285_M1_Fra10ac1	3.0072	4.6805	4.2417	3.7854	3.8254	3.4044	3.9337	3.2228	3.5219	3.4627	1.0817	0.1133	0.3986	0.3994
Rcn2	_EALLGVQEDVDEYVK_.2	#N/A	4.9670	5.2407	5.4626	5.9502	5.1753	4.5430	5.3400	5.2010	4.7937	5.4487	1.0052	0.0076	0.9210	0.0357
Rcn2	_RDPTANEDPEWILVEK_.3	#N/A	4.0719	4.1806	3.7988	4.3547	4.4088	4.5832	3.1227	3.8123	4.6669	4.1791	1.0729	0.1016	0.3558	0.4488
Rcn2	_VIDFDENTALDDTEEGSFR_.2	#N/A	4.4746	3.9178	4.5038	5.0884	4.7942	4.1449	4.3668	3.4664	3.1150	4.4857	1.1630	0.2178	0.1039	0.9833
Rcn2	_LSEEEILENQDLFLTSEATDYGR_.3	#N/A	3.2068	4.0120	3.8936	4.1087	3.8333	3.8962	4.2570	4.3933	3.1915	4.3633	0.9442	-0.0829	0.4423	0.3543
Rcn2	_HYAMQEAK_.2	#N/A	4.2996	2.9266	3.4855	3.9882	3.4252	4.3043	3.7029	4.1303	3.7789	3.9552	0.9605	-0.0581	0.6122	0.2131
	SSS[Phospho (STY)]ESPSDNQVFQATR.2	Q8BP99_S63_M1_	3.8954	3.1998	5.5546	3.5840	3.0211	5.3827	3.5683	3.0336	4.1830	3.5510	1.1457	0.1963	0.4026	0.3951
Daam1	_ESS[Phospho (STY)]EESGEFDDLVSALR_.2	Q8BPM0_S1026_M1_Daam1	3.7644	3.0688	4.4512	3.5511	2.8901	3.3385	3.6993	3.4501	3.7492	3.2283	0.9940	-0.0086	0.9459	0.0242
Dmxl2	_LDHELS[Phospho (STY)]LDR_.2	#N/A	9.4673	8.9137	8.6380	9.4928	7.2624	8.7036	9.5417	9.3112	9.1154	8.6845	0.9545	-0.0672	0.3712	0.4304
Dmxl2	_KQS[Phospho (STY)]EVEADLGYPGGK_.2	#N/A	8.4022	8.1441	9.0234	8.3464	3.3617	7.9523	9.1672	8.6833	8.4842	8.0822	0.8761	-0.1908	0.3506	0.4552
Dmxl2	_NLAS[Phospho (STY)]PEGTLATLGLK_.2	Q8BPN8_S1856_M1_Dmxl2	5.9653	8.2764	8.4497	8.6408	4.8864	3.6449	7.2182	8.4921	7.4207	5.5624	0.9262	-0.1106	0.6663	0.1763
Dmxl2	_ESKSS[Phospho (STY)]DDIDYR_.2	#N/A	7.1182	4.2929	4.2840	6.9933	3.6940	6.4049	7.5713	7.3898	3.4725	6.6673	0.8708	-0.1996	0.4781	0.3205
Dmxl2	_YLDEYNPGIFR_.2	#N/A	5.6715	5.4583	6.2454	5.3102	4.8730	5.1446	5.7510	5.5769	4.8814	5.5184	1.0034	0.0049	0.9496	0.0225
Dmxl2	_LVYSQPLDLPEAVEVIR_.2	#N/A	5.1008	4.9852	5.1163	3.9253	4.0153	5.1183	4.8804	4.6512	4.8945	4.7875	0.9806	-0.0283	0.7619	0.1181
Dmxl2	_ALLT[Phospho (STY)]PQDEEC[Carbamidomethyl (C)]ADGDTEVDVIAEQLK_.3	Q8BPN8_T2017_M1_Dmxl2	4.3133	7.0512	3.4992	4.0019	3.4389	4.2905	3.7166	7.4173	3.7652	3.9689	0.9397	-0.0897	0.7804	0.1077
Dmxl2	_FGNVDADS[Phospho (STY)]PVEETIQDHSALK_.3	#N/A	6.8234	7.7966	8.6107	11.0971	8.0135	7.4046	7.2223	11.1398	8.8245	8.0014	0.9425	-0.0855	0.6320	0.1993
Dmxl2	_M[Oxidation (M)]KLDHELS[Phospho (STY)]LDR_.3	#N/A	7.2678	7.1549	5.4834	8.3089	6.5792	7.4174	6.9236	7.5587	7.9672	7.8193	0.9297	-0.1052	0.3318	0.4791

Dmxl2	_VDPIGPLSYTGGVSELAR_.2	#N/A	6.1399	6.4799	5.5542	4.5239	5.4513	6.1736	6.4812	5.8235	5.9823	6.0192	0.9414	-0.0871	0.3726	0.4287
Dmxl2	_RQS[Phospho (STY)]ESIAAPPVASEDM[Oxidation (M)]DKHR_.4	#N/A	7.1342	6.6595	8.1488	4.0581	5.9229	7.0229	7.3501	7.1630	7.1379	6.8934	0.9096	-0.1367	0.3934	0.4051
Dmxl2	_QLDHES[Phospho (STY)]DDADREDEER_.3	#N/A	5.1761	4.2892	5.4905	4.7846	4.2058	4.3291	4.6949	5.2492	4.8659	3.9736	1.0035	0.0051	0.9625	0.0166
Dmxl2	_LYQAVVDAR_.2	#N/A	4.2629	5.2341	4.5760	4.9211	2.9397	3.6849	3.2217	5.3337	4.2268	4.2516	1.0027	0.0038	0.9840	0.0070
Dmxl2	_VS[Phospho (STY)]VDSNLFVYSK_.2	Q8BPN8_S1141_M1_Dmxl2	3.9677	6.5647	7.3304	6.7844	6.3633	6.6744	7.7980	7.6559	7.3930	6.8685	0.8455	-0.2422	0.1026	0.9889
Dmxl2	_RQS[Phospho (STY)]ESIAAPPVASEDMDK_.2	#N/A	3.7923	3.7185	4.2703	4.2414	3.2800	3.2495	3.6382	3.2706	3.8678	3.3933	1.0610	0.0855	0.4118	0.3853
Dmxl2	_RKQS[Phospho (STY)]EVEADLGYPGGK_.3	#N/A	5.7856	4.7639	3.9796	3.5234	2.9178	3.3108	3.6716	5.5281	3.7769	3.2006	1.0006	0.0009	0.9971	0.0013
Dmxl2	_SS[Phospho (STY)]DDIDYR_.2	#N/A	3.4180	4.2024	4.3662	3.8502	4.0523	3.9408	4.0238	5.3925	5.5445	3.4728	0.8618	-0.2145	0.1829	0.7379
Dmxl2	_LDHELSLDRES[Phospho (STY)]EAGTGSSEHEDGEREGS[Phospho (STY)]PR_.4	Q8BPN8_S456_M2_Dmxl2	3.6399	3.7025	3.3735	4.4181	3.5239	4.2056	3.0656	4.1161	4.3829	4.0954	0.9733	-0.0390	0.7454	0.1276
Dmxl2	_ESEAGTGSSEHEDGEREGS[Phospho (STY)]PR_.3	Q8BPN8_S473_M1_Dmxl2	4.1577	4.1900	4.6203	4.4542	4.5438	4.1302	4.4574	4.5782	4.3986	3.8565	1.0062	0.0089	0.8773	0.0569
Dmxl2	_NIGAGVMQIAISQDNR_.2	#N/A	5.3240	4.1645	4.4124	3.9562	3.9858	3.7437	4.1044	3.0450	3.3440	3.6335	1.2075	0.2720	0.0628	1.2022
Dmxl2	_VGSVLDSRVS[Phospho (STY)]VDS[Phospho (STY)]NLFVYSK_.3	#N/A	3.1488	4.0774	3.8356	4.3382	3.7753	4.1598	4.0529	3.7802	3.4288	4.3052	0.9993	-0.0010	0.9922	0.0034
Dmxl2	_QLDHES[Phospho (STY)]DDADREDEERSQDER_.4	#N/A	3.5008	4.0223	3.5093	4.3820	3.6881	3.8279	3.9688	4.0079	4.3987	4.1457	0.9253	-0.1120	0.1413	0.8500
Sgsm1	_C[Carbamidomethyl (C)]SSGAS[Phospho (STY)]LDSHLHR_.3	#N/A	3.5791	3.9055	4.0815	3.4520	3.5877	3.7401	4.2559	4.4169	5.1268	3.9902	0.8374	-0.2560	0.0127	1.8968
Sgsm1	_QDS[Phospho (STY)]PTKRPALC[Carbamidomethyl (C)]IQK_.3	Q8BPQ7_S215_M1_Sgsm1	4.3545	4.3725	3.5405	4.0431	3.4802	4.2493	3.7578	4.0753	3.7239	2.5142	1.1390	0.1877	0.1891	0.7232
Sgsm1	_RHS[Phospho (STY)]S[Phospho (STY)]GSM[Oxidation (M)]DDRPSISAR_.3	#N/A	5.5974	5.2490	3.8584	4.6655	3.5544	5.5161	3.5635	5.4638	5.8268	5.2838	0.9415	-0.0869	0.6347	0.1974
Sgsm1	_HSSGS[Phospho (STY)]M[Oxidation (M)]DDRPSISAR_.2	#N/A	3.4871	3.7688	3.9085	4.1662	3.6204	3.8557	3.9758	3.6584	3.5511	4.0813	0.9959	-0.0059	0.9235	0.0346
Sgsm1	_SPQGSSESTSS[Phospho (STY)]DKEDDEATDYVFR_.3	Q8BPQ7_S407_M1_Sgsm1	4.1526	3.1898	3.8863	3.4300	3.2963	2.7357	3.5783	3.5711	3.8702	3.1073	0.9764	-0.0344	0.7793	0.1083
Elmo1	_IAFDAESEPNNSSGS[Phospho (STY)]M[Oxidation (M)]EK_.2	#N/A	5.3888	3.6740	4.0408	6.8225	3.5149	3.8377	3.6206	3.6638	7.2194	7.2172	0.8372	-0.2563	0.4254	0.3712
Ppp1r18	_SC[Carbamidomethyl (C)]LVKGS[Phospho (STY)]PER_.2	Q8BQ30_S510_M1_Ppp1r18	6.9619	7.0372	7.8154	7.8131	6.8647	7.2752	7.3361	6.7204	7.0694	6.8939	1.0413	0.0585	0.2627	0.5806
Fam102b	_SSS[Phospho (STY)]FSEFC[Carbamidomethyl (C)]HR_.2	Q8BQS4_S197_M1_Fam102b	3.0373	7.6112	7.4598	8.5597	7.1686	8.2263	8.3540	7.7989	8.2058	7.9940	0.8668	-0.2063	0.3267	0.4859
Fam102b	_C[Carbamidomethyl (C)]PVKQDS[Phospho (STY)]VESQLKR_.3	Q8BQS4_S267_M1_Fam102b	5.4354	5.6774	6.1932	4.4181	5.4318	3.8411	3.0655	5.8687	5.9480	4.0954	1.0889	0.1229	0.5699	0.2442
Fam102b	_SAS[Phospho (STY)]VPDELGAWGHSR_.2	Q8BQS4_S162_M1_Fam102b	4.1048	3.3602	3.8385	3.5546	3.2485	3.3761	3.2693	3.0631	4.2125	3.5216	1.0182	0.0260	0.8119	0.0905
Ralgapb	_SDS[Phospho (STY)]APPTPVNR_.2	Q8BQZ4_S359_M1_Ralgapb	11.0912	8.8404	11.6293	10.8789	10.0103	10.9531	11.6527	11.3862	11.1300	10.9643	0.9365	-0.0946	0.2066	0.6848
Bicd1	_SGS[Phospho (STY)]LKGPDDPR_.2	Q8BR07_S548_M1_Bicd1	3.8874	7.6577	8.2546	3.7720	7.0997	3.5935	3.4867	4.3465	3.9951	7.7708	1.1655	0.2210	0.5653	0.2477
Fam177a1	_RVIHFVSGET[Phospho (STY)]M[Oxidation (M)]EEY[Phospho (STY)]STDEDEVDGLDKK_.4	Q8BR63_T60_M2_Fam177a1	4.1021	3.8322	3.9410	3.6406	3.7063	3.6600	3.9852	3.6870	3.0763	3.5175	1.0693	0.0967	0.1987	0.7019
Palm2	_EES[Phospho (STY)]LATDPAPGTQK_.2	Q8BR92_S355_M1_Palm2	8.4692	7.8006	7.6756	8.5867	7.4749	8.2226	8.3182	7.8172	7.8050	8.2385	0.9992	-0.0012	0.9804	0.0086
Palm2	_LEQEIQALESEESQISAK_.2	#N/A	6.6866	6.0814	6.8255	6.9885	5.0574	5.8944	6.7764	5.8664	5.8416	6.1593	1.0154	0.0220	0.8226	0.0848
Palm2	_AELVLIDEDDEKS[Phospho (STY)]LREK_.3	#N/A	8.2654	8.8293	8.6592	9.9200	8.8148	9.2288	9.0177	9.2848	9.3234	9.1790	0.9730	-0.0395	0.4231	0.3736
Palm2	_AELVLIDEDDEKS[Phospho (STY)]LR_.2	#N/A	7.0557	7.7722	7.1456	9.8950	8.1530	8.3392	8.1276	9.0559	8.6530	8.6600	0.9346	-0.0976	0.3355	0.4743
Scfd1	_VNLEESTGVENS[Phospho (STY)]PAGARPK_.2	#N/A	3.3190	4.5599	4.0058	5.5648	3.9455	3.7921	4.0760	5.8638	3.3897	4.6510	0.9339	-0.0987	0.6187	0.2085
Rell2	_SWGS[Phospho (STY)]GGGQEPGGSQAAGGGQPR_.2	Q8BRJ3_S195_M1_Rell2	3.9377	3.2885	3.1236	3.6263	3.1768	3.4478	3.3410	5.8767	4.1408	3.5933	0.8102	-0.3037	0.1310	0.8828
Rell2	_YGLHEHRDGS[Phospho (STY)]PTDR_.3	Q8BRJ3_S187_M1_Rell2	3.6546	6.1362	4.3414	3.3432	4.2811	3.1647	4.5587	6.2158	4.4239	4.7917	0.8311	-0.2669	0.2251	0.6477
Rell2	_GS[Phospho (STY)]EPDDAQLQPPEDDDVNEDTVER_.3	Q8BRJ3_S52_M1_Rell2	4.3410	3.0014	4.0747	6.1440	2.8227	3.4059	3.7667	3.3827	3.6818	3.2958	1.1227	0.1669	0.5106	0.2919
Prkaa2	_SC[Carbamidomethyl (C)]S[Phospho (STY)]AAGLHR_.2	Q8BRK8_S491_M1_Prkaa2	4.2059	3.1365	3.9395	3.4833	3.3496	5.9919	6.4632	6.2164	6.4422	3.1606	0.7212	-0.4714	0.0983	1.0075
Prkaa2	_TSC[Carbamidomethyl (C)]GS[Phospho (STY)]PNYAAPEVISGR_.2	Q8BRK8_S176_M1_Prkaa2	4.2397	3.1027	3.9734	3.5172	3.3835	3.3046	3.6654	3.4840	3.7831	3.1945	1.0156	0.0223	0.8262	0.0829
Prkaa2	_MPPLIADS[Phospho (STY)]PK_.2	Q8BRK8_S377_M1_Prkaa2	3.8599	3.1153	3.5936	3.1373	3.0036	3.6210	3.2856	3.8638	4.1629	4.3155	0.8673	-0.2054	0.0741	1.1299
Clasp2	_ESSRDTS[Phospho (STY)]PVR_.2	Q8BRT1_S547_M1_Clasp2	8.2842	8.5901	9.4874	8.6479	7.4317	8.4228	8.9546	9.2841	8.8320	8.3856	0.9564	-0.0644	0.3248	0.4883
Clasp2	_AQT[Phospho (STY)]GSAGADPTADVSGQS_.2	#N/A	3.7465	3.5959	3.4802	4.5248	2.8903	3.7343	3.1722	3.9772	4.2763	4.2021	0.9373	-0.0934	0.4859	0.3135
Clasp2	_RAQTGS[Phospho (STY)]AGADPTADVSGQS_.2	Q8BRT1_S1273_M1_Clasp2	5.9379	6.0036	4.2233	6.2998	4.9779	5.7612	6.7116	6.5206	6.1096	6.0597	0.8714	-0.1985	0.0864	1.0634
Clasp2	_VLTTTALSTVSSGAQR_.2	#N/A	4.1969	4.5426	4.5831	3.5849	2.8965	4.1090	3.9616	4.2636	4.6757	4.4141	0.9207	-0.1192	0.3543	0.4506
Clasp2	_MVSQSQPGS[Phospho (STY)]R_.2	Q8BRT1_S465_M1_Clasp2	7.9320	6.8269	3.2545	8.3877	6.3529	3.0344	7.5167	6.6059	6.5104	6.9381	0.8654	-0.2086	0.4579	0.3392
Clasp2	_SSSSS[Phospho (STY)]QESLNRPFSSK_.2	#N/A	4.2047	3.4869	3.6350	6.5437	3.3386	5.7243	3.3271	6.3155	5.9620	5.7951	0.8391	-0.2532	0.3505	0.4553
Clasp2	_SRS[Phospho (STY)]DIDVNAAAGAK_.2	Q8BRT1_S376_M1_Clasp2	8.5006	8.3990	3.9213	8.4496	7.4884	8.5564	8.7903	8.6024	8.4984	8.3558	0.8821	-0.1809	0.3110	0.5073
Clasp2	_IPRPSVS[Phospho (STY)]QGC[Carbamidomethyl (C)]S[Phospho (STY)]R_.2	Q8BRT1_S531_M2_Clasp2	3.9867	6.1077	3.8943	3.6753	3.8341	3.9653	4.1117	6.3961	6.2100	3.6423	0.8338	-0.2623	0.2801	0.5527
Clasp2	_S[Phospho (STY)]QEDMSEPVR_.2	Q8BRT1_S947_M1_Clasp2	6.4384	3.5171	4.3959	6.8454	2.8348	3.3939	3.1124	3.2199	4.3693	3.3647	1.2998	0.3783	0.2701	0.5685
Clasp2	_EILSDDKHDWDQR_.3	#N/A	4.4428	3.5974	4.3156	3.3174	4.7657	3.1389	4.5329	4.9814	2.9488	3.6627	0.9747	-0.0369	0.8418	0.0748
Clasp2	_IC[Carbamidomethyl (C)]DYKS[Phospho (STY)]FDDEESVDGNRPSSAASAFK_.3	Q8BRT1_S14_M1_Clasp2	3.6759	2.9803	4.0958	3.6396	2.8016	3.4270	3.7878	3.3616	3.6607	3.3168	0.9731	-0.0394	0.7246	0.1399
Kiaa1671	_RDES[Phospho (STY)]DEEPPRVER_.2	#N/A	9.2274	9.2548	9.8587	9.3740	9.0305	8.7532	9.0074	9.6233	9.4418	9.2310	0.9918	-0.0118	0.7333	0.1347
She	_KNS[Phospho (STY)]AAELGASK_.2	Q8BSD5_S80_M1_She	3.4141	4.5059	4.1009	4.6036	4.0406	4.3734	4.3183	4.1058	3.1635	3.9707	1.0729	0.1015	0.3685	0.4336
Ppfia2	_C[Carbamidomethyl (C)]ETS[Phospho (STY)]PPPTPR_.2	Q8BSS9_S774_M1_Ppfia2	10.2874	11.0583	11.5864	11.2690	10.6751	11.1274	11.3096	11.0047	11.0026	3.3516	1.2000	0.2630	0.2712	0.5667
Ppfia2	_QAQS[Phospho (STY)]PSGVSSEVEVLK_.2	#N/A	8.4093	8.6489	9.4671	9.0562	8.0319	8.8996	8.8912	8.5889	8.5420	8.7182	1.0077	0.0111	0.8064	0.0934
Ppfia2	_S[Phospho (STY)]LGDHEWNR_.2	#N/A	5.1883	5.3895	4.2299	5.9821	4.2196	5.3686	5.5254	5.9485	5.4517	5.7980	0.8912	-0.1661	0.1334	0.8747
Ppfia2	_VKS[Phospho (STY)]LGDHEWNR_.2	#N/A	4.1156	4.8046	3.5636	5.2940	4.0370	4.5421	5.0077	4.4235	4.8271	4.1123	0.9565	-0.0642	0.5860	0.2321
Ppfia2	_RQAQS[Phospho (STY)]PSGVSSEVEVLK_.3	#N/A	3.0573	4.1689	3.7441	4.2468	3.6838	4.0683	3.9614	3.8717	3.5203	4.2138	0.9837	-0.0238	0.8070	0.0931
Ppfia2	_VAS[Phospho (STY)]VS[Phospho (STY)]LEGLNLAR_.2	Q8BSS9_S687_M2_Ppfia2	3.9062	3.7781	3.3267	7.8149	3.6664	3.8471	4.0888	6.8527	3.4988	3.9216	0.9563	-0.0645	0.8537	0.0687
Ppfia2	_KMVSSEGS[Phospho (STY)]TES[Phospho (STY)]EHLEGMEAGQK_.3	Q8BSS9_S236_M2_Ppfia2	3.4979	4.3031	4.2738	3.8176	4.1244	3.6051	3.9659	3.1836	3.4826	3.4949	1.1147	0.1567	0.1002	0.9990
Tcp11l1	_VGRPHS[Phospho (STY)]SPAR_.2	Q8BTG3_S55_M1_Tcp11l1	5.0044	3.6274	5.0993	4.4933	2.8587	5.3391	3.1407	4.0087	4.9394	4.1705	1.0834	0.1155	0.5720	0.2426
Ndrg4	_LDPTTTTFLK_.2	#N/A	5.7238	6.1240	5.7607	5.6457	5.5709	5.2240	5.8397	6.2641	5.5694	5.0313	0.9998	-0.0003	0.9961	0.0017
Ndrg4	_TAS[Phospho (STY)]LTSASSVDGSR_.2	Q8BTG7_S317_M1_Ndrg4	6.8738	4.2619	6.5573	4.2840	6.3066	3.9752	6.5488	4.2181	5.9546	3.9612	1.0398	0.0563	0.8142	0.0893
Ndrg4	_LSGGAVPSAS[Phospho (STY)]MTR_.2	Q8BTG7_S306_M1_Ndrg4	7.3527	5.1748	4.2744	7.0322	3.9646	4.0614	5.8544	3.8506	3.3394	3.9480	1.2500	0.3219	0.2676	0.5725
Ndrg4	_RLS[Phospho (STY)]GGAVPS[Phospho (STY)]ASMTR_.2	Q8BTG7_S304_M2_Ndrg4	3.9808	3.8888	4.0257	7.5859	4.9026	3.1469	5.5882	4.4604	4.8997	4.8312	0.9279	-0.1079	0.6763	0.1698
Ndrg4	_DLDINRPGTVPNAK_.2	#N/A	3.3534	5.3668	3.9289	4.1317	3.9979	4.1537	4.2799	4.4926	4.7157	4.9379	0.9021	-0.1487	0.2403	0.6192
Srrm2	_SSS[Phospho (STY)]PVTELTAR_.2	Q8BTI8_S1068_M1_Srrm2	12.8648	12.5957	13.1995	12.9170	11.9343	12.5555	13.2582	12.6699	12.7211	12.6910	0.9877	-0.0178	0.5433	0.2649
Srrm2	_S[Phospho (STY)]RT[Phospho (STY)]PPVTR_.2	Q8BTI8_S1878_M2_Srrm2	13.1800	12.9571	13.1488	13.3089	12.5656	12.9557	13.4992	13.1561	13.1836	13.0988	0.9837	-0.0236	0.1919	0.7168
Srrm2	_S[Phospho (STY)]RTS[Phospho (STY)]PVTR_.2	Q8BTI8_S1924_M2_Srrm2	10.4966	10.3040	10.6490	10.3025	9.6265	10.4541	10.4983	10.4423	10.2080	10.4643	0.9906	-0.0136	0.6201	0.2076
Srrm2	_S[Phospho (STY)]RT[Phospho (STY)]PPAIR_.2	Q8BTI8_S1972_M2_Srrm2	12.6972	12.5046	12.6911	13.0358	12.0808	12.4537	13.0475	12.6338	12.7466	12.6584	0.9848	-0.0221	0.3083	0.5110
Srrm2	_SST[Phospho (STY)]PPRQS[Phospho (STY)]PSR_.2	Q8BTI8_S882_M2_Srrm2	11.3928	11.5830	11.8284	11.9068	10.7805	11.5215	11.8964	11.7363	11.7014	11.5463	0.9814	-0.0271	0.3363	0.4732
Srrm2	_VSS[Phospho (STY)]PVLETVQQR_.2	Q8BTI8_S1360_M1_Srrm2	12.5711	12.5613	12.9756	13.1416	11.5247	12.1475	13.0840	12.6705	12.4301	12.3443	0.9885	-0.0167	0.6685	0.1749
Srrm2	_S[Phospho (STY)]RS[Phospho (STY)]PLAIR_.2	Q8BTI8_S1996_M2_Srrm2	12.2181	12.1585	12.4747	12.5964	11.6449	12.1632	12.7656	12.1454	12.2815	12.2110	0.9885	-0.0166	0.5029	0.2985
Srrm2	_S[Phospho (STY)]RTS[Phospho (STY)]PAPWK_.2	#N/A	9.3648	8.9756	9.2212	9.6376	5.9007	9.0073	9.7174	8.9004	9.1676	8.9195	0.9464	-0.0795	0.5156	0.2877
Srrm2	_AAETPAVASC[Carbamidomethyl (C)]WSGPQVS[Phospho (STY)]PEHK_.3	#N/A	10.0245	9.9787	10.4587	10.6644	9.0179	8.5601	10.4776	10.1425	9.9217	9.7428	0.9715	-0.0417	0.5321	0.2740
Srrm2	_SSS[Phospho (STY)]PVTELTARS[Phospho (STY)]PVKQDK_.3	#N/A	3.9595	9.2593	10.1891	9.6054	8.6020	9.3531	3.5899	9.4410	10.3514	9.4118	1.0361	0.0512	0.8653	0.0628
Srrm2	_SRHS[Phospho (STY)]PRLS[Phospho (STY)]R_.3	#N/A	8.9986	8.7898	9.3962	9.1367	8.8782	8.8833	9.2452	9.0371	9.2617	7.5803	1.0265	0.0377	0.5113	0.2914
Srrm2	_S[Phospho (STY)]PVPSAFSDQSR_.2	Q8BTI8_S2404_M1_Srrm2	10.4125	9.7362	9.9511	10.2935	9.2197	9.8826	10.5309	9.7680	10.0136	9.9794	0.9844	-0.0227	0.5511	0.2588
Srrm2	_S[Phospho (STY)]RTS[Phospho (STY)]PVSR_.2	Q8BTI8_S1849_M2_Srrm2	5.7901	7.3454	7.4494	6.3352	6.6054	6.0643	4.1020	7.9748	7.1402	5.6862	1.0598	0.0838	0.6356	0.1968
Srrm2	_S[Phospho (STY)]RS[Phospho (STY)]PQRPGWSR_.3	#N/A	8.7793	8.6924	9.1433	9.1182	8.5380	8.8782	9.0570	8.9527	9.0349	8.7895	0.9888	-0.0162	0.4660	0.3316
Srrm2	_S[Phospho (STY)]RS[Phospho (STY)]IS[Phospho (STY)]PC[Carbamidomethyl (C)]PK_.2	Q8BTI8_S924_M3_Srrm2	8.6677	8.5388	8.8575	9.0082	7.2894	8.6245	9.1645	8.6186	8.5811	8.6686	0.9703	-0.0436	0.4557	0.3414
Srrm2	_NSGPVSEVNTGFS[Phospho (STY)]PEVK_.2	Q8BTI8_S1305_M1_Srrm2	8.7092	8.6212	8.8233	9.2019	7.8339	8.5249	9.2720	8.6324	8.5251	8.6867	0.9818	-0.0265	0.5633	0.2493

Srrm2	_ERAPS[Phospho (STY)]PASR_.2	Q8BTI8_S2381_M1_Srrm2	9.3632	9.5758	10.1528	9.7615	9.0749	9.5078	9.9215	9.8652	9.5051	9.4783	0.9876	-0.0180	0.5805	0.2362
Srrm2	_ALPQT[Phospho (STY)]PR_.2	Q8BTI8_T1448_M1_Srrm2	9.3458	9.6609	10.2272	9.7606	8.7607	9.2283	9.9612	9.6511	9.3605	9.4026	0.9899	-0.0146	0.7383	0.1318
Srrm2	_DKFS[Phospho (STY)]PTQDRPESSTVLK_.3	Q8BTI8_S1151_M1_Srrm2	9.2387	9.0302	9.8891	9.5826	8.3779	9.1145	9.3745	9.0806	9.0931	9.0140	1.0071	0.0102	0.8167	0.0879
Srrm2	_S[Phospho (STY)]RT[Phospho (STY)]PLISR_.2	Q8BTI8_T1836_M2_Srrm2	9.1547	9.0741	9.2835	9.5745	8.5854	8.9222	9.5532	9.1526	9.1343	8.9571	0.9891	-0.0158	0.6256	0.2037
Srrm2	_RVPS[Phospho (STY)]PTPVPK_.2	#N/A	8.7917	8.5421	9.4090	3.4991	3.9293	3.3004	4.1761	3.3400	3.3305	3.3005	1.7658	0.8203	0.1110	0.9546
Srrm2	_REIS[Phospho (STY)]S[Phospho (STY)]SPTSK_.2	Q8BTI8_S453_M2_Srrm2	3.3958	7.9370	8.4666	7.9808	7.0674	3.2032	8.5921	8.4748	7.8289	3.3773	0.8972	-0.1565	0.6558	0.1832
Srrm2	_SGAGS[Phospho (STY)]PPGKR_.2	Q8BTI8_S1179_M1_Srrm2	8.8207	3.6668	4.2461	3.2479	3.5551	7.6351	8.9881	8.8010	3.0183	8.7927	0.7021	-0.5103	0.2268	0.6444
Srrm2	_T[Phospho (STY)]PPVALSSSR_.2	Q8BTI8_T2056_M1_Srrm2	8.5145	3.6743	3.4017	8.6051	7.4488	8.1112	8.7763	8.1491	8.0026	8.1040	0.8024	-0.3177	0.2269	0.6442
Srrm2	_S[Phospho (STY)]RSRT[Phospho (STY)]S[Phospho (STY)]PAPWK_.2	Q8BTI8_S1808_M3_Srrm2	7.0071	3.8380	3.8974	7.3857	7.2477	6.8828	3.6420	3.6518	3.6243	3.8369	1.6383	0.7122	0.0262	1.5810
Srrm2	_VPS[Phospho (STY)]PTPVPK_.2	#N/A	9.1321	7.7796	8.1290	8.0511	3.4997	8.4293	3.0898	7.7314	8.2997	4.0296	1.2965	0.3746	0.2728	0.5641
Srrm2	_GHS[Phospho (STY)]RS[Phospho (STY)]RS[Phospho (STY)]PQWR_.3	Q8BTI8_S503_M3_Srrm2	7.8045	7.3997	8.1020	7.4337	6.6395	7.5646	7.6583	7.1860	7.9452	7.6622	0.9839	-0.0234	0.6745	0.1710
Srrm2	_S[Phospho (STY)]RS[Phospho (STY)]RTS[Phospho (STY)]PVSR_.2	Q8BTI8_S1844_M3_Srrm2	6.9912	6.3083	3.5734	7.1857	4.2279	3.8976	6.8834	7.1520	3.6910	4.0430	0.9856	-0.0209	0.9453	0.0244
Srrm2	_S[Phospho (STY)]RS[Phospho (STY)]RS[Phospho (STY)]LVR_.2	Q8BTI8_S700_M3_Srrm2	7.3624	7.1391	7.5503	7.3581	6.5799	7.0633	7.5931	7.3232	6.9691	7.1883	0.9872	-0.0186	0.6575	0.1821
Srrm2	_SSS[Phospho (STY)]PVTELTARS[Phospho (STY)]PVK_.2	#N/A	3.6519	6.8136	3.8638	7.1904	6.0230	6.5790	3.7505	6.9579	6.9262	3.6234	1.0701	0.0977	0.7397	0.1309
Srrm2	_SRS[Phospho (STY)]T[Phospho (STY)]T[Phospho (STY)]PAPK_.2	Q8BTI8_S250_M3_Srrm2	6.5083	3.7615	6.8868	6.6212	3.5496	3.9223	6.1795	5.8350	6.0750	3.3606	0.9712	-0.0421	0.8788	0.0561
Srrm2	_TS[Phospho (STY)]PAPWKR_.2	#N/A	6.2160	6.6884	3.8405	6.8483	4.1193	3.8150	6.9637	3.8166	6.3791	6.3940	0.8924	-0.1643	0.5175	0.2861
Srrm2	_ARS[Phospho (STY)]RT[Phospho (STY)]PPSAPSQSR_.3	#N/A	8.2346	8.1380	8.8445	8.3319	7.4430	8.2838	8.6398	8.3232	8.4139	8.0321	0.9833	-0.0243	0.5928	0.2271
Srrm2	_RKET[Phospho (STY)]PS[Phospho (STY)]PR_.2	#N/A	8.3735	8.1319	8.6152	7.7065	3.7393	6.7002	8.8193	8.4610	7.6107	7.8851	0.8800	-0.1844	0.3350	0.4750
Srrm2	_HSGST[Phospho (STY)]SPYLK_.2	Q8BTI8_T963_M1_Srrm2	3.9307	3.2351	7.9264	3.3847	3.0564	3.1722	3.5330	3.6164	3.9155	3.0620	1.1659	0.2214	0.5645	0.2483
Srrm2	_GRS[Phospho (STY)]PS[Phospho (STY)]PKPR_.2	Q8BTI8_S1718_M2_Srrm2	7.4316	7.3296	7.8219	7.2573	3.0355	5.6098	8.0821	7.8030	6.3360	7.2834	0.8696	-0.2016	0.3546	0.4503
Srrm2	_NNKS[Phospho (STY)]VT[Phospho (STY)]PQR_.2	#N/A	7.2657	6.9734	6.4532	7.6035	4.1460	5.7700	7.3932	7.4788	6.4889	6.3666	0.9187	-0.1223	0.4352	0.3613
Srrm2	_VSS[Phospho (STY)]PVLETVQQRT[Phospho (STY)]PSR_.3	Q8BTI8_S1360_M2_Srrm2	4.2617	2.9644	3.4477	3.9503	2.8527	3.7719	3.6650	4.1681	3.8167	3.9173	0.9100	-0.1361	0.2715	0.5663
Srrm2	_TS[Phospho (STY)]PLM[Oxidation (M)]LDR_.2	#N/A	8.8372	7.2829	4.0503	10.2890	6.9341	8.5148	10.4181	3.8509	4.1106	7.2418	1.1945	0.2564	0.4679	0.3299
Srrm2	_SLSYS[Phospho (STY)]PVER_.2	Q8BTI8_S2648_M1_Srrm2	7.8069	9.2233	8.3649	9.5295	6.9605	9.2180	9.8529	9.3947	9.3776	9.4114	0.8957	-0.1589	0.0911	1.0405
Srrm2	_S[Phospho (STY)]RTS[Phospho (STY)]PAPWKR_.2	#N/A	7.6887	7.8931	3.9660	8.2910	7.5031	8.0286	8.3264	7.9147	8.0836	7.9391	0.8962	-0.1582	0.3429	0.4649
Srrm2	_GRS[Phospho (STY)]RT[Phospho (STY)]PLTSR_.2	#N/A	9.0653	8.9895	9.4853	9.2205	8.5553	9.0260	9.3236	8.9980	9.2436	9.0655	0.9890	-0.0160	0.5635	0.2491
Srrm2	_RSS[Phospho (STY)]SELSPEVVEK_.2	Q8BTI8_S1339_M1_Srrm2	6.4203	6.5554	6.7953	6.6846	5.9927	6.4292	7.1358	6.5400	6.2763	6.5012	0.9798	-0.0295	0.5295	0.2762
Srrm2	_HAS[Phospho (STY)]S[Phospho (STY)]SPESLKPTPAPGSR_.3	Q8BTI8_S434_M2_Srrm2	5.9243	6.2489	3.9677	6.6294	5.4637	6.0663	6.5923	6.3024	6.2047	6.2143	0.9033	-0.1467	0.2421	0.6160
Srrm2	_LKSGM[Oxidation (M)]S[Phospho (STY)]PEQSK_.2	#N/A	3.9840	3.2815	3.4773	7.2111	6.9540	3.4548	7.3473	6.9122	7.1533	6.3495	0.6811	-0.5541	0.0491	1.3089
Srrm2	_GRS[Phospho (STY)]EC[Carbamidomethyl (C)]DS[Phospho (STY)]S[Phospho (STY)]PEPK_.2	Q8BTI8_S1434_M3_Srrm2	4.9263	4.6746	6.1169	5.1492	5.3555	5.0487	5.6116	5.9963	4.6050	5.5188	0.9593	-0.0599	0.5394	0.2681
Srrm2	_SSSELS[Phospho (STY)]PEVVEK_.2	Q8BTI8_S1343_M1_Srrm2	3.9686	3.9648	6.3852	3.6158	6.3960	3.4171	5.9055	3.2235	6.5362	3.1911	0.9810	-0.0277	0.9303	0.0314
Srrm2	_SM[Oxidation (M)]LQT[Phospho (STY)]PPDQNLSGSK_.2	#N/A	8.8779	8.2096	8.7177	8.4401	7.5840	6.7368	6.8229	8.1428	8.2474	8.6111	1.0174	0.0249	0.7947	0.0998
Srrm2	_PSM[Oxidation (M)]S[Phospho (STY)]PTPLDR_.2	#N/A	3.7977	7.0824	3.5314	6.9381	6.4887	7.1833	7.5899	7.3313	3.5827	7.3387	0.9035	-0.1465	0.6035	0.2193
Srrm2	_S[Phospho (STY)]PVKQDKSEISTDPK_.2	#N/A	3.8359	5.3442	5.7134	5.2967	5.1963	5.5690	5.8142	5.6597	5.7584	5.4112	0.9114	-0.1339	0.1917	0.7174
Srrm2	_VSSPVLETVQQRT[Phospho (STY)]PS[Phospho (STY)]RER_.3	Q8BTI8_S1372_M2_Srrm2	2.9466	6.7006	4.1811	6.8631	4.2172	3.5123	6.8238	7.2359	6.1012	6.1930	0.7190	-0.4760	0.0659	1.1808
Srrm2	_S[Phospho (STY)]RT[Phospho (STY)]PLLPR_.2	Q8BTI8_S1984_M2_Srrm2	8.2256	5.7709	10.5726	3.9098	11.0950	11.6787	12.2104	11.5652	3.8573	4.9663	1.0481	0.0678	0.8718	0.0596
Srrm2	_S[Phospho (STY)]RAS[Phospho (STY)]PVSR_.2	#N/A	7.7421	7.5508	7.8979	7.9233	5.9507	6.6277	8.0152	7.6637	6.5479	7.5237	0.9791	-0.0305	0.7551	0.1220
Srrm2	_QSHSESS[Phospho (STY)]PDGEVK_.2	Q8BTI8_S851_M1_Srrm2	3.6580	3.9492	7.0178	6.7641	3.7987	3.6402	3.8249	6.9343	3.5047	6.4218	0.9291	-0.1061	0.7430	0.1290
Srrm2	_AQS[Phospho (STY)]GTDS[Phospho (STY)]S[Phospho (STY)]PEHKIPAPR_.2	#N/A	7.3666	8.0181	3.7635	3.9765	6.7161	7.1529	8.9372	7.2577	7.8768	3.2888	0.9014	-0.1498	0.6307	0.2002
Srrm2	_NHSGS[Phospho (STY)]RT[Phospho (STY)]PPVALSSSR_.3	Q8BTI8_S2054_M2_Srrm2	7.5911	6.3950	3.8204	7.9306	6.5960	7.7120	5.4694	7.8110	6.1714	3.9107	1.1427	0.1925	0.4330	0.3635
Srrm2	_SLS[Phospho (STY)]GSS[Phospho (STY)]PC[Carbamidomethyl (C)]PK_.2	#N/A	3.5794	3.7182	6.3644	5.7642	6.1827	6.3642	6.3348	4.0766	3.4425	7.0540	1.0195	0.0279	0.9183	0.0370
Srrm2	_SQTS[Phospho (STY)]PKGS[Phospho (STY)]LSR_.2	Q8BTI8_S1058_M2_Srrm2	4.3737	6.4842	3.5596	6.4339	5.7994	4.4721	6.3947	6.4548	3.7048	4.0293	1.0080	0.0115	0.9627	0.0165
Srrm2	_EISSS[Phospho (STY)]PTSK_.2	Q8BTI8_S454_M1_Srrm2	4.0551	3.3105	3.7888	3.6042	3.1988	3.4258	3.3189	3.0134	4.1628	3.5712	1.0134	0.0193	0.8560	0.0675
Srrm2	_SRSGS[Phospho (STY)]S[Phospho (STY)]PGLR_.2	Q8BTI8_S1399_M2_Srrm2	3.0959	5.2968	4.1125	3.7063	5.8037	3.5063	4.3412	6.1842	6.2109	3.9451	0.8227	-0.2816	0.2398	0.6201
Srrm2	_S[Phospho (STY)]VT[Phospho (STY)]PQRER_.2	#N/A	5.3632	4.2982	3.3772	6.0109	2.9233	3.7013	5.3223	5.2378	5.2581	5.7210	0.7946	-0.3316	0.1110	0.9547
Srrm2	_SAVRPS[Phospho (STY)]PS[Phospho (STY)]PER_.2	Q8BTI8_S349_M2_Srrm2	10.6730	10.7897	11.1915	11.0778	10.2878	10.7900	11.1439	10.8906	10.7783	10.7556	0.9917	-0.0120	0.6239	0.2049
Srrm2	_RQPS[Phospho (STY)]PQPS[Phospho (STY)]PR_.2	Q8BTI8_S2656_M2_Srrm2	9.6175	9.1638	9.2495	9.4783	8.2996	9.8044	9.5159	9.1573	9.8876	9.0879	0.9848	-0.0221	0.6530	0.1851
Srrm2	_S[Phospho (STY)]PQRPGWSR_.2	Q8BTI8_S533_M1_Srrm2	4.0349	5.8118	3.2208	5.9244	3.0796	3.5450	3.4382	4.3950	4.0436	5.8849	0.9615	-0.0567	0.8304	0.0807
Srrm2	_GGS[Phospho (STY)]GYHS[Phospho (STY)]RS[Phospho (STY)]PTRQESSR_.3	#N/A	3.0750	4.2278	4.4132	3.9570	4.0801	3.8790	4.1053	3.9175	3.0311	3.4899	1.0833	0.1154	0.3473	0.4593
Srrm2	_S[Phospho (STY)]RS[Phospho (STY)]RT[Phospho (STY)]PLLPR_.2	#N/A	4.3604	9.2279	3.8476	9.4760	8.7002	9.3466	9.9206	9.4009	9.3888	9.3237	0.7880	-0.3437	0.1748	0.7575
Srrm2	_SS[Phospho (STY)]RSS[Phospho (STY)]PELTR_.2	#N/A	8.1333	8.2596	4.1437	8.5027	3.7636	8.1368	8.7276	8.2313	8.2648	8.2410	0.8156	-0.2941	0.2136	0.6703
Srrm2	_S[Phospho (STY)]LTRS[Phospho (STY)]PPAIR_.2	Q8BTI8_S2019_M2_Srrm2	7.5247	10.8962	3.5246	11.6591	10.7929	11.3127	11.5346	11.3143	11.1210	11.4263	0.8181	-0.2896	0.2410	0.6181
Srrm2	_S[Phospho (STY)]RS[Phospho (STY)]RT[Phospho (STY)]PLISR_.2	#N/A	9.5734	9.6040	10.0836	10.0023	9.1055	9.6303	10.1276	9.7316	9.6841	9.6240	0.9872	-0.0186	0.5493	0.2602
Srrm2	_S[Phospho (STY)]RS[Phospho (STY)]RS[Phospho (STY)]PLAIR_.2	#N/A	7.3207	7.7856	3.3883	7.8028	7.1986	7.7974	8.1072	7.6475	7.9379	7.9446	0.8701	-0.2007	0.2804	0.5523
Srrm2	_KET[Phospho (STY)]PS[Phospho (STY)]PR_.2	#N/A	4.0312	3.3112	3.7649	6.6399	3.1325	5.6479	6.8880	3.6925	5.6498	2.9860	0.9203	-0.1198	0.7140	0.1463
Srrm2	_SRT[Phospho (STY)]PPSAPSQSR_.2	Q8BTI8_T2362_M1_Srrm2	9.0771	9.1618	7.6558	9.6256	8.7033	9.0606	9.1775	9.3234	9.2272	9.0519	0.9658	-0.0502	0.3879	0.4113
Srrm2	_RGS[Phospho (STY)]RSS[Phospho (STY)]IEPK_.2	Q8BTI8_S1631_M2_Srrm2	8.6285	8.6576	4.3343	3.6339	7.8535	3.5722	9.0112	3.2049	8.6336	8.7602	0.8258	-0.2761	0.4687	0.3291
Srrm2	_SSRRS[Phospho (STY)]S[Phospho (STY)]SELS[Phospho (STY)]PEVVEK_.2	#N/A	3.7622	5.3402	3.7606	6.0830	3.4799	3.7604	3.6571	4.4016	3.8875	3.9636	1.0973	0.1339	0.5109	0.2917
Srrm2	_S[Phospho (STY)]RS[Phospho (STY)]RAS[Phospho (STY)]PVSR_.2	Q8BTI8_S1864_M3_Srrm2	6.5153	6.6607	6.8293	6.9905	6.5116	7.0716	7.0129	6.7470	6.9364	6.8035	0.9837	-0.0236	0.4191	0.3777
Srrm2	_SEQPLSQVLPS[Phospho (STY)]LSPEHK_.2	#N/A	3.2684	4.0246	3.5677	7.9274	3.9129	3.8020	7.4405	6.9459	3.2546	3.7239	0.8270	-0.2741	0.4744	0.3239
Srrm2	_S[Phospho (STY)]RT[Phospho (STY)]PLTSR_.2	#N/A	9.8508	9.9352	10.5021	10.2868	9.5900	10.0488	10.4665	10.2203	9.9671	10.0531	0.9861	-0.0201	0.4712	0.3268
Srrm2	_RRS[Phospho (STY)]PS[Phospho (STY)]VS[Phospho (STY)]S[Phospho (STY)]PEPTEK_.2	Q8BTI8_S1683_M3_Srrm2	3.4265	3.9159	3.1602	4.2048	3.7373	5.4274	7.3757	7.2209	6.0583	4.8502	0.6240	-0.6804	0.0047	2.3306
Srrm2	_S[Phospho (STY)]SRSS[Phospho (STY)]PELTRK_.2	#N/A	4.0936	3.3690	3.6023	3.5735	3.2291	3.2052	3.5091	4.0662	3.9704	3.4110	0.9393	-0.0904	0.3161	0.5001
Srrm2	_DTPQT[Phospho (STY)]PSR_.2	Q8BTI8_T1428_M1_Srrm2	3.8370	3.1413	3.9348	3.4785	2.9626	3.2660	3.6268	3.5226	3.8217	3.1558	0.9731	-0.0394	0.6859	0.1637
Srrm2	_S[Phospho (STY)]RS[Phospho (STY)]RT[Phospho (STY)]PPVTR_.2	#N/A	10.2624	10.1546	10.6726	10.3941	9.3772	10.2069	3.6178	10.4598	10.2054	10.1891	1.1810	0.2400	0.2769	0.5576
Srrm2	_SRS[Phospho (STY)]PQWR_.2	Q8BTI8_S507_M1_Srrm2	3.7024	3.6610	7.6761	8.2922	6.8890	3.9257	3.8189	4.0770	3.7256	4.0712	1.4506	0.5367	0.1482	0.8292
Srrm2	_S[Phospho (STY)]RS[Phospho (STY)]RT[Phospho (STY)]PPAIR_.2	Q8BTI8_S1970_M3_Srrm2	10.6367	10.4487	11.0386	11.1198	10.1081	10.7518	11.0886	10.8100	10.7638	10.7517	0.9844	-0.0227	0.4259	0.3707
Srrm2	_S[Phospho (STY)]RS[Phospho (STY)]RTS[Phospho (STY)]PVTR_.2	Q8BTI8_S1927_M3_Srrm2	8.0828	7.9532	9.2811	8.5821	3.7397	8.3614	7.9879	7.6807	7.7942	3.6349	1.1317	0.1785	0.5141	0.2890
Srrm2	_ELSHS[Phospho (STY)]PPRENSFESSLEFK_.3	Q8BTI8_S1278_M1_Srrm2	4.0414	4.8569	5.5162	3.8528	3.1796	3.3648	3.8531	5.1586	3.8164	5.1798	0.9186	-0.1226	0.5236	0.2810
Srrm2	_NNKS[Phospho (STY)]VT[Phospho (STY)]PQRER_.2	#N/A	6.5469	6.5361	6.8839	6.9598	5.6164	6.7439	7.1222	6.9873	6.5750	6.4422	0.9655	-0.0506	0.4279	0.3687
Srrm2	_QSSS[Phospho (STY)]PYEDKDKK_.2	Q8BTI8_S333_M1_Srrm2	3.9418	3.4006	3.6755	3.2192	3.2219	3.0067	5.8869	3.7819	5.7827	4.3974	0.6874	-0.5408	0.0084	2.0761
Srrm2	_ARS[Phospho (STY)]HS[Phospho (STY)]PS[Phospho (STY)]SPER_.2	Q8BTI8_S1453_M3_Srrm2	6.8493	3.5717	6.9297	7.0048	5.5486	5.0447	6.9784	3.4075	6.4083	6.8142	0.9869	-0.0190	0.9382	0.0277
Srrm2	_WGKS[Phospho (STY)]RS[Phospho (STY)]PQR_.2	Q8BTI8_S522_M2_Srrm2	4.5487	5.4507	5.3677	5.1493	4.3474	4.8036	4.3532	5.6920	5.2459	5.1208	0.9690	-0.0455	0.6317	0.1995
Srrm2	_SEC[Carbamidomethyl (C)]DS[Phospho (STY)]S[Phospho (STY)]PEPK_.2	#N/A	4.3483	4.2652	3.4221	3.9347	4.1535	3.9729	4.1409	4.1340	3.8527	4.0057	0.9957	-0.0062	0.9252	0.0337
Srrm2	_SSS[Phospho (STY)]AS[Phospho (STY)]PELKDGLPR_.2	Q8BTI8_S1378_M2_Srrm2	3.8791	7.8776	4.5659	8.3133	3.2354	7.9736	8.2971	7.8795	7.8970	7.7882	0.7500	-0.4150	0.1324	0.8782
Srrm2	_SM[Oxidation (M)]LQT[Phospho (STY)]PPDQNLS[Phospho (STY)]GSKSPC[Carbamidomethyl (C)]PQK_.[illegible]	Q8BTI8_S980_M2_Srrm2	4.3680	2.9744	4.1017	3.6454	3.5117	3.1129	3.7937	5.3068	3.6548	3.3227	0.9004	-0.1514	0.3946	0.4039
Srrm2	_SGS[Phospho (STY)]S[Phospho (STY)]PEM[Oxidation (M)]KDKPR_.2	#N/A	5.2283	4.8925	5.1949	5.8712	4.1735	4.6297	5.3462	5.0324	5.3343	4.8567	0.9720	-0.0410	0.6553	0.1836
Srrm2	_RSSRS[Phospho (STY)]S[Phospho (STY)]PELTR_.2	#N/A	3.4653	5.7792	6.2291	4.0742	3.6473	3.4578	3.7006	6.2004	3.8186	5.7662	0.9119	-0.1331	0.6122	0.2131
Srrm2	_GGSGYHS[Phospho (STY)]RS[Phospho (STY)]PTR_.2	#N/A	4.7169	3.8201	5.7647	5.8924	5.3953	5.4992	5.6006	5.6126	5.7003	5.4456	0.9269	-0.1094	0.3389	0.4699

Srrm2	_SGMS[Phospho (STY)]PEQSK_.2	#N/A	3.6855	3.6569	3.4191	4.4637	3.4783	4.2512	3.1112	4.0383	4.3373	4.1410	0.9792	-0.0303	0.7984	0.0978
Srrm2	_SGT[Phospho (STY)]PPRPGSVTNMQADEC[Carbamidomethyl (C)]TAT[Phospho (STY)]PQR_.3	Q8BTI8_T823_M2_Srrm2	3.2144	3.9706	4.4489	3.9927	3.8589	4.2665	4.1409	3.0085	3.3076	3.6699	1.1209	0.1646	0.1792	0.7467
Srrm2	_SGSSQELDGKPSAS[Phospho (STY)]PQER_.2	Q8BTI8_S1508_M1_Srrm2	3.7206	3.0250	4.0511	3.5949	2.8463	3.3823	3.7431	3.6778	3.7054	3.2722	0.6739	-0.5694	0.2136	0.6703
Srrm2	_MELGT[Phospho (STY)]PLR_.2	Q8BTI8_T955_M1_Srrm2	2.9542	3.7594	3.6410	4.1437	3.5807	4.1488	3.8583	3.9748	3.6234	4.1107	0.9519	-0.0711	0.4574	0.3397
Srrm2	_S[Phospho (STY)]RS[Phospho (STY)]RAS[Phospho (STY)]PATHR_.2	Q8BTI8_S1820_M3_Srrm2	4.9212	4.0731	4.0958	4.1046	3.7431	4.1956	4.0229	5.1869	3.4428	4.9027	0.9544	-0.0673	0.6080	0.2161
Srrm2	_SS[Phospho (STY)]PELTR_.2	#N/A	2.9165	3.6727	4.1510	3.6947	3.5610	3.0636	3.8430	3.3064	3.6055	3.3720	0.9938	-0.0089	0.9327	0.0303
Srrm2	_RGGSGYHS[Phospho (STY)]RS[Phospho (STY)]PTR_.3	#N/A	3.2295	3.9857	4.4640	4.0077	3.8740	4.2514	4.1446	2.9934	3.2925	3.6850	1.1246	0.1695	0.1694	0.7710
Srrm2	_C[Carbamidomethyl (C)]RS[Phospho (STY)]PGMLEPLGSAR_.3	Q8BTI8_S2084_M1_Srrm2	3.6566	3.5695	4.3434	6.8812	2.7823	3.4463	4.5608	3.2724	4.4218	3.3123	1.0569	0.0798	0.7855	0.1049
Srrm2	_SGS[Phospho (STY)]SPGLR_.2	Q8BTI8_S1399_M1_Srrm2	3.6482	3.6942	3.3819	4.4265	2.7919	4.2140	3.0739	4.0755	4.3746	4.1038	0.9452	-0.0813	0.5836	0.2339
Srrm2	_MS[Phospho (STY)]C[Carbamidomethyl (C)]FS[Phospho (STY)]RPSM[Oxidation (M)]SPTPLDR_.2	Q8BTI8_S2067_M2_Srrm2	3.4854	4.2906	4.1722	3.8301	4.1120	3.6175	3.9783	3.1711	3.7837	3.5073	1.0853	0.1181	0.1918	0.7172
Srrm2	_TPAAAAAMNLAS[Phospho (STY)]PR_.2	Q8BTI8_S2224_M1_Srrm2	3.7692	3.5732	3.5029	3.8859	3.3945	4.3350	3.1949	3.9545	4.2536	4.2248	0.9582	-0.0617	0.5488	0.2606
Srrm2	_S[Phospho (STY)]RS[Phospho (STY)]RT[Phospho (STY)]PAR_.2	Q8BTI8_S570_M3_Srrm2	3.6139	3.4154	4.3007	3.3025	3.5006	3.1240	4.5180	3.3152	2.9637	3.2695	1.0075	0.0107	0.9407	0.0266
Cpsf7	_SSS[Phospho (STY)]TEPPPPVR_.2	Q8BTV2_S48_M1_Cpsf7	10.8165	10.5288	3.9706	4.4733	10.0717	3.8191	10.9815	3.6452	10.8867	4.4403	0.9722	-0.0407	0.9326	0.0303
Cpsf7	_DSSDSADGRAT[Phospho (STY)]PSENLVPSSAR_.3	Q8BTV2_T203_M1_Cpsf7	7.2760	6.4571	7.5389	7.3947	6.7064	3.6733	3.8154	7.0573	7.2799	7.2120	1.0263	0.0374	0.8713	0.0598
Pak4	_RES[Phospho (STY)]PPPPAR_.2	Q8BTW9_S104_M1_Pak4	7.9137	6.7678	7.3377	8.1406	3.4645	8.1348	7.8656	5.9976	6.7685	8.0208	0.9716	-0.0415	0.8425	0.0744
Gmppb	_LYSGPGIVGNVLVDPSAR_.2	#N/A	3.7636	3.9675	3.2027	3.5450	4.0390	3.3394	4.5626	4.4642	4.3148	3.7773	0.8512	-0.2324	0.0206	1.6868
Tox4	_LSTTPS[Phospho (STY)]PT[Phospho (STY)]NSLHEDGVDDFRR_.3	Q8BU11_S178_M2_Tox4	3.7906	3.4356	4.4774	3.4792	2.9163	3.3124	3.1939	3.1384	4.2878	3.4462	1.0148	0.0212	0.8828	0.0541
Sec62	_EDSKKEETPGT[Phospho (STY)]PK_.2	#N/A	3.6473	4.3116	4.0308	3.5411	3.5756	3.8988	3.8946	4.6294	3.6797	3.7332	0.9623	-0.0554	0.5369	0.2701
Sec62	_SQHS[Phospho (STY)]SGNGNDFEM[Oxidation (M)]ITK_.2	Q8BU14_S356_M1_Sec62	3.8749	3.1792	4.5617	3.4406	3.0005	3.2281	5.3482	3.5605	5.5944	3.1179	0.8053	-0.3124	0.1744	0.7584
Sec62	_EELEQQT[Phospho (STY)]DGDC[Carbamidomethyl (C)]DEEDDDKDGEVPK_.3	Q8BU14_T375_M1_Sec62	4.3289	3.0135	4.0626	3.6064	3.4726	3.1520	3.7546	3.3948	3.6939	3.2836	1.0210	0.0300	0.7947	0.0998
Hook3	_LNQSDSIEDPNS[Phospho (STY)]PAGR_.2	#N/A	9.1222	8.9290	9.3935	9.6676	8.5985	8.7503	9.2672	8.7019	8.9455	9.0015	1.0109	0.0156	0.6750	0.1707
Hook3	_LNQSDSIEDPNS[Phospho (STY)]PAGRR_.2	#N/A	6.9048	6.9661	7.6694	7.4457	6.5940	7.1583	7.1289	7.1627	7.1371	7.1420	0.9973	-0.0040	0.9237	0.0345
Plekha1	_RLS[Phospho (STY)]NPC[Carbamidomethyl (C)]IQR_.2	Q8BUL6_S308_M1_Plekha1	6.4420	7.3298	7.1522	7.6514	6.0819	6.8462	7.2236	7.2907	6.9173	7.1533	0.9680	-0.0470	0.4727	0.3254
Ptpn7	_AQS[Phospho (STY)]QEDSDYINANYIR_.2	Q8BUM3_S143_M1_Ptpn7	3.0763	3.8815	3.7631	5.5268	3.7028	4.0267	4.3875	4.9829	3.0610	3.9165	0.9778	-0.0324	0.8673	0.0618
Foxj3	_VTLYNADQDGSDS[Phospho (STY)]PR_.2	Q8BUR3_S223_M1_Foxj3	6.4631	7.2961	7.2638	7.4957	7.3311	7.6826	8.1139	7.0027	7.3149	7.5170	0.9690	-0.0454	0.4359	0.3606
Gphn	_ASHS[Phospho (STY)]AVDITK_.2	Q8BUV3_S338_M1_Gphn	7.4011	7.7684	8.0867	8.0462	6.8713	7.6903	7.6126	7.7935	7.8347	8.2182	0.9719	-0.0411	0.4078	0.3896
Gphn	_IPDSIISR_.2	#N/A	5.9372	5.8250	5.9876	5.5183	6.2451	6.2897	5.7489	6.2481	5.5605	6.1062	1.0087	0.0124	0.7960	0.0991
Gphn	_VLAQDVYAK_.2	#N/A	5.5495	5.8735	4.4574	4.9155	6.2607	5.5392	5.4888	4.9144	5.8319	5.6214	0.9942	-0.0083	0.9337	0.0298
Gphn	_DTASLSTTPSES[Phospho (STY)]PR_.2	#N/A	11.3523	11.3889	11.8052	12.3778	11.1247	11.9767	11.7493	11.3243	11.6496	12.0117	0.9989	-0.0016	0.9624	0.0166
Gphn	_DLVQDPSLLGGTISAYK_.2	#N/A	4.7278	5.1799	5.8096	5.7308	5.5846	5.6939	5.1188	4.9560	5.1060	6.0151	1.0293	0.0417	0.6027	0.2199
Gphn	_VGVLTVSDSC[Carbamidomethyl (C)]FR_.2	#N/A	3.7612	5.1001	3.4949	5.5035	4.9489	5.5119	5.6613	5.2890	4.9238	5.1493	0.8981	-0.1551	0.2822	0.5495
Gphn	_DTASLSTT[Phospho (STY)]PSES[Phospho (STY)]PRAQATSR_.3	#N/A	4.1124	3.2016	4.0278	3.6420	3.2447	3.4621	3.5115	3.5786	4.1628	3.6090	0.9730	-0.0395	0.6750	0.1707
Gphn	_LSTAS[Phospho (STY)]C[Carbamidomethyl (C)]PT[Phospho (STY)]PK_.2	Q8BUV3_T286_M2_Gphn	7.9669	8.0400	8.2687	8.1144	6.4908	8.1324	7.7517	8.0260	7.2666	7.8326	1.0151	0.0216	0.7570	0.1209
Gphn	_C[Carbamidomethyl (C)]SS[Phospho (STY)]KENILR_.2	Q8BUV3_S328_M1_Gphn	3.3619	8.4895	8.8103	8.7494	8.0860	8.7551	4.1828	5.8861	8.4907	8.6453	1.1334	0.1807	0.5316	0.2744
Gphn	_AQATSRLS[Phospho (STY)]TAS[Phospho (STY)]C[Carbamidomethyl (C)]PT[Phospho (STY)]PK_.2	Q8BUV3_T286_M3_Gphn	6.7148	5.9174	6.2434	7.5522	6.7689	6.4940	5.6033	6.0384	6.9400	6.3261	1.0623	0.0872	0.3129	0.5046
Gphn	_EVHDELEDLPS[Phospho (STY)]PPPPLS[Phospho (STY)]PPPTTSPHK_.4	#N/A	3.8820	6.2075	3.8878	8.7835	7.1214	5.4815	5.8705	8.1157	7.0519	5.4824	0.8890	-0.1698	0.5154	0.2878
Gphn	_VKEVHDELEDLPS[Phospho (STY)]PPPPLS[Phospho (STY)]PPPT[Phospho (STY)]TSPHKQTEDK_.5	Q8BUV3_T198_M3_Gphn	3.5713	3.6769	4.0974	3.9299	3.5209	3.7333	3.8926	3.5029	3.6458	4.0269	0.9968	-0.0046	0.9361	0.0287
Gphn	_VKEVHDELEDLPS[Phospho (STY)]PPPPLS[Phospho (STY)]PPPTTSPHK_.4	#N/A	3.6663	2.9706	4.3531	6.4798	2.7919	3.4367	3.7975	3.2628	3.6510	3.3219	1.1258	0.1710	0.5458	0.2630
Lsm11	_DSS[Phospho (STY)]LTLTR_.2	Q8BUV6_S220_M1_Lsm11	3.6127	4.4179	4.2995	3.7028	4.2392	3.4903	3.8511	3.3163	2.9649	3.2683	1.1822	0.2414	0.0416	1.3806
Lsm11	_S[Phospho (STY)]VPSSLQASAR_.2	Q8BUV6_S281_M1_Lsm11	4.3739	3.6782	3.3978	4.4424	3.4996	4.2299	3.0899	5.2346	4.3586	4.1197	0.9372	-0.0935	0.5492	0.2603
Gpr107	_KVS[Phospho (STY)]NGAVEPQGSWEGTA_.2	Q8BUV8_S537_M1_Gpr107	7.1679	7.4430	7.9714	7.6142	6.2186	6.8326	7.8152	7.6952	7.5696	6.9707	0.9594	-0.0597	0.4088	0.3885
Gprasp2	_QLQIQIDNQNDPEEGQ_.2	#N/A	3.6976	4.5364	3.5851	5.4451	4.2467	5.6734	4.9360	4.8458	4.5965	3.9926	0.9865	-0.0196	0.8996	0.0459
Atp6v1h	_QLQSEQPQTAAARS[Phospho (STY)]_.2	Q8BVE3_S483_M1_Atp6v1h	8.7480	8.8987	9.4099	8.6701	7.9230	8.6969	9.0647	9.1200	9.0660	8.7093	0.9705	-0.0433	0.3291	0.4827
Atp6v1h	_GAVDAAVPTNIIAAK_.2	#N/A	8.4569	8.3721	9.1918	8.3557	8.2717	8.2389	8.8561	8.5375	8.4839	7.9613	1.0025	0.0037	0.9289	0.0320
Atp6v1h	_LEWSPVHK_.2	#N/A	5.8335	6.1255	6.7576	6.3727	5.3782	5.7481	6.1367	6.8694	6.1999	6.0547	0.9558	-0.0652	0.3644	0.4384
Atp6v1h	_LLEVSDDPQVLAVAAHDVGEYVR_.3	#N/A	6.1997	5.6823	5.7700	5.7166	4.6836	5.5501	5.4762	6.2044	5.6203	4.9932	1.0048	0.0069	0.9356	0.0289
Atp6v1h	_LGESVQDLSSFDEYSSELK_.2	#N/A	5.7954	5.3472	6.0625	6.2122	4.1653	5.6081	6.5997	5.7234	5.3522	5.8017	0.9425	-0.0854	0.4550	0.3420
Atp6v1h	_QLQSEQPQTAAAR_.2	#N/A	6.7653	6.5139	3.4289	6.7769	6.7372	6.9382	6.7789	6.9211	7.1091	6.8789	0.8947	-0.1605	0.3263	0.4864
Atp6v1h	_YNIIPVLSDILQESVK_.2	#N/A	5.5284	5.1949	4.1009	5.9063	4.2928	3.1136	4.9565	6.1789	5.2365	5.0992	0.8736	-0.1949	0.2719	0.5656
Atp6v1h	_QLENLEQQKYDDEDISEDIK_.3	#N/A	3.4518	4.9105	4.3546	5.2941	4.1708	3.3852	5.1932	5.1129	4.4555	2.7283	0.9745	-0.0372	0.8569	0.0671
Atp6v1h	_RSS[Phospho (STY)]EDKQEM[Oxidation (M)]LQTEGSQC[Carbamidomethyl (C)]AK_.3	#N/A	3.2562	3.7566	3.9684	4.2907	3.3961	4.0966	4.5348	4.9354	3.2524	3.6817	0.9252	-0.1122	0.4283	0.3683
Atp6v1h	_VIEQLGGK_.2	#N/A	4.1235	4.2035	3.1132	4.1578	4.4117	4.4405	3.3172	3.7428	3.9375	4.7248	1.0367	0.0521	0.6835	0.1652
Nsd2	_SSS[Phospho (STY)]AENQEGDPGTDK_.2	Q8BVE8_S408_M1_Nsd2	7.5618	3.6347	7.6557	4.4859	3.4561	8.0604	7.8972	7.8149	4.3151	7.6629	0.8392	-0.2530	0.4181	0.3787
Dpp9	tyl (Protein N-term)]C[Carbamidomethyl (C)]SGVS[Phospho (STY)]PVEQVAAGDM[Oxidation (M)]DDTA	#N/A	3.4873	3.6081	3.9724	6.6959	5.0403	5.9743	6.0685	6.3016	6.0090	6.1097	0.7834	-0.3521	0.0875	1.0582
Qdpr	_EGGLLTLAGAK_.2	#N/A	7.2478	7.7399	7.2035	6.8828	6.9173	7.0579	6.9869	7.2191	7.4526	7.3393	0.9897	-0.0149	0.6865	0.1634
Qdpr	_VDAILC[Carbamidomethyl (C)]VAGGWAGGNAK_.2	#N/A	4.0970	3.3524	3.8307	6.3913	3.2407	5.6901	6.6589	3.0553	6.2852	6.1567	0.8004	-0.3211	0.2718	0.5657
Qdpr	_AALDGTPGMIGYGM[Oxidation (M)]AK_.2	#N/A	4.3019	3.5574	4.0356	5.8793	3.4457	3.1789	3.7276	3.4218	3.7208	3.2567	1.1514	0.2034	0.3224	0.4915
Qdpr	_MTDSFTEQADQVTADVGK_.2	#N/A	5.7142	4.5570	4.3532	7.2762	5.0165	4.3622	5.2496	3.1042	4.7671	4.8453	1.1607	0.2150	0.3262	0.4866
Qdpr	_GAVHQLC[Carbamidomethyl (C)]QSLAGK_.3	#N/A	3.2376	4.9203	3.2646	3.2369	3.7089	3.0243	3.3851	3.7643	4.6088	4.4717	0.8787	-0.1865	0.2804	0.5522
Qdpr	_QSM[Oxidation (M)]WTSTISSHLATK_.2	#N/A	3.8605	3.4208	3.7425	3.7715	2.6756	3.8878	3.4141	3.8424	3.7852	3.6261	0.9708	-0.0428	0.6779	0.1688
Stx16	_S[Phospho (STY)]PPKWVDGVDEIQYDVGR_.3	#N/A	6.1655	6.2844	6.5983	6.6510	5.4423	5.7740	5.7544	6.5042	6.1217	5.8664	1.0150	0.0215	0.7488	0.1256
Stx16	_QLLAEQVSS[Phospho (STY)]HTTSSPLHSR_.3	Q8BVI5_S30_M1_Stx16	4.1836	3.4390	3.9173	3.4610	3.3273	3.2973	3.6093	3.5401	3.8392	3.1383	1.0205	0.0293	0.7474	0.1265
Stx16	_SIAAELDELADDR_.2	#N/A	3.1734	4.1991	4.4079	5.3129	3.6989	3.5512	4.1000	5.4212	3.3787	3.6290	0.9819	-0.0264	0.8908	0.0502
Snx17	_VTSSVPLPS[Phospho (STY)]GGTSSPSR_.2	Q8BVL3_S331_M1_Snx17	3.7881	8.5842	4.1503	3.9047	3.3040	3.7098	7.0418	3.5668	3.6769	5.7381	0.9136	-0.1303	0.7305	0.1364
Snx17	_SPPLLES[Phospho (STY)]PDASR_.2	Q8BVL3_S421_M1_Snx17	6.8966	7.3402	7.3935	6.0421	6.6804	7.7455	7.6967	4.0038	7.5764	8.0181	1.0282	0.0402	0.8177	0.0874
Snx17	_VLVTVLTSDQTEDVLEAVAAK_.2	#N/A	3.6289	2.9332	4.3157	2.4488	4.2554	3.4741	3.8746	1.9072	2.9487	3.7405	1.1256	0.1707	0.4706	0.3273
Snx17	_SDSQQAVKS[Phospho (STY)]PPLLES[Phospho (STY)]PDASR_.3	#N/A	3.4087	4.1650	3.1424	4.1870	5.2437	6.4896	4.3353	6.4999	6.1746	6.4434	0.7572	-0.4013	0.0955	1.0198
Jakmip1	_HTS[Phospho (STY)]LNDLSLTR_.2	Q8BVL9_S382_M1_Jakmip1	7.3998	3.7444	6.8840	8.2689	6.3766	7.0099	4.3859	7.3602	7.2882	7.3531	1.0026	0.0037	0.9865	0.0059
Ppme1	_QC[Carbamidomethyl (C)]EGITS[Phospho (STY)]PEGSK_.2	Q8BVQ5_S243_M1_Ppme1	12.2225	12.4848	12.8523	3.3557	11.7631	12.3664	12.6956	12.3879	12.3255	12.3600	0.8713	-0.1988	0.4194	0.3774
Ppme1	_SIVEGIIEEEEEDEEGSESVNKR_.3	#N/A	8.1435	8.0433	9.0790	9.3544	7.3485	8.5559	8.8997	7.8113	8.0669	8.3191	1.0177	0.0253	0.7346	0.1339
Ppme1	_SLENAIEWSVK_.2	#N/A	2.9885	4.3328	4.2230	3.1701	3.9802	4.3784	4.3856	4.3430	4.4021	4.4239	0.8762	-0.1906	0.1205	0.9190
Ppme1	_FAEPIGGFQC[Carbamidomethyl (C)]VFPGC[Carbamidomethyl (C)]_.2	#N/A	5.0377	5.0237	4.0597	3.3687	3.0725	3.1562	5.8621	5.8019	5.3803	3.0460	0.7871	-0.3455	0.1656	0.7810
Maob	_YVDLGGSYVGPTQNR_.2	#N/A	5.8482	6.1947	6.5624	4.4649	5.8597	4.2524	6.0529	6.6387	6.1824	5.9045	0.8928	-0.1636	0.2200	0.6575
Maob	_FIGGSGQVSER_.2	#N/A	4.1208	3.2216	3.8545	3.3982	3.2645	3.3601	3.5465	3.6029	3.9020	3.0755	1.0014	0.0020	0.9836	0.0072
Camk1d	_DLKPENLLYYSQDEESK_.3	#N/A	4.6599	4.4573	3.1745	3.9068	3.3889	4.0401	4.2702	3.9671	3.5485	4.1714	0.9871	-0.0187	0.8773	0.0569
Aldh5a1	_AAYDAFNSWK_.2	#N/A	6.9768	7.1955	7.2570	7.0647	7.4569	7.4917	7.2218	7.2680	7.4958	7.2069	0.9921	-0.0114	0.6377	0.1954
Aldh5a1	_EVGEVLC[Carbamidomethyl (C)]TDPLVSK_.2	#N/A	6.9794	6.8301	6.8405	7.1151	7.2169	6.9112	7.2794	6.7583	7.0959	6.6510	1.0052	0.0075	0.8034	0.0951
Aldh5a1	_GIHDSFVTK_.2	#N/A	6.0785	6.9662	7.1301	6.4730	7.3091	6.4430	6.2062	6.6995	6.1529	6.6932	1.0459	0.0647	0.3047	0.5162
Aldh5a1	_NAGQTC[Carbamidomethyl (C)]VC[Carbamidomethyl (C)]SNR_.2	#N/A	6.6753	6.8077	6.7921	7.3113	6.5336	6.3816	6.6264	6.5718	6.9134	6.2027	1.0261	0.0372	0.4145	0.3825

Aldh5a1	_ISFTGSTATGK_.2	#N/A	6.2878	6.5225	6.1741	6.5743	6.5925	6.3432	5.8467	6.6524	6.1197	6.6661	1.0150	0.0214	0.6192	0.2081
Aldh5a1	_LGTVADC[Carbamidomethyl (C)]GVPEAR_.2	#N/A	6.2880	6.6869	6.2178	6.7279	6.2721	5.7785	6.3427	6.5241	6.1661	6.0306	1.0100	0.0144	0.7586	0.1200
Aldh5a1	_WLPAPATFPVYDPASGAK_.2	#N/A	7.8539	8.1890	8.1411	7.9450	8.0837	8.2058	7.8831	7.9903	7.9694	8.0340	1.0126	0.0181	0.2248	0.6482
Aldh5a1	_DM[Oxidation (M)]LC[Carbamidomethyl (C)]ITEETFGPLAPVIK_.2	#N/A	5.7169	5.4173	4.7880	5.1344	5.3410	5.4331	6.0606	5.8152	4.7239	5.4456	0.9626	-0.0550	0.4838	0.3153
Aldh5a1	_VAEQLEVGM[Oxidation (M)]VGVNEGLISSVEC[Carbamidomethyl (C)]PFGGVK_.3	#N/A	4.5044	4.9289	5.3778	5.5843	3.2980	5.2186	5.1087	4.9214	4.8263	4.3792	1.0020	0.0029	0.9830	0.0075
Aldh5a1	_YGIDEYLEVK_.2	#N/A	4.5457	4.5657	4.8391	4.0608	4.6966	4.7880	3.7754	5.0896	4.4894	4.3185	1.0372	0.0527	0.5411	0.2667
Aldh5a1	_QPVGVAAIITPWNFPSAM[Oxidation (M)]ITR_.2	#N/A	3.6576	4.2848	4.2957	3.8395	3.4492	4.0181	2.0238	3.6067	3.4305	3.6448	1.2354	0.3050	0.0664	1.1780
Aldh5a1	_HQSGGNFFEPTLLSNVTR_.2	#N/A	2.8982	2.8386	4.2585	3.0189	3.5427	1.9437	3.4335	3.3573	1.9024	3.2274	1.0347	0.0492	0.8375	0.0770
Arrb1	_DDKDEEDDGTGS[Phospho (STY)]PHLNNR_.2	#N/A	4.2166	4.6337	4.5917	5.2901	3.6275	4.2385	4.7842	4.4338	4.4419	3.5491	1.0304	0.0432	0.7194	0.1430
Arrb1	_GM[Oxidation (M)]KDDKDEEDDGTGS[Phospho (STY)]PHLNNR_.4	#N/A	5.6580	5.4680	6.6562	5.4362	6.0964	5.7543	4.2110	5.9121	6.7073	5.8427	1.0312	0.0443	0.7208	0.1422
Phka2	_S[Phospho (STY)]LNLVDSPQPLLK_.2	Q8BWJ3_S729_M1_Phka2	4.3035	4.8878	5.2221	4.6193	2.8602	3.3684	3.7292	5.8718	5.5010	3.2582	0.9172	-0.1246	0.5977	0.2235
Gprin3	_S[Phospho (STY)]VSTS[Phospho (STY)]PSILPAYLK_.2	Q8BWS5_S327_M2_Gprin3	10.7613	10.6034	11.7628	11.4855	10.7300	10.7849	10.9904	10.7578	10.6768	10.5416	1.0260	0.0371	0.3057	0.5147
Acaa2	_LEDTLWAGLTDQHVK_.2	#N/A	4.0601	4.2846	5.2199	5.4796	4.7021	5.3537	5.0878	4.4978	3.8474	4.6885	1.0706	0.0984	0.4065	0.3909
Acaa2	_VGVPTETGALTLNR_.2	#N/A	5.6164	5.6474	6.1703	5.0479	6.1178	5.8827	5.4702	6.2621	5.9136	5.8761	0.9773	-0.0331	0.6030	0.2197
Acaa2	_TNVSGGAIALGHPLGGSGSR_.2	#N/A	3.3764	3.8324	4.4964	4.2666	4.1990	4.0324	4.0484	3.8599	4.2357	4.1235	0.9919	-0.0118	0.8787	0.0562
Acaa2	_DFSATDLTEFAAR_.2	#N/A	5.2426	5.1759	4.0837	4.8627	5.4418	5.3458	5.0963	5.0208	5.3050	5.4885	0.9613	-0.0569	0.4743	0.3239
Acaa2	_YALQSQQR_.2	#N/A	3.1639	3.9691	3.1070	4.1516	5.9659	3.9391	4.2999	5.6789	3.1486	5.5987	0.8650	-0.2093	0.3999	0.3980
Acaa2	_VPPETIDSVIVGNVM[Oxidation (M)]QSSSDAAYLAR_.3	#N/A	3.6259	2.7990	3.7314	4.7281	3.4220	3.5239	3.9879	3.6257	3.5839	3.4476	0.9937	-0.0091	0.9467	0.0238
Gemin5	_APSQPPS[Phospho (STY)]PTEER_.2	Q8BX17_S1414_M1_Gemin5	3.7199	3.6225	3.4536	4.4982	7.6853	4.2857	9.0404	8.7583	8.6266	4.1755	0.5940	-0.7515	0.0350	1.4556
Vps13c	_EVST[Phospho (STY)]PQDVHTTQGVPAAR_.3	Q8BX70_T1397_M1_Vps13c	3.7787	5.6037	4.0220	3.2568	3.7803	3.2421	5.9814	3.5776	3.6437	3.9255	0.9218	-0.1174	0.6114	0.2137
Vps13c	_LS[Phospho (STY)]FHNDSFR_.2	Q8BX70_S1956_M1_Vps13c	6.8779	3.5095	4.4035	3.4053	2.8424	3.2268	3.1200	6.4226	6.0858	3.3723	0.8514	-0.2321	0.5098	0.2926
Vps13c	_ILLENLGEASSQPS[Phospho (STY)]PTQYAQEAAR_.3	Q8BX70_S1891_M1_Vps13c	6.4409	6.9196	7.4914	3.6444	5.5528	6.3424	6.7113	7.2982	7.0896	3.4684	0.9875	-0.0181	0.9402	0.0268
Vps13c	_SST[Phospho (STY)]PS[Phospho (STY)]PERQVASIPVLSGGTK_.3	Q8BX70_S841_M2_Vps13c	3.2518	4.0080	4.4863	6.7824	3.8963	4.2291	4.1223	2.9711	3.2702	3.7073	1.2628	0.3367	0.1946	0.7109
Vps13c	_TANAS[Phospho (STY)]LEEIIDK_.2	Q8BX70_S736_M1_Vps13c	3.5088	4.3140	4.1956	7.0031	4.1353	3.5942	3.9550	3.4202	3.0688	3.1644	1.3105	0.3901	0.1571	0.8038
Slco2b1	_KVS[Phospho (STY)]VEPQDSHQDAQPR_.3	#N/A	4.4430	3.9429	4.8958	4.5339	2.9669	4.0054	5.2145	4.4771	4.8181	3.5026	0.9174	-0.1243	0.4310	0.3656
Agap1	_VFQDVAQK_.2	#N/A	4.2900	4.5586	5.4106	4.8299	4.1453	4.3631	3.6031	4.4578	4.6419	4.0312	1.0995	0.1368	0.2015	0.6958
Agap1	_ATSAC[Carbamidomethyl (C)]APISS[Phospho (STY)]PK_.2	Q8BXK8_S422_M1_Agap1	7.3035	7.1508	7.3021	7.8035	3.7751	7.2847	7.4760	7.2437	7.3238	7.1458	0.9277	-0.1082	0.5071	0.2949
Agap1	_LDPPPS[Phospho (STY)]PHANR_.2	Q8BXK8_S521_M1_Agap1	10.4944	10.9928	11.0711	11.3417	9.9769	10.8334	11.1018	10.8485	10.5667	10.5857	1.0009	0.0013	0.9728	0.0120
Agap1	_YLTGTYVQEES[Phospho (STY)]PEGGR_.2	Q8BXK8_S101_M1_Agap1	3.2754	4.0630	4.2640	4.0578	3.9111	3.9833	4.2060	5.4225	3.2425	4.1210	0.9241	-0.1138	0.4375	0.3591
Fam53c	_GNAGGGGPQVPQQS[Phospho (STY)]PPKR_.2	#N/A	7.1358	6.9455	7.6512	8.1053	6.9277	7.0535	6.9264	7.0994	7.0272	7.1254	1.0367	0.0520	0.3212	0.4933
Fam53c	_SRS[Phospho (STY)]QPC[Carbamidomethyl (C)]DLDAR_.2	Q8BXQ8_S273_M1_Fam53c	6.4668	4.2289	3.2063	4.2509	4.1172	4.0083	6.9986	6.5001	3.0493	3.9282	0.8556	-0.2250	0.4562	0.3408
Fam53c	_S[Phospho (STY)]LSVPVDLSR_.2	Q8BXQ8_S120_M1_Fam53c	4.0716	3.3760	3.2576	3.2438	3.1973	3.0313	3.3921	3.7573	4.0564	3.7273	0.9008	-0.1507	0.1231	0.9097
Slc7a14	_QNSEALIANDELDC[Carbamidomethyl (C)]S[Phospho (STY)]PE_.2	#N/A	7.5726	9.5612	7.3446	10.6956	9.2188	8.4331	9.4594	10.6012	10.4320	9.9168	0.8715	-0.1984	0.0932	1.0305
Slc7a14	_HKQNSEALIANDELDC[Carbamidomethyl (C)]S[Phospho (STY)]PE_.2	#N/A	4.0935	7.5526	5.7650	8.6766	7.3858	7.9577	7.2803	8.8640	8.4421	8.2969	0.8400	-0.2516	0.1807	0.7430
Slc7a14	Phospho (STY)]C[Carbamidomethyl (C)]SPVS[Phospho (STY)]EGEEFSSPATNTC[Carbamidomethyl (C)]GA	Q8BXR1_S468_M2_Slc7a14	3.6579	2.9622	4.1139	3.6576	2.7835	3.4451	3.8059	3.3435	3.6426	3.3349	0.9731	-0.0394	0.7312	0.1360
Slc7a14	_NLPS[Phospho (STY)]LGDNEM[Oxidation (M)]LIGK_.2	#N/A	3.5681	6.6413	7.6003	7.2708	5.2790	6.6000	6.7005	7.1282	6.4838	6.6247	0.9147	-0.1286	0.4784	0.3202
Nalcn	_GKS[Phospho (STY)]LETLTQDHSNTVR_.3	Q8BXR5_S781_M1_Nalcn	4.1598	5.5626	3.3458	5.9670	3.2855	2.9431	3.5631	6.9223	5.7623	3.8154	0.8395	-0.2524	0.4017	0.3962
Osbpl6	_TAS[Phospho (STY)]SSTEPSVSR_.2	Q8BXR9_S44_M1_Osbpl6	10.2558	10.1174	10.5964	4.4985	9.2801	10.2564	3.1459	10.3306	10.2863	4.1757	1.3125	0.3923	0.2919	0.5348
Osbpl6	_[Acetyl (Protein N-term)]S[Phospho (STY)]SDEKGIS[Phospho (STY)]PAHK_.2	Q8BXR9_S9_M2_Osbpl6	4.5621	4.7785	3.9941	4.3002	4.0087	4.1217	4.0149	5.3043	4.7204	4.7082	0.9162	-0.1263	0.1757	0.7552
Rgs8	_RLS[Phospho (STY)]TEEATR_.2	Q8BXT1_S46_M1_Rgs8	4.0732	5.0662	5.7255	5.4541	3.4709	4.8326	4.3378	5.3363	4.5322	4.3605	1.0277	0.0395	0.7928	0.1008
Rgs8	_SDS[Phospho (STY)]C[Carbamidomethyl (C)]SDFTAILPDKPNR_.2	Q8BXT1_S26_M1_Rgs8	3.0382	3.8434	3.7250	4.2277	3.6647	4.0648	4.4255	6.1146	3.5394	4.1947	0.8232	-0.2808	0.1322	0.8788
Fgd2	_TPEGPDEEDVS[Phospho (STY)]D_.2	Q8BY35_S654_M1_Fgd2	7.0120	7.4881	7.8868	7.7376	7.3455	7.3413	7.7603	7.4909	7.6902	7.5099	0.9810	-0.0276	0.4149	0.3820
Usp47	_STETSDFENIES[Phospho (STY)]PLNER_.2	Q8BY87_S911_M1_Usp47	3.5303	6.6722	6.6369	8.2407	7.0860	7.1314	7.2412	7.8462	7.5482	7.5042	0.8692	-0.2022	0.2621	0.5815
Usp47	_LFVLLPEQS[Phospho (STY)]PGSYSK_.2	Q8BY87_S832_M1_Usp47	3.2818	4.0381	6.2492	6.9078	3.9264	4.1990	4.4761	6.4933	5.3168	4.3445	0.9243	-0.1136	0.6535	0.1847
Usp47	_ELEQHIQTS[Phospho (STY)]DPENFQSEER_.3	Q8BY87_S934_M1_Usp47	3.2758	4.0666	4.5103	4.0541	3.8879	3.8416	4.2024	4.9482	3.2461	3.7314	0.9770	-0.0335	0.7993	0.0973
Sowahd	_PC[Carbamidomethyl (C)]HY[Phospho (STY)]LRPDAS[Phospho (STY)]LNLR_.2	Q8BY98_S236_M2_Sowahd	3.2293	7.9880	3.8957	4.1363	8.2064	7.8525	9.1244	8.0847	8.8440	8.2784	0.6856	-0.5445	0.0575	1.2403
Sowahd	_PDAS[Phospho (STY)]LNLR_.2	Q8BY98_S236_M1_Sowahd	4.2788	3.2199	3.8562	4.6146	3.0412	3.1875	3.5483	3.6012	3.9002	3.0773	1.0476	0.0670	0.6521	0.1857
Tbc1d17	_YLLLASS[Phospho (STY)]PQDSR_.2	Q8BYH7_S176_M1_Tbc1d17	3.8819	3.8307	3.7123	4.2900	3.6520	4.0775	4.4383	3.9035	3.0102	4.1819	1.0062	0.0089	0.9327	0.0303
Tnr	_LILNYSPR_.2	#N/A	8.4880	8.6022	8.6312	8.1618	8.1365	7.8845	8.7602	8.4728	8.2928	8.5743	0.9756	-0.0356	0.2616	0.5824
Tnr	_LYPATEYEISLNSVR_.2	#N/A	8.0595	8.2835	8.7056	8.4577	8.2988	8.3616	8.4925	8.0288	8.4458	8.4225	1.0016	0.0024	0.9233	0.0347
Tnr	_ASGPIDHYR_.2	#N/A	7.8942	7.9760	8.0359	8.3074	8.4143	7.9407	7.8044	7.8353	7.7565	7.8647	1.0358	0.0507	0.0355	1.4494
Tnr	_YEVSISAVR_.2	#N/A	7.6626	7.3749	7.7673	7.5170	7.7962	7.7849	7.0198	7.3620	7.7138	7.7768	1.0244	0.0348	0.2977	0.5262
Tnr	_YGLVGGEGGK_.2	#N/A	7.4480	7.8244	7.7326	7.6446	7.5861	7.0829	7.1738	7.9071	7.2620	7.8539	1.0005	0.0007	0.9852	0.0065
Tnr	_SSLTSTVFTTGGR_.2	#N/A	7.0762	7.4358	7.0960	7.4628	7.1901	7.3026	6.5636	6.9932	7.3661	7.2770	1.0299	0.0425	0.2409	0.6181
Tnr	_AAIENYVLTYK_.2	#N/A	7.1039	7.1043	7.3290	7.2821	7.0056	7.3872	7.3251	7.0616	7.1189	6.9291	1.0131	0.0188	0.3827	0.4172
Tnr	_QSALISWQPPR_.2	#N/A	6.9782	6.8487	5.3895	6.9857	6.9395	7.5297	6.5362	7.0999	7.0452	7.0788	0.9767	-0.0340	0.6850	0.1643
Tnr	_ELIVDAEDTWIR_.2	#N/A	6.9812	6.8737	7.2650	7.3040	6.8985	7.0892	7.2793	6.4751	6.8496	7.2546	1.0149	0.0214	0.5743	0.2409
Tnr	_LDSSVVPNTVTEFAITR_.2	#N/A	6.1712	6.4401	6.5977	6.9451	6.6070	6.9154	6.1743	6.6128	6.4027	6.4853	1.0302	0.0430	0.2763	0.5587
Tnr	_TATSLDLEWDNSEAEAQEYK_.2	#N/A	6.3837	6.6665	7.5361	7.7164	6.1943	6.6469	7.0471	6.4430	6.1387	6.5553	1.0476	0.0670	0.4015	0.3963
Tnr	_DGQEAVFAYYDK_.2	#N/A	6.3930	6.3145	6.6529	6.7015	5.3832	5.8207	6.8506	6.4050	6.4030	6.7998	0.9390	-0.0908	0.1862	0.7300
Tnr	_VATHLSTPQGLQFK_.3	#N/A	6.2236	6.4365	6.2824	6.5736	6.6256	6.3288	6.1762	6.4512	6.2117	6.7141	1.0037	0.0053	0.8597	0.0656
Tnr	_DKEEDM[Oxidation (M)]LEVLLDATKR_.3	#N/A	7.6488	7.6130	8.0315	7.9847	7.4017	7.6643	7.8229	7.2533	7.6872	7.5472	1.0193	0.0276	0.3752	0.4257
Tnr	_SPPTSASVSTVIDGPTQILVR_.2	#N/A	5.0702	4.8271	4.9897	5.7833	5.0839	5.3641	5.2462	5.2434	5.4136	5.4610	0.9710	-0.0424	0.4137	0.3834
Tnr	_GHEFSIPFVEM[Oxidation (M)]K_.2	#N/A	6.3335	6.9436	6.8455	6.9479	6.9415	7.0153	7.0513	6.8944	6.6292	6.6353	1.0052	0.0075	0.8231	0.0846
Tnr	_LEGLSENTDYTVLLQAAQEATR_.3	#N/A	5.3458	6.0470	6.5064	6.4644	5.4962	5.9551	6.0412	5.4342	5.3953	6.6821	1.0137	0.0197	0.8192	0.0866
Tnr	_QSIPATM[Oxidation (M)]NAR_.2	#N/A	6.2639	6.2737	6.3523	6.3776	6.6448	6.2730	6.0566	6.1082	6.4338	5.8947	1.0393	0.0557	0.0718	1.1440
Tnr	_KELIVDAEDTWIR_.3	#N/A	4.4161	4.0177	4.4960	4.5579	4.8712	4.2194	4.1126	4.6149	4.3939	5.0608	0.9745	-0.0372	0.6092	0.2152
Tnr	_VVYSTLAGEQYHEVLVPK_.2	#N/A	3.8905	4.1640	5.1597	3.9437	4.1468	3.8090	4.4737	4.0461	4.3630	4.5323	0.9614	-0.0568	0.5478	0.2614
Tnr	_VGFGNLEDEFWLGLDNIHR_.3	#N/A	4.0236	4.2603	4.0785	5.0536	2.5532	4.4696	3.7705	4.6663	3.6780	3.2996	1.0570	0.0799	0.6612	0.1797
Tnr	_DVSDTVAFVEWTPPR_.2	#N/A	7.1094	7.1089	7.7451	7.1652	7.4365	7.4622	7.6291	7.2700	7.4318	7.3038	0.9904	-0.0139	0.6389	0.1946
Tnr	_ITFTPSSGISSEVTVPR_.2	#N/A	6.8726	6.9288	7.1861	10.0462	7.1647	6.9785	7.0476	8.5537	7.0334	7.0922	1.0132	0.0189	0.8921	0.0496
Tnr	_C[Carbamidomethyl (C)]LNAC[Carbamidomethyl (C)]SGR_.2	#N/A	3.3244	4.8064	4.5589	4.1027	4.6297	3.8902	4.2509	4.3993	3.1975	3.7800	1.0798	0.1108	0.4065	0.3909
Tnr	_DSLTVSWSSPVAPFDYYR_.2	#N/A	4.2573	3.0851	3.9910	3.5347	4.0697	4.3642	3.6830	4.8189	4.9581	4.5576	0.8622	-0.2139	0.1013	0.9945
Tnr	_LQVYC[Carbamidomethyl (C)]DM[Oxidation (M)]TTDGGGWIVFQR_.2	#N/A	4.4540	3.2643	4.5630	5.2146	3.1168	5.1375	3.4402	3.9708	4.0989	3.8273	1.1193	0.1626	0.3645	0.4382
Tnr	_DGQEAVFAYYDKFAVEDSR_.3	#N/A	3.8560	3.1603	4.5428	3.5446	2.9817	3.2470	3.2593	3.0730	4.2225	3.5116	1.0110	0.0158	0.9155	0.0384
Nlgn3	_RQEPLRQPS[Phospho (STY)]PQR_.3	#N/A	8.5817	8.3315	9.0897	8.8322	8.0112	8.5692	8.7237	8.6359	8.7101	8.4904	0.9918	-0.0119	0.7282	0.1378
Nlgn3	_QEPLRQPS[Phospho (STY)]PQR_.2	#N/A	9.7978	9.8299	10.3255	10.1320	9.4928	9.9908	10.1856	9.9638	9.9669	9.8730	0.9931	-0.0100	0.6708	0.1734
Nlgn3	_LTALPDYTLTLR_.2	#N/A	4.5603	5.1123	3.5572	4.7499	4.8211	5.3212	5.2452	4.4935	3.7072	4.4484	1.0477	0.0672	0.6087	0.2156
Fsd1l	_SENGMTGSTSS[Phospho (STY)]LNNVTQ_.2	Q8BYN5_S501_M1_Fsd1l	3.3426	3.8835	4.0294	4.5321	3.7718	4.3536	4.2468	3.5864	3.2350	2.9983	1.1333	0.1806	0.1633	0.7870

Cadps2	_PVSPSPSVLS[Phospho (STY)]EGR_.2	Q8BYR5_S68_M1_Cadps2	7.4602	7.0428	7.1219	6.9603	5.0122	7.1950	7.4619	7.2203	6.9396	7.0574	0.9482	-0.0767	0.4468	0.3499
Arhgap25	_SKDAPIS[Phospho (STY)]PPAQK_.2	#N/A	9.4456	9.2651	9.2573	9.4972	8.8590	9.3276	9.5802	9.2065	8.8707	9.3837	1.0016	0.0023	0.9298	0.0316
Arhgap25	_S[Phospho (STY)]SVGWDATEDPPLSR_.2	Q8BYW1_S379_M1_Arhgap25	4.0685	3.2739	3.8022	3.3460	3.2123	3.1334	3.4942	3.6552	3.9542	3.0233	0.9833	-0.0243	0.8160	0.0883
Arhgap25	_DAPIS[Phospho (STY)]PPAQK_.2	#N/A	7.8399	7.0446	3.8445	8.7298	7.5683	8.8835	7.8840	8.5497	8.3082	8.1612	0.8897	-0.1686	0.3652	0.4375
Znf609	_TNS[Phospho (STY)]M[Oxidation (M)]GSATGPLPGTK_.2	Q8BZ47_S467_M1_Znf609	3.8830	6.9238	7.3728	7.2231	3.0267	7.2622	7.7100	3.8407	4.1398	4.3386	1.1880	0.2485	0.4657	0.3319
Znf609	_LVEPHS[Phospho (STY)]PSPSSK_.2	Q8BZ47_S575_M1_Znf609	4.1604	3.4158	4.4972	3.4990	6.2284	3.3205	3.2137	3.1187	4.2681	6.5589	0.9760	-0.0350	0.9062	0.0428
Znf609	_AEADKIYSFTDNAPS[Phospho (STY)]PSIGGSSR_.3	Q8BZ47_S803_M1_Znf609	4.2507	2.9755	3.4366	3.9393	3.3764	3.7608	3.6540	4.1792	3.8278	3.9063	0.9310	-0.1032	0.3085	0.5107
Znf609	_APS[Phospho (STY)]LTDLVK_.2	Q8BZ47_S1057_M1_Znf609	3.2271	3.9834	4.4616	4.0054	3.8717	4.2538	4.1536	5.5790	3.2949	3.6827	0.9496	-0.0746	0.6520	0.1858
Znf609	_FC[Carbamidomethyl (C)]DS[Phospho (STY)]PTSDLEMR_.2	Q8BZ47_S358_M1_Znf609	4.1140	3.4184	3.3000	3.8026	3.2397	2.9890	3.3498	4.3158	3.9644	3.7696	0.9032	-0.1469	0.1942	0.7117
Dnm3	_S[Phospho (STY)]PPPSPTTQR_.2	#N/A	12.9033	12.6768	13.0177	13.1369	12.2668	12.8017	13.1835	13.0030	12.9738	13.0517	0.9807	-0.0282	0.1552	0.8091
Dnm3	_ENC[Carbamidomethyl (C)]LILAVTPANTDLANSDALK_.2	#N/A	4.1005	4.2249	4.8961	4.8179	4.4725	8.0857	4.2415	8.0447	4.7262	3.9337	0.9739	-0.0382	0.9014	0.0451
Dnm3	_STIGNQVIR_.2	#N/A	6.1057	6.2255	5.1448	5.9259	5.9829	5.7420	5.1612	5.8131	5.6940	5.6832	1.0477	0.0672	0.2745	0.5614
Dnm3	_VPVGDQPPDIEYQIR_.2	#N/A	6.8299	6.7109	7.1984	7.3457	6.4460	6.8607	7.0769	6.7248	6.7571	4.2859	1.1107	0.1514	0.2370	0.6252
Dnm3	_FLELAC[Carbamidomethyl (C)]DSQEDVDSWK_.2	#N/A	5.0402	5.0063	6.0991	3.8659	4.8427	3.6875	6.2561	5.4028	4.8359	5.1334	0.8798	-0.1848	0.2389	0.6218
Dnm3	_RS[Phospho (STY)]PPPSPTTQR_.2	#N/A	11.4610	11.4729	11.8651	11.7192	10.5944	11.1282	11.5144	11.5418	11.5701	11.1072	0.9948	-0.0076	0.8158	0.0884
Dnm3	_APPSVPSR_.2	#N/A	4.3120	3.6164	3.4597	5.0051	5.9664	4.6225	3.1517	3.9977	5.3440	4.1816	1.0787	0.1094	0.5953	0.2253
Dnm3	_LQGQLLSIEHEVEAFK_.3	#N/A	2.4215	3.6532	3.5348	4.0374	3.4745	4.2550	3.7521	3.0977	2.9302	4.0044	1.0338	0.0480	0.7686	0.1143
Dnm3	_RS[Phospho (STY)]PPPS[Phospho (STY)]PTTQRR_.3	#N/A	3.0363	3.8415	3.7231	4.2792	3.6628	4.0666	4.4274	4.2228	3.0211	3.9565	0.9645	-0.0521	0.6833	0.1654
Tigar	_IIQGQGVDAPLSETGFR_.2	#N/A	3.6032	4.3595	3.3369	4.0561	4.8477	5.0257	3.7708	4.7339	4.1693	4.0588	1.0052	0.0074	0.9557	0.0197
Pogz	_SLDAEPSVPSAAKPS[Phospho (STY)]SPEK_.2	Q8BZH4_S421_M1_Pogz	6.1762	5.9500	6.5853	7.0570	3.4666	4.0983	6.9727	3.9073	6.5452	6.7145	0.9206	-0.1194	0.6188	0.2085
	SDLAVSNISPPS[Phospho (STY)]PDSK.2	Q8BZJ8_S111_M1_	7.0080	7.3816	3.6259	8.0090	8.0776	3.9342	3.5847	6.2169	7.6517	7.7195	1.0073	0.0105	0.9722	0.0122
Dock10	_DM[Oxidation (M)]SQS[Phospho (STY)]PTSSFVR_.2	#N/A	5.7240	3.8619	4.3496	3.3514	3.6832	3.4456	5.5004	3.2716	7.2513	3.3184	0.8416	-0.2489	0.4104	0.3868
Dock10	_ASLASLDS[Phospho (STY)]NPSTTEK_.2	Q8BZN6_S1295_M1_Dock10	6.1825	3.9501	5.5156	6.8232	3.7714	6.5312	3.6719	7.1163	6.9475	5.7875	0.9288	-0.1065	0.6610	0.1798
Dock10	_HATS[Phospho (STY)]VDTSFSK_.2	Q8BZN6_S1257_M1_Dock10	4.2715	4.0438	4.5331	4.0769	3.8651	3.8644	4.2252	4.2693	3.2233	3.7542	1.0623	0.0873	0.3322	0.4786
Dock10	_EKDM[Oxidation (M)]SQS[Phospho (STY)]PTSSFVR_.2	#N/A	3.2496	4.0279	3.4878	4.2263	3.8861	4.0509	4.3482	4.1055	3.3196	4.0610	0.9654	-0.0509	0.6116	0.2135
Ncbp3	_AEAPAGPALGLPS[Phospho (STY)]PEVESGLER_.2	Q8BZR9_S25_M1_Ncbp3	3.8253	3.1296	4.5121	3.5139	2.9509	3.2777	3.2286	3.1038	4.2532	3.4809	1.0052	0.0075	0.9600	0.0177
Sowahb	_VAGPDPS[Phospho (STY)]PEDSPNQR_.2	Q8BZW2_S132_M1_Sowahb	7.6099	7.6158	7.9978	4.0276	3.4646	7.5660	7.9972	7.7902	3.7395	3.9945	1.0850	0.1177	0.7293	0.1371
Dzank1	_FTQGLEDQLS[Phospho (STY)]PPGNR_.2	Q8C008_S768_M1_Dzank1	10.2465	10.9162	11.0858	11.8081	10.2309	10.8763	11.0978	11.0545	10.8767	10.8977	0.9890	-0.0160	0.7000	0.1549
Pak5	_S[Phospho (STY)]LVGTPYWM[Oxidation (M)]APEVISR_.2	#N/A	5.4731	7.7964	8.1298	9.0812	5.9042	5.9214	7.0797	7.9324	7.2610	3.8676	1.0789	0.1096	0.6336	0.1982
Pak5	_ESPPTPDQGAAS[Phospho (STY)]R_.2	Q8C015_S114_M1_Pak5	4.0468	8.9998	3.2327	3.7354	3.0677	3.5569	3.4501	8.6701	5.5747	3.7024	0.8300	-0.2688	0.5601	0.2518
Pak5	_ASSSS[Phospho (STY)]PLDYSFQLTPSR_.2	Q8C015_S226_M1_Pak5	3.3357	3.9087	4.0225	3.9073	3.7611	3.4923	4.2399	3.6020	3.2506	3.8743	0.9990	-0.0014	0.9866	0.0059
Pak5	_SNS[Phospho (STY)]LRKES[Phospho (STY)]PPT[Phospho (STY)]PDQGAASR_.3	Q8C015_S99_M3_Pak5	4.1336	4.6093	3.9857	4.4229	3.6064	3.4885	5.9235	3.6437	4.5814	4.6231	0.8611	-0.2158	0.1710	0.7670
Pak5	_KES[Phospho (STY)]PPT[Phospho (STY)]PDQGAASR_.2	Q8C015_T107_M2_Pak5	9.4256	9.6191	9.8350	10.0385	9.1273	9.6210	9.8469	9.6132	9.7297	9.4412	0.9952	-0.0070	0.7970	0.0985
Pak5	_SSYLHQTS[Phospho (STY)]PQPAMR_.2	#N/A	5.2086	3.8048	3.9713	6.2296	3.4255	3.9222	3.4595	3.7427	4.0741	3.9639	1.1619	0.2165	0.2993	0.5239
Pak5	_AQMVFS[Phospho (STY)]PPLSGSDTYPR_.2	Q8C015_S341_M1_Pak5	3.8584	3.4840	3.5921	3.1359	3.3053	4.4242	3.2841	3.8653	4.1644	4.3140	0.9300	-0.1048	0.3816	0.4184
Lrrc4c	_M[Oxidation (M)]NS[Phospho (STY)]KDNVQETQI_.2	#N/A	9.9388	9.8087	10.5188	10.1335	8.9954	9.8918	10.2310	9.9860	9.9720	9.8746	0.9866	-0.0195	0.6229	0.2056
Arhgef10	_NDLSSS[Phospho (STY)]SGS[Phospho (STY)]LNLSHGSSSLEHR_.3	Q8C033_S1259_M2_Arhgef10	3.8030	3.5394	3.5367	4.5813	2.9468	3.6778	3.2288	3.9207	4.2197	4.2586	0.9421	-0.0860	0.5122	0.2905
Arhgef10	_T[Phospho (STY)]RSLVSQDHR_.3	Q8C033_T353_M1_Arhgef10	4.8496	3.3299	4.5831	3.5849	3.0220	3.2067	3.2996	3.0328	4.1822	3.5519	1.0700	0.0976	0.5883	0.2304
Rps6ka5	_TLQPSNPTEGSNPDTLFQFS[Phospho (STY)]D_.2	Q8C050_S862_M1_Rps6ka5	3.8744	3.1788	4.5612	5.7995	3.0001	3.2286	3.5894	7.4821	6.9809	3.5300	0.7303	-0.4534	0.1860	0.7305
Map1s	_RPVVTTHDLEAPS[Phospho (STY)]RANS[Phospho (STY)]QDSLASR_.4	Q8C052_S458_M2_Map1s	3.8358	5.5900	4.5226	3.5244	4.4925	5.4978	5.5454	5.8786	5.6558	3.4914	0.8900	-0.1681	0.3838	0.4159
Map1s	_ANS[Phospho (STY)]QDSLASR_.2	Q8C052_S462_M1_Map1s	11.2486	11.2829	11.3112	11.2767	10.9782	11.2027	11.6174	11.3893	11.3132	11.2408	0.9848	-0.0221	0.0895	1.0480
Camkk2	_GGPC[Carbamidomethyl (C)]VESWGAPAPGS[Phospho (STY)]PPR_.2	Q8C078_S572_M1_Camkk2	11.0841	10.8867	11.2010	11.7626	9.9477	10.6352	11.6202	10.8867	10.8914	10.7559	0.9892	-0.0156	0.7402	0.1306
Camkk2	_S[Phospho (STY)]FGNPFEGSR_.2	#N/A	11.7766	11.3368	11.6126	12.0202	10.9302	11.5422	11.8734	11.3874	11.7736	11.7175	0.9870	-0.0188	0.4888	0.3109
Camkk2	_SLS[Phospho (STY)]APGNLLTK_.2	Q8C078_S511_M1_Camkk2	9.0695	2.9696	9.6264	9.8124	7.7523	8.9484	9.1434	9.1023	8.9658	8.8247	0.8913	-0.1660	0.4790	0.3196
Camkk2	_DQPPEADGQELPLEAS[Phospho (STY)]DPESR_.2	#N/A	3.5671	7.8814	3.8156	6.6645	5.1675	6.0265	5.0646	3.7574	8.4044	8.2445	0.8669	-0.2060	0.5181	0.2856
Camkk2	C[Carbamidomethyl (C)]IC[Carbamidomethyl (C)]PSLSY[Phospho (STY)]SPASS[Phospho (STY)]PQSSPR_.	Q8C078_S133_M2_Camkk2	2.9691	3.7743	3.6559	6.1253	3.5956	4.1339	4.4947	4.1556	2.9538	4.0237	1.0347	0.0491	0.8311	0.0803
Camkk2	_KRS[Phospho (STY)]FGNPFEGSR_.3	#N/A	4.7012	6.5006	6.6003	7.4436	2.9227	3.3059	5.5480	6.5911	5.6647	4.7410	0.9307	-0.1036	0.7079	0.1500
Camkk2	_KMS[Phospho (STY)]LQEPSQGGPASSSNSLDMNGR_.3	Q8C078_S99_M1_Camkk2	3.9139	3.2183	4.6007	3.6025	3.0396	3.1891	3.3172	3.0151	4.1645	3.5695	1.0220	0.0314	0.8336	0.0790
Strip1	_AAS[Phospho (STY)]PPASASDLIEQQQKR_.3	#N/A	4.1873	3.1551	3.9210	3.4647	2.9765	7.2958	3.6130	3.5364	3.8355	3.1420	1.1798	0.2385	0.4631	0.3344
Strip1	_AAS[Phospho (STY)]PPASASDLIEQQQK_.2	#N/A	11.9281	11.5780	12.3578	11.7613	10.3858	11.4549	12.4675	11.9116	11.7148	11.5427	0.9722	-0.0407	0.4012	0.3967
Snx16	_SSS[Phospho (STY)]FGSVSTSSTSSK_.2	Q8C080_S29_M1_Snx16	10.0082	9.8936	10.3660	10.6500	9.1473	9.9790	9.9473	9.8153	9.7950	10.1058	1.0092	0.0133	0.7395	0.1311
Snx16	_EFLC[Carbamidomethyl (C)]LDDPPGPFDS[Phospho (STY)]LEESR_.2	Q8C080_S222_M1_Snx16	3.9039	3.1594	3.6376	3.1814	3.0477	3.5769	3.4701	3.8198	4.1188	4.3595	0.8670	-0.2059	0.0519	1.2851
Ccser1	_GES[Phospho (STY)]PLAHAGEPALR_.2	#N/A	5.1126	4.7363	5.7185	5.5051	4.4225	4.8811	5.5536	5.0503	4.0314	4.6153	1.0520	0.0731	0.5023	0.2991
Farsa	_SLQALGEVIEAELR_.2	#N/A	3.7430	4.0790	4.4979	4.0417	3.5380	4.0517	4.1899	3.5955	4.0970	3.8088	1.0176	0.0252	0.7373	0.1323
Farsa	_LQLVQAGQAEK_.2	#N/A	3.2284	3.1206	4.4629	4.7172	3.9824	4.2525	4.6838	2.9945	4.7010	3.6840	0.9863	-0.0200	0.9092	0.0413
Arhgap12	_ATT[Phospho (STY)]PPNQGRPDS[Phospho (STY)]PVYANLQELK_.3	Q8C0D4_S238_M2_Arhgap12	7.7659	4.0174	4.4956	10.0165	3.9057	8.1611	4.1877	9.2309	3.2608	3.7167	1.2539	0.3264	0.4730	0.3251
Arhgap12	_TSFSQEQSC[Carbamidomethyl (C)]DS[Phospho (STY)]AGEGSER_.2	Q8C0D4_S199_M1_Arhgap12	3.8823	4.1438	4.6475	5.1121	3.9677	4.5718	4.0608	4.5022	4.3040	4.7961	0.9936	-0.0093	0.9195	0.0365
Arhgap12	_IQQDSES[Phospho (STY)]GDELSSSSTEQM[Oxidation (M)]R_.2	#N/A	3.9138	4.1168	4.2000	4.5589	3.9271	4.2447	3.3179	4.3731	4.4283	4.1900	1.0203	0.0290	0.7363	0.1329
Vps26b	_TPGQLSDNNSR_.2	#N/A	6.9330	6.9258	6.5162	6.7922	5.8381	6.3408	6.9501	7.1298	6.7244	6.7860	0.9507	-0.0729	0.1741	0.7591
Vps26b	_SM[Oxidation (M)]S[Phospho (STY)]HQAAIASQR_.2	#N/A	9.7364	9.2921	9.7198	9.9589	8.4723	9.4703	9.7229	9.5221	9.4795	9.5100	0.9878	-0.0178	0.6797	0.1677
Vps26b	_KSM[Oxidation (M)]S[Phospho (STY)]HQAAIASQR_.3	#N/A	5.2610	4.4363	5.7785	5.6101	3.8783	3.7282	4.0183	4.7285	3.4334	4.4985	1.1469	0.1977	0.2618	0.5821
Vps26b	_TPGQLSDNNSRQ_.2	#N/A	5.6833	6.6445	3.9172	6.5046	5.9263	6.3492	6.4039	6.8674	6.3032	6.0933	0.9097	-0.1365	0.3063	0.5139
Vps26b	_ETTGTGPNVYHENDTIAK_.3	#N/A	4.2905	3.5949	3.4765	4.5258	3.4162	4.3133	3.1732	4.1393	4.2753	3.5869	1.0376	0.0532	0.6699	0.1740
Agps	_AASAAGAS[Phospho (STY)]PAATPAAPESGTIPK_.2	Q8C0I1_S57_M1_Agps	4.7754	4.5546	3.3020	5.0891	4.2932	4.7266	4.7156	4.6964	4.5306	4.7083	0.9558	-0.0652	0.5381	0.2691
Sowahc	_FC[Carbamidomethyl (C)]TGDS[Phospho (STY)]PPLEAK_.2	Q8C0J6_S82_M1_Sowahc	3.9782	9.1786	9.9440	9.8114	3.1039	9.2561	9.6096	9.0806	4.1002	9.3726	0.9384	-0.0917	0.8015	0.0961
Sowahc	_NLVGALDEDDGDS[Phospho (STY)]PAAR_.2	Q8C0J6_S391_M1_Sowahc	3.3722	3.9702	4.1134	7.1363	3.7916	3.9379	4.2987	5.3526	5.3791	6.1930	0.8268	-0.2744	0.2633	0.5795
Tmx4	_EDGAHPADTQDVVEDALR_.3	#N/A	4.3690	4.5873	3.4927	4.8116	4.4222	3.7218	3.6150	5.0193	4.5258	4.2146	0.9748	-0.0369	0.7626	0.1177
Gpcpd1	_VKLTLEGLEEDEDDDDDKVS[Phospho (STY)]PTVLHK_.4	#N/A	3.3719	4.1282	4.6064	4.1502	4.0165	4.1090	4.2984	4.3518	3.1500	3.8275	1.0401	0.0567	0.6146	0.2114
Gpcpd1	_LTLEGLEEDEDDDDDKVS[Phospho (STY)]PTVLHK_.3	#N/A	3.8632	3.5796	3.6718	5.2728	3.5782	3.9456	3.7923	3.9837	3.8034	3.9768	1.0247	0.0352	0.7804	0.1077
Wdr59	_AVS[Phospho (STY)]PTEPTPR_.2	Q8C0M0_S564_M1_Wdr59	2.9413	3.7465	3.6281	8.6743	3.5678	8.3517	8.8162	8.5393	8.3503	8.5780	0.6011	-0.7344	0.0340	1.4681
Wdr59	_ETEHISSPWGESS[Phospho (STY)]PEELR_.3	Q8C0M0_S840_M1_Wdr59	3.1249	5.5516	3.1459	6.6530	3.7515	6.2554	4.3388	6.4114	6.5002	6.2895	0.8066	-0.3100	0.2472	0.6070
Wdr59	_LYSGS[Phospho (STY)]PTR_.2	Q8C0M0_S603_M1_Wdr59	3.4425	3.8999	3.1762	4.2208	4.0871	4.0384	4.3691	4.2812	3.0794	3.8981	0.9754	-0.0359	0.7666	0.1155
Asrgl1	_LALFHVEQGK_.2	#N/A	5.6163	6.4474	6.4943	6.3414	6.2831	6.0856	6.3896	6.2049	6.1502	6.3867	0.9886	-0.0165	0.6900	0.1611
Asrgl1	_DLSAGAVSAVR_.2	#N/A	5.8712	5.8562	6.3240	5.9509	6.3857	3.8642	5.7675	5.6403	6.1839	5.6052	0.9844	-0.0227	0.8573	0.0669
Asrgl1	_GNLAYATSTGGIVNK_.2	#N/A	5.1982	5.0415	4.3819	4.8512	6.2887	5.0820	5.2441	5.2351	5.9121	5.8394	0.9250	-0.1125	0.2722	0.5652
Asrgl1	_FAEDMGIPQVPVEK_.2	#N/A	6.4907	4.2282	4.1098	7.4013	6.4389	3.6799	4.0407	4.9560	4.8288	3.5697	1.2398	0.3101	0.2500	0.6020
Asrgl1	_TVEEAAQLALDYMK_.2	#N/A	3.9237	3.2281	3.1097	3.6124	3.0494	3.1792	3.3270	3.0053	4.1547	3.5794	0.9527	-0.0698	0.5407	0.2670
Rapgef5	_HTVDEYS[Phospho (STY)]PQKK_.2	Q8C0Q9_S445_M1_Rapgef5	2.9278	4.4146	4.1623	3.7061	4.2360	3.4935	4.2029	3.2951	3.5942	3.3833	1.0565	0.0793	0.5500	0.2597
Sipa1l1	_TLS[Phospho (STY)]DESIYSSQR_.2	Q8C0T5_S1564_M1_Sipa1l1	12.9537	13.1840	13.6210	13.4849	12.4422	13.1751	13.5885	13.1712	13.1566	13.1354	0.9910	-0.0130	0.6179	0.2091

Sipa1l1	_LIDLES[Phospho (STY)]PTPESQK_.2	#N/A	10.5744	10.6705	11.2693	11.1995	9.2500	10.8608	11.1176	10.7483	10.7487	10.7540	0.9811	-0.0275	0.6062	0.2174
Sipa1l1	_QDPVVHLS[Phospho (STY)]PNK_.2	Q8C0T5_S1249_M1_Sipa1l1	8.6702	8.6856	8.7836	8.8718	7.6660	8.6829	8.7503	8.6632	8.5852	8.4007	0.9954	-0.0067	0.8693	0.0608
Sipa1l1	_DLQAS[Phospho (STY)]PKPTSK_.2	Q8C0T5_S1507_M1_Sipa1l1	4.0816	6.3541	5.3539	3.3590	5.1113	3.1465	3.5073	3.6422	3.9412	3.0363	1.2933	0.3711	0.1526	0.8164
Sipa1l1	_SIS[Phospho (STY)]SDGRPLER_.2	Q8C0T5_S1116_M1_Sipa1l1	3.5410	4.2973	3.2747	4.1183	4.1856	3.9398	3.8330	4.1827	7.3886	3.9966	0.8026	-0.3172	0.2097	0.6784
Sipa1l1	_KLIDLES[Phospho (STY)]PTPESQK_.2	#N/A	3.5233	6.1047	7.1054	3.2119	4.1498	6.2482	4.4274	5.9911	5.8682	6.7935	0.8765	-0.1902	0.4601	0.3372
Sipa1l1	_GSGFS[Phospho (STY)]LDVIDGPISQR_.2	Q8C0T5_S258_M1_Sipa1l1	5.9773	5.8385	3.9197	5.9167	4.6603	3.0659	5.1279	6.0143	6.1536	5.6548	0.8534	-0.2288	0.2310	0.6364
Sipa1l1	_EYGS[Phospho (STY)]TSSIDK_.2	Q8C0T5_S208_M1_Sipa1l1	3.0250	4.3174	4.2595	3.8033	3.6696	2.9550	3.9516	3.1979	3.4969	3.4806	1.0396	0.0560	0.6764	0.1698
Sipa1l1	_RSPAS[Phospho (STY)]IDR_.2	Q8C0T5_S1184_M1_Sipa1l1	3.9616	3.2646	3.1475	3.6502	3.1529	3.4717	3.3649	4.4683	4.1169	3.6172	0.8843	-0.1774	0.1158	0.9363
Sipa1l1	_HS[Phospho (STY)]AS[Phospho (STY)]PVVFSSAR_.2	Q8C0T5_S1410_M2_Sipa1l1	3.3606	6.2556	4.5951	6.8740	4.0051	6.7410	5.9527	6.0818	6.1645	6.5213	0.8584	-0.2202	0.2926	0.5337
Sipa1l1	_SKS[Phospho (STY)]ETGDSSIFR_.2	#N/A	4.0699	7.3119	5.8727	7.7870	3.0939	3.1348	3.4956	3.6539	3.9529	3.0246	1.4757	0.5614	0.1555	0.8084
Sipa1l1	_SKS[Phospho (STY)]ETGDSSIFRK_.2	#N/A	3.8890	3.3771	5.1182	4.2837	3.1612	3.4693	3.9838	3.3570	4.5353	3.9952	0.9787	-0.0311	0.8439	0.0737
Sipa1l1	_SYSSKDPS[Phospho (STY)]PTLASK_.2	Q8C0T5_S1712_M1_Sipa1l1	3.6679	3.4492	7.5387	7.7221	3.4382	6.9258	7.3553	3.4736	4.8402	3.2492	1.1538	0.2064	0.5974	0.2237
Sipa1l1	_QKS[Phospho (STY)]M[Oxidation (M)]PEGFGVSR_.2	#N/A	4.3546	3.6590	3.4171	4.4617	3.4803	6.3050	3.1091	6.4716	6.6660	4.1390	0.8397	-0.2520	0.3844	0.4152
Sipa1l1	_SLHGEFSAS[Phospho (STY)]DSSLTDIQETR_.3	Q8C0T5_S1626_M1_Sipa1l1	3.4251	4.2061	3.8646	4.0483	4.0606	3.8801	3.9943	3.8307	3.2689	3.7256	1.0565	0.0793	0.2909	0.5362
Sipa1l1	_S[Phospho (STY)]MPEGFGVSR_.2	#N/A	2.9025	2.9391	4.1370	3.6807	3.5470	3.0776	3.8290	3.3204	3.6195	3.3580	0.9572	-0.0631	0.5880	0.2306
Tlk1	_ISDYFEYQGGNGSS[Phospho (STY)]PVR_.2	Q8C0V0_S159_M1_Tlk1	3.7405	3.0449	4.4273	6.0133	2.8662	3.3624	3.1438	5.7567	4.3379	3.3962	0.9400	-0.0893	0.7507	0.1245
Tlk1	_LSTS[Phospho (STY)]PTPGSAAAAR_.2	Q8C0V0_S22_M1_Tlk1	3.9134	3.2177	4.6002	3.4021	3.0390	3.1896	7.6162	3.5990	3.8981	3.0794	0.7828	-0.3533	0.2909	0.5363
Frmd6	_HSLS[Phospho (STY)]LDDIR_.2	Q8C0V9_S544_M1_Frmd6	3.9804	3.2847	3.1663	3.6690	3.1061	3.1226	3.4834	7.1583	4.0981	6.7779	0.6298	-0.6669	0.0301	1.5211
Frmd6	_LRGQS[Phospho (STY)]TDS[Phospho (STY)]LPQTIC[Carbamidomethyl (C)]R_.2	Q8C0V9_S522_M2_Frmd6	3.6505	3.5757	4.3373	3.3391	4.2770	3.1606	4.5546	3.2785	4.4280	3.3061	0.9567	-0.0638	0.6606	0.1801
Ppp4r4	_SQS[Phospho (STY)]FNNQAFHAK_.2	Q8C0Y0_S777_M1_Ppp4r4	7.8393	7.1739	7.6731	7.7968	5.9719	7.3837	8.1751	7.7184	7.4751	7.6688	0.9416	-0.0868	0.2639	0.5786
Ldlrap1	_RDS[Phospho (STY)]TPSLK_.2	Q8C142_S198_M1_Ldlrap1	3.8776	3.3063	3.8538	3.4647	4.6955	3.3560	5.9169	3.3702	4.0798	4.2584	0.8531	-0.2292	0.2332	0.6322
Slc39a6	_YDSQLS[Phospho (STY)]SNEEK_.2	Q8C145_S488_M1_Slc39a6	4.0120	3.3164	3.1980	3.3035	3.1377	3.0909	3.4517	4.4178	4.6661	4.6273	0.7791	-0.3600	0.0103	1.9865
Cpne1	_NC[Carbamidomethyl (C)]SS[Phospho (STY)]PEFSK_.2	Q8C166_S54_M1_Cpne1	7.2143	7.0604	7.8824	7.7015	6.8975	6.9234	7.6886	7.2824	3.0726	3.9049	1.3267	0.4079	0.0954	1.0206
Cpne1	_APPT[Phospho (STY)]PGKGPAQAPQA_.2	Q8C166_T525_M1_Cpne1	6.8286	6.4463	3.4573	7.2944	5.8505	6.8727	3.1493	6.3422	6.6046	6.2698	1.0954	0.1315	0.5940	0.2262
Cpne1	_FAAQAAQQR_.2	#N/A	3.6594	4.4157	3.3931	3.9999	6.5442	3.8215	3.7146	4.1185	6.2708	5.8077	0.8649	-0.2093	0.4063	0.3911
Myo9a	_DWNES[Phospho (STY)]PVR_.2	Q8C170_S1950_M1_Myo9a	7.9876	7.3946	7.7268	7.4558	5.8067	7.4057	7.8209	7.7420	7.6532	7.3095	0.9561	-0.0648	0.4270	0.3695
Myo9a	_GNYPSPSS[Phospho (STY)]PVIVR_.2	Q8C170_S2296_M1_Myo9a	7.5013	3.1536	7.7235	3.7612	3.1983	7.0687	8.0266	4.3573	7.6760	6.9160	0.8009	-0.3203	0.3385	0.4704
Myo9a	_S[Phospho (STY)]LEDLHQK_.2	Q8C170_S1259_M1_Myo9a	5.7645	5.4078	4.1771	5.6104	4.2212	5.4894	5.3108	5.6252	5.2861	4.8333	0.9711	-0.0423	0.7053	0.1517
Myo9a	_S[Phospho (STY)]LEILQR_.2	Q8C170_S755_M1_Myo9a	4.1992	3.5035	3.3851	4.6172	3.3248	4.4046	4.7591	3.8848	6.1097	4.2945	0.8202	-0.2860	0.1120	0.9508
Myo9a	_RQS[Phospho (STY)]GTDLQEDVIVR_.2	Q8C170_S1243_M1_Myo9a	4.2243	3.5287	3.4103	3.9130	3.3500	4.3795	3.2394	4.2055	3.8541	3.8800	1.0016	0.0024	0.9825	0.0077
Myo9a	_AAS[Phospho (STY)]AC[Carbamidomethyl (C)]EAQGTEGPLGQAK_.2	Q8C170_S2458_M1_Myo9a	3.2303	4.0355	3.9172	4.3081	3.8569	3.8726	4.1345	3.6987	3.3473	4.3868	0.9944	-0.0081	0.9348	0.0293
Thop1	_WDLSAQQIR_.2	#N/A	4.1334	5.3117	3.3194	4.2075	5.2103	5.6259	4.3696	5.2077	5.4168	3.9737	0.9774	-0.0330	0.8439	0.0737
Thop1	_EELGGLPEDFLSSLEK_.2	#N/A	4.8162	4.6736	4.4428	5.0728	4.1212	4.4556	3.1593	3.9667	4.8875	4.7518	1.0968	0.1333	0.2903	0.5372
Thop1	_TSQTVATFLDELAQK_.2	#N/A	3.0542	4.9656	5.6551	4.9079	3.6988	5.0404	5.5380	4.8498	4.7569	5.3530	0.8886	-0.1704	0.3028	0.5188
Thop1	_VDQVLHTQTDADPAEEYAR_.3	#N/A	5.1239	3.2400	3.8361	3.3798	5.6672	3.3785	3.5281	5.5565	3.9204	3.0571	1.0221	0.0315	0.8995	0.0460
Thop1	_NLNEDTTFLPFTR_.2	#N/A	4.1023	3.1239	3.2883	3.7909	3.0122	4.6883	4.9952	4.3275	5.0757	3.7579	0.8080	-0.3076	0.0676	1.1703
Thop1	_LLEEAFNC[Carbamidomethyl (C)]R_.2	#N/A	3.1999	4.0051	3.8867	4.3894	4.2272	3.9031	4.1040	4.8478	3.3777	4.3564	0.9434	-0.0841	0.4815	0.3174
Camsap2	_SVS[Phospho (STY)]NEGLTLNNSR_.2	Q8C1B1_S439_M1_Camsap2	11.4967	11.4706	11.8347	11.5382	9.8593	11.4588	11.7756	11.5016	11.4896	11.3759	0.9775	-0.0328	0.5006	0.3005
Camsap2	_LNQSS[Phospho (STY)]PDNLTDTK_.2	Q8C1B1_S573_M1_Camsap2	10.0380	9.9404	10.6349	10.1811	8.9072	9.9096	10.5001	9.9208	10.0013	10.0391	0.9822	-0.0259	0.5749	0.2404
Camsap2	_WLKS[Phospho (STY)]PTTPVDPER_.2	Q8C1B1_S836_M1_Camsap2	3.1509	6.7847	3.8377	3.4304	3.7775	3.9520	4.0551	6.6284	3.4267	4.3074	0.9355	-0.0963	0.7366	0.1328
Camsap2	_EQQAWVIS[Phospho (STY)]PPQPSPQK_.2	Q8C1B1_S905_M1_Camsap2	3.8778	3.1332	7.7083	4.2575	3.0215	5.6187	8.0713	8.0312	7.2180	6.0575	0.6267	-0.6741	0.0239	1.6222
Camsap2	_ADVSVEKLDGES[Phospho (STY)]DKEQFDDDQK_.3	#N/A	3.3186	4.0238	4.5531	4.0969	3.9632	3.8843	4.2451	2.5078	3.2033	3.7742	1.1575	0.2110	0.1705	0.7682
Camsap2	_SES[Phospho (STY)]VEGFLSPSR_.2	Q8C1B1_S1285_M1_Camsap2	7.8154	8.8994	7.9871	9.2457	6.0576	8.0263	3.6656	7.9981	7.8668	9.1354	1.1170	0.1597	0.4699	0.3280
Camsap2	_SKS[Phospho (STY)]LADIK_.2	Q8C1B1_S818_M1_Camsap2	3.7492	3.2198	4.2916	4.1826	3.5097	3.1149	4.5089	3.3242	2.9728	3.2604	1.0459	0.0647	0.6711	0.1732
Camsap2	_SSS[Phospho (STY)]MSYVDGFIGTWPK_.2	Q8C1B1_S391_M1_Camsap2	3.2517	3.7581	3.4363	4.2054	3.8892	4.1519	3.9245	4.1114	3.3733	4.0236	0.9803	-0.0287	0.7543	0.1224
Camsap2	_DHIES[Phospho (STY)]PK_.2	Q8C1B1_S1248_M1_Camsap2	4.4156	3.7339	4.1364	4.4280	3.5140	3.0895	4.6400	4.0740	4.5501	3.1679	0.9460	-0.0801	0.5772	0.2387
Camsap2	_LDGES[Phospho (STY)]DKEQFDDDQK_.2	#N/A	5.5704	4.1064	3.2179	3.2835	3.4353	2.8178	3.4318	4.5627	3.9533	4.1594	0.9284	-0.1071	0.6072	0.2167
Septin11	_SYELQESNVR_.2	#N/A	7.8678	7.8103	8.0238	8.1084	8.0271	8.0387	8.0348	7.4968	7.6661	7.9570	1.0245	0.0349	0.1364	0.8653
Septin11	_ELEEEVSNFQK_.2	#N/A	7.7668	7.6747	7.4908	7.7730	7.6100	7.5090	7.6656	6.9478	7.1163	7.4380	1.0474	0.0668	0.0407	1.3905
Septin11	_STSQGFC[Carbamidomethyl (C)]FNILC[Carbamidomethyl (C)]VGETGIGK_.2	#N/A	5.2491	5.6512	6.1101	6.6920	5.6609	6.0282	5.8963	4.9687	5.4269	5.8186	1.0671	0.0937	0.2570	0.5901
Septin11	_[Acetyl (Protein N-term)]AVAVGRPS[Phospho (STY)]NEELR_.2	Q8C1B7_S9_M1_Septin11	6.4582	6.4916	6.4127	6.9678	6.2585	6.2715	6.6409	6.2463	6.4873	6.8823	0.9867	-0.0194	0.6191	0.2082
Septin11	_FESDPATHNEPGVR_.3	#N/A	6.3661	6.3906	3.2900	6.4619	6.7654	6.6866	3.7397	6.4912	6.5661	6.2418	1.0406	0.0574	0.7941	0.1001
Septin11	_SLFNYHDTR_.2	#N/A	7.3185	7.5874	4.3039	3.8477	6.9098	3.6352	7.3297	7.4363	3.4525	3.5250	1.0303	0.0430	0.9023	0.0446
Septin11	_AAAQLLQSQAQQSGAQQTK_.2	#N/A	6.5254	6.6419	6.9877	7.1696	6.4383	6.5041	6.5019	6.1421	6.3455	5.8020	1.0828	0.1148	0.0290	1.5376
Septin11	_KKELEEEVSNFQK_.3	#N/A	6.2822	4.1215	4.0839	4.3280	5.1262	6.4250	5.5578	5.0147	5.6437	5.2786	0.9418	-0.0865	0.5906	0.2287
Septin11	_NLSLSGHVGFDSLPDQLVNK_.3	#N/A	5.8765	5.4136	5.2484	5.8557	5.4917	5.5629	5.7877	5.2969	6.1359	5.8768	0.9654	-0.0507	0.3196	0.4954
Septin11	_LTIVDTVGFGDQINK_.2	#N/A	3.3326	4.0889	4.5671	4.3267	3.9772	4.1482	4.0414	4.3911	4.8178	3.7882	0.9563	-0.0645	0.5201	0.2839
Iws1	_EAEDS[Phospho (STY)]DS[Phospho (STY)]DDNIKR_.2	Q8C1D8_S458_M2_Iws1	6.6130	5.8808	6.2160	6.2712	4.1034	5.1422	6.1103	6.3471	6.2703	6.2239	0.9145	-0.1290	0.2948	0.5304
Iws1	_VVC[Carbamidomethyl (C)]DADDS[Phospho (STY)]DSDVVSDK_.2	Q8C1D8_S366_M1_Iws1	3.4202	5.3932	4.3788	3.7000	5.2589	5.7227	6.3069	5.5739	3.1421	5.4433	0.9080	-0.1393	0.5362	0.2707
Iws1	_IS[Phospho (STY)]DS[Phospho (STY)]ESEELPKPR_.2	Q8C1D8_S183_M2_Iws1	2.9409	5.5747	4.0756	7.5285	4.6002	5.0553	4.5017	5.8675	5.4034	6.6382	0.8857	-0.1750	0.4804	0.3184
Iws1	_KAAVLS[Phospho (STY)]DS[Phospho (STY)]EDDAGNASAK_.2	#N/A	4.8517	5.4121	3.2551	3.2463	3.1948	5.3326	5.9032	5.6159	4.7161	5.2000	0.7866	-0.3462	0.0907	1.0423
Iws1	_IDS[Phospho (STY)]DDDEEKEGDEEK_.2	#N/A	3.5836	3.5882	3.8373	4.4910	3.1846	3.4401	2.9839	3.2993	4.0477	2.9956	1.1068	0.1464	0.2725	0.5647
Iws1	_AAVLS[Phospho (STY)]DS[Phospho (STY)]EDDAGNASAK_.2	#N/A	7.5202	7.4783	6.2222	8.1436	6.9940	7.6141	8.2151	7.6813	7.6939	7.5710	0.9407	-0.0881	0.2272	0.6436
Iws1	_ETTVAS[Phospho (STY)]DS[Phospho (STY)]EEEAGKEESSVK_.2	#N/A	4.0724	3.9551	3.8786	4.3688	3.2928	3.8585	3.6262	4.4866	3.6783	3.9216	0.9939	-0.0088	0.9228	0.0349
Iws1	_GLHS[Phospho (STY)]S[Phospho (STY)]DS[Phospho (STY)]EEEPKR_.2	#N/A	4.7321	4.9243	5.5568	4.9299	3.6013	5.1207	5.0888	5.0608	5.4016	4.8805	0.9418	-0.0864	0.4147	0.3823
Iws1	_AAVLS[Phospho (STY)]DS[Phospho (STY)]EDDAGNASAKK_.2	#N/A	4.2077	3.5121	3.3937	4.7381	3.3334	4.3961	3.2560	4.9227	4.8256	3.8634	0.9320	-0.1016	0.5310	0.2749
Iws1	_QKIDS[Phospho (STY)]DDDEEKEGDEEK_.2	#N/A	3.5752	3.1790	3.9217	2.8350	3.4055	2.7906	3.4308	3.8799	3.8542	3.6336	0.8878	-0.1717	0.1204	0.9194
Iws1	_IDS[Phospho (STY)]DDDEEKEGDEEKVAK_.3	#N/A	3.4182	4.1745	4.9298	4.1965	4.0628	4.0627	4.3447	4.1347	3.6702	2.8653	1.1031	0.1416	0.3104	0.5081
Iws1	_KGLHS[Phospho (STY)]S[Phospho (STY)]DS[Phospho (STY)]EEEPKR_.2	#N/A	3.4955	4.2517	3.2291	4.1639	4.1400	3.9854	3.8786	3.4069	3.6032	3.9510	1.0452	0.0638	0.4955	0.3050
Vash1	_GTGPPS[Phospho (STY)]PTKDR_.2	Q8C1W1_S323_M1_Vash1	7.4505	3.4982	3.9764	3.4166	3.3865	3.2381	3.1313	3.2010	4.3504	7.6403	0.9084	-0.1386	0.7312	0.1360
Ccz1	_TFS[Phospho (STY)]PSKPAK_.2	Q8C1Y8_S74_M1_Ccz1	7.9880	7.4866	8.2805	3.9720	7.0155	7.7055	8.4103	8.0523	7.9334	7.6746	0.8824	-0.1805	0.2809	0.5515
Ccz1	_HIEPELAGRDS[Phospho (STY)]PVR_.3	Q8C1Y8_S264_M1_Ccz1	7.1329	6.3588	6.8645	7.0503	4.9543	6.4658	7.0713	6.6265	6.7609	7.0323	0.9416	-0.0869	0.3664	0.4360
Rbm14	_TRLS[Phospho (STY)]PPR_.2	Q8C2Q3_S582_M1_Rbm14	11.1324	11.1928	11.6337	11.6330	11.1639	11.1330	11.3318	11.1829	11.1589	10.6114	1.0220	0.0314	0.2078	0.6824
Rbm14	_DRS[Phospho (STY)]PLRR_.2	Q8C2Q3_S215_M1_Rbm14	8.4521	8.4080	8.8108	9.1275	8.0022	8.5307	8.9053	8.5432	8.5663	8.5248	0.9908	-0.0134	0.7116	0.1478
Rbm14	_S[Phospho (STY)]PTKSSLDYR_.2	Q8C2Q3_S627_M1_Rbm14	6.6350	7.4617	7.9784	6.7365	7.2122	7.3070	7.0381	6.8477	7.1285	6.6310	1.0449	0.0634	0.2775	0.5567
Rbm14	_QPT[Phospho (STY)]PPFFGR_.2	Q8C2Q3_T206_M1_Rbm14	3.8711	11.6972	12.4226	12.6810	8.8656	11.9106	12.1299	11.9261	11.9201	11.8622	0.8563	-0.2238	0.3523	0.4531
Rbm14	_LSESQLSFR_.2	#N/A	4.8430	4.0568	4.5201	4.6417	5.1880	4.4094	4.1284	3.9047	4.4929	4.4663	1.0541	0.0761	0.4086	0.3887
Rbm14	_RLS[Phospho (STY)]ESQLSFR_.2	Q8C2Q3_S618_M1_Rbm14	3.0649	5.3053	3.7517	6.0177	3.6914	4.0381	3.9690	3.8641	3.5127	5.9915	0.9947	-0.0076	0.9755	0.0108
Rbm14	_IFVGNVSAAC[Carbamidomethyl (C)]TSQELR_.2	#N/A	2.8393	3.3051	3.1867	3.6893	3.0128	3.1023	3.4040	4.4291	4.0777	3.6563	0.8195	-0.2872	0.0166	1.7788

Suco	_SKS[Phospho (STY)]LQFTGK_.2	Q8C341_S1077_M1_Suco	6.5738	6.4702	7.0196	6.2918	5.7835	3.9040	6.9170	6.9148	6.3838	6.3068	0.9060	-0.1425	0.3132	0.5041
Layn	_KQS[Phospho (STY)]EADLAEPRPDLK_.3	Q8C351_S299_M1_Layn	5.7368	5.4736	5.7753	5.7503	4.9720	5.6600	5.6369	5.6920	5.1653	5.3294	1.0193	0.0276	0.5869	0.2314
Layn	_QENS[Phospho (STY)]PNLDVYNVIR_.2	Q8C351_S286_M1_Layn	3.5509	3.6753	4.2377	3.2395	4.1774	3.0610	4.4550	4.8033	5.5259	3.2065	0.8131	-0.2985	0.1016	0.9931
Mettl3	_S[Phospho (STY)]DSPVPTAPTSSGPKPSTTSVAPELATDPELEKK_.4	Q8C3P7_S48_M1_Mettl3	3.9159	3.6756	3.3366	4.1223	3.8189	4.1835	3.9740	5.9302	3.4893	3.9317	0.8871	-0.1729	0.3172	0.4986
Shisa7	_EHLLS[Phospho (STY)]PPR_.2	Q8C3Q5_S451_M1_Shisa7	11.1730	10.4181	10.6550	11.1517	9.8135	10.7562	11.2045	10.7049	10.5267	10.0949	1.0027	0.0039	0.9304	0.0313
Shisa7	_SSS[Phospho (STY)]LTPGLGGPDSM[Oxidation (M)]APR_.2	#N/A	10.7844	5.9248	11.2648	11.0273	9.2558	10.7055	6.6424	10.6621	3.0930	10.3831	1.2771	0.3528	0.2588	0.5870
Shisa7	_RVMS[Phospho (STY)]QEHLLGDGSR_.3	Q8C3Q5_S405_M1_Shisa7	5.8663	4.1988	4.0804	6.7806	4.0201	3.7094	4.0702	4.6583	4.5923	3.0492	1.1670	0.2228	0.3571	0.4472
Shisa7	_EHLLS[Phospho (STY)]PPRS[Phospho (STY)]PALPPDPTTR_.3	Q8C3Q5_S451_M2_Shisa7	4.3221	3.6265	3.5081	5.9168	3.4478	5.7945	3.7254	5.7003	6.8013	6.6998	0.7739	-0.3697	0.1491	0.8265
Shisa7	_LVSQEHLLLSS[Phospho (STY)]PEALR_.2	#N/A	4.3302	3.6110	4.9902	6.3040	4.2609	3.8050	3.4381	6.0636	3.7210	3.6870	1.0764	0.1062	0.6566	0.1827
Tango6	_EAIS[Phospho (STY)]DDEDEALYQK_.2	Q8C3S2_S561_M1_Tango6	4.0129	3.2683	3.7466	7.2415	6.2999	3.4680	3.4386	7.1519	6.9339	6.8878	0.7657	-0.3852	0.2319	0.6347
	SSS[Phospho (STY)]LDALGPAR.2	Q8C3W1_S176_M1_	10.6507	4.1067	11.0323	10.8682	10.2912	10.8324	11.0346	10.2325	10.5679	10.9174	0.9010	-0.1504	0.4698	0.3281
Gpr158	_SAS[Phospho (STY)]AHNLSSEK_.2	Q8C419_S881_M1_Gpr158	9.5360	9.4716	9.5859	9.4371	7.1463	9.1739	9.1848	9.4337	9.1023	9.3123	0.9784	-0.0315	0.6924	0.1597
Gpr158	_SLS[Phospho (STY)]VIASAK_.2	Q8C419_S904_M1_Gpr158	7.8208	3.5805	7.8660	6.8809	3.4688	7.6110	3.7508	7.8580	7.2726	7.4958	0.9409	-0.0879	0.7743	0.1111
Gpr158	_LKEDS[Phospho (STY)]EAESTESVPLVC[Carbamidomethyl (C)]K_.2	#N/A	5.3689	3.3866	3.8265	6.5612	3.2534	3.3049	3.6456	6.6439	6.5595	4.0827	0.8186	-0.2888	0.3424	0.4654
Gpr158	_EQTVQDDLQWYQALVR_.2	#N/A	3.7061	3.4000	3.7995	3.5935	3.8788	4.4199	4.5729	2.7932	4.1736	4.6049	0.9414	-0.0871	0.5513	0.2586
Gpr158	_EES[Phospho (STY)]LKNR_.2	Q8C419_S806_M1_Gpr158	6.6022	6.2874	3.1558	6.1093	4.0666	4.0588	4.3486	4.3016	3.0999	3.8776	1.2917	0.3693	0.1808	0.7428
Gpr158	_VS[Phospho (STY)]LPTANPDVSSGITQIK_.2	Q8C419_S976_M1_Gpr158	3.9286	3.1841	3.6623	5.6012	3.0724	3.5522	3.4454	3.7951	4.0941	4.3842	0.9755	-0.0358	0.8511	0.0700
Gpr158	_S[Phospho (STY)]IDKTEVC[Carbamidomethyl (C)]PWEIHSQSLLEDENR_.3	Q8C419_S1059_M1_Gpr158	3.9084	3.8290	3.7868	3.8366	3.8241	3.6486	3.7364	3.7781	3.7350	3.6972	1.0184	0.0264	0.1731	0.7617
Gpr158	_QC[Carbamidomethyl (C)]S[Phospho (STY)]KEDKEGTDHSAAK_.3	Q8C419_S770_M1_Gpr158	3.3761	4.1323	3.1097	4.1543	4.0206	4.1048	3.9980	4.3477	3.1459	3.8316	0.9962	-0.0055	0.9634	0.0162
Pex5l	_KGS[Phospho (STY)]PGLTR_.2	#N/A	10.8019	10.9763	11.1408	11.2968	10.2146	10.6895	11.0814	10.9318	10.8444	11.0628	0.9884	-0.0168	0.5441	0.2643
Pex5l	_S[Phospho (STY)]PVDSSVLEGVK_.2	#N/A	9.0082	8.4416	8.6040	3.2628	3.1291	8.6620	9.2478	8.3235	8.7489	8.8226	0.7798	-0.3588	0.2206	0.6564
Pex5l	_TGS[Phospho (STY)]KELLWSAEHR_.2	#N/A	5.8196	5.7362	6.7706	6.4222	4.7362	5.3143	6.2961	5.8169	6.7140	5.8216	0.9412	-0.0874	0.4048	0.3928
Pex5l	_SALNSES[Phospho (STY)]ASELELVAPAQAR_.2	Q8C437_S172_M1_Pex5l	4.2288	3.4843	7.5889	8.5542	6.6437	3.2520	3.6545	3.4949	7.5853	3.1836	1.2558	0.3286	0.4425	0.3541
Pex5l	_NKKGS[Phospho (STY)]PGLTR_.2	#N/A	5.6428	7.4489	8.1731	3.2821	3.1591	6.5449	6.5492	4.3965	6.7544	6.1680	0.9567	-0.0639	0.8286	0.0816
Pex5l	_S[Phospho (STY)]QPELSTGK_.2	Q8C437_S157_M1_Pex5l	8.4988	8.1343	4.3907	8.2679	3.4105	7.2068	3.1072	3.2251	4.3745	8.6034	1.3778	0.4624	0.2634	0.5794
Pex5l	_GPETS[Phospho (STY)]SLDLDIQTQLEK_.2	Q8C437_S105_M1_Pex5l	3.9339	3.1893	3.6676	3.2113	3.0776	3.5470	3.3596	3.7898	4.0889	4.3895	0.8799	-0.1846	0.0895	1.0481
Pex5l	_KGS[Phospho (STY)]PGLTRR_.2	#N/A	3.3155	5.6169	6.5358	4.3438	3.9601	4.1654	4.0585	6.0469	3.4232	5.1979	0.9946	-0.0078	0.9743	0.0113
Pex5l	_NHS[Phospho (STY)]LEEEFER_.2	#N/A	8.2919	8.6469	9.7891	9.1098	7.7850	8.6310	8.7297	8.2994	8.4649	9.1075	1.0068	0.0097	0.8806	0.0552
Pex5l	_M[Oxidation (M)]SKS[Phospho (STY)]PVDSSVLEGVK_.2	#N/A	6.8026	6.0002	7.0469	7.7889	4.9830	3.6928	6.2484	6.1865	5.8725	3.5826	1.1060	0.1453	0.5445	0.2640
Pex5l	_RM[Oxidation (M)]S[Phospho (STY)]KS[Phospho (STY)]PVDSSVLEGVK_.3	#N/A	3.5681	5.4291	4.1887	5.5732	5.2391	5.7577	5.1972	5.4495	5.4138	5.9883	0.8997	-0.1525	0.2701	0.5684
Tmem145	_VVTM[Oxidation (M)]AEPGAAS[Phospho (STY)]PPPPAR_.2	#N/A	7.9033	7.9716	8.7022	8.3362	6.6699	7.6827	8.5978	8.4328	8.0483	7.7675	0.9593	-0.0599	0.4088	0.3885
Fbxl3	_GGRDS[Phospho (STY)]DQDS[Phospho (STY)]AEEGTAEKPK_.3	Q8C4V4_S12_M2_Fbxl3	3.1816	3.9868	3.8684	4.3711	3.8081	3.9214	4.2822	3.7474	3.3960	4.3380	0.9785	-0.0313	0.7578	0.1204
Mb21d2	_GSTTSIPS[Phospho (STY)]PQSDGGDPNQPDDR_.2	Q8C525_S376_M1_Mb21d2	3.8181	3.8954	3.5518	3.9175	3.8506	3.7006	5.6378	3.9435	3.5636	5.0270	0.8340	-0.2618	0.0860	1.0656
Cnot2	_TNSMSSSGLGS[Phospho (STY)]PNR_.2	Q8C5L3_S165_M1_Cnot2	7.6290	6.0323	3.3054	8.5076	5.6578	2.9835	6.3543	5.2956	5.2082	6.4147	0.9773	-0.0332	0.9125	0.0398
Cwc22	_GGGQSS[Phospho (STY)]PQEEPTWK_.2	Q8C5N3_S106_M1_Cwc22	3.5624	8.5171	8.8564	8.8312	7.2550	3.5406	9.3858	8.8195	8.6103	8.2940	0.7702	-0.3767	0.1638	0.7856
Clmn	_IKES[Phospho (STY)]PSEQGSR_.2	Q8C5W0_S317_M1_Clmn	7.5053	7.6068	8.3695	8.0288	7.2474	7.4009	7.9057	7.8920	7.8024	7.1505	1.0007	0.0010	0.9837	0.0072
Clmn	_NSHS[Phospho (STY)]DSSIYIR_.2	Q8C5W0_S925_M1_Clmn	7.9374	7.7095	8.4628	7.6487	6.4637	3.5547	8.2194	7.6682	7.4562	7.8972	0.8915	-0.1657	0.3862	0.4132
Clmn	_SNS[Phospho (STY)]LPVKK_.2	Q8C5W0_S419_M1_Clmn	7.4529	7.1899	7.6671	3.1556	6.4618	7.6050	7.8443	3.4621	7.7848	7.0840	1.0069	0.0099	0.9713	0.0126
Clmn	_VFVC[Carbamidomethyl (C)]DQLESPT[Phospho (STY)]GFC[Carbamidomethyl (C)]LDSAPSHK_.3	Q8C5W0_T360_M1_Clmn	3.9954	3.2998	3.1814	3.3201	3.1211	3.1076	3.4684	3.6811	3.9801	2.9974	0.9450	-0.0816	0.4349	0.3616
Tbcel	_YS[Phospho (STY)]PENFPYR_.2	Q8C5W3_S18_M1_Tbcel	7.4101	6.8929	7.4709	7.4467	6.7819	7.1400	7.8361	3.6696	3.6658	7.4541	1.2712	0.3462	0.1349	0.8698
Spata33	_AS[Phospho (STY)]NETLISYGIPDNDEQR_.2	Q8C624_S87_M1_Spata33	3.4949	3.7312	4.1817	3.1836	6.0747	3.6080	4.3991	3.4341	5.8430	3.1505	0.9617	-0.0563	0.8282	0.0818
Rtkn	_RPSDS[Phospho (STY)]VQPAQHSPC[Carbamidomethyl (C)]R_.3	Q8C6B2_S108_M1_Rtkn	3.4704	3.7175	4.7026	4.0720	3.8916	4.2742	4.1764	3.9921	3.7476	3.6817	1.0313	0.0444	0.6241	0.2048
Rtkn	_TFS[Phospho (STY)]LDAAPADHSLGPSR_.2	#N/A	4.0873	4.2433	3.7623	3.6690	4.1372	4.1879	4.0810	4.9736	4.4437	4.1442	0.9102	-0.1358	0.0855	1.0680
Wdr26	_RLS[Phospho (STY)]QSDEDVIR_.2	#N/A	3.1049	4.1212	7.2341	4.2944	6.1721	6.3869	4.0091	3.8241	3.4727	5.7173	1.2263	0.2943	0.3202	0.4946
Wdr26	_LSQS[Phospho (STY)]DEDVIR_.2	Q8C6G8_S103_M1_Wdr26	4.3235	4.4035	3.5095	4.0121	6.1129	6.7867	3.7268	6.3662	6.2911	3.9791	0.9543	-0.0675	0.7955	0.0994
Wdr26	_KRLS[Phospho (STY)]QSDEDVIR_.3	#N/A	3.8012	3.0566	6.9347	3.8582	6.2068	6.2134	6.0676	3.9226	6.6289	4.2567	0.9603	-0.0584	0.8381	0.0767
Usp20	_EAQPPS[Phospho (STY)]PR_.2	Q8C6M1_S369_M1_Usp20	9.3847	9.5252	9.7720	9.4563	8.5098	9.3337	9.7149	9.7853	9.6235	9.4019	0.9687	-0.0458	0.2253	0.6471
Usp20	_LSEQDS[Phospho (STY)]PPPSHPLK_.2	Q8C6M1_S112_M1_Usp20	8.1258	8.3668	8.6450	3.8107	7.5599	3.6322	3.5253	8.4801	3.9564	3.7776	1.3557	0.4390	0.2794	0.5537
Usp20	_KFS[Phospho (STY)]WGQQR_.2	Q8C6M1_S334_M1_Usp20	6.9839	6.8359	6.7775	6.4563	2.8528	6.5405	6.9475	4.0146	6.5582	6.1888	1.0248	0.0354	0.8823	0.0544
Usp20	_AVPIAVADEGESES[Phospho (STY)]EDDDLKPR_.3	Q8C6M1_S134_M1_Usp20	3.8394	3.4514	3.9654	8.0189	3.5627	3.5617	3.8437	8.1079	3.8105	3.5659	0.9106	-0.1351	0.7396	0.1310
Usp20	_STS[Phospho (STY)]PC[Carbamidomethyl (C)]QTPEPDNEAHIR_.3	#N/A	3.8124	3.6824	3.9626	6.2025	3.6714	4.8159	3.8069	3.5753	3.5798	3.0583	1.2433	0.3142	0.1430	0.8445
Usp20	_LSSSPPRAS[Phospho (STY)]PVR_.2	Q8C6M1_S415_M1_Usp20	3.5130	4.3182	4.1998	3.2016	4.1395	3.5900	3.9508	4.7732	3.0646	3.1686	1.0235	0.0334	0.8277	0.0821
Fam126b	_TAITTAS[Phospho (STY)]IRR_.2	Q8C729_S321_M1_Fam126b	2.9131	2.9284	4.1476	6.5512	4.2506	3.4789	3.8397	3.3098	3.6088	6.9582	0.9133	-0.1309	0.7008	0.1544
Fam126b	_VEVT[Phospho (STY)]PTVPR_.2	Q8C729_T306_M1_Fam126b	4.0392	3.3435	6.9863	3.2763	3.1649	3.0638	3.4246	3.7249	4.0239	2.9536	1.1266	0.1720	0.5882	0.2305
Fam126b	_NSLPFDAPDSS[Phospho (STY)]QEGQK_.2	Q8C729_S294_M1_Fam126b	7.3905	8.1082	8.4742	3.5615	7.1461	7.4705	8.8884	8.2478	8.3300	8.2552	0.8333	-0.2631	0.1611	0.7928
Fam126b	_TNS[Phospho (STY)]LSLIR_.2	Q8C729_S442_M1_Fam126b	6.9586	3.1912	3.2209	6.7950	3.0795	3.5451	6.5951	6.5260	6.5849	3.6905	0.7634	-0.3895	0.2490	0.6038
Fam126b	_YSTIS[Phospho (STY)]LQEDR_.2	Q8C729_S491_M1_Fam126b	3.1963	4.1461	4.4308	3.9746	3.9675	3.7620	4.1228	3.0266	3.3257	3.6518	1.1079	0.1479	0.2135	0.6705
Fam126b	_S[Phospho (STY)]AKDKETALAIK_.3	Q8C729_S383_M1_Fam126b	3.9093	3.1648	3.6430	3.7500	3.0531	3.5715	3.4647	2.6500	3.4531	3.7170	1.0584	0.0819	0.4619	0.3354
Fam110b	_SKS[Phospho (STY)]DLSDR_.2	Q8C739_S257_M1_Fam110b	8.9607	8.9559	9.1577	4.1217	4.1822	9.0109	8.9107	8.7846	9.0549	9.1784	0.8237	-0.2799	0.2529	0.5971
Fam110b	_ANS[Phospho (STY)]DIISLNFR_.2	Q8C739_S297_M1_Fam110b	4.0146	3.2700	3.7483	8.1739	6.4401	3.4663	3.3595	7.6298	7.1250	2.9693	0.9206	-0.1194	0.7732	0.1117
Fam110b	_VYPTPGHGS[Phospho (STY)]PQESSSHVSR_.3	Q8C739_S172_M1_Fam110b	3.1960	4.0012	3.8828	4.3854	7.2527	3.9070	4.2678	6.5564	3.3816	4.3524	0.9565	-0.0642	0.8289	0.0815
Fam110b	_VAAMKS[Phospho (STY)]PEADQVEPAC[Carbamidomethyl (C)]GVSR_.3	#N/A	4.2370	4.2317	3.9999	5.4661	4.0848	3.8654	4.2173	3.6473	3.2959	3.8012	1.1534	0.2059	0.1233	0.9092
Cstf2t	_QGGGQPSSFS[Phospho (STY)]PGQSQVTPQDQEK_.3	Q8C7E9_S579_M1_Cstf2t	4.8945	4.1136	4.4633	4.0071	3.9349	5.5616	4.1554	5.0676	5.5888	3.6844	0.9723	-0.0406	0.7916	0.1015
Trim9	_NILVQTPESES[Phospho (STY)]PQSR_.2	Q8C7M3_S46_M1_Trim9	10.4476	10.4546	11.0843	10.7265	9.6714	10.6128	10.9202	10.3197	10.3206	10.4794	0.9990	-0.0014	0.9694	0.0135
Trim9	_RAS[Phospho (STY)]GSGVSDYDYLDLDK_.2	#N/A	3.6292	3.8223	3.8708	6.3723	5.0802	5.5816	5.9733	6.0614	5.8720	4.9159	0.8283	-0.2718	0.1476	0.8310
Srsf12	_S[Phospho (STY)]KS[Phospho (STY)]LPKR_.2	Q8C8K3_S187_M2_Srsf12	9.1367	8.5076	8.6579	8.9866	8.2492	8.5905	9.1553	8.9374	9.1656	8.7827	0.9642	-0.0525	0.1128	0.9477
Srsf12	_HC[Carbamidomethyl (C)]DSIARS[Phospho (STY)]PC[Carbamidomethyl (C)]KS[Phospho (STY)]PR_.3	#N/A	4.8020	4.6109	4.9595	4.9331	3.9935	4.1204	4.1607	4.3243	4.7322	4.2302	1.0477	0.0672	0.4038	0.3938
Srsf12	_QSTSLRQS[Phospho (STY)]RT[Phospho (STY)]PR_.3	Q8C8K3_T174_M2_Srsf12	3.7548	3.8892	4.0893	4.0337	3.7571	3.5845	4.0378	3.6809	4.3088	3.8241	0.9719	-0.0412	0.4648	0.3328
Ank2	_VGT[Phospho (STY)]PTDIHSEK_.2	#N/A	11.3539	10.8355	10.8858	11.3144	10.0802	10.7192	11.5912	11.3067	11.2073	11.1497	0.9603	-0.0584	0.1096	0.9600
Ank2	_S[Phospho (STY)]YIETETESR_.2	#N/A	11.9822	11.8472	12.2622	12.2469	11.2635	11.8583	12.2496	12.0196	11.8975	11.8469	0.9922	-0.0113	0.6524	0.1855
Ank2	_SS[Phospho (STY)]PEDVVVTNSSNR_.2	Q8C8R3_S2924_M1_Ank2	5.5144	5.5377	8.7690	8.7123	4.9964	8.4015	8.9701	8.3929	8.4529	8.5259	0.8140	-0.2969	0.1241	0.9061
Ank2	_TEWQPS[Phospho (STY)]PTDIPLQK_.2	Q8C8R3_S3315_M1_Ank2	9.2181	4.0543	3.8587	10.4890	3.7984	9.6030	10.0335	10.1318	9.8573	9.6106	0.6900	-0.5353	0.1008	0.9967
Ank2	_QPPIS[Phospho (STY)]PTSK_.2	Q8C8R3_S1936_M1_Ank2	3.9963	3.2517	3.7300	11.0682	3.1400	3.4846	3.3778	3.7274	4.0265	2.9510	1.3573	0.4407	0.4520	0.3448
Ank2	_DSLESS[Phospho (STY)]PVEPK_.2	Q8C8R3_S2431_M1_Ank2	3.3440	8.3383	8.7111	8.9018	7.9463	8.4383	9.2342	8.3767	3.1780	8.5871	1.0367	0.0520	0.8661	0.0624
Ank2	_NERHS[Phospho (STY)]PVSSLK_.2	#N/A	8.0193	7.9855	8.1572	3.6728	5.8742	7.8923	8.3828	8.3252	7.9703	7.9619	0.8497	-0.2350	0.2238	0.6502
Ank2	_TQDQQEYYVTT[Phospho (STY)]PGAEVEDPQK_.3	Q8C8R3_T3718_M1_Ank2	6.9764	6.8220	7.2970	7.2874	3.5466	7.2428	6.0878	3.3611	7.1698	7.1435	1.0990	0.1362	0.5848	0.2330
Ank2	_LSPVS[Phospho (STY)]PSAK_.2	Q8C8R3_S1819_M1_Ank2	7.9227	3.4220	8.0717	7.5951	3.5800	7.5960	7.6728	7.9538	7.9395	8.0298	0.8057	-0.3116	0.2144	0.6687
Ank2	_VVTEEVTTTTTTITEK_.2	#N/A	6.5156	7.5912	8.4341	8.9766	6.1948	7.0992	7.2211	8.2254	6.2994	5.9329	1.0793	0.1101	0.4469	0.3497
Ank2	_HTPVS[Phospho (STY)]PSSK_.2	#N/A	3.1005	4.1256	3.7873	4.2900	3.7270	4.0024	8.5901	8.8146	3.4771	8.3750	0.5248	-0.9300	0.0100	1.9998

Ank2	_ATFSPIVTLEPR_.2	#N/A	6.7558	7.0194	7.4656	6.8153	6.6848	6.9039	6.7753	7.1094	7.0014	6.8051	1.0026	0.0037	0.9117	0.0401
Ank2	_AREEQTPLHIASR_.3	#N/A	6.5047	6.5750	5.5450	6.5792	6.9443	6.5706	7.4177	6.8952	6.8714	6.8148	0.9219	-0.1173	0.0731	1.1358
Ank2	_FWLIDC[Carbamidomethyl (C)]R_.2	#N/A	6.4405	6.6324	6.6775	6.6659	5.7729	6.2150	6.2629	6.5418	6.3477	6.2375	1.0084	0.0120	0.7857	0.1048
Ank2	_Y[Phospho (STY)]SLEGGR_.2	Q8C8R3_Y889_M1_Ank2	3.3125	6.3878	6.3976	3.9543	3.5185	3.7528	4.5362	6.7618	3.8538	3.7302	0.9647	-0.0518	0.8610	0.0650
Ank2	_HS[Phospho (STY)]PVSSLK_.2	#N/A	6.0447	5.0258	4.0486	6.2778	4.8522	3.3799	6.0103	6.0832	5.5762	5.9769	0.8353	-0.2596	0.1305	0.8844
Ank2	_HTPVS[Phospho (STY)]PSSKNEK_.2	#N/A	5.2441	6.0482	4.0692	3.6130	5.0434	3.1454	6.0658	5.9217	5.9610	3.2902	0.8526	-0.2300	0.3415	0.4666
Ank2	_ERAEGFES[Phospho (STY)]ESEDGATKPK_.3	#N/A	6.1613	3.0534	4.4358	3.4376	2.8747	6.0152	3.1523	6.5572	4.3294	3.4046	0.9928	-0.0104	0.9750	0.0110
Ank2	_SVYSGQDDES[Phospho (STY)]PESS[Phospho (STY)]PEEQK_.2	Q8C8R3_S3226_M2_Ank2	5.6958	5.3568	5.8545	3.8335	4.7141	5.4962	5.8438	5.0140	5.3371	5.2379	0.9627	-0.0548	0.6403	0.1936
Ank2	_GS[Phospho (STY)]PIVQEPEEASEPK_.2	#N/A	7.3802	5.7470	4.2655	7.1892	5.4569	6.4626	7.3212	6.6839	6.4871	6.3400	0.9069	-0.1410	0.3445	0.4628
Ank2	QPETC[Carbamidomethyl (C)]DGHGC[Carbamidomethyl (C)]ETVSPSNSATPVSLGVQS[Phospho (STY)]PEI	Q8C8R3_S2755_M1_Ank2	6.1125	5.6743	5.7848	6.5264	5.2538	5.8589	6.3200	4.1442	5.9789	5.8309	1.0539	0.0757	0.5163	0.2871
Ank2	_ATS[Phospho (STY)]PLIEETPIGSIK_.2	#N/A	3.4186	9.2048	9.3508	9.5781	4.0452	9.2346	9.2634	9.3254	9.1673	8.8496	0.8165	-0.2925	0.2911	0.5360
Ank2	_ATAVPDSLC[Carbamidomethyl (C)]K_.2	#N/A	6.3016	6.0537	6.3979	6.6045	6.5959	5.6989	7.0840	6.7338	6.3277	6.1105	0.9560	-0.0649	0.2758	0.5594
Ank2	_HLPVS[Phospho (STY)]PGK_.2	#N/A	6.9234	6.1116	5.8134	6.5577	4.8178	6.4040	6.3683	6.4408	6.2118	6.4012	0.9369	-0.0940	0.3074	0.5123
Ank2	_ELEEEWVIVS[Phospho (STY)]DEEIQEAK_.2	Q8C8R3_S1562_M1_Ank2	8.6973	8.5944	9.8999	10.2406	8.7948	9.3261	9.8375	9.9698	9.5775	9.4389	0.9539	-0.0680	0.2528	0.5973
Ank2	_GADSGLLTEPVIR_.2	#N/A	5.6093	4.2413	5.3609	4.2347	4.9067	5.2259	5.3687	4.5674	5.0660	5.0875	0.9816	-0.0268	0.7829	0.1063
Ank2	_TLEQQENFSEVAR_.2	#N/A	4.6722	4.2433	4.5330	4.8606	4.6797	4.4670	5.1673	4.5414	4.2322	4.6324	0.9855	-0.0211	0.7295	0.1370
Ank2	_KTS[Phospho (STY)]LVIVESTDDQSQVFER_.3	#N/A	9.2458	3.6694	3.7874	3.7706	8.1720	9.2067	6.5477	3.9632	9.2840	3.7376	1.0723	0.1008	0.8167	0.0880
Ank2	_TEKHPPVS[Phospho (STY)]PGKTEK_.2	#N/A	6.2811	7.1595	7.2699	5.1027	6.2883	6.3600	5.8270	6.8006	6.1465	6.6041	1.0104	0.0149	0.8836	0.0537
Ank2	_GAAVDFTAR_.2	#N/A	4.5709	4.2207	4.9381	4.2586	4.4043	4.4071	4.3003	4.9332	5.1741	5.0834	0.9167	-0.1256	0.0835	1.0781
Ank2	_TDADADSWSEIREDDAAFEAR_.3	#N/A	5.3839	5.2357	6.3481	5.8646	3.7096	5.5723	5.2385	5.1534	4.3099	5.1233	1.0799	0.1109	0.4409	0.3557
Ank2	_EGHVGLVQELLGR_.3	#N/A	3.4962	4.6718	4.0583	3.5931	3.1163	3.5008	4.5500	4.2944	3.8742	4.8642	0.8507	-0.2333	0.0778	1.1091
Ank2	_ARS[Phospho (STY)]YIETETESR_.2	#N/A	4.9184	8.0301	8.3888	8.4071	7.8626	8.0436	8.5077	8.0638	7.8406	7.7550	0.9461	-0.0799	0.5499	0.2597
Ank2	_ESPC[Carbamidomethyl (C)]RDSLES[Phospho (STY)]S[Phospho (STY)]PVEPK_.2	Q8C8R3_S2431_M2_Ank2	4.2100	4.7456	3.5974	4.8740	3.2318	3.9324	3.5411	4.4206	4.5286	4.3193	0.9753	-0.0361	0.7876	0.1037
Ank2	.C[Carbamidomethyl (C)]KTPEDIS[Phospho (STY)]T[Phospho (STY)]PPEGTKPC[Carbamidomethyl (C)]LQ	Q8C8R3_S3744_M2_Ank2	3.8873	3.1222	3.6410	3.6892	3.0310	3.6177	3.5898	6.8883	3.8811	3.5494	0.7813	-0.3560	0.1771	0.7519
Ank2	_GSPIVQEPEEASEPKEESS[Phospho (STY)]PR_.3	#N/A	12.7126	11.7990	7.5362	11.4639	7.2439	11.7268	8.2316	11.3267	12.0958	11.8269	0.9580	-0.0619	0.7537	0.1228
Ank2	_TERHS[Phospho (STY)]PVFSGKPEK_.3	#N/A	6.7454	6.6311	7.0007	7.1317	5.7072	6.4343	7.1047	7.0490	6.7679	4.1500	1.0543	0.0763	0.5968	0.2242
Ank2	_GSPIVQEPEEASEPKEESS[Phospho (STY)]PRK_.3	#N/A	4.4525	3.7215	4.0244	5.9803	3.4345	3.7100	3.8587	3.5634	3.8625	3.9644	1.1071	0.1468	0.4189	0.3779
Ank2	_TELKKGS[Phospho (STY)]S[Phospho (STY)]EESVDEDR_.2	#N/A	4.0227	1.3122	3.2238	4.1422	2.4161	3.9908	2.3570	4.2336	3.0318	4.0911	0.9289	-0.1064	0.7275	0.1381
Ank2	_NEKLS[Phospho (STY)]PVS[Phospho (STY)]PSAK_.2	#N/A	11.4806	11.5499	11.8397	11.9092	10.5065	11.5310	11.9043	11.6868	11.5556	11.5144	0.9832	-0.0244	0.4830	0.3160
Ank2	_HLPGS[Phospho (STY)]PSIR_.2	Q8C8R3_S1900_M1_Ank2	4.3284	3.5838	4.0620	3.6058	3.4721	3.1525	3.7541	3.3954	3.6944	3.2831	1.0479	0.0674	0.4916	0.3084
Ank2	_NERHS[Phospho (STY)]PVSSLKTER_.4	#N/A	3.9308	3.4116	3.6645	3.2082	3.0745	3.5501	3.3565	3.7929	4.0920	4.3863	0.8890	-0.1697	0.1028	0.9879
Ank2	_MDIVHLLETNTEPLQER_.3	#N/A	5.7947	3.8295	3.4538	5.8191	2.8466	3.7780	5.1161	3.7412	3.8106	3.9054	1.0266	0.0379	0.8769	0.0571
Ank2	_KSDSNAS[Phospho (STY)]FLR_.2	#N/A	6.7787	6.7643	8.7304	8.3726	7.2180	6.8062	7.4332	8.3210	6.9122	8.3271	0.9608	-0.0576	0.5810	0.2358
Ank2	_S[Phospho (STY)]PLSPDTPSSEEVSYEVTPK_.2	Q8C8R3_S2493_M1_Ank2	3.7232	3.5610	3.8929	5.8516	3.7834	3.5653	4.2367	5.5245	3.2920	3.5179	0.9807	-0.0281	0.8977	0.0469
Ank2	_ETTKVGT[Phospho (STY)]PTDIHSEK_.2	#N/A	3.7181	4.8383	3.8028	3.5518	3.5000	3.8655	4.0201	5.3297	4.4852	4.8502	0.8305	-0.2680	0.0446	1.3506
Ank2	_IVDEQGDM[Oxidation (M)]DFQIS[Phospho (STY)]PDRK_.2	#N/A	6.5876	7.5902	8.0705	8.4700	7.1734	8.1595	7.6966	8.8995	8.1662	8.0569	0.9355	-0.0963	0.2326	0.6334
Ank2	_HPPVS[Phospho (STY)]PGKTEK_.2	#N/A	4.0022	8.4710	8.6506	8.5009	3.6218	8.3372	4.1396	8.5056	8.1566	8.4570	0.9475	-0.0778	0.8035	0.0950
Ank2	_THPAVS[Phospho (STY)]PSSK_.2	Q8C8R3_S1777_M1_Ank2	4.5975	8.8777	9.0852	4.0829	4.3965	8.7992	3.4735	4.7974	8.7107	8.6697	1.0354	0.0502	0.8947	0.0483
Ank2	_VSSSSS[Phospho (STY)]ESEPELTQLSK_.2	Q8C8R3_S2624_M1_Ank2	3.3757	3.6576	3.6978	4.3111	3.4944	3.9228	3.8515	4.0991	3.7230	4.0792	0.9505	-0.0732	0.3240	0.4895
Ank2	_HLPVS[Phospho (STY)]PGKTEK_.2	#N/A	7.7931	3.8274	8.3923	8.1112	3.7157	3.7595	8.1389	3.5303	3.8908	7.5318	1.0278	0.0395	0.9197	0.0363
Ank2	_KGS[Phospho (STY)]S[Phospho (STY)]EESVDEDR_.2	#N/A	6.2887	5.6425	5.4054	6.0155	5.5293	3.8381	6.7448	4.6656	6.3521	6.1608	0.8480	-0.2379	0.0604	1.2187
Ank2	_ATS[Phospho (STY)]PLIEETPIGSIKDK_.2	#N/A	4.7161	5.2394	5.8165	3.9367	3.6333	5.5107	3.9137	4.2712	3.6749	3.7461	1.2326	0.3017	0.0838	1.0765
Ank2	_AEGFES[Phospho (STY)]ESEDGATKPK_.2	#N/A	4.7665	3.0797	3.6046	3.5955	3.0830	4.4572	5.0315	5.1736	4.6064	4.4970	0.7798	-0.3587	0.0237	1.6261
Ank2	_SDSNAS[Phospho (STY)]FLR_.2	#N/A	8.9517	7.9592	9.4280	8.4609	8.8315	8.6153	9.6451	8.6450	8.5992	9.7645	0.9503	-0.0736	0.2338	0.6312
Ank2	_IVDEQGDM[Oxidation (M)]DFQIS[Phospho (STY)]PDR_.2	#N/A	5.9866	6.8979	6.7801	7.6028	6.0619	7.3780	7.5933	8.1023	7.2195	7.5114	0.8919	-0.1650	0.0559	1.2526
Ank2	_SKS[Phospho (STY)]ESDASSLDAK_.2	Q8C8R3_S3343_M1_Ank2	11.0633	11.2528	11.7873	11.4628	10.6739	11.1486	11.5425	11.4301	11.2366	11.1323	0.9908	-0.0133	0.6268	0.2028
Ank2	_STSSSGRPGTS[Phospho (STY)]PTR_.2	Q8C8R3_S3411_M1_Ank2	9.1929	9.2128	10.0167	9.6252	8.7698	9.3814	9.5916	9.6588	9.3098	9.2565	0.9907	-0.0134	0.7136	0.1466
Ank2	_RFT[Phospho (STY)]PEEEMFK_.2	#N/A	3.0310	4.3114	4.2655	5.9814	3.6756	2.9490	3.9576	3.1919	3.4909	3.4866	1.1427	0.1924	0.4129	0.3842
Ank2	_SHLVNEVPVLAS[Phospho (STY)]PDLLSEVSEM[Oxidation (M)]K_.3	Q8C8R3_S1467_M1_Ank2	3.5036	5.3728	5.7349	6.0469	4.1482	4.0694	4.4302	5.8226	3.0183	3.9592	1.1173	0.1600	0.4929	0.3072
Ank2	_ELDFTEEQIHQIR_.3	#N/A	4.3763	3.6317	4.1099	3.6537	3.5200	3.1046	3.8020	4.7546	3.6465	3.1993	0.9694	-0.0449	0.7420	0.1296
Ank2	_HSS[Phospho (STY)]LSSSAKPER_.2	Q8C8R3_S1793_M1_Ank2	5.0742	3.4288	4.8527	4.9708	3.9803	5.2819	4.1873	3.7896	3.8743	3.7801	1.1767	0.2347	0.1059	0.9749
Ank2	_KGS[Phospho (STY)]S[Phospho (STY)]EESVDEDRGLVPEPLPTAK_.3	#N/A	3.7783	3.5517	3.9732	9.1288	7.6612	3.3938	3.9856	8.6679	7.7751	3.0570	0.8938	-0.1620	0.7195	0.1430
Ank2	_HEGLAETPETSPESLSFS[Phospho (STY)]PK_.3	Q8C8R3_S2213_M1_Ank2	3.5098	4.9143	4.1966	6.0608	4.1363	5.5935	4.4139	3.4195	3.0681	5.8801	1.0492	0.0693	0.7747	0.1109
Ank2	_VGT[Phospho (STY)]PTDIHSEKELPITNDITDSSQK_.3	#N/A	3.2863	5.2828	5.4507	6.3467	3.9198	3.0821	4.9390	5.7184	3.0057	3.2276	1.0802	0.1113	0.7014	0.1540
Ank2	_SGHDQVVELLLER_.2	#N/A	3.8653	3.8626	3.7571	4.6752	3.7086	3.9688	3.3495	3.9425	4.4559	3.6289	1.0335	0.0475	0.6352	0.1971
Ank2	_ESES[Phospho (STY)]DQEPEEEIGMTSEK_.2	Q8C8R3_S1428_M1_Ank2	5.7117	6.8270	3.8860	9.7722	3.5114	4.4116	7.9985	6.6471	6.6779	7.1536	0.7988	-0.3242	0.2757	0.5596
Ank2	_TPEDIST[Phospho (STY)]PPEGTKPC[Carbamidomethyl (C)]LQTPVTSER_.3	Q8C8R3_T3745_M1_Ank2	3.0468	4.2956	5.3875	3.8250	4.1170	3.6125	3.9733	3.1761	3.9033	3.5023	1.1123	0.1535	0.3676	0.4346
Ank2	_ASSDVM[Oxidation (M)]LNGFGGDAPTLR_.2	#N/A	3.8985	4.2579	4.5853	3.4170	5.0603	3.2045	3.5653	3.5842	4.9416	3.0943	1.0722	0.1006	0.5820	0.2351
Ank2	_NS[Phospho (STY)]IEFFEEISDEASK_.2	Q8C8R3_S3427_M1_Ank2	3.1868	3.9431	4.4213	4.4726	3.8313	4.2941	4.1873	3.0361	3.2945	3.6423	1.1369	0.1852	0.1638	0.7857
Ank2	_IENPNSLQDQSHALLK_.3	#N/A	2.7718	4.2044	3.3809	3.8835	4.7393	3.7051	3.5982	4.6926	4.6092	4.7826	0.8553	-0.2256	0.1562	0.8063
Ank2	_HSPVFSGKPEKHS[Phospho (STY)]PGS[Phospho (STY)]PSTK_.3	Q8C8R3_S1839_M2_Ank2	6.6251	4.3133	3.5997	5.3747	4.2016	5.7968	6.6150	5.9752	5.7940	4.0693	0.8881	-0.1712	0.4102	0.3870
Ank2	_KRFT[Phospho (STY)]PEEEMFK_.3	#N/A	4.3944	3.6498	4.1281	5.6445	3.5381	3.0865	3.8201	3.3293	3.6284	3.3491	1.1534	0.2059	0.2791	0.5543
Ank2	_KS[Phospho (STY)]EEQIGEAK_.2	Q8C8R3_S2217_M1_Ank2	3.5219	3.7218	4.2001	3.6302	3.7263	3.4609	3.9315	4.1104	2.9450	4.1222	0.9822	-0.0258	0.8024	0.0956
Ank2	_TEGDSPAAALS[Phospho (STY)]PQMHQEPVQQDFSGK_.3	#N/A	5.8338	5.9557	6.3733	7.7624	3.9423	4.9519	5.9051	5.5434	5.3235	4.4590	1.0934	0.1288	0.5013	0.2999
Ank2	_SAALLLQNDHNADVQSK_.3	#N/A	3.5964	3.6731	3.8725	4.5992	3.6062	3.9985	3.1233	3.7396	3.8085	3.6076	1.0900	0.1243	0.2000	0.6991
Ank2	_NEKLS[Phospho (STY)]PVS[Phospho (STY)]PSAKTER_.2	#N/A	3.4522	4.2529	5.0489	4.0226	4.1115	3.8272	3.9701	3.6422	3.5296	3.8522	1.0989	0.1361	0.2270	0.6440
Ank2	_DPDGS[Phospho (STY)]AEDDSLEQTSLMESSGK_.2	#N/A	3.1755	3.9317	4.4100	4.4839	3.8200	4.3054	4.1986	3.6346	3.2832	3.6310	1.0907	0.1252	0.2859	0.5438
Ank2	_TAEGTEPKPQGAIRS[Phospho (STY)]PQGLELPLPNR_.4	#N/A	2.9698	7.7663	7.6800	8.8626	3.5964	4.1331	3.8740	7.6752	3.6078	4.1263	1.2103	0.2754	0.5192	0.2847
Ank2	_EIASPSS[Phospho (STY)]PVK_.2	Q8C8R3_S2827_M1_Ank2	4.3300	3.5855	4.0637	3.6075	3.4738	3.1508	3.7558	3.3937	3.6927	3.2848	1.0482	0.0679	0.4896	0.3102
Ank2	_LLRDPDGS[Phospho (STY)]AEDDSLEQTSLMESSGK_.3	#N/A	3.7641	3.7857	4.1481	6.3869	3.4383	4.0784	3.7804	4.0300	3.6187	3.6475	1.1321	0.1790	0.3924	0.4063
Ank2	_HTPVSPSS[Phospho (STY)]KNEKLS[Phospho (STY)]PVS[Phospho (STY)]PSAK_.3	Q8C8R3_S1810_M3_Ank2	4.3122	4.0633	3.4982	5.1738	3.1479	4.0829	3.7155	4.1176	3.7662	3.9679	1.0397	0.0562	0.6829	0.1656
Ank2	_S[Phospho (STY)]PQGLELPLPNR_.2	#N/A	3.2932	3.9329	3.9800	12.3230	3.9198	3.8097	4.1974	11.6345	3.2844	4.4497	0.8843	-0.1774	0.7789	0.1085
Ank2	_VVLKSDT[Phospho (STY)]QQS[Phospho (STY)]EDNNE_.2	Q8C8R3_S3893_M2_Ank2	2.9662	4.0718	4.2690	4.0062	3.3246	4.0206	4.1544	3.3199	4.0281	3.3894	1.0144	0.0206	0.8678	0.0616
Ank2	_FQVSAATESR_.2	#N/A	3.5302	4.2865	3.2639	4.1291	4.1748	3.9507	5.7840	5.0945	3.6379	4.0961	0.8358	-0.2587	0.1172	0.9310
Ank2	_TPTEEGT[Phospho (STY)]PTSEQNPFLFQEGK_.3	Q8C8R3_T3050_M1_Ank2	4.2126	3.5170	3.3986	3.9013	3.0578	4.3912	3.2511	4.2172	3.8658	3.8682	0.9857	-0.0207	0.8635	0.0637
Ank2	_ELGDDS[Phospho (STY)]LPSSQFLDGM[Oxidation (M)]NYLR_.2	Q8C8R3_S874_M1_Ank2	3.5162	3.5452	3.9834	3.2285	3.4035	3.0332	4.0114	3.5954	3.6500	3.9195	0.9097	-0.1365	0.0989	1.0048
Ank2	_HPPVS[Phospho (STY)]PGK_.2	#N/A	4.2854	3.3456	3.4714	4.5309	3.4111	4.3184	3.1784	3.9711	3.6100	3.9410	1.0595	0.0834	0.5023	0.2990
Ank2	_DSEVLSPMADESLAVSHKDS[Phospho (STY)]LEAS[Phospho (STY)]PVLEDNSSHK_.4	Q8C8R3_S2395_M2_Ank2	3.4946	3.7316	4.1814	3.1832	4.1211	3.6084	4.3987	3.4344	3.0830	3.1502	1.0579	0.0811	0.5287	0.2768
Ank2	_PQGAIRS[Phospho (STY)]PQGLELPLPNR_.3	#N/A	4.2193	3.1231	3.9530	1.4981	3.3631	3.2615	3.6451	3.5044	3.8034	3.1741	0.9164	-0.1260	0.5689	0.2450

Elfn1	_AS[Phospho (STY)]PSIWER_.2	Q8C8T7_S773_M1_Elfn1	8.9469	8.9221	9.1263	8.6899	7.5506	8.6763	8.9582	9.0715	8.5590	3.5877	1.1469	0.1977	0.3372	0.4721
Elfn1	_AVAAAAGS[Phospho (STY)]LKK_.2	Q8C8T7_S460_M1_Elfn1	8.0007	7.6062	7.8711	7.3763	6.4339	7.4472	7.9125	7.9799	7.3811	7.6679	0.9639	-0.0531	0.3835	0.4162
Ppfibp1	_SSS[Phospho (STY)]LGNLKK_.2	Q8C8U0_S435_M1_Ppfibp1	7.5686	3.6815	3.5631	8.1784	7.0345	7.9299	7.9431	8.2136	3.7013	7.9312	0.9106	-0.1352	0.6654	0.1769
Tmem163	_ISES[Phospho (STY)]GQFSDGLEDR_.2	Q8C996_S56_M1_Tmem163	5.6863	6.4373	3.7900	7.2489	5.3725	6.3821	6.8676	7.2331	6.6338	6.6186	0.8510	-0.2327	0.1377	0.8611
Tmem163	_GHAPSTAAPAPSPAPM[Oxidation (M)]SSS[Phospho (STY)]VQSDEER_.3	#N/A	4.2835	3.3919	3.7608	5.2365	5.6728	3.8704	5.5504	5.5468	4.6482	3.6414	0.9015	-0.1496	0.4361	0.3604
Tmem163	_GHAPSTAAPAPSPAPM[Oxidation (M)]SSSVQS[Phospho (STY)]DEERQPR_.4	#N/A	7.0808	6.8488	3.9948	4.2613	5.7133	3.8012	4.2433	3.4641	6.9601	3.6024	1.1567	0.2101	0.4894	0.3103
Tmem163	_S[Phospho (STY)]PPGPGVPRPPPR_.3	Q8C996_S11_M1_Tmem163	4.2640	5.0727	3.5078	6.2039	3.3896	4.3398	4.7547	4.8565	4.2487	4.2297	0.9869	-0.0191	0.9169	0.0377
Dido1	_GLS[Phospho (STY)]PSYLGGPR_.2	Q8C9B9_S1840_M1_Dido1	3.1230	4.2194	4.3576	3.9013	4.0407	4.3657	4.0496	3.0998	3.3989	5.5103	1.0797	0.1106	0.6585	0.1814
Strip2	_AAS[Phospho (STY)]PPSYTLDGESQLAPPPSK_.2	#N/A	8.7899	3.6497	4.2593	9.4680	3.9661	4.0969	9.2258	9.4665	8.9730	8.0600	0.6388	-0.6467	0.0480	1.3190
Strip2	_QDS[Phospho (STY)]LDIYNER_.2	#N/A	9.4222	9.1567	9.5365	9.3741	7.8531	9.3614	9.6795	9.4568	9.2044	9.3579	0.9674	-0.0478	0.3814	0.4187
Strip2	_QLLTKQDS[Phospho (STY)]LDIYNER_.3	#N/A	4.0349	3.3392	3.2208	3.7235	3.1606	3.0681	3.4382	4.3950	4.0436	3.6905	0.8799	-0.1845	0.1021	0.9911
Shisa4	_DLTLLITER_.2	#N/A	4.4173	4.7572	5.0320	5.3937	4.1271	4.5551	4.5596	4.9023	5.0255	4.8860	0.9732	-0.0391	0.6095	0.2150
Pde10a	_ST[Phospho (STY)]PEKLNVK_.2	Q8CA95_T780_M1_Pde10a	6.5860	5.4291	3.2726	5.4925	3.2124	5.1355	5.8576	6.4242	3.9918	5.9617	0.8733	-0.1954	0.4077	0.3896
Pde10a	_AYLSLHPQVLDEFVSESVSAETVEK_.3	#N/A	4.2339	3.0320	5.3685	5.4254	3.1224	4.6771	3.4000	4.3589	3.9828	3.6523	1.1199	0.1633	0.4356	0.3609
Pde10a	_EVDLYTGYTTR_.2	#N/A	3.2892	4.0944	3.9760	4.0263	3.9157	3.8138	5.6161	5.9360	3.2739	3.7036	0.8317	-0.2659	0.1938	0.7127
Pgm2l1	_IVLATDPDADRLAVAELQENGR_.3	#N/A	4.9283	5.0568	5.1649	5.6949	4.9559	4.6308	5.4961	5.5530	4.4627	4.1926	1.0296	0.0421	0.6707	0.1735
Pgm2l1	_LIDALIENFLEPSK_.2	#N/A	4.6369	4.7862	4.2970	4.6104	4.5973	5.3910	5.5816	4.5948	5.1800	4.4694	0.9523	-0.0706	0.4182	0.3786
Pgm2l1	_DITTGYDSSQPNKK_.2	#N/A	4.5963	5.1984	4.0598	3.6036	4.3111	4.3234	4.3373	4.2252	3.5487	3.2809	1.1301	0.1765	0.1788	0.7477
Pgm2l1	_AVAGVMITAS[Phospho (STY)]HNR_.2	Q8CAA7_S175_M1_Pgm2l1	3.7877	3.0431	3.5214	6.6917	2.9314	3.6932	3.5864	4.2468	3.8954	3.8387	1.0136	0.0195	0.9430	0.0255
Podxl2	_RDPEDS[Phospho (STY)]DVFEEDTHL_.2	#N/A	3.3789	3.7833	4.0657	3.9135	3.6350	3.6119	4.4262	4.6715	3.2385	3.8535	0.9219	-0.1173	0.2940	0.5317
Nhsl1	_SLS[Phospho (STY)]FSGPR_.2	Q8CAF4_S1482_M1_Nhsl1	4.2356	3.5400	3.4216	4.5807	3.3613	4.3682	3.2282	4.8402	5.4707	5.0768	0.8418	-0.2484	0.1608	0.7936
Nhsl1	_KNS[Phospho (STY)]GAGAVLFSEPSAQEQR_.3	#N/A	5.2635	4.5325	5.1036	4.2994	4.1272	5.5304	4.0140	5.4747	3.5348	4.7970	1.0795	0.1104	0.4495	0.3472
Nhsl1	_NHS[Phospho (STY)]PS[Phospho (STY)]PPVTPTSAAPNLASPK_.3	Q8CAF4_S1373_M2_Nhsl1	3.2768	4.0331	4.5113	4.3826	3.9214	4.2041	4.0972	3.6883	3.3845	4.5285	1.0332	0.0471	0.6735	0.1716
Nhsl1	_SEPSADSPDSPSSC[Carbamidomethyl (C)]S[Phospho (STY)]PNK_.2	Q8CAF4_S1459_M1_Nhsl1	3.9971	3.2525	3.7307	3.2745	3.1408	3.4838	3.4228	3.7267	4.0257	2.9518	0.9853	-0.0213	0.8389	0.0763
Shfl	_FHGKVS[Phospho (STY)]PK_.2	Q8CAF4...	9.3236	9.7953	10.0233	10.2202	9.9921	9.7375	9.7509	9.6373	9.6031	9.8297	1.0148	0.0212	0.4057	0.3917
Shfl	_EKFHGKVS[Phospho (STY)]PK_.2	#N/A	5.5977	4.8781	4.2107	5.9565	5.4781	5.4876	4.4279	5.2994	5.0528	4.9027	1.0706	0.0984	0.3496	0.4564
Immt	_SLEDALNR_.2	#N/A	8.3571	8.1598	8.8188	8.4646	8.1361	8.2981	7.9951	8.6567	8.5103	8.5973	0.9920	-0.0116	0.7104	0.1485
Immt	_SEIQAEQDR_.2	#N/A	7.5347	7.6587	7.6105	7.5357	7.8268	7.5529	7.7433	7.6799	7.6708	7.4525	0.9978	-0.0032	0.8317	0.0801
Immt	_ELDSITPDITPGWK_.2	#N/A	7.2636	7.4752	7.2793	7.5559	7.6292	7.6730	7.8460	7.3976	7.5256	7.0605	1.0029	0.0042	0.8918	0.0497
Immt	_LSEQELEFR_.2	#N/A	7.4460	7.5501	7.2373	7.4129	6.8194	7.4287	7.1684	7.0876	7.1887	7.2860	1.0185	0.0265	0.3639	0.4390
Immt	_AVDEAADALLK_.2	#N/A	6.3643	6.6662	6.5700	6.1322	6.7993	6.4318	6.3397	6.9320	6.5495	6.5726	0.9842	-0.0230	0.5195	0.2844
Immt	_GVYSEETLR_.2	#N/A	6.3250	6.7497	6.0202	6.6467	6.6421	6.8181	5.0841	6.2212	6.6244	6.8889	1.0530	0.0745	0.3754	0.4255
Immt	_TSSAEM[Oxidation (M)]PTIPLGSAVEAIR_.2	#N/A	6.2407	6.3859	6.5231	6.5899	6.2495	6.4948	6.3728	6.3955	6.3812	6.1537	1.0139	0.0200	0.3431	0.4646
Immt	_EIAGATPHITAAEGR_.3	#N/A	6.3436	5.7790	5.8115	6.0465	6.4009	6.4303	5.5903	5.8616	6.0680	6.0514	1.0411	0.0582	0.2036	0.6912
Immt	_TSSVTLQTITAQNAAVQAVK_.2	#N/A	5.6809	5.3077	5.7529	4.4157	4.4377	4.7668	5.6757	4.8102	4.4134	4.0930	1.0658	0.0919	0.4677	0.3300
Immt	_QAAAHTDHLR_.2	#N/A	5.7387	4.6324	3.6050	5.2816	5.2190	4.9862	5.2610	4.3543	5.0976	5.3017	0.9814	-0.0271	0.8276	0.0822
Immt	_STSETTEEAFSSSVR_.2	#N/A	4.2172	6.5183	6.4819	7.2337	5.8849	6.1129	6.6395	6.3938	6.0560	6.3344	0.9558	-0.0653	0.6093	0.2152
Immt	_QHIELALEK_.2	#N/A	4.1889	4.8458	4.5826	5.5974	5.2300	5.1929	4.9999	5.2237	4.9523	5.4816	0.9565	-0.0642	0.4382	0.3583
Immt	_TFDSAVAK_.2	#N/A	5.0438	4.5226	3.7333	4.9489	5.3931	5.1172	4.0961	4.1061	4.0232	5.0872	1.1074	0.1472	0.2353	0.6284
Immt	_VVSQYHELVVQAR_.2	#N/A	4.4001	4.4426	5.0724	5.0609	4.6895	4.1924	4.9513	3.9170	4.5487	3.9375	1.0701	0.0978	0.2958	0.5289
Immt	_GIEQAVQSHAVAEEEAR_.3	#N/A	5.9141	5.3465	6.4166	5.0602	5.2688	5.3888	5.2878	5.9329	5.3312	5.1282	1.0269	0.0383	0.6316	0.1996
Immt	_RIDQLNR_.2	#N/A	5.1656	3.2881	3.1697	3.6724	2.7538	3.1192	3.8982	3.6971	4.4206	3.4358	0.8625	-0.2133	0.2541	0.5950
Immt	_LSTDDLNSLIAHAHR_.3	#N/A	5.9324	6.0373	6.1723	6.3171	5.8736	5.6888	6.0768	6.1213	6.0139	5.7598	1.0018	0.0026	0.9371	0.0282
Immt	_ISSVSEVMK_.2	#N/A	4.8138	3.0321	3.5104	5.4194	4.3885	3.7042	3.2024	3.9470	4.2461	4.2323	1.0609	0.0852	0.6431	0.1917
Immt	_SQEQM[Oxidation (M)]DSFTLDINTAYAR_.2	#N/A	3.3902	3.8360	4.0770	4.5796	4.0167	4.4011	4.2943	3.6140	3.1874	3.6579	1.0981	0.1350	0.2334	0.6319
Immt	_YSTSSSSGLTAGK_.2	#N/A	3.2669	3.9593	3.9537	4.4563	2.8739	4.2779	4.1710	3.6621	3.3982	4.4233	0.9704	-0.0433	0.7570	0.1209
Immt	_VQEQELKYEFEQGLSEK_.3	#N/A	4.1171	3.5755	3.3031	3.1984	3.2428	2.9859	3.3466	4.3127	4.1018	3.7727	0.8765	-0.1902	0.1051	0.9783
Acat2	_VAVLSQNR_.2	#N/A	5.1832	5.6903	4.3659	5.2631	6.1756	5.2977	5.1704	5.7117	5.7661	6.1254	0.9360	-0.0953	0.3203	0.4945
Acat2	[Carbamidomethyl (C)]LAAQSIAM[Oxidation (M)]GDSTIVVAGGM[Oxidation (M)]ENM[Oxidation (M)]	#N/A	4.4012	3.7260	4.7422	3.2076	3.7479	4.1514	3.9447	4.5050	3.1763	3.9856	1.0239	0.0340	0.7988	0.0975
Acat2	_VNIDGGAIALGHPLGASGC[Carbamidomethyl (C)]R_.3	#N/A	3.5696	4.6875	4.2564	5.2674	4.2347	3.5334	4.4737	4.8617	3.0080	4.1595	1.0321	0.0456	0.7821	0.1067
Eloa	_SYS[Phospho (STY)]PEHR_.2	Q8CB77_S125_M1_Eloa	4.2860	7.7534	8.3078	3.5635	8.2866	8.0948	8.3077	3.4377	7.9754	8.0550	0.9671	-0.0483	0.8785	0.0563
Eloa	_S[Phospho (STY)]PEEDQEPIISHQKPGK_.3	Q8CB77_S195_M1_Eloa	5.7539	7.5495	5.4711	6.7475	5.3336	5.3920	6.0956	5.5705	5.4563	5.7613	1.0036	0.0051	0.9457	0.0242
Wdr37	_SHS[Phospho (STY)]LSIR_.2	#N/A	7.8356	7.6416	7.8327	8.1148	3.6666	7.6351	7.3937	7.8904	7.5064	7.6973	0.9933	-0.0097	0.8798	0.0556
Wdr37	_STLLELFGQIER_.2	#N/A	4.9422	3.9947	3.9064	4.2048	4.8279	4.7177	4.9008	4.9792	4.6913	5.0173	0.9051	-0.1439	0.0858	1.0665
Wdr37	_ASHSTSQLS[Phospho (STY)]QK_.2	Q8CBE3_S119_M1_Wdr37	7.0234	6.6032	6.9006	7.2445	6.0056	7.1556	6.9437	6.7527	6.8525	6.6271	1.0041	0.0060	0.9095	0.0412
Wdr37	_KSHS[Phospho (STY)]LSIR_.2	#N/A	6.6650	6.5476	3.4664	3.9690	2.8340	6.6467	6.5551	6.1661	6.7074	6.7273	0.7679	-0.3810	0.1370	0.8634
Wdr37	_DPSIHSVNVFQGHTDTVTSAVFTVGDNVVSGSDDR_.4	#N/A	4.0542	3.1720	3.2401	3.7428	3.1799	3.0488	3.4575	4.3757	4.0243	3.7098	0.8752	-0.1922	0.0955	1.0198
Ephb1	_YLQDDTS[Phospho (STY)]DPTYTSSLGGK_.2	Q8CBF3_S774_M1_Ephb1	3.7803	5.4051	4.1824	5.6894	3.7276	4.8700	5.5908	5.4768	3.6055	5.2806	0.9240	-0.1141	0.5204	0.2836
Mfsd6	_VAILT[Phospho (STY)]DDEEEQKRK_.3	#N/A	7.4870	6.8187	7.2798	7.2807	5.5203	6.6572	7.4768	7.5463	6.6430	6.7001	0.9646	-0.0520	0.5613	0.2508
Mfsd6	_[Acetyl (Protein N-term)]AADDKVAILT[Phospho (STY)]DDEEEQKR_.3	#N/A	3.5158	5.4958	4.2026	6.7793	5.8210	6.7145	4.4199	6.8002	6.9929	6.5763	0.8748	-0.1930	0.3750	0.4259
Mfsd6	_[Acetyl (Protein N-term)]AADDKVAILT[Phospho (STY)]DDEEEQK_.2	#N/A	3.2860	3.9401	3.9728	6.2144	5.4641	4.2970	4.1902	6.6237	6.3942	5.8645	0.7852	-0.3489	0.1177	0.9292
Mfsd6	_IPVPSSPVPIAT[Phospho (STY)]IDLVQQQTEDVM[Oxidation (M)]PR_.3	Q8CBH5_T653_M1_Mfsd6	4.1582	3.4136	4.4993	3.5011	3.3019	3.3227	3.2158	3.1165	4.2659	3.4681	1.0520	0.0731	0.5941	0.2261
Mfsd6	_VAILT[Phospho (STY)]DDEEEQK_.2	#N/A	3.0372	3.8424	3.7240	4.2783	3.6637	4.8623	4.4265	4.7161	3.0219	4.1937	0.9540	-0.0680	0.6736	0.1716
Abi1	_TNPPTQKPPS[Phospho (STY)]PPVSGR_.2	#N/A	10.5473	11.5341	11.8565	11.8196	10.5721	10.8313	11.2983	11.5204	11.0941	10.6673	1.0043	0.0063	0.8916	0.0498
Abi1	_NDDGWFEGVC[Carbamidomethyl (C)]NR_.2	#N/A	8.3696	8.2208	8.3104	8.4887	7.9719	8.0201	8.4374	7.9727	8.0527	8.3134	1.0044	0.0064	0.7942	0.1001
Abi1	_TAS[Phospho (STY)]LNQRPR_.2	Q8CBW3_S231_M1_Abi1	6.8588	4.2704	4.1520	6.9802	6.4028	7.2464	7.1070	7.5766	6.8776	6.9335	0.8402	-0.2512	0.1555	0.8083
Abi1	_HNS[Phospho (STY)]TTSSTSSGGYR_.2	Q8CBW3_S296_M1_Abi1	4.9574	3.7743	4.1386	4.6773	3.6276	4.5736	4.4700	4.2331	4.9270	5.3018	0.9067	-0.1413	0.2170	0.6635
Abi1	_ALIESYQNLTR_.2	#N/A	5.9891	6.0734	5.8107	6.6564	6.2816	6.2617	3.2435	5.8106	6.2292	5.8022	1.1721	0.2292	0.1441	0.8414
Abi1	_LGSQHS[Phospho (STY)]PGR_.2	Q8CBW3_S225_M1_Abi1	8.2028	8.3611	8.4087	8.5792	7.3294	8.0926	8.4777	8.1510	7.9848	8.4357	0.9879	-0.0176	0.6924	0.1597
Abi1	_KNDDGWFEGVC[Carbamidomethyl (C)]NR_.3	#N/A	3.0234	5.4220	3.7102	5.3336	5.1547	5.4974	4.8625	5.4793	5.2945	5.1022	0.9046	-0.1446	0.3933	0.4053
Abi1	_[Acetyl (Protein N-term)]AELQM[Oxidation (M)]LLEEEIPSGKR_.2	#N/A	4.0957	3.9574	3.2817	3.2198	5.3575	4.9926	3.3681	3.7814	4.8038	3.5123	1.0736	0.1024	0.5957	0.2250
Abi1	_DKDDELSFK_.2	#N/A	4.9470	4.0655	3.8037	4.7865	4.4220	4.2523	4.3468	4.3529	3.1017	4.6075	1.0676	0.0944	0.4476	0.3491
Chic1	_VVS[Phospho (STY)]EEHLR_.2	Q8CBW7_S80_M1_Chic1	5.3493	4.8183	5.5051	5.0799	3.9870	4.1715	5.3815	5.6802	4.4969	4.7253	0.9502	-0.0737	0.5306	0.2753
Tmem63c	_S[Phospho (STY)]SPSEVSLEAER_.2	Q8CBX0_S88_M1_Tmem63c	4.3548	2.9876	4.0885	3.6323	2.8089	3.4197	3.7805	3.3689	3.6679	3.3096	1.0048	0.0069	0.9593	0.0180
Tmem63c	_LALLIHNDS[Phospho (STY)]LTSLIYGEQSEK_.3	Q8CBX0_S75_M1_Tmem63c	3.6477	3.6947	3.3813	4.4259	3.5161	4.2134	3.0734	4.0761	4.3751	4.1032	0.9760	-0.0350	0.7693	0.1139
Samd4a	_AYSS[Phospho (STY)]PSTTPEVR_.2	Q8CBY1_S420_M1_Samd4a	7.4951	3.9100	4.3882	3.9320	3.7983	4.3272	4.0803	7.5254	7.3530	3.6093	0.8227	-0.2815	0.3885	0.4106
Dctn4	_LLQPDLQPVSASQLYPR_.2	#N/A	4.8927	5.1083	4.5361	5.3613	5.3439	5.3034	5.5211	4.6141	4.9096	5.2984	1.0010	0.0015	0.9827	0.0076
Dctn4	_IQLVAVNYIPEVR_.2	#N/A	4.6974	5.2840	3.9140	4.7156	4.0577	4.4269	4.6463	4.3498	5.1764	3.8295	1.0034	0.0049	0.9647	0.0156
Dctn4	_LIEYYQQLAQK_.2	#N/A	3.0071	3.5707	3.6227	4.2268	3.5624	4.1670	4.5278	3.9150	3.1940	3.3859	0.9833	-0.0244	0.8536	0.0687

Cacnb2	_SGGNSSS[Phospho (STY)]SLGDIVPSSR_.2	Q8CC27_S202_M1_Cacnb2	3.7726	7.8672	3.5063	8.4667	3.8207	7.6932	8.2651	3.9279	3.9089	4.2212	1.1523	0.2045	0.6143	0.2116
Cacnb2	_SAS[Phospho (STY)]QAEEEPC[Carbamidomethyl (C)]LEPVKK_.2	#N/A	6.5503	3.3902	4.5228	3.5246	2.9617	3.2670	3.2393	7.3543	4.2424	3.4916	0.8809	-0.1830	0.6053	0.2180
Cacnb2	_SAS[Phospho (STY)]QAEEEPC[Carbamidomethyl (C)]LEPVK_.2	#N/A	3.3932	4.1984	4.0800	4.5827	4.0198	3.7097	4.2974	5.3358	3.1844	3.0488	1.1367	0.1849	0.1528	0.8160
Synpo	_VASLS[Phospho (STY)]PAR_.2	Q8CC35_S765_M1_Synpo	10.9356	10.8727	11.2789	11.2682	10.1255	11.0258	10.6747	10.8540	3.1162	11.2429	1.2169	0.2832	0.2478	0.6059
Synpo	_VRSPPSY[Phospho (STY)]STLYPSSDPKPSHLK_.4	Q8CC35_Y571_M1_Synpo	8.2604	8.4311	8.7953	9.8863	7.4964	8.9120	3.6752	8.1960	8.4092	8.3940	1.2039	0.2677	0.1845	0.7341
Synpo	_VTPNPDLLDLVQTADEK_.2	#N/A	8.0103	7.9779	8.1740	8.1615	8.4299	8.1408	7.9125	7.8154	7.9957	8.0234	1.0268	0.0381	0.0455	1.3418
Synpo	_ASPAAAEEAVPEWASC[Carbamidomethyl (C)]LK_.2	#N/A	7.3847	6.9213	6.9102	7.5487	7.2488	7.1620	7.2404	6.7077	6.8344	7.1682	1.0298	0.0424	0.2411	0.6179
Synpo	_YTTNAPGGFR_.2	#N/A	7.0260	7.2685	7.0113	7.5418	7.8237	7.2366	6.8410	7.1468	7.2828	7.3753	1.0219	0.0312	0.4222	0.3745
Synpo	_HPSPQS[Phospho (STY)]PSR_.2	Q8CC35_S230_M1_Synpo	3.2575	4.2433	6.1587	3.8777	3.8920	4.0884	4.0260	3.1237	5.6675	6.0115	0.9035	-0.1464	0.5564	0.2546
Synpo	_VAS[Phospho (STY)]EEEVPLVVYLK_.2	#N/A	12.2372	12.1263	12.6079	12.7377	12.0646	12.3858	12.5174	12.1005	12.3080	12.2748	1.0049	0.0070	0.7062	0.1511
Synpo	_SSPGLYTAPVQDSLQPTAVS[Phospho (STY)]PTYSSDISPVSPSR_.3	Q8CC35_S882_M1_Synpo	4.0223	4.6863	6.5483	8.4293	4.0570	4.4743	4.5958	5.5801	6.0854	4.1362	1.0530	0.0745	0.7869	0.1041
Synpo	_LLGQRS[Phospho (STY)]PVLER_.2	#N/A	6.6901	7.4507	7.7581	8.4409	6.2162	7.0723	7.3615	7.3532	6.7488	6.7974	1.0292	0.0415	0.6407	0.1933
Synpo	_PSLYALS[Phospho (STY)]PVKEPAK_.2	#N/A	6.0135	6.0853	4.1398	6.6766	4.0795	6.0542	6.0295	5.8500	5.8477	5.7845	0.9371	-0.0937	0.5335	0.2729
Synpo	_ASPAAAEEAVPEWASC[Carbamidomethyl (C)]LKS[Phospho (STY)]PR_.2	Q8CC35_S689_M1_Synpo	2.9083	2.9333	4.1428	6.5519	3.5529	3.0718	3.8348	4.6546	3.8383	3.3639	0.9840	-0.0233	0.9350	0.0292
Synpo	_C[Carbamidomethyl (C)]PS[Phospho (STY)]PTM[Oxidation (M)]SLPSSWK_.2	#N/A	8.0187	9.3172	9.6105	10.9816	7.8752	8.3785	9.1802	9.2278	8.6938	8.1606	1.0244	0.0347	0.7442	0.1283
Synpo	_AAS[Phospho (STY)]PAKPSSLDLVPNLPR_.3	#N/A	3.7989	4.0000	3.9233	10.4705	3.8213	3.9946	3.6153	8.2393	3.9402	3.8845	1.0166	0.0237	0.9612	0.0172
Synpo	_PKPNQNLSEASGK_.3	#N/A	3.4180	3.0119	4.0364	3.7366	3.3400	3.7244	3.6176	4.2156	3.9761	3.3648	0.9344	-0.0979	0.3243	0.4890
Synpo	_ENAALLTANGLHLSQSR_.2	#N/A	2.4529	4.2596	3.6533	4.1560	1.8373	3.9775	3.8707	3.9625	2.9875	4.1230	0.9073	-0.1404	0.5469	0.2621
Synpo	_DRAS[Phospho (STY)]PAAAEEAVPEWASC[Carbamidomethyl (C)]LK_.3	#N/A	3.2061	3.9343	3.9793	4.4820	3.8413	4.1078	4.1967	9.2667	3.2857	3.8711	0.7614	-0.3932	0.3018	0.5203
Synpo	_AWAPPASSMADRS[Phospho (STY)]PQPQR_.3	Q8CC35_S512_M1_Synpo	4.1246	3.4290	3.3106	7.2361	3.2503	2.9784	3.3392	3.8103	4.1093	4.3690	1.0378	0.0536	0.8642	0.0634
Rybp	_S[Phospho (STY)]ST[Phospho (STY)]PKGDMSAVNDESF_.2	Q8CCI5_T215_M2_Rybp	3.2595	4.0419	4.4157	4.9460	3.8945	4.0614	4.1627	3.0418	3.3408	3.6368	1.1573	0.2107	0.1423	0.8469
Amer2	_TVPLVDSEGGS[Phospho (STY)]GR_.2	Q8CCJ4_S340_M1_Amer2	10.5270	10.3860	11.0214	3.5937	10.1140	3.3811	10.8483	10.1659	10.4377	10.5224	0.7786	-0.3610	0.2472	0.6069
Amer2	_IC[Carbamidomethyl (C)]LEAGS[Phospho (STY)]PTGSGDQSSR_.2	Q8CCJ4_S280_M1_Amer2	7.7249	7.7841	7.9321	8.7392	8.0056	7.8383	8.1917	8.0684	8.1763	8.2849	0.9785	-0.0314	0.3902	0.4087
Amer2	_ASAVPDPSSVDPPS[Phospho (STY)]DPSADR_.2	Q8CCJ4_S356_M1_Amer2	5.3116	6.8948	6.3357	7.6482	6.7746	7.3047	7.4545	7.0704	7.0857	7.0523	0.9366	-0.0945	0.3172	0.4986
Amer2	_DMSS[Phospho (STY)]VKR_.2	Q8CCJ4_S488_M1_Amer2	3.9356	3.1910	3.6693	3.7238	3.0793	3.5453	3.4384	4.3947	4.0433	3.6908	0.9055	-0.1432	0.1564	0.8058
Amer2	_AAGPGNLVLPGS[Phospho (STY)]LTAS[Phospho (STY)]LEC[Carbamidomethyl (C)]VKEEPPR_.3	Q8CCJ4_S223_M2_Amer2	3.2388	4.1037	4.4733	4.0170	3.9250	3.8045	4.1653	2.9841	3.2832	3.6943	1.1119	0.1531	0.2101	0.6775
Amer2	_RPDS[Phospho (STY)]PGQDASR_.2	Q8CCJ4_S244_M1_Amer2	9.8472	10.2375	10.7440	10.7506	10.1006	10.5388	10.2180	10.2660	10.3453	10.2561	1.0096	0.0138	0.6165	0.2101
Amer2	_S[Phospho (STY)]GGTMPSIFGVK_.2	Q8CCJ4_S71_M1_Amer2	3.9896	3.2450	3.1671	7.3887	3.1333	3.4913	3.3845	4.4487	4.0973	3.6368	1.0456	0.0643	0.8429	0.0742
Amer2	_HAAGEPAGGEQAPAS[Phospho (STY)]AESAPER_.3	Q8CCJ4_S266_M1_Amer2	3.3997	3.8264	4.0865	4.5892	3.1542	4.4107	4.3039	3.5293	3.1779	3.0554	1.1122	0.1534	0.3087	0.5105
Bcas3	_VKS[Phospho (STY)]PPQISPSK_.2	Q8CCN5_S565_M1_Bcas3	3.3477	6.6483	4.5822	4.1260	3.8160	3.9135	4.2743	4.3760	3.1742	8.0159	0.8882	-0.1710	0.6034	0.2194
Bcas3	_EGS[Phospho (STY)]IETLSNSSGSTSGSIPR_.2	Q8CCN5_S886_M1_Bcas3	5.9197	5.6432	4.1771	6.4198	3.5872	5.7973	5.4207	6.0531	5.4887	5.5582	0.9338	-0.0988	0.5376	0.2695
Bcas3	_DVVGS[Phospho (STY)]GTELQR_.2	Q8CCN5_S877_M1_Bcas3	2.9896	3.7948	3.2813	4.3259	5.9882	4.1134	4.4741	4.1761	6.9985	4.0032	0.8309	-0.2673	0.3148	0.5019
Bcas3	_LADAM[Oxidation (M)]AES[Phospho (STY)]PSR_.2	#N/A	3.5538	3.8406	3.4300	6.1515	3.7067	3.6250	3.7029	4.2312	3.8798	3.8997	1.0313	0.0444	0.8247	0.0837
Bcas3	_LTSQDS[Phospho (STY)]YNNFTNNNPGNPR_.2	Q8CCN5_S503_M1_Bcas3	5.3349	5.7613	5.0611	5.5176	4.0119	5.5525	3.5247	3.7722	4.9083	5.5848	1.1707	0.2273	0.1676	0.7758
Bcas3	_LADAM[Oxidation (M)]AES[Phospho (STY)]PSRDVVGSGTELQR_.3	#N/A	3.9531	3.2731	3.1391	3.6417	3.0788	3.1499	3.3564	2.9759	4.1253	6.6705	0.7876	-0.3444	0.2184	0.6607
Bcas3	_HGSY[Phospho (STY)]DSLASDHSGQEDEEWLSQVEIVTHTGPHR_.5	Q8CCN5_Y707_M1_Bcas3	3.9583	5.4974	3.6919	3.2357	3.1020	5.5119	3.4158	5.7348	4.0645	4.4138	0.9453	-0.0811	0.7297	0.1368
Bcas3	_C[Carbamidomethyl (C)]SPVPGLS[Phospho (STY)]SPSGS[Phospho (STY)]PLHGK_.2	Q8CCN5_S486_M2_Bcas3	3.3605	3.8657	4.0473	4.5499	3.7540	4.3715	4.2646	3.5685	3.2171	3.0161	1.1350	0.1827	0.1628	0.7884
Pabpn1	_QMNMS[Phospho (STY)]PPPGNAGPVIMSLEEK_.3	Q8CCS6_S146_M1_Pabpn1	3.6524	3.5738	4.3392	3.3410	4.2789	3.4506	4.5565	3.2767	4.4261	3.3080	0.9694	-0.0449	0.7431	0.1290
Tceal5	_RPAEDYVPR_.2	#N/A	7.7950	7.1150	7.8185	7.0134	7.5430	7.6657	7.1137	7.7112	7.7829	7.0677	1.0098	0.0141	0.7616	0.1182
Tceal5	_DAQDALVPR_.2	#N/A	6.1790	6.4913	5.7429	5.7313	7.2226	6.3218	5.7937	6.2550	6.2102	5.8526	1.0421	0.0595	0.4195	0.3773
Tceal5	_TDRGTDDS[Phospho (STY)]PKNSQEDLQDR_.3	#N/A	5.8178	6.6729	7.4968	6.9953	6.9808	6.6637	6.4481	4.4658	6.3661	6.3424	1.0571	0.0801	0.2358	0.6274
Tceal5	_GTDDS[Phospho (STY)]PKNSQEDLQDR_.2	#N/A	10.3275	10.5408	11.4532	11.1425	10.7469	10.6108	10.7105	10.5611	10.6555	10.5753	1.0168	0.0240	0.4318	0.3648
Tceal5	_M[Oxidation (M)]GGFHWVPR_.2	#N/A	5.3979	6.9541	7.1118	5.5072	6.4969	5.7107	4.2262	6.5777	3.5155	6.1705	1.2097	0.2746	0.1644	0.7841
Tceal5	_ADEDKS[Phospho (STY)]DAEGKPAR_.2	Q8CCT4_S35_M1_Tceal5	3.8941	3.3933	3.5607	4.6053	2.9708	3.6538	1.5047	3.2384	4.7281	2.9114	1.1887	0.2493	0.3536	0.4515
Inpp5f	_S[Phospho (STY)]PSADSIHTR_.2	Q8CDA1_S941_M1_Inpp5f	7.1521	3.6830	3.1948	7.5228	5.8026	7.0684	7.4516	7.4223	7.4605	7.2839	0.7748	-0.3681	0.1204	0.9193
Inpp5f	_GLES[Phospho (STY)]PLKK_.2	Q8CDA1_S936_M1_Inpp5f	7.3093	6.6819	7.7791	7.3679	6.4193	6.9231	7.4590	7.0659	6.6910	6.8167	1.0103	0.0147	0.8088	0.0922
Inpp5f	_IIPS[Phospho (STY)]PDDSK_.2	Q8CDA1_S123_M1_Inpp5f	7.6682	3.3002	7.1266	3.6146	3.1885	7.2865	3.3293	7.3579	7.1542	3.5816	1.0016	0.0022	0.9954	0.0020
Inpp5f	_VQKS[Phospho (STY)]PAEPEAVNEIQQNELK_.3	Q8CDA1_S1103_M1_Inpp5f	5.2861	5.6471	6.1939	5.8170	4.4102	5.2790	6.2785	5.7486	5.3945	5.8174	0.9362	-0.0952	0.3112	0.5070
Inpp5f	_SQS[Phospho (STY)]ASSIDVSTHAPSEAAAGPGSELGK_.3	Q8CDA1_S908_M1_Inpp5f	5.5868	3.2400	3.1216	5.9823	3.0613	3.1673	3.3389	2.9934	4.1428	5.0038	1.0405	0.0573	0.8463	0.0725
Inpp5f	_GLES[Phospho (STY)]PLKKS[Phospho (STY)]PSADSIHTR_.3	#N/A	5.8577	5.8017	6.0872	6.3027	5.1025	5.9921	6.1358	5.6279	5.2906	5.3127	1.0475	0.0669	0.3388	0.4700
Inpp5f	_KSPS[Phospho (STY)]ADSIHTR_.2	#N/A	5.9300	6.1238	7.0619	6.1709	5.6876	5.9014	6.4674	5.9621	5.6088	5.3569	1.0508	0.0715	0.3667	0.4357
Vcpip1	_ESS[Phospho (STY)]PSHGLLK_.2	#N/A	8.7381	4.1854	9.3388	9.2592	8.0673	8.8006	3.9449	3.8882	8.6139	8.8965	1.2729	0.3481	0.2789	0.5545
Vcpip1	_DGPSS[Phospho (STY)]APATPTK_.2	Q8CDG3_S756_M1_Vcpip1	9.9246	9.7675	9.9101	10.3574	8.6102	9.8049	9.8860	9.5411	9.6607	9.8050	1.0006	0.0009	0.9850	0.0066
Vcpip1	_TVS[Phospho (STY)]PSTIR_.2	Q8CDG3_S746_M1_Vcpip1	9.3893	3.0939	9.4515	9.4852	8.4490	9.1635	8.9390	9.1481	8.9458	9.2584	0.9007	-0.1509	0.5036	0.2979
Vcpip1	_APYS[Phospho (STY)]PTTSK_.2	Q8CDG3_S767_M1_Vcpip1	3.7652	3.5472	3.9601	3.4100	7.1002	7.9107	7.7810	3.2501	8.0214	3.3770	0.8826	-0.1802	0.6650	0.1772
Vcpip1	_S[Phospho (STY)]RESS[Phospho (STY)]PSHGLLK_.2	Q8CDG3_S993_M2_Vcpip1	3.5576	3.8120	5.0947	4.7311	3.3806	3.2780	4.0330	3.7433	4.0508	3.4813	1.0388	0.0549	0.7205	0.1424
Fam160b1	_TS[Phospho (STY)]PDHPKNDGKTEVHK_.3	Q8CDM8_S552_M1_Fam160b1	4.1657	3.4211	3.8994	3.4936	3.3094	3.3152	3.2083	3.1240	4.2734	3.1204	1.0493	0.0694	0.5702	0.2440
Txnl1	_QHLENDPGS[Phospho (STY)]NEDADIPK_.2	#N/A	5.8977	5.6385	5.9324	6.2271	4.6962	5.4702	5.8826	5.4683	5.6881	5.9522	0.9819	-0.0264	0.7242	0.1401
Txnl1	_IKQHLENDPGS[Phospho (STY)]NEDADIPK_.3	#N/A	5.4699	6.0386	6.6233	6.7539	5.9938	6.5399	6.1197	6.0996	6.3088	6.4361	0.9993	-0.0010	0.9866	0.0059
Txnl1	_FQNVNSVTLFVQSNQGEEETTR_.3	#N/A	3.5304	4.2867	3.6263	4.1289	4.1750	3.6022	3.8436	3.9895	3.6381	4.5422	0.9721	-0.0409	0.6418	0.1926
Hectd2	_DLS[Phospho (STY)]PGAPPAVAAAAPEER_.2	Q8CDU6_S9_M1_Hectd2	6.4260	3.6135	7.3536	6.5078	6.0001	6.9230	3.1546	6.6346	5.9695	4.1845	1.2310	0.2998	0.2478	0.6058
Scyl2	_RGS[Phospho (STY)]LTLEEK_.2	#N/A	5.9949	3.5353	4.3776	6.2453	3.4236	3.2010	5.8920	6.0410	5.8679	3.3465	0.8442	-0.2444	0.3645	0.4383
Scyl2	_GS[Phospho (STY)]LTLEEK_.2	#N/A	4.2467	3.5021	3.9803	3.4127	3.3904	3.2342	3.1274	3.2049	4.3544	3.3797	1.0316	0.0449	0.7223	0.1413
Nedd4l	_TS[Phospho (STY)]PQELSEEVSR_.2	Q8CFI0_S332_M1_Nedd4l	3.8241	3.4058	4.5109	3.5127	6.5498	3.3343	3.2274	3.1086	7.8938	3.4797	0.9463	-0.0796	0.8359	0.0779
Nedd4l	_DT[Phospho (STY)]LSNPQSPQPSPYNSPKPQHK_.3	Q8CFI0_T502_M1_Nedd4l	4.8421	4.0804	3.8746	3.8727	3.4948	3.6853	4.6550	4.3182	3.9635	4.3642	0.9190	-0.1218	0.2214	0.6549
Nedd4l	_SLS[Phospho (STY)]SPTVTLSAPLEGAKDS[Phospho (STY)]PIRR_.3	Q8CFI0_S477_M2_Nedd4l	3.1171	3.9223	3.8039	4.1984	3.7436	3.9858	4.3466	5.7648	3.1019	4.2736	0.8681	-0.2040	0.2548	0.5938
Nedd4l	_DTLS[Phospho (STY)]NPQSPQPSPYNSPK_.2	#N/A	3.7121	2.9675	3.4457	4.4903	2.8558	3.7688	3.1378	4.0117	4.3107	4.1676	0.9061	-0.1423	0.3500	0.4560
Mef2c	_NS[Phospho (STY)]PGLLVSPGNLNK_.2	Q8CFN5_S222_M1_Mef2c	8.0764	8.8347	8.7315	9.7605	7.8158	8.1010	8.7522	8.5966	8.4974	3.7858	1.1546	0.2074	0.2951	0.5300
Mef2c	_PSPDERES[Phospho (STY)]PSVKR_.3	Q8CFN5_S460_M1_Mef2c	2.9155	3.6717	4.1500	3.6937	3.5600	3.0646	3.8420	3.3074	3.1044	3.3710	1.0302	0.0430	0.7042	0.1523
Mef2c	_S[Phospho (STY)]PPPMNLGMNNR_.2	#N/A	7.6290	4.0240	3.4922	7.9401	2.9023	3.7290	3.1843	3.9719	4.2709	4.2141	1.2666	0.3410	0.3924	0.4062
Nrn1	VC[Carbamidomethyl (C)]TYWEDFHSC[Carbamidomethyl (C)]TVTALTDC[Carbamidomethyl (C)]QEGAK	#N/A	2.9514	3.8499	4.1859	5.5502	3.1878	3.0287	5.1700	3.8281	4.7999	4.1653	0.8445	-0.2439	0.2470	0.6073
Akr7a2	_ALQTTYGTNAPR_.2	#N/A	3.5407	4.8838	3.2744	5.7291	6.2496	3.9401	5.6113	5.6040	5.4338	5.2517	0.8407	-0.2503	0.1998	0.6995
Akr7a2	_VDLFYLHAPDHSTPVEETLR_.3	#N/A	3.9570	3.3504	4.1527	3.7871	3.5861	3.8908	3.4009	4.0071	4.2788	3.3198	1.0095	0.0137	0.8828	0.0541
Tp53bp2	_IPRPLS[Phospho (STY)]PTK_.2	Q8CG79_S697_M1_Tp53bp2	6.3929	6.6400	3.6557	4.4026	4.0690	6.3631	7.0263	3.9215	3.9750	6.4350	0.9840	-0.0233	0.9299	0.0316
Tp53bp2	_KNQS[Phospho (STY)]SEDILR_.2	Q8CG79_S479_M1_Tp53bp2	3.2157	4.0209	3.9025	5.4931	3.8422	3.8873	4.1198	4.8242	3.3619	4.3721	0.9738	-0.0383	0.8167	0.0879
Ppm1f	_IAEELVAVAR_.2	#N/A	3.7436	3.2496	3.4773	4.5219	2.8874	3.7373	3.0601	3.9801	3.4775	2.9789	1.0678	0.0946	0.5154	0.2879
Tns2	_GPLDGSPY[Phospho (STY)]AQVQR_.2	Q8CGB6_Y483_M1_Tns2	6.4727	4.3382	3.3156	5.8739	4.2265	3.8989	3.7921	6.1093	4.4408	4.0375	1.0202	0.0288	0.9043	0.0437
Tns2	_APHSC[Carbamidomethyl (C)]GS[Phospho (STY)]PSEGR_.2	Q8CGB6_S811_M1_Tns2	4.3100	3.6144	3.4960	3.9987	3.4357	4.2938	3.7133	4.1198	5.5284	3.9657	0.8906	-0.1671	0.2481	0.6054
Tns2	_HLPGS[Phospho (STY)]GQQPSPPAR_.2	Q8CGB6_S1082_M1_Tns2	7.7035	7.5192	3.6606	3.8917	3.6427	3.6999	8.0197	3.7228	3.6786	3.7519	1.0472	0.0666	0.8693	0.0608

Tns2	_SFS[Phospho (STY)]LDPLMER_.2	Q8CGB6_S120_M1_Tns2	4.2011	3.5055	3.3871	5.3388	3.3268	4.4027	3.2627	3.8868	4.1858	4.2925	1.0307	0.0437	0.7916	0.1015
Tns2	_GYPSPGAHS[Phospho (STY)]PR_.2	Q8CGB6_S825_M1_Tns2	2.9669	4.3755	4.2015	3.7452	4.1968	3.5327	3.8935	3.2559	6.4807	3.4225	0.8999	-0.1522	0.5317	0.2743
Tns2	_WDS[Phospho (STY)]YENFNQHHEDSVDGALAHTR_.4	Q8CGB6_S455_M1_Tns2	3.8910	3.1954	3.8807	3.4245	3.0167	3.2120	3.5728	3.5767	3.8757	3.1018	0.9731	-0.0394	0.6874	0.1628
Rgs12	_TLPDSQQVPSS[Phospho (STY)]PASK_.2	Q8CGE9_S850_M1_Rgs12	11.7648	3.3217	3.7544	12.3756	3.1430	11.8478	12.3214	11.9155	11.9102	11.8655	0.6416	-0.6403	0.1117	0.9520
Rgs12	_S[Phospho (STY)]LNEDVGEEDSEKK_.2	#N/A	6.2191	6.2413	6.3824	7.2897	5.8978	6.4087	6.8163	6.6749	6.5866	6.4157	0.9673	-0.0480	0.4084	0.3890
Rgs12	_S[Phospho (STY)]LNEDVGEEDSEK_.2	#N/A	8.7220	8.4119	8.8249	9.0508	4.1748	8.7854	8.9221	8.4470	8.6316	8.6197	0.9237	-0.1145	0.5128	0.2900
Rgs12	_QNSAVNSS[Phospho (STY)]PR_.2	Q8CGE9_S1130_M1_Rgs12	5.3938	4.8584	5.3109	3.6795	4.6086	4.3316	4.6018	5.0759	5.2789	4.0172	0.9902	-0.0141	0.9098	0.0410
Rgs12	_ESQGSVSS[Phospho (STY)]AGSLDLSEAC[Carbamidomethyl (C)]R_.2	Q8CGE9_S940_M1_Rgs12	3.9107	3.4317	3.6444	3.1882	5.5915	3.5702	3.3364	3.8130	4.1121	4.3663	0.9955	-0.0065	0.9715	0.0125
Rgs12	_LPAGSSELALSS[Phospho (STY)]PPPVK_.2	Q8CGE9_S1233_M1_Rgs12	4.3157	3.0267	4.0494	3.5931	3.4594	3.3806	3.7414	3.4081	3.7071	3.2704	1.0299	0.0426	0.6907	0.1607
Rgs12	_SKS[Phospho (STY)]GRS[Phospho (STY)]LNEDVGEEDSEK_.2	Q8CGE9_S876_M2_Rgs12	3.0385	2.6060	4.2730	3.8168	3.6830	2.9416	3.9650	3.1844	3.4835	3.4940	0.9608	-0.0578	0.6992	0.1554
Rgs12	_HSISS[Phospho (STY)]DHSNVSTPK_.2	Q8CGE9_S859_M1_Rgs12	3.4135	3.8127	4.1003	4.6029	3.7010	4.4244	4.3176	3.5156	3.1642	3.0691	1.1401	0.1891	0.1644	0.7842
Wdr47	_RIS[Phospho (STY)]DLGNK_.2	Q8CGF6_S332_M1_Wdr47	5.5088	3.1973	6.6970	3.4226	5.3892	5.9018	5.5785	6.6720	3.8777	6.2402	0.8976	-0.1559	0.5265	0.2786
Wdr47	_LSPYPSS[Phospho (STY)]PMR_.2	Q8CGF6_S297_M1_Wdr47	4.0002	3.3045	3.1861	3.3153	3.1258	3.1028	3.4636	4.4297	4.0783	3.6558	0.8547	-0.2265	0.0471	1.3272
Wdr47	_RPQS[Phospho (STY)]ADAYM[Oxidation (M)]TR_.2	#N/A	4.6846	4.7959	5.4603	3.3865	3.4041	4.5502	4.0155	4.9392	3.4674	3.2091	1.1209	0.1647	0.3887	0.4104
Wdr47	_YQQHLEQK_.2	#N/A	3.0125	3.6305	3.0028	4.4902	2.8556	4.2777	3.1376	4.0118	4.3109	4.1675	0.9073	-0.1403	0.4096	0.3877
Fam193a	_LILASS[Phospho (STY)]PQPK_.2	Q8CGI1_S1151_M1_Fam193a	6.4565	3.3539	7.1158	3.5609	2.9980	3.2307	3.2756	3.0567	4.2061	3.5279	1.2662	0.3405	0.3551	0.4496
Lonp1	_VLEFIAVSQLR_.2	#N/A	5.4747	3.8082	4.2183	4.6740	4.4314	5.2308	4.7506	4.0906	4.9554	4.2736	1.0270	0.0384	0.7414	0.1299
Lonp1	_LALLDNHSSEFNVTR_.3	#N/A	3.1850	3.9412	4.4195	3.9633	3.8295	2.9099	4.1115	2.2241	3.3370	3.6405	1.1141	0.1559	0.3985	0.3996
Gnal	_VLTSGIFETR_.2	#N/A	5.9344	5.9330	5.5605	5.5285	4.7687	5.5697	5.4381	6.0519	5.7532	3.2649	1.0823	0.1141	0.4614	0.3359
Gnal	_ESLDLFESIWNNR_.2	#N/A	6.1032	5.8657	6.1246	5.6835	4.0671	2.9507	6.0193	5.8205	5.1066	5.7641	0.9040	-0.1456	0.4521	0.3448
Gnal	_IDSVSLVDYTPTDQDLLR_.2	#N/A	4.0038	4.5618	4.9312	4.7560	3.3612	4.3730	5.1289	4.5583	4.1458	3.1955	1.0174	0.0249	0.8691	0.0609
Gnal	_SNEYQLIDC[Carbamidomethyl (C)]AQYFLER_.2	#N/A	3.1065	2.4630	3.7933	4.2090	3.7330	3.9965	4.3573	3.8225	3.4711	3.6934	0.9255	-0.1117	0.4537	0.3432
Adgrb2	_LS[Phospho (STY)]LDEDEEPK_.2	Q8CGM1_S1269_M1_Adgrb2	6.4898	5.2580	4.5281	5.6838	5.0722	5.8569	5.9989	6.5719	6.4551	6.4169	0.8618	-0.2146	0.0412	1.3855
Bcor	_TDVLC[Carbamidomethyl (C)]TGEDEDC[Carbamidomethyl (C)]QAAS[Phospho (STY)]PLQK_.2	Q8CGN4_S1348_M1_Bcor	4.3107	2.8338	4.0444	4.6801	4.0890	3.7281	3.7364	4.0152	3.5520	4.0131	1.0309	0.0440	0.7318	0.1356
Bcor	_DGGS[Phospho (STY)]PPLLEK_.2	Q8CGN4_S423_M1_Bcor	4.1174	3.4218	3.3034	3.8061	3.2431	5.6598	6.2247	5.0898	3.9610	3.7731	0.8243	-0.2788	0.2318	0.6349
H2bu1	_PDPS[Phospho (STY)]KSAPAPK_.2	Q8CGP0_S5_M1_H2bu1	4.3867	3.6911	3.3850	4.4296	3.5124	4.2171	3.0770	4.0724	4.3715	4.1069	1.0077	0.0110	0.9289	0.0321
H2bu1	_ESYSIYVYK_.2	#N/A	3.6908	2.9463	3.4245	3.9685	4.7501	5.6550	3.6832	4.9324	4.9435	4.8522	0.8848	-0.1766	0.3683	0.4338
Calcoco1	_LPSYGLC[Carbamidomethyl (C)]ESGNTSSS[Phospho (STY)]PPGPR_.2	Q8CGU1_S563_M1_Calcoco1	3.8828	6.5538	3.7363	4.2389	3.4306	3.8454	6.7344	6.9254	6.4796	6.7873	0.6360	-0.6529	0.0031	2.5037
Tph2	_RRS[Phospho (STY)]S[Phospho (STY)]EVEIFVDC[Carbamidomethyl (C)]EC[Carbamidomethyl (C)]GK_.3	Q8CGV2_S101_M2_Tph2	3.0736	3.8298	4.3081	5.4546	4.1982	4.4073	4.3005	5.5691	3.4484	3.5291	1.0000	0.0001	0.9998	0.0001
Tph2	_GLS[Phospho (STY)]LDSAVPEDHQLLGSLTQNK_.3	Q8CGV2_S19_M1_Tph2	3.1464	5.0456	4.3809	6.1895	3.7910	4.3345	4.2276	3.0765	4.9196	3.6020	1.1327	0.1797	0.4232	0.3734
Ogt	_DSGNIPEAIASYR_.2	#N/A	6.5613	6.7108	6.6302	6.8228	6.7186	6.5842	6.7432	6.5253	6.2938	6.6895	1.0165	0.0236	0.2834	0.5476
Ogt	_AIQINPAFADAHSNLASIHK_.3	#N/A	6.0753	5.6054	6.0290	5.8777	5.2517	5.5050	6.1534	5.6540	6.0643	4.9074	1.0051	0.0074	0.9191	0.0366
Ogt	_SDLGNLLK_.2	#N/A	4.0019	2.8954	4.2717	3.2087	3.4960	2.9429	2.8877	3.1857	4.4050	4.7694	0.9101	-0.1358	0.4806	0.3182
Sugp1	_DIDASPS[Phospho (STY)]PLSVQDLK_.2	Q8CH02_S409_M1_Sugp1	4.3589	4.3681	3.5449	4.0476	3.4846	4.2449	3.7622	6.6363	6.6052	6.6940	0.6765	-0.5637	0.0133	1.8766
Sugp1	_ALQQHQHGYDS[Phospho (STY)]DEEVDSELGTWEHQLR_.4	Q8CH02_S483_M1_Sugp1	3.5025	3.9520	4.0098	3.8175	4.1376	3.8129	4.2272	3.6548	3.3034	3.7845	1.0346	0.0491	0.5094	0.2929
Sugp2	_GRS[Phospho (STY)]LNIADQEGTLLGK_.3	#N/A	6.6271	6.6753	7.0797	7.4936	5.6188	6.6604	6.8794	7.0029	6.9996	6.8813	0.9642	-0.0526	0.4600	0.3372
Sltm	_DVQDAIAQS[Phospho (STY)]PEK_.2	#N/A	10.7985	10.4744	10.8591	11.3264	10.4270	10.5723	11.1675	10.4505	10.6167	10.7569	0.9995	-0.0007	0.9817	0.0080
Sltm	_S[Phospho (STY)]PGHM[Oxidation (M)]VILNQTK_.2	#N/A	6.7127	6.0647	6.3797	7.8992	5.8309	6.3758	6.8898	6.6266	6.2362	6.5581	0.9949	-0.0074	0.9325	0.0304
Sltm	_DVQDAIAQS[Phospho (STY)]PEKEAK_.2	#N/A	10.1411	10.3247	11.0040	11.0554	9.9179	10.2816	10.7974	10.2928	10.1287	10.3108	1.0069	0.0099	0.7935	0.1005
Sltm	_ISS[Phospho (STY)]KS[Phospho (STY)]PGHM[Oxidation (M)]VILNQTK_.3	#N/A	3.3629	3.5864	4.2119	5.0914	3.4494	3.5265	3.7914	3.6251	3.6861	3.7468	1.0428	0.0605	0.6551	0.1837
Sltm	_AGAGMITQHSSTAS[Phospho (STY)]PVNR_.2	#N/A	3.9257	3.2729	4.1961	3.8286	3.0942	3.1406	3.3406	3.4423	4.1282	4.0504	0.9561	-0.0647	0.5830	0.2343
Trim32	_EM[Oxidation (M)]DM[Oxidation (M)]S[Phospho (STY)]PEEVAPSPR_.2	#N/A	9.1971	11.0990	11.7625	10.9478	10.5967	11.2107	11.5065	11.0342	11.1154	11.0511	0.9665	-0.0492	0.4338	0.3627
Trim32	_EMDMSPEEVAPS[Phospho (STY)]PRAS[Phospho (STY)]PAK_.2	#N/A	3.1180	3.9232	4.2040	4.1658	3.7445	3.9903	4.1057	7.7085	3.3801	4.0005	0.8039	-0.3149	0.2752	0.5604
Nav1	_SIGS[Phospho (STY)]PESTPK_.2	Q8CH77_S762_M1_Nav1	7.4268	7.6616	3.9608	3.5046	6.8426	3.2920	7.3038	7.5907	7.7033	7.7460	0.7182	-0.4776	0.0781	1.1072
Nav1	_S[Phospho (STY)]LTNLSFLTDSEKK_.2	#N/A	3.1744	4.8764	3.8612	6.4721	4.7362	3.9285	4.2893	5.9548	5.6326	3.8183	0.9156	-0.1272	0.5761	0.2395
Nav1	_SS[Phospho (STY)]PLSWR_.2	Q8CH77_S315_M1_Nav1	5.6681	3.0670	3.5452	3.8478	2.9553	3.6693	3.5625	3.9122	3.9193	5.5638	0.8945	-0.1609	0.4873	0.3122
Nav1	_LYHLPPPS[Phospho (STY)]VGPHSTASPPEDR_.3	Q8CH77_S1816_M1_Nav1	3.1522	4.0739	3.8390	4.3417	3.7788	4.1632	4.0564	3.7768	3.4254	4.3087	0.9999	-0.0001	0.9990	0.0004
Nav1	_S[Phospho (STY)]LTNLSFLTDSEK_.2	#N/A	3.2732	3.7294	3.9600	5.3684	4.9964	4.7699	4.4711	5.1359	4.7961	3.7242	0.9598	-0.0592	0.7139	0.1464
Nav1	_RSS[Phospho (STY)]TIVLR_.2	Q8CH77_S455_M1_Nav1	3.7177	3.5084	4.4045	5.5503	2.8434	5.4284	5.8497	3.2113	4.3607	3.3734	1.0103	0.0148	0.9543	0.0203
Nav1	_VNSNS[Phospho (STY)]LDLPSSSDTHASK_.3	#N/A	5.1676	4.6762	3.7778	5.8351	3.7017	4.8842	5.2534	4.9391	3.6823	3.4278	1.0805	0.1117	0.5456	0.2631
Nav1	_LELVES[Phospho (STY)]LDSDEVDLK_.2	Q8CH77_S380_M1_Nav1	3.0944	3.8507	4.3289	4.5649	3.7390	4.3864	4.2796	5.9919	3.2021	3.5500	0.9385	-0.0916	0.6549	0.1838
Nav1	_SVQPEVELS[Phospho (STY)]GGSGSGGDEGADESR_.2	Q8CH77_S16_M1_Nav1	3.3158	4.1209	4.0026	4.0306	3.9423	3.8181	4.1789	3.5544	4.6607	4.7463	0.9035	-0.1463	0.1536	0.8135
Nav1	_SLAESGLNWFS[Phospho (STY)]ESEEK_.2	Q8CH77_S477_M1_Nav1	3.1124	4.2300	4.3469	5.0980	3.7570	3.6782	4.0390	3.1105	3.4095	3.5680	1.1431	0.1929	0.2202	0.6572
Nav1	_APEAAVS[Phospho (STY)]DDGKS[Phospho (STY)]DDELLSSK_.2	#N/A	3.4634	3.8948	4.3170	3.7909	3.7162	3.6032	3.9806	3.2877	3.6951	3.3781	1.0592	0.0830	0.3098	0.5089
Nav1	_ERPES[Phospho (STY)]C[Carbamidomethyl (C)]DDASK_.2	Q8CH77_S510_M1_Nav1	3.5648	3.7776	3.2985	4.3431	3.5989	4.1306	4.4914	4.1589	2.9571	4.0204	0.9689	-0.0455	0.7213	0.1419
Nav1	_SGYMS[Phospho (STY)]DSDLMGK_.2	Q8CH77_S394_M1_Nav1	4.2292	3.1132	3.9629	3.5066	2.9345	3.2941	3.6549	3.4945	3.7936	3.1839	0.9929	-0.0102	0.9301	0.0315
Nav1	_SLAESGLNWFS[Phospho (STY)]ES[Phospho (STY)]EEKTPK_.3	Q8CH77_S477_M2_Nav1	3.6654	3.6770	3.3990	4.4437	3.4984	4.2311	3.0911	4.0584	4.3574	4.1209	0.9775	-0.0328	0.7827	0.1064
Synj1	_EFGGVGAPPS[Phospho (STY)]PGVAR_.2	Q8CHC4_S1147_M1_Synj1	10.7606	10.5021	11.0495	10.1609	8.8541	10.2442	10.9162	10.3763	10.2582	10.4971	0.9762	-0.0347	0.5552	0.2556
Synj1	_TNC[Carbamidomethyl (C)]LDC[Carbamidomethyl (C)]LDR_.2	#N/A	7.3609	6.8553	7.0584	6.6140	7.0591	6.9781	7.2133	7.1134	7.2145	6.8679	0.9839	-0.0235	0.4425	0.3541
Synj1	_REIEAPKS[Phospho (STY)]PGTAR_.3	#N/A	5.1400	3.7965	7.3087	5.7987	3.9369	3.8859	4.8646	6.4111	3.6505	5.2083	0.9889	-0.0161	0.9489	0.0228
Synj1	_IYAGTGALEGK_.2	#N/A	6.5480	6.1167	6.2383	5.7137	6.0208	6.6412	6.4916	6.7373	6.9568	6.4861	0.9318	-0.1019	0.0497	1.3040
Synj1	_GFEANAPAFDR_.2	#N/A	7.4465	7.1996	7.0444	6.7137	6.3847	6.5323	7.2032	7.5199	6.8713	6.9742	0.9643	-0.0525	0.3155	0.5010
Synj1	_AGVISAPQSQAR_.2	#N/A	6.3825	6.6359	4.2145	6.2344	6.4783	6.5470	7.0667	7.0229	6.1979	6.5017	0.9081	-0.1390	0.2540	0.5952
Synj1	_IDLPNEEVK_.2	#N/A	6.5801	6.5879	7.1159	6.2770	5.9908	5.8566	5.9396	6.8780	6.3822	6.2648	1.0055	0.0080	0.9032	0.0442
Synj1	_QIVVNLLGSK_.2	#N/A	6.1090	5.6142	3.5129	5.9378	5.3882	5.2255	6.3775	6.1244	5.4278	5.2754	0.9132	-0.1309	0.3626	0.4405
Synj1	_EVIAVQGPPDGTVLVSIK_.2	#N/A	4.4526	3.5638	4.2695	5.1708	4.7990	4.4391	5.1676	5.3823	4.8993	5.1029	0.8659	-0.2077	0.0430	1.3667
Synj1	_QQNWDSLIAGDQLINQK_.2	#N/A	5.2259	4.2555	3.2329	4.2775	3.8469	5.0568	4.0914	4.6327	4.1215	4.6753	0.9853	-0.0213	0.8763	0.0573
Synj1	_TPGPPSSQGS[Phospho (STY)]PVDTQPAAQK_.2	#N/A	10.7350	10.8153	11.3315	11.0477	9.9018	10.7258	11.2980	10.8193	10.8090	10.7382	0.9857	-0.0208	0.5711	0.2433
Synj1	_EIEAPKS[Phospho (STY)]PGTAR_.2	#N/A	9.7665	10.1417	10.2361	10.1672	8.3216	9.8300	9.7748	9.9857	9.8402	9.8877	0.9870	-0.0189	0.7381	0.1319
Synj1	_VSEQTLQSASSK_.2	#N/A	4.1684	6.6989	6.6652	6.5039	6.5592	6.4038	6.8498	6.6338	4.2761	6.6263	1.0115	0.0165	0.9223	0.0351
Synj1	_SAEDLDLLNASFQDESK_.2	#N/A	5.9756	6.0503	6.1517	5.5963	5.8331	5.6257	6.6829	6.6976	6.4171	6.1085	0.9067	-0.1413	0.0053	2.2722
Synj1	_ERNEDFVEIAR_.3	#N/A	4.7352	4.8886	3.2355	4.4132	4.1318	5.0306	4.4284	5.3241	5.1129	3.9574	0.9363	-0.0950	0.4940	0.3063
Synj1	_NEDFVEIAR_.2	#N/A	5.6976	3.6190	5.9319	5.2005	5.6901	5.1616	3.7893	5.7197	5.7955	3.3183	1.1205	0.1642	0.4234	0.3732
Synj1	_VTSTEFISLR_.2	#N/A	4.6135	3.5230	3.4046	4.5977	3.3443	3.6160	3.9426	3.9131	4.2667	3.8743	0.9627	-0.0549	0.6439	0.1912
Synj1	_SSAQESTFFDDALIDELLR_.2	#N/A	3.3010	3.1314	3.7171	3.2608	3.1271	3.4975	2.2672	3.4896	4.0394	4.4390	0.9383	-0.0919	0.5902	0.2290
Synj1	_NQPS[Phospho (STY)]PQAGLAGPGPAGYGAAR_.2	Q8CHC4_S1175_M1_Synj1	7.0410	7.3238	3.3380	7.0658	3.2777	6.6299	7.2575	6.9194	3.9264	6.6855	0.9326	-0.1007	0.7269	0.1385
Synj1	_T[Phospho (STY)]SPC[Carbamidomethyl (C)]QS[Phospho (STY)]PTVPEYSAPSLPIRPSR_.3	Q8CHC4_S1053_M2_Synj1	3.5622	4.3185	3.2959	4.3405	4.2068	3.9187	3.8118	5.6766	2.9597	4.0178	0.9572	-0.0631	0.7340	0.1343
Synj1	_QEAIDVLLLGNTLNSDLADK_.2	#N/A	3.5302	3.5912	4.3218	4.1559	4.2615	3.1451	3.2331	2.8961	3.8651	3.2906	1.1545	0.2072	0.1168	0.9325
Synj1	_TPAWTDR_.2	#N/A	4.0927	3.3970	3.2787	3.2228	3.2184	3.0103	3.3711	4.4776	4.0315	4.4009	0.8279	-0.2724	0.0353	1.4528

Synj1	_HLEEEMS[Phospho (STY)]LEK_.2	Q8CHC4_S982_M1_Synj1	3.8638	3.3624	4.5506	3.5524	2.9895	3.2392	3.2671	3.0652	4.2147	3.5194	1.0217	0.0310	0.8304	0.0807
Synj1	_IDLPNEEVKELIR_.3	#N/A	4.2561	3.0863	3.9898	3.5335	2.9077	3.3210	3.6818	3.4676	2.5497	3.2108	1.0893	0.1234	0.4077	0.3897
Synj1	_FLDYGFFYFDGSEVQR_.2	#N/A	3.2241	4.1183	4.4586	4.0023	3.8686	3.7898	4.1506	2.9988	3.2979	3.6796	1.1072	0.1469	0.2239	0.6500
Rapgef2	_RSS[Phospho (STY)]FLNAK_.2	Q8CHG7_S960_M1_Rapgef2	10.1166	10.0321	10.5899	10.4950	3.6297	9.9977	10.4790	10.3144	10.2163	10.0566	0.8906	-0.1671	0.4412	0.3554
Rapgef2	_SETS[Phospho (STY)]PVAPR_.2	Q8CHG7_S1022_M1_Rapgef2	8.8598	8.6300	9.3393	8.7684	7.3508	8.5422	8.5527	8.5484	8.6563	8.6010	0.9991	-0.0013	0.9821	0.0078
Rapgef2	_HIPAALPVSGTLSSS[Phospho (STY)]NPDLLQSHHR_.4	Q8CHG7_S585_M1_Rapgef2	3.5902	8.6364	8.4792	4.3685	4.2348	3.8906	3.7838	4.1335	4.4325	4.0458	1.3499	0.4329	0.2693	0.5698
Rapgef2	_ILS[Phospho (STY)]LSEEGSLER_.2	Q8CHG7_S1088_M1_Rapgef2	3.2533	3.9729	3.9401	4.4427	3.8798	3.8497	4.1574	5.4893	5.6232	6.0397	0.7301	-0.4538	0.0051	2.2965
Rapgef2	_QAEDTISNASSQLSS[Phospho (STY)]PPTS[Phospho (STY)]PQSSPR_.3	#N/A	3.5016	6.8989	4.1884	7.0580	3.6956	6.7846	3.9980	3.5243	6.6771	3.6666	1.1988	0.2616	0.4259	0.3707
Rapgef2	_SSIVSNSS[Phospho (STY)]FDSVPVSLHDERR_.3	#N/A	3.4202	3.9222	3.1539	4.1985	4.0648	4.0607	4.3468	4.3035	3.1017	3.8758	0.9735	-0.0388	0.7487	0.1257
Rapgef2	_S[Phospho (STY)]LGSLSQGSANATVLDVAQTGGHK_.3	Q8CHG7_S930_M1_Rapgef2	4.0850	7.0801	7.2813	8.1267	3.2107	3.0179	3.3787	6.9614	6.3909	4.8047	1.0154	0.0221	0.9517	0.0215
Rapgef2	_SSIVSNSS[Phospho (STY)]FDSVPVSLHDER_.3	#N/A	3.4960	3.8464	3.2297	4.2743	3.6677	4.0618	4.4226	4.2277	3.0259	3.9516	0.9631	-0.0543	0.6561	0.1830
Rapgef2	_NNM[Oxidation (M)]ETETLC[Carbamidomethyl (C)]S[Phospho (STY)]DEDAQELLR_.2	Q8CHG7_S698_M1_Rapgef2	3.9345	3.2917	3.1204	4.3544	3.1800	3.4446	3.3378	4.5247	4.1440	3.5901	0.9115	-0.1336	0.3227	0.4912
Septin8	_EFLSELQR_.2	#N/A	7.8132	7.6738	7.5072	7.1507	7.3174	7.2581	7.6176	7.7394	7.3185	7.2725	0.9955	-0.0065	0.8378	0.0769
Septin8	_ELEEETNAFNC[Carbamidomethyl (C)]R_.2	#N/A	6.5738	6.1575	5.8648	6.5764	6.5082	6.6590	6.7700	6.1144	6.9841	7.0305	0.9502	-0.0737	0.1844	0.7342
Septin8	_SLFDYHDTR_.2	#N/A	4.0653	6.3444	6.6830	6.7015	6.3269	6.4120	6.7820	6.5510	6.4688	6.7461	0.9174	-0.1244	0.3194	0.4957
Septin8	_SVTQGFSFNILC[Carbamidomethyl (C)]VGETGIGK_.2	#N/A	4.1554	4.2860	4.7866	5.6678	4.4641	4.4341	5.0998	4.6821	4.5552	4.0383	1.0084	0.0120	0.9099	0.0410
Septin8	_LTIVDAVGFGDQINK_.2	#N/A	5.3076	4.2451	5.3040	4.4585	4.2958	3.7358	4.0984	3.3551	4.9209	5.3668	1.0276	0.0393	0.8035	0.0950
Septin8	_[Acetyl (Protein N-term)]AATDLERVS[Phospho (STY)]NAEPEPR_.2	#N/A	9.4485	9.3499	9.7729	10.0314	9.3714	9.6344	9.9402	9.4080	9.5914	9.8667	0.9897	-0.0150	0.5661	0.2471
Septin8	_SLSLGGHVGFDSLPDQLVSK_.3	#N/A	5.7662	5.6923	5.7487	6.1032	5.4110	5.7135	6.0292	5.8718	5.8454	5.8489	0.9729	-0.0396	0.2128	0.6721
Ep400	_S[Phospho (STY)]SPVNRPSSATNK_.2	Q8CHI8_S720_M1_Ep400	3.2943	3.9318	3.9811	4.4838	3.8201	4.3053	4.1985	3.6347	3.2833	4.4508	1.0199	0.0285	0.8008	0.0965
Phc3	_ISQRDPS[Phospho (STY)]PESK_.2	Q8CHP6_S261_M1_Phc3	8.2411	8.8658	9.4810	9.1773	8.8636	8.9904	8.9639	2.9705	8.7713	8.8639	1.2089	0.2737	0.2286	0.6408
Phc3	_KGGS[Phospho (STY)]PGLESR_.2	Q8CHP6_S269_M1_Phc3	7.5891	7.6799	7.8383	7.7777	7.2414	3.3197	7.7078	3.5625	3.8616	7.6280	1.2140	0.2798	0.3686	0.4334
Phc3	_M[Oxidation (M)]DRT[Phospho (STY)]PPPPTLS[Phospho (STY)]PAAVTVGR_.3	#N/A	4.1728	6.1410	3.5809	7.2461	5.2092	3.2053	6.0230	6.0769	3.7536	5.9628	0.9032	-0.1470	0.5804	0.2363
Pgp	_FIFDC[Carbamidomethyl (C)]VSQEYGINPER_.2	#N/A	6.2038	6.2405	6.2944	6.2424	6.3546	6.0860	6.5086	5.8643	5.9037	5.6034	1.0447	0.0631	0.1303	0.8852
Pgp	_GETAVPGAPETLR_.2	#N/A	7.3475	7.3557	7.3090	7.2190	7.5105	7.2868	7.4724	7.0982	7.2657	7.5616	0.9985	-0.0022	0.9088	0.0415
Pgp	_FIAGTGC[Carbamidomethyl (C)]LVR_.2	#N/A	6.0106	2.6486	5.1175	6.3363	5.8097	6.1463	5.4730	5.8261	5.6003	5.8045	0.9417	-0.0867	0.6536	0.1847
Pgp	_TILTLTGVSSLEDVK_.2	#N/A	4.5925	3.7054	5.3569	3.5805	4.6390	5.1286	4.8686	4.1784	3.9779	4.9421	1.0019	0.0028	0.9838	0.0071
Pgp	_SNQESDC[Carbamidomethyl (C)]MFK_.2	#N/A	3.6148	4.3710	3.3484	4.3930	4.2593	3.8661	4.5413	4.1090	4.4080	4.0703	0.9284	-0.1072	0.2368	0.6256
Fbxo4	_GAGS[Phospho (STY)]PPPASDWGR_.2	Q8CHQ0_S11_M1_Fbxo4	10.8836	10.7585	11.3294	11.0951	10.3870	10.8379	11.0877	10.7585	10.8250	9.8975	1.0225	0.0321	0.3852	0.4144
Ngef	_TKFVS[Phospho (STY)]FTSR_.2	#N/A	3.1369	9.1400	9.7885	9.3682	7.2385	8.7210	9.2095	9.4112	9.0776	8.7368	0.8672	-0.2056	0.3711	0.4306
Ngef	_FVS[Phospho (STY)]FTSR_.2	#N/A	9.8935	9.1389	9.7820	9.3961	7.4978	9.3352	8.7022	9.7953	9.7147	9.6222	0.9699	-0.0441	0.5749	0.2404
Ngef	_MEDPQRS[Phospho (STY)]QNKDR_.2	#N/A	6.5000	5.2293	5.1592	7.1118	5.1794	5.0550	4.9330	5.2744	5.1426	5.5941	1.0897	0.1239	0.3372	0.4722
Ngef	_VEELEDQGQTLANVFILR_.2	#N/A	2.4205	4.2300	3.9672	4.4950	3.2597	4.0072	3.9003	4.2500	3.7686	3.0318	0.9979	-0.0030	0.9863	0.0060
Ngef	_MEDPQRS[Phospho (STY)]QNK_.2	#N/A	6.9147	4.9865	4.2915	7.0499	5.4885	4.4239	5.5896	5.1787	5.3510	5.6602	1.0149	0.0213	0.8997	0.0459
Epn2	_TTS[Phospho (STY)]PDLFESQSLTSASSK_.2	#N/A	8.7595	8.5718	8.7719	9.3596	8.2619	4.1133	8.9495	8.6020	8.8482	8.8188	0.9055	-0.1431	0.4225	0.3742
Epn2	_TTS[Phospho (STY)]PDLFESQSLTSASSKPSSAR_.3	#N/A	3.4875	8.6692	9.1885	10.4321	7.2924	8.3121	3.8865	8.9949	3.5952	7.4408	1.3207	0.4013	0.2686	0.5708
Epn2	_AGGS[Phospho (STY)]PASYHGSTSPR_.2	Q8CHU3_S192_M1_Epn2	6.6996	7.2123	7.3723	7.4756	6.1951	6.8395	7.2730	6.7462	6.8068	6.7050	1.0121	0.0173	0.7644	0.1167
Epn2	_GSS[Phospho (STY)]QPNLSTSYSEQEYGK_.2	Q8CHU3_S173_M1_Epn2	8.5338	3.5573	8.6666	9.1302	8.1972	8.9905	8.7669	3.5267	8.5351	8.8274	1.0583	0.0817	0.7798	0.1080
Epn2	_NSDPWAASQQPASNAGK_.2	#N/A	5.3576	3.0256	4.0505	3.5943	4.6005	3.2439	3.7426	5.1956	4.7693	3.2716	0.9373	-0.0934	0.6539	0.1845
Eif2b5	_AGS[Phospho (STY)]PQLDDIR_.2	Q8CHW4_S540_M1_Eif2b5	11.7225	12.0232	12.1408	12.0270	10.8728	11.7484	12.7037	12.1560	12.0421	11.9472	0.9626	-0.0550	0.1316	0.8808
Rftn2	_NSS[Phospho (STY)]PSYANPK_.2	Q8CHX7_S228_M1_Rftn2	2.9695	3.7258	4.2041	3.7478	3.6141	3.0105	3.8961	3.2533	3.5524	3.4251	1.0038	0.0055	0.9605	0.0175
Rftn2	_S[Phospho (STY)]LGLDTNASQAAGGR_.2	Q8CHX7_S429_M1_Rftn2	3.5160	4.2723	3.2497	4.1433	4.1606	3.9648	3.8580	5.4333	3.6237	5.8605	0.8276	-0.2731	0.1364	0.8650
Rftn2	_PVTWNSAAQT[Phospho (STY)]PERK_.3	#N/A	6.2779	5.2083	5.4509	6.5205	5.3724	5.2798	5.8465	6.3941	6.0747	5.8935	0.9393	-0.0903	0.2621	0.5816
Rftn2	_PVTWNSAAQT[Phospho (STY)]PER_.2	#N/A	6.9947	6.1787	6.9613	7.0021	5.5849	6.4340	6.9313	6.6769	6.4723	6.4957	0.9822	-0.0259	0.7088	0.1495
Rftn2	_EERPAQSDS[Phospho (STY)]FSGFSSSDSVLR_.3	Q8CHX7_S469_M1_Rftn2	3.7472	3.3717	3.8536	3.6340	3.4818	3.5357	4.1047	3.9303	3.4792	3.6010	0.9537	-0.0683	0.2646	0.5774
Gatad2a	_RPPS[Phospho (STY)]PDVIVLS[Phospho (STY)]DSEQPSSPR_.3	Q8CHY6_S103_M2_Gatad2a	4.3880	4.3390	3.5739	4.0766	4.2273	3.8981	3.7913	4.0419	3.6905	4.0436	1.0493	0.0695	0.2970	0.5272
Flywch1	_SHC[Carbamidomethyl (C)]HS[Phospho (STY)]PDIEGLQAR_.3	Q8CI03_S294_M1_Flywch1	4.0806	6.6977	7.3910	3.3581	6.4205	3.1455	3.5063	3.6431	7.0998	6.7148	0.9888	-0.0163	0.9628	0.0165
Slain2	_S[Phospho (STY)]LPNLSR_.2	Q8CI08_S414_M1_Slain2	8.5590	8.2853	8.9924	8.6576	7.8479	8.4047	8.1081	8.2385	8.1825	8.4482	1.0259	0.0369	0.3274	0.4849
Slain2	_RTS[Phospho (STY)]SEDLR_.2	Q8CI08_S87_M1_Slain2	3.0894	3.8946	3.7762	4.2788	3.7159	4.0136	3.9935	3.8396	3.4882	5.7684	0.8882	-0.1711	0.3210	0.4936
Gnl3	_ELS[Phospho (STY)]PEQSTAGKPSDGSSALDR_.3	Q8CI11_S505_M1_Gnl3	5.9384	4.2686	3.2461	4.1470	4.1569	5.3898	3.8617	4.2114	3.6201	5.8799	1.0299	0.0425	0.8425	0.0744
Pdlim5	_RGS[Phospho (STY)]QGDIK_.2	Q8CI51_S228_M1_Pdlim5	8.1316	7.5109	7.9557	7.7370	3.8983	4.2271	7.8447	7.1784	6.3615	8.4740	0.8811	-0.1827	0.4278	0.3688
Bag4	_S[Phospho (STY)]PGNSPTPMSR_.2	Q8CI61_S175_M1_Bag4	5.6255	4.2440	4.3329	7.5226	4.0653	3.6641	4.0249	3.1245	3.4236	3.5540	1.3900	0.4751	0.1036	0.9846
Vps50	_SVS[Phospho (STY)]RETLK_.2	Q8CI71_S585_M1_Vps50	8.1387	7.8395	8.4226	8.0013	6.1187	7.8958	8.3085	8.3151	7.8043	7.8707	0.9581	-0.0618	0.4558	0.3412
Vps50	_KKS[Phospho (STY)]DYSLNK_.2	Q8CI71_S595_M1_Vps50	3.0545	4.9141	4.2890	5.2754	3.6991	4.4264	5.3931	5.0944	5.3190	3.0710	0.9061	-0.1422	0.4818	0.3171
Vps50	_SAYQDYDS[Phospho (STY)]DSDVPEELKR_.2	#N/A	3.9009	6.7130	3.9459	8.5083	6.7297	3.6565	3.8251	6.9263	7.9138	8.2906	0.8274	-0.2734	0.3986	0.3994
Vps50	_FLEQSRS[Phospho (STY)]PSVSPSK_.2	#N/A	6.9875	7.1388	8.3397	7.4933	3.6888	3.7227	8.8093	3.6666	6.1314	5.6142	1.0286	0.0407	0.8992	0.0461
Vps50	_SAYQDY[Phospho (STY)]DSDSDVPEELK_.2	Q8CI71_Y557_M1_Vps50	4.1585	3.4629	3.3445	3.1570	3.2842	2.9444	3.3052	3.8442	4.1433	4.3351	0.8682	-0.2039	0.0995	1.0024
Vps50	_S[Phospho (STY)]PSVSPSK_.2	#N/A	3.0788	3.8840	3.7656	4.2683	3.7053	4.0241	3.9830	3.8502	3.4988	4.2353	0.9733	-0.0391	0.6735	0.1717
Dis3l2	_RPGLEKAS[Phospho (STY)]DEEPED_.2	Q8CI75_S864_M1_Dis3l2	10.7993	11.0166	11.6031	11.1871	9.6831	10.6404	11.3207	11.2325	10.7234	10.7962	0.9822	-0.0260	0.5923	0.2275
Pygb	_QKQIS[Phospho (STY)]VR_.2	Q8CI94_S15_M1_Pygb	11.3226	11.6965	12.4489	11.6333	11.0645	11.6072	11.5496	11.6944	12.0391	11.2446	0.9997	-0.0004	0.9911	0.0039
Pygb	_[Acetyl (Protein N-term)]AKPLTDSER_.2	#N/A	7.8724	8.2759	7.9118	8.2486	8.1393	8.0642	7.9893	8.3391	7.9278	7.8661	1.0068	0.0098	0.6601	0.1804
Pygb	_GIAGLGDVAEVR_.2	#N/A	6.9251	7.3381	7.2699	7.2985	6.8133	7.0377	7.2406	7.3028	7.1392	7.0423	0.9906	-0.0136	0.5918	0.2279
Pygb	_NLAENISR_.2	#N/A	7.0205	7.2213	7.3468	6.9460	7.3039	6.8859	6.7930	7.5975	7.2341	6.9738	0.9960	-0.0058	0.8696	0.0607
Pygb	_VIFLENYR_.2	#N/A	7.5273	7.2843	7.5412	7.0918	6.2870	7.2242	6.6317	7.2621	7.2122	7.3421	1.0066	0.0096	0.8645	0.0632
Pygb	_VEDVEALDQK_.2	#N/A	6.5516	6.4026	7.0236	6.5304	6.5073	6.3829	6.5287	6.5946	6.6097	6.0957	1.0169	0.0242	0.4970	0.3036
Pygb	_DYFFALAHTVR_.2	#N/A	6.2677	6.8659	6.1013	5.9274	5.4096	5.8734	6.5340	6.7186	6.2146	5.9319	0.9566	-0.0640	0.3578	0.4464
Pygb	_DFYELEPEKFQNK_.2	#N/A	6.5648	6.4187	5.9101	5.9226	5.1328	5.8485	6.7086	5.5549	6.0290	6.1983	0.9744	-0.0373	0.6372	0.1957
Pygb	_LLSLVDDEAFIR_.2	#N/A	6.1506	5.4579	5.3432	6.5475	6.0147	5.5996	6.0206	6.1693	5.9932	5.9360	0.9706	-0.0431	0.4808	0.3180
Pygb	_TC[Carbamidomethyl (C)]AYTNHTVLPEALER_.3	#N/A	5.8228	5.6475	6.0229	5.2198	5.4593	5.6718	5.2011	6.0756	5.9156	5.6564	0.9875	-0.0182	0.7395	0.1310
Pygb	_YEFGIFNQK_.2	#N/A	6.1062	5.7080	5.7140	5.8193	4.6367	5.5760	5.4698	6.2750	5.4486	4.6284	1.0253	0.0360	0.7182	0.1437
Pygb	_QAVDQISSGFFSPK_.2	#N/A	5.6707	4.7657	5.7172	5.9650	5.3437	5.6690	4.8952	5.0488	4.9549	4.7109	1.1263	0.1717	0.0234	1.6315
Pygb	_LVTSIGDVVNHDPVVGDR_.3	#N/A	6.6138	6.7700	7.0722	7.0159	6.4882	6.9388	6.6255	6.7403	6.8690	6.6946	1.0125	0.0179	0.5218	0.2825
Pygb	_HLDHVAALFPGDVDR_.3	#N/A	7.8577	7.7601	7.6517	7.5031	6.7408	7.0954	7.6319	7.6962	7.4532	7.6451	0.9774	-0.0330	0.4659	0.3317
Pygb	_IVNGWQVEEADDWLR_.2	#N/A	3.2734	4.4075	3.4387	4.0420	3.6716	3.7831	4.4778	4.9590	3.6446	3.7350	0.8966	-0.1574	0.2175	0.6626
Pygb	_LLSLVDDEAFIRDVAK_.3	#N/A	4.3165	4.7557	4.4260	4.2819	3.5641	4.2873	3.7198	4.8587	5.3474	4.3421	0.9354	-0.0964	0.4112	0.3860
Pygb	_DFNVGDYIEAVLDR_.2	#N/A	4.0687	4.1816	4.1868	4.0408	3.5968	3.5180	3.8788	4.7341	4.5714	2.3961	1.0095	0.0136	0.9361	0.0287
Pygb	_VIPAADLSQQISTAGTEASGTGNM[Oxidation (M)]K_.3	#N/A	5.0459	5.2210	4.6018	5.2529	5.2611	5.2688	4.3298	5.2382	4.9280	4.7438	1.0621	0.0869	0.1756	0.7556
Pygb	_VFADYEAYIQC[Carbamidomethyl (C)]QAQVDR_.2	#N/A	4.7859	3.4956	3.8720	4.3531	3.8270	4.2984	4.8048	3.9366	3.2901	3.3198	1.0697	0.0972	0.4863	0.3131
Pygb	_HLEIIYAINQR_.2	#N/A	4.3742	4.2697	3.1645	4.2091	2.5133	3.5130	4.3574	3.6532	3.4544	2.8525	1.0264	0.0376	0.8394	0.0760

Pygb	_NNTVNTMR_.2	#N/A	3.7877	3.0921	4.4745	3.4764	2.9134	3.3152	3.1910	3.1413	4.2907	3.4434	0.9981	-0.0028	0.9854	0.0064
Pygb	_WLLLC[Carbamidomethyl (C)]NPGLAEIIVER_.2	#N/A	3.9958	3.3002	3.7759	3.3196	3.1215	3.1071	3.4679	3.6815	3.9806	2.9969	0.9731	-0.0394	0.7117	0.1477
Osbpl11	_SFSLASSGNS[Phospho (STY)]PISQR_.2	Q8CI95_S186_M1_Osbpl11	6.4059	6.8828	7.2671	7.5228	5.4298	6.2729	3.9433	6.7267	6.7054	6.3083	1.1198	0.1632	0.3097	0.5091
Clip4	_SWS[Phospho (STY)]SSTTAGGLEGTVK_.2	Q8CI96_S608_M1_Clip4	3.3165	4.5388	4.5510	4.0947	3.9610	4.1644	4.2430	4.4073	3.2055	5.1124	0.9676	-0.0476	0.7320	0.1355
Tmem230	_LAS[Phospho (STY)]TDDGYIDLQFK_.2	Q8CIB6_S24_M1_Tmem230	3.7698	3.0741	4.4566	3.4584	2.8955	3.3332	3.1731	6.6632	3.4087	3.4254	0.7963	-0.3286	0.2311	0.6362
Copa	_NLS[Phospho (STY)]PGAVESDVR_.2	Q8CIE6_S173_M1_Copa	3.0375	8.7714	4.2720	3.8158	3.6821	2.9425	9.7768	9.0231	3.4844	8.8949	0.5671	-0.8184	0.0680	1.1672
Pak2	_[Acetyl (Protein N-term)]S[Phospho (STY)]DNGELEDKPPAPPVR_.2	Q8CIN4_S2_M1_Pak2	3.9286	8.6870	3.1146	9.1147	8.4170	8.6920	8.9398	8.4846	8.5973	8.7057	0.8054	-0.3122	0.2573	0.5896
Mark4	_RS[Phospho (STY)]PTSTGDTELKEER_.3	Q8CIP4_S438_M1_Mark4	8.1781	7.9678	8.8787	7.7298	7.3806	8.1204	7.8375	8.1101	8.4643	7.7517	1.0002	0.0003	0.9955	0.0019
Mark4	_KAS[Phospho (STY)]C[Carbamidomethyl (C)]SAVGSGSR_.2	Q8CIP4_S458_M1_Mark4	4.4686	4.6359	3.9341	3.4779	3.3442	3.2804	3.1736	5.0638	5.0202	4.3678	0.8753	-0.1922	0.2661	0.5749
Dock3	_GS[Phospho (STY)]PSLPDKYR_.2	Q8CIQ7_S1763_M1_Dock3	8.5575	8.3469	3.4699	4.5145	3.4275	4.3020	3.1619	3.9875	8.0899	8.1696	0.9289	-0.1063	0.8019	0.0959
Dock3	_GNVLASHSPMS[Phospho (STY)]PENIK_.2	#N/A	5.5744	3.9807	4.0019	6.2861	3.9416	3.8575	4.2192	3.6836	3.3321	3.7501	1.2298	0.2984	0.1596	0.7969
Mical3	_GTSQVSS[Phospho (STY)]PSQPPEK_.2	Q8CJ19_S1154_M1_Mical3	6.6197	10.6894	11.2595	11.1722	9.8207	10.7454	11.4076	11.1602	10.7482	10.7556	0.9123	-0.1325	0.3169	0.4991
Mical3	_RKT[Phospho (STY)]SQSEEEEPPR_.3	Q8CJ19_T684_M1_Mical3	3.9957	6.0889	3.1817	3.5269	3.1214	3.1074	3.4474	6.7527	4.0191	3.6513	0.8588	-0.2195	0.4782	0.3204
Mical3	_QVGVFS[Phospho (STY)]PAR_.2	Q8CJ19_S1167_M1_Mical3	8.0906	7.6179	8.2969	7.8014	6.1679	7.6199	8.1584	8.0602	7.7759	7.7328	0.9580	-0.0618	0.4206	0.3761
Mical3	_GRS[Phospho (STY)]EEELEASK_.2	Q8CJ19_S977_M1_Mical3	7.7128	8.0108	8.0368	7.8005	6.5662	7.5781	7.8954	7.7244	7.7889	7.5318	0.9848	-0.0221	0.6899	0.1612
Mical3	_SVFS[Phospho (STY)]GYKK_.2	Q8CJ19_S1736_M1_Mical3	3.8439	7.1671	4.5307	7.3109	2.9696	3.2591	3.6198	6.8421	3.8286	6.4432	0.9351	-0.0969	0.7846	0.1053
Mical3	_KTS[Phospho (STY)]QSEEEPPR_.2	#N/A	2.9284	7.3460	4.1629	3.2301	3.5729	3.0517	4.4457	7.7543	3.0361	3.1971	0.8786	-0.1868	0.6578	0.1819
Mical3	_S[Phospho (STY)]PGTEEAKSPLATK_.2	Q8CJ19_S1171_M1_Mical3	7.4600	7.1344	6.7362	6.7313	4.8737	6.9389	6.0725	7.3130	7.2271	6.1085	0.9948	-0.0075	0.9503	0.0221
Mical3	_AVLS[Phospho (STY)]PIR_.2	Q8CJ19_S1216_M1_Mical3	8.5155	7.6504	8.6976	7.8459	6.1377	8.1327	7.6936	8.3871	8.1422	8.0728	0.9698	-0.0443	0.6263	0.2032
Mical3	_SAGDQPPLLTPKS[Phospho (STY)]PS[Phospho (STY)]DKELR_.3	Q8CJ19_S1404_M2_Mical3	2.9658	4.3766	4.2004	8.3517	6.2908	3.5316	3.8924	8.3113	6.9274	7.7358	0.7374	-0.4395	0.2078	0.6824
Mical3	_QVGVFSPARS[Phospho (STY)]PGT[Phospho (STY)]EEAK_.3	Q8CJ19_T1174_M2_Mical3	3.6658	3.7759	3.9702	4.0471	3.8325	3.8485	4.8295	3.9131	4.9265	4.2254	0.8621	-0.2141	0.0165	1.7820
Mical3	_VKS[Phospho (STY)]PEEPLFPTPLLLR_.3	Q8CJ19_S1187_M1_Mical3	4.0158	6.0056	3.7494	7.2304	3.1595	3.4651	3.4415	6.1111	4.0070	2.9705	1.1142	0.1560	0.6487	0.1880
Mical3	_RQLS[Phospho (STY)]FSEDSDLSSDDILER_.3	Q8CJ19_S1783_M1_Mical3	2.9229	3.6792	4.1575	3.2356	3.5675	3.0571	4.4511	3.3821	3.0307	3.2026	0.9773	-0.0332	0.8220	0.0852
Crocc	_GFSGQRT[Phospho (STY)]PT[Phospho (STY)]PPRHS[Phospho (STY)]PGR_.3	Q8CJ40_T494_M3_Crocc	6.2239	7.1947	7.1034	7.5607	7.0605	4.1012	7.2111	7.4304	6.8534	8.3357	0.8771	-0.1893	0.2243	0.6493
Crocc	_EAPAGGSGDGLS[Phospho (STY)]SPSPLEYSPR_.2	Q8CJ40_S1468_M1_Crocc	6.8566	6.8504	4.3435	4.3626	3.8636	4.1698	4.2763	4.0254	6.8457	3.4656	1.0905	0.1250	0.6608	0.1799
Crocc	_DLAQAALS[Phospho (STY)]DTESGVQLSSSER_.2	Q8CJ40_S464_M1_Crocc	3.6133	3.3188	4.2190	3.5039	4.2398	3.3061	4.2101	4.7427	3.3277	3.3132	0.9491	-0.0753	0.5891	0.2298
Crocc	_TPTPPRHS[Phospho (STY)]PGR_.3	Q8CJ40_S501_M1_Crocc	3.7631	3.8457	3.9928	3.9183	4.0359	3.6768	3.8751	3.7721	3.6465	3.7416	1.0301	0.0428	0.1912	0.7186
Cmtm4	_TES[Phospho (STY)]RDVDSRPEIQR_.3	Q8CJ61_S194_M1_Cmtm4	3.9455	3.9117	4.0678	4.1586	3.7608	3.4701	4.0403	3.4735	3.4586	3.1712	1.0989	0.1361	0.1038	0.9840
Syn3	_SPGS[Phospho (STY)]PQLSR_.2	Q8JZP2_S483_M1_Syn3	11.1909	11.5216	11.8998	11.7040	10.6275	11.5593	11.4206	11.3148	11.2955	11.3550	1.0062	0.0090	0.7680	0.1146
Syn3	_LSPQGQQPVS[Phospho (STY)]PQSGSPQQQR_.3	#N/A	8.7356	9.0895	9.0853	9.4988	7.6818	8.8598	8.8572	8.9798	8.7098	8.6259	1.0036	0.0052	0.9232	0.0347
Syn3	_SQS[Phospho (STY)]LTNSLSTSDTSHR_.2	#N/A	8.3638	8.1436	8.4433	8.6926	7.3075	8.4930	8.3343	8.1922	8.5405	8.2261	0.9901	-0.0144	0.7570	0.1209
Syn3	_STS[Phospho (STY)]QQGEEPQK_.2	Q8JZP2_S519_M1_Syn3	8.9813	8.8335	8.9405	4.0703	3.6273	9.0948	8.3219	8.8524	8.8957	9.0423	0.8268	-0.2743	0.2947	0.5305
Syn3	_SAS[Phospho (STY)]PHPHLNK_.2	Q8JZP2_S530_M1_Syn3	3.4735	3.8689	3.2072	4.2518	3.6902	4.0393	4.4001	4.2502	3.0484	3.9291	0.9611	-0.0572	0.6355	0.1969
Gas2l1	_TGTFS[Phospho (STY)]PQR_.2	Q8JZP9_S292_M1_Gas2l1	7.1415	4.0340	7.7330	7.1728	3.8553	3.8742	4.2350	7.5959	7.0653	7.0897	0.8674	-0.2052	0.4708	0.3271
Gas2l1	_GSPTPSPRPGS[Phospho (STY)]PVPGSER_.3	Q8JZP9_S306_M1_Gas2l1	3.5621	5.1643	5.9865	5.4147	5.0649	3.8006	3.8135	3.9051	3.8618	3.6143	1.2721	0.3472	0.0668	1.1751
Afg3l2	_NLETLQQELGIEGENR_.2	#N/A	3.4944	3.8480	3.2281	4.2727	5.3939	4.0602	4.4210	5.6579	3.0275	3.9500	0.9497	-0.0745	0.7214	0.1418
Eif3b	_GHPSAGAEEEGGS[Phospho (STY)]DGS[Phospho (STY)]AAEAEPR_.2	#N/A	6.8432	6.9160	7.2546	7.4001	6.8572	7.2337	7.3864	7.1508	7.4115	6.8077	0.9854	-0.0212	0.5430	0.2652
Eif3b	_AKPAAQSEEETATS[Phospho (STY)]PAAS[Phospho (STY)]PTPQSAER_.3	Q8JZQ9_S75_M2_Eif3b	10.4281	10.7216	11.1658	11.3299	10.3347	10.7750	11.0234	10.8493	10.8745	10.6075	0.9957	-0.0062	0.8333	0.0792
Eif3b	_SPS[Phospho (STY)]QEPSAPGK_.2	Q8JZQ9_S90_M1_Eif3b	7.9401	7.2836	3.7580	4.1452	6.9281	3.4777	8.0845	7.0864	4.0238	3.8224	0.9712	-0.0421	0.9041	0.0438
Slc4a8	_KLLSS[Phospho (STY)]PGK_.2	Q8JZR6_S1046_M1_Slc4a8	8.3231	8.4665	9.0458	3.5153	3.3816	3.2430	8.9100	8.9127	3.7849	8.3334	0.8010	-0.3201	0.4235	0.3732
Slc4a8	_ALS[Phospho (STY)]INSGNTK_.2	Q8JZR6_S1076_M1_Slc4a8	5.8954	6.0136	4.3521	3.3539	2.7910	5.5465	5.7559	5.7183	5.4392	5.1435	0.8449	-0.2432	0.2627	0.5805
Slc4a8	_DGQTVS[Phospho (STY)]PQSATNLEVK_.2	Q8JZR6_S259_M1_Slc4a8	3.6970	3.5292	4.3838	3.3856	3.4175	5.8738	6.9830	3.2320	6.0098	3.3526	0.8270	-0.2740	0.3725	0.4288
Slc4a8	_KQS[Phospho (STY)]DPHSMDR_.2	Q8JZR6_S246_M1_Slc4a8	3.2946	3.9315	3.9814	4.6944	3.8198	4.3056	4.1988	3.6344	3.2830	4.4511	1.0290	0.0412	0.7329	0.1350
Lin7a	_EQNS[Phospho (STY)]PIYISR_.2	Q8JZS0_S130_M1_Lin7a	9.3229	9.3484	9.8001	9.7410	9.2029	9.4825	9.5815	9.3023	9.3833	9.4589	1.0055	0.0078	0.7056	0.1514
Lin7a	_ATVAAFAASEGHSHPR_.3	#N/A	5.8908	5.3647	3.4301	6.3993	6.1986	5.3511	3.1222	5.7391	5.3551	4.1520	1.1845	0.2442	0.2744	0.5617
Lin7a	_VLQSEFC[Carbamidomethyl (C)]TAIR_.2	#N/A	3.5449	4.3012	3.2786	4.9811	5.1254	4.8705	4.4714	4.9209	2.9771	4.0005	1.0630	0.0881	0.6321	0.1992
Lin7a	_VLEEMEAR_.2	#N/A	4.0389	3.1873	3.2249	5.2278	3.0756	3.5490	3.4422	4.3910	4.0395	3.7672	0.9507	-0.0729	0.6777	0.1690
Lin7a	_QQQQLLIQQQQQQQQQPQQNHM[Oxidation (M)]S_.3	#N/A	3.5714	3.6954	4.4925	4.0252	3.3486	3.5106	3.8714	3.0460	3.1434	3.5865	1.1061	0.1455	0.2053	0.6876
Lin7a	_LQESGEVPVHK_.3	#N/A	3.4956	4.3008	4.1824	3.1842	4.1221	3.6073	4.3998	3.4334	3.0820	3.1512	1.0850	0.1177	0.3945	0.4040
Cpne5	_DIVQFVPFR_.2	#N/A	7.6582	8.0949	7.7121	7.9705	7.6000	7.8328	7.4140	7.7995	7.8578	7.6597	1.0167	0.0240	0.3344	0.4758
Cpne5	cetyl (Protein N-term)]M[Oxidation (M)]EQPEDM[Oxidation (M)]AS[Phospho (STY)]LSEFDSLAGSIPATK	Q8JZW4_S9_M1_Cpne5	3.0710	3.8762	3.7578	4.2604	3.6975	4.0320	4.3928	3.8580	3.5066	4.2274	0.9465	-0.0793	0.4323	0.3642
Cpne5	_FDLYDVDSKS[Phospho (STY)]PDLSK_.2	#N/A	3.8283	6.5676	3.5943	7.7802	6.7126	4.1963	7.5504	7.0739	7.0025	7.0759	0.7590	-0.3978	0.0958	1.0188
Cpne5	RPPPAAPAQS[Phospho (STY)]PPQS[Phospho (STY)]PAHS[Phospho (STY)]PPGSPVHT[Phospho (STY)]HL	Q8JZW4_T591_M3_Cpne5	3.2627	9.2572	3.7950	10.9226	9.8511	8.8162	5.9248	10.1732	9.8448	10.5777	0.8380	-0.2550	0.4531	0.3438
Cpne5	IRPRPPPAAPAQS[Phospho (STY)]PPQS[Phospho (STY)]PAHS[Phospho (STY)]PPGS[Phospho (STY)]PVHT	#N/A	4.2510	3.3437	3.9847	7.3633	3.3947	3.2305	3.3963	5.0839	4.1248	3.2631	1.0742	0.1033	0.7431	0.1289
Sh2d5	_LGNPYC[Carbamidomethyl (C)]S[Phospho (STY)]PTLVR_.2	Q8JZW5_S237_M1_Sh2d5	10.0059	3.0774	4.4598	3.4616	2.8987	8.7663	3.1763	3.1560	4.3054	3.4286	1.5484	0.6307	0.2650	0.5767
Sh2d5	_RAS[Phospho (STY)]DC[Carbamidomethyl (C)]GPAPYRPR_.3	Q8JZW5_S12_M1_Sh2d5	4.8732	4.4025	5.6326	5.3075	4.5548	3.5057	4.9719	5.5366	2.9803	3.2529	1.1260	0.1712	0.4254	0.3712
Rbm17	_RPDPDS[Phospho (STY)]DEDEDYER_.2	#N/A	3.6820	3.5442	4.3688	3.3706	7.0169	3.1921	3.0853	3.2470	4.3965	7.4332	0.9241	-0.1139	0.7585	0.1200
Rbm17	_RPDPDS[Phospho (STY)]DEDEDYERER_.3	#N/A	6.8035	6.7231	7.5786	6.8082	3.7023	4.4232	6.9587	3.5168	7.3676	6.9051	0.9708	-0.0427	0.8694	0.0608
Adgrl2	_S[Phospho (STY)]ENEDIYYK_.2	Q8JZZ7_S1428_M1_Adgrl2	6.3876	5.7218	3.7854	4.2880	3.6935	5.2527	5.7994	5.1824	5.6180	5.7636	0.8684	-0.2036	0.2353	0.6285
Adgrl2	_GNS[Phospho (STY)]DGYIIPINK_.2	Q8JZZ7_S1458_M1_Adgrl2	3.1320	10.3290	4.3665	10.7864	9.3955	10.4786	10.7519	10.6648	10.5889	10.4937	0.7606	-0.3948	0.1821	0.7397
Adgrl2	_S[Phospho (STY)]MPNLGAGR_.2	Q8JZZ7_S1437_M1_Adgrl2	4.5716	3.9179	3.9951	4.7650	3.9348	4.3193	4.2124	3.6207	3.2693	2.9639	1.2087	0.2735	0.0308	1.5119
Spata2	_S[Phospho (STY)]VDAYDSYWESR_.2	Q8K004_S247_M1_Spata2	9.2220	9.6810	9.4896	10.0301	8.5730	9.7048	9.8731	10.0094	10.0053	9.8122	0.9522	-0.0707	0.1062	0.9740
Fnbp1l	_TIS[Phospho (STY)]DGTISAAK_.2	Q8K012_S295_M1_Fnbp1l	10.1402	10.2420	10.7009	10.4181	9.3529	10.1852	10.0987	9.9709	9.8989	10.2384	1.0121	0.0173	0.6231	0.2054
Fnbp1l	_ES[Phospho (STY)]PEGSYTDDANQEVR_.2	#N/A	6.4320	6.2468	6.2150	7.5081	3.3808	6.6964	7.0275	6.3036	4.2399	6.9289	0.9926	-0.0107	0.9605	0.0175
Fnbp1l	_HSSDINHLVTQGRES[Phospho (STY)]PEGSYTDDANQEVR_.4	#N/A	3.4827	5.0562	4.1695	5.9172	4.1092	3.9444	4.0221	3.4897	3.4665	3.1629	1.2578	0.3308	0.0900	1.0458
Bclaf1	_EVQS[Phospho (STY)]PEQVK_.2	#N/A	10.5564	10.5110	10.7652	10.8509	9.9758	10.3104	11.0057	10.7443	10.5798	10.5252	0.9796	-0.0298	0.2675	0.5727
Bclaf1	_QKS[Phospho (STY)]PEIHR_.2	#N/A	9.9534	10.1117	10.7913	10.3531	10.0795	9.9960	10.3982	10.3124	10.3561	10.0326	0.9941	-0.0085	0.7357	0.1333
Bclaf1	_KEVQS[Phospho (STY)]PEQVK_.2	#N/A	8.8659	8.7058	9.0729	9.0516	8.2465	8.5758	9.2278	9.0559	8.6568	8.8185	0.9791	-0.0304	0.3523	0.4531
Bclaf1	_KAEGEPQEES[Phospho (STY)]PLKSK_.2	#N/A	6.9115	7.4998	9.0343	8.0134	7.6904	8.1538	7.7227	7.6893	7.1457	8.1260	1.0278	0.0395	0.6065	0.2172
Bclaf1	_KAEGEPQEES[Phospho (STY)]PLK_.2	#N/A	8.6282	8.0861	8.6870	8.6864	7.4083	7.9925	8.6743	8.5772	8.3584	8.3139	0.9725	-0.0402	0.4160	0.3809
Bclaf1	_SATSGDIWPGLSAYDNS[Phospho (STY)]PR_.2	Q8K019_S221_M1_Bclaf1	3.0092	3.7654	4.2437	9.0253	3.6537	2.9709	9.0448	3.2137	3.5128	3.4647	0.9242	-0.1137	0.8276	0.0822
Bclaf1	_AEGEPQEES[Phospho (STY)]PLKSK_.2	#N/A	4.2106	7.4554	7.6184	3.8992	3.3363	4.3932	3.2532	4.2193	3.8679	7.1869	1.1124	0.1536	0.6718	0.1727
Bclaf1	_TIT[Phospho (STY)]PQNAPR_.2	Q8K019_T305_M1_Bclaf1	7.4723	7.8534	7.9853	7.8120	6.9545	7.4426	8.1626	8.0408	7.4533	3.8045	1.1051	0.1441	0.4146	0.3824
Bclaf1	_KEVQS[Phospho (STY)]PEQVKSEK_.3	#N/A	4.7330	5.4116	6.3038	3.8531	5.8392	5.9375	3.5038	5.4452	5.8071	5.3785	1.0621	0.0869	0.6268	0.2029
Bclaf1	_RIDIS[Phospho (STY)]PSALR_.2	Q8K019_S656_M1_Bclaf1	3.0274	3.7837	4.2619	3.8057	3.6720	2.9526	3.9540	3.1955	3.4945	7.1739	0.8046	-0.3137	0.2916	0.5352
Bclaf1	_ELFDYS[Phospho (STY)]PPLHK_.2	#N/A	4.2972	3.0452	4.0309	8.3548	7.6462	8.5485	3.7229	8.7565	8.8082	3.2520	0.9759	-0.0352	0.9346	0.0294
Bclaf1	_AEGEPQEES[Phospho (STY)]PLK_.2	#N/A	7.7355	6.3842	3.4298	7.4180	5.7507	6.4785	7.3743	7.0024	7.1655	7.0910	0.8660	-0.2075	0.2580	0.5884
Bclaf1	_QKS[Phospho (STY)]PEIHRR_.2	#N/A	5.1749	5.8484	6.6299	5.5418	3.2163	5.1522	7.2849	6.0499	5.7485	5.9324	0.8412	-0.2495	0.1615	0.7919
Bclaf1	_S[Phospho (STY)]PEIHRR_.2	#N/A	6.1580	5.3470	4.4385	3.4403	3.3628	3.2618	6.4948	6.0347	4.3268	5.7914	0.7656	-0.3853	0.1016	0.9932

Bclaf1	_FHDS[Phospho (STY)]EGDDTEETEDYR_.2	#N/A	6.3653	5.1472	6.0919	6.0821	5.5785	5.8948	5.4667	6.3794	6.5480	5.9793	0.9617	-0.0563	0.4478	0.3489
Bclaf1	_YS[Phospho (STY)]PSQNS[Phospho (STY)]PIHHIPSR_.3	#N/A	8.3077	7.4255	6.9026	7.1704	6.1284	7.5857	6.9763	3.7419	7.2312	3.9600	1.3242	0.4052	0.0653	1.1853
Bclaf1	_NTPSQHSHSIQHS[Phospho (STY)]PER_.3	Q8K019_S267_M1_Bclaf1	7.7765	7.6554	8.2951	8.0150	7.2283	7.4697	7.9474	8.0476	7.8757	7.5295	0.9860	-0.0204	0.6209	0.2070
Bclaf1	_LKELFDYS[Phospho (STY)]PPLHK_.3	#N/A	3.9536	7.3464	7.8426	8.2865	5.5987	6.3571	6.7542	7.6916	7.1297	3.7502	1.0368	0.0521	0.8346	0.0785
Bclaf1	_SIFREES[Phospho (STY)]PLR_.2	#N/A	9.0894	9.2567	10.0251	9.4536	8.6070	9.2080	9.8852	9.4907	9.3473	9.3392	0.9745	-0.0372	0.3749	0.4260
Bclaf1	_QKFHDS[Phospho (STY)]EGDDTEETEDYR_.3	#N/A	5.7786	5.4246	6.2138	5.8124	5.3194	5.3680	5.2567	5.5437	5.5452	5.1083	1.0539	0.0758	0.1794	0.7461
Bclaf1	_EKSIFREES[Phospho (STY)]PLR_.3	#N/A	4.8336	5.8896	6.3309	3.8934	4.9838	5.8780	5.6966	5.6504	5.3875	5.7176	0.9445	-0.0824	0.5198	0.2842
Bclaf1	_EVQS[Phospho (STY)]PEQVKSEK_.2	#N/A	8.7382	9.3555	10.1057	9.2846	9.0031	9.2049	9.5356	9.6341	9.0792	9.0397	0.9957	-0.0062	0.8832	0.0540
Bclaf1	_YS[Phospho (STY)]PSQNS[Phospho (STY)]PIHHIPSRR_.3	#N/A	8.2214	8.1706	4.1605	8.4116	3.5706	7.9891	8.3665	3.3851	3.0337	7.8897	1.1914	0.2527	0.5189	0.2850
Bclaf1	_GRADGDWDDQEVLDYFS[Phospho (STY)]DKESAK_.4	#N/A	3.2417	4.0264	4.1857	3.1484	3.8758	3.3562	4.2059	3.3574	3.1179	3.2904	1.0418	0.0591	0.6357	0.1967
Bclaf1	_ADGDWDDQEVLDYFS[Phospho (STY)]DKESAK_.3	#N/A	3.3301	4.0123	4.5646	4.1084	3.9747	4.1507	4.2567	4.3936	3.1918	3.7857	1.0298	0.0424	0.7043	0.1522
Bclaf1	_SQEEPKDTFEHDPSES[Phospho (STY)]IDEFNK_.3	Q8K019_S198_M1_Bclaf1	4.2523	3.5078	3.9860	3.5298	3.3961	3.2285	3.6780	3.4714	3.7704	3.2071	1.0335	0.0476	0.6073	0.2166
Bclaf1	_LRC[Carbamidomethyl (C)]DS[Phospho (STY)]ADLR_.2	Q8K019_S688_M1_Bclaf1	3.0541	3.8104	4.2886	3.8324	3.6987	4.4268	3.9806	3.1688	3.4679	3.5096	1.0906	0.1252	0.2884	0.5400
Scamp1	_TVQTAAANAASTAATSAAQNAFK_.3	#N/A	3.5344	5.1302	5.1579	5.3302	4.4706	4.5176	5.4411	5.1939	5.2676	4.8692	0.9032	-0.1469	0.1934	0.7135
Scamp1	_EMQNLS[Phospho (STY)]QHGR_.2	Q8K021_S112_M1_Scamp1	5.8455	3.2127	3.6909	6.2262	3.6465	3.5236	4.9798	2.7230	4.0655	4.4128	1.0772	0.1073	0.6961	0.1573
Stard8	_SLS[Phospho (STY)]IESLC[Carbamidomethyl (C)]PDEGR_.2	Q8K031_S234_M1_Stard8	7.7443	3.6702	8.1287	4.0925	3.5585	7.9475	3.8072	4.1035	8.0037	7.9143	0.9832	-0.0245	0.9478	0.0233
	IQPQLPDEDGNHS[Phospho (STY)]DKEDEQPQVVVLKK.4	#N/A	3.1905	7.8297	4.5812	9.1917	8.1096	7.6125	6.9190	8.1140	7.7119	7.6318	0.8892	-0.1695	0.5044	0.2973
	KIQPQLPDEDGNHS[Phospho (STY)]DKEDEQPQVVVLK.4	#N/A	3.0565	4.1697	3.7433	5.7643	5.0662	4.7784	3.9606	5.0348	5.0375	5.1668	0.9229	-0.1158	0.5154	0.2878
	IQPQLPDEDGNHS[Phospho (STY)]DKEDEQPQVVVLK.4	#N/A	3.6740	3.6121	3.7793	6.8641	5.7512	5.3754	3.7419	5.5906	6.2515	5.6251	0.9133	-0.1308	0.5879	0.2307
	NSSLLSFDS[Phospho (STY)]EDENE.2	Q8K039_S150_M1_	3.7791	3.7197	4.2539	7.7983	6.2585	3.7456	3.9459	7.2324	3.5899	3.4749	1.0800	0.1111	0.7538	0.1228
Samd14	_S[Phospho (STY)]LDEDEPPPSPLAR_.2	Q8K070_S108_M1_Samd14	11.5205	12.2128	11.9123	12.6082	11.2331	12.4245	12.4203	12.1622	12.2425	12.4186	0.9735	-0.0387	0.2730	0.5638
Samd14	_VTDGC[Carbamidomethyl (C)]GS[Phospho (STY)]PLHR_.2	Q8K070_S77_M1_Samd14	6.9128	7.2906	6.6928	6.8886	6.2740	6.1396	8.1461	6.7527	6.4047	6.7911	0.9539	-0.0681	0.4125	0.3846
Samd14	_HEGFS[Phospho (STY)]PK_.2	Q8K070_S267_M1_Samd14	4.8563	4.8909	5.8154	5.6730	4.4068	4.8503	4.3183	3.5218	5.5727	5.0169	1.1030	0.1415	0.3198	0.4951
Samd14	_AEPHSEDDSRDAS[Phospho (STY)]PPEPASPTIGLDKK_.4	#N/A	6.1104	5.2216	3.7729	3.7127	6.9224	6.7280	6.6556	6.9262	6.7139	3.9685	0.8921	-0.1648	0.4939	0.3064
Samd14	_GSASSGSTTSPT[Phospho (STY)]C[Carbamidomethyl (C)]SPK_.2	Q8K070_T258_M1_Samd14	3.4221	4.0105	3.9585	3.8971	3.5440	3.7120	4.0390	3.5885	3.6919	4.1445	0.9719	-0.0411	0.5194	0.2845
Samd14	_DAS[Phospho (STY)]PPEPASPTIGLDKK_.2	#N/A	3.5974	4.0407	3.9754	3.9057	3.7973	3.6519	3.8507	3.5990	3.7105	3.6799	1.0318	0.0452	0.2708	0.5673
Samd14	_DAS[Phospho (STY)]PPEPASPTIGLDK_.2	#N/A	4.3596	2.9828	4.0933	3.6371	8.7023	3.4245	9.5212	9.0501	3.6632	3.3143	0.7097	-0.4946	0.3086	0.5107
Samd14	_DSASSAEDGEGS[Phospho (STY)]DGPGGK_.2	Q8K070_S64_M1_Samd14	3.8388	3.3707	3.7212	4.1285	3.0028	3.4421	3.4133	3.7520	4.2499	3.8058	0.9419	-0.0864	0.3901	0.4089
Znf536	_GSGS[Phospho (STY)]DQESQSVSR_.2	Q8K083_S678_M1_Znf536	2.8969	3.6532	4.2598	3.2616	5.5665	3.0832	4.4771	3.3560	5.6167	3.2286	0.9082	-0.1389	0.5854	0.2326
Cdk17	_RLS[Phospho (STY)]LPADIR_.2	Q8K0D0_S146_M1_Cdk17	7.2650	6.9532	7.3040	7.8832	5.5879	7.5281	7.7044	7.5618	7.3285	7.3776	0.9458	-0.0804	0.3533	0.4519
Cdk17	_RAS[Phospho (STY)]LSEIGFGK_.2	Q8K0D0_S180_M1_Cdk17	3.1226	3.8789	4.3571	7.5509	3.7672	6.7958	7.1511	6.9546	6.8880	7.0560	0.7005	-0.5136	0.0529	1.2765
Cdk17	_RIS[Phospho (STY)]M[Oxidation (M)]EDLNKR_.3	#N/A	5.6939	4.9379	5.9987	5.3777	4.5454	5.6887	5.3232	5.3332	5.9144	4.5450	0.9759	-0.0352	0.6674	0.1756
Cdk17	_RIS[Phospho (STY)]MEDLNK_.2	#N/A	5.6440	4.8730	3.9197	6.8489	3.3298	3.2948	3.1880	3.1443	3.5857	3.4403	1.3929	0.4781	0.1083	0.9654
Cdk17	_MGSDGESDQASGTSSDEVQS[Phospho (STY)]PTGVC[Carbamidomethyl (C)]LR_.3	Q8K0D0_S122_M1_Cdk17	3.2388	3.9950	4.4733	4.0171	3.8833	4.2421	4.1353	2.9841	3.2832	3.6943	1.1279	0.1736	0.1598	0.7963
Cdk17	_LQISS[Phospho (STY)]PPFDQPMSR_.2	Q8K0D0_S165_M1_Cdk17	3.9244	3.3017	3.1104	7.0709	3.1900	3.4346	3.3277	3.0046	4.1540	3.5801	1.1390	0.1878	0.5599	0.2519
Phyhip	_ISWAM[Oxidation (M)]EDSDLER_.2	#N/A	6.7427	6.6413	7.1769	6.4775	6.3017	7.2462	6.9963	7.0135	7.1000	6.7220	0.9722	-0.0407	0.3692	0.4328
Phyhip	_EYFQHAR_.2	#N/A	6.7093	6.2515	6.0602	6.2530	6.4589	6.0965	6.1069	6.6181	6.3753	6.1651	0.9982	-0.0026	0.9427	0.0256
Phyhip	_THC[Carbamidomethyl (C)]GNVLQPYLK_.3	#N/A	4.7997	4.3498	5.4714	3.6743	4.5758	4.5107	4.1664	4.8770	4.8321	5.1228	0.9608	-0.0576	0.5995	0.2222
Phyhip	_FLTC[Carbamidomethyl (C)]SVEDGELIFR_.2	#N/A	5.8504	2.9577	3.4360	3.9571	5.1963	5.5511	3.6718	4.9090	5.3932	3.9241	1.0038	0.0054	0.9811	0.0083
Phyhip	_[Acetyl (Protein N-term)]M[Oxidation (M)]ELLSTPHSIEINNITC[Carbamidomethyl (C)]DSFR_.3	#N/A	3.5967	4.3529	3.5600	4.0627	4.2412	4.0847	3.7774	4.2351	4.8750	3.9790	0.9446	-0.0822	0.3834	0.4164
Erich5	_LSSERDS[Phospho (STY)]PGAPQAGTM[Oxidation (M)]K_.2	#N/A	3.7596	4.8803	4.8749	3.9766	4.7812	4.6198	3.6913	4.2237	3.7945	4.1423	1.1310	0.1776	0.0909	1.0415
Rtn1	_HQAQVDQYLGLVR_.3	#N/A	6.4478	5.8299	6.5804	5.6984	5.6680	6.3691	6.3688	6.3397	6.3982	6.1486	0.9660	-0.0500	0.3457	0.4613
Rtn1	_THINTVVAK_.2	#N/A	7.0660	7.2088	7.7404	6.7027	7.0831	7.2612	7.5786	7.4100	7.2705	7.0103	0.9808	-0.0279	0.4965	0.3041
Rtn1	_TDEGHPFK_.2	#N/A	7.3093	7.2027	6.5800	6.7248	7.0874	6.9439	7.1960	7.3444	7.1048	7.0776	0.9713	-0.0420	0.2102	0.6773
Rtn1	_VSLVTAPVK_.2	#N/A	4.4071	4.8690	5.5371	3.9358	4.9677	5.7049	4.0173	5.8007	5.2745	5.4883	0.9530	-0.0694	0.6139	0.2119
Rtn1	_APNQPAPVEGK_.2	#N/A	7.1527	7.2637	7.0215	6.5064	7.3469	6.8361	6.9892	7.2123	6.7878	6.9312	1.0059	0.0085	0.8181	0.0872
Rtn1	_ELC[Carbamidomethyl (C)]SGPAR_.2	#N/A	5.2498	5.0010	4.3177	4.8380	5.8734	4.9273	4.0098	5.5781	5.0918	5.4699	0.9994	-0.0008	0.9943	0.0025
Rtn1	_EQDS[Phospho (STY)]PPM[Oxidation (M)]KPGALDAIR_.2	#N/A	9.7028	9.2077	9.5474	10.1042	8.4539	9.0779	10.1146	9.7481	9.4663	9.3355	0.9672	-0.0481	0.3530	0.4522
Rtn1	_[Acetyl (Protein N-term)]AAPPDLQDEPLSLGS[Phospho (STY)]PGSQWFGGR_.3	Q8K0T0_S16_M1_Rtn1	3.7955	6.1313	4.4823	7.4418	3.3190	3.3057	3.1988	6.4213	4.2829	3.4511	1.0939	0.1295	0.7050	0.1518
Rtn1	_REQDS[Phospho (STY)]PPM[Oxidation (M)]KPGALDAIR_.3	#N/A	5.5860	5.8939	6.0384	5.7390	5.4924	5.5147	6.2177	6.3938	6.0537	3.4659	1.0322	0.0457	0.7583	0.1202
Rtn1	_GDGEDEATAVMGAR_.2	#N/A	6.4010	3.9998	3.9132	6.8841	3.8529	3.8766	4.1305	3.7026	3.3512	4.3829	1.2388	0.3090	0.2485	0.6047
Rtn1	_GS[Phospho (STY)]VSEDELIAAIK_.2	Q8K0T0_S350_M1_Rtn1	3.8765	3.6623	4.0007	4.0934	3.4836	3.6059	3.9667	5.6181	3.5265	3.9660	0.8870	-0.1729	0.2455	0.6100
Unc13c	_NAWLSPNDS[Phospho (STY)]DRELESDLSR_.3	Q8K0T7_S450_M1_Unc13c	3.7113	3.0156	4.3981	3.6042	2.8369	3.3917	3.7525	3.3969	3.6960	3.2815	0.9890	-0.0159	0.8997	0.0459
Hspa12a	_ETAPTSTYSS[Phospho (STY)]PAR_.2	Q8K0U4_S24_M1_Hspa12a	11.5446	11.6042	12.0069	11.8870	10.3749	11.5725	11.6208	11.5956	11.5956	11.5030	0.9931	-0.0101	0.7938	0.1003
Hspa12a	_LDLTGS[Phospho (STY)]GGTAVPAR_.2	Q8K0U4_S631_M1_Hspa12a	8.7377	8.3942	8.5597	9.1630	7.8754	8.6609	9.1064	8.8538	5.9440	8.8118	1.0472	0.0666	0.5567	0.2544
Hspa12a	_IIIPQDVGLTILK_.2	#N/A	7.3800	7.1744	6.9609	7.2703	7.2681	7.1628	7.1951	7.4059	7.3746	7.2539	0.9857	-0.0208	0.2406	0.6188
Hspa12a	_TPTTILLTPER_.2	#N/A	7.4329	7.3775	7.3708	7.4829	7.3912	7.3253	7.1996	7.5098	7.6997	6.8443	1.0114	0.0164	0.5949	0.2256
Hspa12a	_[Acetyl (Protein N-term)]ADKEAGGGDAGPR_.2	#N/A	6.7908	7.1832	6.8871	7.3214	7.1426	7.1941	6.8750	6.9131	7.1099	7.1251	1.0115	0.0165	0.5050	0.2967
Hspa12a	_ELSDQAGSDFENSDVR_.2	#N/A	6.8157	6.2369	6.4967	6.5065	6.1765	6.2858	6.8565	6.4176	6.3687	6.8664	0.9687	-0.0459	0.2348	0.6292
Hspa12a	_GAVLFGLDPAVIK_.2	#N/A	6.3922	6.8472	6.6578	5.8669	6.6466	6.7461	6.5496	6.9198	6.7560	7.1518	0.9535	-0.0687	0.1646	0.7835
Hspa12a	_IFGEDFIEQFK_.2	#N/A	5.8889	5.9857	5.9045	5.3111	5.3391	5.4772	6.0658	5.6282	5.3503	5.9644	0.9824	-0.0256	0.6330	0.1986
Hspa12a	_WEGGDPGVSNQK_.2	#N/A	6.7195	6.0841	6.7482	6.9793	6.7774	6.9258	6.8177	6.9163	6.6131	6.4994	0.9991	-0.0013	0.9747	0.0111
Hspa12a	_ATAVDITTSK_.2	#N/A	5.3210	4.5736	4.3852	3.8804	5.6434	5.3708	4.5108	5.9008	5.6546	5.1700	0.9159	-0.1268	0.3248	0.4884
Hspa12a	_FISADQSVALGELVK_.2	#N/A	3.9376	3.2727	3.6713	2.5958	3.5903	3.4711	3.4364	4.4023	3.1823	2.8708	0.9857	-0.0208	0.8909	0.0502
Hspa12a	_ATGGPYGSLGVDYEFEK_.2	#N/A	4.1444	6.3029	3.8781	3.4219	5.9625	3.3364	3.5702	6.0183	3.8783	5.9305	0.9296	-0.1054	0.6948	0.1582
Hspa12a	_DFYHDLDPSEAK_.3	#N/A	5.4500	4.8224	5.7256	4.0386	4.3009	5.4262	3.7533	5.8942	4.9201	5.1003	1.0089	0.0127	0.9318	0.0307
Hspa12a	_EIQTIMQFGDTEIK_.2	#N/A	3.3654	4.1706	4.0522	5.6709	3.9920	3.7375	5.1363	3.0511	3.3502	3.6273	1.0985	0.1356	0.5127	0.2901
Hspa12a	_WSS[Phospho (STY)]QGMLR_.2	Q8K0U4_S437_M1_Hspa12a	4.3736	3.6780	3.5596	6.8935	3.4993	4.2302	3.7769	4.0562	3.7048	4.0293	1.1235	0.1680	0.4876	0.3119
Hspa12a	_TNPLNITLPFSFIDYYK_.2	#N/A	3.3280	1.4437	4.0148	3.2242	3.7864	2.0981	4.2322	2.4969	3.2496	2.9836	0.9204	-0.1197	0.6725	0.1723
Hspa12a	_M[Oxidation (M)]SPDAM[Oxidation (M)]NALFKPTIDSIIEHLR_.4	#N/A	4.2487	3.0938	3.9823	3.5261	3.3924	2.1894	3.6744	2.4343	3.7741	3.2034	1.0409	0.0579	0.7698	0.1136
Cnot3	_STDSEVSQS[Phospho (STY)]PAK_.2	Q8K0V4_S299_M1_Cnot3	8.9034	8.8440	9.5680	9.4279	8.4913	9.0360	9.3567	9.0517	9.2303	8.9575	0.9886	-0.0165	0.6409	0.1932
Rnf208	_KGS[Phospho (STY)]SELGFPR_.2	Q8K0W3_S103_M1_Rnf208	7.3129	7.1673	7.1572	6.7136	4.0411	4.0844	4.3231	4.3272	3.1254	3.8521	1.5561	0.6379	0.0314	1.5025
Fez1	_GLS[Phospho (STY)]LQSNR_.2	Q8K0X8_S298_M1_Fez1	6.9387	6.3013	3.8854	3.4292	3.0120	6.4396	5.5103	3.5720	3.8711	3.1064	1.2456	0.3168	0.3441	0.4633
Fez1	_S[Phospho (STY)]M[Oxidation (M)]EDLVNEFDEK_.2	#N/A	3.7066	8.8386	7.6315	9.8066	8.5165	8.9649	9.0149	9.1780	9.1286	9.1218	0.8683	-0.2038	0.3116	0.5065
Plekho2	_SSS[Phospho (STY)]LGDLLR_.2	Q8K124_S395_M1_Plekho2	4.3134	3.0290	4.0470	3.5908	2.8504	3.3783	3.7391	3.4104	3.7094	3.2681	1.0009	0.0013	0.9922	0.0034
Pdxk	_AEAGEGQKPSPAQLELR_.3	#N/A	8.5655	8.8272	9.2389	8.8986	8.9595	8.6494	8.6347	8.5444	8.7107	8.5869	1.0275	0.0392	0.0952	1.0213
Pdxk	_VLSIQSHVVR_.2	#N/A	7.1027	7.3421	7.3769	7.9410	7.7368	7.5640	7.4971	7.5826	7.5905	7.3630	1.0003	0.0004	0.9890	0.0048
Pdxk	_DKSFLAM[Oxidation (M)]VVDIVR_.2	#N/A	4.7165	4.4520	3.5472	4.4762	4.0702	4.3556	4.8510	3.7180	3.9602	3.7407	1.0497	0.0700	0.5160	0.2874
Pdxk	_VVPVADIITPNQFEAELLSGR_.2	#N/A	5.7563	5.5536	6.0718	6.4676	6.0021	5.5786	5.9860	5.9247	5.4320	5.6350	1.0280	0.0398	0.4559	0.3412

Pdxk	_VNDVNKYDYVLTGYTR_.3	#N/A	7.7125	7.6311	7.3544	7.7541	7.3858	7.8205	7.3580	7.1025	7.2998	7.5593	1.0382	0.0540	0.0548	1.2613
Pdxk	_SQELHELYEGLK_.2	#N/A	6.1110	6.0331	5.9829	6.1866	5.9973	5.9182	5.9439	5.5027	5.1454	5.9055	1.0736	0.1024	0.0297	1.5269
Pdxk	_DKVVPVADIITPNQFEAELLSGR_.3	#N/A	4.9605	5.2066	5.3115	4.1918	5.6851	4.0312	5.6418	5.0625	5.2693	5.2933	0.9212	-0.1184	0.2619	0.5819
Pdxk	_SFLAM[Oxidation (M)]VVDIVR_.2	#N/A	4.2375	4.1367	3.5095	3.4875	4.6422	4.3209	5.0821	4.4399	4.8028	4.2402	0.8738	-0.1946	0.0693	1.1594
Pdxk	_KPDGSTVTQR_.2	#N/A	6.7873	6.5976	7.3913	7.1146	7.4916	7.6810	4.4470	6.9667	6.4153	5.7274	1.2187	0.2854	0.0279	1.5543
Pdxk	_TVSAMQHVLQR_.2	#N/A	5.0449	3.8092	3.9470	6.7077	4.7914	3.2676	3.9372	3.9062	3.7477	4.6240	1.1334	0.1807	0.4252	0.3714
Pdxk	_DIEDPEIVVQATVL_.2	#N/A	2.9125	3.7177	3.5993	4.3597	3.8985	4.1904	4.9264	4.0165	3.6651	3.8577	0.9182	-0.1231	0.3536	0.4515
Cc2d1a	_LANHDEGS[Phospho (STY)]DDEEEETPKK_.3	#N/A	6.9822	6.0285	7.0464	6.7977	6.3051	6.1933	6.4458	6.9554	6.9934	6.5350	0.9742	-0.0377	0.5060	0.2959
Cc2d1a	_LANHDEGS[Phospho (STY)]DDEEEETPKKQNTPAASTTQLK_.4	#N/A	3.4440	4.2492	6.5204	3.1326	4.0705	3.6589	4.3482	3.4850	3.1336	3.0996	1.1885	0.2491	0.3462	0.4607
Cc2d1a	_SSPS[Phospho (STY)]KAPPSGPAPAGK_.2	Q8K1A6_S461_M1_Cc2d1a	3.2619	4.0671	3.9487	4.4513	3.8884	3.8411	4.1660	3.6672	3.3157	4.4183	1.0046	0.0066	0.9501	0.0222
Cc2d1a	_LANHDEGS[Phospho (STY)]DDEEEETPK_.2	#N/A	3.4522	4.9777	5.2668	4.3497	4.4434	4.9225	3.8595	4.7347	4.7095	4.9750	0.9998	-0.0003	0.9981	0.0008
Enho	_SADVDS[Phospho (STY)]LSESSPNSSPGPC[Carbamidomethyl (C)]PEK_.2	Q8K1D8_S43_M1_Enho	2.9239	3.6802	4.1584	3.7022	3.8264	4.7748	3.8505	4.6933	3.5980	3.3795	0.9907	-0.0134	0.9279	0.0325
Tcp11l2	_QC[Carbamidomethyl (C)]VSEDQQS[Phospho (STY)]DSESSR_.2	Q8K1H7_S16_M1_Tcp11l2	3.4285	3.7976	4.1153	4.6180	4.0550	3.6744	3.3895	3.5005	2.3910	3.0841	1.2772	0.3530	0.0180	1.7452
Sde2	_EDGIDAVEVAADRPGS[Phospho (STY)]PR_.2	#N/A	5.1522	5.0227	3.6311	6.0478	5.1068	5.0863	5.5813	6.0079	5.6647	5.8491	0.8670	-0.2058	0.0934	1.0296
Ppp1r11	_AFGESSTESDEDEEEGC[Carbamidomethyl (C)]S[Phospho (STY)]HK_.3	Q8K1L5_S91_M1_Ppp1r11	4.1053	3.2871	3.2913	3.5168	5.4016	3.3248	3.4209	4.0969	4.8725	4.5999	0.8996	-0.1526	0.4141	0.3829
Dnm1l	_ELPSAGS[Phospho (STY)]RDK_.2	Q8K1M6_S535_M1_Dnm1l	9.3908	9.3252	9.2977	3.6571	3.0941	9.2546	8.8636	9.3048	9.1918	9.2720	0.8011	-0.3199	0.2796	0.5535
Dnm1l	_SQLDINNKK_.2	#N/A	6.6041	6.5492	6.6159	7.1581	6.5686	6.4505	6.7432	6.9060	6.5732	6.9113	0.9815	-0.0270	0.4063	0.3912
Dnm1l	_LYTDFDEIRQEIENETER_.3	#N/A	6.3966	6.1881	6.5368	6.5204	5.7576	5.9978	5.2959	6.2960	6.4925	6.5112	1.0137	0.0196	0.7703	0.1134
Dnm1l	_DTLQSELVGQLYK_.2	#N/A	6.3751	6.4619	6.1408	6.0410	6.2279	5.9279	5.8753	5.6273	6.0666	5.8222	1.0595	0.0834	0.0239	1.6214
Dnm1l	_VFSPNVVNLTLVDLPGM[Oxidation (M)]TK_.2	#N/A	6.2193	4.6728	5.0033	5.5915	5.8063	5.6511	5.5065	5.4256	5.6170	5.4270	0.9994	-0.0009	0.9912	0.0039
Dnm1l	_ALQGASQIIAEIR_.2	#N/A	5.2869	5.4379	4.7366	4.2470	5.1069	5.0617	5.1066	4.7703	4.8424	5.4667	0.9867	-0.0193	0.7973	0.0984
Dnm1l	_SVTDSIRDEYAFLQK_.3	#N/A	6.4632	5.7835	3.5612	6.5762	6.3686	6.7731	6.3476	6.0135	6.3043	6.7291	0.9326	-0.1006	0.5130	0.2899
Dnm1l	_SQLDINNK_.2	#N/A	6.8193	6.6127	7.1260	7.2095	6.5208	6.7335	6.8800	6.6694	6.9240	6.8036	1.0026	0.0037	0.9078	0.0420
Dnm1l	_GHAVNLLDVPVPVAR_.2	#N/A	4.6225	4.8397	4.2935	5.2023	4.3101	4.4480	4.7679	5.1568	4.5805	4.0009	0.9985	-0.0022	0.9788	0.0093
Dnm1l	_RLEEPSLR_.2	#N/A	5.0691	4.4197	5.0736	4.7228	4.5461	4.6470	3.1590	4.3236	4.3227	3.4113	1.2477	0.3192	0.0094	2.0263
Dnm1l	_VPSALAPASQEPPPAASAEADGK_.2	#N/A	4.9300	4.8706	3.9064	3.4502	4.8354	3.2376	3.5984	4.8684	4.7994	4.0559	0.9710	-0.0424	0.7924	0.1011
Dnm1l	_DC[Carbamidomethyl (C)]EVIER_.2	#N/A	5.2773	3.0402	4.4226	3.5797	5.6025	4.6700	3.7279	4.6703	4.8317	3.3915	1.0666	0.0930	0.6418	0.1926
Dnm1l	_SKPIPIM[Oxidation (M)]PAS[Phospho (STY)]PQK_.2	#N/A	7.3276	8.3097	7.0882	9.4935	3.8888	7.8362	7.2972	8.1472	7.3478	6.6381	0.9954	-0.0066	0.9739	0.0115
Dnm1l	_TLESVDPLGGLNTIDILTAIR_.2	#N/A	3.3532	4.2350	4.5452	4.6710	4.2636	4.0886	4.4637	4.5123	4.2773	3.5180	1.0000	0.0000	0.9998	0.0001
Dnm1l	_SSLLDDLLTESEDM[Oxidation (M)]AQR_.2	#N/A	5.5230	5.6135	5.9378	5.4506	5.4979	5.7662	5.6561	5.4957	5.6823	5.5444	1.0066	0.0095	0.7261	0.1390
Dnm1l	_LQDVFNTVGADIIQLPQIVVVGTQSSGK_.3	#N/A	3.1300	0.4684	3.8168	4.3195	3.7566	4.1410	4.0342	2.5343	3.3536	4.2865	0.9211	-0.1185	0.7335	0.1346
Dnm1l	_YIETSELC[Carbamidomethyl (C)]GGAR_.2	#N/A	3.9837	3.2881	3.1697	3.6724	3.1094	3.1192	3.3870	4.4461	4.0947	3.6394	0.8712	-0.1990	0.0912	1.0400
Dnm1l	_IIQHC[Carbamidomethyl (C)]SNYSTQELLR_.3	#N/A	4.2615	3.3499	3.7627	3.7473	3.6221	3.9228	3.6767	3.8827	4.0268	3.6676	0.9906	-0.0136	0.8387	0.0764
Dnm1l	_IC[Carbamidomethyl (C)]YIFHETFGR_.2	#N/A	4.1751	4.0639	4.1056	4.0133	4.3461	3.8218	3.9119	3.5629	3.2404	3.8750	1.1206	0.1643	0.0203	1.6927
Dnm1l	_FATEYC[Carbamidomethyl (C)]NTIEGTAK_.2	#N/A	4.3901	3.4635	4.1238	3.6675	3.5338	3.0908	3.8158	3.1216	3.6327	3.3448	1.0669	0.0935	0.4126	0.3845
Dnm1l	_FISNPNSIILAVTAANTDM[Oxidation (M)]ATSEALK_.3	#N/A	3.6755	2.9799	4.3623	3.3641	2.8012	3.4274	3.0788	3.2535	4.4029	2.2381	1.0591	0.0829	0.6822	0.1661
Phldb2	_RLS[Phospho (STY)]AGTTVADVQK_.2	Q8K1N2_S465_M1_Phldb2	3.9264	3.4160	3.6601	3.2038	3.2374	2.9913	3.3521	3.7973	4.0964	4.3819	0.8717	-0.1980	0.0756	1.1213
Spire2	_DRS[Phospho (STY)]FSEHDLAQLR_.3	Q8K1S6_S443_M1_Spire2	3.8319	3.3728	5.0547	4.2458	4.0393	4.9254	3.3706	4.0517	4.1918	4.7761	1.0360	0.0510	0.7228	0.1410
Wdr11	_VYISSPHSS[Phospho (STY)]PAHNK_.2	#N/A	5.5882	4.9494	5.4295	4.6184	4.5893	3.9579	4.0341	4.7728	4.5562	3.6035	1.1447	0.1950	0.1364	0.8651
Coq9	_YTDQS[Phospho (STY)]GEEEEDYESEEQLQHR_.3	Q8K1Z0_S81_M1_Coq9	4.0412	5.9183	5.6918	6.2970	6.1076	6.2824	6.1082	5.3586	6.3039	6.0191	0.9623	-0.0555	0.6428	0.1919
Coq9	_LVQLGQAEK_.2	#N/A	4.7773	4.6561	3.8946	4.8385	5.1205	5.0761	5.1213	4.0793	5.2049	4.9276	0.9781	-0.0320	0.7371	0.1325
	GRDYC[Carbamidomethyl (C)]S[Phospho (STY)]EEEDIT.2	Q8K207_S115_M1_	10.0520	10.3856	11.0047	10.6916	10.3065	10.3705	10.6055	10.1391	10.2052	10.4497	1.0115	0.0164	0.5501	0.2595
	ANM[Oxidation (M)]HIS[Phospho (STY)]ESQEFFR.3	Q8K207_S93_M1_	2.9158	2.9257	4.1503	3.6941	4.2479	3.4816	3.8424	3.3071	3.6061	3.3714	1.0106	0.0152	0.9066	0.0426
Adgrg1	_LPISSGS[Phospho (STY)]TSSSR_.2	Q8K209_S681_M1_Adgrg1	3.1422	3.8985	4.3767	4.5171	3.7868	4.3386	4.2318	3.6014	3.2499	2.9833	1.1403	0.1894	0.1821	0.7397
Adgrg1	_NNSDS[Phospho (STY)]AKLPISSGSTSSSR_.3	#N/A	4.0844	3.3614	4.4914	3.4932	3.2202	3.2192	3.5054	3.1297	4.2792	3.4602	1.0143	0.0205	0.8801	0.0555
Pacs1	_QLS[Phospho (STY)]KPLSER_.2	Q8K212_S517_M1_Pacs1	10.2365	10.1294	10.2143	10.3999	8.3671	10.1321	9.9925	10.1914	10.2169	9.9893	0.9817	-0.0266	0.6515	0.1861
Pacs1	_DVSVPVAEIK_.2	#N/A	6.3107	6.1728	5.7971	5.9699	5.5719	6.0106	6.2693	5.8686	4.8173	5.3959	1.0688	0.0960	0.2092	0.6795
Pacs1	_S[Phospho (STY)]TPLKER_.2	Q8K212_S508_M1_Pacs1	3.7937	3.0981	4.4805	3.4823	2.9194	3.3093	5.2942	3.1353	4.2847	3.4493	0.8696	-0.2016	0.3020	0.5199
Pacs1	_ADLQGSAS[Phospho (STY)]PSKVEGTHTPR_.3	Q8K212_S493_M1_Pacs1	4.3666	5.5871	4.1003	3.6441	3.5103	3.1143	3.7923	5.7355	3.6562	3.3213	0.9824	-0.0256	0.9097	0.0411
Pacs1	_VAAVGSQSYLSSILR_.2	#N/A	4.3662	3.6141	3.9013	4.4040	3.8410	4.2255	3.3672	3.9265	3.4674	0.9999	1.3804	0.4651	0.0744	1.1285
Pacs1	_DTTS[Phospho (STY)]PM[Oxidation (M)]ELAALEK_.2	#N/A	7.2343	3.9399	3.5842	4.1838	3.9768	3.9589	7.4945	6.9786	3.2250	7.3837	0.7144	-0.4852	0.1314	0.8814
Pacs1	_YSSTFLDSAWR_.2	#N/A	5.3228	5.3549	4.7433	5.7680	4.6576	5.0283	3.3280	3.8215	4.1426	4.2773	1.3220	0.4028	0.0017	2.7694
Pacs1	_TNS[Phospho (STY)]SDSERSPDLGHSTQIPR_.3	Q8K212_S526_M1_Pacs1	7.0243	5.9866	8.0832	6.0143	5.8561	7.2136	7.5476	7.5919	7.3608	7.3571	0.8971	-0.1566	0.1317	0.8803
Pacs1	_GSGVAQS[Phospho (STY)]PQQQPQQQPPQPQQPTPPK_.3	Q8K212_S28_M1_Pacs1	3.9315	4.5301	4.1504	5.6658	3.5530	3.6689	3.7100	3.4084	3.8506	3.7664	1.1537	0.2062	0.1984	0.7024
Pacs1	_GS[Phospho (STY)]LGKDTTSPM[Oxidation (M)]ELAALEK_.3	Q8K212_S421_M1_Pacs1	3.4765	4.2817	4.1633	3.8390	4.1030	3.5024	3.9873	3.4525	3.1011	3.6948	1.0942	0.1299	0.1838	0.7357
Arfip2	_HPSHSTS[Phospho (STY)]PSGPGDEVAR_.3	Q8K221_S76_M1_Arfip2	5.1793	4.7959	4.1566	3.9156	4.9855	5.0130	3.6303	5.0141	3.9548	5.4494	1.0359	0.0509	0.7156	0.1453
Fcho1	_GHS[Phospho (STY)]PSC[Carbamidomethyl (C)]LAR_.2	Q8K285_S628_M1_Fcho1	3.1386	3.8949	4.3732	4.5207	3.7832	4.3422	4.2354	3.5978	3.2464	3.5942	1.0928	0.1280	0.3032	0.5183
Mtmr3	_RSS[Phospho (STY)]DPSLNEK_.2	Q8K296_S633_M1_Mtmr3	8.9484	9.1441	9.6737	9.3483	8.5405	4.2062	9.4820	9.4058	9.1731	8.8555	0.9004	-0.1513	0.4071	0.3903
Anln	_DSNLSAAS[Phospho (STY)]PKPGK_.2	Q8K298_S637_M1_Anln	10.0992	10.3316	10.8567	10.9002	10.0989	10.4042	10.6761	10.2001	10.2888	10.4201	1.0050	0.0072	0.7996	0.0971
Anln	_AES[Phospho (STY)]ADS[Phospho (STY)]LGSEDR_.2	#N/A	7.9042	7.6673	3.8108	8.2355	7.4718	8.0513	8.2049	8.0260	8.4116	7.9861	0.8815	-0.1820	0.2932	0.5329
Anln	_AESADS[Phospho (STY)]LGSEDRDLLYSIDAYR_.3	Q8K298_S656_M1_Anln	3.1834	4.2303	4.0855	4.3471	4.6419	4.4928	4.0515	3.7270	3.7330	3.6546	1.0981	0.1350	0.2109	0.6759
Anln	_KDS[Phospho (STY)]LGPDKK_.2	Q8K298_S887_M1_Anln	3.6327	3.6323	4.9797	3.9277	3.7546	3.9749	4.6270	4.5420	3.4049	3.8032	0.9730	-0.0395	0.7594	0.1195
Anln	_LNQVIVDIR_.2	#N/A	3.6911	2.9954	4.3779	3.6244	-0.0451	3.4119	3.7727	3.2379	3.6758	3.4723	0.8502	-0.2342	0.5274	0.2779
Anln	_EVEMS[Phospho (STY)]VDDEDINSSR_.2	Q8K298_S548_M1_Anln	3.8759	3.1802	4.5627	3.5645	3.0016	3.2271	3.5879	3.0531	4.2026	3.5315	0.9930	-0.0101	0.9444	0.0248
Sdha	_GEGGILINSQGER_.2	#N/A	8.5282	8.4140	8.6076	8.6679	8.4423	8.6586	8.7347	8.3765	8.6602	8.6432	0.9941	-0.0085	0.5605	0.2514
Sdha	_NTVIATGGYGR_.2	#N/A	7.5932	7.5996	7.5381	8.1981	8.1504	7.7617	7.7474	7.5867	7.8756	7.7319	1.0092	0.0133	0.6620	0.1791
Sdha	_ISQLYGDLK_.2	#N/A	7.1757	6.4940	6.5510	7.3501	6.7871	6.9448	6.3731	6.7436	7.3275	6.6014	1.0181	0.0259	0.6190	0.2083
Sdha	_AAFGLSEAGFNTAC[Carbamidomethyl (C)]LTK_.2	#N/A	6.2243	5.4498	6.3179	5.4533	5.6861	5.3140	5.6430	4.8287	5.0447	5.8140	1.0766	0.1064	0.1930	0.7144
Sdha	_VGSVLQEGC[Carbamidomethyl (C)]EK_.2	#N/A	7.7523	7.8545	7.6371	8.1368	7.7413	7.6574	7.8003	7.8627	7.7085	7.9897	0.9944	-0.0081	0.6876	0.1627
Sdha	_VSDAISTQYPVVDHEFDAVVVGAGGAGLR_.3	#N/A	5.7705	5.6114	3.6944	5.6823	5.6091	5.7062	5.5606	5.1655	5.9085	5.6415	0.9599	-0.0591	0.6187	0.2085
Sdha	_LGANSLLDLVVFGR_.2	#N/A	5.1926	5.7088	4.8018	5.2964	4.7035	5.0419	5.6878	5.7079	5.5622	4.7332	0.9449	-0.0817	0.2860	0.5436
Sdha	_WHFYDTVK_.2	#N/A	5.5016	5.6089	5.0819	5.2187	5.5006	5.0374	5.4435	5.6721	5.5390	5.0545	0.9811	-0.0275	0.5461	0.2627
Sdha	_AFGGQSLK_.2	#N/A	4.3005	5.3882	5.2884	4.3123	5.4386	4.1471	3.6775	6.1375	5.6722	5.3067	0.9258	-0.1113	0.4850	0.3143
Sdha	_SM[Oxidation (M)]TLEIR_.2	#N/A	5.2737	4.5072	4.2722	3.5540	5.0135	4.4910	3.2352	5.5210	5.2677	6.2399	0.8920	-0.1650	0.3841	0.4155
Sdha	_SM[Oxidation (M)]QNHAAVFR_.2	#N/A	5.6024	6.4630	6.3455	4.0027	6.6521	6.5445	6.0221	6.4233	6.4945	5.9200	0.9550	-0.0665	0.6127	0.2127
Sdha	_ANAGEESVM[Oxidation (M)]NLDK_.2	#N/A	6.0166	5.5896	6.0931	6.1323	6.0136	6.2421	5.9709	5.7878	5.5597	5.9068	1.0359	0.0508	0.1619	0.7907
Sdha	_GVIALC[Carbamidomethyl (C)]IEDGSIHR_.2	#N/A	4.3170	5.1606	4.8096	4.9791	4.8716	4.3371	5.0517	4.7467	4.6652	4.9375	0.9785	-0.0314	0.5969	0.2241
Sdha	_VTLEYRPVIDK_.2	#N/A	5.8507	6.1904	5.6410	6.3835	6.2113	6.2022	5.8528	6.2695	6.1889	6.4346	0.9828	-0.0251	0.5503	0.2594
Sdha	_VDEYDYSKPIQGQQK_.3	#N/A	4.8291	4.1871	3.1645	3.5853	4.4523	3.9426	3.9432	3.9126	3.5385	3.8353	1.0576	0.0808	0.5051	0.2966

Sdha	_TYFSC[Carbamidomethyl (C)]TSAHTSTGDGTAM[Oxidation (M)]VTR_.3	#N/A	3.3588	4.1640	4.0456	5.6696	3.9853	3.7442	4.1050	3.5702	3.2188	3.0144	1.1968	0.2591	0.1637	0.7860
Sdha	_TGHSLLHTLYGR_.3	#N/A	5.5175	5.4814	3.1247	5.2843	3.0645	5.2380	3.5250	4.3595	5.2798	5.4674	0.9915	-0.0123	0.9564	0.0194
Sdha	_SHTVAAQGGINAALGNMEEDNWR_.3	#N/A	4.9532	5.4531	3.3166	8.1196	4.2275	3.8979	3.7911	4.0421	3.6907	4.0434	1.2834	0.3599	0.2428	0.6148
Sdha	_HVNGQDQIVPGLYAC[Carbamidomethyl (C)]GEAAC[Carbamidomethyl (C)]ASVHGANR_.4	#N/A	5.2637	3.8937	4.0308	3.9072	5.0395	4.4865	4.2482	3.5965	5.4784	4.8620	0.9759	-0.0351	0.8108	0.0911
Hacd3	_EGS[Phospho (STY)]PETLTNLKK_.2	#N/A	3.0164	3.8216	7.1879	4.2059	3.6430	6.2623	7.1589	3.9126	6.7173	6.2552	0.7802	-0.3582	0.2302	0.6379
Hacd3	_EGS[Phospho (STY)]PETLTNLK_.2	#N/A	4.2669	6.8086	3.4529	3.9556	3.3926	4.3369	3.6702	4.1629	3.8115	3.9226	1.1226	0.1668	0.4824	0.3166
Hacd3	_WLDES[Phospho (STY)]DAEM[Oxidation (M)]ELR_.2	#N/A	7.9718	8.3547	7.4551	9.5699	7.1625	8.6040	9.1353	9.3248	9.6004	8.8897	0.8862	-0.1743	0.0506	1.2958
Edc3	_SQDVAIS[Phospho (STY)]PQQQQC[Carbamidomethyl (C)]SK_.2	Q8K2D3_S131_M1_Edc3	8.8101	9.2247	9.3118	9.9481	8.3973	9.3057	9.3584	9.1126	8.9977	8.9599	1.0065	0.0093	0.8361	0.0777
Edc3	_HNS[Phospho (STY)]WSSSSR_.2	Q8K2D3_S161_M1_Edc3	6.5590	5.7205	6.3003	6.6452	3.4585	5.6511	5.8402	3.4089	6.2488	3.2695	1.2197	0.2865	0.2686	0.5710
Brd3	_SES[Phospho (STY)]PPPLSEPK_.2	Q8K2F0_S262_M1_Brd3	10.6078	10.5776	11.2232	10.7140	9.5412	10.5705	11.1901	10.7212	10.5411	10.5234	0.9809	-0.0278	0.5212	0.2830
Lsm14a	_SS[Phospho (STY)]PQLDPLR_.2	#N/A	10.1872	10.0609	4.4669	10.5569	9.8652	3.9096	10.6776	9.9086	10.2787	10.3065	0.7942	-0.3324	0.2187	0.6601
Lsm14a	_SFFDNIS[Phospho (STY)]C[Carbamidomethyl (C)]DDNRER_.2	#N/A	3.5282	3.8887	3.3866	3.7704	3.7556	3.5793	3.6630	4.6774	4.6515	3.6356	0.8784	-0.1870	0.0782	1.1070
Lsm14a	_SS[Phospho (STY)]PQLDPLRK_.2	#N/A	10.2417	9.9559	4.1050	10.5593	9.6439	10.0925	10.4773	9.8708	9.9424	10.1658	0.8997	-0.1525	0.4450	0.3516
Lsm14a	_S[Phospho (STY)]PVPARPLPPTSQK_.2	Q8K2F8_S216_M1_Lsm14a	3.2843	8.4363	4.5188	8.2099	3.9289	4.1966	4.2109	7.1356	3.2376	7.2577	0.9943	-0.0083	0.9829	0.0075
Pphln1	_DAS[Phospho (STY)]PSSSSAVASSK_.2	Q8K2H1_S219_M1_Pphln1	10.7340	10.9931	11.3248	11.4131	10.9900	11.0423	10.9852	10.9063	10.7978	10.9727	1.0153	0.0220	0.2373	0.6247
Pphln1	_DAPFFRES[Phospho (STY)]PVGR_.2	#N/A	6.5522	7.1077	7.5402	7.7722	6.8234	7.3032	7.5253	7.1039	6.7958	7.2940	1.0005	0.0007	0.9900	0.0044
Pphln1	_DAPFFRES[Phospho (STY)]PVGRK_.3	#N/A	3.2379	3.7259	4.4724	4.0822	3.2853	4.2430	4.1644	3.4318	3.2841	3.6934	1.0543	0.0762	0.5312	0.2747
Otud6b	_[Acetyl (Protein N-term)]MEEVVAEELDDEEQLVR_.2	#N/A	3.9727	3.6339	4.2790	6.6822	3.4758	3.1024	4.4964	3.3368	2.9854	3.8952	1.1393	0.1882	0.4888	0.3109
Fntb	_PVPGFEEC[Carbamidomethyl (C)]EDEVTS[Phospho (STY)]DPATD_.2	Q8K2I1_S432_M1_Fntb	2.9789	3.7351	4.2134	3.1797	3.6234	3.0012	4.3952	6.8267	3.0866	3.4344	0.7790	-0.3604	0.2058	0.6866
Agfg1	_GTPSQS[Phospho (STY)]PVVGR_.2	Q8K2K6_S181_M1_Agfg1	10.3213	10.5475	10.8752	11.0699	9.5894	10.6607	10.3950	10.2641	10.2333	10.5195	1.0152	0.0218	0.5761	0.2395
Agfg1	_SSS[Phospho (STY)]ADFGTFSTSQSHQTASTVSK_.3	Q8K2K6_S293_M1_Agfg1	4.1926	3.1498	3.9262	3.4700	2.9712	3.2575	3.6183	3.5312	3.8302	3.1473	0.9895	-0.0153	0.8926	0.0493
Agfg1	_SISM[Oxidation (M)]TTFTQQEIEFLQK_.2	#N/A	3.0149	3.1884	3.9612	3.7932	4.1488	3.5807	3.9415	3.2080	3.5070	3.4705	1.0234	0.0334	0.7545	0.1223
Trappc12	_LSSLTAPVGEKS[Phospho (STY)]PDSTSPSYSTR_.3	Q8K2L8_S309_M1_Trappc12	7.1064	6.9889	7.5841	7.7162	4.8974	6.7862	7.0638	7.5082	7.3893	6.7999	0.9522	-0.0707	0.5387	0.2687
Slc38a1	_S[Phospho (STY)]LTNSHLEK_.2	#N/A	7.8107	7.6068	8.2071	7.8601	6.2428	7.4352	8.0361	8.6938	7.7307	3.3923	1.0810	0.1123	0.5939	0.2263
Slc38a1	_RS[Phospho (STY)]LTNSHLEK_.2	#N/A	6.0984	6.2769	6.9060	6.3661	5.1702	6.3398	7.0662	7.0083	6.4855	6.2351	0.9245	-0.1133	0.1658	0.7804
Slc38a1	_S[Phospho (STY)]LTNSHLEKR_.2	#N/A	6.0631	6.6426	6.7056	4.4718	4.9353	6.3199	6.4812	6.5413	5.5933	5.7789	0.9603	-0.0585	0.6496	0.1873
Slc38a1	_RS[Phospho (STY)]LTNS[Phospho (STY)]HLEKR_.3	#N/A	3.6290	3.6893	3.8357	4.0078	3.5489	4.3962	3.8542	3.7264	3.7689	3.8045	1.0165	0.0237	0.7074	0.1503
Ipo11	_NFEDSS[Phospho (STY)]PETLEAHK_.2	Q8K2V6_S343_M1_Ipo11	6.0741	6.4921	4.4848	6.0255	3.8949	4.2306	7.0267	2.9726	6.4526	3.7059	1.0319	0.0453	0.8731	0.0590
Obi1	_LKAEVDNRS[Phospho (STY)]PQK_.2	#N/A	3.9331	4.0498	4.8937	4.4802	3.6315	3.7917	3.4817	3.8400	4.3316	4.1008	1.0486	0.0685	0.5145	0.2886
Obi1	_SVEVEGPSKS[Phospho (STY)]PQGR_.2	Q8K2Y0_S566_M1_Obi1	5.8202	6.0154	4.2127	6.5579	3.4338	6.3388	6.2363	3.9938	6.0757	6.0753	0.9645	-0.0522	0.8037	0.0949
Ccm2	_TAQDPGIS[Phospho (STY)]PSQSLC[Carbamidomethyl (C)]AESSR_.2	Q8K2Y9_S164_M1_Ccm2	8.3506	8.6672	8.6157	9.0532	7.8966	8.7365	8.6831	8.6399	8.7196	8.5178	0.9900	-0.0146	0.6811	0.1668
Ccm2	_GIITDS[Phospho (STY)]FGR_.2	Q8K2Y9_S393_M1_Ccm2	4.0960	9.2688	3.2820	3.7846	8.4089	9.3851	3.4993	9.5455	3.9824	9.4399	0.9628	-0.0546	0.9048	0.0434
Matr3	_RDS[Phospho (STY)]FDDR_.2	#N/A	8.5994	8.3404	8.3418	8.8857	8.5859	8.1672	8.5925	8.4878	8.4389	8.5436	0.9966	-0.0049	0.8330	0.0794
Matr3	_S[Phospho (STY)]ATREPPYR_.2	#N/A	8.8091	8.5079	7.9456	9.4399	8.1204	7.6866	8.7969	8.5916	4.3282	4.1501	1.3018	0.3805	0.1053	0.9775
Matr3	_ITPENLPQILLQLK_.2	#N/A	6.9782	7.0327	6.9295	6.7469	6.9480	6.6888	6.2169	6.5188	6.8493	7.1104	1.0320	0.0454	0.2421	0.6161
Matr3	_GDTDQASNILASFGLSAR_.2	#N/A	6.4496	5.8527	6.3514	5.5697	6.2170	5.9211	6.1631	5.2639	5.4819	6.1900	1.0494	0.0696	0.2927	0.5336
Matr3	_IGPYQPNVPVGIDYVIPK_.2	#N/A	5.9735	5.9049	6.1758	6.6023	5.4610	5.7659	6.1218	6.0712	5.6268	5.9297	1.0073	0.0105	0.8465	0.0724
Matr3	_KRS[Phospho (STY)]YS[Phospho (STY)]PDGK_.2	#N/A	3.1167	6.3811	3.8035	4.1988	3.7432	3.9863	4.3471	6.1253	6.7542	3.8761	0.7970	-0.3273	0.2138	0.6699
Matr3	_S[Phospho (STY)]QESGYYDR_.2	Q8K310_S208_M1_Matr3	7.8482	7.3898	3.7762	8.0068	6.8983	4.0136	7.8330	7.4368	7.5791	7.5505	0.8319	-0.2656	0.2297	0.6389
Matr3	_TES[Phospho (STY)]PAEGKEQEEK_.2	#N/A	7.0158	6.6551	7.2551	4.5881	4.7162	7.0401	7.3363	6.9729	7.2560	7.2607	0.8620	-0.2143	0.1518	0.8189
Matr3	_SFQQSSLGR_.2	#N/A	6.1952	6.0307	6.1656	6.8522	6.3666	6.3414	5.5463	5.9284	6.6310	5.8972	1.0541	0.0760	0.1984	0.7024
Matr3	_TEEGPTLSYGR_.2	#N/A	6.5475	6.9059	3.6778	6.4313	7.0830	7.1592	6.6879	6.4027	6.5619	6.7536	0.9544	-0.0673	0.6677	0.1754
Matr3	_TDAQKT[Phospho (STY)]ESPAEGK_.2	Q8K310_T617_M1_Matr3	3.5551	3.5663	5.3531	4.9502	3.4289	3.9097	4.2950	3.3744	3.6735	3.7710	1.0923	0.1274	0.4556	0.3414
Matr3	_RDS[Phospho (STY)]FDDRGPSLNPVLDYDHGSR_.4	#N/A	4.0609	9.3947	9.8949	11.2809	9.8834	9.2909	9.7102	10.7250	10.3188	9.8401	0.8836	-0.1785	0.3875	0.4117
Matr3	_RSYS[Phospho (STY)]PDGK_.2	#N/A	3.6716	2.9759	4.3584	3.3602	2.7973	3.4314	3.0749	3.2574	4.4069	3.3272	0.9761	-0.0349	0.8256	0.0832
Matr3	_SYS[Phospho (STY)]PDGKES[Phospho (STY)]PSDKK_.2	#N/A	10.6540	10.8039	11.3677	11.3438	10.9307	10.9060	10.9297	10.9281	11.0229	10.8998	1.0051	0.0073	0.7196	0.1429
Matr3	_TDAQKTES[Phospho (STY)]PAEGKEQEEK_.3	#N/A	6.0785	6.9214	3.3909	4.4355	6.6763	6.3947	6.6245	6.4256	6.6828	6.3910	0.8650	-0.2092	0.2578	0.5887
Matr3	_DDWEEKR_.2	#N/A	6.9147	5.7747	6.4026	6.5770	6.7191	4.3690	6.1269	6.4038	6.7445	6.5804	0.9478	-0.0774	0.5130	0.2899
Matr3	_LC[Carbamidomethyl (C)]SLFYTNEEVAK_.2	#N/A	4.4562	3.0661	4.4486	3.4504	4.7687	4.4819	3.1651	5.0701	5.3331	5.2570	0.8737	-0.1948	0.3005	0.5221
Matr3	_SFQQSSLGRDS[Phospho (STY)]QGHGR_.3	Q8K310_S14_M1_Matr3	3.4101	5.2465	5.4240	5.2751	4.0547	4.7088	4.9294	4.3136	3.1118	3.8657	1.1557	0.2088	0.2506	0.6010
Matr3	_S[Phospho (STY)]ATREPPYRVPR_.3	#N/A	4.1184	3.3739	3.8521	3.5409	3.2622	3.3624	3.2556	3.0767	4.2262	3.0732	1.0520	0.0731	0.5402	0.2675
Matr3	_RRT[Phospho (STY)]EEGPTLSYGR_.3	Q8K310_T150_M1_Matr3	2.9618	4.6267	3.6486	5.8176	5.5770	3.9728	5.4580	5.2958	3.6158	5.3002	0.9017	-0.1493	0.4890	0.3107
Matr3	_SYS[Phospho (STY)]PDGKESPSDK_.2	#N/A	7.4054	7.6307	8.0620	7.9828	7.4944	7.6669	7.6724	7.8032	7.6908	7.7541	0.9970	-0.0043	0.8697	0.0606
Matr3	_DSFDDRGPSLNPVLDYDHGSR_.4	#N/A	4.2768	4.2231	3.9011	3.9424	4.7689	5.1065	3.3380	4.1469	4.1993	4.8880	1.0547	0.0769	0.5343	0.2722
Matr3	_VVHIMDFQR_.2	#N/A	4.8157	4.2739	4.3030	6.0430	3.7131	4.4124	3.9950	3.1544	3.4534	3.5241	1.3006	0.3792	0.0379	1.4216
Matr3	_VIHLSNLPHSGYSDSAVLK_.3	#N/A	6.1119	6.5596	6.8047	6.0356	5.8646	6.2866	5.6131	6.6184	6.3745	6.2995	1.0082	0.0117	0.8423	0.0745
Matr3	_GPSLNPVLDYDHGSR_.3	#N/A	3.8490	3.4277	3.5827	4.6273	3.2282	4.4147	3.2747	3.6711	4.4274	3.6094	1.0292	0.0415	0.7588	0.1199
Matr3	_DLSAAGIGLLAAATQSLSM[Oxidation (M)]PASLGR_.2	#N/A	4.0147	3.2115	3.2007	3.7033	3.1404	3.5249	3.4180	4.4151	4.0637	3.6703	0.8906	-0.1672	0.1240	0.9066
Champ1	_AVLPAS[Phospho (STY)]PEPR_.2	Q8K327_S532_M1_Champ1	7.7742	7.4561	8.4638	8.3465	6.9471	7.9085	8.1537	7.4146	7.7883	7.7075	1.0064	0.0093	0.8768	0.0571
Champ1	_KPSSS[Phospho (STY)]PDLWK_.2	Q8K327_S434_M1_Champ1	4.2986	3.6030	6.8260	3.9872	3.4243	4.3052	3.7019	4.1312	3.7798	3.9542	1.1325	0.1795	0.4412	0.3554
Champ1	_TAS[Phospho (STY)]PEPR_.2	Q8K327_S265_M1_Champ1	5.8979	5.8105	3.7177	5.5710	3.1797	3.0490	3.4098	3.7397	4.0387	4.4396	1.1614	0.2159	0.4042	0.3934
Champ1	_DSQENS[Phospho (STY)]DAELSSSEYIR_.2	Q8K327_S617_M1_Champ1	3.3424	7.1758	4.0293	8.4457	7.5000	7.8703	7.5666	7.8371	7.9474	8.1096	0.8129	-0.2988	0.2183	0.6609
Champ1	_SSC[Carbamidomethyl (C)]GS[Phospho (STY)]PPDLWK_.2	Q8K327_S464_M1_Champ1	3.6553	7.2057	4.3421	3.3439	2.7810	3.4477	3.8085	3.2737	4.4231	3.3109	1.1148	0.1568	0.6258	0.2036
Champ1	_RPGPPLS[Phospho (STY)]PEIR_.2	#N/A	3.1643	5.7765	4.3989	5.9743	4.1042	3.7301	4.0909	5.4980	4.8602	4.4130	0.9595	-0.0596	0.7673	0.1150
Champ1	_EQPSKDTESGKS[Phospho (STY)]PSPPER_.3	Q8K327_S108_M1_Champ1	3.5863	3.7410	3.7572	3.7325	2.7112	3.7597	2.5722	4.1223	4.1729	4.3482	0.9327	-0.1005	0.5296	0.2761
Champ1	_LM[Oxidation (M)]EALES[Phospho (STY)]PLEEQQI_.2	Q8K327_S795_M1_Champ1	3.1430	3.9482	3.8298	5.1008	3.7695	3.9600	4.3208	5.1534	3.1277	3.8498	0.9625	-0.0552	0.7495	0.1252
Champ1	_GQES[Phospho (STY)]SS[Phospho (STY)]DQEQVDVESIDFSK_.2	Q8K327_S641_M2_Champ1	3.5458	4.3510	4.2326	3.2344	4.1723	3.5572	3.9180	3.3833	3.0318	3.2014	1.1375	0.1859	0.1347	0.8707
Champ1	_LKKDS[Phospho (STY)]QENS[Phospho (STY)]DAELSSSEYIR_.3	Q8K327_S613_M2_Champ1	3.5458	4.3510	4.2326	3.7697	4.1723	3.5572	3.9180	3.2315	3.5305	3.2014	1.1348	0.1824	0.0713	1.1467
Ssh3	_QAS[Phospho (STY)]VDDSREEDKA_.2	Q8K330_S639_M1_Ssh3	5.0204	4.3218	6.5674	5.9163	5.5985	5.7988	5.5723	5.4641	3.5013	4.9543	1.1363	0.1843	0.2594	0.5860
Ssh3	_S[Phospho (STY)]PPASGHSTPVGPTQDR_.2	#N/A	3.2019	3.8138	4.1547	4.2252	3.7021	3.8401	6.1931	3.3956	3.3692	3.2635	0.9427	-0.0851	0.7058	0.1513
Atat1	_SSS[Phospho (STY)]LGNSPDR_.2	Q8K341_S272_M1_Atat1	3.7274	3.0318	6.7636	7.2315	2.8531	3.3756	3.7364	7.1247	6.9678	7.1785	0.7193	-0.4753	0.1827	0.7382
Atat1	_LLLATDPGGS[Phospho (STY)]PAQR_.2	Q8K341_S315_M1_Atat1	9.5630	9.6415	10.1374	10.0967	9.0138	9.3493	10.0559	9.5281	9.3852	9.6024	0.9990	-0.0014	0.9712	0.0127
Atat1	_RAT[Phospho (STY)]PPAHPPPR_.3	Q8K341_T261_M1_Atat1	2.9669	4.2593	3.6537	3.4879	3.5934	3.9779	3.8710	3.9621	3.6107	3.0583	1.0085	0.0123	0.9137	0.0392
Sash3	_SSS[Phospho (STY)]FKDFAK_.2	Q8K352_S27_M1_Sash3	5.4887	3.0526	4.0235	3.5672	3.4335	3.1911	3.7155	3.4339	6.0216	3.2445	0.9242	-0.1137	0.6622	0.1790
Cbr3	_FHQLDIDDPQSIR_.3	#N/A	7.8134	7.4076	7.9059	6.6575	7.8109	7.5762	7.9380	6.4432	6.3182	7.7834	1.0573	0.0804	0.3533	0.4519
Cbr3	_EYGGLNVLVNNAGIAFR_.2	#N/A	3.0061	3.8113	3.6929	4.3094	3.0715	4.0969	4.4577	3.9229	2.9908	4.1625	0.9437	-0.0837	0.5697	0.2443
Dennd1a	_S[Phospho (STY)]LEDLRAPK_.2	Q8K382_S592_M1_Dennd1a	10.1560	10.4682	11.0181	10.6272	9.1977	10.1935	10.6830	10.5904	10.2377	10.1792	0.9860	-0.0203	0.6698	0.1740
Dennd1a	_RPKS[Phospho (STY)]NITVEGR_.3	Q8K382_S510_M1_Dennd1a	3.0651	3.8703	3.7519	4.2545	3.6916	5.6556	5.0343	3.8639	3.5125	4.2215	0.9736	-0.0386	0.8367	0.0774
Dennd1a	_TM[Oxidation (M)]PS[Phospho (STY)]PQAETQDPR_.2	#N/A	9.4828	8.9056	9.3884	9.8360	8.0306	4.0225	9.4880	9.1640	8.2724	8.5442	0.9335	-0.0993	0.6167	0.2100

Dennd1a	_ESDSAEGDETES[Phospho (STY)]PEQLVR_.2	Q8K382_S546_M1_Dennd1a	3.0755	4.9274	4.3155	5.3234	4.8181	3.6836	5.4923	5.4940	4.0651	5.3079	0.8561	-0.2242	0.1887	0.7242
Plcl2	_RS[Phospho (STY)]LEAIPEK_.2	#N/A	9.7689	9.1515	9.4326	9.7440	8.0568	9.3009	9.8333	9.5595	9.5722	9.4117	0.9633	-0.0539	0.3158	0.5006
Plcl2	_S[Phospho (STY)]LEAIPEKASDENGD_.2	#N/A	8.3852	9.4211	9.5651	9.9277	8.5058	9.7088	10.2765	9.7225	9.6637	9.5665	0.9434	-0.0840	0.1556	0.8081
Plcl2	_LSSNC[Carbamidomethyl (C)]SGVEGDVT[Phospho (STY)]DEDEGAEM[Oxidation (M)]SQR_.3	#N/A	5.6907	6.4102	3.5685	7.2330	5.7014	6.5345	6.8445	6.8810	6.6910	6.5938	0.8673	-0.2054	0.2025	0.6936
Plcl2	_S[Phospho (STY)]LEAIPEK_.2	#N/A	3.4470	3.7791	4.1338	5.5556	4.0735	5.4537	5.4073	6.0764	6.1507	5.6246	0.7579	-0.3999	0.0178	1.7508
Plcl2	_TPS[Phospho (STY)]PAAGPVPR_.2	Q8K394_S87_M1_Plcl2	5.3277	4.3488	4.2304	3.2322	4.1701	3.5594	4.4477	3.3855	4.1241	3.1992	1.0939	0.1294	0.4402	0.3564
Plcl2	_RS[Phospho (STY)]LEAIPEKASDENGD_.2	#N/A	3.1148	5.5468	6.2435	5.8816	3.7509	5.8348	4.3117	5.7997	5.4946	5.4997	0.9594	-0.0598	0.7708	0.1131
Mepce	_ALNAET[Phospho (STY)]PKSS[Phospho (STY)]PLPAK_.2	Q8K3A9_S192_M2_Mepce	3.4162	8.2180	8.3656	8.3463	5.7568	8.2468	8.5339	8.1877	7.8933	8.1459	0.8618	-0.2146	0.3138	0.5033
Swi5	_ELYPDFDLNLND_.2	#N/A	3.9087	4.4104	4.9634	3.7102	4.2933	3.4977	2.4725	3.2909	3.9873	3.3875	1.2576	0.3307	0.0501	1.2997
Ahi1	_ERS[Phospho (STY)]PPLTPK_.2	Q8K3E5_S974_M1_Ahi1	9.4747	9.7942	9.9269	9.8714	3.7184	9.6459	9.4241	3.5329	9.3849	3.0517	1.3765	0.4610	0.2393	0.6211
Ahi1	_GKTPTADDS[Phospho (STY)]DDSR_.2	#N/A	2.9687	3.7739	3.6555	4.6241	3.5952	4.5375	4.4951	5.1927	2.9534	4.6795	0.8912	-0.1661	0.3711	0.4305
Ahi1	_NLSM[Oxidation (M)]TS[Phospho (STY)]PPPGPAK_.2	#N/A	10.0790	10.0621	10.2733	9.4729	9.0136	10.0312	9.8359	9.7143	9.6353	9.5774	1.0135	0.0194	0.6114	0.2137
Ahi1	_AVSNDNEDTNGDGVHEITSRDS[Phospho (STY)]PVHPK_.4	Q8K3E5_S181_M1_Ahi1	6.6454	3.6643	3.4118	6.6338	5.8484	7.1390	6.6121	6.4478	6.5784	6.7129	0.8435	-0.2455	0.2482	0.6051
Ahi1	_GKTPTADDS[Phospho (STY)]DDSREK_.2	#N/A	5.5348	6.2201	6.2642	6.2160	5.3483	6.1730	5.8840	3.8496	5.8097	5.5771	1.1286	0.1746	0.1556	0.8079
Ahi1	_TPTADDS[Phospho (STY)]DDSREK_.2	#N/A	5.9981	6.8344	4.5459	6.7048	2.9848	6.8298	6.6966	6.6582	6.5557	6.2533	0.8637	-0.2113	0.3009	0.5216
Ahi1	_TPTADDS[Phospho (STY)]DDSR_.2	#N/A	3.5030	4.3082	4.1898	3.8124	4.1296	3.5999	3.9607	3.1887	3.4878	3.4897	1.1110	0.1519	0.1012	0.9950
Ahi1	_VKERS[Phospho (STY)]PPLT[Phospho (STY)]PK_.3	#N/A	3.5768	4.3331	3.3105	4.0826	4.2214	3.9041	3.7972	4.0359	3.6845	4.0496	1.0033	0.0048	0.9532	0.0208
Ahi1	_ERS[Phospho (STY)]PPLT[Phospho (STY)]PKEK_.3	#N/A	4.3432	3.6476	3.5292	4.0318	3.4689	4.2606	3.1205	4.0866	3.7352	3.9988	1.0388	0.0549	0.5927	0.2271
Kcnq3	_GSAFTYPSQQS[Phospho (STY)]PR_.2	Q8K3F6_S599_M1_Kcnq3	9.7306	9.3912	10.2616	9.2911	8.1372	9.2892	10.2933	9.8616	9.4281	9.4692	0.9577	-0.0623	0.3225	0.4914
Kcnq3	_T[Phospho (STY)]PQGIGLLAK_.2	Q8K3F6_T82_M1_Kcnq3	3.1004	3.9056	3.7873	4.2150	3.7270	4.0025	4.3633	4.2870	3.0852	4.2569	0.9479	-0.0772	0.5192	0.2846
Vrk3	_DLNSSFET[Phospho (STY)]SPK_.2	Q8K3G5_T58_M1_Vrk3	6.2079	5.8043	4.5299	6.4419	5.6538	3.6598	4.0206	6.0237	6.2541	3.6363	1.0801	0.1112	0.6151	0.2110
Appl2	_YVLLNDQADDTGGS[Phospho (STY)]PSENR_.2	Q8K3G9_S651_M1_Appl2	3.3264	5.4016	5.5514	5.9246	4.9769	4.1565	6.0295	5.5165	5.4430	4.0105	0.9314	-0.1026	0.5675	0.2460
Appl1	_SESNLSSVC[Carbamidomethyl (C)]YIFESNNEGEK_.2	#N/A	3.0450	4.0071	4.2991	5.3360	3.8998	3.8485	5.6037	3.4361	5.3743	4.2015	0.8751	-0.1925	0.3248	0.4883
Appl1	_VNQSALEAVTPS[Phospho (STY)]PSFQQR_.2	Q8K3H0_S401_M1_Appl1	4.5240	5.9867	5.6755	4.3262	4.3834	5.0166	5.2565	6.0666	5.4817	4.8035	0.9229	-0.1158	0.3455	0.4616
Appl1	_EVFQIASNDHDAAINR_.3	#N/A	3.2408	3.9854	3.9276	4.4303	3.8673	4.2518	4.1449	3.6882	3.3368	4.3973	1.0151	0.0216	0.8390	0.0763
Myrip	_GLPS[Phospho (STY)]PGAHPR_.2	Q8K3I4_S540_M1_Myrip	7.5744	7.6160	7.9231	7.6534	6.4727	7.6412	7.6321	6.8769	7.0919	7.3881	1.0321	0.0456	0.4464	0.3503
Myrip	_GSLS[Phospho (STY)]SEENNQGVQEELK_.2	Q8K3I4_S618_M1_Myrip	3.5972	6.1665	4.2840	3.2858	3.5173	6.0214	4.5013	3.3319	2.9804	6.1853	1.0539	0.0757	0.7999	0.0970
Glcci1	_SAS[Phospho (STY)]WGSADQLK_.2	#N/A	10.3298	9.9884	10.0979	10.4206	9.2514	10.0895	10.4378	9.7174	10.1001	10.2198	0.9912	-0.0128	0.7230	0.1409
Glcci1	_S[Phospho (STY)]PGS[Phospho (STY)]PVC[Carbamidomethyl (C)]R_.2	Q8K3I9_S136_M2_Glcci1	8.0374	8.1127	7.9125	8.4321	7.1780	8.0899	7.8907	7.7364	7.7453	8.0292	1.0140	0.0201	0.6324	0.1990
Glcci1	_VPC[Carbamidomethyl (C)]NVEGIS[Phospho (STY)]PELEK_.2	Q8K3I9_S293_M1_Glcci1	8.3361	8.5155	8.6214	8.5088	7.5900	8.1442	9.1146	8.4160	8.5424	8.3782	0.9621	-0.0558	0.2041	0.6901
Glcci1	_SAS[Phospho (STY)]WGSADQLKEIAK_.2	#N/A	4.2111	3.1313	7.9743	7.2193	2.9527	7.0658	7.2950	6.9397	6.7711	7.1014	0.7722	-0.3730	0.2015	0.6958
Glcci1	_S[Phospho (STY)]IDTQTPSVQER_.2	Q8K3I9_S335_M1_Glcci1	9.5505	9.5421	9.9239	10.2037	8.4877	9.6718	9.8647	9.5004	9.5204	9.7586	0.9899	-0.0147	0.7581	0.1203
Glcci1	_STAGS[Phospho (STY)]PPAAAGSGTGPAGSC[Carbamidomethyl (C)]APAAGAGR_.2	#N/A	3.2041	4.0524	3.8909	7.7306	3.8306	3.8998	4.1860	3.7259	4.5113	4.3605	1.0569	0.0799	0.7855	0.1049
Glcci1	_TSS[Phospho (STY)]LDTITGPYLTGQWPR_.2	Q8K3I9_S163_M1_Glcci1	3.7399	3.0442	4.4267	7.1421	2.8656	3.3631	3.7239	3.4256	3.7246	3.2529	1.1600	0.2142	0.5090	0.2933
Ndufs8	_EPATINYPFEK_.2	#N/A	9.0531	9.1363	9.0648	9.2196	9.2809	9.1923	8.9763	8.8745	8.7949	8.9862	1.0281	0.0399	0.0026	2.5809
Ndufs8	_LC[Carbamidomethyl (C)]EAIC[Carbamidomethyl (C)]PAQAITIEAEPR_.2	#N/A	6.3232	6.8453	6.2248	7.0331	7.0979	7.1171	6.4875	6.4045	6.4547	6.9034	1.0322	0.0457	0.3724	0.4289
Ndufs8	_GLGM[Oxidation (M)]TLSYLFR_.2	#N/A	5.8854	6.2415	6.0792	5.8011	6.6656	6.3346	6.4635	6.3482	6.9138	6.3279	0.9470	-0.0786	0.1139	0.9436
Ndufs8	_RYPSGEER_.2	#N/A	5.4403	4.9225	5.3420	5.3693	6.2016	5.6046	5.3570	4.4380	4.9686	5.3852	1.0879	0.1216	0.1478	0.8304
Ndufs8	_ILM[Oxidation (M)]WTELIR_.2	#N/A	7.1533	7.2944	6.0443	6.9590	7.4373	6.8049	6.1814	7.0639	7.1304	6.3710	1.0392	0.0555	0.4325	0.3640
Ndufs8	_YDIDMTK_.2	#N/A	4.0293	3.3131	3.7630	3.3068	3.7615	3.4515	3.4551	3.6944	3.9934	2.9841	1.0205	0.0293	0.7557	0.1216
Optn	_T[Phospho (STY)]SDSDQQTYLFQR_.2	Q8K3K8_T529_M1_Optn	3.3906	3.8355	4.0774	4.5801	3.7238	4.4016	4.2948	3.5384	3.1870	3.0462	1.1379	0.1864	0.1632	0.7874
Minar1	_SWS[Phospho (STY)]LNTEEVPDFER_.2	Q8K3V7_S370_M1_Minar1	4.0431	3.2986	3.7768	3.3206	5.4316	3.4378	6.1494	3.6806	6.3555	2.9979	0.8100	-0.3040	0.2830	0.5482
Babam2	_[Acetyl (Protein N-term)]M[Oxidation (M)]S[Phospho (STY)]PEIALNR_.2	#N/A	5.9104	6.2553	6.7779	6.4494	5.0796	6.1224	6.3693	6.2464	6.1935	6.0289	0.9822	-0.0259	0.7237	0.1404
Casc3	_FGSS[Phospho (STY)]PQRDPNWIGDR_.3	Q8K3W3_S263_M1_Casc3	6.6153	2.9696	4.1065	3.6503	2.7909	3.4378	3.7986	6.2446	5.9450	5.9805	0.7153	-0.4834	0.1009	0.9963
Casc3	_GTVTGERQS[Phospho (STY)]GDGQESTEPVENK_.3	Q8K3W3_S145_M1_Casc3	4.6133	3.9300	4.9044	4.0855	4.7839	4.7860	4.5181	3.3828	3.0314	3.2019	1.2784	0.3543	0.0195	1.7106
Irf2bpl	_NSSS[Phospho (STY)]PVSPASVPGQR_.2	Q8K3X4_S638_M1_Irf2bpl	7.8908	9.3013	9.8763	9.9733	9.2411	9.5467	9.7713	9.2678	9.2642	9.5792	0.9825	-0.0255	0.6871	0.1630
Irf2bpl	_S[Phospho (STY)]PGPPPPVGVK_.2	Q8K3X4_S69_M1_Irf2bpl	8.5456	7.5054	7.6133	9.1968	7.3612	7.1159	8.5857	7.0715	8.2785	8.0821	0.9857	-0.0208	0.8192	0.0866
Irf2bpl	_RKAS[Phospho (STY)]PEPPDSAESALK_.3	#N/A	3.1922	7.1163	7.1407	6.9787	7.0983	6.6660	6.2846	4.3787	3.1770	6.5155	1.2508	0.3229	0.2449	0.6110
Irf2bpl	_RNS[Phospho (STY)]SS[Phospho (STY)]PVS[Phospho (STY)]PASVPGQR_.2	Q8K3X4_S638_M3_Irf2bpl	7.2313	7.7811	7.3514	8.3516	7.7500	6.5009	7.8746	7.4215	7.3934	7.8896	0.9803	-0.0287	0.6675	0.1755
Irf2bpl	_KAS[Phospho (STY)]PEPPDSAESALK_.2	#N/A	8.1415	8.3306	8.9480	9.1230	7.9555	8.1932	8.7536	8.2561	8.3433	8.2621	1.0053	0.0077	0.8667	0.0621
Irf2bpl	_RPGS[Phospho (STY)]VSSTDQER_.2	Q8K3X4_S313_M1_Irf2bpl	4.3587	4.1223	4.8486	3.6361	4.5521	4.3359	3.9215	4.6676	3.5424	5.0183	1.0050	0.0072	0.9511	0.0218
Pom121	_SGPTSS[Phospho (STY)]PFSSPASSR_.2	Q8K3Z9_S409_M1_Pom121	6.8269	3.5234	7.0661	6.7809	5.6324	5.1615	7.2975	3.7037	6.5746	6.6441	0.9632	-0.0542	0.8181	0.0872
Pom121	_SQT[Phospho (STY)]PERPAKK_.2	Q8K3Z9_T421_M1_Pom121	3.3203	4.0766	3.8364	4.3390	3.9649	4.1605	5.7516	3.7795	3.4280	4.3060	0.9151	-0.1281	0.4313	0.3652
Stxbp5	_SSS[Phospho (STY)]VTS[Phospho (STY)]IDKESR_.2	Q8K400_S783_M2_Stxbp5	9.9091	9.9363	10.5196	10.1328	8.8172	7.8989	10.3611	10.1252	9.9325	9.6088	0.9529	-0.0696	0.3883	0.4108
Stxbp5	_QPS[Phospho (STY)]GAGLC[Carbamidomethyl (C)]DITEGTVVPEDR_.2	Q8K400_S693_M1_Stxbp5	3.9955	3.2307	8.0633	3.6841	3.1212	8.6055	3.3988	4.4344	4.0830	3.6511	1.3147	0.3948	0.3729	0.4285
Stxbp5	_RPVSVS[Phospho (STY)]PSSSQEISENQYAVIC[Carbamidomethyl (C)]SEK_.3	Q8K400_S903_M1_Stxbp5	4.2259	3.9651	3.6896	3.5666	3.3599	3.8888	3.5239	3.6679	3.9962	3.6005	1.0231	0.0330	0.6429	0.1919
Mta1	_SAPVINNGS[Phospho (STY)]PTILGK_.2	Q8K4B0_S576_M1_Mta1	4.9934	3.0141	4.3966	3.6057	4.0958	3.3932	3.7540	3.3955	3.6945	5.1171	0.9815	-0.0269	0.8810	0.0550
Mta1	_NNM[Oxidation (M)]S[Phospho (STY)]PHGIPAR_.2	#N/A	8.4345	8.3996	8.9044	9.1345	7.9829	8.1912	8.7086	8.4980	8.4519	8.3597	1.0004	0.0006	0.9890	0.0048
Mta1	_ATVVNGTGTPGQS[Phospho (STY)]PGAGR_.2	Q8K4B0_S386_M1_Mta1	4.0284	3.2838	3.7621	6.3195	3.1721	3.4525	3.4541	3.6954	3.9944	2.9831	1.1335	0.1807	0.4719	0.3261
Ablim1	_QSLGES[Phospho (STY)]PR_.2	Q8K4G5_S470_M1_Ablim1	9.0877	9.2952	9.4757	9.6700	8.9055	9.4417	9.2924	8.9854	9.0921	9.3186	1.0153	0.0219	0.3928	0.4058
Ablim1	_TS[Phospho (STY)]SESIYSR_.2	Q8K4G5_S396_M1_Ablim1	7.5501	3.9167	4.1272	7.7520	3.5372	3.4742	6.9574	6.9666	3.6545	3.3482	0.9671	-0.0483	0.8980	0.0467
Ablim1	_STS[Phospho (STY)]QGSINSPVYSR_.2	Q8K4G5_S496_M1_Ablim1	11.3050	11.2857	11.8299	11.6874	10.8132	11.4519	11.4248	10.9323	11.1747	11.4367	1.0136	0.0196	0.4760	0.3224
Ablim1	_TSS[Phospho (STY)]LPGYGK_.2	Q8K4G5_S738_M1_Ablim1	7.6427	4.1877	4.3893	7.3978	3.7993	7.7816	5.7998	3.0682	7.4207	3.6103	1.1792	0.2378	0.5001	0.3010
Ablim1	idomethyl (C)]LC[Carbamidomethyl (C)]QLC[Carbamidomethyl (C)]AQPM[Oxidation (M)]SSS[Phosphc	#N/A	8.0287	5.1504	8.5684	8.8719	7.8624	8.0949	8.9156	7.9625	3.4863	8.0867	1.0914	0.1262	0.5983	0.2231
Ablim1	_RSS[Phospho (STY)]GREEDEEELLR_.3	#N/A	4.3911	3.4537	3.6473	4.6397	3.6694	3.9569	3.2475	3.5543	4.2177	3.9368	1.0590	0.0827	0.4707	0.3273
Ablim1	_TLS[Phospho (STY)]PTPSAEGYQDVR_.2	#N/A	11.2803	11.3656	11.8786	11.9528	11.1632	11.5815	11.8120	11.2675	11.4707	11.6239	0.9994	-0.0008	0.9732	0.0118
Ablim1	_SS[Phospho (STY)]GREEDEEELLR_.3	#N/A	3.1094	4.2331	4.3439	3.8877	3.9219	4.0549	4.0359	4.1973	3.4126	3.5650	1.0322	0.0457	0.6592	0.1810
Ablim1	_YDS[Phospho (STY)]PLHSASHAPSSK_.3	#N/A	4.8622	3.9047	5.2805	4.5009	4.7656	5.2706	4.2533	3.4751	3.5098	3.1623	1.3233	0.4042	0.0068	2.1699
Ablim1	_QERQS[Phospho (STY)]LGES[Phospho (STY)]PR_.2	#N/A	3.5825	3.7665	4.2693	1.4336	3.6465	4.6205	2.3206	3.4250	2.9951	3.7097	1.1415	0.1910	0.4951	0.3053
Ablim1	_TLS[Phospho (STY)]PTPSAEGYQDVRDR_.3	#N/A	3.2264	3.9826	6.7819	4.4330	3.8709	4.2545	4.1477	3.6855	3.3341	3.6819	1.1920	0.2533	0.2977	0.5263
Ablim1	_HSYTPTTSR_.2	#N/A	3.9875	3.2918	3.1734	3.3280	3.1132	3.1155	3.4763	3.6732	3.9722	3.0053	0.9443	-0.0827	0.4234	0.3732
Ablim1	_GVS[Phospho (STY)]MPNMLEPK_.2	Q8K4G5_S789_M1_Ablim1	3.2153	3.9716	4.4498	3.9936	3.8599	4.2656	4.1418	3.0076	3.3067	3.6709	1.1211	0.1649	0.1788	0.7476
Arhgef6	_KAS[Phospho (STY)]EEEYVIR_.2	#N/A	6.5828	6.7173	6.8570	6.8550	4.9480	6.6553	3.8483	6.7824	6.9077	6.7443	1.0602	0.0843	0.6143	0.2116
Arhgef6	_EIKPSERPLS[Phospho (STY)]PK_.2	#N/A	6.2411	6.1866	6.8668	4.1423	4.4951	6.1386	6.6451	6.6645	6.5110	3.9927	0.9538	-0.0682	0.7270	0.1385
Arhgef6	_KAS[Phospho (STY)]EEEYVIRK_.2	#N/A	4.1957	4.7036	5.3162	3.9031	3.6979	4.0316	3.8804	4.7569	3.5240	3.6059	1.0929	0.1282	0.3618	0.4415
Arhgef6	_KDS[Phospho (STY)]VPQVLLPEEEKLIIEETR_.3	Q8K4I3_S679_M1_Arhgef6	4.1904	3.4947	3.3763	4.6260	3.3161	4.4134	3.2734	3.2977	3.7641	4.3032	1.0665	0.0928	0.5075	0.2946
Arhgef6	_MS[Phospho (STY)]GFMYQGK_.2	Q8K4I3_S487_M1_Arhgef6	4.9468	2.9794	3.4327	3.9354	3.3724	4.3570	3.6501	4.1831	3.8317	3.9024	0.9860	-0.0203	0.8895	0.0509
Mrtfa	_FGSTGST[Phospho (STY)]PPVSPTPSER_.2	Q8K4J6_T488_M1_Mrtfa	6.9468	6.9564	7.6047	7.5816	3.6755	6.5783	7.6379	3.7644	6.8923	6.8636	1.0426	0.0601	0.7980	0.0980
Mrtfa	_AQQPAPASS[Phospho (STY)]PVKR_.2	Q8K4J6_S606_M1_Mrtfa	3.6654	3.6770	3.3991	4.4437	3.4983	4.2312	3.0911	4.0583	4.3573	4.1210	0.9775	-0.0328	0.7827	0.1064

Ddx54	_ALPSFPTSEC[Carbamidomethyl (C)]VS[Phospho (STY)]DVEPDTR_.2	Q8K4L0_S74_M1_Ddx54	3.0191	3.7753	4.1376	3.1394	3.6636	2.9610	4.3550	3.4782	3.1268	3.1064	0.9809	-0.0279	0.8465	0.0724
Ddx54	_IDDRDS[Phospho (STY)]EEEGPSNQR_.2	Q8K4L0_S774_M1_Ddx54	3.7438	2.9993	3.4775	4.5221	2.8876	3.7370	3.6302	3.9799	4.2789	4.1994	0.8854	-0.1756	0.1928	0.7148
Wdr33	_[Acetyl (Protein N-term)]ATEIGS[Phospho (STY)]PPR_.2	Q8K4P0_S7_M1_Wdr33	5.9768	8.7049	4.3345	8.4874	4.2743	8.4294	4.5519	3.2813	4.4307	3.3034	1.7219	0.7840	0.0352	1.4536
Wdr33	_SSS[Phospho (STY)]LDGDHHDGYHR_.3	Q8K4P0_S1273_M1_Wdr33	3.1618	3.9670	3.8486	4.3513	3.7884	3.9411	4.3019	4.2873	3.4158	2.5433	1.0566	0.0795	0.6081	0.2160
Wdr33	_DAPRPDHPPHDGHS[Phospho (STY)]PASR_.4	Q8K4P0_S1204_M1_Wdr33	5.8558	3.4821	3.5940	3.1378	3.3034	4.4261	3.2861	3.8634	4.1624	4.3159	1.0153	0.0218	0.9171	0.0376
Hecw1	_STS[Phospho (STY)]DTDLVTSDSR_.2	Q8K4P8_S68_M1_Hecw1	10.8538	10.8197	11.2910	11.0872	9.0675	10.4978	10.8998	10.7838	10.5658	10.6193	0.9893	-0.0155	0.7884	0.1033
Hecw1	_SYS[Phospho (STY)]AGEASEVSR_.2	Q8K4P8_S1073_M1_Hecw1	8.6964	8.5125	8.8383	8.5829	6.8054	8.6281	8.8035	8.6571	8.6711	8.6457	0.9597	-0.0594	0.3950	0.4034
Hecw1	_EGSLS[Phospho (STY)]PVNSQK_.2	#N/A	8.4000	8.6150	8.5217	8.5293	7.0020	7.8077	8.8286	8.4505	7.9354	8.2585	0.9734	-0.0388	0.5482	0.2611
Hecw1	_GST[Phospho (STY)]TEEEDGLEEESTLK_.2	Q8K4P8_T579_M1_Hecw1	4.0277	3.2314	3.5305	3.5123	3.9660	3.1759	4.9061	3.3337	4.0308	3.3530	0.9150	-0.1281	0.3646	0.4381
Hecw1	_REGSLS[Phospho (STY)]PVNSQK_.2	#N/A	4.7512	3.9073	5.2576	4.3652	3.7286	4.6945	4.3077	5.4687	5.1534	4.1981	0.9307	-0.1036	0.4110	0.3861
Hecw1	_C[Carbamidomethyl (C)]SPC[Carbamidomethyl (C)]SS[Phospho (STY)]PQNSPGLQR_.2	Q8K4P8_S1203_M1_Hecw1	3.6314	3.7110	3.3650	4.4097	4.2759	3.8495	4.5579	4.0924	4.3914	4.0869	0.9046	-0.1446	0.1045	0.9807
Hecw1	_APS[Phospho (STY)]PYRR_.2	Q8K4P8_S1219_M1_Hecw1	3.0525	3.8088	4.2870	3.8308	3.6970	4.4284	3.9790	3.1704	3.4695	3.5080	1.0903	0.1248	0.2900	0.5376
Hecw1	_SGS[Phospho (STY)]VHQMEQLNR_.2	Q8K4P8_S871_M1_Hecw1	4.2474	3.5517	3.4333	3.9360	3.3730	4.3564	3.6507	4.1825	3.8311	3.9030	0.9806	-0.0283	0.7553	0.1219
Rptor	_SVSS[Phospho (STY)]YGNIR_.2	Q8K4Q0_S722_M1_Rptor	9.0282	9.0524	9.3378	3.8785	7.6903	8.8550	9.5870	9.3563	3.4217	8.8993	1.0202	0.0288	0.9229	0.0349
Rptor	_ILDTSSLTQSAPAS[Phospho (STY)]PTNK_.2	Q8K4Q0_S863_M1_Rptor	7.6749	8.9664	9.1622	9.6890	6.1715	8.0469	8.9951	9.3111	8.4601	7.4368	0.9689	-0.0455	0.7240	0.1403
Pitpnc1	_KKS[Phospho (STY)]APETLTLPDPEK_.3	#N/A	8.2424	7.3406	7.6787	7.7137	5.6490	7.7330	4.1980	3.6352	7.4527	4.4503	1.4983	0.5834	0.0170	1.7697
Pitpnc1	_S[Phospho (STY)]APSSAPSTPLSTDAPEFLSIPK_.2	Q8K4R4_S270_M1_Pitpnc1	3.5806	5.0001	3.3830	4.1465	3.6170	3.9597	4.1927	4.2484	3.9298	3.9059	0.9702	-0.0437	0.7025	0.1534
Pitpnc1	_YEDNKGS[Phospho (STY)]NDSIFDSEAK_.3	#N/A	5.0988	4.8257	3.5985	5.3610	5.2384	5.5768	5.7584	5.7529	5.7898	6.2850	0.8395	-0.2525	0.0358	1.4455
Pitpnc1	_S[Phospho (STY)]APETLTLPDPEKK_.2	#N/A	2.9226	3.6788	9.6318	3.7009	3.5671	9.7269	4.4515	9.5968	9.6630	3.3781	0.8177	-0.2903	0.5738	0.2412
Naxe	_GNPSGIQPDLLISLTAPK_.2	#N/A	6.6873	7.0600	7.6933	6.9115	7.3396	7.3940	7.3248	7.1264	6.8917	7.2469	1.0047	0.0067	0.8714	0.0598
Naxe	_YHYLGGR_.2	#N/A	6.7542	7.1186	6.7094	6.8527	7.1164	6.8994	7.1160	6.7862	6.6407	6.9182	1.0063	0.0090	0.7282	0.1377
Naxe	_SPPTVLVIC[Carbamidomethyl (C)]GPGNNGGDGLVC[Carbamidomethyl (C)]AR_.2	#N/A	5.5566	5.8413	5.8452	6.5914	5.2733	5.1668	5.5902	5.6056	5.5252	5.8256	1.0134	0.0193	0.7841	0.1056
Naxe	_RGS[Phospho (STY)]ETMAGAAVK_.2	Q8K4Z3_S43_M1_Naxe	8.3520	7.7477	4.3648	9.8292	6.7419	3.4249	7.9704	6.3116	6.1347	7.2249	0.9758	-0.0353	0.9002	0.0456
Slc8a2	_GIS[Phospho (STY)]ALLLNQGNGDK_.2	#N/A	8.1311	7.8967	8.2859	8.3719	6.2811	7.9151	8.4862	8.2747	8.1504	7.4918	0.9646	-0.0521	0.5233	0.2813
Slc8a2	_S[Phospho (STY)]GIIIGAEGDPPK_.2	Q8K596_S266_M1_Slc8a2	6.4467	6.4715	3.3559	7.1698	2.9445	6.4570	7.1882	7.1561	7.1107	7.1508	0.7655	-0.3856	0.1096	0.9604
Slc8a2	_C[Carbamidomethyl (C)]QPGVLLPVWEPEDPSLGDK_.2	#N/A	3.4936	4.2444	4.1804	3.1822	4.1454	3.1667	4.3977	4.6420	4.6776	4.5892	0.8162	-0.2930	0.0134	1.8736
Slc8a2	_TNLALVIGTHSWR_.2	#N/A	3.4641	3.9755	4.6014	4.1451	3.8004	3.9326	2.3330	4.3569	2.0083	2.6650	1.4033	0.4888	0.0363	1.4406
Slc8a2	_GIS[Phospho (STY)]ALLLNQGNGDKK_.2	#N/A	3.7648	3.5776	3.4985	4.5431	2.9086	3.7160	3.1906	3.9589	4.2579	4.2204	0.9389	-0.0910	0.4936	0.3066
Dcaf8	_GHGHS[Phospho (STY)]DEEDEEQPR_.2	#N/A	4.8289	5.1294	5.4137	5.1617	5.5037	5.2354	5.2384	5.2905	5.2970	4.9786	1.0021	0.0031	0.9369	0.0283
Flcn	_AHSPAEGASSESSS[Phospho (STY)]PGPK_.2	Q8QZS3_S73_M1_Flcn	2.9385	4.4039	4.1730	3.7167	4.2253	3.5042	3.8650	3.2844	3.5835	3.3940	1.0836	0.1158	0.3517	0.4539
Acat1	_FASEITPITISVK_.2	#N/A	8.4227	8.4322	8.3514	8.6976	8.6049	8.5940	8.6336	8.4608	8.5317	8.6946	0.9927	-0.0106	0.4525	0.3444
Acat1	_QEQDTYALSSYTR_.2	#N/A	7.9710	7.8037	7.8790	8.3654	8.4262	8.4521	8.2355	7.7861	8.0114	8.1177	1.0139	0.0199	0.5267	0.2784
Acat1	_QATLGAGLPISTPC[Carbamidomethyl (C)]TTVNK_.2	#N/A	7.2540	7.0645	7.2526	7.5275	7.9368	7.7390	7.3163	7.2918	7.2588	7.2739	1.0243	0.0347	0.3271	0.4853
Acat1	_LGTAAIQGAIEK_.2	#N/A	6.6434	6.9888	6.7837	7.4691	7.2551	7.0892	7.3656	7.5592	6.9364	7.4368	0.9609	-0.0575	0.1662	0.7792
Acat1	_TPIGSFLGSLASQPATK_.2	#N/A	6.8882	7.2222	7.3972	7.0245	7.0483	6.8652	6.5731	6.8582	6.3626	7.1222	1.0513	0.0722	0.0730	1.1367
Acat1	_SKEAWDAGK_.2	#N/A	6.7306	6.8665	5.8447	6.7486	7.0178	6.8138	6.3089	6.4055	6.3674	6.8393	1.0293	0.0417	0.4394	0.3572
Acat1	_M[Oxidation (M)]LEIDPQK_.2	#N/A	5.5701	6.3478	7.0839	5.9733	6.6341	6.6014	6.1854	6.5117	6.2254	6.5000	1.0020	0.0029	0.9648	0.0156
Acat1	_LEDLIVK_.2	#N/A	5.9857	5.0994	3.1935	5.8009	5.9156	5.5725	2.0563	5.2745	5.4557	5.9836	1.1212	0.1650	0.5403	0.2674
Acat1	_EVYM[Oxidation (M)]GNVIQGGEGQAPTR_.2	#N/A	5.8228	5.7743	6.0425	6.2405	6.1418	6.1646	5.9751	6.0649	6.0577	5.9357	1.0038	0.0054	0.8274	0.0823
Acat1	_GATPYGGVK_.2	#N/A	5.0395	5.2098	4.9742	5.3550	4.1073	5.5313	3.9113	5.2197	3.0592	5.1103	1.1644	0.2196	0.1772	0.7514
Acat1	_ENGTITAANASTLNDGAAALVLM[Oxidation (M)]TAEAAQR_.3	#N/A	3.7081	3.8578	3.2725	4.3171	4.6189	3.7564	4.4656	3.5468	4.5740	4.2208	0.9334	-0.0995	0.3845	0.4152
Acat1	_DGLTDVYNK_.2	#N/A	5.7303	3.7213	3.6029	5.2626	5.1685	4.7541	3.8202	5.4158	3.6615	5.9108	1.0010	0.0014	0.9944	0.0024
Acat1	_EEVKEVYMGNVIQGGEGQAPTR_.3	#N/A	4.4588	4.2365	4.1181	6.1602	3.2231	3.6716	4.0324	3.4977	3.1463	3.0869	1.2530	0.3254	0.1469	0.8328
Acat1	_IAAFADAAVDPIDFPLAPAYAVPK_.3	#N/A	4.3837	3.6881	3.3880	4.4326	3.3414	3.6877	3.0800	4.0694	4.3685	4.1099	0.9778	-0.0324	0.8002	0.0968
Ccsap	_AHS[Phospho (STY)]VDVEK_.2	#N/A	10.8889	10.7818	11.2863	11.4957	10.7908	11.0104	11.0488	10.9184	10.9857	11.1859	1.0007	0.0010	0.9620	0.0168
Ccsap	_ETDGAPS[Phospho (STY)]GPGPR_.2	Q8QZT2_S129_M1_Ccsap	8.1918	3.9381	8.9576	4.3223	8.6876	3.9701	8.5720	8.6000	8.5060	4.2893	0.8469	-0.2398	0.4747	0.3235
Ccsap	_GALGEPS[Phospho (STY)]APS[Phospho (STY)]AREEEQPVGER_.3	Q8QZT2_S76_M2_Ccsap	3.5342	5.9090	4.2211	6.4509	5.9492	6.1079	5.9234	6.1285	6.0170	6.1410	0.8859	-0.1747	0.2878	0.5409
Ccsap	_QRAHS[Phospho (STY)]VDVEK_.3	#N/A	4.4961	4.5115	5.4980	4.9749	4.4710	4.9828	4.6736	3.0423	5.0394	4.9602	1.0858	0.1187	0.4003	0.3976
Ccsap	_QMDMGS[Phospho (STY)]QK_.2	Q8QZT2_S180_M1_Ccsap	3.0899	3.2824	3.7767	4.2794	3.7164	4.1009	3.9941	3.8391	3.4877	4.2464	0.9527	-0.0699	0.5072	0.2949
Stk32c	_GSSAAAPPSS[Phospho (STY)]PPPGR_.2	#N/A	14.3627	14.5192	15.1260	14.7553	13.9140	14.5123	14.7967	14.6921	14.5372	14.3886	0.9951	-0.0071	0.7491	0.1255
Stk32c	_RGS[Phospho (STY)]SAAAPPSS[Phospho (STY)]PPPGR_.2	Q8QZV4_S18_M2_Stk32c	6.3017	4.2583	7.8197	3.9824	7.3110	3.7842	3.9289	3.9769	7.4014	3.7711	1.1691	0.2254	0.5023	0.2990
Stk32c	_M[Oxidation (M)]S[Phospho (STY)]MSSISSGSAR_.2	#N/A	5.1938	3.3538	3.4897	4.2291	3.2007	4.0309	3.4204	4.0484	4.1952	4.0853	0.9947	-0.0077	0.9598	0.0178
Fam161a	_SS[Phospho (STY)]NPKPR_.2	Q8QZV6_S384_M1_Fam161a	3.2982	4.1033	3.9850	4.0173	3.9247	3.8048	4.1656	3.6309	3.2829	2.9538	1.0990	0.1362	0.2069	0.6842
Mapre2	_SDKDLETQVIQLNEQVHSLK_.3	#N/A	5.4141	5.2076	5.0484	5.4534	4.9675	5.4787	5.5251	5.1593	4.8569	5.7291	0.9895	-0.0153	0.7756	0.1104
Mapre2	_LALEGVEK_.2	#N/A	6.5792	7.0157	3.2203	6.8514	3.5396	3.0686	3.4846	6.8822	6.8566	6.6712	0.8447	-0.2435	0.4588	0.3384
Mapre2	_SSPASKPGSTPSRPSS[Phospho (STY)]AK_.3	Q8R001_S222_M1_Mapre2	4.2382	3.2847	3.4242	4.2959	3.1336	4.0967	3.4555	4.1917	4.0372	3.8938	0.9617	-0.0563	0.6261	0.2034
Mapre2	_EIELLC[Carbamidomethyl (C)]QEHGQENDDLVQR_.3	#N/A	5.4587	4.4512	5.3947	4.6814	4.3918	4.7256	5.0454	3.9745	4.6356	4.6786	1.0583	0.0817	0.3925	0.4062
Mapre2	_SHHANS[Phospho (STY)]PTAGAAK_.2	Q8R001_S199_M1_Mapre2	6.6032	4.4221	4.3037	6.1466	4.2435	3.4860	4.5211	6.5320	6.4363	6.1809	0.8226	-0.2818	0.1863	0.7299
Blmh	_NLVHSGATK_.2	#N/A	5.2454	3.7294	3.3466	4.3912	4.2575	3.8679	4.5395	4.1108	4.4098	4.0685	0.9667	-0.0488	0.6932	0.1591
Blmh	_VENSWGEDHGHK_.3	#N/A	3.5339	4.2901	3.2676	4.1255	4.1784	2.3266	3.8402	3.9930	3.6416	4.0925	0.9303	-0.1043	0.5073	0.2948
Gspt1	_ENLSAAFSR_.2	#N/A	4.2920	3.3789	3.6972	3.2409	4.4642	3.0284	4.9840	3.7602	4.5077	4.1169	0.8483	-0.2373	0.1070	0.9708
Itpka	_DTLEISDFFR_.2	#N/A	4.1501	4.4408	4.9872	3.5092	4.6946	5.1169	3.2239	3.8421	4.8744	4.6744	1.0793	0.1101	0.4634	0.3341
Itpka	_TTPLPDGQILDHR_.2	#N/A	5.3851	5.4872	4.5421	5.5742	5.4740	5.3641	5.9774	5.3470	5.0994	4.5291	1.0126	0.0181	0.8345	0.0786
Hnrnpl	_SSSGLLEWDSK_.2	#N/A	6.8395	6.6031	7.1436	6.9806	6.9082	6.7464	6.4902	6.5903	6.6580	7.3170	1.0157	0.0225	0.5633	0.2492
Hnrnpl	_FSTPEQAAK_.2	#N/A	7.0815	6.9777	6.9858	7.0585	7.5885	6.8000	6.6138	7.1404	7.1092	7.4833	0.9993	-0.0010	0.9816	0.0081
Hnrnpl	_IEYAKPTR_.2	#N/A	6.8093	7.2932	6.6200	6.7274	7.5228	6.5368	6.9367	6.8606	6.6691	6.4522	1.0280	0.0399	0.4162	0.3807
Hnrnpl	_VFNVFC[Carbamidomethyl (C)]LYGNVEK_.2	#N/A	4.9894	5.2296	5.0303	5.2877	5.8506	5.7758	5.3822	5.4178	4.7869	5.8888	0.9984	-0.0022	0.9751	0.0109
Hnrnpl	_YYGGGNEGGR_.2	#N/A	6.0370	5.9296	6.3978	6.0218	6.4275	6.4114	6.1795	6.3639	5.9567	6.0034	1.0128	0.0183	0.5882	0.2305
Hnrnpl	_NDQDTWDYTNPNLSGQGDPGSNPNKR_.3	#N/A	3.2982	4.0545	4.5328	4.3611	3.9428	4.1826	4.0758	3.7574	1.0158	4.3281	1.2331	0.3022	0.2686	0.5709
Hnrnpl	_ISRPGDS[Phospho (STY)]DDSR_.2	Q8R081_S182_M1_Hnrnpl	6.4418	6.2421	6.9788	7.0787	6.0850	6.4734	6.6919	4.1114	6.6798	6.4579	1.0943	0.1301	0.3225	0.4914
Hnrnpl	_ASLNGADIYSGC[Carbamidomethyl (C)]C[Carbamidomethyl (C)]TLK_.2	#N/A	3.5280	6.0100	3.2617	6.9648	5.6817	3.9528	4.4546	4.1957	5.9176	5.9633	0.9546	-0.0670	0.7934	0.1005
Hnrnpl	_LC[Carbamidomethyl (C)]FSTAQHAS_.2	#N/A	3.3675	4.1727	4.0543	3.9480	3.9940	3.7355	4.0963	3.0532	3.3522	4.8569	1.0102	0.0146	0.9144	0.0389
Hnrnpl	_SKPGAAMVEMADGYAVDR_.3	#N/A	3.5336	3.3446	3.7314	3.2752	3.1660	3.0627	3.4235	3.7260	4.0250	2.9525	0.9492	-0.0752	0.4383	0.3582
Gtf2f2	_HYQTEEKS[Phospho (STY)]D_.2	Q8R0A0_S248_M1_Gtf2f2	5.3280	3.7381	6.8653	5.7391	5.5355	2.9982	6.0574	5.5479	3.0895	6.9197	0.9316	-0.1022	0.7135	0.1466
Zbtb44	_DGSIS[Phospho (STY)]PVSSEC[Carbamidomethyl (C)]SAVER_.2	Q8R0A2_S161_M1_Zbtb44	5.7958	3.6532	5.2671	5.9114	5.6853	5.6423	6.5380	5.9107	5.6342	5.8802	0.8890	-0.1697	0.1852	0.7324
Tceal3	_[Acetyl (Protein N-term)]MEEVRGENEGKLEK_.3	#N/A	4.4805	3.9703	3.8338	5.5350	5.0066	3.6129	3.7752	3.7449	3.6917	3.9505	1.1625	0.2172	0.1470	0.8327
Kiaa0513	_SANSWLAEK_.2	#N/A	3.8889	3.8804	5.3756	3.9555	4.2938	3.9667	4.9206	4.0863	4.2263	4.0188	0.9800	-0.0291	0.8060	0.0936
Kiaa0513	_TVTM[Oxidation (M)]IS[Phospho (STY)]PEDEQK_.2	#N/A	8.7566	8.6940	9.2129	9.3473	8.1343	8.7825	9.3779	9.2103	8.9140	8.9168	0.9689	-0.0456	0.2665	0.5743
Kiaa0513	_FWNAAFFDAVHC[Carbamidomethyl (C)]ER_.2	#N/A	4.3997	3.8335	3.9990	4.5017	1.7343	4.3232	4.0091	3.0191	2.9324	3.3315	1.1431	0.1930	0.4251	0.3715

Kiaa0513	_QAVIGNLDEEQYK_.2	#N/A	4.3041	4.4457	5.0202	4.0249	4.3014	3.8123	4.1497	3.9125	3.4284	4.5541	1.0765	0.1064	0.3063	0.5138
Kiaa0513	_TVTM[Oxidation (M)]IS[Phospho (STY)]PEDEQKGEK_.2	#N/A	10.2340	10.4035	11.0816	10.8059	9.5617	10.2226	10.8153	10.5915	10.2758	10.2495	0.9906	-0.0136	0.7432	0.1289
Kiaa0513	_S[Phospho (STY)]SSNESFSSNQSADSAPDEETLALR_.3	Q8R0A7_S72_M1_Kiaa0513	3.1727	3.9290	4.4072	4.4867	3.8173	4.3082	4.2013	3.0502	3.2804	3.6283	1.1356	0.1835	0.1723	0.7636
Kiaa0513	_IFSGGEDLDQEEK_.2	#N/A	3.1370	3.8933	4.3716	3.9153	3.7816	4.3438	4.0636	3.0858	3.3849	3.5926	1.1063	0.1457	0.2192	0.6591
Fahd1	_IITLEEGDLILTGTPK_.2	#N/A	3.3894	3.7777	3.9215	3.1905	3.9342	4.0915	2.7493	4.3343	3.9067	3.7410	1.0094	0.0135	0.9165	0.0379
Tada3	_ESGADGASTS[Phospho (STY)]PR_.2	Q8R0L9_S298_M1_Tada3	3.8144	3.4118	4.5012	3.5030	2.9401	3.2886	3.2177	6.0675	4.2640	7.0146	0.6957	-0.5235	0.0649	1.1880
Aamdc	_ETGTEHS[Phospho (STY)]PGVQPADVK_.2	#N/A	5.8545	5.9883	4.1584	3.7705	5.8774	6.0937	6.0080	3.3560	3.6550	3.3794	1.2905	0.3679	0.1426	0.8459
Atf7	_TDSVIIADQTPT[Phospho (STY)]PTR_.2	Q8R0S1_T53_M1_Atf7	7.0805	7.3305	7.4237	4.0200	6.7612	7.6372	7.6745	7.1443	7.0443	7.3559	0.9184	-0.1228	0.4183	0.3785
Iqsec1	_SLGGQQGS[Phospho (STY)]PK_.2	Q8R0S2_S415_M1_Iqsec1	11.4670	11.6486	11.8664	11.4561	10.5966	11.2335	11.8357	11.6541	11.5252	11.2934	0.9828	-0.0250	0.4324	0.3641
Iqsec1	_LQHSTS[Phospho (STY)]VLR_.2	#N/A	8.9068	8.4110	9.0847	8.9752	7.8509	8.7418	9.2127	8.9119	8.8968	8.8007	0.9672	-0.0481	0.2661	0.5750
Iqsec1	_SALSSS[Phospho (STY)]LRDLSEAGKR_.3	#N/A	4.2386	3.8937	3.4245	7.8949	6.0096	6.7878	7.6546	4.1949	3.8435	6.9610	0.9490	-0.0755	0.8144	0.0892
Iqsec1	_M[Oxidation (M)]QFS[Phospho (STY)]FEGPEK_.2	#N/A	11.0658	10.9261	11.4384	11.2636	9.7936	10.8744	11.4536	10.8944	10.9248	10.9940	0.9844	-0.0227	0.5952	0.2253
Iqsec1	_SALSSS[Phospho (STY)]LR_.2	#N/A	3.2945	4.0508	4.5290	4.0728	3.9391	4.1864	4.2210	4.4292	7.0418	6.4050	0.7263	-0.4614	0.0346	1.4603
Iqsec1	_SLAES[Phospho (STY)]IDDALNC[Carbamidomethyl (C)]R_.2	Q8R0S2_S251_M1_Iqsec1	3.8312	3.0866	3.5649	7.9706	6.5144	3.6497	3.5428	4.2903	6.8090	6.5118	0.9019	-0.1490	0.6764	0.1698
Iqsec1	_ETLIGIYER_.2	#N/A	3.4608	3.1346	4.3399	2.8706	4.0584	3.3193	4.0319	5.4187	5.2557	4.7432	0.7261	-0.4617	0.0078	2.1074
Iqsec1	_VLINFNAPNPQDR_.2	#N/A	5.1504	4.2562	3.2282	4.2728	4.5868	4.9639	5.6588	5.1140	4.8492	5.2824	0.8438	-0.2451	0.0600	1.2218
Iqsec1	_ETRNS[Phospho (STY)]WDSPAFSNDVIR_.3	#N/A	3.6211	5.3839	4.8643	4.1552	4.6899	5.5531	4.8606	4.1468	5.7389	4.0320	1.0036	0.0051	0.9734	0.0117
Iqsec1	_LQHSTS[Phospho (STY)]VLRK_.2	#N/A	3.4192	4.2244	4.3525	3.8963	4.0457	3.6838	4.0446	3.1049	3.4039	3.5736	1.1147	0.1567	0.1235	0.9083
Iqsec1	_SALSSS[Phospho (STY)]LRDLSEAGK_.2	#N/A	3.7296	3.7553	3.6236	5.2071	3.8737	3.7246	5.6573	5.4684	5.4277	5.7228	0.7157	-0.4826	0.0010	3.0075
Iqsec1	_GVDDGEDIPR_.2	#N/A	3.9023	4.2643	5.2427	3.5909	4.9955	5.3505	3.2077	4.9175	4.9492	4.7287	1.0240	0.0343	0.8365	0.0775
Iqsec1	_S[Phospho (STY)]LHSEEVPASDTAR_.2	Q8R0S2_S260_M1_Iqsec1	6.8373	6.7123	4.2699	4.6240	6.5842	6.5238	7.5788	3.4945	3.1431	7.1999	1.1067	0.1462	0.6187	0.2085
Iqsec1	_STPS[Phospho (STY)]LERPEPR_.2	Q8R0S2_S362_M1_Iqsec1	2.9060	3.6623	4.1406	3.6843	3.5506	3.0740	3.8326	7.4141	6.9407	3.3616	0.6502	-0.6210	0.0587	1.2311
Iqsec1	_LRSS[Phospho (STY)]MSENR_.2	Q8R0S2_S159_M1_Iqsec1	4.3173	3.6216	3.5032	4.0059	3.2989	4.2866	3.1465	4.1126	3.7612	3.9729	1.0242	0.0345	0.7504	0.1247
Iqsec1	_SLS[Phospho (STY)]ESYELSSDLQDK_.2	Q8R0S2_S106_M1_Iqsec1	4.1176	3.2248	3.8513	3.3951	3.2614	3.1826	3.5434	3.6061	7.1198	3.0724	0.8086	-0.3066	0.3100	0.5087
Iqsec1	_NSWDS[Phospho (STY)]PAFSNDVIR_.2	Q8R0S2_S513_M1_Iqsec1	3.6442	3.5447	3.8738	10.7558	3.4759	4.0398	3.9330	10.3670	3.7655	3.6226	0.9017	-0.1493	0.7931	0.1007
Iqsec1	_SRS[Phospho (STY)]LS[Phospho (STY)]ESYELSSDLQDK_.2	#N/A	3.8941	3.2000	3.8010	6.5693	3.0435	3.3365	3.4931	3.6727	3.9718	4.1113	1.0027	0.0039	0.9843	0.0069
Cacnb4	_HSNHSTENS[Phospho (STY)]PIER_.2	#N/A	5.9826	5.5871	6.4589	6.2520	5.2351	5.9812	5.4299	5.4809	5.6763	5.7900	1.0575	0.0807	0.2105	0.6768
Cacnb4	_HSNHS[Phospho (STY)]TENS[Phospho (STY)]PIERR_.3	#N/A	4.2274	3.4811	3.9573	3.8298	3.6447	3.7939	3.8805	4.4104	4.2214	3.5857	0.9498	-0.0743	0.3309	0.4804
Cacnb4	_ATHTSSS[Phospho (STY)]TPM[Oxidation (M)]T[Phospho (STY)]PLLGR_.2	#N/A	7.5075	3.4591	5.0339	3.4123	7.3173	8.3280	4.7254	7.7341	3.8129	5.0769	1.0947	0.1306	0.7050	0.1518
Cacnb4	_SNTRSS[Phospho (STY)]LAEVQSEIER_.3	Q8R0S4_S287_M1_Cacnb4	3.9016	3.2060	4.5884	3.5903	3.0273	3.2013	3.3049	3.0274	4.1768	5.2111	0.9124	-0.1322	0.5026	0.2988
Cacnb4	_SDGS[Phospho (STY)]TTSTS[Phospho (STY)]FILR_.2	Q8R0S4_S38_M2_Cacnb4	3.3172	3.9095	3.1676	5.5478	3.9538	3.9843	4.3605	4.2909	5.0971	3.8895	0.9026	-0.1478	0.3919	0.4069
Cacnb4	_NRGS[Phospho (STY)]PGGC[Carbamidomethyl (C)]SHDSR_.2	#N/A	4.7905	3.4026	3.8808	5.6165	3.2909	3.4074	5.2587	4.9729	3.9493	3.8766	0.9004	-0.1514	0.4418	0.3547
Cacnb4	_NRLS[Phospho (STY)]SSSQHSR_.2	Q8R0S4_S474_M1_Cacnb4	3.3725	3.9846	2.8845	4.7792	3.9915	3.7713	2.4038	4.1422	3.3497	3.4844	1.1352	0.1829	0.3263	0.4863
Aldh1l1	_ILPNVPEVEDSTDFFK_.2	#N/A	5.8859	5.5130	5.8917	5.5615	5.2159	5.5591	6.0828	5.8767	5.8004	6.1041	0.9394	-0.0902	0.0354	1.4505
Aldh1l1	_LIAEGTAPR_.2	#N/A	5.7981	6.3772	6.2276	5.2888	6.3527	5.5375	5.2740	6.7707	5.8495	5.9973	0.9929	-0.0103	0.9028	0.0444
Aldh1l1	_KVS[Phospho (STY)]LELGGK_.2	Q8R0Y6_S671_M1_Aldh1l1	5.5054	5.5720	5.4654	6.0901	4.2318	5.4838	5.1171	5.5218	5.4768	5.6968	0.9887	-0.0164	0.8556	0.0677
Aldh1l1	_KLVEYC[Carbamidomethyl (C)]QR_.2	#N/A	6.3554	5.8741	5.8798	5.9858	5.9067	6.3219	7.0379	6.4885	6.6472	6.2804	0.9154	-0.1275	0.0112	1.9498
Aldh1l1	_EC[Carbamidomethyl (C)]DVLPDDTVSTLYNR_.2	#N/A	4.2493	3.8593	3.9742	3.5180	4.8708	4.2554	3.8100	4.3636	5.0043	5.0681	0.9035	-0.1465	0.2204	0.6567
Aldh1l1	_AGLILFGNDDR_.2	#N/A	5.6326	5.2399	3.9202	6.1154	4.4919	5.6130	5.6910	5.7624	5.5373	3.1413	1.0270	0.0384	0.8398	0.0758
Aldh1l1	_DLGEAALNEYLR_.2	#N/A	4.9450	5.2937	3.7519	5.2906	5.0656	4.5551	4.8434	5.1952	4.4081	4.7913	1.0327	0.0465	0.6577	0.1820
Aldh1l1	_ANATEFGLASGVFTR_.2	#N/A	4.7003	2.6161	3.2851	3.7878	3.7720	4.6253	3.5024	4.3307	4.1045	3.7548	0.9681	-0.0468	0.7785	0.1087
Aldh1l1	_GSASSALELTEEELATAEAVR_.2	#N/A	4.6053	4.2862	4.8813	5.2039	3.7933	3.4823	5.2265	4.5930	4.5579	4.7824	0.9135	-0.1306	0.2768	0.5578
Aldh1l1	_LRGEDGESEC[Carbamidomethyl (C)]VINYVEK_.3	#N/A	4.9343	3.6898	3.2385	4.2831	3.5803	4.1581	4.0839	4.2189	3.0171	4.4092	1.0123	0.0177	0.9055	0.0431
Aldh1l1	_IAVIGQSLFGQEVYC[Carbamidomethyl (C)]QLR_.2	#N/A	3.3096	4.0658	2.6694	3.6170	3.1601	4.1713	4.0645	4.4142	3.2124	3.6878	0.9100	-0.1360	0.3588	0.4451
Aldh1l1	_TYSTINPTDGSVIC[Carbamidomethyl (C)]QVSLAQVSDVDK_.3	#N/A	3.2397	3.9960	4.4743	3.9868	3.8843	4.2411	4.1343	3.6989	3.3474	3.6953	1.0676	0.0944	0.3404	0.4680
Aldh1l1	_DTNHGPQNHEAHLR_.4	#N/A	3.7036	3.5226	4.3904	3.3922	1.1132	3.3994	3.1069	3.2254	4.3749	3.3592	0.9252	-0.1122	0.6789	0.1682
Vps37c	_LALES[Phospho (STY)]PEVQDLQLER_.2	Q8R105_S29_M1_Vps37c	3.2228	3.9791	4.4574	7.3122	3.8674	4.2580	4.1512	6.3360	3.3305	3.6784	1.0325	0.0461	0.8792	0.0559
Flad1	_C[Carbamidomethyl (C)]LS[Phospho (STY)]PGGHPVYR_.2	#N/A	3.5830	5.0621	3.8169	4.5579	3.3532	4.1192	3.8494	3.7713	3.8315	3.7374	1.0750	0.1043	0.4087	0.3886
Apeh	_ALDVSAS[Phospho (STY)]DEEM[Oxidation (M)]ARPK_.2	#N/A	4.6287	4.9730	4.2702	5.7293	3.4289	5.4311	5.1797	5.0406	5.0412	5.1766	0.9284	-0.1072	0.4151	0.3819
Bud13	_VRHDT[Phospho (STY)]PDLS[Phospho (STY)]PPRR_.3	#N/A	6.4757	5.8318	6.4215	6.7471	5.4779	6.4392	5.4357	6.8973	6.4115	6.4566	0.9892	-0.0157	0.8481	0.0715
Bud13	_HDT[Phospho (STY)]PDPS[Phospho (STY)]PPRR_.2	#N/A	5.8652	5.6337	6.7127	3.9841	5.1698	5.3783	5.4079	6.1979	5.9439	6.3213	0.9145	-0.1290	0.3237	0.4898
Bud13	_AHHES[Phospho (STY)]PDLEHLK_.3	Q8R149_S297_M1_Bud13	3.4349	3.6537	3.4224	4.4670	3.4750	4.2545	3.1144	4.0350	4.3341	4.1443	1.0077	0.0110	0.9280	0.0324
Bud13	_HDLDAS[Phospho (STY)]PPRK_.3	Q8R149_S238_M1_Bud13	3.4113	4.2165	4.0981	3.9041	4.0379	3.6916	1.3675	3.0970	3.3961	3.5814	1.3610	0.4447	0.0426	1.3701
Bud13	_HDT[Phospho (STY)]PDLS[Phospho (STY)]PPRR_.3	#N/A	3.3036	3.9225	3.9904	4.4931	3.8108	4.3146	4.2078	3.6254	3.2740	2.9592	1.1296	0.1759	0.1666	0.7784
Bud13	_VRHDT[Phospho (STY)]PDLS[Phospho (STY)]PPR_.3	#N/A	3.6296	2.9339	4.3164	3.3182	4.2561	3.4734	3.8342	4.0797	2.9480	3.2852	1.0333	0.0473	0.7401	0.1307
Bphl	_TDFAPQLQSLNK_.2	#N/A	7.0854	6.6692	7.0059	7.1085	7.3458	7.1060	7.3642	6.6405	6.6533	6.7531	1.0293	0.0416	0.2885	0.5398
Bphl	_FTLVAWDPR_.2	#N/A	5.3951	5.7104	4.9644	6.1163	5.6651	5.7867	3.7615	5.4431	3.6870	5.7145	1.2053	0.2694	0.0767	1.1152
Bphl	_TC[Carbamidomethyl (C)]EDWVDGISQFK_.2	#N/A	4.8888	4.9667	4.4263	3.9701	5.2380	4.8084	4.1184	4.1872	3.8906	5.4332	1.0701	0.0978	0.4136	0.3835
Bphl	_FADEFNR_.2	#N/A	2.6970	2.6394	3.1059	3.7725	2.9671	3.3515	3.2447	2.1225	2.3493	3.4970	1.1019	0.1399	0.4287	0.3679
Bphl	_QVSLLGWSDGGITALIAAAK_.2	#N/A	3.0368	2.8834	3.7236	4.2787	3.6633	4.0662	4.4270	4.2233	3.0215	1.4878	1.0969	0.1334	0.6122	0.2131
Syngr3	_AGAAFDPVSFAR_.2	#N/A	8.9692	8.7445	8.5751	8.6001	8.2217	8.3781	9.0909	8.9356	8.5435	8.4699	0.9796	-0.0297	0.3493	0.4568
Syngr3	_FQQISSVR_.2	#N/A	5.9925	5.7091	5.5653	5.9058	6.1209	6.3155	6.0480	6.0131	5.9419	5.6638	1.0031	0.0044	0.9096	0.0411
Syngr3	_TTPGPGTAQAGDAAR_.2	#N/A	8.2127	8.0597	8.3376	8.1144	7.7665	7.8308	8.3613	8.3796	7.9617	7.8662	0.9891	-0.0158	0.5807	0.2361
Syngr3	_[Acetyl (Protein N-term)]MEGASFGAGR_.2	#N/A	3.4616	3.6048	3.6586	4.9769	3.3161	3.9828	3.8760	3.1902	3.7484	3.3361	1.0836	0.1159	0.4013	0.3965
Dock7	_SLSNS[Phospho (STY)]NPDISGTPTSPDDEVR_.2	Q8R1A4_S900_M1_Dock7	3.2208	4.1216	4.4553	3.9991	3.9429	3.7866	4.1474	3.0021	7.9188	7.8681	0.6838	-0.5483	0.1147	0.9404
Dock7	_SNS[Phospho (STY)]WVNTGPK_.2	Q8R1A4_S929_M1_Dock7	8.6322	8.4363	8.8825	8.3240	3.3209	8.1800	8.7126	8.7457	8.4080	8.1279	0.8977	-0.1557	0.4484	0.3484
Dock7	_SPS[Phospho (STY)]GSAFGSQENLR_.2	Q8R1A4_S1422_M1_Dock7	7.6216	6.9301	7.6013	3.8857	3.5435	3.6866	7.5755	3.8395	3.7867	7.1924	0.9904	-0.0139	0.9686	0.0139
Dock7	_KGS[Phospho (STY)]WSER_.2	Q8R1A4_S432_M1_Dock7	3.7006	2.9560	3.4343	3.9588	2.8443	3.7803	3.6734	4.1597	3.8083	3.9258	0.8854	-0.1756	0.1083	0.9655
Dock7	_TTS[Phospho (STY)]GDDAC[Carbamidomethyl (C)]NLTSFR_.2	Q8R1A4_S452_M1_Dock7	3.0954	3.5351	4.0279	4.2374	3.7292	5.1765	4.1723	4.1510	5.6026	4.0121	0.9522	-0.0706	0.6972	0.1567
Dock7	_RNS[Phospho (STY)]SLVGR_.2	Q8R1A4_S439_M1_Dock7	4.1583	4.3812	3.8920	3.5010	3.3021	4.7082	3.2157	3.5654	3.8644	3.1131	1.1601	0.2143	0.1074	0.9690
Dock7	_M[Oxidation (M)]NS[Phospho (STY)]LTFKK_.2	#N/A	3.7826	3.6846	6.1619	5.3593	3.5729	5.4327	6.0618	6.0572	5.4372	3.3839	0.8912	-0.1661	0.4748	0.3234
Dock7	_SMS[Phospho (STY)]IDDTPR_.2	Q8R1A4_S182_M1_Dock7	3.2554	4.0117	4.4899	6.6313	3.9000	4.2255	5.6483	3.7145	3.3631	4.3709	1.0339	0.0480	0.8442	0.0735
Stac2	_SKS[Phospho (STY)]VENFFLR_.2	Q8R1B0_S48_M1_Stac2	4.2708	3.5262	4.3868	7.7624	3.4145	3.2101	3.1033	3.2291	4.3785	3.3556	1.2593	0.3326	0.3397	0.4689
Eif3c	_QPLLLS[Phospho (STY)]EDEEDTKR_.2	#N/A	8.5909	8.6137	9.0035	8.1811	6.9065	9.0394	8.8284	9.4376	9.2551	9.0663	0.9172	-0.1247	0.1067	0.9719
Cplx3	_NETDESQIQLAGGDVELPR_.2	#N/A	4.2440	3.7599	4.2068	3.1549	4.4960	5.0549	4.9167	4.3958	3.6952	4.6861	0.9388	-0.0911	0.5084	0.2938
Mettl22	_AAS[Phospho (STY)]DPNPAEPAR_.2	#N/A	8.5668	8.4171	8.7584	8.6248	8.0067	8.5607	8.8135	8.5896	8.5207	8.1916	0.9953	-0.0067	0.8183	0.0871
Mettl22	_AAS[Phospho (STY)]DPNPAEPARDK_.2	#N/A	5.7731	5.9692	6.7271	5.6447	5.8352	5.7036	5.9920	5.7530	6.0854	5.4296	1.0219	0.0312	0.6030	0.2197
Hid1	_TGS[Phospho (STY)]QEGTSM[Oxidation (M)]EGSRPAAPAEPGTLK_.3	#N/A	4.5894	3.8171	6.9018	3.7930	3.5229	6.0627	6.6902	6.9739	3.6738	3.5786	0.9143	-0.1292	0.6722	0.1725
Hid1	_TGS[Phospho (STY)]QEGTSMEGSR_.2	#N/A	3.6097	4.4149	4.2965	3.7057	4.2363	3.4932	3.8540	3.3193	3.5945	3.2654	1.1286	0.1745	0.0865	1.0632

Uqcr10	_AFDQGADAIYEHINEGK_.2	#N/A	5.5401	5.2205	5.3561	4.3848	4.8487	4.7494	5.0988	5.1311	5.2957	5.4546	0.9564	-0.0642	0.3485	0.4578
Dync1li1	_NVLLLGEDGAGK_.2	#N/A	6.6702	6.9967	6.7851	7.1586	6.8553	6.4942	6.7229	6.5634	6.9714	6.7622	1.0106	0.0152	0.6153	0.2109
Dync1li1	_DAVFIPAGWDNDKK_.2	#N/A	6.6596	6.6753	6.6773	7.1554	6.5745	6.8795	6.4553	6.2968	6.5684	6.4116	1.0524	0.0737	0.0211	1.6767
Dync1li1	_FSLDALSLR_.2	#N/A	6.3539	6.2400	5.6434	6.0793	6.1103	6.0328	5.8600	6.4982	6.3254	6.4861	0.9657	-0.0503	0.2429	0.6146
Dync1li1	_KPASVSPTTPTS[Phospho (STY)]PTEGEAS_.2	Q8R1Q8_S516_M1_Dync1li1	12.8225	13.0644	13.6079	13.1619	12.6410	12.7843	13.3896	12.9400	13.0527	13.0117	0.9935	-0.0094	0.6740	0.1713
Dync1li1	_DFQEYVEPGEDFPAS[Phospho (STY)]PQRR_.3	#N/A	11.8942	12.0445	12.7251	12.9672	11.5858	12.2473	12.1710	12.4784	12.4144	12.2939	0.9923	-0.0112	0.7335	0.1346
Dync1li1	_DFQEYVEPGEDFPAS[Phospho (STY)]PQR_.2	#N/A	12.3239	12.2075	12.3863	13.3124	12.1637	12.4631	12.6609	12.5560	12.5458	12.4907	0.9931	-0.0101	0.7001	0.1549
Dync1li1	_IPAVVVEK_.2	#N/A	3.8227	4.2365	4.1264	4.7373	4.0063	3.4660	3.5154	4.3189	4.8754	4.8392	0.9267	-0.1097	0.3611	0.4424
Dync1li1	_SVSS[Phospho (STY)]NVASVSPIPAGSK_.2	Q8R1Q8_S415_M1_Dync1li1	3.6664	2.9708	4.3532	3.3550	2.7921	3.4366	5.4448	3.2626	4.4120	3.3220	0.8342	-0.2615	0.2077	0.6826
Dync1li1	_VPGGS[Phospho (STY)]PRT[Phospho (STY)]PNR_.2	Q8R1Q8_S405_M2_Dync1li1	3.5591	3.6671	4.2459	3.2477	3.5554	3.0692	4.4632	3.3699	3.0185	3.2147	1.0116	0.0166	0.9044	0.0437
Dync1li1	_GLEYLYLNVHDEDRDDQTR_.3	#N/A	4.0622	3.3666	3.2482	3.7508	3.9266	3.0408	4.2380	4.3676	4.0162	3.7178	0.8729	-0.1961	0.0591	1.2284
Dync1li1	_NIDLVYK_.2	#N/A	3.7395	3.0438	4.4263	3.4281	2.8652	3.3635	3.1428	2.0818	4.3390	1.1081	1.3035	0.3824	0.2267	0.6446
Stard7	_ATAPS[Phospho (STY)]PERK_.2	Q8R1R3_S356_M1_Stard7	3.9236	6.5819	3.1096	3.6123	5.6617	4.4338	3.3269	5.9691	4.1548	6.7377	0.8692	-0.2022	0.5061	0.2958
Chmp7	_VS[Phospho (STY)]PVNDVDVGVYQLM[Oxidation (M)]QSEQLLSR_.3	#N/A	3.7709	4.3589	4.8282	5.3481	4.1415	3.4088	4.0106	4.4767	4.5574	3.8093	1.0228	0.0325	0.8104	0.0913
Spart	_S[Phospho (STY)]PEQESVSTAPQR_.2	#N/A	7.9008	7.5960	7.3645	8.5728	7.2026	7.8299	8.0579	7.7521	7.6065	7.9515	0.9876	-0.0181	0.7174	0.1442
Spart	_DAC[Carbamidomethyl (C)]KKS[Phospho (STY)]PEQESVSTAPQR_.3	#N/A	6.1071	5.6875	6.1079	6.3960	5.2148	4.2208	3.7863	5.5424	4.9380	5.6003	1.1320	0.1789	0.2487	0.6044
Spart	_KS[Phospho (STY)]PEQESVSTAPQR_.2	#N/A	4.2385	4.8194	5.6597	5.8142	4.6479	5.1155	3.6642	5.7823	4.8880	4.8954	1.0503	0.0708	0.6153	0.2109
Aagab	_VASAESC[Carbamidomethyl (C)]HSEQQEPS[Phospho (STY)]PTAER_.3	Q8R2R3_S211_M1_Aagab	6.2590	4.3639	4.2130	6.6673	6.1113	3.5443	6.5914	6.9683	6.9530	6.0785	0.7812	-0.3563	0.0669	1.1748
Aagab	_AFWM[Oxidation (M)]AIGGDRDEIEGLSS[Phospho (STY)]DDEH_.3	Q8R2R3_S312_M1_Aagab	3.5770	3.7655	3.3106	4.3552	3.5868	4.1427	4.5035	4.1468	2.9450	4.0325	0.9700	-0.0440	0.7329	0.1350
Actr8	_RIPVS[Phospho (STY)]PEQTR_.2	Q8R2S9_S132_M1_Actr8	3.1376	6.4879	4.3721	3.9158	3.7821	4.3433	4.0641	3.0853	3.3844	3.5931	1.2288	0.2973	0.2194	0.6588
Tmcc3	_PGS[Phospho (STY)]DTALTVDR_.2	Q8R310_S4_M1_Tmcc3	5.7329	5.9946	6.8633	3.6422	3.5085	3.1161	3.7904	6.2261	5.4366	5.8678	0.9023	-0.1483	0.5826	0.2346
Ubqln1	_ALSNLESIPGGYNALR_.2	#N/A	6.3961	6.7038	6.5522	6.2597	6.8160	6.1542	6.4916	6.5677	6.6311	6.4446	1.0276	0.0393	0.4469	0.3498
Ubqln1	_EANLQALIATGGDINAAIER_.2	#N/A	4.4551	2.8263	4.1616	4.9397	2.9844	3.6370	3.7888	3.6955	4.3779	4.7757	0.9218	-0.1175	0.5097	0.2927
Ubqln1	_FQQLEQLSAM[Oxidation (M)]GFLNR_.2	#N/A	3.6928	4.5807	4.3796	3.6227	4.0477	3.4101	3.7680	3.2362	3.6776	3.3484	1.1277	0.1734	0.1174	0.9303
Ubqln1	_[Acetyl (Protein N-term)]AESAESGGPPGAQDSAADGGPAEPK_.2	#N/A	3.8947	3.4477	3.6284	4.1286	3.0384	3.5862	3.3204	3.8290	4.1281	4.3503	0.9267	-0.1098	0.3030	0.5185
Pspc1	_LFVGNLPTDITEEDFK_.2	#N/A	3.5954	3.8630	4.2822	3.8426	3.5190	4.5741	2.9959	3.3336	3.9249	3.6482	1.1353	0.1831	0.1096	0.9600
Pspc1	_FAQPGTFEFEYASR_.2	#N/A	3.8435	5.5986	3.9282	3.4720	6.1618	5.6842	5.0455	6.0651	6.1697	5.7342	0.8310	-0.2670	0.1580	0.8014
Ccdc12	_GQEDSLAS[Phospho (STY)]AVDATTGQEAC[Carbamidomethyl (C)]DSD_.2	Q8R344_S152_M1_Ccdc12	4.0108	3.8523	3.9619	4.5651	3.9016	3.6631	4.1793	4.2527	3.4143	3.6664	1.0295	0.0419	0.6224	0.2060
Rab11fip5	_TYS[Phospho (STY)]DEASQLR_.2	#N/A	9.5503	9.5300	10.0289	10.0093	8.4657	9.7528	9.6698	9.3329	9.4201	9.7308	1.0019	0.0027	0.9547	0.0201
Rab11fip5	_S[Phospho (STY)]PSHSSWLSTEGGR_.2	#N/A	7.8160	7.8669	4.2162	8.3067	3.3018	7.9593	7.9780	7.8398	7.6565	8.0404	0.8349	-0.2603	0.2826	0.5488
Rab11fip5	_RTYS[Phospho (STY)]DEASQLR_.2	#N/A	4.8345	5.4432	4.5667	5.8201	4.4787	3.7368	3.6299	5.2248	5.6467	3.8822	1.0473	0.0667	0.7000	0.1549
Rab11fip5	_GSPSLGAS[Phospho (STY)]PHHSSTGEEK_.3	#N/A	4.5427	6.8306	3.9633	4.4478	3.6676	3.5622	5.9351	6.7272	3.8813	4.7739	0.8448	-0.2433	0.3265	0.4861
Rab11fip5	_DKPRS[Phospho (STY)]PFSK_.2	Q8R361_S188_M1_Rab11fip5	4.0434	5.8640	3.7770	3.3208	3.1204	3.1083	3.4691	3.6804	3.9794	2.9981	1.0964	0.1328	0.5583	0.2531
Rab11fip5	_ATVALDEVFR_.2	#N/A	4.4427	3.3613	3.5622	4.0648	4.1443	4.2276	3.0875	4.0536	3.7022	4.0318	1.0668	0.0933	0.3963	0.4019
Rab11fip5	_RGS[Phospho (STY)]VGEKGS[Phospho (STY)]PSLGASPHHSSTGEEK_.4	Q8R361_S474_M2_Rab11fip5	3.2907	3.9355	3.9775	4.4802	3.9172	3.8123	4.1948	3.6383	3.2869	4.4472	1.0027	0.0039	0.9717	0.0125
Igsf8	_VLPDELQVSAAPPGPR_.2	#N/A	8.3990	8.1126	8.3275	8.0833	7.9804	7.8621	8.7360	8.3567	8.2765	8.1948	0.9686	-0.0460	0.0979	1.0093
Igsf8	_AIPEAPVGR_.2	#N/A	7.0359	7.3483	7.5849	6.5484	7.2812	6.5910	7.0675	7.2672	7.0475	6.8328	1.0016	0.0023	0.9618	0.0169
Igsf8	_LVAQLDTEGIGSLGPGYEDR_.2	#N/A	5.4704	5.7615	5.5371	5.6262	4.9125	5.5978	5.8284	5.7899	5.2662	6.0220	0.9577	-0.0624	0.2559	0.5919
Igsf8	_DSQFSYAVFGPR_.2	#N/A	5.5304	6.4055	6.2635	6.1493	5.4624	5.9183	6.4258	5.8553	5.3868	5.9773	1.0074	0.0106	0.8714	0.0598
Igsf8	_VASGDLQVQR_.2	#N/A	5.8713	6.0309	6.1269	6.0616	5.7536	6.0923	6.4455	6.2281	6.2704	6.0437	0.9588	-0.0607	0.0314	1.5035
Igsf8	_ATLQEVVGLR_.2	#N/A	4.8801	5.6360	3.6822	4.5808	5.0664	4.3347	3.3743	5.2999	5.9989	5.6180	0.9259	-0.1111	0.5305	0.2753
Igsf8	_HAAYSVGWEM[Oxidation (M)]APAGAPGPGR_.3	#N/A	5.2241	4.2563	4.1379	3.1397	4.8829	3.6518	6.1295	6.0533	5.5656	5.1952	0.7349	-0.4443	0.0075	2.1243
Igsf8	_LEAARPADAGTYR_.3	#N/A	4.1106	3.7334	4.1795	3.1813	4.8664	3.7129	4.3969	3.4363	3.0849	4.2487	1.0455	0.0641	0.6644	0.1776
Igsf8	_LTVHEGQELALGC[Carbamidomethyl (C)]LAQTK_.3	#N/A	3.8128	3.4133	4.4996	3.5014	3.3016	4.6659	3.2161	4.6333	4.2656	3.4684	0.9923	-0.0112	0.9414	0.0262
Igsf8	_AVLAHVDVQTLSSQLAVTVGPGER_.3	#N/A	2.8984	2.9432	4.1160	3.6767	3.5430	3.0816	3.8250	3.3245	3.6235	3.3540	0.9560	-0.0649	0.5725	0.2422
Igsf8	_EEGVVLEAVAWLAGGTVYR_.2	#N/A	4.0275	3.1986	3.2135	3.7162	3.0869	3.5377	3.4308	4.4023	4.0509	3.6832	0.8899	-0.1682	0.1255	0.9014
Clmp	_LVKPSSSSSGS[Phospho (STY)]R_.2	Q8R373_S293_M1_Clmp	3.0857	3.8909	3.7725	4.2298	3.7122	4.0173	4.3781	4.2722	3.0704	4.2421	0.9484	-0.0765	0.5307	0.2752
Mcmbp	_VS[Phospho (STY)]PSTSYTPSR_.2	Q8R3C0_S154_M1_Mcmbp	7.8757	7.6328	8.1216	8.2034	7.6214	7.9720	8.1513	8.0772	7.5590	7.8007	1.1461	0.1967	0.2605	0.5843
Tbc1d13	_SGVTNMSS[Phospho (STY)]PHK_.2	Q8R3D1_S184_M1_Tbc1d13	4.8920	4.3159	4.1975	3.8048	4.1373	3.5922	3.9530	3.4183	3.0669	3.1663	1.2221	0.2894	0.0263	1.5802
Mcat	_DSS[Phospho (STY)]VAEEGAQEAVAR_.2	Q8R3F5_S41_M1_Mcat	5.9681	5.9247	4.4373	3.4391	2.8761	3.3525	3.1538	3.1786	4.3280	5.7982	1.0531	0.0746	0.8031	0.0952
Mboat2	_RTS[Phospho (STY)]TQENVHLSQAK_.3	Q8R3I2_S472_M1_Mboat2	3.6890	3.5372	4.3758	3.3776	4.7937	3.1992	3.0923	3.2400	4.8394	3.3446	1.0888	0.1227	0.4512	0.3456
Cluap1	_KPEPLDES[Phospho (STY)]DNDF_.2	#N/A	3.1371	3.9423	3.8239	6.6645	5.9069	5.0119	4.3267	6.9818	7.2724	6.6109	0.7539	-0.4076	0.1132	0.9462
Cluap1	_IRKPEPLDES[Phospho (STY)]DNDF_.2	#N/A	4.2013	5.7867	3.3873	7.1465	5.4211	5.4208	5.3364	6.7028	3.6618	6.1854	0.9176	-0.1240	0.6083	0.2159
Ppp6r2	_NVPGLAAPSS[Phospho (STY)]PTQK_.2	Q8R3Q2_S670_M1_Ppp6r2	12.1669	12.0225	12.6223	12.1271	10.5679	11.8701	12.6046	11.8359	12.0267	11.6334	0.9893	-0.0156	0.7509	0.1244
Ppp6r2	_VGTEGLVDS[Phospho (STY)]FSQGLER_.2	Q8R3Q2_S289_M1_Ppp6r2	4.2262	3.4817	3.9599	3.5037	3.3700	3.2546	3.6520	3.4975	3.7965	3.1810	1.0286	0.0407	0.6569	0.1825
Exoc1	_LTGSTSS[Phospho (STY)]LNK_.2	Q8R3S6_S473_M1_Exoc1	6.5092	6.6330	6.7162	6.5813	4.4683	3.7666	6.5315	6.9838	5.0791	6.1532	0.9341	-0.0984	0.5970	0.2240
Sh3glb2	_GKVPVTYLELLS[Phospho (STY)]_.2	#N/A	6.4288	7.7002	8.0354	8.8552	7.7846	7.1853	7.4903	7.8577	7.7322	7.0500	1.0176	0.0251	0.7709	0.1130
Sh3glb2	_QTEVLLQPNPSAR_.2	#N/A	6.9374	7.4138	7.5747	7.3157	7.6968	7.1469	7.2075	7.1186	7.4628	7.1513	1.0155	0.0223	0.4885	0.3111
Sh3glb2	_NFLEGDWK_.2	#N/A	6.4508	6.4058	6.5410	6.4187	6.1597	6.7290	6.1553	6.2074	6.2042	6.6467	1.0234	0.0334	0.2949	0.5304
Sh3glb2	_VAQTEFDR_.2	#N/A	6.1659	5.9429	5.9023	6.1000	6.6654	5.9653	4.0620	5.8271	6.2077	6.2872	1.0943	0.1300	0.2614	0.5827
Sh3glb2	_VEEFLYEK_.2	#N/A	5.5769	4.1522	4.7604	4.9619	5.2080	4.6474	4.2295	4.9098	5.1152	4.7440	1.0284	0.0404	0.6556	0.1834
Sh3glb2	_SQTTYYAQC[Carbamidomethyl (C)]YR_.2	#N/A	6.0478	5.8981	4.4960	3.5063	6.0397	6.1608	6.0320	5.1293	5.8432	5.7861	0.9404	-0.0886	0.5760	0.2396
Sh3glb2	_LASDAGIFFTR_.2	#N/A	3.8961	4.2464	2.7073	4.1188	4.0834	3.4511	4.9856	4.4335	3.5456	3.9020	0.8895	-0.1690	0.2631	0.5799
Sh3glb2	_DFIHTASLSFLTPLR_.2	#N/A	3.7600	3.6719	3.5410	3.7061	3.4067	3.9158	2.9085	4.2202	4.0857	3.8730	0.9722	-0.0407	0.6871	0.1630
Sh3glb2	_TELDAHFENLLAR_.2	#N/A	4.7881	3.7478	4.3231	4.2882	3.9300	4.1742	4.2427	3.7014	4.0629	3.4546	1.0888	0.1227	0.1755	0.7558
Sh3glb2	_RLDLDAC[Carbamidomethyl (C)]K_.2	#N/A	6.6422	6.5718	6.2945	6.7384	7.1926	6.2655	6.4978	6.5137	6.8602	6.4568	1.0054	0.0077	0.8556	0.0677
Sh3glb2	_LLLEGISSTHVNHLR_.3	#N/A	3.4584	5.2463	3.1920	4.2366	6.3678	6.2055	3.9157	4.5617	3.0636	5.4230	1.1281	0.1740	0.5167	0.2867
Sh3glb2	_AVQFTEEK_.2	#N/A	4.3365	3.5919	4.3210	3.3229	3.9165	3.1444	4.5384	4.5379	3.4618	3.3771	0.9481	-0.0769	0.5845	0.2332
Sh3glb2	_VPVTYLELLS[Phospho (STY)]_.2	#N/A	3.3806	4.1369	3.1143	4.7753	4.0252	4.1003	4.3072	4.3596	3.1413	3.8362	1.0028	0.0041	0.9773	0.0100
Irf2bp1	_NVAEALGHS[Phospho (STY)]PKDPGGGGGSVR_.3	#N/A	7.5955	7.1786	8.1664	8.2513	7.3282	7.6269	3.5379	7.4217	7.1528	7.3915	1.2063	0.2706	0.1310	0.8829
Irf2bp1	_KAS[Phospho (STY)]PEPEGETAGK_.2	#N/A	6.7347	6.9339	7.6174	7.4115	7.0590	7.3075	7.2132	6.9691	6.9315	6.8904	1.0252	0.0359	0.3470	0.4596
Irf2bp1	_NVAEALGHS[Phospho (STY)]PK_.2	#N/A	5.2600	4.4790	3.4403	5.5970	4.0442	5.0847	5.2193	4.3896	4.3405	5.4947	0.9568	-0.0638	0.6702	0.1738
Irf2bp1	_AGGAS[Phospho (STY)]PAASSTTQPPAQHR_.2	#N/A	5.5676	5.6946	6.2320	3.6140	3.0469	5.8285	5.6376	5.5991	5.3564	5.6856	0.8972	-0.1565	0.4226	0.3741
Irf2bp1	_RKAS[Phospho (STY)]PEPEGETAGK_.2	#N/A	3.9324	3.7330	4.8828	3.9315	3.5738	3.7368	3.8708	3.7152	3.6141	3.5881	1.0725	0.1010	0.3058	0.5145
Irf2bp1	_S[Phospho (STY)]PGPPALKHPTSK_.3	#N/A	3.4581	6.2555	3.6429	5.3498	3.5873	4.1589	3.6498	4.8827	4.5026	5.3155	0.9610	-0.0574	0.7890	0.1029
Irf2bp1	_S[Phospho (STY)]PGPPALK_.2	#N/A	3.7834	3.0389	3.5171	3.8759	2.9271	3.6975	3.5906	4.2425	3.8911	4.2390	0.8703	-0.2004	0.0619	1.2085
Cacnb1	_QDT[Phospho (STY)]FDADTPGSR_.2	Q8R3Z5_T499_M1_Cacnb1	7.5250	6.8742	4.0052	3.5489	6.9621	7.1736	7.0253	6.7208	6.8708	7.2904	0.8621	-0.2140	0.3164	0.4998
Cacnb1	_SDGS[Phospho (STY)]TSSDTTSNSFVR_.2	Q8R3Z5_S44_M1_Cacnb1	6.1579	7.0080	7.1995	6.4314	6.4651	6.9805	7.2214	6.5636	6.4825	6.9294	0.9865	-0.0197	0.7215	0.1417
Cacnb1	_LSSSKS[Phospho (STY)]GDNSSSSLGDVVTGTR_.3	#N/A	4.0114	6.2852	3.7451	3.4614	3.1719	5.7706	3.3822	6.4474	4.0844	4.0848	0.9795	-0.0298	0.9161	0.0380

Cacnb1	_S[Phospho (STY)]GDNSSSSLGDVVTGTR_.2	#N/A	8.0604	8.1939	7.5870	8.7465	6.7967	8.3259	8.4189	8.2315	6.3269	8.4755	1.0113	0.0161	0.8726	0.0592
Cacnb1	_QGS[Phospho (STY)]WEDEEDYEEEM[Oxidation (M)]TDNR_.2	#N/A	3.1849	6.2334	5.3238	7.0802	5.3577	6.4210	6.0504	6.2976	7.0614	6.0875	0.8786	-0.1868	0.3124	0.5052
Cacnb1	_ATHPPSS[Phospho (STY)]TPPNPLLNR_.3	Q8R3Z5_S417_M1_Cacnb1	3.6558	5.6694	3.3894	4.0039	3.7262	3.8254	3.7186	4.1149	5.1779	3.9709	0.9528	-0.0698	0.6931	0.1592
Cacnb1	_ALS[Phospho (STY)]RQDT[Phospho (STY)]FDADTPGSR_.2	#N/A	3.8305	5.2253	4.0261	5.3619	4.4904	5.5060	5.7136	5.3607	5.4239	5.2485	0.8719	-0.1978	0.1017	0.9929
Cacnb1	_GPYPPSQEIPM[Oxidation (M)]EVFDPS[Phospho (STY)]PQGK_.3	#N/A	3.7547	5.3899	3.4013	6.7576	4.3496	5.4332	3.6186	5.7799	5.5960	3.8709	1.0278	0.0396	0.8703	0.0603
Micos13	_DSWNSGIISVM[Oxidation (M)]SALSVAPSK_.2	#N/A	3.3549	5.3062	6.2993	5.1089	4.2820	5.1572	5.1628	5.0530	3.2227	5.7523	1.0251	0.0358	0.8616	0.0647
Micos13	_GSVAGGAVYLVYDQELLGPSDK_.2	#N/A	4.6902	4.3634	4.4535	4.5254	4.0779	4.9613	4.1455	4.1238	4.5201	3.6000	1.1012	0.1390	0.0884	1.0538
Cadm4	_VWSVASTVR_.2	#N/A	5.1810	4.9034	4.2834	4.8740	1.9335	5.0702	4.3251	4.8432	5.1461	5.5414	0.8812	-0.1825	0.3992	0.3988
Cadm4	_GSYLTHEAS[Phospho (STY)]GLDEQGEAR_.3	#N/A	4.7544	4.1110	4.2219	4.6730	3.7979	4.1361	4.4282	5.5396	5.3023	4.5340	0.8650	-0.2093	0.0482	1.3170
Cadm4	_FQLEEFSPR_.2	#N/A	4.3527	5.9629	5.8239	5.3174	5.0601	3.1281	6.4351	5.8311	2.9596	5.0518	0.9746	-0.0371	0.8774	0.0568
Cadm4	_LHQYDGSIVVIQNPAR_.3	#N/A	4.0794	2.9925	3.4708	4.5154	2.8808	3.7438	3.1628	4.1923	4.2857	4.1927	0.9130	-0.1314	0.3964	0.4018
Purg	_RAS[Phospho (STY)]GEEQEC[Carbamidomethyl (C)]LD_.2	Q8R4E6_S342_M1_Purg	10.6191	10.6233	11.0757	11.1806	10.4616	10.7438	10.9060	10.8681	10.8784	10.6827	0.9954	-0.0066	0.7484	0.1259
Purg	_DALVQLIEDYGEGDIEER_.2	#N/A	5.1906	5.3483	6.7354	6.3507	5.3807	5.5575	5.7751	5.3691	5.2247	4.0191	1.1302	0.1766	0.1686	0.7731
Purg	_HSAPS[Phospho (STY)]PPVSVGS[Phospho (STY)]EEHPHSVLK_.3	#N/A	6.6261	6.5598	6.4805	6.6218	4.1501	7.1112	6.5382	6.5385	8.2608	3.9246	0.9909	-0.0131	0.9502	0.0222
Purg	_QKHSAPS[Phospho (STY)]PPVS[Phospho (STY)]VGS[Phospho (STY)]EEHPHSVLK_.4	#N/A	3.8861	5.6231	3.3166	6.0098	5.6800	5.5978	4.0965	6.7392	6.5234	5.9069	0.8629	-0.2128	0.3148	0.5020
Arhgef12	_SEGVQDAEPQSLVGS[Phospho (STY)]PSTR_.2	Q8R4H2_S341_M1_Arhgef12	8.7114	8.5573	8.8588	8.8585	3.8074	8.6679	8.9199	8.5820	8.6298	2.9626	1.0875	0.1211	0.6888	0.1619
Arhgef12	_TGTGDIATC[Carbamidomethyl (C)]DS[Phospho (STY)]PR_.2	Q8R4H2_S1327_M1_Arhgef12	7.3400	6.9098	7.4213	7.5177	5.7482	6.6028	7.5549	7.2418	6.6024	7.1006	0.9717	-0.0414	0.6075	0.2165
Arhgef12	_ITS[Phospho (STY)]PVLVGEENNVVHNQK_.3	#N/A	3.4878	5.6299	3.6592	6.5914	3.3798	5.6261	3.6590	5.8651	3.9020	5.7228	0.9878	-0.0176	0.9467	0.0238
Arhgef12	_HLSTPSSVS[Phospho (STY)]PEPQDPAK_.2	Q8R4H2_S637_M1_Arhgef12	3.6172	4.3735	3.3509	4.3955	4.2618	3.8636	4.5438	4.1065	4.4055	4.0728	0.9288	-0.1066	0.2395	0.6208
Arhgef12	_TDWSSGDASRPSSDSADS[Phospho (STY)]PK_.3	Q8R4H2_S309_M1_Arhgef12	3.0948	3.8511	4.3293	3.8731	3.7394	4.3861	4.2792	3.1281	3.4271	3.5504	1.0786	0.1092	0.3842	0.4154
Arhgef12	_VEHHDLSVAGLQS[Phospho (STY)]PDR_.3	Q8R4H2_S1176_M1_Arhgef12	5.3229	4.8531	3.7951	4.2978	4.1566	3.9946	4.3554	5.6311	5.4173	5.4929	0.8429	-0.2466	0.0595	1.2253
Arhgef12	_SSS[Phospho (STY)]HDFDPTDSSSK_.2	#N/A	3.1342	7.2569	3.8210	4.1813	3.7607	7.4493	4.3296	7.4593	3.1189	7.1639	0.8942	-0.1614	0.6620	0.1792
Arhgef12	_SSS[Phospho (STY)]HDFDPTDSSSKK_.2	#N/A	4.5298	4.0399	4.1132	5.2409	3.7543	4.2067	4.0999	3.5121	4.3598	3.9561	1.0834	0.1156	0.2999	0.5231
Gtf2a1l	_LLPGNELR_.2	#N/A	6.8682	7.5117	7.4149	6.1396	7.8934	7.0771	6.9159	7.3002	7.6608	7.1991	0.9837	-0.0236	0.7327	0.1351
Neto1	_GTGATADFADVAEDFENY[Phospho (STY)]HK_.3	Q8R4I7_Y417_M1_Neto1	4.3013	3.6056	3.4872	3.1412	3.4270	4.3025	3.1625	3.5868	3.7772	3.9569	1.0248	0.0354	0.7568	0.1210
Clybl	_VDC[Carbamidomethyl (C)]AVLDC[Carbamidomethyl (C)]EDGVAENK_.2	#N/A	4.2151	3.1273	3.9488	3.4926	3.3588	3.2658	4.4447	3.5086	3.8077	3.1698	0.9559	-0.0651	0.6032	0.2195
Nup35	_TLGT[Phospho (STY)]PTQSGSTPR_.2	Q8R4R6_T272_M1_Nup35	6.4382	6.5920	7.0089	7.1156	6.9181	6.7817	3.2060	6.0617	6.3976	6.6664	1.2196	0.2864	0.0949	1.0229
Ppp1r14c	_KLS[Phospho (STY)]PPQKK_.2	Q8R4S0_S157_M1_Ppp1r14c	7.0337	6.5236	7.1100	7.9571	7.2363	7.1806	6.4809	7.4974	6.4174	7.1519	1.0416	0.0588	0.3879	0.4113
Cys1	_LAPAVS[Phospho (STY)]PEKK_.2	Q8R4T1_S95_M1_Cys1	7.0850	7.5046	8.0837	8.0425	7.0313	7.5478	7.6517	7.4174	7.7264	7.9723	0.9814	-0.0270	0.5791	0.2373
Cys1	_GNPEDSC[Carbamidomethyl (C)]ASEAPGNS[Phospho (STY)]PK_.2	Q8R4T1_S116_M1_Cys1	6.3825	5.4950	4.5340	7.0189	5.6818	5.9014	4.0745	6.6105	5.8892	5.7490	1.0457	0.0644	0.6839	0.1650
Luzp1	_ELS[Phospho (STY)]PQHKR_.2	#N/A	8.2165	8.9151	9.1461	9.0959	7.6822	8.7469	8.6694	8.6227	8.5330	8.3750	1.0098	0.0141	0.7854	0.1049
Luzp1	_KGS[Phospho (STY)]LDYLK_.2	#N/A	5.9543	5.5445	4.2419	6.3399	4.1816	3.5479	5.8794	5.6455	5.7387	3.4377	0.9600	-0.0589	0.7857	0.1047
Luzp1	_RKGS[Phospho (STY)]LDYLK_.2	#N/A	3.2784	6.7037	4.5129	6.6073	5.2060	5.6190	4.2049	5.8458	6.1555	6.5549	0.9351	-0.0967	0.6488	0.1879
Luzp1	_ELS[Phospho (STY)]PQHK_.2	#N/A	6.1655	5.5875	5.4174	2.5607	3.2719	4.4390	4.9525	5.6789	4.9760	3.5397	0.9555	-0.0657	0.7980	0.0980
Luzp1	_NQRPFS[Phospho (STY)]PR_.2	Q8R4U7_S746_M1_Luzp1	5.9686	5.6466	6.2737	7.5898	2.9549	6.6649	6.3989	6.5650	5.6087	6.3520	0.9388	-0.0911	0.6535	0.1848
Luzp1	_S[Phospho (STY)]QENILQGFSLPNK_.2	Q8R4U7_S612_M1_Luzp1	3.1964	3.9527	4.4309	7.5272	3.8410	4.2845	4.1229	3.0265	5.7738	3.6520	1.0953	0.1313	0.6747	0.1709
Luzp1	_EFALS[Phospho (STY)]NEHYSLSSK_.2	#N/A	4.6469	4.1000	3.5122	5.3819	2.6743	4.5636	4.4386	4.7891	5.2026	4.9731	0.8548	-0.2264	0.1980	0.7033
Luzp1	_EKPDS[Phospho (STY)]DDDLDIESFVTAK_.2	#N/A	3.8281	3.8824	3.2545	4.0009	3.7469	3.5838	3.7156	4.2832	3.7914	3.8523	0.9503	-0.0736	0.2826	0.5489
Luzp1	_TFS[Phospho (STY)]DSTHVSVPNDIVGK_.2	Q8R4U7_S513_M1_Luzp1	3.4554	4.2117	3.1891	4.2337	4.1000	4.0255	4.3820	4.2683	3.0665	3.9110	0.9903	-0.0140	0.9104	0.0408
Lrrc8c	_SQS[Phospho (STY)]LKS[Phospho (STY)]IPEK_.2	#N/A	9.9369	9.6742	10.2433	9.7730	8.6428	9.5815	10.3168	9.8764	9.8736	9.8585	0.9660	-0.0499	0.2761	0.5589
Lrrc8c	_SGT[Phospho (STY)]IQSGPEGNLVR_.2	Q8R502_T198_M1_Lrrc8c	3.7224	5.2555	4.2651	3.8685	3.5600	3.6696	3.8587	3.8622	3.7890	5.8691	0.9337	-0.0989	0.5926	0.2272
Lrrc8c	_S[Phospho (STY)]QSLKS[Phospho (STY)]IPEKFVVDK_.2	Q8R502_S210_M2_Lrrc8c	3.8324	3.1590	3.6648	3.2714	2.9581	3.2793	3.5600	3.4931	3.9358	3.4453	0.9314	-0.1026	0.2251	0.6476
Mib2	_RVS[Phospho (STY)]ADGQPFQR_.2	#N/A	8.2955	8.2976	8.9863	9.0430	7.7085	8.5338	8.6989	8.4599	8.1947	8.4431	1.0033	0.0048	0.9182	0.0371
Mib2	_HGS[Phospho (STY)]PSTPDR_.2	Q8R516_S44_M1_Mib2	3.4455	6.0684	4.1323	6.4030	4.0720	3.6575	4.0183	6.6487	5.8385	6.5155	0.8044	-0.3139	0.2009	0.6970
Mib2	_VS[Phospho (STY)]ADGQPFQR_.2	#N/A	3.8522	3.1077	3.3045	3.8071	2.9960	3.6286	3.5218	4.3114	3.9599	3.7741	0.8863	-0.1741	0.0881	1.0548
Sh3kbp1	_S[Phospho (STY)]IEVENDFLPVEK_.2	Q8R550_S274_M1_Sh3kbp1	8.6621	8.5927	8.8367	9.6647	8.0798	9.0112	8.3932	9.0791	8.7891	9.1816	0.9940	-0.0086	0.8652	0.0629
Sh3kbp1	_ELS[Phospho (STY)]GESDELGISQDEQLSK_.2	Q8R550_S156_M1_Sh3kbp1	5.6176	4.1536	3.7598	7.5813	6.5982	6.2297	4.0856	6.9214	7.4910	4.0918	1.0016	0.0024	0.9931	0.0030
Sh3kbp1	_ASS[Phospho (STY)]PSLFSTEGKPK_.2	Q8R550_S631_M1_Sh3kbp1	9.8228	10.1696	10.5495	10.3377	9.5305	9.8746	10.2854	3.5292	9.4766	9.8046	1.2143	0.2802	0.2006	0.6976
Otud7a	_AAGGAAS[Phospho (STY)]PGPGGAR_.2	Q8R554_S749_M1_Otud7a	7.4803	7.8190	8.3962	8.3983	6.5317	7.6751	7.6010	7.8010	7.7954	7.9073	0.9924	-0.0111	0.8720	0.0595
Otud7a	_AS[Phospho (STY)]PGPTAAQPTQLVLK_.2	Q8R554_S711_M1_Otud7a	4.2230	3.0032	3.4090	3.9116	3.3487	4.3808	3.6263	4.2069	3.8554	3.8786	0.9540	-0.0680	0.5577	0.2536
Snap47	_ELLLSQPGTAANIPSHVTR_.3	#N/A	5.4268	5.0165	5.4856	5.2149	4.8982	5.0542	5.2532	4.7776	4.8970	5.1872	1.0306	0.0435	0.3355	0.4743
Snap47	_IELLEDALVLR_.2	#N/A	4.2981	4.3198	4.6570	4.3798	3.8069	2.5287	4.2215	4.2911	3.5174	4.5183	0.9665	-0.0492	0.7546	0.1223
Snap47	_ELPTGGQEGEQLQLQK_.2	#N/A	5.7805	5.9897	6.2240	6.6637	5.5106	6.0354	3.6334	6.7385	6.5574	4.6625	1.0318	0.0452	0.7713	0.1128
Prpsap2	_LGIAVIHGEAQDAESDLVDGRHS[Phospho (STY)]PPM[Oxidation (M)]VR_.4	#N/A	3.8912	8.3079	3.6249	8.9483	3.0349	6.4298	7.4413	3.8325	7.6603	6.6807	0.8911	-0.1664	0.6494	0.1875
Prpsap2	_HS[Phospho (STY)]PPMVR_.2	#N/A	3.6221	3.6041	4.3089	6.8718	4.2486	3.4809	4.5262	3.3069	2.9555	3.2777	1.2387	0.3088	0.2703	0.5681
Apbb1ip	_S[Phospho (STY)]SDTC[Carbamidomethyl (C)]GSPALPSK_.2	Q8R5A3_S531_M1_Apbb1ip	7.9402	3.6999	3.3762	4.4208	2.7862	3.8384	3.0682	4.0812	4.3803	4.0981	1.1118	0.1529	0.6637	0.1780
Apbb1ip	_RSSDT[Phospho (STY)]C[Carbamidomethyl (C)]GSPALPSK_.2	Q8R5A3_T534_M1_Apbb1ip	3.8303	3.5121	3.5640	4.6086	3.3334	4.3961	3.2560	3.8934	4.1925	4.2859	0.9916	-0.0122	0.9210	0.0357
Apbb1ip	_QGLGNHS[Phospho (STY)]PGATR_.2	Q8R5A3_S507_M1_Apbb1ip	5.9658	4.0518	6.2110	6.1323	3.8731	3.8564	4.1507	5.7170	3.3310	4.4030	1.1397	0.1886	0.4213	0.3754
Tbc1d22a	_SVS[Phospho (STY)]ESHTPC[Carbamidomethyl (C)]PSESTGDTVPLQR_.3	Q8R5A6_S144_M1_Tbc1d22a	8.5595	8.2798	9.1593	3.5538	3.2493	3.3753	8.6036	3.0639	4.2133	3.5208	1.2431	0.3139	0.5289	0.2766
Actr1b	_AGFAGDQIPK_.2	#N/A	7.7291	7.8660	8.0100	7.5598	7.0371	7.4712	7.6101	7.6009	7.5561	7.4354	1.0082	0.0117	0.7384	0.1317
Actr1b	_VQYTLPDGSTLDVGPAR_.2	#N/A	6.8596	5.9542	6.8083	6.9351	6.1953	6.2698	6.5894	6.4748	6.1491	6.4954	1.0119	0.0171	0.7420	0.1296
Actr1b	_TLFSNIVLSGGSTLFK_.2	#N/A	6.0989	5.9825	6.0069	5.7938	5.5088	5.7098	6.2037	6.1420	6.3985	6.3570	0.9322	-0.1012	0.0084	2.0741
Actr1b	_DQLQTFSEEHPVLLTEAPLNPSK_.3	#N/A	6.1888	5.2581	5.7773	6.2401	5.2592	5.7952	6.2515	5.7598	5.6156	5.3084	1.0034	0.0048	0.9445	0.0248
Actr1b	_TTGVVLDSGDGVTHAVPIYEGFAM[Oxidation (M)]PHSIM[Oxidation (M)]R_.3	#N/A	4.1036	4.6216	5.4992	4.5794	4.4113	4.5848	4.9463	4.7761	4.7545	4.6861	0.9671	-0.0482	0.5330	0.2733
Actr1b	_AC[Carbamidomethyl (C)]YLSINPQKDEALETEK_.3	#N/A	5.1308	5.1939	3.3035	4.8109	2.9970	3.6276	4.6860	4.3677	3.9609	3.7731	0.9953	-0.0068	0.9711	0.0127
Actr1b	_KEYEEDGSR_.2	#N/A	2.9469	4.3955	4.1814	3.7251	3.5914	3.5126	3.8734	3.2760	3.5751	3.4024	1.0549	0.0771	0.5115	0.2912
Usp15	_S[Phospho (STY)]PGASNFSTLPK_.2	Q8R5H1_S229_M1_Usp15	3.8188	6.9863	3.5525	7.1577	2.9626	3.6620	6.9187	3.9049	6.7160	6.9795	0.7651	-0.3862	0.2347	0.6296
Usp15	_WYYFDDSSVSTASEDQIVSK_.2	#N/A	3.3221	3.7535	4.7306	6.4918	3.5748	4.4950	3.8524	3.9148	4.3126	4.1048	1.0861	0.1192	0.5766	0.2391
Wasf1	_IENDVATILSR_.2	#N/A	6.5632	7.2259	7.4649	7.1476	7.4609	7.1234	7.0496	7.2048	7.0980	7.1283	1.0062	0.0089	0.8022	0.0957
Wasf1	_LAQGPELAEDDADLLHK_.3	#N/A	6.0546	4.5361	5.5224	5.3261	5.4847	5.4856	5.5215	5.7270	5.1941	6.2707	0.9513	-0.0721	0.3952	0.4032
Wasf1	_SSTIQDQQLFDRK_.2	#N/A	3.2919	3.2673	3.9390	4.4551	3.3352	3.8494	3.8122	3.8893	3.9304	3.7398	0.9601	-0.0587	0.5498	0.2598
Arl6ip5	_AWDDFFPGSDR_.2	#N/A	6.7955	6.9983	7.2182	6.6159	6.2601	6.8158	7.2038	7.3030	6.3719	6.8380	0.9790	-0.0306	0.5559	0.2550
Arl6ip5	_TPMGIILDALEQQEDNINK_.2	#N/A	2.9829	4.3595	4.2174	4.6942	4.1808	3.5487	3.9095	3.2400	3.5390	3.4385	1.1318	0.1786	0.2031	0.6922
Cadm1	_SDDSVIQLLNPNR_.2	#N/A	7.7644	7.3383	6.9409	8.0550	6.7986	7.1126	8.0627	7.2421	7.2264	7.7566	0.9687	-0.0459	0.4485	0.3482
Cadm1	_C[Carbamidomethyl (C)]EASNIVGK_.2	#N/A	3.4839	3.0224	4.1321	3.8718	4.1358	3.5990	4.4940	1.6822	4.3890	3.7229	1.0379	0.0537	0.8151	0.0888
Cadm1	_GTYFTHEAK_.2	#N/A	5.7221	5.4376	3.5154	5.9098	5.7364	5.8829	5.1238	5.9378	4.2410	5.8035	1.0172	0.0246	0.8756	0.0577
Cadm1	_GADDAADADTAIINAEGGQNNSEEKKEYFI_.3	#N/A	3.4434	4.1997	3.1771	2.4085	4.0880	1.6075	4.3699	4.2803	3.0786	3.8990	0.8073	-0.3089	0.2162	0.6651
Tmx1	_VEEEQEADEEDVS[Phospho (STY)]EEEAEDREGASK_.3	#N/A	3.4933	4.6094	4.1801	5.0957	4.0734	3.4212	4.5991	5.1149	4.4579	3.1489	0.9573	-0.0629	0.7024	0.1534
Tmx1	_KVEEEQEADEEDVS[Phospho (STY)]EEEAEDR_.3	#N/A	3.7242	3.5020	4.4110	3.4128	3.3903	3.2343	3.1275	3.2049	4.3543	3.3798	1.0272	0.0388	0.7663	0.1156

Cops8	_SANSELGGIWSVGQR_.2	#N/A	5.1564	4.5702	6.1513	5.2152	5.3367	5.1603	5.4795	5.3391	5.1626	5.7025	0.9712	-0.0421	0.5867	0.2316
Cops8	_GVLEQGWQADSTTR_.2	#N/A	4.1561	3.4115	3.8897	4.3261	4.6448	5.1561	3.2180	3.5677	4.9826	3.1108	1.1463	0.1970	0.2707	0.5674
Slc6a8	_GLTTLT[Phospho (STY)]PVS[Phospho (STY)]ESSK_.2	Q8VBW1_S628_M2_Slc6a8	3.4400	3.9024	3.1736	4.2183	3.7238	5.8134	4.3665	5.7039	5.6947	3.8955	0.8230	-0.2810	0.1870	0.7282
Nae1	_LLQSVGQAPESISEK_.2	#N/A	4.2673	4.8269	4.0605	4.5316	4.7288	4.7906	3.1265	4.4625	4.6963	4.5267	1.0788	0.1095	0.3414	0.4668
Mpdz	_DNSQTPAVPAPDLEPIPSTS[Phospho (STY)]R_.2	Q8VBX6_S1590_M1_Mpdz	3.9962	3.3006	3.1822	3.3193	3.1219	3.1067	3.4675	3.6819	3.9810	2.9965	0.9451	-0.0815	0.4361	0.3604
Camkk1	_KFS[Phospho (STY)]LQER_.2	Q8VBY2_S74_M1_Camkk1	6.3091	5.9466	6.5103	6.0053	5.1231	5.6857	5.9070	5.7129	6.0804	6.0158	1.0002	0.0002	0.9970	0.0013
Camkk1	_S[Phospho (STY)]FGNPFEPQAR_.2	#N/A	3.0784	12.1813	12.1550	13.0465	11.5980	12.5139	12.8794	12.2847	12.6095	12.5314	0.8558	-0.2247	0.3768	0.4239
Camkk1	_EGC[Carbamidomethyl (C)]GEGC[Carbamidomethyl (C)]KS[Phospho (STY)]PELPGVQEDEAAS_.2	#N/A	3.4732	6.9500	6.7033	7.6795	5.4460	6.3757	7.4027	6.9818	6.8530	6.9002	0.8678	-0.2045	0.2576	0.5890
Camkk1	_SM[Oxidation (M)]S[Phospho (STY)]APGSLLMK_.2	#N/A	3.7283	3.7396	4.1853	7.8458	3.5953	3.8781	6.2230	3.3433	3.6424	5.1755	0.9781	-0.0320	0.9221	0.0352
Camkk1	_KRS[Phospho (STY)]FGNPFEPQAR_.3	#N/A	4.2740	2.9521	3.4600	7.2395	2.8404	3.7842	3.6773	5.8884	3.8044	3.9296	0.9461	-0.0800	0.8082	0.0925
Ccdc9	_S[Phospho (STY)]M[Oxidation (M)]EKENM[Oxidation (M)]AVEEK_.2	#N/A	4.3871	4.5230	5.1541	5.2367	4.1542	5.0154	4.6053	4.1080	4.5569	8.8389	0.8585	-0.2201	0.4134	0.3836
Rapgef3	_LENGC[Carbamidomethyl (C)]GNVS[Phospho (STY)]PQTK_.2	Q8VCC8_S531_M1_Rapgef3	3.1706	3.9268	4.4051	4.4888	3.8151	4.3103	4.2035	3.6297	3.2783	2.9549	1.1430	0.1928	0.1652	0.7820
Reep2	_TETS[Phospho (STY)]EDDLGDKAPK_.2	#N/A	6.0679	4.2116	4.1265	6.4319	3.6690	6.1758	6.6057	3.9881	3.3970	3.2323	1.1877	0.2481	0.3877	0.4115
Reep2	_TETS[Phospho (STY)]EDDLGDKAPKR_.3	#N/A	3.3732	4.7384	3.1069	4.1515	3.7905	3.9390	5.7973	5.3277	3.1487	3.8288	0.8507	-0.2333	0.2725	0.5646
Reep2	_TETS[Phospho (STY)]EDDLGDK_.2	#N/A	3.5493	4.3056	3.2830	4.3276	4.1939	3.9315	4.4759	4.1744	2.9726	4.0049	1.0064	0.0092	0.9433	0.0254
Gpn1	_GTLDEEDEEADS[Phospho (STY)]DTDDIDHR_.3	#N/A	3.9680	5.2874	3.5079	6.0220	4.7307	6.2800	4.6918	6.0531	5.8375	5.4754	0.9005	-0.1511	0.3965	0.4018
Gpn1	_GNAS[Phospho (STY)]PVLDPSDLILTR_.2	Q8VCE2_S314_M1_Gpn1	2.9224	5.2383	4.2343	5.8871	3.5670	3.0576	5.4010	5.3723	3.0301	3.2031	0.9764	-0.0345	0.9036	0.0440
Rfxap	_[Acetyl (Protein N-term)]MEAQAVPEGSGPSTAS[Phospho (STY)]PR_.2	Q8VCG9_S16_M1_Rfxap	3.9729	3.3695	3.7066	3.2504	3.1166	3.5080	3.3986	3.7508	4.0499	4.4285	0.8926	-0.1639	0.1129	0.9472
Pex19	_AKPS[Phospho (STY)]PEHAPTISAPDASGPQK_.3	#N/A	9.3228	9.3038	9.9073	10.2084	9.2495	9.4066	9.7705	9.0702	9.2751	9.4180	1.0195	0.0279	0.4552	0.3418
Pex19	_IC[Carbamidomethyl (C)]EQFEAET[Phospho (STY)]PTDSEATQR_.2	#N/A	3.5647	5.0200	3.4305	3.9332	3.3702	3.8563	3.6479	4.2238	3.8724	4.2753	0.9645	-0.0522	0.6803	0.1673
Pex19	_AKPS[Phospho (STY)]PEHAPTISAPDASGPQKR_.4	#N/A	3.3362	4.1414	4.0230	4.5257	3.9627	3.7668	4.2403	3.5928	3.2414	2.9918	1.1259	0.1711	0.1711	0.7669
Plppr2	_LSPWEDLSQAPTM[Oxidation (M)]DS[Phospho (STY)]PLEK_.2	#N/A	3.6464	5.1487	3.9589	3.8542	3.8307	3.6566	3.8300	6.6166	5.5330	3.6645	0.8177	-0.2903	0.1934	0.7135
Gripap1	_KQES[Phospho (STY)]FC[Carbamidomethyl (C)]R_.2	#N/A	6.5343	6.4689	6.5825	7.0777	6.3722	6.3499	6.6571	6.4213	6.6742	7.0454	0.9798	-0.0294	0.4497	0.3470
Gripap1	_EC[Carbamidomethyl (C)]VGS[Phospho (STY)]PDPDLPEGAN_.2	#N/A	8.8219	12.0789	11.2250	12.5603	11.5439	12.1041	12.5096	11.6307	12.0307	12.2695	0.9405	-0.0886	0.3334	0.4770
Gripap1	_LSKEC[Carbamidomethyl (C)]VGS[Phospho (STY)]PDPDLPEGAN_.2	#N/A	2.9073	7.7652	4.1418	3.2512	3.5519	7.4022	4.4667	6.9279	7.1442	3.2182	0.8892	-0.1694	0.6651	0.1771
Gripap1	_TQTGDSSS[Phospho (STY)]VSSFSYR_.2	Q8VD04_S631_M1_Gripap1	3.9005	3.0981	3.9412	5.8853	2.9550	3.4785	3.6091	4.9521	4.1976	3.5562	0.9504	-0.0734	0.7449	0.1279
Gripap1	_KKQES[Phospho (STY)]FC[Carbamidomethyl (C)]R_.2	#N/A	4.0353	3.3085	3.1782	5.1208	3.9713	3.8107	4.3728	4.2034	3.9007	4.3730	0.9268	-0.1097	0.4213	0.3754
Gripap1	_TGLEELVLSEM[Oxidation (M)]NS[Phospho (STY)]PSR_.2	#N/A	3.8048	7.2862	3.9050	6.3865	4.1245	4.4082	4.8467	3.7192	6.5147	7.2816	0.8918	-0.1652	0.5567	0.2544
Znf385a	_QPGS[Phospho (STY)]PSPPSVPESGQGVTK_.2	Q8VD12_S160_M1_Znf385a	4.2562	2.9700	3.4421	3.9448	3.3818	4.3476	3.6595	4.1737	3.8223	3.9118	0.9568	-0.0637	0.5794	0.2371
Sgtb	_SFS[Phospho (STY)]SSADEHS_.2	#N/A	7.4891	7.5859	7.6719	7.8147	6.1520	7.5792	7.3079	6.9565	7.4923	7.8650	0.9969	-0.0045	0.9481	0.0232
Sgtb	_SRS[Phospho (STY)]FSSSADEHS_.2	Q8VD33_S295_M1_Sgtb	3.0348	6.6456	4.2693	7.0921	6.6580	3.6006	3.9614	6.8356	6.6327	6.7998	0.8612	-0.2155	0.4528	0.3441
Sgtb	_NDIRPLSNS[Phospho (STY)]VPEDVGK_.3	Q8VD33_S77_M1_Sgtb	4.4189	3.3718	4.5412	3.5430	3.2601	3.3645	3.2577	3.0746	4.2241	3.5100	1.0663	0.0927	0.5288	0.2767
Sgip1	_TGS[Phospho (STY)]PLTVATGNDQAATEAK_.2	Q8VD37_S265_M1_Sgip1	11.3982	11.3203	12.0278	11.8716	10.5993	11.2523	11.8392	11.2103	11.2193	11.3289	1.0011	0.0015	0.9670	0.0146
Sgip1	_AT[Phospho (STY)]PPPPPPPTYR_.2	#N/A	12.5925	12.5338	12.4686	13.1454	11.6888	12.2672	12.9582	12.2537	12.4150	12.5243	0.9929	-0.0102	0.7514	0.1241
Sgip1	_RST[Phospho (STY)]PT[Phospho (STY)]PELTSK_.2	#N/A	10.7479	10.9653	11.2715	11.4192	10.1156	10.8773	11.0515	10.7048	10.7238	10.8400	1.0064	0.0092	0.7817	0.1070
Sgip1	_TVPAT[Phospho (STY)]PPR_.2	Q8VD37_T259_M1_Sgip1	11.3871	11.4731	3.3379	4.3825	11.5864	11.4862	11.9308	4.1195	4.4186	11.7649	1.1097	0.1501	0.7480	0.1261
Sgip1	_TVPATPPRT[Phospho (STY)]GS[Phospho (STY)]PLTVATGNDQAATEAK_.3	#N/A	9.0072	9.6549	9.4690	8.3604	3.2398	3.7683	3.5196	9.5462	4.1085	9.1112	1.1033	0.1418	0.7384	0.1317
Sgip1	_RS[Phospho (STY)]PGAIKR_.2	#N/A	9.4965	10.1294	10.4833	10.4065	9.0612	10.1179	9.6797	9.8703	10.0258	9.8129	1.0104	0.0149	0.7338	0.1344
Sgip1	_STPT[Phospho (STY)]PELTSK_.2	#N/A	9.2590	9.3454	9.8100	9.8720	8.3170	9.1732	9.1649	3.0778	9.2853	9.3848	1.2029	0.2665	0.2482	0.6051
Sgip1	_NLS[Phospho (STY)]SEEVAR_.2	#N/A	9.3847	9.2046	3.8434	9.7438	8.3757	9.3133	9.1981	9.1866	9.3962	9.4410	0.8931	-0.1631	0.4090	0.3882
Sgip1	_VAEQTFIKDDYLETLSS[Phospho (STY)]PK_.3	#N/A	7.7043	7.3371	7.2295	8.0195	6.6394	4.8749	7.6197	7.4645	3.8130	7.7580	1.0456	0.0643	0.7567	0.1211
Sgip1	_S[Phospho (STY)]PGAIKR_.2	#N/A	8.8832	8.8713	8.8454	9.5055	8.0597	9.0621	8.5025	8.9183	8.9377	9.0650	1.0017	0.0025	0.9541	0.0204
Sgip1	_HVPS[Phospho (STY)]PLNLEEVQKK_.3	#N/A	8.3372	8.7644	9.3351	9.0813	7.5262	8.5212	8.3764	8.2759	8.2456	8.3279	1.0346	0.0491	0.4028	0.3950
Sgip1	_RS[Phospho (STY)]PGAIK_.2	#N/A	8.4378	8.1721	8.3116	8.4248	6.6748	7.9724	8.1476	8.0692	7.9149	8.2551	0.9879	-0.0175	0.7855	0.1048
Sgip1	_RNLS[Phospho (STY)]SEEVARPR_.3	#N/A	3.1843	4.1582	4.4188	6.6816	3.8288	6.2507	4.1108	6.4137	3.3377	3.6398	1.0864	0.1196	0.6857	0.1639
Sgip1	_VAEQTFIKDDYLETLSS[Phospho (STY)]PKEC[Carbamidomethyl (C)]GLGQR_.4	#N/A	3.9241	3.4183	3.6578	7.5169	3.0678	2.9890	3.3498	6.8921	4.0987	4.3796	0.8751	-0.1924	0.5980	0.2233
Sgip1	_TVVSS[Phospho (STY)]PGPGSGSGTGTTSGASSPAR_.2	Q8VD37_S424_M1_Sgip1	5.5561	6.0387	6.0733	6.9256	3.3542	5.6049	6.5128	6.1233	3.8499	5.7185	1.0074	0.0106	0.9589	0.0182
Sgip1	_ITGEM[Oxidation (M)]VLSFPAGITR_.2	#N/A	3.6199	5.7694	6.5999	4.3982	6.0043	6.0548	6.2927	6.0170	6.0154	6.0890	0.8860	-0.1746	0.2692	0.5700
Sgip1	_IEKLPS[Phospho (STY)]ISDLDSIFGPVLS[Phospho (STY)]PK_.3	Q8VD37_S287_M2_Sgip1	3.2349	4.0147	8.3217	9.2307	3.8709	4.0638	6.2020	5.8616	7.2266	3.7892	0.9456	-0.0807	0.8337	0.0790
Sgip1	_YNS[Phospho (STY)]PELDEEGYSIRPEEPGSTK_.3	Q8VD37_S78_M1_Sgip1	3.7521	3.6090	3.8257	6.5272	3.3379	5.6927	3.6846	3.8525	5.8911	7.0154	0.8721	-0.1974	0.5013	0.2999
Sgip1	_KVAEQTFIKDDYLETLSS[Phospho (STY)]PK_.3	#N/A	3.8285	4.0653	3.5856	4.2456	3.5254	4.2043	4.2175	4.0304	4.0905	4.0553	0.9538	-0.0682	0.2762	0.5587
Sgip1	_IPDISQK_.2	#N/A	4.5517	4.2118	3.1892	4.3677	5.4192	4.6027	4.2532	4.2682	4.9787	5.2238	0.9379	-0.0925	0.5058	0.2961
Sgip1	_NLS[Phospho (STY)]SEEVARPR_.2	#N/A	10.7346	11.0864	11.7582	11.6617	10.5262	11.0607	11.0896	10.9056	10.9788	10.9537	1.0142	0.0204	0.5536	0.2568
Sgip1	_EC[Carbamidomethyl (C)]GLGQRAT[Phospho (STY)]PPPPPPPTYR_.3	#N/A	4.1011	3.1250	3.2871	3.7898	3.0133	3.6113	5.6661	6.3415	5.1587	3.7568	0.6668	-0.5847	0.0069	2.1609
Sgip1	_DDYLETLSS[Phospho (STY)]PKEC[Carbamidomethyl (C)]GLGQR_.3	#N/A	3.7587	3.0142	3.4924	3.9006	2.9025	3.7221	3.6153	4.7428	3.8665	3.8676	0.8613	-0.2154	0.0890	1.0508
Sgip1	_WVHFSDAS[Phospho (STY)]PEHVT[Phospho (STY)]PELTPR_.3	#N/A	10.1569	10.4778	10.5979	11.9882	10.4854	10.5538	10.5848	11.0737	10.8493	10.4910	0.9963	-0.0053	0.9112	0.0404
Sgip1	_HVPS[Phospho (STY)]PLNLEEVQK_.2	#N/A	7.4240	7.3617	7.8084	8.0104	6.3014	7.3880	7.6762	7.4081	7.0471	7.2098	1.0064	0.0092	0.8869	0.0521
Sgip1	_ASIGNIALSPSPVR_.2	#N/A	6.3970	6.3026	6.5540	6.8893	6.7574	6.6137	6.6809	6.5686	6.3436	5.9116	1.0329	0.0466	0.2648	0.5771
Sgip1	_DDYLETLSS[Phospho (STY)]PK_.2	#N/A	4.3515	3.6558	3.5374	4.0401	3.4772	4.2523	3.7548	4.0784	3.7270	4.0071	0.9984	-0.0023	0.9771	0.0101
Sgip1	_ASIGNIALS[Phospho (STY)]PS[Phospho (STY)]PVRK_.2	Q8VD37_S149_M2_Sgip1	2.9757	3.7320	4.2102	5.3865	3.6331	3.0043	3.9023	3.2472	3.5462	3.4313	1.0826	0.1146	0.5525	0.2577
Sgip1	_ASIGNIALS[Phospho (STY)]PS[Phospho (STY)]PVRKS[Phospho (STY)]PR_.3	Q8VD37_S149_M3_Sgip1	2.9682	3.7734	3.3027	5.6975	3.5947	4.1348	4.4956	4.1547	2.9529	4.0246	1.0013	0.0018	0.9931	0.0030
Sgip1	_YNTDAM[Oxidation (M)]STAVALNNVQFLVPIDGGVTK_.3	#N/A	4.1385	3.4429	3.6332	3.1770	3.2642	2.9644	3.3252	3.8242	4.1233	4.6792	0.8618	-0.2146	0.1106	0.9562
Sgip1	_EQVVT[Phospho (STY)]PPAASDIPADSPAPAPPGPTGSAGPPGPPGPR_.4	Q8VD37_T335_M1_Sgip1	4.3434	6.5489	3.5294	8.2476	3.4691	5.0890	3.7467	6.6077	3.7351	3.9990	1.1509	0.2028	0.5544	0.2562
Sgip1	_GPS[Phospho (STY)]PLTM[Oxidation (M)]GAQDTLPVAAAFTETVNAYFK_.3	Q8VD37_S528_M1_Sgip1	3.5006	1.0735	3.2343	2.0073	4.1452	3.9803	3.8734	3.9597	3.6083	3.9562	0.7768	-0.3644	0.2025	0.6935
Sgip1	_LPS[Phospho (STY)]ISDLDSIFGPVLSPK_.2	#N/A	4.0513	3.1749	3.2372	1.9905	3.0632	3.5614	3.4546	4.3786	4.0272	3.7069	0.8170	-0.2915	0.1001	0.9996
Sgip1	_GKHFY[Phospho (STY)]SS[Phospho (STY)]S[Phospho (STY)]ES[Phospho (STY)]EEEEESHKK_.3	Q8VD37_S104_M3_Sgip1	4.2913	3.0511	4.0250	3.5687	3.4350	3.1896	3.7170	3.4324	3.7315	3.2460	1.0175	0.0250	0.8200	0.0862
Tspyl4	_GLEDAPS[Phospho (STY)]PSKGGDAPSAPVAADDSSK_.3	#N/A	6.5100	5.9258	3.4801	3.8357	6.1983	3.7791	6.0403	4.0219	5.6699	6.3455	0.8626	-0.2133	0.3792	0.4212
Tspyl4	_NGC[Carbamidomethyl (C)]QLEGPHS[Phospho (STY)]PAKPK_.2	#N/A	4.0453	4.2643	3.6992	5.0468	4.1720	4.5385	4.3365	4.6497	4.9995	4.6943	0.9196	-0.1210	0.1836	0.7362
Tspyl4	_GLEDAPS[Phospho (STY)]PSK_.2	#N/A	4.2327	3.4882	3.9664	3.5102	3.3764	3.2482	3.6584	3.4910	3.7901	3.1874	1.0298	0.0424	0.6441	0.1910
Myh9	_VVFQEFR_.2	#N/A	3.4782	4.4717	5.5772	5.5559	3.4364	4.9141	4.7735	4.6265	3.6141	4.5295	1.0425	0.0600	0.7347	0.1339
Myh9	_KGTGDC[Carbamidomethyl (C)]S[Phospho (STY)]DEEVDGKADGADAK_.3	#N/A	6.0117	5.9253	6.6959	6.0560	4.9832	5.5243	6.5680	6.3814	6.1794	5.7496	0.9432	-0.0844	0.3070	0.5129
Myh9	_GTGDC[Carbamidomethyl (C)]S[Phospho (STY)]DEEVDGK_.2	#N/A	3.5731	3.6531	4.2599	3.2617	4.1996	3.0833	4.4773	3.3559	3.0045	3.2287	1.0441	0.0623	0.6750	0.1707
Myh9	_GTGDC[Carbamidomethyl (C)]S[Phospho (STY)]DEEVDGKADGADAK_.2	#N/A	5.1732	4.7301	5.8272	5.7768	3.5864	3.0382	5.8223	3.2811	4.8171	4.4719	1.0197	0.0281	0.9030	0.0443
Myh9	_LQQELDDLLVDLDHQR_.3	#N/A	3.6516	3.5746	4.3384	3.3402	2.8043	3.4514	4.5558	4.0859	4.4268	4.1377	0.8199	-0.2865	0.0206	1.6859
Myh9	_KGTGDC[Carbamidomethyl (C)]S[Phospho (STY)]DEEVDGK_.2	#N/A	4.7235	3.8751	4.0486	5.0488	3.6403	3.6187	3.1257	4.6606	4.4244	4.0358	1.0240	0.0342	0.8152	0.0887
Myh9	_KQELEEIC[Carbamidomethyl (C)]HDLEAR_.3	#N/A	4.1657	4.5548	4.4918	3.4936	4.4489	3.3152	5.3857	4.4173	4.2734	4.2945	0.8880	-0.1714	0.1770	0.7520
Myh9	_DFSALESQLQDTQELLQEENR_.3	#N/A	3.1779	4.4453	4.5938	5.8036	3.8044	3.9251	5.2343	4.7533	3.1626	3.8149	1.0119	0.0170	0.9336	0.0298
Myh9	_ANLQIDQINTDLNLER_.2	#N/A	4.2848	2.9414	3.4708	3.9734	3.4105	4.3190	4.5765	4.1450	3.7936	3.9404	0.9075	-0.1401	0.2554	0.5929
Myh9	_ELETQISELQEDLESER_.2	#N/A	4.2796	4.2700	3.4655	3.9682	3.4053	4.3242	3.6829	3.8016	3.7989	3.9352	1.0388	0.0549	0.5183	0.2854

Phip	_TAFYNEDDS[Phospho (STY)]EEEQR_.2	Q8VDD9_S1783_M1_Phip	6.9686	7.0430	7.6288	7.7831	6.1329	6.9760	8.0221	7.6710	7.3731	6.9440	0.9448	-0.0819	0.2702	0.5683
Phip	_VLS[Phospho (STY)]DS[Phospho (STY)]EEEEKDADVPGTSTR_.2	#N/A	3.1905	5.6772	4.0740	5.5422	4.6326	3.4165	6.1658	6.3346	5.1842	3.0428	0.8534	-0.2287	0.3721	0.4293
Phip	_KVLS[Phospho (STY)]DS[Phospho (STY)]EEEEKDADVPGTSTR_.3	#N/A	3.9039	3.4385	3.6376	6.1053	5.7764	2.9688	3.3296	4.5541	4.1189	4.3594	1.0525	0.0738	0.7671	0.1151
Parn	_TLS[Phospho (STY)]PDPR_.2	Q8VDG3_S556_M1_Parn	6.0667	5.5499	4.5739	3.5757	4.4963	3.3973	3.2904	3.0419	4.1913	5.1945	1.1732	0.2304	0.3344	0.4758
Hdlbp	_ETDPGS[Phospho (STY)]PR_.2	#N/A	2.9301	9.2253	8.3857	9.3352	3.5746	7.5690	8.3632	9.5488	8.1373	3.3856	0.9291	-0.1062	0.7805	0.1076
Hdlbp	_EAKETDPGS[Phospho (STY)]PR_.2	#N/A	4.3497	7.7189	8.7671	7.4240	7.7595	7.8834	7.5078	7.6163	7.2066	7.2238	0.9903	-0.0140	0.9293	0.0318
Hdlbp	_ETDPGS[Phospho (STY)]PRR_.2	#N/A	4.2898	7.0072	3.4758	7.4434	3.4155	7.0394	3.6931	4.1400	7.0403	7.1878	0.9873	-0.0185	0.9556	0.0197
Hdlbp	_VATLNS[Phospho (STY)]EEENDPPTYK_.2	Q8VDJ3_S31_M1_Hdlbp	7.9717	7.9767	4.0735	8.8630	3.7315	8.2658	8.4404	8.1208	8.3378	8.4080	0.8911	-0.1663	0.3302	0.4813
Zgpat	_GKS[Phospho (STY)]LDQC[Carbamidomethyl (C)]AEILQK_.2	Q8VDM1_S353_M1_Zgpat	3.7071	4.0773	3.8866	4.2647	3.2137	3.6484	3.1933	3.6133	4.3277	4.6783	0.9612	-0.0571	0.6498	0.1872
Zgpat	_LLSTVDQES[Phospho (STY)]PAQEDAEYLAFQK_.3	Q8VDM1_S70_M1_Zgpat	4.2946	2.9315	3.4806	3.9833	4.8765	4.6371	3.6979	4.1352	3.7838	2.1441	1.1726	0.2297	0.2788	0.5547
Psmd2	_TPVQSQQPSATT[Phospho (STY)]PSGADEK_.2	#N/A	7.9466	3.9081	8.0259	8.3394	7.3928	7.8611	8.5638	8.2616	8.0634	8.0560	0.8797	-0.1849	0.2796	0.5535
Psmd2	_DKTPVQSQQPSATT[Phospho (STY)]PSGADEK_.3	#N/A	3.2150	5.3747	3.1979	5.8533	4.6519	4.0900	5.6757	5.7016	3.0608	5.3327	0.8896	-0.1687	0.4892	0.3105
Psmd2	_TITGFQTHTTPVLLAHGER_.3	#N/A	4.5006	3.7513	5.7548	4.1355	3.7765	4.5846	4.8722	3.7442	4.6197	4.1906	1.0139	0.0199	0.8908	0.0502
Psmd2	_FGGS[Phospho (STY)]GSQVDSAR_.2	Q8VDM4_S361_M1_Psmd2	5.3170	3.9491	4.1813	6.3316	3.9489	6.0206	6.1892	4.2540	6.1038	4.0329	0.9637	-0.0534	0.8005	0.0966
Psmd2	_LVGSQEELASWGHEYVR_.3	#N/A	3.6063	4.3626	3.3400	4.3846	4.2509	3.8746	4.5329	4.1174	4.4165	4.0619	0.9271	-0.1093	0.2282	0.6417
Hnrnpul1	_GRS[Phospho (STY)]PQPPAEEDEDDFDDTLVAIDTYNC[Carbamidomethyl (C)]DLHFK_.4	#N/A	4.2415	7.0395	3.4274	7.4522	6.7795	7.1057	6.6294	7.2955	7.0789	7.1912	0.8499	-0.2346	0.2633	0.5795
Hnrnpul1	_NYILDQTNVYGS[Phospho (STY)]AQR_.2	Q8VDM6_S513_M1_Hnrnpul1	6.5333	6.5819	7.2250	3.2220	5.5767	6.6517	7.2355	6.9301	6.9877	6.2576	0.8705	-0.2001	0.2739	0.5624
Hnrnpul1	_S[Phospho (STY)]PQPPAEEDEDDFDDTLVAIDTYNC[Carbamidomethyl (C)]DLHFK_.3	#N/A	3.9516	4.8499	3.6853	6.0397	3.0954	3.5292	5.6075	5.4298	4.0711	4.4072	0.8592	-0.2190	0.3040	0.5171
Atp1a1	_S[Phospho (STY)]PDFTNENPLETR_.2	Q8VDN2_S228_M1_Atp1a1	12.3075	11.8700	12.2529	12.1269	10.9480	11.8867	12.8598	12.1129	12.1681	12.0329	0.9679	-0.0471	0.2196	0.6585
Atp1a1	_IVEIPFNSTNK_.2	#N/A	10.4074	10.2019	10.4846	10.0400	9.8862	10.0456	10.5228	10.3559	10.2174	10.0234	0.9901	-0.0144	0.5007	0.3004
Atp1a1	_LNIPVNQVNPR_.2	#N/A	9.7792	9.5152	10.1790	9.4967	9.3434	9.2039	9.9411	9.7022	9.6675	9.2309	0.9949	-0.0074	0.8229	0.0846
Atp1a1	_YHTEIVFAR_.2	#N/A	7.5855	7.0549	7.4329	7.4690	6.7225	7.2364	7.8981	7.7576	7.6905	7.5230	0.9395	-0.0901	0.0279	1.5540
Atp1a1	_LSLDELHR_.2	#N/A	8.7871	8.6608	8.5822	8.3797	8.4313	8.1485	8.9497	8.8680	8.7486	8.3804	0.9727	-0.0399	0.1570	0.8040
Atp1a1	_AVFQANQENLPILK_.2	#N/A	8.2076	7.8453	8.0901	8.1629	7.5468	8.1587	8.2835	8.1333	8.0391	7.9906	0.9865	-0.0196	0.4591	0.3381
Atp1a1	_TSATWFALSR_.2	#N/A	7.5539	7.6855	7.8845	7.7922	7.2319	7.5382	8.1414	8.2582	7.9105	7.5629	0.9556	-0.0655	0.0693	1.1591
Atp1a1	_GIVVYTGDR_.2	#N/A	8.1198	7.6072	8.2886	7.2384	7.2959	7.9799	6.7460	8.0456	8.2911	7.7135	1.0073	0.0104	0.8770	0.0570
Atp1a1	_YQLSIHK_.2	#N/A	7.5451	6.8285	7.1907	7.2212	6.9765	6.8750	7.6002	7.2942	7.0364	7.0668	0.9802	-0.0288	0.4276	0.3690
Atp1a1	_KYGTDLSR_.2	#N/A	7.4835	6.9955	7.2154	6.3534	6.9062	6.9828	7.6175	7.3079	7.4622	7.0108	0.9510	-0.0725	0.1363	0.8656
Atp1a1	_C[Carbamidomethyl (C)]SSILLHGK_.2	#N/A	5.6911	6.2771	5.1448	5.5268	6.2787	5.5572	5.6128	6.1399	6.5338	5.5408	0.9646	-0.0520	0.4951	0.3053
Atp1a1	_YGTDLSR_.2	#N/A	5.7655	5.5671	5.4918	3.8982	4.9117	4.8074	4.9348	5.9483	5.9692	5.8806	0.8927	-0.1637	0.1693	0.7712
Atp1a1	_NIAFFSTNC[Carbamidomethyl (C)]VEGTAR_.2	#N/A	6.4619	4.3806	6.7387	4.7541	4.4168	5.7263	6.8626	5.3124	6.0517	6.1769	0.8873	-0.1726	0.2764	0.5584
Atp1a1	_IISANGC[Carbamidomethyl (C)]K_.2	#N/A	6.0834	5.2782	3.8257	4.6604	6.3160	5.1248	5.6030	5.9305	6.0455	4.8865	0.9285	-0.1070	0.4553	0.3417
Atp1a1	_AVAGDASESALLK_.2	#N/A	5.8406	6.4456	6.0608	6.3837	6.0730	6.0305	7.2753	7.4179	5.8888	6.3162	0.9129	-0.1314	0.1008	0.9965
Atp1a1	_DM[Oxidation (M)]DELKK_.2	#N/A	6.0095	5.1940	5.1399	5.4838	5.6860	4.7816	4.8455	5.4617	5.0168	4.5154	1.0852	0.1180	0.1584	0.8003
Atp1a1	_EVS[Phospho (STY)]MDDHK_.2	#N/A	7.2301	4.7425	3.9869	7.5811	5.6727	3.8029	6.3177	5.0693	5.4325	5.2732	0.9963	-0.0054	0.9814	0.0081
Atp1a1	_WVNDVEDSYGQQWTYEQR_.2	#N/A	5.0736	4.7848	6.1264	5.3552	3.0431	3.7679	5.1883	4.7461	4.7154	4.2434	0.9933	-0.0096	0.9587	0.0183
Atp1a1	_M[Oxidation (M)]SINAEDVVVGDLVEVK_.2	#N/A	6.9617	7.2230	7.7668	7.3333	5.9544	7.4411	7.7850	7.2933	7.1966	7.3435	0.9607	-0.0579	0.4110	0.3861
Atp1a1	_DM[Oxidation (M)]TSEELDDILR_.2	#N/A	6.8124	6.1597	6.4559	6.2618	6.1466	6.3908	7.0334	6.5804	6.2772	6.6575	0.9599	-0.0590	0.1708	0.7676
Atp1a1	_ADIGVAM[Oxidation (M)]GIVGSDVSK_.2	#N/A	5.3845	5.3121	5.7602	5.2099	5.1823	5.6975	5.0965	5.7753	5.8579	5.3225	0.9839	-0.0234	0.6557	0.1833
Atp1a1	_EQPLDEELKDAFQNAYLELGGLGER_.3	#N/A	3.7509	3.5915	3.4846	4.7480	2.8946	3.4602	4.3523	4.7843	4.6818	4.2065	0.8111	-0.3021	0.0321	1.4930
Atp1a1	_DKYEPAAVSEHGDKK_.3	#N/A	5.9531	5.2950	5.2018	5.0694	4.9696	5.6489	5.5569	5.7357	5.7919	5.2350	0.9599	-0.0590	0.3294	0.4822
Atp1a1	_EVS[Phospho (STY)]M[Oxidation (M)]DDHKLSLDELHR_.3	#N/A	4.7934	6.0072	4.0771	6.1899	3.5181	5.6369	6.4213	6.5161	4.3171	5.7040	0.8776	-0.1884	0.3351	0.4748
Atp1a1	_AC[Carbamidomethyl (C)]VVHGSDLKDM[Oxidation (M)]TSEELDDILR_.4	#N/A	3.8679	4.4002	4.1608	4.6746	4.0730	4.4755	3.2823	4.8352	4.6737	4.7929	0.9725	-0.0402	0.7248	0.1398
Atp1a1	_M[Oxidation (M)]TVAHM[Oxidation (M)]WFDNQIHEADTTENQSGVSFDK_.4	#N/A	5.7168	6.3639	7.4836	6.0974	2.8032	5.9415	6.0839	6.6486	6.2836	4.1150	0.9916	-0.0121	0.9592	0.0181
Atp1a1	_EVS[Phospho (STY)]M[Oxidation (M)]DDHKLS[Phospho (STY)]LDELHRK_.3	#N/A	4.1293	4.2973	3.9006	4.1398	3.3993	4.2349	4.6287	4.5217	3.7806	4.2008	0.9379	-0.0925	0.2737	0.5627
Ccdc92	_KSLS[Phospho (STY)]APLHPEFEEVYR_.3	#N/A	4.2835	3.3087	3.6685	4.7684	2.9757	4.3203	3.1803	3.7648	4.2682	4.2102	1.0082	0.0118	0.9391	0.0273
Ccdc92	_SATGQHS[Phospho (STY)]PAR_.2	Q8VDN4_S261_M1_Ccdc92	3.9616	3.2171	3.6953	3.2391	3.1054	3.5192	3.3874	3.7621	4.0611	4.4172	0.8847	-0.1768	0.0989	1.0047
Ccdc92	_SLS[Phospho (STY)]APLHPEFEEVYR_.2	#N/A	3.5759	3.5878	3.7004	3.7934	3.3947	3.8359	3.6741	3.8403	3.8269	4.3040	0.9327	-0.1006	0.0887	1.0518
Mical1	_VS[Phospho (STY)]PVPSPSQPAR_.2	Q8VDP3_S777_M1_Mical1	8.0445	7.3752	3.5543	7.1331	6.4917	7.4811	3.7717	4.1350	6.7591	7.6149	1.1992	0.2621	0.3489	0.4573
Ccar2	_EAAPDTGAEPS[Phospho (STY)]PEDSDPTYSSK_.2	Q8VDP4_S298_M1_Ccar2	6.2443	6.1951	6.6516	7.1597	5.6252	6.2834	7.0473	6.4212	6.4225	6.6084	0.9600	-0.0589	0.3815	0.4185
Ccar2	_HDLS[Phospho (STY)]PYR_.2	Q8VDP4_S222_M1_Ccar2	7.6267	7.6582	7.9022	7.8843	7.1118	7.4590	7.6737	7.4346	3.3356	7.5607	1.1701	0.2266	0.2287	0.6407
Ccar2	_RPSS[Phospho (STY)]GGEEEEKAR_.2	#N/A	3.9294	3.2208	4.0312	3.5515	3.0768	3.3574	3.4819	4.0800	3.9916	3.3923	0.9442	-0.0829	0.4105	0.3867
Ccar2	_S[Phospho (STY)]VAS[Phospho (STY)]NQSEM[Oxidation (M)]EYSSLQDM[Oxidation (M)]PK_.2	#N/A	4.6238	6.4690	4.2466	5.9494	5.5999	6.4365	6.8549	6.4043	6.6276	6.3266	0.8475	-0.2387	0.0734	1.1342
Ptgr2	_VVGIC[Carbamidomethyl (C)]GTQEK_.2	#N/A	3.3929	4.4159	3.1266	4.1712	5.2578	3.9587	4.3195	4.3308	3.1290	4.8664	0.9741	-0.0378	0.8300	0.0809
Sirt2	_LLDELTLEGVTR_.2	#N/A	7.5635	8.0904	7.8271	8.0489	7.8558	7.9245	8.1749	7.7016	8.0148	7.9527	0.9905	-0.0138	0.5565	0.2545
Sirt2	_IFSEATPR_.2	#N/A	7.7149	7.9198	7.4864	7.7387	8.3837	7.9319	7.5808	7.9447	7.9446	8.1629	0.9942	-0.0084	0.8076	0.0928
Sirt2	_NLFTQTLGLGSQK_.2	#N/A	7.4926	7.8030	7.7129	7.5390	7.6199	7.8307	7.4291	7.8582	7.4878	7.6267	1.0087	0.0125	0.5419	0.2660
Sirt2	_SPSTGLYANLEK_.2	#N/A	7.2089	7.1229	6.8193	7.2370	6.8743	7.2035	7.0184	6.8454	6.5838	6.9715	1.0325	0.0462	0.1034	0.9854
Sirt2	_ELEDLVR_.2	#N/A	5.6739	5.3784	5.5846	4.5603	5.8513	5.7983	3.5701	5.9428	6.3021	5.7792	1.0141	0.0201	0.8930	0.0491
Sirt2	_C[Carbamidomethyl (C)]EQC[Carbamidomethyl (C)]QSVVKPDIVFFGENLPSR_.3	#N/A	5.1600	4.8112	4.7778	5.8967	4.9628	4.9363	5.1980	4.5365	5.1954	4.7186	1.0364	0.0515	0.4970	0.3036
Sirt2	_VAGLEPQDLVEAHGTFYTSHC[Carbamidomethyl (C)]VNTSC[Carbamidomethyl (C)]R_.4	#N/A	6.3028	6.2453	6.4229	6.5287	5.7324	6.1234	6.0616	3.3054	6.4410	6.1653	1.1334	0.1806	0.2549	0.5937
Sirt2	_EHANIDAQSGSQAPNPSTTISPGK_.3	#N/A	5.5980	5.7394	5.7082	7.0004	5.8087	5.6761	5.3327	5.7337	5.8750	5.7532	1.0437	0.0618	0.4132	0.3839
Sirt2	_EHANIDAQSGSQAPNPSTTISPGKS[Phospho (STY)]PPPAK_.4	Q8VDQ8_S372_M1_Sirt2	3.4886	3.8869	4.1754	7.7068	3.6854	6.6133	5.9134	3.4662	3.7386	4.5902	1.1127	0.1541	0.6335	0.1983
Sirt2	_FFSC[Carbamidomethyl (C)]M[Oxidation (M)]QSDFSK_.2	#N/A	3.7085	3.6339	3.4422	4.4868	5.0247	3.7956	3.1343	4.0152	4.3142	4.1641	1.0277	0.0395	0.7799	0.1079
Kri1	_YVDEDNS[Phospho (STY)]DGETVDHR_.2	Q8VDQ9_S142_M1_Kri1	5.5985	5.5696	6.5076	5.7165	4.6886	5.7026	6.2832	5.6134	6.0323	5.1590	0.9755	-0.0358	0.7000	0.1549
Caap1	_ILEGDNGLDS[Phospho (STY)]DMEEEADDGC[Carbamidomethyl (C)]K_.2	Q8VDY9_S183_M1_Caap1	4.0445	3.1816	3.2305	6.2419	2.7807	3.5547	3.4478	2.5215	4.0339	3.7001	1.1206	0.1643	0.5647	0.2482
Caap1	_SVNEILGLAESS[Phospho (STY)]PKEPK_.2	Q8VDY9_S307_M1_Caap1	3.6634	5.0812	5.7897	5.7528	4.9527	4.9613	3.6992	4.1251	3.7737	3.9988	1.2909	0.3684	0.0223	1.6509
Zdhhc5	_GLGS[Phospho (STY)]PEPGTTAPYLGR_.2	Q8VDZ4_S621_M1_Zdhhc5	8.5611	8.4905	9.1951	8.7792	2.9369	8.0787	8.8948	8.8346	8.1161	7.9909	0.9071	-0.1406	0.5355	0.2713
Zdhhc5	_LSRGDS[Phospho (STY)]LKEPTSIADSSR_.3	#N/A	4.1157	5.0645	3.5664	5.9844	3.2413	5.3024	5.6055	5.7365	4.8195	4.7466	0.8697	-0.2015	0.2773	0.5571
Zdhhc5	_SIGSASPGPGQPPLSS[Phospho (STY)]PTR_.2	Q8VDZ4_S694_M1_Zdhhc5	4.1310	3.2114	3.8647	9.1261	3.0327	3.1959	3.5567	8.5815	3.8918	7.2742	0.7599	-0.3962	0.3925	0.4062
Zdhhc5	_VLC[Carbamidomethyl (C)]SS[Phospho (STY)]PAPR_.2	Q8VDZ4_S247_M1_Zdhhc5	3.0106	4.3318	4.2452	3.7889	4.1531	3.5764	5.4006	3.2123	3.5113	5.6625	0.8660	-0.2075	0.3196	0.4954
Zdhhc5	_GDS[Phospho (STY)]LKEPTSIADSSR_.2	#N/A	8.4622	8.2241	8.7785	8.4490	6.7378	8.0422	8.5680	8.4239	8.2285	8.0770	0.9749	-0.0366	0.5948	0.2257
Zdhhc5	_SEPS[Phospho (STY)]LEPESFRS[Phospho (STY)]PTFGK_.2	Q8VDZ4_S401_M2_Zdhhc5	4.1427	3.4471	4.1343	3.8314	3.2684	3.8148	3.5461	3.6408	3.9847	3.7984	1.0082	0.0118	0.8801	0.0554
Zdhhc5	_SFHFDPLSSGS[Phospho (STY)]R_.2	Q8VDZ4_S425_M1_Zdhhc5	3.9454	7.5727	7.1569	7.4632	3.0891	7.0667	3.4286	3.7783	4.0774	4.4010	1.5426	0.6254	0.0722	1.1412
Smg8	_LYEVAIDGKEEDLGS[Phospho (STY)]PTGELTSK_.3	Q8VE18_S469_M1_Smg8	3.3323	3.8938	4.0191	4.5218	3.4470	4.2838	5.0144	4.5167	3.2453	2.9879	0.9937	-0.0091	0.9578	0.0187
Mios	_GFSQYGVSGSPT[Phospho (STY)]K_.2	Q8VE19_T768_M1_Mios	9.6392	9.5057	3.3723	9.7648	9.0430	9.3848	3.5896	9.4986	9.5092	8.8977	1.0734	0.1022	0.7438	0.1286
Mios	_QYTEDNDQKS[Phospho (STY)]PGNK_.2	Q8VE19_S444_M1_Mios	3.4498	5.1044	3.7609	4.7269	4.7347	4.9973	4.5654	4.3162	4.9847	2.7292	1.0755	0.1051	0.5664	0.2469
Gdap1l1	_DNIISDYDQIIDYVER_.2	#N/A	5.9237	6.0634	7.2995	6.7020	5.5219	6.2198	6.4822	6.2852	6.2635	6.4522	0.9871	-0.0188	0.8052	0.0941
Gdap1l1	_LDHEEEPQLSEPYLSK_.3	#N/A	6.1956	5.3514	4.0759	5.0368	4.8953	4.6957	5.9064	6.1212	5.1314	5.4469	0.8921	-0.1647	0.1676	0.7756

Gdap1l1	_LNLGEEVPVIIHR_.3	#N/A	4.6520	3.5598	3.9121	3.5702	4.0888	5.1078	4.5523	4.7143	5.0649	4.2864	0.8913	-0.1660	0.1761	0.7543
Gdap1l1	_TFTGEHVVALM[Oxidation (M)]PEAGS[Phospho (STY)]PQHAR_.3	#N/A	4.1852	4.1858	3.9189	5.7689	3.3290	3.2978	3.1910	4.8580	4.5459	3.7088	1.0094	0.0135	0.9462	0.0240
Uba5	_KTEDSASEVTVEDSGESLEDLMAR_.3	#N/A	4.2028	3.9651	3.1110	5.7709	4.0218	4.1036	4.3038	3.4646	3.1447	3.8328	1.0740	0.1029	0.5748	0.2405
Ogfrl1	_IS[Phospho (STY)]PGASHVNSK_.2	#N/A	7.0617	6.9859	6.6638	7.0019	6.7728	6.8463	7.3502	7.2352	6.9230	6.6622	0.9781	-0.0319	0.3222	0.4919
Ogfrl1	_EPGEEADKPS[Phospho (STY)]PEPGSGDPKPR_.3	Q8VE52_S382_M1_Ogfrl1	5.1386	4.8923	6.2988	5.9960	2.8504	3.3782	5.5308	5.5039	6.0525	3.5502	0.9224	-0.1165	0.6429	0.1919
Ogfrl1	_AS[Phospho (STY)]PVPEDHAEAAGAEQGGESTEGNAKPK_.4	#N/A	3.9274	3.9993	4.4252	4.0478	3.3319	3.5949	3.1564	3.6632	4.3112	3.8314	1.0394	0.0557	0.6011	0.2211
Ogfrl1	_DSAADQSDS[Phospho (STY)]PPEK_.2	Q8VE52_S406_M1_Ogfrl1	2.9698	3.7261	4.1869	4.2435	3.6144	3.0103	4.4043	3.4289	3.0775	3.5844	1.0004	0.0006	0.9970	0.0013
Ogfrl1	_DSKIS[Phospho (STY)]PGASHVNSK_.3	#N/A	3.6278	2.9322	4.3146	3.3164	4.2543	3.4752	4.5320	3.3012	2.9498	3.2834	1.0389	0.0551	0.7347	0.1339
Taf12	_IAGTPGT[Phospho (STY)]GGRLSPENNQVLTK_.3	Q8VE65_T46_M1_Taf12	3.8125	3.5780	3.6898	4.1924	3.6357	4.0140	5.0844	3.9722	3.6208	4.1594	0.9076	-0.1398	0.1957	0.7083
Pdcd10	_IPDEINDR_.2	#N/A	5.9064	5.0139	5.1527	5.6616	5.7520	5.1367	5.1561	5.3860	5.2129	5.7344	1.0121	0.0173	0.7749	0.1108
Fam114a2	_AETSLGIPS[Phospho (STY)]PTEISAEVK_.2	Q8VE88_S119_M1_Fam114a2	3.2166	9.0238	3.9035	4.4061	8.4382	4.2276	9.0652	9.0895	9.1044	8.9185	0.6121	-0.7082	0.0257	1.5896
Ccdc115	_ADAQT[Phospho (STY)]PEEVGPSEASLR_.2	Q8VE99_T90_M1_Ccdc115	6.1346	6.1991	7.0386	7.8426	6.8500	6.4166	7.2848	6.5976	6.9480	6.9535	0.9713	-0.0419	0.5824	0.2348
Grk5	_INSNHINSNSTGS[Phospho (STY)]S_.2	Q8VEB1_S589_M1_Grk5	5.7115	6.0309	6.0174	3.3377	5.6499	5.7745	5.5743	3.6635	5.8451	5.5777	1.0494	0.0696	0.7095	0.1491
MACIR	_S[Phospho (STY)]LDYLNLDK_.2	Q8VEB3_S167_M1_MACIR	3.4196	4.1758	3.1533	4.2398	4.0641	4.0613	3.9545	3.8787	4.8170	3.8751	0.9325	-0.1009	0.3691	0.4328
Bloc1s4	_[Acetyl (Protein N-term)]MEEGPAVGTLS[Phospho (STY)]R_.2	Q8VED2_S11_M1_Bloc1s4	4.3759	2.9665	4.1096	3.6534	3.5196	3.1050	3.8016	3.3478	3.6469	3.3306	1.0255	0.0363	0.7686	0.1143
Lgalsl	_IQTLSAIDTIK_.2	#N/A	6.4040	6.8954	6.8800	6.7434	6.8021	6.5382	6.3693	6.1929	6.4654	6.4998	1.0515	0.0725	0.0210	1.6780
Lgalsl	_[Acetyl (Protein N-term)]AGS[Phospho (STY)]VADSDAVVK_.2	Q8VED9_S4_M1_Lgalsl	12.0494	12.2112	12.5393	12.5981	12.1853	12.4340	12.7462	11.9918	12.3608	12.4211	0.9965	-0.0051	0.7984	0.0978
Lgalsl	_GEEQSAIPYFPFIPDQPFR_.2	#N/A	4.5878	4.5481	5.2422	5.7696	5.2722	5.2074	4.8821	5.3444	5.1049	5.3204	0.9887	-0.0164	0.8224	0.0849
Lgalsl	_VFVDGHQLFDFYHR_.3	#N/A	4.1516	5.3729	4.0567	4.8419	4.5083	4.2579	4.6851	4.7237	4.5039	5.1194	0.9524	-0.0704	0.4340	0.3625
Lgalsl	_LDDGHLNNS[Phospho (STY)]LGS[Phospho (STY)]PVQADVYFPR_.3	Q8VED9_S22_M2_Lgalsl	3.1770	4.4138	4.0690	9.4453	3.8701	4.8806	4.5282	8.5338	5.5880	3.6609	0.8921	-0.1647	0.6843	0.1647
Lgalsl	_[Acetyl (Protein N-term)]AGSVADSDAVVKLDDGHLNNSLGS[Phospho (STY)]PVQADVYFPR_.4	#N/A	2.9995	4.2267	5.9202	4.1890	3.9649	4.0105	3.9037	3.9295	3.5781	4.1560	1.0839	0.1163	0.5259	0.2791
Rpe	:thyl (C)]AEAGANM[Oxidation (M)]IVS[Phospho (STY)]GS[Phospho (STY)]AIM[Oxidation (M)]RS[Phospl	Q8VEE0_S196_M3_Rpe	4.1401	3.0860	3.3261	3.8288	3.2658	2.9628	3.5434	4.2897	3.9383	3.7958	0.8826	-0.1802	0.1206	0.9185
Gramd1a	_S[Phospho (STY)]SPSSSPSLR_.2	Q8VEF1_S11_M1_Gramd1a	3.9177	3.9358	4.0717	3.7160	3.7827	3.4489	7.5275	7.1688	3.6017	3.1664	0.7104	-0.4933	0.1282	0.8920
Arl8a	_EIC[Carbamidomethyl (C)]C[Carbamidomethyl (C)]YSISC[Carbamidomethyl (C)]K_.2	#N/A	5.3051	4.3887	5.7251	5.4789	4.9640	4.9412	5.4934	5.2587	5.0323	5.3596	0.9712	-0.0421	0.5648	0.2481
Arl8a	_M[Oxidation (M)]NLSAIQDR_.2	#N/A	5.6826	5.5608	5.9251	5.0496	5.6454	6.0328	5.7672	5.9168	6.2451	5.7687	0.9536	-0.0686	0.1997	0.6996
Arl8a	_DLAGALDEK_.2	#N/A	3.4165	4.1728	3.1502	4.1948	4.0611	4.0644	4.3430	4.3072	3.1055	4.5314	0.9439	-0.0833	0.5245	0.2802
Vps4a	_ENQSEGKGS[Phospho (STY)]DS[Phospho (STY)]DSEGDNPEK_.2	Q8VEJ9_S95_M2_Vps4a	3.3946	4.0838	4.0814	4.5841	3.7199	4.4056	3.8471	3.5344	3.1830	3.0502	1.1884	0.2490	0.0414	1.3834
Tmem30a	_EIEIDYTGTEPSS[Phospho (STY)]PC[Carbamidomethyl (C)]NK_.2	Q8VEK0_S89_M1_Tmem30a	4.0804	3.2294	3.6585	3.7601	4.1278	3.5712	3.6231	3.9613	3.8637	3.7949	0.9809	-0.0278	0.6966	0.1570
Hnrnpu	_DLPEHAVLK_.2	#N/A	8.1057	8.2127	8.2220	8.2408	8.4900	8.5503	8.1734	8.1424	8.4547	8.5870	0.9957	-0.0062	0.7800	0.1079
Hnrnpu	_SSGPTSLFAVTVAPPGAR_.2	#N/A	8.7704	8.0471	8.5363	8.3296	8.2915	8.4040	8.4989	8.2719	8.5407	8.1024	0.9872	-0.0186	0.4924	0.3076
Hnrnpu	_DIDIHEVR_.2	#N/A	7.4499	7.4579	7.6910	7.7899	7.4176	7.5089	7.1250	7.3801	7.3643	7.3743	1.0330	0.0469	0.0299	1.5241
Hnrnpu	_LNTLLQR_.2	#N/A	7.1962	7.0841	7.3420	7.0173	7.5351	7.0132	7.2656	6.7250	7.3674	6.8710	1.0199	0.0285	0.4082	0.3891
Hnrnpu	_EKPYFPIPEDC[Carbamidomethyl (C)]TFIQNVPLEDR_.3	#N/A	6.7658	7.0384	7.0265	7.1577	6.9158	6.5128	7.0383	7.0398	6.5650	6.5780	1.0143	0.0205	0.5577	0.2536
Hnrnpu	_NFILDQTNVSAAAQR_.2	#N/A	6.5423	5.7128	6.7445	5.9011	6.3403	6.4011	6.4133	5.7105	6.0003	6.8256	1.0058	0.0084	0.8993	0.0461
Hnrnpu	_LLEQYKEESKK_.3	#N/A	5.8546	5.8551	5.9217	5.2344	6.6488	6.3759	5.1744	5.7192	6.3249	5.6881	1.0445	0.0629	0.4361	0.3605
Hnrnpu	KS[Phospho (STY)]PQPPVEEEDEHFDDTVVC[Carbamidomethyl (C)]LDTYNC[Carbamidomethyl (C)]DLHFK	#N/A	8.9519	9.0360	9.7719	9.7270	8.5286	8.8343	9.4281	9.2904	9.2892	9.1643	0.9837	-0.0237	0.5756	0.2399
Hnrnpu	S[Phospho (STY)]PQPPVEEEDEHFDDTVVC[Carbamidomethyl (C)]LDTYNC[Carbamidomethyl (C)]DLHFK_	#N/A	3.8448	5.4294	4.5316	3.4707	2.9705	5.4962	3.6189	3.0842	6.2155	3.5005	1.0453	0.0639	0.8165	0.0881
Hnrnpu	_GYFEYIEENKYSR_.2	#N/A	5.1021	4.2511	4.2695	3.2713	4.5157	4.9369	4.9018	4.6623	4.3004	5.0732	0.9275	-0.1086	0.3641	0.4388
Hnrnpu	_[Acetyl (Protein N-term)]SSS[Phospho (STY)]PVNVKK_.2	#N/A	12.3371	12.6979	13.3092	13.0154	12.5107	12.7955	12.8663	12.6119	12.6163	12.6314	1.0076	0.0109	0.6172	0.2096
Hnrnpu	_FIEIAAR_.2	#N/A	3.9051	3.8226	3.6388	3.7741	3.6296	3.5692	3.3308	5.5412	4.2972	5.2172	0.8753	-0.1922	0.3026	0.5191
Hnrnpu	_[Acetyl (Protein N-term)]SSS[Phospho (STY)]PVNVK_.2	#N/A	3.9963	3.7461	3.7300	3.9899	3.8562	3.6183	4.1381	3.8612	3.3755	3.6672	1.0166	0.0237	0.6865	0.1633
Hnrnpu	_NGQDLGVAFK_.2	#N/A	3.3862	4.1914	4.0730	2.8217	4.1397	3.7168	4.0776	3.0719	3.3709	3.0418	1.0976	0.1344	0.3503	0.4556
Hnrnpu	_VSELKEELKK_.3	#N/A	3.4309	4.2360	4.1177	4.6203	1.8176	3.6721	4.3350	3.4982	3.1468	2.1340	1.1130	0.1545	0.5671	0.2464
Hnrnpu	_PAMEPGNGS[Phospho (STY)]LDLGGDAAGR_.2	#N/A	3.4129	4.1691	3.1465	6.4970	4.0574	4.0680	3.9612	4.3109	3.1091	3.8684	1.1083	0.1483	0.5356	0.2712
Hnrnpu	_LQAALDNEAGGRPAMEPGNGS[Phospho (STY)]LDLGGDAAGR_.3	#N/A	3.4715	4.2278	3.6636	4.2144	3.4563	4.0360	3.9291	3.9307	3.5792	4.2429	0.9807	-0.0281	0.7309	0.1361
Hnrnpu	_LSASSLTM[Oxidation (M)]ESFAFLWAGGR_.2	#N/A	3.5616	3.6646	4.2484	3.2502	4.1881	3.5414	4.4657	3.3675	3.0161	3.2172	1.0642	0.0898	0.5081	0.2941
Rem2	_GHAGGQRPEPSS[Phospho (STY)]PDGPAPPTRR_.4	#N/A	3.8602	3.7051	4.1834	3.7271	3.5934	3.0332	4.1226	3.2760	3.5751	4.9883	0.9231	-0.1154	0.4118	0.3853
Rem2	_QAS[Phospho (STY)]PLGTPTPEADTTLLK_.2	Q8VEL9_S27_M1_Rem2	8.0047	7.8043	8.3131	8.6017	6.9092	8.1018	7.9241	7.8283	7.3537	7.8824	1.0269	0.0383	0.5255	0.2794
Rem2	_GHAGGQRPEPSS[Phospho (STY)]PDGPAPPTR_.3	#N/A	8.0460	8.0371	8.1823	8.4633	3.5380	7.7635	7.9070	7.9303	7.4747	8.0089	0.9372	-0.0936	0.6229	0.2056
Rem2	_RGS[Phospho (STY)]M[Oxidation (M)]PVPYK_.2	#N/A	5.8983	4.2470	3.9025	4.4052	4.7106	3.8337	6.1061	5.9653	6.1422	6.4265	0.7304	-0.4532	0.0030	2.5298
Rem2	_SRS[Phospho (STY)]C[Carbamidomethyl (C)]HDLSVL_.2	Q8VEL9_S334_M1_Rem2	3.3834	3.9355	3.9978	4.3365	3.7927	4.1419	4.2750	3.9972	3.2086	3.8389	1.0265	0.0377	0.6890	0.1618
Rem2	_RRGS[Phospho (STY)]M[Oxidation (M)]PVPYK_.3	#N/A	2.9899	3.7461	4.2244	3.7681	3.6344	2.9902	3.9164	3.2330	3.5321	3.4454	1.0077	0.0110	0.9230	0.0348
Rem2	_GS[Phospho (STY)]MPVPYK_.2	#N/A	3.7961	3.1004	4.4829	3.5194	2.9218	3.3069	3.6677	3.4818	3.7808	3.1967	0.9970	-0.0043	0.9733	0.0117
Slc25a3	_IQTQPGYANTLR_.2	#N/A	10.2186	10.1673	10.3658	9.6214	10.1682	9.8332	10.4800	10.2740	10.1061	9.9538	0.9862	-0.0201	0.4243	0.3723
Slc25a3	_GSTASQVLQR_.2	#N/A	8.3511	8.3714	8.5609	8.2431	8.2173	8.2595	8.5572	8.7038	8.5331	8.3227	0.9771	-0.0334	0.0611	1.2139
Slc25a3	_EEGLNAFYK_.2	#N/A	6.0881	5.6761	5.2457	5.4274	5.8863	5.1923	5.7454	6.2503	6.3567	6.6049	0.8953	-0.1596	0.0228	1.6414
Slc25a3	_FGFYEVFK_.2	#N/A	5.2199	5.0501	5.2207	4.9144	3.9305	4.6519	5.0093	5.1355	5.0132	4.4525	0.9854	-0.0212	0.8037	0.0949
Slc25a3	_GIFNGFSITLK_.2	#N/A	5.9652	4.0641	5.6144	4.7982	4.0736	4.9326	5.3366	3.4071	5.3392	5.1484	1.0208	0.0298	0.8584	0.0663
Slc25a3	_MYKEEGLNAFYK_.2	#N/A	6.4148	6.1132	3.3256	7.1149	5.5945	2.9633	6.1964	5.5403	5.4900	5.6623	0.9182	-0.1231	0.6103	0.2144
Slc25a3	_YYALC[Carbamidomethyl (C)]GFGGVLSC[Carbamidomethyl (C)]GLTHTAVVPLDLVK_.3	#N/A	3.7970	3.1014	4.4838	3.5184	2.9227	3.3059	3.6667	3.4827	3.7818	3.4527	0.9793	-0.0302	0.8064	0.0934
Rbm39	_YRS[Phospho (STY)]PYSGPK_.2	Q8VH51_S97_M1_Rbm39	7.9840	8.2193	8.7295	8.5048	7.4131	7.8766	8.7367	8.4867	8.3020	8.1238	0.9654	-0.0508	0.2981	0.5257
Rbm39	_DKS[Phospho (STY)]PVREPIDNLTPEER_.3	#N/A	10.1055	9.9213	10.7141	10.6136	9.2823	9.9598	10.6203	10.0814	10.1743	9.7488	0.9944	-0.0081	0.8555	0.0678
Rbm39	_S[Phospho (STY)]KS[Phospho (STY)]PFRK_.2	Q8VH51_S127_M2_Rbm39	9.5261	9.1227	9.4576	9.3554	8.5168	9.2163	9.8389	9.4150	9.6501	9.1069	0.9681	-0.0468	0.2135	0.6705
Amot	_DTTVISHS[Phospho (STY)]PNTSYDTALEAR_.3	Q8VHG2_S693_M1_Amot	3.4779	3.5504	4.0357	3.6448	5.7728	6.3612	4.1284	5.8108	6.0779	6.3085	0.8016	-0.3191	0.1800	0.7448
Agap3	_IEDLALQNQIR_.2	#N/A	5.7393	5.7120	5.7042	6.1486	6.2685	5.6562	6.4683	5.8511	5.9619	5.6712	0.9805	-0.0284	0.5588	0.2527
Wasf3	_LSQSVHHGASSEGSLS[Phospho (STY)]PDTR_.3	Q8VHI6_S235_M1_Wasf3	4.0879	3.7028	4.0728	4.1181	3.5507	4.0396	4.0964	3.9673	3.4292	4.3559	0.9915	-0.0123	0.8690	0.0610
Mark1	_SAAYNGPPAS[Phospho (STY)]PSHETGAFAHAR_.3	Q8VHJ5_S633_M1_Mark1	8.3622	8.5005	3.3530	4.3976	4.2639	8.0632	4.5459	8.6268	7.9413	7.9410	0.8476	-0.2385	0.4580	0.3392
Mark1	_RGT[Phospho (STY)]STGIISK_.2	Q8VHJ5_T648_M1_Mark1	4.0876	3.1385	7.0692	3.7763	3.2133	3.5978	3.4909	4.3422	3.9908	3.7433	1.0656	0.0917	0.7475	0.1264
Mark1	_SRPSS[Phospho (STY)]DLNNSTLQSPAHLK_.3	Q8VHJ5_S394_M1_Mark1	3.8359	6.6367	6.5252	7.3595	3.4190	5.4190	6.2772	5.8281	5.8403	6.7185	0.9764	-0.0344	0.8930	0.0492
Mark1	_RDPS[Phospho (STY)]EGEASGR_.2	Q8VHJ5_S666_M1_Mark1	3.7744	3.0298	7.3424	6.4596	2.9181	6.3842	3.2001	3.9493	6.9565	6.2167	0.9811	-0.0275	0.9396	0.0271
Mark1	_KPPEFEGGESLS[Phospho (STY)]SGSLC[Carbamidomethyl (C)]QR_.3	#N/A	3.2611	3.9637	4.4956	4.0394	5.8217	4.1230	4.2367	3.8627	7.0224	6.2337	0.8024	-0.3176	0.1949	0.7103
Mark1	_STAS[Phospho (STY)]PSNNVYSGGSMAR_.2	Q8VHJ5_S488_M1_Mark1	4.0013	3.3057	3.1873	3.3142	3.1270	3.1016	3.4624	4.4285	3.4147	3.6569	0.8928	-0.1637	0.1514	0.8198
Mark1	_RPQANS[Phospho (STY)]VESEQKEEWGK_.3	Q8VHJ5_S444_M1_Mark1	3.0812	3.8375	4.3157	4.5781	3.7258	4.3997	4.2928	3.5403	3.1889	3.0443	1.1345	0.1821	0.2239	0.6500
Mark1	_SIS[Phospho (STY)]ANQK_.2	Q8VHJ5_S414_M1_Mark1	3.0172	4.3252	4.2518	3.7955	4.1465	3.5830	3.9438	3.2056	3.5047	3.4728	1.0910	0.1257	0.2867	0.5425
Caskin2	_SPS[Phospho (STY)]QES[Phospho (STY)]IGAR_.2	Q8VHK1_S696_M2_Caskin2	7.1565	3.8252	7.2209	4.5904	3.7135	7.1838	4.3051	7.1336	7.1636	7.5920	0.8574	-0.2219	0.4099	0.3874
Caskin2	_T[Phospho (STY)]SPSLTPTR_.2	Q8VHK1_T840_M1_Caskin2	3.1609	4.0652	3.8477	4.3504	3.7875	3.9420	4.0651	3.7681	3.4167	4.3174	0.9916	-0.0122	0.9002	0.0457
Setd7	_IAYVYPDQR_.2	#N/A	5.2811	6.1309	6.0851	6.2899	6.4866	6.1530	6.0602	5.7120	5.9516	6.1951	1.0153	0.0219	0.6966	0.1570
Setd7	_VYVADSLISSAGEGLFSK_.2	#N/A	3.4041	4.0563	5.2142	3.9143	3.6040	4.4129	5.0905	4.2377	4.0994	4.7235	0.9037	-0.1460	0.2799	0.5529
Setd7	_SGPEAPEWYQVELK_.2	#N/A	5.6410	5.8670	4.2477	6.5707	5.3643	5.9705	3.9397	5.5628	4.8346	5.8276	1.1129	0.1543	0.3053	0.5153

Setd7	_AFQATQQK_.2	#N/A	3.9090	4.1784	3.7346	4.2373	4.1952	4.0588	3.9519	3.8812	3.5298	4.9637	0.9928	-0.0105	0.9137	0.0392
Setd7	_AVEAEEELTVAYGYDHS[Phospho (STY)]PPGK_.3	Q8VHL1_S340_M1_Setd7	3.9698	3.3726	3.7035	3.2472	3.1135	3.5111	3.3955	3.7540	4.0530	3.5084	0.9479	-0.0771	0.3598	0.4440
Ppp1r16b	_RAS[Phospho (STY)]LSDR_.2	Q8VHQ3_S350_M1_Ppp1r16b	3.5025	3.7236	5.2457	3.1911	4.1290	3.0127	4.4067	3.4265	3.0751	3.1581	1.0808	0.1121	0.5694	0.2445
Ppp1r16b	_EQS[Phospho (STY)]PQTLLELKR_.3	Q8VHQ3_S477_M1_Ppp1r16b	4.2663	3.3136	3.7919	4.8458	3.2019	3.4227	3.3158	4.4899	3.9646	3.0129	1.0301	0.0428	0.7971	0.0985
Rab40c	_[Acetyl (Protein N-term)]GTQGS[Phospho (STY)]PVKSYDYLLK_.2	Q8VHQ4_S6_M1_Rab40c	5.0987	2.9655	3.4466	3.9493	3.3863	3.7708	3.6640	5.1973	3.8178	5.2999	0.8387	-0.2538	0.1926	0.7154
Gatad2b	_LQQQAALS[Phospho (STY)]PTTAPAVSSVSK_.2	Q8VHR5_S487_M1_Gatad2b	3.0447	6.4391	4.2792	7.7777	4.1190	3.6105	5.8154	3.1782	5.2249	3.5003	1.1013	0.1392	0.6843	0.1647
Gatad2b	_S[Phospho (STY)]LDPADERDDVLAKR_.3	#N/A	3.9959	6.7491	3.7295	7.4154	6.6566	6.9434	7.3099	6.6714	6.7288	7.2441	0.8464	-0.2406	0.2339	0.6309
Gatad2b	_GRLT[Phospho (STY)]PSPDIIVLS[Phospho (STY)]DNEASSPR_.3	#N/A	3.5929	5.3925	3.8152	3.6388	3.3807	3.8293	4.8751	3.8267	3.8748	4.0484	0.9483	-0.0765	0.6225	0.2058
Gatad2b	_VSS[Phospho (STY)]PLPS[Phospho (STY)]PSAM[Oxidation (M)]SDAANSQAAAK_.2	#N/A	3.8837	3.7163	3.7852	4.2879	3.7249	4.3637	4.9560	4.9022	3.5438	3.5394	0.9351	-0.0969	0.4542	0.3427
Gatad2b	_LTPSPDIIVLS[Phospho (STY)]DNEASS[Phospho (STY)]PR_.2	#N/A	3.8753	3.9177	3.7993	4.6033	3.7390	4.0750	3.6977	3.7206	3.7740	3.7957	1.0679	0.0948	0.1556	0.8079
Gatad2b	_S[Phospho (STY)]LDPADERDDVLAK_.2	#N/A	3.6315	4.3878	7.9156	4.0278	4.2761	3.8494	3.7425	8.0502	3.7392	3.9948	0.9590	-0.0605	0.8685	0.0612
Cacng8	_AGGGAGGS[Phospho (STY)]GGSGPSAILR_.2	Q8VHW2_S251_M1_Cacng8	6.8310	5.6669	7.0593	6.8286	4.9859	6.1835	7.3881	7.7794	7.4804	7.2908	0.8363	-0.2580	0.0196	1.7086
Cacng8	_DAS[Phospho (STY)]PGGPGGPGFASTDISM[Oxidation (M)]YTLSR_.3	#N/A	4.1536	4.5120	4.9930	3.6660	3.2555	3.8587	3.5673	4.8055	5.0047	3.5256	0.9639	-0.0531	0.7400	0.1308
Cacng8	_SSS[Phospho (STY)]RGS[Phospho (STY)]SEASPSR_.2	Q8VHW2_S277_M2_Cacng8	3.4422	4.2168	4.1290	3.9778	4.0669	3.9232	4.3463	3.5103	3.1589	3.9448	1.0586	0.0822	0.4015	0.3963
Satb2	_SGS[Phospho (STY)]PDVKGPPPVK_.2	Q8VI24_S20_M1_Satb2	7.5794	3.8354	3.2407	4.2853	7.2729	4.0728	4.4336	7.9595	3.0149	3.9626	1.0424	0.0598	0.8770	0.0570
Satb2	_ES[Phospho (STY)]SPPREEAPPPPPTEDSC[Carbamidomethyl (C)]AK_.3	Q8VI24_S593_M1_Satb2	4.0916	3.2131	3.5508	3.6090	2.9921	3.6394	5.7471	4.0076	3.9702	4.1636	0.7862	-0.3470	0.0396	1.4023
Satb2	_SES[Phospho (STY)]PC[Carbamidomethyl (C)]LRDS[Phospho (STY)]PDRR_.2	#N/A	4.1010	3.4054	4.0876	4.4895	3.2267	3.8552	4.8482	3.6813	4.0300	4.4970	0.9054	-0.1433	0.2362	0.6266
Pxn	_YAHQQPPS[Phospho (STY)]PLPVYSSSAK_.3	Q8VI36_S83_M1_Pxn	6.1589	5.7046	6.5752	6.9048	5.9670	3.5150	6.1727	4.4768	6.0082	5.8309	1.0324	0.0460	0.7972	0.0985
Lrp4	_RGS[Phospho (STY)]LPDTGWK_.2	#N/A	3.2592	4.0832	6.6303	7.1369	3.9045	6.2644	7.5378	4.4645	6.6902	6.5497	0.8261	-0.2756	0.2989	0.5245
Lrp4	_GS[Phospho (STY)]LPDTGWK_.2	#N/A	3.3533	4.1096	3.8034	4.3060	3.9979	4.1275	4.0207	6.2889	3.4611	5.4813	0.8206	-0.2852	0.1500	0.8240
Pcnx3	_SQAPLDLSLSPDVSSEAS[Phospho (STY)]PAR_.2	Q8VI59_S1980_M1_Pcnx3	3.6147	3.7278	3.3483	4.3929	4.2592	3.8662	4.5412	4.1091	4.4081	4.0702	0.9033	-0.1467	0.0983	1.0073
Nr3c2	_SSVSS[Phospho (STY)]PANLNNSR_.2	Q8VII8_S299_M1_Nr3c2	7.1276	7.1662	7.4210	7.6961	6.4836	6.8519	7.3593	7.4688	4.2376	6.7315	1.1047	0.1436	0.3187	0.4966
Nr3c2	_DAEYTYDQQNQQGS[Phospho (STY)]LSPAK_.2	Q8VII8_S127_M1_Nr3c2	3.9205	4.1491	5.2566	4.1700	3.9704	3.7592	4.8450	3.5852	3.4961	3.5164	1.0890	0.1230	0.3897	0.4092
Sfpq	_YGEPGEVFINK_.2	#N/A	7.2822	7.5623	7.6480	7.4046	7.2230	7.3073	7.8088	7.3497	7.3283	7.4907	0.9880	-0.0174	0.4849	0.3143
Sfpq	_LFVGNLPADITEDEFKR_.3	#N/A	6.6037	6.9493	6.8806	7.1122	6.8561	6.9399	7.0903	6.8088	6.9120	7.0238	0.9902	-0.0143	0.5053	0.2964
Sfpq	_FATHAAALSVR_.2	#N/A	5.9230	5.8156	5.8979	6.4787	6.5220	6.0816	6.1912	5.8475	6.0246	6.0605	1.0147	0.0211	0.6084	0.2158
Sfpq	_FGQGGAGPVGGQGPR_.2	#N/A	6.1636	6.7451	3.1547	6.7339	6.8656	6.1746	6.7059	6.7461	6.5930	6.9653	0.8845	-0.1770	0.3129	0.5045
Sfpq	_PVIVEPLEQLDDEDGLPEK_.2	#N/A	5.9263	5.5752	5.1650	6.3647	5.5211	5.3991	5.7686	5.9466	3.9839	5.3575	1.0749	0.1042	0.3675	0.4347
Sfpq	_LFVGNLPADITEDEFK_.2	#N/A	4.6296	4.0679	3.8451	4.3477	5.2420	4.1693	4.0624	3.8910	4.6345	5.2158	0.9849	-0.0220	0.8511	0.0700
Ciapin1	_EPVETAEVNNDK_.2	#N/A	6.4559	5.3206	4.7651	6.5069	5.3758	5.7570	5.5564	5.3602	6.0416	6.1359	0.9867	-0.0193	0.8451	0.0731
Ciapin1	_EALS[Phospho (STY)]PEEVQSVQEHLGYHSDSLR_.3	Q8WTY4_S136_M1_Ciapin1	3.5901	6.0842	3.3238	6.3499	3.5737	5.2057	5.8650	5.8546	5.5364	5.2635	0.8327	-0.2642	0.2181	0.6613
Wdr13	_YGPLS[Phospho (STY)]EPGSAR_.2	Q91V09_S70_M1_Wdr13	9.2657	9.0055	9.5288	9.2578	8.3910	9.0570	9.3954	8.8590	8.8092	8.9713	1.0084	0.0121	0.7442	0.1283
Wdr13	_LVVHEGSPVTSISAR_.3	#N/A	4.3185	4.1808	4.1288	5.6085	4.7667	4.0201	3.9668	4.3572	4.3221	4.6059	1.0443	0.0625	0.5715	0.2430
Wdr13	_LLWAGDDR_.2	#N/A	3.4265	3.5515	3.3793	4.5539	3.5773	3.1717	3.2704	3.6887	4.5873	4.3161	0.9103	-0.1355	0.3287	0.4832
Wdr13	_VLALSFDAPGR_.2	#N/A	3.8405	3.6241	3.5742	7.4664	4.2703	4.0861	3.2662	4.1699	4.1823	4.2961	1.1253	0.1703	0.5422	0.2659
Wdr13	_[Acetyl (Protein N-term)]AAVWQQVLAVDAR_.2	#N/A	3.8731	3.1286	3.6068	3.7862	3.0169	3.6077	3.5009	4.3323	3.9809	3.7532	0.9002	-0.1518	0.1253	0.9020
Acot7	_Y[Phospho (STY)]EAQKLER_.2	Q91V12_Y195_M1_Acot7	7.4082	7.7306	8.1019	7.5748	7.5768	7.5212	7.6100	7.6800	7.5886	7.2289	1.0167	0.0238	0.4211	0.3757
Acot7	_VLEVPPIVYLR_.2	#N/A	7.9426	7.8095	7.9170	7.6430	7.6528	7.7905	7.7741	8.0932	7.9739	8.0321	0.9779	-0.0322	0.0715	1.1456
Acot7	_LM[Oxidation (M)]DEVAGIVAAR_.2	#N/A	7.0366	7.2143	7.2694	7.5418	7.2934	7.3244	7.3326	7.6335	7.4159	7.4783	0.9752	-0.0362	0.0944	1.0248
Acot7	_SM[Oxidation (M)]EIEVLVDADPVVDNSQK_.2	#N/A	5.3935	5.1642	3.1563	3.6589	4.6081	5.7864	5.6853	5.1568	6.0272	4.9902	0.8098	-0.3044	0.0908	1.0419
Acot7	_AASAFFTYVSLNQEGK_.2	#N/A	3.4275	4.5597	5.6651	5.0810	4.6299	3.6755	4.3316	4.0220	4.9234	5.3671	0.9668	-0.0487	0.7610	0.1186
Acot7	_M[Oxidation (M)]EEAGAIISTR_.2	#N/A	5.2941	5.4565	5.6982	5.0489	6.6295	6.1801	5.3298	6.0327	5.8122	6.3731	0.9713	-0.0420	0.6394	0.1942
Acot7	_TNIVTASVDAINFHDK_.2	#N/A	5.7449	5.5849	5.9240	5.5939	5.3063	5.4114	5.9219	5.7353	5.9263	5.3058	0.9776	-0.0327	0.4514	0.3454
Acot7	_M[Oxidation (M)]TFTSNK_.2	#N/A	2.9029	2.9386	4.1374	3.6812	3.5475	3.0771	3.8295	3.3200	3.6190	3.3585	0.9573	-0.0630	0.5886	0.2302
Slc12a5	_EIQSITDESR_.2	#N/A	7.8655	7.6082	7.8701	7.4529	7.4711	7.2541	7.7911	7.9114	7.6105	7.3336	0.9903	-0.0141	0.6526	0.1853
Slc12a5	_ENLWSSYLTK_.2	#N/A	7.1993	6.9145	7.0921	6.9113	6.4860	6.8411	7.5171	7.0696	7.2045	6.7100	0.9694	-0.0448	0.2657	0.5757
Slc12a5	_LNEVIVNK_.2	#N/A	6.1527	5.7270	6.6221	5.5962	5.8187	6.0452	6.1915	6.1445	6.1952	5.3982	1.0019	0.0027	0.9641	0.0159
Slc12a5	_VDQDQNVVHPQLLSLTSQLK_.3	#N/A	5.1195	4.9304	5.1952	4.6223	4.0621	5.0823	5.3496	5.1656	4.6268	4.2135	0.9993	-0.0011	0.9907	0.0040
Slc12a5	_LVLLNM[Oxidation (M)]PGPR_.2	#N/A	6.5037	6.0880	6.9717	4.6924	6.1798	6.6721	6.8399	7.0210	6.7758	6.6153	0.9078	-0.1396	0.1669	0.7776
Slc12a5	_GFC[Carbamidomethyl (C)]QVVISSNLR_.2	#N/A	5.9628	5.8866	5.6290	5.6971	4.8562	5.2452	6.0274	5.4751	5.5455	5.8701	0.9680	-0.0469	0.4643	0.3332
Slc12a5	_NKGPS[Phospho (STY)]PVSSEGIK_.2	Q91V14_S1044_M1_Slc12a5	3.9020	3.4404	3.6356	3.1794	3.0457	3.5789	6.2193	3.8218	4.1208	4.3575	0.7481	-0.4187	0.0340	1.4689
Slc12a5	_DLTTFLYHLR_.2	#N/A	4.4937	5.3222	5.6710	4.9745	3.7488	4.4260	4.8991	4.8561	5.1099	4.4575	0.9880	-0.0174	0.8792	0.0559
Slc12a5	_HGFDVC[Carbamidomethyl (C)]AK_.2	#N/A	5.0720	4.9698	3.7802	3.9627	4.7303	3.7606	4.9272	5.0304	3.9733	5.0956	0.9207	-0.1192	0.3437	0.4638
Slc12a5	_ESS[Phospho (STY)]PFINSTDTEK_.2	Q91V14_S49_M1_Slc12a5	9.6594	9.2456	9.7081	9.2378	8.1213	9.3737	9.9702	9.6939	9.2253	9.3309	0.9654	-0.0508	0.3369	0.4724
Slc12a5	_EIQSITDES[Phospho (STY)]RGSIRR_.3	#N/A	6.7311	3.4400	4.3454	5.1919	3.3022	7.0845	3.5825	5.1937	3.9670	7.1700	1.0075	0.0108	0.9722	0.0123
Slc12a5	_EREIQSITDESRGS[Phospho (STY)]IR_.3	#N/A	3.9792	3.2835	3.1651	6.9383	3.1049	6.2923	3.4846	6.5436	6.0450	5.9883	0.8088	-0.3062	0.3351	0.4748
Slc12a5	_EIQSITDESRGS[Phospho (STY)]IR_.2	#N/A	5.3306	5.7327	5.6801	5.8807	3.8296	5.8746	5.1055	5.9083	5.5788	5.8588	0.9599	-0.0590	0.6139	0.2119
Slc12a5	_ESSPFINS[Phospho (STY)]TDTEKGR_.2	Q91V14_S54_M1_Slc12a5	4.3085	3.6129	3.4945	3.9972	3.4342	4.2953	3.7118	4.1213	3.7699	3.9642	0.9911	-0.0129	0.8760	0.0575
Slc12a5	_GLTIVGSVLEGTFLDNHPQAQR_.3	#N/A	4.0782	3.6093	3.4737	4.5183	3.7578	4.3058	3.0271	2.9971	4.6743	5.0800	1.0032	0.0046	0.9798	0.0089
Slc12a5	_TLVMEQR_.2	#N/A	3.8733	3.3529	4.5601	5.6548	2.9989	3.3834	3.2766	3.0558	3.0183	3.5289	1.2331	0.3023	0.1807	0.7431
Slc12a5	_ITAEVEVVEM[Oxidation (M)]HESDISAYTYEK_.3	#N/A	4.5415	5.0116	3.8319	4.4563	3.7717	5.1850	5.0236	3.8788	4.4084	3.6244	1.0549	0.0771	0.5633	0.2493
Slc12a5	_NVSMFPGNPER_.2	#N/A	3.9877	3.2920	3.1736	3.3278	3.1133	3.1153	3.4761	3.6733	3.9724	3.0051	0.9443	-0.0827	0.4237	0.3730
Slc12a5	_IFTVAQM[Oxidation (M)]DDNSIQM[Oxidation (M)]K_.2	#N/A	3.7691	3.0735	4.4559	3.4578	2.8948	3.3338	3.6946	3.1599	4.3093	3.4248	0.9590	-0.0604	0.6763	0.1698
Nrbp2	_EDES[Phospho (STY)]EDESDILEESPC[Carbamidomethyl (C)]GR_.2	Q91V36_S20_M1_Nrbp2	2.9005	3.7057	3.5873	7.1180	3.5270	4.2024	3.0624	6.1177	4.3861	4.0923	0.9454	-0.0810	0.7990	0.0975
Nrbp2	_AKT[Phospho (STY)]PT[Phospho (STY)]PEPFDSETR_.2	#N/A	7.0607	7.9489	3.9170	8.4176	5.6916	7.9279	7.7914	7.7869	7.6696	7.6066	0.8851	-0.1761	0.3429	0.4649
Nrbp2	_AKT[Phospho (STY)]PT[Phospho (STY)]PEPFDSETRK_.3	#N/A	4.2549	3.5592	3.4408	3.9435	3.3806	4.3489	3.2089	4.1750	4.6332	3.9105	0.9597	-0.0594	0.6282	0.2019
Rab14	_NLTNPNTVIILIGNK_.2	#N/A	5.9810	5.6732	6.4697	4.7447	5.7322	5.9191	6.0917	6.4732	6.2033	6.3248	0.9171	-0.1248	0.1185	0.9264
Rab14	_IYQNIQDGSLDLNAAESGVQHKPSAPQGGR_.4	#N/A	5.1629	4.0939	4.0246	5.1910	4.3469	3.1899	3.0831	4.9231	4.8697	4.1358	1.0193	0.0275	0.8776	0.0567
Rab14	_STYNHLSSWLTDAR_.3	#N/A	5.5384	5.0788	5.5248	5.6410	4.5991	4.8520	5.6581	5.8458	4.1327	5.1038	1.0040	0.0057	0.9575	0.0189
Rab14	_QFAEENGLLFLEASAK_.2	#N/A	4.3954	4.3316	3.5814	4.0840	4.2199	3.9055	3.7987	4.0345	3.6831	3.9704	1.0554	0.0778	0.2358	0.6275
Rab14	_FM[Oxidation (M)]ADC[Carbamidomethyl (C)]PHTIGVEFGTR_.3	#N/A	4.0411	3.2966	3.7748	3.6182	3.1849	3.4397	3.3329	3.6826	3.9816	2.9959	1.0174	0.0249	0.8012	0.0963
Rab14	_TGENVEDAFLEAAKK_.3	#N/A	3.1446	3.9008	4.0121	4.5148	3.7891	4.3363	4.2295	3.6037	2.2276	2.9810	1.2114	0.2767	0.1376	0.8614
Sfxn3	_ELQVGIPVTDEAGQR_.2	#N/A	9.2412	8.9396	9.4066	9.0640	8.5514	9.0797	9.5272	9.2099	9.0927	9.0331	0.9817	-0.0266	0.3568	0.4476
Sfxn3	_NLLLSGEQLEASR_.2	#N/A	7.7666	7.7775	7.8511	7.9310	7.3628	7.7045	7.8825	8.1009	7.5564	7.6557	0.9915	-0.0124	0.6449	0.1905
Sfxn3	_NIVQNYR_.2	#N/A	7.6391	7.8293	7.8188	7.4086	7.8151	7.2935	7.4376	7.8129	7.5905	7.3738	1.0106	0.0153	0.5865	0.2317
Sfxn3	_HFFTVTDPR_.2	#N/A	6.7312	6.9220	5.8994	6.1953	6.3050	6.7323	6.8674	6.7394	6.6908	6.5884	0.9617	-0.0563	0.2457	0.6097
Sfxn3	_[Acetyl (Protein N-term)]GDLPLNINIQEPR_.2	#N/A	6.6239	5.9517	5.4235	5.7140	6.7561	6.7679	6.9187	6.9997	6.1631	5.7358	0.9616	-0.0566	0.5359	0.2709
Sfxn3	_SSIHVTR_.2	#N/A	6.2395	5.6327	5.4836	5.7858	5.8274	5.6126	5.8310	5.9709	5.5903	5.5300	1.0058	0.0083	0.8392	0.0761
Sfxn3	_AGVATPGLTEDQLWR_.2	#N/A	6.1880	6.1890	6.7042	6.7635	5.3598	5.5133	6.5413	6.3858	6.1339	5.3030	1.0047	0.0068	0.9402	0.0268

Sfxn3	_QGIFQVVISR_.2	#N/A	5.0125	3.5604	4.2974	3.6982	3.4487	4.1541	4.5445	5.0731	4.2836	4.0651	0.8969	-0.1570	0.2165	0.6645
Sfxn3	_YVYDSAFHPDTGEK_.2	#N/A	6.9705	6.7996	7.2155	6.4256	6.1619	6.6397	7.1552	6.5459	6.6758	6.9557	0.9808	-0.0279	0.5708	0.2435
Sfxn3	_AQIQAQNPSIDVVYYNK_.2	#N/A	5.5338	5.4706	5.9518	5.9884	5.0548	5.7036	5.9100	6.0447	5.5766	5.3294	0.9828	-0.0250	0.6655	0.1768
Sfxn3	_SGDAPITVQQLGTAYVSATTGAVATALGLK_.3	#N/A	3.8508	3.4203	4.5376	4.3399	2.9765	3.2522	3.6130	2.5741	3.5023	2.3689	1.2372	0.3070	0.1149	0.9395
Acly	_TAS[Phospho (STY)]FSESR_.2	#N/A	11.7076	11.5594	11.7173	11.8985	10.1206	11.4914	11.4373	11.5996	11.5749	11.4736	0.9908	-0.0133	0.7595	0.1195
Acly	_TAS[Phospho (STY)]FSESRADEVAPAKK_.3	#N/A	7.5356	7.9929	8.6331	8.2513	7.1800	8.1814	7.9516	7.7301	7.8716	7.8660	1.0137	0.0196	0.7010	0.1543
Acly	_EAGVFVPR_.2	#N/A	6.6150	6.6298	6.1840	6.4318	6.4187	6.0277	6.1527	6.9724	6.4081	6.5186	0.9803	-0.0287	0.5004	0.3007
Acly	_TIAIIAEGIPEALTR_.2	#N/A	5.8728	5.9186	6.0586	6.1965	5.4821	6.0760	5.9439	5.8962	6.0498	5.8839	0.9984	-0.0023	0.9449	0.0246
Acly	_LNTEDIKR_.2	#N/A	5.4896	5.5881	5.6086	5.9753	6.0619	3.7594	5.8464	5.8181	5.5225	5.4427	0.9569	-0.0635	0.5927	0.2271
Acly	_DLVSSLTSGLLTIGDR_.2	#N/A	4.1058	3.3970	4.6226	3.2179	4.3328	4.2219	4.0294	3.7957	3.1968	4.1730	1.0485	0.0683	0.5916	0.2280
Acly	_DGVYILDLAAK_.2	#N/A	4.3691	4.4406	4.2089	4.9827	4.1486	4.9572	4.4262	4.4585	3.0556	4.6645	1.0883	0.1221	0.3207	0.4940
Acly	_TAS[Phospho (STY)]FSESRADEVAPAK_.2	#N/A	3.1191	3.8754	4.3536	4.5402	3.7637	4.3618	4.2549	3.5782	3.2268	3.0064	1.1381	0.1867	0.1971	0.7053
Acly	_LGLVGVNLSLDGVK_.2	#N/A	5.6166	4.8360	3.2379	4.2825	5.3061	4.0699	5.4023	5.3091	3.0178	3.9597	1.0307	0.0437	0.8359	0.0778
Acly	_SINNPDMR_.2	#N/A	3.5466	4.3518	4.2334	3.2353	4.1732	3.5563	4.4508	3.3824	3.0310	3.2023	1.0947	0.1305	0.3629	0.4403
Acly	_SAYDSTM[Oxidation (M)]ETM[Oxidation (M)]NYAQIR_.2	#N/A	3.2338	3.9924	3.9206	4.4233	3.8603	4.2448	4.1379	3.6952	3.3438	4.3903	1.0139	0.0199	0.8506	0.0703
Ppp1r14a	_GPGGS[Phospho (STY)]PSGLQK_.2	#N/A	10.2344	10.6386	10.7079	10.8842	10.9192	10.2941	10.9076	10.1708	10.2316	10.4590	1.0164	0.0234	0.4146	0.3823
Ppp1r14a	_QPGFPQPSPSDDPS[Phospho (STY)]LSPRQDR_.3	Q91VC7_S134_M1_Ppp1r14a	3.9129	3.1684	3.6466	3.1904	3.0567	3.5679	3.3387	3.8108	4.1098	4.3685	0.8763	-0.1904	0.0839	1.0763
Ppp1r14a	_QPGFPQPSPSDDPSLS[Phospho (STY)]PR_.2	Q91VC7_S136_M1_Ppp1r14a	9.2641	9.3717	3.8796	9.8497	9.0106	9.4610	9.8071	9.0221	9.1461	9.4389	0.9058	-0.1427	0.4713	0.3267
Ppp1r14a	_ARGPGGS[Phospho (STY)]PSGLQK_.2	#N/A	8.8035	9.4485	9.7610	9.5819	9.6170	9.1529	9.6046	3.7585	8.8074	9.1331	1.2004	0.2635	0.1899	0.7215
Ppp1r14a	_GPGGS[Phospho (STY)]PSGLQKR_.2	#N/A	4.1146	8.2669	8.5741	7.9695	8.4630	2.9883	3.3491	7.7594	7.3006	7.5992	1.0350	0.0496	0.8850	0.0531
Ndufs1	_VAVTPPGLAR_.2	#N/A	8.8182	9.0951	9.0322	9.0024	9.3686	8.8351	8.8066	9.0203	8.9080	8.9637	1.0113	0.0162	0.3820	0.4179
Ndufs1	_FEAPLFNAR_.2	#N/A	8.8564	9.0471	8.7362	8.9109	8.9823	8.8248	8.8460	9.0448	8.7744	8.8661	1.0011	0.0016	0.8930	0.0491
Ndufs1	_ILQDIASGR_.2	#N/A	8.2790	8.5318	8.8698	8.3287	8.6667	8.2816	8.3099	8.6576	8.4228	8.2116	1.0110	0.0158	0.5406	0.2671
Ndufs1	_FASEIAGVDDLGTTGR_.2	#N/A	7.9109	7.9870	8.6336	8.5725	8.2076	7.8342	8.3029	8.0194	7.9972	7.8635	1.0180	0.0258	0.4661	0.3315
Ndufs1	_GWNILTNSEK_.2	#N/A	7.8858	7.9325	7.9962	7.9319	7.8265	8.0768	8.2783	8.0102	8.0177	7.8693	0.9873	-0.0185	0.2407	0.6185
Ndufs1	_AVTEGAQAVEEPSIC[Carbamidomethyl (C)]_.2	#N/A	7.2926	7.4027	7.3365	7.5448	7.3974	7.2885	7.7881	7.3036	7.2970	7.4597	0.9886	-0.0165	0.4339	0.3626
Ndufs1	_VALIGSPVDLTYR_.2	#N/A	7.1499	7.8037	7.8711	7.8275	7.3497	7.1427	7.5653	7.8308	7.5157	7.2096	0.9992	-0.0012	0.9764	0.0104
Ndufs1	_SATYVNTEGR_.2	#N/A	7.1465	7.2712	7.2455	7.5155	7.5829	7.3545	7.3487	7.3431	7.2618	7.2986	1.0054	0.0078	0.6606	0.1801
Ndufs1	_GNDM[Oxidation (M)]QVGTYIEK_.2	#N/A	7.1928	7.2911	7.1645	6.7851	7.1136	7.5105	7.3364	7.0244	7.1473	7.7050	0.9826	-0.0253	0.4733	0.3249
Ndufs1	_YDHLGDSPK_.2	#N/A	7.1431	7.1845	6.7627	7.4324	7.6475	7.1986	7.2177	7.3797	6.9803	7.1069	1.0079	0.0114	0.7406	0.1304
Ndufs1	_ALSEIAGITLPYDTLDQVR_.2	#N/A	7.2375	7.2831	6.8788	7.5758	7.2064	7.4316	7.2407	7.3134	7.3850	7.1781	0.9986	-0.0021	0.9359	0.0288
Ndufs1	_LSVAGNC[Carbamidomethyl (C)]R_.2	#N/A	6.7994	7.1032	7.0244	7.2299	7.7610	7.6480	7.2804	7.3142	7.3856	6.9692	0.9825	-0.0254	0.5468	0.2622
Ndufs1	_GLLTYTSWEDALSR_.2	#N/A	6.4337	6.3783	6.9666	6.7900	6.8859	6.7942	6.9810	5.9186	7.0718	6.6851	1.0066	0.0095	0.8601	0.0655
Ndufs1	_SWLHNDLK_.2	#N/A	6.4259	6.7446	6.4053	6.5083	6.9787	6.6122	5.8367	6.9312	6.4830	6.1827	1.0400	0.0565	0.2710	0.5670
Ndufs1	_HSFC[Carbamidomethyl (C)]EVLK_.2	#N/A	6.4806	6.3045	6.0696	6.5756	6.6164	6.2260	6.1073	6.2124	6.4892	6.1547	1.0221	0.0315	0.3151	0.5016
Ndufs1	_IASQVAALDLGYKPGVEAIR_.3	#N/A	5.7533	5.6538	5.6505	6.0787	5.4690	5.3151	5.2351	5.6538	5.4793	5.6593	1.0266	0.0379	0.3708	0.4309
Ndufs1	_M[Oxidation (M)]LFLLGADGGC[Carbamidomethyl (C)]ITR_.2	#N/A	6.2202	5.0065	5.5955	5.9689	5.7334	5.4817	5.4670	4.7303	5.6926	5.5655	1.0566	0.0795	0.2991	0.5241
Ndufs1	_VTTGVAAEWK_.2	#N/A	6.1472	5.8002	3.3271	6.7841	6.5023	6.0875	6.5304	6.1436	6.2001	6.5471	0.9086	-0.1382	0.3922	0.4065
Ndufs1	_MC[Carbamidomethyl (C)]LVEIEK_.2	#N/A	5.8785	4.8187	3.6885	6.6697	4.2793	4.1013	5.2467	4.3399	4.4851	4.9377	1.0323	0.0459	0.8070	0.0931
Ndufs1	_DFYM[Oxidation (M)]TDSISR_.2	#N/A	6.6817	6.6264	6.5965	6.0256	6.4562	6.6437	6.6143	6.5289	6.3582	6.9086	0.9852	-0.0215	0.5484	0.2609
Ndufs1	_LTEPMVR_.2	#N/A	4.6926	3.5066	4.4064	5.0056	4.6650	3.3834	3.1229	3.2094	2.8514	3.5194	1.3466	0.4293	0.0157	1.8051
Ndufs1	_NRLEEVSPNLVR_.3	#N/A	6.8201	6.7571	6.9132	6.3774	6.9381	6.9978	6.3641	6.6449	6.8961	5.9794	1.0509	0.0716	0.1264	0.8983
Ndufs1	_M[Oxidation (M)]HEDINEEWISDK_.3	#N/A	3.5899	3.4935	3.2461	3.7488	3.1858	4.3691	3.4635	4.3756	4.3137	4.0962	0.8876	-0.1720	0.1343	0.8719
Ndufs1	_VAGM[Oxidation (M)]LQNFEGNAVAAIAGGLVDAEALVALK_.3	#N/A	3.7162	2.9716	3.4499	4.4945	2.8599	3.7647	3.1419	4.0075	4.3066	4.1718	0.9068	-0.1411	0.3524	0.4530
Ndufs1	_SNYLLNTTIAGVEEADVVLLVGTNPR_.3	#N/A	3.4217	3.8045	4.1085	4.6111	4.0482	3.6813	4.3258	3.5073	3.1559	3.0773	1.1221	0.1662	0.2010	0.6969
Rnh1	_[Acetyl (Protein N-term)]S[Phospho (STY)]LDIQC[Carbamidomethyl (C)]EQLSDAR_.2	Q91VI7_S2_M1_Rnh1	7.4925	8.8187	9.2098	10.1021	9.1536	9.5361	9.2763	9.2947	9.7164	9.6429	0.9546	-0.0670	0.3742	0.4269
Rnh1	_ELSLASNELKDEGAR_.3	#N/A	5.4804	4.8337	3.5864	4.0726	5.1783	4.8287	3.5213	4.2585	5.0867	5.4174	1.0202	0.0289	0.8562	0.0674
Rnh1	_LLC[Carbamidomethyl (C)]EGLQDPQC[Carbamidomethyl (C)]R_.2	#N/A	3.1511	4.7590	3.1198	5.7245	6.0175	3.9519	4.3127	5.4862	5.0333	3.8417	0.9541	-0.0679	0.7681	0.1146
Itm2c	_AAASGPASASAPAAEILLT[Phospho (STY)]PAR_.2	Q91VK4_T39_M1_Itm2c	4.1186	4.2469	3.8523	5.5109	3.2624	3.3622	3.2554	4.0232	3.9041	3.0734	1.1389	0.1876	0.3083	0.5111
Terf2ip	_YLLGNAPVS[Phospho (STY)]PSSQK_.2	Q91VL8_S200_M1_Terf2ip	8.5407	4.2272	9.1508	9.0353	8.1234	8.8747	4.3261	3.5071	3.1556	8.7237	1.6217	0.6975	0.0601	1.2214
Zbtb1	_LST[Phospho (STY)]PKER_.2	Q91VL9_T203_M1_Zbtb1	12.9191	13.1249	13.5920	13.6379	12.6964	13.3454	13.2154	13.1809	13.2611	13.3479	0.9976	-0.0035	0.8726	0.0592
Rbmxl1	_DVYLS[Phospho (STY)]PR_.2	#N/A	3.2566	3.9696	3.9434	4.4460	10.4206	4.2676	7.5287	3.6724	3.3210	10.9602	0.7928	-0.3350	0.5214	0.2828
Rbmxl1	_RDVYLS[Phospho (STY)]PR_.2	#N/A	4.2431	7.5125	3.9767	3.5205	7.3765	7.5584	7.8061	7.7139	7.8981	7.8925	0.7279	-0.4581	0.0670	1.1736
Rbmxl1	_[Acetyl (Protein N-term)]VEADRPGK_.2	#N/A	6.9644	6.9705	6.5395	7.0917	7.3953	7.0213	6.7238	6.7129	7.1903	7.2787	1.0030	0.0043	0.9132	0.0394
Rbmxl1	_LFIGGLNTETNEK_.2	#N/A	5.8761	5.8773	5.9402	6.8486	6.1528	6.2111	5.7854	5.7998	5.6449	5.9406	1.0619	0.0866	0.1032	0.9863
Rbmxl1	_ST[Phospho (STY)]PSGPVR_.2	#N/A	4.0349	3.3201	3.4632	4.4931	3.1677	4.0621	3.1406	4.0090	6.3990	6.6892	0.7425	-0.4295	0.1173	0.9308
Rbmxl1	_GFAFVTFESPADAK_.2	#N/A	5.7981	5.0536	6.3454	6.1613	6.2021	6.1652	5.6202	5.5987	5.8889	5.6507	1.0465	0.0656	0.3190	0.4961
Rbmxl1	_VEQATKPS[Phospho (STY)]FESGR_.2	Q91VM5_S88_M1_Rbmxl1	6.1512	5.3678	6.7118	4.1398	6.0567	6.1410	4.0125	4.3622	3.1604	3.8171	1.5011	0.5860	0.0050	2.3010
Rbmxl1	_DVYLS[Phospho (STY)]PRDDGYSTK_.2	#N/A	9.0534	9.7392	10.5016	10.0391	9.4541	9.7319	10.0430	9.7270	9.4732	9.7094	1.0015	0.0022	0.9566	0.0192
Rbmxl1	_REPLPSR_.2	#N/A	3.3242	4.8966	4.5587	4.3352	4.7527	4.6656	4.0499	5.1021	3.6667	4.0044	1.0514	0.0724	0.5855	0.2325
Rbmxl1	_DSYESYGNSR_.2	#N/A	6.3088	3.0427	6.5579	6.6429	6.5760	6.4704	6.2169	6.4274	6.6331	6.0707	0.9363	-0.0950	0.5959	0.2248
Rbmxl1	_RST[Phospho (STY)]PSGPVR_.2	#N/A	9.7767	9.9750	10.5351	10.3656	9.9012	9.9743	10.0627	9.9636	9.9031	10.0328	1.0098	0.0140	0.5423	0.2658
Rbmxl1	_DYSHS[Phospho (STY)]SSRDDYPSR_.3	Q91VM5_S246_M1_Rbmxl1	3.7824	3.5892	3.5161	3.8961	3.3798	3.6836	4.0444	3.9706	3.6050	4.2503	0.9177	-0.1238	0.0514	1.2886
Rbmxl1	_DRDYSDHPSGGSYR_.3	#N/A	5.0174	3.7776	3.7582	4.0014	5.6599	4.5521	4.5265	4.0430	5.1431	4.2114	0.9956	-0.0064	0.9646	0.0156
Rbmxl1	_SDLYSSGR_.2	#N/A	4.2782	2.9480	3.4642	3.9668	2.8363	3.7883	3.6815	4.1517	3.8002	3.9338	0.9114	-0.1339	0.2879	0.5408
Ppa2	_ILGTLALIDQSETDWK_.2	#N/A	4.5244	3.4531	3.9313	4.3113	3.8997	4.8573	3.4617	3.8460	3.6275	4.9531	1.0480	0.0677	0.6195	0.2080
Ppa2	_AFALDVINSAHER_.2	#N/A	3.8700	3.0780	4.4029	1.7745	5.1734	3.1864	3.9409	2.4576	3.7221	4.0071	1.0139	0.0199	0.9433	0.0253
Chchd6	_LSES[Phospho (STY)]VVNR_.2	Q91VN4_S33_M1_Chchd6	3.7544	3.5880	3.4880	4.5326	4.2635	5.2628	3.1801	5.3482	4.2684	4.2099	0.9757	-0.0355	0.8390	0.0762
Chchd6	_KKAS[Phospho (STY)]LTHEQQSAR_.3	#N/A	4.2972	5.1933	5.8444	5.7258	5.4318	5.2257	4.8300	5.8514	5.2576	5.4844	0.9870	-0.0188	0.8375	0.0770
Chchd6	_AYQHC[Carbamidomethyl (C)]VSTAR_.2	#N/A	4.6523	4.8448	4.3982	5.3076	5.7120	4.9706	5.2475	5.2264	4.5471	3.8728	1.0545	0.0766	0.4863	0.3131
Chchd6	_KAS[Phospho (STY)]LTHEQQSAR_.3	#N/A	3.1854	3.9906	3.8722	4.3748	1.9623	3.9176	3.5720	3.7437	3.3922	4.3418	0.9437	-0.0837	0.6652	0.1770
Chchd6	_AS[Phospho (STY)]LTHEQQSAR_.2	#N/A	3.7464	3.7605	3.8398	4.1898	2.9491	3.7398	3.7955	4.2687	4.0163	3.7641	0.9351	-0.0967	0.2917	0.5351
Chchd6	_LSSQQFHEAASK_.2	#N/A	3.5565	4.9779	4.2433	5.6672	5.6893	3.5464	4.4607	4.9788	4.8258	4.9725	0.9593	-0.0600	0.7102	0.1486
Atp5f1c	_HLIIGVSSDR_.2	#N/A	7.8291	8.2399	8.2565	8.8045	8.3760	8.5840	8.2036	8.6131	8.6458	8.3022	0.9890	-0.0160	0.6402	0.1937
Atp5f1c	_ADIKAPEDK_.2	#N/A	7.7282	7.5211	8.0515	7.7421	7.9901	8.2028	7.4181	7.7705	7.8402	7.4180	1.0343	0.0486	0.1335	0.8746
Atp5f1c	_NEVAALTAAGK_.2	#N/A	7.3002	7.1697	6.9142	7.5391	6.5639	6.8556	6.5188	7.6842	6.7370	6.7053	0.9970	-0.0044	0.9567	0.0192
Atp5f1c	_VYGTGSLALYEK_.2	#N/A	6.4682	6.4993	6.9017	6.4591	6.7836	7.0849	6.7037	6.5310	6.9657	6.2731	1.0123	0.0176	0.6593	0.1809
Atp5f1c	_ELIEIISGAAALD_.2	#N/A	5.6312	6.3392	6.5493	6.5048	5.6819	6.2814	5.4898	5.5438	5.6943	5.5614	1.0588	0.0824	0.2664	0.5745
Atp5f1c	_NASDM[Oxidation (M)]IDKLTLTFNR_.3	#N/A	5.9612	6.2982	6.5839	5.9496	6.3282	6.5076	5.7887	6.1541	6.4142	6.4687	1.0105	0.0150	0.7322	0.1354

Atp5f1c	_EVMIVGVGEK_.2	#N/A	7.0557	5.8092	3.1684	8.6240	6.0857	3.4925	5.9851	5.5144	5.6068	6.1142	0.9829	-0.0249	0.9286	0.0322
Atp5f1c	_THSDQFLVSFK_.2	#N/A	6.7952	6.7658	6.9193	7.2305	6.8628	7.1705	6.8726	6.8326	6.6684	7.2208	1.0085	0.0122	0.6774	0.1692
Atp5f1c	_GLC[Carbamidomethyl (C)]GAIHSSVAK_.2	#N/A	3.5099	6.2953	3.2435	6.9141	6.2897	5.3481	5.0038	5.8699	3.0121	3.9654	1.1801	0.2390	0.4128	0.3842
Atp5f1c	_MTAMDNASK_.2	#N/A	4.6022	3.5174	3.9956	5.8877	3.4057	3.2189	3.1121	3.4618	3.7608	3.2167	1.2116	0.2769	0.2099	0.6780
Atp5f1c	_NASDMIDK_.2	#N/A	3.1991	4.1433	4.4336	3.9774	3.8436	3.7648	4.1256	3.0238	3.3229	3.6546	1.1025	0.1407	0.2360	0.6271
Ddx1	_ALIVEPSR_.2	#N/A	5.6627	5.5810	5.3961	6.0705	5.6683	5.9566	5.4027	5.6280	5.3371	5.1774	1.0624	0.0874	0.0512	1.2906
Ddx1	_ELAEQTLNNVK_.2	#N/A	7.0328	6.6828	7.3978	7.1070	6.6303	6.4926	7.0202	6.4359	6.1106	6.7137	1.0488	0.0687	0.2071	0.6839
Ddx1	_DQLSVLDNGVDIVVGTPGR_.2	#N/A	4.7805	3.1215	3.9384	5.3510	4.0881	3.2859	5.0748	4.5286	4.3590	4.4057	0.8916	-0.1655	0.3072	0.5125
Ddx1	_DNTRPGANS[Phospho (STY)]PEM[Oxidation (M)]WSEAIK_.3	#N/A	4.0136	4.0294	5.5496	5.5829	4.5400	5.4382	5.2803	4.8056	4.4188	4.4127	1.0274	0.0390	0.7642	0.1168
Ddx1	_FLVLDEADGLLSQGYSDFINR_.2	#N/A	0.9569	3.3945	3.6816	2.8339	3.2158	3.0128	3.3736	1.8056	2.2732	4.4035	0.9613	-0.0569	0.8696	0.0607
Map1lc3a	_IPVIIER_.2	#N/A	8.8070	8.6894	8.9929	8.3598	8.9362	8.8384	7.9753	8.8425	8.7904	8.7262	1.0218	0.0311	0.3742	0.4269
Map1lc3a	_QLPVLDK_.2	#N/A	6.9515	7.3709	6.8712	6.7837	7.6513	6.7558	7.0324	7.3902	7.6682	7.0787	0.9687	-0.0459	0.3290	0.4828
Map1lc3a	_PS[Phospho (STY)]DRPFK_.2	Q91VR7_S3_M1_Map1lc3a	5.2120	4.6795	4.4355	4.9856	5.1588	4.9401	5.3680	5.2027	5.2842	5.4057	0.9223	-0.1168	0.0287	1.5428
Map1lc3a	_FLVPDHVNM[Oxidation (M)]SELVK_.3	#N/A	5.3378	4.9291	5.0881	5.3053	5.1481	5.2523	4.4187	4.7985	4.6628	5.1594	1.0876	0.1211	0.0208	1.6817
Brk1	_EIHQDWANR_.2	#N/A	6.9685	7.0978	6.8595	7.3805	7.4599	7.3146	6.8575	6.9962	7.4454	6.9990	1.0149	0.0214	0.5260	0.2790
Brk1	_EYIEIITSSIK_.2	#N/A	5.4095	5.0190	5.7554	5.9533	6.1187	5.6443	5.1700	5.2634	6.1478	5.4524	1.0257	0.0366	0.6098	0.2148
Brk1	_RIEYIEAR_.2	#N/A	5.3974	4.9617	4.9876	5.4920	5.2814	5.5961	5.5978	5.1795	5.6742	4.6511	1.0020	0.0028	0.9649	0.0155
Brk1	_ISDFLNSFDM[Oxidation (M)]SC[Carbamidomethyl (C)]R_.2	#N/A	4.3546	5.2586	5.6197	4.0432	5.0578	5.1088	5.9954	5.5037	6.3587	4.7524	0.8681	-0.2040	0.1050	0.9786
Brk1	_VTKGETLT[Phospho (STY)]_.2	Q91VR8_T75_M1_Brk1	4.1178	3.3732	3.8515	3.3952	3.2615	3.3631	3.5435	3.6059	3.9050	3.0725	1.0081	0.0117	0.9002	0.0456
Ptov1	_SRS[Phospho (STY)]WPGGPR_.2	Q91VU8_S36_M1_Ptov1	7.3320	7.0462	3.4474	3.9501	3.3871	4.3424	7.8895	7.6780	7.4724	3.9171	0.7297	-0.4547	0.1619	0.7907
Sh3bgrl3	_SQQSEVTR_.2	#N/A	6.6890	6.8333	6.6850	7.0137	8.1341	7.0437	6.4212	6.6702	6.7128	7.0925	1.0509	0.0716	0.2853	0.5446
Sh3bgrl3	_VYSTSVTGSR_.2	#N/A	7.9343	8.1227	8.4649	8.5049	9.0828	8.5298	8.1782	8.2945	8.4149	8.2405	1.0191	0.0272	0.4650	0.3326
Sh3bgrl3	_IQYQLVDISQDNALRDEM[Oxidation (M)]R_.3	#N/A	6.3140	5.4970	5.3713	6.4812	6.0243	5.1626	6.1020	5.7028	5.5627	5.9094	0.9981	-0.0027	0.9712	0.0127
Sh3bgrl3	_IQYQLVDISQDNALR_.2	#N/A	4.1992	3.1745	4.0788	4.8621	4.1032	4.3080	4.0718	3.5370	4.0114	4.9972	0.9920	-0.0116	0.9301	0.0315
Golga4	_TSS[Phospho (STY)]FTDQLDDVTPNR_.2	Q91VW5_S41_M1_Golga4	4.2105	6.4603	4.0591	7.4075	5.4429	3.2881	4.0405	6.8149	6.7752	6.9418	0.8375	-0.2559	0.3370	0.4724
Ubap2	_NS[Phospho (STY)]VEEWTTEDWTEDLSETK_.2	Q91VX2_S254_M1_Ubap2	3.6218	3.9881	3.8088	3.6721	3.6718	3.6916	3.4884	3.6910	4.0846	3.7407	0.9977	-0.0034	0.9433	0.0254
Smap1	_LLYEANLPENFR_.2	#N/A	6.7388	7.1642	7.4220	7.5047	7.5312	7.4447	7.1497	7.1843	7.2101	7.1930	1.0162	0.0232	0.4756	0.3227
Ncoa5	_SS[Phospho (STY)]ADSLPGPISR_.2	Q91W39_S378_M1_Ncoa5	9.0763	3.9056	9.0918	4.0284	3.7717	9.0358	3.7431	8.7071	8.9894	8.9773	0.8528	-0.2297	0.5443	0.2642
Ncoa5	_[Acetyl (Protein N-term)]M[Oxidation (M)]NTAPSRPS[Phospho (STY)]PTR_.2	#N/A	7.6975	3.0454	8.5185	8.3265	7.5381	3.3834	8.2414	8.1517	7.9552	7.5611	0.8046	-0.3137	0.2603	0.5845
Ncoa5	_DRS[Phospho (STY)]PIRGS[Phospho (STY)]PR_.2	#N/A	9.6228	9.7796	9.8023	10.0833	9.5716	9.6932	9.7772	9.6915	9.5013	9.9256	1.0036	0.0052	0.7718	0.1125
Ncoa5	_LLRSS[Phospho (STY)]ADS[Phospho (STY)]LPGPISR_.3	#N/A	3.7274	3.9041	4.4142	3.4161	3.6934	3.4025	3.9710	3.2286	3.7175	3.3831	1.0516	0.0726	0.4494	0.3474
Csde1	_EAFGFIER_.2	#N/A	5.9847	6.3215	5.2139	5.3813	5.8943	5.5636	5.5545	5.9359	5.0025	6.3353	1.0034	0.0049	0.9511	0.0218
Csde1	_ATNIEVLSNTFQFTNEAR_.2	#N/A	3.9820	4.1610	3.6594	4.7278	4.6209	4.8872	4.7910	4.4392	4.6424	4.4797	0.9459	-0.0803	0.3553	0.4494
Csde1	_LLGRNS[Phospho (STY)]NSK_.2	Q91W50_S514_M1_Csde1	3.2889	5.5704	3.9757	5.3602	3.8256	5.7947	4.1930	3.6401	3.2887	5.5805	1.1102	0.1509	0.5133	0.2896
Csde1	_SPAAPGQS[Phospho (STY)]PTGSVC[Carbamidomethyl (C)]YER_.2	Q91W50_S123_M1_Csde1	7.2980	7.7760	3.7793	8.2632	7.3186	3.7484	7.6407	7.5695	7.6854	3.7093	0.9568	-0.0637	0.8306	0.0806
Ubl7	_DM[Oxidation (M)]PGGFLFDGLS[Phospho (STY)]DDEDDFHPSTR_.3	#N/A	3.2851	3.4040	3.7849	4.0634	6.3549	3.7150	4.2117	6.1444	5.6872	3.7407	0.8292	-0.2702	0.2855	0.5444
Ube2e2	_VDDS[Phospho (STY)]PSTSGGSSDGDQR_.2	Q91W82_S11_M1_Ube2e2	3.8127	3.7915	4.2532	3.9319	3.6398	3.5899	4.0218	4.0567	3.4995	3.4680	1.0199	0.0285	0.6813	0.1666
Cdc42ep1	_RSDS[Phospho (STY)]LLSFR_.2	#N/A	8.3942	8.3098	8.9245	8.8125	7.8832	8.4591	8.8703	8.0639	8.1535	8.5905	1.0053	0.0076	0.8595	0.0658
Cdc42ep1	_AS[Phospho (STY)]WGASR_.2	Q91W92_S332_M1_Cdc42ep1	3.3879	4.1441	3.1215	4.1662	4.0324	4.0930	3.9862	4.3359	3.1341	3.8434	0.9998	-0.0003	0.9983	0.0008
Cdc42ep1	_SDS[Phospho (STY)]LLSFR_.2	#N/A	3.1669	4.0593	3.8537	4.3563	3.7934	3.9361	4.0710	3.7621	3.4107	4.3233	0.9921	-0.0115	0.9065	0.0426
Plpp7	_RQS[Phospho (STY)]QQLPEEDC[Carbamidomethyl (C)]M[Oxidation (M)]QLNPSFK_.3	#N/A	4.6798	4.9094	6.1633	6.3795	4.0218	4.4673	5.8516	5.6711	5.0111	3.8328	1.0023	0.0034	0.9848	0.0066
Setd3	_S[Phospho (STY)]GTGATATVSPK_.2	Q91WC0_S12_M1_Setd3	6.4910	3.2514	4.1998	3.4820	3.1104	6.0989	3.1967	6.2819	5.6573	6.4178	0.8238	-0.2797	0.3536	0.4515
Acsl6	_LVILM[Oxidation (M)]EPFEDALR_.2	#N/A	4.6819	5.4110	5.0477	4.4204	4.8713	4.1214	5.7267	5.3471	5.2518	4.0112	0.9360	-0.0954	0.4118	0.3853
Acsl6	_LPELSDLGQFFR_.2	#N/A	4.9017	5.3984	3.7426	4.6915	3.1527	3.4720	5.5561	5.8228	4.1166	4.7812	0.8338	-0.2623	0.1637	0.7860
Acsl6	_VGFFQGDIR_.2	#N/A	3.6894	4.9788	3.4231	4.4677	3.6906	3.8715	5.1291	5.2287	5.0007	4.1450	0.8245	-0.2784	0.0436	1.3602
Acsl6	_LVDAEELNYWTC[Carbamidomethyl (C)]K_.2	#N/A	4.6158	4.7983	3.1442	5.5751	4.0551	3.6039	3.9635	3.8696	3.5182	4.6966	1.0715	0.0996	0.5737	0.2413
Acsl6	_EALDSDGWLHTGDIGK_.3	#N/A	2.8966	3.6529	4.1311	3.2619	3.5412	3.0835	4.4775	3.3557	3.0043	3.2289	0.9748	-0.0369	0.8033	0.0951
Ndufs2	_VLFGEITR_.2	#N/A	7.3063	7.2450	6.9325	7.0084	7.2333	6.9881	6.9126	7.3155	7.0833	7.1079	1.0020	0.0029	0.8958	0.0478
Ndufs2	_IIEQC[Carbamidomethyl (C)]LNK_.2	#N/A	6.7532	7.1642	6.9990	6.9742	7.2802	6.6833	6.8474	6.9763	6.8384	6.6445	1.0218	0.0312	0.2816	0.5504
Ndufs2	_TQPYDVYDQVEFDVPIGSR_.2	#N/A	6.9083	7.0589	6.4706	6.8158	6.6515	7.0887	6.4887	6.6132	6.6967	7.2599	1.0100	0.0144	0.7196	0.1429
Ndufs2	_TYLQALPYFDR_.2	#N/A	6.6072	6.5184	6.7732	6.7764	6.3272	6.4276	6.6899	7.0760	6.8302	6.8218	0.9587	-0.0608	0.0367	1.4350
Ndufs2	_GSGIQWDLR_.2	#N/A	6.5942	6.6014	6.4201	6.6121	6.5790	6.5300	6.7481	6.6713	6.7682	6.0852	0.9982	-0.0027	0.9302	0.0314
Ndufs2	_APGFAHLAGLDK_.2	#N/A	6.6503	6.5598	6.0914	6.1616	6.1172	3.6065	6.2155	6.5336	5.9096	6.5003	0.9324	-0.1010	0.4907	0.3092
Ndufs2	_M[Oxidation (M)]FEFYER_.2	#N/A	4.7999	5.0481	5.4117	3.2556	3.9308	5.2028	5.3824	4.9566	5.8916	5.4111	0.8517	-0.2316	0.1151	0.9388
Ndufs2	_TVDIGVVTAEDALNYGFSGVM[Oxidation (M)]LR_.3	#N/A	4.1130	4.5241	5.9633	5.2962	3.6760	4.2946	3.4056	4.4276	4.8885	4.8797	1.0555	0.0779	0.6445	0.1908
Ndufs2	_IDEVEEM[Oxidation (M)]LTNNR_.2	#N/A	8.9342	8.4869	9.0148	9.3523	7.9168	8.3490	9.0163	8.4553	8.3677	8.7481	1.0033	0.0048	0.9234	0.0346
Ndufs2	_AVTNM[Oxidation (M)]TLNFGPQHPAAHGVLR_.4	#N/A	4.4584	4.1192	3.8386	4.8310	4.0516	4.8510	3.7127	5.1730	4.9669	4.5495	0.9473	-0.0780	0.4898	0.3100
Ndufs2	_TSM[Oxidation (M)]ESLIHHFK_.3	#N/A	4.1277	3.2424	3.4073	4.2010	3.1114	3.7793	3.1448	3.7372	4.1292	4.1846	0.9594	-0.0597	0.6238	0.2049
Ndufs2	_LDYVSM[Oxidation (M)]M[Oxidation (M)]C[Carbamidomethyl (C)]NEQAYSIAVEK_.2	#N/A	4.2408	3.5452	3.5309	4.5755	3.3665	4.3630	3.2229	3.9265	4.2256	4.2528	1.0077	0.0110	0.9284	0.0323
Prkag2	_IYASSS[Phospho (STY)]PPDTGQR_.2	Q91WG5_S196_M1_Prkag2	4.0988	11.1098	11.5469	11.5761	10.5614	11.2301	11.5698	11.2076	11.1759	11.1844	0.8880	-0.1714	0.4222	0.3745
Prkag2	_PSS[Phospho (STY)]PVS[Phospho (STY)]APVRPK_.2	Q91WG5_S87_M2_Prkag2	6.8437	3.1161	3.2960	6.5636	3.2358	6.4275	3.5134	4.3198	3.9684	3.7657	1.2626	0.3364	0.3189	0.4964
Prkag2	_SSS[Phospho (STY)]KESSPNSNPSTSPGGIR_.2	Q91WG5_S131_M1_Prkag2	3.3178	4.0246	4.5523	4.0961	3.8459	3.8836	4.2444	4.4059	3.2041	3.7734	1.0119	0.0170	0.8791	0.0560
Dgkg	_[Acetyl (Protein N-term)]S[Phospho (STY)]EEQWVSLSSEEFDQLQK_.2	Q91WG7_S2_M1_Dgkg	3.5377	4.2940	3.2714	4.3160	4.1823	3.9432	3.8363	4.1860	5.8599	3.9933	0.8781	-0.1876	0.2423	0.6157
Dgkg	_EGASSSEPNVSDYNS[Phospho (STY)]DNAAK_.2	Q91WG7_S107_M1_Dgkg	3.6297	3.8894	4.0454	5.8379	3.7385	3.5158	3.7374	3.4519	3.7509	4.1711	1.0878	0.1214	0.4895	0.3103
Dgkg	_QET[Phospho (STY)]PDHPK_.2	Q91WG7_T87_M1_Dgkg	3.1321	3.9373	3.8189	4.3216	3.7586	3.9709	4.3317	3.7969	3.4455	4.2885	0.9641	-0.0528	0.5993	0.2223
Itfg2	_DTGS[Phospho (STY)]PPASEEATGDSR_.2	Q91WI7_S219_M1_Itfg2	8.4240	8.2077	8.2706	8.8383	8.0234	8.4628	4.3403	8.5810	8.3640	8.4428	1.1264	0.1717	0.2894	0.5385
Lrrfip2	_SDKQYAENYTRPSS[Phospho (STY)]R_.3	Q91WK0_S92_M1_Lrrfip2	3.9309	4.6839	3.1168	3.6195	3.0565	3.1721	3.3342	4.7135	4.1476	3.5865	0.9116	-0.1335	0.4128	0.3843
Eif3h	_DFS[Phospho (STY)]PEALKK_.2	#N/A	6.8197	6.7356	6.4185	6.3322	6.0070	6.5730	6.6814	6.7501	7.0255	6.7953	0.9513	-0.0721	0.0749	1.1255
Eif3h	_LFM[Oxidation (M)]AQALQEYNN_.2	#N/A	3.6051	5.8092	6.2672	7.3004	4.4459	5.0044	5.2322	5.9541	5.1328	4.8131	1.0232	0.0330	0.8664	0.0623
Eif3h	_EKDFS[Phospho (STY)]PEALKK_.3	#N/A	4.1731	3.4775	3.3591	3.1424	3.2988	4.4307	3.2907	3.8588	4.1578	4.3205	0.9334	-0.0994	0.4431	0.3535
Gcsh	_SC[Carbamidomethyl (C)]YEDGWLIK_.2	#N/A	4.6215	5.0028	4.3143	5.3791	5.3739	4.5964	4.0063	5.1789	4.6472	4.9964	1.0370	0.0524	0.5827	0.2345
Rrp9	_RLPVS[Phospho (STY)]PVAGS_.2	Q91WM3_S470_M1_Rrp9	7.4347	7.2018	8.0373	7.6679	6.8672	6.9916	7.6277	7.3928	7.1998	7.3848	0.9953	-0.0067	0.8866	0.0523
Yars1	_APWELLELR_.2	#N/A	5.2478	4.5604	4.3853	5.2637	4.1748	5.1393	4.3725	5.1619	4.8868	4.3575	1.0214	0.0306	0.7385	0.1316
Yars1	_TSYYENVIK_.2	#N/A	5.0202	4.5719	4.7887	4.6924	5.3440	5.4895	4.9545	5.6092	5.0122	4.7755	0.9797	-0.0296	0.6733	0.1718
Cisd1	_VVHAFDM[Oxidation (M)]EDLGDK_.2	#N/A	6.7861	6.7722	6.6497	6.6094	6.7036	7.1621	7.0677	6.9575	6.8744	7.1718	0.9662	-0.0496	0.0704	1.1525
Cisd1	_AM[Oxidation (M)]VNLQIQK_.2	#N/A	4.8808	6.2691	3.9365	5.9670	5.0920	6.0636	5.3821	6.1258	5.3180	6.1477	0.9347	-0.0975	0.4671	0.3306
Cisd1	_HNEETGDNVGPLIIK_.2	#N/A	5.8992	5.7056	6.1271	6.3091	5.7918	5.9076	6.2156	6.0075	5.9365	5.6402	1.0011	0.0016	0.9644	0.0157
Dtnbp1	_STPGVS[Phospho (STY)]DDSDQC[Carbamidomethyl (C)]DSTQDI_.2	Q91WZ8_S340_M1_Dtnbp1	3.6042	6.4214	3.3379	4.0551	4.2488	3.8767	3.7698	4.0633	3.7119	4.0221	1.0939	0.1295	0.5391	0.2683
S1pr5	_S[Phospho (STY)]PSAAGPSGGGLR_.2	#N/A	3.6458	8.3192	8.3657	8.3416	7.3351	8.2928	9.2330	3.2233	4.0031	8.0302	1.2060	0.2702	0.4279	0.3687

S1pr5	_S[Phospho (STY)]PSAAGPSGGGLRR_.2	#N/A	3.6937	3.8735	7.4005	6.3417	2.8281	3.6199	3.4259	3.7967	4.0958	3.7302	1.2297	0.2983	0.3792	0.4211
Zfand2b	_TLPSSSS[Phospho (STY)]PSR_.2	Q91X58_S173_M1_Zfand2b	3.0434	4.2990	4.2779	3.8217	4.1204	3.6091	3.9699	3.1795	3.4786	3.4989	1.0935	0.1289	0.2694	0.5696
Ncald	_IYGNFFPYGDASK_.2	#N/A	7.5852	7.6908	3.8635	7.4970	7.8379	7.2869	8.1409	7.6981	7.8245	7.4851	0.8938	-0.1620	0.3243	0.4890
Ncald	_AEM[Oxidation (M)]LEIVQAIYK_.2	#N/A	5.7896	5.2865	5.5011	5.7727	5.7226	5.5681	5.4555	5.3066	6.3217	5.6513	0.9864	-0.0197	0.7143	0.1461
Golm1	_LQEEVPS[Phospho (STY)]EEQM[Oxidation (M)]PQEK_.2	#N/A	6.5061	6.3347	6.9496	6.9964	5.7522	6.1826	6.8932	6.5577	6.2526	6.0050	1.0041	0.0059	0.9285	0.0322
Trex1	_AC[Carbamidomethyl (C)]S[Phospho (STY)]PGASEITGLSK_.2	Q91XB0_S78_M1_Trex1	8.2544	3.6171	8.2501	8.5579	8.1893	8.3505	7.7772	3.3200	8.2073	8.2979	1.0540	0.0758	0.7915	0.1016
Dap	_[Acetyl (Protein N-term)]SS[Phospho (STY)]PPEGKLETK_.2	Q91XC8_S3_M1_Dap	3.8412	10.3014	11.0349	10.3770	2.9669	10.3248	10.3556	9.8378	9.9955	10.0113	0.8100	-0.3039	0.3408	0.4675
Pnpo	_S[Phospho (STY)]SQIGAVVSR_.2	Q91XF0_S164_M1_Pnpo	3.2366	4.0418	3.9234	4.4261	3.8632	7.1589	4.1408	6.3929	6.7742	6.5394	0.7450	-0.4246	0.1140	0.9433
Pnpo	_FFTNYESR_.2	#N/A	6.4242	6.1561	5.7490	6.3870	6.4276	5.3812	5.5769	5.5875	5.9115	6.0304	1.0538	0.0756	0.2292	0.6399
Pnpo	_GLATGDS[Phospho (STY)]PLGPM[Oxidation (M)]THHGEEDWVYER_.4	#N/A	3.9566	3.5437	3.5252	4.1861	3.6425	3.4729	3.8113	4.4268	3.6387	3.6551	0.9583	-0.0614	0.4568	0.3403
Osbpl1a	_ITM[Oxidation (M)]PVIFNEPLSFLQR_.2	#N/A	2.9330	3.9610	3.7358	3.6572	3.4626	3.9449	3.7925	4.5184	3.6625	4.0645	0.9018	-0.1491	0.1483	0.8289
Osbpl1a	_M[Oxidation (M)]QC[Carbamidomethyl (C)]VAAFAVSAVASQWER_.2	#N/A	3.8858	3.4566	3.6195	3.1632	3.2779	3.8817	3.3115	3.8379	4.1370	4.3414	0.9080	-0.1393	0.1647	0.7833
Dlg2	_AIS[Phospho (STY)]LEGEPR_.2	#N/A	12.1731	11.3517	12.5146	11.9730	10.4303	11.6190	11.8864	11.8623	11.8649	11.8210	0.9847	-0.0223	0.6410	0.1931
Dlg2	_LC[Carbamidomethyl (C)]DKPAS[Phospho (STY)]PR_.2	Q91XM9_S360_M1_Dlg2	11.1812	11.3193	11.7466	11.3934	10.4693	10.8205	11.5280	11.4188	11.1666	11.0724	0.9875	-0.0182	0.5797	0.2368
Dlg2	_QPS[Phospho (STY)]VTLQR_.2	Q91XM9_S406_M1_Dlg2	9.7168	9.3428	3.7257	9.9271	3.6655	4.0640	9.4165	11.3601	11.5538	11.3160	0.6177	-0.6950	0.0384	1.4160
Dlg2	_GPELVHVS[Phospho (STY)]EK_.2	Q91XM9_S45_M1_Dlg2	9.6672	9.1126	9.7712	9.6219	8.3904	9.2436	9.8361	9.7151	9.4185	9.3363	0.9712	-0.0421	0.3527	0.4526
Dlg2	_GQEDLILSYEPVTR_.2	#N/A	7.3410	7.1700	7.6491	7.4747	7.0034	7.1522	7.3581	7.1207	7.2888	7.1702	1.0088	0.0127	0.6317	0.1995
Dlg2	_LVIEEQSGPFIWIPSK_.2	#N/A	5.4975	6.0474	5.7666	5.8610	5.9616	5.8421	5.4906	5.4206	6.2057	5.9546	1.0107	0.0153	0.7377	0.1321
Dlg2	_AIS[Phospho (STY)]LEGEPRK_.2	#N/A	9.2971	9.9239	10.7849	9.9649	8.6843	9.9588	10.1174	9.9485	9.9499	9.9094	0.9787	-0.0310	0.5767	0.2391
Dlg2	_DYHFVISR_.2	#N/A	6.1007	6.2316	6.3139	6.7133	6.0098	6.2247	6.4421	6.2732	6.2149	6.3076	0.9931	-0.0100	0.7458	0.1274
Dlg2	_YQDEDGPHDHS[Phospho (STY)]LPR_.3	#N/A	9.4747	9.0244	9.9605	9.4796	8.4796	9.0767	9.6344	9.5214	9.4748	9.1070	0.9804	-0.0286	0.5179	0.2857
Dlg2	_YGDILHVINASDDEWWQAR_.3	#N/A	4.4093	4.7218	4.8164	6.2018	4.5894	3.1791	5.1843	4.2357	4.5573	4.7937	0.9915	-0.0123	0.9406	0.0266
Dlg2	_TSLPPIS[Phospho (STY)]PGR_.2	Q91XM9_S323_M1_Dlg2	10.0224	3.8380	10.4077	4.2827	3.6593	9.9259	9.5998	10.4975	9.2483	3.9600	0.8434	-0.2457	0.5507	0.2591
Dlg2	_TSLPPIS[Phospho (STY)]PGRYS[Phospho (STY)]PIPK_.2	#N/A	3.6532	3.6268	4.2346	8.2920	3.8574	3.7552	4.1598	7.4600	3.9955	6.9619	0.8096	-0.3047	0.3899	0.4091
Dlg2	_HYS[Phospho (STY)]PVEC[Carbamidomethyl (C)]DK_.2	Q91XM9_S365_M1_Dlg2	9.3551	9.3492	9.6564	9.5964	8.6289	9.0115	9.6205	9.5024	9.3677	9.2938	0.9810	-0.0277	0.4068	0.3906
Dlg2	_NLSQIENVHGYVLQSHIS[Phospho (STY)]PLK_.3	Q91XM9_S65_M1_Dlg2	4.3735	7.0107	3.5595	8.7113	5.4226	5.6396	7.2857	7.8215	7.3620	4.0291	0.8734	-0.1952	0.4943	0.3060
Dlg2	_FIEAGQYNDNLYGTSVQSVR_.2	#N/A	4.3937	4.3441	4.5209	3.1768	3.6950	4.2489	4.3733	3.7895	4.4085	4.4578	0.9544	-0.0673	0.5264	0.2787
Dlg2	_DSGLPSQGLSFK_.2	#N/A	6.6883	6.4579	6.7901	6.5593	6.7337	6.8381	6.7945	6.6942	6.3660	6.5976	1.0098	0.0141	0.5478	0.2614
Dlg2	_SKDS[Phospho (STY)]GLPSQGLSFK_.2	Q91XM9_S553_M1_Dlg2	3.8271	3.6877	7.6840	7.2597	6.0703	3.7300	7.6400	6.8773	6.8000	6.9654	0.7604	-0.3952	0.1162	0.9348
Mtmr4	_TSS[Phospho (STY)]DPNLNNHSQEVR_.2	Q91XS1_S629_M1_Mtmr4	3.7471	3.4791	5.5392	5.2408	3.3674	3.2572	3.1504	3.1819	4.3314	3.4027	1.1674	0.2233	0.3252	0.4879
Wrnip1	_AKGPS[Phospho (STY)]PPGAK_.2	Q91XU0_S65_M1_Wrnip1	8.7873	8.7994	9.1602	8.9009	7.9042	8.7159	9.0477	7.9639	8.1704	8.8016	1.0254	0.0361	0.4883	0.3113
Wrnip1	_RPAAAAAAGSAS[Phospho (STY)]PR_.2	#N/A	3.7953	7.2523	6.4248	8.3697	5.7860	6.7502	5.9057	6.2755	6.5483	6.5869	1.0106	0.0153	0.9347	0.0293
Wrnip1	_RLS[Phospho (STY)]ESSALK_.2	Q91XU0_S75_M1_Wrnip1	7.1183	6.6912	6.9700	3.7128	3.0902	6.7244	7.1836	6.5509	7.4031	7.0981	0.8100	-0.3040	0.1897	0.7220
Pip4k2c	_FAIDDHDYLVSLTR_.3	#N/A	5.4547	5.0575	4.7264	5.3743	4.5806	5.2191	5.1772	5.5615	5.0368	5.2538	0.9641	-0.0527	0.3735	0.4277
Pip4k2c	_ENLPSHFK_.2	#N/A	3.4984	5.2185	4.2569	5.7398	6.1592	3.9824	4.6634	4.2253	5.5434	3.9540	1.0463	0.0653	0.7340	0.1343
Basp1	_AEGAGTEEEGT[Phospho (STY)]PK_.2	#N/A	11.3664	11.2661	11.4231	11.8857	11.5975	11.3181	11.5389	11.1860	11.3637	11.4586	1.0078	0.0113	0.5182	0.2855
Basp1	_ETPAASEAPSSAAK_.2	#N/A	10.9727	10.8021	11.6162	11.1740	11.3033	11.0327	10.6624	10.9933	11.0403	10.9123	1.0228	0.0325	0.1600	0.7959
Basp1	_AGEAS[Phospho (STY)]AES[Phospho (STY)]TGAADGAAPEEGEAK_.2	Q91XV3_S131_M2_Basp1	10.0980	9.6202	10.7004	11.0927	10.5552	10.4247	10.6652	10.2274	10.3055	10.4336	1.0007	0.0010	0.9793	0.0091
Basp1	_ESEPQAAADATEVK_.2	#N/A	11.5077	11.4424	11.1699	11.8436	12.2459	11.5946	11.3457	11.2545	11.3758	11.5458	1.0223	0.0318	0.2304	0.6376
Basp1	_KTEAPAAAGPEAK_.2	#N/A	10.1646	10.4590	10.5297	10.6559	11.1394	10.7636	10.1212	10.2236	10.1931	10.4641	1.0359	0.0509	0.0707	1.1504
Basp1	_APAPAAPAAAEPQAEAPAAAASS[Phospho (STY)]EQSVAVK_.3	#N/A	6.2397	4.7180	6.6637	7.8955	6.3584	6.3770	4.7518	6.9041	6.2047	7.2377	1.0161	0.0230	0.8854	0.0529
Basp1	_GYNVNDEK_.2	#N/A	7.4908	7.2793	7.6932	7.3614	8.2874	7.1062	6.3494	7.1693	7.5151	7.6555	1.0508	0.0714	0.2806	0.5520
Basp1	_TEAPAAAGPEAK_.2	#N/A	7.2565	7.3047	7.1099	7.5347	8.0272	7.4100	7.1629	7.3520	7.3665	7.2446	1.0218	0.0312	0.3723	0.4291
Basp1	_DAADGEAKAEEK_.2	#N/A	6.5296	6.3513	5.9840	6.3768	7.9777	6.8086	6.1385	6.3427	6.4042	6.5791	1.0479	0.0676	0.4228	0.3739
Basp1	_AAAAKEEAPK_.2	#N/A	5.0364	4.6261	4.9172	5.3827	5.8854	5.3338	4.7999	4.5618	4.6013	5.8231	1.0506	0.0712	0.4617	0.3356
Basp1	_SDAAPAASDSKPSSAEPAPSSK_.3	#N/A	9.4986	9.8294	10.1214	10.1057	10.9557	9.8371	9.6210	9.6748	10.1249	10.1266	1.0173	0.0248	0.5516	0.2584
Basp1	_SDAAPAASDSKPSSAEPAPSSKET[Phospho (STY)]PAASEAPSSAAK_.4	Q91XV3_T183_M1_Basp1	4.4644	4.6376	4.0048	5.4058	5.0922	4.0735	4.2052	4.3843	4.4520	4.9755	1.0242	0.0344	0.7364	0.1329
Basp1	_APAPAAPAAAEPQAEAPAAAASS[Phospho (STY)]EQSVAVKE_.3	#N/A	11.1611	11.5399	11.0558	12.3679	11.0511	11.6277	11.6953	11.4545	11.5047	11.6094	0.9915	-0.0124	0.7156	0.1453
Basp1	_GYNVNDEKAK_.2	#N/A	5.4177	6.2254	5.9072	5.9129	6.6079	5.9911	5.4035	5.6943	5.8240	6.0892	1.0448	0.0632	0.2958	0.5290
Basp1	_ESTEEKPKDAADGEAK_.2	#N/A	4.1697	4.5287	4.6037	4.0552	5.4628	4.7143	4.5771	3.9682	4.5444	4.4235	1.0481	0.0678	0.4697	0.3282
Basp1	_KAEGAGTEEEGT[Phospho (STY)]PKESEPQAAADATEVK_.4	#N/A	9.7477	9.5648	10.1472	10.3502	9.9999	8.0623	9.6176	9.7482	9.9020	9.9240	0.9844	-0.0226	0.7285	0.1376
Basp1	_AEGAGTEEEGT[Phospho (STY)]PKESEPQAAADATEVK_.3	#N/A	11.0448	11.2788	11.9416	11.9292	11.2662	11.2575	11.5596	11.1935	11.3153	11.2367	1.0112	0.0161	0.5558	0.2551
Basp1	_AEPEKS[Phospho (STY)]EGAAEEQPEPAPAPEQEAAAPGPAAGGEAPK_.4	#N/A	10.8974	10.9004	11.4018	11.5507	11.2993	11.1933	11.3276	10.5899	10.9789	11.1376	1.0180	0.0258	0.3116	0.5064
Basp1	_DAADGEAKAEEKEADK_.3	#N/A	5.5663	5.2570	5.5432	5.7455	4.9495	5.8931	6.3526	5.7288	5.9124	5.8156	0.9227	-0.1160	0.0559	1.2525
Basp1	_AGEASAESTGAADGAAPEEGEAKKTEAPAAAGPEAK_.4	#N/A	4.3383	4.0680	4.2362	4.6974	4.4817	4.7786	3.9282	3.6967	5.0052	5.0690	1.0019	0.0028	0.9789	0.0093
Basp1	_EADKAAAAKEEAPK_.2	#N/A	5.5141	5.7396	5.9240	6.0034	6.2293	5.8144	5.5550	5.5406	5.4821	5.8759	1.0459	0.0647	0.1091	0.9621
Basp1	_KAEGAGTEEEGT[Phospho (STY)]PK_.2	#N/A	8.1424	8.2058	7.9081	8.6757	8.7247	8.4225	8.2860	8.0784	8.2383	8.3986	1.0117	0.0167	0.5898	0.2293
Basp1	_AEEKEADKAAAAK_.2	#N/A	4.8509	4.6433	4.7703	4.3777	5.8894	5.6847	4.6949	4.4868	5.5505	4.9923	1.0213	0.0304	0.7779	0.1091
Basp1	_ESEPQAAADATEVKESTEEKPK_.3	#N/A	8.8919	8.3243	8.7595	9.5428	9.1280	8.4464	8.5751	8.1510	8.4181	8.2174	1.0610	0.0854	0.0683	1.1658
Basp1	_KKGYNVNDEK_.2	#N/A	3.7813	4.1508	3.7622	3.8896	5.2304	4.0276	4.9262	3.2266	3.5022	4.2318	1.0425	0.0600	0.6944	0.1584
Basp1	_DKKAEGAGT[Phospho (STY)]EEEGTPK_.2	#N/A	4.0621	3.2485	4.3145	4.2721	3.0986	3.3915	3.4754	3.6263	4.0042	4.1276	0.9797	-0.0295	0.8061	0.0936
Basp1	_EEAPKAEPEKS[Phospho (STY)]EGAAEEQPEPAPAPEQEAAAPGPAAGGEAPK_.4	#N/A	3.6613	3.6982	3.5609	4.0635	3.5648	3.7675	3.7782	4.1462	3.7948	4.0305	0.9446	-0.0822	0.1041	0.9826
Basp1	_SEGAAEEQPEPAPAPEQEAAAPGPAAGGEAPK_.3	#N/A	3.7053	3.5208	4.3922	3.3940	3.4091	3.2155	3.1087	3.2237	4.3731	3.3610	1.0255	0.0363	0.7821	0.1067
Mapk8	_TAGTSFM[Oxidation (M)]M[Oxidation (M)]T[Phospho (STY)]PY[Phospho (STY)]VVTR_.2	#N/A	3.2991	8.6528	4.1807	9.2591	3.9313	3.9842	4.2103	3.4610	3.2977	8.8117	1.1225	0.1668	0.7314	0.1358
Vill	_GSQDS[Phospho (STY)]PENELGLDLR_.2	Q91YD6_S766_M1_Vill	3.3968	4.2020	4.0836	4.5862	4.0233	3.7062	4.3009	3.5323	3.1808	3.0524	1.1374	0.1857	0.1519	0.8186
Wasl	_VIY[Phospho (STY)]DFIEK_.2	Q91YD9_Y253_M1_Wasl	4.1087	5.0444	5.5899	5.6024	4.3117	5.0414	4.6242	5.2917	5.2064	5.7867	0.9469	-0.0787	0.4764	0.3221
Rbm5	_DYDS[Phospho (STY)]PERER_.2	#N/A	3.0439	4.2985	4.2784	3.8222	4.1198	3.6097	6.8262	3.1790	3.4780	6.6282	0.7681	-0.3806	0.1909	0.7192
Rbm5	_YDDYRDYDS[Phospho (STY)]PERER_.3	#N/A	4.3188	4.4082	3.5048	4.0074	3.4445	4.2850	3.7221	4.1111	5.1878	3.9744	0.9402	-0.0890	0.4701	0.3278
Rbm5	_GLVAAYSGDS[Phospho (STY)]DNEEELVER_.2	Q91YE7_S624_M1_Rbm5	3.7168	3.4508	4.0848	8.3908	6.0077	3.7928	3.8174	7.6629	3.7404	3.3075	1.0594	0.0833	0.8345	0.0786
Synpo2	_AQS[Phospho (STY)]PTPSLPASWK_.2	Q91YE8_S895_M1_Synpo2	4.1757	9.0035	9.0758	9.5213	8.3408	8.5989	3.6015	3.5480	8.5175	8.7328	1.3311	0.4126	0.2238	0.6502
Uckl1	_YFGTDAVPDGS[Phospho (STY)]DDDEAATVG_.2	Q91YL3_S539_M1_Uckl1	6.2028	3.5982	3.4779	11.4664	9.6034	3.7366	3.1700	10.9741	9.4033	7.2863	0.8235	-0.2802	0.5551	0.2556
Arhgap35	_NEEENIY[Phospho (STY)]SVPHDSTQGK_.2	#N/A	7.5137	7.5578	8.0349	7.7670	6.5769	7.3613	7.5654	7.5169	7.5975	7.4205	0.9925	-0.0109	0.8300	0.0809
Arhgap35	_TSFSVGS[Phospho (STY)]DDELGPIRK_.2	#N/A	4.3241	3.5796	4.0578	4.2302	3.4679	3.1571	3.9760	5.8965	3.3385	3.2663	0.9232	-0.1153	0.5745	0.2407
Arhgap35	_NQKNS[Phospho (STY)]LSDLNIDR_.2	#N/A	4.0362	3.3136	4.5182	3.5200	3.1718	3.2644	4.5964	3.0989	4.5751	5.1623	0.8346	-0.2608	0.1413	0.8498
Arhgap35	_NIIEATHM[Oxidation (M)]YDNVAEAC[Carbamidomethyl (C)]STTEEVFNS[Phospho (STY)]PR_.3	#N/A	4.9878	6.3168	6.6206	6.6325	4.0232	4.7155	5.2445	6.5235	5.3149	4.6815	1.0199	0.0284	0.8713	0.0598
Arhgap35	_TSFSVGS[Phospho (STY)]DDELGPIR_.2	#N/A	3.5563	8.5677	8.5087	9.9995	8.2725	8.4926	8.8048	9.7248	9.0980	8.4030	0.8770	-0.1894	0.3645	0.4382
Arhgap35	_AQSNGSGNGS[Phospho (STY)]DSEMDTSSLER_.2	Q91YM2_S1134_M1_Arhgap35	3.3526	3.8736	4.0394	4.5420	3.9791	3.7504	4.2567	3.5764	3.2250	3.0082	1.1155	0.1577	0.2035	0.6914
Nln	_EAAS[Phospho (STY)]PLQAM[Oxidation (M)]SSYTAAGR_.2	#N/A	4.3160	4.3570	4.6540	5.6195	3.6053	4.3509	3.8997	4.7213	3.6368	3.7515	1.1203	0.1639	0.2487	0.6042
Csdc2	_DLPS[Phospho (STY)]PLPTKR_.2	#N/A	3.4353	9.1596	9.1124	9.3475	4.0798	9.2313	8.4655	8.3749	8.3423	3.8908	1.0173	0.0248	0.9426	0.0257

Csdc2	_[Acetyl (Protein N-term)]TSESTLPPVVPPLHS[Phospho (STY)]PK_.2	Q91YQ3_S16_M1_Csdc2	3.1826	6.9730	6.2764	7.8137	5.8347	6.4988	5.7943	6.6921	6.1360	5.7401	1.0010	0.0014	0.9945	0.0024
Csdc2	_DLPS[Phospho (STY)]PLPTK_.2	#N/A	2.9670	3.7232	4.2015	3.1916	3.6115	3.0131	4.4071	3.4261	3.0747	3.1586	0.9814	-0.0270	0.8545	0.0683
Csdc2	_AS[Phospho (STY)]AGPVFK_.2	Q91YQ3_S65_M1_Csdc2	4.1728	3.4771	3.3587	3.8614	3.2985	4.4310	3.5761	4.2571	3.9057	2.8489	1.0328	0.0466	0.7309	0.1362
Rpn1	_VTAEVVLVHPGGGSTSR_.2	#N/A	3.7059	3.2972	3.4396	4.4842	3.2936	3.7273	3.5363	4.1411	4.3169	4.1614	0.9057	-0.1429	0.1896	0.7223
Rpn1	_NIQVDSPYDISR_.2	#N/A	4.2405	4.6658	3.4265	4.5758	4.3626	4.3633	3.2232	4.1893	3.8379	5.0471	1.0486	0.0685	0.6130	0.2125
Twf1	_SPLLEIVER_.2	#N/A	6.0613	5.8064	5.6841	5.7309	5.7969	6.1437	6.2069	5.9517	6.1835	5.7655	0.9741	-0.0379	0.2504	0.6014
Twf1	_YLLSQSS[Phospho (STY)]PAPLTAAEEELR_.2	Q91YR1_S143_M1_Twf1	3.1098	3.8341	5.7208	6.2449	5.3675	2.8592	6.0994	5.6399	5.9922	5.6544	0.7736	-0.3704	0.1115	0.9528
Ndufv1	_GPDWILGEM[Oxidation (M)]K_.2	#N/A	8.0293	8.4249	8.4427	7.6454	7.9527	8.1137	8.0307	8.0608	7.9773	7.7348	1.0189	0.0271	0.3882	0.4110
Ndufv1	_IFTNLYGR_.2	#N/A	7.7752	8.0285	7.8678	7.4386	7.9816	7.8656	7.3106	8.0082	7.9292	8.0146	1.0014	0.0020	0.9523	0.0212
Ndufv1	_LVEGC[Carbamidomethyl (C)]LVGGR_.2	#N/A	7.4430	7.2135	7.2628	7.6226	7.8022	7.3292	7.3465	7.4168	7.0560	7.3476	1.0211	0.0301	0.2789	0.5546
Ndufv1	_EAYEAGLIGK_.2	#N/A	6.9340	7.2157	7.6629	7.4740	7.5499	7.4135	6.9651	7.2577	7.3005	7.6283	1.0120	0.0171	0.6255	0.2038
Ndufv1	_NAC[Carbamidomethyl (C)]GSDYDFDVFVVR_.2	#N/A	6.7701	6.9496	7.4561	7.2870	6.6938	6.6940	7.0465	6.6683	6.7718	7.1166	1.0108	0.0155	0.7007	0.1545
Ndufv1	_YLVVNADEGEPGTC[Carbamidomethyl (C)]K_.2	#N/A	6.2055	6.4022	5.6764	7.1303	6.6296	6.7136	6.5536	6.5588	5.9640	6.2100	1.0218	0.0312	0.6320	0.1993
Ndufv1	_GAGAYIC[Carbamidomethyl (C)]GEETALIESIEGK_.2	#N/A	6.3606	6.4358	7.3108	7.5789	6.1320	6.4247	6.9127	6.5882	6.2619	6.5983	1.0177	0.0254	0.7227	0.1410
Ndufv1	_HFRPELEDR_.3	#N/A	7.5092	5.6294	6.7821	6.6861	6.8525	6.7778	7.0296	6.3705	6.7672	5.7410	1.0354	0.0502	0.5637	0.2489
Ndufv1	_GEFYNEASNLQVAIR_.2	#N/A	6.2901	2.7964	6.2346	5.9678	5.6840	5.7775	6.2892	5.4264	6.3895	6.1948	0.8985	-0.1544	0.4033	0.3944
Ndufv1	_GDARPAEIDSLWEISK_.3	#N/A	6.1388	5.8594	6.0452	5.1667	5.5664	6.2039	3.9579	5.3984	5.6616	5.7923	1.1206	0.1643	0.1473	0.8317
Ndufv1	_YLVVNADEGEPGTC[Carbamidomethyl (C)]KDR_.2	#N/A	5.3840	5.6635	5.8524	6.0448	5.7840	6.0407	5.5784	5.9664	5.7196	5.6506	1.0116	0.0166	0.6579	0.1818
Ndufv1	_KTSFGSLKDEDR_.3	#N/A	6.6922	4.1932	3.7198	5.6554	5.9683	6.5476	5.7305	5.5970	5.9819	5.9961	0.9376	-0.0930	0.5827	0.2346
Ndufv1	_EGVDWMNK_.2	#N/A	4.3099	4.1534	3.5341	4.8754	2.9442	3.6804	1.6971	3.9233	1.8977	4.2560	1.3305	0.4119	0.1609	0.7936
Ndufv1	_HESC[Carbamidomethyl (C)]GQC[Carbamidomethyl (C)]TPC[Carbamidomethyl (C)]R_.2	#N/A	3.1495	4.0766	3.8363	4.3390	3.7771	3.9534	4.0537	3.7795	3.4281	4.3060	0.9906	-0.0136	0.8887	0.0513
Ndufv1	_HAGGVTGGWDNLLAVIPGGSSTPLIPK_.3	#N/A	3.1901	0.7747	3.8769	4.1697	1.7370	4.2011	4.0943	3.7389	3.3875	4.3466	0.7687	-0.3795	0.2621	0.5816
Ndufv1	_TSFGSLKDEDR_.3	#N/A	3.6134	4.4186	4.3002	3.7021	4.2399	3.4896	3.8504	3.2990	3.5981	3.3794	1.1214	0.1653	0.0964	1.0160
Ndufv1	_QIEGHTIC[Carbamidomethyl (C)]ALGDGAAWPVQGLIR_.3	#N/A	3.1957	3.6541	3.8825	4.1198	3.8222	3.9072	4.2680	4.3822	3.1805	3.7971	0.9633	-0.0539	0.6094	0.2151
Ythdf2	_DGLNDDDFEPYLS[Phospho (STY)]PQAR_.2	Q91YT7_S39_M1_Ythdf2	8.2050	3.2547	7.6472	3.2767	3.1430	7.5034	3.3748	3.7245	7.9222	2.9540	1.2250	0.2928	0.5381	0.2691
Ptbp2	_DFGNSPLHR_.2	#N/A	4.0298	4.8385	3.2158	5.6814	5.7455	3.0731	3.4339	3.7155	4.0146	2.9629	1.2545	0.3272	0.1893	0.7228
Ptbp2	_LALDGQNIYNAC[Carbamidomethyl (C)]C[Carbamidomethyl (C)]TLR_.2	#N/A	4.3527	3.6570	3.5386	4.4637	3.4784	4.5366	3.1111	4.0384	4.3374	4.0083	1.0337	0.0479	0.7011	0.1542
Ttc1	_AAEAHPQDDHVEEEC[Carbamidomethyl (C)]FHDC[Carbamidomethyl (C)]SASFEEEQPGAHVAGSK_.5	#N/A	5.8717	4.2057	4.3712	3.9150	4.0271	3.7024	6.0663	3.0862	3.3853	3.5922	1.0785	0.1090	0.6508	0.1865
Ttc1	_QDSSTGSYSINFVQNPNNNR_.2	#N/A	3.4126	3.9298	3.1463	6.1489	4.0572	3.9784	4.3392	4.3111	3.1093	3.8682	1.0525	0.0739	0.7356	0.1334
Grhpr	_LLDAAGANLR_.2	#N/A	6.3215	6.4933	6.5457	6.3814	6.8058	6.1701	5.6592	6.4483	6.6833	5.9077	1.0451	0.0636	0.2364	0.6263
Grhpr	_VFVTGPLPAEGR_.2	#N/A	5.7489	6.5704	5.4211	5.5226	5.8331	5.8021	6.2785	5.4789	5.9646	6.0972	0.9767	-0.0339	0.5898	0.2293
Grhpr	_NTM[Oxidation (M)]SLLAANNLLAGLR_.2	#N/A	5.0173	5.1286	5.3100	4.4212	5.1056	4.8749	5.0946	4.7228	4.4394	5.3075	1.0177	0.0253	0.7033	0.1528
Grhpr	_VISTLSVGVDHLALDEIK_.3	#N/A	4.0979	3.1283	3.2838	3.7865	3.0166	3.6080	3.5012	1.8081	3.9806	3.4980	1.0907	0.1252	0.5217	0.2825
Srgap2	_S[Phospho (STY)]PDSTANVR_.2	#N/A	9.3837	9.0777	9.0981	9.4564	2.8079	9.1372	9.3664	9.0000	9.1745	9.0999	0.8908	-0.1668	0.4774	0.3211
Srgap2	_ELEGPIYS[Phospho (STY)]R_.2	Q91Z67_S691_M1_Srgap2	8.5065	8.0396	4.3262	8.6763	4.0720	8.1066	8.8106	3.1312	4.3022	8.2642	1.1769	0.2350	0.5388	0.2685
Srgap2	_QTPRS[Phospho (STY)]PDSTANVR_.3	#N/A	4.0845	3.3889	3.2705	3.2310	3.2102	3.0185	3.3793	3.7702	4.0692	4.4091	0.8619	-0.2145	0.0686	1.1634
Srgap2	_KQDS[Phospho (STY)]SQAIPLVVESC[Carbamidomethyl (C)]IR_.2	Q91Z67_S500_M1_Srgap2	3.7775	3.0329	3.5112	6.7151	2.9212	3.7034	3.5965	6.2904	4.2453	4.2331	0.8589	-0.2194	0.4691	0.3287
Srgap1	_GLNNDS[Phospho (STY)]PERR_.2	Q91Z69_S883_M1_Srgap1	5.8883	5.8592	3.9788	6.8196	3.9185	5.3725	4.1718	5.1023	3.2767	3.7008	1.3060	0.3852	0.0981	1.0083
Srgap1	_DMNS[Phospho (STY)]PTDR_.2	Q91Z69_S812_M1_Srgap1	3.1919	4.0343	3.8787	5.6353	3.9226	4.2029	4.0960	3.7371	3.3857	4.3483	1.0649	0.0907	0.5857	0.2323
Ugp2	_IYTFNQSR_.2	#N/A	5.7454	5.6320	5.0114	6.1612	6.0243	5.6736	5.5852	6.2439	5.8394	5.5440	0.9836	-0.0238	0.7026	0.1533
Ugp2	_AM[Oxidation (M)]S[Phospho (STY)]QDGASQFQEVILQELELSVK_.3	Q91ZJ5_S13_M1_Ugp2	3.7256	3.0300	4.4124	3.5899	2.8513	3.3773	3.7381	3.2034	3.7104	3.3812	0.9970	-0.0043	0.9731	0.0119
Ugp2	_NENTFLDLTVQQIEHLNK_.3	#N/A	3.8573	3.1616	4.5441	3.4582	2.9830	3.2457	3.6065	3.5430	3.8420	3.1355	1.0028	0.0040	0.9757	0.0107
Sh2b1	_ALANDGTS[Phospho (STY)]PGER_.2	Q91ZM2_S219_M1_Sh2b1	3.6462	7.4475	3.3799	7.7313	2.7899	4.2120	3.0719	4.0775	4.3766	4.1018	1.2459	0.3172	0.4178	0.3790
Sh2b1	_S[Phospho (STY)]SEDLAGPLPSSVPSSTTSSKPK_.3	Q91ZM2_S126_M1_Sh2b1	3.3129	4.0295	4.5474	4.0912	3.8508	3.8787	4.2395	7.8076	3.2090	3.7685	0.8309	-0.2673	0.3697	0.4322
Ndfip2	_DDFS[Phospho (STY)]DVEQLR_.2	Q91ZP6_S195_M1_Ndfip2	3.3202	6.1528	4.5547	7.2013	5.0744	4.1607	4.0539	6.8947	6.7966	6.5627	0.8355	-0.2593	0.2965	0.5279
Necab2	_TLS[Phospho (STY)]FDLQQR_.2	Q91ZP9_S271_M1_Necab2	7.3606	3.3200	3.2016	8.7129	7.5774	3.0873	8.2623	7.7270	8.0030	8.1304	0.6903	-0.5348	0.0990	1.0046
Necab2	_VYEGGSNVDQFVTR_.2	#N/A	4.8177	4.1104	3.8026	4.3052	4.0397	4.1267	4.0199	3.8133	3.4618	4.2722	1.0793	0.1101	0.2010	0.6967
Dst	_SES[Phospho (STY)]NSSITATQPTLAK_.2	Q91ZU6_S7186_M1_Dst	8.7484	8.8627	9.1274	8.8946	7.2713	8.7191	9.4427	8.9469	8.8912	8.7561	0.9550	-0.0664	0.2944	0.5310
Dst	_QKS[Phospho (STY)]FSEDVISHK_.3	#N/A	6.5287	3.7817	6.4026	3.9911	3.4592	3.8729	4.8300	6.2300	3.9737	3.9581	0.9841	-0.0231	0.9300	0.0315
Dst	_LLDPEDVDVSS[Phospho (STY)]PDEK_.2	Q91ZU6_S237_M1_Dst	3.7165	3.5741	3.6886	10.3566	8.6880	4.0128	3.9059	10.5949	10.0857	9.7311	0.6612	-0.5968	0.1808	0.7429
Dst	_AM[Oxidation (M)]VESQQKS[Phospho (STY)]PVKR_.3	#N/A	4.8816	5.0920	5.7713	4.9111	4.6483	5.0113	4.8891	5.2101	4.9997	5.0023	1.0054	0.0078	0.8961	0.0477
Dst	_AMVESQQKS[Phospho (STY)]PVK_.2	#N/A	5.0332	3.2297	3.7080	3.2518	3.1180	3.5066	3.4000	3.7494	4.0485	4.4299	0.9320	-0.1016	0.5289	0.2766
Dst	_QLS[Phospho (STY)]LLDSR_.2	Q91ZU6_S5407_M1_Dst	4.2755	2.9506	3.4615	3.9642	3.4012	4.4283	3.6789	4.1543	3.8029	3.9312	0.9585	-0.0612	0.5934	0.2266
Mia2	_DNLGNSNVPDS[Phospho (STY)]PIPAEC[Carbamidomethyl (C)]EAAGR_.2	Q91ZV0_S1271_M1_Mia2	2.9002	4.3259	3.5870	4.0897	4.2142	3.9112	3.8044	4.0288	3.6774	4.0567	0.9862	-0.0201	0.8518	0.0696
Mia2	_SLS[Phospho (STY)]PPWEQDRR_.2	Q91ZV0_S1190_M1_Mia2	3.9558	3.3866	10.1685	3.2332	8.4247	3.0207	7.9860	3.7679	9.9351	9.3633	0.6911	-0.5331	0.2497	0.6027
Lrp1	_IDLETGENR_.2	#N/A	5.2413	3.3996	5.4086	5.2132	4.7727	4.9128	4.9699	5.1290	4.9690	4.5637	0.9830	-0.0247	0.8353	0.0781
Lrp1	_AVTDEEPFLIFANR_.2	#N/A	5.2367	5.4343	4.3588	3.9025	5.4593	5.8475	4.0508	5.4682	4.9410	3.5798	1.1175	0.1603	0.3276	0.4846
Lrp1	_AEGSEYQVLYIADDNEIR_.2	#N/A	4.7943	4.0525	5.2827	4.8828	3.9972	4.5622	5.2717	5.0211	3.9641	4.1403	0.9991	-0.0013	0.9913	0.0038
Lrp1	_HS[Phospho (STY)]LAS[Phospho (STY)]TDEKR_.2	Q91ZX7_S4521_M2_Lrp1	6.3491	4.1319	4.8371	6.1525	4.6657	6.0006	6.1464	6.0490	6.1233	6.1356	0.8761	-0.1908	0.1476	0.8310
Lrp1	_IFFSDIHFGNIQQINDDGSGR_.3	#N/A	3.8374	3.1418	4.5242	3.4781	2.9631	3.2655	3.6263	3.0916	3.8222	3.4821	1.0084	0.0121	0.9279	0.0325
Sncb	_EGVVAAAEK_.2	#N/A	9.0549	8.9298	9.3932	8.9541	9.3633	8.9333	7.7113	9.0449	9.0409	9.1689	1.0416	0.0587	0.2519	0.5989
Sncb	_TKEQASHLGGAVFSGAGNIAAATGLVK_.3	#N/A	6.8888	7.1737	7.8975	7.7147	7.4309	7.4331	7.2358	7.1354	7.5255	7.8340	0.9987	-0.0019	0.9668	0.0146
Sncb	_QGVTEAAEK_.2	#N/A	8.0982	8.3058	8.3507	8.0550	8.5772	8.0182	8.0632	8.2508	8.0324	8.0764	1.0159	0.0227	0.3039	0.5173
Sncb	_TKQGVTEAAEK_.2	#N/A	6.9294	7.2631	7.0081	7.5460	8.2596	8.0465	7.2394	7.0999	7.0134	7.3932	1.0448	0.0633	0.2944	0.5311
Sncb	_TKEGVLYVGSK_.2	#N/A	8.3144	8.6155	8.8326	8.6159	9.1196	8.7236	8.6723	8.1641	8.8000	8.5180	1.0193	0.0276	0.3718	0.4297
Sncb	_EGVLYVGSK_.2	#N/A	9.8580	9.3984	9.8442	9.7003	9.4310	9.6138	9.1860	9.2635	4.1037	9.7499	1.1938	0.2556	0.1767	0.7529
Sncb	_EQASHLGGAVFSGAGNIAAATGLVK_.3	#N/A	6.6038	6.1412	6.6792	6.3913	5.6651	6.2858	6.0512	6.0035	6.2066	6.4177	1.0202	0.0289	0.5529	0.2574
Sncb	_TSGVVQGVASVAEK_.2	#N/A	8.2797	8.9324	8.5680	9.5282	9.5418	9.2157	8.7533	8.8908	9.0358	8.9520	1.0116	0.0166	0.7100	0.1487
Kcnh5	_KTDS[Phospho (STY)]C[Carbamidomethyl (C)]DSGITK_.2	Q920E3_S863_M1_Kcnh5	4.6596	4.5828	5.7826	3.7316	3.0715	3.5531	3.4463	4.3869	4.9848	3.7522	1.0212	0.0302	0.8810	0.0550
Kcnh5	_AGEARS[Phospho (STY)]PLEHS[Phospho (STY)]PSQADVK_.3	Q920E3_S883_M2_Kcnh5	3.5013	3.8411	3.2350	4.2796	3.6624	4.0671	3.0480	4.2224	3.0206	3.9569	1.0568	0.0797	0.5350	0.2717
Fdps	_YEEDSYNR_.2	#N/A	2.5280	2.8777	3.9294	3.7484	3.8692	3.8603	4.3173	3.6864	3.3350	4.0499	0.9017	-0.1493	0.3148	0.5020
Wdr7	_TYQVPPVQPAS[Phospho (STY)]PGSHNALK_.3	#N/A	10.9933	10.6112	11.4364	11.9602	8.4093	10.3130	9.5724	12.2510	10.7388	9.8945	1.0006	0.0009	0.9938	0.0027
Wdr7	_GPITAVSFAPDGR_.2	#N/A	6.1532	5.4282	3.3626	5.6858	5.6786	5.7317	6.3215	6.1437	5.5788	5.6939	0.8998	-0.1523	0.2926	0.5338
Wdr7	_ARDS[Phospho (STY)]PPPSSNIVQGQIK_.3	Q920I9_S935_M1_Wdr7	6.1584	3.0021	3.4100	3.9126	3.3497	3.7342	3.6273	5.5746	3.8544	3.8796	0.9277	-0.1083	0.6650	0.1772
Wdr7	_LPASC[Carbamidomethyl (C)]LPASDSFR_.2	#N/A	5.8230	4.1132	4.5914	4.1352	5.8921	5.6405	6.1832	5.9939	5.9933	3.8125	0.9157	-0.1270	0.4764	0.3220
Wdr7	_LAC[Carbamidomethyl (C)]THTGIQFYQFSVGNQQEGR_.3	#N/A	3.4494	3.7768	4.1362	3.1380	4.0759	2.9595	4.3535	3.4796	3.1282	3.1050	1.0207	0.0295	0.8358	0.0779
Wdr7	_RQS[Phospho (STY)]REDSDPEYR_.3	Q920I9_S772_M1_Wdr7	3.5625	3.6637	4.2493	3.2511	4.1890	3.5405	4.4666	3.3665	3.0151	3.2181	1.0643	0.0899	0.5079	0.2943
Syt17	_RIS[Phospho (STY)]SLDSR_.2	Q920M7_S106_M1_Syt17	3.7713	5.6010	6.7797	6.4561	5.0794	6.2721	6.4292	6.2319	6.3287	6.1090	0.9020	-0.1488	0.3118	0.5062
Msi2	_[Acetyl (Protein N-term)]M[Oxidation (M)]EANGS[Phospho (STY)]PGTSGSANDSQHDPGK_.2	Q920Q6_S6_M1_Msi2	3.1476	3.9039	4.3821	4.5117	3.7922	4.3333	4.2264	3.6067	3.4751	3.6032	1.0762	0.1059	0.3597	0.4441

Ivns1abp	_NS[Phospho (STY)]PQSS[Phospho (STY)]PTSTPK_.2	Q920Q8_S322_M2_Ivns1abp	6.0974	7.6812	6.1376	6.5117	7.5103	5.8241	7.5034	5.7189	5.5418	8.0446	0.9888	-0.0163	0.9092	0.0413
Als2	_QHKDS[Phospho (STY)]PEILSR_.3	#N/A	3.6465	5.2324	4.3333	5.4404	4.3051	3.4564	4.5507	3.2825	4.4319	3.3022	1.1312	0.1778	0.3319	0.4790
Als2	_QLGC[Carbamidomethyl (C)]ES[Phospho (STY)]PGQGEVWK_.2	Q920R0_S1302_M1_Als2	5.8843	5.7445	7.1120	6.1767	4.4216	4.9946	6.5348	5.7750	4.8410	4.4703	1.0587	0.0822	0.6135	0.2122
Als2	_RQHKDS[Phospho (STY)]PEILSR_.3	#N/A	3.5144	4.2707	3.2481	4.1449	4.1590	3.9665	3.8596	3.9735	3.6221	4.1119	0.9980	-0.0029	0.9729	0.0119
Als2	_SSS[Phospho (STY)]LM[Oxidation (M)]DIREEELEGGSR_.3	#N/A	3.5272	3.8958	4.0781	4.6493	3.7841	3.9447	3.8378	3.9939	3.7969	3.5973	1.0455	0.0643	0.4174	0.3795
Als2	_RLS[Phospho (STY)]LPGLLSQVS[Phospho (STY)]PR_.2	Q920R0_S477_M2_Als2	3.2179	4.0231	3.9047	4.4074	3.8444	3.8851	4.1220	3.9192	3.3597	4.3744	0.9839	-0.0234	0.8135	0.0897
Gjc3	_S[Phospho (STY)]KDTTDELSVVEAK_.2	Q921C1_S253_M1_Gjc3	3.5759	4.3321	3.5808	4.0835	4.2204	3.9050	3.7982	4.0350	3.6836	4.0505	1.0149	0.0213	0.7545	0.1223
Brwd1	_KS[Phospho (STY)]SSDKESNLQVTQK_.3	Q921C3_S1888_M1_Brwd1	3.9460	3.2504	3.8257	3.3695	3.0717	3.1570	3.5177	3.0396	3.9307	3.0468	1.0157	0.0224	0.8381	0.0767
Brwd1	_RGPEVEGS[Phospho (STY)]PVSEALR_.3	Q921C3_S1830_M1_Brwd1	3.9112	3.2156	4.5980	3.4043	3.0369	4.2344	3.5526	3.0178	4.1672	3.5668	1.0440	0.0621	0.6777	0.1690
Bicd2	_QTS[Phospho (STY)]LDNEKDR_.2	Q921C5_S411_M1_Bicd2	3.0622	3.8185	4.2968	4.5971	3.7068	4.4186	4.3118	3.5214	3.1700	3.0633	1.1327	0.1798	0.2382	0.6230
Bicd2	_S[Phospho (STY)]PVLLPK_.2	Q921C5_S578_M1_Bicd2	4.1816	3.0446	3.3675	3.8702	2.9329	3.6917	3.5849	4.2483	3.8969	3.8372	0.9031	-0.1470	0.2013	0.6962
Tardbp	_LPNSKQS[Phospho (STY)]PDEPLR_.2	#N/A	4.1632	3.1227	3.6472	3.6621	3.2979	3.5154	8.0775	3.9542	7.8321	3.5127	0.6105	-0.7118	0.0495	1.3058
Tardbp	_FGGNPGGFGNQGGFGNSR_.2	#N/A	3.8832	3.4592	3.6169	6.1439	3.2805	2.9481	3.3089	3.8405	4.1396	5.5506	0.9237	-0.1145	0.6575	0.1821
Tardbp	_GISVHISNAEPK_.3	#N/A	3.7445	2.9999	3.4782	3.9148	2.8882	3.7364	3.6295	3.9792	3.8522	4.2001	0.8838	-0.1782	0.0906	1.0427
Hnrnpll	_TEEGEIVYS[Phospho (STY)]AEESENR_.2	#N/A	3.6117	4.3680	3.3454	4.0476	4.2563	5.6800	3.7623	4.0708	3.7194	4.0673	1.0802	0.1113	0.4797	0.3190
Hnrnpll	_LKTEEGEIVYS[Phospho (STY)]AEESENR_.3	#N/A	3.3657	4.5699	3.5785	5.2535	4.0103	4.1180	4.2486	5.2402	2.9271	4.5553	0.9780	-0.0321	0.8617	0.0647
Hnrnpll	_ITRPGNTDDPSGGNK_.2	#N/A	3.5582	3.7273	3.8273	3.9128	3.9728	3.5005	4.4740	3.8090	3.8913	3.8089	0.9384	-0.0917	0.1626	0.7889
Sh3bp4	_SYS[Phospho (STY)]LSELSVLQAK_.2	Q921I6_S245_M1_Sh3bp4	3.9682	3.2725	3.1541	3.3473	3.0939	3.1348	5.4987	3.6539	3.9529	3.0246	0.8254	-0.2768	0.1546	0.8108
Rheb	_IILEAEKIDGAAS[Phospho (STY)]QGK_.2	Q921J2_S175_M1_Rheb	4.1282	4.7100	3.8619	4.4632	3.2720	4.2119	3.5539	3.5955	3.8945	4.5083	1.0565	0.0793	0.4981	0.3027
Rheb	_ALAESWNAAFLESSAK_.2	#N/A	3.1001	3.8564	4.3346	4.5593	3.7447	3.9746	4.2739	3.5592	3.8097	3.0254	1.0712	0.0993	0.4527	0.3442
Cyrib	_INNVPAEGENEVNNELANR_.2	#N/A	8.3302	8.1683	8.3906	8.2383	7.2706	8.1475	8.7666	8.1911	8.1843	8.0218	0.9759	-0.0352	0.4413	0.3553
Cyrib	_DAEGILEDLQSYR_.2	#N/A	7.3691	7.3221	7.3497	7.0461	6.9005	7.4003	7.7315	7.4629	7.2609	7.1910	0.9757	-0.0355	0.2415	0.6172
Cyrib	_EAIQHPADEKLQEK_.3	#N/A	4.0767	3.3149	5.1163	3.3050	4.4973	3.8892	3.4533	5.0152	4.1154	4.4384	0.9478	-0.0774	0.6276	0.2023
Rin1	_RLS[Phospho (STY)]ADGSLGR_.2	Q921Q7_S446_M1_Rin1	6.0177	4.3424	3.5705	4.0732	3.5103	4.2192	3.7879	5.8435	3.6939	4.0402	0.9879	-0.0175	0.9340	0.0296
Rin1	_EKPSTDPLY[Phospho (STY)]DTPDTR_.2	#N/A	5.9344	7.4351	3.9083	6.9978	2.9633	7.0533	6.8550	7.4912	7.4584	6.4015	0.8105	-0.3031	0.2061	0.6860
Rin1	_SM[Oxidation (M)]S[Phospho (STY)]SAFC[Carbamidomethyl (C)]SLLAPER_.2	#N/A	3.9665	3.3876	4.1414	7.8258	3.2494	3.2181	4.1431	3.4020	3.4234	3.2305	1.2108	0.2760	0.4383	0.3582
Rin1	_ALPLS[Phospho (STY)]PAQELQR_.2	Q921Q7_S590_M1_Rin1	3.4299	4.1862	3.1636	4.2082	4.0745	4.0510	4.3565	4.2938	3.0920	3.8855	0.9860	-0.0204	0.8694	0.0608
Dars1	_NNAYLAQS[Phospho (STY)]PQLYK_.2	Q922B2_S249_M1_Dars1	7.6809	7.2086	7.7912	7.9539	6.7485	7.7423	7.9228	7.1653	3.7226	7.5684	1.1404	0.1896	0.2809	0.5514
Dars1	_FLEPTLR_.2	#N/A	4.8505	5.6335	5.2260	5.1661	5.2593	4.6798	3.8091	5.1931	4.8984	4.8851	1.0936	0.1291	0.1747	0.7576
Dars1	_IISAASEGGANVFTVSYFK_.2	#N/A	2.2473	3.6167	3.3156	4.3602	3.5818	4.1476	4.5084	4.1418	4.4409	2.6730	0.8995	-0.1528	0.4595	0.3377
Ppp6r3	_C[Carbamidomethyl (C)]TAPLTPSSS[Phospho (STY)]PEQR_.2	Q922D4_S824_M1_Ppp6r3	3.2800	4.0852	3.9668	4.4694	3.9065	5.7814	4.1972	3.6494	6.0949	4.0808	0.9429	-0.0849	0.6832	0.1655
Ppp6r3	_IQQFDDGGS[Phospho (STY)]DEEDIWEEK_.2	Q922D4_S588_M1_Ppp6r3	3.2944	7.0963	4.2475	9.0376	7.1234	6.0267	4.7050	8.7823	7.8886	7.2389	0.8580	-0.2210	0.4482	0.3485
Mthfd1	_TPVPSDIAISR_.2	#N/A	5.4954	5.3137	6.3694	5.1472	5.8070	5.8221	5.3046	5.6784	5.1608	5.7141	1.0356	0.0505	0.4562	0.3408
Mthfd1	_LDIDPETITWQR_.2	#N/A	4.1243	4.0417	4.5352	4.4864	3.9453	4.1802	4.2273	4.4230	3.2212	4.1756	1.0516	0.0726	0.4232	0.3734
Clip1	_TASESISNLSEAGS[Phospho (STY)]VK_.2	Q922J3_S203_M1_Clip1	8.0672	8.4215	8.5025	8.8867	7.2947	8.4691	8.2988	8.1138	8.2126	8.5150	0.9986	-0.0020	0.9692	0.0136
Clip1	_TASESISNLS[Phospho (STY)]EAGS[Phospho (STY)]VKKGER_.3	#N/A	3.9149	5.0180	3.8016	4.8426	3.7413	4.7319	4.3490	4.4425	3.0995	4.1191	1.0847	0.1174	0.4034	0.3943
Clip1	_S[Phospho (STY)]VEELQLK_.2	Q922J3_S910_M1_Clip1	3.4700	4.2752	4.1568	4.7734	4.0965	3.4991	3.9938	4.8773	5.0008	4.8165	0.8658	-0.2079	0.0786	1.1043
Tspan2	_HVQSM[Oxidation (M)]YEEAYSDYLKDR_.3	#N/A	3.6664	6.1098	6.5292	4.4447	3.4973	4.8833	6.4163	5.9091	5.1991	4.1220	0.8972	-0.1565	0.4787	0.3199
	KSPSGPVKS[Phospho (STY)]PPLS[Phospho (STY)]PVGTTPVK.3	Q922M7_S189_M2_	2.9518	3.7081	4.1863	7.0650	4.4520	3.0282	4.4222	5.3327	6.0431	3.4074	0.8814	-0.1821	0.5420	0.2660
Glyr1	_KLS[Phospho (STY)]LSEGK_.2	Q922P9_S130_M1_Glyr1	4.5580	4.9712	4.7249	5.0729	2.8479	3.7767	5.1851	5.2424	4.3186	4.1597	0.9151	-0.1280	0.4380	0.3586
Lrrc59	_LQQLPADFGR_.2	#N/A	4.6477	3.7064	3.5880	4.8288	5.0435	4.2018	5.5112	5.4439	3.6764	4.0576	0.9280	-0.1078	0.5062	0.2957
Lrrc59	_LVNLQHLDLLNNR_.2	#N/A	3.7871	3.4885	3.5208	3.5235	1.5976	4.3529	4.0354	3.9366	3.0939	3.5262	0.9261	-0.1108	0.6079	0.2161
Pdia6	_GSFSEQGINEFLR_.2	#N/A	5.8066	5.9792	6.2042	6.0927	6.1656	6.3190	5.8738	5.9198	6.0840	5.8138	1.0290	0.0412	0.1343	0.8719
Pdia6	_LAAVDATVNQVLASR_.2	#N/A	4.0449	4.1205	4.7882	4.7276	4.5586	4.9213	4.3428	4.2356	4.4748	4.6518	1.0227	0.0324	0.6266	0.2030
Pde2a	_VETVYTYLLDGESR_.2	#N/A	5.7640	5.3090	5.7001	3.6927	4.7218	5.5976	5.8031	6.0009	5.8247	5.9550	0.8702	-0.2005	0.0995	1.0022
Pde2a	_DIILATDLAHHLR_.3	#N/A	4.3740	4.3213	3.5600	4.4423	3.5168	4.2298	3.0898	3.6444	3.7709	4.0771	1.1175	0.1603	0.1508	0.8216
Pde2a	_RPEAVQNTS[Phospho (STY)]VDASEDQKDEK_.3	Q922S4_S204_M1_Pde2a	4.8651	3.2263	4.0569	3.2850	2.9078	4.4747	4.4649	3.5587	4.2582	3.3655	0.9721	-0.0408	0.8145	0.0891
Pde2a	_GLPS[Phospho (STY)]NNSLDFLDEEYEVPDLDGTR_.3	Q922S4_S908_M1_Pde2a	3.9227	3.9574	3.6564	3.9686	3.8457	3.6280	3.9795	3.7428	3.7883	3.7539	1.0036	0.0051	0.8820	0.0546
Pde2a	_LLHDGIQPVAAIDSNFANFTYTPR_.3	#N/A	3.2985	2.9276	4.5330	4.0768	3.8652	3.8643	4.2251	4.4252	3.5949	1.1670	1.1216	0.1656	0.5540	0.2565
Pde2a	_SVLAALYSSEGSVM[Oxidation (M)]ER_.2	#N/A	4.6798	4.3069	3.6920	6.1414	4.1093	4.0162	3.9093	3.9238	3.5724	4.1617	1.1539	0.2066	0.2246	0.6486
Pde2a	_SQQYPAAR_.2	#N/A	3.7177	3.6247	3.4514	4.4960	2.8614	4.0043	3.1434	4.0060	4.3051	4.1733	0.9451	-0.0814	0.5545	0.2561
Prpf3	_WDEQTSNTKGDDDEES[Phospho (STY)]DEEAVKK_.3	#N/A	3.6711	3.6713	3.4048	4.4494	2.0988	4.2369	3.0968	4.0526	4.3517	4.1267	0.9185	-0.1226	0.5227	0.2818
Prpf3	_GDDDEES[Phospho (STY)]DEEAVKK_.2	#N/A	4.0520	3.3914	4.2639	4.8728	3.3674	4.7779	4.5126	4.9088	4.0980	4.5279	0.9134	-0.1308	0.3057	0.5146
Ubxn1	_SS[Phospho (STY)]PPATDPGPVPSSPSQEPPTKR_.3	Q922Y1_S188_M1_Ubxn1	3.4559	3.7951	4.1427	3.8688	3.6627	3.6772	3.7670	3.7467	3.7061	3.7305	1.0079	0.0113	0.8098	0.0916
Dbr1	_NQAIYAAVDDGDAS[Phospho (STY)]AE_.2	Q923B1_S548_M1_Dbr1	3.1768	3.9820	3.8636	4.3663	4.7096	3.9262	4.0810	5.1142	5.2349	4.5715	0.8429	-0.2466	0.0580	1.2369
Dbr1	_C[Carbamidomethyl (C)]GETVES[Phospho (STY)]GDEKDLAK_.2	#N/A	4.3972	3.5195	4.0187	4.1068	3.1464	3.9387	3.1801	3.6098	4.2862	3.8813	1.0308	0.0438	0.7050	0.1518
Wbp11	_RRDEDM[Oxidation (M)]LYS[Phospho (STY)]PELAQR_.3	#N/A	5.7233	3.8702	3.8637	3.6023	3.6915	3.3897	6.9491	3.4535	3.7526	3.2796	0.9231	-0.1155	0.6909	0.1606
Ppp1r16a	_SVS[Phospho (STY)]YHLSPEENSAPDALVR_.3	Q923M0_S432_M1_Ppp1r16a	5.1891	3.6825	3.6462	5.2892	3.1595	3.5949	3.6450	3.5509	4.9259	5.4489	0.9319	-0.1017	0.6285	0.2017
Ppp1r16a	_RVS[Phospho (STY)]LTHR_.2	Q923M0_S353_M1_Ppp1r16a	4.3485	3.2228	4.6052	3.6070	3.0441	3.1845	3.5453	3.0106	4.0262	3.5740	1.0366	0.0519	0.7377	0.1321
Stard13	_KKGDDS[Phospho (STY)]DEEDLC[Carbamidomethyl (C)]ISNK_.3	Q923Q2_S133_M1_Stard13	5.2496	5.4990	3.6636	4.7143	4.4708	5.2074	5.3019	5.5656	4.0929	4.3855	0.9926	-0.0107	0.9378	0.0279
Camk2g	_QET[Phospho (STY)]VEC[Carbamidomethyl (C)]LR_.2	Q923T9_T287_M1_Camk2g	7.6722	6.3243	6.3237	7.4198	6.2833	7.3386	7.4152	6.9429	7.1751	7.1337	0.9619	-0.0560	0.4448	0.3519
Camk2g	_FTDDYQLFEELGK_.2	#N/A	6.6029	5.9848	6.5756	6.2940	6.0146	6.3501	6.8609	6.3331	6.4042	6.4755	0.9671	-0.0483	0.2272	0.6436
Camk2g	_FYFENLLSK_.2	#N/A	5.5757	6.0212	3.3285	5.8891	5.4967	6.0434	6.0629	5.8780	5.5591	5.1135	0.9538	-0.0682	0.6501	0.1870
Camk2g	_GST[Phospho (STY)]ESC[Carbamidomethyl (C)]NTTTEDEDLK_.2	Q923T9_T382_M1_Camk2g	3.6086	4.6807	4.1377	3.9321	3.7315	3.8756	3.9148	4.0413	3.5487	3.7111	1.0501	0.0705	0.3971	0.4011
Camk2g	_SLLNKKS[Phospho (STY)]DGGVK_.2	#N/A	4.9855	5.2016	5.4256	5.2084	4.0901	3.7258	4.5510	4.6168	5.0849	3.8654	1.0537	0.0755	0.5656	0.2475
Camk2g	_ITEQLIEAINNGDFEAYTK_.2	#N/A	5.1914	5.2423	4.9903	5.5578	4.7189	5.0567	4.9516	5.0250	5.4576	5.1825	0.9946	-0.0078	0.8724	0.0593
Camk2g	_WLNVHYHC[Carbamidomethyl (C)]SGAPAAPLQ_.3	#N/A	3.9340	4.6234	3.9613	4.4639	4.5729	4.6433	5.0928	4.3195	4.7739	4.8407	0.9179	-0.1235	0.1020	0.9916
Camk2g	_KTS[Phospho (STY)]TQEYAAK_.2	Q923T9_S36_M1_Camk2g	3.7392	2.9947	3.4729	4.5175	2.8830	3.7416	3.6348	3.9845	4.2835	4.1948	0.8841	-0.1776	0.1882	0.7253
Camk2g	_S[Phospho (STY)]SSSVHLM[Oxidation (M)]PQSNNK_.2	#N/A	3.7148	3.8578	4.4389	5.8028	3.6791	3.4161	3.7769	3.4005	3.6995	3.3059	1.1709	0.2276	0.2213	0.6550
Camk2g	_GSTES[Phospho (STY)]C[Carbamidomethyl (C)]NTTTEDEDLKVR_.2	#N/A	3.9208	3.7586	3.5872	3.7872	3.4835	4.1050	3.9743	4.3000	3.7473	3.6892	0.9608	-0.0577	0.3592	0.4447
Gprc5b	_SAVGFSNGS[Phospho (STY)]LEQR_.2	Q923Z0_S355_M1_Gprc5b	5.9359	5.9947	3.8445	6.3363	4.6064	5.0280	6.8935	5.6355	5.2868	5.9773	0.8895	-0.1689	0.2758	0.5593
Cic	_KNS[Phospho (STY)]TDLDSAPEDPT[Phospho (STY)]SPK_.2	Q924A2_S2299_M2_Cic	3.2232	3.9795	8.4974	4.0015	7.9831	4.2576	4.1508	2.9996	3.2987	3.6788	1.5073	0.5920	0.1692	0.7716
Cic	_NSTDLDSAPEDPTS[Phospho (STY)]PK_.2	Q924A2_S2311_M1_Cic	3.2688	4.0740	7.2638	4.0467	7.5885	7.4225	4.1950	7.2472	7.2349	3.7240	1.0019	0.0027	0.9937	0.0028
Cic	_TQS[Phospho (STY)]LSALPK_.2	Q924A2_S1080_M1_Cic	4.1927	4.8480	3.3787	5.6525	3.3184	5.3811	4.9032	5.1534	5.4355	3.8484	0.9228	-0.1159	0.5363	0.2706
Cic	_S[Phospho (STY)]SSEAKPASLGLAGGHK_.3	Q924A2_S1184_M1_Cic	3.0645	4.0867	4.0668	4.0509	3.7006	4.2337	3.9805	3.5795	3.4922	3.8922	1.0351	0.0497	0.5983	0.2231
Cic	_SS[Phospho (STY)]PPPPLPAEERPGTK_.2	Q924A2_S2148_M1_Cic	3.3975	10.5710	3.1312	4.1758	10.2883	3.9633	11.3997	4.3262	10.4707	10.1396	0.6518	-0.6174	0.1881	0.7256
Cic	_EGS[Phospho (STY)]TEFDWGDETSR_.2	Q924A2_S553_M1_Cic	3.7065	5.7756	3.4402	3.9528	5.2043	3.7744	3.6675	6.3312	6.2373	4.1621	0.8450	-0.2430	0.3102	0.5083
Cic	_AQSVS[Phospho (STY)]PVQATPSGGSAQLLPGK_.2	Q924A2_S1809_M1_Cic	3.3742	4.1305	3.7825	7.6485	4.0188	4.1066	3.9998	3.8334	3.4819	4.2521	1.1589	0.2128	0.4660	0.3316
Cic	_AAS[Phospho (STY)]EDMTS[Phospho (STY)]DEER_.2	#N/A	5.0460	3.9409	3.8555	5.9017	4.2745	3.8001	3.9075	3.7335	3.8408	3.4846	1.1946	0.2565	0.1325	0.8778

Wac	_QQSHDPVS[Phospho (STY)]PR_.2	#N/A	8.6208	8.3994	8.5343	8.9288	8.2863	8.5142	8.4965	8.4533	8.4310	8.3937	1.0123	0.0176	0.3880	0.4112
Wac	_DAADPS[Phospho (STY)]PPNK_.2	#N/A	8.7424	8.8421	8.9907	9.1716	8.5774	8.6399	8.9905	8.0301	8.8935	8.9823	1.0118	0.0170	0.6459	0.1898
Wac	_QGPVSHSATQQPVTADKQQSHDPVS[Phospho (STY)]PR_.4	#N/A	3.8111	3.1154	4.4979	3.4997	4.6724	3.2919	3.6527	3.1179	4.2674	3.4667	1.0520	0.0731	0.6323	0.1991
Wac	_MRDAADPS[Phospho (STY)]PPNK_.2	#N/A	3.8744	7.2185	3.6080	9.2963	3.2894	2.9393	3.3001	3.8494	4.1484	4.3299	1.2894	0.3667	0.4225	0.3741
Wac	_S[Phospho (STY)]NS[Phospho (STY)]PENKYSDSTGHNK_.2	Q924H7_S62_M2_Wac	3.7749	3.7160	3.5842	4.0868	3.6043	3.9083	3.8015	4.0421	3.6907	4.0538	0.9697	-0.0443	0.3584	0.4456
Wac	_QGPVS[Phospho (STY)]HS[Phospho (STY)]ATQQPVT[Phospho (STY)]ADK_.2	Q924H7_S491_M3_Wac	8.5187	4.0941	4.5724	4.1161	3.9824	4.1430	4.0362	4.3859	3.1841	3.7934	1.2739	0.3493	0.2902	0.5372
Mta3	_HAELSGS[Phospho (STY)]PLK_.2	Q924K8_S516_M1_Mta3	7.3578	7.1515	7.8007	7.6601	6.3571	7.2337	7.4369	7.4514	7.2727	7.1912	0.9894	-0.0154	0.7754	0.1104
Mta3	_S[Phospho (STY)]PPSLQTPATK_.2	Q924K8_S547_M1_Mta3	6.9995	4.1017	3.8113	4.3140	5.9059	6.7006	6.9324	6.5766	3.4531	6.2126	0.9157	-0.1270	0.6220	0.2062
Mta3	_M[Oxidation (M)]PTQSDEEKS[Phospho (STY)]PS[Phospho (STY)]PTAEDPR_.2	#N/A	4.0690	3.9575	4.1878	5.7615	3.8284	4.1913	3.7943	3.9142	3.7935	4.7561	1.0660	0.0921	0.5295	0.2761
Mpi	_ISQQVFDGNNMEDIYGK_.2	#N/A	4.3243	4.0057	3.8873	5.6411	3.8271	3.9024	4.2632	4.3871	3.1853	3.7922	1.0916	0.1264	0.4148	0.3822
Slc12a6	_IDDIPGLSDTS[Phospho (STY)]PDLSSR_.2	Q924N4_S32_M1_Slc12a6	9.4853	8.7638	4.3619	3.6706	3.7591	3.4714	9.3212	3.1822	9.3244	8.7195	0.7314	-0.4513	0.2972	0.5270
Slc12a6	_LTSIGS[Phospho (STY)]DEDEETETYQEK_.2	Q924N4_S1032_M1_Slc12a6	3.5628	7.8669	7.4185	8.2129	6.9706	7.9841	8.6594	8.4340	8.2366	8.0309	0.8396	-0.2522	0.1736	0.7604
Spred2	_TISS[Phospho (STY)]PTSC[Carbamidomethyl (C)]EHR_.2	Q924S7_S168_M1_Spred2	7.9231	7.6341	4.3679	7.8581	6.6357	7.0634	8.1245	8.0914	7.7575	3.3367	1.0126	0.0181	0.9420	0.0259
Spred1	_HVS[Phospho (STY)]FQDEDEIVR_.2	Q924S8_S239_M1_Spred1	3.5670	4.2813	3.3007	4.3089	4.1077	4.2838	4.1406	5.0342	4.4205	5.1264	0.8493	-0.2357	0.0414	1.3831
Trim66	_SVSS[Phospho (STY)]PGSGPAAESLGTR_.2	Q924W6_S767_M1_Trim66	7.6220	7.9292	8.3947	8.3540	7.2779	8.0186	4.0750	7.8411	7.9746	7.6534	1.1520	0.2041	0.2134	0.6707
Trps1	_GS[Phospho (STY)]SERGSPIEK_.2	Q925H1_S1061_M1_Trps1	3.7404	2.9958	3.4741	6.3578	2.8841	3.7405	3.6336	4.1995	3.8481	3.8859	0.9932	-0.0098	0.9690	0.0137
Trps1	_HPNYS[Phospho (STY)]PPGS[Phospho (STY)]PIEK_.2	Q925H1_S1081_M2_Trps1	2.9800	3.7852	3.6669	4.1695	3.6066	4.1229	3.8842	3.9490	3.5976	4.1365	0.9563	-0.0644	0.4943	0.3060
Atad3	_TAGTLLGEGFR_.2	#N/A	5.2084	5.3432	5.2941	5.3591	6.1173	4.9579	3.5996	4.9846	5.6664	5.2054	1.1061	0.1455	0.2374	0.6245
Med1	_SQT[Phospho (STY)]PPGVATPPIPK_.2	Q925J9_T1051_M1_Med1	8.8150	3.2169	9.1531	9.5402	3.1052	8.9266	9.4726	8.9663	4.0691	3.6649	1.0891	0.1231	0.7775	0.1093
Med1	_NSSQTGGKPGSS[Phospho (STY)]PITK_.2	Q925J9_S1158_M1_Med1	3.7081	5.6414	3.4417	3.9513	4.8945	5.4796	3.6660	4.0157	3.8158	4.1636	1.1543	0.2070	0.2525	0.5978
Med1	_HDGGS[Phospho (STY)]PSIK_.2	Q925J9_S1403_M1_Med1	3.7488	3.5936	3.4825	4.5271	4.8330	4.3145	5.7348	3.9749	4.2740	4.2043	0.8980	-0.1552	0.3061	0.5141
Sfxn5	_EAVQLLEDYK_.2	#N/A	6.1612	5.7674	5.3779	5.6674	5.4575	5.7409	5.9210	6.0678	5.9418	6.0065	0.9517	-0.0714	0.0790	1.1024
Sfxn5	_HALLETALTR_.2	#N/A	5.6306	5.5631	4.3269	5.0086	4.3809	4.7313	5.1577	5.0534	5.3692	4.6801	0.9753	-0.0360	0.6982	0.1560
Sfxn5	_YGELEEGIDVLDADGNLVGSSK_.2	#N/A	5.3144	4.8902	5.3428	5.5346	4.1111	4.3788	4.9295	5.5918	4.7444	3.9215	1.0275	0.0391	0.7504	0.1247
Sfxn5	_HFLDIIDPR_.2	#N/A	4.5204	5.5673	4.3405	5.4304	3.4808	4.5290	5.3085	5.5124	5.1548	5.1655	0.8788	-0.1864	0.1454	0.8374
Sfxn5	_FQQTSFYGR_.2	#N/A	6.5243	7.0512	6.9811	7.0840	3.9597	6.3175	4.0589	7.0559	6.5548	4.3511	1.1479	0.1990	0.3697	0.4321
Grip1	_RQS[Phospho (STY)]IPEEFK_.2	Q925T6_S43_M1_Grip1	9.1456	8.9480	9.5459	9.5759	8.1759	9.0433	9.5400	9.0291	9.0228	9.0123	0.9914	-0.0124	0.7860	0.1046
Grip1	_SLYSTS[Phospho (STY)]PR_.2	Q925T6_S432_M1_Grip1	4.1977	7.7538	3.3837	8.0028	3.3234	4.4061	7.6120	7.7381	3.8807	3.8533	0.8972	-0.1565	0.6818	0.1663
Grip1	_TSLS[Phospho (STY)]PVPKPR_.2	Q925T6_S846_M1_Grip1	5.9257	7.0182	4.0548	3.5986	3.4648	3.1598	4.5538	3.4026	3.7017	3.2758	1.2152	0.2812	0.3615	0.4418
Grip1	_QAS[Phospho (STY)]FQER_.2	Q925T6_S952_M1_Grip1	5.3333	4.4860	5.0647	5.4135	4.9384	5.2822	4.6359	5.3628	5.3282	4.6704	1.0174	0.0249	0.7219	0.1415
Grip1	_QTDAQSASS[Phospho (STY)]PK_.2	#N/A	3.2797	3.9465	3.9665	4.4691	3.9062	4.2906	4.1838	3.6494	3.2979	4.4361	1.0217	0.0310	0.7786	0.1087
Grip1	_KQTDAQSASS[Phospho (STY)]PK_.2	#N/A	3.9619	3.2643	3.1479	3.6505	3.0876	3.4720	3.3652	4.4680	4.1166	3.6175	0.8815	-0.1819	0.1134	0.9452
Taldo1	_SYEPQEDPGVK_.2	#N/A	4.7774	5.5117	4.0527	5.5631	5.4513	5.5094	4.7078	4.9824	3.7038	5.5757	1.0847	0.1173	0.3865	0.4129
Taldo1	_WLHNEDQM[Oxidation (M)]AVEK_.3	#N/A	3.5244	4.8094	4.1997	4.1997	5.4480	4.8533	3.2080	3.1243	4.7852	4.5219	1.1524	0.2046	0.2534	0.5961
Taldo1	_LSSTWEGIQAGK_.2	#N/A	6.0552	6.1565	3.7859	6.3946	6.7776	6.8728	6.3395	5.9792	6.6615	6.5596	0.9408	-0.0880	0.5410	0.2668
Taldo1	_M[Oxidation (M)]ESALDQLK_.2	#N/A	5.0246	4.8333	5.3280	3.9294	5.1028	5.3933	4.0322	4.6276	5.0383	5.3521	1.0363	0.0514	0.6382	0.1950
Taldo1	_[Acetyl (Protein N-term)]SGS[Phospho (STY)]PVKR_.2	#N/A	13.0636	13.2089	13.6700	13.3750	12.3082	13.0801	13.3037	13.1993	13.1349	13.1843	0.9933	-0.0096	0.7172	0.1444
Taldo1	_TIVM[Oxidation (M)]GAS[Phospho (STY)]FR_.2	Q93092_S237_M1_Taldo1	10.4668	10.3714	11.1734	10.5300	10.1182	10.7200	10.8553	10.5964	10.6834	10.6475	0.9876	-0.0180	0.5018	0.2994
Hnrnpab	_IFVGGLNPEATEEK_.2	#N/A	7.0638	7.0499	6.7346	7.1992	7.2786	6.2362	6.6901	6.7914	6.3781	6.7646	1.0407	0.0576	0.2349	0.6292
Hnrnpab	_EVYQQQQYGSGGR_.2	#N/A	5.1571	4.8200	3.1783	3.6810	5.8140	5.4129	5.2214	4.6467	5.4949	5.4553	0.8987	-0.1541	0.3647	0.4381
Hnrnpab	_EYFGQFGEIEAIELPIDPK_.2	#N/A	4.2379	4.5522	5.5143	5.2019	4.1612	3.9642	4.0868	4.2210	4.1104	4.1362	1.1128	0.1542	0.1801	0.7445
Myo5a	_AIS[Phospho (STY)]PTSATSSGR_.2	#N/A	14.0474	13.7083	14.1242	13.8788	12.5988	13.4055	14.4280	14.1461	13.8084	13.7384	0.9713	-0.0420	0.2368	0.6257
Myo5a	_SAPEVTAPGAPAYR_.2	#N/A	7.3005	7.5177	3.9451	7.1909	7.4305	7.2802	7.9052	7.4647	7.4436	7.2082	0.9030	-0.1472	0.3415	0.4667
Myo5a	_YQNLLNEFSR_.2	#N/A	6.2512	6.0667	5.9065	5.5884	5.1793	5.8421	5.9830	5.9548	5.0940	5.0956	1.0495	0.0697	0.3532	0.4520
Myo5a	_M[Oxidation (M)]LPELFQDDEKAIS[Phospho (STY)]PTSATSSGR_.3	#N/A	7.3462	9.1119	9.2707	9.8413	6.6927	7.6191	7.8739	9.4473	8.0789	7.3023	1.0169	0.0242	0.8560	0.0675
Myo5a	_AC[Carbamidomethyl (C)]GVLETIR_.2	#N/A	5.5163	5.2566	4.2743	4.3069	4.7979	4.3389	3.1989	5.5870	4.9234	5.0147	1.0144	0.0207	0.8940	0.0487
Myo5a	_QLELDLNDER_.2	#N/A	5.4135	4.2712	4.0369	5.4505	3.9766	4.3610	5.1896	5.4392	4.6403	4.4933	0.9280	-0.1078	0.3834	0.4163
Myo5a	_ETLEPLIQAAQLLQVK_.2	#N/A	4.6448	4.7868	3.5571	3.7389	4.0349	3.3269	3.2492	4.1879	4.0486	4.2790	1.0187	0.0267	0.8406	0.0754
Myo5a	_TSS[Phospho (STY)]IADEGTYTLDSILR_.2	Q99104_S1650_M1_Myo5a	7.3944	7.5608	4.1586	7.2067	3.3407	7.0385	4.0649	7.5842	3.2186	7.0341	1.1171	0.1597	0.6289	0.2015
Myo5a	_VLNLYTPVNEFEER_.2	#N/A	6.2382	3.9461	6.2117	5.5469	3.7674	3.9620	6.2698	6.4018	5.4120	5.5808	0.8359	-0.2586	0.1643	0.7844
Myo5a	_SHENEAEALRGEIQSLKEENNR_.4	#N/A	5.3449	6.4999	7.1494	6.3900	3.9533	6.5178	6.8460	5.7706	6.4685	5.2862	0.9808	-0.0280	0.8613	0.0648
Myo5a	_LHIGMENK_.2	#N/A	3.8226	3.1299	6.1258	5.2140	2.9512	3.2775	3.2288	3.1035	4.2529	3.4811	1.1621	0.2168	0.4329	0.3636
Myo5a	_YNVSQLEEWLR_.2	#N/A	4.6861	3.9646	3.1115	4.1561	3.7859	3.9436	4.0797	4.9598	4.9286	4.5961	0.8492	-0.2358	0.0524	1.2804
Myo5a	_YFATVSGSASEANVEEK_.2	#N/A	3.7798	3.0352	3.5135	4.5581	2.9235	3.7011	3.2055	4.6077	4.2430	4.2354	0.8803	-0.1840	0.2402	0.6194
Myo5a	_IGELEVGQMENIS[Phospho (STY)]PGQIIDEPIRPVNIPR_.3	Q99104_S1450_M1_Myo5a	3.5376	3.6885	4.2244	3.2262	4.1642	3.5653	4.4418	3.3914	3.0400	3.1932	1.0619	0.0867	0.5143	0.2888
Myo5a	_VLMEQLTSVSEELDVR_.2	#N/A	2.9960	4.2302	3.6828	4.1854	4.1185	4.0070	3.9001	3.9330	3.5816	4.1524	0.9944	-0.0081	0.9346	0.0294
Cacna1d	_SES[Phospho (STY)]GDEQFPTIC[Carbamidomethyl (C)]R_.2	Q99246_S1833_M1_Cacna1d	3.0937	6.4702	4.0714	4.0104	5.1792	6.2656	6.9519	6.6345	3.4569	6.4733	0.8247	-0.2781	0.3062	0.5140
Cacna1d	_QT[Phospho (STY)]VLSWQAAIDAAR_.2	Q99246_T49_M1_Cacna1d	4.2192	3.0069	3.4052	3.9079	2.8952	3.7294	3.6225	4.2106	3.8592	3.8749	0.9063	-0.1419	0.2320	0.6345
Cacna1d	_RQS[Phospho (STY)]S[Phospho (STY)]QDDVLPSPALPHR_.3	Q99246_S1950_M2_Cacna1d	3.3278	4.0840	4.5623	4.1060	3.9723	4.1531	4.0463	5.8167	3.1942	3.7833	0.9582	-0.0616	0.7294	0.1370
Cacna1d	_YS[Phospho (STY)]PSHSTR_.2	Q99246_S1991_M1_Cacna1d	3.8073	3.5351	3.5409	4.5856	3.3565	4.3730	3.2330	3.9165	4.2155	4.2628	0.9896	-0.0151	0.9014	0.0451
Cacna1d	_RIS[Phospho (STY)]ELNMK_.2	Q99246_S869_M1_Cacna1d	2.9605	3.7168	4.1950	4.0558	3.6051	3.0195	3.8870	3.2624	3.5615	3.4161	1.0171	0.0245	0.8362	0.0777
Sec14l2	_MNQQGADT[Phospho (STY)]PK_.2	Q99J08_T401_M1_Sec14l2	4.9512	4.2174	4.0990	6.7210	1.7680	3.6908	4.3163	4.2614	3.1654	2.7928	1.1671	0.2229	0.5098	0.2926
Wdr77	_KDT[Phospho (STY)]PPPLVPPAAR_.2	#N/A	7.8413	8.0613	7.8344	8.3078	7.4908	8.0191	8.3972	7.4433	7.4843	8.0520	1.0104	0.0149	0.7321	0.1354
Wdr77	_YEHDDIVSTVTVLSSGTQAVSGSK_.3	#N/A	3.1623	3.9675	3.1086	4.1532	3.2078	3.9407	4.3015	4.3488	3.1470	3.8305	0.9189	-0.1221	0.3635	0.4395
Ctu1	_AGATC[Carbamidomethyl (C)]VSQC[Carbamidomethyl (C)]DLS[Phospho (STY)]PVAE_.2	Q99J10_S416_M1_Ctu1	5.6305	6.5182	4.5028	6.8172	5.1590	6.2736	6.0552	6.4974	6.3106	6.2550	0.9263	-0.1104	0.3401	0.4683
Ctu1	_AGATC[Carbamidomethyl (C)]VSQC[Carbamidomethyl (C)]DLS[Phospho (STY)]PGNGEDR_.2	Q99J10_S378_M1_Ctu1	3.6138	3.9791	4.4573	4.0011	3.8674	4.2581	4.1494	3.0001	3.2991	3.6784	1.1409	0.1902	0.0793	1.1008
Mcoln1	_SELQAYIEQC[Carbamidomethyl (C)]QDS[Phospho (STY)]PTSGK_.2	Q99J21_S547_M1_Mcoln1	3.3022	9.4887	4.5368	10.4936	8.9936	4.1786	10.2678	10.6111	10.1811	9.8037	0.6688	-0.5804	0.0698	1.1561
Thumpd1	_[Acetyl (Protein N-term)]ATTAQQS[Phospho (STY)]PQPVAGKR_.2	#N/A	8.0336	8.7312	9.4078	8.9564	8.1447	8.7859	8.7681	8.3645	8.4906	8.3797	1.0207	0.0295	0.5371	0.2699
Thumpd1	_FIDKDQQPS[Phospho (STY)]GS[Phospho (STY)]EGEDDDAEAALKK_.3	#N/A	3.8290	9.4881	3.7382	10.0375	9.3405	9.2390	9.0251	9.5180	9.7475	9.0296	0.8159	-0.2936	0.2931	0.5330
Thumpd1	_[Acetyl (Protein N-term)]ATTAQQS[Phospho (STY)]PQPVAGK_.2	#N/A	12.2686	12.5185	12.9215	13.0334	12.2119	12.5551	12.8257	12.3756	12.4914	12.5403	1.0021	0.0031	0.8902	0.0505
Thumpd1	_DQQPS[Phospho (STY)]GS[Phospho (STY)]EGEDDDAEAALKK_.3	#N/A	3.5097	3.7777	5.3262	4.8265	4.5903	4.7091	4.8399	3.8797	4.6137	4.4978	0.9997	-0.0004	0.9976	0.0010
Thumpd1	_FIDKDQQPS[Phospho (STY)]GS[Phospho (STY)]EGEDDDAEAALK_.3	#N/A	3.9455	3.2807	3.1315	3.4962	3.0712	3.4556	3.3488	3.5200	2.4658	3.6011	1.0504	0.0709	0.5524	0.2577
Nrbp1	_[Acetyl (Protein N-term)]S[Phospho (STY)]EGESQTVVSSGSDPK_.2	Q99J45_S2_M1_Nrbp1	8.8931	8.9168	8.7755	9.8892	8.9868	9.1992	9.2023	9.1636	9.6018	9.0618	0.9841	-0.0231	0.5359	0.2709
Nrbp1	_TPT[Phospho (STY)]PEPAEVETR_.2	Q99J45_T433_M1_Nrbp1	2.9986	4.2275	3.6854	4.1881	3.6252	7.9189	8.5032	7.6868	3.5790	7.9000	0.6420	-0.6394	0.0860	1.0654
Nans	_GSDHSASLEPGELAELVR_.3	#N/A	4.8212	4.3644	3.7962	5.1189	4.7050	3.5899	3.4126	2.6594	3.3401	4.6157	1.2544	0.3270	0.0796	1.0991
Nptxr	_AELLLLQSTAEQLR_.2	#N/A	4.3357	5.0547	5.3726	5.0076	5.4393	3.1513	5.2852	5.3032	4.8861	4.7700	0.9340	-0.0986	0.4833	0.3158
Nptxr	_FLC[Carbamidomethyl (C)]TPLAAAC[Carbamidomethyl (C)]PSGAEQGDAAGER_.2	#N/A	3.0482	4.1780	3.7350	4.2376	4.0663	4.0591	3.9523	3.8809	3.5295	4.2046	0.9989	-0.0016	0.9864	0.0059
Mpst	_HIPGAAFFDIDR_.2	#N/A	5.2613	5.4559	6.8364	5.8496	5.8875	4.3530	4.7945	5.5343	5.7076	5.6626	1.0337	0.0478	0.6964	0.1571
Mpst	_ALVSAQWVAEALK_.2	#N/A	2.9085	5.5163	4.0941	4.9804	5.0038	5.0526	4.8500	4.9959	5.4526	4.5676	0.9247	-0.1129	0.4795	0.3193

Mpst	_AQPEHIISEGR_.3	#N/A	3.6595	3.9359	3.6786	3.2224	3.7760	3.8313	3.3706	3.7788	2.6757	4.3389	1.0404	0.0571	0.6511	0.1864
Mpst	_FQGTQPEPR_.2	#N/A	4.2572	6.3204	3.9909	6.6893	6.6946	5.9217	3.6830	3.4665	3.7655	3.2120	1.5986	0.6768	0.0098	2.0071
Mpst	_THEDILENLDAR_.3	#N/A	3.9800	3.2843	3.1659	3.6686	4.2237	4.5120	3.3833	3.4209	4.5713	3.6356	1.0141	0.0202	0.8829	0.0541
Mpst	_HWLNQNLPISSGK_.2	#N/A	4.1467	3.0794	3.3327	3.8354	4.7505	3.6569	3.5501	4.2831	5.0980	3.8024	0.9084	-0.1386	0.3738	0.4274
Mpst	_DGIEPGHIPGSVNIPFTEFLTNEGLEK_.3	#N/A	3.6462	4.4025	3.3799	4.7260	2.7899	3.8347	2.8336	4.0775	3.7539	4.1018	1.0284	0.0404	0.8122	0.0903
Mpst	_AFGHHSVSLLDGGFR_.3	#N/A	6.1551	2.9630	3.4413	3.9518	6.0146	3.7733	3.6664	4.1667	3.8153	4.1632	1.1088	0.1491	0.5568	0.2543
Stoml2	_ILEPGLNVLIPVLDR_.2	#N/A	4.9477	5.4976	5.6268	5.3048	5.3165	5.2488	5.1379	4.5779	5.3412	4.9725	1.0632	0.0884	0.1072	0.9698
Stoml2	_NTVILFVPQQEAWVVER_.2	#N/A	3.5748	4.2182	3.8791	3.4229	4.2199	2.5284	3.5711	3.8904	3.4615	3.1001	1.0384	0.0544	0.7092	0.1493
Stoml2	_APVPGAQNSSQSR_.2	#N/A	3.7532	3.0576	4.4400	6.6489	4.8811	3.3498	3.7106	3.1758	3.7379	3.4088	1.2414	0.3119	0.2467	0.6078
Pacsin3	_DLQQSIEAAS[Phospho (STY)]DEEDLR_.2	Q99JB8_S276_M1_Pacsin3	3.1847	3.9409	4.4192	4.4747	3.8292	4.2962	4.1894	3.6438	3.2924	4.4417	1.0340	0.0482	0.6921	0.1598
Pacsin3	_DGTAPPPQSPSSPGSGQDEDWS[Phospho (STY)]DEESPR_.3	Q99JB8_S354_M1_Pacsin3	4.1769	3.1656	3.9105	5.4743	3.3206	3.9857	3.6026	3.5469	3.8459	3.1316	1.1342	0.1816	0.3079	0.5117
Pacsin3	_GGRS[Phospho (STY)]PDEVTLTSIVPTR_.3	Q99JB8_S319_M1_Pacsin3	3.3448	4.1011	4.5793	5.7641	3.9894	4.1361	4.0292	3.8039	3.4525	4.2815	1.1098	0.1503	0.3568	0.4476
Pacsin3	_ALYDYAGQEADELS[Phospho (STY)]FR_.2	Q99JB8_S383_M1_Pacsin3	3.0111	4.2150	3.6979	4.2006	4.1033	4.0221	3.9153	3.9179	3.5665	4.1676	0.9957	-0.0062	0.9493	0.0226
Psip1	_QSNASS[Phospho (STY)]DVEVEEK_.2	#N/A	10.4015	10.2991	10.6470	11.0462	9.9832	10.4086	10.7098	10.1835	10.1474	10.4779	1.0082	0.0117	0.6979	0.1562
Psip1	_NLAKPGVTSTS[Phospho (STY)]DS[Phospho (STY)]EDEDDQEGEKK_.3	#N/A	3.7037	4.9893	4.3905	6.1732	3.5923	3.2138	3.1070	4.9795	4.7309	3.3593	1.0741	0.1031	0.6682	0.1751
Psip1	_FSSQQASTK_.2	#N/A	3.0801	4.0614	4.3146	4.1804	5.5221	4.8448	4.7720	3.4836	5.1949	4.6203	0.9593	-0.0599	0.7266	0.1387
Psip1	_QVDTEEAGM[Oxidation (M)]VTAATASNVKAS[Phospho (STY)]PK_.3	#N/A	5.7213	5.8535	6.6094	7.3255	5.9686	6.1458	5.7896	6.5545	6.0310	5.6260	1.0451	0.0636	0.4573	0.3398
Psip1	_NLAKPGVTSTS[Phospho (STY)]DS[Phospho (STY)]EDEDDQEGEK_.3	#N/A	3.4868	5.5186	3.2205	4.1726	6.0910	5.4272	3.8873	3.9459	5.3267	5.1497	1.0165	0.0236	0.9142	0.0390
Psip1	_QSNASS[Phospho (STY)]DVEVEEKETNVSK_.2	#N/A	3.2927	3.7958	2.7781	4.0710	3.8711	3.8584	4.2192	4.4310	3.2293	3.7482	0.9243	-0.1136	0.3892	0.4098
Psip1	_PGVTSTS[Phospho (STY)]DS[Phospho (STY)]EDEDDQEGEK_.2	#N/A	3.8386	3.3876	4.5254	5.8930	4.4919	3.3487	3.2419	3.0904	4.2398	3.4942	1.2079	0.2725	0.2031	0.6922
Psip1	_QVDTEEAGM[Oxidation (M)]VTAATASNVKAS[Phospho (STY)]PKR_.3	#N/A	4.7299	5.7677	6.5414	6.0937	4.8803	4.3628	4.5364	5.8961	5.2138	4.3539	1.0792	0.1099	0.4684	0.3294
Psip1	_ETNVSKEDT[Phospho (STY)]DQEK_.2	#N/A	5.7050	4.8566	3.7600	4.2626	4.0413	4.0841	4.7502	4.2424	3.5044	4.2296	1.0645	0.0902	0.5357	0.2711
Psip1	_EDTDQEEKAS[Phospho (STY)]NEDVTK_.2	#N/A	3.3470	3.7607	4.1921	4.3133	3.6748	3.6808	4.0079	3.7186	3.5146	3.5126	1.0379	0.0536	0.5165	0.2869
Psip1	_NFQAAHR_.2	#N/A	3.5863	4.3426	3.3200	4.9818	5.0699	3.8945	3.7877	4.3808	3.6941	4.0400	1.0562	0.0789	0.5819	0.2351
Psip1	_ETNVSKEDT[Phospho (STY)]DQEEKASNEDVTK_.3	#N/A	3.7326	3.7335	3.7826	2.7975	3.9905	3.3283	3.8985	3.4978	3.2160	3.7609	0.9910	-0.0131	0.9000	0.0458
Psip1	_QSNASS[Phospho (STY)]DVEVEEKETNVSKEDTDQEEK_.4	#N/A	3.5841	4.3403	3.3177	4.3624	4.2286	3.8968	4.5106	4.5850	4.4387	4.0396	0.9002	-0.1517	0.1062	0.9739
Gpr37l1	_LLGDVSC[Carbamidomethyl (C)]R_.2	#N/A	6.5391	6.5687	6.6067	6.2143	6.2367	6.5272	6.5443	6.7019	6.1179	5.7543	1.0004	0.0006	0.9927	0.0032
Gpr37l1	_ES[Phospho (STY)]PPLLPLGTPC[Carbamidomethyl (C)]_.2	#N/A	9.8747	9.1773	9.1155	9.6670	8.5281	9.1806	10.1323	9.5966	9.5306	9.2834	0.9607	-0.0578	0.2122	0.6733
Gpr37l1	_PRES[Phospho (STY)]PPLLPLGTPC[Carbamidomethyl (C)]_.2	#N/A	4.0069	6.7678	3.7406	7.4081	3.1506	3.4740	7.2260	7.1510	4.0159	2.9616	0.8912	-0.1661	0.6606	0.1801
Klhl22	_SLLHEQPRGT[Phospho (STY)]PNR_.2	#N/A	4.5706	5.4159	5.8738	3.9411	3.7578	3.7286	5.0650	4.8657	4.0714	4.7682	0.9692	-0.0451	0.7784	0.1088
Cdc26	_EDVEGVGTS[Phospho (STY)]DGEGAAGLSSDPK_.2	#N/A	3.8429	4.4403	3.9066	3.9114	3.9363	3.7181	3.8316	3.7083	3.6348	4.7214	0.9963	-0.0054	0.9519	0.0214
Cdc26	_QKEDVEGVGTS[Phospho (STY)]DGEGAAGLSSDPK_.3	#N/A	3.7621	3.8922	4.0918	3.6356	3.5019	3.3480	3.7839	3.3687	3.4678	3.3129	1.0487	0.0686	0.3257	0.4871
Homer3	_SQS[Phospho (STY)]ADTPGPTER_.2	Q99JP6_S159_M1_Homer3	7.8816	8.1370	8.8086	8.7871	8.3750	8.2610	8.2571	7.8854	8.6816	8.1527	1.0159	0.0227	0.5820	0.2351
Ggt7	_LPSSSSEM[Oxidation (M)]GSQDGS[Phospho (STY)]PLRETR_.3	#N/A	5.8810	6.5071	3.5476	4.2001	3.3261	6.4446	3.4522	3.9172	6.2151	6.6638	0.9847	-0.0223	0.9388	0.0274
Ggt7	_LPSSSSEM[Oxidation (M)]GSQDGS[Phospho (STY)]PLR_.2	#N/A	5.6606	5.7037	5.4747	6.4880	5.3212	5.4752	5.9338	5.5076	5.0362	5.4258	1.0386	0.0546	0.4355	0.3610
Ggt7	_KDEDAFLGDPDT[Phospho (STY)]DPDSFLK_.3	Q99JP7_T56_M1_Ggt7	4.3026	3.0398	4.0363	3.5800	3.4463	3.3675	3.7283	3.4212	3.7202	3.2573	1.0275	0.0391	0.7118	0.1477
Sfxn1	_NILLTNEQLENAR_.2	#N/A	5.3438	5.7237	5.9165	4.6685	5.1838	4.1951	5.3471	5.9339	5.1365	4.4626	0.9908	-0.0134	0.9090	0.0414
Sfxn1	_QAITQVVISR_.2	#N/A	4.6089	4.9425	4.0611	4.2623	3.9767	4.1682	5.0737	4.6810	5.0870	3.8488	0.9281	-0.1077	0.2904	0.5369
Sfxn1	_ASHFFTVTDPR_.3	#N/A	2.2729	3.4842	3.9624	3.4306	3.3725	3.2521	3.1453	3.4950	1.6596	3.1835	1.1480	0.1991	0.3540	0.4510
Gorasp2	_ADASSLTVDVTS[Phospho (STY)]PASK_.2	Q99JX3_S418_M1_Gorasp2	9.2719	9.4554	9.5335	10.2776	8.7424	9.5439	9.5111	9.3453	9.3297	9.7264	0.9992	-0.0011	0.9785	0.0094
Gorasp2	_LYVYNTDTDNC[Carbamidomethyl (C)]R_.2	#N/A	5.0583	5.3602	4.1363	5.8341	4.9404	5.4074	5.3860	4.7288	4.6566	5.1417	1.0290	0.0413	0.6672	0.1758
Eif3m	_[Acetyl (Protein N-term)]SVPAFIDISEEDQAAELR_.2	#N/A	5.3067	5.6085	6.4278	5.0136	4.2767	5.2335	5.3452	4.4203	5.8286	5.1255	1.0253	0.0361	0.7674	0.1150
Hadhb	_AALSGLLHR_.2	#N/A	6.3622	6.8325	6.9761	6.8035	6.9635	6.4298	7.0804	6.9941	6.6274	6.9531	0.9731	-0.0393	0.2711	0.5669
Hadhb	_DFIYVSQDPK_.2	#N/A	6.6080	5.6646	6.0312	6.5820	5.9621	6.3851	6.2050	5.7300	5.5641	5.8480	1.0632	0.0884	0.1341	0.8726
Hadhb	_NIVVVEGVR_.2	#N/A	6.2370	5.1549	3.6062	6.1918	6.0305	6.5739	5.4700	5.7792	6.4461	6.4197	0.9343	-0.0981	0.5229	0.2816
Hadhb	_DVVDYIIFGTVIQEVK_.2	#N/A	3.0163	4.3261	4.2509	2.9581	2.7252	3.5821	3.9429	3.2066	3.5056	3.4719	0.9843	-0.0228	0.8855	0.0528
Plpp3	_EILSPVDIIDR_.2	#N/A	5.4016	5.9776	5.3876	4.5553	5.0827	5.4449	6.2498	6.2107	5.7632	5.4418	0.8972	-0.1565	0.0645	1.1905
Plpp3	_NGGS[Phospho (STY)]PALNNNPR_.2	Q99JY8_S19_M1_Plpp3	9.1018	7.0763	7.9289	7.4669	5.9644	6.2220	8.9945	8.9096	6.5697	7.1828	0.9216	-0.1179	0.4392	0.3574
Actr3	_DYEEIGPSIC[Carbamidomethyl (C)]R_.2	#N/A	7.5417	7.3830	7.8052	8.0088	7.7863	7.6352	7.7983	7.2809	7.6438	7.7943	1.0084	0.0120	0.6770	0.1694
Actr3	_AEPEDHYFLLTEPPLNTPENR_.3	#N/A	6.8619	7.9196	7.8635	7.9057	6.1153	6.6572	7.3284	7.7909	6.4941	5.6114	1.0609	0.0853	0.4728	0.3253
Actr3	_GVDDLDFFIGDEAIEKPTYATK_.3	#N/A	4.4155	5.7147	5.8335	4.8490	5.5271	5.8079	5.3528	5.1123	6.1196	6.0270	0.9478	-0.0773	0.4348	0.3618
Actr3	_YSYVC[Carbamidomethyl (C)]PDLVK_.2	#N/A	6.8232	6.9327	3.9253	6.9645	6.9589	6.4020	6.7959	6.5277	6.8006	6.7124	0.9441	-0.0829	0.5586	0.2529
Actr3	_QYTGVNAISK_.2	#N/A	5.0015	5.4263	4.4920	6.1477	6.3698	5.3530	5.2362	5.1756	5.8105	5.9789	0.9846	-0.0223	0.8333	0.0792
Actr3	_DITYFIQQLLR_.2	#N/A	3.7889	5.0335	4.4757	5.8070	4.6154	5.3713	5.4045	5.1893	5.2748	5.1441	0.9230	-0.1156	0.3020	0.5200
Actr3	_LPAC[Carbamidomethyl (C)]VVDC[Carbamidomethyl (C)]GTGYTK_.2	#N/A	6.0077	6.7395	3.8860	6.3944	7.0652	4.2102	4.1033	6.7717	6.5285	5.8252	0.9845	-0.0225	0.9168	0.0377
Actr3	_HNPVFGVM[Oxidation (M)]S[Phospho (STY)]_.2	#N/A	11.3004	11.4417	12.0706	11.4050	10.7178	11.4531	11.8915	11.4140	11.5578	11.3769	0.9860	-0.0204	0.5166	0.2868
Actr3	_TLTGTVIDSGDGVTHVIPVAEGYVIGSC[Carbamidomethyl (C)]IK_.3	#N/A	2.9993	3.8045	4.0220	4.2278	3.7231	4.3247	4.4644	2.8278	2.1732	4.1558	1.1307	0.1772	0.3936	0.4049
Arfgap2	_VSNQS[Phospho (STY)]FTEIER_.2	Q99K28_S239_M1_Arfgap2	3.9772	6.5836	7.6343	3.2546	5.9002	6.4887	3.3969	7.0958	6.8652	6.3241	0.9526	-0.0701	0.8037	0.0949
Arfgap2	_AIS[Phospho (STY)]SDM[Oxidation (M)]FFGR_.2	#N/A	4.4245	4.7018	5.1487	5.2704	3.8545	5.3846	5.6932	5.1157	5.0834	5.0501	0.9163	-0.1261	0.2127	0.6721
Arfgap2	_HGTDLWIDSM[Oxidation (M)]NSAPSHS[Phospho (STY)]PEKK_.4	#N/A	5.3126	3.7976	6.7789	6.9991	3.4927	3.4388	4.0023	6.8524	5.9070	3.4270	0.9847	-0.0222	0.9431	0.0254
Arfgap2	_[Acetyl (Protein N-term)]AAS[Phospho (STY)]PSKTEIQTIFK_.2	Q99K28_S4_M1_Arfgap2	3.2841	4.0893	3.9709	5.8793	3.9107	3.8188	4.1883	4.7985	3.2935	4.4406	0.9949	-0.0074	0.9682	0.0140
Arfgap2	_LAYQELQIDR_.2	#N/A	3.8487	3.1531	4.5355	3.5373	2.9744	3.2543	3.6151	3.0803	4.2297	3.5043	0.9842	-0.0229	0.8740	0.0585
Eps8l2	_YWGPAS[Phospho (STY)]PTHK_.2	Q99K30_S573_M1_Eps8l2	4.0468	3.3022	3.1099	3.6125	8.6501	8.7324	3.3272	8.5171	8.7624	8.9335	0.7099	-0.4944	0.2535	0.5960
Eps8l2	_RAEDSYTSQHTS[Phospho (STY)]PESEGAPHL_.3	Q99K30_S720_M1_Eps8l2	3.1224	4.3078	4.3570	3.9007	3.7670	4.3584	4.0490	3.1004	5.0950	4.9804	0.9217	-0.1177	0.4687	0.3291
Eps8l2	_RRES[Phospho (STY)]QDEEPR_.2	Q99K30_S242_M1_Eps8l2	3.4220	4.2272	4.1088	3.8935	4.0485	3.6810	4.0418	3.4665	3.1556	3.0776	1.1343	0.1818	0.0808	1.0928
Usp11	_QSQTSSSDTPAS[Phospho (STY)]PVSSSTPNSDIM[Oxidation (M)]DIN_.3	#N/A	3.6925	3.6179	3.4995	4.3932	3.4392	5.1273	3.5785	3.9409	3.9369	3.9691	1.0273	0.0388	0.7701	0.1135
Nono	_AVVIVDDR_.2	#N/A	7.3582	7.1530	7.6708	7.2662	7.3452	7.2465	6.1521	6.7993	7.3313	7.7938	1.0457	0.0645	0.3065	0.5136
Nono	_AGEVFIHK_.2	#N/A	4.5988	3.8440	5.3642	4.3830	3.9567	4.0016	4.9262	4.4868	4.2198	4.2822	0.9731	-0.0394	0.7133	0.1467
Nono	_VELDNMPLR_.2	#N/A	4.0897	3.3452	3.8234	5.8426	4.4452	3.3912	3.2843	3.0480	4.1974	3.5404	1.1816	0.2407	0.2471	0.6071
Nono	_FAC[Carbamidomethyl (C)]HSASLTVR_.3	#N/A	3.9547	3.3877	3.6884	3.2322	1.9817	3.5261	3.3804	3.7690	4.0680	4.4103	0.8434	-0.2457	0.1576	0.8025
Rragc	_SC[Carbamidomethyl (C)]SHQTS[Phospho (STY)]APSLK_.2	Q99K70_S381_M1_Rragc	5.6321	3.3785	3.2601	5.0914	4.6845	3.0288	5.9700	4.3557	5.3873	5.4078	0.7915	-0.3374	0.1134	0.9455
Rragc	_[Acetyl (Protein N-term)]SLQYGAEETPLAGS[Phospho (STY)]YGAADSFPK_.3	Q99K70_S15_M1_Rragc	3.4404	5.4255	3.1741	4.2187	3.7233	4.0061	4.3669	4.2833	3.0816	3.8960	1.0233	0.0332	0.8502	0.0705
Rragc	_MSPNETLFLEST[Phospho (STY)]NK_.2	Q99K70_T95_M1_Rragc	3.8473	3.8115	4.0882	4.0085	3.6329	4.1384	3.9677	4.3083	3.5605	3.8301	1.0011	0.0016	0.9780	0.0097
Med24	_LLS[Phospho (STY)]SSDDDANILSSPTDR_.2	Q99K74_S860_M1_Med24	3.6678	3.5876	3.7574	7.0044	4.5295	3.5505	3.7511	6.5740	7.0458	6.2209	0.7375	-0.4393	0.1242	0.9059
Psat1	_ELLAVPNNYK_.2	#N/A	8.2431	8.3168	8.1782	8.3025	7.7927	8.0135	8.5741	8.2295	7.9650	8.2491	0.9863	-0.0199	0.4513	0.3455
Psat1	_ASLYNAVTTEDVEK_.2	#N/A	6.7387	6.8013	7.1176	7.0930	6.6698	6.8414	6.9663	6.8931	6.2032	7.0483	1.0146	0.0210	0.5976	0.2236
Psat1	_QVVNFGPGPAK_.2	#N/A	5.8810	6.3117	6.1872	6.1942	6.5035	5.6200	5.8683	6.2526	5.8203	6.0459	1.0199	0.0285	0.5236	0.2810
Psat1	_EC[Carbamidomethyl (C)]PSVLDYK_.2	#N/A	6.1788	5.8278	3.8746	3.4184	6.6916	5.3256	6.4639	6.2640	6.6191	3.0957	0.9303	-0.1043	0.6887	0.1620
Psat1	_DDLLGFSLR_.2	#N/A	5.3066	5.1435	3.9658	3.5096	4.5192	5.0186	3.1419	4.6272	5.1286	4.9016	1.0286	0.0407	0.8086	0.0923

Psat1	_LPHSVLLEIQK_.2	#N/A	3.8594	5.0216	4.5410	4.8934	4.5429	4.3175	4.4124	4.3368	3.6841	4.9601	1.0416	0.0588	0.5586	0.2529
Psat1	_SQM[Oxidation (M)]IYEIIDNSQGFYVC[Carbamidomethyl (C)]PVER_.3	#N/A	3.2268	3.9830	5.7168	5.7183	3.8713	4.2541	3.2815	2.9961	3.2952	5.3036	1.1997	0.2626	0.3021	0.5198
Psat1	_SADYVVTGAWSAK_.2	#N/A	3.8253	3.5171	3.5590	5.4068	3.3384	4.3911	3.2510	3.8984	4.1975	4.2809	1.0254	0.0362	0.8262	0.0829
Hagh	_ALLEVLGR_.2	#N/A	4.6670	6.2393	3.5652	5.1241	5.7921	5.3483	3.6382	5.2504	6.1420	5.6638	0.9902	-0.0143	0.9388	0.0274
Hagh	_HVEPGNAAIQEK_.2	#N/A	6.1221	5.4506	5.8173	5.7832	6.1901	6.3980	6.1643	4.9377	5.5105	5.5105	1.0776	0.1079	0.1422	0.8471
Hagh	_TVQQHAGETDPVTTM[Oxidation (M)]R_.2	#N/A	3.6526	3.6291	2.3394	4.4309	4.0292	3.9167	3.4821	4.2759	3.6956	3.7471	0.9648	-0.0517	0.7386	0.1316
Hagh	_VTHLSTLQVGSLSVK_.3	#N/A	2.5893	3.8927	3.4672	4.2292	3.2954	3.3425	4.3774	4.1154	2.6398	4.0429	0.9145	-0.1290	0.4638	0.3337
Rbm10	_GLVAAYSGES[Phospho (STY)]DS[Phospho (STY)]EEEQER_.2	Q99KG3_S736_M2_Rbm10	3.7175	8.4557	3.4512	9.7313	8.8009	8.8315	6.2380	8.7512	8.9649	9.3631	0.8602	-0.2173	0.4706	0.3274
Rbm10	_LASDDRPS[Phospho (STY)]PPR_.2	Q99KG3_S723_M1_Rbm10	9.6941	10.0431	10.6898	10.5308	9.5926	9.8529	9.9963	9.7554	10.0514	10.0538	1.0103	0.0148	0.6746	0.1709
Stk24	_[Acetyl (Protein N-term)]AHS[Phospho (STY)]PVQSGLPGMQNLK_.2	Q99KH8_S4_M1_Stk24	4.2764	3.0660	4.0101	9.1327	3.4202	3.3414	3.7022	3.4473	3.7463	3.2312	1.2858	0.3627	0.4151	0.3819
Aco2	_SQFTITPGSEQIR_.2	#N/A	10.5834	10.6134	10.8563	10.9080	10.7978	10.7085	10.7909	10.5813	10.5566	10.5284	1.0123	0.0176	0.1514	0.8198
Aco2	_NAVTQEFGPVPDTAR_.2	#N/A	10.7304	10.6356	10.9103	10.9011	10.6210	10.5757	10.8856	10.4947	10.6049	10.5394	1.0092	0.0132	0.3642	0.4386
Aco2	_VDVSPTSQR_.2	#N/A	9.2592	9.5685	9.6431	9.6797	9.6541	9.4482	9.4533	9.4719	9.3526	9.5185	1.0099	0.0141	0.3168	0.4992
Aco2	_SDFDPGQDTYQHPPK_.3	#N/A	9.3477	9.3377	9.1351	9.7163	9.4306	9.1946	9.3656	9.3025	9.1733	9.3486	1.0068	0.0097	0.5841	0.2335
Aco2	_NTIVTSYNR_.2	#N/A	10.0467	10.2095	10.3702	10.4210	10.5151	10.1746	10.1725	10.2695	10.3826	10.1959	1.0034	0.0048	0.7316	0.1357
Aco2	_FNPETDFLTGK_.2	#N/A	10.0855	10.2744	10.4391	10.2787	10.1411	10.1122	10.4019	10.2046	9.9341	10.1781	1.0041	0.0060	0.6910	0.1605
Aco2	_EHAALEPR_.2	#N/A	9.9489	9.8357	9.6833	9.9155	10.0967	9.8102	9.5549	9.7392	9.7321	9.8344	1.0171	0.0245	0.0861	1.0649
Aco2	_IVYGHLDDPANQEIER_.3	#N/A	8.9838	8.8475	8.9084	9.1491	8.7470	9.0089	8.8862	8.6901	8.5233	8.7628	1.0258	0.0368	0.0418	1.3788
Aco2	_DFAPGKPLK_.2	#N/A	9.6704	10.0001	9.3054	9.8197	10.1672	9.3549	9.7691	9.7375	9.8445	9.5892	0.9984	-0.0023	0.9343	0.0295
Aco2	_FKLEAPDADELPR_.2	#N/A	8.3714	8.2554	8.2913	8.4724	8.4203	8.3642	8.4723	8.1601	8.2510	8.3424	1.0067	0.0097	0.4251	0.3715
Aco2	_LEAPDADELPR_.2	#N/A	9.2532	8.5191	8.4961	8.8132	9.0447	8.7463	9.0287	8.7177	8.7068	9.1283	0.9906	-0.0136	0.6441	0.1911
Aco2	_LTGSLSGWTSPK_.2	#N/A	8.2789	8.5144	8.8053	9.0684	8.9105	8.7074	8.5880	8.7232	8.5538	8.7579	1.0068	0.0097	0.7069	0.1506
Aco2	_EGWPLDIR_.2	#N/A	8.4246	8.5125	7.7178	8.8205	8.0304	8.0011	8.5946	8.4563	8.1033	8.6817	0.9754	-0.0359	0.3919	0.4068
Aco2	_IHETNLK_.2	#N/A	7.9962	7.5489	7.3558	7.5599	7.9536	7.6465	7.0165	7.8604	7.5566	7.7958	1.0158	0.0226	0.5633	0.2493
Aco2	_DVGGIVLANAC[Carbamidomethyl (C)]GPC[Carbamidomethyl (C)]IGQWDR_.2	#N/A	7.4526	7.5922	6.9482	7.6999	7.6550	7.1755	7.5968	7.2510	7.4217	6.6703	1.0257	0.0365	0.4239	0.3728
Aco2	_QGLLPLTFADPSDYNK_.2	#N/A	7.3814	7.4279	7.5140	7.5266	6.9230	7.1022	7.6947	7.2550	7.4772	7.4968	0.9775	-0.0328	0.2755	0.5598
Aco2	_DGYAQILR_.2	#N/A	8.0151	7.2679	7.6508	7.0005	7.3427	7.6627	5.7255	7.6933	7.8524	8.0622	1.0214	0.0305	0.7451	0.1278
Aco2	_QALAHGLK_.2	#N/A	6.5144	6.2344	6.5006	6.6647	7.1469	6.4505	5.8744	6.7000	6.8376	6.8462	1.0032	0.0045	0.9339	0.0297
Aco2	_DVGGIVLANAC[Carbamidomethyl (C)]GPC[Carbamidomethyl (C)]IGQWDRK_.3	#N/A	5.6377	6.0360	6.6767	6.6617	6.6544	5.9697	5.7935	6.2456	6.1353	6.0839	1.0343	0.0487	0.4145	0.3825
Aco2	_TDIANLAEEFK_.2	#N/A	6.4609	5.5455	4.6814	6.3699	5.8053	6.1270	5.6901	5.7785	5.5242	6.1436	1.0082	0.0118	0.8960	0.0477
Aco2	_NDANPETHAFVTSPEIVTALAIAGTLK_.3	#N/A	6.3033	5.7390	5.7891	6.5547	5.1318	5.7157	5.6444	5.7585	5.7165	5.8349	1.0233	0.0332	0.6169	0.2098
Aco2	_WVVIGDENYGEGSSR_.2	#N/A	8.4496	8.5941	9.2285	9.5443	8.4440	8.8446	8.9563	8.8422	8.7589	8.7383	1.0030	0.0044	0.9110	0.0405
Aco2	_VAGILTVK_.2	#N/A	5.5717	4.7362	6.1568	5.2735	5.6744	6.0085	3.5551	6.0228	6.3518	6.0533	1.0135	0.0194	0.9008	0.0454
Aco2	_AKDINQEVYNFLATAGAK_.3	#N/A	6.9453	7.0326	7.4227	7.2738	6.6539	7.1106	7.0061	7.1818	7.0198	7.3376	0.9911	-0.0128	0.6843	0.1647
Aco2	_GHLDNISNNLLIGAINIENGK_.3	#N/A	6.9463	6.4031	6.9048	7.4659	5.9924	6.4349	6.2900	6.5118	6.6468	6.9942	1.0122	0.0175	0.7879	0.1035
Aco2	_C[Carbamidomethyl (C)]TTDHISAAGPWLK_.2	#N/A	5.1049	5.6179	4.3460	6.0634	6.1470	5.9270	6.2057	5.8267	5.6816	5.9727	0.9346	-0.0976	0.3200	0.4948
Aco2	_VAM[Oxidation (M)]SHFEPSEYIR_.3	#N/A	7.5128	7.7984	7.9327	7.7882	8.1212	7.5943	7.3701	7.8601	7.8381	7.6289	1.0152	0.0218	0.4419	0.3547
Aco2	_VGLIGSC[Carbamidomethyl (C)]TNSSYEDM[Oxidation (M)]GR_.2	#N/A	5.2592	5.2596	3.4401	5.6383	5.1149	4.9861	3.7290	5.3677	5.1363	5.0665	1.0259	0.0368	0.8057	0.0938
Aco2	_VAM[Oxidation (M)]QDATAQM[Oxidation (M)]AM[Oxidation (M)]LQFISSGLPK_.3	#N/A	4.8913	4.7122	5.6597	4.4877	4.9189	4.8487	5.5428	5.3068	5.0105	4.8983	0.9480	-0.0770	0.2794	0.5538
Aco2	_SDFDPGQDTYQHPPKDSSGQR_.4	#N/A	3.3794	6.1245	4.0662	5.3746	5.8567	4.5418	4.0844	5.5803	5.6672	5.4945	0.9684	-0.0463	0.7995	0.0972
Aco2	_DINQEVYNFLATAGAK_.2	#N/A	6.7643	6.5776	6.8642	6.8166	6.6716	6.9644	6.6932	6.2924	6.6575	6.7545	1.0268	0.0382	0.1412	0.8501
Aco2	_VAVPSTIHC[Carbamidomethyl (C)]DHLIEAQVGGEK_.3	#N/A	7.0933	6.7370	7.0839	6.4827	7.0374	6.5647	6.8907	6.2572	6.7486	6.7766	1.0247	0.0352	0.3831	0.4167
Aco2	_VAVPSTIHC[Carbamidomethyl (C)]DHLIEAQVGGEKDLR_.4	#N/A	3.6840	2.9884	4.3708	3.3726	2.8097	3.4189	3.0873	7.3742	4.3944	3.3396	0.7564	-0.4028	0.2172	0.6632
Aco2	_HPNGTQETILLNHTFNETQIEWFR_.4	#N/A	2.9454	4.3970	4.1799	3.7237	4.2183	3.5112	3.8720	3.2775	3.5765	3.4010	1.0842	0.1167	0.3453	0.4618
Ubxn2a	_AKS[Phospho (STY)]VEVDNK_.2	Q99KJ0_S154_M1_Ubxn2a	5.9381	6.8521	6.4054	6.8698	5.4309	6.9455	6.7556	6.7069	6.0482	6.3064	0.9927	-0.0106	0.8927	0.0493
Ubxn2a	_C[Carbamidomethyl (C)]LS[Phospho (STY)]PTEQKK_.2	Q99KJ0_S54_M1_Ubxn2a	3.6043	3.7381	3.3379	4.3826	4.2488	4.1700	4.5308	4.4325	4.4185	4.0598	0.8975	-0.1560	0.0839	1.0764
Dctn2	_YADLPGIAR_.2	#N/A	7.4222	7.9130	7.7741	7.0319	7.8649	7.5405	7.3830	7.4957	7.6596	7.7672	1.0019	0.0028	0.9379	0.0278
Dctn2	_WSPVASTLPELVQR_.2	#N/A	6.7218	7.1420	6.9075	7.4278	7.4573	7.3852	7.4819	7.0571	7.1623	7.2216	0.9921	-0.0114	0.7475	0.1264
Dctn2	_LLGPDAAINLADPDGALAK_.2	#N/A	5.9256	6.3150	6.5225	6.7689	6.6271	6.5196	6.3473	6.5361	6.3416	6.5973	0.9986	-0.0020	0.9556	0.0197
Dctn2	_QQLVASHLEK_.2	#N/A	5.8769	6.4279	6.2250	6.1836	6.8064	6.3787	5.9811	6.1604	6.0329	6.0216	1.0442	0.0624	0.1339	0.8732
Dctn2	_ASVEDADTQNK_.2	#N/A	6.1070	5.7612	5.4032	6.1738	6.4759	5.4761	5.9973	5.4843	6.2450	6.2743	0.9832	-0.0244	0.7085	0.1496
Dctn2	_VSALDLAVLDQVEAR_.2	#N/A	5.0232	4.7337	5.2965	3.7701	5.5733	5.0241	4.7020	4.2803	5.4883	5.2520	0.9945	-0.0080	0.9454	0.0244
Dctn2	_LLHEVQELTTEVEK_.3	#N/A	6.1252	5.2884	3.8069	6.1267	6.2088	6.0005	4.5361	5.6545	5.7583	6.6687	0.9891	-0.0158	0.9194	0.0365
Dctn2	_ENLATVEGNFASIDAR_.2	#N/A	6.0390	5.9259	6.4231	6.2905	6.0382	5.7572	6.2801	5.4935	5.7889	6.0203	1.0311	0.0442	0.3412	0.4670
Dctn2	_LTELEATVR_.2	#N/A	4.0694	3.3721	3.8352	4.1714	4.4358	4.8953	3.3960	5.3032	5.1163	5.5195	0.8544	-0.2271	0.1700	0.7696
Dctn2	_DNTALLTQVQTTM[Oxidation (M)]R_.2	#N/A	5.6804	6.3540	6.3650	5.6209	5.9324	6.1404	6.4149	5.7069	6.0080	6.3095	0.9846	-0.0224	0.6628	0.1786
Dctn2	_VHQLYETIQR_.2	#N/A	4.8109	4.1487	3.4425	4.8783	5.0335	4.6475	4.9727	4.8332	4.2409	4.7994	0.9537	-0.0684	0.5282	0.2772
Dctn2	_GLDFSDR_.2	#N/A	3.4929	3.3370	4.4964	4.1591	4.1574	4.2575	3.2924	3.0399	4.7913	3.8402	1.0648	0.0906	0.5477	0.2615
Dctn2	_TGYES[Phospho (STY)]GDYEM[Oxidation (M)]LGEGLGVK_.2	Q99KJ8_S83_M1_Dctn2	3.6709	2.9264	3.4046	5.2604	2.8147	3.8099	3.0967	4.0528	4.3518	4.1265	0.9337	-0.0990	0.6192	0.2082
Hars2	_DLS[Phospho (STY)]PQQMVVR_.2	Q99KK9_S66_M1_Hars2	8.9779	7.1216	4.3603	10.1939	7.4630	3.1836	8.3685	6.7604	5.6158	7.8045	0.9644	-0.0523	0.8651	0.0629
Clint1	_TIDLGAAAHYTGDKAS[Phospho (STY)]PDQNASTHTPQSSAK_.4	Q99KN9_S307_M1_Clint1	4.0041	3.5995	4.2133	4.1817	3.4557	3.8555	3.5413	4.2256	3.9691	4.0256	0.9859	-0.0204	0.7827	0.1064
Cryl1	_[Acetyl (Protein N-term)]ASPAAGGVVIVGSGLIGR_.2	#N/A	3.0226	3.6101	3.1559	3.4564	3.2859	3.0878	3.9205	1.5663	4.0019	4.1790	0.9569	-0.0635	0.7773	0.1094
Nampt	_SYS[Phospho (STY)]FDEVRK_.2	Q99KQ4_S472_M1_Nampt	7.0200	7.5528	7.8284	7.9184	3.5553	7.4434	7.9795	7.5624	7.7431	7.5950	0.8920	-0.1648	0.3564	0.4481
Rnf34	_GLGSGDDLDSSS[Phospho (STY)]LNSSR_.2	Q99KR6_S163_M1_Rnf34	3.9106	3.1660	3.6443	3.7487	3.8888	4.6784	5.3629	5.2018	4.5303	4.4643	0.7852	-0.3489	0.0095	2.0226
Rnf34	_AS[Phospho (STY)]LSDLSSLEEVEGMSVR_.2	Q99KR6_S258_M1_Rnf34	3.3912	3.9512	3.1249	4.1695	4.4816	4.0896	4.3178	4.3325	3.1307	3.8468	0.9900	-0.0144	0.9124	0.0398
Vmp1	_DQHNGS[Phospho (STY)]LTDPSSVHEK_.3	Q99KU0_S23_M1_Vmp1	5.7247	5.3881	5.6750	5.0001	5.1443	4.7668	4.8144	5.8817	3.6143	3.5055	1.1862	0.2463	0.1250	0.9030
Mlf2	_LAIQGPEDS[Phospho (STY)]PSR_.2	Q99KX1_S237_M1_Mlf2	12.8082	13.0776	13.7782	13.3905	13.0174	14.0935	13.3837	12.6342	13.1222	13.2428	1.0202	0.0289	0.3756	0.4253
Mlf2	_DSDS[Phospho (STY)]GLEQMSIGHHIR_.3	Q99KX1_S145_M1_Mlf2	3.6657	3.6420	3.7809	4.4937	3.4544	3.8655	4.2023	3.8360	4.0087	3.9075	0.9570	-0.0634	0.4022	0.3955
Gak	_EPETGLDNTS[Phospho (STY)]PK_.2	Q99KY4_S809_M1_Gak	3.1068	3.9120	3.7936	6.0498	5.5499	3.9961	4.3569	4.2934	3.0916	6.8918	0.9448	-0.0819	0.7723	0.1122
Znf639	_YFDNKDDDS[Phospho (STY)]DPETANDLPK_.3	Q99KZ6_S60_M1_Znf639	3.3528	3.9896	4.5873	5.5173	3.8109	3.9186	4.2794	6.0822	3.1691	6.2417	0.8489	-0.2364	0.3180	0.4975
Pagr1a	_DLFSLDSEGPSPTS[Phospho (STY)]PPLR_.2	Q99L02_S236_M1_Pagr1a	7.7150	7.9264	3.5981	8.4557	7.8844	7.6616	8.0621	7.5893	7.4048	7.5358	0.9423	-0.0857	0.6446	0.1907
Dhrs1	_VLPSC[Carbamidomethyl (C)]DLAR_.2	#N/A	4.1931	3.0331	3.3790	4.2207	5.6614	5.0175	3.5964	4.2368	3.8854	5.2298	1.0032	0.0047	0.9816	0.0081
Dhrs1	_ATAQEAQSLGGR_.2	#N/A	5.5698	3.2373	3.8387	3.3825	5.4436	3.1700	3.5308	3.6187	3.9177	5.8159	0.9730	-0.0394	0.8764	0.0573
Hibadh	_DLGLAQDSATSTK_.2	#N/A	6.0178	5.7319	6.3626	5.8312	6.2645	5.9850	6.5117	6.2120	6.1793	6.1184	0.9643	-0.0524	0.1552	0.8092
Hibadh	_TPILLGSLAHQIYR_.2	#N/A	4.4194	3.2999	4.0955	3.8940	5.3233	4.7338	3.9362	4.6627	3.7879	3.9868	1.0491	0.0691	0.6198	0.2077
Gmpr2	_VTQQVNPIFSNS[Phospho (STY)]Q_.2	Q99L27_S347_M1_Gmpr2	5.5279	5.5415	3.5370	5.8035	4.8950	5.5525	5.8596	3.9492	5.4918	5.7373	0.9778	-0.0323	0.8388	0.0763
Cds2	_AETAPLPTSVDDT[Phospho (STY)]PEVLNR_.2	Q99L43_T50_M1_Cds2	4.0822	3.3377	3.8159	7.9753	3.2260	3.3986	6.0252	7.7543	7.0311	3.0370	0.7222	-0.4694	0.2190	0.6595
Cds2	_LDGETAS[Phospho (STY)]DSESR_.2	#N/A	9.8893	9.1983	9.3674	9.4785	8.6177	9.2038	9.9662	9.6115	9.6664	9.4791	0.9599	-0.0591	0.1266	0.8977
Cds2	_ESESEAKLDGETAS[Phospho (STY)]DSESR_.2	#N/A	4.0614	3.3470	3.2474	3.4445	3.1871	4.1441	3.3554	4.3168	4.1100	3.7819	0.9180	-0.1235	0.2736	0.5628

Cds2	_EDAPPEDKES[Phospho (STY)]ESEAK_.2	Q99L43_S20_M1_Cds2	3.9744	3.2787	3.1603	3.6630	3.1000	3.1286	3.4894	4.4555	4.1041	3.6300	0.8634	-0.2120	0.0667	1.1762
Cds2	_ALS[Phospho (STY)]NLSSR_.2	Q99L43_S59_M1_Cds2	6.4903	5.7751	4.1114	3.6552	3.5215	6.1110	3.8035	6.1953	3.6450	3.3325	1.1649	0.2203	0.4363	0.3602
Cds2	_EDAPPEDKESESEAKLDGETAS[Phospho (STY)]DSESR_.4	#N/A	5.6530	3.9441	4.2486	3.2504	3.5700	3.5500	3.6225	5.9538	3.9072	3.2174	0.9667	-0.0489	0.8366	0.0775
Eif2s2	_DYTYEELLNR_.2	#N/A	5.2742	5.3302	5.6622	5.2767	5.0661	5.7288	5.3112	5.4431	5.5099	5.6030	0.9859	-0.0205	0.5932	0.2268
Eif2s2	_[Acetyl (Protein N-term)]S[Phospho (STY)]GDEM[Oxidation (M)]IFDPTM[Oxidation (M)]SKK_.2	#N/A	9.1138	9.2760	10.2759	9.7187	9.1487	9.5420	9.6378	9.3616	9.4858	9.4305	1.0035	0.0051	0.8875	0.0518
Eif2s2	_[Acetyl (Protein N-term)]S[Phospho (STY)]GDEM[Oxidation (M)]IFDPTM[Oxidation (M)]SK_.2	#N/A	13.4816	13.7975	14.5640	13.5273	13.4361	14.1901	14.3015	13.7249	13.9289	13.9174	0.9903	-0.0141	0.6046	0.2185
Eif2s2	_KKDAS[Phospho (STY)]DDLDDLNFFNQK_.3	Q99L45_S67_M1_Eif2s2	3.2214	4.0266	3.9083	4.4109	3.8480	3.8815	4.2423	3.7076	3.3562	4.3779	0.9903	-0.0141	0.8916	0.0498
Eif2s2	_IFDIDEAEEAIKDVK_.3	#N/A	3.7259	1.1705	3.4596	3.3320	2.8697	3.7550	3.6481	3.9978	3.8336	4.1815	0.7795	-0.3593	0.1266	0.8975
Eif2s2	_EYVTC[Carbamidomethyl (C)]HTC[Carbamidomethyl (C)]R_.2	#N/A	4.0780	3.2644	3.8117	3.3554	3.2217	3.1429	3.5037	3.6457	3.9448	3.0327	0.9851	-0.0217	0.8344	0.0786
Eif2s2	_IES[Phospho (STY)]DAQEPAEPEDDLDIMLGNK_.3	Q99L45_S105_M1_Eif2s2	3.3327	4.0890	4.5673	4.1110	3.9773	4.1481	4.0413	4.3910	3.1892	3.7883	1.0481	0.0677	0.5356	0.2711
St13	_AIEINPDSAQPYK_.2	#N/A	7.5765	7.7741	8.1667	7.4439	7.9307	7.4887	7.7885	7.5236	7.5417	7.6524	1.0136	0.0195	0.5138	0.2892
St13	_GAAIEALNDGELQK_.2	#N/A	5.4936	6.0334	5.6101	6.1904	5.6379	5.1777	5.2830	5.7896	5.0505	5.8538	1.0357	0.0506	0.4431	0.3535
St13	_AIDLFTDAIK_.2	#N/A	4.5844	5.9500	5.6282	6.0141	6.5741	6.5174	5.3842	5.9028	6.3585	6.2052	0.9858	-0.0206	0.8411	0.0752
St13	_KGAAIEALNDGELQK_.3	#N/A	4.7333	4.0773	3.4306	5.2604	4.7118	3.7840	3.1226	4.5021	4.1731	4.9450	1.0352	0.0499	0.7598	0.1193
St13	_QDPSVLHTEEMR_.3	#N/A	4.2807	2.9454	3.4667	4.7698	2.8337	3.7909	3.6840	4.1491	2.5352	3.6764	1.0484	0.0682	0.7281	0.1378
Sntb1	_GSPVSEIGWETPPPES[Phospho (STY)]PR_.2	#N/A	7.0953	6.7066	7.5089	8.5612	6.4603	7.2600	7.3849	7.6194	3.7811	7.2349	1.1169	0.1595	0.3772	0.4235
Sntb1	_KGSPVSEIGWETPPPES[Phospho (STY)]PR_.3	#N/A	3.8298	3.5361	3.2780	5.4125	3.8077	4.6595	4.0981	5.5142	4.9278	5.4752	0.8168	-0.2919	0.0948	1.0232
Mcrs1	_GDQVLNFS[Phospho (STY)]DAEDLIDDSK_.2	#N/A	3.0095	4.2167	3.6963	4.1990	3.6360	4.0935	3.9136	3.9195	3.5681	4.1660	0.9786	-0.0312	0.7535	0.1229
Mcrs1	_GDQVLNFS[Phospho (STY)]DAEDLIDDSKLK_.3	#N/A	3.2491	4.0053	4.4836	4.0273	3.8936	4.2318	4.1756	2.9738	3.2729	3.7046	1.1274	0.1730	0.1671	0.7770
Dnttip1	_QAEEESHRGS[Phospho (STY)]PIPK_.2	Q99LB0_S160_M1_Dnttip1	3.9182	3.2226	3.9276	4.7862	2.6536	4.3678	1.8205	3.9305	3.6979	3.1431	1.2111	0.2764	0.2553	0.5930
Mat2b	_VLITGATGLLGR_.2	#N/A	6.1454	5.1862	6.0398	5.7814	5.9276	5.9716	6.2084	6.2151	5.8575	5.6493	0.9765	-0.0343	0.5158	0.2875
Mat2b	_AVLENNLGAAVLR_.2	#N/A	4.0683	4.2133	5.0261	4.2553	5.3926	4.1852	4.3836	3.3602	4.6224	3.1808	1.1638	0.2189	0.1495	0.8254
Ndufa10	_LQSWLYASR_.2	#N/A	6.7248	7.1286	7.1509	7.8981	7.2334	7.4276	7.1383	7.4993	7.1970	7.3674	0.9945	-0.0079	0.8527	0.0692
Ndufa10	_QDDWTFHYLR_.2	#N/A	5.5593	5.9289	6.1408	5.8331	5.7307	6.2503	6.4299	5.9401	6.1617	6.0868	0.9598	-0.0592	0.1474	0.8316
Ndufa10	_VITVDGNIC[Carbamidomethyl (C)]SGK_.2	#N/A	6.0097	5.8299	5.4842	6.1777	6.3596	6.0094	6.2737	6.2191	6.2184	5.9592	0.9693	-0.0449	0.2806	0.5520
Ndufa10	_VTSAYLQDIENAYKK_.3	#N/A	5.2045	5.4539	3.7979	4.3044	5.3503	5.1306	3.4899	5.3634	5.0861	5.2232	1.0173	0.0248	0.8684	0.0613
Ndufa10	_LLQYADALEHLLSTGQGVVLER_.3	#N/A	4.1290	4.3334	3.3150	3.8177	3.3832	2.9739	3.3347	4.3008	3.9494	3.7847	0.9132	-0.1311	0.2369	0.6254
Ndufa10	_VTSAYLQDIENAYK_.2	#N/A	3.9155	3.2199	4.6023	3.6041	5.7303	3.1874	3.3188	3.0135	4.1629	3.5711	1.1498	0.2013	0.3536	0.4515
Ndufa10	_TEVLNYTTIPVYLPEITIGAHQGSR_.3	#N/A	3.2299	4.0351	3.9167	4.0856	3.8564	3.8731	4.2339	3.6992	3.2146	4.3863	0.9869	-0.0190	0.8523	0.0694
Ndufa10	_YAPGYNAEVGDK_.2	#N/A	3.0022	3.7584	4.2367	3.7804	3.6467	2.9779	3.9287	3.2207	7.6790	3.4577	0.7803	-0.3580	0.2786	0.5551
Etfa	_LNVAPVSDIIEIK_.2	#N/A	6.3753	6.6615	6.2940	6.9081	6.8057	6.8020	5.8562	6.4710	6.1888	6.8559	1.0470	0.0663	0.1957	0.7085
Etfa	_GLLPEELTPLILETQK_.2	#N/A	5.3223	5.6136	5.1959	5.8814	6.0452	5.4882	6.0878	6.3034	5.9906	6.1630	0.9112	-0.1342	0.0140	1.8536
Etfa	_TIYAGNALC[Carbamidomethyl (C)]TVK_.2	#N/A	4.9476	5.5346	4.7920	4.0656	5.6078	5.4471	5.0054	5.2890	5.4309	5.4025	0.9591	-0.0603	0.5071	0.2949
Etfa	_VLVAQHDAYK_.2	#N/A	4.8282	5.5326	4.9914	3.1568	6.1109	5.3447	4.8194	4.5715	4.1435	4.8170	1.0886	0.1224	0.4643	0.3332
Etfa	_APSSSSVGISEWLDQK_.2	#N/A	3.2165	4.7275	4.9420	4.6952	5.3080	5.0397	3.8776	3.0064	4.5380	4.9680	1.1360	0.1840	0.3031	0.5184
Etfa	_AAVDAGFVPNDM[Oxidation (M)]QVGQTGK_.2	#N/A	6.4208	6.8710	7.1335	6.9712	6.9121	7.0160	6.7895	6.5246	6.5839	6.9996	1.0242	0.0346	0.3141	0.5029
Etfa	_SPDTFVR_.2	#N/A	3.5211	5.4175	3.4494	4.6697	5.2578	4.8173	3.1415	4.1974	4.4689	4.1713	1.1320	0.1789	0.3142	0.5028
Etfa	_VVPEMTEILK_.2	#N/A	4.5110	4.0770	3.8653	5.8454	3.2754	3.3492	4.7905	3.5921	3.4260	3.0864	1.1155	0.1577	0.4689	0.3289
Etfa	_SDRPELTGAK_.2	#N/A	5.0622	5.2895	4.2609	5.1511	4.2006	3.5289	4.3360	4.0769	3.0035	5.2970	1.0966	0.1331	0.4550	0.3420
Etfa	_GTSFEAAATSGGSASSEK_.2	#N/A	3.0271	3.8323	3.7139	6.3367	3.6536	5.1896	4.4367	4.2136	3.0118	3.9657	1.0986	0.1357	0.5832	0.2342
Etfa	_LLYDLADQLHAAVGASR_.2	#N/A	4.3476	3.6520	3.5336	4.0362	3.4733	4.2562	3.7509	4.0823	3.7308	4.3433	0.9764	-0.0344	0.6891	0.1617
Gps1	_SPPREGS[Phospho (STY)]QGELTPANSQSR_.3	#N/A	9.6221	9.6025	10.4519	9.6868	8.8137	9.4377	9.4610	9.8367	9.6769	9.6047	0.9956	-0.0064	0.8813	0.0549
Gps1	_EGS[Phospho (STY)]QGELTPANSQSR_.2	#N/A	11.7168	11.2912	11.4535	11.9418	10.6766	11.4107	11.9113	11.5418	11.5488	11.5611	0.9806	-0.0282	0.3581	0.4459
Gps1	_LFLELEPQVR_.2	#N/A	4.9947	5.2612	4.5909	4.6984	4.8888	5.1471	4.9978	5.3691	5.0538	4.7788	0.9763	-0.0346	0.4835	0.3156
Gps1	_GERDS[Phospho (STY)]QTQAILTK_.2	Q99LD4_S220_M1_Gps1	3.4666	4.2228	3.2002	4.2449	4.1111	4.0143	4.3931	4.2572	3.0554	5.0159	0.9273	-0.1088	0.4622	0.3352
Rtcb	_NLDFQDVLDK_.2	#N/A	5.2492	3.9148	4.5059	3.9559	4.9932	4.8009	4.8019	4.4157	3.6412	4.6534	1.0438	0.0619	0.5947	0.2257
Rtcb	_MLQADPNKVS[Phospho (STY)]PR_.2	Q99LF4_S208_M1_Rtcb	1.4981	3.9338	4.4121	4.4818	3.8221	4.3033	4.1965	3.6367	3.2853	4.4488	0.9615	-0.0567	0.8131	0.0899
Tmem51	_AETETS[Phospho (STY)]PGHAPDR_.2	Q99LG1_S178_M1_Tmem51	4.0003	3.3047	3.1863	3.8602	3.1260	4.0150	3.4634	4.4295	4.0781	3.6559	0.9169	-0.1252	0.2703	0.5681
Ptdss1	_TYSEC[Carbamidomethyl (C)]EDGTYS[Phospho (STY)]PEISWHHGK_.3	Q99LH2_S425_M1_Ptdss1	3.7410	3.6014	3.4747	4.5193	4.3095	4.3068	3.1668	3.9827	4.2817	4.1966	1.0218	0.0311	0.7843	0.1055
Sh3bp5l	_TVAS[Phospho (STY)]DLQK_.2	Q99LH9_S342_M1_Sh3bp5l	8.4968	4.2443	8.7987	8.9799	4.1326	8.2905	8.3000	3.9472	8.0857	8.5080	0.9926	-0.0107	0.9719	0.0124
Sh3bp5l	_RGS[Phospho (STY)]DIGVR_.2	Q99LH9_S377_M1_Sh3bp5l	8.0055	7.8882	8.3727	8.3776	7.3756	8.1357	8.3926	8.1597	8.0771	8.1586	0.9791	-0.0304	0.4129	0.3841
Sh3bp5l	_GLSDHAS[Phospho (STY)]LDGQELGAQSR_.2	#N/A	5.6513	4.6102	6.3447	6.4486	5.5813	3.6899	5.6649	5.7780	4.4251	6.4408	0.9660	-0.0499	0.7731	0.1117
Sh3bp5l	_TVAS[Phospho (STY)]DLQKC[Carbamidomethyl (C)]DS[Phospho (STY)]VEHLR_.3	#N/A	3.8102	3.0907	4.1060	6.0943	2.9447	3.4979	3.6091	5.7488	5.3985	5.9947	0.7564	-0.4028	0.1195	0.9227
Clcc1	_FHSGNKS[Phospho (STY)]PEVLR_.3	Q99LI2_S433_M1_Clcc1	3.2230	4.1194	4.4575	6.5172	3.9407	6.7825	6.6856	6.6106	6.8440	6.6214	0.7234	-0.4671	0.0383	1.4172
Clcc1	_KEVSPDS[Phospho (STY)]SEAEELSDC[Carbamidomethyl (C)]LHR_.3	#N/A	3.2433	4.0485	4.5284	4.0722	5.6185	3.8596	4.2204	4.4299	3.2281	3.7494	1.0823	0.1141	0.5034	0.2981
Clcc1	_EVSPDS[Phospho (STY)]SEAEELSDC[Carbamidomethyl (C)]LHR_.3	#N/A	3.9111	3.7466	3.6447	3.9165	3.7880	3.6843	3.8140	3.6993	4.7794	4.6057	0.8952	-0.1597	0.0833	1.0792
Cstf3	_RPNEDS[Phospho (STY)]DEDEEKGAVVPPVHDIYR_.4	#N/A	3.6383	4.0466	3.9022	9.5073	8.2821	8.1563	3.5685	9.1010	9.1319	8.9892	0.8126	-0.2993	0.4319	0.3646
Hgs	_ALQNAVSTFVNR_.2	#N/A	5.5820	5.7689	3.1296	6.5902	6.3004	5.9613	5.7823	6.3648	6.1859	6.2435	0.9042	-0.1453	0.3859	0.4135
Hgs	_ASSTTELPPEYLTSPLSQQS[Phospho (STY)]QLPPK_.3	Q99LI8_S246_M1_Hgs	3.7475	3.0519	4.4344	3.4362	2.8732	3.3554	3.7162	3.1815	4.4309	3.4032	0.9522	-0.0707	0.6270	0.2027
Cttnbp2nl	_FQSQADQDQQASGLQS[Phospho (STY)]PPSR_.2	Q99LJ0_S481_M1_Cttnbp2nl	6.2488	6.2682	6.1239	7.1857	6.4334	5.0005	7.0581	4.3507	3.1490	5.5303	1.2366	0.3063	0.1525	0.8167
Cttnbp2nl	_DLS[Phospho (STY)]PTLLDNSAAK_.2	Q99LJ0_S488_M1_Cttnbp2nl	3.2894	8.3862	8.4392	9.5013	8.1406	8.6748	8.7406	8.8162	8.8288	8.8864	0.8776	-0.1884	0.3706	0.4311
Pwp1	_GLSVSGPC[Carbamidomethyl (C)]GSRS[Phospho (STY)]PQQTPMES_.2	Q99LL5_S493_M1_Pwp1	6.0773	4.3781	4.2597	8.1606	4.1994	3.5301	3.8909	3.3561	3.0047	3.2285	1.5136	0.5980	0.0881	1.0552
Pwp1	_GKLQEEGGS[Phospho (STY)]EEEEAGNPSEDGM[Oxidation (M)]QSGPTQAPPR_.3	Q99LL5_S49_M1_Pwp1	3.5015	3.8409	3.2351	4.2798	3.6623	4.0672	4.4280	4.2223	3.0205	3.9570	0.9635	-0.0536	0.6611	0.1797
Grpel1	_DLLEVADILEK_.2	#N/A	6.0636	5.9779	5.2414	5.7601	6.0273	5.8006	5.6373	5.1606	5.6828	5.7605	1.0452	0.0638	0.2201	0.6575
Park7	_VTVAGLAGKDPVQC[Carbamidomethyl (C)]SR_.3	#N/A	6.0721	6.5305	5.9078	6.3685	7.0597	6.2989	6.1560	6.1594	6.4572	6.1013	1.0248	0.0354	0.4928	0.3073
Park7	_VTTHPLAK_.2	#N/A	6.3708	7.2141	6.7914	6.8239	7.7952	6.8429	6.5121	7.4798	7.2133	7.2589	0.9799	-0.0293	0.6434	0.1915
Park7	_DVMIC[Carbamidomethyl (C)]PDTSLEDAK_.2	#N/A	6.7848	4.3343	3.6641	7.3642	5.9332	4.1256	5.9105	3.6522	4.7160	5.6389	1.0780	0.1083	0.6717	0.1729
Park7	_VEKDGLILTSR_.2	#N/A	6.3924	6.9578	7.3689	6.8813	7.0422	7.1945	7.0732	6.7029	6.6813	6.9196	1.0188	0.0269	0.5078	0.2943
Park7	_TQGPYDVVVLPGGNLGAQNLSESPM[Oxidation (M)]VK_.3	#N/A	6.0359	5.7183	6.4643	5.9277	6.3168	6.4006	6.1578	6.1265	6.0090	6.1777	1.0043	0.0062	0.8692	0.0609
Park7	_GAEEM[Oxidation (M)]ETVIPVDVMR_.2	#N/A	5.7536	5.7346	4.3028	7.7734	6.4467	3.3173	5.2302	4.4641	4.8153	5.5925	1.1053	0.1444	0.5394	0.2681
Ndufs5	_PFLDIQK_.2	#N/A	6.2802	6.5524	6.4605	6.2240	7.0374	6.1886	6.3118	6.2644	6.4477	6.8390	0.9987	-0.0019	0.9657	0.0152
Ndufs5	_IEFDDFEEC[Carbamidomethyl (C)]LLR_.2	#N/A	6.2173	5.9838	6.1917	6.3021	6.7366	6.1337	5.8553	5.8079	5.9769	6.7515	1.0267	0.0381	0.4760	0.3224
Ndufs5	_EC[Carbamidomethyl (C)]KIEFDDFEEC[Carbamidomethyl (C)]LLR_.3	#N/A	4.7678	4.5458	5.5888	6.6845	5.3594	5.5657	4.7929	4.6676	5.0740	5.8742	1.0620	0.0868	0.4928	0.3074
Ndufs5	_HFM[Oxidation (M)]FLSAEQPYK_.2	#N/A	4.2793	6.5442	7.0090	3.9679	5.8192	6.6395	6.3698	4.1506	6.2372	6.4862	0.9826	-0.0253	0.9016	0.0450
Ndufs5	_EWIEC[Carbamidomethyl (C)]AHGIGGTR_.3	#N/A	5.1963	4.8754	4.6504	6.1190	6.3361	5.7940	5.2569	5.4028	5.6096	5.8096	0.9956	-0.0064	0.9479	0.0232
	RGS[Phospho (STY)]FEAGR.2	#N/A	6.2026	6.4235	6.6052	6.4941	4.7883	5.7719	6.8085	6.6540	6.8100	6.3698	0.9080	-0.1393	0.1256	0.9011
	RGS[Phospho (STY)]FEAGRR.2	#N/A	5.8685	6.6675	5.2458	6.2141	5.6517	6.0447	6.8125	6.7838	5.9845	6.2928	0.9197	-0.1208	0.1159	0.9359
Rnps1	_S[Phospho (STY)]PS[Phospho (STY)]PKPTK_.2	#N/A	8.3518	3.2840	3.7622	7.1850	3.1723	6.1583	7.5703	7.9650	6.0767	7.6800	0.7263	-0.4613	0.1277	0.8937
Rnps1	_RS[Phospho (STY)]PS[Phospho (STY)]PKPTK_.2	#N/A	3.3536	4.6049	4.0404	4.5431	3.9801	3.7494	4.2577	5.6298	3.2240	3.0092	1.0037	0.0054	0.9781	0.0096

Rnps1	_APS[Phospho (STY)]PTKR_.2	#N/A	4.1775	3.4329	3.9112	3.4549	3.3212	3.3034	3.6032	3.5462	3.8453	3.1322	1.0194	0.0277	0.7610	0.1186
Rnps1	_SSTRAPS[Phospho (STY)]PTK_.2	#N/A	3.3297	4.0860	3.8270	4.3296	3.9743	4.1511	4.0443	3.7889	3.4375	4.2966	1.0149	0.0213	0.8071	0.0931
Epdr1	_ALVSYDGLNQR_.2	#N/A	7.1313	7.1825	6.9418	7.2229	7.3378	7.1710	7.0671	7.3313	6.9246	6.8410	1.0175	0.0251	0.2847	0.5456
Epdr1	_DC[Carbamidomethyl (C)]YPVQETFIR_.2	#N/A	7.0627	6.6459	6.3569	7.1061	7.0339	6.9362	6.8138	6.6143	6.7811	6.8804	1.0125	0.0179	0.6055	0.2179
Epdr1	_SYETWIGVYTAK_.2	#N/A	5.3615	5.4670	3.2667	6.0696	5.4645	3.5909	6.1080	5.7181	5.4592	5.6668	0.8487	-0.2366	0.1833	0.7368
Epdr1	_QVLYQQSSGHNNR_.2	#N/A	3.6657	5.0385	4.4124	5.4052	5.5333	3.4923	3.6607	5.2431	3.9054	3.5031	1.1258	0.1710	0.3754	0.4255
Scrt1	_GGASGPATPAPPQLS[Phospho (STY)]PVQA_.2	Q99M85_S344_M1_Scrt1	7.3553	7.5134	8.1696	8.1353	6.5377	4.1079	8.0937	7.6101	7.2149	7.2394	0.9244	-0.1133	0.4939	0.3064
Dnaja3	_VTIPVPAGVEDGQTVR_.2	#N/A	6.2228	6.1632	6.7021	6.3278	7.1341	7.0836	6.4551	6.2752	6.5034	6.7754	1.0159	0.0227	0.6725	0.1723
Grk2	_NKPRS[Phospho (STY)]PVVELSK_.3	#N/A	5.8326	5.5746	3.6030	5.5590	4.7525	5.5345	5.6741	4.0401	3.6887	6.0737	1.0562	0.0789	0.6770	0.1694
Grk2	_SLLEGLLQR_.2	#N/A	4.2010	4.4432	3.8821	3.7250	4.4645	5.0633	3.2256	4.7385	4.0036	4.4849	1.0446	0.0629	0.6234	0.2053
Grk2	_GEVNAADAFDIGS[Phospho (STY)]FDEEDTK_.2	Q99MK8_S487_M1_Grk2	3.9902	3.4882	3.7239	4.1664	3.6029	3.6503	3.6965	3.8696	3.8389	3.6396	1.0024	0.0035	0.9486	0.0229
Grk2	_EAQQLVQR_.2	#N/A	3.3973	4.1536	3.7594	4.2620	4.0419	4.1569	2.6312	3.8565	2.5095	4.4053	1.1824	0.2417	0.1682	0.7742
Grk2	_S[Phospho (STY)]PVVELSK_.2	#N/A	4.3702	4.0858	3.8272	4.3298	3.9741	4.1513	4.0445	3.7887	3.4372	4.2968	1.0594	0.0833	0.2342	0.6304
Kars1	_ETAATTETPESTEASPS[Phospho (STY)]V_.2	Q99MN1_S594_M1_Kars1	3.5829	3.7596	3.3165	8.4927	4.2274	7.9727	4.5094	4.1409	4.4399	4.0384	1.2202	0.2872	0.4562	0.3408
Pccb	_DTSYLFITGPEVVK_.2	#N/A	5.2694	4.9683	3.3252	4.8875	5.5570	5.5166	4.3056	4.9126	5.0536	5.1373	1.0141	0.0202	0.8825	0.0543
Pccb	_EFFNFLPLSSQDPAPIR_.2	#N/A	4.3280	4.9876	4.4112	5.1109	4.3088	4.3208	4.2618	4.9589	5.1538	4.2198	0.9848	-0.0221	0.7977	0.0982
Pccb	_IM[Oxidation (M)]DQAITVGAPVIGLNDSGGAR_.2	#N/A	4.0199	3.6683	4.5944	3.2465	4.0523	3.0680	4.0097	4.8414	3.0198	3.2135	1.0010	0.0015	0.9933	0.0029
Pccb	_IQEGVESLAGYADIFLR_.2	#N/A	3.6825	3.6349	4.2781	3.2799	3.3132	3.9347	4.4955	2.9025	2.9863	4.3164	1.0033	0.0047	0.9759	0.0106
Brap	_EQSESVNTAPESPS[Phospho (STY)]K_.2	Q99MP8_S118_M1_Brap	7.9032	7.1923	7.7424	8.0764	6.9726	7.5859	7.8119	7.3568	7.8034	7.6060	0.9914	-0.0125	0.7842	0.1056
Brap	_SREQSESVNTAPES[Phospho (STY)]PSK_.2	#N/A	3.6632	3.9076	5.4945	3.9444	4.6171	3.6770	3.9492	3.7697	5.1588	4.6931	0.9601	-0.0588	0.7045	0.1521
Col25a1	_GEPGS[Phospho (STY)]PGAAGQSGLPGPK_.2	Q99MQ5_S265_M1_Col25a1	4.2863	8.6297	4.0200	9.4212	3.4301	8.6051	8.8774	3.4374	8.4395	3.2411	1.0667	0.0931	0.8350	0.0783
Gigyf1	_S[Phospho (STY)]IEEGDGAFGR_.2	Q99MR1_S137_M1_Gigyf1	9.4912	9.3212	9.8525	9.8685	8.7096	9.6116	9.0952	9.1802	9.5437	9.7066	1.0101	0.0144	0.7135	0.1466
Gigyf1	_ISS[Phospho (STY)]PPGPPGDLEDEEGLK_.2	Q99MR1_S413_M1_Gigyf1	3.9200	3.1755	3.2367	10.6315	3.0638	3.5608	10.3099	9.7984	4.0277	3.7063	0.6606	-0.5982	0.2877	0.5410
Gigyf1	_SQS[Phospho (STY)]WDDRGER_.2	Q99MR1_S157_M1_Gigyf1	6.4087	6.7696	7.4261	7.2948	3.7568	4.3686	6.5701	7.0345	6.7082	3.5678	1.0057	0.0082	0.9743	0.0113
Gigyf1	_STS[Phospho (STY)]PDGGPR_.2	Q99MR1_S228_M1_Gigyf1	3.2344	7.8695	3.9212	7.8276	3.8610	6.3545	4.1386	3.6946	3.3432	4.3909	1.4162	0.5020	0.1749	0.7572
Gigyf1	_GLEEEEEEEEPS[Phospho (STY)]EGVDEERPEAGGK_.3	Q99MR1_S343_M1_Gigyf1	3.5702	4.3754	4.2570	3.7453	4.1967	3.5328	3.8936	3.3588	3.0074	3.2258	1.1705	0.2271	0.0447	1.3492
Slc12a9	_[Acetyl (Protein N-term)]ASESS[Phospho (STY)]PLLAYR_.2	Q99MR3_S6_M1_Slc12a9	9.1030	8.6256	9.3288	8.3942	7.6876	8.8626	4.5369	8.8925	8.9793	9.0119	1.1033	0.1419	0.4052	0.3923
Srrt	_[Acetyl (Protein N-term)]GDS[Phospho (STY)]DDEYDRR_.2	#N/A	8.6357	8.8322	9.4322	9.2019	8.6946	9.1241	9.2769	8.6592	8.9504	8.8475	1.0060	0.0086	0.7868	0.1042
Srrt	_ERFS[Phospho (STY)]PPRHELS[Phospho (STY)]PPQK_.3	#N/A	9.2856	6.6457	6.3469	9.4033	7.3959	7.3774	7.4775	9.2552	3.9156	8.5039	1.0624	0.0873	0.7025	0.1533
Srrt	_HELS[Phospho (STY)]PPQKR_.2	Q99MR6_S74_M1_Srrt	8.2055	8.0084	4.1148	8.7317	7.8110	7.5662	8.6254	7.9355	8.2990	7.5622	0.9137	-0.1302	0.4430	0.3536
Srrt	_FS[Phospho (STY)]PPRHELS[Phospho (STY)]PPQKR_.3	#N/A	6.7397	5.5860	3.4032	5.6417	5.6672	5.8673	6.4599	4.0542	3.7772	5.7586	1.0941	0.1298	0.5543	0.2562
Srrt	_[Acetyl (Protein N-term)]GDS[Phospho (STY)]DDEYDR_.2	#N/A	6.6947	7.2384	3.4519	6.8918	7.3524	3.7627	6.7657	7.6623	4.3046	4.1738	1.0300	0.0427	0.8847	0.0532
Srrt	_ERFS[Phospho (STY)]PPR_.2	Q99MR6_S67_M1_Srrt	3.0588	3.8640	3.7456	8.8590	3.6853	8.0036	7.6121	7.1254	3.0435	8.0788	0.8048	-0.3134	0.4481	0.3486
Srrt	_LREC[Carbamidomethyl (C)]ELS[Phospho (STY)]PGVNR_.3	Q99MR6_S492_M1_Srrt	3.2359	3.9903	3.9227	4.4253	3.8624	3.8671	4.1400	3.6931	3.3417	4.3923	0.9980	-0.0029	0.9773	0.0100
Tmem121b	_GSWVS[Phospho (STY)]PSR_.2	Q99MX7_S521_M1_Tmem121b	3.4268	4.1830	8.2266	4.2326	4.0713	4.0541	3.9473	3.8859	7.9106	7.5437	0.8071	-0.3091	0.3949	0.4035
Tmem121b	_GHLENEGGPHGYVNTLAVASQN_.3	#N/A	4.0980	3.3535	3.8317	3.5613	3.2418	3.3828	3.2760	2.9125	4.2057	3.5283	1.0280	0.0399	0.7300	0.1367
Cadm3	_GNPVPQQYVWVK_.2	#N/A	7.7226	7.0766	7.6647	7.3445	7.0162	7.2480	7.1343	7.4150	7.1991	7.3207	1.0108	0.0154	0.6349	0.1973
Cadm3	_LLLHC[Carbamidomethyl (C)]EGR_.2	#N/A	5.6044	6.1754	5.3669	4.3898	5.0913	5.3837	5.3069	6.3890	5.7368	6.2546	0.9009	-0.1505	0.1411	0.8506
Cadm3	_GTYLTHEAK_.2	#N/A	4.9321	3.5549	3.5212	5.5306	5.4514	5.0332	5.3174	5.6527	5.5150	5.9900	0.8312	-0.2667	0.0817	1.0879
Cadm3	_GTYLTHEAKGS[Phospho (STY)]DDAPDADTAIINAEGGQSGGDDKK_.4	#N/A	3.3612	4.1174	4.5957	4.1395	4.0057	4.1197	4.2877	4.3626	5.1410	3.8167	0.9215	-0.1179	0.2784	0.5553
Cadm3	_GSDDAPDADTAIINAEGGQS[Phospho (STY)]GGDDKK_.3	Q99N28_S386_M1_Cadm3	3.6657	4.1723	3.3175	4.3560	3.6842	3.8266	3.7198	4.6559	3.7734	3.9143	0.9555	-0.0657	0.5068	0.2952
Cadm3	_EDDGANIVC[Carbamidomethyl (C)]SVNHESLK_.2	#N/A	4.1304	4.0199	3.3164	3.8190	4.0825	3.8689	4.9702	4.4530	4.5988	4.3101	0.8450	-0.2429	0.0056	2.2543
Cadm3	_GSDDAPDADTAIINAEGGQSGGDDKKEYFI_.3	#N/A	3.3296	4.0858	4.5641	4.3298	3.9741	4.1513	4.0445	4.3942	3.1924	3.7851	1.0567	0.0795	0.4771	0.3214
Sytl2	_LALQYVPEPS[Phospho (STY)]PGK_.2	#N/A	10.6841	10.4963	11.2867	11.0189	9.9699	10.6443	11.2181	10.5704	10.6151	10.4947	0.9962	-0.0056	0.8805	0.0553
Sytl2	_LALQYVPEPS[Phospho (STY)]PGKK_.3	#N/A	3.4976	3.8448	3.2313	4.2759	3.6661	4.0634	4.4242	4.2261	3.0243	3.9532	0.9632	-0.0541	0.6576	0.1821
Raf1	_SAS[Phospho (STY)]EPSLHR_.2	Q99N57_S621_M1_Raf1	8.6752	8.3651	9.4265	8.8699	3.4941	9.0041	3.7717	4.0614	9.2686	8.9737	1.2230	0.2904	0.4022	0.3955
Raf1	_AAHTEDINAC[Carbamidomethyl (C)]TLTTS[Phospho (STY)]PR_.2	#N/A	4.1342	3.5794	3.5721	3.9387	3.2674	3.9629	3.1947	3.9681	4.4790	4.1752	0.9464	-0.0794	0.4590	0.3382
Raf1	_RAS[Phospho (STY)]DDGKLTDSSK_.2	#N/A	3.9106	3.9140	4.0782	4.6220	4.2592	4.1697	4.3177	4.3924	4.6442	4.0516	0.9558	-0.0653	0.2805	0.5521
Sf3b1	_GGDSIGET[Phospho (STY)]PT[Phospho (STY)]PGASK_.2	Q99NB9_T326_M2_Sf3b1	7.5580	3.5163	7.4665	4.6044	3.3376	4.3919	3.2518	7.5834	7.0196	7.0876	0.8252	-0.2771	0.4091	0.3882
Sf3b1	_AKGSET[Phospho (STY)]PGAT[Phospho (STY)]PGSK_.2	#N/A	4.1396	3.1185	3.2936	4.1886	3.0068	3.6178	3.5109	4.3222	4.0829	3.7633	0.9084	-0.1386	0.2603	0.5845
Sf3b1	_WDETPASQMGGST[Phospho (STY)]PVLTPGK_.2	Q99NB9_T350_M1_Sf3b1	3.5679	2.1688	4.2547	3.2565	3.5466	3.0781	4.4720	3.3611	3.0097	3.2235	0.9418	-0.0864	0.6517	0.1859
Sf3b1	_GSET[Phospho (STY)]PGAT[Phospho (STY)]PGSK_.2	#N/A	3.2575	4.0849	4.4920	5.0623	3.9021	3.8233	4.1841	4.4662	3.2644	3.7131	1.0504	0.0709	0.6170	0.2097
Sf3b1	_DT[Phospho (STY)]PGHGSGWAETPR_.2	Q99NB9_T303_M1_Sf3b1	4.6384	3.2279	3.1843	5.3195	3.1240	3.1047	3.4016	5.5791	4.0801	3.6539	0.9013	-0.1499	0.5281	0.2773
Sf3b1	_TM[Oxidation (M)]IIS[Phospho (STY)]PERLDPFADGGKT[Phospho (STY)]PDPK_.3	#N/A	4.2358	4.7225	5.5466	5.5510	3.8862	4.7421	4.3720	5.6878	4.7017	4.5639	0.9895	-0.0152	0.9059	0.0429
Sf3b1	_KLSSWDQAET[Phospho (STY)]PGHT[Phospho (STY)]PSLR_.3	Q99NB9_T223_M2_Sf3b1	3.1314	4.2110	4.3659	3.9097	1.0658	3.6971	4.0579	4.1483	3.3906	3.4638	0.9022	-0.1485	0.5821	0.2350
Sf3b1	_RWDQTADQT[Phospho (STY)]PGAT[Phospho (STY)]PK_.2	Q99NB9_T207_M2_Sf3b1	4.1920	3.4961	3.5799	4.6245	3.3175	4.4120	3.2720	3.8775	4.1765	4.3018	1.0077	0.0111	0.9298	0.0316
Rims1	_QGS[Phospho (STY)]PTQSPPADTSFGSR_.2	Q99NE5_S1023_M1_Rims1	9.3886	10.0780	10.3769	10.3632	8.4265	10.0441	10.6721	10.2040	8.7671	9.9211	0.9887	-0.0164	0.8294	0.0813
Rims1	_GRS[Phospho (STY)]QDYPDRLEK_.3	#N/A	10.7888	10.7951	11.3837	11.4524	10.2377	11.0369	10.8989	10.9155	11.0323	10.9119	1.0009	0.0012	0.9683	0.0140
Rims1	_S[Phospho (STY)]LDEIHPTRR_.3	#N/A	11.0617	10.9358	11.6194	11.1881	10.1575	11.0707	10.6476	11.1112	11.1267	11.1240	1.0003	0.0004	0.9906	0.0041
Rims1	_S[Phospho (STY)]LDEIHPTR_.2	#N/A	11.9120	11.4661	11.8311	12.1915	10.5327	11.4977	11.7817	11.6559	11.6781	11.7018	0.9887	-0.0164	0.6653	0.1770
Rims1	_SGS[Phospho (STY)]IEQASLVVEER_.2	Q99NE5_S1051_M1_Rims1	8.4735	8.0650	8.4161	8.8930	2.8706	8.3087	8.4612	8.7220	8.3890	8.3942	0.8838	-0.1783	0.4227	0.3740
Rims1	_ETS[Phospho (STY)]PISSHPVTWQPSK_.2	#N/A	6.9896	3.7138	8.1081	8.1048	6.2766	7.1235	7.7066	7.4033	7.5911	6.8639	0.9091	-0.1375	0.4499	0.3469
Rims1	_GRS[Phospho (STY)]QDYPDR_.2	#N/A	8.6474	8.1460	8.2581	8.8820	7.6821	8.4162	8.3543	8.4026	8.5576	8.3620	0.9904	-0.0139	0.7194	0.1430
Rims1	_S[Phospho (STY)]QDYPDRLEK_.2	#N/A	8.4241	7.8968	7.5439	8.5565	3.4389	8.0861	7.9508	3.2535	8.1148	3.2499	1.2981	0.3764	0.2864	0.5430
Rims1	_ETS[Phospho (STY)]PISSHPVTWQPSKEGDR_.3	#N/A	3.7412	3.4850	8.2654	3.4298	6.2366	3.3617	3.1445	6.8978	6.6055	3.3968	0.9485	-0.0762	0.8491	0.0711
Rims1	_IQIQHAS[Phospho (STY)]PENDR_.2	Q99NE5_S927_M1_Rims1	6.6205	6.2866	6.9693	6.7202	5.6322	6.7634	6.5099	6.5381	5.9328	6.4333	1.0228	0.0326	0.6046	0.2185
Rims1	_SS[Phospho (STY)]DSDVSDVSAISR_.2	Q99NE5_S1111_M1_Rims1	4.1484	8.1065	4.0418	3.7023	3.4130	8.3947	9.0945	8.5902	8.5562	5.8887	0.6600	-0.5995	0.0700	1.1549
Rims1	_SASTNC[Carbamidomethyl (C)]LRPDTSLHS[Phospho (STY)]PER_.3	#N/A	3.4508	3.8916	5.6389	5.5181	3.2398	4.8388	4.7393	5.2504	4.3715	4.8897	0.9204	-0.1196	0.5083	0.2939
Rims1	_ASQSS[Phospho (STY)]LESSSGPPC[Carbamidomethyl (C)]IR_.2	#N/A	9.7635	9.4251	10.2667	9.9714	8.7232	9.4860	10.2841	9.6389	9.5418	9.5399	0.9851	-0.0216	0.6486	0.1880
Rims1	_RAS[Phospho (STY)]QSSLESSSGPPC[Carbamidomethyl (C)]IR_.3	#N/A	3.8086	5.5339	4.0894	3.6564	9.5413	4.6729	3.5409	4.7927	5.4598	10.6601	0.8534	-0.2287	0.6084	0.2158
Rims1	_IPESSHPPLESSSS[Phospho (STY)]SFESQK_.3	Q99NE5_S547_M1_Rims1	3.3786	7.5270	4.0088	4.2956	7.0630	8.1360	4.2984	8.2497	3.1769	7.6825	0.9800	-0.0291	0.9374	0.0281
Rims1	_SASTNC[Carbamidomethyl (C)]LRPDTSLHS[Phospho (STY)]PERER_.4	#N/A	3.5163	3.7098	4.2031	5.5775	4.7397	5.1790	4.4205	6.0900	3.0613	3.1720	1.0721	0.1004	0.6767	0.1696
Rims1	_S[Phospho (STY)]AEC[Carbamidomethyl (C)]LHM[Oxidation (M)]TSELQPSLDR_.3	#N/A	6.1577	6.2580	3.3893	7.4132	3.2733	6.3676	4.1369	6.9745	6.9625	6.7273	0.8833	-0.1791	0.5040	0.2976
Rims1	_FKQTTGSGSS[Phospho (STY)]QELDHEQYSK_.3	Q99NE5_S1082_M1_Rims1	4.1978	3.1446	3.9315	3.4752	2.9659	4.3230	3.6235	5.0028	3.8250	3.1525	0.9416	-0.0869	0.6049	0.2183
Rims1	_S[Phospho (STY)]TETGM[Oxidation (M)]AAEMR_.2	Q99NE5_S1240_M1_Rims1	3.5038	5.3274	4.4446	3.8204	3.4594	3.8719	5.7555	4.8358	3.5117	3.5477	0.9226	-0.1162	0.5602	0.2517
Rims1	_STSQLS[Phospho (STY)]QTESGHKK_.2	Q99NE5_S1224_M1_Rims1	4.0646	3.1616	3.2505	3.7532	3.1903	3.0384	3.4679	4.3653	4.0139	3.7202	0.8761	-0.1908	0.0975	1.0108
Rims1	_SC[Carbamidomethyl (C)]DNASAKS[Phospho (STY)]SDSDVSDVSAISR_.3	#N/A	3.9761	3.3663	3.7098	5.9236	3.1876	3.0410	3.4018	3.7476	4.0467	4.4317	0.9899	-0.0147	0.9464	0.0239
Rims1	_SRS[Phospho (STY)]SEHSSIR_.2	Q99NE5_S988_M1_Rims1	4.0392	3.3436	3.7325	3.2762	3.1649	3.0637	3.4245	3.7249	4.0240	2.9535	0.9731	-0.0394	0.7267	0.1386
Rims1	_QPSRESTDGSINSY[Phospho (STY)]SSEGNLIFPGVR_.3	Q99NE5_Y1268_M1_Rims1	4.3414	4.3856	3.5274	7.3255	4.2739	3.8516	3.7447	4.0884	4.9092	3.9970	1.1034	0.1420	0.5676	0.2459

Rims1	_S[Phospho (STY)]VS[Phospho (STY)]PHRGDDQGRPR_.3	Q99NE5_S788_M2_Rims1	4.2761	2.8816	4.0097	2.6473	1.7253	3.2048	3.7018	3.4477	3.2311	3.2308	0.9181	-0.1233	0.5811	0.2357
Rims1	_S[Phospho (STY)]RSVS[Phospho (STY)]PHR_.2	Q99NE5_S786_M2_Rims1	3.5393	3.6869	4.2261	3.2279	3.5752	3.0494	4.4434	5.3704	3.0383	3.1949	0.8851	-0.1761	0.3678	0.4344
Rims1	_LQTHDESSLPLPQPS[Phospho (STY)]PFMPR_.3	Q99NE5_S716_M1_Rims1	4.3349	3.0075	4.0686	6.9196	2.8288	3.3998	3.7606	3.3888	3.6879	3.2896	1.1590	0.2129	0.4881	0.3115
Nsmf	_QGS[Phospho (STY)]KEC[Carbamidomethyl (C)]PGC[Carbamidomethyl (C)]AQLVPGPSSR_.3	#N/A	7.3823	7.2993	7.2229	7.8290	3.8476	6.1707	4.4261	6.7749	7.0539	7.1606	1.0427	0.0603	0.7723	0.1122
Nsmf	_M[Oxidation (M)]YS[Phospho (STY)]VDGVSDDVPIR_.2	#N/A	6.9550	7.2246	4.1454	9.1924	5.9546	7.4796	3.6935	8.0436	8.0649	7.6598	0.9941	-0.0085	0.9741	0.0114
Nsmf	_SWS[Phospho (STY)]DPTPMK_.2	Q99NF2_S294_M1_Nsmf	6.1105	3.3723	3.6994	5.6907	3.2494	3.2603	3.9001	3.9258	3.9483	4.6439	1.0307	0.0436	0.8585	0.0663
Nsmf	_ERS[Phospho (STY)]FS[Phospho (STY)]RS[Phospho (STY)]WSDPTPMK_.2	#N/A	3.3777	3.4073	3.5389	4.0386	3.2538	3.9798	3.7533	4.0856	3.7342	4.0056	0.9242	-0.1138	0.1448	0.8394
Cep41	_LEDNDS[Phospho (STY)]AT[Phospho (STY)]SEADAEIAAK_.2	Q99NF3_T98_M2_Cep41	3.5063	5.3298	3.7675	5.8663	3.7199	3.5598	4.2134	5.6210	3.8989	3.7301	0.9830	-0.0247	0.9086	0.0416
Cep41	_GGQNLPTSC[Carbamidomethyl (C)]PASHSS[Phospho (STY)]PR_.3	Q99NF3_S358_M1_Cep41	3.7300	2.9854	3.4636	4.5083	2.8737	3.7509	3.1557	3.9938	4.2928	4.1855	0.9091	-0.1374	0.3606	0.4430
Ankrd17	_NS[Phospho (STY)]PLDC[Carbamidomethyl (C)]GSASPNK_.2	Q99NH0_S2055_M1_Ankrd17	6.3222	3.1484	3.2637	6.9842	5.6387	5.4039	6.1095	6.2113	4.0007	5.7953	0.9272	-0.1090	0.6693	0.1744
Ankrd17	_EHYPVSSPSS[Phospho (STY)]PS[Phospho (STY)]PPAQPGGVSR_.3	Q99NH0_S2041_M2_Ankrd17	4.2257	8.8138	3.9594	8.9475	8.4281	8.9924	9.5064	8.9961	8.9099	9.0562	0.7928	-0.3350	0.1687	0.7729
Pard3	_RSSDPALTGLS[Phospho (STY)]TSVSDNNFSSEEPSR_.3	Q99NH2_S152_M1_Pard3	3.5694	3.6568	4.2562	3.2580	3.5451	3.0795	4.4735	3.3597	3.0082	3.2250	1.0126	0.0180	0.8969	0.0472
Pard3	_NNYSPGRFS[Phospho (STY)]PDSHC[Carbamidomethyl (C)]VANR_.3	Q99NH2_S383_M1_Pard3	3.2334	3.9927	3.9202	4.4229	3.8810	4.2444	4.1376	4.6456	3.3442	4.3899	0.9564	-0.0644	0.5706	0.2437
Hspbp1	_LLQTC[Carbamidomethyl (C)]FSS[Phospho (STY)]PTDDSM[Oxidation (M)]DR_.2	#N/A	3.1911	3.9963	3.8779	8.8224	7.2556	8.2801	8.7474	8.6789	8.3827	4.7930	0.7717	-0.3739	0.2719	0.5657
Rtn4	_KAQIITEKTS[Phospho (STY)]PK_.2	#N/A	10.1267	10.6000	11.1046	10.6629	9.5945	10.2161	10.8161	10.7563	10.3255	10.0419	0.9904	-0.0139	0.7481	0.1260
Rtn4	_GSGS[Phospho (STY)]VDETLFALPAASEPVIPSSAEK_.3	Q99P72_S167_M1_Rtn4	3.7757	3.5667	3.5094	4.5540	2.9194	3.7052	3.2014	3.9480	4.2471	4.2312	0.9398	-0.0896	0.4985	0.3023
Rtn4	_LPS[Phospho (STY)]DTEKEDR_.2	#N/A	3.2203	8.1381	8.7669	4.4097	3.8468	8.2241	4.1244	8.7559	3.3573	4.3767	1.1838	0.2435	0.5729	0.2420
Rtn4	_HQAQIDHYLGLANK_.3	#N/A	7.2291	7.2201	7.6663	7.0350	6.7557	6.7702	7.4892	7.5919	7.3719	6.9918	0.9662	-0.0495	0.2551	0.5933
Rtn4	_[Acetyl (Protein N-term)]M[Oxidation (M)]EDIDQSSLVSSSADS[Phospho (STY)]PPRPPPAFK_.3	#N/A	9.1904	7.1940	10.9590	11.3351	9.4408	9.4919	10.1633	10.6460	10.1812	9.3946	0.9510	-0.0724	0.5435	0.2648
Rtn4	_TSNPFLVAIHDSEADYVTTDNLSK_.3	#N/A	3.5107	4.6332	4.7394	5.0059	4.6093	4.3357	4.9480	3.1106	4.8797	4.4025	1.0316	0.0449	0.7569	0.1210
Rtn4	_LSTEPS[Phospho (STY)]PEFSNYSEIAK_.2	Q99P72_S690_M1_Rtn4	8.0115	8.5590	3.3393	9.0540	5.0701	8.5852	8.8507	9.0201	8.6484	8.5977	0.8091	-0.3056	0.1979	0.7035
Rtn4	_GPLPAAPPT[Phospho (STY)]APERQPSWER_.3	Q99P72_T98_M1_Rtn4	2.9086	3.7138	3.3623	8.3035	4.9248	4.1944	7.0183	4.0951	6.5822	5.5413	0.7863	-0.3468	0.3013	0.5211
Rtn4	_EAEEKLPS[Phospho (STY)]DTEKEDR_.2	#N/A	6.6736	8.2250	8.9570	8.0174	6.6901	7.9395	8.5988	8.5859	8.1097	8.0805	0.9289	-0.1064	0.2464	0.6084
Rtn4	_VVKEDGVM[Oxidation (M)]S[Phospho (STY)]PEK_.2	#N/A	9.8087	9.5615	9.5796	9.9943	8.7881	9.5091	10.1199	9.7866	9.4878	9.7116	0.9758	-0.0353	0.3425	0.4653
Rtn4	_DDS[Phospho (STY)]PKEYTDLEVSNK_.2	#N/A	8.1851	8.9827	8.2612	9.5676	8.0201	8.9392	9.5272	9.3125	8.9441	8.9755	0.9423	-0.0858	0.1418	0.8482
Rtn4	_RRGS[Phospho (STY)]GSVDETLFALPAASEPVIPSSAEK_.3	#N/A	7.0473	9.5529	9.6174	10.5700	7.4235	7.4637	8.9402	9.8161	8.8092	4.0172	1.0908	0.1254	0.5914	0.2281
Rtn4	_EHGYLGNLSAVASTEGTIEETLNEASR_.3	#N/A	4.1651	4.6153	4.3456	3.8537	3.0504	3.3291	3.5684	4.3808	3.6345	3.8207	1.0109	0.0157	0.9049	0.0434
Rtn4	_RGS[Phospho (STY)]GSVDETLFALPAASEPVIPSSAEK_.3	#N/A	5.1648	9.8983	4.1876	10.4366	3.1067	8.8933	3.6641	10.1501	9.4265	8.5806	0.8734	-0.1954	0.6263	0.2032
Rtn4	_AQIITEKTS[Phospho (STY)]PK_.2	#N/A	4.2939	2.9910	10.5258	10.1600	9.1931	9.7894	10.3847	10.3314	3.7586	9.9714	0.9087	-0.1381	0.7197	0.1428
Rtn4	_AYLESEVAISEELVQK_.2	#N/A	5.8495	6.1251	7.2816	6.5446	4.7551	6.6141	6.4576	5.6338	5.5894	6.0130	1.0458	0.0647	0.5759	0.2396
Rtn4	_ESLTEVSETVTQHK_.2	#N/A	4.6753	4.4974	4.7427	3.8015	3.5612	4.3963	4.3724	4.4040	3.9569	4.2831	1.0059	0.0085	0.9259	0.0334
Rtn4	_SKDKEDLVC[Carbamidomethyl (C)]SAALHNPQES[Phospho (STY)]PATLTK_.4	Q99P72_S329_M1_Rtn4	4.1993	3.0269	3.3852	3.8879	3.3249	4.4045	3.6026	4.2306	3.8792	3.8549	0.9519	-0.0710	0.5441	0.2643
Rtn4	_EDGVMS[Phospho (STY)]PEK_.2	#N/A	6.4059	3.7077	4.2052	6.5037	3.5960	3.0286	4.4226	3.4106	3.0592	3.1741	1.3008	0.3794	0.2257	0.6465
Rtn4	_LSAS[Phospho (STY)]PQEVGKPYLESFQPNLHITK_.3	Q99P72_S768_M1_Rtn4	3.2329	3.9892	4.4674	4.4265	3.8775	4.2480	4.1411	2.9900	3.3406	3.6885	1.1413	0.1907	0.1392	0.8564
Rtn4	_ESAEFSVLEYSEM[Oxidation (M)]GSSFNGS[Phospho (STY)]PK_.3	Q99P72_S291_M1_Rtn4	3.5589	4.3152	3.2926	4.3372	4.2035	3.9220	4.4855	4.1648	2.9630	4.0145	1.0080	0.0115	0.9292	0.0319
Nup155	_AAPQS[Phospho (STY)]PSVPK_.2	Q99P88_S992_M1_Nup155	4.3278	4.3992	3.5137	4.0164	2.7867	3.8379	3.7311	4.1021	9.3021	3.9834	0.7223	-0.4693	0.2212	0.6553
Ogfr	_IALNLEGC[Carbamidomethyl (C)]ALS[Phospho (STY)]PTSQEPR_.2	Q99PG2_S403_M1_Ogfr	5.0378	6.3940	6.6508	7.4131	5.1587	3.7492	6.6007	6.8605	3.2238	5.9087	1.0151	0.0217	0.9302	0.0314
Lpin2	_S[Phospho (STY)]DSELEVKPSESLLR_.2	#N/A	3.3234	4.1286	4.0414	7.9554	3.9499	6.2286	4.1699	7.1096	3.3081	3.9201	1.0672	0.0938	0.7898	0.1025
Ntm	_VTVNYPPYISEAK_.2	#N/A	8.3156	8.2260	8.2770	8.3792	8.1528	7.9937	8.0448	8.3382	8.1738	7.7839	1.0172	0.0246	0.2649	0.5769
Ntm	_AVGFVSEDEYLEIQGITR_.2	#N/A	5.8787	6.2794	7.2596	6.9473	5.5351	6.5105	6.2140	6.4165	5.8455	5.9158	1.0498	0.0701	0.4054	0.3922
Ntm	_EQSGEYEC[Carbamidomethyl (C)]SASNDVAAPVVR_.2	#N/A	6.2796	6.0997	6.6287	7.5314	6.2649	6.7819	6.3361	7.0725	6.1910	6.7363	1.0021	0.0030	0.9658	0.0151
Ntm	_GTGVPVGQK_.2	#N/A	5.9242	6.3530	6.7665	6.4708	6.2114	6.1536	6.2703	6.5403	6.0231	6.1815	1.0095	0.0136	0.7367	0.1327
Ntm	_VHLIVQVSPK_.2	#N/A	6.1302	5.1508	6.1015	4.2109	4.2861	4.6877	6.2530	5.0811	5.4215	5.6908	0.9079	-0.1395	0.3129	0.5047
Ntm	_STILYAGNDK_.2	#N/A	7.9979	6.8563	7.6783	7.3918	7.8476	7.0142	7.3033	7.4878	7.3527	7.4835	1.0078	0.0112	0.8145	0.0891
Ntm	_AMDNVTVR_.2	#N/A	5.8607	3.4747	3.6014	5.8571	3.2960	6.7017	3.2934	3.8560	4.1551	4.3233	1.2282	0.2966	0.2907	0.5365
Rrbp1	_LASS[Phospho (STY)]PKDR_.2	Q99PL5_S135_M1_Rrbp1	3.2166	3.9729	7.5877	4.4427	3.8612	4.2642	4.1574	7.3688	3.3244	6.5026	0.8538	-0.2281	0.4944	0.3059
Ubxn6	_GKS[Phospho (STY)]PQLALR_.2	Q99PL6_S36_M1_Ubxn6	11.1017	11.3272	11.5523	11.6699	10.0556	11.2109	10.9285	10.9168	10.9955	11.0843	1.0156	0.0224	0.5782	0.2379
Ubxn6	_EALQNDWLPFELR_.2	#N/A	5.5244	5.6282	5.4902	4.0009	6.0042	5.7242	6.4919	5.6799	5.7273	5.3864	0.9268	-0.1096	0.3251	0.4879
Ubxn6	_SVLRPELLAAIEQLS_.2	#N/A	3.2085	3.9648	4.4431	4.4508	3.8531	4.2723	4.1655	3.6677	3.3162	4.4178	1.0361	0.0511	0.6633	0.1783
Znf318	_RAS[Phospho (STY)]PS[Phospho (STY)]PPR_.2	#N/A	9.5114	9.5582	9.4394	10.0352	8.9871	9.4970	9.8596	9.3220	9.4507	9.4948	0.9972	-0.0041	0.8920	0.0496
Znf318	_RGS[Phospho (STY)]PS[Phospho (STY)]PPR_.2	Q99PP2_S79_M2_Znf318	9.4759	9.6389	10.1433	10.1937	8.8187	9.4467	9.8435	9.6852	9.7048	9.5971	0.9909	-0.0132	0.7462	0.1272
Znf318	_DQVVGGNVS[Phospho (STY)]PR_.2	Q99PP2_S2206_M1_Znf318	8.1944	7.9483	7.8936	8.8735	7.9750	7.9691	8.4826	8.1520	8.1190	8.1175	0.9908	-0.0133	0.7195	0.1430
Znf318	_WAS[Phospho (STY)]PS[Phospho (STY)]PPR_.2	Q99PP2_S69_M2_Znf318	7.7025	8.6777	6.5276	9.1929	7.9767	8.3203	9.0311	7.4796	8.7935	8.6630	0.9499	-0.0742	0.4555	0.3416
Znf318	_LGS[Phospho (STY)]PVDGLQDM[Oxidation (M)]DRDDLTDDSVFTR_.3	Q99PP2_S205_M1_Znf318	3.8983	3.3279	4.5851	3.5869	3.0240	3.2047	3.3016	2.1477	4.1801	3.5539	1.0936	0.1291	0.5068	0.2951
Znf318	_S[Phospho (STY)]PGLC[Carbamidomethyl (C)]SDSSLEESLR_.2	Q99PP2_S167_M1_Znf318	3.9956	3.2510	3.7292	3.6638	3.1393	3.4853	3.3785	4.4547	4.1033	3.6308	0.9106	-0.1350	0.2015	0.6958
Znf318	_RAS[Phospho (STY)]PS[Phospho (STY)]PPRGR_.3	#N/A	3.4972	3.8452	3.2309	4.2755	4.1418	3.9836	4.4238	4.2265	3.0247	3.9528	0.9801	-0.0291	0.8125	0.0902
Trim33	_LKS[Phospho (STY)]DERPVHIK_.3	Q99PP7_S1134_M1_Trim33	4.7632	4.2714	4.7033	4.2054	4.4344	3.4079	4.2054	3.0313	4.6451	4.7825	1.0316	0.0448	0.7519	0.1239
Arhgdia	_VAVSADPNVPNVIVTR_.2	#N/A	9.4338	9.6279	9.9235	9.8677	9.6989	9.8445	9.7015	9.5693	9.6917	9.6415	1.0085	0.0122	0.4226	0.3741
Arhgdia	_[Acetyl (Protein N-term)]AEQEPTAEQLAQIAAENEEDEHSVNY[Phospho (STY)]KPPAQK_.4	Q99PT1_Y27_M1_Arhgdia	5.4857	6.9133	6.9850	7.4064	6.5591	6.8481	6.4332	6.9593	6.7738	6.9645	0.9877	-0.0178	0.8174	0.0876
Arhgdia	_TDYM[Oxidation (M)]VGSYGPR_.2	#N/A	7.8105	7.4537	7.5493	7.3614	8.0747	7.8865	7.7347	7.8142	7.7948	7.8880	0.9848	-0.0221	0.4333	0.3632
Arhgdia	_S[Phospho (STY)]QEIQELDKDDESLRK_.3	#N/A	8.3545	8.3706	8.8670	9.3878	8.0785	8.6114	8.4621	8.7863	8.4975	8.7421	0.9988	-0.0017	0.9674	0.0144
Arhgdia	_LTLVC[Carbamidomethyl (C)]STAPGPLELDLTGDLESFKK_.3	#N/A	3.9602	3.9815	4.0965	4.7039	5.0218	4.1517	4.4496	5.0270	5.2556	4.8161	0.8838	-0.1782	0.0618	1.2091
Arhgdia	_YIQHTYR_.2	#N/A	7.1327	7.3894	6.9878	7.1869	7.6032	6.9572	6.9444	7.3593	7.2515	7.0936	1.0066	0.0095	0.7531	0.1231
Arhgdia	_SIQEIQELDKDDESLR_.3	#N/A	6.3258	5.9947	6.4183	7.0434	5.7628	5.5790	5.9497	6.3922	6.0529	6.2327	1.0049	0.0071	0.9161	0.0380
Arhgdia	_IDKTDYM[Oxidation (M)]VGSYGPR_.3	#N/A	7.3335	7.8373	8.2627	7.2616	7.1300	6.9338	7.1575	7.9785	7.1224	7.2387	1.0116	0.0166	0.7824	0.1066
Arhgdia	_AEEYEFLTPM[Oxidation (M)]EEAPK_.2	#N/A	8.3211	8.1779	8.6426	8.5202	8.3262	8.6454	8.4063	8.0446	8.0928	8.4863	1.0220	0.0314	0.2053	0.6877
Acsbg1	_ES[Phospho (STY)]PSHGLELSAPEK_.2	Q99PU5_S53_M1_Acsbg1	3.9987	3.3031	3.1847	3.3168	3.1244	3.1042	3.4650	4.4311	3.9835	3.6544	0.8597	-0.2181	0.0523	1.2812
Acsbg1	_AAS[Phospho (STY)]LDGAEEALWTTR_.2	Q99PU5_S70_M1_Acsbg1	4.2835	3.5879	3.4882	7.5224	3.4092	4.3203	7.4578	7.2323	5.0124	4.2101	0.7419	-0.4307	0.1696	0.7706
Acsbg1	_DDQQGASLGTSQDNSQTSSLIDGQTLSK_.3	#N/A	3.4241	4.2574	4.5852	6.1225	4.0686	3.5140	5.3486	4.7130	3.0979	3.8796	1.0162	0.0231	0.9162	0.0380
Dhx30	_GGS[Phospho (STY)]FEM[Oxidation (M)]TDDDSAIR_.2	#N/A	6.4125	5.6843	6.8418	6.8815	5.1851	5.7798	6.3276	6.0763	5.9067	6.2812	0.9972	-0.0040	0.9631	0.0163
Bcl11b	_ELAGNSST[Phospho (STY)]PPPVS[Phospho (STY)]PGR_.2	Q99PV8_S381_M2_Bcl11b	8.6563	9.5680	8.8576	10.1574	9.1179	9.4545	9.4403	9.1580	9.1130	9.6512	0.9959	-0.0060	0.8988	0.0464
Bcl11b	_FST[Phospho (STY)]PPGDLLDGGLSGR_.2	Q99PV8_T744_M1_Bcl11b	4.3525	8.6758	4.0862	9.8417	8.1634	7.9027	8.7856	9.1684	8.2452	7.5225	0.8505	-0.2336	0.3422	0.4657
Bcl11b	_SDDGLSAAS[Phospho (STY)]SPEPGTSELPGDLK_.2	Q99PV8_S495_M1_Bcl11b	4.0484	5.9914	3.6130	8.3428	6.2690	6.0890	7.1355	7.8148	6.4522	6.3881	0.8241	-0.2791	0.2171	0.6634
Bcl11b	_VLDKSS[Phospho (STY)]PPPSSR_.2	Q99PV8_S96_M1_Bcl11b	8.9982	3.6630	4.2500	3.2518	9.5323	3.0733	9.9643	3.3658	3.0144	3.2188	1.1167	0.1592	0.7853	0.1050
Bcl11b	_VSEPVEIGIQVTPDEDDHLLS[Phospho (STY)]PTK_.3	Q99PV8_S128_M1_Bcl11b	4.3424	3.5979	4.0761	3.6199	3.4862	3.1384	3.7682	3.3813	3.6803	3.2972	1.0505	0.0711	0.4748	0.3234
Bcl11b	_SPFLST[Phospho (STY)]PPLPPMPAGT[Phospho (STY)]PPPQPPAK_.3	Q99PV8_T406_M2_Bcl11b	3.7426	3.0469	4.4294	6.9407	2.8682	3.3604	3.7212	3.1865	3.7273	3.3982	1.1586	0.2124	0.4959	0.3046
Ndufa5	_TTGLVGLAVC[Carbamidomethyl (C)]DTPHER_.2	#N/A	4.9124	4.8466	4.4956	5.4483	5.4053	4.3849	5.5824	4.9304	4.5964	5.1368	0.9860	-0.0204	0.7909	0.1019
Ndufa5	_KYTEQITNEK_.2	#N/A	4.2699	4.0869	3.9685	5.3840	4.6289	4.2843	4.1821	4.4682	3.7094	4.3332	1.0632	0.0884	0.3950	0.4034
Ndufa5	_LEALLQGGEVEEVILQAEK_.2	#N/A	3.6482	4.2581	3.5483	4.3484	3.2138	3.5714	3.3039	3.0284	4.1778	3.5562	1.0706	0.0984	0.4296	0.3670

Cox6c	_AGIFQSAK_.2	#N/A	5.6658	4.6114	5.9059	5.0930	5.4519	5.7279	4.0777	5.6713	5.6410	5.2789	1.0469	0.0661	0.5459	0.2629
Cox6c	_NYDSM[Oxidation (M)]KDFEEMRK_.3	#N/A	5.9626	4.0589	4.5474	5.2341	3.8981	4.0211	4.2394	4.4110	3.2092	3.7684	1.1826	0.2420	0.1679	0.7749
Cox6c	_AYAEFYR_.2	#N/A	3.6173	6.9162	3.3510	6.1734	4.2619	3.8636	3.2804	7.5512	7.3277	7.4276	0.7343	-0.4455	0.1665	0.7786
Cox6c	_NYDSMKDFEEMR_.3	#N/A	3.7107	3.0151	4.0610	3.6048	2.8364	3.3922	3.7530	3.3964	3.6955	3.2820	0.9731	-0.0394	0.7126	0.1472
Tomm22	_[Acetyl (Protein N-term)]AAAVAAAGAGEPLS[Phospho (STY)]PEELLPK_.2	#N/A	10.7653	12.2672	12.6897	13.0191	11.3005	11.2970	11.4892	12.4848	11.9533	11.0702	1.0120	0.0171	0.7928	0.1008
Tomm22	_LWGLTEM[Oxidation (M)]FPER_.2	#N/A	3.2336	5.1491	4.4681	4.4258	5.3196	5.2081	4.1405	5.4157	5.6642	5.1917	0.9081	-0.1391	0.3582	0.4458
Tomm22	_LQM[Oxidation (M)]EQQQQLQQR_.2	#N/A	3.6889	4.4251	4.6753	4.6342	3.9773	4.2056	4.4596	4.5347	4.5120	4.2922	0.9591	-0.0602	0.3945	0.4040
Tomm22	_AEAEKAEEELEEDDDDELDETLSER_.3	#N/A	4.1824	3.4378	3.9160	4.3032	3.3261	3.2985	3.1917	3.1406	4.2901	3.4440	1.0647	0.0904	0.4849	0.3143
Atp5mg	_VELVPPTPAEIPTAIQSVK_.2	#N/A	9.9745	9.9292	9.8853	10.0667	9.8061	9.8334	10.0734	10.1384	9.9445	9.7225	0.9946	-0.0078	0.5556	0.2553
Atp5mg	_LATFWHYAK_.2	#N/A	7.8626	7.7985	7.9919	7.8371	6.9070	7.5705	7.7923	7.9200	7.7431	7.3725	0.9941	-0.0086	0.8418	0.0748
	TPS[Phospho (STY)]PGEQQQVQEVK.2	#N/A	10.3239	10.4550	3.8517	10.9892	11.2248	10.5749	10.8789	10.4545	10.5389	10.6661	0.8999	-0.1522	0.4814	0.3175
	TPS[Phospho (STY)]PGEQQQVQEVKK.3	#N/A	3.1635	4.0627	3.8503	4.3529	5.3472	4.1745	4.0676	3.7656	3.4141	4.3199	1.0685	0.0956	0.5191	0.2847
Glo1	_RFEELGVK_.2	#N/A	6.8239	7.7514	7.5497	7.4552	7.9966	7.2260	7.2746	7.2131	7.5726	7.0637	1.0256	0.0364	0.4332	0.3633
Glo1	_GLAFIQDPDGYWIEILNPNK_.2	#N/A	5.8318	6.1443	5.4117	6.6766	5.6059	5.7695	6.1012	5.8086	5.9200	5.3269	1.0203	0.0290	0.6681	0.1752
Glo1	_DFLLQQTM[Oxidation (M)]LR_.2	#N/A	6.5706	6.6998	5.7765	6.2918	6.9522	7.0673	6.5419	6.5320	6.7969	3.7175	1.1124	0.1536	0.3199	0.4950
Glo1	_FEELGVK_.2	#N/A	4.1657	4.8085	4.7066	2.9846	4.3485	4.5235	3.8424	4.3326	4.5587	4.3341	0.9975	-0.0036	0.9769	0.0101
Glo1	_SLDFYTR_.2	#N/A	4.8716	4.0226	5.7640	4.2102	4.7162	5.0463	4.2463	4.3926	5.0015	4.8512	1.0322	0.0457	0.6810	0.1669
Glo1	_GFGHIGIAVPDVYSAC[Carbamidomethyl (C)]K_.2	#N/A	4.6138	3.6109	4.4557	3.6705	3.9426	3.7869	4.7976	2.0172	5.0722	3.6767	1.0315	0.0447	0.8405	0.0754
Mgst3	_IASGLGLAWIIGR_.2	#N/A	4.5948	4.2511	3.6512	3.7418	3.0613	3.9212	3.4565	3.8284	4.0012	4.3731	0.9886	-0.0165	0.8887	0.0513
Mgst3	_VLYAYGYYTGDPSK_.2	#N/A	3.2962	4.0525	3.8605	4.3631	3.7835	4.1846	4.0778	4.0912	3.4040	3.9885	1.0085	0.0122	0.8893	0.0509
Glod4	_VTLAVSDLQK_.2	#N/A	6.4554	6.6653	6.7694	7.2459	6.7200	6.8702	6.9687	6.6859	6.6673	6.6975	1.0049	0.0070	0.8274	0.0823
Glod4	_VAEGIFETEAPGGYK_.2	#N/A	6.7030	6.6871	6.9201	7.3239	6.6587	7.1651	6.4096	6.7410	6.4306	6.8983	1.0438	0.0618	0.1295	0.8878
Glod4	_FQTVHFFR_.2	#N/A	6.2282	6.5922	6.6672	6.6075	6.2982	6.3628	6.6113	6.5317	6.1386	6.6337	0.9970	-0.0043	0.8858	0.0527
Glod4	_SPSQSDPVLK_.2	#N/A	6.1940	6.1557	6.4415	6.5441	6.5450	6.4282	5.6845	6.4874	6.0207	6.0258	1.0545	0.0766	0.0682	1.1664
Glod4	_IYEQDEEKQR_.2	#N/A	4.4621	5.1900	5.3570	4.4929	4.8485	4.8224	4.0991	5.2663	5.0500	4.8583	1.0091	0.0130	0.8765	0.0572
Glod4	_ALLGYADNQC[Carbamidomethyl (C)]K_.2	#N/A	5.0712	5.6228	5.7994	5.9633	5.7449	5.2666	5.4090	5.8649	5.6118	5.6523	0.9900	-0.0145	0.7727	0.1120
Glod4	_FYLQDRS[Phospho (STY)]PSQSDPVLK_.3	#N/A	4.0211	3.6967	4.0146	6.0423	4.5515	5.5904	5.3863	5.3387	3.7839	5.8070	0.9161	-0.1265	0.4976	0.3032
Glod4	_ESHSILTPLVSLDTPGK_.3	#N/A	5.1809	5.0968	4.8876	5.5679	5.3987	5.5074	4.3605	4.9380	5.4359	5.5402	1.0404	0.0571	0.4408	0.3558
Glod4	_DVLGM[Oxidation (M)]QVLR_.2	#N/A	4.6367	4.2306	4.5951	5.0995	8.1277	7.7360	4.7450	5.2009	6.1929	4.8080	1.0957	0.1318	0.6009	0.2212
Glod4	_LELQGIQGAVDHAAAFGR_.3	#N/A	5.3242	5.1302	5.4100	5.5779	5.5282	5.4254	5.2074	5.0263	4.8756	5.5599	1.0449	0.0634	0.1413	0.8499
Glod4	_LLDDAMEADKSDEWFATR_.3	#N/A	3.6134	3.7290	3.3471	5.0667	4.2580	3.8675	4.5400	4.1103	4.4094	4.0690	0.9295	-0.1055	0.3800	0.4203
Arpc5	_ALAAGGVGSIVR_.2	#N/A	6.3218	6.7963	6.4833	7.0643	6.9713	6.7000	6.3803	6.7064	6.9415	6.5037	1.0135	0.0194	0.6203	0.2074
Lap3	_QVIDC[Carbamidomethyl (C)]QLADVNNLGK_.2	#N/A	7.5096	7.2361	7.9821	8.0272	7.5473	6.7325	7.8963	7.4401	7.3640	7.4105	0.9971	-0.0042	0.9363	0.0286
Lap3	_ANKPGDVVR_.2	#N/A	6.5349	6.3959	6.4931	6.7589	7.1011	6.9085	6.2647	6.5454	5.7852	6.3161	1.0756	0.1052	0.0364	1.4386
Lap3	_ADM[Oxidation (M)]GGAATIC[Carbamidomethyl (C)]SAIVSAAK_.2	#N/A	5.4225	5.3882	5.8541	5.2682	5.5068	5.4153	5.9181	4.8295	4.8081	6.3187	1.0013	0.0019	0.9824	0.0077
Lap3	_SAGAC[Carbamidomethyl (C)]TAAAFLR_.2	#N/A	5.0018	5.3899	4.0808	5.4928	5.8240	5.3476	5.0316	4.6705	5.2019	5.3421	1.0253	0.0360	0.7074	0.1503
Lap3	_DKDDDLPQFTSAGESFNK_.3	#N/A	3.1914	5.8667	3.8782	6.7147	5.9913	6.3461	4.0955	6.1148	5.6126	6.3123	0.9634	-0.0538	0.8145	0.0891
Lap3	_TLIEFLLR_.2	#N/A	3.0485	5.2159	3.4045	4.1213	1.3138	3.6005	3.9613	4.6492	4.5490	4.6194	0.7764	-0.3652	0.1766	0.7531
Lap3	_FAEIIEK_.2	#N/A	3.2591	3.9671	3.9459	4.4485	3.3513	4.2701	4.1632	2.9403	3.3185	2.1234	1.2351	0.3046	0.1134	0.9453
Lap3	_S[Phospho (STY)]WIEEQEMGSFLSVAK_.2	Q9CPY7_S238_M1_Lap3	3.6436	3.6988	3.3773	4.4219	3.5201	4.2094	3.0693	4.0801	4.3792	4.0992	0.9757	-0.0356	0.7663	0.1156
Mid1ip1	_T[Phospho (STY)]TPAPS[Phospho (STY)]PGSANESFFAPSR_.2	Q9CQ20_S71_M2_Mid1ip1	3.4556	4.2608	4.3161	3.8599	4.0821	7.2382	4.0082	3.1413	3.4403	3.5372	1.2842	0.3609	0.1944	0.7112
Lamtor1	_LLLDPSST[Phospho (STY)]PTK_.2	#N/A	3.8098	3.9926	9.3641	9.3909	3.5679	8.8982	3.8479	8.9826	9.0315	3.7505	1.0157	0.0225	0.9596	0.0179
Lamtor1	_KLLLDPSST[Phospho (STY)]PTK_.2	#N/A	10.2622	10.0993	10.9114	10.4382	9.7171	10.0496	10.6258	10.2143	2.9505	10.1250	1.2084	0.2732	0.2648	0.5771
Ndufc2	_DHDMFGYIK_.2	#N/A	5.3477	2.6822	4.0872	4.3685	3.7141	4.4114	4.3045	3.5286	3.1772	1.4496	1.3168	0.3970	0.1706	0.7681
Ndufc2	_TYAEILEPFHPVR_.2	#N/A	6.4497	5.7745	6.1274	6.1605	5.7229	5.5805	6.4231	6.3691	6.4374	5.5720	0.9627	-0.0548	0.3564	0.4480
Pgls	_DLPAAAAPAGPASFAR_.2	#N/A	6.8894	6.6364	6.4802	4.0793	7.4244	6.6647	6.8296	7.0477	6.6158	6.8152	0.9319	-0.1017	0.4603	0.3369
Pgls	_LPIPDSQVLTINPALPVEDAAEDYAR_.3	#N/A	4.0669	3.7963	3.8005	3.3443	5.3944	4.6727	3.4926	3.6569	4.3880	3.0216	1.1482	0.1994	0.2531	0.5967
Pgls	_TGALC[Carbamidomethyl (C)]WFLDEAAAR_.2	#N/A	3.0479	4.1782	3.7347	4.2374	3.6744	4.0589	3.9521	2.6793	3.5297	4.2044	1.0642	0.0898	0.5263	0.2788
Decr1	_VAGHPDVVINNAAGNFISPSER_.3	#N/A	5.7378	5.9722	5.5630	5.6831	4.5792	5.0842	5.5945	5.0593	5.6246	5.7617	0.9867	-0.0194	0.8056	0.0939
Uqcrq	_HVISYSLSPFEQR_.3	#N/A	4.5933	4.8297	4.5740	5.5831	5.2861	4.8602	4.3592	5.1937	5.5540	5.0720	0.9821	-0.0261	0.7592	0.1196
Uqcrq	_EFGNLAR_.2	#N/A	6.2953	6.2576	4.4479	5.6621	5.6905	5.8853	4.8036	6.4316	6.1986	6.0674	0.9713	-0.0421	0.7166	0.1447
Uqcrq	_AFPSYFSK_.2	#N/A	5.9347	6.3637	6.6058	5.7416	6.2999	6.0514	5.6814	6.5349	5.8465	3.4408	1.1470	0.1979	0.1918	0.7171
Uqcrq	_KNPAMYENDK_.2	#N/A	5.7706	4.6671	4.0784	6.3242	5.0679	3.4097	5.9706	3.3790	4.9702	3.2995	1.1093	0.1497	0.5390	0.2684
Ndufa2	_AHPNLPILIR_.2	#N/A	7.6639	8.0272	7.9979	8.0457	7.8567	7.3707	7.4969	7.9309	8.0331	7.8977	0.9984	-0.0023	0.9406	0.0266
Ndufa2	_TVSLNNLSADEVTR_.2	#N/A	6.9740	6.4770	7.1397	7.0370	6.9921	7.2266	6.9084	6.8168	6.4773	6.7727	1.0342	0.0485	0.1691	0.7720
Ndufa2	_EC[Carbamidomethyl (C)]SEVQPK_.2	#N/A	5.1055	5.1554	4.4679	4.2671	5.7123	4.6205	3.9818	4.7714	5.1483	4.9505	1.0372	0.0526	0.6201	0.2075
Ndufa2	_YAFGQEK_.2	#N/A	5.2168	5.7933	5.1680	4.4106	5.0905	5.3065	4.1127	5.5873	5.3322	5.6952	0.9966	-0.0049	0.9630	0.0164
Ndufa2	_AM[Oxidation (M)]QNVLSGK_.2	#N/A	5.1807	5.1142	4.9289	3.8498	5.8713	5.7845	5.4244	5.6636	5.3926	5.5074	0.9317	-0.1020	0.3464	0.4604
Mien1	_RAS[Phospho (STY)]NGEPVEK_.2	Q9CQ86_S97_M1_Mien1	3.4704	2.1863	3.2041	3.7432	3.9752	4.0573	3.7730	3.5793	2.3409	3.3320	1.0562	0.0789	0.6837	0.1652
Cuta	_TQSSLVPALTEFVR_.2	#N/A	5.8112	4.9151	5.5863	4.9347	6.5560	6.0592	4.5236	4.9596	5.6463	5.7750	1.0799	0.1109	0.3299	0.4816
Fis1	_GLLQTEPQNNQAK_.2	#N/A	6.4761	6.1886	7.1333	4.0200	6.3591	3.8074	6.7394	6.6669	6.4567	6.3227	0.8652	-0.2089	0.2527	0.5974
Fis1	_[Acetyl (Protein N-term)]M[Oxidation (M)]EAVLNELVSVEDLK_.2	#N/A	4.4521	4.8956	4.9223	4.8490	4.7222	5.3748	4.7340	4.7069	4.3419	5.1634	1.0280	0.0399	0.5321	0.2740
Fis1	_KFQSEQAAGS[Phospho (STY)]VSK_.2	Q9CQ92_S29_M1_Fis1	3.1031	4.1231	3.7899	4.2925	4.0114	3.9329	4.0072	3.8259	3.4745	4.2595	0.9958	-0.0061	0.9493	0.0226
Fis1	_STQFEYAWC[Carbamidomethyl (C)]LVR_.2	#N/A	3.7135	3.3999	3.6762	3.2200	3.2212	3.0074	3.3682	3.7812	4.0803	4.3981	0.8633	-0.2120	0.0445	1.3515
Sdhb	_LQDPFSVYR_.2	#N/A	8.4129	8.7792	8.5645	8.6782	8.7084	8.4963	8.6935	8.6233	8.6961	8.6281	0.9938	-0.0090	0.4818	0.3172
Sdhb	_DLVPDLSNFYAQYK_.2	#N/A	7.8120	8.1917	8.4647	8.5158	8.5512	8.4440	8.3245	8.1235	8.1127	8.2381	1.0159	0.0227	0.4109	0.3862
Sdhb	_QQYLQSIEDR_.2	#N/A	8.1149	7.8272	8.0695	8.2905	8.1344	8.0084	8.0358	7.6442	7.8899	8.0166	1.0225	0.0321	0.1319	0.8796
Sdhb	_RIDTDLSK_.2	#N/A	6.1308	5.9793	6.0330	6.1075	6.6382	5.8924	6.0027	5.8607	5.5439	5.8525	1.0542	0.0762	0.0759	1.1195
Sdhb	_WDPDKTGDKPR_.2	#N/A	6.8633	6.2222	4.7693	6.6665	7.1439	6.8937	6.3609	6.1962	6.4465	6.5821	1.0047	0.0068	0.9481	0.0231
Sdhb	_MQTYEVDLNK_.2	#N/A	5.9389	4.5150	4.2290	6.8686	5.5699	3.5602	5.1451	4.6352	3.5275	4.8852	1.1243	0.1690	0.4339	0.3626
Sdhb	_SIEPYLK_.2	#N/A	4.1102	2.6590	3.1804	4.2250	3.0738	2.2084	4.3733	2.0227	3.3305	4.0830	0.9393	-0.0903	0.7273	0.1383
Sdhb	_IKNEVDSTLTFR_.2	#N/A	3.3860	4.1423	3.1197	2.5279	4.0306	3.4003	4.3126	4.3377	3.1359	3.8416	0.8791	-0.1860	0.2456	0.6098
Sdhb	_QQYLQSIEDREK_.3	#N/A	4.1164	3.1097	3.3024	3.8051	2.8408	2.3946	3.5198	1.4436	3.9620	3.7721	1.0275	0.0391	0.8807	0.0552
Cisd2	_VVNEINIEDLC[Carbamidomethyl (C)]LTK_.2	#N/A	4.0723	3.3767	3.2583	3.7610	3.1980	4.3991	3.3914	4.3575	4.0061	3.7280	0.9501	-0.0739	0.5327	0.2736
Bzw1	_NAEEES[Phospho (STY)]ESEAEEGD_.2	Q9CQC6_S411_M1_Bzw1	7.2263	4.2307	3.2081	9.2148	7.1215	4.0065	9.5048	4.2493	3.5821	3.9300	1.0975	0.1342	0.7597	0.1194
Ndufb4	_VSHIEDPALIR_.2	#N/A	6.8328	6.7749	6.7072	7.1917	7.1409	6.6861	6.7579	6.8534	7.1624	6.6303	1.0055	0.0080	0.7993	0.0973
Ndufb4	_EYLLQYNDPK_.2	#N/A	7.1335	6.7187	7.0817	7.1636	6.9062	7.3301	7.0474	6.5929	6.4041	6.9689	1.0448	0.0632	0.1008	0.9965
Ndufb4	_EYLLQYNDPKR_.3	#N/A	6.4035	6.1150	6.7490	6.4273	6.4494	6.7474	6.3857	6.3462	5.9765	6.4279	1.0315	0.0447	0.2158	0.6659
Ndufb4	_YKPAPLATLPSTLDPAEYDVSPETR_.3	#N/A	5.1006	5.5232	5.9345	6.5290	5.5634	5.8066	5.8490	5.3159	5.2830	5.2336	1.0595	0.0834	0.2666	0.5741

Rab5a	_QASPNIVIALSGNK_.2	#N/A	4.9919	4.1574	5.2384	5.2479	4.3147	4.2973	4.9033	4.9354	4.9461	5.1885	0.9428	-0.0849	0.3101	0.5086
Rab5a	_GAQAAIVVYDITNEESFAR_.2	#N/A	4.2857	3.5412	4.0194	4.5803	3.4295	3.1951	3.0883	3.2440	4.3934	3.2238	1.1016	0.1397	0.3602	0.4434
Rgs10	_WASS[Phospho (STY)]LENLLEDPEGVQR_.2	Q9CQE5_S41_M1_Rgs10	6.5663	4.0181	3.8949	7.1912	7.0082	7.0357	6.5935	6.7468	7.0080	4.3646	0.9635	-0.0537	0.8145	0.0891
RTRAF	_INEAIVAVQAIIADPK_.2	#N/A	3.4398	5.0064	3.1734	4.2196	3.4968	4.0411	3.9343	3.2846	3.7907	4.1866	1.0256	0.0364	0.8015	0.0961
Nudt21	_LPGGELNPGEDEVEGLKR_.3	#N/A	5.9191	6.7459	7.0564	6.9121	6.2684	6.9095	6.5967	6.2423	6.2772	6.2369	1.0469	0.0661	0.2457	0.6097
Mtres1	_EADEEDS[Phospho (STY)]DEETSYPER_.2	Q9CQF4_S106_M1_Mtres1	11.3921	11.5212	11.6611	12.1987	11.4244	11.5669	11.8047	11.4246	11.4818	11.6926	1.0023	0.0033	0.8779	0.0566
Ndufb5	_NFYDGPEK_.2	#N/A	6.5057	6.4554	6.4451	5.7480	5.8345	5.7851	5.6905	6.9497	5.9780	6.5768	0.9730	-0.0394	0.5809	0.2359
Ndufb5	_EFIDHSPK_.2	#N/A	5.8201	5.5799	4.7114	3.2336	5.6531	3.5247	5.4960	5.1206	4.6811	4.9315	0.9400	-0.0893	0.6239	0.2049
Ndufb5	_TLAILQIESEKAELR_.2	#N/A	3.8378	4.2266	4.8916	3.8700	3.3593	3.7973	4.4548	4.1576	4.0903	4.0149	0.9564	-0.0643	0.5261	0.2789
Gmfb	_NTEDLTEEWLR_.2	#N/A	7.2323	7.0716	7.0669	7.4029	7.1290	7.3860	6.9506	7.0787	7.0446	7.2716	1.0181	0.0259	0.2086	0.6806
Gmfb	_LVQTAELTK_.2	#N/A	5.7747	5.5316	6.4229	6.0662	6.8956	5.9157	5.3887	6.0424	6.2784	6.5023	1.0080	0.0114	0.8819	0.0546
Gmfb	_LVVLDEELEGVSPDELKDELPER_.3	#N/A	4.4342	4.9719	5.2421	5.2336	5.0581	4.2752	4.2784	5.3575	4.7189	4.6885	1.0228	0.0325	0.7042	0.1523
Gmfb	_ETHNAAIIM[Oxidation (M)]K_.2	#N/A	4.2032	3.7595	3.8287	3.1553	5.1691	5.3368	4.5495	4.0374	4.5692	3.1223	1.0424	0.0599	0.7446	0.1281
Gmfb	_[Acetyl (Protein N-term)]SESLVVC[Carbamidomethyl (C)]DVAEDLVEK_.2	#N/A	9.5182	10.0859	11.3560	11.0698	9.8425	10.6789	10.0441	9.8327	9.4893	9.9599	1.0604	0.0846	0.1588	0.7992
Cotl1	_KAGGANYDAQS[Phospho (STY)]E_.2	#N/A	7.8014	8.0317	8.1896	8.3287	8.1162	7.5739	8.4036	7.8487	8.2439	4.1727	1.1172	0.1598	0.3284	0.4836
Cotl1	_EFVISDRK_.2	#N/A	6.9083	7.3780	7.5138	7.3389	7.4361	7.4108	7.0562	7.1153	7.4706	7.1754	1.0176	0.0251	0.3649	0.4379
Cotl1	_ELEEDFIR_.2	#N/A	7.1235	6.6896	6.2941	6.7737	6.9907	7.3353	5.4415	6.9306	6.8176	7.1842	1.0416	0.0588	0.4697	0.3282
Cotl1	_EVVQNFAK_.2	#N/A	4.6480	5.7144	4.5964	5.0315	6.0330	5.2975	3.5755	5.7565	6.2340	4.6682	1.0319	0.0454	0.7778	0.1092
Cotl1	_EFVISDR_.2	#N/A	6.5393	5.6685	5.9140	5.6203	5.6873	6.1588	4.2018	5.8622	6.4665	5.9507	1.0553	0.0777	0.4896	0.3101
Cotl1	_YDGATIVPGDQGADYQHFIQQC[Carbamidomethyl (C)]TDDVR_.3	#N/A	1.4475	3.4949	3.9732	3.4199	3.3832	3.2414	3.1346	3.1978	4.3472	3.3869	0.8986	-0.1543	0.4953	0.3051
Cotl1	_AGGANYDAQS[Phospho (STY)]E_.2	#N/A	9.8004	9.8166	9.8936	9.8938	9.2110	8.7629	9.9271	9.6436	9.6504	9.7043	0.9827	-0.0252	0.5123	0.2905
Cotl1	_FALITWIGEDVSGLQR_.2	#N/A	4.9367	4.4688	3.7104	4.2796	4.2713	3.8884	4.4600	4.0201	5.0812	4.4942	0.9436	-0.0838	0.3905	0.4083
Cotl1	_FTTGDAMSK_.2	#N/A	3.0172	4.2089	3.7040	4.6762	4.8441	4.0282	3.9214	3.9118	3.5604	4.1737	1.0483	0.0681	0.6108	0.2141
Denr	_LTVENS[Phospho (STY)]PK_.2	#N/A	7.0542	6.4970	4.5893	7.3101	5.9787	3.2005	6.9457	6.9001	4.1760	7.0312	0.9215	-0.1179	0.6291	0.2013
Denr	_WPEVDDDSIEDLGEVKK_.3	#N/A	5.1810	5.1009	4.5531	6.1109	5.4150	5.9476	5.5057	5.0616	5.5879	5.4401	0.9974	-0.0038	0.9646	0.0156
Denr	_LTVENS[Phospho (STY)]PKQETGITEGQGPVGEEEEK_.3	#N/A	4.2128	3.4682	3.9465	3.4466	3.3565	3.2681	3.1612	3.1711	4.3205	5.1719	0.9141	-0.1295	0.4531	0.3438
Ndufb9	_HLESWC[Carbamidomethyl (C)]IHR_.3	#N/A	5.1175	5.2737	5.7348	6.3438	4.3925	5.3608	3.3763	6.1939	5.2012	5.6892	1.0499	0.0703	0.6755	0.1704
Ndufb9	_VPEWC[Carbamidomethyl (C)]LDYWHPSEK_.3	#N/A	3.8723	3.0363	4.0397	5.0724	5.2632	4.9349	3.7318	3.4177	3.7167	5.1894	1.0887	0.1226	0.5302	0.2756
Ndufb9	_ARFEEHKNEK_.3	#N/A	3.7919	3.0037	4.3862	3.3880	2.8251	3.9613	3.1027	3.2296	4.6518	3.3550	0.9929	-0.0103	0.9528	0.0210
Ndufb9	_EAEEEFWQNQHPQPYIFPDSPGGTSFER_.3	#N/A	3.8318	3.5106	3.5655	4.6101	3.3319	4.3976	3.2576	3.8919	4.1909	4.2874	0.9917	-0.0120	0.9223	0.0351
Nicn1	_S[Phospho (STY)]PSLAFPK_.2	Q9CQM0_S142_M1_Nicn1	6.0209	3.8156	3.6972	4.1715	3.6369	5.7507	4.4534	3.9186	5.9007	4.1669	0.9795	-0.0299	0.8888	0.0512
Txndc17	_SWC[Carbamidomethyl (C)]PDC[Carbamidomethyl (C)]VEAEPVIR_.2	#N/A	6.4294	6.8774	6.7259	6.7345	7.2169	6.2777	6.8546	6.1507	6.4005	6.4972	1.0362	0.0513	0.2856	0.5442
Txndc17	_[Acetyl (Protein N-term)]ATFEEVSVLGFEEFDK_.2	#N/A	4.8957	5.7305	7.0620	6.8959	5.9054	6.4700	5.9003	4.2623	5.6923	5.3516	1.1619	0.2165	0.1275	0.8945
Txndc17	_LVESEC[Carbamidomethyl (C)]C[Carbamidomethyl (C)]QSSLVEM[Oxidation (M)]IFSED_.2	#N/A	5.3909	5.0820	5.9853	7.2033	5.1209	5.1274	5.4639	5.2366	6.4417	5.0900	1.1064	0.1459	0.2582	0.5881
Glrx3	_HNIQFSSFDIFSDEEVR_.3	#N/A	4.8656	3.4389	5.7577	5.2107	4.0354	4.8026	3.5378	4.1192	4.2558	5.3466	1.0858	0.1188	0.4953	0.3052
Glrx3	_[Acetyl (Protein N-term)]AAGAAEAGEAAVAVVEVGSAQQFEELLR_.3	#N/A	4.0316	3.2870	3.7652	4.4771	3.0926	3.4493	2.4278	2.1876	4.1393	3.5948	1.1932	0.2548	0.2238	0.6502
Trap1	_AFLEALQNQAETSSK_.2	#N/A	3.9427	3.6745	3.6763	3.2201	3.4168	3.0076	3.3684	4.2328	4.0801	4.3982	0.8681	-0.2041	0.0668	1.1750
Trap1	_GVVDSEDIPLNLS[Phospho (STY)]R_.2	Q9CQN1_S403_M1_Trap1	4.0087	3.2175	3.1946	7.8853	3.1058	3.5188	3.4120	7.4911	4.0698	3.6643	0.8918	-0.1652	0.6881	0.1623
Tomm6	_NLSDIDLM[Oxidation (M)]APQPGV_.2	#N/A	4.1822	3.9105	3.7921	4.2948	5.2417	5.5535	4.0095	4.5926	5.0309	5.0998	0.9600	-0.0589	0.6683	0.1750
Trappc2	_FAMNPFYEPNS[Phospho (STY)]PIR_.2	Q9CQP2_S119_M1_Trappc2	3.6635	4.1713	3.3972	7.2592	4.1698	3.8174	3.7105	4.1226	3.7712	3.9628	1.1339	0.1813	0.4963	0.3042
Atp5pb	_HYLFDVQR_.2	#N/A	8.8468	8.9786	9.2050	8.8860	8.4613	8.8849	9.0692	9.1936	8.9657	8.6779	0.9889	-0.0161	0.5281	0.2773
Atp5pb	_IAQLEEVK_.2	#N/A	7.5319	7.5616	7.6502	7.0355	7.5507	7.6416	6.7003	7.8326	7.8161	7.6530	0.9993	-0.0010	0.9834	0.0073
Atp5pb	_AQQALVQK_.2	#N/A	6.5824	6.9641	6.8545	6.5288	6.7991	6.3245	6.1066	6.6833	6.8421	6.7930	1.0105	0.0151	0.7120	0.1475
Atp5pb	_QIQDAIDMEK_.2	#N/A	6.3530	4.8745	3.8738	6.6900	5.3842	3.9160	4.9894	3.3813	3.3906	5.2025	1.2219	0.2891	0.2294	0.6394
Atp5pb	_SISVQQEKETIAK_.2	#N/A	5.4786	5.8761	6.4696	5.8371	5.0524	5.7177	5.6715	5.6489	5.0106	4.6194	1.0957	0.1318	0.1493	0.8260
Atp5pb	_RKEEEHM[Oxidation (M)]IDWVEK_.4	#N/A	7.2057	5.1345	3.7746	4.1628	4.5217	5.9726	6.1892	5.5769	5.7090	5.4322	0.8956	-0.1591	0.3945	0.4039
Atp5pb	_KEEEHMIDWVEK_.3	#N/A	3.9123	3.1678	3.6460	3.1898	3.0561	3.5686	3.3380	3.8114	4.1105	4.3679	0.8762	-0.1906	0.0837	1.0771
Atp5pb	_LDYHISVQNM[Oxidation (M)]M[Oxidation (M)]R_.3	#N/A	4.3216	4.4054	3.5076	4.0103	2.1859	4.2822	3.7250	4.1082	3.7568	3.9773	0.9727	-0.0400	0.8149	0.0889
Rps21	_TYGIC[Carbamidomethyl (C)]GAIR_.2	#N/A	5.6917	6.2900	5.4600	6.4489	6.7574	5.8681	5.8470	6.0221	6.2205	5.7370	1.0217	0.0310	0.6391	0.1944
Rps21	_[Acetyl (Protein N-term)]M[Oxidation (M)]QNDAGEFVDLYVPR_.2	#N/A	3.6008	3.7811	4.2498	4.2687	2.5947	4.3319	4.2526	3.6298	3.7924	3.9558	0.9736	-0.0386	0.7784	0.1088
Rps21	_DHASIQM[Oxidation (M)]NVAEVDR_.2	#N/A	3.6405	3.6099	3.7683	3.8646	4.1881	4.8181	3.7438	3.7716	5.2036	4.5717	0.9211	-0.1186	0.3752	0.4258
Acot13	_VTLVSAAPEK_.2	#N/A	7.6612	7.9905	8.3025	7.8757	7.9379	6.5246	6.5149	7.7288	6.8070	6.6354	1.1147	0.1567	0.0724	1.1402
Acot13	_TLAFASVDLTNK_.2	#N/A	5.9856	6.1771	5.7252	7.1932	6.6734	6.2069	6.2555	5.9725	5.7145	6.3342	1.0425	0.0600	0.4016	0.3962
Acot13	_[Acetyl (Protein N-term)]SSMTQNLR_.2	#N/A	3.2846	4.0578	4.5191	4.0629	0.3837	4.1963	4.2111	4.4391	3.2374	3.7402	0.8747	-0.1931	0.5642	0.2486
Ppp6c	_APLDLDKYVEIAR_.3	#N/A	2.9908	3.0126	4.2254	2.6671	3.0196	2.9892	3.5580	3.2634	3.5311	3.2848	0.9242	-0.1138	0.3895	0.4095
Slc25a46	_SFGSGTELGHWVTT[Phospho (STY)]PPDIPGSR_.3	Q9CQS4_T45_M1_Slc25a46	4.1666	5.8873	6.1636	7.5262	3.3103	3.3143	3.5923	3.5572	3.8562	4.4359	1.3111	0.3908	0.2166	0.6644
Sec61b	_PGPTPSGTNVGSSGRS[Phospho (STY)]PSK_.2	#N/A	6.6428	6.0439	6.8238	6.3348	5.2878	6.1741	6.5034	6.6043	6.7621	5.3965	0.9844	-0.0227	0.7961	0.0990
Sec61b	_S[Phospho (STY)]PSKAVAAR_.2	#N/A	3.4032	3.8230	5.1029	3.6627	4.0297	3.6998	4.3073	3.5259	3.1744	3.0588	1.1243	0.1690	0.2814	0.5506
Rer1	_VDPSLM[Oxidation (M)]EDS[Phospho (STY)]DDGPSLPTK_.2	#N/A	3.6895	3.7188	3.7248	7.1983	3.5640	3.9209	4.1919	8.1112	3.4784	7.3977	0.7425	-0.4295	0.2357	0.6277
Rer1	_[Acetyl (Protein N-term)]S[Phospho (STY)]EGDSVGDSVHGKPSVVYR_.3	Q9CQU3_S2_M1_Rer1	2.9972	3.8024	3.6840	4.3183	3.6237	4.1058	4.4666	4.1837	2.9819	4.1536	0.9515	-0.0717	0.5987	0.2228
Zwint	_NQS[Phospho (STY)]YLQLLC[Carbamidomethyl (C)]SLQNK_.2	Q9CQU5_S216_M1_Zwint	4.2138	4.3233	3.3998	5.8936	2.9006	3.7240	3.6171	3.8899	3.8646	3.8694	1.0697	0.0972	0.6299	0.2007
Retreg3	_AM[Oxidation (M)]S[Phospho (STY)]GSWER_.2	#N/A	6.8391	6.6756	7.4307	6.5443	5.3787	6.8065	7.4582	7.3175	7.0968	6.9752	0.9169	-0.1252	0.1305	0.8843
Retreg3	_GQTPLT[Phospho (STY)]EGSEDLDGHSDPEES[Phospho (STY)]FAR_.3	Q9CQV4_S325_M2_Retreg3	3.5694	3.7150	3.7649	5.4657	3.7064	3.7142	4.0535	5.0631	3.5440	3.5401	0.9850	-0.0218	0.8994	0.0460
Map1lc3b	_S[Phospho (STY)]FEQRVEDVR_.2	Q9CQV6_S12_M1_Map1lc3b	7.5706	8.3899	8.6574	8.7436	8.5602	8.1738	8.5021	8.3161	8.4515	8.1407	0.9996	-0.0006	0.9887	0.0049
Dnajc19	_EAALILGVS[Phospho (STY)]PTANK_.2	Q9CQV7_S70_M1_Dnajc19	3.8149	3.1193	4.5017	3.5006	2.9406	3.2880	3.6488	3.5006	3.7996	3.4705	0.9785	-0.0313	0.7996	0.0971
Ywhab	_DSTLIM[Oxidation (M)]QLLR_.2	#N/A	10.2856	9.7964	9.5232	10.2517	9.9818	9.7059	10.0111	9.6807	9.7745	9.9140	1.0080	0.0115	0.6472	0.1890
Ywhab	_YLSEVASGENK_.2	#N/A	10.1674	10.2806	10.4125	10.5327	10.3377	10.4211	10.5455	10.3671	10.4084	10.3793	0.9936	-0.0092	0.3855	0.4140
Ywhab	_LAEQAER_.2	#N/A	8.9345	8.8263	9.0034	8.5154	9.6819	8.7431	7.5253	9.0126	9.3538	9.2292	1.0194	0.0278	0.6748	0.1708
Ywhab	_YLILNATQAESK_.2	#N/A	8.1008	7.6517	8.0333	8.5883	8.2332	8.3738	8.0629	8.2312	8.4611	8.4004	0.9849	-0.0220	0.5014	0.2999
Ywhab	_AVTEQGHELSNEER_.3	#N/A	7.3024	7.3264	7.6090	7.4270	7.8937	7.4126	6.7895	7.0956	7.3445	7.1658	1.0558	0.0784	0.0265	1.5770
Ywhab	_AKLAEQAER_.2	#N/A	7.8299	7.7440	8.2302	7.5000	7.8867	8.2066	7.9767	7.7502	7.9109	7.9802	0.9994	-0.0009	0.9744	0.0113
Ywhab	_QTTVSNSQQAYQEAFEISKK_.3	#N/A	7.1583	7.4075	8.2703	7.5138	7.2334	7.3219	7.2475	7.1753	7.5963	7.3128	1.0206	0.0295	0.5096	0.2927
Ywhab	_QTTVSNSQQAYQEAFEISK_.2	#N/A	5.9217	5.5386	6.2958	6.1442	5.5808	5.7809	6.4545	5.4143	6.1787	5.9330	0.9803	-0.0287	0.6265	0.2031
Ywhab	_DNLTLWTSENQGDEGDAGEGEN_.2	#N/A	12.9611	13.1466	14.0091	14.0034	13.2157	13.4572	13.8182	13.3885	13.5225	13.3661	0.9957	-0.0062	0.8165	0.0881
Ywhab	_EM[Oxidation (M)]QPTHPIR_.2	#N/A	10.3622	10.7367	10.5716	10.8402	11.1492	10.4194	10.3569	10.7210	10.5469	10.4860	1.0145	0.0207	0.3726	0.4288
Ywhab	_M[Oxidation (M)]KGDYFR_.2	#N/A	8.5288	8.5751	8.4069	8.8874	9.0191	8.3076	8.4613	8.6229	8.5254	8.5465	1.0096	0.0138	0.5853	0.2326
Ywhab	_VISS[Phospho (STY)]IEQK_.2	Q9CQV8_S66_M1_Ywhab	3.9486	3.2040	8.4751	7.8809	7.7285	7.4265	7.4874	3.7751	4.0742	7.8308	1.1126	0.1539	0.6618	0.1793
Ywhab	_[Acetyl (Protein N-term)]T[Phospho (STY)]M[Oxidation (M)]DKSELVQK_.2	#N/A	6.8039	7.2033	7.4083	7.2465	6.8353	7.2223	7.1419	6.9147	7.0860	7.1072	1.0081	0.0117	0.6718	0.1728
Ywhab	_KEM[Oxidation (M)]QPTHPIR_.3	#N/A	7.2783	7.1990	7.2979	6.4856	8.6570	7.9255	7.0829	7.4422	8.2525	7.6460	0.9826	-0.0253	0.7632	0.1173

Ywhab	_TAFDEAIAELDTLNEESYK_.2	#N/A	7.6299	7.3887	7.2563	8.1996	7.0329	7.5140	7.6944	7.1593	7.1169	7.4035	1.0218	0.0311	0.5048	0.2968
Ywhab	_YDDM[Oxidation (M)]AAAM[Oxidation (M)]K_.2	#N/A	4.8417	4.8061	4.4562	5.2841	6.0868	4.3209	4.7787	5.1558	5.6644	4.9442	0.9669	-0.0485	0.6517	0.1860
Ywhab	_NLLSVAYK_.2	#N/A	10.2738	10.1138	10.4176	9.7717	10.4250	10.4692	8.4008	10.5754	10.6523	10.5957	1.0188	0.0269	0.6899	0.1612
Ywhab	_IEAELQDIC[Carbamidomethyl (C)]NDVLELLDK_.2	#N/A	3.2628	3.9634	3.9496	3.2566	3.8517	4.2738	4.1669	2.5284	0.9827	4.4192	1.2431	0.3140	0.3016	0.5205
Arl8b	_DLPNALDEK_.2	#N/A	6.4436	6.5107	6.6943	6.5455	5.7299	6.4556	6.6804	6.6070	6.5997	6.7483	0.9606	-0.0580	0.1726	0.7631
Cyb5b	_[Acetyl (Protein N-term)]AT[Phospho (STY)]PEASGSGEKVEGSEPSVTYYR_.3	#N/A	6.8833	7.2454	7.8061	7.4717	6.8364	7.4831	7.3945	7.6307	7.2642	7.7463	0.9705	-0.0431	0.3247	0.4885
Cyb5b	_RNS[Phospho (STY)]AEETWM[Oxidation (M)]VIHGR_.3	#N/A	4.8582	5.0594	6.0004	5.8521	4.9476	5.0125	5.6244	5.7111	5.2258	5.5532	0.9565	-0.0641	0.3979	0.4003
Cyb5b	_FLSEHPGGEEVLLEQAGADATESFEDVGHSPDAR_.4	#N/A	5.4980	6.2254	6.7234	7.9388	5.8035	6.3886	6.4927	6.4435	6.1042	6.6913	0.9995	-0.0007	0.9943	0.0025
Cyb5b	_[Acetyl (Protein N-term)]AT[Phospho (STY)]PEASGSGEK_.2	#N/A	8.9336	8.7137	8.6274	9.0030	7.9172	9.0551	8.9925	8.6826	3.8790	9.0217	1.1392	0.1881	0.3259	0.4869
Cyb5b	_NSAEETWM[Oxidation (M)]VIHGR_.2	#N/A	3.3684	4.0385	3.8856	3.8054	3.9874	4.5212	3.7649	3.6702	3.7630	4.0572	1.0316	0.0449	0.5667	0.2466
Mrps36	_LSASEALGSAALPSHSSAISQHSK_.3	#N/A	5.3205	5.5334	3.8581	5.9534	6.1986	5.5143	5.6428	5.3711	5.3390	5.4156	0.9916	-0.0122	0.9163	0.0379
Ramac	_RPPES[Phospho (STY)]PPIVEEWNSR_.3	#N/A	6.1057	8.7095	6.8345	9.5349	8.5416	9.1123	6.7659	8.6948	8.2340	8.8387	1.0008	0.0011	0.9937	0.0027
Ramac	_[Acetyl (Protein N-term)]S[Phospho (STY)]DTSEEIPNFEEMFASR_.2	Q9CQY2_S2_M1_Ramac	4.1708	3.4262	3.9045	3.4482	3.3145	3.3460	3.5965	5.5630	3.8879	3.9506	0.8476	-0.2386	0.1393	0.8561
Hsbp1	_TM[Oxidation (M)]QDITLVVETLLQQM[Oxidation (M)]QDK_.3	#N/A	3.1330	4.0903	4.4866	3.7872	4.1173	3.8178	4.1786	2.6092	4.3179	3.7077	1.0546	0.0766	0.6133	0.2123
Hsbp1	_NIADLM[Oxidation (M)]TQAGVEELDPENKIPTAQKS[Phospho (STY)]_.3	Q9CQZ1_S76_M1_Hsbp1	4.3839	4.2241	4.2809	3.8399	3.2038	4.2199	3.9300	3.7764	4.3687	4.1097	0.9949	-0.0074	0.9352	0.0291
Hsbp1	_NIADLMTQAGVEELDPENKIPTAQK_.3	#N/A	3.4705	4.3390	3.5740	4.9219	3.5377	3.8981	3.7913	4.0419	3.6904	4.0436	1.0167	0.0239	0.8341	0.0788
Ndufa6	_QAAAAAASTSVKPIFSR_.2	#N/A	4.3788	4.0867	3.8421	5.2183	4.5831	4.4856	3.5519	4.0978	4.4429	4.1772	1.0897	0.1240	0.2326	0.6333
Ndufa6	_NAHVTDPR_.2	#N/A	5.6809	6.0338	4.3378	5.5202	6.7850	5.2754	5.4543	5.4575	3.4187	3.5588	1.2534	0.3258	0.1011	0.9954
Ndufa6	_MELQETIK_.2	#N/A	3.9912	3.2538	3.1254	4.3700	3.5995	3.1635	3.3428	2.9681	2.0806	3.5951	1.1960	0.2582	0.1490	0.8267
Ndufa6	_VVDLLVIK_.2	#N/A	2.0724	3.0525	3.5308	2.6611	1.9434	1.0399	3.5769	2.4387	1.6357	1.1262	1.0861	0.1192	0.7680	0.1146
Ndufa6	_EVPNTVHLM[Oxidation (M)]QLDITVK_.2	#N/A	3.8492	3.1047	3.5829	4.6275	2.9930	3.6316	3.5248	8.2416	4.1735	4.3048	0.7175	-0.4789	0.1509	0.8212
Ndufb3	_MELPDYR_.2	#N/A	6.6322	4.8778	3.2602	6.7284	5.4551	3.9543	4.7839	4.5259	4.8703	3.9821	1.1345	0.1821	0.4293	0.3672
Psmd9	_[Acetyl (Protein N-term)]S[Phospho (STY)]GEDVPHR_.2	Q9CR00_S2_M1_Psmd9	8.8117	8.4097	8.4308	8.8832	8.6943	8.9205	8.5550	8.3061	8.7166	9.1032	1.0025	0.0036	0.9052	0.0432
Psmd9	_ADVDLYQVR_.2	#N/A	3.6197	6.1123	6.5557	5.5106	5.8448	5.7399	6.1580	5.4412	5.9584	6.0292	0.9436	-0.0838	0.5504	0.2593
Psmd9	_LASNS[Phospho (STY)]PVLPQAFAR_.2	Q9CR00_S128_M1_Psmd9	3.7482	7.5308	3.4819	9.5317	2.8919	3.7327	3.1739	3.9755	4.2746	4.2037	1.3189	0.3993	0.3986	0.3995
Ppid	_VFFDVDIGGER_.2	#N/A	5.3303	5.3408	4.2948	6.2775	5.7668	4.7965	5.7399	5.7801	6.3645	5.9446	0.8899	-0.1684	0.1174	0.9304
Ppid	_ILLISEDLK_.2	#N/A	4.6979	5.1423	3.8029	4.1994	3.8929	4.6947	4.3477	4.8177	4.6662	3.9037	0.9935	-0.0094	0.9288	0.0321
Ppid	_AIQAELLK_.2	#N/A	3.3066	4.1632	5.0019	4.6726	5.2140	5.1408	4.2164	4.5843	4.4782	4.2623	1.0451	0.0637	0.6177	0.2092
Ppid	_IVLELFADIVPK_.2	#N/A	3.6312	3.5949	4.3180	3.3199	1.1320	3.1414	4.5354	3.2978	2.9464	3.2869	0.9070	-0.1408	0.6115	0.2136
Ppid	_EYDQALADLKK_.3	#N/A	3.2080	3.9643	4.4425	3.9863	3.8526	4.2728	4.1346	3.0149	3.3139	3.6636	1.1197	0.1631	0.1817	0.7406
Ppid	_AQEIAPGDK_.2	#N/A	4.1662	3.1762	3.8999	3.4437	3.3100	3.3146	3.5919	3.5575	3.8565	3.1210	1.0057	0.0082	0.9337	0.0298
Ppid	_[Acetyl (Protein N-term)]SHASPAAKPSNSK_.3	#N/A	3.6006	3.6256	4.2874	3.2892	3.5139	3.1107	4.5047	3.3284	2.9770	3.2562	1.0155	0.0222	0.8753	0.0578
Ndufab1	_LYDKIDPEK_.2	#N/A	8.5604	8.8554	8.8197	9.1991	9.3909	8.9184	8.9171	8.9670	8.7572	8.8124	1.0106	0.0152	0.5620	0.2503
Ndufab1	_LM[Oxidation (M)]C[Carbamidomethyl (C)]PQEIVDYIADKK_.3	#N/A	8.1532	8.3745	8.0979	8.3653	8.4941	8.4088	8.1433	8.3207	8.3417	8.2456	1.0064	0.0092	0.5596	0.2521
Dph2	_PALPLPVGS[Phospho (STY)]PSSQPESLER_.2	Q9CR25_S188_M1_Dph2	5.7942	6.4906	3.1678	7.8393	4.7882	3.9999	4.3607	6.7175	3.0878	3.8897	1.1845	0.2443	0.4580	0.3392
Vta1	_YAGSALQYEDVGTAVQNLQK_.2	#N/A	4.6826	5.0123	5.5557	4.8440	3.7380	3.6582	2.3030	4.3406	3.8249	2.5832	1.4042	0.4898	0.0410	1.3876
Vta1	_LTPEDFAR_.2	#N/A	3.6603	2.9646	4.3471	3.3489	4.4857	3.3427	3.0636	3.2687	3.1317	3.3159	1.1606	0.2149	0.1330	0.8760
Ccdc43	_DSLRDES[Phospho (STY)]QR_.2	Q9CR29_S186_M1_Ccdc43	7.2427	7.4296	4.4853	7.8261	3.9129	3.8166	4.1774	7.7357	3.2711	7.0340	1.0416	0.0588	0.8627	0.0641
Ccdc43	_AALLAQYADVT[Phospho (STY)]DEEDEADKKDDAGASTANVSSDR_.4	Q9CR29_T137_M1_Ccdc43	4.3501	2.9923	4.0838	3.6276	2.8136	3.4151	3.7759	3.3736	3.6726	3.3049	1.0043	0.0063	0.9629	0.0164
Atp6v1g1	_[Acetyl (Protein N-term)]ASQSQGIQQLLQAEK_.2	#N/A	5.9768	6.2295	5.6739	6.2184	6.9313	6.4465	6.2736	5.5048	6.0667	6.2498	1.0369	0.0523	0.4169	0.3800
Atp6v1g1	_[Acetyl (Protein N-term)]ASQSQGIQQLLQAEKR_.3	#N/A	4.9469	5.0305	5.6061	5.3368	5.1443	5.5990	3.7473	4.4798	4.6193	3.3475	1.3035	0.3824	0.0023	2.6412
Rpl14	_AAILKAS[Phospho (STY)]PK_.2	Q9CR57_S139_M1_Rpl14	11.9201	12.0223	12.9079	12.1973	11.6790	11.8659	12.4402	12.6203	12.0304	11.9811	0.9862	-0.0200	0.5217	0.2826
Rpl14	_LVAIVDVIDQNR_.2	#N/A	6.1537	5.9384	6.8819	6.4863	5.8161	5.9881	6.3626	6.2642	5.7562	5.8671	1.0244	0.0348	0.5480	0.2613
Rpl14	_AAIAAAAAAAAAK_.2	#N/A	1.4009	3.3489	3.8271	3.5659	3.2372	3.3875	3.2806	3.0517	1.6685	3.5329	1.0848	0.1174	0.6703	0.1738
Ndufb7	_DYC[Carbamidomethyl (C)]AHYLIR_.2	#N/A	7.4995	7.2349	7.7245	8.0978	7.6793	7.7640	7.5646	7.6776	7.1291	7.2445	1.0355	0.0503	0.1802	0.7442
Ndufb7	_DSFPNFLAC[Carbamidomethyl (C)]K_.2	#N/A	6.4330	6.0888	6.3110	6.4397	6.2346	5.6126	5.7563	6.2324	5.9055	6.3066	1.0225	0.0322	0.4916	0.3084
Ndufb7	_VAQGQGEGEVGPEVAL_.2	#N/A	3.1651	6.4947	6.0578	6.5765	6.2987	6.5599	4.2089	6.5092	5.8843	6.3955	1.0190	0.0272	0.8947	0.0483
Ndufb7	_HEQHDWDYC[Carbamidomethyl (C)]EHLDYVK_.4	#N/A	4.6455	4.0681	3.4744	3.9771	3.4141	4.3153	3.6918	4.1414	3.7900	5.0801	0.9537	-0.0684	0.5948	0.2256
Slc25a11	_[Acetyl (Protein N-term)]AATASPGAGR_.2	#N/A	7.5064	8.0990	7.7999	7.6702	7.2507	7.6168	7.8303	7.9844	7.3259	7.6567	0.9945	-0.0079	0.8239	0.0841
Slc25a11	_NVFNALVR_.2	#N/A	7.7118	7.6676	8.0342	7.0493	6.9575	7.3824	7.6138	8.0303	7.9137	7.9396	0.9483	-0.0766	0.1062	0.9738
Slc25a11	_TSFHALTSILK_.2	#N/A	6.3203	6.1760	6.6832	5.6931	5.1945	5.6312	5.8249	6.0459	5.8529	5.9423	1.0056	0.0081	0.9081	0.0419
Slc25a11	_LTGADGTPPGFLLK_.2	#N/A	6.5631	7.1524	3.2412	5.6459	6.4541	6.2855	7.1300	7.2784	6.3552	6.2834	0.8711	-0.1990	0.2703	0.5682
Slc25a11	_AVVVNAAQLASYSQSK_.2	#N/A	5.9363	3.1797	3.6579	3.2017	4.5367	5.5101	5.2741	4.4795	5.5726	5.2399	0.8435	-0.2455	0.2401	0.6196
Slc25a11	_M[Oxidation (M)]IDGKPEYK_.2	#N/A	4.1274	4.4033	3.3030	2.3207	4.4729	4.5448	4.0279	4.7243	4.3133	4.5428	0.8773	-0.1888	0.2795	0.5536
Slc25a11	_MQLSGEGAK_.2	#N/A	5.9924	4.1026	4.5934	6.6117	5.0605	3.1963	4.7740	5.0463	4.7190	4.3263	1.0445	0.0628	0.7542	0.1225
Slc25a11	_LGIYTVLFER_.2	#N/A	4.5492	5.2225	4.2057	3.7495	4.1925	3.5370	5.3061	5.7651	3.5507	4.5538	0.8850	-0.1762	0.2932	0.5329
Slc25a11	_ALIGM[Oxidation (M)]TAGATGAFVGTPAEVALIR_.2	#N/A	3.6216	4.1760	4.7041	3.1304	3.8169	3.7866	4.4984	4.5559	3.3532	3.5108	0.9731	-0.0393	0.7796	0.1081
Slc25a11	_QATYTTTR_.2	#N/A	3.5153	3.7108	4.2021	3.2039	3.5991	3.0255	4.4195	4.2628	3.0623	2.9958	0.9614	-0.0568	0.7083	0.1498
Slc25a11	_FLFGGLAGM[Oxidation (M)]GATVFVQPLDLVK_.2	#N/A	3.5634	3.6627	4.2502	3.2520	3.5510	3.0736	2.2525	3.3656	3.0142	3.2190	1.2012	0.2644	0.0694	1.1583
Uqcrfs1	_SGPFAPVLSATSR_.2	#N/A	9.1546	9.3178	9.5526	9.4538	9.7004	9.4765	9.3388	9.3956	9.3168	9.3495	1.0099	0.0142	0.3699	0.4319
Uqcrfs1	_LSDIPEGK_.2	#N/A	7.8992	7.9855	8.1162	7.3818	8.3049	8.0441	6.6683	8.1727	8.1407	7.9739	1.0280	0.0398	0.5255	0.2795
Uqcrfs1	_GVAGALRPLLQGAVPAASEPPVLDVK_.3	#N/A	7.1643	7.3320	7.3708	7.3223	7.4547	7.4619	7.6620	7.2713	7.3418	7.2513	0.9959	-0.0060	0.7524	0.1235
Uqcrfs1	_RAEVLDSTK_.2	#N/A	7.0164	7.3052	6.9369	7.0138	7.2855	7.2510	6.7501	7.2246	6.7537	7.0217	1.0284	0.0405	0.1480	0.8296
Uqcrfs1	_VPDFSDYR_.2	#N/A	7.0092	6.5021	6.4624	6.6745	6.7501	6.2364	6.6653	6.4281	6.8053	6.2837	1.0092	0.0132	0.7242	0.1401
Uqcrfs1	_DPQHDLDR_.2	#N/A	6.6982	6.0573	5.7988	6.0347	7.0424	6.0871	6.1913	6.1331	6.2012	6.1110	1.0207	0.0295	0.6156	0.2107
Uqcrfs1	_PLVATVGLNVPASVR_.2	#N/A	5.9692	3.7197	5.7590	4.6604	5.5231	5.4394	5.3804	5.0569	5.4912	5.4701	0.9680	-0.0469	0.7055	0.1515
Uqcrfs1	_EIDQEAAVEVSQLRDPQHDLDR_.3	#N/A	6.6623	6.4685	7.2159	6.7858	6.2915	6.6960	6.5443	6.3937	6.7715	6.6965	1.0129	0.0185	0.6370	0.1959
Uqcrfs1	_NVVSQFVSSM[Oxidation (M)]SASADVLAM[Oxidation (M)]SK_.2	#N/A	5.2768	5.6943	6.4948	5.8439	5.8038	6.1693	5.7190	5.6776	6.6357	6.1066	0.9744	-0.0374	0.5913	0.2282
Uqcrfs1	_GPAPLNLEVPAYEFTSDDVVVVG_.2	#N/A	4.1951	2.9534	3.7079	5.0403	4.3060	4.0818	4.4426	3.9052	4.1844	4.4292	0.9545	-0.0672	0.6130	0.2126
Uqcrfs1	_EIDQEAAVEVSQLR_.3	#N/A	4.3723	3.5225	3.5583	4.0609	3.4980	3.8824	3.7756	4.0576	3.7061	4.0279	0.9805	-0.0285	0.7050	0.1518
Carhsp1	_GNVVPS[Phospho (STY)]PLPTR_.2	Q9CR86_S42_M1_Carhsp1	11.8068	11.6047	11.8038	12.4317	10.9062	11.6921	3.1014	11.2418	11.4150	11.6729	1.2511	0.3232	0.1968	0.7060
Carhsp1	_TFS[Phospho (STY)]ATVR_.2	Q9CR86_S53_M1_Carhsp1	8.4737	7.3997	8.7268	4.2393	7.9019	4.0267	4.3875	8.3784	3.0610	8.7748	1.1048	0.1437	0.6908	0.1606
Carhsp1	_DRS[Phospho (STY)]PSPLR_.2	#N/A	10.6507	10.8636	11.1348	11.3689	10.6635	10.9475	10.9674	10.6011	10.6999	10.9010	1.0135	0.0194	0.3812	0.4189
Carhsp1	_TRDRS[Phospho (STY)]PSPLR_.2	#N/A	4.4396	5.0936	3.6899	5.7748	4.3386	5.2143	5.2870	3.6984	4.5208	5.4071	1.0064	0.0092	0.9526	0.0211
Carhsp1	_DRS[Phospho (STY)]PS[Phospho (STY)]PLRGNVVPS[Phospho (STY)]PLPTR_.3	Q9CR86_S31_M3_Carhsp1	3.8382	3.7682	4.0105	9.7140	6.3988	4.3132	3.7825	6.1215	5.1234	3.9357	1.0190	0.0272	0.9466	0.0238
Carhsp1	_HETWSGHVISN_.2	#N/A	4.0427	3.2981	3.7763	3.6167	3.1864	6.3054	3.3314	3.0009	4.1504	3.5837	1.1482	0.1993	0.4247	0.3719
Necap1	_ASGTGGLSLLPPPPGGK_.2	#N/A	8.6111	8.7979	8.9157	9.0429	9.0052	9.0580	8.6701	8.6829	8.6065	8.7001	1.0277	0.0395	0.0284	1.5473
Necap1	_LDQPDWTGR_.2	#N/A	7.9841	7.9459	7.6012	8.2185	8.4181	7.9304	7.7059	7.8960	7.8365	7.8949	1.0234	0.0333	0.2468	0.6076
Necap1	_LSIGNITAK_.2	#N/A	7.4892	7.6416	8.0357	7.7309	7.6437	7.7330	7.2764	7.7051	7.5321	7.4582	1.0293	0.0416	0.0959	1.0183

Necap1	_VSGELFAQAPVEQYPGIAVETVTDSSR_.3	#N/A	4.4163	4.4686	5.8940	5.0883	5.8253	5.0127	4.3537	4.5690	5.4507	5.0640	1.0531	0.0747	0.5151	0.2881
Necap1	_SAFIGIGFTDR_.2	#N/A	6.4371	4.9722	3.5753	5.8800	5.9816	5.9842	5.9189	5.4561	3.9494	6.1176	1.0208	0.0296	0.8702	0.0604
Necap1	_ASDWKLDQPDWTGR_.3	#N/A	4.8414	5.0002	5.8852	4.5293	5.4696	3.0469	4.4409	4.7607	5.4644	5.5595	0.9484	-0.0764	0.6448	0.1906
Necap1	_ESQEMDNRPK_.2	#N/A	4.0583	4.2257	3.6873	5.2951	4.1140	4.0115	3.9046	3.9285	3.5771	4.1569	1.0874	0.1209	0.2848	0.5455
Fam136a	_C[Carbamidomethyl (C)]HAPLAQAQALVTSELER_.3	#N/A	4.0746	4.7928	3.8083	4.2982	5.1934	4.4768	4.5678	3.6491	5.1568	4.4790	0.9950	-0.0073	0.9509	0.0219
Golph3	_AAGGGGGS[Phospho (STY)]GEDEAQSR_.2	Q9CRA5_S35_M1_Golph3	6.1664	7.2615	7.3028	7.2335	5.0259	4.6169	6.9706	6.1145	5.5661	6.9119	0.9808	-0.0280	0.8591	0.0660
Exosc5	_ADANLLTDTGTESS[Phospho (STY)]PR_.2	Q9CRA8_S20_M1_Exosc5	3.1554	3.9117	4.3899	3.9337	3.8000	4.3255	4.0819	6.1590	3.3666	6.4644	0.7811	-0.3565	0.1268	0.8969
Snx24	_AESC[Carbamidomethyl (C)]GS[Phospho (STY)]FDETESEESSK_.2	Q9CRB0_S116_M1_Snx24	5.0806	3.9051	4.6534	5.7874	4.6590	5.2124	5.1098	5.0813	5.2538	5.0803	0.9516	-0.0716	0.4686	0.3292
Tppp3	_ALEELATKR_.2	#N/A	6.1027	6.6818	6.1681	5.4070	6.8533	6.6464	6.3675	5.8768	6.0755	5.7947	1.0467	0.0658	0.3612	0.4422
Tppp3	_[Acetyl (Protein N-term)]AASTDIAGLEESFRK_.2	#N/A	5.2743	6.0436	6.3347	6.3792	6.5104	6.4176	5.9821	5.9469	6.0543	6.3850	1.0111	0.0160	0.7916	0.1015
Tppp3	_AVTGTDVDIVFSK_.2	#N/A	4.3804	4.3213	4.2867	5.1021	3.9040	3.2809	4.1862	2.9933	3.2623	3.7766	1.1851	0.2450	0.1144	0.9415
Chchd3	_LSENVIDR_.2	#N/A	5.9989	6.2065	6.7404	5.9994	6.4599	5.8406	6.0397	6.0952	5.8466	5.7517	1.0462	0.0652	0.1734	0.7609
Chchd3	_YSSVYGASVSDEDLK_.2	#N/A	4.6943	5.8186	5.4648	7.3754	6.5281	6.4004	4.5485	6.8250	5.1454	5.7780	1.0848	0.1174	0.4619	0.3355
Chchd3	_YEYHPVC[Carbamidomethyl (C)]ADLQTK_.3	#N/A	5.4354	5.3620	3.2544	5.5902	5.6077	4.2143	3.1133	5.3497	4.3519	3.9362	1.1766	0.2346	0.2675	0.5726
Ociad1	_FDSNVSGQSSFGT[Phospho (STY)]SPAADNIEK_.2	Q9CRD0_T146_M1_Ociad1	3.3810	4.0299	4.0414	4.1593	4.0255	4.0125	4.3075	4.3431	3.1413	3.8365	1.0088	0.0127	0.8992	0.0462
Ociad1	_RSS[Phospho (STY)]PPGHYTQKPK_.3	Q9CRD0_S123_M1_Ociad1	3.5313	3.6520	3.6157	4.1184	3.4373	3.8047	5.0875	4.0464	3.6949	4.0854	0.8734	-0.1953	0.0799	1.0974
Dbndd2	_TSS[Phospho (STY)]LSSDSSNLR_.2	Q9CRD4_S117_M1_Dbndd2	7.9288	8.3047	3.9868	8.7691	8.6118	6.7694	8.7686	8.5298	8.6184	8.4687	0.8603	-0.2172	0.2317	0.6351
Filip1	_ENGPS[Phospho (STY)]APQEK_.2	#N/A	9.0632	8.9798	8.6534	9.5499	8.3867	9.0112	9.1685	8.8254	9.0115	9.1717	0.9886	-0.0166	0.6383	0.1950
Pard3b	_ASS[Phospho (STY)]PEGEEPASPQQSK_.2	Q9CSB4_S338_M1_Pard3b	3.9534	3.2577	3.1393	3.3621	3.0790	3.1496	3.5104	3.6390	3.9381	3.6090	0.9046	-0.1447	0.0886	1.0524
Snw1	_GPPS[Phospho (STY)]PPAPVM[Oxidation (M)]HS[Phospho (STY)]PSR_.2	#N/A	9.1139	9.2619	3.7367	10.1118	9.2167	9.3212	9.5779	8.9791	9.2089	9.2975	0.9131	-0.1312	0.5207	0.2834
Snw1	_GPPS[Phospho (STY)]PPAPVM[Oxidation (M)]HSPS[Phospho (STY)]RK_.3	#N/A	6.9754	4.8617	4.1458	8.1310	5.7526	3.6384	3.6800	8.1275	3.7991	3.5507	1.1660	0.2215	0.5407	0.2670
Ranbp3	_TSS[Phospho (STY)]LTHSEEK_.2	#N/A	8.5606	8.8608	9.2959	9.2089	8.7158	8.9700	8.7970	8.6216	8.8247	8.7535	1.0213	0.0304	0.2460	0.6090
Ranbp3	_S[Phospho (STY)]PSESAEETHTLEEKVPQK_.3	#N/A	3.5744	3.8771	3.8182	3.7371	5.6265	3.3902	3.6970	3.9628	3.8330	6.0221	0.9144	-0.1291	0.5507	0.2591
Ranbp3	_VLS[Phospho (STY)]PPKLNEANSDTSR_.3	Q9CT10_S257_M1_Ranbp3	8.1066	7.2572	3.9530	7.8140	7.2090	7.4544	7.7931	6.9579	7.9291	6.8989	0.9420	-0.0862	0.6074	0.2165
Ranbp3	_S[Phospho (STY)]PSESAEETHTLEEK_.2	#N/A	4.8286	5.0362	3.9046	6.0756	5.6809	5.2653	5.4822	5.3086	6.2570	5.1427	0.9251	-0.1124	0.3617	0.4417
Ranbp3	_ERTSS[Phospho (STY)]LTHSEEK_.2	#N/A	3.2840	4.0892	6.6870	4.4734	5.2714	3.8190	6.0479	5.7948	5.9469	4.4404	0.8284	-0.2715	0.2039	0.6905
Ranbp3	_SAGSSSPEAGEDS[Phospho (STY)]DHEDGNYC[Carbamidomethyl (C)]PPVKR_.4	Q9CT10_S40_M1_Ranbp3	3.2313	4.0581	4.2302	4.2203	3.6809	4.1791	4.0723	4.1342	3.3232	4.0634	1.0090	0.0129	0.8941	0.0486
Arpc2	_ASHTAPQVLFSHR_.3	#N/A	7.2002	7.3247	7.3900	6.8137	7.4081	7.2041	7.3088	6.8990	7.6671	7.0889	0.9976	-0.0035	0.9215	0.0355
Arpc2	_NC[Carbamidomethyl (C)]FASVFEK_.2	#N/A	6.5240	6.5124	5.5831	6.1128	6.3115	6.0327	5.8484	6.2177	6.1045	6.0806	1.0192	0.0275	0.5585	0.2530
Arpc2	_DYLHYHIK_.2	#N/A	6.3763	6.1036	7.0123	6.1088	5.7238	6.6677	5.9995	6.2791	6.4893	5.8465	1.0290	0.0413	0.5115	0.2912
Arpc2	_M[Oxidation (M)]ILLEVNNR_.2	#N/A	7.2896	7.4824	7.7917	8.1799	7.3732	7.6169	7.9348	7.7916	7.6307	7.5968	0.9850	-0.0218	0.5310	0.2749
Arpc2	_IIEETLALK_.2	#N/A	3.2391	3.7093	4.2036	5.8036	5.0817	4.5422	4.4210	5.3651	5.7657	4.6223	0.8783	-0.1871	0.2849	0.5453
Arpc2	_ELQAHGADELLKR_.3	#N/A	4.7921	5.0608	5.5663	5.1484	4.5433	5.0142	4.8083	5.2416	5.1429	5.1805	0.9858	-0.0207	0.7159	0.1451
Arpc2	_VFM[Oxidation (M)]QEFK_.2	#N/A	3.9981	5.0240	5.1537	4.8209	4.5028	4.5837	3.4032	4.9656	5.1079	4.6675	1.0318	0.0452	0.7091	0.1493
Arpc2	_DNTINLIHTFR_.2	#N/A	6.6662	7.0205	7.2437	7.2381	6.7147	6.9720	6.9829	6.8969	7.3529	7.0569	0.9863	-0.0198	0.5343	0.2722
Arpc2	_YFQFQEEGKEGENR_.2	#N/A	3.3636	3.7749	5.5219	4.3081	3.6632	3.8651	3.7583	4.0131	4.0265	4.1197	1.0260	0.0370	0.8012	0.0963
Smc3	_SLQSLEASLHAM[Oxidation (M)]ESTRES[Phospho (STY)]LK_.3	Q9CW03_S776_M1_Smc3	3.2422	8.1061	4.4767	4.4172	6.6661	7.5816	7.1621	8.6553	7.9146	3.6977	0.8383	-0.2545	0.4278	0.3687
Smc3	_GDVEGSQS[Phospho (STY)]QDEGEGSGESER_.2	#N/A	3.6952	2.9506	3.4288	4.4735	2.8389	3.7857	3.1209	4.0286	4.3276	4.1507	0.9032	-0.1469	0.3407	0.4677
Smc3	_KGDVEGSQS[Phospho (STY)]QDEGEGSGESER_.3	#N/A	3.3988	3.9436	3.1325	4.1771	4.0434	4.0821	4.3253	4.3249	3.1232	3.8544	0.9717	-0.0415	0.7323	0.1353
Ppp1r3g	_SFS[Phospho (STY)]LPADPILQAAK_.2	Q9CW07_S81_M1_Ppp1r3g	7.7611	9.3060	9.6780	10.5150	8.4040	8.8488	8.8055	4.1871	8.8362	8.3523	1.2041	0.2680	0.1688	0.7727
Raver1	_LLS[Phospho (STY)]PIASNR_.2	Q9CW46_S576_M1_Raver1	2.9105	5.7861	5.1572	7.4734	4.5695	5.4185	6.1653	5.9960	5.2980	5.2585	0.9190	-0.1219	0.5743	0.2409
Golga1	_ESVASM[Oxidation (M)]GADS[Phospho (STY)]GDDFASDGSSSR_.2	#N/A	4.0289	3.3073	4.1130	3.4167	3.1536	3.2171	3.4305	3.5884	4.3387	3.2269	0.9707	-0.0428	0.7209	0.1421
Golga1	_GSIRPS[Phospho (STY)]IS[Phospho (STY)]NPR_.2	Q9CW79_S749_M2_Golga1	4.2032	3.0229	3.3892	3.8919	2.9112	3.7134	3.6065	4.2266	3.8752	3.8589	0.9050	-0.1441	0.2184	0.6607
Mtfr1l	_NAS[Phospho (STY)]VPNLR_.2	Q9CWE0_S100_M1_Mtfr1l	9.4802	8.4646	9.7438	9.5290	8.7281	9.4936	9.2893	9.1574	9.7471	9.3299	0.9850	-0.0219	0.6294	0.2011
Mtfr1l	_AS[Phospho (STY)]FETLPNISDLC[Carbamidomethyl (C)]LK_.2	Q9CWE0_S38_M1_Mtfr1l	3.0270	8.6686	6.6681	7.9265	6.1050	5.8768	6.4577	7.0746	6.5204	5.7975	0.9406	-0.0884	0.6710	0.1733
Mtfr1l	_TTC[Carbamidomethyl (C)]SS[Phospho (STY)]S[Phospho (STY)]EEDDC[Carbamidomethyl (C)]ISLSK_.2	Q9CWE0_S222_M2_Mtfr1l	3.7138	3.5186	4.0461	6.0073	5.4766	3.9801	5.1304	5.2120	3.7969	5.8096	0.8937	-0.1621	0.4191	0.3777
Mtfr1l	_ASS[Phospho (STY)]FADM[Oxidation (M)]M[Oxidation (M)]GILK_.2	#N/A	6.6809	9.0786	4.1837	10.0351	7.3607	3.7762	3.8522	8.8889	3.7465	3.7075	1.3573	0.4407	0.3033	0.5181
Mtfr1l	_TTELQDELSHLR_.3	#N/A	3.6415	3.5847	4.3283	3.3301	4.2680	3.4615	4.5456	3.2876	4.4370	3.2971	0.9684	-0.0463	0.7342	0.1342
Tubb2b	_INVYYNEATGNK_.2	#N/A	9.2233	8.8902	9.3199	9.5807	9.2913	9.1547	9.1205	9.0461	9.1330	9.3481	1.0089	0.0128	0.5366	0.2703
Cd2bp2	_GTGRPNS[Phospho (STY)]PQRLDR_.3	#N/A	7.9124	7.6331	3.5105	8.3923	8.3672	8.0403	7.8057	7.8335	7.8461	4.2324	1.0548	0.0770	0.7587	0.1199
Cd2bp2	_GTGRPNS[Phospho (STY)]PQR_.2	#N/A	6.7708	6.9756	5.9706	4.4834	6.6794	4.2708	7.1425	6.9564	6.7821	4.1606	0.9358	-0.0957	0.6398	0.1939
Cd2bp2	_GKHSLDS[Phospho (STY)]DEEDDDEEGSSK_.2	Q9CWK3_S49_M1_Cd2bp2	3.0448	3.8500	3.7316	4.2707	3.6713	4.0582	4.4190	4.2313	3.0295	3.9480	0.9652	-0.0511	0.6861	0.1636
Snx2	_SIS[Phospho (STY)]APVIFDR_.2	Q9CWK8_S119_M1_Snx2	6.7876	3.3374	7.4450	3.7216	3.1587	6.9103	7.1348	7.1025	4.0454	6.5719	0.8412	-0.2495	0.4275	0.3690
Snx2	_ELILSSEPS[Phospho (STY)]PAVT[Phospho (STY)]PVTPTTLIAPR_.3	Q9CWK8_S97_M2_Snx2	3.0052	4.2210	3.6920	4.1946	3.6317	4.0161	3.9093	3.9239	3.5725	4.1616	0.9747	-0.0369	0.7073	0.1504
Ctnnbl1	_EYAENIGDGRS[Phospho (STY)]PEFR_.3	Q9CWL8_S545_M1_Ctnnbl1	5.1306	3.4861	3.5900	3.1338	3.0001	4.4221	3.2820	5.4923	5.0211	4.6348	0.8234	-0.2804	0.1866	0.7290
Pfdn1	_LADIQIEQLNR_.2	#N/A	6.7938	6.3751	6.4963	7.1287	7.0019	7.1630	6.6040	6.7308	6.5587	6.7410	1.0252	0.0359	0.3602	0.4434
Ddah1	_S[Phospho (STY)]QGEEVDFAR_.2	Q9CWS0_S33_M1_Ddah1	8.6923	8.6536	9.2845	9.1266	8.7169	9.0123	8.7720	8.4632	8.7945	8.8761	1.0215	0.0307	0.2527	0.5975
Ddah1	_[Acetyl (Protein N-term)]AGLGHPSAFGR_.2	#N/A	9.0290	9.2659	9.3210	9.1155	9.5415	8.8436	8.9856	9.2459	9.0449	9.0559	1.0113	0.0163	0.4586	0.3386
Ddah1	_GAEILADTFKDYAVSTVPVADSLHLK_.3	#N/A	5.8104	6.4487	7.3235	6.9625	5.6876	6.2743	6.2860	6.0117	6.2550	6.7446	1.0148	0.0212	0.7947	0.0998
Ddah1	_TPEEYPESAK_.2	#N/A	5.7731	5.3872	5.9303	5.2940	6.8656	5.1597	5.4076	5.6706	6.3664	5.2103	1.0126	0.0180	0.8548	0.0681
Ddah1	_GAEILADTFK_.2	#N/A	6.4053	5.6394	6.1551	6.8955	5.7859	6.1004	2.8838	6.0831	6.3069	6.1247	1.1210	0.1648	0.3597	0.4441
Ddah1	_EFFVGLSK_.2	#N/A	5.6437	6.1025	5.3411	6.2849	5.6938	5.5097	5.1968	6.1200	5.6967	6.1306	0.9959	-0.0059	0.9286	0.0322
Ddah1	_LQLNIVEM[Oxidation (M)]KDENATLDGGDVLFTGR_.3	#N/A	5.6775	3.5526	3.5235	6.0256	5.3840	6.4757	5.7848	6.1107	6.6207	6.6635	0.8112	-0.3019	0.1133	0.9457
Ddah1	_SFC[Carbamidomethyl (C)]SM[Oxidation (M)]AGPNLIAIGSSESAQK_.2	#N/A	6.0664	5.6134	6.1372	3.7027	6.2123	6.1619	5.8106	5.9323	3.5975	5.9855	1.0596	0.0835	0.6517	0.1860
Ddah1	_QHELYVGVLGSK_.2	#N/A	3.6541	4.1139	4.1147	3.6519	4.1750	3.9055	3.3666	3.5173	4.0235	3.9551	1.0593	0.0831	0.2463	0.6085
Ddah1	_DYAVSTVPVADSLHLK_.3	#N/A	3.3653	4.1705	4.0521	4.5547	3.9918	3.7377	4.0985	3.5637	3.2123	3.0209	1.1453	0.1957	0.1066	0.9723
	LLPEGEETVES[Phospho (STY)]DDDKDER.3	Q9CWU4_S156_M1_	3.8066	3.7323	4.2080	3.7517	6.4556	3.5064	3.9000	3.2804	3.5795	3.4290	1.1963	0.2585	0.2603	0.5845
Ddx47	_[Acetyl (Protein N-term)]AADEEPDSPS[Phospho (STY)]GALQTAAEEEETK_.3	Q9CWX9_S11_M1_Ddx47	4.0643	5.1148	3.2502	3.7529	6.3698	3.5744	3.4676	7.1410	6.5333	3.7199	0.8349	-0.2603	0.3954	0.4030
Rpain	_[Acetyl (Protein N-term)]AESSGS[Phospho (STY)]PHR_.2	Q9CWY9_S7_M1_Rpain	3.7771	3.0814	4.4639	3.4657	2.9028	3.3259	3.6867	6.1787	4.3014	3.4327	0.7961	-0.3290	0.1527	0.8161
Rbm8a	_GFGS[Phospho (STY)]EEGSR_.2	Q9CWZ3_S42_M1_Rbm8a	7.9799	7.6844	8.2845	7.9854	8.0237	3.5819	7.7913	3.8247	8.0216	8.1140	1.0459	0.0648	0.8031	0.0952
Rbm8a	_M[Oxidation (M)]REDYDS[Phospho (STY)]VEQDGDEPGPQR_.3	#N/A	3.2843	5.0149	4.1727	4.9654	5.1088	5.1060	4.9255	3.4692	5.1936	5.2833	0.9769	-0.0338	0.8340	0.0789
Rbm8a	_[Acetyl (Protein N-term)]ADVLDLHEAGGEDFAM[Oxidation (M)]DEDGDES[Phospho (STY)]IHK_.3	Q9CWZ3_S24_M1_Rbm8a	3.8044	3.1087	3.9673	3.5111	2.9301	3.2986	3.6594	3.4901	3.7891	1.0628	1.1454	0.1959	0.4522	0.3446
Napg	_LIENVDPEK_.2	#N/A	7.5383	7.6354	7.9924	7.8031	8.0051	7.8827	6.9328	7.7209	7.9392	8.0632	1.0190	0.0271	0.5323	0.2739
Napg	_AVQLYQQTANVFENEER_.2	#N/A	6.9768	7.3959	7.4238	7.5914	6.5983	7.2539	7.3651	6.8906	6.9784	7.1037	1.0173	0.0247	0.5620	0.2502
Napg	_INEGLEHLAK_.2	#N/A	6.6199	6.7290	6.8614	6.9694	7.2350	6.6400	6.6229	6.9848	6.6738	6.0918	1.0378	0.0535	0.2241	0.6496
Napg	_FDEAALSIQK_.2	#N/A	5.2291	5.5428	5.1045	6.2662	6.0209	5.8779	5.5012	5.5622	5.8578	5.8399	0.9971	-0.0042	0.9474	0.0235
Napg	_QAVELLGK_.2	#N/A	4.6753	4.7822	5.0930	4.8764	5.1666	5.0121	4.6184	5.4182	4.2478	5.0886	1.0188	0.0269	0.6968	0.1569
Napg	_LPEAVQLIEK_.2	#N/A	7.4695	7.0626	3.2482	7.3747	7.3285	7.0391	7.0668	7.4626	7.1678	6.9281	0.9205	-0.1196	0.5191	0.2847
Napg	_TIAQVLVHLHR_.3	#N/A	5.9101	6.9803	7.0122	6.9585	7.3350	7.1330	6.7950	6.7909	7.2756	7.0728	0.9863	-0.0198	0.7340	0.1343

Napg	_WKPDYDSAASEYGK_.2	#N/A	4.0951	5.6744	3.5924	4.3680	5.8317	4.7961	3.3306	4.2712	3.9596	5.6270	1.0999	0.1373	0.4899	0.3099
Napg	_YMDNDYAK_.2	#N/A	3.8815	3.3446	4.5683	4.1946	3.2329	3.3917	3.2848	3.0475	4.1969	3.5371	1.0718	0.1000	0.4753	0.3230
Napg	_KS[Phospho (STY)]PAT[Phospho (STY)]PQAKPDGAAGMAAEEEEDEYSGGLC[Carbamidomethyl (C)]_.3	#N/A	3.2303	4.0355	3.9171	4.4197	3.8568	3.8727	4.1344	3.6987	3.3473	4.3867	0.9992	-0.0012	0.9910	0.0039
Napg	_KKS[Phospho (STY)]PAT[Phospho (STY)]PQAK_.2	#N/A	3.4302	4.1865	3.1639	4.2085	4.0748	4.0507	4.3568	4.2935	3.0917	3.8858	0.9860	-0.0203	0.8699	0.0605
Ist1	_IVADQLC[Carbamidomethyl (C)]AK_.2	#N/A	4.0075	3.3119	3.1935	3.6961	4.5175	3.0954	3.4108	4.4223	4.6141	3.6631	0.9030	-0.1472	0.3128	0.5047
Ist1	_ELDSGLAESVSTLIWAAPR_.2	#N/A	4.1544	5.5828	5.9961	3.1611	3.2801	2.9486	3.3094	4.2755	5.4870	6.0326	0.8767	-0.1899	0.4964	0.3042
Sugt1	_ALEQNPDDAQYYC[Carbamidomethyl (C)]QR_.2	#N/A	5.1600	4.9438	3.3378	5.4980	5.3045	5.4060	4.5307	5.0055	4.4199	5.3153	1.0257	0.0366	0.7878	0.1036
Sugt1	_[Acetyl (Protein N-term)]AAAAAGPASSQR_.2	#N/A	4.6538	4.5379	3.3008	4.3454	4.8107	5.2643	4.2676	4.1566	5.0941	5.1228	0.9625	-0.0552	0.6688	0.1747
Sugt1	_LFQQIYSDGSDEVKR_.3	#N/A	4.3467	3.6022	4.0804	3.6242	3.9953	4.8874	3.7725	3.3770	3.6760	3.3015	1.1579	0.2115	0.0663	1.1788
D10Wsu102e	_IEILDS[Phospho (STY)]PASK_.2	Q9CX66_S177_M1_D10Wsu102e	4.1705	3.1719	3.9042	4.9624	3.3142	3.3104	4.7894	4.3944	3.8523	4.7003	0.8583	-0.2205	0.1449	0.8389
D10Wsu102e	_SDS[Phospho (STY)]KEEDSPEESSR_.2	Q9CX66_S125_M1_D10Wsu102e	4.3535	3.6578	3.5394	3.5139	3.4792	4.2503	3.1103	4.0392	4.3382	4.1401	0.9724	-0.0404	0.7242	0.1401
Rgs19	_LQPLPSC[Carbamidomethyl (C)]EVC[Carbamidomethyl (C)]TPPS[Phospho (STY)]PK_.2	Q9CX84_S80_M1_Rgs19	8.5050	9.0928	9.2393	3.9478	7.2323	3.7353	9.4675	9.3788	8.6847	3.0233	0.9110	-0.1345	0.7116	0.1478
Rgs19	_LMHS[Phospho (STY)]PTGR_.2	Q9CX84_S97_M1_Rgs19	3.7349	3.4913	4.4217	3.4235	2.8606	3.3681	3.1382	3.1941	4.3436	3.3905	1.0095	0.0136	0.9250	0.0339
Hnrnpa0	_LFIGGLNVQTSESGLR_.2	#N/A	4.7155	5.5997	6.3641	5.2887	5.8108	6.0394	5.6412	5.2859	6.0192	5.7570	0.9930	-0.0101	0.9050	0.0433
Hnrnpa0	_GDVAEGDLIEHFSQFGAVEK_.3	#N/A	4.4234	3.1226	4.1371	4.9937	2.2373	4.6888	3.7553	3.5449	4.6900	4.2128	0.9711	-0.0423	0.8432	0.0741
Hnrnpa0	_SGGGGGGGGSWGGR_.2	#N/A	3.2951	4.0514	4.5296	4.3643	3.9397	4.1715	4.0790	3.7542	4.2170	4.3313	0.9910	-0.0130	0.8824	0.0543
Bcl7a	_QENSSNSS[Phospho (STY)]PAPETNPPVPSDGTEAK_.3	Q9CXE2_S114_M1_Bcl7a	4.0129	3.3295	3.7466	3.2903	3.1509	3.0778	3.4386	3.7108	4.0099	2.9676	0.9725	-0.0402	0.7145	0.1460
Bcl7a	_MKLEASQQNS[Phospho (STY)]EEM[Oxidation (M)]_.2	Q9CXE2_S207_M1_Bcl7a	2.9380	4.2882	3.6248	4.1274	4.7203	3.9489	3.8421	3.9911	3.6397	4.1523	1.0090	0.0129	0.9166	0.0378
Tbc1d15	_DDS[Phospho (STY)]PTQTLASPNAC[Carbamidomethyl (C)]R_.2	Q9CXF4_S655_M1_Tbc1d15	7.0745	6.3546	3.8107	7.0956	6.2418	3.7377	4.0280	6.8047	6.6763	3.5580	1.0859	0.1189	0.6752	0.1706
Rbm33	_SIVNTS[Phospho (STY)]PPC[Carbamidomethyl (C)]R_.2	Q9CXK9_S792_M1_Rbm33	8.0406	3.8250	4.0879	4.5906	7.4495	3.7018	4.3053	7.4374	3.1765	3.0567	1.1755	0.2332	0.5542	0.2563
Rbm33	_ANS[Phospho (STY)]GGGDGPHVSSK_.2	Q9CXK9_S1032_M1_Rbm33	7.7461	8.1144	8.3040	8.8142	7.8288	8.1262	8.4004	8.0667	8.3486	8.0050	0.9940	-0.0087	0.8197	0.0863
Rbm33	_DIKEES[Phospho (STY)]DEEDDDDEESGR_.2	Q9CXK9_S243_M1_Rbm33	3.5569	4.0015	3.2449	3.1685	3.5350	2.3683	3.0256	3.0972	2.6670	4.2602	1.0153	0.0219	0.9014	0.0451
Gng10	_[Acetyl (Protein N-term)]SSGASVS[Phospho (STY)]ALQR_.2	Q9CXP8_S8_M1_Gng10	7.9095	7.9543	8.5998	8.1890	8.1109	8.1234	9.0213	7.8373	8.7108	8.0724	0.9688	-0.0458	0.3257	0.4872
Cenpv	_LLLDTFEYQGLVK_.2	#N/A	4.3150	3.8144	4.4235	4.3923	4.6747	4.7625	4.9161	3.4239	3.3330	4.9314	1.0593	0.0830	0.5478	0.2614
Cenpv	_SVVTEEFNGS[Phospho (STY)]DWER_.2	Q9CXS4_S234_M1_Cenpv	3.3179	4.0742	4.5524	6.1650	4.2228	4.1630	4.0561	4.4863	3.2040	3.7735	1.1381	0.1867	0.3405	0.4679
Cenpv	_SNPGGFGIAPHC[Carbamidomethyl (C)]LDEGTVR_.2	#N/A	3.1993	3.9794	4.1951	4.1908	3.8352	4.1002	4.1151	3.4329	3.3534	4.0356	1.0488	0.0688	0.4835	0.3156
Cenpv	_QRLS[Phospho (STY)]FEGAAK_.2	Q9CXS4_S105_M1_Cenpv	3.6737	3.5525	4.3605	3.3623	2.7994	3.4293	3.0770	3.2553	4.4048	3.3293	1.0037	0.0053	0.9714	0.0126
Cacybp	_WDYLTQVEK_.2	#N/A	4.8009	5.1761	3.1593	5.1593	5.1813	4.8228	4.2892	4.4120	4.9185	5.0051	1.0130	0.0186	0.8902	0.0505
Cacybp	_[Acetyl (Protein N-term)]ASVLEELQKDLEEVK_.2	#N/A	3.9264	3.2192	3.6601	2.3579	1.3541	2.3888	3.5716	2.8033	2.2548	4.3820	0.8662	-0.2072	0.4979	0.3029
Rpl11	_VLEQLTGQTPVFSK_.2	#N/A	6.3646	5.9336	6.5911	5.8450	6.2642	6.6490	6.1537	5.6636	6.3432	5.9850	1.0395	0.0558	0.2765	0.5582
Rpl11	_YDGIILPGK_.2	#N/A	5.6028	6.5248	6.5283	5.7855	5.7688	5.4020	6.3182	6.1439	6.2970	5.9010	0.9627	-0.0548	0.3953	0.4030
Rpl11	_[Acetyl (Protein N-term)]AQDQGEKENPM[Oxidation (M)]R_.2	#N/A	3.2039	3.4885	3.9668	3.5106	4.2843	3.2478	3.6588	3.4906	3.7897	3.1878	1.0241	0.0344	0.7315	0.1358
Ilf2	_VLQSALAAIR_.2	#N/A	4.2804	5.3310	4.9330	5.2851	4.4642	4.4115	5.3058	5.3941	4.9629	4.9371	0.9290	-0.1063	0.1867	0.7290
Ilf2	_NQDLAPNSAEQASILSLVTK_.2	#N/A	4.4407	3.6577	3.4184	4.8917	4.4218	5.3768	5.4547	4.0390	5.3705	4.3880	0.9075	-0.1400	0.3695	0.4324
Ndufs4	_EDAIAFAEK_.2	#N/A	7.3711	6.9362	6.7129	7.2092	7.6889	7.6783	6.9124	6.9783	7.0258	7.5131	1.0223	0.0319	0.5073	0.2947
Ndufs4	_DTQLITVDEK_.2	#N/A	6.8420	6.4768	6.7398	7.4147	7.1098	6.9724	7.0176	6.4094	7.2941	6.9744	1.0003	0.0004	0.9928	0.0031
Ndufs4	_LDITTLTGVPEEHIK_.2	#N/A	5.6172	5.8317	5.1499	5.9377	5.6137	5.4802	5.9049	5.7569	5.6561	5.5992	0.9783	-0.0316	0.4351	0.3615
Ndufs4	_NNMQSGVNNTK_.2	#N/A	4.6329	5.6929	5.2744	6.0794	4.2353	3.6117	4.1247	5.6637	3.8068	4.4716	1.0895	0.1237	0.5004	0.3007
Ndufs4	_DTQLITVDEKLDITTLTGVPEEHIK_.3	#N/A	4.8982	5.0413	3.7018	7.2126	4.7253	4.0880	5.5941	5.7152	5.1241	4.1714	0.9599	-0.0591	0.7699	0.1136
Ndufs4	_NNMQSGVNNTKK_.2	#N/A	2.4598	2.9752	3.4369	4.5545	3.5027	3.7611	3.6543	4.1789	3.8275	3.9066	0.8861	-0.1745	0.2684	0.5713
Tecr	_S[Phospho (STY)]LKDEDVLQK_.2	Q9CY27_S58_M1_Tecr	5.6106	5.5292	4.1518	5.4781	4.5764	5.2459	5.5816	6.0719	5.6840	4.6403	0.9280	-0.1079	0.3374	0.4718
Chtop	_GHLDAELDAYM[Oxidation (M)]AQTDPETND_.2	#N/A	6.0142	5.5350	6.7758	4.3865	5.7338	5.7574	6.5997	6.2733	5.7603	6.0778	0.9227	-0.1160	0.2885	0.5399
Serbp1	_SAAQAAAQTNSNAAGK_.2	#N/A	4.7004	5.4120	4.8711	5.9252	5.4239	5.4015	4.1605	4.3137	4.8635	5.1761	1.1427	0.1925	0.0540	1.2677
Serbp1	_GGSGSHNWGTVKDELTES[Phospho (STY)]PK_.3	#N/A	5.7161	5.1749	4.5037	6.3592	5.2539	3.2860	3.6468	5.4113	5.4460	3.4726	1.1234	0.1679	0.4444	0.3522
Serbp1	_EFDRHS[Phospho (STY)]GSDR_.2	Q9CY58_S197_M1_Serbp1	4.2882	4.0091	3.4741	4.5282	5.0204	4.3156	3.1756	4.1695	4.4547	3.9438	1.0855	0.1184	0.3527	0.4527
Serbp1	_TDKSSAS[Phospho (STY)]APDVDDPEAFPALA_.2	#N/A	4.2422	3.1002	6.7601	3.5196	2.9215	3.3071	3.6679	6.9648	3.7806	3.1969	0.9029	-0.1473	0.6811	0.1668
Serbp1	_SSAS[Phospho (STY)]APDVDDPEAFPALA_.2	#N/A	3.2400	3.8616	3.9268	4.4294	3.7163	4.2232	4.3390	7.1273	3.3541	3.7576	0.8396	-0.2522	0.3221	0.4921
Serbp1	_DELTES[Phospho (STY)]PKYIQK_.2	#N/A	3.6269	4.3831	3.5298	6.9980	4.2714	3.8540	6.1977	6.1046	3.7346	3.9995	0.8872	-0.1727	0.5217	0.2826
Serbp1	_FDQLFDDES[Phospho (STY)]DPFEVLK_.2	Q9CY58_S25_M1_Serbp1	3.1144	4.1118	3.8012	4.3038	5.6841	4.1254	4.0185	4.4974	4.9123	4.2708	0.9470	-0.0786	0.6209	0.2070
Serbp1	_GGSGSHNWGTVKDELTES[Phospho (STY)]PKYIQK_.4	#N/A	3.5399	4.2962	3.2736	4.3182	4.1845	3.9410	3.8341	4.1838	2.9820	3.9955	1.0471	0.0664	0.5775	0.2385
Serbp1	_SKS[Phospho (STY)]EEAHAEDSVM[Oxidation (M)]DHHFR_.4	#N/A	9.4532	9.0165	8.1794	10.0191	7.9030	3.9528	8.2276	9.2800	7.7467	8.5795	0.9561	-0.0647	0.7526	0.1234
Serbp1	_S[Phospho (STY)]EEAHAEDSVMDHHFR_.3	#N/A	3.5029	3.6116	3.7054	3.8920	3.4548	3.8093	3.9629	3.9530	3.6016	4.1751	0.9336	-0.0991	0.0771	1.1130
Blvra	_SGS[Phospho (STY)]LEEVPNVGVNK_.2	Q9CY64_S236_M1_Blvra	9.8646	9.7788	10.3835	10.2054	9.1569	9.7411	10.3571	9.6685	9.8082	9.8290	0.9939	-0.0088	0.8132	0.0898
Blvra	_SQEVDVAYIC[Carbamidomethyl (C)]TESSSHEDYIR_.3	#N/A	6.6774	6.9158	8.0714	7.3955	5.3804	6.5112	7.2438	6.1848	6.4787	5.7559	1.0638	0.0893	0.4571	0.3400
Blvra	_QISLEDALR_.2	#N/A	4.7239	3.1103	4.4927	4.1551	4.7659	3.9850	3.6578	3.4916	3.7907	3.7219	1.1473	0.1983	0.1299	0.8863
Blvra	_DQDIFIQK_.2	#N/A	3.7700	3.6220	3.2119	4.5453	3.8348	4.7640	4.7317	4.4039	4.2934	3.6815	0.9253	-0.1120	0.3820	0.4179
Tomm34	_VPS[Phospho (STY)]AGDVER_.2	Q9CYG7_S186_M1_Tomm34	7.1411	6.9785	6.3939	7.8140	6.8351	7.3565	7.4763	6.9036	6.6780	7.2372	1.0018	0.0026	0.9651	0.0154
Tomm34	_TVLQIDNSVASALEGINR_.2	#N/A	5.1617	4.4319	3.9977	4.0854	2.8917	4.2578	4.9228	3.1872	4.4735	4.2107	0.9855	-0.0211	0.9014	0.0451
Tomm34	_VSDS[Phospho (STY)]VEQLR_.2	Q9CYG7_S8_M1_Tomm34	4.2176	3.5220	5.3470	4.5987	3.3433	4.3862	3.2461	5.5235	3.8608	3.8732	1.0266	0.0379	0.8433	0.0740
Tomm34	_SSLSDISSLLQIEPR_.2	#N/A	3.6626	4.4189	3.3963	3.9967	2.8064	3.8182	3.7114	4.1218	3.5681	4.1182	0.9493	-0.0750	0.5331	0.2732
Tomm34	_YSESLLC[Carbamidomethyl (C)]SSLESATYSNR_.2	#N/A	3.6051	4.3614	3.3388	4.0542	4.2497	3.8757	3.7689	4.0643	3.7128	4.0607	1.0032	0.0046	0.9545	0.0202
Prxl2a	_LDELGVPLYAVVK_.2	#N/A	6.1323	5.9241	6.4392	6.0803	6.0494	5.5620	5.8169	6.2746	6.2648	5.7969	0.9988	-0.0017	0.9697	0.0134
	LTQNRGDS[Phospho (STY)]DDGR.2	Q9CYI0_S55_M1_	3.2942	3.3645	4.4775	4.0213	3.9207	3.8088	3.4596	2.9799	3.2789	3.8373	1.1256	0.1707	0.1475	0.8313
	S[Phospho (STY)]LGNDVK.2	Q9CYI0_S186_M1_	4.1993	3.4548	3.9330	3.4600	3.3430	3.2816	3.1747	3.1576	4.3070	3.1540	1.0474	0.0669	0.5932	0.2268
Luc7l	_RS[Phospho (STY)]EEKEAGEI_.2	Q9CYI4_S363_M1_Luc7l	8.2611	8.3868	8.6556	8.7570	7.6254	8.2341	8.6583	8.1857	7.8783	8.3080	1.0076	0.0109	0.8020	0.0958
Luc7l	_ERS[Phospho (STY)]LREESWEYGR_.3	#N/A	3.6234	4.2227	4.3737	3.6343	3.2229	4.4858	4.1199	4.2225	3.7509	3.6506	0.9978	-0.0032	0.9756	0.0107
Luc7l	_S[Phospho (STY)]LREESWEYGR_.2	#N/A	3.4491	3.7771	4.1359	5.3914	3.6654	5.1066	4.3532	3.4799	3.1285	3.1047	1.2098	0.2747	0.1567	0.8048
Atp6ap2	_QENTQSPYNLAYK_.2	#N/A	5.2190	4.9212	5.9283	5.0541	5.0392	5.5346	6.7111	5.7596	5.8063	5.6681	0.8825	-0.1804	0.0330	1.4809
Ssbp1	_S[Phospho (STY)]GDSEVYQMGDVSQK_.2	Q9CYR0_S67_M1_Ssbp1	5.6536	4.3323	4.2139	3.2157	4.7427	3.5759	4.4312	3.4020	3.0505	3.1827	1.2196	0.2865	0.1653	0.7816
Cap2	_THTPSPTS[Phospho (STY)]PK_.2	#N/A	12.8597	12.5640	12.9822	13.2724	12.4953	12.5242	12.7955	12.6677	12.8635	12.9129	0.9979	-0.0030	0.8738	0.0586
Cap2	_IQEIQTFR_.2	#N/A	7.6049	7.6525	7.6044	7.6154	7.6534	7.5023	7.4158	7.3744	7.5480	7.2646	1.0277	0.0394	0.0054	2.2677
Cap2	_LEQLSAGLDGPPR_.2	#N/A	6.8831	7.0137	7.1521	7.6318	7.3828	7.0110	7.3566	7.0431	7.4292	7.1940	0.9894	-0.0153	0.6407	0.1933
Cap2	_TEGC[Carbamidomethyl (C)]HLYLSEDALDC[Carbamidomethyl (C)]EIVSAK_.3	#N/A	6.3756	6.6734	6.5573	7.6157	5.5413	5.8686	7.0565	5.8593	5.9309	6.0946	1.0326	0.0463	0.6475	0.1888
Cap2	_LINSM[Oxidation (M)]VAEFLK_.2	#N/A	4.5989	4.9476	4.9677	4.5339	4.6264	3.9403	5.1184	4.9913	5.0990	5.2289	0.9008	-0.1507	0.0312	1.5063
Cap2	_SNS[Phospho (STY)]PQKHTPVLELEGK_.3	#N/A	8.6740	9.0479	10.0062	9.2616	8.5804	9.0021	9.0370	9.0382	8.7949	8.7969	1.0200	0.0286	0.5224	0.2820
Cap2	_S[Phospho (STY)]NSPQKHTPVLELEGKK_.3	#N/A	3.7931	3.6528	3.9235	3.6818	3.5260	3.6017	3.5042	3.7439	3.8567	3.5303	1.0103	0.0148	0.7131	0.1469
Cap2	_HTPVLELEGKK_.3	#N/A	3.2595	3.1874	4.1879	3.3124	4.2180	4.5602	2.6549	3.8489	3.9940	3.6561	1.0704	0.0981	0.5399	0.2677
Cap2	_AQGQIRS[Phospho (STY)]PTKTHTPSPTS[Phospho (STY)]PK_.3	#N/A	9.7686	10.5215	11.3998	10.7407	10.2689	10.5964	10.4463	10.5613	4.2191	4.2592	1.4311	0.5171	0.0606	1.2174
Cap2	_EM[Oxidation (M)]NDAATFYTNR_.2	#N/A	5.2559	4.7840	5.9445	5.3258	5.5334	5.2411	5.4117	5.4155	5.3410	5.2492	0.9987	-0.0019	0.9729	0.0120

Cap2	_THTPS[Phospho (STY)]PTS[Phospho (STY)]PKSNSPQK_.2	#N/A	5.6696	7.4660	4.0729	6.7368	3.7104	6.6281	6.4786	6.9140	8.2050	7.7602	0.7785	-0.3612	0.0886	1.0524
Cap2	_S[Phospho (STY)]PTKTHTPSPTS[Phospho (STY)]PK_.2	#N/A	8.0871	8.2998	4.0214	8.4140	7.9317	6.2433	6.5637	6.1633	6.0601	8.3910	1.0547	0.0768	0.7152	0.1456
Cap2	_VEYQEDRNDLVISETELK_.3	#N/A	5.3966	6.5092	8.0210	6.7293	6.0787	4.4289	5.8747	5.1629	5.9378	5.6156	1.0967	0.1332	0.4181	0.3787
Cap2	_SSEM[Oxidation (M)]NVLVPQDDDYREFPIPEQFK_.3	#N/A	5.2149	5.4273	5.6630	5.9504	4.7087	4.9798	5.7504	5.3127	5.1972	4.8084	1.0108	0.0155	0.8428	0.0743
Cap2	_AQGQIRS[Phospho (STY)]PTK_.2	#N/A	11.8500	11.9746	12.3847	12.3969	11.7672	11.9335	12.1577	11.9997	11.9125	11.8870	1.0052	0.0074	0.6852	0.1642
Cap2	_SALFAQLNQGEAITK_.2	#N/A	5.4344	4.3493	6.0328	4.8626	5.3359	4.8252	5.5276	5.3931	5.2921	4.7481	0.9809	-0.0279	0.7683	0.1145
Cap2	_VLAGDVETHAEM[Oxidation (M)]VHGAFQAQR_.3	#N/A	3.6760	5.9764	3.4096	3.7889	5.3270	5.7376	3.9372	5.2425	3.7112	5.8819	0.9913	-0.0125	0.9562	0.0194
Cap2	_TIWDGSK_.2	#N/A	3.4151	3.9273	3.1487	4.1934	4.0596	3.6033	4.3416	4.3087	3.1069	4.2920	0.9283	-0.1074	0.3883	0.4109
Hdhd3	_DVVLHTFR_.2	#N/A	5.6050	4.8291	5.6098	5.3309	5.9883	5.6251	4.3278	5.8865	5.3848	5.4828	1.0432	0.0610	0.5091	0.2932
Tpd52l2	_NSATFKS[Phospho (STY)]FEDR_.2	#N/A	9.9425	9.9925	10.8255	10.8084	9.4767	10.1074	10.1390	9.9232	10.1625	10.1552	1.0096	0.0138	0.7320	0.1355
Tpd52l2	_S[Phospho (STY)]WHDVQVSTAYVK_.2	Q9CYZ2_S96_M1_Tpd52l2	3.7550	3.5874	3.4887	4.5333	5.8577	4.3208	3.1808	3.9687	5.9391	4.2106	0.9844	-0.0227	0.9191	0.0366
Tpd52l2	rotein N-term)]M[Oxidation (M)]DSASQDINLNSPNKGVLS[Phospho (STY)]DFM[Oxidation (M)]TDVPVDI	Q9CYZ2_S19_M1_Tpd52l2	4.1032	3.8350	4.0679	5.5210	3.2288	3.6515	3.7536	3.7700	3.7122	3.6984	1.0896	0.1237	0.4257	0.3709
Tpd52l2	_[Acetyl (Protein N-term)]MDSASQDINLNS[Phospho (STY)]PNK_.2	#N/A	7.3029	5.4129	3.5559	8.5334	5.6276	4.2784	6.7009	4.5140	5.2326	6.0073	1.0305	0.0434	0.8706	0.0602
Tpd52l2	_ENGSDNLPPS[Phospho (STY)]PGSGDQTLPDHAPF_.3	Q9CYZ2_S206_M1_Tpd52l2	9.4035	9.2104	9.5615	9.6727	8.2668	10.0985	9.7459	3.5345	8.8781	9.6904	1.0511	0.0719	0.8211	0.0856
Tpd52l2	_S[Phospho (STY)]FEDRVGTIK_.2	#N/A	3.5921	4.3484	3.3258	4.3704	4.2367	3.8887	3.7819	4.1316	4.4306	4.0477	0.9664	-0.0493	0.5898	0.2293
Tpd52l2	_HSIS[Phospho (STY)]M[Oxidation (M)]PVM[Oxidation (M)]R_.2	Q9CYZ2_S168_M1_Tpd52l2	2.9577	3.7139	4.1922	3.2008	3.6022	3.0224	4.4164	3.4168	4.3273	4.9387	0.8066	-0.3100	0.0451	1.3461
Dbndd1	_[Acetyl (Protein N-term)]M[Oxidation (M)]ES[Phospho (STY)]PEGAPGEIVK_.2	#N/A	7.9069	8.7185	8.7596	9.4101	8.6792	8.9470	9.1194	8.4614	8.8848	9.0339	0.9845	-0.0226	0.6287	0.2016
Uqcrc1	_VYEEDAVPGLTPC[Carbamidomethyl (C)]R_.2	#N/A	10.7192	10.6056	11.0548	11.0100	10.8317	10.6679	10.8852	10.4598	10.5241	10.5745	1.0192	0.0275	0.1281	0.8923
Uqcrc1	_TDLTDYLNR_.2	#N/A	10.7116	10.7232	10.9780	10.8574	10.3221	10.6792	10.8986	10.5549	10.5838	10.6271	1.0043	0.0062	0.7318	0.1356
Uqcrc1	_NALVSHLDGTTPVC[Carbamidomethyl (C)]EDIGR_.3	#N/A	7.5862	8.0315	8.4115	8.1656	8.7395	8.2114	7.7828	7.6417	8.3032	8.4308	1.0188	0.0269	0.5599	0.2519
Uqcrc1	_LC[Carbamidomethyl (C)]TSATESEVTR_.2	#N/A	7.4484	8.0739	8.2036	7.9739	8.1422	8.0167	7.7255	7.8818	7.8517	8.3015	1.0046	0.0066	0.8364	0.0776
Uqcrc1	_EHTAYLIK_.2	#N/A	7.9235	7.7475	8.3245	7.8153	8.0295	8.0240	7.7412	7.7985	7.7222	7.9291	1.0230	0.0329	0.1420	0.8476
Uqcrc1	_IPLAEWESR_.2	#N/A	7.1767	7.1089	7.0488	7.0063	7.5036	6.9035	6.9693	7.3231	6.9597	7.4264	0.9937	-0.0091	0.7600	0.1192
Uqcrc1	_RIPLAEWESR_.2	#N/A	7.0492	7.2641	7.1254	6.6530	6.9440	7.1214	7.0580	6.9915	6.8094	7.3676	0.9957	-0.0062	0.8350	0.0783
Uqcrc1	_VVELLADIVQNSSLEDSQIEKER_.3	#N/A	6.3792	6.7962	7.7351	6.8392	6.1135	7.1396	7.1877	6.4984	6.0947	7.3858	1.0307	0.0437	0.6406	0.1934
Uqcrc1	_NRPGNALEK_.2	#N/A	6.4563	6.5312	6.2367	6.5721	7.0719	6.1597	5.7631	6.1403	6.3927	6.2930	1.0581	0.0815	0.1081	0.9664
Uqcrc1	_NNGAGYFLEHLAFK_.2	#N/A	5.3549	5.0739	4.7243	4.3818	4.8216	4.0714	5.5127	5.5408	5.4650	5.6815	0.8537	-0.2282	0.0094	2.0260
Uqcrc1	_IQEVDAQM[Oxidation (M)]LR_.2	#N/A	8.1742	8.6538	8.9058	8.3505	8.0130	8.7522	7.8225	7.5407	7.7353	7.7278	1.0997	0.1371	0.0032	2.4974
Uqcrc1	_SLLTYGR_.2	#N/A	6.8500	5.6883	4.0542	5.5702	6.5460	6.3202	3.2794	6.9136	6.8963	6.6380	0.9842	-0.0230	0.9167	0.0378
Uqcrc1	_YFYDQC[Carbamidomethyl (C)]PAVAGYGPIEQLPDYNR_.3	#N/A	5.5313	7.0225	6.8526	7.5214	5.9112	6.1611	6.1686	7.0267	6.3398	5.9440	1.0205	0.0292	0.7679	0.1147
Uqcrc1	_SGM[Oxidation (M)]FWLR_.2	#N/A	5.9975	6.2342	5.7583	5.5201	5.6606	6.7892	3.4633	6.8354	5.5217	6.1047	1.0934	0.1288	0.4327	0.3638
Uqcrc1	_FTGSEIR_.2	#N/A	6.9115	6.9010	6.3959	5.8511	7.6357	6.8524	3.2045	7.1457	7.1898	7.4722	1.0807	0.1120	0.5737	0.2413
Uqcrc1	_M[Oxidation (M)]VLAAAGGVEHQQLLDLAQK_.3	#N/A	6.1381	6.0109	6.7458	5.8112	5.7851	6.0018	6.1216	5.7150	5.7893	6.0566	1.0273	0.0388	0.4331	0.3634
Uqcrc1	_VVELLADIVQNSSLEDSQIEK_.2	#N/A	4.3629	4.6683	5.1660	5.9262	4.2606	3.9188	5.3499	4.2911	4.5351	5.2351	0.9720	-0.0409	0.7569	0.1209
Uqcrc1	_VASEQSSHATC[Carbamidomethyl (C)]TVGVWIDAGSR_.3	#N/A	4.1850	4.3515	3.5615	4.0642	4.7049	2.8119	3.6344	4.0543	3.7029	4.0312	1.0235	0.0336	0.8034	0.0951
Uqcrc1	_EVESIGAHLNAYSTR_.2	#N/A	4.0207	4.2409	4.2553	4.1195	4.0954	4.5532	3.9595	3.5813	3.5070	3.8861	1.1287	0.1747	0.0062	2.2092
Ola1	_IGIVGLPNVGK_.2	#N/A	5.8325	6.4391	6.2419	5.7686	5.6357	6.0026	6.0457	6.2619	5.3687	6.1314	1.0058	0.0084	0.8790	0.0560
Ola1	_IPAFLNVVDIAGLVK_.2	#N/A	3.4138	3.9438	4.4220	4.4719	3.2614	4.2258	2.8561	1.7571	2.3613	3.2716	1.5446	0.6272	0.0054	2.2702
Ola1	_NYIVEDGDIIFFK_.2	#N/A	3.7648	5.7002	3.4985	4.5431	3.8319	5.4181	5.0736	3.8764	4.5195	4.6276	0.9857	-0.0208	0.9020	0.0448
Naxd	_DDLLLNNVR_.2	#N/A	7.5024	7.2932	7.4848	8.1036	6.9795	6.9294	6.6075	7.0514	7.4800	7.5093	1.0307	0.0437	0.4481	0.3486
Naxd	_SYSPELIVHPVLDSSNAVEEVEK_.3	#N/A	5.2833	4.1874	4.8843	5.4968	4.4511	4.7388	5.2990	5.0831	4.5103	5.0826	0.9693	-0.0450	0.6049	0.2183
Naxd	_LSQALGNITVVQK_.2	#N/A	4.6507	4.4810	3.6105	4.6707	4.8612	5.2456	4.1726	4.3510	4.2241	4.2045	1.0823	0.1140	0.2481	0.6054
Naxd	_AILTPNHVEFSR_.3	#N/A	3.2965	3.9297	3.9833	5.0776	4.5831	4.3586	4.2006	4.4122	3.6584	4.0617	1.0298	0.0423	0.7277	0.1380
Naxd	_STTTTDMITEVGTAFSR_.2	#N/A	3.6404	2.9448	4.3272	5.9732	4.2669	3.4626	3.8233	3.2886	4.4380	3.2960	1.1054	0.1445	0.5184	0.2854
Nsfl1c	_S[Phospho (STY)]PGETSKPRPFAGGGYR_.3	#N/A	8.8690	8.6365	8.9116	9.2761	8.2289	8.8769	8.2795	8.6446	8.6042	8.8645	1.0234	0.0334	0.3457	0.4613
Nsfl1c	_EANLLNAVIVQR_.2	#N/A	6.6004	6.3753	6.1018	6.6717	6.8855	6.2865	6.5799	6.5148	6.4574	6.2337	1.0063	0.0090	0.8036	0.0950
Nsfl1c	_SYQDPSNAQFLESIR_.2	#N/A	6.1342	6.2667	6.3012	6.6165	6.7724	6.5221	5.7713	5.6647	6.0099	5.7008	1.1121	0.1533	0.0015	2.8212
Nsfl1c	_DLIHDQDEEEEEEEGQR_.3	#N/A	5.9241	5.5377	6.8830	6.7339	6.2797	5.9273	5.8155	5.9785	5.4911	5.6636	1.0423	0.0597	0.4519	0.3450
Nsfl1c	_ASSSILINEAEPTTNIQIR_.2	#N/A	6.0061	4.9754	5.9859	5.7406	6.2096	6.0452	5.1972	5.6006	5.9227	5.9750	1.0270	0.0385	0.5817	0.2353
Nsfl1c	_RHS[Phospho (STY)]GQDVHVVLK_.3	#N/A	9.2727	9.3892	9.8145	9.9570	9.6442	10.1921	9.6285	9.4468	9.8401	10.2984	0.9906	-0.0136	0.6989	0.1556
Nsfl1c	_S[Phospho (STY)]PNELVDDLFK_.2	#N/A	7.2148	6.4973	6.6707	7.4662	7.1421	6.8354	7.2394	6.5559	6.8866	7.3620	0.9943	-0.0082	0.8697	0.0606
Nsfl1c	_RRHS[Phospho (STY)]GQDVHVVLK_.3	#N/A	7.1573	8.4199	3.8364	10.2410	6.5640	7.5405	7.6997	8.5612	8.0562	7.6706	0.9120	-0.1329	0.5385	0.2688
Nsfl1c	_VTKS[Phospho (STY)]PGETSKPR_.2	#N/A	5.2673	6.0449	5.6037	6.1336	4.4504	5.8492	5.1984	5.4150	4.0062	5.3734	1.1120	0.1532	0.2141	0.6693
Nsfl1c	_TGFSLDNGDLR_.2	#N/A	5.8182	5.8156	3.9263	6.2400	5.6118	3.2883	5.6485	5.2989	5.7920	5.5953	0.9164	-0.1260	0.4728	0.3253
Nsfl1c	_FYAGGSER_.2	#N/A	5.8873	5.5981	5.2735	5.2199	6.3345	5.8337	3.0993	5.8562	5.9595	5.9556	1.0907	0.1253	0.4514	0.3454
Nsfl1c	_S[Phospho (STY)]PGETSKPR_.2	#N/A	4.2756	4.7615	4.0093	3.5530	2.8881	3.3405	4.9616	3.4481	3.7472	3.2303	0.9890	-0.0159	0.9295	0.0317
Nsfl1c	_HS[Phospho (STY)]GQDVHVVLK_.2	#N/A	4.3592	4.5475	4.0810	4.8934	3.8494	4.8085	4.0948	3.8317	3.8285	4.6837	1.0763	0.1061	0.2663	0.5746
Nsfl1c	_KKS[Phospho (STY)]PNELVDDLFK_.2	#N/A	3.6350	3.8025	3.8829	11.2349	3.6435	3.6636	3.7989	3.8706	3.5192	3.7782	1.3302	0.4116	0.4531	0.3438
Antxr1	_EVPPPPVEES[Phospho (STY)]EEEDDDGLPK_.2	Q9CZ52_S360_M1_Antxr1	3.1626	3.9678	3.8494	8.9713	7.7331	7.9414	8.7729	9.4670	8.8945	9.1132	0.6552	-0.6099	0.0435	1.3616
Cep97	_QLM[Oxidation (M)]SQS[Phospho (STY)]QDEELSPLAAVETR_.2	#N/A	3.1437	3.9489	3.8305	4.4187	3.7702	3.9780	4.3558	3.9171	3.1258	4.5526	0.9650	-0.0514	0.6813	0.1667
Sdhc	_HLLWDLGK_.2	#N/A	5.4289	5.1301	3.7715	4.5973	4.4102	4.0956	5.1835	5.2539	4.5423	4.1690	0.9551	-0.0663	0.5862	0.2320
Thumpd2	_LLQGS[Phospho (STY)]PEQGEAVTR_.2	#N/A	3.8116	3.5308	4.9538	5.7867	4.5923	3.6692	5.6189	5.5766	5.7927	5.2185	0.7909	-0.3385	0.0358	1.4458
Thumpd2	_DHGESQEDKLLQGS[Phospho (STY)]PEQGEAVTR_.3	#N/A	3.6670	3.5591	4.3538	3.3557	2.7927	1.6025	3.0703	3.2620	2.6456	3.3226	1.0477	0.0672	0.7744	0.1110
Scrn1	_AVIES[Phospho (STY)]DEEQGR_.2	Q9CZC8_S360_M1_Scrn1	9.2748	9.5564	9.9600	9.7910	9.3568	9.4590	9.7173	9.3091	9.4070	9.7629	1.0018	0.0026	0.9173	0.0375
Scrn1	_THAIVISR_.2	#N/A	6.1698	5.3201	5.7133	5.6951	5.8699	6.1385	5.3702	5.7190	6.0159	4.9564	1.0548	0.0770	0.2476	0.6063
Scrn1	_FQEKPDR_.2	#N/A	6.6085	7.6711	6.4031	8.2104	7.3015	7.7924	6.9098	6.7808	6.4949	7.2046	1.0706	0.0985	0.2368	0.6256
Scrn1	_VEC[Carbamidomethyl (C)]TYISIDQVPR_.2	#N/A	5.7884	5.5488	3.6282	5.6648	5.7260	5.7707	5.1469	5.6985	5.1062	5.7205	0.9883	-0.0170	0.8920	0.0496
Scrn1	_DKASGVC[Carbamidomethyl (C)]IDSESFLTTASIVSVLPQNR_.3	#N/A	6.4512	8.2265	8.3245	3.4571	7.2622	7.2830	7.9072	7.8085	7.9135	6.9734	0.8933	-0.1628	0.4070	0.3904
Scrn1	_AQS[Phospho (STY)]PC[Carbamidomethyl (C)]FGDDDPAK_.2	#N/A	7.0525	6.1408	5.9784	7.3907	5.9412	6.3227	6.9396	6.6858	6.5728	7.0817	0.9488	-0.0758	0.3112	0.5070
Scrn1	_ASGVC[Carbamidomethyl (C)]IDSESFLTTASIVSVLPQNR_.3	#N/A	3.6364	3.7919	4.3232	5.4940	4.2629	4.1402	5.1057	4.1211	4.4696	3.8318	0.9755	-0.0358	0.7946	0.0998
Scrn1	_SSPC[Carbamidomethyl (C)]IHYFTGTPDPSR_.3	#N/A	6.7672	6.8711	6.9644	7.2817	6.8338	6.5618	6.8120	6.6221	6.7416	6.4629	1.0331	0.0470	0.1436	0.8428
Scrn1	_PAWLWGAEM[Oxidation (M)]GANEHGVC[Carbamidomethyl (C)]IANEAINAR_.3	#N/A	4.4031	4.2881	4.0856	5.5064	3.3604	3.8347	4.4829	4.5895	4.6033	4.3909	0.9402	-0.0890	0.4856	0.3137
Scrn1	_AQS[Phospho (STY)]PC[Carbamidomethyl (C)]FGDDDPAKKEPR_.3	#N/A	6.9316	7.0958	6.9829	7.5630	6.9462	7.1279	7.2667	7.5645	7.2103	7.4399	0.9644	-0.0523	0.0913	1.0394
Scrn1	_EPAAETEALLGM[Oxidation (M)]DLVR_.2	#N/A	6.5167	6.7466	6.8828	6.8440	6.8194	6.7370	7.0230	6.5576	6.4805	6.6878	1.0105	0.0151	0.5597	0.2520
Scrn1	_TMLELEK_.2	#N/A	3.9023	3.3239	4.5891	3.5909	3.0280	3.2007	3.3056	3.0267	2.5056	3.5579	1.1636	0.2185	0.1764	0.7535
Scrn1	_DEVQEVVYFPAVDHDAESK_.3	#N/A	3.6893	2.9447	3.4230	3.9701	2.8330	3.7916	3.6847	4.1484	3.7970	4.1449	0.8728	-0.1963	0.0849	1.0708
Mxra7	_YS[Phospho (STY)]PGQLR_.2	Q9CZH7_S118_M1_Mxra7	3.2328	3.9891	8.1912	8.2743	3.8774	7.9208	4.1412	7.8903	7.9335	3.6884	1.0002	0.0002	0.9995	0.0002
Mxra7	_VAEPEES[Phospho (STY)]EAEEPAAEGR_.2	Q9CZH7_S79_M1_Mxra7	10.2828	10.1514	10.6478	10.6888	9.3963	10.1529	10.6438	10.2258	10.1800	10.0849	0.9938	-0.0090	0.8114	0.0908
Hspa12b	_SPVPS[Phospho (STY)]PPGSPR_.2	Q9CZJ2_S25_M1_Hspa12b	4.0130	9.7342	10.5879	9.7574	7.8730	3.4679	3.3611	9.9106	9.7856	3.6134	1.1357	0.1835	0.6849	0.1644

Hspa12b	_RSS[Phospho (STY)]VNLVK_.2	Q9CZJ2_S434_M1_Hspa12b	4.2308	3.1116	7.2934	3.5082	5.9979	6.8046	6.9055	7.3487	7.2686	7.0893	0.7211	-0.4718	0.0594	1.2259
Hspa12b	_TQESC[Carbamidomethyl (C)]GIAPLTPS[Phospho (STY)]QSPKPEAR_.3	Q9CZJ2_S44_M1_Hspa12b	6.8605	6.1339	4.3363	5.9535	5.0637	6.1634	6.6690	6.4796	6.1270	6.6922	0.8860	-0.1746	0.1551	0.8094
Hspa12b	_RS[Phospho (STY)]IDSS[Phospho (STY)]FR_.2	#N/A	4.7361	4.0057	4.7263	3.8082	4.1426	3.5807	4.4202	4.2306	3.0628	3.1717	1.1197	0.1631	0.2624	0.5810
Hspa12b	_S[Phospho (STY)]IDSS[Phospho (STY)]FR_.2	#N/A	3.5660	3.7124	4.2528	4.0558	3.6007	3.8773	3.7705	5.0034	3.8650	4.0228	0.9229	-0.1158	0.2579	0.5885
Rpl15	_SLQSVAEER_.2	#N/A	5.7994	5.9937	6.0660	4.9050	5.7341	5.9017	4.5460	5.8982	6.3451	5.5253	1.0277	0.0395	0.6884	0.1621
Rpl15	_NTLQLHR_.2	#N/A	4.8881	5.6373	4.4548	5.0846	5.9100	5.1615	4.5249	5.2143	5.2864	3.4236	1.1251	0.1701	0.2193	0.6590
Rpl15	_VLNSYWVGEDSTYK_.2	#N/A	4.0238	5.1764	3.2098	3.7124	3.0907	3.5339	3.4271	4.4061	5.2570	3.6794	0.9043	-0.1451	0.4501	0.3467
Shisa9	_AVGNSDGDWAVATLKS[Phospho (STY)]PK_.3	Q9CZN4_S296_M1_Shisa9	5.2935	4.8021	4.4600	5.8152	3.8700	5.1680	4.1486	2.9975	3.2965	3.6810	1.3882	0.4732	0.0090	2.0457
Shisa9	_AFS[Phospho (STY)]PEHGPAQQNGQK_.2	Q9CZN4_S337_M1_Shisa9	7.5705	7.3508	3.8884	8.0906	6.6533	7.2515	7.9515	7.8811	7.3679	7.2697	0.8928	-0.1636	0.3247	0.4886
Aldh1b1	_TFVEESIYR_.2	#N/A	2.9855	2.7357	4.2200	4.5270	3.6300	2.9946	4.3886	3.2374	3.5365	3.4410	0.9629	-0.0545	0.7581	0.1203
Rab3b	_LLAEQLGFDFFEASAK_.2	#N/A	4.0392	3.0566	3.8820	4.0091	3.2920	3.3326	3.5740	4.5356	3.8745	4.0365	0.8993	-0.1531	0.1719	0.7648
Rab3b	_M[Oxidation (M)]SDS[Phospho (STY)]M[Oxidation (M)]DTDPSVLGASK_.2	#N/A	6.6795	3.0270	7.7554	4.7852	3.1425	7.6859	8.1434	8.0632	3.7917	6.8392	0.8216	-0.2834	0.4067	0.3907
Rab3b	VDAIC[Carbamidomethyl (C)]DKM[Oxidation (M)]S[Phospho (STY)]DSM[Oxidation (M)]DTDPSVLGASK_	Q9CZT8_S188_M1_Rab3b	3.0101	4.2161	3.6969	4.1996	3.6366	4.0929	3.9143	6.5082	3.5675	4.1666	0.8391	-0.2531	0.2424	0.6154
Cs	_VVPGYGHAVLR_.2	#N/A	10.2946	10.6302	10.6596	10.4680	10.7158	10.3219	10.5691	10.8252	10.5306	10.3099	0.9959	-0.0060	0.7343	0.1341
Cs	_GLVYETSVLDPDEGIR_.2	#N/A	10.3558	10.0257	10.6064	10.4698	9.9864	10.3859	10.4538	10.1187	10.1936	10.3010	1.0037	0.0054	0.7900	0.1024
Cs	_EGSSIGAIDSR_.2	#N/A	8.8601	8.7999	8.4185	8.7641	7.9482	8.2303	8.5485	8.4660	8.2506	8.8207	0.9979	-0.0030	0.9337	0.0298
Cs	_ALGFPLERPK_.2	#N/A	9.9570	10.4155	10.4493	9.9821	10.1916	9.9219	10.2533	10.2550	10.2211	10.0817	0.9951	-0.0071	0.7002	0.1548
Cs	_IVPNILLEQGK_.2	#N/A	9.5985	9.8044	9.8509	9.3773	9.5179	9.5359	9.7618	9.8950	9.7509	9.4126	0.9906	-0.0136	0.4812	0.3177
Cs	_GYSIPEC[Carbamidomethyl (C)]QK_.2	#N/A	9.1683	9.2118	9.4526	9.2056	9.3377	9.1254	9.1355	9.1126	9.2311	9.2194	1.0082	0.0118	0.2887	0.5395
Cs	_DYIWNTLNSGR_.2	#N/A	9.3560	9.1223	9.1525	9.4109	8.9650	9.1150	9.1752	9.1917	9.2139	9.1771	0.9997	-0.0004	0.9771	0.0101
Cs	_LVAQLYK_.2	#N/A	7.3908	7.1100	6.8496	6.6071	6.9053	7.0277	5.0849	7.4281	7.5000	7.2795	1.0232	0.0332	0.7490	0.1255
Cs	_EVGKDVSDEKLR_.3	#N/A	6.5227	6.4743	6.4007	6.0985	7.1301	6.7079	6.5674	6.5310	7.0854	6.9033	0.9681	-0.0468	0.3233	0.4904
Cs	_EVGKDVSDEK_.2	#N/A	6.6723	6.4679	5.7907	6.1965	6.7785	6.4017	6.4812	6.5161	6.4199	6.5374	0.9840	-0.0233	0.5848	0.2330
Cs	_ALGVLAQLIWSR_.2	#N/A	4.8065	5.5873	5.2674	5.2208	5.3922	6.3610	6.0724	6.2339	6.1567	5.6031	0.9040	-0.1455	0.0799	1.0972
Cs	_AYAEGM[Oxidation (M)]NR_.2	#N/A	6.8870	6.9381	6.0146	7.1635	7.9510	7.0158	6.3643	6.6829	7.0440	6.7850	1.0411	0.0581	0.4341	0.3625
Cs	_LDWSHNFTNM[Oxidation (M)]LGYTDPQFTELM[Oxidation (M)]R_.3	#N/A	4.4699	4.0569	4.9448	4.6955	4.0187	4.7886	4.7404	4.9640	5.8088	3.5701	0.9423	-0.0857	0.5260	0.2790
Cs	_AALPSHVVTM[Oxidation (M)]LDNFPTNLHPM[Oxidation (M)]SQLSAAITALNSESNFAR_.4	#N/A	5.6415	6.2013	5.6850	6.8177	4.7736	5.5093	6.8189	6.5426	6.1354	6.1349	0.9007	-0.1509	0.1283	0.8918
Cs	_TVVGQITVDM[Oxidation (M)]M[Oxidation (M)]YGGM[Oxidation (M)]R_.2	#N/A	6.0078	6.1941	7.3846	5.4352	5.6601	6.2318	6.0025	6.4084	6.4150	6.0876	0.9878	-0.0177	0.8363	0.0776
Cs	_DVLSNLIPK_.2	#N/A	3.3420	3.8842	4.0288	4.5314	3.9685	4.3529	4.2461	3.5871	3.2356	2.9976	1.1426	0.1923	0.1343	0.8720
Cs	_YWELIYEDC[Carbamidomethyl (C)]M[Oxidation (M)]DLIAK_.2	#N/A	4.1851	2.1926	3.9187	2.7102	3.3288	3.2958	3.1890	3.1433	4.2928	3.4932	0.9270	-0.1094	0.5674	0.2461
Cs	_SMSTDGLMK_.2	#N/A	3.7072	3.6352	3.4409	4.4855	2.8509	3.7737	3.1329	4.0166	4.3156	4.1627	0.9339	-0.0986	0.4718	0.3262
Tomm70	_NVDLSTFYQNR_.2	#N/A	7.3643	7.4791	7.7323	7.9114	7.4403	7.4706	7.6471	7.6565	7.5434	7.6236	0.9933	-0.0098	0.6503	0.1869
Tomm70	_AAAFEQLQK_.2	#N/A	7.1029	7.2610	7.4424	7.1950	7.1799	6.8794	7.2352	6.9986	6.5910	6.7176	1.0423	0.0597	0.0851	1.0699
Tomm70	_SYFSSFTDDIISQPM[Oxidation (M)]LK_.2	#N/A	6.8333	6.6439	6.9891	6.7642	6.8942	6.9028	6.4730	6.7716	6.6777	6.7773	1.0244	0.0348	0.0868	1.0616
Tomm70	_FALAQAQK_.2	#N/A	6.2535	6.2673	6.0846	6.0808	6.3658	5.8089	5.5253	6.3704	6.1301	6.0649	1.0201	0.0287	0.5059	0.2960
Tomm70	_YEQAIQC[Carbamidomethyl (C)]YTEAISLC[Carbamidomethyl (C)]PTEK_.2	#N/A	5.1092	4.3288	5.4196	5.9632	4.7124	4.4871	4.6386	4.9603	4.4192	4.6633	1.0713	0.0994	0.3390	0.4698
Tomm70	_ATFYLLIGSANAAKPDLDK_.2	#N/A	4.6026	4.3333	5.5513	5.5074	4.8148	5.1547	5.3503	5.6763	4.3744	5.1988	0.9697	-0.0444	0.6532	0.1850
Tomm70	_C[Carbamidomethyl (C)]IDLEPDNATTYVHK_.3	#N/A	5.8596	3.5581	3.2394	4.4343	5.6338	5.2263	3.8683	4.9976	3.6795	5.0239	1.0606	0.0849	0.6821	0.1662
Tomm70	_GDASGLKR_.2	#N/A	5.6062	3.4514	5.2086	4.8392	5.7145	5.4186	5.3442	5.2432	4.9722	5.2574	0.9684	-0.0463	0.7135	0.1466
Tomm70	_YM[Oxidation (M)]AEALLLR_.2	#N/A	5.2917	5.5719	4.9051	5.9224	4.7044	5.0506	5.3216	5.4713	5.6192	4.9645	0.9807	-0.0281	0.6966	0.1570
Tomm70	_C[Carbamidomethyl (C)]AEGYALYAQALTDQQQFGK_.2	#N/A	3.6556	3.4896	3.5011	4.0038	3.7514	4.1932	3.7185	4.1147	4.8191	4.6106	0.8726	-0.1966	0.0536	1.2707
Tomm70	_AS[Phospho (STY)]PALGSGHHDGSGDSLEM[Oxidation (M)]SSLDR_.3	#N/A	9.7024	9.4879	9.5827	10.6679	9.0217	9.2871	9.5618	9.6438	9.6269	9.2562	1.0108	0.0155	0.7381	0.1319
Tomm70	_SDEDKDKEGEALEVK_.3	#N/A	4.2575	3.5129	3.9911	3.6761	3.4012	3.2234	3.1166	3.4663	4.5289	3.2122	1.0268	0.0382	0.7740	0.1112
Tomm70	_WKEVAQDC[Carbamidomethyl (C)]TK_.2	#N/A	3.9475	3.2518	3.1334	3.3680	5.2649	3.1555	3.5163	3.6331	3.9322	3.0453	1.0439	0.0620	0.7378	0.1321
Tomm70	_GFEEIIK_.2	#N/A	1.2679	3.0472	3.3649	3.8676	3.3047	4.4248	3.5823	4.2509	3.8995	3.8346	0.8255	-0.2766	0.2580	0.5884
Tomm70	_QAYTANNSSQVQAAMK_.2	#N/A	3.8149	3.4113	4.5017	3.5035	3.2996	3.3250	3.2182	3.1141	4.2636	3.4705	1.0358	0.0508	0.6968	0.1569
Tomm70	_NSERKT[Phospho (STY)]PEGR_.2	Q9CZW5_T88_M1_Tomm70	4.3250	3.5804	4.0586	3.3344	3.4687	3.1559	4.5499	3.2832	4.4327	3.3014	0.9389	-0.0910	0.5239	0.2807
Tomm70	_EC[Carbamidomethyl (C)]LEDVTAVC[Carbamidomethyl (C)]ILEGFQNEQSM[Oxidation (M)]LLADK_.3	#N/A	3.4600	4.2652	4.1468	3.8555	4.0866	3.6429	4.0037	3.1457	3.4448	3.1157	1.1406	0.1898	0.0676	1.1704
Rps19	_DVNQQEFVR_.2	#N/A	7.3711	7.6472	7.4805	7.2345	7.5994	7.4121	6.8078	7.3889	7.3322	7.3100	1.0344	0.0487	0.0974	1.0114
Rps19	_ELAPYDENWFYTR_.2	#N/A	6.7985	6.5839	6.3347	6.3528	6.8535	6.3554	6.6343	6.4249	6.2669	6.3304	1.0206	0.0295	0.3592	0.4447
Rps19	_IAGQVAAANK_.2	#N/A	4.3235	4.3570	4.2386	3.4703	5.2723	3.8410	4.4613	3.3772	4.5364	2.9340	1.1106	0.1513	0.3658	0.4367
Rps19	_GGAGVGSMTK_.2	#N/A	3.2788	4.0636	4.5134	4.0571	4.4820	3.8446	4.2054	4.4449	3.2431	3.7344	1.0340	0.0483	0.6826	0.1658
Emc4	_S[Phospho (STY)]DRGSGQGDSLYPVGYLDK_.3	Q9CZX9_S32_M1_Emc4	3.1078	3.8641	4.3423	4.5516	3.7524	4.3731	4.2662	3.1151	4.6707	3.5634	1.0243	0.0346	0.8135	0.0897
Ube2v1	_LPQPPEGQC[Carbamidomethyl (C)]YS[Phospho (STY)]N_.2	Q9CZY3_S146_M1_Ube2v1	3.6946	2.9501	3.4283	4.4729	2.8384	8.4564	3.1204	4.0291	8.8742	4.1502	0.8539	-0.2278	0.6342	0.1978
Ube2v1	_VNM[Oxidation (M)]SGVSSSNGVVDPR_.2	#N/A	3.8267	5.3254	4.2794	4.4218	5.4199	5.2434	3.6777	4.9509	4.4800	4.6891	1.0682	0.0951	0.4727	0.3254
Nt5c3a	_DNSNIILLGDS[Phospho (STY)]QGDLR_.2	Q9D020_S273_M1_Nt5c3a	3.5483	4.3535	4.2234	7.8558	6.4591	5.6811	3.9154	6.6516	3.5331	3.4444	1.2206	0.2875	0.3720	0.4294
Ssbp3	_NS[Phospho (STY)]PNNISGISNPPGTPR_.2	Q9D032_S347_M1_Ssbp3	2.9918	4.3506	4.2263	7.7712	4.1719	5.6287	3.9184	3.2311	5.7291	3.4474	1.1899	0.2509	0.4410	0.3556
Pdhb	_VLEDNSVPQVK_.2	#N/A	9.8564	9.7503	10.0507	9.9525	10.0461	9.7302	9.8185	9.6373	9.6367	9.8247	1.0173	0.0248	0.0786	1.1046
Pdhb	_VVSPWNSEDAK_.2	#N/A	9.1915	9.1730	9.3323	9.4464	9.4468	9.2395	8.9771	9.1228	9.1808	9.3083	1.0172	0.0247	0.0938	1.0278
Pdhb	_EGIEC[Carbamidomethyl (C)]EVINLR_.2	#N/A	8.6507	8.6408	8.2076	8.8542	8.1075	8.1897	8.6898	8.3373	8.4264	8.5504	0.9930	-0.0101	0.7362	0.1330
Pdhb	_VFLLGEEVAQYDGAYK_.2	#N/A	8.3093	8.2374	8.6512	8.3946	8.2760	8.6561	8.5347	8.0404	8.3309	8.4494	1.0098	0.0141	0.5409	0.2669
Pdhb	_DFLIPIGK_.2	#N/A	6.5825	6.5425	5.7703	6.6271	6.9152	7.1446	5.6475	6.9537	7.0058	7.0497	0.9899	-0.0146	0.8563	0.0674
Pdhb	_TIRPM[Oxidation (M)]DIEAIEASVM[Oxidation (M)]K_.3	#N/A	7.5186	7.8864	7.9920	7.6377	7.4645	7.7956	7.3314	7.7789	8.0182	8.0449	0.9900	-0.0144	0.6581	0.1817
Pdhb	_IM[Oxidation (M)]EGPAFNFLDAPAVR_.2	#N/A	6.8198	7.0940	7.2722	7.3018	6.6319	6.8332	6.6667	6.9674	6.9787	6.8730	1.0176	0.0251	0.4445	0.3522
Pdhb	_TYYM[Oxidation (M)]SAGLQPVPIVFR_.2	#N/A	8.1594	8.1409	8.4530	8.2732	8.2283	8.2145	8.1352	8.0334	8.2022	8.2883	1.0098	0.0141	0.2952	0.5299
Pdhb	_EAINQGM[Oxidation (M)]DEELERDEK_.3	#N/A	6.3876	6.7304	6.9160	6.9838	6.8485	6.5522	5.8418	6.5632	6.7541	6.8482	1.0361	0.0511	0.3062	0.5140
Pdhb	_PVGHC[Carbamidomethyl (C)]LEAAAVLSK_.3	#N/A	2.7493	3.5861	3.4677	4.5346	4.5091	4.3221	3.1821	3.9674	4.2664	4.2119	0.9884	-0.0169	0.9154	0.0384
Mdm1	_ASS[Phospho (STY)]QDGLETLKK_.2	Q9D067_S556_M1_Mdm1	8.0716	4.3298	4.2471	8.4981	7.8308	8.3763	8.3426	8.2641	8.1518	8.4371	0.8305	-0.2679	0.2121	0.6734
Mdm1	_VQGTHFS[Phospho (STY)]R_.2	Q9D067_S357_M1_Mdm1	3.4612	4.2664	4.1480	3.1498	4.0877	3.6418	4.3844	3.4678	3.1164	3.1168	1.0770	0.1070	0.4318	0.3648
Mdm1	_RAS[Phospho (STY)]S[Phospho (STY)]QDGLETLKK_.3	Q9D067_S555_M2_Mdm1	3.4353	3.7909	4.1221	4.6248	4.0618	2.9455	4.3395	4.2321	3.1423	3.0909	1.0348	0.0494	0.7561	0.1214
Mgrn1	_TQSKS[Phospho (STY)]PDSTLR_.2	Q9D074_S428_M1_Mgrn1	3.1441	8.4976	8.7302	8.7443	8.0041	8.3338	8.5345	8.5349	8.3666	2.9814	1.0663	0.0927	0.7700	0.1135
Mgrn1	_ESSS[Phospho (STY)]PESFGTEEGDEPSLK_.2	Q9D074_S470_M1_Mgrn1	3.8374	3.4195	4.1516	4.0709	2.9717	3.4827	5.3783	3.8008	4.0999	3.7482	0.8588	-0.2196	0.1511	0.8207
Mgrn1	_YKEDADS[Phospho (STY)]PTEDGEKPR_.2	#N/A	4.4136	4.4789	5.0813	4.3862	4.2240	3.5752	3.9526	4.4860	3.8760	3.7181	1.0877	0.1213	0.2446	0.6116
Srsf9	_GRDS[Phospho (STY)]PYQSR_.2	Q9D0B0_S205_M1_Srsf9	3.8780	3.1334	3.2787	3.7814	3.0217	3.6029	6.2449	4.3371	3.9857	3.7484	0.7533	-0.4087	0.0480	1.3187
Srsf9	_GS[Phospho (STY)]PHYFS[Phospho (STY)]PFRPY_.2	#N/A	3.8575	6.5325	6.8152	7.5829	7.0307	6.7756	7.0421	6.7988	6.8676	6.9235	0.9312	-0.1029	0.4979	0.3028
Srsf9	_STS[Phospho (STY)]YGYSR_.2	Q9D0B0_S190_M1_Srsf9	3.4502	4.2064	3.1838	4.2284	4.0947	4.0307	4.3767	4.2736	3.0718	3.9057	0.9894	-0.0153	0.9019	0.0448
Tstd3	_EWVTYEISEEKQES_.2	#N/A	5.4613	5.6992	6.6721	6.8152	5.6109	6.7251	6.2157	4.3960	5.1804	5.3474	1.1663	0.2220	0.0797	1.0988
Pbdc1	_GADS[Phospho (STY)]GGEKEEGANR_.2	#N/A	4.4756	4.3647	3.1902	6.0642	5.5470	4.0223	4.3831	3.8183	3.0654	5.1708	1.1220	0.1660	0.4545	0.3425
Pbdc1	_GADS[Phospho (STY)]GGEKEEGANREGEK_.3	#N/A	2.9567	4.2695	3.6435	4.1461	3.5832	4.1463	3.9587	3.9724	3.8874	4.1131	0.9518	-0.0713	0.4765	0.3220

Hnrnpm	_AFITNIPFDVK_.2	#N/A	6.7150	6.7132	6.3960	6.6710	6.0536	6.7082	6.4058	6.6433	6.2313	6.3660	1.0205	0.0292	0.4171	0.3798
Hnrnpm	_INEILSNALK_.2	#N/A	5.2229	5.7916	5.0145	5.3066	5.7793	5.7784	5.3643	5.4803	5.7404	5.3362	1.0003	0.0005	0.9923	0.0034
Hnrnpm	_MGLS[Phospho (STY)]MDR_.2	Q9D0E1_S527_M1_Hnrnpm	3.9010	3.2053	3.8708	3.4145	3.0266	3.2020	3.5628	3.5866	3.8857	3.0918	0.9731	-0.0394	0.6886	0.1620
Hnrnpm	_MGLAMGGAGGAS[Phospho (STY)]FDR_.2	Q9D0E1_S617_M1_Hnrnpm	4.2710	3.5265	4.0047	3.3883	3.4148	3.2098	3.1030	3.2293	4.3788	3.2258	1.0435	0.0615	0.6444	0.1908
Hnrnpm	_VGS[Phospho (STY)]EIER_.2	Q9D0E1_S431_M1_Hnrnpm	3.3693	4.1745	4.0561	4.5587	3.9958	3.7337	4.0945	3.5598	3.2083	3.0249	1.1467	0.1975	0.1036	0.9848
Hnrnpm	_MGS[Phospho (STY)]GVER_.2	Q9D0E1_S512_M1_Hnrnpm	4.3477	3.6520	3.5336	4.4687	3.4734	4.2561	3.1161	4.0334	4.3324	1.0397	1.2635	0.3374	0.2274	0.6433
Lysmd1	_S[Phospho (STY)]YGSLVQSSC[Carbamidomethyl (C)]SPVR_.2	Q9D0E3_S23_M1_Lysmd1	3.8959	3.3303	5.6754	3.5845	3.0216	3.2071	3.2992	3.0331	4.1825	3.5515	1.0765	0.1064	0.6278	0.2022
Lysmd1	_DLFNGLDS[Phospho (STY)]EEENDGEEEVRPSKDEIGSSSGR_.4	Q9D0E3_S99_M1_Lysmd1	4.1426	3.4469	3.3285	3.8312	3.2683	2.9604	3.5459	4.2873	3.9359	3.7982	0.8984	-0.1546	0.1503	0.8231
Pgm1	_YDYEEVEAEGANK_.2	#N/A	10.0391	9.8301	10.5818	10.0383	9.5837	10.0894	10.4530	9.7907	10.0508	10.1727	0.9911	-0.0129	0.6667	0.1761
Pgm1	_ADNFEYSDPVDGSISK_.2	#N/A	7.0701	7.1155	7.6606	7.1911	6.7852	7.2896	7.2196	6.9329	7.2592	7.2094	1.0042	0.0060	0.8543	0.0684
Pgm1	_ELLSGPNR_.2	#N/A	6.5270	6.9325	6.8767	7.0250	6.8752	6.7584	6.5498	6.8119	6.8821	6.6898	1.0147	0.0211	0.3749	0.4260
Pgm1	_TQAYPDQKPGTSGLR_.3	#N/A	6.3836	5.8398	6.0290	6.1316	6.5587	6.0599	5.6533	6.3339	6.5820	6.4286	0.9868	-0.0191	0.7049	0.1519
Pgm1	_IALYETPTGWK_.2	#N/A	6.6336	6.5315	6.6039	6.4040	6.3966	6.2913	6.4246	6.3981	5.9964	6.7827	1.0119	0.0171	0.6110	0.2140
Pgm1	_LIFADGSR_.2	#N/A	5.5628	5.3623	4.4182	5.5630	5.1744	5.2674	4.8360	6.1736	5.4024	5.6013	0.9494	-0.0750	0.3914	0.4073
Pgm1	_QQFDLENK_.2	#N/A	5.0695	5.2759	4.5823	5.6025	4.7872	5.4617	5.0715	4.7237	5.3329	4.9328	1.0229	0.0326	0.6241	0.2047
Pgm1	_AIGGIILTAS[Phospho (STY)]HNPGGPNGDFGIK_.3	Q9D0F9_S117_M1_Pgm1	4.0167	3.3257	7.3056	9.2411	5.0540	3.0816	4.9775	7.7121	4.0061	2.9714	1.0856	0.1184	0.7846	0.1054
Pgm1	_VDLGVLGK_.2	#N/A	4.2863	4.3038	3.3641	3.7706	3.6684	3.9699	2.8120	4.0577	4.0911	5.0084	0.9753	-0.0360	0.8130	0.0899
Pgm1	_INQDPQVM[Oxidation (M)]LAPLISIALK_.2	#N/A	3.2432	3.9150	3.2567	4.2430	4.0797	5.0191	4.3135	4.6228	4.5319	4.3877	0.8870	-0.1730	0.1817	0.7405
Pgm1	_QEATLVVGGDGR_.2	#N/A	2.9509	6.3636	4.1854	3.2076	3.5955	3.0291	4.4231	3.4100	3.0586	6.7451	0.8819	-0.1812	0.5916	0.2280
Pgm1	_FFGNLM[Oxidation (M)]DASK_.2	#N/A	4.3552	3.9110	3.4166	4.4612	4.8085	4.2486	4.1759	4.0409	4.4813	4.3077	0.9879	-0.0175	0.8469	0.0722
Pgm1	_FNISNGGPAPEAITDK_.2	#N/A	4.8563	4.8228	4.0850	3.9173	5.5781	3.7047	4.0655	3.0839	3.3830	3.9409	1.2420	0.3127	0.0637	1.1960
Pgm1	_DLEALMLDR_.2	#N/A	3.8054	3.0609	3.5391	3.8539	2.9492	3.6754	3.5686	4.2646	3.9132	3.8209	0.8944	-0.1611	0.1084	0.9652
Pgm1	_LYIDSYEKDVAK_.3	#N/A	3.8671	3.3591	4.5539	3.5557	3.2474	3.3772	3.2704	3.0620	4.2114	3.5227	1.0408	0.0577	0.6640	0.1778
Stx17	_LTSS[Phospho (STY)]C[Carbamidomethyl (C)]PDLPSQSDK_.2	Q9D0I4_S288_M1_Stx17	8.3684	7.9778	8.2004	8.5197	3.5715	3.6886	3.8444	8.0601	3.8114	8.0676	1.1304	0.1768	0.6328	0.1987
Ptms	_[Acetyl (Protein N-term)]S[Phospho (STY)]EKSVEAAAELSAK_.2	Q9D0J8_S2_M1_Ptms	3.6636	6.0096	6.6078	8.6408	7.6193	6.3259	6.6458	6.7547	7.3328	7.4548	0.9192	-0.1215	0.5330	0.2733
Ptms	_TAEEEDEADPKR_.3	#N/A	3.8799	3.9350	3.5899	4.3525	3.5069	4.1562	3.6028	6.3498	3.9999	4.1742	0.8614	-0.2153	0.2576	0.5890
Oxct1	_FYTDPVEAVK_.2	#N/A	8.6454	8.3186	8.2299	8.4729	8.7320	7.9729	8.5400	8.4798	8.3788	8.2924	0.9967	-0.0047	0.8588	0.0661
Oxct1	_GVFDVDKK_.2	#N/A	8.2413	8.1174	8.5080	8.7204	8.8066	8.1781	8.2872	8.3503	8.4304	8.5796	1.0020	0.0029	0.9179	0.0372
Oxct1	_DGSVAIASKPR_.2	#N/A	8.0085	8.0401	8.3895	8.4104	8.2861	8.0976	7.9585	8.0523	7.9788	8.1395	1.0216	0.0308	0.1119	0.9513
Oxct1	_YGDLANWM[Oxidation (M)]IPGK_.2	#N/A	7.7815	7.9213	7.9357	7.2972	7.4725	7.6369	7.6728	8.1494	7.7310	7.8406	0.9778	-0.0324	0.2932	0.5329
Oxct1	_AGGAGVPAFYTSTGYGTLVQEGGSPIK_.3	#N/A	4.5938	3.4361	5.5271	4.6174	4.2871	4.7978	5.0383	4.4333	4.2126	4.9909	0.9731	-0.0393	0.7523	0.1236
Oxct1	_GM[Oxidation (M)]GGAM[Oxidation (M)]DLVSSSK_.2	#N/A	6.5143	6.7763	7.1043	6.4935	6.9116	6.7852	6.6648	6.6260	6.4337	6.7661	0.9962	-0.0055	0.9349	0.0292
Oxct1	_C[Carbamidomethyl (C)]TLPLTGK_.2	#N/A	3.5795	5.1304	3.3132	4.0798	6.2680	4.2460	4.6653	5.0385	5.7032	5.2582	0.8587	-0.2198	0.2469	0.6074
Oxct1	_QFLSGELEVELTPQGTLAER_.2	#N/A	2.9877	5.3894	5.5201	6.0688	5.2624	4.1153	3.8918	5.3388	5.5113	5.2055	0.9807	-0.0281	0.8855	0.0528
Oxct1	_DLTAVSNNAGVDNFGLGLLLR_.2	#N/A	5.4850	5.3727	5.7268	6.2771	5.5053	5.4368	5.6687	5.6114	5.2349	5.7493	1.0122	0.0175	0.7357	0.1333
Oxct1	_ETVTVLPGASFFSSDESFAMIR_.2	#N/A	3.7913	3.0468	3.5250	6.3487	3.4468	3.6895	3.2171	3.9324	4.2314	3.1073	1.0974	0.1340	0.5995	0.2222
Oxct1	_STGC[Carbamidomethyl (C)]DFAVSPNLM[Oxidation (M)]PM[Oxidation (M)]QQIST_.2	#N/A	5.0383	5.5826	5.3174	4.6190	5.8967	5.8237	5.7707	5.5838	5.5527	5.6597	0.9535	-0.0686	0.3312	0.4799
Oxct1	_VVVTMEHSAK_.2	#N/A	2.9835	4.3589	4.2180	5.1956	3.9224	2.9966	4.7023	3.2394	3.5385	2.7317	1.1106	0.1513	0.4925	0.3076
Oxct1	_M[Oxidation (M)]ISSYVGENAEFER_.2	#N/A	5.2202	4.7729	5.1920	4.2468	4.3465	3.9744	5.1995	4.0877	4.7286	4.6423	0.9916	-0.0121	0.9058	0.0430
Armc10	_S[Phospho (STY)]AEDLTDGSYDDILNAEQLKK_.3	#N/A	2.9253	3.7305	9.7311	9.6273	3.5518	4.1777	4.5385	9.9221	3.6523	4.0817	1.0136	0.0194	0.9708	0.0129
Armc10	_S[Phospho (STY)]AEDLTDGSYDDILNAEQLK_.2	#N/A	8.8990	8.9782	9.0858	4.2251	3.7169	4.0126	9.5702	9.7365	9.4238	8.8927	0.6896	-0.5362	0.0719	1.1432
Armc10	_LLYLLESTDDPVITEK_.2	#N/A	2.5236	1.7964	3.9812	4.4838	3.5772	4.3053	3.5250	2.9917	3.4063	4.4508	0.9586	-0.0610	0.8092	0.0920
Rnmt	_ASVASDPES[Phospho (STY)]PPGGNEPAAASGQR_.2	#N/A	10.2404	10.0878	10.8305	10.9814	9.9918	10.4444	10.6780	10.3541	10.4584	10.2880	0.9985	-0.0021	0.9455	0.0243
Rnmt	_GDVS[Phospho (STY)]EDEPSLGR_.2	Q9D0L8_S100_M1_Rnmt	11.2390	11.2223	11.7970	11.5471	10.3139	11.4177	11.2018	11.1227	11.2291	11.2078	1.0059	0.0085	0.8066	0.0933
Rnmt	_EFGEDLVEQNSSYVQDS[Phospho (STY)]PSKK_.3	#N/A	3.2408	3.9971	4.4753	4.0191	3.8854	6.1349	4.1674	6.2402	3.2811	3.6964	0.9875	-0.0181	0.9421	0.0259
Rnmt	_EFGEDLVEQNSSYVQDS[Phospho (STY)]PSKKR_.3	#N/A	3.3393	6.3217	6.8091	6.3403	5.9501	3.7810	4.1418	3.8850	3.3240	5.8455	1.2616	0.3352	0.2307	0.6370
Rnmt	_EFGEDLVEQNSSYVQDS[Phospho (STY)]PSK_.2	#N/A	6.5771	3.6171	8.9081	9.2283	8.4752	9.0210	9.2047	8.7311	8.9829	8.6680	0.8585	-0.2201	0.2950	0.5302
Rnmt	_LPENT[Phospho (STY)]PPC[Carbamidomethyl (C)]QQVDQPK_.2	Q9D0L8_T34_M1_Rnmt	3.7125	2.9679	3.4442	3.9469	6.1386	3.7684	3.6616	4.1716	3.8202	3.9139	1.0269	0.0383	0.8588	0.0661
Cyc1	_HLVGVC[Carbamidomethyl (C)]YTEEEAK_.2	#N/A	4.8727	4.9667	4.3514	5.5834	4.7991	4.9273	4.8463	5.1134	5.2508	5.1131	0.9677	-0.0474	0.4624	0.3349
Cyc1	_LSDYFKPYPNPEAAR_.3	#N/A	7.7527	7.2672	8.2134	7.7690	7.3448	7.9125	4.2157	7.9695	7.6371	7.4074	1.1326	0.1796	0.2409	0.6182
Cyc1	_HGGEDYVFSLLTGYC[Carbamidomethyl (C)]EPPTGVSLR_.3	#N/A	4.8830	5.1683	3.1822	5.3609	4.7044	5.2685	4.4909	5.3094	4.8883	4.5926	0.9877	-0.0178	0.8963	0.0476
Cyc1	_GLLSSLDHTSIR_.2	#N/A	6.7132	6.6857	6.5288	6.5748	6.6574	6.5616	6.5876	6.5117	6.2010	6.7489	1.0166	0.0237	0.3056	0.5148
Cyc1	_DVATFLR_.2	#N/A	7.0623	5.9939	5.8628	4.0310	6.2449	6.5902	3.9809	6.0573	7.3011	6.8659	0.9856	-0.0209	0.9148	0.0387
Cyc1	_ALAEEVEVQDGPNDDGEM[Oxidation (M)]FM[Oxidation (M)]RPGK_.3	#N/A	4.3329	5.0384	5.4754	4.3974	4.6658	5.1415	5.1970	4.6485	4.9990	4.5783	0.9972	-0.0041	0.9585	0.0184
Cyc1	_WASEPEHDHRK_.3	#N/A	3.7976	3.0531	3.5313	4.5759	4.0275	3.6832	3.2233	3.9261	4.3290	4.2532	0.9606	-0.0579	0.6489	0.1878
Dynll2	_DIAAYIKK_.2	#N/A	6.3860	7.3873	7.5512	6.8133	6.5574	7.0898	6.7961	6.9782	6.9447	6.9533	1.0067	0.0096	0.8508	0.0702
Dynll2	_NFGSYVTHETK_.2	#N/A	6.2487	6.1685	5.7044	6.3181	6.3439	5.6059	6.2841	6.3459	5.9387	5.8753	0.9925	-0.0109	0.8150	0.0888
Dynll2	_YNPTWHC[Carbamidomethyl (C)]IVGR_.2	#N/A	8.9600	8.9515	8.8707	8.6954	8.6734	8.4765	9.1203	8.9196	8.6060	8.4950	0.9984	-0.0023	0.9277	0.0326
Dynll2	_NADM[Oxidation (M)]SEDMQQDAVDC[Carbamidomethyl (C)]ATQAMEK_.3	#N/A	4.2160	3.4961	4.0139	3.6928	3.3507	3.9328	3.4075	3.7852	4.1109	3.6598	1.0115	0.0164	0.8387	0.0764
Hint2	_ISQAEEDDQQLLGHLLLVAK_.3	#N/A	3.6551	3.4544	4.3419	3.2685	3.6307	3.1652	4.5592	3.2740	4.4234	3.3107	0.9214	-0.1181	0.4047	0.3929
Snu13	_QQIQSIQQS[Phospho (STY)]IER_.2	Q9D0T1_S122_M1_Snu13	3.2337	4.2511	3.8001	4.0120	4.8323	3.6103	4.3631	3.8532	4.4435	3.5555	0.9760	-0.0350	0.7741	0.1112
Ebag9	_KLS[Phospho (STY)]GDQITLPTTVDYSSVPK_.2	#N/A	3.1279	3.9863	4.1128	7.2800	3.8746	4.2588	4.1520	6.8624	3.3377	3.7888	0.9790	-0.0306	0.9241	0.0343
Fxyd6	_APGDEEAQVENLITTNAAEPQKAEN_.3	#N/A	10.1696	10.8017	11.9110	11.1283	9.1148	10.2686	11.1149	10.9237	10.4205	10.3998	0.9861	-0.0202	0.7759	0.1102
Fxyd6	_APGDEEAQVENLITTNAAEPQK_.2	#N/A	2.2291	0.7633	3.1628	3.7463	4.0737	4.0517	1.5017	3.5336	1.7994	3.8847	1.1211	0.1650	0.7011	0.1542
Ints12	_SS[Phospho (STY)]PITVQTSK_.2	Q9D168_S127_M1_Ints12	7.8812	3.3902	8.6984	8.9232	8.2932	8.3379	3.3818	8.1985	4.0706	8.1897	1.2730	0.3483	0.3031	0.5183
Ints12	_SVS[Phospho (STY)]C[Carbamidomethyl (C)]DNVSK_.2	Q9D168_S377_M1_Ints12	6.4740	6.3022	6.0520	6.3277	5.9086	4.1352	4.2722	6.0356	5.9389	3.8013	1.1705	0.2271	0.2148	0.6680
Gatd3a	_GVTEAHVDQK_.2	#N/A	6.9300	6.9735	6.8915	6.9499	7.2945	7.0181	6.2545	6.7830	6.8367	6.5581	1.0608	0.0851	0.0141	1.8506
Gatd3a	_ITSLAQLNAANHDAAIFPGGFGAAK_.3	#N/A	6.0156	6.6871	6.3717	6.9649	6.8414	6.4801	6.9325	6.4487	6.8881	6.8194	0.9687	-0.0459	0.3126	0.5050
Gatd3a	_NLSTFAVDGK_.2	#N/A	6.1589	6.7330	7.5736	6.4604	6.5461	6.5626	5.3753	6.7005	6.5568	6.2113	1.0743	0.1034	0.2109	0.6760
Gatd3a	_NVLELTGK_.2	#N/A	3.8675	4.5934	5.0507	4.8484	5.4217	5.4771	4.0444	5.6251	5.8248	5.7582	0.9178	-0.1237	0.3642	0.4387
Gatd3a	_VNKEVER_.2	#N/A	3.9472	3.9535	3.3602	3.7825	5.3969	3.8544	3.7995	4.0367	4.2480	4.4143	0.9817	-0.0266	0.8435	0.0739
Gatd3a	_NVLAESAR_.2	#N/A	4.4887	3.6393	4.2883	3.3102	5.4187	3.0977	3.4585	4.6653	4.6284	5.2108	0.8997	-0.1524	0.4197	0.3771
Gatd3a	_GGAEVQIFAPDVPQMHVIDHTK_.3	#N/A	3.0130	3.6509	4.2475	6.0048	3.6575	2.9671	4.0117	3.2099	3.5090	3.4685	1.1053	0.1444	0.5435	0.2648
Cndp2	_TVFGVEPDLTR_.2	#N/A	7.0582	7.1524	7.3263	7.5299	7.5776	7.2965	7.4593	6.9846	6.9905	7.2184	1.0224	0.0319	0.2755	0.5599
Cndp2	_AVFQYIDENQDR_.2	#N/A	7.2911	7.4588	7.4608	7.4584	6.9935	7.5774	7.7464	6.9648	7.2288	7.3218	1.0079	0.0114	0.7368	0.1326
Cndp2	_TGQEIPVNLR_.2	#N/A	6.8443	7.1102	6.8297	7.0391	7.2757	6.6340	6.8015	6.8261	6.8600	6.9293	1.0148	0.0212	0.4214	0.3753
Cndp2	_LNYIEGTK_.2	#N/A	4.4219	3.1593	4.4315	3.7832	7.0763	4.7431	3.3219	6.5870	4.6535	4.0499	0.9891	-0.0157	0.9556	0.0197
Cndp2	_FAELQSPNK_.2	#N/A	3.9243	6.2887	3.1103	6.1467	6.1673	6.1215	3.3276	5.5460	5.7426	5.9051	1.0317	0.0451	0.8546	0.0682
Cndp2	_EGGSIPVTLTFQEATGK_.2	#N/A	5.6169	5.4273	4.4088	6.2407	5.4188	5.7862	6.3765	4.9286	5.8414	6.0403	0.9459	-0.0802	0.4502	0.3466

Tbcb	_YEIS[Phospho (STY)]PEAYER_.2	Q9D1E6_S110_M1_Tbcb	8.9310	8.7368	8.9141	9.2700	8.2139	8.7726	9.0354	8.8130	8.9368	8.7797	0.9905	-0.0138	0.6547	0.1840
Tbcb	_AQQEAEAAQR_.2	#N/A	3.2655	4.3010	3.9523	4.1376	4.9306	4.2765	4.0437	4.6890	3.8491	4.2205	0.9865	-0.0196	0.8600	0.0655
Tbcb	_AQASAISVGSR_.2	#N/A	3.1735	4.0527	3.8603	4.3629	3.8202	4.4547	4.0776	3.7555	4.7337	4.3299	0.9360	-0.0954	0.3732	0.4280
Akt1s1	_SSDEENGPPSS[Phospho (STY)]PDLDR_.2	Q9D1F4_S213_M1_Akt1s1	7.3260	7.8671	7.9564	8.3084	7.4736	7.5974	8.1478	7.4811	8.1721	7.8246	0.9808	-0.0279	0.5178	0.2858
Akt1s1	_EDEEEDEDEPTETETS[Phospho (STY)]GER_.2	Q9D1F4_S117_M1_Akt1s1	7.6884	8.0760	4.5118	8.8441	7.8525	7.9769	8.7057	3.5107	7.8360	7.8783	1.0729	0.1015	0.6846	0.1646
Akt1s1	_LNT[Phospho (STY)]SDFQK_.2	Q9D1F4_T247_M1_Akt1s1	6.1040	5.1761	5.6893	6.1825	3.3106	5.6318	5.1918	5.5320	5.4460	3.1216	1.1091	0.1494	0.4773	0.3212
Akt1s1	_TEARSS[Phospho (STY)]DEENGPPSS[Phospho (STY)]PDLDR_.3	#N/A	6.1113	5.5121	3.5239	5.9237	3.6767	5.6904	5.5755	5.4195	4.2881	4.2288	1.0400	0.0566	0.7740	0.1113
Akt1s1	_AATATRPPGPPPAPQPPS[Phospho (STY)]PAPS[Phospho (STY)]PPPRPALAR_.4	Q9D1F4_S88_M2_Akt1s1	4.2948	2.9313	3.4808	7.8386	3.4205	3.8050	3.6981	4.1350	3.7836	3.9504	1.1036	0.1423	0.6722	0.1725
Rab1b	_EFADSLGVPFLETSAK_.2	#N/A	7.2351	7.1271	7.1709	7.1852	6.5106	6.6103	7.5802	7.0857	6.7255	7.1845	0.9761	-0.0349	0.4512	0.3456
Rab1b	_[Acetyl (Protein N-term)]M[Oxidation (M)]NPEYDYLFK_.2	#N/A	4.5533	5.4971	5.5982	3.8585	4.8164	5.3668	5.8926	5.5071	4.8663	5.4937	0.9096	-0.1366	0.2341	0.6306
Rab1b	_GAHGIIVVYDVTDQESYANVK_.3	#N/A	3.3670	5.4728	4.6015	5.3147	3.7968	3.9327	4.2935	4.4908	4.2541	3.9297	1.0406	0.0574	0.7101	0.1487
Rab1b	_MGPGAAS[Phospho (STY)]GGERPNLK_.2	#N/A	5.1176	3.8702	3.9044	5.3143	3.7585	3.7147	3.8472	3.8712	3.8707	3.6351	1.1245	0.1693	0.2428	0.6147
Lrrc57	_SLSLNNNK_.2	#N/A	4.8858	6.6839	3.2974	7.0866	4.5644	6.7656	4.6433	4.8645	2.9582	4.0193	1.3460	0.4287	0.1311	0.8825
Atp6v1f	_DTTINEIEDTFR_.2	#N/A	8.3587	8.2059	8.2994	8.5798	8.8318	8.8661	8.6093	8.1624	8.4817	8.6714	1.0050	0.0072	0.8082	0.0925
Atp6v1f	_DDIGIILINQYIAEM[Oxidation (M)]VR_.2	#N/A	3.5497	3.9132	4.2201	4.0965	4.9423	4.2812	5.0374	4.0757	3.6574	3.9635	0.9961	-0.0056	0.9622	0.0168
Atp6v1f	_GM[Oxidation (M)]FTAEDLR_.2	#N/A	6.7922	7.0306	7.4972	6.7302	7.4090	6.9546	6.2088	6.7367	6.6016	7.0457	1.0633	0.0885	0.0829	1.0815
Atp6v1f	_LIAVIGDEDTVTGFLLGGIGELNK_.3	#N/A	3.3829	2.9506	3.1166	3.1029	3.7808	3.9487	3.1938	3.7200	3.7592	3.8385	0.9318	-0.1019	0.3259	0.4870
	LLSVTPTS[Phospho (STY)]EGYSIK.2	Q9D1K7_S99_M1_	3.6812	3.5449	4.3680	3.3698	3.4332	3.1914	3.0845	3.2478	7.1452	3.3368	0.8560	-0.2244	0.4731	0.3251
Chchd2	_LC[Carbamidomethyl (C)]EGFNEVLR_.2	#N/A	5.6780	4.9948	4.0308	4.9580	5.0214	3.7590	4.1198	3.0296	5.1508	3.6488	1.1889	0.2496	0.1759	0.7547
Sec13	_GQGSVSASITEGQQNEQ_.2	#N/A	5.9168	3.2591	3.1407	7.3421	5.4188	5.9536	3.5091	6.8928	7.0981	3.0381	1.0073	0.0105	0.9760	0.0106
Kat8	_S[Phospho (STY)]PGRVS[Phospho (STY)]PPTPAR_.2	Q9D1P2_S37_M2_Kat8	9.6580	4.3248	9.0267	10.0158	4.2131	9.5602	10.2606	9.3041	2.9534	9.6051	0.9712	-0.0421	0.9085	0.0417
Rpl34	_AFLIEEQK_.2	#N/A	5.2962	5.3432	6.0583	6.2188	5.9761	5.3418	5.9354	5.0595	5.0879	5.3587	1.0644	0.0901	0.2349	0.6291
Rpl34	_SAC[Carbamidomethyl (C)]GVC[Carbamidomethyl (C)]PGR_.2	#N/A	3.8429	4.7984	4.5297	5.3750	4.8962	4.9320	4.9614	4.8428	4.2356	3.8469	1.0576	0.0807	0.4630	0.3345
Rpl34	_LSYNTASNK_.2	#N/A	2.9797	4.3627	4.2142	3.7580	4.0934	3.0003	3.9063	3.2432	3.5422	3.4353	1.0575	0.0806	0.5560	0.2549
Lingo1	_S[Phospho (STY)]DAGISSADAPR_.2	#N/A	9.1451	8.8291	9.0601	9.2239	7.9151	8.8524	9.3595	9.1198	9.0106	9.1985	0.9635	-0.0536	0.2193	0.6589
Lingo1	_HNIEIEYVPRKS[Phospho (STY)]DAGISSADAPR_.4	#N/A	3.0882	6.4141	7.2346	4.0783	6.5005	4.1940	6.3273	7.9544	7.3653	6.8499	0.7372	-0.4400	0.0705	1.1519
Lingo1	_FVAVPEGIPTETR_.2	#N/A	6.2994	6.3181	6.7604	5.5931	5.7892	5.7672	6.1982	6.0986	5.9438	5.8417	1.0112	0.0160	0.7820	0.1068
Lingo1	_LTVFPDGTLEVR_.2	#N/A	4.1744	4.7809	5.7064	3.8630	3.3001	4.6972	5.1294	4.6098	5.1257	3.8300	0.9458	-0.0804	0.6192	0.2081
Lingo1	_KS[Phospho (STY)]DAGISSADAPR_.2	#N/A	9.0692	9.0950	9.5315	9.4460	8.0967	9.1071	9.5585	9.3054	8.9646	9.0886	0.9814	-0.0271	0.5570	0.2542
Nol3	_ASEQEEVGGPEGS[Phospho (STY)]EALQPR_.2	Q9D1X0_S142_M1_Nol3	5.1702	5.6752	5.3668	6.4563	4.9475	5.3388	3.2785	5.0126	5.7101	5.5397	1.1243	0.1690	0.2730	0.5639
Nol3	_LVETLQADSGLLLDALVAR_.2	#N/A	3.6868	3.5124	4.1897	3.9102	3.7424	3.7317	3.6248	3.5569	3.9242	3.8772	1.0133	0.0190	0.7281	0.1378
Bcl9	_IPVEGPLS[Phospho (STY)]PSR_.2	Q9D219_S686_M1_Bcl9	8.2714	4.3895	7.9123	4.0262	7.7824	3.8477	8.5816	7.6510	8.0663	8.5270	0.7358	-0.4426	0.0867	1.0622
Bcl9	_SST[Phospho (STY)]PSHGQTPAPEPISAQK_.2	Q9D219_T155_M1_Bcl9	3.9155	5.0662	4.6023	5.6774	3.1990	3.4256	3.3188	3.0135	4.1629	3.5711	1.2269	0.2950	0.1714	0.7661
Bcl9	_FSNQGKPGGSASQSQPS[Phospho (STY)]PC[Carbamidomethyl (C)]DSK_.3	Q9D219_S62_M1_Bcl9	3.8210	3.5215	3.5546	4.5992	2.9647	3.6599	3.2467	3.9028	4.2018	4.2765	0.9437	-0.0837	0.5222	0.2822
Toe1	_AADS[Phospho (STY)]DDDVPPVPTPSDGGVNK_.2	Q9D2E2_S5_M1_Toe1	3.9312	3.1866	3.6649	3.2086	8.3416	7.1204	3.4428	3.7925	4.0916	4.3868	1.2496	0.3214	0.4199	0.3769
Dlst	_VEGGTPLFTLR_.2	#N/A	8.2337	8.4357	7.7094	8.5269	8.5910	8.3741	8.5299	8.3893	8.3904	8.5634	0.9815	-0.0269	0.3771	0.4235
Dlst	_AKPAETPAPAHK_.3	#N/A	5.9864	4.8513	5.2575	5.4075	6.6222	6.0323	5.7136	5.1368	5.6598	6.0360	1.0100	0.0144	0.8788	0.0561
Dlst	_NVETM[Oxidation (M)]NYADIER_.2	#N/A	4.9061	4.9539	4.0694	5.5989	5.3823	5.4260	4.9840	5.2079	5.1199	5.6552	0.9646	-0.0520	0.5609	0.2511
Dlst	_ASAFALQEQPVVNAVIDDATK_.2	#N/A	6.4254	6.8302	6.6336	7.0926	6.9335	6.7251	6.8737	6.6043	6.6716	6.5944	1.0131	0.0187	0.5203	0.2838
Dlst	_TINELGEK_.2	#N/A	5.7581	5.6792	3.5984	4.8700	6.6094	6.1911	4.5521	5.9709	5.8236	5.5996	0.9935	-0.0094	0.9547	0.0202
Dlst	_NDVITVQTPAFAESVTEGDVR_.2	#N/A	4.1978	3.0283	3.3838	5.2305	3.3235	4.4060	3.6011	4.2320	3.8806	3.8535	1.0094	0.0135	0.9355	0.0290
Ube2v2	_LPQPPEGQTYNN_.2	#N/A	11.6495	11.4717	12.0652	11.7636	11.3999	11.6211	11.9930	11.2917	11.5431	11.6905	1.0028	0.0040	0.8518	0.0697
Ube2v2	_LLEELEEGQK_.2	#N/A	8.4946	8.5474	8.2243	8.5132	8.5764	8.6416	8.4710	8.4032	8.4642	8.5595	1.0030	0.0043	0.7556	0.1217
Ube2v2	_VILQELR_.2	#N/A	8.2794	8.2318	7.6710	7.9239	8.5240	8.1693	7.0607	8.3250	8.4275	8.2255	1.0154	0.0221	0.6858	0.1638
Ube2v2	_WTGM[Oxidation (M)]IIGPPR_.2	#N/A	7.2777	7.5333	7.4952	6.8893	7.7156	7.9329	7.3470	7.5058	7.6680	7.5463	0.9943	-0.0082	0.8291	0.0814
Ube2v2	_YPEAPPSVR_.2	#N/A	8.4502	8.6608	4.1122	8.3127	9.1431	7.5061	8.1187	8.5675	8.5667	8.5011	0.9122	-0.1326	0.4536	0.3433
Ube2v2	_ENM[Oxidation (M)]KLPQPPEGQTYNN_.2	#N/A	9.6655	9.9000	10.7361	10.4046	9.6847	9.9904	10.1505	9.8464	9.8845	9.9016	1.0118	0.0170	0.6127	0.2128
Ube2v2	_VEC[Carbamidomethyl (C)]GS[Phospho (STY)]KYPEAPPSVR_.3	Q9D2M8_S71_M1_Ube2v2	2.8991	3.6554	4.1337	3.6774	3.5437	3.0809	3.8257	3.3238	3.6228	3.3547	0.9906	-0.0137	0.8956	0.0479
Ube2v2	_GVGDGTVSWGLEDDEDM[Oxidation (M)]TLTR_.2	#N/A	5.0397	5.2087	5.5193	5.9152	5.0599	5.3062	5.3346	4.7820	4.4319	5.3597	1.0732	0.1020	0.1763	0.7537
Dtna	_SS[Phospho (STY)]PSHTISR_.2	Q9D2N4_S568_M1_Dtna	4.3179	3.5982	3.8013	3.8173	3.4526	3.8265	3.4630	3.7987	3.7328	3.6682	1.0373	0.0528	0.4191	0.3777
Mobp	_HQPAAS[Phospho (STY)]PVVVR_.2	Q9D2P8_S85_M1_Mobp	11.4783	11.5532	12.0878	11.4978	10.7044	11.2751	12.1809	11.8248	11.4996	11.3992	0.9750	-0.0366	0.3057	0.5147
Mobp	_ATS[Phospho (STY)]PQRPK_.2	#N/A	9.3245	9.3820	9.5958	8.8213	2.8257	8.4324	9.9975	9.5996	4.3408	9.1241	0.9756	-0.0357	0.9078	0.0420
Mobp	_GGS[Phospho (STY)]PTRAPR_.2	Q9D2P8_S162_M1_Mobp	8.3657	9.2783	9.9208	3.6362	7.7888	8.6466	9.3982	9.2456	8.6658	9.1563	0.8709	-0.1994	0.3347	0.4754
Mobp	_S[Phospho (STY)]PPRPAKPR_.2	Q9D2P8_S107_M1_Mobp	8.7286	8.7236	8.5020	8.9947	8.1892	8.3973	9.3521	9.0884	8.7216	8.5843	0.9611	-0.0572	0.1215	0.9154
Mobp	_RATS[Phospho (STY)]PQRPK_.2	#N/A	3.8683	8.1031	4.5551	3.5569	2.9939	3.2348	3.2716	3.0609	7.2435	3.5239	1.0258	0.0368	0.9317	0.0307
Mobp	_SS[Phospho (STY)]PLRGPGTSR_.2	Q9D2P8_S150_M1_Mobp	6.0910	6.4707	6.9311	6.6226	6.0575	4.3499	4.2431	6.5065	6.7407	6.2554	1.0254	0.0362	0.8224	0.0849
Mobp	_S[Phospho (STY)]PLM[Oxidation (M)]PAKPR_.2	#N/A	8.5046	7.9749	7.1351	8.7418	6.7595	7.3864	8.7713	7.8771	7.6930	7.9254	0.9608	-0.0577	0.4966	0.3040
Hist3h2ba	_PEPSRST[Phospho (STY)]PAPK_.2	Q9D2U9_T8_M1_Hist3h2ba	7.7105	8.0416	8.5465	8.2255	7.7459	4.1094	8.2117	7.9609	3.1505	7.9569	1.0845	0.1171	0.6643	0.1776
Coro7	_IWDLQTGAER_.2	#N/A	4.4564	4.9474	4.1953	4.7964	4.2456	5.0501	4.6110	4.7642	3.7656	4.7738	1.0305	0.0433	0.6224	0.2059
Coro7	_S[Phospho (STY)]LQSLLGPSSK_.2	Q9D2V7_S459_M1_Coro7	4.0919	7.9369	3.2779	8.9491	3.2176	8.0392	8.2569	7.8220	7.7256	3.7476	0.8593	-0.2188	0.5596	0.2521
Coro7	_EDPLPQDS[Phospho (STY)]FEGVDEDEWD_.2	#N/A	3.9257	3.3005	3.1116	3.6143	3.0513	3.4358	3.3290	3.0034	4.1528	3.5813	0.9687	-0.0459	0.6770	0.1694
Coro7	_LGNREDPLPQDS[Phospho (STY)]FEGVDEDEWD_.3	#N/A	3.4304	3.7957	4.1172	4.6199	4.0570	3.6725	4.3346	3.4986	3.1472	3.0861	1.1229	0.1672	0.2013	0.6961
Acp1	_SPIAEAVFR_.2	#N/A	7.1951	7.4320	7.2275	6.7680	7.2355	7.6656	6.8988	7.4375	7.5067	7.4118	0.9918	-0.0118	0.7584	0.1201
Acp1	_SVLFVC[Carbamidomethyl (C)]LGNIC[Carbamidomethyl (C)]R_.2	#N/A	5.7719	4.9765	4.3398	5.8543	5.9029	5.1878	6.0384	5.4127	5.7078	5.4122	0.9461	-0.0799	0.3945	0.4040
Lamp5	_VHFVYDSSEK_.2	#N/A	5.1835	3.0700	4.0061	3.5498	5.1604	3.3373	3.6981	6.1208	3.7504	3.2271	0.9648	-0.0517	0.8373	0.0771
Rufy3	_NYVEELNR_.2	#N/A	7.1050	7.4891	7.4276	7.6528	7.1350	7.3599	7.2903	7.4357	7.5196	7.3256	0.9958	-0.0061	0.7936	0.1004
Rufy3	_TAEGQALSEAR_.2	#N/A	6.2616	6.4097	6.2427	6.9279	6.8218	6.8666	6.8114	6.4160	6.1824	6.5720	1.0143	0.0205	0.6431	0.1917
Rufy3	_GLIESALNLGR_.2	#N/A	6.1231	6.2312	5.8823	6.1629	6.0348	6.5718	6.1845	6.0800	6.5073	6.2578	0.9857	-0.0208	0.5349	0.2718
Rufy3	_LQSSDLGVK_.2	#N/A	2.8975	4.4186	5.0901	4.2828	4.8224	4.1137	3.3363	4.1134	3.5080	4.8464	1.0809	0.1123	0.5187	0.2851
Rufy3	_VS[Phospho (STY)]M[Oxidation (M)]DGEWLC[Carbamidomethyl (C)]LR_.2	#N/A	6.6442	6.6554	7.0200	7.4917	6.2938	6.7748	6.9211	7.0662	7.0714	6.9833	0.9719	-0.0412	0.3743	0.4268
Rufy3	]SALT[Phospho (STY)]PPTDM[Oxidation (M)]PT[Phospho (STY)]PTTDKITQAAM[Oxidation (M)]ETIYLC[C	#N/A	2.9065	3.6628	4.1410	3.6848	3.5511	3.0736	3.8330	3.3164	3.6155	3.3621	0.9919	-0.0117	0.9114	0.0403
Rufy3	_[Acetyl (Protein N-term)]SALT[Phospho (STY)]PPTDM[Oxidation (M)]PTPTTDK_.2	#N/A	11.1414	10.9433	11.4880	10.9881	10.4153	10.9937	11.6612	10.8263	11.0539	10.8784	0.9901	-0.0144	0.6498	0.1872
Rufy3	_DGNS[Phospho (STY)]SKGSEGDGQITAILDQK_.3	Q9D394_S247_M1_Rufy3	3.7948	3.0503	3.5285	3.8645	2.9386	3.6861	3.5792	4.2539	3.9025	3.8315	0.8934	-0.1625	0.1071	0.9704
Rufy3	_GEDLDSQVGVIDFSM[Oxidation (M)]YLK_.2	#N/A	3.9441	3.2820	3.1301	3.6328	3.1703	4.7617	3.7893	2.9849	4.1343	3.5998	1.0073	0.0105	0.9451	0.0245
Rufy3	_VKEESSYLLESNR_.3	#N/A	4.0947	3.3991	3.2807	3.7833	3.2204	3.0083	3.4980	4.3351	3.9837	3.7503	0.8902	-0.1678	0.1236	0.9079
Atp5f1d	_IEANEALVK_.2	#N/A	9.0882	9.2059	9.3923	9.2771	9.8291	9.4122	8.9132	9.3054	9.3680	9.5743	1.0083	0.0119	0.6623	0.1790
Atp5f1d	_IEANEALVKALE_.2	#N/A	4.7560	5.0518	3.7646	5.0762	5.0375	4.4206	5.6879	4.8302	4.1900	5.1419	0.9440	-0.0832	0.4637	0.3338
Atp5f1d	_AQSELSGAADEAAR_.2	#N/A	8.8048	8.8236	8.9721	9.2583	9.3864	9.2163	8.8389	8.8202	8.9263	9.1175	1.0169	0.0242	0.2971	0.5272
Mfap3l	_SES[Phospho (STY)]PTADSDASSLHEQPQQIAIK_.3	Q9D3X9_S298_M1_Mfap3l	3.3586	4.1149	3.8718	3.9212	3.2819	3.4705	4.0849	5.3564	3.8823	3.6897	0.8628	-0.2129	0.1295	0.8876

Dlgap1	_GC[Carbamidomethyl (C)]S[Phospho (STY)]QDDEC[Carbamidomethyl (C)]VSLR_.2	#N/A	11.8835	11.7195	12.1762	12.2536	11.3587	11.5763	12.3462	11.6349	11.9265	11.9306	0.9890	-0.0160	0.5508	0.2590
Dlgap1	_SHS[Phospho (STY)]LEGPSK_.2	Q9D415_S154_M1_Dlgap1	8.8037	9.1404	9.8200	9.5089	3.2665	9.1216	9.2295	9.1733	8.9568	9.4753	0.8988	-0.1539	0.4834	0.3157
Dlgap1	_ERS[Phospho (STY)]LESSQR_.2	Q9D415_S947_M1_Dlgap1	9.6860	9.8482	10.2330	10.2233	9.6940	9.9649	9.8397	9.8517	9.9034	9.9729	1.0050	0.0072	0.7066	0.1508
Dlgap1	_SLDSLDPAGLLTS[Phospho (STY)]PK_.2	Q9D415_S437_M1_Dlgap1	4.1076	8.5822	8.8909	9.2950	3.0751	8.2719	8.3946	8.6340	8.2851	7.9103	0.8472	-0.2392	0.3874	0.4118
Dlgap1	_AVSEVS[Phospho (STY)]INR_.2	Q9D415_S421_M1_Dlgap1	8.7327	8.4900	8.7495	8.9983	8.1427	8.6804	8.6507	8.4933	7.7689	8.7482	1.0258	0.0367	0.3718	0.4297
Dlgap1	_AS[Phospho (STY)]PDESQTLR_.2	#N/A	7.7651	3.4123	3.8906	8.0353	7.5176	7.6787	3.2171	7.8185	7.8917	7.4159	0.9692	-0.0451	0.8890	0.0511
Dlgap1	_SRNES[Phospho (STY)]YM[Oxidation (M)]R_.2	#N/A	3.2658	4.3074	3.4884	4.9924	3.6948	4.0349	4.1827	4.0879	3.3159	4.0657	1.0130	0.0186	0.8902	0.0505
Dlgap1	_AM[Oxidation (M)]GDEDS[Phospho (STY)]GDS[Phospho (STY)]DTS[Phospho (STY)]PKPSPK_.2	#N/A	7.1612	6.1329	8.4687	7.5627	7.4826	7.8655	8.0710	8.0960	7.9284	6.0333	0.9885	-0.0167	0.8813	0.0549
Dlgap1	_GSVNGGKAS[Phospho (STY)]PDESQTLR_.2	#N/A	3.7036	5.2400	3.8742	4.0125	4.8018	3.6428	5.3080	4.9181	4.7546	4.4438	0.8675	-0.2051	0.1141	0.9427
Dlgap1	_AIEKGC[Carbamidomethyl (C)]S[Phospho (STY)]QDDEC[Carbamidomethyl (C)]VSLR_.3	#N/A	3.6862	3.5399	4.3730	3.3748	4.4819	3.1964	3.0895	3.2428	4.3922	3.3418	1.0736	0.1024	0.4911	0.3088
Dlgap1	_GDMISQSGLSNS[Phospho (STY)]TESLDSM[Oxidation (M)]K_.2	Q9D415_S605_M1_Dlgap1	3.9289	3.8513	3.8965	5.2790	3.7106	3.3183	4.1720	3.8601	4.1368	3.8991	0.9951	-0.0070	0.9563	0.0194
Dlgap1	_QNS[Phospho (STY)]ATESAESIEIYIPEAQTR_.3	Q9D415_S973_M1_Dlgap1	3.5218	4.3270	4.2086	3.7937	4.1483	3.5812	3.9420	7.7268	3.0558	3.1774	0.8781	-0.1875	0.5562	0.2548
Dlgap1	_C[Carbamidomethyl (C)]LS[Phospho (STY)]IGIQVDDAEEPEK_.2	Q9D415_S666_M1_Dlgap1	3.3029	4.1081	3.9897	4.0126	3.9294	3.8001	4.1609	3.6261	3.2747	2.9585	1.1004	0.1381	0.1995	0.7000
GOLGA7B	_RSAS[Phospho (STY)]LATK_.2	Q9D428_S16_M1_GOLGA7B	3.5473	4.3525	4.2341	3.7682	4.1738	3.5557	3.9165	3.2330	5.0473	3.2029	1.0230	0.0329	0.8246	0.0837
GOLGA7B	_DYS[Phospho (STY)]DGTIC[Carbamidomethyl (C)]QFQTK_.2	Q9D428_S28_M1_GOLGA7B	3.5067	7.7742	3.6500	4.1526	4.1513	3.9741	3.8673	7.4791	7.1836	7.0730	0.7085	-0.4972	0.1165	0.9337
Clvs1	_SQS[Phospho (STY)]VVEAGTLK_.2	#N/A	7.7436	7.3068	6.8355	7.3606	6.0712	7.3808	3.5061	7.1519	7.5488	7.4826	1.1081	0.1481	0.4262	0.3704
Clvs1	_HTC[Carbamidomethyl (C)]S[Phospho (STY)]NLER_.2	Q9D4C9_S313_M1_Clvs1	3.9443	3.0459	3.7443	3.7263	2.9019	5.1324	4.4073	3.9055	3.8168	3.5670	0.9554	-0.0658	0.6942	0.1585
Clvs1	_SQS[Phospho (STY)]VVEAGTLKHEEK_.2	#N/A	4.0655	4.4887	3.4767	4.3302	3.2538	4.2817	3.5209	3.8523	4.0001	4.5309	1.0017	0.0024	0.9829	0.0075
Clvs1	_EC[Carbamidomethyl (C)]S[Phospho (STY)]PKPMK_.2	#N/A	4.7631	2.5966	4.1622	5.0241	4.1019	3.6276	3.9884	3.1611	3.4601	3.5174	1.1456	0.1961	0.2990	0.5244
Clvs1	_EC[Carbamidomethyl (C)]S[Phospho (STY)]PKPMKR_.2	#N/A	5.8884	3.1393	3.6176	7.0540	3.0276	3.5970	3.3096	3.8398	4.1389	4.8968	1.0843	0.1168	0.7124	0.1473
Plpp6	_ASDS[Phospho (STY)]PVHR_.2	Q9D4F2_S60_M1_Plpp6	3.1622	3.9185	4.3967	4.4971	3.8068	4.3187	4.2118	4.2299	3.2699	3.6178	1.0481	0.0678	0.5755	0.2399
Exoc2	_ASNTADTLFQEVLGR_.2	#N/A	2.9071	3.7122	3.3638	5.1191	3.5336	4.1959	4.5567	4.0386	4.3926	4.0857	0.8915	-0.1657	0.2858	0.5440
Exoc2	_GSS[Phospho (STY)]FQSGRDDTWR_.2	Q9D4H1_S432_M1_Exoc2	3.1424	3.8987	4.3769	4.5170	3.7870	4.3385	4.2316	3.0805	3.2501	3.5980	1.1328	0.1799	0.1922	0.7162
Phf14	_NSADDEELT[Phospho (STY)]NDS[Phospho (STY)]LTLSQSK_.2	Q9D4H9_S283_M2_Phf14	3.9970	3.3014	3.1830	5.8640	3.1227	3.1060	3.4003	4.4329	5.6824	3.6526	0.8766	-0.1900	0.4618	0.3356
Wdr20	_SNS[Phospho (STY)]LPHSAVSNAASK_.2	#N/A	7.8173	8.0551	8.1708	8.4970	6.9992	7.7657	8.0729	7.7774	7.8081	7.7453	1.0042	0.0061	0.9034	0.0441
Atad1	_EYVNSTSEES[Phospho (STY)]HDEDEIRPVQQQDLHR_.4	Q9D5T0_S322_M1_Atad1	3.7345	3.4916	4.4213	3.4232	3.3799	3.2447	3.1378	3.1945	4.3439	3.3902	1.0282	0.0402	0.7577	0.1205
Rab11fip1	_ESS[Phospho (STY)]PSNSPSPQGFR_.2	Q9D620_S340_M1_Rab11fip1	6.7610	7.2467	4.3818	3.9256	4.0164	3.7131	7.7143	3.0756	3.3746	3.6029	1.1273	0.1729	0.6445	0.1908
Lratd1	_GPPGC[Carbamidomethyl (C)]SPC[Carbamidomethyl (C)]PES[Phospho (STY)]PSR_.2	Q9D650_S67_M1_Lratd1	6.4098	3.8410	4.0720	4.5746	4.0117	5.2783	6.3222	6.2897	3.1924	3.0408	0.9972	-0.0041	0.9883	0.0051
Ttc9b	_C[Carbamidomethyl (C)]SLQREDS[Phospho (STY)]DGGTGGPAR_.2	#N/A	3.8709	4.8610	4.8744	5.0938	4.5786	5.3002	4.6516	4.5782	5.0997	4.8360	0.9941	-0.0085	0.9197	0.0364
Ttc9b	_EDS[Phospho (STY)]DGGTGGPAR_.2	#N/A	5.9112	8.5444	4.5606	8.3446	3.2407	3.3839	6.9397	7.6497	4.2047	8.0232	0.8449	-0.2432	0.4738	0.3244
Tubb4a	_IREEFPDR_.2	#N/A	10.3127	10.2970	10.2141	10.4762	10.4909	10.3009	10.2602	10.2000	10.2648	10.2231	1.0109	0.0156	0.0887	1.0522
Tubb4a	_GHYTEGAELVDAVLDVVR_.3	#N/A	8.0899	8.0943	8.0330	8.7346	7.8240	8.3427	8.0965	8.1333	8.4615	8.3208	0.9919	-0.0117	0.7125	0.1472
Tubb4a	_GHYTEGAELVDAVLDVVRK_.3	#N/A	6.8696	7.7190	8.6573	8.3313	7.6041	7.5632	7.6238	8.0387	7.6730	7.6335	1.0063	0.0090	0.8871	0.0520
Tubb4a	_EVDEQM[Oxidation (M)]LSVQSK_.2	#N/A	9.3294	9.2745	9.2526	9.8219	9.1964	9.5723	9.3602	9.1532	9.4182	9.5266	1.0046	0.0067	0.7616	0.1183
Tubb4a	_M[Oxidation (M)]AATFIGNSTAIQELFK_.2	#N/A	8.7802	8.9121	9.9696	10.1986	8.9426	9.3699	9.1680	9.0075	9.0159	9.4635	1.0217	0.0309	0.5480	0.2612
Tubb4a	_INVYYNEATGGNYVPR_.2	#N/A	7.8482	7.8969	8.4493	8.0805	8.0335	8.0238	8.0628	7.7113	7.9029	7.8558	1.0218	0.0312	0.1977	0.7040
Lurap1	_RGS[Phospho (STY)]WDSLPDTSSTDR_.2	Q9D6I9_S126_M1_Lurap1	4.0614	3.2255	3.2474	7.1842	6.0750	3.3739	3.4647	6.6748	4.0183	6.8901	0.8605	-0.2168	0.5278	0.2775
Ndufb8	_GGDPTKEPEPVVHYDI_.3	#N/A	6.5396	6.5958	6.7929	6.4621	5.9548	6.4476	6.2387	6.5516	6.1224	6.1233	1.0330	0.0468	0.2425	0.6152
Ndufb8	_QYPYNNLYLER_.2	#N/A	7.4258	7.4190	7.2947	6.9226	6.7035	7.2084	7.2881	7.0441	6.5830	6.7769	1.0346	0.0490	0.2479	0.6058
Ndufb8	_DPWYQWDHSELR_.2	#N/A	5.8065	5.0148	4.0971	3.2959	5.9949	4.3265	5.6698	5.8537	4.0690	5.4734	0.9031	-0.1471	0.4359	0.3606
Ndufv2	_DSDSILETLQR_.2	#N/A	9.6659	9.7607	10.0163	10.0597	9.9487	9.8234	9.8546	9.5009	9.6139	9.7755	1.0199	0.0284	0.0918	1.0370
Ndufv2	_DIEEIIDELK_.2	#N/A	8.4086	8.4662	8.2695	8.4560	8.6847	8.3943	8.4332	8.1446	8.1486	8.7306	1.0098	0.0141	0.5457	0.2631
Ndufv2	_DTPENNPDTPFDFTPENYKR_.3	#N/A	7.6769	7.6678	7.8574	8.1265	7.8321	8.0519	7.8437	7.5067	7.4530	7.6081	1.0350	0.0496	0.0546	1.2626
Ndufv2	_DTPENNPDTPFDFTPENYK_.2	#N/A	6.9252	6.4526	6.1645	7.1991	6.9862	6.4021	6.7775	6.4576	6.4156	6.5937	1.0194	0.0277	0.5726	0.2421
Ndufv2	_NYPEGHQAAAVLPVLDLAQR_.3	#N/A	7.3976	7.6042	7.7923	8.1279	7.7862	7.7532	7.3814	7.3820	7.2863	7.4443	1.0502	0.0706	0.0187	1.7289
Ndufv2	_FC[Carbamidomethyl (C)]C[Carbamidomethyl (C)]EPAGGLTSLTEPPKGPGFGVQAGL_.3	#N/A	2.5169	3.7337	4.9695	3.9106	4.3480	4.3486	4.0589	3.0906	3.3896	4.7365	1.0399	0.0564	0.7745	0.1110
Ndufv2	_VYEVATFYTM[Oxidation (M)]YNR_.2	#N/A	2.9825	6.0790	6.7629	3.7608	6.3957	6.9141	3.9091	3.2404	5.5756	6.4543	1.1434	0.1934	0.5260	0.2790
Ndufv2	_YHIQVC[Carbamidomethyl (C)]TTTPC[Carbamidomethyl (C)]M[Oxidation (M)]LR_.3	#N/A	3.1401	3.8964	4.3747	4.5192	6.4699	4.3407	4.2339	3.0827	4.7134	5.9444	0.9918	-0.0118	0.9613	0.0172
Slc25a22	_LAANDFFR_.2	#N/A	8.3501	8.4688	8.3459	7.9105	7.9385	7.9576	8.3589	8.6730	8.1383	7.9748	0.9850	-0.0218	0.4990	0.3019
Slc25a22	_HEGPSAFLK_.2	#N/A	6.5921	7.3425	6.8577	6.6928	6.8149	6.5247	6.4464	7.4470	7.1415	6.7591	0.9792	-0.0303	0.5450	0.2636
Slc25a22	_NHGIAGLYK_.2	#N/A	6.7377	6.5760	6.2997	6.2109	6.6226	6.1305	6.6065	6.8353	6.5251	6.4035	0.9753	-0.0361	0.2954	0.5296
Slc25a22	_GVNEDTYSGFLDC[Carbamidomethyl (C)]AR_.2	#N/A	7.7972	7.3875	7.8917	7.6951	7.3926	7.2301	7.8941	7.5895	7.1718	7.1388	1.0157	0.0225	0.5676	0.2459
Slc25a22	_SEGYFGMYR_.2	#N/A	6.3892	5.4325	3.8728	6.7493	5.0084	3.3417	5.4243	4.2746	4.6119	2.4316	1.2262	0.2942	0.2980	0.5257
Slc25a22	_LINGGIAGLIGVTC[Carbamidomethyl (C)]VFPIDLAK_.2	#N/A	3.0716	3.8768	3.7584	4.2611	3.6981	4.0314	3.9758	3.8574	3.5060	4.2281	0.9720	-0.0409	0.6588	0.1813
Slc25a22	_SPFYVSFLAGC[Carbamidomethyl (C)]VAGSAAAVAVNPC[Carbamidomethyl (C)]DVVK_.3	#N/A	3.5724	3.7700	3.3061	4.3507	3.5913	4.1382	4.4990	1.5902	2.9495	4.0280	1.1596	0.2137	0.3692	0.4327
Idh3a	_APIQWEER_.2	#N/A	9.9266	10.0669	9.5577	10.2437	10.1646	9.9329	10.0091	9.6899	9.7658	9.6953	1.0196	0.0280	0.1995	0.7000
Idh3a	_TPYTDVNIVTIR_.2	#N/A	8.9478	9.1798	9.4046	9.4742	9.2110	9.4007	9.3128	9.0851	9.1589	9.0044	1.0142	0.0203	0.2842	0.5463
Idh3a	_NVTAIQGPGGK_.2	#N/A	8.8781	9.0757	9.3608	9.2844	9.6029	9.0881	9.0370	9.1712	9.2229	9.0683	1.0099	0.0142	0.5221	0.2822
Idh3a	_LITEEASKR_.2	#N/A	8.7422	9.1649	9.1280	9.2134	9.3995	9.0776	8.9244	9.0399	9.1254	8.9952	1.0111	0.0159	0.4118	0.3853
Idh3a	_IAEFAFEYAR_.2	#N/A	8.7009	8.8891	8.8997	8.9910	8.9444	9.0548	9.1290	8.7398	9.0088	8.8891	0.9968	-0.0046	0.7610	0.1186
Idh3a	_SNVTAVHK_.2	#N/A	7.9032	7.9636	7.3180	7.5524	8.4881	7.7609	7.5970	7.8472	7.8743	8.0278	0.9993	-0.0010	0.9803	0.0087
Idh3a	_ENTEGEYSGIEHVIVDGVVQSIK_.3	#N/A	7.6792	7.8927	9.1954	9.4420	7.3956	7.7232	8.1208	7.8655	7.8429	8.8069	1.0076	0.0110	0.8994	0.0460
Idh3a	_C[Carbamidomethyl (C)]SDFTEEIC[Carbamidomethyl (C)]R_.2	#N/A	7.0706	6.7661	6.4656	7.3709	7.3996	7.4123	7.0165	6.9665	7.2057	7.5448	0.9857	-0.0207	0.6620	0.1792
Idh3a	_IEAAC[Carbamidomethyl (C)]FATIK_.2	#N/A	6.4248	6.0243	5.7933	7.1863	7.0093	6.7080	5.8673	7.2011	6.5823	6.2733	1.0067	0.0096	0.9065	0.0427
Idh3a	_EVAENC[Carbamidomethyl (C)]KDIK_.2	#N/A	5.2416	5.6608	6.5147	5.9630	6.1918	5.4272	5.8243	6.0317	5.9404	5.5928	0.9976	-0.0035	0.9575	0.0189
Idh3a	_M[Oxidation (M)]SDGLFLQK_.2	#N/A	7.3665	7.3226	7.4462	7.2489	7.5728	7.2668	7.3329	7.3367	7.1920	7.3585	1.0090	0.0129	0.3707	0.4310
Idh3a	_HM[Oxidation (M)]GLFDHAAK_.2	#N/A	6.4609	6.8166	6.7239	6.1834	7.4779	6.3497	6.5087	6.8336	6.2437	7.0393	1.0019	0.0027	0.9648	0.0156
Idh3a	_TPIAAGHPSM[Oxidation (M)]NLLLR_.2	#N/A	6.9475	7.5415	7.6967	6.8126	7.1778	7.2618	7.2980	7.4148	7.4874	7.3212	0.9809	-0.0278	0.4472	0.3495
Idh3a	_C[Carbamidomethyl (C)]SDFTEEIC[Carbamidomethyl (C)]RR_.2	#N/A	3.2828	4.0880	3.8411	3.8975	4.0275	3.8779	4.4737	3.8184	3.6942	3.6725	0.9798	-0.0294	0.7159	0.1452
Mrrf	_ALVEDIISLEEVDEDM[Oxidation (M)]K_.2	#N/A	4.2243	4.1304	5.5053	5.6748	5.0639	5.5649	4.0904	5.1881	4.9380	5.3435	1.0281	0.0399	0.7500	0.1250
Borcs6	_TLS[Phospho (STY)]GEEEAESVGVSSR_.2	Q9D6W8_S43_M1_Borcs6	8.4538	9.0302	9.2265	10.1753	8.6821	9.4427	9.2449	9.4352	9.3891	9.5267	0.9755	-0.0358	0.4845	0.3147
Borcs6	_S[Phospho (STY)]LDGLSGAC[Carbamidomethyl (C)]GGGGSSSSGETGAGGGR_.2	#N/A	5.8175	5.5670	5.3399	5.4556	4.7179	5.4979	5.7482	5.4996	5.0615	5.8646	0.9740	-0.0380	0.5571	0.2541
Borcs6	_S[Phospho (STY)]LDGLSGAC[Carbamidomethyl (C)]GGGGSSSSGETGAGGGRR_.3	#N/A	6.1444	4.4068	4.2884	3.2902	4.2281	5.8780	6.0667	6.0896	5.9681	3.2572	0.8804	-0.1838	0.4376	0.3589
Borcs6	_RGS[Phospho (STY)]PGGVEMNVELPQQEGDDDDDEDEEAAAGR_.3	Q9D6W8_S130_M1_Borcs6	4.3566	3.6120	4.3010	3.3028	3.5003	3.1243	4.5183	3.3148	2.9634	3.2698	1.0520	0.0731	0.6413	0.1930
Nop56	_EEVAS[Phospho (STY)]EPEEAASPTTPK_.2	#N/A	7.7004	7.8916	7.6368	8.3473	7.0178	7.7119	8.4356	8.0548	7.9371	7.6514	0.9623	-0.0554	0.2686	0.5708
Nop56	_SSPKEEVAS[Phospho (STY)]EPEEAASPTTPK_.3	#N/A	7.2281	7.2270	7.5002	7.4639	6.1575	7.3199	7.0900	7.7114	7.4400	5.7917	1.0201	0.0288	0.7465	0.1270
Nop56	_EELAS[Phospho (STY)]DLEEM[Oxidation (M)]ATSSAK_.2	#N/A	4.4865	5.3847	3.3723	7.6069	6.2845	6.1365	3.2117	7.6610	6.7518	7.2440	0.8919	-0.1650	0.5602	0.2516
Nop56	_KSSPKEEVAS[Phospho (STY)]EPEEAASPTTPK_.3	#N/A	3.9440	3.4328	4.9357	3.8481	4.0498	4.8989	5.1160	5.1374	4.4328	4.0073	0.8955	-0.1593	0.2322	0.6341
Nop56	_APKEELAS[Phospho (STY)]DLEEM[Oxidation (M)]ATSSAK_.2	#N/A	3.6184	3.3442	4.3052	5.2866	3.2072	3.2790	4.1847	6.2277	4.8271	5.4117	0.7438	-0.4270	0.0398	1.4003

Nop56	_KAPKEELAS[Phospho (STY)]DLEEMATSSAK_.3	#N/A	3.7163	3.8727	3.5567	4.0594	3.7359	4.2548	3.7741	4.0829	3.7315	4.0264	0.9903	-0.0140	0.8043	0.0946
Rpap3	_AVDNPPRGS[Phospho (STY)]PK_.2	Q9D706_S429_M1_Rpap3	8.5421	8.5724	8.5613	8.7773	8.3352	8.4959	8.6242	8.2189	8.4925	8.7654	1.0026	0.0037	0.8551	0.0680
Fam219a	_NGSM[Oxidation (M)]GS[Phospho (STY)]PVNQQPK_.2	#N/A	7.7793	7.9438	7.7829	7.6604	7.3050	7.8578	8.0007	7.7766	7.7345	8.0953	0.9772	-0.0333	0.2146	0.6684
Prps1	_ENISEWR_.2	#N/A	6.7204	6.3816	7.0193	6.2443	7.0087	6.6348	5.9021	6.7918	6.9126	6.9305	1.0051	0.0074	0.8966	0.0474
Prps1	_VYAILTHGIFSGPAISR_.3	#N/A	3.5279	3.4455	3.8294	3.7114	3.0856	3.9805	3.2125	3.4929	4.1169	3.9392	0.9746	-0.0371	0.6944	0.1584
Prps1	_INNAC[Carbamidomethyl (C)]FEAVVVTNTIPQEDK_.2	#N/A	3.6000	6.1559	4.2868	3.2886	4.2265	5.7658	4.6211	3.3290	5.8484	3.2556	0.9661	-0.0497	0.8611	0.0650
Prps1	_IQVIDISM[Oxidation (M)]ILAEAIR_.2	#N/A	4.3107	3.6151	3.4967	3.9994	3.4364	4.2931	3.7140	2.9231	3.7677	3.9664	1.0740	0.1030	0.3562	0.4483
Prps1	_VAILVDDMADTC[Carbamidomethyl (C)]GTIC[Carbamidomethyl (C)]HAADK_.3	#N/A	4.1570	3.4613	3.3429	5.1787	3.2827	2.9460	3.3068	4.2729	3.9215	3.8126	0.9738	-0.0383	0.8271	0.0824
Prps1	_IFSGSSHQDLSQK_.2	#N/A	3.0694	4.2343	4.3040	3.8477	4.1870	4.4114	3.9960	3.1535	4.4525	3.5250	1.1351	0.1829	0.1374	0.8619
Iscu	_ELS[Phospho (STY)]APAR_.2	Q9D7P6_S30_M1_Iscu	9.3418	9.0428	9.6299	9.4716	9.5571	9.4035	8.8353	9.2426	9.6572	9.5585	1.0090	0.0130	0.6525	0.1854
Iscu	_AAS[Phospho (STY)]ALLLRS[Phospho (STY)]PR_.2	#N/A	8.8040	9.5560	9.4274	10.3124	9.6413	9.8268	9.8903	9.6471	9.9408	9.8885	0.9749	-0.0367	0.3663	0.4361
Iscu	_RAAS[Phospho (STY)]ALLLRS[Phospho (STY)]PR_.3	#N/A	3.2136	5.5226	4.2279	6.3694	5.4379	5.5561	5.6675	6.1699	5.9172	5.9853	0.8517	-0.2316	0.1701	0.7692
Iscu	_RAAS[Phospho (STY)]ALLLR_.2	Q9D7P6_S15_M1_Iscu	4.1649	3.4204	3.8986	3.4424	3.3087	3.3159	3.5906	3.5588	3.8578	3.1197	1.0170	0.0243	0.7897	0.1026
Rpl22l1	_YFQISQDEDGS[Phospho (STY)]ESED_.2	Q9D7S7_S118_M1_Rpl22l1	4.0329	7.5084	3.4640	7.5710	6.0870	8.6203	6.2337	8.6704	8.3674	8.5403	0.7813	-0.3560	0.1707	0.7678
Chmp5	_NKDGVLVDEFGLPQIPAS_.2	#N/A	2.9888	4.0372	4.1678	3.1697	4.3807	2.9912	4.3852	3.4480	4.5700	3.1367	0.9325	-0.1009	0.5570	0.2541
Lysmd2	_[Acetyl (Protein N-term)]ADLS[Phospho (STY)]PAPALR_.2	Q9D7V2_S5_M1_Lysmd2	7.0133	7.5189	7.2967	7.7046	6.0615	7.4924	7.1101	6.9194	6.4034	7.4760	1.0292	0.0416	0.5781	0.2380
Lysmd2	_AHRPSAPS[Phospho (STY)]PPPR_.3	#N/A	6.6892	6.6669	7.5244	6.5559	3.3530	6.7909	3.3558	6.7835	7.2557	6.5694	1.0454	0.0641	0.7977	0.0981
Lysmd2	_STS[Phospho (STY)]EPEEAELSLSLAR_.2	Q9D7V2_S33_M1_Lysmd2	3.0458	3.8020	4.2803	3.8241	3.6903	2.9343	3.9723	5.8540	3.4762	3.5013	0.8560	-0.2243	0.2748	0.5609
Kctd4	_HNS[Phospho (STY)]VEDAEQGK_.2	#N/A	7.3627	7.2266	7.4245	7.5624	6.8511	7.1777	7.4304	7.3069	7.2032	7.5161	0.9869	-0.0191	0.5007	0.3004
Kctd4	_HNS[Phospho (STY)]VEDAEQGKNC[Carbamidomethyl (C)]K_.2	#N/A	3.2382	3.5108	3.4720	3.2199	3.7169	3.3642	3.5641	3.0889	3.6393	3.9345	0.9617	-0.0564	0.4411	0.3555
Dusp3	_VYVGNASVAQDITQLQK_.2	#N/A	5.3065	4.8358	4.9884	5.3539	5.1594	5.4848	5.3304	4.9268	4.7286	4.9232	1.0424	0.0599	0.2217	0.6542
Dusp3	_ANDTQEFNLSAYFER_.2	#N/A	5.7675	4.0766	5.7991	5.1281	5.3515	5.3672	5.5020	5.2845	5.0433	5.4111	0.9883	-0.0169	0.8563	0.0674
Dusp3	_EIGPNDGFLAQLC[Carbamidomethyl (C)]QLNDR_.2	#N/A	4.9531	4.9912	5.1524	5.6422	5.1981	4.7427	5.1344	5.4383	5.1904	4.6674	1.0011	0.0016	0.9783	0.0095
Dusp3	_LGITHVLNAAEGR_.3	#N/A	4.6611	4.5815	4.3498	4.0530	4.0484	4.0209	4.3691	3.2170	3.9869	3.5709	1.1320	0.1789	0.0768	1.1146
Ggct	_GKIS[Phospho (STY)]DEM[Oxidation (M)]EDIIK_.2	#N/A	3.5600	3.2482	3.9126	4.6208	3.6677	4.3751	4.3757	4.2305	4.1897	3.7905	0.9399	-0.0894	0.4028	0.3949
Ppa1	_AAPFTLEYR_.2	#N/A	7.4662	7.7274	7.8523	7.4595	7.8692	7.6966	7.4765	7.4127	7.4168	7.4892	1.0308	0.0438	0.0391	1.4074
Ppa1	_VIAINVDDPDAANYKDISDVER_.3	#N/A	6.0308	6.0412	7.2550	6.6695	6.9095	6.1069	6.7047	5.7993	6.2823	6.1700	1.0422	0.0596	0.4113	0.3858
Ppa1	_HTGC[Carbamidomethyl (C)]C[Carbamidomethyl (C)]GDNDPIDVC[Carbamidomethyl (C)]EIGSK_.3	#N/A	6.8358	5.7839	5.8801	6.8952	6.3192	5.7500	6.3223	5.8620	6.1124	6.5672	1.0045	0.0065	0.9259	0.0334
Ppa1	_VLGILAM[Oxidation (M)]IDEGETDWK_.2	#N/A	4.9136	4.3473	3.6822	4.8773	4.5498	4.7618	4.7276	4.4051	4.1606	5.0214	0.9876	-0.0180	0.8443	0.0735
Ppa1	_STHDYWK_.2	#N/A	4.3325	4.7001	4.3509	4.8471	5.6897	4.8857	3.7530	4.7395	4.1279	4.7855	1.1033	0.1418	0.1990	0.7012
Fip1l1	_ERDHS[Phospho (STY)]PTPSVFNSDEER_.3	#N/A	6.5541	6.1903	6.5784	7.4221	6.2751	6.4220	6.6242	6.7737	6.2884	6.2220	1.0149	0.0213	0.7082	0.1498
Fip1l1	_ERDHS[Phospho (STY)]PTPSVFNSDEERYR_.4	#N/A	6.7164	7.0472	7.7537	8.3530	6.6632	7.1219	6.8916	7.7060	7.1052	6.6433	1.0267	0.0380	0.6316	0.1996
Fip1l1	_AES[Phospho (STY)]PDLR_.2	Q9D824_S280_M1_Fip1l1	3.6759	2.9803	4.3627	3.6395	2.8016	3.4270	3.7878	3.3616	3.6607	2.2804	1.0637	0.0891	0.6100	0.2147
Aplf	_TTGASQLQGS[Phospho (STY)]PEITK_.2	Q9D842_S149_M1_Aplf	3.6764	3.6660	3.4101	6.8869	6.2100	3.8045	7.0665	6.3616	6.1758	4.1320	0.7767	-0.3645	0.1892	0.7231
Aplf	_RLS[Phospho (STY)]PSGNSK_.2	Q9D842_S238_M1_Aplf	3.9473	3.2788	3.1333	3.6359	3.0730	3.1556	3.3506	2.9817	4.1311	3.6029	0.9585	-0.0611	0.5905	0.2288
Uqcrb	_WYYNAAGFNK_.2	#N/A	7.7829	8.0561	7.7216	8.1090	8.2519	8.0425	7.9256	7.8714	8.0887	7.9466	1.0045	0.0065	0.7516	0.1240
Uqcrb	_LPEDLYNDR_.2	#N/A	8.0816	8.2625	8.2966	8.2557	8.5972	8.3476	8.1588	7.8346	8.0685	8.4448	1.0222	0.0316	0.2080	0.6820
Uqcrb	_DDTLHETEDVKEAIR_.3	#N/A	7.2454	6.8616	7.7942	7.3833	7.7530	7.3727	6.7937	6.6508	7.2904	6.7389	1.0776	0.1079	0.0344	1.4633
Uqcrb	_DDTLHETEDVK_.2	#N/A	6.7551	6.7246	6.4561	7.0692	7.2852	6.8788	6.5823	6.2373	6.2567	7.0355	1.0511	0.0719	0.1486	0.8279
Uqcrb	_RLPEDLYNDR_.2	#N/A	7.4787	6.9716	7.0575	6.4534	5.9893	7.5544	7.8158	7.5118	7.2488	8.0292	0.9041	-0.1455	0.0606	1.2173
Uqcrb	_YEEDKFYLEPYLK_.2	#N/A	6.3823	6.2037	6.4397	6.9689	6.3577	6.5184	6.4885	5.9221	6.4994	6.4159	1.0232	0.0331	0.4187	0.3781
Fam241b	_SS[Phospho (STY)]SSQGFFNR_.2	Q9D882_S27_M1_Fam241b	3.5791	6.9690	7.4331	4.0106	6.4123	3.9989	3.5406	3.4917	4.0545	3.7631	1.4547	0.5407	0.0928	1.0324
U2af1	_AVIDLNNR_.2	#N/A	8.0121	7.7856	8.5280	8.1632	7.6682	7.5751	8.4566	7.9179	7.8258	7.9780	0.9889	-0.0161	0.6858	0.1638
Itpa	_IDLPEYQGEPDEISIQK_.2	#N/A	7.3657	7.2489	6.4675	7.4582	6.9979	7.0484	7.1954	6.7191	6.9130	6.9895	1.0206	0.0295	0.4885	0.3111
Itpa	_IVFVTGNAK_.2	#N/A	3.2261	2.3877	3.6293	4.5424	2.9079	2.8134	0.8946	3.9596	4.2586	3.5863	1.0240	0.0343	0.9185	0.0369
Itpa	_LQEYFSVAAGAGDH_.2	#N/A	4.2589	3.5143	3.9926	3.4004	3.4026	3.2220	5.7832	3.2172	5.6718	3.3674	0.8053	-0.3124	0.1771	0.7517
Arpc5l	_ALAVGGLGSIIR_.2	#N/A	7.4512	7.9243	7.7598	7.8330	7.7830	7.8618	7.6156	7.9175	7.5809	7.7334	1.0074	0.0106	0.5983	0.2231
Arpc5l	_AFHAALRNS[Phospho (STY)]PINTK_.3	#N/A	3.9086	6.5686	3.6423	8.0119	5.0202	3.6056	3.6221	7.0064	4.1475	4.9539	1.0393	0.0556	0.8641	0.0635
Arpc5l	_NS[Phospho (STY)]PINTK_.2	#N/A	3.2458	7.0017	4.4803	4.4136	3.8903	7.0297	4.1283	7.3656	3.3535	7.8695	0.8822	-0.1808	0.5980	0.2233
Arpc5l	_SSEIEQAVQSLDR_.2	#N/A	7.0320	7.1405	7.2101	7.3080	7.4910	7.3916	7.4977	6.9702	7.1930	7.3696	1.0006	0.0009	0.9715	0.0126
Chmp4b	_GGPTPQEAIQR_.2	#N/A	6.0681	6.5330	3.5040	6.8065	7.1382	6.4055	6.0149	6.0076	6.6147	6.7485	0.9574	-0.0628	0.7039	0.1525
Chmp4b	_KIEQELTAAK_.2	#N/A	3.3784	3.7456	4.1852	4.5066	5.2556	4.4011	3.6196	3.7818	4.5555	3.5206	1.0972	0.1338	0.3520	0.4534
Chmp4b	_QLAQIDGTLSTIEFQR_.2	#N/A	4.1013	3.7679	5.2911	4.3528	4.3314	3.9838	4.5011	3.9663	4.1845	3.6761	1.0546	0.0766	0.4845	0.3147
Ndufa11	_FFESYHEVPDGTQC[Carbamidomethyl (C)]HR_.3	#N/A	6.7321	6.1957	3.3195	6.1450	6.2123	6.5101	6.7489	6.3048	6.4444	3.7892	1.0053	0.0076	0.9718	0.0124
Ndufa11	_EKPDDPLNYFIGGC[Carbamidomethyl (C)]AGGLTLGAR_.3	#N/A	5.5045	4.7646	4.0671	5.7115	4.2612	4.4976	5.3823	5.1828	4.8936	4.9359	0.9416	-0.0868	0.4248	0.3719
Rpl4	_ILKS[Phospho (STY)]PEIQR_.2	Q9D8E6_S295_M1_Rpl4	6.7332	6.8785	7.3335	7.2203	6.6475	6.3130	7.3512	6.7220	6.5291	6.4156	1.0148	0.0212	0.7044	0.1522
Rpl4	_FC[Carbamidomethyl (C)]IWTESAFR_.2	#N/A	4.6777	5.8883	4.0140	3.5578	4.6398	4.8483	5.3195	5.2079	3.7425	4.9132	0.9601	-0.0588	0.7096	0.1490
Rpl4	_QPYAVSELAGHQTSAESWGTGR_.3	#N/A	2.9758	3.8902	4.0227	4.5254	3.7785	4.3469	4.2401	3.5931	3.2417	2.9915	1.1156	0.1579	0.2788	0.5548
Rpl4	_SGQGAFGNM[Oxidation (M)]C[Carbamidomethyl (C)]R_.2	#N/A	4.8925	3.8774	3.7590	4.2617	3.6988	4.0307	3.9764	3.8568	3.5054	4.2287	1.0501	0.0705	0.4679	0.3298
P2yr13	_TAGSSEDHHS[Phospho (STY)]S[Phospho (STY)]QTDNITLA_.2	Q9D8I2_S328_M2_P2yr13	4.2084	3.1340	3.9420	3.4858	3.3521	3.2725	3.6341	3.5154	3.8144	3.1631	1.0096	0.0139	0.8906	0.0503
Eef1g	_STFVLDEFKR_.2	#N/A	7.2838	7.5053	7.6181	7.6548	7.1063	7.7708	7.9369	7.7403	7.7779	7.2329	0.9763	-0.0347	0.3296	0.4820
Eef1g	_[Acetyl (Protein N-term)]AAGTLYTYPENWR_.2	#N/A	7.1445	7.1871	7.5518	7.1586	6.7602	7.0498	7.2116	7.2491	7.2123	6.7156	1.0063	0.0091	0.7921	0.1012
Eef1g	_TFLVGER_.2	#N/A	6.8371	5.9766	6.2000	5.4650	5.9740	5.9223	6.1478	6.7451	6.3818	6.4179	0.9439	-0.0834	0.1858	0.7310
Eef1g	_QVLEPSFR_.2	#N/A	6.8137	7.3008	7.0329	6.1007	6.6677	6.4408	6.5300	6.8227	6.7298	6.3665	1.0172	0.0246	0.6368	0.1960
Eef1g	_ILGLLDTHLK_.2	#N/A	6.2900	7.1339	7.0981	6.9483	6.0610	5.8416	6.3578	7.0213	6.6588	6.7131	0.9812	-0.0274	0.6947	0.1582
Eef1g	_WFLTC[Carbamidomethyl (C)]INQPQFR_.2	#N/A	6.3302	5.6416	3.2135	6.2911	5.5640	5.7151	6.1679	6.0449	5.8799	5.8932	0.9104	-0.1354	0.3883	0.4108
Eef1g	_ALIAAQYSGAQVR_.2	#N/A	4.7923	4.5891	3.5465	5.6947	4.9069	4.4394	4.9529	5.0867	4.9921	4.7803	0.9411	-0.0875	0.4418	0.3548
Eef1g	_KLDPGSEETQTLVR_.2	#N/A	4.7800	5.0926	4.9669	5.0330	4.4322	4.3901	4.7201	4.9800	4.4740	4.8212	1.0071	0.0102	0.8543	0.0684
Eef1g	_KFAESQPK_.2	#N/A	4.6957	4.0976	4.4793	4.0231	5.0055	3.8105	4.8613	4.7587	4.2141	5.1030	0.9192	-0.1215	0.2017	0.6953
Eef1g	_QATENAKEEVKR_.3	#N/A	3.3138	2.7401	3.2630	3.7657	3.2027	3.3367	3.4804	3.0111	3.8753	3.7327	0.9278	-0.1081	0.2901	0.5375
Arfgap3	_LTNTS[Phospho (STY)]FTEIEK_.2	Q9D8S3_S241_M1_Arfgap3	3.5973	6.0958	3.3310	4.3756	4.2419	6.3049	4.5239	4.1264	4.4255	4.0529	1.0877	0.1213	0.5784	0.2378
Arfgap3	_SSS[Phospho (STY)]FSSWDDGADSYWK_.2	Q9D8S3_S374_M1_Arfgap3	4.2581	3.5624	3.4440	5.6652	3.3838	4.3457	3.2057	5.1395	3.8204	4.9812	0.9588	-0.0608	0.7666	0.1154
Bola1	_FEGM[Oxidation (M)]S[Phospho (STY)]PLQR_.2	#N/A	7.3696	7.2765	7.7119	8.2339	6.3183	7.3082	7.1524	6.7795	6.9835	7.4521	1.0392	0.0555	0.4379	0.3586
Bola1	_ENPQLDIS[Phospho (STY)]PPC[Carbamidomethyl (C)]LGGSK_.2	Q9D8S9_S123_M1_Bola1	3.6519	7.0747	4.3387	3.3406	4.2785	3.4510	4.5561	3.2771	7.7457	3.3076	0.9225	-0.1163	0.7469	0.1267
Bola1	_AKLEQALS[Phospho (STY)]PEVLER_.3	Q9D8S9_S45_M1_Bola1	3.4546	3.8878	3.1883	4.2329	3.7091	4.0204	4.3812	4.2691	3.0673	3.8089	0.9658	-0.0502	0.6758	0.1702
Slirp	_ALHGAQTS[Phospho (STY)]DEER_.2	Q9D8T7_S105_M1_Slirp	6.6758	6.3706	4.3422	6.8704	6.8553	6.6738	4.1837	6.6152	6.6611	6.6972	1.0428	0.0605	0.7204	0.1424
Snx5	_[Acetyl (Protein N-term)]AAVPELLEQQEEDR_.2	#N/A	6.6198	6.3379	7.1179	6.4280	5.8442	6.3365	7.2270	5.9289	6.7041	6.6950	0.9712	-0.0422	0.5410	0.2668
Snx5	_[Acetyl (Protein N-term)]AAVPELLEQQEEDRSK_.2	#N/A	2.9781	3.3391	4.3242	4.9262	4.3932	3.5855	2.6532	2.9593	4.0195	3.9300	1.1575	0.2110	0.2841	0.5465
Ociad2	_FHSFEDQLR_.2	#N/A	5.8465	5.9175	6.6759	6.6374	6.6765	6.2173	6.5384	6.6188	6.6710	6.4587	0.9630	-0.0544	0.2611	0.5832

Ociad2	_[Acetyl (Protein N-term)]ASVSTHGNQEK_.2	#N/A	3.7428	3.8331	3.1376	4.3369	4.7960	3.9747	3.9372	3.0037	3.9960	4.0937	1.0566	0.0794	0.5604	0.2515
Ociad2	_GAGFGPEHNR_.2	#N/A	5.9829	5.9571	6.5920	5.9567	6.3159	6.4490	6.0327	6.5942	6.8905	5.5493	0.9908	-0.0134	0.8393	0.0761
Efhd2	_ADLNQGIGEPQS[Phospho (STY)]PSR_.2	#N/A	9.8256	9.7485	9.9181	9.9727	9.0097	9.9677	9.6301	9.2770	9.6870	9.8453	1.0932	0.1286	0.1851	0.7326
Efhd2	_RADLNQGIGEPQS[Phospho (STY)]PSR_.3	#N/A	7.1957	5.0700	5.1250	7.1397	6.1799	5.4915	6.4780	4.9428	7.1177	5.1184	1.0202	0.0288	0.8577	0.0667
Efhd2	_VQAINVSSR_.2	#N/A	8.1415	8.5735	8.7149	8.5629	8.9617	8.5870	8.0637	8.2570	8.6035	8.2707	1.0351	0.0498	0.1104	0.9569
Efhd2	_LSEIDVSTEGVK_.2	#N/A	6.5018	6.9714	6.8262	7.0769	7.2263	7.3817	7.1959	6.5889	6.9682	7.0562	1.0065	0.0093	0.8173	0.0876
Efhd2	_RADLNQGIGEPQS[Phospho (STY)]PSRR_.3	#N/A	4.3471	7.2825	7.7326	7.5929	3.4908	7.1923	4.2005	6.8991	7.0786	3.2920	1.1687	0.2249	0.4758	0.3225
Efhd2	_ADLNQGIGEPQS[Phospho (STY)]PSRR_.3	#N/A	6.1227	6.5304	6.5965	3.7318	5.5359	6.1651	5.5010	3.4811	4.0987	6.3976	1.1870	0.2474	0.2634	0.5794
Efhd2	_[Acetyl (Protein N-term)]ATDELASKLS[Phospho (STY)]R_.2	Q9D8Y0_S11_M1_Efhd2	3.7798	6.6610	4.1480	7.3713	6.8065	6.8736	6.6186	5.3431	5.7464	6.2847	0.9903	-0.0141	0.9454	0.0244
Efhd2	_ELQSTFK_.2	#N/A	4.1875	3.5879	3.3735	3.8761	3.6226	4.4163	3.5908	4.2424	3.8909	3.8431	0.9877	-0.0178	0.8391	0.0762
Efhd2	_SMIQEVDEDFDSK_.2	#N/A	3.0005	4.3419	4.2350	3.7788	3.6451	2.9795	3.9271	3.2224	3.5214	3.4561	1.0373	0.0528	0.6913	0.1603
Borcs5	_GLLSGQTS[Phospho (STY)]PTNAK_.2	#N/A	3.5744	3.8194	10.0075	10.1248	9.2586	9.8572	10.0305	4.2007	9.7051	9.8012	0.9217	-0.1177	0.7456	0.1275
Borcs5	_GLLSGQTS[Phospho (STY)]PTNAKLEK_.2	#N/A	3.5574	4.6625	3.2911	6.4370	5.7175	6.2760	6.3592	4.1663	2.9646	3.6672	1.0657	0.0917	0.7540	0.1226
Rwdd2a	_KLS[Phospho (STY)]DEPSVQAK_.2	#N/A	3.1820	4.1604	4.4165	4.7421	3.9817	3.7477	4.1085	3.0409	3.3400	3.6376	1.1435	0.1934	0.1637	0.7860
Rwdd2a	_KLS[Phospho (STY)]DEPSVQAKPVK_.2	#N/A	4.0908	5.7328	3.2767	6.4961	5.7354	3.6009	6.4490	4.3391	3.9877	3.7464	1.0414	0.0585	0.8248	0.0837
Fam135b	_SVSAPQIS[Phospho (STY)]SPEESAEGADTIK_.2	Q9DAI6_S775_M1_Fam135b	3.9543	4.5928	5.4992	6.3492	3.3063	3.2981	3.9432	5.8613	4.1519	3.3622	1.0393	0.0557	0.8281	0.0819
Fam135b	_NTAGFSEDLDPSSKENS[Phospho (STY)]PPR_.3	Q9DAI6_S805_M1_Fam135b	2.9653	3.7215	4.1998	5.6437	3.6098	3.0148	3.8918	3.2576	3.5567	3.4208	1.0927	0.1279	0.5434	0.2649
Gng12	_TASTNS[Phospho (STY)]IAQAR_.2	Q9DAS9_S10_M1_Gng12	8.5560	8.4510	9.0708	8.8349	8.4578	8.5973	8.7207	8.6084	8.6457	8.5808	1.0026	0.0037	0.8643	0.0633
Gng12	_SDPLLM[Oxidation (M)]GIPTSENPFK_.2	#N/A	7.1211	7.2694	7.4956	7.3558	6.9185	7.0135	7.2550	7.2661	7.3161	6.8880	1.0020	0.0029	0.9182	0.0371
Gng12	_LEASIER_.2	#N/A	5.8496	6.1869	6.1008	5.5896	6.0634	6.2799	4.9731	6.3405	6.2683	5.7791	1.0294	0.0417	0.5575	0.2538
Gng12	_ASADLM[Oxidation (M)]SYC[Carbamidomethyl (C)]EEHAR_.2	#N/A	3.8068	3.8643	3.9852	4.1851	3.7096	3.5313	3.8998	3.2956	3.7747	3.3216	1.0767	0.1066	0.1413	0.8499
Mrgbp	_VLTANSNPSS[Phospho (STY)]PSAAK_.2	Q9DAT2_S195_M1_Mrgbp	5.8914	5.6624	6.0422	4.0897	6.6436	3.6471	3.7608	5.7589	6.4929	6.0677	0.9655	-0.0507	0.8110	0.0910
Zfyve19	_WS[Phospho (STY)]PPQNYK_.2	Q9DAZ9_S69_M1_Zfyve19	8.0657	3.8520	3.2241	4.2687	8.0284	8.2238	8.0962	7.8003	7.3383	3.9460	0.8747	-0.1931	0.5703	0.2439
Zfyve19	_VTLQDYHLPDS[Phospho (STY)]DEDEETAIQR_.3	Q9DAZ9_S280_M1_Zfyve19	3.0003	4.3421	4.2349	7.8342	3.6449	2.9797	3.9269	6.7067	3.9301	3.4559	0.9633	-0.0540	0.8834	0.0538
Zfyve19	_TNS[Phospho (STY)]QGQASQSLEEEKYK_.2	Q9DAZ9_S219_M1_Zfyve19	4.3469	2.9807	3.5328	4.1763	3.4725	4.2569	3.1169	1.9154	3.2943	4.0025	1.2310	0.2999	0.1460	0.8358
Zfyve19	_EHQTS[Phospho (STY)]PYHPR_.2	Q9DAZ9_S378_M1_Zfyve19	4.6204	3.4224	3.7756	3.5415	5.0676	3.0399	4.0616	4.1152	3.5792	3.5704	1.0208	0.0297	0.8515	0.0698
Napa	_YEELFPAFSDSR_.2	#N/A	8.5092	8.6016	8.2125	8.9428	8.5893	8.5547	8.4994	8.5537	8.3992	8.2836	1.0159	0.0228	0.3244	0.4889
Napa	_TIQGDEEDLR_.2	#N/A	7.1100	7.1504	7.2669	7.5122	7.3860	7.4204	7.2329	7.3137	7.1323	6.6942	1.0302	0.0430	0.1536	0.8136
Napa	_VAGYAAQLEQYQK_.2	#N/A	6.3997	5.8653	5.8226	7.5488	7.2077	6.2115	6.3773	6.6108	6.1248	6.3467	1.0227	0.0324	0.7090	0.1494
Napa	_NSQSFFSGLFGGSSK_.2	#N/A	5.0164	5.2321	5.1238	5.6512	5.3844	4.7288	5.0505	4.8985	5.6349	5.3134	0.9933	-0.0097	0.8696	0.0607
Napa	_LLEAHEEQNVDSYTEAVKEYDSISR_.4	#N/A	5.0503	5.5687	6.0117	5.9107	4.6005	4.8385	4.9888	4.2542	5.3108	5.2726	1.0753	0.1048	0.3245	0.4887
Napa	_AIDIYEQVGTSAM[Oxidation (M)]DSPLLK_.2	#N/A	4.1285	4.2029	4.0898	5.6933	4.6667	4.7918	4.5515	5.1496	5.1491	4.5322	0.9484	-0.0765	0.4855	0.3138
Napa	_LLEAHEEQNVDSYTEAVK_.3	#N/A	5.7138	4.8432	5.0770	5.4489	4.1012	4.7322	5.5418	4.2311	5.0074	4.8801	1.0144	0.0207	0.8488	0.0712
Napa	_AIEIYTDMGR_.2	#N/A	4.0285	3.1977	3.2144	4.6624	3.1542	3.0745	3.4318	4.4014	4.0500	3.6841	0.9135	-0.1305	0.3894	0.4096
Napa	_HDAATC[Carbamidomethyl (C)]FVDAGNAFK_.2	#N/A	3.9766	3.2320	3.7103	5.3173	3.1203	3.5043	3.3974	3.7471	4.0462	4.4322	0.9755	-0.0357	0.8351	0.0783
Mrpl12	_NYVQGINLVQAK_.2	#N/A	4.6641	4.5885	5.1014	5.8376	4.8279	4.5679	4.6691	4.9014	5.2004	4.5004	1.0235	0.0336	0.6915	0.1602
Mrpl12	_LTEAKPVDK_.2	#N/A	4.7652	5.3834	5.4118	5.3840	6.2252	5.8939	5.5203	5.1689	4.5281	5.3879	1.0697	0.0973	0.2788	0.5547
Atp5po	_LVRPPVQVYGIEGR_.3	#N/A	9.9627	10.4282	11.1191	10.5229	10.5260	10.4907	10.4341	10.2739	10.7213	10.6886	0.9980	-0.0029	0.9205	0.0360
Atp5po	_VSLAVLNPYIK_.2	#N/A	9.0013	9.3813	9.2419	9.4259	9.2398	9.0044	9.1309	9.4715	9.2569	9.3454	0.9908	-0.0133	0.4533	0.3436
Atp5po	_SFLSPNQILK_.2	#N/A	8.8267	9.1825	9.1213	9.1391	9.0783	8.7167	8.9783	9.3357	8.9375	9.1138	0.9911	-0.0128	0.5232	0.2813
Atp5po	_YATALYSAASK_.2	#N/A	8.0891	8.3177	8.7384	8.2903	8.5712	8.1215	7.7899	8.3234	7.9995	8.1039	1.0373	0.0529	0.0916	1.0380
Atp5po	_SLNDITKR_.2	#N/A	7.4086	7.7146	7.9631	7.8368	8.1590	7.5413	7.4913	7.8035	7.8938	7.2441	1.0213	0.0305	0.4014	0.3964
Atp5po	_EKFSPLTANLM[Oxidation (M)]NLLAENGR_.3	#N/A	7.6525	7.8142	8.0594	7.6140	7.5741	7.8971	7.6674	7.8769	7.9475	7.8300	0.9921	-0.0115	0.5796	0.2369
Atp5po	_FSPLTANLM[Oxidation (M)]NLLAENGR_.2	#N/A	6.5610	6.0685	6.0006	6.4587	6.3303	6.1964	6.3491	6.4993	6.2695	6.4195	0.9820	-0.0262	0.3613	0.4421
Atp5po	_LGNTQGIISAFSTIM[Oxidation (M)]SVHR_.3	#N/A	6.0041	5.6462	6.2718	6.3427	5.2105	5.5980	5.5807	5.7671	5.5406	6.2516	1.0105	0.0150	0.8204	0.0860
Atp5po	_TDPSIMGGMIVR_.2	#N/A	3.5548	4.3600	4.2416	7.7201	5.4378	3.5482	3.2361	3.2405	3.5395	3.4380	1.4302	0.5162	0.1122	0.9500
Atp5po	_GEVPC[Carbamidomethyl (C)]TVTTASPLDDAVLSELK_.2	#N/A	6.8653	6.7176	6.6176	7.1529	7.1457	6.5459	6.7015	6.8090	6.6361	6.6398	1.0215	0.0307	0.3246	0.4886
Atp5po	_YVDMSAK_.2	#N/A	3.6426	2.9469	4.1292	3.6729	4.2691	3.4604	3.8212	3.3283	3.6273	3.3502	1.0439	0.0620	0.5680	0.2456
Slc25a18	_IQLQDAGR_.2	#N/A	8.3304	8.3876	8.1330	8.2463	8.3012	8.2789	8.3892	8.5308	8.4154	8.1658	0.9886	-0.0166	0.2360	0.6271
Slc25a18	_GAAVNLTLVTPEK_.2	#N/A	7.2611	7.0021	7.1356	7.6709	6.8942	7.6574	7.2262	7.5500	7.3534	7.2159	0.9910	-0.0131	0.7211	0.1420
Slc25a18	_LAANDFLR_.2	#N/A	5.2693	4.9243	5.6393	5.5793	5.2940	5.2006	5.6025	5.5063	5.7342	5.6409	0.9461	-0.0800	0.0605	1.2185
Slc25a18	_ASFTHSFVAGC[Carbamidomethyl (C)]TAGSVAAVAVTPLDVLK_.3	#N/A	4.3241	3.6284	3.5100	4.4923	3.4498	4.2797	3.1397	4.1058	3.7544	3.9797	1.0541	0.0760	0.5082	0.2940
Pabir1	_VSTTTDSPVS[Phospho (STY)]PAQAASPFIPVDELSSK_.3	Q9DB52_S267_M1_Pabir1	3.3148	5.7231	6.3263	6.7366	3.9594	4.1661	4.2413	5.7728	3.2071	4.9091	1.1114	0.1524	0.5626	0.2498
Pabir1	_RIDFIPVS[Phospho (STY)]PAPS[Phospho (STY)]PTR_.2	Q9DB52_S140_M2_Pabir1	2.9284	4.4140	4.1630	8.9486	6.9334	3.4942	3.8550	7.4610	7.1888	3.3840	0.9406	-0.0884	0.8288	0.0816
Pabir1	_RNS[Phospho (STY)]TTFPSR_.2	Q9DB52_S59_M1_Pabir1	4.2626	3.5180	3.9963	3.3968	3.4063	3.2183	3.1114	3.2209	4.3703	3.3638	1.0331	0.0470	0.7154	0.1454
Pabir1	_SNS[Phospho (STY)]APLIHGLSDSSPVFQAEAPSAR_.3	Q9DB52_S34_M1_Pabir1	3.7640	3.6179	3.5120	6.9835	3.7067	3.6236	4.0030	3.9993	3.5964	3.5320	1.1107	0.1514	0.5686	0.2452
Prxl2b	_SILDQHDVR_.2	#N/A	5.5464	5.9183	5.8216	5.6595	6.2943	5.0645	5.2458	5.0544	5.5021	6.0254	1.0477	0.0673	0.3588	0.4451
Prxl2b	_WIAQDLSNLR_.2	#N/A	5.4260	5.8009	5.2016	5.4725	5.6644	5.6854	5.4542	5.4552	5.4549	5.3506	1.0208	0.0297	0.3459	0.4610
Fundc1	_NPPPQDYES[Phospho (STY)]DDESYEVLDLTEYAR_.3	Q9DB70_S13_M1_Fundc1	3.3987	3.7445	4.4434	3.8426	5.8443	4.0060	4.3135	3.7061	4.9691	4.9463	0.9397	-0.0898	0.6055	0.2179
Cdip1	_[Acetyl (Protein N-term)]S[Phospho (STY)]NEPPPPYPGGPTAPLLEEK_.2	Q9DB75_S2_M1_Cdip1	3.6141	3.6121	4.3009	3.3027	3.5004	3.1242	4.5182	6.7837	2.9635	3.2697	0.8157	-0.2939	0.2919	0.5347
Uqcrc2	_TSAAPGGVPLQPQDLEFTK_.2	#N/A	9.2038	9.0633	8.9442	9.1674	9.1388	9.1583	9.2671	9.0279	8.9697	8.8297	1.0099	0.0142	0.3353	0.4746
Uqcrc2	_AVAQGNLSSADVQAAK_.2	#N/A	7.2104	7.8985	7.6270	8.2664	7.6579	8.0663	8.1743	8.0087	8.0069	8.1814	0.9623	-0.0554	0.1548	0.8103
Uqcrc2	_GNNTTSLLSQSVAK_.2	#N/A	8.8451	8.9339	8.8456	8.9009	8.9998	9.1538	9.1379	8.6628	9.0732	8.7463	1.0047	0.0067	0.7174	0.1443
Uqcrc2	_AVAFQNSQTR_.2	#N/A	8.2716	8.6241	8.6532	8.8656	8.8269	8.4957	8.3245	8.5191	8.3477	8.3689	1.0277	0.0395	0.0825	1.0838
Uqcrc2	_NALANPLYC[Carbamidomethyl (C)]PDYR_.2	#N/A	7.7860	7.8782	8.0103	7.8708	7.7492	7.5185	7.8651	7.5382	7.8472	7.5808	1.0122	0.0175	0.4104	0.3868
Uqcrc2	_YRGGEIR_.2	#N/A	7.3269	7.3484	6.0168	7.9018	6.3418	6.5821	6.9426	7.3358	6.4274	7.5392	0.9799	-0.0292	0.7413	0.1300
Uqcrc2	_WEVAALR_.2	#N/A	5.8007	5.4149	5.6220	5.6437	5.5977	5.7541	4.2557	5.7408	6.2316	5.7016	1.0285	0.0406	0.6614	0.1796
Uqcrc2	_LPNGLVIASLENYAPLSR_.2	#N/A	5.8445	5.8527	4.5811	5.3462	6.0042	5.3015	5.3964	5.4432	5.8392	6.1334	0.9623	-0.0554	0.4994	0.3015
Uqcrc2	_YEDSNNLGTSHLLR_.2	#N/A	7.6714	7.6875	7.7974	7.7788	7.6149	7.6274	7.6573	7.6870	7.7846	7.5112	1.0047	0.0068	0.5585	0.2530
Uqcrc2	_M[Oxidation (M)]ALVGLGVSHSVLK_.2	#N/A	7.2566	7.3576	7.8069	6.9546	6.9182	7.4142	7.5022	7.4902	7.5656	7.5042	0.9693	-0.0450	0.2068	0.6845
Uqcrc2	_ENM[Oxidation (M)]AYTVEGIR_.2	#N/A	7.3552	7.0958	6.3387	8.0313	6.6039	7.1822	6.8664	7.3478	6.4151	6.7433	1.0377	0.0534	0.4691	0.3287
Uqcrc2	_IIENLHDVAYK_.2	#N/A	6.8930	7.5860	7.6385	7.3511	7.4291	7.1471	7.4663	7.2616	7.3041	7.1914	1.0048	0.0069	0.8223	0.0850
Uqcrc2	_SM[Oxidation (M)]AASGNLGHTPFLDEL_.2	#N/A	7.0995	6.2443	6.8214	6.6230	6.8303	6.8564	6.7671	6.2394	6.4295	6.5892	1.0268	0.0382	0.2718	0.5658
Uqcrc2	_QVAEQFLNM[Oxidation (M)]R_.2	#N/A	6.6235	6.3752	6.1961	6.6075	6.2098	6.6074	6.3423	5.9591	6.1435	6.2868	1.0410	0.0580	0.0746	1.1274
Uqcrc2	_SQLKIDK_.2	#N/A	4.6888	4.4622	5.4978	5.3926	4.0399	5.2759	4.8859	5.6220	5.1821	4.1222	0.9879	-0.0176	0.8810	0.0550
Uqcrc2	_LASSLTTK_.2	#N/A	3.9435	4.0093	3.6771	3.2209	3.2203	3.0084	3.3692	4.3981	5.3775	4.4427	0.7990	-0.3237	0.0527	1.2780
Uqcrc2	_ITSEELHYFVQNHFTSAR_.3	#N/A	6.7365	6.9804	7.3933	7.0860	6.5414	6.6540	6.6072	6.4771	6.7519	6.8793	1.0104	0.0150	0.8306	0.0806
Smg9	_WKEPGSSGPQNLS[Phospho (STY)]GPGGR_.3	Q9DB90_S32_M1_Smg9	4.3934	3.6489	4.1271	3.2659	3.5372	3.0874	4.4814	3.3303	3.6293	3.3482	0.9944	-0.0081	0.9521	0.0213
Selenoo	_LEQLS[Phospho (STY)]PSDLQR_.2	Q9DBC0_S544_M1_Selenoo	4.1749	3.4793	3.3609	3.1406	3.3006	4.4289	3.2888	3.8606	4.1597	3.8305	0.9637	-0.0533	0.6630	0.1785

Cmtr1	_HLS[Phospho (STY)]STSDDEPLSSVNHAAK_.3	Q9DBC3_S27_M1_Cmtr1	3.2247	6.0873	4.1782	6.0282	4.9358	5.9435	4.1034	6.5236	3.3689	6.0967	1.0086	0.0123	0.9608	0.0173
Prkar1a	_EDEIS[Phospho (STY)]PPPPNPVVK_.2	#N/A	7.4044	7.2687	6.5432	7.9112	6.1709	7.4493	8.0919	7.1653	7.5667	7.2914	0.9463	-0.0796	0.3020	0.5199
Prkar1a	_LTVADALEPVQFEDGQK_.2	#N/A	6.4298	6.5693	6.4289	6.6025	6.3417	6.6526	6.2179	6.0620	6.2995	6.3758	1.0425	0.0601	0.0116	1.9342
Prkar1a	_TDSREDEIS[Phospho (STY)]PPPPNPVVK_.3	#N/A	10.3752	10.9632	11.0693	11.5721	10.4937	10.6048	11.3039	10.4048	10.5639	10.3666	1.0175	0.0250	0.5324	0.2738
Aldh7a1	_FQDEEEVFEWNNEVK_.2	#N/A	4.2878	5.7174	5.8879	5.5524	5.2351	5.6115	4.5062	5.0235	5.2290	5.2852	1.0740	0.1030	0.2887	0.5395
Aldh7a1	_GEVITTYC[Carbamidomethyl (C)]PANNEPIAR_.2	#N/A	5.0171	5.0909	5.7615	5.3965	5.1249	5.3053	4.8834	5.1749	5.4767	4.8296	1.0376	0.0533	0.3256	0.4873
Aldh7a1	_VNLLSFTGSTQVGK_.2	#N/A	3.9469	2.9953	3.4168	3.9195	4.6521	3.7410	4.6095	4.1990	3.3372	4.2211	0.9235	-0.1149	0.4038	0.3939
Ap2b1	_DIPNENELQFQIK_.2	#N/A	8.9053	8.6211	9.0485	8.7581	8.6858	8.9473	9.3035	9.2339	9.0970	8.8358	0.9682	-0.0466	0.0388	1.4116
Ap2b1	_IQPGNPNYTLSLK_.2	#N/A	8.4602	8.3952	8.3307	8.4203	8.0068	8.1900	8.3363	8.6204	8.2763	8.1508	0.9946	-0.0079	0.7099	0.1488
Ap2b1	_LLSTDPVTAK_.2	#N/A	7.9855	7.9626	8.0782	7.3345	7.8476	7.5599	7.9771	8.1220	7.9944	7.5509	0.9853	-0.0214	0.5288	0.2767
Ap2b1	_LQNNNVYTIAK_.2	#N/A	7.3086	7.2279	7.0766	7.3427	6.9761	6.6235	7.2733	7.3376	7.5559	7.1781	0.9668	-0.0487	0.1456	0.8367
Ap2b1	_QVFLATWK_.2	#N/A	6.0733	6.3586	6.5104	6.1376	5.5970	6.2625	5.5197	6.3532	5.9024	5.8186	1.0438	0.0618	0.2560	0.5918
Ap2b1	_VNYVVQEAIVVIR_.2	#N/A	5.1973	4.6874	5.4272	4.9489	4.1505	4.7389	5.4815	5.1938	5.1729	5.1794	0.9242	-0.1137	0.1281	0.8925
Ap2b1	_APEVSQYIYQVYDSILKN_.3	#N/A	3.1408	3.6098	4.7268	5.0040	2.7015	3.8519	4.0694	3.9902	4.1253	4.4090	0.9254	-0.1118	0.5211	0.2831
Ap2b1	_DSDYYNM[Oxidation (M)]LLK_.2	#N/A	6.4129	6.3548	6.4780	6.2905	6.0940	6.0612	6.6370	6.7020	6.3175	6.0057	0.9792	-0.0304	0.4099	0.3873
Ap2b1	_KPSETQELVQQVLSLATQDSDNPDLR_.3	#N/A	4.8127	4.4237	4.5143	4.7084	1.2951	4.2011	5.1320	4.1925	3.2422	3.8982	0.9700	-0.0440	0.8732	0.0589
Ap2b1	_LHDINAQM[Oxidation (M)]VEDQGFLDSLR_.3	#N/A	6.9169	6.4262	6.4594	6.4895	5.9624	6.3715	6.9606	6.8284	6.5991	6.6371	0.9528	-0.0697	0.0963	1.0165
Ap2b1	_LAPPLVTLLSGEPEVQYVALR_.2	#N/A	5.2858	4.9954	5.1185	4.8342	4.6646	4.4976	3.6915	5.6202	5.0822	4.7413	1.0242	0.0344	0.7527	0.1234
Ap2b1	_GLEISGTFTHR_.2	#N/A	6.7385	5.9644	6.0213	5.6314	6.2880	3.7553	6.0520	5.4754	3.1095	3.0033	1.3000	0.3785	0.1449	0.8390
Ap2b1	_EC[Carbamidomethyl (C)]HLNADTVSSK_.2	#N/A	5.9663	5.7700	5.1614	5.3800	5.7045	5.8991	6.1327	5.8302	5.8662	6.3020	0.9360	-0.0954	0.0679	1.1683
Ap2b1	_DLIADSNPM[Oxidation (M)]VVANAVAALSEISESHPNSNLLDLNPQNINK_.4	#N/A	4.0521	3.8108	3.7196	4.7428	3.1778	3.0508	4.2388	4.0010	4.0369	4.4415	0.8994	-0.1530	0.2311	0.6362
Ap2b1	_APEVSQYIYQVYDSILK_.2	#N/A	3.5774	4.3826	4.1943	3.7381	4.2039	3.5256	3.8864	3.2631	3.5621	3.4154	1.1147	0.1567	0.0960	1.0176
Ap2b1	_LTNGIWILAELR_.2	#N/A	3.7765	2.9896	4.4633	3.4652	2.9022	3.3264	3.1798	3.2243	2.4319	3.4322	1.1370	0.1852	0.2523	0.5980
Rpn2	_YHVPVVVVPEGSTSDTQEQAILR_.3	#N/A	4.3935	4.8669	4.3191	3.8628	3.7291	3.9408	3.7723	4.6761	4.5156	3.5401	1.0144	0.0206	0.8521	0.0695
Wwp2	_TATAASEQS[Phospho (STY)]PGAR_.2	Q9DBH0_S211_M1_Wwp2	7.3822	7.8026	8.0956	8.0609	6.7974	7.3900	7.4388	7.6060	7.4069	7.4906	1.0137	0.0196	0.6991	0.1555
Pgam1	_ALPFWNEEIVPQIK_.2	#N/A	10.9051	11.1909	11.2480	11.0939	11.2049	11.0453	10.9893	10.9739	11.0132	11.0734	1.0093	0.0133	0.1666	0.7784
Pgam1	_VLIAAHGNSLR_.2	#N/A	9.6434	10.3307	10.0203	10.4293	10.2090	10.1035	10.1616	10.1797	10.0536	10.0833	1.0003	0.0004	0.9831	0.0074
Pgam1	_HYGGLTGLNK_.2	#N/A	8.4740	8.6735	8.8886	8.9696	9.0404	8.7889	8.6164	8.7593	8.8223	8.7446	1.0080	0.0116	0.5466	0.2623
Pgam1	_YADLTEDQLPSC[Carbamidomethyl (C)]ESLKDTIAR_.3	#N/A	8.6174	8.9152	9.0590	9.3270	9.3707	9.2404	9.2240	8.9167	8.9259	8.8597	1.0119	0.0170	0.5243	0.2805
Pgam1	_FSGWYDADLSPAGHEEAK_.2	#N/A	6.5928	6.2990	6.2682	5.9660	5.9964	6.6104	5.8094	5.9696	5.9765	6.4885	1.0376	0.0532	0.2500	0.6021
Pgam1	_AM[Oxidation (M)]EAVAAQGK_.2	#N/A	7.2369	7.4133	7.2554	7.6111	8.3411	7.4731	7.1738	7.6640	7.7015	7.6200	1.0020	0.0029	0.9486	0.0229
Pgam1	_YADLTEDQLPSC[Carbamidomethyl (C)]ESLK_.2	#N/A	6.5598	5.8852	6.4339	6.6926	6.6495	6.3491	6.0811	3.5437	6.1853	5.9008	1.1844	0.2441	0.0910	1.0408
Pgam1	_HGES[Phospho (STY)]AWNLENR_.2	Q9DBJ1_S14_M1_Pgam1	8.1166	8.1603	8.7281	8.5188	7.7438	8.2656	8.3723	7.9323	8.0120	8.2203	1.0149	0.0214	0.5439	0.2645
Pgam1	_KAM[Oxidation (M)]EAVAAQGK_.2	#N/A	5.3128	5.2987	5.5976	5.3859	6.1791	5.6930	5.0801	4.6568	5.0801	5.0650	1.1222	0.1663	0.0126	1.8992
Pgam1	_SYDVPPPPM[Oxidation (M)]EPDHPFYSNISK_.3	#N/A	9.4717	9.5660	9.9332	9.8934	9.8590	9.7598	9.5680	9.3546	9.4770	9.2537	1.0355	0.0503	0.0165	1.7828
Pgam1	_DAGYEFDIC[Carbamidomethyl (C)]FTSVQK_.2	#N/A	4.7749	5.8038	3.7728	5.2103	4.0285	4.0970	4.6295	3.8430	4.1934	4.4247	1.0800	0.1111	0.4436	0.3530
Pgam1	_FSGWYDADLSPAGHEEAKR_.3	#N/A	7.0974	7.0869	7.4585	7.7677	8.0511	7.8406	7.3823	7.3632	8.0647	7.8124	0.9862	-0.0200	0.6801	0.1675
Pgam1	_HLEGLSEEAIM[Oxidation (M)]ELNLPTGIPIVYELDK_.3	#N/A	3.8971	3.3290	4.5839	1.1721	3.0228	3.2058	3.3004	3.0319	4.1813	3.5528	0.9105	-0.1353	0.6232	0.2054
Pgam1	_TLWTVLDAIDQMWLPVVR_.2	#N/A	3.7161	3.5101	4.4029	3.4047	2.8417	3.2262	3.1194	3.2130	4.3624	3.3717	1.0001	0.0001	0.9992	0.0003
Acadsb	_YAIGSLNEGR_.2	#N/A	5.5381	5.5063	4.2583	5.4604	5.4655	5.6927	3.9503	5.7142	6.0835	3.4793	1.1068	0.1464	0.3977	0.4005
Acadsb	_IFDFQGLQHQVAQVATQLEATR_.3	#N/A	3.3260	3.9001	4.0128	4.5155	3.7884	4.3370	4.2302	3.6030	3.2516	2.9816	1.1318	0.1786	0.1643	0.7843
Gdap2	_ENGPYFASYPPS[Phospho (STY)]PDL_.2	Q9DBL2_S495_M1_Gdap2	9.4952	8.8661	4.0349	8.5509	8.4820	3.1797	8.8225	3.4225	9.2595	8.7388	0.9392	-0.0904	0.8025	0.0955
P33monox	_EEKHPASAQSTPSST[Phospho (STY)]PHAS[Phospho (STY)]PK_.3	#N/A	4.7174	3.6753	6.0662	3.8839	6.0613	6.1860	4.0321	5.7597	5.7192	6.3300	0.9337	-0.0989	0.6234	0.2052
P33monox	_HPASAQSTPSSTPHAS[Phospho (STY)]PK_.3	#N/A	6.1089	4.9220	4.7027	5.0444	4.4594	5.7427	6.1446	5.4597	5.7645	6.0927	0.8803	-0.1839	0.0778	1.1090
Ddx59	_EGEGSLRPSS[Phospho (STY)]PQR_.2	Q9DBN9_S150_M1_Ddx59	3.1872	3.9435	4.4218	3.9655	3.8318	4.2936	4.1868	3.0356	3.3347	3.6428	1.1100	0.1506	0.2208	0.6559
Cmpk1	_NQDNLQGWNK_.2	#N/A	8.3580	8.5784	8.9027	9.0104	8.7082	8.6159	8.7742	8.3449	8.4086	8.6610	1.0174	0.0248	0.3343	0.4758
Cmpk1	_FLIDGFPR_.2	#N/A	6.7468	7.9919	6.9876	7.7998	7.3573	6.9799	7.2961	7.6104	6.9882	6.9730	1.0130	0.0186	0.7470	0.1267
Cmpk1	_IVPVEITISLLK_.2	#N/A	5.6060	5.3488	5.0276	5.3696	5.3355	5.4920	5.5794	5.4131	5.8998	5.9461	0.9393	-0.0903	0.0404	1.3934
Cmpk1	_IQTYLESTKPIIDLYEEM[Oxidation (M)]GK_.3	#N/A	4.2201	5.0972	5.9037	5.5061	5.3070	5.9375	3.2436	5.4773	5.4910	5.6092	1.0753	0.1048	0.5207	0.2834
Cmpk1	_SVDEVFGEVVK_.2	#N/A	6.0697	6.2103	6.7265	6.9173	6.4002	6.5122	6.4465	6.1959	6.5025	6.2112	1.0211	0.0301	0.4621	0.3353
Cmpk1	_YGYTHLSAGELLR_.3	#N/A	3.7518	3.6697	4.0470	5.1179	4.3498	4.2524	3.9619	3.5958	3.9085	4.0340	1.0834	0.1155	0.2789	0.5546
Cmpk1	_KIDASKS[Phospho (STY)]VDEVFGEVVK_.3	#N/A	4.0252	3.3172	3.7589	3.3303	3.1690	3.4556	3.4510	3.6985	3.9975	2.9800	0.9937	-0.0092	0.9269	0.0330
Cmpk1	_SDDNRESLEK_.2	#N/A	3.9865	3.3559	3.7201	3.2639	3.1773	1.3343	3.4122	3.7373	4.0363	4.4420	0.8036	-0.3154	0.1693	0.7712
Cmpk1	_KNPDSQYGELIEK_.3	#N/A	4.2716	2.9546	3.4576	3.9602	3.3973	4.3322	3.6749	4.1583	3.8069	3.9272	0.9581	-0.0617	0.5905	0.2288
Akap8	_ADS[Phospho (STY)]DGDLS[Phospho (STY)]ENDDGAGDLR_.2	Q9DBR0_S320_M2_Akap8	4.3740	6.1360	3.5600	6.8199	6.2280	6.8712	6.9997	6.3033	6.2436	6.7378	0.8621	-0.2141	0.2406	0.6186
Akap8	_S[Phospho (STY)]IEDKTR_.2	Q9DBR0_S634_M1_Akap8	4.8700	3.4046	3.2862	3.2152	3.2260	3.0027	4.3924	3.7860	5.1327	3.7559	0.8205	-0.2855	0.1146	0.9409
Akap8	_SNS[Phospho (STY)]STFIR_.2	Q9DBR0_S201_M1_Akap8	3.8113	3.0668	3.5450	3.8480	2.9551	3.6696	3.5627	4.2704	3.9190	3.8150	0.8949	-0.1603	0.1092	0.9617
Xrn2	_AEDS[Phospho (STY)]DS[Phospho (STY)]EPEPEDNVR_.2	#N/A	8.0317	8.3090	7.9558	8.2671	7.4876	8.2271	8.6231	8.1279	8.2887	7.8174	0.9796	-0.0298	0.4370	0.3596
Xrn2	_NS[Phospho (STY)]PGC[Carbamidomethyl (C)]QVASNPR_.2	Q9DBR1_S448_M1_Xrn2	7.6558	8.1357	8.0506	8.2668	7.6323	7.8694	7.9399	7.8614	7.8899	8.0651	0.9995	-0.0007	0.9775	0.0099
Xrn2	_RKAEDS[Phospho (STY)]DS[Phospho (STY)]EPEPEDNVR_.2	#N/A	4.3900	4.4770	4.8963	5.2302	3.8755	4.5511	4.8113	4.9011	4.3738	4.5243	0.9822	-0.0258	0.7542	0.1225
Xrn2	_KAEDS[Phospho (STY)]DS[Phospho (STY)]EPEPEDNVR_.2	#N/A	7.6625	7.8120	8.2732	8.1623	7.2286	7.7163	8.1302	7.9381	7.8769	7.7760	0.9847	-0.0222	0.5629	0.2496
Fam13c	_AGTPAHES[Phospho (STY)]PQSSIFR_.2	#N/A	5.9698	6.2508	4.0055	7.2365	4.1536	6.4269	6.7799	6.1012	3.7343	5.7799	1.0134	0.0192	0.9314	0.0309
Fam13c	_SEAVGPEPGS[Phospho (STY)]S[Phospho (STY)]GEETSDAVVPEKR_.3	Q9DBR2_S405_M2_Fam13c	2.9816	6.1266	4.2147	6.7427	6.1281	6.3540	6.2257	6.1923	6.6207	3.8231	0.9491	-0.0754	0.7575	0.1206
Ppp1r12a	_SAS[Phospho (STY)]YSYLEDR_.2	Q9DBR7_S909_M1_Ppp1r12a	7.9880	3.9764	8.6910	4.4392	7.2855	8.3837	4.1539	8.1418	7.9612	8.1948	0.9552	-0.0662	0.8138	0.0895
Ppp1r12a	_T[Phospho (STY)]GSYGALAEISASK_.2	Q9DBR7_T443_M1_Ppp1r12a	5.8288	6.1270	6.0898	3.3096	5.1508	3.3501	5.8822	6.2858	6.0227	6.2169	0.8155	-0.2943	0.1358	0.8672
Ppp1r12a	_DKKS[Phospho (STY)]PLIESTANMENNQPQK_.3	#N/A	7.0645	4.1121	4.5903	8.4480	4.0004	4.1251	5.4420	4.3679	3.1662	6.0481	1.1333	0.1805	0.5765	0.2392
Ppp1r12a	_IS[Phospho (STY)]PKEEER_.2	Q9DBR7_S422_M1_Ppp1r12a	10.3804	10.5723	11.1759	3.4025	10.3899	10.4392	10.5499	10.6860	10.2328	10.4591	0.8961	-0.1582	0.4908	0.3091
Ppp1r12a	_STGVSFWTQDS[Phospho (STY)]DENEQER_.2	Q9DBR7_S861_M1_Ppp1r12a	3.1499	3.9551	3.8367	4.3394	4.4028	3.9531	4.3139	6.4535	3.4277	4.3063	0.8517	-0.2316	0.2542	0.5949
Ppp1r12a	_SPLIESTANM[Oxidation (M)]ENNQPQK_.2	#N/A	4.2697	5.0711	5.7755	6.0627	4.3074	5.0901	5.4880	4.6142	4.6691	4.0045	1.0857	0.1186	0.3933	0.4053
Ppp1r12a	_KTGS[Phospho (STY)]YGALAEISASK_.2	#N/A	3.6600	3.5662	4.3468	5.9634	2.7857	3.4430	3.0633	3.2690	4.4184	3.3156	1.1263	0.1716	0.4881	0.3115
Ppp1r12a	_STGVSFWTQDSDENEQERQS[Phospho (STY)]DTEDGSSKR_.4	Q9DBR7_S870_M1_Ppp1r12a	3.7695	5.0722	4.0501	7.1591	5.7157	3.4928	3.9200	6.6374	6.0378	5.9162	0.8665	-0.2067	0.4040	0.3936
Ppp1r12a	_RIS[Phospho (STY)]EMEEELK_.2	Q9DBR7_S994_M1_Ppp1r12a	3.7524	3.5900	3.4861	4.1383	3.4113	4.3182	3.1781	3.9713	4.2704	4.2080	0.9682	-0.0466	0.6616	0.1794
Tprg1l	_YDFISLQC[Carbamidomethyl (C)]QQVVR_.2	#N/A	5.4752	5.5170	4.1501	5.6335	5.1492	4.9218	5.5598	3.3074	4.7830	3.3711	1.2082	0.2728	0.1267	0.8972
Tprg1l	_DTVDSAGTSPT[Phospho (STY)]AVLAAGEDAGAGRPGAGTPLR_.3	Q9DBS2_T16_M1_Tprg1l	3.8425	3.0426	3.5762	4.6208	2.9863	3.6383	3.2682	3.8812	4.1802	4.2981	0.9260	-0.1109	0.4394	0.3572
Klc4	_VSS[Phospho (STY)]PTVNTTLK_.2	Q9DBS5_S460_M1_Klc4	8.5441	8.3067	8.8536	8.6228	6.8600	8.4482	3.1209	8.1695	8.3977	8.4860	1.1745	0.2320	0.2953	0.5297
Klc4	_RSS[Phospho (STY)]ELLVR_.2	Q9DBS5_S566_M1_Klc4	5.6552	3.5150	3.5611	4.6057	3.3363	5.1578	3.2531	3.8963	4.1954	5.3712	1.0302	0.0429	0.8410	0.0752
Klc4	_S[Phospho (STY)]SELLVR_.2	Q9DBS5_S565_M1_Klc4	3.0797	3.8360	4.3142	3.8580	3.7243	4.4012	4.0063	3.1432	3.4422	3.5353	1.0955	0.1315	0.2638	0.5788
Klc4	_AAS[Phospho (STY)]LNYLNQPNAAPLQVSR_.2	Q9DBS5_S590_M1_Klc4	3.9327	3.1881	3.6663	7.1761	3.0764	3.5482	3.4414	4.3918	4.0404	4.3882	1.0080	0.0115	0.9688	0.0138
Osbpl3	_ALVHQLS[Phospho (STY)]NESR_.2	Q9DBS9_S405_M1_Osbpl3	8.5159	8.1785	8.8150	3.4407	7.4069	8.0820	8.7034	8.1817	8.2985	8.2532	0.8860	-0.1746	0.3797	0.4206

Osbpl3	_QDS[Phospho (STY)]WEVVEGLR_.2	Q9DBS9_S33_M1_Osbpl3	3.5885	4.3937	4.2753	5.8871	3.4244	3.5144	4.4927	5.5017	5.1664	4.9436	0.8318	-0.2657	0.1317	0.8804
Ampd2	_S[Phospho (STY)]LPGNAPC[Carbamidomethyl (C)]LK_.2	Q9DBT5_S19_M1_Ampd2	9.3321	8.3218	7.3524	8.9342	3.4332	8.7050	6.0820	8.5774	9.2909	9.0692	0.9303	-0.1042	0.6602	0.1804
Ampd2	_QIS[Phospho (STY)]QDVKLEPDILLR_.3	#N/A	6.1094	6.7837	7.0457	7.4618	5.5571	6.2310	5.9416	6.8755	6.9061	6.0712	1.0128	0.0184	0.8443	0.0735
Ampd2	_S[Phospho (STY)]LAESELR_.2	Q9DBT5_S53_M1_Ampd2	2.9398	6.5104	3.6266	6.7415	3.5663	6.9144	3.8439	3.9892	7.0111	4.0962	1.0665	0.0928	0.7859	0.1047
Ampd2	_TDS[Phospho (STY)]DSDLQLYK_.2	Q9DBT5_S109_M1_Ampd2	4.3428	3.6471	3.5287	4.4736	4.7316	5.5763	6.6732	5.5987	5.7523	6.3517	0.7193	-0.4753	0.0042	2.3765
Ampd2	_SAPYEFPEES[Phospho (STY)]PIEQLEER_.2	Q9DBT5_S70_M1_Ampd2	4.2795	3.5839	3.4655	3.9682	3.4052	4.3243	3.1842	4.1503	3.7989	3.9351	1.0187	0.0268	0.7971	0.0985
Ampd2	_QIS[Phospho (STY)]QDVK_.2	#N/A	3.7311	3.0355	4.4179	3.4197	2.8568	3.3718	3.1344	3.1979	4.3473	3.3867	0.9874	-0.0183	0.9050	0.0434
Riok3	_AASFLKDDGS[Phospho (STY)]PPVLSAD_.2	Q9DBU3_S512_M1_Riok3	6.2873	6.2081	6.9835	6.9507	6.0972	6.5618	6.9542	6.2627	6.4908	6.3309	1.0008	0.0011	0.9829	0.0075
Pdcl	_NSATC[Carbamidomethyl (C)]HSEDS[Phospho (STY)]DLEID_.2	Q9DBX2_S296_M1_Pdcl	7.6784	9.2134	6.2300	9.7431	8.9190	9.2764	9.6896	9.1778	9.3321	9.4795	0.8327	-0.2642	0.2340	0.6308
Nvl	_ESLPLDLS[Phospho (STY)]DDQSNSK_.2	Q9DBY8_S190_M1_Nvl	3.8553	3.1108	3.5890	9.9343	8.7888	3.6255	3.5187	9.2017	9.3671	8.7308	0.7118	-0.4905	0.2772	0.5572
Nebl	_QSFTTVADTPENLR_.2	#N/A	5.9749	5.9714	6.6259	6.4561	6.8403	6.1703	6.3632	5.9043	6.0558	6.2486	1.0320	0.0455	0.3537	0.4514
Nebl	_GVQPHVVEMDR_.3	#N/A	3.0794	4.1467	3.7662	4.2689	4.0350	4.0904	3.9836	3.8496	3.4982	4.2359	1.0015	0.0022	0.9817	0.0080
Plxdc2	_RGS[Phospho (STY)]GHPAYAEVEPVGEK_.3	Q9DC11_S507_M1_Plxdc2	5.4809	4.2297	4.1113	4.6140	4.0510	3.6784	4.3287	3.5045	3.1531	3.0801	1.2401	0.3104	0.0631	1.2000
Dcaf6	_DGEQS[Phospho (STY)]PNVSLM[Oxidation (M)]QR_.2	#N/A	7.1415	7.4435	8.0047	6.7041	6.3899	7.2424	7.8242	7.2551	7.4344	7.3141	0.9594	-0.0598	0.3517	0.4538
Dcaf6	_DSALQDTDDS[Phospho (STY)]DDDPVLIPGAR_.2	Q9DC22_S673_M1_Dcaf6	4.0822	3.2602	3.8159	3.3597	3.2260	3.1471	3.5079	8.8491	3.9406	3.0370	0.7203	-0.4733	0.2482	0.6052
Csnk1d	_GAPVNVS[Phospho (STY)]SSDLTGR_.2	Q9DC28_S382_M1_Csnk1d	3.8621	3.3641	4.5489	3.5507	3.2524	3.3722	3.2654	3.0669	7.9064	3.5177	0.8241	-0.2791	0.4348	0.3617
Abcb6	_AIQASLAK_.2	#N/A	6.1958	6.6328	6.7005	5.3253	6.8947	5.7196	3.8921	6.2105	6.6516	6.6247	1.0685	0.0955	0.5294	0.2763
Hmg20a	_S[Phospho (STY)]PLTGYVR_.2	#N/A	7.5676	7.3212	6.4454	3.8812	7.2385	7.1053	6.9073	4.2373	3.8859	7.3210	1.1799	0.2387	0.3418	0.4662
Hmg20a	_DSNAPKS[Phospho (STY)]PLTGYVR_.3	#N/A	3.1959	4.0011	3.8827	4.3854	3.8224	3.9070	4.2678	3.7331	4.7320	4.7330	0.8853	-0.1757	0.1028	0.9879
Gnai3	_ISQTNYIPTQQDVLR_.2	#N/A	5.8892	5.4999	5.5511	5.7249	5.1342	4.8485	6.2911	5.3672	5.4967	6.0521	0.9379	-0.0925	0.2074	0.6832
Gnai3	_EYQLNDSASYYLNDLDR_.2	#N/A	3.8917	4.1344	3.1118	6.2384	4.0227	4.1028	6.5665	4.3456	6.3708	3.8337	0.8051	-0.3127	0.2162	0.6651
Ndufa9	_NFDFEDVFVNIPR_.2	#N/A	6.9701	6.8055	6.6180	6.1438	6.3974	6.4291	6.7179	6.3081	6.3939	6.8305	0.9997	-0.0004	0.9918	0.0036
Ndufa9	_QPVYVADVSK_.2	#N/A	6.7927	6.4640	6.8408	6.4482	6.4959	6.4543	6.8092	6.5150	6.3301	6.4411	1.0090	0.0129	0.6467	0.1893
Ndufa9	_M[Oxidation (M)]GSQVIIPYR_.2	#N/A	6.9157	6.7387	7.0097	5.3803	6.2288	6.4248	6.9633	7.6185	6.6695	6.5848	0.9268	-0.1097	0.1930	0.7143
Ndufa9	_SVFPEAIIIRPSDIFGR_.3	#N/A	5.9398	5.1919	5.4608	5.3247	4.5283	5.8912	5.2290	5.4529	6.3100	5.5050	0.9583	-0.0615	0.4892	0.3105
Ndufa9	_SSVSGVVATVFGATGFLGR_.2	#N/A	4.3951	3.6506	4.1288	3.6726	3.5389	2.3689	1.8089	2.2037	2.4863	3.0732	1.5152	0.5995	0.0176	1.7552
Ndufa9	_AVQHSNVVINLIGR_.3	#N/A	5.5400	5.5380	6.0802	5.2171	5.0480	5.3295	4.8652	5.3183	5.5136	5.0802	1.0509	0.0716	0.2521	0.5984
Ndufa9	_IHISDVM[Oxidation (M)]PTDLPGLEDLGVQPTPLELK_.3	#N/A	3.3235	3.6482	4.6610	4.3359	2.4633	4.1574	4.0827	4.2817	3.2883	3.2027	1.0137	0.0197	0.9146	0.0387
Ndufa9	_GIVNATKDPDAVGK_.2	#N/A	5.2926	5.3024	4.8399	4.8433	4.7525	4.8628	5.4171	5.1489	5.0060	4.8435	0.9762	-0.0348	0.4651	0.3324
Ndufa9	_LM[Oxidation (M)]GDLGQLTFLEWDAR_.2	#N/A	4.7717	2.9674	5.8049	3.3517	2.7887	4.8242	3.0664	4.2856	4.4154	4.1850	1.0242	0.0346	0.8887	0.0512
Ndufa9	_WLSSEIEETKPAK_.3	#N/A	3.0372	3.4379	3.9161	3.4769	3.3262	3.2984	3.1916	1.9042	4.2901	3.4439	1.0648	0.0906	0.6318	0.1994
Ndufa9	_C[Carbamidomethyl (C)]DVYDIMHLR_.3	#N/A	3.1315	3.8878	4.3660	3.9098	3.7761	4.3494	4.0580	3.0914	3.3904	3.5871	1.1052	0.1444	0.2229	0.6518
Ndufs7	_LDDLINWAR_.2	#N/A	7.6642	7.8231	7.5556	7.4537	7.4396	7.6921	7.9362	7.7822	7.5761	7.4822	0.9884	-0.0169	0.4436	0.3530
Ndufs7	_SRAEYVVTK_.2	#N/A	6.1838	6.3703	6.4230	6.0848	7.1548	5.7915	6.4111	6.7454	6.4438	6.0869	0.9864	-0.0197	0.7446	0.1281
Ndufs7	_KVYDQM[Oxidation (M)]PEPR_.2	#N/A	5.0762	5.0704	5.2328	4.4478	4.5098	3.4784	5.0742	4.0835	5.0229	4.7285	0.9807	-0.0282	0.8154	0.0887
Ndufs7	_QADVM[Oxidation (M)]IVAGTLTNK_.2	#N/A	5.3675	5.9974	7.0525	6.0885	6.0724	6.4169	5.5479	5.9626	5.6828	6.5456	1.0389	0.0551	0.5061	0.2958
Hmgn3	_TES[Phospho (STY)]IEKEGE_.2	#N/A	3.8137	3.0958	3.3008	3.8034	2.9477	3.5156	3.5181	3.6867	3.9852	3.7704	0.9125	-0.1321	0.1401	0.8537
Arpp21	_AWSS[Phospho (STY)]TDSDSSNR_.2	Q9DCB4_S362_M1_Arpp21	7.4965	7.2705	7.8708	8.0112	6.9481	7.6306	7.5488	7.2423	7.4242	3.6975	1.1636	0.2186	0.2013	0.6962
Arpp21	_TGS[Phospho (STY)]ESSSSAGSSGSLSR_.2	Q9DCB4_S408_M1_Arpp21	6.4658	6.5316	6.6820	7.0895	3.2535	6.5995	6.5475	6.6415	6.5820	6.6727	0.9233	-0.1152	0.5020	0.2993
Arpp21	_SES[Phospho (STY)]LDEEEKLELQRR_.3	#N/A	3.6519	2.9563	4.3387	3.6636	4.2784	3.4511	3.8119	7.3976	3.6366	3.3075	0.8204	-0.2856	0.3400	0.4686
Arpp21	_SES[Phospho (STY)]LDEEEKLELQR_.2	#N/A	3.6783	4.0247	7.8198	9.1961	3.4382	7.9193	7.7324	8.5795	8.4951	7.8920	0.7355	-0.4432	0.1409	0.8512
Arpp21	_QSS[Phospho (STY)]SETELRWPDHQR_.3	Q9DCB4_S346_M1_Arpp21	3.3090	4.1142	3.9958	4.0065	3.9355	3.7939	4.1547	2.9947	3.2938	2.9646	1.1513	0.2033	0.0915	1.0386
Arpp21	_TAS[Phospho (STY)]FGGITVLTR_.2	Q9DCB4_S381_M1_Arpp21	3.5881	3.6381	4.2749	7.4972	4.2146	3.0983	4.4923	3.3409	2.9895	3.2437	1.2470	0.3185	0.3374	0.4719
Arpp21	_DNS[Phospho (STY)]S[Phospho (STY)]IDKEDNQNR_.2	Q9DCB4_S256_M2_Arpp21	3.3649	4.1701	4.0517	4.5543	3.9914	3.7381	4.0989	3.5642	3.2127	3.0205	1.1452	0.1956	0.1069	0.9711
Tomm20	_IVS[Phospho (STY)]AQSLAEDDVE_.2	Q9DCC8_S135_M1_Tomm20	5.7151	7.0542	4.5651	7.9354	5.0758	5.3889	7.8816	7.5726	7.7108	7.7665	0.7702	-0.3767	0.0267	1.5736
Pgd	_LVPLLDTGDIIIDGGNSEYR_.2	#N/A	5.4586	5.0242	5.4190	5.0566	4.3631	5.3047	4.5552	5.7307	3.7031	3.2744	1.1827	0.2421	0.1359	0.8669
Pgd	_VDDFLANEAK_.2	#N/A	5.3143	3.5400	5.3156	4.5806	5.1433	4.3681	5.6898	5.5915	5.3277	5.6850	0.8451	-0.2428	0.0440	1.3565
Pgd	_NPELQNLLLDDFFK_.2	#N/A	3.5809	3.5717	3.3146	4.3592	2.0600	3.9000	3.7931	3.6423	4.4419	3.0176	0.9303	-0.1042	0.5871	0.2313
Pgd	_GILFVGSGVSGGEEGAR_.2	#N/A	4.2727	3.8426	3.6369	3.1806	3.9277	2.9681	3.3289	3.2105	4.1196	3.8663	1.0019	0.0027	0.9826	0.0076
Tjap1	_KDS[Phospho (STY)]LTQAQEQGTVLS_.2	Q9DCD5_S527_M1_Tjap1	3.9411	5.9579	3.1270	6.0869	3.0668	5.6766	6.5874	2.9879	3.9258	6.1983	0.9427	-0.0851	0.7855	0.1049
Ssr3	_KLS[Phospho (STY)]EADNR_.2	#N/A	6.1591	6.1268	6.8681	6.0057	6.2282	5.7147	6.6028	6.7024	6.4380	3.5520	1.0618	0.0865	0.5840	0.2336
Ssr3	_KLS[Phospho (STY)]EADNRK_.2	#N/A	4.2346	4.4412	4.8695	4.7858	2.8226	1.6652	4.9873	4.3805	4.7487	3.2419	0.8764	-0.1904	0.4790	0.3196
Ssr3	_QQS[Phospho (STY)]EEDLLLQDFSR_.2	Q9DCF9_S11_M1_Ssr3	4.3650	7.5209	3.5510	7.9473	7.1654	4.2388	3.0987	4.0507	8.3491	4.1286	1.1816	0.2408	0.5303	0.2755
Eif3f	_TC[Carbamidomethyl (C)]FS[Phospho (STY)]PNR_.2	Q9DCH4_S262_M1_Eif3f	9.7065	9.6909	9.7638	10.3713	9.6402	9.6784	9.8502	9.4556	9.5250	9.7738	1.0163	0.0233	0.3567	0.4477
Eif3f	_VIGLSSDLQQVGGASAR_.2	#N/A	4.3485	3.6039	4.0821	3.6259	3.4922	3.1324	4.5264	3.3753	4.7941	3.3032	0.9286	-0.1069	0.4705	0.3275
Ndufa8	_ARPEPNPVIEGDLKPAK_.3	#N/A	9.3881	9.2431	9.7461	9.5663	9.7491	9.4501	9.5143	9.1823	9.2004	9.2558	1.0254	0.0361	0.0849	1.0712
Ndufa8	_TDRPLPENPYHSR_.3	#N/A	7.5319	7.2108	6.8168	7.4941	8.2526	7.7407	6.9749	7.1477	7.5703	7.3800	1.0330	0.0468	0.3975	0.4006
Ndufa8	_FDQC[Carbamidomethyl (C)]VLDK_.2	#N/A	6.5958	6.6953	6.2532	6.7274	7.0633	6.2242	6.2649	6.5575	5.7908	6.6650	1.0433	0.0612	0.2558	0.5921
Ndufa8	_PGIVELPTLEELKVEEVK_.3	#N/A	5.8144	5.8830	5.3786	6.2983	6.1384	5.9197	5.1892	5.9697	5.5248	5.9462	1.0438	0.0619	0.2884	0.5400
Ndufa8	_LVNGC[Carbamidomethyl (C)]ALNFFR_.2	#N/A	5.2210	5.4247	4.9312	5.6576	5.0694	5.1270	3.1870	5.7941	4.9702	5.5878	1.0724	0.1009	0.4885	0.3111
Ndufa8	_AAAHHYGAQC[Carbamidomethyl (C)]DK_.3	#N/A	3.3892	4.1944	4.0760	4.5786	4.0157	3.7138	4.2933	3.5398	3.1884	3.0448	1.1359	0.1839	0.1538	0.8130
Ppp1r2	_TSAAS[Phospho (STY)]PPVVPSAEQPRPIVEEELSKK_.4	#N/A	4.3268	3.6312	9.2773	4.4895	7.8315	4.2770	3.1370	8.3261	4.3115	4.1668	1.1311	0.1777	0.6727	0.1722
Ppp1r2	_TSAAS[Phospho (STY)]PPVVPSAEQPRPIVEEELSK_.3	#N/A	7.1969	7.5567	3.9655	8.3292	6.9438	7.3347	7.6917	7.3518	7.3830	4.4351	1.0257	0.0366	0.8645	0.0633
Ppp1r2	_EQESS[Phospho (STY)]GEEDNDLSPEER_.2	#N/A	7.3489	10.1868	10.3507	11.4736	10.3988	10.1598	10.2478	10.1945	9.1461	9.7660	1.0150	0.0215	0.8452	0.0730
Ppp1r2	_TREQES[Phospho (STY)]S[Phospho (STY)]GEEDNDLSPEER_.2	#N/A	8.2244	8.6821	9.0334	9.4272	9.1511	8.7998	8.5057	8.6788	9.0295	8.4863	1.0244	0.0347	0.3952	0.4032
Paics	_EIVLADVIDNDSWR_.2	#N/A	4.6664	5.5716	5.3546	5.7049	5.2174	5.4836	6.0204	4.8147	5.7289	4.6627	1.0050	0.0072	0.9371	0.0282
Paics	_DDANNDPQWSEEQLIAAK_.2	#N/A	4.6797	4.1666	3.9424	5.4239	3.5850	3.7978	5.2110	4.3527	4.5658	4.6404	0.9091	-0.1375	0.2892	0.5387
Slc38a3	_S[Phospho (STY)]PSKEPHFTDFEGK_.3	#N/A	7.7934	6.0470	7.8113	7.5579	5.7395	7.3798	7.8181	8.2124	7.8065	5.9843	0.9463	-0.0797	0.5318	0.2742
Slc38a3	_HLEGLLPVGVPTTDTQR_.3	#N/A	6.4542	6.5756	6.2533	5.9784	5.3331	6.0370	6.7429	6.4967	5.9456	6.1942	0.9622	-0.0555	0.3919	0.4069
Slc38a3	_AYEQLGYR_.2	#N/A	4.9236	4.6793	4.7724	4.6670	3.0322	4.7296	4.8604	5.5580	4.1719	3.5622	0.9844	-0.0227	0.8903	0.0505
Ap3s1	_FYQPY[Phospho (STY)]SEDTQQQIIR_.2	Q9DCR2_Y23_M1_Ap3s1	4.4966	4.1332	3.9462	5.0815	4.2528	4.7315	4.4660	5.2175	5.3576	4.8924	0.8910	-0.1665	0.0742	1.1298
Mettl26	_IS[Phospho (STY)]PQSNVDFDLTLR_.2	Q9DCS2_S149_M1_Mettl26	8.4472	8.5009	9.1567	9.4698	8.4910	8.4408	8.9037	8.9508	8.9493	8.4803	0.9921	-0.0115	0.7828	0.1064
Mettl26	_DTVLLEELGQASGLVLER_.2	#N/A	3.9408	3.1410	3.5766	4.2335	3.0016	3.9008	3.7939	3.0594	3.6947	3.4698	1.0365	0.0517	0.6584	0.1815
Ndufb10	_PDSWDKDVYPEPPSR_.3	#N/A	9.0334	8.9007	9.2502	9.1941	9.3697	9.1789	8.9587	8.8784	8.9502	9.0226	1.0226	0.0322	0.0499	1.3020
Ndufb10	_YLDLGAYYSAR_.2	#N/A	6.9608	6.9613	6.6665	7.0670	7.1678	6.5553	6.9585	6.7148	7.1315	6.7546	1.0010	0.0014	0.9643	0.0158
Ndufb10	_AYDLVVDWPVTLVR_.2	#N/A	6.8683	6.4389	6.5574	6.4635	7.0138	6.4229	6.4446	6.4519	6.8722	6.4742	1.0102	0.0146	0.6731	0.1719
Ndufb10	_RVPDITEC[Carbamidomethyl (C)]K_.2	#N/A	5.8432	5.7012	4.8402	5.9021	6.5139	6.1080	6.0849	5.9605	5.2941	5.4788	1.0199	0.0284	0.7325	0.1352
Ndufb10	_EGDVLC[Carbamidomethyl (C)]IYEAEM[Oxidation (M)]QWR_.2	#N/A	3.6337	4.5142	5.5867	5.0579	4.0207	5.2312	4.7792	3.9020	4.7239	3.4019	1.1124	0.1537	0.3400	0.4685
Ndufb10	_DFKVDQEIM[Oxidation (M)]NIIQER_.3	#N/A	6.1937	5.8426	6.8110	5.6633	5.9771	6.5478	5.4857	5.3072	6.0405	6.4204	1.0618	0.0865	0.2669	0.5736

Ndufb10	_VDQEIMNIIQER_.2	#N/A	5.4701	5.1294	3.5387	6.9015	5.5576	4.2510	3.1110	4.0385	4.5310	4.8713	1.2425	0.3133	0.1699	0.7698
Ndufs3	_SLADLTAVDVPTR_.2	#N/A	8.6025	8.7503	8.8201	8.8745	8.7039	8.8059	8.6260	8.5671	8.5313	8.6189	1.0202	0.0289	0.0107	1.9702
Ndufs3	_VVAEPVELAQEFR_.2	#N/A	8.3186	8.3075	8.6453	8.1221	8.5565	8.1941	8.5515	8.3393	8.5055	8.3693	0.9900	-0.0144	0.4740	0.3242
Ndufs3	_DFPLTGYVELR_.2	#N/A	7.7732	8.0553	7.8531	7.9651	8.0865	7.8390	7.7322	7.8372	8.1492	7.6674	1.0105	0.0150	0.4616	0.3358
Ndufs3	_YDDEVKR_.2	#N/A	7.2504	6.9732	7.4791	7.4374	7.6648	7.2437	6.5699	6.6138	7.4368	7.3049	1.0516	0.0726	0.1354	0.8685
Ndufs3	_DHTNAQFK_.2	#N/A	5.6230	5.0738	5.7407	5.0530	6.0433	5.3095	5.6801	5.5566	5.8887	5.3625	0.9737	-0.0385	0.5195	0.2844
Ndufs3	_FEIVYNLLSLR_.2	#N/A	5.6838	4.8154	5.1243	4.8089	3.9818	4.2487	5.2561	5.0622	4.8483	5.4139	0.9285	-0.1071	0.2912	0.5357
Ndufs3	_LEAGDKKPETK_.2	#N/A	5.3164	5.2072	5.6266	4.8172	5.2369	5.1918	5.0750	5.6085	4.9206	5.5218	0.9908	-0.0134	0.8012	0.0963
Ndufs3	_QLSAFGEYVAEILPK_.2	#N/A	4.4561	4.6928	4.4056	4.6812	5.4577	4.7952	5.1822	4.4279	5.1827	4.5742	0.9807	-0.0282	0.7166	0.1447
Ndufs3	_ILTDYGFEGHPFR_.2	#N/A	5.7025	5.4168	5.4572	6.1257	6.1898	5.8381	5.7265	5.8408	5.8811	5.9129	0.9911	-0.0129	0.7671	0.1152
Ndufs3	_FDLNSPWEAFPAYR_.2	#N/A	5.1556	4.4272	3.3492	3.1522	4.6521	4.8651	4.6795	5.1751	5.7170	4.5845	0.8798	-0.1847	0.2855	0.5443
Bap18	_VYEDSGIPLPAES[Phospho (STY)]PK_.2	Q9DCT6_S96_M1_Bap18	8.8816	8.3352	8.8393	9.1557	8.7020	9.0085	9.2800	8.5846	8.7545	8.9790	0.9911	-0.0129	0.6836	0.1652
Crip2	_GVNTGAVGSYIYDKDPEGTVQP_.2	#N/A	6.4879	6.4494	6.6779	6.5042	6.8940	6.9433	7.0063	5.9846	6.2670	6.7238	1.0252	0.0360	0.4607	0.3366
Crip2	_KTS[Phospho (STY)]GPPKGPSK_.2	Q9DCT8_S104_M1_Crip2	3.7778	6.2365	6.0551	6.4848	6.2326	3.3251	5.9337	6.0194	5.9749	6.4416	0.8785	-0.1869	0.3369	0.4725
Crip2	_TVYFAEK_.2	#N/A	2.8122	3.6849	3.4265	3.2200	3.9136	4.4519	3.6812	3.0113	3.2660	3.6468	1.0540	0.0758	0.5789	0.2374
Rmdn1	_VEEILAQADYLYESGETEK_.2	#N/A	5.1756	5.8569	6.0586	6.5806	4.8646	5.3273	5.1815	4.7510	4.8396	5.3759	1.1205	0.1641	0.1161	0.9352
Etfb	_EIDGGLETLR_.2	#N/A	7.0728	6.9912	7.1796	7.0240	7.2323	6.8023	6.8234	7.0286	7.3440	7.2259	0.9922	-0.0112	0.6564	0.1829
Etfb	_GIHVEIPGAQAESLGPLQVAR_.3	#N/A	6.2650	6.8296	6.4689	6.9819	6.1114	6.3085	6.4961	6.9609	5.1019	5.7431	1.0689	0.0962	0.2884	0.5400
Etfb	_EIIAVSC[Carbamidomethyl (C)]GPSQC[Carbamidomethyl (C)]QETIR_.2	#N/A	4.9948	5.2540	5.4334	5.4213	5.3228	5.7709	4.7867	5.1594	4.8228	4.0861	1.1384	0.1870	0.0182	1.7405
Etfb	_AGDLGVDLTSK_.2	#N/A	5.6126	5.5530	4.4674	6.1837	5.8589	5.8251	4.1412	5.7257	3.2891	5.3671	1.2057	0.2699	0.1136	0.9447
Etfb	_TALAMGADR_.2	#N/A	4.5948	0.9321	3.4407	4.9721	4.0796	4.3491	3.2091	2.6058	4.2394	3.9103	1.0679	0.0947	0.7749	0.1108
Atp5pd	_SC[Carbamidomethyl (C)]AEFVSGSQLR_.2	#N/A	7.7806	8.0564	8.1092	8.7206	9.0469	7.9604	7.9240	8.0737	8.1393	7.9050	1.0335	0.0476	0.3237	0.4898
Atp5pd	_LASLSEKPPAIDWAYYR_.3	#N/A	7.3673	7.4947	7.1249	7.8018	7.7400	7.5848	7.2471	7.4311	7.5653	7.5151	1.0107	0.0153	0.5839	0.2336
Atp5pd	_ANVAKPGLVDDFEK_.2	#N/A	7.3207	7.2875	7.3793	7.6825	7.8372	7.4408	7.3998	7.2138	7.0679	7.1983	1.0376	0.0532	0.0610	1.2149
Atp5pd	_ANVAKPGLVDDFEKK_.3	#N/A	8.1919	8.3362	8.5687	8.2992	8.6303	8.3612	8.0863	8.2372	7.9666	8.1724	1.0348	0.0493	0.0198	1.7033
Atp5pd	_SWNETFHAR_.2	#N/A	7.5587	7.9520	7.7735	8.3577	8.4682	8.1780	7.6811	7.9563	7.7713	8.0684	1.0227	0.0324	0.3784	0.4220
Atp5pd	_IPVPEDKYTALVDQEEKEDVK_.3	#N/A	7.7135	7.7575	8.6414	7.3492	8.1348	8.2037	8.3264	7.0710	7.5287	8.1341	1.0260	0.0370	0.5513	0.2586
Atp5pd	_YTALVDQEEKEDVK_.2	#N/A	4.7145	5.0856	4.1837	4.6580	5.3154	3.9457	4.4993	4.4319	4.3825	4.7135	1.0319	0.0453	0.6109	0.2140
Atp5pd	_TIDWVSFVEVM[Oxidation (M)]PQNQK_.2	#N/A	7.4095	7.5289	8.0901	7.7950	8.0605	8.7464	7.8497	7.4866	7.7723	7.9513	1.0223	0.0319	0.5204	0.2837
Atp5pd	_NIIPFDQM[Oxidation (M)]TIDDLNEIFPETK_.3	#N/A	4.5180	5.6339	4.3980	6.1539	5.0636	6.1831	5.2473	5.4626	5.0477	5.0378	1.0243	0.0346	0.7657	0.1159
Atp5pd	_PGLVDDFEK_.2	#N/A	4.5137	4.0925	3.8205	3.7542	5.0822	4.1608	4.8954	4.0457	4.6644	4.2901	0.9471	-0.0784	0.4437	0.3529
Apoo	_IDELSLYSVPEGQSK_.2	#N/A	8.3601	8.3325	8.6696	8.5428	8.3521	8.6113	8.5317	8.2574	8.4143	8.2627	1.0133	0.0191	0.2591	0.5865
Apoo	_LYDWGLR_.2	#N/A	7.1347	7.3382	7.3882	6.7371	7.0292	6.8178	6.8134	7.1535	7.3576	6.8499	1.0043	0.0063	0.8615	0.0647
Apoo	_TQLEENISQLR_.2	#N/A	6.8034	6.9613	7.2739	6.8237	6.9999	6.7912	6.6166	6.8594	6.8048	6.6566	1.0309	0.0439	0.0823	1.0848
Apoo	_GYIVIEDLWK_.2	#N/A	5.3114	5.7577	3.9850	5.8005	5.5896	5.5500	4.6962	5.7108	5.9860	5.9021	0.9567	-0.0639	0.5817	0.2353
Dtd1	_SASS[Phospho (STY)]GAEGDVSSER_.2	#N/A	7.0199	6.4869	6.2827	7.1867	6.4437	6.7263	6.6608	6.7029	6.9645	3.2595	1.1347	0.1823	0.3021	0.5198
Dtd1	_SASS[Phospho (STY)]GAEGDVSSEREP_.2	#N/A	10.1635	10.4151	10.8406	10.8490	10.0591	10.4905	10.5967	10.3379	10.2872	10.3276	1.0079	0.0114	0.6558	0.1832
Dtd1	_ASVTVGGEQISAIGR_.2	#N/A	5.0846	4.0160	3.8969	4.3996	4.7980	4.8154	4.9411	3.7189	3.3675	4.3666	1.0984	0.1354	0.3049	0.5158
Dtd1	_AKGPSESS[Phospho (STY)]KER_.2	Q9DD18_S182_M1_Dtd1	2.9412	3.6975	4.1757	3.7195	3.5857	3.0389	3.8677	3.2817	3.5808	3.3967	0.9985	-0.0022	0.9839	0.0070
Tsc1	_GGFDS[Phospho (STY)]PFYR_.2	Q9EP53_S502_M1_Tsc1	3.8470	6.1637	4.5338	6.7603	3.2675	3.3572	3.2503	3.0820	6.4929	6.6714	0.9550	-0.0664	0.8452	0.0731
Wdr4	_SPFPGS[Phospho (STY)]PEQTKK_.2	Q9EP82_S397_M1_Wdr4	7.8058	3.7180	8.2966	8.2900	3.6063	3.0183	4.4123	3.4208	7.7574	3.1638	1.2347	0.3042	0.5007	0.3004
Senp3	_NHLS[Phospho (STY)]PQEGGATPQVPSPC[Carbamidomethyl (C)]C[Carbamidomethyl (C)]R_.3	Q9EP97_S163_M1_Senp3	3.4907	4.2469	3.2244	4.2690	4.1352	3.9902	6.7453	4.2331	3.0313	6.0437	0.7765	-0.3650	0.1506	0.8222
Senp3	_GS[Phospho (STY)]PPVPSGPPMEEDGLR_.2	Q9EP97_S206_M1_Senp3	4.3237	3.5791	4.0573	3.3357	3.4674	3.1572	4.5512	3.2819	4.4314	3.3027	0.9387	-0.0912	0.5229	0.2816
Pycard	_EIHPYLVM[Oxidation (M)]DLEQS[Phospho (STY)]_.2	#N/A	6.9914	7.3900	7.8691	7.9827	8.4839	7.9817	7.5866	7.7757	7.9265	7.6598	1.0059	0.0085	0.8702	0.0604
Parva	_[Acetyl (Protein N-term)]ATS[Phospho (STY)]PQKSPLVPK_.2	Q9EPC1_S4_M1_Parva	6.4995	6.1898	5.4989	5.4053	3.3821	5.3038	6.9899	4.9785	5.0858	5.8644	0.9390	-0.0909	0.6135	0.2122
Parva	_SPTPKS[Phospho (STY)]PPSR_.2	Q9EPC1_S19_M1_Parva	7.9594	7.8691	8.8899	3.3044	3.7901	7.6886	8.0674	3.5137	7.9811	7.9287	0.9579	-0.0620	0.8528	0.0692
Atp13a1	_DS[Phospho (STY)]PVLSNSGPR_.2	Q9EPE9_S896_M1_Atp13a1	7.9169	2.9965	3.4748	3.9183	2.8848	7.2254	8.1639	3.9826	7.1338	7.5026	0.7073	-0.4995	0.1869	0.7284
Arfgap1	_S[Phospho (STY)]PSSDSWTC[Carbamidomethyl (C)]ADASTGR_.2	#N/A	9.8322	9.7436	9.7639	7.5250	8.7906	9.6265	9.6328	9.3122	9.5659	7.2030	1.0319	0.0454	0.6740	0.1713
Arfgap1	_AAPSTADEGWDNQNW_.2	#N/A	6.9393	6.9964	7.5480	7.7365	7.3794	7.5176	7.8976	7.1047	7.0908	7.6582	0.9886	-0.0166	0.7197	0.1428
Arfgap1	_TLQFTAHR_.2	#N/A	6.1310	6.9626	6.5811	7.0786	7.3046	6.9775	6.0415	6.8556	7.0999	6.8817	1.0178	0.0254	0.6827	0.1658
Arfgap1	_NSNSDGWESWEGASGEGR_.2	#N/A	4.5910	7.7934	4.5127	7.9800	6.9221	7.1501	5.3230	7.1520	7.1258	6.9383	0.9784	-0.0315	0.8726	0.0592
Arfgap1	_KAAPSTADEGWDNQNW_.2	#N/A	6.9967	6.7768	6.9730	7.0332	6.1171	7.1087	7.7954	7.0611	7.1645	7.0653	0.9399	-0.0895	0.0985	1.0065
Arfgap1	_AKS[Phospho (STY)]PSSDSWTC[Carbamidomethyl (C)]ADASTGR_.3	#N/A	6.9541	6.6992	3.4835	6.0396	3.7602	5.5318	3.6680	6.3980	6.7044	7.2680	0.8095	-0.3048	0.1373	0.8624
Arfgap1	_SS[Phospho (STY)]DSWDVWGSGSASNNK_.2	#N/A	3.2355	3.9917	4.0723	10.9685	10.8923	4.2570	10.1315	10.0975	3.3399	3.5605	0.9195	-0.1211	0.8261	0.0830
Arfgap1	_RS[Phospho (STY)]SDSWDVWGSGSASNNK_.3	#N/A	3.5652	3.7079	3.6950	3.9595	3.5715	3.9907	4.0029	4.0491	3.5854	3.7210	0.9762	-0.0347	0.5008	0.3003
Arfgap1	_AFEDWLNDDLGSYQGAQENR_.2	#N/A	6.2969	5.6232	5.6396	6.8552	5.7549	5.5581	6.1647	5.8395	6.1049	6.5057	0.9677	-0.0474	0.5047	0.2970
Arfgap1	_DVTTFFSGK_.2	#N/A	4.3237	3.3883	4.9675	4.6019	4.7762	3.3483	3.7091	3.8015	4.7615	4.7437	0.9954	-0.0067	0.9643	0.0158
Arfgap1	_AKS[Phospho (STY)]PSSDS[Phospho (STY)]WTC[Carbamidomethyl (C)]ADASTGRR_.3	#N/A	3.7431	3.9933	3.5263	4.0289	3.4660	4.1419	3.5362	4.0929	3.7415	3.9959	0.9935	-0.0094	0.8894	0.0509
Arfgap1	_GLGVHLSFVR_.2	#N/A	5.6242	5.1922	4.3129	4.1600	5.7887	5.7468	4.5929	5.1499	5.6417	4.1396	1.0525	0.0739	0.5875	0.2310
Arfgap1	_S[Phospho (STY)]PSSDSWTC[Carbamidomethyl (C)]ADASTGRR_.2	#N/A	3.3932	6.0816	4.1415	6.6416	3.4981	5.5139	5.9926	5.7514	6.2828	3.6733	0.8992	-0.1532	0.5378	0.2694
Arfgap1	_FGSQASQK_.2	#N/A	3.5647	3.6615	4.2515	3.6555	4.4114	3.5383	5.0624	4.4714	4.3599	4.4664	0.8381	-0.2547	0.0126	1.9003
Arfgap1	_EWSLESSPAQNWTPPQPK_.2	#N/A	5.4471	5.7597	5.3052	5.8974	5.7399	5.8485	5.8325	5.2545	5.3539	5.3588	1.0397	0.0562	0.2089	0.6801
Ipo7	_ETENDDLTNVIQK_.2	#N/A	5.6390	5.1843	5.1249	3.5709	3.7444	5.2419	5.0513	5.6442	4.9116	4.3643	0.9515	-0.0717	0.6341	0.1979
Nbea	_RQFS[Phospho (STY)]PGPR_.2	#N/A	10.9270	10.7799	11.3828	10.6090	9.5373	10.4296	11.2666	11.2370	10.9138	10.5174	0.9661	-0.0498	0.3105	0.5079
Nbea	_TPLENVPGNLS[Phospho (STY)]PIKDPDR_.2	#N/A	8.4956	8.8828	9.4663	9.6237	7.2221	8.4912	9.1497	9.6505	8.7765	8.5875	0.9619	-0.0560	0.4928	0.3073
Nbea	_QFS[Phospho (STY)]PGPR_.2	#N/A	9.6139	9.4778	9.9731	9.1953	7.8852	9.3414	9.7872	9.6337	9.3884	9.3446	0.9695	-0.0446	0.4615	0.3358
Nbea	_AVPNVDAGSIIS[Phospho (STY)]DTERSDDGKESGK_.3	Q9EPN1_S1252_M1_Nbea	3.2798	6.2086	4.3029	3.6834	8.0404	8.4496	8.8027	10.4357	9.0526	5.8413	0.6634	-0.5921	0.0704	1.1527
Nbea	_EIEDLSQSQS[Phospho (STY)]PES[Phospho (STY)]ETDYPVSTDTR_.3	Q9EPN1_S1001_M2_Nbea	3.7031	3.4980	4.0604	3.8094	3.3607	3.5803	3.8105	3.5452	3.9526	3.7126	0.9769	-0.0337	0.5623	0.2500
Nbea	_SQAIVNQNS[Phospho (STY)]ETELMLEGDDDAVSLLQEK_.3	Q9EPN1_S2114_M1_Nbea	3.3299	4.0554	3.8778	4.6978	3.6556	3.8956	4.2564	3.7502	3.2350	3.7854	1.0431	0.0609	0.5863	0.2318
Tcf20	_AGSS[Phospho (STY)]PTQGAQNEAPR_.2	Q9EPQ8_S588_M1_Tcf20	8.0919	8.2109	8.3566	8.7798	7.6124	8.0635	8.6237	8.2413	7.7470	8.1727	0.9987	-0.0018	0.9670	0.0146
Tcf20	_LSTS[Phospho (STY)]PATR_.2	Q9EPQ8_S603_M1_Tcf20	4.1947	3.4501	3.9284	3.4722	3.3384	3.2862	3.6204	5.6984	3.8281	3.1494	0.8865	-0.1738	0.3637	0.4393
Tcf20	_GSQEDDPAASQRPPSNS[Phospho (STY)]GVK_.3	Q9EPQ8_S671_M1_Tcf20	3.7242	4.2145	3.7360	3.7487	4.0680	3.8797	4.3477	3.9626	4.0949	3.5296	0.9778	-0.0324	0.6181	0.2089
Tcf20	_LSTS[Phospho (STY)]PATRDEAAS[Phospho (STY)]PGAK_.3	Q9EPQ8_S603_M2_Tcf20	4.2783	3.5827	3.4643	3.9669	3.4040	4.3255	3.6816	4.1515	3.8001	3.9339	0.9859	-0.0205	0.8127	0.0901
Slc23a2	_SSLAETLDSTGS[Phospho (STY)]LDPQR_.2	Q9EPR4_S81_M1_Slc23a2	7.7918	8.2483	8.9670	10.0759	8.6138	7.9143	8.6936	8.4406	8.9401	8.0180	1.0092	0.0133	0.8677	0.0617
Inpp4a	_AS[Phospho (STY)]PTSTEEEQVMLR_.2	Q9EPW0_S488_M1_Inpp4a	2.9468	3.7520	3.6336	4.1363	3.5734	4.1561	3.8510	3.9822	3.6308	4.1033	0.9506	-0.0730	0.4477	0.3490
Aldh6a1	_VNAGDQPGADLGPLITPQAK_.2	#N/A	7.2340	6.8072	7.2366	7.4912	7.5693	7.4564	6.9274	7.0746	7.1554	6.9185	1.0399	0.0565	0.0965	1.0157
Aldh6a1	_TLADAEGDVFR_.2	#N/A	6.8176	6.7554	7.3960	6.4505	6.8141	7.2529	6.9709	6.7887	6.5829	6.3969	1.0343	0.0487	0.2912	0.5358
Aldh6a1	_EGASILLDGR_.2	#N/A	5.5515	2.2061	3.9616	4.8885	4.7238	5.0003	5.4363	5.6400	4.9277	5.2722	0.8251	-0.2774	0.1699	0.7698
Aldh6a1	_AISFVGSNQAGEYIFER_.2	#N/A	5.1330	5.3094	5.8447	4.4857	4.5938	5.0905	4.5975	3.6328	4.3536	5.1187	1.1470	0.1979	0.1014	0.9938

Aldh6a1	_DMDLYSYR_.2	#N/A	2.9420	3.7472	3.3289	4.3735	3.5685	4.1610	4.5218	4.1285	4.4276	4.0508	0.8610	-0.2160	0.0696	1.1572
Pold3	_VDLS[Phospho (STY)]DEEAKETEHLK_.3	Q9EQ28_S306_M1_Pold3	4.3380	5.5405	3.5239	5.9265	5.5655	4.2658	3.7413	4.0919	5.6186	3.9936	1.1143	0.1562	0.4225	0.3742
Dpysl5	_IPHGVSGVQDR_.2	#N/A	7.0064	7.3211	7.1328	7.5086	7.6994	7.0143	7.3725	7.1808	7.0854	7.1151	1.0128	0.0183	0.5633	0.2492
Dpysl5	_ILNLYPR_.2	#N/A	6.6473	7.1499	7.2338	6.5918	6.8214	6.0002	6.9722	7.1456	6.7664	6.8505	0.9722	-0.0407	0.4390	0.3575
Dpysl5	_VVNDDC[Carbamidomethyl (C)]THEADVYIESGIIQQVGR_.3	#N/A	6.2215	6.7977	7.2725	7.2538	6.5153	6.6579	6.9770	6.1462	6.5642	6.6862	1.0293	0.0416	0.4659	0.3317
Dpysl5	_FVAVTSSNAAK_.2	#N/A	6.0152	6.6263	6.2011	6.7197	6.7975	6.5432	6.6086	6.5171	6.4152	6.4168	0.9991	-0.0013	0.9731	0.0118
Dpysl5	_AEM[Oxidation (M)]ETLVR_.2	#N/A	5.7007	5.8755	5.4015	4.9022	6.5617	6.0822	5.2188	5.8131	6.0622	5.9498	0.9988	-0.0018	0.9834	0.0073
Dpysl5	_C[Carbamidomethyl (C)]HGVPLVTISR_.2	#N/A	5.5993	6.1652	4.3829	6.4719	5.8978	6.0308	5.3374	6.0868	5.4069	5.4209	1.0350	0.0497	0.6382	0.1951
Dpysl5	_EM[Oxidation (M)]GT[Phospho (STY)]PLADT[Phospho (STY)]PTRPVTR_.2	#N/A	11.3656	11.8271	12.6257	12.0117	11.3556	12.0391	12.1443	11.8293	11.9270	11.9415	0.9925	-0.0109	0.7282	0.1378
Dpysl5	_KEM[Oxidation (M)]GT[Phospho (STY)]PLADT[Phospho (STY)]PTRPVTR_.3	#N/A	6.5678	6.6747	3.9457	6.7121	5.6160	6.9198	6.8077	6.2226	6.6955	6.5841	0.9233	-0.1152	0.4163	0.3805
Dpysl5	_DLHESSFSLSGS[Phospho (STY)]QIDDHVPKR_.4	#N/A	3.7068	7.1054	8.1182	8.1720	3.7559	6.7156	4.2949	6.8988	5.2544	6.5058	1.0913	0.1260	0.6589	0.1812
Dpysl5	_ELM[Oxidation (M)]IPGGAK_.2	#N/A	6.3736	6.5663	6.5030	6.1393	6.8052	6.1524	6.0158	6.2632	6.4349	6.2876	1.0277	0.0394	0.2758	0.5594
Dpysl5	_VVYENGVFM[Oxidation (M)]C[Carbamidomethyl (C)]AEGTGK_.2	#N/A	4.2155	5.1409	3.4015	5.1464	4.5779	5.3243	3.6188	5.3577	5.4060	4.9445	0.9592	-0.0602	0.7029	0.1531
Dpysl5	_M[Oxidation (M)]SVVWER_.2	#N/A	4.9091	5.2231	5.9977	4.7611	5.3014	4.2774	4.6627	5.0739	4.8972	4.7854	1.0460	0.0649	0.4837	0.3154
Dpysl5	_DLHESSFSLSGS[Phospho (STY)]QIDDHVPK_.3	#N/A	6.0931	6.2032	6.5700	7.3945	5.9028	6.3383	6.2464	6.6903	5.9625	6.5746	1.0076	0.0109	0.8755	0.0578
Dpysl5	_EALDLGITGPEGIEISHPEELEAEATHR_.3	#N/A	3.7896	3.7110	4.7226	4.6099	3.7328	4.2075	3.7488	4.5315	3.8788	4.7187	0.9785	-0.0313	0.7700	0.1135
Dpysl5	_LDTNTSTYLM[Oxidation (M)]SLLANDTLNIVASDHRPFTTK_.4	#N/A	3.4438	4.2001	3.1775	3.5296	4.0884	4.0126	3.9302	4.2799	3.1321	3.6534	0.9981	-0.0027	0.9812	0.0083
Dpysl5	_DSELYQVFHAC[Carbamidomethyl (C)]R_.2	#N/A	4.9112	4.9543	3.9426	5.9670	5.2597	4.8080	5.0202	4.7566	5.1697	4.9891	0.9980	-0.0029	0.9772	0.0100
Dpysl5	_TISASTQVQGGDFNLYENM[Oxidation (M)]R_.2	#N/A	4.1631	3.4674	3.3490	3.8517	3.2888	4.3513	3.9872	4.2668	3.5917	4.8066	0.8996	-0.1526	0.2069	0.6841
Dpysl5	_AMGKEDFTK_.2	#N/A	2.9518	4.3906	4.1863	3.7301	3.5963	3.0283	3.8783	3.2711	3.5702	3.4073	1.0327	0.0464	0.7247	0.1398
Dpysl5	_THC[Carbamidomethyl (C)]PIYLVNVSSISAGDVIAAAK_.3	#N/A	3.5711	4.3274	3.3048	3.4101	4.2157	3.9098	3.8029	4.1526	2.9508	4.0267	1.0151	0.0217	0.8577	0.0667
Dpysl5	_TPYLGDVAIVVHPGK_.3	#N/A	3.5640	3.6622	4.2508	3.2526	1.1137	3.5390	4.4681	3.3650	3.0136	3.2196	0.9186	-0.1225	0.6523	0.1855
Cert1	_FVQKPYS[Phospho (STY)]R_.2	Q9EQG9_S373_M1_Cert1	3.6697	4.4259	3.4033	7.5686	2.8134	3.8112	3.7044	4.1288	6.7403	3.9567	0.9243	-0.1135	0.7424	0.1294
Cert1	_SSS[Phospho (STY)]M[Oxidation (M)]SS[Phospho (STY)]IDLVSASDDVHR_.2	Q9EQG9_S377_M2_Cert1	3.7994	3.5205	4.3380	3.7890	3.3759	3.6362	3.6325	3.5387	3.9279	3.6843	1.0128	0.0183	0.8018	0.0959
Vps35	_RES[Phospho (STY)]PESEGPIYEGLIL_.2	#N/A	9.5694	9.2969	9.6720	9.6515	8.7783	9.1602	9.8286	9.6931	9.5273	9.3001	0.9757	-0.0354	0.2772	0.5572
Vps35	_SRRES[Phospho (STY)]PESEGPIYEGLIL_.3	#N/A	8.9092	3.9725	3.8294	8.7479	8.1419	4.1050	8.0678	9.4815	9.3422	8.7467	0.7053	-0.5036	0.0832	1.0801
Vps35	_IREDLPNLESSEETEQINK_.3	#N/A	6.8585	6.3059	6.9057	6.4698	5.4746	6.3611	6.6433	6.6534	6.0864	6.5921	0.9849	-0.0219	0.7403	0.1306
Vps35	_AELAELPLR_.2	#N/A	6.6839	6.7042	6.2806	5.9773	5.5996	5.6959	6.4785	6.8972	6.2303	6.3730	0.9480	-0.0771	0.2455	0.6100
Vps35	_SDDPDQQYLILNTAR_.2	#N/A	6.1509	5.4342	6.4017	5.6064	5.8095	6.2472	6.3707	6.3426	5.9879	6.2135	0.9539	-0.0681	0.2041	0.6901
Vps35	_LSQLEGVNVER_.2	#N/A	5.7927	5.2228	3.8892	5.9188	4.8952	3.2205	5.9974	5.5374	5.4306	4.9305	0.8811	-0.1826	0.2896	0.5383
Vps35	_IPVDTYNNILTVLK_.2	#N/A	5.1937	5.1637	4.7666	5.1361	3.6691	5.0448	5.5088	5.3212	4.3846	5.4321	0.9355	-0.0961	0.3887	0.4104
Vps35	_LFDIFSQQVATVIQSR_.2	#N/A	3.9467	4.1009	5.4225	4.0198	3.0546	4.6059	4.8013	4.7365	4.1057	4.7216	0.9130	-0.1314	0.3698	0.4320
Vps35	_PTTQQS[Phospho (STY)]PQDEQEKLLDEAIQAVK_.3	#N/A	3.4568	7.1175	7.8361	7.4992	6.2819	3.8916	6.3651	7.6214	6.9894	6.5808	0.9689	-0.0455	0.8754	0.0578
Vps35	_LLDEAIQAVK_.2	#N/A	4.5928	3.4422	3.1276	3.3886	4.5373	3.1613	3.5221	3.9561	4.1368	4.6046	0.9145	-0.1289	0.3970	0.4012
Vps35	_PTTQQS[Phospho (STY)]PQDEQEK_.2	#N/A	9.1954	8.6862	8.7668	9.0281	8.2297	8.9466	9.3373	9.0228	9.0160	8.8854	0.9717	-0.0414	0.2096	0.6785
Vps35	_LNLEHIATSSAVSK_.2	#N/A	3.3081	3.7438	4.2865	3.8302	3.6281	3.8528	3.8633	3.9543	4.0464	3.6508	0.9732	-0.0391	0.5725	0.2422
Vps35	_ES[Phospho (STY)]PESEGPIYEGLIL_.2	#N/A	3.7907	3.2282	4.0439	3.7241	3.1612	3.9822	3.4388	3.7535	3.5608	3.6911	1.0122	0.0175	0.8312	0.0803
Vps35	_TQC[Carbamidomethyl (C)]ALAASK_.2	#N/A	3.0331	4.3093	4.2676	3.8114	3.6777	2.9469	3.9597	5.7657	3.4888	3.4887	0.8799	-0.1845	0.3650	0.4377
Magi3	_SGS[Phospho (STY)]PKLDPSEVYLK_.2	#N/A	3.9164	6.6819	7.3662	7.2641	3.4947	6.4846	7.0509	6.8432	3.5224	6.1076	0.9978	-0.0032	0.9907	0.0041
Magi3	_GGPC[Carbamidomethyl (C)]S[Phospho (STY)]PTK_.2	Q9EQJ9_S662_M1_Magi3	2.9969	4.2292	3.6837	4.1864	4.1175	4.0079	3.9011	3.9321	3.5807	4.1534	0.9945	-0.0080	0.9355	0.0290
Magi3	_QPEDESHQAFSQNGS[Phospho (STY)]PR_.3	#N/A	3.7838	4.3521	3.5636	3.5681	3.5034	4.2289	7.1689	4.0549	3.7035	4.0333	0.8087	-0.3063	0.2130	0.6717
Slc19a2	_KQEDPSSS[Phospho (STY)]PQASTS_.2	Q9EQN9_S492_M1_Slc19a2	4.1531	6.6888	7.4177	3.4306	3.0106	3.2180	7.5571	7.0859	7.1833	6.7272	0.6518	-0.6174	0.0357	1.4476
Ehd4	_DKPVYDELFYTLS[Phospho (STY)]PINGK_.3	Q9EQP2_S459_M1_Ehd4	4.8488	4.8144	4.3441	4.5990	3.9984	4.7468	4.2645	4.6750	5.0931	4.3556	0.9916	-0.0121	0.8682	0.0614
Oga	_LENEGS[Phospho (STY)]DEDIETDVLYSPQM[Oxidation (M)]ALK_.3	#N/A	3.4916	3.5543	3.6733	4.3845	3.3994	3.8432	3.8778	6.5710	3.7857	4.2614	0.8055	-0.3121	0.1410	0.8507
Syt13	_PAVTAPEVINYADYTLETTEESAAPAS[Phospho (STY)]PQAQSDSR_.4	Q9EQT6_S113_M1_Syt13	6.2076	6.1843	4.4195	6.6014	5.9028	3.3703	3.7311	6.8107	6.4185	6.4826	0.9295	-0.1054	0.6435	0.1915
Set	_IDFYFDENPYFENK_.2	#N/A	6.6347	6.6067	6.9955	6.3349	6.4090	6.8992	7.0699	6.3313	6.4464	6.5517	1.0071	0.0102	0.8069	0.0932
Set	_VEVTEFEDIK_.2	#N/A	5.7236	4.8106	5.9816	6.2508	5.9262	5.8518	5.9889	5.4810	5.1638	6.2296	1.0073	0.0105	0.8987	0.0464
Set	_EFHLNESGDPSSK_.2	#N/A	4.7039	5.2565	5.6853	5.9887	5.9111	5.9321	5.5768	5.3775	5.0619	5.5851	1.0332	0.0471	0.5344	0.2721
Rims2	_TGSVQTS[Phospho (STY)]PSSTPGTGR_.2	Q9EQZ7_S1095_M1_Rims2	9.1122	9.1835	9.4945	9.4190	8.1351	8.7213	9.5576	9.1176	8.4983	8.9397	0.9981	-0.0028	0.9569	0.0191
Rims2	_TSNHS[Phospho (STY)]PPTPR_.2	Q9EQZ7_S472_M1_Rims2	9.8574	10.1462	10.4604	10.3457	9.6238	9.2740	10.2856	2.9755	10.0175	9.8775	1.2005	0.2637	0.2757	0.5596
Rims2	_SWS[Phospho (STY)]PSAPPPQR_.2	Q9EQZ7_S1039_M1_Rims2	9.8921	9.8817	3.1518	3.3496	3.0915	10.0056	3.4979	9.9115	9.7000	9.6482	0.8013	-0.3196	0.4904	0.3094
Rims2	_ASQSS[Phospho (STY)]LESSTGPSYSR_.2	Q9EQZ7_S1518_M1_Rims2	8.2498	8.2933	8.3249	8.9068	3.8158	8.0841	8.8772	8.3491	7.8499	8.0505	0.9192	-0.1216	0.5123	0.2905
Rims2	_RHS[Phospho (STY)]DVSLANAELEDSR_.3	Q9EQZ7_S409_M1_Rims2	7.2455	7.7058	7.7089	8.1325	7.2789	7.7891	7.8079	7.8467	7.8488	7.6047	0.9828	-0.0250	0.4717	0.3263
Rims2	_YRS[Phospho (STY)]DPNLAR_.2	Q9EQZ7_S375_M1_Rims2	7.9955	3.3774	8.9472	3.5374	7.3298	3.3589	8.2532	3.6017	4.2297	3.0767	1.2019	0.2654	0.5679	0.2457
Rims2	_SPS[Phospho (STY)]DYADRR_.2	Q9EQZ7_S311_M1_Rims2	3.2040	6.3328	6.3469	3.9823	5.6495	5.9040	6.4294	6.1427	6.2696	3.6596	0.9309	-0.1033	0.6604	0.1802
Rims2	_S[Phospho (STY)]ASQLSQTEGGGKK_.2	Q9EQZ7_S1285_M1_Rims2	3.6664	4.4227	3.4001	4.4447	2.8101	3.8145	3.7076	4.0573	4.3564	4.1220	0.9259	-0.1111	0.3964	0.4019
Rims2	_NDGSQS[Phospho (STY)]DTAVGALGTSGK_.2	Q9EQZ7_S1251_M1_Rims2	6.7609	7.0379	7.2207	4.1033	3.8388	3.8907	7.4442	7.3893	3.1970	7.0083	0.8747	-0.1931	0.5257	0.2793
Rims2	_IPDSTHAQLESSSSSFES[Phospho (STY)]QK_.3	Q9EQZ7_S788_M1_Rims2	4.3034	3.5588	4.0371	3.5808	3.4471	3.1775	3.7291	3.4203	3.7194	3.2581	1.0431	0.0609	0.5249	0.2799
Rims2	_RAS[Phospho (STY)]QSS[Phospho (STY)]LESSTGPSYSR_.2	Q9EQZ7_S1515_M2_Rims2	3.7441	4.9712	4.0744	3.3540	3.5120	4.5234	4.0705	3.4658	3.4804	4.3429	1.0495	0.0697	0.6139	0.2119
Rims2	_RADS[Phospho (STY)]LRK_.2	Q9EQZ7_S493_M1_Rims2	3.8056	3.5282	3.4099	3.9125	3.3496	3.1605	3.3575	1.3439	3.8546	2.7848	1.2443	0.3153	0.1650	0.7824
Rims2	_RQLHGES[Phospho (STY)]PTR_.2	Q9EQZ7_S965_M1_Rims2	3.8900	3.8003	4.0528	3.6913	3.6216	3.4787	3.8395	3.2555	3.6312	3.1451	1.0830	0.1151	0.1187	0.9256
Rims2	_NDS[Phospho (STY)]LSSDQSESVRPPPPPRPHK_.3	Q9EQZ7_S522_M1_Rims2	3.3355	4.0069	4.5700	4.1138	3.8282	3.9012	4.2620	4.3882	3.1864	3.7911	1.0134	0.0192	0.8647	0.0631
Rims2	_RS[Phospho (STY)]PIPLDRPDMR_.3	Q9EQZ7_S479_M1_Rims2	2.4469	4.1385	4.0201	2.1526	3.9598	3.7697	4.2374	3.5957	3.2443	2.9889	0.9710	-0.0425	0.8422	0.0746
Stx12	_ELGSLPLPLSASEQR_.2	#N/A	4.4309	4.5482	4.6509	4.8326	4.4466	4.5567	5.1456	4.6115	4.7669	4.3371	0.9708	-0.0427	0.3958	0.4025
Stx12	_AGSRLS[Phospho (STY)]AEDR_.2	Q9ER00_S142_M1_Stx12	6.1823	2.9529	4.3353	3.3371	4.2750	3.4545	6.0159	5.8930	4.4299	5.5689	0.7467	-0.4214	0.0679	1.1681
Stx12	_DFNSIIQTC[Carbamidomethyl (C)]SGNIQR_.2	#N/A	5.3246	5.1167	6.0422	4.5764	4.7505	4.9719	6.0303	4.1899	4.5391	3.8956	1.1001	0.1376	0.3390	0.4699
Stx12	_LQENLQQLQHSTNQLAK_.3	#N/A	4.8811	4.3648	4.2464	5.2361	4.9005	3.5434	4.7444	5.3001	4.8520	4.7739	0.9209	-0.1189	0.2672	0.5732
Stx12	_ISQATAQIK_.2	#N/A	3.8053	2.8582	5.3785	2.6194	3.4489	4.0412	3.9659	4.5421	3.5159	4.2182	0.9092	-0.1373	0.5085	0.2937
Stx12	_QLEADILDVNQIFK_.2	#N/A	3.4159	3.3464	4.1027	4.6054	4.0424	3.6870	4.3201	3.5131	3.1617	3.0715	1.0995	0.1369	0.3216	0.4927
Stx12	_LM[Oxidation (M)]NDFSSALNNFQVVQR_.2	#N/A	3.1783	2.7398	4.4128	3.9565	3.8228	3.7440	4.1048	2.2874	3.3437	1.8270	1.2600	0.3335	0.1767	0.7529
Stx12	_NPGPSGPQPR_.2	#N/A	4.3207	4.4064	3.5066	4.0093	3.4463	4.2832	3.7240	4.1092	3.7578	3.9763	1.0266	0.0379	0.6617	0.1793
Kcnh7	_HVS[Phospho (STY)]DPGLPGK_.2	Q9ER47_S1188_M1_Kcnh7	9.2029	9.1766	9.9605	9.2948	8.0527	9.1358	9.6408	9.5490	9.5317	9.1504	0.9651	-0.0513	0.3384	0.4706
Kcnh7	_KQS[Phospho (STY)]LPQEDPDVVVIDSSK_.2	#N/A	6.7112	5.9880	6.4999	6.0941	4.2144	6.1010	6.5878	6.5197	6.2023	5.9439	0.9400	-0.0892	0.4418	0.3548
Kcnh7	_SSLLGSTS[Phospho (STY)]DSNLNK_.2	Q9ER47_S319_M1_Kcnh7	3.3736	3.9688	3.1073	4.1519	3.7901	3.9394	7.3369	4.3501	3.1483	6.5438	0.6964	-0.5221	0.0743	1.1287
Kcnh7	_SFS[Phospho (STY)]PSSQC[Carbamidomethyl (C)]PEFLDLEK_.2	Q9ER47_S1103_M1_Kcnh7	4.3112	4.4158	3.4972	3.9998	4.3469	3.8213	3.7145	4.1187	3.7672	3.9668	1.0056	0.0081	0.9232	0.0347
Kcnh7	_RLS[Phospho (STY)]FES[Phospho (STY)]EGEKDFSK_.3	#N/A	4.0900	3.3454	3.8237	4.0983	3.2337	3.4496	4.0060	3.6924	3.9915	3.8374	0.9463	-0.0796	0.3351	0.4748
Kcnh7	_LNTAS[Phospho (STY)]EDNLTSLLK_.2	Q9ER47_S1135_M1_Kcnh7	4.1076	3.4120	3.2936	3.7962	3.2333	2.9954	3.5109	4.3222	3.9708	3.7632	0.8924	-0.1643	0.1299	0.8864
Kcnh7	_SQSVNDS[Phospho (STY)]EGDTGK_.2	Q9ER47_S878_M1_Kcnh7	3.9737	3.2524	3.1597	3.6624	3.0994	3.4839	3.3770	4.4561	4.1047	3.6294	0.8835	-0.1786	0.1153	0.9383
Osbpl5	_ETMSGQTTFLGS[Phospho (STY)]PDSR_.2	Q9ER64_S746_M1_Osbpl5	3.1285	4.0976	3.8153	5.4543	3.7551	3.9744	4.0327	3.8005	3.4491	4.2850	1.0374	0.0530	0.7361	0.1330

Osbpl5	_ENAEDS[Phospho (STY)]DAETQDHSR_.2	Q9ER64_S311_M1_Osbpl5	3.0168	4.2094	3.7036	3.9888	4.0977	4.0277	3.9209	3.9123	3.5608	4.1732	0.9869	-0.0191	0.8395	0.0760
Wtap	_EGS[Phospho (STY)]TPEDDFPSSSGNGNK_.2	Q9ER69_S297_M1_Wtap	3.4818	4.2870	4.1686	6.7898	4.1084	3.6211	4.3860	3.4472	3.0958	3.1375	1.2539	0.3264	0.2138	0.6699
Cars1	_VQPQWS[Phospho (STY)]PPAGTEPC[Carbamidomethyl (C)]R_.2	Q9ER72_S102_M1_Cars1	3.7549	3.5418	9.5008	9.8795	2.8806	8.5932	9.5845	9.3153	9.2885	9.1773	0.6807	-0.5550	0.1136	0.9445
Cars1	_ALQEGEGDLSIS[Phospho (STY)]ADR_.2	Q9ER72_S390_M1_Cars1	3.6494	3.6930	3.3831	4.4277	2.7932	3.8314	3.0751	4.0743	4.3734	4.1050	0.9290	-0.1062	0.4572	0.3399
Tfip11	_TTQSLQDFPVADS[Phospho (STY)]EEEAEEEFQK_.3	Q9ERA6_S211_M1_Tfip11	2.9942	3.7505	4.2288	3.7725	3.6388	2.9858	3.9208	6.1064	3.5277	3.4498	0.8378	-0.2553	0.2489	0.6040
Tfip11	_EEATYGVWAERDS[Phospho (STY)]DEERPSFGGK_.3	Q9ERA6_S60_M1_Tfip11	3.7824	3.7239	3.6868	4.1895	3.6265	3.4516	3.9041	4.3790	3.0403	4.1565	0.9673	-0.0480	0.6439	0.1912
Tfip11	_GAAEEADS[Phospho (STY)]EDS[Phospho (STY)]DAEEKPVKQEDFPK_.3	Q9ERA6_S96_M2_Tfip11	3.7407	3.6017	3.4744	3.6872	2.8844	2.4059	3.1664	3.9830	4.2821	2.3367	0.9585	-0.0612	0.7541	0.1226
Ralgps2	_EDLAGPDIGAS[Phospho (STY)]PQGGR_.2	Q9ERD6_S308_M1_Ralgps2	6.3386	6.2222	3.1882	6.4321	3.1279	6.1052	6.4048	4.4277	6.3651	5.8716	0.9078	-0.1395	0.5714	0.2430
Tubb3	_YLTVATVFR_.2	#N/A	10.3435	10.6260	10.8861	10.7290	10.3605	10.5226	10.3034	10.6023	10.5880	10.5707	1.0059	0.0085	0.6260	0.2034
Tubb3	_VREEYPDR_.2	#N/A	9.0431	8.7669	8.6761	8.6617	9.1877	8.7364	8.6596	8.9332	8.7715	8.6776	1.0097	0.0139	0.5049	0.2968
Tubb3	_M[Oxidation (M)]SSTFIGNSTAIQELFKR_.3	#N/A	6.3583	6.7987	8.2216	6.7040	6.0950	7.0813	6.0475	6.3716	6.7202	7.3332	1.0390	0.0553	0.5711	0.2433
Tubb3	_EVDEQM[Oxidation (M)]LAIQSK_.2	#N/A	9.5651	9.7213	9.8353	9.9031	9.4616	9.8445	9.5268	9.5338	9.6722	9.8469	1.0080	0.0115	0.4938	0.3064
Tubb3	_LHFFM[Oxidation (M)]PGFAPLTAR_.3	#N/A	8.4655	8.6269	8.3565	7.9511	8.0850	8.3635	8.1993	8.5459	8.3978	8.1037	0.9996	-0.0006	0.9814	0.0081
Tubb3	_MSSTFIGNSTAIQELFK_.2	#N/A	9.9466	10.0613	11.5864	10.7587	10.2401	10.7178	10.5380	10.2445	10.3600	10.9824	1.0020	0.0028	0.9525	0.0211
Tubb3	_ISVYYNEASSHK_.2	#N/A	6.7883	6.9800	6.6796	7.4923	6.9374	7.1220	6.6468	7.2431	6.9325	7.1842	0.9998	-0.0003	0.9928	0.0031
Tubb3	_LATPTYGDLNHLVSATM[Oxidation (M)]SGVTTSLR_.3	#N/A	5.5512	5.6654	6.0619	5.9697	5.8079	6.2043	5.3040	5.9611	5.8149	6.2091	1.0093	0.0134	0.7890	0.1029
Tubb3	_ISVYYNEASS[Phospho (STY)]HKYVPR_.3	Q9ERD7_S56_M1_Tubb3	3.4005	3.8256	4.0874	4.5900	3.7139	4.4115	4.3047	3.5285	3.1771	3.0562	1.1388	0.1876	0.1636	0.7863
Tubb3	_VAVC[Carbamidomethyl (C)]DIPPR_.2	#N/A	2.9090	4.3171	3.5958	4.0985	9.4809	3.9200	3.8132	4.0200	8.9626	4.0655	0.9051	-0.1439	0.7602	0.1191
Tubb3	_FWEVISDEHGIDPSGNYVGDSDLQLER_.3	#N/A	3.9004	3.4420	3.6341	3.1779	3.2633	2.9654	3.3262	3.8233	4.1223	4.3560	0.8695	-0.2017	0.0714	1.1462
Tubb3	omethyl (C)]ENC[Carbamidomethyl (C)]DC[Carbamidomethyl (C)]LQGFQLTHSLGGGTGSGM[Oxidation (	#N/A	3.2242	4.0850	3.9110	4.4137	3.8508	3.8820	4.1158	3.7081	3.3567	4.3807	1.0011	0.0015	0.9880	0.0053
Tlnrd1	_[Acetyl (Protein N-term)]ASGS[Phospho (STY)]AGKPTGEAASPAPGSAVGGASSQPR_.3	Q9ERE8_S5_M1_Tlnrd1	3.2734	4.0786	3.9602	4.4629	3.8999	3.8296	4.1775	3.6556	3.3042	4.4299	1.0066	0.0095	0.9297	0.0317
Mapk8ip2	_MISEGSS[Phospho (STY)]PIR_.2	Q9ERE9_S370_M1_Mapk8ip2	4.1870	5.7635	3.9207	3.4645	2.9767	3.2520	6.1393	5.3567	3.8357	3.1418	0.9433	-0.0842	0.7574	0.1207
Mapk8ip2	_SSQELSS[Phospho (STY)]PGSDSEDAGGAR_.2	Q9ERE9_S254_M1_Mapk8ip2	4.1759	3.4313	3.9096	3.4534	3.3196	3.3050	3.1981	3.5478	3.8469	3.1306	1.0490	0.0691	0.4764	0.3220
Wdr61	_ENIETVVTGSLDDLVK_.2	#N/A	4.0424	3.3468	3.2284	4.6029	3.7941	3.0606	3.4457	4.3874	3.6259	4.4710	0.9238	-0.1143	0.4261	0.3705
Lima1	_ELS[Phospho (STY)]VEEQIK_.2	Q9ERG0_S735_M1_Lima1	12.1265	10.0047	3.2537	8.7789	4.1646	8.2091	11.1534	10.1461	3.6277	3.9756	1.0734	0.1022	0.8271	0.0825
Lima1	_TSS[Phospho (STY)]LPESSPSK_.2	Q9ERG0_S367_M1_Lima1	6.6812	3.4669	3.6092	7.6004	3.0192	3.6054	3.3012	3.8482	4.1473	4.3311	1.1937	0.2554	0.4761	0.3223
Strn3	_SLLGLSNSEPNGS[Phospho (STY)]VEAK_.2	Q9ERG2_S214_M1_Strn3	7.8293	7.9264	8.3192	8.4825	7.4971	8.1882	8.4037	7.6936	8.1171	8.1593	0.9935	-0.0095	0.8139	0.0894
Strn3	_NLEQILNGGES[Phospho (STY)]PKQK_.2	#N/A	3.5470	3.6278	4.4913	3.7988	3.4492	3.6348	9.1517	8.5466	8.7677	3.5962	0.5001	-0.9999	0.0074	2.1311
Strn3	_VVSHPTLPVTITAHEDR_.4	#N/A	3.3086	3.9176	3.9954	2.6384	3.9351	4.3195	4.2127	3.6205	3.2690	2.9642	1.0481	0.0678	0.6647	0.1774
Strn3	_NLEQILNGGES[Phospho (STY)]PK_.2	#N/A	4.1258	4.1512	4.0328	8.9436	3.9725	3.7570	4.1178	3.5830	3.2316	3.0016	1.3867	0.4716	0.2349	0.6292
Cse1l	_LLQTDDEEEAGLLELLK_.2	#N/A	4.3708	0.3126	3.6875	4.1676	2.3515	3.9550	4.3158	3.3986	3.1327	3.2569	0.8908	-0.1669	0.6524	0.1855
Cse1l	_IVEDEPNKIC[Carbamidomethyl (C)]EADR_.3	#N/A	3.3013	2.6707	3.1534	3.6560	3.0931	3.4776	3.3707	4.4624	4.1110	3.6230	0.8288	-0.2710	0.0337	1.4721
Znf704	_VGESDC[Carbamidomethyl (C)]S[Phospho (STY)]DGEEDFYYTEIK_.2	Q9ERQ3_S381_M1_Znf704	2.9372	4.4052	4.1717	3.7155	4.2266	3.5029	3.8637	3.2857	3.5848	3.3927	1.0835	0.1156	0.3529	0.4524
Ndel1	_S[Phospho (STY)]APSS[Phospho (STY)]PTLDC[Carbamidomethyl (C)]EK_.2	#N/A	11.8477	12.0399	12.0056	12.2430	12.1060	11.9777	11.9543	11.7889	11.8994	12.1275	1.0079	0.0113	0.3140	0.5030
Ndel1	_GTENSFPS[Phospho (STY)]PK_.2	Q9ERR1_S231_M1_Ndel1	9.4393	9.8536	10.2571	10.2068	9.2728	9.7135	9.8430	9.4810	9.3873	9.6278	1.0215	0.0306	0.3724	0.4289
Ndel1	_KSAPS[Phospho (STY)]SPTLDC[Carbamidomethyl (C)]EK_.2	Q9ERR1_S197_M1_Ndel1	3.4062	4.1474	6.4327	3.9861	3.9688	3.7735	5.6889	3.8990	3.3270	3.6633	1.0341	0.0483	0.8434	0.0740
Ndel1	_M[Oxidation (M)]DSAVQASLS[Phospho (STY)]LPAT[Phospho (STY)]PVGK_.2	#N/A	6.8220	5.8210	7.9055	8.0640	8.2691	7.2158	7.3558	8.4450	8.5573	7.5424	0.9216	-0.1178	0.2732	0.5635
Ndel1	_ISALNIVGDLLR_.2	#N/A	3.6361	4.7858	3.9170	3.4608	3.5776	3.2482	3.6978	4.0178	4.6217	3.1381	0.9747	-0.0370	0.7980	0.0980
Ndufa13	_LLIEDLEAR_.2	#N/A	8.7840	8.7026	8.9390	8.6554	8.2020	8.5842	8.9971	9.0169	8.6833	8.7306	0.9760	-0.0350	0.1795	0.7459
Ndufa13	_VGESVFHTTR_.2	#N/A	6.6836	6.8379	6.8517	7.0011	6.9147	6.8446	6.9035	7.0094	6.5444	6.6719	1.0108	0.0155	0.4818	0.3171
Ndufa13	_IALM[Oxidation (M)]PLFQAEK_.2	#N/A	6.5788	6.1724	6.0341	5.9038	5.5924	5.4972	6.0896	6.3988	6.0807	5.5429	0.9892	-0.0156	0.7990	0.0975
Ndufa13	_ENLEEEAIIM[Oxidation (M)]K_.2	#N/A	5.4663	5.8284	3.9584	5.0205	5.6708	6.2998	3.6505	6.6763	4.9631	5.7854	1.0200	0.0285	0.8765	0.0573
Plekha3	_SAS[Phospho (STY)]HPGSC[Carbamidomethyl (C)]SSER_.2	Q9ERS4_S211_M1_Plekha3	3.5369	3.6892	4.2237	4.8142	4.1634	3.5660	4.4411	4.5304	3.0407	3.1925	1.0520	0.0732	0.6374	0.1956
Ppp1r1a	_STLSM[Oxidation (M)]S[Phospho (STY)]PR_.2	#N/A	10.5131	10.8129	11.0385	11.1645	10.6296	10.7597	10.5208	10.5545	10.5995	10.7444	1.0203	0.0289	0.1396	0.8550
Ppp1r1a	_[Acetyl (Protein N-term)]MEPDNS[Phospho (STY)]PRK_.2	#N/A	6.6682	4.6456	4.5158	8.3338	5.2974	3.8470	4.9417	4.4377	3.2407	5.6553	1.2150	0.2810	0.3263	0.4864
Ppp1r1a	_[Acetyl (Protein N-term)]MEPDNS[Phospho (STY)]PR_.2	#N/A	5.9596	3.8243	4.1479	7.0189	5.2932	3.9232	3.7065	4.1661	4.1531	4.7764	1.1970	0.2594	0.2596	0.5857
Ppp1r1a	_TEDSSAHMLPLDSQGASLV_.2	#N/A	3.2467	4.0519	4.5251	4.0688	3.1524	3.8563	4.2171	4.4332	3.2314	3.7461	0.9769	-0.0337	0.7988	0.0976
Ppp1r1a	_EPDNS[Phospho (STY)]PR_.2	#N/A	3.8751	3.4673	3.6088	3.1526	3.2886	2.9400	3.3008	3.8486	4.1477	4.3307	0.8674	-0.2053	0.0687	1.1631
Ranbp2	_SHETDGGS[Phospho (STY)]AHGDEEDDGPHFEPVVPLPDKIEVK_.5	Q9ERU9_S1154_M1_Ranbp2	3.4121	3.8140	4.0989	4.6016	4.0387	3.6908	4.3163	3.5169	5.5853	3.0678	0.9566	-0.0640	0.7210	0.1421
Ranbp2	_YIASVQGSAPS[Phospho (STY)]PR_.2	Q9ERU9_S21_M1_Ranbp2	5.8942	5.9124	5.8722	6.6703	4.2224	5.5608	5.9446	3.3332	5.5914	6.4761	1.0660	0.0923	0.6207	0.2071
Ranbp2	_HSTPS[Phospho (STY)]PTKYSLS[Phospho (STY)]PSK_.2	Q9ERU9_S781_M2_Ranbp2	3.5801	7.0092	3.3137	4.3583	3.5837	4.1458	4.5066	4.1437	2.9419	4.0356	1.1087	0.1489	0.5854	0.2326
Ranbp2	_SALS[Phospho (STY)]PSKSPAK_.2	Q9ERU9_S2113_M1_Ranbp2	3.7089	5.2682	3.8844	3.8353	3.7674	5.2510	4.4831	5.8137	5.6168	3.6665	0.8756	-0.1917	0.3040	0.5171
Ranbp2	_LNSNNSAS[Phospho (STY)]PHR_.2	Q9ERU9_S837_M1_Ranbp2	3.8293	3.3968	4.5162	4.2094	2.9550	3.2736	3.2327	4.7507	4.2491	3.4850	0.9408	-0.0880	0.5862	0.2319
Ranbp2	_HSTPS[Phospho (STY)]PTK_.2	Q9ERU9_S781_M1_Ranbp2	4.1489	3.4043	3.8826	3.4263	3.2926	3.3320	3.5746	3.5748	3.8739	3.1036	1.0140	0.0200	0.8269	0.0825
Ranbp2	_NRPGYVS[Phospho (STY)]EEEEDDEYEM[Oxidation (M)]AVK_.3	Q9ERU9_S2505_M1_Ranbp2	3.8154	3.1197	3.9564	3.5001	2.9410	3.2876	3.6484	3.5010	3.8001	3.1774	0.9731	-0.0394	0.6878	0.1626
Mkrn2	_KQLS[Phospho (STY)]SEGTVR_.2	Q9ERV1_S365_M1_Mkrn2	3.3929	4.1492	3.1266	4.1712	4.0375	4.0880	4.2817	4.7620	4.6558	4.7877	0.8282	-0.2720	0.0125	1.9037
Mkrn2	_VNNPGGC[Carbamidomethyl (C)]SDPQTS[Phospho (STY)]PEMKPHSYLDAIR_.4	Q9ERV1_S139_M1_Mkrn2	3.3441	3.9983	4.5786	5.9943	3.9887	4.1368	4.2706	4.3796	3.1779	3.7997	1.1109	0.1517	0.4191	0.3777
Chrm3	_SC[Carbamidomethyl (C)]SSY[Phospho (STY)]ELQQQGTKR_.2	Q9ERZ3_Y292_M1_Chrm3	4.1988	3.4543	5.2011	3.4763	3.3426	3.2820	4.6339	3.5249	3.8239	3.1536	1.0110	0.0158	0.9294	0.0318
Chrm3	_SC[Carbamidomethyl (C)]S[Phospho (STY)]SYELQQQGTK_.2	Q9ERZ3_S290_M1_Chrm3	3.6565	4.9629	3.4927	3.9954	4.3324	3.9015	5.4845	4.1448	3.7934	3.9624	0.8989	-0.1538	0.3250	0.4881
Chrm2	_KEPVANQDPVS[Phospho (STY)]PSLVQGR_.3	#N/A	3.7966	6.6153	7.8906	7.0243	3.7475	6.6173	6.8994	7.4665	7.2052	5.9058	0.8660	-0.2076	0.3524	0.4529
Chrm2	_EPVANQDPVS[Phospho (STY)]PSLVQGR_.2	#N/A	2.9674	8.2975	4.2020	3.7457	3.6120	8.1358	3.8940	8.5932	3.5545	3.4230	1.0604	0.0846	0.8564	0.0673
Chrm2	_EKKEPVANQDPVS[Phospho (STY)]PSLVQGR_.3	#N/A	4.0581	3.1681	3.2440	3.7467	3.1837	3.5682	3.4614	4.3718	4.0204	3.7137	0.8980	-0.1552	0.1383	0.8592
Ube4b	_LAGGQTSQPTTPLTS[Phospho (STY)]PQR_.2	Q9ES00_S31_M1_Ube4b	6.2478	3.4220	3.3036	3.8063	3.2433	2.9853	3.3461	6.5090	6.5529	3.7733	0.7601	-0.3958	0.2249	0.6480
Ube4b	_TVEDLKNNESQWKDS[Phospho (STY)]PLATR_.3	#N/A	4.2120	4.4357	3.5449	4.0475	4.2564	4.3296	3.7622	5.4343	3.7195	4.1927	0.9674	-0.0478	0.7050	0.1518
Ube4b	_HLLNS[Phospho (STY)]PTDPFNR_.2	Q9ES00_S1136_M1_Ube4b	2.9540	3.7592	3.6408	6.6392	3.5805	4.1490	3.8581	3.9751	3.6236	4.1104	1.0587	0.0824	0.7398	0.1309
Ube4b	_SQSSEGVSSLSSS[Phospho (STY)]PSNSLETQSQSLSR_.3	Q9ES00_S88_M1_Ube4b	3.9346	3.2390	5.1390	3.8910	3.6456	4.6661	3.6033	4.8875	4.1841	3.6394	1.0018	0.0026	0.9868	0.0058
Ube4b	_SQS[Phospho (STY)]M[Oxidation (M)]DIDGVSC[Carbamidomethyl (C)]EK_.2	#N/A	6.8161	6.2202	7.1392	7.0044	5.4734	6.5657	7.0188	6.3663	6.4625	6.7058	0.9847	-0.0223	0.7684	0.1144
Ube4b	_NNESQWKDS[Phospho (STY)]PLATR_.2	#N/A	3.4934	3.9630	3.2271	4.3194	4.5074	4.1234	5.9069	5.4512	5.6649	4.1439	0.7444	-0.4259	0.0096	2.0183
Arhgef7	_KPS[Phospho (STY)]DEEFAVR_.2	#N/A	11.3586	11.2158	11.7667	11.8224	10.1464	11.2931	11.5387	11.2091	11.1997	11.2766	0.9966	-0.0050	0.9048	0.0434
Arhgef7	_EDLSKS[Phospho (STY)]PK_.2	Q9ES28_S655_M1_Arhgef7	8.3834	9.0277	9.1589	9.3011	7.5927	8.8979	8.5893	8.9563	8.5894	8.6076	1.0048	0.0068	0.9045	0.0436
Arhgef7	_EIKPSEKPVS[Phospho (STY)]PK_.2	#N/A	10.5499	10.6885	11.3066	11.1799	10.3194	10.6777	10.9662	10.8657	10.7945	10.6433	0.9972	-0.0041	0.8836	0.0537
Arhgef7	_M[Oxidation (M)]S[Phospho (STY)]GFIYQGK_.2	#N/A	9.7633	9.8869	10.3606	9.6804	9.5276	9.9093	10.0009	9.3576	9.7585	9.5734	1.0033	0.0048	0.9030	0.0443
Arhgef7	_SFDS[Phospho (STY)]LGSQSSHSR_.2	Q9ES28_S135_M1_Arhgef7	5.3166	3.6147	6.1157	6.1565	3.3628	6.0012	4.4597	5.6067	4.6685	4.5328	1.0576	0.0808	0.6971	0.1567
Arhgef7	_LETFDANDLYQGQNFNK_.2	#N/A	4.7915	3.9384	5.5303	4.6281	4.2185	3.6177	3.8011	4.7935	4.6374	4.7483	0.9909	-0.0132	0.9196	0.0364
Arhgef7	_IKS[Phospho (STY)]FDSLGSQSSHSR_.3	Q9ES28_S132_M1_Arhgef7	4.8649	5.5506	5.0356	4.3861	3.8443	4.5512	3.9846	5.5546	3.7674	5.1708	1.0186	0.0266	0.8555	0.0678
Arhgef7	_S[Phospho (STY)]LDM[Oxidation (M)]TDNTNSQLVVR_.2	#N/A	6.9562	7.9838	6.5482	8.5824	7.0813	8.2971	7.8735	8.2578	8.0194	8.6176	0.9247	-0.1130	0.1974	0.7047
Arhgef7	_KPS[Phospho (STY)]DEEFAVRK_.3	#N/A	8.0913	8.4660	9.1954	8.7944	7.4712	8.5758	8.2335	8.3257	8.3460	8.4629	1.0108	0.0155	0.7756	0.1104
Arhgef7	_S[Phospho (STY)]TAALEEDAQILK_.2	Q9ES28_S682_M1_Arhgef7	3.0530	4.1732	3.7398	4.2424	4.0615	4.0640	3.9571	3.8761	3.5246	4.2094	0.9993	-0.0011	0.9913	0.0038
Arhgef7	_SGTLKS[Phospho (STY)]PPK_.2	Q9ES28_S236_M1_Arhgef7	4.0975	2.2931	3.2835	3.7861	3.0169	3.6077	3.5008	4.3323	3.9809	3.7531	0.8601	-0.2174	0.1639	0.7854

Rtn3	_SEEGHPFK_.2	#N/A	7.2174	7.1886	6.8321	6.1909	7.0802	6.9746	7.0272	7.2319	6.7845	6.3408	1.0099	0.0142	0.7902	0.1023
Rtn3	(Protein N-term)]AESSAATQSPSVSSSSSGAEPSALGGGGGS[Phospho (STY)]PGAC[Carbamidomethyl (C)]P.	Q9ES97_S31_M1_Rtn3	9.5341	10.5725	10.9396	11.1559	8.9861	9.8487	10.4944	10.6515	10.4157	9.6864	0.9865	-0.0196	0.7733	0.1117
Rtn3	_SALDFGQSK_.2	#N/A	3.7614	5.5303	3.4951	5.5367	5.6033	5.4997	5.4115	5.8942	5.3765	4.9942	0.9050	-0.1440	0.3575	0.4468
Rtn3	_ETTGGAPTM[Oxidation (M)]S[Phospho (STY)]PDLEQEQLTIR_.2	#N/A	9.1357	9.1816	9.7809	9.6749	8.0829	9.2925	9.7998	9.6024	9.2945	9.2776	0.9682	-0.0467	0.3783	0.4222
Rtn3	_KLDS[Phospho (STY)]PQGTNK_.2	#N/A	4.0253	3.3297	3.2113	5.7598	4.5482	6.4363	3.4286	4.4045	5.8954	3.6810	1.0458	0.0646	0.8093	0.0919
Rtn3	_TQIDHYVGIAR_.2	#N/A	5.9282	6.5555	6.3820	6.7497	6.1725	6.2998	7.0195	6.5599	6.3602	6.3457	0.9660	-0.0499	0.2793	0.5539
Rtn3	_GIVDKEEGDVLEAVLEK_.3	#N/A	4.6567	3.1308	4.8844	5.7006	3.2211	4.1021	4.6615	5.0186	4.4187	4.4983	0.9211	-0.1185	0.5015	0.2997
Rtn3	_QTFAPQSGPQSSSDILEHTDVK_.3	#N/A	4.1830	3.2113	3.2008	3.7034	3.0996	3.5449	3.4181	4.4150	3.7823	4.2612	0.8794	-0.1854	0.1214	0.9158
Rtn3	_KLDS[Phospho (STY)]PQGTNKDR_.2	#N/A	3.5526	4.3578	4.2394	4.8941	4.1791	3.5504	4.9658	5.4790	4.4881	3.2082	0.9104	-0.1354	0.4080	0.3893
Rtn3	_DQTKSIVEK_.2	#N/A	2.2509	3.4862	3.3678	3.8704	3.3075	4.4220	3.8876	2.8024	3.8966	0.6863	1.2245	0.2921	0.3933	0.4052
Rtn3	_Y[Phospho (STY)]VEDTDGSSPEDLM[Oxidation (M)]AVLTGAEEK_.3	Q9ES97_Y589_M1_Rtn3	3.9406	3.5673	4.1227	3.3521	3.4556	3.1736	3.9650	5.7901	3.8169	3.3191	0.8530	-0.2294	0.2190	0.6596
Rtn3	_S[Phospho (STY)]LEGEVASQVPNTLNEVTPEK_.2	Q9ES97_S529_M1_Rtn3	3.5200	4.2763	3.6367	4.1393	4.1646	3.9608	3.8540	5.2816	3.6277	4.1063	0.9365	-0.0946	0.4411	0.3554
Rtn3	_SEMC[Carbamidomethyl (C)]ENSEQPQAQPET[Phospho (STY)]PTQK_.2	Q9ES97_T524_M1_Rtn3	4.3178	3.5732	4.0514	3.3416	3.4615	3.1631	4.5571	3.4060	4.4255	3.2725	0.9326	-0.1006	0.4701	0.3278
Rtn3	_QFSHTTAALEEVSR_.3	#N/A	4.2709	3.0715	4.0046	3.5483	2.8928	3.3358	3.6966	3.4528	3.7519	3.3450	0.9885	-0.0167	0.8887	0.0512
Nckipsd	_AAPTT[Phospho (STY)]PPPPVK_.2	#N/A	3.9779	3.2482	3.1639	3.6665	3.1365	3.4881	3.3812	4.4519	4.1005	3.6335	0.8857	-0.1752	0.1171	0.9313
Nckipsd	_RAAPTT[Phospho (STY)]PPPPVK_.2	#N/A	3.2691	4.3197	3.8069	4.0474	3.9137	3.5651	3.8809	3.8079	3.3029	3.7247	1.0384	0.0543	0.5280	0.2773
Rb1cc1	_VSTSQAS[Phospho (STY)]PQSAASPR_.2	Q9ESK9_S646_M1_Rb1cc1	7.0022	7.7420	7.9108	7.8069	3.7821	7.0855	8.1928	7.8376	7.2533	7.4929	0.8953	-0.1596	0.3529	0.4523
Rb1cc1	_STELVLS[Phospho (STY)]PDM[Oxidation (M)]PR_.2	#N/A	6.0656	6.1491	3.7829	5.8345	3.6915	6.5093	6.6929	6.5957	6.3087	6.3479	0.8231	-0.2809	0.1141	0.9426
Rb1cc1	_S[Phospho (STY)]MEHVAPDPTGTER_.2	Q9ESK9_S261_M1_Rb1cc1	3.9412	3.2850	3.1271	3.6298	3.1733	3.4513	3.3445	2.9878	4.1373	3.5968	0.9767	-0.0340	0.7495	0.1252
Trim2	_[Acetyl (Protein N-term)]ASEGASIPS[Phospho (STY)]PVVR_.2	Q9ESN6_S10_M1_Trim2	11.4916	11.6590	11.9590	12.0148	10.5578	11.6601	11.9176	11.3028	11.4788	11.6271	0.9979	-0.0031	0.9344	0.0295
Trim2	_VKS[Phospho (STY)]PGSGHVK_.2	Q9ESN6_S440_M1_Trim2	9.9800	10.1234	10.4304	10.3496	9.1810	9.8492	10.2787	9.9607	9.6277	10.0797	0.9999	-0.0002	0.9967	0.0014
Trim2	_VIRS[Phospho (STY)]ADVS[Phospho (STY)]PTTEGVKR_.3	#N/A	7.8154	8.5314	9.3127	8.6217	8.5833	8.4892	8.3551	7.9103	8.0652	8.3414	1.0479	0.0674	0.1675	0.7761
Trim2	_SADVS[Phospho (STY)]PTTEGVK_.2	#N/A	12.6216	12.7967	13.0801	13.2961	12.6136	12.8673	13.0888	12.6671	12.7154	12.9847	1.0012	0.0017	0.9258	0.0335
Trim2	_VLQSQLDTLLQGQESIK_.2	#N/A	4.4926	4.2408	3.5247	4.0500	4.3226	4.2562	3.2167	3.8452	4.4876	5.3279	0.9830	-0.0247	0.8611	0.0649
Trim2	_EGDFTLSLR_.2	#N/A	4.8862	4.0002	3.9008	3.2872	3.7406	3.4443	4.1181	3.7150	4.4078	4.3304	0.9357	-0.0959	0.4191	0.3777
Trim2	_LYDQHIR_.2	#N/A	4.7149	4.2820	3.9283	4.8666	4.7508	4.2525	4.1456	4.8845	3.3361	5.4657	1.0248	0.0353	0.7920	0.1013
Trim2	_VIRS[Phospho (STY)]ADVS[Phospho (STY)]PTTEGVK_.2	#N/A	8.6294	9.6182	9.7945	9.9514	9.4354	9.6601	9.8435	9.3230	9.4764	9.5810	0.9957	-0.0062	0.8751	0.0579
Trim2	_ASIVDDIHSTFDELQK_.3	#N/A	3.9881	3.3543	3.7218	4.0405	3.6243	3.0530	3.4138	4.5768	4.1001	4.4437	0.8783	-0.1873	0.1121	0.9505
Trim2	_LNELADQDFPLHPR_.2	#N/A	4.3803	4.8052	4.5275	5.2703	4.4440	3.8094	5.1042	3.7360	5.2769	4.7263	0.9636	-0.0535	0.6536	0.1847
Trim2	_SADVS[Phospho (STY)]PTTEGVKR_.2	#N/A	9.8378	9.9388	10.2143	10.4461	9.8089	10.0708	10.2804	9.9014	9.8890	10.1714	0.9992	-0.0011	0.9592	0.0181
Trim2	_TGNAYLTAELS[Phospho (STY)]TPDGSVADGEILDNK_.3	Q9ESN6_S370_M1_Trim2	3.7125	3.6404	3.6207	4.1233	3.5604	3.7489	3.8380	4.0654	3.7140	4.0903	0.9510	-0.0725	0.1639	0.7855
Trim2	_RVKS[Phospho (STY)]PGS[Phospho (STY)]GHVK_.2	#N/A	4.4263	4.1901	4.0818	3.6060	2.9853	4.8813	4.9730	4.3815	4.7972	4.2635	0.8750	-0.1926	0.1510	0.8212
Trim2	_RPASMYS[Phospho (STY)]TGK_.2	Q9ESN6_S459_M1_Trim2	4.0095	3.2167	3.1955	3.6981	3.1050	3.5197	3.4128	4.4204	4.0689	3.6651	0.8884	-0.1708	0.1217	0.9148
Trim2	_VFNQEGEFMLK_.2	#N/A	3.4407	4.2459	4.1275	3.8748	4.0672	3.6623	4.0231	3.1263	3.4254	3.5868	1.1024	0.1407	0.1301	0.8858
Mapk8ip3	_LFSSSSS[Phospho (STY)]PPPAK_.2	#N/A	10.3238	4.4138	9.8471	4.0019	2.8012	8.7299	3.7166	4.1166	3.7652	3.9689	1.7180	0.7808	0.1372	0.8626
Mapk8ip3	_STHPS[Phospho (STY)]PEK_.2	Q9ESN9_S755_M1_Mapk8ip3	7.9872	7.5530	7.7683	7.6495	3.0099	6.2070	8.0465	3.0448	3.8690	7.8002	1.1767	0.2348	0.4983	0.3025
Mapk8ip3	_ANKTS[Phospho (STY)]PTSGEGTRPGGIIHVYGDDSSDK_.4	Q9ESN9_S1192_M1_Mapk8ip3	3.7503	3.0057	3.4840	7.2773	2.8940	3.7306	3.6237	3.9734	3.8580	4.2059	1.0277	0.0394	0.9004	0.0455
Mapk8ip3	_QLS[Phospho (STY)]PNGGQEDTR_.2	#N/A	8.4779	8.2281	8.5558	8.3662	6.8965	8.2938	8.5044	8.3085	3.1937	8.2109	1.1534	0.2059	0.3400	0.4686
Mapk8ip3	_S[Phospho (STY)]PTAAGFSQR_.2	Q9ESN9_S603_M1_Mapk8ip3	7.3945	6.3867	4.4427	3.4445	2.8816	3.3470	7.1529	6.9774	4.3225	7.2361	0.7240	-0.4660	0.1423	0.8467
Mapk8ip3	_TGSS[Phospho (STY)]PTQGIVNK_.2	Q9ESN9_S366_M1_Mapk8ip3	11.2412	11.3497	11.7937	11.5129	10.2893	11.1112	11.6514	11.4009	11.1745	11.2601	0.9863	-0.0198	0.5861	0.2320
Mapk8ip3	_YKQLS[Phospho (STY)]PNGGQEDTR_.2	#N/A	5.6484	6.3151	6.3120	6.1716	4.8839	5.9932	4.2620	6.2499	5.8758	4.1682	1.1456	0.1961	0.1810	0.7424
Mapk8ip3	_AKET[Phospho (STY)]PEADATSSR_.2	Q9ESN9_T764_M1_Mapk8ip3	3.8596	3.1151	3.5933	3.1371	3.0034	3.6212	3.2854	3.8304	4.1631	4.3152	0.8691	-0.2024	0.0784	1.1056
Mapk8ip3	_LFSSSSS[Phospho (STY)]PPPAKR_.2	#N/A	4.1740	3.4294	3.9077	3.4514	3.3177	3.3069	3.2000	3.5497	3.8488	3.1287	1.0484	0.0682	0.4821	0.3169
Mapk8ip3	_EYNALHQR_.2	#N/A	3.4594	4.4950	4.5944	3.5962	4.0231	3.4177	3.3109	3.8995	3.8591	3.5632	1.0746	0.1038	0.3713	0.4303
Mapk8ip3	_LMELQEAVR_.2	#N/A	3.0793	4.2631	4.3139	3.8576	3.7239	4.4015	4.0059	3.1435	3.4426	3.5349	1.1156	0.1578	0.1994	0.7002
Eif4enif1	_SS[Phospho (STY)]PVESLKK_.2	Q9EST3_S77_M1_Eif4enif1	7.9905	8.4124	8.9106	8.7686	8.3202	8.2160	8.4584	4.2237	8.3419	8.1689	1.1559	0.2091	0.2078	0.6824
Eif4enif1	_SSS[Phospho (STY)]PVGLAK_.2	Q9EST3_S949_M1_Eif4enif1	3.7264	3.6160	3.4600	7.5171	6.9876	6.9528	5.9706	6.8022	7.1932	4.1819	0.8906	-0.1671	0.5752	0.2402
Eif4enif1	_SSS[Phospho (STY)]LGSTPHEELER_.2	Q9EST3_S352_M1_Eif4enif1	7.1434	7.5616	8.0297	8.2817	7.2459	7.5508	8.0052	7.4466	7.6595	7.7301	0.9903	-0.0141	0.7665	0.1155
Brd4	_IHS[Phospho (STY)]PIIR_.2	Q9ESU6_S1153_M1_Brd4	7.9370	7.9680	8.2973	8.4389	7.3315	7.9873	8.1525	8.0814	7.9057	8.1017	0.9917	-0.0120	0.7462	0.1271
Brd4	_MPDEPEEPVVTVSS[Phospho (STY)]PAVPPPTK_.2	Q9ESU6_S471_M1_Brd4	3.3467	4.1030	4.5812	4.1250	3.9913	4.1342	4.0273	4.3770	3.1752	3.8023	1.0524	0.0737	0.5002	0.3009
Ddx24	_AQAVS[Phospho (STY)]EEEEEEEGQSSSPK_.2	Q9ESV0_S80_M1_Ddx24	3.8113	5.3418	5.4224	4.7999	3.3709	4.7560	5.7209	4.7347	4.5510	5.0243	0.9153	-0.1276	0.3919	0.4068
Agk	_LASFWAQPQDASSR_.2	#N/A	6.8584	6.3375	4.2783	3.8221	6.5325	6.7084	6.0914	6.2865	6.8522	6.4406	0.8969	-0.1569	0.3697	0.4321
Agk	_IPIGFIPLGQTSSLSHTLFAESGNK_.3	#N/A	4.1477	2.0446	3.8814	3.5117	3.2914	3.3332	2.8728	3.5761	2.9783	2.9761	1.0863	0.1194	0.5149	0.2883
Dkc1	_TVLES[Phospho (STY)]GGETGDGDNDTTK_.2	#N/A	7.4903	7.5383	8.1272	7.7332	6.6341	7.6315	8.2491	7.8837	7.6229	7.4565	0.9645	-0.0522	0.3607	0.4429
Dkc1	_KRDSES[Phospho (STY)]ES[Phospho (STY)]DETPTVPQLK_.3	#N/A	3.8513	3.6403	4.1249	8.0295	7.1172	6.6973	3.6468	8.1911	8.0031	7.0223	0.8304	-0.2681	0.4021	0.3957
Dkc1	_VKVVEEM[Oxidation (M)]S[Phospho (STY)]E_.2	#N/A	4.5054	4.7850	5.5664	4.6379	4.6612	5.0501	5.2957	5.0138	4.5334	4.7395	0.9958	-0.0061	0.9341	0.0296
Dkc1	_TVLES[Phospho (STY)]GGETGDGDNDTTKK_.2	#N/A	2.3116	3.2961	3.9061	3.3238	3.1901	3.4346	3.4720	3.3969	3.9765	3.0011	0.9370	-0.0938	0.5011	0.3000
Gtf2i	_ESTSSKS[Phospho (STY)]PPR_.2	Q9ESZ8_S823_M1_Gtf2i	3.8603	3.1647	4.5471	7.4290	2.9860	3.2426	3.6034	3.0687	6.8975	7.1447	0.8120	-0.3005	0.4424	0.3541
Jph3	_TS[Phospho (STY)]INSLR_.2	Q9ET77_S163_M1_Jph3	3.1008	4.2416	4.3354	3.8791	4.0629	6.6067	4.0274	6.8331	6.5496	3.5564	0.8339	-0.2620	0.3613	0.4421
Jph3	_TES[Phospho (STY)]PTTFSWTSHHR_.3	Q9ET77_S578_M1_Jph3	6.2932	5.2577	4.2372	5.9415	3.6472	2.9774	5.8702	3.2203	5.5264	6.2140	0.9074	-0.1401	0.5902	0.2290
Jph3	_SLPVALES[Phospho (STY)]DEETGDELK_.2	Q9ET77_S706_M1_Jph3	8.7856	9.5091	9.9101	10.5063	8.8341	9.6411	9.9025	10.2172	9.8403	9.7730	0.9595	-0.0596	0.2752	0.5603
Jph3	_S[Phospho (STY)]PLRTS[Phospho (STY)]INSLR_.2	Q9ET77_S158_M2_Jph3	3.6013	3.7412	3.3349	4.3795	4.2458	6.3722	4.5278	4.1225	4.4215	4.0568	0.9993	-0.0010	0.9959	0.0018
Jph3	_QVS[Phospho (STY)]VDEER_.2	#N/A	8.8789	8.6896	9.0190	9.0138	8.0578	8.8522	8.9813	8.9702	8.5807	8.8950	0.9882	-0.0172	0.6119	0.2134
Jph3	_EFS[Phospho (STY)]PSFQHR_.2	Q9ET77_S420_M1_Jph3	12.1224	12.0010	12.7567	12.2831	11.2868	11.8074	12.6155	12.2383	12.0180	11.9466	0.9867	-0.0192	0.5755	0.2400
Jph1	_VLEKPPS[Phospho (STY)]PKES[Phospho (STY)]PHFYR_.3	#N/A	9.8489	9.6964	10.0028	10.0260	8.8834	9.4896	10.2541	9.9596	9.6742	9.7684	0.9742	-0.0378	0.3191	0.4961
Jph1	_QNPS[Phospho (STY)]PGAR_.2	#N/A	7.8573	8.0287	8.1223	8.1401	6.3380	6.9300	8.1720	8.0856	7.7509	7.8781	0.9495	-0.0747	0.3357	0.4741
Jph1	_KQNPS[Phospho (STY)]PGAR_.2	#N/A	4.8400	5.0252	4.8876	4.9450	2.9696	3.2591	3.6199	4.4004	4.4331	4.4955	1.0198	0.0283	0.8728	0.0591
Jph1	_QSHS[Phospho (STY)]PQPSS[Phospho (STY)]PEPSK_.2	Q9ET80_S475_M2_Jph1	3.4274	3.4559	3.9342	3.4589	3.3442	3.2804	3.1735	3.1588	4.3082	3.4259	0.9906	-0.0136	0.8957	0.0478
Jph1	_TS[Phospho (STY)]LASLR_.2	Q9ET80_S162_M1_Jph1	2.9024	4.3238	3.5892	4.0918	3.5289	4.2006	3.8065	4.0266	3.6752	4.0588	0.9694	-0.0448	0.6853	0.1641
Jph1	_QS[Phospho (STY)]HSPQPSS[Phospho (STY)]PEPSKK_.3	Q9ET80_S473_M2_Jph1	3.2247	4.0299	3.9115	4.4141	3.8512	3.8783	4.1288	3.7044	3.3529	4.3811	0.9982	-0.0026	0.9800	0.0088
Jph1	_RGS[Phospho (STY)]LLGS[Phospho (STY)]MK_.2	Q9ET80_S216_M2_Jph1	3.2518	4.0081	4.4864	4.0301	3.8964	4.2290	4.1784	2.9710	3.2701	3.7074	1.1280	0.1737	0.1663	0.7792
Rcan1;Rcan3	_QFLIS[Phospho (STY)]PPAS[Phospho (STY)]PPVGWK_.2	Q9JHG6_S161_M2_Rcan1;Rcan3	3.0472	7.8561	4.0600	8.9164	7.0196	3.8308	3.9637	7.7621	7.3584	3.8612	1.0091	0.0130	0.9732	0.0118
Ivd	_FLQENLAPK_.2	#N/A	4.9418	5.2092	4.8755	5.5079	5.4609	5.0340	4.9843	5.6062	4.1786	5.5434	1.0184	0.0263	0.7597	0.1194
Ivd	_LYEIGAGTSEVR_.2	#N/A	5.5571	3.9087	4.3869	4.5069	5.8757	5.1243	4.2216	5.6503	5.1145	4.9004	0.9842	-0.0229	0.8659	0.0625
Rabggta	_ELLLC[Carbamidomethyl (C)]NNR_.2	#N/A	5.2632	5.2091	5.5809	6.2386	5.2894	5.5742	5.6723	4.9294	5.8087	5.3828	1.0142	0.0204	0.7635	0.1172
Rabggta	_LPENVLLR_.2	#N/A	5.3673	5.8684	4.6625	4.9841	5.0767	3.6268	4.9841	5.0811	5.2015	5.2620	0.9608	-0.0577	0.6194	0.2080
Lamtor2	_AQALVQYLEEPLTQVAAS_.2	#N/A	3.0826	3.1752	4.3172	3.8609	1.1250	4.3982	4.0092	3.1403	1.4307	3.5382	1.0980	0.1349	0.7067	0.1507
Palmd	_SGPQC[Carbamidomethyl (C)]SS[Phospho (STY)]PTC[Carbamidomethyl (C)]QEETEDVR_.2	Q9JHU2_S385_M1_Palmd	6.2783	3.2097	6.3896	6.6370	4.7750	3.5266	3.4197	5.7725	5.5916	3.6720	1.1131	0.1546	0.5851	0.2328
Palmd	_SNGPTHTS[Phospho (STY)]PTRPTPQPR_.3	Q9JHU2_S321_M1_Palmd	5.8618	3.5190	3.9973	6.8293	5.6971	3.2173	3.1105	6.0002	5.7872	3.2183	1.0717	0.0999	0.7481	0.1261

Palmd	_MELS[Phospho (STY)]PSRAS[Phospho (STY)]PGK_.2	Q9JHU2_S370_M2_Palmd	6.5005	3.1568	3.9815	7.1456	5.4111	4.8310	5.5097	4.7459	4.8595	5.6349	0.9968	-0.0046	0.9839	0.0070
Dync1h1	_LVQT[Phospho (STY)]PLTDR_.2	Q9JHU4_T1880_M1_Dync1h1	10.3163	9.8952	10.1040	5.1737	8.1134	9.8305	5.3047	9.9513	5.5590	9.9904	1.1564	0.2096	0.4308	0.3657
Dync1h1	_VTDFGDKVEDPTFLNQLQSGVNR_.3	#N/A	7.3421	7.2953	7.8442	7.1290	5.8315	6.7431	7.6820	7.6370	7.0921	6.9107	0.9591	-0.0602	0.4561	0.3409
Dync1h1	_VAAPDVVVPTLDTVR_.2	#N/A	6.4624	6.4772	6.8542	6.3646	6.3819	6.2139	7.3220	7.1213	6.4356	6.3601	0.9485	-0.0763	0.1503	0.8231
Dync1h1	_EQPWVSVQPR_.2	#N/A	6.4479	6.6345	6.5054	5.8695	5.9208	6.1765	7.0415	7.0181	6.2102	6.3406	0.9408	-0.0880	0.1369	0.8635
Dync1h1	_EYQTQLIQR_.2	#N/A	7.0276	6.4371	6.6446	6.1414	6.2420	6.4021	7.0967	6.5926	6.8559	6.0126	0.9764	-0.0345	0.5404	0.2673
Dync1h1	_SLLQALNEVK_.2	#N/A	5.9361	6.3973	6.7717	5.8916	5.3446	5.5016	5.5871	6.4192	5.9542	6.0624	0.9947	-0.0077	0.9194	0.0365
Dync1h1	_LLNTFLER_.2	#N/A	5.6918	6.2417	6.2210	5.3865	4.5239	5.6923	6.3422	6.2087	6.0236	5.4918	0.9351	-0.0968	0.3047	0.5162
Dync1h1	_VTFVNFTVTR_.2	#N/A	5.7177	5.9349	6.0588	5.2745	5.5063	5.8193	5.7632	6.0143	6.2428	6.2255	0.9434	-0.0840	0.0809	1.0922
Dync1h1	_QNLDGLLNQLK_.2	#N/A	5.2075	5.0769	3.9529	4.2451	3.5052	4.5032	5.2730	5.0248	4.3547	3.8066	0.9567	-0.0638	0.6510	0.1864
Dync1h1	_EWTDGLFTHVLR_.2	#N/A	4.8296	4.0871	5.0911	3.6115	3.6811	3.7362	4.3474	4.9016	3.6897	3.9590	0.9878	-0.0177	0.8969	0.0473
Dync1h1	_TFSEILNR_.2	#N/A	5.1775	5.8383	5.8598	4.5676	4.9755	4.9743	5.6353	5.9791	5.5002	5.4521	0.9274	-0.1087	0.1821	0.7397
Dync1h1	_LNTQEIFDDWAR_.2	#N/A	5.4120	4.8540	3.8681	5.5486	4.8585	3.3465	5.8875	4.6854	4.8135	3.0891	1.0063	0.0090	0.9647	0.0156
Dync1h1	_LSLSNAISTVLPLTQLR_.2	#N/A	4.6274	4.3607	4.2647	3.9418	2.5758	3.7072	4.4895	4.5718	4.5768	4.1717	0.8788	-0.1863	0.1937	0.7129
Dync1h1	_FLSDPQVHTVLVER_.3	#N/A	5.7053	5.4730	4.4410	3.8366	4.4241	5.0260	5.4267	5.7527	5.2831	4.9468	0.9001	-0.1518	0.2030	0.6925
Dync1h1	_IQGLTVEQAEAVVR_.2	#N/A	3.8533	3.1190	5.1872	3.5033	2.9404	3.4771	5.2125	4.4663	3.1475	3.6265	0.8947	-0.1605	0.4511	0.3458
Dync1h1	_LQGATC[Carbamidomethyl (C)]SNNK_.2	#N/A	4.4588	4.0300	3.3390	4.6090	4.2178	3.9967	4.5884	5.4020	4.4670	4.2572	0.8782	-0.1875	0.0956	1.0193
Dync1h1	_SAC[Carbamidomethyl (C)]DTVDTWLDDTAK_.2	#N/A	4.6527	4.2015	3.8026	4.3052	4.3683	3.6686	4.0199	4.3072	5.1874	4.2722	0.9370	-0.0939	0.3391	0.4697
Dync1h1	_EVWNTYELDLVNYQNK_.2	#N/A	5.3600	4.3755	5.4380	4.7243	3.1422	3.0865	5.3915	4.8327	5.0776	4.4459	0.8820	-0.1811	0.3232	0.4905
Dync1h1	_VQVALEELQDLK_.2	#N/A	3.4126	4.3802	3.7441	4.2468	3.9780	4.0683	3.9615	4.6601	4.9107	4.2138	0.8952	-0.1597	0.0960	1.0176
Dync1h1	_FNYGFEYLGVQDK_.2	#N/A	4.4424	3.7947	3.6763	4.3260	3.3143	4.5557	5.0285	4.9029	4.6492	4.4463	0.8448	-0.2434	0.0268	1.5721
Dync1h1	_ESPEVLLTLDILK_.2	#N/A	4.6263	3.4126	3.1246	3.7926	4.0355	3.8191	4.7370	4.2133	4.2744	4.3619	0.8647	-0.2097	0.0667	1.1758
Dync1h1	_LKQDGDSFR_.2	#N/A	5.9615	3.0047	5.7507	5.0505	4.8210	5.5230	5.9875	5.7861	3.6851	5.3739	0.9636	-0.0535	0.7896	0.1026
Dync1h1	_LQGEFQLR_.2	#N/A	4.7556	4.5950	4.9855	4.5583	3.7456	4.7487	4.8631	5.9914	4.9069	4.5575	0.8986	-0.1542	0.1581	0.8012
Dync1h1	_IFTIESAR_.2	#N/A	2.2952	3.2799	3.7581	3.3019	5.1501	5.5201	3.4502	3.6993	3.9983	4.7664	0.9763	-0.0346	0.8908	0.0502
Dync1h1	_ENFIPTIVNFSAEEISDAIR_.2	#N/A	4.0305	3.2859	3.7642	4.8156	3.1742	3.4504	3.3436	4.3698	4.5327	2.9852	0.9857	-0.0207	0.9030	0.0443
Dync1h1	_M[Oxidation (M)]LSAVSQQVQC[Carbamidomethyl (C)]IQEALR_.2	#N/A	4.0080	4.0003	5.0329	3.4150	3.6768	3.6209	4.2740	4.5749	4.2445	4.1928	0.9161	-0.1264	0.2624	0.5811
Dync1h1	_TDS[Phospho (STY)]TSDGRPAWMR_.2	#N/A	8.3375	3.7435	4.1527	8.6415	3.3853	3.6950	3.8421	3.8761	3.8670	3.6326	1.3999	0.4853	0.2612	0.5830
Dync1h1	_SPLVM[Oxidation (M)]DVLNIQGVQR_.2	#N/A	3.6754	4.1719	4.4216	3.3640	2.0979	4.1225	5.2382	4.2530	4.0853	3.4905	0.8536	-0.2283	0.2628	0.5803
Dync1h1	_IAFIM[Oxidation (M)]DESNVLDSGFLER_.2	#N/A	3.9691	3.6236	3.9962	3.6902	3.1129	3.5118	4.3801	3.4614	3.0531	4.4247	0.9532	-0.0691	0.5872	0.2312
Dync1h1	_DHLYGTLDPNTR_.2	#N/A	5.7468	3.6260	3.4501	4.4947	3.4473	4.2822	5.0433	5.3895	4.3064	5.2084	0.8371	-0.2565	0.1362	0.8657
Dync1h1	_DVLLVAQGEM[Oxidation (M)]ALEEFLK_.2	#N/A	4.1018	3.3572	3.8355	3.5575	3.2455	3.3791	3.2722	3.1026	4.2095	3.0565	1.0496	0.0699	0.5508	0.2590
Dync1h1	_QLQNISQAAASGGAK_.2	#N/A	4.9698	3.0462	3.3659	3.8686	4.2966	4.8093	3.5832	4.5634	3.8985	3.8356	1.0225	0.0321	0.8401	0.0757
Dync1h1	_IQFVGAC[Carbamidomethyl (C)]NPPTDPGR_.2	#N/A	3.8102	3.1145	4.4970	3.5053	2.9359	3.2928	3.6536	6.3296	3.7949	3.1826	0.8316	-0.2661	0.2898	0.5379
Dync1h1	_FTQDTQPHYIYSPR_.3	#N/A	3.9905	3.3519	3.7242	3.2679	3.1732	4.4873	3.4162	4.0894	3.9261	4.4461	0.9235	-0.1148	0.3545	0.4503
Dync1h1	_LEDDAKDNQQK_.2	#N/A	3.1916	3.9479	4.4261	3.9699	3.6993	4.2893	4.1824	3.0313	3.3303	3.6472	1.1051	0.1442	0.2457	0.6095
Dync1h1	_VAEVLFDAADANAIEEVNLAYENVK_.3	#N/A	4.2169	3.4724	3.9506	3.4944	3.3607	3.2639	3.6426	3.5068	3.8059	3.1717	1.0268	0.0382	0.6757	0.1702
Dync1h1	_TFSSIPVSRIC[Carbamidomethyl (C)]K_.2	#N/A	3.1740	3.9792	4.5977	4.1415	3.8005	3.9290	4.2898	4.3605	1.6333	1.7584	1.3078	0.3871	0.1892	0.7230
Dync1h1	_EAIVNSC[Carbamidomethyl (C)]VFVHQTLHQANAR_.3	#N/A	4.1800	3.9173	4.1618	4.2034	4.0697	3.9909	5.2890	4.2986	3.0968	3.8807	0.9869	-0.0190	0.8861	0.0525
Dync1h1	_LALESIC[Carbamidomethyl (C)]LLLGESTTDWK_.2	#N/A	2.9845	3.7408	4.2190	3.7628	3.6291	2.9955	3.9111	3.2384	3.5374	3.0267	1.0370	0.0524	0.6724	0.1724
Isyna1	_SC[Carbamidomethyl (C)]IENIFR_.2	#N/A	5.2040	3.3476	4.3172	4.5282	5.0921	4.3497	6.5104	6.4847	4.6077	4.7277	0.8013	-0.3197	0.0736	1.1329
Isyna1	_VIVLWTANTER_.2	#N/A	4.2850	2.9411	3.4710	3.9737	4.5746	4.3188	3.6883	4.2513	4.3480	3.9407	0.9680	-0.0469	0.7087	0.1495
Isyna1	_HVFVGGDDFK_.2	#N/A	4.6799	4.4609	3.2567	4.3479	4.3081	4.8907	3.8510	4.2007	2.9989	4.2071	1.1336	0.1809	0.2016	0.6955
Nit2	_AVDNQVYVATASPAR_.2	#N/A	3.5879	3.3871	3.8653	3.4091	4.7128	3.8901	3.5574	3.9807	3.8506	3.0864	1.0525	0.0738	0.5394	0.2681
Nit2	_TLS[Phospho (STY)]PGDSFSTFDTPYC[Carbamidomethyl (C)]K_.2	Q9JHW2_S133_M1_Nit2	2.9639	3.7691	3.6507	5.3485	3.5905	4.1390	3.8681	4.1235	3.6137	4.1204	0.9946	-0.0078	0.9611	0.0172
Nit2	_FAELAQIYAQR_.2	#N/A	3.6847	2.9402	3.4184	4.4630	2.8284	3.7962	3.1104	4.0390	4.3381	4.1403	0.9014	-0.1497	0.3353	0.4746
Stk3	_ELEEEENS[Phospho (STY)]DEDELDSHTM[Oxidation (M)]VK_.3	#N/A	3.4595	4.1436	3.6355	5.2598	4.0454	4.5230	4.3220	5.2433	4.6234	5.3717	0.8543	-0.2271	0.1026	0.9887
Nudt3	_LVGIFENQER_.2	#N/A	7.5418	7.6002	7.8605	7.5100	7.2445	7.6677	7.4797	7.4151	7.4748	7.3868	1.0177	0.0253	0.2450	0.6109
Nudt3	_SESEEEVLLVSSSR_.2	#N/A	5.2003	7.6231	8.1067	8.8064	7.6147	7.6968	7.9670	5.3790	8.0289	7.9923	1.0226	0.0323	0.8424	0.0745
Nudt3	_WIVPGGGM[Oxidation (M)]EPEEEPSVAAVR_.2	#N/A	5.2299	4.9786	5.6673	5.5807	5.0864	5.5289	5.4810	5.3656	4.9728	5.2423	1.0152	0.0217	0.6496	0.1874
Rnf14	_LTYHGLS[Phospho (STY)]PC[Carbamidomethyl (C)]K_.2	#N/A	8.7818	8.4082	8.6227	8.6446	7.4005	8.0316	9.0657	8.4154	8.2260	8.1502	0.9823	-0.0257	0.6446	0.1907
Rnf14	_LTYHGLS[Phospho (STY)]PC[Carbamidomethyl (C)]KVTAEK_.3	#N/A	4.2502	2.9760	3.4361	3.9388	2.8643	3.7603	3.6535	4.1797	3.8283	3.3438	0.9430	-0.0846	0.5120	0.2907
Actn2	_ETADTDTAEQVIASFR_.2	#N/A	3.6090	5.0514	5.1829	4.6773	4.7069	5.2821	5.4797	4.8228	4.8616	5.5393	0.9180	-0.1234	0.2568	0.5903
Actn2	_ISSSNPYSTVTM[Oxidation (M)]DELR_.2	#N/A	5.1522	6.0042	6.0979	4.8495	5.2807	5.8498	5.8557	5.9040	5.8282	5.5417	0.9579	-0.0620	0.3934	0.4052
Actn2	_ATLPEADGER_.2	#N/A	6.3824	3.1695	3.2426	6.8357	6.5286	6.2433	3.4600	6.8628	6.4053	6.1898	0.9426	-0.0854	0.7650	0.1163
Actn2	_AC[Carbamidomethyl (C)]LISM[Oxidation (M)]GYDLGEAEFAR_.2	#N/A	6.1463	5.6856	6.2380	4.1694	5.1414	4.3921	6.3307	5.5676	5.4096	5.5026	0.9286	-0.1069	0.4203	0.3764
Actn2	_HTNYTMEHIR_.3	#N/A	3.4715	3.4421	4.4709	4.5084	3.8028	3.2942	3.1874	3.1449	1.0356	3.4397	1.4181	0.5040	0.0615	1.2108
Actn2	_VEQIAAIAQELNELDYHDAVNVNDR_.3	#N/A	3.9725	3.2279	3.7061	3.6869	3.1162	3.5084	3.4016	4.4316	4.0802	4.4280	0.8656	-0.2082	0.0610	1.2149
Sgpp1	_NS[Phospho (STY)]LTGEEGELVK_.2	#N/A	3.2643	3.9619	3.9511	4.4537	3.8908	7.6952	4.1684	3.6648	3.3133	4.4207	1.1656	0.2210	0.4633	0.3342
Sgpp1	_RNS[Phospho (STY)]LTGEEGELVK_.2	#N/A	6.9045	7.0430	7.4996	4.1236	3.8184	6.9614	4.2719	4.3784	3.1766	6.9073	1.2936	0.3713	0.2232	0.6513
Lgi1	_DNALC[Carbamidomethyl (C)]ENAR_.2	#N/A	7.4128	7.6211	7.3425	7.9157	7.6917	7.7080	7.6049	7.5877	7.7317	7.7791	0.9921	-0.0114	0.6076	0.2164
Lgi1	_FQELNVQAPR_.2	#N/A	7.1362	6.6707	7.2836	6.9940	6.3859	6.5077	6.9157	6.7286	6.9828	6.7429	0.9981	-0.0027	0.9481	0.0232
Lgi1	_GLDSLTNVDLR_.2	#N/A	6.5564	6.1649	6.4654	6.1257	6.3121	6.4852	6.8134	6.1982	6.4099	6.4244	0.9830	-0.0247	0.4440	0.3527
Lgi1	_DFDC[Carbamidomethyl (C)]IITEFAK_.2	#N/A	6.2157	6.2149	6.7111	6.2800	6.0332	6.2557	6.5954	6.5548	6.1622	6.3375	0.9801	-0.0289	0.3903	0.4086
Lgi1	_SLIHLSLANNNLQTLPK_.3	#N/A	5.1033	4.7411	5.3148	5.1452	3.2619	4.1963	4.3362	4.4509	4.9353	5.2297	0.9766	-0.0342	0.8031	0.0952
Lgi1	_IQDIEVLK_.2	#N/A	5.6570	4.9955	5.7301	5.5462	5.3798	5.6691	4.3303	5.5236	4.9604	5.3409	1.0908	0.1254	0.1061	0.9745
Lgi1	_GNAFNC[Carbamidomethyl (C)]DC[Carbamidomethyl (C)]K_.2	#N/A	4.7118	4.8046	4.5459	3.5478	4.5172	4.4878	4.6331	4.9482	5.1101	4.6498	0.9174	-0.1244	0.1467	0.8337
Lgi1	_KPNDIETFK_.2	#N/A	3.6716	4.2889	3.8631	4.0347	3.8694	3.6421	4.0029	4.0186	3.1310	3.1041	1.0928	0.1281	0.2022	0.6943
Lgi1	_WGGSSFQDIQR_.2	#N/A	3.0244	5.1397	3.7112	5.5013	4.0901	4.0354	3.9285	4.7826	5.0047	4.1808	0.9500	-0.0740	0.6720	0.1727
Lgi1	_DTDVEYLEIARPPLALR_.2	#N/A	3.4845	3.4136	4.1519	4.0572	3.2513	3.8446	3.3651	3.8834	3.4909	3.1321	1.0671	0.0937	0.3330	0.4776
Lgi1	_C[Carbamidomethyl (C)]PAVC[Carbamidomethyl (C)]TC[Carbamidomethyl (C)]SK_.2	#N/A	4.1325	3.3879	3.8662	3.5269	3.2762	3.3484	3.2416	3.0908	4.2402	3.0872	1.0512	0.0720	0.5487	0.2606
Sh2b2	_S[Phospho (STY)]SEDVSAHAATK_.2	Q9JID9_S106_M1_Sh2b2	3.9318	3.2116	5.7323	3.5637	4.9277	3.3701	3.4887	5.7878	5.8323	3.4081	0.8906	-0.1671	0.5141	0.2889
Praf2	_ALDDFVLGSAR_.2	#N/A	5.6916	5.2850	5.7149	4.7971	5.3180	5.4377	5.9562	6.0792	5.3861	5.4771	0.9388	-0.0912	0.1480	0.8299
Praf2	_TPMGLLLEALGQEQEAGS_.2	#N/A	3.3484	4.1536	4.0352	4.6166	3.9750	3.7545	4.1153	3.5806	3.2292	3.0040	1.1431	0.1929	0.1217	0.9146
Inpp5e	_TLS[Phospho (STY)]LDDKGWR_.2	Q9JII1_S89_M1_Inpp5e	3.3728	4.1291	3.1065	4.2866	6.1505	4.1081	4.0012	7.8181	3.4805	7.3952	0.7389	-0.4366	0.1931	0.7141
Inpp5e	_FRGS[Phospho (STY)]QEDLTVQNGASPC[Carbamidomethyl (C)]R_.3	Q9JII1_S103_M1_Inpp5e	3.5096	3.8328	3.2432	5.8335	3.6542	4.0753	4.4361	4.2142	3.0124	3.9651	1.0302	0.0429	0.8313	0.0802
Dazap1	_LFVGGLDWSTTQETLR_.2	#N/A	4.4023	3.9904	4.5865	4.1303	4.1269	4.3434	4.3352	3.6392	4.4912	3.8076	1.0479	0.0675	0.3506	0.4551
Dazap1	_IFVGGIPHNC[Carbamidomethyl (C)]GETELR_.3	#N/A	4.0458	4.8588	6.0626	5.0419	4.2940	4.5589	3.4491	4.0160	3.0189	4.6619	1.2704	0.3453	0.0566	1.2471
Dazap1	_SYFSQYGEVVDC[Carbamidomethyl (C)]VIM[Oxidation (M)]K_.2	#N/A	3.5140	3.8285	5.1674	5.4012	3.5218	3.5759	4.4405	4.2098	3.3259	4.0591	1.0397	0.0562	0.7513	0.1242

Akr1a1	_DAGHPLYPFNDPY_.2	#N/A	7.2025	7.1360	7.4136	7.7407	7.2505	7.5860	7.5647	7.4634	7.4422	7.9663	0.9710	-0.0425	0.1912	0.7184
Akr1a1	_[Acetyl (Protein N-term)]TASSVLLHTGQK_.2	#N/A	6.9127	7.5984	7.4597	7.6367	7.5536	7.4285	7.2320	7.2587	7.1443	7.1910	1.0312	0.0444	0.1399	0.8541
Akr1a1	_ALGLSNFNSR_.2	#N/A	7.1877	7.2559	7.4438	7.0140	7.1311	7.2910	7.1445	7.2061	7.0455	7.0574	1.0151	0.0216	0.2207	0.6561
Akr1a1	_GLEVTAYSPLGSSDR_.2	#N/A	6.3090	6.5519	6.7495	7.3350	6.8497	6.8257	6.4861	6.4636	6.4186	6.8934	1.0312	0.0443	0.3260	0.4868
Akr1a1	_ILQNIQVFDFTFSPEEM[Oxidation (M)]K_.2	#N/A	4.1636	3.7080	4.5843	4.3630	4.0711	4.1240	4.3870	3.9300	4.7345	4.5635	0.9467	-0.0790	0.2816	0.5504
Akr1a1	_HALSAGYR_.2	#N/A	6.1097	6.3112	6.2168	6.6437	6.7558	6.2152	5.8784	5.9518	6.1931	5.9755	1.0626	0.0876	0.0310	1.5084
Akr1a1	_HPDEPVLLEEPVVLALAEK_.3	#N/A	4.1765	3.7945	4.3830	4.4215	3.8424	4.3017	4.0484	3.5815	4.0755	4.0089	1.0572	0.0803	0.2153	0.6669
Akr1a1	_EELFVTSK_.2	#N/A	1.8187	3.6213	4.0996	3.6433	2.3632	2.8985	4.5090	3.3578	3.6569	3.3206	0.8284	-0.2717	0.2341	0.6306
Akr1a1	_YDSTHYK_.2	#N/A	4.3180	3.5734	4.0517	3.5954	3.4617	3.1629	4.5569	3.4058	3.7048	3.2727	0.9890	-0.0160	0.8984	0.0465
Ddx21	_EIITEEPS[Phospho (STY)]EEEADM[Oxidation (M)]PKPK_.3	#N/A	3.5945	3.5036	3.8151	4.5636	5.1563	4.0802	3.8408	5.6433	3.8108	3.7592	0.9661	-0.0498	0.7742	0.1111
Sv2a	_GGLS[Phospho (STY)]DGEGPPGGR_.2	#N/A	14.1870	14.0115	14.4018	14.3173	13.0765	13.8440	14.5697	14.2706	14.0778	13.9971	0.9820	-0.0262	0.3654	0.4372
Sv2a	_HLQAVDYAAR_.2	#N/A	7.4383	7.3692	7.7324	7.7459	7.5076	7.1565	7.5418	7.7703	7.4180	7.3626	0.9958	-0.0061	0.8215	0.0854
Sv2a	_GGLS[Phospho (STY)]DGEGPPGGRGEAQR_.2	#N/A	8.2803	8.4078	9.2801	8.4840	7.7690	8.3339	8.5399	8.6861	8.5371	8.3999	0.9866	-0.0195	0.6627	0.1787
Sv2a	_M[Oxidation (M)]ADGAPLAGVR_.2	#N/A	7.7171	7.9907	7.5640	7.4386	7.7226	7.2138	7.7942	7.8123	7.7123	7.4071	0.9805	-0.0283	0.4239	0.3727
Sv2a	_FEEEDDDDDFPAPADGYYR_.2	#N/A	7.2031	7.0572	4.2508	7.9284	6.9443	7.0355	7.4915	8.0614	7.7175	7.4353	0.8776	-0.1884	0.1906	0.7199
Sv2a	_VFSVTHIK_.2	#N/A	6.8593	6.4962	6.8111	6.1775	5.7704	6.0829	6.8124	6.9803	6.4269	5.8003	0.9787	-0.0311	0.6582	0.1816
Sv2a	_DREELAQQYETILR_.3	#N/A	6.2051	5.8057	5.5006	5.2452	4.7681	5.6761	5.4984	5.4358	5.3016	6.0234	0.9944	-0.0082	0.9139	0.0391
Sv2a	_[Acetyl (Protein N-term)]MEEGFRDR_.2	#N/A	4.8039	3.3079	3.9875	5.4085	3.7652	3.8023	3.1259	3.6283	3.2769	4.1397	1.1797	0.2384	0.1808	0.7428
Sv2a	_GGQYFNDK_.2	#N/A	4.4496	3.7037	3.1545	3.6572	3.9515	3.6428	3.3718	4.5946	4.4234	3.8840	0.9242	-0.1138	0.3466	0.4602
Sv2a	_VQDEYSR_.2	#N/A	3.4394	3.3861	4.0135	3.9888	3.5204	3.7763	4.1371	2.8830	3.3114	3.6661	1.0537	0.0755	0.4797	0.3190
Sv2a	_TIHQEDELIEIQSDTGTWYQR_.3	#N/A	7.0015	6.9718	8.1050	8.0385	5.1462	7.1761	7.6471	7.1397	6.1904	6.4934	1.0299	0.0425	0.7426	0.1292
Acin1	_TAQVPS[Phospho (STY)]PPR_.2	#N/A	13.3880	13.2341	13.7056	13.7508	12.6658	13.1832	13.6337	13.3224	13.2599	13.3042	0.9956	-0.0064	0.7906	0.1020
Acin1	_TAQVPS[Phospho (STY)]PPRGK_.2	#N/A	10.9096	11.9637	12.9511	12.2037	11.2944	11.8187	11.7917	11.9419	11.4662	11.5130	1.0153	0.0219	0.6464	0.1895
Acin1	_LQPEQGS[Phospho (STY)]PK_.2	Q9JIX8_S729_M1_Acin1	11.4489	11.5154	11.9447	11.6828	10.9518	11.4110	11.5903	11.6906	11.5309	11.4047	0.9947	-0.0077	0.7329	0.1350
Acin1	_SLS[Phospho (STY)]PLSGTTDTK_.2	#N/A	10.2866	10.1113	10.5219	10.6148	9.4113	10.2076	10.5872	10.2248	10.0548	10.2387	0.9918	-0.0119	0.7299	0.1367
Acin1	_GVQAGNS[Phospho (STY)]DTEGGQPGR_.2	#N/A	7.9430	3.2017	8.4674	8.5124	7.6196	8.2390	8.1732	8.1443	3.8821	8.1757	1.0334	0.0473	0.8648	0.0631
Acin1	_SSSFSEEKGES[Phospho (STY)]DDEKPR_.3	#N/A	5.5630	5.3714	6.3907	6.0367	5.9700	6.0326	5.5791	6.3787	6.1766	5.8414	0.9833	-0.0242	0.6808	0.1670
Acin1	_KS[Phospho (STY)]LS[Phospho (STY)]PGVSR_.2	Q9JIX8_S655_M2_Acin1	6.0339	6.1958	6.4666	6.8973	5.8442	6.1235	6.6834	6.3820	4.2279	3.5061	1.2039	0.2677	0.1414	0.8497
Acin1	_S[Phospho (STY)]RST[Phospho (STY)]PVRDR_.2	Q9JIX8_S1326_M2_Acin1	5.1613	4.6151	5.2067	5.3460	3.6477	4.9208	3.9993	4.4789	5.4573	5.0615	1.0141	0.0202	0.8738	0.0586
Acin1	_KIS[Phospho (STY)]VVSATK_.2	Q9JIX8_S825_M1_Acin1	4.3376	4.3826	4.5380	4.9132	3.3869	4.8495	3.1309	4.6078	4.3508	4.0018	1.0941	0.1297	0.3475	0.4591
Acin1	_SLS[Phospho (STY)]PLSGTTDTKAES[Phospho (STY)]PAGR_.2	#N/A	4.7649	6.2968	5.5814	6.6828	6.1090	6.9193	4.1116	3.9245	4.0891	6.2992	1.3154	0.3955	0.0420	1.3767
Acin1	_SQS[Phospho (STY)]PLPEDLEK_.2	Q9JIX8_S389_M1_Acin1	3.9569	3.2205	3.8858	9.6478	8.2955	3.2946	3.9456	9.1289	7.8438	3.3792	0.8863	-0.1742	0.7155	0.1454
Acin1	_HLS[Phospho (STY)]HPEPEQQHVIQR_.3	#N/A	5.7693	6.1700	6.3744	5.8988	5.0835	5.4343	5.8793	5.8120	5.3940	5.7456	1.0141	0.0203	0.7612	0.1185
Acin1	_GVQAGNS[Phospho (STY)]DTEGGQPGRK_.2	#N/A	3.8776	3.1330	3.6113	3.1551	3.0213	3.6033	4.7303	3.8461	4.1452	4.3332	0.7975	-0.3265	0.0054	2.2710
Plekho1	_AKDPPQS[Phospho (STY)]PPDSESEQLLLETER_.3	Q9JIY0_S341_M1_Plekho1	3.7359	3.4903	4.4227	3.4245	2.8616	3.3671	3.1392	3.1931	4.3425	3.3915	1.0096	0.0138	0.9242	0.0342
Kcnip2	_DLDGS[Phospho (STY)]YDQLTGHPPGPSKK_.3	#N/A	8.4728	8.5471	9.1257	9.4860	8.5723	9.1570	8.8610	8.8473	2.9667	9.1647	1.1922	0.2536	0.2681	0.5717
Kcnip2	_DLDGS[Phospho (STY)]YDQLTGHPPGPSK_.2	#N/A	3.5286	7.5473	7.4532	8.8760	7.4344	8.3301	8.2174	7.9868	7.9233	8.2040	0.8901	-0.1679	0.3843	0.4153
Rprm	_RPS[Phospho (STY)]KEVEAVVVGPY_.2	Q9JJ72_S98_M1_Rprm	8.5554	8.6656	8.7873	9.4531	8.0946	9.1050	9.2551	9.2693	8.9795	8.8529	0.9656	-0.0505	0.2504	0.6014
Tamalin	_NFTQS[Phospho (STY)]PEQQR_.2	#N/A	9.5354	9.5724	9.9441	10.0558	9.3011	9.6211	9.8504	9.7425	9.6784	9.7003	0.9927	-0.0106	0.6381	0.1951
Tamalin	_FIPGLNRS[Phospho (STY)]LEEEESQL_.2	#N/A	8.4624	9.0239	9.9449	9.1353	8.7318	8.9328	9.1848	9.3849	8.8015	8.7209	1.0017	0.0025	0.9581	0.0186
Tamalin	_NFTQS[Phospho (STY)]PEQQRK_.2	#N/A	3.5347	4.2910	3.2684	4.3130	4.1793	3.9462	7.2081	4.1890	6.8148	3.9903	0.7066	-0.5010	0.0499	1.3017
Tamalin	_S[Phospho (STY)]LEEEESQL_.2	#N/A	3.8751	6.1191	4.5619	6.8860	5.9987	7.0736	6.8141	6.9198	6.9971	6.9193	0.8322	-0.2651	0.1135	0.9451
Rilpl1	_T[Phospho (STY)]SPQPESGIKR_.2	Q9JJC6_T348_M1_Rilpl1	3.2825	4.0599	4.5171	4.0608	3.9271	3.8483	7.5429	4.4412	3.2394	3.7381	0.8331	-0.2634	0.3463	0.4605
Rilpl1	_[Acetyl (Protein N-term)]M[Oxidation (M)]EEPLGS[Phospho (STY)]PPAALSALEK_.2	#N/A	9.5986	9.8426	10.3473	10.3709	9.6253	9.9783	11.1608	9.8048	9.9854	10.0108	0.9727	-0.0400	0.3784	0.4221
Tacc2	_VQNS[Phospho (STY)]PPVGR_.2	Q9JJG0_S419_M1_Tacc2	11.5498	11.7336	12.0411	12.3365	11.5569	11.8189	11.8295	11.6038	11.6298	11.8609	1.0092	0.0133	0.5268	0.2783
Tacc2	_FNS[Phospho (STY)]PSEELDYR_.2	Q9JJG0_S714_M1_Tacc2	7.6047	8.0144	7.9836	8.6492	7.4224	8.0779	8.7452	7.9458	8.0308	8.2393	0.9658	-0.0502	0.3095	0.5093
Tacc2	_TRPPS[Phospho (STY)]LK_.2	#N/A	7.3552	7.9926	8.0884	8.1749	7.3088	4.3234	4.2165	7.6545	7.5659	7.7474	1.0605	0.0847	0.6945	0.1583
Tacc2	_KTRPPS[Phospho (STY)]LK_.2	#N/A	5.0902	3.3141	3.7924	3.3361	3.2024	5.4637	3.4844	3.6650	3.9641	5.1986	0.9890	-0.0160	0.9413	0.0263
Tacc2	_LDNTPASPPRS[Phospho (STY)]PT[Phospho (STY)]EPSDTPIAK_.3	Q9JJG0_T516_M2_Tacc2	3.3525	4.7173	3.5426	6.6499	3.9900	3.9746	3.8418	3.9966	6.8580	6.3367	0.8313	-0.2666	0.3397	0.4689
Tacc2	_ETQQEPGEES[Phospho (STY)]PVPSEEHLAPETK_.3	Q9JJG0_S354_M1_Tacc2	4.9148	5.4626	5.8117	7.0872	4.8597	4.2864	3.8990	5.5515	5.7594	3.8154	1.1361	0.1841	0.3466	0.4602
Lancl2	_SGNYPSSLSNETDR_.2	#N/A	6.4512	6.2625	5.8891	6.2415	5.0488	5.7741	5.6643	6.5278	5.1303	5.3344	1.0495	0.0697	0.4525	0.3444
Lancl2	_SFPNPFPDYEAAASAAGLAAGSAEETGR_.3	#N/A	4.7680	4.1784	3.1558	4.6719	2.3380	3.5969	2.5669	3.5365	4.4280	3.8777	1.0507	0.0713	0.7575	0.1206
Sh3bgrl	_GDYDAFFEAR_.2	#N/A	5.1425	5.7858	6.2303	6.5519	6.1742	6.5655	4.7345	5.9649	6.1892	5.7892	1.0448	0.0632	0.5015	0.2997
Sh3bgrl	_QQDVLC[Carbamidomethyl (C)]FLEANK_.2	#N/A	4.0054	4.6223	4.1165	5.1984	5.0834	4.2887	5.0155	4.7319	4.4722	4.5130	0.9721	-0.0408	0.6470	0.1891
Pfn2	_SQGGEPTYNVAVGR_.2	#N/A	9.3894	9.2270	9.6868	9.7602	9.8796	9.3607	9.4683	9.3931	9.4206	9.4699	1.0119	0.0171	0.4230	0.3736
Pfn2	_DSLYVDGDC[Carbamidomethyl (C)]TM[Oxidation (M)]DIR_.2	#N/A	7.3081	7.7852	8.3623	8.2331	8.1181	8.3396	7.7124	8.0803	7.9781	7.6690	1.0209	0.0299	0.4824	0.3166
Pfn2	_EGVHGGGLNKK_.2	#N/A	6.8420	6.1611	5.1478	5.9618	5.1641	6.3799	6.5208	6.1050	6.0449	6.2113	0.9554	-0.0659	0.4567	0.3404
Pfn2	_VLVFVMGK_.2	#N/A	4.5674	2.6019	3.3058	4.7367	3.8905	3.9087	3.5988	4.0313	2.6426	4.0542	1.0708	0.0986	0.6135	0.2122
Pfn2	DNLM[Oxidation (M)]C[Carbamidomethyl (C)]DGC[Carbamidomethyl (C)]C[Carbamidomethyl (C)]QEA	#N/A	3.6993	2.6102	4.3861	4.4529	2.8250	3.2094	3.1026	3.2297	4.3791	1.3603	1.1698	0.2263	0.4421	0.3545
Cacng4	_ASSSS[Phospho (STY)]PYAR_.2	Q9JJV4_S228_M1_Cacng4	6.1294	3.9098	6.5199	4.5058	3.7981	4.3273	4.2205	3.6127	3.2612	2.9720	1.3835	0.4683	0.0655	1.1839
Cacng4	_STEAS[Phospho (STY)]PSRDAS[Phospho (STY)]PVGLK_.2	#N/A	4.5251	4.0134	5.1061	5.6030	4.5894	5.6765	5.7877	5.7465	5.0598	3.7796	0.9657	-0.0503	0.7360	0.1331
Cacng4	_DAS[Phospho (STY)]PVGLK_.2	Q9JJV4_S259_M1_Cacng4	4.6154	5.4418	3.8441	4.3468	3.7839	3.9456	4.8239	3.7717	3.4203	4.3138	1.1340	0.1815	0.3869	0.4124
Cacng3	_SRDLS[Phospho (STY)]PISK_.2	#N/A	10.3259	10.3431	10.8811	10.4423	9.3957	10.1363	10.9637	10.6721	10.4852	10.2059	0.9690	-0.0454	0.2743	0.5617
Cacng3	_S[Phospho (STY)]HSELLK_.2	Q9JJV5_S211_M1_Cacng3	6.6670	5.7106	5.7592	4.5689	4.0059	3.7235	4.2836	6.3198	3.1982	6.0795	1.0206	0.0294	0.9052	0.0432
Cacng3	_DLS[Phospho (STY)]PISK_.2	#N/A	3.8426	3.1470	4.5295	3.5313	2.9683	5.9140	3.2460	3.0864	6.8271	3.4983	0.9578	-0.0622	0.8500	0.0706
Smpd3	_DGDSGS[Phospho (STY)]LGSPSASR_.2	Q9JJY3_S289_M1_Smpd3	9.0187	3.9410	7.5746	4.0190	5.4880	8.1735	8.6857	7.6364	6.9716	8.9096	0.7911	-0.3380	0.1894	0.7225
Smpd3	_TAS[Phospho (STY)]VEYKGDGGR_.2	Q9JJY3_S208_M1_Smpd3	3.6719	6.5537	4.3587	6.1404	5.5372	3.4311	3.0752	6.9944	6.4965	3.3275	0.9951	-0.0071	0.9820	0.0079
Smpd3	_IY[Phospho (STY)]IDSPTNTSISAASFSSLVSPQGGDGSR_.3	Q9JJY3_Y170_M1_Smpd3	3.4846	6.0724	4.1714	7.2753	4.4388	3.6184	4.3887	6.3846	3.0930	3.1402	1.1392	0.1880	0.5636	0.2490
Ddx20	_DSS[Phospho (STY)]PGSLPK_.2	Q9JJY4_S569_M1_Ddx20	3.9812	5.7608	3.1755	3.6781	3.1250	3.4997	3.3928	4.4403	6.2512	3.6451	0.8731	-0.1957	0.4541	0.3429
Ddx20	_AAMHTYSSPTVATQS[Phospho (STY)]PK_.2	Q9JJY4_S472_M1_Ddx20	4.1676	3.0585	3.3536	3.8563	3.2933	2.9353	5.3595	4.2622	3.9108	3.8233	0.7938	-0.3332	0.0420	1.3773
Tuba8	_ES[Phospho (STY)]IDLVLDR_.2	Q9JJZ2_S114_M1_Tuba8	5.3511	4.8911	3.4446	5.0404	5.1165	5.4596	5.9597	5.3350	5.4684	5.4792	0.8783	-0.1872	0.1212	0.9165
Ube2j1	_NTSMS[Phospho (STY)]PR_.2	Q9JJZ4_S251_M1_Ube2j1	3.8484	3.1038	3.5820	6.3374	2.9921	3.6325	3.5257	4.3075	4.2381	3.7780	0.9883	-0.0169	0.9446	0.0248
Klf13	_ADLES[Phospho (STY)]PQRK_.2	#N/A	5.8723	4.8828	3.5232	6.4895	3.4630	6.0296	5.9960	4.0230	6.1603	5.9621	0.9111	-0.1343	0.5454	0.2633
Klf13	_SRADLES[Phospho (STY)]PQRK_.3	#N/A	4.1022	3.1240	3.2882	3.7908	3.2279	3.0008	3.5055	4.3277	3.9762	3.7578	0.8794	-0.1855	0.1071	0.9703
Pdk2	_TLSQFTDALVTIR_.2	#N/A	3.8446	3.8954	3.6561	4.1588	3.8997	4.1337	4.1306	3.9597	3.6083	3.7334	1.0190	0.0272	0.5968	0.2242
Kcnq5	_SPSTDITAEGS[Phospho (STY)]PTK_.2	#N/A	7.4312	3.4377	7.6733	7.3048	6.1253	3.3385	7.9738	7.5405	7.1027	6.7900	0.8005	-0.3210	0.1978	0.7038
Kcnq5	_SWS[Phospho (STY)]FNDR_.2	Q9JK45_S467_M1_Kcnq5	3.2918	4.0506	4.5263	7.1916	3.8719	7.2953	4.2183	4.4319	3.2302	3.7474	1.2895	0.3668	0.2537	0.5957
Kcnq5	_RSPSTDITAEGS[Phospho (STY)]PTK_.2	#N/A	2.9137	4.3237	4.7467	4.1032	4.2007	4.1762	6.1180	4.0153	3.6639	4.6437	0.8844	-0.1772	0.3449	0.4622
Uchl3	_FLENYDAIR_.2	#N/A	6.2632	5.8547	5.5570	6.2727	6.5232	5.9956	6.4606	6.6125	5.8663	6.4787	0.9564	-0.0643	0.2440	0.6126

Uchl3	_WLPLEANPEVTNQFLK_.2	#N/A	5.2090	5.5016	4.5286	4.8833	5.7765	6.0797	5.6889	5.0802	5.4749	6.1013	0.9541	-0.0678	0.4695	0.3284
Uchl3	_FLEESVSM[Oxidation (M)]S[Phospho (STY)]PEER_.2	#N/A	7.0504	7.9967	8.3283	8.0470	7.6994	8.1947	8.1968	8.0730	7.8894	7.9995	0.9809	-0.0278	0.5411	0.2667
Uchl3	_TSDETLLEDAIEVC[Carbamidomethyl (C)]KK_.3	#N/A	3.2987	4.0550	3.8580	4.3606	3.9433	3.6814	4.0753	3.7579	3.4065	4.3276	0.9934	-0.0095	0.9181	0.0371
Ybx3	_S[Phospho (STY)]RPLNAVSQDGK_.2	Q9JKB3_S328_M1_Ybx3	7.4410	7.1673	8.1299	8.5244	7.2831	7.7842	7.7752	7.4794	7.5102	7.9702	1.0049	0.0071	0.8973	0.0470
Ybx3	_[Acetyl (Protein N-term)]S[Phospho (STY)]EAGEATTGGTTLPQAAADAPAAAPPDPAPK_.3	Q9JKB3_S2_M1_Ybx3	3.2981	4.0544	4.5326	4.3612	3.9427	4.1828	4.0759	3.7572	3.4058	4.3282	1.0437	0.0617	0.5464	0.2625
Cend1	_PAPTVPAAPSS[Phospho (STY)]PDATSEPK_.2	#N/A	9.3153	9.0145	3.9617	9.5779	8.2306	3.9205	9.7386	9.1731	8.9775	3.8103	0.9258	-0.1113	0.7448	0.1279
Cend1	_DTQAEKQEQAAAPGPAATK_.3	#N/A	7.6326	8.2360	8.4866	7.9782	8.2046	7.7902	7.9393	8.1767	7.8782	7.8707	1.0111	0.0159	0.6177	0.2092
Cend1	_QEQAAAPGPAATK_.2	#N/A	7.8993	7.6674	7.0493	7.6575	8.2391	7.7345	7.5500	7.4722	7.6376	7.6840	1.0161	0.0230	0.5631	0.2494
Cend1	_DTQAEKQEQAAAPGPAATKK_.3	#N/A	6.6595	6.8626	7.0947	6.4168	6.7854	7.1925	6.9404	6.8651	6.5675	6.6422	1.0121	0.0173	0.6268	0.2028
Cend1	_VPQATAEAK_.2	#N/A	7.0403	6.2867	6.0890	5.9704	6.8850	6.6571	5.7673	6.3532	6.5865	6.9510	1.0115	0.0165	0.8109	0.0911
Cend1	_SASS[Phospho (STY)]PKPDTK_.2	#N/A	8.2546	7.6164	8.1064	7.8001	7.3059	7.5862	8.3036	8.1821	7.5013	8.1311	0.9687	-0.0459	0.3050	0.5158
Cend1	_SASS[Phospho (STY)]PKPDTKVPQATAEAK_.3	#N/A	11.7069	12.4127	13.6052	12.4232	12.1380	12.4989	12.4082	12.3792	12.2947	8.3089	1.0984	0.1354	0.2320	0.6345
Cend1	_GPGDGAEEDESNTGGR_.2	#N/A	5.0731	4.9669	4.8425	4.9950	4.5959	4.6400	5.0746	5.3664	5.1808	5.2424	0.9303	-0.1043	0.0114	1.9444
Cend1	_ADPVLLNNHSNLKPAPTVPAAPSS[Phospho (STY)]PDATSEPKGPGDGAEEDESNTGGR_.5	#N/A	6.7702	8.3993	9.1748	9.3652	7.0555	7.5046	4.3015	7.9859	8.0372	7.3836	1.1614	0.2158	0.2486	0.6045
Cend1	_ADPVLLNNHSNLKPAPTVPAAPSS[Phospho (STY)]PDATSEPK_.4	#N/A	3.9364	9.8158	9.8820	11.0994	9.4151	7.9536	3.3621	9.7872	8.2698	9.9328	1.1079	0.1478	0.6467	0.1893
Cend1	_GKS[Phospho (STY)]ASSPKPDTK_.2	Q9JKC6_S7_M1_Cend1	3.7214	3.0257	4.4082	3.7260	2.8471	3.3816	3.1247	3.2076	4.3571	3.3770	1.0005	0.0007	0.9963	0.0016
Scamp5	_AQEEWTTGAWK_.2	#N/A	8.2930	7.8541	8.5745	7.8453	7.4413	7.8965	8.5554	8.2070	8.1445	7.9741	0.9713	-0.0420	0.3221	0.4920
Scamp5	HVQQAAQNAAM[Oxidation (M)]GAAQGAM[Oxidation (M)]NQPQTQYSATPNYTYSNEM[Oxidation (M)	#N/A	3.7549	3.4712	4.4417	3.4435	2.8806	3.2651	3.1582	3.1741	4.3235	3.4105	1.0075	0.0107	0.9413	0.0263
Nectin1	_AGPLGGSS[Phospho (STY)]YEEEEEEGGGGGER_.3	#N/A	3.6023	3.5852	4.2891	4.3441	3.4580	3.6610	3.6931	5.6659	4.0597	3.7973	0.8442	-0.2444	0.2549	0.5936
Nectin1	_AGPLGGSS[Phospho (STY)]YEEEEEEGGGGGERK_.3	#N/A	4.0498	3.3542	4.1895	3.2765	3.1755	3.9403	3.4248	3.4938	4.0346	3.9973	0.9804	-0.0286	0.7852	0.1050
Nectin1	_AGIPQHHPPM[Oxidation (M)]AQNLQYPDDS[Phospho (STY)]DDEKK_.4	#N/A	3.8563	3.6021	3.9169	3.9474	8.3924	9.1874	3.7705	9.7306	3.7782	4.0206	1.0298	0.0424	0.9301	0.0315
Stx6	_[Acetyl (Protein N-term)]S[Phospho (STY)]M[Oxidation (M)]EDPFFVVK_.2	#N/A	7.6484	7.3031	7.4003	6.9946	5.8362	7.5039	6.8619	7.5105	7.3639	7.5412	0.9720	-0.0410	0.5856	0.2324
Tmod2	_ANDPSLQEVNLNNIK_.2	#N/A	9.1740	9.6330	10.1754	10.3315	9.2964	9.4593	9.6440	9.4281	9.3321	9.3426	1.0256	0.0365	0.3597	0.4441
Tmod2	_SLETNTHVK_.2	#N/A	6.1801	6.2390	6.1084	6.2784	7.0509	6.5835	5.7797	6.5517	5.9947	6.1311	1.0879	0.1215	0.0324	1.4900
Tmod2	_SNDPVALAFAEM[Oxidation (M)]LK_.2	#N/A	4.6820	5.2662	4.8189	4.7348	5.2798	5.3364	5.7327	5.1869	5.1143	5.0959	0.9503	-0.0736	0.2173	0.6629
Tmod2	_NIDEDELLGK_.2	#N/A	5.7373	5.0587	3.4742	5.6628	5.6604	5.7971	4.5083	4.8699	5.4785	5.3242	1.0370	0.0524	0.7145	0.1460
Tmod2	_QLENVLDDLDPESATLPAGFR_.2	#N/A	4.7872	5.3490	4.7145	5.8867	5.8646	5.2580	5.3812	5.3436	5.1603	5.3402	1.0007	0.0010	0.9889	0.0049
Tmod2	_ENDTLTEIK_.2	#N/A	6.3643	6.2061	4.3397	6.1684	6.6802	6.1764	5.2997	5.8367	5.9697	6.1742	1.0291	0.0413	0.7163	0.1449
Tmod2	_DREDFVPFTGEK_.2	#N/A	5.9852	5.8736	5.6415	6.0995	6.1859	5.9219	5.9913	5.8228	6.1923	6.1478	0.9855	-0.0210	0.4792	0.3195
Tmod2	_SLNVESNFITGTGILALVEALR_.2	#N/A	2.9260	3.6823	4.1605	3.2325	3.5706	3.0541	4.4481	1.0231	3.0337	3.1995	1.1748	0.2324	0.4239	0.3728
Ndufaf3	_VTGAALIPPPGETALASLGQASE_.2	#N/A	5.1861	5.9964	4.5967	6.1267	4.0068	4.1187	5.7912	3.8213	5.4878	6.0816	0.9452	-0.0813	0.6503	0.1869
Nova1	_QTLPSSPT[Phospho (STY)]TTK_.2	Q9JKN6_T156_M1_Nova1	7.9419	8.0852	8.5318	8.7374	8.1107	8.3975	8.3882	7.9362	8.2531	8.4103	0.9156	-0.1272	0.4756	0.3228
Nova1	_IKQTLPSS[Phospho (STY)]PTTTK_.2	#N/A	3.6980	3.5282	8.2455	3.3866	3.4165	7.6336	3.1013	3.2310	7.5391	3.3536	1.1576	0.2111	0.6525	0.1854
Nova1	_TLVEYQELTGAR_.2	#N/A	3.6810	3.5452	4.3678	3.3696	4.5153	3.4220	3.0843	4.1184	4.3975	3.3366	1.0221	0.0316	0.8214	0.0855
Chrac1	_REEEEDNEDDGS[Phospho (STY)]DLGEALA_.2	Q9JKP8_S122_M1_Chrac1	3.5188	4.3240	4.2056	7.1058	6.3294	6.9887	4.4229	6.3458	7.1003	6.6294	0.8837	-0.1784	0.4651	0.3324
Hyou1	_DAVIYPILVEFTR_.2	#N/A	5.0396	4.6216	3.5734	4.5483	5.0617	4.5150	4.6129	3.8042	4.6216	4.6224	1.0328	0.0465	0.6627	0.1787
Hyou1	_VLQLINDNTATALSYGVFR_.2	#N/A	3.9328	4.6464	5.5360	5.1024	3.5936	3.8200	3.6214	3.3646	4.6431	4.3930	1.1081	0.1481	0.3792	0.4212
Hyou1	_VESVFETLVEDSPEEESTLTK_.2	#N/A	4.0856	2.8578	3.2716	3.6003	3.2113	3.0173	3.4890	4.3442	3.9928	3.7413	0.8584	-0.2203	0.0740	1.1310
Ldb3	_ASSEGAQGSVS[Phospho (STY)]PK_.2	Q9JKS4_S179_M1_Ldb3	5.1265	4.8409	5.2339	5.8099	4.8069	4.2782	5.5338	5.5590	3.3193	4.7920	1.0448	0.0632	0.6726	0.1722
Habp4	_SLAASGAQQPDS[Phospho (STY)]PGGPQPPGQK_.2	#N/A	9.1809	9.1010	9.5059	9.9323	8.9104	9.3720	9.6390	9.1195	9.1302	9.4105	1.0010	0.0014	0.9669	0.0146
Habp4	_AFDS[Phospho (STY)]FDQR_.2	Q9JKS5_S206_M1_Habp4	5.6804	5.7553	6.1399	7.1932	4.7018	6.6895	5.7344	6.3402	6.4876	6.7955	0.9507	-0.0730	0.5299	0.2758
Habp4	_KSLAASGAQQPDS[Phospho (STY)]PGGPQPPGQK_.3	#N/A	8.5366	8.8173	9.1755	9.6605	8.4968	8.6737	8.6322	8.6728	8.5519	8.5640	1.0335	0.0475	0.2459	0.6093
Habp4	_S[Phospho (STY)]PAVASGHRPGAGGR_.3	Q9JKS5_S74_M1_Habp4	3.8016	3.5408	3.5353	4.5799	3.3621	4.3674	3.2274	3.9221	4.2211	4.2572	0.9891	-0.0158	0.8965	0.0474
Habp4	_RGEQQGWNDNR_.3	#N/A	2.3204	3.0793	3.9968	3.5406	2.4478	3.2177	3.6888	3.4606	3.7597	3.2179	0.8779	-0.1879	0.2409	0.6182
Adrm1	_DKKDEEEDM[Oxidation (M)]S[Phospho (STY)]LD_.2	#N/A	7.6244	7.1937	6.5711	8.4560	7.3736	6.9825	7.2988	6.9335	6.3605	6.9639	1.0693	0.0967	0.2237	0.6504
Adrm1	_SQS[Phospho (STY)]AAVTPSSSTSSAR_.2	Q9JKV1_S213_M1_Adrm1	5.8399	5.7965	5.0369	4.1168	5.3747	5.9975	5.1629	3.9897	5.4207	5.7893	1.0530	0.0745	0.5840	0.2336
Nudt5	_ESTESS[Phospho (STY)]PGKHLVTSEELISEGK_.3	Q9JKX6_S10_M1_Nudt5	5.6371	3.1781	4.5605	3.5623	2.9994	5.9160	3.2770	3.0553	4.2047	3.5293	1.2253	0.2931	0.2731	0.5636
Fmn2	_GATADDSGGGS[Phospho (STY)]PVLAAK_.2	Q9JL04_S493_M1_Fmn2	8.6163	8.5912	9.2426	8.8145	8.0125	8.6851	9.0033	8.4971	8.7165	8.3918	1.0009	0.0014	0.9724	0.0122
Fmn2	_SSILES[Phospho (STY)]PKK_.2	Q9JL04_S629_M1_Fmn2	10.1504	9.9971	10.5846	10.4936	9.6574	10.0304	10.5820	10.3081	10.2171	10.1837	0.9835	-0.0240	0.3932	0.4053
Fmn2	_LPEEEPS[Phospho (STY)]PKDVDTEPK_.2	Q9JL04_S614_M1_Fmn2	3.6421	2.9465	7.5853	7.3827	6.3595	3.4608	6.9682	7.3118	6.8844	7.1000	0.7401	-0.4343	0.1277	0.8939
Fmn2	_GS[Phospho (STY)]PGEEWVPEVEEASQR_.2	#N/A	10.2024	10.0174	10.4067	11.2444	10.1119	10.3816	10.6615	10.7142	10.5972	10.4502	0.9800	-0.0291	0.3853	0.4142
Fmn2	_SIQTS[Phospho (STY)]PTEEGR_.2	Q9JL04_S724_M1_Fmn2	10.8933	11.1398	11.6521	11.4586	10.4904	11.0011	11.3714	11.1309	11.0482	11.1226	0.9944	-0.0081	0.7825	0.1065
Fmn2	_S[Phospho (STY)]ADWTEELGVR_.2	Q9JL04_S459_M1_Fmn2	2.9086	3.6649	4.2481	3.2499	3.5532	3.0714	4.4654	3.3677	3.0163	3.2169	0.9809	-0.0279	0.8549	0.0681
Fmn2	_QLSSPNHSPS[Phospho (STY)]QSPNQSPR_.3	Q9JL04_S411_M1_Fmn2	4.0123	3.5022	4.3556	4.1157	4.0503	3.9630	3.9507	3.1849	3.5089	3.4581	1.1345	0.1821	0.0372	1.4295
Fmnl1	_SKPLDQSVEDLS[Phospho (STY)]K_.2	Q9JL26_S189_M1_Fmnl1	3.6209	7.8929	4.5598	7.9633	6.2239	7.8152	3.8520	7.7453	7.5810	7.6452	0.9463	-0.0796	0.7759	0.1102
Fmnl1	_EAAADTSGREEPPTPKS[Phospho (STY)]PPK_.3	Q9JL26_S1021_M1_Fmnl1	6.8173	6.8491	7.4737	7.1735	4.0660	6.7058	7.4222	7.4259	7.2581	7.2117	0.8888	-0.1701	0.2319	0.6347
Fmnl1	_GPGDVVSIEILPGAAAT[Phospho (STY)]PSGDDAQAPR_.3	Q9JL26_T509_M1_Fmnl1	3.3216	4.1268	4.4501	5.7593	3.9482	3.7813	4.1421	5.2274	4.9312	3.6711	0.9417	-0.0866	0.6231	0.2055
Gltp	_[Acetyl (Protein N-term)]ALLAEHLLKPLPADR_.3	#N/A	4.1797	5.4801	5.5768	5.4492	5.3592	4.0572	5.3118	5.9261	5.7197	5.3415	0.9000	-0.1521	0.1768	0.7525
Mpp6	_IDSILEVVQTGR_.2	#N/A	6.4474	5.5383	6.1841	6.4648	6.3700	6.1465	6.0216	6.0128	5.9745	5.7780	1.0412	0.0583	0.2162	0.6652
Mpp6	_VENNDLVIAR_.2	#N/A	5.3750	5.5835	5.3946	5.1633	5.8660	5.7064	5.0082	5.2307	5.4617	4.9763	1.0669	0.0934	0.0592	1.2275
Mpp6	_NAEFDRHEIQIYEEVAK_.3	#N/A	3.1993	3.9617	4.4338	4.5001	3.8439	4.2816	3.5283	4.1278	3.9465	3.4937	1.0696	0.0971	0.3703	0.4315
Mpp6	_YLEHGEYEGNLYGTK_.3	#N/A	2.9421	3.6984	4.1766	3.2164	3.5867	3.0379	4.4319	3.4013	3.0498	3.1834	0.9791	-0.0305	0.8360	0.0778
Mpp5	_NLDLAS[Phospho (STY)]PEEYPK_.2	Q9JLB2_S25_M1_Mpp5	8.4323	8.6610	8.6691	9.3059	8.0424	8.6392	9.0395	8.5873	8.6330	8.6497	0.9883	-0.0170	0.6617	0.1793
Cubn	_VNVT[Phospho (STY)]NEFKSSGNTM[Oxidation (M)]K_.3	Q9JLB4_T2533_M1_Cubn	3.9641	8.6316	3.6782	9.0596	3.7860	9.3482	3.6122	10.2304	9.6620	4.1478	0.9274	-0.1087	0.8097	0.0917
Sacs	_DSAPTTPTS[Phospho (STY)]PTEFLTPGLR_.2	Q9JLC8_S4266_M1_Sacs	3.3244	4.0133	5.5633	6.1656	4.3344	3.8610	4.2436	3.7050	4.7451	3.5610	1.1181	0.1611	0.4441	0.3525
Dclk1	_SPSPSPTS[Phospho (STY)]PGSLR_.2	#N/A	3.3314	11.2311	11.8853	11.6510	10.5375	11.3105	11.6252	11.2934	11.3667	11.2776	0.8771	-0.1891	0.4296	0.3669
Dclk1	_DIKPENLLVYEHQDGSK_.3	#N/A	7.2288	7.2765	7.7058	7.3469	6.3575	7.4570	7.7542	7.1414	7.2098	6.8605	0.9982	-0.0025	0.9646	0.0157
Dclk1	_SFEALLADLTR_.2	#N/A	6.1728	6.0983	6.1883	5.9796	6.1084	5.3274	6.2381	6.3972	6.4923	6.0762	0.9489	-0.0756	0.1153	0.9381
Dclk1	_S[Phospho (STY)]PASTSSVNGTPGSQLSTPR_.2	#N/A	3.0021	4.2241	3.6889	4.1915	3.6286	4.0131	3.9062	3.9269	3.5755	4.1585	0.9742	-0.0377	0.7018	0.1538
Dclk1	_TLSDNVNLPQGVR_.2	#N/A	7.5192	7.2314	7.5523	7.4057	7.3770	7.2513	7.4468	7.1882	7.5520	7.1055	1.0091	0.0130	0.5536	0.2568
Dclk1	_NVNPNWSVNVK_.2	#N/A	5.9333	5.1242	6.2307	5.8934	5.6231	5.5660	5.9724	6.1407	5.0466	6.0890	0.9856	-0.0209	0.7736	0.1115
Dclk1	_REESEEGFQIPATITER_.3	#N/A	6.9150	8.8908	8.6217	9.8741	8.4829	9.0920	8.1104	9.7274	9.5412	9.2717	0.9436	-0.0837	0.3960	0.4023
Dclk1	_TIGDGNFAVVK_.2	#N/A	5.6675	6.0820	6.3275	6.1463	6.1149	6.2276	6.3111	6.2383	5.7058	5.8426	1.0116	0.0166	0.6826	0.1659
Dclk1	_GIVYAISPDR_.2	#N/A	6.0524	5.7059	4.1748	5.7583	6.2061	6.0647	6.2920	6.0460	6.1029	5.5336	0.9444	-0.0825	0.4344	0.3621
Dclk1	_SKS[Phospho (STY)]PASTSSVNGTPGSQLSTPR_.3	#N/A	3.5039	3.7826	4.0340	3.9321	3.6975	4.9477	3.9877	8.5871	8.0118	4.1805	0.6433	-0.6365	0.0584	1.2336
Dclk1	_SPSPSPTS[Phospho (STY)]PGSLRK_.2	#N/A	10.8768	11.3698	11.8050	11.5482	10.6082	11.1008	11.3656	11.2629	11.0354	11.0117	1.0044	0.0063	0.8399	0.0758
Dclk1	_SGKS[Phospho (STY)]PSPSPT[Phospho (STY)]SPGSLR_.2	#N/A	6.1795	3.8265	3.5840	4.0139	3.6478	4.1190	4.5561	3.9797	3.6148	4.2061	1.0341	0.0483	0.7967	0.0987
Dclk1	_DM[Oxidation (M)]ELEHFDERDK_.3	#N/A	3.2019	3.2653	4.4364	4.0025	4.3795	4.6065	4.2091	4.1366	4.7104	3.9366	0.9373	-0.0933	0.4545	0.3424
Dclk1	_AQPAPPELNSESEDYS[Phospho (STY)]PSSSETVR_.3	Q9JLM8_S742_M1_Dclk1	7.9326	8.5196	9.1134	9.3835	8.0480	8.5033	8.9178	8.6745	9.2393	7.7692	0.9923	-0.0112	0.8664	0.0623

Dclk1	_ISQHGGS[Phospho (STY)]STSLSSTK_.2	#N/A	9.5478	9.2147	10.0867	9.9089	8.7319	9.6213	9.4061	9.3064	9.1584	9.2257	1.0264	0.0375	0.3629	0.4402
Dclk1	_VC[Carbamidomethyl (C)]SS[Phospho (STY)]M[Oxidation (M)]DENDGPGEGDELGRR_.3	#N/A	3.8484	3.5180	4.0144	5.4786	3.7320	5.7126	5.3979	6.2567	5.9972	5.9721	0.7423	-0.4299	0.0172	1.7649
Dclk1	_VC[Carbamidomethyl (C)]SS[Phospho (STY)]M[Oxidation (M)]DENDGPGEGDELGR_.2	#N/A	6.6734	7.0204	7.3949	8.2104	3.7951	7.2427	7.2671	7.2519	3.4929	6.7955	1.0840	0.1164	0.6359	0.1966
Dclk1	_SGKS[Phospho (STY)]PSPSPTS[Phospho (STY)]PGSLRK_.3	#N/A	3.8981	3.5621	3.7848	5.1445	4.0966	5.0115	4.2446	4.8933	5.5353	3.6940	0.9255	-0.1117	0.4814	0.3175
Dclk1	_TLQTLSSEK_.2	#N/A	4.1794	3.0059	4.2101	4.0350	4.3514	3.2443	3.6051	4.8917	4.8001	4.6467	0.8555	-0.2252	0.1202	0.9201
Dclk1	_EES[Phospho (STY)]EEGFQIPATITER_.2	#N/A	4.0248	3.3176	3.7585	9.8520	3.1389	3.0897	3.4505	9.5211	3.9980	2.9795	0.9084	-0.1387	0.8065	0.0934
Dclk1	_[Acetyl (Protein N-term)]S[Phospho (STY)]FGRDMELEHFDERDK_.3	#N/A	3.3581	3.8681	4.0449	4.0599	3.9846	3.7449	4.2622	3.5709	3.2195	3.0137	1.0929	0.1282	0.2367	0.6258
Dclk1	_[Acetyl (Protein N-term)]S[Phospho (STY)]FGRDMELEHFDER_.3	#N/A	3.6339	3.5923	4.3207	3.3225	3.4806	3.1440	4.5380	3.2951	2.9437	3.2895	1.0187	0.0267	0.8538	0.0686
Cd2ap	_S[Phospho (STY)]VDLDAFVAR_.2	Q9JLQ0_S458_M1_Cd2ap	4.3439	6.0465	4.0776	7.6514	5.8660	5.8013	3.7696	7.0206	5.6920	6.3984	0.9844	-0.0226	0.9205	0.0360
Higd1a	_[Acetyl (Protein N-term)]STNTDLSLSSYDEGQGSK_.2	#N/A	4.0168	4.5470	5.0788	4.8235	3.4747	4.4157	4.5479	4.8770	4.2691	4.3704	0.9727	-0.0400	0.7036	0.1527
Bag3	_SQS[Phospho (STY)]PAASDC[Carbamidomethyl (C)]SSSSSSASLPSSGR_.2	Q9JLV1_S179_M1_Bag3	5.4528	5.8382	5.2930	4.0342	3.6532	5.9827	6.0922	5.3989	3.5399	5.8150	0.9675	-0.0476	0.8075	0.0928
Bag3	_AASPFRS[Phospho (STY)]PVR_.2	Q9JLV1_S274_M1_Bag3	4.5131	3.2342	3.7124	3.2562	3.1225	3.5022	3.3953	3.7450	4.0441	4.4343	0.9109	-0.1346	0.3020	0.5200
Cul3	_FLQESFNNDR_.2	#N/A	4.2807	3.5361	4.0143	3.3787	3.4244	2.8903	7.3545	6.8742	4.3884	3.3457	0.6534	-0.6140	0.0455	1.3416
Auh	_LAIIPGGGGTQR_.2	#N/A	7.9513	8.0864	8.5220	8.5560	8.1558	8.1291	7.9807	8.4295	7.9321	8.0292	1.0174	0.0248	0.3910	0.4079
Auh	_HLEEENR_.2	#N/A	5.3428	6.9276	4.3172	7.0100	7.6767	6.8782	5.1995	4.9390	5.6125	5.2307	1.2122	0.2777	0.1266	0.8976
Auh	_EFLPQGPVAM[Oxidation (M)]R_.2	#N/A	6.8296	7.0295	6.9662	6.8512	7.1857	7.1459	7.3432	7.2130	6.9916	7.3467	0.9692	-0.0451	0.0571	1.2433
Auh	_SEVPGIFC[Carbamidomethyl (C)]AGADLK_.2	#N/A	3.2902	4.0954	3.9770	5.3924	4.9965	5.6284	4.1943	5.1075	5.1866	4.7442	0.9491	-0.0754	0.6377	0.1954
Auh	_AVGLISHVLEQNQEGDAAYRK_.3	#N/A	4.8006	4.8291	5.0465	3.8020	4.9170	5.0352	4.9835	4.5497	5.4318	5.0901	0.9451	-0.0815	0.3535	0.4516
Auh	_AVGLISHVLEQNQEGDAAYR_.3	#N/A	3.6473	3.8531	7.4523	4.7648	2.7734	4.0843	4.4805	4.2362	4.9441	4.1825	0.9929	-0.0103	0.9708	0.0129
Rabgef1	_QTS[Phospho (STY)]IETDR_.2	Q9JM13_S132_M1_Rabgef1	10.5538	10.6312	3.6835	11.0019	9.9674	10.7817	10.7220	10.6403	10.5698	10.7083	0.8852	-0.1759	0.4231	0.3736
Rabgef1	_YM[Oxidation (M)]SGQTS[Phospho (STY)]PR_.2	#N/A	8.2200	7.9685	7.8681	8.4173	8.3117	7.9399	7.8914	7.7861	8.1298	7.9181	1.0239	0.0341	0.1779	0.7498
Rabgef1	_LDAQSLNLSQEDFDRYM[Oxidation (M)]S[Phospho (STY)]GQTSPR_.3	#N/A	3.8130	3.4365	3.7943	4.0029	3.3075	3.6799	3.8339	3.8186	3.6684	3.9056	0.9647	-0.0518	0.3570	0.4473
Nt5c	_DTEVFIC[Carbamidomethyl (C)]TT[Phospho (STY)]PLLK_.2	#N/A	3.4341	3.7921	5.4558	4.6236	4.0606	6.0900	6.2720	5.3355	3.1435	3.0897	1.0260	0.0370	0.8912	0.0500
Nt5c	_WVEQNLGPEFVER_.2	#N/A	5.7617	5.7218	4.5472	6.7492	6.3331	6.1391	6.0390	5.9720	6.4004	5.1982	0.9954	-0.0066	0.9518	0.0214
Nt5c	_EM[Oxidation (M)]NDM[Oxidation (M)]KDTEVFIC[Carbamidomethyl (C)]TT[Phospho (STY)]PLLK_.3	#N/A	4.1486	5.3599	5.5166	5.6259	4.7710	3.5376	5.0785	4.7987	4.8620	4.8910	0.9835	-0.0240	0.8557	0.0677
Nt5c	_VLVDM[Oxidation (M)]DGVLADFESGLLQGFR_.2	#N/A	3.2072	3.9635	4.4418	4.4521	3.8518	4.2736	4.1668	2.8614	3.3149	3.6628	1.1514	0.2034	0.1419	0.8480
Mink1	_SQS[Phospho (STY)]LQDQPTR_.2	Q9JM52_S603_M1_Mink1	11.4659	11.4134	11.6088	11.5689	9.7649	11.4364	11.3278	11.5229	11.4778	11.3816	0.9809	-0.0278	0.5672	0.2463
Mink1	_LDSS[Phospho (STY)]PVLSPGNK_.2	Q9JM52_S746_M1_Mink1	11.5055	11.7373	11.8722	11.9131	10.1916	11.5436	11.9226	11.7999	11.5024	11.4841	0.9814	-0.0270	0.5411	0.2667
Mink1	_SDSVLPASHGHLPQAGS[Phospho (STY)]LER_.3	#N/A	11.3403	11.6396	11.9093	12.0198	9.2883	11.3115	11.4510	11.6935	11.3914	11.1043	0.9861	-0.0202	0.7686	0.1143
Mink1	_S[Phospho (STY)]DPGWER_.2	Q9JM52_S706_M1_Mink1	6.1609	5.0864	5.9582	6.2137	2.9569	3.2717	5.3734	6.0249	6.2333	6.3130	0.8255	-0.2767	0.2127	0.6722
Mink1	_QQNS[Phospho (STY)]PLAK_.2	Q9JM52_S537_M1_Mink1	4.2227	7.3949	7.4304	6.9375	4.8352	6.9788	3.6260	4.2071	3.8557	6.5665	1.3804	0.4651	0.0879	1.0560
Mink1	_GQS[Phospho (STY)]PPTKDGGSDYQSR_.2	Q9JM52_S903_M1_Mink1	7.6711	4.4190	8.7833	7.8880	7.5317	3.8182	8.1192	8.5684	7.9499	7.7390	0.8259	-0.2759	0.2175	0.6626
Mink1	_QQQQLQQQQQR_.2	#N/A	2.7038	3.6710	4.3047	3.3065	3.5412	3.4851	3.9834	4.3124	2.9597	3.2735	0.9641	-0.0527	0.7284	0.1376
Mink1	_VGASTKLDSS[Phospho (STY)]PVLS[Phospho (STY)]PGNK_.2	#N/A	3.2542	5.4437	3.9410	4.4436	3.8807	3.8488	4.1583	5.5494	3.6683	4.6471	0.9178	-0.1238	0.4754	0.3229
Kcnj10	_VAS[Phospho (STY)]PSGLR_.2	Q9JM63_S338_M1_Kcnj10	2.9472	4.2790	3.6340	7.4973	4.1673	3.9582	3.8513	3.9818	3.6304	7.6597	0.9232	-0.1152	0.7495	0.1252
Arpc3	_AYLQQLR_.2	#N/A	7.3754	7.5668	7.9140	7.1559	7.5802	7.0911	7.0327	7.6759	7.4525	7.5193	1.0037	0.0053	0.8898	0.0507
Arpc3	_DTDIVDEAIYYFK_.2	#N/A	6.2152	5.3566	5.4138	6.1455	6.1845	6.0433	6.0908	5.8978	5.9241	5.9464	0.9880	-0.0174	0.7370	0.1325
Arpc3	_LIGNMALLPLR_.2	#N/A	5.7573	4.4098	3.5706	6.6308	4.8771	4.2192	4.5631	3.9936	2.9911	4.3749	1.2337	0.3029	0.1795	0.7460
Arpc3	_ETKDTDIVDEAIYYFK_.3	#N/A	3.8129	4.1034	4.5658	3.8088	3.3651	2.8674	3.1913	4.0318	3.3458	3.4091	1.0742	0.1033	0.4590	0.3382
Arpc3	_PAYHSSLM[Oxidation (M)]DPDTK_.3	#N/A	4.9465	3.6703	3.9623	4.0914	5.6292	4.8356	3.8061	4.1247	3.7733	5.6839	1.0404	0.0571	0.7442	0.1283
Arpc3	_VFDPQSDKPSK_.3	#N/A	4.2557	3.5600	3.4417	3.9443	3.3951	4.3481	3.2081	4.1742	3.7683	3.9113	1.0156	0.0223	0.8318	0.0800
Arl6ip4	_S[Phospho (STY)]AGEDNDGPVLTDEQK_.2	Q9JM93_S140_M1_Arl6ip4	7.3196	7.6920	8.1611	7.8286	6.3960	7.5510	8.3019	7.6527	7.7587	7.4708	0.9609	-0.0575	0.3964	0.4019
Cdc42ep4	_SLSSS[Phospho (STY)]PVKK_.2	Q9JM96_S140_M1_Cdc42ep4	9.2496	9.4830	9.9225	9.8838	9.5531	6.7546	9.5874	9.3400	9.3778	9.4456	0.9686	-0.0461	0.6409	0.1932
Cdc42ep4	_EADDES[Phospho (STY)]LDEQASASK_.2	#N/A	8.2742	7.9224	8.4228	9.1689	7.0552	9.0749	8.1637	8.5056	7.8128	8.5180	1.0084	0.0121	0.8725	0.0592
Cdc42ep4	_DQWGS[Phospho (STY)]EEEEEAGGYR_.2	#N/A	3.5362	5.9156	3.2699	7.7375	6.2712	6.8483	6.9786	6.9783	7.3079	7.5216	0.7777	-0.3628	0.1234	0.9088
Cdc42ep4	_AREADDES[Phospho (STY)]LDEQASASK_.2	#N/A	7.6632	3.9087	7.5781	8.2453	3.3048	8.2406	7.2082	3.6115	7.2759	3.2543	1.2159	0.2821	0.4481	0.3486
Cdc42ep4	_DQWGS[Phospho (STY)]EEEEEAGGYRDK_.3	#N/A	3.0526	4.2337	4.0361	4.0533	3.9272	5.7357	3.9679	6.7526	5.8160	3.9046	0.8166	-0.2923	0.2249	0.6480
Cdc42ep4	_NAMS[Phospho (STY)]LPQLNEK_.2	Q9JM96_S116_M1_Cdc42ep4	4.0133	3.2687	3.1434	3.6460	3.1570	3.4676	3.3607	2.9716	4.1210	3.6130	0.9809	-0.0279	0.7999	0.0969
Usp14	_VNQQPNANDKNS[Phospho (STY)]PPKEIK_.3	Q9JMA1_S393_M1_Usp14	9.3076	9.1685	9.9315	10.0282	9.2093	9.4566	9.6312	9.6887	9.3405	9.3551	1.0014	0.0020	0.9504	0.0221
Usp14	_AS[Phospho (STY)]GEM[Oxidation (M)]ASAQYITAALR_.2	#N/A	5.4297	8.4626	8.8564	9.4421	3.9070	7.8704	8.3181	8.5733	8.1936	3.9998	1.0078	0.0112	0.9688	0.0138
Usp14	_QSPTLQR_.2	#N/A	4.4952	4.6428	2.7339	3.7667	5.6376	3.1219	4.7954	3.3648	3.6638	3.3137	1.0745	0.1037	0.6570	0.1824
Usp14	_LEAIEDDSGRETDSSSAPAVT[Phospho (STY)]PSK_.3	Q9JMA1_T235_M1_Usp14	3.8737	3.3524	4.5606	4.9879	2.9994	3.2292	3.2771	4.2680	4.2047	3.5294	1.0037	0.0053	0.9758	0.0106
Qtrt1	_[Acetyl (Protein N-term)]AAVGS[Phospho (STY)]PGSLESAPR_.2	Q9JMA2_S6_M1_Qtrt1	7.7421	7.7075	7.9928	7.9300	7.2082	3.2745	8.4569	7.2407	8.0160	3.4200	1.0284	0.0404	0.8863	0.0524
Dnaja4	_LISQAYEVLSDPK_.2	#N/A	3.9268	3.4156	3.6605	4.4679	3.2369	2.9917	3.3525	4.7524	4.8143	4.3824	0.8361	-0.2582	0.0989	1.0046
Stard10	_AGGAGGEGS[Phospho (STY)]DDDTSLT_.2	Q9JMD3_S284_M1_Stard10	11.2867	11.5021	11.6702	11.9826	10.8637	11.4593	11.7960	11.2709	11.3259	11.5686	0.9974	-0.0037	0.8935	0.0489
Ap3b2	_AFYGS[Phospho (STY)]EEDEAK_.2	#N/A	8.9613	8.5790	8.8887	4.2869	7.4899	4.0055	4.0016	3.8316	3.4802	4.2539	1.8077	0.8542	0.0287	1.5415
Ap3b2	_QLIVPSEQGGALSR_.2	#N/A	5.3071	5.0553	5.2450	4.9788	5.4618	3.4236	5.2703	5.6285	4.7412	4.3548	0.9826	-0.0253	0.8495	0.0709
Ap3b2	_ATGYQELPDWPEEAPDPSVR_.2	#N/A	5.1138	5.1287	3.3637	4.4084	4.9323	4.9681	5.3810	5.2248	5.2647	4.8814	0.8968	-0.1572	0.1743	0.7587
Ap3b2	_AFYGS[Phospho (STY)]EEDEAKGPGSEEAATAALPAR_.3	#N/A	8.9299	8.8363	9.3784	10.2538	8.2512	8.6914	8.8390	10.1298	8.8136	8.3418	1.0029	0.0041	0.9571	0.0190
Ap3b2	_TQFLS[Phospho (STY)]PTQNESLLEENPEK_.2	Q9JME5_S253_M1_Ap3b2	3.2785	4.0837	3.9653	4.4679	3.9050	3.8245	4.1853	3.6505	3.9123	4.4349	0.9691	-0.0453	0.6150	0.2111
Ap3b2	_LEVLTNLANETNIPTVLR_.2	#N/A	4.0525	3.8258	3.3062	3.3481	3.9012	2.9828	4.0265	4.8679	3.9582	4.1810	0.8382	-0.2546	0.0338	1.4715
Ap3b2	_TTLVAGSVVM[Oxidation (M)]AFEEVC[Carbamidomethyl (C)]PER_.2	#N/A	4.2693	3.5737	3.4553	3.9579	3.3950	4.3345	3.6726	4.1605	3.8091	3.9249	0.9844	-0.0227	0.7952	0.0995
Ap3b2	_GGGS[Phospho (STY)]ES[Phospho (STY)]EQS[Phospho (STY)]EEEDEKKK_.2	Q9JME5_S731_M3_Ap3b2	4.2195	3.5239	3.4055	3.9081	3.3452	4.3843	3.2443	4.2103	3.8589	3.8751	1.0002	0.0002	0.9984	0.0007
Tmub1	_EEAPGAES[Phospho (STY)]PSLR_.2	Q9JMG3_S71_M1_Tmub1	4.2387	3.4941	7.9923	3.4207	3.3824	3.2422	3.1353	3.1970	4.3464	3.1934	1.2385	0.3086	0.4185	0.3783
Hdgfl3	_GFNEGLWEIENNPGVK_.2	#N/A	5.5689	5.3615	6.2794	5.9854	5.8707	5.9357	6.4527	5.5689	5.6609	5.2759	1.0164	0.0234	0.7257	0.1392
Hdgfl3	_S[Phospho (STY)]PGDEDDKDC[Carbamidomethyl (C)]KEEENK_.2	#N/A	3.5157	3.7640	4.2025	3.8167	2.6881	3.6407	3.8112	3.9077	3.8584	3.6138	0.9491	-0.0753	0.4834	0.3157
Hdgfl3	_FTGYQTIQQSSSETEGEGGNTADAS[Phospho (STY)]S[Phospho (STY)]EEEGDRVEDGKGK_.4	#N/A	3.3230	4.0792	4.5575	4.1012	3.9675	4.1579	4.2495	4.4008	3.1990	3.7785	1.0318	0.0451	0.6863	0.1635
Hdgfl3	_FTGYQTIQQSSSETEGEGGNTADAS[Phospho (STY)]S[Phospho (STY)]EEEGDRVEDGK_.4	#N/A	4.0365	3.1896	3.2225	3.7252	3.0779	3.5467	3.4398	4.3933	4.0419	3.6922	0.8907	-0.1670	0.1277	0.8937
Txnrd1	_VLVLDFVTPTPLGTR_.2	#N/A	4.8084	4.8672	3.2528	4.2689	5.0398	5.7094	4.6977	4.6107	5.3199	5.2768	0.9360	-0.0954	0.4978	0.3029
Txnrd1	_VTAQSTNSEETIEGEFNTVLLAVGR_.3	#N/A	4.3256	2.4516	4.0593	4.1372	3.4693	3.1660	3.7513	2.6301	3.6972	3.3701	1.0712	0.0992	0.5824	0.2348
Txnrd1	_VVYENAYGR_.2	#N/A	3.0895	4.2529	4.3240	3.8678	4.0743	3.6552	4.0160	3.1334	3.4325	3.5450	1.0978	0.1347	0.2445	0.6117
Myo18a	_RFS[Phospho (STY)]FSQR_.2	Q9JMH9_S140_M1_Myo18a	7.3441	7.2754	3.6227	7.2245	6.0392	2.9539	3.3147	7.5333	7.3057	7.2745	0.9035	-0.1465	0.6464	0.1895
Myo18a	_S[Phospho (STY)]LAHADEAR_.2	Q9JMH9_S865_M1_Myo18a	7.4209	6.7551	7.2186	7.1049	5.4146	6.3379	6.9031	7.0832	7.4013	6.8603	0.9500	-0.0740	0.3914	0.4073
Myo18a	_FSHSYLSDS[Phospho (STY)]DTEAK_.2	#N/A	8.3400	8.7721	6.7233	9.1699	7.8354	8.7281	9.5389	8.9990	8.9532	8.8004	0.9106	-0.1352	0.1198	0.9216
Myo18a	_SSSPTSHWKPLAPDPS[Phospho (STY)]DDEHDPVDSISRPR_.5	Q9JMH9_S2016_M1_Myo18a	4.2903	2.9359	3.4762	3.9789	3.4160	4.3135	3.6936	4.1396	3.7882	3.9459	0.9597	-0.0593	0.6048	0.2184
Myo18a	_NELRLS[Phospho (STY)]SDRLETR_.3	Q9JMH9_S1279_M1_Myo18a	3.0006	3.2331	3.6874	4.1901	3.6271	4.1024	3.9047	3.9284	3.5770	4.1571	0.9353	-0.0965	0.3554	0.4493
Myo18a	_VASGSDLHLTDIDSDSNR_.3	#N/A	4.9008	4.2212	3.6769	4.4922	3.9697	4.4765	3.6989	4.7303	4.5085	4.8963	0.9621	-0.0557	0.5939	0.2263
Myo18a	_NKGPSKAPS[Phospho (STY)]DDGS[Phospho (STY)]LK_.2	Q9JMH9_S1994_M2_Myo18a	3.7502	3.4165	6.0111	3.6322	3.4984	3.1312	3.7804	6.1403	5.6611	3.3094	0.8272	-0.2737	0.3186	0.4967
Myo18a	_FSHSYLSDS[Phospho (STY)]DTEAKLTETSA_.3	#N/A	3.4631	3.7295	4.1704	3.9027	3.5805	3.6675	4.0287	4.4324	3.5599	4.1411	0.9287	-0.1068	0.1731	0.7617

Myo18a	_NKLEGDS[Phospho (STY)]DVDS[Phospho (STY)]ELEDRVDGVK_.3	Q9JMH9_S1966_M2_Myo18a	3.4598	4.2650	4.1466	3.1484	4.0863	3.6432	4.3639	4.1419	3.1178	3.1154	1.0290	0.0412	0.7655	0.1161
Myo18a	_APS[Phospho (STY)]DDGSLK_.2	Q9JMH9_S1994_M1_Myo18a	3.4512	3.8912	3.1849	4.2295	3.7125	4.0170	4.3778	4.2725	3.0707	6.4720	0.8240	-0.2793	0.2130	0.6716
Csnk1e	_GAPANVS[Phospho (STY)]SSDLTGR_.2	Q9JMK2_S389_M1_Csnk1e	3.5481	3.7943	3.2818	4.3264	3.6156	4.1139	4.4747	4.1756	2.9738	4.0037	0.9675	-0.0476	0.7054	0.1516
Csnk1e	_IQQTGNTS[Phospho (STY)]PR_.2	Q9JMK2_S363_M1_Csnk1e	8.6299	8.7800	9.0332	8.8304	7.7173	8.5467	8.8070	8.7098	10.0894	8.5635	0.9499	-0.0741	0.2489	0.6039
Glrx	_[Acetyl (Protein N-term)]AQEFVNC[Carbamidomethyl (C)]K_.2	#N/A	5.5678	6.2069	6.6599	6.2359	6.2890	5.1840	5.7874	6.0147	5.7515	5.4873	1.0458	0.0646	0.3886	0.4105
Rhoa	_LVIVGDGAC[Carbamidomethyl (C)]GK_.2	#N/A	8.0112	8.0588	8.3384	7.8049	7.8976	7.6438	7.9404	7.9985	7.6442	7.5594	1.0223	0.0318	0.2766	0.5582
Rhoa	_TC[Carbamidomethyl (C)]LLIVFSK_.2	#N/A	4.5225	5.4259	4.4979	4.3039	5.4268	4.9288	5.7376	5.5826	5.1867	4.7005	0.9150	-0.1282	0.1837	0.7358
Rhoa	_M[Oxidation (M)]KQEPVKPEEGR_.3	#N/A	5.9270	5.9445	7.0116	4.5461	6.4403	6.8972	5.7753	6.3218	6.8205	6.3386	0.9705	-0.0432	0.7133	0.1467
Rhoa	_EVFEMATR_.2	#N/A	6.1152	4.5557	3.7029	6.8801	5.1312	3.0342	4.8730	3.8361	4.1670	4.8355	1.1073	0.1471	0.5522	0.2579
Rhoa	_QVELALWDTAGQEDYDR_.2	#N/A	6.6595	6.3939	6.8588	6.6866	5.7949	6.3807	7.1732	6.4962	6.5883	6.5297	0.9650	-0.0514	0.3366	0.4729
Psma6	_AINQGGLTSVAVR_.2	#N/A	5.8316	6.1629	5.6490	6.3213	6.3963	5.7512	5.5313	6.3237	6.0683	6.3766	0.9907	-0.0134	0.8064	0.0935
Psma6	_LLDSSTVTHLFK_.2	#N/A	4.8328	5.4284	5.1414	5.0241	5.0180	5.5040	5.5913	4.5729	5.0770	4.5481	1.0426	0.0602	0.3974	0.4008
Psma6	_HITIFSPEGR_.2	#N/A	6.8588	6.5554	6.3530	6.5615	6.1649	6.1857	6.6754	6.2366	6.1764	6.5058	1.0442	0.0624	0.2498	0.6025
Psma6	_YEAANWK_.2	#N/A	4.4318	4.5507	3.1901	4.3705	5.2199	4.5723	3.9020	4.7002	4.6023	4.2914	1.0035	0.0050	0.9679	0.0142
Psma6	_ITESIGC[Carbamidomethyl (C)]VM[Oxidation (M)]TGM[Oxidation (M)]TADSR_.2	#N/A	5.2411	3.9783	3.8599	4.1424	3.7996	3.9299	4.2907	4.3596	3.1578	4.3295	1.0308	0.0437	0.7398	0.1309
Psma6	_ILTEAEIDAHLVALAERD_.3	#N/A	3.4822	4.2874	4.1690	3.1708	4.1087	3.6208	3.9815	3.4468	3.0954	3.1378	1.1145	0.1564	0.1991	0.7009
Dkk3	_PAFVGSHDHS[Phospho (STY)]EESQLPR_.3	#N/A	7.3064	7.6677	7.1601	8.3373	8.2880	7.9714	7.9690	7.1286	8.0438	8.1830	0.9946	-0.0079	0.8958	0.0478
Hapln1	_LLVEAEQAK_.2	#N/A	6.7236	6.6847	6.8972	7.0270	6.9847	6.7595	6.8735	6.4074	6.7465	7.2692	1.0032	0.0046	0.8934	0.0489
Hapln1	_GGSDNDASLVITDLTLEDYGR_.2	#N/A	5.4137	5.3509	5.0660	6.6620	5.2212	5.5898	5.7429	5.1359	3.5815	4.8975	1.1470	0.1978	0.1637	0.7860
Hapln1	_EPC[Carbamidomethyl (C)]GGQNTVPGVR_.2	#N/A	7.5323	6.9042	3.4116	8.0463	7.8695	6.3569	7.7025	7.7968	7.3816	7.6011	0.8775	-0.1886	0.3207	0.4938
Hapln1	_DPTAFGSGIHK_.2	#N/A	6.1423	5.7098	6.3665	6.4526	6.8344	5.7571	6.1396	6.1032	6.7295	6.1693	0.9881	-0.0173	0.7718	0.1125
Hapln1	_YNLNFHEAR_.2	#N/A	5.6212	5.5216	6.6703	6.1006	6.3158	3.3966	2.4541	5.7989	6.3941	6.6739	1.0514	0.0723	0.7854	0.1049
Hapln1	_C[Carbamidomethyl (C)]SPTEAAVR_.2	#N/A	5.6847	5.4283	4.4466	5.2139	5.7173	5.5586	5.5997	5.2092	5.5230	5.5989	0.9743	-0.0376	0.5931	0.2269
Hapln1	_C[Carbamidomethyl (C)]DAGWLADGSVR_.2	#N/A	5.3863	4.9686	3.8264	5.0488	5.5272	5.2753	5.1362	3.7894	4.9149	5.5472	1.0327	0.0464	0.7231	0.1408
Hapln1	_LTSDYLR_.2	#N/A	4.8968	4.6211	5.1598	3.2201	5.1619	4.8619	4.4530	4.8138	4.7843	5.0718	0.9734	-0.0389	0.7506	0.1246
Prep	_VFLDPNTLSDDGTVALR_.2	#N/A	4.2854	2.9408	3.4713	3.9740	5.1476	3.7955	3.6887	5.4493	3.7931	3.9410	0.9331	-0.0999	0.5906	0.2287
Pin1	_SGEEDFESLASQFSDC[Carbamidomethyl (C)]SSAK_.2	#N/A	5.5435	6.0117	5.6106	6.6329	4.5775	5.4246	6.0861	5.5015	5.0371	5.5593	1.0158	0.0226	0.8265	0.0828
Pin1	_TGEM[Oxidation (M)]SGPVFTDSGIHIILR_.3	#N/A	4.2959	2.5499	4.1205	3.7812	3.9409	3.6028	3.9698	3.1045	4.5069	3.6857	0.9734	-0.0389	0.8022	0.0957
Ptk2b	_RNS[Phospho (STY)]LPQIPTLNLEAR_.3	#N/A	5.4734	7.9205	7.6508	9.5120	4.8395	3.6072	6.7482	8.2391	7.0508	5.0938	0.9584	-0.0614	0.8259	0.0831
Ptk2b	_NLLDAVDQAK_.2	#N/A	5.4835	5.0634	5.3590	4.8299	5.2647	4.7948	4.6343	5.0460	4.2924	3.8480	1.1520	0.2042	0.0257	1.5902
Ptk2b	_MSGVSEPLSRVK_.2	#N/A	5.3982	5.5731	5.9482	4.5257	4.4516	4.7748	5.1788	4.7468	5.1657	3.8523	1.0794	0.1102	0.3730	0.4283
Ptk2b	_SHLSESC[Carbamidomethyl (C)]SIES[Phospho (STY)]DIYAEIPDETLR_.3	Q9QVP9_S399_M1_Ptk2b	3.0161	3.7723	4.2506	3.1425	3.6606	2.9640	4.3580	3.4752	3.1238	3.1095	0.9861	-0.0202	0.8919	0.0497
Ptk2b	_NS[Phospho (STY)]LPQIPTLNLEAR_.2	#N/A	3.9794	3.2348	3.7130	3.2568	3.1231	3.5015	3.3947	3.7444	4.0434	4.4349	0.8883	-0.1709	0.1094	0.9608
Srcin1	_LPPSS[Phospho (STY)]PQK_.2	Q9QWI6_S540_M1_Srcin1	12.8144	3.2364	12.9545	13.1776	12.5736	12.8259	12.9592	12.7436	12.8152	12.8977	0.8763	-0.1905	0.4516	0.3452
Srcin1	_KAES[Phospho (STY)]EELEVQKPQVK_.3	#N/A	9.6690	9.7858	10.4554	10.5080	9.2312	10.1072	9.6916	9.9809	9.9415	10.1168	1.0027	0.0039	0.9209	0.0358
Srcin1	_AES[Phospho (STY)]EELEVQKPQVK_.2	#N/A	9.6760	9.7095	9.9196	10.1850	9.0537	10.0128	10.1348	9.7828	9.9106	10.1108	0.9774	-0.0329	0.3189	0.4963
Srcin1	_RFS[Phospho (STY)]NVGLVHTSER_.3	#N/A	6.8765	7.4566	7.9643	9.3275	3.2507	6.8099	7.6479	8.3663	7.4700	7.4581	0.8981	-0.1550	0.4748	0.3235
Srcin1	_AVS[Phospho (STY)]VEAAERDWEEKR_.3	#N/A	7.2771	6.9548	7.6522	7.4892	6.0726	6.9929	7.3430	7.2984	7.4060	6.8960	0.9775	-0.0328	0.6027	0.2199
Srcin1	_AVS[Phospho (STY)]VEAAER_.2	#N/A	6.8669	6.2226	5.6080	6.6512	6.3164	6.5454	6.5417	6.6023	6.7452	6.9137	0.9504	-0.0734	0.1920	0.7167
Srcin1	_GVTDTLAQIR_.2	#N/A	6.5355	6.6281	5.7729	6.9045	6.6139	6.6985	6.6716	6.4504	6.1067	6.6464	1.0088	0.0126	0.8062	0.0936
Srcin1	_TLGGGGGGGS[Phospho (STY)]GGRR_.2	Q9QWI6_S73_M1_Srcin1	6.1541	6.7323	5.3572	6.5849	5.5557	7.1894	6.3656	6.4838	5.8429	6.0439	1.0126	0.0181	0.8421	0.0746
Srcin1	_YLNDEELITQQLNDLEK_.2	#N/A	5.4625	5.4447	5.4239	5.7081	5.5129	5.5774	5.9655	5.1078	4.3992	4.9486	1.0815	0.1131	0.1510	0.8210
Srcin1	_AAQGPAGS[Phospho (STY)]PDKGK_.2	#N/A	8.4539	8.7471	9.0603	9.0592	7.4414	8.4199	8.7496	8.7520	8.1606	8.6149	0.9955	-0.0066	0.9079	0.0420
Srcin1	_FSNVGLVHTS[Phospho (STY)]ERR_.3	#N/A	4.1967	6.6268	3.9304	7.4302	5.5424	3.2616	3.6224	6.7748	6.2334	6.1507	0.9068	-0.1411	0.6124	0.2130
Srcin1	_EVSGPNETSS[Phospho (STY)]PGSEKPSGSR_.3	#N/A	6.7227	6.4293	7.4424	8.0176	6.5162	7.7719	3.7065	7.6265	7.1073	3.9059	1.2799	0.3560	0.1166	0.9335
Srcin1	_QLGETLTELK_.2	#N/A	5.6810	5.9657	3.1226	6.0860	6.0231	5.1299	5.5434	5.0661	5.1274	5.3926	1.0099	0.0142	0.9313	0.0309
Srcin1	_GEGLYADPYGLLHEGR_.3	#N/A	5.7324	6.0288	5.5432	5.4771	5.4454	5.6999	5.0366	5.6017	5.5494	5.5598	1.0400	0.0566	0.1934	0.7136
Srcin1	_QIASLTGLVQSALLR_.2	#N/A	4.3878	3.7738	4.1910	4.0747	4.1641	4.0873	3.7879	3.9440	2.4146	4.1960	1.1471	0.1980	0.1506	0.8221
Srcin1	_DSGSSSVFAES[Phospho (STY)]PGGK_.2	#N/A	11.7521	11.9224	12.5026	12.1821	11.6536	11.9277	12.2867	11.5757	11.6924	11.8659	1.0114	0.0163	0.5191	0.2847
Srcin1	_KDS[Phospho (STY)]GSSSVFAES[Phospho (STY)]PGGK_.2	Q9QWI6_S588_M2_Srcin1	9.9644	10.1921	10.5922	9.7873	9.7263	10.2098	10.6203	10.0314	10.1216	10.1088	0.9861	-0.0202	0.4888	0.3109
Srcin1	_LSYAGGRPPSYAGS[Phospho (STY)]PVHHAAER_.3	#N/A	6.7169	7.5512	8.3704	8.8765	6.6927	6.4481	3.5035	7.5193	7.2720	7.0013	1.1769	0.2350	0.2518	0.5989
Srcin1	_HTQGAQPGLADQAAK_.2	#N/A	4.2456	4.6411	3.1844	4.1283	4.6079	4.0748	4.9443	3.7995	3.7283	4.3566	0.9857	-0.0208	0.8680	0.0615
Srcin1	_S[Phospho (STY)]SPPM[Oxidation (M)]LSADDAEYPR_.2	#N/A	3.8099	4.6230	3.5435	7.4563	4.7277	3.7122	7.6442	3.9522	6.9730	4.2654	0.8137	-0.2974	0.3413	0.4669
Srcin1	_AALTQYSAK_.2	#N/A	4.4975	2.9964	3.4157	4.1933	5.4127	4.3481	3.6330	4.6441	4.0919	4.9459	0.9573	-0.0629	0.7170	0.1445
Srcin1	_GS[Phospho (STY)]DELTVPR_.2	#N/A	3.0250	6.9672	7.8374	7.9038	7.6739	3.9151	4.2759	7.2840	7.0870	7.6444	0.9464	-0.0795	0.7880	0.1035
Srcin1	_GLQNSASDLR_.2	#N/A	4.3903	6.3313	3.3814	6.4196	6.6788	6.6138	3.0734	6.4048	6.6019	6.3759	1.0039	0.0056	0.9827	0.0076
Srcin1	_RQEDPLQR_.2	#N/A	6.1360	4.0568	3.8562	6.3269	6.3454	6.3727	4.0735	6.5215	5.8633	6.2108	0.9732	-0.0391	0.8459	0.0727
Srcin1	_RGS[Phospho (STY)]DELTVPR_.2	#N/A	10.8307	10.7871	11.4706	11.2937	10.1763	10.7922	11.2941	10.6724	10.8570	10.9152	0.9961	-0.0057	0.8700	0.0605
Srcin1	_S[Phospho (STY)]PGVLFLQFGEETR_.2	Q9QWI6_S247_M1_Srcin1	4.2225	3.4779	4.4350	3.4368	2.5389	3.2584	3.1515	3.2828	4.3302	4.2447	0.9492	-0.0753	0.6677	0.1754
Srcin1	_S[Phospho (STY)]FPSSHGLTTTR_.2	Q9QWI6_S1079_M1_Srcin1	8.4638	3.9917	3.9213	8.4446	3.8800	4.2455	4.1386	3.6945	3.3431	4.3909	1.4110	0.4967	0.2149	0.6677
Srcin1	_GEPDPERS[Phospho (STY)]SPPM[Oxidation (M)]LSADDAEYPR_.3	#N/A	4.1086	5.2225	3.6967	3.5238	3.0050	3.3721	3.5421	3.9350	3.9592	8.5091	0.7664	-0.3839	0.2831	0.5481
Srcin1	_RFS[Phospho (STY)]NVGLVHT[Phospho (STY)]SERR_.3	Q9QWI6_T86_M2_Srcin1	3.9562	3.0564	3.9768	5.4274	3.0819	4.6624	3.3595	5.2192	4.9751	5.3254	0.8532	-0.2291	0.2758	0.5594
Srcin1	_QVDEGM[Oxidation (M)]WPPPNNLLNQS[Phospho (STY)]PK_.2	#N/A	7.3989	8.5545	8.7348	10.1097	7.1661	7.8173	7.9598	8.6984	8.1348	7.4706	1.0286	0.0407	0.7046	0.1521
Srcin1	_LKAAQGPAGS[Phospho (STY)]PDKGK_.3	#N/A	4.1059	5.0607	4.9675	4.8756	4.6964	3.6452	5.0727	3.4348	5.5157	4.6079	0.9787	-0.0311	0.8330	0.0794
Srcin1	_S[Phospho (STY)]PNTAILIKDEAR_.2	#N/A	8.3235	7.8745	7.8303	8.7558	7.4534	8.0450	8.5943	8.1270	8.2690	8.3005	0.9669	-0.0486	0.2875	0.5413
Srcin1	_AAQGPAGS[Phospho (STY)]PDK_.2	#N/A	6.0013	5.1355	3.4750	3.9777	2.8254	3.7992	3.6923	4.1408	3.7894	3.9447	1.0798	0.1108	0.6175	0.2094
Srcin1	_LGGAPTGQGVSPSPSAILER_.2	#N/A	4.1042	4.0903	4.0531	4.5933	3.8179	3.9506	4.6797	4.3287	3.4821	4.0831	0.9899	-0.0146	0.8662	0.0624
Srcin1	_VEGSNGAAT[Phospho (STY)]PSAPVC[Carbamidomethyl (C)]GSGSK_.2	Q9QWI6_T675_M1_Srcin1	3.6499	3.6956	3.5811	3.9644	3.5208	4.7790	3.8336	5.4832	3.7747	6.0151	0.8092	-0.3055	0.1133	0.9458
Srcin1	_S[Phospho (STY)]S[Phospho (STY)]GATPVSGPPPPSASSTPAGQPTAVSR_.3	Q9QWI6_S687_M2_Srcin1	4.0149	3.6710	3.9085	7.4834	3.9117	4.2475	4.5306	5.4811	3.8669	3.7210	1.0317	0.0451	0.8670	0.0620
Srcin1	_TEKPSKS[Phospho (STY)]PPPPPPR_.3	Q9QWI6_S1070_M1_Srcin1	3.1034	3.8597	4.3380	3.8817	3.7480	5.6329	4.2706	3.1195	3.4185	3.5590	1.1398	0.1888	0.3220	0.4921
Srcin1	_S[Phospho (STY)]PNTAILIK_.2	#N/A	3.0458	3.8021	4.2804	4.6135	3.6904	2.9342	6.1818	3.5050	3.1536	3.0797	0.9366	-0.0945	0.7185	0.1436
Srcin1	_FRQS[Phospho (STY)]LPLSR_.2	Q9QWI6_S233_M1_Srcin1	4.2466	3.5021	3.9803	3.4127	3.3904	3.2342	3.1274	3.2049	4.3543	3.2014	1.0448	0.0633	0.6269	0.2028
Srcin1	_DAFMDHLK_.2	#N/A	3.7447	3.0490	4.4315	5.2821	3.4030	3.3583	3.7191	3.4304	3.7294	3.2481	1.0981	0.1350	0.4495	0.3473
Srcin1	_HTVIAAQS[Phospho (STY)]LEALSGLQK_.3	#N/A	4.1484	4.0336	4.3065	5.1604	3.2553	2.7510	3.8144	4.3370	3.8184	3.6895	1.0071	0.0102	0.9519	0.0214
Srcin1	_EMVYASRES[Phospho (STY)]S[Phospho (STY)]PTR_.2	Q9QWI6_S356_M2_Srcin1	3.7403	2.9957	3.4740	4.5186	2.8840	3.7406	3.1660	3.9834	4.2825	4.1959	0.9109	-0.1346	0.3671	0.4352
Srcin1	_FSNVGLVHTSER_.2	#N/A	3.5593	3.8878	4.1804	3.9259	4.2751	4.1820	4.3662	3.8895	3.8397	4.0358	0.9923	-0.0112	0.8554	0.0678
Srcin1	_KVAAETDFSK_.2	#N/A	4.3120	3.5675	4.3455	3.3473	5.5471	3.1688	3.0620	3.2703	2.5479	2.2328	1.4571	0.5431	0.0315	1.5012
Srcin1	_LADVSAPSGGPPPPHSPY[Phospho (STY)]SGPPSRGSPVR_.3	Q9QWI6_Y561_M1_Srcin1	3.3559	4.2932	3.5463	5.3472	4.1815	3.9496	4.2736	4.0046	4.0363	4.1321	1.0002	0.0002	0.9987	0.0006
Srcin1	_QVDEGMWPPPNNLLNQS[Phospho (STY)]PKK_.3	#N/A	3.8031	3.5393	3.5368	6.7079	2.9468	3.6778	3.2288	3.9206	4.2197	4.2586	1.0328	0.0466	0.8611	0.0649

Srcin1	_RQVDEGMWPPPNNLLNQS[Phospho (STY)]PK_.3	#N/A	3.3464	4.1026	4.5809	9.8177	3.9909	4.1345	4.0277	3.8055	3.4541	4.2800	1.2836	0.3602	0.3959	0.4025
Asap1	_SHTGDLS[Phospho (STY)]PNVQSR_.2	Q9QWY8_S1045_M1_Asap1	6.1843	4.3866	7.1918	4.0290	3.4661	3.8506	3.7437	6.6317	3.7380	7.0232	0.9181	-0.1233	0.6876	0.1627
Asap1	_TLS[Phospho (STY)]DPPSPLPHGPPNK_.2	#N/A	9.3202	9.7339	10.2158	10.4059	8.8408	9.4424	9.9981	9.8649	9.6251	9.5310	0.9903	-0.0141	0.7671	0.1152
Asap1	_SPRPQSFC[Carbamidomethyl (C)]HSS[Phospho (STY)]S[Phospho (STY)]ISPQDK_.2	#N/A	3.5010	9.0426	3.5847	9.6567	3.8297	9.5439	3.7717	3.8845	7.7296	3.8975	1.3538	0.4370	0.3689	0.4331
Asap1	_QDEM[Oxidation (M)]DES[Phospho (STY)]DDDLDDKPSPIKK_.3	#N/A	4.3377	5.1903	4.7927	7.2152	4.8050	6.4847	5.4696	6.6658	6.7437	5.5170	0.8970	-0.1568	0.3535	0.4516
Asap1	_QDEMDES[Phospho (STY)]DDDLDDKPSPIK_.2	#N/A	4.2822	3.3118	3.4681	4.8839	3.4079	4.3217	3.6855	4.1478	3.7963	4.8942	0.9552	-0.0661	0.6494	0.1875
Son	_ESAQAVAVALS[Phospho (STY)]PK_.2	#N/A	8.5972	8.8748	8.6570	9.7925	8.1392	8.8459	9.5715	9.0757	9.1243	9.2439	0.9529	-0.0696	0.1739	0.7598
Son	_S[Phospho (STY)]RT[Phospho (STY)]PLRR_.2	Q9QX47_S2038_M2_Son	8.1988	7.9415	8.3443	8.2565	7.7280	8.3113	8.5018	8.2087	8.3704	8.2090	0.9769	-0.0337	0.1959	0.7079
Son	_SAAS[Phospho (STY)]PVVISIPER_.2	Q9QX47_S1794_M1_Son	2.9017	7.2950	7.8967	7.4697	6.4583	7.2227	8.2538	7.4542	7.3611	7.3694	0.8595	-0.2184	0.2959	0.5288
Son	_FS[Phospho (STY)]RS[Phospho (STY)]PIR_.2	#N/A	6.2121	5.8204	3.5218	6.4503	4.8194	5.8214	5.9167	4.1548	6.1948	5.3236	1.0080	0.0115	0.9494	0.0226
Son	_RFS[Phospho (STY)]RS[Phospho (STY)]PIR_.2	#N/A	5.6974	6.8471	4.1620	7.4130	4.1017	6.5754	7.0720	6.2314	3.1024	5.9246	1.0388	0.0550	0.8320	0.0799
Son	_DVEDSLPIKESAQAVAVALS[Phospho (STY)]PK_.3	#N/A	3.7439	5.3119	4.6640	5.7260	4.9486	4.1331	3.6301	5.7826	4.6448	4.2890	1.0366	0.0519	0.7539	0.1227
Son	_RRS[Phospho (STY)]FS[Phospho (STY)]IS[Phospho (STY)]PVR_.2	#N/A	3.2879	7.9475	4.5224	8.5956	7.6361	7.9492	5.7526	8.4503	8.4872	8.5153	0.8532	-0.2290	0.3831	0.4166
Chm	_ESTVLGDSEEPS[Phospho (STY)]E_.2	Q9QXG2_S664_M1_Chm	9.7107	9.9547	10.1113	10.3260	9.5344	10.0869	10.6701	9.8977	10.0908	10.0070	0.9791	-0.0305	0.3207	0.4938
Chm	_VQDNTET[Phospho (STY)]PKR_.2	#N/A	3.9667	3.4499	3.7004	4.0183	3.8846	3.5884	4.1665	3.8312	4.1303	3.6956	0.9525	-0.0702	0.2355	0.6281
Acss2	_GWS[Phospho (STY)]PPPEVR_.2	#N/A	12.8352	12.1974	12.5757	12.4256	11.1826	12.0819	13.1047	12.3666	12.3467	12.3038	0.9749	-0.0366	0.3684	0.4337
Acss2	_VRGWS[Phospho (STY)]PPPEVR_.2	#N/A	9.1869	9.1220	9.8424	9.3276	7.7268	8.7166	9.7923	9.5301	9.1675	9.1304	0.9556	-0.0656	0.3107	0.5077
Acss2	_AELGM[Oxidation (M)]NDS[Phospho (STY)]PSQSPPVK_.2	#N/A	8.6518	6.4529	9.2037	8.2028	7.7578	6.8448	9.5672	9.1706	7.2211	8.7824	0.9041	-0.1455	0.2519	0.5987
Kif21b	_GATDTS[Phospho (STY)]PLTR_.2	Q9QXL1_S1217_M1_Kif21b	6.4271	5.7876	3.2429	4.1501	5.0269	3.9717	3.8648	6.7386	3.0127	3.9648	1.0847	0.1174	0.6852	0.1642
Kif21b	_AVS[Phospho (STY)]AEC[Carbamidomethyl (C)]LGPPLDSSTK_.2	Q9QXL1_S1150_M1_Kif21b	7.8511	7.9189	8.4043	8.0813	3.7743	7.9201	7.9620	8.3560	7.5836	7.6005	0.9301	-0.1045	0.5599	0.2519
Kif21b	_TVS[Phospho (STY)]LPTR_.2	Q9QXL1_S1198_M1_Kif21b	4.2184	3.0077	3.4044	3.9070	2.8960	3.7286	4.5224	4.2114	3.8600	3.8740	0.8567	-0.2231	0.0785	1.1049
Kif21a	_VC[Carbamidomethyl (C)]PQTDAENATDNK_.2	#N/A	8.0679	7.8420	8.2574	8.2629	7.6300	7.7889	8.4117	8.0209	8.1908	8.0330	0.9768	-0.0338	0.2477	0.6060
Kif21a	_DQVLQNLGSVESYSEEK_.2	#N/A	6.5551	6.5713	7.4627	6.6289	5.6572	6.4619	7.0103	6.2620	6.2831	6.2915	1.0146	0.0209	0.7797	0.1081
Kif21a	_ITQLVSEQANQVLAR_.2	#N/A	5.4991	5.8358	5.4719	4.9875	5.2577	5.1543	5.5322	5.2486	5.0253	5.2136	1.0215	0.0306	0.5348	0.2718
Kif21a	_HSDSGASETSLS[Phospho (STY)]PPSS[Phospho (STY)]PPSRPR_.3	Q9QXL2_S1273_M2_Kif21a	2.9305	4.4119	4.1650	3.7088	3.5751	3.4963	3.8571	3.2924	3.5914	3.3861	1.0518	0.0728	0.5390	0.2684
Kif21a	_TTASTQM[Oxidation (M)]NVQSS[Phospho (STY)]R_.2	Q9QXL2_S224_M1_Kif21a	4.9999	5.9140	6.0963	5.4395	5.5450	5.5805	5.9655	5.8962	6.5202	5.3491	0.9432	-0.0843	0.2509	0.6005
Kif21a	_AHNLQDGQLSDTGDLGEDIAS[Phospho (STY)]N_.2	#N/A	4.3409	5.5519	4.7816	7.6524	6.4022	4.0861	5.1226	7.6149	6.8988	6.2308	0.8457	-0.2417	0.2543	0.5946
Kif21a	_LISESS[Phospho (STY)]PM[Oxidation (M)]NEFETLTAK_.2	#N/A	6.9302	5.9338	6.4970	7.4459	4.8750	5.7737	6.9539	6.4460	6.3464	6.1000	0.9661	-0.0497	0.6650	0.1772
Kif21a	_MTISNMEADMNR_.2	#N/A	3.4276	3.7985	4.1144	4.6171	3.6868	2.9378	4.3318	3.5014	3.1500	3.0833	1.0703	0.0980	0.5239	0.2807
Jmy	_KSQAWAEGGS[Phospho (STY)]PR_.2	Q9QXM1_S108_M1_Jmy	6.5508	6.5881	7.4852	6.6784	5.9617	6.9469	7.0395	6.6467	6.3814	6.4931	1.0093	0.0133	0.8313	0.0803
Jmy	_GPES[Phospho (STY)]PLRS[Phospho (STY)]PAR_.2	Q9QXM1_S130_M2_Jmy	5.8973	5.8163	4.0870	3.9488	3.7035	5.6307	5.8809	5.5004	5.1513	5.3474	0.8862	-0.1744	0.2836	0.5473
Jmy	_IKEASPES[Phospho (STY)]EDEEEALPC[Carbamidomethyl (C)]TDWEN_.3	Q9QXM1_S969_M1_Jmy	4.3770	3.6814	3.3947	4.4393	3.5027	4.2268	3.0867	4.0627	4.3618	4.1166	1.0077	0.0110	0.9286	0.0322
Pde7b	_RGS[Phospho (STY)]GQDLAGPAPETLEQTEGATP_.3	#N/A	6.9923	6.6913	7.1829	7.1663	3.7918	7.0580	7.0213	6.8003	6.7010	7.0650	0.9396	-0.0899	0.5582	0.2532
Pde7b	_GSGQDLAGPAPETLEQTEGAT[Phospho (STY)]P_.2	Q9QXQ1_T445_M1_Pde7b	2.9082	4.5103	4.1427	6.3576	5.5464	5.8226	3.8348	6.7400	6.4644	6.3343	0.8354	-0.2595	0.2852	0.5448
Pde7b	_RRGS[Phospho (STY)]GQDLAGPAPETLEQTEGATP_.3	#N/A	2.9187	4.5167	3.6055	4.3968	3.5452	4.1842	4.5450	4.1052	4.6469	4.0741	0.8891	-0.1696	0.1868	0.7286
Plec	_AQLEPVAS[Phospho (STY)]PAK_.2	#N/A	12.1805	11.9958	12.5679	12.0616	10.7703	11.7474	12.7979	12.2860	12.0332	11.9029	0.9700	-0.0440	0.3217	0.4925
Plec	_NDESQLS[Phospho (STY)]PATR_.2	#N/A	9.9006	9.5951	9.9078	4.2281	4.0944	4.0311	10.1249	9.8887	9.7723	9.6065	0.7067	-0.5009	0.1072	0.9698
Plec	_RAS[Phospho (STY)]FAEK_.2	Q9QXS1_S1729_M1_Plec	7.6443	7.7272	8.1897	7.5289	5.6818	7.2904	7.7968	8.2031	7.9568	7.4513	0.9353	-0.0965	0.3006	0.5220
Plec	_AQLEPVAS[Phospho (STY)]PAKKPK_.2	#N/A	6.9105	7.6053	8.8927	7.0715	6.2261	7.6085	7.4318	7.9277	7.4824	6.9870	0.9904	-0.0139	0.8861	0.0525
Plec	_LPVDVAYQR_.2	#N/A	5.6140	5.5247	3.7536	5.3581	5.8020	4.9972	5.8186	6.0228	5.0141	5.5401	0.9243	-0.1136	0.3410	0.4673
Plec	_ARNDESQLS[Phospho (STY)]PATR_.2	#N/A	8.2536	8.3544	8.9521	8.4387	7.5248	8.3838	8.9448	8.6612	4.1175	8.3485	1.1064	0.1459	0.4154	0.3816
Plec	_SSS[Phospho (STY)]VGSSSSYPISSAGPR_.2	Q9QXS1_S4393_M1_Plec	10.4942	10.1565	11.0165	10.3946	9.0782	10.2355	10.9055	10.4058	10.4085	10.2867	0.9741	-0.0379	0.4515	0.3453
Plec	_WQAVLAQTDVR_.2	#N/A	4.1038	4.6124	3.5309	4.0335	3.4749	3.8551	3.7482	4.3972	4.1091	4.0005	0.9683	-0.0464	0.6048	0.2184
Plec	_DAPDGPSVEAEPEYTFEGLR_.2	#N/A	5.5267	3.6664	3.4096	4.4542	4.3840	4.2417	5.6712	5.5964	4.0600	4.1839	0.8775	-0.1885	0.2766	0.5581
Plec	_LTVDEAVR_.2	#N/A	5.7400	3.0293	5.6276	5.4794	2.9176	3.7070	3.6002	6.4260	2.3220	4.9091	1.0238	0.0339	0.9190	0.0367
Plec	_GYYSPYSVSGSGSTAGS[Phospho (STY)]R_.2	Q9QXS1_S4633_M1_Plec	5.8452	4.4028	4.1741	3.7179	3.5841	3.5053	3.8661	3.2833	3.5824	3.3951	1.1906	0.2517	0.1803	0.7440
Plec	_VLALPEPSPAAPTLR_.2	#N/A	5.3124	4.9428	4.4405	3.9843	3.9578	3.7717	5.0417	3.0169	4.8012	5.0785	0.9815	-0.0269	0.8724	0.0593
Plec	_LLDAQLATGGIVDPR_.2	#N/A	3.1961	4.1463	4.4306	3.5513	1.1345	3.7619	4.1227	4.3102	2.5937	3.4414	0.9317	-0.1020	0.7242	0.1402
Plec	_FLEVQYLTGGLIEPDTPGR_.2	#N/A	3.9354	4.0337	4.4090	3.4108	4.3905	3.3807	3.7415	3.2068	4.3562	4.5601	0.9901	-0.0144	0.9092	0.0413
Plec	_VYHDPSTQEPVTYSQLQQR_.3	#N/A	3.2375	4.0427	3.9243	4.1533	4.3058	3.8654	4.2262	3.6915	4.3819	4.7539	0.9198	-0.1206	0.2204	0.6567
Plec	_LLDAQLSTGGIVDPSK_.2	#N/A	4.2553	3.0871	3.9889	3.5327	3.3990	3.2256	3.6810	3.4685	3.7675	3.2100	1.0141	0.0202	0.8481	0.0716
Plec	_FLEGTSC[Carbamidomethyl (C)]IAGVFVDATK_.2	#N/A	3.0100	3.9026	3.6968	3.9953	3.6365	4.6013	3.9141	3.9190	3.8563	3.2875	1.0168	0.0240	0.8347	0.0785
Plec	_AQAEVEGLGK_.2	#N/A	2.9874	3.7436	4.2219	3.7657	3.6319	4.4174	3.9139	3.2355	3.5346	3.4429	1.0744	0.1036	0.3735	0.4278
Plec	_TEVVETTQVYTEEETRR_.3	#N/A	3.8984	3.1539	3.6321	3.1759	3.0422	3.5824	3.4756	4.6539	4.1243	4.3540	0.8223	-0.2823	0.0229	1.6408
Plec	_EEELQQTLQQEQNMLDR_.2	#N/A	2.9099	4.3163	3.5967	4.0994	3.5364	4.1931	3.8140	4.0191	3.6677	4.0664	0.9701	-0.0439	0.6890	0.1618
Dbn1	_[Acetyl (Protein N-term)]AGVSFSGHR_.2	#N/A	7.5946	7.6886	6.9516	8.0923	7.9863	6.7322	7.8438	6.8681	6.7584	6.8862	1.0590	0.0827	0.2603	0.5846
Dbn1	_YVLINWVGEDVPDAR_.2	#N/A	7.0755	6.3908	6.9488	6.9960	7.1933	7.1816	6.9686	6.7746	6.8108	6.9605	1.0125	0.0179	0.5997	0.2221
Dbn1	_KQQS[Phospho (STY)]LEAEEAK_.2	#N/A	6.3488	5.8716	6.4286	7.0778	5.8993	5.8289	6.7100	6.4374	6.1140	6.7027	0.9617	-0.0563	0.3856	0.4138
Dbn1	_EESAADWALYTYEDGSDDLK_.2	#N/A	4.6390	5.6660	5.1708	6.8816	5.1551	5.4715	5.5490	5.0689	5.3563	4.8435	1.0563	0.0790	0.4932	0.3070
Dbn1	_LSS[Phospho (STY)]PVLHR_.2	Q9QXS6_S142_M1_Dbn1	13.5039	13.7226	14.2751	14.0479	13.3138	13.6949	13.8122	13.6696	13.6583	13.6741	1.0041	0.0059	0.7652	0.1162
Dbn1	_EGTQASEGYFSQS[Phospho (STY)]QEEEFAQSEEPC[Carbamidomethyl (C)]AK_.3	Q9QXS6_S658_M1_Dbn1	5.1186	5.1957	4.9448	7.3907	4.5960	5.9016	6.8519	4.7465	4.8749	4.4939	1.0539	0.0758	0.6843	0.1648
Dbn1	_ASDSGPSSSSSSSSS[Phospho (STY)]PPR_.2	#N/A	11.5485	11.4536	11.6494	12.0368	11.2954	11.5120	11.6803	11.4147	11.4788	11.6687	1.0019	0.0027	0.8781	0.0564
Dbn1	_EQFWEQAK_.2	#N/A	7.3600	7.3304	7.4880	7.5472	7.5709	7.3756	7.1246	7.3375	7.1746	7.3450	1.0276	0.0393	0.0199	1.7008
Dbn1	_LREDENAEPVGTTYQK_.3	#N/A	6.2274	6.5175	6.4287	6.0769	6.4218	6.3825	5.5795	5.8411	5.8855	6.4594	1.0675	0.0942	0.0441	1.3557
Dbn1	_M[Oxidation (M)]APTPIPTR_.2	#N/A	3.3102	7.5046	7.4063	4.0053	7.9259	7.4664	7.2040	7.5677	7.6252	7.2306	0.8465	-0.2404	0.3088	0.5103
Dbn1	_SPS[Phospho (STY)]DSSTASTPIAEQIER_.2	Q9QXS6_S387_M1_Dbn1	5.9383	8.1719	8.2098	9.0550	7.3487	8.2883	8.2513	7.9517	8.2384	8.4864	0.9518	-0.0712	0.4956	0.3049
Dbn1	_ASDSGPSSSSSSSSS[Phospho (STY)]PPRTPFPYITC[Carbamidomethyl (C)]HR_.4	#N/A	3.7709	3.0264	3.5046	3.8884	2.9147	3.7099	3.6031	4.2301	3.8786	3.8554	0.8914	-0.1659	0.1053	0.9775
Dbn1	_LELLAAYEEVIR_.2	#N/A	4.7819	4.3892	4.2709	3.7314	4.5038	5.0256	4.4831	3.2697	4.9952	4.3374	1.0419	0.0593	0.6378	0.1953
Dbn1	_SESEVEEAAAIIAQRPDNPR_.3	#N/A	3.2709	4.9232	3.9577	4.4604	3.8975	4.6498	4.1751	4.9649	4.4215	4.1386	0.9476	-0.0776	0.5168	0.2867
Dbn1	_LAASGEGGLQELSGHFENQK_.3	#N/A	5.9493	5.1945	5.9680	5.7092	6.2712	6.0126	5.6542	5.8763	5.8259	6.0080	1.0017	0.0024	0.9619	0.0169
Dbn1	_KSESEVEEAAAIIAQRPDNPR_.3	#N/A	3.8484	3.6146	4.7121	4.2108	4.4057	4.1265	4.8264	3.9168	4.1683	4.3481	0.9625	-0.0552	0.5365	0.2705
Dbn1	_KQQS[Phospho (STY)]LEAEEAKR_.3	#N/A	3.7848	3.2263	4.3883	2.9158	2.5847	3.4732	3.0561	3.8038	3.8263	3.6543	0.9471	-0.0784	0.6100	0.2147
Dbn1	_EREQQIEEHR_.3	#N/A	2.5431	3.3546	3.7215	1.5380	3.8206	3.9315	3.4135	3.7359	4.0350	2.6142	0.9136	-0.1304	0.5934	0.2266
Dbn1	_VAEFFQGVDVIVNASSVEDIDAGAIGQR_.3	#N/A	3.3956	3.9468	3.1292	4.1739	4.0401	3.9613	4.3221	4.3282	3.1264	3.8511	0.9661	-0.0498	0.6781	0.1687
Cnpy2	_NGES[Phospho (STY)]SELDLQGIR_.2	Q9QXT0_S115_M1_Cnpy2	4.2528	3.2676	3.9865	3.5160	3.1277	3.3199	3.4728	3.3006	4.0488	3.3393	1.0107	0.0154	0.8896	0.0508
Cnpy2	_SEAHLTELLEEVC[Carbamidomethyl (C)]DR_.3	#N/A	4.3486	4.0243	3.7101	5.0211	4.0709	4.4079	4.1245	3.4159	3.3323	4.4319	1.1144	0.1562	0.1985	0.7022
Cnpy2	_SQDLHC[Carbamidomethyl (C)]GAC[Carbamidomethyl (C)]R_.3	#N/A	3.8564	3.4860	3.5900	3.1338	2.0116	3.6245	3.2821	3.8674	4.1664	4.3119	0.8405	-0.2507	0.1440	0.8417
Pcsk1n	_ILTGS[Phospho (STY)]SEPEAAPAPR_.2	Q9QXV0_S204_M1_Pcsk1n	11.0686	11.3036	11.8749	11.5012	11.4263	11.4939	11.4965	10.9171	11.3059	11.2285	1.0185	0.0264	0.2452	0.6104
Pcsk1n	_ALAHLLEAER_.2	#N/A	7.6802	8.2176	7.9128	7.7235	8.4897	8.0693	8.0043	7.7518	7.7664	7.6715	1.0278	0.0396	0.2311	0.6362

Pcsk1n	_VLAQLLR_.2	#N/A	7.2454	7.5704	8.1540	6.9170	6.3691	7.1918	6.4726	7.1404	6.7022	7.3425	1.0473	0.0666	0.3710	0.4306
Pcsk1n	_LDPAALAAQLVPAPAAAPR_.2	#N/A	6.4596	6.4109	6.1407	5.9748	6.3919	6.5519	6.1514	5.9042	6.1243	6.2565	1.0348	0.0493	0.1296	0.8875
Pcsk1n	_AEAQEAEDQQAR_.2	#N/A	5.3099	6.2513	5.1613	6.0708	6.8285	6.6188	5.3124	5.4314	5.8908	6.2930	1.0538	0.0756	0.4517	0.3452
Pcsk1n	_ASDPPLAPDDDPDAPAAQLAR_.2	#N/A	7.1928	7.5636	7.4073	7.3544	7.7905	7.7117	7.3356	6.8237	7.2748	7.4351	1.0396	0.0561	0.1071	0.9704
Pcsk1n	_ARAEAQEAEDQQAR_.2	#N/A	2.7921	6.2919	3.7226	3.2663	3.1748	3.0538	5.8046	3.7348	4.0339	4.4445	0.8252	-0.2772	0.3271	0.4853
Cbx8	_VDDKPSS[Phospho (STY)]PGDSSK_.2	Q9QXV1_S164_M1_Cbx8	3.1064	4.1197	6.1164	4.2959	3.7329	3.9965	5.8379	5.9230	6.0987	4.2629	0.7645	-0.3875	0.0679	1.1684
Cbx8	_RQDS[Phospho (STY)]DLVQYGVTSPSSAEASSK_.3	Q9QXV1_S229_M1_Cbx8	4.3545	5.1053	4.0882	3.6320	5.0921	3.1263	4.5203	3.3692	5.7775	3.3093	0.9974	-0.0037	0.9861	0.0061
Slc7a8	_PTPVKDPDS[Phospho (STY)]EEQP_.2	Q9QXW9_S527_M1_Slc7a8	8.3316	7.9209	3.2797	3.7824	7.3148	3.0092	9.0380	8.4376	8.1454	3.7494	0.7636	-0.3892	0.3080	0.5115
Slc7a8	_NHPGSDTS[Phospho (STY)]PEAEASSGGGGVALKK_.3	#N/A	9.4197	9.7369	10.0888	10.1188	8.7574	9.2041	8.3899	10.1197	9.6697	9.6352	1.0106	0.0153	0.8076	0.0928
Slc7a8	_NHPGSDTS[Phospho (STY)]PEAEASSGGGGVALK_.3	#N/A	6.6334	3.7874	3.3202	5.7170	3.5541	3.6927	3.4793	6.3853	3.8860	3.9741	1.0044	0.0064	0.9827	0.0076
Ehd3	_SKLPNS[Phospho (STY)]VLGK_.2	Q9QXY6_S479_M1_Ehd3	8.5526	8.8640	9.5088	9.3233	8.0337	8.4227	8.8520	8.6342	8.4813	8.6806	1.0141	0.0202	0.6869	0.1631
Ehd3	_LLPLEEYYR_.2	#N/A	6.0000	6.1154	5.8056	6.2480	6.2557	5.8852	6.2277	6.0863	6.1857	6.4773	0.9692	-0.0452	0.1353	0.8689
Ehd3	_ELVNNLAEIYGR_.2	#N/A	5.4767	5.4772	5.2583	5.8337	5.9343	3.4154	5.9801	6.0001	5.2975	5.8517	0.9049	-0.1441	0.2941	0.5316
Ehd3	_IVNTPEVIR_.2	#N/A	6.4338	6.3832	6.0246	5.8119	6.7942	6.3827	5.5467	6.2342	6.4358	5.8617	1.0474	0.0668	0.2586	0.5874
Ehd3	_DKPM[Oxidation (M)]YDEIFYTLS[Phospho (STY)]PVDGK_.3	#N/A	7.3734	8.3837	8.0478	7.6666	7.2796	7.8117	8.4678	7.9557	7.8800	8.3368	0.9510	-0.0724	0.1350	0.8695
Ehd3	_EHQIS[Phospho (STY)]PGDFPNLKR_.3	#N/A	3.8817	6.8731	3.6153	8.0930	5.8937	6.2445	6.5066	7.1771	6.7814	6.6200	0.8517	-0.2316	0.2934	0.5325
Ehd3	_LNAFGNAFLNR_.2	#N/A	3.6804	5.1208	3.4141	4.7252	3.9069	4.4611	4.5561	5.4723	4.5714	4.6850	0.8749	-0.1928	0.1490	0.8269
Ehd3	_DIQS[Phospho (STY)]LPR_.2	Q9QXY6_S284_M1_Ehd3	3.9475	3.2787	3.1334	3.6361	3.1670	3.4576	3.3508	3.9979	4.8520	4.8127	0.8080	-0.3076	0.0367	1.4353
Ehd3	_EHQIS[Phospho (STY)]PGDFPNLK_.3	#N/A	3.2919	3.9342	3.9787	3.3175	3.9185	4.3029	4.1961	3.6371	3.2857	4.4484	0.9740	-0.0380	0.7383	0.1318
Ehd3	_IGPEPTTDSFIAVM[Oxidation (M)]QGDVEGIIPGNALVVDPK_.3	#N/A	3.7805	5.8121	4.4673	3.5350	2.9062	3.3224	3.6832	5.0340	3.7652	3.2123	1.0120	0.0171	0.9410	0.0264
Macf1	_LLDAEDVDVPS[Phospho (STY)]PDEK_.2	Q9QXZ0_S280_M1_Macf1	11.1491	12.2403	11.9129	12.2895	11.0749	11.9172	12.6766	12.5104	12.2899	12.1776	0.9477	-0.0775	0.0516	1.2873
Macf1	_S[Phospho (STY)]QDSVLDPAER_.2	#N/A	3.6007	4.4059	4.2875	8.7844	4.2272	8.4838	4.5049	8.8674	8.7425	8.7177	0.7306	-0.4528	0.1940	0.7123
Macf1	_AFVESQQKS[Phospho (STY)]PGKR_.3	#N/A	7.1195	7.5308	7.8841	6.8707	5.4332	6.9187	7.0705	7.4618	6.8560	6.7652	0.9888	-0.0163	0.8643	0.0633
Macf1	_DTQDIVHDLES[Phospho (STY)]PGIDPSIIK_.3	#N/A	6.6823	5.6660	6.4579	7.0847	4.4777	5.2171	7.0154	7.2154	6.4167	5.9179	0.8930	-0.1632	0.2362	0.6267
Macf1	_RQGS[Phospho (STY)]FSEDVISHK_.3	#N/A	7.7060	3.5061	8.5561	7.6068	3.3944	3.2302	8.0206	7.8088	3.7721	3.2054	0.9938	-0.0089	0.9835	0.0072
Macf1	_AFVESQQKS[Phospho (STY)]PGK_.2	#N/A	6.7671	6.4799	6.9896	6.9954	4.9034	6.2039	6.5923	6.9051	6.3380	6.3067	0.9777	-0.0325	0.7364	0.1329
Macf1	_EAEEELAASGGQSPT[Phospho (STY)]GEQIPQFQQR_.3	Q9QXZ0_T5553_M1_Macf1	3.1401	3.9453	3.8269	4.2396	3.7667	3.9630	4.1758	4.0976	3.3045	4.2966	0.9609	-0.0575	0.5656	0.2475
Macf1	_AFLAELEQNS[Phospho (STY)]PK_.2	Q9QXZ0_S4483_M1_Macf1	3.6915	8.9504	9.7322	9.4493	8.0804	9.3113	10.1235	9.7342	9.5195	9.2270	0.8499	-0.2346	0.2524	0.5980
Macf1	_RKS[Phospho (STY)]QDSVLDPAER_.3	#N/A	3.5784	4.3347	6.8655	4.3567	4.2230	5.9981	4.5049	4.1453	2.9436	4.0340	1.2523	0.3246	0.1933	0.7139
Macf1	_QGS[Phospho (STY)]FSEDVISHK_.2	#N/A	7.9670	7.8911	8.6015	8.0495	3.8968	7.6460	8.7059	8.2269	8.0795	8.0552	0.8881	-0.1712	0.3262	0.4865
Macf1	_AM[Oxidation (M)]DATLS[Phospho (STY)]PTKTETVK_.2	#N/A	6.6899	6.6102	3.6250	5.5750	5.5864	6.1776	7.4269	3.5732	3.7091	6.2851	1.0880	0.1217	0.6401	0.1938
Macf1	_LNSQES[Phospho (STY)]DEEPQISDVPHISK_.3	Q9QXZ0_S3085_M1_Macf1	3.6876	5.2232	4.0671	5.2633	4.4347	5.2265	3.9282	5.8335	5.0316	3.7626	1.0025	0.0035	0.9830	0.0074
Ulk2	_TPLPTIIGS[Phospho (STY)]PTK_.2	Q9QY01_S600_M1_Ulk2	5.6347	4.1066	3.8063	4.3090	3.7461	3.9834	5.9870	6.3205	5.2545	4.2760	0.7811	-0.3564	0.0460	1.3376
Myo9b	_AQDKPES[Phospho (STY)]PSGSTQIQR_.2	Q9QY06_S1264_M1_Myo9b	3.0510	3.8073	4.1057	4.6083	3.6956	4.4298	4.3230	4.2171	3.1587	3.0745	1.0694	0.0968	0.5296	0.2761
Plxnb3	_TNS[Phospho (STY)]LLLR_.2	Q9QY40_S1747_M1_Plxnb3	3.4014	4.1577	3.1351	4.2579	4.0460	4.0795	3.9726	3.8605	3.5091	4.2249	0.9883	-0.0170	0.8669	0.0620
Vapb	_SLTSPLDDTEVKK_.2	#N/A	4.5925	4.5060	4.7238	5.6664	4.8009	4.7843	5.8861	4.9112	4.4925	4.9836	0.9561	-0.0648	0.4997	0.3013
Vapb	_SLTS[Phospho (STY)]PLDDTEVK_.2	#N/A	4.2822	2.9440	3.4682	3.9708	3.4079	3.7923	3.6855	4.2497	3.7963	3.9378	0.9303	-0.1043	0.3232	0.4905
Nup210	_ELAS[Phospho (STY)]PLALT[Phospho (STY)]PR_.2	Q9QY81_S1839_M2_Nup210	3.7422	3.6002	3.4759	6.9375	6.0879	4.3080	3.1679	7.3349	6.8893	4.1978	0.8693	-0.2021	0.5381	0.2692
Tomm40	_FVNWQVDGEYR_.2	#N/A	3.0702	4.2722	4.3047	3.8485	3.7148	4.3718	3.9968	5.4943	4.9622	3.5258	0.8744	-0.1936	0.2316	0.6353
Tomm40	_MAIQTQQSK_.2	#N/A	3.7378	3.7954	3.6770	4.7396	3.6168	4.1127	4.4735	3.9388	3.5874	4.1467	0.9777	-0.0325	0.7400	0.1308
Add3	_TEEVLS[Phospho (STY)]PDGSPSK_.2	#N/A	9.4698	9.2885	9.6370	8.5974	9.1067	8.3967	9.0946	8.5123	8.5658	9.4585	1.0196	0.0280	0.5854	0.2325
Add3	_EQDHIIIIPR_.3	#N/A	4.7986	4.5371	3.1449	4.1287	3.6374	4.2085	4.0737	4.1500	4.0464	4.5228	0.9709	-0.0427	0.7118	0.1477
Add3	_TLDNLGYR_.2	#N/A	5.7248	5.4382	3.4719	4.7819	6.3398	5.5060	5.4582	6.1262	6.8465	5.5318	0.8698	-0.2013	0.2047	0.6889
Add3	_TEEVLSPDGSPSKS[Phospho (STY)]PSKK_.3	#N/A	6.9939	8.5343	8.9945	8.8729	7.8716	8.6569	8.5924	6.6059	8.4372	8.7477	1.0278	0.0395	0.6953	0.1578
Add3	_TEEVLSPDGSPSKS[Phospho (STY)]PSK_.2	#N/A	9.1473	9.4024	9.8951	10.2632	8.7725	9.5198	9.6274	10.3044	10.5357	3.8511	1.1073	0.1470	0.4939	0.3063
Add3	_GLSFSEATASTLVK_.2	#N/A	3.8743	1.6650	4.5611	4.4563	3.0571	4.1364	3.5894	4.1821	3.4695	3.5300	0.9817	-0.0267	0.9094	0.0412
Add3	_SPERTEEVLSPDGSPSKS[Phospho (STY)]PSK_.3	#N/A	6.7639	7.6476	8.5983	6.2089	3.4615	7.4173	7.1641	7.2010	7.4599	3.8207	1.0423	0.0598	0.8170	0.0878
Add3	_[Acetyl (Protein N-term)]SSDTSPAVVTT[Phospho (STY)]PPPPSM[Oxidation (M)]PHK_.2	#N/A	7.1278	4.0240	3.6372	7.6241	6.3484	4.0285	4.0106	7.6288	3.5182	4.1069	1.1347	0.1824	0.5954	0.2252
Add3	_SDVEIPATVTAFSFEDDSAPLS[Phospho (STY)]PLK_.3	#N/A	3.0872	3.8434	4.3217	6.3184	3.7317	4.3937	4.2869	3.5463	4.4629	3.0383	1.1171	0.1598	0.4901	0.3097
Add3	_HKSDVEIPATVTAFSFEDDSAPLS[Phospho (STY)]PLK_.3	#N/A	3.7417	4.5210	4.6812	3.9579	3.6362	3.5985	3.6725	5.3112	4.9143	4.4047	0.8792	-0.1858	0.1710	0.7671
Add3	_SPERTEEVLS[Phospho (STY)]PDGSPSK_.2	#N/A	3.6759	3.6818	5.9417	5.6749	3.5446	4.9330	5.4936	5.4157	4.2233	4.0804	0.9525	-0.0701	0.7262	0.1389
Add3	_NMS[Phospho (STY)]PDLR_.2	#N/A	5.3056	3.8677	3.6520	5.8395	2.6481	4.0559	4.3508	3.5283	3.9701	4.2304	1.0518	0.0729	0.7416	0.1298
Add2	_STPAS[Phospho (STY)]PVQS[Phospho (STY)]PSK_.2	#N/A	14.4161	14.5442	14.9116	15.0907	14.6274	14.5721	15.0871	14.6016	14.5592	14.7204	0.9967	-0.0047	0.7712	0.1128
Add2	_DIATEKPGS[Phospho (STY)]PVK_.2	#N/A	9.0636	8.5430	8.3029	9.6305	8.8520	8.6864	9.3678	8.8151	8.8163	9.0953	0.9804	-0.0286	0.5127	0.2901
Add2	_VNVADEVQR_.2	#N/A	8.1040	8.2861	7.6096	8.1503	8.3204	8.3172	8.0184	8.1281	8.0671	8.1559	1.0048	0.0069	0.7891	0.1029
Add2	_FSEDDPEYLR_.2	#N/A	8.1482	8.1601	7.7745	8.1035	8.1011	7.8398	7.8579	7.6835	7.9445	8.2033	1.0125	0.0179	0.4387	0.3578
Add2	_WLNTPNTYLR_.2	#N/A	7.0163	7.2047	7.1064	7.4888	7.0856	6.9387	6.9685	7.5107	7.2304	7.0993	0.9914	-0.0125	0.6560	0.1831
Add2	_VNILGEVVEK_.2	#N/A	6.6072	6.5304	6.6363	7.2710	7.3067	6.8611	6.9656	7.0552	7.0787	6.7843	0.9853	-0.0213	0.5922	0.2275
Add2	_GVSC[Carbamidomethyl (C)]SEVTASSLIK_.2	#N/A	5.5694	5.4000	6.6146	6.5451	6.2475	6.1539	5.4755	5.9030	5.9902	5.8323	1.0497	0.0700	0.3189	0.4964
Add2	_EQDHFLISPK_.2	#N/A	6.5216	6.1120	6.0094	6.2494	6.2160	6.2302	6.3682	5.8767	5.9139	6.1807	1.0227	0.0324	0.3083	0.5110
Add2	_SAGPQSQLLASVIAEK_.2	#N/A	6.0617	6.0128	6.2638	5.6111	5.9795	6.0140	6.0696	5.6481	5.7297	5.7137	1.0346	0.0490	0.1668	0.7778
Add2	_[Acetyl (Protein N-term)]SEDTVPEAAS[Phospho (STY)]PPPSQGQHYFDR_.3	Q9QYB8_S11_M1_Add2	15.4301	15.3583	15.9144	15.9186	14.8555	15.5337	15.8619	15.3105	15.4807	15.5170	0.9974	-0.0038	0.8592	0.0659
Add2	_TESVTSGPLSPEGS[Phospho (STY)]PSKS[Phospho (STY)]PSK_.2	#N/A	4.5742	5.2897	3.5163	6.3286	7.2681	5.4232	6.4274	5.5524	7.3854	7.5264	0.8032	-0.3161	0.1206	0.9186
Add2	_TESVTSGPLS[Phospho (STY)]PEGSPSK_.2	#N/A	7.2291	6.4352	6.9686	7.7796	6.9845	6.5440	7.6615	6.8252	7.2884	6.8023	0.9784	-0.0315	0.6179	0.2091
Add2	_LEQEQEGEKDIATEKPGS[Phospho (STY)]PVK_.3	#N/A	7.4792	6.6573	3.4939	3.5332	7.3475	6.6896	7.6510	4.0804	7.0336	7.1831	0.9044	-0.1450	0.6023	0.2202
Add2	_HPFVQEK_.2	#N/A	4.9166	5.9387	3.8896	5.6413	5.9439	4.9993	5.7077	5.4691	5.3487	5.4164	0.9519	-0.0711	0.5362	0.2707
Add2	_TESVTSGPLSPEGSPS[Phospho (STY)]KS[Phospho (STY)]PSKK_.3	#N/A	7.8869	3.6664	7.5049	8.2330	7.9936	3.2681	7.5340	8.0306	7.5595	7.7978	0.8312	-0.2668	0.3012	0.5211
Add2	_SPAVS[Phospho (STY)]PSKTSEDTK_.2	#N/A	10.0076	10.0527	10.4338	10.5997	9.9630	10.0803	10.4156	10.0569	10.1217	10.1572	1.0002	0.0002	0.9912	0.0038
Add2	_NMGS[Phospho (STY)]PRPK_.2	#N/A	10.0022	7.9830	10.2133	10.9967	7.4175	5.2727	9.8373	7.1432	7.5645	10.0732	0.9992	-0.0012	0.9957	0.0019
Add2	_AGTKS[Phospho (STY)]PAVS[Phospho (STY)]PSK_.2	#N/A	11.7048	11.6002	11.5148	12.1576	10.3736	11.4494	11.8872	11.5833	11.4901	11.7573	0.9818	-0.0265	0.5117	0.2909
Add2	_IENPNQFVPLYTDPQEVLDM[Oxidation (M)]R_.3	#N/A	3.6761	5.0525	4.3629	4.2970	4.6568	4.7808	4.4091	4.7959	4.4023	4.9531	0.9636	-0.0536	0.5451	0.2636
Add2	_AGTKS[Phospho (STY)]PAVS[Phospho (STY)]PSKTSEDTK_.2	#N/A	10.0630	10.1119	10.5040	10.5688	9.4187	10.2151	10.2679	10.0970	10.1777	10.2904	0.9940	-0.0087	0.7810	0.1074
Add2	_STPAS[Phospho (STY)]PVQS[Phospho (STY)]PSKAGTK_.2	#N/A	9.6058	10.7832	11.4727	10.6369	10.9766	10.5405	10.9013	10.5553	10.3749	10.4563	1.0092	0.0132	0.7741	0.1112
Add2	_SPS[Phospho (STY)]TESQLMSK_.2	#N/A	8.0743	6.8099	4.1701	8.9788	6.9478	3.7708	7.3582	6.1022	3.9431	7.2098	1.0496	0.0699	0.8109	0.0911
Add2	_S[Phospho (STY)]PAVS[Phospho (STY)]PSKTSEDTKK_.2	#N/A	8.4772	8.4762	8.5033	9.0722	8.6368	8.6541	8.7530	8.7102	8.8034	8.6421	0.9896	-0.0150	0.4694	0.3285
Add2	_DIATEKPGS[Phospho (STY)]PVKS[Phospho (STY)]TPAS[Phospho (STY)]PVQS[Phospho (STY)]PSK_.3	#N/A	3.1843	3.7323	3.9450	3.8258	3.8289	4.1963	4.4451	4.3223	3.3948	4.2395	0.9232	-0.1153	0.2515	0.5994
Add2	_DKTESVTSGPLS[Phospho (STY)]PEGS[Phospho (STY)]PSK_.2	#N/A	4.3737	4.5352	3.7065	4.0348	3.4926	4.6340	4.9119	4.7245	4.1280	4.3725	0.9107	-0.1349	0.1780	0.7497
Add2	_DKTESVTSGPLSPEGSPS[Phospho (STY)]KSPSK_.3	#N/A	3.8363	5.9050	3.5700	6.7650	5.6952	5.7617	6.1192	3.9580	3.7890	5.6919	1.0749	0.1041	0.6589	0.1812
Add2	_SRS[Phospho (STY)]PSTESQLM[Oxidation (M)]SK_.2	#N/A	8.1818	8.6472	9.0625	9.3906	7.3769	8.0505	8.4010	8.5988	7.9727	7.9375	1.0272	0.0388	0.5862	0.2319

Add2	_GLGQM[Oxidation (M)]TTNADTDGDSYKDKTESVTSGPLS[Phospho (STY)]PEGSPSK_.4	#N/A	3.5575	3.9436	3.9774	5.3480	4.6332	3.9693	4.7613	3.6464	4.5408	4.6779	0.9618	-0.0562	0.6748	0.1708
Add2	_C[Carbamidomethyl (C)]LGPTC[Carbamidomethyl (C)]K_.2	#N/A	3.9159	3.8708	3.2053	4.2499	4.4052	4.0374	4.3982	2.9984	3.0503	4.6600	1.0452	0.0638	0.6853	0.1641
Add2	_NHGM[Oxidation (M)]VALGDTVEEAFYK_.3	#N/A	3.9659	5.0620	6.1116	4.3410	4.7258	3.2139	3.1070	3.2253	4.2297	4.8196	1.1884	0.2491	0.2621	0.5815
Add2	_SPAVS[Phospho (STY)]PSK_.2	#N/A	3.4227	3.8723	5.3326	4.3999	4.8229	5.5209	4.3393	3.9325	5.6292	4.2348	1.0062	0.0089	0.9580	0.0186
Add2	_SSSGMPIR_.2	#N/A	3.5909	3.6353	4.2777	3.2795	4.2174	3.5121	4.4950	3.3381	2.9867	3.2465	1.0670	0.0935	0.5021	0.2992
Add2	_LGEHEFEALMR_.3	#N/A	3.4136	1.9426	4.1004	4.6031	3.7008	4.4246	4.3178	3.5154	3.1640	3.0693	1.0515	0.0724	0.7470	0.1267
Add2	_SAGPQSQLLASVIAEKS[Phospho (STY)]R_.3	#N/A	3.6467	4.4030	3.3804	6.2947	2.7904	3.8342	3.0724	4.0770	4.3761	4.1023	1.0387	0.0548	0.8247	0.0837
Add1	_AAVVTS[Phospho (STY)]PPPTTAPHK_.2	#N/A	13.8570	14.0752	14.5332	14.4064	13.5106	13.8893	14.3193	13.7176	13.9530	13.8320	1.0064	0.0093	0.6922	0.1597
Add1	_TPS[Phospho (STY)]FLKK_.2	Q9QYC0_S724_M1_Add1	12.8762	12.8345	13.2135	13.1623	11.9201	12.7131	12.9303	12.7496	12.8419	12.9421	0.9938	-0.0089	0.7503	0.1248
Add1	_AAVVTS[Phospho (STY)]PPPTTAPHKER_.3	#N/A	9.8676	10.8501	11.9712	10.7790	10.3156	10.7124	10.5699	10.8603	10.6814	10.4497	1.0102	0.0147	0.7730	0.1118
Add1	_GS[Phospho (STY)]EENLDETR_.2	#N/A	9.1450	8.9881	9.2630	9.3162	8.4883	8.9154	9.0259	8.9612	8.9466	9.2866	0.9961	-0.0057	0.8355	0.0781
Add1	_LEEDLPQEPTS[Phospho (STY)]R_.2	Q9QYC0_S628_M1_Add1	9.7857	3.0840	3.5622	3.8308	2.9723	9.1777	3.5455	3.8952	4.1942	9.4556	1.0246	0.0350	0.9491	0.0227
Add1	_VNLQGDIVDR_.2	#N/A	8.1251	8.2299	7.9372	8.0460	7.9780	8.1935	8.0960	8.0877	8.2469	8.2187	0.9905	-0.0137	0.2899	0.5378
Add1	_TSTSAVPNLFVPLNTNPK_.2	#N/A	7.9333	7.4548	7.8954	7.3955	7.7309	7.6316	7.5728	7.8943	7.9586	7.7694	0.9839	-0.0233	0.3700	0.4317
Add1	_TLASAGGPDNLVLLDPGK_.2	#N/A	7.5777	7.1937	6.6516	7.4282	7.4404	7.5888	7.4224	7.1741	7.4510	7.5250	0.9892	-0.0156	0.6865	0.1633
Add1	_YFDRVDENNPEYLR_.3	#N/A	7.8444	7.7305	8.1119	7.7831	7.3768	7.8523	7.6762	7.5183	7.7446	7.7819	1.0134	0.0192	0.4519	0.3450
Add1	_GSDSIAYDKGEK_.2	#N/A	6.9771	7.1739	7.1932	7.3940	7.2685	7.3092	7.0959	7.0277	7.0099	7.1116	1.0224	0.0319	0.0702	1.1539
Add1	_SLVQGELVTASK_.2	#N/A	6.6715	6.9080	6.8237	6.8954	7.0752	6.9105	6.8877	6.8323	7.0480	6.8016	0.9983	-0.0024	0.8875	0.0518
Add1	_SPGT[Phospho (STY)]PAGEGSGSPPK_.2	#N/A	12.5658	12.4218	12.6956	12.8874	12.1314	12.5645	12.8234	12.5289	12.5596	12.6348	0.9927	-0.0106	0.5300	0.2757
Add1	_EKS[Phospho (STY)]PPDQSAVPNT[Phospho (STY)]PPSTPVK_.3	#N/A	8.2723	7.8603	3.8593	9.0266	9.3070	8.3224	9.4905	9.2170	8.0144	9.9308	0.8485	-0.2371	0.2290	0.6402
Add1	_EYQPHVIVSTTGPNPFNTLTDR_.3	#N/A	5.1048	4.3437	4.3769	5.3830	5.1079	4.8826	5.0354	5.2519	4.9989	5.1934	0.9505	-0.0732	0.2851	0.5450
Add1	_M[Oxidation (M)]LDNLGYR_.2	#N/A	6.3454	6.7595	5.7428	7.0677	6.7954	5.9249	6.0566	6.3024	6.7510	6.5084	1.0054	0.0078	0.9086	0.0416
Add1	_KQKGS[Phospho (STY)]EENLDETR_.3	#N/A	8.7874	8.5017	9.3644	8.8075	8.8201	8.7807	8.5739	8.7635	9.1687	8.8306	1.0011	0.0015	0.9581	0.0186
Add1	_SPPDQSAVPNT[Phospho (STY)]PPSTPVK_.2	#N/A	7.5757	9.6829	9.8323	10.5193	9.2701	3.7223	7.6606	9.7784	10.0388	9.6954	0.9075	-0.1400	0.5428	0.2654
Add1	_SRS[Phospho (STY)]PGT[Phospho (STY)]PAGEGSGSPPK_.2	#N/A	11.4576	11.6716	11.9876	12.1798	11.4249	11.5682	11.9420	11.7132	11.5081	11.6166	1.0017	0.0025	0.9104	0.0408
Add1	_WQIGEQEFEALM[Oxidation (M)]R_.2	#N/A	5.5242	5.3350	4.4492	3.4510	4.2471	3.2725	5.1267	4.5291	5.4871	4.6210	0.8864	-0.1739	0.3007	0.5219
Add1	_YSDVEVPAS[Phospho (STY)]VTGHSFASDGDSGTC[Carbamidomethyl (C)]SPLRHSFQK_.4	Q9QYC0_S415_M1_Add1	4.3206	4.4064	3.5065	4.0092	2.7939	3.8307	3.7239	4.1093	3.7579	3.9762	0.9793	-0.0302	0.8034	0.0951
Add1	_GDDASEEGQNGSS[Phospho (STY)]PK_.2	#N/A	9.0922	8.3534	8.0335	8.4963	7.0401	7.8060	9.0295	8.5299	7.4663	9.9225	0.9313	-0.1027	0.2972	0.5270
Add1	_WLHSGRGDDASEEGQNGSS[Phospho (STY)]PK_.3	#N/A	7.3002	7.5663	8.4745	7.7805	7.7296	8.0504	7.4541	7.7021	7.9910	7.6193	1.0163	0.0233	0.5937	0.2264
Add1	_QKGS[Phospho (STY)]EENLDETR_.2	#N/A	9.9036	10.0688	10.6796	10.5081	9.8266	10.2070	10.3070	10.2309	10.1152	10.0553	1.0021	0.0031	0.9053	0.0432
Add1	_YSDVEVPASVTGHSFASDGDSGTC[Carbamidomethyl (C)]S[Phospho (STY)]PLR_.3	#N/A	10.2912	10.6019	11.3238	11.6253	10.2181	10.4090	10.7583	10.7451	10.7924	10.2867	1.0093	0.0134	0.7603	0.1190
Add1	_SPPDQSAVPNT[Phospho (STY)]PPST[Phospho (STY)]PVKLEEDLPQEPTSR_.4	#N/A	6.0026	7.3204	6.5335	7.8396	6.1237	4.3596	4.0308	7.5514	7.1910	7.0492	0.9857	-0.0208	0.9201	0.0362
Add1	_NMAPDLR_.2	#N/A	3.9251	1.3672	4.0909	2.8073	2.8065	3.4222	3.7830	3.3665	3.6655	3.3120	0.8692	-0.2022	0.3960	0.4023
Add1	_KYSDVEVPASVTGHSFASDGDSGTC[Carbamidomethyl (C)]S[Phospho (STY)]PLR_.4	#N/A	3.0827	4.8227	4.3172	4.5766	3.7273	4.1195	4.2913	3.5419	5.1498	3.0428	1.0253	0.0360	0.8397	0.0759
Add1	_EKSPPDQS[Phospho (STY)]AVPNT[Phospho (STY)]PPSTPVKLEEDLPQEPTSR_.4	Q9QYC0_S605_M2_Add1	3.5287	3.7022	3.6905	3.8205	3.5235	4.2361	3.6850	4.7095	3.8615	4.2839	0.9070	-0.1409	0.1265	0.8980
Add1	_IREQNLQDIK_.3	#N/A	3.3795	3.9629	3.1132	4.1578	3.7842	3.9453	4.3061	4.3442	3.1424	3.8351	0.9531	-0.0693	0.5587	0.2528
Pcnx1	_SSS[Phospho (STY)]LIHR_.2	Q9QYC1_S551_M1_Pcnx1	5.1746	3.9954	5.4854	4.5459	3.7476	4.6958	4.1349	3.6982	3.3468	3.6947	1.2390	0.3092	0.0401	1.3974
Bcl11a	_GC[Carbamidomethyl (C)]S[Phospho (STY)]PGESASGGLSK_.2	#N/A	9.6477	9.6305	10.1714	10.4402	9.6504	9.6040	10.0125	9.7089	9.7609	9.7685	1.0046	0.0066	0.8205	0.0859
Bcl11a	_GC[Carbamidomethyl (C)]S[Phospho (STY)]PGESASGGLSKK_.2	#N/A	6.8571	4.2650	3.6479	6.9637	3.5877	7.1049	7.3291	3.9679	3.6165	4.1176	1.1359	0.1838	0.5794	0.2370
Bcl11a	_IYLESEHGS[Phospho (STY)]PLTPR_.3	#N/A	5.7250	5.6472	6.1043	4.3728	5.6248	6.2573	5.2995	5.9468	5.6179	5.7619	0.9939	-0.0089	0.9249	0.0339
Bcl11a	_FST[Phospho (STY)]PPGELDGGISGR_.2	Q9QYE3_T701_M1_Bcl11a	8.8974	3.1700	3.2421	9.1790	3.0583	8.7730	9.0183	8.3030	4.0223	3.7118	0.9664	-0.0493	0.9171	0.0376
Bcl11a	_ELAGNTSS[Phospho (STY)]PPLS[Phospho (STY)]PGRPSPMQR_.3	Q9QYE3_S328_M2_Bcl11a	3.8000	3.0555	3.5337	4.5783	2.9438	3.6809	3.5740	3.9237	4.2228	4.2556	0.9010	-0.1504	0.2595	0.5859
Bcl11a	_GVDKPPS[Phospho (STY)]PSPIEMK_.2	Q9QYE3_S86_M1_Bcl11a	6.4272	3.4029	3.8811	3.4249	3.2912	3.3335	3.2266	3.5763	3.8754	3.1021	1.1495	0.2010	0.4452	0.3515
Bcl11a	_LLLGS[Phospho (STY)]PSSLS[Phospho (STY)]PFSK_.2	Q9QYE3_S625_M2_Bcl11a	4.0287	3.1975	3.2147	3.7173	3.1544	3.0743	3.4320	4.4012	4.0497	3.6843	0.8731	-0.1958	0.0917	1.0376
Ndrg3	_LDPINTTLLK_.2	#N/A	6.6777	6.6266	6.1365	6.4283	6.9181	6.9050	6.4280	7.2454	6.8021	6.7160	0.9732	-0.0393	0.3939	0.4047
Ndrg3	_DLEIERPILGQNDNR_.3	#N/A	9.8750	9.6383	10.3360	10.0872	9.2088	9.7345	10.2651	9.6418	9.7587	9.9498	0.9909	-0.0133	0.6985	0.1558
Ndrg3	_THSTSSS[Phospho (STY)]IGSGESPFSR_.2	#N/A	7.6501	6.5694	6.9821	7.2452	5.5986	3.7223	7.6263	7.0268	7.0726	7.0951	0.8736	-0.1949	0.2558	0.5920
Ndrg3	_S[Phospho (STY)]RT[Phospho (STY)]HSTSSSIGSGESPFSR_.3	Q9QYF9_T329_M2_Ndrg3	4.2226	3.4039	3.9563	4.3642	3.3664	3.3070	3.6483	3.5498	3.8489	3.7259	1.0208	0.0297	0.7618	0.1182
Ndrg3	_HQTMEVS[Phospho (STY)]C[Carbamidomethyl (C)]_.2	Q9QYF9_S374_M1_Ndrg3	5.9071	4.3058	3.6072	6.5269	3.5469	4.1826	5.2236	4.0086	3.6572	4.2131	1.0944	0.1302	0.5736	0.2414
Ndrg3	_LHIAQDINQENLQLFLGSYNGR_.3	#N/A	4.2911	3.8583	3.4771	3.9797	2.6846	4.3127	3.6944	4.1387	3.7873	4.1354	0.9564	-0.0643	0.6129	0.2126
Ndrg3	_SVTSNQSDGTQESC[Carbamidomethyl (C)]ES[Phospho (STY)]PDVLDR_.3	Q9QYF9_S361_M1_Ndrg3	3.0879	4.1382	3.7748	2.1393	4.0265	4.0989	3.9921	3.8411	3.4897	4.2444	0.9107	-0.1350	0.4346	0.3619
Ndrg3	_C[Carbamidomethyl (C)]STLLVVGDNSPAVEAVVEC[Carbamidomethyl (C)]NSR_.3	#N/A	3.0746	4.2678	4.3091	3.8529	4.0892	3.6403	4.0011	3.1483	3.4474	3.5301	1.0964	0.1328	0.2517	0.5990
Ndrg2	_SC[Carbamidomethyl (C)]FQPLFR_.2	#N/A	8.3448	8.7140	8.4008	8.2741	8.5750	8.2361	8.2772	8.6321	8.3901	8.4221	0.9993	-0.0011	0.9565	0.0193
Ndrg2	_[Acetyl (Protein N-term)]AELQEVQITEEKPLLPGQTPETAK_.3	#N/A	5.8671	6.0542	6.4792	6.2589	6.4137	6.7216	6.0233	6.0779	6.0933	6.3367	1.0271	0.0386	0.3463	0.4606
Ndrg2	_GIIQHAPNLENIELYWNSYNNR_.3	#N/A	7.2740	7.6037	7.5235	8.4104	7.6027	7.4618	7.5053	7.6188	7.6430	7.6713	1.0048	0.0069	0.8621	0.0644
Ndrg2	_LDPTQTSFLK_.2	#N/A	7.4287	7.5664	7.7778	7.5101	7.9583	7.2745	7.8359	7.4855	7.5664	7.6257	0.9944	-0.0080	0.7680	0.1146
Ndrg2	_GWM[Oxidation (M)]DWAAHK_.2	#N/A	6.0918	7.0123	7.0097	5.7785	6.1192	6.3936	6.5876	6.7802	5.9435	6.5042	0.9918	-0.0119	0.8628	0.0641
Ndrg2	_YALNHPDTVEGLVLINIDPNAK_.3	#N/A	6.2475	6.4270	5.9503	6.1664	6.0600	6.2771	6.3239	6.0878	6.3985	6.4607	0.9795	-0.0299	0.2622	0.5814
Ndrg2	_M[Oxidation (M)]ADSGGQPQLTQPGK_.2	#N/A	7.3545	7.8366	7.3738	7.3390	7.9396	7.6756	7.5100	7.6470	7.6794	7.7039	0.9936	-0.0092	0.7381	0.1319
Ndrg2	_TAS[Phospho (STY)]LTSAASIDGSR_.2	Q9QYG0_S332_M1_Ndrg2	12.0632	12.7060	12.7895	13.4332	12.6559	12.9932	13.0121	12.9894	12.7989	12.9702	0.9869	-0.0190	0.4869	0.3125
Ndrg2	_FGDM[Oxidation (M)]QEIIQNFVR_.2	#N/A	6.0491	6.5117	6.5428	7.0012	5.9333	5.9077	7.4775	6.3941	6.0303	7.1965	0.9335	-0.0992	0.2307	0.6369
Ndrg2	_TASLTSAAS[Phospho (STY)]IDGS[Phospho (STY)]RSR_.2	#N/A	3.7810	3.4481	3.8012	4.8638	5.4269	4.9117	3.8143	3.8506	6.3493	3.8952	0.9765	-0.0343	0.8733	0.0588
Ndrg2	_SRT[Phospho (STY)]AS[Phospho (STY)]LTSAASIDGSR_.2	#N/A	5.4044	7.8620	6.8695	9.6368	8.4757	9.2390	8.9174	9.0344	8.8925	9.2709	0.8766	-0.1900	0.2050	0.6883
Ndrg2	_TLSQSS[Phospho (STY)]ESGTLPSGPPGHTM[Oxidation (M)]EVSC[Carbamidomethyl (C)]_.3	#N/A	8.7732	3.8318	4.0456	7.8704	8.1100	8.3049	8.9969	8.0737	8.8317	8.0735	0.8032	-0.3161	0.1899	0.7215
Ndrg2	Phospho (STY)]RT[Phospho (STY)]LSQSSESGTLPSGPPGHTM[Oxidation (M)]EVSC[Carbamidomethyl (C)]	Q9QYG0_S346_M2_Ndrg2	3.6739	3.8465	3.2048	4.0937	3.8641	3.7567	7.2362	7.4983	7.1010	5.8384	0.5406	-0.8874	0.0000	4.9584
Ndrg2	_LSRS[Phospho (STY)]RT[Phospho (STY)]AS[Phospho (STY)]LTSAASIDGSR_.3	#N/A	4.0312	3.3315	3.7649	4.1621	3.1749	3.4563	3.4569	5.4569	4.0119	3.8394	0.8717	-0.1981	0.2164	0.6648
Ndrg2	_LTGLTSSIPDM[Oxidation (M)]ILGHLFSQEELSGNSELIQK_.3	#N/A	3.7707	3.0751	4.4575	3.4593	2.8964	3.3323	3.1740	3.1583	4.3077	3.4263	0.9949	-0.0074	0.9609	0.0173
Ndrg2	_C[Carbamidomethyl (C)]PVMLVVGDQAPHEDAVVEC[Carbamidomethyl (C)]NSK_.3	#N/A	3.0155	3.8207	3.7023	4.2049	3.6420	4.0875	3.9196	3.9136	3.5621	4.1719	0.9624	-0.0553	0.5521	0.2580
Dnajb12	_SAS[Phospho (STY)]DEDLKK_.2	Q9QYI4_S123_M1_Dnajb12	3.9195	3.2239	3.8522	3.3960	3.0452	3.1834	3.5442	3.6052	3.9042	3.0733	0.9731	-0.0394	0.6916	0.1601
Dnajb2	_GEAAKLS[Phospho (STY)]PSSEEK_.2	#N/A	8.4524	8.8417	9.2880	9.5339	8.8288	8.8379	8.9960	8.8808	8.8639	8.9681	1.0041	0.0059	0.8580	0.0665
Dnajb2	_SAS[Phospho (STY)]PDDIKK_.2	Q9QYI5_S15_M1_Dnajb2	6.5739	3.9948	7.4955	4.4209	3.8831	4.2424	4.1356	6.8511	6.8043	6.8343	0.8287	-0.2711	0.2983	0.5254
Dnajb2	_LS[Phospho (STY)]PSSEEK_.2	#N/A	6.0787	3.9717	4.6053	5.6891	6.0922	3.9365	5.6612	5.0916	5.1261	3.8263	1.0276	0.0393	0.8271	0.0824
Dnajb2	_GEAAKLS[Phospho (STY)]PS[Phospho (STY)]SEEKASR_.2	#N/A	3.7844	3.5580	7.8533	4.5627	2.9281	3.6965	3.2101	7.0481	4.2384	4.2400	0.9387	-0.0912	0.8033	0.0951
Dnajb2	_S[Phospho (STY)]PEEVFR_.2	Q9QYI5_S98_M1_Dnajb2	3.2354	3.9907	3.9222	4.4249	3.8620	3.8675	4.1396	2.6484	3.3422	4.3919	1.0698	0.0973	0.5118	0.2909
Dnaja2	_VIEPGC[Carbamidomethyl (C)]VR_.2	#N/A	6.8711	7.0333	6.9721	7.4502	8.0272	6.8226	6.7229	7.2548	7.2307	7.2618	1.0110	0.0158	0.7693	0.1139
Dnaja2	_EHEVFQR_.2	#N/A	6.5295	5.6337	5.1570	6.4973	6.2722	6.0042	5.9566	5.6081	5.6701	6.5241	1.0128	0.0183	0.8185	0.0870
Dnaja2	_VSLEDLYNGK_.2	#N/A	6.1931	5.6356	6.4242	6.2849	5.9854	6.7022	6.0274	5.8764	6.2402	6.5625	1.0045	0.0064	0.9038	0.0439
Dnaja2	_YGEQGLR_.2	#N/A	6.2038	6.2822	3.5108	5.8211	6.2731	6.0942	5.5784	6.2725	5.9731	5.8695	0.9619	-0.0561	0.6998	0.1550
Dnaja2	_NVLC[Carbamidomethyl (C)]SAC[Carbamidomethyl (C)]SGQGGK_.2	#N/A	4.4773	5.0597	3.7393	5.2221	5.0680	4.5937	4.5732	3.8125	4.1179	5.3919	1.0491	0.0691	0.5901	0.2291

Dnaja2	_DGNDLHM[Oxidation (M)]TYK_.2	#N/A	6.7303	5.9391	5.4099	3.6548	5.6323	6.1645	5.8938	6.5200	4.9297	3.3321	1.0812	0.1126	0.5995	0.2222
Dnaja2	_ITFTGEADQAPGVEPGDIVLLLQEK_.3	#N/A	3.5461	3.6800	4.2329	3.2347	3.5683	3.0563	4.4503	3.3829	3.4917	3.2017	0.9784	-0.0316	0.8020	0.0958
Dnaja2	_IGLVEALC[Carbamidomethyl (C)]GFQFTFK_.2	#N/A	4.2333	3.4887	3.9669	3.4261	3.3770	3.2476	3.1408	3.4905	4.3410	3.1880	1.0235	0.0335	0.7854	0.1049
Dnajb1	_GAS[Phospho (STY)]DDEIKR_.2	Q9QYJ3_S16_M1_Dnajb1	7.6793	7.8484	7.8844	8.4755	7.9665	7.9253	7.4574	7.7657	7.9317	7.7764	1.0298	0.0424	0.1840	0.7351
Pnck	_HSHPGLGTSQS[Phospho (STY)]PKW_.3	#N/A	2.9615	4.3809	4.1961	3.7398	3.6061	3.0185	3.8881	3.2613	5.1312	3.4171	0.9302	-0.1044	0.5586	0.2529
Pnck	_KLGQS[Phospho (STY)]PEGEEASR_.2	Q9QYK9_S316_M1_Pnck	3.1414	4.2010	6.1842	6.2118	5.6481	6.4133	4.0679	6.1499	6.7800	3.5970	1.0294	0.0418	0.8728	0.0591
Pnck	_HSHPGLGTSQS[Phospho (STY)]PK_.3	#N/A	3.4603	3.8821	3.1939	4.2386	4.1048	4.0260	4.3868	4.2635	3.0617	3.9158	0.9771	-0.0334	0.7834	0.1060
Map1a	_VPSAPGQES[Phospho (STY)]PVPDTKST[Phospho (STY)]PPTR_.3	#N/A	4.1347	3.6465	10.0270	11.2490	9.1943	7.3207	9.6283	7.8112	10.7558	3.7903	0.9498	-0.0742	0.8467	0.0723
Map1a	_GEPVPAWEGKS[Phospho (STY)]PEQEVR_.2	#N/A	10.9583	10.1996	10.6156	11.0398	9.8348	10.7967	10.4796	10.4647	10.7941	10.9836	0.9900	-0.0144	0.6928	0.1594
Map1a	_EAELT[Phospho (STY)]PENIAAAR_.2	Q9QYR6_T633_M1_Map1a	9.5609	9.6309	9.7033	10.0393	9.3642	9.6356	10.0221	9.5510	9.6663	9.7434	0.9908	-0.0134	0.5324	0.2738
Map1a	_EQDVVQGWR_.2	#N/A	8.3682	8.5298	8.7520	8.6651	8.6901	8.0848	8.5524	8.3234	8.3761	8.5509	1.0076	0.0109	0.6505	0.1868
Map1a	_VLFPGNAPQNK_.2	#N/A	8.5505	8.6987	8.8587	8.6911	8.7621	8.2617	8.5219	8.7507	8.5282	8.0913	1.0194	0.0277	0.3140	0.5030
Map1a	_ASYYVVSGNDPANGEPSR_.2	#N/A	8.0857	7.7159	8.6410	8.4399	8.3150	7.0387	8.2282	8.0851	8.2274	7.4951	1.0038	0.0055	0.9285	0.0322
Map1a	_GVEQQDGAVPEK_.2	#N/A	7.9350	7.9752	8.3223	7.9527	8.2650	8.1274	7.9701	7.6741	7.6129	7.9836	1.0366	0.0519	0.0379	1.4211
Map1a	_DLAAGAVPANLKPSK_.2	#N/A	7.8703	8.1869	7.7135	8.1047	8.3693	8.3308	8.0245	7.8361	8.0325	8.1008	1.0122	0.0175	0.5049	0.2968
Map1a	_SSRPDTLLSSEQRPGK_.3	#N/A	7.6407	7.7024	7.7788	8.1406	8.6369	8.0115	7.6431	7.6669	7.9405	7.6677	1.0331	0.0469	0.2322	0.6342
Map1a	_GAALQQTQAPEPR_.2	#N/A	7.1471	7.4844	7.5269	7.8828	7.9368	7.4775	7.5175	7.4514	7.6180	7.7967	0.9974	-0.0038	0.9043	0.0437
Map1a	_GAAPPPAASGHR_.2	#N/A	6.7009	7.7442	7.4728	7.6471	7.9655	7.4574	7.1549	7.4435	7.1349	7.4652	1.0272	0.0387	0.4178	0.3790
Map1a	_EVVPDFPEDK_.2	#N/A	7.0523	7.2765	6.9749	7.4365	7.5550	7.4975	7.5685	7.2885	7.0596	7.3149	0.9988	-0.0018	0.9524	0.0212
Map1a	_DTAVELGKGPEPK_.2	#N/A	7.2750	7.0784	7.4756	7.6750	7.3745	7.3095	7.3309	6.9992	7.2676	7.1347	1.0253	0.0360	0.1621	0.7901
Map1a	_NRAPDS[Phospho (STY)]GAEVER_.2	#N/A	6.5263	6.7833	7.0156	7.2622	6.6358	7.0441	7.3599	7.0564	6.6087	7.0149	0.9812	-0.0274	0.4999	0.3012
Map1a	_DLEQKDEHLELR_.3	#N/A	7.0867	6.6899	7.4128	7.0661	7.4348	7.3158	6.2043	6.7837	6.7577	7.3764	1.0571	0.0801	0.1422	0.8470
Map1a	_LGIQAEPLYR_.2	#N/A	7.0251	7.2586	7.1758	7.1392	6.2747	6.6901	6.8346	7.2730	6.8013	7.2478	0.9841	-0.0231	0.6202	0.2075
Map1a	_LDM[Oxidation (M)]YVLNPVK_.2	#N/A	6.7966	6.7353	6.2223	6.0505	6.8035	6.8047	6.9933	6.9446	6.8436	6.9990	0.9458	-0.0804	0.0649	1.1876
Map1a	_EGEGGAGAPDSSS[Phospho (STY)]FSSK_.2	Q9QYR6_S1900_M1_Map1a	5.2815	4.9993	3.7726	5.9684	3.7976	4.7725	5.6563	6.0652	3.5660	6.1420	0.8895	-0.1690	0.3888	0.4103
Map1a	_DFQEPLPQK_.2	#N/A	6.4526	6.0365	6.0643	6.6415	6.3366	6.6809	6.0487	6.1758	6.6574	6.8041	0.9918	-0.0119	0.8008	0.0965
Map1a	_NEPTTPSWLAEIPPWVPK_.2	#N/A	5.9822	6.7480	6.2645	6.5586	6.5231	6.0317	6.3633	6.2215	5.9865	6.6351	1.0079	0.0113	0.8006	0.0966
Map1a	_DAEQTEPEQREPTPYPDER_.3	#N/A	5.8885	6.5490	6.6382	6.6209	6.3969	6.5997	6.0133	6.3997	6.0554	6.5347	1.0317	0.0450	0.2996	0.5235
Map1a	_VRTES[Phospho (STY)]SEALK_.2	#N/A	7.0787	6.8116	6.9431	3.7360	3.6023	3.0223	7.0990	7.0375	3.5642	3.4133	0.9849	-0.0219	0.9520	0.0214
Map1a	_AQWGENLQVTLIPTHDTEVTR_.3	#N/A	5.2903	4.7133	6.0293	5.5127	5.7791	5.3958	5.6197	5.4359	4.7581	6.3433	0.9845	-0.0225	0.8102	0.0914
Map1a	_S[Phospho (STY)]PTPGKGPVDR_.2	#N/A	13.1724	13.4185	13.7695	13.7282	13.3738	13.5366	13.5390	13.4081	13.3594	13.4324	1.0048	0.0070	0.6000	0.2218
Map1a	_ELSSEPRT[Phospho (STY)]PPAQK_.2	#N/A	10.8680	11.2012	11.6091	11.5703	10.5963	11.1480	11.4240	11.1414	11.1062	11.1136	0.9972	-0.0040	0.8867	0.0522
Map1a	_ALGLEESPEEEGK_.2	#N/A	10.7285	11.0774	11.0041	11.5976	10.6703	10.9897	11.5764	10.9824	11.0921	11.2263	0.9815	-0.0270	0.3216	0.4926
Map1a	_ADS[Phospho (STY)]KESLK_.2	#N/A	10.3444	10.3454	10.8351	10.6623	10.0471	10.5094	10.6490	10.3876	10.4502	10.5903	0.9941	-0.0085	0.6880	0.1624
Map1a	_ALGLEES[Phospho (STY)]PEEEGKAR_.2	#N/A	9.1796	9.9480	10.4800	10.1567	9.5260	9.8542	10.1082	9.7829	9.7773	9.8470	0.9978	-0.0031	0.9317	0.0307
Map1a	_ALALVPGTPTR_.2	#N/A	7.2496	9.7996	7.5583	10.3134	8.5691	9.3518	9.7962	9.6384	9.4431	9.3384	0.9218	-0.1175	0.2725	0.5646
Map1a	_GEKELSSEPRT[Phospho (STY)]PPAQK_.3	#N/A	7.0460	7.2715	8.2275	3.9219	7.0689	8.2246	8.2837	7.1505	7.6181	8.0941	0.8939	-0.1619	0.3502	0.4557
Map1a	_SHWDDGTNDS[Phospho (STY)]DLEKGAR_.3	#N/A	6.2570	7.5881	8.9436	7.8109	7.2816	8.0179	7.2113	7.4282	6.9007	7.2689	1.0621	0.0870	0.3589	0.4450
Map1a	_APEHIIPQPTQTDR_.2	#N/A	6.9168	7.1073	6.1371	7.1403	7.4866	6.9299	7.0395	6.9818	7.0146	6.8107	0.9988	-0.0018	0.9714	0.0126
Map1a	_AKPAS[Phospho (STY)]PARR_.2	#N/A	7.6043	3.2918	8.0061	7.0657	7.2102	6.9909	8.0858	7.8270	6.9475	7.7063	0.8761	-0.1908	0.3203	0.4944
Map1a	_QLS[Phospho (STY)]PESLGTLQFGELSLGK_.2	#N/A	5.5124	8.5168	8.1947	9.6151	6.7536	6.5298	7.5372	8.0968	7.3875	5.5857	1.0515	0.0725	0.6875	0.1627
Map1a	_ESTFLDEGPNEQEITPLQHTPR_.3	#N/A	6.4432	6.7082	6.4222	6.7226	5.6234	5.6108	6.7230	6.2279	6.0156	6.1670	0.9955	-0.0065	0.9239	0.0344
Map1a	_VLVLEDR_.2	#N/A	6.7969	6.5996	6.9309	6.8756	6.5774	6.6322	6.3199	6.9638	6.7238	6.9580	0.9991	-0.0013	0.9678	0.0142
Map1a	_EKETWEER_.2	#N/A	6.0520	6.1435	6.0448	6.1722	6.3405	6.2530	6.4122	6.0159	5.6996	5.8419	1.0293	0.0416	0.2312	0.6359
Map1a	_VVSNTIEPLTLFHK_.2	#N/A	4.7185	4.4093	4.7066	4.7489	4.9654	4.8803	4.0028	4.8391	5.0359	5.1493	0.9961	-0.0057	0.9367	0.0284
Map1a	_APSLDSSLPQLPSPSSPGAPLLSNLPR_.3	#N/A	5.2696	5.3715	3.4458	5.4430	5.6623	5.3560	5.8693	4.8633	5.0821	5.7113	0.9461	-0.0800	0.5448	0.2637
Map1a	_SPQAQDTLGSLAGGQTGC[Carbamidomethyl (C)]TIQLLPEQDK_.3	#N/A	5.8583	9.8070	9.3599	11.0272	7.9356	8.2064	8.9991	9.6191	9.1261	7.8560	0.9774	-0.0330	0.8392	0.0761
Map1a	_IDSVLLTHIGADNLPGINGLLQR_.3	#N/A	5.6104	5.3277	5.6140	5.5907	5.1390	4.9867	4.4933	5.1685	5.9228	5.0755	1.0413	0.0583	0.4530	0.3439
Map1a	_C[Carbamidomethyl (C)]LS[Phospho (STY)]PDDSTVK_.2	Q9QYR6_S981_M1_Map1a	11.6998	12.0054	12.7362	12.3627	11.7351	12.0156	12.2283	11.9629	11.9471	11.9342	1.0062	0.0089	0.7318	0.1356
Map1a	_SEPQDFQEDS[Phospho (STY)]WGDTK_.2	Q9QYR6_S1023_M1_Map1a	10.1452	11.8626	11.5670	12.5949	11.6242	11.9464	12.3336	11.9430	11.9299	12.1181	0.9621	-0.0557	0.3084	0.5108
Map1a	_AKPAS[Phospho (STY)]PAR_.2	#N/A	10.8123	3.2539	10.8576	10.3294	3.7170	10.2988	11.0253	10.5746	3.1801	10.6680	0.9266	-0.1100	0.7932	0.1006
Map1a	_YPVATQK_.2	#N/A	5.6270	5.5407	4.7364	5.7418	6.1060	5.5125	3.5315	5.6683	5.5824	5.6759	1.0840	0.1163	0.3938	0.4047
Map1a	_TQEPAEPQKDELLGFTDQSFS[Phospho (STY)]PEDAESLSVLSVVSPDTAK_.4	Q9QYR6_S1133_M1_Map1a	3.8679	4.0514	3.9759	4.8955	3.4594	3.1232	4.4942	3.4961	3.7595	4.2870	0.9717	-0.0415	0.7589	0.1198
Map1a	_NLISPELGVVFFNVPDK_.2	#N/A	4.9061	4.9575	4.9862	5.2314	4.6268	3.6502	5.3645	4.5825	4.1921	4.4366	1.0178	0.0254	0.8195	0.0865
Map1a	_ETS[Phospho (STY)]PTRGEPVPAWEGK_.3	#N/A	12.9766	12.7130	13.9813	13.4554	12.4184	12.8443	12.8101	13.1192	12.8704	12.9583	1.0097	0.0139	0.6798	0.1676
Map1a	_ELALSS[Phospho (STY)]PEDLTQDFEELKR_.3	#N/A	4.8793	7.2991	7.7416	6.2467	7.1743	4.1245	6.2612	7.7813	7.6788	6.0832	0.8983	-0.1547	0.4157	0.3812
Map1a	_EM[Oxidation (M)]TLDQKS[Phospho (STY)]PEKAK_.2	#N/A	9.5234	9.9866	10.6155	10.3986	9.6706	9.7859	9.9803	9.9992	9.9211	9.8719	1.0054	0.0078	0.8134	0.0897
Map1a	_ETS[Phospho (STY)]PTRGEPVPAWEGKSPEQEVR_.4	#N/A	6.4097	4.0716	9.5960	8.5236	3.9411	4.0653	4.1313	8.4871	7.7351	7.6218	0.8724	-0.1970	0.5650	0.2479
Map1a	_HRADS[Phospho (STY)]KESLK_.2	#N/A	7.7069	5.0933	8.2699	7.9244	6.0096	7.3318	7.9931	7.7756	7.1040	7.9777	0.9149	-0.1284	0.3444	0.4630
Map1a	_TES[Phospho (STY)]SEALKAEK_.2	#N/A	6.3017	6.2636	6.5291	6.8891	6.1655	6.2910	6.6478	3.9656	6.3635	6.5212	1.0906	0.1251	0.3386	0.4703
Map1a	_ADS[Phospho (STY)]KES[Phospho (STY)]LKAAPK_.2	#N/A	10.6744	10.9411	11.5037	11.3783	10.5911	11.0279	11.1909	10.9513	10.8152	10.9436	1.0040	0.0058	0.8283	0.0818
Map1a	_S[Phospho (STY)]PPC[Carbamidomethyl (C)]EDFSVTGESEK_.2	#N/A	6.0099	5.7886	3.7505	7.7045	6.0297	6.3986	4.4000	6.4726	6.1127	6.5423	1.0111	0.0159	0.9341	0.0296
Map1a	_STPSQVTSEEKDGHS[Phospho (STY)]PM[Oxidation (M)]SK_.3	#N/A	10.4417	7.4210	11.1447	11.1772	10.3584	10.6915	10.7635	10.5192	6.9469	3.4828	1.2873	0.3643	0.1767	0.7527
Map1a	_GFKS[Phospho (STY)]PPC[Carbamidomethyl (C)]EDFSVTGESEK_.3	#N/A	7.0274	6.4733	6.4461	8.5525	6.4442	6.6561	6.7195	7.3432	7.3603	7.4709	0.9598	-0.0592	0.5295	0.2761
Map1a	_LSSFATSVAEDQSVASLTAPQTEETGK_.3	#N/A	5.4635	5.2766	5.7976	6.6701	5.4675	4.9577	5.2274	5.0558	5.7076	5.7778	1.0300	0.0427	0.6359	0.1966
Map1a	_TPS[Phospho (STY)]PKEAGR_.2	Q9QYR6_S2066_M1_Map1a	5.3206	7.3509	7.2347	5.3962	4.4451	7.4273	6.0483	7.0452	7.0502	6.3821	0.9343	-0.0980	0.5456	0.2632
Map1a	_NRAPDS[Phospho (STY)]GAEVEREK_.3	#N/A	5.2688	5.1085	4.5153	5.4443	5.5767	5.3551	5.0478	5.9752	5.8228	5.3353	0.9398	-0.0896	0.2288	0.6405
Map1a	_SSLLLDTVTSIPSSR_.2	#N/A	6.3809	6.4931	6.7517	6.7123	7.5376	6.4145	6.5946	8.0961	6.4420	6.4030	0.9755	-0.0358	0.6758	0.1702
Map1a	_VPSAPGQES[Phospho (STY)]PVPDTK_.2	#N/A	13.3662	13.4840	14.1270	14.0449	12.8859	13.4952	13.8876	13.3990	13.4483	13.5208	1.0002	0.0003	0.9900	0.0044
Map1a	_SHWDDGTNDS[Phospho (STY)]DLEK_.2	#N/A	9.0416	9.0417	9.1893	9.6493	8.2567	9.1458	9.5045	8.8957	9.0625	9.2103	0.9875	-0.0181	0.6622	0.1790
Map1a	_VRTES[Phospho (STY)]SEALKAEK_.3	#N/A	4.9333	8.4153	8.8186	8.8988	8.1958	8.3125	8.6405	8.4452	5.6804	8.5169	1.0138	0.0198	0.9120	0.0400
Map1a	_APDS[Phospho (STY)]GAEVEREK_.2	#N/A	4.9689	5.9186	6.2059	6.3197	4.0135	5.6029	6.3165	5.5796	5.6157	6.0251	0.9355	-0.0961	0.4418	0.3547
Map1a	_AELEEM[Oxidation (M)]EEVHPS[Phospho (STY)]DEEEEETK_.3	#N/A	3.6154	3.7761	3.8589	6.0196	3.6174	3.6839	3.8182	3.7084	2.9371	3.7118	1.1556	0.2086	0.3109	0.5074
Map1a	_EM[Oxidation (M)]TLDQKS[Phospho (STY)]PEK_.2	#N/A	11.6592	11.5739	11.8222	12.2966	11.3528	11.5741	11.9711	11.7079	11.6212	11.7160	0.9965	-0.0050	0.8217	0.0853
Map1a	_S[Phospho (STY)]PC[Carbamidomethyl (C)]T[Phospho (STY)]PKEQQLHK_.2	#N/A	8.1109	8.5042	8.4250	8.8528	7.3875	8.3269	8.2509	8.1808	8.3028	8.3088	1.0009	0.0012	0.9786	0.0094
Map1a	_WLAES[Phospho (STY)]PVGLPPEEEDK_.2	#N/A	8.8615	3.4122	3.2938	9.1381	3.2335	2.9951	8.8751	8.7430	8.7874	9.0757	0.5812	-0.7828	0.0406	1.3913
Map1a	_TRHDEYLEVTK_.3	#N/A	5.5525	4.6698	4.2187	5.1372	5.1933	5.0791	3.9319	4.2972	4.6950	3.1875	1.2352	0.3047	0.0260	1.5856
Map1a	_SPFEIIS[Phospho (STY)]PPAS[Phospho (STY)]PPEMTGQR_.2	#N/A	12.7814	12.8743	11.0459	14.2081	11.3388	12.3570	12.9602	13.3801	11.8547	11.6282	0.9983	-0.0025	0.9753	0.0109
Map1a	_QLS[Phospho (STY)]PESLGTLQFGELSLGKEEK_.3	#N/A	6.1070	7.4496	8.1763	7.9696	6.8931	6.7542	6.4573	7.2833	7.0781	6.6056	1.0538	0.0756	0.4156	0.3813

Map1a	_S[Phospho (STY)]PESLSSPAM[Oxidation (M)]EDLAM[Oxidation (M)]EWGGK_.2	#N/A	5.2211	5.9092	5.9255	5.9198	3.7332	6.3872	6.1375	7.0574	5.9311	6.4166	0.8638	-0.2112	0.1342	0.8722
Map1a	_EKEGAALDQDNR_.2	#N/A	6.0256	6.4436	6.1673	6.5816	5.8059	5.9720	6.5392	6.1506	6.3104	6.4904	0.9676	-0.0476	0.2494	0.6030
Map1a	_GKDLYLEDQGLAEKDK_.3	#N/A	5.5870	5.5648	6.8063	6.3352	5.8956	6.0979	5.7686	5.8131	5.8578	5.5363	1.0529	0.0744	0.2571	0.5899
Map1a	_KVAELEEEQS[Phospho (STY)]QGSSSYSDWVK_.3	#N/A	6.7604	7.2391	8.5624	7.9319	6.8735	7.4027	6.9841	7.2156	7.1449	7.2577	1.0435	0.0614	0.4014	0.3964
Map1a	_VAELEEEQS[Phospho (STY)]QGSSSYSDWVK_.2	#N/A	8.9339	8.8790	9.6754	9.6309	8.7237	9.1475	9.4754	8.6799	8.9734	9.0407	1.0136	0.0195	0.6268	0.2029
Map1a	_M[Oxidation (M)]AS[Phospho (STY)]PPPSGPPSAAHTPFHQSPVEEK_.4	#N/A	8.7955	9.5213	10.4649	9.9699	9.0745	9.3200	9.1898	10.1562	9.9627	9.6497	0.9779	-0.0322	0.5589	0.2527
Map1a	_PASPALSEGS[Phospho (STY)]S[Phospho (STY)]SEATTPVISSVAER_.3	#N/A	3.6955	3.4364	3.7551	4.2100	7.1663	3.9577	3.8369	4.7209	6.6309	5.2378	0.8558	-0.2247	0.4106	0.3865
Map1a	_QEPEPGPNVEPSFT[Phospho (STY)]PPAVPPR_.2	Q9QYR6_T2031_M1_Map1a	8.6635	4.2865	3.8223	10.2903	6.7072	3.8198	4.4185	9.0936	7.9254	7.0044	0.8811	-0.1826	0.6140	0.2118
Map1a	_VPEVTESHTTR_.2	#N/A	7.1160	7.3023	7.4393	7.4325	7.7201	7.4752	7.2959	7.3961	7.2168	7.4930	1.0087	0.0125	0.5863	0.2319
Map1a	_APDS[Phospho (STY)]GAEVER_.2	#N/A	7.1145	6.3968	6.7297	7.1871	6.5379	6.8916	6.8449	6.6005	7.1345	6.9325	0.9900	-0.0144	0.7171	0.1444
Map1a	_DEALDEENKPGGQQDK_.2	#N/A	4.0239	4.1386	4.3642	5.4021	3.6820	3.6714	4.2587	4.5951	4.3836	4.7688	0.9361	-0.0953	0.4205	0.3762
Map1a	_GKDLYLEDQGLAEK_.3	#N/A	4.3229	4.5418	3.3197	4.9803	4.8562	4.9740	4.5125	5.0877	3.9117	4.2442	1.0135	0.0194	0.8778	0.0566
Map1a	_S[Phospho (STY)]APC[Carbamidomethyl (C)]GSLAFSGDR_.2	Q9QYR6_S2162_M1_Map1a	9.6527	9.7548	9.7148	10.3740	9.1252	9.6454	9.9240	9.7308	9.9618	9.7846	0.9859	-0.0205	0.5219	0.2824
Map1a	_EES[Phospho (STY)]EPEVKEDVIEK_.2	#N/A	6.9915	6.7818	6.6491	8.0042	6.5524	7.2270	7.6093	7.2685	7.3009	7.2461	0.9562	-0.0645	0.2844	0.5461
Map1a	_QEATPRS[Phospho (STY)]PC[Carbamidomethyl (C)]T[Phospho (STY)]PKEQQLHK_.3	#N/A	3.9365	6.8236	3.1225	7.3427	6.1309	7.1489	6.7848	6.8372	4.1419	6.1195	0.9632	-0.0542	0.8377	0.0769
Map1a	_WLAESPVGLPPEEEDKLTR_.3	#N/A	4.9709	6.1514	3.9022	10.7186	5.9168	6.3501	5.5249	10.1256	5.7962	5.5516	0.9386	-0.0915	0.7874	0.1038
Map1a	_EKEVVPDFPEDK_.2	#N/A	6.7704	6.5518	6.8870	6.8450	6.6533	6.9300	6.7624	6.5469	6.5597	6.5975	1.0236	0.0337	0.1002	0.9991
Map1a	_HMQEALK_.2	#N/A	4.9338	3.0974	3.5757	3.9203	2.9580	3.6389	3.8479	3.8818	4.1808	4.2975	0.9100	-0.1361	0.3551	0.4496
Map1a	_TEATQGLDYVPSAGTIS[Phospho (STY)]PTSSLEEDK_.3	#N/A	6.6419	5.9601	6.1243	7.4313	6.5462	6.5225	6.7488	6.5581	6.6241	6.3776	0.9940	-0.0087	0.8870	0.0521
Map1a	_APISLSQDPSPPLNGSTTSC[Carbamidomethyl (C)]GPDRRT[Phospho (STY)]PS[Phospho (STY)]PK_.4	#N/A	3.0444	4.2980	4.2789	3.8227	6.2670	3.6101	3.9709	10.4242	6.8993	9.5205	0.5478	-0.8683	0.0261	1.5837
Map1a	_RS[Phospho (STY)]PT[Phospho (STY)]PGKGPVDR_.2	#N/A	5.9364	5.1886	5.4831	6.1316	6.2744	5.5122	5.9967	5.0873	5.1390	5.3499	1.0670	0.0935	0.2212	0.6553
Map1a	_EAELTPENIAAAREES[Phospho (STY)]EPEVKEDVIEK_.4	#N/A	3.8756	3.6659	4.2751	3.8189	3.4525	3.8788	3.9671	8.1072	3.5113	3.4961	0.8024	-0.3176	0.3227	0.4912
Map1a	_KHS[Phospho (STY)]PGEITGPGGHFM[Oxidation (M)]TSDSSLTK_.4	#N/A	3.5196	5.7069	4.2812	7.2412	5.1406	5.0930	3.9684	6.5599	5.9629	5.6673	0.9321	-0.1014	0.6444	0.1908
Map1a	_S[Phospho (STY)]IEEAC[Carbamidomethyl (C)]LTLQHLNR_.2	Q9QYR6_S155_M1_Map1a	4.0806	3.2182	3.3864	5.4888	4.1305	3.3655	3.5017	4.4104	4.7792	4.3665	0.9251	-0.1123	0.5274	0.2778
Map1a	_EAELTPENIAAAREES[Phospho (STY)]EPEVK_.3	#N/A	3.8138	3.0693	3.5475	4.5921	2.9576	3.6670	3.5602	3.9099	5.8139	4.2694	0.8222	-0.2825	0.1531	0.8152
Map1a	_GDSALFAVNGFNILVDGGSDR_.2	#N/A	3.2318	4.0369	3.9186	5.1591	3.8583	3.8712	4.1359	4.3614	2.2855	3.3362	1.1368	0.1850	0.3538	0.4512
Map1a	_QEATPRS[Phospho (STY)]PC[Carbamidomethyl (C)]T[Phospho (STY)]PK_.2	#N/A	7.2505	7.9147	7.4058	7.6785	6.2834	7.3356	7.3939	7.3160	7.4835	7.4813	0.9855	-0.0210	0.7188	0.1434
Map1a	_DQILSEK_.2	#N/A	3.1435	3.0831	4.0666	3.6104	7.4169	3.3978	3.7586	3.3908	3.0619	3.2877	1.2208	0.2878	0.4074	0.3900
Map1a	_PRYS[Phospho (STY)]EER_.2	Q9QYR6_S1694_M1_Map1a	3.8275	3.0829	3.3292	3.8319	2.9712	3.6534	3.5466	4.2866	3.9352	3.7989	0.8863	-0.1741	0.0877	1.0570
Map1a	_ELALS[Phospho (STY)]S[Phospho (STY)]PEDLTQDFEELK_.2	#N/A	3.2583	4.0635	3.9451	4.0572	3.8848	3.8446	4.2054	3.6707	3.3193	4.4148	0.9846	-0.0225	0.8153	0.0887
Map1a	_SPEQEVR_.2	#N/A	3.1686	4.1016	3.8554	4.3580	4.4414	3.9394	4.0727	4.0895	3.6680	4.1803	0.9937	-0.0091	0.9222	0.0352
Map1a	_APDS[Phospho (STY)]GAEVEREKETWEER_.3	#N/A	4.0866	3.2558	3.8203	4.5613	3.0771	3.1515	3.5123	3.6371	3.9362	3.0413	1.0360	0.0510	0.7170	0.1445
Map1a	_AELEEM[Oxidation (M)]EEVHPS[Phospho (STY)]DEEEEETKAESFYQK_.4	#N/A	3.5018	5.3788	6.4691	3.9188	6.1110	3.7210	4.0818	3.5470	3.9239	3.8858	1.2566	0.3296	0.1772	0.7515
Map1a	_DSVMHQKDEALDEENKPGGQQDK_.4	#N/A	4.3281	3.4223	3.3695	4.9386	3.7950	3.9630	3.3000	4.9508	3.9086	3.8392	0.9924	-0.0110	0.9426	0.0257
Map1a	_TEATQGLDYVPSAGTIS[Phospho (STY)]PTSSLEEDKGFK_.4	#N/A	3.4697	5.1979	5.8626	4.4420	3.4481	3.8340	3.7271	5.5250	3.9405	4.2735	1.0021	0.0030	0.9881	0.0052
Map1a	_DEAS[Phospho (STY)]EEKEQVLEQK_.2	#N/A	2.9837	4.3587	4.2182	4.9824	4.1801	3.5494	3.9102	3.2392	6.3541	2.5056	1.0108	0.0155	0.9555	0.0198
Map1a	_FPTSTYDLSGPEGPGPFEASQSAESAVPASSSK_.3	#N/A	4.2164	2.6400	3.9501	3.4429	3.3602	3.2644	3.1576	1.5439	4.3242	3.4099	1.1191	0.1623	0.5107	0.2919
Map1a	_SSFLEDK_.2	#N/A	2.9704	3.7756	3.3004	4.3450	3.5970	4.1325	4.4933	4.1570	2.9552	4.0223	0.9437	-0.0837	0.5689	0.2450
Map1a	_DRDITLQQDAYWK_.3	#N/A	3.2815	4.0867	3.9683	4.4710	3.9080	3.8214	4.1822	3.6475	3.2961	4.4380	1.0082	0.0118	0.9134	0.0394
Map1a	_EQKDEAS[Phospho (STY)]EEKEQVLEQK_.3	#N/A	3.7749	3.0793	4.4617	3.5405	2.9006	3.3280	3.6888	3.4606	3.7597	3.2178	0.9950	-0.0072	0.9551	0.0200
Map1a	_DKT[Phospho (STY)]PEEKDK_.2	Q9QYR6_T1472_M1_Map1a	4.1899	3.4943	3.3759	4.6264	3.3156	4.4139	3.2738	3.8756	4.1747	3.8455	1.0291	0.0413	0.7463	0.1271
Pclo	_EHKENPELVDDLS[Phospho (STY)]PR_.3	#N/A	8.7552	9.5485	9.7557	9.6130	8.7679	9.5559	9.6889	9.3037	9.3787	9.3913	0.9886	-0.0166	0.6630	0.1785
Pclo	_ENPELVDDLS[Phospho (STY)]PR_.2	#N/A	9.1957	10.1332	9.9310	10.8495	9.3971	10.3267	10.6085	10.1819	10.3256	10.4826	0.9589	-0.0606	0.2177	0.6621
Pclo	_TQPQKVS[Phospho (STY)]PEQPQDQQK_.2	#N/A	8.1944	8.5742	9.4012	9.1098	8.8124	8.8116	9.3001	8.6301	9.0614	9.0887	0.9775	-0.0328	0.4236	0.3730
Pclo	_SPVPSQQAS[Phospho (STY)]PK_.2	#N/A	9.6110	9.3066	9.2909	9.9015	8.9905	9.4465	9.7250	9.1753	9.2627	9.7012	0.9956	-0.0063	0.8367	0.0774
Pclo	_SHSSQNSQQS[Phospho (STY)]PKPSVIK_.3	#N/A	7.5665	7.0188	7.9721	7.7979	6.7590	5.4980	7.4224	7.3178	7.3408	7.4670	0.9614	-0.0568	0.5578	0.2535
Pclo	_AKS[Phospho (STY)]PGVDPK_.2	Q9QYX7_S4544_M1_Pclo	7.9512	8.3162	8.5991	8.4043	6.4821	8.1093	8.0761	7.9256	7.7159	8.0144	1.0055	0.0080	0.9143	0.0389
Pclo	_SLVSVLPEKKPS[Phospho (STY)]EEEK_.3	Q9QYX7_S1149_M1_Pclo	7.5336	7.7358	8.0650	8.5603	7.0164	7.4733	8.0614	7.9336	7.7099	7.6672	0.9857	-0.0208	0.7003	0.1547
Pclo	_SPVPSQQAS[Phospho (STY)]PKKELPSK_.3	#N/A	7.7704	7.5989	8.2534	8.7889	7.3752	4.2886	4.1818	7.8287	7.3977	7.9808	1.0728	0.1014	0.6549	0.1838
Pclo	_S[Phospho (STY)]QEVTDFLAPLQTSSR_.2	Q9QYX7_S4016_M1_Pclo	6.6697	7.7817	3.8781	8.9711	6.7931	6.2177	7.4361	8.1941	3.8783	6.8760	1.0186	0.0265	0.9176	0.0373
Pclo	_KSS[Phospho (STY)]TS[Phospho (STY)]FDDDAGR_.2	#N/A	7.2738	7.0907	7.1830	7.5656	5.8481	6.9480	6.9650	7.0546	7.1842	7.3398	0.9788	-0.0309	0.6391	0.1944
Pclo	_SEGITKPSLQQPS[Phospho (STY)]PK_.2	#N/A	9.0681	9.0094	9.7032	9.5781	8.4034	9.2057	8.9538	8.8368	8.8777	9.0771	1.0252	0.0359	0.3766	0.4241
Pclo	_SQPTT[Phospho (STY)]PQETVTGK_.2	Q9QYX7_T860_M1_Pclo	9.3178	3.2033	3.2101	8.9708	3.1633	10.0072	8.9515	8.4723	8.7357	10.1771	0.6948	-0.5252	0.1575	0.8027
Pclo	_ITQHFSTEEFEDEYVTDYTR_.3	#N/A	3.1663	7.4286	8.0696	8.5956	6.7507	7.4573	7.6342	7.2256	6.8398	7.4848	0.9473	-0.0782	0.7102	0.1486
Pclo	_ELPSKQDS[Phospho (STY)]PKAPESK_.2	#N/A	3.8187	3.7718	3.6972	6.6664	3.6321	4.3194	3.9146	6.0218	3.6142	4.1669	0.9748	-0.0369	0.8833	0.0539
Pclo	_YNS[Phospho (STY)]LDLR_.2	Q9QYX7_S3936_M1_Pclo	2.9142	2.9274	4.1487	3.6925	6.5016	3.0658	3.8407	3.3087	3.6077	6.8362	0.8810	-0.1828	0.5991	0.2225
Pclo	_SSTS[Phospho (STY)]FDDDAGRR_.2	#N/A	3.1336	3.9388	3.1373	4.1819	3.7601	3.9694	4.3302	4.3201	3.1183	3.8592	0.9437	-0.0837	0.5120	0.2907
Pclo	_KTQPQKVS[Phospho (STY)]PEQPQDQQK_.3	#N/A	6.9899	7.1568	7.7881	7.7993	7.1344	7.5179	7.7879	7.4277	7.4777	7.2294	0.9889	-0.0161	0.6919	0.1599
Pclo	_DSFSQESSPSS[Phospho (STY)]PSDLAK_.2	#N/A	9.8982	10.1680	10.6031	10.4108	9.6599	10.1969	10.5454	9.8429	10.0592	10.1484	1.0007	0.0010	0.9734	0.0117
Pclo	_KIVDSGVQT[Phospho (STY)]DDEETADR_.2	#N/A	5.2119	3.7032	5.1087	6.4405	3.6429	3.8292	5.8156	3.6885	5.5625	3.8280	0.9857	-0.0208	0.9280	0.0325
Pclo	_FS[Phospho (STY)]PIQESR_.2	#N/A	6.5083	5.7331	6.4378	6.6483	6.2320	5.9802	6.6664	6.0671	6.1322	6.4606	0.9882	-0.0172	0.7292	0.1371
Pclo	_VYKLPTAVSLYS[Phospho (STY)]PTDEQSVM[Oxidation (M)]QK_.3	#N/A	4.1479	3.4522	7.2492	3.8365	5.3502	2.9551	3.3159	7.1988	6.2834	4.6278	0.8398	-0.2518	0.4387	0.3579
Pclo	_SSTS[Phospho (STY)]FDDDAGR_.2	#N/A	3.7433	3.0476	9.0541	3.4319	2.8689	3.3597	3.1466	3.1858	4.3352	3.3989	1.2088	0.2736	0.5672	0.2462
Pclo	_ASYDSVEDSSESENS[Phospho (STY)]PVAR_.2	#N/A	6.1229	6.7627	6.0613	7.3098	7.1031	6.2072	7.3249	5.8324	6.0386	5.9829	1.0476	0.0671	0.4621	0.3352
Pclo	_TQT[Phospho (STY)]PSETR_.2	Q9QYX7_T1390_M1_Pclo	2.9862	4.2400	3.6730	4.1756	4.1283	8.2590	3.8903	8.1435	3.5914	7.9560	0.7764	-0.3652	0.3639	0.4390
Pclo	_AMPRPAS[Phospho (STY)]DSK_.2	Q9QYX7_S769_M1_Pclo	3.3949	3.9475	3.1286	8.4706	4.0395	4.0860	4.3215	4.3288	3.1270	3.8505	1.1547	0.2075	0.5750	0.2403
Pclo	_DQFGSSHS[Phospho (STY)]LPEVQQHM[Oxidation (M)]R_.3	#N/A	6.9376	8.4645	8.2730	7.6684	8.1730	9.1288	8.8194	9.1549	8.8398	8.8356	0.9097	-0.1366	0.0698	1.1562
Pclo	_LPAQQPGPQTAAK_.2	#N/A	5.9489	5.2282	4.3492	6.4023	6.9070	6.6376	5.6334	6.1202	6.0710	6.3038	0.9801	-0.0290	0.8185	0.0870
Pclo	_SHGPLLPTIEDSS[Phospho (STY)]EEEELREEEELLKEQEK_.4	Q9QYX7_S1709_M1_Pclo	3.8745	4.8594	4.0853	4.0196	3.6921	4.2575	3.9723	7.1655	5.6185	5.9613	0.7274	-0.4591	0.0245	1.6100
Pclo	_SSS[Phospho (STY)]EGHLR_.2	Q9QYX7_S4814_M1_Pclo	3.5637	4.3688	4.2505	3.2523	4.1902	3.5393	4.4678	3.3654	3.0140	3.2193	1.0979	0.1347	0.3539	0.4511
Pclo	_DQGKSEGITKPSLQQPS[Phospho (STY)]PK_.3	#N/A	3.3002	4.0422	4.5347	4.0784	3.8636	3.9647	4.2267	4.4236	3.2218	5.1482	0.9316	-0.1022	0.4591	0.3381
Pclo	_SM[Oxidation (M)]S[Phospho (STY)]DPKPLSPTADESSR_.2	#N/A	8.5728	8.7150	9.7806	9.1841	8.8599	8.8486	8.9150	8.8571	8.7959	8.5685	1.0238	0.0340	0.3899	0.4091
Pclo	_SPVPSQQAS[Phospho (STY)]PKK_.2	#N/A	9.1455	9.0156	8.8989	9.6172	8.7260	9.1599	9.1533	8.8459	8.8932	9.3314	1.0042	0.0060	0.8378	0.0769
Pclo	_KDS[Phospho (STY)]FSQESSPSS[Phospho (STY)]PSDLAK_.2	Q9QYX7_S1332_M2_Pclo	3.9487	3.6516	3.8425	3.4864	3.4894	6.1654	3.8927	3.8457	3.6966	6.1525	0.9319	-0.1018	0.6804	0.1672
Pclo	_GSVPAAAAES[Phospho (STY)]PSM[Oxidation (M)]HR_.2	#N/A	9.0278	9.2136	9.3014	9.4273	7.7965	9.1999	8.8935	8.9249	8.7535	8.9123	1.0139	0.0199	0.6997	0.1551
Pclo	_DISISEEEIKESQEK_.2	#N/A	4.2910	2.4380	1.9634	1.6374	3.4347	3.1899	2.8283	2.2622	3.7318	1.7489	1.0692	0.0966	0.7715	0.1126
Pclo	_TPPSNLSPIEDAS[Phospho (STY)]PTEELR_.2	Q9QYX7_S1772_M1_Pclo	7.6705	8.0602	7.8055	12.1549	10.0744	8.1060	7.1698	11.3113	10.1480	9.5586	0.9405	-0.0885	0.6326	0.1989
Pclo	_M[Oxidation (M)]SKFS[Phospho (STY)]PIQESR_.2	#N/A	5.8496	7.3718	8.0630	8.0300	6.8904	7.3816	7.8157	7.0084	7.3914	7.3057	0.9843	-0.0228	0.7990	0.0974
Pclo	_HSWHDEDDETFDES[Phospho (STY)]PELK_.3	#N/A	4.0148	5.9760	3.7293	7.8513	6.1461	6.5933	5.1518	7.4044	6.5801	7.0100	0.8748	-0.1929	0.3865	0.4128

Pclo	_ADPTVQLAPS[Phospho (STY)]PPKS[Phospho (STY)]PK_.2	#N/A	8.6709	9.4060	9.5248	9.7175	8.4758	9.2887	9.8079	9.1454	9.3288	9.4816	0.9724	-0.0403	0.3731	0.4282
Pclo	_IVDSGVQT[Phospho (STY)]DDEETADR_.2	#N/A	9.5870	9.4551	9.9672	10.3241	8.8433	9.5541	9.7994	9.6475	9.6258	9.6334	0.9943	-0.0082	0.8370	0.0773
Pclo	_LPTAVSLY[Phospho (STY)]SPTDEQSVM[Oxidation (M)]QK_.2	#N/A	6.5767	8.7493	4.0815	8.5739	6.5856	7.7192	7.2686	8.1879	7.4991	7.5649	0.9237	-0.1146	0.5333	0.2730
Pclo	_ERPKT[Phospho (STY)]PPS[Phospho (STY)]NLSPIEDAS[Phospho (STY)]PTEELR_.3	#N/A	3.7082	3.9390	3.4917	7.6243	5.1656	3.5766	3.4670	4.0818	4.9563	4.0520	1.1075	0.1473	0.6177	0.2092
Pclo	_KSS[Phospho (STY)]TS[Phospho (STY)]FDDDAGRR_.2	#N/A	6.1349	6.1148	5.9330	3.8254	3.4637	3.5497	6.2354	6.1356	5.9774	6.1011	0.7913	-0.3376	0.1014	0.9941
Pclo	_T[Phospho (STY)]PPAVATDQK_.2	Q9QYX7_T1120_M1_Pclo	3.4432	4.2484	4.1300	3.1319	4.0698	3.6597	4.3474	3.4858	3.1344	3.0989	1.0750	0.1044	0.4356	0.3609
Pclo	_GAHAHSGPTSAGSSSVPS[Phospho (STY)]PGQPGS[Phospho (STY)]PSVSK_.3	Q9QYX7_S4592_M2_Pclo	4.2452	6.7419	6.4197	8.8382	5.2859	4.8350	6.0996	7.1299	5.7087	4.0502	1.0546	0.0767	0.7581	0.1203
Pclo	_TSQS[Phospho (STY)]FPPTGDTITPLDSK_.2	Q9QYX7_S748_M1_Pclo	3.9914	7.3107	3.7251	3.2688	3.1351	3.4895	9.3168	8.0307	8.0215	8.6640	0.4882	-1.0346	0.0008	3.0731
Pclo	_ETGDGIILEVLDAYK_.2	#N/A	4.0758	3.3079	4.6051	2.4570	3.1962	3.4284	3.3216	3.8589	4.1602	3.5739	0.9418	-0.0865	0.6078	0.2162
Pclo	_AYLQGVAEDRDY[Phospho (STY)]M[Oxidation (M)]SDSEVSSTRPSR_.3	#N/A	3.9470	7.1446	3.4939	7.1727	6.4748	7.1948	7.3126	7.0328	6.9488	3.6788	0.9458	-0.0804	0.7680	0.1147
Pclo	_S[Phospho (STY)]RSHGIFPDPSK_.3	Q9QYX7_S4782_M1_Pclo	4.1202	3.4246	3.3062	3.8088	3.2459	2.9827	3.3435	4.3096	3.9582	3.7758	0.9050	-0.1440	0.2038	0.6909
Pclo	_GSVPAAAAES[Phospho (STY)]PSMHRK_.2	#N/A	2.9646	2.9869	3.8651	5.2917	3.2751	3.3495	3.5571	3.5923	3.8914	3.0861	1.0256	0.0365	0.8517	0.0697
Pclo	_S[Phospho (STY)]RTDFKEEYK_.2	#N/A	3.5050	4.2663	3.8393	4.0457	3.6812	3.5690	3.9265	3.7806	3.6923	3.9345	0.9959	-0.0059	0.9227	0.0349
Pclo	_KTTLYFDEEPELEM[Oxidation (M)]ESLTDS[Phospho (STY)]PEDR_.3	#N/A	2.9051	2.9365	4.1396	6.6597	5.4754	3.4709	3.7900	7.0947	6.2506	3.3607	0.8323	-0.2649	0.4402	0.3563
Pclo	_QAELDEEEKEIDAK_.2	#N/A	1.2139	3.6620	3.4141	4.4587	1.7403	4.2462	3.1061	4.0433	0.8625	4.1360	1.0282	0.0401	0.9275	0.0327
Pclo	_RASYDSVEDSSESENS[Phospho (STY)]PVAR_.3	#N/A	3.1762	3.9814	5.1908	4.2137	4.7413	3.9280	4.1446	4.1328	4.2179	4.0543	1.0164	0.0235	0.8543	0.0684
Pclo	_SIS[Phospho (STY)]M[Oxidation (M)]EQLMK_.2	Q9QYX7_S4706_M1_Pclo	3.5785	4.4436	4.2653	5.7495	4.2050	3.0887	4.6855	3.3505	2.9991	3.2341	1.1835	0.2430	0.2684	0.5712
Pclo	_DYMS[Phospho (STY)]DSEVSSTRPSR_.2	#N/A	4.4119	3.3691	3.3950	5.1718	3.2924	3.8357	3.3501	4.1624	4.1260	4.0074	1.0003	0.0004	0.9977	0.0010
Pclo	_DIAFIMDDFQHAM[Oxidation (M)]S[Phospho (STY)]DSEAYHLR_.3	Q9QYX7_S4358_M1_Pclo	3.1253	3.8816	4.3598	5.9443	3.7699	4.3556	4.0519	3.0976	3.3966	3.5809	1.2004	0.2635	0.2031	0.6923
Pclo	_AEEDS[Phospho (STY)]MEDPYELK_.2	Q9QYX7_S4042_M1_Pclo	4.0256	3.2005	3.2116	3.7142	3.1513	3.0773	3.4289	4.4042	4.0528	3.6812	0.8728	-0.1963	0.0913	1.0394
Pclo	_RHS[Phospho (STY)]WHDEDDETFDESPELK_.4	Q9QYX7_S1563_M1_Pclo	3.5568	3.7432	4.4802	3.7670	3.4651	3.8666	3.8820	4.5511	3.3375	5.3993	0.8883	-0.1708	0.2609	0.5835
Pclo	_SPSTIS[Phospho (STY)]LK_.2	Q9QYX7_S131_M1_Pclo	3.8362	3.5062	3.5699	4.6145	3.3275	4.4020	3.2620	3.8875	4.1865	4.2918	0.9921	-0.0115	0.9261	0.0334
Pclo	_TSVAQTHLEDAGAAIAAAEAAVQQLR_.3	#N/A	3.0064	4.3361	4.2409	3.7846	4.1574	3.5721	2.6738	2.2774	3.5156	1.2280	1.5883	0.6675	0.0140	1.8539
Pclo	_TTLYFDEEPELEM[Oxidation (M)]ESLTDS[Phospho (STY)]PEDR_.3	#N/A	3.7020	2.9574	3.4356	3.9574	2.8457	3.7789	3.6721	4.1611	3.8097	3.9244	0.8855	-0.1754	0.1081	0.9661
Pclo	_SPS[Phospho (STY)]TIS[Phospho (STY)]LKESK_.2	Q9QYX7_S128_M2_Pclo	3.6582	2.9625	4.3450	3.6573	2.7838	3.4448	3.8056	3.3438	3.6429	3.3346	0.9840	-0.0233	0.8553	0.0679
Pclo	_GGSLGC[Carbamidomethyl (C)]QTETDPDTQS[Phospho (STY)]PPYMGATSPPK_.3	Q9QYX7_S3506_M1_Pclo	4.1440	3.4484	3.3300	4.9191	3.2697	2.9590	3.3198	3.8297	4.1287	4.3496	0.9415	-0.0870	0.5915	0.2281
Pclo	_KSVDTSVQT[Phospho (STY)]DDEDQDEWDM[Oxidation (M)]PSR_.3	#N/A	4.0356	3.2910	3.5109	3.4484	3.1793	3.4459	3.3391	4.1173	3.5920	3.3513	0.9681	-0.0467	0.5973	0.2238
Pclo	_LPS[Phospho (STY)]PT[Phospho (STY)]SPLSPHSNK_.2	Q9QYX7_S2489_M2_Pclo	2.9654	3.7217	4.2000	3.7437	3.6100	3.0146	4.4086	3.2574	3.5565	3.4210	0.9677	-0.0474	0.7174	0.1442
Pclo	_LPSPTSPLSPHSNKS[Phospho (STY)]S[Phospho (STY)]PR_.3	Q9QYX7_S2501_M2_Pclo	2.9332	3.7384	3.3377	4.3823	3.5597	4.1698	4.5306	4.1197	4.4188	4.0596	0.8610	-0.2160	0.0718	1.1440
Pclo	_TPPSNLS[Phospho (STY)]PIEDAS[Phospho (STY)]PTEELRQAAEM[Oxidation (M)]EELHR_.4	#N/A	4.3852	3.6896	3.5712	4.4311	3.5109	4.2186	3.0785	4.0709	4.3700	4.1084	1.0156	0.0223	0.8501	0.0705
Pclo	_KQIIEM[Oxidation (M)]SADEDAS[Phospho (STY)]GSEDEEFIR_.3	Q9QYX7_S1505_M1_Pclo	3.3266	3.8995	4.0134	4.5161	3.7878	4.3376	4.2308	3.6024	3.2510	2.9823	1.1318	0.1787	0.1643	0.7844
Pclo	_SSSEGHLRS[Phospho (STY)]HGPS[Phospho (STY)]R_.3	Q9QYX7_S4820_M2_Pclo	4.2654	3.0770	3.9991	3.5428	2.8983	3.3303	3.6911	3.4583	3.7574	3.2201	0.9963	-0.0053	0.9652	0.0154
Gab1	_SYS[Phospho (STY)]HDVLPK_.2	Q9QYY0_S266_M1_Gab1	4.3247	4.4023	3.5107	4.0133	3.4504	4.2791	3.7280	6.6969	3.7537	3.9803	0.8804	-0.1838	0.3989	0.3991
Gab1	_SSS[Phospho (STY)]LEGFHSQYK_.2	Q9QYY0_S420_M1_Gab1	6.7691	3.6458	4.2672	3.2690	4.2069	3.0905	4.4845	3.3486	2.9972	3.2360	1.1966	0.2590	0.3733	0.4280
Gab1	_SVLTAGGVSGEELDENYVPM[Oxidation (M)]NPNS[Phospho (STY)]PPR_.3	#N/A	3.2589	4.1357	3.7774	5.6254	3.8954	4.0409	3.9948	3.8386	3.4872	5.8376	0.9610	-0.0574	0.7813	0.1072
Spast	_SVS[Phospho (STY)]PQTLEAYIR_.2	Q9QYY8_S595_M1_Spast	8.5076	8.6551	8.8694	3.8694	8.1335	8.5355	8.9622	8.6386	8.5704	8.3840	0.8985	-0.1544	0.3994	0.3986
Spast	_SSGTAPAPAS[Phospho (STY)]PS[Phospho (STY)]PPEPGPGGEAESVR_.3	Q9QYY8_S101_M2_Spast	4.0243	3.2019	3.2102	5.6079	3.1500	3.5344	4.7756	4.4056	4.0542	3.6799	0.8958	-0.1588	0.4208	0.3759
Tollip	_GPVYIGELPQDFLR_.2	#N/A	6.4832	6.5599	6.4715	6.5815	6.5692	6.4257	6.8764	6.6530	6.6676	6.6197	0.9718	-0.0413	0.0100	1.9984
Tollip	_LSITVVQAK_.2	#N/A	4.7305	4.5538	5.3794	5.8239	5.4727	5.0984	4.0092	5.2585	4.3617	5.4178	1.0871	0.1205	0.2876	0.5412
Tollip	_QGQVEDEWYSLSGR_.2	#N/A	4.5648	5.9098	3.4777	3.9803	3.4174	6.0741	3.6950	6.4128	3.7867	3.9473	1.0247	0.0352	0.8928	0.0493
Nagk	_[Acetyl (Protein N-term)]AALYGGVEGGGTR_.2	#N/A	2.9034	3.6597	4.1379	4.4042	4.4358	4.9151	3.8300	3.3195	3.6185	4.6838	1.0552	0.0775	0.6323	0.1991
Nagk	_LLIEELR_.2	#N/A	3.1021	2.4328	3.6489	4.1515	3.4336	3.8274	3.8662	3.9670	3.0981	4.1185	0.9124	-0.1323	0.3829	0.4169
Nagk	_SLGLSLS[Phospho (STY)]GGEQEDAVR_.2	Q9QZ08_S76_M1_Nagk	4.1296	3.3851	3.8633	3.4071	3.2734	3.3513	3.5553	3.5941	3.8932	3.0843	1.0103	0.0148	0.8723	0.0593
Nfu1	_IRPTVQEDGGDVIYR_.3	#N/A	3.2936	4.0488	4.5281	4.9229	4.7003	3.4055	4.2201	2.7536	3.2284	3.6348	1.1997	0.2626	0.1445	0.8401
Nfu1	_GFEDGIVR_.2	#N/A	3.1549	4.1875	4.3895	3.9332	3.7995	4.3259	4.0815	3.0680	3.3670	3.6105	1.1227	0.1670	0.1711	0.7667
Vps29	_IQHILC[Carbamidomethyl (C)]TGNLC[Carbamidomethyl (C)]TK_.3	#N/A	5.4368	5.7741	5.6639	6.1278	5.3412	5.7895	5.0002	6.0832	5.8404	5.7723	1.0026	0.0038	0.9509	0.0219
Vps29	_VVTVGQFK_.2	#N/A	5.5873	5.9002	5.9343	6.2209	5.9285	5.9127	4.7651	5.9758	5.9065	5.4577	1.0702	0.0978	0.1490	0.8267
Vps29	_TLAGDVHIVR_.2	#N/A	5.3931	5.7080	5.3061	6.2797	6.3629	6.1299	5.9729	6.2682	6.0858	6.2719	0.9534	-0.0688	0.2685	0.5710
Vps29	_FEAFEHENK_.2	#N/A	5.7289	4.5364	4.1909	5.9386	5.7450	5.4230	4.8072	5.1978	5.0869	3.4119	1.1372	0.1854	0.2335	0.6316
Rgs20	_ARAS[Phospho (STY)]PSASPADPGLPEGSER_.3	Q9QZB1_S13_M1_Rgs20	6.5160	6.1794	3.7745	3.5897	3.3242	5.8902	6.0016	6.4856	4.9173	5.8130	0.8406	-0.2506	0.2752	0.5604
Rgs20	_NQEDQRPQRAS[Phospho (STY)]HEIR_.3	Q9QZB1_S86_M1_Rgs20	2.7340	4.3801	3.5684	2.6877	2.9245	3.8570	4.5504	3.5459	3.3637	2.3553	0.9724	-0.0403	0.8536	0.0687
Actr10	_RAFS[Phospho (STY)]TEK_.2	Q9QZB7_S414_M1_Actr10	3.3975	4.1794	4.4484	3.9922	4.0327	3.9171	4.1405	3.8955	7.1481	3.6695	0.8475	-0.2387	0.3146	0.5022
Actr10	_DSVVEILFEQDNEEK_.2	#N/A	5.4696	3.0031	6.1933	3.3873	5.0417	5.6084	6.0231	4.7208	5.1809	5.1167	0.9094	-0.1370	0.5112	0.2914
Plxnc1	_KQS[Phospho (STY)]QQLELLESELRK_.3	Q9QZC2_S984_M1_Plxnc1	3.4425	5.3676	6.2739	6.3826	3.6720	4.7950	5.3601	6.2932	5.7846	3.0981	0.9717	-0.0413	0.8687	0.0611
Ercc4	_TSS[Phospho (STY)]PEVQETKK_.2	Q9QZD4_S363_M1_Ercc4	6.9753	3.9964	6.8543	4.4192	5.6306	6.4814	6.9349	6.9338	6.3850	6.6927	0.8500	-0.2344	0.1633	0.7871
Chml	_S[Phospho (STY)]SPLEASATPADSLDSASLPK_.2	Q9QZD5_S122_M1_Chml	4.0489	3.1772	3.2349	5.5097	3.0655	3.5591	3.4522	4.3809	4.0295	3.7046	0.9676	-0.0474	0.8069	0.0932
Chml	_LVVQDS[Phospho (STY)]IEQPKR_.2	#N/A	5.6430	5.8923	5.6784	6.4423	4.3266	6.0181	5.9366	5.8624	6.0936	5.9888	0.9492	-0.0753	0.4334	0.3631
Serinc1	_LTLTS[Phospho (STY)]DESTLIEDGNGR_.2	Q9QZI8_S348_M1_Serinc1	4.8482	3.4841	4.0056	3.6800	3.3460	3.7058	3.5164	6.1341	4.0010	4.0588	0.8684	-0.2036	0.3088	0.5103
Serinc1	_SDGS[Phospho (STY)]LDDGDGIHR_.2	Q9QZI8_S364_M1_Serinc1	10.2123	10.0879	10.7307	10.1444	9.0452	9.8907	10.6523	10.4026	10.0616	9.9400	0.9761	-0.0349	0.4510	0.3458
Dkkl1	_GGAREDS[Phospho (STY)]LEDGVHIPQHAK_.4	Q9QZL9_S200_M1_Dkkl1	3.1163	3.1058	4.3508	3.8946	3.7609	3.3624	4.0428	3.1066	1.6636	2.9579	1.2228	0.2902	0.1929	0.7146
Ubqln2	_GPAAAPGAAS[Phospho (STY)]PPAEPK_.2	Q9QZM0_S25_M1_Ubqln2	13.0367	13.3551	13.9724	13.8035	13.2311	13.3503	13.5576	13.0565	13.1564	13.3786	1.0129	0.0184	0.4231	0.3736
Ubqln2	_EKEEFAVPENSTVQQFK_.3	#N/A	7.0046	7.0959	7.2969	7.0496	7.4213	7.1862	6.8864	6.8554	6.8855	7.4884	1.0209	0.0298	0.3438	0.4637
Ubqln2	_SQTDQLVLIFAGK_.2	#N/A	4.7407	4.9710	4.1462	5.2082	5.3911	4.9315	5.1428	4.8212	4.9124	4.4518	1.0137	0.0196	0.7968	0.0987
Ubqln2	_NQDLALSNLESIPGGYNALR_.2	#N/A	5.2949	6.0731	5.2866	6.1559	6.2059	6.2786	5.0530	6.0175	5.8481	5.5746	1.0461	0.0650	0.3964	0.4019
Ubqln2	_NPEISHLLNNPDIM[Oxidation (M)]R_.3	#N/A	4.1544	3.0711	3.7151	3.7022	4.1981	4.3616	4.9134	4.5975	4.6420	4.1096	0.8470	-0.2396	0.0349	1.4571
Afdn	_YVLSSQHRPDIS[Phospho (STY)]PTER_.3	Q9QZQ1_S655_M1_Afdn	9.4026	9.6265	9.9860	9.6496	8.4208	9.1701	9.9819	9.8037	3.8196	9.3801	1.1370	0.1852	0.3752	0.4257
Afdn	_S[Phospho (STY)]QEELREEK_.2	#N/A	8.7712	8.3994	8.8328	8.6300	7.2808	8.3165	8.9329	8.5870	8.5286	8.4059	0.9719	-0.0411	0.4507	0.3462
Afdn	_S[Phospho (STY)]QDADLPGSSGAPENLTFK_.2	Q9QZQ1_S1770_M1_Afdn	2.9787	3.7350	7.7343	9.0296	6.8963	8.2819	7.7278	8.5964	8.4595	8.1436	0.7826	-0.3536	0.2014	0.6959
Afdn	_TSS[Phospho (STY)]VVTLEVAK_.2	Q9QZQ1_S1083_M1_Afdn	8.2735	4.3174	8.4948	7.9316	7.1190	8.2669	8.6556	8.1687	8.2429	8.2791	0.8877	-0.1718	0.2833	0.5477
Afdn	_TQVLS[Phospho (STY)]PDSLFTAK_.2	Q9QZQ1_S1719_M1_Afdn	7.2737	3.0398	4.0363	3.5800	6.0784	7.5935	7.8549	3.4211	7.1653	7.1337	0.8238	-0.2797	0.4049	0.3927
Afdn	_SSPNVANQPPS[Phospho (STY)]PGGKGPYTSGTAAK_.3	#N/A	7.4008	8.0523	4.0194	4.5221	6.7420	7.3357	7.3513	8.6121	7.8148	7.1842	0.8198	-0.2867	0.1546	0.8108
Afdn	_ADHRS[Phospho (STY)]SPNVANQPPS[Phospho (STY)]PGGK_.3	#N/A	6.2114	5.7821	5.4919	4.2074	3.5667	4.6143	4.5887	3.6439	3.5499	4.8550	1.1970	0.2595	0.1952	0.7095
Afdn	_SSPNVANQPPS[Phospho (STY)]PGGK_.2	#N/A	10.5194	10.2847	10.8053	10.7706	9.3525	10.2261	10.9082	10.6183	10.3412	10.4261	0.9766	-0.0341	0.4191	0.3777
Afdn	_VTRS[Phospho (STY)]QEELREEK_.3	#N/A	3.4636	4.2688	6.3740	5.3345	4.0902	5.0269	4.0001	5.8487	5.4229	3.5291	1.0127	0.0181	0.9332	0.0300
Afdn	_LPYLVELS[Phospho (STY)]PGR_.2	Q9QZQ1_S391_M1_Afdn	3.4310	3.7952	4.1178	4.6204	2.9189	2.9411	4.3351	5.4563	3.1466	3.0866	0.9079	-0.1393	0.5300	0.2757
Afdn	_TIS[Phospho (STY)]NPEVVMK_.2	Q9QZQ1_S216_M1_Afdn	5.7437	3.3407	3.2223	6.6174	3.1621	3.0666	3.4397	4.3935	4.0421	4.6378	1.0155	0.0221	0.9403	0.0268
Afdn	_EYFTFPASKS[Phospho (STY)]QDR_.2	#N/A	6.1815	6.1057	3.9232	4.4258	3.8629	3.9606	6.3188	6.6573	6.4693	6.2091	0.7396	-0.4353	0.0187	1.7277
Macroh2a1	_EFVEAVLELR_.2	#N/A	4.9735	3.1528	3.2593	4.2048	4.8612	4.6871	4.3849	4.3565	4.0073	3.7289	1.0171	0.0244	0.8740	0.0585
Macroh2a1	_AASADSTTEGT[Phospho (STY)]PTDGFTVLSTK_.2	Q9QZQ8_T178_M1_Macroh2a1	3.4160	3.9973	4.0173	5.1757	3.6277	4.6293	4.2530	3.9066	3.7584	4.2354	1.0261	0.0372	0.7689	0.1141

Hipk2	_AVC[Carbamidomethyl (C)]STY[Phospho (STY)]LQSR_.2	Q9QZR5_Y361_M1_Hipk2	7.5849	7.2803	7.6016	7.3305	6.8735	7.0022	7.8720	7.2857	3.0693	7.6362	1.1257	0.1709	0.3991	0.3990
Hipk2	_NVSTC[Carbamidomethyl (C)]EVTSSQAISS[Phospho (STY)]PQR_.2	Q9QZR5_S827_M1_Hipk2	2.9877	3.7929	3.6745	4.1772	3.6143	4.1152	4.4760	3.9413	3.5899	4.1442	0.9230	-0.1156	0.2720	0.5654
Rnf4	_LRQDHADSC[Carbamidomethyl (C)]VVS[Phospho (STY)]S[Phospho (STY)]DDEELSR_.3	Q9QZS2_S98_M2_Rnf4	3.3548	3.8714	4.0416	4.5442	3.9568	3.7482	4.0395	3.5743	3.2228	3.0104	1.1322	0.1792	0.1243	0.9055
Numb	_TNPS[Phospho (STY)]PTNPFSSDLQK_.2	Q9QZS3_S636_M1_Numb	9.3498	9.2815	9.5524	9.5626	8.1523	9.3976	9.1668	9.0399	9.0099	9.1131	1.0147	0.0211	0.6381	0.1951
Numb	_RTPS[Phospho (STY)]EADR_.2	Q9QZS3_S440_M1_Numb	3.5935	3.7489	3.3272	4.3718	3.5702	4.1593	4.5201	4.1302	4.4293	4.0491	0.8863	-0.1742	0.0582	1.2354
Sh2d3c	_TASPAS[Phospho (STY)]PSGSK_.2	Q9QZS8_S341_M1_Sh2d3c	2.9061	3.7113	3.5929	4.0956	3.5327	4.1968	4.5576	4.0229	3.6715	4.0626	0.9004	-0.1513	0.1800	0.7446
Sh2d3c	_VHAPSATPSTSAQPAS[Phospho (STY)]PVAR_.3	Q9QZS8_S435_M1_Sh2d3c	5.2276	4.4222	3.3996	4.4443	2.8097	3.8149	3.0917	4.0578	4.3568	4.1215	1.0289	0.0411	0.8241	0.0840
Sh2d3c	_S[Phospho (STY)]ITMTDGLTTDK_.2	Q9QZS8_S354_M1_Sh2d3c	3.2098	4.1326	4.4443	3.9881	3.8544	4.2711	4.1363	3.0131	3.3122	3.6654	1.1279	0.1736	0.1601	0.7956
Atp11c	_RAS[Phospho (STY)]DSLSAR_.2	Q9QZW0_S1105_M1_Atp11c	3.8772	3.4652	3.6108	3.1546	3.0209	2.9421	3.3029	3.8466	4.1456	4.3327	0.8562	-0.2240	0.0612	1.2136
Srr	_GLIPDT[Phospho (STY)]PEEKPK_.2	Q9QZX7_T71_M1_Srr	8.8021	9.6329	9.5975	10.0784	9.1705	9.8362	10.2284	9.6913	9.8429	10.0059	0.9575	-0.0627	0.1331	0.8757
Srr	_DLVDDVFTVTEDEIK_.2	#N/A	7.6919	6.9293	8.1104	7.7595	7.1325	7.9185	7.3013	6.8408	7.0218	7.4595	1.0607	0.0851	0.1327	0.8771
Srr	_VYAAEPSNADDC[Carbamidomethyl (C)]YQSK_.2	#N/A	6.1756	4.0352	6.1878	7.1208	6.3486	6.1023	6.7979	6.6635	6.2422	6.2242	0.9249	-0.1127	0.3925	0.4062
Srr	_LAIQAYGASIVYC[Carbamidomethyl (C)]DPSDESR_.2	#N/A	2.9496	2.4777	4.4140	3.9577	3.9843	3.9336	4.1060	3.3862	3.3425	3.6350	1.0006	0.0008	0.9961	0.0017
Zranb2	_S[Phospho (STY)]RPSS[Phospho (STY)]PAVR_.2	#N/A	10.0636	9.9473	10.4084	10.1522	9.2820	9.8908	10.3177	10.8175	9.9515	9.8881	0.9720	-0.0409	0.2961	0.5286
Zranb2	_YNLDAS[Phospho (STY)]EEEDSNK_.2	#N/A	6.6574	5.8029	6.0914	6.7131	4.7260	5.9505	4.0037	6.1510	5.9184	5.6134	1.1049	0.1439	0.3166	0.4995
Zranb2	_EES[Phospho (STY)]DGEYDEFGR_.2	#N/A	3.0860	8.5676	3.7728	9.0081	3.7125	8.8250	9.3677	8.7990	9.2635	9.0135	0.6763	-0.5642	0.0823	1.0845
Zranb2	_YNLDAS[Phospho (STY)]EEEDSNKKK_.2	#N/A	4.4555	4.9921	4.7306	4.7040	4.0221	4.7363	4.4741	4.8592	4.9509	4.5130	0.9803	-0.0287	0.6476	0.1887
Zranb2	_S[Phospho (STY)]RPSS[Phospho (STY)]PAVRK_.2	#N/A	3.3339	3.9646	3.5667	6.2672	3.9705	3.9681	4.2506	5.4473	3.2392	6.2327	0.8719	-0.1978	0.4366	0.3599
Zranb2	_ENVEYIEREES[Phospho (STY)]DGEYDEFGR_.3	#N/A	4.2776	2.9486	3.4636	6.3725	3.4033	3.7878	3.6809	4.9039	3.2328	3.9332	1.0265	0.0378	0.8824	0.0543
Zranb2	_SRS[Phospho (STY)]RPSS[Phospho (STY)]PAVR_.2	#N/A	3.8922	3.4439	3.8117	3.4459	3.6038	3.2283	3.8790	3.8831	5.7771	3.3326	0.8466	-0.2402	0.1815	0.7411
Zranb2	_ENVEYIEREES[Phospho (STY)]DGEYDEFGRK_.3	#N/A	3.1904	6.7929	4.5813	8.8978	7.2930	6.9112	6.5414	8.4653	7.9250	7.2973	0.8307	-0.2676	0.2763	0.5585
Zranb2	_YNLDAS[Phospho (STY)]EEEDSNKK_.2	#N/A	3.7630	6.0381	5.7086	7.2324	2.9068	5.8982	6.2691	6.3312	6.0217	3.8633	0.9353	-0.0964	0.7098	0.1489
Zranb2	_PS[Phospho (STY)]S[Phospho (STY)]PAVRK_.2	#N/A	3.4996	3.8964	3.7780	4.2243	3.7177	4.0118	4.3726	4.2822	3.0759	4.2476	0.9650	-0.0515	0.6240	0.2048
Prkab1	_S[Phospho (STY)]QNNFVAILDLPEGEHQYK_.3	#N/A	7.2666	7.3298	7.7494	8.1738	6.9205	7.2828	7.2878	7.6581	7.5380	7.2937	1.0013	0.0018	0.9692	0.0136
Pex14	_[Acetyl (Protein N-term)]ASSEQAEQPNQPSS[Phospho (STY)]PPGSENVVPR_.3	Q9R0A0_S15_M1_Pex14	4.1686	3.1338	3.6889	8.1504	7.0460	7.5501	7.6474	7.7025	7.7701	7.6475	0.7310	-0.4520	0.1025	0.9891
Zeb2	_AYLQSITPQGYSDS[Phospho (STY)]EER_.2	Q9R0G7_S1124_M1_Zeb2	3.7028	3.7956	3.9735	3.6712	3.6169	3.4586	3.8194	3.4254	3.7244	4.1000	0.9830	-0.0248	0.6611	0.1797
Zeb2	_SNT[Phospho (STY)]PS[Phospho (STY)]PLNLSSTSSK_.2	Q9R0G7_T782_M2_Zeb2	3.5639	6.0293	3.6963	6.6042	3.3951	3.8346	4.0190	3.9934	6.1270	6.5407	0.8744	-0.1937	0.4897	0.3100
Zeb2	_S[Phospho (STY)]DHEEDNMEDGME_.2	#N/A	3.5157	4.2940	3.7886	4.1985	3.5068	3.7852	3.9262	3.6801	3.5700	3.5602	1.0445	0.0628	0.3963	0.4019
Zeb2	_VYQYSNSRS[Phospho (STY)]PSLER_.3	Q9R0G7_S705_M1_Zeb2	3.9391	3.1945	3.6728	3.7202	3.0828	3.5418	3.4349	4.3982	3.7938	3.6872	0.9208	-0.1191	0.2281	0.6418
Atp2b2	_TSPVEGLPGTAPDLEK_.2	#N/A	8.8747	8.7106	9.1054	8.8706	8.5568	8.5008	9.1568	8.8744	8.6720	8.4118	0.9990	-0.0015	0.9592	0.0181
Atp2b2	_EIPDPSSINAK_.2	#N/A	8.4373	8.1484	8.8688	7.6035	8.0711	7.7336	8.4897	8.3708	8.3684	8.1579	0.9757	-0.0355	0.4281	0.3685
Atp2b2	_ILSGAGEAR_.2	#N/A	6.9819	7.3219	7.0153	6.5364	6.9471	6.4801	7.1546	7.6777	7.2549	6.9979	0.9463	-0.0797	0.0851	1.0700
Atp2b2	_GIIDSTHTEQR_.2	#N/A	7.1324	6.7232	7.4028	6.5204	6.8164	6.9439	7.3779	7.3482	7.2476	7.0268	0.9549	-0.0666	0.0928	1.0324
Atp2b2	_GASEIVLKK_.2	#N/A	4.8464	4.5668	4.5500	3.3029	3.7652	5.5640	5.2272	4.8037	5.0268	3.7263	0.9439	-0.0833	0.6030	0.2197
Atp2b2	_YGDLLPADGLFIQGNDLK_.2	#N/A	6.0831	6.2736	7.0606	6.5446	5.4932	5.7881	7.0014	7.0967	6.5606	6.3183	0.9204	-0.1197	0.1307	0.8837
Atp2b2	_NNSAIDS[Phospho (STY)]GINLTTDTSK_.2	Q9R0K7_S1170_M1_Atp2b2	6.1753	6.0366	5.8668	6.4569	4.6766	5.9000	6.3535	6.5487	5.4170	6.0980	0.9587	-0.0609	0.5140	0.2890
Atp2b2	_AFRSS[Phospho (STY)]LYEGLEKPESR_.3	Q9R0K7_S1105_M1_Atp2b2	3.3171	3.9091	4.0039	4.5066	3.9436	3.7859	4.2212	6.6859	3.2605	2.9727	0.9127	-0.1318	0.6050	0.2182
Atp2b2	_AGQVVQIPVAEIVVGDIAQIK_.2	#N/A	5.6743	4.7990	3.7285	4.4361	4.1819	4.2318	5.4947	5.2376	6.7545	4.8599	0.8070	-0.3093	0.0514	1.2889
Atp2b2	_IDESSLTGESDQVR_.2	#N/A	7.9380	7.5129	3.7879	7.0605	7.1318	6.8790	3.4799	3.6695	7.2695	7.4789	1.2272	0.2954	0.3119	0.5060
Atp2b2	_MPDESFR_.2	#N/A	5.7066	3.1592	4.5031	5.2295	4.6071	4.2123	3.7269	4.9879	3.3499	4.2734	1.1188	0.1619	0.3858	0.4136
Atp2b2	_EASDIILTDDNFSSIVK_.2	#N/A	7.0466	6.8875	7.4084	7.3063	5.8115	6.8610	7.4898	6.9300	6.7711	6.9571	0.9787	-0.0311	0.6480	0.1884
Atp2b2	_NES[Phospho (STY)]SHGGEFGC[Carbamidomethyl (C)]TM[Oxidation (M)]EELR_.3	#N/A	3.8439	3.4781	3.9261	6.9958	5.0193	5.5130	3.8751	5.9947	5.9398	5.3258	0.9077	-0.1397	0.5499	0.2597
Atp2b2	_T[Phospho (STY)]SIHNFM[Oxidation (M)]AHPEFR_.3	#N/A	5.1902	7.9182	5.0728	9.0819	5.1354	4.9302	7.8869	5.5952	7.7871	6.9078	0.8832	-0.1792	0.4398	0.3567
Atp2b2	_QNSS[Phospho (STY)]PPSSLNK_.2	#N/A	10.9993	10.4453	10.8148	10.8958	9.7126	10.5227	11.2873	10.7515	10.7309	10.6227	0.9739	-0.0381	0.3202	0.4945
Atp2b2	_IDESSLTGESDQVRK_.2	#N/A	4.8777	4.9631	5.7229	4.5569	4.3753	4.7150	5.1322	5.0001	4.7682	5.1532	0.9711	-0.0423	0.5775	0.2384
Atp2b2	_TLELLVNAIAINSAYTTK_.2	#N/A	3.8075	4.1021	3.7485	4.3135	1.9000	4.1350	3.3886	3.8050	4.2001	4.2805	0.9360	-0.0954	0.6177	0.2092
Atp2b2	_VIEPMAC[Carbamidomethyl (C)]DGLR_.2	#N/A	6.2584	4.3471	3.3245	6.0448	4.2354	3.8901	4.5173	4.1329	4.4320	4.0464	1.0937	0.1292	0.5329	0.2733
Atp2b2	_IEDSQPHIPLIDDTDLEEDAALKQNSS[Phospho (STY)]PPSSLNK_.4	#N/A	8.8108	3.9796	4.4578	9.6513	3.8679	8.6452	9.2673	9.9505	9.2289	8.5768	0.7097	-0.4947	0.0934	1.0297
Atp2b2	_TEC[Carbamidomethyl (C)]GLLGFVLDLR_.2	#N/A	3.5260	4.3312	4.2128	3.7895	4.1526	3.5769	4.5747	3.4643	3.0516	3.1816	1.1019	0.1399	0.2970	0.5272
Atp2b2	_EEIPEEELNEDVEEIDHAER_.3	#N/A	3.8909	3.3353	4.5777	7.0784	3.2236	3.4010	3.2942	6.4434	4.1875	3.5465	0.9733	-0.0391	0.9043	0.0437
Atp2b2	_LTQKEEIPEEELNEDVEEIDHAER_.4	#N/A	4.1794	3.0844	3.6867	4.5098	3.3149	3.1190	3.2531	5.1443	4.2319	3.9597	0.8799	-0.1846	0.2813	0.5508
Atp2b2	_M[Oxidation (M)]TVVQAYVGDVHYK_.3	#N/A	3.9723	2.0395	3.1583	3.6609	2.2873	3.1306	3.4914	4.4575	4.1061	2.3462	0.8448	-0.2433	0.3236	0.4900
Pcm1	_SNRPVS[Phospho (STY)]ADGNYRPLAK_.3	Q9R0L6_S957_M1_Pcm1	7.0477	7.1828	7.8276	7.9990	6.7574	6.8927	7.4634	7.2666	7.2000	7.6289	0.9858	-0.0207	0.7087	0.1495
Pcm1	_VTNAIS[Phospho (STY)]PESSPGVGR_.2	Q9R0L6_S65_M1_Pcm1	8.6178	8.1651	9.0578	8.5936	8.0421	8.6737	8.9389	8.2802	8.5519	8.6702	0.9901	-0.0144	0.7049	0.1519
Pcm1	_ILEGDHGS[Phospho (STY)]PAGEIDDEDKDKDETETVK_.4	Q9R0L6_S1729_M1_Pcm1	3.7447	3.0490	4.4315	3.5708	2.8704	3.3583	3.7191	3.4304	3.7294	5.0182	0.8817	-0.1816	0.2730	0.5639
Pcm1	_RS[Phospho (STY)]S[Phospho (STY)]LVDEAPEDEEFEQK_.2	Q9R0L6_S644_M2_Pcm1	3.4547	3.9534	3.6998	5.7327	4.7862	3.9772	4.3380	4.7438	5.0929	5.2175	0.8802	-0.1841	0.2416	0.6169
Pcm1	_HIS[Phospho (STY)]ESDER_.2	Q9R0L6_S1432_M1_Pcm1	2.3005	4.3942	4.1828	3.7265	3.5928	3.5140	3.8748	3.2746	3.5737	3.4038	1.0246	0.0350	0.8285	0.0817
Pcm1	_NVRS[Phospho (STY)]DVS[Phospho (STY)]DQEEDEESER_.2	Q9R0L6_S1766_M2_Pcm1	2.8165	3.3927	4.0107	3.1821	2.3273	3.2039	3.0970	3.2353	4.3847	3.3494	0.8973	-0.1563	0.3577	0.4465
Pcm1	_KAS[Phospho (STY)]AQASLASK_.2	Q9R0L6_S1280_M1_Pcm1	4.3489	3.6043	4.0826	3.6263	3.4926	3.1320	3.7746	3.3749	3.6739	3.3036	1.0517	0.0728	0.4676	0.3301
Celsr2	_STLTSSYNC[Carbamidomethyl (C)]PS[Phospho (STY)]PYADGR_.2	Q9R0M0_S2648_M1_Celsr2	4.3530	2.9894	4.0867	3.6305	3.4968	3.4179	3.7787	3.3707	3.6698	3.3078	1.0370	0.0524	0.6360	0.1965
Syt11	_DYGEELRS[Phospho (STY)]PM[Oxidation (M)]TSLT[Phospho (STY)]PGESK_.3	Q9R0N3_T139_M2_Syt11	3.5390	4.3270	3.9862	3.5150	3.7897	3.6959	4.0747	3.6956	3.3442	3.4820	1.0438	0.0618	0.4501	0.3467
Syt7	_NSLETVGT[Phospho (STY)]PDSGR_.2	#N/A	9.4825	9.1667	9.6457	9.5398	8.3844	9.0876	9.8665	9.4778	9.2808	9.1661	0.9757	-0.0356	0.4117	0.3854
Syt7	_NSLETVGT[Phospho (STY)]PDS[Phospho (STY)]GRGRGEK_.3	#N/A	5.6959	5.6155	6.3130	6.0669	5.8191	6.1918	5.5240	6.2949	6.2443	5.6656	1.0031	0.0044	0.9339	0.0297
Syt7	_YKNSLETVGT[Phospho (STY)]PDS[Phospho (STY)]GRGR_.3	#N/A	6.6141	6.2767	6.0822	6.3318	5.8290	6.5478	7.1626	7.1959	6.8633	6.4813	0.9068	-0.1411	0.0118	1.9296
Syt7	_YKNSLETVGT[Phospho (STY)]PDSGR_.2	#N/A	5.1464	5.9024	5.1589	5.9447	6.0805	5.0428	6.2940	7.1787	5.5098	4.3004	0.9528	-0.0698	0.6249	0.2042
Syt7	_VLYLQVLDYDR_.2	#N/A	4.2460	2.8586	5.0350	3.6531	3.0902	3.1385	5.4582	4.2403	4.1140	2.1471	0.9199	-0.1205	0.6548	0.1839
Syt7	_NSLETVGT[Phospho (STY)]PDS[Phospho (STY)]GRGR_.2	#N/A	8.4827	8.2488	8.3391	8.5626	7.4673	8.1777	9.0053	8.7616	8.5381	8.5032	0.9438	-0.0834	0.0571	1.2430
Syt6	_S[Phospho (STY)]VDGDDAKSEAAK_.2	#N/A	6.2437	4.1735	7.2544	7.3922	6.6342	6.5494	6.8040	6.8003	3.2093	6.7366	1.0827	0.1147	0.6118	0.2134
Syt6	_EASSPSSANPASETLQS[Phospho (STY)]PSSR_.2	Q9R0N8_S108_M1_Syt6	4.0607	3.2817	6.7304	3.3381	3.2044	3.1256	6.7433	4.6685	3.9621	5.9652	0.6840	-0.5479	0.0689	1.1619
Syt6	_QKS[Phospho (STY)]VDGDDAKSEAAK_.2	#N/A	4.3544	4.6301	5.9244	5.1822	5.5051	4.7694	4.0735	4.3821	5.1737	5.0233	1.0853	0.1181	0.3063	0.5138
Syt6	_QKS[Phospho (STY)]VDGDDAK_.2	#N/A	4.2133	3.1291	3.9470	3.4908	3.3570	3.2782	3.6390	3.5104	3.8095	6.8438	0.8020	-0.3184	0.2235	0.6507
Esd	_AFSGYLGPDESK_.2	#N/A	7.2629	7.0454	3.7779	7.1855	7.1213	7.0267	7.3688	7.1673	6.6012	7.3547	0.9224	-0.1166	0.4607	0.3366
Esd	_M[Oxidation (M)]YSYVTEELPQLINANFPVDPQR_.3	#N/A	3.5732	3.5958	4.2600	3.7423	4.4346	4.9905	3.8905	3.2589	4.4933	5.3150	0.9670	-0.0485	0.7638	0.1170
Esd	_AYDATC[Carbamidomethyl (C)]LVK_.2	#N/A	3.7227	3.0271	4.4095	3.4113	5.2686	3.3803	3.1260	3.2063	4.3557	5.1061	0.9801	-0.0290	0.8930	0.0491
Smap	_SAS[Phospho (STY)]PDDDLGSSNWEAADLGNEERK_.3	#N/A	4.0628	3.1634	3.2488	3.7514	3.1885	3.5729	3.4661	4.3671	4.0156	3.7184	0.8988	-0.1539	0.1402	0.8531
Smap	_SAS[Phospho (STY)]PDDDLGSSNWEAADLGNEER_.3	#N/A	2.9941	4.2321	3.6809	4.1835	3.6206	4.1089	3.8982	3.9349	3.5835	4.1505	0.9773	-0.0332	0.7416	0.1298
Dstn	_[Acetyl (Protein N-term)]AS[Phospho (STY)]GVQVADEVC[Carbamidomethyl (C)]R_.2	Q9R0P5_S3_M1_Dstn	12.5076	12.3686	13.0546	12.9872	12.0790	12.7403	13.1143	12.3693	12.6750	12.7158	0.9925	-0.0109	0.6840	0.1649
Dstn	_YALYDASFETK_.2	#N/A	7.3883	7.2945	7.2319	7.8378	7.9703	7.6111	6.9233	7.4617	7.7511	7.5169	1.0192	0.0275	0.5110	0.2916
Dstn	_HEYQANGPEDLNR_.2	#N/A	6.6476	6.7929	6.7512	7.1328	6.7733	6.6163	6.8120	6.6762	6.8233	6.6276	1.0076	0.0109	0.6300	0.2007

Dstn	_EILVGDVGATITDPFK_.2	#N/A	5.4860	4.8403	3.1783	5.2115	5.0081	5.4892	5.4399	5.0053	4.9513	5.6702	0.9245	-0.1133	0.4156	0.3813
Dstn	_HFVGM[Oxidation (M)]LPEKDC[Carbamidomethyl (C)]R_.3	#N/A	5.7768	5.6644	3.7626	5.9724	6.3531	5.9961	7.0093	5.4053	6.4427	5.5700	0.9150	-0.1282	0.3794	0.4209
Dstn	_C[Carbamidomethyl (C)]IVVEEGKEILVGDVGATITDPFK_.3	#N/A	3.0498	4.2926	3.9514	3.8281	3.6944	4.4310	4.0962	3.1731	3.4721	3.5054	1.0878	0.1215	0.3174	0.4984
Uchl1	_M[Oxidation (M)]PFPVNHGASSEDSLLQDAAK_.3	#N/A	9.2274	9.2710	9.3282	9.5767	9.3603	9.2942	9.1155	9.0305	8.9272	9.0053	1.0359	0.0508	0.0017	2.7658
Uchl1	_LGVAGQWR_.2	#N/A	9.4209	10.0374	9.7017	9.6704	9.4471	9.4153	9.6889	9.8467	9.3983	9.6736	0.9962	-0.0055	0.8069	0.0932
Uchl1	_QFLSETEK_.2	#N/A	9.0897	9.4626	9.6195	9.3835	10.0681	9.4505	9.1953	9.2763	9.4701	9.5922	1.0137	0.0197	0.4937	0.3066
Uchl1	_NEAIQAAHDSVAQEGQC[Carbamidomethyl (C)]R_.3	#N/A	7.8654	7.8370	8.0812	8.4587	8.5275	7.7911	7.9295	7.6055	7.9030	8.0838	1.0270	0.0385	0.2807	0.5518
Uchl1	_FSAVALC[Carbamidomethyl (C)]K_.2	#N/A	7.8111	7.6677	7.5323	7.4694	7.8426	7.6713	7.3383	7.3429	7.8649	7.8906	1.0074	0.0107	0.7045	0.1521
Uchl1	_QFLSETEKLS[Phospho (STY)]PEDR_.2	#N/A	7.0376	7.5032	8.1532	7.9940	7.7312	7.8638	7.4197	7.2077	7.5128	7.7995	1.0306	0.0435	0.3399	0.4686
Uchl1	_QTIGNSC[Carbamidomethyl (C)]GTIGLIHAVANNQDKLEFEDGSVLK_.4	#N/A	5.1001	4.5329	5.7502	5.0466	4.2484	3.7025	4.1084	4.6699	5.0351	4.9286	1.0095	0.0137	0.9146	0.0388
Uchl1	_C[Carbamidomethyl (C)]FEKNEAIQAAHDSVAQEGQC[Carbamidomethyl (C)]R_.4	#N/A	6.2205	6.6997	6.4931	6.5993	7.1287	6.2238	6.1673	6.2550	6.2762	6.5004	1.0414	0.0586	0.1898	0.7216
Uchl1	_QIEELKGQEVSPK_.2	#N/A	7.1227	7.4423	7.7063	7.6595	7.8849	7.7614	7.0467	7.1683	7.2429	7.4952	1.0494	0.0696	0.0539	1.2687
Arpc1a	_TLESSIQGLR_.2	#N/A	8.1522	7.8928	8.6315	8.4626	8.2723	8.0406	7.7689	8.3053	8.4229	7.7230	1.0232	0.0331	0.3754	0.4255
Arpc1a	_WSPLENK_.2	#N/A	5.7649	6.3572	5.9003	5.6831	5.5980	5.9930	6.0400	6.7310	6.1781	5.5309	0.9612	-0.0570	0.3511	0.4545
Arpc1a	_[Acetyl (Protein N-term)]SLHQFLLEPITC[Carbamidomethyl (C)]HAWNR_.3	#N/A	4.3615	4.9409	4.9220	5.8769	4.8112	4.9578	4.1520	5.4360	4.9674	4.7418	1.0319	0.0454	0.6518	0.1859
Arpc1a	_FC[Carbamidomethyl (C)]TTGIDGAM[Oxidation (M)]TIWDFK_.2	#N/A	5.8998	6.0537	6.1362	5.8091	5.5840	5.4590	6.5367	5.9689	5.9142	5.8325	0.9605	-0.0581	0.2312	0.6360
Arpc1a	_NAYVWSQK_.2	#N/A	4.7148	5.2695	5.5688	3.1611	5.3151	5.0666	4.3766	5.4746	5.4042	5.4424	0.9372	-0.0936	0.5286	0.2769
Arpc1a	_IVTC[Carbamidomethyl (C)]GADR_.2	#N/A	5.1282	3.8363	4.0767	4.5793	5.9862	3.7131	5.1994	5.6356	5.8877	4.8174	0.8455	-0.2420	0.1243	0.9057
Arpc1a	_EVDEKPASTPWGSK_.2	#N/A	5.8811	5.9347	6.0794	6.0517	5.6973	5.9471	6.3219	5.9466	5.8752	5.7688	0.9923	-0.0112	0.7056	0.1515
Arpc1a	_SVQVSTLR_.2	#N/A	4.2365	8.7917	3.3771	3.8797	3.7980	4.1063	3.5944	4.2388	4.1669	3.9904	1.1753	0.2330	0.5198	0.2842
Arpc1a	_NM[Oxidation (M)]S[Phospho (STY)]AMER_.2	#N/A	3.3248	4.6208	4.1251	4.1090	3.6345	4.1652	4.0387	3.5214	4.0368	3.9504	1.0282	0.0402	0.6739	0.1714
Arpc1a	_LAWVSHDSTVSVADASK_.2	#N/A	2.7162	3.9694	4.3276	4.3481	3.9814	4.2763	4.2612	3.1757	3.3685	3.4492	1.1046	0.1436	0.3422	0.4657
Arpc1a	_EHNGHITGIDWAPK_.3	#N/A	4.0791	3.3834	3.2650	3.7677	3.2048	3.0239	3.3847	4.3508	3.9994	3.7347	0.8931	-0.1631	0.1478	0.8302
Arpc1a	_LISVC[Carbamidomethyl (C)]YFESENDWWVSK_.2	#N/A	3.1458	4.0803	3.8326	4.3353	3.7723	3.9571	4.0500	1.8406	3.4318	4.3023	1.1315	0.1782	0.3806	0.4195
Ptges3	_HLNEIDLFHC[Carbamidomethyl (C)]IDPNDSK_.3	#N/A	7.7046	7.7366	7.8342	8.7044	7.8310	7.7758	8.0350	7.7804	7.7469	7.8995	1.0083	0.0120	0.7532	0.1231
Ptges3	_DWEDDSDEDM[Oxidation (M)]SNFDR_.2	#N/A	7.2207	12.2057	8.5217	12.5189	12.0636	12.3929	12.7491	12.2232	12.2751	12.4052	0.8717	-0.1981	0.2179	0.6617
Ptges3	_SILC[Carbamidomethyl (C)]C[Carbamidomethyl (C)]LR_.2	#N/A	6.9001	3.3718	7.1296	3.3938	7.8345	3.3645	5.9595	7.4682	7.4120	7.3390	0.7569	-0.4018	0.1719	0.7648
Ptges3	_LTFSC[Carbamidomethyl (C)]LGGSDNFK_.2	#N/A	5.5018	5.9366	5.5293	6.7059	6.4094	5.9130	6.0118	5.8393	5.9604	5.7633	1.0179	0.0256	0.6819	0.1663
Ptges3	_KGESGQSWPR_.2	#N/A	4.8516	4.8483	4.0661	5.1680	4.8677	4.7930	5.5527	5.1918	4.1334	5.2727	0.9460	-0.0800	0.4052	0.3923
Srsf10	_S[Phospho (STY)]RS[Phospho (STY)]FDYNYRR_.3	#N/A	8.0969	8.1684	7.8793	8.2795	7.9383	8.3308	8.3241	8.3039	8.4945	8.1043	0.9770	-0.0336	0.1258	0.9003
Srsf10	_S[Phospho (STY)]RS[Phospho (STY)]FDYNYR_.2	#N/A	8.5441	9.5769	8.0631	9.9902	8.7555	9.4376	10.0415	9.6793	9.6200	9.6625	0.9293	-0.1058	0.1055	0.9768
Srsf10	_S[Phospho (STY)]FDYNYR_.2	#N/A	3.7048	3.6376	3.4384	6.4423	5.1940	6.1792	3.1305	6.6783	6.9578	6.3489	0.8247	-0.2780	0.3347	0.4754
Srsf10	_SRS[Phospho (STY)]WT[Phospho (STY)]SPK_.2	Q9R0U0_S253_M2_Srsf10	3.8606	3.1649	3.7425	3.4815	3.8275	3.2689	4.0864	3.9553	3.7981	3.5311	0.9258	-0.1112	0.1493	0.8259
Srsf10	_S[Phospho (STY)]RS[Phospho (STY)]RS[Phospho (STY)]FDYNYR_.2	#N/A	4.0882	6.3159	4.3064	7.0525	5.6413	6.5461	6.5541	6.4938	6.5467	3.3840	0.9850	-0.0218	0.9244	0.0341
Srsf10	_S[Phospho (STY)]RS[Phospho (STY)]RS[Phospho (STY)]YER_.2	Q9R0U0_S119_M3_Srsf10	4.2062	3.1362	6.5516	3.4837	3.3499	3.2747	5.6455	3.5175	3.8166	3.1609	0.9914	-0.0125	0.9663	0.0149
Srsf10	_S[Phospho (STY)]RS[Phospho (STY)]HSDNDRFK_.3	Q9R0U0_S156_M2_Srsf10	4.2309	3.1115	3.9645	3.5083	3.3746	3.2500	3.6566	3.4929	3.7919	3.1856	1.0118	0.0169	0.8694	0.0608
Rpgr	_SS[Phospho (STY)]SSEILGGNDPTSK_.2	Q9R0X5_S913_M1_Rpgr	4.0074	3.2629	3.7411	7.8189	3.1512	3.4734	3.4331	3.7163	4.0153	2.9622	1.2012	0.2645	0.4645	0.3330
Ak1	_IIFVVGGPGSGK_.2	#N/A	8.8298	9.2638	9.4723	9.4455	9.4664	9.3970	9.2517	9.0775	9.3056	9.3381	1.0075	0.0108	0.6220	0.2062
Ak1	_VDSSNGFLIDGYPR_.2	#N/A	7.2207	7.3605	6.3312	7.6447	7.5699	7.0199	7.3519	7.2084	7.4405	6.7294	1.0012	0.0017	0.9758	0.0106
Ak1	_GELVPLDTVLDM[Oxidation (M)]LR_.2	#N/A	6.0657	6.6303	6.0960	6.5578	7.2287	6.9139	6.4615	6.9221	6.9118	6.5556	0.9805	-0.0284	0.6161	0.2104
Ak1	_RLETYYNATEPVISFYDKR_.3	#N/A	6.0497	6.5920	6.7673	7.3570	6.9008	6.6943	6.7653	6.4732	6.6131	6.4581	1.0227	0.0324	0.5244	0.2803
Ak1	_VDDNEETIKK_.2	#N/A	5.6864	5.6275	5.9538	5.7574	6.2926	5.7560	5.8879	5.0971	5.1415	5.5830	1.0771	0.1071	0.0639	1.1946
Ak1	_LETYYNATEPVISFYDKR_.3	#N/A	5.2269	4.6153	5.1294	5.0117	5.5283	5.5598	5.0098	5.2588	5.4433	5.1852	0.9912	-0.0127	0.8186	0.0869
Ak1	_YGYTHLSTGDLLR_.3	#N/A	5.3142	5.2160	5.1389	4.4285	5.3048	5.2623	5.5490	5.0654	5.0264	4.9949	0.9907	-0.0135	0.8178	0.0874
Ak1	_AEVSSGSER_.2	#N/A	5.7596	4.0809	3.8321	5.1594	3.9692	4.6410	5.8281	5.3608	3.4323	5.8808	0.8923	-0.1643	0.3842	0.4154
Dlc1	_SEDS[Phospho (STY)]DEDEPC[Carbamidomethyl (C)]AISGK_.2	#N/A	7.8905	7.6109	8.3588	8.5094	7.2614	7.7089	8.2181	7.9573	7.8973	7.7823	0.9907	-0.0134	0.7765	0.1099
Dlc1	_QDPIPGS[Phospho (STY)]PDNSR_.2	Q9R0Z9_S129_M1_Dlc1	3.6416	3.5846	4.3284	3.3302	7.9579	8.5909	8.9238	3.2874	8.6471	8.3780	0.7168	-0.4804	0.2359	0.6273
Dlc1	_FSDEGDSDSALDSVSPC[Carbamidomethyl (C)]PSS[Phospho (STY)]PK_.2	Q9R0Z9_S510_M1_Dlc1	4.0615	4.9096	4.0580	3.6018	4.4971	3.2671	3.7500	3.3995	3.6985	3.2791	1.1512	0.2032	0.1287	0.8905
Dlc1	_RSEDS[Phospho (STY)]DEDEPC[Carbamidomethyl (C)]AISGK_.2	#N/A	4.1735	1.2150	3.3594	3.8621	3.2991	4.4304	3.2903	4.2564	3.9050	3.8291	0.8874	-0.1724	0.5004	0.3007
Dlc1	_KRSEDS[Phospho (STY)]DEDEPC[Carbamidomethyl (C)]AISGK_.3	#N/A	3.0019	3.9259	3.3374	4.1207	3.7876	3.3254	3.6051	4.0074	3.6560	3.9240	0.9434	-0.0841	0.3805	0.4197
Gda	_YTFPTEQR_.2	#N/A	8.6609	8.7222	8.6461	8.6904	8.7328	8.8385	8.4241	8.6785	8.6678	8.8023	1.0083	0.0120	0.3458	0.4612
Gda	_TPPLALVFR_.2	#N/A	8.4696	8.8672	8.1762	8.3038	8.3662	8.4515	8.3831	8.7404	8.7614	8.5385	0.9806	-0.0282	0.2660	0.5751
Gda	_DHLLGVSDSGK_.2	#N/A	8.5733	8.4229	8.6801	8.5502	8.6782	8.5326	8.3754	8.2757	8.6615	8.5447	1.0128	0.0184	0.2333	0.6321
Gda	_STDVAEEVYTR_.2	#N/A	7.7871	7.7002	7.8981	7.8909	8.1101	8.0378	7.6496	7.9410	7.9815	8.0376	1.0002	0.0003	0.9880	0.0052
Gda	_IVFLEESSQQEK_.2	#N/A	6.9514	7.2194	7.0217	8.0143	7.4173	7.7374	7.3223	7.4477	7.0050	7.8489	0.9983	-0.0024	0.9622	0.0167
Gda	_NIEEVYVGGK_.2	#N/A	7.0838	6.8539	6.3099	7.2827	6.8927	7.0967	7.1187	6.1672	6.9571	7.2594	1.0064	0.0093	0.8680	0.0615
Gda	_EFDALLINPR_.2	#N/A	6.3518	6.3556	6.0627	6.7335	6.4861	6.3361	6.5442	6.4885	6.6074	6.2876	0.9855	-0.0211	0.4704	0.3275
Gda	_FLYLGDDR_.2	#N/A	6.4761	5.9720	5.7767	6.2540	5.6987	6.3449	6.4606	6.3767	6.1804	6.6521	0.9485	-0.0762	0.1035	0.9851
Gda	_ETTEESVKETER_.2	#N/A	3.9394	4.2354	4.0480	4.8840	5.1248	5.5048	4.6177	4.0612	5.0877	4.5878	1.0060	0.0086	0.9417	0.0261
Gda	_GTFVHSTWTC[Carbamidomethyl (C)]PM[Oxidation (M)]EVLR_.3	#N/A	6.1183	5.8583	3.1935	6.2488	6.3170	6.2571	5.8016	6.7213	6.2554	6.0221	0.9138	-0.1301	0.4297	0.3668
Gda	_FVSEM[Oxidation (M)]LQK_.2	#N/A	3.2438	4.9677	4.0341	4.6244	5.0887	4.2687	4.4209	4.6891	4.4963	4.0886	0.9881	-0.0172	0.8884	0.0514
Gda	_NYTDVYDKNNLLTNK_.2	#N/A	5.2909	4.7507	4.6977	5.3416	5.3700	5.0386	5.2413	5.3751	5.2691	5.3779	0.9559	-0.0650	0.1720	0.7644
Gda	_TVM[Oxidation (M)]AHGC[Carbamidomethyl (C)]YLSEEELNIFSER_.3	#N/A	5.4393	5.8076	6.1163	6.1179	5.3070	5.7612	5.5690	5.3236	5.3036	5.7079	1.0515	0.0725	0.1723	0.7636
Gda	_FTLSC[Carbamidomethyl (C)]TETLM[Oxidation (M)]SELGNIAK_.2	#N/A	3.8497	2.8987	4.6989	4.3146	3.7843	3.8396	3.8874	4.4194	3.6157	4.0945	0.9734	-0.0389	0.7596	0.1194
Gda	_AVM[Oxidation (M)]VSNVLLINK_.2	#N/A	4.4590	3.3152	3.7608	3.3046	3.1709	3.4537	3.4529	3.6966	5.4163	2.9819	0.9204	-0.1197	0.5412	0.2666
Gda	_ASDSPIDLFYGDFVGDISEAVIQK_.3	#N/A	3.1049	3.9101	3.8683	5.2546	3.7314	4.3335	4.3589	3.8241	3.4727	4.1968	1.0178	0.0255	0.8636	0.0637
Gda	_IGLGTDVAGGYSYSM[Oxidation (M)]LDAIR_.2	#N/A	3.4516	4.0632	3.8673	4.3700	3.8070	3.7691	4.0847	3.7485	3.3971	3.8088	1.0341	0.0484	0.5247	0.2801
Gda	_THDLYIQSHISENREEIEAVK_.4	#N/A	3.3334	4.1386	4.4383	3.9820	3.9600	3.7695	4.1303	3.0191	3.3182	3.6593	1.1147	0.1567	0.1677	0.7755
Foxo1	_ASLQSGQEGPGDS[Phospho (STY)]PGSQFSK_.2	Q9R1E0_S284_M1_Foxo1	4.7635	3.4791	5.8676	5.6493	5.1481	5.2650	5.3009	5.0888	5.5918	5.7057	0.9275	-0.1086	0.4066	0.3908
Cetn2	_M[Oxidation (M)]S[Phospho (STY)]PKPELTEDQKQEIR_.3	Q9R1K9_S20_M1_Cetn2	3.2223	5.0770	4.4568	4.0005	3.8668	4.2586	5.6861	3.0006	5.4404	4.5291	0.8891	-0.1695	0.3952	0.4031
Mast1	_SRS[Phospho (STY)]LS[Phospho (STY)]PGR_.2	Q9R1L5_S161_M2_Mast1	10.6758	10.5579	10.8000	10.6588	9.7036	10.5436	10.8813	10.8545	10.7000	10.6333	0.9742	-0.0376	0.2185	0.6606
Mast1	_HFGS[Phospho (STY)]TESITDEDGGRR_.3	#N/A	3.5924	4.3487	3.5643	4.0669	4.2370	3.8884	3.7816	4.0516	6.8762	4.0339	0.8429	-0.2466	0.2568	0.5904
Mast1	_LEEQDS[Phospho (STY)]GGSNTPEQDDTSEGR_.2	Q9R1L5_S346_M1_Mast1	5.0953	5.0519	3.1922	4.8263	3.2018	4.0243	5.2595	5.1040	4.1803	4.3004	0.8983	-0.1547	0.3689	0.4330
Mast1	_GGS[Phospho (STY)]PEIKR_.2	Q9R1L5_S772_M1_Mast1	4.8431	4.8883	6.1070	4.1689	3.7732	4.7626	5.5468	5.3057	4.7665	3.8461	0.9776	-0.0327	0.8338	0.0789
Mast1	_DSS[Phospho (STY)]PSRDYSPAVSGLR_.3	Q9R1L5_S954_M1_Mast1	4.1597	3.1827	3.8934	3.4372	3.3034	3.2246	3.5854	3.5640	3.8631	3.1144	1.0005	0.0007	0.9943	0.0025
Mast1	_AALS[Phospho (STY)]PVQEHETGRR_.3	#N/A	5.3286	5.3837	5.9753	5.0160	3.1127	3.5122	5.6452	5.8827	5.7438	5.7379	0.8208	-0.2850	0.1154	0.9377
Mast1	_AALS[Phospho (STY)]PVQEHETGR_.2	#N/A	4.5332	5.1582	5.1619	4.1485	4.2272	5.1514	4.5970	5.6159	3.8354	4.6231	1.0133	0.0191	0.8740	0.0585
Mast1	_HFGS[Phospho (STY)]TESITDEDGGR_.2	#N/A	3.8478	3.1521	4.5346	3.5364	2.9735	3.2552	3.2511	3.0813	4.2307	3.5034	1.0095	0.0136	0.9273	0.0328
Mast1	_S[Phospho (STY)]PITIQR_.2	Q9R1L5_S968_M1_Mast1	3.7564	7.6965	4.4432	3.4450	2.8821	3.3466	3.1597	3.1726	7.8413	8.0112	0.7684	-0.3801	0.3889	0.4102

Mast1	_FSASEASFLEGEAS[Phospho (STY)]PPLGAR_.2	Q9R1L5_S791_M1_Mast1	3.1842	4.1582	4.4187	7.1497	3.8288	4.2967	4.1108	7.3034	3.3377	3.6398	0.9800	-0.0291	0.9295	0.0318
Psma4	_TTIFSPEGR_.2	#N/A	7.3352	7.4522	7.7170	7.4916	7.6065	7.5915	7.1323	7.5430	7.3775	7.5840	1.0166	0.0238	0.2793	0.5539
Psma4	_LLDEVFFSEK_.2	#N/A	5.9347	5.6164	6.5701	6.2775	5.8225	6.3014	5.9944	6.1315	5.8716	6.1488	1.0084	0.0120	0.7953	0.0995
Psma4	_QAYTQFGGK_.2	#N/A	4.0359	3.3065	3.7696	3.3133	2.6695	3.1008	3.4616	3.6878	3.0093	5.2386	0.8744	-0.1936	0.3197	0.4953
Psmb3	_LYIGLAGLATDVQTVAQR_.3	#N/A	4.3448	3.6596	3.7866	4.8055	4.0522	4.2503	4.0039	4.2472	3.5689	4.2998	1.0298	0.0423	0.6434	0.1915
Psmb3	_DAVSGM[Oxidation (M)]GVIVHVIEK_.3	#N/A	2.9555	3.7118	4.1901	3.2030	3.6001	1.4482	4.4185	3.4147	3.0633	3.1700	0.9056	-0.1430	0.5587	0.2528
Psmb2	_FILNLPTFSVR_.2	#N/A	6.4103	6.4871	6.3059	6.3590	6.6650	6.3206	6.7476	6.8363	6.7474	6.5569	0.9558	-0.0653	0.0072	2.1413
Psmb2	_YYTPTISR_.2	#N/A	6.6299	6.8907	3.7040	7.2910	6.8433	6.6391	6.5670	6.7786	6.7805	7.3171	0.9231	-0.1155	0.4603	0.3370
Psmb2	_[Acetyl (Protein N-term)]M[Oxidation (M)]EYLIGIQGPDYVLVASDR_.2	#N/A	3.9619	3.3805	3.6956	3.2394	3.1056	4.2653	3.3876	3.8408	4.0609	4.4175	0.9188	-0.1221	0.2963	0.5283
Psma1	_NQYDNDVTVWSPQGR_.2	#N/A	7.8867	7.7870	8.0820	8.4097	7.8057	7.9590	8.0434	7.8228	7.7584	7.9524	1.0119	0.0171	0.4872	0.3123
Psma1	_ETLPAEQDLTTK_.2	#N/A	7.5615	7.3238	7.5952	7.6954	7.8449	7.5232	7.8163	7.2565	7.3873	7.8323	1.0023	0.0033	0.9078	0.0420
Psma1	_TQIPTQR_.2	#N/A	3.6442	6.7285	6.4215	6.4310	6.8366	6.2863	5.7438	6.2009	6.5437	6.6735	0.9630	-0.0543	0.7235	0.1406
Psma1	_AQPS[Phospho (STY)]QAAEEPAEK_.2	#N/A	3.8596	3.1150	3.2971	2.2770	3.0033	2.8433	3.5145	4.3187	3.3122	3.7668	0.8224	-0.2821	0.0702	1.1536
Psma1	_AQPS[Phospho (STY)]QAAEEPAEKADEPMEH_.3	#N/A	3.6098	3.3234	4.2966	3.5006	3.9322	3.3028	4.2055	3.3232	3.3308	3.3096	1.0335	0.0475	0.6647	0.1774
Psma1	_IHQIEYAMEAVK_.2	#N/A	3.2656	4.0768	4.5001	4.0439	3.8982	3.8313	4.1921	4.4581	3.2564	3.7211	1.0074	0.0107	0.9237	0.0345
Tagln3	_GFS[Phospho (STY)]EEQLR_.2	#N/A	8.7825	8.1756	8.7840	8.6443	8.0704	8.6967	7.9041	8.6310	8.8384	9.0040	0.9920	-0.0116	0.7911	0.1018
Tagln3	_LINSLYPPGQEPIPK_.2	#N/A	9.4516	9.3059	9.7535	9.3590	9.8625	9.2381	9.4584	9.4479	8.1674	9.5595	1.0368	0.0521	0.2825	0.5489
Tagln3	_RGFS[Phospho (STY)]EEQLR_.2	#N/A	8.7997	8.5804	9.1973	9.2049	7.3583	8.7332	9.0307	8.7487	8.6762	8.9806	0.9759	-0.0352	0.5628	0.2497
Tagln3	_GEPSWFHR_.2	#N/A	7.7133	7.6226	7.2068	8.2740	8.0040	8.1717	7.7669	7.8247	8.2209	8.3499	0.9741	-0.0379	0.3962	0.4021
Tagln3	_TTDIFQTVDLWEGK_.2	#N/A	7.2949	6.9853	8.2020	7.6133	7.3409	7.3902	7.4715	7.2804	7.5707	7.1445	1.0142	0.0203	0.6523	0.1855
Tagln3	_GPSYGLSR_.2	#N/A	7.1870	7.2790	6.5097	7.4318	7.8213	7.2865	6.3933	7.3985	7.3318	7.2058	1.0240	0.0343	0.5676	0.2459
Tagln3	_TLM[Oxidation (M)]ALGSVAVTK_.2	#N/A	5.9440	6.5279	6.0271	6.1620	6.7933	6.4162	5.8541	6.4548	6.7241	6.3022	0.9965	-0.0050	0.9225	0.0350
Tagln3	_AAEVYGVR_.2	#N/A	6.7366	6.5325	7.0633	6.7631	7.5543	6.9726	5.2362	7.1557	7.0839	7.1327	1.0428	0.0605	0.5118	0.2909
Tagln3	_WLM[Oxidation (M)]DGTVLC[Carbamidomethyl (C)]K_.2	#N/A	6.1756	6.9419	3.6709	6.6184	6.7350	6.7863	6.6293	7.0373	6.9737	6.8281	0.8963	-0.1580	0.2972	0.5269
Tagln3	_LVDWIILQC[Carbamidomethyl (C)]AEDIEHPPPGR_.3	#N/A	5.4303	5.2563	3.5517	5.6278	5.1340	3.9385	5.5157	5.1388	4.9689	5.7962	0.9007	-0.1509	0.2839	0.5468
Tagln3	_QM[Oxidation (M)]EQISQFLK_.2	#N/A	6.0916	6.5457	6.8495	6.7087	7.1439	6.7075	6.3996	6.7056	6.8740	6.5630	1.0059	0.0084	0.8470	0.0721
Tagln3	_QGQNVIGLQMGSNK_.2	#N/A	3.1267	4.4693	3.8135	6.1866	3.7533	3.9762	4.3370	3.8023	3.1115	4.2832	1.0869	0.1202	0.5789	0.2374
Atp6ap1	_FY[Phospho (STY)]PVSAR_.2	Q9R1Q9_Y326_M1_Atp6ap1	7.5853	7.1850	7.9667	7.6034	6.4660	7.0402	7.6546	7.6052	7.5110	7.5221	0.9649	-0.0515	0.3556	0.4490
Atp6ap1	_Y[Phospho (STY)]WFAMER_.2	Q9R1Q9_Y332_M1_Atp6ap1	4.1561	3.4115	4.5014	5.0846	3.2998	3.3248	3.2179	3.1144	4.2638	3.4702	1.1270	0.1724	0.3294	0.4823
Trim3	_S[Phospho (STY)]PGGPGSHVR_.2	#N/A	9.4883	9.5754	9.7708	9.9557	9.0084	9.5955	9.6542	9.5388	9.5170	9.5799	0.9993	-0.0010	0.9683	0.0140
Trim3	_TGS[Phospho (STY)]AELC[Carbamidomethyl (C)]AEITGPDGVR_.2	Q9R1R2_S359_M1_Trim3	3.7518	3.8907	6.7284	7.1400	6.0698	6.1074	3.7801	3.3738	3.6729	6.5965	1.2890	0.3663	0.2207	0.6562
Trim3	_EDS[Phospho (STY)]PGPEVQPMDK_.2	#N/A	8.1995	6.7811	4.0188	9.3182	6.7646	3.3500	7.6563	3.4387	6.5381	7.1402	1.0342	0.0486	0.8835	0.0538
Trim3	_ALRPGDLPPS[Phospho (STY)]PDDVKR_.3	#N/A	4.0275	7.0561	3.2134	8.6821	5.5842	6.2103	7.6299	8.3257	6.8843	7.6150	0.7612	-0.3936	0.1201	0.9204
Trim3	_VKS[Phospho (STY)]PGGPGSHVR_.2	#N/A	8.5561	8.7232	8.9643	9.0993	8.1887	8.7069	8.7152	8.5856	8.5340	8.6918	1.0087	0.0124	0.6659	0.1766
Trim3	_REDS[Phospho (STY)]PGPEVQPMDK_.2	#N/A	7.3509	6.5796	4.5979	9.0579	6.1013	3.3629	6.6552	5.3146	5.5996	6.3879	1.0310	0.0441	0.8649	0.0630
Trim3	_ALRPGDLPPS[Phospho (STY)]PDDVK_.2	#N/A	3.7667	3.0453	4.0259	3.6028	2.8791	3.5314	5.6865	5.4762	5.4598	3.5698	0.6884	-0.5386	0.0082	2.0880
Septin6	_RNEFLGELQK_.2	#N/A	7.2796	6.7525	7.1370	6.8970	7.1119	7.0133	7.1989	7.0360	7.1018	6.9561	0.9942	-0.0085	0.7011	0.1542
Septin6	_SDAISKS[Phospho (STY)]ELAK_.2	Q9R1T4_S192_M1_Septin6	6.8440	3.3234	7.5600	7.5246	6.6493	7.3773	7.4558	7.0761	7.1100	6.8381	0.9194	-0.1212	0.5111	0.2915
Septin6	_EAELKEAEKELHEK_.3	#N/A	6.8101	6.0369	7.3699	6.5564	6.5451	7.1748	7.5111	6.2934	6.7796	5.8997	1.0193	0.0276	0.7372	0.1324
Septin6	_NEFLGELQK_.2	#N/A	6.6392	5.9333	5.5147	6.2605	6.2010	6.1749	6.4142	6.2795	5.8424	6.1891	0.9902	-0.0142	0.7840	0.1057
Septin6	_AAAELLQSQGSQAGGS[Phospho (STY)]QTLK_.2	Q9R1T4_S416_M1_Septin6	4.7832	5.3145	3.5377	5.6702	4.3043	4.9567	4.5197	4.3549	4.8462	4.7843	1.0291	0.0414	0.7433	0.1288
Septin6	_STLM[Oxidation (M)]DTLFNTK_.2	#N/A	6.7653	6.1199	6.1507	6.9815	6.1288	6.5625	6.4826	5.9999	5.9912	6.5318	1.0320	0.0455	0.3968	0.4014
Septin6	_VNMEDLR_.2	#N/A	6.1911	4.7122	3.4901	6.3605	5.8478	3.7245	3.6176	3.4013	4.2391	4.1514	1.3120	0.3918	0.1077	0.9677
Septin6	_SVSQGFC[Carbamidomethyl (C)]FNILC[Carbamidomethyl (C)]VGETGLGK_.2	#N/A	4.7672	3.0436	4.0325	6.2617	4.4262	4.6939	5.9090	4.7541	4.7956	5.7738	0.8548	-0.2263	0.2282	0.6416
Septin6	_TVPLAGHVGFDSLPDQLVNK_.3	#N/A	6.9729	7.3870	7.3714	7.4788	6.1938	5.8417	6.5683	7.2001	6.8201	6.6642	1.0090	0.0129	0.8734	0.0588
Septin6	_DTDPDSKPFSLQETYEAK_.3	#N/A	4.5865	4.3655	3.6489	5.0001	4.6684	4.4582	3.5097	4.1945	4.1902	5.0160	1.0537	0.0755	0.5174	0.2862
Septin6	_QVGEDC[Carbamidomethyl (C)]R_.2	#N/A	2.9499	4.1203	3.5674	3.2086	5.2932	3.0302	4.4242	3.4090	3.0576	3.4054	1.0338	0.0480	0.8192	0.0866
Adam22	_SNS[Phospho (STY)]TETLSPAK_.2	Q9R1V6_S855_M1_Adam22	10.0385	10.1152	10.4366	10.3049	9.7026	9.9390	10.5766	10.3678	10.1077	10.2822	0.9764	-0.0345	0.1520	0.8182
Adam22	_SPSSS[Phospho (STY)]TGSIASSR_.2	Q9R1V6_S868_M1_Adam22	8.8755	9.0965	9.4640	9.2437	8.7715	3.8306	9.6605	8.5634	8.9816	7.5613	0.9450	-0.0816	0.6902	0.1610
Adam22	_FAISENPLITLR_.2	#N/A	5.7374	4.9579	4.9362	5.0090	5.1061	4.4292	6.0773	5.8599	5.2231	5.0308	0.9065	-0.1416	0.1132	0.9461
Adam22	_FQPLGTVC[Carbamidomethyl (C)]R_.2	#N/A	6.6246	4.2612	4.3158	6.4980	6.4046	6.5729	6.3424	6.4746	3.4407	6.0411	1.0367	0.0520	0.8088	0.0922
Adam22	_KLAS[Phospho (STY)]GEC[Carbamidomethyl (C)]K_.2	Q9R1V6_S387_M1_Adam22	6.5348	6.4845	6.1949	6.3683	4.0255	4.4099	6.8951	6.3205	6.2986	3.0546	1.0049	0.0070	0.9765	0.0103
Adam22	_S[Phospho (STY)]NSTETLSPAKS[Phospho (STY)]PSSSTGSIASSR_.3	Q9R1V6_S853_M2_Adam22	3.6793	3.8544	3.7579	4.0299	3.8274	3.7060	5.5428	3.9203	3.6658	3.3905	0.9223	-0.1166	0.4319	0.3646
Adam22	_DVLC[Carbamidomethyl (C)]GYLLC[Carbamidomethyl (C)]TNIGNIPR_.2	#N/A	3.0744	4.2680	4.3089	3.8526	2.8037	3.6401	3.9837	3.3235	3.4476	3.5299	1.0243	0.0346	0.8021	0.0958
Adam22	_LSVVYTNTYAK_.2	#N/A	3.7836	3.1647	3.5173	5.2594	4.6025	4.0550	3.5904	5.0605	3.8913	5.2500	0.9136	-0.1304	0.4717	0.3264
Adam22	_SNS[Phospho (STY)]WQGNMGGNK_.2	#N/A	11.8630	10.5050	8.8241	12.5664	10.1484	7.4446	11.3248	9.9910	10.1528	10.6374	0.9714	-0.0419	0.7698	0.1136
Adam22	_SNGLSHS[Phospho (STY)]WSERIPDTK_.3	#N/A	3.0121	3.8173	5.4997	5.8765	3.6387	4.0908	4.4516	4.1987	2.9969	3.9806	1.1064	0.1458	0.5251	0.2798
Adam22	_SNS[Phospho (STY)]WQGNM[Oxidation (M)]GGNKK_.2	#N/A	8.4825	8.1534	8.6152	9.1085	7.4342	3.4079	8.7416	8.4579	8.0428	8.1368	0.9028	-0.1475	0.4729	0.3252
Adam22	_ADAVHLFSGSQFESSR_.3	#N/A	3.0150	3.6220	3.7018	4.2045	3.6415	4.0879	3.9192	3.9140	3.5626	4.1185	0.9571	-0.0633	0.4956	0.3049
Adam22	_SNGLSHS[Phospho (STY)]WSER_.2	#N/A	8.2773	7.2910	8.1080	7.2823	5.9120	7.7118	7.4567	7.1664	8.0749	6.8025	1.0075	0.0108	0.9116	0.0402
Adam23	_DNQC[Carbamidomethyl (C)]QYIWGTK_.2	#N/A	6.4756	5.8692	6.1893	6.2551	5.6403	5.7155	6.7377	6.0216	5.9563	6.1111	0.9706	-0.0431	0.4331	0.3634
Adam23	_QDGYSC[Carbamidomethyl (C)]NQNQGR_.2	#N/A	3.3447	3.9977	4.5792	4.1229	3.9892	3.9104	4.2712	4.3791	3.1773	3.8002	1.0214	0.0306	0.7847	0.1053
Abcc5	_S[Phospho (STY)]LEC[Carbamidomethyl (C)]QDALETAAR_.2	Q9R1X5_S43_M1_Abcc5	6.7788	6.7622	5.7412	6.4487	5.3423	6.1761	7.2889	6.4837	6.9065	6.9043	0.9003	-0.1515	0.0649	1.1881
Abcc5	_NATLAWDSSHSSIQNS[Phospho (STY)]PKLT[Phospho (STY)]PK_.3	Q9R1X5_S509_M2_Abcc5	3.4022	4.2074	4.0890	3.9133	4.0287	3.6779	4.9079	6.4443	3.3869	3.5906	0.8481	-0.2377	0.2634	0.5794
Abcc5	_T[Phospho (STY)]RSLEC[Carbamidomethyl (C)]QDALETAAR_.3	Q9R1X5_T41_M1_Abcc5	4.0388	3.2942	3.7724	3.3162	3.1825	3.4421	3.4645	3.6850	3.9840	2.9935	0.9932	-0.0098	0.9213	0.0356
Pts	_LHS[Phospho (STY)]PSLSDEENLR_.3	Q9R1Z7_S27_M1_Pts	4.3662	5.4375	3.5522	4.0548	5.9966	6.6163	3.0976	5.7626	6.1400	6.3803	0.9362	-0.0952	0.7011	0.1542
Pts	_VFETDNNIVVYKGE_.2	#N/A	3.2400	4.9669	4.5318	4.0755	5.9128	5.1693	4.2238	4.4265	5.1669	3.6710	0.9011	-0.1502	0.4346	0.3619
Tfeb	_RS[Phospho (STY)]SFSM[Oxidation (M)]EEGDVL_.2	Q9R210_S465_M1_Tfeb	3.7526	3.0080	6.4156	5.9372	5.2104	3.7283	6.4037	5.7550	6.1610	4.2081	0.8301	-0.2686	0.2672	0.5732
Khdrbs3	_FLIPDYNDEIR_.2	#N/A	5.6164	5.7290	4.3330	6.3971	5.8201	6.3219	5.3917	3.1244	3.4234	3.5541	1.4723	0.5581	0.0110	1.9583
Hebp1	_FATVEVTDKPVDEALR_.3	#N/A	5.7415	6.5185	6.0803	6.2068	6.8364	6.6529	6.1207	5.8224	6.4034	6.7337	1.0111	0.0159	0.7947	0.0998
Hebp1	_IPNQFQGS[Phospho (STY)]PPAPSDESVK_.2	#N/A	6.8178	5.8471	3.2620	7.5598	4.1728	6.4144	6.8330	4.1955	2.9937	3.9838	1.2616	0.3352	0.2990	0.5243
Hebp1	_IPNQFQGS[Phospho (STY)]PPAPSDESVKIEER_.3	#N/A	3.0335	3.8447	4.0045	4.0436	3.6980	3.5885	3.9483	8.3217	3.2564	3.9011	0.7623	-0.3917	0.2546	0.5942
Hebp1	_EADYVAHATQLR_.2	#N/A	4.9646	6.1787	5.7367	5.6736	6.0229	5.9708	5.7211	5.3225	5.6126	5.7378	1.0285	0.0405	0.5129	0.2900
Myo1c	_HLGY[Phospho (STY)]KPEEY[Phospho (STY)]K_.2	Q9WTI7_Y703_M2_Myo1c	6.0393	6.5518	7.2415	6.8649	6.8053	7.2428	6.9744	6.4533	6.3306	6.5443	1.0327	0.0465	0.4269	0.3697
Myo1c	_DAES[Phospho (STY)]PSWR_.2	Q9WTI7_S408_M1_Myo1c	3.5382	3.6880	4.2250	3.2268	3.5763	3.0484	4.4423	3.3908	3.0394	3.1938	1.0096	0.0138	0.9198	0.0363
Stk11	_IDS[Phospho (STY)]TEVIYQPR_.2	Q9WTK7_S31_M1_Stk11	11.6317	11.5990	11.9441	11.9166	10.7982	11.8367	11.9186	11.3692	11.7886	11.8897	0.9897	-0.0149	0.6298	0.2008
Lypla2	_DLAILQC[Carbamidomethyl (C)]HGELDPM[Oxidation (M)]VPVR_.3	#N/A	3.7529	4.8991	5.0123	4.9455	4.8114	5.1146	4.6214	4.2392	4.9627	5.0844	1.0061	0.0088	0.9245	0.0341
Cdh22	_GPSS[Phospho (STY)]PEPDFSVFR_.2	Q9WTP5_S724_M1_Cdh22	3.3079	4.0641	4.5424	7.2630	3.9524	4.1730	4.2344	4.4159	3.2141	3.7634	1.1647	0.2200	0.4097	0.3876
Ak3	_TVGIDDLTGEPLIQR_.2	#N/A	6.4346	6.5524	5.8941	6.7866	6.7084	6.9172	6.5565	6.3379	6.4636	6.5334	1.0117	0.0168	0.6985	0.1558

Ak3	_HLSSGDLLR_.2	#N/A	5.2464	4.7956	3.5641	5.3414	6.1273	5.1031	3.5944	5.4996	4.7931	5.3906	1.0436	0.0616	0.7132	0.1468
Ak3	_EDDKPETVIKR_.3	#N/A	4.7474	4.0800	3.4922	4.5368	4.9050	3.9109	2.1536	4.1064	4.6807	4.2141	1.1293	0.1755	0.3750	0.4260
Ak3	_TLTQC[Carbamidomethyl (C)]SWLLDGFPR_.2	#N/A	5.6624	4.8956	3.9455	4.0568	4.6959	3.8442	4.2050	4.4035	3.7532	3.7340	1.1225	0.1667	0.2339	0.6310
Ak3	_GVLETFSGTETNK_.2	#N/A	3.5304	4.5853	3.2641	4.3087	5.5990	5.6412	4.4570	4.1933	2.9915	5.6375	1.0390	0.0551	0.8073	0.0930
Akap12	_EGITPWAS[Phospho (STY)]FK_.2	#N/A	7.4094	7.0418	6.5998	7.1709	5.2367	3.5084	7.4263	6.4110	6.3816	4.4280	0.9999	-0.0001	0.9995	0.0002
Akap12	_GPSEAPQEAEAEEGAT[Phospho (STY)]SDGEKKR_.3	Q9WTQ5_T583_M1_Akap12	4.8930	8.8966	9.1919	8.3398	7.7925	7.6078	7.3483	8.1785	5.1090	7.8724	1.2505	0.3225	0.2155	0.6665
Akap12	_GPSEAPQEAEAEEGATS[Phospho (STY)]DGEK_.2	#N/A	7.3043	7.3333	3.5569	7.9405	7.2296	7.3317	7.3997	6.9638	7.0354	7.1970	0.9488	-0.0759	0.6673	0.1757
Akap12	_AEADASGNLTKES[Phospho (STY)]PDTNGPK_.2	#N/A	5.7533	6.1959	5.6372	5.4818	5.0974	6.1367	5.7749	5.0522	5.0078	5.9827	1.0482	0.0679	0.3888	0.4102
Akap12	_EGITPWAS[Phospho (STY)]FKK_.2	#N/A	4.1237	8.2312	8.5260	8.6696	3.2674	3.3572	8.6601	8.0745	7.5263	8.2903	0.7409	-0.4327	0.1679	0.7750
Akap12	_EVC[Carbamidomethyl (C)]VSGGDHTQLTDLS[Phospho (STY)]PEEK_.3	#N/A	3.5754	3.8265	3.7001	7.5424	3.5349	5.8144	3.7065	6.8108	6.0871	4.7598	0.8735	-0.1951	0.5208	0.2833
Akap12	_EKEPTKPLES[Phospho (STY)]PTS[Phospho (STY)]PVSNETTSSFK_.3	#N/A	3.3078	4.2487	3.9946	7.1704	3.9344	3.8465	3.9531	4.6099	3.5121	3.8910	0.9945	-0.0080	0.9785	0.0094
Akap12	_VELPLEDQVGDLEALS[Phospho (STY)]EK_.2	#N/A	4.3507	4.3763	3.5367	6.1455	5.5280	4.2531	3.7540	5.5445	5.6957	4.0063	0.9891	-0.0158	0.9366	0.0285
Akap12	_EPTKPLES[Phospho (STY)]PTS[Phospho (STY)]PVSNETTSSFKK_.3	#N/A	3.1074	3.8637	4.3419	3.8857	3.7520	4.3735	4.0340	3.1155	3.4145	3.5630	1.1007	0.1384	0.2405	0.6189
Akap12	_SATLSSTESTAS[Phospho (STY)]GM[Oxidation (M)]QDEVR_.2	#N/A	3.3952	3.7626	4.0820	6.8144	6.5701	6.6129	4.0232	6.0314	6.0894	3.5951	1.0550	0.0772	0.7876	0.1037
Akap12	_EPT[Phospho (STY)]KPLES[Phospho (STY)]PTSPVSNETTSSFK_.3	Q9WTQ5_T265_M2_Akap12	3.6174	3.7098	4.1295	3.9818	3.6558	3.7855	3.9733	3.9746	3.5206	3.7810	1.0002	0.0003	0.9947	0.0023
Akap12	_LS[Phospho (STY)]ADYEKVELPLEDQVGDLEALSEK_.3	Q9WTQ5_S350_M1_Akap12	4.0581	3.3428	3.4926	3.6822	3.1912	4.0386	4.2175	4.0464	4.0383	4.0941	0.8866	-0.1736	0.0373	1.4280
Akap12	_C[Carbamidomethyl (C)]QETESNEEQSIS[Phospho (STY)]PEKR_.2	#N/A	3.6188	3.5886	3.7812	4.6781	3.4060	3.4910	4.2936	4.2424	4.6763	3.7963	0.8844	-0.1772	0.1146	0.9407
Akap12	_TKEVC[Carbamidomethyl (C)]VSGGDHTQLTDLS[Phospho (STY)]PEEK_.3	#N/A	4.0145	3.3279	3.7481	3.2919	3.1493	3.0794	3.4402	3.7093	4.0083	2.9692	0.9727	-0.0400	0.7158	0.1452
Akap12	_AEDSGAEQLASEIEPSREESWVS[Phospho (STY)]IKK_.4	#N/A	3.3457	3.7837	3.9113	4.7277	3.6913	4.2435	4.2723	3.7408	3.2080	3.8013	1.0519	0.0730	0.5333	0.2731
Akap12	_HPEGIVSEVEM[Oxidation (M)]LSS[Phospho (STY)]QER_.3	#N/A	3.7575	3.6409	3.9234	4.0574	3.5292	3.9488	3.8419	3.6170	3.6324	3.0149	1.0802	0.1113	0.1434	0.8434
Akap12	_RPS[Phospho (STY)]ES[Phospho (STY)]DKEELDK_.2	Q9WTQ5_S613_M2_Akap12	4.3506	3.6549	3.5365	4.0392	3.4762	4.2532	3.7539	4.0793	3.7279	4.0062	0.9983	-0.0025	0.9748	0.0111
Trpv2	_LETS[Phospho (STY)]DGDEEGSAEVNK_.2	Q9WTR1_S15_M1_Trpv2	8.4352	8.4258	8.7338	8.5339	7.1321	8.0934	9.1178	8.8143	8.2988	8.3576	0.9513	-0.0721	0.2388	0.6219
Trpv2	_NFS[Phospho (STY)]PQIK_.2	Q9WTR1_S47_M1_Trpv2	6.1184	5.6476	3.8870	4.9390	3.8268	5.6473	5.7167	5.9548	5.3608	5.4748	0.8906	-0.1672	0.2584	0.5877
Trpv2	_NSAS[Phospho (STY)]EEDHLPLQVLQSH_.3	Q9WTR1_S743_M1_Trpv2	4.1168	3.4212	3.3028	4.5048	3.2425	2.9861	3.3469	3.8800	3.9616	3.7724	0.9614	-0.0569	0.6610	0.1798
Cdh13	_INENTGSVSVTR_.2	#N/A	4.5563	3.8409	4.7332	3.5336	7.3811	4.6188	3.6818	7.3334	4.6853	5.1437	1.1082	0.1482	0.5522	0.2579
Cdh13	_ETIATYQLYVETTDASGK_.2	#N/A	3.6302	6.2982	3.3638	4.4085	4.2747	6.1883	7.1406	4.0936	4.3926	4.0857	0.9525	-0.0702	0.7948	0.0998
Tenm1	_ANS[Phospho (STY)]ALS[Phospho (STY)]LTDTDHER_.2	Q9WTS4_S142_M2_Tenm1	3.2471	4.0953	4.4816	4.0254	3.9166	3.8129	4.0178	2.9758	3.2748	3.7027	1.1251	0.1701	0.1511	0.8207
Atp6v1g2	_[Acetyl (Protein N-term)]ASQTQGIQQLLQAEK_.2	#N/A	8.6272	8.3086	8.4753	8.6692	8.5935	8.2940	8.6201	8.0969	8.2625	8.4247	1.0172	0.0246	0.2711	0.5669
Atp6v1g2	_[Acetyl (Protein N-term)]ASQTQGIQQLLQAEKR_.3	#N/A	6.8517	7.2611	7.4109	7.3360	7.0200	7.0721	6.9444	6.7965	6.9068	7.0463	1.0340	0.0482	0.0773	1.1120
Atp6v1g2	_EREQEFQSK_.2	#N/A	6.3391	6.6596	7.1020	6.4692	6.8478	6.4472	5.5958	6.2913	5.7588	6.5566	1.0981	0.1350	0.0328	1.4835
Atp6v1g2	_VLAQLLGM[Oxidation (M)]VC[Carbamidomethyl (C)]EVRPQVHPNYR_.3	#N/A	6.3507	5.7427	6.7014	6.4420	5.6334	5.8686	6.1029	6.0748	6.1154	6.0256	1.0071	0.0103	0.8493	0.0709
Atp6v1g2	_QVQGM[Oxidation (M)]QSSQQR_.2	#N/A	5.4410	3.8180	4.2888	5.5334	5.6514	5.3639	4.0233	4.2913	5.0000	3.8809	1.1668	0.2226	0.1400	0.8540
Atp6v1g2	_EEAQM[Oxidation (M)]EVEQYRR_.3	#N/A	6.5840	6.7084	7.4398	6.3556	8.0557	7.4391	6.3174	7.4125	6.6346	7.0749	1.0346	0.0490	0.5527	0.2575
Atp6v1g2	_QQAAM[Oxidation (M)]GS[Phospho (STY)]QGNLSAEVEQATR_.2	#N/A	3.8263	4.3935	3.6249	5.1427	3.9323	4.6953	3.9837	4.1614	4.5472	4.4928	0.9937	-0.0091	0.9335	0.0299
Atp6v1g2	_QQAAM[Oxidation (M)]GSQGNLSAEVEQATRR_.3	#N/A	4.2608	4.2846	5.0558	3.9457	4.5554	4.1682	3.2120	4.5629	4.7734	3.8730	1.0665	0.0929	0.4482	0.3485
Atp6v1g2	_EEAQMEVEQYR_.2	#N/A	3.7601	3.5823	3.4937	4.5383	3.4037	4.3258	3.1858	3.9637	4.2627	4.2156	0.9856	-0.0209	0.8607	0.0652
Phf2	_RPSASS[Phospho (STY)]PNNTAAK_.2	Q9WTU0_S1060_M1_Phf2	3.9322	3.1877	3.6659	3.2097	3.0760	3.5487	3.4418	3.7915	4.0906	4.3878	0.8749	-0.1928	0.0686	1.1639
Phf2	_DSDYVYPSLES[Phospho (STY)]DEDNPVFK_.2	Q9WTU0_S876_M1_Phf2	3.5910	3.8061	3.7375	3.8525	3.4442	3.6006	3.7236	3.8002	5.1373	4.3351	0.8642	-0.2106	0.0645	1.1905
Phf2	_RKGS[Phospho (STY)]DDAPYSPTAR_.3	Q9WTU0_S893_M1_Phf2	3.2802	4.0854	3.9670	4.0352	3.9068	3.8227	4.1835	3.6488	4.6041	4.4367	0.9126	-0.1320	0.1367	0.8641
Scn8a	_ELS[Phospho (STY)]EGEEKGDPEK_.2	Q9WTU3_S504_M1_Scn8a	2.9932	6.1028	3.6800	6.5033	3.6197	5.2148	6.6921	6.3547	6.4384	5.8548	0.7396	-0.4351	0.0607	1.2168
Scn8a	_VFKS[Phospho (STY)]ES[Phospho (STY)]EDGMR_.2	#N/A	4.9382	3.5453	3.4269	5.8892	3.3667	4.3628	3.6443	4.1889	3.8375	3.8966	1.0933	0.1287	0.5083	0.2938
Scn8a	_FRDPGSENEFADDEHS[Phospho (STY)]T[Phospho (STY)]VEESEGRR_.4	Q9WTU3_S589_M2_Scn8a	4.0682	3.1580	3.2541	3.7568	3.0463	5.0595	3.4715	4.3617	4.0103	3.7238	0.9568	-0.0637	0.6978	0.1563
Scn8a	_VFKS[Phospho (STY)]ES[Phospho (STY)]EDGMRR_.3	#N/A	3.3530	3.8732	4.0398	4.5425	3.9795	3.7500	4.2571	3.5760	3.2246	3.0086	1.1156	0.1578	0.2035	0.6915
Mapk9	_NYVENRPK_.2	#N/A	5.6012	3.1623	3.2498	5.2819	5.6068	5.2519	5.4851	5.7345	5.2879	5.7363	0.8438	-0.2450	0.1850	0.7329
Mapk9	[illegible][Carbamidomethyl (C)]TNFM[Oxidation (M)]M[Oxidation (M)]T[Phospho (STY)]PY[Phospho (STY)]VV[illegible]	#N/A	4.1992	3.1776	3.7885	5.5567	3.3375	5.1618	3.6044	5.4680	3.8580	3.7499	1.0080	0.0115	0.9571	0.0190
Mapk9	_DYIDQWNK_.2	#N/A	3.8764	3.1808	3.8953	3.4391	3.0021	3.2266	3.5874	3.5621	3.8611	3.1164	0.9731	-0.0394	0.6862	0.1636
Prkra	_EDS[Phospho (STY)]GTFSLGK_.2	#N/A	9.8878	9.3707	10.0117	9.9603	8.9317	9.6973	9.5244	9.4592	9.7138	9.8277	1.0012	0.0018	0.9587	0.0183
Prkra	_AEAPPLQREDS[Phospho (STY)]GTFSLGK_.3	#N/A	5.6654	4.0377	4.1866	4.0148	3.9260	3.8022	4.1671	3.9508	5.9152	3.8057	0.9579	-0.0620	0.7303	0.1365
Prkra	_HRAEAPPLQREDS[Phospho (STY)]GTFSLGK_.4	#N/A	3.7588	6.5155	3.4925	8.3783	2.9025	3.7221	3.6152	3.9649	4.2640	4.2144	1.1944	0.2562	0.5012	0.3000
Skp1	_GKT[Phospho (STY)]PEEIRK_.2	#N/A	11.3845	11.6140	12.1956	12.1156	11.7864	11.7816	11.8637	11.5820	11.5881	11.7308	1.0104	0.0149	0.4791	0.3196
Skp1	_GKT[Phospho (STY)]PEEIR_.2	#N/A	9.6518	9.5191	9.9364	9.7207	9.6554	9.7257	9.7704	9.5510	9.6282	9.6790	1.0046	0.0066	0.5891	0.2298
Skp1	_TDDIPVWDQEFLK_.2	#N/A	8.4739	8.0762	8.0585	8.3251	8.6415	8.4288	8.3680	7.6958	8.1929	8.3318	1.0229	0.0327	0.3035	0.5179
Skp1	_T[Phospho (STY)]PEEIRK_.2	#N/A	7.2152	6.8828	7.2182	7.5795	6.8612	6.7297	7.1250	6.7325	6.7524	7.1402	1.0207	0.0296	0.4573	0.3398
Skp1	_RTDDIPVWDQEFLK_.3	#N/A	4.9363	6.8096	6.0465	7.3919	6.5367	4.9418	5.8073	6.1691	5.5791	5.0353	1.0819	0.1136	0.4219	0.3748
Skp1	_LQSSDGEIFEVDVEIAK_.2	#N/A	6.8339	6.8337	8.0386	8.0417	6.9278	7.4803	7.4827	6.3296	6.8980	7.4883	1.0439	0.0620	0.4246	0.3720
Ptpn4	_S[Phospho (STY)]PSKPLAR_.2	Q9WU22_S358_M1_Ptpn4	7.9581	6.9220	4.3992	7.5850	2.8381	7.4023	7.1727	7.7973	3.6971	7.4337	0.9477	-0.0775	0.7995	0.0972
Ptpn4	_NSS[Phospho (STY)]FTQEATR_.2	Q9WU22_S403_M1_Ptpn4	4.1342	6.4794	3.8679	6.8559	5.5200	6.9480	6.7212	4.4968	6.4166	3.0889	0.9918	-0.0119	0.9631	0.0163
Pfdn5	_IQPALQEK_.2	#N/A	5.3135	6.0025	5.7567	5.2260	6.6896	5.6336	5.0125	5.4549	5.7863	3.9445	1.1427	0.1925	0.1228	0.9106
Pfdn5	_NQLDQEVEFLSTSIAQLK_.3	#N/A	3.9808	3.9988	3.1759	3.6785	2.8427	3.5001	3.3932	4.4399	4.1805	3.6455	0.9069	-0.1410	0.2570	0.5900
Lemd3	_VLLGFSSDES[Phospho (STY)]DVEASPR_.2	Q9WU40_S140_M1_Lemd3	3.3706	3.4334	4.0574	3.9170	3.2548	3.8210	3.8254	3.6967	3.6408	3.7723	0.9755	-0.0358	0.6093	0.2152
Ncor2	_EGT[Phospho (STY)]PPPPPPPR_.2	Q9WU42_T1350_M1_Ncor2	9.2287	9.5198	9.4688	10.2037	8.8397	9.5059	9.8425	9.7404	9.4635	9.4649	0.9827	-0.0252	0.5075	0.2945
Ncor2	_S[Phospho (STY)]PGNTSQPPAFFSK_.2	Q9WU42_S2215_M1_Ncor2	8.6164	7.9587	7.7968	8.8595	7.4464	8.1574	8.8755	8.0382	8.2541	8.3970	0.9700	-0.0440	0.4287	0.3679
Ncor2	_SDHALTS[Phospho (STY)]PGGGGK_.2	Q9WU42_S2351_M1_Ncor2	8.0673	3.4353	8.7582	9.0535	8.2348	8.7337	8.6621	8.5568	8.4264	3.4465	1.0606	0.0849	0.7737	0.1114
Ncor2	_TDDT[Phospho (STY)]S[Phospho (STY)]GEDNDEKEAVASK_.2	#N/A	3.9270	3.2314	3.1130	5.0551	4.7641	3.1760	3.3303	4.8715	4.3769	3.9701	0.9373	-0.0934	0.6230	0.2055
Ncor2	_PASHTHQHS[Phospho (STY)]PIS[Phospho (STY)]PR_.3	Q9WU42_S1816_M2_Ncor2	3.5189	4.8339	4.5765	5.9661	3.9865	4.7712	5.2225	4.3818	5.0650	4.7084	0.9514	-0.0719	0.6162	0.2103
Ncor2	_LEPVS[Phospho (STY)]PPS[Phospho (STY)]PPHADPELELAPSR_.3	Q9WU42_S149_M2_Ncor2	3.0376	3.7939	4.2721	9.9997	3.6822	2.9425	3.9641	9.5132	3.4844	3.4932	0.9037	-0.1461	0.7908	0.1019
Ncor2	_EKTDDT[Phospho (STY)]S[Phospho (STY)]GEDNDEKEAVASK_.2	#N/A	4.1995	5.3988	3.6975	5.9573	3.2110	3.4448	3.6146	5.3363	3.8557	3.5310	1.0572	0.0803	0.7327	0.1351
Pdcd6ip	_LLDEEEATDNDLR_.2	#N/A	6.4368	6.0769	6.1723	6.2737	6.3015	6.3198	6.5647	5.8913	6.1623	6.5947	0.9937	-0.0091	0.7952	0.0995
Pdcd6ip	_STSVVEQGGIQTVDQLIK_.2	#N/A	3.3036	4.2512	5.5552	4.0330	3.5917	4.4626	4.6358	4.1006	4.1822	4.6414	0.9566	-0.0640	0.6611	0.1797
Pdcd6ip	_LANQAADYFGDAFK_.2	#N/A	3.8247	4.7531	4.5115	3.5133	3.3552	3.3348	4.1407	3.1044	4.5220	3.9936	0.9853	-0.0214	0.8859	0.0526
Pdcd6ip	_FYNELTEILVR_.2	#N/A	3.9958	4.3742	3.7295	2.4826	3.1395	3.4851	3.4215	4.5501	3.5506	4.4514	0.8851	-0.1761	0.2979	0.5259
Pdcd6ip	_NIQVSHQEFSK_.2	#N/A	1.1068	4.5600	4.5184	3.5203	2.9573	3.3418	3.2349	3.5550	4.2468	3.4873	0.9182	-0.1231	0.6692	0.1745
Pfkp	_[Acetyl (Protein N-term)]S[Phospho (STY)]DLDSSSSSAYPK_.2	Q9WUA3_S2_M1_Pfkp	8.0276	8.0128	8.1200	8.3080	7.4238	8.6457	8.9141	8.3752	8.3824	8.4214	0.9491	-0.0753	0.0961	1.0172
Pfkp	_DLLFQPVAELK_.2	#N/A	6.6825	6.0783	6.9982	6.1932	6.8146	6.3994	6.5058	6.8820	6.7992	6.3483	0.9840	-0.0233	0.6265	0.2031
Pfkp	_YLEHLSGDGK_.2	#N/A	6.4715	6.3871	7.2291	6.2226	6.1823	6.4863	6.2367	6.5997	6.6754	6.3429	1.0051	0.0073	0.8802	0.0554
Pfkp	_NVLGHM[Oxidation (M)]QQGGAPSPFDR_.3	#N/A	6.6672	6.9400	6.6935	6.1043	6.7977	6.9112	7.0031	6.6634	6.7027	6.6712	0.9890	-0.0160	0.6706	0.1735
Pfkp	_EIGWADVGGWTGQGGSILGTK_.2	#N/A	5.3169	5.0651	5.4157	5.6191	4.8535	4.8976	5.9482	4.9610	4.8086	4.8638	1.0096	0.0137	0.8567	0.0672
Pfkp	_FVSDDSIC[Carbamidomethyl (C)]VLGIC[Carbamidomethyl (C)]K_.2	#N/A	5.9765	5.7519	3.6345	5.0762	5.8376	2.1866	5.6778	5.8967	5.8404	5.4310	0.8306	-0.2678	0.2511	0.6002
Pfkp	_LNIIIVSEGAIDM[Oxidation (M)]QNKPITSEK_.3	#N/A	4.5130	3.6091	5.6911	4.6090	4.1486	4.3407	5.9546	5.1904	4.6412	4.3511	0.8909	-0.1666	0.2558	0.5920

Pfkp	_KVTDFEHR_.2	#N/A	6.4562	4.4148	3.3922	5.4230	5.1519	5.8880	6.4886	6.0570	6.1076	5.3970	0.8517	-0.2315	0.1659	0.7801
Pfkp	_IIEVVDAIM[Oxidation (M)]TTAQSHQR_.2	#N/A	4.4041	4.1034	4.2185	4.4919	3.8176	3.8787	3.3501	3.8322	3.4762	4.1068	1.1249	0.1698	0.0454	1.3434
Pfkp	_LGITNLC[Carbamidomethyl (C)]VIGGDGSLTGANLFR_.2	#N/A	2.4482	1.8401	4.5614	2.3208	0.3985	1.2830	4.2534	1.2167	3.1951	2.4467	0.7711	-0.3751	0.4887	0.3109
Pfkp	_WDC[Carbamidomethyl (C)]VSSILQVGGTIIGSAR_.2	#N/A	3.7699	3.9146	3.7962	3.7450	3.7359	3.3600	3.7964	3.2259	3.3891	3.4256	1.0755	0.1049	0.0896	1.0478
Rbck1	_ALLS[Phospho (STY)]PEDYQR_.2	Q9WUB0_S344_M1_Rbck1	8.2114	8.5903	8.7609	9.1701	3.5014	8.4247	8.6875	8.5418	8.4406	8.6515	0.9063	-0.1419	0.4789	0.3197
Pygm	_LITAIGDVVNHDPAVGDR_.3	#N/A	5.1272	5.4862	4.5781	4.5945	4.2281	5.4050	5.3497	5.3478	4.8565	5.1284	0.9483	-0.0766	0.3610	0.4424
Pygm	_IGEDYISDLDQLRK_.3	#N/A	4.2436	3.9473	3.9773	5.1238	3.1934	3.2373	4.5410	4.7477	3.8041	3.5230	0.9518	-0.0712	0.6548	0.1839
Pygm	_[Acetyl (Protein N-term)]SRPLSDQDKR_.3	#N/A	2.3209	3.7205	4.1987	3.7425	3.6088	3.0158	3.8908	3.2587	3.5577	2.7785	1.0187	0.0268	0.8745	0.0582
Ly6h	_QC[Carbamidomethyl (C)]QPTDTVC[Carbamidomethyl (C)]ASVR_.2	#N/A	6.5889	6.8455	6.5711	6.4272	7.0291	6.1683	6.4942	6.2658	6.6482	6.1128	1.0352	0.0500	0.2501	0.6019
Stub1	_WNS[Phospho (STY)]IEER_.2	Q9WUD1_S150_M1_Stub1	6.5224	6.1541	6.2576	6.4393	6.0125	6.4490	6.0461	5.9709	6.1461	6.4912	1.0231	0.0329	0.3261	0.4866
Stub1	_LGTGGGGS[Phospho (STY)]PDKSPSAQELK_.2	#N/A	7.8831	8.0674	8.4191	8.6360	7.1074	7.3996	8.2904	7.9783	7.8490	8.1235	0.9824	-0.0256	0.6583	0.1816
Casp8ap2	_KNS[Phospho (STY)]PGVSHSSQK_.2	Q9WUF3_S1818_M1_Casp8ap2	9.3343	9.3811	9.9373	9.6533	8.2508	9.2518	9.7192	9.6118	9.3272	9.2267	0.9821	-0.0261	0.5948	0.2257
Tmem115	_TDS[Phospho (STY)]PLPLEEASTPPGK_.2	Q9WUH1_S320_M1_Tmem115	6.9929	5.4538	3.4286	7.0296	3.1268	4.1030	7.4181	4.1105	6.7637	6.0884	0.8240	-0.2793	0.3373	0.4719
Eif4h	_EALTYDGALLGDR_.2	#N/A	6.9780	6.2935	6.2919	7.0165	7.0863	6.2251	6.5389	6.2405	6.1470	6.8599	1.0313	0.0445	0.4387	0.3578
Eif4h	_[Acetyl (Protein N-term)]ADFDTYDDR_.2	#N/A	5.9488	6.8127	6.8701	6.6532	6.9351	6.8671	7.0349	5.6759	6.4648	6.6255	1.0358	0.0508	0.4550	0.3420
Eif4h	_TVATPLNQVANPNSAIFGGAR_.2	#N/A	4.5034	4.7483	4.5460	4.8828	5.0122	4.4149	3.7657	4.5349	4.0185	3.5962	1.1774	0.2356	0.0080	2.0964
Eif4h	_GSNM[Oxidation (M)]DFREPTEEER_.3	#N/A	5.0601	4.3637	5.5876	5.2760	6.0558	5.2878	3.6803	4.3947	4.9779	5.2013	1.1552	0.2081	0.1095	0.9607
Eif4h	_DKDTDKFK_.2	#N/A	4.8285	4.6699	3.1588	4.0895	5.4959	5.1139	4.9369	4.0953	4.1056	5.0187	1.0045	0.0064	0.9666	0.0148
Eif4h	_AYSSFGGGR_.2	#N/A	4.1720	3.8682	4.0448	4.5474	3.9696	3.7450	4.2621	3.5710	4.5575	4.2659	0.9745	-0.0373	0.6405	0.1935
Zbtb18	_TVAS[Phospho (STY)]PC[Carbamidomethyl (C)]SSTESLSQR_.2	Q9WUK6_S214_M1_Zbtb18	6.9143	3.0226	4.4050	3.4069	2.8439	3.3847	7.3257	3.2108	7.0324	3.3739	0.7633	-0.3897	0.3252	0.4879
Arl3	_ILLLGLDNAGK_.2	#N/A	6.1160	6.6995	6.1587	6.6452	5.9942	6.1768	6.4112	6.5037	6.2991	5.8491	1.0052	0.0075	0.8680	0.0615
Arl3	_LNVWDIGGQR_.2	#N/A	5.5137	6.0074	5.7171	6.3339	6.5228	5.4719	6.1180	5.6282	5.6381	5.9552	1.0159	0.0228	0.7110	0.1481
Arl3	_QDLLTAAPASEIAEGLNLHTIR_.3	#N/A	3.1776	3.9339	4.4121	4.9718	3.8222	4.5649	4.1964	3.0453	3.8881	3.6332	1.1236	0.1682	0.2608	0.5837
Coro1b	_LEEQLGR_.2	#N/A	4.7783	4.7483	3.9773	4.7164	5.9120	5.3754	4.9980	5.5539	4.8889	4.8700	0.9685	-0.0461	0.6687	0.1748
Coro1b	_VTWDSTFC[Carbamidomethyl (C)]AVNPK_.2	#N/A	3.3531	4.1583	4.0399	4.5425	3.9796	3.7499	4.2572	3.5759	3.5755	3.0087	1.1016	0.1396	0.2376	0.6242
Coro1c	_NDQC[Carbamidomethyl (C)]YDDIR_.2	#N/A	5.1370	5.1266	5.2451	4.9163	4.5555	5.6056	4.4803	5.0825	5.0825	4.4127	1.0699	0.0975	0.1855	0.7318
Coro1c	_VTWDSSFC[Carbamidomethyl (C)]AVNPR_.2	#N/A	3.7966	3.5458	3.5303	2.5809	3.6743	3.6843	4.6413	3.9745	4.2262	4.2120	0.8136	-0.2976	0.0137	1.8648
Suclg1	_LIGPNC[Carbamidomethyl (C)]PGVINPGEC[Carbamidomethyl (C)]K_.2	#N/A	9.0606	9.3616	9.5916	9.5669	9.1658	8.9723	9.2314	9.0328	9.1024	8.9219	1.0236	0.0337	0.1720	0.7645
Suclg1	_HLGLPVFNTVK_.2	#N/A	7.7781	8.0337	8.2349	8.1186	8.0886	8.0720	7.5143	8.2804	7.9154	8.1255	1.0120	0.0172	0.5486	0.2608
Suclg1	_LVGGTTPGK_.2	#N/A	6.5046	7.2843	6.8546	6.9331	7.6726	6.9872	6.0679	7.1165	7.3685	7.2399	1.0131	0.0188	0.7770	0.1096
Suclg1	_IIC[Carbamidomethyl (C)]QGFTGK_.2	#N/A	6.5173	6.3300	6.6009	6.8096	7.4299	6.5903	5.7347	6.5306	7.0000	6.9280	1.0251	0.0358	0.5999	0.2219
Suclg1	_MGHAGAIIAGGK_.2	#N/A	5.6397	5.2198	4.4611	6.1567	4.7523	3.4249	5.4804	4.7619	6.0264	5.6656	0.9013	-0.1499	0.3398	0.4688
Suclg1	_ISALQSAGVVVSM[Oxidation (M)]SPAQLGTTIYK_.3	#N/A	3.1110	3.9162	3.1599	3.6222	3.7375	3.9920	4.3528	4.2975	3.0957	3.7275	0.9280	-0.1078	0.3819	0.4180
Suclg1	_QGTFHSQQALEYGTK_.3	#N/A	4.7949	4.3243	4.2059	4.2826	4.1456	3.5839	3.9447	3.4099	3.0585	3.9870	1.1730	0.2302	0.0470	1.3280
Eci2	_ASQQDFENALNQVK_.2	#N/A	3.7400	4.3539	4.0458	3.1669	4.7523	2.6712	4.1047	3.4943	3.5907	3.6337	1.0223	0.0318	0.8443	0.0735
Ak4	_IAQNFGLQHLSSGHLLR_.3	#N/A	5.1512	3.7513	4.2295	4.0712	4.3902	4.6492	5.2929	5.7177	3.5270	5.3126	0.8814	-0.1822	0.2354	0.6282
Tnip1	_YLQDQLS[Phospho (STY)]PLTR_.2	Q9WUU8_S416_M1_Tnip1	6.8057	7.1905	4.3784	3.9222	6.7912	6.8555	7.3930	6.9499	6.7807	6.9218	0.8544	-0.2270	0.2064	0.6853
Gabbr1	_HPPT[Phospho (STY)]PPDPSGGLPR_.2	#N/A	8.8914	3.2738	9.5523	4.2831	3.1276	8.4317	3.4263	5.6084	8.7602	4.1520	1.1409	0.1902	0.6780	0.1688
Gabbr1	_RHPPT[Phospho (STY)]PPDPSGGLPR_.3	#N/A	3.3461	7.0093	4.5806	6.8034	3.9907	4.1348	6.6202	4.3776	3.1758	4.0466	1.0927	0.1279	0.6777	0.1690
Nfat5	_RSS[Phospho (STY)]PIFQTTK_.2	Q9WV30_S618_M1_Nfat5	6.0814	4.3881	3.5248	4.0275	4.8398	4.2649	6.0919	4.0910	3.7396	3.9945	1.0093	0.0134	0.9478	0.0233
Mpp2	_TLVLIGAQGVGR_.2	#N/A	5.8393	5.6268	5.6013	6.1323	6.0431	5.6883	5.0503	6.0144	5.7881	6.0275	1.0178	0.0254	0.6480	0.1884
Mpp2	_VEGGELVIAR_.2	#N/A	5.8842	5.5087	4.7268	5.8769	5.9824	5.8612	5.5863	5.7945	6.0348	5.8493	0.9697	-0.0444	0.5070	0.2950
Mpp2	_DNNLELVQEILR_.2	#N/A	5.5230	5.3966	5.5724	6.0212	6.4027	5.5457	5.9733	5.4071	4.7336	5.8081	1.0480	0.0676	0.3974	0.4008
Mpp2	_GIM[Oxidation (M)]ES[Phospho (STY)]PIVR_.2	#N/A	9.5971	8.7866	9.7592	10.0903	6.4957	9.2529	8.7757	9.1901	9.0147	9.3220	0.9913	-0.0126	0.9097	0.0411
Mpp2	_DLAELAEQSS[Phospho (STY)]TAAELAR_.2	Q9WV34_S86_M1_Mpp2	3.9117	3.1672	3.6454	3.7476	6.0254	3.5691	6.8132	3.8120	4.1110	4.3673	0.8399	-0.2518	0.3391	0.4697
Mpp2	_EGQGYSFVSR_.2	#N/A	6.7289	6.1274	3.6485	6.3063	6.2462	6.1047	6.5604	5.9007	6.0763	6.3876	0.9405	-0.0885	0.5383	0.2690
Mpp2	_DLELT[Phospho (STY)]PTSGTLC[Carbamidomethyl (C)]GSLSGK_.2	Q9WV34_T303_M1_Mpp2	3.0601	3.8653	3.7469	4.2554	3.6867	4.0428	4.4036	6.2934	3.0449	3.9326	0.8546	-0.2267	0.3006	0.5221
Mpp2	_ALQELLR_.2	#N/A	4.6881	4.1132	3.5319	4.6140	4.2827	4.3306	3.1178	4.8703	4.6327	4.4677	0.9972	-0.0041	0.9753	0.0108
Mpp2	_TYET[Phospho (STY)]PPPS[Phospho (STY)]PGLDPTFSNQPVPPDAVR_.3	Q9WV34_T117_M2_Mpp2	3.6408	3.3937	3.4562	3.8160	3.2328	7.6868	3.6456	9.7963	3.8414	4.0186	0.7895	-0.3410	0.4675	0.3302
Mpp2	_ILPSYQEPHLPR_.3	#N/A	3.4279	5.3879	5.7904	4.9047	5.2754	4.9486	4.3591	5.0173	5.3385	3.4810	1.0894	0.1236	0.4627	0.3347
Mpp2	_NAEFDRHELLIYEEVAR_.4	#N/A	3.4627	4.2190	3.1964	4.2410	4.1073	4.0182	3.9113	3.5859	3.6072	3.9183	1.0315	0.0448	0.6277	0.2022
Mpp2	_YLEHGEYEGNLYGTR_.3	#N/A	4.1644	3.4687	3.3503	3.1511	3.2900	2.9386	3.2994	3.8500	4.1491	4.3293	0.8687	-0.2031	0.1025	0.9893
Vapa	_VAHSDKPGS[Phospho (STY)]TSAVSFR_.3	Q9WV55_S214_M1_Vapa	3.3404	4.0967	4.5749	5.5624	3.9850	4.1405	4.4673	4.6264	3.4481	3.7960	1.0487	0.0686	0.6619	0.1792
Gsk3b	_IQAAAS[Phospho (STY)]PPANATAASDTNAGDR_.2	#N/A	8.9528	8.9622	9.4645	9.5681	7.7608	8.6947	9.5177	9.4802	9.0424	8.7929	0.9666	-0.0490	0.4168	0.3801
Gsk3b	_GQTNNAASASASNST_.2	#N/A	4.9362	5.9035	4.3967	4.9518	3.7690	5.0683	5.1271	5.9653	5.1539	6.0057	0.8696	-0.2016	0.1165	0.9335
Gsk3b	_VTTVVATPGQGPDRPQEVSYTDTK_.3	#N/A	4.2284	4.1424	4.0241	4.5041	4.9209	4.9762	4.5538	4.0659	5.0206	4.7405	0.9719	-0.0411	0.6341	0.1978
Dmtn	_GNS[Phospho (STY)]LPC[Carbamidomethyl (C)]VLEQK_.2	#N/A	14.0214	13.6430	13.9746	14.2792	12.9761	13.7851	14.0740	13.4144	13.6539	13.8576	1.0022	0.0031	0.9095	0.0412
Dmtn	_ESVGGS[Phospho (STY)]PQSK_.2	#N/A	7.4739	11.5273	11.8058	11.7312	11.0155	11.5517	11.3997	11.2582	11.0189	11.4616	0.9616	-0.0565	0.6274	0.2024
Dmtn	_HLS[Phospho (STY)]AEDFSR_.2	Q9WV69_S372_M1_Dmtn	10.9599	11.1226	11.5495	11.7756	10.3109	11.0749	11.2583	10.9844	11.0669	11.3954	0.9961	-0.0057	0.8745	0.0582
Dmtn	_GAEEEEEEEDDDS[Phospho (STY)]EEEIK_.2	#N/A	11.0424	11.3079	11.4506	12.0751	11.1770	11.3468	11.6118	11.2426	11.0448	11.4045	1.0065	0.0094	0.7295	0.1370
Dmtn	_QPLTS[Phospho (STY)]PGSVSSSR_.2	Q9WV69_S11_M1_Dmtn	9.9868	10.1163	10.5503	10.7087	9.8220	10.2557	10.1932	9.7833	10.0531	10.2150	1.0178	0.0254	0.3728	0.4286
Dmtn	_SREC[Carbamidomethyl (C)]SLS[Phospho (STY)]PK_.2	#N/A	8.3047	8.8186	8.9230	8.9921	8.0240	8.5713	8.7242	8.1131	8.5592	8.8180	1.0061	0.0087	0.8272	0.0824
Dmtn	_TLGIISQASTPR_.2	#N/A	6.9761	6.9435	6.8339	7.1887	7.0799	7.0062	6.5474	6.8086	6.5486	7.4099	1.0258	0.0367	0.3364	0.4732
Dmtn	_DSSVPGS[Phospho (STY)]PSSIVAK_.2	Q9WV69_S26_M1_Dmtn	12.8467	9.3038	13.5033	13.3850	8.8741	13.0656	13.2455	12.7329	13.0005	12.9962	0.9104	-0.1354	0.3189	0.4964
Dmtn	_SSS[Phospho (STY)]LPSYGR_.2	Q9WV69_S289_M1_Dmtn	12.3266	12.3270	13.0049	6.5665	7.3156	12.5756	12.1177	12.1963	12.4793	12.5771	0.8658	-0.2079	0.2995	0.5236
Dmtn	_M[Oxidation (M)]DRGNS[Phospho (STY)]LPC[Carbamidomethyl (C)]VLEQK_.3	#N/A	8.5645	8.6368	9.5111	9.0072	8.0833	8.9838	8.0663	8.8290	8.6148	8.8955	1.0228	0.0326	0.5159	0.2874
Dmtn	_EC[Carbamidomethyl (C)]SLS[Phospho (STY)]PK_.2	#N/A	9.4684	3.1600	9.5233	3.7548	8.9825	9.0862	7.8620	4.3637	9.4584	9.5607	0.9383	-0.0919	0.7969	0.0986
Dmtn	_STS[Phospho (STY)]PPPSPEVWAESR_.2	Q9WV69_S92_M1_Dmtn	15.6680	15.6669	16.4255	16.1770	15.5099	15.8985	16.2225	15.5322	15.7333	15.9052	1.0027	0.0039	0.8460	0.0726
Dmtn	_KGAEEEEEEEDDDS[Phospho (STY)]EEEIK_.2	#N/A	7.6322	7.5041	7.7420	8.2299	7.3778	7.6365	7.6077	7.5231	6.5265	7.7525	1.0455	0.0642	0.2455	0.6099
Dmtn	_GAEEEEEEEDDDS[Phospho (STY)]EEEIKAIR_.3	#N/A	4.3362	5.1044	3.5221	5.6581	5.2698	6.1493	3.1276	4.6897	5.2091	5.0102	1.1103	0.1510	0.4384	0.3582
Dmtn	_QRESVGGS[Phospho (STY)]PQSK_.2	#N/A	6.1595	6.3591	6.8139	9.3174	8.6065	6.7083	6.7753	8.5819	6.5954	6.7456	1.0213	0.0304	0.8463	0.0725
Dmtn	_TRS[Phospho (STY)]LPDR_.2	#N/A	9.3728	9.5723	9.9278	4.0793	9.6527	9.6169	9.7664	9.5890	9.4791	9.7281	0.9028	-0.1475	0.4429	0.3537
Dmtn	_S[Phospho (STY)]LPDRTPFHTSLHSGTSK_.3	#N/A	3.6150	6.0311	3.8509	7.3850	3.4514	3.9011	4.5494	5.8727	3.7936	3.5959	1.0568	0.0797	0.7905	0.1021
Dmtn	_VFAM[Oxidation (M)]S[Phospho (STY)]PEEFGK_.2	Q9WV69_S383_M1_Dmtn	5.1774	5.4830	5.7879	5.9250	5.6966	5.4219	5.4870	6.1527	6.5515	6.6511	0.8988	-0.1540	0.0367	1.4355
Dmtn	_MDNQVLGYK_.2	#N/A	3.4351	3.7911	4.1219	4.8965	4.0616	3.6679	4.3393	3.4939	3.1425	3.1640	1.1303	0.1768	0.2139	0.6699
Dmtn	_LQS[Phospho (STY)]TEFSPSGSEAGSPGLQNGEGQR_.3	Q9WV69_S303_M1_Dmtn	3.1342	4.0907	4.1187	4.0477	3.9120	3.8300	4.1908	3.4821	3.4157	3.9849	1.0231	0.0330	0.7272	0.1384
Dmtn	_KGAEEEEEEEDDDS[Phospho (STY)]EEEIKAIR_.3	#N/A	3.9744	2.2288	3.7081	3.0753	3.1181	3.0393	3.4001	1.9348	4.0484	4.4300	0.9239	-0.1141	0.6358	0.1967
Asb3	_S[Phospho (STY)]VDVADNR_.2	#N/A	7.5448	7.2594	7.5471	7.4274	5.9535	7.1822	6.6630	7.3149	7.2212	7.0302	1.0135	0.0193	0.7803	0.1077
Asb3	_GRS[Phospho (STY)]VDVADNR_.2	#N/A	5.1032	5.1084	5.7034	5.8094	4.1955	4.8839	5.7334	5.4224	5.7401	5.2050	0.9292	-0.1060	0.2520	0.5986

Stxbp4	_SSS[Phospho (STY)]PLDRDPAFR_.2	Q9WV89_S12_M1_Stxbp4	3.7113	3.0157	4.3981	3.6042	2.8370	3.3917	3.7525	3.3970	3.6960	3.2815	0.9890	-0.0159	0.8997	0.0459
Epb41l3	_VESTSVGSIS[Phospho (STY)]PGGAK_.2	Q9WV92_S804_M1_Epb41l3	10.9193	10.9245	11.3085	11.1935	9.7673	11.0636	11.0311	10.6459	10.7278	10.9106	1.0031	0.0045	0.9106	0.0407
Epb41l3	_ETEIT[Phospho (STY)]PEDGED_.2	#N/A	9.5591	9.6981	9.9639	10.3405	9.3583	9.7859	10.0037	9.6459	3.2072	9.6807	1.2028	0.2664	0.2439	0.6128
Epb41l3	_DSVS[Phospho (STY)]AAEVGTGQYATTK_.2	Q9WV92_S470_M1_Epb41l3	7.0900	6.8860	6.6356	7.6749	6.4996	7.1483	7.4229	7.0314	7.1530	7.3284	0.9662	-0.0497	0.3084	0.5108
Epb41l3	_KKAEEAT[Phospho (STY)]PVTALR_.3	#N/A	7.7850	7.6488	8.5231	7.6563	3.8382	8.1971	7.5762	7.5388	7.6578	3.1729	1.1215	0.1655	0.5420	0.2660
Epb41l3	_RLS[Phospho (STY)]TSPVR_.2	Q9WV92_S599_M1_Epb41l3	7.5295	8.4616	7.9964	3.7319	3.5982	3.8917	8.1935	3.2988	8.8795	7.4975	0.8422	-0.2477	0.5002	0.3008
Epb41l3	_EVPVVHTETK_.2	#N/A	7.4900	7.2388	7.5398	7.3824	7.8287	7.2196	7.2946	7.1310	7.4813	7.4469	1.0152	0.0217	0.4218	0.3749
Epb41l3	_AEEAT[Phospho (STY)]PVTALR_.2	#N/A	8.0574	3.9594	3.1167	4.1613	4.0276	7.8086	4.3096	7.1403	7.4906	7.8740	0.7740	-0.3696	0.2688	0.5706
Epb41l3	_ASALIDRPAPYFER_.3	#N/A	7.4124	7.1647	7.1973	7.3426	7.0826	7.1571	7.6524	7.2780	7.7958	7.2898	0.9630	-0.0544	0.0511	1.2912
Epb41l3	_SHFESSTVR_.2	#N/A	6.8801	6.5881	6.7611	6.6292	7.2272	6.7452	6.4019	6.4876	6.8090	6.5192	1.0382	0.0542	0.1050	0.9790
Epb41l3	_LM[Oxidation (M)]DGSEILSLLESAR_.2	#N/A	6.2338	6.0442	6.8883	5.8301	6.3237	6.5333	6.2948	6.1218	6.3587	6.5883	0.9950	-0.0073	0.8795	0.0558
Epb41l3	_KAEEAT[Phospho (STY)]PVTALR_.2	#N/A	3.5360	7.7951	4.2228	3.7795	4.1625	3.5670	3.9278	7.6472	3.5207	3.1916	0.9866	-0.0195	0.9594	0.0180
Epb41l3	_DEVDAAEST[Phospho (STY)]PTDR_.2	#N/A	6.8057	4.0024	3.8840	4.3867	3.8237	3.9058	4.1013	3.7318	6.5844	4.3536	0.9521	-0.0708	0.7810	0.1074
Epb41l3	_TEPVEAEVESTPHPQPLS[Phospho (STY)]TEK_.3	Q9WV92_S686_M1_Epb41l3	8.4177	4.2967	3.6163	8.5825	4.1850	3.9404	3.8336	3.9996	3.6481	4.0859	1.4149	0.5007	0.2126	0.6724
Epb41l3	_QLEYQQFEDDKLS[Phospho (STY)]QR_.3	#N/A	7.2654	6.7358	6.7947	7.8775	4.9381	7.2121	6.4552	7.1112	6.8832	7.2448	0.9827	-0.0252	0.8268	0.0826
Epb41l3	_TFLETSTETALTNEWEK_.2	#N/A	5.0567	4.5398	4.3923	4.6983	4.1019	3.9219	4.7977	4.9491	3.5962	4.0345	1.0247	0.0352	0.7518	0.1239
Epb41l3	_VLQETILVEER_.2	#N/A	6.1812	5.9186	6.4799	5.7846	5.1808	5.7060	5.9264	5.7928	5.9395	5.9193	0.9967	-0.0047	0.9345	0.0294
Epb41l3	_ELEDKVIELHK_.2	#N/A	4.8874	4.4467	5.2041	4.8217	5.1515	5.1613	4.8145	5.0608	5.0339	5.2470	0.9814	-0.0271	0.5841	0.2335
Epb41l3	_LSTS[Phospho (STY)]PVR_.2	Q9WV92_S601_M1_Epb41l3	4.2858	3.5412	4.0195	3.5632	3.4295	3.1951	3.0882	3.4379	3.7370	3.2405	1.0878	0.1214	0.2401	0.6196
Epb41l3	_EIGDKDRDFAAAAAK_.3	#N/A	4.2809	5.9135	6.1855	6.3257	5.9871	6.2387	5.1249	6.3608	6.0068	3.2357	1.1235	0.1680	0.3731	0.4282
Epb41l3	_VVVHKETEIT[Phospho (STY)]PEDGED_.2	#N/A	8.2557	9.0116	10.4008	9.2815	8.7272	9.0248	9.0009	9.0565	8.8878	8.6714	1.0239	0.0341	0.5844	0.2333
Epb41l3	_S[Phospho (STY)]SSS[Phospho (STY)]KLSR_.2	#N/A	5.8340	3.7931	3.7080	3.8515	3.9435	4.0290	4.0710	4.6885	3.9100	5.3467	0.9310	-0.1032	0.5421	0.2659
Epb41l3	_FAPNHTK_.2	#N/A	4.3106	5.1564	4.2459	4.3459	4.7181	3.5438	5.1555	4.7035	4.5454	5.1197	0.8987	-0.1540	0.1369	0.8635
Epb41l3	_GKEGSS[Phospho (STY)]VTEAAK_.2	Q9WV92_S740_M1_Epb41l3	4.1990	3.4544	3.9327	3.4765	3.3567	3.3166	3.8776	3.5247	3.8238	3.1537	1.0077	0.0111	0.9052	0.0432
Epb41l3	_QQPALEQFPEAAAHST[Phospho (STY)]PVKR_.4	Q9WV92_T64_M1_Epb41l3	3.3784	4.1347	3.7783	4.2809	4.0230	4.1025	3.9956	4.8984	4.5310	4.2479	0.8939	-0.1618	0.0717	1.1446
Epb41l3	_TITYESSQVDPGADLEPGVLM[Oxidation (M)]SAQTITSETTSTTTTTHITK_.4	#N/A	4.2203	4.7403	5.8969	5.7254	4.5771	4.8581	6.0178	3.7488	4.1120	4.6670	1.0791	0.1098	0.5002	0.3008
Epb41l3	_GIS[Phospho (STY)]QTNLITTVT[Phospho (STY)]PEKK_.2	#N/A	9.1481	9.2084	9.6810	9.8437	7.8061	9.2953	9.4254	9.2728	9.2252	9.1123	0.9897	-0.0149	0.8041	0.0947
Epb41l3	_SSSSKLS[Phospho (STY)]RS[Phospho (STY)]PLK_.2	#N/A	6.2456	6.0884	6.3206	6.7101	5.3745	6.2573	6.2515	6.3169	6.6796	6.1402	0.9715	-0.0417	0.4771	0.3214
Epb41l3	_VTLLDGSEYGC[Carbamidomethyl (C)]DVDKR_.3	#N/A	5.9660	5.2434	6.0052	5.3164	5.6158	5.8286	5.1759	5.4124	5.7394	5.7693	1.0250	0.0357	0.5106	0.2919
Epb41l3	_LSRS[Phospho (STY)]PLK_.2	Q9WV92_S107_M1_Epb41l3	4.0734	7.6863	7.9609	7.5923	6.9188	7.5465	7.8194	7.5292	3.8846	7.6788	1.0349	0.0495	0.8295	0.0812
Epb41l3	_GISQTNLITTVT[Phospho (STY)]PEK_.2	#N/A	6.8436	6.4681	6.5849	10.3223	5.9226	6.8240	7.0772	9.0658	6.8247	6.7200	0.9648	-0.0516	0.7837	0.1059
Epb41l3	_QLEYQQFEDDKLSQRS[Phospho (STY)]S[Phospho (STY)]SSK_.3	#N/A	4.3818	3.6862	3.5678	6.2668	3.5075	4.2220	3.7851	4.0481	3.6966	4.0374	1.0977	0.1345	0.4981	0.3026
Epb41l3	_DEVDAAEST[Phospho (STY)]PTDRR_.2	#N/A	5.5690	3.8880	3.9056	3.7888	3.8453	3.5944	3.7243	3.8507	3.7551	3.6264	1.0961	0.1324	0.3646	0.4382
Epb41l3	_NWLDPAK_.2	#N/A	4.2643	3.5197	3.9980	3.5417	3.4080	3.6928	3.6900	3.7785	4.8017	3.2190	0.9652	-0.0512	0.6773	0.1692
Epb41l3	_EQHPDMSVTK_.2	#N/A	4.7725	4.2117	4.0933	4.5959	4.4607	3.6965	4.3106	3.5225	3.1711	3.0621	1.2242	0.2919	0.0295	1.5297
Epb41l3	_IVITGDADIDHDQALAQAIK_.3	#N/A	2.9461	3.0287	4.2106	3.2124	4.4468	5.0844	4.4279	3.4052	4.5574	4.2909	0.9163	-0.1260	0.5016	0.2996
Epb41l3	_S[Phospho (STY)]LDGASVSENHEIYM[Oxidation (M)]K_.2	#N/A	8.1031	8.6195	8.4363	9.4437	8.2403	8.9721	8.7686	9.1595	9.1466	8.9177	0.9597	-0.0593	0.2096	0.6787
Epb41l3	_AEEERVEEEDRR_.3	#N/A	1.0725	3.4524	3.9306	3.4744	2.9742	3.2839	3.1771	3.5268	3.8258	1.8942	0.9760	-0.0351	0.9063	0.0427
Epb41l3	_TFLETSTETALTNEWEKR_.3	#N/A	3.4659	3.3292	5.0671	3.2906	3.1505	3.0781	3.4389	3.7105	4.3414	4.8188	0.8740	-0.1943	0.2911	0.5360
Epb41l3	_VC[Carbamidomethyl (C)]VEHHTFFR_.3	#N/A	2.7829	3.4849	4.4281	3.4299	2.8670	2.9585	3.1446	3.1877	2.9974	3.3969	1.0451	0.0637	0.6662	0.1764
Timm9	_LTETC[Carbamidomethyl (C)]FLDC[Carbamidomethyl (C)]VK_.2	#N/A	6.0339	6.0887	5.3264	6.3527	6.5246	6.0771	5.3866	6.2542	5.8175	6.1493	1.0280	0.0399	0.5428	0.2654
Timm9	_EFLGTYNK_.2	#N/A	5.4087	5.1780	5.7881	5.0316	5.7844	5.9683	4.1564	5.4387	5.9065	5.6018	1.0475	0.0670	0.5064	0.2955
Timm9	_EVKPEEVTC[Carbamidomethyl (C)]SEHC[Carbamidomethyl (C)]LQK_.3	#N/A	5.5248	5.4670	4.3274	5.9542	6.0197	5.3879	5.4483	5.4628	5.1165	5.0208	1.0351	0.0498	0.5831	0.2343
Timm9	_FQEYHIQQNEALAAK_.2	#N/A	4.1819	3.1605	3.9156	3.4593	3.3256	3.2990	3.6076	3.5418	3.8409	3.1366	1.0072	0.0103	0.9172	0.0375
Timm8a1	_FIDTSQFILNR_.2	#N/A	5.4221	5.2579	6.1978	6.1328	6.0373	5.5498	6.0379	4.2211	5.3581	4.9661	1.1206	0.1643	0.1268	0.8969
Timm8a1	_SKPVFSESLS[Phospho (STY)]D_.2	Q9WVA2_S96_M1_Timm8a1	4.2398	5.7535	3.9735	6.8948	6.6973	3.2410	6.7369	3.4839	3.7829	6.7016	0.9917	-0.0120	0.9684	0.0139
Timm8a1	_LDS[Phospho (STY)]RAEAC[Carbamidomethyl (C)]FVNC[Carbamidomethyl (C)]VER_.3	Q9WVA2_S57_M1_Timm8a1	3.9047	3.2090	4.5915	3.5933	3.0304	3.1983	3.3080	3.5068	2.4281	3.5603	1.1209	0.1647	0.3188	0.4965
Bub3	_VAVEYLDPS[Phospho (STY)]PEVQK_.2	Q9WVA3_S211_M1_Bub3	3.2361	8.2540	4.4706	8.6285	7.9266	8.3823	8.6002	3.6952	3.3438	8.2443	1.1416	0.1911	0.6197	0.2078
Tagln2	_NFS[Phospho (STY)]DNQLQEGK_.2	Q9WVA4_S163_M1_Tagln2	6.4117	6.8906	7.0236	6.3012	7.2174	7.0155	7.2585	6.5510	6.6616	6.8438	0.9973	-0.0040	0.9355	0.0289
Pacsin2	_LDSGQVGLYPANYVEAIQ_.2	#N/A	4.7911	4.9581	4.2848	5.2508	5.3039	5.1725	3.9768	4.9462	5.1209	3.5058	1.1305	0.1770	0.1531	0.8149
Pacsin2	_AYAQQLTEWAR_.2	#N/A	5.5321	5.3187	6.1127	4.4965	5.9157	5.2184	4.2112	5.8515	3.2705	2.9627	1.3334	0.4151	0.0505	1.2967
Msl3	_SSS[Phospho (STY)]PIPLTPSK_.2	Q9WVG9_S404_M1_Msl3	8.0793	7.4280	3.1545	7.9733	4.0654	7.5471	4.3474	7.9023	3.1011	3.8764	1.3262	0.4073	0.2928	0.5335
Foxo3	_WPGS[Phospho (STY)]PTSR_.2	Q9WVH4_S293_M1_Foxo3	3.4723	4.2285	3.2059	7.9136	4.1168	4.0086	3.9018	7.4262	7.3871	3.9278	0.7934	-0.3340	0.3531	0.4521
Mapk8ip1	_PHPSISEEDEGFDC[Carbamidomethyl (C)]LSS[Phospho (STY)]PER_.3	#N/A	3.7781	3.5574	3.2238	5.4724	3.7494	4.3466	4.0300	4.3211	3.6462	4.4289	0.9792	-0.0303	0.8480	0.0716
Psmd13	_GSIDEVDKR_.2	#N/A	3.5000	4.3052	5.2144	3.1886	4.2941	4.5514	4.4041	3.4290	4.5923	5.3801	0.9380	-0.0923	0.5890	0.2299
Ehd1	_DKPTYDEIFYTLS[Phospho (STY)]PVNGK_.3	#N/A	3.1704	3.9290	5.8001	7.3567	6.4395	5.9732	6.9835	7.2057	6.6407	6.5180	0.7964	-0.3285	0.1268	0.8968
Magi2	_S[Phospho (STY)]SFPDSTEAFDPR_.2	Q9WVQ1_S725_M1_Magi2	9.2157	9.9049	4.3798	3.9236	3.7899	4.3356	4.0719	3.0776	3.3766	3.6009	1.6776	0.7464	0.1387	0.8578
Magi2	_GGFFS[Phospho (STY)]PWK_.2	Q9WVQ1_S685_M1_Magi2	7.0127	7.1892	6.8057	7.2372	6.0430	6.9934	4.9481	7.1185	7.0444	6.8615	1.0596	0.0835	0.4307	0.3658
Magi2	_IIPQEELNS[Phospho (STY)]PTSAPSSEK_.2	Q9WVQ1_S1013_M1_Magi2	11.3375	11.4983	12.0767	11.9020	11.2600	11.4375	11.9294	11.3803	11.4942	11.5832	0.9990	-0.0014	0.9539	0.0205
Magi2	_M[Oxidation (M)]DS[Phospho (STY)]SGPDYKELDVHLR_.3	Q9WVQ1_S768_M1_Magi2	3.2885	4.0448	4.5231	5.8041	4.4486	4.1923	4.0855	3.7477	5.2589	4.3378	1.0060	0.0086	0.9590	0.0182
Magi2	_GAQGFGFTIADS[Phospho (STY)]PTGQR_.2	Q9WVQ1_S621_M1_Magi2	3.6174	6.3661	4.3042	7.9231	4.2439	3.4856	4.5216	6.9436	2.9602	6.7179	0.9440	-0.0831	0.8074	0.0929
Magi2	_SPGSVSTHHSS[Phospho (STY)]PR_.2	#N/A	5.3557	5.0914	4.9836	5.8826	4.4958	5.4496	6.0268	5.3317	4.4297	5.7542	0.9674	-0.0479	0.6437	0.1913
Magi2	_NKS[Phospho (STY)]VTNMEK_.2	Q9WVQ1_S219_M1_Magi2	3.6665	2.9709	4.3533	3.6490	2.7922	3.4365	3.7973	3.3522	3.6512	3.3263	0.9848	-0.0221	0.8621	0.0644
Fxr2	_AGPGDRDPPS[Phospho (STY)]RGEESR_.3	Q9WVR4_S477_M1_Fxr2	3.6430	6.3606	6.7025	3.6725	4.2695	6.2801	3.8208	3.3287	3.6277	3.3498	1.4595	0.5455	0.0600	1.2221
Fxr2	_TDGS[Phospho (STY)]ISGDRQPVTVADYISR_.3	Q9WVR4_S602_M1_Fxr2	3.8731	4.7718	5.0926	5.2643	3.4583	4.5924	3.7112	4.7103	4.5402	4.2810	1.0460	0.0648	0.6336	0.1982
Ncdn	_EAEPDLLAVLR_.2	#N/A	7.8792	7.7453	7.4356	7.3392	7.1298	7.6249	7.8070	7.9837	7.7638	7.5310	0.9684	-0.0463	0.1614	0.7921
Ncdn	_IFDAVGFTFPNR_.2	#N/A	7.5556	7.5805	7.4060	6.5325	6.9660	7.3151	7.8512	7.4901	7.3309	7.3307	0.9634	-0.0538	0.2639	0.5786
Ncdn	_IPILSTFLTAR_.2	#N/A	7.3043	7.6345	7.5664	6.8133	6.9371	7.1721	7.3649	7.8047	7.3938	7.2232	0.9720	-0.0410	0.3165	0.4996
Ncdn	_GLSEDFQR_.2	#N/A	7.0868	6.8804	7.4722	6.2226	7.0203	7.0289	6.3783	7.3193	7.2471	6.9892	0.9955	-0.0065	0.9092	0.0413
Ncdn	_EVC[Carbamidomethyl (C)]QLLPFLVR_.2	#N/A	7.0639	6.9464	6.7419	6.5036	6.4693	6.3745	6.9610	7.2528	6.7120	6.4684	0.9759	-0.0352	0.4214	0.3753
Ncdn	_EGAPSLLC[Carbamidomethyl (C)]K_.2	#N/A	6.7472	6.8688	5.7634	5.7055	6.0648	5.1859	7.0171	6.6013	6.3422	6.4927	0.9157	-0.1270	0.1502	0.8233
Ncdn	_DLQAGLAR_.2	#N/A	5.3840	4.9311	5.4654	5.0135	5.5732	5.5778	4.1562	5.9777	5.5707	6.0885	0.9772	-0.0332	0.7525	0.1235
Ncdn	_TLYEEAEEASDISQQVANLAISPTT[Phospho (STY)]PGPSWPGDALR_.4	Q9Z0E0_T451_M1_Ncdn	3.8227	3.9089	4.2071	3.7509	3.6172	3.4734	3.6591	5.7664	3.5713	3.4282	0.9246	-0.1131	0.5179	0.2857
Ncdn	_NDSEQFAALLLVTK_.2	#N/A	3.9335	3.6999	3.1194	4.0274	3.7939	4.3971	3.3368	3.6580	4.7905	4.1000	0.9640	-0.0528	0.6761	0.1700
Ncdn	_EAPDGC[Carbamidomethyl (C)]PDHVLR_.2	#N/A	7.1203	6.9002	3.3541	6.6560	6.7281	6.3779	7.0331	7.3389	6.8244	6.9157	0.8807	-0.1833	0.2811	0.5512
Ncdn	_SM[Oxidation (M)]IDDTYQC[Carbamidomethyl (C)]LTAVAGTPR_.2	#N/A	4.8077	5.0089	5.4391	4.8082	4.1577	4.8074	4.9151	4.9253	4.9265	4.1692	1.0220	0.0314	0.6975	0.1565
Ncdn	_LQAGEETASHYR_.2	#N/A	5.0600	4.3599	3.7404	4.6645	4.2747	4.3291	4.4333	4.2331	4.4109	4.2399	1.0174	0.0249	0.7491	0.1255
Ncdn	_LLSTS[Phospho (STY)]PALQGTPASR_.2	Q9Z0E0_S583_M1_Ncdn	3.4101	3.8161	4.0969	4.5995	4.0366	3.6929	4.3142	6.7466	3.1675	3.0657	0.9118	-0.1333	0.6045	0.2186
Ncdn	_EAIGAVIHYLLQVGPEK_.2	#N/A	2.9275	4.2986	3.6143	4.1170	4.1869	3.9385	1.2833	4.0015	3.6501	4.0840	1.1820	0.2413	0.3414	0.4667

Slc22a5	_DGEES[Phospho (STY)]PTVLK_.2	Q9Z0E8_S548_M1_Slc22a5	3.4471	4.2033	3.1807	4.2254	4.0916	4.0338	3.9270	4.2767	3.0749	3.9026	1.0180	0.0258	0.8263	0.0828
Adam17	_S[Phospho (STY)]FEDLTDHPVTR_.2	Q9Z0F8_S794_M1_Adam17	4.1979	5.4501	3.3839	6.7091	6.4173	6.2070	6.5556	5.9431	6.3164	5.9758	0.8704	-0.2003	0.2784	0.5553
Zbtb22	_ASENQS[Phospho (STY)]PSSSNYFSPR_.2	Q9Z0G7_S203_M1_Zbtb22	6.6820	6.3297	4.5069	3.4954	6.5726	3.2829	6.9719	3.5058	6.6786	6.7866	0.8595	-0.2184	0.4409	0.3557
Celf2	_ELFEPYGAVYQINVLR_.2	#N/A	4.2177	4.4310	3.9682	5.7582	5.5261	5.4061	5.7367	5.2554	5.2615	6.0709	0.8752	-0.1923	0.1369	0.8635
Celf2	_SWS[Phospho (STY)]EKELK_.2	Q9Z0H4_S52_M1_Celf2	7.2326	4.2788	7.4540	7.8701	7.0826	7.0690	7.3694	7.1446	3.6302	7.3377	1.0723	0.1007	0.6497	0.1873
Celf2	_VM[Oxidation (M)]FSPFGQIEEC[Carbamidomethyl (C)]R_.2	#N/A	4.8836	5.3798	5.7710	6.1980	5.3695	5.3845	5.4764	5.5284	5.4442	5.4542	1.0040	0.0058	0.9255	0.0336
Clip2	_IGFPSTS[Phospho (STY)]PAK_.2	Q9Z0H8_S295_M1_Clip2	10.3557	10.3235	10.6570	10.4053	8.9218	9.9247	10.5132	10.0864	9.9401	10.2736	0.9897	-0.0150	0.7595	0.1195
Clip2	_QAS[Phospho (STY)]GPSSSGAATTVSEKPGPK_.3	#N/A	6.9022	3.4738	7.7245	7.3536	6.6273	6.7329	6.5073	7.1214	6.6877	6.7900	0.9546	-0.0670	0.7052	0.1517
Clip2	_YSLIDPAS[Phospho (STY)]PPELLK_.2	Q9Z0H8_S980_M1_Clip2	6.7233	3.7569	6.6911	3.1579	3.6452	2.9794	3.4734	3.4598	3.1083	3.1249	1.2775	0.3533	0.3191	0.4961
Clip2	_TGNESGSNLS[Phospho (STY)]DSGSVK_.2	Q9Z0H8_S208_M1_Clip2	7.1644	6.9926	7.6331	7.9206	6.4716	6.9781	7.3317	7.1528	7.1077	6.9305	1.0088	0.0126	0.8230	0.0846
Clip2	_EGS[Phospho (STY)]PLHK_.2	Q9Z0H8_S43_M1_Clip2	3.3682	4.4296	4.6787	4.2046	3.7375	3.9920	4.2762	4.3675	3.5783	4.2674	0.9869	-0.0190	0.8532	0.0689
Clip2	_VLLLEANRHS[Phospho (STY)]PGPER_.3	#N/A	7.5962	8.0993	8.7128	9.0378	7.3814	7.9073	8.0071	8.2118	8.0379	7.7907	1.0138	0.0198	0.7494	0.1253
Clip2	_QQHIEQLLAER_.3	#N/A	3.1000	3.2899	4.3345	3.7093	4.0662	4.4591	4.2741	3.1229	3.4220	4.0751	1.0277	0.0394	0.7793	0.1083
Clip2	_TGNESGSNLS[Phospho (STY)]DSGS[Phospho (STY)]VKR_.2	#N/A	4.1175	3.2249	3.8512	3.3950	3.0462	3.1825	3.5433	3.6062	3.9052	3.0723	0.9824	-0.0256	0.8135	0.0896
Nos1	_VTQPLGT[Phospho (STY)]PTK_.2	Q9Z0J4_T128_M1_Nos1	3.9912	8.4368	3.7249	8.7590	3.1349	3.4897	3.4169	3.7325	4.0316	4.4468	1.3453	0.4279	0.3439	0.4636
Nos1	_KSS[Phospho (STY)]GDGPDLR_.2	Q9Z0J4_S858_M1_Nos1	7.3679	6.9610	4.1225	7.3824	4.5011	7.0524	7.4407	5.2604	4.9291	6.9215	1.0152	0.0218	0.9206	0.0359
Nos1	_GNVPVVLNNPYSENEQSPASGKQS[Phospho (STY)]PTK_.3	Q9Z0J4_S287_M1_Nos1	3.8494	3.3768	4.5362	3.5380	2.9750	3.3595	3.2527	4.7956	4.2291	3.5050	0.9139	-0.1299	0.4088	0.3885
Palm	_SETLVNAQQT[Phospho (STY)]PLGT[Phospho (STY)]PKENR_.2	#N/A	9.2213	9.9439	10.4139	10.4583	9.3186	10.3203	10.0457	9.8789	9.7646	9.9923	1.0026	0.0037	0.9310	0.0311
Palm	_EPAPLNGS[Phospho (STY)]AAELPATK_.2	Q9Z0P4_S345_M1_Palm	9.8508	4.3553	9.4551	10.2131	3.6316	9.7697	3.9136	3.2358	9.7741	9.7339	1.1823	0.2416	0.5779	0.2381
Palm	_VLGLQDTIK_.2	#N/A	7.1316	7.4766	7.1149	7.4386	7.4628	7.2938	6.9770	7.4391	7.0251	7.2266	1.0213	0.0304	0.2346	0.6297
Palm	_GLAEDVTR_.2	#N/A	6.0494	6.4468	7.1493	6.4600	6.9121	6.0075	4.4175	6.1877	6.4834	6.6318	1.0968	0.1333	0.2542	0.5947
Palm	_GLEESITR_.2	#N/A	5.7489	5.7947	6.1562	6.0058	6.3866	6.7410	3.3320	6.3389	6.0445	6.4013	1.1103	0.1509	0.3493	0.4568
Palm	_ADEVTLS[Phospho (STY)]EAGSTAGPAEPR_.2	Q9Z0P4_S244_M1_Palm	5.7855	5.9901	5.9133	7.4760	5.5771	6.7994	4.4198	6.4625	6.1331	6.5286	1.0630	0.0882	0.5120	0.2907
Palm	_SETLVNAQQT[Phospho (STY)]PLGT[Phospho (STY)]PK_.2	#N/A	12.5100	12.1088	12.1618	12.8797	11.6027	12.3963	12.6610	11.9982	12.2668	12.5119	0.9933	-0.0097	0.7468	0.1268
Palm	_EIDVLEFGESAPAASK_.2	#N/A	6.8641	6.3617	7.1386	7.4355	6.8535	6.7955	7.1789	6.5306	6.8268	7.0998	0.9999	-0.0002	0.9969	0.0013
Palm	_EENQTGPTTT[Phospho (STY)]PS[Phospho (STY)]DTQDLDM[Oxidation (M)]K_.2	#N/A	8.5874	9.2784	7.1165	9.9630	9.2148	9.2948	9.8236	8.9578	9.0803	9.4593	0.9549	-0.0666	0.4446	0.3520
Palm	_[Acetyl (Protein N-term)]M[Oxidation (M)]EVLATDTAS[Phospho (STY)]QQER_.2	#N/A	8.3105	9.0181	9.4428	8.8259	8.9429	9.3640	9.0495	8.7979	8.8079	8.9478	1.0094	0.0134	0.7075	0.1502
Palm	_AELVVIEDAATPR_.2	#N/A	6.6339	6.4669	6.7917	6.9555	6.9757	6.8225	6.8365	6.8615	6.6854	6.9261	0.9922	-0.0113	0.6346	0.1975
Palm	_EENQTGPTTTPS[Phospho (STY)]DT[Phospho (STY)]QDLDM[Oxidation (M)]KKPR_.3	Q9Z0P4_T367_M2_Palm	3.5475	4.3038	3.2812	4.3258	5.4328	3.9334	3.8265	4.1762	2.9744	4.0031	1.1048	0.1437	0.3986	0.3995
Palm	_ENSAAPSPGRPQSAS[Phospho (STY)]PAKEEQK_.3	#N/A	10.2803	10.1323	10.5314	10.9530	10.4613	10.2067	10.5473	10.3317	10.2629	10.3738	1.0047	0.0067	0.7688	0.1142
Palm	_TST[Phospho (STY)]PVRS[Phospho (STY)]PGGSTMM[Oxidation (M)]K_.2	#N/A	9.7447	9.1090	3.7889	8.0360	8.4797	6.0746	9.1294	5.2204	3.6600	9.2367	1.1068	0.1463	0.6591	0.1811
Palm	_WLLEGTPSSASEGDEDMRK_.3	#N/A	3.3415	4.0009	4.5761	7.0888	3.8222	3.9073	4.2681	4.3822	3.1804	3.7971	1.1406	0.1897	0.4684	0.3294
Palm	_PQSAS[Phospho (STY)]PAKEEQK_.2	#N/A	3.4565	4.2640	3.7123	4.2541	3.4619	3.8181	3.5186	3.8674	3.5524	4.2887	1.0055	0.0080	0.9302	0.0314
Palm	_EEQKSETLVNAQQT[Phospho (STY)]PLGT[Phospho (STY)]PKENR_.3	#N/A	3.8180	2.2417	3.3387	3.6027	2.9617	2.2237	3.5561	4.2771	3.9257	2.1514	0.8716	-0.1982	0.4038	0.3939
Palm	_ENSAAPS[Phospho (STY)]PGRPQSASPAK_.2	Q9Z0P4_S116_M1_Palm	4.2292	4.7771	4.3640	4.3528	3.6346	4.1099	3.6205	3.1753	4.0913	3.4568	1.1837	0.2433	0.0270	1.5679
Palm	_S[Phospho (STY)]PGGSTMMK_.2	#N/A	4.2291	3.1133	3.9628	3.5066	3.3728	3.2940	3.6548	3.4946	3.7937	3.1838	1.0136	0.0195	0.8475	0.0719
Twf2	_ETIELVHTEPTNVAQLPSR_.3	#N/A	5.8048	5.4643	5.8696	6.2688	5.8516	5.6933	5.8280	5.7221	5.9937	5.9131	0.9934	-0.0096	0.7919	0.1013
Twf2	_VIIEDEQLVLGASQEPVGR_.2	#N/A	3.9537	3.7615	3.5028	4.0055	2.7196	4.2445	5.0835	4.3108	3.0794	4.0143	0.8971	-0.1566	0.3508	0.4550
Twf2	_AVLPLLDAQEPC[Carbamidomethyl (C)]YLLFR_.2	#N/A	3.8023	5.0252	3.8549	4.0488	2.9533	3.6658	4.0266	3.6532	3.5257	3.0928	1.0887	0.1226	0.4219	0.3748
Sufu	_KDS[Phospho (STY)]LGSDSSTAIIPHELIR_.3	Q9Z0P7_S346_M1_Sufu	3.8063	3.4198	4.4931	3.4950	3.3081	3.3165	3.2096	3.1227	4.2721	3.4619	1.0350	0.0497	0.7028	0.1532
Sufu	_RLS[Phospho (STY)]GKDTEQIR_.3	Q9Z0P7_S301_M1_Sufu	4.3289	3.0135	4.0626	3.6064	2.8348	3.3938	3.7546	3.3948	3.6939	3.2837	1.0023	0.0034	0.9794	0.0090
Itsn1	_LPEEPSS[Phospho (STY)]EDEQQPEK_.2	#N/A	5.8188	9.3881	9.6996	10.1348	8.9748	9.5095	9.7457	6.1010	9.3772	9.4781	1.0283	0.0402	0.8211	0.0856
Itsn1	_STS[Phospho (STY)]IDTGPTESPASLK_.2	#N/A	8.3216	3.8594	8.4895	9.1739	6.7081	8.4824	8.2914	8.5428	8.5347	3.9386	1.0244	0.0348	0.8973	0.0471
Itsn1	_QRS[Phospho (STY)]LEAAR_.2	Q9Z0R4_S623_M1_Itsn1	7.6427	7.2985	8.5309	8.0026	6.9313	7.6020	7.5913	7.5776	7.3981	7.7725	1.0110	0.0157	0.7814	0.1071
Itsn1	_QVQQNS[Phospho (STY)]LHR_.2	Q9Z0R4_S558_M1_Itsn1	7.5426	7.4555	7.8426	7.8995	7.2590	7.7541	7.4956	7.1431	7.3713	7.0581	1.0493	0.0695	0.0436	1.3602
Itsn1	_STS[Phospho (STY)]IDTGPTESPASLKR_.2	#N/A	6.1150	6.3052	6.7600	6.9480	4.4435	6.7762	6.3233	6.5240	6.4849	6.5856	0.9607	-0.0579	0.6067	0.2170
Itsn1	_KSTS[Phospho (STY)]IDTGPTESPASLKR_.3	#N/A	3.8094	3.5330	3.5431	4.5877	3.3543	4.3752	3.2352	3.9143	4.2133	4.2650	0.9898	-0.0148	0.9032	0.0442
Itsn1	_SAFTPATATGSSPS[Phospho (STY)]PVLGQGEK_.2	Q9Z0R4_S897_M1_Itsn1	6.6594	6.7541	3.4659	8.1101	3.4050	6.6863	6.8573	6.5495	6.4294	6.1918	0.8985	-0.1543	0.5274	0.2779
Itsn1	_LLS[Phospho (STY)]PGTSKIT[Phospho (STY)]PTELPK_.2	#N/A	7.7073	7.1949	7.4165	7.7024	6.1427	7.3198	7.2642	7.2220	7.2379	6.9319	0.9884	-0.0169	0.7840	0.1057
Itsn1	_LPEEPSS[Phospho (STY)]EDEQQPEKK_.2	#N/A	7.6836	7.9781	8.1101	3.2624	7.1768	3.0840	7.9356	3.3552	3.0038	3.2294	1.4188	0.5047	0.2661	0.5750
Itsn1	_SGS[Phospho (STY)]GM[Oxidation (M)]SVISSSSVDQR_.2	#N/A	8.0030	8.5245	3.9788	8.8612	7.3210	8.4990	8.6524	8.2709	8.1662	8.4405	0.8985	-0.1545	0.3883	0.4109
Itsn1	_LLS[Phospho (STY)]PGTSK_.2	Q9Z0R4_S1130_M1_Itsn1	6.2136	3.2893	6.4604	3.6254	5.2561	3.4470	3.3401	2.9922	4.1416	3.5924	1.3409	0.4232	0.1538	0.8130
Itsn1	_SNPYC[Carbamidomethyl (C)]EVTM[Oxidation (M)]GS[Phospho (STY)]QC[Carbamidomethyl (C)]HITK_.	#N/A	4.0442	4.0330	4.4163	4.2626	3.8856	4.0702	5.3922	3.0414	3.2944	4.8109	0.9961	-0.0056	0.9731	0.0118
Itsn1	_DQFS[Phospho (STY)]PDDFLGR_.2	Q9Z0R4_S1663_M1_Itsn1	4.3873	3.6427	4.1210	3.6647	3.5310	3.6527	3.8130	3.3364	3.6355	3.3420	1.0854	0.1182	0.1638	0.7858
Itsn1	_AQS[Phospho (STY)]FDVASAPPAAEWAVPQSSR_.2	Q9Z0R4_S203_M1_Itsn1	3.8783	3.1827	4.5651	3.4371	3.0040	3.2246	3.5854	3.5640	3.8631	3.1144	1.0048	0.0069	0.9592	0.0181
Bpnt1	_NYEYYASHVPESVK_.2	#N/A	6.0940	5.8158	6.0992	6.3224	6.1019	6.3936	6.1692	6.4372	5.8912	6.2712	0.9912	-0.0127	0.7038	0.1525
Bpnt1	_IIQLIEGK_.2	#N/A	3.7476	3.0603	3.3519	4.2663	3.7287	4.7150	3.5692	3.8453	5.1899	4.1443	0.9103	-0.1356	0.3925	0.4061
Bpnt1	_LTDIHGNALQYNK_.3	#N/A	4.0295	4.5890	4.3687	3.3070	5.2273	5.4786	3.4552	4.1442	5.6232	2.9843	1.1106	0.1514	0.4829	0.3161
Bpnt1	_SHSNQLVTDC[Carbamidomethyl (C)]ISAMNPDTVLR_.3	#N/A	3.8237	3.2856	3.9498	3.4936	4.1764	3.2965	3.6419	3.5079	4.6445	4.6007	0.8956	-0.1590	0.1981	0.7031
Bpnt1	_LVQM[Oxidation (M)]SIC[Carbamidomethyl (C)]SSLAR_.2	#N/A	3.9926	3.3498	3.7263	3.2700	3.1363	3.4883	3.4183	3.7312	4.0302	4.4481	0.8943	-0.1612	0.1196	0.9222
Bpnt1	_[Acetyl (Protein N-term)]ASSHTVLMR_.2	#N/A	3.5347	3.8077	3.2684	2.1585	3.6290	4.1005	4.4613	4.1890	2.9872	3.9903	0.8745	-0.1935	0.2854	0.5446
Xpr1	_VLIEDT[Phospho (STY)]DDEANT_.2	Q9Z0U0_T689_M1_Xpr1	3.9423	5.4308	3.6760	6.1932	4.0628	5.9453	7.0939	6.2736	4.0804	5.8955	0.8354	-0.2595	0.2404	0.6191
Tjp2	_GSYGS[Phospho (STY)]DPEEEEYR_.2	#N/A	8.1835	7.8797	7.7718	8.7760	7.8826	8.0834	8.6756	7.7458	7.7534	8.2106	1.0000	0.0000	0.9995	0.0002
Tjp2	_AAS[Phospho (STY)]RDQLR_.2	Q9Z0U1_S960_M1_Tjp2	4.0739	4.0042	5.2362	5.3927	4.9660	4.6864	5.3740	4.3592	5.0751	5.6309	0.9250	-0.1125	0.3296	0.4821
Tjp2	_SNLPATAGSEIPGGST[Phospho (STY)]K_.2	Q9Z0U1_T1009_M1_Tjp2	5.9955	4.0255	3.7182	4.4183	4.0728	6.2046	6.8657	3.7284	6.2284	3.7226	0.9227	-0.1161	0.6542	0.1843
Tjp2	_S[Phospho (STY)]IDRDYDR_.2	#N/A	4.1864	3.1560	3.9201	5.7124	3.7643	5.3636	3.6122	3.5373	4.4331	5.1600	1.0394	0.0557	0.7861	0.1045
Tjp2	_S[Phospho (STY)]IDRDYDRDYER_.3	#N/A	6.4914	6.7013	6.1623	5.8754	3.1628	6.1660	6.7023	6.5977	6.1099	6.5634	0.8870	-0.1729	0.3079	0.5115
Tjp2	_S[Phospho (STY)]QNREDSFDYSK_.2	Q9Z0U1_S982_M1_Tjp2	3.8450	6.3961	5.8151	4.3179	6.0408	6.3349	6.2117	6.5048	3.8297	6.3076	0.9553	-0.0659	0.7424	0.1294
Tjp2	_S[Phospho (STY)]RGRS[Phospho (STY)]LER_.2	Q9Z0U1_S169_M2_Tjp2	3.7940	3.6668	4.7089	4.2328	3.8931	3.8669	3.5543	3.3974	3.4793	3.0664	1.1934	0.2551	0.0156	1.8074
Tjp2	_GRS[Phospho (STY)]IDRDYDR_.3	#N/A	2.9167	3.7219	3.6035	4.3988	4.0636	3.6284	2.4425	3.3040	4.4022	3.2583	1.1105	0.1512	0.3899	0.4090
Tjp2	_KVQVAPLQGS[Phospho (STY)]PPLSHDDR_.3	#N/A	7.0798	8.5850	8.8275	9.7469	7.1973	7.6340	8.2397	8.3058	8.0520	7.7496	1.0113	0.0163	0.8706	0.0602
Tjp2	_EHLRS[Phospho (STY)]RS[Phospho (STY)]PS[Phospho (STY)]PESR_.3	#N/A	4.8384	4.5678	6.4869	4.8904	3.2904	4.9163	5.3537	6.2939	5.7181	5.2118	0.8560	-0.2243	0.1806	0.7432
Tjp2	_DAS[Phospho (STY)]PPPAFKPEPPK_.2	#N/A	11.0871	11.1390	11.5484	11.7615	10.8450	11.2020	11.5801	11.0587	11.0929	11.1493	1.0039	0.0056	0.8290	0.0814
Tjp2	_SRS[Phospho (STY)]PS[Phospho (STY)]PESR_.2	Q9Z0U1_S265_M2_Tjp2	6.7756	6.9672	6.6677	3.7620	3.1325	3.9532	7.1978	6.8034	3.7832	7.2061	0.8339	-0.2621	0.3791	0.4213
Tjp2	_RQQYS[Phospho (STY)]DQDYHS[Phospho (STY)]S[Phospho (STY)]TEKLK_.3	Q9Z0U1_S404_M3_Tjp2	3.3274	3.8799	4.0384	5.0464	3.7682	3.7565	4.2558	3.5827	3.2313	3.9020	1.0605	0.0848	0.5264	0.2787
Tjp2	_VQVAPLQGS[Phospho (STY)]PPLSHDDR_.3	#N/A	6.6694	3.5371	6.1210	6.5434	5.3402	6.4938	3.9604	6.5928	6.6092	6.6198	0.9729	-0.0397	0.8463	0.0725
Tjp2	_GSYGS[Phospho (STY)]DPEEEEYRQQLAAHSK_.3	#N/A	8.4520	9.4648	9.8006	10.0838	9.2570	9.6893	9.6591	9.5705	9.5137	9.7104	0.9838	-0.0235	0.6088	0.2155
Tjp2	_M[Oxidation (M)]SYLTAM[Oxidation (M)]GADYLS[Phospho (STY)]C[Carbamidomethyl (C)]DSR_.2	#N/A	3.7470	3.4621	4.0244	4.2838	4.0491	3.6406	3.9816	3.7660	4.0651	3.5106	1.0097	0.0139	0.8451	0.0731
Tjp2	_SYHEAYEPDYGGGYS[Phospho (STY)]PSYDR_.3	Q9Z0U1_S239_M1_Tjp2	2.9402	4.7576	3.9241	3.8779	3.9456	3.8580	3.8554	3.6755	3.6097	3.7915	1.0404	0.0572	0.6255	0.2037

Tjp2	_ERPS[Phospho (STY)]SREETSGR_.3	Q9Z0U1_S420_M1_Tjp2	3.3808	3.3898	4.2911	3.6417	3.2523	3.2255	3.7900	3.3616	3.6607	3.3190	0.9993	-0.0011	0.9910	0.0039
Tjp2	_HSSHDMLSHS[Phospho (STY)]WEGNRER_.4	#N/A	4.0926	3.1336	3.2786	3.7812	3.0219	3.6028	3.4959	4.3372	3.5327	3.7482	0.9223	-0.1166	0.2930	0.5332
Tjp2	_HSSHDMLSHS[Phospho (STY)]WEGNR_.3	#N/A	3.3577	3.9847	4.5922	4.1360	3.8060	3.9235	4.2843	4.3660	3.1642	3.8133	1.0153	0.0219	0.8473	0.0720
Tjp2	_RQQYS[Phospho (STY)]DQDYHSSTEK_.3	Q9Z0U1_S404_M1_Tjp2	3.1548	4.0714	3.8416	4.3442	3.9597	4.1657	4.0589	3.7743	3.4228	4.3112	1.0080	0.0115	0.9076	0.0421
Tjp2	_SRHS[Phospho (STY)]S[Phospho (STY)]HDMLSHSWEGNR_.4	Q9Z0U1_S139_M2_Tjp2	3.8876	3.3386	4.5744	3.5762	3.0132	3.2154	3.2909	3.0415	4.1909	3.5432	1.0240	0.0342	0.8147	0.0890
Tjp2	_LAGGNDVGIFVAGIQEGTSAEQEGLQEGDQILK_.3	#N/A	3.2630	4.0192	4.4975	4.3964	3.9075	4.2179	4.1111	4.4608	3.2590	3.7185	1.0419	0.0592	0.6082	0.2159
Kcnd2	_GS[Phospho (STY)]VQELSTIQIR_.2	Q9Z0V2_S552_M1_Kcnd2	8.6235	7.8956	8.4088	8.5752	7.3519	8.0847	9.1399	8.9363	8.4833	8.1613	0.9397	-0.0898	0.1222	0.9129
Kcnd2	_TPLSNS[Phospho (STY)]RSS[Phospho (STY)]LNAK_.2	Q9Z0V2_S575_M2_Kcnd2	3.7339	8.2324	4.1364	3.7616	3.9999	3.5725	3.6279	7.3166	3.7872	7.0425	0.8400	-0.2515	0.4942	0.3061
Kcnd2	_IPNANVS[Phospho (STY)]GSHR_.2	Q9Z0V2_S546_M1_Kcnd2	3.3619	7.0527	8.1280	3.9536	3.9884	3.7411	4.1019	7.7791	3.3466	3.6309	1.0685	0.0956	0.8121	0.0904
Ngfr	_ADIVESLC[Carbamidomethyl (C)]SESTATS[Phospho (STY)]PV_.2	Q9Z0W1_S425_M1_Ngfr	3.1077	4.1185	3.7945	4.2971	3.7342	3.9953	4.0118	4.1268	3.4700	4.2641	0.9680	-0.0469	0.6285	0.2017
Aifm1	_AIASATEGGSVPQIR_.2	#N/A	4.2124	4.3697	3.9460	5.3452	4.6686	3.2773	3.6381	4.7539	3.8104	4.8458	1.0096	0.0139	0.9265	0.0331
Aifm1	_SITVIGGGFLGSELAC[Carbamidomethyl (C)]ALGR_.2	#N/A	3.1570	4.1854	4.3915	3.9353	3.8016	4.3239	4.0835	3.0659	2.6539	3.6126	1.1824	0.2417	0.1091	0.9620
Dctn3	_[Acetyl (Protein N-term)]AALTDVQR_.2	#N/A	6.9666	6.7746	6.4002	6.3925	6.9536	6.7152	6.5945	6.8697	6.6486	6.9415	0.9907	-0.0135	0.6773	0.1692
Dctn3	_VQVALGNIASK_.2	#N/A	4.7286	4.6892	5.0289	5.6153	4.9958	5.0460	5.7672	4.8971	4.6811	5.1052	0.9813	-0.0272	0.7141	0.1463
Dctn3	_LAQIHIQQQDQC[Carbamidomethyl (C)]VAITEESK_.3	#N/A	4.1115	5.5456	3.2974	3.8001	3.2371	4.5954	3.5148	4.3184	3.9670	3.7671	1.0529	0.0744	0.6697	0.1741
Pard6a	_LTGPSSVGPGPTDPDS[Phospho (STY)]DDDSSDLVIENR_.3	Q9Z101_S278_M1_Pard6a	4.2504	3.0920	3.9841	9.0492	2.9133	3.3153	3.6761	8.3363	3.7724	3.2051	0.9340	-0.0985	0.8416	0.0749
Adnp	_LM[Oxidation (M)]HDAS[Phospho (STY)]DS[Phospho (STY)]EVDQDDVVEWK_.3	#N/A	3.8604	3.6856	3.7341	5.8147	4.2139	3.9642	3.6587	5.3774	3.7953	3.8305	1.0112	0.0161	0.9310	0.0311
Hnrnpdl	_VFVGGLS[Phospho (STY)]PDTSEEQIK_.2	Q9Z130_S122_M1_Hnrnpdl	3.6301	4.3863	3.3637	4.4083	4.2746	6.0061	4.5566	4.9237	4.3927	4.0856	0.9677	-0.0473	0.7751	0.1106
Hnrnpdl	_DLTEYLSR_.2	#N/A	4.6556	3.5326	5.5164	4.7041	5.3681	5.9459	3.7776	5.0140	5.1388	4.9982	1.0468	0.0660	0.6715	0.1729
Hnrnpdl	_YHQIGSGK_.2	#N/A	4.3558	4.6780	3.4188	3.4986	4.9314	5.1706	3.6744	4.0243	4.4239	4.4443	1.0484	0.0682	0.6320	0.1993
Hnrnpdl	_[Acetyl (Protein N-term)]MEDM[Oxidation (M)]NEYSNIEEFAEGSK_.2	#N/A	3.9205	3.6669	3.9772	5.8177	3.4607	3.5055	3.6175	3.5879	3.7339	3.6684	1.1112	0.1521	0.3966	0.4016
Cpne6	_NNLNPSWEPFR_.2	#N/A	6.6658	6.7240	6.4834	6.4552	6.6837	6.7553	6.9519	6.6597	6.7891	6.9320	0.9700	-0.0440	0.0410	1.3876
Cpne6	_LLDGDDGPLR_.2	#N/A	6.5393	6.9574	6.6281	6.5181	6.9436	6.1587	7.2763	6.1693	6.5816	6.7449	0.9897	-0.0149	0.7788	0.1086
Cpne6	_SC[Carbamidomethyl (C)]SS[Phospho (STY)]PVFSR_.2	Q9Z140_S68_M1_Cpne6	12.4192	12.5397	13.0212	13.0319	12.2037	12.4111	12.8379	12.5133	12.4896	12.5850	0.9998	-0.0002	0.9918	0.0036
Cpne6	_C[Carbamidomethyl (C)]VLAEVPR_.2	#N/A	3.5431	5.3524	4.0920	3.9025	4.4470	4.7784	4.2354	5.4380	4.6203	3.1987	0.9953	-0.0068	0.9679	0.0142
Cpne6	_PSTPAM[Oxidation (M)]TPSPS[Phospho (STY)]P_.2	#N/A	11.6505	11.6385	12.2130	11.9998	11.6839	11.8125	12.2757	11.7175	11.9113	11.8323	0.9915	-0.0123	0.5231	0.2814
Cpne6	_SSQSLHC[Carbamidomethyl (C)]LS[Phospho (STY)]PR_.2	#N/A	10.2439	10.3932	10.9833	10.7393	10.2512	10.4375	10.8032	10.5849	10.5621	10.4096	0.9923	-0.0112	0.6295	0.2010
Cpne6	[Acetyl (Protein N-term)]S[Phospho (STY)]DPEM[Oxidation (M)]GWVPEPPAM[Oxidation (M)]TLGASR_	#N/A	3.2180	7.5254	7.8018	8.8403	6.5338	6.2115	6.5430	9.4749	7.4385	6.3595	0.8973	-0.1563	0.5210	0.2831
Cpne6	_LYSDEQWVEVER_.2	#N/A	5.9442	3.5334	3.5427	4.5873	2.9528	5.1076	3.2347	5.6114	6.4611	5.7683	0.8119	-0.3006	0.2515	0.5995
Cpne6	_YSVLLVLTDGVVSDM[Oxidation (M)]AETR_.2	#N/A	3.8786	3.1829	4.5654	3.5672	3.0043	4.7543	3.2819	3.0504	4.1999	4.2239	1.0370	0.0524	0.7635	0.1172
Cpne6	_TEVLRSC[Carbamidomethyl (C)]S[Phospho (STY)]S[Phospho (STY)]PVFSR_.2	Q9Z140_S67_M2_Cpne6	4.1664	3.4218	3.9001	3.4929	3.3101	3.3145	3.2076	3.1247	5.3262	3.4599	0.9527	-0.0698	0.7011	0.1542
Cpne6	_VLAIEYFFEEK_.2	#N/A	3.7010	3.0053	4.3878	3.6145	2.8267	3.4020	3.7628	3.3866	3.6857	3.7537	0.9568	-0.0638	0.6045	0.2186
Sumo3	_VAGQDGSVVQFK_.2	#N/A	6.5102	6.9497	6.9453	7.1878	7.3949	7.0111	6.6035	6.3353	6.9951	6.5940	1.0555	0.0779	0.0823	1.0844
Sumo3	_[Acetyl (Protein N-term)]S[Phospho (STY)]EEKPKEGVK_.2	Q9Z172_S2_M1_Sumo3	4.6040	5.4090	6.2811	5.1671	5.3116	5.1572	5.6883	5.2464	5.7210	5.3389	0.9678	-0.0472	0.5649	0.2481
Eya4	_NNPS[Phospho (STY)]PPPDSDLER_.2	Q9Z191_S338_M1_Eya4	8.4250	3.3803	3.6958	9.8236	3.1059	3.5187	3.3879	3.7616	4.0606	8.9778	1.0551	0.0773	0.8839	0.0536
Plcb1	_NDSIPQEDFTPDVYR_.2	#N/A	8.1950	8.2283	8.8425	8.4972	8.1617	8.4203	8.7450	8.1481	8.1544	8.5199	0.9999	-0.0002	0.9954	0.0020
Plcb1	_YNEIQNDYLR_.2	#N/A	7.0186	7.0197	7.5968	7.2800	7.0836	7.3232	7.4090	7.2523	7.1409	7.0882	0.9997	-0.0005	0.9861	0.0061
Plcb1	_WDDDSTIVTPIILR_.2	#N/A	6.9386	6.3123	6.8758	6.5284	6.7262	7.4207	6.6756	6.2530	6.6260	6.7503	1.0341	0.0484	0.3243	0.4891
Plcb1	_SYIQEVVQYIK_.2	#N/A	5.2968	5.1912	4.3372	4.6669	4.9821	5.1458	5.0117	4.9022	5.1157	5.7470	0.9504	-0.0734	0.3140	0.5031
Plcb1	_SPVEFVEYNK_.2	#N/A	7.2255	6.9568	5.8467	7.3956	6.7496	6.7575	7.5389	7.0109	7.0325	7.3869	0.9420	-0.0863	0.1927	0.7150
Plcb1	_EVIEAIAEC[Carbamidomethyl (C)]AFK_.2	#N/A	5.9987	6.0420	4.0320	6.4427	5.7152	5.4962	6.4883	5.7303	5.6706	5.4699	0.9625	-0.0551	0.6503	0.1869
Plcb1	_LQVTPEGR_.2	#N/A	5.1485	5.0270	5.7931	5.6270	5.8149	5.8362	5.7489	6.1407	5.6029	5.4609	0.9656	-0.0505	0.3911	0.4077
Plcb1	_LIFGDALLM[Oxidation (M)]EPLEK_.2	#N/A	5.0856	4.9459	3.7602	2.8392	4.8310	5.3066	4.7591	4.9858	4.5432	4.6838	0.9406	-0.0883	0.5849	0.2329
Plcb1	_EADPGETS[Phospho (STY)]SEAPSETR_.2	Q9Z1B3_S848_M1_Plcb1	7.7867	7.5721	7.4984	8.4002	6.9928	7.5542	3.7842	7.8267	7.7242	3.8247	1.3185	0.3989	0.0844	1.0739
Plcb1	_VGTYVEVDM[Oxidation (M)]FGLPVDTR_.2	#N/A	5.9132	5.8413	3.1670	3.6696	5.2377	5.0108	3.3843	4.6787	5.6403	5.4255	1.0051	0.0073	0.9733	0.0117
Plcb1	_TSPFPILLSFENHVDSPK_.2	#N/A	5.3387	4.5656	4.3684	4.4106	4.6709	4.5007	4.2043	4.6661	4.3807	3.3369	1.1195	0.1628	0.1269	0.8967
Plcb1	_SEPSS[Phospho (STY)]PDHGSSAIEQDLAALDAEM[Oxidation (M)]TQK_.3	#N/A	11.1182	11.1072	11.4587	8.6186	10.5347	11.1598	11.4004	11.2333	11.1691	11.1443	0.9492	-0.0752	0.3183	0.4971
Plcb1	_VNLKS[Phospho (STY)]PSSEEIERENPGR_.3	#N/A	4.0139	5.6256	4.0264	3.6888	3.1396	5.8213	3.3183	3.6328	3.9744	3.5781	1.2096	0.2745	0.2152	0.6671
Plcb1	_YVNLM[Oxidation (M)]EQR_.2	#N/A	3.3589	6.3440	4.5934	5.7501	6.1287	5.8204	5.2596	6.3216	5.5996	5.9791	0.9210	-0.1187	0.4749	0.3234
Plcb1	_SPS[Phospho (STY)]SEEIER_.2	#N/A	8.8843	8.0750	3.8713	8.9430	7.1844	8.3168	8.2574	8.3674	3.4035	8.7588	1.0485	0.0683	0.8093	0.0919
Plcb1	_ELLDVGNIGHLEQR_.2	#N/A	4.0121	3.0911	3.9974	3.2117	3.8178	3.8731	3.7172	3.5223	4.7152	4.3368	0.9004	-0.1514	0.2153	0.6670
Plcb1	_KSEPSSPDHGSS[Phospho (STY)]AIEQDLAALDAEM[Oxidation (M)]TQK_.4	#N/A	3.6712	4.0382	7.3014	3.9786	3.8986	3.7019	4.0129	7.2228	6.8430	3.6986	0.8140	-0.2969	0.3525	0.4529
Plcb1	_FESFEISK_.2	#N/A	4.5056	4.1322	4.2190	5.3417	4.8371	2.9956	3.9110	3.4086	4.9595	5.3963	0.9818	-0.0265	0.8861	0.0525
Plcb1	_C[Carbamidomethyl (C)]VELDC[Carbamidomethyl (C)]WK_.2	#N/A	5.0373	4.0054	3.6826	4.1852	4.4631	2.9853	3.5964	3.9228	4.7533	4.1522	0.9887	-0.0164	0.9117	0.0401
Plcb1	_VNLKS[Phospho (STY)]PSSEEIER_.2	#N/A	7.9784	8.5527	8.1742	9.4400	7.5034	9.1890	9.2573	8.8327	9.0656	9.1736	0.9329	-0.1002	0.1511	0.8206
Plcb1	_LTDVAEEC[Carbamidomethyl (C)]QNNQLKK_.2	#N/A	3.2492	3.6848	3.4502	3.9003	3.4194	2.6165	3.5260	3.3229	3.1195	3.9367	0.9742	-0.0377	0.7429	0.1291
Plcb1	_KKSEPSSPDHGS[Phospho (STY)]S[Phospho (STY)]AIEQDLAALDAEM[Oxidation (M)]TQK_.4	#N/A	8.9396	7.7877	3.3960	4.2800	7.3870	3.8034	7.0167	4.1236	3.7867	7.8533	1.0417	0.0589	0.8748	0.0581
Plcb1	_SPS[Phospho (STY)]SEEIERENPGR_.2	#N/A	3.8388	3.7945	4.3091	3.8528	3.6440	3.5884	4.0011	4.0017	3.4997	3.5301	1.0212	0.0303	0.6532	0.1850
Plcb1	_DYVPDTYADVIEALSNPIR_.2	#N/A	4.7858	4.7551	3.7504	4.8023	4.5258	4.3923	3.9677	4.6943	4.4450	4.1284	1.0448	0.0632	0.4487	0.3480
Plcb1	_SPS[Phospho (STY)]SEEIERENPGREFDTPL_.3	#N/A	3.4496	4.2059	3.1833	4.2098	4.0942	4.0313	3.9245	4.2741	3.0724	3.9051	1.0180	0.0257	0.8259	0.0831
Plcb1	_VFLNNLC[Carbamidomethyl (C)]PRPEIDNIFSEFGAK_.3	#N/A	3.1601	4.0661	2.5676	3.5863	3.1804	3.6429	3.8841	3.9323	3.4175	3.5306	0.9122	-0.1325	0.2837	0.5471
Eif3g	_GIPLPTGDTS[Phospho (STY)]PEPELLPGDPLPPPK_.3	Q9Z1D1_S42_M1_Eif3g	3.8313	3.9660	7.2097	4.2018	3.9671	3.8994	4.0973	4.1763	3.9657	3.9752	1.1132	0.1547	0.5179	0.2858
Gys1	_HSS[Phospho (STY)]PHQS[Phospho (STY)]EDEEEPR_.2	#N/A	4.0774	3.3818	3.9118	3.4839	3.2031	3.5735	3.4639	3.9404	3.9984	3.8440	0.9458	-0.0803	0.3168	0.4993
Atp6v1c1	_GVTQIDNDLK_.2	#N/A	7.9374	7.8914	8.2773	7.6094	7.3904	7.5918	7.7171	7.9782	7.8116	7.9053	0.9911	-0.0129	0.6867	0.1632
Atp6v1c1	_[Acetyl (Protein N-term)]TEFWLISAPGEK_.2	#N/A	7.3000	7.0965	7.3742	6.3326	6.6390	7.0545	7.4905	7.0183	7.1994	7.1045	0.9671	-0.0483	0.3147	0.5021
Atp6v1c1	_GNLQNLER_.2	#N/A	6.5880	7.0330	7.4223	6.6269	6.7307	6.8082	6.9149	7.4075	6.9629	7.0590	0.9693	-0.0451	0.2676	0.5725
Atp6v1c1	_VGTLDVLVGLSDELAK_.2	#N/A	6.4156	5.9043	5.3161	4.8098	3.7825	4.9700	5.7366	5.8508	5.8739	5.7429	0.8963	-0.1579	0.2354	0.6282
Atp6v1c1	_IDC[Carbamidomethyl (C)]NLLEFK_.2	#N/A	5.0943	5.3866	4.2699	4.9445	5.1049	4.4132	5.7539	5.3581	5.1055	5.0257	0.9168	-0.1253	0.1239	0.9070
Atp6v1c1	_SSNVLSEDQDSYLC[Carbamidomethyl (C)]NVTLFR_.2	#N/A	4.8960	4.7202	3.9342	5.4527	4.4331	4.2780	5.0944	5.2291	4.7881	5.5795	0.8930	-0.1633	0.1019	0.9920
Atp6v1c1	_ASAYNNLK_.2	#N/A	5.5920	5.6333	6.0361	4.7529	5.5031	5.8361	6.2726	6.0473	5.5775	5.3780	0.9553	-0.0659	0.3758	0.4251
Atp6v1c1	_NNNLAVSSK_.2	#N/A	4.9593	5.0039	4.9565	5.3463	4.7914	4.3037	4.6087	5.2019	4.4567	5.0502	1.0133	0.0190	0.7814	0.1072
Atp6v1c1	_KAVDDFR_.2	#N/A	6.1318	6.0836	3.6871	5.4316	5.5140	5.7149	6.1725	6.3737	5.7930	5.9572	0.8935	-0.1625	0.2058	0.6866
Atp6v1c1	_VQENLLASGVDLVTYITR_.3	#N/A	4.7128	4.1615	5.1172	4.8387	3.7191	4.6060	4.1804	4.2463	4.6030	4.3223	1.0433	0.0612	0.5027	0.2987
Atp6v1c1	_FNIPDLK_.2	#N/A	3.1402	4.6384	3.6880	3.1569	2.4028	3.0772	4.0668	2.4815	4.3363	3.6961	0.9192	-0.1216	0.5735	0.2414
Atp6v1c1	_NISEIIAK_.2	#N/A	2.9282	3.7334	3.3427	1.5110	3.5547	1.7182	4.5356	2.9479	2.3942	2.3204	0.9175	-0.1242	0.7028	0.1532
Atp6v1c1	_TC[Carbamidomethyl (C)]QQTWEK_.2	#N/A	3.3663	4.5062	4.0531	3.9492	6.6357	3.7367	4.0975	3.0520	3.3510	3.0219	1.2940	0.3719	0.1525	0.8166
Atp6v1c1	_SRAS[Phospho (STY)]AYNNLK_.2	Q9Z1G3_S141_M1_Atp6v1c1	4.2375	3.5419	3.4235	3.9261	3.3632	4.3663	3.6408	4.1923	3.8409	3.8931	0.9789	-0.0307	0.7386	0.1316
Atp6v1c1	_LDAFVEGVVK_.2	#N/A	3.5781	2.5719	4.1937	3.7374	4.2046	3.5249	3.8857	3.2638	3.5628	3.4147	1.0293	0.0416	0.7572	0.1208
Atp6v1c1	_VAQYMADVLEDSKDK_.3	#N/A	3.6528	2.9572	4.3396	3.3414	4.2793	3.4502	4.5569	3.2762	4.4256	3.3084	0.9430	-0.0846	0.5860	0.2321

Atp6v0a1	_DLNPDVNVFQR_.2	#N/A	8.6112	8.5922	8.7496	8.1517	8.1666	8.1874	8.9666	8.6846	8.4510	8.3103	0.9775	-0.0328	0.3104	0.5081
Atp6v0a1	_FLPFSFEHIR_.2	#N/A	6.5799	7.2834	6.6231	6.3273	6.2328	6.3564	7.4183	7.6041	6.9790	6.5708	0.9194	-0.1213	0.0634	1.1979
Atp6v0a1	_HLGTLNFGGIR_.2	#N/A	7.1522	7.2549	7.1871	6.4849	6.2641	6.7327	7.5271	7.2910	6.6412	6.8033	0.9689	-0.0456	0.4350	0.3615
Atp6v0a1	_LGFVAGVINR_.2	#N/A	6.7746	6.2557	6.3909	6.6837	5.6147	5.9608	6.5726	7.1259	6.3372	6.5439	0.9451	-0.0815	0.1995	0.7001
Atp6v0a1	_NFLELTELK_.2	#N/A	6.2041	6.3312	6.2903	5.8553	5.6152	5.9026	5.8232	6.8494	6.8373	6.6946	0.9209	-0.1188	0.0652	1.1860
Atp6v0a1	_FYTGTGFK_.2	#N/A	6.4597	6.1114	6.0767	5.3338	5.5925	5.5443	4.7819	6.7206	6.7188	6.0983	0.9627	-0.0549	0.6057	0.2178
Atp6v0a1	_GTEHSGSTVPSILNR_.2	#N/A	5.0526	5.3043	4.3242	4.9117	5.1254	5.0076	5.3271	5.5426	5.3994	4.4515	0.9564	-0.0643	0.4098	0.3874
Atp6v0a1	_KANIPIMDTGENPEVPFPR_.3	#N/A	5.6805	4.5292	3.8879	6.3181	4.8357	3.3267	5.2170	4.4096	3.7070	4.0698	1.0947	0.1306	0.5253	0.2796
Atp6v0a1	_ANIPIM[Oxidation (M)]DTGENPEVPFPR_.2	#N/A	6.8607	6.9792	6.6165	6.1786	6.1007	6.7221	6.7591	6.9906	6.5553	6.1607	0.9939	-0.0088	0.8663	0.0623
Atp6v0a1	_LHWVEFQNK_.2	#N/A	3.1199	5.4964	3.8067	5.5935	4.5087	3.9831	6.1513	5.6185	4.4176	5.0438	0.8324	-0.2647	0.1639	0.7854
Atp6v0a1	_IDDLQM[Oxidation (M)]VLNQTEDHR_.3	#N/A	5.0883	5.0209	4.7288	4.2499	3.5032	4.8723	4.8726	5.9798	5.2226	5.2801	0.8574	-0.2220	0.0667	1.1762
Atp6v0a1	_QAEIENPLEDPVTGDYVHK_.3	#N/A	7.0182	6.4227	6.7951	6.5440	6.3286	6.9420	6.7191	6.7536	6.5449	6.3214	1.0137	0.0197	0.5985	0.2229
Atp6v0a1	_FTHGFQNIVDAYGIGTYR_.3	#N/A	6.1512	6.3529	6.9131	6.2651	5.0315	6.0695	6.6112	6.9723	6.1890	6.2343	0.9429	-0.0848	0.3143	0.5026
Atp6v0a1	_AIYHTLNLC[Carbamidomethyl (C)]NIDVTQK_.2	#N/A	3.5826	3.6089	5.0077	3.4921	2.6305	2.9821	3.1567	2.4535	3.5658	3.7765	1.0965	0.1329	0.5285	0.2770
Atp6v0a1	_ASLYPC[Carbamidomethyl (C)]PETPQER_.2	#N/A	8.1409	8.3830	8.3650	7.7674	7.7532	7.6856	8.1276	8.2590	8.0218	7.9497	0.9909	-0.0132	0.6792	0.1680
Atp6v0a1	_EINTNQEALKR_.2	#N/A	5.1551	5.0394	4.6375	5.1261	4.8595	5.0188	5.5828	5.2951	3.9411	5.3887	0.9843	-0.0228	0.8065	0.0934
Atp6v0a1	_DM[Oxidation (M)]IDLEANFEKIENELK_.3	#N/A	3.1212	4.0769	4.3557	3.8995	3.1147	4.3596	4.0478	4.2529	4.3880	4.6177	0.8832	-0.1792	0.1382	0.8594
Atp6v0a1	_M[Oxidation (M)]QTNQTPPTYNK_.2	#N/A	6.0734	4.9197	4.1839	6.4630	4.5720	6.2753	6.5002	7.3649	4.1306	3.2522	1.0193	0.0276	0.9133	0.0394
Atp6v0a1	_DMIDLEANFEK_.2	#N/A	3.8430	3.4994	3.5767	4.6213	3.3207	4.4087	3.2687	3.8807	4.1798	4.2986	0.9927	-0.0106	0.9317	0.0307
Atp6v0a1	_ERIPTFER_.2	#N/A	6.4402	3.6190	3.4571	4.5017	2.8672	6.2387	7.2068	4.0003	4.2993	4.1790	0.9186	-0.1225	0.6924	0.1596
Atp6v0a1	_SVFIIFFQGDQLK_.2	#N/A	3.8563	3.4861	3.5899	3.1337	3.0000	4.4220	3.2820	3.8675	1.7642	4.3118	1.0832	0.1153	0.6059	0.2176
Nek4	_SPAS[Phospho (STY)]LEGHT[Phospho (STY)]GK_.2	Q9Z1J2_S343_M2_Nek4	9.3694	9.7225	4.0233	10.2262	9.7286	5.0705	6.2350	3.7414	3.7834	5.5659	1.6607	0.7318	0.0629	1.2013
Nek4	_RSSDGGDGEGS[Phospho (STY)]ELVKPLYPSNK_.3	Q9Z1J2_S438_M1_Nek4	3.9086	3.3771	4.1981	3.4071	3.2309	3.2142	3.3231	5.4344	4.1422	4.5538	0.8150	-0.2952	0.0805	1.0943
Apc2	_VSS[Phospho (STY)]PEDSPAGTPQR_.2	Q9Z1K7_S2143_M1_Apc2	7.4756	7.3403	4.1276	7.8753	3.6736	2.9510	7.9898	7.9802	7.4236	7.2263	0.7281	-0.4577	0.1061	0.9744
Apc2	_RGS[Phospho (STY)]DGEARPLPR_.3	Q9Z1K7_S2099_M1_Apc2	5.4296	4.8845	3.5787	4.0814	3.5184	5.5851	5.9502	5.5471	5.7912	5.4878	0.7926	-0.3354	0.0374	1.4267
Apc2	_APS[Phospho (STY)]PEQQR_.2	Q9Z1K7_S1805_M1_Apc2	6.8437	3.5165	4.3965	3.3983	3.4048	3.2198	6.4720	7.1520	4.3688	5.9103	0.6911	-0.5330	0.0620	1.2075
Apc2	_FHAPALGPEPAART[Phospho (STY)]PEGS[Phospho (STY)]PVHGSGPSK_.4	Q9Z1K7_S108_M2_Apc2	3.4690	6.8524	3.2027	6.5693	5.8290	6.3585	6.9131	7.0640	6.9501	6.6536	0.7803	-0.3579	0.1054	0.9773
Ddx39b	_GLAITFVSDENDAK_.2	#N/A	4.3720	4.8532	3.5579	5.1460	5.2343	4.5226	5.9583	5.2052	5.1790	5.0330	0.8635	-0.2118	0.0743	1.1288
Ddx39b	_DVQEIFR_.2	#N/A	3.4200	3.9985	4.5391	3.6486	3.4140	3.4361	3.7969	4.7358	4.4484	3.3259	0.9181	-0.1233	0.3557	0.4489
Ndufa7	_NWASGQDLQAK_.2	#N/A	8.0078	7.9107	7.8370	7.9302	8.2922	7.9462	7.9500	7.7131	8.0058	8.0716	1.0066	0.0095	0.6227	0.2058
Ndufa7	_LSNNYYC[Carbamidomethyl (C)]TR_.2	#N/A	6.4110	6.7282	5.9343	6.6149	7.2167	6.4963	6.3018	6.9600	6.5090	6.6542	0.9940	-0.0086	0.8741	0.0585
Ndufa7	_AAESSAM[Oxidation (M)]AATEK_.2	#N/A	5.6388	6.4924	6.1585	6.0917	6.7388	6.6486	6.2057	5.9229	6.4208	5.9436	1.0280	0.0399	0.4779	0.3207
Ndufa7	_YQEIAKR_.2	#N/A	5.6348	6.4153	6.2033	5.5761	6.5659	6.1034	5.1477	6.1669	5.7309	5.6166	1.0737	0.1026	0.1530	0.8154
Vars1	_DINLDVNR_.2	#N/A	3.8832	3.1876	3.8885	3.4323	3.0089	3.2197	3.5805	4.1227	4.1166	3.1096	0.9208	-0.1191	0.3065	0.5135
Vars1	_[Acetyl (Protein N-term)]SILYVSPHPDAFPSLR_.2	#N/A	3.7463	4.9206	4.4331	3.5692	2.8720	3.3566	3.7174	5.0350	3.7311	3.2465	0.9705	-0.0433	0.8172	0.0877
Vars1	_LSATVTEAFVR_.2	#N/A	3.8081	3.1124	4.4949	3.4967	2.9338	3.2949	3.2114	3.1209	4.2704	3.4637	1.0019	0.0028	0.9850	0.0065
Bag6	_PLTS[Phospho (STY)]PESLSR_.2	#N/A	8.9881	8.0964	8.4799	4.0681	3.8739	8.4128	8.8960	4.4339	8.5867	8.6755	0.9135	-0.1305	0.6648	0.1773
Bag6	_LQEDPNYS[Phospho (STY)]PQR_.2	#N/A	4.0763	6.1960	5.9899	6.6199	3.2020	3.0267	7.0038	4.3536	6.9176	6.8117	0.7736	-0.3703	0.1786	0.7481
Bag6	_ESFSLVQVQPGVDIIR_.2	#N/A	3.3217	3.7503	4.0085	4.3162	4.1013	3.7813	3.5999	3.6073	3.7611	2.9773	1.1129	0.1543	0.1159	0.9359
Bag6	_TSPEPQRENAS[Phospho (STY)]PAPGTTAEEAM[Oxidation (M)]SR_.3	#N/A	6.6018	6.9266	7.8753	7.4552	6.4546	6.5959	7.0490	7.1386	6.9995	4.3195	1.0954	0.1314	0.3512	0.4544
Bag6	_LQEDPNYS[Phospho (STY)]PQRFPNAHR_.3	#N/A	9.8335	9.5032	10.4344	4.3903	8.9492	4.2118	9.9196	3.7282	9.7359	4.3573	1.1372	0.1855	0.6395	0.1941
Bag6	_AAGARPLTS[Phospho (STY)]PESLSR_.2	#N/A	3.8331	3.4357	3.5668	3.9892	3.2859	3.9496	3.2865	3.9007	4.1998	4.9962	0.8977	-0.1557	0.2232	0.6513
Bag6	_ENAS[Phospho (STY)]PAPGTTAEEAMSR_.2	#N/A	6.1772	4.8454	3.1797	6.9198	4.6545	3.6576	5.2354	4.0470	3.6341	4.7155	1.1129	0.1543	0.5430	0.2652
Rasgrp1	_SNHVLAQMDHGDS[Phospho (STY)]A_.2	#N/A	6.5690	4.0097	4.1977	3.9186	4.6296	3.7354	6.3279	5.4013	5.1270	5.3797	0.8113	-0.3017	0.1064	0.9729
Septin3	_VVNIIPVIAK_.2	#N/A	8.4468	8.5969	8.0297	8.2352	8.4100	8.2178	8.1390	8.4446	8.6397	8.4805	0.9877	-0.0178	0.4564	0.3406
Septin3	_STLVNTLFK_.2	#N/A	7.5041	7.9105	7.8261	7.4825	7.5449	7.5018	7.3395	7.7879	7.5929	7.7456	1.0016	0.0022	0.9272	0.0328
Septin3	_KASS[Phospho (STY)]WNR_.2	Q9Z1S5_S92_M1_Septin3	3.9838	3.2882	3.1698	3.6725	7.0260	3.1191	3.3871	7.4238	7.3773	7.1399	0.6385	-0.6472	0.0691	1.1607
Septin3	_THLQDLK_.2	#N/A	6.6822	6.8279	6.2555	7.2630	6.8514	6.1818	6.2700	6.5675	6.7998	6.7997	1.0102	0.0147	0.7753	0.1105
Septin3	_YINEQYEK_.2	#N/A	6.1982	6.1962	5.8974	5.8613	6.7371	5.8205	4.9946	6.5082	5.7216	6.2343	1.0433	0.0611	0.4463	0.3504
Septin3	_EVTHNIHYETYR_.3	#N/A	5.0461	5.2084	5.1777	5.1119	6.0807	6.5971	6.0654	5.6621	6.3054	6.2039	0.9138	-0.1300	0.1712	0.7665
Septin3	_LTVIDTPGFGDQINNENC[Carbamidomethyl (C)]WEPIEK_.3	#N/A	3.1670	3.9058	4.6692	4.9947	3.6098	3.1013	2.8114	4.3378	2.6560	4.3235	1.1064	0.1459	0.5067	0.2953
Septin3	_ADTM[Oxidation (M)]TLEEK_.2	#N/A	5.3986	5.7227	5.6693	5.6925	5.6576	6.1345	5.4885	5.8940	5.6438	5.4527	1.0165	0.0236	0.5399	0.2677
Septin3	_AIGHVIEEGGVK_.2	#N/A	5.7297	6.2019	6.2018	6.6632	6.4778	6.4921	6.4328	6.1544	5.9801	6.1727	1.0177	0.0253	0.5684	0.2453
Septin3	_ASSWNREEK_.2	#N/A	3.3442	3.4574	3.9357	3.4795	3.3457	3.2789	3.1720	2.8963	3.8208	3.6823	1.0238	0.0339	0.7108	0.1483
Gab2	_ASS[Phospho (STY)]C[Carbamidomethyl (C)]ETYEYPAR_.2	Q9Z1S8_S402_M1_Gab2	9.4084	9.6501	10.1426	10.0834	8.9986	9.4166	9.8159	9.3965	9.5853	9.6864	0.9995	-0.0007	0.9854	0.0064
Gab2	_SAS[Phospho (STY)]FSQGTR_.2	Q9Z1S8_S211_M1_Gab2	5.1236	4.9401	3.7969	4.2996	3.7366	6.0591	4.3537	3.8189	5.7412	5.7143	0.9495	-0.0747	0.6885	0.1621
Gab2	_SSLTGS[Phospho (STY)]ETDNEDVYTFK_.2	Q9Z1S8_S282_M1_Gab2	4.2052	3.1372	3.9389	5.2845	3.3489	5.0304	3.6309	3.5185	3.8176	3.1599	1.1772	0.2353	0.2079	0.6820
Ap3b1	_EDGGLEDNEKNFYES[Phospho (STY)]EEEEEEKEK_.4	#N/A	3.3730	4.1782	4.0598	3.9425	3.9995	3.7300	4.0908	3.0586	3.3577	3.6198	1.0987	0.1359	0.1638	0.7858
Ap3b1	_NFYES[Phospho (STY)]EEEEEEKEK_.2	#N/A	3.9908	4.6637	3.1659	4.6604	3.1346	3.4900	3.3832	4.8108	4.9216	3.8006	0.9106	-0.1351	0.4403	0.3562
Pikfyve	_SAS[Phospho (STY)]ITNLSLDR_.2	Q9Z1T6_S307_M1_Pikfyve	8.1106	7.5518	8.2868	4.1459	6.1903	7.8752	8.6777	8.0899	7.8795	8.0683	0.8591	-0.2190	0.2008	0.6972
Pikfyve	_[Acetyl (Protein N-term)]ATDDKSS[Phospho (STY)]PTLDSANDLPR_.2	Q9Z1T6_S8_M1_Pikfyve	5.8181	5.9917	6.5007	6.6941	5.7188	3.6539	4.0837	6.3663	6.1942	6.1670	1.0047	0.0068	0.9705	0.0130
Pikfyve	_SGSPMVPSYETSVS[Phospho (STY)]PQANR_.2	Q9Z1T6_S329_M1_Pikfyve	4.0513	3.3556	3.2372	3.2642	3.1770	3.0517	3.4125	3.7370	4.0360	4.4423	0.8590	-0.2192	0.0593	1.2268
Stk39	_T[Phospho (STY)]PDIAQR_.2	#N/A	12.2018	11.6643	11.6508	12.0186	10.1585	11.6473	11.3903	11.5707	11.8539	11.9713	0.9881	-0.0173	0.7251	0.1396
Stk39	_RVPGSS[Phospho (STY)]GHLHK_.3	Q9Z1W9_S383_M1_Stk39	4.2035	4.1379	4.0427	5.5052	5.0398	3.7280	4.0613	3.3001	5.9954	5.9049	0.9226	-0.1162	0.5738	0.2413
Stk39	_TEDGDWEWS[Phospho (STY)]DDEM[Oxidation (M)]DEKSEEGK_.3	#N/A	4.0254	4.3990	3.8934	6.0486	4.1838	5.3067	3.3939	4.7522	5.4243	4.3022	1.0391	0.0553	0.7589	0.1198
Stk39	_TEDGDWEWS[Phospho (STY)]DDEM[Oxidation (M)]DEK_.2	#N/A	3.4931	3.9199	4.0947	9.3167	7.4851	5.9852	4.3163	8.3689	8.2524	8.2138	0.7843	-0.3505	0.3006	0.5220
Stk39	_LLTRT[Phospho (STY)]PDIAQR_.2	#N/A	3.3277	3.9472	6.3582	5.8753	3.4399	4.2883	3.7203	5.8182	3.8040	4.1947	1.0354	0.0502	0.8431	0.0741
Ptdss2	_VAGGSGSES[Phospho (STY)]PLLK_.2	#N/A	8.8234	8.2258	9.0584	8.0801	7.2039	8.4279	9.1439	8.7640	8.3772	8.4340	0.9566	-0.0640	0.3268	0.4857
Ptdss2	_RVAGGSGSES[Phospho (STY)]PLLK_.2	#N/A	4.2496	2.9766	3.4355	3.9382	2.8649	3.7597	3.6529	4.1803	3.8289	3.9052	0.9089	-0.1377	0.2597	0.5855
Ilf3	_RPM[Oxidation (M)]EEDGEEKS[Phospho (STY)]PSK_.3	#N/A	3.1656	3.6583	3.7199	3.9438	4.1453	3.8200	4.0921	3.9167	3.3950	3.6211	0.9963	-0.0054	0.9479	0.0233
Strap	_TVDFTQDSNYLLTGGQDK_.2	#N/A	5.6294	5.6294	6.1209	6.3230	5.5926	6.3489	5.5842	4.1774	5.4507	5.1183	1.1688	0.2251	0.0250	1.6017
Strap	_C[Carbamidomethyl (C)]VLPEEDS[Phospho (STY)]GELAKPK_.2	#N/A	5.1455	5.6459	4.1760	6.4932	5.4541	6.0166	6.4058	5.6987	5.6460	6.2422	0.9150	-0.1281	0.2727	0.5644
Hnrnpc	_GFAFVQYVNER_.2	#N/A	6.5838	6.2605	7.1438	6.3819	6.5365	6.4923	6.8593	6.5847	6.2655	6.7652	0.9921	-0.0114	0.7874	0.1038
Hnrnpc	_VFIGNLNTLVVK_.2	#N/A	6.0424	6.0055	6.1917	5.8956	6.1753	5.4767	5.6117	5.6249	5.6728	6.4975	1.0193	0.0275	0.6174	0.2095
Hnrnpc	_M[Oxidation (M)]IAGQVLDINLAAEPK_.2	#N/A	3.4258	4.2310	3.5140	4.6153	3.7774	3.8692	4.3300	2.9227	3.3107	2.9954	1.1522	0.2043	0.1724	0.7634
Hnrnpc	_SEEEQSSASVK_.2	#N/A	3.6655	4.4860	3.3992	3.4173	4.6097	4.5823	3.7085	3.1620	3.5704	4.2389	1.0972	0.1338	0.3376	0.4716
Hnrnpc	_QADLSFSS[Phospho (STY)]PVEM[Oxidation (M)]K_.2	#N/A	6.8014	6.4100	3.7086	3.6408	3.6483	6.8531	7.1496	5.8391	3.7374	6.9390	0.8750	-0.1926	0.5011	0.3001
Hnrnpc	_NEKS[Phospho (STY)]EEEQSSASVKK_.3	#N/A	4.3802	3.6356	4.1138	3.9545	3.5239	3.1007	4.4947	3.3436	2.9870	3.2463	1.0759	0.1055	0.4710	0.3269
Hnrnpc	_NEKS[Phospho (STY)]EEEQSSASVK_.2	#N/A	5.6579	5.9035	6.4473	6.5497	6.0503	6.1509	6.3109	6.1028	5.6574	5.8494	1.0245	0.0349	0.4943	0.3060

Hnrnpc	_SEEEQSSAS[Phospho (STY)]VKKDETNVK_.3	#N/A	3.7874	3.0428	3.5211	4.5657	2.9311	3.6935	3.5866	3.9363	4.2354	3.7145	0.9282	-0.1076	0.4122	0.3848
Hnrnpc	_SDVEAIFSK_.2	#N/A	4.2703	3.5747	3.4563	2.7307	3.3960	4.0131	3.6736	3.6358	4.0451	4.3255	0.9116	-0.1335	0.2850	0.5451
Dpp6	_KHEDESEAWLHR_.3	#N/A	7.8897	4.4895	6.9660	5.7631	6.4539	7.5938	8.2731	6.0127	6.2218	6.7581	0.9574	-0.0628	0.7118	0.1476
Dpp6	_IYFLSTEDLPR_.2	#N/A	5.6836	5.2204	6.1163	4.4025	4.1795	5.4920	5.7451	6.0189	5.4088	5.0965	0.9309	-0.1034	0.3815	0.4185
Dpp6	_HLYSANTVDDFNR_.2	#N/A	4.3156	4.4379	4.2759	4.3608	3.3419	3.0993	4.9355	4.5700	4.6111	3.9140	0.8811	-0.1825	0.1592	0.7980
Fxyd1	_TGEPDEEEGTFRSS[Phospho (STY)]IR_.3	Q9Z239_S83_M1_Fxyd1	3.7677	3.5603	3.9386	4.2381	3.3817	3.9525	3.7310	3.6742	3.8127	4.0343	0.9983	-0.0025	0.9698	0.0133
Snapin	_[Acetyl (Protein N-term)]AAAGSAAVSGAGT[Phospho (STY)]PVAGPTGR_.2	Q9Z266_T14_M1_Snapin	3.5614	7.5671	4.7524	8.3519	6.5795	7.7810	7.1830	7.4345	7.1118	7.6385	0.8761	-0.1908	0.3756	0.4253
Snapin	_AMLDSGVYPPGS[Phospho (STY)]PSK_.2	Q9Z266_S133_M1_Snapin	8.3956	3.4792	3.9574	9.3166	3.3675	3.2571	3.1503	3.5000	4.3315	3.1785	1.4959	0.5810	0.2566	0.5907
Snapin	_VVLVNNILQNAQER_.2	#N/A	3.3438	4.3396	3.9666	4.4693	4.8088	4.2908	4.1839	3.6492	4.0351	4.4363	1.0312	0.0443	0.6700	0.1739
Rasal1	_RIS[Phospho (STY)]FKGT[Phospho (STY)]PTEEQVR_.3	Q9Z268_S396_M2_Rasal1	3.7553	4.2255	3.4890	3.9040	2.7182	3.5985	3.6187	3.9684	5.0112	4.2109	0.8603	-0.2171	0.1315	0.8811
Rasal1	_LLEDSS[Phospho (STY)]LDTEVDPGR_.2	Q9Z268_S749_M1_Rasal1	4.3784	2.9640	4.1121	3.6559	2.7853	3.4433	3.8041	3.3453	3.6443	3.3332	1.0070	0.0101	0.9423	0.0258
Baz1b	_LADDEGDS[Phospho (STY)]DSESVGQSR_.2	Q9Z277_S1464_M1_Baz1b	7.6556	7.4265	8.2385	3.9165	6.3150	6.1527	8.4491	8.0243	7.4939	3.7967	0.9534	-0.0689	0.7865	0.1043
Baz1b	_EENVLGKVDR_.2	#N/A	7.5030	7.3055	7.2764	7.3490	6.7686	7.3279	7.6750	7.5212	7.3993	7.2460	0.9725	-0.0403	0.1999	0.6991
Pdpk1	_ANS[Phospho (STY)]FVGTAQYVSPELLTEK_.2	#N/A	9.3443	10.1468	10.4137	10.8545	9.1279	9.4681	9.7841	10.2386	9.8693	9.2296	1.0115	0.0165	0.7783	0.1089
Ate1	_KEGS[Phospho (STY)]KEFIHPQSIEEK_.3	#N/A	6.2217	3.2499	7.2160	7.0024	5.0187	6.1260	6.6874	6.6696	6.5024	3.0472	1.0138	0.0198	0.9407	0.0265
Ate1	_EFIHPQS[Phospho (STY)]IEEK_.2	Q9Z2A5_S174_M1_Ate1	5.6561	6.0420	6.0863	6.3338	4.9861	5.5318	6.6119	6.5143	5.8414	5.2561	0.9532	-0.0691	0.4445	0.3521
Ate1	_EGS[Phospho (STY)]KEFIHPQSIEEK_.3	#N/A	6.4424	5.8528	6.5731	6.9289	4.8710	6.2865	6.3685	6.3584	5.8724	6.0541	0.9993	-0.0010	0.9914	0.0038
Rps6ka4	_LEPVYSPAGS[Phospho (STY)]PPPGDPR_.2	Q9Z2B9_S347_M1_Rps6ka4	4.2632	3.5675	3.4491	3.9518	3.3888	4.3406	3.2006	4.1667	3.8153	3.9188	1.0136	0.0195	0.8523	0.0694
Rps6ka4	_RGS[Phospho (STY)]PVPASSGR_.2	Q9Z2B9_S745_M1_Rps6ka4	3.3362	7.0019	4.5707	4.3232	3.9808	4.1447	4.0378	7.1245	3.4439	3.7918	0.9913	-0.0126	0.9669	0.0146
Mtmr1	_GSS[Phospho (STY)]PTHSATPVHTSV_.2	Q9Z2C4_S657_M1_Mtmr1	11.7191	11.7750	12.3536	11.9452	10.9393	11.6108	12.2149	11.9711	11.8278	11.6629	0.9836	-0.0238	0.4643	0.3332
Mtmr1	_HPSIETLDS[Phospho (STY)]PTGSHVEWC[Carbamidomethyl (C)]K_.3	Q9Z2C4_S53_M1_Mtmr1	4.8483	3.6981	3.9796	3.8709	4.5721	3.5361	3.8438	4.4801	6.9112	3.6831	0.8636	-0.2116	0.3475	0.4590
Mtmr7	_SPS[Phospho (STY)]QGDEDSALILTQDNLK_.2	#N/A	4.0574	3.5158	6.8791	7.9852	3.5291	3.8364	3.8170	7.7613	3.5827	3.4771	1.0660	0.0922	0.8175	0.0875
Mtmr7	_S[Phospho (STY)]PSGGEHAPSEDSGKDR_.3	#N/A	3.6301	4.3863	3.3637	4.0293	4.2746	3.8508	3.7440	4.0892	3.7378	4.0856	1.0021	0.0031	0.9698	0.0133
Mtmr7	_S[Phospho (STY)]PSGGEHAPSEDSGK_.2	#N/A	6.0155	3.4701	3.3517	3.8544	3.2914	5.9653	3.5690	4.2641	3.9127	3.8214	1.1112	0.1522	0.5396	0.2679
Mtmr2	_AS[Phospho (STY)]SPAQC[Carbamidomethyl (C)]VTPVQTVV_.2	Q9Z2D1_S630_M1_Mtmr2	8.2294	3.3135	3.7917	8.4237	7.1541	3.4228	3.3160	8.3558	4.1657	3.0128	1.2143	0.2801	0.5443	0.2641
Mtmr2	_SSS[Phospho (STY)]C[Carbamidomethyl (C)]ESLGAQLPAAR_.2	Q9Z2D1_S6_M1_Mtmr2	10.2219	10.0592	10.5696	10.3426	8.9601	9.9512	10.7073	10.1991	10.3777	10.3231	0.9630	-0.0543	0.2347	0.6295
Mtmr2	_SASAISSDSISTSADNFS[Phospho (STY)]PDLR_.2	Q9Z2D1_S58_M1_Mtmr2	3.3496	4.1059	4.5841	4.1279	3.9942	4.1313	4.0244	4.3741	3.1723	3.8052	1.0533	0.0749	0.4930	0.3072
Mecp2	_AETSESSGSAPAVPEASAS[Phospho (STY)]PK_.2	#N/A	12.3171	12.1682	12.7861	12.9492	12.0902	12.3589	12.9040	12.3041	12.1435	12.4008	1.0006	0.0008	0.9758	0.0106
Mecp2	_VLEKS[Phospho (STY)]PGK_.2	Q9Z2D6_S216_M1_Mecp2	4.1849	4.3466	3.0576	3.7754	3.1758	3.5777	4.1604	3.6666	3.2142	2.8273	1.0632	0.0884	0.5488	0.2606
Mecp2	_GGS[Phospho (STY)]LESDGC[Carbamidomethyl (C)]PKEPAK_.2	Q9Z2D6_S421_M1_Mecp2	6.1728	6.4626	6.1920	7.0219	6.4854	6.2667	6.7386	4.7826	4.4155	6.3474	1.1548	0.2077	0.1102	0.9580
Mecp2	_EGKHEPLQPSAHHSAEPAEAGKAETSESSGSAPAVPEASAS[Phospho (STY)]PK_.5	#N/A	4.3487	5.0860	4.3089	3.3107	3.4924	3.1322	4.5262	5.0569	4.8758	3.2777	0.8900	-0.1681	0.3577	0.4465
Mecp2	_VGDTSLDPNDFDFTVTGR_.2	#N/A	4.5713	4.5598	3.3476	4.8882	4.8291	3.8670	3.7602	5.1187	4.4089	4.0694	1.0010	0.0015	0.9909	0.0040
Mecp2	_MPFQAS[Phospho (STY)]PGGK_.2	Q9Z2D6_S229_M1_Mecp2	5.7436	4.3875	4.2691	6.2806	5.1006	3.5207	3.8815	3.2680	3.5670	3.2379	1.3999	0.4853	0.0312	1.5058
Mbd3	_VRYDS[Phospho (STY)]SNQVK_.2	Q9Z2D8_S85_M1_Mbd3	4.0878	3.2546	3.8215	3.3653	3.0759	3.1528	3.5136	3.6359	3.9349	4.4967	0.8882	-0.1711	0.1446	0.8397
Mbd3	_YLGGS[Phospho (STY)]M[Oxidation (M)]DLSTFDFR_.2	#N/A	3.1064	4.1223	3.7932	4.2958	3.9372	3.9982	4.0105	3.8251	3.4737	4.2628	0.9955	-0.0065	0.9454	0.0244
Mbd2	_AADTEEVDIDM[Oxidation (M)]DS[Phospho (STY)]GDEA_.2	#N/A	4.2351	9.4996	3.9688	9.6089	9.3318	9.8069	9.7055	9.3255	9.3677	9.5525	0.8160	-0.2934	0.2609	0.5835
Bnip3l	_ILLDAQHES[Phospho (STY)]GQSSSR_.2	#N/A	5.2241	3.7989	3.7112	5.6211	5.8110	5.3803	5.6843	5.5054	5.7813	5.5144	0.8760	-0.1910	0.1818	0.7403
Bnip3l	_DHSSQS[Phospho (STY)]EEEVVEGEKEVEALKK_.4	#N/A	3.6467	4.0977	3.7658	4.1503	3.8757	6.5156	3.9917	3.8727	6.5932	3.8860	0.9468	-0.0789	0.7580	0.1204
Rgs6	_SVYGVTDETQSQS[Phospho (STY)]PVHIPSQPIR_.3	#N/A	7.6565	7.3582	7.8615	8.5434	5.4577	7.1917	7.1482	7.2720	7.4685	7.1636	1.0113	0.0161	0.8819	0.0546
Rgs6	_KSVYGVTDETQSQS[Phospho (STY)]PVHIPSQPIRK_.4	#N/A	3.1461	3.9513	3.8329	8.1515	5.1231	3.9569	4.0502	7.3311	3.4315	4.5846	0.9679	-0.0471	0.8953	0.0480
Rgs6	_SVYGVTDETQSQS[Phospho (STY)]PVHIPSQPIRK_.3	#N/A	3.8351	7.5371	8.4079	5.1173	3.7449	6.9898	7.1521	3.6975	7.5148	6.9933	0.9368	-0.0942	0.7532	0.1231
Rgs6	_KSVYGVTDETQSQS[Phospho (STY)]PVHIPSQPIR_.3	#N/A	3.2408	4.0460	3.9276	4.4303	3.8674	3.8621	4.1450	3.6882	3.3368	4.3973	1.0010	0.0014	0.9888	0.0049
Epb41l1	_ETDPS[Phospho (STY)]PEER_.2	#N/A	13.0175	13.0603	13.4968	13.2279	12.3831	13.1164	13.0881	13.1087	13.0879	12.9509	0.9993	-0.0009	0.9652	0.0154
Epb41l1	_LVS[Phospho (STY)]PEPPPK_.2	Q9Z2H5_S378_M1_Epb41l1	11.8412	11.4987	11.8095	11.8715	10.9994	11.5931	11.9658	11.6905	11.5933	11.6354	0.9898	-0.0147	0.5301	0.2756
Epb41l1	_S[Phospho (STY)]LPELDR_.2	#N/A	11.0361	10.3492	10.7225	10.8537	8.8631	10.5434	9.8727	10.6621	10.7975	10.6931	0.9894	-0.0154	0.8042	0.0946
Epb41l1	_S[Phospho (STY)]LDGAEFSR_.2	#N/A	10.0242	9.4247	9.4669	9.7713	9.0930	9.7653	9.5842	9.6677	9.9832	9.9777	0.9783	-0.0316	0.2889	0.5392
Epb41l1	_IKELKPEQETT[Phospho (STY)]PR_.2	#N/A	8.0779	3.8762	7.4169	7.2826	7.0764	8.1481	8.1127	8.2049	6.9280	6.8834	0.9266	-0.1099	0.5367	0.2703
Epb41l1	_S[Phospho (STY)]LPELDRDK_.2	#N/A	9.2264	9.1993	9.1673	9.5417	7.5135	8.9944	9.1884	9.4425	8.7077	9.0313	0.9833	-0.0243	0.7051	0.1518
Epb41l1	_AGLREGS[Phospho (STY)]EEK_.2	Q9Z2H5_S564_M1_Epb41l1	8.0576	7.8581	7.7825	8.1624	6.7811	7.6740	7.9724	7.9534	7.9135	7.8619	0.9740	-0.0380	0.4381	0.3585
Epb41l1	_VTLLDASEYEC[Carbamidomethyl (C)]EVEK_.2	#N/A	6.7154	6.3199	7.3206	7.3581	6.1410	6.7978	6.9975	6.5103	6.5337	6.9879	1.0027	0.0039	0.9495	0.0225
Epb41l1	_ETDPS[Phospho (STY)]PEERDK_.2	#N/A	2.9168	7.9208	3.6036	4.3987	7.8220	8.1859	7.8915	4.1033	4.4024	7.7260	0.9630	-0.0543	0.8841	0.0535
Epb41l1	_DNHLAIER_.2	#N/A	6.6482	6.2538	6.4153	6.5565	6.0782	6.6419	6.3066	6.5627	6.4842	6.2737	1.0040	0.0057	0.8487	0.0713
Epb41l1	_FAPNQTR_.2	#N/A	5.8355	6.2364	6.1803	5.9711	6.1905	5.9501	5.5068	6.0083	6.2975	6.1490	1.0117	0.0168	0.6707	0.1735
Epb41l1	_KAQEETPQQPEAAAAVTT[Phospho (STY)]PVT[Phospho (STY)]PAGHSHPETNSNEK_.4	#N/A	4.0672	3.3226	3.8008	3.3446	3.2109	3.4137	3.3069	3.6566	3.9556	3.0219	1.0119	0.0170	0.8640	0.0635
Epb41l1	_HQAS[Phospho (STY)]INELKR_.2	#N/A	7.9210	7.3253	8.3098	7.8986	6.5333	7.7302	7.3945	8.0041	8.0019	7.6870	0.9804	-0.0285	0.6636	0.1781
Epb41l1	_S[Phospho (STY)]EAEEGEVR_.2	#N/A	8.0962	7.4925	7.5414	7.8056	7.4695	7.6165	8.1013	7.6070	7.6944	7.9140	0.9797	-0.0296	0.3246	0.4887
Epb41l1	_S[Phospho (STY)]EAEEGEVRT[Phospho (STY)]PTK_.2	Q9Z2H5_T475_M2_Epb41l1	6.2866	6.4501	6.0701	6.8997	5.8708	6.0600	3.1190	6.9250	6.0613	6.8774	1.0918	0.1267	0.4930	0.3072
Epb41l1	_AQEETPQQPEAAAAVT[Phospho (STY)]T[Phospho (STY)]PVTPAGHSHPETNSNEK_.4	#N/A	4.0001	6.3979	5.3596	6.6325	5.3774	6.6686	4.7530	6.4059	3.8396	5.3676	1.1272	0.1728	0.3687	0.4333
Epb41l1	_RLPSSPAS[Phospho (STY)]PS[Phospho (STY)]PKGT[Phospho (STY)]PEK_.2	#N/A	7.9752	7.5376	6.3318	8.1943	6.0296	7.7947	7.7245	7.7289	7.6126	7.6918	0.9507	-0.0729	0.4368	0.3597
Epb41l1	_GAC[Carbamidomethyl (C)]ST[Phospho (STY)]PEM[Oxidation (M)]PQFESVK_.2	#N/A	9.0575	8.3809	8.7251	9.0849	3.5728	8.4989	9.1618	8.4957	8.5530	8.4992	0.9089	-0.1379	0.4911	0.3089
Epb41l1	_GAAAM[Oxidation (M)]IPGPQTVATEIR_.2	#N/A	6.2074	6.7118	6.7810	3.4805	6.1785	6.3244	3.1952	6.1214	6.7195	6.4309	1.0589	0.0825	0.7236	0.1405
Epb41l1	_LPSSPAS[Phospho (STY)]PS[Phospho (STY)]PK_.2	#N/A	8.2682	8.4715	8.7324	8.8968	8.1310	8.4236	8.7529	8.4400	8.5888	8.4695	0.9912	-0.0128	0.6414	0.1929
Epb41l1	_IIITGDEDVDQDQALALAIK_.2	#N/A	4.1131	4.6567	4.6101	5.3303	3.3227	4.4271	3.6825	4.4269	4.3439	4.1581	1.0619	0.0867	0.5006	0.3005
Epb41l1	_GPSSQEDESGGLEDS[Phospho (STY)]PDR_.2	Q9Z2H5_S677_M1_Epb41l1	7.7305	6.8863	7.2332	7.5891	7.1156	7.5694	7.9492	7.5888	7.7802	7.5538	0.9528	-0.0697	0.0803	1.0953
Epb41l1	_SLS[Phospho (STY)]PIIGK_.2	Q9Z2H5_S782_M1_Epb41l1	7.1104	6.5397	3.9544	6.5189	3.8468	4.2786	4.1718	3.6614	3.3100	4.4241	1.3811	0.4658	0.0974	1.0117
Epb41l1	_RLPSSPAS[Phospho (STY)]PS[Phospho (STY)]PK_.2	#N/A	11.1912	11.2801	11.5178	11.6829	10.6701	11.1553	11.6193	11.4313	11.4074	11.2340	0.9848	-0.0221	0.3848	0.4147
Epb41l1	_EDDAES[Phospho (STY)]GGRRS[Phospho (STY)]EAEEGEVR_.3	#N/A	4.9474	4.1938	3.9457	4.9090	4.0319	3.8937	4.7458	4.9579	5.0703	3.9775	0.9216	-0.1178	0.2748	0.5610
Epb41l1	_DVLTSTYGATAETLSTSTTTHVTK_.3	#N/A	2.8451	3.9359	3.9254	4.1069	3.9010	4.9976	3.1256	4.3338	4.2854	4.4752	0.9746	-0.0371	0.8158	0.0884
Epb41l1	_RS[Phospho (STY)]EAEEGEVR_.2	#N/A	6.5333	4.3660	4.2109	3.7547	5.4559	6.4638	5.6229	6.6713	3.5456	5.9644	0.9412	-0.0874	0.7028	0.1532
Epb41l1	_SLDGAEFSRPAS[Phospho (STY)]VSENHDAGPEGDKR_.4	Q9Z2H5_S441_M1_Epb41l1	3.5356	3.8361	3.5400	4.1614	3.7037	6.6102	3.9844	6.8043	4.0943	4.1776	0.8879	-0.1715	0.5287	0.2768
Epb41l1	_LPSSPASPS[Phospho (STY)]PKGT[Phospho (STY)]PEK_.2	#N/A	3.7530	4.4781	3.6263	5.6269	3.5246	5.0714	4.8008	3.9105	4.0519	4.9477	0.9817	-0.0266	0.8719	0.0595
Epb41l1	_RSEAEEGEVRT[Phospho (STY)]PTK_.3	#N/A	6.8482	5.9830	3.9239	6.5234	6.0822	4.7549	4.1412	6.4183	7.0170	3.6427	1.0718	0.1001	0.6732	0.1719
Epb41l1	_DYSEADGLS[Phospho (STY)]ERTT[Phospho (STY)]PSK_.2	Q9Z2H5_S75_M2_Epb41l1	2.9282	3.7334	3.6150	4.1177	3.5547	4.1747	4.5355	4.0008	3.6494	4.0847	0.9065	-0.1416	0.1999	0.6992
Epb41l1	_ELKPEQETT[Phospho (STY)]PR_.2	#N/A	11.9986	11.9477	12.3495	12.2427	10.5471	11.8469	11.9708	11.9902	11.9564	11.9052	0.9888	-0.0162	0.6990	0.1555
Epb41l1	_AETMTVSSLAIR_.2	#N/A	3.2630	4.0682	3.9498	5.9384	3.8895	3.8400	4.2008	4.4495	3.2477	4.4194	1.0193	0.0276	0.8829	0.0541
Epb41l1	_LPSSPASPS[Phospho (STY)]PKGT[Phospho (STY)]PEKASER_.3	#N/A	3.5616	3.7808	6.7783	6.7853	4.2061	3.9193	4.4881	5.9646	2.9604	4.0171	1.1104	0.1511	0.6149	0.2112
Epb41l1	_SLPELDRDKS[Phospho (STY)]DSETEGLVFAQDLK_.3	#N/A	3.8871	3.7066	4.2014	4.0506	3.5280	3.8274	3.5596	5.2855	4.0208	3.8004	0.9281	-0.1077	0.3874	0.4119
Epb41l1	_DKS[Phospho (STY)]DS[Phospho (STY)]ETEGLVFAQDLK_.2	#N/A	3.4227	3.8201	4.0283	4.3524	3.6878	3.9260	4.3666	7.6542	3.7413	3.6558	0.7978	-0.3259	0.2356	0.6278
Epb41l1	_APES[Phospho (STY)]DMGDEDQDQER_.2	Q9Z2H5_S578_M1_Epb41l1	3.8739	3.3523	4.5607	3.5625	2.9996	3.2291	3.2772	3.0551	4.2045	3.5295	1.0227	0.0324	0.8236	0.0843

Epb41l1	_VSAADSTQVDGGTPMVK_.2	#N/A	3.4569	4.2132	3.6998	4.2024	4.1015	4.0240	3.9171	5.6174	3.5646	4.1694	0.9149	-0.1284	0.3725	0.4289
Epb41l1	_PASVSENHDAGPEGDKREDDAES[Phospho (STY)]GGR_.4	#N/A	3.1952	4.0310	3.8820	4.3847	3.8217	4.2062	4.0993	3.7338	3.3824	4.3517	1.0073	0.0105	0.9187	0.0368
Letm1	_LLELQSIGTNNFLR_.2	#N/A	6.0325	5.8901	6.3765	6.0861	5.6327	6.0239	6.0943	5.8494	5.7636	5.7719	1.0234	0.0333	0.3516	0.4540
Letm1	_VAEVEGEKVDNK_.2	#N/A	6.0740	5.7429	6.1202	5.7801	5.6229	5.8763	6.5041	5.6351	5.2129	5.2517	1.0387	0.0547	0.4202	0.3765
Letm1	_LISLTSALDDNKDGNINIDDLVK_.3	#N/A	3.9635	2.5562	3.1932	3.9945	4.0713	4.6483	3.2657	5.1169	3.8934	3.6628	0.9380	-0.0923	0.6298	0.2008
Letm1	_FLQDTIEEMALK_.2	#N/A	4.1134	3.1128	3.2994	3.8020	3.2391	2.9896	3.5167	4.3165	3.9650	3.7690	0.8803	-0.1839	0.1106	0.9561
Sucla2	_INFDS[Phospho (STY)]NSAYR_.2	Q9Z2I9_S279_M1_Sucla2	8.9876	8.9035	9.5845	9.1864	8.5720	9.0797	9.3776	8.8214	9.1027	8.9199	0.9997	-0.0005	0.9877	0.0054
Sucla2	_EQAVTLAQK_.2	#N/A	7.7866	7.6526	7.5256	7.7679	8.1439	8.0982	7.7467	7.7443	7.8148	7.9033	1.0034	0.0050	0.8395	0.0760
Sucla2	_IVFSPEEAK_.2	#N/A	8.0318	7.2915	7.5425	7.2009	7.4953	7.4341	6.9486	8.0748	7.8433	7.9258	0.9742	-0.0377	0.4492	0.3475
Sucla2	_ILAC[Carbamidomethyl (C)]DDLDEAAK_.2	#N/A	7.2477	7.3608	7.2135	7.8190	7.1841	7.4001	7.4099	7.0506	7.2268	7.3764	1.0144	0.0207	0.4640	0.3335
Sucla2	_IC[Carbamidomethyl (C)]NQVLVC[Carbamidomethyl (C)]ER_.2	#N/A	6.6420	6.6401	5.8779	7.3855	6.9916	6.7144	6.5237	7.0398	6.7843	6.9928	0.9815	-0.0270	0.6522	0.1856
Sucla2	_LSEIVTLAK_.2	#N/A	6.0027	5.4219	5.9751	6.2450	5.9211	6.0536	5.4321	6.6297	5.9670	6.4838	0.9687	-0.0458	0.4774	0.3211
Sucla2	_LHGGTPANFLDVGGGATVQQVTEAFK_.3	#N/A	5.6622	5.5658	6.7126	6.5078	5.8514	6.0187	6.3361	5.9487	5.9155	6.5331	0.9789	-0.0307	0.6359	0.1966
Sucla2	_SSDEAYAIAK_.2	#N/A	5.4826	4.5682	3.8223	5.7071	5.9071	5.1611	4.1242	5.4231	5.1455	5.3170	1.0211	0.0301	0.8260	0.0830
Sucla2	_IFDLQDWSQEDER_.2	#N/A	5.8341	5.6060	4.2420	3.2438	5.1661	3.5478	6.3117	5.3986	5.5069	5.6121	0.8071	-0.3091	0.0939	1.0274
Sucla2	_VQAILVNIFGGIM[Oxidation (M)]R_.2	#N/A	4.1301	4.0340	3.7224	3.0164	3.1750	3.2845	3.4144	3.7350	4.6775	4.1223	0.8929	-0.1634	0.2206	0.6564
Sucla2	_AVSSQM[Oxidation (M)]IGQK_.2	#N/A	4.7733	4.9291	5.4133	4.1620	5.3337	4.8161	4.6384	5.1566	4.7099	4.6565	1.0238	0.0340	0.6601	0.1804
Sucla2	_C[Carbamidomethyl (C)]DVIAQGIVM[Oxidation (M)]AVK_.2	#N/A	5.0213	5.3650	4.1447	4.1068	5.1548	5.3312	5.3488	5.2510	5.0633	5.3945	0.9220	-0.1171	0.2091	0.6797
Sucla2	_YDATMVEINPM[Oxidation (M)]VEDSDGK_.2	#N/A	3.7601	3.1594	3.7649	4.0391	3.1749	3.4632	3.5404	3.7060	3.9272	3.8552	0.9476	-0.0777	0.3382	0.4708
Sucla2	_VLC[Carbamidomethyl (C)]MDAK_.2	#N/A	3.6197	4.3760	3.5370	4.0396	4.2643	3.8612	3.7543	4.0788	3.7274	4.0066	1.0149	0.0213	0.7648	0.1164
Ubl3	_EFLFS[Phospho (STY)]PNDSASDIAK_.2	Q9Z2M6_S27_M1_Ubl3	3.2507	4.0559	3.9375	4.4402	3.8773	3.8522	4.2130	5.8823	3.3269	4.4072	0.8755	-0.1918	0.2647	0.5772
Actl6a	_EGS[Phospho (STY)]PANWK_.2	#N/A	6.8076	6.5146	6.5118	6.6003	5.8073	4.4011	6.8123	6.4990	6.5943	7.4997	0.8914	-0.1659	0.1700	0.7696
Actl6a	_EGS[Phospho (STY)]PANWKR_.2	#N/A	4.5411	4.8790	4.1672	5.2956	5.0812	4.3839	4.7147	5.5626	5.5878	5.1318	0.9001	-0.1519	0.0927	1.0330
Knop1	_ATS[Phospho (STY)]PSNNVDEVQIPEISLSK_.2	Q9Z2Q2_S57_M1_Knop1	2.9507	3.7559	3.6375	4.1401	3.5772	4.1523	4.5131	5.2641	3.6269	4.1071	0.8000	-0.3219	0.1057	0.9761
Septin5	_ADC[Carbamidomethyl (C)]LVPSEIR_.2	#N/A	9.3890	9.2306	9.2494	9.0818	9.4597	9.1240	9.3812	9.0855	9.2251	9.4289	0.9974	-0.0038	0.8075	0.0929
Septin5	_IREEIDK_.2	#N/A	8.9494	8.4548	8.4032	8.9173	9.1700	8.7801	8.6564	8.5822	8.6996	8.6285	1.0159	0.0228	0.3978	0.4004
Septin5	_VNIVPLIAK_.2	#N/A	7.6594	8.2070	7.7611	7.8504	8.1115	7.8290	7.7187	8.3346	8.2123	7.9087	0.9825	-0.0254	0.3908	0.4080
Septin5	_KLLSAEER_.2	#N/A	7.7813	7.7691	7.9573	7.9909	8.2355	7.7477	7.7746	7.9183	7.8113	7.8445	1.0098	0.0140	0.4623	0.3351
Septin5	_INQTVEILK_.2	#N/A	7.8183	7.8963	7.5290	7.8228	7.6995	7.5988	7.6132	7.6202	7.6021	7.6658	1.0134	0.0192	0.2043	0.6898
Septin5	_HTVDIEEK_.2	#N/A	7.4814	7.3342	7.2815	7.3767	7.7658	7.4876	7.3355	7.2134	7.2278	7.7314	1.0105	0.0151	0.5680	0.2457
Septin5	_DVTC[Carbamidomethyl (C)]DVHYENYR_.2	#N/A	7.1086	6.4542	6.5730	7.3231	7.2978	7.1970	7.5052	6.9452	6.8796	7.2811	0.9776	-0.0327	0.4980	0.3028
Septin5	_M[Oxidation (M)]KDEELR_.2	#N/A	6.4594	6.4253	6.9730	6.1875	7.3586	7.0322	6.5166	6.8086	6.6723	6.9302	1.0011	0.0016	0.9761	0.0105
Septin5	_LATPEDKQDIDKQYVGFATLPNQVHR_.4	#N/A	6.7791	8.2027	8.5482	7.3921	6.9868	7.0345	7.1212	7.1481	7.9616	6.8827	1.0292	0.0415	0.6209	0.2069
Septin5	_QYVGFATLPNQVHR_.2	#N/A	6.4576	6.3497	6.1426	6.6846	6.6792	6.6762	6.3663	6.0463	6.9077	6.6500	1.0009	0.0013	0.9762	0.0105
Septin5	_SKLAT[Phospho (STY)]PEDKQDIDK_.2	#N/A	3.4284	5.8274	3.1621	4.2067	4.0730	4.0606	4.3550	4.2962	3.0945	3.8840	1.0560	0.0787	0.6888	0.1619
Septin5	_STLVHSLFLTDLYKDR_.3	#N/A	5.2613	5.8719	4.4387	5.9749	5.3198	6.2981	4.5010	5.2309	5.3207	5.8500	1.0578	0.0810	0.4766	0.3218
Septin5	_M[Oxidation (M)]ES[Phospho (STY)]PIPILPLPTPDAETEK_.2	#N/A	9.5252	10.0566	10.3196	10.3872	8.9853	9.8932	9.9015	9.9607	9.6439	9.5277	1.0105	0.0151	0.7258	0.1392
Septin5	_STLVHSLFLTDLYK_.2	#N/A	4.6451	4.4092	3.4947	4.9832	4.4124	4.7090	4.6722	4.8097	4.5455	4.6449	0.9516	-0.0715	0.4177	0.3792
Septin5	_LYPWGIVEVENQAHC[Carbamidomethyl (C)]DFVK_.2	#N/A	5.8586	4.2764	4.9560	5.3215	4.7493	3.8944	5.0948	4.9429	6.3188	6.6920	0.8404	-0.2508	0.1030	0.9870
Septin5	_FGIHVYQFPEC[Carbamidomethyl (C)]DSDEDEDFKQQDR_.4	#N/A	7.5466	6.8959	8.9423	6.0049	10.4102	7.3168	7.6951	11.0651	10.7706	10.1466	0.7917	-0.3370	0.0743	1.1291
Septin5	_ESAPFAVIGSNTVVEAK_.2	#N/A	7.3498	7.1116	7.5784	7.7486	7.2023	7.2529	7.2903	6.9521	7.3717	7.4529	1.0148	0.0211	0.4994	0.3015
Septin5	_LAT[Phospho (STY)]PEDKQDIDK_.2	#N/A	8.7167	8.7281	8.9773	9.4299	8.1841	8.7751	9.0040	8.6265	8.6093	8.8146	1.0044	0.0063	0.8659	0.0625
Septin5	_AHC[Carbamidomethyl (C)]IQQM[Oxidation (M)]TSK_.2	#N/A	6.1564	5.3926	5.5423	6.6665	6.7784	5.7049	5.8181	5.3842	6.1604	5.5665	1.0537	0.0755	0.3758	0.4251
Septin5	_ADC[Carbamidomethyl (C)]LVPSEIRK_.3	#N/A	3.2563	5.9670	4.4908	4.0346	4.0456	4.2245	4.1829	4.8155	5.9326	5.2061	0.8614	-0.2153	0.2342	0.6304
Septin5	_THMHDLK_.2	#N/A	4.3479	3.4545	3.3361	3.8910	3.4023	2.9528	3.5535	4.2797	3.9283	5.3600	0.8327	-0.2642	0.1077	0.9677
Septin5	_THM[Oxidation (M)]HDLKDVTC[Carbamidomethyl (C)]DVHYENYR_.4	#N/A	5.4740	6.3197	7.6284	3.4358	6.0890	6.7692	3.5841	6.9819	6.5707	6.4686	1.0087	0.0125	0.9584	0.0184
Septin5	_FGIHVYQFPEC[Carbamidomethyl (C)]DS[Phospho (STY)]DEDEDFK_.3	#N/A	2.9795	4.2466	3.6663	4.1690	3.6061	4.1234	3.8837	3.9495	3.5981	4.1360	0.9760	-0.0350	0.7310	0.1361
Septin5	_MKDEELRR_.2	#N/A	3.5063	4.2779	4.2991	3.5210	4.0992	3.6303	4.1273	3.1583	3.4574	3.5201	1.0906	0.1252	0.2335	0.6316
Tsc22d3	_LSPEEPAPEAPET[Phospho (STY)]PETPEAPGGSAV_.3	Q9Z2S7_T125_M1_Tsc22d3	3.3221	4.0906	3.8344	7.3937	4.0490	3.8878	4.2486	6.7261	4.3437	3.7776	0.9279	-0.1080	0.7173	0.1443
Psma7	_ALLEVVQSGGK_.2	#N/A	6.2054	6.5820	6.6184	6.5990	6.8687	6.9292	6.4830	6.9037	6.7917	6.4830	0.9953	-0.0068	0.8452	0.0731
Psma7	_ILNPEEIEK_.2	#N/A	7.3091	6.8042	3.9253	7.2721	7.2140	7.2918	7.2831	7.3910	7.0145	7.5358	0.9083	-0.1388	0.3600	0.4437
Psma7	_NYTDDAIETDDLTIK_.2	#N/A	2.9421	6.4247	4.1767	6.5532	5.9333	6.3386	6.2470	5.7976	6.1500	6.4030	0.8773	-0.1889	0.3537	0.4514
Psma7	_YVAEIEKEKEENEK_.3	#N/A	6.9573	6.9337	7.2168	6.8087	7.1834	6.7299	7.1213	6.3771	6.7793	7.1884	1.0153	0.0219	0.5708	0.2435
Psma7	_AITVFSPDGHLFQVEYAQEAVKK_.3	#N/A	4.4571	4.9667	5.1254	4.7841	5.1279	5.0733	5.6183	5.0135	5.1965	4.2580	0.9802	-0.0288	0.7144	0.1460
Psma5	_GPQLFHM[Oxidation (M)]DPSGTFVQC[Carbamidomethyl (C)]DAR_.3	#N/A	5.7730	5.7325	5.6990	5.6825	5.8999	6.5312	5.8288	5.5463	5.7481	6.1174	1.0131	0.0188	0.6997	0.1551
Psma5	_GVNTFS[Phospho (STY)]PEGR_.2	Q9Z2U1_S16_M1_Psma5	7.1769	6.2689	7.3664	6.4369	6.9315	7.2397	6.2442	6.3391	6.4379	6.2934	1.0908	0.1254	0.0390	1.4088
Psma5	_ITS[Phospho (STY)]PLM[Oxidation (M)]EPSSIEK_.2	#N/A	9.6237	9.5544	10.1774	10.0778	9.2122	9.6778	9.9598	9.2269	9.3912	9.6453	1.0172	0.0247	0.4779	0.3207
Psma5	_LFQVEYAIEAIK_.2	#N/A	3.6325	4.9529	3.3661	4.4107	5.1187	5.0625	5.5752	5.2989	5.2141	5.8716	0.8058	-0.3115	0.0307	1.5128
Psma5	_EELEEVIKDI_.2	#N/A	3.6420	2.9463	4.9953	4.9440	5.2755	4.5267	5.0828	4.3917	5.2646	3.2976	0.9732	-0.0392	0.8406	0.0754
Psma5	_AIGSASEGAQSSLQEVYHK_.3	#N/A	2.2401	3.7900	3.9538	4.1693	5.3613	3.7528	4.3175	6.0862	3.3133	3.2116	0.9163	-0.1261	0.6420	0.1925
Psma5	_PFGVALLFGGVDEK_.2	#N/A	3.6649	3.5613	4.3517	3.3535	2.7906	3.1750	3.0682	3.2641	4.4136	3.3205	0.9904	-0.0139	0.9276	0.0326
Hdac6	_AVLAQGQS[Phospho (STY)]SEQAAK_.2	Q9Z2V5_S909_M1_Hdac6	6.5981	4.3682	3.5447	6.0628	4.2565	3.8689	3.7621	4.0711	3.7197	4.0144	1.2290	0.2975	0.2006	0.6976
Hdac6	_HNPQS[Phospho (STY)]PLQESSATLK_.2	Q9Z2V5_S21_M1_Hdac6	6.5500	4.2089	3.1863	4.2309	4.0972	4.0282	4.3792	6.1148	3.0693	3.9082	1.0036	0.0052	0.9842	0.0069
Hdac5	_KTAS[Phospho (STY)]EPNLK_.2	Q9Z2V6_S250_M1_Hdac5	7.6536	7.5581	4.3236	7.7472	7.3661	7.7259	4.0157	3.1338	3.4328	3.5447	1.9997	0.9998	0.0010	2.9881
Hdac5	_DEDGES[Phospho (STY)]GPDEGPDLEESSAGYKK_.3	#N/A	3.4570	3.7691	4.1438	3.1457	4.0836	4.7803	4.1190	5.0420	3.1206	3.1126	1.0125	0.0179	0.9209	0.0358
Hdac5	_DEDGES[Phospho (STY)]GPDEGPDLEESSAGYK_.2	#N/A	3.9313	3.4111	3.6650	5.3929	4.1213	3.5495	3.3570	5.1779	4.7123	4.3869	0.9100	-0.1360	0.4319	0.3646
Hdac5	_RSS[Phospho (STY)]PLLR_.2	Q9Z2V6_S270_M1_Hdac5	7.3951	7.5182	3.4505	4.4951	3.4469	7.6161	7.2522	4.0069	6.8818	4.1724	1.0135	0.0194	0.9537	0.0206
Hdac5	_VAERRS[Phospho (STY)]S[Phospho (STY)]PLLR_.3	Q9Z2V6_S269_M2_Hdac5	5.8796	5.1168	4.1440	5.0791	5.3507	5.8204	4.3613	5.7736	3.1204	5.8458	1.0956	0.1317	0.4729	0.3252
Hdac5	_TQS[Phospho (STY)]SPAAPGSMK_.2	Q9Z2V6_S650_M1_Hdac5	3.0007	3.7569	4.2352	3.7789	3.6452	2.9794	4.3734	3.2222	3.5213	3.4562	0.9788	-0.0309	0.8151	0.0888
Dnpep	_ETAC[Carbamidomethyl (C)]TTGVLQTLTLFK_.2	#N/A	3.3601	4.1164	4.5946	4.2993	2.6324	4.1208	4.0139	2.6060	3.4678	3.8157	1.1088	0.1490	0.4192	0.3775
Dnpep	_SPSPFHVVAEC[Carbamidomethyl (C)]R_.3	#N/A	2.9932	3.7494	4.2277	3.7714	3.6377	2.9869	4.3809	3.2297	3.5288	3.4487	0.9764	-0.0344	0.7940	0.1002
Hnrnpf	_ITGEAFVQFASQELAEK_.2	#N/A	4.4093	3.7650	5.1701	3.2396	4.1094	2.9710	3.9356	3.2138	3.0331	4.7041	1.0598	0.0837	0.6734	0.1717
Homer1	_LTAALLESTANVK_.2	#N/A	6.6261	6.1368	7.0283	6.9262	6.8812	6.2487	6.7066	6.7007	6.7097	6.7626	0.9883	-0.0170	0.6911	0.1605
Homer1	_LQEVEIR_.2	#N/A	6.8362	6.3640	7.1819	6.9792	6.9315	6.6544	5.9646	6.6405	6.9079	7.0166	1.0290	0.0412	0.4400	0.3565
Homer1	_FGQWADSR_.2	#N/A	6.0335	6.0902	6.5967	6.0514	6.4819	5.6914	6.2366	6.0517	6.1566	6.4473	0.9895	-0.0153	0.7271	0.1384
Homer1	_IFELTELR_.2	#N/A	5.8265	6.0121	4.8011	6.7190	5.9352	6.5330	4.8548	6.1944	6.2480	6.3945	1.0081	0.0117	0.9164	0.0379
Homer1	_TELNQTVQELEETLK_.2	#N/A	5.5408	4.6363	3.7010	5.7702	5.4121	5.3955	5.2137	5.2183	5.2137	5.6906	0.9516	-0.0716	0.5428	0.2654
Homer1	_[Acetyl (Protein N-term)]GEQPIFSTR_.2	#N/A	7.4104	7.0540	4.5787	7.3294	7.5961	7.3233	7.1512	7.3665	7.3360	7.0410	0.9527	-0.0699	0.5758	0.2397
Homer1	_TLLEILDGK_.2	#N/A	6.3504	6.2817	4.5108	5.8377	6.1540	6.1576	5.8734	6.4698	5.9518	6.0054	0.9682	-0.0466	0.6159	0.2105

Homer1	_AIINSTITPNM[Oxidation (M)]TFTK_.2	#N/A	5.5031	5.9879	6.6014	3.2326	6.7073	5.4948	6.0249	6.0552	6.6708	6.6429	0.8802	-0.1841	0.2830	0.5482
Homer1	_TQALSHASS[Phospho (STY)]AISK_.2	Q9Z2Y3_S189_M1_Homer1	4.8663	4.6189	4.4653	4.3941	4.7971	4.5338	4.6999	5.4424	4.2246	4.3464	0.9859	-0.0204	0.7881	0.1034
Homer1	_IISLDGSK_.2	#N/A	3.1455	3.7405	5.8713	4.2092	4.2903	4.2144	3.5872	4.8853	4.2713	4.7679	0.9697	-0.0444	0.8044	0.0945
Homer1	_NKDLEGQLSDLEQR_.3	#N/A	5.7084	6.2036	5.7626	6.8881	6.2581	6.3044	6.2905	6.1719	6.2907	6.2843	0.9885	-0.0166	0.7526	0.1234
Homer1	_AEPTQNALPFPHSAGDR_.3	#N/A	5.9155	6.4444	5.8375	6.5824	6.6927	5.7966	6.0587	5.9000	5.8189	6.4601	1.0251	0.0358	0.5369	0.2701
Homer1	_HWEAELATLK_.2	#N/A	4.1275	4.0900	4.2522	4.7703	2.9252	3.7950	3.2029	3.9789	4.2789	4.3603	1.0096	0.0138	0.9217	0.0354
Homer1	_DLEGQLS[Phospho (STY)]DLEQR_.2	#N/A	3.3133	3.9129	4.0001	5.7020	3.8012	4.3243	4.2174	3.6157	3.2643	2.9689	1.1874	0.2478	0.1965	0.7067
Homer1	_NKDLEGQLS[Phospho (STY)]DLEQRLEK_.3	#N/A	4.1195	3.2229	3.8532	3.3969	3.0442	3.1844	3.5452	3.4062	3.9033	3.0742	0.9965	-0.0050	0.9636	0.0161
Homer1	_ANTVYGLGFSSEHHLSK_.3	#N/A	5.0406	3.8867	4.3650	4.5289	3.7750	4.3504	7.3392	3.5896	3.2381	2.9951	1.0079	0.0114	0.9688	0.0137
Homer1	_HAVTVSYFYDSTR_.2	#N/A	3.9104	3.4320	3.6441	3.1879	3.0541	1.3449	3.3361	3.8133	4.1124	4.3660	0.7923	-0.3358	0.1411	0.8504
Plpbp	_IGS[Phospho (STY)]TIFGER_.2	Q9Z2Y8_S244_M1_Plpbp	4.4600	5.2517	4.2144	5.4069	5.3452	4.4863	4.0800	5.1225	4.4981	5.0083	1.0392	0.0555	0.5942	0.2261
Plpbp	_KGPTEPLK_.2	#N/A	3.8579	4.2439	3.7439	3.0222	4.1111	3.0751	3.4359	3.4426	4.2544	2.9649	1.0429	0.0606	0.6668	0.1760
Kcnq2	_GKGS[Phospho (STY)]PQAQTVR_.2	Q9Z351_S429_M1_Kcnq2	8.1926	8.4584	8.6869	8.4490	7.0248	7.9995	8.8961	8.7435	8.2721	8.3067	0.9510	-0.0725	0.2358	0.6274
Kcnq2	_RSPSADQSLDDS[Phospho (STY)]PSKVPK_.3	#N/A	5.7658	6.1205	6.6049	6.8267	5.1810	6.4105	6.4643	6.3053	5.8892	3.7920	1.0960	0.1323	0.3785	0.4219
Kcnq2	_SPS[Phospho (STY)]ADQSLDDSPSK_.2	Q9Z351_S440_M1_Kcnq2	6.2643	6.0874	3.4612	6.1830	4.1140	5.5811	6.8177	6.1484	5.5523	5.7496	0.8706	-0.1999	0.2621	0.5816
Kcnq2	_SWS[Phospho (STY)]FGDR_.2	Q9Z351_S457_M1_Kcnq2	3.2633	7.5405	4.4979	4.0416	5.8470	8.1324	4.1899	4.4604	7.8968	7.9619	0.9064	-0.1418	0.6704	0.1737
Kcnq2	_YPQT[Phospho (STY)]WNGR_.2	Q9Z351_T287_M1_Kcnq2	3.5448	3.6814	4.2316	3.2334	4.1713	3.5582	4.4489	3.3843	3.0328	3.2004	1.0626	0.0876	0.5123	0.2905
Kcnq2	_S[Phospho (STY)]PSADQSLDDSPSKVPK_.2	#N/A	6.1610	7.1629	4.9541	3.7634	3.4039	6.9663	7.6653	7.4175	6.0307	3.5543	0.8760	-0.1911	0.5102	0.2923
Kcnq2	_QNS[Phospho (STY)]EEASLPGEDIVEDNK_.2	Q9Z351_S479_M1_Kcnq2	5.9007	3.7452	6.4956	7.4980	5.6365	3.6391	7.3483	7.4449	7.5586	7.2515	0.7412	-0.4320	0.0401	1.3969
Kcnq2	_EPEPAPPYHS[Phospho (STY)]PEDSR_.2	#N/A	4.9469	5.5990	3.8856	5.4271	3.8511	3.9284	4.1514	3.7982	3.9025	4.7102	1.1125	0.1538	0.3271	0.4853
Kcnq2	_EPEPAPPYHS[Phospho (STY)]PEDSRDHADK_.3	#N/A	3.4143	4.1705	3.1479	6.4207	4.0588	4.0666	4.3408	4.3095	3.1077	3.8698	1.0784	0.1089	0.6413	0.1929
Kcnq2	_RS[Phospho (STY)]PSADQSLDDSPSK_.2	#N/A	5.5738	3.4825	4.0051	6.0385	3.3708	3.2849	6.0367	4.0184	5.5348	3.0838	0.9195	-0.1211	0.6582	0.1817
Osbpl10	_SSPGSVAAS[Phospho (STY)]PSGGGGR_.2	S4R1M9_S66_M1_Osbpl10	8.3483	7.5725	3.7538	8.3058	3.9709	8.3871	8.0125	8.0545	7.4699	3.8350	0.9825	-0.0255	0.9335	0.0299
Osbpl10	_ATSAGSS[Phospho (STY)]PSC[Carbamidomethyl (C)]SLAGR_.2	S4R1M9_S32_M1_Osbpl10	5.4915	5.2162	4.3047	3.5335	5.0463	6.0855	6.2198	3.3583	3.7677	5.1516	1.0696	0.0971	0.6551	0.1837
