# Supplementary material for: The Chronic Effects of a Single Low-Intensity Blast Exposure on Phosphoproteome Networks and Cognitive Function Influenced by Mutant Tau Overexpression
Source: Int J Mol Sci. 2024 Mar 15;25(6):3338. doi: 10.3390/ijms25063338 (PMC10970429; doi:10.3390/ijms25063338)
Supplement: Supplementary file 1 [file ijms-25-03338-s001.zip › Supplementary Table S1b- MS2 significant phosphopeptides_non-carriers.pdf]

Supplementary Table S1b- MS2 significant phosphopeptides (sheet = 'Non-carrier sig LIB vs sham')

PG.Genes	PG.ProteinGroups	PG.ProteinDescriptions	EG.PrecursorId	Expression Ratio	LOG2FC	pvalue	(-) Log10 p value
Bsn	O88737	Protein bassoon	_YLGQGLQYGS[Phospho (STY)]FTDLR_.2	0.5899	-0.7614	0.0004	3.4300
Elavl1	P70372	ELAV-like protein 1	_VLVDQTTGLSR_.2	1.3599	0.4435	0.0004	3.4217
Atp6v1g1	Q9CR51	V-type proton ATPase subunit G 1	_[Acetyl (Protein N-term)]ASQSQGIQQLLQAEKR_.3	2.3902	1.2571	0.0006	3.2594
S100a13	P97352	Protein S100-A13	_KGS[Phospho (STY)]LNINEFK_.2	0.6593	-0.6009	0.0008	3.1109
Uqcrc1	Q9CZ13	Cytochrome b-c1 complex subunit 1, mitochondrial	_NNGAGYFLEHLAFK_.2	0.5814	-0.7825	0.0009	3.0228
Rasa3	Q60790	Ras GTPase-activating protein 3	_T[Phospho (STY)]RYGSQEHPIGDK_.3	0.6164	-0.6980	0.0010	3.0119
Lrrc7	Q80TE7	Leucine-rich repeat-containing protein 7	_GVIAISKS[Phospho (STY)]TER_.2	0.2314	-2.1113	0.0013	2.8848
Dlg2	Q91XM9	Disks large homolog 2	_QPS[Phospho (STY)]VTLQR_.2	0.1803	-2.4719	0.0016	2.7913
Nsfl1c	Q9CZ44	NSFL1 cofactor p47	_SYQDPSNAQFLESIR_.2	1.5537	0.6357	0.0017	2.7772
Syngap1	F6SEU4	Ras/Rap GTPase-activating protein SynGAP	_S[Phospho (STY)]VSM[Oxidation (M)]LDLQGDGPGGR_.2	0.1824	-2.4550	0.0018	2.7353
Uba1	Q02053	Ubiquitin-like modifier-activating enzyme 1	_SLPASLVEPDFVM[Oxidation (M)]TDFAK_.2	0.7045	-0.5052	0.0020	2.6997
Iqsec1	Q8R0S2	IQ motif and SEC7 domain-containing protein 1	_SALSSS[Phospho (STY)]LRDLSEAGK_.2	0.4005	-1.3202	0.0022	2.6587
Tesk1	O70146	Dual specificity testis-specific protein kinase 1	_S[Phospho (STY)]LPSSPELPR_.2	0.0803	-3.6384	0.0023	2.6396
Actr1b	Q8R5C5	Beta-centractin	_TLFSNIVLSGGSTLFK_.2	0.7544	-0.4066	0.0024	2.6242
Actr2	P61161	Actin-related protein 2	_HIVLSGGSTM[Oxidation (M)]YPGLPSR_.2	0.6066	-0.7211	0.0024	2.6222
Npy	P57774	Pro-neuropeptide Y	_SSPETLISDLLM[Oxidation (M)]KES[Phospho (STY)]TENAPR_.3	0.1513	-2.7244	0.0025	2.6032
Ccnk	O88874	Cyclin-K	_AVVVS[Phospho (STY)]PKEENK_.2	0.7232	-0.4676	0.0026	2.5817
Pacs1	Q8K212	Phosphofurin acidic cluster sorting protein 1	_YSSTFLDSAWR_.2	2.3509	1.2332	0.0026	2.5807
Nefh	P19246	Neurofilament heavy polypeptide	_AVAS[Phospho (STY)]EEETPAK_.2	4.6469	2.2163	0.0027	2.5645
Iqsec1	Q8R0S2	IQ motif and SEC7 domain-containing protein 1	_ETLIGIYER_.2	0.3862	-1.3727	0.0027	2.5618
Anp32a	O35381	Acidic leucine-rich nuclear phosphoprotein 32 family member A	_NRT[Phospho (STY)]PSDVK_.2	0.1136	-3.1374	0.0030	2.5295
Igsf8	Q8R366	Immunoglobulin superfamily member 8	_HAAYSVGWEM[Oxidation (M)]APAGAPGPGR_.3	0.3995	-1.3237	0.0031	2.5088
Agfg2	Q80WC7	Arf-GAP domain and FG repeat-containing protein 2	_ELGGC[Carbamidomethyl (C)]SQAGNR_.2	0.7590	-0.3978	0.0033	2.4856
Uqcrc1	Q9CZ13	Cytochrome b-c1 complex subunit 1, mitochondrial	_IQEVDAQM[Oxidation (M)]LR_.2	1.7506	0.8078	0.0034	2.4722
Zfr	O88532	Zinc finger RNA-binding protein	_DS[Phospho (STY)]DGVDGFEAEGK_.2	0.1873	-2.4164	0.0036	2.4454
Dclk1	Q9JLM8	Serine/threonine-protein kinase DCLK1	_VC[Carbamidomethyl (C)]SS[Phospho (STY)]M[Oxidation (M)]DENDGPGEGDELGRR_.3	0.4018	-1.3153	0.0037	2.4324
Plcl2	Q8K394	Inactive phospholipase C-like protein 2	_S[Phospho (STY)]LEAIPEK_.2	0.4221	-1.2443	0.0038	2.4223
Uqcrc1	Q9CZ13	Cytochrome b-c1 complex subunit 1, mitochondrial	_EVESIGAHLNAYSTR_.2	1.5011	0.5860	0.0040	2.4032
Fam114a2	Q8VE88	Protein FAM114A2	_AETSLGIPS[Phospho (STY)]PTEISAEVK_.2	0.2880	-1.7958	0.0043	2.3637
Dync2i2	Q5U4F6	Cytoplasmic dynein 2 intermediate chain 2	_VGS[Phospho (STY)]AGAAALAAGGAGGAER_.2	0.5210	-0.9407	0.0050	2.3009
Etfa	Q99LC5	Electron transfer flavoprotein subunit alpha, mitochondrial	_GLLPEELTPLILETQK_.2	0.7040	-0.5063	0.0050	2.3002
Map7d2	A2AG50	MAP7 domain-containing protein 2	_RLSS[Phospho (STY)]PVK_.2	0.1281	-2.9650	0.0053	2.2790
Ap2a2	P17427	AP-2 complex subunit alpha-2	_YGGTFQNVSVK_.2	0.4839	-1.0472	0.0053	2.2751
Myh10	Q61879	Myosin-10	_DLEAQIEAANK_.2	0.4569	-1.1300	0.0054	2.2713
Tppp	Q7TQD2	Tubulin polymerization-promoting protein	_DC[Carbamidomethyl (C)]HVIDGK_.2	0.2662	-1.9095	0.0054	2.2685
Eif4g1	Q6NZJ6	Eukaryotic translation initiation factor 4 gamma 1	_EATLPPVS[Phospho (STY)]PPK_.2	0.1657	-2.5933	0.0056	2.2531
Atp5md	Q78IK2	ATP synthase membrane subunit DAPIT, mitochondrial	_AGAESDGQFQFTGIK_.2	0.7086	-0.4970	0.0056	2.2503
Acin1	Q9JIX8	Apoptotic chromatin condensation inducer in the nucleus	_GVQAGNS[Phospho (STY)]DTEGGQPGRK_.2	0.5708	-0.8089	0.0057	2.2455
Coro1c	Q9WUM4	Coronin-1C	_VTWDSSFC[Carbamidomethyl (C)]AVNPR_.2	0.5208	-0.9412	0.0058	2.2329
Rbmxl1	Q91VM5	RNA binding motif protein, X-linked-like-1	_VEQATKPS[Phospho (STY)]FESGR_.2	4.5335	2.1806	0.0061	2.2165
Cdc42bpb	Q7TT50	Serine/threonine-protein kinase MRCK beta	_SM[Oxidation (M)]S[Phospho (STY)]DPDQDFDKEPDSDSTK_.2	0.6080	-0.7180	0.0061	2.2112
Fasn	P19096	Fatty acid synthase	_TLEAVQDLLEQGR_.2	0.5935	-0.7526	0.0062	2.2044
Slc39a6	Q8C145	Zinc transporter ZIP6	_YDSQLS[Phospho (STY)]SNEEK_.2	0.5202	-0.9429	0.0064	2.1932
Kcnb2	A6H8H5	Potassium voltage-gated channel subfamily B member 2	_S[Phospho (STY)]M[Oxidation (M)]ELIDVAVEK_.2	0.5139	-0.9604	0.0065	2.1895
Psma5	Q9Z2U1	Proteasome subunit alpha type-5	_LFQVEYAIEAIK_.2	0.5311	-0.9129	0.0065	2.1888
Atp1a2	Q6PIE5	Sodium/potassium-transporting ATPase subunit alpha-2	_YQLSIHER_.2	0.7152	-0.4835	0.0067	2.1750
Ctnnd1	P30999	Catenin delta-1	_S[Phospho (STY)]M[Oxidation (M)]GYDDLDYGM[Oxidation (M)]M[Oxidation (M)]SDYGTAR_.2	0.4032	-1.3105	0.0069	2.1629
Ola1	Q9CZ30	Obg-like ATPase 1	_IPAFLNVVDIAGLVK_.2	2.3226	1.2157	0.0070	2.1552
Apc2	Q9Z1K7	Adenomatous polyposis coli protein 2	_RGS[Phospho (STY)]DGEARPLPR_.3	0.4891	-1.0318	0.0071	2.1510
Psmb4	P99026	Proteasome subunit beta type-4	_VNDSTM[Oxidation (M)]LGASGDYADFQYLK_.2	1.3758	0.4602	0.0071	2.1485
Cltc	Q68FD5	Clathrin heavy chain 1	_VGYTPDWIFLLR_.2	0.4690	-1.0924	0.0072	2.1429
Aldoa	P05064	Fructose-bisphosphate aldolase A	_VNPC[Carbamidomethyl (C)]IGGVILFHETLYQK_.2	0.7110	-0.4922	0.0074	2.1335
Ubqln2	Q9QZM0	Ubiquilin-2	_NPEISHLLNNPDIM[Oxidation (M)]R_.3	0.6059	-0.7228	0.0074	2.1308
Gpd1l	Q3ULJ0	Glycerol-3-phosphate dehydrogenase 1-like protein	_FC[Carbamidomethyl (C)]ETTIGSK_.2	2.2083	1.1429	0.0075	2.1267
Grin2b	Q01097	Glutamate receptor ionotropic, NMDA 2B	_QHSY[Phospho (STY)]DTFVDLQKEEAALAPR_.3	0.6034	-0.7289	0.0075	2.1230
Dnm1l	Q8K1M6	Dynamin-1-like protein	_IC[Carbamidomethyl (C)]YIFHETFGR_.2	1.4426	0.5286	0.0077	2.1163
Cadps	Q80TJ1	Calcium-dependent secretion activator 1	_LQKEEEER_.2	1.5339	0.6172	0.0078	2.1079
Pclo	Q9QYX7	Protein piccolo	_TSVAQTHLEDAGAAIAAAEAAVQQLR_.3	2.3104	1.2081	0.0078	2.1053

Trappc9	Q3U0M1	Trafficking protein particle complex subunit 9	_VESRPTNPS[Phospho (STY)]EGS[Phospho (STY)]KTGDLGHVK_.3	0.3473	-1.5256	0.0079	2.1001
Sptan1	P16546	Spectrin alpha chain, non-erythrocytic 1	_HALLEADVAAHQDR_.3	1.5510	0.6332	0.0081	2.0934
Prkcg	P63318	Protein kinase C gamma type	_GSDELYAIK_.2	0.6141	-0.7035	0.0082	2.0856
Rbm14	Q8C2Q3	RNA-binding protein 14	_IFVGNVSAAC[Carbamidomethyl (C)]TSQELR_.2	0.5845	-0.7747	0.0086	2.0672
Rbmxl1	Q91VM5	RNA binding motif protein, X-linked-like-1	_RDVYLS[Phospho (STY)]PR_.2	0.4101	-1.2861	0.0086	2.0667
Cap2	Q9CYT6	Adenylyl cyclase-associated protein 2	_LINSM[Oxidation (M)]VAEFLK_.2	0.6677	-0.5826	0.0087	2.0590
Hdgf	P51859	Hepatoma-derived growth factor	_GFSEGLWEIENNPTVK_.2	1.7512	0.8083	0.0087	2.0582
Ndrg2	Q9QYG0	Protein NDRG2	_S[Phospho (STY)]RT[Phospho (STY)]LSQSSESGTLPSGPPGHTM[Oxidation (M)]EVSC[Carbamidomethyl (C)]_.3	0.1261	-2.9874	0.0089	2.0521
Ldha	P06151	L-lactate dehydrogenase A chain	_DLADELALVDVM[Oxidation (M)]EDKLK_.2	0.7282	-0.4577	0.0089	2.0503
Bud13	Q8R149	BUD13 homolog	_HDLDAS[Phospho (STY)]PPRK_.3	1.7534	0.8101	0.0092	2.0381
Etfb	Q9DCW4	Electron transfer flavoprotein subunit beta	_EIIAVSC[Carbamidomethyl (C)]GPSQC[Carbamidomethyl (C)]QETIR_.2	1.5218	0.6058	0.0092	2.0363
Scg2	Q03517	Secretogranin-2	_VAWIPDVESR_.2	1.3358	0.4178	0.0094	2.0273
Prkcg	P63318	Protein kinase C gamma type	_SVPSLC[Carbamidomethyl (C)]GVDHTER_.2	0.7357	-0.4428	0.0094	2.0266
Ywhag	P61982	14-3-3 protein gamma	_NC[Carbamidomethyl (C)]SETQYESK_.2	1.3070	0.3862	0.0094	2.0257
Sptbn1	Q62261	Spectrin beta chain, non-erythrocytic 1	_QAEAFLNNQEYVLAHTEM[Oxidation (M)]PTTLEGAEAAIK_.3	0.6973	-0.5201	0.0097	2.0129
Cct6a	P80317	T-complex protein 1 subunit zeta	_QADLYISEGLHPR_.2	0.7361	-0.4421	0.0099	2.0040
Slc20a2	Q80UP8	Sodium-dependent phosphate transporter 2	_AS[Phospho (STY)]DESLRK_.2	2.9919	1.5811	0.0099	2.0039
Map6d1	Q14BB9	MAP6 domain-containing protein 1	_TVYVLPVGDADAAVVATTSYR_.2	2.1293	1.0903	0.0100	2.0019
Myh10	Q61879	Myosin-10	_LVQEQGSHSK_.2	1.9379	0.9545	0.0100	1.9992
Eef1a1	P10126	Elongation factor 1-alpha 1	_M[Oxidation (M)]DSTEPPYSQK_.2	1.3028	0.3816	0.0101	1.9960
Cntn2	Q61330	Contactin-2	_TTGPGGDGIPAEVHIVR_.3	2.2086	1.1431	0.0103	1.9876
Gstm5	P48774	Glutathione S-transferase Mu 5	_SM[Oxidation (M)]VLGYWDIR_.2	0.7055	-0.5033	0.0103	1.9855
Gng3	P63216	Guanine nucleotide-binding protein G(I)/G(S)/G(O) subunit gamma-3	_IEASLC[Carbamidomethyl (C)]R_.2	0.6626	-0.5937	0.0104	1.9819
Dnajc21	E9Q8D0	DnaJ homolog subfamily C member 21	_YEKEFGDGS[Phospho (STY)]DENEVEDQEPR_.3	2.1021	1.0719	0.0106	1.9745
Gpd1l	Q3ULJ0	Glycerol-3-phosphate dehydrogenase 1-like protein	_KLTDIINNDHENVK_.3	1.7205	0.7828	0.0107	1.9724
Fam171b	Q14CH0	Protein FAM171B	_KSTVEDFEANTS[Phospho (STY)]PTK_.2	0.4105	-1.2845	0.0107	1.9687
Tuba3a	P05214	Tubulin alpha-3 chain	_LIGQIVSSITASLR_.2	0.5948	-0.7494	0.0108	1.9677
Map1lc3a	Q91VR7	Microtubule-associated proteins 1A/1B light chain 3A	_FLVPDHVNM[Oxidation (M)]SELVK_.3	1.3130	0.3929	0.0110	1.9575
Frrs1l	B1AXV0	DOMON domain-containing protein FRRS1L	_NPARDEEGVFENNR_.3	0.7125	-0.4890	0.0113	1.9480
Srrm3	Q80WV7	Serine/arginine repetitive matrix protein 3	_RRDS[Phospho (STY)]PSFMEPR_.3	0.6721	-0.5733	0.0114	1.9430
Map2	P20357	Microtubule-associated protein 2	_IDLSHVTSK_.2	1.5723	0.6529	0.0116	1.9370
Myh10	Q61879	Myosin-10	_IAEC[Carbamidomethyl (C)]SSQLAEEEEKAK_.2	1.6637	0.7344	0.0118	1.9267
Dnm1	P39053	Dynamin-1	_LREEM[Oxidation (M)]ER_.2	1.8929	0.9206	0.0119	1.9255
Dmtn	Q9WV69	Dematin	_VFAM[Oxidation (M)]S[Phospho (STY)]PEEFGK_.2	0.5778	-0.7914	0.0122	1.9149
Rab3c	P62823	Ras-related protein Rab-3C	_LVDIIC[Carbamidomethyl (C)]DK_.2	0.3929	-1.3476	0.0122	1.9134
Slc25a3	Q8VEM8	Phosphate carrier protein, mitochondrial	_EEGLNAFYK_.2	0.6488	-0.6241	0.0124	1.9083
Shisa9	Q9CZN4	Protein shisa-9	_AVGNSDGDWAVATLKS[Phospho (STY)]PK_.3	2.8232	1.4973	0.0127	1.8977
Dock9	Q8BIK4	Dedicator of cytokinesis protein 9	_GSLISTDS[Phospho (STY)]GNSLPDRNPEK_.3	0.6760	-0.5648	0.0127	1.8961
Rps26	P62855	40S ribosomal protein S26	_DISEASVFDAYVLPK_.2	1.7415	0.8003	0.0128	1.8937
Clip3	B9EHT4	CAP-Gly domain-containing linker protein 3	_SPSS[Phospho (STY)]PSLGSLQQR_.2	0.3997	-1.3231	0.0128	1.8930
Prkacb	P68181	cAMP-dependent protein kinase catalytic subunit beta	_ILQAVEFPFLVR_.2	0.5713	-0.8076	0.0128	1.8912
Pygb	Q8CI94	Glycogen phosphorylase, brain form	_QAVDQISSGFFSPK_.2	1.5682	0.6491	0.0129	1.8894
Sorbs1	Q62417	Sorbin and SH3 domain-containing protein 1	_YS[Phospho (STY)]FSDDTK_.2	1.5923	0.6711	0.0129	1.8881
Aldh1l1	Q8R0Y6	Cytosolic 10-formyltetrahydrofolate dehydrogenase	_ILPNVPEVEDSTDFFK_.2	0.7425	-0.4295	0.0131	1.8825
Sh3gl1	Q62419	Endophilin-A2	_ITAS[Phospho (STY)]SSFR_.2	1.5095	0.5941	0.0131	1.8820
Ank2	Q8C8R3	Ankyrin-2	_AEGFES[Phospho (STY)]ESEDGATKPK_.2	0.5358	-0.9001	0.0131	1.8819
Dnm1l	Q8K1M6	Dynamin-1-like protein	_RLEEPSLR_.2	1.6464	0.7193	0.0132	1.8801
Rpsa	P14206	40S ribosomal protein SA	_LLVVTDPR_.2	1.3554	0.4387	0.0132	1.8785
Cltc	Q68FD5	Clathrin heavy chain 1	_LLEM[Oxidation (M)]NLM[Oxidation (M)]HAPQVADAILGNQM[Oxidation (M)]FTHYDR_.4	0.5706	-0.8095	0.0132	1.8782
Ppig	A2AR02	Peptidyl-prolyl cis-trans isomerase G	_HM[Oxidation (M)]S[Phospho (STY)]ES[Phospho (STY)]PNRKVEK_.3	1.5762	0.6565	0.0138	1.8614
Plec	Q9QXS1	Plectin	_TEVVETTQVYTEETRR_.3	0.5994	-0.7385	0.0138	1.8608
Actn1	Q7TPR4	Alpha-actinin-1	_DGLGFC[Carbamidomethyl (C)]ALIHR_.2	0.5628	-0.8292	0.0141	1.8518
Rack1	P68040	Receptor of activated protein C kinase 1	_FSPNSSNPIIVSC[Carbamidomethyl (C)]GWDK_.2	1.3289	0.4102	0.0142	1.8470
Pgam1	Q9DBJ1	Phosphoglycerate mutase 1	_KAM[Oxidation (M)]EAVAAQGK_.2	1.5714	0.6521	0.0143	1.8458
Pitpnc1	Q8K4R4	Cytoplasmic phosphatidylinositol transfer protein 1	_KKS[Phospho (STY)]APETLTLPDPEK_.3	4.0070	2.0025	0.0143	1.8453
Atp6v1e1	P50518	V-type proton ATPase subunit E 1	_IMEYYEK_.2	1.8198	0.8638	0.0144	1.8412
Mia3	Q8BI84	Transport and Golgi organization protein 1 homolog	_DETAAQTPGS[Phospho (STY)]GEAVLSK_.2	0.3974	-1.3312	0.0146	1.8362
Kcnj3	P63250	G protein-activated inward rectifier potassium channel 1	_ERHNS[Phospho (STY)]VEC[Carbamidomethyl (C)]LDGLDDISTK_.3	0.5129	-0.9632	0.0148	1.8306
U2surp	Q6NV83	U2 snRNP-associated SURP motif-containing protein	_VKS[Phospho (STY)]PS[Phospho (STY)]PK_.2	1.6537	0.7257	0.0149	1.8261
Myh10	Q61879	Myosin-10	_LDPHLVLDQLR_.2	0.6038	-0.7279	0.0152	1.8194
Caap1	Q8VDY9	Caspase activity and apoptosis inhibitor 1	_SVNEILGLAESS[Phospho (STY)]PKEPK_.2	2.4994	1.3216	0.0154	1.8132
Ywhae	P62259	14-3-3 protein epsilon	_M[Oxidation (M)]KGDYHR_.2	0.1924	-2.3780	0.0155	1.8109

Pde1b	Q01065	Calcium/calmodulin-dependent 3',5'-cyclic nucleotide phosphodiesterase 1B	_TIVFELLTR_.2	0.7203	-0.4734	0.0155	1.8090
Rps20	P60867	40S ribosomal protein S20	_DTGKTPVEPEVAIHR_.3	1.3186	0.3990	0.0156	1.8065
Map6	Q7TSJ2	Microtubule-associated protein 6	_M[Oxidation (M)]VHETSYSAQFK_.3	0.6237	-0.6811	0.0157	1.8055
Fmn2	Q9JL04	Formin-2	_QLSSPNHSPS[Phospho (STY)]QSPNQSPR_.3	1.4508	0.5369	0.0158	1.8005
Atp1a3	Q6PIC6	Sodium/potassium-transporting ATPase subunit alpha-3	_YQLSIHETEDPNDNR_.3	0.7582	-0.3994	0.0159	1.8000
Glul	P15105	Glutamine synthetase	_AMREENGLK_.2	1.7857	0.8365	0.0162	1.7894
Calm1;Calm2;Calm3	P0DP26;P0DP27;P0DP28	Calmodulin-1;Calmodulin-2;Calmodulin-3	_DGNGYISAAELR_.2	0.4398	-1.1850	0.0164	1.7861
Pclo	Q9QYX7	Protein piccolo	_TSQS[Phospho (STY)]FPPTGDTITPLDSK_.2	0.1146	-3.1254	0.0169	1.7730
Eif2s2	Q99L45	Eukaryotic translation initiation factor 2 subunit 2	_IFDIDEAEEAIKDVK_.3	0.5984	-0.7408	0.0170	1.7708
Cadm3	Q99N28	Cell adhesion molecule 3	_EDDGANIVC[Carbamidomethyl (C)]SVNHESLK_.2	0.6585	-0.6028	0.0170	1.7697
Ubr5	Q80TP3	E3 ubiquitin-protein ligase UBR5	_WLDGAS[Phospho (STY)]FDNER_.2	0.4638	-1.1086	0.0170	1.7688
Mpst	Q99J99	3-mercaptopyruvate sulfurtransferase	_FQGTQPEPR_.2	5.3201	2.4115	0.0170	1.7687
Ywhab	Q9CQV8	14-3-3 protein beta/alpha	_AVTEQGHELSNEER_.3	1.3344	0.4162	0.0171	1.7679
Cbx1	P83917	Chromobox protein homolog 1	_ADS[Phospho (STY)]DSEDKGEESKPK_.2	0.5926	-0.7548	0.0172	1.7654
Nav3	Q80TN7	Neuron navigator 3	_KTS[Phospho (STY)]LDGSQNQDDVVLHVSSK_.3	0.6872	-0.5411	0.0172	1.7639
Nr4a1;Nr4a2;Nr4a3	P12813;Q06219;Q9QZB6	ubfamily 4 group A member 1;Nuclear receptor subfamily 4 group A member 2;Nuclear receptor subfamily 4	_TDS[Phospho (STY)]LKGR_.2	0.2866	-1.8031	0.0176	1.7537
Hk1	P17710	Hexokinase-1	_IDEAVLITWTK_.2	0.7440	-0.4267	0.0176	1.7537
Map6	Q7TSJ2	Microtubule-associated protein 6	_S[Phospho (STY)]LYSEPFKEC[Carbamidomethyl (C)]PK_.2	1.5792	0.6592	0.0179	1.7483
Dlgap2	Q8BJ42	Disks large-associated protein 2	_EAEENDLLEDILGK_.2	2.1439	1.1003	0.0180	1.7445
Ap2m1	P84091	AP-2 complex subunit mu	_LS[Phospho (STY)]KFDSER_.2	0.6901	-0.5351	0.0180	1.7437
Hspa5	P20029	Endoplasmic reticulum chaperone BiP	_SQIFSTASDNQPTVTIK_.2	1.3218	0.4025	0.0183	1.7377
Septin3	Q9Z1S5	Neuronal-specific septin-3	_KASS[Phospho (STY)]WNR_.2	0.2266	-2.1418	0.0184	1.7355
Dmwd	Q08274	Dystrophia myotonica WD repeat-containing protein	_GGSGGNSS[Phospho (STY)]NDKLSGPAPR_.2	1.3009	0.3796	0.0186	1.7312
Strap	Q9Z1Z2	Serine-threonine kinase receptor-associated protein	_TVDFTQDSNYLLTGGQDK_.2	1.6549	0.7268	0.0186	1.7301
Cse1l	Q9ERK4	Exportin-2	_IVEDEPNKIC[Carbamidomethyl (C)]EADR_.3	0.6161	-0.6988	0.0186	1.7299
Dlg3	P70175	Disks large homolog 3	_FIEAGQFNDNLYGTSIQSVR_.2	1.5494	0.6317	0.0191	1.7195
Syngap1	F6SEU4	Ras/Rap GTPase-activating protein SynGAP	_S[Phospho (STY)]QPMVLR_.2	0.6891	-0.5373	0.0192	1.7176
Tjp2	Q9Z0U1	Tight junction protein ZO-2	_S[Phospho (STY)]RGRS[Phospho (STY)]LER_.2	1.6506	0.7230	0.0192	1.7165
Gja1	P23242	Gap junction alpha-1 protein	_PRPDDLEI_.2	0.5015	-0.9956	0.0193	1.7151
Akap5	D3YVF0	A-kinase anchor protein 5	_KATVGQAEEATIDHTEK_.3	2.3946	1.2598	0.0194	1.7122
Arhgef12	Q8R4H2	Rho guanine nucleotide exchange factor 12	_VEHHDLSVAGLQS[Phospho (STY)]PDR_.3	0.5442	-0.8778	0.0195	1.7099
Irf2bp1	Q8R3Y8	Interferon regulatory factor 2-binding protein 1	_S[Phospho (STY)]PGPPALK_.2	0.6904	-0.5346	0.0195	1.7093
Hspa12b	Q9CZJ2	Heat shock 70 kDa protein 12B	_RSS[Phospho (STY)]VNLVK_.2	0.4342	-1.2035	0.0196	1.7078
Srrm2	Q8BTI8	Serine/arginine repetitive matrix protein 2	_S[Phospho (STY)]RSRT[Phospho (STY)]S[Phospho (STY)]PAPWK_.2	7.9197	2.9855	0.0196	1.7076
Vcp	Q01853	Transitional endoplasmic reticulum ATPase	_EAVC[Carbamidomethyl (C)]IVLSDDTC[Carbamidomethyl (C)]SDEKIR_.3	0.6920	-0.5312	0.0196	1.7069
Ube2m	P61082	NEDD8-conjugating enzyme Ubc12	_DINELNLPK_.2	1.9388	0.9552	0.0197	1.7065
Crmp1	P97427	Dihydropyrimidinase-related protein 1	_NLHQSNFSLSGAQIDDNNPRR_.4	1.6112	0.6881	0.0197	1.7055
Epb41l3	Q9WV92	Band 4.1-like protein 3	_EQHPDMSVTK_.2	1.7157	0.7788	0.0197	1.7054
Srrm1	Q52KI8	Serine/arginine repetitive matrix protein 1	_AS[Phospho (STY)]PS[Phospho (STY)]PPPKR_.2	0.3650	-1.4542	0.0197	1.7047
Trap1	Q9CQN1	Heat shock protein 75 kDa, mitochondrial	_AFLEALQNQAETSSK_.2	0.6587	-0.6023	0.0198	1.7033
Vim	P20152	Vimentin	_DNLAEDIMR_.2	1.5628	0.6441	0.0198	1.7025
Rnf208	Q8K0W3	RING finger protein 208	_KGS[Phospho (STY)]SELGFPR_.2	6.7879	2.7630	0.0200	1.7000
Scg5	P12961	Neuroendocrine protein 7B2	_DFSEDQGYPDPPNPC[Carbamidomethyl (C)]PLGK_.2	1.8763	0.9079	0.0201	1.6979
Hnrnpa2b1	O88569	Heterogeneous nuclear ribonucleoproteins A2/B1	_EES[Phospho (STY)]GKPGAHVTVK_.2	3.6517	1.8686	0.0202	1.6952
Sgsm1	Q8BPQ7	Small G protein signaling modulator 1	_C[Carbamidomethyl (C)]SSGAS[Phospho (STY)]LDSHLHR_.3	0.6016	-0.7331	0.0203	1.6919
Larp1	Q6ZQ58	La-related protein 1	_TASISSS[Phospho (STY)]PSEGTPAVGSYGC[Carbamidomethyl (C)]TPQSLPK_.3	0.6515	-0.6182	0.0204	1.6909
Fis1	Q9CQ92	Mitochondrial fission 1 protein	_STQFEYAWC[Carbamidomethyl (C)]LVR_.2	0.6683	-0.5815	0.0204	1.6893
Ywhaz	P63101	14-3-3 protein zeta/delta	_VVSSIEQK_.2	1.3849	0.4698	0.0206	1.6855
Fkbp15	Q6P9Q6	FK506-binding protein 15	_[Acetyl (Protein N-term)]MFGAGDEDDTDFLS[Phospho (STY)]PSGGAK_.2	0.6791	-0.5582	0.0207	1.6843
Tcp11l2	Q8K1H7	T-complex protein 11-like protein 2	_QC[Carbamidomethyl (C)]VSEDQQS[Phospho (STY)]DSESSR_.2	1.7271	0.7884	0.0208	1.6809
Adgrl2	Q8JZZ7	Adhesion G protein-coupled receptor L2	_S[Phospho (STY)]MPNLGAGR_.2	1.6795	0.7480	0.0210	1.6781
Ablim3	Q69ZX8	Actin-binding LIM protein 3	_SS[Phospho (STY)]PYHSQLDVR_.2	4.2392	2.0838	0.0210	1.6769
Synj1	Q8CHC4	Synaptojanin-1	_TPAWTDR_.2	0.6145	-0.7026	0.0211	1.6764
Sec23ip	Q6NZC7	SEC23-interacting protein	_EM[Oxidation (M)]AS[Phospho (STY)]PSSESNESKR_.2	1.7974	0.8459	0.0211	1.6748
Gpd1l	Q3ULJ0	Glycerol-3-phosphate dehydrogenase 1-like protein	_VC[Carbamidomethyl (C)]IVGSGNWGSAVAK_.2	0.6917	-0.5319	0.0214	1.6687
Gpr162	Q3UN16	Probable G-protein coupled receptor 162	_RLS[Phospho (STY)]HDETNIFSTPR_.3	0.5519	-0.8576	0.0215	1.6681
Ndufs1	Q91VD9	NADH-ubiquinone oxidoreductase 75 kDa subunit, mitochondrial	_LTEPMVR_.2	2.1133	1.0795	0.0217	1.6639
L1cam	P11627	Neural cell adhesion molecule L1	_PM[Oxidation (M)]KDETFGEY[Phospho (STY)]RSLES[Phospho (STY)]DNEEK_.3	0.6180	-0.6943	0.0217	1.6628
Map1a	Q9QYR6	Microtubule-associated protein 1A	_LDM[Oxidation (M)]YVLNPVK_.2	0.7293	-0.4554	0.0219	1.6598
Rims4	P60191	Regulating synaptic membrane exocytosis protein 4	_QASQLS[Phospho (STY)]LESTVGPC[Carbamidomethyl (C)]GERS_.2	2.2060	1.1415	0.0224	1.6501
Ganab	Q8BHN3	Neutral alpha-glucosidase AB	_DAVHYGGWEHR_.3	0.5964	-0.7456	0.0224	1.6492
Cct4	P80315	T-complex protein 1 subunit delta	_VIDPATATSVDLR_.2	1.3203	0.4009	0.0224	1.6491

Zranb2	Q9R020	Zinc finger Ran-binding domain-containing protein 2	_EES[Phospho (STY)]DGEYDEFGR_.2	0.4209	-1.2483	0.0226	1.6458
Lmtk2	Q3TYD6	Serine/threonine-protein kinase LMTK2	_HSGTSPQASPALLTEEGS[Phospho (STY)]PTAPTDPILKPEETK_.4	0.3894	-1.3608	0.0226	1.6453
Suclg1	Q9WUM5	Succinate--CoA ligase [ADP/GDP-forming] subunit alpha, mitochondrial	_QGTFHSQQALEYGTK_.3	1.6301	0.7050	0.0229	1.6411
Atp5f1a	Q03265	ATP synthase subunit alpha, mitochondrial	_LTDADAMK_.2	1.5864	0.6658	0.0230	1.6390
Ehbp1	Q69ZW3	EH domain-binding protein 1	_SRS[Phospho (STY)]LEC[Carbamidomethyl (C)]R_.2	0.6128	-0.7065	0.0230	1.6387
Cltc	Q68FD5	Clathrin heavy chain 1	_SVDPTLALSVYLR_.2	0.7672	-0.3823	0.0232	1.6340
Apoe	P08226	Apolipoprotein E	_NEVHTM[Oxidation (M)]LGQS[Phospho (STY)]TEEIR_.2	1.7311	0.7917	0.0232	1.6338
Bcr	Q6PAJ1	Breakpoint cluster region protein	_ASAPRPPPAPADGADPAPVEESEARPDGEGS[Phospho (STY)]PSK_.4	0.4263	-1.2302	0.0235	1.6284
Trim3	Q9R1R2	Tripartite motif-containing protein 3	_ALRPGDLPPS[Phospho (STY)]PDDVK_.2	0.3506	-1.5120	0.0235	1.6282
MAPT	P10636	Microtubule-associated protein tau	_IPAKT[Phospho (STY)]PPAPKT[Phospho (STY)]PPSSGEPPK_.3	0.6127	-0.7068	0.0236	1.6273
Sowahd	Q8BY98	Ankyrin repeat domain-containing protein SOWAHD	_PC[Carbamidomethyl (C)]HY[Phospho (STY)]LRPDAS[Phospho (STY)]LNLR_.2	0.3761	-1.4107	0.0236	1.6268
Sugp1	Q8CH02	SURP and G-patch domain-containing protein 1	_DIDASPS[Phospho (STY)]PLSVQDLK_.2	0.2480	-2.0117	0.0237	1.6246
Prdx6	O08709	Peroxiredoxin-6	_VVFIFGPDK_.2	1.5233	0.6072	0.0238	1.6242
Prkcg	P63318	Protein kinase C gamma type	_M[Oxidation (M)]GPSSSPIPSPS[Phospho (STY)]PS[Phospho (STY)]PTDSKR_.3	0.5156	-0.9557	0.0238	1.6240
Ripor1	Q68FE6	Rho family-interacting cell polarization regulator 1	_HTSS[Phospho (STY)]PEVVAEDR_.2	4.6545	2.2186	0.0238	1.6237
Actn4	P57780	Alpha-actinin-4	_VLAVNQENEHLMEDYER_.3	2.0139	1.0100	0.0239	1.6223
Pdxk	Q8K183	Pyridoxal kinase	_KPDGSTVTQR_.2	1.8698	0.9029	0.0239	1.6209
Cacna1a	P97445	Voltage-dependent P/Q-type calcium channel subunit alpha-1A	_AWPGS[Phospho (STY)]PER_.2	0.4468	-1.1624	0.0239	1.6207
Ap2a1	P17426	AP-2 complex subunit alpha-1	_DFLTPPLLSVR_.2	0.7295	-0.4550	0.0240	1.6197
Snx16	Q8C080	Sorting nexin-16	_EFLC[Carbamidomethyl (C)]LDDPPGPFDS[Phospho (STY)]LEESR_.2	0.6847	-0.5465	0.0240	1.6195
Rbm15b	Q6PHZ5	Putative RNA-binding protein 15B	_RNS[Phospho (STY)]LEGYSR_.2	0.5614	-0.8328	0.0242	1.6163
Arid1a	A2BH40	AT-rich interactive domain-containing protein 1A	_SNS[Phospho (STY)]VGIQDAFPDGSDPTFQK_.2	0.2060	-2.2792	0.0243	1.6139
Tbc1d9b	Q5SVR0	TBC1 domain family member 9B	_VFQKNS[Phospho (STY)]PM[Oxidation (M)]EDLGAK_.2	0.4939	-1.0176	0.0243	1.6137
Lrrc7	Q80TE7	Leucine-rich repeat-containing protein 7	_S[Phospho (STY)]QSVPMLDDEMLM[Oxidation (M)]YGSSK_.2	1.3415	0.4238	0.0245	1.6105
Lsm11	Q8BUV6	U7 snRNA-associated Sm-like protein LSm11	_DSS[Phospho (STY)]LTLTR_.2	1.6054	0.6829	0.0246	1.6084
Calcoco1	Q8CGU1	Calcium-binding and coiled-coil domain-containing protein 1	_LPSYGLC[Carbamidomethyl (C)]ESGNTSSS[Phospho (STY)]PPGPR_.2	0.3124	-1.6787	0.0246	1.6083
Tbc1d13	Q8R3D1	TBC1 domain family member 13	_SGVTNMSS[Phospho (STY)]PHK_.2	1.7758	0.8285	0.0249	1.6032
Vat1l	Q80TB8	Synaptic vesicle membrane protein VAT-1 homolog-like	_TPLVPGFEC[Carbamidomethyl (C)]SGIVEALGDSVK_.2	1.3759	0.4604	0.0250	1.6017
Dop1b	Q3UHQ6	Protein dopey-2	_S[Phospho (STY)]DLSEEDLPYYVDLPDR_.2	1.5874	0.6667	0.0251	1.6011
Palm	Q9Z0P4	Paralemmin-1	_ENSAAPS[Phospho (STY)]PGRPQSASPAK_.2	1.5362	0.6193	0.0251	1.6002
Cox5a	P12787	Cytochrome c oxidase subunit 5A, mitochondrial	_GM[Oxidation (M)]NTLVGYDLVPEPK_.2	1.6131	0.6899	0.0252	1.5992
Tardbp	Q921F2	TAR DNA-binding protein 43	_LPNSKQS[Phospho (STY)]PDEPLR_.2	0.0841	-3.5726	0.0252	1.5990
Map1a	Q9QYR6	Microtubule-associated protein 1A	_WLAES[Phospho (STY)]PVGLPPEEEDK_.2	0.3364	-1.5717	0.0252	1.5978
Wdr59	Q8C0M0	GATOR complex protein WDR59	_AVS[Phospho (STY)]PTEPTPR_.2	0.3572	-1.4851	0.0253	1.5972
Bcl9	Q9D219	B-cell CLL/lymphoma 9 protein	_IPVEGPLS[Phospho (STY)]PSR_.2	0.4230	-1.2411	0.0254	1.5959
Uqcrb	Q9D855	Cytochrome b-c1 complex subunit 7	_RLPEDLYNDR_.2	0.6354	-0.6542	0.0254	1.5958
Rpl6	P47911	60S ribosomal protein L6	_AGS[Phospho (STY)]DAAASRPR_.2	0.6600	-0.5995	0.0255	1.5933
Gpr162	Q3UN16	Probable G-protein coupled receptor 162	_RSS[Phospho (STY)]LDGSESAK_.2	0.2976	-1.7487	0.0257	1.5908
Uqcrb	Q9D855	Cytochrome b-c1 complex subunit 7	_DDTLHETEDVKEAIR_.3	1.4494	0.5355	0.0258	1.5875
Septin11	Q8C1B7	Septin-11	_AAAQLLQSQAQQSGAQQTK_.2	1.4152	0.5010	0.0259	1.5859
Bsn	O88737	Protein bassoon	_SPSVS[Phospho (STY)]PDR_.2	1.7066	0.7711	0.0260	1.5845
Atp1a3	Q6PIC6	Sodium/potassium-transporting ATPase subunit alpha-3	_ADIGVAM[Oxidation (M)]GIAGSDVSK_.2	0.7375	-0.4392	0.0261	1.5838
Gigyf1	Q99MR1	GRB10-interacting GYF protein 1	_GLEEEEEEEEPS[Phospho (STY)]EGVDEERPEAGGK_.3	1.5538	0.6358	0.0263	1.5808
Cul3	Q9JLV5	Cullin-3	_FLQESFNNDR_.2	0.1517	-2.7212	0.0263	1.5799
Zdhhc5	Q8VDZ4	Palmitoyltransferase ZDHHC5	_SFHFDPLSSGS[Phospho (STY)]R_.2	7.0602	2.8197	0.0264	1.5786
Slc12a2	P55012	Solute carrier family 12 member 2	_PLGPTPS[Phospho (STY)]QSR_.2	0.1222	-3.0332	0.0265	1.5771
H4c1	P62806	Histone H4	_DNIQGITK_.2	0.6584	-0.6030	0.0267	1.5742
Abl2	Q4JIM5	Tyrosine-protein kinase ABL2	_DKS[Phospho (STY)]PSSLLEDAK_.2	0.1359	-2.8799	0.0269	1.5699
Hcn1	O88704	Potassium/sodium hyperpolarization-activated cyclic nucleotide-gated channel 1	_KNS[Phospho (STY)]ILLQK_.2	0.5842	-0.7754	0.0270	1.5689
Begain	Q68EF6	Brain-enriched guanylate kinase-associated protein	_SADALAGYAAS[Phospho (STY)]DGDGDRLR_.2	0.5077	-0.9779	0.0270	1.5685
Wdr47	Q8CGF6	WD repeat-containing protein 47	_LSPYPSS[Phospho (STY)]PMR_.2	0.6495	-0.6226	0.0270	1.5680
Hsp90ab1	P11499	Heat shock protein HSP 90-beta	_APFDLFENK_.2	0.6494	-0.6228	0.0273	1.5644
Nefm	P08553	Neurofilament medium polypeptide	_EEIAEYRR_.2	1.7510	0.8082	0.0273	1.5639
Atrx	Q61687	Transcriptional regulator ATRX	_TTSTSNPS[Phospho (STY)]S[Phospho (STY)]PAPDWYK_.2	0.6732	-0.5709	0.0274	1.5624
Map1b	P14873	Microtubule-associated protein 1B	_SSM[Oxidation (M)]LFDTM[Oxidation (M)]QHHLALNR_.3	0.3658	-1.4510	0.0274	1.5623
Prpf4b	Q61136	Serine/threonine-protein kinase PRP4 homolog	_S[Phospho (STY)]KS[Phospho (STY)]PPLR_.2	0.6671	-0.5840	0.0274	1.5618
Gls	D3Z7P3	Glutaminase kidney isoform, mitochondrial	_ILQEYQVQYTPQGDSDDGK_.2	1.5310	0.6145	0.0275	1.5613
Adgrl3	Q80TS3	Adhesion G protein-coupled receptor L3	_GSSDGFIVPPNKDGAS[Phospho (STY)]PEGTSK_.3	0.4025	-1.3128	0.0275	1.5605
Atp6v1g2	Q9WTT4	V-type proton ATPase subunit G 2	_EREQEFQSK_.2	1.4593	0.5453	0.0275	1.5605
Phb2	O35129	Prohibitin-2	_AQFLVEK_.2	0.6449	-0.6329	0.0275	1.5602
Eif4h	Q9WUK2	Eukaryotic translation initiation factor 4H	_TVATPLNQVANPNSAIFGGAR_.2	1.4422	0.5282	0.0276	1.5594
Pds5b	Q4VA53	Sister chromatid cohesion protein PDS5 homolog B	_M[Oxidation (M)]ETVSNASSSSNPSS[Phospho (STY)]PGR_.2	0.5640	-0.8263	0.0276	1.5591

Trappc9	Q3U0M1	Trafficking protein particle complex subunit 9	_FNFESVPES[Phospho (STY)]PGEK_.2	0.3908	-1.3556	0.0277	1.5568
Rnf34	Q99KR6	E3 ubiquitin-protein ligase RNF34	_GLGSGDDLDSSS[Phospho (STY)]LNSSR_.2	0.5386	-0.8926	0.0278	1.5564
Dclk1	Q9JLM8	Serine/threonine-protein kinase DCLK1	_SKS[Phospho (STY)]PASTSSVNGTPGSQLSTPR_.3	0.0810	-3.6266	0.0279	1.5550
Nek4	Q9Z1J2	Serine/threonine-protein kinase Nek4	_SPAS[Phospho (STY)]LEGHT[Phospho (STY)]GK_.2	16.9901	4.0866	0.0281	1.5513
Grm5	Q3UVX5	Metabotropic glutamate receptor 5	_DSVDSGSTTPNS[Phospho (STY)]PVSESALC[Carbamidomethyl (C)]IPSSPK_.3	0.2703	-1.8876	0.0282	1.5499
Prkaa2	Q8BRK8	5'-AMP-activated protein kinase catalytic subunit alpha-2	_MPPLIADS[Phospho (STY)]PK_.2	0.6755	-0.5660	0.0282	1.5496
Gap43	P06837	Neuromodulin	_AGDAPSEEK_.2	0.7455	-0.4238	0.0282	1.5491
Acap2	Q6ZQK5	Arf-GAP with coiled-coil, ANK repeat and PH domain-containing protein 2	_YSALLS[Phospho (STY)]PSEQEK_.2	0.4540	-1.1392	0.0282	1.5491
Ank2	Q8C8R3	Ankyrin-2	_GAAVDFTAR_.2	0.7054	-0.5035	0.0284	1.5471
Strn3	Q9ERG2	Striatin-3	_NLEQILNGGES[Phospho (STY)]PKQK_.2	0.0496	-4.3321	0.0284	1.5467
Vps4a	Q8VEJ9	Vacuolar protein sorting-associated protein 4A	_ENQSEGKGS[Phospho (STY)]DS[Phospho (STY)]DSEGDNPEK_.2	1.5897	0.6688	0.0284	1.5462
Thop1	Q8C1A5	Thimet oligopeptidase	_NLNEDTTFLPFTR_.2	0.5298	-0.9164	0.0284	1.5460
Idh2	P54071	Isocitrate dehydrogenase [NADP], mitochondrial	_FAQTLEK_.2	0.6689	-0.5800	0.0284	1.5460
Scrn1	Q9CZC8	Secernin-1	_DEVQEVVYFPAVDHDAESK_.3	0.6938	-0.5274	0.0285	1.5450
Atp11c	Q9QZW0	Phospholipid-transporting ATPase 11C	_RAS[Phospho (STY)]DSLSAR_.2	0.6692	-0.5794	0.0286	1.5439
Ank2	Q8C8R3	Ankyrin-2	_HTPVS[Phospho (STY)]PSSK_.2	0.0622	-4.0062	0.0286	1.5430
Arhgdia	Q99PT1	Rho GDP-dissociation inhibitor 1	_LTLVC[Carbamidomethyl (C)]STAPGPLELDLTGDLESFKK_.3	0.6591	-0.6014	0.0287	1.5428
Prdx6	O08709	Peroxiredoxin-6	_PGGLLLGDEAPNFEANTTIGR_.2	1.3069	0.3861	0.0288	1.5403
Pacsin1	Q61644	Protein kinase C and casein kinase substrate in neurons protein 1	_GPQYGSLER_.2	1.3254	0.4064	0.0288	1.5402
Sec16a	E9QAT4	Protein transport protein Sec16A	_RSS[Phospho (STY)]LS[Phospho (STY)]SHSHQSQIYR_.3	0.6939	-0.5272	0.0291	1.5367
Dclk1	Q9JLM8	Serine/threonine-protein kinase DCLK1	_TLQTLSSEK_.2	0.6062	-0.7221	0.0292	1.5349
Dync1li1	Q8R1Q8	Cytoplasmic dynein 1 light intermediate chain 1	_GLEYLYLNVHDEDRDDQTR_.3	0.7122	-0.4896	0.0293	1.5332
Adra2a	Q01338	Alpha-2A adrenergic receptor	_TRAS[Phospho (STY)]QVKPGDS[Phospho (STY)]LPR_.3	1.5464	0.6289	0.0293	1.5325
Fam171a2	A2A699	Protein FAM171A2	_S[Phospho (STY)]VEGPGGLEPSLDEYRR_.3	0.4941	-1.0173	0.0295	1.5302
Ap2a1	P17426	AP-2 complex subunit alpha-1	_AADLLYAM[Oxidation (M)]C[Carbamidomethyl (C)]DR_.2	0.7464	-0.4220	0.0296	1.5292
Rpl18	P35980	60S ribosomal protein L18	_ILTFDQLALESPK_.2	0.5194	-0.9450	0.0296	1.5281
Ddx3y	Q62095	ATP-dependent RNA helicase DDX3Y	_DLLDLLVEAK_.2	0.7335	-0.4472	0.0297	1.5277
Astn1	Q61137	Astrotactin-1	_GNSPSDES[Phospho (STY)]EERER_.2	0.6846	-0.5467	0.0297	1.5270
Kiaa1549	Q68FD9	UPF0606 protein KIAA1549	_LITTDS[Phospho (STY)]DGTYK_.2	0.4760	-1.0709	0.0298	1.5263
Ubxn2a	Q99KJ0	UBX domain-containing protein 2A	_C[Carbamidomethyl (C)]LS[Phospho (STY)]PTEQKK_.2	0.7272	-0.4596	0.0298	1.5262
Ptprd	Q64487	Receptor-type tyrosine-protein phosphatase delta	_KSS[Phospho (STY)]LPNSK_.2	0.4469	-1.1620	0.0298	1.5256
Snph	Q80U23	Syntaphilin	_EEGTGESAGGS[Phospho (STY)]PARS[Phospho (STY)]LTR_.2	0.4436	-1.1726	0.0300	1.5230
Cbr1	P48758	Carbonyl reductase [NADPH] 1	_GVHAEEGWPNSAYGVTK_.2	2.3901	1.2571	0.0302	1.5205
Slc20a2	Q80UP8	Sodium-dependent phosphate transporter 2	_AS[Phospho (STY)]DES[Phospho (STY)]LRKVQEAESPGFK_.3	0.6806	-0.5551	0.0302	1.5193
Cap2	Q9CYT6	Adenylyl cyclase-associated protein 2	_THTPS[Phospho (STY)]PTS[Phospho (STY)]PKSNSPQK_.2	0.4038	-1.3084	0.0303	1.5181
Cacna1e	Q61290	Voltage-dependent R-type calcium channel subunit alpha-1E	_QGT[Phospho (STY)]PVPASGPAAAYK_.2	0.5170	-0.9518	0.0304	1.5175
Ap3b2	Q9JME5	AP-3 complex subunit beta-2	_LEVLTNLANETNIPTVLR_.2	0.6359	-0.6532	0.0305	1.5162
Iqsec1	Q8R0S2	IQ motif and SEC7 domain-containing protein 1	_VLINFNAPNPQDR_.2	0.6241	-0.6801	0.0305	1.5159
Myh10	Q61879	Myosin-10	_LQQLFNHTM[Oxidation (M)]FILEQEEYQR_.3	0.6213	-0.6866	0.0306	1.5144
Mycbp2	Q7TPH6	E3 ubiquitin-protein ligase MYCBP2	_STS[Phospho (STY)]PKPKPVPK_.2	1.6601	0.7313	0.0307	1.5132
Sptbn1	Q62261	Spectrin beta chain, non-erythrocytic 1	_FM[Oxidation (M)]ELLEPLSER_.2	0.7667	-0.3832	0.0308	1.5120
Sptan1	P16546	Spectrin alpha chain, non-erythrocytic 1	_M[Oxidation (M)]M[Oxidation (M)]LDHC[Carbamidomethyl (C)]LELQLFHR_.3	0.6789	-0.5588	0.0308	1.5118
Tardbp	Q921F2	TAR DNA-binding protein 43	_GISVHISNAEPK_.3	0.7148	-0.4844	0.0308	1.5108
Skt	A2AQ25	Sickle tail protein	_HQEETAHAPGQPLHC[Carbamidomethyl (C)]STGS[Phospho (STY)]PGDVK_.4	6.3718	2.6717	0.0309	1.5099
Kcnb1	Q03717	Potassium voltage-gated channel subfamily B member 1	_S[Phospho (STY)]IEM[Oxidation (M)]M[Oxidation (M)]DIVVEK_.2	0.6580	-0.6039	0.0310	1.5093
Psma5	Q9Z2U1	Proteasome subunit alpha type-5	_GVNTFS[Phospho (STY)]PEGR_.2	1.5070	0.5917	0.0310	1.5086
Unc80	Q8BLN6	Protein unc-80 homolog	_VAS[Phospho (STY)]IQS[Phospho (STY)]EPGQQNVLLQQPLGR_.3	0.7132	-0.4877	0.0311	1.5075
Acsbg1	Q99PU5	Long-chain-fatty-acid--CoA ligase ACSBG1	_ES[Phospho (STY)]PSHGLELSAPEK_.2	0.6578	-0.6043	0.0311	1.5073
M6pr	P24668	Cation-dependent mannose-6-phosphate receptor	_GVGDDQLGEES[Phospho (STY)]EERDDHLLPM[Oxidation (M)]_.3	0.4788	-1.0626	0.0311	1.5068
Uqcrc2	Q9DB77	Cytochrome b-c1 complex subunit 2, mitochondrial	_LASSLTTK_.2	0.4901	-1.0288	0.0312	1.5061
	Q99M08	Uncharacterized protein C4orf3 homolog	_RGS[Phospho (STY)]FEAGR_.2	0.6825	-0.5510	0.0312	1.5054
Map1a	Q9QYR6	Microtubule-associated protein 1A	_TRHDEYLEVTK_.3	1.7016	0.7669	0.0312	1.5054
Git1	Q68FF6	ARF GTPase-activating protein GIT1	_SAVPFLPVNPEYSATR_.2	1.4652	0.5511	0.0313	1.5045
Eef1b	O70251	Elongation factor 1-beta	_SIQADGLVWGSSK_.2	2.4936	1.3182	0.0314	1.5024
U2surp	Q6NV83	U2 snRNP-associated SURP motif-containing protein	_RVKS[Phospho (STY)]PS[Phospho (STY)]PK_.2	0.3899	-1.3588	0.0316	1.5008
Mtcl1	Q3UHU5	Microtubule cross-linking factor 1	_GRS[Phospho (STY)]PS[Phospho (STY)]PLGVGSETFR_.3	0.7224	-0.4691	0.0317	1.4995
Ppp1r1a	Q9ERT9	Protein phosphatase 1 regulatory subunit 1A	_EPDNS[Phospho (STY)]PR_.2	0.6866	-0.5425	0.0317	1.4992
Frmd4a	Q8BIE6	FERM domain-containing protein 4A	_SVDIS[Phospho (STY)]PTR_.2	0.1370	-2.8674	0.0317	1.4991
Syt7	Q9R0N7	Synaptotagmin-7	_NSLETVGT[Phospho (STY)]PDS[Phospho (STY)]GRGR_.2	0.7440	-0.4266	0.0318	1.4980
Ktn1	Q61595	Kinectin	_DLLT[Phospho (STY)]ELQK_.2	1.6223	0.6981	0.0319	1.4965
Dclk1	Q9JLM8	Serine/threonine-protein kinase DCLK1	_SFEALLADLTR_.2	0.7693	-0.3784	0.0319	1.4957
Tfpt	Q3U1J1	TCF3 fusion partner homolog	_EGLS[Phospho (STY)]PSQR_.2	0.3332	-1.5855	0.0320	1.4948

Hnrnpa2b1	O88569	Heterogeneous nuclear ribonucleoproteins A2/B1	_LTDC[Carbamidomethyl (C)]VVMR_.2	2.0078	1.0056	0.0323	1.4913
Pank4	Q80YV4	4'-phosphopantetheine phosphatase	_VRS[Phospho (STY)]FDHPGK_.2	0.7630	-0.3903	0.0324	1.4895
Cdk16	Q04735	Cyclin-dependent kinase 16	_KIS[Phospho (STY)]TEDINKR_.2	0.7259	-0.4622	0.0324	1.4889
Pdha1	P35486	Pyruvate dehydrogenase E1 component subunit alpha, somatic form, mitochondrial	_M[Oxidation (M)]VNSNLASVEELKEIDVEVRK_.4	0.7448	-0.4251	0.0325	1.4887
Rb1cc1	Q9ESK9	RB1-inducible coiled-coil protein 1	_STELVLS[Phospho (STY)]PDM[Oxidation (M)]PR_.2	0.5798	-0.7865	0.0331	1.4807
Tomm70	Q9CZW5	Mitochondrial import receptor subunit TOM70	_C[Carbamidomethyl (C)]AEGYALYAQALTDQQQFGK_.2	0.6708	-0.5761	0.0331	1.4803
Pcnx1	Q9QYC1	Pecanex-like protein 1	_SSS[Phospho (STY)]LIHR_.2	2.1086	1.0763	0.0332	1.4786
Actn2	Q9JI91	Alpha-actinin-2	_VEQIAAIAQELNELDYHDAVNVNDR_.3	0.6838	-0.5483	0.0335	1.4744
Gpsm1	Q6IR34	G-protein-signaling modulator 1	_AAQSSVTAS[Phospho (STY)]PQTEEFFDLIASSQSR_.3	0.6877	-0.5401	0.0336	1.4733
Akap12	Q9WTQ5	A-kinase anchor protein 12	_LS[Phospho (STY)]ADYEKVELPLEDQVGDLEALSEK_.3	0.7659	-0.3847	0.0336	1.4731
Cltc	Q68FD5	Clathrin heavy chain 1	_AM[Oxidation (M)]LSANIR_.2	0.6695	-0.5788	0.0337	1.4724
Kif1a	P33173	Kinesin-like protein KIF1A	_AASVSS[Phospho (STY)]LHER_.2	0.1930	-2.3736	0.0338	1.4708
Atp6v0a1	Q9Z1G4	V-type proton ATPase 116 kDa subunit a1	_NFLELTELK_.2	0.6990	-0.5167	0.0338	1.4707
Syngap1	F6SEU4	Ras/Rap GTPase-activating protein SynGAP	_LLNDISTALR_.2	0.7257	-0.4626	0.0340	1.4687
Nfic	P70255	Nuclear factor 1 C-type	_NWTEDIEGGISS[Phospho (STY)]PVKK_.2	0.5614	-0.8329	0.0341	1.4676
Atp2b1	G5E829	Plasma membrane calcium-transporting ATPase 1	_ADVGFAM[Oxidation (M)]GIAGTDVAK_.2	0.6927	-0.5298	0.0341	1.4672
Tubb3	Q9ERD7	Tubulin beta-3 chain	_FWEVISDEHGIDPSGNYVGDSDLQLER_.3	0.6887	-0.5381	0.0342	1.4662
Prkar1b	P12849	cAMP-dependent protein kinase type I-beta regulatory subunit	_[Acetyl (Protein N-term)]ASPS[Phospho (STY)]C[Carbamidomethyl (C)]FHSEDEDSLK_.2	0.7238	-0.4664	0.0342	1.4662
Tpm3	P21107	Tropomyosin alpha-3 chain	_AADAEAEVASLNRR_.2	0.7637	-0.3890	0.0342	1.4659
Vta1	Q9CR26	Vacuolar protein sorting-associated protein VTA1 homolog	_YAGSALQYEDVGTAVQNLQK_.2	2.4693	1.3041	0.0342	1.4658
Ran	P62827	GTP-binding nuclear protein Ran	_YVATLGVEVHPLVFHTNR_.3	0.5681	-0.8158	0.0343	1.4652
Sgip1	Q8VD37	SH3-containing GRB2-like protein 3-interacting protein 1	_EC[Carbamidomethyl (C)]GLGQRAT[Phospho (STY)]PPPPPPPTYR_.3	0.2932	-1.7698	0.0343	1.4645
Gabbr2	Q80T41	Gamma-aminobutyric acid type B receptor subunit 2	_FTQFQDSR_.2	0.5370	-0.8971	0.0343	1.4644
Sorbs2	Q3UTJ2	Sorbin and SH3 domain-containing protein 2	_RFS[Phospho (STY)]IESLLEEETQVR_.3	1.5679	0.6488	0.0343	1.4642
Dync1h1	Q9JHU4	Cytoplasmic dynein 1 heavy chain 1	_FNYGFEYLGVQDK_.2	0.6653	-0.5879	0.0344	1.4637
Isyna1	Q9JHU9	Inositol-3-phosphate synthase 1	_SC[Carbamidomethyl (C)]IENIFR_.2	0.3260	-1.6169	0.0345	1.4628
Dock4	P59764	Dedicator of cytokinesis protein 4	_NSAPASM[Oxidation (M)]S[Phospho (STY)]PDGTR_.2	2.5127	1.3293	0.0346	1.4615
Cap1	P40124	Adenylyl cyclase-associated protein 1	_C[Carbamidomethyl (C)]VNTTLQIK_.2	1.6152	0.6917	0.0347	1.4603
Grin2a	P35436	Glutamate receptor ionotropic, NMDA 2A	_YLPEEVAHS[Phospho (STY)]DIS[Phospho (STY)]ETSSR_.2	0.2941	-1.7654	0.0347	1.4601
Palm3	A2TJV2	Paralemmin-3	_QGTSS[Phospho (STY)]PELPTWVK_.2	0.6893	-0.5368	0.0348	1.4585
Dync1h1	Q9JHU4	Cytoplasmic dynein 1 heavy chain 1	_ESPEVLLTLDILK_.2	0.6729	-0.5714	0.0349	1.4576
Dmxl2	Q8BPN8	DmX-like protein 2	_VS[Phospho (STY)]VDSNLFVYSK_.2	0.5697	-0.8117	0.0349	1.4575
Sipa1l3	G3X9J0	Signal-induced proliferation-associated 1-like protein 3	_QDAAGKDS[Phospho (STY)]PNR_.2	0.7189	-0.4762	0.0349	1.4571
Cds2	Q99L43	Phosphatidate cytidylyltransferase 2	_EDAPPEDKES[Phospho (STY)]ESEAK_.2	0.6631	-0.5927	0.0349	1.4569
Slc4a10	Q5DTL9	Sodium-driven chloride bicarbonate exchanger	_ESRFPSKS[Phospho (STY)]SPS_.2	0.7189	-0.4761	0.0350	1.4560
Cltc	Q68FD5	Clathrin heavy chain 1	_AHTM[Oxidation (M)]TDDVTFWK_.2	0.3848	-1.3779	0.0350	1.4558
Stim2	P83093	Stromal interaction molecule 2	_DELS[Phospho (STY)]LEDSSR_.2	0.4187	-1.2561	0.0351	1.4553
Dctn1	O08788	Dynactin subunit 1	_AFLQGGQEATDIALLLR_.2	1.4052	0.4908	0.0351	1.4543
Huwe1	Q7TMY8	E3 ubiquitin-protein ligase HUWE1	_DLS[Phospho (STY)]MSEEDQMMR_.2	0.6894	-0.5367	0.0352	1.4539
Usp6nl	Q80XC3	USP6 N-terminal-like protein	_SVGRPS[Phospho (STY)]PK_.2	0.5355	-0.9010	0.0352	1.4534
Tufm	Q8BFR5	Elongation factor Tu, mitochondrial	_DKPHVNVGTIGHVDHGK_.4	1.3713	0.4556	0.0352	1.4533
Tuba8	Q9JJZ2	Tubulin alpha-8 chain	_ES[Phospho (STY)]IDLVLDR_.2	0.6741	-0.5689	0.0352	1.4532
Nsf	P46460	Vesicle-fusing ATPase	_VEVDMEK_.2	0.7033	-0.5079	0.0354	1.4514
Srrm2	Q8BTI8	Serine/arginine repetitive matrix protein 2	_QSSS[Phospho (STY)]PYEDKDKK_.2	0.3288	-1.6046	0.0355	1.4502
Fth1	P09528	Ferritin heavy chain	_QNYHQDAEAAINR_.3	0.6915	-0.5321	0.0357	1.4470
Stmn2	P55821	Stathmin-2	_NKELQVELS[Phospho (STY)]G_.2	0.7340	-0.4462	0.0358	1.4466
Ampd2	Q9DBT5	AMP deaminase 2	_TDS[Phospho (STY)]DSDLQLYK_.2	0.3911	-1.3543	0.0358	1.4460
Dlg4	Q62108	Disks large homolog 4	_RVHS[Phospho (STY)]DS[Phospho (STY)]ETDDIGFIPSKR_.3	0.3844	-1.3793	0.0359	1.4455
Phf2	Q9WTU0	Lysine-specific demethylase PHF2	_RPSASS[Phospho (STY)]PNNTAAK_.2	0.6999	-0.5148	0.0359	1.4448
Map1a	Q9QYR6	Microtubule-associated protein 1A	_PRYS[Phospho (STY)]EER_.2	0.7190	-0.4759	0.0361	1.4422
Bnip3	O55003	BCL2/adenovirus E1B 19 kDa protein-interacting protein 3	_SSHC[Carbamidomethyl (C)]DS[Phospho (STY)]PPR_.2	0.5824	-0.7798	0.0361	1.4419
Actr2	P61161	Actin-related protein 2	_GYAFNHSADFETVR_.3	1.5380	0.6210	0.0362	1.4413
Pip5k1c	O70161	Phosphatidylinositol 4-phosphate 5-kinase type-1 gamma	_RTQS[Phospho (STY)]SGQDGRPQEEPHAEDLQK_.4	3.9051	1.9653	0.0365	1.4380
Pacs1	Q8K212	Phosphofurin acidic cluster sorting protein 1	_VAAVGSQSYLSSILR_.2	1.6216	0.6974	0.0366	1.4367
Tomm20	Q9DCC8	Mitochondrial import receptor subunit TOM20 homolog	_IVS[Phospho (STY)]AQSLAEDDVE_.2	0.4549	-1.1364	0.0366	1.4366
Dnaja1	P63037	DnaJ homolog subfamily A member 1	_TIVITSHPGQIVK_.2	1.3999	0.4853	0.0366	1.4361
Macf1	Q9QXZ0	Microtubule-actin cross-linking factor 1	_LLDAEDVDVPS[Phospho (STY)]PDEK_.2	0.6940	-0.5269	0.0367	1.4356
Nme2	Q01768	Nucleoside diphosphate kinase B	_SC[Carbamidomethyl (C)]AHDWVYE_.2	1.3653	0.4493	0.0367	1.4355
Vsnl1	P62761	Visinin-like protein 1	_VEM[Oxidation (M)]LEIIEAIYK_.2	0.5879	-0.7663	0.0367	1.4352
Pikfyve	Q9Z1T6	1-phosphatidylinositol 3-phosphate 5-kinase	_SGSPMVPSYETSVS[Phospho (STY)]PQANR_.2	0.6640	-0.5908	0.0367	1.4349
Cct8	P42932	T-complex protein 1 subunit theta	_HFSGLEEAVYR_.2	0.6086	-0.7163	0.0369	1.4324
Mapk8ip3	Q9ESN9	C-Jun-amino-terminal kinase-interacting protein 3	_AKET[Phospho (STY)]PEADATSSR_.2	0.6919	-0.5314	0.0373	1.4287

Ehd3	Q9QXY6	EH domain-containing protein 3	_DIQS[Phospho (STY)]LPR_.2	0.5604	-0.8354	0.0373	1.4284
Rab10	P61027	Ras-related protein Rab-10	_EPNSENVDISS[Phospho (STY)]GGGVTGWK_.2	0.3150	-1.6667	0.0373	1.4284
Camk2d	Q6PHZ2	Calcium/calmodulin-dependent protein kinase type II subunit delta	_IC[Carbamidomethyl (C)]DPGLTAFEPEALGNLVEGM[Oxidation (M)]DFHR_.3	3.1896	1.6734	0.0374	1.4272
Ppp1r13b	Q62415	Apoptosis-stimulating of p53 protein 1	_VNGTSSPQS[Phospho (STY)]PLSTSGR_.2	0.7191	-0.4757	0.0374	1.4270
Pitpnc1	Q8K4R4	Cytoplasmic phosphatidylinositol transfer protein 1	_YEDNKGS[Phospho (STY)]NDSIFDSEAK_.3	0.6143	-0.7030	0.0375	1.4258
Kiaa1522	A2A7S8	Uncharacterized protein KIAA1522	_M[Oxidation (M)]GS[Phospho (STY)]PGSDPQK_.2	0.6907	-0.5338	0.0375	1.4257
Phldb2	Q8K1N2	Pleckstrin homology-like domain family B member 2	_RLS[Phospho (STY)]AGTTVADVQK_.2	0.6908	-0.5336	0.0377	1.4232
Ppp2cb	P62715	Serine/threonine-protein phosphatase 2A catalytic subunit beta isoform	_AHQLVM[Oxidation (M)]EGYNWC[Carbamidomethyl (C)]HDR_.3	0.5102	-0.9707	0.0378	1.4229
C2cd5	Q7TPS5	C2 domain-containing protein 5	_SQSESS[Phospho (STY)]DEVTELDLSHGK_.3	0.6789	-0.5587	0.0378	1.4229
Slc25a12	Q8BH59	Calcium-binding mitochondrial carrier protein Aralar1	_NIFLQYASTEVDGEHYM[Oxidation (M)]TPEDFVQR_.3	0.6949	-0.5251	0.0378	1.4226
Ctr9	Q62018	RNA polymerase-associated protein CTR9 homolog	_NSNS[Phospho (STY)]DS[Phospho (STY)]DDDERPNR_.2	1.4393	0.5253	0.0378	1.4225
Cacna1e	Q61290	Voltage-dependent R-type calcium channel subunit alpha-1E	_DSSDEHC[Carbamidomethyl (C)]VDISSVGT[Phospho (STY)]PLAR_.3	0.7240	-0.4660	0.0379	1.4216
Hsp90ab1	P11499	Heat shock protein HSP 90-beta	_YHTSQSGDEM[Oxidation (M)]TSLSEYVSR_.3	1.9387	0.9551	0.0379	1.4214
Champ1	Q8K327	Chromosome alignment-maintaining phosphoprotein 1	_LKKDS[Phospho (STY)]QENS[Phospho (STY)]DAELSSSEYIR_.3	1.4589	0.5448	0.0379	1.4212
Gap43	P06837	Neuromodulin	_AEDGPAKEEPK_.2	1.3892	0.4742	0.0379	1.4211
Phrf1	A6H619	PHD and RING finger domain-containing protein 1	_S[Phospho (STY)]RS[Phospho (STY)]PS[Phospho (STY)]LEHRPR_.3	0.6910	-0.5333	0.0380	1.4204
Mdh1	P14152	Malate dehydrogenase, cytoplasmic	_VLVTGAAGQIAYSLLYSIGNGSVFGK_.3	0.6703	-0.5772	0.0380	1.4202
Cct4	P80315	T-complex protein 1 subunit delta	_AFADAM[Oxidation (M)]EVIPSTLAENAGLNPISTVTELR_.3	0.6704	-0.5770	0.0380	1.4197
Cltb	Q6IRU5	Clathrin light chain B	_DLEEWNQR_.2	1.3138	0.3938	0.0381	1.4188
Plppr3	Q7TPB0	Phospholipid phosphatase-related protein type 3	_GLGLPDEAS[Phospho (STY)]PVHLR_.2	0.6940	-0.5270	0.0382	1.4182
Sv2b	Q8BG39	Synaptic vesicle glycoprotein 2B	_QVHDTNMR_.2	2.2039	1.1401	0.0382	1.4179
Scn3b	Q8BHK2	Sodium channel subunit beta-3	_AEEAAQENASDY[Phospho (STY)]LAIPSENK_.2	0.2983	-1.7452	0.0382	1.4176
Mast1	Q9R1L5	Microtubule-associated serine/threonine-protein kinase 1	_AALS[Phospho (STY)]PVQEHETGRR_.3	0.5896	-0.7622	0.0383	1.4170
Ina	P46660	Alpha-internexin	_HSAEVAGYQDSIGQLESDLR_.3	1.3430	0.4254	0.0383	1.4164
Cct7	P80313	T-complex protein 1 subunit eta	_INALTAASEAAC[Carbamidomethyl (C)]LIVSVDETIK_.3	0.7242	-0.4656	0.0384	1.4160
Cdk18	Q04899	Cyclin-dependent kinase 18	_FS[Phospho (STY)]MEDLNK_.2	0.7227	-0.4685	0.0384	1.4156
Wdtc1	Q80ZK9	WD and tetratricopeptide repeats protein 1	_GC[Carbamidomethyl (C)]IS[Phospho (STY)]PQVELPPYLER_.2	0.7223	-0.4694	0.0384	1.4152
Pacs1	Q8K212	Phosphofurin acidic cluster sorting protein 1	_DTTS[Phospho (STY)]PM[Oxidation (M)]ELAALEK_.2	0.2937	-1.7676	0.0385	1.4143
Mib2	Q8R516	E3 ubiquitin-protein ligase MIB2	_VS[Phospho (STY)]ADGQPFQR_.2	0.7192	-0.4755	0.0386	1.4131
Ctnnd2	O35927	Catenin delta-2	_NS[Phospho (STY)]YGAPAEDIK_.2	0.7028	-0.5087	0.0388	1.4115
Dbn1	Q9QXS6	Drebrin	_ASDSGPSSSSSSSSS[Phospho (STY)]PPRTPFPYITC[Carbamidomethyl (C)]HR_.4	0.7282	-0.4576	0.0390	1.4089
Shank1	D3YZU1	SH3 and multiple ankyrin repeat domains protein 1	_THS[Phospho (STY)]PATS[Phospho (STY)]EDEER_.2	0.7192	-0.4755	0.0390	1.4087
Eif5b	Q05D44	Eukaryotic translation initiation factor 5B	_IEPIPGES[Phospho (STY)]PK_.2	0.7641	-0.3881	0.0391	1.4083
Abcf1	Q6P542	ATP-binding cassette sub-family F member 1	_GGNVFEALIQDDS[Phospho (STY)]EEEEEEENR_.3	0.7192	-0.4755	0.0391	1.4078
Nsf	P46460	Vesicle-fusing ATPase	_FLALM[Oxidation (M)]REEGAS[Phospho (STY)]PLDFD_.2	0.6907	-0.5338	0.0392	1.4073
Atp5pb	Q9CQQ7	ATP synthase F(0) complex subunit B1, mitochondrial	_KEEEHMIDWVEK_.3	0.6971	-0.5206	0.0392	1.4066
Psmb5	O55234	Proteasome subunit beta type-5	_AIYQATYR_.2	0.6547	-0.6111	0.0393	1.4057
Rbbp6	P97868	E3 ubiquitin-protein ligase RBBP6	_YHS[Phospho (STY)]RS[Phospho (STY)]RS[Phospho (STY)]PQAFR_.3	0.5381	-0.8941	0.0393	1.4056
Scg2	Q03517	Secretogranin-2	_VPSPVS[Phospho (STY)]SEDDLQEEEQLEQAIK_.3	0.6916	-0.5320	0.0393	1.4055
Ppp1r14a	Q91VC7	Protein phosphatase 1 regulatory subunit 14A	_QPGFPQPSPSDDPS[Phospho (STY)]LSPRQDR_.3	0.6973	-0.5201	0.0394	1.4046
Calr	P14211	Calreticulin	_VHVIFNYK_.2	0.5893	-0.7629	0.0395	1.4034
Tomm70	Q9CZW5	Mitochondrial import receptor subunit TOM70	_EC[Carbamidomethyl (C)]LEDVTAVC[Carbamidomethyl (C)]ILEGFQNEQSM[Oxidation (M)]LLADK_.3	1.4290	0.5150	0.0395	1.4033
P33monox	Q9DBN4	Putative monooxygenase p33MONOX	_HPASAQSTPSSTPHAS[Phospho (STY)]PK_.3	0.6231	-0.6824	0.0395	1.4030
Dennd4b	Q3U1Y4	DENN domain-containing protein 4B	_SGS[Phospho (STY)]LGSAR_.2	0.7300	-0.4539	0.0397	1.4014
Prkar2b	P31324	cAMP-dependent protein kinase type II-beta regulatory subunit	_NLDPEQM[Oxidation (M)]SQVLDAMFEK_.2	0.6978	-0.5191	0.0397	1.4010
Gnao1	P18872	Guanine nucleotide-binding protein G(o) subunit alpha	_PVVYSNTIQSLAAIVR_.2	0.7169	-0.4801	0.0398	1.3997
Lsm14a	Q8K2F8	Protein LSM14 homolog A	_SFFDNIS[Phospho (STY)]C[Carbamidomethyl (C)]DDNRER_.2	0.6621	-0.5950	0.0399	1.3992
Grin2b	Q01097	Glutamate receptor ionotropic, NMDA 2B	_RQHS[Phospho (STY)]YDTFVDLQK_.3	0.5031	-0.9911	0.0399	1.3992
Pclo	Q9QYX7	Protein piccolo	_TTLYFDEEPELEM[Oxidation (M)]ESLTDS[Phospho (STY)]PEDR_.3	0.7167	-0.4807	0.0400	1.3985
Dock7	Q8R1A4	Dedicator of cytokinesis protein 7	_KGS[Phospho (STY)]WSER_.2	0.7164	-0.4811	0.0401	1.3974
Thumpd2	Q9CZB3	THUMP domain-containing protein 2	_LLQGS[Phospho (STY)]PEQGEAVTR_.2	0.5534	-0.8537	0.0401	1.3970
Myh9	Q8VDD5	Myosin-9	_LQQELDDLLVDLDHQR_.3	0.6577	-0.6044	0.0401	1.3966
Scn2a	B1AWN6	Sodium channel protein type 2 subunit alpha	_AM[Oxidation (M)]SM[Oxidation (M)]ASILT[Phospho (STY)]NT[Phospho (STY)]M[Oxidation (M)]EELEESR_.2	0.2022	-2.3064	0.0403	1.3947
Rbmxl1	Q91VM5	RNA binding motif protein, X-linked-like-1	_ST[Phospho (STY)]PSGPVR_.2	0.2610	-1.9381	0.0404	1.3939
Homer1	Q9Z2Y3	Homer protein homolog 1	_HAVTVSYFYDSTR_.2	0.6198	-0.6901	0.0404	1.3937
Prickle1	Q3U5C7	Prickle-like protein 1	_KS[Phospho (STY)]RSDNALNLVTER_.3	0.6819	-0.5524	0.0404	1.3932
Stip1	Q60864	Stress-induced-phosphoprotein 1	_ALDLDSSC[Carbamidomethyl (C)]K_.2	1.3616	0.4453	0.0406	1.3914
Rufy3	Q9D394	Protein RUFY3	_DGNS[Phospho (STY)]SKGSEGDGQITAILDQK_.3	0.7322	-0.4497	0.0408	1.3893
Copa	Q8CIE6	Coatomer subunit alpha	_NLS[Phospho (STY)]PGAVESDVR_.2	0.1891	-2.4029	0.0408	1.3891
Slc3a2	P10852	4F2 cell-surface antigen heavy chain	_NQKDEINETDLK_.3	0.7325	-0.4492	0.0410	1.3876
Cpeb4	Q7TN98	Cytoplasmic polyadenylation element-binding protein 4	_QQLSPS[Phospho (STY)]PGQEAGILPETEK_.2	2.4761	1.3081	0.0411	1.3859
Ank2	Q8C8R3	Ankyrin-2	_ETTKVGT[Phospho (STY)]PTDIHSEK_.2	0.5848	-0.7741	0.0414	1.3834

Swi5	Q8K3D3	DNA repair protein SWI5 homolog	_ELYPDFDLNLND_.2	1.8604	0.8956	0.0414	1.3825
Rem2	Q8VEL9	GTP-binding protein REM 2	_RGS[Phospho (STY)]M[Oxidation (M)]PVPYK_.2	0.4351	-1.2007	0.0415	1.3816
Nefh	P19246	Neurofilament heavy polypeptide	_SPAEPKS[Phospho (STY)]PAEPKS[Phospho (STY)]PAEAK_.2	1.4335	0.5195	0.0416	1.3810
Camk4	P08414	Calcium/calmodulin-dependent protein kinase type IV	_ASS[Phospho (STY)]DPPSTQDAK_.2	0.7005	-0.5134	0.0417	1.3802
Actr2	P61161	Actin-related protein 2	_LALETTVLVESYTLPDGR_.2	0.6841	-0.5477	0.0417	1.3798
Dnajc5	P60904	DnaJ homolog subfamily C member 5	_EINNAHAILTDATK_.2	0.6505	-0.6204	0.0418	1.3790
Pgm1	Q9D0F9	Phosphoglucomutase-1	_DLEALMLDR_.2	0.7340	-0.4461	0.0420	1.3765
Hspa8	P63017	Heat shock cognate 71 kDa protein	_YKAEDEKQR_.2	1.5124	0.5968	0.0422	1.3743
H3-5;H3-3a	P02301;P84244	Histone H3.3C;Histone H3.3	_FQSAAIGALQEASEAYLVGLFEDTNLC[Carbamidomethyl (C)]AIHAK_.3	1.4200	0.5059	0.0423	1.3739
Wdfy3	Q6VNB8	WD repeat and FYVE domain-containing protein 3	_SQS[Phospho (STY)]EYC[Carbamidomethyl (C)]NVGTK_.2	0.5520	-0.8572	0.0424	1.3729
Ndufa9	Q9DC69	NADH dehydrogenase [ubiquinone] 1 alpha subcomplex subunit 9, mitochondrial	_SSVSGVVATVFGATGFLGR_.2	1.9729	0.9803	0.0425	1.3718
Srgap2	Q91Z67	SLIT-ROBO Rho GTPase-activating protein 2	_QTPRS[Phospho (STY)]PDSTANVR_.3	0.6745	-0.5682	0.0427	1.3699
Ank2	Q8C8R3	Ankyrin-2	_VGT[Phospho (STY)]PTDIHSEK_.2	0.7352	-0.4438	0.0428	1.3688
Akap8	Q9DBR0	A-kinase anchor protein 8	_SNS[Phospho (STY)]STFIR_.2	0.7350	-0.4442	0.0428	1.3684
Gapvd1	Q6PAR5	GTPase-activating protein and VPS9 domain-containing protein 1	_LQELESC[Carbamidomethyl (C)]SGLGS[Phospho (STY)]TSDDTDVR_.2	1.4184	0.5043	0.0428	1.3682
Dctn2	Q99KJ8	Dynactin subunit 2	_LTELEATVR_.2	0.5584	-0.8405	0.0428	1.3681
Uba1	Q02053	Ubiquitin-like modifier-activating enzyme 1	_QFLDYFK_.2	0.6972	-0.5203	0.0429	1.3678
Rab10	P61027	Ras-related protein Rab-10	_ANINIEK_.2	0.6738	-0.5697	0.0429	1.3671
Lap3	Q9CPY7	Cytosol aminopeptidase	_ANKPGDVVR_.2	1.3294	0.4108	0.0430	1.3663
Map1b	P14873	Microtubule-associated protein 1B	_EEC[Carbamidomethyl (C)]PRPMSIS[Phospho (STY)]PPDFSPK_.3	1.4835	0.5690	0.0430	1.3662
Nefh	P19246	Neurofilament heavy polypeptide	_VKS[Phospho (STY)]EEMIKVVEK_.3	1.4332	0.5193	0.0430	1.3660
Clec2l	P0C7M9	C-type lectin domain family 2 member L	_PRS[Phospho (STY)]PAEAEAR_.2	0.7594	-0.3970	0.0431	1.3658
Hid1	Q8R1F6	Protein HID1	_TGS[Phospho (STY)]QEGTSMEGSR_.2	1.4764	0.5621	0.0431	1.3657
Pfkp	Q9WUA3	ATP-dependent 6-phosphofructokinase, platelet type	_IIEVVDAIM[Oxidation (M)]TTAQSHQR_.2	1.3332	0.4149	0.0432	1.3650
Sgip1	Q8VD37	SH3-containing GRB2-like protein 3-interacting protein 1	_LPS[Phospho (STY)]ISDLDSIFGPVLSPK_.2	0.6346	-0.6562	0.0432	1.3648
Ank2	Q8C8R3	Ankyrin-2	_AREEQTPLHIASR_.3	0.7202	-0.4735	0.0432	1.3648
Atp2b1	G5E829	Plasma membrane calcium-transporting ATPase 1	_SSLYEGLEKPES[Phospho (STY)]R_.2	0.6601	-0.5993	0.0434	1.3626
Kif1b	Q60575	Kinesin-like protein KIF1B	_SGLS[Phospho (STY)]LEELR_.2	0.5271	-0.9238	0.0434	1.3626
Hyou1	Q9JKR6	Hypoxia up-regulated protein 1	_VESVFETLVEDSPEEESTLTK_.2	0.6786	-0.5593	0.0435	1.3614
Ak1	Q9R0Y5	Adenylate kinase isoenzyme 1	_VDDNEETIKK_.2	1.3501	0.4330	0.0436	1.3605
Znf365	Q8BG89	Protein ZNF365	_MVEAVDRT[Phospho (STY)]IEK_.2	0.7091	-0.4960	0.0436	1.3605
Pcsk1n	Q9QXV0	ProSAAS	_ASDPPLAPDDDPDAPAAQLAR_.2	1.3029	0.3817	0.0440	1.3561
Map1a	Q9QYR6	Microtubule-associated protein 1A	_APISLSQDPSPPLNGSTTSC[Carbamidomethyl (C)]GPDRRT[Phospho (STY)]PS[Phospho (STY)]PK_.4	0.0464	-4.4291	0.0441	1.3555
Pgk1	P09411	Phosphoglycerate kinase 1	_IQLINNM[Oxidation (M)]LDK_.2	0.7018	-0.5109	0.0443	1.3538
Gapvd1	Q6PAR5	GTPase-activating protein and VPS9 domain-containing protein 1	_FSLC[Carbamidomethyl (C)]S[Phospho (STY)]DNLEGISEGPSNR_.2	0.7160	-0.4820	0.0444	1.3530
Tubb2a	Q7TMM9	Tubulin beta-2A chain	_INVYYNEAAGNKYVPR_.3	1.4116	0.4974	0.0444	1.3528
Slc8a2	Q8K596	Sodium/calcium exchanger 2	_S[Phospho (STY)]GIIIGAEGDPPK_.2	0.5409	-0.8867	0.0444	1.3527
Iqsec2	Q5DU25	IQ motif and SEC7 domain-containing protein 2	_M[Oxidation (M)]QFS[Phospho (STY)]FEEYEK_.2	0.7298	-0.4545	0.0445	1.3518
Snap25	P60879	Synaptosomal-associated protein 25	_ADQLADESLESTRR_.2	1.3135	0.3934	0.0445	1.3517
Dlgap2	Q8BJ42	Disks large-associated protein 2	_C[Carbamidomethyl (C)]SS[Phospho (STY)]IGVQDSEFPDHQPYPR_.3	0.4221	-1.2444	0.0447	1.3500
Eif4b	Q8BGD9	Eukaryotic translation initiation factor 4B	_RGDDSFGDKYR_.3	1.6692	0.7391	0.0447	1.3498
Gigyf2	Q6Y7W8	GRB10-interacting GYF protein 2	_QQQQQQQQQQQHQQSNR_.3	0.7167	-0.4806	0.0448	1.3488
Aldoa	P05064	Fructose-bisphosphate aldolase A	_VDKGVVPLAGTNGETTTQGLDGLSER_.3	1.3015	0.3802	0.0448	1.3483
Rapgef2	Q8CHG7	Rap guanine nucleotide exchange factor 2	_ILS[Phospho (STY)]LSEEGSLER_.2	0.4068	-1.2976	0.0449	1.3478
Marcks	P26645	Myristoylated alanine-rich C-kinase substrate	_GEATAERPGEAAVASS[Phospho (STY)]PSK_.2	1.3243	0.4052	0.0449	1.3478
Adra2c	Q01337	Alpha-2C adrenergic receptor	_TEVEPDES[Phospho (STY)]SAAER_.2	0.7373	-0.4396	0.0450	1.3466
Ahsa1	Q8BK64	Activator of 90 kDa heat shock protein ATPase homolog 1	_QTFGYGAR_.2	0.6676	-0.5828	0.0450	1.3464
Cdk17	Q8K0D0	Cyclin-dependent kinase 17	_RAS[Phospho (STY)]LSEIGFGK_.2	0.4209	-1.2486	0.0451	1.3458
Tmsb10	Q6ZWY8	Thymosin beta-10	_[Acetyl (Protein N-term)]ADKPDM[Oxidation (M)]GEIASFDK_.2	1.4911	0.5764	0.0451	1.3454
Spen	Q62504	Msx2-interacting protein	_DLEPGEVPS[Phospho (STY)]DS[Phospho (STY)]DEDAEHR_.2	0.4963	-1.0107	0.0452	1.3446
Stip1	Q60864	Stress-induced-phosphoprotein 1	_IGNSYFKEEK_.2	1.5385	0.6215	0.0453	1.3437
Tgoln1;Tgoln2	Q62313;Q62314	Trans-Golgi network integral membrane protein 1;Trans-Golgi network integral membrane protein 2	_DADS[Phospho (STY)]GDSQNPPNQPSK_.2	2.9766	1.5737	0.0456	1.3411
Hnrnpa2b1	O88569	Heterogeneous nuclear ribonucleoproteins A2/B1	_GGSDGYGSGR_.2	0.6942	-0.5265	0.0458	1.3395
Plppr3	Q7TPB0	Phospholipid phosphatase-related protein type 3	_RAS[Phospho (STY)]VDVDLLAPR_.2	0.6513	-0.6186	0.0459	1.3378
Wdr1	O88342	WD repeat-containing protein 1	_DYSGQGVVK_.2	1.3124	0.3923	0.0460	1.3371
Pex5l	Q8C437	PEX5-related protein	_GPETS[Phospho (STY)]SLDLDIQTQLEK_.2	0.7060	-0.5022	0.0460	1.3369
Cntn1	P12960	Contactin-1	_IFNIQLEDEGLYEC[Carbamidomethyl (C)]EAENIR_.3	0.5458	-0.8736	0.0460	1.3369
Pkp4	Q68FH0	Plakophilin-4	_PSPIYISSYSS[Phospho (STY)]PAR_.2	0.4036	-1.3090	0.0462	1.3357
Nfrkb	Q6PIJ4	Nuclear factor related to kappa-B-binding protein	_SPS[Phospho (STY)]PAVPLR_.2	1.4460	0.5321	0.0463	1.3343
Gpi	P06745	Glucose-6-phosphate isomerase	_ILLANFLAQTEALM[Oxidation (M)]K_.2	0.7456	-0.4235	0.0463	1.3341
Stt3b	Q3TDQ1	Dolichyl-diphosphooligosaccharide--protein glycosyltransferase subunit STT3B	_ENPPVEDS[Phospho (STY)]S[Phospho (STY)]DEDDKRNPGNLYDK_.3	0.4276	-1.2257	0.0465	1.3326
Adgrb1	Q3UHD1	Adhesion G protein-coupled receptor B1	_NENVATLSVSS[Phospho (STY)]LER_.2	0.5993	-0.7386	0.0466	1.3316

Uba1	Q02053	Ubiquitin-like modifier-activating enzyme 1	_IYDDDFFQNLDGVANALDNIDAR_.3	1.4238	0.5098	0.0466	1.3315
Ndufv1	Q91YT0	NADH dehydrogenase [ubiquinone] flavoprotein 1, mitochondrial	_TSFGSLKDEDR_.3	1.4614	0.5474	0.0467	1.3306
Dnmt1	P13864	DNA (cytosine-5)-methyltransferase 1	_S[Phospho (STY)]KSDSDTLSVETSPSSVATR_.3	1.5378	0.6209	0.0467	1.3306
Znf106	O88466	Zinc finger protein 106	_ATHVPENS[Phospho (STY)]DTEQDVFTAKPAR_.3	0.6749	-0.5673	0.0467	1.3305
Kctd12	Q6WVG3	BTB/POZ domain-containing protein KCTD12	_MFTQQQPQELAR_.2	1.4627	0.5486	0.0468	1.3301
Dnm1l	Q8K1M6	Dynamin-1-like protein	_YIETSELC[Carbamidomethyl (C)]GGAR_.2	0.6751	-0.5668	0.0468	1.3300
Slc8a2	Q8K596	Sodium/calcium exchanger 2	_C[Carbamidomethyl (C)]QPGVLLPVWEPEDPSLGDK_.2	0.6551	-0.6102	0.0469	1.3288
Pip5k1c	O70161	Phosphatidylinositol 4-phosphate 5-kinase type-1 gamma	_ITVQVEPVC[Carbamidomethyl (C)]GVGVVPK_.2	1.3059	0.3851	0.0469	1.3285
Ube4b	Q9ES00	Ubiquitin conjugation factor E4 B	_NNESQWKDS[Phospho (STY)]PLATR_.2	0.4390	-1.1877	0.0470	1.3279
Tuba1b	P05213	Tubulin alpha-1B chain	_EDM[Oxidation (M)]AALEKDYEEVGVDSVEGEGEEEGEEY_.3	0.7186	-0.4768	0.0470	1.3278
Gja1	P23242	Gap junction alpha-1 protein	_C[Carbamidomethyl (C)]NTQQPGC[Carbamidomethyl (C)]ENVC[Carbamidomethyl (C)]YDK_.2	1.3962	0.4815	0.0470	1.3276
Srrm2	Q8BTI8	Serine/arginine repetitive matrix protein 2	_RRS[Phospho (STY)]PS[Phospho (STY)]VS[Phospho (STY)]S[Phospho (STY)]PEPTEK_.2	0.2162	-2.2094	0.0470	1.3275
Iqsec2	Q5DU25	IQ motif and SEC7 domain-containing protein 2	_AAHLPLLTIEPPSDSS[Phospho (STY)]VDLSDR_.3	1.4140	0.4998	0.0471	1.3266
Mark2	Q05512	Serine/threonine-protein kinase MARK2	_GRNS[Phospho (STY)]ATSADEQPHIGNYR_.3	0.7073	-0.4995	0.0471	1.3265
Dync1h1	Q9JHU4	Cytoplasmic dynein 1 heavy chain 1	_LQGATC[Carbamidomethyl (C)]SNNK_.2	0.6371	-0.6505	0.0471	1.3265
Dmxl2	Q8BPN8	DmX-like protein 2	_SS[Phospho (STY)]DDIDYR_.2	0.4838	-1.0475	0.0472	1.3264
Aak1	Q3UHJ0	AP2-associated protein kinase 1	_REQGSSGLGSGSS[Phospho (STY)]GGGGSSSGLGSGYIGR_.3	0.6921	-0.5309	0.0473	1.3254
Dctn1	O08788	Dynactin subunit 1	_LNQLSTHTHVVDITR_.3	1.4217	0.5076	0.0473	1.3249
Pde2a	Q922S4	cGMP-dependent 3',5'-cyclic phosphodiesterase	_VETVYTYLLDGESR_.2	0.6833	-0.5495	0.0474	1.3246
Ap2b1	Q9DBG3	AP-2 complex subunit beta	_APEVSQYIYQVYDSILK_.2	1.4224	0.5083	0.0474	1.3243
Hivep3	A2A884	Transcription factor HIVEP3	_RSS[Phospho (STY)]VESPK_.2	1.4228	0.5087	0.0474	1.3239
Pip5k1c	O70161	Phosphatidylinositol 4-phosphate 5-kinase type-1 gamma	_IM[Oxidation (M)]DYSLLLGVHNIDQQER_.3	1.4084	0.4940	0.0476	1.3225
Ahcyl2	Q68FL4	Putative adenosylhomocysteinase 3	_TPELTWER_.2	1.5697	0.6505	0.0476	1.3222
Aldoc	P05063	Fructose-bisphosphate aldolase C	_ISDRTPSALAILENANVLAR_.3	1.3455	0.4281	0.0477	1.3216
Ctu1	Q99J10	Cytoplasmic tRNA 2-thiolation protein 1	_AGATC[Carbamidomethyl (C)]VSQC[Carbamidomethyl (C)]DLS[Phospho (STY)]PGNGEDR_.2	1.4355	0.5215	0.0477	1.3211
Sv2b	Q8BG39	Synaptic vesicle glycoprotein 2B	_YFQDEEYKS[Phospho (STY)]K_.2	0.7334	-0.4473	0.0479	1.3200
Ttc3	O88196	E3 ubiquitin-protein ligase TTC3	_KQDS[Phospho (STY)]DEM[Oxidation (M)]PFGC[Carbamidomethyl (C)]IK_.2	0.6180	-0.6943	0.0479	1.3195
Cyfip2	Q5SQX6	Cytoplasmic FMR1-interacting protein 2	_YAPLHLVPLIER_.2	0.5972	-0.7437	0.0479	1.3194
Tpt1	P63028	Translationally-controlled tumor protein	_LEEQKPER_.2	1.8302	0.8720	0.0480	1.3189
Clasp1	Q80TV8	CLIP-associating protein 1	_RS[Phospho (STY)]PTGSTASR_.2	1.3902	0.4753	0.0482	1.3172
Prps1	Q9D7G0	Ribose-phosphate pyrophosphokinase 1	_IFSGSSHQDLSQK_.2	1.4900	0.5753	0.0482	1.3167
Atp1a2	Q6PIE5	Sodium/potassium-transporting ATPase subunit alpha-2	_LC[Carbamidomethyl (C)]FVGLM[Oxidation (M)]SM[Oxidation (M)]IDPPR_.2	0.5977	-0.7426	0.0483	1.3163
Pja1	O55176	E3 ubiquitin-protein ligase Praja-1	_EQPQSSS[Phospho (STY)]GESWELLPGKEELER_.3	0.6919	-0.5314	0.0483	1.3156
Acot7	Q91V12	Cytosolic acyl coenzyme A thioester hydrolase	_SM[Oxidation (M)]EIEVLVDADPVVDNSQK_.2	0.5607	-0.8348	0.0484	1.3156
Acin1	Q9JIX8	Apoptotic chromatin condensation inducer in the nucleus	_SLS[Phospho (STY)]PLSGTTDTKAES[Phospho (STY)]PAGR_.2	2.3991	1.2625	0.0485	1.3141
Srrm2	Q8BTI8	Serine/arginine repetitive matrix protein 2	_LKSGM[Oxidation (M)]S[Phospho (STY)]PEQSK_.2	0.4251	-1.2342	0.0486	1.3138
Sumo3	Q9Z172	Small ubiquitin-related modifier 3	_VAGQDGSVVQFK_.2	1.3103	0.3899	0.0486	1.3133
Psma3	O70435	Proteasome subunit alpha type-3	_AVENSSTAIGIR_.2	1.6558	0.7275	0.0486	1.3133
Eps8l2	Q99K30	Epidermal growth factor receptor kinase substrate 8-like protein 2	_RRES[Phospho (STY)]QDEEPR_.2	1.3964	0.4817	0.0488	1.3115
Arf3;Arf1	P61205;P84078	ADP-ribosylation factor 3;ADP-ribosylation factor 1	_HYFQNTQGLIFVVDSNDR_.3	0.5056	-0.9838	0.0488	1.3112
Chgb	P16014	Secretogranin-1	_WASS[Phospho (STY)]REDAGAPVEDSQGQTK_.3	1.9595	0.9705	0.0489	1.3105
Slc25a12	Q8BH59	Calcium-binding mitochondrial carrier protein Aralar1	_FTLGSVAGAVGATAVYPIDLVK_.2	1.3795	0.4641	0.0489	1.3104
C2cd3	Q52KB6	C2 domain-containing protein 3	_SDVGTSELPS[Phospho (STY)]EDDGVEPSR_.2	0.4615	-1.1155	0.0490	1.3101
Ccar2	Q8VDP4	Cell cycle and apoptosis regulator protein 2	_S[Phospho (STY)]VAS[Phospho (STY)]NQSEM[Oxidation (M)]EYSSLQDM[Oxidation (M)]PK_.2	0.6118	-0.7089	0.0490	1.3095
Sucla2	Q9Z2I9	Succinate--CoA ligase [ADP-forming] subunit beta, mitochondrial	_IFDLQDWSQEDER_.2	0.5508	-0.8605	0.0491	1.3088
Cdk13	Q69ZA1	Cyclin-dependent kinase 13	_S[Phospho (STY)]RS[Phospho (STY)]PYS[Phospho (STY)]PVLRR_.3	0.7197	-0.4745	0.0492	1.3083
Atp6v1c1	Q9Z1G3	V-type proton ATPase subunit C 1	_IDC[Carbamidomethyl (C)]NLLEFK_.2	0.6929	-0.5292	0.0492	1.3082
Apc	Q61315	Adenomatous polyposis coli protein	_SFHVEDTPVC[Carbamidomethyl (C)]FS[Phospho (STY)]R_.3	1.3940	0.4792	0.0492	1.3077
Hivep2	Q3UHF7	Transcription factor HIVEP2	_LADLEHSSGESS[Phospho (STY)]FESTGTGLSR_.3	1.3933	0.4786	0.0493	1.3069
Htt	P42859	Huntingtin	_DEVAEAS[Phospho (STY)]DPESKPC[Carbamidomethyl (C)]R_.2	0.7198	-0.4743	0.0493	1.3068
Fam117b	Q3U3E2	Protein FAM117B	_S[Phospho (STY)]PEQGRPSAEKR_.3	0.7409	-0.4327	0.0494	1.3067
Mapre3	Q6PER3	Microtubule-associated protein RP/EB family member 3	_LIGTAVPQRTS[Phospho (STY)]PTGPK_.2	0.3012	-1.7314	0.0496	1.3046
Synj1	Q8CHC4	Synaptojanin-1	_IYAGTGALEGK_.2	0.7643	-0.3878	0.0496	1.3045
Ap1b1	O35643	AP-1 complex subunit beta-1	_VIASMTVGK_.2	0.6692	-0.5795	0.0496	1.3041
Runx1t1	Q61909	Protein CBFA2T1	_QQS[Phospho (STY)]PVNPDPVALDAHR_.3	1.3697	0.4539	0.0498	1.3032
Septin5	Q9Z2Q6	Septin-5	_LYPWGIVEVENQAHC[Carbamidomethyl (C)]DFVK_.2	0.4923	-1.0223	0.0498	1.3030
Armt1	A6H630	Damage-control phosphatase ARMT1	_[Acetyl (Protein N-term)]AES[Phospho (STY)]PAFLSAK_.2	0.6206	-0.6884	0.0499	1.3019
Lyst	P97412	Lysosomal-trafficking regulator	_QPEPFS[Phospho (STY)]PR_.2	0.5886	-0.7646	0.0499	1.3016
