# Supplementary material for: The Chronic Effects of a Single Low-Intensity Blast Exposure on Phosphoproteome Networks and Cognitive Function Influenced by Mutant Tau Overexpression
Source: Int J Mol Sci. 2024 Mar 15;25(6):3338. doi: 10.3390/ijms25063338 (PMC10970429; doi:10.3390/ijms25063338)
Supplement: Supplementary file 1 [file ijms-25-03338-s001.zip › Supplementary Table S1b- MS2 significant phosphopeptides_rTg4510.pdf]

Supplementary Table S1b- MS2 significant phosphopeptides (sheet = 'rTg4510 sig LIB vs sham')

PG.Genes	PG.ProteinGroups	PG.ProteinDescriptions	EG.PrecursorId	Phos site	Expression Ratio	LOG2FC	pvalue	(-) Log10 p value
Ttbk1	Q6PCN3	Tau-tubulin kinase 1	_RVNS[Phospho (STY)]PESER_.2	#N/A	0.0698	-3.8405	0.0000	6.3618
Hepacam	Q640R3	Hepatocyte cell adhesion molecule	_SEADTLPRS[Phospho (STY)]GEQER_.2	#N/A	2.3820	1.2522	0.0000	4.3240
Vdac3	Q60931	Voltage-dependent anion-selective channel protein 3	_WNTDNTLGTEISWENK_.2	#N/A	0.4164	-1.2640	0.0001	3.8572
Tubb4b	P68372	Tubulin beta-4B chain	_TAVC[Carbamidomethyl (C)]DIPPR_.2	#N/A	0.6352	-0.6547	0.0002	3.6608
Hnrnpc	Q9Z204	Heterogeneous nuclear ribonucleoproteins C1/C2	_QADLSFSS[Phospho (STY)]PVEM[Oxidation (M)]K_.2	#N/A	0.2549	-1.9720	0.0003	3.5822
Smarcc2	Q6PDG5	SWI/SNF complex subunit SMARCC2	_TLTDEVNS[Phospho (STY)]PDS[Phospho (STY)]DRRDK_.3	#N/A	0.7359	-0.4424	0.0003	3.5386
Gria2	P23819	Glutamate receptor 2	_YTSALTYDAVQVM[Oxidation (M)]TEAFR_.2	#N/A	1.8506	0.8880	0.0003	3.4836
Adgrl3	Q80TS3	Adhesion G protein-coupled receptor L3	_GSSDGFIVPPNKDGAS[Phospho (STY)]PEGTSK_.3	#N/A	0.2666	-1.9074	0.0004	3.3682
Ank3	G5E8K5	Ankyrin-3	_TEEPVS[Phospho (STY)]PLTAYQK_.2	G5E8K5_S1883_M1_Ank3	8.1876	3.0334	0.0006	3.2461
Epb41l3	Q9WV92	Band 4.1-like protein 3	_SSSSKLS[Phospho (STY)]RS[Phospho (STY)]PLK_.2	#N/A	1.3275	0.4087	0.0006	3.2144
Cntn2	Q61330	Contactin-2	_TTGPGGDGIPAEVHIVR_.3	#N/A	0.4419	-1.1782	0.0007	3.1712
Zc3h13	E9Q784	Zinc finger CCCH domain-containing protein 13	_SKLS[Phospho (STY)]PS[Phospho (STY)]PSLRK_.2	#N/A	0.2158	-2.2124	0.0008	3.1110
Psip1	Q99JF8	PC4 and SFRS1-interacting protein	_FSSQQASTK_.2	#N/A	0.3788	-1.4006	0.0011	2.9717
Cnp	P16330	2',3'-cyclic-nucleotide 3'-phosphodiesterase	_M[Oxidation (M)]VSADAYK_.2	#N/A	0.6769	-0.5630	0.0014	2.8475
Cct7	P80313	T-complex protein 1 subunit eta	_ATISNDGATILK_.2	#N/A	1.7170	0.7799	0.0015	2.8162
Fyb1	O35601	FYN-binding protein 1	_VAGQSS[Phospho (STY)]PSGIQSR_.2	O35601_S28_M1_Fyb1	0.2375	-2.0738	0.0017	2.7805
Cyb5a	P56395	Cytochrome b5	_[Acetyl (Protein N-term)]AGQS[Phospho (STY)]DKDVK_.2	P56395_S5_M1_Cyb5a	0.6005	-0.7358	0.0020	2.7067
Hsp90aa1	P07901	Heat shock protein HSP 90-alpha	_HGLEVIYM[Oxidation (M)]IEPIDEYC[Carbamidomethyl (C)]VQQLK_.3	#N/A	0.4754	-1.0727	0.0021	2.6736
Rabep1	O35551	Rab GTPase-binding effector protein 1	_RAQS[Phospho (STY)]TDS[Phospho (STY)]LGTSSSLQSK_.2	#N/A	1.7132	0.7767	0.0022	2.6502
Hsp90b1	P08113	Endoplasmin	_LISLTDENALAGNEELTVK_.2	#N/A	1.9566	0.9684	0.0024	2.6266
Eno2	P17183	Gamma-enolase	_NYPVVSIEDPFDQDDWAAWSK_.3	#N/A	2.1240	1.0868	0.0024	2.6176
Scn1a	A2APX8	Sodium channel protein type 1 subunit alpha	_QAS[Phospho (STY)]FTYNK_.2	A2APX8_S1939_M1_Scn1a	4.5433	2.1837	0.0029	2.5432
Prrt2	E9PUL5	Proline-rich transmembrane protein 2	_AELAPETTETPVETPETVQATDLSLNPEEGSK_.3	#N/A	0.4005	-1.3200	0.0029	2.5427
Pdha1	P35486	Pyruvate dehydrogenase E1 component subunit alpha, somatic form, mitochondrial	_AAASTDYYKR_.2	#N/A	0.6736	-0.5701	0.0029	2.5423
Pclo	Q9QYX7	Protein piccolo	_LPAQQPGPQTAAK_.2	#N/A	0.2916	-1.7777	0.0030	2.5263
Gtf2f2	Q8R0A0	General transcription factor IIF subunit 2	_HYQTEEKS[Phospho (STY)]D_.2	Q8R0A0_S248_M1_Gtf2f2	0.3302	-1.5987	0.0031	2.5139
Shank2	Q80Z38	SH3 and multiple ankyrin repeat domains protein 2	_QSNVEDS[Phospho (STY)]PEK_.2	#N/A	4.9765	2.3151	0.0031	2.5041
Ubap2l	Q80X50	Ubiquitin-associated protein 2-like	_YPSSISSS[Phospho (STY)]PQKDLTQAK_.2	#N/A	2.9965	1.5833	0.0032	2.4957
Ndufs1	Q91VD9	NADH-ubiquinone oxidoreductase 75 kDa subunit, mitochondrial	_HSFC[Carbamidomethyl (C)]EVLK_.2	#N/A	1.4834	0.5689	0.0034	2.4624
Samm50	Q8BGH2	Sorting and assembly machinery component 50 homolog	_LELNYC[Carbamidomethyl (C)]IPM[Oxidation (M)]GVQGGDR_.2	#N/A	0.4366	-1.1958	0.0035	2.4613
Srsf3	P84104	Serine/arginine-rich splicing factor 3	_VYVGNLGNNGNK_.2	#N/A	0.2509	-1.9949	0.0035	2.4580
Dennd4b	Q3U1Y4	DENN domain-containing protein 4B	_S[Phospho (STY)]APSS[Phospho (STY)]PAPR_.2	Q3U1Y4_S732_M2_Dennd4b	3.9846	1.9944	0.0036	2.4496
Sgip1	Q8VD37	SH3-containing GRB2-like protein 3-interacting protein 1	_KVAEQTFIKDDYLETLSS[Phospho (STY)]PK_.3	#N/A	1.6840	0.7519	0.0036	2.4392
Kpnb1	P70168	Importin subunit beta-1	_[Acetyl (Protein N-term)]M[Oxidation (M)]ELITILEK_.2	#N/A	0.6113	-0.7102	0.0037	2.4303
Srrm1	Q52KI8	Serine/arginine repetitive matrix protein 1	_RWQS[Phospho (STY)]PVTK_.2	#N/A	0.1629	-2.6177	0.0038	2.4198
Pde1b	Q01065	Calcium/calmodulin-dependent 3',5'-cyclic nucleotide phosphodiesterase 1B	_QILDTEDELRELR_.3	#N/A	1.9354	0.9527	0.0038	2.4179
Pafah1b1	P63005	Platelet-activating factor acetylhydrolase IB subunit beta	_FILSC[Carbamidomethyl (C)]ADDK_.2	#N/A	0.7444	-0.4259	0.0040	2.4031
Rab14	Q91V41	Ras-related protein Rab-14	_QFAEENGLLFLEASAK_.2	#N/A	1.6222	0.6979	0.0040	2.4013
Sugt1	Q9CX34	Protein SGT1 homolog	_ALEQNPDDAQYYC[Carbamidomethyl (C)]QR_.2	#N/A	0.5925	-0.7552	0.0040	2.3966
Ppia	P17742	Peptidyl-prolyl cis-trans isomerase A	_VKEGM[Oxidation (M)]NIVEAM[Oxidation (M)]ER_.3	#N/A	0.6390	-0.6461	0.0041	2.3873
Hdgfl2	Q3UMU9	Hepatoma-derived growth factor-related protein 2	_AQEDGQDS[Phospho (STY)]EDGPR_.2	Q3UMU9_S620_M1_Hdgfl2	0.3336	-1.5839	0.0042	2.3796
Tjp2	Q9Z0U1	Tight junction protein ZO-2	_GRS[Phospho (STY)]IDRDYDR_.3	#N/A	1.5091	0.5937	0.0043	2.3674
Eci1	P42125	Enoyl-CoA delta isomerase 1, mitochondrial	_EADIQNFTSFISK_.2	#N/A	1.5592	0.6408	0.0044	2.3596
Srrm2	Q8BTI8	Serine/arginine repetitive matrix protein 2	_SGSSQELDGKPSAS[Phospho (STY)]PQER_.2	Q8BTI8_S1508_M1_Srrm2	0.0123	-6.3438	0.0044	2.3587
Syncrip	Q7TMK9	Heterogeneous nuclear ribonucleoprotein Q	_TGYTLDVTTGQR_.2	#N/A	0.2688	-1.8951	0.0045	2.3444
Prnp	P04925	Major prion protein	_PM[Oxidation (M)]IHFGNDWEDR_.3	#N/A	0.5764	-0.7950	0.0047	2.3322
Tpm3	P21107	Tropomyosin alpha-3 chain	_TEERAELAESK_.2	#N/A	1.6208	0.6967	0.0048	2.3208
Atg2b	Q80XK6	Autophagy-related protein 2 homolog B	_S[Phospho (STY)]SPVC[Carbamidomethyl (C)]STAPVETEPK_.2	Q80XK6_S239_M1_Atg2b	1.7650	0.8197	0.0048	2.3188
Ssr3	Q9DCF9	Translocon-associated protein subunit gamma	_QQS[Phospho (STY)]EEDLLLQDFSR_.2	Q9DCF9_S11_M1_Ssr3	0.1160	-3.1083	0.0049	2.3088
Flt1	P35969	Vascular endothelial growth factor receptor 1	_DGFADPHFHSGS[Phospho (STY)]SDDVR_.3	P35969_S1207_M1_Flt1	1.7968	0.8454	0.0051	2.2954
Vipas39	Q8BGQ1	Spermatogenesis-defective protein 39 homolog	_ETAGS[Phospho (STY)]SGSTPEGR_.2	Q8BGQ1_S77_M1_Vipas39	1.7973	0.8458	0.0051	2.2907
Mtmr7	Q9Z2C9	Myotubularin-related protein 7	_S[Phospho (STY)]PSGGEHAPSEDSGKDR_.3	#N/A	1.7981	0.8465	0.0052	2.2816
Chchd6	Q91VN4	MICOS complex subunit Mic25	_KKAS[Phospho (STY)]LTHEQQSAR_.3	#N/A	0.4718	-1.0836	0.0053	2.2797
Gatad2b	Q8VHR5	Transcriptional repressor p66-beta	_S[Phospho (STY)]LDPADERDDVLAK_.2	#N/A	1.7984	0.8467	0.0053	2.2785
Cttn	Q60598	Src substrate cortactin	_YGIDKDKVDK_.2	#N/A	1.7076	0.7720	0.0053	2.2774
Ap3b2	Q9JME5	AP-3 complex subunit beta-2	_QLIVPSEQGGALSR_.2	#N/A	0.6730	-0.5714	0.0053	2.2733
Ndufb8	Q9D6J5	NADH dehydrogenase [ubiquinone] 1 beta subcomplex subunit 8, mitochondrial	_DPWYQWDHSELR_.2	#N/A	2.1523	1.1059	0.0054	2.2642
Eif4g1	Q6NZJ6	Eukaryotic translation initiation factor 4 gamma 1	_AAS[Phospho (STY)]LTEDR_.2	#N/A	1.8378	0.8780	0.0056	2.2547
Arpc2	Q9CVB6	Actin-related protein 2/3 complex subunit 2	_YFQFQEEGKEGENR_.2	#N/A	1.6471	0.7199	0.0056	2.2503
Sptbn1	Q62261	Spectrin beta chain, non-erythrocytic 1	_RRPPS[Phospho (STY)]PDPNTK_.2	#N/A	0.0401	-4.6386	0.0057	2.2440
Apoe	P08226	Apolipoprotein E	_DAEDLQKR_.2	#N/A	1.8015	0.8492	0.0057	2.2412
Ank2	Q8C8R3	Ankyrin-2	_HTPVSPSS[Phospho (STY)]KNEKLS[Phospho (STY)]PVS[Phospho (STY)]PSAK_.3	Q8C8R3_S1810_M3_Ank2	1.5821	0.6619	0.0058	2.2353
Scp2	P32020	Sterol carrier protein 2	_GSVLPNSDKK_.2	#N/A	0.3998	-1.3228	0.0059	2.2319
Smn1	P97801	Survival motor neuron protein	_RGT[Phospho (STY)]GQSDDSDIWDDTALIK_.3	P97801_T22_M1_Smn1	1.6162	0.6926	0.0059	2.2288
Oxr1	Q4KMM3	Oxidation resistance protein 1	_DQQGQELKRDS[Phospho (STY)]ETEVEELR_.3	#N/A	1.7574	0.8134	0.0059	2.2270
MAPT	P10636	Microtubule-associated protein tau	_SKIGS[Phospho (STY)]TENLK_.2	#N/A	2.5561	1.3540	0.0059	2.2267
Scrn1	Q9CZC8	Secernin-1	_DEVQEVVYFPAVDHDAESK_.3	#N/A	1.9367	0.9536	0.0060	2.2193
Tfip11	Q9ERA6	Tuftelin-interacting protein 11	_TTQSLQDFPVADS[Phospho (STY)]EEEAEEEFQK_.3	Q9ERA6_S211_M1_Tfip11	0.6578	-0.6042	0.0062	2.2107
Slc16a7	O70451	Monocarboxylate transporter 2	_VGS[Phospho (STY)]RHDS[Phospho (STY)]TLKK_.2	#N/A	2.3437	1.2288	0.0063	2.1993
Ubqln1	Q8R317	Ubiquilin-1	_FQQQLEQLSAM[Oxidation (M)]GFLNR_.2	#N/A	0.6109	-0.7109	0.0065	2.1897
Map1b	P14873	Microtubule-associated protein 1B	_TLEVVSPSQSVTGSAGHTPYYQSPTDEK_.3	#N/A	1.8478	0.8858	0.0065	2.1851
Fam171a2	A2A699	Protein FAM171A2	_RDS[Phospho (STY)]LTSPEDELGAEVGDEAGDK_.3	#N/A	1.8060	0.8528	0.0066	2.1811
Agap2	Q3UHD9	Arf-GAP with GTPase, ANK repeat and PH domain-containing protein 2	_SGNS[Phospho (STY)]LNKEWK_.2	Q3UHD9_S684_M1_Agap2	0.3512	-1.5095	0.0067	2.1745
Ncan	P55066	Neurocan core protein	_HLQAAFEDGFDNC[Carbamidomethyl (C)]DAGWLSDR_.3	#N/A	0.4223	-1.2437	0.0069	2.1619

Nsf	P46460	Vesicle-fusing ATPase	_VEVDMEK_.2	#N/A	1.8074	0.8539	0.0069	2.1608
Hecw1	Q8K4P8	E3 ubiquitin-protein ligase HECW1	_C[Carbamidomethyl (C)]SPC[Carbamidomethyl (C)]SS[Phospho (STY)]PQNSPGLQR_.2	Q8K4P8_S1203_M1_Hecw1	1.6771	0.7459	0.0070	2.1565
Vcp	Q01853	Transitional endoplasmic reticulum ATPase	_AIANEC[Carbamidomethyl (C)]QANFISIK_.2	#N/A	1.6139	0.6905	0.0071	2.1496
Rpl34	Q9D1R9	60S ribosomal protein L34	_SAC[Carbamidomethyl (C)]GVC[Carbamidomethyl (C)]PGR_.2	#N/A	0.5361	-0.8994	0.0073	2.1377
Smc3	Q9CW03	Structural maintenance of chromosomes protein 3	_GDVEGSQS[Phospho (STY)]QDEGEGSGESER_.2	#N/A	1.8711	0.9039	0.0073	2.1364
Lrrc7	Q80TE7	Leucine-rich repeat-containing protein 7	_S[Phospho (STY)]REQQPYEGNINK_.2	#N/A	1.5699	0.6507	0.0074	2.1316
Sec16a	E9QAT4	Protein transport protein Sec16A	_RSS[Phospho (STY)]LS[Phospho (STY)]SHSHQSQIYR_.3	E9QAT4_S1384_M2_Sec16a	1.8094	0.8555	0.0074	2.1315
Sorbs1	Q62417	Sorbin and SH3 domain-containing protein 1	_DIS[Phospho (STY)]PEEIDLKNEPWYK_.3	#N/A	1.5570	0.6387	0.0075	2.1267
Mia3	Q8BI84	Transport and Golgi organization protein 1 homolog	_EEAKPAS[Phospho (STY)]PSSVQDR_.2	#N/A	1.7087	0.7729	0.0075	2.1248
Srrm1	Q52KI8	Serine/arginine repetitive matrix protein 1	_LS[Phospho (STY)]PSAS[Phospho (STY)]PPR_.2	#N/A	1.8103	0.8562	0.0076	2.1169
Ctnna2	Q61301	Catenin alpha-2	_SKLDAEVAK_.2	#N/A	0.4258	-1.2317	0.0077	2.1143
Ndufaf3	Q9JKL4	NADH dehydrogenase [ubiquinone] 1 alpha subcomplex assembly factor 3	_VTGAALIPPPGETALASLGQASE_.2	#N/A	0.5718	-0.8064	0.0077	2.1132
Naca	P70670;Q60817	olypeptide-associated complex subunit alpha, muscle-specific form;Nascent polypeptide-associated complex sub	_DIELVMSQANVSR_.2	#N/A	1.8107	0.8566	0.0078	2.1104
Map1a	Q9QYR6	Microtubule-associated protein 1A	_AKPAS[Phospho (STY)]PAR_.2	#N/A	1.4377	0.5237	0.0079	2.1020
Kcnh7	Q9ER47	Potassium voltage-gated channel subfamily H member 7	_SFS[Phospho (STY)]PSSQC[Carbamidomethyl (C)]PEFLDLEK_.2	Q9ER47_S1103_M1_Kcnh7	1.7094	0.7735	0.0079	2.0999
Itsn1	Q9Z0R4	Intersectin-1	_SGS[Phospho (STY)]GM[Oxidation (M)]SVISSSSVDQR_.2	#N/A	2.4167	1.2731	0.0080	2.0992
Parva	Q9EPC1	Alpha-parvin	_[Acetyl (Protein N-term)]ATS[Phospho (STY)]PQKSPLVPK_.2	Q9EPC1_S4_M1_Parva	2.3086	1.2070	0.0080	2.0957
Alcam	Q61490	CD166 antigen	_VLQPVEGEVAILFK_.2	#N/A	0.6376	-0.6492	0.0081	2.0900
Cars1	Q9ER72	Cysteine--tRNA ligase, cytoplasmic	_ALQEGEGDLSIS[Phospho (STY)]ADR_.2	Q9ER72_S390_M1_Cars1	1.6624	0.7333	0.0081	2.0892
C2cd4c	Q5HZI2	C2 calcium-dependent domain-containing protein 4C	_RAT[Phospho (STY)]PEPGPESGQAPR_.3	Q5HZI2_T282_M1_C2cd4c	1.7413	0.8002	0.0082	2.0869
Gpr37l1	Q99JG2	G-protein coupled receptor 37-like 1	_PRES[Phospho (STY)]PPLLPLGTPC[Carbamidomethyl (C)]_.2	#N/A	0.1220	-3.0349	0.0082	2.0865
Cep170b	Q80U49	Centrosomal protein of 170 kDa protein B	_AGS[Phospho (STY)]FTGPSDSETAPAR_.2	Q80U49_S1166_M1_Cep170b	1.4096	0.4953	0.0082	2.0850
Ube2m	P61082	NEDD8-conjugating enzyme Ubc12	_TC[Carbamidomethyl (C)]DISFSDPDDLLNFK_.2	#N/A	0.6743	-0.5684	0.0082	2.0844
Pclo	Q9QYX7	Protein piccolo	_TTLYFDEEPELEM[Oxidation (M)]ESLTDS[Phospho (STY)]PEDR_.3	#N/A	1.8126	0.8580	0.0083	2.0814
Gnao1	P18872	Guanine nucleotide-binding protein G(o) subunit alpha	_PVVYSNTIQSLAAIVR_.2	#N/A	1.8129	0.8583	0.0084	2.0763
Psap	Q61207	Prosaposin	_TVVTEAGNLLK_.2	#N/A	1.3519	0.4350	0.0085	2.0691
Cap2	Q9CYT6	Adenylyl cyclase-associated protein 2	_SSEM[Oxidation (M)]NVLVPQDDDYREFPIPEQFK_.3	#N/A	0.6899	-0.5355	0.0087	2.0627
Cops4	O88544	COP9 signalosome complex subunit 4	_ISQTAPEWTAQAM[Oxidation (M)]EAQM[Oxidation (M)]AQ_.2	#N/A	0.3856	-1.3748	0.0087	2.0587
Add2	Q9QYB8	Beta-adducin	_SAGPQSQLLASVIAEKS[Phospho (STY)]R_.3	#N/A	1.7048	0.7696	0.0089	2.0500
Acat1	Q8QZT1	Acetyl-CoA acetyltransferase, mitochondrial	_IAAFADAAVDPIDFPLAPAYAVPK_.3	#N/A	1.5684	0.6493	0.0090	2.0462
Pclo	Q9QYX7	Protein piccolo	_SPVPSQQAS[Phospho (STY)]PKK_.2	#N/A	1.3806	0.4653	0.0090	2.0446
Sgip1	Q8VD37	SH3-containing GRB2-like protein 3-interacting protein 1	_RS[Phospho (STY)]PGAIK_.2	#N/A	1.5616	0.6430	0.0091	2.0420
Akt1s1	Q9D1F4	Proline-rich AKT1 substrate 1	_AATATRPPGPPPAPQPPS[Phospho (STY)]PAPS[Phospho (STY)]PPPRPALAR_.4	Q9D1F4_S88_M2_Akt1s1	1.7110	0.7748	0.0092	2.0351
Syn1	O88935	Synapsin-1	_QAS[Phospho (STY)]QAGPGPR_.2	#N/A	1.7123	0.7759	0.0093	2.0338
Arfgap1	Q9EPJ9	ADP-ribosylation factor GTPase-activating protein 1	_TLQFTAHR_.2	#N/A	0.7422	-0.4302	0.0094	2.0273
Wasf1	Q8R5H6	Wiskott-Aldrich syndrome protein family member 1	_SSTIQDQQLFDRK_.2	#N/A	1.5445	0.6272	0.0096	2.0194
Fsd1	Q7TPM6	Fibronectin type III and SPRY domain-containing protein 1	_LDGSTSHQNLR_.2	#N/A	0.5850	-0.7734	0.0096	2.0183
Cpne1	Q8C166	Copine-1	_APPT[Phospho (STY)]PGKGPAQAPQA_.2	Q8C166_T525_M1_Cpne1	3.3994	1.7653	0.0096	2.0179
Myo18a	Q9JMH9	Unconventional myosin-XVIIIa	_SSSPTSHWKPLAPDPS[Phospho (STY)]DDEHDPVDSISRPR_.5	Q9JMH9_S2016_M1_Myo18a	1.7114	0.7752	0.0096	2.0168
Taldo1	Q93092	Transaldolase	_M[Oxidation (M)]ESALDQLK_.2	#N/A	0.6455	-0.6314	0.0097	2.0134
Cbarp	Q66L44	Voltage-dependent calcium channel beta subunit-associated regulatory protein	_ASS[Phospho (STY)]LDTRGS[Phospho (STY)]PK_.2	#N/A	1.3877	0.4727	0.0097	2.0113
Rbmxl1	Q91VM5	RNA binding motif protein, X-linked-like-1	_SDLYSSGR_.2	#N/A	1.7404	0.7994	0.0098	2.0107
Tjp2	Q9Z0U1	Tight junction protein ZO-2	_ERPS[Phospho (STY)]SREETSGR_.3	Q9Z0U1_S420_M1_Tjp2	0.7426	-0.4294	0.0100	1.9983
Kif2a	P28740	Kinesin-like protein KIF2A	_DFRGS[Phospho (STY)]LDYRPLTTADPIDEHR_.4	P28740_S206_M1_Kif2a	1.6443	0.7175	0.0101	1.9970
Stx1a	O35526	Syntaxin-1A	_LKS[Phospho (STY)]IEQSIEQEEGLNR_.3	O35526_S95_M1_Stx1a	1.3214	0.4021	0.0101	1.9949
Syngap1	F6SEU4	Ras/Rap GTPase-activating protein SynGAP	_GKS[Phospho (STY)]QQLTVSAAQK_.2	#N/A	2.0961	1.0677	0.0102	1.9920
Smg6	P61406	Telomerase-binding protein EST1A	_NWS[Phospho (STY)]GC[Carbamidomethyl (C)]GEGEQK_.2	P61406_S331_M1_Smg6	2.1935	1.1332	0.0103	1.9878
Speg	Q62407	Striated muscle-specific serine/threonine-protein kinase	_AAS[Phospho (STY)]VELPQRR_.2	#N/A	1.3612	0.4449	0.0103	1.9865
Rad23b	P54728	UV excision repair protein RAD23 homolog B	_QIIQQNPSLLPALLQQIGR_.3	#N/A	1.5698	0.6505	0.0104	1.9847
Vapb	Q9QY76	Vesicle-associated membrane protein-associated protein B	_SLTS[Phospho (STY)]PLDDTEVK_.2	#N/A	1.7122	0.7759	0.0104	1.9842
Acot13	Q9CQR4	Acyl-coenzyme A thioesterase 13	_TLAFASVDLTNK_.2	#N/A	1.4059	0.4915	0.0105	1.9790
Actr3b	Q641P0	Actin-related protein 3B	_LSQELSGGR_.2	#N/A	1.5251	0.6090	0.0106	1.9756
Ina	P46660	Alpha-internexin	_VSSAGLSLK_.2	#N/A	0.6252	-0.6777	0.0106	1.9740
Creg2	Q8BGC9	Protein CREG2	_RES[Phospho (STY)]GS[Phospho (STY)]SEASPGPR_.2	Q8BGC9_S109_M2_Creg2	1.7339	0.7940	0.0106	1.9738
Chd2	E9PZM4	Chromodomain-helicase-DNA-binding protein 2	_SPLDHRS[Phospho (STY)]PLER_.3	E9PZM4_S1806_M1_Chd2	0.6214	-0.6864	0.0107	1.9721
Ptpn3	A2ALK8	Tyrosine-protein phosphatase non-receptor type 3	_SSSSVSPSSNAPGS[Phospho (STY)]C[Carbamidomethyl (C)]SPDGVDQR_.2	A2ALK8_S467_M1_Ptpn3	1.5032	0.5880	0.0107	1.9694
Ndufs1	Q91VD9	NADH-ubiquinone oxidoreductase 75 kDa subunit, mitochondrial	_VAGM[Oxidation (M)]LQNFEGNAVAAIAGGLVDAEALVALK_.3	#N/A	2.0957	1.0674	0.0108	1.9686
Atp6v1c1	Q9Z1G3	V-type proton ATPase subunit C 1	_LDAFVEGVVK_.2	#N/A	0.6214	-0.6863	0.0108	1.9669
Cit	P49025	Citron Rho-interacting kinase	_DKMNS[Phospho (STY)]PGLQSK_.2	#N/A	0.7433	-0.4279	0.0109	1.9630
Cend1	Q9JKC6	Cell cycle exit and neuronal differentiation protein 1	_SASS[Phospho (STY)]PKPDTK_.2	#N/A	1.6325	0.7071	0.0110	1.9585
Mettl26	Q9DCS2	Methyltransferase-like 26	_DTVLLEELGQASGLVLER_.2	#N/A	1.3730	0.4573	0.0110	1.9574
Dctn2	Q99KJ8	Dynactin subunit 2	_TGYES[Phospho (STY)]GDYEM[Oxidation (M)]LGEGLGVK_.2	Q99KJ8_S83_M1_Dctn2	1.6940	0.7605	0.0110	1.9571
Cbx3	P23198	Chromobox protein homolog 3	_KSLS[Phospho (STY)]DSESDDSKSK_.2	#N/A	1.6549	0.7268	0.0112	1.9527
Kansl3	A2RSY1	KAT8 regulatory NSL complex subunit 3	_GSRPAS[Phospho (STY)]PAAR_.2	A2RSY1_S515_M1_Kansl3	0.6216	-0.6860	0.0112	1.9522
Ndufs4	Q9CXZ1	NADH dehydrogenase [ubiquinone] iron-sulfur protein 4, mitochondrial	_NNMQSGVNNTKK_.2	#N/A	1.8131	0.8585	0.0113	1.9470
Tpm1	P58771	Tropomyosin alpha-1 chain	_C[Carbamidomethyl (C)]AELEEELKTVTNNLK_.3	#N/A	1.6359	0.7101	0.0113	1.9459
Borcs5	Q9D920	BLOC-1-related complex subunit 5	_GLLSGQTS[Phospho (STY)]PTNAKLEK_.2	#N/A	3.9173	1.9698	0.0114	1.9420
Glod4	Q9CPV4	Glyoxalase domain-containing protein 4	_LELQGIQGAVDHAAAFGR_.3	#N/A	1.3219	0.4026	0.0114	1.9416
Gigyf1	Q99MR1	GRB10-interacting GYF protein 1	_GLEEEEEEEEPS[Phospho (STY)]EGVDEERPEAGGK_.3	Q99MR1_S343_M1_Gigyf1	0.6217	-0.6857	0.0115	1.9390
Slc38a3	Q9DCP2	Sodium-coupled neutral amino acid transporter 3	_AYEQLGYR_.2	#N/A	0.5446	-0.8768	0.0116	1.9339
Acat2	Q8CAY6	Acetyl-CoA acetyltransferase, cytosolic	_VAVLSQNR_.2	#N/A	0.6467	-0.6289	0.0117	1.9331
Scg2	Q03517	Secretogranin-2	_VPSPVSSEDDLQEEEQLEQAIKEHLGPGS[Phospho (STY)]SQEM[Oxidation (M)]ER_.4	#N/A	1.6883	0.7556	0.0117	1.9327
Srrm2	Q8BTI8	Serine/arginine repetitive matrix protein 2	_VSS[Phospho (STY)]PVLETVQQRT[Phospho (STY)]PSR_.3	Q8BTI8_S1360_M2_Srrm2	1.7343	0.7944	0.0117	1.9316
Exoc1	Q8R3S6	Exocyst complex component 1	_LTGSTSS[Phospho (STY)]LNK_.2	Q8R3S6_S473_M1_Exoc1	2.0928	1.0655	0.0117	1.9304
Rab3c	P62823	Ras-related protein Rab-3C	_TYSWDNAQVILAGNK_.2	#N/A	1.3144	0.3944	0.0118	1.9293
Cobll1	Q3UMF0	Cordon-bleu protein-like 1	_QSS[Phospho (STY)]LTFQSSDPEHVR_.2	#N/A	0.6218	-0.6855	0.0118	1.9292
Stub1	Q9WUD1	STIP1 homology and U box-containing protein 1	_WNS[Phospho (STY)]IEER_.2	Q9WUD1_S150_M1_Stub1	0.5447	-0.8764	0.0119	1.9239
Tubb4b	P68372	Tubulin beta-4B chain	_TLKLTTPTYGDLNHLVSATM[Oxidation (M)]SGVTTC[Carbamidomethyl (C)]LR_.3	#N/A	0.4443	-1.1704	0.0122	1.9132
Rps20	P60867	40S ribosomal protein S20	_TPVEPEVAIHR_.3	#N/A	2.4748	1.3073	0.0123	1.9116

Cs	Q9CZU6	Citrate synthase, mitochondrial	_EVGKDVSDEKLR_.3	#N/A	0.6311	-0.6642	0.0123	1.9093
Psmd9	Q9CR00	26S proteasome non-ATPase regulatory subunit 9	_ADVDLYQVR_.2	#N/A	0.6112	-0.7102	0.0127	1.8976
Actr1b	Q8R5C5	Beta-centractin	_AC[Carbamidomethyl (C)]YLSINPQKDEALETEK_.3	#N/A	0.4483	-1.1573	0.0127	1.8964
Clasp2	Q8BRT1	CLIP-associating protein 2	_IPRPSVS[Phospho (STY)]QGC[Carbamidomethyl (C)]S[Phospho (STY)]R_.2	Q8BRT1_S531_M2_Clasp2	0.2229	-2.1656	0.0127	1.8963
Dlst	Q9D2G2	hydrolipoyllysine-residue succinyltransferase component of 2-oxoglutarate dehydrogenase complex, mitochondr	_TINELGEK_.2	#N/A	0.3698	-1.4354	0.0127	1.8961
Nsmf	Q99NF2	NMDA receptor synaptonuclear signaling and neuronal migration factor	_ERS[Phospho (STY)]FS[Phospho (STY)]RS[Phospho (STY)]WSDPTPMK_.2	#N/A	1.6295	0.7044	0.0128	1.8930
Ywhaz	P63101	14-3-3 protein zeta/delta	_KGIVDQSQQAYQEAFEISK_.3	#N/A	1.5444	0.6271	0.0128	1.8929
Map4k4	P97820	Mitogen-activated protein kinase kinase kinase kinase 4	_DSPLQGGGQQNSQAGQR_.2	#N/A	0.5563	-0.8462	0.0128	1.8918
Ptprz1	B9EKR1	Receptor-type tyrosine-protein phosphatase zeta	_QSPINIDEDLTQVNVNLK_.2	#N/A	1.4799	0.5655	0.0129	1.8892
Lmnb1	P14733	Lamin-B1	_LSPS[Phospho (STY)]PSSR_.2	P14733_S394_M1_Lmnb1	0.2765	-1.8545	0.0129	1.8891
Lxn	P70202	Latexin	_AASVAENC[Carbamidomethyl (C)]INYQQGTPHK_.2	#N/A	1.5616	0.6430	0.0130	1.8861
Faf1	P54731	FAS-associated factor 1	_TPS[Phospho (STY)]GEFLER_.2	P54731_S581_M1_Faf1	0.6222	-0.6845	0.0130	1.8859
Uba1	Q02053	Ubiquitin-like modifier-activating enzyme 1	_IYDDDFFQNLDGVANALDNIDAR_.3	#N/A	0.6047	-0.7258	0.0131	1.8812
Znf385a	Q8VD12	Zinc finger protein 385A	_QPGS[Phospho (STY)]PSPPSVPESGQGVTK_.2	Q8VD12_S160_M1_Znf385a	1.7148	0.7780	0.0132	1.8790
Chtop	Q9CY57	Chromatin target of PRMT1 protein	_GHLDAELDAYM[Oxidation (M)]AQTDPETND_.2	#N/A	0.5249	-0.9299	0.0133	1.8777
Hivep3	A2A884	Transcription factor HIVEP3	_RSS[Phospho (STY)]VESPK_.2	A2A884_S489_M1_Hivep3	0.6554	-0.6096	0.0133	1.8776
Tufm	Q8BFR5	Elongation factor Tu, mitochondrial	_DKPHVNVGTIGHVDHGK_.4	#N/A	0.6223	-0.6843	0.0133	1.8774
Icam5	Q60625	Intercellular adhesion molecule 5	_SFVGEPPR_.2	#N/A	0.4611	-1.1168	0.0134	1.8726
Gls	D3Z7P3	Glutaminase kidney isoform, mitochondrial	_ILQEYQVQYTPQGDSDDGK_.2	#N/A	0.6224	-0.6842	0.0135	1.8693
Epb41l1	Q9Z2H5	Band 4.1-like protein 1	_LPSSPASPS[Phospho (STY)]PKGT[Phospho (STY)]PEKASER_.3	#N/A	12.9846	3.6987	0.0136	1.8674
Ogdh	Q60597	2-oxoglutarate dehydrogenase, mitochondrial	_ELEQIFC[Carbamidomethyl (C)]QFDSK_.2	#N/A	1.7738	0.8268	0.0137	1.8624
Nefh	P19246	Neurofilament heavy polypeptide	_VKS[Phospho (STY)]EEMIKVVEK_.3	#N/A	0.6224	-0.6840	0.0138	1.8609
Slc25a11	Q9CR62	Mitochondrial 2-oxoglutarate/malate carrier protein	_M[Oxidation (M)]IDGKPEYK_.2	#N/A	0.6230	-0.6828	0.0138	1.8605
Znf609	Q8BZ47	Zinc finger protein 609	_AEADKIYSFTDNAPS[Phospho (STY)]PSIGGSSR_.3	Q8BZ47_S803_M1_Znf609	1.7153	0.7785	0.0139	1.8569
Camk2g	Q923T9	Calcium/calmodulin-dependent protein kinase type II subunit gamma	_QET[Phospho (STY)]VEC[Carbamidomethyl (C)]LR_.2	Q923T9_T287_M1_Camk2g	1.6788	0.7474	0.0139	1.8560
Cd99l2	Q8BIF0	CD99 antigen-like protein 2	_QETQS[Phospho (STY)]AEPPPPEPPRI_.2	Q8BIF0_S226_M1_Cd99l2	0.6225	-0.6839	0.0140	1.8546
Cltc	Q68FD5	Clathrin heavy chain 1	_LEKHELIEFR_.3	#N/A	0.6765	-0.5638	0.0140	1.8538
Champ1	Q8K327	Chromosome alignment-maintaining phosphoprotein 1	_LKKDS[Phospho (STY)]QENS[Phospho (STY)]DAELSSSEYIR_.3	Q8K327_S613_M2_Champ1	0.6225	-0.6838	0.0141	1.8502
Champ1	Q8K327	Chromosome alignment-maintaining phosphoprotein 1	_GQES[Phospho (STY)]SS[Phospho (STY)]DQEQVDVESIDFSK_.2	Q8K327_S641_M2_Champ1	0.6225	-0.6838	0.0141	1.8501
Map1a	Q9QYR6	Microtubule-associated protein 1A	_AKPAS[Phospho (STY)]PARR_.2	#N/A	1.7492	0.8067	0.0141	1.8496
Camk2g	Q923T9	Calcium/calmodulin-dependent protein kinase type II subunit gamma	_KTS[Phospho (STY)]TQEYAAK_.2	Q923T9_S36_M1_Camk2g	1.8131	0.8585	0.0142	1.8480
Ncoa7	Q6DFV7	Nuclear receptor coactivator 7	_YYS[Phospho (STY)]VDDNQNK_.2	Q6DFV7_S92_M1_Ncoa7	4.9702	2.3133	0.0142	1.8467
Srrm4	Q8BKA3	Serine/arginine repetitive matrix protein 4	_SRGQEKGS[Phospho (STY)]PGGDLSK_.3	#N/A	1.6169	0.6932	0.0144	1.8420
Eci2	Q9WUR2	Enoyl-CoA delta isomerase 2	_ASQQDFENALNQVK_.2	#N/A	1.8638	0.8983	0.0144	1.8405
Ina	P46660	Alpha-internexin	_EEIHEYRR_.2	#N/A	0.6958	-0.5232	0.0144	1.8402
Sptbn1	Q62261	Spectrin beta chain, non-erythrocytic 1	_HDTSASTQS[Phospho (STY)]T[Phospho (STY)]PASSR_.2	Q62261_T2319_M2_Sptbn1	1.4420	0.5281	0.0147	1.8326
Obi1	Q8K2Y0	ORC ubiquitin ligase 1	_LKAEVDNRS[Phospho (STY)]PQK_.2	#N/A	2.3606	1.2392	0.0148	1.8293
Cadm2	Q8BLQ9	Cell adhesion molecule 2	_GAEDAPDADTAIINAEGS[Phospho (STY)]QVNAEEKK_.3	#N/A	1.9006	0.9264	0.0149	1.8275
Tbr1	Q64336	T-box brain protein 1	_RIS[Phospho (STY)]PADTPVSESSSPLK_.3	Q64336_S640_M1_Tbr1	1.6720	0.7415	0.0150	1.8238
Hnrnpa2b1	O88569	Heterogeneous nuclear ribonucleoproteins A2/B1	_GFGDGYNGYGGGPGGGNFGGS[Phospho (STY)]PGYGGGR_.3	O88569_S259_M1_Hnrnpa2b1	1.6189	0.6950	0.0150	1.8235
Srrm2	Q8BTI8	Serine/arginine repetitive matrix protein 2	_S[Phospho (STY)]RS[Phospho (STY)]RTS[Phospho (STY)]PVTR_.2	Q8BTI8_S1927_M3_Srrm2	2.8693	1.5207	0.0150	1.8234
Dclk1	Q9JLM8	Serine/threonine-protein kinase DCLK1	_TLQTLSSEK_.2	#N/A	0.5473	-0.8695	0.0150	1.8227
Glul	P15105	Glutamine synthetase	_Y[Phospho (STY)]NRKPAETNLR_.3	#N/A	0.7417	-0.4311	0.0153	1.8155
Tanc2	A2A690	Protein TANC2	_SQS[Phospho (STY)]ASYYPVC[Carbamidomethyl (C)]HSK_.2	A2A690_S1726_M1_Tanc2	1.7237	0.7855	0.0153	1.8142
Trappc2	Q9CQP2	Trafficking protein particle complex subunit 2	_FAMNPFYEPNS[Phospho (STY)]PIR_.2	Q9CQP2_S119_M1_Trappc2	2.0438	1.0313	0.0153	1.8141
Glod4	Q9CPV4	Glyoxalase domain-containing protein 4	_FYLQDRS[Phospho (STY)]PSQSDPVLK_.3	#N/A	0.6282	-0.6707	0.0156	1.8074
Cct8	P42932	T-complex protein 1 subunit theta	_KDWDDDQND_.2	#N/A	0.7146	-0.4847	0.0156	1.8064
Wwc2	Q6NXJ0	Protein WWC2	_LNRSDS[Phospho (STY)]DSSTLAK_.2	#N/A	1.6421	0.7155	0.0157	1.8048
Nrgn	P60761	Neurogranin	_KGPGPGGPGGAGGAR_.2	#N/A	1.3255	0.4065	0.0157	1.8046
St13	Q99L47	Hsc70-interacting protein	_QDPSVLHTEEMR_.3	#N/A	1.9226	0.9430	0.0158	1.8020
Nefh	P19246	Neurofilament heavy polypeptide	_SAQEEITEYRR_.3	#N/A	1.6477	0.7205	0.0161	1.7942
Wars1	P32921	Tryptophan--tRNA ligase, cytoplasmic	_DLTLEQAYSYTVENAK_.2	#N/A	1.6085	0.6857	0.0161	1.7924
Arpp21	Q9DCB4	cAMP-regulated phosphoprotein 21	_AWSS[Phospho (STY)]TDSDSSNR_.2	Q9DCB4_S362_M1_Arpp21	1.4188	0.5047	0.0162	1.7910
Sh3glb2	Q8R3V5	Endophilin-B2	_RLDLDAC[Carbamidomethyl (C)]K_.2	#N/A	0.3692	-1.4376	0.0163	1.7883
Prkce	P16054	Protein kinase C epsilon type	_SKS[Phospho (STY)]APTS[Phospho (STY)]PC[Carbamidomethyl (C)]DQELKELENNIR_.3	#N/A	1.7232	0.7851	0.0163	1.7883
Zswim8	Q3UHH1	Zinc finger SWIM domain-containing protein 8	_KQS[Phospho (STY)]AGPNS[Phospho (STY)]PTGGGGGGGSGGTR_.2	#N/A	0.6791	-0.5584	0.0164	1.7856
Ap2a2	P17427	AP-2 complex subunit alpha-2	_LSTVASTDILATVLEEM[Oxidation (M)]PPFPER_.3	#N/A	1.5152	0.5995	0.0164	1.7849
Igsf8	Q8R366	Immunoglobulin superfamily member 8	_AVLAHVDVQTLSSQLAVTVGPGER_.3	#N/A	0.6725	-0.5723	0.0164	1.7848
Retreg2	Q6NS82	Reticulophagy regulator 2	_SAPPAGDEPLAETES[Phospho (STY)]ESEAELAGFSPVVDVK_.3	Q6NS82_S276_M1_Retreg2	1.5366	0.6197	0.0165	1.7817
Mapk4	Q6P5G0	Mitogen-activated protein kinase 4	_AGST[Phospho (STY)]PLAEDVQVDPR_.2	Q6P5G0_T383_M1_Mapk4	0.4488	-1.1560	0.0168	1.7756
Ganab	Q8BHN3	Neutral alpha-glucosidase AB	_DAVHYGGWEHR_.3	#N/A	1.6410	0.7146	0.0168	1.7734
Uhrf1bp1l	A2RSJ4	UHRF1-binding protein 1-like	_SS[Phospho (STY)]PEISLR_.2	A2RSJ4_S1250_M1_Uhrf1bp1l	0.4664	-1.1003	0.0171	1.7658
Rims2	Q9EQZ7	Regulating synaptic membrane exocytosis protein 2	_RADS[Phospho (STY)]LRK_.2	Q9EQZ7_S493_M1_Rims2	2.6427	1.4020	0.0173	1.7630
Ap3b2	Q9JME5	AP-3 complex subunit beta-2	_TTLVAGSVVM[Oxidation (M)]AFEEVC[Carbamidomethyl (C)]PER_.2	#N/A	1.8898	0.9183	0.0173	1.7622
Fmnl1	Q9JL26	Formin-like protein 1	_SKPLDQSVEDLS[Phospho (STY)]K_.2	Q9JL26_S189_M1_Fmnl1	1.8435	0.8825	0.0174	1.7595
Ogfrl1	Q8VE52	Opioid growth factor receptor-like protein 1	_DSAADQSDS[Phospho (STY)]PPEK_.2	Q8VE52_S406_M1_Ogfrl1	0.6412	-0.6411	0.0179	1.7463
Ranbp2	Q9ERU9	E3 SUMO-protein ligase RanBP2	_NRPGYVS[Phospho (STY)]EEEEDDEDYEM[Oxidation (M)]AVK_.3	Q9ERU9_S2505_M1_Ranbp2	0.7280	-0.4579	0.0181	1.7424
Cacna1d	Q99246	Voltage-dependent L-type calcium channel subunit alpha-1D	_QT[Phospho (STY)]VLSWQAAIDAAR_.2	Q99246_T49_M1_Cacna1d	1.7185	0.7811	0.0185	1.7329
Abl1	P00520	Tyrosine-protein kinase ABL1	_SC[Carbamidomethyl (C)]S[Phospho (STY)]ASC[Carbamidomethyl (C)]M[Oxidation (M)]PHGAR_.2	#N/A	1.6739	0.7433	0.0186	1.7310
Arhgap23	Q69ZH9	Rho GTPase-activating protein 23	_ARS[Phospho (STY)]DDYLSR_.2	Q69ZH9_S351_M1_Arhgap23	1.4220	0.5080	0.0186	1.7307
Atrx	Q61687	Transcriptional regulator ATRX	_VC[Carbamidomethyl (C)]DQTSKFS[Phospho (STY)]PK_.2	Q61687_S315_M1_Atrx	0.4474	-1.1604	0.0187	1.7277
Dctn4	Q8CBY8	Dynactin subunit 4	_LIEYYQQLAQK_.2	#N/A	1.8039	0.8511	0.0187	1.7274
Hnrnpk	P61979	Heterogeneous nuclear ribonucleoprotein K	_GGDLM[Oxidation (M)]AYDRR_.2	#N/A	0.4608	-1.1179	0.0190	1.7215
Agap1	Q8BXK8	Arf-GAP with GTPase, ANK repeat and PH domain-containing protein 1	_VFQDVAQK_.2	#N/A	1.5428	0.6256	0.0191	1.7183
Safb	D3YXK2	Scaffold attachment factor B1	_SVVS[Phospho (STY)]FDKVK_.2	D3YXK2_S626_M1_Safb	1.9536	0.9661	0.0192	1.7173
Iqsec1	Q8R0S2	IQ motif and SEC7 domain-containing protein 1	_VLINFNAPNPQDR_.2	#N/A	0.4979	-1.0059	0.0193	1.7155
Spata18	Q0P557	Mitochondria-eating protein	_SNS[Phospho (STY)]PC[Carbamidomethyl (C)]TTVAK_.2	Q0P557_S273_M1_Spata18	0.4072	-1.2963	0.0193	1.7148
Limd2	Q8BGB5	LIM domain-containing protein 2	_SKS[Phospho (STY)]FSLR_.2	Q8BGB5_S30_M1_Limd2	1.3230	0.4038	0.0193	1.7145
Ptdss2	Q9Z1X2	Phosphatidylserine synthase 2	_RVAGGSGSES[Phospho (STY)]PLLK_.2	#N/A	1.6511	0.7234	0.0194	1.7127

Rala	P63321	Ras-related protein Ral-A	_VIM[Oxidation (M)]VGSGGVGK_.2	#N/A	0.7477	-0.4195	0.0194	1.7124
Caskin1	Q6P9K8	Caskin-1	_RRAS[Phospho (STY)]DLAGSVDTGSAGSVK_.3	#N/A	0.4954	-1.0132	0.0194	1.7111
Dck	P43346	Deoxycytidine kinase	_FC[Carbamidomethyl (C)]PS[Phospho (STY)]PSTSSEGTR_.2	P43346_S11_M1_Dck	6.0431	2.5953	0.0195	1.7109
Camk2b	P28652	Calcium/calmodulin-dependent protein kinase type II subunit beta	_QET[Phospho (STY)]VEC[Carbamidomethyl (C)]LKK_.2	#N/A	2.0944	1.0665	0.0197	1.7065
Mctp1	E9PV86	Multiple C2 and transmembrane domain-containing protein 1	_AAS[Phospho (STY)]PSFQAR_.2	E9PV86_S16_M1_Mctp1	6.1820	2.6281	0.0200	1.7000
Igsf8	Q8R366	Immunoglobulin superfamily member 8	_HAAYSVGWEM[Oxidation (M)]APAGAPGPGR_.3	#N/A	0.4406	-1.1825	0.0200	1.6995
Tpi1	P17751	Triosephosphate isomerase	_ELASQPDVDGFLVGGASLKPEFVDIINAK_.3	#N/A	0.5057	-0.9837	0.0200	1.6991
Rnf14	Q9JI90	E3 ubiquitin-protein ligase RNF14	_LTYHGLS[Phospho (STY)]PC[Carbamidomethyl (C)]KVTAEK_.3	#N/A	1.8895	0.9180	0.0200	1.6991
Adam22	Q9R1V6	Disintegrin and metalloproteinase domain-containing protein 22	_KLAS[Phospho (STY)]GEC[Carbamidomethyl (C)]K_.2	Q9R1V6_S387_M1_Adam22	0.6322	-0.6616	0.0200	1.6987
Sptan1	P16546	Spectrin alpha chain, non-erythrocytic 1	_TKQDEVNAAWQR_.3	#N/A	0.4252	-1.2338	0.0200	1.6980
Mars1	Q68FL6	Methionine--tRNA ligase, cytoplasmic	_NNSELLNNLGNFINR_.2	#N/A	0.5976	-0.7428	0.0200	1.6980
Pde7b	Q9QXQ1	cAMP-specific 3',5'-cyclic phosphodiesterase 7B	_GSGQDLAGPAPETLEQTEGAT[Phospho (STY)]P_.2	Q9QXQ1_T445_M1_Pde7b	0.2454	-2.0265	0.0202	1.6951
Ptprz1	B9EKR1	Receptor-type tyrosine-protein phosphatase zeta	_QSPINIDEDLTQVNVNLKK_.3	#N/A	1.3356	0.4175	0.0202	1.6949
Tbr1	Q64336	T-box brain protein 1	_SKLS[Phospho (STY)]PVLDGVS[Phospho (STY)]ELRHSFDGSAADR_.4	Q64336_S84_M2_Tbr1	0.6582	-0.6035	0.0204	1.6902
Arfgap2	Q99K28	ADP-ribosylation factor GTPase-activating protein 2	_LAYQELQIDR_.2	#N/A	0.6976	-0.5195	0.0205	1.6875
Fasn	P19096	Fatty acid synthase	_TLEAVQDLLEQGR_.2	#N/A	0.6449	-0.6328	0.0208	1.6816
Atp1a3	Q6PIC6	Sodium/potassium-transporting ATPase subunit alpha-3	_KADIGVAM[Oxidation (M)]GIAGSDVSK_.2	#N/A	0.5528	-0.8552	0.0209	1.6807
Dnajc5	P60904	DnaJ homolog subfamily C member 5	_QRS[Phospho (STY)]LS[Phospho (STY)]TSGESLYHVLGLDK_.3	P60904_S10_M2_Dnajc5	0.6826	-0.5509	0.0209	1.6791
Rassf5	Q5EBH1	Ras association domain-containing protein 5	_S[Phospho (STY)]PESTLTPTLNQNVC[Carbamidomethyl (C)]K_.2	Q5EBH1_S177_M1_Rassf5	2.2680	1.1814	0.0210	1.6769
Hnrnpul1	Q8VDM6	Heterogeneous nuclear ribonucleoprotein U-like protein 1	_NYILDQTNVYGS[Phospho (STY)]AQR_.2	Q8VDM6_S513_M1_Hnrnpul1	2.0621	1.0441	0.0211	1.6763
Srrm2	Q8BTI8	Serine/arginine repetitive matrix protein 2	_VSSPVLETVQQRT[Phospho (STY)]PS[Phospho (STY)]RER_.3	Q8BTI8_S1372_M2_Srrm2	0.3080	-1.6990	0.0211	1.6754
Gap43	P06837	Neuromodulin	_QDEGKEDPEADQEHA_.2	#N/A	0.4773	-1.0669	0.0211	1.6751
Atn1	O35126	Atrophin-1	_TAS[Phospho (STY)]PPGPPQYSK_.2	O35126_S617_M1_Atn1	0.4190	-1.2550	0.0214	1.6705
Pdxk	Q8K183	Pyridoxal kinase	_KPDGSTVTQR_.2	#N/A	1.9500	0.9634	0.0214	1.6688
Srrm1	Q52KI8	Serine/arginine repetitive matrix protein 1	_VSHS[Phospho (STY)]PPPK_.2	Q52KI8_S657_M1_Srrm1	5.6731	2.5041	0.0214	1.6686
Actn1	Q7TPR4	Alpha-actinin-1	_LLETIDQLYLEYAK_.2	#N/A	1.5177	0.6019	0.0219	1.6605
Camk2a	P11798	Calcium/calmodulin-dependent protein kinase type II subunit alpha	_QET[Phospho (STY)]VDC[Carbamidomethyl (C)]LK_.2	#N/A	4.8904	2.2900	0.0219	1.6602
Anxa11	P97384	Annexin A11	_SDTSGHFQR_.2	#N/A	0.6064	-0.7216	0.0219	1.6594
Rab1A	P62821	Ras-related protein Rab-1A	_IQST[Phospho (STY)]PVK_.2	P62821_T195_M1_Rab1A	2.4522	1.2941	0.0220	1.6572
Cep170b	Q80U49	Centrosomal protein of 170 kDa protein B	_SQS[Phospho (STY)]FTHTPPADPK_.2	Q80U49_S421_M1_Cep170b	6.3521	2.6672	0.0223	1.6524
Gdap1l1	Q8VE33	Ganglioside-induced differentiation-associated protein 1-like 1	_LDHEEEPQLSEPYLSK_.3	#N/A	0.6313	-0.6636	0.0224	1.6502
Atp6v1b2	P62814	V-type proton ATPase subunit B, brain isoform	_TVYETLDIGWQLLR_.2	#N/A	1.6051	0.6827	0.0225	1.6481
Zeb2	Q9R0G7	Zinc finger E-box-binding homeobox 2	_S[Phospho (STY)]DHEEDNMEDGME_.2	#N/A	1.4700	0.5559	0.0225	1.6476
Wdr7	Q920I9	WD repeat-containing protein 7	_RQS[Phospho (STY)]REDSDPEYR_.3	Q920I9_S772_M1_Wdr7	0.6561	-0.6081	0.0225	1.6475
Sorbs1	Q62417	Sorbin and SH3 domain-containing protein 1	_SKS[Phospho (STY)]EM[Oxidation (M)]NYIEGEK_.2	#N/A	0.4718	-1.0838	0.0225	1.6469
Pgam1	Q9DBJ1	Phosphoglycerate mutase 1	_FSGWYDADLSPAGHEEAK_.2	#N/A	1.3307	0.4122	0.0226	1.6461
Rab11fip5	Q8R361	Rab11 family-interacting protein 5	_RTYS[Phospho (STY)]DEASQLR_.2	#N/A	2.7503	1.4596	0.0226	1.6452
Vars1	Q9Z1Q9	Valine--tRNA ligase	_DINLDVNR_.2	#N/A	0.5654	-0.8225	0.0227	1.6447
Kif2a	P28740	Kinesin-like protein KIF2A	_LIDIGNSC[Carbamidomethyl (C)]R_.2	#N/A	0.4390	-1.1877	0.0227	1.6436
H1-2	P15864	Histone H1.2	_ASGPPVSELITK_.2	#N/A	0.5293	-0.9177	0.0229	1.6411
Ndrg1	Q62433	Protein NDRG1	_SRT[Phospho (STY)]AS[Phospho (STY)]GSSVTSLEGTR_.2	#N/A	0.5406	-0.8874	0.0230	1.6375
Ptpra	P18052	Receptor-type tyrosine-protein phosphatase alpha	_QAGSHSNS[Phospho (STY)]FR_.2	P18052_S180_M1_Ptpra	0.7617	-0.3927	0.0230	1.6374
Peak1	Q69Z38	Inactive tyrosine-protein kinase PEAK1	_SLFTSQSSGEGEAHQTTES[Phospho (STY)]PT[Phospho (STY)]AK_.3	Q69Z38_S836_M2_Peak1	0.6371	-0.6504	0.0231	1.6366
Caskin1	Q6P9K8	Caskin-1	_SLPQS[Phospho (STY)]PTHR_.2	Q6P9K8_S828_M1_Caskin1	6.0388	2.5943	0.0231	1.6362
Wdr33	Q8K4P0	pre-mRNA 3' end processing protein WDR33	_[Acetyl (Protein N-term)]ATEIGS[Phospho (STY)]PPR_.2	Q8K4P0_S7_M1_Wdr33	0.1211	-3.0456	0.0233	1.6319
Tomm34	Q9CYG7	Mitochondrial import receptor subunit TOM34	_SSLSDISSLLQIEPR_.2	#N/A	1.6687	0.7387	0.0234	1.6303
Ppp3cb	P48453	Serine/threonine-protein phosphatase 2B catalytic subunit beta isoform	_VDEEIALR_.2	#N/A	1.6547	0.7266	0.0237	1.6260
Gpd1l	Q3ULJ0	Glycerol-3-phosphate dehydrogenase 1-like protein	_VC[Carbamidomethyl (C)]IVGSGNWGSAVAK_.2	#N/A	2.0334	1.0239	0.0238	1.6234
Setd2	E9Q5F9	Histone-lysine N-methyltransferase SETD2	_TGS[Phospho (STY)]PTPSNQLNDSPTFK_.2	E9Q5F9_S614_M1_Setd2	1.7755	0.8282	0.0238	1.6230
Usp11	Q99K46	Ubiquitin carboxyl-terminal hydrolase 11	_QSQTSSSDTPAS[Phospho (STY)]PVSSSTPNSDIM[Oxidation (M)]DIN_.3	#N/A	1.7624	0.8175	0.0239	1.6213
Add2	Q9QYB8	Beta-adducin	_SPAVS[Phospho (STY)]PSK_.2	#N/A	2.5515	1.3513	0.0240	1.6190
Nefh	P19246	Neurofilament heavy polypeptide	_IPSISTHIK_.2	#N/A	1.4062	0.4918	0.0241	1.6176
Synj1	Q8CHC4	Synaptojanin-1	_EIEAPKS[Phospho (STY)]PGTAR_.2	#N/A	1.8558	0.8920	0.0241	1.6172
Naa15	Q80UM3	N-alpha-acetyltransferase 15, NatA auxiliary subunit	_ITVNGDS[Phospho (STY)]SAETEELANEI_.2	Q80UM3_S854_M1_Naa15	1.3866	0.4715	0.0242	1.6166
Setd7	Q8VHL1	Histone-lysine N-methyltransferase SETD7	_IAYVYPDQR_.2	#N/A	1.8549	0.8914	0.0242	1.6163
Pold3	Q9EQ28	DNA polymerase delta subunit 3	_VDLS[Phospho (STY)]DEEAKETEHLK_.3	Q9EQ28_S306_M1_Pold3	0.4019	-1.3152	0.0243	1.6143
Pkp4	Q68FH0	Plakophilin-4	_S[Phospho (STY)]YEDPYC[Carbamidomethyl (C)]DDR_.2	Q68FH0_S1136_M1_Pkp4	2.0300	1.0215	0.0245	1.6103
Dmtn	Q9WV69	Dematin	_ESVGGS[Phospho (STY)]PQSK_.2	#N/A	1.3356	0.4175	0.0248	1.6057
Psmd3	P14685	26S proteasome non-ATPase regulatory subunit 3	_LQLDS[Phospho (STY)]PEDAEFIVAK_.2	P14685_S426_M1_Psmd3	0.6708	-0.5761	0.0249	1.6046
Amph	Q7TQF7	Amphiphysin	_LYEVM[Oxidation (M)]TK_.2	#N/A	1.6585	0.7299	0.0249	1.6042
Fry	E9Q8I9	Protein furry homolog	_LLLPGSSPS[Phospho (STY)]S[Phospho (STY)]PEDEVKDR_.2	E9Q8I9_S1382_M2_Fry	1.5102	0.5947	0.0249	1.6034
Scn3b	Q8BHK2	Sodium channel subunit beta-3	_AEEAAQENASDY[Phospho (STY)]LAIPSENK_.2	Q8BHK2_Y198_M1_Scn3b	0.2607	-1.9395	0.0249	1.6030
Ubqln1	Q8R317	Ubiquilin-1	_ALSNLESIPGGYNALR_.2	#N/A	1.5529	0.6350	0.0250	1.6021
Ermn	Q5EBJ4	Ermin	_YNTISYR_.2	#N/A	0.7287	-0.4566	0.0250	1.6021
Nefh	P19246	Neurofilament heavy polypeptide	_S[Phospho (STY)]LEGEAAALR_.2	P19246_S122_M1_Nefh	0.4641	-1.1075	0.0250	1.6014
Stxbp5l	Q5DQR4	Syntaxin-binding protein 5-like	_EEIQESLTSDYFQIC[Carbamidomethyl (C)]K_.2	#N/A	1.7936	0.8428	0.0253	1.5970
Ndufs2	Q91WD5	NADH dehydrogenase [ubiquinone] iron-sulfur protein 2, mitochondrial	_TSM[Oxidation (M)]ESLIHHFK_.3	#N/A	1.7298	0.7906	0.0256	1.5913
Arpp21	Q9DCB4	cAMP-regulated phosphoprotein 21	_TGS[Phospho (STY)]ESSSSAGSSGSLSR_.2	Q9DCB4_S408_M1_Arpp21	1.5286	0.6122	0.0256	1.5910
Wac	Q924H7	WW domain-containing adapter protein with coiled-coil	_S[Phospho (STY)]NS[Phospho (STY)]PENKYSDSTGHNK_.2	Q924H7_S62_M2_Wac	1.4886	0.5740	0.0257	1.5903
Dpysl5	Q9EQF6	Dihydropyrimidinase-related protein 5	_THC[Carbamidomethyl (C)]PIYLVNVSSISAGDVIAAAK_.3	#N/A	1.7715	0.8250	0.0257	1.5900
Glul	P15105	Glutamine synthetase	_QM[Oxidation (M)]YM[Oxidation (M)]SLPQGEK_.2	#N/A	0.5559	-0.8472	0.0258	1.5886
Rhoa	Q9QUI0	Transforming protein RhoA	_M[Oxidation (M)]KQEPVKPEEGR_.3	#N/A	0.5696	-0.8120	0.0258	1.5878
Trim2	Q9ESN6	Tripartite motif-containing protein 2	_ASIVDDIHSTFDELQK_.3	#N/A	0.5149	-0.9575	0.0259	1.5872
Sptbn1	Q62261	Spectrin beta chain, non-erythrocytic 1	_EAIC[Carbamidomethyl (C)]EVALDYKK_.3	#N/A	1.5375	0.6206	0.0259	1.5869
Tnr	Q8BYI9	Tenascin-R	_DGQEAVFAYYDKFAVEDSR_.3	#N/A	0.6938	-0.5274	0.0261	1.5835
Kcnq5	Q9JK45	Potassium voltage-gated channel subfamily KQT member 5	_RSPSTDITAEGS[Phospho (STY)]PTK_.2	#N/A	3.3339	1.7372	0.0262	1.5825
Hpca	P84075	Neuron-specific calcium-binding protein hippocalcin	_TFDTNSDGTIDFR_.2	#N/A	0.4253	-1.2333	0.0264	1.5791
Sptbn1	Q62261	Spectrin beta chain, non-erythrocytic 1	_DLVAIEAK_.2	#N/A	0.4819	-1.0532	0.0265	1.5773
Chl1	P70232	Neural cell adhesion molecule L1-like protein	_DETFGEYS[Phospho (STY)]DSDEKPLKGS[Phospho (STY)]LR_.3	P70232_S1148_M2_Chl1	1.3802	0.4649	0.0266	1.5754

Lingo1	Q9D1T0	Leucine-rich repeat and immunoglobulin-like domain-containing nogo receptor-interacting protein 1	_S[Phospho (STY)]DAGISSADAPR_.2	#N/A	1.3044	0.3834	0.0266	1.5748
Nos1	Q9Z0J4	Nitric oxide synthase, brain	_KSS[Phospho (STY)]GDGPDLR_.2	Q9Z0J4_S858_M1_Nos1	2.4359	1.2844	0.0267	1.5732
Ociad2	Q9D8W7	OCIA domain-containing protein 2	_FHSFEDQLR_.2	#N/A	1.6955	0.7617	0.0269	1.5702
Dpp6	Q9Z218	Dipeptidyl aminopeptidase-like protein 6	_HLYSANTVDDFNR_.2	#N/A	0.6098	-0.7136	0.0270	1.5681
Dlgap3	Q6PFD5	Disks large-associated protein 3	_WRPSIGVQVETIS[Phospho (STY)]DSDTENR_.3	Q6PFD5_S641_M1_Dlgap3	0.5168	-0.9523	0.0271	1.5674
Dcx	O88809	Neuronal migration protein doublecortin	_GNPSAAAGPKAS[Phospho (STY)]PTPQK_.2	O88809_S287_M1_Dcx	0.6862	-0.5433	0.0271	1.5673
Eepd1	Q3TGW2	Endonuclease/exonuclease/phosphatase family domain-containing protein 1	_GNSAQHS[Phospho (STY)]PSSLRR_.3	#N/A	0.2958	-1.7574	0.0271	1.5668
Ndrg3	Q9QYF9	Protein NDRG3	_S[Phospho (STY)]RT[Phospho (STY)]HSTSSSIGSGESPFSR_.3	Q9QYF9_T329_M2_Ndrg3	0.6244	-0.6795	0.0272	1.5662
Vps26b	Q8C0E2	Vacuolar protein sorting-associated protein 26B	_ETTGTGPNVYHENDTIAK_.3	#N/A	1.6108	0.6878	0.0273	1.5631
Ncbp3	Q8BZR9	Nuclear cap-binding protein subunit 3	_AEAPAGPALGLPS[Phospho (STY)]PEVESGLER_.2	Q8BZR9_S25_M1_Ncbp3	0.7255	-0.4630	0.0274	1.5629
Phyhipl	Q8BGT8	Phytanoyl-CoA hydroxylase-interacting protein-like	_ISWDMDSK_.2	#N/A	1.6052	0.6827	0.0275	1.5610
Ermn	Q5EBJ4	Ermin	_SRPVDILHS[Phospho (STY)]K_.3	Q5EBJ4_S166_M1_Ermn	0.6883	-0.5389	0.0275	1.5603
Bsn	O88737	Protein bassoon	_YSS[Phospho (STY)]VSNIYSDHR_.2	O88737_S2068_M1_Bsn	1.8226	0.8660	0.0276	1.5598
Pccb	Q99MN9	Propionyl-CoA carboxylase beta chain, mitochondrial	_IM[Oxidation (M)]DQAITVGAPVIGLNDSGGAR_.2	#N/A	0.7151	-0.4838	0.0277	1.5572
Pitpnm1	O35954	Membrane-associated phosphatidylinositol transfer protein 1	_C[Carbamidomethyl (C)]NTGSEGPEAQT[Phospho (STY)]PGK_.2	O35954_T270_M1_Pitpnm1	1.6797	0.7482	0.0278	1.5557
Smpd3	Q9JJY3	Sphingomyelin phosphodiesterase 3	_TAS[Phospho (STY)]VEYKGDGGR_.2	Q9JJY3_S208_M1_Smpd3	2.2849	1.1922	0.0280	1.5535
Cpe	Q00493	Carboxypeptidase E	_YIGNM[Oxidation (M)]HGNEAVGR_.3	#N/A	0.5597	-0.8372	0.0280	1.5525
Nefm	P08553	Neurofilament medium polypeptide	_KAES[Phospho (STY)]PVKEK_.2	#N/A	1.3942	0.4794	0.0281	1.5509
Dnm3	Q8BZ98	Dynamin-3	_LQGQLLSIEHEVEAFK_.3	#N/A	1.6896	0.7566	0.0281	1.5507
Prkacb	P68181	cAMP-dependent protein kinase catalytic subunit beta	_ATEQYYAMK_.2	#N/A	0.6905	-0.5343	0.0284	1.5463
Vcpip1	Q8CDG3	Deubiquitinating protein VCPIP1	_S[Phospho (STY)]RESS[Phospho (STY)]PSHGLLK_.2	Q8CDG3_S993_M2_Vcpip1	0.7131	-0.4879	0.0286	1.5441
Pdcd4	Q61823	Programmed cell death protein 4	_SGVAVPTS[Phospho (STY)]PKGR_.2	#N/A	0.3430	-1.5435	0.0286	1.5440
Ak1	Q9R0Y5	Adenylate kinase isoenzyme 1	_AEVSSGSER_.2	#N/A	0.4986	-1.0039	0.0287	1.5421
Znf318	Q99PP2	Zinc finger protein 318	_RAS[Phospho (STY)]PS[Phospho (STY)]PPRGR_.3	#N/A	1.7009	0.7663	0.0287	1.5416
Mast1	Q9R1L5	Microtubule-associated serine/threonine-protein kinase 1	_HFGS[Phospho (STY)]TESITDEDGGR_.2	#N/A	0.6187	-0.6926	0.0288	1.5413
Tra2b	P62996	Transformer-2 protein homolog beta	_S[Phospho (STY)]RS[Phospho (STY)]YS[Phospho (STY)]RDYR_.2	P62996_S87_M3_Tra2b	0.3582	-1.4810	0.0289	1.5398
Gda	Q9R111	Guanine deaminase	_AVM[Oxidation (M)]VSNVLLINK_.2	#N/A	0.3728	-1.4236	0.0289	1.5388
Arhgef7	Q9ES28	Rho guanine nucleotide exchange factor 7	_LETFDANDLYQGQNFNK_.2	#N/A	1.5517	0.6338	0.0290	1.5381
Cct6a	P80317	T-complex protein 1 subunit zeta	_VLAQNSGFDLQETLVK_.2	#N/A	1.9146	0.9370	0.0290	1.5370
Mtx2	O88441	Metaxin-2	_[Acetyl (Protein N-term)]SLVAEAFVSQIAATEPWPENATLYQQLR_.3	#N/A	0.6805	-0.5553	0.0292	1.5354
Lin7c	O88952	Protein lin-7 homolog C	_VLQSEFC[Carbamidomethyl (C)]NAVR_.2	#N/A	1.3246	0.4056	0.0292	1.5347
Atat1	Q8K341	Alpha-tubulin N-acetyltransferase 1	_SSS[Phospho (STY)]LGNSPDR_.2	Q8K341_S272_M1_Atat1	4.3407	2.1179	0.0292	1.5345
Ppp4r3a	Q6P2K6	Serine/threonine-protein phosphatase 4 regulatory subunit 3A	_TNLSGRQS[Phospho (STY)]PSFK_.2	Q6P2K6_S728_M1_Ppp4r3a	2.1568	1.1089	0.0292	1.5341
Kcnab2	P62482	Voltage-gated potassium channel subunit beta-2	_LQLEYVDVVFANRPDPNTPM[Oxidation (M)]EETVR_.3	#N/A	0.6977	-0.5194	0.0294	1.5317
Pgrmc1	O55022	Membrane-associated progesterone receptor component 1	_GDQPGASGDNDDDEPPPLPR_.2	#N/A	0.7449	-0.4248	0.0296	1.5288
Aco2	Q99KI0	Aconitate hydratase, mitochondrial	_HPNGTQETILLNHTFNETQIEWFR_.4	#N/A	0.6534	-0.6140	0.0297	1.5273
Fkbp15	Q6P9Q6	FK506-binding protein 15	_VTS[Phospho (STY)]LEEELTDLR_.2	Q6P9Q6_S722_M1_Fkbp15	0.6534	-0.6139	0.0297	1.5265
Pdcd5	P56812	Programmed cell death protein 5	_KVM[Oxidation (M)]DS[Phospho (STY)]DEDDADY_.2	#N/A	1.7967	0.8454	0.0298	1.5252
Sgta	Q8BJU0	Small glutamine-rich tetratricopeptide repeat-containing protein alpha	_APDRT[Phospho (STY)]PPSEEDSAEAER_.3	#N/A	1.8975	0.9241	0.0299	1.5245
Pcm1	Q9R0L6	Pericentriolar material 1 protein	_HIS[Phospho (STY)]ESDER_.2	Q9R0L6_S1432_M1_Pcm1	0.6535	-0.6137	0.0300	1.5229
Cic	Q924A2	Protein capicua homolog	_SS[Phospho (STY)]PPPPLPAEERPGTK_.2	Q924A2_S2148_M1_Cic	5.4391	2.4434	0.0301	1.5207
Stambpl1	Q76N33	AMSH-like protease	_SDGSNFANYS[Phospho (STY)]PPVNR_.2	Q76N33_S242_M1_Stambpl1	1.7472	0.8050	0.0302	1.5194
Napb	P28663	Beta-soluble NSF attachment protein	_LLEAHEEQNSEAYTEAVK_.3	#N/A	1.6082	0.6855	0.0303	1.5187
Actn1	Q7TPR4	Alpha-actinin-1	_AIM[Oxidation (M)]TYVSSFYHAFSGAQK_.2	#N/A	1.4152	0.5010	0.0303	1.5186
Znf608	Q56A10	Zinc finger protein 608	_DRHS[Phospho (STY)]PFSQR_.3	Q56A10_S1452_M1_Znf608	0.5875	-0.7673	0.0304	1.5170
Bcar1	Q61140	Breast cancer anti-estrogen resistance protein 1	_GS[Phospho (STY)]PGFTPEDLDR_.2	Q61140_S572_M1_Bcar1	0.6477	-0.6266	0.0306	1.5148
Esd	Q9R0P3	S-formylglutathione hydrolase	_AYDATC[Carbamidomethyl (C)]LVK_.2	#N/A	0.4377	-1.1920	0.0306	1.5140
Vps35	Q9EQH3	Vacuolar protein sorting-associated protein 35	_LFDIFSQQVATVIQSR_.2	#N/A	0.6458	-0.6308	0.0306	1.5139
Pdia6	Q922R8	Protein disulfide-isomerase A6	_LAAVDATVNQVLASR_.2	#N/A	1.8643	0.8986	0.0307	1.5134
Rad23b	P54728	UV excision repair protein RAD23 homolog B	_NENLAANFLLQQNFDED_.2	#N/A	0.5387	-0.8925	0.0307	1.5132
Elfn1	Q8C8T7	Protein ELFN1	_AVAAAAGS[Phospho (STY)]LKK_.2	Q8C8T7_S460_M1_Elfn1	1.3730	0.4573	0.0307	1.5125
Add2	Q9QYB8	Beta-adducin	_EQDHFLISPK_.2	#N/A	1.4609	0.5469	0.0307	1.5124
Prkaca	P05132	cAMP-dependent protein kinase catalytic subunit alpha	_WETPSQNTAQLDQFDR_.2	#N/A	2.7659	1.4678	0.0309	1.5104
Top2b	Q64511	DNA topoisomerase 2-beta	_IVETINSDS[Phospho (STY)]DSEFGIPK_.2	#N/A	1.3269	0.4080	0.0310	1.5080
Speg	Q62407	Striated muscle-specific serine/threonine-protein kinase	_SRS[Phospho (STY)]VQDLR_.2	Q62407_S2396_M1_Speg	9.2145	3.2039	0.0311	1.5073
Usp34	Q6ZQ93	Ubiquitin carboxyl-terminal hydrolase 34	_DSSIIDPGTEQDLPS[Phospho (STY)]PENSSVK_.2	Q6ZQ93_S3443_M1_Usp34	2.7453	1.4570	0.0314	1.5030
Setd2	E9Q5F9	Histone-lysine N-methyltransferase SETD2	_RRGS[Phospho (STY)]LS[Phospho (STY)]PPSSAYER_.3	#N/A	0.7034	-0.5077	0.0317	1.4991
Dpysl4	O35098	Dihydropyrimidinase-related protein 4	_C[Carbamidomethyl (C)]QC[Carbamidomethyl (C)]TDGQIYEIFSLIR_.2	#N/A	0.5737	-0.8018	0.0317	1.4991
Ppp1r1b	Q60829	Protein phosphatase 1 regulatory subunit 1B	_ELGYPQEDDEEDEDEEEDEEEDSQAEVLK_.3	#N/A	0.7143	-0.4855	0.0318	1.4976
Epb41l2	O70318	Band 4.1-like protein 2	_VTEGTIREEQEEC[Carbamidomethyl (C)]DEELEEEPGQGAK_.3	#N/A	0.6879	-0.5397	0.0318	1.4970
Ap1b1	O35643	AP-1 complex subunit beta-1	_C[Carbamidomethyl (C)]LKDEDPYVRK_.3	#N/A	0.6069	-0.7206	0.0320	1.4954
Dpysl5	Q9EQF6	Dihydropyrimidinase-related protein 5	_TPYLGDVAIVVHPGK_.3	#N/A	0.6837	-0.5486	0.0321	1.4940
Otud7a	Q8R554	OTU domain-containing protein 7A	_AS[Phospho (STY)]PGPTAAQPTQLVLK_.2	Q8R554_S711_M1_Otud7a	1.6305	0.7053	0.0322	1.4921
Npm1	Q61937	Nucleophosmin	_C[Carbamidomethyl (C)]GSGPVHISGQHLVAVEEDAES[Phospho (STY)]EDEDEEDVKLLGM[Oxidation (M)]SGK_.5	Q61937_S125_M1_Npm1	1.6162	0.6926	0.0323	1.4914
Ppfia2	Q8BSS9	Liprin-alpha-2	_S[Phospho (STY)]LGDHEWNR_.2	#N/A	1.6080	0.6853	0.0323	1.4914
Tmem38a	Q3TMP8	Trimeric intracellular cation channel type A	_AKEELGEGS[Phospho (STY)]RK_.3	#N/A	1.6150	0.6916	0.0323	1.4912
Ripor2	Q80U16	Rho family-interacting cell polarization regulator 2	_SQS[Phospho (STY)]FAGFSGLQER_.2	Q80U16_S46_M1_Ripor2	0.3428	-1.5444	0.0323	1.4903
Pkp4	Q68FH0	Plakophilin-4	_GLYPGSSKPSPIY[Phospho (STY)]ISSYS[Phospho (STY)]SPAR_.3	Q68FH0_S1098_M2_Pkp4	0.3631	-1.4614	0.0323	1.4902
Lzts3	A2AHG0	Leucine zipper putative tumor suppressor 3	_LERIES[Phospho (STY)]TEI_.2	A2AHG0_S697_M1_Lzts3	0.6544	-0.6118	0.0324	1.4894
Sh3glb2	Q8R3V5	Endophilin-B2	_SQTTYYAQC[Carbamidomethyl (C)]YR_.2	#N/A	0.4659	-1.1020	0.0324	1.4892
Gnl1	P36916	Guanine nucleotide-binding protein-like 1	_GLQDGLRS[Phospho (STY)]SS[Phospho (STY)]NSR_.2	P36916_S32_M2_Gnl1	0.7185	-0.4769	0.0326	1.4869
Ywhaz	P63101	14-3-3 protein zeta/delta	_TAFDEAIAELDTLSEESYKDSTLIMQLLR_.3	#N/A	0.6843	-0.5473	0.0326	1.4865
Otud4	B2RRE7	OTU domain-containing protein 4	_LQRPKEES[Phospho (STY)]S[Phospho (STY)]EDENEVSNILR_.3	#N/A	0.7220	-0.4699	0.0327	1.4850
Mical3	Q8CJ19	[F-actin]-monooxygenase MICAL3	_SVFS[Phospho (STY)]GYKK_.2	Q8CJ19_S1736_M1_Mical3	2.2348	1.1601	0.0327	1.4850
Cmpk1	Q9DBP5	UMP-CMP kinase	_KNPDSQYGELIEK_.3	#N/A	1.5908	0.6698	0.0328	1.4839
Ubap2l	Q80X50	Ubiquitin-associated protein 2-like	_NQDEC[Carbamidomethyl (C)]VIALHDC[Carbamidomethyl (C)]NGDVNR_.3	#N/A	1.3056	0.3847	0.0328	1.4835
Pnn	O35691	Pinin	_RGFS[Phospho (STY)]DSGGGPPAK_.2	#N/A	1.5898	0.6688	0.0329	1.4834
Anxa5	P48036	Annexin A5	_DLVDDLKSELTGK_.2	#N/A	0.7347	-0.4449	0.0329	1.4829
Dst	Q91ZU6	Dystonin	_QLS[Phospho (STY)]LLDSR_.2	Q91ZU6_S5407_M1_Dst	1.5876	0.6668	0.0329	1.4823

Arpp21	Q9DCB4	cAMP-regulated phosphoprotein 21	_TAS[Phospho (STY)]FGGITVLTR_.2	Q9DCB4_S381_M1_Arpp21	0.2600	-1.9436	0.0329	1.4823
Rpl35a	O55142	60S ribosomal protein L35a	_IEGVYAR_.2	#N/A	0.5410	-0.8864	0.0330	1.4816
Lcp1	Q61233	Plastin-2	_GS[Phospho (STY)]VSDEEMMELR_.2	Q61233_S5_M1_Lcp1	1.5855	0.6649	0.0330	1.4812
Pitpnm1	O35954	Membrane-associated phosphatidylinositol transfer protein 1	_SSYSSQHGGGVS[Phospho (STY)]PQS[Phospho (STY)]LSEWR_.3	O35954_S326_M2_Pitpnm1	0.6546	-0.6113	0.0331	1.4807
Camkk2	Q8C078	Calcium/calmodulin-dependent protein kinase kinase 2	_KMS[Phospho (STY)]LQEPSQGGPASSSNSLDMNGR_.3	Q8C078_S99_M1_Camkk2	0.7145	-0.4850	0.0331	1.4804
Gnl1	P36916	Guanine nucleotide-binding protein-like 1	_ERREEQTDT[Phospho (STY)]S[Phospho (STY)]DGESVTHHIR_.4	#N/A	0.1827	-2.4522	0.0331	1.4800
Septin7	O55131	Septin-7	_NLEAQHKELEEK_.2	#N/A	0.5994	-0.7385	0.0333	1.4772
Idh3a	Q9D6R2	Isocitrate dehydrogenase [NAD] subunit alpha, mitochondrial	_EVAENC[Carbamidomethyl (C)]KDIK_.2	#N/A	0.7358	-0.4427	0.0334	1.4758
Gatd3a	Q9D172	Glutamine amidotransferase-like class 1 domain-containing protein 3A, mitochondrial	_VNKEVER_.2	#N/A	0.6094	-0.7146	0.0335	1.4755
Npr2	Q6VVW5	Atrial natriuretic peptide receptor 2	_GSS[Phospho (STY)]YGSLM[Oxidation (M)]TAHGK_.2	Q6VVW5_S523_M1_Npr2	0.2390	-2.0648	0.0338	1.4714
Map1b	P14873	Microtubule-associated protein 1B	_ESVVS[Phospho (STY)]GDDR_.2	#N/A	0.3075	-1.7014	0.0338	1.4712
Tjp2	Q9Z0U1	Tight junction protein ZO-2	_HSSHDMLSHS[Phospho (STY)]WEGNR_.3	#N/A	1.7357	0.7955	0.0338	1.4712
Ak3	Q9WTP7	GTP:AMP phosphotransferase AK3, mitochondrial	_GVLETFSGTETNK_.2	#N/A	2.3584	1.2378	0.0339	1.4698
Rpl18a	P62717	60S ribosomal protein L18a	_DLTTAGAVTQC[Carbamidomethyl (C)]YR_.2	#N/A	1.5426	0.6254	0.0339	1.4694
Ckmt1	P30275	Creatine kinase U-type, mitochondrial	_GLSLPPAC[Carbamidomethyl (C)]TR_.2	#N/A	4.6285	2.2105	0.0339	1.4692
Rcn2	Q8BP92	Reticulocalbin-2	_HYAMQEAK_.2	#N/A	1.5681	0.6491	0.0339	1.4692
Nav1	Q8CH77	Neuron navigator 1	_SLAESGLNWFS[Phospho (STY)]ES[Phospho (STY)]EEKTPK_.3	Q8CH77_S477_M2_Nav1	1.5435	0.6262	0.0340	1.4691
Aars1	Q8BGQ7	Alanine--tRNA ligase, cytoplasmic	_M[Oxidation (M)]ALELLTQEFGIPVER_.2	#N/A	0.4140	-1.2724	0.0340	1.4688
Ssh1	Q76I79	Protein phosphatase Slingshot homolog 1	_SDS[Phospho (STY)]LPQVEELEK_.2	Q76I79_S585_M1_Ssh1	3.9035	1.9648	0.0340	1.4682
Celsr2	Q9R0M0	Cadherin EGF LAG seven-pass G-type receptor 2	_STLTSSYNC[Carbamidomethyl (C)]PS[Phospho (STY)]PYADGR_.2	Q9R0M0_S2648_M1_Celsr2	0.5829	-0.7787	0.0340	1.4680
Hecw1	Q8K4P8	E3 ubiquitin-protein ligase HECW1	_SYS[Phospho (STY)]AGEASEVSR_.2	Q8K4P8_S1073_M1_Hecw1	1.4312	0.5172	0.0340	1.4680
Bin1	O08539	Myc box-dependent-interacting protein 1	_IAENNDLLWM[Oxidation (M)]DYHQK_.3	#N/A	1.3680	0.4521	0.0341	1.4679
Atxn2	O70305	Ataxin-2	_MSSEGPPRM[Oxidation (M)]S[Phospho (STY)]PK_.2	O70305_S593_M1_Atxn2	1.8187	0.8629	0.0341	1.4679
Rpl34	Q9D1R9	60S ribosomal protein L34	_LSYNTASNK_.2	#N/A	0.6549	-0.6107	0.0341	1.4678
Psma6	Q9QUM9	Proteasome subunit alpha type-6	_HITIFSPEGR_.2	#N/A	0.4349	-1.2014	0.0342	1.4665
Cplx1	P63040	Complexin-1	_EAEAQAAM[Oxidation (M)]EANSEGS[Phospho (STY)]LTRPK_.2	#N/A	2.0642	1.0456	0.0342	1.4662
Gnb5	P62881	Guanine nucleotide-binding protein subunit beta-5	_VSPDGTAFC[Carbamidomethyl (C)]SGSWDHTLR_.2	#N/A	0.5351	-0.9021	0.0343	1.4651
Tmem63c	Q8CBX0	Calcium permeable stress-gated cation channel 1	_LALLIHNDS[Phospho (STY)]LTSLIYGEQSEK_.3	Q8CBX0_S75_M1_Tmem63c	1.5456	0.6282	0.0343	1.4647
Rps3a	P97351	40S ribosomal protein S3a	_EVQTNDLKEVVNK_.3	#N/A	1.5459	0.6284	0.0344	1.4638
Arhgap21	Q6DFV3	Rho GTPase-activating protein 21	_KGS[Phospho (STY)]SSEVFSDAAR_.2	Q6DFV3_S917_M1_Arhgap21	0.5256	-0.9280	0.0345	1.4622
Rpl18	P35980	60S ribosomal protein L18	_ILTFDQLALESPK_.2	#N/A	0.6130	-0.7061	0.0345	1.4618
Atp8a1	P70704	Phospholipid-transporting ATPase IA	_SESLQQNLLHGYAFSQDENGIVSQSEVIR_.3	#N/A	0.7107	-0.4927	0.0345	1.4617
Gigyf2	Q6Y7W8	GRB10-interacting GYF protein 2	_ALSSGGSITS[Phospho (STY)]PPLSPALPK_.2	Q6Y7W8_S26_M1_Gigyf2	0.7020	-0.5106	0.0345	1.4617
Atxn7l3	A2AWT3	Ataxin-7-like protein 3	_LQWDGSSDLS[Phospho (STY)]PSDSGSSK_.2	A2AWT3_S281_M1_Atxn7l3	0.3810	-1.3923	0.0346	1.4615
Dmxl2	Q8BPN8	DmX-like protein 2	_LDHELSLDRES[Phospho (STY)]EAGTGSSEHEDGEREGS[Phospho (STY)]PR_.4	Q8BPN8_S456_M2_Dmxl2	1.5465	0.6290	0.0346	1.4612
Cryab	P23927	Alpha-crystallin B chain	_HEERQDEHGFISR_.4	#N/A	1.5589	0.6405	0.0346	1.4607
Map1a	Q9QYR6	Microtubule-associated protein 1A	_DEAS[Phospho (STY)]EEKEQVLEQK_.2	#N/A	0.6550	-0.6103	0.0347	1.4601
Thrap3	Q569Z6	Thyroid hormone receptor-associated protein 3	_S[Phospho (STY)]YS[Phospho (STY)]PAHNR_.2	Q569Z6_S55_M2_Thrap3	0.3039	-1.7183	0.0347	1.4601
Ccser2	Q3UHI0	Serine-rich coiled-coil domain-containing protein 2	_HTSGNNLIS[Phospho (STY)]PDTDYR_.2	#N/A	0.2614	-1.9359	0.0349	1.4567
Palm	Q9Z0P4	Paralemmin-1	_PQSAS[Phospho (STY)]PAKEEQK_.2	#N/A	1.4287	0.5147	0.0350	1.4560
Pbx2	O35984	Pre-B-cell leukemia transcription factor 2	_TAVSVAQGGHS[Phospho (STY)]R_.2	O35984_S330_M1_Pbx2	1.9897	0.9925	0.0350	1.4553
Cpne6	Q9Z140	Copine-6	_YSVLLVLTDGVVSDM[Oxidation (M)]AETR_.2	#N/A	0.6806	-0.5552	0.0351	1.4548
Apc	Q61315	Adenomatous polyposis coli protein	_T[Phospho (STY)]GSSSSILSASSESSEK_.2	Q61315_T2567_M1_Apc	1.5377	0.6207	0.0351	1.4548
Syt3	O35681	Synaptotagmin-3	_KLHFS[Phospho (STY)]VYDFDR_.3	#N/A	0.1973	-2.3413	0.0351	1.4546
Dhx30	Q99PU8	ATP-dependent RNA helicase DHX30	_GGS[Phospho (STY)]FEM[Oxidation (M)]TDDDSAIR_.2	#N/A	1.7296	0.7905	0.0351	1.4543
Ak1	Q9R0Y5	Adenylate kinase isoenzyme 1	_LETYYNATEPVISFYDKR_.3	#N/A	1.4484	0.5345	0.0352	1.4535
Atp5pb	Q9CQQ7	ATP synthase F(0) complex subunit B1, mitochondrial	_LDYHISVQNM[Oxidation (M)]M[Oxidation (M)]R_.3	#N/A	1.5503	0.6326	0.0354	1.4514
Klc1	O88447	Kinesin light chain 1	_RS[Phospho (STY)]RES[Phospho (STY)]LNMDVVK_.3	#N/A	0.6553	-0.6099	0.0354	1.4507
Plcl2	Q8K394	Inactive phospholipase C-like protein 2	_TPS[Phospho (STY)]PAAGPVPR_.2	Q8K394_S87_M1_Plcl2	0.6764	-0.5640	0.0354	1.4506
Sptbn1	Q62261	Spectrin beta chain, non-erythrocytic 1	_DVSSVELLM[Oxidation (M)]NNHQGIK_.2	#N/A	0.7367	-0.4409	0.0356	1.4489
Wdr7	Q920I9	WD repeat-containing protein 7	_LPASC[Carbamidomethyl (C)]LPASDSFR_.2	#N/A	0.3916	-1.3527	0.0356	1.4487
Kcnab2	P62482	Voltage-gated potassium channel subunit beta-2	_QTGS[Phospho (STY)]PGM[Oxidation (M)]IYSTR_.2	#N/A	1.8859	0.9152	0.0356	1.4485
Eef2	P58252	Elongation factor 2	_YFDPANGK_.2	#N/A	0.3992	-1.3249	0.0357	1.4479
Apeh	Q8R146	Acylamino-acid-releasing enzyme	_ALDVSAS[Phospho (STY)]DEEM[Oxidation (M)]ARPK_.2	#N/A	0.6691	-0.5796	0.0357	1.4470
Arhgef17	Q80U35	Rho guanine nucleotide exchange factor 17	_SLS[Phospho (STY)]DPIPQR_.2	Q80U35_S728_M1_Arhgef17	0.2295	-2.1235	0.0358	1.4462
Agap1	Q8BXK8	Arf-GAP with GTPase, ANK repeat and PH domain-containing protein 1	_ATSAC[Carbamidomethyl (C)]APISS[Phospho (STY)]PK_.2	Q8BXK8_S422_M1_Agap1	4.0272	2.0098	0.0359	1.4450
Cnnm3	Q32NY4	Metal transporter CNNM3	_IPVYEEER_.2	#N/A	0.5194	-0.9452	0.0359	1.4449
Mphosph8	Q3TYA6	M-phase phosphoprotein 8	_GTVAVGDS[Phospho (STY)]EEDGEDVFEVER_.2	Q3TYA6_S51_M1_Mphosph8	0.7365	-0.4412	0.0360	1.4440
Rnh1	Q91VI7	Ribonuclease inhibitor	_ELSLASNELKDEGAR_.3	#N/A	0.5148	-0.9580	0.0360	1.4436
Dbnl	Q62418	Drebrin-like protein	_VAGTGEGGLEELVEELNSGK_.2	#N/A	0.7087	-0.4967	0.0362	1.4412
Pdhb	Q9D051	Pyruvate dehydrogenase E1 component subunit beta, mitochondrial	_EAINQGM[Oxidation (M)]DEELERDEK_.3	#N/A	0.7507	-0.4137	0.0363	1.4405
Cpne1	Q8C166	Copine-1	_FAAQAAQQR_.2	#N/A	4.8839	2.2880	0.0363	1.4398
Ndufa5	Q9CPP6	NADH dehydrogenase [ubiquinone] 1 alpha subcomplex subunit 5	_LEALLQGGEVEEVILQAEK_.2	#N/A	0.7147	-0.4845	0.0363	1.4395
Elavl2	Q60899	ELAV-like protein 2	_FANNPSQK_.2	#N/A	1.5692	0.6500	0.0364	1.4387
Rbx1	P62878	E3 ubiquitin-protein ligase RBX1	_QVC[Carbamidomethyl (C)]PLDNR_.2	#N/A	0.5454	-0.8746	0.0364	1.4386
Rbbp6	P97868	E3 ubiquitin-protein ligase RBBP6	_PSANREDFS[Phospho (STY)]PER_.2	#N/A	0.4450	-1.1681	0.0365	1.4383
Pacs1	Q8K212	Phosphofurin acidic cluster sorting protein 1	_S[Phospho (STY)]TPLKER_.2	Q8K212_S508_M1_Pacs1	0.7237	-0.4666	0.0365	1.4382
Samm50	Q8BGH2	Sorting and assembly machinery component 50 homolog	_SSLSHAM[Oxidation (M)]VIDSR_.2	#N/A	1.5581	0.6398	0.0365	1.4377
Cct3	P80318	T-complex protein 1 subunit gamma	_NLQDAM[Oxidation (M)]QVC[Carbamidomethyl (C)]R_.2	#N/A	0.3339	-1.5824	0.0365	1.4377
Ank3	G5E8K5	Ankyrin-3	_NEDLAELLNGM[Oxidation (M)]DEELDS[Phospho (STY)]PEELGTKR_.3	#N/A	1.5559	0.6378	0.0365	1.4373
Akap5	D3YVF0	A-kinase anchor protein 5	_QFLISM[Oxidation (M)]ENEQVGVFANDSDFEGR_.3	#N/A	1.6526	0.7248	0.0367	1.4353
Armcx3	Q8BHS6	Armadillo repeat-containing X-linked protein 3	_RAS[Phospho (STY)]PNSDDTVLSPQELQK_.3	Q8BHS6_S110_M1_Armcx3	1.6042	0.6819	0.0368	1.4338
Tcf20	Q9EPQ8	Transcription factor 20	_LSTS[Phospho (STY)]PATRDEAAS[Phospho (STY)]PGAK_.3	Q9EPQ8_S603_M2_Tcf20	1.5420	0.6248	0.0369	1.4335
Myh9	Q8VDD5	Myosin-9	_ELETQISELQEDLESER_.2	#N/A	1.5410	0.6239	0.0369	1.4331
Abat	P61922	4-aminobutyrate aminotransferase, mitochondrial	_TLLTGLLDLQAQYPQFISR_.2	#N/A	0.7233	-0.4673	0.0369	1.4328
Gfap	P03995	Glial fibrillary acidic protein	_GTNES[Phospho (STY)]LER_.2	P03995_S302_M1_Gfap	1.5510	0.6332	0.0369	1.4326
Cit	P49025	Citron Rho-interacting kinase	_S[Phospho (STY)]PEHQPSAMSLLAPPSSR_.3	P49025_S1348_M1_Cit	1.5352	0.6184	0.0370	1.4316
Pde4a	O89084	cAMP-specific 3',5'-cyclic phosphodiesterase 4A	_QS[Phospho (STY)]PSPTLEEEPGVLSDPALPDK_.2	O89084_S672_M1_Pde4a	1.5350	0.6182	0.0370	1.4313
Pkm	P52480	Pyruvate kinase PKM	_LNFSHGTHEYHAETIK_.3	#N/A	1.8210	0.8647	0.0371	1.4312

Tox4	Q8BU11	TOX high mobility group box family member 4	_LSTTPS[Phospho (STY)]PT[Phospho (STY)]NSLHEDGVDDFRR_.3	Q8BU11_S178_M2_Tox4	0.7252	-0.4636	0.0371	1.4308
Sowaha	Q8BLS7	Ankyrin repeat domain-containing protein SOWAHA	_QRS[Phospho (STY)]EEPSPR_.2	Q8BLS7_S242_M1_Sowaha	1.7502	0.8075	0.0372	1.4296
Bcas3	Q8CCN5	Breast carcinoma-amplified sequence 3 homolog	_VKS[Phospho (STY)]PPQISPSK_.2	Q8CCN5_S565_M1_Bcas3	13.4173	3.7460	0.0372	1.4296
Samm50	Q8BGH2	Sorting and assembly machinery component 50 homolog	_DVVVQHVHFDGLGR_.3	#N/A	1.5314	0.6149	0.0372	1.4293
H1-2	P15864	Histone H1.2	_ALAAAGYDVEKNNSR_.2	#N/A	1.3597	0.4433	0.0374	1.4276
Znf148	Q61624	Zinc finger protein 148	_AGMTSS[Phospho (STY)]PDATTGQTFG_.2	Q61624_S784_M1_Znf148	1.3800	0.4646	0.0375	1.4261
Dock7	Q8R1A4	Dedicator of cytokinesis protein 7	_KGS[Phospho (STY)]WSER_.2	Q8R1A4_S432_M1_Dock7	2.2021	1.1388	0.0375	1.4258
Slc25a3	Q8VEM8	Phosphate carrier protein, mitochondrial	_YYALC[Carbamidomethyl (C)]GFGGVLSC[Carbamidomethyl (C)]GLTHTAVVPLDLVK_.3	#N/A	0.7404	-0.4336	0.0375	1.4255
Rem2	Q8VEL9	GTP-binding protein REM 2	_GS[Phospho (STY)]MPVPYK_.2	#N/A	0.7405	-0.4334	0.0375	1.4255
Fam171a2	A2A699	Protein FAM171A2	_TGLC[Carbamidomethyl (C)]SPEDNS[Phospho (STY)]LTPLLDEVVAPEGR_.3	A2A699_S744_M1_Fam171a2	0.5758	-0.7964	0.0376	1.4253
Rbm8a	Q9CWZ3	RNA-binding protein 8A	_[Acetyl (Protein N-term)]ADVLDLHEAGGEDFAM[Oxidation (M)]DEDGDES[Phospho (STY)]IHK_.3	Q9CWZ3_S24_M1_Rbm8a	0.7396	-0.4352	0.0376	1.4249
Grin2b	Q01097	Glutamate receptor ionotropic, NMDA 2B	_AGNLYDIS[Phospho (STY)]EDNSLQELDQPAAPVAVSSNASTTK_.3	Q01097_S1255_M1_Grin2b	0.7412	-0.4321	0.0376	1.4248
Ermn	Q5EBJ4	Ermin	_EDSPLGS[Phospho (STY)]PGSQPGTPDEQPVFGK_.3	#N/A	0.6056	-0.7236	0.0376	1.4245
Plch2	A2AP18	1-phosphatidylinositol 4,5-bisphosphate phosphodiesterase eta-2	_IRDC[Carbamidomethyl (C)]EDPNDFSVSTLS[Phospho (STY)]PSGK_.3	A2AP18_S619_M1_Plch2	0.7414	-0.4318	0.0376	1.4244
Rims1	Q99NE5	Regulating synaptic membrane exocytosis protein 1	_S[Phospho (STY)]RSVS[Phospho (STY)]PHR_.2	Q99NE5_S786_M2_Rims1	0.7130	-0.4880	0.0378	1.4228
Cand1	Q6ZQ38	Cullin-associated NEDD8-dissociated protein 1	_ITSEALLVTQQLVK_.2	#N/A	1.3919	0.4770	0.0378	1.4224
Agap1	Q8BXK8	Arf-GAP with GTPase, ANK repeat and PH domain-containing protein 1	_LDPPPS[Phospho (STY)]PHANR_.2	Q8BXK8_S521_M1_Agap1	1.3501	0.4331	0.0378	1.4222
Dnajc19	Q9CQV7	Mitochondrial import inner membrane translocase subunit TIM14	_EAALILGVS[Phospho (STY)]PTANK_.2	Q9CQV7_S70_M1_Dnajc19	0.7384	-0.4376	0.0379	1.4215
Ehd3	Q9QXY6	EH domain-containing protein 3	_IGPEPTTDSFIAVM[Oxidation (M)]QGDVEGIIPGNALVVDPK_.3	#N/A	0.7423	-0.4299	0.0379	1.4212
Snn	P61807	Stannin	_LM[Oxidation (M)]TANS[Phospho (STY)]PEVHG_.2	#N/A	1.3911	0.4762	0.0381	1.4190
Tns2	Q8CGB6	Tensin-2	_APHSC[Carbamidomethyl (C)]GS[Phospho (STY)]PSEGR_.2	Q8CGB6_S811_M1_Tns2	1.5163	0.6006	0.0381	1.4189
Map1a	Q9QYR6	Microtubule-associated protein 1A	_WLAESPVGLPPEEEDKLTR_.3	#N/A	0.4958	-1.0121	0.0381	1.4188
Tpd52l2	Q9CYZ2	Tumor protein D54	_S[Phospho (STY)]FEDRVGTIK_.2	#N/A	1.7244	0.7861	0.0382	1.4181
Map1a	Q9QYR6	Microtubule-associated protein 1A	_EQKDEAS[Phospho (STY)]EEKEQVLEQK_.3	#N/A	0.7430	-0.4286	0.0382	1.4180
Cltc	Q68FD5	Clathrin heavy chain 1	_NNRPSEGPLQTR_.2	#N/A	0.5811	-0.7833	0.0382	1.4180
Dmxl2	Q8BPN8	DmX-like protein 2	_ALLT[Phospho (STY)]PQDEEC[Carbamidomethyl (C)]ADGDTEVDVIAEQLK_.3	Q8BPN8_T2017_M1_Dmxl2	1.5137	0.5981	0.0383	1.4168
Hnrnph1	O35737	Heterogeneous nuclear ribonucleoprotein H	_EGRPSGEAFVELES[Phospho (STY)]EDEVK_.3	O35737_S63_M1_Hnrnph1	1.5732	0.6537	0.0384	1.4158
Sash3	Q8K352	SAM and SH3 domain-containing protein 3	_SSS[Phospho (STY)]FKDFAK_.2	Q8K352_S27_M1_Sash3	0.7200	-0.4740	0.0384	1.4158
Akap1	O08715	A-kinase anchor protein 1, mitochondrial	_S[Phospho (STY)]ESSGNLPSVADTR_.2	#N/A	1.5247	0.6085	0.0385	1.4151
Slc12a5	Q91V14	Solute carrier family 12 member 5	_IFTVAQM[Oxidation (M)]DDNSIQM[Oxidation (M)]K_.2	#N/A	0.7436	-0.4274	0.0386	1.4137
	Q3UE31	Uncharacterized protein KIAA0930 homolog	_SHS[Phospho (STY)]ANDSEEFFREDDSGADLHNATNLR_.4	Q3UE31_S324_M1_	0.7368	-0.4407	0.0387	1.4127
Srsf5	O35326	Serine/arginine-rich splicing factor 5	_VS[Phospho (STY)]WQDLK_.2	O35326_S120_M1_Srsf5	1.6947	0.7610	0.0387	1.4123
Etfb	Q9DCW4	Electron transfer flavoprotein subunit beta	_EIIAVSC[Carbamidomethyl (C)]GPSQC[Carbamidomethyl (C)]QETIR_.2	#N/A	0.7041	-0.5061	0.0387	1.4119
Snx6	Q6P8X1	Sorting nexin-6	_[Acetyl (Protein N-term)]M[Oxidation (M)]EGLDDGPDFLS[Phospho (STY)]EEDR_.2	Q6P8X1_S13_M1_Snx6	0.7439	-0.4268	0.0388	1.4116
Daam1	Q8BPM0	Disheveled-associated activator of morphogenesis 1	_ESS[Phospho (STY)]EESGEFDDLVSALR_.2	Q8BPM0_S1026_M1_Daam1	0.7442	-0.4263	0.0389	1.4096
Hint1	P70349	Histidine triad nucleotide-binding protein 1	_C[Carbamidomethyl (C)]AADLGLK_.2	#N/A	0.6989	-0.5168	0.0390	1.4090
Alpl	P09242	Alkaline phosphatase, tissue-nonspecific isozyme	_TYNTNAQVPDS[Phospho (STY)]AGTATAYLC[Carbamidomethyl (C)]GVK_.3	P09242_S110_M1_Alpl	0.6693	-0.5793	0.0390	1.4088
Mef2c	Q8CFN5	Myocyte-specific enhancer factor 2C	_PSPDERES[Phospho (STY)]PSVKR_.3	Q8CFN5_S460_M1_Mef2c	0.6591	-0.6015	0.0390	1.4087
Scamp3	O35609	Secretory carrier-associated membrane protein 3	_TAAANAAAGAAENAFR_.2	#N/A	0.4524	-1.1444	0.0390	1.4086
Ildr2	B5TVM2	Immunoglobulin-like domain-containing receptor 2	_GWTYS[Phospho (STY)]PAPAR_.2	B5TVM2_S511_M1_Ildr2	23.9086	4.5795	0.0390	1.4084
Ddost	O54734	Dolichyl-diphosphooligosaccharide--protein glycosyltransferase 48 kDa subunit	_TLVLLDNLNVR_.2	#N/A	0.5735	-0.8022	0.0391	1.4084
Magi3	Q9EQJ9	Membrane-associated guanylate kinase, WW and PDZ domain-containing protein 3	_QPEDESHQAFSQNGS[Phospho (STY)]PR_.3	#N/A	1.4821	0.5676	0.0391	1.4078
Stoml2	Q99JB2	Stomatin-like protein 2, mitochondrial	_APVPGAQNSSQSR_.2	#N/A	0.6407	-0.6422	0.0391	1.4076
Thop1	Q8C1A5	Thimet oligopeptidase	_VDQVLHTQTDADPAEEYAR_.3	#N/A	1.9496	0.9632	0.0392	1.4072
Acly	Q91V92	ATP-citrate synthase	_SINNPDMR_.2	#N/A	0.6905	-0.5343	0.0392	1.4065
Prdx1	P35700	Peroxiredoxin-1	_IGYPAPNFK_.2	#N/A	0.7354	-0.4435	0.0392	1.4063
Prrc2b	Q7TPM1	Protein PRRC2B	_LKFS[Phospho (STY)]DDEDEEDVVK_.2	#N/A	0.6532	-0.6145	0.0393	1.4054
Srrm2	Q8BTI8	Serine/arginine repetitive matrix protein 2	_DTPQT[Phospho (STY)]PSR_.2	Q8BTI8_T1428_M1_Srrm2	0.7358	-0.4426	0.0393	1.4051
Ndrg3	Q9QYF9	Protein NDRG3	_LHIAQDINQENLQLFLGSYNGR_.3	#N/A	1.5077	0.5923	0.0394	1.4051
Lrp1	Q91ZX7	Prolow-density lipoprotein receptor-related protein 1	_IFFSDIHFGNIQQINDDGSGR_.3	#N/A	0.7358	-0.4427	0.0394	1.4046
Gnb4	P29387	Guanine nucleotide-binding protein subunit beta-4	_SGVLAGHDNR_.2	#N/A	0.0347	-4.8492	0.0395	1.4038
Skt	A2AQ25	Sickle tail protein	_VSS[Phospho (STY)]EDPGPTPQTR_.2	A2AQ25_S1087_M1_Skt	1.4878	0.5732	0.0395	1.4033
Aagab	Q8R2R3	Alpha- and gamma-adaptin-binding protein p34	_AFWM[Oxidation (M)]AIGGDRDEIEGLSS[Phospho (STY)]DDEH_.3	Q8R2R3_S312_M1_Aagab	1.5541	0.6361	0.0396	1.4023
Pccb	Q99MN9	Propionyl-CoA carboxylase beta chain, mitochondrial	_EFFNFLPLSSQDPAPIR_.2	#N/A	1.4030	0.4885	0.0396	1.4022
Msi1	Q61474	RNA-binding protein Musashi homolog 1	_[Acetyl (Protein N-term)]M[Oxidation (M)]ETDAPQPGLAS[Phospho (STY)]PDSPHDPC[Carbamidomethyl (C)]K_.2	#N/A	1.3802	0.4649	0.0397	1.4014
Hrh1	P70174	Histamine H1 receptor	_RIS[Phospho (STY)]ETSEDQTLVDR_.2	P70174_S344_M1_Hrh1	1.4964	0.5815	0.0397	1.4013
Slc32a1	O35633	Vesicular inhibitory amino acid transporter	_LTNVATSVSNK_.2	#N/A	0.6010	-0.7345	0.0399	1.3990
Omg	Q63912	Oligodendrocyte-myelin glycoprotein	_AHVIGTPC[Carbamidomethyl (C)]SK_.2	#N/A	1.3104	0.3900	0.0399	1.3990
Pgm2l1	Q8CAA7	Glucose 1,6-bisphosphate synthase	_DITTGYDSSQPNKK_.2	#N/A	0.6248	-0.6785	0.0399	1.3985
Ptpa	P58389	Serine/threonine-protein phosphatase 2A activator	_FGSLLPIHPVTSG_.2	#N/A	0.6839	-0.5482	0.0402	1.3959
Sptbn1	Q62261	Spectrin beta chain, non-erythrocytic 1	_IQEKVDSIDDR_.3	#N/A	0.7131	-0.4878	0.0402	1.3955
Hspd1	P63038	60 kDa heat shock protein, mitochondrial	_DM[Oxidation (M)]AIATGGAVFGEEGLNLNLEDVQAHDLGK_.3	#N/A	1.5137	0.5981	0.0402	1.3954
Atp6v1a	P50516	V-type proton ATPase catalytic subunit A	_EASIYTGITLSEYFR_.2	#N/A	0.7015	-0.5115	0.0403	1.3947
Gad1	P48318	Glutamate decarboxylase 1	_ERQS[Phospho (STY)]SKNLLSC[Carbamidomethyl (C)]ENSDQGAR_.3	#N/A	1.4910	0.5763	0.0403	1.3943
Cavin2	Q63918	Caveolae-associated protein 2	_S[Phospho (STY)]SPFKVS[Phospho (STY)]PLSFGR_.2	Q63918_S287_M2_Cavin2	0.6559	-0.6085	0.0404	1.3936
Ap2a1	P17426	AP-2 complex subunit alpha-1	_AVEYLTLSSVASTDVLATVLEEM[Oxidation (M)]PPFPER_.3	#N/A	0.7037	-0.5069	0.0405	1.3928
Nfia	Q02780	Nuclear factor 1 A-type	_S[Phospho (STY)]PGSGSQSSGWHEVEPGLPSPSTLK_.3	Q02780_S323_M1_Nfia	0.7455	-0.4238	0.0407	1.3906
Sars1	P26638	Serine--tRNA ligase, cytoplasmic	_EIGNLLHPSVPISNDEDADNKVER_.4	#N/A	1.4900	0.5753	0.0407	1.3904
Arhgef2	Q60875	Rho guanine nucleotide exchange factor 2	_LES[Phospho (STY)]FESLRGER_.2	#N/A	7.7845	2.9606	0.0408	1.3898
Sphkap	Q6NSW3	A-kinase anchor protein SPHKAP	_RAS[Phospho (STY)]TDLGK_.2	Q6NSW3_S841_M1_Sphkap	0.7045	-0.5053	0.0408	1.3898
Hgs	Q99LI8	Hepatocyte growth factor-regulated tyrosine kinase substrate	_ASSTTELPPEYLTSPLSQQS[Phospho (STY)]QLPPK_.3	Q99LI8_S246_M1_Hgs	0.7461	-0.4226	0.0408	1.3896
Wdr44	Q6NVE8	WD repeat-containing protein 44	_S[Phospho (STY)]NSGRELTDEEILASVMIK_.3	Q6NVE8_S344_M1_Wdr44	0.7341	-0.4459	0.0409	1.3882
Rims3	Q80U57	Regulating synaptic membrane exocytosis protein 3	_QGSRESTDGSTNS[Phospho (STY)]NSSEGTFIFPTR_.3	Q80U57_S114_M1_Rims3	0.7341	-0.4459	0.0409	1.3881
Akap12	Q9WTQ5	A-kinase anchor protein 12	_RPS[Phospho (STY)]ES[Phospho (STY)]DKEEELDK_.2	Q9WTQ5_S613_M2_Akap12	1.4886	0.5740	0.0410	1.3872
Chp1	P61022	Calcineurin B homologous protein 1	_ETGFSHSQITR_.3	#N/A	0.7463	-0.4222	0.0410	1.3870
Pcm1	Q9R0L6	Pericentriolar material 1 protein	_ILEGDHGS[Phospho (STY)]PAGEIDDEDKDKDETETVK_.4	Q9R0L6_S1729_M1_Pcm1	0.7464	-0.4219	0.0412	1.3854
Ppp5c	Q60676	Serine/threonine-protein phosphatase 5	_GNHETDNMNQIYGFEGEVK_.3	#N/A	1.4879	0.5733	0.0412	1.3854
Marcks	P26645	Myristoylated alanine-rich C-kinase substrate	_AEDGAAPS[Phospho (STY)]PSSET[Phospho (STY)]PKKK_.2	#N/A	0.4393	-1.1867	0.0412	1.3853
Actn1	Q7TPR4	Alpha-actinin-1	_IDQLEC[Carbamidomethyl (C)]DHQLIQEALIFDNK_.3	#N/A	0.6468	-0.6286	0.0412	1.3853

Ranbp2	Q9ERU9	E3 SUMO-protein ligase RanBP2	_SALS[Phospho (STY)]PSKSPAK_.2	Q9ERU9_S2113_M1_Ranbp2	2.3066	1.2058	0.0413	1.3837
Scn4b	Q7M729	Sodium channel subunit beta-4	_EC[Carbamidomethyl (C)]LVSSSGNDNTENGLPGSK_.2	#N/A	1.6856	0.7532	0.0414	1.3832
Cnksr2	Q80YA9	Connector enhancer of kinase suppressor of ras 2	_GSES[Phospho (STY)]PNSFLDQEYRK_.2	#N/A	2.9093	1.5407	0.0414	1.3825
Sybu	Q8BHS8	Syntabulin	_SGSS[Phospho (STY)]PPS[Phospho (STY)]PR_.2	Q8BHS8_S199_M2_Sybu	0.7064	-0.5014	0.0415	1.3823
Bcl11b	Q99PV8	B-cell lymphoma/leukemia 11B	_SPFLST[Phospho (STY)]PPLPPMPAGT[Phospho (STY)]PPPQPPAK_.3	Q99PV8_T406_M2_Bcl11b	0.7467	-0.4215	0.0415	1.3822
Gria2	P23819	Glutamate receptor 2	_ADIAIAPLTITLVR_.2	#N/A	1.3381	0.4202	0.0415	1.3819
Tmcc2	Q80W04	Transmembrane and coiled-coil domains protein 2	_GS[Phospho (STY)]PHLLR_.2	Q80W04_S189_M1_Tmcc2	1.5659	0.6470	0.0416	1.3811
Ugp2	Q91ZJ5	UTP--glucose-1-phosphate uridylyltransferase	_NENTFLDLTVQQIEHLNK_.3	#N/A	0.7335	-0.4472	0.0417	1.3800
Lmtk3	Q5XJV6	Serine/threonine-protein kinase LMTK3	_AQRPPEGM[Oxidation (M)]S[Phospho (STY)]PELPPR_.3	#N/A	0.5929	-0.7541	0.0417	1.3796
Rtn4	Q99P72	Reticulon-4	_ESAEFSVLEYSEM[Oxidation (M)]GSSFNGS[Phospho (STY)]PK_.3	Q99P72_S291_M1_Rtn4	1.7369	0.7965	0.0417	1.3796
Ndufb5	Q9CQH3	NADH dehydrogenase [ubiquinone] 1 beta subcomplex subunit 5, mitochondrial	_NFYDGPEK_.2	#N/A	0.6788	-0.5590	0.0418	1.3793
Glcci1	Q8K3I9	Glucocorticoid-induced transcript 1 protein	_TSS[Phospho (STY)]LDTITGPYLTGQWPR_.2	Q8K3I9_S163_M1_Glcci1	0.7470	-0.4209	0.0419	1.3780
Atp6v1c1	Q9Z1G3	V-type proton ATPase subunit C 1	_SRAS[Phospho (STY)]AYNNLK_.2	Q9Z1G3_S141_M1_Atp6v1c1	1.5251	0.6089	0.0420	1.3767
Syngap1	F6SEU4	Ras/Rap GTPase-activating protein SynGAP	_TVS[Phospho (STY)]VPVEGRPHGEHEYHLGR_.4	#N/A	0.6885	-0.5385	0.0421	1.3755
Ppid	Q9CR16	Peptidyl-prolyl cis-trans isomerase D	_[Acetyl (Protein N-term)]SHASPAAKPSNSK_.3	#N/A	0.7147	-0.4846	0.0422	1.3746
Pde1c	Q64338	Calcium/calmodulin-dependent 3',5'-cyclic nucleotide phosphodiesterase 1C	_[Acetyl (Protein N-term)]MES[Phospho (STY)]PTKEIEEFESNSLK_.3	Q64338_S3_M1_Pde1c	0.7330	-0.4482	0.0423	1.3740
Cttn	Q60598	Src substrate cortactin	_HC[Carbamidomethyl (C)]S[Phospho (STY)]QVDSVR_.2	Q60598_S113_M1_Cttn	0.6403	-0.6432	0.0424	1.3730
Prickle2	Q80Y24	Prickle-like protein 2	_S[Phospho (STY)]FQESLGQGSR_.2	#N/A	1.4499	0.5360	0.0424	1.3721
Arhgef2	Q60875	Rho guanine nucleotide exchange factor 2	_EAQELGS[Phospho (STY)]PEDR_.2	#N/A	1.4136	0.4993	0.0425	1.3720
Stim2	P83093	Stromal interaction molecule 2	_S[Phospho (STY)]MIFSPASR_.2	P83093_S661_M1_Stim2	0.6670	-0.5843	0.0425	1.3718
Tecr	Q9CY27	Very-long-chain enoyl-CoA reductase	_S[Phospho (STY)]LKDEDVLQK_.2	Q9CY27_S58_M1_Tecr	1.6419	0.7153	0.0425	1.3717
Chgb	P16014	Secretogranin-1	_ADEFPDFY[Phospho (STY)]DS[Phospho (STY)]EEQM[Oxidation (M)]GPHQEANDEK_.3	P16014_S626_M2_Chgb	1.4831	0.5686	0.0426	1.3710
Bola1	Q9D8S9	BolA-like protein 1	_FEGM[Oxidation (M)]S[Phospho (STY)]PLQR_.2	#N/A	1.8563	0.8925	0.0426	1.3707
Vars1	Q9Z1Q9	Valine--tRNA ligase	_LSATVTEAFVR_.2	#N/A	0.7588	-0.3982	0.0426	1.3706
Stxbp1	O08599	Syntaxin-binding protein 1	_LDAYKADDPTMGEGPDK_.3	#N/A	1.5093	0.5939	0.0427	1.3700
Camk4	P08414	Calcium/calmodulin-dependent protein kinase type IV	_DSTDLLGKK_.2	#N/A	1.3319	0.4135	0.0427	1.3694
Pgm2	Q7TSV4	Phosphoglucomutase-2	_[Acetyl (Protein N-term)]AAAT[Phospho (STY)]PTETPAPEGSGLGMDAR_.2	Q7TSV4_T5_M1_Pgm2	0.7476	-0.4197	0.0428	1.3688
Osbpl6	Q8BXR9	Oxysterol-binding protein-related protein 6	_TAS[Phospho (STY)]SSTEPSVSR_.2	Q8BXR9_S44_M1_Osbpl6	1.4089	0.4946	0.0428	1.3681
Tuba1b	P05213	Tubulin alpha-1B chain	_RAFVHWYVGEGMEEGEFSEAR_.3	#N/A	0.7478	-0.4194	0.0430	1.3665
Cacnb2	Q8CC27	Voltage-dependent L-type calcium channel subunit beta-2	_SGGNSSS[Phospho (STY)]SLGDIVPSSR_.2	Q8CC27_S202_M1_Cacnb2	0.0951	-3.3940	0.0430	1.3663
Dync1h1	Q9JHU4	Cytoplasmic dynein 1 heavy chain 1	_LALESIC[Carbamidomethyl (C)]LLLGESTTDWK_.2	#N/A	0.7087	-0.4967	0.0430	1.3660
Son	Q9QX47	Protein SON	_RFS[Phospho (STY)]RS[Phospho (STY)]PIR_.2	#N/A	2.0965	1.0680	0.0431	1.3659
Calcoco1	Q8CGU1	Calcium-binding and coiled-coil domain-containing protein 1	_LPSYGLC[Carbamidomethyl (C)]ESGNTSSS[Phospho (STY)]PPGPR_.2	Q8CGU1_S563_M1_Calcoco1	4.8836	2.2879	0.0431	1.3657
Kcnq2	Q9Z351	Potassium voltage-gated channel subfamily KQT member 2	_YPQT[Phospho (STY)]WNGR_.2	Q9Z351_T287_M1_Kcnq2	0.6934	-0.5282	0.0431	1.3655
Gab2	Q9Z1S8	GRB2-associated-binding protein 2	_ASS[Phospho (STY)]C[Carbamidomethyl (C)]ETYEYPAR_.2	Q9Z1S8_S402_M1_Gab2	1.7624	0.8175	0.0431	1.3653
Kiaa1109	A2AAE1	Transmembrane protein KIAA1109	_SLDQDS[Phospho (STY)]PSKK_.2	A2AAE1_S3835_M1_Kiaa1109	0.6702	-0.5772	0.0432	1.3648
Hspa12a	Q8K0U4	Heat shock 70 kDa protein 12A	_WSS[Phospho (STY)]QGMLR_.2	Q8K0U4_S437_M1_Hspa12a	1.4813	0.5669	0.0432	1.3648
Abl2	Q4JIM5	Tyrosine-protein kinase ABL2	_STQASSGS[Phospho (STY)]PALPR_.2	Q4JIM5_S621_M1_Abl2	1.3522	0.4353	0.0432	1.3647
Rbm26	Q6NZN0	RNA-binding protein 26	_RLNHS[Phospho (STY)]PPQSSSR_.3	#N/A	0.4862	-1.0404	0.0432	1.3646
Atp2a2	O55143	Sarcoplasmic/endoplasmic reticulum calcium ATPase 2	_TASEM[Oxidation (M)]VLADDNFSTIVAAVEEGR_.3	#N/A	1.4117	0.4975	0.0432	1.3645
Tnrc6b	Q8BKI2	Trinucleotide repeat-containing gene 6B protein	_SSSS[Phospho (STY)]AGSEVGGQSTGSNHK_.2	Q8BKI2_S601_M1_Tnrc6b	0.7479	-0.4191	0.0432	1.3643
Hspa2	P17156	Heat shock-related 70 kDa protein 2	_QTQTFTTYSDNQSSVLVQVYEGER_.3	#N/A	0.7322	-0.4496	0.0433	1.3640
Cadps	Q80TJ1	Calcium-dependent secretion activator 1	_SLTSNLPNVNLPNVNLPK_.2	#N/A	0.6048	-0.7255	0.0433	1.3637
Zfyve26	Q5DU37	Zinc finger FYVE domain-containing protein 26	_GPLGLRS[Phospho (STY)]PSES[Phospho (STY)]PQHIAATER_.3	Q5DU37_S605_M2_Zfyve26	0.7480	-0.4189	0.0433	1.3630
Top2b	Q64511	DNA topoisomerase 2-beta	_KTSFDQDS[Phospho (STY)]DVDIFPSDFTSEPPALPR_.3	Q64511_S1568_M1_Top2b	0.7098	-0.4946	0.0434	1.3624
Ccdc85a	Q5SP85	Coiled-coil domain-containing protein 85A	_STS[Phospho (STY)]PEHLHKPR_.2	#N/A	2.4914	1.3169	0.0434	1.3624
Hecw1	Q8K4P8	E3 ubiquitin-protein ligase HECW1	_APS[Phospho (STY)]PYRR_.2	Q8K4P8_S1219_M1_Hecw1	0.7080	-0.4982	0.0435	1.3617
Bclaf1	Q8K019	Bcl-2-associated transcription factor 1	_LRC[Carbamidomethyl (C)]DS[Phospho (STY)]ADLR_.2	Q8K019_S688_M1_Bclaf1	0.7076	-0.4989	0.0435	1.3614
Nagk	Q9QZ08	N-acetyl-D-glucosamine kinase	_[Acetyl (Protein N-term)]AALYGGVEGGGTR_.2	#N/A	0.5210	-0.9406	0.0435	1.3610
Ndrg2	Q9QYG0	Protein NDRG2	_LTGLTSSIPDM[Oxidation (M)]ILGHLFSQEELSGNSELIQK_.3	#N/A	0.7495	-0.4161	0.0436	1.3602
Calb2	Q08331	Calretinin	_EM[Oxidation (M)]NIQQLTTYR_.2	#N/A	0.7508	-0.4134	0.0437	1.3597
Srcin1	Q9QWI6	SRC kinase signaling inhibitor 1	_LGGAPTGQGVSPSPSAILER_.2	#N/A	2.0920	1.0649	0.0437	1.3595
Atp6v0d1	P51863	V-type proton ATPase subunit d 1	_AGVLSQADYLNLVQC[Carbamidomethyl (C)]ETLEDLK_.3	#N/A	0.7140	-0.4860	0.0438	1.3590
Ccser2	Q3UHI0	Serine-rich coiled-coil domain-containing protein 2	_SSS[Phospho (STY)]GESLAQSPDNAK_.2	Q3UHI0_S198_M1_Ccser2	1.5341	0.6174	0.0438	1.3585
Tmem230	Q8CIB6	Transmembrane protein 230	_LAS[Phospho (STY)]TDDGYIDLQFK_.2	Q8CIB6_S24_M1_Tmem230	0.7498	-0.4155	0.0439	1.3577
Kmt2d	Q6PDK2	Histone-lysine N-methyltransferase 2D	_LC[Carbamidomethyl (C)]PQPEEPYLS[Phospho (STY)]PQPEEPR_.2	Q6PDK2_S727_M1_Kmt2d	0.2509	-1.9949	0.0439	1.3575
Coa6	Q8BGD8	Cytochrome c oxidase assembly factor 6 homolog	_EKFEAGGFQSSQSTENS_.2	#N/A	2.5558	1.3537	0.0440	1.3568
Isoc2a	P85094	Isochorismatase domain-containing protein 2A	_QSGAFLATSESLILQLVR_.2	#N/A	0.7107	-0.4927	0.0440	1.3565
Stx17	Q9D0I4	Syntaxin-17	_LTSS[Phospho (STY)]C[Carbamidomethyl (C)]PDLPSQSDK_.2	Q9D0I4_S288_M1_Stx17	5.6961	2.5100	0.0442	1.3548
Prpf3	Q922U1	U4/U6 small nuclear ribonucleoprotein Prp3	_WDEQTSNTKGDDDEES[Phospho (STY)]DEEAVKK_.3	#N/A	1.7365	0.7962	0.0442	1.3541
Prrt3	Q6PE13	Proline-rich transmembrane protein 3	_S[Phospho (STY)]IDAALFR_.2	Q6PE13_S806_M1_Prrt3	0.7163	-0.4813	0.0443	1.3539
Iqsec2	Q5DU25	IQ motif and SEC7 domain-containing protein 2	_AAHLPLLTIEPPSDSS[Phospho (STY)]VDLSDR_.3	Q5DU25_S593_M1_Iqsec2	0.5409	-0.8865	0.0443	1.3538
Arfgef2	A2A5R2	Brefeldin A-inhibited guanine nucleotide-exchange protein 2	_HLDVDLDRQS[Phospho (STY)]LSSIDR_.3	A2A5R2_S1518_M1_Arfgef2	0.7315	-0.4510	0.0443	1.3537
Atxn1	P54254	Ataxin-1	_EAS[Phospho (STY)]PSTLNDK_.2	P54254_S382_M1_Atxn1	0.4111	-1.2823	0.0443	1.3535
Calr	P14211	Calreticulin	_VHVIFNYK_.2	#N/A	0.6589	-0.6019	0.0443	1.3534
Ugp2	Q91ZJ5	UTP--glucose-1-phosphate uridylyltransferase	_AM[Oxidation (M)]S[Phospho (STY)]QDGASQFQEVILQELELSVK_.3	Q91ZJ5_S13_M1_Ugp2	0.7486	-0.4177	0.0444	1.3528
Cdk14	O35495	Cyclin-dependent kinase 14	_TLS[Phospho (STY)]ESFSR_.2	O35495_S24_M1_Cdk14	0.7314	-0.4513	0.0445	1.3515
Washc2	Q6PGL7	WASH complex subunit 2	_EGLLPASDQEAGGPSDIFSSSS[Phospho (STY)]PLDK_.3	Q6PGL7_S783_M1_Washc2	1.4927	0.5780	0.0446	1.3506
Nipsnap1	O55125	Protein NipSnap homolog 1	_IQFHNVKPEC[Carbamidomethyl (C)]LDAYNSLTEAVLPK_.4	#N/A	0.7487	-0.4175	0.0446	1.3505
Cadm2	Q8BLQ9	Cell adhesion molecule 2	_GAEDAPDADTAIINAEGS[Phospho (STY)]QVNAEEK_.3	#N/A	1.6072	0.6845	0.0447	1.3501
Gapdh	P16858	Glyceraldehyde-3-phosphate dehydrogenase	_LEKPAKYDDIK_.3	#N/A	0.5219	-0.9383	0.0448	1.3490
Map7d1	A2AJI0	MAP7 domain-containing protein 1	_LSTGSELS[Phospho (STY)]PK_.2	A2AJI0_S460_M1_Map7d1	0.7489	-0.4172	0.0448	1.3483
Ppp1r14c	Q8R4S0	Protein phosphatase 1 regulatory subunit 14C	_KLS[Phospho (STY)]PPQKK_.2	Q8R4S0_S157_M1_Ppp1r14c	0.6741	-0.5689	0.0449	1.3480
Septin2	P42208	Septin-2	_STLINSLFLTDLYPER_.2	#N/A	0.5468	-0.8708	0.0449	1.3476
Ipo5	Q8BKC5	Importin-5	_HIVENAVQK_.2	#N/A	1.4337	0.5197	0.0449	1.3474
Hspa8	P63017	Heat shock cognate 71 kDa protein	_GRLS[Phospho (STY)]KEDIER_.3	P63017_S511_M1_Hspa8	1.3371	0.4191	0.0449	1.3473
Sh3bgrl3	Q91VW3	SH3 domain-binding glutamic acid-rich-like protein 3	_VYSTSVTGSR_.2	#N/A	0.3942	-1.3430	0.0451	1.3463
Itsn1	Q9Z0R4	Intersectin-1	_AQS[Phospho (STY)]FDVASAPPAAEWAVPQSSR_.2	Q9Z0R4_S203_M1_Itsn1	0.7310	-0.4520	0.0451	1.3458
Dlc1	Q9R0Z9	Rho GTPase-activating protein 7	_KRSEDS[Phospho (STY)]DEDEPC[Carbamidomethyl (C)]AISGK_.3	#N/A	1.4344	0.5204	0.0452	1.3449

Napb	P28663	Beta-soluble NSF attachment protein	_AIAHYEQSADYYK_.3	#N/A	1.4903	0.5756	0.0452	1.3448
Trim28	Q62318	Transcription intermediary factor 1-beta	_VS[Phospho (STY)]LERLDLDLTSDSQPPVFK_.3	Q62318_S489_M1_Trim28	0.7524	-0.4105	0.0452	1.3445
Ranbp2	Q9ERU9	E3 SUMO-protein ligase RanBP2	_LNSNNSAS[Phospho (STY)]PHR_.2	Q9ERU9_S837_M1_Ranbp2	0.6159	-0.6993	0.0455	1.3422
Tmod2	Q9JKK7	Tropomodulin-2	_DREDFVPFTGEK_.2	#N/A	0.7407	-0.4331	0.0456	1.3414
Atp5f1c	Q91VR2	ATP synthase subunit gamma, mitochondrial	_ELIEIISGAAALD_.2	#N/A	1.4053	0.4909	0.0456	1.3411
Pfkm	P47857	ATP-dependent 6-phosphofructokinase, muscle type	_SSYLNIVGLVGSIDNDFC[Carbamidomethyl (C)]GTDM[Oxidation (M)]TIGTDSALHR_.3	#N/A	1.5369	0.6200	0.0458	1.3396
Adgrl1	Q80TR1	Adhesion G protein-coupled receptor L1	_TDTLTEYASWEDYVAAR_.2	#N/A	0.7520	-0.4112	0.0458	1.3388
Tbc1d5	Q80XQ2	TBC1 domain family member 5	_EFTGS[Phospho (STY)]PPPSATKK_.2	Q80XQ2_S578_M1_Tbc1d5	9.8818	3.3048	0.0459	1.3382
Hecw1	Q8K4P8	E3 ubiquitin-protein ligase HECW1	_SGS[Phospho (STY)]VHQMEQLNR_.2	Q8K4P8_S871_M1_Hecw1	1.6693	0.7393	0.0459	1.3379
Rbmxl1	Q91VM5	RNA binding motif protein, X-linked-like-1	_DRDYSDHPSGGSYR_.3	#N/A	0.5660	-0.8212	0.0460	1.3370
Ppp3r1	Q63810	Calcineurin subunit B type 1	_KLDLDNSGSLSVEEFM[Oxidation (M)]SLPELQQNPLVQR_.3	#N/A	0.7495	-0.4159	0.0461	1.3359
Snap25	P60879	Synaptosomal-associated protein 25	_HM[Oxidation (M)]ALDM[Oxidation (M)]GNEIDTQNR_.3	#N/A	0.6708	-0.5759	0.0462	1.3357
Rem2	Q8VEL9	GTP-binding protein REM 2	_RRGS[Phospho (STY)]M[Oxidation (M)]PVPYK_.3	#N/A	0.7213	-0.4713	0.0463	1.3347
Eps15l1	Q60902	Epidermal growth factor receptor substrate 15-like 1	_GIDPPQVLS[Phospho (STY)]PDM[Oxidation (M)]VPPSER_.2	Q60902_S353_M1_Eps15l1	0.4538	-1.1399	0.0463	1.3341
Relch	Q148V7	RAB11-binding protein RELCH	_AGSISTLDSLDFARY[Phospho (STY)]SDDGNRETDER_.3	Q148V7_Y192_M1_Relch	0.6089	-0.7158	0.0464	1.3337
Phactr1	Q2M3X8	Phosphatase and actin regulator 1	_RLS[Phospho (STY)]QRPTAEELEQR_.3	Q2M3X8_S467_M1_Phactr1	1.4837	0.5692	0.0465	1.3327
Atg4c	Q811C2	Cysteine protease ATG4C	_DFDFTSTAAS[Phospho (STY)]EEDLFSEDERK_.3	Q811C2_S434_M1_Atg4c	1.4736	0.5594	0.0465	1.3322
Arl6ip5	Q8R5J9	PRA1 family protein 3	_TPMGIILDALEQQEDNINK_.2	#N/A	0.6803	-0.5557	0.0465	1.3321
Icam5	Q60625	Intercellular adhesion molecule 5	_LDDLDC[Carbamidomethyl (C)]PR_.2	#N/A	0.7621	-0.3920	0.0466	1.3319
Ppig	A2AR02	Peptidyl-prolyl cis-trans isomerase G	_EQS[Phospho (STY)]PVSK_.2	#N/A	0.6154	-0.7005	0.0466	1.3318
Necab2	Q91ZP9	N-terminal EF-hand calcium-binding protein 2	_TLS[Phospho (STY)]FDLQQR_.2	Q91ZP9_S271_M1_Necab2	0.1253	-2.9969	0.0466	1.3314
Rap2b	P61226	Ras-related protein Rap-2b	_SALTVQFVTGSFIEK_.2	#N/A	0.6061	-0.7223	0.0467	1.3311
Pnpo	Q91XF0	Pyridoxine-5'-phosphate oxidase	_GLATGDS[Phospho (STY)]PLGPM[Oxidation (M)]THHGEEDWVYER_.4	#N/A	1.3566	0.4400	0.0467	1.3303
Kcnma1	Q08460	Calcium-activated potassium channel subunit alpha-1	_LEDEQPPTLS[Phospho (STY)]PK_.2	#N/A	0.4716	-1.0844	0.0468	1.3301
Cltc	Q68FD5	Clathrin heavy chain 1	_YIQAAC[Carbamidomethyl (C)]K_.2	#N/A	0.5737	-0.8017	0.0468	1.3294
Rab3b	Q9CZT8	Ras-related protein Rab-3B	_LLAEQLGFDFFEASAK_.2	#N/A	1.4783	0.5639	0.0469	1.3291
Hnrnpa1	P49312	Heterogeneous nuclear ribonucleoprotein A1	_GFAFVTFDDHDSVDK_.2	#N/A	1.5706	0.6513	0.0469	1.3286
Cep170b	Q80U49	Centrosomal protein of 170 kDa protein B	_ESPLS[Phospho (STY)]PPTVPDPGGATPGSAR_.2	Q80U49_S981_M1_Cep170b	0.4673	-1.0975	0.0470	1.3282
Homer1	Q9Z2Y3	Homer protein homolog 1	_TELNQTVQELEETLK_.2	#N/A	1.5600	0.6416	0.0470	1.3282
Ctnna2	Q61301	Catenin alpha-2	_LVYDGVR_.2	#N/A	0.5860	-0.7710	0.0471	1.3273
Vat1l	Q80TB8	Synaptic vesicle membrane protein VAT-1 homolog-like	_DSVTHLFDR_.2	#N/A	0.6083	-0.7173	0.0471	1.3268
Myh9	Q8VDD5	Myosin-9	_GTGDC[Carbamidomethyl (C)]S[Phospho (STY)]DEEVDGK_.2	#N/A	0.7432	-0.4282	0.0472	1.3265
Hk1	P17710	Hexokinase-1	_GAAM[Oxidation (M)]VTAVAYR_.2	#N/A	1.3871	0.4720	0.0473	1.3252
Prkcb	P68404	Protein kinase C beta type	_QPVELT[Phospho (STY)]PTDK_.2	P68404_T642_M1_Prkcb	0.0697	-3.8436	0.0473	1.3251
Wdr1	O88342	WD repeat-containing protein 1	_LATGSDDNC[Carbamidomethyl (C)]AAFFEGPPFK_.2	#N/A	0.5275	-0.9227	0.0473	1.3247
Sgip1	Q8VD37	SH3-containing GRB2-like protein 3-interacting protein 1	_RNLS[Phospho (STY)]SEEVARPR_.3	#N/A	4.0908	2.0324	0.0474	1.3238
Rims2	Q9EQZ7	Regulating synaptic membrane exocytosis protein 2	_TSNHS[Phospho (STY)]PPTPR_.2	Q9EQZ7_S472_M1_Rims2	2.4144	1.2717	0.0475	1.3234
Brsk2	Q69Z98	Serine/threonine-protein kinase BRSK2	_S[Phospho (STY)]ISGASSGLSTS[Phospho (STY)]PLSSPR_.2	Q69Z98_S413_M2_Brsk2	0.2633	-1.9254	0.0475	1.3232
Stxbp4	Q9WV89	Syntaxin-binding protein 4	_SSS[Phospho (STY)]PLDRDPAFR_.2	Q9WV89_S12_M1_Stxbp4	0.7502	-0.4146	0.0475	1.3229
Snip1	Q8BIZ6	Smad nuclear-interacting protein 1	_HRS[Phospho (STY)]GDALTTVVVK_.3	#N/A	1.7784	0.8305	0.0476	1.3228
Grm5	Q3UVX5	Metabotropic glutamate receptor 5	_GS[Phospho (STY)]SGETLR_.2	#N/A	0.7436	-0.4274	0.0476	1.3224
Cox6c	Q9CPQ1	Cytochrome c oxidase subunit 6C	_NYDSMKDFEEMR_.3	#N/A	0.7503	-0.4145	0.0477	1.3217
Nsf	P46460	Vesicle-fusing ATPase	_KNIDSNPYDTDK_.2	#N/A	0.4841	-1.0466	0.0477	1.3215
Prkce	P16054	Protein kinase C epsilon type	_SAPTS[Phospho (STY)]PC[Carbamidomethyl (C)]DQELKELENNIRK_.3	#N/A	0.1857	-2.4292	0.0478	1.3209
Aldh1b1	Q9CZS1	Aldehyde dehydrogenase X, mitochondrial	_TFVEESIYR_.2	#N/A	0.7349	-0.4443	0.0478	1.3204
Mast1	Q9R1L5	Microtubule-associated serine/threonine-protein kinase 1	_S[Phospho (STY)]PITIQR_.2	Q9R1L5_S968_M1_Mast1	0.7541	-0.4072	0.0478	1.3202
Fez1	Q8K0X8	Fasciculation and elongation protein zeta-1	_GLS[Phospho (STY)]LQSNR_.2	Q8K0X8_S298_M1_Fez1	0.1641	-2.6075	0.0479	1.3200
Dclk1	Q9JLM8	Serine/threonine-protein kinase DCLK1	_[Acetyl (Protein N-term)]S[Phospho (STY)]FGRDMELEHFDER_.3	#N/A	0.7156	-0.4828	0.0479	1.3197
Plcb1	Q9Z1B3	1-phosphatidylinositol 4,5-bisphosphate phosphodiesterase beta-1	_LQVTPEGR_.2	#N/A	0.6744	-0.5682	0.0479	1.3196
Psmb5	O55234	Proteasome subunit beta type-5	_DAYSGGAVNLYHVR_.2	#N/A	1.7145	0.7778	0.0479	1.3194
Kiaa1107	Q80TK0	AP2-interacting clathrin-endocytosis protein	_SSVSSRQS[Phospho (STY)]DENVTR_.2	Q80TK0_S353_M1_Kiaa1107	0.7195	-0.4749	0.0480	1.3188
Aldh6a1	Q9EQ20	Methylmalonate-semialdehyde dehydrogenase [acylating], mitochondrial	_DMDLYSYR_.2	#N/A	1.5003	0.5853	0.0481	1.3178
Znrf2	Q71FD5	E3 ubiquitin-protein ligase ZNRF2	_AYS[Phospho (STY)]GSDLPSGTGSGGGGADGAR_.2	Q71FD5_S18_M1_Znrf2	17.9883	4.1690	0.0482	1.3173
Fabp3	P11404	Fatty acid-binding protein, heart	_NGDTITIK_.2	#N/A	1.4472	0.5332	0.0482	1.3170
Plec	Q9QXS1	Plectin	_EEELQQTLQQEQNMLDR_.2	#N/A	1.4793	0.5649	0.0482	1.3169
Myo5a	Q99104	Unconventional myosin-Va	_QLELDLNDER_.2	#N/A	0.6442	-0.6344	0.0482	1.3168
Dcx	O88809	Neuronal migration protein doublecortin	_SKQS[Phospho (STY)]PISTPTS[Phospho (STY)]PGSLRK_.3	O88809_S332_M2_Dcx	0.7416	-0.4313	0.0482	1.3166
Oxct1	Q9D0K2	Succinyl-CoA:3-ketoacid coenzyme A transferase 1, mitochondrial	_GM[Oxidation (M)]GGAM[Oxidation (M)]DLVSSSK_.2	#N/A	0.5701	-0.8108	0.0484	1.3154
Isoc2a	P85094	Isochorismatase domain-containing protein 2A	_DASHPQFK_.2	#N/A	0.6907	-0.5338	0.0484	1.3151
Rhob	P62746	Rho-related GTP-binding protein RhoB	_IQAYDYLEC[Carbamidomethyl (C)]SAK_.2	#N/A	1.4961	0.5812	0.0484	1.3149
Kif2a	P28740	Kinesin-like protein KIF2A	_AVFQESIR_.2	#N/A	0.6980	-0.5188	0.0486	1.3137
Vps50	Q8CI71	Syndetin	_KKS[Phospho (STY)]DYSLNK_.2	Q8CI71_S595_M1_Vps50	0.3998	-1.3225	0.0487	1.3126
Cc2d1a	Q8K1A6	Coiled-coil and C2 domain-containing protein 1A	_LANHDEGS[Phospho (STY)]DDEEEETPKK_.3	#N/A	0.7688	-0.3794	0.0487	1.3124
Ahcyl2	Q68FL4	Putative adenosylhomocysteinase 3	_QVVVC[Carbamidomethyl (C)]GYGEVGK_.2	#N/A	0.7508	-0.4134	0.0489	1.3106
Qdpr	Q8BVI4	Dihydropteridine reductase	_VDAILC[Carbamidomethyl (C)]VAGGWAGGNAK_.2	#N/A	1.9116	0.9347	0.0491	1.3092
Eml1	Q05BC3	Echinoderm microtubule-associated protein-like 1	_TSSS[Phospho (STY)]ERVS[Phospho (STY)]PGGR_.2	Q05BC3_S135_M2_Eml1	1.5377	0.6207	0.0492	1.3078
Sirt2	Q8VDQ8	NAD-dependent protein deacetylase sirtuin-2	_FFSC[Carbamidomethyl (C)]M[Oxidation (M)]QSDFSK_.2	#N/A	1.7318	0.7923	0.0493	1.3070
Ptpn12	P35831	Tyrosine-protein phosphatase non-receptor type 12	_DQITKS[Phospho (STY)]PAEVTDIGFGNR_.3	#N/A	0.6725	-0.5723	0.0494	1.3066
Atl1	Q8BH66	Atlastin-1	_VAVLLM[Oxidation (M)]DTQGTFDSQSTLR_.2	#N/A	1.9197	0.9409	0.0494	1.3064
Tiam1	Q60610	T-lymphoma invasion and metastasis-inducing protein 1	_RSNATNSSYS[Phospho (STY)]PPTGR_.2	#N/A	0.0581	-4.1060	0.0494	1.3060
Ndufs2	Q91WD5	NADH dehydrogenase [ubiquinone] iron-sulfur protein 2, mitochondrial	_APGFAHLAGLDK_.2	#N/A	1.3006	0.3792	0.0494	1.3059
Dlg4	Q62108	Disks large homolog 4	_EQLMNSSLGSGT[Phospho (STY)]ASLRS[Phospho (STY)]NPKR_.3	Q62108_S425_M2_Dlg4	1.5384	0.6214	0.0496	1.3046
Taldo1	Q93092	Transaldolase	_WLHNEDQM[Oxidation (M)]AVEK_.3	#N/A	0.5845	-0.7748	0.0496	1.3042
Napb	P28663	Beta-soluble NSF attachment protein	_ASHS[Phospho (STY)]FLR_.2	P28663_S26_M1_Napb	0.7512	-0.4128	0.0497	1.3037
Sptan1	P16546	Spectrin alpha chain, non-erythrocytic 1	_ASAFNSWFENAEEDLTDPVR_.2	#N/A	1.4414	0.5274	0.0497	1.3034
Arvcf	P98203	Armadillo repeat protein deleted in velo-cardio-facial syndrome homolog	_NFDT[Phospho (STY)]LDLPK_.2	P98203_T643_M1_Arvcf	1.4823	0.5678	0.0498	1.3032
Tkt	P40142	Transketolase	_ISSDLDGHPVPK_.3	#N/A	1.5197	0.6038	0.0498	1.3024
Wdr47	Q8CGF6	WD repeat-containing protein 47	_YQQHLEQK_.2	#N/A	1.4800	0.5656	0.0499	1.3022
Ncor1	Q60974	Nuclear receptor corepressor 1	_YS[Phospho (STY)]PESQSQTVLHPR_.2	Q60974_S2116_M1_Ncor1	1.4827	0.5682	0.0499	1.3020
