# Supplementary material for: The Chronic Effects of a Single Low-Intensity Blast Exposure on Phosphoproteome Networks and Cognitive Function Influenced by Mutant Tau Overexpression
Source: Int J Mol Sci. 2024 Mar 15;25(6):3338. doi: 10.3390/ijms25063338 (PMC10970429; doi:10.3390/ijms25063338)
Supplement: Supplementary file 1 [file ijms-25-03338-s001.zip › Supplementary Table S2- Cogwall data.pdf]

Supplementary Table S2. Cogwall data

Group	0-24hrs			25-48hrs		
	Left D1	Middle D1	Right D1	Left D2	Middle D2	Right D2
Blast NC 1	320	137	85	562	58	15
Blast NC 2	627	240	91	1000	35	14
Blast NC 3	385	124	90	576	64	27
Blast NC 4	463	82	58	655	22	12
Blast NC 5	308	86	91	721	52	22
Blast NC 6	384	70	29	666	28	12
Blast rTg4510 1	695	189	62	830	103	44
Blast rTg4510 2	670	112	116	920	66	41
Blast rTg4510 3	242	95	67	450	97	36
Blast rTg4510 4	1095	255	166	1728	198	374
Blast rTg4510 5	375	153	92	740	109	12
Sham NC 1	306	159	94	610	43	31
Sham NC 2	751	134	176	995	102	87
Sham NC 3	509	132	105	761	31	16
Sham NC 4	503	102	74	690	69	12
Sham rTg4510 1	345	175	109	755	84	21
Sham rTg4510 2	585	183	99	879	162	31
Sham rTg4510 3	660	174	71	970	44	15
Sham rTg4510 4	965	241	127	997	117	68
Sham rTg4510 5	581	186	98	855	73	30

Learning Index (cumulative)	Learning Level
0.589266932	5
0.597600658	5
0.441660639	4
0.365651183	3
0.543733385	4
0.296623441	3
0.229734204	2
0.299421196	3
0.345719478	3
0.058017667	1
0.509254056	4
0.688813566	5
0.265097626	3
0.519052106	4
0.308292694	3
0.658834621	5
0.290444404	3
0.426762023	4
0.23910927	2
0.441616084	4
