# Supplementary material for: The Chronic Effects of a Single Low-Intensity Blast Exposure on Phosphoproteome Networks and Cognitive Function Influenced by Mutant Tau Overexpression
Source: Int J Mol Sci. 2024 Mar 15;25(6):3338. doi: 10.3390/ijms25063338 (PMC10970429; doi:10.3390/ijms25063338)
Supplement: Supplementary file 1 [file ijms-25-03338-s001.zip › Supplementary Table S4- Standard Peptide screening results.pdf]

Supplementary Table S4. Standard Peptide screening results

PhosPepNames	PearsonCorrelation	Phos site
QAS[Phospho (STY)]LDGLQQLR.2	-0.435532965	A1A5B6_S560_M1_Tbc1d25
GQSQLS[Phospho (STY)]NPTDDSWK.2	-0.253155967	A2A5R2_S1535_M1_Arfgef2
ERGS[Phospho (STY)]PVSGR.2	0.031730342	A2A5R2_S276_M1_Arfgef2
PQSPVIQATAGS[Phospho (STY)]PK.2	-0.055790115	A2A5R2_S227_M1_Arfgef2
QSLS[Phospho (STY)]SADNLEPDVQGHQVAAR.3	0.26961245	A2A5R2_S355_M1_Arfgef2
ELEKPM[Oxidation (M)]QSKPQS[Phospho (STY)]PVIQAT[Phospho (STY)]AGSPK.3	-0.156981257	A2A5R2_S218_M2_Arfgef2
HLDVDLDRQS[Phospho (STY)]LSSIDR.3	-0.091736215	A2A5R2_S1518_M1_Arfgef2
FS[Phospho (STY)]PPPVGGQSK.2	-0.00085794	A2A690_S1521_M1_Tanc2
QGQTS[Phospho (STY)]PIKPK.2	0.118939186	A2A690_S1981_M1_Tanc2
SC[Carbamidomethyl (C)]DELSPVS[Phospho (STY)]PTQGGYPSEPTR.2	0.170221241	A2A690_S1831_M1_Tanc2
EYPS[Phospho (STY)]PPPSPLR.2	-0.110855563	A2A690_S1534_M1_Tanc2
AS[Phospho (STY)]PPAESMSVYR.2	0.134465916	A2A690_S1549_M1_Tanc2
QIASDS[Phospho (STY)]PHAS[Phospho (STY)]PK.2	-0.226392502	A2A690_S400_M2_Tanc2
DC[Carbamidomethyl (C)]SYGAVTS[Phospho (STY)]PTSTLESR.2	0.111200597	A2A690_S169_M1_Tanc2
SQS[Phospho (STY)]ASYYPVC[Carbamidomethyl (C)]HSK.2	0.066336872	A2A690_S1726_M1_Tanc2
SNSSVGS[Phospho (STY)]PTR.2	-0.220065918	A2A690_S1480_M1_Tanc2
SSS[Phospho (STY)]QLGSPDVSHLIR.2	-0.218590358	A2A690_S1744_M1_Tanc2
PSQGLPVIQS[Phospho (STY)]PPSS[Phospho (STY)]PAHR.3	-0.147153569	A2A690_S1446_M2_Tanc2
[Acetyl (Protein N-term)]MFRNS[Phospho (STY)]LK.2	-0.212209003	A2A690_S5_M1_Tanc2
S[Phospho (STY)]VEGPGGLEPSLDEYR.2	0.070661014	A2A699_S445_M1_Fam171a2
S[Phospho (STY)]VEGPGGLEPSLDEYRR.3	0.043642478	A2A699_S445_M1_Fam171a2
TGLC[Carbamidomethyl (C)]SPEDNS[Phospho (STY)]LTPLLDEVVAPEGR.3	0.01593021	A2A699_S744_M1_Fam171a2
RDS[Phospho (STY)]LTSPEDELGAEVGDEAGDKK.3	0.087158463	A2A699_S785_M1_Fam171a2
RDS[Phospho (STY)]LTSPEDELGAEVGDEAGDK.3	0.089289566	A2A699_S785_M1_Fam171a2
DS[Phospho (STY)]LTSPEDELGAEVGDEAGDKK.3	0.003110529	A2A699_S785_M1_Fam171a2
IYRS[Phospho (STY)]QS[Phospho (STY)]PHYFQSGR.3	-0.18119354	A2A6A1_S1037_M2_Gpatch8
SEPPGSGS[Phospho (STY)]PAPPR.2	-0.058507595	A2A6A1_S735_M1_Gpatch8
SQS[Phospho (STY)]PHYFQSGR.2	-0.175348301	A2A6A1_S1039_M1_Gpatch8
KGS[Phospho (STY)]WGHES[Phospho (STY)]PEERR.3	-0.082378511	A2A6A1_S1013_M2_Gpatch8
SC[Carbamidomethyl (C)]S[Phospho (STY)]DTALNAIVAK.2	0.014935243	A2A6T1_S318_M1_Cdr2l
DLLGGEES[Phospho (STY)]PGEGK.2	0.214296404	A2A6T1_S407_M1_Cdr2l
AAHQES[Phospho (STY)]DNENEIQIQNQLKK.3	-0.201445477	A2A791_S121_M1_Zmym4
ENS[Phospho (STY)]KETFSGK.2	-0.275057912	A2A791_S161_M1_Zmym4
AAHQES[Phospho (STY)]DNENEIQIQNQLK.3	0.236003549	A2A791_S121_M1_Zmym4
GS[Phospho (STY)]PSGGSTAETSDTASIR.2	-0.123393278	A2A7S8_S402_M1_Kiaa1522
MGS[Phospho (STY)]PGSDPQKK.2	-0.327641555	A2A7S8_S1003_M1_Kiaa1522
GLAVAPAS[Phospho (STY)]PGK.2	0.112068132	A2A7S8_S543_M1_Kiaa1522
LQLERPVS[Phospho (STY)]PEAQADLQR.3	-0.147073175	A2A7S8_S909_M1_Kiaa1522
M[Oxidation (M)]GS[Phospho (STY)]PGSDPQK.2	-0.087722951	A2A7S8_S1003_M1_Kiaa1522
GSYS[Phospho (STY)]FDDHVADPEVPSR.2	-0.220574082	A2A884_S555_M1_Hivep3
RSS[Phospho (STY)]VESPK.2	-0.337414681	A2A884_S489_M1_Hivep3
VVFENEQDNNS[Phospho (STY)]LTK.2	-0.278940253	A2AAE1_S2755_M1_Kiaa1109
SDENVLDS[Phospho (STY)]PK.2	-0.301030036	A2AAE1_S1355_M1_Kiaa1109
TLKTES[Phospho (STY)]PNASR.2	-0.395006073	A2AAE1_S3894_M1_Kiaa1109

ALETIPIT[Phospho (STY)]PIER.2	-0.259991344	A2AAE1_T3996_M1_Kiaa1109
YTAGSAS[Phospho (STY)]PTPTFK.2	0.028112507	A2AAE1_S2603_M1_Kiaa1109
SLDQDS[Phospho (STY)]PSKK.2	-0.136428781	A2AAE1_S3835_M1_Kiaa1109
SSETFGPAGVRS[Phospho (STY)]PTEPTC[Carbamidomethyl (C)]R.3	-0.052295231	A2AAE1_S2737_M1_Kiaa1109
AS[Phospho (STY)]PGSSSYLK.2	0.407703412	A2AAE1_S3196_M1_Kiaa1109
S[Phospho (STY)]PTEPTC[Carbamidomethyl (C)]R.2	-0.174888812	A2AAE1_S2737_M1_Kiaa1109
SDENVLDS[Phospho (STY)]PKQR.2	-0.186500833	A2AAE1_S1355_M1_Kiaa1109
MVPETGIPAEC[Carbamidomethyl (C)]QS[Phospho (STY)]GQK.2	-0.295020925	A2AAE1_S628_M1_Kiaa1109
RDDGQASVPTEISGTS[Phospho (STY)]PVS[Phospho (STY)]PNTQDK.3	-0.025370189	A2AAE1_S1287_M2_Kiaa1109
KES[Phospho (STY)]IIKSEEELLER.3	-0.207955878	A2AAY5_S528_M1_Sh3pxd2b
LRPAKS[Phospho (STY)]QEK.2	-0.129696203	A2AAY5_S672_M1_Sh3pxd2b
AES[Phospho (STY)]PKQVDDPPEPVYANVER.3	-0.133058412	A2AB59_S215_M1_Arhgap27
KSS[Phospho (STY)]QDS[Phospho (STY)]DTPAQASPPEEK.2	0.002208889	A2AB59_S459_M2_Arhgap27
TQAQHSSPGEM[Oxidation (M)]ASS[Phospho (STY)]PQGLDNPALLR.3	-0.166551871	A2ADY9_S128_M1_Ddi2
SNS[Phospho (STY)]SEALLVDR.2	-0.366775273	A2AEV7_S357_M1_Ccdc120
VVITQS[Phospho (STY)]PGK.2	0.213038452	A2AF53_S333_M1_Tmem185a;Tmem185b
EES[Phospho (STY)]LEKR.2	-0.247548974	A2AG50_S401_M1_Map7d2
RLSS[Phospho (STY)]PVK.2	-0.055102309	A2AG50_S332_M1_Map7d2
VATSAASGGHGS[Phospho (STY)]PLR.2	-0.278028477	A2AG50_S315_M1_Map7d2
EGAPGQQSTVLPREES[Phospho (STY)]LEKR.3	-0.467825137	A2AG50_S401_M1_Map7d2
SSYKSS[Phospho (STY)]PTR.2	-0.312762549	A2AG50_S264_M1_Map7d2
S[Phospho (STY)]NSLDDSTEDVQSM[Oxidation (M)]DVSPVSK.2	0.083021672	A2AG50_S683_M1_Map7d2
NLIEGFNS[Phospho (STY)]PGQETTLNTFC[Carbamidomethyl (C)].2	-0.353588624	A2AG50_S770_M1_Map7d2
TAKPTYLGS[Phospho (STY)]PVK.2	-0.245213352	A2AG50_S357_M1_Map7d2
HLSSSTVAISYS[Phospho (STY)]PDR.2	0.12206038	A2AG50_S248_M1_Map7d2
SYVNRS[Phospho (STY)]PS[Phospho (STY)]PNVKPVEK.3	-0.095624206	A2AG58_S78_M2_Bclaf3
EVKPPAKEK.2	-0.107702083	#N/A
TSS[Phospho (STY)]PQPSTSR.2	0.06183461	A2AH22_S1206_M1_Ambra1
S[Phospho (STY)]PSDPGGELPEK.2	-0.138503504	A2AHC3_S1133_M1_Camsap1
VES[Phospho (STY)]LEALPILSR.2	-0.311738281	A2AHC3_S1407_M1_Camsap1
TDVS[Phospho (STY)]PPSPQMPR.2	0.254596723	A2AHC3_S550_M1_Camsap1
SVHREES[Phospho (STY)]YSDSGTK.3	-0.306060448	A2AHC3_S1359_M1_Camsap1
LERIES[Phospho (STY)]TEI.2	-0.179598438	A2AHG0_S697_M1_Lzts3
VHHSGS[Phospho (STY)]IQS[Phospho (STY)]FKQQK.3	-0.333111808	A2AHL1_S10_M2_Ano3
S[Phospho (STY)]LPC[Carbamidomethyl (C)]LAQSYAHSK.2	-0.391178995	A2AHL1_S36_M1_Ano3
VHHSGS[Phospho (STY)]IQSFK.3	-0.192369115	A2AHL1_S7_M1_Ano3
SFLSEPSS[Phospho (STY)]PGR.2	-0.23939333	A2AIV2_S1578_M1_Virma
HADGEKEDQFNGS[Phospho (STY)]PPRPQPR.4	0.221829023	A2AIV2_S173_M1_Virma
SC[Carbamidomethyl (C)]S[Phospho (STY)]VPLPSNHGEVDELR.3	0.231527803	A2AJ88_S379_M1_Pnpla7
S[Phospho (STY)]PAQAPEPAAEGLGR.2	-0.127811308	A2AJA9_S593_M1_Ajm1
PYLSEEPPRPS[Phospho (STY)]PR.2	-0.285025802	A2AJA9_S319_M1_Ajm1
ELATHAAPLGS[Phospho (STY)]YAR.3	-0.116624809	A2AJA9_S794_M1_Ajm1
GRS[Phospho (STY)]YENLLGR.2	0.048012129	A2AJA9_S468_M1_Ajm1
S[Phospho (STY)]WDNILAPGPR.2	-0.200159945	A2AJA9_S448_M1_Ajm1
S[Phospho (STY)]YENLLGR.2	-0.40382124	A2AJA9_S468_M1_Ajm1
ASQGLATAPAS[Phospho (STY)]PPVLQR.2	-0.413055793	A2AJA9_S109_M1_Ajm1
YAALSLSETSLT[Phospho (STY)]EK.2	-0.114656478	A2AJA9_T514_M1_Ajm1

S[Phospho (STY)]SPPWADWGPR.2	0.006911209	A2AJA9_S397_M1_Ajm1
RYAALSLSET[Phospho (STY)]S[Phospho (STY)]LTEK.2	-0.20162085	A2AJA9_S512_M2_Ajm1
VALAAGS[Phospho (STY)]PTRPPPAR.2	-0.208185563	A2AJA9_S854_M1_Ajm1
HVALS[Phospho (STY)]RT[Phospho (STY)]PT[Phospho (STY)]PSDLYC[Carbamidomethyl (C)]TDPR.3	0.181775989	A2AJA9_S236_M3_Ajm1
RSS[Phospho (STY)]QPS[Phospho (STY)]PTTVPASDSPPAK.2	-0.341443224	A2AJI0_S115_M2_Map7d1
SSQPS[Phospho (STY)]PTTVPASDSPPAK.2	-0.307490773	A2AJI0_S118_M1_Map7d1
SSATLWNS[Phospho (STY)]PSR.2	0.140304525	A2AJI0_S282_M1_Map7d1
RKPGAGGS[Phospho (STY)]PALAR.3	-0.572860775	A2AJI0_S401_M1_Map7d1
KPGAGGS[Phospho (STY)]PALAR.2	-0.124618672	A2AJI0_S401_M1_Map7d1
SAS[Phospho (STY)]ASPLTPC[Carbamidomethyl (C)]SAPR.2	-0.462444526	A2AJI0_S368_M1_Map7d1
S[Phospho (STY)]RGPT[Phospho (STY)]PTATGPR.2	-0.305933931	A2AJI0_S95_M2_Map7d1
AAEEKEPAAPAS[Phospho (STY)]PAPSPVPSPTPAQPQK.3	-0.198771516	A2AJI0_S544_M1_Map7d1
GPT[Phospho (STY)]PTATGPR.2	0.06828448	A2AJI0_T99_M1_Map7d1
LSTGSELS[Phospho (STY)]PK.2	-0.246439807	A2AJI0_S460_M1_Map7d1
QRS[Phospho (STY)]PIALPVK.2	-0.133518034	A2AJT4_S211_M1_Pnisr
SNRSS[Phospho (STY)]IER.2	-0.089727051	A2AJT4_S601_M1_Pnisr
SSSSVSPSSNAPGS[Phospho (STY)]C[Carbamidomethyl (C)]SPDGVDQR.2	0.252101255	A2ALK8_S467_M1_Ptpn3
S[Phospho (STY)]LS[Phospho (STY)]VERLETK.2	0.379731875	A2ALK8_S357_M2_Ptpn3
RS[Phospho (STY)]LS[Phospho (STY)]VER.2	0.129913324	A2ALK8_S357_M2_Ptpn3
RS[Phospho (STY)]LS[Phospho (STY)]VERLETK.2	0.248065681	A2ALK8_S357_M2_Ptpn3
RES[Phospho (STY)]PPAGQK.2	-0.271564563	A2ALS5_S515_M1_Rap1gap
DDVPFFGPPLPDPAVFR.2	0.066717712	#N/A
RSS[Phospho (STY)]AIGIENIQEVQEK.2	-0.041705382	A2ALS5_S499_M1_Rap1gap
TEVLQGFS[Phospho (STY)]R.2	0.188196896	A2ALS5_S566_M1_Rap1gap
SENSSTQS[Phospho (STY)]S[Phospho (STY)]PEM[Oxidation (M)]PTTK.2	0.144564706	A2ALS5_S541_M2_Rap1gap
S[Phospho (STY)]SAIGIENIQEVQEK.2	0.253200148	A2ALS5_S498_M1_Rap1gap
LPYTEGDAQQLQR.2	0.133234277	#N/A
SSAIGIENIQEVQEKRES[Phospho (STY)]PPAGQK.3	-0.392498426	A2ALS5_S515_M1_Rap1gap
TPDS[Phospho (STY)]GHVSQEPK.2	0.059422249	A2ALS5_S525_M1_Rap1gap
SQS[Phospho (STY)]M[Oxidation (M)]DAM[Oxidation (M)]GLSNK.2	-0.05905648	A2ALS5_S441_M1_Rap1gap
RES[Phospho (STY)]PPAGQKT[Phospho (STY)]PDSGHVSQEPK.3	-0.035355675	A2ALS5_S515_M2_Rap1gap
DSC[Carbamidomethyl (C)]SS[Phospho (STY)]PPSLNYGK.2	-0.247053251	A2ALU4_S1165_M1_Shroom2
IVHSES[Phospho (STY)]QPEKESR.2	-0.544621634	A2ALU4_S1089_M1_Shroom2
VENALNNLDDNPSPGDR.2	-0.373277363	#N/A
SPS[Phospho (STY)]PQFAPQK.2	0.118581385	A2ALU4_S1043_M1_Shroom2
HVTLPSS[Phospho (STY)]PR.2	-0.001081422	A2AN08_S2716_M1_Ubr4
TKEGVGS[Phospho (STY)]PK.2	-0.315486948	A2AN08_S457_M1_Ubr4
TLSDVEDQKELAS[Phospho (STY)]PVSPELR.3	-0.278837535	A2AN08_S178_M1_Ubr4
T[Phospho (STY)]GSTSSKEDDYESDAATIVQK.3	0.082592711	A2AN08_T360_M1_Ubr4
GTKPGS[Phospho (STY)]LDSHAAGQPLPR.3	-0.325105133	A2AP18_S987_M1_Plch2
VAS[Phospho (STY)]VEEGDETLDSPGSQSR.2	-0.335485364	A2AP18_S676_M1_Plch2
KVAS[Phospho (STY)]VEEGDETLDSPGSQSR.3	-0.23783375	A2AP18_S676_M1_Plch2
IRDC[Carbamidomethyl (C)]EDPNDFSVSTLS[Phospho (STY)]PSGK.3	-0.23230545	A2AP18_S619_M1_Plch2
KLPANISEDAEEGEVS[Phospho (STY)]DEDS[Phospho (STY)]ADEM[Oxidation (M)]EDDC[Carbamidomethyl (C)]K.3	0.180626838	A2AP18_S561_M2_Plch2
S[Phospho (STY)]IEDLHR.2	-0.35451446	A2APV2_S171_M1_Fmnl2
LLEQEALM[Oxidation (M)]EQQDAKS[Phospho (STY)]PSHK.3	-0.208982194	A2APV2_S1015_M1_Fmnl2
QAS[Phospho (STY)]FTYNK.2	0.239338198	A2APX8_S1939_M1_Scn1a

FS[Phospho (STY)]IEGNR.2	0.037310346	A2APX8_S537_M1_Scn1a
NSNLS[Phospho (STY)]QTSR.2	-0.16892278	A2APX8_S623_M1_Scn1a
RNS[Phospho (STY)]NLSQTSR.2	0.146529368	A2APX8_S620_M1_Scn1a
RDS[Phospho (STY)]LFVPR.2	-0.289286477	A2APX8_S607_M1_Scn1a
QQEAAQQAAATTAS[Phospho (STY)]EHSR.3	-0.289148967	A2APX8_S467_M1_Scn1a
RYS[Phospho (STY)]S[Phospho (STY)]PHQSLLSIR.3	-0.409347282	A2APX8_S550_M2_Scn1a
VSS[Phospho (STY)]EDPGPTPQTR.2	-0.093543637	A2AQ25_S1087_M1_Skt
NRPGS[Phospho (STY)]LDK.2	-0.356034274	A2AQ25_S1762_M1_Skt
IPALS[Phospho (STY)]PSSGK.2	0.042193021	A2AQ25_S1808_M1_Skt
NVY[Phospho (STY)]YELNDVR.2	0.119246598	A2AQ25_Y244_M1_Skt
IGGKS[Phospho (STY)]PPPPPPPPR.3	-0.192380679	A2AQ25_S1049_M1_Skt
KYPDSHLPTLGSKT[Phospho (STY)]PPAS[Phospho (STY)]PHR.4	-0.302083587	A2AQ25_S474_M2_Skt
RQEQLS[Phospho (STY)]PEGTLPASR.2	-0.065467806	A2AQ25_S1647_M1_Skt
HQEETAHAPGQPLHC[Carbamidomethyl (C)]STGS[Phospho (STY)]PGDVK.4	-0.015626118	A2AQ25_S869_M1_Skt
KAGGDC[Carbamidomethyl (C)]KPTS[Phospho (STY)]PSLPASK.3	-0.452888149	A2AQ25_S1796_M1_Skt
AAPTTSSSSSPPS[Phospho (STY)]PAS[Phospho (STY)]PTSLNQGAR.3	-0.246366544	A2AQ25_S1902_M2_Skt
T[Phospho (STY)]PPASPHR.2	0.062292852	A2AQ25_T470_M1_Skt
SIS[Phospho (STY)]PSPSAILER.2	-0.217809047	A2AQ25_S361_M1_Skt
S[Phospho (STY)]PPPPPPPPRR.2	-0.07066763	A2AQ25_S1049_M1_Skt
FQS[Phospho (STY)]PPHAGK.2	0.033007843	A2AQ25_S1868_M1_Skt
GDAAGDKLELSEDS[Phospho (STY)]PNS[Phospho (STY)]EQELDK.3	0.31100954	A2AQ25_S1035_M2_Skt
NEGFYADPYLYHEGR.3	-0.408268503	#N/A
ADREQS[Phospho (STY)]PVSK.2	-0.437470408	A2AR02_S685_M1_Ppig
FRRS[Phospho (STY)]ET[Phospho (STY)]PPHWR.3	-0.31628019	A2AR02_S354_M2_Ppig
S[Phospho (STY)]ET[Phospho (STY)]PPHWR.2	0.178613395	A2AR02_S354_M2_Ppig
SELNEIKENQRS[Phospho (STY)]PVR.3	-0.32290666	A2AR02_S395_M1_Ppig
HM[Oxidation (M)]S[Phospho (STY)]ES[Phospho (STY)]PNRKVEK.3	-0.321006844	A2AR02_S411_M2_Ppig
HMS[Phospho (STY)]ES[Phospho (STY)]PNR.2	-0.00456923	A2AR02_S411_M2_Ppig
KADREQS[Phospho (STY)]PVSK.2	0.22919744	A2AR02_S685_M1_Ppig
ENQRS[Phospho (STY)]PVR.2	-0.293103569	A2AR02_S395_M1_Ppig
RS[Phospho (STY)]ET[Phospho (STY)]PPHWR.2	-0.269164979	A2AR02_S354_M2_Ppig
S[Phospho (STY)]PPKADDKER.2	0.001936413	A2AR02_S288_M1_Ppig
T[Phospho (STY)]RSPVEKENQK.2	-0.038633558	A2AR02_T712_M1_Ppig
EQS[Phospho (STY)]PVSK.2	-0.269119326	A2AR02_S685_M1_Ppig
ALQTS[Phospho (STY)]PQPVEGTGLPR.2	-0.241455793	A2ARP1_S959_M1_Ppip5k1
TDPGS[Phospho (STY)]IENLC[Carbamidomethyl (C)]PGK.2	-0.510232271	A2ARP1_S939_M1_Ppip5k1
TLHS[Phospho (STY)]PPLQLR.2	0.036590974	A2ARP1_S1147_M1_Ppip5k1
GSNSDS[Phospho (STY)]PPGHPAPAPS[Phospho (STY)]PERR.3	-0.071105058	A2ARS0_S193_M2_Ankrd63
GSNSDS[Phospho (STY)]PPGHPAPAPSPER.2	-0.274456329	A2ARS0_S193_M1_Ankrd63
KTTAS[Phospho (STY)]PSVR.2	0.063661569	A2AWA9_S486_M1_Rabgap1
GVSDEDT[Phospho (STY)]DEEKETLK.2	0.062917554	A2AWA9_T991_M1_Rabgap1
QGDETPSTNNGS[Phospho (STY)]DDEKTGLK.2	-0.132853579	A2AWA9_S42_M1_Rabgap1
TTAS[Phospho (STY)]PSVR.2	0.114426671	A2AWA9_S486_M1_Rabgap1
LQWDGSSDLS[Phospho (STY)]PSDSGSSK.2	-0.066165776	A2AWT3_S281_M1_Atxn7l3
PYVPC[Carbamidomethyl (C)]PTPPRK.2	-0.195370206	#N/A
IELLPS[Phospho (STY)]R.2	-0.268378807	A2BH40_S1866_M1_Arid1a
SNS[Phospho (STY)]VGIQDAFPDGSDPTFQK.2	0.256615063	A2BH40_S1185_M1_Arid1a

SHHAPM[Oxidation (M)]S[Phospho (STY)]PGSSGGGGQPLAR.3	-0.158580532	A2BH40_S365_M1_Arid1a
GPS[Phospho (STY)]PSPVGSPASVAQSR.2	0.098010687	A2BH40_S697_M1_Arid1a
NPQMPQYTSPQPGS[Phospho (STY)]ALSPR.2	-0.115096907	A2BH40_S770_M1_Arid1a
SC[Carbamidomethyl (C)]S[Phospho (STY)]WHTLR.2	-0.25038205	A2CG49_S2374_M1_Kalrn
ATAAAESSDGS[Phospho (STY)]IKK.2	0.231215701	A2CG49_S2368_M1_Kalrn
TVSKS[Phospho (STY)]PER.2	0.152914464	A2CG63_S778_M1_Arid4b
SAS[Phospho (STY)]DTNLQK.2	-0.258104601	A2RSJ4_S952_M1_Uhrf1bp1l
SS[Phospho (STY)]PEISLR.2	-0.014908797	A2RSJ4_S1250_M1_Uhrf1bp1l
DVASTDS[Phospho (STY)]DSVLNYR.2	-0.182683033	A2RSJ4_S1006_M1_Uhrf1bp1l
SGS[Phospho (STY)]GEMTSEGSHTK.2	0.25445237	A2RSJ4_S988_M1_Uhrf1bp1l
NLGS[Phospho (STY)]PPKSPT[Phospho (STY)]HASPQHTQTEK.3	-0.237880382	A2RSJ4_S414_M2_Uhrf1bp1l
EEKQEHLTDS[Phospho (STY)]PVALGPER.3	0.269019596	A2RSQ0_S822_M1_Dennd5b
QEHLTDS[Phospho (STY)]PVALGPER.2	0.114533706	A2RSQ0_S822_M1_Dennd5b
QC[Carbamidomethyl (C)]RT[Phospho (STY)]PPQQKS[Phospho (STY)]PTTTR.3	-0.206684099	A2RSQ0_S1068_M2_Dennd5b
LQSDVLATGPANNNR.2	-0.245047958	#N/A
GSRPAS[Phospho (STY)]PAAR.2	-0.287119532	A2RSY1_S515_M1_Kansl3
TSGAKDDVS[Phospho (STY)]PEEQGK.2	-0.18426415	A2TJV2_S601_M1_Palm3
TGGEGS[Phospho (STY)]LDEEAEGSKK.2	-0.697506978	A2TJV2_S565_M1_Palm3
QGTSS[Phospho (STY)]PELPTWVK.2	-0.005253907	A2TJV2_S270_M1_Palm3
RSS[Phospho (STY)]S[Phospho (STY)]PAELELKDDLQQAHGR.4	-0.274277615	A3KGS3_S819_M2_Ralgapa2
DVYTIFDAEVESTS[Phospho (STY)]PK.2	-0.305661383	A6H5Z3_S261_M1_Exoc6b
EVS[Phospho (STY)]PAPATQGESR.2	-0.306911996	A6H619_S1201_M1_Phrf1
S[Phospho (STY)]RS[Phospho (STY)]PS[Phospho (STY)]LEHRPR.3	-0.082149219	A6H619_S1169_M3_Phrf1
[Acetyl (Protein N-term)]AES[Phospho (STY)]PAFLSAK.2	-0.025924711	A6H630_S4_M1_Armt1
DSVDDNHLS[Phospho (STY)]PSR.2	-0.029052357	A6H8H5_S488_M1_Kcnb2
S[Phospho (STY)]M[Oxidation (M)]ELIDVAVEK.2	-0.1003136	A6H8H5_S461_M1_Kcnb2
RNS[Phospho (STY)]APVSVSAVR.2	-0.385870114	A6X8Z5_S1163_M1_Arhgap31
S[Phospho (STY)]MDSLC[Carbamidomethyl (C)]SVPVEGK.2	-0.576263454	A6X8Z5_S343_M1_Arhgap31
SS[Phospho (STY)]PPPLSGASEVDAGELGSER.2	-0.166177867	A6X919_S23_M1_Dpy19l1
ETS[Phospho (STY)]LDLEPMAESEEQANPC[Carbamidomethyl (C)]R.2	0.247891717	A9Z1V5_S782_M1_Vwa5b1
SPQGLGAFTAVVR.2	-0.229165697	#N/A
QVNSTSSPS[Phospho (STY)]PEHLLAEDR.3	-0.393842518	B1AVZ0_S25_M1_Uprt
SAS[Phospho (STY)]EDS[Phospho (STY)]IRKK.2	-0.228011794	B1AWN6_S528_M2_Scn2a
KVSS[Phospho (STY)]IYKK.2	-0.409360635	B1AWN6_S1931_M1_Scn2a
KSAS[Phospho (STY)]EDSIR.2	-0.147719646	B1AWN6_S528_M1_Scn2a
FS[Phospho (STY)]LEGSR.2	-0.2976281	B1AWN6_S540_M1_Scn2a
S[Phospho (STY)]AS[Phospho (STY)]EDSIRK.2	-0.167622939	B1AWN6_S526_M2_Scn2a
QQEEAQAAAAAASAES[Phospho (STY)]R.2	0.064775201	B1AWN6_S471_M1_Scn2a
LSSKS[Phospho (STY)]EKELK.2	-0.16719865	B1AWN6_S497_M1_Scn2a
GSLFS[Phospho (STY)]PR.2	-0.252043611	B1AWN6_S568_M1_Scn2a
[Acetyl (Protein N-term)]AQSVLVPPGPDSFR.2	0.274093006	#N/A
KQQEEAQAAAAAASAES[Phospho (STY)]R.3	-0.073649174	B1AWN6_S471_M1_Scn2a
GFRFS[Phospho (STY)]LEGSR.2	0.051798004	B1AWN6_S540_M1_Scn2a
VKDIGS[Phospho (STY)]ENDFADDEHSTFEDNDSRR.4	-0.11255854	B1AWN6_S589_M1_Scn2a
KSAS[Phospho (STY)]EDS[Phospho (STY)]IRK.2	-0.133818867	B1AWN6_S528_M2_Scn2a
DFSGAGGIGVFS[Phospho (STY)]ESSSVASK.2	-0.258957299	B1AWN6_S484_M1_Scn2a
AM[Oxidation (M)]SM[Oxidation (M)]ASILT[Phospho (STY)]NT[Phospho (STY)]M[Oxidation (M)]EELEESR.2	-0.1271879	B1AWN6_T713_M2_Scn2a

RPS[Phospho (STY)]NVSQASR.2	0.015167049	B1AWN6_S623_M1_Scn2a
SSSYHVS[Phospho (STY)]M[Oxidation (M)]DLLEDPTSR.3	-0.247953625	B1AWN6_S692_M1_Scn2a
RFS[Phospho (STY)]S[Phospho (STY)]PHQSLLSIR.3	-0.001891409	B1AWN6_S553_M2_Scn2a
TLDFDPLLSPAS[Phospho (STY)]PK.2	-0.022180243	B1AXD8_S21_M1_Akirin2
IIAQFS[Phospho (STY)]KDYEPTDNPST.2	-0.081743262	B1AXH1_S1208_M1_Nhsl2
SKS[Phospho (STY)]IS[Phospho (STY)]LKK.2	0.077645398	B1AXH1_S557_M2_Nhsl2
ATTPSQLS[Phospho (STY)]IEVEAR.2	-0.085732958	B1AXH1_S690_M1_Nhsl2
RIIAQFS[Phospho (STY)]KDYEPTDNPST.3	0.070163022	B1AXH1_S1208_M1_Nhsl2
S[Phospho (STY)]LSVPTDSGVTSVDYDEEQK.2	-0.006867856	B1AXH1_S497_M1_Nhsl2
DQGHSSS[Phospho (STY)]PTGSLAR.2	0.310581302	B1AXH1_S301_M1_Nhsl2
NPARDEEGVFENNR.3	0.207704513	#N/A
S[Phospho (STY)]PQGFFQSSNK.2	-0.164232396	B1AY10_S81_M1_Nfx1
EHS[Phospho (STY)]PSESEKEVVIADPR.3	0.317606829	B1AY10_S147_M1_Nfx1
AQGLSEQT[Phospho (STY)]SDTSSLESVAR.2	-0.311826795	B1AY10_T125_M1_Nfx1
NYSSS[Phospho (STY)]PPC[Carbamidomethyl (C)]HLPR.2	-0.07585534	B1AY10_S51_M1_Nfx1
VSDQNS[Phospho (STY)]PVLPK.2	-0.24068555	B1AY13_S2044_M1_Usp24
NMDQGGGGS[Phospho (STY)]PR.2	0.251144091	B1AY13_S1940_M1_Usp24
TIS[Phospho (STY)]AQDTLAYATALLNEK.2	-0.07437202	B1AY13_S2558_M1_Usp24
VSDQNS[Phospho (STY)]PVLPKK.2	-0.252961864	B1AY13_S2044_M1_Usp24
SSPSSPS[Phospho (STY)]PTLGR.2	-0.211878167	B1AZA5_S329_M1_Tmem245
GGPAEAPS[Phospho (STY)]PR.2	-0.093582212	B1AZA5_S12_M1_Tmem245
GGPAEAPS[Phospho (STY)]PRGS[Phospho (STY)]PRPESR.3	-0.132088441	B1AZA5_S12_M2_Tmem245
KIDSHPS[Phospho (STY)]PSHSSTVK.3	0.385353585	B1AZI6_S1417_M1_Thoc2
ATQQS[Phospho (STY)]LGEQSNPR.2	0.050441777	B1AZP2_S405_M1_Dlgap4
QNS[Phospho (STY)]ATESADSIEIYVPEAQTR.2	-0.136074041	B1AZP2_S973_M1_Dlgap4
ANSWQLVET[Phospho (STY)]PEK.2	-0.308145078	B1AZP2_T915_M1_Dlgap4
SNISGWWS[Phospho (STY)]SDDNLDGEGGAFR.2	-0.203779215	B1AZP2_S206_M1_Dlgap4
ANSWQLVET[Phospho (STY)]PEKR.2	-0.379692544	B1AZP2_T915_M1_Dlgap4
APS[Phospho (STY)]M[Oxidation (M)]EGTAGK.2	0.236818718	B1AZP2_S156_M1_Dlgap4
ATQQS[Phospho (STY)]LGEQS[Phospho (STY)]NPRR.2	-0.22887278	B1AZP2_S405_M2_Dlgap4
SLPDC[Carbamidomethyl (C)]TSHPNSIS[Phospho (STY)]IDAGPR.3	0.105280846	B1AZP2_S744_M1_Dlgap4
AM[Oxidation (M)]GDEDS[Phospho (STY)]DESGGGS[Phospho (STY)]PKPSPK.2	-0.27269134	B1AZP2_S377_M2_Dlgap4
S[Phospho (STY)]LDRLDSVDM[Oxidation (M)]LLPSK.2	-0.2085244	B1AZP2_S415_M1_Dlgap4
RS[Phospho (STY)]LDRLDS[Phospho (STY)]VDM[Oxidation (M)]LLPSK.3	0.204344499	#N/A
RDT[Phospho (STY)]DS[Phospho (STY)]DTQDANDSSC[Carbamidomethyl (C)]K.2	-0.349702258	B1AZP2_S716_M2_Dlgap4
IHRAS[Phospho (STY)]DPGLPAEEPK.3	-0.484799345	B2RQC6_S1859_M1_Cad
ADGIPNDSS[Phospho (STY)]DSEM[Oxidation (M)]EDR.2	0.474531659	B2RR83_S1105_M1_Ythdc2
STDSSSYPSPC[Carbamidomethyl (C)]AS[Phospho (STY)]PS[Phospho (STY)]PPSSGK.2	0.166376469	B2RR83_S1282_M2_Ythdc2
TAADVVS[Phospho (STY)]PGANSVDR.2	-0.194855769	B2RRE7_S1000_M1_Otud4
EES[Phospho (STY)]S[Phospho (STY)]EDENEVSNILR.2	-0.094053966	B2RRE7_S1016_M2_Otud4
LQRPKEES[Phospho (STY)]S[Phospho (STY)]EDENEVSNILR.3	-0.266282554	B2RRE7_S1016_M2_Otud4
LQDPNNPAIIR.3	-0.094403646	#N/A
DSGVQAC[Carbamidomethyl (C)]FNR.2	-0.136940836	#N/A
IAQPNYIPTQQDVLR.2	-0.385382032	#N/A
EYQLNDSAAYYLNDLDR.2	-0.1532509	#N/A
IDFGDSAR.2	-0.176434608	#N/A
SREYQLNDSAAYYLNDLDR.3	-0.344389277	#N/A

AVVYSNTIQSIIAIIR.2	-0.301515349	#N/A
VVGT[Phospho (STY)]PGGSEVGQR.2	-0.121378593	B2RUJ5_T333_M1_Apba1
QDIS[Phospho (STY)]PTR.2	-0.248991997	B2RUJ5_S391_M1_Apba1
APT[Phospho (STY)]PGGGHPDS[Phospho (STY)]PGLPAPAGQQQR.3	-0.29332715	B2RUJ5_S317_M2_Apba1
M[Oxidation (M)]DS[Phospho (STY)]YEQEEDIDQIVAEVK.2	0.423334873	B2RUJ5_S267_M1_Apba1
QSM[Oxidation (M)]SSQS[Phospho (STY)]LDK.2	-0.000969638	B2RUJ5_S289_M1_Apba1
SNS[Phospho (STY)]QENVEASHPSQDGKR.3	-0.583122987	B2RUJ5_S573_M1_Apba1
SNS[Phospho (STY)]QENVEASHPSQDGK.2	-0.428416503	B2RUJ5_S573_M1_Apba1
SDGES[Phospho (STY)]DSPEKEAEFAPYPR.3	-0.168618561	B2RUJ5_S250_M1_Apba1
SAS[Phospho (STY)]TESGFHNHTDTAEGDVLAAAR.3	0.230981389	B2RUJ5_S84_M1_Apba1
LHHY[Phospho (STY)]DERSDGESDS[Phospho (STY)]PEKEAEFAPYPR.4	-0.142225441	B2RUJ5_Y242_M2_Apba1
AAEDM[Oxidation (M)]PEAEQDLERAPT[Phospho (STY)]PGGGHPDS[Phospho (STY)]PGLPAPAGQQQR.4	0.092956511	B2RUJ5_S317_M2_Apba1
LHHYDERS[Phospho (STY)]DGESDSPEK.3	-0.026667083	B2RUJ5_S246_M1_Apba1
[Acetyl (Protein N-term)]ADS[Phospho (STY)]LDEFIEEQK.2	-0.176529342	B2RX88_S4_M1_Cspp1
S[Phospho (STY)]LQLVDSR.2	-0.233259613	B2RY04_S1765_M1_Dock5
LGASNS[Phospho (STY)]PGQPNSVK.2	-0.246451898	B2RY56_S672_M1_Rbm25
LGASNS[Phospho (STY)]PGQPNSVKR.2	-0.30794816	B2RY56_S672_M1_Rbm25
GWTYS[Phospho (STY)]PAPAR.2	-0.092710197	B5TVM2_S511_M1_Ildr2
GGS[Phospho (STY)]LETPSK.2	-0.215170892	B5TVM2_S562_M1_Ildr2
AHGAFYQDGS[Phospho (STY)]LDEYYGR.2	-0.294378379	B5TVM2_S487_M1_Ildr2
SPSS[Phospho (STY)]PSLGSLQQR.2	0.15356491	B9EHT4_S402_M1_Clip3
KKS[Phospho (STY)]PS[Phospho (STY)]SPSLGSLQQR.3	0.219892471	B9EHT4_S399_M2_Clip3
VQTELKEETQPLSHS[Phospho (STY)]PK.3	-0.161076127	B9EJ80_S515_M1_Pdzd8
VQTELKEETQPLSHS[Phospho (STY)]PKR.4	0.180236417	B9EJ80_S515_M1_Pdzd8
KES[Phospho (STY)]LKVQK.2	-0.111813202	B9EJ86_S120_M1_Osbpl8
GYSSPEPDIQDS[Phospho (STY)]SGSEAQSVKPSTR.3	0.342797558	B9EJ86_S807_M1_Osbpl8
DVS[Phospho (STY)]PTSRDNLVAK.2	0.067460225	B9EJA2_S472_M1_Cttnbp2
DVS[Phospho (STY)]PTSR.2	0.008203012	B9EJA2_S472_M1_Cttnbp2
QSSHS[Phospho (STY)]DLGPSPPTALPSSANR.3	-0.446373444	B9EJA2_S362_M1_Cttnbp2
LSLGS[Phospho (STY)]DDEADLVK.2	-0.165117471	B9EJA2_S1510_M1_Cttnbp2
FQGNANDPDQNGNNTQS[Phospho (STY)]PPSR.2	-0.17204542	B9EJA2_S465_M1_Cttnbp2
LASISVATEGPVTR.2	-0.133196311	#N/A
YQY[Phospho (STY)]LNTR.2	0.084364045	B9EJX3_Y114_M1_ATp6v1fnb
SVSEINS[Phospho (STY)]DDELPGK.2	0.17052862	B9EKI3_S340_M1_Tmf1
EVSADVS[Phospho (STY)]EEENFLTDFK.2	-0.154177497	B9EKR1_S576_M1_Ptprz1
FAVLYQPLAGNDQAK.2	-0.218852557	#N/A
FSSFEEAVK.2	-0.233046937	#N/A
FSEQGLLK.2	0.139206788	#N/A
QSPINIDEDLTQVNVNLKK.3	-0.190548209	#N/A
QSPINIDEDLTQVNVNLK.2	0.184039184	#N/A
DTDGVLETDDTGIAPGSPR.2	0.23088718	#N/A
ASLENTFIHNTGK.2	-0.299967812	#N/A
C[Carbamidomethyl (C)]MSC[Carbamidomethyl (C)]LPYRES[Phospho (STY)]R.2	0.178569082	#N/A
C[Carbamidomethyl (C)]MSC[Carbamidomethyl (C)]LPYRES[Phospho (STY)]REK.3	-0.359376284	#N/A
IVEPEVVGES[Phospho (STY)]DSEVEGDAWR.3	0.178591221	C0HKD8_S116_M1_Mfap1a;Mfap1b
SLAALDALNTDDENDEEEY[Phospho (STY)]EAWK.3	-0.363654439	C0HKD8_Y276_M1_Mfap1a;Mfap1b
KIVEPEVVGES[Phospho (STY)]DS[Phospho (STY)]EVEGDAWR.3	-0.533857493	C0HKD8_S116_M2_Mfap1a;Mfap1b

RPDYAPM[Oxidation (M)]ES[Phospho (STY)]S[Phospho (STY)]DEEDEEFQFIK.3	-0.016209217	C0HKD8_S52_M2_Mfap1a;Mfap1b
NDEELNKLLGR.2	-0.356200222	#N/A
REDYQEILDS[Phospho (STY)]PIK.2	-0.027716358	D3YVE8_S409_M1_Slc35g2
EDYQEILDS[Phospho (STY)]PIK.2	-0.02107422	D3YVE8_S409_M1_Slc35g2
GPVAAS[Phospho (STY)]PQK.2	0.030479289	D3YVF0_S22_M1_Akap5
VTVDHAEEATVGQAEEATVGQAEK.3	-0.48370571	#N/A
VTVDQAEEATVGQAEEATVGHTEK.3	-0.380358253	#N/A
VQGEADDLEIK.2	0.159140656	#N/A
LSQAEETTVAQAK.2	-0.019289119	#N/A
ASLLESSAAGS[Phospho (STY)]PR.2	-0.311812323	D3YVF0_S251_M1_Akap5
ALSQAGEATAGHPEEATVIQAESQAK.3	-0.068414564	#N/A
QPAGAWAS[Phospho (STY)]IK.2	-0.216604389	D3YVF0_S79_M1_Akap5
SSAIQM[Oxidation (M)]GT[Phospho (STY)]PELEKETK.2	-0.524925296	D3YVF0_T220_M1_Akap5
DEKGPVAAS[Phospho (STY)]PQKER.2	-0.395499806	D3YVF0_S22_M1_Akap5
AQTQPDDQAIQAGS[Phospho (STY)]TQGLQEGVLVR.3	0.354770178	D3YVF0_S171_M1_Akap5
GPVAAS[Phospho (STY)]PQKER.2	-0.21783314	D3YVF0_S22_M1_Akap5
KATVGQAEEATIDHTEK.3	0.0086434	#N/A
TSEQYETLLIETASSLVK.2	-0.330226739	#N/A
DEKGPVAAS[Phospho (STY)]PQK.2	-0.083925143	D3YVF0_S22_M1_Akap5
SSAIQMGT[Phospho (STY)]PELEK.2	0.300983941	D3YVF0_T220_M1_Akap5
HTPEAGGS[Phospho (STY)]GQR.2	-0.017081737	D3YVF0_S68_M1_Akap5
EATVVGQAEEATVDRAEEATVGQAEEATVGHTEK.3	-0.116105129	#N/A
METS[Phospho (STY)]VSEIQVETK.2	0.004451892	D3YVF0_S4_M1_Akap5
TAEETKKHT[Phospho (STY)]PEAGGSGQR.3	-0.327117051	D3YVF0_T62_M1_Akap5
VTVDQAEETTVGQAEEATVGQAGEAILSQAK.3	0.001385778	#N/A
METSVSEIQVETKDEKGPVAAS[Phospho (STY)]PQK.3	0.454906438	D3YVF0_S22_M1_Akap5
SGETALGQAEEAAVGQADKR.2	-0.365247743	#N/A
QFLISM[Oxidation (M)]ENEQVGVFANDSDFEGR.3	0.312075354	#N/A
VKKPQNS[Phospho (STY)]LK.2	-0.222015775	D3YWQ0_S328_M1_Dgki
VPLHLES[Phospho (STY)]PHEER.3	-0.521142483	D3YXJ0_S1067_M1_Dgkh
KVS[Phospho (STY)]TSGQMR.2	-0.057810484	D3YXJ0_S53_M1_Dgkh
AAAATAPPS[Phospho (STY)]PGPAQPGPR.2	-0.210832742	D3YXK1_S150_M1_Samd1
YLGGS[Phospho (STY)]GGASGR.2	0.04265267	D3YXK1_S242_M1_Samd1
APTAALS[Phospho (STY)]PEPQDSK.2	-0.087605788	D3YXK2_S366_M1_Safb
APTAALS[Phospho (STY)]PEPQDSKEDVK.2	-0.415774352	D3YXK2_S366_M1_Safb
SVVS[Phospho (STY)]FDKVK.2	0.168468415	D3YXK2_S626_M1_Safb
APTAALS[Phospho (STY)]PEPQDSKEDVKK.3	-0.398123025	D3YXK2_S366_M1_Safb
[Acetyl (Protein N-term)]AETLSGLGDASAAGAAAVSSAAS[Phospho (STY)]ETGTRR.3	0.099252903	D3YXK2_S24_M1_Safb
[Acetyl (Protein N-term)]AETLSGLGDASAAGAAAVSSAASETGTR.3	0.058369814	#N/A
AIEDEGGNPDEIEVTSEC[Carbamidomethyl (C)]NK.2	0.025079668	#N/A
MSS[Phospho (STY)]VEEDSDTKR.2	-0.593908977	D3YXK2_S406_M1_Safb
SEPVKEEGS[Phospho (STY)]ELEQPFAQATSSVGPDR.3	-0.300442251	D3YXK2_S235_M1_Safb
RPYEVDGR.2	0.06574937	#N/A
SSS[Phospho (STY)]PDKFK.2	-0.34272035	D3YZP9_S412_M1_Ccdc6
RSSS[Phospho (STY)]PDKFK.2	-0.307200968	D3YZP9_S412_M1_Ccdc6
LDQPVSAPPS[Phospho (STY)]PR.2	-0.51039039	D3YZP9_S237_M1_Ccdc6
QLS[Phospho (STY)]ESESSLEM[Oxidation (M)]DDER.2	0.095940157	D3YZP9_S316_M1_Ccdc6

EQDVVPFQES[Phospho (STY)]PK.2	-0.156942798	D3YZU1_S413_M1_Shank1
S[Phospho (STY)]IDEGM[Oxidation (M)]FSAEPYLR.2	-0.330251313	D3YZU1_S1291_M1_Shank1
GSST[Phospho (STY)]EDGPGVPPPSPR.2	0.236658353	D3YZU1_T1518_M1_Shank1
EQDVVPFQES[Phospho (STY)]PKYAAR.2	-0.472822276	D3YZU1_S413_M1_Shank1
DGPAGGTGGSGGPGGS[Phospho (STY)]LGSR.2	-0.284303508	D3YZU1_S540_M1_Shank1
NHREQDVVPFQES[Phospho (STY)]PK.3	-0.326435558	D3YZU1_S413_M1_Shank1
GFFATESS[Phospho (STY)]FDPHHR.2	0.243657297	D3YZU1_S869_M1_Shank1
ALDPASPLGLALAAR.2	-0.434969948	#N/A
QKS[Phospho (STY)]IGAAEDDRPYLAPPAMK.3	0.256051215	D3YZU1_S898_M1_Shank1
AGLGS[Phospho (STY)]QEK.2	-0.147049365	D3YZU1_S1434_M1_Shank1
GGSGGS[Phospho (STY)]TDSHHGGASYIPER.3	0.058706531	D3YZU1_S1907_M1_Shank1
GRPSS[Phospho (STY)]SGTPR.2	0.095233175	D3YZU1_S519_M1_Shank1
S[Phospho (STY)]IGAAEDDRPYLAPPAM[Oxidation (M)]K.3	-0.047994118	D3YZU1_S898_M1_Shank1
THS[Phospho (STY)]PATS[Phospho (STY)]EDEER.2	0.344891096	D3YZU1_S4_M2_Shank1
HS[Phospho (STY)]KSIDEGMFSAEPYLR.3	-0.078432876	D3YZU1_S1289_M1_Shank1
VGWFPSDC[Carbamidomethyl (C)]LEEVANRS[Phospho (STY)]QEGR.3	0.065976237	D3YZU1_S615_M1_Shank1
LESGGS[Phospho (STY)]SGGYGAYAAGSR.2	0.051665189	D3YZU1_S1310_M1_Shank1
RPHS[Phospho (STY)]PDPGAQPAPPR.3	-0.24497265	D3Z2R5_S13_M1_Selenon
ASGSGSC[Carbamidomethyl (C)]NAPGS[Phospho (STY)]PEGSSQLC[Carbamidomethyl (C)]SPR.2	0.065013675	D3Z6Q9_S430_M1_Bin2
AS[Phospho (STY)]PDASSNPEPAETR.2	0.320846788	D3Z6Q9_S443_M1_Bin2
IEKGDVS[Phospho (STY)]EEEDFR.2	-0.060887657	D3Z7H4_S274_M1_Gsg1l
VADYIPQLAK.2	0.122305982	#N/A
VLSPEAVR.2	0.219189931	#N/A
LTLQTTSDGVM[Oxidation (M)]LDKDLFK.3	-0.110744302	#N/A
DSPGETDAFGNSEGK.2	0.268235406	#N/A
C[Carbamidomethyl (C)]VQSNIVLLTQAFR.2	0.213338592	#N/A
YAIAVNDLGTEYVHR.2	-0.511599874	#N/A
M[Oxidation (M)]AGNEYVGFSNATFQSER.2	-0.434097879	#N/A
ILQEYQVQYTPQGDSDDGKGNQTVHK.3	0.317763923	#N/A
TALHVAAAEGHVEVVK.2	-0.092845078	#N/A
FALSAMDMEQR.2	-0.467872998	#N/A
SVINLLFAAYTGDVSALR.3	-0.029726208	#N/A
ILQEYQVQYTPQGDSDDGK.2	-0.205241596	#N/A
AS[Phospho (STY)]PSPSPSEAR.2	0.00952271	E9PUL5_S98_M1_Prrt2
AHGGHPGS[Phospho (STY)]PRGS[Phospho (STY)]LSR.3	-0.357200825	E9PUL5_S244_M2_Prrt2
HPS[Phospho (STY)]SQLAGPGVEGGEGTQKPR.3	-0.616732495	E9PUL5_S254_M1_Prrt2
T[Phospho (STY)]PPANGAPPR.2	0.013874494	E9PUL5_T214_M1_Prrt2
QENGAVVPLQAGDGEEGPAPQPHS[Phospho (STY)]PPSTK.3	-0.276573009	E9PUL5_S208_M1_Prrt2
[Acetyl (Protein N-term)]AASS[Phospho (STY)]SQVSEMK.2	0.217542625	E9PUL5_S5_M1_Prrt2
AELAPETTETPVETPETVQATDLSLNPEEGSKAS[Phospho (STY)]PSPSPSEAR.4	-0.152215792	E9PUL5_S98_M1_Prrt2
AHGGHPGS[Phospho (STY)]PR.2	-0.303353514	E9PUL5_S244_M1_Prrt2
AELAPETTETPVETPETVQATDLSLNPEEGSK.3	-0.357616063	#N/A
SLS[Phospho (STY)]EPEELAR.2	0.196412314	E9PV82_S119_M1_Fam53a
AAS[Phospho (STY)]PSFQAR.2	-0.028297019	E9PV86_S16_M1_Mctp1
GSSTATGTGGVT[Phospho (STY)]PPGGR.2	-0.124500034	E9PV86_T145_M1_Mctp1
S[Phospho (STY)]GS[Phospho (STY)]PAPETTNESVPFAQHSSLDSR.3	0.249099374	E9PYH6_S481_M2_Setd1a
TSS[Phospho (STY)]FHLLR.2	-0.271532	E9PYL2_S1070_M1_Prr12

TEDEEFLIQHLLQAPS[Phospho (STY)]PPR.3	0.08438699	E9PYL2_S648_M1_Prr12
ASLAC[Carbamidomethyl (C)]SPLGGGEPS[Phospho (STY)]PGAGEPSK.2	0.001032565	E9PYL2_S338_M1_Prr12
NLETLPSFS[Phospho (STY)]S[Phospho (STY)]DEEDSVAK.2	-0.017688237	E9PYL2_S1372_M2_Prr12
TGS[Phospho (STY)]PELAAR.2	-0.045649881	E9PZ19_S1215_M1_Igsf9b
AEAEAEATT[Phospho (STY)]PIELISR.2	0.205518076	E9PZ19_T845_M1_Igsf9b
SLES[Phospho (STY)]PLSSGKVS[Phospho (STY)]PESIR.2	-0.383217008	E9PZ19_S775_M2_Igsf9b
APS[Phospho (STY)]ESS[Phospho (STY)]DDQGQPAAK.2	-0.646448848	E9PZ19_S794_M2_Igsf9b
LKDEHGLEPAS[Phospho (STY)]PR.3	-0.419941218	E9PZM4_S1365_M1_Chd2
KQES[Phospho (STY)]S[Phospho (STY)]DDDDDDDEAPKR.2	-0.106139396	E9PZM4_S207_M2_Chd2
SPLDHRS[Phospho (STY)]PLER.3	-0.193925347	E9PZM4_S1806_M1_Chd2
LEEPPELNRQS[Phospho (STY)]PNPR.3	-0.219988802	E9Q1P8_S169_M1_Irf2bp2
S[Phospho (STY)]PTGAQPAAAKPPPLSAK.2	-0.394872336	E9Q1P8_S71_M1_Irf2bp2
RKPS[Phospho (STY)]PEPEGEVGPPK.3	-0.025158504	E9Q1P8_S343_M1_Irf2bp2
S[Phospho (STY)]PADSLSSAAGASELSAEGAGK.2	-0.487520928	E9Q1P8_S250_M1_Irf2bp2
DTLLALHQHGHS[Phospho (STY)]GPFESK.3	-0.282990828	E9Q1P8_S300_M1_Irf2bp2
NSSS[Phospho (STY)]PPSPSSMNQR.2	-0.300887813	E9Q1P8_S440_M1_Irf2bp2
RYDS[Phospho (STY)]DDDLPR.2	0.098941898	E9Q343_S351_M1_Gpr137c
TSSVSSISQVS[Phospho (STY)]PER.2	-0.215481492	E9Q3L2_S268_M1_Pi4ka
KTSSVSSISQVS[Phospho (STY)]PER.2	-0.052730132	E9Q3L2_S268_M1_Pi4ka
EAAVPEEEGGT[Phospho (STY)]PEKEISIEDAK.3	-0.139743821	E9Q401_T1864_M1_Ryr2
EAAVPEEEGGT[Phospho (STY)]PEK.2	-0.004108288	E9Q401_T1864_M1_Ryr2
SNC[Carbamidomethyl (C)]YM[Oxidation (M)]VC[Carbamidomethyl (C)]AGESM[Oxidation (M)]S[Phospho (STY)]PGQGR.2	-0.038492099	#N/A
IAEDPSRDGPS[Phospho (STY)]PTSGSSK.2	-0.196388636	E9Q401_S2362_M1_Ryr2
DGPS[Phospho (STY)]PTSGSSK.2	0.239721838	E9Q401_S2362_M1_Ryr2
ISQTSQVS[Phospho (STY)]IDAAHGYSPR.3	-0.268109449	E9Q401_S2813_M1_Ryr2
APS[Phospho (STY)]PASFQR.2	-0.344269131	E9Q4N7_S1550_M1_Arid1b
SS[Phospho (STY)]PALAAPDASVDPK.2	-0.458908879	E9Q4N7_S1757_M1_Arid1b
RLS[Phospho (STY)]GNEYVFTK.2	-0.132279833	E9Q4S1_S497_M1_Pde8b
IHRDS[Phospho (STY)]GDNSQTEPHSFR.3	-0.36259767	E9Q4S1_S377_M1_Pde8b
FVNSINKPLAAESEGS[Phospho (STY)]DC[Carbamidomethyl (C)]EC[Carbamidomethyl (C)]NPTGK.3	0.328350012	E9Q4S1_S734_M1_Pde8b
ASS[Phospho (STY)]PFRR.2	0.201413238	E9Q5C9_S626_M1_Nolc1
GGSISVQVNSVKFDS[Phospho (STY)]E.2	-0.028286376	E9Q5C9_S701_M1_Nolc1
AAKES[Phospho (STY)]EEEEEEEETEEK.2	-0.180482073	E9Q5C9_S563_M1_Nolc1
TGS[Phospho (STY)]PTPSNQLNDSPTFK.2	-0.038656646	E9Q5F9_S614_M1_Setd2
RGS[Phospho (STY)]LS[Phospho (STY)]PPSSAYER.2	0.381827246	E9Q5F9_S2053_M2_Setd2
RRGS[Phospho (STY)]LS[Phospho (STY)]PPSSAYER.3	-0.283854826	E9Q5F9_S2053_M2_Setd2
AKS[Phospho (STY)]PT[Phospho (STY)]PDGSER.2	-0.123787423	E9Q5K9_S146_M2_Ythdc1
RAKS[Phospho (STY)]PT[Phospho (STY)]PDGSER.2	-0.173411631	E9Q5K9_S146_M2_Ythdc1
GIS[Phospho (STY)]PIVFDR.2	-0.135714468	E9Q5K9_S309_M1_Ythdc1
S[Phospho (STY)]LDDLSAPPHHR.2	-0.357584029	E9Q6B2_S247_M1_Ccdc85c
EVS[Phospho (STY)]PPGAR.2	-0.096204234	E9Q6J5_S3010_M1_Bod1l
TETDITTVEQSS[Phospho (STY)]PSGK.2	-0.440861308	E9Q6J5_S2841_M1_Bod1l
YYS[Phospho (STY)]DSDDELTVEQR.2	0.084089524	E9Q6J5_S480_M1_Bod1l
AQLS[Phospho (STY)]PSVKR.2	-0.206584249	E9Q6J5_S3000_M1_Bod1l
KLS[Phospho (STY)]SQPSTDVSTDKER.3	-0.504429499	E9Q6J5_S236_M1_Bod1l
GSDDVLVSGEVPEC[Carbamidomethyl (C)]EVGHM[Oxidation (M)]S[Phospho (STY)]PR.3	-0.110843366	E9Q6J5_S2001_M1_Bod1l
LPVSSS[Phospho (STY)]TSNLHVDR.2	-0.061032139	E9Q6P5_S160_M1_Ttc7b

SC[Carbamidomethyl (C)]YNLTNPSDSGRGS[Phospho (STY)]SLLDR.3	0.07449792	E9Q6P5_S629_M1_Ttc7b
GS[Phospho (STY)]SLLDR.2	0.018510823	E9Q6P5_S629_M1_Ttc7b
VEQALSEVASS[Phospho (STY)]LQSSAPK.2	-0.098053936	E9Q6P5_S678_M1_Ttc7b
[Acetyl (Protein N-term)]S[Phospho (STY)]DTEENPLEESR.2	0.176211902	E9Q743_S2_M1_Cfap251
NTEEPSS[Phospho (STY)]PVRK.2	-0.194983406	E9Q784_S110_M1_Zc3h13
SKLS[Phospho (STY)]PS[Phospho (STY)]PSLR.2	0.132612159	E9Q784_S207_M2_Zc3h13
S[Phospho (STY)]PERPTGDLR.2	-0.048909424	E9Q784_S77_M1_Zc3h13
EVS[Phospho (STY)]PEVVR.2	-0.175221721	E9Q784_S198_M1_Zc3h13
DQRPSS[Phospho (STY)]PIR.2	0.038750749	E9Q784_S704_M1_Zc3h13
AAVVAS[Phospho (STY)]PLLDQQR.2	-0.091592215	E9Q784_S242_M1_Zc3h13
SLS[Phospho (STY)]PSHLTEDR.2	-0.206975308	E9Q784_S953_M1_Zc3h13
S[Phospho (STY)]KGDS[Phospho (STY)]DVS[Phospho (STY)]DEEAAPQNK.2	-0.07862454	E9Q784_S1086_M3_Zc3h13
RS[Phospho (STY)]PERPTGDLR.3	-0.25462919	E9Q784_S77_M1_Zc3h13
SSS[Phospho (STY)]PGQHHS[Phospho (STY)]PLSSR.2	-0.399400594	E9Q784_S318_M2_Zc3h13
SKLS[Phospho (STY)]PS[Phospho (STY)]PSLRK.2	-0.174710885	E9Q784_S207_M2_Zc3h13
LRS[Phospho (STY)]PSNDSAHR.2	-0.05465481	E9Q784_S1273_M1_Zc3h13
S[Phospho (STY)]AS[Phospho (STY)]PYPTHC[Carbamidomethyl (C)]LSS[Phospho (STY)]PQR.2	-0.369384973	E9Q784_S369_M3_Zc3h13
SQS[Phospho (STY)]C[Carbamidomethyl (C)]SDTVQDR.2	0.10888747	E9Q7F2_S279_M1_Rnf169
TQPDGTSVPGEPAS[Phospho (STY)]PISQR.2	0.242204143	E9Q7G0_S1739_M1_Numa1
VSSETHQGPGT[Phospho (STY)]PESK.2	-0.421135614	E9Q7G0_T1982_M1_Numa1
LQES[Phospho (STY)]PAPEKGEVLGDALQLDTLK.3	-0.136625193	E9Q7G0_S398_M1_Numa1
YEKEFGDGS[Phospho (STY)]DENEVEDQEPR.3	-0.157413394	E9Q8D0_S283_M1_Dnajc21
SFS[Phospho (STY)]VPKK.2	-0.090634304	E9Q8I9_S1991_M1_Fry
NSDLFTVLS[Phospho (STY)]R.2	-0.210670586	E9Q8I9_S1936_M1_Fry
AT[Phospho (STY)]FAGGSRDGVITC[Carbamidomethyl (C)]QPGDSEEK.3	0.063486485	E9Q8I9_T2810_M1_Fry
LLLPGSSPS[Phospho (STY)]S[Phospho (STY)]PEDEVKDR.2	0.416526324	E9Q8I9_S1382_M2_Fry
SSS[Phospho (STY)]PDLSSSSK.2	-0.120090911	E9Q8I9_S1940_M1_Fry
S[Phospho (STY)]LDSLDKC[Carbamidomethyl (C)]DM[Oxidation (M)]QILEER.3	-0.011418403	E9Q8I9_S2495_M1_Fry
EQENMDDTNS[Phospho (STY)]EQQFR.2	0.333358977	E9Q8I9_S2459_M1_Fry
NFS[Phospho (STY)]FAKK.2	0.19526132	E9Q9D5_S147_M1_Rabl2
STHS[Phospho (STY)]LPSR.2	0.450943763	E9QAT4_S1377_M1_Sec16a
APS[Phospho (STY)]LTSDSEGKKPAQAVK.3	-0.060818034	E9QAT4_S2101_M1_Sec16a
ASHYS[Phospho (STY)]DQLAPR.2	0.024874595	E9QAT4_S1245_M1_Sec16a
S[Phospho (STY)]IHS[Phospho (STY)]EHSAR.2	-0.067891563	E9QAT4_S1362_M2_Sec16a
FTGS[Phospho (STY)]FDDDAEIHRDPYGEEADRR.4	-0.430496935	E9QAT4_S1342_M1_Sec16a
RSS[Phospho (STY)]LS[Phospho (STY)]SHSHQSQIYR.3	0.131341742	E9QAT4_S1384_M2_Sec16a
LPS[Phospho (STY)]PTKEKPPPPPPGGGK.3	0.053315779	F6SEU4_S788_M1_Syngap1
S[Phospho (STY)]QPKLDR.2	-0.246167165	F6SEU4_S146_M1_Syngap1
TKS[Phospho (STY)]QPKLDR.2	-0.075868248	F6SEU4_S146_M1_Syngap1
FRS[Phospho (STY)]ADHDR.2	-0.242246661	F6SEU4_S165_M1_Syngap1
LLNDISTALR.2	0.276483806	#N/A
ALYESEENC[Carbamidomethyl (C)]EVDPIK.2	-0.017592694	#N/A
GKS[Phospho (STY)]QQLTVSAAQKPR.3	-0.38119128	F6SEU4_S1070_M1_Syngap1
EFAEYVTNHYR.2	-0.164438152	#N/A
QQS[Phospho (STY)]LSKEGS[Phospho (STY)]IGGSGGSGGGGGGGLKPSITK.3	-0.269735617	F6SEU4_S1105_M2_Syngap1
EYEEEIHS[Phospho (STY)]LKER.2	-0.402502304	F6SEU4_S1201_M1_Syngap1
SSPAYC[Carbamidomethyl (C)]TSSS[Phospho (STY)]DITEPEQK.2	-0.095928289	F6SEU4_S825_M1_Syngap1

TVS[Phospho (STY)]VPVEGR.2	0.188848293	F6SEU4_S87_M1_Syngap1
DLNSS[Phospho (STY)]IDLQSFM[Oxidation (M)]AR.2	-0.236589654	F6SEU4_S766_M1_Syngap1
ILM[Oxidation (M)]QYQAR.2	-0.278175777	#N/A
EGS[Phospho (STY)]IGGSGGSGGGGGGGLKPSITK.3	-0.014739694	F6SEU4_S1111_M1_Syngap1
GKS[Phospho (STY)]QQLTVSAAQK.2	0.088043121	F6SEU4_S1070_M1_Syngap1
QY[Phospho (STY)]SMEAAPAAPFR.2	-0.254414536	F6SEU4_Y116_M1_Syngap1
VKEYEEEIHS[Phospho (STY)]LKER.3	0.141015933	F6SEU4_S1201_M1_Syngap1
LSQM[Oxidation (M)]GVTTDGVPAQQLR.2	0.227923613	#N/A
S[Phospho (STY)]VSM[Oxidation (M)]LDLQGDGPGGR.2	-0.064984266	F6SEU4_S840_M1_Syngap1
DAIGEFIR.2	-0.297493555	#N/A
RLLSQEEQTSK.2	-0.160596291	#N/A
GPS[Phospho (STY)]AEM[Oxidation (M)]QGYMMR.2	0.2666657	F6SEU4_S752_M1_Syngap1
GLNSS[Phospho (STY)]M[Oxidation (M)]DMAR.2	0.11030826	F6SEU4_S780_M1_Syngap1
C[Carbamidomethyl (C)]TAS[Phospho (STY)]SLAEHQANLR.2	0.303563542	F6SEU4_S534_M1_Syngap1
QHS[Phospho (STY)]QTPSTLNPTM[Oxidation (M)]PASER.3	-0.180761242	F6SEU4_S1135_M1_Syngap1
S[Phospho (STY)]MDESRLDR.2	0.441344851	F6SEU4_S1183_M1_Syngap1
TVS[Phospho (STY)]VPVEGRPHGEHEYHLGR.4	-0.319120381	F6SEU4_S87_M1_Syngap1
EYSKS[Phospho (STY)]M[Oxidation (M)]DESRLDR.3	0.244938245	F6SEU4_S1183_M1_Syngap1
EYSKS[Phospho (STY)]MDESR.2	-0.18427986	F6SEU4_S1183_M1_Syngap1
LS[Phospho (STY)]QGSGSSITAAGMR.2	0.240168525	F6SEU4_S892_M1_Syngap1
VKEYEEEIHS[Phospho (STY)]LK.3	-0.609479283	F6SEU4_S1201_M1_Syngap1
TVAWVSNM[Oxidation (M)]PHLS[Phospho (STY)]ADIESAHIER.3	-0.305433085	F6SEU4_S1162_M1_Syngap1
S[Phospho (STY)]QPMVLR.2	0.271446685	F6SEU4_S743_M1_Syngap1
QTPQAPQS[Phospho (STY)]PR.2	-0.177717776	F6ZDS4_S2141_M1_Tpr
TDGFAEAIHS[Phospho (STY)]PQVAGVPR.3	-0.22540726	F6ZDS4_S2223_M1_Tpr
GAILSEEELAAM[Oxidation (M)]S[Phospho (STY)]PTAAAVAK.2	0.066213557	F6ZDS4_S453_M1_Tpr
LGAPENSGIST[Phospho (STY)]LER.2	-0.216333547	F8VPU2_T24_M1_Farp1
AC[Carbamidomethyl (C)]TLELGPHQS[Phospho (STY)]PALPK.2	-0.443223493	F8VPU2_S427_M1_Farp1
SPDEATAADQES[Phospho (STY)]EDDLSASR.2	-0.079104516	F8VPU2_S892_M1_Farp1
QSPQSASLTFGEGTES[Phospho (STY)]PGGQSC[Carbamidomethyl (C)]QQAK.3	-0.034506434	F8VPU2_S403_M1_Farp1
SNGPTPELLASS[Phospho (STY)]PPDNK.2	0.230682239	F8VPU2_S875_M1_Farp1
AC[Carbamidomethyl (C)]TLELGPHQS[Phospho (STY)]PALPKS[Phospho (STY)]PPGSK.3	-0.38632885	F8VPU2_S427_M2_Farp1
GAGS[Phospho (STY)]PTPLPTK.2	-0.030492724	F8VPZ9_S1427_M1_Bicra
TLS[Phospho (STY)]DESLC[Carbamidomethyl (C)]SGR.2	-0.439600664	G3X9J0_S1538_M1_Sipa1l3
SSS[Phospho (STY)]EITLSEC[Carbamidomethyl (C)]DVEEPGDPR.2	-0.549077079	G3X9J0_S166_M1_Sipa1l3
EQSNPS[Phospho (STY)]PSQDTDGVK.2	0.067135783	G3X9J0_S94_M1_Sipa1l3
S[Phospho (STY)]KDVEFQDGWPR.2	-0.081994952	G3X9J0_S140_M1_Sipa1l3
QDAAGKDS[Phospho (STY)]PNR.2	0.329611333	G3X9J0_S1248_M1_Sipa1l3
LSVSS[Phospho (STY)]NDTQESGNSSGPSPGAK.2	-0.442727831	G3X9K3_S394_M1_Arfgef1
GREGS[Phospho (STY)]LTGTK.2	-0.184916768	G3X9K3_S1076_M1_Arfgef1
S[Phospho (STY)]VDIHDSIQPR.2	-0.30240835	G3X9K3_S1566_M1_Arfgef1
QQQHLLQS[Phospho (STY)]PVSHHEPESPHLR.5	0.322060954	G3X9K3_S234_M1_Arfgef1
TLS[Phospho (STY)]FDTSK.2	-0.202630012	G3XA57_S277_M1_Rab11fip2
NSS[Phospho (STY)]PPPSPNK.2	-0.065924399	G5E829_S1178_M1_Atp2b1
SATSSSPGS[Phospho (STY)]PLHSLETSL.2	-0.352252562	G5E829_S1211_M1_Atp2b1
EASDIILTDDNFTSIVK.2	-0.223172671	#N/A
VYTFNSVR.2	-0.018496821	#N/A

TSPNEGLSGNPADLER.2	0.012273311	#N/A
GIIDSTVSEQR.2	-0.149097198	#N/A
QVVAVTGDGTNDGPALK.2	-0.148655877	#N/A
YGDLLPADGILIQGNDLK.2	-0.324209241	#N/A
IDESSLTGESDHVKK.3	-0.184606432	#N/A
ADVGFAM[Oxidation (M)]GIAGTDVAK.2	-0.224746129	#N/A
EANHDGDFGITLTELR.3	-0.021515853	#N/A
KQDGAIENR.2	-0.036497245	#N/A
NS[Phospho (STY)]LKEANHDGDFGITLTELR.3	-0.184569949	G5E829_S17_M1_Atp2b1
TIC[Carbamidomethyl (C)]LAFR.2	0.291736551	#N/A
M[Oxidation (M)]VTGDNINTAR.2	-0.03037507	#N/A
TVIEPM[Oxidation (M)]ASEGLR.2	0.251344022	#N/A
TGTLTMNR.2	0.104875183	#N/A
KADVGFAM[Oxidation (M)]GIAGTDVAK.3	-0.135177436	#N/A
FFDIDSGR.2	0.327942871	#N/A
QVVAVTGDGTNDGPALKK.2	0.157509785	#N/A
IQESYGDVYGIC[Carbamidomethyl (C)]TK.2	0.072496829	#N/A
SSLYEGLEKPES[Phospho (STY)]R.2	-0.305145584	G5E829_S1137_M1_Atp2b1
EEIPEEELAEDVEEIDHAER.3	0.144996605	#N/A
SSPTDKHTLVK.2	0.097611315	#N/A
NVYDSISK.2	-0.328885954	#N/A
[Acetyl (Protein N-term)]GDMANNSVAYS[Phospho (STY)]GVKNSLK.2	0.095739178	G5E829_S12_M1_Atp2b1
HLDAC[Carbamidomethyl (C)]ETM[Oxidation (M)]GNATAIC[Carbamidomethyl (C)]SDK.3	-0.116736209	#N/A
MTVVQAYINEK.2	-0.224041749	#N/A
IEDS[Phospho (STY)]EPHIPLIDDTDAEDDAPTKR.3	0.267675118	G5E829_S1155_M1_Atp2b1
AQDGAAMEMQPLKS[Phospho (STY)]EEGGDGDEKDK.3	-0.195051182	G5E829_S338_M1_Atp2b1
SES[Phospho (STY)]PPAELPSLR.2	0.180482177	G5E870_S312_M1_Trip12
AQTAPTKTS[Phospho (STY)]PR.2	-0.092273783	G5E870_S1460_M1_Trip12
DDSLDLS[Phospho (STY)]PQGR.2	0.611076254	G5E870_S1024_M1_Trip12
AAS[Phospho (STY)]KDTISNNR.2	0.080437225	G5E870_S1111_M1_Trip12
SAS[Phospho (STY)]PDYNR.2	-0.027269645	G5E870_S77_M1_Trip12
DFPQYFAVVSR.2	-0.223560466	#N/A
EFDS[Phospho (STY)]DSLR.2	-0.277160141	G5E8K5_S958_M1_Ank3
TEEPVS[Phospho (STY)]PLTAYQK.2	0.301822047	G5E8K5_S1883_M1_Ank3
NEDLAELLNGM[Oxidation (M)]DEELDS[Phospho (STY)]PEELGTKR.3	-0.000311821	G5E8K5_S1114_M1_Ank3
YSYLTEPSMS[Phospho (STY)]PQSPC[Carbamidomethyl (C)]ER.2	-0.127171858	G5E8K5_S1470_M1_Ank3
IRDTS[Phospho (STY)]QEPC[Carbamidomethyl (C)]GR.2	-0.320527926	G5E8K5_S1406_M1_Ank3
NEDLAELLNGM[Oxidation (M)]DEELDS[Phospho (STY)]PEELGTK.3	0.342789867	G5E8K5_S1114_M1_Ank3
GETALHM[Oxidation (M)]AAR.2	0.261848142	#N/A
RQS[Phospho (STY)]FASLALR.2	0.060935366	G5E8K5_S1451_M1_Ank3
ADIVQQLLQQGASPNAATTSGYTPLHLAAR.3	0.041444204	#N/A
VALLLLDQGAS[Phospho (STY)]PHAAAK.2	-0.212785318	G5E8K5_S606_M1_Ank3
VHGEPAS[Phospho (STY)]DLS[Phospho (STY)]DID.2	0.196529311	G5E8P1_S1052_M2_Brd1
FVVTDGGITR.2	-0.026025891	#N/A
SYDYQSVC[Carbamidomethyl (C)]DPGAAPK.2	0.575578205	#N/A
IIM[Oxidation (M)]LFTDGGEER.2	0.447552496	#N/A
QLVEIAAR.2	-0.194256108	#N/A

VFTFSVGQHNYDR.3	-0.363625755	#N/A
VNHEPEPASGAS[Phospho (STY)]PGATIPK.2	-0.105185782	O08539_S324_M1_Bin1
GVFPENFTER.2	0.234104472	#N/A
LNQNLNDVLVSLEK.2	-0.022032651	#N/A
VQAQHDYTATDTDELQLK.3	-0.271916291	#N/A
GNKS[Phospho (STY)]PS[Phospho (STY)]PPPDGSPAATPEIR.3	-0.098494101	#N/A
NQAEEELIK.2	0.13117979	#N/A
ADETKDEQFEQC[Carbamidomethyl (C)]VQNFNK.3	-0.253416831	#N/A
SPS[Phospho (STY)]PPPDGSPAATPEIR.2	-0.18357261	O08539_S298_M1_Bin1
LVDQALLTM[Oxidation (M)]DTYLGQFPDIK.3	-0.232434962	#N/A
VNHEPEPASGASPGATIPKS[Phospho (STY)]PSQLR.3	-0.332514325	O08539_S332_M1_Bin1
AGDVVLVIPFQNPEEQDEGWLM[Oxidation (M)]GVK.3	0.063099805	#N/A
AQPSDNAPEKGNKS[Phospho (STY)]PS[Phospho (STY)]PPPDGSPAATPEIR.4	-0.218212284	#N/A
HHYESLQTAK.2	-0.084884642	#N/A
AAPQWC[Carbamidomethyl (C)]QGK.2	-0.05522987	#N/A
IAENNDLLWM[Oxidation (M)]DYHQK.3	-0.05409468	#N/A
QHGSNTFTVK.2	0.232222379	#N/A
LQAHLVAQTNLLR.3	-0.345438827	#N/A
ESDWNQHKELEK.3	-0.063961295	#N/A
VGFYVNTFQSIAGLEENFHK.3	0.018151604	#N/A
NLGS[Phospho (STY)]INTELQDVQR.2	-0.237754442	O08547_S137_M1_Sec22b
DLQQYQSQAK.2	0.650672818	#N/A
GSPLVVISQGK.2	0.008945348	#N/A
M[Oxidation (M)]VIPGGIDVHTR.2	-0.084803562	#N/A
SAAEVIAQAR.2	0.153187584	#N/A
QIGENLIVPGGVK.2	0.055956635	#N/A
VFNLYPR.2	0.223907153	#N/A
KPFPDFVYK.2	0.060539443	#N/A
IVLEDGTLHVTEGSGR.3	0.064988287	#N/A
ILDLGITGPEGHVLSRPEEVEAEAVNR.4	-0.181753211	#N/A
GTVVYGEPITASLGTDGSHYWSK.3	0.131397504	#N/A
SITIANQTNC[Carbamidomethyl (C)]PLYVTK.2	-0.039128873	#N/A
AVGKDNFTLIPEGTNGTEER.3	-0.371837987	#N/A
TIEAHSR.2	0.157670242	#N/A
ITS[Phospho (STY)]DRLLIK.2	-0.169055111	O08553_S14_M1_Dpysl2
NIPRIT[Phospho (STY)]SDR.2	-0.31376181	O08553_T13_M1_Dpysl2
TS[Phospho (STY)]PAKQQAPPVR.2	-0.156403494	O08553_S522_M1_Dpysl2
DNFTLIPEGTNGTEER.2	-0.274053059	#N/A
ISVGSDADLVIWDPDSVK.2	0.126376193	#N/A
M[Oxidation (M)]SVIWDK.2	0.250253282	#N/A
GIQEEM[Oxidation (M)]EALVK.2	0.304935426	#N/A
TVT[Phospho (STY)]PASS[Phospho (STY)]AKTS[Phospho (STY)]PAK.2	0.02706613	#N/A
TVT[Phospho (STY)]PASS[Phospho (STY)]AK.2	-0.095097807	#N/A
TVT[Phospho (STY)]PASS[Phospho (STY)]AKTS[Phospho (STY)]PAKQQAPPVR.3	0.205280537	#N/A
FQM[Oxidation (M)]PDQGM[Oxidation (M)]TSADDFFQGTK.2	-0.204220214	#N/A
DHGVNSFLVYM[Oxidation (M)]AFK.2	-0.04353073	#N/A
NLHQSGFSLSGAQIDDNIPR.3	-0.266483992	#N/A

THNSALEYNIFEGM[Oxidation (M)]EC[Carbamidomethyl (C)]R.3	-0.110296354	#N/A
FQLTDSQIYEVLSVIR.3	-0.368194146	#N/A
M[Oxidation (M)]DENQFVAVTSTNAAK.2	0.039085636	#N/A
IVNDDQSFYADIYM[Oxidation (M)]EDGLIK.2	-0.511974842	#N/A
NLHQSGFSLSGAQIDDNIPRR.4	-0.374068817	#N/A
GLYDGPVC[Carbamidomethyl (C)]EVSVT[Phospho (STY)]PK.2	0.017957161	O08553_T509_M1_Dpysl2
GLYDGPVC[Carbamidomethyl (C)]EVSVTPKT[Phospho (STY)]VT[Phospho (STY)]PASSAK.3	0.245374752	O08553_T512_M2_Dpysl2
DIGAIAQVHAENGDIIAEEQQR.3	-0.392246361	#N/A
DRFQLTDSQIYEVLSVIR.3	-0.489084937	#N/A
SC[Carbamidomethyl (C)]C[Carbamidomethyl (C)]DYSLHVDITEWHK.3	-0.44664077	#N/A
_GLYDGPVC[Carbamidomethyl (C)]EVSVT[Phospho (STY)]PKT[Phospho (STY)]VT[Phospho (STY)]PAS[Phospho (STY)]S[Phospho (STY)]AKT	0.11946588	#N/A
GRIS[Phospho (STY)]VGSDADLVIWDPDSVK.3	-0.187473002	O08553_S402_M1_Dpysl2
AALAGGTTM[Oxidation (M)]IIDHVVPEPGTSLLAAFDQWR.3	-0.377193024	#N/A
AAAFVTSPPLSPDPTTPDFLNSLLSC[Carbamidomethyl (C)]GDLQVTGSAHC[Carbamidomethyl (C)]TFNTAQK.4	-0.168016088	#N/A
FTQSYDYLT[Phospho (STY)]DEEER.2	0.169332391	O08576_T215_M1_Rundc3a
SNEC[Carbamidomethyl (C)]LVSDSPEGSPALS[Phospho (STY)]PS.2	0.116038058	O08576_S444_M1_Rundc3a
DDIFSS[Phospho (STY)]LEEEGKDR.2	0.339660706	O08579_S142_M1_Emd
QSEPFVASQSPPY[Phospho (STY)]EDGSADH.2	-0.186331506	O08581_Y329_M1_Kcnk1
LVLVS[Phospho (STY)]PTSEQYDSLLR.2	-0.269009963	O08582_S69_M1_Gtpbp1
SRS[Phospho (STY)]PVDSPVPASM[Oxidation (M)]FAPEPSS[Phospho (STY)]PGAAR.3	-0.189487693	O08582_S25_M2_Gtpbp1
SPVDSPVPASM[Oxidation (M)]FAPEPSS[Phospho (STY)]PGAAR.3	-0.161526357	O08582_S25_M1_Gtpbp1
LLQTTNNS[Phospho (STY)]PMNSKPQQIK.3	0.06681035	O08582_S580_M1_Gtpbp1
QQLS[Phospho (STY)]AEELDAQLDAYNAR.2	0.087813926	O08583_S237_M1_Alyref
LQS[Phospho (STY)]EPESIR.2	0.022739325	O08585_S104_M1_Clta
LEALDANSR.2	0.044115326	#N/A
ELEEWYAR.2	-0.034579266	#N/A
VADEAFYK.2	-0.027078406	#N/A
LC[Carbamidomethyl (C)]DFNPK.2	0.564491196	#N/A
KQEAEWK.2	0.086814019	#N/A
QDEQLQK.2	0.011929061	#N/A
WREEQTER.2	-0.125475655	#N/A
AIKELEEWYAR.2	-0.208707735	#N/A
LQS[Phospho (STY)]EPESIRK.2	-0.263772074	O08585_S104_M1_Clta
NNIDDVVR.2	0.043466718	#N/A
VENGS[Phospho (STY)]LC[Carbamidomethyl (C)]DQEIDSIC[Carbamidomethyl (C)]SIER.2	-0.09617522	O08586_S294_M1_Pten
KLNKTDEEISS[Phospho (STY)].2	-0.076729527	O08599_S594_M1_Stxbp1
SSAS[Phospho (STY)]FSTTAVSAR.2	-0.462856801	O08599_S509_M1_Stxbp1
LNKTDEEISS[Phospho (STY)].2	-0.397071225	O08599_S594_M1_Stxbp1
SQLLILDR.2	-0.308832928	#N/A
HYPYISTR.2	-0.037834737	#N/A
ISEQTYQLSR.2	-0.202464545	#N/A
NGITEENLNK.2	-0.087720114	#N/A
YETSGIGEAR.2	0.121883001	#N/A
HIAEVSQEVTR.2	0.50927031	#N/A
DNALLAQLIQDK.2	0.366380187	#N/A
AIVPILLDANVSTYDK.2	-0.0641294	#N/A
NKAPGEYR.2	-0.013151189	#N/A

LLDTLKK.2	0.480803354	#N/A
EPLPSLEAVYLITPSEK.2	-0.185138879	#N/A
ADDPTM[Oxidation (M)]GEGPDK.2	-0.316391301	#N/A
EVLLDEDDDLWIALR.2	0.18265206	#N/A
IM[Oxidation (M)]HDVIKK.2	-0.31748837	#N/A
SLKDFSSSK.2	-0.190059109	#N/A
LAEQIATLC[Carbamidomethyl (C)]ATLK.2	0.011873628	#N/A
DNALLAQLIQDKLDAYK.2	-0.115006528	#N/A
AAHVFFTDSC[Carbamidomethyl (C)]PDALFNELVK.3	-0.278698361	#N/A
HYQGTVDK.2	0.017140721	#N/A
VLVVDQLSM[Oxidation (M)]R.2	-0.300509561	#N/A
REPLPSLEAVYLITPSEK.3	-0.106275949	#N/A
VEQDLAM[Oxidation (M)]GTDAEGEK.2	-0.263638589	#N/A
SVHSLISDFKDPPTAK.3	-0.454965147	#N/A
M[Oxidation (M)]LSSC[Carbamidomethyl (C)]C[Carbamidomethyl (C)]K.2	0.311125209	#N/A
DFSSSKR.2	-0.010248548	#N/A
KM[Oxidation (M)]PQYQK.2	0.414553388	#N/A
IM[Oxidation (M)]HDVIK.2	0.186525728	#N/A
C[Carbamidomethyl (C)]AYEVTQANGK.2	0.146245844	#N/A
M[Oxidation (M)]TDIM[Oxidation (M)]TEGITIVEDINKR.3	-0.553598134	#N/A
WEVLIGSTHILTPQK.2	-0.331393728	#N/A
M[Oxidation (M)]TDIM[Oxidation (M)]TEGITIVEDINK.2	-0.535232521	#N/A
DIM[Oxidation (M)]EDTIEDKLDTK.2	-0.041989906	#N/A
DIMEDTIEDK.2	0.470302066	#N/A
YSTHLHLAEDC[Carbamidomethyl (C)]M[Oxidation (M)]K.3	0.030366473	#N/A
LIQHAQIPPEDSEIITNMAHLGVPIVTDSTLR.4	0.166104053	#N/A
VEQDLAMGTDAEGEKIKDPMR.3	0.47146455	#N/A
GFDPSSPVLHELTFQAM[Oxidation (M)]SYDLLPIENDVYK.3	-0.311318937	#N/A
LDAYKADDPTMGEGPDK.3	0.313248128	#N/A
DLS[Phospho (STY)]QMLKK.2	-0.194593886	O08599_S328_M1_Stxbp1
VIENTDGS[Phospho (STY)]EEEM[Oxidation (M)]DAR.2	-0.118080076	O08638_S1954_M1_Myh11
GTGYTEQLQQYSS[Phospho (STY)]PGLGVK.2	-0.271828498	O08644_S630_M1_Ephb6
ELESGTEES[Phospho (STY)]DEEPTPSPR.2	0.235281529	O08648_S454_M1_Map3k4
LES[Phospho (STY)]EEDSIGWGTADC[Carbamidomethyl (C)]GPEASR.2	0.388448496	O08648_S492_M1_Map3k4
AGVAS[Phospho (STY)]PVQR.2	-0.2729979	O08672_S643_M1_Kifc2
LSILYPATTGR.2	0.14137518	#N/A
LPFPIIDDK.2	0.483922961	#N/A
VVFIFGPDKK.2	-0.139684955	#N/A
DINAYNGETPTEK.2	0.085306012	#N/A
VVDSLQLTGTKPVATPVDWK.3	-0.141517047	#N/A
DLAILLGM[Oxidation (M)]LDPVEK.2	0.21955899	#N/A
NFDEILR.2	0.174692	#N/A
DFTPVC[Carbamidomethyl (C)]TTELGR.2	-0.258998849	#N/A
PGGLLLGDEAPNFEANTTIGR.2	-0.029088319	#N/A
VVFIFGPDK.2	0.145062294	#N/A
LIALSIDSVEDHLAWSK.2	-0.062989435	#N/A
KGESVM[Oxidation (M)]VVPTLSEEEAK.3	-0.143957623	#N/A

LPFPIIDDKGR.2	-0.364621907	#N/A
FHDFLGDSWGILFSHPR.3	-0.195272498	#N/A
RRS[Phospho (STY)]ESSGNLPSVADTR.3	0.164880719	O08715_S101_M1_Akap1
S[Phospho (STY)]ESSGNLPSVADTR.2	0.002980787	O08715_S101_M1_Akap1
NETLGGTC[Carbamidomethyl (C)]LNVGC[Carbamidomethyl (C)]IPSK.2	0.08269943	#N/A
GIEIPEVR.2	-0.009680786	#N/A
IPNIYAIGDVVAGPM[Oxidation (M)]LAHK.3	-0.146475247	#N/A
ADGSTQVIDTK.2	0.068352598	#N/A
EANLAAAFGKPINF.2	-0.205067903	#N/A
IDVSVEAASGGK.2	-0.071390744	#N/A
VC[Carbamidomethyl (C)]HAHPTLSEAFR.3	0.161271664	#N/A
ALTGGIAHLFK.2	-0.101216388	#N/A
SEEQLKEEGIEFK.2	-0.331253118	#N/A
RPFTQNLGLEELGIELDPK.3	-0.428846806	#N/A
LGADVTAVEFLGHVGGIGIDM[Oxidation (M)]EISK.3	-0.040782042	#N/A
AEVITC[Carbamidomethyl (C)]DVLLVC[Carbamidomethyl (C)]IGR.2	-0.002390515	#N/A
VLGAHILGPGAGEM[Oxidation (M)]VNEAALALEYGASC[Carbamidomethyl (C)]EDIAR.3	0.088192146	#N/A
NFLASQVPFPSR.2	0.205557715	#N/A
VVTIAPGLFATPLLTTLPEK.2	-0.258070556	#N/A
LVAGEM[Oxidation (M)]GQNEPDQGGQR.2	-0.127214691	#N/A
GVIINTASVAAFEGQVGQAAYSASK.3	-0.273691869	#N/A
S[Phospho (STY)]PGESQSEDIEASR.2	-0.09058743	O08759_S8_M1_Ube3a
NLDFQALEETTEYDGGYTR.2	0.143963812	#N/A
AASAPAKES[Phospho (STY)]PK.2	-0.262178035	O08784_S794_M1_Tcof1
NSS[Phospho (STY)]PAVPAPTPEGVQAVNTTK.2	-0.044691789	O08784_S853_M1_Tcof1
KLS[Phospho (STY)]GDLEAGAPK.2	0.050437414	O08784_S1191_M1_Tcof1
AASAPAKES[Phospho (STY)]PKK.2	0.06194463	O08784_S794_M1_Tcof1
DSAS[Phospho (STY)]PIQKK.2	0.137265649	O08784_S1303_M1_Tcof1
SAEPLANTVLASET[Phospho (STY)]EEEGNAQALGPTAK.3	-0.19779822	O08784_T171_M1_Tcof1
ASAVS[Phospho (STY)]PEKAPMTSK.2	0.172546207	O08784_S1216_M1_Tcof1
LDETQTLLR.2	0.281627115	#N/A
ASEQIYGSPSSSPYEC[Carbamidomethyl (C)]LR.2	-0.172160653	#N/A
QLTAHLQDVNR.3	0.252825767	#N/A
QSQIQVFEDGADTTS[Phospho (STY)]PETPDSSASK.3	0.105291789	O08788_S105_M1_Dctn1
AFLQGGQEATDIALLLR.2	0.10638836	#N/A
LVLTQEQLHQLHSR.3	-0.04484733	#N/A
LHISQLQHENSILR.3	-0.259210635	#N/A
LNQLSTHTHVVDITR.3	-0.132929209	#N/A
VTFSC[Carbamidomethyl (C)]AAGLGQR.2	0.289120528	#N/A
VEAAQETVADYQQTIK.2	-0.414084045	#N/A
SLEDQVETLR.2	-0.13245514	#N/A
YEQGTGC[Carbamidomethyl (C)]WQGPNR.2	0.066808161	#N/A
RHES[Phospho (STY)]DEDS[Phospho (STY)]LGSSGR.2	-0.410145139	O08796_S469_M2_Eef2k
VES[Phospho (STY)]PGTYQQDPWAMTDEEK.2	-0.442191937	O08915_S159_M1_Aip
VTGEVLDILSR.2	-0.107107227	#N/A
DIHDDQDYLHSLGK.3	-0.035230514	#N/A
VASSDLVNM[Oxidation (M)]GISVVSYTLK.2	-0.036409965	#N/A

S[Phospho (STY)]PPVMVAGGR.2	-0.058253161	O08917_S19_M1_Flot1
ITLVSSGSGTM[Oxidation (M)]GAAK.2	0.167759384	#N/A
RTPS[Phospho (STY)]EAER.2	0.36514059	O08919_S411_M1_Numbl
AEAAAAPAVAPGPAQPGHVS[Phospho (STY)]PTPATTS[Phospho (STY)]PGEKGEAGTPVAAGTTAAAIPR.4	0.115077521	O08919_S270_M2_Numbl
SVLWVSADGLR.2	0.520831445	#N/A
KAEAAAAPAVAPGPAQPGHVS[Phospho (STY)]PT[Phospho (STY)]PATTSPGEK.4	0.209130792	O08919_T265_M2_Numbl
KAEAAAAPAVAPGPAQPGHVS[Phospho (STY)]PT[Phospho (STY)]PATTSPGEKGEAGTPVAAGTTAAAIPR.4	-0.250161879	O08919_T265_M2_Numbl
TPS[Phospho (STY)]EAER.2	-0.196465434	O08919_S411_M1_Numbl
EGS[Phospho (STY)]FRLS[Phospho (STY)]GGGRPAER.3	0.049577747	O08919_S224_M2_Numbl
[Acetyl (Protein N-term)]SLYPS[Phospho (STY)]LEDLKVDK.2	0.125973632	O08992_S6_M1_Sdcbp
AVSYLGPK.2	-0.043305743	#N/A
VC[Carbamidomethyl (C)]IDSEHSSDTLLATLNK.3	0.018320087	#N/A
S[Phospho (STY)]AGELPGATSDTNDIDTTAASRPGPAR.3	-0.155436701	O09039_S129_M1_Sh2b3
ATWGDGGDNS[Phospho (STY)]PSNVVSK.2	-0.221380669	O09044_S110_M1_Snap23
NM[Oxidation (M)]QNVEHVPLTLDR.3	-0.197629104	#N/A
GAVYSFDPVGSYQR.2	0.295068187	#N/A
AGGSASAM[Oxidation (M)]LQPLLDNQVGFK.2	0.064051845	#N/A
DVYTGDALR.2	0.157698501	#N/A
DVFISAAER.2	-0.087576645	#N/A
LSEGFSIHTR.2	-0.324720446	#N/A
IAC[Carbamidomethyl (C)]EEEFS[Phospho (STY)]DS[Phospho (STY)]DEEGEGGRK.2	-0.165610028	O09106_S421_M2_Hdac1
MLPHAPGVQM[Oxidation (M)]QAIPEDAIPEES[Phospho (STY)]GDEDEEDPDKR.4	0.017042783	#N/A
AVIAPSGVEK.2	0.019325615	#N/A
QREPTMQWQEDPEPEDENVYAK.3	0.17919823	#N/A
AQGLTEEDIVFLPQPDK.2	0.551086951	#N/A
SDFS[Phospho (STY)]DLEQSVK.2	0.15376927	O09126_S782_M1_Sema4d
VYNVTQHAVGIIVNK.3	-0.021496982	#N/A
TLNEWSSQISPDLVR.2	-0.082809849	#N/A
LYADS[Phospho (STY)]DS[Phospho (STY)]GEDSDKR.2	0.263783235	O35071_S674_M2_Kif1c
RNS[Phospho (STY)]LDGGSR.2	-0.410560824	O35071_S1031_M1_Kif1c
VVLEDGNLLVTPGAGR.2	-0.007986524	#N/A
GVPTVVISQGR.2	0.021682963	#N/A
QIGENLIVPGGIK.2	0.138049782	#N/A
M[Oxidation (M)]DENEFVAVTSTNAAK.2	0.420082818	#N/A
QANC[Carbamidomethyl (C)]PLYVTK.2	0.137882893	#N/A
VAVGSDADLVIWNPR.2	0.177921533	#N/A
DLGAVAQVHAENGDIVEEEQKR.3	-0.317942579	#N/A
TIDAHGLM[Oxidation (M)]VLPGGVDVHTR.3	-0.340802652	#N/A
NLHQSGFSLSGS[Phospho (STY)]QADDHIAR.3	-0.16568552	O35098_S544_M1_Dpysl4
GAADMVAQAK.2	0.532740834	#N/A
[Acetyl (Protein N-term)]S[Phospho (STY)]FQGKKS[Phospho (STY)]IPR.2	-0.328010425	O35098_S2_M2_Dpysl4
C[Carbamidomethyl (C)]QC[Carbamidomethyl (C)]TDGQIYEIFSLIR.2	-0.183287345	#N/A
SHNLNVEYNIFEGVEC[Carbamidomethyl (C)]R.3	-0.26120855	#N/A
TLFLGIPDENFEDHS[Phospho (STY)]APPSPEEK.3	-0.092394925	O35099_S1036_M1_Map3k5
TAS[Phospho (STY)]PPGPPQYSK.2	-0.127041426	O35126_S617_M1_Atn1
RAPS[Phospho (STY)]PGSYK.2	0.35781671	O35126_S630_M1_Atn1
SEEIS[Phospho (STY)]ESESEETSAPK.2	0.163004226	O35126_S77_M1_Atn1

GRAS[Phospho (STY)]PGGVSTSSSDGKAEK.3	-0.164743003	O35126_S34_M1_Atn1
TEQELPRPQS[Phospho (STY)]PSDLDSLDGR.3	0.224957266	O35126_S101_M1_Atn1
TGT[Phospho (STY)]PPGYR.2	0.181352614	O35126_T654_M1_Atn1
GRAS[Phospho (STY)]PGGVSTSSSDGK.2	0.035664072	O35126_S34_M1_Atn1
PHVMS[Phospho (STY)]PGNR.2	0.129353568	O35126_S881_M1_Atn1
QEPAEEYEPPES[Phospho (STY)]PVPPARS[Phospho (STY)]PS[Phospho (STY)]PPPK.3	-0.467250525	O35126_S724_M3_Atn1
VLPSIVNEVLK.2	-0.000134866	#N/A
ESVFTVEGGHR.2	0.339793977	#N/A
FNASQLITQR.2	-0.166130667	#N/A
LGLDYEER.2	-0.028610015	#N/A
LLLGAGAVAYGVR.2	-0.352347859	#N/A
IGGVQQDTILAEGLHFR.3	-0.507685638	#N/A
DLQM[Oxidation (M)]VNISLR.2	0.126037417	#N/A
QVAQQEAQR.2	0.260096863	#N/A
AQVSLLIR.2	-0.270792236	#N/A
IYLTADNLVLNLQDESFTR.2	0.084720133	#N/A
IPWFQYPIIYDIR.2	0.08127652	#N/A
AQFLVEK.2	-0.163628548	#N/A
ASGSPDPTISWFR.2	-0.06685392	#N/A
AAYLKDGS[Phospho (STY)]KEPIVEM[Oxidation (M)]R.3	-0.401387319	O35136_S765_M1_Ncam2
IEIFQTLPVR.2	-0.08340249	#N/A
ITNHEDGS[Phospho (STY)]PVNEPNETTPLTEPEKLPLKEENGK.4	0.095824074	O35136_S786_M1_Ncam2
DGS[Phospho (STY)]KEPIVEMR.2	0.300196751	O35136_S765_M1_Ncam2
LTIYNANIEDAGIYR.2	-0.074947317	#N/A
ITNHEDGS[Phospho (STY)]PVNEPNETTPLTEPEK.3	0.06429004	O35136_S786_M1_Ncam2
GSNTELTVR.2	0.168069983	#N/A
TKEQLAALR.2	0.282660544	#N/A
EAGGAFGKR.2	-0.084894718	#N/A
EQLAALR.2	0.11504613	#N/A
EKAEEDRYFR.3	-0.136551555	#N/A
AEEDRYFR.2	0.217957029	#N/A
KHHEDEIDHHSKEIER.4	-0.253434897	#N/A
KHHEDEIDHHSK.3	-0.301264182	#N/A
HHEDEIDHHSK.3	-0.025260599	#N/A
NS[Phospho (STY)]PMTISR.2	0.073514339	O35144_S399_M1_Terf2
DLVLANLASPSS[Phospho (STY)]PAHK.2	-0.503575102	O35144_S367_M1_Terf2
SLGS[Phospho (STY)]EEINPVHTDNFQER.3	-0.104763556	O35188_S262_M1_Cx3cl1
IPAGLENR.2	0.304039926	#N/A
FFPLEAWQIGK.2	0.514094792	#N/A
PFVELETNLPASR.2	-0.008966303	#N/A
STEPC[Carbamidomethyl (C)]AHLLVSSIGVVGTAEQNR.3	-0.348576437	#N/A
THSASFFK.2	0.379826177	#N/A
LC[Carbamidomethyl (C)]AATATILDKPEDR.3	0.029359303	#N/A
FLTEELSLDQDR.2	0.225185973	#N/A
SGLLNSTS[Phospho (STY)]DSDLVR.2	0.070598768	O35219_S322_M1_Kcnh2
DTNM[Oxidation (M)]IPGS[Phospho (STY)]PGS[Phospho (STY)]AELESGFNR.2	-0.255687818	O35219_S873_M2_Kcnh2
RAS[Phospho (STY)]S[Phospho (STY)]ADDIEAMR.2	-0.061960023	O35219_S285_M2_Kcnh2

VNVDIINFGEEEVNTEK.2	-0.322429811	#N/A
EKVNVDIINFGEEEVNTEK.3	-0.463599946	#N/A
AAAASAAEAGIAT[Phospho (STY)]PGTEDSDDALLK.2	0.037120298	O35226_T250_M1_Psmd4
RAAAASAAEAGIAT[Phospho (STY)]PGTEDSDDALLK.3	0.2411736	O35226_T250_M1_Psmd4
SLDDS[Phospho (STY)]EEEDDEDSGHSSR.2	0.033861853	O35245_S810_M1_Pkd2
QYS[Phospho (STY)]QHGLDGK.2	0.019288383	O35250_S283_M1_Exoc7
AVEYFQDNSPDS[Phospho (STY)]PELNK.2	0.276733963	O35250_S133_M1_Exoc7
GGGSGGGDES[Phospho (STY)]EGEEVDED.2	0.054650812	O35295_S316_M1_Purb
VSEVKPSYR.2	0.213319013	#N/A
RGGGSGGGDES[Phospho (STY)]EGEEVDED.2	-0.171010506	O35295_S316_M1_Purb
GGGGGGGGGPGGFQPAPR.2	-0.009924869	#N/A
DSLGDFIEHYAQLGPSS[Phospho (STY)]PEQLAAGAEEGGGPRR.4	0.143155147	O35295_S113_M1_Purb
[Acetyl (Protein N-term)]ADGDSGS[Phospho (STY)]ERGGGGGGGGGPGGFQPAPR.3	-0.002616344	O35295_S8_M1_Purb
DSLGDFIEHYAQLGPSS[Phospho (STY)]PEQLAAGAEEGGGPR.4	-0.5203922	O35295_S113_M1_Purb
GGGGGGGGPGGEQETQELASK.2	0.021567263	#N/A
[Acetyl (Protein N-term)]ADGDS[Phospho (STY)]GSER.2	-0.329491406	O35295_S6_M1_Purb
S[Phospho (STY)]GS[Phospho (STY)]RS[Phospho (STY)]PVPEK.2	-0.10468434	O35326_S226_M3_Srsf5
SKS[Phospho (STY)]PASVDR.2	-0.020493791	O35326_S247_M1_Srsf5
VS[Phospho (STY)]WQDLK.2	0.114704479	O35326_S120_M1_Srsf5
NVPQEDIC[Carbamidomethyl (C)]EDS[Phospho (STY)]DIDGDYR.2	0.017063456	O35343_S60_M1_Kpna4
EAAWAISNLTISGR.2	0.063737498	#N/A
VQNTSLEAIVQNASSDNQGIQLSAVQAAR.3	-0.079248514	#N/A
NVPQEESLEDS[Phospho (STY)]DVDADFK.2	0.110450422	O35344_S60_M1_Kpna3
[Acetyl (Protein N-term)]M[Oxidation (M)]ETM[Oxidation (M)]AS[Phospho (STY)]PGKDNYR.2	-0.427199119	#N/A
GSS[Phospho (STY)]QLDVNEEVEALIVK.2	-0.054163359	O35379_S290_M1_Abcc1
KLELSENR.2	-0.204894734	#N/A
ELVLDNC[Carbamidomethyl (C)]K.2	0.146109304	#N/A
NRT[Phospho (STY)]PSDVK.2	0.130016977	O35381_T15_M1_Anp32a
SLDLFNC[Carbamidomethyl (C)]EVTNLNAYR.2	0.079396545	#N/A
ISGDLEVLAEK.2	-0.071092134	#N/A
HLNLSGNK.2	0.013910159	#N/A
LLPQVM[Oxidation (M)]YLDGYDR.2	-0.081103417	#N/A
KREPDDEGEEDD.2	-0.050589779	#N/A
S[Phospho (STY)]GELQGGPDDNLIEGGGTK.2	0.021279695	O35382_S469_M1_Exoc4
GGS[Phospho (STY)]LGLGGGGGMGPR.2	0.474623114	O35393_S274_M1_Efnb3
LFVVPTDESQAR.2	-0.434788945	#N/A
ALLNVVDSAR.2	-0.17989527	#N/A
SQLEAVFLR.2	0.20001354	#N/A
S[Phospho (STY)]QENPNTYSEDLDV.2	0.174487314	O35417_S235_M1_Pdyn
SYPS[Phospho (STY)]SELR.2	-0.177226179	O35454_S684_M1_Clcn6
ES[Phospho (STY)]RTPEELTILGETQEEEDEILPR.3	-0.625398862	O35454_S23_M1_Clcn6
VLAQQGEYSEAIPILR.2	0.241152747	#N/A
S[Phospho (STY)]RS[Phospho (STY)]HDRIPYQR.3	-0.102270578	O35492_S197_M2_Clk3
YRS[Phospho (STY)]PEPDPYLSYR.3	0.018368382	O35492_S157_M1_Clk3
ADS[Phospho (STY)]YEKLEK.2	-0.399863286	O35495_S134_M1_Cdk14
SSSAGKES[Phospho (STY)]PKVR.2	-0.301122366	O35495_S112_M1_Cdk14
HSSPSS[Phospho (STY)]PTS[Phospho (STY)]PK.2	-0.210824511	O35495_S123_M2_Cdk14

RVHS[Phospho (STY)]ENNAC[Carbamidomethyl (C)]INFK.3	-0.203380766	O35495_S95_M1_Cdk14
VHS[Phospho (STY)]ENNAC[Carbamidomethyl (C)]INFK.2	-0.498241835	O35495_S95_M1_Cdk14
FGKADS[Phospho (STY)]YEKLEK.2	0.115780124	O35495_S134_M1_Cdk14
RHS[Phospho (STY)]SPSS[Phospho (STY)]PTSPK.2	-0.081823793	O35495_S119_M2_Cdk14
TLS[Phospho (STY)]ESFSR.2	-0.180073075	O35495_S24_M1_Cdk14
HSSPSSPT[Phospho (STY)]S[Phospho (STY)]PKFGK.3	0.251909081	O35495_S126_M2_Cdk14
HSAILAS[Phospho (STY)]PNPDEK.2	-0.286440622	O35526_S64_M1_Stx1a
QALSEIETR.2	-0.195825346	#N/A
IEYNVEHAVDYVER.2	-0.142227939	#N/A
FM[Oxidation (M)]DEFFEQVEEIR.2	-0.239223203	#N/A
TAKDS[Phospho (STY)]DDDDDVTVTVDR.2	-0.347543116	O35526_S14_M1_Stx1a
KHSAILAS[Phospho (STY)]PNPDEK.3	-0.151068736	O35526_S64_M1_Stx1a
TAKDS[Phospho (STY)]DDDDDVTVTVDRDR.3	-0.226475337	O35526_S14_M1_Stx1a
SIEQSIEQEEGLNR.2	-0.212379712	#N/A
DS[Phospho (STY)]DDDDDVTVTVDRDR.2	-0.005344743	O35526_S14_M1_Stx1a
TKEELEELMSDIKK.3	0.117135302	#N/A
LKS[Phospho (STY)]IEQSIEQEEGLNR.3	-0.228098978	O35526_S95_M1_Stx1a
KFVEVM[Oxidation (M)]SEYNATQSDYR.3	-0.144296951	#N/A
FVEVM[Oxidation (M)]SEYNATQSDYR.2	-0.37488984	#N/A
AQS[Phospho (STY)]TDSLGTSSSLQSK.2	-0.478452748	O35551_S407_M1_Rabep1
RAQS[Phospho (STY)]TDS[Phospho (STY)]LGTSSSLQSK.2	0.091214687	O35551_S407_M2_Rabep1
QNAVLQAAQDDLGHLR.3	0.366541936	#N/A
VAGQSS[Phospho (STY)]PSGIQSR.2	-0.227846215	O35601_S28_M1_Fyb1
TTAVEIDYDS[Phospho (STY)]LKR.2	-0.03449335	O35601_S561_M1_Fyb1
LNINSS[Phospho (STY)]PDEHEPLLR.2	0.22748516	O35607_S863_M1_Bmpr2
LNINSS[Phospho (STY)]PDEHEPLLRR.3	-0.130484618	O35607_S863_M1_Bmpr2
SVS[Phospho (STY)]PTVNPMSTAMQNER.2	0.066364969	O35607_S515_M1_Bmpr2
NLKES[Phospho (STY)]SDENLMEHSLK.3	0.175477674	O35607_S680_M1_Bmpr2
EVDKNLKES[Phospho (STY)]S[Phospho (STY)]DENLMEHSLK.3	-0.315819631	O35607_S680_M2_Bmpr2
KLS[Phospho (STY)]PTEPR.2	-0.081772713	O35609_S78_M1_Scamp3
LS[Phospho (STY)]PTEPR.2	-0.29847352	O35609_S78_M1_Scamp3
TAAANAAAGAAENAFR.2	-0.017412342	#N/A
AGS[Phospho (STY)]PVSATEEKPK.2	-0.021927234	O35615_S286_M1_Zfpm1
GSEGSQS[Phospho (STY)]PGSSVDDAEDDPSR.2	0.110661999	O35615_S681_M1_Zfpm1
APRS[Phospho (STY)]PS[Phospho (STY)]PAPENTPSDPADQGAR.3	-0.272154292	O35615_S925_M2_Zfpm1
TIS[Phospho (STY)]APVVRPK.2	0.174124571	O35618_S367_M1_Mdm4
TVGHSVVS[Phospho (STY)]PQDTVQR.2	-0.386220582	O35621_S242_M1_Pmm1
SLS[Phospho (STY)]IEIGHEVK.2	-0.131377277	O35623_S50_M1_Bet1
GGAPLPPSGS[Phospho (STY)]KDQAVGAGGEFGGHDKPK.3	-0.006694888	O35633_S97_M1_Slc32a1
SLFQEGSR.2	-0.180258451	#N/A
GGAPLPPSGS[Phospho (STY)]K.2	-0.018033653	O35633_S97_M1_Slc32a1
LTNVATSVSNK.2	-0.114338216	#N/A
ILIAC[Carbamidomethyl (C)]LYEENEDGEVVR.2	-0.331555079	#N/A
DC[Carbamidomethyl (C)]EDPNPLIR.2	0.100870353	#N/A
DC[Carbamidomethyl (C)]PLNTEAASNK.2	0.029115965	#N/A
VDKITEYLC[Carbamidomethyl (C)]EPLRK.3	-0.131080299	#N/A
YNDPIYVK.2	-0.104914046	#N/A

KGEIFELK.2	-0.27849501	#N/A
LASQANIAQVLAELK.2	0.159353953	#N/A
VIASMTVGK.2	-0.154997242	#N/A
NINLIVQK.2	-0.195025818	#N/A
EYATEVDVDFVR.2	0.251315914	#N/A
LSHANSAVVLSAVK.2	0.368387314	#N/A
LQSSNIFTVAK.2	0.256627686	#N/A
LVYLYLM[Oxidation (M)]NYAK.2	-0.101537364	#N/A
AAM[Oxidation (M)]IWIVGEYAER.2	-0.101245396	#N/A
NVEGQDM[Oxidation (M)]LYQSLK.2	0.026943786	#N/A
APEVSQHVYQAYETILKN.3	-0.314382607	#N/A
ITEYLC[Carbamidomethyl (C)]EPLRK.2	0.224256816	#N/A
TAAVC[Carbamidomethyl (C)]VAK.2	-0.211973256	#N/A
DDREAQSIC[Carbamidomethyl (C)]ER.3	-0.454430786	#N/A
C[Carbamidomethyl (C)]LKDEDPYVRK.3	0.102423841	#N/A
KPTETQELVQQVLSLATQDSDNPDLR.3	-0.38357487	#N/A
C[Carbamidomethyl (C)]LKDEDPYVR.3	-0.007166003	#N/A
AFVEFLTDEIKEEK.2	0.180081338	#N/A
ATHS[Phospho (STY)]PSAREEEGPSGAT.2	-0.111968982	O35668_S615_M1_Hap1
NKS[Phospho (STY)]EVDLYNSDPLVC[Carbamidomethyl (C)]R.2	-0.21794936	O35678_S189_M1_Mgll
LTLPFLLLQGSADR.2	0.433092615	#N/A
IDSSVLSR.2	-0.015851521	#N/A
MPEASS[Phospho (STY)]PR.2	-0.486728052	O35678_S6_M1_Mgll
VC[Carbamidomethyl (C)]FGIQLLNAVAR.2	-0.274632964	#N/A
ALIFVSHGAGEHC[Carbamidomethyl (C)]GR.3	0.582166197	#N/A
GLSEKENS[Phospho (STY)]E.2	-0.365939056	O35681_S586_M1_Syt3
KLHFS[Phospho (STY)]VYDFDR.3	0.110049641	O35681_S382_M1_Syt3
LHFS[Phospho (STY)]VYDFDR.2	0.072012109	O35681_S382_M1_Syt3
GGGS[Phospho (STY)]GEAGAPC[Carbamidomethyl (C)]GR.2	-0.122039397	O35681_S290_M1_Syt3
LQLEIDQK.2	0.253953305	#N/A
WTQTLAELDLAVPFR.2	0.057560257	#N/A
GFS[Phospho (STY)]DSGGGPPAK.2	-0.288663873	O35691_S66_M1_Pnn
RGFS[Phospho (STY)]DSGGGPPAK.2	0.190414869	O35691_S66_M1_Pnn
SLS[Phospho (STY)]PGKENINSQEVEK.2	0.477137808	O35691_S442_M1_Pnn
QES[Phospho (STY)]DPEDDDVKKPALQSSVVATSK.3	0.155254923	O35691_S100_M1_Pnn
C[Carbamidomethyl (C)]VDGNQLS[Phospho (STY)]PVGEPK.2	0.027872939	O35711_S405_M1_Ppfibp2
T[Phospho (STY)]QSGNFNTDAPGM[Oxidation (M)]AEFR.2	0.114267526	O35711_T516_M1_Ppfibp2
VHIEIGPDGR.2	0.226560775	#N/A
GLPWSC[Carbamidomethyl (C)]SADEVQR.2	-0.067652473	#N/A
STGEAFVQFASQEIAEK.2	-0.401215913	#N/A
ATENDIYNFFSPLNPVR.2	0.070206314	#N/A
HTGPNS[Phospho (STY)]PDTANDGFVR.2	-0.52723376	O35737_S104_M1_Hnrnph1
THYDPPRK.2	0.320601686	#N/A
THYDPPR.2	-0.29307396	#N/A
DLNYC[Carbamidomethyl (C)]FSGM[Oxidation (M)]SDHR.2	0.297610624	#N/A
EGRPSGEAFVELES[Phospho (STY)]EDEVK.3	0.332617725	O35737_S63_M1_Hnrnph1
TSEDTSSGS[Phospho (STY)]PPK.2	-0.240258629	O35841_S464_M1_Api5

TSEDTSSGS[Phospho (STY)]PPKK.2	-0.386609493	O35841_S464_M1_Api5
TSEDTSSGS[Phospho (STY)]PPKKSPGGPK.3	-0.172828575	O35841_S464_M1_Api5
SEEETS[Phospho (STY)]PLVTHQNPAGPVAIAPELESK.3	-0.237478068	O35874_S507_M1_Slc1a4
EQFVEFR.2	-0.103294599	#N/A
GGSPLTTTQGGS[Phospho (STY)]PTK.2	-0.116061123	O35927_S273_M1_Ctnnd2
GGS[Phospho (STY)]APEGAAYAAPR.2	-0.24502672	O35927_S282_M1_Ctnnd2
SPS[Phospho (STY)]IDSIQK.2	-0.089892728	O35927_S531_M1_Ctnnd2
SASAPAS[Phospho (STY)]PR.2	-0.372083056	O35927_S1098_M1_Ctnnd2
ALQS[Phospho (STY)]PEHHIDPIYEDR.3	-0.448505957	O35927_S412_M1_Ctnnd2
T[Phospho (STY)]GSQHGPQNAAAATFQR.3	0.016309024	O35927_T469_M1_Ctnnd2
TSTAPSS[Phospho (STY)]PGVDSVPLQR.2	-0.487990364	O35927_S458_M1_Ctnnd2
NS[Phospho (STY)]YGAPAEDIK.2	0.20608482	O35927_S1149_M1_Ctnnd2
TPSIS[Phospho (STY)]PVR.2	-0.203884262	O35927_S1082_M1_Ctnnd2
NSSLS[Phospho (STY)]PGLNTSNGDGSETETTSAILASVK.3	-0.400749385	O35927_S32_M1_Ctnnd2
TPSIS[Phospho (STY)]PVRVS[Phospho (STY)]PNNR.2	0.111180466	O35927_S1082_M2_Ctnnd2
QLQYC[Carbamidomethyl (C)]ASVDS[Phospho (STY)]PYSK.2	-0.022011828	O35927_S511_M1_Ctnnd2
SLSQS[Phospho (STY)]QGDPLPPAHTGTFR.2	0.019938614	O35927_S437_M1_Ctnnd2
SPS[Phospho (STY)]IDSIQKDPR.2	-0.585653666	O35927_S531_M1_Ctnnd2
SGPALPPEGTLARS[Phospho (STY)]PS[Phospho (STY)]IDSIQK.3	0.006018315	#N/A
AS[Phospho (STY)]YSSQHGHLAPELR.3	0.241448284	O35927_S395_M1_Ctnnd2
SGPALPPEGTLARS[Phospho (STY)]PS[Phospho (STY)]IDSIQKDPR.3	0.186386638	#N/A
AAGQSFSQGTTGR.2	-0.230210667	#N/A
C[Carbamidomethyl (C)]NTGSEGPEAQT[Phospho (STY)]PGK.2	7.88E-05	O35954_T270_M1_Pitpnm1
PGTSTAGT[Phospho (STY)]PDGPEAPPGPDASPDAS[Phospho (STY)]FGK.3	-0.084190336	O35954_S304_M2_Pitpnm1
SSYSSQHGGGVS[Phospho (STY)]PQS[Phospho (STY)]LSEWR.3	-0.200707487	O35954_S326_M2_Pitpnm1
RGS[Phospho (STY)]M[Oxidation (M)]NNEM[Oxidation (M)]LSPEVGPVRDPLADGVEVLGR.4	-0.430901627	O35954_S593_M1_Pitpnm1
S[Phospho (STY)]M[Oxidation (M)]VQHYYR.2	0.047056974	O35963_S98_M1_Rab33b
[Acetyl (Protein N-term)]SSSAAS[Phospho (STY)]PLFAPGEDC[Carbamidomethyl (C)]GPAWR.2	-0.464131555	O35969_S7_M1_Gamt
TAVSVAQGGHS[Phospho (STY)]R.2	-0.119409178	O35984_S330_M1_Pbx2
ATEEPSGTGS[Phospho (STY)]DELIK.2	0.270277007	O54724_S42_M1_Cavin1
IIGAVDQIQLTQAQLEER.2	-0.062701339	#N/A
TLVLLDNLNVR.2	-0.050641841	#N/A
MVKGS[Phospho (STY)]IDR.2	-0.148825782	O54774_S9_M1_Ap3d1
NAEAVKS[Phospho (STY)]PEKEGVLGVEK.3	-0.154435972	O54774_S825_M1_Ap3d1
RHSSLPTES[Phospho (STY)]DEDIAPAQR.3	-0.352053694	O54774_S760_M1_Ap3d1
HSSLPTES[Phospho (STY)]DEDIAPAQR.3	-0.444953435	O54774_S760_M1_Ap3d1
NAEAVKS[Phospho (STY)]PEK.2	-0.113184239	O54774_S825_M1_Ap3d1
DLILQC[Carbamidomethyl (C)]LDDKDESIR.3	0.214139242	#N/A
EQAADTEAAQEVTQLLVER.2	-0.193202306	#N/A
NLQDLVR.2	0.052056628	#N/A
LEDPDPGVQSAAVNVIC[Carbamidomethyl (C)]ELAR.2	-0.183742375	#N/A
VDIITEEMPENALPS[Phospho (STY)]DEDDKDPNDPYR.4	0.290467871	O54774_S784_M1_Ap3d1
SSS[Phospho (STY)]SERPEPQQK.2	-0.314106848	O54781_S10_M1_Srpk2
TVSASS[Phospho (STY)]TGDLPK.2	0.051744313	O54781_S490_M1_Srpk2
AADLEDT[Phospho (STY)]T[Phospho (STY)]EEETAK.2	0.102184178	O54781_T331_M2_Srpk2
[Acetyl (Protein N-term)]S[Phospho (STY)]VNS[Phospho (STY)]EKSSSSERPEPQQK.2	-0.232417114	O54781_S2_M2_Srpk2
NTSYAS[Phospho (STY)]FER.2	-0.060710142	O54784_S316_M1_Dapk3

RS[Phospho (STY)]PLPGEPQRPK.3	-0.326554333	O54786_S314_M1_Dffa
S[Phospho (STY)]WHDM[Oxidation (M)]QTTTAYK.2	-0.022943216	O54818_S86_M1_Tpd52l1
S[Phospho (STY)]WHDM[Oxidation (M)]QTTTAYKK.3	-0.428740987	O54818_S86_M1_Tpd52l1
NSSTFKS[Phospho (STY)]FEER.2	-0.212376826	O54818_S149_M1_Tpd52l1
GC[Carbamidomethyl (C)]LAS[Phospho (STY)]PVFAR.2	-0.48523989	O54828_S588_M1_Rgs9
FLESEFSSENLR.2	0.012267078	#N/A
SHS[Phospho (STY)]PTHTPTPETKPPTEDELHQQIK.4	0.038201241	O54829_S241_M1_Rgs7
SSAYQELLQAK.2	0.03637025	#N/A
YTFEDAQEHIYK.2	-0.202668199	#N/A
S[Phospho (STY)]VYGLQNDIR.2	0.185642069	O54829_S229_M1_Rgs7
EVIC[Carbamidomethyl (C)]QVLYLGNVIDYK.2	-0.678225937	#N/A
RQS[Phospho (STY)]SSYEDPWKITDEQR.3	-0.079343631	O54916_S272_M1_Reps1
RQS[Phospho (STY)]SSYEDPWK.2	-0.016817677	O54916_S272_M1_Reps1
SSS[Phospho (STY)]LDMNR.2	0.14913901	O54916_S561_M1_Reps1
LKS[Phospho (STY)]EDELRPDVDEHTQK.3	-0.313103939	O54916_S708_M1_Reps1
KGPGS[Phospho (STY)]HDAVQPR.3	-0.237822917	O54916_S145_M1_Reps1
LIDLEDSADVGEQPGEVGY[Phospho (STY)]SGSPAEAPPSK.3	0.361528667	O54916_Y389_M1_Reps1
TLS[Phospho (STY)]M[Oxidation (M)]IEEEIR.2	0.267637866	O54931_S740_M1_Akap2
KGS[Phospho (STY)]ITEYTATEEK.2	-0.533598461	O54940_S114_M1_Bnip2
VDYYEVLGVQR.2	-0.193999885	#N/A
DC[Carbamidomethyl (C)]FGC[Carbamidomethyl (C)]LR.2	0.142436064	#N/A
SSSLLIPPLEAALANFSK.2	0.092802779	#N/A
LVYDSWSSGK.2	-0.035523016	#N/A
QAVLYVDSR.2	-0.057313559	#N/A
FASTVQGDVR.2	0.263808427	#N/A
SLGM[Oxidation (M)]AVEDLVAAK.2	-0.06965965	#N/A
GFLGVM[Oxidation (M)]PAYSAAEDALTTK.2	-0.094617394	#N/A
TAAVSAIATK.2	0.335912521	#N/A
RAPAFLSAEEVQDHLR.3	-0.264420689	#N/A
GPDGGVM[Oxidation (M)]QPVR.2	0.322849601	#N/A
APAFLSAEEVQDHLR.2	-0.134562608	#N/A
ELDDELMR.2	0.33180856	#N/A
LEETLPVIR.2	0.337432291	#N/A
LLNFPTIVER.2	-0.062340688	#N/A
ESVLIISTDPAHNISDAFDQK.3	0.203583412	#N/A
VITSDRSS[Phospho (STY)]EVGTDEALDDTQK.3	-0.088724755	O54988_S543_M1_Slk
ATLEQPETDEVEQVSESNS[Phospho (STY)]IEELER.3	-0.195546788	O54988_S647_M1_Slk
LENLPDTQDQQTVDVNSVSEENENNR.3	0.175474558	#N/A
DQNLPQILEESRS[Phospho (STY)]E.2	-0.255110852	O54991_S1384_M1_Cntnap1
AVATQGAFNSWDWVTR.2	0.149740013	#N/A
NSTLS[Phospho (STY)]EEDYIER.2	-0.233239224	O55003_S88_M1_Bnip3
SSHC[Carbamidomethyl (C)]DS[Phospho (STY)]PPR.2	0.13318601	O55003_S60_M1_Bnip3
AEIDSHS[Phospho (STY)]FGEK.2	-0.042206551	O55003_S79_M1_Bnip3
SQT[Phospho (STY)]PQDTNRAEIDSHSFGEK.3	0.048365726	O55003_T66_M1_Bnip3
ILLDAQHES[Phospho (STY)]GR.2	-0.250743519	O55003_S48_M1_Bnip3
ESLS[Phospho (STY)]WGTQR.2	0.07428838	O55017_S1929_M1_Cacna1b
SHS[Phospho (STY)]KETPGADTQVR.3	-0.389077813	O55017_S892_M1_Cacna1b

AS[Phospho (STY)]C[Carbamidomethyl (C)]EALYSEM[Oxidation (M)]DPEER.2	-0.030062294	O55017_S783_M1_Cacna1b
GLS[Phospho (STY)]EHNALLQK.2	0.021172171	O55017_S2221_M1_Cacna1b
RGS[Phospho (STY)]PEEATER.2	-0.037943509	O55017_S915_M1_Cacna1b
IGS[Phospho (STY)]DPYLGQR.2	-0.290411885	O55017_S2244_M1_Cacna1b
RGS[Phospho (STY)]PEEATEREPR.3	0.18417148	O55017_S915_M1_Cacna1b
RSHS[Phospho (STY)]KETPGADTQVR.3	-0.174102573	O55017_S892_M1_Cacna1b
S[Phospho (STY)]LEKGPSLSVDPEGAPSTAAGPGLPHGEGSTAC[Carbamidomethyl (C)]R.4	0.194231143	O55017_S2056_M1_Cacna1b
EVAEVS[Phospho (STY)]PM[Oxidation (M)]S[Phospho (STY)]AANISIAAR.2	0.229213227	O55017_S745_M2_Cacna1b
LQNLRAS[Phospho (STY)]C[Carbamidomethyl (C)]EALYSEM[Oxidation (M)]DPEER.3	-0.404474291	O55017_S783_M1_Cacna1b
GDQPGASGDNDDDEPPPLPR.2	-0.027059144	#N/A
RFDGVQDPR.2	0.047358152	#N/A
FYGPEGPYGVFAGR.2	0.339159402	#N/A
EGEEPTVYS[Phospho (STY)]DDEEPKDETAR.3	-0.269241003	O55022_S181_M1_Pgrmc1
LLKEGEEPTVYSDDEEPKDETAR.4	0.09266571	#N/A
DFTPAELR.2	0.026461083	#N/A
EGEEPTVYS[Phospho (STY)]DDEEPKDETARK.4	-0.663451372	O55022_S181_M1_Pgrmc1
SLLVTELGSSR.2	0.016396272	#N/A
SS[Phospho (STY)]PADLVTVTDQKVEK.2	-0.005288523	O55023_S38_M1_Impa1
EIEIIPLQR.2	0.357482703	#N/A
LC[Carbamidomethyl (C)]SIPIHGIR.2	-0.081000654	#N/A
LQVSQQEDITK.2	0.20601772	#N/A
S[Phospho (STY)]SPADLVTVTDQK.2	-0.052293318	O55023_S37_M1_Impa1
IIAANSITLAK.2	0.498905522	#N/A
[Acetyl (Protein N-term)]ADPWQEC[Carbamidomethyl (C)]M[Oxidation (M)]DYAVILAR.2	0.334166908	#N/A
S[Phospho (STY)]TSATDTHHVELAR.3	0.115940701	O55028_S31_M1_Bckdk
DNNQFASASLDR.2	-0.149605821	#N/A
DSESS[Phospho (STY)]PSDFSVSLK.2	-0.15513266	O55033_S316_M1_Nck2
DAS[Phospho (STY)]PTPSTDAEYPANGSGADR.2	-0.166693004	O55033_S90_M1_Nck2
EQVTNVGGAVVTGVTAVAQK.2	-0.290777358	#N/A
AKEGVVAAAEK.2	0.080299196	#N/A
TVEGAGNIAAATGFVK.2	0.113211274	#N/A
EGVLYVGSKT[Phospho (STY)]K.2	0.203997781	O55042_T44_M1_Snca
TKEGVVHGVTTVAEK.3	-0.094025887	#N/A
TKEQVTNVGGAVVTGVTAVAQK.3	-0.626163976	#N/A
TVEGAGNIAAATGFVKK.2	-0.269140356	#N/A
EGVVHGVTTVAEK.2	0.154698585	#N/A
[Acetyl (Protein N-term)]S[Phospho (STY)]LDM[Oxidation (M)]KEHPDAEVQK.2	-0.080155524	O55060_S2_M1_Tpmt
AVQQYLEETQNK.2	-0.304469786	#N/A
TEEVEVES[Phospho (STY)]EEDPILEHPPENPVK.3	-0.169681926	O55091_S137_M1_Impact
KKTEEVEVES[Phospho (STY)]EEDPILEHPPENPVK.4	0.10614519	O55091_S137_M1_Impact
KTEEVEVES[Phospho (STY)]EEDPILEHPPENPVK.3	-0.340310311	O55091_S137_M1_Impact
AGGAFDPYTLVR.2	-0.088937996	#N/A
LPEQELSR.2	0.086791802	#N/A
FLESAAADVS[Phospho (STY)]DEDEDEDTDGR.2	0.252812031	O55106_S245_M1_Strn
EALKEFDFLVTSEEGDNESR.3	-0.145256452	#N/A
FYDNNTGK.2	0.176841592	#N/A
VISHPTLPISITAHEDR.3	-0.165320081	#N/A

AGPNIYELR.2	-0.159702785	#N/A
GWDENVYYTVPLVR.2	0.20222409	#N/A
LKPGTM[Oxidation (M)]IEWGNNWAR.3	0.17246548	#N/A
KETSNLYK.2	-0.053878256	#N/A
DSEGSWFR.2	0.244268544	#N/A
IQFHNVKPEC[Carbamidomethyl (C)]LDAYNSLTEAVLPK.4	-0.231537543	#N/A
PGPNIYELR.2	-0.330888053	#N/A
LKENQEFVNFR.3	-0.132996642	#N/A
IC[Carbamidomethyl (C)]QEVLPK.2	0.246871849	#N/A
KETSSLYK.2	-0.082111292	#N/A
LQFHNVKPEC[Carbamidomethyl (C)]LDAYNK.3	-0.131913444	#N/A
SDMLLSR.2	0.210367812	#N/A
ILEQQNSSRT[Phospho (STY)]LEK.2	-0.330669365	O55131_T425_M1_Septin7
LKDSEAELQR.2	-0.100404129	#N/A
FEDYLNAESR.2	0.124284379	#N/A
NLEGYVGFANLPNQVYR.2	-0.27660613	#N/A
ANWEAQQR.2	0.321828184	#N/A
ADTLTPEEC[Carbamidomethyl (C)]QQFK.2	0.199828707	#N/A
ADTLTPEEC[Carbamidomethyl (C)]QQFKK.2	0.210753612	#N/A
LAAVTYNGVDNNK.2	-0.309023129	#N/A
STLINSLFLTDLYSPEYPGPSHR.3	0.083383022	#N/A
IYEFPETDDEEENKLVK.3	-0.324835554	#N/A
DVTNNVHYENYR.2	0.056369839	#N/A
GQLTKS[Phospho (STY)]PLAQM[Oxidation (M)]EEERR.3	-0.332070221	#N/A
IYEFPETDDEEENK.2	-0.144410557	#N/A
S[Phospho (STY)]PLAQM[Oxidation (M)]EEER.2	0.105919675	#N/A
NLEAQHKELEEK.2	-0.233792291	#N/A
KTVQVEQSK.2	0.167210397	#N/A
THMQDLK.2	0.224664978	#N/A
S[Phospho (STY)]PLAQM[Oxidation (M)]EEERR.2	-0.00695953	#N/A
ILEQQNSSR.2	0.145582478	#N/A
QFEEEKANWEAQQR.2	-0.346371615	#N/A
YRADS[Phospho (STY)]HGELDLAR.3	-0.140323715	O55137_S56_M1_Acot1
QLGSEC[Carbamidomethyl (C)]LHVS[Phospho (STY)]PK.2	-0.049126827	O55137_S416_M1_Acot1
IEGVYAR.2	-0.132857393	#N/A
NNTVTPGGKPNK.2	0.209388425	#N/A
EFDELSPS[Phospho (STY)]AQR.2	-0.315840855	O55143_S663_M1_Atp2a2
WGS[Phospho (STY)]NELPAEEGK.2	-0.3036179	O55143_S38_M1_Atp2a2
VGEATETALTC[Carbamidomethyl (C)]LVEK.2	0.070223712	#N/A
VDQSILTGESVSVIK.2	-0.028657179	#N/A
NM[Oxidation (M)]LFSGTNIAAGK.2	-0.408478697	#N/A
EWGS[Phospho (STY)]GSDTLR.2	0.07622881	O55143_S553_M1_Atp2a2
VIM[Oxidation (M)]ITGDNK.2	-0.156386418	#N/A
SLPSVETLGC[Carbamidomethyl (C)]TSVIC[Carbamidomethyl (C)]SDK.2	0.2680838	#N/A
ANAC[Carbamidomethyl (C)]NSVIK.2	0.102365812	#N/A
IGIFGQDEDVTSK.2	0.073401814	#N/A
DIVPGDIVEIAVGDKVPADIR.3	0.137099497	#N/A

IEVASSVK.2	-0.038937477	#N/A
MNVFDTELK.2	0.069294559	#N/A
NYLEQPGKEC[Carbamidomethyl (C)]VQPATK.3	-0.248091028	#N/A
GTAVAIC[Carbamidomethyl (C)]R.2	0.036783656	#N/A
TASEM[Oxidation (M)]VLADDNFSTIVAAVEEGR.3	-0.217880528	#N/A
TGTLTTNQMSVC[Carbamidomethyl (C)]R.2	0.16175409	#N/A
IVEFLQSFDEITAM[Oxidation (M)]TGDGVNDAPALK.3	-0.096088477	#N/A
IVEFLQSFDEITAM[Oxidation (M)]TGDGVNDAPALKK.3	-0.586114515	#N/A
YETNLTFVGC[Carbamidomethyl (C)]VGM[Oxidation (M)]LDPPR.2	0.497191249	#N/A
IRDEMVATEQER.3	-0.260678238	#N/A
SRS[Phospho (STY)]PFASTR.2	-0.270788686	O55176_S32_M1_Pja1
EQPQSSS[Phospho (STY)]GESWELLPGKEELER.3	-0.089821986	O55176_S341_M1_Pja1
FTSVNHDAKEEC[Carbamidomethyl (C)]GKVES[Phospho (STY)]PPAAR.4	0.229075896	O55176_S137_M1_Pja1
VFFDT[Phospho (STY)]DDDDDVPHSTSR.2	0.101872104	O55176_T231_M1_Pja1
YYEEDS[Phospho (STY)]DSDKEWM[Oxidation (M)]AALR.3	-0.259986839	O55176_S317_M1_Pja1
GNNS[Phospho (STY)]LER.2	0.002568519	O55176_S175_M1_Pja1
GPGLYYVDSEGNR.2	-0.35106589	#N/A
RGPGLYYVDSEGNR.3	-0.06447847	#N/A
AIYQATYR.2	0.427883834	#N/A
ATAGAYIASQTVK.2	0.222155344	#N/A
VEEAYDLAR.2	0.391817119	#N/A
DAYSGGAVNLYHVR.2	0.096548971	#N/A
FLHGVIVAADSR.3	0.553449321	#N/A
AEENNSGVESSGYGS[Phospho (STY)]PGPTWDR.2	0.484713791	O70133_S136_M1_Dhx9
DFVNYLVR.2	-0.200845972	#N/A
YTQVGPDHNR.2	0.259285646	#N/A
S[Phospho (STY)]LPSSPELPR.2	-0.099349317	O70146_S439_M1_Tesk1
SSSLKSS[Phospho (STY)]PSK.2	-0.199144275	O70161_S453_M1_Pip5k1c
SPS[Phospho (STY)]DTSEQPR.2	-0.104632359	O70161_S540_M1_Pip5k1c
ITVQVEPVC[Carbamidomethyl (C)]GVGVVPK.2	-0.592622868	#N/A
RTQS[Phospho (STY)]SGQDGRPQEEPHAEDLQK.4	-0.147644425	O70161_S554_M1_Pip5k1c
SSS[Phospho (STY)]LKSS[Phospho (STY)]PSKK.2	-0.319991441	O70161_S449_M2_Pip5k1c
TQS[Phospho (STY)]SGQDGRPQEEPHAEDLQK.3	-0.323213008	O70161_S554_M1_Pip5k1c
DC[Carbamidomethyl (C)]LVLESFK.2	0.102206979	#N/A
IM[Oxidation (M)]DYSLLLGVHNIDQQER.3	-0.5591246	#N/A
SWVYSPLHYSARPASDGESDT.3	-0.064879656	#N/A
DAS[Phospho (STY)]LEELQKR.2	-0.427286845	O70166_S81_M1_Stmn3
DAS[Phospho (STY)]LEELQK.2	-0.244578028	O70166_S81_M1_Stmn3
ALEENNNFSR.2	-0.584598147	#N/A
KDAS[Phospho (STY)]LEELQK.2	-0.121240823	O70166_S81_M1_Stmn3
SPSDLSPES[Phospho (STY)]PVLSSPPK.2	-0.234893967	O70166_S68_M1_Stmn3
ASGQS[Phospho (STY)]FEVILK.2	0.028664855	O70166_S53_M1_Stmn3
RAS[Phospho (STY)]GQS[Phospho (STY)]FEVILK.2	0.208813369	O70166_S50_M2_Stmn3
RKDAS[Phospho (STY)]LEELQK.2	0.326583627	O70166_S81_M1_Stmn3
KDAS[Phospho (STY)]LEELQKR.3	0.074498192	O70166_S81_M1_Stmn3
NKEQREEMS[Phospho (STY)]G.2	-0.416665653	O70166_S179_M1_Stmn3
IYIDDNNKK.2	0.086815536	#N/A

DVEFLAQLK.2	-0.076268738	#N/A
FGIDDQDFQNSLTR.2	0.192728154	#N/A
C[Carbamidomethyl (C)]TC[Carbamidomethyl (C)]KEPS[Phospho (STY)]PVS[Phospho (STY)]PITVLK.3	0.128563201	O70174_S540_M2_Chrna4
IFHTVTTTDDPVIR.3	-0.365498794	#N/A
TPAGLQVLNDYLADK.2	0.222303058	#N/A
SIQADGLVWGSSK.2	-0.12570569	#N/A
DDDDIDLFGS[Phospho (STY)]DDEEESEEAKK.3	0.198830567	O70251_S106_M1_Eef1b
YGPSS[Phospho (STY)]VEDTTGSGAADAK.2	0.202264864	O70251_S83_M1_Eef1b
YGPSSVEDTTGSGAADAKDDDDIDLFGS[Phospho (STY)]DDEEESEEAK.4	-0.360255252	O70251_S106_M1_Eef1b
DDDDIDLFGS[Phospho (STY)]DDEEESEEAK.2	0.10625534	O70251_S106_M1_Eef1b
QDC[Carbamidomethyl (C)]C[Carbamidomethyl (C)]GNC[Carbamidomethyl (C)]S[Phospho (STY)]DS[Phospho (STY)]EEELPTRL.2	0.229812714	O70293_S566_M2_Grk6
DVLDIEQFS[Phospho (STY)]TVK.2	-0.288375177	O70293_S484_M1_Grk6
DNS[Phospho (STY)]EEFLKR.2	-0.396023189	O70305_S400_M1_Atxn2
VALENDDRS[Phospho (STY)]EEEKYTAVQR.3	-0.20252164	O70305_S435_M1_Atxn2
ASETS[Phospho (STY)]PSFSK.2	-0.255948723	O70305_S741_M1_Atxn2
QNS[Phospho (STY)]PAGSKENVK.2	0.133265602	O70305_S727_M1_Atxn2
TNS[Phospho (STY)]PSIS[Phospho (STY)]PSMLSNAEHKR.3	-0.693305609	O70305_S832_M2_Atxn2
TNS[Phospho (STY)]PSIS[Phospho (STY)]PSMLSNAEHK.2	-0.096659361	O70305_S832_M2_Atxn2
MSSEGPPRM[Oxidation (M)]S[Phospho (STY)]PK.2	0.279909068	O70305_S593_M1_Atxn2
DSFIDSSSSSSNC[Carbamidomethyl (C)]TSGSSK.2	0.175062134	#N/A
GM[Oxidation (M)]S[Phospho (STY)]PVVSEHRK.3	0.050495558	O70305_S753_M1_Atxn2
SGLS[Phospho (STY)]PANDTGAK.2	-0.223893912	O70310_S47_M1_Nmt1
SGLS[Phospho (STY)]PANDTGAKK.2	-0.542693387	O70310_S47_M1_Nmt1
GS[Phospho (STY)]PGGDLGAK.2	-0.209225659	O70311_S38_M1_Nmt2
EKPNSGGTKS[Phospho (STY)]DS[Phospho (STY)]AS[Phospho (STY)]DSQEIK.2	-0.005237765	O70311_S66_M3_Nmt2
QRS[Phospho (STY)]YNLVVAK.2	0.065957522	O70318_S86_M1_Epb41l2
[Acetyl (Protein N-term)]TTEVGS[Phospho (STY)]ASEVKK.2	0.0112525	O70318_S7_M1_Epb41l2
LVS[Phospho (STY)]PEQPPK.2	0.116779881	O70318_S492_M1_Epb41l2
EVQTSELKAEVAS[Phospho (STY)]QK.2	0.150132616	O70318_S201_M1_Epb41l2
EM[Oxidation (M)]S[Phospho (STY)]PGHGPGETR.2	-0.276095972	O70318_S698_M1_Epb41l2
EPTQADVEDQILGKEES[Phospho (STY)]LPEEESR.3	0.223030154	O70318_S116_M1_Epb41l2
S[Phospho (STY)]YNLVVAK.2	0.042788393	O70318_S86_M1_Epb41l2
IVITGDAALDHDQALAQAIR.3	0.142840799	#N/A
AKEVENEQTPVS[Phospho (STY)]EPEEEKGSQPGPPVER.4	0.144591225	O70318_S38_M1_Epb41l2
GLSPAQADSQFLENAK.2	0.389949302	#N/A
VTEGTIREEQEEC[Carbamidomethyl (C)]DEELEEEPGQGAK.3	-0.420192064	#N/A
KEPTQADVEDQILGKEES[Phospho (STY)]LPEEESR.4	-0.292639355	O70318_S116_M1_Epb41l2
ILAFPC[Carbamidomethyl (C)]NQFGR.2	-0.344761472	#N/A
YAEC[Carbamidomethyl (C)]GLR.2	-0.102135794	#N/A
IC[Carbamidomethyl (C)]VNGDDAHPLWK.3	0.091098423	#N/A
TDVNYTQLVDLHAR.2	-0.081581744	#N/A
QHS[Phospho (STY)]PGSTDSLSNDSQR.2	-0.166523166	O70374_S400_M1_Cbfa2t2
IEQNLQS[Phospho (STY)]PTQQQTAR.2	0.202661891	O70405_S450_M1_Ulk1
VPS[Phospho (STY)]PQGADVR.2	0.096124197	O70405_S521_M1_Ulk1
DIREEAEK.2	0.047500746	#N/A
SNFGYNIPLK.2	0.29158128	#N/A
[Acetyl (Protein N-term)]SSIGTGYDLSASTFSPDGR.2	0.194610166	#N/A

AVENSSTAIGIR.2	-0.222336239	#N/A
ESLKEEDES[Phospho (STY)]DDDNM[Oxidation (M)].2	0.026922108	O70435_S250_M1_Psma3
YAKESLKEEDES[Phospho (STY)]DDDNM[Oxidation (M)].2	-0.199065573	O70435_S250_M1_Psma3
EFGSLPTT[Phospho (STY)]PSEQR.2	-0.266836413	O70439_T79_M1_Stx7
TLNQLGT[Phospho (STY)]PQDSPELR.2	-0.056275619	O70439_T41_M1_Stx7
[Acetyl (Protein N-term)]S[Phospho (STY)]YTPGIGGDSAQLAQR.2	-0.570377708	O70439_S2_M1_Stx7
ITQC[Carbamidomethyl (C)]SVEIQR.2	0.108121656	#N/A
SSEYHLEDNAAYYLNDLER.3	-0.488013165	#N/A
QFEDLNR.2	-0.136019938	#N/A
IAAPDYIPTVEDILR.2	-0.019127782	#N/A
GQNTYEEAAVYIQR.2	-0.422423533	#N/A
LWADPGAQAC[Carbamidomethyl (C)]FGR.2	-0.369263473	#N/A
VGS[Phospho (STY)]RHDS[Phospho (STY)]TLKK.2	0.03793373	O70451_S214_M2_Slc16a7
VGS[Phospho (STY)]RHDS[Phospho (STY)]TLK.2	0.050309748	O70451_S214_M2_Slc16a7
NLLEDDS[Phospho (STY)]DEEEDFFLR.2	0.039528957	O70480_S30_M1_Vamp4
RNLLEDDS[Phospho (STY)]DEEEDFFLR.3	-0.10906567	O70480_S30_M1_Vamp4
MDVS[Phospho (STY)]PEPPLAPQR.2	0.318648752	O70481_S15_M1_Ubr1
FTTYEIR.2	0.123353574	#N/A
YSDFEWLR.2	0.170792349	#N/A
[Acetyl (Protein N-term)]AETVADTRR.2	0.113986081	#N/A
[Acetyl (Protein N-term)]S[Phospho (STY)]DTAVADTRR.2	0.413086489	O70493_S2_M1_Snx12
GDEGIFEESFIEER.2	-0.372387394	#N/A
FTTYEVR.2	0.138376478	#N/A
RYS[Phospho (STY)]DFEWLK.2	-0.024722872	O70493_S73_M1_Snx12
IAGHPLAQNER.3	-0.111015383	#N/A
S[Phospho (STY)]PQAAQPPPPLPGAR.2	-0.094957925	O70507_S921_M1_Hcn4
GGLS[Phospho (STY)]PPGHSPGPPR.2	-0.11212399	O70507_S1011_M1_Hcn4
RAS[Phospho (STY)]PHS[Phospho (STY)]S[Phospho (STY)]GESVAAFSLYPR.3	-0.280732776	O70507_S1090_M3_Hcn4
ADT[Phospho (STY)]PS[Phospho (STY)]GDEQEPNGALDSK.2	0.164477706	O70551_S450_M2_Srpk1
EINGISVANQTVEQLQK.2	-0.390843962	#N/A
TQSSSC[Carbamidomethyl (C)]ERDS[Phospho (STY)]PSTSR.2	-0.451225084	O70589_S582_M1_Cask
GAVSAEQVIAGFNR.2	-0.237988512	#N/A
IIETLSQQLQAK.2	0.243743288	#N/A
EFS[Phospho (STY)]EDEEEETTSQDWGTPR.2	0.242114503	O70622_S44_M1_Rtn2
SSQS[Phospho (STY)]PPLAAAGPSAR.2	0.213891233	O88196_S1903_M1_Ttc3
RNSDS[Phospho (STY)]AGPFAVPDHLR.3	-0.113324341	O88196_S1062_M1_Ttc3
GASQVS[Phospho (STY)]PSEQS[Phospho (STY)]PEADEK.2	-0.137691931	O88196_S1794_M2_Ttc3
KQDS[Phospho (STY)]DEM[Oxidation (M)]PFGC[Carbamidomethyl (C)]IK.2	-0.238077071	O88196_S1009_M1_Ttc3
IIIYSSGGQVK.2	-0.390742161	#N/A
EVLASS[Phospho (STY)]QEALVVTER.2	-0.313643181	O88273_S56_M1_Grem2
MNLSKS[Phospho (STY)]PTK.2	-0.377173391	O88291_S270_M1_Znf326
S[Phospho (STY)]PTKTDPKNEEEEK.2	-0.418164978	O88291_S270_M1_Znf326
M[Oxidation (M)]NLSKS[Phospho (STY)]PTKTDPK.2	0.207097185	O88291_S270_M1_Znf326
S[Phospho (STY)]AAEELELTVLK.2	-0.632019951	O88327_S373_M1_Ctnnal1
VFASLPQVER.2	-0.196231071	#N/A
YAPSGFYIASGDISGK.2	-0.084977135	#N/A
YEYQPFAGK.2	0.088663157	#N/A

IAVVGEGR.2	0.168577103	#N/A
SIQC[Carbamidomethyl (C)]LTVHR.2	0.302896142	#N/A
DYSGQGVVK.2	-0.116769755	#N/A
DIAWTEDSKR.2	0.017026864	#N/A
GPVTDVAYSHDGAFLAVC[Carbamidomethyl (C)]DASK.3	-0.312653718	#N/A
VINSVDIK.2	0.229763514	#N/A
LATGSDDNC[Carbamidomethyl (C)]AAFFEGPPFK.2	-0.323992212	#N/A
VC[Carbamidomethyl (C)]ALGESK.2	0.036234536	#N/A
VYSILASTLKDEGK.2	-0.007564849	#N/A
AHDGGIYAISWSPDSTHLLSASGDK.3	-0.158168208	#N/A
VVTVFSVADGYSENNVFYGHHAK.3	-0.596730162	#N/A
GDHFLYTNGK.2	0.142254169	#N/A
M[Oxidation (M)]TVNESEQLVSC[Carbamidomethyl (C)]SMDDTVR.2	-0.248625878	#N/A
SYIYSGSHDGHINYWDSETGENDSFSGK.4	-0.349430044	#N/A
NIDNPAIADIYTEHAHQVVVAK.3	-0.143346201	#N/A
GSLDS[Phospho (STY)]DNDDSDC[Carbamidomethyl (C)]PYSEK.2	0.301858482	O88343_S1029_M1_Slc4a4
NLTSSS[Phospho (STY)]LNDISDKPEKDQLK.3	-0.182204202	O88343_S257_M1_Slc4a4
LQQAVM[Oxidation (M)]LGALTEVPVPTR.2	-0.174752885	#N/A
NLTSSS[Phospho (STY)]LNDISDKPEK.2	0.170376183	O88343_S257_M1_Slc4a4
LADYYPINSDFK.2	0.218764593	#N/A
MFSNPDNGS[Phospho (STY)]PAM[Oxidation (M)]THR.2	0.345595835	#N/A
ISENYSDKS[Phospho (STY)]DVENADESSSSILKPLISPAAER.4	-0.350983105	O88343_S68_M1_Slc4a4
KGS[Phospho (STY)]LDS[Phospho (STY)]DNDDSDC[Carbamidomethyl (C)]PYSEK.2	0.035676918	O88343_S1026_M2_Slc4a4
KKGS[Phospho (STY)]LDS[Phospho (STY)]DNDDSDC[Carbamidomethyl (C)]PYSEK.3	-0.384686641	O88343_S1026_M2_Slc4a4
TLDQVLEDVDQC[Carbamidomethyl (C)]C[Carbamidomethyl (C)]QALSQR.3	-0.011320626	#N/A
[Acetyl (Protein N-term)]SLVAEAFVSQIAATEPWPENATLYQQLR.3	-0.245825213	#N/A
VDS[Phospho (STY)]PTVTTTLK.2	-0.151539128	O88447_S459_M1_Klc1
DAANLLNDALAIR.2	0.080412741	#N/A
S[Phospho (STY)]RESLNM[Oxidation (M)]DVVK.2	-0.175395767	O88447_S520_M1_Klc1
AC[Carbamidomethyl (C)]KVDS[Phospho (STY)]PTVTTTLK.2	-0.115672305	O88447_S459_M1_Klc1
S[Phospho (STY)]RES[Phospho (STY)]LNMDVVKYESGPDGGEEA.3	-0.058146394	O88447_S520_M2_Klc1
RS[Phospho (STY)]RES[Phospho (STY)]LNMDVVK.3	-0.474182873	O88447_S520_M2_Klc1
VDS[Phospho (STY)]PTVNTTLR.2	0.064706144	O88448_S443_M1_Klc2
ASS[Phospho (STY)]LNFLNK.2	-0.457063926	O88448_S575_M1_Klc2
YEVAVPLC[Carbamidomethyl (C)]K.2	-0.176181695	#N/A
S[Phospho (STY)]GS[Phospho (STY)]FGKLR.2	-0.46514347	O88448_S537_M2_Klc2
AC[Carbamidomethyl (C)]KVDS[Phospho (STY)]PTVNTTLR.2	-0.047907373	O88448_S443_M1_Klc2
TLHNLVIQYASQGR.2	-0.359809366	#N/A
RSS[Phospho (STY)]EMLVR.2	0.470293082	O88448_S551_M1_Klc2
YADESGNM[Oxidation (M)]DFDNFISC[Carbamidomethyl (C)]LVR.2	0.018292123	#N/A
ILGGVISAISEAAAQYNPEPPPPR.3	-0.356350722	#N/A
ATHVPENS[Phospho (STY)]DTEQDVFTAKPAR.3	0.343640237	O88466_S1381_M1_Znf106
IGHDS[Phospho (STY)]ELENQEK.2	-0.048813566	O88485_S180_M1_Dync1i1
VTQVDFLPR.2	-0.157405726	#N/A
ANRADS[Phospho (STY)]EEEGAVELAA.2	-0.266321071	O88485_S618_M1_Dync1i1
KEPVQDDS[Phospho (STY)]DLDR.2	-0.018574841	O88485_S50_M1_Dync1i1
ALAEDSDIFFDYSGR.2	-0.075830015	#N/A

ETQTPLATHQS[Phospho (STY)]EEDEEDEEMVEPK.3	-0.146424227	O88485_S162_M1_Dync1i1
SVSTPSDAGS[Phospho (STY)]QDSGDLGPLTR.2	-0.194583572	O88485_S94_M1_Dync1i1
[Acetyl (Protein N-term)]SDKSDLKAELER.3	-0.154829357	#N/A
TLQWDTDPSVLQLQSDSELGR.2	-0.252240021	#N/A
LDLWNLNSDTEVPTASVAIEGASALNR.3	-0.100744418	#N/A
SVSTPSEAGS[Phospho (STY)]QDSGDGAVGSR.2	-0.361722952	O88487_S95_M1_Dync1i2
TLAEINANR.2	-0.451348955	#N/A
EIAVGDSEGQIVIYDVGEQIAVPR.3	-0.515790355	#N/A
DLEDKEGEIQAGAK.2	0.107414787	#N/A
EAEALLQSM[Oxidation (M)]GLTTDSPIVPPPM[Oxidation (M)]S[Phospho (STY)]PSSK.3	0.033160789	O88487_S81_M1_Dync1i2
AAS[Phospho (STY)]PQEVTPQADEK.2	-0.192587847	O88491_S2369_M1_Nsd1
RFS[Phospho (STY)]GNEYILATK.2	0.508729824	O88502_S452_M1_Pde8a
SEPQPEEGS[Phospho (STY)]PAAGQK.2	-0.045340192	O88508_S102_M1_Dnmt3a
S[Phospho (STY)]PPEEPPDFC[Carbamidomethyl (C)]C[Carbamidomethyl (C)]PK.2	-0.223091367	O88522_S380_M1_Ikbkg
RS[Phospho (STY)]PPEEPPDFC[Carbamidomethyl (C)]C[Carbamidomethyl (C)]PK.2	-0.016142312	O88522_S380_M1_Ikbkg
ETIPLQESTLYTEDR.2	-0.131029776	#N/A
RDS[Phospho (STY)]DGVDGFEAEGKK.3	-0.366785178	O88532_S1054_M1_Zfr
RRDS[Phospho (STY)]DGVDGFEAEGK.3	-0.377759361	O88532_S1054_M1_Zfr
RDS[Phospho (STY)]DGVDGFEAEGK.2	-0.023080724	O88532_S1054_M1_Zfr
DS[Phospho (STY)]DGVDGFEAEGK.2	0.006648791	O88532_S1054_M1_Zfr
[Acetyl (Protein N-term)]ASALEQFVNSVR.2	0.123712885	#N/A
YTSQIVGR.2	0.192043276	#N/A
SM[Oxidation (M)]GS[Phospho (STY)]QEDDSGNKPSSYS.2	-0.372163576	O88543_S410_M1_Cops3
YATDTFAGLC[Carbamidomethyl (C)]HQLTNALVER.3	-0.164607241	#N/A
NAAQVLVGIPLETGQK.2	0.179949461	#N/A
ATTADGSSILDR.2	0.240153799	#N/A
LYLEDDDPVQAEAYINR.2	0.302922583	#N/A
VISFEEQVASIR.2	-0.030813215	#N/A
ISQTAPEWTAQAM[Oxidation (M)]EAQM[Oxidation (M)]AQ.2	-0.285662168	#N/A
IASQMITEGR.2	0.357552684	#N/A
GGGGNFGPGPGSNFR.2	0.143227874	#N/A
GGNFGFGDSR.2	-0.11706142	#N/A
IDTIEIITDR.2	0.060939309	#N/A
EDTEEHHLR.2	0.369965776	#N/A
GFGFVTFDDHDPVDK.2	-0.113272016	#N/A
NYYEQWGK.2	-0.097153113	#N/A
PHS[Phospho (STY)]IDGR.2	-0.201943613	O88569_S85_M1_Hnrnpa2b1
YHTINGHNAEVR.3	0.374137806	#N/A
QEMQEVQSSR.2	0.320684619	#N/A
LTDC[Carbamidomethyl (C)]VVMR.2	0.094816224	#N/A
DYFEEYGKIDTIEIITDR.3	-0.34085992	#N/A
DYFEEYGK.2	-0.029744814	#N/A
EES[Phospho (STY)]GKPGAHVTVK.2	-0.059099285	O88569_S102_M1_Hnrnpa2b1
LFIGGLSFETTEESLR.2	-0.32912841	#N/A
EESGKPGAHVTVKK.3	-0.025254227	#N/A
GFGDGYNGYGGGPGGGNFGGS[Phospho (STY)]PGYGGGR.3	0.136458398	O88569_S259_M1_Hnrnpa2b1
NM[Oxidation (M)]GGPYGGGNYGPGGSGGS[Phospho (STY)]GGYGGR.3	-0.535582235	O88569_S344_M1_Hnrnpa2b1

GGSDGYGSGR.2	-0.345107658	#N/A
AVYQGPGSS[Phospho (STY)]PVKS.2	-0.328154952	O88587_S261_M1_Comt
DAS[Phospho (STY)]PVGVK.2	-0.224331025	O88602_S253_M1_Cacng2
STEPSHS[Phospho (STY)]RDAS[Phospho (STY)]PVGVK.3	-0.37050418	O88602_S249_M2_Cacng2
LLQTSNITK.2	0.068584758	#N/A
STM[Oxidation (M)]VGTPYWM[Oxidation (M)]APEVVTR.2	-0.536962037	#N/A
ALYLIATNGTPELQNPEK.2	-0.018911218	#N/A
EC[Carbamidomethyl (C)]LQALEFLHSNQVIHR.3	-0.475911169	#N/A
SAEDYNS[Phospho (STY)]SNTLNVK.2	-0.335358841	O88643_S155_M1_Pak1
DVATSPIS[Phospho (STY)]PTENNTTPPDALTR.2	-0.002890957	O88643_S223_M1_Pak1
MSDEEILEK.2	0.510595087	#N/A
ELIINEILVM[Oxidation (M)]R.2	0.400854412	#N/A
SDNILLGM[Oxidation (M)]DGSVK.2	0.009333429	#N/A
LTDFGFC[Carbamidomethyl (C)]AQITPEQSK.2	0.540009514	#N/A
SVIEPLPVTPTR.2	0.005600207	#N/A
LLQTSNITKS[Phospho (STY)]EQK.2	-0.254639918	O88643_S115_M1_Pak1
[Acetyl (Protein N-term)]S[Phospho (STY)]NNGVDIQDKPPAPPMR.2	-0.264884677	O88643_S2_M1_Pak1
IGQGASGTVYTAMDVATGQEVAIK.3	0.236037129	#N/A
VDILDPALLR.2	0.476135072	#N/A
DSYLILETLPTEYDSR.2	-0.055183109	#N/A
QTYFLPVIGLVDAEK.2	0.26793351	#N/A
M[Oxidation (M)]ATVWDEAEQDGIGEEVLK.2	-0.16933452	#N/A
[Acetyl (Protein N-term)]M[Oxidation (M)]QEMNLLPTPES[Phospho (STY)]PVTR.2	0.218195365	O88685_S12_M1_Psmc3
TSPYGVPGS[Phospho (STY)]PATR.2	-0.395997839	O88703_S771_M1_Hcn2
GEPQC[Carbamidomethyl (C)]S[Phospho (STY)]PEGPAR.2	-0.112666706	O88703_S90_M1_Hcn2
PLVGPLALGS[Phospho (STY)]PR.2	-0.237222935	O88703_S726_M1_Hcn2
GPKVS[Phospho (STY)]FSC[Carbamidomethyl (C)]R.2	0.22455848	O88703_S101_M1_Hcn2
GSQAS[Phospho (STY)]FLQR.2	-0.282571394	O88703_S134_M1_Hcn2
TAAPS[Phospho (STY)]PDRR.2	-0.13450865	O88703_S834_M1_Hcn2
DSAS[Phospho (STY)]PGAASGLDPLDSAR.2	-0.447770371	O88703_S842_M1_Hcn2
LC[Carbamidomethyl (C)]S[Phospho (STY)]RDS[Phospho (STY)]AC[Carbamidomethyl (C)]TPGAAK.2	-0.190741899	O88703_S64_M2_Hcn2
GAASGPSAAEEAGS[Phospho (STY)]EEAGPAGEPR.2	0.393329036	O88703_S119_M1_Hcn2
RDS[Phospho (STY)]ASPGAASGLDPLDSAR.3	0.256171483	O88703_S840_M1_Hcn2
TAAPS[Phospho (STY)]PDRRDS[Phospho (STY)]AS[Phospho (STY)]PGAASGLDPLDSAR.3	0.027181011	O88703_S834_M3_Hcn2
RAPPGPLPPAAS[Phospho (STY)]PGPPAAS[Phospho (STY)]PPAAPS[Phospho (STY)]SPR.3	0.273758508	O88703_S743_M3_Hcn2
LGT[Phospho (STY)]PPGGGAAGK.2	-0.230062216	O88704_T39_M1_Hcn1
RLGT[Phospho (STY)]PPGGGAAGK.2	-0.189245558	O88704_T39_M1_Hcn1
VDGGGGEEPAGS[Phospho (STY)]FEDAEGPR.2	-0.340807127	O88704_S69_M1_Hcn1
KNS[Phospho (STY)]ILLQK.2	-0.136774746	O88704_S588_M1_Hcn1
VDGGGGEEPAGS[Phospho (STY)]FEDAEGPRR.3	-0.279227467	O88704_S69_M1_Hcn1
QMSS[Phospho (STY)]GAIPPNR.2	-0.345592836	O88704_S868_M1_Hcn1
KRS[Phospho (STY)]EPS[Phospho (STY)]PGSSGGVM[Oxidation (M)]EQHLVQHDR.4	-0.257592561	O88705_S558_M2_Hcn3
PLVALLDGR.2	0.245577704	#N/A
IGSGFDNIDIK.2	-0.178591595	#N/A
GAALDVHESEPFSFSQGPLK.3	0.231882213	#N/A
VGQAVALR.2	-0.311145081	#N/A
TTWLHQALR.2	-0.222119539	#N/A

VQSVEQIR.2	-0.083075335	#N/A
DVATVAFC[Carbamidomethyl (C)]DAQSTQEIHEK.3	-0.435284994	#N/A
VLNEAVGALM[Oxidation (M)]YHTITLTR.2	0.176008455	#N/A
LSSSSATLLNS[Phospho (STY)]PDR.2	-0.24598984	O88735_S209_M1_Map7
SLS[Phospho (STY)]DPKPLS[Phospho (STY)]PTAEESAKER.3	-0.252813051	O88737_S2860_M2_Bsn
GEEES[Phospho (STY)]EEDSYDPR.2	-0.463877269	O88737_S3301_M1_Bsn
DYLS[Phospho (STY)]DSELNQLR.2	0.022076395	O88737_S3022_M1_Bsn
DAC[Carbamidomethyl (C)]EPES[Phospho (STY)]GPDPSTVR.2	-0.125545436	O88737_S2578_M1_Bsn
DST[Phospho (STY)]PTASESQR.2	0.240275988	O88737_T3254_M1_Bsn
ATSVPGPTQATAPPEVGR.2	-0.027461996	#N/A
DPEPPEPLTFR.2	0.107011152	#N/A
AS[Phospho (STY)]PQAKPLR.2	-0.521474749	O88737_S600_M1_Bsn
ELQELQTIK.2	-0.181575042	#N/A
AQGPSGQEAES[Phospho (STY)]PR.2	-0.188602383	O88737_S121_M1_Bsn
YLELGITQR.2	0.142160048	#N/A
SQASEEES[Phospho (STY)]PVS[Phospho (STY)]PLGRPR.2	-0.235289122	#N/A
QTS[Phospho (STY)]LADLEQK.2	-0.077625176	O88737_S3158_M1_Bsn
SHGPLLPTIEDSS[Phospho (STY)]EEEELREEEELLREQEK.4	-0.247286614	O88737_S1051_M1_Bsn
LRDAC[Carbamidomethyl (C)]EPES[Phospho (STY)]GPDPSTVR.3	0.003997447	O88737_S2578_M1_Bsn
SQAS[Phospho (STY)]EEESPVSPLGR.2	-0.347949567	O88737_S3512_M1_Bsn
IADSSVQT[Phospho (STY)]DDEEGEGR.2	-0.417336897	O88737_T2595_M1_Bsn
EKPLSGGDSEVGAPQPSR.3	-0.103190939	#N/A
ASPQTTKAS[Phospho (STY)]PQAK.2	-0.093286968	O88737_S600_M1_Bsn
SLS[Phospho (STY)]DPKPLSPTAEESAK.2	-0.308863637	O88737_S2860_M1_Bsn
SADC[Carbamidomethyl (C)]SVQT[Phospho (STY)]DDEDNADWEQPVRR.3	0.096552646	O88737_T2622_M1_Bsn
AQGPSGQEAES[Phospho (STY)]PRR.2	-0.277603219	O88737_S121_M1_Bsn
YLGQGLQYGS[Phospho (STY)]FTDLR.2	0.376558715	O88737_S2046_M1_Bsn
QVEQAVQTAPYR.2	0.265656226	#N/A
GLGEHRDYLS[Phospho (STY)]DSELNQLR.3	0.044014368	O88737_S3022_M1_Bsn
RIADSS[Phospho (STY)]VQTDDEEGEGR.2	-0.253308339	O88737_S2592_M1_Bsn
EQQDTAESS[Phospho (STY)]DDFGSQLR.2	-0.059964228	O88737_S818_M1_Bsn
LLEGSLGEPAPLPLPTPQQPPAGVPHR.3	0.170525612	#N/A
SQQQLHS[Phospho (STY)]PALS[Phospho (STY)]PAHSPAK.3	-0.319136899	O88737_S241_M2_Bsn
SSC[Carbamidomethyl (C)]SEYS[Phospho (STY)]PSPSLDSEAETLDGGPTR.3	-0.308423973	O88737_S1137_M1_Bsn
EPELEM[Oxidation (M)]ESLTGS[Phospho (STY)]PEDR.2	0.044345794	O88737_S980_M1_Bsn
SQGS[Phospho (STY)]FEYQDTQDHDYGGR.3	-0.287375067	O88737_S1236_M1_Bsn
SHGPLLPTIEDSS[Phospho (STY)]EEEELREEEELLR.4	-0.258684104	O88737_S1051_M1_Bsn
SSS[Phospho (STY)]SAVEDQKR.2	-0.145906347	O88737_S767_M1_Bsn
MASAYS[Phospho (STY)]GEK.2	0.04128775	O88737_S3460_M1_Bsn
LQPQSQPTTR.2	0.158008138	#N/A
QASATAPGRES[Phospho (STY)]PR.2	-0.238649656	O88737_S105_M1_Bsn
ASPQATKAS[Phospho (STY)]PQTTK.2	0.092441084	O88737_S593_M1_Bsn
GYM[Oxidation (M)]TPT[Phospho (STY)]SPAGSER.2	0.31885077	O88737_T1406_M1_Bsn
LLDTSFAS[Phospho (STY)]SER.2	-0.216707818	O88737_S2822_M1_Bsn
GPGATQSGPR.2	0.151398913	#N/A
DSAVDLSSLK.2	-0.036443525	#N/A
SKTPPSNLSPIEDAS[Phospho (STY)]PTEELR.3	-0.113241648	O88737_S1114_M1_Bsn

DAC[Carbamidomethyl (C)]EPES[Phospho (STY)]GPDPSTVRR.3	0.084652591	O88737_S2578_M1_Bsn
LHSS[Phospho (STY)]PVSSTLTSK.2	-0.047958474	O88737_S1370_M1_Bsn
HSYS[Phospho (STY)]LGFADGR.2	0.316750096	O88737_S2029_M1_Bsn
QPQTQQQQQQQQQQGLGQQAPQQAPSQAR.3	-0.423811625	#N/A
SPQVLYS[Phospho (STY)]PVS[Phospho (STY)]PLSPHR.2	-0.074965619	O88737_S2805_M2_Bsn
QAAPGPQQSQPPSSR.2	0.052470879	#N/A
YSS[Phospho (STY)]VSNIYSDHR.2	0.221284515	O88737_S2068_M1_Bsn
AS[Phospho (STY)]GAGGPPR.2	0.019385101	O88737_S2295_M1_Bsn
LQLEQIQQLQQQLQLQLEEQK.3	-0.196279039	#N/A
ISKFS[Phospho (STY)]PIEEAK.2	-0.067504742	O88737_S3382_M1_Bsn
ASPQATKAS[Phospho (STY)]PQATK.2	-0.2743793	O88737_S579_M1_Bsn
QREQQDTAESS[Phospho (STY)]DDFGSQLR.3	-0.11105198	O88737_S818_M1_Bsn
LYSSM[Oxidation (M)]SDT[Phospho (STY)]NLAEAGLNYHAQR.3	-0.409010239	O88737_T1992_M1_Bsn
HREEEQLLVQR.3	0.435935762	#N/A
GYSYFTGSS[Phospho (STY)]PPLSPSTPS[Phospho (STY)]ESPTFSPGK.3	-0.223909683	O88737_S1482_M2_Bsn
VTQHFAK.2	0.441455371	#N/A
SQQQLHS[Phospho (STY)]PALS[Phospho (STY)]PAHS[Phospho (STY)]PAKQPLGKPEQER.4	-0.069582554	O88737_S241_M3_Bsn
SPSVS[Phospho (STY)]PDR.2	-0.177462456	O88737_S142_M1_Bsn
SSVSQSPAPTYPSDSHYTSLEQNVPR.3	-0.020840999	#N/A
HDY[Phospho (STY)]VEDSS[Phospho (STY)]EGGLSPLPPQPPAR.3	-0.138638683	O88737_S834_M2_Bsn
GEHSSTLPASTPSYTSGTSPTSLSSLEEDSDSSPS[Phospho (STY)]RR.4	0.128091342	O88737_S1021_M1_Bsn
FS[Phospho (STY)]PIEEAK.2	-0.021874515	O88737_S3382_M1_Bsn
ASQPVAESTPAGLGAAVYEEILQTSQSIAR.3	0.334151578	#N/A
GPANYNTC[Carbamidomethyl (C)]TAC[Carbamidomethyl (C)]K.2	0.152624996	#N/A
S[Phospho (STY)]KSQQQLHSPALSPAHSPAK.4	0.003099226	O88737_S233_M1_Bsn
ADTQAQPQMQGR.2	-0.199255372	#N/A
SDS[Phospho (STY)]VTGHTSQK.2	-0.135981497	O88738_S493_M1_Birc6
ISFVIPC[Carbamidomethyl (C)]NNH.2	-0.342659148	#N/A
QQS[Phospho (STY)]TGAIPATQAR.2	-0.104351875	O88746_S405_M1_Tom1
QNS[Phospho (STY)]VSSNTSQR.2	-0.254386348	O88746_S180_M1_Tom1
GSS[Phospho (STY)]LADQRK.2	-0.008225756	O88746_S376_M1_Tom1
AADRLPNLAS[Phospho (STY)]PSAEGPPRPSPGTAPR.4	0.126444126	O88746_S462_M1_Tom1
SKQS[Phospho (STY)]PISTPTS[Phospho (STY)]PGSLRK.3	-0.23289885	O88809_S332_M2_Dcx
GNPSAAAGPKAS[Phospho (STY)]PTPQK.2	-0.309550502	O88809_S287_M1_Dcx
ATDFVVPGPGK.2	0.188104431	#N/A
ALEDVC[Carbamidomethyl (C)]IETIEAGFM[Oxidation (M)]TK.2	-0.434767964	#N/A
TVEAEAAHGTVTR.2	0.166802329	#N/A
HAYGDQYR.2	0.221814379	#N/A
QSSLAEPVS[Phospho (STY)]PSKR.2	-0.108388819	O88845_S187_M1_Akap10
ASVAVHS[Phospho (STY)]PQK.2	-0.278274646	O88845_S52_M1_Akap10
NSC[Carbamidomethyl (C)]SS[Phospho (STY)]PLRELSEK.2	0.184286727	O88845_S281_M1_Akap10
[Acetyl (Protein N-term)]SS[Phospho (STY)]PERDEGTPVPDSR.2	0.18089183	O88852_S3_M1_Tspyl1
KPS[Phospho (STY)]PQPS[Phospho (STY)]PPR.2	-0.307917076	O88874_S325_M2_Ccnk
AVVVS[Phospho (STY)]PKEENK.2	-0.180211154	O88874_S341_M1_Ccnk
M[Oxidation (M)]S[Phospho (STY)]PMGTASGSNSPTSDSASVQR.2	0.031368959	O88878_S48_M1_Zfand5
TTGTLPS[Phospho (STY)]PQNFK.2	-0.365327937	O88910_S307_M1_Mpp3
QAS[Phospho (STY)]ISGPAPTK.2	-0.258091772	O88935_S568_M1_Syn1

SLKPDFVLIR.2	-0.25908183	#N/A
TYATAEPFIDAK.2	-0.090668032	#N/A
ASGAPPGGQQR.2	0.229230823	#N/A
VLLVIDEPHTDWAK.2	-0.199737712	#N/A
TGPPTTQQPR.2	0.229043027	#N/A
VDNQHDFQDIASVVALTK.3	-0.001977471	#N/A
KLGTEEFPLIDQTFYPNHK.3	-0.203417705	#N/A
IHGEIDIK.2	-0.031053721	#N/A
QGPPQKPPGPAGPTR.3	0.168040054	#N/A
EM[Oxidation (M)]LSSTTYPVVVK.2	-0.232214782	#N/A
QAGAPQATR.2	0.049836667	#N/A
M[Oxidation (M)]TQALPR.2	0.184933485	#N/A
QPQRDAS[Phospho (STY)]PGR.2	-0.219251301	O88935_S427_M1_Syn1
LGTEEFPLIDQTFYPNHK.3	-0.309902374	#N/A
ASTAAPVASPAAPSPGSSGGGGFFSSLSNAVK.3	-0.136453169	#N/A
DAS[Phospho (STY)]PGRGSHSQSSSPGALTLGR.3	-0.411467871	O88935_S427_M1_Syn1
VKVDNQHDFQDIASVVALTK.3	-0.596097418	#N/A
QLIVELVVNK.2	-0.028849518	#N/A
TSVSGNWK.2	0.008654992	#N/A
GSHSQSSS[Phospho (STY)]PGALTLGR.2	-0.262103481	O88935_S438_M1_Syn1
KIHGEIDIK.2	-0.082820546	#N/A
SQSLTNAFNLPEPAPPRPSLSQDEVKAETIR.4	-0.31051157	#N/A
QTTAAAAATFSEQVGGGSGGAGR.2	-0.198609692	#N/A
PQPPPPPPSAAS[Phospho (STY)]PGATPGSATASAER.3	-0.362172037	O88935_S39_M1_Syn1
QAS[Phospho (STY)]QAGPGPR.2	-0.078591189	O88935_S605_M1_Syn1
LPSPT[Phospho (STY)]AAPQQSASQATPVTQGQGR.3	-0.298201425	O88935_T512_M1_Syn1
QHAFSMAR.2	0.479928904	#N/A
SQSLTNAFNLPEPAPPRPSLSQDEVK.3	0.180817996	#N/A
RLS[Phospho (STY)]DSNFM[Oxidation (M)]ANLPNGYM[Oxidation (M)]TDLQR.3	0.10208438	O88935_S9_M1_Syn1
PVAGGPGAPPAARPPASPS[Phospho (STY)]PQR.3	-0.196354541	O88935_S553_M1_Syn1
QSRPVAGGPGAPPAARPPASPS[Phospho (STY)]PQR.4	-0.262566362	O88935_S553_M1_Syn1
DHIIEVVGSSM[Oxidation (M)]PLIGDHQDEDK.3	-0.344445872	#N/A
VEQAEFSDLNLVAHANGGFSVDM[Oxidation (M)]EVLR.3	-0.007081549	#N/A
LWVDTC[Carbamidomethyl (C)]SEIFGGLDIC[Carbamidomethyl (C)]AVEALHGK.3	0.058204265	#N/A
HFKDEEEDEEEAS[Phospho (STY)]PDGSGR.3	-0.016356732	O88939_S537_M1_Zbtb7a
[Acetyl (Protein N-term)]AALVEPLGLER.2	0.289169735	#N/A
QQHHSYTS[Phospho (STY)]LESRG.3	-0.400882535	O88951_S202_M1_Lin7b
QQHHSY[Phospho (STY)]TSLESR.3	-0.427660737	O88951_Y200_M1_Lin7b
VLQSEFC[Carbamidomethyl (C)]NAVR.2	0.195489258	#N/A
KSLLSHEFQDET[Phospho (STY)]DT[Phospho (STY)]EEETLYSSKH.4	-0.184288587	O89001_T1365_M2_Cpd
SLLSHEFQDET[Phospho (STY)]DT[Phospho (STY)]EEETLYSSKH.3	0.057825195	O89001_T1365_M2_Cpd
SLLSHEFQDET[Phospho (STY)]DT[Phospho (STY)]EEETLYSSK.3	-0.179903004	O89001_T1365_M2_Cpd
DYTGEDVTPENFLAVLR.2	0.283136979	#N/A
AAS[Phospho (STY)]QGSESPLLPTQR.2	-0.123811527	O89032_S1029_M1_Sh3pxd2a
EAEENPVGAC[Carbamidomethyl (C)]ESQGS[Phospho (STY)]PLK.2	0.234186694	O89032_S546_M1_Sh3pxd2a
GSSGDSDS[Phospho (STY)]PGSSSLSLAVK.2	0.096327691	O89032_S643_M1_Sh3pxd2a
SAS[Phospho (STY)]DAGIRDTPK.2	0.099646104	O89032_S721_M1_Sh3pxd2a

RIS[Phospho (STY)]PASSLQR.2	0.039354211	O89032_S592_M1_Sh3pxd2a
NSS[Phospho (STY)]FSTAR.2	-0.155454991	O89032_S1008_M1_Sh3pxd2a
PEDVNPPK.2	-0.493785016	#N/A
ATPEPSGTPSSDTVSR.2	0.271681122	#N/A
DGALIC[Carbamidomethyl (C)]TSC[Carbamidomethyl (C)]R.2	-0.238250952	#N/A
ILTTGFSR.2	0.166476911	#N/A
QVALWDTK.2	0.153915058	#N/A
DAGPLLISLK.2	0.424033419	#N/A
HVFGQPAK.2	0.381873166	#N/A
VSQTTWDSGFC[Carbamidomethyl (C)]AVNPK.2	0.307714945	#N/A
LDRLEETVQAK.2	0.325892108	#N/A
NLNAIVQK.2	-0.156353641	#N/A
KSDLFQEDLYPPTAGPDPALTAEEWLGGR.3	0.137503571	#N/A
APPVPGAVSGGS[Phospho (STY)]GEVDELFDVK.2	0.032035602	O89079_S13_M1_Cope
S[Phospho (STY)]PLGGPPSVC[Carbamidomethyl (C)]K.2	-0.308240453	O89084_S204_M1_Pde4a
GYS[Phospho (STY)]DSAER.2	0.002107826	O89084_S49_M1_Pde4a
SLS[Phospho (STY)]LSLPGPR.2	0.252433756	O89084_S13_M1_Pde4a
QS[Phospho (STY)]PSPTLEEEPGVLSDPALPDK.2	0.288889182	O89084_S672_M1_Pde4a
S[Phospho (STY)]GNQVSEYISNTFLDK.2	0.316829907	O89084_S274_M1_Pde4a
TLSPS[Phospho (STY)]EEAQGLLGLPSM[Oxidation (M)]AAEVEAPR.3	-0.412365375	O89084_S788_M1_Pde4a
SDS[Phospho (STY)]DYDMSPK.2	-0.1559778	O89084_S147_M1_Pde4a
YDSRPGGYGYGYGR.3	0.0326126	#N/A
YSGGNYRDNYDN.2	-0.114573405	#N/A
SLAENYFDSTGR.2	-0.417214985	#N/A
IPQSHIQQIC[Carbamidomethyl (C)]ENILTSGENLSR.3	-0.138101697	#N/A
DVNYGWLIR.2	-0.088246038	#N/A
VVLPM[Oxidation (M)]ELPIR.2	0.069463305	#N/A
LLEVDNR.2	-0.318913408	#N/A
ILYM[Oxidation (M)]M[Oxidation (M)]DEINNPVLTVK.2	-0.410148485	#N/A
M[Oxidation (M)]LISSEDVLHSWAVPSLGLK.3	-0.327940317	#N/A
VIGGDDLSTLTGK.2	0.445240828	#N/A
NVLIVEDIIDTGK.2	0.120723176	#N/A
SIPM[Oxidation (M)]TVDFIR.2	0.04122304	#N/A
EM[Oxidation (M)]GGHHIVALC[Carbamidomethyl (C)]VLK.3	5.13E-05	#N/A
DLNHVC[Carbamidomethyl (C)]VISETGK.2	0.121483001	#N/A
SC[Carbamidomethyl (C)]S[Phospho (STY)]ASC[Carbamidomethyl (C)]M[Oxidation (M)]PHGAR.2	-0.092697275	P00520_S717_M1_Abl1
DFPIANGDR.2	0.162486657	#N/A
GGPLSDSYR.2	0.103280606	#N/A
VLEALHSIK.2	0.151863109	#N/A
[Acetyl (Protein N-term)]S[Phospho (STY)]HHWGYSK.2	-0.455097812	P00920_S2_M1_Ca2
AVQQPDGLAVLGIFLK.2	0.112605998	#N/A
IGPASQGLQK.2	0.159569355	#N/A
SIVNNGHSFNVEFDDSQDNAVLK.3	-0.334073347	#N/A
QSPVDIDTATAQHDPALQPLLISYDK.3	0.351270245	#N/A
YAAELHLVHWNTK.3	-0.053810084	#N/A
TLNFNEEGDAEEAM[Oxidation (M)]VDNWRPAQPLK.3	-0.32781695	#N/A
EPITVSSEQM[Oxidation (M)]SHFR.2	0.005225608	#N/A

HNGPENWHKDFPIANGDR.4	-0.062058535	#N/A
VTLSNQPYIK.2	0.155268203	#N/A
DNSIQHEFSLTR.2	0.183330449	#N/A
HVLSGTLGIPEHTYR.3	-0.168511522	#N/A
VTSLTAC[Carbamidomethyl (C)]LVNQNLR.2	0.229230193	#N/A
M[Oxidation (M)]FASFPTTK.2	0.526847684	#N/A
FLASVSTVLTSK.2	0.460006939	#N/A
VDPVNFK.2	0.566234938	#N/A
VLSGEDKSNIK.2	0.083333157	#N/A
IGGHGAEYGAEALER.3	0.447555175	#N/A
TYFPHFDVSHGSAQVK.3	0.430947069	#N/A
LLVVYPWTQR.2	0.442627087	#N/A
LHVDPENFR.2	0.488948416	#N/A
DFTPAAQAAFQK.2	0.462937812	#N/A
VHLTDAEK.2	0.427617224	#N/A
VNSDEVGGEALGR.2	0.478763373	#N/A
VITAFNDGLNHLDSLK.3	0.458684238	#N/A
GTFASLSELHC[Carbamidomethyl (C)]DK.2	0.411243258	#N/A
YFDSFGDLSSASAIM[Oxidation (M)]GNAK.2	0.143154006	#N/A
VNPDEVGGEALGR.2	0.443008277	#N/A
YRPGTVALR.2	0.049017248	#N/A
RVTIM[Oxidation (M)]PK.2	-0.020663517	#N/A
STELLIR.2	-0.039954718	#N/A
EIAQDFK.2	0.16577165	#N/A
EIAQDFKTDLR.2	0.343039053	#N/A
FQSAAIGALQEASEAYLVGLFEDTNLC[Carbamidomethyl (C)]AIHAK.3	-0.470407209	#N/A
SC[Carbamidomethyl (C)]C[Carbamidomethyl (C)]SC[Carbamidomethyl (C)]C[Carbamidomethyl (C)]PVGC[Carbamidomethyl (C)]SK.2	0.113272997	#N/A
ANPYEC[Carbamidomethyl (C)]GFDPTSSAR.2	-0.195313198	#N/A
DNNVLQK.2	0.054537252	#N/A
IYLPHSLPQQ.2	-0.020241062	#N/A
FADLTDAASR.2	0.230113069	#N/A
LADVYQAELR.2	0.144234575	#N/A
ITIPVQTFSNLQIR.2	0.139483841	#N/A
ESASYQEALAR.2	0.242072695	#N/A
FLEQQNK.2	0.198396484	#N/A
KLLEGEENR.2	0.25354526	#N/A
ALAAELNQLR.2	0.189856173	#N/A
QEADEATLAR.2	0.278289347	#N/A
HLQEYQDLLNVK.2	-0.025134064	#N/A
KVESLEEEIQFLR.3	-0.037198253	#N/A
LEAENNLAAYR.2	0.399728379	#N/A
S[Phospho (STY)]YASETVVR.2	0.024853412	P03995_S12_M1_Gfap
QLQALTC[Carbamidomethyl (C)]DLESLR.2	0.379797617	#N/A
DGEVIKDSK.2	0.257108593	#N/A
QKLQDETNLR.2	-0.020538833	#N/A
MTPPLPAR.2	0.375548646	#N/A
DNFAQDLGTLR.2	-0.016897455	#N/A

LRLDQLTANSAR.2	0.394225475	#N/A
LEVERDNFAQDLGTLR.3	-0.288671188	#N/A
FASYIEK.2	-0.1766868	#N/A
TQYEAVATSNM[Oxidation (M)]QETEEWYR.2	-0.192373593	#N/A
EQLAQQQVHVEM[Oxidation (M)]DVAKPDLTAALR.3	-0.158820856	#N/A
AEMMELNDR.2	0.489754135	#N/A
LEEEGQSLKEEMAR.2	0.221852006	#N/A
LALDIEIATYR.2	0.107279028	#N/A
GTNES[Phospho (STY)]LER.2	0.298547913	P03995_S302_M1_Gfap
FFS[Phospho (STY)]GDRGAPK.2	-0.431963001	P04370_S178_M1_Mbp
TQDENPVVHFFK.2	-0.253893162	#N/A
LFS[Phospho (STY)]RDAPGR.2	-0.438532579	P04370_S96_M1_Mbp
NIVTPRT[Phospho (STY)]PPPSQGK.2	-0.206528005	P04370_T229_M1_Mbp
DTGILDS[Phospho (STY)]IGR.2	0.259609058	P04370_S172_M1_Mbp
TTHYGS[Phospho (STY)]LPQK.2	0.090639219	P04370_S201_M1_Mbp
HRDTGILDSIGR.3	0.103330956	#N/A
TTHYGSLPQKS[Phospho (STY)]QHGR.3	0.029244875	P04370_S206_M1_Mbp
DSRSGS[Phospho (STY)]PMAR.2	-0.083601559	P04370_S245_M1_Mbp
S[Phospho (STY)]KYLATASTM[Oxidation (M)]DHAR.2	0.453482175	P04370_S144_M1_Mbp
YLATASTM[Oxidation (M)]DHAR.2	0.464944447	#N/A
T[Phospho (STY)]PPPSQGK.2	-0.418775547	P04370_T229_M1_Mbp
S[Phospho (STY)]QHGRTQDENPVVHFFK.4	0.152635994	P04370_S206_M1_Mbp
SGS[Phospho (STY)]PM[Oxidation (M)]AR.2	-0.446125219	P04370_S245_M1_Mbp
YPGQGSPGGNR.2	-0.043829042	#N/A
VVEQM[Oxidation (M)]C[Carbamidomethyl (C)]VTQYQK.2	0.033441474	#N/A
PM[Oxidation (M)]IHFGNDWEDR.3	0.097996463	#N/A
ESQAYYDGRR.2	-0.20592427	#N/A
HVAGAAAAGAVVGGLGGYM[Oxidation (M)]LGSAM[Oxidation (M)]SR.3	-0.427420347	#N/A
DDNGVPFVR.2	0.163122102	#N/A
QVLFSADDR.2	0.167190356	#N/A
ELSDIALR.2	0.004094279	#N/A
ALQASALNAWR.2	0.446095538	#N/A
DNAGAATEEFIK.2	0.21293587	#N/A
PHSYPALSAEQKK.3	-0.121634666	#N/A
LSQIGVENTEENR.2	0.185696216	#N/A
VDKGVVPLAGTDGETTTQGLDGLLER.3	-0.079321692	#N/A
ISDRTPSALAILENANVLAR.3	-0.309361716	#N/A
YEGSGDGGAAAQSLYIANHAY.2	0.031664148	#N/A
TPSALAILENANVLAR.2	0.080643558	#N/A
PHSYPALSAEQK.2	0.293727769	#N/A
TVPPAVPGVTFLSGGQSEEEASLNLNAINR.3	-0.502907214	#N/A
GILAADESVGS[Phospho (STY)]M[Oxidation (M)]AK.2	-0.076230058	P05063_S39_M1_Aldoc
GVVPLAGTDGETTTQGLDGLLER.2	0.190189046	#N/A
YSPEEIAM[Oxidation (M)]ATVTALR.2	0.143087229	#N/A
AEM[Oxidation (M)]NGLAAQGR.2	0.381479037	#N/A
LSQIGVENTEENRR.3	-0.006598898	#N/A
RLS[Phospho (STY)]QIGVENTEENRR.3	-0.293001999	P05063_S45_M1_Aldoc

DNAGAATEEFIKR.2	0.190544831	#N/A
GILAADESVGS[Phospho (STY)]M[Oxidation (M)]AKR.2	-0.104035321	P05063_S39_M1_Aldoc
GILAADESTGS[Phospho (STY)]IAK.2	0.243835652	P05064_S39_M1_Aldoa
ELSDIAHR.2	0.214287289	#N/A
ALANSLAC[Carbamidomethyl (C)]QGK.2	0.158147902	#N/A
PHPYPALTPEQK.2	0.065136902	#N/A
DGADFAKWR.2	-0.179764653	#N/A
AAQEEYIKR.2	0.003719231	#N/A
LQSIGTENTEENR.2	0.075021543	#N/A
GVVPLAGTNGETTTQGLDGLSER.2	0.162097436	#N/A
YASIC[Carbamidomethyl (C)]QQNGIVPIVEPEILPDGDHDLKR.4	0.185240002	#N/A
PHPYPALTPEQKK.3	-0.236773929	#N/A
IGEHTPSALAIM[Oxidation (M)]ENANVLAR.3	0.050703807	#N/A
KELSDIAHR.2	-0.031638629	#N/A
VDKGVVPLAGTNGETTTQGLDGLSER.3	-0.055863903	#N/A
KDGADFAK.2	-0.066177737	#N/A
AAQEEYIK.2	0.13795386	#N/A
GGVVGIKVDK.2	-0.551340404	#N/A
QLLLTADDR.2	0.203071481	#N/A
LQSIGTENTEENRR.3	-0.186360702	#N/A
SKGGVVGIK.2	0.06676437	#N/A
ALQASALK.2	-0.070267908	#N/A
ADDGRPFPQVIK.2	0.276197456	#N/A
GILAADESTGS[Phospho (STY)]IAKR.2	-0.140073345	P05064_S39_M1_Aldoa
ALSDHHVYLEGTLLKPNM[Oxidation (M)]VTPGHAC[Carbamidomethyl (C)]TQK.5	-0.058767753	#N/A
FSNEEIAM[Oxidation (M)]ATVTALR.2	-0.33105949	#N/A
C[Carbamidomethyl (C)]QYVTEK.2	-0.072477248	#N/A
RLQSIGTENTEENRR.3	-0.16526793	#N/A
YTPSGQSGAAASESLFISNHAY.2	-0.262308157	#N/A
VNPC[Carbamidomethyl (C)]IGGVILFHETLYQK.2	-0.108997159	#N/A
TVPPAVTGVTFLSGGQSEEEASINLNAINK.3	-0.455870359	#N/A
FSNEEIAMATVTALRR.3	0.340443621	#N/A
FKGPGDTSNFDDYEEEEIRVS[Phospho (STY)]INEK.4	0.117165639	P05132_S339_M1_Prkaca
WETPSQNTAQLDQFDR.2	0.14495341	#N/A
HIYLLPSGR.2	-0.308649773	#N/A
ITWSNPPAQGAR.2	-0.242594072	#N/A
NFGLYNER.2	-0.239727061	#N/A
VGGVQSLGGTGALR.2	0.246697947	#N/A
IVAATLSDPELFK.2	0.317393781	#N/A
LVLGDNSLAIR.2	0.13380145	#N/A
DIRPYC[Carbamidomethyl (C)]YWDAEKR.3	-0.048214793	#N/A
QVEYLVNEK.2	-0.154100437	#N/A
APPSVFAQVPQAPPVLVFK.2	0.024276706	#N/A
NTPIYVSSPTWENHNAVFSAAGFK.3	-0.291724147	#N/A
IGADFLGR.2	-0.15649059	#N/A
VNLGVGAYR.2	0.335155344	#N/A
VGNLTVVGK.2	-0.046440956	#N/A

IANDNSLNHEYLPILGLAEFR.3	0.21561306	#N/A
IVAATLSDPELFKEWK.3	0.165121735	#N/A
QIAAVM[Oxidation (M)]QR.2	0.519268205	#N/A
INM[Oxidation (M)]C[Carbamidomethyl (C)]GLTTK.2	0.217749332	#N/A
TPGTWSHITEQIGM[Oxidation (M)]FSFTGLNPK.3	-0.28619525	#N/A
TDESQPWVLPVVR.2	0.265558939	#N/A
FLFPFFDSAYQGFASGDLEK.2	-0.084943562	#N/A
LTADFRDDPDPR.2	-0.120338749	#N/A
DIRPYC[Carbamidomethyl (C)]YWDAEK.3	-0.211516944	#N/A
NLDYVATSIHEAVTK.2	-0.342803155	#N/A
WYNGTDNK.2	-0.005197815	#N/A
VLSQMEK.2	0.307206782	#N/A
YFVSEGFELFC[Carbamidomethyl (C)]AQSFSK.2	-0.080413197	#N/A
IAATILTSPDLR.2	0.150657986	#N/A
ASAELALGENNEVLK.2	-0.413822788	#N/A
TQLVSNLKK.2	0.206221932	#N/A
DDNGKPYVLPSVR.2	-0.139695779	#N/A
PSWGNHTPIFR.3	0.010466556	#N/A
FVTVQTISGTGALR.2	-0.291987525	#N/A
VGAFTVVC[Carbamidomethyl (C)]K.2	-0.042620701	#N/A
VGASFLQR.2	-0.063796337	#N/A
NLDKEYLPIGGLAEFC[Carbamidomethyl (C)]K.3	-0.078180743	#N/A
KQWLQEVK.2	-0.309200981	#N/A
KAEAQIAAK.2	0.276722289	#N/A
QWLQEVK.2	-0.070461208	#N/A
TQLVSNLK.2	0.271030406	#N/A
TC[Carbamidomethyl (C)]GFDFSGALEDISK.2	0.281739629	#N/A
M[Oxidation (M)]NLGVGAYR.2	-0.071289378	#N/A
DAGM[Oxidation (M)]QLQGYR.2	-0.066144373	#N/A
IAATILTSPDLRK.2	0.102619756	#N/A
NM[Oxidation (M)]GLYGER.2	0.296600853	#N/A
EYLPIGGLAEFC[Carbamidomethyl (C)]K.2	0.545577319	#N/A
EFSVYMTK.2	0.342490734	#N/A
IPEQSVLLLHAC[Carbamidomethyl (C)]AHNPTGVDPRPEQWK.5	0.170922757	#N/A
ISVAGVTSGNVGYLAHAIHQVTK.3	-0.198462296	#N/A
EIIDLVLDR.2	0.031252261	#N/A
VGINYQPPTVVPGGDLAK.2	-0.423701262	#N/A
IHFPLATYAPVISAEK.2	-0.148675815	#N/A
LSVDYGKK.2	-0.050317532	#N/A
DVNAAIATIK.2	-0.013278575	#N/A
QLFHPEQLITGK.2	0.02344821	#N/A
SIQFVDWC[Carbamidomethyl (C)]PTGFK.2	-0.010918204	#N/A
QLFHPEQLITGKEDAANNYAR.4	-0.285448999	#N/A
EDAANNYAR.2	0.078700082	#N/A
RNLDIERPTYTNLNR.3	-0.121320124	#N/A
DVNAAIATIKTK.2	-0.095630092	#N/A
YM[Oxidation (M)]AC[Carbamidomethyl (C)]C[Carbamidomethyl (C)]LLYR.2	-0.071912844	#N/A

AYHEQLSVAEITNAC[Carbamidomethyl (C)]FEPANQM[Oxidation (M)]VK.3	-0.360399543	#N/A
DYEEVGVDSVEGEGEEEGEEY.2	-0.047207694	#N/A
FDGALNVDLTEFQTNLVPYPR.3	-0.005418553	#N/A
LDHKFDLM[Oxidation (M)]YAK.2	0.022158587	#N/A
NLDIERPTYTNLNR.3	-0.221360492	#N/A
AVFVDLEPTVIDEVR.2	-0.029501492	#N/A
EDMAALEK.2	0.314081228	#N/A
TIGGGDDS[Phospho (STY)]FNTFFSETGAGK.2	0.0972225	P05213_S48_M1_Tuba1b
LISQIVSSITASLR.2	0.000868095	#N/A
FDLM[Oxidation (M)]YAK.2	-0.039629845	#N/A
AFVHWYVGEGM[Oxidation (M)]EEGEFSEAR.3	-0.287363295	#N/A
AVC[Carbamidomethyl (C)]M[Oxidation (M)]LSNTTAIAEAWAR.3	-0.046044842	#N/A
GDVVPKDVNAAIATIK.2	-0.024782073	#N/A
AFVHWYVGEGM[Oxidation (M)]EEGEFSEAREDM[Oxidation (M)]AALEK.4	-0.115375092	#N/A
LS[Phospho (STY)]VDYGK.2	-0.066909627	P05213_S158_M1_Tuba1b
LADQC[Carbamidomethyl (C)]TGLQGFLVFHSFGGGTGSGFTSLLM[Oxidation (M)]ER.3	0.258807026	#N/A
EDM[Oxidation (M)]AALEKDYEEVGVDSVEGEGEEEGEEY.3	0.078337127	#N/A
RAFVHWYVGEGMEEGEFSEAR.3	-0.159848956	#N/A
TIQFVDWC[Carbamidomethyl (C)]PTGFK.2	0.089621264	#N/A
LIGQIVSSITASLR.2	0.143817674	#N/A
S[Phospho (STY)]LEPSENVHGAGGAFPASQTPSKPASADGHR.4	0.004736833	P05480_S17_M1_Src
LFGGFNSSDTVTS[Phospho (STY)]PQR.2	-0.382571059	P05480_S74_M1_Src
VNS[Phospho (STY)]VGSSASSTQPLLVHEDA.2	-0.199845504	P05532_S962_M1_Kit
LAS[Phospho (STY)]PELER.2	-0.366099385	P05627_S73_M1_Jun;Jund
VTLTPEEEAR.2	-0.000527569	#N/A
LVIITAGAR.2	-0.057956521	#N/A
SADTLWGIQK.2	-0.20918079	#N/A
VIGSGC[Carbamidomethyl (C)]NLDSAR.2	-0.142347116	#N/A
QVVDSAYEVIK.2	-0.291138472	#N/A
DQLIVNLLK.2	0.329791948	#N/A
DYC[Carbamidomethyl (C)]VTANSK.2	-0.003217167	#N/A
DQLIVNLLKEEQAPQNK.3	-0.275507817	#N/A
DLADELALVDVM[Oxidation (M)]EDK.2	0.139078714	#N/A
GEM[Oxidation (M)]M[Oxidation (M)]DLQHGSLFLK.3	0.355377656	#N/A
SLNPELGTDADKEQWK.2	-0.047946289	#N/A
DLADELALVDVM[Oxidation (M)]EDKLK.2	-0.247468965	#N/A
LLIVSNPVDILTYVAWK.2	-0.293811081	#N/A
SNT[Phospho (STY)]PIKVDGK.2	-0.340735082	P06745_T109_M1_Gpi
ELFEADPER.2	-0.208929786	#N/A
INYTENR.2	-0.042638643	#N/A
AVLHVALR.2	0.147645545	#N/A
VFEGNRPTNSIVFTK.3	-0.170508829	#N/A
GKLPEEARK.2	-0.347496051	#N/A
HFVALSTNTAK.2	0.326112868	#N/A
TLASLSPETSLFIIASK.2	-0.197290831	#N/A
EWFLEAAKDPSAVAK.2	0.090125143	#N/A
NRS[Phospho (STY)]NTPIK.2	-0.044765852	P06745_S107_M1_Gpi

LRELFEADPER.2	-0.340385102	#N/A
NRS[Phospho (STY)]NTPIKVDGK.3	-0.329348644	P06745_S107_M1_Gpi
EWFLEAAK.2	0.073522733	#N/A
DVM[Oxidation (M)]PEVNR.2	0.412145471	#N/A
TFTTQETITNAETAK.2	0.31133645	#N/A
TFTTQETITNAETAKEWFLEAAK.3	-0.714040487	#N/A
VWFVSNIDGTHIAK.2	-0.272607712	#N/A
M[Oxidation (M)]KS[Phospho (STY)]FC[Carbamidomethyl (C)]QR.2	0.278235246	P06745_S131_M1_Gpi
KIEPELEGSSAVTSHDSSTNGLISFIK.4	-0.141653276	#N/A
EVM[Oxidation (M)]QM[Oxidation (M)]LVELAK.2	-0.389599145	#N/A
ILLANFLAQTEALM[Oxidation (M)]K.2	0.330509802	#N/A
FAAYFQQGDMESNGK.2	0.306796302	#N/A
SPEDLEK.2	-0.475749622	#N/A
DNM[Oxidation (M)]FSGSK.2	-0.192798014	#N/A
ELQAAGKS[Phospho (STY)]PEDLEK.2	0.198366361	P06745_S455_M1_Gpi
NRNS[Phospho (STY)]NVVPYDFNR.2	-0.369285192	P06800_S964_M1_Ptprc
AIFASGSPFDPVTLPDGR.2	-0.073270624	#N/A
EGDGSATTDAAPATS[Phospho (STY)]PK.2	-0.182910433	#N/A
EKDDAPVADGVEKK.3	-0.231445297	#N/A
AEDGPAKEEPK.2	0.196680798	#N/A
KGDAPAAEAEAK.2	0.474408239	#N/A
KGEGDAAPSEEK.2	0.148414364	#N/A
EGDGSATTDAAPATS[Phospho (STY)]PKAEEPSK.3	-0.24697033	#N/A
KGEGDAAPSEEKAGS[Phospho (STY)]AETESAAK.2	-0.297072909	P06837_S128_M1_Gap43
QADVPAAVTDAAATT[Phospho (STY)]PAAEDAATK.2	-0.372304679	P06837_T172_M1_Gap43
NDEDQKIEQDGVKPEDK.3	-0.002214897	#N/A
KEGDGSATTDAAPATS[Phospho (STY)]PK.2	-0.039888336	#N/A
ATTDNS[Phospho (STY)]PSSKAEDGPAKEEPK.3	0.017605825	P06837_S142_M1_Gap43
QDEGKEDPEADQEHA.2	-0.052912686	#N/A
AGS[Phospho (STY)]AETESAAK.2	0.245827751	P06837_S128_M1_Gap43
AEDGPAKEEPKQADVPAAVTDAAATT[Phospho (STY)]PAAEDAATK.4	-0.302207863	P06837_T172_M1_Gap43
AAQPPTETAESSQAEEEKDAVDEAKPK.4	0.056672975	#N/A
EGDGSATTDAAPATS[Phospho (STY)]PKAEEPSKAGDAPSEEK.4	-0.526514065	#N/A
AGDAPS[Phospho (STY)]EEKKGEGDAAPSEEK.3	-0.24058365	P06837_S110_M1_Gap43
KEGDGSATTDAAPATS[Phospho (STY)]PKAEEPSK.3	-0.111688004	#N/A
ATTDNS[Phospho (STY)]PSSKAEDGPAK.2	0.054308389	P06837_S142_M1_Gap43
AGSAETESAAKATTDNS[Phospho (STY)]PSSK.2	-0.101165499	P06837_S142_M1_Gap43
GEGDAAPS[Phospho (STY)]EEKAGSAETESAAK.2	0.304510619	P06837_S122_M1_Gap43
AGDAPSEEK.2	0.459114735	#N/A
AEEPSKAGDAPSEEK.3	-0.042285974	#N/A
ESARQDEGKEDPEADQEHA.3	-0.182509086	#N/A
LVQEVTDFAK.2	0.347676047	#N/A
APQVSTPTLVEAAR.2	0.200368483	#N/A
QTALAELVK.2	-0.135194404	#N/A
TC[Carbamidomethyl (C)]VADESAANC[Carbamidomethyl (C)]DK.2	0.487617617	#N/A
DQVANSAFVER.2	0.210145378	#N/A
NPDDITNEEYGEFYK.2	-0.16063092	#N/A

LGIHEDSQNR.2	0.040981887	#N/A
ELISNSSDALDK.2	0.299437254	#N/A
DKEVS[Phospho (STY)]DDEAEEK.2	-0.303921673	P07901_S231_M1_Hsp90aa1
HIYFITGETK.2	-0.036615225	#N/A
ELHINLIPSK.2	0.374389494	#N/A
ELHINLIPSKQDR.3	0.054973877	#N/A
APFDLFENR.2	-0.257011559	#N/A
RAPFDLFENR.2	0.008843104	#N/A
ESDDKPEIEDVGS[Phospho (STY)]DEEEEEK.2	0.275348478	P07901_S263_M1_Hsp90aa1
ESDDKPEIEDVGS[Phospho (STY)]DEEEEEKK.3	-0.35021839	P07901_S263_M1_Hsp90aa1
ELISNSSDALDKIR.2	-0.473303439	#N/A
HGLEVIYM[Oxidation (M)]IEPIDEYC[Carbamidomethyl (C)]VQQLK.3	-0.075489672	#N/A
DKEVS[Phospho (STY)]DDEAEEKEEK.2	-0.094091774	P07901_S231_M1_Hsp90aa1
FYEQFSK.2	-0.447723121	#N/A
SLTNDWEEHLAVK.2	0.230266811	#N/A
HLEINPDHSIIETLR.3	-0.188731972	#N/A
DNSTM[Oxidation (M)]GYMAAK.2	0.549951318	#N/A
LVTSPC[Carbamidomethyl (C)]C[Carbamidomethyl (C)]IVTSTYGWTANM[Oxidation (M)]ER.3	-0.499785613	#N/A
KHLEINPDHSIIETLR.4	-0.044096221	#N/A
ERDKEVS[Phospho (STY)]DDEAEEK.2	-0.158411184	P07901_S231_M1_Hsp90aa1
LGLGIDEDDPTVDDTSAAVTEEMPPLEGDDDTSR.3	0.176054127	#N/A
LGIHEDSQNRK.3	-0.006574103	#N/A
SILFVPTSAPR.2	-0.146286079	#N/A
TFEINPR.2	0.210668237	#N/A
FAFQAEVNR.2	0.123095897	#N/A
GLFDEYGSK.2	0.323523543	#N/A
TDDEVVQREEEAIQLDGLNASQIR.3	-0.315862572	#N/A
GVVDSDDLPLNVSR.2	0.218589223	#N/A
SGTSEFLNK.2	0.107278897	#N/A
DISTNYYASQK.2	-0.144549807	#N/A
SGYLLPDTK.2	0.001861616	#N/A
NLLHVTDTGVGM[Oxidation (M)]TR.2	-0.328110252	#N/A
FQSSHHSTDITSLDQYVER.3	-0.410882996	#N/A
LISLTDENALAGNEELTVK.2	-0.005646025	#N/A
ELEEQLGPVAEETR.2	-0.231822071	#N/A
LQAEIFQAR.2	0.417069056	#N/A
LGPLVEQGR.2	0.021934544	#N/A
DRLEEVR.2	-0.119760658	#N/A
IQASVATNPIITPVAQENQ.2	-0.432183801	#N/A
NEVHTM[Oxidation (M)]LGQS[Phospho (STY)]TEEIR.2	-0.368246339	P08226_S139_M1_Apoe
LGADMEDLR.2	0.3806172	#N/A
DAEDLQKR.2	0.173159174	#N/A
SKMEEQTQQIR.2	-0.240234503	#N/A
DGVANVSIEDR.2	-0.126625346	#N/A
HVGDLGNVTAGK.2	0.28488722	#N/A
VISLSGEHSIIGR.2	0.269280764	#N/A
TM[Oxidation (M)]VVHEK.2	0.178094376	#N/A

LAC[Carbamidomethyl (C)]GVIGIAQ.2	0.249739497	#N/A
GDGPVQGTIHFEQK.2	0.310986781	#N/A
KHGGPADEER.2	0.241184082	#N/A
QDDLGKGGNEESTK.2	0.002725463	#N/A
GC[Carbamidomethyl (C)]DVVVIPAGVPR.2	-0.009052892	#N/A
VDFPQDQLATLTGR.2	0.149894155	#N/A
GYLGPEQLPDC[Carbamidomethyl (C)]LK.2	0.439212608	#N/A
IFGVTTLDIVR.2	0.026127781	#N/A
M[Oxidation (M)]IAEAIPELK.2	0.137493282	#N/A
TIIPLISQC[Carbamidomethyl (C)]TPK.2	0.351035151	#N/A
IQEAGTEVVK.2	0.009630297	#N/A
ETEC[Carbamidomethyl (C)]TYFSTPLLLGK.2	0.079844284	#N/A
VNVPVIGGHAGK.2	-0.238131539	#N/A
EGVVEC[Carbamidomethyl (C)]SFVQSK.2	0.000197669	#N/A
VAVLGASGGIGQPLSLLLK.2	-0.135507418	#N/A
ANTFVAELK.2	0.088105187	#N/A
KGEDFVK.2	-0.314500129	#N/A
LTLYDIAHTPGVAADLSHIETR.3	-0.13760134	#N/A
HGVYNPNK.2	0.056918284	#N/A
NSPLVS[Phospho (STY)]R.2	0.342096932	P08249_S51_M1_Mdh2
FVFSLVDAM[Oxidation (M)]NGK.2	0.193089087	#N/A
AGAGSATLSM[Oxidation (M)]AYAGAR.2	-0.121814263	#N/A
ITPFEEK.2	-0.085484477	#N/A
ASS[Phospho (STY)]DPPSTQDAK.2	0.448679715	P08414_S353_M1_Camk4
DSTDLLGKK.2	0.328665374	#N/A
RDS[Phospho (STY)]EEKLK.2	-0.126644519	P08414_S433_M1_Camk4
TEIGVLLR.2	-0.038799759	#N/A
LGS[Phospho (STY)]ASSSHTSIQENHK.3	-0.199515879	P08414_S337_M1_Camk4
AQLQDLNDR.2	-0.056735044	#N/A
EYQDLLNVK.2	0.006801816	#N/A
KLLEGEETR.2	-0.352809242	#N/A
VHELEQQNK.2	0.032686237	#N/A
YEEEVLSR.2	0.25783625	#N/A
ALYEQEIR.2	-0.047081246	#N/A
FTVLTESAAK.2	0.124009806	#N/A
TLEIEAC[Carbamidomethyl (C)]R.2	-0.002982658	#N/A
VLEAELLVLR.2	0.314761277	#N/A
KGADEAALAR.2	0.017786547	#N/A
[Acetyl (Protein N-term)]SSFGYDPYFSTSYK.2	0.064259632	#N/A
FASFIER.2	0.139491075	#N/A
AAKDEVSESR.2	0.257124481	#N/A
EGLEETLR.2	0.321539471	#N/A
KEES[Phospho (STY)]AGEEQVAK.2	-0.033774927	P08551_S532_M1_Nefl
YLKEYQDLLNVK.2	-0.446247182	#N/A
AEEAKDEPPS[Phospho (STY)]EGEAEEEEK.2	-0.138043054	P08551_S473_M1_Nefl
LLEGEETR.2	0.187655659	#N/A
SFPAYYTSHVQEEQTEVEETIEATK.3	-0.262558878	#N/A

EES[Phospho (STY)]AGEEQVAK.2	0.226362257	P08551_S532_M1_Nefl
IDSLM[Oxidation (M)]DEIAFLKK.3	-0.205135923	#N/A
QALQGEREGLEETLR.3	-0.281673099	#N/A
RYVETPR.2	0.149532812	#N/A
NM[Oxidation (M)]QNAEEWFK.2	-0.126575787	#N/A
ESEEEKKEES[Phospho (STY)]AGEEQVAK.3	-0.190021529	P08551_S532_M1_Nefl
IDSLM[Oxidation (M)]DEIAFLK.2	-0.173441347	#N/A
SAYSGLQSSSYLM[Oxidation (M)]SAR.2	-0.082284016	#N/A
M[Oxidation (M)]ALDIEIAAYR.2	0.068228083	#N/A
AAKDEVSESRR.3	-0.082049249	#N/A
SAYSSYSAPVSSSLSVR.2	-0.099166528	#N/A
LSFTSVGSITSGYSQSSQVFGR.2	-0.35317423	#N/A
SYSSSSGSLM[Oxidation (M)]PSLENLDLSQVAAISNDLK.3	-0.420565433	#N/A
TQEKAQLQDLNDR.3	0.062740343	#N/A
YEEEVLSREDAEGR.3	0.0320842	#N/A
AEEAKDEPPS[Phospho (STY)]EGEAEEEEKEK.3	-0.140013723	P08551_S473_M1_Nefl
S[Phospho (STY)]PVEEVKPKPEAK.3	-0.103495943	P08553_S610_M1_Nefm
GVVTNGLDVS[Phospho (STY)]PAEEK.2	-0.098252404	P08553_S769_M1_Nefm
AES[Phospho (STY)]PVKEK.2	-0.237413583	P08553_S669_M1_Nefm
QLS[Phospho (STY)]DIEER.2	-0.119473172	P08553_S344_M1_Nefm
VHYLEQQNK.2	0.190059301	#N/A
EIEAEIQALR.2	0.158493611	#N/A
[Acetyl (Protein N-term)]SYTLDSLGNPSAYR.2	0.315821625	#N/A
VQSLQDEVAFLR.2	0.22681517	#N/A
FSTFSGSITGPLYTHR.2	-0.10902033	#N/A
LTEAAEQNK.2	0.275856424	#N/A
SAKEEIAEYR.2	0.180263702	#N/A
AVEEVITISK.2	0.44919213	#N/A
SAKEEIAEYRR.3	-0.214511239	#N/A
VTSHAIVK.2	0.299628741	#N/A
LRDDTEAAIR.2	0.274497136	#N/A
SNHEEEVADLLAQIQASHITVER.3	-0.044299649	#N/A
GS[Phospho (STY)]PSTVSSSYKR.2	0.396705832	P08553_S44_M1_Nefm
KGEDS[Phospho (STY)]SDDKVVVTK.2	-0.030249792	P08553_S779_M1_Nefm
KAES[Phospho (STY)]PVKEK.2	0.038560237	P08553_S669_M1_Nefm
EEEPEAEKS[Phospho (STY)]PVKS[Phospho (STY)]PEAK.3	-0.040878108	P08553_S502_M2_Nefm
HNHDLSSYQDTIQQLENELR.3	0.27165209	#N/A
VSGS[Phospho (STY)]PSSGFR.2	-0.108452336	P08553_S30_M1_Nefm
SIELESVR.2	-0.187546635	#N/A
VKEKAEEEGGS[Phospho (STY)]EEEGSDR.3	-0.075307552	#N/A
ATLEMVNHEK.2	0.250057398	#N/A
S[Phospho (STY)]PVEEVKPK.2	0.103686858	P08553_S610_M1_Nefm
SNEKEQLQGLNDR.2	-0.191032782	#N/A
EEEKGVVTNGLDVS[Phospho (STY)]PAEEK.3	0.002523417	P08553_S769_M1_Nefm
GS[Phospho (STY)]PSTVSSSYK.2	0.100763578	P08553_S44_M1_Nefm
AEEEGGS[Phospho (STY)]EEEGSDRSPQESK.3	0.086142897	#N/A
EEIAEYRR.2	0.026162245	#N/A

GTKES[Phospho (STY)]LER.2	0.421871216	P08553_S338_M1_Nefm
ITS[Phospho (STY)]EGGDGATK.2	-0.084976725	P08553_S795_M1_Nefm
QASHAQLGDAYDQEIR.3	0.402435871	#N/A
EEKPQPQEK.2	0.356975504	#N/A
AQVQLDSDHLEEDIHR.3	-0.150475062	#N/A
FAGYIEK.2	-0.168498798	#N/A
LTEAAEQNKEAIR.2	0.348284612	#N/A
LS[Phospho (STY)]RSNEKEQLQGLNDR.3	-0.081018437	P08553_S95_M1_Nefm
TDISTALK.2	-0.27548765	#N/A
EVTKES[Phospho (STY)]PKEEK.2	0.089249954	P08553_S645_M1_Nefm
AEEEGGS[Phospho (STY)]EEEGSDR.2	0.120954127	#N/A
VEEHEETFEEK.2	0.36166253	#N/A
SPVKS[Phospho (STY)]PEAK.2	-0.05865991	P08553_S506_M1_Nefm
EKAEEEGGS[Phospho (STY)]EEEGSDR.2	0.010283626	#N/A
QPSVTISSK.2	0.091306169	#N/A
AKS[Phospho (STY)]PMPK.2	0.111748046	P08553_S605_M1_Nefm
EGSS[Phospho (STY)]EKDEGEQEEEEGETEAEGEGEEAEAKEEK.4	-0.1022071	P08553_S551_M1_Nefm
SVTVTQKVEEHEETFEEK.3	-0.19022114	#N/A
AEEEGGS[Phospho (STY)]EEEGSDRSPQESKK.3	0.128814812	#N/A
EKAEEEGGS[Phospho (STY)]EEEGSDRSPQESK.3	-0.071671629	#N/A
SQLEC[Carbamidomethyl (C)]HSDQNMHQAEEWFK.3	-0.283182151	#N/A
DTKEEKPQPQEK.3	-0.291733909	#N/A
LFDSIC[Carbamidomethyl (C)]NNK.2	0.156693	#N/A
IAQSDYIPTQQDVLR.2	-0.140416841	#N/A
EYQLNDSAAYYLNDLER.2	-0.067631989	#N/A
AVVYSNTIQSIM[Oxidation (M)]AIVK.2	-0.362856991	#N/A
NLREDGEK.2	0.099100848	#N/A
NNLKDC[Carbamidomethyl (C)]GLF.2	-0.08689876	#N/A
M[Oxidation (M)]FDVGGQR.2	-0.012348338	#N/A
YDEAASYIQSK.2	0.32259397	#N/A
SREYQLNDSAAYYLNDLER.3	-0.285377302	#N/A
IIHEDGYSEEEC[Carbamidomethyl (C)]R.3	-0.178194405	#N/A
TTGIVETHFTFK.2	-0.135327326	#N/A
MFDVGGQRS[Phospho (STY)]ER.2	0.020423838	P08752_S207_M1_Gnai2
QLFALSC[Carbamidomethyl (C)]AAEEQGM[Oxidation (M)]LPEDLSGVIR.3	0.500961268	#N/A
LWADHGVQAC[Carbamidomethyl (C)]FGR.3	-0.288655809	#N/A
EIYTHFTC[Carbamidomethyl (C)]ATDTK.3	0.164328434	#N/A
AMGNLQIDFADPQR.2	-0.130291828	#N/A
NVQFVFDAVTDVIIK.2	0.212956132	#N/A
YSPTS[Phospho (STY)]PTYSPTTPK.2	-0.092916503	P08775_S1878_M1_Polr2a
YSPTS[Phospho (STY)]PTYSPTSPK.2	0.045741798	P08775_S1913_M1_Polr2a
YTPTSPSYSPSS[Phospho (STY)]PEYTPASPK.2	-0.097187582	P08775_S1850_M1_Polr2a
EADDIVNWLK.2	0.390086157	#N/A
NNFEGEITK.2	0.10853112	#N/A
TGPAATTLSDTAAAESLVDSSEVTVIGFFK.3	-0.002382331	#N/A
TVIDYNGER.2	0.024502258	#N/A
ILFIFIDSDHTDNQR.3	0.046197783	#N/A

NLQSLDPSHR.2	-0.031484379	#N/A
TYNTNAQVPDS[Phospho (STY)]AGTATAYLC[Carbamidomethyl (C)]GVK.3	-0.409682278	P09242_S110_M1_Alpl
NDLAVVDVR.2	0.074156079	#N/A
LELQGSNSR.2	-0.158608093	#N/A
ETLEEVFEK.2	0.266667826	#N/A
VEGSEPTTPFNLFIGNLNPNK.2	0.105415415	#N/A
NLS[Phospho (STY)]FNITEDELK.2	0.247486674	P09405_S403_M1_Ncl
GLS[Phospho (STY)]EDTTEETLK.2	0.102987016	P09405_S577_M1_Ncl
NNQITNNQR.2	0.041898062	#N/A
LTLDKLDVK.2	0.000709478	#N/A
FHVEEEGKGK.2	0.194416119	#N/A
ITLPVDFVTADKFDENAK.2	-0.255858115	#N/A
YSLEPVAAELK.2	0.121071142	#N/A
GC[Carbamidomethyl (C)]ITIIGGGDTATC[Carbamidomethyl (C)]C[Carbamidomethyl (C)]AK.2	-0.005401246	#N/A
LGDVYVNDAFGTAHR.3	0.002507345	#N/A
FC[Carbamidomethyl (C)]LDNGAK.2	0.05328952	#N/A
FHVEEEGK.2	0.028901892	#N/A
KYAEAVGR.2	-0.071774274	#N/A
IQLINNM[Oxidation (M)]LDK.2	0.233033518	#N/A
ELNYFAK.2	-0.243356607	#N/A
VNEM[Oxidation (M)]IIGGGM[Oxidation (M)]AFTFLK.2	0.364610261	#N/A
AEPAKIDAFR.2	-0.293725093	#N/A
VLNNM[Oxidation (M)]EIGTSLYDEEGAK.2	0.141992494	#N/A
SLM[Oxidation (M)]DEVVK.2	0.175750817	#N/A
VDFNVPMK.2	0.176051267	#N/A
ALESPERPFLAILGGAK.3	0.105048827	#N/A
AHSSM[Oxidation (M)]VGVNLPQK.2	0.359785806	#N/A
DC[Carbamidomethyl (C)]VGPEVENAC[Carbamidomethyl (C)]ANPAAGTVILLENLR.3	0.19457236	#N/A
VSHVSTGGGASLELLEGK.3	0.101528334	#N/A
WNTEDKVSHVSTGGGASLELLEGK.3	-0.03733744	#N/A
QIVWNGPVGVFEWEAFAR.2	-0.187798914	#N/A
[Acetyl (Protein N-term)]TTAS[Phospho (STY)]PSQVR.2	-0.056317479	P09528_S5_M1_Fth1
QNYHQDAEAAINR.3	0.179125088	#N/A
GELLEAIKR.2	0.227382843	#N/A
NVRPDYLK.2	0.211754125	#N/A
GDVTTQVALQPALK.2	0.318421203	#N/A
AIWNVINWENVTER.2	-0.221199789	#N/A
YHEALAK.2	0.156790172	#N/A
GELLEAIK.2	0.05087139	#N/A
HHAAYVNNLNATEEK.3	0.282130143	#N/A
FNGGGHINHTIFWTNLSPK.3	-0.176745159	#N/A
DFGSFEK.2	-0.314170696	#N/A
LGGS[Phospho (STY)]PTNGNSAAPSPPESEP.2	-0.263233947	P0C027_S148_M1_Nudt10;Nudt11
LKLGGSPT[Phospho (STY)]NGNSAAPSPPESEP.2	-0.19440801	P0C027_T150_M1_Nudt10;Nudt11
SEREDEVLLVSSSR.3	-0.141740697	#N/A
T[Phospho (STY)]GDPANLNRLR.2	0.031946467	P0C090_T341_M1_Rc3h2
MHQVM[Oxidation (M)]S[Phospho (STY)]IEEVER.2	0.14367686	P0C0A3_S119_M1_Chmp6

ATIAGGGVIPHIHK.3	-0.032492368	#N/A
GDEELDSLIK.2	-0.57627287	#N/A
SGS[Phospho (STY)]KENVQETQI.2	0.023493229	P0C192_S700_M1_Lrrc4b
RRANS[Phospho (STY)]C[Carbamidomethyl (C)]S[Phospho (STY)]I.2	-0.079238449	P0C5J4_S358_M2_Gpr52
GSS[Phospho (STY)]PQVLPR.2	-0.377893283	P0C673_S378_M1_Igsf11
AQLVS[Phospho (STY)]PPAPPTK.2	-0.277110916	P0C7L0_S211_M1_Wipf3
TAVAPPPLPGANNSGSET[Phospho (STY)]PPPLPPK.3	-0.218490424	P0C7L0_T338_M1_Wipf3
SGS[Phospho (STY)]GYEGSTSWK.2	-0.569216744	P0C7M9_S47_M1_Clec2l
S[Phospho (STY)]PAEAEAR.2	-0.198212821	P0C7M9_S29_M1_Clec2l
PRS[Phospho (STY)]PAEAEAR.2	0.066972889	P0C7M9_S29_M1_Clec2l
PAPAAPRPRS[Phospho (STY)]PAEAEAR.3	-0.251558219	P0C7M9_S29_M1_Clec2l
DTTEDS[Phospho (STY)]ITEDDKRR.3	-0.111491723	P0C913_S28_M1_
DTTEDS[Phospho (STY)]ITEDDKR.2	0.193946274	P0C913_S28_M1_
TLSDYNIQK.2	0.026703698	#N/A
ESTLHLVLR.2	0.286132129	#N/A
TITLEVEPSDTIENVK.2	0.169235319	#N/A
QLEDGRTLS[Phospho (STY)]DYNIQK.3	-0.283051484	P0CG49_S57_M1_Ubb;Ubc;Rps27a;Uba52
FNVGTDDIAIEESNAIINDGK.2	-0.106084454	#N/A
DEGS[Phospho (STY)]YHVDESR.2	0.313096957	P0DI97_S423_M1_Nrxn1
YPAGNNDNER.2	0.207734508	#N/A
NYIS[Phospho (STY)]NSAQSNGAVVK.2	0.044359894	P0DI97_S434_M1_Nrxn1
TLFQLLR.2	-0.088706405	#N/A
ELRPNEEVTWK.2	-0.064134399	#N/A
EAFSLFDK.2	0.267646563	#N/A
EAFSLFDKDGDGTITTK.3	-0.306105095	#N/A
ELGTVMR.2	0.324686301	#N/A
VFDKDGNGYISAAELR.3	0.164127073	#N/A
DGNGYISAAELR.2	-0.095024864	#N/A
M[Oxidation (M)]KDTDSEEEIREAFR.3	0.213208373	#N/A
DTDSEEEIREAFR.2	0.239227646	#N/A
M[Oxidation (M)]KDTDSEEEIR.2	0.165583858	#N/A
[Acetyl (Protein N-term)]ADQLTEEQIAEFKEAFSLFDKDGDGTITTK.3	-0.311542492	#N/A
[Acetyl (Protein N-term)]ADQLTEEQIAEFK.2	0.068401661	#N/A
DTDSEEEIR.2	-0.381054274	#N/A
IGGIGTVPVGR.2	-0.073258706	#N/A
RYEEIVK.2	-0.031540655	#N/A
LPLQDVYK.2	0.156066696	#N/A
QLIVGVNK.2	-0.022136113	#N/A
QTVAVGVIK.2	0.219345961	#N/A
STTTGHLIYK.2	0.117364256	#N/A
TIEKFEK.2	0.087178618	#N/A
EHALLAYTLGVK.2	-0.253454828	#N/A
M[Oxidation (M)]DSTEPPYSQK.2	0.553439876	#N/A
VETGVLKPGM[Oxidation (M)]VVTFAPVNVTTEVK.3	0.232644665	#N/A
YYVTIIDAPGHR.2	-0.174376805	#N/A
DGSASGTTLLEALDC[Carbamidomethyl (C)]ILPPTRPTDKPLR.3	-0.139806222	#N/A
NMITGTSQADC[Carbamidomethyl (C)]AVLIVAAGVGEFEAGISK.3	-0.447391196	#N/A

THINIVVIGHVDSGK.3	0.323193827	#N/A
DAAQSS[Phospho (STY)]PAFGDR.2	-0.175208739	P10518_S215_M1_Alad
DAAQSS[Phospho (STY)]PAFGDRR.2	-0.21592045	P10518_S215_M1_Alad
EQWSNC[Carbamidomethyl (C)]PTIGQIR.2	0.259425758	#N/A
GENHC[Carbamidomethyl (C)]GIESEIVAGIPR.3	0.535097447	#N/A
SC[Carbamidomethyl (C)]EAGYSPSYKEDK.2	0.279344295	#N/A
DQGSC[Carbamidomethyl (C)]GSC[Carbamidomethyl (C)]WAFGAVEAISDR.2	-0.286423277	#N/A
NGPVEGAFTVFSDFLTYK.2	0.043518672	#N/A
EIMAEIYK.2	-0.234872486	#N/A
LQAEAPHIVVGTPGR.3	-0.147536393	#N/A
ETQALVLAPTR.2	-0.136651999	#N/A
SGYSSPGS[Phospho (STY)]PGTPGSR.2	0.066095707	P10636_S519_M1_MAPT
SKIGS[Phospho (STY)]TENLK.2	0.29897526	P10636_S579_M1_MAPT
TDHGAEIVYK.2	0.093691152	#N/A
LDLSNVQSK.2	0.049856608	#N/A
SEKLDFKDR.2	0.032192965	#N/A
HVPGGGSVQIVYKPVDLSK.3	0.179737518	#N/A
SEKLDFK.2	0.081899281	#N/A
VQIINKK.2	0.098089243	#N/A
LQTAPVPM[Oxidation (M)]PDLK.2	0.104808955	#N/A
T[Phospho (STY)]PPKSPSSAK.2	0.059963568	P10636_T548_M1_MAPT
IGS[Phospho (STY)]LDNITHVPGGGNK.2	-0.121580672	P10636_S673_M1_MAPT
IGSTENLK.2	0.015027691	#N/A
VAVVRT[Phospho (STY)]PPKS[Phospho (STY)]PSSAK.2	-0.088134942	P10636_S552_M2_MAPT
TPS[Phospho (STY)]LPTPPTR.2	0.001003541	P10636_S531_M1_MAPT
IGS[Phospho (STY)]LDNITHVPGGGNKK.3	-0.110264385	P10636_S673_M1_MAPT
SRT[Phospho (STY)]PS[Phospho (STY)]LPTPPTREPK.3	0.117273744	#N/A
AKTDHGAEIVYKS[Phospho (STY)]PVVS[Phospho (STY)]GDTSPR.4	0.011205357	#N/A
IGS[Phospho (STY)]TENLKHQPGGGK.3	-0.128267056	P10636_S579_M1_MAPT
SPVVS[Phospho (STY)]GDTS[Phospho (STY)]PR.2	-0.03558995	#N/A
TDHGAEIVYKS[Phospho (STY)]PVVSGDTS[Phospho (STY)]PR.3	0.148836827	#N/A
TPS[Phospho (STY)]LPTPPTREPK.2	-0.034118588	P10636_S531_M1_MAPT
AKTDHGAEIVYK.2	0.111741295	#N/A
SRT[Phospho (STY)]PS[Phospho (STY)]LPTPPTR.2	0.205733255	#N/A
KLDLSNVQSK.2	0.044966003	#N/A
IPAKT[Phospho (STY)]PPAPK.2	0.057459222	P10636_T492_M1_MAPT
T[Phospho (STY)]PPSSGEPPK.2	0.060660952	P10636_T498_M1_MAPT
TPPAPKT[Phospho (STY)]PPSSGEPPK.2	0.016675696	P10636_T498_M1_MAPT
SKIGS[Phospho (STY)]TENLKHQPGGGK.3	-0.19928527	P10636_S579_M1_MAPT
DQGGYTM[Oxidation (M)]HQDQEGDTDAGLK.3	0.019825394	#N/A
QEFEVM[Oxidation (M)]EDHAGTYGLGDR.3	0.017128115	#N/A
VQS[Phospho (STY)]KIGS[Phospho (STY)]LDNITHVPGGGNK.3	0.049592273	P10636_S669_M2_MAPT
SGDRSGYSSPGS[Phospho (STY)]PGTPGSR.3	-0.178362318	P10636_S519_M1_MAPT
IPAKT[Phospho (STY)]PPAPKT[Phospho (STY)]PPSSGEPPK.3	0.04036925	P10636_T492_M2_MAPT
TPPAPKT[Phospho (STY)]PPSSGEPPKSGDR.3	0.063722011	P10636_T498_M1_MAPT
C[Carbamidomethyl (C)]GSLGNIHHKPGGGQVEVK.3	0.072246532	#N/A
T[Phospho (STY)]PPSSGEPPKSGDR.2	0.112188041	P10636_T498_M1_MAPT

HLSNVSSTGS[Phospho (STY)]IDM[Oxidation (M)]VDSPQLATLADEVSASLAK.4	0.213844488	#N/A
QEFEVMEDHAGTYGLGDRK.3	0.044678898	#N/A
IPAKTT[Phospho (STY)]PS[Phospho (STY)]PK.2	-0.018796459	P10637_S470_M2_Mapt
IPAKTT[Phospho (STY)]PS[Phospho (STY)]PKT[Phospho (STY)]PPGSGEPPK.2	-0.227629781	P10637_S470_M3_Mapt
T[Phospho (STY)]PPKSPSASK.2	0.028294094	P10637_T523_M1_Mapt
ESPPQPPADDGAEEPGS[Phospho (STY)]ETSDAK.2	-0.113213244	P10637_S50_M1_Mapt
VAVVRT[Phospho (STY)]PPKS[Phospho (STY)]PSASK.2	-0.09593295	P10637_S527_M2_Mapt
TTPS[Phospho (STY)]PKT[Phospho (STY)]PPGSGEPPKSGER.3	0.232655697	P10637_S470_M2_Mapt
QEFDTM[Oxidation (M)]EDHAGDYTLLQDQEGDM[Oxidation (M)]DHGLK.4	-0.215642443	#N/A
TTPS[Phospho (STY)]PKT[Phospho (STY)]PPGSGEPPK.2	-0.190097262	P10637_S470_M2_Mapt
ST[Phospho (STY)]PTAEDVTAPLVDER.2	0.209501277	P10637_T58_M1_Mapt
T[Phospho (STY)]PPGSGEPPK.2	0.221726218	P10637_T473_M1_Mapt
SGERS[Phospho (STY)]GYSS[Phospho (STY)]PGSPGTPGSR.2	-0.386028115	P10637_S487_M2_Mapt
T[Phospho (STY)]PPGSGEPPKSGER.2	-0.085461954	P10637_T473_M1_Mapt
EAFQEALAAAGDK.2	0.111403749	#N/A
VGEFSGANK.2	0.49124907	#N/A
VKLIESK.2	-0.189123954	#N/A
VGEFSGANKEK.2	-0.081387575	#N/A
ITQSNAILR.2	-0.024430102	#N/A
YIATPIFSK.2	0.314958087	#N/A
PM[Oxidation (M)]ILGYWNVR.2	-0.002151807	#N/A
SQWLNEK.2	-0.13941502	#N/A
C[Carbamidomethyl (C)]LDAFPNLR.2	0.245879103	#N/A
RPWFAGDK.2	0.515519106	#N/A
LYSEFLGK.2	0.120629033	#N/A
YTM[Oxidation (M)]GDAPDFDR.2	0.136407306	#N/A
ADIVENQVM[Oxidation (M)]DTR.2	0.168066161	#N/A
LGLDFPNLPYLIDGSHK.3	-0.044715976	#N/A
MLLEYTDSSYDEK.2	0.213871479	#N/A
M[Oxidation (M)]LLEYTDSSYDEKR.2	0.498437512	#N/A
IRADIVENQVM[Oxidation (M)]DTR.3	-0.150782139	#N/A
VTYVDFLAYDILDQYR.2	-0.046268075	#N/A
EPAISSQNS[Phospho (STY)]PEAR.2	0.278825569	P10711_S100_M1_Tcea1
KKEPAISSQNS[Phospho (STY)]PEAR.3	0.007393506	P10711_S100_M1_Tcea1
DTYVSSFPR.2	0.340800709	#N/A
LLLSTDSAR.2	-0.353315852	#N/A
GQNEDPGSLLTQFR.2	-0.225942238	#N/A
LGASNLPAGISLPASAK.2	0.127611195	#N/A
IGDLQAFVGR.2	0.140071343	#N/A
QINPTLGSQEDFKDLLQSAK.3	0.12021113	#N/A
EALSSWLQDGVDGFQFR.2	-0.055638935	#N/A
SHLEYLSTLK.2	-0.018260166	#N/A
HWDQNER.2	-0.171828668	#N/A
DAGGIAGLK.2	-0.057881004	#N/A
NQKDEINETDLK.3	0.228640278	#N/A
LLLPGELAK.2	0.231110253	#N/A
ESYSVYVYK.2	0.057856512	#N/A

EIQTAVR.2	-0.070033113	#N/A
KESYSVYVYK.2	-0.240150309	#N/A
AM[Oxidation (M)]GIM[Oxidation (M)]NSFVNDIFER.2	-0.044810908	#N/A
QVHPDTGISSK.2	0.092259195	#N/A
VGENADSQIK.2	0.177470934	#N/A
[Acetyl (Protein N-term)]TENS[Phospho (STY)]TSAPAAKPK.2	-0.127596814	P10922_S5_M1_H1-0
GVGASGSFR.2	0.039695062	#N/A
EQISDIDDAVR.2	-0.005163891	#N/A
QSS[Phospho (STY)]SSRDDNMFQIGK.3	0.157578015	P11031_S56_M1_Sub1
TIDIEPDIETLLSQQSGNS.2	-0.363686306	#N/A
SLGVGFATR.2	0.228409017	#N/A
[Acetyl (Protein N-term)]ADAFVGTWK.2	0.349574626	#N/A
SLVTLDGGK.2	0.066242857	#N/A
WNGQETTLTR.2	0.249528592	#N/A
LILTLTHGSVVSTR.2	-0.33439972	#N/A
NGDTITIK.2	0.5790669	#N/A
IEDVGS[Phospho (STY)]DEEDDSGK.2	0.06390695	P11499_S255_M1_Hsp90ab1
TKPIWTR.2	0.334016197	#N/A
ADLINNLGTIAK.2	0.261933407	#N/A
IDIIPNPQER.2	0.448945213	#N/A
YIDQEELNK.2	0.341796174	#N/A
EDQTEYLEER.2	0.10508138	#N/A
ELISNASDALDK.2	-0.23146562	#N/A
SIYYITGESK.2	-0.04713747	#N/A
EGLELPEDEEEKKK.3	0.183879738	#N/A
NPDDITQEEYGEFYK.2	0.246032853	#N/A
ADHGEPIGR.2	-0.097656979	#N/A
EQVANSAFVER.2	0.07426908	#N/A
ALLFIPR.2	0.276037916	#N/A
EIS[Phospho (STY)]DDEAEEEK.2	0.037418379	P11499_S226_M1_Hsp90ab1
YESLTDPSK.2	-0.098511567	#N/A
EKEIS[Phospho (STY)]DDEAEEEK.2	-0.120107405	P11499_S226_M1_Hsp90ab1
EKYIDQEELNK.2	-0.106520002	#N/A
VFIM[Oxidation (M)]DSC[Carbamidomethyl (C)]DELIPEYLNFIR.2	-0.417856155	#N/A
GVVDSEDLPLNIS[Phospho (STY)]R.2	-0.019038806	P11499_S391_M1_Hsp90ab1
APFDLFENK.2	0.242569279	#N/A
LGIHEDSTNR.2	-0.03419932	#N/A
ELISNASDALDKIR.2	-0.316924484	#N/A
EKEIS[Phospho (STY)]DDEAEEEKGEK.2	-0.377667223	P11499_S226_M1_Hsp90ab1
EIS[Phospho (STY)]DDEAEEEKGEK.2	-0.206674852	P11499_S226_M1_Hsp90ab1
AKFENLC[Carbamidomethyl (C)]K.2	-0.442990863	#N/A
IEDVGS[Phospho (STY)]DEEDDSGKDK.2	-0.214162378	P11499_S255_M1_Hsp90ab1
FYEAFSK.2	0.048192915	#N/A
IEDVGS[Phospho (STY)]DEEDDSGKDKK.3	-0.365845812	P11499_S255_M1_Hsp90ab1
EM[Oxidation (M)]LQQSK.2	0.024545274	#N/A
YESLTDPSKLDSGK.2	-0.008889141	#N/A
SLTNDWEDHLAVK.2	0.081388692	#N/A

HLEINPDHPIVETLR.3	0.11546274	#N/A
YHTSQSGDEM[Oxidation (M)]TSLSEYVSR.3	-0.220602286	#N/A
HNDDEQYAWESSAGGSFTVR.3	-0.283731677	#N/A
EGLELPEDEEEKK.3	-0.096208587	#N/A
VILHLKEDQTEYLEER.3	-0.071296679	#N/A
KHLEINPDHPIVETLR.4	0.068987409	#N/A
LGIHEDSTNRR.3	0.064500348	#N/A
LGLGIDEDEVTAEEPSAAVPDEIPPLEGDEDASR.3	0.126828396	#N/A
SDSS[Phospho (STY)]QPMLLR.2	0.169690805	P11531_S3617_M1_Dmd
NAPGKPMREDT[Phospho (STY)]M.2	0.310785887	P11531_T3677_M1_Dmd
SLES[Phospho (STY)]DNEEK.2	-0.009806391	P11627_S1184_M1_L1cam
EKEAAGGNDSSGAT[Phospho (STY)]SPINPAVALE.2	-0.030222773	P11627_T1250_M1_L1cam
TLQLLNVGEEDDGEYTC[Carbamidomethyl (C)]LAENSLGSAR.3	-0.194522635	#N/A
AQLLVVGSPGPVPHLELSDR.3	-0.078018201	#N/A
EAAGGNDSSGATS[Phospho (STY)]PINPAVALE.2	0.147011478	P11627_S1251_M1_L1cam
DETFGEYRS[Phospho (STY)]LES[Phospho (STY)]DNEEK.2	0.162754665	#N/A
PM[Oxidation (M)]KDETFGEY[Phospho (STY)]RSLES[Phospho (STY)]DNEEK.3	-0.157569113	P11627_Y1179_M2_L1cam
DKEDTQVDS[Phospho (STY)]EARPMK.3	-0.087354847	P11627_S1166_M1_L1cam
LVALQGQSLILEC[Carbamidomethyl (C)]IAEGFPTPTIK.3	-0.173009034	#N/A
ITQYLDAGGIPR.2	-0.08315224	#N/A
HPWIS[Phospho (STY)]HR.2	0.19179492	P11798_S272_M1_Camk2a
FTEEYQLFEELGK.2	-0.127575157	#N/A
VLAGQEYAAK.2	-0.05169407	#N/A
WQIVHFHR.2	-0.12785608	#N/A
FYFENLWSR.2	-0.278482931	#N/A
ITAAEALK.2	0.118739186	#N/A
ESSES[Phospho (STY)]TNT[Phospho (STY)]T[Phospho (STY)]IEDEDTKVR.2	0.168864642	#N/A
M[Oxidation (M)]C[Carbamidomethyl (C)]DPGM[Oxidation (M)]TAFEPEALGNLVEGLDFHR.3	-0.060447616	#N/A
ESS[Phospho (STY)]ESTNTTIEDEDTK.2	-0.086464963	#N/A
NFS[Phospho (STY)]GGKSGGNK.2	0.099956991	P11798_S314_M1_Camk2a
[Acetyl (Protein N-term)]ATITC[Carbamidomethyl (C)]TR.2	0.080289504	#N/A
S[Phospho (STY)]TVASC[Carbamidomethyl (C)]M[Oxidation (M)]HR.2	0.502025274	P11798_S275_M1_Camk2a
NDGVKES[Phospho (STY)]SESTNTTIEDEDTK.2	-0.441225267	P11798_S330_M1_Camk2a
VTEQLIEAISNGDFESYTK.2	-0.403410726	#N/A
QETVDC[Carbamidomethyl (C)]LKK.2	0.357883754	#N/A
QET[Phospho (STY)]VDC[Carbamidomethyl (C)]LK.2	0.365326308	P11798_T286_M1_Camk2a
SGAPSVLPH.2	-0.077422381	#N/A
NDGVKESS[Phospho (STY)]ESTNTTIEDEDTKVR.3	0.14594817	#N/A
KNDGVKESS[Phospho (STY)]ESTNTTIEDEDTK.3	-0.305808039	#N/A
VDGKTS[Phospho (STY)]PVQSK.2	0.001252768	P11862_S283_M1_Gas2
SLLVIPNTLAVNAAQDSTDLVAK.2	-0.328172556	#N/A
SSFGPVGLDK.2	-0.068617426	#N/A
LLEVEHPAAK.2	-0.061362304	#N/A
ILATGANVILTTGGIDDM[Oxidation (M)]YLK.2	-0.06487211	#N/A
YINENLIINTDELGR.2	0.146130837	#N/A
FATEAAITILR.2	-0.138482342	#N/A
IC[Carbamidomethyl (C)]DDELILIK.2	0.196724279	#N/A

DDKHGS[Phospho (STY)]YENAVHSGALDD.3	-0.633937176	P11983_S544_M1_Tcp1
M[Oxidation (M)]LVDDIGDVTITNDGATILK.2	-0.525837976	#N/A
HGSYENAVHS[Phospho (STY)]GALDD.2	-0.083394398	P11983_S551_M1_Tcp1
DC[Carbamidomethyl (C)]LINAAK.2	0.201086849	#N/A
SLHDALC[Carbamidomethyl (C)]VVK.2	-0.20309849	#N/A
IAC[Carbamidomethyl (C)]LDFSLQK.2	-0.460273384	#N/A
SQNVM[Oxidation (M)]AAASIANIVK.2	-0.366140957	#N/A
VES[Phospho (STY)]LEQEAANER.2	-0.056699202	P12023_S441_M1_App
C[Carbamidomethyl (C)]LVGEFVSDALLVPDK.2	0.234795251	#N/A
QYTSIHHGVVEVDAAVT[Phospho (STY)]PEER.3	-0.326077983	P12023_T743_M1_App
TDEHVIDQGDDGDNFYVIER.3	-0.270999664	#N/A
SVGQYDNR.2	0.267723258	#N/A
AATIIATSEGSLWGLDR.2	-0.19174455	#N/A
GTYDILVTK.2	-0.096294209	#N/A
M[Oxidation (M)]FGS[Phospho (STY)]NLDLM[Oxidation (M)]DPGQ.2	-0.069511732	P12367_S392_M1_Prkar2a
NISHYEEQLVK.2	0.153960556	#N/A
RVS[Phospho (STY)]VC[Carbamidomethyl (C)]AETFNPDEEEEDNDPR.3	0.187789621	P12367_S96_M1_Prkar2a
VSVC[Carbamidomethyl (C)]AETFNPDEEEEDNDPR.2	-0.704901856	#N/A
RTLS[Phospho (STY)]IDKGF.2	-0.522714001	P12382_S775_M1_Pfkl
RTLS[Phospho (STY)]IDK.2	-0.288996302	P12382_S775_M1_Pfkl
GGTPSAFDR.2	-0.359910077	#N/A
VTVLGHVQR.2	-0.061292946	#N/A
TNVLGHLQQGGAPTPFDR.3	-0.544640514	#N/A
SFENNWK.2	0.01040112	#N/A
VFANAPDSAC[Carbamidomethyl (C)]VIGLR.2	-0.20102498	#N/A
TLS[Phospho (STY)]IDKGF.2	-0.45839037	P12382_S775_M1_Pfkl
TFVLEVM[Oxidation (M)]GR.2	-0.096636776	#N/A
NEWGSLLEELVK.2	-0.231624061	#N/A
AIGVLTSGGDAQGM[Oxidation (M)]NAAVR.2	-0.043948548	#N/A
IM[Oxidation (M)]EVIDAITTTAQSHQR.2	-0.330024198	#N/A
TGISEGHTVYIVHDGFEGLAK.3	0.229523855	#N/A
SQPGAEGSPES[Phospho (STY)]PPGR.2	-0.348373294	P12657_S254_M1_Chrm1
RPGS[Phospho (STY)]VHRT[Phospho (STY)]PSR.3	-0.165618381	P12657_S451_M2_Chrm1
TDLALILSAGDN.2	-0.326229573	#N/A
SFVDQYGQR.2	0.190984955	#N/A
ELQNLIQELLQAR.2	0.156213582	#N/A
NIMALSDGGK.2	0.020519021	#N/A
LNDFASAVR.2	0.113922944	#N/A
ILEVVKDK.2	0.060331887	#N/A
GM[Oxidation (M)]NTLVGYDLVPEPK.2	0.298222701	#N/A
RLNDFASAVR.2	-0.263431016	#N/A
TDS[Phospho (STY)]LKGR.2	0.26444898	P12813_S344_M1_Nr4a1;Nr4a2;Nr4a3
LSDQFHDILIR.2	0.499486325	#N/A
YITDWQNVFR.2	0.372486072	#N/A
NVLFSHLDDNER.3	0.004179038	#N/A
S[Phospho (STY)]PNEEYVEVGR.2	-0.151093553	P12849_S307_M1_Prkar1b
[Acetyl (Protein N-term)]ASPS[Phospho (STY)]C[Carbamidomethyl (C)]FHSEDEDSLK.2	0.392269831	P12849_S5_M1_Prkar1b

SNSQC[Carbamidomethyl (C)]DSHDEEIS[Phospho (STY)]PTPPNPVVK.3	-0.159392085	P12849_S83_M1_Prkar1b
GGVSAEVYTEEDAVSYVR.2	-0.45402265	#N/A
IVVQGEPGDDFYIITEGTASVLQR.3	0.540964779	#N/A
NDGGIYTC[Carbamidomethyl (C)]FAENNR.2	-0.154785463	#N/A
M[Oxidation (M)]NNGDVDLTNDR.2	-0.110150475	#N/A
GPPGPPGGLR.2	0.392627897	#N/A
IKTDGAAPNVAPSDVGGGGGTNR.3	-0.130645601	#N/A
VTVTNPDTGR.2	0.286353444	#N/A
FIPLIPIPER.2	0.094987852	#N/A
IVESYQIR.2	0.068013561	#N/A
ELTITWAPLSR.2	0.019863143	#N/A
VQVTSQEYSAR.2	-0.11133793	#N/A
TDGAAPNVAPSDVGGGGGTNR.2	0.011293962	#N/A
DGEYVVEVR.2	-0.244088806	#N/A
GM[Oxidation (M)]VLLC[Carbamidomethyl (C)]DPPYHFPDDLSYR.3	-0.05834751	#N/A
HSIEVPIPR.2	-0.210861411	#N/A
ILYRPDGQHDGK.3	-0.155535702	#N/A
AHSDGGDGVVSQVK.2	-0.305736744	#N/A
VVATNTLGTGEPSIPSNR.2	0.277403215	#N/A
ILALAPTFEM[Oxidation (M)]NPM[Oxidation (M)]K.2	-0.00312439	#N/A
GSDNHSPISK.2	-0.269936951	#N/A
STEATLSFGYLDPFPPEERPEVK.3	-0.19532363	#N/A
VLEPM[Oxidation (M)]PSTAEISTSGAVLK.2	-0.251706723	#N/A
TDPPIIEGNMESAK.2	0.265880797	#N/A
KVTVTNPDTGR.2	-0.161749677	#N/A
IFNIQLEDEGLYEC[Carbamidomethyl (C)]EAENIR.3	-0.016139534	#N/A
FVSQTNGNLYIANVESSDR.2	-0.344005256	#N/A
VLSSSEISVHWK.2	-0.236542441	#N/A
DAGVYYC[Carbamidomethyl (C)]LASNNYGM[Oxidation (M)]VR.2	-0.475287955	#N/A
TADDGC[Carbamidomethyl (C)]LENAPDT[Phospho (STY)]AEFSR.2	0.158158085	P12961_T137_M1_Scg5
KSVPHFS[Phospho (STY)]EEEKEAE.3	-0.422561763	P12961_S205_M1_Scg5
SVPHFS[Phospho (STY)]EEEKEAE.2	-0.107453008	P12961_S205_M1_Scg5
DFSEDQGYPDPPNPC[Carbamidomethyl (C)]PLGK.2	-0.118608857	#N/A
QTATQLLK.2	-0.049578825	#N/A
NFGIGQDIQPK.2	0.143301451	#N/A
HVVPNEVVVQR.2	-0.229692214	#N/A
DSQEEEKTEALTSAK.2	-0.318011868	#N/A
NPPEAATAPAS[Phospho (STY)]PK.2	-0.316523422	P13595_S1005_M1_Ncam1
GVTASSSS[Phospho (STY)]PASAPK.2	-0.242721456	P13595_S887_M1_Ncam1
FFLC[Carbamidomethyl (C)]QVAGDAK.2	0.122348936	#N/A
GLGEISAATEFK.2	0.163233229	#N/A
ALASEWKPEIR.2	0.007743791	#N/A
NAPTPQEFK.2	-0.068053285	#N/A
AGEQDASIHLK.2	-0.043618449	#N/A
FIVLSNNYLQIR.2	-0.20443199	#N/A
NPPEAATAPAS[Phospho (STY)]PKSK.2	0.037824068	P13595_S1005_M1_Ncam1
VEPYSSTAQVQFDEPEATGGVPILK.3	0.120117201	#N/A

NVDKNDEAEYVC[Carbamidomethyl (C)]IAENK.3	-0.088302409	#N/A
AAHFVFR.2	0.510256118	#N/A
AAFSKDES[Phospho (STY)]KEPIVEVR.3	-0.369106034	P13595_S774_M1_Ncam1
GPDPEPTQPGTVKNPPEAATAPAS[Phospho (STY)]PK.3	0.105526536	P13595_S1005_M1_Ncam1
DES[Phospho (STY)]KEPIVEVR.2	-0.216966068	P13595_S774_M1_Ncam1
EANM[Oxidation (M)]EGIVTIM[Oxidation (M)]GLKPETR.3	-0.493141371	#N/A
C[Carbamidomethyl (C)]VVTAEDGTQSEATVNVK.2	-0.332001039	#N/A
LEGQM[Oxidation (M)]GEDGNSIK.2	0.05310177	#N/A
ASPAPT[Phospho (STY)]PTPAGAASPLAAVAAPATDAPQAK.3	-0.345415556	P13595_T950_M1_Ncam1
DGEPIENEEEDDEKHIFSDDSSELTIR.4	-0.365196989	#N/A
SLGEESWHFK.2	0.007036381	#N/A
IGQESLEFILVQADTPSSPSIDR.3	0.181988133	#N/A
LPSGSDHVMLK.2	0.205413553	#N/A
TEEERT[Phospho (STY)]PNHDGGK.2	0.022422389	P13595_T788_M1_Ncam1
LISEVIGER.2	-0.291165425	#N/A
DLM[Oxidation (M)]QTPNFR.2	-0.02693992	#N/A
EADDDEEADDDVSEM[Oxidation (M)]PS[Phospho (STY)]PK.2	0.166648227	P13864_S717_M1_Dnmt1
S[Phospho (STY)]KSDSDTLSVETSPSSVATR.3	-0.455041666	P13864_S138_M1_Dnmt1
YNPNVLPVQC[Carbamidomethyl (C)]TGK.2	0.031518619	#N/A
SYEAYVLNIIR.2	0.061081509	#N/A
AYGENIGYSEK.2	-0.181909175	#N/A
AKEEGSWKK.2	-0.165093183	#N/A
EGKPC[Carbamidomethyl (C)]IIIK.2	-0.081965294	#N/A
FIWNSEK.2	0.013967678	#N/A
AKEEGSWK.2	-0.222872808	#N/A
VAPPGLTQIPQIQK.2	-0.057653207	#N/A
AYGENIGYSEKDR.2	-0.111145342	#N/A
KFIWNSEK.2	-0.065399174	#N/A
YKDSAQKDDM[Oxidation (M)]IFEDC[Carbamidomethyl (C)]GNVPSEPK.4	-0.313917117	#N/A
DDM[Oxidation (M)]IFEDC[Carbamidomethyl (C)]GNVPSEPK.2	0.094252235	#N/A
DSAQKDDM[Oxidation (M)]IFEDC[Carbamidomethyl (C)]GNVPSEPK.3	-0.276232594	#N/A
TEISFRPNDPK.2	-0.053278881	#N/A
GDINHER.2	-0.319456351	#N/A
TGVAPIIDVVR.2	-0.122595509	#N/A
LWTLVSEQTR.2	0.420979085	#N/A
LLEPVLLLGK.2	-0.35097054	#N/A
DILIQYDR.2	0.284520472	#N/A
TATAVAHC[Carbamidomethyl (C)]K.2	0.325100536	#N/A
EIKDILIQYDR.3	0.308700312	#N/A
FVEGLPINDFSR.2	0.103327325	#N/A
GEFITTVQQR.2	0.183406656	#N/A
VIVVGNPANTNC[Carbamidomethyl (C)]LTASK.2	-0.172279257	#N/A
ENFSC[Carbamidomethyl (C)]LTR.2	0.333439428	#N/A
DLDVAVLVGSM[Oxidation (M)]PR.2	0.392769392	#N/A
LGVTADDVK.2	-0.021924613	#N/A
EVGVYEALKDDSWLK.2	-0.050285051	#N/A
SQGTALEK.2	0.202426703	#N/A

ELTEEKETAFEFLSSA.2	-0.304017564	#N/A
NVIIWGNHSSTQYPDVNHAK.3	-0.09629335	#N/A
KLS[Phospho (STY)]SAM[Oxidation (M)]SAAK.2	0.148125013	P14152_S241_M1_Mdh1
EKM[Oxidation (M)]DLTAK.2	0.179934906	#N/A
IFKS[Phospho (STY)]QGTALEK.2	0.458423353	P14152_S111_M1_Mdh1
LSSAMSAAK.2	-0.14233264	#N/A
VLVTGAAGQIAYSLLYSIGNGSVFGK.3	0.04846542	#N/A
YVDIAIPC[Carbamidomethyl (C)]NNK.2	0.087371876	#N/A
FAAATGATPIAGR.2	0.009877665	#N/A
AIVAIENPADVSVISSR.2	0.236174507	#N/A
LLVVTDPR.2	-0.091137315	#N/A
[Acetyl (Protein N-term)]SGALDVLQM[Oxidation (M)]KEEDVLK.2	0.007388756	#N/A
ADHQPLTEASYVNLPTIALC[Carbamidomethyl (C)]NTDSPLR.3	0.021223477	#N/A
EQFLDGDAWTNR.2	-0.111617114	#N/A
LFPSGLDQK.2	-0.202312904	#N/A
FYGDLEKDK.2	-0.036791598	#N/A
QIDNPDYK.2	0.209144963	#N/A
FEPFSNK.2	0.288314619	#N/A
IDDPTDSKPEDWDKPEHIPDPDAK.4	0.048787895	#N/A
DKGLQTSQDAR.2	-0.163042088	#N/A
VHVIFNYK.2	-0.268943563	#N/A
HEQNIDC[Carbamidomethyl (C)]GGGYVK.2	0.253273883	#N/A
IKDPDAAKPEDWDER.3	0.01731907	#N/A
LKEEEEDKK.2	-0.188899651	#N/A
DM[Oxidation (M)]HGDSEYNIM[Oxidation (M)]FGPDIC[Carbamidomethyl (C)]GPGTK.3	-0.26335559	#N/A
FLNVTPNVEVNVEC[Carbamidomethyl (C)]R.2	-0.403352287	#N/A
SC[Carbamidomethyl (C)]GQVVEEWK.2	0.248241024	#N/A
NDVC[Carbamidomethyl (C)]RPGR.2	0.024016106	#N/A
INAANIATDDERDK.2	0.340471201	#N/A
EFVWNPR.2	-0.122631149	#N/A
YYEQPDNGVLNYPK.2	0.092451727	#N/A
SC[Carbamidomethyl (C)]GQVVEEWKEFVWNPR.3	-0.535988626	#N/A
INAANIATDDERDKFAGR.3	-0.179126404	#N/A
TQLGDC[Carbamidomethyl (C)]SGIGDPTHYGYSTGQPC[Carbamidomethyl (C)]VFIK.3	-0.326334805	#N/A
QLS[Phospho (STY)]SGVSEIR.2	0.055104039	P14602_S86_M1_Hspb1
SPS[Phospho (STY)]WEPFR.2	0.024810895	P14602_S15_M1_Hspb1
SVFPEQANNNEWAR.2	-0.491195345	#N/A
LQLDS[Phospho (STY)]PEDAEFIVAK.2	-0.330986365	P14685_S426_M1_Psmd3
[Acetyl (Protein N-term)]ATAT[Phospho (STY)]PVQQQR.2	0.469746998	P14733_T5_M1_Lmnb1
ASAPATPLS[Phospho (STY)]PTR.2	-0.426649849	P14733_S24_M1_Lmnb1
LKLS[Phospho (STY)]PSPSSR.2	-0.0676054	P14733_S392_M1_Lmnb1
LSPS[Phospho (STY)]PSSR.2	-0.283126193	P14733_S394_M1_Lmnb1
NQNSWGTGEDVK.2	-0.008678687	#N/A
AGSRAS[Phospho (STY)]APAT[Phospho (STY)]PLSPTR.2	-0.047508833	P14733_S17_M2_Lmnb1
SEIDLLNIR.2	0.141714936	#N/A
DLESDIIGDTSGHFQK.2	-0.204636641	#N/A
TLIEILATR.2	0.305046187	#N/A

SLEDALSSDTSGHFR.2	0.11648664	#N/A
M[Oxidation (M)]LVVLLQGTR.2	-0.105019737	#N/A
ENDDVVSEDLVQQDVQDLYEAGELK.3	-0.335070594	#N/A
GIGTDEATIIDIVTHR.2	-0.363667344	#N/A
WGTDEAQFIYILGNR.2	-0.156088103	#N/A
ILISLATGNR.2	-0.080215866	#N/A
AINEAYKEDYHK.3	-0.133037969	#N/A
IAQGAMYR.2	0.281881888	#N/A
IIQLLDDYPK.2	0.252011213	#N/A
GHLENNPALEK.2	0.113962059	#N/A
TSFFQALGITTK.2	-0.116569242	#N/A
C[Carbamidomethyl (C)]FIVGADNVGSK.2	0.200003257	#N/A
AFLADPSAFAAAAPAAAATTAAPAAAAAPAK.3	0.370030101	#N/A
EDLTEIR.2	0.343700535	#N/A
AEAKEES[Phospho (STY)]EESDEDM[Oxidation (M)]GFGLFD.2	0.150621558	P14869_S304_M1_Rplp0
DLTGQVPT[Phospho (STY)]PPVK.2	-0.398867366	P14873_T527_M1_Map1b
VLS[Phospho (STY)]PLRS[Phospho (STY)]PPLLGSESPYEDFLSADSK.3	-0.392495428	P14873_S1391_M2_Map1b
SKPLAAS[Phospho (STY)]PKPGALK.2	-0.372404423	P14873_S2267_M1_Map1b
T[Phospho (STY)]PEEGGYSYEISEK.2	-0.049859981	P14873_T1945_M1_Map1b
QPGIQS[Phospho (STY)]PSR.2	0.085785661	P14873_S1073_M1_Map1b
C[Carbamidomethyl (C)]YTTEKKS[Phospho (STY)]PSEAR.3	-0.405932498	P14873_S2068_M1_Map1b
SSYYVVSGNDPAAEEPSR.2	-0.094948766	#N/A
AVLDALLEGK.2	0.073887008	#N/A
VLFPGNSTQYNILEGLEK.2	-0.300154393	#N/A
FSPEVPGQK.2	0.441912116	#N/A
T[Phospho (STY)]PEDGGYTC[Carbamidomethyl (C)]EITEK.2	-0.281582158	P14873_T1928_M1_Map1b
AIGNIELGIR.2	0.18334525	#N/A
SVGNTIEPVILFQK.2	-0.119663595	#N/A
DVSDERLS[Phospho (STY)]PAK.2	0.02573285	P14873_S1247_M1_Map1b
TPEVSGYTYEK.2	0.012368714	#N/A
QGFPDRES[Phospho (STY)]PVSDLTSTGLYQDK.3	0.359140455	P14873_S1438_M1_Map1b
TTEAAATAVGTAATTAAVVAAAGIAASGPVK.3	-0.287884968	#N/A
SWDTNLIEC[Carbamidomethyl (C)]NLDQELK.2	0.153997107	#N/A
SVNFSLTPNEIK.2	0.022759946	#N/A
DAYC[Carbamidomethyl (C)]S[Phospho (STY)]EEKELK.2	-0.096153851	P14873_S1225_M1_Map1b
SPSLSPSPPS[Phospho (STY)]PIEK.2	-0.326222283	P14873_S1260_M1_Map1b
SDIS[Phospho (STY)]PLTPR.2	-0.381728705	P14873_S1781_M1_Map1b
S[Phospho (STY)]PPLLGSESPYEDFLSADSK.2	-0.071478624	P14873_S1395_M1_Map1b
TIKS[Phospho (STY)]PC[Carbamidomethyl (C)]DSGYSYETIEK.2	-0.346134369	P14873_S1911_M1_Map1b
ESVVS[Phospho (STY)]GDDR.2	-0.225123838	P14873_S992_M1_Map1b
ADS[Phospho (STY)]RESLKPATK.3	0.101714853	P14873_S541_M1_Map1b
TIKT[Phospho (STY)]PEDGGYTC[Carbamidomethyl (C)]EITEK.2	-0.222713074	P14873_T1928_M1_Map1b
ASLTLFC[Carbamidomethyl (C)]PEEGDWK.2	-0.375786212	#N/A
KLGGDVS[Phospho (STY)]PTQIDVSQFGSFK.3	0.007918355	P14873_S1497_M1_Map1b
THDVGGYYYEK.2	-0.081488259	#N/A
SVSPGVTQAVVEEHC[Carbamidomethyl (C)]AS[Phospho (STY)]PEEK.3	-0.471920519	P14873_S1307_M1_Map1b
FEDEGAGFEESS[Phospho (STY)]ETGDYEEK.2	0.168719367	P14873_S934_M1_Map1b

SPC[Carbamidomethyl (C)]DSGYSYETIEK.2	-0.166272583	#N/A
AAEAGVTEEQYGYLGTSAK.2	-0.453811345	#N/A
HNLQDFINIK.2	0.253040129	#N/A
LSPAKS[Phospho (STY)]PS[Phospho (STY)]LSPSPPSPIEK.2	-0.241173851	P14873_S1251_M2_Map1b
TTRT[Phospho (STY)]PEEGGYSYEISEK.3	-0.005250215	P14873_T1945_M1_Map1b
EVSSKEEQS[Phospho (STY)]PVK.2	0.18438431	P14873_S614_M1_Map1b
ESS[Phospho (STY)]PLYSPGFSDSTSAAK.2	-0.297115075	P14873_S1789_M1_Map1b
ETAAAHQASS[Phospho (STY)]SPPIDAATAEPYGFR.3	-0.107734009	P14873_S1814_M1_Map1b
TTRS[Phospho (STY)]PDTSAYC[Carbamidomethyl (C)]YETM[Oxidation (M)]EK.2	-0.201377223	#N/A
RES[Phospho (STY)]VVS[Phospho (STY)]GDDR.2	-0.030617124	P14873_S989_M2_Map1b
GEAEQS[Phospho (STY)]EEEGEEEDKAEDAR.3	0.007620818	P14873_S1013_M1_Map1b
VDSILLTHIGDDNLPGINSM[Oxidation (M)]LQR.3	-0.380410963	#N/A
ASLSPM[Oxidation (M)]DEPVPDSES[Phospho (STY)]PVEK.2	-0.523675148	P14873_S1384_M1_Map1b
SQGSTSNS[Phospho (STY)]DWM[Oxidation (M)]K.2	0.499015181	P14873_S343_M1_Map1b
DLTTSS[Phospho (STY)]VEKDSGGK.2	-0.277592424	P14873_S1851_M1_Map1b
TPQASTYSYETSDR.2	-0.289269413	#N/A
TTEAAATAVGTAATTAAVVAAAGIAASGPVKELEAER.3	-0.313070375	#N/A
[Acetyl (Protein N-term)]ATVVVEATEPEPSGSIGNPAASTS[Phospho (STY)]PSLSHR.3	-0.228115168	P14873_S25_M1_Map1b
RSES[Phospho (STY)]PFEGK.2	-0.571292121	P14873_S1422_M1_Map1b
TESKPS[Phospho (STY)]VTEK.2	0.019371078	P14873_S601_M1_Map1b
SLM[Oxidation (M)]S[Phospho (STY)]S[Phospho (STY)]PEDLTK.2	0.132286059	P14873_S828_M2_Map1b
SPSLSPSPPS[Phospho (STY)]PIEKTPLGER.3	-0.377894964	P14873_S1260_M1_Map1b
LGGDVS[Phospho (STY)]PTQIDVSQFGSFK.2	-0.103684231	P14873_S1497_M1_Map1b
KES[Phospho (STY)]KEETPEVTK.2	-0.433679064	P14873_S561_M1_Map1b
MSISEGTVS[Phospho (STY)]DK.2	0.231728734	P14873_S1523_M1_Map1b
VQSLEGEKLS[Phospho (STY)]PK.2	-0.187215455	P14873_S1775_M1_Map1b
EKRES[Phospho (STY)]VVSGDDR.2	-0.041331845	P14873_S989_M1_Map1b
HM[Oxidation (M)]DPPPAPMQDRS[Phospho (STY)]PS[Phospho (STY)]PR.3	0.154287027	P14873_S2205_M2_Map1b
SSHLPTEVTEKPQAVPVSFEFSEAKDENER.4	-0.078431066	#N/A
EVSSKEEQS[Phospho (STY)]PVKAEVAEK.2	-0.142569452	P14873_S614_M1_Map1b
SRT[Phospho (STY)]PVQDHR.2	-0.087778234	P14873_T1629_M1_Map1b
LM[Oxidation (M)]ITDAAR.2	-0.359967489	#N/A
LEMYVLNPVK.2	0.176948606	#N/A
KLGGDVS[Phospho (STY)]PTQIDVSQFGSFKEDTK.3	-0.357230939	P14873_S1497_M1_Map1b
T[Phospho (STY)]PVQDHR.2	-0.368020004	P14873_T1629_M1_Map1b
NVDVEFFK.2	-0.466824622	#N/A
ITSFPESESYSYETSTK.2	0.022351916	#N/A
VQSLEGEKLS[Phospho (STY)]PKS[Phospho (STY)]DISPLTPR.3	0.155070756	P14873_S1775_M2_Map1b
EEQS[Phospho (STY)]PVKAEVAEK.2	-0.031298073	P14873_S614_M1_Map1b
S[Phospho (STY)]PDTSAYC[Carbamidomethyl (C)]YETMEK.2	0.271153951	#N/A
TSDVETM[Oxidation (M)]SSQSALALDER.2	-0.25779348	#N/A
EWYQETHEK.2	0.123598663	#N/A
SDVLETVVLINPSDEAVSTEVR.2	-0.254643722	#N/A
ESVVS[Phospho (STY)]GDDRAEEDM[Oxidation (M)]DDVLEK.3	0.119098055	P14873_S992_M1_Map1b
AEEDM[Oxidation (M)]DDVLEKGEAEQS[Phospho (STY)]EEEGEEEDKAEDAR.4	0.067256576	P14873_S1013_M1_Map1b
PMSIS[Phospho (STY)]PPDFS[Phospho (STY)]PK.2	0.108047442	P14873_S1616_M2_Map1b
QGFPDRES[Phospho (STY)]PVSDLTSTGLYQDKQEEK.4	0.269453098	P14873_S1438_M1_Map1b

AETEEAEEPEEDGEDNASGSASK.2	-0.041794583	#N/A
LGGDVS[Phospho (STY)]PTQIDVSQFGSFKEDTK.3	-0.078452313	P14873_S1497_M1_Map1b
DYNASASTIS[Phospho (STY)]PPSSMEEDKFSK.3	0.059591488	#N/A
DVM[Oxidation (M)]SDETNNEETES[Phospho (STY)]PSQEFVNITK.3	-0.446208269	P14873_S1151_M1_Map1b
IAELEEERS[Phospho (STY)]QGSTSNSDWM[Oxidation (M)]K.3	-0.056756118	P14873_S336_M1_Map1b
SSM[Oxidation (M)]LFDTM[Oxidation (M)]QHHLALNR.3	-0.12352268	#N/A
ASAELDIKDVS[Phospho (STY)]DER.2	0.161754531	P14873_S1242_M1_Map1b
GEAEQS[Phospho (STY)]EEEGEEEDK.2	0.433265517	P14873_S1013_M1_Map1b
TAKS[Phospho (STY)]RT[Phospho (STY)]PVQDHR.3	-0.14589978	P14873_S1627_M2_Map1b
ASAELDIKDVS[Phospho (STY)]DERLSPAK.3	-0.150043283	P14873_S1242_M1_Map1b
DENERAS[Phospho (STY)]LS[Phospho (STY)]PM[Oxidation (M)]DEPVPDSESPVEK.3	-0.320244485	P14873_S1371_M2_Map1b
ELEAERS[Phospho (STY)]LM[Oxidation (M)]S[Phospho (STY)]S[Phospho (STY)]PEDLTK.2	0.156649897	P14873_S825_M3_Map1b
TDATDGKDYNASASTIS[Phospho (STY)]PPSSM[Oxidation (M)]EEDKFSK.4	-0.122529365	#N/A
QQDLNIM[Oxidation (M)]VLASSSTVVM[Oxidation (M)]QDESFPAC[Carbamidomethyl (C)]K.3	0.446765267	#N/A
SEQSSM[Oxidation (M)]SIEFGQES[Phospho (STY)]PEHSLAMDFSR.3	0.165877313	P14873_S1649_M1_Map1b
KIAELEEERS[Phospho (STY)]QGSTSNSDWM[Oxidation (M)]K.3	-0.041865486	P14873_S336_M1_Map1b
RES[Phospho (STY)]VVS[Phospho (STY)]GDDRAEEDMDDVLEK.3	0.024970367	P14873_S989_M2_Map1b
DIKPQLELIEDEEK.3	-0.44547692	#N/A
NLISPDLGVVFLNVPENLKDPEPNIK.3	-0.299982253	#N/A
AQWGSNM[Oxidation (M)]QVTLIPTHDSEVM[Oxidation (M)]R.3	0.030728706	#N/A
EEC[Carbamidomethyl (C)]PRPMSIS[Phospho (STY)]PPDFSPK.3	0.013054621	P14873_S1616_M1_Map1b
TLEVVSPSQSVTGSAGHTPYYQSPTDEK.3	0.069041774	#N/A
SDISPLTPRES[Phospho (STY)]S[Phospho (STY)]PLYSPGFSDSTSAAK.3	-0.021588517	P14873_S1788_M2_Map1b
HSPTEDDES[Phospho (STY)]AKAEADVHLK.3	0.187906268	P14873_S974_M1_Map1b
DIVEAHYR.2	0.069459316	#N/A
[Acetyl (Protein N-term)]ATSASSHLNK.2	0.151849192	#N/A
KPAETNLR.2	0.216179231	#N/A
TC[Carbamidomethyl (C)]LLNETGDEPFQYKN.2	-0.300892337	#N/A
LVLC[Carbamidomethyl (C)]EVFK.2	0.349369432	#N/A
TLDC[Carbamidomethyl (C)]EPK.2	0.199135106	#N/A
TC[Carbamidomethyl (C)]LLNETGDEPFQYK.2	-0.049868064	#N/A
KGYFEDR.2	-0.012775412	#N/A
AC[Carbamidomethyl (C)]LYAGVK.2	0.199215814	#N/A
C[Carbamidomethyl (C)]IEEAIDK.2	-0.074280235	#N/A
M[Oxidation (M)]GDHLWIAR.2	-0.251025648	#N/A
VQAM[Oxidation (M)]YIWVDGTGEGLR.2	-0.178988572	#N/A
ITGTNAEVM[Oxidation (M)]PAQWEFQIGPC[Carbamidomethyl (C)]EGIR.3	-0.514778621	#N/A
QM[Oxidation (M)]YM[Oxidation (M)]SLPQGEK.2	-0.287246094	#N/A
Y[Phospho (STY)]NRKPAETNLR.3	-0.301609578	P15105_Y104_M1_Glul
RPSANC[Carbamidomethyl (C)]DPYAVTEAIVR.3	0.311511777	#N/A
AMREENGLK.2	-0.203322847	#N/A
C[Carbamidomethyl (C)]IEEAIDKLSK.2	-0.123473742	#N/A
RLTGFHETSNINDFSAGVANR.4	-0.04515052	#N/A
LTGFHETSNINDFSAGVANR.3	0.409490617	#N/A
VC[Carbamidomethyl (C)]EDFGVIATFDPK.2	-0.078728827	#N/A
SAAPHPGDIGDFINEGLK.3	-0.178590158	#N/A
IWC[Carbamidomethyl (C)]TEPSPGIVAFPR.2	0.173632159	#N/A

LLGSGS[Phospho (STY)]PSSSAR.2	0.284597574	P15331_S57_M1_Prph
LAWLAEPDAHS[Phospho (STY)]HFDYDPR.3	-0.242728921	P15388_S44_M1_Kcnc1
HIPRPPQLGS[Phospho (STY)]PNYC[Carbamidomethyl (C)]K.3	-0.092858335	P15388_S468_M1_Kcnc1
ILS[Phospho (STY)]PVDSGNK.2	0.105620245	P15508_S1218_M1_Sptb
LLTS[Phospho (STY)]PDVSYDEAR.2	0.083797177	P15508_S1289_M1_Sptb
NIIHGSDSVK.2	-0.44585307	#N/A
FLQASEDLLK.2	0.244383359	#N/A
YM[Oxidation (M)]HSGPVVAM[Oxidation (M)]VWEGLNVVK.3	-0.210173804	#N/A
FLQASEDLLKEHYTDLK.3	-0.063936005	#N/A
EISLWFQPEELVEYK.2	-0.11025356	#N/A
SC[Carbamidomethyl (C)]AQNWIYE.2	0.035087124	#N/A
[Acetyl (Protein N-term)]S[Phospho (STY)]EAAPAAPAAAPPAEK.2	-0.134074923	P15864_S2_M1_H1-2
ALAAAGYDVEK.2	0.106998481	#N/A
GTGASGSFK.2	-0.131875561	#N/A
ASGPPVSELITK.2	-0.055868819	#N/A
[Acetyl (Protein N-term)]S[Phospho (STY)]EAAPAAPAAAPPAEKAPAK.2	-0.539504927	P15864_S2_M1_H1-2
ALAAAGYDVEKNNSR.2	0.297361119	#N/A
KDS[Phospho (STY)]EEEVSLLGNQDIEEGNSR.3	0.056459183	P15920_S695_M1_Atp6v0a2
IYHS[Phospho (STY)]EER.2	-0.082730033	P16014_S155_M1_Chgb
WASS[Phospho (STY)]REDAGAPVEDSQGQTK.3	0.005199346	P16014_S100_M1_Chgb
SSYEGHPLS[Phospho (STY)]EER.2	0.074194155	P16014_S308_M1_Chgb
WWQQEEQLGPEES[Phospho (STY)]REEVRFPDR.4	-0.054813362	P16014_S493_M1_Chgb
ES[Phospho (STY)]KEADVATVR.2	0.27745548	P16014_S318_M1_Chgb
EGVDDQESLRPSNQQAS[Phospho (STY)]KEAK.3	-0.34890696	P16014_S147_M1_Chgb
AS[Phospho (STY)]EEEPEYGEESR.2	-0.321228757	P16014_S342_M1_Chgb
WTEGGGHS[Phospho (STY)]R.2	-0.066489103	P16014_S129_M1_Chgb
EGVDDQESLRPSNQQAS[Phospho (STY)]K.3	-0.368528715	P16014_S147_M1_Chgb
KHIEDS[Phospho (STY)]GEKPNTFSNK.3	-0.432341463	P16014_S190_M1_Chgb
WWQQEEQLGPEES[Phospho (STY)]REEVR.3	0.163563187	P16014_S493_M1_Chgb
SGSNKSS[Phospho (STY)]YEGHPLSEER.3	0.161002239	P16014_S301_M1_Chgb
S[Phospho (STY)]HHLAHYRASEEEPEYGEESR.4	-0.126978586	P16014_S333_M1_Chgb
GRGS[Phospho (STY)]EEDRAPR.2	0.217914081	P16014_S365_M1_Chgb
EDAGAPVEDSQGQTK.2	-0.312907139	#N/A
WTEGGGHS[Phospho (STY)]REGVDDQESLRPSNQQASK.4	-0.133198123	P16014_S129_M1_Chgb
SSYEGHPLS[Phospho (STY)]EERRPSPK.3	0.355963586	P16014_S308_M1_Chgb
ADAHS[Phospho (STY)]M[Oxidation (M)]ELEEK.2	0.180043797	P16014_S220_M1_Chgb
SEES[Phospho (STY)]QEREYKR.3	-0.135621619	P16014_S378_M1_Chgb
ADAHS[Phospho (STY)]MELEEKTHS[Phospho (STY)]R.2	-0.399344219	P16014_S220_M2_Chgb
HIEDS[Phospho (STY)]GEKPNTFSNK.3	0.025052607	P16014_S190_M1_Chgb
ADEFPDFY[Phospho (STY)]DS[Phospho (STY)]EEQM[Oxidation (M)]GPHQEANDEK.3	0.423546623	P16014_S626_M2_Chgb
LAAGAESPQPASGNS[Phospho (STY)]PSEDDR.2	-0.09153883	P16054_S337_M1_Prkce
SAPTS[Phospho (STY)]PC[Carbamidomethyl (C)]DQELK.2	0.608997709	P16054_S350_M1_Prkce
ALS[Phospho (STY)]FDNRGEEHR.3	-0.34302582	P16054_S368_M1_Prkce
KALS[Phospho (STY)]FDNR.2	-0.157207776	P16054_S368_M1_Prkce
ASSATDGQLAS[Phospho (STY)]PGENGEVRPGQAK.3	-0.268743451	P16054_S388_M1_Prkce
KQET[Phospho (STY)]PDEVGSQR.2	-0.350478663	P16054_T228_M1_Prkce
SAPTS[Phospho (STY)]PC[Carbamidomethyl (C)]DQELKELENNIRK.3	0.098011066	P16054_S350_M1_Prkce

ALS[Phospho (STY)]FDNR.2	-0.494578956	P16054_S368_M1_Prkce
SKS[Phospho (STY)]APTS[Phospho (STY)]PC[Carbamidomethyl (C)]DQELK.2	-0.02905793	#N/A
KLAAGAESPQPASGNS[Phospho (STY)]PSEDDR.3	-0.014737588	P16054_S337_M1_Prkce
LAAGAESPQPASGNS[Phospho (STY)]PSEDDRSK.3	-0.385014181	P16054_S337_M1_Prkce
KALS[Phospho (STY)]FDNRGEEHR.3	-0.242368586	P16054_S368_M1_Prkce
SAPTS[Phospho (STY)]PC[Carbamidomethyl (C)]DQELKELENNIR.3	-0.173239778	P16054_S350_M1_Prkce
ASSATDGQLAS[Phospho (STY)]PGENGEVRPGQAKR.3	-0.217781912	P16054_S388_M1_Prkce
DVILQDDDVDC[Carbamidomethyl (C)]TMTEK.2	-0.353589305	#N/A
SKS[Phospho (STY)]APTS[Phospho (STY)]PC[Carbamidomethyl (C)]DQELKELENNIR.3	0.191625962	#N/A
LIASVADDEAAVPNNK.2	-0.179305812	#N/A
FIIPQIVK.2	0.109744019	#N/A
LKDDEVAQLR.2	0.037534877	#N/A
IVVVTAGVR.2	0.316951883	#N/A
SADTLWDIQK.2	-0.321075361	#N/A
GLTSVINQK.2	0.021360061	#N/A
LKDDEVAQLRK.3	-0.460071351	#N/A
SLADELALVDVLEDK.2	0.126681588	#N/A
EKLIASVADDEAAVPNNK.3	-0.302960808	#N/A
KSADTLWDIQK.2	-0.244894034	#N/A
M[Oxidation (M)]VVDSAYEVIK.2	-0.137522053	#N/A
IHPVSTM[Oxidation (M)]VK.2	-0.086994701	#N/A
IVADKDYSVTANSK.2	-0.063143362	#N/A
SLADELALVDVLEDKLK.2	-0.066598302	#N/A
ITVVGVGQVGM[Oxidation (M)]AC[Carbamidomethyl (C)]AISILGK.2	-0.294230151	#N/A
GEM[Oxidation (M)]M[Oxidation (M)]DLQHGSLFLQTPK.3	-0.164238424	#N/A
GM[Oxidation (M)]YGIENEVFLSLPC[Carbamidomethyl (C)]ILNAR.3	0.154019498	#N/A
KSS[Phospho (STY)]VEGLEPAENK.2	-0.321062655	P16254_S45_M1_Srp14
ALPSVGLQSDQS[Phospho (STY)]PQR.2	-0.273477539	P16283_S198_M1_Slc4a3
SYS[Phospho (STY)]ERDFEFHR.3	0.319533562	P16283_S67_M1_Slc4a3
FSIGS[Phospho (STY)]DEDDSPGLPVK.2	0.007013202	P16283_S170_M1_Slc4a3
AKFSIGS[Phospho (STY)]DEDDSPGLPVK.2	0.04282361	P16283_S170_M1_Slc4a3
SRPWS[Phospho (STY)]PSASYDLR.2	-0.097255046	P16283_S224_M1_Slc4a3
GS[Phospho (STY)]PSGLAPILR.2	0.029362481	P16283_S296_M1_Slc4a3
IQGGRGS[Phospho (STY)]PSGLAPILR.3	-0.44980638	P16283_S296_M1_Slc4a3
NQWQLS[Phospho (STY)]ADDLKK.2	-0.20472733	P16330_S169_M1_Cnp
HFISGDEPK.2	0.208487841	#N/A
KS[Phospho (STY)]HTFLPK.2	0.451136049	P16330_S9_M1_Cnp
LDEDLAGYC[Carbamidomethyl (C)]R.2	0.009666734	#N/A
ADFSEAYKR.2	0.110594854	#N/A
LSISALFVTPK.2	0.291848099	#N/A
AAGAEEYAQQEVVK.2	0.031127726	#N/A
AAGAEEYAQQEVVKR.2	-0.027540622	#N/A
KAGQVFLEELGNHK.3	-0.18676219	#N/A
AIFTGYYGK.2	-0.174068553	#N/A
RPPGVLHC[Carbamidomethyl (C)]TTK.3	0.351299768	#N/A
EKPELQFPFLQDEDTVATLHEC[Carbamidomethyl (C)]K.4	-0.183707013	#N/A
LDC[Carbamidomethyl (C)]AQLK.2	0.0725519	#N/A

GKLY[Phospho (STY)]SLGK.2	0.242075542	P16330_Y372_M1_Cnp
GGSQGEAVGELPR.2	-0.160677932	#N/A
VLVLDDTNHER.2	0.047205026	#N/A
[Acetyl (Protein N-term)]M[Oxidation (M)]NTS[Phospho (STY)]FTRK.2	0.083603745	P16330_S4_M1_Cnp
NQWQLSADDLK.2	0.053571516	#N/A
EKNQWQLSADDLKK.3	0.085893339	#N/A
S[Phospho (STY)]HTFLPK.2	0.257268618	P16330_S9_M1_Cnp
RLDEDLAGYC[Carbamidomethyl (C)]R.3	0.178574088	#N/A
AGQVFLEELGNHK.2	-0.05061268	#N/A
AHVTLGC[Carbamidomethyl (C)]AADVQPVQTGLDLLDILQQVK.3	-0.249346336	#N/A
EKLELVSYFGK.2	-0.004468717	#N/A
ADFSEAYK.2	0.019514748	#N/A
SSETLRK.2	0.103030697	#N/A
VLVLDDTNHERER.3	-0.175344067	#N/A
HFISGDEPKEK.2	0.030217396	#N/A
M[Oxidation (M)]VSADAYK.2	-0.183471139	#N/A
LELVSYFGK.2	0.345181603	#N/A
LDQLFEM[Oxidation (M)]ADQYQYQVVLVEPK.3	-0.412663557	#N/A
[Acetyl (Protein N-term)]M[Oxidation (M)]NTS[Phospho (STY)]FTR.2	0.161754613	P16330_S4_M1_Cnp
EKNQWQLSADDLK.3	0.271627907	#N/A
ERLDQLFEM[Oxidation (M)]ADQYQYQVVLVEPK.3	-0.604926965	#N/A
IDIVENR.2	0.252010158	#N/A
RQVEIAQR.2	0.489002317	#N/A
TTSLELFM[Oxidation (M)]YLNEVAGK.2	-0.093190342	#N/A
S[Phospho (STY)]LQQLAEER.2	-0.228029528	P16546_S1217_M1_Sptan1
KLDPAQSAS[Phospho (STY)]R.2	0.147443583	P16546_S1031_M1_Sptan1
EANQQQQFNR.2	-0.153070653	#N/A
EKEPIAASTNR.2	0.021654361	#N/A
DLTGVQNLR.2	0.254774658	#N/A
LSDDNTIGQEEIQQR.2	-0.354303627	#N/A
SSEEIESAFR.2	0.121839432	#N/A
ELPTAFDYVEFTR.2	0.18574024	#N/A
LFGAAEVQR.2	0.000415584	#N/A
LLVSSEDYGR.2	-0.419907979	#N/A
EELYQNLTR.2	0.030905405	#N/A
LIQEQHPEEELIK.2	0.126236865	#N/A
ADVVESWIGEK.2	0.035848894	#N/A
WTQLLANSATR.2	-0.139709112	#N/A
EANELQQWITEK.2	0.122729617	#N/A
DLAALEDKVK.2	0.032227742	#N/A
DVEDEETWIR.2	-0.440003656	#N/A
DLASVQALLR.2	0.268187529	#N/A
DLSSVQTLLTK.2	0.003227952	#N/A
ITALDEFATK.2	-0.112319249	#N/A
VLETAEDIQER.2	0.072461594	#N/A
WRS[Phospho (STY)]LQQLAEER.3	-0.360333369	P16546_S1217_M1_Sptan1
TATDEAYKDPSNLQGK.2	-0.141337092	#N/A

GLVSSDELAK.2	0.354321635	#N/A
LLEAQSHFR.2	0.059411216	#N/A
KFEEFQTDLAAHEER.3	-0.216201043	#N/A
IDGITIQAR.2	0.338056365	#N/A
LIDVNHYAK.2	0.250753469	#N/A
VNEVSQFAAK.2	0.589656678	#N/A
REELITNWEQIR.3	-0.412869543	#N/A
VNSLGETAQR.2	0.058199895	#N/A
QKLEDSYR.2	-0.135726461	#N/A
LAQFVEHWK.2	0.015369818	#N/A
DVDEIEAWISEK.2	0.060183446	#N/A
LDPAQSAS[Phospho (STY)]RENLLEEQGSIALR.3	0.347735685	P16546_S1031_M1_Sptan1
DVDETIGWIK.2	-0.002754893	#N/A
NTTGVTEEALK.2	0.074469895	#N/A
M[Oxidation (M)]NEVISLWK.2	-0.324311526	#N/A
LSILSEER.2	0.063448305	#N/A
LGESQTLQQFSR.2	-0.405564504	#N/A
LDENSAFLQFNWK.2	-0.202379948	#N/A
QDEVNAAWQR.2	0.314904553	#N/A
DLTNVQNLQK.2	0.038344948	#N/A
KLLEATELK.2	0.389195969	#N/A
EELITNWEQIR.2	0.086531869	#N/A
KVEDLFLTFAK.2	-0.116055005	#N/A
LGDSHDLQR.2	0.175356937	#N/A
KFDDFQK.2	0.115352125	#N/A
LLEATELK.2	-0.178603263	#N/A
QWELLLEK.2	0.368705728	#N/A
VNDVC[Carbamidomethyl (C)]TNGQDLIK.2	0.084092888	#N/A
DLM[Oxidation (M)]SWINGIR.2	0.063355902	#N/A
C[Carbamidomethyl (C)]TELNQAWTSLGK.2	0.235138517	#N/A
LAALADQWQFLVQK.2	-0.128287717	#N/A
IAALQAFADQLIAVDHYAK.3	0.046460374	#N/A
KLSDDNTIGQEEIQQR.3	0.034758547	#N/A
QVEELYQSLLELGEK.2	0.396701278	#N/A
DVTGAEALLER.2	-0.413474805	#N/A
EAIVTSEELGQDLEHVEVLQK.3	-0.257167001	#N/A
HQALQAEIAGHEPR.3	0.24855743	#N/A
LQQSHPLSASQIQVK.2	0.375062638	#N/A
ENLLEEQGSIALR.2	0.207035138	#N/A
EAHDAFR.2	0.125863881	#N/A
TALLELWELR.2	0.178002643	#N/A
RQQVLDR.2	-0.065977899	#N/A
TALLELWELRR.2	0.165393327	#N/A
LKDLNSQADSLM[Oxidation (M)]TSSAFDTSQVK.3	-0.319898334	#N/A
DAEELEKWIQEK.2	-0.051789828	#N/A
C[Carbamidomethyl (C)]NSLEEIK.2	0.225907881	#N/A
KHEGFER.2	-0.078781992	#N/A

VAEDLESEGLM[Oxidation (M)]AEEVQAVQQQEVYGAM[Oxidation (M)]PR.3	-0.602046567	#N/A
ALINADELANDVAGAEALLDR.2	-0.41350577	#N/A
LNHQEFK.2	0.248765243	#N/A
M[Oxidation (M)]M[Oxidation (M)]LDHC[Carbamidomethyl (C)]LELQLFHR.3	-0.420033429	#N/A
GEIDAHEDSFK.2	0.137600497	#N/A
DTEQVDNWM[Oxidation (M)]SK.2	-0.23758782	#N/A
GVIDM[Oxidation (M)]GNSLIER.2	0.246832277	#N/A
DLAALGDKVNSLGETAQR.3	-0.011074433	#N/A
AQLADSFHLQQFFR.3	0.189502697	#N/A
QQVAPM[Oxidation (M)]DDETGKELVLALYDYQEKS[Phospho (STY)]PR.3	0.040765877	P16546_S982_M1_Sptan1
SADESGQALLAASHYASDEVR.3	-0.264841922	#N/A
DLIGVQNLLK.2	0.449845777	#N/A
DC[Carbamidomethyl (C)]EQAENWM[Oxidation (M)]AAR.2	0.577753892	#N/A
DSDELKSWVNEK.2	-0.28972127	#N/A
AINVQEEK.2	0.385580797	#N/A
SSLSSAQADFNQLAELDR.2	-0.316934727	#N/A
GLVSSDELAKDVTGAEALLER.3	-0.330534417	#N/A
ASAFNSWFENAEEDLTDPVR.2	-0.114815265	#N/A
LIQSHPESAEDLKEK.3	0.279224465	#N/A
DM[Oxidation (M)]DDEESWIK.2	0.251753829	#N/A
EKLDILDQERTDLEK.3	-0.212409571	#N/A
M[Oxidation (M)]QHNLEQQIQAR.3	0.057006698	#N/A
DLNSQADSLM[Oxidation (M)]TSSAFDTSQVK.2	-0.22089691	#N/A
DLTSWVTEMK.2	0.388432128	#N/A
M[Oxidation (M)]TLVASEDYGDTLAAIQGLLK.2	-0.464769888	#N/A
ADVVESWIGEKENSLK.2	0.028972886	#N/A
QQNFNTGIK.2	0.380398411	#N/A
YTEHSTVGLAQQWDQLDQLGM[Oxidation (M)]R.3	-0.568546864	#N/A
LDETGNLM[Oxidation (M)]ISEGHFASETIR.3	-0.265029288	#N/A
RNEVLDR.2	0.043923866	#N/A
KLLVSSEDYGR.2	0.106935392	#N/A
EC[Carbamidomethyl (C)]EDVM[Oxidation (M)]DWINDK.2	-0.353306393	#N/A
EAFLNTEDKGDSLDSVEALIK.3	-0.036145704	#N/A
KLDPAQSAS[Phospho (STY)]RENLLEEQGSIALR.3	-0.262756024	P16546_S1031_M1_Sptan1
ELVLALYDYQEKS[Phospho (STY)]PR.2	0.328105546	P16546_S982_M1_Sptan1
SLGYDLPM[Oxidation (M)]VEEGEPDPEFEAILDTVDPNR.3	0.229955149	#N/A
DEADS[Phospho (STY)]KTAS[Phospho (STY)]PWK.2	-0.418291667	P16546_S1186_M2_Sptan1
DLAALEDK.2	0.082374784	#N/A
LQVASDENYKDPTNLQGK.3	0.13030929	#N/A
YEALKEPM[Oxidation (M)]VAR.2	0.164837422	#N/A
EAALTNEEVGADLEQVEVLQK.2	0.194706489	#N/A
SQLLGSAHEVQR.2	0.387125405	#N/A
EKEPIVGSTDYGKDEDSAEALLK.3	-0.090074913	#N/A
QQVAPM[Oxidation (M)]DDETGKELVLALYDYQEK.3	-0.47283367	#N/A
TYLLDGSC[Carbamidomethyl (C)]MVEESGTLESQLEATK.3	0.290870064	#N/A
NQALNTDNYGHDLASVQALQR.3	-0.483738278	#N/A
NNHHEENISSK.2	-0.249777975	#N/A

EKLDILDQER.2	-0.174372269	#N/A
DADETKEWIEEK.2	-0.025017597	#N/A
HQEHKGEIDAHEDSFK.4	-0.196979508	#N/A
QFQDAGHFDAENIKK.3	-0.089279538	#N/A
KIEDLGAAM[Oxidation (M)]EEALILDNK.3	-0.283807027	#N/A
TKQDEVNAAWQR.3	-0.254303842	#N/A
QEFAQHANAFHQWIQETR.3	-0.36478204	#N/A
EKLSILSEER.2	-0.166059986	#N/A
SADESGQALLAASHYASDEVREK.3	-0.40548364	#N/A
GNAM[Oxidation (M)]VEEGHFAAEDVK.2	-0.206801635	#N/A
EQLMASDDFGR.2	0.399477231	#N/A
QETFDAGLQAFQQEGIANITALK.3	0.275194124	#N/A
HALLEADVAAHQDR.3	-0.46051854	#N/A
HQLLEADISAHEDR.3	-0.41341622	#N/A
LIQNNHYAM[Oxidation (M)]EDVATR.3	0.336647221	#N/A
KHEAFETDFTVHK.3	-0.186472072	#N/A
AKLGDSHDLQR.3	0.031620088	#N/A
DMDDEESWIKEK.2	0.19926397	#N/A
LM[Oxidation (M)]VHTVATFNSIK.2	0.392123991	#N/A
AGTFQAFEQFGQQLLAHGHYASPEIK.3	0.144223081	#N/A
HQAFEAELHANADR.3	0.017230594	#N/A
LIQNNHYAMEDVATRR.3	-0.043492339	#N/A
HEAFETDFTVHK.3	-0.020546401	#N/A
QETFDAGLQAFQQEGIANITALKDQLLAAK.3	0.177472734	#N/A
EKEQLMASDDFGR.3	-0.23273928	#N/A
IEDLGAAMEEALILDNK.2	0.296504051	#N/A
RQDLEDSLQAQQYFADANEAESWM[Oxidation (M)]R.3	-0.395439506	#N/A
KHEAFETDFTVHKDR.4	-0.453099341	#N/A
GAAQNIIPASTGAAK.2	-0.081114681	#N/A
VPTPNVSVVDLTC[Carbamidomethyl (C)]R.2	0.014746617	#N/A
VIPELNGK.2	0.118015334	#N/A
LTGM[Oxidation (M)]AFR.2	-0.041408787	#N/A
VGVNGFGR.2	0.158068324	#N/A
LVINGKPITIFQER.3	0.066769796	#N/A
PITIFQER.2	0.315648498	#N/A
VVKQASEGPLK.2	0.365988943	#N/A
VIISAPSADAPM[Oxidation (M)]FVM[Oxidation (M)]GVNHEKYDNSLK.4	-0.455071528	#N/A
PITIFQERDPTNIK.3	-0.081325634	#N/A
WGEAGAEYVVESTGVFTTM[Oxidation (M)]EK.2	-0.305712691	#N/A
VIISAPSADAPM[Oxidation (M)]FVM[Oxidation (M)]GVNHEK.3	0.163071507	#N/A
RVIISAPSADAPM[Oxidation (M)]FVM[Oxidation (M)]GVNHEK.3	-0.387202194	#N/A
LISWYDNEYGYSNR.2	-0.476029343	#N/A
DPTNIKWGEAGAEYVVESTGVFTTM[Oxidation (M)]EK.3	-0.365940565	#N/A
VVDLM[Oxidation (M)]AYM[Oxidation (M)]ASK.2	0.223495254	#N/A
VVDLM[Oxidation (M)]AYM[Oxidation (M)]ASKE.2	0.062415089	#N/A
QASEGPLK.2	-0.159680603	#N/A
LEKPAKYDDIK.3	-0.26937198	#N/A

IVSNASC[Carbamidomethyl (C)]TTNC[Carbamidomethyl (C)]LAPLAK.2	0.0524072	#N/A
TVDGPSGKLWR.2	-0.227504529	#N/A
VIHDNFGIVEGLM[Oxidation (M)]TTVHAITATQK.4	-0.172189367	#N/A
FNGTVKAENGK.2	0.279595382	#N/A
VEIVAINDPFIDLNYMVYMFQYDSTHGK.3	-0.048166476	#N/A
NDSVIVADQTPT[Phospho (STY)]PTR.2	-0.155020986	P16951_T53_M1_Atf2
SKIEEPSVVETTHQDS[Phospho (STY)]PLPHPESTTSDEK.4	-0.158621579	P16951_S118_M1_Atf2
[Acetyl (Protein N-term)]SESGS[Phospho (STY)]KSSQPLASK.2	-0.123026824	P17095_S6_M1_Hmga1
KLEKEEEEGISQES[Phospho (STY)]S[Phospho (STY)]EEEQ.2	-0.191292202	P17095_S102_M2_Hmga1
IHSEEQS[Phospho (STY)]EEED.2	-0.298793699	P17141_S590_M1_Zfp37
DAGTITGLNVLR.2	0.070302329	#N/A
LYQGGPGGGGSSGGPTIEEVD.2	0.146097671	#N/A
QTQTFTTYSDNQSSVLVQVYEGER.3	0.04162889	#N/A
YITPDQLADLYK.2	0.014701404	#N/A
GVSQAVEHINK.2	0.174886075	#N/A
GNPTVEVDLYTAK.2	-0.094735861	#N/A
TIAPALVSK.2	0.122272909	#N/A
SC[Carbamidomethyl (C)]NC[Carbamidomethyl (C)]LLLK.2	0.252890732	#N/A
IGAEVYHNLK.2	0.112640961	#N/A
KVNVVEQEK.2	0.171761833	#N/A
IEEELGSK.2	-0.069652591	#N/A
DATNVGDEGGFAPNILENKEALELLK.3	-0.281380117	#N/A
VNVVEQEK.2	0.138644003	#N/A
HIADLAGNPEVILPVPAFNVINGGSHAGNK.4	0.008189601	#N/A
AAVPSGASTGIYEALELRDNDK.3	-0.281563731	#N/A
SGKYDLDFK.2	0.110722703	#N/A
KVNVVEQEKIDK.2	0.1968287	#N/A
IEEELGSKAK.2	0.407050873	#N/A
DATNVGDEGGFAPNILENK.2	-0.413958984	#N/A
LAM[Oxidation (M)]QEFM[Oxidation (M)]ILPVGASSFR.2	-0.320230484	#N/A
YGKDATNVGDEGGFAPNILENK.3	-0.146517097	#N/A
FGANAILGVSLAVC[Carbamidomethyl (C)]K.2	0.357352912	#N/A
SGKYDLDFKS[Phospho (STY)]PDDPSR.3	-0.382790453	P17182_S263_M1_Eno1
LM[Oxidation (M)]IEM[Oxidation (M)]DGTENK.2	-0.369820868	#N/A
AAVPSGASTGIYEALELR.2	-0.152513943	#N/A
YDLDFKS[Phospho (STY)]PDDPSR.2	0.032499178	P17182_S263_M1_Eno1
SGETEDTFIADLVVGLC[Carbamidomethyl (C)]TGQIK.2	-0.018986428	#N/A
FTASAGIQVVGDDLTVTNPK.2	-0.395870725	#N/A
SFVQNYPVVSIEDPFDQDDWGAWQK.3	-0.28518357	#N/A
VNVVEQEKIDK.2	0.372001555	#N/A
LAQSNGWGVM[Oxidation (M)]VSHR.2	0.078548229	#N/A
FTASAGIQVVGDDLTVTNPKR.3	-0.054558201	#N/A
AGYTDQVVIGM[Oxidation (M)]DVAASEFYR.2	-0.087952469	#N/A
IDKLMIEM[Oxidation (M)]DGTENK.3	0.250087361	#N/A
EALELLK.2	0.109128486	#N/A
IEEELGDEAR.2	0.131412162	#N/A
VNQIGSVTEAIQAC[Carbamidomethyl (C)]K.2	0.029296459	#N/A

AVDHINSR.2	0.195085369	#N/A
YITGDQLGALYQDFVR.2	-0.201741826	#N/A
DGKYDLDFK.2	-0.299042296	#N/A
AC[Carbamidomethyl (C)]NC[Carbamidomethyl (C)]LLLK.2	0.374801542	#N/A
LGAEVYHTLK.2	0.069842506	#N/A
DATNVGDEGGFAPNILENSEALELVK.3	-0.109872265	#N/A
FAGHNFR.2	0.243768818	#N/A
LDNLM[Oxidation (M)]LELDGTENK.2	-0.018690696	#N/A
DKYGKDATNVGDEGGFAPNILENSEALELVK.4	-0.086325538	#N/A
[Acetyl (Protein N-term)]S[Phospho (STY)]IEKIWAR.2	-0.161360044	P17183_S2_M1_Eno2
DGKYDLDFKS[Phospho (STY)]PADPSR.3	-0.15690057	P17183_S263_M1_Eno2
IAPALISSGISVVEQEK.2	0.007414674	#N/A
HIAQLAGNSDLILPVPAFNVINGGSHAGNK.3	0.172077919	#N/A
M[Oxidation (M)]VIGM[Oxidation (M)]DVAASEFYR.2	-0.420610177	#N/A
LAM[Oxidation (M)]QEFM[Oxidation (M)]ILPVGAESFR.3	-0.065178905	#N/A
YDLDFKS[Phospho (STY)]PADPSR.2	-0.197472189	P17183_S263_M1_Eno2
IAPALISSGISVVEQEKLDNLM[Oxidation (M)]LELDGTENK.4	-0.220199563	#N/A
FTANVGIQIVGDDLTVTNPK.2	-0.351748255	#N/A
LAQENGWGVM[Oxidation (M)]VSHR.3	-0.305015786	#N/A
NYPVVSIEDPFDQDDWAAWSK.3	0.378362588	#N/A
NADVELQQR.2	0.084210991	#N/A
DFLTPPLLSVR.2	0.070364426	#N/A
LLQC[Carbamidomethyl (C)]YPPPEDAAVK.2	-0.121547848	#N/A
GLAVFISDIR.2	-0.12091552	#N/A
QLSLPLQEAQK.2	0.163154822	#N/A
HLC[Carbamidomethyl (C)]ELLAQQF.2	0.068518311	#N/A
QSAALC[Carbamidomethyl (C)]LLR.2	0.183625791	#N/A
ATIQGVLR.2	0.051454532	#N/A
LVEC[Carbamidomethyl (C)]LETVLNK.2	-0.075488824	#N/A
NSGVLFENQLLQIGVK.2	-0.334213375	#N/A
THIDTVINALK.2	-0.133887975	#N/A
YGGTAQSLTLK.2	0.232684601	#N/A
IAGDYVSEEVWYR.2	-0.131506643	#N/A
TSKEPVSR.2	-0.232624837	#N/A
LLGFGSALLDNVDPNPENFVGAGIIQTK.3	0.232926456	#N/A
AC[Carbamidomethyl (C)]NQLGQFLQHR.3	-0.055617515	#N/A
VGGYILGEFGNLIAGDPR.2	0.084863928	#N/A
AADLLYAM[Oxidation (M)]C[Carbamidomethyl (C)]DR.2	-0.266490778	#N/A
FFQPTEM[Oxidation (M)]AAQDFFQR.2	-0.042812633	#N/A
ILVAGDSMDSVK.2	0.121845617	#N/A
ANHPMDAEVTK.2	0.401688612	#N/A
YLALESM[Oxidation (M)]C[Carbamidomethyl (C)]TLASSEFSHEAVK.3	-0.471830676	#N/A
TSVQFQNFLPTVVHPGDLQTQLAVQTK.3	0.173627002	#N/A
ASPDLVPMGEWTAR.2	0.436480343	#N/A
VAAQVDGGAQVQQVLNIEC[Carbamidomethyl (C)]LR.2	-0.008053401	#N/A
LEPNAQAQMYR.2	0.258655114	#N/A
AVEYLTLSSVASTDVLATVLEEM[Oxidation (M)]PPFPER.3	-0.498846432	#N/A

TTQIGC[Carbamidomethyl (C)]LLR.2	0.16223974	#N/A
LLQC[Carbamidomethyl (C)]YPPPDPAVR.2	0.171397443	#N/A
ATIQDVLR.2	0.044368173	#N/A
QLSNPQQEVQNIFK.2	0.106114068	#N/A
SSPLIQFNLLHSK.2	-0.029728584	#N/A
KGPSTVTDLEETKR.3	-0.126158398	#N/A
LTEC[Carbamidomethyl (C)]LETILNK.2	-0.148625164	#N/A
FFQPTEMASQDFFQR.2	-0.303060664	#N/A
THIETVINALK.2	0.114103804	#N/A
YGGTFQNVSVK.2	-0.180639864	#N/A
LSTVASTDILATVLEEM[Oxidation (M)]PPFPER.3	0.095767104	#N/A
TVFEALQAPAC[Carbamidomethyl (C)]HENLVK.3	-0.286330967	#N/A
VIQIVINR.2	-0.176232295	#N/A
AVDLLYAMC[Carbamidomethyl (C)]DR.2	0.195061491	#N/A
TSPDLVPMGDWTSR.2	0.217227641	#N/A
ILVAGDTMDSVK.2	-0.018600171	#N/A
SHYEEGPGK.2	-0.193041846	#N/A
NLPFSVENK.2	-0.008578476	#N/A
FLSQIESDR.2	-0.232837913	#N/A
ITPELLTR.2	0.017851308	#N/A
ESLLFEGR.2	-0.026377567	#N/A
HIDLVEGDEGR.2	-0.309021953	#N/A
ASGVEGADVVK.2	-0.249379475	#N/A
LVPDSDVR.2	0.040191439	#N/A
GAALITAVGVR.2	0.055158483	#N/A
SIPDGTEHGDFLALDLGGTNFR.3	-0.201890189	#N/A
ATDC[Carbamidomethyl (C)]VGHDVATLLR.3	-0.033186949	#N/A
TTVGVDGSLYK.2	0.244575	#N/A
GDFIALDLGGSSFR.2	0.089815843	#N/A
LSDEILIDILTR.2	-0.057865395	#N/A
VVDEYSLNSGK.2	0.364200395	#N/A
RLVPDSDVR.2	0.263794999	#N/A
IDEAVLITWTK.2	0.080266833	#N/A
GQISEPLK.2	0.121696198	#N/A
AILQQLGLNSTC[Carbamidomethyl (C)]DDSILVK.2	-0.220176425	#N/A
NILIDFTK.2	0.119606241	#N/A
M[Oxidation (M)]HPQYSR.2	-0.137230468	#N/A
M[Oxidation (M)]LPTFVR.2	0.020640528	#N/A
TDFDKVVDEYSLNSGK.3	-0.338019267	#N/A
M[Oxidation (M)]ISGM[Oxidation (M)]YLGEIVR.2	-0.400423383	#N/A
IM[Oxidation (M)]HQTVK.2	-0.081769965	#N/A
GAAM[Oxidation (M)]VTAVAYR.2	0.365644031	#N/A
LPVGFTFSFPC[Carbamidomethyl (C)]R.2	0.071721349	#N/A
DYNPTASVK.2	0.222104002	#N/A
C[Carbamidomethyl (C)]TVSFLLSEDGSGK.2	-0.033119283	#N/A
QIEETLSHFR.2	-0.058509066	#N/A
FTTSDVAAIETDKEGVQNAK.3	0.272213182	#N/A

SANLVAATLGAILNR.2	-0.21405042	#N/A
M[Oxidation (M)]C[Carbamidomethyl (C)]INTEWGAFGDDGSLEDIR.2	-0.046090217	#N/A
FLLSESGSGK.2	-0.158977415	#N/A
LGVEPSHDDC[Carbamidomethyl (C)]VSVQHVC[Carbamidomethyl (C)]TIVSFR.4	0.005710986	#N/A
QALMEVK.2	0.006197753	#N/A
AAQLC[Carbamidomethyl (C)]GAGMAAVVEK.2	0.043872825	#N/A
TVC[Carbamidomethyl (C)]GVVSK.2	-0.178498742	#N/A
SEMEMGLR.2	-0.414988102	#N/A
SIYGEKFEDENFILK.3	-0.166692379	#N/A
VSFELFADK.2	0.253237878	#N/A
KITISDC[Carbamidomethyl (C)]GQL.2	0.126890843	#N/A
VNPTVFFDITADDEPLGR.2	-0.185802608	#N/A
VSFELFADKVPK.2	-0.209376139	#N/A
IIPGFMC[Carbamidomethyl (C)]QGGDFTR.2	0.318564775	#N/A
HTGPGILSM[Oxidation (M)]ANAGPNTNGSQFFIC[Carbamidomethyl (C)]TAK.3	-0.446893575	#N/A
FEDENFILK.2	-0.015946864	#N/A
EGM[Oxidation (M)]NIVEAMER.2	0.410666109	#N/A
VKEGM[Oxidation (M)]NIVEAM[Oxidation (M)]ER.3	-0.393210501	#N/A
TEWLDGK.2	-0.056608833	#N/A
[Acetyl (Protein N-term)]M[Oxidation (M)]VNPTVFFDITADDEPLGR.2	-0.085108288	#N/A
VVLAYEPVWAIGTGK.2	0.108843945	#N/A
IIYGGSVTGATC[Carbamidomethyl (C)]K.2	0.262006676	#N/A
SNVNDGVAQSTR.2	0.078808975	#N/A
FFVGGNWK.2	-0.196615099	#N/A
IAVAAQNC[Carbamidomethyl (C)]YK.2	0.451722554	#N/A
VSHALAEGLGVIAC[Carbamidomethyl (C)]IGEK.3	-0.103145028	#N/A
VIADNVKDWSK.2	-0.120132182	#N/A
DLGATWVVLGHSER.2	0.232636155	#N/A
VVFEQTK.2	-0.00560653	#N/A
VTNGAFTGEISPGM[Oxidation (M)]IK.2	0.105317468	#N/A
TATPQQAQEVHEK.2	0.478491395	#N/A
HVFGESDELIGQK.2	0.026186731	#N/A
ELASQPDVDGFLVGGASLKPEFVDIINAK.3	-0.298516391	#N/A
C[Carbamidomethyl (C)]LGELIC[Carbamidomethyl (C)]TLNAANVPAGTEVVC[Carbamidomethyl (C)]APPTAYIDFAR.3	-0.186276659	#N/A
RHVFGESDELIGQK.3	0.271950312	#N/A
TFDEIASGFR.2	0.049484243	#N/A
AKS[Phospho (STY)]VLKK.2	-0.113812702	P17809_S226_M1_Slc2a1
VTILELFR.2	0.110139373	#N/A
QGGASQSDKT[Phospho (STY)]PEELFHPLGADSQV.3	0.204918142	P17809_T478_M1_Slc2a1
IINEPTAAAIAYGLDR.2	0.193817037	#N/A
NQVALNPQNTVFDAK.2	0.390600539	#N/A
ATAGDTHLGGEDFDNR.3	-0.07576126	#N/A
QAGSHSNS[Phospho (STY)]FR.2	0.343049792	P18052_S180_M1_Ptpra
VVQEYIDAFSDY[Phospho (STY)]ANFK.2	-0.412611447	P18052_Y825_M1_Ptpra
VSSLPTVYLK.2	0.192669102	#N/A
TTEPVSELLK.2	0.085573787	#N/A
QPGIVFVAAK.2	0.376433866	#N/A

QIFGEATK.2	0.141884859	#N/A
ILDIAC[Carbamidomethyl (C)]WVHHK.3	0.219289141	#N/A
AIGAVPLIQGEYMIPC[Carbamidomethyl (C)]EK.2	0.068176621	#N/A
YIVDADDR.2	-0.137548355	#N/A
SEINVEGPPR.2	-0.243174302	#N/A
SGEYSC[Carbamidomethyl (C)]IFLPEPVGR.2	0.287359351	#N/A
YVVVSTPEK.2	0.005564833	#N/A
VLQEDTLPDLHTK.3	-0.230174492	#N/A
NLS[Phospho (STY)]LPFILHEK.2	-0.319803277	P18581_S645_M1_Slc7a2
NQS[Phospho (STY)]PVLEPVGR.2	-0.402399774	P18654_S715_M1_Rps6ka3
DLKPENILLDEEGHIK.3	0.051369163	#N/A
[Acetyl (Protein N-term)]AS[Phospho (STY)]GVAVSDGVIK.2	-0.424113609	P18760_S3_M1_Cfl1
YALYDATYETK.2	0.00404255	#N/A
KSSTPEEVK.2	0.144424152	#N/A
LGGSAVISLEGKPL.2	-0.154102979	#N/A
SSTPEEVKK.2	0.167258148	#N/A
EILVGDVGQTVDDPYTTFVK.2	0.309910327	#N/A
M[Oxidation (M)]LPDKDC[Carbamidomethyl (C)]R.2	0.393469426	#N/A
NIILEEGKEILVGDVGQTVDDPYTTFVK.3	-0.223908272	#N/A
NIILEEGK.2	0.075868175	#N/A
AVLFC[Carbamidomethyl (C)]LSEDKK.2	-0.067010019	#N/A
[Acetyl (Protein N-term)]ASGVAVSDGVIKVFNDM[Oxidation (M)]K.2	-0.127998758	#N/A
KEDLVFIFWAPENAPLK.3	0.133774309	#N/A
HELQANC[Carbamidomethyl (C)]YEEVKDR.3	0.298999654	#N/A
SST[Phospho (STY)]PEEVKKR.2	0.562930916	P18760_T25_M1_Cfl1
KSST[Phospho (STY)]PEEVKK.2	-0.042220678	P18760_T25_M1_Cfl1
LTGIKHELQANC[Carbamidomethyl (C)]YEEVKDR.4	-0.124833462	#N/A
MIYASSK.2	0.262706833	#N/A
IGAGDYQPTEQDILR.2	-0.319408602	#N/A
LFDVGGQR.2	-0.166592427	#N/A
NLKEDGISAAK.2	-0.250388538	#N/A
LWGDSGIQEC[Carbamidomethyl (C)]FNR.2	-0.304518172	#N/A
EYQLNDSAK.2	-0.122281341	#N/A
KDLFGEK.2	-0.070923462	#N/A
YYLDSLDR.2	-0.222825588	#N/A
M[Oxidation (M)]VC[Carbamidomethyl (C)]DVVSR.2	0.114466285	#N/A
IIHEDGFSGEDVK.3	-0.061824596	#N/A
QYKPVVYSNTIQSLAAIVR.3	-0.3933139	#N/A
LFDVGGQRS[Phospho (STY)]ER.2	-0.407232989	P18872_S207_M1_Gnao1
M[Oxidation (M)]HESLM[Oxidation (M)]LFDSIC[Carbamidomethyl (C)]NNK.3	-0.412440941	#N/A
M[Oxidation (M)]EDTEPFSAELLSAM[Oxidation (M)]MR.2	0.273354411	#N/A
AM[Oxidation (M)]DTLGVEYGDKER.2	0.290131318	#N/A
NLKEDGIS[Phospho (STY)]AAKDVK.3	-0.467449695	P18872_S29_M1_Gnao1
SREYQLNDSAK.2	-0.316193037	#N/A
SPLTIC[Carbamidomethyl (C)]FPEYPGSNTYEDAAAYIQTQFESK.3	0.365652951	#N/A
PVVYSNTIQSLAAIVR.2	0.145595463	#N/A
SRS[Phospho (STY)]DTSLK.2	0.023603235	P19096_S2201_M1_Fasn

VQEVQQVSTNK.2	0.038785954	#N/A
VTAIYIDPATHR.2	0.107630452	#N/A
TLEAVQDLLEQGR.2	0.01772528	#N/A
GYTVLGVEGR.2	0.379485263	#N/A
LFDHPEVPTPPESASVSR.3	0.339864175	#N/A
PPYTIVYFPVR.2	0.635517918	#N/A
YVTLIYTNYENGK.2	0.16853765	#N/A
MLLADQGQSWK.2	0.340516802	#N/A
EAAQM[Oxidation (M)]DM[Oxidation (M)]VNDGVEDLR.2	-0.143787774	#N/A
ALPGHLKPFETLLSQNQGGK.3	-0.362203463	#N/A
FEDGDLTLYQSNAILR.2	-0.171762182	#N/A
EAAQMDMVNDGVEDLRGK.3	0.106465292	#N/A
AKPLDVKS[Phospho (STY)]PEAQTPVQEEAK.3	-0.026423673	P19246_S834_M1_Nefh
S[Phospho (STY)]PGEAKS[Phospho (STY)]PAEAK.2	0.097562633	#N/A
SPAEAKS[Phospho (STY)]PIEVK.2	0.051823374	P19246_S763_M1_Nefh
S[Phospho (STY)]PAEAKS[Phospho (STY)]PAAVK.2	0.165221123	#N/A
S[Phospho (STY)]PGEAKS[Phospho (STY)]PAAVK.2	0.053108987	P19246_S715_M2_Nefh
TSVSSVSAS[Phospho (STY)]PSR.2	-0.373564925	P19246_S61_M1_Nefh
S[Phospho (STY)]PAEAKS[Phospho (STY)]PGEAK.2	0.086710766	P19246_S541_M2_Nefh
SPIEVKS[Phospho (STY)]PEK.2	0.044472885	P19246_S769_M1_Nefh
SEKEQLQALNDR.2	-0.023004629	#N/A
S[Phospho (STY)]PGEAKS[Phospho (STY)]PAEPK.2	0.058872634	#N/A
S[Phospho (STY)]PSEAKS[Phospho (STY)]PAEAK.2	-0.166157428	#N/A
S[Phospho (STY)]PVKEDIKPPAEAK.3	0.135430978	P19246_S795_M1_Nefh
S[Phospho (STY)]PAEAKS[Phospho (STY)]PAEPK.2	0.023448558	#N/A
EQLQALNDR.2	0.305631062	#N/A
AVAS[Phospho (STY)]EEETPAK.2	-0.060754111	P19246_S965_M1_Nefh
S[Phospho (STY)]PVKEEVK.2	0.194975711	P19246_S888_M1_Nefh
IPSISTHIK.2	0.049660387	#N/A
HQADIASYQDAIQQLDSELR.3	-0.21900304	#N/A
S[Phospho (STY)]PEAQTPVQEEAK.2	0.149156294	P19246_S834_M1_Nefh
SPIEVKS[Phospho (STY)]PEKAK.2	-0.175420936	P19246_S769_M1_Nefh
S[Phospho (STY)]PAEAKS[Phospho (STY)]PEK.2	-0.073355693	P19246_S783_M2_Nefh
C[Carbamidomethyl (C)]DVTSALR.2	0.296546801	#N/A
SPAEAKS[Phospho (STY)]PAEAKS[Phospho (STY)]PIEVK.3	-0.081592985	#N/A
S[Phospho (STY)]LEGEAAALR.2	-0.400714879	P19246_S122_M1_Nefh
S[Phospho (STY)]PEKAKS[Phospho (STY)]PVK.2	0.071309008	P19246_S789_M2_Nefh
VKEEAKS[Phospho (STY)]PGEAK.2	-0.178132851	P19246_S523_M1_Nefh
SPAAVKS[Phospho (STY)]PAEAKS[Phospho (STY)]PAAVK.2	-0.13146042	#N/A
SPAEPKS[Phospho (STY)]PAEAKS[Phospho (STY)]PAEVK.2	-0.08619911	P19246_S691_M2_Nefh
SPAEAKS[Phospho (STY)]PAEVKS[Phospho (STY)]PAEAK.2	-0.207371918	#N/A
ETVIVEGQTEEIR.2	0.09606668	#N/A
SPAEAKS[Phospho (STY)]PIEVKS[Phospho (STY)]PEK.2	0.003518682	P19246_S769_M2_Nefh
SPAEPKS[Phospho (STY)]PAEAKS[Phospho (STY)]PAEPK.2	0.174271879	#N/A
SPAEAKS[Phospho (STY)]PAEAKS[Phospho (STY)]PAEVK.2	-0.046866968	P19246_S667_M2_Nefh
SPAEAKS[Phospho (STY)]PATVKS[Phospho (STY)]PGEAK.2	-0.166825023	P19246_S643_M2_Nefh
AQALQEEC[Carbamidomethyl (C)]GYLR.2	0.627630582	#N/A

SPGEAKS[Phospho (STY)]PAAVKS[Phospho (STY)]PAEAK.2	-0.006349758	#N/A
SPGEAKS[Phospho (STY)]PAEPKS[Phospho (STY)]PAEAK.2	0.043180594	P19246_S685_M2_Nefh
SPSEAKS[Phospho (STY)]PAEAKS[Phospho (STY)]PAEAK.2	-0.181788134	#N/A
SPAEVKS[Phospho (STY)]PAEAKS[Phospho (STY)]PAEVK.2	0.02182712	P19246_S709_M2_Nefh
S[Phospho (STY)]PGEAKS[Phospho (STY)]PAEAKS[Phospho (STY)]PAEAK.2	0.11111561	P19246_S745_M3_Nefh
SPAEAKS[Phospho (STY)]PAEAKS[Phospho (STY)]PAEAK.2	0.022568115	P19246_S613_M2_Nefh
S[Phospho (STY)]PAEAKS[Phospho (STY)]PGEAKS[Phospho (STY)]PGEAK.2	0.208731438	#N/A
SPAEAKS[Phospho (STY)]PAEVKS[Phospho (STY)]PGEAK.2	0.0463558	#N/A
S[Phospho (STY)]PGEAKS[Phospho (STY)]PAEPKS[Phospho (STY)]PAEPK.2	0.068099602	#N/A
SPAEAKS[Phospho (STY)]PAEAK.2	-0.113015975	P19246_S607_M1_Nefh
S[Phospho (STY)]PGEAKS[Phospho (STY)]PGEAKS[Phospho (STY)]PAEAK.2	0.159752268	P19246_S523_M3_Nefh
S[Phospho (STY)]PGEAKS[Phospho (STY)]PAEAKS[Phospho (STY)]PGEAK.2	0.167387523	#N/A
SPATVKS[Phospho (STY)]PGEAK.2	-0.157339099	P19246_S589_M1_Nefh
VKS[Phospho (STY)]EEMIK.2	0.078719669	P19246_S438_M1_Nefh
S[Phospho (STY)]PAEAKS[Phospho (STY)]PATVK.2	-0.02656594	P19246_S631_M2_Nefh
SPGEAKS[Phospho (STY)]PSEAK.2	-0.075455083	P19246_S595_M1_Nefh
SPAEPKS[Phospho (STY)]PAEPKS[Phospho (STY)]PAEAK.2	-0.106894892	#N/A
SPAEAKS[Phospho (STY)]PAAVKS[Phospho (STY)]PGEAK.2	-0.111247688	#N/A
SPAEVKS[Phospho (STY)]PGEAKS[Phospho (STY)]PAEPK.2	-0.144753804	P19246_S685_M2_Nefh
TPVKEGAKS[Phospho (STY)]PAEAK.2	0.281580924	P19246_S783_M1_Nefh
SPAEVKS[Phospho (STY)]PGEAK.2	-0.093197542	P19246_S679_M1_Nefh
S[Phospho (STY)]PGEAKS[Phospho (STY)]PGEAK.2	-0.029902244	P19246_S523_M2_Nefh
S[Phospho (STY)]PAEAKS[Phospho (STY)]PAEVK.2	0.09560107	P19246_S667_M2_Nefh
SPAAVKS[Phospho (STY)]PAEAK.2	0.118378942	P19246_S727_M1_Nefh
S[Phospho (STY)]PAEVKS[Phospho (STY)]PAEAK.2	0.109188723	#N/A
S[Phospho (STY)]PAEPKS[Phospho (STY)]PAEAK.2	0.099801813	#N/A
SPAAVKS[Phospho (STY)]PGEAK.2	0.035681183	P19246_S739_M1_Nefh
S[Phospho (STY)]PAEPKS[Phospho (STY)]PATVK.2	0.169370567	#N/A
SPATVKS[Phospho (STY)]PGEAKS[Phospho (STY)]PSEAK.2	-0.097635078	#N/A
SPAEVKS[Phospho (STY)]PGEAKS[Phospho (STY)]PAAVK.2	0.249517089	P19246_S715_M2_Nefh
SPAEAKS[Phospho (STY)]PAEAKS[Phospho (STY)]PATVK.2	-0.045058711	P19246_S631_M2_Nefh
EEVKS[Phospho (STY)]PVKEEVK.2	-0.274300103	P19246_S888_M1_Nefh
AKT[Phospho (STY)]PVKEGAK.2	0.040221895	P19246_T775_M1_Nefh
HPTDIRPPEQVKS[Phospho (STY)]PAK.3	0.18317486	P19246_S859_M1_Nefh
SPAEAKS[Phospho (STY)]PAEPKS[Phospho (STY)]PATVK.2	0.247178025	#N/A
S[Phospho (STY)]PGEAKS[Phospho (STY)]PSEAKS[Phospho (STY)]PAEAK.2	-0.368268873	#N/A
S[Phospho (STY)]PGEAKS[Phospho (STY)]PGEAKS[Phospho (STY)]PGEAK.2	-0.115077729	#N/A
SPAEPKS[Phospho (STY)]PAEPK.2	0.064624615	P19246_S565_M1_Nefh
S[Phospho (STY)]PAEPKS[Phospho (STY)]PATVKS[Phospho (STY)]PGEAK.2	-0.26664821	#N/A
QREEAEAAAR.2	-0.251186466	#N/A
EGAKS[Phospho (STY)]PAEAKS[Phospho (STY)]PEK.2	0.151319142	P19246_S783_M2_Nefh
S[Phospho (STY)]PVKEGAKPPEK.2	0.254764602	P19246_S815_M1_Nefh
S[Phospho (STY)]PGEAKS[Phospho (STY)]PGEAKS[Phospho (STY)]PAEPK.2	0.214651421	#N/A
S[Phospho (STY)]PAEAKS[Phospho (STY)]PEKAK.2	-0.322860576	P19246_S783_M2_Nefh
LEQEHLLEDIAHVR.3	-0.170276843	#N/A
SPAAVKS[Phospho (STY)]PGEAKS[Phospho (STY)]PGEAK.3	-0.494868599	P19246_S745_M2_Nefh
EEAKS[Phospho (STY)]PGEAKS[Phospho (STY)]PGEAK.2	0.355697714	P19246_S523_M2_Nefh

SAAGSSSGFHSWAR.2	0.344344772	#N/A
VKS[Phospho (STY)]EEMIKVVEK.3	-0.256110541	P19246_S438_M1_Nefh
EGAKS[Phospho (STY)]PAEAK.2	0.195704032	P19246_S783_M1_Nefh
AKS[Phospho (STY)]PEKEEAK.2	0.395495714	P19246_S867_M1_Nefh
VTEGVTEEEDKEAQGQEGEEAEEGEEKEEEEGAAATS[Phospho (STY)]PPAEEAASPEK.5	-0.327459151	P19246_S500_M1_Nefh
EDIKPPAEAKS[Phospho (STY)]PEK.3	0.030253201	P19246_S809_M1_Nefh
SEKETVIVEGQTEEIR.3	-0.350667658	#N/A
AQLEGHAVQSTLQSEEWFR.3	-0.305728115	#N/A
PPAEAKS[Phospho (STY)]PEK.2	-0.006252425	P19246_S809_M1_Nefh
SAQEEITEYRR.3	0.192633766	#N/A
YQAVTATLEEK.2	0.196715175	#N/A
SLS[Phospho (STY)]EQPVVDTATATEQAK.2	-0.030189845	P19426_S51_M1_Nelfe
SM[Oxidation (M)]S[Phospho (STY)]ADEDLQEPSR.2	-0.530882105	P19426_S115_M1_Nelfe
EDPNLVPSISNK.2	0.294465784	#N/A
AASGTKEDPNLVPSISNK.3	-0.150525796	#N/A
EIM[Oxidation (M)]IAAQK.2	0.087647332	#N/A
LVPHQMAH.2	0.515255109	#N/A
AASGTKEDPNLVPSISNKR.3	-0.087406386	#N/A
EDPNLVPSISNKR.2	0.177296625	#N/A
C[Carbamidomethyl (C)]PNC[Carbamidomethyl (C)]GTHYK.2	0.157695753	#N/A
SEDYAFPTYADR.2	-0.155328727	#N/A
ANPIQGFSAK.2	0.0359255	#N/A
ADWSSLSR.2	0.042611734	#N/A
EKADWSSLSR.2	-0.169020869	#N/A
SEDYAFPTYADRR.2	-0.391804753	#N/A
IQFNESFAEM[Oxidation (M)]NR.2	-0.051902475	#N/A
DWVAM[Oxidation (M)]QTK.2	0.0751393	#N/A
DYPLPDVAHVTM[Oxidation (M)]LSASQK.3	-0.473835405	#N/A
QPS[Phospho (STY)]IELPSMAVASTK.2	0.309734964	P19973_S243_M1_Lsp1
ITITNDQNR.2	-0.016313277	#N/A
ITPSYVAFTPEGER.2	0.411985071	#N/A
LTPEEIER.2	0.182378134	#N/A
NQLTSNPENTVFDAK.2	-0.067838761	#N/A
SQIFSTASDNQPTVTIK.2	-0.047338117	#N/A
TWNDPSVQQDIK.2	-0.231209233	#N/A
VYEGERPLTK.2	0.296722007	#N/A
NELESYAYSLK.2	-0.088580986	#N/A
ELEEIVQPIISK.2	-0.212378166	#N/A
NTVVPTKK.2	-0.120421461	#N/A
IEIESFFEGEDFSETLTR.2	-0.286614905	#N/A
VTHAVVTVPAYFNDAQR.3	-0.246620343	#N/A
NQLTSNPENTVFDAKR.3	0.087213987	#N/A
IINEPTAAAIAYGLDKR.3	-0.048986835	#N/A
TFAPEEISAM[Oxidation (M)]VLTK.2	0.45764186	#N/A
KSDIDEIVLVGGSTR.3	0.233810337	#N/A
ALSSQHQAR.2	0.074090906	#N/A
IEWLESHQDADIEDFK.3	-0.058059337	#N/A

VLEDSDLK.2	0.140326914	#N/A
DAGTIAGLNVMR.2	0.156176705	#N/A
LYGSGGPPPT[Phospho (STY)]GEEDTSEKDEL.2	-0.049238876	P20029_T644_M1_Hspa5
AKFEELNM[Oxidation (M)]DLFR.3	-0.512207474	#N/A
NPLPSKETIEQEK.3	0.107821057	#N/A
ETIEQEKQAGES[Phospho (STY)].2	-0.119096195	P20065_S50_M1_Tmsb4x
TETQEKNPLPSKETIEQEK.3	-0.024531455	#N/A
KTETQEKNPLPSK.3	0.276825874	#N/A
TET[Phospho (STY)]QEKNPLPSK.2	0.054484232	P20065_T29_M1_Tmsb4x
GLFIIDPNGVVK.2	0.011801772	#N/A
HLSVNDLPVGR.2	0.367596976	#N/A
DYGVLLESAGIALR.2	0.060430538	#N/A
GTAVVNGEFK.2	0.264327822	#N/A
ELSLDDFK.2	0.250813105	#N/A
VELQELNDR.2	0.021432175	#N/A
FADLSEAANR.2	0.093082829	#N/A
NLQEAEEWYK.2	-0.071524956	#N/A
QDVDNAS[Phospho (STY)]LAR.2	0.067279941	P20152_S214_M1_Vim
TNEKVELQELNDR.3	-0.398607512	#N/A
ISLPLPTFSSLNLR.2	0.326004253	#N/A
ILLAELEQLK.2	0.284809377	#N/A
SLYSSS[Phospho (STY)]PGGAYVTR.2	0.081708278	P20152_S56_M1_Vim
QQYESVAAK.2	0.264730451	#N/A
EM[Oxidation (M)]EENFALEAANYQDTIGR.2	-0.365788719	#N/A
FANYIDK.2	-0.180071932	#N/A
SYVTTSTR.2	-0.34299006	#N/A
DNLAEDIMR.2	-0.452896903	#N/A
ETNLESLPLVDTHSK.3	-0.372227791	#N/A
SVSSSSYR.2	-0.214387884	#N/A
ARVDHGAEIITQS[Phospho (STY)]PSR.3	-0.259900755	P20357_S1783_M1_Map2
IST[Phospho (STY)]PERK.2	-0.369655892	P20357_T1445_M1_Map2
KSEVQAHS[Phospho (STY)]PSRK.3	-0.139235757	P20357_S1485_M1_Map2
GLSS[Phospho (STY)]VPEVAEVEPTTK.2	-0.033339401	P20357_S1023_M1_Map2
SSVAS[Phospho (STY)]PRR.2	-0.151056674	P20357_S1791_M1_Map2
DGS[Phospho (STY)]PDAPATPEK.2	-0.056908605	P20357_S1352_M1_Map2
GRIST[Phospho (STY)]PER.2	-0.122826097	P20357_T1445_M1_Map2
FAAPAQPEEER.2	0.275470521	#N/A
LINQPLPDLK.2	0.001211794	#N/A
NKDDLTLSR.2	0.127530859	#N/A
AEPSQLDIK.2	0.010630018	#N/A
ADQGLDFAATK.2	0.288475171	#N/A
ELITTKDTS[Phospho (STY)]PEK.2	-0.343832293	P20357_S1013_M1_Map2
VKDEFTAEK.2	0.064721584	#N/A
EIQGLFEEK.2	-0.384184643	#N/A
VKIESVK.2	0.082973415	#N/A
ETSAPSVQEPTLTETEPQTK.2	0.053003636	#N/A
SDTLQISDLLVSESR.2	0.013757744	#N/A

GGQVQIVTK.2	-0.286715535	#N/A
SKIGS[Phospho (STY)]TDNIK.2	-0.075558197	P20357_S1680_M1_Map2
VTDGISKS[Phospho (STY)]PEK.2	-0.204593615	P20357_S1539_M1_Map2
GSAQESLDTIS[Phospho (STY)]PK.2	-0.408832906	P20357_S608_M1_Map2
TPGTPGT[Phospho (STY)]PSYPR.2	-0.23220536	P20357_T1623_M1_Map2
VAIIRT[Phospho (STY)]PPKS[Phospho (STY)]PATPK.2	-0.281230838	P20357_S1654_M2_Map2
LAS[Phospho (STY)]VSADAEVAR.2	0.33995656	P20357_S823_M1_Map2
T[Phospho (STY)]PPKSPATPK.2	-0.030616224	P20357_T1650_M1_Map2
KETS[Phospho (STY)]PETSLIQDEVALK.2	-0.325660334	P20357_S1161_M1_Map2
TTAASGDLAQAPGAFK.2	-0.064526265	#N/A
STVS[Phospho (STY)]IEEAVAK.2	-0.154469276	P20357_S447_M1_Map2
SILTEQLETIPKEER.2	0.152327048	#N/A
SILTEQLETIPK.2	0.395689969	#N/A
SGILVPSEK.2	-0.244107158	#N/A
VELFGLGITYDQASTK.2	0.140820298	#N/A
IDLSHVTSK.2	-0.169648141	#N/A
KSEVQAHS[Phospho (STY)]PSR.2	-0.188284654	P20357_S1485_M1_Map2
STELGSDYYELSDSR.2	0.256754533	#N/A
DLATDLSLIEVK.2	0.017444622	#N/A
VTS[Phospho (STY)]EPEAVSERR.2	-0.027092296	P20357_S520_M1_Map2
SEVQAHS[Phospho (STY)]PSR.2	-0.162014578	P20357_S1485_M1_Map2
ELITTKDTS[Phospho (STY)]PEKTEK.3	-0.49318694	P20357_S1013_M1_Map2
DGS[Phospho (STY)]PDAPATPEKEEVAFSEYK.3	-0.057949656	P20357_S1352_M1_Map2
VTS[Phospho (STY)]EPEAVSER.2	0.013657007	P20357_S520_M1_Map2
DWFIEM[Oxidation (M)]PTESK.2	0.205591162	#N/A
VDHGAEIITQS[Phospho (STY)]PSR.2	-0.294571564	P20357_S1783_M1_Map2
VTDGISKS[Phospho (STY)]PEKR.2	-0.063528838	P20357_S1539_M1_Map2
VDHGAEIITQSPSRSS[Phospho (STY)]VAS[Phospho (STY)]PR.3	-0.081045815	P20357_S1788_M2_Map2
S[Phospho (STY)]GILVPSEKK.2	-0.157826251	P20357_S1635_M1_Map2
ETS[Phospho (STY)]PETSLIQDEVALK.2	0.028859184	P20357_S1161_M1_Map2
EKDVLEDIPR.2	0.288498019	#N/A
VSDFGQM[Oxidation (M)]ASGM[Oxidation (M)]NVDAGK.2	-0.126205081	#N/A
M[Oxidation (M)]PSKPGEDFEHAALVPDTSK.4	-0.457165029	#N/A
YFETSALKEDM[Oxidation (M)]TR.2	0.131075503	#N/A
ESS[Phospho (STY)]KDEEPLKDK.2	-0.24021065	P20357_S365_M1_Map2
TGVIQTS[Phospho (STY)]TEQSFSK.2	-0.168066531	P20357_S472_M1_Map2
VGS[Phospho (STY)]LDNAHHVPGGGNVK.3	-0.358331568	P20357_S1743_M1_Map2
SEVQAHS[Phospho (STY)]PSRK.2	-0.253089629	P20357_S1485_M1_Map2
NQHDEKELQAK.2	-0.082392612	#N/A
GVSGDREENS[Phospho (STY)]FSLNSSISSAR.3	-0.34654867	P20357_S1567_M1_Map2
SGTSTPTTPGSTAITPGT[Phospho (STY)]PPSYSSR.3	-0.286269541	P20357_T1609_M1_Map2
GRIST[Phospho (STY)]PERK.2	0.083316517	P20357_T1445_M1_Map2
AGKS[Phospho (STY)]GTSTPTTPGSTAITPGTPPSY[Phospho (STY)]SSR.3	-0.049109777	P20357_S1592_M2_Map2
VTGGQTIQVETSSESPFPAK.2	0.072686154	#N/A
MFTIDPK.2	0.308514825	#N/A
EAS[Phospho (STY)]PPTSADK.2	-0.231811457	P20357_S938_M1_Map2
KANDKLDTVLEK.3	0.427175349	#N/A

VKDEFTAEKEAS[Phospho (STY)]PPTSADK.3	-0.311409714	P20357_S938_M1_Map2
TGVIQTSTEQS[Phospho (STY)]FSKEDQK.3	0.070442152	P20357_S476_M1_Map2
DKVTDGISKS[Phospho (STY)]PEK.2	0.091188621	P20357_S1539_M1_Map2
IVQVVTAEAVAVLKGEQEK.2	-0.430588542	#N/A
TPGT[Phospho (STY)]PKS[Phospho (STY)]GILVPSEK.2	-0.049474882	P20357_S1635_M2_Map2
APHWTSASLTEAAAHPHS[Phospho (STY)]PEM[Oxidation (M)]K.3	-0.1822189	#N/A
EEFVETC[Carbamidomethyl (C)]PGELK.2	0.495774207	#N/A
LEGAGSATIAEVEM[Oxidation (M)]PFYEDK.2	0.099756508	#N/A
DQPAALPLAAEETANLPPS[Phospho (STY)]PPPS[Phospho (STY)]PASEQTATVEEDLLTASK.4	0.022797296	P20357_S136_M2_Map2
TPGTPGTPSYPRT[Phospho (STY)]PGT[Phospho (STY)]PK.2	-0.15409184	P20357_T1629_M2_Map2
ARVDHGAEIITQS[Phospho (STY)]PSRSS[Phospho (STY)]VAS[Phospho (STY)]PR.3	-0.368273508	#N/A
IGSTDNIK.2	0.09785413	#N/A
YFETSALK.2	-0.072701811	#N/A
GHDLSPLASDILTNTSGSM[Oxidation (M)]DEGDDY[Phospho (STY)]LPPTTPAVEK.4	-0.017204067	P20357_Y746_M1_Map2
SM[Oxidation (M)]SINLPM[Oxidation (M)]SC[Carbamidomethyl (C)]LDSIALGFNFGR.3	0.222492451	#N/A
LSNVSS[Phospho (STY)]SGSINLLESPQLATLAEDVTAALAK.3	-0.011148573	P20357_S1800_M1_Map2
APHWTSASLTEAAAHPHS[Phospho (STY)]PEM[Oxidation (M)]KDQGGAGEGLSR.5	-0.342117751	#N/A
ASQPSPPAQEAGYSTLAQSYTPDHPSELPEEPSS[Phospho (STY)]PQER.4	0.071557222	P20357_S655_M1_Map2
KDEWGLAAPIS[Phospho (STY)]PGPLTPM[Oxidation (M)]R.3	-0.320939879	P20357_S283_M1_Map2
DTS[Phospho (STY)]PEKTEK.2	-0.259533222	P20357_S1013_M1_Map2
ANDKLDTVLEKS[Phospho (STY)]EEHIDSK.3	-0.329616563	P20357_S969_M1_Map2
ESEEM[Oxidation (M)]GGKVELFGLGITYDQASTK.3	0.234438969	#N/A
YTVPLPSPVQDSENLSGESGSFYEGTDDKVR.3	-0.506769826	#N/A
IVQVVTAEAVAVLK.2	0.261468213	#N/A
VIS[Phospho (STY)]PSEDR.2	-0.034732716	P20444_S319_M1_Prkca
[Acetyl (Protein N-term)]ADVYPANDSTASQDVANR.2	-0.054043404	#N/A
VIS[Phospho (STY)]PSEDRKQPSNNLDR.3	-0.410087988	P20444_S319_M1_Prkca
VIS[Phospho (STY)]PSEDRK.2	0.087675583	P20444_S319_M1_Prkca
LTDFNFLM[Oxidation (M)]VLGK.2	0.22454561	#N/A
DVVIQDDDVEC[Carbamidomethyl (C)]TM[Oxidation (M)]VEK.2	-0.085121068	#N/A
T[Phospho (STY)]FC[Carbamidomethyl (C)]GTPDYIAPEIIAYQPYGK.3	-0.032911428	P20444_T497_M1_Prkca
LKPS[Phospho (STY)]DKDR.2	-0.217042105	P20444_S234_M1_Prkca
LSVEIWDWDR.2	-0.147660583	#N/A
NLIPM[Oxidation (M)]DPNGLSDPYVK.2	-0.359828723	#N/A
DVVIQDDDVEC[Carbamidomethyl (C)]TM[Oxidation (M)]VEKR.3	-0.368766095	#N/A
KDVVIQDDDVEC[Carbamidomethyl (C)]TMVEK.3	-0.340290653	#N/A
LLGDLGLR.2	-0.012327138	#N/A
NLYGTQSLELPFQGAHR.2	-0.126749521	#N/A
TQVVHESFQGR.2	0.232861134	#N/A
SGLLLTSILTIR.2	0.050751647	#N/A
GESPELDLSYSHSDLGK.2	0.32769095	#N/A
NVTESSSFS[Phospho (STY)]GGDNPHVLYSPEFR.3	-0.231643604	P20917_S549_M1_Mag
LREDEGTWVQVSLLHFVPTR.3	-0.307091468	#N/A
IQLVEEELDR.2	0.038377818	#N/A
AADAEAEVASLNR.2	0.04182909	#N/A
KYEEVAR.2	0.235992827	#N/A
HIAEEADR.2	0.129022644	#N/A

YSQKEDKYEEEIK.3	-0.207370406	#N/A
KLVIIEGDLER.2	0.166250276	#N/A
IQLVEEELDRAQER.3	-0.353980151	#N/A
LEEAEKAADESER.2	0.054835617	#N/A
KLVIIEGDLERTEER.3	-0.452686055	#N/A
C[Carbamidomethyl (C)]SELEEELKNVTNNLK.2	-0.407189863	#N/A
ALKDEEKM[Oxidation (M)]ELQEIQLK.3	-0.422795148	#N/A
AADAEAEVASLNRR.2	0.119601525	#N/A
TEERAELAESK.2	0.310841872	#N/A
LVIIEGDLERTEER.3	-0.189074074	#N/A
RIQLVEEELDR.2	0.029036432	#N/A
HIAEEADRKYEEVAR.4	0.078616035	#N/A
AISDELDHALNDMTS[Phospho (STY)]I.2	0.035365082	P21107_S284_M1_Tpm3
LLLLGTGESGK.2	-0.223315918	#N/A
IATVGYLPTQQDVLR.2	0.100420256	#N/A
DTILQLNLK.2	0.414277522	#N/A
MVDVGGQR.2	-0.020840798	#N/A
TLWSDPGVQEC[Carbamidomethyl (C)]YDR.2	0.433196368	#N/A
VADPSYLPTQQDVLR.2	-0.185006249	#N/A
YYLNDLDR.2	0.113330921	#N/A
VSAFENPYVDAIK.2	-0.031159389	#N/A
[Acetyl (Protein N-term)]TLESIMAC[Carbamidomethyl (C)]C[Carbamidomethyl (C)]LSEEAK.2	0.279212738	#N/A
EVDVEKVS[Phospho (STY)]AFENPYVDAIK.2	-0.116079119	P21279_S122_M1_Gnaq
VPTTGIIEYPFDLQSVIFR.2	-0.326106975	#N/A
S[Phospho (STY)]TRKS[Phospho (STY)]IC[Carbamidomethyl (C)]GPHDQDR.3	0.114389153	P21447_S663_M2_Abcb1a
S[Phospho (STY)]IC[Carbamidomethyl (C)]GPHDQDR.2	-0.091261221	P21447_S667_M1_Abcb1a
DSGSS[Phospho (STY)]LIR.2	-0.172114654	P21447_S657_M1_Abcb1a
ALDFAVSEYNK.2	0.295784997	#N/A
SQT[Phospho (STY)]NWGPGESFR.2	0.178686605	P21619_T528_M1_Lmnb2
RRLETEDTSGS[Phospho (STY)]PSR.3	-0.052147488	P21619_S427_M1_Lmnb2
RLETEDTSGS[Phospho (STY)]PSR.2	0.033307986	P21619_S427_M1_Lmnb2
LETEDTSGS[Phospho (STY)]PSR.2	0.135661388	P21619_S427_M1_Lmnb2
ATSSSSSSS[Phospho (STY)]GVGM[Oxidation (M)]SVGQGR.2	-0.455071711	P21619_S403_M1_Lmnb2
WLEETDSSSGS[Phospho (STY)]PTNLDK.2	0.244348328	P21952_S324_M1_Pou3f1
SDDS[Phospho (STY)]NGIENNVPR.2	-0.057899096	P21995_S312_M1_Emb
FAESLPSDEEGENYS[Phospho (STY)]K.2	0.010231541	P22005_S252_M1_Penk
ELLGTGDNR.2	-0.197689482	#N/A
FAESLPSDEEGENY[Phospho (STY)]SKEVPEIEK.3	0.276453277	P22005_Y251_M1_Penk
FAESLPSDEEGENYS[Phospho (STY)]KEVPEIEKR.3	-0.150292366	P22005_S252_M1_Penk
LVRPGDINFLAC[Carbamidomethyl (C)]TLEC[Carbamidomethyl (C)]EGQLPSFK.3	0.200627887	#N/A
DADEGDTLANS[Phospho (STY)]SDLLK.2	0.098835384	P22005_S153_M1_Penk
VPEGDVTVILNNLLEGYDNK.2	-0.434967248	#N/A
NLIM[Oxidation (M)]SGYS[Phospho (STY)]PEHDVSGISDPFLQVR.3	0.481579118	P22892_S232_M1_Ap1g1
LTWHSC[Carbamidomethyl (C)]PEDEAQ.2	-0.253957622	#N/A
KVEEAEPEEFVVEK.2	-0.221852557	#N/A
SLS[Phospho (STY)]DSESDDSKSK.2	-0.226959971	P23198_S95_M1_Cbx3
KSLS[Phospho (STY)]DSESDDSK.2	-0.12527304	P23198_S95_M1_Cbx3

SLS[Phospho (STY)]DSESDDSK.2	0.02158376	P23198_S95_M1_Cbx3
RKS[Phospho (STY)]LS[Phospho (STY)]DSESDDSK.2	-0.064912986	P23198_S93_M2_Cbx3
KSLS[Phospho (STY)]DSESDDSKSK.2	0.329290859	P23198_S95_M1_Cbx3
C[Carbamidomethyl (C)]PQIVIAFYEER.2	-0.571054516	#N/A
ASS[Phospho (STY)]RPRPDDLEI.2	-0.321615019	P23242_S373_M1_Gja1
PRPDDLEI.2	0.245447244	#N/A
LVTGDRNNS[Phospho (STY)]S[Phospho (STY)]C[Carbamidomethyl (C)]RNYNK.3	-0.368883347	P23242_S296_M2_Gja1
QASEQNWANYSAEQNR.2	-0.161421205	#N/A
SDPY[Phospho (STY)]HATTGPLSPSK.2	0.152825555	P23242_Y247_M1_Gja1
LVTGDRNNS[Phospho (STY)]SC[Carbamidomethyl (C)]R.2	0.058672548	P23242_S296_M1_Gja1
VAAGHELQPLAIVDQRPSS[Phospho (STY)]RAS[Phospho (STY)]S[Phospho (STY)]R.4	-0.185064878	P23242_S365_M3_Gja1
C[Carbamidomethyl (C)]NTQQPGC[Carbamidomethyl (C)]ENVC[Carbamidomethyl (C)]YDK.2	-0.256742948	#N/A
VELS[Phospho (STY)]EEELK.2	0.181115133	P23475_S558_M1_Xrcc6
ELVDDSITNVK.2	-0.229534863	#N/A
ALDVGSGSGILTAC[Carbamidomethyl (C)]FAR.2	-0.072488734	#N/A
LILPVGPAGGNQM[Oxidation (M)]LEQYDKLQDGSVK.3	-0.492736279	#N/A
VFEVM[Oxidation (M)]LATDR.2	-0.098815649	#N/A
SGGASHSELIHNLR.3	0.0241754	#N/A
TDKVFEVM[Oxidation (M)]LATDR.3	-0.484356565	#N/A
ELVDDSITNVKK.3	0.521456311	#N/A
ADIAIAPLTITLVR.2	0.338247093	#N/A
NAQNINPSSSQNS[Phospho (STY)]QNFATYK.2	-0.277383661	P23819_S863_M1_Gria2
YAYLLESTM[Oxidation (M)]NEYIEQR.2	-0.207567873	#N/A
YTSALTYDAVQVM[Oxidation (M)]TEAFR.2	0.247645169	#N/A
VLGDVIEVHGK.2	-0.062681095	#N/A
[Acetyl (Protein N-term)]M[Oxidation (M)]DIAIHHPWIR.3	-0.173765921	#N/A
QDEHGFISR.2	0.165052835	#N/A
FSVNLDVK.2	-0.398838664	#N/A
APSWIDTGLSEMR.2	0.224339409	#N/A
HEERQDEHGFISR.4	0.291533136	#N/A
LTDIQYGR.2	0.163182044	#N/A
HLTM[Oxidation (M)]DDLATALEGNR.3	0.066731973	#N/A
QSILELAQQWGEFK.2	-0.189986385	#N/A
TVDNFVALATGEK.2	-0.033283253	#N/A
VLEGMDVVR.2	-0.000433021	#N/A
DTNGSQFFITTVK.2	-0.0856894	#N/A
VYFDLQIGDESVGR.2	-0.344004651	#N/A
FLQPGSQR.2	-0.12227881	#N/A
EKEESYDLILSR.3	-0.03410102	#N/A
PTPSASS[Phospho (STY)]PQPK.2	-0.237157287	P24529_S8_M1_Th
RQS[Phospho (STY)]LIEDARK.3	-0.269706135	P24529_S40_M1_Th
RQS[Phospho (STY)]LIEDAR.2	-0.1564655	P24529_S40_M1_Th
QAEAVTS[Phospho (STY)]PR.2	-0.331338466	P24529_S31_M1_Th
RAVS[Phospho (STY)]EQDTK.2	-0.12525008	P24529_S19_M1_Th
FEVPSGDLAALLSSVR.2	0.135259814	#N/A
FDPYTLAIDVLDS[Phospho (STY)]PHTIR.2	0.167550432	P24529_S472_M1_Th
SLEGVQDELHTLTQALSAIS.2	0.001235363	#N/A

VFETFEAK.2	0.293944669	#N/A
GMGS[Phospho (STY)]LDAMDK.2	0.385952372	P24547_S416_M1_Impdh2
[Acetyl (Protein N-term)]SS[Phospho (STY)]PAQPAVPAPLADLK.2	-0.374977513	P24549_S3_M1_Aldh1a1
GFFVQPTVFSNVTDEM[Oxidation (M)]R.2	-0.265021133	#N/A
IHGQTIPSDGDIFTYTR.3	0.304490851	#N/A
VFANAYLSDLGGC[Carbamidomethyl (C)]IK.2	0.1068624	#N/A
VTLELGGK.2	-0.182938572	#N/A
VAFTGSTQVGK.2	0.232947398	#N/A
SFESTVGQGSDTYSYIFR.2	0.214161598	#N/A
GVGDDQLGEES[Phospho (STY)]EERDDHLLPM[Oxidation (M)].3	0.341533554	P24668_S268_M1_M6pr
S[Phospho (STY)]PLRPPR.2	-0.026864832	P24788_S234_M1_Cdk11b
DLLSDLQDIS[Phospho (STY)]DSER.2	0.469235898	P24788_S270_M1_Cdk11b
TYSYLTPDLWK.2	0.183160885	#N/A
AEDKEWIPVTK.3	-0.371541652	#N/A
VLVQNAAGS[Phospho (STY)]QEK.2	-0.279064572	P26039_S2040_M1_Tln1
APDFVFYAPR.2	0.007536065	#N/A
IGFPWSEIR.2	-0.181085904	#N/A
NISFNDKK.2	-0.411294796	#N/A
QLLTLSNELSQAR.2	0.525356518	#N/A
IAQDLEM[Oxidation (M)]YGINYFEIK.2	-0.273217787	#N/A
RKEDEVEEWQHR.3	-0.290677417	#N/A
GTDLWLGVDALGLNIYEK.2	0.343178386	#N/A
LLDGYDNR.2	-0.313406866	#N/A
SAAAPSASSTPTAIAS[Phospho (STY)]PK.2	-0.141741677	P26049_S433_M1_Gabra3
SRTS[Phospho (STY)]VQTEDDQLIAGQSAR.3	0.268118624	P26231_S655_M1_Ctnna1
QDLLAYLQR.2	-0.015586106	#N/A
EYAQVFR.2	0.237364861	#N/A
TS[Phospho (STY)]VQTEDDQLIAGQSAR.2	-0.126597305	P26231_S655_M1_Ctnna1
TPEELDDSDFET[Phospho (STY)]EDFDVR.2	0.015961207	P26231_T645_M1_Ctnna1
LESIISGAALM[Oxidation (M)]ADSSC[Carbamidomethyl (C)]TR.2	-0.650234966	#N/A
GWRPSS[Phospho (STY)]REDS[Phospho (STY)]VEAR.2	0.020017583	P26339_S410_M2_Chga
DDGQSDS[Phospho (STY)]QAVDGDGK.2	-0.539711043	P26339_S282_M1_Chga
[Acetyl (Protein N-term)]SDAAVDTSSEITTK.2	-0.00915456	#N/A
VAEDDEDDDVDTKK.2	0.392703236	#N/A
RVAEDDEDDDVDTKK.3	0.177239914	#N/A
GAKEEHGGLIRS[Phospho (STY)]PR.3	-0.519601066	P26369_S79_M1_U2af2
EEHGGLIRS[Phospho (STY)]PR.3	-0.18166243	P26369_S79_M1_U2af2
[Acetyl (Protein N-term)]S[Phospho (STY)]DFDEFER.2	-0.017002714	P26369_S2_M1_U2af2
NYTDNELEK.2	0.33919289	#N/A
TAAYVNAIEK.2	0.147070782	#N/A
YSTDVSVDEVK.2	-0.003787573	#N/A
C[Carbamidomethyl (C)]AVVDVPFGGAK.2	0.184159855	#N/A
YNLGLDLR.2	0.300332634	#N/A
HGGTIPVVPTAEFQDR.2	-0.365885266	#N/A
NLNHVSYGR.2	0.069508376	#N/A
C[Carbamidomethyl (C)]VGVGESDGSIWNPDGIDPK.2	0.064654166	#N/A
RDDGSWEVIEGYR.3	0.309297094	#N/A

M[Oxidation (M)]VEGFFDR.2	-0.04522544	#N/A
GFIGPGIDVPAPDM[Oxidation (M)]STGER.2	-0.165193907	#N/A
DDGSWEVIEGYR.2	0.057938597	#N/A
IIAEGANGPTTPEADKIFLER.3	-0.071282869	#N/A
TFVVQGFGNVGLHSM[Oxidation (M)]R.2	-0.132254362	#N/A
IIAEGANGPTTPEADK.2	-0.001175252	#N/A
GASIVEDKLVEDLK.2	0.106645958	#N/A
ALASLMTYK.2	0.385029221	#N/A
DSNYHLLM[Oxidation (M)]SVQESLER.3	-0.364345865	#N/A
VYEGSILEADC[Carbamidomethyl (C)]DILIPAASEK.2	-0.316774073	#N/A
DIVHSGLAYTM[Oxidation (M)]ER.3	0.288418018	#N/A
FTMELAK.2	-0.295427251	#N/A
SVVALHNLINNK.2	-0.233368345	#N/A
LLIDEAIQK.2	0.061773887	#N/A
VLDLDLFR.2	0.066642805	#N/A
YLIATSEQPIAALHR.3	-0.106373448	#N/A
TIC[Carbamidomethyl (C)]AILENYQAEK.2	-0.041553247	#N/A
YAGLSTC[Carbamidomethyl (C)]FR.2	0.281150951	#N/A
IEQFVYSSPHDNK.2	-0.115959903	#N/A
EVPLENQLQS[Phospho (STY)]M[Oxidation (M)]EVTEA.2	-0.210773513	P26638_S506_M1_Sars1
ELVSC[Carbamidomethyl (C)]SNC[Carbamidomethyl (C)]TDYQAR.2	0.505244092	#N/A
AKEVPLENQLQS[Phospho (STY)]MEVTEA.2	-0.405263103	P26638_S506_M1_Sars1
EIGNLLHPSVPISNDEDADNKVER.4	0.43124251	#N/A
AEDGAAPS[Phospho (STY)]PSSETPK.2	-0.114447681	P26645_S138_M1_Marcks
LSGFS[Phospho (STY)]FKK.2	-0.425236616	P26645_S163_M1_Marcks
GEATAERPGEAAVASS[Phospho (STY)]PSK.2	-0.022623304	P26645_S27_M1_Marcks
VNGDAS[Phospho (STY)]PAAAEPGAK.2	-0.02965963	P26645_S46_M1_Marcks
FS[Phospho (STY)]FKKS[Phospho (STY)]FK.2	-0.246961328	P26645_S152_M2_Marcks
LSGFS[Phospho (STY)]FKKS[Phospho (STY)]K.2	-0.213339601	P26645_S163_M2_Marcks
DEAAAATEPGAGAADKEAAEAEPAEPSS[Phospho (STY)]PAAEAEGASASSTSSPK.4	-0.06923714	P26645_S113_M1_Marcks
ESGEGAEAEGATAEGAK.2	0.165963479	#N/A
PGEAAVASS[Phospho (STY)]PSK.2	-0.12074244	P26645_S27_M1_Marcks
SKKES[Phospho (STY)]GEGAEAEGATAEGAK.3	-0.115132793	#N/A
AEDGAAPS[Phospho (STY)]PSSET[Phospho (STY)]PKKK.2	-0.216158267	#N/A
EAAEAEPAEPSS[Phospho (STY)]PAAEAEGASASSTSSPK.3	-0.077140284	P26645_S113_M1_Marcks
S[Phospho (STY)]FKLS[Phospho (STY)]GFSFK.2	0.045536705	P26645_S156_M2_Marcks
AEDGAAPSPSSET[Phospho (STY)]PKK.2	-0.086974604	P26645_T143_M1_Marcks
GEATAERPGEAAVASS[Phospho (STY)]PSKANGQENGHVK.4	-0.104508566	P26645_S27_M1_Marcks
LSGFS[Phospho (STY)]FK.2	-0.110050864	P26645_S163_M1_Marcks
TAAKGEATAERPGEAAVASS[Phospho (STY)]PSK.3	0.175085379	P26645_S27_M1_Marcks
KES[Phospho (STY)]GEGAEAEGATAEGAKDEAAAAAGGEGAAAPGEQAGGAGAEGAAGGEPR.4	-0.344116715	#N/A
ESGEGAEAEGATAEGAKDEAAAAAGGEGAAAPGEQAGGAGAEGAAGGEPR.4	0.015244116	#N/A
KES[Phospho (STY)]GEGAEAEGATAEGAK.2	-0.11593584	#N/A
GVQVETISPGDGR.2	-0.325294514	#N/A
GWEEGVAQM[Oxidation (M)]SVGQR.2	0.100169158	#N/A
SKVGS[Phospho (STY)]TENIK.2	-0.377719761	P27546_S914_M1_Map4
SAS[Phospho (STY)]ADLSR.2	0.327835729	P27546_S828_M1_Map4

LATTVS[Phospho (STY)]APDLK.2	-0.165065538	P27546_S901_M1_Map4
AAVGVTGNDITTPPNKEPPPS[Phospho (STY)]PEK.3	0.07226981	P27546_S667_M1_Map4
DM[Oxidation (M)]S[Phospho (STY)]PSAETEAPLAK.2	-0.136928768	P27546_S517_M1_Map4
TEFIPLLDGDEK.2	0.275149126	#N/A
NTT[Phospho (STY)]PTGAAPPAGM[Oxidation (M)]TSTR.2	-0.204610321	P27546_T847_M1_Map4
ATS[Phospho (STY)]PSTLVSTGPSSR.2	-0.206339563	P27546_S785_M1_Map4
DM[Oxidation (M)]S[Phospho (STY)]PLPESEVTLGK.2	-0.294693472	P27546_S475_M1_Map4
GQSTVPPC[Carbamidomethyl (C)]TAS[Phospho (STY)]PEPVK.2	-0.062965142	P27546_S598_M1_Map4
SKVGST[Phospho (STY)]ENIKHQPGGGR.3	0.006211778	P27546_T915_M1_Map4
TTSASS[Phospho (STY)]VKR.2	-0.370516539	P27546_S841_M1_Map4
VGS[Phospho (STY)]TENIKHQPGGGR.3	-0.191900949	P27546_S914_M1_Map4
DM[Oxidation (M)]S[Phospho (STY)]PLPESEVTLGKDVVILPETK.3	-0.271285805	P27546_S475_M1_Map4
VGS[Phospho (STY)]LDNVGHLPAGGAVK.2	-0.13674793	P27546_S1046_M1_Map4
VAEFNNVT[Phospho (STY)]PLS[Phospho (STY)]EEEVTSVK.2	0.25343576	P27546_S506_M2_Map4
AAVGVTGNDITT[Phospho (STY)]PPNKEPPPS[Phospho (STY)]PEKK.3	-0.388376626	P27546_S667_M2_Map4
DFMAALEAEPYDDIVGETVEK.2	0.202127002	#N/A
S[Phospho (STY)]PATTLPK.2	0.237868179	P27546_S798_M1_Map4
VGS[Phospho (STY)]TENIK.2	-0.538070041	P27546_S914_M1_Map4
AAEQM[Oxidation (M)]STLPIDAPS[Phospho (STY)]PLENLEQK.3	0.141525592	P27546_S617_M1_Map4
LTESLNIFETIVNNR.2	-0.144740177	#N/A
ALWEDSGIQNAYDR.2	0.474060418	#N/A
NNASTDYDLSDK.2	0.004031631	#N/A
LLGGVTIAQGGVLPNIQAVLLPK.2	0.290064846	#N/A
LLYGDAPKS[Phospho (STY)]PR.2	0.104798796	P27671_S718_M1_Rasgrf1
AGS[Phospho (STY)]ADDEDSDIKK.2	0.041302919	P27671_S199_M1_Rasgrf1
SEPIPESNEGPVK.2	0.18705939	#N/A
LAPEYEAAATR.2	0.058027345	#N/A
EATNPPIIQEEKPK.2	0.155877218	#N/A
ELNDFISYLQR.2	-0.007391663	#N/A
DLFSDGHSEFLK.2	-0.185222315	#N/A
TADGIVSHLK.2	0.156699314	#N/A
VDC[Carbamidomethyl (C)]TANTNTC[Carbamidomethyl (C)]NK.2	-0.023834448	#N/A
FISDKDASVVGFFR.3	0.064310751	#N/A
DLLTAYYDVDYEK.2	-0.038662447	#N/A
TFSHELSDFGLESTTGEVPVVAIR.3	0.060524453	#N/A
QAGPASVPLR.2	0.249909593	#N/A
FVM[Oxidation (M)]QEEFSR.2	0.312953181	#N/A
FAHTNIESLVK.2	0.083587127	#N/A
DGEEAGAYDGPR.2	0.24269843	#N/A
MDATANDVPSPYEVK.2	0.318082556	#N/A
SAS[Phospho (STY)]EPSLNR.2	-0.194211953	P28028_S714_M1_Braf
NNPKS[Phospho (STY)]PQKPIVR.3	-0.437767525	P28028_S135_M1_Braf
S[Phospho (STY)]PQKPIVR.2	-0.211879461	P28028_S135_M1_Braf
SSS[Phospho (STY)]APNVHINTIEPVNIDDLIR.3	0.068737	P28028_S348_M1_Braf
RDS[Phospho (STY)]SDDWEIPDGQITVGQR.3	0.135171298	P28028_S431_M1_Braf
AGFQTEDFSLYAC[Carbamidomethyl (C)]AS[Phospho (STY)]PK.2	0.038589342	P28028_S735_M1_Braf
DS[Phospho (STY)]SDDWEIPDGQITVGQR.2	-0.521163238	P28028_S431_M1_Braf

SC[Carbamidomethyl (C)]C[Carbamidomethyl (C)]SC[Carbamidomethyl (C)]C[Carbamidomethyl (C)]PAGC[Carbamidomethyl (C)]EK.2	0.038631719	#N/A
GEEGAKAEAEK.2	0.119240362	#N/A
DC[Carbamidomethyl (C)]VC[Carbamidomethyl (C)]KGEEGAK.2	0.308622235	#N/A
_[Acetyl (Protein N-term)]M[Oxidation (M)]DPETC[Carbamidomethyl (C)]PC[Carbamidomethyl (C)]PTGGSC[Carbamidomethyl (C)]TC[Ca	0.340925227	#N/A
DC[Carbamidomethyl (C)]VC[Carbamidomethyl (C)]KGEEGAKAEAEK.3	0.045264209	#N/A
KGS[Phospho (STY)]GFGHR.2	-0.021553227	P28230_S233_M1_Gjb1
VILQDFTGVPAVVDFAAM[Oxidation (M)]R.2	0.336256161	#N/A
AAADDGEEPKS[Phospho (STY)]EPETKK.3	-0.220069643	P28352_S18_M1_Apex1
IDPSAPLDK.2	0.233034812	#N/A
AAVAWEAGKPLSIEEIEVAPPK.3	0.043588472	#N/A
EFGASEC[Carbamidomethyl (C)]ISPQDFS[Phospho (STY)]K.2	-0.192154405	P28474_S247_M1_Adh5
AKEFGASEC[Carbamidomethyl (C)]ISPQDFS[Phospho (STY)]K.3	0.054040843	P28474_S247_M1_Adh5
LVSEYMSK.2	-0.359511875	#N/A
SIQEVLVEM[Oxidation (M)]TDGGVDYSFEC[Carbamidomethyl (C)]IGNVK.3	0.185591081	#N/A
YAPTTTPS[Phospho (STY)]PEDGFEVQPLHPDK.3	-0.560206254	P28571_S659_M1_Slc6a9
VVDLLATDADIVSR.2	0.284551196	#N/A
IC[Carbamidomethyl (C)]DLLSDFDEFSAR.2	-0.047572332	#N/A
ESSDS[Phospho (STY)]TNTTIEDEDAK.2	0.155542018	P28652_S397_M1_Camk2b
FTDEYQLYEDIGK.2	-0.171030731	#N/A
HPWVC[Carbamidomethyl (C)]QR.2	0.136942597	#N/A
ITAHEALK.2	0.20815775	#N/A
LC[Carbamidomethyl (C)]TGHEYAAK.2	0.192772505	#N/A
ADGVKPQTNS[Phospho (STY)]TK.2	-0.085809514	P28652_S358_M1_Camk2b
FYFENLLAK.2	0.201585162	#N/A
AGAYDFPSPEWDTVTPEAK.2	0.064697409	#N/A
NLINQM[Oxidation (M)]LTINPAK.2	0.101152457	#N/A
QET[Phospho (STY)]VEC[Carbamidomethyl (C)]LKK.2	0.385353869	P28652_T287_M1_Camk2b
GAILTTM[Oxidation (M)]LATR.2	0.26879743	#N/A
WQNVHFHC[Carbamidomethyl (C)]SGAPVAPLQ.3	0.145395189	#N/A
LTQYIDGQGR.2	0.238640592	#N/A
QET[Phospho (STY)]VEC[Carbamidomethyl (C)]LK.2	0.27292586	P28652_T287_M1_Camk2b
ESSDST[Phospho (STY)]NT[Phospho (STY)]T[Phospho (STY)]IEDEDAKAR.2	-0.179735292	P28652_T398_M3_Camk2b
DLKPENLLLASK.2	-0.150882395	#N/A
NSSAITS[Phospho (STY)]PK.2	-0.359947129	P28652_S367_M1_Camk2b
LTQYIDGQGRPR.2	0.198911857	#N/A
S[Phospho (STY)]TVASMMHR.2	0.506574533	P28652_S276_M1_Camk2b
IC[Carbamidomethyl (C)]DPGLTSFEPEALGNLVEGM[Oxidation (M)]DFHR.3	0.166199698	#N/A
TTEQLIEAVNNGDFEAYAK.2	-0.519804693	#N/A
KADGVKPQTNS[Phospho (STY)]TK.2	-0.355214161	P28652_S358_M1_Camk2b
QTTAPATMSTAASGTTM[Oxidation (M)]GLVEQAK.2	-0.28244148	#N/A
LDGLVDTPTGYIESLPK.2	-0.100008773	#N/A
NVDLLSDMVQEHDEPILK.3	-0.23062834	#N/A
TWS[Phospho (STY)]EKDLR.2	-0.194932948	P28659_S28_M1_Celf1
NNNQQLAQLQK.2	0.315729781	#N/A
NLSADQWR.2	0.188901738	#N/A
AAEDLFVNIR.2	-0.390058897	#N/A
SVLQPSQQK.2	0.058349553	#N/A

NAYHAVYK.2	-0.510142044	#N/A
LVVENVDVLTQM[Oxidation (M)]R.2	0.083128901	#N/A
ASLSLADHR.2	0.442111228	#N/A
PQS[Phospho (STY)]PDLC[Carbamidomethyl (C)]DDDVEFR.2	-0.196726161	P28661_S68_M1_Septin4
PRS[Phospho (STY)]PWGK.2	-0.083643073	P28661_S107_M1_Septin4
ESIPFAVIGSNTVVEAR.2	0.03376622	#N/A
VARPQILEPRPQS[Phospho (STY)]PDLC[Carbamidomethyl (C)]DDDVEFR.4	-0.286380746	P28661_S68_M1_Septin4
PQILEPRPQS[Phospho (STY)]PDLC[Carbamidomethyl (C)]DDDVEFR.3	-0.206660377	P28661_S68_M1_Septin4
IYQFPDC[Carbamidomethyl (C)]DS[Phospho (STY)]DEDEDFKLQDQALK.3	0.077849919	P28661_S325_M1_Septin4
IYQFPDC[Carbamidomethyl (C)]DS[Phospho (STY)]DEDEDFK.2	0.002313085	P28661_S325_M1_Septin4
STLVNSLFLTDLYR.2	0.174086445	#N/A
HFLEDS[Phospho (STY)]SDDAELSK.2	0.083656611	P28661_S28_M1_Septin4
LDPY[Phospho (STY)]DSSEDDKEYVGFATLPNQVHR.3	-0.1971778	P28661_Y115_M1_Septin4
VAAYAAQLEQYQK.2	0.00654872	#N/A
NWSAAGNAFC[Carbamidomethyl (C)]QAAK.2	-0.152953661	#N/A
KADPQEAINC[Carbamidomethyl (C)]LNAAIDIYTDM[Oxidation (M)]GR.3	-0.34632763	#N/A
YEEM[Oxidation (M)]FPAFTDSR.2	-0.274757112	#N/A
AALC[Carbamidomethyl (C)]HFIVDELNAK.3	-0.140172596	#N/A
SIQGDGEGDGDLK.2	-0.390901229	#N/A
HDSATSFVDAGNAYK.2	-0.434472117	#N/A
LDQWLTTM[Oxidation (M)]LLR.2	0.033078851	#N/A
LLEAHEEQNSEAYTEAVK.3	-0.197822653	#N/A
ASHS[Phospho (STY)]FLR.2	-0.111529387	P28663_S26_M1_Napb
AIEIYEQVGANTM[Oxidation (M)]DNPLLK.2	-0.079787459	#N/A
LHMQLQSK.2	0.292699593	#N/A
IEEAC[Carbamidomethyl (C)]EMYTR.2	0.249134956	#N/A
LLEAHEEQNSEAYTEAVKEFDSISR.4	-0.121741472	#N/A
AIAHYEQSADYYKGEESNSSANK.3	-0.334130888	#N/A
EAVQLMAEAEKR.2	0.419251447	#N/A
KLLEAHEEQNSEAYTEAVK.3	-0.218105441	#N/A
AIAHYEQSADYYK.3	0.393419475	#N/A
AAAT[Phospho (STY)]PESQEPQAK.2	-0.093656922	P28667_T148_M1_Marcksl1
GDVTAEEAAGAS[Phospho (STY)]PAK.2	-0.188114801	P28667_S22_M1_Marcksl1
LSGLS[Phospho (STY)]FKR.2	-0.425238845	P28667_S104_M1_Marcksl1
GEVAPKET[Phospho (STY)]PK.2	-0.061305992	P28667_T85_M1_Marcksl1
LSGLS[Phospho (STY)]FK.2	-0.252012869	P28667_S104_M1_Marcksl1
GEVAPKET[Phospho (STY)]PKK.2	-0.068759796	P28667_T85_M1_Marcksl1
SVELDS[Phospho (STY)]DDGGGSAAQK.2	-0.173883771	P28738_S820_M1_Kif5c
LYLVDLAGSEK.2	-0.050385407	#N/A
ILQDSLGGNC[Carbamidomethyl (C)]R.2	0.357082078	#N/A
SLEPC[Carbamidomethyl (C)]DNT[Phospho (STY)]PIIDNITPVVDGISAEK.3	-0.125102312	P28738_T396_M1_Kif5c
SLSALGNVISALAEGTK.2	-0.199035536	#N/A
ISFLENNLEQLTK.2	-0.36393363	#N/A
TTIVIC[Carbamidomethyl (C)]C[Carbamidomethyl (C)]SPSVFNEAETK.2	-0.23035929	#N/A
HVAVTNMNEHSSR.3	-0.339192438	#N/A
GSLDYRPLTTADPIDEHR.3	-0.182455954	#N/A
PSQLPEQSSSAQQNGSVSDIS[Phospho (STY)]PVQAAK.3	-0.134601414	P28740_S139_M1_Kif2a

YLENQTFR.2	0.055170626	#N/A
IDILTELR.2	0.306786099	#N/A
LIDIGNSC[Carbamidomethyl (C)]R.2	-0.277307839	#N/A
DFRGS[Phospho (STY)]LDYRPLTTADPIDEHR.4	0.168947159	P28740_S206_M1_Kif2a
AVFQESIR.2	-0.203206512	#N/A
S[Phospho (STY)]AKPETVIDSLLQ.2	-0.002657019	P28741_S689_M1_Kif3a
KQT[Phospho (STY)]PVPDKK.2	-0.111047196	P28741_T635_M1_Kif3a
LLAEALNQVTQR.2	0.123577011	#N/A
SPSDYSNFDPEFLNEKPQLS[Phospho (STY)]FSDK.3	-0.460269262	P28867_S643_M1_Prkcd
FSPAGPILSIR.2	-0.181699167	#N/A
SLGYAYVNFQQPADAER.2	-0.225077753	#N/A
ALYDTFSAFGNILSC[Carbamidomethyl (C)]K.2	0.18109511	#N/A
SKVDEAVAVLQAHQAK.3	-0.144333015	#N/A
NLDDGIDDERLR.3	-0.287677483	#N/A
SGVLAGHDNR.2	-0.255385142	#N/A
QEFSS[Phospho (STY)]EEM[Oxidation (M)]TK.2	0.004293643	P29533_S166_M1_Vcam1
EIEIDIEPTDKVER.2	-0.069353958	#N/A
VM[Oxidation (M)]HTQC[Carbamidomethyl (C)]HSTPDS[Phospho (STY)]AEDVR.3	0.107222119	#N/A
VM[Oxidation (M)]HTQC[Carbamidomethyl (C)]HSTPDS[Phospho (STY)]AEDVRK.4	-0.323767357	#N/A
LPSDVVTSVR.2	0.368878677	#N/A
IAIAALEVLEEENLAENADK.2	-0.382149656	#N/A
IVFADGNFWGR.2	0.432965591	#N/A
AFYNNVLGEYEEYITK.2	0.25323091	#N/A
KTEQGPPSSEYIFER.3	0.286540477	#N/A
LYPPSAEYPDLR.2	0.035718813	#N/A
VVVDALSGLK.2	0.245509829	#N/A
TTPTGWTLDQC[Carbamidomethyl (C)]IQTGVDNPGHPFIK.3	-0.197251866	#N/A
GWEFM[Oxidation (M)]WNER.2	-0.111676493	#N/A
LYPPSAEYPDLRK.3	0.01390714	#N/A
TVGM[Oxidation (M)]VAGDEETYEVFAELFDPVIQER.3	-0.121843796	#N/A
HTTDLDASK.2	0.019208034	#N/A
GIWHNNEK.2	0.288150572	#N/A
HNGYDPR.2	0.102817538	#N/A
HTTDLDAS[Phospho (STY)]KIR.2	-0.174506283	P30275_S148_M1_Ckmt1
GLSLPPAC[Carbamidomethyl (C)]TR.2	0.431322201	#N/A
GLKEVEK.2	-0.180299749	#N/A
HNNC[Carbamidomethyl (C)]M[Oxidation (M)]ASHLTPAVYAR.3	-0.061787182	#N/A
GTGGVDTAATGSVFDISNLDR.2	-0.16044206	#N/A
GWEFMWNERLGYILTC[Carbamidomethyl (C)]PSNLGTGLR.3	0.252823266	#N/A
SFLIWVNEEDHTR.2	0.269883086	#N/A
LGYILTC[Carbamidomethyl (C)]PSNLGTGLR.2	-0.18116198	#N/A
VVVDALSGLKGDLAGR.2	-0.031629272	#N/A
SGYFDER.2	-0.04596948	#N/A
IRS[Phospho (STY)]GYFDER.2	-0.191760781	P30275_S152_M1_Ckmt1
RGTGGVDTAATGSVFDISNLDR.3	-0.137101619	#N/A
TC[Carbamidomethyl (C)]DEGSS[Phospho (STY)]PSKPK.2	-0.555534147	P30415_S1060_M1_Nktr
TQLAVC[Carbamidomethyl (C)]QQR.2	0.08104412	#N/A

AYS[Phospho (STY)]VEDFQPENLESGGVFR.2	-0.339241317	P30873_S364_M1_Sstr1
VGGSS[Phospho (STY)]VDLHR.2	-0.180003408	P30999_S269_M1_Ctnnd1
HYEDGYPGGSDNYGS[Phospho (STY)]LSR.3	-0.428665744	P30999_S230_M1_Ctnnd1
GSLAS[Phospho (STY)]LDSLRK.2	-0.100641841	P30999_S349_M1_Ctnnd1
SQSSHSY[Phospho (STY)]DDSTLPLIDR.3	-0.030027386	P30999_Y865_M1_Ctnnd1
GS[Phospho (STY)]LASLDSLR.2	0.235248764	P30999_S346_M1_Ctnnd1
S[Phospho (STY)]M[Oxidation (M)]GYDDLDYGM[Oxidation (M)]M[Oxidation (M)]SDYGTAR.2	-0.011944027	P30999_S288_M1_Ctnnd1
TLDRS[Phospho (STY)]GDLGDMEPLK.3	-0.627663992	P30999_S920_M1_Ctnnd1
AATITATSPGALWGLDR.2	0.019758886	#N/A
TWGDAGAAAGGGIPSK.2	0.242788059	#N/A
C[Carbamidomethyl (C)]VGNYDNR.2	0.127826257	#N/A
ASVC[Carbamidomethyl (C)]AEAYNPDEEEDDAESR.2	-0.155779494	#N/A
AASAHAIGTVK.2	0.132674708	#N/A
VYNDGEQIIAQGDLADSFFIVESGEVK.3	-0.187038906	#N/A
LQQENER.2	-0.107344436	#N/A
GSFGELALM[Oxidation (M)]YNTPR.2	0.119102946	#N/A
VVDVIGTK.2	0.33141576	#N/A
EGEHVIDQGDDGDNFYVIDR.3	-0.355015037	#N/A
GTFDIYVK.2	-0.321604858	#N/A
GVNFAEEPM[Oxidation (M)]RS[Phospho (STY)]DSENGEEEEAAEAGAFNAPVINR.4	0.21041039	P31324_S83_M1_Prkar2b
HQPADLLEFALQHFTR.3	-0.409995842	#N/A
NLDPEQM[Oxidation (M)]SQVLDAMFEK.2	0.322861388	#N/A
RAS[Phospho (STY)]VC[Carbamidomethyl (C)]AEAYNPDEEEDDAESR.3	-0.108215524	P31324_S112_M1_Prkar2b
C[Carbamidomethyl (C)]LAMDVQAFER.2	0.086251233	#N/A
WLEEADSSSGS[Phospho (STY)]PTSIDK.2	-0.336206176	P31360_S343_M1_Pou3f2
WLEEADSSTGS[Phospho (STY)]PTSIDK.2	-0.067555973	P31361_S390_M1_Pou3f3;Pou3f4
QC[Carbamidomethyl (C)]DNPWNTDR.2	-0.0781135	#N/A
AGDLPDRDTWK.2	-0.320749713	#N/A
VADGQIS[Phospho (STY)]TEVSEAPVASDKPK.3	-0.140399512	P31648_S14_M1_Slc6a1
GS[Phospho (STY)]ETLGGGGGGAAGTR.2	-0.44362527	P31650_S21_M1_Slc6a11
LTVPSADLK.2	-0.142420107	#N/A
AAEEARGS[Phospho (STY)]ETLGGGGGGAAGTR.3	0.221942244	P31650_S21_M1_Slc6a11
VKGDGTISAITEK.3	-0.098542246	#N/A
SGS[Phospho (STY)]PSDNSGAEEM[Oxidation (M)]EVSLAKPK.3	-0.290469972	P31750_S124_M1_Akt1
AKWDSWNK.2	0.03748012	#N/A
TYVEKVDELKK.3	-0.029547164	#N/A
[Acetyl (Protein N-term)]SQAEFDKAAEEVKR.3	-0.233100835	#N/A
TQPTDEEM[Oxidation (M)]LFIYSHFK.3	-0.026670757	#N/A
QATVGDVNTDRPGLLDLK.3	-0.052015647	#N/A
[Acetyl (Protein N-term)]SQAEFDKAAEEVK.2	-0.146607865	#N/A
TYVEKVDELK.2	0.050664955	#N/A
DVKPSNILVNSR.2	0.056826093	#N/A
KLEELELDEQQR.3	0.059142515	#N/A
IPEQILGK.2	0.063136689	#N/A
LEAFLTQK.2	0.188545062	#N/A
ISELGAGNGGVVFK.2	-0.364734858	#N/A
LPSGVFSLEFQDFVNK.2	-0.103593899	#N/A

LC[Carbamidomethyl (C)]DFGVSGQLIDSM[Oxidation (M)]ANSFVGTR.2	-0.275746925	#N/A
KLEELELDEQQRK.3	-0.05983474	#N/A
VGELKDDDFEK.3	0.103417951	#N/A
LEELELDEQQR.2	0.482787868	#N/A
LQGTHYSVQSDIWSM[Oxidation (M)]GLSLVEM[Oxidation (M)]AVGR.3	-0.378746602	#N/A
KLEEEGEQFVKK.3	0.059548587	#N/A
GSVLPNSDKK.2	-0.170951846	#N/A
EATWVVDVK.2	0.087234949	#N/A
EEDQATEILQR.2	-0.271572456	#N/A
QVTVLELFR.2	-0.014195101	#N/A
LWGTSDVVQEIQEM[Oxidation (M)]KDESVR.3	-0.068937173	#N/A
TFEDIAR.2	0.059358603	#N/A
S[Phospho (STY)]PSRPLPEVTDEYKNDVK.3	-0.491876623	P32067_S92_M1_Ssb
KFAS[Phospho (STY)]IAR.2	-0.439022283	P32211_S379_M1_Chrm4
LVVVGAGGVGK.2	0.559134799	#N/A
SYGIPFIETSAK.2	0.282902274	#N/A
TGEGFLC[Carbamidomethyl (C)]VFAINNTK.2	-0.416139715	#N/A
QVVIDGETC[Carbamidomethyl (C)]LLDILDTAGQEEYSAM[Oxidation (M)]R.3	-0.01033911	#N/A
SFEDIHHYR.3	-0.167926808	#N/A
ASEDFVDPWTVR.2	0.420358673	#N/A
TLIDVLQPLIAEHQAR.2	-0.106169526	#N/A
DLTLEQAYSYTVENAK.2	0.192397966	#N/A
C[Carbamidomethyl (C)]NAIINEDPNNK.2	-0.169559008	#N/A
AASVSS[Phospho (STY)]LHER.2	-0.24032321	P33173_S419_M1_Kif1a
NLFGSGS[Phospho (STY)]LR.2	-0.330167141	P33173_S1317_M1_Kif1a
DVLS[Phospho (STY)]PLRPSR.2	-0.346123426	P33173_S1093_M1_Kif1a
SGTS[Phospho (STY)]QEELR.2	-0.268453746	P33173_S937_M1_Kif1a
KTPS[Phospho (STY)]PVR.2	0.297808277	P33173_S1553_M1_Kif1a
ISLVDLAGSER.2	-0.046422924	#N/A
FQSESC[Carbamidomethyl (C)]PVVGMS[Phospho (STY)]R.2	0.081753876	P33173_S932_M1_Kif1a
IS[Phospho (STY)]FDDQHFEK.2	0.016945411	P33173_S912_M1_Kif1a
S[Phospho (STY)]DSLILDHQWELEK.2	0.211320337	P33173_S1373_M1_Kif1a
VTGVYELSLC[Carbamidomethyl (C)]HVADAGS[Phospho (STY)]PGM[Oxidation (M)]QR.3	-0.422477017	P33173_S1342_M1_Kif1a
TPQPC[Carbamidomethyl (C)]S[Phospho (STY)]RPAS[Phospho (STY)]PEPELLPELDSK.3	0.105667738	P33173_S1533_M2_Kif1a
PASPEPELLPELDSKKT[Phospho (STY)]PS[Phospho (STY)]PVR.4	-0.104668655	P33173_S1553_M2_Kif1a
PAS[Phospho (STY)]PEPELLPELDSK.2	-0.116614807	P33173_S1537_M1_Kif1a
FLNAENAQK.2	0.118938525	#N/A
FASENDLPEWK.2	0.156900404	#N/A
TLEEDEEELFK.2	-0.084487419	#N/A
VAEKLEALSVR.3	-0.141468037	#N/A
YMEDSTY[Phospho (STY)]YK.2	-0.128978333	P34152_Y576_M1_Ptk2
LQPQEIS[Phospho (STY)]PPPTANLDR.2	-0.354265971	P34152_S910_M1_Ptk2
LHISPDR.2	-0.036631989	#N/A
LLC[Carbamidomethyl (C)]GLLSDR.2	-0.089545915	#N/A
ASVPEGFLSELTQQLAQATGK.2	0.078670606	#N/A
PM[Oxidation (M)]FIVNTNVPR.2	0.130693731	#N/A
PAQYIAVHVVPDQLM[Oxidation (M)]TFSGTNDPC[Carbamidomethyl (C)]ALC[Carbamidomethyl (C)]SLHSIGK.4	-0.529769671	#N/A

VYINYYDM[Oxidation (M)]NAANVGWNGSTFA.2	-0.203279305	#N/A
IQNTGDY[Phospho (STY)]YDLYGGEK.2	-0.140255914	P35235_Y62_M1_Ptpn11
ESQSHPGDFVLSVR.3	0.032180299	#N/A
FEIWDTAGQER.2	0.171996173	#N/A
GVDLQESNPASR.2	-0.070035183	#N/A
LVLLGESAVGK.2	0.019339169	#N/A
QASPNIVIALAGNK.2	-0.299817047	#N/A
YHSLAPM[Oxidation (M)]YYR.3	0.374273095	#N/A
GAQAAIVVYDITNTDTFAR.2	-0.345603692	#N/A
AVEFQEAQAYADDNSLLFM[Oxidation (M)]ETSAK.3	-0.178030258	#N/A
[Acetyl (Protein N-term)]S[Phospho (STY)]AGGDFGNPLRK.2	-0.348059387	P35279_S2_M1_Rab6a
TDLADKR.2	0.08772642	#N/A
LVFLGEQSVGK.2	0.084415324	#N/A
SREDMIDIK.2	-0.044808536	#N/A
VAAALPGMESTQDR.2	0.034873474	#N/A
DSNGAILVYDVTDEDSFQK.2	-0.508736027	#N/A
VNLAIWDTAGQER.2	-0.450098324	#N/A
FNS[Phospho (STY)]ITSAYYR.2	-0.154607993	P35283_S105_M1_Rab12
IIQTPGLWESENQNK.2	-0.498794663	#N/A
LAIWDTAGQER.2	0.348808355	#N/A
TC[Carbamidomethyl (C)]DGVQC[Carbamidomethyl (C)]AFEELVEK.2	-0.090512851	#N/A
AMSIPTS[Phospho (STY)]PTR.2	0.004294543	P35347_S400_M1_Crhr1;Crhr2
S[Phospho (STY)]IHREPGSYAGR.3	-0.398770344	P35363_S298_M1_Htr2a
QHS[Phospho (STY)]YDNILDKPR.3	-0.010921235	P35436_S1291_M1_Grin2a
YLPEEVAHS[Phospho (STY)]DIS[Phospho (STY)]ETSSR.2	0.216842928	P35436_S1059_M2_Grin2a
KMPS[Phospho (STY)]IESDV.2	0.45935592	P35436_S1459_M1_Grin2a
SM[Oxidation (M)]ESLRQDS[Phospho (STY)]LNQNPVSQR.3	-0.260019496	P35436_S1025_M1_Grin2a
SIS[Phospho (STY)]LKDR.2	-0.110801053	P35436_S1312_M1_Grin2a
DKGS[Phospho (STY)]PHSEGSDRYR.3	-0.500760175	P35436_S1198_M1_Grin2a
SPDFNLTGS[Phospho (STY)]QSNM[Oxidation (M)]LK.2	-0.329915238	P35436_S890_M1_Grin2a
QDS[Phospho (STY)]LNQNPVSQR.2	0.022561635	P35436_S1025_M1_Grin2a
KKS[Phospho (STY)]PDFNLTGS[Phospho (STY)]QSNMLK.3	-0.090987994	P35436_S882_M2_Grin2a
STASYC[Carbamidomethyl (C)]SRDS[Phospho (STY)]R.2	-0.226021603	P35436_S1416_M1_Grin2a
LEEGPPVTTVLTR.2	-0.190246567	#N/A
AILAELTGR.2	0.078312922	#N/A
FAAAYC[Carbamidomethyl (C)]R.2	0.009223583	#N/A
TREEIQEVR.2	0.134826162	#N/A
AAASTDYYKR.2	-0.425122998	#N/A
VDGM[Oxidation (M)]DILC[Carbamidomethyl (C)]VR.2	0.349040265	#N/A
YGM[Oxidation (M)]GTSVER.2	0.018211128	#N/A
NFYGGNGIVGAQVPLGAGIALAC[Carbamidomethyl (C)]K.3	0.034372898	#N/A
GPILM[Oxidation (M)]ELQTYR.2	0.447856345	#N/A
M[Oxidation (M)]VNSNLASVEELKEIDVEVR.3	-0.22526237	#N/A
YHGHS[Phospho (STY)]M[Oxidation (M)]SDPGVSYR.3	-0.189841741	P35486_S293_M1_Pdha1
LPC[Carbamidomethyl (C)]IFIC[Carbamidomethyl (C)]ENNR.2	-0.068501386	#N/A
YHGHSMSDPGVS[Phospho (STY)]YRT[Phospho (STY)]REEIQEVR.4	-0.296789089	P35486_T303_M2_Pdha1
M[Oxidation (M)]VNSNLASVEELKEIDVEVRK.4	0.345017818	#N/A

ASLQNLLSASQAR.2	0.189356484	#N/A
ASSIVVSGTPIR.2	0.044943991	#N/A
APVPTGEVYFADSFDR.2	-0.10614953	#N/A
TYFTDKK.2	-0.223284246	#N/A
AEEDEILNRS[Phospho (STY)]PR.2	-0.310434109	P35564_S582_M1_Canx
QKS[Phospho (STY)]DAEEDGVTGS[Phospho (STY)]QDEEDSKPK.3	-0.234107239	P35564_S553_M2_Canx
AEEDEILNR.2	0.29010543	#N/A
SDAEEDGVTGS[Phospho (STY)]QDEEDSKPK.3	-0.106385362	P35564_S563_M1_Canx
VVDDWANDGWGLK.2	-0.201673563	#N/A
[Acetyl (Protein N-term)]AS[Phospho (STY)]PPDTDGFSDVRK.2	-0.071527407	P35569_S3_M1_Irs1
[Acetyl (Protein N-term)]AS[Phospho (STY)]PPDTDGFSDVR.2	-0.034914573	P35569_S3_M1_Irs1
VLFDNTGR.2	0.023287897	#N/A
IGYPAPNFK.2	-0.305639364	#N/A
TIAQDYGVLK.2	0.260556439	#N/A
LVQAFQFTDK.2	0.204724892	#N/A
ADEGISFR.2	-0.033082494	#N/A
SKEYFSK.2	0.145738111	#N/A
GLFIIDDK.2	0.270479025	#N/A
ATAVM[Oxidation (M)]PDGQFK.2	0.388180771	#N/A
DISLSEYK.2	-0.133714963	#N/A
SVDEIIR.2	-0.148341092	#N/A
LNC[Carbamidomethyl (C)]QVIGASVDSHFC[Carbamidomethyl (C)]HLAWINTPK.3	-0.061158559	#N/A
QFYDQALQQAVM[Oxidation (M)]DDDANNAK.3	-0.33378609	#N/A
NSLC[Carbamidomethyl (C)]PSGGNILTPLLQQDC[Carbamidomethyl (C)]HQK.3	-0.062916201	#N/A
TFHETLNC[Carbamidomethyl (C)]C[Carbamidomethyl (C)]GSNALTTLTTTILR.3	-0.147783198	#N/A
QFGIVTIGEEK.2	-0.024928398	#N/A
IC[Carbamidomethyl (C)]TASENFLR.2	-0.050381715	#N/A
KIC[Carbamidomethyl (C)]TASENFLR.2	-0.169399207	#N/A
SKEEQELHDIHSTR.3	0.120068961	#N/A
M[Oxidation (M)]QKYEDIK.2	-0.408119045	#N/A
QFGIVTIGEEKK.2	0.050383762	#N/A
DLYGDFK.2	-0.089418686	#N/A
EEQELHDIHSTRS[Phospho (STY)]K.3	-0.27773697	P35802_S270_M1_Gpm6a
S[Phospho (STY)]KERLNAYT.2	-0.097212782	P35802_S270_M1_Gpm6a
SKEEQELHDIHSTRS[Phospho (STY)]K.3	0.028781412	P35802_S270_M1_Gpm6a
EEQELHDIHSTRS[Phospho (STY)]KER.3	-0.206535009	P35802_S270_M1_Gpm6a
YEDIKS[Phospho (STY)]KEEQELHDIHSTR.4	-0.299804489	P35802_S256_M1_Gpm6a
EEQELHDIHSTR.3	0.14560005	#N/A
[Acetyl (Protein N-term)]MEENMEEGQTQK.2	0.254745821	#N/A
AKEEQELQDIQS[Phospho (STY)]R.2	-0.154078095	P35803_S318_M1_Gpm6b
EEQELQDIQS[Phospho (STY)]R.2	-0.072952	P35803_S318_M1_Gpm6b
QYGIIPWNAFPGK.2	-0.209971034	#N/A
ELHGEFK.2	-0.228065341	#N/A
S[Phospho (STY)]KEQLNSYT.2	-0.377396076	P35803_S320_M1_Gpm6b
MQAYQDIK.2	0.219273035	#N/A
[Acetyl (Protein N-term)]M[Oxidation (M)]KPAM[Oxidation (M)]ETAAEENTEQS[Phospho (STY)]QER.2	0.078612936	P35803_S16_M1_Gpm6b
DADVSEES[Phospho (STY)]PPPLPER.2	0.289315689	P35831_S673_M1_Ptpn12

NLS[Phospho (STY)]FEIKK.2	0.078137403	P35831_S434_M1_Ptpn12
S[Phospho (STY)]PAEVTDIGFGNR.2	-0.197595031	P35831_S748_M1_Ptpn12
DQITKS[Phospho (STY)]PAEVTDIGFGNR.3	-0.379882293	P35831_S748_M1_Ptpn12
NLS[Phospho (STY)]FEIK.2	-0.25953002	P35831_S434_M1_Ptpn12
DGFADPHFHSGS[Phospho (STY)]SDDVR.3	0.238296309	P35969_S1207_M1_Flt1
EILGTAQSVGC[Carbamidomethyl (C)]NVDGR.2	-0.046733163	#N/A
QAQIEVVPSASALIIK.2	0.189307323	#N/A
HSGNITFDEIVNIAR.3	-0.293596024	#N/A
ILTFDQLALESPK.2	-0.000296305	#N/A
SQDIYLR.2	0.04720731	#N/A
LVFLGLDNAGK.2	0.203357239	#N/A
SPS[Phospho (STY)]PGRREEDGDELAR.3	-0.587960333	P36552_S101_M1_Cpox
GLQDGLRS[Phospho (STY)]SS[Phospho (STY)]NSR.2	-0.329793232	P36916_S32_M2_Gnl1
REEQTDTS[Phospho (STY)]DGESVTHHIR.4	-0.276073813	P36916_S51_M1_Gnl1
EEQTDTS[Phospho (STY)]DGESVTHHIR.3	-0.109981966	P36916_S51_M1_Gnl1
ERREEQTDT[Phospho (STY)]S[Phospho (STY)]DGESVTHHIR.4	-0.081391862	#N/A
EVYQPGSVLDFPR.2	0.138132834	#N/A
LLEAGDFIC[Carbamidomethyl (C)]QALNR.2	0.510597313	#N/A
EVSVFGAVSELFTR.2	-0.502111924	#N/A
VKQEPPS[Phospho (STY)]PPHS[Phospho (STY)]PR.2	0.005928837	P38532_S303_M2_Hsf1
DAGQISGLNVLR.2	0.215131232	#N/A
NAVITVPAYFNDSQR.2	-0.056552246	#N/A
TTPSVVAFTADGER.2	0.095281378	#N/A
VQQTVQDLFGR.2	0.013864069	#N/A
AQFEGIVTDLIK.2	0.333013903	#N/A
NTTIPTKK.2	-0.108474197	#N/A
VINEPTAAALAYGLDK.2	0.319659258	#N/A
QAVTNPNNTFYATK.2	0.548094599	#N/A
QAASSLQQASLK.2	-0.073488741	#N/A
STNGDTFLGGEDFDQALLR.2	0.274282145	#N/A
EQQIVIQSSGGLSKDDIENM[Oxidation (M)]VK.3	-0.370742961	#N/A
LLGQFTLIGIPPAPR.2	-0.250465378	#N/A
ETAENYLGHTAK.2	-0.12655824	#N/A
ALLAGKDSETGENIR.2	-0.273482291	#N/A
ETGVDLTKDNM[Oxidation (M)]ALQR.3	-0.243214287	#N/A
S[Phospho (STY)]DIGEVILVGGMT[Phospho (STY)]R.2	-0.154129122	P38647_S378_M2_Hspa9
AM[Oxidation (M)]QDAEVSK.2	-0.010832953	#N/A
RYDDPEVQKDTK.3	-0.047850994	#N/A
M[Oxidation (M)]EEFKDQLPADEC[Carbamidomethyl (C)]NK.3	-0.001215072	#N/A
AM[Oxidation (M)]QDAEVSKSDIGEVILVGGMTR.3	0.167815759	#N/A
ASNGDAWVEAHGK.2	0.269742469	#N/A
VEAVNM[Oxidation (M)]AEGIIHDTETK.3	-0.345973394	#N/A
VINEPTAAALAYGLDKSEDK.3	-0.49649963	#N/A
TIAPC[Carbamidomethyl (C)]QK.2	0.003852581	#N/A
MKETAENYLGHTAK.3	-0.193483831	#N/A
GAVVGIDLGTTNSC[Carbamidomethyl (C)]VAVM[Oxidation (M)]EGK.2	0.027314341	#N/A
SQVFSTAADGQTQVEIK.2	0.031613506	#N/A

S[Phospho (STY)]PTSSPTPQR.2	-0.189925614	P39053_S774_M1_Dnm1
GISPVPINLR.2	0.177034092	#N/A
SSVLENFVGR.2	-0.163234252	#N/A
VPVGDQPPDIEFQIR.2	-0.202194124	#N/A
DITAALAAER.2	0.182744151	#N/A
VLNQQLTNHIR.2	0.252104426	#N/A
VYSPHVLNLTLVDLPGM[Oxidation (M)]TK.3	-0.580833921	#N/A
IVTTHIR.2	0.046897032	#N/A
FFLSHPSYR.2	0.019767867	#N/A
NFRPDDPAR.2	0.533907285	#N/A
GYIGVVNR.2	-0.02433296	#N/A
LQDAFSAIGQNADLDLPQIAVVGGQSAGK.3	0.091194053	#N/A
NVYKDYR.2	-0.21750287	#N/A
C[Carbamidomethyl (C)]VDM[Oxidation (M)]VISELISTVR.2	-0.128844548	#N/A
GM[Oxidation (M)]EDLIPLVNR.2	0.195963103	#N/A
KKTS[Phospho (STY)]GNQDEILVIR.3	0.290295579	P39053_S512_M1_Dnm1
EVDEYKNFRPDDPAR.3	0.313130914	#N/A
M[Oxidation (M)]GTPYLQK.2	-0.397134108	#N/A
FTDFEEVR.2	0.237008114	#N/A
ALLQM[Oxidation (M)]VQQFAVDFEKR.3	0.03432256	#N/A
LDLM[Oxidation (M)]DEGTDAR.2	0.177728045	#N/A
NLVDSYM[Oxidation (M)]AIVNK.2	-0.330026829	#N/A
LREEM[Oxidation (M)]ER.2	0.357270419	#N/A
EISYAIK.2	0.288632238	#N/A
KDITAALAAER.2	-0.174200746	#N/A
TSGNQDEILVIR.2	0.216425149	#N/A
ENC[Carbamidomethyl (C)]LILAVSPANSDLANSDALK.2	-0.222211284	#N/A
RSPTSS[Phospho (STY)]PTPQR.2	-0.314940317	P39053_S778_M1_Dnm1
LEIEAETDRVTGTNK.2	-0.152963022	#N/A
S[Phospho (STY)]PTSS[Phospho (STY)]PTPQRR.2	-0.282293977	P39053_S778_M2_Dnm1
IEGSGDQIDTYELSGGAR.2	-0.11309402	#N/A
KTSGNQDEILVIR.3	-0.12686336	#N/A
RS[Phospho (STY)]PT[Phospho (STY)]SSPTPQRR.2	0.092392248	P39053_T776_M2_Dnm1
YM[Oxidation (M)]LSVDNLK.2	0.225686089	#N/A
TIM[Oxidation (M)]HLM[Oxidation (M)]INNTK.2	-0.016896153	#N/A
LQSQLLSIEKEVDEYK.3	-0.5269822	#N/A
LDLM[Oxidation (M)]DEGTDARDVLENK.3	-0.154363912	#N/A
ALLQM[Oxidation (M)]VQQFAVDFEK.2	0.201612053	#N/A
HIFALFNTEQR.2	-0.17599079	#N/A
RIEGSGDQIDTYELSGGAR.3	-0.13741013	#N/A
QLELAC[Carbamidomethyl (C)]ETQEEVDSWK.2	0.058653972	#N/A
RPLVLQLVNSTTEYAEFLHC[Carbamidomethyl (C)]K.3	0.09629576	#N/A
EALSIIGDINTTTVSTPM[Oxidation (M)]PPPVDDSWLQVQSVPAGR.4	0.055140886	#N/A
EFIFSELLANLYSC[Carbamidomethyl (C)]GDQNTLM[Oxidation (M)]EESAEQAQR.3	-0.116728554	#N/A
ASETEENGSDSFMHSMDPQLER.3	0.184167027	#N/A
ERS[Phospho (STY)]LS[Phospho (STY)]PR.2	0.079049636	P39447_S166_M2_Tjp1
AVPVS[Phospho (STY)]PSAVEEDEDEDGHTVVATAR.3	-0.511985803	P39447_S1614_M1_Tjp1

EIS[Phospho (STY)]QDSLAAR.2	-0.15312643	P39447_S212_M1_Tjp1
S[Phospho (STY)]VAS[Phospho (STY)]SQPAKPTK.2	-0.212632113	P39447_S175_M2_Tjp1
ADGAT[Phospho (STY)]SDDLDLHDDR.2	0.219527153	P39447_T809_M1_Tjp1
KS[Phospho (STY)]REDLSAQPVQTK.3	-0.001227553	P39447_S617_M1_Tjp1
LSYLSAPGSEYSM[Oxidation (M)]YSTDS[Phospho (STY)]R.2	-0.091688896	P39447_S837_M1_Tjp1
DDISEIQSLAS[Phospho (STY)]DHS[Phospho (STY)]GR.2	-0.249966293	P39447_S297_M2_Tjp1
SAS[Phospho (STY)]LENKK.2	-0.576533991	P39447_S1277_M1_Tjp1
S[Phospho (STY)]REDLSAQPVQTK.2	-0.262292514	P39447_S617_M1_Tjp1
IDS[Phospho (STY)]PGLKPASQQK.2	0.408486947	P39447_S912_M1_Tjp1
ATLLNVPDLSDSIHS[Phospho (STY)]ANAS[Phospho (STY)]ER.3	-0.079303346	P39447_S280_M2_Tjp1
VQIPVSHPDPEPVS[Phospho (STY)]DNEDDSYDEEVHDPR.4	0.151219338	P39447_S125_M1_Tjp1
KVQIPVSHPDPEPVS[Phospho (STY)]DNEDDSY[Phospho (STY)]DEEVHDPR.4	-0.088511193	P39447_S125_M2_Tjp1
QAS[Phospho (STY)]RDLEQPSYR.2	-0.329903352	P39447_S1051_M1_Tjp1
DGS[Phospho (STY)]LNQSSGYR.2	-0.083802088	P39688_S21_M1_Fyn
GAY[Phospho (STY)]SLSIR.2	-0.317732185	P39688_Y185_M1_Fyn
WTAPEAALYGR.2	-0.059707225	#N/A
LT[Phospho (STY)]EERDGSLNQSSGYR.3	-0.28286256	P39688_T15_M1_Fyn
ALLATASQC[Carbamidomethyl (C)]QQPAGNK.2	0.053901107	#N/A
NSLDC[Carbamidomethyl (C)]EIVSAK.2	-0.236112753	#N/A
VPTISINK.2	-0.031195111	#N/A
KEPALLELEGKK.3	-0.266470676	#N/A
TDGC[Carbamidomethyl (C)]HAYLSK.2	0.00705347	#N/A
INSITVDNC[Carbamidomethyl (C)]KK.2	0.278825353	#N/A
SALFAQINQGESITHALK.2	-0.169132518	#N/A
LSDLLAPISEQIQEVITFR.2	-0.182979885	#N/A
TLWNGQK.2	0.279000776	#N/A
[Acetyl (Protein N-term)]ADMQNLVER.2	0.133150616	#N/A
LEAVSHTSDM[Oxidation (M)]HC[Carbamidomethyl (C)]GYGDS[Phospho (STY)]PSK.3	-0.364135682	#N/A
C[Carbamidomethyl (C)]VNTTLQIK.2	0.442368485	#N/A
VENQENVSNLVIDDTELK.2	-0.176635238	#N/A
EM[Oxidation (M)]NDAAM[Oxidation (M)]FYTNR.2	0.161579049	#N/A
PQTS[Phospho (STY)]PSPKPATK.2	-0.082129083	P40124_S307_M1_Cap1
EFHTTGLAWSK.3	-0.069561331	#N/A
VLDPFTIKPLDR.2	-0.126112371	#N/A
LAVSQVPR.2	0.150417683	#N/A
LDNLVAIFDINR.2	0.179901645	#N/A
LGQSDPAPLQHQVDIYQK.3	-0.066756321	#N/A
HQPTAIIAK.2	0.025710873	#N/A
IIALDGDTK.2	0.102880837	#N/A
NSTFSELFK.2	-0.128362539	#N/A
NM[Oxidation (M)]AEQIIQEIYSQVQSK.2	0.059467468	#N/A
ILATPPQEDAPSVDIANIR.2	0.144325783	#N/A
TVPFC[Carbamidomethyl (C)]STFAAFFTR.2	0.42512041	#N/A
SVPM[Oxidation (M)]STVFYPSDGVATEK.2	-0.111345708	#N/A
M[Oxidation (M)]PTPPSYK.2	-0.128734103	#N/A
TSRPENAIIYSNNEDFQVGQAK.3	-0.255594815	#N/A
C[Carbamidomethyl (C)]EAFGWHTIIVDGHSVEELC[Carbamidomethyl (C)]K.3	0.010461662	#N/A

ISSDLDGHPVPK.3	0.206510298	#N/A
ILTVEDHYYEGGIGEAVSAAVVGEPGVTVTR.3	-0.071246598	#N/A
VYC[Carbamidomethyl (C)]M[Oxidation (M)]LGDGEVSEGSVWEAM[Oxidation (M)]AFAGIYK.3	-0.579593793	#N/A
FES[Phospho (STY)]PDSQASAEQPEM.2	0.099742509	P40336_S315_M1_Vps26a
LFTETEKEANS[Phospho (STY)]PESLK.2	-0.006889709	P40749_S135_M1_Syt4
QTYSTEPNNLK.2	0.000816274	#N/A
[Acetyl (Protein N-term)]SAHLQWMVVR.2	-0.159997103	#N/A
VSEEDQS[Phospho (STY)]LENS[Phospho (STY)]EADVK.2	-0.151566205	P41234_S1327_M2_Abca2
QPAEPGTSQEPGLASS[Phospho (STY)]PSGC[Carbamidomethyl (C)]PR.2	0.240709033	P41234_S1238_M1_Abca2
VSEEDQS[Phospho (STY)]LENS[Phospho (STY)]EADVKESR.2	-0.156020964	P41234_S1327_M2_Abca2
DADSS[Phospho (STY)]VEKQVALLK.2	0.0518919	P41539_S76_M1_Tac1
EADIQNFTSFISK.2	0.235537265	#N/A
QS[Phospho (STY)]PGPALAR.2	-0.123145023	P42128_S87_M1_Foxk1
EGS[Phospho (STY)]PIPHDPDLGSK.2	-0.432495085	P42128_S431_M1_Foxk1
S[Phospho (STY)]APASPT[Phospho (STY)]HPGLM[Oxidation (M)]S[Phospho (STY)]PR.2	0.228433334	P42128_S402_M3_Foxk1
LTVVDTPGYGDAINC[Carbamidomethyl (C)]R.2	0.141218028	#N/A
TVQIEASTVEIEER.2	-0.537902702	#N/A
TIISYIDEQFER.2	-0.285887218	#N/A
IYHLPDAES[Phospho (STY)]DEDEDFKEQTR.3	-0.035927815	P42208_S218_M1_Septin2
STLINSLFLTDLYPER.2	-0.150806806	#N/A
IYHLPDAES[Phospho (STY)]DEDEDFK.2	0.11126268	P42208_S218_M1_Septin2
QQPTQFINPETPGYVGFANLPNQVHR.3	0.166570597	#N/A
AEITSQESQISSYEEELLK.2	-0.208207914	#N/A
NITGSS[Phospho (STY)]PVADFSAIK.2	-0.441818711	P42567_S324_M1_Eps15
VSEVKPTYR.2	0.072432041	#N/A
IAEVGAGGNK.2	-0.026450943	#N/A
FFFDVGSNK.2	0.293434436	#N/A
LIDDYGVEEEPAELPEGTSLTVDNKR.3	0.105894388	#N/A
GPGLGSTQGQTIALPAQGLIEFR.3	0.133854513	#N/A
VDIQNKR.2	0.147200623	#N/A
NSITVPYK.2	0.163253702	#N/A
YSEEMKK.2	0.033001816	#N/A
RHS[Phospho (STY)]LSC[Carbamidomethyl (C)]TK.2	-0.068812628	P42859_S1841_M1_Htt
S[Phospho (STY)]GEEEDSGSAAQLGMC[Carbamidomethyl (C)]NR.2	0.356772775	P42859_S1853_M1_Htt
DEVAEAS[Phospho (STY)]DPESKPC[Carbamidomethyl (C)]R.2	-0.124107415	P42859_S637_M1_Htt
SGS[Phospho (STY)]IVELLAGGGSS[Phospho (STY)]C[Carbamidomethyl (C)]SPVLSR.2	-0.053713844	P42859_S398_M2_Htt
SLNPQKS[Phospho (STY)]GEEEDSGSAAQLGM[Oxidation (M)]C[Carbamidomethyl (C)]NR.3	-0.006724008	P42859_S1853_M1_Htt
EFC[Carbamidomethyl (C)]IPTSSTIEQQNS[Phospho (STY)]AR.2	-0.334085876	P42866_S363_M1_Oprm1
KDWDDDQND.2	-0.233466869	#N/A
DVDEVSSLLR.2	-0.064667903	#N/A
FAEAFEAIPR.2	0.238187487	#N/A
LATNAAVTVLR.2	0.231750203	#N/A
AVDDGVNTFK.2	-0.419567292	#N/A
LFVTNDAATILR.2	-0.493514911	#N/A
LYSVHQEGNK.2	0.207366119	#N/A
EDGAISTIVLR.2	0.235604072	#N/A
AIAGTGANVIVTGGK.2	0.235079813	#N/A

HEKEDGAISTIVLR.3	0.237443381	#N/A
GSTDNLM[Oxidation (M)]DDIER.2	0.27101221	#N/A
QYGSETFLAK.2	0.341376825	#N/A
HFSGLEEAVYR.2	0.107277335	#N/A
SADC[Carbamidomethyl (C)]S[Phospho (STY)]VEEEPWK.2	0.216328314	P43006_S562_M1_Slc1a2
NLFPENLVQAC[Carbamidomethyl (C)]FQQIQTVTK.2	-0.025879422	#N/A
KNDEVSSLDAFLDLIR.3	-0.043642976	#N/A
[Acetyl (Protein N-term)]ASTEGANNM[Oxidation (M)]PK.2	0.269705913	#N/A
TSVNVVGDSFGAGIVYHLSK.2	-0.459125529	#N/A
SELDTIDSQHR.2	-0.089261881	#N/A
C[Carbamidomethyl (C)]LEDNLGIDKR.2	-0.06568115	#N/A
SADC[Carbamidomethyl (C)]S[Phospho (STY)]VEEEPWKR.2	-0.133784304	P43006_S562_M1_Slc1a2
M[Oxidation (M)]QEDIEMTK.2	0.338575334	#N/A
TQSIYDDKNHR.2	-0.112466061	#N/A
M[Oxidation (M)]HDSHLS[Phospho (STY)]SDEPK.2	-0.107179655	P43006_S24_M1_Slc1a2
ESNS[Phospho (STY)]NQC[Carbamidomethyl (C)]VYAAHNSVVIDEC[Carbamidomethyl (C)]K.3	0.133316976	P43006_S532_M1_Slc1a2
NDEVSSLDAFLDLIR.2	-0.226044306	#N/A
TQSIYDDK.2	-0.098023958	#N/A
M[Oxidation (M)]HDSHLS[Phospho (STY)]S[Phospho (STY)]DEPKHR.3	-0.422332945	#N/A
DLEVVAR.2	-0.10749812	#N/A
NHRESNS[Phospho (STY)]NQC[Carbamidomethyl (C)]VYAAHNSVVIDEC[Carbamidomethyl (C)]K.4	-0.193858521	P43006_S532_M1_Slc1a2
TQSIY[Phospho (STY)]DDKNHRESNSNQC[Carbamidomethyl (C)]VYAAHNSVVIDEC[Carbamidomethyl (C)]K.5	-0.624562514	P43006_Y522_M1_Slc1a2
HEEHERPPFVAYPHLR.4	-0.027302635	#N/A
ALTYFVALPGVGVSM[Oxidation (M)]LNVFLK.2	-0.259378404	#N/A
[Acetyl (Protein N-term)]S[Phospho (STY)]ETAPAAPAAPAPAEK.2	-0.024914268	P43274_S2_M1_H1-4
TSGPPVSELITK.2	0.24837065	#N/A
[Acetyl (Protein N-term)]S[Phospho (STY)]ETAPAAPAAPAPAEKTPVK.2	0.051392161	P43274_S2_M1_H1-4
[Acetyl (Protein N-term)]S[Phospho (STY)]ETAPAETAAPAPVEK.2	0.165134189	P43276_S2_M1_H1-5
[Acetyl (Protein N-term)]SETAPAETAAPAPVEKS[Phospho (STY)]PAKK.3	-0.175999449	P43276_S18_M1_H1-5
ALAAGGYDVEK.2	-0.121483098	#N/A
[Acetyl (Protein N-term)]SETAPAETAAPAPVEKS[Phospho (STY)]PAK.2	-0.277630179	P43276_S18_M1_H1-5
[Acetyl (Protein N-term)]S[Phospho (STY)]ETAPAAPAAPAPVEK.2	-0.320625806	P43277_S2_M1_H1-3
[Acetyl (Protein N-term)]SETAPAAPAAPAPVEKT[Phospho (STY)]PVK.2	0.34239402	P43277_T18_M1_H1-3
FC[Carbamidomethyl (C)]PS[Phospho (STY)]PSTSSEGTR.2	0.200328	P43346_S11_M1_Dck
EQLQPHENGEGNS[Phospho (STY)]ET.2	-0.063520562	P43406_S1042_M1_Itgav
YNNQLATR.2	-0.243964541	#N/A
TIGVSNFNPLQIER.2	0.416808903	#N/A
[Acetyl (Protein N-term)]ASHLELNNGTK.2	0.109512344	#N/A
TTAQVLIR.2	0.428403207	#N/A
VAIDLGYR.2	0.276187774	#N/A
SPPGQVTEAVK.2	0.345365154	#N/A
LIEYC[Carbamidomethyl (C)]HSK.2	-0.206961581	#N/A
HIDC[Carbamidomethyl (C)]AQVYQNEK.2	-0.083200217	#N/A
[Acetyl (Protein N-term)]AS[Phospho (STY)]GVTVNDEVIK.2	0.137310749	P45591_S3_M1_Cfl2
QIIVEEAK.2	-0.255830238	#N/A
QILVGDIGDTVEDPYTSFVK.2	-0.327974355	#N/A
LLPLNDC[Carbamidomethyl (C)]R.2	0.140496054	#N/A

HEWQVNGLDDIKDR.3	0.107718794	#N/A
LVIPSELGYGER.2	-0.123094941	#N/A
IPGGATLVFEVELLK.2	-0.072538443	#N/A
GSGEEPAT[Phospho (STY)]PSRK.2	-0.393809133	P46061_T411_M1_Rangap1
VFVGYNSTGAELR.2	-0.16262315	#N/A
HDIIGEFK.2	-0.113047881	#N/A
TLNPVFNEQFTFK.2	-0.06146367	#N/A
VPYSELGGK.2	-0.287506119	#N/A
LTVVILEAK.2	0.035959922	#N/A
M[Oxidation (M)]DVGGLSDPYVK.2	0.246163066	#N/A
DDDAETGLT[Phospho (STY)]DGEEKEEPKEEEK.3	0.1295143	P46096_T128_M1_Syt1
HWSDM[Oxidation (M)]LANPR.2	0.258557213	#N/A
DLQSAEKEEQEK.2	-0.218277991	#N/A
VFLLPDK.2	-0.09894975	#N/A
VQVVVTVLDYDK.2	-0.116743631	#N/A
TLVM[Oxidation (M)]AVYDFDR.2	-0.006831093	#N/A
VPM[Oxidation (M)]NTVDFGHVTEEWR.3	-0.134870197	#N/A
NTLNPYYNESFS[Phospho (STY)]FEVPFEQIQK.3	-0.362667033	P46096_S344_M1_Syt1
VSNGS[Phospho (STY)]PSLER.2	-0.355084571	P46414_S10_M1_Cdkn1b
TEENVSDGS[Phospho (STY)]PNAGTVEQTPK.2	0.315294061	P46414_S178_M1_Cdkn1b
VLAQES[Phospho (STY)]QDVSGSR.2	0.191359881	P46414_S106_M1_Cdkn1b
GILLYGPPGC[Carbamidomethyl (C)]GK.2	-0.044618406	#N/A
LLDYVPIGPR.2	0.08399829	#N/A
YVGESEANIR.2	0.155344857	#N/A
VLDDGELLVQQTK.2	-0.262160545	#N/A
SQLSC[Carbamidomethyl (C)]VVVDDIER.2	-0.090126876	#N/A
NFSGAELEGLVR.2	-0.122404113	#N/A
LLIIGTTSR.2	-0.247454299	#N/A
NIDSNPYDTDK.2	0.279148762	#N/A
KLLIIGTTSR.2	0.160016495	#N/A
IAEESNFPFIK.2	-0.232883579	#N/A
WGDPVTR.2	-0.129374214	#N/A
VVNGPEILNK.2	0.226213601	#N/A
AENSSLNLIGK.2	0.004338781	#N/A
AESLQVTR.2	0.069196891	#N/A
TTIAQQVK.2	-0.255139863	#N/A
TKENRQS[Phospho (STY)]IINPDWNFEK.3	-0.378861366	P46460_S207_M1_Nsf
YVGESEANIRK.2	0.065664703	#N/A
M[Oxidation (M)]GIGGLDKEFSDIFR.3	0.050730194	#N/A
LQILHIHTAR.3	-0.257259985	#N/A
KIFDDAYK.2	0.256849468	#N/A
IEVGLVVGNSQVAFEK.2	0.194077968	#N/A
QC[Carbamidomethyl (C)]IGTM[Oxidation (M)]TIEIDFLQK.2	0.16901025	#N/A
M[Oxidation (M)]EIGLPDEK.2	-0.062723417	#N/A
DIEAM[Oxidation (M)]DPSILK.2	0.242495206	#N/A
TPLVSVLLEGPPHSGK.2	-0.007055543	#N/A
IDGVEQLNNILVIGM[Oxidation (M)]TNRPDLIDEALLRPGR.4	-0.003572793	#N/A

KLFADAEEEQR.3	0.494974426	#N/A
M[Oxidation (M)]IGFSETAK.2	0.472897712	#N/A
AAQSTAMNR.2	0.290810647	#N/A
EFSDIFR.2	-0.147742537	#N/A
GHQLLSADVDIK.2	-0.201905645	#N/A
ENRQS[Phospho (STY)]IINPDWNFEK.3	-0.063470624	P46460_S207_M1_Nsf
KLFADAEEEQRR.3	0.29647347	#N/A
QSIINPDWNFEK.2	0.355694725	#N/A
GSM[Oxidation (M)]AGSTGVHDTVVNQLLSK.3	-0.497367793	#N/A
VEVDM[Oxidation (M)]EKAESLQVTR.3	-0.302803676	#N/A
C[Carbamidomethyl (C)]PTDELSLSNC[Carbamidomethyl (C)]AVVNEK.2	0.057482435	#N/A
KNIDSNPYDTDK.2	0.134444508	#N/A
LFADAEEEQRR.2	0.082396604	#N/A
FLALM[Oxidation (M)]REEGAS[Phospho (STY)]PLDFD.2	-0.080404391	P46460_S739_M1_Nsf
EEGAS[Phospho (STY)]PLDFD.1	-0.407811283	P46460_S739_M1_Nsf
DFQSGQHVM[Oxidation (M)]VR.2	0.047086266	#N/A
VEVDMEK.2	0.126437822	#N/A
GNDS[Phospho (STY)]DGEAESDDPEK.2	-0.31376972	P46467_S102_M1_Vps4b
GNDS[Phospho (STY)]DGEAESDDPEKKK.2	0.053187499	P46467_S102_M1_Vps4b
TM[Oxidation (M)]LELINQLDGFDPR.2	-0.30678802	#N/A
FVVDLSDQVAPTDIEEGM[Oxidation (M)]R.2	0.364502729	#N/A
AQIWDTAGQER.2	-0.189870072	#N/A
VVLIGDSGVGK.2	0.06511882	#N/A
[Acetyl (Protein N-term)]GTRDDEYDYLFK.2	-0.267284311	#N/A
NNLSFIETSALDSTNVEEAFK.2	-0.241159186	#N/A
HLTYENVER.2	-0.333127271	#N/A
NILTEIYR.2	-0.25411101	#N/A
STIGVEFATR.2	-0.060054269	#N/A
AITSAYYR.2	-0.164393893	#N/A
NEFNLESK.2	-0.524347219	#N/A
AAHDES[Phospho (STY)]PGNNVVDISVPPTTDGQR.3	-0.411027168	P46638_S190_M1_Rab11b
TNEKEQLQGLNDR.3	-0.120598993	#N/A
DGLAEEVQR.2	-0.017630767	#N/A
VHQLETQNR.2	0.204432384	#N/A
ALEAELAALR.2	0.207012738	#N/A
QILELEER.2	0.095687585	#N/A
AQLEEASSAR.2	0.252973974	#N/A
TIEIEGLR.2	0.149936731	#N/A
FANLNEQAAR.2	0.41260092	#N/A
NLQSAEEWYK.2	0.042812578	#N/A
KVESLLDELAFVR.3	0.080292396	#N/A
RLPASDGLDLSQAAAR.3	0.095083249	#N/A
DVDGATLAR.2	-0.090117615	#N/A
LPASDGLDLSQAAAR.2	0.318440687	#N/A
AQALLER.2	0.012297038	#N/A
FAVFIEK.2	0.179441364	#N/A
AQYESLAAK.2	0.001535101	#N/A

VGELFQR.2	0.499238806	#N/A
KVFGDSSR.2	0.183286805	#N/A
LSGPGGSGSFR.2	0.404588878	#N/A
VESLLDELAFVR.2	0.183962672	#N/A
SNVASTAAC[Carbamidomethyl (C)]SSASSLGLGLAYR.2	-0.504615595	#N/A
GANES[Phospho (STY)]LER.2	-0.072127249	P46660_S335_M1_Ina
[Acetyl (Protein N-term)]SFGSEHYLC[Carbamidomethyl (C)]SASSYR.2	-0.284409837	#N/A
[Acetyl (Protein N-term)]SFGSEHYLC[Carbamidomethyl (C)]SAS[Phospho (STY)]SYRK.3	-0.172658505	P46660_S13_M1_Ina
EEIHEYRR.2	-0.047379302	#N/A
VSSAGLSLK.2	-0.29940221	#N/A
VGEGFEETLGEAVISTK.2	-0.256256803	#N/A
KEEEEEEEEASKEVSK.3	-0.221541323	#N/A
SATEESTSS[Phospho (STY)]SQKM.2	-0.092960436	P46660_S497_M1_Ina
HSAEVAGYQDSIGQLESDLR.3	-0.203224531	#N/A
ILSSTASK.2	0.254073316	#N/A
VVDLLAQDADIVC[Carbamidomethyl (C)]R.2	-0.357488051	#N/A
ELPVNAQNYVR.2	0.608282642	#N/A
RQIS[Phospho (STY)]EDVDGPDNR.2	0.068167012	P46935_S309_M1_Nedd4
QIS[Phospho (STY)]EDVDGPDNR.2	-0.249933537	P46935_S309_M1_Nedd4
RPSPDDDLT[Phospho (STY)]DEDNDDM[Oxidation (M)]QLQAQR.3	0.248243962	P46935_T287_M1_Nedd4
QAS[Phospho (STY)]TDAGTAGALTPQHVR.2	0.163946278	P46938_S94_M1_Yap1
GDS[Phospho (STY)]ETDLEALFNAVM[Oxidation (M)]NPK.2	0.157273367	P46938_S46_M1_Yap1
EAGALDSLPGIPLATS[Phospho (STY)]SEDLEKS.2	0.098835259	P47212_S117_M1_Gal
LHLLPGASK.2	0.015475435	#N/A
KQEELTDEEKEIINR.3	0.094204867	#N/A
TKPQQPAGEPATQEQPTPESR.3	-0.165038406	#N/A
TGPAGGFQAAPHTAAPYSQAAPAR.3	0.102723822	#N/A
DSEGWDHAHGGGTGDTSR.3	-0.210002335	#N/A
WHQLQNENHVSS[Phospho (STY)]D.2	-0.162553824	P47708_S680_M1_Rph3a
RANS[Phospho (STY)]VQAAR.2	-0.487429537	P47708_S271_M1_Rph3a
DSEGWDHAHGGGTGDTSRS[Phospho (STY)]PAGLR.4	-0.080664401	P47708_S262_M1_Rph3a
ANS[Phospho (STY)]VQAAR.2	0.254436062	P47708_S271_M1_Rph3a
ISVC[Carbamidomethyl (C)]DEDKFGHNEFIGETR.3	-0.304999721	#N/A
TIPIDGDFFSYTR.2	0.103080236	#N/A
AAFQLGSPWR.2	-0.241368415	#N/A
VVGNPFDSR.2	-0.188317	#N/A
TFVQENVYDEFVER.2	-0.444050226	#N/A
VAEQTPLTALYVANLIK.2	-0.117148557	#N/A
TEQGPQVDETQFK.2	0.053206969	#N/A
TIEEVVGR.2	0.190738607	#N/A
LLC[Carbamidomethyl (C)]GGGAAADR.2	0.671215252	#N/A
ELGEYGLQAYTEVK.2	-0.217709662	#N/A
LADLIER.2	-0.302111449	#N/A
EATDPRPYEAENAIESWR.3	-0.189010816	#N/A
LLLNNDNLLR.2	0.406827651	#N/A
FTVTPSTTQVVGILK.2	0.314861856	#N/A
EHYPNGVC[Carbamidomethyl (C)]TVYGK.2	-0.091031611	#N/A

TSVETALR.2	-0.111037013	#N/A
EGAAHAFAQYNLDQFTPVK.3	-0.347963205	#N/A
[Acetyl (Protein N-term)]ADLEEQLSDEEKVR.2	-0.217791954	#N/A
[Acetyl (Protein N-term)]ADLEEQLSDEEK.2	0.000724504	#N/A
FIIHAPPGEFNEVFNDVR.3	0.060829126	#N/A
DIQDSLTVSNEVQTAK.2	-0.202936922	#N/A
IVEAAENEYQTAISENYQTMSDTTFK.3	0.000296329	#N/A
DYLLC[Carbamidomethyl (C)]DYNR.2	-0.260314239	#N/A
STLNEIYFGK.2	0.02486567	#N/A
ELS[Phospho (STY)]QVLTQR.2	-0.471136887	P47757_S263_M1_Capzb
GC[Carbamidomethyl (C)]WDSIHVVEVQEK.2	-0.299729638	#N/A
KLEVEANNAFDQYR.3	-0.352600307	#N/A
LEVEANNAFDQYR.2	0.179075586	#N/A
SGSGTM[Oxidation (M)]NLGGSLTR.2	-0.239075424	#N/A
[Acetyl (Protein N-term)]SDQQLDC[Carbamidomethyl (C)]ALDLMR.2	0.132918771	#N/A
ALLTPVAIAAGR.2	-0.241061846	#N/A
LC[Carbamidomethyl (C)]DFGISGQLVDSIAK.2	0.041748913	#N/A
LRTHS[Phospho (STY)]IESSGK.3	-0.243877299	P47809_S78_M1_Map2k4
YVYSVLDDVIPEEILGK.2	0.043957451	#N/A
FTLNPNTTGVQNPHIER.3	0.242385214	#N/A
THS[Phospho (STY)]IESSGK.2	0.020496876	P47809_S78_M1_Map2k4
LKIS[Phospho (STY)]PEQHWDFTAEDLKDLGEIGR.4	-0.058122883	P47809_S88_M1_Map2k4
ILDQM[Oxidation (M)]PATPSS[Phospho (STY)]PM[Oxidation (M)]YVD.2	-0.343834917	P47809_S392_M1_Map2k4
HTDDEM[Oxidation (M)]TGY[Phospho (STY)]VATR.2	-0.313685667	P47811_Y182_M1_Mapk14
VDS[Phospho (STY)]TTC[Carbamidomethyl (C)]LFPVEEK.2	0.145116152	P47856_S259_M1_Gfpt1
KRS[Phospho (STY)]GEAAV.2	-0.333575215	P47857_S775_M1_Pfkm
ALVFQPVTELKDQTDFEHR.3	-0.31890744	#N/A
RFDEAIK.2	-0.145006259	#N/A
DLQVNVEHLVQK.2	-0.433123687	#N/A
GQIEEAGWSYVGGWTGQGGSK.2	-0.377183699	#N/A
ALVFQPVTELK.2	0.18299353	#N/A
NLEQISANITK.2	0.108107425	#N/A
IVEIVDAITTTAQSHQR.2	-0.327928541	#N/A
SEWSDLLNDLQK.2	0.272030864	#N/A
GITNLC[Carbamidomethyl (C)]VIGGDGSLTGADTFR.2	-0.174416299	#N/A
IFANTPDSGC[Carbamidomethyl (C)]VLGM[Oxidation (M)]R.2	0.065502117	#N/A
VLVVHDGFEGLAK.2	-0.090914707	#N/A
AIAVLTSGGDAQGM[Oxidation (M)]NAAVR.2	0.029915711	#N/A
NVLGHM[Oxidation (M)]QQGGSPTPFDR.3	-0.48504864	#N/A
LNIIIVAEGAIDK.2	-0.286833321	#N/A
SSYLNIVGLVGSIDNDFC[Carbamidomethyl (C)]GTDM[Oxidation (M)]TIGTDSALHR.3	0.473197245	#N/A
VFFVHEGYQGLVDGGEHIR.3	-0.099084207	#N/A
EATWESVSM[Oxidation (M)]M[Oxidation (M)]LQLGGTVIGSAR.3	-0.492116885	#N/A
ELS[Phospho (STY)]AERPLNEQIAEAEADK.3	-0.02697076	P47867_S40_M1_Scg3
AVPQLQGYLR.2	-0.248530102	#N/A
QLDSGLLLVTGPLVINR.2	0.194261879	#N/A
SSITPGTVLIILTGR.2	0.162391585	#N/A

KAGS[Phospho (STY)]DAAASRPR.2	-0.18125046	P47911_S21_M1_Rpl6
HQEGEIFDTEKEKYEITEQR.4	-0.429516008	#N/A
AGS[Phospho (STY)]DAAASRPR.2	0.188625023	P47911_S21_M1_Rpl6
S[Phospho (STY)]PPTSGLQSLR.2	-0.252813306	P47930_S200_M1_Fosl2
Y[Phospho (STY)]PSPPVGSVSAPNLPTAEENLEYVR.3	0.331013007	P47941_Y105_M1_Crkl
IFDPQNPDDNE.2	-0.240511991	#N/A
DSS[Phospho (STY)]TC[Carbamidomethyl (C)]PGDYVLSVSENSR.2	0.239339072	P47941_S42_M1_Crkl
AAGVSVEPFWPGLFAK.2	0.203975446	#N/A
ALANVNIGSLIC[Carbamidomethyl (C)]NVGAGGPAPAAGAAPAGGAAPSTAAAPAEEK.4	-0.388571582	#N/A
KEES[Phospho (STY)]EES[Phospho (STY)]EDDM[Oxidation (M)]GFGLFD.2	0.112968938	P47955_S101_M2_Rplp1
EFNAEVHRK.2	0.061336845	#N/A
VGLTNYAAAYC[Carbamidomethyl (C)]TGLLLAR.2	-0.131689625	#N/A
YLMEEDEDAYKK.3	-0.513733722	#N/A
VDTWFNQPAR.2	-0.112786967	#N/A
LATQLTGPVM[Oxidation (M)]PIR.2	-0.232307395	#N/A
GFSLEELR.2	-0.193371319	#N/A
KGDSS[Phospho (STY)]AEELK.2	-0.074386888	P47963_S140_M1_Rpl13
TIGISVDPR.2	0.517746077	#N/A
STESLQANVQR.2	0.214868062	#N/A
EVC[Carbamidomethyl (C)]GFAPYER.2	-0.101969048	#N/A
GTVTDFPGFDGR.2	-0.270031968	#N/A
VLTEIIASR.2	-0.190728767	#N/A
SEIDLFNIR.2	0.046006691	#N/A
GLGTDEDSILNLLTSR.2	0.18227219	#N/A
GAGTDDHTLIR.2	-0.139863498	#N/A
QEIAQEFK.2	0.44149036	#N/A
ADAEVLRK.2	-0.089675718	#N/A
DLVDDLKSELTGK.2	0.023873783	#N/A
ETSGNLEQLLLAVVK.2	0.041887486	#N/A
LYDAYELK.2	-0.232224859	#N/A
TPEELSAIK.2	-0.347392958	#N/A
S[Phospho (STY)]LDGAAAAESTDR.2	-0.39458658	P48193_S543_M1_Epb41
LSTHS[Phospho (STY)]PFR.2	0.087102265	P48193_S706_M1_Epb41
RAS[Phospho (STY)]RS[Phospho (STY)]LDGAAAAESTDR.3	0.031872084	P48193_S541_M2_Epb41
GVPDS[Phospho (STY)]PERR.2	-0.318342034	P48318_S527_M1_Gad1
ANSVTWNPHK.2	0.349542985	#N/A
GTVGFENQINK.2	0.108213876	#N/A
NLLS[Phospho (STY)]C[Carbamidomethyl (C)]ENSDQGAR.2	-0.285122744	P48318_S77_M1_Gad1
TNS[Phospho (STY)]LEEKSR.2	-0.457828156	P48318_S55_M1_Gad1
ERQS[Phospho (STY)]SKNLLSC[Carbamidomethyl (C)]ENSDQGAR.3	0.319202034	P48318_S71_M1_Gad1
QSS[Phospho (STY)]KNLLSC[Carbamidomethyl (C)]ENSDQGAR.3	-0.482915919	P48318_S72_M1_Gad1
M[Oxidation (M)]M[Oxidation (M)]GVPLQC[Carbamidomethyl (C)]SALLVR.2	-0.340132956	#N/A
HYDLSYDTGDK.2	0.117463043	#N/A
M[Oxidation (M)]VISNPAATHQDIDFLIEEIER.3	-0.206047247	#N/A
[Acetyl (Protein N-term)]ASPGSGFWSFGS[Phospho (STY)]EDGSADPENPGTAR.3	-0.099393233	P48320_S13_M1_Gad2
DRAS[Phospho (STY)]PESLDLTR.2	-0.060553222	P48437_S511_M1_Prox1
GMSHS[Phospho (STY)]PSVALR.2	0.13847954	P48437_S179_M1_Prox1

GFS[Phospho (STY)]PPHR.2	-0.490883381	P48453_S472_M1_Ppp3cb
LFEVGGSPANTR.2	-0.381879196	#N/A
IC[Carbamidomethyl (C)]S[Phospho (STY)]FEEAK.2	-0.236247265	P48453_S479_M1_Ppp3cb
YLFLGDYVDR.2	-0.307724563	#N/A
EESESVLTLK.2	0.095816001	#N/A
AHEAQDAGYR.2	-0.088162516	#N/A
IINEGAAILR.2	0.191444631	#N/A
YENNVM[Oxidation (M)]NIR.2	0.349928945	#N/A
HLTEYFTFK.2	-0.416186652	#N/A
IC[Carbamidomethyl (C)]S[Phospho (STY)]FEEAKGLDR.2	-0.479907038	P48453_S479_M1_Ppp3cb
VDEEIALR.2	0.241495728	#N/A
IRS[Phospho (STY)]FEEAR.2	-0.037575565	P48455_S462_M1_Ppp3cc
[Acetyl (Protein N-term)]AQENAAFS[Phospho (STY)]PGSEEPPR.2	-0.30849025	P48543_S9_M1_Kcnj9
KLES[Phospho (STY)]SESR.2	0.010523398	P48678_S423_M1_Lmna
LADALQELR.2	0.210247741	#N/A
LQEKEDLQELNDR.3	0.110907454	#N/A
SGAQASSTPLS[Phospho (STY)]PTR.2	-0.300235856	P48678_S22_M1_Lmna
LSPS[Phospho (STY)]PTSQR.2	-0.269107729	P48678_S392_M1_Lmna
SS[Phospho (STY)]FSQHAR.2	0.1942743	P48678_S429_M1_Lmna
GSHC[Carbamidomethyl (C)]S[Phospho (STY)]GSGDPAEYNLR.2	0.193035742	P48678_S573_M1_Lmna
NSNLVGAAHEELQQSR.2	-0.400382432	#N/A
SVGGSGGGS[Phospho (STY)]FGDNLVTR.2	0.413986454	P48678_S637_M1_Lmna
LRLS[Phospho (STY)]PS[Phospho (STY)]PTSQR.2	-0.403655488	P48678_S390_M2_Lmna
MQQQLDEYQELLDIK.2	0.01571227	#N/A
NKS[Phospho (STY)]NEDQSM[Oxidation (M)]GNWQIR.3	-0.394381266	P48678_S458_M1_Lmna
ASS[Phospho (STY)]HSSQSQGGGSVTK.2	-0.298071568	P48678_S404_M1_Lmna
AQHEDQVEQYKK.3	0.138359278	#N/A
LEAALGEAK.2	0.088454169	#N/A
S[Phospho (STY)]FDDPIVQTER.2	-0.342564727	P48722_S74_M1_Hspa4l
LHGRS[Phospho (STY)]FDDPIVQTER.3	-0.349121466	P48722_S74_M1_Hspa4l
QLT[Phospho (STY)]QDLLNSYIENEGK.2	-0.201539652	P48722_T593_M1_Hspa4l
SGGIETIANEYSDR.2	-0.025520287	#N/A
VLATTFDPYLGGR.2	0.108560395	#N/A
SIDLPIQSSLYR.2	-0.059491446	#N/A
SQIVTNVR.2	0.351685442	#N/A
LSLTQDPVVK.2	-0.282524498	#N/A
DKLGTVYEK.2	0.462453557	#N/A
QDLPSLDEKPR.3	0.291222921	#N/A
AQFEQLC[Carbamidomethyl (C)]ASLLAR.2	-0.077783287	#N/A
C[Carbamidomethyl (C)]HAEHT[Phospho (STY)]PEEEIDHTGAK.3	0.032446486	P48722_T545_M1_Hspa4l
GSSQHTDS[Phospho (STY)]GEMEVD.2	0.093955889	P48722_S832_M1_Hspa4l
FITPEDMNK.2	0.292669476	#N/A
TSFEEGTGEC[Carbamidomethyl (C)]EVFSK.2	-0.007529636	#N/A
SVM[Oxidation (M)]AAAQVAGLNC[Carbamidomethyl (C)]LR.2	-0.20052662	#N/A
NHPAPFSK.2	-0.164596064	#N/A
TGSEHNGPMDGQSGSET[Phospho (STY)]SPDPPK.3	-0.009723325	P48722_T818_M1_Hspa4l
[Acetyl (Protein N-term)]SVVGIDLGFLNC[Carbamidomethyl (C)]YIAVAR.2	-0.270410712	#N/A

RNS[Phospho (STY)]EIDELK.2	0.091898062	P48725_S1437_M1_Pcnt
LQAEGLSPR.2	0.282882251	#N/A
ILLNAC[Carbamidomethyl (C)]C[Carbamidomethyl (C)]PGWVR.2	0.294026084	#N/A
FVEDTKK.2	0.389807708	#N/A
FHQLDIDNPQSIR.3	-0.152587059	#N/A
GVHAEEGWPNSAYGVTK.2	-0.009835689	#N/A
[Acetyl (Protein N-term)]SS[Phospho (STY)]SRPVALVTGANK.2	0.263105996	P48758_S3_M1_Cbr1
FSGDVVLAAR.2	-0.402041377	#N/A
GGASDALLYR.2	0.103148565	#N/A
LFQEDNGM[Oxidation (M)]PVHLK.2	0.074200889	#N/A
IAAFLQSDR.2	0.161235394	#N/A
YIC[Carbamidomethyl (C)]GEAPDYDR.2	0.122380223	#N/A
SM[Oxidation (M)]VLGYWDIR.2	0.158694746	#N/A
SQWLDVK.2	0.489684682	#N/A
LTFVDFLTYDVLDQNR.2	0.161081041	#N/A
M[Oxidation (M)]LLEFTDTSYEEKR.3	0.235554563	#N/A
QIFLGGVDR.2	-0.068309479	#N/A
EQGFLSFWR.2	-0.103922684	#N/A
EFNGLGDC[Carbamidomethyl (C)]LTK.2	0.019718267	#N/A
[Acetyl (Protein N-term)]GDQALSFLK.2	0.329337911	#N/A
DFLAGGIAAAVSK.2	0.139242505	#N/A
AAYFGVYDTAK.2	-0.115955548	#N/A
LAADVGKGS[Phospho (STY)]SQR.2	0.127794569	P48962_S149_M1_Slc25a4
IAKDEGANAFFK.3	0.194823194	#N/A
GADIM[Oxidation (M)]YTGTLDC[Carbamidomethyl (C)]WR.2	-0.012626667	#N/A
DEGANAFFK.2	-0.023806104	#N/A
GSS[Phospho (STY)]QREFNGLGDC[Carbamidomethyl (C)]LTK.3	0.085490916	P48962_S150_M1_Slc25a4
RVS[Phospho (STY)]EVEAVLSQK.2	-0.137692431	P49025_S479_M1_Cit
MNS[Phospho (STY)]PGLQSK.2	0.307900479	P49025_S1458_M1_Cit
DKS[Phospho (STY)]PGRPLER.2	-0.232752396	P49025_S1999_M1_Cit
DKMNS[Phospho (STY)]PGLQSK.2	-0.422106502	P49025_S1458_M1_Cit
ERS[Phospho (STY)]PGRLFEDSSR.3	0.062194171	P49025_S2021_M1_Cit
S[Phospho (STY)]PEHQPSAMSLLAPPSSR.3	0.103536566	P49025_S1348_M1_Cit
VVLGDS[Phospho (STY)]VDGGGADQLGGVAEVR.2	-0.201460198	P49070_S88_M1_Camlg
IMGFHRPGS[Phospho (STY)]GS[Phospho (STY)]EEENQTK.3	0.046735412	P49070_S53_M2_Camlg
GFAFVTFDDHDSVDKIVIQK.3	-0.27180267	#N/A
SES[Phospho (STY)]PKEPEQLRK.2	-0.369354131	P49312_S6_M1_Hnrnpa1
LFIGGLSFETTDESLR.2	-0.090063944	#N/A
EDSQRPGAHLTVK.3	0.054827809	#N/A
IEVIEIM[Oxidation (M)]TDR.2	0.06905046	#N/A
[Acetyl (Protein N-term)]S[Phospho (STY)]KSES[Phospho (STY)]PKEPEQLR.2	0.240162317	P49312_S2_M2_Hnrnpa1
SES[Phospho (STY)]PKEPEQLR.2	-0.241453596	P49312_S6_M1_Hnrnpa1
NQGGYGGSSSSSSYGSGR.2	-0.039758842	#N/A
GFAFVTFDDHDSVDK.2	0.090078962	#N/A
GFGFVTYATVEEVDAAM[Oxidation (M)]NAR.2	-0.055208573	#N/A
SHFEQWGTLTDC[Carbamidomethyl (C)]VVM[Oxidation (M)]R.3	-0.196271381	#N/A
YHTVNGHNC[Carbamidomethyl (C)]EVR.3	0.269482951	#N/A

DYFEQYGK.2	-0.28711781	#N/A
TLADVLVQEVIK.2	0.158136704	#N/A
NVIEAVYNR.2	0.038850881	#N/A
ALGDFDYK.2	-0.128741853	#N/A
GPTEQLVSPEPEVHDIER.3	0.210285403	#N/A
LNPYKNDDTDSAS[Phospho (STY)]TDDM[Oxidation (M)]W.2	-0.075869924	P49443_S377_M1_Ppm1a
TS[Phospho (STY)]PSSSPASLSR.2	0.359382852	P49586_S343_M1_Pcyt1a
AVTC[Carbamidomethyl (C)]DIS[Phospho (STY)]EDEED.2	-0.380888413	P49586_S362_M1_Pcyt1a
QS[Phospho (STY)]PSSS[Phospho (STY)]PTHER.2	0.309426623	P49586_S319_M2_Pcyt1a
SPS[Phospho (STY)]PSFR.2	-0.337701832	P49586_S331_M1_Pcyt1a
MLQAIS[Phospho (STY)]PK.2	-0.065183037	P49586_S315_M1_Pcyt1a
HIGLVYSGM[Oxidation (M)]GPDYR.3	0.052300297	#N/A
LAQQYYLVYQEPIPTAQLVQR.3	-0.336802205	#N/A
LVQIEYALAAVAGGAPSVGIK.3	0.32224561	#N/A
YNEDLELEDAIHTAILTLK.3	-0.143412723	#N/A
AAVQELSGSILT[Phospho (STY)]SEDPEER.2	-0.205043247	P49769_T370_M1_Psen1
NNDPELEEVNLNNIR.2	-0.246270883	#N/A
AM[Oxidation (M)]VALIDVFHQYSGR.2	-0.278986933	#N/A
S[Phospho (STY)]VDEVNYWDK.2	-0.056148726	P50136_S344_M1_Bckdha
IGHHS[Phospho (STY)]TSDDSSAYR.2	0.175725063	P50136_S334_M1_Bckdha
S[Phospho (STY)]VDEVNYWDKQDHPISR.3	-0.017089475	P50136_S344_M1_Bckdha
IGHHS[Phospho (STY)]TSDDSSAY[Phospho (STY)]RSVDEVNYWDKQDHPISR.5	-0.019762703	P50136_S334_M2_Bckdha
NNGYAISTPTSEQYR.2	-0.110776919	#N/A
EDPLIIPVPASENPFR.2	0.05578912	#N/A
VSQAASDLLAYC[Carbamidomethyl (C)]EAHVR.2	-0.037066523	#N/A
EGMSNNSTT[Phospho (STY)]S[Phospho (STY)]ISQAR.2	0.033631151	P50153_S12_M2_Gng4
VAVVAGYGDVGK.2	0.036682693	#N/A
VADIGLAAWGR.2	0.173876839	#N/A
VPAINVNDSVTK.2	-0.05366629	#N/A
KLDEAVAEAHLGK.3	-0.205226669	#N/A
GISEETTTGVHNLYK.2	0.148000608	#N/A
KQNDVFGEADQ.2	-0.150811321	#N/A
TDDYLDQPC[Carbamidomethyl (C)]LETINR.2	-0.277861733	#N/A
QLIC[Carbamidomethyl (C)]DPSYIPDR.2	-0.209640241	#N/A
IIC[Carbamidomethyl (C)]ILSHPIK.2	0.036003409	#N/A
FLVFVANFDENDPK.2	0.28854218	#N/A
VVEGSFVYK.2	-0.131346137	#N/A
NPYYGGESSSITPLEELYKR.3	-0.067173873	#N/A
NPYYGGESSSITPLEELYK.2	0.365763497	#N/A
M[Oxidation (M)]LLYTEVTR.2	-0.1938345	#N/A
TFEGVDPQNTSM[Oxidation (M)]R.2	0.005041967	#N/A
QNDVFGEADQ.2	-0.024212823	#N/A
YIAIASTTVETAEPEKEVEPALELLEPIDQK.3	-0.143038368	#N/A
VPSTETEALASNLM[Oxidation (M)]GM[Oxidation (M)]FEK.3	-0.387375285	#N/A
KSDIYVC[Carbamidomethyl (C)]M[Oxidation (M)]ISYAHNVAAQGK.3	-0.344093642	#N/A
FDLGQDVIDFTGHALALYR.3	-0.093147229	#N/A
LPANHPLLTGQR.2	0.383445144	#N/A

TVISQSLSK.2	0.281078458	#N/A
VGHSELVGEIIR.2	-0.213606665	#N/A
LIKDDFLQQNGYTPYDR.3	-0.037979247	#N/A
YSNSDVIIYVGC[Carbamidomethyl (C)]GER.2	-0.341553327	#N/A
DIKWEFIPSK.2	-0.276067218	#N/A
VGSHITGGDIYGIVNENSLIK.3	-0.397968824	#N/A
GVNVSALSR.2	0.068111665	#N/A
AVETTAQSDNK.2	0.313000566	#N/A
ALDEYYDKHFTEFVPLR.3	-0.71882775	#N/A
EILQEEEDLAEIVQLVGK.2	-0.346926218	#N/A
HFTEFVPLR.2	-0.034222764	#N/A
AKEILQEEEDLAEIVQLVGK.3	-0.321849068	#N/A
IKADYAQLLEDM[Oxidation (M)]QNAFR.3	-0.455549534	#N/A
QVRPVTEK.2	-0.055676939	#N/A
LAEM[Oxidation (M)]PADSGYPAYLGAR.2	0.510845473	#N/A
TALVANTSNM[Oxidation (M)]PVAAR.2	0.385185613	#N/A
LASFYER.2	-0.110443117	#N/A
ITWSIIR.2	0.132711106	#N/A
RAVETTAQSDNK.2	0.275672695	#N/A
GNEM[Oxidation (M)]SEVLR.2	0.333279253	#N/A
ALDEYYDK.2	-0.103157231	#N/A
EHM[Oxidation (M)]GEILYK.2	-0.013124011	#N/A
LEGDM[Oxidation (M)]ATIQVYEETSGVSVGDPVLR.3	-0.260922804	#N/A
DFPELTM[Oxidation (M)]EVDGK.2	-0.05712068	#N/A
FSMVQVWPVR.2	0.16262336	#N/A
FKDPVKDGEAK.3	-0.269234043	#N/A
DDFLQQNGYTPYDR.2	0.50532844	#N/A
ADYAQLLEDM[Oxidation (M)]QNAFR.2	-0.066472993	#N/A
ADYAQLLEDM[Oxidation (M)]QNAFRS[Phospho (STY)]LED.2	-0.17923701	P50516_S614_M1_Atp6v1a
EASIYTGITLSEYFR.2	-0.053689562	#N/A
TVGM[Oxidation (M)]LSNM[Oxidation (M)]ISFYDMAR.2	0.292700833	#N/A
ASLAETDKITLEVAK.3	0.004441299	#N/A
GSVTYIAPPGNYDASDVVLELEFEGVK.3	-0.105803452	#N/A
GALFGANANR.2	0.237328605	#N/A
KQDFPLVK.2	-0.240680225	#N/A
[Acetyl (Protein N-term)]ALSDADVQK.2	0.25847565	#N/A
VSNTLESR.2	-0.00944562	#N/A
AEEEFNIEK.2	-0.249811604	#N/A
DDLITDLLNEAK.2	0.253161456	#N/A
LDLIAQQM[Oxidation (M)]M[Oxidation (M)]PEVR.2	-0.334969811	#N/A
ARDDLITDLLNEAK.2	-0.206010812	#N/A
IQM[Oxidation (M)]SNLM[Oxidation (M)]NQAR.2	-0.142352756	#N/A
IMEYYEK.2	-0.091707501	#N/A
IKVSNT[Phospho (STY)]LESR.2	0.268871093	P50518_T195_M1_Atp6v1e1
SGELAVQALDQFATVVEAK.2	0.252029404	#N/A
[Acetyl (Protein N-term)]S[Phospho (STY)]GEDEQQEQTIAEDLVVTK.2	0.461709411	P50580_S2_M1_Pa2g4
EQKPFTPAS[Phospho (STY)]PVQSTPSKPSDK.3	-0.425660451	P51125_S219_M1_Cast

SNDTSQT[Phospho (STY)]PPGETVPR.2	0.160189873	P51125_T479_M1_Cast
S[Phospho (STY)]NEQM[Oxidation (M)]VSEKPSESK.2	-0.458107724	P51125_S165_M1_Cast
SLSES[Phospho (STY)]ELIGELSADFDR.2	0.313240786	P51125_S446_M1_Cast
NNIPYFETSAK.2	-0.2632995	#N/A
VIILGDSGVGK.2	0.102074343	#N/A
FQSLGVAFYR.2	-0.287751435	#N/A
EAINVEQAFQTIAR.2	-0.501660449	#N/A
ATIGADFLTK.2	0.420071363	#N/A
TLDSWRDEFLIQASPR.3	-0.172807581	#N/A
LVTM[Oxidation (M)]QIWDTAGQER.2	-0.298531925	#N/A
LPANALLGEENK.2	0.199236098	#N/A
AQDTAELFFEDVR.2	0.08199234	#N/A
THIC[Carbamidomethyl (C)]VTR.2	0.087850356	#N/A
IFSS[Phospho (STY)]EHDIFR.2	-0.129303752	P51174_S55_M1_Acadl
TILSNQTVDIPENVEITLK.2	0.178616244	#N/A
FLDGIYVSEK.2	0.064254709	#N/A
RNNS[Phospho (STY)]ISEAK.2	0.017558961	P51432_S1105_M1_Plcb3
VDSEGIS[Phospho (STY)]PNR.2	-0.130952165	P51660_S308_M1_Hsd17b4
VVLVTGAGGGLGR.2	-0.119478876	#N/A
IDVVVNNAGILR.2	0.271915488	#N/A
ISDEDWDIIHR.3	-0.199104278	#N/A
ASLGS[Phospho (STY)]DDSTQAK.2	-0.00089483	P51830_S1295_M1_Adcy9
GQGTAS[Phospho (STY)]PGSVSDLAQTVK.2	-0.25737099	P51830_S610_M1_Adcy9
AIEKDS[Phospho (STY)]C[Carbamidomethyl (C)]EDIGVEEASELSK.3	0.18077414	P51830_S1332_M1_Adcy9
QGDEES[Phospho (STY)]ENSVKR.2	-0.291710221	P51830_S354_M1_Adcy9
IIADDLMKQGDEES[Phospho (STY)]ENS[Phospho (STY)]VKR.3	0.028794228	P51830_S354_M2_Adcy9
QIEINTISASFGGLASR.2	-0.153632979	#N/A
AGDVLEDS[Phospho (STY)]PK.2	-0.173427798	P51859_S165_M1_Hdgf
RAGDVLEDS[Phospho (STY)]PK.2	-0.130757845	P51859_S165_M1_Hdgf
AGDVLEDS[Phospho (STY)]PKRPK.2	-0.510706207	P51859_S165_M1_Hdgf
GSAEGSS[Phospho (STY)]DEEGKLVIDEPAKEK.3	-0.02051565	P51859_S133_M1_Hdgf
GSAEGSS[Phospho (STY)]DEEGKLVIDEPAK.3	0.097261151	P51859_S133_M1_Hdgf
GFSEGLWEIENNPTVK.2	-0.411133027	#N/A
KGSAEGS[Phospho (STY)]S[Phospho (STY)]DEEGKLVIDEPAK.3	0.246491898	#N/A
SC[Carbamidomethyl (C)]AAEPEVEPEAHEGDGDKKGSAEGS[Phospho (STY)]S[Phospho (STY)]DEEGK.4	-0.056466737	#N/A
LYPEGLAQLAR.2	-0.095405887	#N/A
NVADYYPEYK.2	-0.253440465	#N/A
FFEHEVK.2	0.193373351	#N/A
NIVWIAEC[Carbamidomethyl (C)]IAQR.2	-0.132587528	#N/A
ADDYEQVK.2	-0.068565748	#N/A
AYLESFYK.2	0.015929051	#N/A
LKEQEC[Carbamidomethyl (C)]R.2	0.017635316	#N/A
LLFEGAGSNPGDK.2	-0.104405571	#N/A
AFIITINSFGTELSKEDR.2	-0.221455114	#N/A
LHLQSTDYGNFLANEASPLTVSVIDDK.3	-0.409200235	#N/A
LLFEGAGSNPGDKTLEDR.3	-0.477007784	#N/A
FC[Carbamidomethyl (C)]TLLGGTTADAM[Oxidation (M)]C[Carbamidomethyl (C)]PILEFEADRR.3	-0.037382571	#N/A

AGVLSQADYLNLVQC[Carbamidomethyl (C)]ETLEDLK.3	-0.538026768	#N/A
NTEINFQLGEEFEETSIDDR.2	-0.333217749	#N/A
LTDSQNFDEYMK.2	-0.162239773	#N/A
GNLANVIR.2	-0.051868426	#N/A
TAVAPIER.2	-0.076064444	#N/A
YFPTQALNFAFK.2	-0.107291386	#N/A
EQGVLSFWR.2	-0.115673262	#N/A
LLLQVQHASK.2	0.243094371	#N/A
QIFLGGVDKR.2	-0.029727712	#N/A
DFLAGGVAAAISK.2	0.053399535	#N/A
GAWSNVLR.2	0.062022742	#N/A
GLGDC[Carbamidomethyl (C)]LVK.2	0.091966212	#N/A
AAYFGIYDTAK.2	-0.216571431	#N/A
QIFLGGVDK.2	0.267833637	#N/A
[Acetyl (Protein N-term)]TDAAVSFAK.2	0.125392824	#N/A
GM[Oxidation (M)]GGAFVLVLYDEIKK.2	-0.160226673	#N/A
MM[Oxidation (M)]MQSGR.2	-0.269095034	#N/A
GTDIMYTGTLDC[Carbamidomethyl (C)]WR.2	0.156064512	#N/A
GM[Oxidation (M)]GGAFVLVLYDEIK.2	-0.006370188	#N/A
TC[Carbamidomethyl (C)]S[Phospho (STY)]LPDLSK.2	-0.36877844	P51954_S997_M1_Nek1
SSDVPLPLELLET[Phospho (STY)]GGSPSK.2	0.619243325	P51954_T615_M1_Nek1
EQPGDEYS[Phospho (STY)]EEEESVLK.2	0.111289225	P51954_S1071_M1_Nek1
VLDASWYSPGTR.2	0.244684599	#N/A
LLS[Phospho (STY)]PTHEK.2	0.098042643	P52479_S541_M1_Usp10
C[Carbamidomethyl (C)]S[Phospho (STY)]PPVPSPLASEK.2	-0.144257047	P52479_S359_M1_Usp10
TC[Carbamidomethyl (C)]DS[Phospho (STY)]PQNPVDFISGPVPDSPFPR.3	-0.390146306	P52479_S208_M1_Usp10
LDIDSAPITAR.2	0.076498031	#N/A
NTGIIC[Carbamidomethyl (C)]TIGPASR.2	-0.022019945	#N/A
GDYPLEAVR.2	-0.136323204	#N/A
APIIAVTR.2	0.230314128	#N/A
DAVLNAWAEDVDLR.2	-0.189843491	#N/A
IENHEGVR.2	0.175028802	#N/A
IYVDDGLISLQVK.2	-0.087128859	#N/A
C[Carbamidomethyl (C)]DENILWLDYK.2	0.011476872	#N/A
GVNLPGAAVDLPAVSEK.2	0.031341823	#N/A
KAADVHEVR.2	-0.067047841	#N/A
EKGADFLVTEVENGGSLGSK.3	-0.138386894	#N/A
AADVHEVRK.2	0.070911022	#N/A
AADVHEVR.2	0.254969045	#N/A
GDLGIEIPAEK.2	-0.420427949	#N/A
M[Oxidation (M)]QHLIAR.2	0.409617616	#N/A
ITLDNAYM[Oxidation (M)]EK.2	-0.422338239	#N/A
GVNLPGAAVDLPAVSEKDIQDLK.3	-0.131159822	#N/A
GADFLVTEVENGGSLGSKK.3	-0.454340454	#N/A
VNLAM[Oxidation (M)]DVGK.2	-0.206288024	#N/A
AEGSDVANAVLDGADC[Carbamidomethyl (C)]IM[Oxidation (M)]LSGETAK.3	0.164985305	#N/A
EATESFASDPILYR.2	0.378176696	#N/A

AEGSDVANAVLDGADC[Carbamidomethyl (C)]IM[Oxidation (M)]LSGETAKGDYPLEAVR.3	-0.071567502	#N/A
FGVEQDVDM[Oxidation (M)]VFASFIR.2	-0.306943079	#N/A
EATESFASDPILYRPVAVALDTK.3	-0.223705557	#N/A
LAPITSDPTEAAAVGAVEASFK.2	-0.458393264	#N/A
GSGTAEVELKK.2	-0.090528826	#N/A
EATESFASDPILYRPVAVALDTKGPEIR.4	-0.481283472	#N/A
RFDEILEASDGIM[Oxidation (M)]VAR.3	-0.098744765	#N/A
GADFLVTEVENGGSLGSK.2	-0.186377694	#N/A
LNFSHGTHEYHAETIK.3	0.069537696	#N/A
GSGTAEVELK.2	0.009548883	#N/A
FDEILEASDGIMVAR.2	0.282290529	#N/A
TGTC[Carbamidomethyl (C)]GYC[Carbamidomethyl (C)]GLQFK.2	-0.091017499	#N/A
EVNENFAIDLIAQQPVNEVEHR.3	-0.022006748	#N/A
ITHTGQVYDEK.2	0.165312504	#N/A
VYINLDKETK.2	0.205166265	#N/A
IIAC[Carbamidomethyl (C)]DGGGGALGHPK.2	0.112933488	#N/A
QKEVNENFAIDLIAQQPVNEVEHR.4	0.490036648	#N/A
ALRFSY[Phospho (STY)]HLEGS[Phospho (STY)]QPR.2	0.011486899	P52734_S98_M2_Fgd1
SLS[Phospho (STY)]LDPGQSLEPHPEGPQR.3	-0.338390349	P52734_S116_M1_Fgd1
LLNSTNRDDEDT[Phospho (STY)]PPNSPNVDLGK.3	-0.072817454	P52734_T710_M1_Fgd1
QES[Phospho (STY)]VELTVQQK.2	-0.068944879	P52734_S364_M1_Fgd1
AAYQVAALPR.2	0.2469672	#N/A
APAAIGPYSQAVQVDR.2	0.120707651	#N/A
AAGC[Carbamidomethyl (C)]DFNNVVK.2	-0.072247984	#N/A
TYFQGSLPAR.2	0.18176573	#N/A
TTVLLADM[Oxidation (M)]NDFGTVNEIYK.2	-0.499851372	#N/A
TIYISGQVGLDPSSGQLVPGGVVEEAK.3	0.062800395	#N/A
KPAQETEETSSQES[Phospho (STY)]AEED.2	-0.043279338	P52927_S104_M1_Hmga2
LSDIGEGIR.2	0.165679061	#N/A
AAS[Phospho (STY)]REEPIEWEF.2	0.175922925	P53564_S1506_M1_Cux1
SC[Carbamidomethyl (C)]S[Phospho (STY)]IDTSPGTSSLASPASQR.2	-0.251505621	P53668_S298_M1_Limk1
VQGVDPGC[Carbamidomethyl (C)]M[Oxidation (M)]S[Phospho (STY)]PDVK.2	-0.009121157	P53668_S210_M1_Limk1
SEPSGS[Phospho (STY)]PPAPAHSR.2	-0.001913362	P53783_S325_M1_Sox1
LFTNFHR.2	0.20025742	#N/A
LEPEAWK.2	0.165932215	#N/A
VENFIHK.2	0.204155299	#N/A
VILPVSVDEYQVGQLYSVAEASK.3	0.128500453	#N/A
NETGGGEGVEVLVNEPYEKDDGEKGQYTHK.4	0.03129791	#N/A
HVEAIYIDIADR.2	-0.267298477	#N/A
MLAPEGALNIHEK.3	0.312238588	#N/A
SDANTDLIGGS[Phospho (STY)]PK.2	-0.194843726	P53986_S230_M1_Slc16a1
SKES[Phospho (STY)]LQEAGK.2	0.09796186	P53986_S213_M1_Slc16a1
EGKEDEAS[Phospho (STY)]T[Phospho (STY)]DVDEKPK.2	-0.077328935	P53986_S461_M2_Slc16a1
LKS[Phospho (STY)]KES[Phospho (STY)]LQEAGK.2	-0.138915179	P53986_S210_M2_Slc16a1
AAQSPQQHSSGDPTEEES[Phospho (STY)]PV.2	-0.228268288	P53986_S491_M1_Slc16a1
SDANTDLIGGS[Phospho (STY)]PKGEK.2	-0.197410923	P53986_S230_M1_Slc16a1
S[Phospho (STY)]KESLQEAGKSDANTDLIGGSPK.3	-0.001747835	P53986_S210_M1_Slc16a1

YIIIGDTGVGK.2	-0.195468142	#N/A
TASNVEEAFINTAK.2	-0.159530084	#N/A
IQEGVFDINNEANGIK.2	-0.184767675	#N/A
LQIWDTAGQESFR.2	-0.150959868	#N/A
DTFNHLTTWLEDAR.3	-0.224982884	#N/A
SFDFEGSLS[Phospho (STY)]PVIAPK.2	-0.102828833	P53995_S688_M1_Anapc1
ATDFVVDR.2	0.112592103	#N/A
GKLDGNQDLIR.2	-0.552062865	#N/A
LNEHFLNTTDFLDTIK.3	-0.195893583	#N/A
YFDLGLPNR.2	0.410925444	#N/A
DQTNDQVTIDSALATQK.2	0.44578825	#N/A
TIEAEAAHGTVTR.2	0.059172075	#N/A
FAQTLEK.2	0.40268915	#N/A
DKHVEEVRK.2	0.00872564	#N/A
EHEKEVLQK.2	-0.140800332	#N/A
SKESVPDFPLS[Phospho (STY)]PPKK.3	-0.135805783	P54227_S38_M1_Stmn1
SHEAEVLK.2	-0.45873203	#N/A
ESVPDFPLS[Phospho (STY)]PPKKK.3	-0.278618651	P54227_S38_M1_Stmn1
ASGQAFELILS[Phospho (STY)]PR.2	-0.40476965	P54227_S25_M1_Stmn1
SKESVPDFPLS[Phospho (STY)]PPK.2	-0.035319359	P54227_S38_M1_Stmn1
RKS[Phospho (STY)]HEAEVLK.2	-0.42737549	P54227_S63_M1_Stmn1
KSHEAEVLK.2	0.082639098	#N/A
NKES[Phospho (STY)]KDPADETEAD.2	-0.013352567	P54227_S139_M1_Stmn1
REHEKEVLQK.3	-0.277542839	#N/A
[Acetyl (Protein N-term)]ASSDIQVKELEKR.3	-0.074249373	#N/A
RAS[Phospho (STY)]GQAFELILS[Phospho (STY)]PR.2	-0.103738381	P54227_S16_M2_Stmn1
[Acetyl (Protein N-term)]ASSDIQVK.2	-0.316442875	#N/A
ESVPDFPLS[Phospho (STY)]PPK.2	-0.253407522	P54227_S38_M1_Stmn1
EAS[Phospho (STY)]PSTLNDK.2	0.124501773	P54254_S382_M1_Atxn1
RWS[Phospho (STY)]APETR.2	-0.514787755	P54254_S751_M1_Atxn1
WS[Phospho (STY)]APETR.2	-0.251139031	P54254_S751_M1_Atxn1
ALS[Phospho (STY)]AGLDYSPPSAPR.2	-0.141639081	P54254_S81_M1_Atxn1
HGS[Phospho (STY)]AGTSGEHGLQGM[Oxidation (M)]GLHK.3	-0.168753632	P54254_S62_M1_Atxn1
RRWS[Phospho (STY)]APETR.3	0.211084257	P54254_S751_M1_Atxn1
SAAVSASS[Phospho (STY)]PEAK.2	-0.106801661	P54276_S63_M1_Msh6
VEEHS[Phospho (STY)]PLER.2	-0.349661572	P54285_S393_M1_Cacnb3
RS[Phospho (STY)]PPPSLAK.2	0.023381689	P54285_S152_M1_Cacnb3
LLAQDS[Phospho (STY)]EHDHNDR.3	-0.252877268	P54285_S464_M1_Cacnb3
QAWTGSS[Phospho (STY)]QR.2	-0.616028701	P54285_S418_M1_Cacnb3
DSLMPS[Phospho (STY)]DEASESSR.2	0.125272341	P54285_S403_M1_Cacnb3
SVS[Phospho (STY)]EAALAQPEGLLGTDTLK.2	-0.420145195	P54310_S559_M1_Lipe
TTS[Phospho (STY)]PTAESVRPTESMR.3	0.298695313	P54310_S542_M1_Lipe
NENLAANFLLSQNFDDE.2	0.001390584	#N/A
IDIDPEETVK.2	-0.187861683	#N/A
ASFNNPDR.2	0.018879092	#N/A
TLQQQTFK.2	0.291399701	#N/A
NENLAANFLLQQNFDED.2	-0.310965723	#N/A

QIIQQNPSLLPALLQQIGR.3	0.441363876	#N/A
RTS[Phospho (STY)]PVQTR.2	-0.342352453	P54731_S269_M1_Faf1
TPS[Phospho (STY)]GEFLER.2	0.043407698	P54731_S581_M1_Faf1
FLEDDPSDPTYTSS[Phospho (STY)]LGGK.2	-0.178836309	P54754_S790_M1_Ephb3
FLEDDTS[Phospho (STY)]DPTYTSALGGK.2	0.257767105	P54763_S776_M1_Ephb2
ENAPAIIFIDEIDAIATK.2	-0.094295753	#N/A
LQQELEFLEVQEEYIKDEQK.3	-0.234254836	#N/A
GEGTPAAGDGLGRPLGPTPS[Phospho (STY)]QSR.3	0.246068952	P55012_S74_M1_Slc12a2
PLGPTPS[Phospho (STY)]QSR.2	0.192858802	P55012_S74_M1_Slc12a2
KES[Phospho (STY)]KGPIVPLNVADQK.3	-0.274305118	P55012_S987_M1_Slc12a2
S[Phospho (STY)]PGTKDVVVNVDYSK.2	-0.188828878	P55012_S937_M1_Slc12a2
ELGGEVFYVGPAR.2	-0.153282291	#N/A
LSSATIAAPR.2	-0.017425302	#N/A
FDAYC[Carbamidomethyl (C)]FR.2	-0.00952944	#N/A
VSLPAYPR.2	-0.288853755	#N/A
YPIQTPR.2	0.443547174	#N/A
AALAELVALPC[Carbamidomethyl (C)]FFTLQPR.2	0.011695039	#N/A
YPITQSR.2	0.376280943	#N/A
YQC[Carbamidomethyl (C)]DEGFSQHR.2	-0.159206671	#N/A
ASDSGLYR.2	-0.130551322	#N/A
YNVHATVR.2	0.06750382	#N/A
DFQWTDNTGLQYENWR.2	-0.147430133	#N/A
C[Carbamidomethyl (C)]GGPAPGVR.2	0.104307981	#N/A
HLQAAFEDGFDNC[Carbamidomethyl (C)]DAGWLSDR.3	-0.345973932	#N/A
FINSFGHENSWIGLNDR.3	-0.088972016	#N/A
QDLPILVAK.2	0.288663431	#N/A
AQGM[Oxidation (M)]PTLTSTS[Phospho (STY)]SEGHPEPK.3	-0.112027791	P55066_S869_M1_Ncan
GSYM[Oxidation (M)]EVEDNR.2	-0.418319157	#N/A
AEQPTESGS[Phospho (STY)]PEVHC[Carbamidomethyl (C)]GER.2	-0.481164449	P55144_S808_M1_Tyro3
ERTEADLPKPTS[Phospho (STY)]PK.3	-0.440267311	P55194_S535_M1_Sh3bp1
DNHSQADHS[Phospho (STY)]PLTTAAPFSR.3	-0.209323559	P55194_S262_M1_Sh3bp1
KLEGNS[Phospho (STY)]PQGS[Phospho (STY)]SHGVK.2	0.095822971	P55258_S181_M2_Rab8a
LEGNS[Phospho (STY)]PQGS[Phospho (STY)]SHGVK.2	0.021566695	P55258_S181_M2_Rab8a
VAQWLIQEPHK.2	-0.365844067	#N/A
YAAENNR.2	-0.401607332	#N/A
LADLYGS[Phospho (STY)]KDTFDDDS.2	-0.230320112	P55288_S788_M1_Cdh11
PGLRPAPNS[Phospho (STY)]VDVDDFINTR.3	-0.012614088	P55288_S714_M1_Cdh11
IQEYNVLLDTLSR.2	-0.142189404	#N/A
RES[Phospho (STY)]GEEFR.2	-0.115619968	P55302_S53_M1_Lrpap1
DLS[Phospho (STY)]LEEIQK.2	-0.458512385	P55821_S80_M1_Stmn2
DLS[Phospho (STY)]LEEIQKK.2	-0.360198626	P55821_S80_M1_Stmn2
KLEAAEER.2	-0.066620788	#N/A
KDLS[Phospho (STY)]LEEIQK.2	-0.117119224	P55821_S80_M1_Stmn2
KKDLS[Phospho (STY)]LEEIQK.2	-0.211914497	P55821_S80_M1_Stmn2
RKS[Phospho (STY)]QEAQVLK.2	-0.407202084	P55821_S97_M1_Stmn2
RAS[Phospho (STY)]GQAFELILKPPS[Phospho (STY)]PISEAPR.3	0.006632352	P55821_S50_M2_Stmn2
AS[Phospho (STY)]GQAFELILKPPS[Phospho (STY)]PISEAPR.3	0.141833756	P55821_S50_M2_Stmn2

NKELQVELS[Phospho (STY)]G.2	0.148869529	P55821_S178_M1_Stmn2
KDLS[Phospho (STY)]LEEIQKK.3	0.414912787	P55821_S80_M1_Stmn2
GFKEEGS[Phospho (STY)]PDR.2	-0.374597307	P55937_S60_M1_Golga3
LHNQNS[Phospho (STY)]VPR.2	-0.003539154	P55937_S1479_M1_Golga3
LHNQNS[Phospho (STY)]VPRDGLGQ.2	-0.36784848	P55937_S1479_M1_Golga3
DFTPSGIAGAFR.2	0.192372871	#N/A
EAGS[Phospho (STY)]EAESSSADPGPGR.2	-0.169702639	P56183_S434_M1_Rrp1
VTS[Phospho (STY)]PEKAEEAK.2	0.035454617	P56212_S23_M1_Arpp19
YFDS[Phospho (STY)]GDYNM[Oxidation (M)]AK.2	-0.38373403	P56212_S62_M1_Arpp19
KPS[Phospho (STY)]LVASK.2	0.493681881	P56212_S104_M1_Arpp19
GQKYFDS[Phospho (STY)]GDYNM[Oxidation (M)]AK.2	-0.137156377	P56212_S62_M1_Arpp19
SVDYEVFGTVQGVC[Carbamidomethyl (C)]FR.2	-0.220421472	#N/A
NTS[Phospho (STY)]KGTVTGQVQGPEEK.2	-0.262343928	P56375_S51_M1_Acyp2
VQGVFFR.2	0.252650549	#N/A
[Acetyl (Protein N-term)]AEGDTLVSVDYEIFGK.2	-0.123914069	#N/A
VIANLDYSDFQIVK.2	-0.042815441	#N/A
FM[Oxidation (M)]QQWLETR.2	0.223663794	#N/A
LGLVGWVQNTDR.2	0.0416041	#N/A
GTVQGQLQGPVSK.2	-0.054006519	#N/A
MFQTLIQK.2	0.369816509	#N/A
EETGIEASQLTIIEGFR.2	0.091441422	#N/A
ATLQEGHQFLC[Carbamidomethyl (C)]ST[Phospho (STY)]PA.2	-0.448708686	P56380_T145_M1_Nudt2
QAGLSYIR.2	0.044453475	#N/A
DALKTEFK.2	-0.083779798	#N/A
FSQIC[Carbamidomethyl (C)]AK.2	0.062035752	#N/A
ALDIYSAVDDAS[Phospho (STY)]HEK.2	-0.013596543	P56388_S48_M1_Cartpt
ALDIYSAVDDAS[Phospho (STY)]HEKELPR.3	-0.008207831	P56388_S48_M1_Cartpt
SLC[Carbamidomethyl (C)]PVSWVSAWDDR.2	0.044364801	#N/A
IAEGTFPGKI.2	-0.011507238	#N/A
NC[Carbamidomethyl (C)]WQNYLDFHR.3	0.114897472	#N/A
IAEGTFPGK.2	0.015376149	#N/A
[Acetyl (Protein N-term)]AEDIKTK.2	-0.174309426	#N/A
FPNQNQTK.2	0.099199868	#N/A
GGDVSVC[Carbamidomethyl (C)]EWYR.2	0.047609747	#N/A
APSFHDK.2	0.007246677	#N/A
FLEEHPGGEEVLR.3	-0.007536515	#N/A
YYTLEEIQK.2	0.260898468	#N/A
EQAGGDATENFEDVGHSTDAR.3	0.14467736	#N/A
[Acetyl (Protein N-term)]AGQS[Phospho (STY)]DKDVK.2	-0.27716476	P56395_S5_M1_Cyb5a
TYIIGELHPDDR.2	0.442845156	#N/A
GTGLQPGEEELPDIAPPLVT[Phospho (STY)]PDEPK.3	-0.336973693	P56399_T623_M1_Usp5
ATNNS[Phospho (STY)]LER.2	0.041525871	P56399_S751_M1_Usp5
DGLGGLPDIVR.2	-0.039181422	#N/A
IFQNAPTDPTQDFSTQVAK.2	-0.029472401	#N/A
IVILPDYLEIAR.2	0.070227945	#N/A
WVIYNDQK.2	-0.278764897	#N/A
[Acetyl (Protein N-term)]AELSEEALLSVLPTIR.2	0.219775792	#N/A

DLGYIYFYQR.2	0.023216457	#N/A
ENLWLNLTDGSILC[Carbamidomethyl (C)]GR.2	0.058134423	#N/A
SSENPNEVFR.2	0.164614913	#N/A
ETGYPLAVK.2	0.019618916	#N/A
AVDWIFSHIDDLDAEAAM[Oxidation (M)]DIS[Phospho (STY)]EGR.3	-0.156721821	P56399_S775_M1_Usp5
QAEEEKVPLPELVR.3	-0.321282943	#N/A
GHPEFSTNR.2	0.278602439	#N/A
YFDGSGGNNHAVEHYR.3	0.11592491	#N/A
FASFPDYLVIQIK.2	0.034637977	#N/A
KQEVQAWDGEVR.3	0.074711448	#N/A
SAAES[Phospho (STY)]ISESVPVGPK.2	-0.048461917	P56399_S783_M1_Usp5
VTSAVEALLSADSASR.2	-0.169390928	#N/A
VLDSGAPIKIPVGPETLGR.3	-0.210658556	#N/A
AIAELGIYPAVDPLDSTSR.2	0.015307481	#N/A
IPVGPETLGR.2	0.341835507	#N/A
LVPLKETIK.2	-0.045086374	#N/A
VVDLLAPYAK.2	0.203011266	#N/A
VALTGLTVAEYFR.2	0.111110945	#N/A
IGLFGGAGVGK.2	0.176109938	#N/A
ADKLAEEHGS.2	0.02533501	#N/A
VLDSGAPIK.2	0.107226084	#N/A
IM[Oxidation (M)]NVIGEPIDER.2	0.005776223	#N/A
TIAM[Oxidation (M)]DGTEGLVR.2	0.233782923	#N/A
TVLIM[Oxidation (M)]ELINNVAK.2	0.277309539	#N/A
IM[Oxidation (M)]DPNIVGNEHYDVAR.3	0.111552396	#N/A
VALVYGQM[Oxidation (M)]NEPPGAR.2	-0.116151477	#N/A
AHGGYSVFAGVGER.3	0.168384809	#N/A
FLSQPFQVAEVFTGHM[Oxidation (M)]GK.3	-0.391717919	#N/A
KGSITSVQAIYVPADDLTDPAPATTFAHLDATTVLSR.4	0.179509219	#N/A
IPSAVGYQPTLATDM[Oxidation (M)]GTM[Oxidation (M)]QER.3	-0.288770779	#N/A
FTQAGSEVSALLGR.2	-0.468741137	#N/A
TREGNDLYHEM[Oxidation (M)]IESGVINLK.4	-0.196234099	#N/A
EGNDLYHEM[Oxidation (M)]IESGVINLK.3	0.126010173	#N/A
DQEGQDVLLFIDNIFR.2	0.045736068	#N/A
IM[Oxidation (M)]NVIGEPIDERGPIK.3	-0.016735749	#N/A
LVLEVAQHLGESTVR.3	-0.114262543	#N/A
QFAPIHAEAPEFIEM[Oxidation (M)]SVEQEILVTGIK.3	0.095841555	#N/A
SLQDIIAILGM[Oxidation (M)]DELSEEDKLTVSR.3	0.155203081	#N/A
GFQQILAGEYDHLPEQAFYM[Oxidation (M)]VGPIEEAVAK.4	-0.210553084	#N/A
IVAVIGAVVDVQFDEGLPPILNALEVQGR.3	-0.043739127	#N/A
TTTNVLGDSLGAGIVEHLSR.2	-0.23212994	#N/A
IVQVTAADAFLDLIR.2	-0.358732796	#N/A
DVEM[Oxidation (M)]GNS[Phospho (STY)]VIEENEMK.2	0.178577365	#N/A
NRDVEMGNS[Phospho (STY)]VIEENEM[Oxidation (M)]K.3	0.270302821	#N/A
DLLQTELSGFLDVQK.2	-0.155165488	#N/A
LNATAS[Phospho (STY)]LEQDKIEPPR.3	-0.386734575	P56695_S32_M1_Wfs1
GITS[Phospho (STY)]ENEAEVK.2	-0.229459481	P56695_S158_M1_Wfs1

LNATAS[Phospho (STY)]LEQDK.2	0.018282613	P56695_S32_M1_Wfs1
[Acetyl (Protein N-term)]MNSGT[Phospho (STY)]PPPSPSGPPPPPAPQPQAR.3	-0.416007411	P56695_T5_M1_Wfs1
KVM[Oxidation (M)]DS[Phospho (STY)]DEDDADY.2	0.287610222	P56812_S119_M1_Pdcd5
[Acetyl (Protein N-term)]ADEELEALRK.2	0.281179297	#N/A
RKVM[Oxidation (M)]DS[Phospho (STY)]DEDDADY.2	-0.218022605	P56812_S119_M1_Pdcd5
VSEQGLIEILEK.2	0.605139017	#N/A
VMDS[Phospho (STY)]DEDDADY.2	0.206247629	P56812_S119_M1_Pdcd5
AAIDWFDGK.2	0.06072423	#N/A
GEATVS[Phospho (STY)]FDDPPSAK.2	-0.11255631	P56959_S333_M1_Fus
HDSEQDNSDNNTIFVQGLGENVTIESVADYFK.3	0.255486247	#N/A
SDS[Phospho (STY)]PPLC[Carbamidomethyl (C)]PAADSR.2	-0.15929827	P56974_S715_M1_Nrg2
SSS[Phospho (STY)]FGNFDR.2	-0.1377754	P57725_S23_M1_Samsn1
FTAGDFSTTVIQNVNK.2	-0.255111533	#N/A
IEIFPSR.2	-0.010204182	#N/A
VNAIEHVIIPR.2	-0.222186317	#N/A
EAAFSLAEAK.2	0.011733684	#N/A
TLAYIITELDER.2	0.425863069	#N/A
SSPETLISDLLM[Oxidation (M)]KES[Phospho (STY)]TENAPR.3	0.024755306	P57774_S82_M1_Npy
ITSLEVENQNLR.2	0.190099838	#N/A
[Acetyl (Protein N-term)]ATNFLAHEK.2	0.546778905	#N/A
ATAPQTQHVS[Phospho (STY)]PM[Oxidation (M)]R.2	-0.045887263	P57776_S133_M1_Eef1d
LSSLEKSS[Phospho (STY)]PTPR.2	-0.095191693	P57776_S119_M1_Eef1d
GATPAEDDEDKDIDLFGSDEEEEDKEAAR.4	0.159349755	#N/A
DIDLFGS[Phospho (STY)]DEEEEDKEAAR.3	0.004921109	P57776_S162_M1_Eef1d
EAILAIHK.2	-0.033818103	#N/A
DGLAFNALIHR.2	0.192195729	#N/A
M[Oxidation (M)]APYQGPDAAPGALDYK.2	-0.281582456	#N/A
AGTQIENIDEDFRDGLK.3	0.200105514	#N/A
IC[Carbamidomethyl (C)]DQWDNLGSLTHSR.3	0.053841663	#N/A
VLAVNQENEHLMEDYER.3	-0.371189631	#N/A
LTAELIEQAAQYTNAVR.2	-0.302867373	#N/A
S[Phospho (STY)]LSASPALGSTK.2	-0.343064456	P58058_S46_M1_Nadk
VFS[Phospho (STY)]GVVS[Phospho (STY)]T[Phospho (STY)]GLK.2	-0.353676888	P58252_S418_M3_Eef2
GEGQLSAAER.2	0.352797977	#N/A
VNFTVDQIR.2	-0.14874596	#N/A
FSVSPVVR.2	0.242658105	#N/A
YEWDVAEAR.2	0.192993477	#N/A
STLTDSLVC[Carbamidomethyl (C)]K.2	0.080449308	#N/A
DLEEDHAC[Carbamidomethyl (C)]IPIKK.3	0.104405354	#N/A
SDPVVSYR.2	-0.109132177	#N/A
TFC[Carbamidomethyl (C)]QLILDPIFK.2	0.243987527	#N/A
ETVSEESNVLC[Carbamidomethyl (C)]LSK.2	-0.20966202	#N/A
AYLPVNESFGFTADLR.2	-0.109847579	#N/A
C[Carbamidomethyl (C)]ELLYEGPPDDEAAM[Oxidation (M)]GIK.2	-0.365342054	#N/A
EGIPALDNFLDKL.2	0.232514119	#N/A
FT[Phospho (STY)]DTRKDEQER.2	0.535116291	P58252_T57_M1_Eef2
VFDAIMNFR.2	0.27139724	#N/A

AGETRFT[Phospho (STY)]DTR.2	0.329844049	P58252_T57_M1_Eef2
ALLELQLEPEELYQTFQR.2	0.04381472	#N/A
YFDPANGK.2	-0.383444905	#N/A
EDLYLKPIQR.3	-0.216073776	#N/A
STAISLFYELSENDLNFIK.2	-0.191990951	#N/A
VVVVGDQSAGK.2	0.080861054	#N/A
EVLEDFAEDGEK.2	-0.348906621	#N/A
ESVEQQADSFK.2	-0.405852663	#N/A
AVEVAWETLQEEFSR.2	-0.245807949	#N/A
EFDLTKEEDLAALR.3	-0.08518748	#N/A
FNLETEWK.2	0.163633648	#N/A
TSVLEM[Oxidation (M)]IAQAR.2	-0.285976075	#N/A
DFFTAGSPGETAFR.2	0.029220538	#N/A
VNDEHPAYLASDEITTVR.3	0.145086715	#N/A
IQQIIEGK.2	0.081900575	#N/A
IDQLQEELLHTQLK.3	-0.30819619	#N/A
LDAFIEALHQEK.2	-0.272950413	#N/A
SIVTDLVSQM[Oxidation (M)]DPHGR.3	-0.401922182	#N/A
LVALLDTLDR.2	-0.110239021	#N/A
FGSLLPIHPVTSG.2	-0.250619437	#N/A
LDQEAENLVATVVPTHLAAAVPEVAVYLK.3	0.460652768	#N/A
S[Phospho (STY)]LELNGAGEPVEGAPR.2	-0.196133246	P58404_S206_M1_Strn4
AS[Phospho (STY)]PGPGGLSGGESLLVK.2	-0.004372526	P58404_S223_M1_Strn4
GSKPSASSAS[Phospho (STY)]LEKR.3	-0.083740258	P58406_S332_M1_Hrh3
VQPHGS[Phospho (STY)]LEQC[Carbamidomethyl (C)]WK.2	-0.089248803	P58406_S439_M1_Hrh3
RDS[Phospho (STY)]SSHEETGASHTLYGHGVC[Carbamidomethyl (C)]K.4	-0.456730228	P58463_S329_M1_Foxp2
AVEPALPQS[Phospho (STY)]PAGGAGAK.2	-0.456141747	P58468_S38_M1_Fam207a
LEFQQQLGEAPGDAS[Phospho (STY)]P.2	0.023599561	P58686_S82_M1_
FS[Phospho (STY)]PVLGR.2	-0.210932328	P58742_S495_M1_Aaas
KLVIIESDLERAEER.3	-0.370707478	#N/A
KLVIIESDLER.3	-0.026276445	#N/A
TVTNNLKS[Phospho (STY)]LEAQAEK.2	0.010178627	P58771_S206_M1_Tpm1
C[Carbamidomethyl (C)]AELEEELKTVTNNLK.3	0.085153585	#N/A
AQKDEEKMEIQEIQLK.3	-0.043761557	#N/A
LVIIESDLERAEER.3	0.311408687	#N/A
EPAAGGS[Phospho (STY)]LSGTR.2	-0.414731526	P58802_S18_M1_Tbc1d10a
ENYEDQEPLVGHES[Phospho (STY)]PITLAAR.3	0.179692828	P58871_S602_M1_Tnks1bp1
LANISVPASES[Phospho (STY)]PR.2	-0.224057263	P58871_S300_M1_Tnks1bp1
VSGAGLS[Phospho (STY)]PSRK.2	0.017960042	P58871_S1131_M1_Tnks1bp1
DS[Phospho (STY)]LGSFSTR.2	-0.109602266	P58871_S866_M1_Tnks1bp1
VPSS[Phospho (STY)]DEEVVEEPQSR.2	-0.018721707	P58871_S1612_M1_Tnks1bp1
VSGAGLSPS[Phospho (STY)]R.2	0.104930754	P58871_S1133_M1_Tnks1bp1
SLSSGFS[Phospho (STY)]PEEAQQQDEEFEK.2	-0.141825643	P58871_S980_M1_Tnks1bp1
WLDDLLAS[Phospho (STY)]PPPNSGSAR.2	-0.183552221	P58871_S692_M1_Tnks1bp1
M[Oxidation (M)]QAESQS[Phospho (STY)]PTNVDLEDKER.2	-0.034734614	P58871_S1063_M1_Tnks1bp1
SSGSLS[Phospho (STY)]PGLETEDPLEAR.2	-0.346074463	P58871_S1375_M1_Tnks1bp1
GEGVSQVGPGT[Phospho (STY)]PPAPESPR.2	-0.014965207	P58871_T533_M1_Tnks1bp1

RFS[Phospho (STY)]EGVLQPPSQDQEK.2	0.115107292	P58871_S429_M1_Tnks1bp1
NM[Oxidation (M)]APGAGC[Carbamidomethyl (C)]S[Phospho (STY)]PGEPR.2	-0.512535945	P58871_S1290_M1_Tnks1bp1
DVGHLEEGASGGLLS[Phospho (STY)]PSTPHSR.3	-0.170054371	P58871_S1022_M1_Tnks1bp1
GYSS[Phospho (STY)]QDAEEQDREFEKR.3	0.051957657	P58871_S912_M1_Tnks1bp1
ASRVPS[Phospho (STY)]S[Phospho (STY)]DEEVVEEPQSR.2	0.08484909	P58871_S1611_M2_Tnks1bp1
RDS[Phospho (STY)]LGSFSTR.2	-0.551878206	P58871_S866_M1_Tnks1bp1
LDS[Phospho (STY)]PPPSPITEASEAAEAAEADSWAVSGR.3	0.161362569	P58871_S496_M1_Tnks1bp1
HS[Phospho (STY)]LGQEVIGIGGSQDESEVPVR.3	-0.177958066	P58871_S796_M1_Tnks1bp1
NRS[Phospho (STY)]AEEGEVTESK.2	-0.121772259	P58871_S1657_M1_Tnks1bp1
S[Phospho (STY)]PPSGSQSLLEGIM[Oxidation (M)]TASSSK.2	-0.370335733	P58871_S1437_M1_Tnks1bp1
DRQS[Phospho (STY)]PSTC[Carbamidomethyl (C)]SEGLLGWAQK.3	-0.290248224	P58871_S713_M1_Tnks1bp1
S[Phospho (STY)]HTGEAAAVR.2	-0.517066384	P59017_S387_M1_Bcl2l13
ALQTILSQPVTYEAYR.2	0.062216041	#N/A
KKSHT[Phospho (STY)]GEAAAVR.3	-0.126623802	P59017_T389_M1_Bcl2l13
[Acetyl (Protein N-term)]ANENHGS[Phospho (STY)]PR.2	-0.125264006	P59114_S8_M1_Pcif1
QLS[Phospho (STY)]EEQPSGNGVK.2	-0.154674933	P59114_S116_M1_Pcif1
AFS[Phospho (STY)]EDEALAQQDSK.2	-0.337149531	P59281_S597_M1_Arhgap39
KPSS[Phospho (STY)]ETDIENWASK.2	0.105866065	P59281_S683_M1_Arhgap39
DGST[Phospho (STY)]GSSLEPELEER.2	-0.395772111	P59281_T133_M1_Arhgap39
QLVYVEQAGS[Phospho (STY)]SPK.2	-0.346349188	P59281_S402_M1_Arhgap39
EDTSSC[Carbamidomethyl (C)]S[Phospho (STY)]PPGVLLEK.2	-0.203687116	P59281_S169_M1_Arhgap39
SVS[Phospho (STY)]DQFYR.2	-0.098823347	P59325_S10_M1_Eif5
AM[Oxidation (M)]GPLVLTEVLFDEK.2	0.182519094	#N/A
GLTLS[Phospho (STY)]DDLER.2	-0.739586976	P59325_S227_M1_Eif5
VETVKS[Phospho (STY)]DNKDDDIDIDAI.2	0.202448955	P59325_S417_M1_Eif5
EEGIIDSSDKEIVAEAER.3	-0.230301199	#N/A
AAGPATS[Phospho (STY)]PPR.2	0.038874934	P59644_S266_M1_Inpp5j
QLS[Phospho (STY)]PTSVGPK.2	-0.247574059	P59644_S171_M1_Inpp5j
SRS[Phospho (STY)]PGLAR.2	0.028995713	P59644_S885_M1_Inpp5j
VKAPSGGPS[Phospho (STY)]PSGR.2	-0.442696488	P59644_S691_M1_Inpp5j
HRS[Phospho (STY)]RS[Phospho (STY)]PGLAR.3	-0.157994665	P59644_S883_M2_Inpp5j
GGSRS[Phospho (STY)]PS[Phospho (STY)]PQSR.2	-0.068442838	P59644_S909_M2_Inpp5j
S[Phospho (STY)]C[Carbamidomethyl (C)]KSELPSSAPGGGGV.2	0.242401577	P59648_S65_M1_Fxyd7
SELPSS[Phospho (STY)]APGGGGV.2	0.221821312	P59648_S73_M1_Fxyd7
ADSRS[Phospho (STY)]ES[Phospho (STY)]PTC[Carbamidomethyl (C)]K.2	-0.482413149	#N/A
SESPTC[Carbamidomethyl (C)]KSC[Carbamidomethyl (C)]KS[Phospho (STY)]ELPSSAPGGGGV.3	-0.052168188	P59648_S68_M1_Fxyd7
KADS[Phospho (STY)]RSES[Phospho (STY)]PTC[Carbamidomethyl (C)]K.2	0.269082088	#N/A
SES[Phospho (STY)]LSNC[Carbamidomethyl (C)]SIGK.2	0.10263186	P59672_S663_M1_Anks1a
SES[Phospho (STY)]LSNC[Carbamidomethyl (C)]SIGKK.2	0.435663775	P59672_S663_M1_Anks1a
GTESTQDAC[Carbamidomethyl (C)]AK.2	-0.177064984	#N/A
S[Phospho (STY)]DPNLSAPEK.2	0.078672007	P59764_S1759_M1_Dock4
NSAPASM[Oxidation (M)]S[Phospho (STY)]PDGTR.2	-0.199896907	P59764_S1629_M1_Dock4
AVNPTPSSWS[Phospho (STY)]LDSGK.2	0.109031478	P59764_S1778_M1_Dock4
AS[Phospho (STY)]PLLSDK.2	-0.125199958	P59764_S1709_M1_Dock4
S[Phospho (STY)]LPVSIC[Carbamidomethyl (C)]R.2	-0.165055589	P59808_S805_M1_Sash1
EQS[Phospho (STY)]DDETEDSVK.2	-0.149168314	P59808_S241_M1_Sash1
AGGS[Phospho (STY)]VESLR.2	-0.363930456	P59808_S500_M1_Sash1

S[Phospho (STY)]C[Carbamidomethyl (C)]ETLEGPEPVESWPR.2	0.301647937	P59808_S813_M1_Sash1
DSGC[Carbamidomethyl (C)]YES[Phospho (STY)]SENLENAK.2	-0.343713696	P59808_S723_M1_Sash1
LKAGGS[Phospho (STY)]VESLR.2	0.103063151	P59808_S500_M1_Sash1
SHS[Phospho (STY)]LDDLQGDADVGK.2	0.072887332	P59808_S831_M1_Sash1
AVLLTAVELLQEYDS[Phospho (STY)]NSDQSGSQEK.3	0.377601641	P59808_S692_M1_Sash1
ELLLQPVTISR.2	-0.096097302	#N/A
VLIEGSINSVR.2	-0.150257529	#N/A
AENFFILR.2	0.124075436	#N/A
IVAEEFLK.2	0.114561916	#N/A
ATLQAALC[Carbamidomethyl (C)]LENFSSQVVER.2	-0.257668504	#N/A
QASQLS[Phospho (STY)]LESTVGPC[Carbamidomethyl (C)]GERS.2	-0.408779441	P60191_S257_M1_Rims4
QASQLS[Phospho (STY)]LESTVGPC[Carbamidomethyl (C)]GER.2	0.12954505	P60191_S257_M1_Rims4
TSASIGSLC[Carbamidomethyl (C)]ADAR.2	0.220545668	#N/A
M[Oxidation (M)]YGVLPWNAFPGK.2	0.03526936	#N/A
LIETYFSK.2	0.017493233	#N/A
VC[Carbamidomethyl (C)]GSNLLSIC[Carbamidomethyl (C)]K.2	0.263193992	#N/A
GLLEC[Carbamidomethyl (C)]C[Carbamidomethyl (C)]AR.2	0.038387468	#N/A
QIFGDYK.2	-0.090437372	#N/A
VC[Carbamidomethyl (C)]HC[Carbamidomethyl (C)]LGK.2	0.286377868	#N/A
WLGHPDK.2	0.060292751	#N/A
GQHQAHSLER.2	-0.012919783	#N/A
GLSATVTGGQK.2	0.131548166	#N/A
LVVPATQC[Carbamidomethyl (C)]GSLIGK.2	0.12731215	#N/A
AITIAGVPQSVTEC[Carbamidomethyl (C)]VK.2	0.15572272	#N/A
IITLTGPTNAIFK.2	0.211534854	#N/A
IANPVEGSSGR.2	0.128982506	#N/A
QGANINEIR.2	-0.573329088	#N/A
QVTITGSAASISLAQYLINAR.2	-0.007578007	#N/A
QIC[Carbamidomethyl (C)]LVM[Oxidation (M)]LETLSQS[Phospho (STY)]PQGR.2	-0.353411058	P60335_S173_M1_Pcbp1
GEGPAVPGDT[Phospho (STY)]PPPTPR.2	-0.421535076	P60469_T714_M1_Ppfia3
LAPPS[Phospho (STY)]PAR.2	-0.438709708	P60469_S683_M1_Ppfia3
QAQS[Phospho (STY)]PGGVSSEVEVLK.2	-0.338396872	P60469_S142_M1_Ppfia3
RGS[Phospho (STY)]ALGPDEAGGELER.3	-0.297207689	P60469_S17_M1_Ppfia3
YSQAPTLPSGAPLDPYGAGS[Phospho (STY)]GR.2	-0.272503354	P60469_S537_M1_Ppfia3
VSSSGLDS[Phospho (STY)]LGR.2	-0.445749852	P60469_S645_M1_Ppfia3
ESVSLAGTPSDETLATDPLGLAK.2	-0.306973149	#N/A
GS[Phospho (STY)]ALGPDEAGGELER.2	0.077429482	P60469_S17_M1_Ppfia3
TAELEEALER.2	-0.280806727	#N/A
S[Phospho (STY)]LPGSALELR.2	-0.21212896	P60469_S508_M1_Ppfia3
M[Oxidation (M)]AQALALQAGS[Phospho (STY)]PEDGAPPR.2	-0.26560166	P60469_S737_M1_Ppfia3
RQAQS[Phospho (STY)]PGGVSSEVEVLK.3	0.18273429	P60469_S142_M1_Ppfia3
QM[Oxidation (M)]S[Phospho (STY)]QLEEELGTAHR.3	-0.414929696	P60469_S264_M1_Ppfia3
WSGAKDESS[Phospho (STY)]KDWDR.3	-0.147193324	P60469_S554_M1_Ppfia3
IVPGAPELLQR.2	0.293260028	#N/A
NTLFVSNNSR.2	0.106055544	#N/A
LAGDPGEDPR.2	0.140337873	#N/A
AEQLFSSALC[Carbamidomethyl (C)]AAR.2	-0.052309516	#N/A

LSGPPDASGAVFVLGGEGLR.2	-0.404154161	#N/A
TPGTGSLAAAVETASGR.2	0.274716527	#N/A
C[Carbamidomethyl (C)]GM[Oxidation (M)]TTVLTLTGVSSLEEAQAYLTAGQR.3	0.16588612	#N/A
AVLVGYDEQFSFSR.2	-0.218232779	#N/A
EDHS[Phospho (STY)]LEHR.2	0.194293969	P60521_S10_M1_Gabarapl2
WM[Oxidation (M)]FKEDHS[Phospho (STY)]LEHR.3	-0.261272601	P60521_S10_M1_Gabarapl2
VAPEEHPVLLTEAPLNPK.3	-0.336941439	#N/A
AGFAGDDAPR.2	-0.055282214	#N/A
DSYVGDEAQSKR.2	-0.162871827	#N/A
IIAPPERK.2	0.152473177	#N/A
AVFPSIVGRPR.2	0.073452935	#N/A
DSYVGDEAQSK.2	-0.016891545	#N/A
AVFPSIVGR.2	0.281323375	#N/A
C[Carbamidomethyl (C)]DVDIRK.2	-0.038147018	#N/A
IKIIAPPER.2	-0.353050829	#N/A
GYSFTTTAER.2	0.260949165	#N/A
TTGIVM[Oxidation (M)]DSGDGVTHTVPIYEGYALPHAILR.3	-0.46152839	#N/A
KDLYANTVLSGGTTM[Oxidation (M)]YPGIADR.3	0.04355076	#N/A
[Acetyl (Protein N-term)]DDDIAALVVDNGSGMC[Carbamidomethyl (C)]K.2	0.239425055	#N/A
[Acetyl (Protein N-term)]DDDIAALVVDNGSGM[Oxidation (M)]C[Carbamidomethyl (C)]KAGFAGDDAPR.3	-0.119352009	#N/A
LC[Carbamidomethyl (C)]YVALDFEQEM[Oxidation (M)]ATAASSSSLEK.3	-0.254029615	#N/A
EKLC[Carbamidomethyl (C)]YVALDFEQEM[Oxidation (M)]ATAASSSSLEK.3	-0.473494118	#N/A
EITALAPSTM[Oxidation (M)]K.2	0.095497873	#N/A
FRC[Carbamidomethyl (C)]PEALFQPSFLGM[Oxidation (M)]ESC[Carbamidomethyl (C)]GIHETTFNSIM[Oxidation (M)]K.4	-0.302178995	#N/A
HQGVM[Oxidation (M)]VGM[Oxidation (M)]GQK.2	0.017405498	#N/A
DLYANTVLSGGTTM[Oxidation (M)]YPGIADR.2	-0.435028324	#N/A
IWHHTFYNELR.3	-0.34074075	#N/A
QEYDESGPSIVHR.2	-0.210630033	#N/A
QEYDESGPSIVHRK.3	-0.392944512	#N/A
DLTDYLM[Oxidation (M)]K.2	-0.423877272	#N/A
C[Carbamidomethyl (C)]PEALFQPSFLGM[Oxidation (M)]ESC[Carbamidomethyl (C)]GIHETTFNSIM[Oxidation (M)]K.3	-0.63549869	#N/A
DLTDYLM[Oxidation (M)]KILTER.2	0.201373026	#N/A
S[Phospho (STY)]YELPDGQVITIGNER.2	-0.014938903	P60710_S239_M1_Actb
KGPGPGGPGGAGGAR.2	0.429165274	#N/A
_[Acetyl (Protein N-term)]M[Oxidation (M)]DC[Carbamidomethyl (C)]C[Carbamidomethyl (C)]TESAC[Carbamidomethyl (C)]SKPDDDILDIF	-0.302148212	#N/A
GPGPGGPGGAGGAR.2	0.035103872	#N/A
IKS[Phospho (STY)]GEC[Carbamidomethyl (C)]GR.2	-0.198381479	P60761_S48_M1_Nrgn
QKPITPETAEK.2	0.064331725	#N/A
WVPEITHHC[Carbamidomethyl (C)]PK.3	0.122782602	#N/A
TPFLLVGTQIDLR.2	0.081525724	#N/A
TC[Carbamidomethyl (C)]LLISYTTNK.2	-0.304573783	#N/A
TPFLLVGTQIDLRDDPSTIEK.3	-0.190801497	#N/A
YVEC[Carbamidomethyl (C)]SALTQK.2	-0.039228301	#N/A
LRPLSYPQTDVFLVC[Carbamidomethyl (C)]FSVVSPSSFENVK.3	-0.005477617	#N/A
S[Phospho (STY)]QGGSYGYR.2	0.017230495	P60824_S146_M1_Cirbp
KSS[Phospho (STY)]LVTSK.2	-0.020277283	P60840_S109_M1_Ensa
[Acetyl (Protein N-term)]S[Phospho (STY)]QKQEEENPAEETGEEK.2	0.475956652	P60840_S2_M1_Ensa

ATQALVLAPTR.2	-0.0878055	#N/A
GYDVIAQAQSGTGK.2	0.424657828	#N/A
GIDVQQVSLVINYDLPTNR.2	0.286115735	#N/A
FGFSQDS[Phospho (STY)]GRGK.2	-0.276235186	P60853_S50_M1_Lzts1
ALSFS[Phospho (STY)]DGGSK.2	-0.331779879	P60853_S233_M1_Lzts1
S[Phospho (STY)]FDEKEFASGQTFEERPR.3	-0.329666992	P60853_S283_M1_Lzts1
DTGKTPVEPEVAIHR.3	0.081417745	#N/A
LIDLHSPSEIVK.2	-0.181716618	#N/A
VC[Carbamidomethyl (C)]ADLIR.2	0.115566389	#N/A
TPVEPEVAIHR.3	-0.113958606	#N/A
RADQLADESLESTRR.3	-0.56875102	#N/A
FC[Carbamidomethyl (C)]GLC[Carbamidomethyl (C)]VC[Carbamidomethyl (C)]PC[Carbamidomethyl (C)]NK.2	0.332325584	#N/A
ADQLADESLESTR.2	0.323214847	#N/A
IEEGM[Oxidation (M)]DQINK.2	0.353627409	#N/A
TLVM[Oxidation (M)]LDEQGEQLER.2	-0.224942863	#N/A
IDEANQR.2	0.098043048	#N/A
EQM[Oxidation (M)]AISGGFIR.2	-0.208490504	#N/A
MLQLVEESK.2	0.388638999	#N/A
AWGNNQDGVVASQPAR.2	-0.040562828	#N/A
RADQLADESLESTR.3	-0.268063951	#N/A
ENEM[Oxidation (M)]DENLEQVSGIIGNLR.2	-0.258994641	#N/A
NELEEMQR.2	0.013041271	#N/A
ADQLADESLESTRR.2	0.0586256	#N/A
HM[Oxidation (M)]ALDM[Oxidation (M)]GNEIDTQNR.3	-0.246486193	#N/A
M[Oxidation (M)]LQLVEESKDAGIR.2	-0.435107869	#N/A
[Acetyl (Protein N-term)]AEDADM[Oxidation (M)]RNELEEMQR.2	-0.287164539	#N/A
VVDEREQM[Oxidation (M)]AISGGFIR.3	-0.25796057	#N/A
EINNAHAILTDATKR.3	0.201900625	#N/A
SLS[Phospho (STY)]TSGESLYHVLGLDKNATSDDIKK.4	-0.201938375	P60904_S10_M1_Dnajc5
NATSDDIKK.2	-0.302436405	#N/A
SLS[Phospho (STY)]TSGESLYHVLGLDK.2	-0.3042665	P60904_S10_M1_Dnajc5
EINNAHAILTDATK.2	0.412513197	#N/A
APEGEETEFYVS[Phospho (STY)]PEDLEAQLQSDER.3	-0.21616067	P60904_S151_M1_Dnajc5
QRS[Phospho (STY)]LS[Phospho (STY)]TSGESLYHVLGLDK.3	-0.298831976	P60904_S10_M2_Dnajc5
YHPDKNPDNPEAADKFK.4	0.028811752	#N/A
GVDLHEQSQQNK.2	-0.255980814	#N/A
GAQAAIVVYDITNQETFAR.2	-0.242042548	#N/A
DELLQVLR.2	-0.142840088	#N/A
IINAFFSEGEDQVNFR.2	0.267287808	#N/A
ETGFSHSQITR.3	-0.183576762	#N/A
AFLTLAEDILR.2	-0.167250804	#N/A
GKGEQIAR.2	0.148486361	#N/A
EPNSENVDISS[Phospho (STY)]GGGVTGWK.2	-0.09654251	P61027_S188_M1_Rab10
KTPVKEPNSENVDISS[Phospho (STY)]GGGVTGWK.3	0.039446952	P61027_S188_M1_Rab10
ANINIEK.2	0.239950002	#N/A
NIDEHANEDVER.2	0.151928376	#N/A
TPVKEPNSENVDISS[Phospho (STY)]GGGVTGWK.3	0.021728334	P61027_S188_M1_Rab10

SSTNVEEAFFTLAR.2	0.213023204	#N/A
SQWSPALTISK.2	0.24241755	#N/A
ELS[Phospho (STY)]DLAR.2	0.127632791	P61079_S11_M1_Ube2d3
ELS[Phospho (STY)]DLQR.2	-0.015948351	P61080_S11_M1_Ube2d1
EAAEVLQNNR.2	-0.236155973	#N/A
GGYIGSTYFER.2	0.160769054	#N/A
DINELNLPK.2	0.02486422	#N/A
TC[Carbamidomethyl (C)]DISFSDPDDLLNFK.2	-0.300464366	#N/A
LLAEPVPGIK.2	0.188716802	#N/A
TNEAQAIETAR.2	0.044766632	#N/A
IYHPNVDK.2	0.216000981	#N/A
WSPALQIR.2	0.218235116	#N/A
YFHVVIAGPQDSPFEGGTFK.3	0.067146459	#N/A
LLAEPVPGIKAEPDESNAR.3	-0.356616675	#N/A
LELFLPEEYPM[Oxidation (M)]AAPK.2	0.075757496	#N/A
AEPDESNAR.2	0.296385352	#N/A
TVLLSIQALLSAPNPDDPLANDVAEQWK.3	-0.411672365	#N/A
ILLTEPPM[Oxidation (M)]NPTK.2	-0.155997554	#N/A
HLWDYTFGPEK.2	-0.084823528	#N/A
DLM[Oxidation (M)]VGDEASELR.2	-0.096786935	#N/A
LC[Carbamidomethyl (C)]YVGYNIEQEQK.2	0.278799694	#N/A
GYAFNHSADFETVR.3	-0.111795123	#N/A
HIVLSGGSTM[Oxidation (M)]YPGLPSR.2	0.071111978	#N/A
LALETTVLVESYTLPDGR.2	-0.284935941	#N/A
AQYYLPDGSTIEIGPSR.2	-0.255197135	#N/A
DQLQTFSEEHPVLLTEAPLNPR.3	0.108932569	#N/A
AC[Carbamidomethyl (C)]YLSINPQKDETLETEK.3	0.077448246	#N/A
KEYEEDGAR.2	0.002113242	#N/A
NISFTVWDVGGQDK.2	-0.17663035	#N/A
DAVLLVFANK.2	0.17555482	#N/A
LGEIVTTIPTIGFNVETVEYK.2	-0.239422549	#N/A
M[Oxidation (M)]LAEDELRDAVLLVFANK.3	0.152723475	#N/A
M[Oxidation (M)]LAEDELR.2	0.234961343	#N/A
QDLPNAM[Oxidation (M)]NAAEITDK.2	0.210848064	#N/A
ILM[Oxidation (M)]VGLDAAGK.2	0.230176189	#N/A
HYFQNTQGLIFVVDSNDRER.3	-0.420521129	#N/A
HYFQNTQGLIFVVDSNDR.3	0.136777559	#N/A
YDPTIEDFYR.2	0.112065712	#N/A
VVVLGSGGVGK.2	0.225098775	#N/A
ALAEEWSC[Carbamidomethyl (C)]PFM[Oxidation (M)]ETSAK.2	0.040814382	#N/A
ASVDELFAEIVR.2	-0.147014983	#N/A
SALTVQFVTGSFIEK.2	-0.490843635	#N/A
EIEVDSSPSVLEILDTAGTEQFASM[Oxidation (M)]R.3	0.277778949	#N/A
FNPFVTSDR.2	0.259061487	#N/A
YVIYIER.2	-0.126903395	#N/A
TKQELEDLTADIKK.3	0.003024293	#N/A
LAIFTDDIK.2	-0.06490126	#N/A

QLEITGR.2	-0.036619729	#N/A
LSEDVEQVKK.2	0.128852839	#N/A
QALNEIETR.2	0.074712805	#N/A
LSEDVEQVK.2	-0.063026998	#N/A
TQHSTLSR.2	0.107261713	#N/A
DHFM[Oxidation (M)]DEFFEQVEEIR.3	-0.351338049	#N/A
TTTNEELEDM[Oxidation (M)]LESGK.2	-0.164845006	#N/A
IEYNVEHSVDYVER.3	-0.148706102	#N/A
SAKDS[Phospho (STY)]DDEEEVVHVDR.3	-0.243092516	P61264_S14_M1_Stx1b
AIEQSIEQEEGLNRS[Phospho (STY)]SADLR.3	-0.235453983	P61264_S108_M1_Stx1b
KQHS[Phospho (STY)]AILAAPNPDEK.3	-0.180461435	P61264_S58_M1_Stx1b
AIEQSIEQEEGLNR.2	-0.098362909	#N/A
LKAIEQSIEQEEGLNR.3	-0.053597652	#N/A
QHSAILAAPNPDEK.2	0.533967972	#N/A
DS[Phospho (STY)]DDEEEVVHVDR.2	-0.100181168	P61264_S14_M1_Stx1b
TQELRS[Phospho (STY)]AKDS[Phospho (STY)]DDEEEVVHVDR.4	-0.178329629	P61264_S10_M2_Stx1b
FVEVM[Oxidation (M)]TEYNATQSK.2	0.042336803	#N/A
KFVEVM[Oxidation (M)]TEYNATQSK.3	0.412158639	#N/A
EPS[Phospho (STY)]LHEIGEK.2	-0.200280213	P61329_S212_M1_Fgf12
VVAIQGVK.2	-0.007623976	#N/A
KS[Phospho (STY)]SGTPTMNGGK.2	0.034237051	P61329_S226_M1_Fgf12
SRKS[Phospho (STY)]S[Phospho (STY)]GTPTMNGGK.2	0.351977031	P61329_S226_M2_Fgf12
YSVDIPLDK.2	-0.050192322	#N/A
NIDDGTSDRPYSHALVAGIDR.3	0.178837173	#N/A
NWS[Phospho (STY)]GC[Carbamidomethyl (C)]GEGEQK.2	0.342587406	P61406_S331_M1_Smg6
LM[Oxidation (M)]TANS[Phospho (STY)]PEVHG.2	-0.119617707	P61807_S83_M1_Snn
GNYLVDVDGNR.2	-0.337802991	#N/A
GTFC[Carbamidomethyl (C)]SFDTPDEAIR.2	-0.357752246	#N/A
GVVLGGC[Carbamidomethyl (C)]GDK.2	0.055721531	#N/A
TVAGIIVEPIQSEGGDNHASDDFFR.3	-0.424201879	#N/A
EDLLNNVAR.2	0.266296151	#N/A
IFNTWLGDPSK.2	0.087244304	#N/A
IDIPSFDWPIAPFPR.2	-0.094303011	#N/A
LKYPLEEFTTDNQQEEAR.3	0.40499009	#N/A
M[Oxidation (M)]LDLYSQISSVPIGYNHPALAK.3	-0.088752487	#N/A
NLLLAEVINIIK.2	0.325781778	#N/A
C[Carbamidomethyl (C)]LEEVEDLIVK.2	0.252763454	#N/A
TLLTGLLDLQAQYPQFISR.2	-0.056486775	#N/A
EEFRPSAPYR.3	0.061543952	#N/A
REDLLNNVAR.2	-0.044386246	#N/A
QLNTIQNAEAVHFFC[Carbamidomethyl (C)]NYEESR.3	-0.384434652	#N/A
TM[Oxidation (M)]GC[Carbamidomethyl (C)]LATTHSK.2	0.391959608	#N/A
VDIEFDYDGPLM[Oxidation (M)]K.2	-0.031387479	#N/A
YPLEEFTTDNQQEEAR.2	0.333889667	#N/A
GM[Oxidation (M)]SQLITMAC[Carbamidomethyl (C)]GSC[Carbamidomethyl (C)]SNENAFK.2	0.318614742	#N/A
VLSVPESTPFTAVLK.2	0.598688093	#N/A
ITLTSDPR.2	-0.02146816	#N/A

LEC[Carbamidomethyl (C)]LLNNNK.2	-0.251866836	#N/A
LALGSFVEEYNNK.2	-0.468083455	#N/A
AIEQADLLQEEDES[Phospho (STY)]PR.2	-0.092674236	P61967_S147_M1_Ap1s1
TDYNASVSVPDSSGPER.2	0.129047294	#N/A
IILDLISESPIK.2	0.068186929	#N/A
GSDFDC[Carbamidomethyl (C)]ELR.2	-0.203022116	#N/A
IITITGTQDQIQNAQYLLQNSVK.3	-0.371295338	#N/A
DYDDM[Oxidation (M)]S[Phospho (STY)]PR.2	-0.448214647	P61979_S284_M1_Hnrnpk
LFQEC[Carbamidomethyl (C)]C[Carbamidomethyl (C)]PHSTDR.3	0.084914558	#N/A
DLAGSIIGK.2	-0.033271772	#N/A
RDYDDM[Oxidation (M)]S[Phospho (STY)]PR.2	0.130256482	P61979_S284_M1_Hnrnpk
RPAEDM[Oxidation (M)]EEEQAFKR.3	0.460838726	#N/A
GSYGDLGGPIITTQVTIPK.2	-0.127843215	#N/A
LLIHQSLAGGIIGVK.3	0.005727083	#N/A
IIPTLEEGLQLPS[Phospho (STY)]PTATSQLPLESDAVEC[Carbamidomethyl (C)]LNYQHYK.4	-0.220392204	P61979_S116_M1_Hnrnpk
NTDEMVELR.2	0.158790489	#N/A
GGDLM[Oxidation (M)]AYDRR.2	-0.192591866	#N/A
ILSISADIETIGEILKK.2	-0.24232712	#N/A
ILSISADIETIGEILK.2	-0.086695314	#N/A
[Acetyl (Protein N-term)]VDREQLVQK.2	-0.075427143	#N/A
MKGDYYR.2	0.328824415	#N/A
AYSEAHEISK.2	0.102005908	#N/A
ATVVESSEKAYSEAHEISK.3	-0.44620785	#N/A
RATVVESSEK.2	0.244440606	#N/A
NC[Carbamidomethyl (C)]SETQYESK.2	0.120395087	#N/A
EHM[Oxidation (M)]QPTHPIR.2	0.076627023	#N/A
NVTELNEPLSNEER.2	-0.309829481	#N/A
YLAEVATGEK.2	0.075615135	#N/A
NC[Carbamidomethyl (C)]SETQYES[Phospho (STY)]KVFYLK.2	-0.18513537	P61982_S119_M1_Ywhag
DNLTLWT[Phospho (STY)]SDQQDDDGGEGNN.2	0.365499163	P61982_T234_M1_Ywhag
TAFDDAIAELDTLNEDSYK.2	-0.278399423	#N/A
YLAEVATGEKR.2	-0.085152452	#N/A
ELEAVC[Carbamidomethyl (C)]QDVLSLLDNYLIK.2	0.175662141	#N/A
VISSIEQKTS[Phospho (STY)]ADGNEKK.2	-0.100967324	P61982_S71_M1_Ywhag
VISSIEQKTS[Phospho (STY)]ADGNEK.2	0.294102964	P61982_S71_M1_Ywhag
FQEQEC[Carbamidomethyl (C)]PPS[Phospho (STY)]PEPTR.2	-0.143736523	#N/A
KFQEQEC[Carbamidomethyl (C)]PPS[Phospho (STY)]PEPTRK.3	-0.230492772	#N/A
FQEQEC[Carbamidomethyl (C)]PPS[Phospho (STY)]PEPTRK.2	0.003985158	#N/A
KFQEQEC[Carbamidomethyl (C)]PPS[Phospho (STY)]PEPTR.2	0.102184575	#N/A
[Acetyl (Protein N-term)]AAAGWRDGS[Phospho (STY)]GQEK.2	-0.17702936	P62071_S10_M1_Rras2
YLDIHER.2	-0.111845285	#N/A
GESVC[Carbamidomethyl (C)]LDR.2	0.169304011	#N/A
EAELSKGESVC[Carbamidomethyl (C)]LDR.2	0.206197178	#N/A
VQIAVANAQELLQR.2	-0.215632083	#N/A
YM[Oxidation (M)]DAWNTVSR.2	-0.186156261	#N/A
LDPGAIMEQVK.2	0.220311277	#N/A
FIDTTLAITGR.2	0.287313072	#N/A

HVVFIAQR.2	0.453480326	#N/A
AIIIFVPVPQLK.2	-0.108025528	#N/A
LLEVQGS[Phospho (STY)]RPGK.2	0.043840299	P62137_S22_M1_Ppp1ca
[Acetyl (Protein N-term)]SDSEKLNLDSIIGR.2	0.291184834	#N/A
[Acetyl (Protein N-term)]ADGELNVDSLITR.2	0.215938558	#N/A
VAEEHAPSIVFIDEIDAIGTK.3	0.365428882	#N/A
AVANQTSATFLR.2	0.185440608	#N/A
APQETYADIGGLDNQIQEIK.2	-0.03548922	#N/A
NDSYTLHK.2	-0.214603153	#N/A
LTPEEEEILNK.2	0.099054147	#N/A
IIDVVYNASNNELVR.2	-0.106656379	#N/A
NC[Carbamidomethyl (C)]IVLIDSTPYR.2	0.053304635	#N/A
ISSLLEEQFQQGK.2	0.013039152	#N/A
LDVGNFSWGSEC[Carbamidomethyl (C)]C[Carbamidomethyl (C)]TR.2	0.277598184	#N/A
IVVNLTGR.2	-0.046221185	#N/A
M[Oxidation (M)]NVLADALK.2	-0.355349589	#N/A
HGYIGEFEIIDDHR.3	0.072587753	#N/A
EALQDVEDENQ.2	-0.11332613	#N/A
YLAEFATGNDR.2	-0.125726066	#N/A
LIC[Carbamidomethyl (C)]C[Carbamidomethyl (C)]DILDVLDK.2	0.311913368	#N/A
VAGM[Oxidation (M)]DVELTVEER.2	-0.357320279	#N/A
EAAENSLVAYK.2	0.096961158	#N/A
IISSIEQK.2	0.021876236	#N/A
LIC[Carbamidomethyl (C)]C[Carbamidomethyl (C)]DILDVLDKHLIPAANTGESK.4	-0.435019222	#N/A
HLIPAANTGESK.2	0.34382967	#N/A
QM[Oxidation (M)]VETELK.2	0.358876862	#N/A
YLAEFATGNDRK.2	-0.023508113	#N/A
AAFDDAIAELDTLSEESYK.2	-0.232680743	#N/A
[Acetyl (Protein N-term)]M[Oxidation (M)]DDREDLVYQAK.2	0.029920084	#N/A
M[Oxidation (M)]KGDYHR.2	-0.027940715	#N/A
KEAAENSLVAYK.2	0.060856062	#N/A
AASDIAM[Oxidation (M)]TELPPTHPIR.2	-0.033651061	#N/A
YDEM[Oxidation (M)]VESM[Oxidation (M)]KK.2	0.204638441	#N/A
EENKGGEDKLK.2	0.035907978	#N/A
IISSIEQKEENK.2	-0.209080507	#N/A
KVAGM[Oxidation (M)]DVELTVEER.3	-0.337046148	#N/A
YDEM[Oxidation (M)]VESMK.2	0.149355753	#N/A
LAEQAERYDEM[Oxidation (M)]VESM[Oxidation (M)]KK.3	-0.406898559	#N/A
IISSIEQKEENKGGEDK.3	0.030574858	#N/A
DNLTLWTSDMQGDGEEQNK.2	0.285688566	#N/A
AAFDDAIAELDTLSEESYKDSTLIMQLLR.3	-0.15698933	#N/A
LAEQAERYDEMVESMK.3	0.027970901	#N/A
IEDVTPIPSDSTR.2	0.168669221	#N/A
TPGPGAQSALR.2	0.068105763	#N/A
ANPFGGASHAK.2	0.302680191	#N/A
VLNTNIDGR.2	-0.264657636	#N/A
IPDWFLNR.2	0.262505315	#N/A

YSQVLANGLDNK.2	-0.013951663	#N/A
LREDLER.2	0.273674081	#N/A
GHQQLYWSHPR.3	-0.138173361	#N/A
DIGFIKLD.2	0.10443553	#N/A
DYLHYIR.2	-0.053604363	#N/A
EAIEGTYIDKK.2	-0.475475957	#N/A
DSHGVAQVR.2	-0.131659129	#N/A
LILIESR.2	0.038530507	#N/A
NNTQVLINC[Carbamidomethyl (C)]R.2	-0.09550528	#N/A
VAQLEQVYIR.2	-0.11172386	#N/A
FLILPDM[Oxidation (M)]LK.2	0.252085274	#N/A
[Acetyl (Protein N-term)]AANATTNPSQLLPLELVDK.2	-0.118804261	#N/A
FNVWDVGGQDK.2	-0.312025762	#N/A
EVIELPLTNPELFQR.2	0.344123986	#N/A
ALQSVGQIVGEVLK.2	-0.33679955	#N/A
ADHDFVVQEDFM[Oxidation (M)]K.3	0.161014429	#N/A
RSS[Phospho (STY)]LVITTK.2	-0.379930255	P62482_S112_M1_Kcnab2
QTGS[Phospho (STY)]PGM[Oxidation (M)]IYSTR.2	0.08418626	P62482_S20_M1_Kcnab2
AEVVLGNIIK.2	0.104773174	#N/A
SS[Phospho (STY)]LVITTK.2	-0.29997778	P62482_S112_M1_Kcnab2
YPESTTGS[Phospho (STY)]PAR.2	0.186426941	P62482_S9_M1_Kcnab2
[Acetyl (Protein N-term)]M[Oxidation (M)]YPESTTGS[Phospho (STY)]PAR.2	0.029913642	P62482_S9_M1_Kcnab2
[Acetyl (Protein N-term)]MYPESTTGS[Phospho (STY)]PARLS[Phospho (STY)]LR.2	-0.148788342	P62482_S14_M2_Kcnab2
NEGVSSVLLGASNAEQLM[Oxidation (M)]ENIGAIQVLPK.3	0.190360877	#N/A
LQLEYVDVVFANRPDPNTPM[Oxidation (M)]EETVR.3	-0.305798655	#N/A
EIGILTTNK.2	0.050196537	#N/A
ALFDSYTNLER.2	-0.07354452	#N/A
VADYC[Carbamidomethyl (C)]ENNYIQSPDK.2	-0.07707052	#N/A
VADYC[Carbamidomethyl (C)]ENNYIQS[Phospho (STY)]PDKQR.3	-0.116817714	P62484_S40_M1_Abi2
NMAPSQQS[Phospho (STY)]PVR.2	0.317092047	P62484_S221_M1_Abi2
HTPPTIGGS[Phospho (STY)]LPYR.2	-0.101053922	P62484_S301_M1_Abi2
[Acetyl (Protein N-term)]AELQM[Oxidation (M)]LLEEEIPGGR.2	0.091635719	#N/A
TTPPTQKPPS[Phospho (STY)]PPM[Oxidation (M)]SGK.2	0.047677241	P62484_S177_M1_Abi2
ENDM[Oxidation (M)]S[Phospho (STY)]PSNNVVPIHVPPTTENKPK.3	-0.337180213	P62492_S190_M1_Rab11a
NSQLEQENNLLK.2	0.053723756	#N/A
EEVEVLK.2	-0.056130344	#N/A
[Acetyl (Protein N-term)]AEVEETLKR.2	0.329646879	#N/A
EIDPQNDLTFLR.2	-0.026741392	#N/A
GVQGIIVVNTEGIPIK.2	-0.06238703	#N/A
RYDEIVK.2	-0.251245898	#N/A
KLEDNPK.2	0.074455138	#N/A
VETGILRPGM[Oxidation (M)]VVTFAPVNITTEVK.3	0.073635079	#N/A
YYITIIDAPGHR.2	-0.037471348	#N/A
EVSAYIKK.2	0.378880226	#N/A
SGDAAIVEM[Oxidation (M)]VPGKPM[Oxidation (M)]C[Carbamidomethyl (C)]VESFSQYPPLGR.3	-0.490415697	#N/A
LSNIFVIGK.2	-0.020617229	#N/A
EC[Carbamidomethyl (C)]LPLIIFLR.2	-0.01925839	#N/A

TDITYPAGFM[Oxidation (M)]DVISIDK.2	-0.415175385	#N/A
FDTGNLC[Carbamidomethyl (C)]M[Oxidation (M)]VTGGANLGR.2	-0.291089383	#N/A
YSFLQFDPAPR.2	-0.062500541	#N/A
NVVTIFSAPNYC[Carbamidomethyl (C)]YR.2	0.033195329	#N/A
ELDQWVEQLNEC[Carbamidomethyl (C)]K.2	-0.200564079	#N/A
GAGYTFGQDISETFNHANGLTLVSR.3	-0.366283771	#N/A
QITQVYGFYDEC[Carbamidomethyl (C)]LR.2	-0.009665931	#N/A
SPDTNYLFM[Oxidation (M)]GDYVDR.2	-0.076327594	#N/A
LQEVPHEGPM[Oxidation (M)]C[Carbamidomethyl (C)]DLLWSDPDDR.3	0.24712264	#N/A
AHQLVM[Oxidation (M)]EGYNWC[Carbamidomethyl (C)]HDR.3	0.01530295	#N/A
SSGEIVYC[Carbamidomethyl (C)]GQVFEKS[Phospho (STY)]PLR.3	0.244026252	P62717_S71_M1_Rpl18a
DLTTAGAVTQC[Carbamidomethyl (C)]YR.2	0.223949926	#N/A
YPIEHGIITNWDDM[Oxidation (M)]EK.3	-0.126118999	#N/A
VAPEEHPTLLTEAPLNPK.2	-0.272314578	#N/A
DLYANNVLSGGTTM[Oxidation (M)]YPGIADR.2	0.441994695	#N/A
FILIQNR.2	-0.148339311	#N/A
HTNFVEFR.2	0.204883795	#N/A
VYTVVDEM[Oxidation (M)]FLAGEIR.2	-0.157826432	#N/A
LVVVGDGAC[Carbamidomethyl (C)]GK.2	0.075244507	#N/A
EVFETATR.2	0.017744225	#N/A
IQAYDYLEC[Carbamidomethyl (C)]SAK.2	0.260045513	#N/A
DLRS[Phospho (STY)]DEHVR.2	-0.051091191	P62746_S123_M1_Rhob
EHTEFTDHELQEWYK.3	-0.134517156	#N/A
LAPDYDALDVANK.2	-0.518741852	#N/A
IRTS[Phospho (STY)]PTFR.2	-0.32260126	P62751_S43_M1_Rpl23a
KLYDIDVAK.2	0.354560636	#N/A
DIPGLTDTTVPR.2	-0.319984008	#N/A
LIEVDDER.2	0.033637882	#N/A
FFPYGDASK.2	0.015149318	#N/A
FAQHAFR.2	0.178994969	#N/A
STEFNEHELK.2	-0.02942434	#N/A
EFIC[Carbamidomethyl (C)]ALSITSR.2	0.118747737	#N/A
NKDDQITLDEFKEAAK.3	-0.351154819	#N/A
LNLEEFQQLYVK.2	0.159877477	#N/A
NGDGTIDFR.2	0.252045553	#N/A
LAPEVM[Oxidation (M)]EDLVK.2	0.308703297	#N/A
MNEDGLTPEQR.2	0.281336464	#N/A
LNWAFNM[Oxidation (M)]YDLDGDGK.2	0.001302684	#N/A
M[Oxidation (M)]DKNKDDQITLDEFK.3	0.330603447	#N/A
VEM[Oxidation (M)]LEIIEAIYK.2	0.257260573	#N/A
TFDKNGDGTIDFR.3	-0.015404634	#N/A
NKDDQITLDEFK.2	0.144915788	#N/A
SDPSIVLLLQC[Carbamidomethyl (C)]DIQK.2	0.125048797	#N/A
GPDGLTALEATDNQAIK.2	-0.16009495	#N/A
GEDVNRT[Phospho (STY)]LEGGR.2	-0.081878159	P62774_T31_M1_Mtpn
NGDLDEVKDYVAK.2	-0.216290128	#N/A
VFLENVIR.2	-0.033661865	#N/A

DAVTYTEHAK.2	-0.066083604	#N/A
ISGLIYEETR.2	-0.003403943	#N/A
TVTAM[Oxidation (M)]DVVYALK.2	0.244888129	#N/A
DNIQGITKPAIR.2	-0.141081929	#N/A
TVTAM[Oxidation (M)]DVVYALKR.2	-0.317979247	#N/A
RIS[Phospho (STY)]GLIYEETR.2	0.262972921	P62806_S48_M1_H4c1
DAVTYTEHAKR.3	-0.009079623	#N/A
DNIQGITK.2	0.024198161	#N/A
NNTYAPTATSY[Phospho (STY)]TPNLAR.2	0.157583932	P62812_Y374_M1_Gabra1
NFITQGPYENR.2	-0.406526265	#N/A
NYLSQPR.2	0.544870949	#N/A
QIYPPINVLPSLSR.2	-0.088847508	#N/A
EQALAVSR.2	0.196373355	#N/A
IPQSTLSEFYPR.2	0.296474592	#N/A
AVVQVFEGTSGIDAK.2	0.120932716	#N/A
GFPGYM[Oxidation (M)]YTDLATIYER.2	-0.333966106	#N/A
SGQVLEVSGSK.2	-0.15884372	#N/A
AVVGEEALTSDDLLYLEFLQK.2	-0.283118403	#N/A
EEVPGRR.2	-0.011053644	#N/A
IPIFSAAGLPHNEIAAQIC[Carbamidomethyl (C)]R.3	0.025026246	#N/A
GPVVLAEDFLDIM[Oxidation (M)]GQPINPQC[Carbamidomethyl (C)]R.3	0.135233086	#N/A
TSC[Carbamidomethyl (C)]EFTGDILR.2	0.353413726	#N/A
TPVSEDMLGR.2	0.326816697	#N/A
KTSC[Carbamidomethyl (C)]EFTGDILR.3	0.169438321	#N/A
IYPEEM[Oxidation (M)]IQTGISAIDGM[Oxidation (M)]NSIAR.3	0.135161282	#N/A
SAIGEGMTR.2	0.136821343	#N/A
YAEIVHLTLPDGTKR.3	-0.499851165	#N/A
LALTTAEFLAYQC[Carbamidomethyl (C)]EK.2	-0.168978904	#N/A
YAEIVHLTLPDGTK.2	0.419418201	#N/A
TVSGVNGPLVILDHVK.3	-0.022607865	#N/A
TVYETLDIGWQLLR.2	0.330083123	#N/A
HVLVILTDM[Oxidation (M)]SSYAEALR.3	-0.306971479	#N/A
SDFEENGSM[Oxidation (M)]DNVC[Carbamidomethyl (C)]LFLNLANDPTIER.3	-0.011287644	#N/A
QWLQEIDR.2	-0.142849883	#N/A
EFADSLGIPFLETSAK.2	0.079852183	#N/A
IQST[Phospho (STY)]PVK.2	0.098202859	P62821_T195_M1_Rab1A
KVVDYTTAK.2	0.203098771	#N/A
TITS[Phospho (STY)]SYYR.2	0.081831168	P62821_S78_M1_Rab1A
GAHGIIVVYDVTDQESFNNVK.3	-0.422959755	#N/A
YASENVNK.2	-0.10687658	#N/A
FADDTYTESYISTIGVDFK.2	-0.343075405	#N/A
QWLQEIDRYAS[Phospho (STY)]ENVNK.3	0.235471988	P62821_S114_M1_Rab1A
MGPGATAGGAEK.2	0.176075449	#N/A
TITTAYYR.2	-0.288049354	#N/A
TYSWDNAQVILAGNK.2	-0.031494384	#N/A
LVDIIC[Carbamidomethyl (C)]DK.2	0.557860132	#N/A
MSESLETDPAITAAK.2	-0.406305128	#N/A

LGEQLGFEFFETSAK.2	-0.164491253	#N/A
DNINVKQTFER.3	0.164646351	#N/A
VC[Carbamidomethyl (C)]ENIPIVLC[Carbamidomethyl (C)]GNK.2	0.234763072	#N/A
LVLVGDGGTGK.2	-0.223155148	#N/A
[Acetyl (Protein N-term)]AAQGEPQVQFK.2	0.10741133	#N/A
NLQYYDISAK.2	0.298540938	#N/A
FNVWDTAGQEK.2	-0.143307635	#N/A
HLTGEFEK.2	0.053506885	#N/A
YVATLGVEVHPLVFHTNR.3	0.021480682	#N/A
ISLGLPVGAVINC[Carbamidomethyl (C)]ADNTGAK.2	-0.286183582	#N/A
GSAITGPVAK.2	-0.172885322	#N/A
YDPTIEDSYR.2	-0.490057509	#N/A
YDPTIEDSYRK.2	0.293323667	#N/A
LVVLGSGGVGK.2	-0.066265374	#N/A
EQGQNLAR.2	-0.122344689	#N/A
INVNEIFYDLVR.2	0.520028482	#N/A
QVEVDC[Carbamidomethyl (C)]QQC[Carbamidomethyl (C)]M[Oxidation (M)]LEILDTAGTEQFTAM[Oxidation (M)]R.3	-0.445265611	#N/A
QWC[Carbamidomethyl (C)]NC[Carbamidomethyl (C)]AFLESSAK.2	-0.583579284	#N/A
TTPDVIFVFGFR.2	-0.078882361	#N/A
LITPAVVSER.2	0.356245482	#N/A
AALQELLSK.2	0.335656865	#N/A
AQVIYTR.2	-0.187117802	#N/A
DISEASVFDAYVLPK.2	0.15962984	#N/A
EGDVLTLLESER.2	-0.312278652	#N/A
TGS[Phospho (STY)]QGQC[Carbamidomethyl (C)]TQVR.2	0.060715289	P62858_S23_M1_Rps28
ADDTFEALR.2	0.087719854	#N/A
ESSTVFELK.2	-0.029685313	#N/A
TTIFTDAK.2	0.27145647	#N/A
VHAIPLR.2	0.259184277	#N/A
LLVSASQDGK.2	0.197639251	#N/A
AGVLAGHDNR.2	-0.068762042	#N/A
LIIWDSYTTNK.2	-0.122972229	#N/A
ELAGHTGYLSC[Carbamidomethyl (C)]C[Carbamidomethyl (C)]R.3	0.417716823	#N/A
LFVSGAC[Carbamidomethyl (C)]DASAK.2	0.287726556	#N/A
KAC[Carbamidomethyl (C)]ADATLSQITNNIDPVGR.3	-0.287221254	#N/A
VSC[Carbamidomethyl (C)]LGVTDDGM[Oxidation (M)]AVATGSWDSFLK.3	-0.411538719	#N/A
LLLAGYDDFNC[Carbamidomethyl (C)]NVWDALK.2	-0.09378251	#N/A
ADQELM[Oxidation (M)]TYSHDNIIC[Carbamidomethyl (C)]GITSVSFSK.3	-0.404698236	#N/A
[Acetyl (Protein N-term)]SELDQLRQEAEQLK.2	-0.164767751	#N/A
[Acetyl (Protein N-term)]SELDQLRQEAEQLKNQIR.3	-0.351254842	#N/A
IYAM[Oxidation (M)]HWGTDSR.2	0.257232507	#N/A
AC[Carbamidomethyl (C)]ADATLSQITNNIDPVGR.2	0.219136684	#N/A
[Acetyl (Protein N-term)]SELDQLR.2	-0.269111737	#N/A
SSWVM[Oxidation (M)]TC[Carbamidomethyl (C)]AYAPSGNYVAC[Carbamidomethyl (C)]GGLDNIC[Carbamidomethyl (C)]SIYNLK.3	0.153249914	#N/A
QTFTGHESDINAIC[Carbamidomethyl (C)]FFPNGNAFATGSDDATC[Carbamidomethyl (C)]R.3	-0.533816554	#N/A
QVC[Carbamidomethyl (C)]PLDNR.2	0.097308115	#N/A
[Acetyl (Protein N-term)]AAAMDVDTPSGTNSGAGK.2	0.603081141	#N/A

TFVSGAC[Carbamidomethyl (C)]DASIK.2	0.081302081	#N/A
KAC[Carbamidomethyl (C)]GDSTLTQITAGLDPVGR.3	0.045498814	#N/A
AC[Carbamidomethyl (C)]GDSTLTQITAGLDPVGR.2	0.036613876	#N/A
[Acetyl (Protein N-term)]S[Phospho (STY)]ELEQLRQEAEQLR.2	-0.323591922	P62880_S2_M1_Gnb2
LLLAGYDDFNC[Carbamidomethyl (C)]NIWDAM[Oxidation (M)]K.2	-0.382918741	#N/A
ELPGHTGYLSC[Carbamidomethyl (C)]C[Carbamidomethyl (C)]R.3	0.337537917	#N/A
[Acetyl (Protein N-term)]SELEQLR.2	0.232094759	#N/A
ADQELLM[Oxidation (M)]YSHDNIIC[Carbamidomethyl (C)]GITSVAFSR.3	-0.144823862	#N/A
QTFIGHESDINAVAFFPNGYAFTTGSDDATC[Carbamidomethyl (C)]R.3	-0.323461294	#N/A
SEAES[Phospho (STY)]LKGK.2	0.242588141	P62881_S63_M1_Gnb5
VIVWDSFTTNK.2	-0.521128779	#N/A
SEAES[Phospho (STY)]LKGKLEEER.2	0.11266057	P62881_S63_M1_Gnb5
SGQC[Carbamidomethyl (C)]VQAFETHESDVNSVR.3	-0.23979893	#N/A
LHDVELHQVAER.3	-0.051133404	#N/A
VSPDGTAFC[Carbamidomethyl (C)]SGSWDHTLR.2	0.022881546	#N/A
VC[Carbamidomethyl (C)]TLAIIDPGDSDIIR.2	-0.159772732	#N/A
LVILANNC[Carbamidomethyl (C)]PALR.2	0.03434431	#N/A
TGPNLHGLFGR.2	-0.130230561	#N/A
TGQAAGFSYTDANK.2	0.194473071	#N/A
GITWGEDTLM[Oxidation (M)]EYLENPKK.3	-0.24617636	#N/A
ADLIAYLK.2	0.277334371	#N/A
KTGQAAGFSYTDANK.3	0.099552267	#N/A
SAINEVVTR.2	0.373136629	#N/A
EYTINIHK.2	-0.192961308	#N/A
LYTLVTYVPVTTFK.2	-0.012516488	#N/A
NLQTVNVDEN.2	0.206034075	#N/A
DEILPTT[Phospho (STY)]PISEQK.2	-0.205627898	P62908_T221_M1_Rps3
AELNEFLTR.2	-0.092047377	#N/A
GLC[Carbamidomethyl (C)]AIAQAESLR.2	0.091727498	#N/A
SYC[Carbamidomethyl (C)]AEIAHNVSSK.2	0.437685522	#N/A
DIIHDPGR.2	-0.155007811	#N/A
AVVGVVAGGGR.2	0.112903125	#N/A
YPDHLHISTS[Phospho (STY)]PC[Carbamidomethyl (C)].2	0.217998339	P62956_S273_M1_Cacng7
LS[Phospho (STY)]DC[Carbamidomethyl (C)]SDYSGQFLQPEAWR.2	0.13586893	P62956_S222_M1_Cacng7
AADPPAENSS[Phospho (STY)]APEAEQGGAE.2	0.012830336	P62960_S312_M1_Ybx1
DSPSVWAAVPGK.2	0.056540992	#N/A
TFVSITPAEVGVLVGK.2	-0.074838774	#N/A
STGGAPTFNVTVTM[Oxidation (M)]TAK.2	0.024732815	#N/A
DSLLQDGEFTM[Oxidation (M)]DLR.2	-0.28079143	#N/A
[Acetyl (Protein N-term)]AGWNAYIDSLM[Oxidation (M)]ADGTC[Carbamidomethyl (C)]QDAAIVGYK.3	0.184708469	#N/A
C[Carbamidomethyl (C)]YEM[Oxidation (M)]ASHLR.2	0.481989149	#N/A
[Acetyl (Protein N-term)]S[Phospho (STY)]DSGEQNYGER.2	-0.194413618	P62996_S2_M1_Tra2b
[Acetyl (Protein N-term)]SDSGEQNYGERES[Phospho (STY)]R.2	-0.008416064	P62996_S14_M1_Tra2b
S[Phospho (STY)]RS[Phospho (STY)]YS[Phospho (STY)]RDYR.2	-0.132318848	P62996_S83_M3_Tra2b
RPHT[Phospho (STY)]PTPGIYMGR.3	-0.003987669	P62996_T201_M1_Tra2b
RHS[Phospho (STY)]HS[Phospho (STY)]HS[Phospho (STY)]PMSTR.3	0.281790601	#N/A
RRHS[Phospho (STY)]HS[Phospho (STY)]HS[Phospho (STY)]PM[Oxidation (M)]STR.3	0.01021138	#N/A

HSHS[Phospho (STY)]HS[Phospho (STY)]PMSTR.2	0.200709799	P62996_S97_M2_Tra2b
C[Carbamidomethyl (C)]VVVGDGAVGK.2	0.247290834	#N/A
YLEC[Carbamidomethyl (C)]SALTQR.2	-0.126534736	#N/A
AVLC[Carbamidomethyl (C)]PPPVK.2	-0.146362349	#N/A
HHC[Carbamidomethyl (C)]PNTPIILVGTK.3	-0.250767022	#N/A
TVFDEAIR.2	0.122203718	#N/A
LDLRDDKDTIEK.3	-0.317800234	#N/A
LTPITYPQGLAM[Oxidation (M)]AK.2	-0.175228654	#N/A
DDKDTIEK.2	-0.159966705	#N/A
LRPLSYPQTDVFLIC[Carbamidomethyl (C)]FSLVSPASFENVR.3	-0.09051476	#N/A
TAPYVVTGSVDQTVK.2	0.381402411	#N/A
VWDYETGDFER.2	-0.103733874	#N/A
KWTS[Phospho (STY)]VIR.2	-0.011350596	P63005_S57_M1_Pafah1b1
FILSC[Carbamidomethyl (C)]ADDK.2	0.055201966	#N/A
YALSGHR.2	0.314602118	#N/A
LWDFQGFEC[Carbamidomethyl (C)]IR.2	-0.286043003	#N/A
QRDELNR.2	-0.285366377	#N/A
GHTDSVQDISFDHSGK.3	-0.477466393	#N/A
M[Oxidation (M)]S[Phospho (STY)]ESLDTADPAVTGAK.2	-0.108591134	P63011_S188_M1_Rab3a
LVDVIC[Carbamidomethyl (C)]EK.2	-0.029142843	#N/A
YADDSFTPAFVSTVGIDFK.2	-0.043014211	#N/A
QLADHLGFEFFEASAK.3	-0.084144838	#N/A
ESSDQNFDYMFK.2	0.416031624	#N/A
LVDVIC[Carbamidomethyl (C)]EKMS[Phospho (STY)]ESLDTADPAVTGAK.3	0.022454989	P63011_S188_M1_Rab3a
VEIIANDQGNR.2	-0.045648777	#N/A
TTPSYVAFTDTER.2	-0.101141406	#N/A
TVTNAVVTVPAYFNDSQR.2	0.065216783	#N/A
LSKEDIER.2	-0.089835211	#N/A
DAGTIAGLNVLR.2	0.202224107	#N/A
IINEPTAAAIAYGLDKK.2	0.2921726	#N/A
GTLDPVEK.2	-0.020100896	#N/A
ITITNDKGR.2	-0.088503795	#N/A
SQIHDIVLVGGSTR.3	0.046507877	#N/A
LLQDFFNGK.2	0.314935552	#N/A
VC[Carbamidomethyl (C)]NPIITK.2	0.655096318	#N/A
FEELNADLFR.2	0.151362028	#N/A
VQVEYKGETK.2	0.003339355	#N/A
EIAEAYLGK.2	0.216990553	#N/A
C[Carbamidomethyl (C)]NEIISWLDK.2	0.100162946	#N/A
YKAEDEKQR.2	-0.0872828	#N/A
SFYPEEVSSM[Oxidation (M)]VLTK.2	0.312045454	#N/A
DIS[Phospho (STY)]ENKR.2	-0.439025761	P63017_S254_M1_Hspa8
ARFEELNADLFR.2	0.10945481	#N/A
NQTAEKEEFEHQQK.3	0.362625173	#N/A
ITITNDK.2	-0.013176432	#N/A
NSLESYAFNM[Oxidation (M)]K.2	-0.060704146	#N/A
GPAVGIDLGTTYSC[Carbamidomethyl (C)]VGVFQHGK.3	-0.154291205	#N/A

NQTAEKEEFEHQQKELEK.4	-0.353235237	#N/A
HWPFM[Oxidation (M)]VVNDAGRPK.3	-0.534338475	#N/A
NQVAM[Oxidation (M)]NPTNTVFDAK.2	-0.024895873	#N/A
SINPDEAVAYGAAVQAAILSGDK.2	-0.030130395	#N/A
FDDAVVQSDM[Oxidation (M)]K.2	0.353996882	#N/A
QTQTFTTYSDNQPGVLIQVYEGER.3	-0.173513483	#N/A
NQVAM[Oxidation (M)]NPTNTVFDAKR.3	-0.170613712	#N/A
GRLS[Phospho (STY)]KEDIER.3	-0.167857955	P63017_S511_M1_Hspa8
STAGDTHLGGEDFDNR.3	-0.181517941	#N/A
RFDDAVVQSDM[Oxidation (M)]K.2	0.498041091	#N/A
M[Oxidation (M)]KEIAEAYLGK.2	-0.018719331	#N/A
MVNHFIAEFK.2	0.3668774	#N/A
IINEPTAAAIAYGLDK.2	-0.013758914	#N/A
MVQEAEKYK.2	0.339570481	#N/A
LDKSQIHDIVLVGGSTR.3	-0.475225439	#N/A
LYQSAGGM[Oxidation (M)]PGGM[Oxidation (M)]PGGFPGGGAPPSGGASSGPTIEEVD.3	-0.257615481	#N/A
YKAEDEK.2	-0.21549379	#N/A
DLISHDELFSDIYK.2	0.047345465	#N/A
LEEQKPER.2	-0.021141854	#N/A
GKLEEQKPER.3	0.166949602	#N/A
EIADGLC[Carbamidomethyl (C)]LEVEGK.2	0.188246838	#N/A
KAADYVR.2	-0.034663936	#N/A
TIVITSHPGQIVK.2	-0.06461355	#N/A
QISQAYEVLADSK.2	0.331449042	#N/A
DHAVFTR.2	0.124802617	#N/A
ITFHGEGDQEPGLEPGDIIIVLDQK.3	0.01552691	#N/A
NVVHQLS[Phospho (STY)]VTLEDLYNGATR.3	-0.130934622	P63037_S112_M1_Dnaja1
VTDALNATR.2	0.169391675	#N/A
APGFGDNRK.2	0.236435979	#N/A
APGFGDNR.2	0.069333885	#N/A
AAVEEGIVLGGGC[Carbamidomethyl (C)]ALLR.2	0.077155621	#N/A
GANPVEIR.2	0.129031263	#N/A
LVQDVANNTNEEAGDGTTTATVLAR.3	-0.191394762	#N/A
GYISPYFINTSK.2	0.08057141	#N/A
ISSVQSIVPALEIANAHR.3	0.063998594	#N/A
LSDGVAVLK.2	-0.140288577	#N/A
VGLQVVAVK.2	0.392428344	#N/A
IQEITEQLDITTSEYEK.2	-0.260087659	#N/A
C[Carbamidomethyl (C)]EFQDAYVLLSEK.2	-0.023702834	#N/A
TVIIEQSWGSPK.2	-0.081152572	#N/A
NAGVEGSLIVEK.2	0.120196978	#N/A
KPLVIIAEDVDGEALSTLVLNR.3	0.140140111	#N/A
SIDLKDK.2	-0.15120181	#N/A
VGEVIVTK.2	-0.176259854	#N/A
IQEITEQLDITTSEYEKEK.3	-0.334199952	#N/A
C[Carbamidomethyl (C)]IPALDSLKPANEDQK.2	0.254827781	#N/A
TLNDELEIIEGM[Oxidation (M)]K.2	0.220642657	#N/A

VGGTSDVEVNEKK.2	0.114004007	#N/A
ALMLQGVDLLADAVAVTM[Oxidation (M)]GPK.3	0.181328528	#N/A
_TALLDAAGVASLLTTAEAVVTEIPKEEKDPGM[Oxidation (M)]GAM[Oxidation (M)]GGM[Oxidation (M)]GGGM[Oxidation (M)]GGGM[Oxidat	-0.165989711	#N/A
ILQSSSEVGYDAM[Oxidation (M)]LGDFVNM[Oxidation (M)]VEK.3	-0.336107143	#N/A
DM[Oxidation (M)]AIATGGAVFGEEGLNLNLEDVQAHDLGK.3	0.062103485	#N/A
KAIPPGC[Carbamidomethyl (C)]GDEPEEEDESILDTVIK.3	-0.257227437	#N/A
EAEAQAAM[Oxidation (M)]EANSEGS[Phospho (STY)]LTRPK.2	0.30165839	P63040_S93_M1_Cplx1
RRDPS[Phospho (STY)]LEEIQK.3	-0.591094704	P63042_S90_M1_Stmn4
DQKLS[Phospho (STY)]ELDDR.2	-0.323961632	P63044_S61_M1_Vamp2
LSELDDR.2	0.035075079	#N/A
ADALQAGASQFETSAAK.2	-0.343010625	#N/A
[Acetyl (Protein N-term)]SATAATVPPAAPAGEGGPPAPPPNLTSNR.3	0.33486254	#N/A
LQQTQAQVDEVVDIM[Oxidation (M)]R.2	-0.23939078	#N/A
LSELDDRADALQAGASQFETSAAK.3	-0.150443577	#N/A
[Acetyl (Protein N-term)]AESEAETPGT[Phospho (STY)]PGEFESK.2	-0.12304695	P63046_T11_M1_Sult4a1
AAVAIQSQFR.2	0.178910419	#N/A
VQEEFDIDM[Oxidation (M)]DAPETER.2	-0.021620386	#N/A
KVQEEFDIDM[Oxidation (M)]DAPETER.3	0.031107342	#N/A
VEC[Carbamidomethyl (C)]GS[Phospho (STY)]DPEENSAR.2	-0.17819301	P63058_S12_M1_Thra
DAVTHIGR.2	0.260735282	#N/A
IAIWTTEC[Carbamidomethyl (C)]ENR.2	-0.040188705	#N/A
NSAIS[Phospho (STY)]FDNSGIQYR.2	0.101734661	P63080_S394_M1_Gabrb3
KQS[Phospho (STY)]MPKEGHGR.3	-0.414530523	P63080_S406_M1_Gabrb3
[Acetyl (Protein N-term)]AAAAAAGPEM[Oxidation (M)]VR.2	-0.183992444	#N/A
GQVFDVGPR.2	-0.010547619	#N/A
NYLLSLPHK.2	0.180418533	#N/A
ELIFEETAR.2	0.32012826	#N/A
LKELIFEETAR.3	0.26927825	#N/A
DVYIVQDLM[Oxidation (M)]ETDLYK.2	-0.013897676	#N/A
VADPDHDHTGFLTEYVATR.3	-0.276168578	#N/A
ISPFEHQTYC[Carbamidomethyl (C)]QR.3	-0.017139376	#N/A
YTNLSYIGEGAYGM[Oxidation (M)]VC[Carbamidomethyl (C)]SAYDNLNK.3	-0.462215936	#N/A
APEIMLNSK.2	-0.044296981	#N/A
YIHSANVLHR.3	-0.018598423	#N/A
GNHEC[Carbamidomethyl (C)]AS[Phospho (STY)]INR.2	-0.004761057	P63087_S129_M1_Ppp1cc
HDLDLIC[Carbamidomethyl (C)]R.2	0.050116068	#N/A
NVQLQENEIR.2	0.123483668	#N/A
IYGFYDEC[Carbamidomethyl (C)]K.2	0.03803918	#N/A
TFTDC[Carbamidomethyl (C)]FNC[Carbamidomethyl (C)]LPIAAIVDEK.2	-0.189145775	#N/A
EIFLSQPILLELEAPLK.2	-0.310832039	#N/A
YPENFFLLR.2	-0.249444253	#N/A
[Acetyl (Protein N-term)]ADIDKLNIDSIIQR.2	0.053444427	#N/A
IFC[Carbamidomethyl (C)]C[Carbamidomethyl (C)]HGGLSPDLQSM[Oxidation (M)]EQIR.3	-0.421735516	#N/A
AHQVVEDGYEFFAK.2	-0.399917216	#N/A
QSLETIC[Carbamidomethyl (C)]LLLAYK.2	0.005756401	#N/A
IC[Carbamidomethyl (C)]GDIHGQYYDLLR.3	0.129646021	#N/A
EKIETELR.2	0.20160314	#N/A

GIVDQSQQAYQEAFEISKK.3	-0.37659828	#N/A
VVSSIEQK.2	0.188678988	#N/A
YLAEVAAGDDK.2	0.223723017	#N/A
FLIPNASQPESK.2	0.02640745	#N/A
[Acetyl (Protein N-term)]M[Oxidation (M)]DKNELVQK.2	0.459441914	#N/A
SVTEQGAELSNEER.2	-0.053703522	#N/A
KGIVDQSQQAYQEAFEISK.3	-0.08012404	#N/A
YDDM[Oxidation (M)]AAC[Carbamidomethyl (C)]M[Oxidation (M)]K.2	0.542548018	#N/A
DIC[Carbamidomethyl (C)]NDVLSLLEK.2	0.360490149	#N/A
TAFDEAIAELDTLSEESYK.2	-0.211767882	#N/A
YLAEVAAGDDKK.2	0.145505609	#N/A
VVSS[Phospho (STY)]IEQKTEGAEKK.3	0.262916878	P63101_S64_M1_Ywhaz
GIVDQSQQAYQEAFEISK.2	0.076808359	#N/A
VVSS[Phospho (STY)]IEQKTEGAEK.2	-0.219171745	P63101_S64_M1_Ywhaz
TAFDEAIAELDTLSEESYKDSTLIMQLLR.3	-0.265464051	#N/A
IPS[Phospho (STY)]S[Phospho (STY)]PDLKK.2	-0.105605914	P63141_S440_M2_Kcna2
ETEGEEQAQYLQVTS[Phospho (STY)]C[Carbamidomethyl (C)]PK.2	-0.133054279	P63141_S434_M1_Kcna2
IPS[Phospho (STY)]S[Phospho (STY)]PDLKKS[Phospho (STY)]R.2	-0.182655265	P63141_S440_M3_Kcna2
SDYMEIQEGVNNS[Phospho (STY)]NEDFREENLK.3	0.276683435	P63141_S468_M1_Kcna2
IPS[Phospho (STY)]S[Phospho (STY)]PDLK.2	-0.610379766	P63141_S440_M2_Kcna2
VSAIVEQSWNDS.2	0.435109929	#N/A
GKFEDMAK.2	-0.340534868	#N/A
M[Oxidation (M)]SSYAFFVQTC[Carbamidomethyl (C)]R.2	-0.373255704	#N/A
[Acetyl (Protein N-term)]S[Phospho (STY)]DQEAKPSTEDLGDK.2	-0.179483165	P63166_S2_M1_Sumo1
[Acetyl (Protein N-term)]S[Phospho (STY)]DQEAKPSTEDLGDKK.2	-0.106309734	P63166_S2_M1_Sumo1
NADM[Oxidation (M)]SEEM[Oxidation (M)]QQDSVEC[Carbamidomethyl (C)]ATQALEK.3	-0.428575893	#N/A
[Acetyl (Protein N-term)]ASNNTAS[Phospho (STY)]IAQAR.2	-0.173651143	P63213_S8_M1_Gng2
EDPLLTPVPASENPFR.2	0.312434806	#N/A
KLVEQLK.2	0.293723577	#N/A
IEASLC[Carbamidomethyl (C)]R.2	0.215698029	#N/A
GETPVNSTM[Oxidation (M)]S[Phospho (STY)]IGQAR.2	-0.207472634	P63216_S12_M1_Gng3
MKGET[Phospho (STY)]PVNSTM[Oxidation (M)]SIGQAR.3	-0.20672635	#N/A
VHLVGIDIFTGK.3	-0.047366531	#N/A
EDLRLPEGDLGK.3	0.179662848	#N/A
EDLRLPEGDLGKEIEQK.3	0.10272367	#N/A
RNDFQLIGIQDGYLSLLQDSGEVR.3	-0.345456655	#N/A
IVEMSTSK.2	0.289980857	#N/A
[Acetyl (Protein N-term)]TDVETTYADFIASGR.2	0.082004769	#N/A
SSTEQSGEAQGEAAK.2	-0.201921419	#N/A
SSTEQSGEAQGEAAKS[Phospho (STY)]ES.2	-0.419999062	P63248_S74_M1_Pkia
ERHNS[Phospho (STY)]VEC[Carbamidomethyl (C)]LDGLDDISTK.3	-0.024848968	P63250_S385_M1_Kcnj3
HNS[Phospho (STY)]VEC[Carbamidomethyl (C)]LDGLDDISTK.2	-0.409200684	P63250_S385_M1_Kcnj3
[Acetyl (Protein N-term)]EEEIAALVIDNGSGM[Oxidation (M)]C[Carbamidomethyl (C)]K.2	-0.352157297	#N/A
[Acetyl (Protein N-term)]EEEIAALVIDNGSGM[Oxidation (M)]C[Carbamidomethyl (C)]KAGFAGDDAPR.3	-0.37645572	#N/A
AAPALT[Phospho (STY)]PPDR.2	-0.34287885	P63318_T655_M1_Prkcg
ENVFPGSTTR.2	-0.025237575	#N/A
DVIVQDDDVDC[Carbamidomethyl (C)]TLVEK.2	-0.29534253	#N/A

RGS[Phospho (STY)]DELYAIK.2	0.23936096	P63318_S373_M1_Prkcg
FEAC[Carbamidomethyl (C)]NYPLELYER.2	-0.096366266	#N/A
C[Carbamidomethyl (C)]FFGAS[Phospho (STY)]PGR.2	-0.370622791	P63318_S342_M1_Prkcg
APVDGWYK.2	-0.039446436	#N/A
LSVEVWDWDR.2	0.197596499	#N/A
S[Phospho (STY)]GENFDKFFTR.2	-0.134151942	P63318_S639_M1_Prkcg
DVIVQDDDVDC[Carbamidomethyl (C)]TLVEKR.3	-0.418045363	#N/A
KDVIVQDDDVDC[Carbamidomethyl (C)]TLVEK.3	0.170662695	#N/A
S[Phospho (STY)]PTSPVPVPVM.2	0.370564947	P63318_S687_M1_Prkcg
SVPSLC[Carbamidomethyl (C)]GVDHTER.2	-0.168399146	#N/A
M[Oxidation (M)]GPSSSPIPSPS[Phospho (STY)]PS[Phospho (STY)]PTDSKR.3	-0.108010338	#N/A
NDFM[Oxidation (M)]GAM[Oxidation (M)]SFGVSELLK.2	-0.328326863	#N/A
APTS[Phospho (STY)]DEIHITVGEAR.2	-0.113677797	P63318_S170_M1_Prkcg
DLKLDNVMLDAEGHIK.3	0.250940813	#N/A
S[Phospho (STY)]VPSLC[Carbamidomethyl (C)]GVDHTERR.3	-0.335990834	P63318_S145_M1_Prkcg
LLNQEEGEYYNVPVADADNC[Carbamidomethyl (C)]SLLQK.3	-0.152579538	#N/A
GSDELYAIK.2	0.342557954	#N/A
LGSGPDGEPTIR.2	0.045297183	#N/A
MGPSSSPIPSPSPS[Phospho (STY)]PTDSK.2	-0.300925184	P63318_S330_M1_Prkcg
VIM[Oxidation (M)]VGSGGVGK.2	-0.416971051	#N/A
ADQWNVNYVETSAK.2	-0.078502682	#N/A
SDLEDKR.2	0.030921891	#N/A
VKEDENVPFLLVGNK.3	0.326807942	#N/A
GQNSLALHK.2	0.310892549	#N/A
VVLDGEEVQIDILDTAGQEDYAAIR.3	-0.27041125	#N/A
TALIHDGLAR.2	0.316001031	#N/A
[Acetyl (Protein N-term)]AEEGIAAGGVM[Oxidation (M)]DVNTALQEVLK.2	-0.031932106	#N/A
DVIEEYFK.2	-0.039613589	#N/A
AEAGAGS[Phospho (STY)]ATEFQFR.2	0.171898919	P63325_S146_M1_Rps10
IAIYELLFK.2	-0.070287301	#N/A
IITEGASILR.2	0.081890649	#N/A
EVFDNDGKPR.2	-0.149660883	#N/A
QTLQSATVEAIEADEAIK.2	-0.341625864	#N/A
GFS[Phospho (STY)]PQHK.2	-0.13670199	P63328_S462_M1_Ppp3ca
ITSFEEAK.2	-0.033549876	#N/A
TQEHFTHNTVR.2	-0.028738955	#N/A
GLTPTGMLPSGVLSGGK.2	0.288454915	#N/A
DAM[Oxidation (M)]PSDANLNSINK.2	0.294353803	#N/A
ALASETNGTDSNGSNSSNIQ.2	-0.191647775	#N/A
LEESVALR.2	0.081709217	#N/A
ITS[Phospho (STY)]FEEAKGLDR.2	0.024936677	P63328_S469_M1_Ppp3ca
VTEMLVNVLNIC[Carbamidomethyl (C)]SDDELGSEEDGFDGATAAAR.3	0.016887551	#N/A
QTLQSATVEAIEADEAIKGFS[Phospho (STY)]PQHK.3	0.259449997	P63328_S462_M1_Ppp3ca
ELDQWIEQLNEC[Carbamidomethyl (C)]K.2	0.057916694	#N/A
VLPSITTEILK.2	0.053000345	#N/A
DLQNVNITLR.2	0.269670595	#N/A
FDAGELITQR.2	-0.085159499	#N/A

IYTSIGEDYDER.2	0.243397862	#N/A
NVPVITGSK.2	-0.07339375	#N/A
NITYLPAGQSVLLQLPQ.2	-0.084588386	#N/A
QVSDDLTER.2	0.106765693	#N/A
AAIISAEGDSK.2	-0.149548487	#N/A
EFTEAVEAK.2	0.500877806	#N/A
AAELIANSLATAGDGLIELR.3	0.023129772	#N/A
YQQGDFGYC[Carbamidomethyl (C)]PR.2	0.25676624	#N/A
GNEFFC[Carbamidomethyl (C)]EVDEDYIQDK.2	-0.134641991	#N/A
ITVTSEVPFSK.2	0.065024498	#N/A
AGNLGGGVVTIER.2	0.154063371	#N/A
YFQINQDEEEEEDED.2	0.141188136	#N/A
TDQVIQSLIALVNDPQPEHPLR.3	-0.346385584	#N/A
NIQVDEANLLTWQGLIVPDNPPYDK.3	0.07742231	#N/A
FSPNSSNPIIVSC[Carbamidomethyl (C)]GWDK.2	0.024920465	#N/A
IIVDELKQEVISTSSK.3	-0.172029993	#N/A
DVLSVAFSSDNR.2	-0.436200223	#N/A
VWNLANC[Carbamidomethyl (C)]K.2	0.175101014	#N/A
YWLC[Carbamidomethyl (C)]AATGPSIK.2	0.307076109	#N/A
AEPPQC[Carbamidomethyl (C)]TSLAWSADGQTLFAGYTDNLVR.3	-0.172011243	#N/A
YTVQDESHSEWVSC[Carbamidomethyl (C)]VR.3	-0.221638833	#N/A
AKEDFLR.2	-0.177865715	#N/A
TLGTGSFGR.2	0.005042082	#N/A
ILQAVEFPFLVR.2	0.003331248	#N/A
TWTLC[Carbamidomethyl (C)]GTPEYLAPEIILSK.2	-0.058672497	#N/A
FRGS[Phospho (STY)]GDTSNFDDYEEEEIR.3	0.108898214	P68181_S322_M1_Prkacb
GS[Phospho (STY)]GDTSNFDDYEEEEIR.2	-0.221863107	P68181_S322_M1_Prkacb
ATEQYYAMK.2	-0.222014625	#N/A
AVTEQGAELSNEER.2	0.05846036	#N/A
EKVESELR.2	0.102968199	#N/A
YLAEVAC[Carbamidomethyl (C)]GDDR.2	-0.242820313	#N/A
YLIANATNPESK.2	0.092577604	#N/A
SIC[Carbamidomethyl (C)]TTVLELLDK.2	0.409494828	#N/A
YLAEVAC[Carbamidomethyl (C)]GDDRK.2	0.22534741	#N/A
KQTIENSQGAYQEAFDISK.3	-0.291481565	#N/A
TAFDEAIAELDTLNEDSYK.2	-0.267580966	#N/A
KQTIENSQGAYQEAFDISKK.3	-0.149283085	#N/A
YDDMATC[Carbamidomethyl (C)]MK.2	0.086484793	#N/A
QTIENSQGAYQEAFDISK.2	0.216321528	#N/A
DVNAAIAAIK.2	0.16452938	#N/A
EIIDPVLDR.2	-0.413754461	#N/A
AVFVDLEPTVIDEIR.2	-0.070389008	#N/A
TIGGGDDSFTTFFC[Carbamidomethyl (C)]ETGAGK.2	-0.039835587	#N/A
FPGQLNADLR.2	0.168986278	#N/A
EVDEQM[Oxidation (M)]LNVQNK.2	-0.188305391	#N/A
AVLVDLEPGTM[Oxidation (M)]DSVR.2	-0.091457292	#N/A
LAVNM[Oxidation (M)]VPFPR.2	0.066393482	#N/A

LHFFM[Oxidation (M)]PGFAPLTSR.3	-0.09724786	#N/A
TAVC[Carbamidomethyl (C)]DIPPR.2	0.089885575	#N/A
FPGQLNADLRK.2	0.080816663	#N/A
IREEYPDR.2	-0.004635006	#N/A
YLTVAAVFR.2	0.000475949	#N/A
GHYTEGAELVDSVLDVVR.2	-0.239775168	#N/A
INVYYNEATGGK.2	0.160883747	#N/A
IM[Oxidation (M)]NTFSVVPSPK.2	-0.052304291	#N/A
ALTVPELTQQM[Oxidation (M)]FDAK.2	-0.001553593	#N/A
M[Oxidation (M)]SATFIGNSTAIQELFKR.3	-0.522777811	#N/A
KLAVNM[Oxidation (M)]VPFPR.3	-0.30148618	#N/A
NM[Oxidation (M)]M[Oxidation (M)]AAC[Carbamidomethyl (C)]DPR.2	0.272827007	#N/A
SGPFGQIFRPDNFVFGQSGAGNNWAK.3	-0.476522673	#N/A
NSSYFVEWIPNNVK.2	-0.089876445	#N/A
GHYTEGAELVDSVLDVVRK.3	-0.477991773	#N/A
LTTPTYGDLNHLVSATMSGVTTC[Carbamidomethyl (C)]LR.3	0.165640137	#N/A
KEAESC[Carbamidomethyl (C)]DC[Carbamidomethyl (C)]LQGFQLTHSLGGGTGSGM[Oxidation (M)]GTLLISK.4	-0.073443335	#N/A
EAESC[Carbamidomethyl (C)]DC[Carbamidomethyl (C)]LQGFQLTHSLGGGTGSGM[Oxidation (M)]GTLLISK.3	-0.071402916	#N/A
FWEVISDEHGIDPTGTYHGDSDLQLER.4	-0.108779982	#N/A
ISEQFTAM[Oxidation (M)]FR.2	-0.252823663	#N/A
TLKLTTPTYGDLNHLVSATM[Oxidation (M)]SGVTTC[Carbamidomethyl (C)]LR.3	-0.264938044	#N/A
MSATFIGNSTAIQELFK.2	-0.27137103	#N/A
[Acetyl (Protein N-term)]ADPAAGPPPS[Phospho (STY)]EGEESTVR.2	-0.050710289	P68404_S11_M1_Prkcb
LIPDPKS[Phospho (STY)]ESK.2	-0.455501375	P68404_S206_M1_Prkcb
ENIWDGVTTK.2	-0.347146797	#N/A
QPVELT[Phospho (STY)]PTDK.2	-0.273289959	P68404_T642_M1_Prkcb
KGTDELYAVK.2	0.118835341	#N/A
C[Carbamidomethyl (C)]SLNPEWNETFR.2	0.186925518	#N/A
LSVEIWDWDLTSR.2	-0.14322916	#N/A
DLKLDNVM[Oxidation (M)]LDSEGHIK.3	-0.295774015	#N/A
AGVDGWFK.2	-0.026266064	#N/A
NLVPM[Oxidation (M)]DPNGLSDPY[Phospho (STY)]VK.2	-0.357839876	P68404_Y195_M1_Prkcb
C[Carbamidomethyl (C)]VM[Oxidation (M)]NVPSLC[Carbamidomethyl (C)]GTDHTER.3	-0.227169392	#N/A
FQLKES[Phospho (STY)]DKDR.3	-0.133431275	P68404_S234_M1_Prkcb
AVTELNEPLSNEDR.2	-0.253716181	#N/A
NSVVEASEAAYK.2	0.04629059	#N/A
YLAEVASGEK.2	-0.118528144	#N/A
YLAEVASGEKK.2	0.041969328	#N/A
NC[Carbamidomethyl (C)]NDFQYESK.2	0.196453938	#N/A
[Acetyl (Protein N-term)]GDREQLLQR.2	0.046294127	#N/A
ELETVC[Carbamidomethyl (C)]NDVLALLDK.2	-0.001001749	#N/A
QAFDDAIAELDTLNEDSYK.2	-0.027248755	#N/A
AVTELNEPLSNEDRNLLSVAYK.3	0.099726758	#N/A
NSVVEASEAAYKEAFEISK.3	-0.417993284	#N/A
EAFEISK.2	-0.509807961	#N/A
DNLTLWTSDQQDEEAGEGN.2	0.164476131	#N/A
YDDMASAMK.2	-0.068317896	#N/A

AAVENLPTFLVELSR.2	-0.006872185	#N/A
LAATNALLNSLEFTK.2	0.078061426	#N/A
VLANPGNSQVAR.2	-0.150693704	#N/A
WLAIDANAR.2	0.112035279	#N/A
SNEILTAIIQGM[Oxidation (M)]R.2	0.388676518	#N/A
[Acetyl (Protein N-term)]M[Oxidation (M)]ELITILEK.2	-0.345140678	#N/A
WHTLTNELPGAVLS[Phospho (STY)]D.2	-0.111718342	P70169_S411_M1_Doc2b
SRS[Phospho (STY)]NS[Phospho (STY)]GLDYIK.2	-0.204448485	P70174_S381_M2_Hrh1
SNS[Phospho (STY)]GLDYIK.2	0.224642679	P70174_S383_M1_Hrh1
RIS[Phospho (STY)]ETSEDQTLVDR.2	0.42298594	P70174_S344_M1_Hrh1
GVTSNTS[Phospho (STY)]DSESSSK.2	0.219548827	P70175_S632_M1_Dlg3
LLAVNNTNLQDVR.2	0.192599781	#N/A
EQMMNSSMS[Phospho (STY)]SGS[Phospho (STY)]GSLR.2	-0.12083718	P70175_S508_M2_Dlg3
FIEAGQFNDNLYGTSIQSVR.2	-0.269669516	#N/A
VNDDLISEFPHK.3	-0.051125981	#N/A
ALFDYDR.2	0.175496358	#N/A
NATHEQAAAALKR.3	-0.203953815	#N/A
VNEVDVSEVVHSR.3	-0.554249423	#N/A
IQALKASPS[Phospho (STY)]K.2	-0.495259762	P70181_S407_M1_Pip5k1b
IPLKPS[Phospho (STY)]PTK.2	-0.097529785	P70182_S443_M1_Pip5k1a
S[Phospho (STY)]GPSGNSC[Carbamidomethyl (C)]TSQLMASGEHR.3	0.264769356	P70182_S458_M1_Pip5k1a
RDS[Phospho (STY)]LPGK.2	-0.340700179	P70188_S102_M1_Kifap3
RDS[Phospho (STY)]LPGKEK.2	-0.157846846	P70188_S102_M1_Kifap3
DGIVLGADTR.2	0.211902734	#N/A
VTPLEIEVLEETVQTM[Oxidation (M)]DTS.2	-0.364962464	#N/A
ATEGMVVADKNC[Carbamidomethyl (C)]SK.2	-0.462833799	#N/A
LFLVQTVQQASK.2	0.292134698	#N/A
FSVEEIIQK.2	-0.03966498	#N/A
AASVAENC[Carbamidomethyl (C)]INYQQGTPHK.2	0.139928302	#N/A
TASS[Phospho (STY)]PDSLR.2	-0.267025195	P70206_S1630_M1_Plxna1
QTSAYNISNS[Phospho (STY)]STFTK.2	-0.570782821	P70206_S1611_M1_Plxna1
LGKDS[Phospho (STY)]PSNK.2	-0.497256215	P70206_S1793_M1_Plxna1
YTGS[Phospho (STY)]PDSLR.2	-0.440395088	P70207_S1630_M1_Plxna2;Plxna4
YTGS[Phospho (STY)]PDS[Phospho (STY)]LRSR.2	-0.468876351	P70207_S1630_M2_Plxna2;Plxna4
S[Phospho (STY)]LSDLTSQK.2	-0.068481296	P70213_S244_M1_Fv1
DETFGEYS[Phospho (STY)]DSDEKPLKGS[Phospho (STY)]LR.3	0.099139047	P70232_S1138_M2_Chl1
SPFNSPS[Phospho (STY)]PQDSPR.2	-0.160737425	P70255_S339_M1_Nfic
NWTEDIEGGISS[Phospho (STY)]PVKK.2	-0.224236077	P70255_S323_M1_Nfic
S[Phospho (STY)]IDDSEMESPVDDVFYPGTGR.2	-0.602179742	P70257_S280_M1_Nfix
S[Phospho (STY)]PAAGSSQSSGWPNDVDAGPASLKK.3	0.017411798	P70257_S301_M1_Nfix
S[Phospho (STY)]PAAGSSQSSGWPNDVDAGPASLK.2	-0.079978769	P70257_S301_M1_Nfix
NS[Phospho (STY)]FTPLSSSNTIR.2	-0.557651639	P70265_S469_M1_Pfkfb2
RNS[Phospho (STY)]FTPLSSSNTIR.3	0.218656577	P70265_S469_M1_Pfkfb2
TDVSNFDEEFTGEAPTLS[Phospho (STY)]PPR.2	-0.072637912	P70268_S920_M1_Pkn1
RSS[Phospho (STY)]VSGISLEDNR.2	-0.080718252	P70271_S119_M1_Pdlim4
S[Phospho (STY)]SVSGISLEDNR.2	-0.257364496	P70271_S118_M1_Pdlim4
IHIDPESQDC[Carbamidomethyl (C)]S[Phospho (STY)]PATSR.2	-0.553190875	P70271_S111_M1_Pdlim4

IAC[Carbamidomethyl (C)]DEEFS[Phospho (STY)]DS[Phospho (STY)]EDEGEGGRR.2	-0.104292086	P70288_S422_M2_Hdac2
M[Oxidation (M)]LPHAPGVQM[Oxidation (M)]QAIPEDAVHEDS[Phospho (STY)]GDEDGEDPDKR.4	0.097125339	#N/A
LYTLVLTDPDAPSR.2	0.086336949	#N/A
VDYAGVTVDELGK.2	-0.03387625	#N/A
FKVETFR.2	0.009688755	#N/A
VLTPTQVM[Oxidation (M)]NRPSSISWDGLDPGK.3	-0.406306426	#N/A
LYEQLSGK.2	0.120138729	#N/A
[Acetyl (Protein N-term)]AADISQWAGPLC[Carbamidomethyl (C)]LQEVDEPPQHALR.3	-0.048871394	#N/A
GNDISSGTVLSDYVGSGPPSGTGLHR.3	0.034365372	#N/A
YVWLVYEQEQPLSC[Carbamidomethyl (C)]DEPILSNK.3	0.030077147	#N/A
YNLGAPVAGTC[Carbamidomethyl (C)]YQAEWDDYVPK.3	-0.198351652	#N/A
EWHHFLVVNM[Oxidation (M)]K.2	-0.589772998	#N/A
AS[Phospho (STY)]PALVAK.2	-0.043680472	P70297_S156_M1_Stam
NTGASSGATS[Phospho (STY)]EESTEAEFC[Carbamidomethyl (C)]R.2	-0.207589808	P70302_S40_M1_Stim1
DLTHS[Phospho (STY)]DSESSLHMSDR.3	0.254863738	P70302_S519_M1_Stim1
IQNGTSGVR.2	-0.054450786	#N/A
NDQWNWDNIR.2	-0.505263754	#N/A
TLTSDVANLANEKEELNNK.3	-0.5181193	#N/A
QIQQLESNNR.2	-0.066554827	#N/A
ENLLLSDS[Phospho (STY)]PPC[Carbamidomethyl (C)]RENDAIQTR.3	-0.031771222	P70336_S425_M1_Rock2
NLLLLANSTEEQQK.2	0.576080725	#N/A
KS[Phospho (STY)]EESQEIQKK.2	-0.057285935	P70336_S439_M1_Rock2
DSDIEQLR.2	-0.472956837	#N/A
AAC[Carbamidomethyl (C)]S[Phospho (STY)]DPGAFMGR.2	0.209209646	P70339_S252_M1_Frat1
AQVAQPGGDTIFGK.2	-0.142200489	#N/A
IIFEDDR.2	0.028751074	#N/A
M[Oxidation (M)]VVNEGADGGQSVYHIHLHVLGGR.4	-0.449082709	#N/A
C[Carbamidomethyl (C)]LAFHDIS[Phospho (STY)]PQAPTHFLVIPK.3	-0.014163577	P70349_S45_M1_Hint1
C[Carbamidomethyl (C)]AADLGLK.2	-0.591156858	#N/A
FIAFSGEGQS[Phospho (STY)]LR.2	-0.211624203	P70362_S299_M1_Ufd1
LLQEGS[Phospho (STY)]PSDITTLSVEPEKK.3	-0.05949399	P70365_S702_M1_Ncoa1
KGS[Phospho (STY)]PC[Carbamidomethyl (C)]DTLASSTEKR.3	0.319395568	P70365_S22_M1_Ncoa1
EHS[Phospho (STY)]GLSPQDDSNSGMSIPR.3	0.216351574	P70365_S369_M1_Ncoa1
QMSSQNS[Phospho (STY)]PSR.2	0.015428303	P70365_S570_M1_Ncoa1
KGS[Phospho (STY)]PC[Carbamidomethyl (C)]DTLASSTEK.2	-0.355507412	P70365_S22_M1_Ncoa1
VLVDQTTGLSR.2	0.180852631	#N/A
FAANPNQNK.2	0.468511904	#N/A
TNLIVNYLPQNM[Oxidation (M)]TQEELR.2	-0.021731896	#N/A
LGS[Phospho (STY)]LSLKK.2	-0.1566995	P70392_S484_M1_Rasgrf2
KFS[Phospho (STY)]S[Phospho (STY)]PPPLAVSR.2	0.286050906	P70392_S725_M2_Rasgrf2
TLNS[Phospho (STY)]C[Carbamidomethyl (C)]KVPQIR.2	-0.380985204	P70392_S633_M1_Rasgrf2
EHLVDGKS[Phospho (STY)]PR.2	0.210253391	P70392_S717_M1_Rasgrf2
KLS[Phospho (STY)]LTSSLNSR.2	-0.022887367	P70392_S745_M1_Rasgrf2
VPS[Phospho (STY)]AT[Phospho (STY)]S[Phospho (STY)]QPEK.2	-0.100027861	P70396_S113_M3_Dlx5
AQENYEGGEEVS[Phospho (STY)]PPQTK.2	-0.044587547	P70398_S2547_M1_Usp9x
STPFIVPS[Phospho (STY)]SPTEQGGR.2	0.178213187	P70399_S381_M1_Tp53bp1
SEDRPSS[Phospho (STY)]PQVSVAAVETK.3	0.176283399	P70399_S268_M1_Tp53bp1

MESLGS[Phospho (STY)]PR.2	0.142618299	P70399_S533_M1_Tp53bp1
AETTEKDAVTEDS[Phospho (STY)]PQPPLPSVR.3	-0.236696703	P70399_S822_M1_Tp53bp1
DAVTEDS[Phospho (STY)]PQPPLPSVR.2	-0.086148184	P70399_S822_M1_Tp53bp1
GGPGKLS[Phospho (STY)]PR.2	-0.370400049	P70399_S1359_M1_Tp53bp1
ENTQIDDTEPLS[Phospho (STY)]PVSNSK.2	0.072558251	P70399_S552_M1_Tp53bp1
KLPTSEEERS[Phospho (STY)]PAK.3	-0.000403315	P70399_S1675_M1_Tp53bp1
ASQEPFS[Phospho (STY)]PAEDVM[Oxidation (M)]ETDLLEGLAANQDRPSK.3	-0.116942845	P70399_S1115_M1_Tp53bp1
EAPKEEC[Carbamidomethyl (C)]PEAM[Oxidation (M)]EVETSVISIDS[Phospho (STY)]PQK.3	-0.187119406	P70399_S719_M1_Tp53bp1
EHHPEEGFS[Phospho (STY)]GSEVEEVPETPC[Carbamidomethyl (C)]GSHR.4	-0.116012673	P70399_S652_M1_Tp53bp1
S[Phospho (STY)]NISSPVTPTAASSSSTTPTR.2	0.094784374	P70399_S1628_M1_Tp53bp1
GSGEKPASAPVDDTES[Phospho (STY)]LHS[Phospho (STY)]QGEEEFEM[Oxidation (M)]PQPPHGHVLHR.5	0.106880675	P70399_S1213_M2_Tp53bp1
TEEDRENTQIDDTEPLS[Phospho (STY)]PVSNSK.3	0.034960491	P70399_S552_M1_Tp53bp1
SEALS[Phospho (STY)]S[Phospho (STY)]VLDQEEAM[Oxidation (M)]DTK.2	0.456911864	P70399_S631_M2_Tp53bp1
ENTEGEYSSLEHESVAGVVESLK.3	-0.4230179	#N/A
LHSYATSIR.2	-0.006932005	#N/A
LGDGLFLQC[Carbamidomethyl (C)]C[Carbamidomethyl (C)]R.2	0.03507295	#N/A
HKDIDILIVR.3	-0.004354698	#N/A
HAC[Carbamidomethyl (C)]VPVDFEEVHVSSNADEEDIR.4	-0.103989664	#N/A
TSLDLYANVIHC[Carbamidomethyl (C)]K.2	0.116475968	#N/A
DIDILIVR.2	-0.320819752	#N/A
AVLASM[Oxidation (M)]DNENM[Oxidation (M)]HTPDIGGQGTTSQAIQDIIR.3	0.108485433	#N/A
LAEMYGGGES[Phospho (STY)]DKDA.2	0.198571882	P70408_S784_M1_Cdh10
TPSVAKS[Phospho (STY)]PEAK.2	0.019411714	P70429_S347_M1_Evl
SNS[Phospho (STY)]VEKPVSSLLSR.2	-0.494407042	P70429_S329_M1_Evl
QVQNGPS[Phospho (STY)]PEEM[Oxidation (M)]DIQR.2	0.03765223	P70429_S130_M1_Evl
KEDESQTEDPSTS[Phospho (STY)]PSPGTR.2	-0.275880554	P70429_S302_M1_Evl
VQRPEDAS[Phospho (STY)]GGSSPSGTSK.2	-0.393804355	P70429_S240_M1_Evl
VKPAGS[Phospho (STY)]VNDVGLDALDLDR.2	-0.342805312	P70429_S367_M1_Evl
SAS[Phospho (STY)]SDTSEELNSQDSPK.2	-0.051350556	P70441_S285_M1_Slc9a3r1
[Acetyl (Protein N-term)]S[Phospho (STY)]ADAAAGEPLPR.2	-0.373302922	P70441_S2_M1_Slc9a3r1
LLVVDPETDER.2	-0.023729024	#N/A
AVDPDSPAEASGLR.2	0.414751487	#N/A
EALVEPASES[Phospho (STY)]PRPALAR.3	-0.388209932	P70441_S275_M1_Slc9a3r1
SEQAEPPAAADTHEAGDQNEAEK.3	-0.073032519	#N/A
LVEVNGENVEKETHQQVVSR.3	-0.080112514	#N/A
SASSDTSEELNSQDS[Phospho (STY)]PKR.2	-0.33606708	P70441_S297_M1_Slc9a3r1
QGDNIS[Phospho (STY)]DDEDEVR.2	-0.2674493	P70452_S15_M1_Stx4
RGS[Phospho (STY)]HPYIDFR.3	-0.098096256	P70453_S58_M1_Pde7a
KVS[Phospho (STY)]KQEEASGGPLAPK.3	-0.173325829	P70460_S235_M1_Vasp
KAESSPNAEPSDEGNS[Phospho (STY)]REHSAGSC[Carbamidomethyl (C)]TNFQC[Carbamidomethyl (C)]K.4	0.09267876	P70663_S411_M1_Sparcl1
HGAGDDY[Phospho (STY)]FIPS[Phospho (STY)]QEFLEAER.2	0.002525133	P70663_S353_M2_Sparcl1
AAGS[Phospho (STY)]KEHIPHTEQQDQEGK.3	0.184137915	P70663_S283_M1_Sparcl1
KAESSPNAEPSDEGNS[Phospho (STY)]R.2	0.205237975	P70663_S411_M1_Sparcl1
HQSEQGNQGQESDSEAEGEDKAAGSKEHIPHT[Phospho (STY)]EQQDQEGK.5	-0.319966579	P70663_T290_M1_Sparcl1
AEKPSALNS[Phospho (STY)]EEETHEQSTEQDK.3	-0.023672999	P70663_S70_M1_Sparcl1
SPASDTYIVFGEAK.2	0.064954407	#N/A
NNSNDIVNAIM[Oxidation (M)]ELTM[Oxidation (M)].2	0.3037374	#N/A

DIELVMSQANVSR.2	0.002615977	#N/A
SSLPHS[Phospho (STY)]QENLPK.2	0.178318926	P70671_S135_M1_Irf3
S[Phospho (STY)]VDSGIYLDSSYK.2	0.125739741	P70677_S26_M1_Casp3
SGSSS[Phospho (STY)]PDSEITELKFPSISQD.2	0.377146154	P70698_S575_M1_Ctps1
SGSSS[Phospho (STY)]PDSEITELK.2	0.24650123	P70698_S575_M1_Ctps1
GC[Carbamidomethyl (C)]RLS[Phospho (STY)]PR.2	-0.384989684	P70698_S562_M1_Ctps1
RTVS[Phospho (STY)]EIR.2	-0.135003794	P70704_S9_M1_Atp8a1
IIYQAASPDEGALVR.2	0.054311991	#N/A
LDGHGTVPLGADQILLR.3	-0.020889504	#N/A
T[Phospho (STY)]SLADQEEVR.2	-0.39013357	P70704_T28_M1_Atp8a1
TDDVS[Phospho (STY)]EKT[Phospho (STY)]S[Phospho (STY)]LADQEEVR.2	0.011745181	P70704_S25_M3_Atp8a1
SESLQQNLLHGYAFSQDENGIVSQSEVIR.3	-0.275603224	#N/A
TC[Carbamidomethyl (C)]TIILR.2	-0.519035561	#N/A
LLDVVHPAAK.2	0.108854984	#N/A
ATISNDGATILK.2	0.509564865	#N/A
LPIGDVATQYFADR.2	0.376658723	#N/A
SLHDAIM[Oxidation (M)]IVR.2	-0.212889612	#N/A
SQDAEVGDGTTSVTLLAAEFLK.2	-0.425912613	#N/A
QLC[Carbamidomethyl (C)]DNAGFDATNILNK.2	-0.138338428	#N/A
TATQLAVNK.2	-0.289862472	#N/A
ALEIIPR.2	-0.375386137	#N/A
INALTAASEAAC[Carbamidomethyl (C)]LIVSVDETIKNPR.3	-0.202934296	#N/A
INALTAASEAAC[Carbamidomethyl (C)]LIVSVDETIK.3	0.334199063	#N/A
GATQQILDEAER.2	-0.055081783	#N/A
[Acetyl (Protein N-term)]ASLSLAPVNIFK.2	0.389506743	#N/A
LSSFIGAIAIGDLVK.2	-0.02514051	#N/A
EALLSSAVDHGSDEAR.2	-0.121235278	#N/A
VQDDEVGDGTTSVTVLAAELLR.2	-0.63480208	#N/A
LAVEAVLR.2	-0.028899124	#N/A
FWQDLM[Oxidation (M)]NIAGTTLSSK.2	-0.081837992	#N/A
NIGVDNPAAK.2	0.029847632	#N/A
M[Oxidation (M)]LPTIIADNAGYDSADLVAQLR.2	-0.342825872	#N/A
LALVTGGEIASTFDHPELVK.3	0.046080217	#N/A
VAEIEHAEKEK.3	-0.16483194	#N/A
IDDVVNTR.2	-0.009131224	#N/A
VIDPATATSVDLR.2	0.009946156	#N/A
LVIEEAER.2	-0.221965389	#N/A
DALSDLALHFLNK.2	0.263797405	#N/A
ALIAGGGAPEIELALR.2	0.546480432	#N/A
AQDIEAGDGTTSVVIIAGSLLDSC[Carbamidomethyl (C)]TK.3	0.037487149	#N/A
ITGC[Carbamidomethyl (C)]TSPGK.2	-0.07303048	#N/A
VANSGITR.2	0.326717613	#N/A
AFADAM[Oxidation (M)]EVIPSTLAENAGLNPISTVTELR.3	0.048421749	#N/A
DVEREDIEFIC[Carbamidomethyl (C)]K.3	-0.447657769	#N/A
VLVDINNPEPLIQTAK.2	0.063795749	#N/A
LGFAGVVQEISFGTTK.2	-0.449875717	#N/A
[Acetyl (Protein N-term)]ASVGTLAFDEYGRPFLIIK.2	-0.144747882	#N/A

TSLGPNGLDK.2	0.135933512	#N/A
WVGGPEIELIAIATGGR.2	-0.137683884	#N/A
ESNPALGIDC[Carbamidomethyl (C)]LHK.2	0.286875335	#N/A
DVDFELIK.2	0.235284712	#N/A
DVDFELIKVEGK.3	-0.122754038	#N/A
GLVLDHGAR.2	0.368423342	#N/A
GIDPFSLDALAK.2	0.322670505	#N/A
IITEGFEAAK.2	0.198923007	#N/A
VLAQNSGFDLQETLVK.2	0.045201948	#N/A
GLQDVLR.2	0.245325528	#N/A
VATAQDDITGDGTTSNVLIIGELLK.3	-0.399126071	#N/A
EMDRETLIDVAR.3	0.006285165	#N/A
QADLYISEGLHPR.2	-0.152889756	#N/A
ISTPVDVNNR.2	-0.38891657	#N/A
AVAQALEVIPR.2	0.130953601	#N/A
IPGGIIEDSC[Carbamidomethyl (C)]VLR.2	0.44072292	#N/A
ELGIWEPLAVK.2	-0.030948372	#N/A
EIQVQHPAAK.2	-0.291464169	#N/A
EILSEVER.2	0.117648362	#N/A
TAVETAVLLLR.2	0.276758681	#N/A
IVLLDSSLEYK.2	0.0614237	#N/A
NVLLDPQLVPGGGASEM[Oxidation (M)]AVAHALTEK.3	-0.51320197	#N/A
TLIQNC[Carbamidomethyl (C)]GASTIR.2	0.274218867	#N/A
WSSLAC[Carbamidomethyl (C)]NIALDAVK.2	0.027377025	#N/A
NLQDAM[Oxidation (M)]QVC[Carbamidomethyl (C)]R.2	-0.011644677	#N/A
QTGAPDAGQE.1	0.24666262	#N/A
KGESQTDIEITREEDFTR.4	-0.105887183	#N/A
ADSVAGAIQSDPAEGS[Phospho (STY)]QESHGR.3	-0.147938994	P80560_S339_M1_Ptprn2
SEQPEEVLSS[Phospho (STY)]EEETAGVEHVR.3	0.055675869	P80560_S423_M1_Ptprn2
INSVSSQFS[Phospho (STY)]DGPM[Oxidation (M)]PS[Phospho (STY)]PSAR.2	0.085588966	P80560_S684_M2_Ptprn2
SLQQNADNEKWFS[Phospho (STY)]LER.3	-0.128191971	P80560_S139_M1_Ptprn2
KSEQPEEVLS[Phospho (STY)]S[Phospho (STY)]EEETAGVEHVR.3	-0.053229961	P80560_S422_M2_Ptprn2
SGRLS[Phospho (STY)]QELDLVSHK.3	0.081102235	P81117_S89_M1_Nucb2
VAS[Phospho (STY)]PTSGLKR.2	0.443624287	P81122_S1089_M1_Irs2
RVS[Phospho (STY)]GDGAQDLDR.2	-0.449371314	P81122_S556_M1_Irs2
HNSAS[Phospho (STY)]VENVSLRK.3	-0.10132638	P81122_S1165_M1_Irs2
TYS[Phospho (STY)]LTTPAR.2	-0.115385024	P81122_S573_M1_Irs2
SNT[Phospho (STY)]PESIAETPPAR.2	-0.020888107	P81122_T517_M1_Irs2
ASSPAESS[Phospho (STY)]PEDSGYMR.2	-0.4135107	P81122_S728_M1_Irs2
QVPQPSSAS[Phospho (STY)]LDEYTLMR.2	0.227158135	P81122_S590_M1_Irs2
GPGTGGDEASAAGGS[Phospho (STY)]PPQPPR.2	-0.509562753	P81122_S66_M1_Irs2
SIVPSS[Phospho (STY)]PQSQR.2	0.112234431	P83093_S523_M1_Stim2
GS[Phospho (STY)]PEC[Carbamidomethyl (C)]VGLTETK.2	0.167300898	P83093_S650_M1_Stim2
GESPVTADVSRGS[Phospho (STY)]PEC[Carbamidomethyl (C)]VGLTETK.3	0.097458973	P83093_S650_M1_Stim2
DELS[Phospho (STY)]LEDSSR.2	-0.144800735	P83093_S631_M1_Stim2
DELSLEDSSRGES[Phospho (STY)]PVTADVSR.3	-0.182803435	P83093_S640_M1_Stim2
RAS[Phospho (STY)]GSAGAAASPSAAAAGER.2	0.002397238	P83093_S28_M1_Stim2

S[Phospho (STY)]MIFSPASR.2	-0.002768518	P83093_S661_M1_Stim2
QNS[Phospho (STY)]DPTSENPPLPTR.2	-0.36142169	P83510_S611_M1_Tnik
TTSIS[Phospho (STY)]PALAR.2	-0.447212568	P83510_S651_M1_Tnik
ANS[Phospho (STY)]KSEGS[Phospho (STY)]PVLPHEPSK.2	0.10250046	#N/A
VRANS[Phospho (STY)]KSEGS[Phospho (STY)]PVLPHEPSK.3	-0.005397481	#N/A
SES[Phospho (STY)]FSISGVQPAR.2	0.206347441	P83510_S541_M1_Tnik
KNS[Phospho (STY)]PGNGSALGPR.2	-0.358217538	P83510_S659_M1_Tnik
SEGS[Phospho (STY)]PVLPHEPSK.2	-0.269314173	P83510_S740_M1_Tnik
VYQTSPT[Phospho (STY)]DEDEEDDESSAAALFTSELLR.3	-0.084922934	P83510_T950_M1_Tnik
AS[Phospho (STY)]NPDLR.2	0.215510516	P83510_S678_M1_Tnik
LGSQPIRAS[Phospho (STY)]NPDLR.3	0.24176428	P83510_S678_M1_Tnik
GTEDGSGSPHS[Phospho (STY)]PPHLC[Carbamidomethyl (C)]SK.3	-0.07512731	P83741_S2027_M1_Wnk1
LEC[Carbamidomethyl (C)]VEPNC[Carbamidomethyl (C)]R.2	0.011260238	#N/A
KKVEEVLEEEEEEYVVEK.3	-0.253445829	#N/A
VEEVLEEEEEEYVVEK.2	-0.575824163	#N/A
ADS[Phospho (STY)]DSEDKGEESKPK.2	0.237653719	P83917_S89_M1_Cbx1
EIPSHVLSK.2	0.041068954	#N/A
LISSDGHEFIVK.2	0.087799791	#N/A
EHALTSGTIK.2	-0.164040359	#N/A
GAKS[Phospho (STY)]DPSIVR.2	-0.271000545	P84075_S175_M1_Hpca
LSLEEFIR.2	0.436539861	#N/A
FAEHVFR.2	0.215811457	#N/A
IYANFFPYGDASK.2	0.180816876	#N/A
ENTEFSELELQEWYK.2	-0.084818894	#N/A
LLQC[Carbamidomethyl (C)]DPSSASQF.2	0.173885435	#N/A
TFDTNSDGTIDFR.2	-0.071796887	#N/A
EEM[Oxidation (M)]LEIVQAIYK.2	-0.064460039	#N/A
LRPEMLQDLR.2	0.237916804	#N/A
DC[Carbamidomethyl (C)]PTGILNVDEFKK.3	-0.130203409	#N/A
MPEDESTPEKR.2	0.154965752	#N/A
VQESADELQK.2	-0.004020317	#N/A
AALEQPC[Carbamidomethyl (C)]EGS[Phospho (STY)]LTRPK.2	-0.009002091	P84086_S93_M1_Cplx2
KAIPAGC[Carbamidomethyl (C)]GDEEEEEEESILDTVLK.3	-0.066865846	#N/A
AIPAGC[Carbamidomethyl (C)]GDEEEEEEESILDTVLK.3	-0.16148307	#N/A
YLPGPLQDMFK.2	0.427031951	#N/A
[Acetyl (Protein N-term)]SHTILLVQPTK.2	0.308503995	#N/A
TYADYESVNEC[Carbamidomethyl (C)]M[Oxidation (M)]EGVC[Carbamidomethyl (C)]K.2	-0.414881293	#N/A
MYEEHLK.2	-0.267345576	#N/A
ADTQTYQPYNK.2	0.213798222	#N/A
TFITQQGIK.2	0.143636242	#N/A
ESQISAEIELLPTNDK.2	-0.001375257	#N/A
VNVIHAR.2	0.113819612	#N/A
DIILPFR.2	0.021645591	#N/A
VYRDDIGR.2	-0.221472789	#N/A
TSFFHVK.2	-0.23147514	#N/A
M[Oxidation (M)]IGGLFIYNHK.2	-0.265117729	#N/A
ASENAIVWK.2	-0.039175228	#N/A

SNIWLAAVTK.2	0.099124299	#N/A
S[Phospho (STY)]PVTNIAR.2	0.139882333	P84091_S45_M1_Ap2m1
SGIYETR.2	-0.135505262	#N/A
QSIAIDDC[Carbamidomethyl (C)]TFHQC[Carbamidomethyl (C)]VR.3	0.043473044	#N/A
LNYSDHDVIK.2	-0.188451428	#N/A
LS[Phospho (STY)]KFDSER.2	0.292092068	P84091_S255_M1_Ap2m1
EEQSQITSQVTGQIGWR.2	-0.006868468	#N/A
EYIPTVFDNYSAQSAVDGR.2	0.08982114	#N/A
YLEC[Carbamidomethyl (C)]SALQQDGVK.2	0.062357729	#N/A
VWLDPNETNEIANANSR.2	-0.480056173	#N/A
LLADQAEAR.2	0.160783709	#N/A
TLS[Phospho (STY)]KEEETKK.2	-0.094574607	P84099_S189_M1_Rpl19
S[Phospho (STY)]RS[Phospho (STY)]LS[Phospho (STY)]RDR.2	-0.45983858	P84104_S126_M3_Srsf3
RRS[Phospho (STY)]PPPR.2	-0.189135028	P84104_S108_M1_Srsf3
NPPGFAFVEFEDPRDAADAVR.3	-0.113335299	#N/A
NPPGFAFVEFEDPR.2	0.13277614	#N/A
ERS[Phospho (STY)]LS[Phospho (STY)]RER.2	-0.041344133	P84104_S138_M2_Srsf3
VYVGNLGNNGNK.2	-0.034355018	#N/A
NHKPS[Phospho (STY)]RS[Phospho (STY)]FSR.3	-0.161443452	P84104_S148_M2_Srsf3
[Acetyl (Protein N-term)]MHRDS[Phospho (STY)]C[Carbamidomethyl (C)]PLDC[Carbamidomethyl (C)]K.2	-0.094999133	P84104_S5_M1_Srsf3
SVS[Phospho (STY)]PPGYAAQTAASPAPR.2	-0.375094423	P84309_S8_M1_Adcy5
SGGAVT[Phospho (STY)]PQQQQR.2	-0.059298849	P84309_T62_M1_Adcy5
S[Phospho (STY)]VELGLEER.2	0.175791195	P84309_S156_M1_Adcy5
DVAAHFLAR.2	-0.251979233	#N/A
ILGDC[Carbamidomethyl (C)]YYC[Carbamidomethyl (C)]VSGLPEAR.2	0.041473289	#N/A
DASHPQFK.2	-0.062754208	#N/A
QSGAFLATSESLILQLVR.2	-0.421977046	#N/A
LIPDGC[Carbamidomethyl (C)]GVK.2	0.242691211	#N/A
LGELYSVGES[Phospho (STY)]DKET.2	-0.090043616	P97291_S795_M1_Cdh8
LNNS[Phospho (STY)]LLSR.2	-0.521111753	P97292_S321_M1_Hrh2
QNPSITWIR.2	-0.237917246	#N/A
IVTSEEVIIR.2	0.202366855	#N/A
AAPDITGHK.2	0.166544931	#N/A
SVGYPHPEWIWR.2	0.103284193	#N/A
VTVNTAYGSNGVSVLR.2	-0.138159284	#N/A
QLWDGAR.2	0.039963284	#N/A
LTLEDSGTYEC[Carbamidomethyl (C)]R.2	0.171913452	#N/A
NGVELTATR.2	0.141853195	#N/A
AEDSGEYHC[Carbamidomethyl (C)]VYHFVSAPK.3	-0.408160226	#N/A
SENKNEGQDAMMYC[Carbamidomethyl (C)]K.3	-0.146715	#N/A
C[Carbamidomethyl (C)]SQAVYAAEK.2	0.279725589	#N/A
GFGFGQGAGALVHSE.2	-0.103567206	#N/A
GYGYGQGAGTLSTDKGESLGIK.3	-0.219786428	#N/A
TVYFAEEVQC[Carbamidomethyl (C)]EGNSFHK.2	-0.184250812	#N/A
HEEAPGHRPTTNPNASK.4	0.090779187	#N/A
LADMYGGM[Oxidation (M)]DS[Phospho (STY)]DKDS.2	0.319043955	P97326_S786_M1_Cdh6
LITEDVQGK.2	-0.117090418	#N/A

TTDGYLLR.2	-0.104862685	#N/A
ADGYEPPVQES[Phospho (STY)]V.2	-0.174221179	P97351_S263_M1_Rps3a
VFEVSLADLQNDEVAFR.2	-0.216223673	#N/A
KDWYDVK.2	0.031769669	#N/A
EVQTNDLKEVVNK.3	0.240512101	#N/A
LIPDSIGKDIEK.3	-0.067824427	#N/A
KGS[Phospho (STY)]LNINEFK.2	0.072471769	P97352_S32_M1_S100a13
SDS[Phospho (STY)]NQYIR.2	-0.054193688	P97366_S689_M1_Evi5
LFIYNPSSGEFLGR.2	-0.02377269	#N/A
FDSGLNKK.2	-0.320390958	#N/A
SAT[Phospho (STY)]PPPAEPASLPQEPPK.2	-0.29935842	P97379_T227_M1_G3bp2
LPNFGFVVFDDSEPVQR.2	0.198271908	#N/A
VDAKPEVQSQPPR.3	-0.472432322	#N/A
HLEELEEKS[Phospho (STY)]AT[Phospho (STY)]PPPAEPASLPQEPPK.3	0.099836446	P97379_S225_M2_G3bp2
GDMEQNDSDNRR.2	-0.16737473	#N/A
DVQELYAAGENR.2	-0.192854128	#N/A
SDTSGHFQR.2	-0.317204198	#N/A
GSEEDPLLS[Phospho (STY)]PVETWK.2	0.073805551	P97393_S1201_M1_Arhgap5
GGIDNPAITS[Phospho (STY)]DQEVDDKK.2	-0.139080787	P97393_S1217_M1_Arhgap5
HRGSEEDPLLS[Phospho (STY)]PVETWK.3	-0.014138259	P97393_S1201_M1_Arhgap5
QPEPFS[Phospho (STY)]PR.2	-0.177891879	P97412_S375_M1_Lyst
RM[Oxidation (M)]S[Phospho (STY)]QEHPSQASEAELAQR.3	-0.508307717	P97412_S2614_M1_Lyst
STANHDSES[Phospho (STY)]PVHS[Phospho (STY)]PSAHR.3	-0.32692124	P97412_S2560_M2_Lyst
MFLAYQDQS[Phospho (STY)]FDIPDR.2	0.366505082	P97412_S3251_M1_Lyst
KS[Phospho (STY)]IPHITSDR.2	-0.055671348	P97427_S8_M1_Crmp1
DNFTLIPEGVNGIEER.2	-0.057760753	#N/A
VFGLHSVSR.2	-0.181518845	#N/A
SAADIIALAR.2	-0.105555808	#N/A
IFNLYPR.2	-0.208162118	#N/A
INC[Carbamidomethyl (C)]PVYITK.2	0.024591099	#N/A
IVFEDGNISVSK.2	-0.046549793	#N/A
KPFPEHLYQR.3	0.205435579	#N/A
AALAGGTTM[Oxidation (M)]IIDHVVPEPGSSLLTSFEK.3	-0.080337162	#N/A
WHEAADTK.2	0.063809432	#N/A
DLYQM[Oxidation (M)]SDSQLYEAFTFLK.2	-0.384154482	#N/A
IINDDQSFYADVYLEDGLIK.2	-0.240562912	#N/A
STVEYNIFEGM[Oxidation (M)]EC[Carbamidomethyl (C)]HGSPLVVISQGK.3	-0.344151056	#N/A
HAAPAPS[Phospho (STY)]AK.2	-0.059972197	P97427_S518_M1_Crmp1
S[Phospho (STY)]IPHITSDR.2	-0.186748701	P97427_S8_M1_Crmp1
GM[Oxidation (M)]YDGPVYEVPAT[Phospho (STY)]PK.2	-0.171690419	P97427_T509_M1_Crmp1
HAAPAPS[Phospho (STY)]AKSS[Phospho (STY)]PSK.2	0.023408336	#N/A
HAAPAPSAKS[Phospho (STY)]S[Phospho (STY)]PSKHQPPPIR.4	-0.086439484	#N/A
M[Oxidation (M)]TVVWDK.2	0.250645555	#N/A
IVAPPGGRS[Phospho (STY)]NITS[Phospho (STY)]LG.2	-0.266394067	P97427_S566_M2_Crmp1
SS[Phospho (STY)]PSKHQPPPIR.2	0.041370082	P97427_S522_M1_Crmp1
EELEVLVQDK.2	0.291762966	#N/A
GLGAVILVHAENGDLIAQEQK.3	-0.008322547	#N/A

NLHQSNFSLS[Phospho (STY)]GAQIDDNNPR.3	-0.411166658	P97427_S542_M1_Crmp1
NLHQSNFSLSGAQIDDNNPRR.4	-0.02913067	#N/A
HQPPPIR.2	-0.019137562	#N/A
IAVGSDADVVIWDPDKM[Oxidation (M)]K.2	-0.135805379	#N/A
ILEM[Oxidation (M)]GITGPEGHALSRPEELEAEAVFR.4	-0.017284037	#N/A
[Acetyl (Protein N-term)]S[Phospho (STY)]HQGKKS[Phospho (STY)]IPHITSDR.3	-0.089981482	P97427_S2_M2_Crmp1
SKS[Phospho (STY)]NPDFLK.2	0.283930736	P97434_S992_M1_Mprip
SKS[Phospho (STY)]NPDFLKK.2	-0.159277972	P97434_S992_M1_Mprip
AEEQLPPLLS[Phospho (STY)]PPS[Phospho (STY)]PSTPHSR.3	0.322825899	P97434_S289_M2_Mprip
DFASEAPTAPLSDAC[Carbamidomethyl (C)]PLS[Phospho (STY)]PHRR.3	0.139428504	P97434_S364_M1_Mprip
AATEALGEKS[Phospho (STY)]PEGTTVSGYDIM[Oxidation (M)]K.3	-0.057313758	P97434_S976_M1_Mprip
DFASEAPTAPLS[Phospho (STY)]DAC[Carbamidomethyl (C)]PLSPHR.3	-0.025635793	P97434_S358_M1_Mprip
RLS[Phospho (STY)]VEIYDK.2	0.123672478	P97438_S348_M1_Kcnk2
DPVPQSGLS[Phospho (STY)]PER.2	-0.261547859	P97441_S66_M1_Slc30a3
DRSS[Phospho (STY)]AGGGLR.2	-0.112481549	P97441_S23_M1_Slc30a3
SLFTEPS[Phospho (STY)]EPLPEEPK.2	0.242945326	P97441_S38_M1_Slc30a3
ESPS[Phospho (STY)]WVTQR.2	-0.24061512	P97445_S1981_M1_Cacna1a
RAWPGS[Phospho (STY)]PER.2	-0.228475245	P97445_S867_M1_Cacna1a
WSRS[Phospho (STY)]PSEGR.2	-0.065919724	P97445_S2200_M1_Cacna1a
M[Oxidation (M)]AGPAAPPGGS[Phospho (STY)]PR.2	-0.110117377	P97445_S2273_M1_Cacna1a
KQNLLAS[Phospho (STY)]R.2	-0.113781812	P97445_S792_M1_Cacna1a
PLVSYS[Phospho (STY)]PAPR.2	-0.07556307	P97445_S2252_M1_Cacna1a
AWPGS[Phospho (STY)]PER.2	0.179069036	P97445_S867_M1_Cacna1a
KQNLLAS[Phospho (STY)]REALYGDAAER.3	-0.261690841	P97445_S792_M1_Cacna1a
GADYTEPDS[Phospho (STY)]PREPPGGAHDPAPR.3	-0.028297484	P97445_S2303_M1_Cacna1a
GADYTEPDSPREPPGGAHDPAPRS[Phospho (STY)]PR.4	0.110731336	P97445_S2318_M1_Cacna1a
EEQNRT[Phospho (STY)]PLM[Oxidation (M)]FQR.3	-0.382853686	P97445_T1935_M1_Cacna1a
GNDLSTISDTS[Phospho (STY)]PM[Oxidation (M)]KR.2	-0.257957746	P97445_S2071_M1_Cacna1a
QGSSSVSGS[Phospho (STY)]PAPSTSGTSTPR.2	-0.365848051	P97445_S2220_M1_Cacna1a
AS[Phospho (STY)]IKS[Phospho (STY)]AKLENSTFFHK.3	-0.046074147	P97445_S459_M2_Cacna1a
EVAEVS[Phospho (STY)]PLS[Phospho (STY)]AANM[Oxidation (M)]SIAVK.2	0.21353739	P97445_S755_M2_Cacna1a
RMAGPAAPPGGS[Phospho (STY)]PR.2	0.056648449	P97445_S2273_M1_Cacna1a
AKEVAEVS[Phospho (STY)]PLS[Phospho (STY)]AANM[Oxidation (M)]SIAVK.3	-0.195254126	P97445_S755_M2_Cacna1a
WSRS[Phospho (STY)]PS[Phospho (STY)]EGREHTTHR.4	-0.149877164	P97445_S2200_M2_Cacna1a
EM[Oxidation (M)]GTDGYSDSEHY[Phospho (STY)]LPMEGQTR.3	-0.418323201	P97445_Y2033_M1_Cacna1a
FEVIDKPQS.2	0.118314096	#N/A
QASGGPVDIGPEYQQDLDRELYK.3	-0.046743563	#N/A
QASGGPVDIGPEYQQDLDR.2	0.040108267	#N/A
GEM[Oxidation (M)]DTFPTFK.2	0.001550335	#N/A
FDDPKFEVIDKPQS.2	-0.143792257	#N/A
VNQAIWLLC[Carbamidomethyl (C)]TGAR.2	-0.112229	#N/A
GSSNSYAIK.2	0.205574102	#N/A
WSTDDVQINDISLQDYIAVK.2	0.175910948	#N/A
PLREPGSSHLGS[Phospho (STY)]PDTAR.3	-0.234876262	P97492_S203_M1_Rgs14
EPGSSHLGS[Phospho (STY)]PDTAR.2	-0.335264146	P97492_S203_M1_Rgs14
S[Phospho (STY)]LGSGESESESRPGK.2	-0.250532805	P97492_S289_M1_Rgs14
S[Phospho (STY)]LPLGVEELGQLPLAEGPC[Carbamidomethyl (C)]GR.2	-0.351276101	P97492_S218_M1_Rgs14

SPLYQEC[Carbamidomethyl (C)]LLAEAEGRPLREPGSSHLGS[Phospho (STY)]PDTAR.4	0.041219506	P97492_S203_M1_Rgs14
GLS[Phospho (STY)]LPDIK.2	-0.352680437	P97492_S339_M1_Rgs14
S[Phospho (STY)]LGSGESESESR.2	-0.358657795	P97492_S289_M1_Rgs14
GLLSQGS[Phospho (STY)]PLSWEETQR.2	-0.443175944	P97494_S8_M1_Gclc
RKPS[Phospho (STY)]PS[Phospho (STY)]PPPPTATESR.3	-0.037545075	P97496_S327_M2_Smarcc1
NEEPVRS[Phospho (STY)]PERR.2	-0.066522573	P97496_S309_M1_Smarcc1
LEEQLS[Phospho (STY)]PR.2	-0.404788949	P97500_S391_M1_Myt1l
KLS[Phospho (STY)]ETNPQDR.2	0.273322974	P97500_S343_M1_Myt1l
M[Oxidation (M)]VEES[Phospho (STY)]DEEVC[Carbamidomethyl (C)]LSSLEC[Carbamidomethyl (C)]LR.2	-0.352714884	P97500_S319_M1_Myt1l
SAFTTS[Phospho (STY)]DVVR.2	-0.2319276	P97772_S853_M1_Grm1
APIASIHS[Phospho (STY)]VDLDLL.2	-0.286327634	P97783_S84_M1_Mllt11
APQS[Phospho (STY)]PTLAPAK.2	-0.181810116	P97792_S332_M1_Cxadr
TQYNQVPSEDFERAPQS[Phospho (STY)]PTLAPAK.3	-0.160742169	P97792_S332_M1_Cxadr
HDQQPAITR.2	0.052396195	#N/A
LLIYSFAGEYVPR.2	-0.163533081	#N/A
PSPPEVSGPADR.2	0.271067474	#N/A
NNM[Oxidation (M)]DFSIR.2	-0.404829198	#N/A
GSTSS[Phospho (STY)]TRLHEPEK.3	0.06475906	P97797_S409_M1_Sirpa
DGQELHPLETTVNPSGK.3	-0.332684715	#N/A
VIC[Carbamidomethyl (C)]EVAHITLDR.2	-0.350841785	#N/A
VPRPEDTLTYADLDMVHLSR.4	-0.408842164	#N/A
NS[Phospho (STY)]QDITPVDNSM[Oxidation (M)]DSNIHQR.3	-0.326436117	P97798_S1225_M1_Neo1
GTGQSDDS[Phospho (STY)]DIWDDTALIK.2	0.116233873	P97801_S28_M1_Smn1
[Acetyl (Protein N-term)]AM[Oxidation (M)]GSGGAGS[Phospho (STY)]EQEDTVLFR.2	-0.393637821	#N/A
[Acetyl (Protein N-term)]M[Oxidation (M)]AM[Oxidation (M)]GSGGAGSEQEDTVLFR.2	-0.42200006	#N/A
RGT[Phospho (STY)]GQSDDSDIWDDTALIK.3	0.150429512	P97801_T22_M1_Smn1
SGLGELILPENEPGSSIM[Oxidation (M)]PGK.2	-0.354514267	#N/A
IYELAAGGTAVGTGLNTR.2	-0.031592176	#N/A
LHDALSAK.2	-0.116077711	#N/A
AAAEVNQEYGLDPK.2	0.296424158	#N/A
YYGAQTVR.2	-0.328322785	#N/A
VEFDTFGELK.2	0.043701531	#N/A
AIEM[Oxidation (M)]LGGELGSK.2	0.418205012	#N/A
MPIPVIQAFGILK.2	0.190249861	#N/A
ETAIELGYLTAEQFDEWVKPK.3	-0.059555671	#N/A
LNDHFPLVVWQTGSGTQTNM[Oxidation (M)]NVNEVISNR.3	0.146042496	#N/A
SQSSNDTFPTAM[Oxidation (M)]HIAAAVEVHK.3	0.164748201	#N/A
THTQDAVPLTLGQEFSGYVQQVQYAMVR.3	0.169625604	#N/A
AASS[Phospho (STY)]PNLSNGETESVK.2	-0.057034048	P97820_S795_M1_Map4k4
TTSRS[Phospho (STY)]PVLSR.2	0.269367653	P97820_S621_M1_Map4k4
RDS[Phospho (STY)]PLQGGGQQNSQAGQR.3	-0.10839904	P97820_S629_M1_Map4k4
SSS[Phospho (STY)]KSEGSPSPR.2	-0.074016777	P97820_S701_M1_Map4k4
DSPLQGGGQQNSQAGQR.2	-0.222206582	#N/A
S[Phospho (STY)]EGSPSPR.2	0.138121896	P97820_S703_M1_Map4k4
SSGGREDSES[Phospho (STY)]PGTQR.2	-0.101822556	P97825_S79_M1_Jpt1
SNS[Phospho (STY)]SEASSGDFLDLK.2	0.213009549	P97825_S87_M1_Jpt1
STS[Phospho (STY)]PAPADVAPAQEDLR.2	-0.3256655	P97855_S231_M1_G3bp1

YQDEVFGGFVTEPQEES[Phospho (STY)]EEEVEEPEER.3	-0.130053162	P97855_S149_M1_G3bp1
KPEKPLFSSTS[Phospho (STY)]PQDSSPR.3	0.284395876	P97863_S328_M1_Nfib
VEGTEIVKPS[Phospho (STY)]PK.2	-0.18831832	P97868_S1179_M1_Rbbp6
SRS[Phospho (STY)]PQAFR.2	0.121837139	P97868_S773_M1_Rbbp6
YHS[Phospho (STY)]RS[Phospho (STY)]RS[Phospho (STY)]PQAFR.3	0.116845537	P97868_S769_M3_Rbbp6
WDKDDFES[Phospho (STY)]EEEDVK.2	0.02834459	P97868_S1329_M1_Rbbp6
PSANREDFS[Phospho (STY)]PER.2	-0.3739059	P97868_S862_M1_Rbbp6
VDGDREKS[Phospho (STY)]PR.2	-0.602908248	P97868_S1053_M1_Rbbp6
S[Phospho (STY)]MVEHYYR.2	0.395293863	P97950_S101_M1_Rab33a
SAS[Phospho (STY)]QDC[Carbamidomethyl (C)]IETTPGAQEGK.2	-0.203321219	P98084_S481_M1_Apba2
SAS[Phospho (STY)]QDC[Carbamidomethyl (C)]IETTPGAQEGKK.2	-0.216310692	P98084_S481_M1_Apba2
DSLSFSNTNNK.2	-0.076320489	#N/A
GDS[Phospho (STY)]DVPEVPPSSDR.2	0.057825849	P98191_S56_M1_Cds1
ATS[Phospho (STY)]VGDQLEAPAR.2	-0.275983741	P98200_S6_M1_Atp8a2
EQS[Phospho (STY)]SDDFC[Carbamidomethyl (C)]R.2	-0.367845236	P98200_S421_M1_Atp8a2
[Acetyl (Protein N-term)]S[Phospho (STY)]RATS[Phospho (STY)]VGDQLEAPAR.2	0.183736886	P98200_S2_M2_Atp8a2
M[Oxidation (M)]TSC[Carbamidomethyl (C)]TNDS[Phospho (STY)]C[Carbamidomethyl (C)]DFNDPR.2	-0.125053248	P98200_S435_M1_Atp8a2
DIPSYGS[Phospho (STY)]LSR.2	0.060244118	P98203_S205_M1_Arvcf
GSLGS[Phospho (STY)]LDR.2	0.296300308	P98203_S337_M1_Arvcf
S[Phospho (STY)]LAADDEGGPDLEPDYSTATR.2	-0.012775836	P98203_S269_M1_Arvcf
RSPS[Phospho (STY)]VDSTR.2	-0.478574194	P98203_S347_M1_Arvcf
S[Phospho (STY)]LPEHFQAEPYGLEDDTR.3	0.013899276	P98203_S251_M1_Arvcf
S[Phospho (STY)]PSVDSTR.2	-0.220641202	P98203_S345_M1_Arvcf
NFDT[Phospho (STY)]LDLPK.2	0.043238931	P98203_T643_M1_Arvcf
ISVYYNEATGGK.2	-0.213788243	#N/A
M[Oxidation (M)]AVTFIGNSTAIQELFKR.3	-0.033662719	#N/A
MAVTFIGNSTAIQELFK.2	0.155049321	#N/A
ALTVPELTQQVFDAK.2	0.009558861	#N/A
FWEVISDEHGIDPTGTYHGDSDLQLDR.4	0.245549738	#N/A
QPVLSQTEAR.2	0.030632237	#N/A
FQIATVTEK.2	-0.305126929	#N/A
AIHSWLTR.2	-0.377531621	#N/A
TQNPM[Oxidation (M)]VTGTSVLGVK.2	0.138859529	#N/A
FDGGVVIAADM[Oxidation (M)]LGSYGSLAR.2	-0.541749603	#N/A
VNDSTM[Oxidation (M)]LGASGDYADFQYLK.2	-0.055505334	#N/A
LASVPAGGAVAVSAAPGSAAPAAGSAPAAAEEK.3	0.349276021	#N/A
NIEDVIAQGVGK.2	-0.418103162	#N/A
YVASYLLAALGGNSSPSAK.2	-0.253919774	#N/A
ILDSVGIEADDDRLNK.2	-0.130825727	#N/A
KEESEES[Phospho (STY)]DDDM[Oxidation (M)]GFGLFD.2	0.283419643	P99027_S105_M1_Rplp2
LASVPAGGAVAVSAAPGSAAPAAGSAPAAAEEKKDEK.4	-0.093004351	#N/A
LELC[Carbamidomethyl (C)]DNR.2	0.128635744	#N/A
EHC[Carbamidomethyl (C)]EQLEK.2	0.145337048	#N/A
ERLELC[Carbamidomethyl (C)]DNR.2	-0.112100729	#N/A
SQTEEDC[Carbamidomethyl (C)]TEELFDFLHAR.3	0.045730907	#N/A
LLADPTGAFGK.2	0.227392516	#N/A
THLPGFVEQAGALK.2	0.016469578	#N/A

VGDAIPSVEVFEGEPGK.2	-0.053640321	#N/A
VGDAIPSVEVFEGEPGKK.2	-0.0664882	#N/A
KVNLAELFK.2	-0.066190029	#N/A
ALNVEPDGTGLTC[Carbamidomethyl (C)]SLAPNILSQL.2	-0.12716121	#N/A
VNLAELFK.2	0.213117379	#N/A
ATDLLLDDSLVSLFGNR.2	0.211573649	#N/A
ATDLLLDDSLVSLFGNRR.3	-0.401593252	#N/A
FSM[Oxidation (M)]VIDNGIVK.2	0.462363585	#N/A
SNAQGIDLNR.2	0.18140911	#N/A
LTASAPGYLAITK.2	-0.092951272	#N/A
IVYVNEK.2	0.284400159	#N/A
YIGNM[Oxidation (M)]HGNEAVGR.3	-0.339177816	#N/A
EALVSVWLQC[Carbamidomethyl (C)]TAISR.2	-0.434203417	#N/A
SHIAQWS[Phospho (STY)]PHT[Phospho (STY)]PPR.3	-0.124475557	Q00560_S665_M2_Il6st
DVAGDIFHQQC[Carbamidomethyl (C)]K.2	-0.250430114	#N/A
S[Phospho (STY)]GDETPGSEAPGDK.2	0.055312039	Q00PI9_S159_M1_Hnrnpul2
DLLVQQASQC[Carbamidomethyl (C)]LSK.2	0.016855802	#N/A
YNVLGAETVLTQM[Oxidation (M)]R.2	-0.452622808	#N/A
NFILDQC[Carbamidomethyl (C)]NVYNSGQR.2	0.092963827	#N/A
AVEEQGDDQDS[Phospho (STY)]EK.2	0.195100259	Q00PI9_S183_M1_Hnrnpul2
S[Phospho (STY)]KSPPPPEEEAKDEEEDQTLVNLDTYTSDLHFQISK.5	-0.015501194	Q00PI9_S224_M1_Hnrnpul2
S[Phospho (STY)]GDETPGSEAPGDKAVEEQGDDQDSEK.3	-0.332351532	Q00PI9_S159_M1_Hnrnpul2
AVEEQGDDQDS[Phospho (STY)]EKS[Phospho (STY)]KPAGS[Phospho (STY)]DGER.3	-0.31314526	Q00PI9_S183_M3_Hnrnpul2
QHEVEIPSPT[Phospho (STY)]QKEK.3	-0.607579974	Q01063_T289_M1_Pde4d
NNFAALTNLQDR.2	-0.402743476	#N/A
SDS[Phospho (STY)]DYDLSPK.2	-0.116821647	Q01063_S136_M1_Pde4d
HSVSEMASNK.2	-0.015962313	#N/A
QHEVEIPS[Phospho (STY)]PTQK.2	0.497986236	Q01063_S287_M1_Pde4d
TALQQLER.2	0.147497755	#N/A
TIVFELLTR.2	0.424522114	#N/A
QILDTEDELR.2	-0.029369805	#N/A
DWLASTFTQQTR.2	-0.423373028	#N/A
S[Phospho (STY)]PPEM[Oxidation (M)]LESDC[Carbamidomethyl (C)]PSPLELK.2	-0.074100685	Q01065_S7_M1_Pde1b
QILDTEDELRELR.3	-0.025476021	#N/A
NLEYTASLLEAVYIDETR.2	-0.416075461	#N/A
S[Phospho (STY)]FTHSDC[Carbamidomethyl (C)]K.2	-0.468448212	Q01097_S940_M1_Grin2b
YPQS[Phospho (STY)]PTNSK.2	-0.310547583	Q01097_S1284_M1_Grin2b
HSQLSDLY[Phospho (STY)]GK.2	-0.386541273	Q01097_Y1039_M1_Grin2b
SDRYS[Phospho (STY)]GHDDLIR.3	-0.087270705	Q01097_S1050_M1_Grin2b
SVS[Phospho (STY)]LKDK.2	0.028372327	Q01097_S1323_M1_Grin2b
RQHS[Phospho (STY)]YDTFVDLQKEEAALAPR.4	-0.245785588	Q01097_S1303_M1_Grin2b
ENS[Phospho (STY)]PHWEHVDLTDIYK.3	0.189364084	Q01097_S1143_M1_Grin2b
RES[Phospho (STY)]S[Phospho (STY)]VYDISEHRR.3	-0.441439592	Q01097_S929_M2_Grin2b
QHSY[Phospho (STY)]DTFVDLQKEEAALAPR.3	0.291255904	Q01097_Y1304_M1_Grin2b
RQHS[Phospho (STY)]YDTFVDLQK.3	-0.063003739	Q01097_S1303_M1_Grin2b
QHSY[Phospho (STY)]DTFVDLQK.2	-0.054203175	Q01097_Y1304_M1_Grin2b
S[Phospho (STY)]DVSDISTHTVTYGNIEGNAAK.3	-0.152589791	Q01097_S1058_M1_Grin2b

NM[Oxidation (M)]ANLSGVNGS[Phospho (STY)]PQSALDFIR.2	-0.089540981	Q01097_S917_M1_Grin2b
AGNLYDIS[Phospho (STY)]EDNSLQELDQPAAPVAVSSNASTTK.3	-0.143790049	Q01097_S1255_M1_Grin2b
ESSVY[Phospho (STY)]DISEHRR.3	-0.05848238	Q01097_Y932_M1_Grin2b
DLYC[Carbamidomethyl (C)]HKS[Phospho (STY)]D.2	0.384480894	Q01147_S326_M1_Creb1
ILNDLS[Phospho (STY)]SDAPGVPR.2	-0.00087518	Q01147_S128_M1_Creb1
TEVEPDES[Phospho (STY)]SAAER.2	0.464307909	Q01337_S286_M1_Adra2c
RGPGAAGPGASGS[Phospho (STY)]GHGEER.3	0.400646565	Q01338_S362_M1_Adra2a
TRAS[Phospho (STY)]QVKPGDS[Phospho (STY)]LPR.3	-0.364744347	Q01338_S339_M2_Adra2a
ASQVKPGDS[Phospho (STY)]LPR.2	-0.146650193	Q01338_S346_M1_Adra2a
NAEPPS[Phospho (STY)]PTPAAHTR.2	-0.511902322	Q01341_S53_M1_Adcy6
GLVGEIIKR.2	-0.110324689	#N/A
GDFC[Carbamidomethyl (C)]IQVGR.2	0.275313703	#N/A
DRPFFPGLVK.2	-0.286986695	#N/A
SC[Carbamidomethyl (C)]AHDWVYE.2	0.157201246	#N/A
GLVGEIIK.2	0.2485721	#N/A
TFIAIKPDGVQR.2	-0.296698042	#N/A
NIIHGSDSVESAEK.2	0.226774529	#N/A
VM[Oxidation (M)]LGETNPADSKPGTIR.3	-0.381835418	#N/A
SITADGES[Phospho (STY)]PPTTK.2	-0.195745336	Q01815_S815_M1_Cacna1c
EAVSAAS[Phospho (STY)]EDDIFR.2	-0.14700993	Q01815_S1691_M1_Cacna1c
TGNNQADTES[Phospho (STY)]PSHEK.2	-0.446066842	Q01815_S1742_M1_Cacna1c
EAVSAAS[Phospho (STY)]EDDIFRR.2	0.182323565	Q01815_S1691_M1_Cacna1c
EVDIGIPDATGR.2	-0.014041106	#N/A
SVS[Phospho (STY)]DNDIR.2	-0.341814168	Q01853_S748_M1_Vcp
LDQLIYIPLPDEK.2	0.092480634	#N/A
GVLFYGPPGC[Carbamidomethyl (C)]GK.2	0.264880282	#N/A
RSVS[Phospho (STY)]DNDIR.2	-0.31346783	Q01853_S748_M1_Vcp
GGNIGDGGGAADR.2	0.097727241	#N/A
WALSQSNPSALR.2	0.098413322	#N/A
M[Oxidation (M)]DELQLFR.2	-0.393844839	#N/A
ETVVEVPQVTWEDIGGLEDVKR.3	0.107986778	#N/A
LGDVISIQPC[Carbamidomethyl (C)]PDVK.2	0.14616752	#N/A
LEILQIHTK.2	0.264166324	#N/A
ESIESEIRR.2	0.060301639	#N/A
AIANEC[Carbamidomethyl (C)]QANFISIK.2	0.41664912	#N/A
SVS[Phospho (STY)]DNDIRK.2	0.241153706	Q01853_S748_M1_Vcp
LAGESESNLR.2	0.213943884	#N/A
QAAPC[Carbamidomethyl (C)]VLFFDELDSIAK.2	0.348785865	#N/A
LIVDEAINEDNSVVSLSQPK.2	-0.279274959	#N/A
ELQELVQYPVEHPDK.2	0.090168899	#N/A
NAPAIIFIDELDAIAPK.2	0.054169344	#N/A
EM[Oxidation (M)]VELPLR.2	0.248810986	#N/A
DHFEEAM[Oxidation (M)]R.2	0.113027779	#N/A
IVSQLLTLM[Oxidation (M)]DGLK.2	0.095596456	#N/A
ESIESEIR.2	-0.011602692	#N/A
EAVC[Carbamidomethyl (C)]IVLSDDTC[Carbamidomethyl (C)]SDEK.2	-0.288952626	#N/A
[Acetyl (Protein N-term)]ASGADSKGDDLSTAILK.2	-0.00833605	#N/A

M[Oxidation (M)]TNGFSGADLTEIC[Carbamidomethyl (C)]QR.2	-0.091262941	#N/A
VINQILTEM[Oxidation (M)]DGM[Oxidation (M)]STK.2	-0.404368958	#N/A
EDEEES[Phospho (STY)]LNEVGYDDIGGC[Carbamidomethyl (C)]R.2	-0.028305166	Q01853_S197_M1_Vcp
LADDVDLEQVANETHGHVGADLAALC[Carbamidomethyl (C)]SEAALQAIR.4	-0.218491735	#N/A
DVDLEFLAK.2	0.354821969	#N/A
EAVC[Carbamidomethyl (C)]IVLSDDTC[Carbamidomethyl (C)]SDEKIR.3	-0.066734497	#N/A
LAGESESNLRK.3	0.292584007	#N/A
AENYDIS[Phospho (STY)]PADR.2	-0.248686995	Q02053_S876_M1_Uba1
DNPGVVTC[Carbamidomethyl (C)]LDEAR.2	-0.290786766	#N/A
RVS[Phospho (STY)]GPDPK.2	-0.313439511	Q02053_S13_M1_Uba1
YDGQVAVFGSDFQEK.2	-0.44925929	#N/A
[Acetyl (Protein N-term)]SSS[Phospho (STY)]PLSKK.2	-0.33420302	Q02053_S4_M1_Uba1
LKSDTAAAAVR.2	-0.221262642	#N/A
QNSLDEDLIR.2	-0.254844544	#N/A
QPAENVNQYLTDSK.2	-0.389826446	#N/A
SLPASLVEPDFVM[Oxidation (M)]TDFAK.2	-0.323676302	#N/A
AAVASLLQSVQVPEFTPK.2	-0.183887316	#N/A
SIPIC[Carbamidomethyl (C)]TLK.2	0.331465029	#N/A
YFLVGAGAIGC[Carbamidomethyl (C)]ELLK.2	-0.268017175	#N/A
NEEDATELVGLAQAVNAR.2	-0.48948826	#N/A
ATLPS[Phospho (STY)]PDKLPGFK.2	-0.230266949	Q02053_S835_M1_Uba1
VGEFC[Carbamidomethyl (C)]HSR.2	0.091746947	#N/A
LAGTQPLEVLEAVQR.2	-0.084405892	#N/A
NGS[Phospho (STY)]EADIDESLYSR.2	-0.051009693	Q02053_S46_M1_Uba1
VVQGHQQLDSYK.2	-0.086929438	#N/A
QFLDYFK.2	0.416114779	#N/A
LDQPM[Oxidation (M)]TEIVSR.2	-0.033011403	#N/A
M[Oxidation (M)]LQTSSVLVSGLR.2	0.056914342	#N/A
NFPNAIEHTLQWAR.2	-0.149532461	#N/A
LAYVAAGDLAPINAFIGGLAAQEVM[Oxidation (M)]K.3	-0.013633241	#N/A
NFAM[Oxidation (M)]IGLGC[Carbamidomethyl (C)]GEGGEVVVTDM[Oxidation (M)]DTIEK.3	0.021047396	#N/A
DEFEGLFK.2	0.014245868	#N/A
QM[Oxidation (M)]NPYIQVTSHQNR.3	0.167583671	#N/A
IHVS[Phospho (STY)]DQELQSANASVDDSRLEELK.3	-0.336333668	Q02053_S810_M1_Uba1
IYDDDFFQNLDGVANALDNIDAR.3	-0.253774213	#N/A
VLGPYTFSIC[Carbamidomethyl (C)]DTSNFSDYIR.2	0.319531549	#N/A
QNSLDEDLIRK.3	0.070581452	#N/A
TS[Phospho (STY)]M[Oxidation (M)]GGTQQQFVEGVR.2	-0.123463724	#N/A
HAVVNLINYQDDAELATR.3	-0.329788091	#N/A
RTS[Phospho (STY)]M[Oxidation (M)]GGTQQQFVEGVR.3	-0.050124231	#N/A
TMQNTNDVETAR.2	-0.172918123	#N/A
GPPETGS[Phospho (STY)]PK.2	-0.128699937	Q02357_S1759_M1_Ank1
LDQVVES[Phospho (STY)]PAIPR.2	-0.302778617	Q02357_S852_M1_Ank1
AEDS[Phospho (STY)]DAIPEWK.2	-0.047016717	Q02357_S1589_M1_Ank1
SVEDEM[Oxidation (M)]DS[Phospho (STY)]PGEEPFYTGQGR.2	0.302210278	Q02780_S310_M1_Nfia
LKS[Phospho (STY)]VEDEM[Oxidation (M)]DS[Phospho (STY)]PGEEPFYTGQGR.3	-0.313677209	Q02780_S303_M2_Nfia
S[Phospho (STY)]PGSGSQSSGWHEVEPGLPSPSTLK.3	-0.196137362	Q02780_S323_M1_Nfia

KVPEQPPELPQLDS[Phospho (STY)]QHL.3	-0.201175527	Q02819_S456_M1_Nucb1
SGKLS[Phospho (STY)]QELDFVSHNVR.3	-0.106271878	Q02819_S85_M1_Nucb1
AQRLS[Phospho (STY)]QETEALGR.2	-0.372849044	Q02819_S368_M1_Nucb1
LSQELDFVSHNVR.3	-0.142377537	#N/A
NPNS[Phospho (STY)]LKR.2	-0.142504908	Q03137_S887_M1_Epha4
IDTIAADESFTQVDIGDR.2	-0.557046803	#N/A
TYVDPFTYEDPNQAVR.2	-0.251385995	#N/A
RYS[Phospho (STY)]DHAGPAIPSVVAYPK.3	-0.29940138	Q03141_S419_M1_Mark3
SRGS[Phospho (STY)]TNLFSK.2	0.112690987	Q03141_S601_M1_Mark3
SAELDASDSSS[Phospho (STY)]SSNLSLAK.2	0.15449408	Q03141_S378_M1_Mark3
IDIMVGM[Oxidation (M)]GYS[Phospho (STY)]QEEIQESLSK.2	0.156559857	Q03141_S341_M1_Mark3
VRPNSDLSNSTGQS[Phospho (STY)]PHHK.3	-0.4259516	Q03141_S400_M1_Mark3
TPVAS[Phospho (STY)]THS[Phospho (STY)]ISSATTPDR.2	-0.575708659	Q03141_S540_M2_Mark3
SFGS[Phospho (STY)]PNR.2	-0.000483848	Q03147_S164_M1_Cdk7
PSS[Phospho (STY)]PVNTPSSQPPAAK.2	0.139201684	Q03173_S355_M1_Enah
S[Phospho (STY)]PVISRPK.2	-0.200204244	Q03173_S740_M1_Enah
NSRPSS[Phospho (STY)]PVNTPSSQPPAAK.3	-0.516054684	Q03173_S355_M1_Enah
AVDSLVPIGR.2	-0.063030494	#N/A
VVDALGNAIDGK.2	0.010627886	#N/A
VLSIGDGIAR.2	0.161714485	#N/A
TGAIVDVPVGEELLGR.2	-0.208970325	#N/A
ELIIGDR.2	-0.261036279	#N/A
LELAQYR.2	0.045540926	#N/A
TSIAIDTIINQK.2	-0.060221258	#N/A
GYLDKLEPSK.2	-0.227065326	#N/A
HALIIYDDLSK.2	-0.137794438	#N/A
ILGADTSVDLEETGR.2	-0.059838607	#N/A
VVDALGNAIDGKGPIGSK.2	-0.167575812	#N/A
STVAQLVK.2	0.075917962	#N/A
RTGAIVDVPVGEELLGR.3	-0.341907132	#N/A
EGDVVKR.2	0.036500991	#N/A
LKEIVTNFLAGFEP.2	-0.020569139	#N/A
RFNDGTDEK.2	-0.218477183	#N/A
EIVTNFLAGFEP.2	-0.193487003	#N/A
EAYPGDVFYLHSR.2	-0.296714508	#N/A
FNDGTDEK.2	0.014726721	#N/A
ISEQSDAK.2	-0.155873008	#N/A
QM[Oxidation (M)]SLLLR.2	-0.293969805	#N/A
RLTDADAM[Oxidation (M)]K.2	0.325457342	#N/A
FNDGTDEKK.2	-0.038272654	#N/A
LYC[Carbamidomethyl (C)]IYVAIGQK.2	0.286991744	#N/A
GM[Oxidation (M)]SLNLEPDNVGVVVFGNDK.2	-0.29632128	#N/A
QGQYSPM[Oxidation (M)]AIEEQVAVIYAGVR.2	-0.267296419	#N/A
EPMQTGIK.2	0.363373735	#N/A
TGTAEM[Oxidation (M)]SSILEER.2	0.120854399	#N/A
FENAFLSHVISQHQSLLGNIR.3	-0.211078806	#N/A
EVAAFAQFGSDLDAATQQLLSR.3	-0.377979764	#N/A

LTDADAMK.2	0.15307045	#N/A
YTIVVSATASDAAPLQYLAPYSGC[Carbamidomethyl (C)]SM[Oxidation (M)]GEYFR.3	-0.377451493	#N/A
NVQAEEMVEFSSGLK.2	-0.360301688	#N/A
S[Phospho (STY)]LEDLSSC[Carbamidomethyl (C)]PR.2	-0.125748714	Q03391_S1258_M1_Grin2d
LAFEDES[Phospho (STY)]PPAPSR.2	0.013665737	Q03391_S1049_M1_Grin2d
RGS[Phospho (STY)]AHFSSLESEV.2	-0.039752716	Q03391_S1313_M1_Grin2d
VAWIPDVESR.2	0.188643922	#N/A
IETQTQEEVRDS[Phospho (STY)]KENTEK.3	-0.039269195	Q03517_S270_M1_Scg2
IETQTQEEVRDS[Phospho (STY)]K.2	-0.067300139	Q03517_S270_M1_Scg2
QAPYENLNDQELGEYLAR.2	0.131163917	#N/A
EHLGPGSS[Phospho (STY)]QEM[Oxidation (M)]ER.2	-0.224916345	Q03517_S556_M1_Scg2
VPSPVS[Phospho (STY)]SEDDLQEEEQLEQAIK.3	-0.115339712	Q03517_S532_M1_Scg2
SPYFPNQYSQDK.2	0.122801889	#N/A
S[Phospho (STY)]GQLGLPDEENRR.2	-0.502212741	Q03517_S287_M1_Scg2
ESKDQLSEDASK.2	0.208324859	#N/A
VPSPVSSEDDLQEEEQLEQAIKEHLGPGS[Phospho (STY)]SQEM[Oxidation (M)]ER.4	0.204390238	Q03517_S555_M1_Scg2
FPLMYEENS[Phospho (STY)]RENPFKR.3	-0.194382575	Q03517_S176_M1_Scg2
NHFESSPLPTS[Phospho (STY)]PK.2	0.022656379	Q03717_S804_M1_Kcnb1
SLHGSTS[Phospho (STY)]PK.2	-0.028073813	Q03717_S782_M1_Kcnb1
VQDNHLS[Phospho (STY)]PNK.2	0.016274783	Q03717_S484_M1_Kcnb1
T[Phospho (STY)]PPRSPEK.2	-0.176513467	Q03717_T732_M1_Kcnb1
TQS[Phospho (STY)]QPILNTK.2	-0.34756995	Q03717_S541_M1_Kcnb1
TEKNHFESSPLPTS[Phospho (STY)]PK.3	0.041118891	Q03717_S804_M1_Kcnb1
VQDNHLS[Phospho (STY)]PNKWK.3	-0.453828382	Q03717_S484_M1_Kcnb1
DKVQDNHLS[Phospho (STY)]PNKWK.3	0.038178663	Q03717_S484_M1_Kcnb1
SSSS[Phospho (STY)]PQHLNVQQLQDMYSK.3	0.00238462	Q03717_S520_M1_Kcnb1
S[Phospho (STY)]IEM[Oxidation (M)]M[Oxidation (M)]DIVVEK.2	-0.405157726	Q03717_S457_M1_Kcnb1
STSS[Phospho (STY)]LPPEPMEIVR.2	-0.098796097	Q03717_S15_M1_Kcnb1
NGS[Phospho (STY)]IVSMNMK.2	-0.27526435	Q03717_S444_M1_Kcnb1
EQGS[Phospho (STY)]PEKAR.2	-0.052641648	Q03717_S511_M1_Kcnb1
QNGGLEDSGS[Phospho (STY)]GDGQM[Oxidation (M)]LC[Carbamidomethyl (C)]VR.2	0.126795064	Q03719_S460_M1_Kcnd1
ETLAQLQQEFQR.2	0.186227001	#N/A
VLTPELYAELR.2	0.111631781	#N/A
FSEVLKR.2	0.176037222	#N/A
DLFDPIIEER.2	0.092280669	#N/A
GIWHNDNK.2	0.189982402	#N/A
LLIEMEQR.2	0.314695259	#N/A
HGGYQPSDEHKTDLNPDNLQGGDDLDPNYVLSSR.5	-0.530581845	#N/A
FC[Carbamidomethyl (C)]TGLTQIETLFK.2	-0.016422872	#N/A
TFLVWINEEDHLR.2	0.01260507	#N/A
HEKFS[Phospho (STY)]EVLKR.3	0.217718658	Q04447_S309_M1_Ckb
NYEFM[Oxidation (M)]WNPHLGYILTC[Carbamidomethyl (C)]PSNLGTGLR.3	0.062171997	#N/A
LEQGQAIDDLM[Oxidation (M)]PAQK.2	0.076600915	#N/A
LAVEALSSLDGDLSGR.2	-0.065834365	#N/A
HEKFS[Phospho (STY)]EVLK.2	-0.468050436	Q04447_S309_M1_Ckb
TDLNPDNLQGGDDLDPNYVLSSR.3	-0.239499725	#N/A
FPAEDEFPDLSSHNNHM[Oxidation (M)]AK.3	0.176581474	#N/A

LGFSEVELVQM[Oxidation (M)]VVDGVK.2	-0.373900702	#N/A
PFSNSHNTQK.2	0.051260482	#N/A
LRFPAEDEFPDLSSHNNHM[Oxidation (M)]AK.4	-0.316453681	#N/A
SMTEAEQQQLIDDHFLFDKPVSPLLLASGM[Oxidation (M)]AR.4	0.353465759	#N/A
GTGGVDTAAVGGVFDVSNADR.2	-0.445550554	#N/A
HGGYQPSDEHK.3	-7.90E-05	#N/A
RGTGGVDTAAVGGVFDVSNADR.3	-0.163696403	#N/A
QAS[Phospho (STY)]PVAFKK.2	-0.26160088	Q04573_S367_M1_Npy1r
QAS[Phospho (STY)]PVAFK.2	-0.194364176	Q04573_S367_M1_Npy1r
TS[Phospho (STY)]LKQAS[Phospho (STY)]PVAFK.2	-0.082785545	Q04573_S362_M2_Npy1r
TS[Phospho (STY)]LKQAS[Phospho (STY)]PVAFKK.3	0.286035702	Q04573_S362_M2_Npy1r
S[Phospho (STY)]FDHLISDTK.2	-0.360408437	Q04690_S2545_M1_Nf1
KVS[Phospho (STY)]VSESNVLLDEEVLTDPK.3	0.117423436	Q04690_S2599_M1_Nf1
SFS[Phospho (STY)]PGSYER.2	0.202588317	Q04690_S2190_M1_Nf1
SSSGLATYS[Phospho (STY)]PPM[Oxidation (M)]GAVSER.2	-0.201256852	Q04690_S866_M1_Nf1
KS[Phospho (STY)]FDHLISDTK.2	-0.357201678	Q04690_S2545_M1_Nf1
SM[Oxidation (M)]S[Phospho (STY)]LDM[Oxidation (M)]GQPSQANTK.2	0.073157982	Q04690_S2525_M1_Nf1
SAGS[Phospho (STY)]FKR.2	-0.542977212	Q04690_S2831_M1_Nf1
GSEGYLAATYPAVGQTS[Phospho (STY)]PR.2	0.138435876	Q04690_S2517_M1_Nf1
KS[Phospho (STY)]M[Oxidation (M)]SLDMGQPSQANTK.2	-0.010833302	Q04690_S2523_M1_Nf1
SMS[Phospho (STY)]LDMGQPSQANTKK.2	0.122964622	Q04690_S2525_M1_Nf1
KIS[Phospho (STY)]TEDINK.2	-0.000303278	Q04735_S110_M1_Cdk16
KIS[Phospho (STY)]TEDINKR.2	-0.112587929	Q04735_S110_M1_Cdk16
QLS[Phospho (STY)]MTLR.2	-0.064129199	Q04735_S12_M1_Cdk16
AS[Phospho (STY)]PGPGELGK.2	-0.08945595	Q04888_S45_M1_Sox10
[Acetyl (Protein N-term)]AEEQDLS[Phospho (STY)]EVELSPVGSEEPR.2	-0.067035463	Q04888_S8_M1_Sox10
RAS[Phospho (STY)]LSDIGFGK.2	-0.029289075	Q04899_S109_M1_Cdk18
RFS[Phospho (STY)]M[Oxidation (M)]EDLNKR.3	-0.183412211	Q04899_S66_M1_Cdk18
RFS[Phospho (STY)]MEDLNK.2	0.278859122	Q04899_S66_M1_Cdk18
AS[Phospho (STY)]LSDIGFGK.2	0.027283203	Q04899_S109_M1_Cdk18
FS[Phospho (STY)]MEDLNK.2	0.334208385	Q04899_S66_M1_Cdk18
SS[Phospho (STY)]DQAVPAIPTSNSYSK.2	-0.202869809	Q05512_S409_M1_Mark2
SRNS[Phospho (STY)]PLLDR.2	-0.234131188	Q05512_S483_M1_Mark2
RSS[Phospho (STY)]DQAVPAIPTSNSYSK.3	-0.213246075	Q05512_S409_M1_Mark2
S[Phospho (STY)]SELEGDTITLKPR.2	0.031909488	Q05512_S365_M1_Mark2
DQQNLPYGVTPAS[Phospho (STY)]PSGHSQGR.3	0.199129487	Q05512_S616_M1_Mark2
NS[Phospho (STY)]PLLDR.2	0.026764725	Q05512_S483_M1_Mark2
PSADLTNSSAPS[Phospho (STY)]PSHK.2	-0.154233564	Q05512_S390_M1_Mark2
GRNS[Phospho (STY)]ATSADEQPHIGNYR.3	0.218333585	Q05512_S40_M1_Mark2
VPVAS[Phospho (STY)]PSAHNISSSSGAPDR.3	-0.434038478	Q05512_S566_M1_Mark2
NSATS[Phospho (STY)]ADEQPHIGNYR.2	-0.362586779	Q05512_S43_M1_Mark2
ELGVGLALR.2	0.160865577	#N/A
[Acetyl (Protein N-term)]ASLKDLEGK.2	-0.187965456	#N/A
M[Oxidation (M)]AAM[Oxidation (M)]AKPDC[Carbamidomethyl (C)]IITC[Carbamidomethyl (C)]DGNNITVK.3	-0.283964897	#N/A
M[Oxidation (M)]IVEC[Carbamidomethyl (C)]VM[Oxidation (M)]NNATC[Carbamidomethyl (C)]TR.2	0.006647591	#N/A
TTVFSC[Carbamidomethyl (C)]NLGEKFDETTADGR.3	-0.236047261	#N/A
TETVC[Carbamidomethyl (C)]TFQDGALVQHQQWDGK.3	-0.374966876	#N/A

KTETVC[Carbamidomethyl (C)]TFQDGALVQHQQWDGK.3	-0.015169776	#N/A
LMESHGFEEYMK.2	-0.141618825	#N/A
HRS[Phospho (STY)]EEENQVNLPK.3	-0.210637623	Q05915_S51_M1_Gch1
ALAVSDLNR.2	-0.410052189	#N/A
DFTATFGPLDSLNTR.2	0.038121886	#N/A
GANAVGYTNYPDNVVFK.2	0.231968752	#N/A
FIGPSPEVVR.2	0.404816734	#N/A
AC[Carbamidomethyl (C)]TELGIR.2	-0.012023095	#N/A
DM[Oxidation (M)]TLEGDDLILEIE.2	-0.039808935	#N/A
VVEIAPATHLDPQLR.2	0.088287284	#N/A
DAHQSLLATR.2	0.473634188	#N/A
VFDYSEYWEGAR.2	0.012274186	#N/A
AEAEAQAEELSFPR.2	0.349616109	#N/A
EGPEGFAR.2	0.538023515	#N/A
VVHSYEELEENYTR.3	-0.185416557	#N/A
ADFAQAC[Carbamidomethyl (C)]QDAGVR.2	-0.375228661	#N/A
LSLS[Phospho (STY)]TNC[Carbamidomethyl (C)]IEK.2	-0.309898644	Q05A62_S56_M1_Dnal1
GQTT[Phospho (STY)]PDTEVADDVGSK.2	0.128563904	Q05AA6_T910_M1_Drp2
EKGQTT[Phospho (STY)]PDTEVADDVGSK.3	-0.111729594	Q05AA6_T910_M1_Drp2
TSSS[Phospho (STY)]ERVS[Phospho (STY)]PGGR.2	0.161788886	Q05BC3_S135_M2_Eml1
TAS[Phospho (STY)]EGS[Phospho (STY)]EAETPEAPK.2	0.18569656	Q05CL8_S253_M2_Larp7
TAS[Phospho (STY)]EGS[Phospho (STY)]EAETPEAPKQPAK.2	-0.293869074	Q05CL8_S253_M2_Larp7
DLEFC[Carbamidomethyl (C)]S[Phospho (STY)]T[Phospho (STY)]EEEKETDR.2	-0.104901192	Q05CL8_S334_M2_Larp7
IEPIPGES[Phospho (STY)]PK.2	-0.148840148	Q05D44_S1164_M1_Eif5b
SVPTVDS[Phospho (STY)]GNEDDDSSFK.2	-0.373913543	Q05D44_S215_M1_Eif5b
TARPNSEAPLS[Phospho (STY)]GSEDADDSNK.3	-0.007416997	Q05D44_S137_M1_Eif5b
KTARPNSEAPLS[Phospho (STY)]GSEDADDSNK.3	-0.132714165	Q05D44_S137_M1_Eif5b
PNSEAPLS[Phospho (STY)]GSEDADDSNK.2	-0.123071194	Q05D44_S137_M1_Eif5b
TSFDENDS[Phospho (STY)]EELEDKDSK.2	-0.318084658	Q05D44_S114_M1_Eif5b
KTS[Phospho (STY)]FDENDS[Phospho (STY)]EELEDKDSK.3	-0.742353431	Q05D44_S108_M2_Eif5b
DVAQIFNNILR.2	-0.41129773	#N/A
VPPVQVSPLIK.2	0.0625785	#N/A
ELAEAQDDSILK.2	0.209791863	#N/A
YSYLKPR.2	0.343920917	#N/A
YSALIIGM[Oxidation (M)]AYGAK.2	-0.034615598	#N/A
S[Phospho (STY)]PPDGQSFR.2	-0.406254853	Q06649_S414_M1_Sh3bp2
ASGIIDTLFQDR.2	-0.0688791	#N/A
VSTVTTHSSDSEVPSR.3	-0.042088474	#N/A
QQSQVLDAMQDSFAR.2	0.035311105	#N/A
SDTSGHFER.2	0.310858052	#N/A
GFGTDEQAIVDVVSNR.2	-0.178756111	#N/A
LYQAGEGR.2	-0.499664713	#N/A
AEALSSLHGDDQDS[Phospho (STY)]EDEVLTVPEVK.3	0.099537776	Q07113_S2401_M1_Igf2r
LVSFHDDS[Phospho (STY)]DEDLLHI.2	-0.386671526	Q07113_S2476_M1_Igf2r
[Acetyl (Protein N-term)]GEHS[Phospho (STY)]PDDNIIFFK.2	0.09727747	Q07243_S5_M1_Mtf1
ESSTNNSVS[Phospho (STY)]PSESLR.2	0.316950765	Q08122_S203_M1_Tle3
DAPTS[Phospho (STY)]PASVASSSSTPSSK.2	0.137895704	Q08122_S286_M1_Tle3

VS[Phospho (STY)]PAHS[Phospho (STY)]PPENGLDK.2	0.066687897	Q08122_S263_M2_Tle3
SVVEGISSQPGSS[Phospho (STY)]PSGTVV.2	0.027011168	Q08274_S659_M1_Dmwd
YHS[Phospho (STY)]LGNISR.2	-0.400262323	Q08274_S537_M1_Dmwd
TLPGT[Phospho (STY)]PGAT[Phospho (STY)]PPASGSSR.2	-0.366622881	Q08274_T457_M2_Dmwd
RYHS[Phospho (STY)]LGNISR.3	-0.491403214	Q08274_S537_M1_Dmwd
AFTDEETEAQAGQASWPRS[Phospho (STY)]PSK.3	0.207303204	Q08274_S643_M1_Dmwd
LGDPDGAGEPPST[Phospho (STY)]PSGLGAGGDR.2	0.036481786	Q08274_T112_M1_Dmwd
GGSGGNSS[Phospho (STY)]NDKLSGPAPR.2	-0.050495953	Q08274_S551_M1_Dmwd
SNS[Phospho (STY)]LPHPAGGGK.2	-0.060132878	Q08274_S487_M1_Dmwd
S[Phospho (STY)]IDLNKPIDKR.3	0.015618989	Q08274_S145_M1_Dmwd
LLPVQENFLLK.2	0.100986097	#N/A
SGYIEANELK.2	-0.273319664	#N/A
EM[Oxidation (M)]NIQQLTTYR.2	-0.30774141	#N/A
ELENFFQELEK.2	0.068282107	#N/A
SVMSLAEAGK.2	-0.31386247	#N/A
GGYST[Phospho (STY)]PQTLANR.2	-0.276541648	Q08460_T1061_M1_Kcnma1
LEDEQPPTLS[Phospho (STY)]PK.2	0.174041156	Q08460_S711_M1_Kcnma1
RLEDEQPPTLS[Phospho (STY)]PK.2	0.030811112	Q08460_S711_M1_Kcnma1
SS[Phospho (STY)]PDNSPVHGMLR.2	0.407668186	Q08460_S924_M1_Kcnma1
DSVSS[Phospho (STY)]VSDVSQYR.2	-0.036585727	Q08509_S58_M1_Eps8
QNSSSS[Phospho (STY)]DSGGSIVR.2	0.393459721	Q08509_S661_M1_Eps8
RKS[Phospho (STY)]QMEEVQDELFQR.3	-0.403292054	Q08509_S684_M1_Eps8
EGINPGYDDY[Phospho (STY)]ADSDEDQHDAYLER.3	0.109066874	Q08943_Y441_M1_Ssrp1
SQTAPEAGNDTGNEDAIGGNVNK.2	-0.046178011	#N/A
TAS[Phospho (STY)]PSPLRPDAPVEK.2	-0.348872254	Q09XV5_S1997_M1_Chd8
GT[Phospho (STY)]PQT[Phospho (STY)]PKDDPVLTLLSNR.3	-0.035415128	Q0GA42_T821_M2_Cnnm1
SDGLRS[Phospho (STY)]PGEVVYLR.3	0.016123264	Q0GA42_S850_M1_Cnnm1
SFSEDAVTDSS[Phospho (STY)]GSGTLPR.2	0.194528754	Q0GNC1_S1226_M1_Inf2
AVLLASDAQAC[Carbamidomethyl (C)]TLEEVVER.2	-0.161254553	#N/A
ALS[Phospho (STY)]LPETLTSK.2	-0.12758641	Q0KK59_S1242_M1_Unc79
VDS[Phospho (STY)]PGKPAPR.2	0.168253011	Q0KK59_S1596_M1_Unc79
FKAFS[Phospho (STY)]GEGQK.2	-0.540756858	Q0KL01_S216_M1_Ubxn2b
STPATC[Carbamidomethyl (C)]RS[Phospho (STY)]PRT[Phospho (STY)]PPHR.3	-0.170375632	Q0KL01_S56_M2_Ubxn2b
AFS[Phospho (STY)]GEGQK.2	-0.163578367	Q0KL01_S216_M1_Ubxn2b
DSDDSAAT[Phospho (STY)]PQDETIEER.2	-0.044243499	Q0KL02_T1824_M1_Trio
NFLNALTS[Phospho (STY)]PIEYQR.2	-0.312665588	Q0KL02_S2282_M1_Trio
SADAGSQKDS[Phospho (STY)]DDSAATPQDETIEER.3	-0.193411939	Q0KL02_S1818_M1_Trio
SARS[Phospho (STY)]PS[Phospho (STY)]PSTGTR.2	-0.403792246	Q0P557_S244_M2_Spata18
SNS[Phospho (STY)]PC[Carbamidomethyl (C)]TTVAK.2	-0.242836342	Q0P557_S273_M1_Spata18
SADS[Phospho (STY)]PPGSSGQALSLAPAER.2	0.000922625	Q0P5V2_S597_M1_Sobp
GS[Phospho (STY)]PPINSLSR.2	0.001866883	Q0P5V9_S408_M1_Slc45a4
S[Phospho (STY)]M[Oxidation (M)]SDLYDLQQR.2	-0.124786899	Q0P5V9_S472_M1_Slc45a4
LGVSVS[Phospho (STY)]PSR.2	0.289036547	Q0P678_S530_M1_Zc3h18
KRPLS[Phospho (STY)]PQSK.2	-0.041665585	Q0P678_S888_M1_Zc3h18
KANLS[Phospho (STY)]PDR.2	-0.374535012	Q0P678_S847_M1_Zc3h18
ANLS[Phospho (STY)]PDRGSR.2	0.301748212	Q0P678_S847_M1_Zc3h18
M[Oxidation (M)]GS[Phospho (STY)]PKPER.2	0.183894487	Q0P678_S863_M1_Zc3h18

ANLS[Phospho (STY)]PDR.2	0.132626615	Q0P678_S847_M1_Zc3h18
S[Phospho (STY)]PQPPSR.2	-0.048756816	Q0P678_S483_M1_Zc3h18
SQDQDS[Phospho (STY)]EAHELSR.2	0.166957049	Q0P678_S80_M1_Zc3h18
VQSQEETRS[Phospho (STY)]DEEDRASEPK.3	0.325376313	Q0P678_S64_M1_Zc3h18
S[Phospho (STY)]LGDASPSAPESVGAR.2	-0.233096996	Q0PHV7_S165_M1_Dact3
GRS[Phospho (STY)]VEQS[Phospho (STY)]PPR.2	-0.23625083	Q0PHV7_S370_M2_Dact3
SQS[Phospho (STY)]ETSLLGR.2	-0.024591651	Q0PHV7_S409_M1_Dact3
AFSFPVS[Phospho (STY)]PER.2	-0.019161198	Q0PHV7_S10_M1_Dact3
PRPPEAS[Phospho (STY)]PPPGGAR.2	-0.257235491	Q0PHV7_S290_M1_Dact3
SFS[Phospho (STY)]APYPTAAAGAETC[Carbamidomethyl (C)]SSAER.2	-0.297452505	Q0PHV7_S188_M1_Dact3
GRS[Phospho (STY)]VEQS[Phospho (STY)]PPRER.3	-0.48924208	Q0PHV7_S370_M2_Dact3
GASSSS[Phospho (STY)]GVVVQVR.2	-0.116960412	Q0VBF8_S29_M1_Stum
DAETAAAAAAVAAADPR.2	-0.278183435	#N/A
SLS[Phospho (STY)]PGGAALGYR.2	-0.373300427	Q0VBL3_S293_M1_Rbm15
LHSYSS[Phospho (STY)]PSTK.2	-0.164092788	Q0VBL3_S127_M1_Rbm15
HLDRS[Phospho (STY)]PESERPR.3	0.099182947	Q0VBL3_S655_M1_Rbm15
S[Phospho (STY)]RS[Phospho (STY)]PLDKDAYAPSSSVVGTSVGSHR.3	0.240913139	Q0VBL3_S256_M2_Rbm15
HC[Carbamidomethyl (C)]T[Phospho (STY)]PS[Phospho (STY)]PDRS[Phospho (STY)]PELSSNR.3	-0.334836976	Q0VBL3_S670_M3_Rbm15
DRT[Phospho (STY)]PPLLYR.2	-0.59683948	Q0VBL3_T567_M1_Rbm15
KSEPKVDSS[Phospho (STY)]PR.2	0.12727496	Q0VBM2_S827_M1_Fam83b
TS[Phospho (STY)]APEPR.2	-0.28791749	Q0VGB7_S63_M1_Ppp4r2
LLQQGLAQVEAGR.2	-0.316429443	#N/A
S[Phospho (STY)]QEEAPGHR.2	-0.259615443	Q0VGU4_S423_M1_Vgf
QNALLFAEEEDGEAGAEDKRS[Phospho (STY)]QEEAPGHR.4	-0.083844041	Q0VGU4_S423_M1_Vgf
SPC[Carbamidomethyl (C)]ETISS[Phospho (STY)]PSSTLESK.2	0.006617246	Q0VGY8_S211_M1_Tanc1
QQGPPPAPANDS[Phospho (STY)]DNEEDAPASSLK.3	-0.076777238	Q0VGY8_S1436_M1_Tanc1
VLGATLSPELIQK.2	0.15476093	#N/A
DNWEELHNR.2	-0.310857495	#N/A
LSVEGFAVDK.2	-0.211666083	#N/A
LGLQNDLFSLAR.2	-0.027639499	#N/A
AGIISTVEVLK.2	0.012634379	#N/A
QILSADLR.2	0.037904926	#N/A
IDFVGELNDK.2	-0.024707121	#N/A
YQGGFLISR.2	0.195166259	#N/A
YAAVTQFEATDAR.2	0.342721282	#N/A
DVFSPIGER.2	0.081406399	#N/A
ETALLIDPK.2	0.104853249	#N/A
DADSIHQYLLQR.2	-0.275701785	#N/A
AFFESHPAPSAER.2	0.196144475	#N/A
S[Phospho (STY)]PVYLTVLK.2	0.184846231	Q11011_S746_M1_Npepps
VALSNMNVIDR.2	0.046796242	#N/A
QM[Oxidation (M)]GFPLIYVEAEQVEDDR.2	-0.4191791	#N/A
ATFDISLVVPK.2	0.197634625	#N/A
EAS[Phospho (STY)]FEGISK.2	-0.198252147	Q11136_S162_M1_Pepd
YAVDDVQYTDEIASVLTSR.2	-0.154753062	#N/A
NYKSS[Phospho (STY)]PEIQEPIKPLEK.3	0.24208188	Q148V7_S244_M1_Relch
YS[Phospho (STY)]DDGNRETDER.2	-0.15376389	Q148V7_S193_M1_Relch

SS[Phospho (STY)]PEIQEPIKPLEK.2	-0.097880836	Q148V7_S244_M1_Relch
AGS[Phospho (STY)]ISTLDSLDFAR.2	-0.281887238	Q148V7_S180_M1_Relch
VVPALITLSSDPEISVR.2	0.091092783	#N/A
AGSISTLDSLDFARY[Phospho (STY)]SDDGNRETDER.3	-0.137663934	Q148V7_Y192_M1_Relch
[Acetyl (Protein N-term)]AAMAPGGGGSGSGVNPFLS[Phospho (STY)]DSDEDDDEVAATEDRR.3	0.396522568	Q148V7_S20_M1_Relch
QES[Phospho (STY)]TETLVVDR.2	0.011429967	Q149C2_S409_M1_Traf3ip1
RQES[Phospho (STY)]TETLVVDR.2	0.156224354	Q149C2_S409_M1_Traf3ip1
NNS[Phospho (STY)]PAPPQPAPVK.2	0.140718323	Q14AX6_S1079_M1_Cdk12
ETTSGTTAEPVKNNS[Phospho (STY)]PAPPQPAPVK.3	-0.070256107	Q14AX6_S1079_M1_Cdk12
RRS[Phospho (STY)]S[Phospho (STY)]SPFLSK.2	0.260105729	Q14AX6_S331_M2_Cdk12
RQS[Phospho (STY)]IS[Phospho (STY)]PPYKEPSAYQSSTR.3	0.143972099	Q14AX6_S273_M2_Cdk12
S[Phospho (STY)]RS[Phospho (STY)]PAYSR.2	0.05883084	Q14AX6_S356_M2_Cdk12
SVLSGDDSTGSEPPLS[Phospho (STY)]PPER.2	-0.340650863	Q14B80_S557_M1_Kcnc2
TELNM[Oxidation (M)]AC[Carbamidomethyl (C)]NSTQS[Phospho (STY)]DTC[Carbamidomethyl (C)]LGK.2	-0.28564656	Q14B80_S526_M1_Kcnc2
AGS[Phospho (STY)]RDAAQERPSGPGGR.3	-0.697561091	Q14BB9_S82_M1_Map6d1
TVYVLPVGDADAAVVATTSYR.2	-0.324863268	#N/A
VVTTHS[Phospho (STY)]SGWDPS[Phospho (STY)]PGASFQVPEVR.3	-0.086010974	Q14BB9_S154_M2_Map6d1
SDVAVPLTLHGYS[Phospho (STY)]DPGSEESGADC[Carbamidomethyl (C)]SVSR.3	0.012379491	Q14BB9_S34_M1_Map6d1
STVEDFEANTS[Phospho (STY)]PTK.2	-0.27546684	Q14CH0_S792_M1_Fam171b
RDS[Phospho (STY)]KTNIWK.2	0.024637427	Q14CH0_S808_M1_Fam171b
DQTTS[Phospho (STY)]TTHINHISSVK.3	-0.228467691	Q14CH0_S405_M1_Fam171b
HILEGGSGVIIEHPGEES[Phospho (STY)]PGRK.3	0.275814149	Q14CH0_S777_M1_Fam171b
KSTVEDFEANTS[Phospho (STY)]PTK.2	-0.19850038	Q14CH0_S792_M1_Fam171b
STVEDFEANTSPT[Phospho (STY)]KK.2	0.227803672	Q14CH0_T794_M1_Fam171b
FQQPPQPEGEEDAS[Phospho (STY)]DGGR.2	-0.150818899	Q1HFZ0_S23_M1_Nsun2
EGVILTNENAAS[Phospho (STY)]PEQPGDEDAK.2	0.290802408	Q1HFZ0_S723_M1_Nsun2
KKEGVILTNENAAS[Phospho (STY)]PEQPGDEDAK.3	0.202614746	Q1HFZ0_S723_M1_Nsun2
RSS[Phospho (STY)]TSSEPTPTVK.2	-0.366068559	Q2KN98_S833_M1_Specc1l
SKS[Phospho (STY)]DNQISDK.2	0.089300999	Q2KN98_S171_M1_Specc1l
KGS[Phospho (STY)]SGNASEVSVAC[Carbamidomethyl (C)]LTER.3	0.295647173	Q2KN98_S385_M1_Specc1l
S[Phospho (STY)]KFANLGR.2	-0.032754536	Q2M3X8_S111_M1_Phactr1
KLS[Phospho (STY)]QRPTVEELR.3	-0.338397102	Q2M3X8_S505_M1_Phactr1
SDSLVPGTHT[Phospho (STY)]PPIR.3	-0.189113538	Q2M3X8_T104_M1_Phactr1
RLS[Phospho (STY)]QRPTAEELEQR.3	0.233941545	Q2M3X8_S467_M1_Phactr1
SKS[Phospho (STY)]DTPYLAEAR.2	-0.198786997	Q2M3X8_S67_M1_Phactr1
SDT[Phospho (STY)]PYLAEAR.2	0.192240177	Q2M3X8_T69_M1_Phactr1
NEQEEQEEKR.2	-0.191683709	#N/A
GEPNVSY[Phospho (STY)]IC[Carbamidomethyl (C)]SR.2	-0.422261909	Q2NL51_Y279_M1_Gsk3a
[Acetyl (Protein N-term)]S[Phospho (STY)]GGGPSGGGPGGSGR.2	-0.14275237	Q2NL51_S2_M1_Gsk3a
T[Phospho (STY)]SSFAEPGGGGGGGGGGPGGSASGPGGTGGGK.3	-0.301784036	Q2NL51_T19_M1_Gsk3a
KSHS[Phospho (STY)]SPSLNPDASPVTAK.3	-0.414038332	Q2PFD7_S1000_M1_Psd3
VLLFQTQSPEEM[Oxidation (M)]QGWINK.2	-0.09855218	#N/A
IGS[Phospho (STY)]TTNPFLDIPHDPNAAVYK.3	-0.381494882	Q2PFD7_S759_M1_Psd3
DHYLEFEK.2	0.101725842	#N/A
SHS[Phospho (STY)]SPSLNPDASPVTAK.2	-0.49562736	Q2PFD7_S1000_M1_Psd3
ALYNS[Phospho (STY)]IKNEK.2	-0.430200585	Q2PFD7_S719_M1_Psd3
INC[Carbamidomethyl (C)]VAAVFSAPPFPAAIGSQK.2	-0.23667551	#N/A

KDHRPET[Phospho (STY)]PGIK.3	0.261489535	Q2PFD7_T1029_M1_Psd3
LEWAVDDEEKKK.3	0.005275247	#N/A
DEYKPEKS[Phospho (STY)]LSDEDLK.2	-0.104971895	Q2PFD7_S825_M1_Psd3
VAAAAGSGPS[Phospho (STY)]PPC[Carbamidomethyl (C)]S[Phospho (STY)]PGHDRER.3	0.053832785	Q2TBE6_S47_M2_Pi4k2a
SAS[Phospho (STY)]ESYTQSFQSR.2	-0.192149268	Q2TBE6_S462_M1_Pi4k2a
[Acetyl (Protein N-term)]M[Oxidation (M)]DETS[Phospho (STY)]PLVS[Phospho (STY)]PER.2	0.175916756	Q2TBE6_S5_M2_Pi4k2a
VAAAAGSGPS[Phospho (STY)]PPC[Carbamidomethyl (C)]S[Phospho (STY)]PGHDR.2	-0.196717769	Q2TBE6_S47_M2_Pi4k2a
M[Oxidation (M)]AEDEES[Phospho (STY)]PGELC[Carbamidomethyl (C)]PDVLYR.2	0.126851579	Q2YDW2_S41_M1_Msto1
DSYIEVLLPLGTDPELR.2	0.142424623	#N/A
IPVYEEER.2	-0.222719504	#N/A
SLS[Phospho (STY)]ELESLKLPAESNEK.2	-0.275310102	Q3B7Z2_S238_M1_Osbp
M[Oxidation (M)]LAES[Phospho (STY)]DDS[Phospho (STY)]GDEESVSQTDK.2	-7.25E-06	Q3B7Z2_S188_M2_Osbp
TGS[Phospho (STY)]NIS[Phospho (STY)]GASSDVSLDEQYK.2	0.205025556	Q3B7Z2_S377_M2_Osbp
SLS[Phospho (STY)]ELESLK.2	-0.103743632	Q3B7Z2_S238_M1_Osbp
GATVLPANPPGS[Phospho (STY)]AGS[Phospho (STY)]GKDQC[Carbamidomethyl (C)]C[Carbamidomethyl (C)]SGK.3	-0.021283641	Q3B7Z2_S333_M2_Osbp
DSGETQASSSTS[Phospho (STY)]PR.2	-0.195455063	Q3KNJ2_S245_M1_Nhej1
ELYELWEEYETQSSEEAK.2	-0.31837981	#N/A
YDFEPYVSNNSWS[Phospho (STY)]PIM[Oxidation (M)]R.2	-0.407529763	Q3TBD2_S568_M1_Arhgap45
T[Phospho (STY)]PDSEAPDVESR.2	-0.209492619	Q3TDD9_T652_M1_Ppp1r21
SGESSSQLS[Phospho (STY)]QEQK.2	-0.186217738	Q3TDD9_S101_M1_Ppp1r21
T[Phospho (STY)]PDSEAPDVESREDLIK.2	-0.317654806	Q3TDD9_T652_M1_Ppp1r21
LTTPATLTLPEIAASGLTR.2	-0.066397329	#N/A
ENPPVEDS[Phospho (STY)]S[Phospho (STY)]DEDDKR.2	0.163446731	Q3TDQ1_S495_M2_Stt3b
ENPPVEDS[Phospho (STY)]S[Phospho (STY)]DEDDKRNPGNLYDK.3	0.297484726	Q3TDQ1_S495_M2_Stt3b
EASYSLIR.2	-0.403237451	#N/A
TLS[Phospho (STY)]PLDR.2	-0.177010421	Q3TEL6_S414_M1_Rnf157
AQAPELQQEEERPGAVGS[Phospho (STY)]PR.3	-0.394172553	Q3TES0_S256_M1_Iqsec3
LQTSQHS[Phospho (STY)]PK.2	-0.207396638	Q3TES0_S1055_M1_Iqsec3
VQTPQS[Phospho (STY)]PHQHPVAPGAIADKEK.4	-0.389416275	Q3TES0_S132_M1_Iqsec3
VQTPQS[Phospho (STY)]PHQHPVAPGAIADK.3	-0.11426473	Q3TES0_S132_M1_Iqsec3
S[Phospho (STY)]PTLSTDTLR.2	-0.001412824	Q3TES0_S646_M1_Iqsec3
GNSAQHS[Phospho (STY)]PSSLR.2	-0.054887553	Q3TGW2_S110_M1_Eepd1
GNSAQHS[Phospho (STY)]PSSLRR.3	-0.126695705	Q3TGW2_S110_M1_Eepd1
EHGPFRS[Phospho (STY)]VEDLVR.3	-0.131316632	Q3TGW2_S173_M1_Eepd1
S[Phospho (STY)]VEDLVR.2	0.079826838	Q3TGW2_S173_M1_Eepd1
LNGTDPEDVIR.2	0.171526475	#N/A
GNFNYIEFTR.2	-0.136293088	#N/A
FTDEEVDELYR.2	-0.009333569	#N/A
ATSNVFAM[Oxidation (M)]FDQSQIQEFK.2	-0.607348676	#N/A
EAFNMIDQNR.2	0.28923922	#N/A
RAEAQALLQDYVSTQS[Phospho (STY)]AEE.2	0.198512325	Q3THG9_S409_M1_Aarsd1
ELS[Phospho (STY)]LDDPEVEQVR.2	0.226604881	Q3THG9_S174_M1_Aarsd1
GAQADHFTESPLS[Phospho (STY)]PGSQVQVR.3	-0.168345545	Q3THG9_S88_M1_Aarsd1
AEAQALLQDYVSTQS[Phospho (STY)]AEE.2	-0.053265605	Q3THG9_S409_M1_Aarsd1
TLNM[Oxidation (M)]TTS[Phospho (STY)]PEEK.2	-0.317991416	Q3THK7_S332_M1_Gmps
TLNMTTS[Phospho (STY)]PEEKR.2	-0.015979783	Q3THK7_S332_M1_Gmps
NFLYDIAGC[Carbamidomethyl (C)]SGNFTVQNR.2	-0.347667344	#N/A

VIC[Carbamidomethyl (C)]AEEPYIC[Carbamidomethyl (C)]K.2	-0.230867565	#N/A
QADFEAHNILR.3	-0.418206348	#N/A
FVIGGPQGDAGLTGR.2	0.080371979	#N/A
TQVTVQYMQDR.2	0.006493928	#N/A
EADEDS[Phospho (STY)]EPEREVR.2	-0.268522051	Q3TIX9_S81_M1_Usp39
EREADEDS[Phospho (STY)]EPEREVR.3	-0.470147676	Q3TIX9_S81_M1_Usp39
EADEDS[Phospho (STY)]EPER.2	-0.178289669	Q3TIX9_S81_M1_Usp39
EREADEDS[Phospho (STY)]EPER.2	-0.32394161	Q3TIX9_S81_M1_Usp39
DSEAGSST[Phospho (STY)]PTTSTR.2	-0.418317119	Q3TKT4_T1390_M1_Smarca4
NFSLLSFGEEAEEEEEEVNR.2	0.344426631	#N/A
S[Phospho (STY)]LEDVKPR.2	-0.439000772	Q3TLH4_S853_M1_Prrc2c
SFS[Phospho (STY)]SQRPGVDR.2	-0.218308497	Q3TLH4_S1516_M1_Prrc2c
SESS[Phospho (STY)]DFEVVPK.2	-0.548283145	Q3TLH4_S1222_M1_Prrc2c
S[Phospho (STY)]VSHGSNHAQNAEEQR.3	0.077641025	Q3TLH4_S899_M1_Prrc2c
SPS[Phospho (STY)]DNSLNTTGIIC[Carbamidomethyl (C)]R.2	-0.004006429	Q3TLI0_S684_M1_Trappc10
AKEELGEGS[Phospho (STY)]R.2	0.195075825	Q3TMP8_S289_M1_Tmem38a
AKEELGEGS[Phospho (STY)]RK.3	0.30216044	Q3TMP8_S289_M1_Tmem38a
VC[Carbamidomethyl (C)]HLGDQLEGVNT[Phospho (STY)]PR.3	-0.27211942	Q3TPX4_T122_M1_Exoc5
ADS[Phospho (STY)]ES[Phospho (STY)]EEDEQESEEVR.2	-0.370951123	Q3TQI7_S15_M2_
VGDTEKPEPERS[Phospho (STY)]PPNR.3	-0.43386319	Q3TQI7_S261_M1_
RRADS[Phospho (STY)]ES[Phospho (STY)]EEDEQESEEVR.2	0.016806967	Q3TQI7_S15_M2_
RADS[Phospho (STY)]ES[Phospho (STY)]EEDEQESEEVR.2	-0.252315934	Q3TQI7_S15_M2_
GKGPEQS[Phospho (STY)]PQGRPVGTTR.3	-0.222849627	Q3TR08_S209_M1_Fndc4
DEGGS[Phospho (STY)]PEGASPSTASEVEEEK.2	-0.387274513	Q3TRM4_S1319_M1_Pnpla6
ESPGGC[Carbamidomethyl (C)]TS[Phospho (STY)]PGSQEK.2	0.222406874	Q3TRR0_S257_M1_Map9
S[Phospho (STY)]PSAATSSHYLGSLK.2	-0.219323109	Q3TRR0_S389_M1_Map9
KQS[Phospho (STY)]LEPDKADHIR.3	-0.276545053	Q3TRR0_S414_M1_Map9
STS[Phospho (STY)]SGETSSGLDADGHFKPSPQPR.3	-0.085067364	Q3TRR0_S160_M1_Map9
RTS[Phospho (STY)]FQDELIR.2	-0.167849336	Q3TRR0_S23_M1_Map9
M[Oxidation (M)]NDFHIS[Phospho (STY)]DDEEKNSPR.3	-0.060783023	Q3TRR0_S79_M1_Map9
QNSPVTS[Phospho (STY)]PGSS[Phospho (STY)]PLAQR.2	0.069508338	Q3TTA7_S480_M2_Cblb
GLS[Phospho (STY)]LTETELEELR.2	-0.246504042	Q3TVA9_S50_M1_Ccdc136
TQS[Phospho (STY)]PGGC[Carbamidomethyl (C)]SVEAVLAR.2	-0.163060419	Q3TVI4_S75_M1_Hexim2
GREPS[Phospho (STY)]SSQPVVPVDVEDQAK.3	0.170917983	Q3TVI8_S164_M1_Pbxip1
LTSVSS[Phospho (STY)]S[Phospho (STY)]VDFDQR.2	0.07714202	Q3TWI9_S114_M2_Tmem63b
SHS[Phospho (STY)]PLPAPPSK.2	-0.428852444	Q3TWW8_S303_M1_Srsf6
LIVENLSSR.2	-0.2432197	#N/A
ARS[Phospho (STY)]MS[Phospho (STY)]PPPK.2	0.073598121	Q3TWW8_S314_M2_Srsf6
S[Phospho (STY)]M[Oxidation (M)]S[Phospho (STY)]PPPK.2	-0.280790413	Q3TWW8_S314_M2_Srsf6
SRS[Phospho (STY)]QS[Phospho (STY)]RS[Phospho (STY)]HS[Phospho (STY)]PLPAPPSK.3	0.020799709	Q3TWW8_S297_M3_Srsf6
ARS[Phospho (STY)]MS[Phospho (STY)]PPPKR.3	-0.024452466	Q3TWW8_S314_M2_Srsf6
TVGT[Phospho (STY)]PIASVPGSTNTGTVPGSEK.2	-0.025406577	Q3TXS7_T273_M1_Psmd1
TPDAS[Phospho (STY)]PEPK.2	0.12401198	Q3TXS7_S315_M1_Psmd1
TASAVAGKT[Phospho (STY)]PDAS[Phospho (STY)]PEPKDQTLK.3	-0.54017531	Q3TXS7_S315_M2_Psmd1
IEEEEQEPEPPEPFEYIDD.2	0.082304353	#N/A
TASAVAGKT[Phospho (STY)]PDAS[Phospho (STY)]PEPK.2	-0.221030473	Q3TXS7_S315_M2_Psmd1
AAVESLGFILFR.2	-0.420596251	#N/A

TPEQC[Carbamidomethyl (C)]PSVVSLLSESYNPHVR.3	-0.119493193	#N/A
WAPPLER.2	-0.071906111	#N/A
DPPVVDC[Carbamidomethyl (C)]TC[Carbamidomethyl (C)]FGLPR.2	-0.058822989	#N/A
YIEDAIGESAK.2	0.142060046	#N/A
GGHVVVQK.2	-0.185598459	#N/A
KYIEDAIGESAK.2	-0.182200127	#N/A
HIM[Oxidation (M)]STTNVR.2	0.121988301	#N/A
QPWAEPEEM[Oxidation (M)]S[Phospho (STY)]EEK.2	-0.218626365	Q3TXX4_S504_M1_Slc17a7
C[Carbamidomethyl (C)]GFVGHDQLAGS[Phospho (STY)]DES[Phospho (STY)]EMEDEAEPPGAPPAPPPSYGATHSTVQPPRPPPPVR.5	-0.55212968	Q3TXX4_S519_M2_Slc17a7
RQEGAETLELS[Phospho (STY)]ADGRPVTTHTR.4	0.167124768	Q3TXX4_S36_M1_Slc17a7
DTDAYSDLS[Phospho (STY)]DGEK.2	0.117436422	Q3TY60_S117_M1_Fam131b
NS[Phospho (STY)]NAYGIGALAK.2	0.114691791	Q3TY60_S47_M1_Fam131b
RNS[Phospho (STY)]NAYGIGALAK.2	-0.354778422	Q3TY60_S47_M1_Fam131b
VSDVTSSGVQSFDEEEGDANN.2	0.082368009	#N/A
DLESLS[Phospho (STY)]PR.2	-0.005304589	Q3TY60_S297_M1_Fam131b
DTDAYSDLS[Phospho (STY)]DGEKEAR.2	-0.296971251	Q3TY60_S117_M1_Fam131b
RDTDAYSDLS[Phospho (STY)]DGEKEAR.3	-0.212304021	Q3TY60_S117_M1_Fam131b
KVSDVTSSGVQS[Phospho (STY)]FDEEEGDANN.2	-0.153637172	Q3TY60_S322_M1_Fam131b
APTIQPQHS[Phospho (STY)]HEAVRR.3	-0.27359638	Q3TY60_S102_M1_Fam131b
RDTDAYSDLS[Phospho (STY)]DGEK.2	0.151366196	Q3TY60_S117_M1_Fam131b
DLESLS[Phospho (STY)]PREDPEM[Oxidation (M)]STALSR.3	0.253586902	Q3TY60_S297_M1_Fam131b
VALS[Phospho (STY)]EEEEEERFER.2	-0.424920262	Q3TY65_S294_M1_Ica1l
ALPRDS[Phospho (STY)]LEGDDFEK.3	-0.323586305	Q3TY65_S316_M1_Ica1l
GKEELSASGKGS[Phospho (STY)]PR.2	-0.247948532	Q3TY86_S36_M1_Aifm3
LPTPQPYPS[Phospho (STY)]PQDC[Carbamidomethyl (C)]VEATVC[Carbamidomethyl (C)]HVK.3	0.086829569	Q3TY86_S63_M1_Aifm3
EELSASGKGS[Phospho (STY)]PR.2	0.233265665	Q3TY86_S36_M1_Aifm3
GTVAVGDS[Phospho (STY)]EEDGEDVFEVER.2	-0.245196396	Q3TYA6_S51_M1_Mphosph8
GQS[Phospho (STY)]PPESVVPEESSDVR.2	-0.483925669	Q3TYD6_S1104_M1_Lmtk2
YFS[Phospho (STY)]PPPPAR.2	-0.047874151	Q3TYD6_S1418_M1_Lmtk2
HSGTSPQASPALLTEEGS[Phospho (STY)]PTAPTDPILKPEETK.4	0.352699551	Q3TYD6_S797_M1_Lmtk2
NAGFTSALLES[Phospho (STY)]PQR.2	-0.440122374	Q3TYD6_S742_M1_Lmtk2
SSS[Phospho (STY)]SLLASPSHIAAK.2	0.104845263	Q3TZZ7_S662_M1_Esyt2
DTSPDKGELVS[Phospho (STY)]DEEEDT.2	0.043722073	Q3U0J8_S959_M1_Tbc1d2b
FNFESVPES[Phospho (STY)]PGEK.2	-0.168091906	Q3U0M1_S953_M1_Trappc9
VESRPTNPS[Phospho (STY)]EGS[Phospho (STY)]KTGDLGHVK.3	0.00303606	Q3U0M1_S840_M2_Trappc9
AAS[Phospho (STY)]GGAALASPGPGSGSGTPTGSGGK.2	0.027739339	Q3U0S6_S286_M1_Rasip1
SVS[Phospho (STY)]ELSLQGR.2	0.205239185	Q3U0S6_S322_M1_Rasip1
GS[Phospho (STY)]PQQIDHAK.2	0.070540652	Q3U0V1_S481_M1_Khsrp
IINDLLQSLR.2	0.04385867	#N/A
SVSLTGAPESVQK.2	0.241782694	#N/A
VQIS[Phospho (STY)]PDSGGLPER.2	-0.280047811	Q3U0V1_S182_M1_Khsrp
IGGDAATTVNNNTPDFGFGGQK.2	-0.046330613	#N/A
SSSS[Phospho (STY)]C[Carbamidomethyl (C)]NDLYATVK.2	-0.002586259	Q3U1F9_S351_M1_Pag1
RPS[Phospho (STY)]EEPEPDYEAIQTLNR.3	-0.317705111	Q3U1F9_S379_M1_Pag1
HSANAESILGT[Phospho (STY)]C[Carbamidomethyl (C)]SDLDEESPPPVPVK.3	0.17623068	Q3U1F9_T245_M1_Pag1
TTATLDPTS[Phospho (STY)]PAPGEGPSGR.2	-0.224896635	Q3U1J1_S180_M1_Tfpt
EGLS[Phospho (STY)]PSQR.2	-0.162272933	Q3U1J1_S167_M1_Tfpt

LLPYPTLAS[Phospho (STY)]PPFD.2	0.215861543	Q3U1J1_S255_M1_Tfpt
ASKIS[Phospho (STY)]S[Phospho (STY)]EDGEETR.2	0.077022724	Q3U1T9_S516_M2_Dennd1b
DGVLS[Phospho (STY)]PSSQAQLSSR.2	-0.291448322	Q3U1V6_S365_M1_Uevld
SS[Phospho (STY)]PPASPTIIPR.2	-0.206924801	Q3U1V8_S541_M1_Map3k9
S[Phospho (STY)]APSS[Phospho (STY)]PAPR.2	0.198721748	Q3U1Y4_S732_M2_Dennd4b
SGS[Phospho (STY)]LGSAR.2	0.475752557	Q3U1Y4_S961_M1_Dennd4b
SAPPS[Phospho (STY)]PPPPGTR.2	0.100967107	Q3U1Z5_S38_M1_Gpsm3
RLS[Phospho (STY)]ADLR.2	-0.201069424	Q3U214_S769_M1_Mast3
RGQS[Phospho (STY)]ADKLGLGTSER.3	-0.431708021	Q3U214_S1237_M1_Mast3
DPLEEIQPLNDLDESQPPAPGS[Phospho (STY)]PESR.3	-0.091455028	Q3U214_S370_M1_Mast3
KQEAVQEVS[Phospho (STY)]FDEEPGPPR.2	-0.166682877	Q3U214_S1289_M1_Mast3
GQS[Phospho (STY)]ADKLGLGTSER.2	-0.392374359	Q3U214_S1237_M1_Mast3
QEAVQEVS[Phospho (STY)]FDEEPGPPR.2	0.076277766	Q3U214_S1289_M1_Mast3
SFS[Phospho (STY)]EDREDGWGQSLGDR.3	-0.137742619	Q3U214_S750_M1_Mast3
S[Phospho (STY)]SESVVDEDGGR.2	-0.043912774	Q3U214_S156_M1_Mast3
HLGS[Phospho (STY)]EDDETNDEESSTEIPQFSSC[Carbamidomethyl (C)]SHR.4	0.075568449	Q3U214_S702_M1_Mast3
VY[Phospho (STY)]SSSEFLAVQPTPTFAER.2	-0.168875553	Q3U214_Y730_M1_Mast3
QQS[Phospho (STY)]LGGSESPGPVPR.2	-0.105784269	Q3U2I3_S510_M1_Fam160a2
ERS[Phospho (STY)]PS[Phospho (STY)]PPAVQC[Carbamidomethyl (C)]K.2	-0.306654277	Q3U3C9_S826_M2_Gse1
YSPDEM[Oxidation (M)]NNS[Phospho (STY)]PNFEEK.2	0.027389112	Q3U3C9_S857_M1_Gse1
RQT[Phospho (STY)]PS[Phospho (STY)]PR.2	-0.025289024	Q3U3C9_S840_M2_Gse1
SSS[Phospho (STY)]WGSTEQLK.2	0.182301037	Q3U3E2_S268_M1_Fam117b
TS[Phospho (STY)]PTVATQTGASVTSTR.2	0.093987272	Q3U3E2_S102_M1_Fam117b
SPS[Phospho (STY)]APVC[Carbamidomethyl (C)]K.2	-0.205623684	Q3U3E2_S190_M1_Fam117b
SSS[Phospho (STY)]WGSTEQLKEIAK.2	-0.14216008	Q3U3E2_S268_M1_Fam117b
RSPS[Phospho (STY)]APVC[Carbamidomethyl (C)]K.2	-0.43191467	Q3U3E2_S190_M1_Fam117b
S[Phospho (STY)]PEQGRPSAEKR.3	-0.018506817	Q3U3E2_S176_M1_Fam117b
SIDTQTPGGADKGS[Phospho (STY)]NNSSR.3	-0.41154752	Q3U3E2_S399_M1_Fam117b
QQLQELGAGS[Phospho (STY)]PGPVTNK.2	-0.448188072	Q3U3N6_S400_M1_Tepsin
S[Phospho (STY)]AESLQSLNSGLC[Carbamidomethyl (C)]PEK.2	-0.404541906	Q3U5C7_S592_M1_Prickle1
NLFQQQS[Phospho (STY)]SEVDPR.2	-0.484796102	Q3U5C7_S433_M1_Prickle1
KS[Phospho (STY)]RSDNALNLVTER.3	-0.284002518	Q3U5C7_S682_M1_Prickle1
SKS[Phospho (STY)]QSRPQQVK.2	-0.164298034	Q3U5C7_S625_M1_Prickle1
S[Phospho (STY)]QSRPQQVK.2	-0.126946756	Q3U5C7_S625_M1_Prickle1
S[Phospho (STY)]RSDNALNLVTER.2	-0.355978313	Q3U5C7_S682_M1_Prickle1
EGS[Phospho (STY)]PAPLEPEPGASQPK.2	-0.216425793	Q3UCQ1_S389_M1_Foxk2
[Acetyl (Protein N-term)]M[Oxidation (M)]EIQSGPQPGS[Phospho (STY)]PGR.2	-0.246292235	#N/A
[Acetyl (Protein N-term)]M[Oxidation (M)]EIQSGPQPGS[Phospho (STY)]PGRAER.2	-0.492045269	#N/A
LEENGDLYPES[Phospho (STY)]P.2	0.021487876	Q3UDP0_S459_M1_Wdr41
LINSELGS[Phospho (STY)]PSR.2	-0.53845351	Q3UDW8_S238_M1_Hgsnat
VTSFS[Phospho (STY)]TPPT[Phospho (STY)]PER.2	-0.275445757	Q3UE31_S289_M2_
SRS[Phospho (STY)]LS[Phospho (STY)]GTGR.2	-0.091527116	Q3UE31_S351_M2_
SLVGS[Phospho (STY)]WLK.2	-0.365106982	Q3UE31_S362_M1_
SHS[Phospho (STY)]ANDSEEFFREDDSGADLHNATNLR.4	-0.246851269	Q3UE31_S324_M1_
SS[Phospho (STY)]VSS[Phospho (STY)]VGAR.2	-0.241240929	Q3UFK8_S21_M2_Frmd8
SHRSS[Phospho (STY)]VSS[Phospho (STY)]VGAR.2	-0.010516472	Q3UFK8_S21_M2_Frmd8
TDWNEDC[Carbamidomethyl (C)]KS[Phospho (STY)]PK.2	-0.341259484	Q3UFT3_S356_M1_Garem1

STC[Carbamidomethyl (C)]APSS[Phospho (STY)]PPTPATLGATIK.2	-0.144037365	Q3UFT3_S493_M1_Garem1
[Acetyl (Protein N-term)]AAAAAAAAAAGDS[Phospho (STY)]DSWDADTFSM[Oxidation (M)]EDPVRK.3	-0.06103986	Q3UGC7_S14_M1_Eif3j1
TFFLEALR.2	-0.050636037	#N/A
FVTNTTK.2	0.140727683	#N/A
S[Phospho (STY)]PTNSSEIFTPAHEENVR.2	0.067476185	Q3UGS4_S21_M1_Mcrip1
EHLS[Phospho (STY)]EPQSLR.2	-0.440212099	Q3UGY8_S1966_M1_Arfgef3
VAAPSSS[Phospho (STY)]PSAEAEYWR.2	-0.045732082	Q3UGY8_S1646_M1_Arfgef3
SGS[Phospho (STY)]TGSSLSVSVR.2	0.041358894	Q3UGY8_S2090_M1_Arfgef3
YSESNFS[Phospho (STY)]VDDQDLSR.2	0.253286776	Q3UGY8_S593_M1_Arfgef3
DGAESSRDS[Phospho (STY)]M[Oxidation (M)]EINEADFR.3	-0.165721734	Q3UGY8_S471_M1_Arfgef3
SDVSDIGS[Phospho (STY)]DNC[Carbamidomethyl (C)]SLADEEQTPR.2	-0.235070284	Q3UGY8_S628_M1_Arfgef3
HSFS[Phospho (STY)]AGPELLR.2	-0.188648539	Q3UGY8_S2074_M1_Arfgef3
LLSPYSPQT[Phospho (STY)]QETDSIGQK.2	-0.13979733	Q3UH60_T56_M1_Dip2b
AGS[Phospho (STY)]LGPETPSR.2	0.065874154	Q3UH66_S1797_M1_Wnk2
QSS[Phospho (STY)]LPGTGGVASDFVK.2	-0.488278206	Q3UH66_S1770_M1_Wnk2
YS[Phospho (STY)]APPDVYLDEIPSSPEVK.2	-0.254966089	Q3UH66_S1713_M1_Wnk2
TSVPESIASAGTGS[Phospho (STY)]PSK.2	0.096047484	Q3UH68_S523_M1_Limch1
S[Phospho (STY)]PEPEATLTFPFLDK.2	0.173771938	Q3UH68_S719_M1_Limch1
QTPS[Phospho (STY)]PDVVLR.2	0.349109869	Q3UH68_S217_M1_Limch1
S[Phospho (STY)]ASQDLIKK.2	0.200275556	Q3UH68_S377_M1_Limch1
SRQT[Phospho (STY)]PS[Phospho (STY)]PDVVLR.2	0.034754303	Q3UH68_S217_M2_Limch1
DDS[Phospho (STY)]FDSLDSFGSR.2	-0.026095317	Q3UH68_S201_M1_Limch1
SAS[Phospho (STY)]QDLIKKEEER.3	-0.145543949	Q3UH68_S379_M1_Limch1
SDSLS[Phospho (STY)]PPR.2	-0.08464399	Q3UH68_S192_M1_Limch1
KETDDIDS[Phospho (STY)]PKR.3	-0.338014295	Q3UH68_S169_M1_Limch1
GSSDGRGS[Phospho (STY)]DSESDLPHR.3	-0.134597535	Q3UH68_S231_M1_Limch1
TINHQM[Oxidation (M)]ES[Phospho (STY)]PGER.2	-0.003736638	Q3UH68_S973_M1_Limch1
HGRDDS[Phospho (STY)]FDSLDSFGSR.3	-0.049417746	Q3UH68_S201_M1_Limch1
LHSQDPLLS[Phospho (STY)]PER.2	-0.307249403	Q3UH99_S434_M1_Shisa6
ETISAIDTS[Phospho (STY)]PKENTPVR.2	0.412799432	Q3UH99_S247_M1_Shisa6
ILSDEQLLS[Phospho (STY)]TER.2	-0.360384425	Q3UH99_S422_M1_Shisa6
YVLTS[Phospho (STY)]PR.2	-0.163427462	Q3UHB8_S90_M1_Ccdc177
SHS[Phospho (STY)]LDSLSR.2	-0.38597531	Q3UHB8_S231_M1_Ccdc177
RTS[Phospho (STY)]PS[Phospho (STY)]PPAR.2	0.027058	Q3UHB8_S209_M2_Ccdc177
NSC[Carbamidomethyl (C)]PAGSASS[Phospho (STY)]APNPLGR.2	-0.075006152	Q3UHB8_S281_M1_Ccdc177
KSHS[Phospho (STY)]LDS[Phospho (STY)]LSR.2	-0.377513983	Q3UHB8_S231_M2_Ccdc177
SFSLGDLSHS[Phospho (STY)]PQTAQHVER.3	0.157827162	Q3UHB8_S310_M1_Ccdc177
DGALSSESGASSSSY[Phospho (STY)]SGESLR.2	0.395609818	Q3UHB8_Y254_M1_Ccdc177
DSSEATGWEEPS[Phospho (STY)]PPSIR.2	-0.116040299	Q3UHC0_S714_M1_Tnrc6c
AET[Phospho (STY)]PPSHQAGTQLNR.3	0.348929605	Q3UHC0_T776_M1_Tnrc6c
QKDSSEATGWEEPS[Phospho (STY)]PPSIR.3	-0.106248279	Q3UHC0_S714_M1_Tnrc6c
GDS[Phospho (STY)]PELKPR.2	-0.343911877	Q3UHC7_S978_M1_Dab2ip
LPS[Phospho (STY)]PTPENK.2	0.030689397	Q3UHC7_S702_M1_Dab2ip
RQMS[Phospho (STY)]LTEK.2	0.125909166	Q3UHC7_S895_M1_Dab2ip
QQSSSSKGDS[Phospho (STY)]PELKPR.3	-0.562727756	Q3UHC7_S978_M1_Dab2ip
LHLHGS[Phospho (STY)]PR.2	-0.123456423	Q3UHD1_S1289_M1_Adgrb1
NENVATLSVSS[Phospho (STY)]LERR.2	-0.204636921	Q3UHD1_S1467_M1_Adgrb1

NENVATLSVSS[Phospho (STY)]LER.2	-0.260866549	Q3UHD1_S1467_M1_Adgrb1
RPS[Phospho (STY)]LPEEEKMK.3	0.285319642	Q3UHD1_S1257_M1_Adgrb1
QLS[Phospho (STY)]EENANLQEYVEK.2	0.011626909	Q3UHD3_S1286_M1_Mtus2
QLS[Phospho (STY)]EEHAVKR.3	-0.240663571	Q3UHD3_S109_M1_Mtus2
SM[Oxidation (M)]S[Phospho (STY)]PVER.2	0.028534175	Q3UHD3_S332_M1_Mtus2
SES[Phospho (STY)]GYGFNVR.2	-0.402744187	Q3UHD6_S49_M1_Snx27
IVKSES[Phospho (STY)]GYGFNVR.2	-0.216517406	Q3UHD6_S49_M1_Snx27
ALS[Phospho (STY)]TDC[Carbamidomethyl (C)]TPSGDLSPLSR.2	-0.20804226	Q3UHD9_S802_M1_Agap2
GSEPRDPGS[Phospho (STY)]PR.2	-0.41242887	Q3UHD9_S54_M1_Agap2
GAGSRLS[Phospho (STY)]WPESEGKPR.3	0.048707504	Q3UHD9_S211_M1_Agap2
RSS[Phospho (STY)]AASLGR.2	-0.191930332	Q3UHD9_S1172_M1_Agap2
ELLGAELRTS[Phospho (STY)]PK.2	0.058591394	Q3UHD9_S382_M1_Agap2
LRTDS[Phospho (STY)]QSEAVAIQAIR.3	-0.013669558	Q3UHD9_S921_M1_Agap2
FLTGS[Phospho (STY)]YQVLEK.2	-0.037198602	Q3UHD9_S424_M1_Agap2
LKS[Phospho (STY)]FGS[Phospho (STY)]LR.2	0.002845282	Q3UHD9_S856_M2_Agap2
SGNS[Phospho (STY)]LNKEWK.2	-0.279033509	Q3UHD9_S684_M1_Agap2
TDS[Phospho (STY)]QSEAVAIQAIR.2	0.034217169	Q3UHD9_S921_M1_Agap2
RTS[Phospho (STY)]LFANR.2	0.095304984	Q3UHD9_S642_M1_Agap2
RRS[Phospho (STY)]S[Phospho (STY)]AASLGR.2	-0.133734979	Q3UHD9_S1171_M2_Agap2
ELTLVLTAIGNDTANR.2	-0.033745079	#N/A
LS[Phospho (STY)]WPESEGKPR.2	0.024559663	Q3UHD9_S211_M1_Agap2
SS[Phospho (STY)]AASLGR.2	-0.273629471	Q3UHD9_S1172_M1_Agap2
AEAAAVAGLSTPGS[Phospho (STY)]LHR.2	-0.040966394	Q3UHD9_S632_M1_Agap2
AVVNSQEWTLSR.2	-0.388185667	#N/A
AQS[Phospho (STY)]QPESDKDHERPLPALSWAR.4	0.094687891	Q3UHE1_S946_M1_Pitpnm3
NVLSDS[Phospho (STY)]VESSDDEFFDAR.2	-0.08670353	Q3UHE1_S27_M1_Pitpnm3
GS[Phospho (STY)]PPLLDAPAS[Phospho (STY)]PPQAPR.2	-0.030603093	Q3UHE1_S495_M2_Pitpnm3
KGS[Phospho (STY)]ISSTQDTPVVVEEDC[Carbamidomethyl (C)]SLASSK.3	0.089008952	Q3UHE1_S295_M1_Pitpnm3
GSHS[Phospho (STY)]FDER.2	0.102518265	Q3UHF7_S565_M1_Hivep2
QASFSGS[Phospho (STY)]PEIAQGEAGVDPSVK.2	0.056332348	Q3UHF7_S1081_M1_Hivep2
ELSLS[Phospho (STY)]TEEGNEPSPEK.2	0.221668763	Q3UHF7_S2415_M1_Hivep2
SPS[Phospho (STY)]LGSEDLPLAADPDK.2	0.029850473	Q3UHF7_S764_M1_Hivep2
EM[Oxidation (M)]S[Phospho (STY)]QGDASPR.2	-0.437809433	Q3UHF7_S2085_M1_Hivep2
LADLEHSSGESS[Phospho (STY)]FESTGTGLSR.3	-0.329557659	Q3UHF7_S947_M1_Hivep2
SES[Phospho (STY)]TEMVAC[Carbamidomethyl (C)]PQDK.2	0.085973655	Q3UHF7_S811_M1_Hivep2
S[Phospho (STY)]IEQAEDAHKK.2	-0.169928995	Q3UHF7_S169_M1_Hivep2
MSTFSS[Phospho (STY)]QEGPR.2	-0.351917372	Q3UHF7_S41_M1_Hivep2
QSTS[Phospho (STY)]EDSLNSTER.2	0.013187282	Q3UHF7_S2295_M1_Hivep2
GWGS[Phospho (STY)]PGRPK.2	0.109486568	Q3UHH1_S1132_M1_Zswim8
QSAGPNS[Phospho (STY)]PTGGGGGGGSGGTR.2	0.072580623	Q3UHH1_S53_M1_Zswim8
RLS[Phospho (STY)]AEGGDK.2	-0.462020702	Q3UHH1_S564_M1_Zswim8
KQS[Phospho (STY)]AGPNS[Phospho (STY)]PTGGGGGGGSGGTR.2	-0.365136617	Q3UHH1_S48_M2_Zswim8
SSS[Phospho (STY)]GESLAQSPDNAK.2	-0.054143224	Q3UHI0_S198_M1_Ccser2
HTSGNNLIS[Phospho (STY)]PDTDYR.2	-0.172652685	Q3UHI0_S487_M1_Ccser2
RGS[Phospho (STY)]AGGVGDRR.2	-0.123392474	Q3UHI4_S21_M1_Tmed8
RGS[Phospho (STY)]AGGVGDR.2	-0.079544234	Q3UHI4_S21_M1_Tmed8
VGSLT[Phospho (STY)]PPSS[Phospho (STY)]PK.2	-0.306584946	Q3UHJ0_S622_M2_Aak1

VQTT[Phospho (STY)]PPPTIQGQK.2	-0.370818034	Q3UHJ0_T604_M1_Aak1
EQGSSGLGSGSS[Phospho (STY)]GGGGSSSGLGSGYIGR.3	-0.208857087	Q3UHJ0_S21_M1_Aak1
LTDPIPTTETSIAPR.2	-0.169479969	#N/A
VAEDEFDPIPVLITK.2	0.134181057	#N/A
AAEDSNLISGFGVAEGSEK.2	-0.180803489	#N/A
DLKVENILLHDR.3	-0.079489836	#N/A
FQNPQAEGVNAVEDEIKK.3	0.031259868	#N/A
SKS[Phospho (STY)]ATTT[Phospho (STY)]PSGS[Phospho (STY)]PR.2	-0.307676136	Q3UHJ0_S668_M3_Aak1
LPSQTESVTSNR.2	0.28937104	#N/A
NSS[Phospho (STY)]GSSESSLPNLAR.2	-0.233928634	Q3UHJ0_S936_M1_Aak1
AQATPSQPLQSSQPK.2	0.289802303	#N/A
SATTTPSGS[Phospho (STY)]PR.2	-0.181072193	Q3UHJ0_S676_M1_Aak1
VGSLT[Phospho (STY)]PPSS[Phospho (STY)]PKTQR.2	-0.107473125	Q3UHJ0_S622_M2_Aak1
REQGSSGLGSGSS[Phospho (STY)]GGGGSSSGLGSGYIGR.3	0.046852719	Q3UHJ0_S21_M1_Aak1
ILSDVTHSAVFGVPASK.2	-0.255000892	#N/A
STQLLQAAAAEASLNK.2	-0.269700755	#N/A
YTTLSYR.2	-0.331727589	#N/A
GHYVLC[Carbamidomethyl (C)]DFGSATNK.3	0.054819394	#N/A
LQTGFTENEVLQIFC[Carbamidomethyl (C)]DTC[Carbamidomethyl (C)]EAVAR.3	0.331471708	#N/A
GSNYHLSDNDAS[Phospho (STY)]DVE.2	-0.138753343	Q3UHK1_S634_M1_Slc2a13
KAS[Phospho (STY)]EDVEYTLR.2	0.170018092	Q3UHK1_S6_M1_Slc2a13
VKGSNYHLS[Phospho (STY)]DNDASDVE.2	-0.140763302	Q3UHK1_S629_M1_Slc2a13
SLSS[Phospho (STY)]LMGER.2	-0.14403415	Q3UHK1_S18_M1_Slc2a13
SLLAAESAAS[Phospho (STY)]LQGAELER.2	-0.217587022	Q3UHK1_S47_M1_Slc2a13
AAAT[Phospho (STY)]PEPAVAQPDSTALEGATGQAPPSSK.3	-0.264678776	Q3UHL1_T470_M1_Camkv
TEEFC[Carbamidomethyl (C)]EIFR.2	0.164981265	#N/A
EPC[Carbamidomethyl (C)]GTPEYLAPEVVGR.2	-0.173526025	#N/A
LENLVYYNR.2	-0.300468097	#N/A
IVISDFHLAK.2	0.00027354	#N/A
AT[Phospho (STY)]PATEESTVPATQSSALPAAK.2	-0.115710975	Q3UHL1_T446_M1_Camkv
EVFDWILDQGYYSER.2	0.236104773	#N/A
SDDIAS[Phospho (STY)]ADR.1	-0.000539755	Q3UHL1_S392_M1_Camkv
ITAEEAISHEWISGNAASDK.3	-0.246031893	#N/A
LMEVEQDQR.2	0.194956383	#N/A
GEEATGC[Carbamidomethyl (C)]AQESQR.2	0.078433313	#N/A
SAT[Phospho (STY)]PATDGR.2	-0.673559068	Q3UHL1_T438_M1_Camkv
NYNQPSEVTDR.2	-0.481716899	#N/A
S[Phospho (STY)]DLSEEDLPYYVDLPDR.2	0.067079043	Q3UHQ6_S1080_M1_Dop1b
AALLAAFQPES[Phospho (STY)]PR.2	-0.15335147	Q3UHQ6_S1167_M1_Dop1b
SEDSGIGLSASS[Phospho (STY)]PELSEHLR.3	-0.35646685	Q3UHQ6_S597_M1_Dop1b
QELGPGQETGTSS[Phospho (STY)]R.2	-0.155071556	Q3UHU5_S1795_M1_Mtcl1
EDVTPPLS[Phospho (STY)]PDDLK.2	0.180624872	Q3UHU5_S1407_M1_Mtcl1
GLPSAGSKEDVT[Phospho (STY)]PPLS[Phospho (STY)]PDDLK.3	-0.0107086	Q3UHU5_S1407_M2_Mtcl1
GRS[Phospho (STY)]PS[Phospho (STY)]PLGVGSETFR.3	0.254732645	Q3UHU5_S1799_M2_Mtcl1
SLDS[Phospho (STY)]DES[Phospho (STY)]EDEDDDYQQK.2	-0.167494437	Q3UHX2_S60_M2_Pdap1
GVEGLIDIENPNR.2	0.106164006	#N/A
M[Oxidation (M)]QSLS[Phospho (STY)]LNK.2	-0.369817502	Q3UHX2_S178_M1_Pdap1

QYTS[Phospho (STY)]PEEIDAQLQAEK.2	0.060662517	Q3UHX2_S19_M1_Pdap1
SLDS[Phospho (STY)]DESEDEDDDYQQKR.2	-0.209904809	#N/A
KSLDS[Phospho (STY)]DES[Phospho (STY)]EDEDDDYQQK.2	0.114587566	Q3UHX2_S60_M2_Pdap1
KSLDS[Phospho (STY)]DESEDEDDDYQQKR.2	-0.411287196	#N/A
LGEQGPEPGPTPPQTPT[Phospho (STY)]PPST[Phospho (STY)]PPLAK.3	-0.015483219	Q3UIA2_T736_M2_Arhgap17
SVDISLGGS[Phospho (STY)]PR.2	-0.14179474	Q3UIL6_S609_M1_Plekha7
KVMS[Phospho (STY)]PLQS[Phospho (STY)]PTK.2	0.011524551	Q3UIL6_S900_M2_Plekha7
HLSGSSS[Phospho (STY)]PPPR.2	0.040303556	Q3UIL6_S482_M1_Plekha7
IEDVM[Oxidation (M)]AGLS[Phospho (STY)]ANKENY[Phospho (STY)]R.3	0.231806999	Q3UIL6_S820_M2_Plekha7
DSQDTSAEQS[Phospho (STY)]DHDDEVASLASASGGFGSK.3	0.001279766	Q3UJB9_S884_M1_Edc4
[Acetyl (Protein N-term)]ASC[Carbamidomethyl (C)]AS[Phospho (STY)]IDIEDATQHLR.2	-0.302507761	Q3UJB9_S6_M1_Edc4
RNS[Phospho (STY)]PVER.2	0.119538167	Q3UJD6_S1258_M1_Usp19
SHS[Phospho (STY)]LPNSLDYAQASER.2	-0.45693308	Q3UJU9_S46_M1_Rmdn3
SLQGLAGEIVGEVR.2	0.358253172	#N/A
AIPGEAGDAAILPS[Phospho (STY)]LSQEGQEK.2	0.049843312	Q3UJU9_S81_M1_Rmdn3
DSDKES[Phospho (STY)]GDAEDEVSC[Carbamidomethyl (C)]ETVR.2	-0.04344185	Q3UJU9_S193_M1_Rmdn3
KDS[Phospho (STY)]LDLDVEAASSPAAAALEEDDSSGR.3	-0.217299009	Q3UJU9_S212_M1_Rmdn3
KLEGQS[Phospho (STY)]PQQVSR.2	-0.326144316	Q3UKC1_S632_M1_Tax1bp1
VPS[Phospho (STY)]WEDNVVC[Carbamidomethyl (C)]SQPAR.2	-0.394072721	Q3UKC1_S693_M1_Tax1bp1
AAS[Phospho (STY)]PVEELLTM[Oxidation (M)]EDEGNSDM[Oxidation (M)]LVVTTK.3	-0.220922929	Q3UKC1_S124_M1_Tax1bp1
LSNAS[Phospho (STY)]PNPNEGVK.2	-0.420452796	Q3UKU1_S501_M1_Ell2
ASS[Phospho (STY)]PPDRIDIFGR.2	-0.020900169	Q3UL36_S75_M1_Arglu1
ERASS[Phospho (STY)]PPDR.2	0.060375729	Q3UL36_S75_M1_Arglu1
ELLQTPNFR.2	0.281303301	#N/A
IC[Carbamidomethyl (C)]DEITGR.2	0.195193897	#N/A
GIDEGPDGLK.2	0.168989268	#N/A
KLTDIINNDHENVK.3	0.069320004	#N/A
FC[Carbamidomethyl (C)]ETTIGSK.2	0.067164507	#N/A
LISDIIR.2	0.311772632	#N/A
LQGPQTSAEVYR.2	-0.410184178	#N/A
VC[Carbamidomethyl (C)]IVGSGNWGSAVAK.2	0.158537989	#N/A
TIEELEKELLNGQK.3	0.135105861	#N/A
ELYLSNNGIEVIEGLENNNK.2	-0.423152702	#N/A
ELDLYDNQIK.2	-0.404340801	#N/A
IEGLQSLVNLR.2	0.018984596	#N/A
QIDATYVR.2	0.207769744	#N/A
SLETVYLER.2	-0.168989053	#N/A
C[Carbamidomethyl (C)]IENLEELQSLR.2	-0.063688935	#N/A
HGGGGIVANLS[Phospho (STY)]EQSLKDGVDR.3	0.10861373	Q3UM45_S45_M1_Ppp1r7
LQNLDALTNLTVLSVQSNR.2	-0.37246269	#N/A
RVES[Phospho (STY)]EES[Phospho (STY)]GDEEGKK.2	0.034992988	#N/A
LTMLDIASNR.2	0.297897912	#N/A
IENISNLHQLQM[Oxidation (M)]LELGSNR.3	-0.101033462	#N/A
KHGGGGIVANLS[Phospho (STY)]EQS[Phospho (STY)]LKDGVDR.3	-0.009408797	Q3UM45_S45_M2_Ppp1r7
VES[Phospho (STY)]EES[Phospho (STY)]GDEEGKK.2	0.112571197	#N/A
VESEES[Phospho (STY)]GDEEGKKHGGGGIVANLSEQSLK.4	0.165136285	Q3UM45_S27_M1_Ppp1r7
HGGGGIVANLS[Phospho (STY)]EQSLK.2	0.450876276	Q3UM45_S45_M1_Ppp1r7

RVES[Phospho (STY)]EES[Phospho (STY)]GDEEGK.2	0.06072481	#N/A
GAGQQQS[Phospho (STY)]QEMM[Oxidation (M)]EVDR.2	0.050605203	#N/A
GAGQQQS[Phospho (STY)]QEM[Oxidation (M)]M[Oxidation (M)]EVDRR.3	-0.194284603	#N/A
DAEDVDLTHYR.3	0.21707852	#N/A
ELDLYDNQIKK.3	-0.172634485	#N/A
GAEDPEEEHELAVDMETINLDR.3	0.027480222	#N/A
VAS[Phospho (STY)]PANVGTLHTLSR.2	-0.299417647	Q3UMB5_S788_M1_Smcr8
SS[Phospho (STY)]VESVLIK.2	0.117504821	Q3UMB5_S420_M1_Smcr8
SDS[Phospho (STY)]QASLTVPLSPHVVR.2	-0.116375903	Q3UMB5_S488_M1_Smcr8
[Acetyl (Protein N-term)]AVDTLS[Phospho (STY)]PDWDFDRVDDGSQK.3	-0.025022835	Q3UMB9_S7_M1_Washc4
RQSS[Phospho (STY)]LTFQSSDPEHVR.3	0.026072108	Q3UMF0_S1215_M1_Cobll1
TLS[Phospho (STY)]SPTGTETNPPK.2	-0.280020416	Q3UMF0_S896_M1_Cobll1
QDS[Phospho (STY)]NPKPKPSNEITR.3	0.022768755	Q3UMF0_S794_M1_Cobll1
ITVEKDPDSALGIS[Phospho (STY)]DGETSPSSK.3	0.148449387	Q3UMF0_S546_M1_Cobll1
QSS[Phospho (STY)]LTFQSSDPEHVR.2	-0.251781428	Q3UMF0_S1215_M1_Cobll1
TETFEDEGAENS[Phospho (STY)]FSR.2	0.189010567	Q3UMF9_S378_M1_Faxc
PSEVPAQS[Phospho (STY)]PTR.2	-0.268481534	Q3UMR0_S634_M1_Ankrd27
VAIYSPDGVR.2	0.313361271	#N/A
DLLSHEDAATLNDVK.3	-0.061728233	#N/A
TGS[Phospho (STY)]TGALGPSER.2	-0.226819403	Q3UMT1_S431_M1_Ppp1r12c
ALNGVSS[Phospho (STY)]PVSSNPK.2	-0.290185983	Q3UMT1_S403_M1_Ppp1r12c
GGSSEELHDS[Phospho (STY)]PR.2	0.037374155	Q3UMU9_S635_M1_Hdgfl2
GGS[Phospho (STY)]S[Phospho (STY)]GEELEDEEPVKK.2	0.038022209	#N/A
TRLAS[Phospho (STY)]ESANDDNEDS.2	-0.328992508	Q3UMU9_S659_M1_Hdgfl2
AERGGS[Phospho (STY)]S[Phospho (STY)]GEELEDEEPVK.2	-0.114395612	#N/A
AQEDGQDS[Phospho (STY)]EDGPR.2	-0.471612919	Q3UMU9_S620_M1_Hdgfl2
LAS[Phospho (STY)]ESANDDNEDS.2	-0.346993725	Q3UMU9_S659_M1_Hdgfl2
GGS[Phospho (STY)]S[Phospho (STY)]GEELEDEEPVKKR.3	-0.325872154	#N/A
GGS[Phospho (STY)]SGEELEDEEPVK.2	0.128260318	Q3UMU9_S366_M1_Hdgfl2
KRS[Phospho (STY)]EGLSLER.3	-0.292931976	Q3UMU9_S450_M1_Hdgfl2
AERGGS[Phospho (STY)]S[Phospho (STY)]GEELEDEEPVKK.3	-0.195539827	#N/A
RLS[Phospho (STY)]HDETNIFSTPR.3	-0.066709453	Q3UN16_S413_M1_Gpr162
RSS[Phospho (STY)]LDGSESAK.2	-0.305763053	Q3UN16_S256_M1_Gpr162
C[Carbamidomethyl (C)]SLTGS[Phospho (STY)]EGSSR.2	0.122034969	Q3UN16_S565_M1_Gpr162
RLS[Phospho (STY)]LGSPDSR.2	-0.216936105	Q3UN16_S539_M1_Gpr162
RC[Carbamidomethyl (C)]S[Phospho (STY)]LT[Phospho (STY)]GSEGSSR.2	-0.174616129	Q3UN16_S561_M2_Gpr162
DLAAAAAQKS[Phospho (STY)]PSAEAAAPPPGPR.3	-0.147716916	Q3UNH4_S691_M1_Gprin1
KAS[Phospho (STY)]LPASEK.2	-0.144624356	Q3UNH4_S468_M1_Gprin1
SLGTWGS[Phospho (STY)]LSAAK.2	-0.047950737	Q3UNH4_S446_M1_Gprin1
RERPGS[Phospho (STY)]TGEGDLVSLR.3	0.032620608	Q3UNH4_S219_M1_Gprin1
VITQIPGATSPGKVETPS[Phospho (STY)]LQK.3	0.488955042	Q3UNH4_S584_M1_Gprin1
VDIVS[Phospho (STY)]PGGDNAGSLR.2	-0.276626733	Q3UNH4_S182_M1_Gprin1
ERPGS[Phospho (STY)]TGEGDLVSLR.2	0.112995163	Q3UNH4_S219_M1_Gprin1
AETVS[Phospho (STY)]PGEVDAM[Oxidation (M)]TLGK.2	-0.297325426	Q3UNH4_S495_M1_Gprin1
KAS[Phospho (STY)]LPASEKTDPLASSK.3	-0.375679301	Q3UNH4_S468_M1_Gprin1
VDIVSPGGDNAGS[Phospho (STY)]LRK.2	-0.311587303	Q3UNH4_S190_M1_Gprin1
NSELVS[Phospho (STY)]PVKPER.2	-0.301600315	Q3UNH4_S369_M1_Gprin1

KTDPEFSGKLT[Phospho (STY)]PGSSGK.3	0.530269531	Q3UNH4_T256_M1_Gprin1
ADSASPS[Phospho (STY)]PR.2	-0.256061291	Q3UNH4_S622_M1_Gprin1
APS[Phospho (STY)]PAPRPPSR.2	-0.249781835	Q3UNH4_S764_M1_Gprin1
SVATGPM[Oxidation (M)]T[Phospho (STY)]PQAAAPPAVPPVFPEVR.3	-0.292177357	Q3UNH4_T795_M1_Gprin1
APS[Phospho (STY)]WDAGAPPPR.2	-0.400779208	Q3UNH4_S714_M1_Gprin1
S[Phospho (STY)]PSAEAAAPPPGPR.2	-0.059614213	Q3UNH4_S691_M1_Gprin1
MDGNS[Phospho (STY)]LKQADSTSTR.2	-0.218675498	Q3UNH4_S110_M1_Gprin1
IQQQLGEEAS[Phospho (STY)]PR.2	-0.388901559	Q3UPH7_S959_M1_Arhgef40
LVTFESVAVPLQQGAEQQR.2	-0.121231197	#N/A
DSDQVAQS[Phospho (STY)]DGEESPAAEEQLLGER.3	0.13869923	Q3UPL0_S526_M1_Sec31a
AQDGSS[Phospho (STY)]PLSLQDLIEK.2	0.245316798	Q3UPL0_S712_M1_Sec31a
VDGPDVALKDSDQVAQS[Phospho (STY)]DGEESPAAEEQLLGER.4	-0.130624139	Q3UPL0_S526_M1_Sec31a
S[Phospho (STY)]TQSLSLQR.2	-0.589621996	Q3UPL5_S217_M1_Ag2
TQPVTFDEIQEVEEEGVS[Phospho (STY)]PM[Oxidation (M)]EEEK.3	0.105328928	Q3UPL5_S196_M1_Ag2
NS[Phospho (STY)]SNEELTK.2	0.101641474	Q3UQN2_S402_M1_Fcho2
HS[Phospho (STY)]PVQMNR.2	0.2320592	Q3UQN2_S394_M1_Fcho2
SGS[Phospho (STY)]PGPLHSVSGYK.2	-0.018946556	Q3UQS8_S728_M1_Rbm20
EKEHC[Carbamidomethyl (C)]DS[Phospho (STY)]EGEADAFDPGKK.3	0.076299658	Q3UQU0_S103_M1_Brd9
EKEHC[Carbamidomethyl (C)]DS[Phospho (STY)]EGEADAFDPGK.3	-0.090342683	Q3UQU0_S103_M1_Brd9
HTAPS[Phospho (STY)]SPSPGTR.2	0.31344019	Q3UR70_S852_M1_Tgfbrap1
HTAPSS[Phospho (STY)]PSPGTRT.2	-0.235050883	Q3UR70_S853_M1_Tgfbrap1
ESDLSDTLS[Phospho (STY)]PSKEK.2	0.43464119	Q3URD3_S469_M1_Slmap
SDVIHAPLPS[Phospho (STY)]PVDK.2	0.249567165	Q3URD3_S148_M1_Slmap
AKESDLSDTLSPS[Phospho (STY)]KEK.2	-0.615507236	Q3URD3_S471_M1_Slmap
HKNS[Phospho (STY)]IDLEYVPR.3	0.014091494	Q3URE9_S576_M1_Lingo2
RSS[Phospho (STY)]IIKDPSNQK.2	0.11886223	Q3USB7_S78_M1_Plcl1
TVS[Phospho (STY)]FSSM[Oxidation (M)]PSEK.2	0.064008925	Q3USB7_S96_M1_Plcl1
S[Phospho (STY)]GVALPGAAGVPADSEAGLLEAAR.2	0.068861703	Q3USB7_S48_M1_Plcl1
RM[Oxidation (M)]S[Phospho (STY)]GDYNGEQK.2	-0.390386984	Q3USB7_S570_M1_Plcl1
KKT[Phospho (STY)]VS[Phospho (STY)]FSSMPSEK.2	0.113950024	Q3USB7_S96_M2_Plcl1
KLPSESDLLEGEVT[Phospho (STY)]DEDEEAEM[Oxidation (M)]SR.3	-0.144702355	Q3USB7_T557_M1_Plcl1
STGVQTS[Phospho (STY)]PDLRK.2	0.287159947	Q3USH1_S108_M1_Insyn2a
STGVQTS[Phospho (STY)]PDLR.2	0.109173268	Q3USH1_S108_M1_Insyn2a
GVS[Phospho (STY)]QEKDGQISSAIVSSVQSK.3	0.11506305	Q3USH5_S903_M1_Sfswap
VKLDDDS[Phospho (STY)]EEDEESR.2	-0.178427051	Q3USH5_S601_M1_Sfswap
RKS[Phospho (STY)]EPAVGPLR.3	-0.328072409	Q3UTJ2_S339_M1_Sorbs2
RGS[Phospho (STY)]LPDNSILHR.3	-0.540181333	Q3UTJ2_S829_M1_Sorbs2
ESQS[Phospho (STY)]PDSAWR.2	-0.41748678	Q3UTJ2_S40_M1_Sorbs2
S[Phospho (STY)]EPAVGPLR.2	-0.154276129	Q3UTJ2_S339_M1_Sorbs2
GAEDYPDPPLPHSYS[Phospho (STY)]SDR.2	-0.144606606	Q3UTJ2_S1097_M1_Sorbs2
VQS[Phospho (STY)]SPNLLAAGR.2	0.469323651	Q3UTJ2_S27_M1_Sorbs2
SFISSSPSS[Phospho (STY)]PSR.2	-0.562142894	Q3UTJ2_S382_M1_Sorbs2
TS[Phospho (STY)]PGRADLPGSSSTFTK.3	-0.314764701	Q3UTJ2_S358_M1_Sorbs2
APHYPGIGPVDESGIPTAIR.3	0.003815412	#N/A
SC[Carbamidomethyl (C)]DDLLNDDC[Carbamidomethyl (C)]DS[Phospho (STY)]FPDPK.2	0.205330718	Q3UTJ2_S474_M1_Sorbs2
GLLHGWSQSS[Phospho (STY)]TEEVPR.2	-0.331893437	Q3UTJ2_S577_M1_Sorbs2
QNAEIWSSTEETVS[Phospho (STY)]PK.2	-0.053684458	Q3UTJ2_S456_M1_Sorbs2

SRS[Phospho (STY)]C[Carbamidomethyl (C)]DDLLNDDC[Carbamidomethyl (C)]DSFPDPK.3	-0.299904521	Q3UTJ2_S463_M1_Sorbs2
GS[Phospho (STY)]LPDNSILHR.2	-0.451783821	Q3UTJ2_S829_M1_Sorbs2
RFS[Phospho (STY)]IESLLEEETQVR.3	-0.471988412	Q3UTJ2_S630_M1_Sorbs2
RRGS[Phospho (STY)]LPDNSILHR.4	-0.445918472	Q3UTJ2_S829_M1_Sorbs2
LLDRS[Phospho (STY)]PSR.2	0.225931998	Q3UTQ8_S306_M1_Cdkl5
SNS[Phospho (STY)]KDIQNLSVGLPR.3	-0.007367107	Q3UTQ8_S343_M1_Cdkl5
NLSEGNNANYTEY[Phospho (STY)]VATR.2	-0.192008495	Q3UTQ8_Y171_M1_Cdkl5
VSS[Phospho (STY)]LPSDSSSGTNHSK.2	-0.009372935	Q3UTQ8_S738_M1_Cdkl5
DLTNNNIPHLLS[Phospho (STY)]PK.2	0.067483417	Q3UTQ8_S407_M1_Cdkl5
KSS[Phospho (STY)]PSSPAVR.2	-0.461044657	Q3UTZ3_S495_M1_Trappc14
SQS[Phospho (STY)]FSHQQPSR.2	-0.108090105	Q3UTZ3_S517_M1_Trappc14
VAS[Phospho (STY)]LQEELQDITPEEEIAYK.2	-0.081812052	Q3UUF8_S260_M1_Ankrd34b
NSGANHY[Phospho (STY)]SSDSQLAEGVTPPTVEDGK.3	0.194468702	Q3UUF8_Y365_M1_Ankrd34b
HQS[Phospho (STY)]IDVK.2	-0.330325526	Q3UUF8_S293_M1_Ankrd34b
RGS[Phospho (STY)]GAFPLDHSLAQSR.3	-0.272574601	Q3UUF8_S426_M1_Ankrd34b
SVS[Phospho (STY)]LSKR.2	-0.276269467	Q3UUG6_S324_M1_Tbc1d24
QKS[Phospho (STY)]VSLSK.2	-0.060915959	Q3UUG6_S322_M1_Tbc1d24
S[Phospho (STY)]KSHEFQLGNR.3	0.149643432	Q3UVC0_S312_M1_Ksr2
TES[Phospho (STY)]VPC[Carbamidomethyl (C)]DINNPVR.2	0.092170287	Q3UVC0_S484_M1_Ksr2
TPNIVTTVT[Phospho (STY)]PPGT[Phospho (STY)]PPMR.2	0.041393791	Q3UVC0_T273_M2_Ksr2
KLS[Phospho (STY)]EASDER.2	-0.242203395	Q3UVG3_S671_M1_Fam91a1
[Acetyl (Protein N-term)]AAAAAVGPGLGSGPGDS[Phospho (STY)]PEGPEADAPER.3	0.114566641	Q3UVL4_S18_M1_Vps51
[Acetyl (Protein N-term)]AAAAAVGPGLGSGPGDS[Phospho (STY)]PEGPEADAPERR.3	-0.130589808	Q3UVL4_S18_M1_Vps51
IDVFS[Phospho (STY)]PVEFNK.2	0.205637207	Q3UVL4_S690_M1_Vps51
SIHS[Phospho (STY)]FELEKR.3	-0.378969252	Q3UVR3_S448_M1_Ttbk2
RGS[Phospho (STY)]SGETLR.2	-0.313759641	Q3UVX5_S870_M1_Grm5
TDDDAPS[Phospho (STY)]LHS[Phospho (STY)]ETAAR.2	-0.112359869	Q3UVX5_S1037_M2_Grm5
SVTWAQNEKST[Phospho (STY)]R.2	-0.306843703	Q3UVX5_T923_M1_Grm5
DSVDSGSTTPNS[Phospho (STY)]PVSESALC[Carbamidomethyl (C)]IPSSPK.3	0.034099274	Q3UVX5_S1173_M1_Grm5
SSS[Phospho (STY)]LVNLWK.2	0.290322301	Q3UVX5_S860_M1_Grm5
S[Phospho (STY)]PS[Phospho (STY)]PISTLSHLAGSAGR.2	-0.019468763	Q3UVX5_S1014_M2_Grm5
GS[Phospho (STY)]SGETLR.2	-0.28790752	Q3UVX5_S870_M1_Grm5
SFGGGTGSGFTSLLM[Oxidation (M)]ER.2	-0.090315299	#N/A
AVC[Carbamidomethyl (C)]M[Oxidation (M)]LSNTTAIVEAWAR.2	0.117133388	#N/A
IKDDIVSS[Phospho (STY)]LQK.2	0.122798227	Q3UXZ6_S58_M1_Fam81a
GGVGAPGS[Phospho (STY)]PSTPPTR.2	-0.470458748	Q3UYC0_S220_M1_Ppm1h
SS[Phospho (STY)]LPNGEGLQLK.2	0.476248648	Q3UYC0_S123_M1_Ppm1h
AGTITST[Phospho (STY)]PNR.2	0.207749002	Q3UYC0_T113_M1_Ppm1h
KAGTITST[Phospho (STY)]PNR.2	-0.090073363	Q3UYC0_T113_M1_Ppm1h
AGTITSTPNRNS[Phospho (STY)]K.2	-0.180005505	Q3UYC0_S118_M1_Ppm1h
RSS[Phospho (STY)]LPNGEGLQLK.2	-0.385481343	Q3UYC0_S123_M1_Ppm1h
GLS[Phospho (STY)]PPHKK.2	-0.384021868	Q3UYG8_S266_M1_Macrod2
GLS[Phospho (STY)]PPHK.2	-0.119152877	Q3UYG8_S266_M1_Macrod2
VIEPPAASC[Carbamidomethyl (C)]PSS[Phospho (STY)]PR.2	0.087605137	Q3UYI5_S510_M1_Rgl3
RGS[Phospho (STY)]GDTSISM[Oxidation (M)]DTEASIR.3	0.099340645	Q3UZ39_S88_M1_Lrrfip1
S[Phospho (STY)]PDANMPEEEGVVR.2	0.128904053	Q3UZA1_S333_M1_Rcsd1
LGQKS[Phospho (STY)]PDANMPEEEGVVR.3	0.402407026	Q3UZA1_S333_M1_Rcsd1

S[Phospho (STY)]KDPGSPQLNQEAMADGVEGTPWSAEKPR.4	-0.527381787	Q3UZA1_S211_M1_Rcsd1
SFSDVLPES[Phospho (STY)]PK.2	0.031468574	Q3V0G7_S455_M1_Garnl3
IC[Carbamidomethyl (C)]LPYS[Phospho (STY)]PTK.2	0.289180251	Q3V0G7_S179_M1_Garnl3
GASAHTS[Phospho (STY)]PQT[Phospho (STY)]PPAR.2	0.364632814	Q3V0G7_S848_M2_Garnl3
ALELEPLELEGSLAGS[Phospho (STY)]PPGLAPPPPPHR.3	0.094430434	Q3V0I2_S68_M1_Prr7
NRTS[Phospho (STY)]VDFK.2	-0.312040115	Q3V1L4_S511_M1_Nt5c2
STS[Phospho (STY)]LKER.2	0.311413531	Q3V3Q7_S408_M1_Pacs2
TES[Phospho (STY)]LVIPSTR.2	-0.020624452	Q3V3Q7_S387_M1_Pacs2
ANS[Phospho (STY)]LDNER.2	0.11988355	Q3V3Q7_S424_M1_Pacs2
ANS[Phospho (STY)]LDNERC[Carbamidomethyl (C)]PDTR.2	-0.070853657	Q3V3Q7_S424_M1_Pacs2
EPSGQPEDS[Phospho (STY)]PEAETSTLDVFTEK.3	-0.037513454	Q3V3Q7_S361_M1_Pacs2
LQRS[Phospho (STY)]PVLK.2	-0.172629723	Q3V3V9_S1242_M1_Carmil2
GPRPDLETS[Phospho (STY)]PGAAAR.2	-0.162963765	Q3V3V9_S1008_M1_Carmil2
AGS[Phospho (STY)]DGDIM[Oxidation (M)]DSSTETPPISIK.2	-0.086494683	#N/A
KAGS[Phospho (STY)]DGDIM[Oxidation (M)]DSSTETPPISIK.2	-0.255366118	#N/A
PLPPYPTEPSSPERSPPS[Phospho (STY)]PAT[Phospho (STY)]DQR.3	0.293680968	Q3V3V9_S1284_M2_Carmil2
TEYVC[Carbamidomethyl (C)]DS[Phospho (STY)]PTEGPSK.2	0.206679518	Q3ZK22_S667_M1_Vezt
DLS[Phospho (STY)]PELAPDEGAR.2	0.022039708	Q497V6_S182_M1_Bahd1
AHS[Phospho (STY)]PQGEGEIPLHR.3	-0.418564202	Q4ACU6_S482_M1_Shank3
S[Phospho (STY)]LGEEPVGGLGSLLDPAK.2	-0.336990519	Q4ACU6_S1510_M1_Shank3
S[Phospho (STY)]KLWGDPVESR.2	-0.025735767	Q4ACU6_S1471_M1_Shank3
STS[Phospho (STY)]M[Oxidation (M)]QDTVR.2	-0.009241952	Q4ACU6_S801_M1_Shank3
S[Phospho (STY)]M[Oxidation (M)]TAELEELASIR.2	-0.08270629	Q4ACU6_S694_M1_Shank3
SAS[Phospho (STY)]DINLKGDQPAASPGPTLR.3	-0.323569048	Q4ACU6_S375_M1_Shank3
RAPSPVKPAS[Phospho (STY)]LER.3	0.168991909	Q4ACU6_S1583_M1_Shank3
S[Phospho (STY)]KSM[Oxidation (M)]TAELEELASIR.3	0.261492525	Q4ACU6_S692_M1_Shank3
TKS[Phospho (STY)]VGEDEKLASLLEGR.3	-0.221141993	Q4ACU6_S781_M1_Shank3
FM[Oxidation (M)]DY[Phospho (STY)]VQLHSTDK.2	-0.040410718	Q4ACU6_Y122_M1_Shank3
LFSSLGELSTISAQR.2	0.321786631	#N/A
S[Phospho (STY)]LGEEPVGGLGSLLDPAKK.2	-0.194855159	Q4ACU6_S1510_M1_Shank3
APS[Phospho (STY)]TTLTLR.2	-0.238300047	Q4ACU6_S685_M1_Shank3
HYTVGSY[Phospho (STY)]DSLTSHSDYVIDDK.3	-0.082026411	Q4ACU6_Y555_M1_Shank3
SAS[Phospho (STY)]DINLK.2	0.487646333	Q4ACU6_S375_M1_Shank3
LDEILAVAAEPTLRPDIADADSR.3	-0.126997679	#N/A
GPRPGSLDY[Phospho (STY)]SSGEGLGLTFGGPSPGPVK.3	-0.437386439	Q4ACU6_Y998_M1_Shank3
SPTPVHS[Phospho (STY)]PDADRPGPLFVDVQTR.3	-0.385062938	Q4ACU6_S1134_M1_Shank3
VPS[Phospho (STY)]PDHR.2	-0.179058175	Q4FK66_S209_M1_Prpf38a
SKS[Phospho (STY)]DPVLHHSEER.3	0.043817812	Q4FZH1_S114_M1_Fam189a2
DKS[Phospho (STY)]PSSLLEDAK.2	-0.064961132	Q4JIM5_S632_M1_Abl2
STQASSGS[Phospho (STY)]PALPR.2	0.0472671	Q4JIM5_S621_M1_Abl2
S[Phospho (STY)]NSTSSM[Oxidation (M)]SSGLPEQDR.2	0.109695325	Q4JIM5_S783_M1_Abl2
TVS[Phospho (STY)]TSSQPEENVDR.2	0.119626547	Q4JIM5_S819_M1_Abl2
[Acetyl (Protein N-term)]M[Oxidation (M)]NVEHEVNLLVEEIHR.3	0.044543394	#N/A
SQS[Phospho (STY)]VDITAPGFNPLGGAGK.2	-0.33738877	Q4KMM3_S15_M1_Oxr1
ESLPIELDQLS[Phospho (STY)]GR.2	0.052610273	Q4KMM3_S294_M1_Oxr1
DTIQQVSQR.2	0.365034602	#N/A
GTIEYTVES[Phospho (STY)]R.2	-0.321439201	Q4KMM3_S104_M1_Oxr1

TSSNPGALSHETGLSGLETATK.3	-0.1988674	#N/A
DSETEVEELRK.2	-0.207906753	#N/A
DQGNDS[Phospho (STY)]ASTAPR.2	-0.025672061	Q4KMM3_S323_M1_Oxr1
DQQGQELKR.2	-0.224188809	#N/A
DQQGQELKRDS[Phospho (STY)]ETEVEELR.3	0.048632931	Q4KMM3_S488_M1_Oxr1
RM[Oxidation (M)]S[Phospho (STY)]FQKPK.2	0.131780528	Q4KMM3_S90_M1_Oxr1
VVSSTS[Phospho (STY)]EEEEAFTEK.2	-0.222709528	Q4KMM3_S204_M1_Oxr1
DSDGQVFGALASEPFK.2	-0.129945725	#N/A
TPIIEEEQNNSANTQK.2	0.106871693	#N/A
FDTTPNELVQLNK.2	-0.231933824	#N/A
KATGVS[Phospho (STY)]AEDADPR.2	-0.563221559	Q4KMM3_S308_M1_Oxr1
RDS[Phospho (STY)]ETEVEELRK.3	0.002292074	Q4KMM3_S488_M1_Oxr1
ADLEPESFRPNLSDPSELLLPDQIEK.3	-0.049040744	#N/A
M[Oxidation (M)]S[Phospho (STY)]FQKPK.2	0.0616174	Q4KMM3_S90_M1_Oxr1
IKESLPIELDQLSGR.3	0.11586452	#N/A
DSLNSIALK.2	-0.230249983	#N/A
KS[Phospho (STY)]QSVDITAPGFNPLGGAGK.3	-0.26792417	Q4KMM3_S13_M1_Oxr1
TFVSQASATM[Oxidation (M)]QQYAQR.2	-0.069396631	#N/A
SSDASSESVQTVSQMEVQSLTATSEAANVPDR.3	0.132765702	#N/A
ARDQGNDS[Phospho (STY)]ASTAPR.2	0.311194455	Q4KMM3_S323_M1_Oxr1
HEYWFAVPQER.2	-0.068101463	#N/A
ALS[Phospho (STY)]PTGSSR.2	-0.109575864	Q4KUS2_S255_M1_Unc13a
ESYSDSM[Oxidation (M)]HS[Phospho (STY)]YEEFSEPR.3	-0.11594106	Q4KUS2_S244_M1_Unc13a
HS[Phospho (STY)]QTDLVSR.2	-0.343543422	Q4QQM4_S14_M1_Trp53i11
KHS[Phospho (STY)]QTDLVSR.2	-0.29061292	Q4QQM4_S14_M1_Trp53i11
APS[Phospho (STY)]PSQPPKK.2	-0.039396692	Q4VA53_S1381_M1_Pds5b
TAS[Phospho (STY)]DSDEQQWPEEK.2	-0.069307409	Q4VA53_S1255_M1_Pds5b
M[Oxidation (M)]ETVSNASSSSNPSS[Phospho (STY)]PGR.2	0.057186302	Q4VA53_S1166_M1_Pds5b
AES[Phospho (STY)]PETSAVESTQSTPQK.2	-0.199285518	Q4VA53_S1356_M1_Pds5b
HKEELLENEDEQNS[Phospho (STY)]PPKK.4	-0.34262723	Q4VA53_S1281_M1_Pds5b
SSS[Phospho (STY)]PVLVEEEPER.2	-0.407780326	Q501J7_S118_M1_Phactr4
QHRES[Phospho (STY)]GEGEEEVADSAR.3	-0.017002138	Q505F5_S293_M1_Lrrc47
KRQS[Phospho (STY)]VSGLHR.3	-0.296315805	Q505F5_S430_M1_Lrrc47
RQS[Phospho (STY)]VSGLHR.2	-0.08386606	Q505F5_S430_M1_Lrrc47
KQHRES[Phospho (STY)]GEGEEEVADSAR.3	-0.54548441	Q505F5_S293_M1_Lrrc47
IVS[Phospho (STY)]PKQEHEDR.2	-0.515682428	Q50H33_S331_M1_Kctd8
RNS[Phospho (STY)]ELFQSLISK.2	-0.056900498	Q50H33_S413_M1_Kctd8
SDVGTSELPS[Phospho (STY)]EDDGVEPSR.2	0.127193147	Q52KB6_S453_M1_C2cd3
EVKAEEKS[Phospho (STY)]PVSINVK.3	-0.204156887	Q52KE7_S358_M1_Ccnl1
QQAS[Phospho (STY)]KSPYNGVR.3	-0.483646387	Q52KE7_S378_M1_Ccnl1
LRPVS[Phospho (STY)]PEEIRR.3	-0.380514884	Q52KF3_S229_M1_Spire1
S[Phospho (STY)]VDKSDEELQFPK.2	0.127757994	Q52KF3_S520_M1_Spire1
RHS[Phospho (STY)]IEKETPTNVR.3	-0.471209029	Q52KF3_S365_M1_Spire1
SRS[Phospho (STY)]VDKSDEELQFPK.3	-0.4758711	Q52KF3_S520_M1_Spire1
APTLAELDSS[Phospho (STY)]DS[Phospho (STY)]EEEKSLHK.3	-0.030396312	Q52KF3_S306_M2_Spire1
HS[Phospho (STY)]IEKETPTNVR.3	0.256037942	Q52KF3_S365_M1_Spire1
RLS[Phospho (STY)]PS[Phospho (STY)]AS[Phospho (STY)]PPR.2	-0.227816162	Q52KI8_S387_M3_Srrm1

TAS[Phospho (STY)]PPPPPKR.2	-0.096191403	Q52KI8_S635_M1_Srrm1
RRT[Phospho (STY)]PS[Phospho (STY)]PPPR.2	-0.166736532	Q52KI8_S602_M2_Srrm1
APQTSS[Phospho (STY)]PPPVR.2	0.173029019	Q52KI8_S714_M1_Srrm1
SRVS[Phospho (STY)]VS[Phospho (STY)]PGR.2	-0.207592459	Q52KI8_S427_M2_Srrm1
AAS[Phospho (STY)]PSPQSVR.2	-0.195302346	Q52KI8_S779_M1_Srrm1
RVS[Phospho (STY)]HS[Phospho (STY)]PPPK.2	-0.014076651	Q52KI8_S655_M2_Srrm1
RRS[Phospho (STY)]PT[Phospho (STY)]PPPR.2	-0.213213171	Q52KI8_S591_M2_Srrm1
S[Phospho (STY)]PS[Phospho (STY)]PPPAR.2	-0.245108157	Q52KI8_S561_M2_Srrm1
RRS[Phospho (STY)]PS[Phospho (STY)]PPPAR.2	-0.243774559	Q52KI8_S561_M2_Srrm1
RYS[Phospho (STY)]PPIQR.2	-0.100561047	Q52KI8_S616_M1_Srrm1
WQS[Phospho (STY)]PVTK.2	-0.308462225	Q52KI8_S550_M1_Srrm1
S[Phospho (STY)]PT[Phospho (STY)]PPPR.2	-0.13121119	Q52KI8_S591_M2_Srrm1
RRS[Phospho (STY)]PS[Phospho (STY)]LSSK.2	-0.368781934	Q52KI8_S672_M2_Srrm1
RAS[Phospho (STY)]PS[Phospho (STY)]PPPK.2	-0.107479005	Q52KI8_S645_M2_Srrm1
S[Phospho (STY)]PELPEPSVR.2	0.31795254	Q52KI8_S220_M1_Srrm1
S[Phospho (STY)]RS[Phospho (STY)]YS[Phospho (STY)]PR.2	-0.06041716	Q52KI8_S304_M3_Srrm1
AAS[Phospho (STY)]PS[Phospho (STY)]PQSVRR.2	-0.259397044	Q52KI8_S779_M2_Srrm1
KAQVS[Phospho (STY)]PQS.2	0.177277378	Q52KI8_S943_M1_Srrm1
AKS[Phospho (STY)]PT[Phospho (STY)]PSLS[Phospho (STY)]PAR.2	0.028096893	Q52KI8_S832_M3_Srrm1
RWQS[Phospho (STY)]PVTK.2	-0.106521259	Q52KI8_S550_M1_Srrm1
QRS[Phospho (STY)]PTVTK.2	-0.161314411	Q52KI8_S664_M1_Srrm1
S[Phospho (STY)]PS[Phospho (STY)]PPPARR.2	-0.19351054	Q52KI8_S561_M2_Srrm1
GAS[Phospho (STY)]AS[Phospho (STY)]PQGRQS[Phospho (STY)]PSPSTRPIR.3	-0.313760436	Q52KI8_S723_M3_Srrm1
RS[Phospho (STY)]PT[Phospho (STY)]PPPR.2	0.337917575	Q52KI8_S591_M2_Srrm1
RS[Phospho (STY)]PS[Phospho (STY)]PPPAR.2	-0.44342381	Q52KI8_S561_M2_Srrm1
APKPEPVPEPKEPS[Phospho (STY)]PEK.3	-0.439744577	Q52KI8_S260_M1_Srrm1
RVS[Phospho (STY)]HS[Phospho (STY)]PPPKQR.3	-0.167126475	Q52KI8_S655_M2_Srrm1
SVS[Phospho (STY)]GS[Phospho (STY)]PEPAAK.2	0.388920935	Q52KI8_S795_M2_Srrm1
TAS[Phospho (STY)]PPPPPK.2	-0.234463833	Q52KI8_S635_M1_Srrm1
AS[Phospho (STY)]PS[Phospho (STY)]PPPKR.2	-0.052120697	Q52KI8_S645_M2_Srrm1
RRS[Phospho (STY)]PS[Phospho (STY)]PAPPPPPPPPPR.3	-0.354907246	#N/A
VSHS[Phospho (STY)]PPPK.2	-0.03823794	Q52KI8_S657_M1_Srrm1
KETES[Phospho (STY)]EAEDDNLDDLER.2	0.34124761	Q52KI8_S915_M1_Srrm1
S[Phospho (STY)]PSPAPPPPPPPPPR.2	0.222810573	Q52KI8_S572_M1_Srrm1
RES[Phospho (STY)]PS[Phospho (STY)]PAPKPR.2	-0.004899511	Q52KI8_S448_M2_Srrm1
EKS[Phospho (STY)]PELPEPSVR.2	-0.444946642	Q52KI8_S220_M1_Srrm1
RHS[Phospho (STY)]PS[Phospho (STY)]PRPR.2	-0.199394519	Q52KI8_S702_M2_Srrm1
QS[Phospho (STY)]PSPSTRPIR.2	0.227426613	Q52KI8_S731_M1_Srrm1
RYS[Phospho (STY)]PS[Phospho (STY)]PPPK.2	-0.125100565	Q52KI8_S624_M2_Srrm1
Y[Phospho (STY)]SPPIQR.2	0.260929749	Q52KI8_Y615_M1_Srrm1
S[Phospho (STY)]RS[Phospho (STY)]PS[Phospho (STY)]PAPEKK.2	-0.030728532	Q52KI8_S207_M3_Srrm1
RVS[Phospho (STY)]RT[Phospho (STY)]PEPK.2	-0.253754021	Q52KI8_S743_M2_Srrm1
TRHS[Phospho (STY)]PTPQQSNR.3	-0.086537634	Q52KI8_S412_M1_Srrm1
RES[Phospho (STY)]PSPAPK.2	-0.173273452	Q52KI8_S448_M1_Srrm1
RAS[Phospho (STY)]PS[Phospho (STY)]PPPKR.2	-0.029401004	Q52KI8_S645_M2_Srrm1
KSRVS[Phospho (STY)]VS[Phospho (STY)]PGR.2	0.25890989	Q52KI8_S427_M2_Srrm1
RRT[Phospho (STY)]AS[Phospho (STY)]PPPPPK.2	-0.529771893	Q52KI8_S635_M2_Srrm1

RYS[Phospho (STY)]PS[Phospho (STY)]PPPKR.2	-0.018948347	Q52KI8_S624_M2_Srrm1
S[Phospho (STY)]PS[Phospho (STY)]PAPPPPPPPPPPRR.3	0.062272896	#N/A
RRAS[Phospho (STY)]PS[Phospho (STY)]PPPK.2	0.104914115	Q52KI8_S645_M2_Srrm1
KVELS[Phospho (STY)]ES[Phospho (STY)]EEDKGSK.2	-0.070730446	Q52KI8_S461_M2_Srrm1
HRPSS[Phospho (STY)]PAT[Phospho (STY)]PPPK.2	-0.35752045	Q52KI8_S401_M2_Srrm1
KPPAPPS[Phospho (STY)]PVQSQS[Phospho (STY)]PSTNWSPAVPAK.3	0.134741825	Q52KI8_S810_M2_Srrm1
KPPAPPS[Phospho (STY)]PVQS[Phospho (STY)]QSPSTNWS[Phospho (STY)]PAVPAKK.4	-0.154477555	Q52KI8_S810_M3_Srrm1
APQTSS[Phospho (STY)]PPPVRR.2	-0.032971425	Q52KI8_S714_M1_Srrm1
RS[Phospho (STY)]PS[Phospho (STY)]PAPPPPPPPPPPR.3	0.229582634	#N/A
LS[Phospho (STY)]PSAS[Phospho (STY)]PPR.2	0.124652425	Q52KI8_S387_M2_Srrm1
VS[Phospho (STY)]VS[Phospho (STY)]PGR.2	-0.539986884	Q52KI8_S427_M2_Srrm1
RGAS[Phospho (STY)]AS[Phospho (STY)]PQGR.2	0.28737443	Q52KI8_S723_M2_Srrm1
EARS[Phospho (STY)]PQPNK.2	-0.107763299	Q52KI8_S694_M1_Srrm1
HS[Phospho (STY)]PS[Phospho (STY)]PRPR.2	-0.155515539	Q52KI8_S702_M2_Srrm1
RRVS[Phospho (STY)]HS[Phospho (STY)]PPPK.3	-0.05862274	Q52KI8_S655_M2_Srrm1
RRS[Phospho (STY)]PS[Phospho (STY)]PR.2	0.121675379	Q52KI8_S609_M2_Srrm1
SSGQESESIPEYTAEEEREDNR.3	0.046738493	#N/A
SSGQESES[Phospho (STY)]IPEYTAEEER.2	-0.450315132	Q52KR3_S2850_M1_Prune2
NLSLTC[Carbamidomethyl (C)]FAGEEPSS[Phospho (STY)]PER.2	0.033256737	Q52KR3_S594_M1_Prune2
DTSLTS[Phospho (STY)]GSPTSDLGQTWNNSK.2	-0.049218041	Q52KR3_S876_M1_Prune2
ASVSDLS[Phospho (STY)]PR.2	-0.295696794	Q569Z6_S243_M1_Thrap3
ERS[Phospho (STY)]PALK.2	-0.325459501	Q569Z6_S248_M1_Thrap3
S[Phospho (STY)]REEEWDPEYTPK.2	-0.377398129	Q569Z6_S860_M1_Thrap3
NKKS[Phospho (STY)]PEIHR.2	-0.18580615	Q569Z6_S669_M1_Thrap3
IDIS[Phospho (STY)]PSTFR.2	-0.414453441	Q569Z6_S679_M1_Thrap3
ERS[Phospho (STY)]PALKS[Phospho (STY)]PLQSVVVR.3	-0.096061355	Q569Z6_S248_M2_Thrap3
S[Phospho (STY)]YS[Phospho (STY)]PAHNR.2	0.113715433	Q569Z6_S53_M2_Thrap3
M[Oxidation (M)]DS[Phospho (STY)]FDEDLAR.2	-0.00477529	Q569Z6_S572_M1_Thrap3
FSGEEGEIEDDESGT[Phospho (STY)]ENREEK.3	-0.311612876	Q569Z6_T937_M1_Thrap3
IDIS[Phospho (STY)]PSTFRK.2	-0.126586032	Q569Z6_S679_M1_Thrap3
S[Phospho (STY)]PRPSPVPK.2	0.073387187	Q569Z6_S264_M1_Thrap3
M[Oxidation (M)]DS[Phospho (STY)]FDEDLARPSGLLAQER.3	-0.022561609	Q569Z6_S572_M1_Thrap3
S[Phospho (STY)]RS[Phospho (STY)]YS[Phospho (STY)]PAHNR.2	-0.454614624	Q569Z6_S51_M3_Thrap3
WAHDKFS[Phospho (STY)]GEEGEIEDDESGTENR.3	-0.198743214	Q569Z6_S924_M1_Thrap3
SS[Phospho (STY)]FSITR.2	0.099991302	Q569Z6_S557_M1_Thrap3
WAHDKFSGEEGEIEDDES[Phospho (STY)]GTENREEK.4	-0.028580704	Q569Z6_S935_M1_Thrap3
FSGEEGEIEDDES[Phospho (STY)]GTENR.2	0.041748447	Q569Z6_S935_M1_Thrap3
EQKLS[Phospho (STY)]TDDLKTEEEGK.3	0.360524631	Q56A07_S192_M1_Scn2b
EQKLS[Phospho (STY)]TDDLK.2	0.072812524	Q56A07_S192_M1_Scn2b
LS[Phospho (STY)]TDDLKTEEEGK.2	0.205498567	Q56A07_S192_M1_Scn2b
YDVSVTLK.2	-0.152353811	#N/A
KHS[Phospho (STY)]PDRQDGPVPK.3	-0.303311509	Q56A08_S348_M1_Gpkow
DRHS[Phospho (STY)]PFSQR.3	-0.475591565	Q56A10_S1452_M1_Znf608
APGS[Phospho (STY)]PGAGNPPGTPK.2	-0.206584985	Q56A10_S626_M1_Znf608
STSFMSVS[Phospho (STY)]PSK.2	-0.001236526	Q570Y9_S265_M1_Deptor
ANS[Phospho (STY)]LPAGTR.2	-0.375547391	Q571I4_S40_M1_Prag1
TLVHSSS[Phospho (STY)]DGHIDPQHTAGK.3	0.293400267	Q571K4_S103_M1_Tab3

YLYM[Oxidation (M)]EYHS[Phospho (STY)]PEDNR.2	-0.003179941	Q571K4_S60_M1_Tab3
SSSSGSDDY[Phospho (STY)]AYTQALLLHQR.2	0.184516351	Q571K4_Y516_M1_Tab3
ETDYPAGEELSES[Phospho (STY)]GQVDK.2	-0.154665296	Q58A65_S815_M1_Spag9
SASQSS[Phospho (STY)]LDKLDQELKEQQK.3	0.136297102	Q58A65_S733_M1_Spag9
SASQSS[Phospho (STY)]LDKLDQELK.2	-0.078566535	Q58A65_S733_M1_Spag9
SQS[Phospho (STY)]GEDESLNQPGPIK.2	-0.389939444	Q5BL07_S1212_M1_Pex1
TPS[Phospho (STY)]QEGC[Carbamidomethyl (C)]QDLTQEQR.2	-0.250133934	Q5BL07_S1182_M1_Pex1
NTTEEVGS[Phospho (STY)]PAKEK.2	-0.188281556	Q5BLK4_S705_M1_Tut7
SSS[Phospho (STY)]ISS[Phospho (STY)]IDKDSK.2	-0.45089619	Q5DQR4_S819_M2_Stxbp5l
RLS[Phospho (STY)]SADVSK.2	0.313552521	Q5DQR4_S770_M1_Stxbp5l
SLSGSTNTVSS[Phospho (STY)]EGVTK.2	-0.124077732	Q5DQR4_S598_M1_Stxbp5l
TSYQS[Phospho (STY)]LTELNDSPVPLELER.2	-0.314242931	Q5DQR4_S727_M1_Stxbp5l
EEIQESLTSDYFQIC[Carbamidomethyl (C)]K.2	-0.142959931	#N/A
SRSS[Phospho (STY)]S[Phospho (STY)]ISS[Phospho (STY)]IDKDSK.2	0.065137124	Q5DQR4_S818_M3_Stxbp5l
FPSKSSPS[Phospho (STY)].2	-0.307440163	Q5DTL9_S1118_M1_Slc4a10
S[Phospho (STY)]FADIGKK.2	-0.230078634	Q5DTL9_S238_M1_Slc4a10
ENS[Phospho (STY)]TVDFSK.2	-0.188595325	Q5DTL9_S280_M1_Slc4a10
NAGQVVS[Phospho (STY)]PQSAPAC[Carbamidomethyl (C)]AENK.2	-0.259521982	Q5DTL9_S261_M1_Slc4a10
GHTS[Phospho (STY)]PC[Carbamidomethyl (C)]GMK.2	0.015163126	Q5DTL9_S297_M1_Slc4a10
ERDSGLEDGRES[Phospho (STY)]PSFDTPSQR.4	-0.707582013	Q5DTL9_S89_M1_Slc4a10
DSGLEDGRES[Phospho (STY)]PSFDTPSQR.3	-0.565308903	Q5DTL9_S89_M1_Slc4a10
DDPSVINIS[Phospho (STY)]DEM[Oxidation (M)]SK.2	0.43524789	Q5DTL9_S1085_M1_Slc4a10
ESRFPSKS[Phospho (STY)]SPS.2	0.269924093	Q5DTL9_S1115_M1_Slc4a10
KQS[Phospho (STY)]EPNSMDK.2	0.360928381	Q5DTL9_S247_M1_Slc4a10
NDVS[Phospho (STY)]RENSTVDFSK.2	-0.256746079	Q5DTL9_S276_M1_Slc4a10
S[Phospho (STY)]FADIGK.2	-0.071581917	Q5DTL9_S238_M1_Slc4a10
ALVVPEPEPDSDS[Phospho (STY)]NQER.2	0.199475267	Q5DTM8_S138_M1_Rnf20
KALVVPEPEPDSDS[Phospho (STY)]NQER.2	-0.448029347	Q5DTM8_S138_M1_Rnf20
S[Phospho (STY)]PAINFHHTPFVDGK.3	0.012190131	Q5DTN8_S622_M1_Jakmip3
RPS[Phospho (STY)]SLNDLDQSQDER.2	-0.125309588	Q5DTN8_S383_M1_Jakmip3
S[Phospho (STY)]LSELADPNPK.2	-0.15246546	Q5DTT2_S720_M1_Psd
FC[Carbamidomethyl (C)]SEGDC[Carbamidomethyl (C)]AIS[Phospho (STY)]PPR.2	-0.015430989	Q5DTT2_S17_M1_Psd
LEASTS[Phospho (STY)]DPLPAGGGSVLPGSR.2	-0.174831056	Q5DTT2_S156_M1_Psd
LALGS[Phospho (STY)]TDTLSNGQK.2	-0.132765281	Q5DTT2_S543_M1_Psd
AQRPGS[Phospho (STY)]ETR.2	0.188477965	Q5DTT2_S1011_M1_Psd
RQS[Phospho (STY)]DPSIR.2	-0.077481251	Q5DTV4_S492_M1_Zc3h12c
DFS[Phospho (STY)]PRPVSR.2	-0.16850937	Q5DTX6_S954_M1_Jcad
AAPLYC[Carbamidomethyl (C)]LSEPRGS[Phospho (STY)]QELTK.3	-0.309671593	Q5DTX6_S980_M1_Jcad
SADS[Phospho (STY)]VEEPDPLK.2	-0.014797479	Q5DTX6_S1248_M1_Jcad
S[Phospho (STY)]PPQHIPNPYLEDPVPR.3	-0.252942619	Q5DTX6_S351_M1_Jcad
GPT[Phospho (STY)]PS[Phospho (STY)]PVNEQSSAFVHSSPR.3	0.085318044	Q5DTX6_S486_M2_Jcad
DQQTQTS[Phospho (STY)]FHEDSK.2	0.008840609	Q5DTX6_S693_M1_Jcad
TAQQPAGLALENVAS[Phospho (STY)]PDRR.3	-0.082541475	Q5DTX6_S888_M1_Jcad
SESLVLATDTNNNDFK.2	0.304818258	#N/A
AEGHASS[Phospho (STY)]PQPQSEGTC[Carbamidomethyl (C)]K.2	-0.099588887	Q5DTX6_S543_M1_Jcad
APQASIC[Carbamidomethyl (C)]SDVDGFPTSQAT[Phospho (STY)]SPEPGKKDEEAK.4	-0.536332469	Q5DTX6_T1164_M1_Jcad
RKS[Phospho (STY)]DLLR.2	-0.471962711	Q5DTY9_S362_M1_Kctd16

TLTSGS[Phospho (STY)]RESNISSKK.3	-0.208528084	Q5DTY9_S372_M1_Kctd16
WSSS[Phospho (STY)]HC[Carbamidomethyl (C)]DC[Carbamidomethyl (C)]C[Carbamidomethyl (C)]C[Carbamidomethyl (C)]K.2	0.286070335	Q5DTY9_S286_M1_Kctd16
TLTSGS[Phospho (STY)]RESNISSK.2	-0.535873716	Q5DTY9_S372_M1_Kctd16
QSPDEFC[Carbamidomethyl (C)]HS[Phospho (STY)]DFEDASQGSDTR.3	0.039100815	Q5DTY9_S137_M1_Kctd16
EKLS[Phospho (STY)]IEEELEK.2	-0.077083382	Q5DTY9_S389_M1_Kctd16
QSNIQT[Phospho (STY)]LDRPIKK.3	-0.07560946	Q5DTY9_T340_M1_Kctd16
QSNIQT[Phospho (STY)]LDRPIK.2	-0.361207758	Q5DTY9_T340_M1_Kctd16
LLAPEDVKQS[Phospho (STY)]PDEFC[Carbamidomethyl (C)]HSDFEDASQGSDTR.4	-0.302934093	Q5DTY9_S130_M1_Kctd16
S[Phospho (STY)]PHIPYK.2	-0.038767672	Q5DU02_S237_M1_Usp22
DPLGARDS[Phospho (STY)]PGR.2	0.050030544	Q5DU25_S82_M1_Iqsec2
HSWDS[Phospho (STY)]PAFNNDVVQR.2	0.116423638	Q5DU25_S734_M1_Iqsec2
SSS[Phospho (STY)]PGAGGGHSTSTSTSPATTLQR.3	0.098045788	Q5DU25_S214_M1_Iqsec2
SS[Phospho (STY)]LEDTYGAGDGLK.2	-0.319008862	Q5DU25_S1114_M1_Iqsec2
LIEAFSQR.2	-0.021827003	#N/A
M[Oxidation (M)]QFS[Phospho (STY)]FEEYEK.2	-0.245526273	Q5DU25_S383_M1_Iqsec2
GALSSS[Phospho (STY)]LR.2	-0.170039598	Q5DU25_S1133_M1_Iqsec2
QLVYEADGC[Carbamidomethyl (C)]S[Phospho (STY)]PHGTLK.2	0.191259336	Q5DU25_S617_M1_Iqsec2
ESQY[Phospho (STY)]QNLR.2	-0.12663231	Q5DU25_Y89_M1_Iqsec2
DPLGARDS[Phospho (STY)]PGRESQYQNLR.3	0.342481784	Q5DU25_S82_M1_Iqsec2
ETRHS[Phospho (STY)]WDSPAFNNDVVQR.3	0.003088071	Q5DU25_S731_M1_Iqsec2
[Acetyl (Protein N-term)]M[Oxidation (M)]EAGSGPPGGPGSES[Phospho (STY)]PNR.2	0.095627588	Q5DU25_S15_M1_Iqsec2
GELHRDPLGARDS[Phospho (STY)]PGR.3	-0.374800789	Q5DU25_S82_M1_Iqsec2
SS[Phospho (STY)]LEDTYGAGDGLKR.2	0.13156865	Q5DU25_S1114_M1_Iqsec2
AAHLPLLTIEPPSDSS[Phospho (STY)]VDLSDR.3	-0.163601235	Q5DU25_S593_M1_Iqsec2
DS[Phospho (STY)]PGRESQYQNLR.2	-0.021649503	Q5DU25_S82_M1_Iqsec2
LRSS[Phospho (STY)]ASESR.2	-0.333234035	Q5DU25_S363_M1_Iqsec2
ALSDSYELS[Phospho (STY)]TDLQDK.2	0.088049794	Q5DU25_S316_M1_Iqsec2
EAS[Phospho (STY)]YQLQDTTGYTAR.2	0.330143082	Q5DU25_S138_M1_Iqsec2
AQNPAYFEGKPAS[Phospho (STY)]LDEGAM[Oxidation (M)]AGAR.3	-0.417077061	Q5DU25_S402_M1_Iqsec2
GALSSS[Phospho (STY)]LRDLSDAGK.2	-0.219649997	Q5DU25_S1133_M1_Iqsec2
RNS[Phospho (STY)]VGS[Phospho (STY)]LDSTIEGSVISSPRPHQR.4	0.083437237	Q5DU25_S1148_M2_Iqsec2
GPLGLRS[Phospho (STY)]PSES[Phospho (STY)]PQHIAATER.3	-0.148465388	Q5DU37_S605_M2_Zfyve26
S[Phospho (STY)]KTLLSTSGGSADIDASK.2	-0.309504519	Q5DU41_S186_M1_Lrrc8b
S[Phospho (STY)]PESTLTPTLNQNVC[Carbamidomethyl (C)]K.2	0.340225756	Q5EBH1_S177_M1_Rassf5
HREGS[Phospho (STY)]PLKEESLAR.3	-0.261681912	Q5EBJ4_S211_M1_Ermn
ILNENPEENLFVVHQAIK.3	0.243034605	#N/A
YNTISYR.2	-0.117355752	#N/A
EDSPLGS[Phospho (STY)]PGSQPGTPDEQPVFGKK.3	-0.481349332	Q5EBJ4_S227_M1_Ermn
EGS[Phospho (STY)]PLKEESLAR.2	-0.258438363	Q5EBJ4_S211_M1_Ermn
EDSPLGS[Phospho (STY)]PGSQPGTPDEQPVFGK.3	-0.326992127	Q5EBJ4_S227_M1_Ermn
SRPVDILHS[Phospho (STY)]K.3	-0.306727917	Q5EBJ4_S166_M1_Ermn
SGS[Phospho (STY)]ISNYR.2	-0.270791646	Q5EG47_S496_M1_Prkaa1
SIDDEITEAKS[Phospho (STY)]GTATPQR.2	0.400726594	Q5EG47_S486_M1_Prkaa1
GADNDGS[Phospho (STY)]GSESGYTTPK.2	-0.102957262	Q5F2E7_S213_M1_Nufip2
SLS[Phospho (STY)]SDEATNPISR.2	-0.17406278	Q5F2E7_S113_M1_Nufip2
KSS[Phospho (STY)]DIKPGLSSK.2	-0.005045379	Q5F2E7_S304_M1_Nufip2
DYEIENQNPLAS[Phospho (STY)]PTNTLLGSAK.3	-0.246737109	Q5F2E7_S626_M1_Nufip2

NDS[Phospho (STY)]WGSFDLR.2	-0.209027115	Q5F2E7_S649_M1_Nufip2
NS[Phospho (STY)]PQALR.2	-0.232265567	Q5F2E8_S965_M1_Taok1
NS[Phospho (STY)]PQALRR.2	-0.311033172	Q5F2E8_S965_M1_Taok1
ASDPQS[Phospho (STY)]PPQVSR.2	-0.178639625	Q5F2E8_S421_M1_Taok1
AGS[Phospho (STY)]LKDPEIAELFFKEDPEK.3	-0.188907669	Q5F2E8_S9_M1_Taok1
AEYVFIVDS[Phospho (STY)]DGEDEATC[Carbamidomethyl (C)]R.2	0.071753557	Q5FW52_S85_M1_Mlip
SSS[Phospho (STY)]PEPVTHLK.2	0.224544446	Q5FWK3_S51_M1_Arhgap1
STQKS[Phospho (STY)]PATAPK.2	-0.30031549	Q5FWK3_S223_M1_Arhgap1
FLLDHQGELFPSTDAQGV.2	-0.050648849	#N/A
SDDSKSS[Phospho (STY)]S[Phospho (STY)]PEPVTHLK.2	-0.23581564	Q5FWK3_S50_M2_Arhgap1
LAS[Phospho (STY)]IDEK.2	-0.164715326	Q5FWK3_S27_M1_Arhgap1
TKEGS[Phospho (STY)]AELVPR.2	0.574366678	Q5GH64_S154_M1_Xkr7
EGS[Phospho (STY)]AELVPR.2	-0.345890268	Q5GH64_S154_M1_Xkr7
GS[Phospho (STY)]GAGSAALC[Carbamidomethyl (C)]LR.2	0.282950059	Q5GH67_S87_M1_Xkr4
TVVSSGSAAGEGEVRPST[Phospho (STY)]PQR.3	0.191423369	Q5GH67_T208_M1_Xkr4
EYTEAS[Phospho (STY)]PLEDKVVELDNIPVR.3	0.030315508	Q5H8C4_S2581_M1_Vps13a
HGS[Phospho (STY)]LSADDSTPDTSPGVR.2	-0.086462804	Q5HZI2_S259_M1_C2cd4c
AQTSYGFATLAES[Phospho (STY)]PHTR.2	0.176008027	Q5HZI2_S153_M1_C2cd4c
ESLFHSEHGALAQVGS[Phospho (STY)]PGAGR.3	-0.419355596	Q5HZI2_S175_M1_C2cd4c
RAT[Phospho (STY)]PEPGPESGQAPR.3	0.046917857	Q5HZI2_T282_M1_C2cd4c
IQPNDGPVFFK.2	-0.503414075	#N/A
VVYTGIYDTEGVAPTK.2	0.15670018	#N/A
IKPTTLQVENISIGGVLVPLELK.3	0.284220775	#N/A
[Acetyl (Protein N-term)]GDLPGLVR.2	0.256270159	#N/A
DHC[Carbamidomethyl (C)]QWGSPFSVIEYEC[Carbamidomethyl (C)]KPNETR.4	0.496990448	#N/A
LLTGSSYK.2	-0.141879582	#N/A
S[Phospho (STY)]PAS[Phospho (STY)]PLNNQGIPTPAQLTK.2	0.006495705	Q5M956_S12_M2_Kctd1
TIS[Phospho (STY)]PPSAVVETDTPPIGK.2	-0.075655511	Q5NBX1_S826_M1_Cobl
AEHLGLSGADS[Phospho (STY)]DEDPAK.2	-0.277542831	Q5NBX1_S272_M1_Cobl
KEPDPS[Phospho (STY)]PPSQDNR.2	0.231560924	Q5NBX1_S676_M1_Cobl
S[Phospho (STY)]SLGNDETDKEK.2	-0.213088157	Q5NBX1_S234_M1_Cobl
KSS[Phospho (STY)]LGNDETDKEK.2	-0.523435999	Q5NBX1_S235_M1_Cobl
S[Phospho (STY)]PPTRKDDAAPNLHSEAR.4	-0.662014034	Q5NBX1_S982_M1_Cobl
GGS[Phospho (STY)]PAPAC[Carbamidomethyl (C)]ESC[Carbamidomethyl (C)]NC[Carbamidomethyl (C)]ADQK.2	0.087331389	Q5NCQ5_S418_M1_Dph1
ALS[Phospho (STY)]PEGALRR.2	-0.032607724	Q5NCX5_S504_M1_Neurl4
ALS[Phospho (STY)]PEGALR.2	0.068979477	Q5NCX5_S504_M1_Neurl4
S[Phospho (STY)]FPLHS[Phospho (STY)]PVAGVAHR.3	0.054956957	Q5NCX5_S904_M2_Neurl4
ESSDNQPPS[Phospho (STY)]PSLPEELK.2	0.237783137	Q5PR69_S992_M1_Crad
ETAQTDS[Phospho (STY)]PQPQER.2	0.544486598	Q5PR69_S487_M1_Crad
VNLS[Phospho (STY)]PVTPAK.2	-0.502651379	Q5PR69_S552_M1_Crad
ESSDNQPPS[Phospho (STY)]PSLPEELKGQK.3	-0.141767211	Q5PR69_S992_M1_Crad
DLPSFLVPGLPS[Phospho (STY)]PQK.2	-0.252698141	Q5PR69_S783_M1_Crad
PASQT[Phospho (STY)]PPSS[Phospho (STY)]PLSK.2	-0.06774436	Q5PR69_S953_M2_Crad
C[Carbamidomethyl (C)]DES[Phospho (STY)]PNREVQSSEAEALAR.3	0.263247277	Q5PR73_S126_M1_Diras2
VAVFGAGGVGK.2	-0.547392389	#N/A
ELFQELLNLEK.2	0.347002149	#N/A
PEQSNDYR.2	0.118576717	#N/A

GTFRES[Phospho (STY)]YIPTVEDTYR.3	-0.048219577	Q5PR73_S35_M1_Diras2
VLLAADS[Phospho (STY)]EEEGDFPSGR.2	-0.056159792	Q5PSV9_S168_M1_Mdc1
IVHSS[Phospho (STY)]PSSDGR.2	-0.069987218	Q5QNQ6_S760_M1_Osbp2
STPLPGPPGS[Phospho (STY)]PR.2	-0.309869021	Q5RJI5_S508_M1_Brsk1
SRLNS[Phospho (STY)]IR.2	0.286895827	Q5RJI5_S555_M1_Brsk1
NSFLGS[Phospho (STY)]PR.2	0.021479883	Q5RJI5_S563_M1_Brsk1
FQVDISSSEGPEPS[Phospho (STY)]PR.2	-0.507625708	Q5RJI5_S676_M1_Brsk1
SSGGT[Phospho (STY)]PLHS[Phospho (STY)]PLHTPR.2	-0.390159766	Q5RJI5_S519_M2_Brsk1
S[Phospho (STY)]PVFSFSPEPGAGDEAR.2	-0.104811564	Q5RJI5_S450_M1_Brsk1
SVSGASTGLSSS[Phospho (STY)]PLSS[Phospho (STY)]PR.2	-0.176889894	Q5RJI5_S443_M2_Brsk1
S[Phospho (STY)]M[Oxidation (M)]EVLSITDAGSGGSPVPTR.2	-0.398463687	Q5RJI5_S399_M1_Brsk1
VDS[Phospho (STY)]PMLSR.2	0.367968595	Q5RJI5_S384_M1_Brsk1
SLPS[Phospho (STY)]NGELDPDVLESM[Oxidation (M)]ASLGC[Carbamidomethyl (C)]FR.3	-0.377329958	Q5RJI5_S311_M1_Brsk1
EESLAGC[Carbamidomethyl (C)]S[Phospho (STY)]LVESQSK.2	-0.063085477	Q5RKR3_S700_M1_Islr2
ASYLES[Phospho (STY)]EKS[Phospho (STY)]YPAR.2	-0.088531333	Q5RKR3_S654_M2_Islr2
SNS[Phospho (STY)]ISVGEVYR.2	-0.117082269	Q5S006_S910_M1_Lrrk2
AGS[Phospho (STY)]PGSEVVTLQQFLEESNK.2	-0.376438394	Q5SNZ0_S1677_M1_Ccdc88a
FYDPS[Phospho (STY)]PPR.2	0.401121709	Q5SNZ0_S1387_M1_Ccdc88a
RT[Phospho (STY)]SIHDFLSK.3	-0.196863116	Q5SNZ0_T1821_M1_Ccdc88a
QKS[Phospho (STY)]LTLTPTR.2	-0.126118949	Q5SNZ0_S1417_M1_Ccdc88a
DT[Phospho (STY)]TSFEDISPQGISDDSSTGSR.2	0.032948516	Q5SNZ0_T1560_M1_Ccdc88a
SVS[Phospho (STY)]GKTPGDFYDRR.3	-0.392205165	Q5SNZ0_S1738_M1_Ccdc88a
VHASRPAS[Phospho (STY)]LDSGR.3	-0.172436344	Q5SNZ0_S1588_M1_Ccdc88a
SSS[Phospho (STY)]QENLLDEVMK.2	0.031114962	Q5SNZ0_S1704_M1_Ccdc88a
S[Phospho (STY)]SPMLQHK.2	0.160967089	Q5SNZ0_S1653_M1_Ccdc88a
STS[Phospho (STY)]PEHLHKPR.2	0.285314793	Q5SP85_S250_M1_Ccdc85a
GTS[Phospho (STY)]PEHLK.2	0.271120559	Q5SP85_S353_M1_Ccdc85a
HALGGS[Phospho (STY)]LEHLPR.3	-0.545050551	Q5SP85_S342_M1_Ccdc85a
ARGTS[Phospho (STY)]PEHLK.2	-0.390663433	Q5SP85_S353_M1_Ccdc85a
HRPGGS[Phospho (STY)]PEHTR.3	-0.199598674	Q5SP85_S320_M1_Ccdc85a
AAGGSAPAAESC[Carbamidomethyl (C)]PSAPAGAST[Phospho (STY)]PTGVDDLSK.3	-0.253818482	Q5SP85_T24_M1_Ccdc85a
GS[Phospho (STY)]PEQQRHPHPGSS[Phospho (STY)]PEVLPK.3	-0.251515537	Q5SP85_S284_M2_Ccdc85a
HASGSGGSGGGS[Phospho (STY)]REGTLR.3	0.309007559	Q5SP85_S380_M1_Ccdc85a
HVLSGS[Phospho (STY)]PEHFQK.2	-0.10652828	Q5SP85_S308_M1_Ccdc85a
HHASGGS[Phospho (STY)]PEHLQKPR.3	-0.200005863	Q5SP85_S228_M1_Ccdc85a
HSGGS[Phospho (STY)]PEHLQK.2	-0.342182031	Q5SP85_S330_M1_Ccdc85a
HPHPGS[Phospho (STY)]SPEVLPK.3	0.149578198	Q5SP85_S295_M1_Ccdc85a
GPS[Phospho (STY)]PEHHKPLC[Carbamidomethyl (C)]K.3	0.170453335	Q5SP85_S273_M1_Ccdc85a
SYLQDPIWR.2	-0.119365154	#N/A
YAPLHLVPLIER.2	-0.310730002	#N/A
FISELAR.2	0.158290059	#N/A
QLQVVPLFGDM[Oxidation (M)]QIELAR.2	-0.11377645	#N/A
SLDQAISR.2	0.151031828	#N/A
SVETDSSTVEHVR.2	-0.166568668	#N/A
LGT[Phospho (STY)]PQQIAIAR.2	-0.307450023	Q5SQX6_T1067_M1_Cyfip2
NAFVTGIAR.2	-0.020118852	#N/A
YSNSEVVTGSGLDSQK.2	0.363945271	#N/A

M[Oxidation (M)]ESVFNQAIR.2	0.121347163	#N/A
EANHNVSAPYGR.2	-0.067093236	#N/A
NNPPTIVQDK.2	-0.338934082	#N/A
FHLLVANR.2	0.060190886	#N/A
TVTYEDPQAVGGLASALDNR.2	-0.140560271	#N/A
GNS[Phospho (STY)]LAEQR.2	-0.288889912	Q5SRX1_S394_M1_Tom1l2
GIEFPM[Oxidation (M)]ADLDALS[Phospho (STY)]PIHTPQR.3	-0.386532884	Q5SRX1_S160_M1_Tom1l2
IVELISR.2	-0.210365235	#N/A
AAETVPDLPS[Phospho (STY)]PPTEAPAPASNTSTR.3	0.066572337	Q5SRX1_S479_M1_Tom1l2
DFIDSVLVK.2	-0.114823096	#N/A
EVM[Oxidation (M)]LALTVLETC[Carbamidomethyl (C)]VK.2	-0.140736109	#N/A
VSNEEVTEELLHVNDDLNNVFLR.3	-0.075391075	#N/A
GDQEEELDRPVSS[Phospho (STY)]PGEAEQK.3	-0.343050626	Q5SSH7_S2407_M1_Zzef1
S[Phospho (STY)]M[Oxidation (M)]EETRPVPTVK.2	0.054193724	Q5SSH7_S1538_M1_Zzef1
S[Phospho (STY)]PEMDKLK.2	0.327225625	Q5SSH7_S240_M1_Zzef1
LLPSSGPC[Carbamidomethyl (C)]VAEVSTAEEPS[Phospho (STY)]PPST[Phospho (STY)]PTR.3	-0.150643786	Q5SSH7_S1515_M2_Zzef1
TSSVVEEHFQGSAS[Phospho (STY)]PTEAATPAAGDR.3	-0.348740459	Q5SSH7_S1475_M1_Zzef1
GQLHGS[Phospho (STY)]S[Phospho (STY)]DES[Phospho (STY)]EVENEAK.2	-0.278858459	Q5SSI6_S114_M3_Utp18
SQGGGDSVS[Phospho (STY)]PTPPEGLAPGVEAGK.2	-0.041003481	Q5SSL4_S72_M1_Abr
GIEEVGIYR.2	-0.431541207	#N/A
ISQNFLSSINEDIDPR.2	0.094681537	#N/A
GPPEGSETMPYIDES[Phospho (STY)]PTMSPQLSAR.3	0.245639284	Q5SSL4_S53_M1_Abr
C[Carbamidomethyl (C)]SQSNNQFQK.2	-0.417031278	#N/A
HSATDKRDS[Phospho (STY)]EEESESTAL.3	-0.158591522	Q5SSM3_S805_M1_Arhgap44
SKELS[Phospho (STY)]PGSGQK.2	0.304371567	Q5SSM3_S598_M1_Arhgap44
RES[Phospho (STY)]PPSAER.2	-0.383000548	Q5SSZ5_S934_M1_Tns3
VTGS[Phospho (STY)]PAEEDEEETK.2	0.014682122	Q5SU73_S171_M1_Coil
VTGS[Phospho (STY)]PAEEDEEETKKK.3	-0.250009604	Q5SU73_S171_M1_Coil
RQEDSGQNEKAS[Phospho (STY)]DLETK.3	0.01544388	Q5SU73_S146_M1_Coil
QEDSGQNEKAS[Phospho (STY)]DLETK.2	-0.095453125	Q5SU73_S146_M1_Coil
NEVNGTSEDIKSEGDTQS[Phospho (STY)]N.2	-0.452318772	Q5SUF2_S431_M1_Luc7l3
SLS[Phospho (STY)]LGDKEISR.2	-0.235897461	Q5SV85_S1067_M1_Synrg
S[Phospho (STY)]GSIDDSFTDFQEM[Oxidation (M)]PASSK.2	-0.254634764	Q5SV85_S465_M1_Synrg
TADSVS[Phospho (STY)]PLEPPTK.2	-0.417590727	Q5SV85_S576_M1_Synrg
QLS[Phospho (STY)]LEGAGLAMEEFKENTSSTK.3	-0.384965085	Q5SV85_S744_M1_Synrg
QLS[Phospho (STY)]LEGAGLAMEEFK.2	-0.536422771	Q5SV85_S744_M1_Synrg
SETSS[Phospho (STY)]NPSS[Phospho (STY)]PEIC[Carbamidomethyl (C)]PNK.2	-0.24292677	Q5SVL6_S590_M2_Rap1gap2
NQSRS[Phospho (STY)]PIK.2	-0.127877178	Q5SVL6_S539_M1_Rap1gap2
TTFS[Phospho (STY)]PPVAAATAK.2	-0.213424519	Q5SVL6_S525_M1_Rap1gap2
NQSRS[Phospho (STY)]PIKR.2	-0.219791873	Q5SVL6_S539_M1_Rap1gap2
TPDGGHSS[Phospho (STY)]QEIK.2	0.052135312	Q5SVL6_S581_M1_Rap1gap2
SHS[Phospho (STY)]M[Oxidation (M)]ETM[Oxidation (M)]VGSQR.2	-0.127746125	Q5SVL6_S488_M1_Rap1gap2
LHS[Phospho (STY)]GSEGQGDSR.2	0.141498378	Q5SVL6_S553_M1_Rap1gap2
LSQSSQDSS[Phospho (STY)]PVR.2	-0.34055832	Q5SVQ0_S59_M1_Kat7
S[Phospho (STY)]SGSETEQVVDFSDR.2	0.206496497	Q5SVQ0_S101_M1_Kat7
GTS[Phospho (STY)]PPDYR.2	0.098722308	Q5SVR0_S997_M1_Tbc1d9b
VFQKNS[Phospho (STY)]PM[Oxidation (M)]EDLGAK.2	0.016668854	Q5SVR0_S464_M1_Tbc1d9b

NS[Phospho (STY)]PMEDLGAK.2	0.325745437	Q5SVR0_S464_M1_Tbc1d9b
AVEDM[Oxidation (M)]GS[Phospho (STY)]PQTAK.2	-0.181967677	Q5SW19_S1300_M1_Cluh
IPHSSS[Phospho (STY)]SENIR.2	0.002103505	Q5SW75_S1217_M1_Ssh2
EM[Oxidation (M)]TTS[Phospho (STY)]ADQIAEVK.2	-0.623218196	Q5SW75_S487_M1_Ssh2
DEDS[Phospho (STY)]LEEDAQR.2	-0.094977688	Q5SWP3_S1268_M1_Nacad
VSLS[Phospho (STY)]PHSTLNPK.2	-0.090572123	Q5SWP3_S1216_M1_Nacad
EFPALEQKDEDS[Phospho (STY)]LEEDAQR.3	0.127416194	Q5SWP3_S1268_M1_Nacad
SSM[Oxidation (M)]S[Phospho (STY)]GLHLVK.2	-0.53771479	Q5SWU9_S79_M1_Acaca
FIIGSVSEDNS[Phospho (STY)]EDEISNLVK.2	0.161480579	Q5SWU9_S29_M1_Acaca
LNRSDS[Phospho (STY)]DSSTLSK.2	-0.078508546	Q5SXA9_S939_M1_Wwc1
LGSS[Phospho (STY)]PTSSC[Carbamidomethyl (C)]NPTPTK.2	0.117458259	Q5SXY1_S133_M1_Specc1
ASS[Phospho (STY)]EDTLNKPGSASSGVAR.2	-0.035279461	Q5SXY1_S55_M1_Specc1
SSKGS[Phospho (STY)]PTGSSPNNASELSLASLTEK.3	-0.229187004	Q5SXY1_S355_M1_Specc1
S[Phospho (STY)]STSLAFESR.2	0.07338782	Q5SXY1_S37_M1_Specc1
T[Phospho (STY)]SPAPSDSATTVK.2	-0.066983027	Q5SXY1_T809_M1_Specc1
KLGSS[Phospho (STY)]PTSSC[Carbamidomethyl (C)]NPTPTK.2	0.052054209	Q5SXY1_S133_M1_Specc1
S[Phospho (STY)]PLSGIPVR.2	-0.01158903	Q5SXY1_S847_M1_Specc1
GSPT[Phospho (STY)]GSS[Phospho (STY)]PNNASELSLASLTEK.2	-0.307005623	Q5SXY1_S360_M2_Specc1
IGELGAPEVWGLS[Phospho (STY)]PK.2	-0.322870863	Q5SZT7_S141_M1_Nkapl;Nkap
ESS[Phospho (STY)]DEDRDSKLPSK.2	0.319682376	Q5U3K5_S638_M1_Rabl6
VAPQQC[Carbamidomethyl (C)]S[Phospho (STY)]EPETK.2	-0.055520277	Q5U3K5_S502_M1_Rabl6
EDLS[Phospho (STY)]DVTDEDTGPAQPPPPSK.2	0.413757855	Q5U3K5_S594_M1_Rabl6
NISLSS[Phospho (STY)]EEEAEGLAGHPR.3	0.162386874	Q5U3K5_S483_M1_Rabl6
FPVREDLS[Phospho (STY)]DVTDEDTGPAQPPPPSK.3	-0.12791271	Q5U3K5_S594_M1_Rabl6
NKPGVYTK.2	0.340958864	#N/A
[Acetyl (Protein N-term)]ATALS[Phospho (STY)]EEELDNEDYYSLLNVR.3	-0.234728865	Q5U458_S6_M1_Dnajc11
RTPAEIR.2	0.392488823	#N/A
VESTS[Phospho (STY)]GSEC[Carbamidomethyl (C)]ENPTK.2	0.329973121	Q5U4C1_S295_M1_Gprasp1
APS[Phospho (STY)]PAPAVS[Phospho (STY)]PK.2	-0.233166033	Q5U4C3_S676_M2_Scaf1
S[Phospho (STY)]AS[Phospho (STY)]PPPAASSSSSSR.2	-0.075882473	Q5U4C3_S577_M2_Scaf1
S[Phospho (STY)]AS[Phospho (STY)]PGPPPAR.2	-0.175593295	Q5U4C3_S491_M2_Scaf1
S[Phospho (STY)]PFLKPDER.2	-0.258685833	Q5U4C3_S821_M1_Scaf1
FDIYDPFHPTDEAYS[Phospho (STY)]PPPAPEQK.3	-0.123458013	Q5U4C3_S240_M1_Scaf1
GTEETSWS[Phospho (STY)]GEER.2	-0.160854469	Q5U4C3_S912_M1_Scaf1
APS[Phospho (STY)]PAPAVS[Phospho (STY)]PKR.2	0.446911482	Q5U4C3_S676_M2_Scaf1
SGDPAPPDS[Phospho (STY)]PSWEAK.2	0.010011112	Q5U4C3_S518_M1_Scaf1
QRS[Phospho (STY)]AS[Phospho (STY)]PGPPPAR.2	0.197008097	Q5U4C3_S491_M2_Scaf1
QRS[Phospho (STY)]GDPAPPDS[Phospho (STY)]PSWEAK.2	0.296000436	Q5U4C3_S510_M2_Scaf1
RRS[Phospho (STY)]AS[Phospho (STY)]PPPAASSSSSSR.3	0.072369545	Q5U4C3_S577_M2_Scaf1
REVLY[Phospho (STY)]DSEGLS[Phospho (STY)]ADER.2	-0.399704008	Q5U4C3_S695_M2_Scaf1
VYTY[Phospho (STY)]IQSR.2	0.083226782	Q5U4C9_Y380_M1_Dyrk2;Dyrk4
VGS[Phospho (STY)]AGAAALAAGGAGGAER.2	-0.089540827	Q5U4F6_S15_M1_Dync2i2
YMGALS[Phospho (STY)]QSR.2	-0.298619497	Q5U5V2_S195_M1_Hykk
TS[Phospho (STY)]PTASVNK.2	0.23572966	Q5XG69_S359_M1_Fam169a
HFQDSEFSSS[Phospho (STY)]QGEDENVAK.3	0.071005284	Q5XG69_S348_M1_Fam169a
TLLGS[Phospho (STY)]SDNVATVSNIEK.2	0.468134902	Q5XG69_S523_M1_Fam169a
TSPDTDSEM[Oxidation (M)]LIDQSPSDDKGHTEENLS[Phospho (STY)]PVSK.4	0.158216041	Q5XG69_S512_M1_Fam169a

AVDS[Phospho (STY)]S[Phospho (STY)]SEEIEVEVPVVDRR.2	-0.418153907	Q5XG69_S630_M2_Fam169a
SQSEEQSEASS[Phospho (STY)]EHLEQFTQSAEK.3	-0.145455361	Q5XG69_S614_M1_Fam169a
KYVIS[Phospho (STY)]DEEEEEDD.2	0.17367075	Q5XJE5_S659_M1_Leo1
YVIS[Phospho (STY)]DEEEEEDD.2	-0.14132766	Q5XJE5_S659_M1_Leo1
LNS[Phospho (STY)]DEEGESSGKR.2	-0.669747217	Q5XJE5_S631_M1_Leo1
KLNS[Phospho (STY)]DEEGESSGKR.3	0.348608692	Q5XJE5_S631_M1_Leo1
[Acetyl (Protein N-term)]ADM[Oxidation (M)]EDLFGS[Phospho (STY)]EAESEAER.2	0.18946749	Q5XJE5_S10_M1_Leo1
KLNS[Phospho (STY)]DEEGESSGK.2	0.365428316	Q5XJE5_S631_M1_Leo1
MQNT[Phospho (STY)]DDEDR.2	-0.099553077	Q5XJE5_T189_M1_Leo1
S[Phospho (STY)]PSVSSEYYIR.2	0.129652456	Q5XJV6_S531_M1_Lmtk3
ALVNGEPM[Oxidation (M)]S[Phospho (STY)]PEAGEK.2	-0.271695243	Q5XJV6_S962_M1_Lmtk3
AQRPPEGM[Oxidation (M)]S[Phospho (STY)]PELPPR.3	-0.265128704	Q5XJV6_S232_M1_Lmtk3
VAENGLES[Phospho (STY)]PEKEER.2	-0.218500457	Q5XJV6_S947_M1_Lmtk3
QGLPS[Phospho (STY)]PPGTPR.2	-0.079431208	Q5Y5T5_S672_M1_Zdhhc8
TPRPGS[Phospho (STY)]AESALSVQR.2	-0.357184736	Q5Y5T5_S335_M1_Zdhhc8
TS[Phospho (STY)]PPTPAMYK.2	-0.131889433	Q5Y5T5_S346_M1_Zdhhc8
MADTGS[Phospho (STY)]PGM[Oxidation (M)]QR.2	0.120517782	Q60575_S1454_M1_Kif1b
IVEGQGQSSEVIS[Phospho (STY)]PPEEVNR.2	-0.013854928	Q60575_S1075_M1_Kif1b
SGLS[Phospho (STY)]LEELR.2	-0.427190533	Q60575_S1057_M1_Kif1b
EFSQVHGSIS[Phospho (STY)]DC[Carbamidomethyl (C)]K.2	0.169680638	Q60575_S1605_M1_Kif1b
GDS[Phospho (STY)]LILEHQWELEK.2	-0.074151846	Q60575_S1487_M1_Kif1b
KLS[Phospho (STY)]NPDIFAPTGK.2	-0.040693775	Q60592_S14_M1_Mast2
SLS[Phospho (STY)]EEKEDHSDGLAGLK.3	-0.156298922	Q60592_S817_M1_Mast2
TQAAGPS[Phospho (STY)]SPPRPPT[Phospho (STY)]PK.2	-0.104252428	Q60596_S445_M2_Xrcc1
SSPYPTDVAR.2	0.042395136	#N/A
LNVLANVIR.2	-0.020019481	#N/A
FEEFLQR.2	0.268933793	#N/A
DVVVDLVC[Carbamidomethyl (C)]YR.2	0.104976761	#N/A
QILLPFR.2	0.325545636	#N/A
IC[Carbamidomethyl (C)]EEAFTR.2	0.046706768	#N/A
THLTELQR.2	-0.052970405	#N/A
ELEQIFC[Carbamidomethyl (C)]QFDSK.2	0.166823884	#N/A
TSFDEM[Oxidation (M)]LPGTHFQR.3	-0.576480192	#N/A
VASSVPVENFTIHGGLSR.3	0.134478751	#N/A
SWDIFFR.2	-0.241858896	#N/A
LVEDHLAVQSLIR.3	-0.151846493	#N/A
VVNAPIFHVNSDDPEAVMYVC[Carbamidomethyl (C)]K.3	0.122543229	#N/A
TKAEQFYC[Carbamidomethyl (C)]GDTEGKK.3	-0.379057768	#N/A
VFHLPTTTFIGGQEPALPLR.3	-0.111713346	#N/A
HHVLHDQNVDKR.3	-0.126071697	#N/A
TVPIEAVTSK.2	0.112633615	#N/A
NAST[Phospho (STY)]FEEVVQVPSAYQK.2	0.370533931	Q60598_T323_M1_Cttn
SAVGFDYQGK.2	-0.011104055	#N/A
HC[Carbamidomethyl (C)]S[Phospho (STY)]QVDSVR.2	0.163308373	Q60598_S113_M1_Cttn
YGVQADRVDK.2	-0.061724663	#N/A
SAVGFEYQGK.2	0.15642823	#N/A
TVQGSGHQEHINIHK.3	0.113380946	#N/A

KQT[Phospho (STY)]PPASPSPQPIEDRPPSSPIYEDAAPFK.4	0.127166372	Q60598_T401_M1_Cttn
VDQSAVGFEYQGK.2	0.053039194	#N/A
SAVGHEYQSK.2	-0.095280284	#N/A
YGIDKDKVDK.2	0.152720145	#N/A
DYSSGFGGK.2	-0.408791137	#N/A
S[Phospho (STY)]PQDRS[Phospho (STY)]PILAEVK.2	-0.350791476	Q60603_S899_M2_Kcnh1
ALGQNPTNAEVLK.2	0.107361605	#N/A
NKDQGTYEDYVEGLR.3	0.02722114	#N/A
EAFQLFDR.2	0.125938052	#N/A
HVLVTLGEK.2	0.113085941	#N/A
ILYSQC[Carbamidomethyl (C)]GDVMR.2	-0.076670899	#N/A
ANS[Phospho (STY)]LGDLYAQK.2	-0.234286903	Q60610_S231_M1_Tiam1
TES[Phospho (STY)]LPSAQQYVPFGGK.2	0.038182787	Q60610_S1407_M1_Tiam1
SNATNSSYS[Phospho (STY)]PPTGR.2	-0.185847594	Q60610_S358_M1_Tiam1
SKS[Phospho (STY)]ADIWR.2	-0.099044103	Q60610_S172_M1_Tiam1
RSNATNSSYS[Phospho (STY)]PPTGR.2	-0.078696234	Q60610_S358_M1_Tiam1
S[Phospho (STY)]LEGIFDDTVPDGK.2	-0.083830531	Q60610_S726_M1_Tiam1
FTIDSDAISASS[Phospho (STY)]PEKEPQQPAGGGDTDR.3	-0.061982397	Q60610_S1466_M1_Tiam1
S[Phospho (STY)]LEGIFDDTVPDGKR.2	-0.124129783	Q60610_S726_M1_Tiam1
LEQNGS[Phospho (STY)]PLGR.2	0.172186056	Q60611_S38_M1_Satb1
LDDLDC[Carbamidomethyl (C)]PR.2	0.1107284	#N/A
SFVGEPPR.2	-0.04544583	#N/A
ASLTLTLLR.2	0.170807645	#N/A
QLM[Oxidation (M)]C[Carbamidomethyl (C)]VVTLGGESR.2	0.007426229	#N/A
C[Carbamidomethyl (C)]TAVNGQGQAVK.2	0.052247065	#N/A
SGKEEVMEGPLR.3	-0.196678684	#N/A
NYVTPVNR.2	0.159056078	#N/A
NQQIFLR.2	0.096479146	#N/A
FNSLNELVDYHR.2	0.190069851	#N/A
ATADDELSFK.2	0.250103104	#N/A
NVVLQTLEGHLR.2	-0.070978641	#N/A
IFVGGLSPDTPEEK.2	-0.375539473	#N/A
YHNVGLSK.2	0.315934194	#N/A
IDASKNEEDEGHSNSS[Phospho (STY)]PR.3	-0.195177274	Q60668_S83_M1_Hnrnpd
IREYFGGFGEVESIELPM[Oxidation (M)]DNK.3	-0.158610348	#N/A
EYFGGFGEVESIELPM[Oxidation (M)]DNK.2	-0.134045952	#N/A
NEEDEGHSNS[Phospho (STY)]SPR.2	-0.086684114	Q60668_S82_M1_Hnrnpd
IDASKNEEDEGHSNS[Phospho (STY)]S[Phospho (STY)]PRHTEAAAAQR.4	-0.395744909	Q60668_S82_M2_Hnrnpd
AEDS[Phospho (STY)]SEGHEEEVLGGRGEK.3	-0.317721664	Q60673_S307_M1_Ptprn
AEDS[Phospho (STY)]SEGHEEEVLGGR.3	0.006384781	Q60673_S307_M1_Ptprn
SVVDSLDIESM[Oxidation (M)]TIEDEYSGPK.2	0.035464175	#N/A
AEGYEVAHGGR.2	0.310609144	#N/A
AFLEENQLDYIIR.2	-0.011558855	#N/A
FYSQAIELNPGNAIYYGNR.2	-0.096851458	#N/A
TQANDYFK.2	-0.131740761	#N/A
TEC[Carbamidomethyl (C)]YGYALGDATR.2	0.480279754	#N/A
GNHETDNMNQIYGFEGEVK.3	0.431883597	#N/A

LAAIQESGVER.2	0.113222731	#N/A
QVLLGDQIPK.2	-0.082659552	#N/A
TTTGSYIANR.2	0.405988164	#N/A
SSSGSWGTGGHPS[Phospho (STY)]PSR.2	-0.17694222	Q60722_S66_M1_Tcf4
NGGQASSS[Phospho (STY)]PNYEGPLHSLQSR.3	0.062950381	Q60722_S371_M1_Tcf4
DLGSHDNLS[Phospho (STY)]PPFVNSR.3	-0.245866593	Q60722_S92_M1_Tcf4
QLYQTLTDYDIR.2	0.084337762	#N/A
FNDILGR.2	0.018287819	#N/A
FVHSENQHLVSPEALDFLDK.4	0.123493905	#N/A
YSEVFEAINITNNEK.2	0.235313069	#N/A
VLGTEDLYDYIDKYNIELDPR.3	-0.418043166	#N/A
TPALVFEHVNNTDFK.2	0.239300565	#N/A
EYWDYESHVVEWGNQDDYQLVR.3	-0.206759705	#N/A
KDDEENYLDLFSHK.3	-0.176723051	#N/A
DPSGAHPSVR.2	0.364133772	#N/A
S[Phospho (STY)]C[Carbamidomethyl (C)]SKDPSGAHPSVR.2	0.060824671	Q60749_S18_M1_Khdrbs1
MGHEPGVAK.2	0.495894595	#N/A
KM[Oxidation (M)]DELGSK.2	0.392851317	#N/A
FYYSSGSSS[Phospho (STY)]PTHAK.2	-0.35732483	Q60771_S198_M1_Cldn11
FYYSSGSSS[Phospho (STY)]PTHAKSAHV.3	-0.011956322	Q60771_S198_M1_Cldn11
KSTGDSQNLGSSS[Phospho (STY)]PGRK.2	-0.232175221	Q60780_S108_M1_Gas7
HETDM[Oxidation (M)]FNQSTVEPVDQLLR.3	0.205801896	#N/A
STGDSQNLGSSS[Phospho (STY)]PGR.2	-0.14974593	Q60780_S108_M1_Gas7
STGDSQNLGSSS[Phospho (STY)]PGRK.2	-0.27306514	Q60780_S108_M1_Gas7
KSTGDSQNLGS[Phospho (STY)]SSPGR.2	-0.062260122	Q60780_S106_M1_Gas7
C[Carbamidomethyl (C)]VDLYNQAQSK.2	-0.337607698	#N/A
YGS[Phospho (STY)]QEHPIGDK.2	0.092092852	Q60790_S809_M1_Rasa3
T[Phospho (STY)]RYGSQEHPIGDK.3	-0.510587871	Q60790_T805_M1_Rasa3
DGNSEDQVEGR.2	-0.012897471	#N/A
ATLSEPGEEPQHPS[Phospho (STY)]PP.2	-0.288518208	Q60829_S192_M1_Ppp1r1b
GLEGPWERPPPLDEPQR.3	-0.084108904	#N/A
GTVGQKPTC[Carbamidomethyl (C)]GR.2	0.364599245	#N/A
VSEHSS[Phospho (STY)]PEEEASPHQR.3	-0.3785752	Q60829_S46_M1_Ppp1r1b
IQFSVPAPPSQLDPR.2	0.021640593	#N/A
GLEGPWERPPPLDEPQRDGNSEDQVEGR.4	-0.105987857	#N/A
ELGYPQEDDEEDEDEEEDEEEDSQAEVLK.3	-0.523699198	#N/A
FYEAELEELDFQGATEESR.2	-0.352350946	#N/A
LAYINPDLALEEK.2	0.168768567	#N/A
LDPQNHVLYSNR.3	0.172147958	#N/A
HDS[Phospho (STY)]PEDVKR.2	-0.194575366	Q60864_S481_M1_Stip1
ALDLDSSC[Carbamidomethyl (C)]K.2	0.056136376	#N/A
DPQALSEHLK.2	0.582280768	#N/A
ALSAGNIDDALQC[Carbamidomethyl (C)]YSEAIK.2	0.052673098	#N/A
DPQALSEHLKNPVIAQK.3	0.133987886	#N/A
TPDVLKK.2	0.42887129	#N/A
HDS[Phospho (STY)]PEDVK.2	-0.327346744	Q60864_S481_M1_Stip1
ELIEQLQNKPSDLGTK.3	0.249437242	#N/A

LM[Oxidation (M)]DVGLIAIR.2	0.616980271	#N/A
HYTEAIKR.2	0.185963966	#N/A
HDS[Phospho (STY)]PEDVKRR.3	-0.32408541	Q60864_S481_M1_Stip1
HYTEAIK.2	-0.157805003	#N/A
S[Phospho (STY)]LLSDPTYR.2	-0.293609038	Q60864_S145_M1_Stip1
LILEQMQK.2	-0.027032265	#N/A
DAIHFYNK.2	0.077638086	#N/A
TYEEGLKHEANNLQLK.3	-0.253765472	#N/A
IGNSYFKEEK.2	-0.177973176	#N/A
NKGNEC[Carbamidomethyl (C)]FQK.2	0.193763994	#N/A
AMDVYQK.2	-0.210477732	#N/A
AMADPEVQQIMSDPAMR.2	-0.06566655	#N/A
YQEVTNNLEFAK.2	-0.039838505	#N/A
HESQVDSVVK.2	0.297782215	#N/A
EAQELGS[Phospho (STY)]PEDR.2	-0.088882291	Q60875_S931_M1_Arhgef2
LS[Phospho (STY)]PPHSPR.2	-0.082006239	Q60875_S955_M1_Arhgef2
LQDSS[Phospho (STY)]DPDTGSEEEVSSR.2	0.299515119	Q60875_S940_M1_Arhgef2
LES[Phospho (STY)]FESLR.2	0.007163929	Q60875_S646_M1_Arhgef2
SLHRPFDDREAQELGS[Phospho (STY)]PEDR.3	0.176552894	Q60875_S931_M1_Arhgef2
LQEIYNR.2	-0.103654438	#N/A
TFNGS[Phospho (STY)]IELC[Carbamidomethyl (C)]R.2	-0.394260822	Q60875_S710_M1_Arhgef2
QILSQS[Phospho (STY)]TDSLNMR.2	0.345318999	Q60875_S174_M1_Arhgef2
S[Phospho (STY)]LPAGDALYLSFNPPQPSR.2	-0.298545575	Q60875_S885_M1_Arhgef2
LES[Phospho (STY)]FESLRGER.2	-0.00376195	Q60875_S646_M1_Arhgef2
SVSTTNIAGHFNDES[Phospho (STY)]PLGLR.3	0.042836252	Q60875_S163_M1_Arhgef2
LQDSSDPDTGSEEEVSSRLS[Phospho (STY)]PPHSPR.3	0.132568351	Q60875_S955_M1_Arhgef2
VSYARPSS[Phospho (STY)]ASIR.2	0.114109404	Q60899_S119_M1_Elavl2
ELEQLFSQYGR.2	0.155412518	#N/A
IEAEEAIK.2	-0.218608287	#N/A
TNQAILSQLYQS[Phospho (STY)]PNR.2	-0.430100845	Q60899_S221_M1_Elavl2
FANNPSQK.2	0.585767195	#N/A
LQQEETQLEQSIQAGR.2	-0.076820486	#N/A
STPSHGSVSSLNSTGS[Phospho (STY)]LS[Phospho (STY)]PK.2	0.123021859	Q60902_S253_M2_Eps15l1
ESDPFHS[Phospho (STY)]SSSDDFFKK.3	0.338135246	Q60902_S669_M1_Eps15l1
GIDPPQVLS[Phospho (STY)]PDM[Oxidation (M)]VPPSER.2	-0.075387269	Q60902_S353_M1_Eps15l1
TVFAGAVPVLPAS[Phospho (STY)]PPPK.2	-0.21959875	Q60902_S229_M1_Eps15l1
TVFAGAVPVLPAS[Phospho (STY)]PPPKDSLR.3	-0.0532496	Q60902_S229_M1_Eps15l1
SEPIS[Phospho (STY)]PPRDR.2	0.186115797	Q60929_S406_M1_Mef2a
YQLDPTASISAK.2	0.128041879	#N/A
[Acetyl (Protein N-term)]AEC[Carbamidomethyl (C)]C[Carbamidomethyl (C)]VPVC[Carbamidomethyl (C)]PR.2	0.299032804	#N/A
SNFAVGYR.2	-0.134959936	#N/A
LTLSALVDGK.2	0.235301723	#N/A
WC[Carbamidomethyl (C)]EYGLTFTEK.2	0.003870378	#N/A
GFGFGLVK.2	0.070404309	#N/A
LTFDTTFSPNTGK.2	0.260760366	#N/A
SFNAGGHK.2	-0.013701979	#N/A
VNNSSLIGVGYTQTLRPGVK.2	-0.194946329	#N/A

LTFDTTFSPNTGKK.2	0.228066869	#N/A
TGDFQLHTNVNNGTEFGGSIYQK.3	-0.012438594	#N/A
WNTDNTLGTEIAIEDQIC[Carbamidomethyl (C)]QGLK.3	0.071695182	#N/A
SC[Carbamidomethyl (C)]SGVEFSTSGSSNTDTGK.2	-0.413875641	#N/A
[Acetyl (Protein N-term)]C[Carbamidomethyl (C)]NT[Phospho (STY)]PTYC[Carbamidomethyl (C)]DLGK.2	-0.513444525	Q60931_T4_M1_Vdac3
LTLDTIFVPNTGK.2	0.112418494	#N/A
LSQNNFALGYK.2	0.341993104	#N/A
LTLDTIFVPNTGKK.2	-0.538353729	#N/A
VC[Carbamidomethyl (C)]NYGLTFTQK.2	-0.302809026	#N/A
NFNAGGHK.2	0.176954865	#N/A
VNNASLIGLGYTQTLRPGVK.2	0.110090321	#N/A
IETSINLAWTAGSNNTR.2	-0.214731036	#N/A
WNTDNTLGTEISWENK.2	-0.058847204	#N/A
YQVDPDAC[Carbamidomethyl (C)]FSAK.2	0.185858858	#N/A
LTFDSSFS[Phospho (STY)]PNTGK.2	0.118569738	Q60932_S117_M1_Vdac1
VTQSNFAVGYK.2	0.086053833	#N/A
WTEYGLTFTEK.2	-0.135036869	#N/A
TDEFQLHTNVNDGTEFGGSIYQK.3	-0.307679363	#N/A
GYGFGLIK.2	-0.132741266	#N/A
SENGLEFTSSGSANTETTK.2	0.120063	#N/A
TKSENGLEFTSSGSANTETTK.3	-0.08430847	#N/A
LTFDSSFS[Phospho (STY)]PNTGKK.2	-0.319498653	Q60932_S117_M1_Vdac1
LETAVNLAWTAGNSNTR.2	0.02134054	#N/A
VNNSSLIGLGYTQTLKPGIK.2	-0.185801325	#N/A
EHINLGC[Carbamidomethyl (C)]DVDFDIAGPSIR.3	0.083750843	#N/A
WNTDNTLGTEITVEDQLAR.2	-0.588833132	#N/A
KLETAVNLAWTAGNSNTR.3	-0.24222089	#N/A
KSFS[Phospho (STY)]QPGLR.2	0.029413416	Q60949_S231_M1_Tbc1d1
QSARPST[Phospho (STY)]PEPDC[Carbamidomethyl (C)]TQLEPTGD.2	-0.367242949	Q60949_T1242_M1_Tbc1d1
LNPSAS[Phospho (STY)]SPNFFK.2	-0.223268626	Q60949_S660_M1_Tbc1d1
SFS[Phospho (STY)]QPGLR.2	-0.016810376	Q60949_S231_M1_Tbc1d1
VPAPPGS[Phospho (STY)]PC[Carbamidomethyl (C)]R.2	-0.083460407	Q60953_S17_M1_Pml
LATSS[Phospho (STY)]PEQSWPSTFK.2	-0.125710005	Q60953_S515_M1_Pml
[Acetyl (Protein N-term)]ADKEAAFDDAVEER.2	-0.618088025	#N/A
AQLS[Phospho (STY)]PGLYDDSSAR.2	-0.262159476	Q60974_S1481_M1_Ncor1
VS[Phospho (STY)]PENLVDK.2	-0.254004399	Q60974_S2134_M1_Ncor1
SHIPSEPYEPIS[Phospho (STY)]PPQGPAVHEK.3	0.180225863	Q60974_S2165_M1_Ncor1
EPAPLLSAQYETLS[Phospho (STY)]DSDD.2	-0.227978036	Q60974_S2449_M1_Ncor1
YS[Phospho (STY)]PESQSQTVLHPR.2	0.101081366	Q60974_S2116_M1_Ncor1
GS[Phospho (STY)]PPAAGGSPSSLK.2	0.021903552	#N/A
RKS[Phospho (STY)]ELPQDPHTEK.3	-0.23928661	Q60996_S497_M1_Ppp2r5c
[Acetyl (Protein N-term)]S[Phospho (STY)]GTNNVAQAR.2	-0.158583291	Q61016_S2_M1_Gng7
KLVEQLR.2	0.157245956	#N/A
NDPLLVGVPASENPFK.2	-0.041601121	#N/A
NDPLLVGVPASENPFKDK.3	0.25599311	#N/A
ASSDLMGYC[Carbamidomethyl (C)]EQHAR.2	0.294614126	#N/A
LLQDSSS[Phospho (STY)]PVDLAK.2	0.459108173	Q61026_S699_M1_Ncoa2

SST[Phospho (STY)]PLPTVSSSAENTR.2	-0.254297223	Q61029_T159_M1_Tmpo
EQGTESRSS[Phospho (STY)]T[Phospho (STY)]PLPTVSSSAENTR.3	-0.296917346	Q61029_S158_M2_Tmpo
QNGSNDSDRYS[Phospho (STY)]DNDEDSKIELK.3	-0.299113113	Q61029_S183_M1_Tmpo
DQGGELLSLR.2	0.000657985	#N/A
IFSIVEQR.2	-0.008642611	#N/A
AALEELVR.2	0.141148959	#N/A
YDLTVPFAR.2	-0.236427818	#N/A
ASAEQIEEEVTK.2	0.324061999	#N/A
TIC[Carbamidomethyl (C)]SSVDKLDK.3	-0.39910273	#N/A
[Acetyl (Protein N-term)]SDS[Phospho (STY)]LDNEEKPPAPPLR.2	-0.081671792	Q61036_S4_M1_Pak3
STS[Phospho (STY)]LNERPK.2	-0.065081128	Q61037_S939_M1_Tsc2
IDIGRLS[Phospho (STY)]PEAK.2	-0.014314028	Q61037_S1412_M1_Tsc2
S[Phospho (STY)]PSGLRPR.2	-0.109785425	Q61037_S1455_M1_Tsc2
SSS[Phospho (STY)]SPELQTLQDILGDLGDK.2	-0.176210523	Q61037_S1388_M1_Tsc2
SQS[Phospho (STY)]GILDGEAATWSATGEESR.2	-0.243422018	Q61037_S1421_M1_Tsc2
SIS[Phospho (STY)]VSEHVVR.2	0.011889865	Q61037_S981_M1_Tsc2
SM[Oxidation (M)]S[Phospho (STY)]GGHGLR.2	-0.22434587	Q61037_S1132_M1_Tsc2
LISS[Phospho (STY)]VDDFTEFV.2	0.28435366	Q61037_S1806_M1_Tsc2
S[Phospho (STY)]LDKAKEEEK.2	-0.24122853	Q61048_S51_M1_Wbp4
THPYS[Phospho (STY)]PK.2	-0.006976584	Q61069_S186_M1_Usf1;Usf2
RPQS[Phospho (STY)]VER.2	-0.293687595	Q61070_S56_M1_Ei24
FPS[Phospho (STY)]PHPS[Phospho (STY)]PAK.2	-0.065363927	Q61070_S326_M2_Ei24
KLEEALST[Phospho (STY)]EGAEDTGNSDK.2	-0.032259091	Q61074_T525_M1_Ppm1g
AQS[Phospho (STY)]YPDNHQEFTDYDNPIFEK.3	-0.111150557	Q61083_S239_M1_Map3k2
AIDILDRS[Phospho (STY)]S[Phospho (STY)]SM[Oxidation (M)]K.2	0.286990543	Q61084_S110_M2_Map3k3
TES[Phospho (STY)]VPSDINNPVDR.2	-0.256158971	Q61097_S392_M1_Ksr1
SKS[Phospho (STY)]HESQLGNR.2	0.028816262	Q61097_S297_M1_Ksr1
VDDDS[Phospho (STY)]LGEFPVSNSR.2	0.086217202	Q61103_S142_M1_Dpf2
QGS[Phospho (STY)]LDIYSTAEGK.2	-0.217936026	Q61120_S282_M1_Shc3
AAS[Phospho (STY)]VEC[Carbamidomethyl (C)]ISPVTPR.2	-0.220706337	Q61120_S354_M1_Shc3
SFS[Phospho (STY)]PKS[Phospho (STY)]PLELGEK.2	0.294660787	Q61127_S159_M2_Nab2
KS[Phospho (STY)]KS[Phospho (STY)]PPLR.2	-0.192376351	Q61136_S240_M2_Prpf4b
KKS[Phospho (STY)]PIVNER.2	-0.329268196	Q61136_S278_M1_Prpf4b
SKDAS[Phospho (STY)]PINRWS[Phospho (STY)]PTR.2	-0.363654595	Q61136_S431_M2_Prpf4b
S[Phospho (STY)]RS[Phospho (STY)]PLLNDR.2	-0.228094661	Q61136_S366_M2_Prpf4b
RRS[Phospho (STY)]LS[Phospho (STY)]PK.2	-0.201716895	Q61136_S354_M2_Prpf4b
S[Phospho (STY)]RS[Phospho (STY)]PLRR.2	-0.13667994	Q61136_S451_M2_Prpf4b
AKS[Phospho (STY)]RS[Phospho (STY)]LER.2	-0.001328704	Q61136_S394_M2_Prpf4b
TLS[Phospho (STY)]PGRR.2	-0.298294591	Q61136_S387_M1_Prpf4b
SKDAS[Phospho (STY)]PINR.2	-0.267408732	Q61136_S431_M1_Prpf4b
S[Phospho (STY)]KS[Phospho (STY)]PVDLR.2	-0.072111085	Q61136_S292_M2_Prpf4b
S[Phospho (STY)]RS[Phospho (STY)]PLLNDRR.2	-0.492604013	Q61136_S366_M2_Prpf4b
DAS[Phospho (STY)]PINRWS[Phospho (STY)]PTR.2	-0.253403789	Q61136_S431_M2_Prpf4b
EVIEASDKEGLS[Phospho (STY)]PAKR.3	-0.30241102	Q61136_S94_M1_Prpf4b
LC[Carbamidomethyl (C)]DFGSASHVADNDITPY[Phospho (STY)]LVSR.3	-0.300303873	Q61136_Y849_M1_Prpf4b
S[Phospho (STY)]KS[Phospho (STY)]PVDLRDK.2	-0.592284552	Q61136_S292_M2_Prpf4b
RS[Phospho (STY)]RS[Phospho (STY)]PLLNDR.3	-0.124737498	Q61136_S366_M2_Prpf4b

S[Phospho (STY)]KS[Phospho (STY)]PPLR.2	0.393989431	Q61136_S240_M2_Prpf4b
VGS[Phospho (STY)]PRS[Phospho (STY)]PVNK.2	-0.329303558	Q61137_S376_M2_Astn1
SAS[Phospho (STY)]AEAANEIHYIPSVLIGGHGR.3	-0.372887679	Q61137_S194_M1_Astn1
GNSPSDES[Phospho (STY)]EERERDPK.3	-0.349207062	Q61137_S906_M1_Astn1
VQGHNS[Phospho (STY)]SGTLSIR.2	0.152055608	Q61137_S227_M1_Astn1
GNSPSDES[Phospho (STY)]EERER.2	0.106941	Q61137_S906_M1_Astn1
GS[Phospho (STY)]PGFTPEDLDR.2	0.102024611	Q61140_S572_M1_Bcar1
TQES[Phospho (STY)]EEDC[Carbamidomethyl (C)]EEDPDR.2	0.35244685	Q61144_S52_M1_Psen2
TSLMSAES[Phospho (STY)]PTSR.2	-0.207017221	Q61144_S25_M1_Psen2
YSS[Phospho (STY)]NGNLETPSKR.2	-0.008344062	Q61146_S369_M1_Ocln
YSS[Phospho (STY)]NGNLETPSK.2	0.053798628	Q61146_S369_M1_Ocln
NVS[Phospho (STY)]AGTQDM[Oxidation (M)]PPPPSDYAER.2	-0.006508254	Q61146_S300_M1_Ocln
SQS[Phospho (STY)]SSQFR.2	0.115633941	Q61151_S32_M1_Ppp2r5e
TPS[Phospho (STY)]EIQFHQVK.2	-0.086461397	Q61161_S328_M1_Map4k2
ASDPHLGTLS[Phospho (STY)]PEDSELETHDMFPDTIHSR.4	-0.637207841	Q61161_S298_M1_Map4k2
[Acetyl (Protein N-term)]M[Oxidation (M)]EGEAVEAIVEESETFIK.2	-0.224286771	#N/A
LDS[Phospho (STY)]PTLSR.2	-0.12302914	Q61165_S697_M1_Slc9a1
IGS[Phospho (STY)]DPLAYEPK.2	-0.263246702	Q61165_S707_M1_Slc9a1
ADLPVITIDPAS[Phospho (STY)]PQSPESVDLVNEELK.3	-0.00975302	Q61165_S727_M1_Slc9a1
ITNYLTVPAHKLDS[Phospho (STY)]PTLSR.3	-0.013386432	Q61165_S697_M1_Slc9a1
C[Carbamidomethyl (C)]LS[Phospho (STY)]DPGPHPEPGEGEPFIPK.3	0.338083145	Q61165_S801_M1_Slc9a1
[Acetyl (Protein N-term)]AVNVYSTSVTSDNLSR.2	-0.179420692	#N/A
NIELIC[Carbamidomethyl (C)]QENEGENDPVLQR.2	-0.107785473	#N/A
LEHEYIQNFK.3	-0.123412794	#N/A
LTVEDLEKER.2	-0.029722799	#N/A
QITVNDLPVGR.2	0.030962103	#N/A
SLSQNYGVLK.2	0.154761615	#N/A
NDEGIAYR.2	-0.047609006	#N/A
EGGLGPLNIPLLADVTK.2	-0.181854932	#N/A
GLFIIDAK.2	0.232873482	#N/A
KEGGLGPLNIPLLADVTK.3	0.047979796	#N/A
SAPDFTATAVVDGAFK.2	0.010518206	#N/A
LGC[Carbamidomethyl (C)]EVLGVSVDSQFTHLAWINTPR.3	-0.19485633	#N/A
VSC[Carbamidomethyl (C)]EVEPPGTSDS[Phospho (STY)]PAAR.2	0.172296014	Q61193_S518_M1_Rgl2
SASC[Carbamidomethyl (C)]GS[Phospho (STY)]PLSGNTGEGTSR.2	0.225383802	Q61193_S622_M1_Rgl2
LLAQDQGQGIPLPETAS.2	-0.009808352	#N/A
IIVLGLLPR.2	0.224782895	#N/A
GEKPNPLR.2	0.097076344	#N/A
[Acetyl (Protein N-term)]SQGDSNPAAIPHAAEDIQGDDR.3	-0.045830904	#N/A
ELFSPLHALNFGIGGDTTR.3	0.166178336	#N/A
[Acetyl (Protein N-term)]SQGDSNPAAIPHAAEDIQGDDRWM[Oxidation (M)]SQHNR.4	-0.539708454	#N/A
GC[Carbamidomethyl (C)]SFLPDPYQK.2	0.114556941	#N/A
SLPC[Carbamidomethyl (C)]DIC[Carbamidomethyl (C)]K.2	0.22214986	#N/A
TVVTEAGNLLK.2	0.263820932	#N/A
LVSDVQTAVK.2	0.365413428	#N/A
LVLYLEHNLEK.2	-0.034021885	#N/A
LGPGVSDIC[Carbamidomethyl (C)]K.2	-0.315107584	#N/A

EVVDSYLPVILDM[Oxidation (M)]IK.2	0.435791724	#N/A
TC[Carbamidomethyl (C)]EWIHDSSLSASC[Carbamidomethyl (C)]K.3	0.374963178	#N/A
IYQY[Phospho (STY)]IQSR.2	-0.280447004	Q61214_Y321_M1_Dyrk1a
FSLVGIAGQDLNEGNR.2	-0.004674501	#N/A
GS[Phospho (STY)]VSDEEMMELR.2	0.26091377	Q61233_S5_M1_Lcp1
EGES[Phospho (STY)]LEDLMK.2	-0.227069332	Q61233_S257_M1_Lcp1
QPSS[Phospho (STY)]PGPQPR.2	-0.161465859	Q61234_S195_M1_Snta1
NSAGGTSVGWDS[Phospho (STY)]PPASPLQR.2	-0.106549696	Q61234_S183_M1_Snta1
YLEIC[Carbamidomethyl (C)]AADGQDAVFLR.2	-0.546980347	#N/A
GPAGEASAS[Phospho (STY)]PPVR.2	0.102425818	Q61235_S90_M1_Sntb2
GLGPPS[Phospho (STY)]PPAPPR.2	-0.048267974	Q61235_S75_M1_Sntb2
GPAGEASAS[Phospho (STY)]PPVRR.3	0.286070025	Q61235_S90_M1_Sntb2
[Acetyl (Protein N-term)]AAS[Phospho (STY)]EDELLLPR.2	-0.211713459	Q61249_S4_M1_Igbp1
AEAYS[Phospho (STY)]PAENR.2	-0.277261508	Q61285_S58_M1_Abcd2
TSS[Phospho (STY)]TNEDEDLNPEQK.2	0.301185218	Q61286_S583_M1_Tcf12
GTQADWES[Phospho (STY)]PER.2	-0.335728809	Q61290_S2073_M1_Cacna1e
QGT[Phospho (STY)]PVPASGPAAAYK.2	-0.113216308	Q61290_T29_M1_Cacna1e
GTQADWES[Phospho (STY)]PERR.2	-0.408469567	Q61290_S2073_M1_Cacna1e
S[Phospho (STY)]YHSSLR.2	-0.069353281	Q61290_S2013_M1_Cacna1e
HLLS[Phospho (STY)]PDVSR.2	-0.115732065	Q61290_S2054_M1_Cacna1e
KHLLS[Phospho (STY)]PDVSR.2	-0.355136795	Q61290_S2054_M1_Cacna1e
AS[Phospho (STY)]IKS[Phospho (STY)]TKVDGASYFR.3	-0.172275654	Q61290_S447_M2_Cacna1e
FGEAVVVGRPGS[Phospho (STY)]GDGDSDQSR.3	0.148859792	Q61290_S15_M1_Cacna1e
DSSDEHC[Carbamidomethyl (C)]VDISSVGT[Phospho (STY)]PLAR.3	-0.230143453	Q61290_T441_M1_Cacna1e
GISQS[Phospho (STY)]EPDLSC[Carbamidomethyl (C)]M[Oxidation (M)]TANM[Oxidation (M)]DK.2	-0.198406643	#N/A
SAS[Phospho (STY)]QERS[Phospho (STY)]LDEGVSVEGEKEHEPHSSHR.4	-0.033302561	Q61290_S944_M2_Cacna1e
TDGEAS[Phospho (STY)]PLKEAETKEEEEEVEK.3	-0.168401827	Q61290_S1099_M1_Cacna1e
S[Phospho (STY)]LDEGVSVEGEKEHEPHSSHR.4	0.029891171	Q61290_S948_M1_Cacna1e
EVS[Phospho (STY)]PM[Oxidation (M)]SAPNM[Oxidation (M)]PSIER.2	-0.22197459	Q61290_S737_M1_Cacna1e
GGS[Phospho (STY)]LKGDIGGLTSALDNQR.3	-0.354345711	Q61290_S856_M1_Cacna1e
HMQMSS[Phospho (STY)]QEALNK.2	-0.406524677	Q61290_S794_M1_Cacna1e
AKEVS[Phospho (STY)]PMSAPNMPSIER.3	0.057620153	Q61290_S737_M1_Cacna1e
VYGTAAVNS[Phospho (STY)]PVVSWK.2	-0.232103178	Q61301_S901_M1_Ctnna2
NATNEQDLANR.2	0.020530672	#N/A
GHTGIGELAAALNEFDNK.3	-0.372999353	#N/A
QVQEAIAGISSAAQATS[Phospho (STY)]PTDEAK.2	-0.420159291	Q61301_S262_M1_Ctnna2
[Acetyl (Protein N-term)]TSATS[Phospho (STY)]PIILK.2	-0.207218812	Q61301_S6_M1_Ctnna2
LVYDGVR.2	-0.0200821	#N/A
IVEEALEAVK.2	-0.080290394	#N/A
LLEPLVTQVTTLVNTSNK.2	0.023680919	#N/A
TPEELEDDSDFEQEDYDVR.2	0.154131933	#N/A
SKLDAEVAK.2	-0.429942377	#N/A
NATM[Oxidation (M)]LYTASQAFLR.2	0.187824272	#N/A
LVEVANLAC[Carbamidomethyl (C)]SISNNEEGVK.2	0.188160104	#N/A
VLTEAVDDITSVDDFLSVSENHILEDVNK.3	-0.294149143	#N/A
HIS[Phospho (STY)]PVQALSEFK.2	-0.224359542	Q61301_S939_M1_Ctnna2
QSVGSGS[Phospho (STY)]PVQTVGLETR.2	-0.215074499	Q61315_S2713_M1_Apc

SELS[Phospho (STY)]PITR.2	-0.085090367	Q61315_S2283_M1_Apc
SGRS[Phospho (STY)]PTGNTPPVIDSVSEK.3	0.201008614	Q61315_S2674_M1_Apc
AVEFSSGAKS[Phospho (STY)]PSK.2	-0.38637485	Q61315_S1359_M1_Apc
HSS[Phospho (STY)]PSGTVAAR.2	-0.059599292	Q61315_S2774_M1_Apc
SPT[Phospho (STY)]GNTPPVIDSVSEK.2	0.200818398	Q61315_T2676_M1_Apc
YSDEQLNSGRQS[Phospho (STY)]PSQNER.3	0.011455003	Q61315_S1040_M1_Apc
ALEAELDAQHLSETFDNIDNLS[Phospho (STY)]PK.3	0.068482992	Q61315_S778_M1_Apc
T[Phospho (STY)]GSSSSILSASSESSEK.2	0.196619494	Q61315_T2567_M1_Apc
QAS[Phospho (STY)]SDSDSILSLK.2	-0.065784743	Q61315_S2129_M1_Apc
LKSESEKQS[Phospho (STY)]PR.2	-0.35308962	Q61315_S2063_M1_Apc
SHS[Phospho (STY)]ES[Phospho (STY)]PSRLPINR.2	-0.212381581	Q61315_S2533_M2_Apc
C[Carbamidomethyl (C)]TSVSS[Phospho (STY)]LDSFESR.2	0.144901695	Q61315_S1391_M1_Apc
S[Phospho (STY)]PPEHYVQETPLVFSR.2	0.027947631	Q61315_S1370_M1_Apc
NDS[Phospho (STY)]LSSLDFDDDDVDLSR.2	0.129713725	Q61315_S1859_M1_Apc
SKT[Phospho (STY)]PPPPPQTVQAK.2	0.412660945	Q61315_T1437_M1_Apc
TPASKS[Phospho (STY)]PSEGPGATTSPR.2	0.106997133	Q61315_S2260_M1_Apc
KPTS[Phospho (STY)]PVKPMPQNTEYR.3	-0.072305363	Q61315_S1772_M1_Apc
SFHVEDTPVC[Carbamidomethyl (C)]FS[Phospho (STY)]R.3	-0.39711582	Q61315_S2021_M1_Apc
GC[Carbamidomethyl (C)]ALQC[Carbamidomethyl (C)]AILSPAFK.2	0.28861797	#N/A
EFSITDVVPYPISLR.2	0.167895225	#N/A
AGGIETIANEYSDR.2	-0.294136202	#N/A
VLATAFDTTLGGR.2	0.508229349	#N/A
QSLTVDPVVK.2	0.460967332	#N/A
NHAAPFS[Phospho (STY)]K.2	-0.307236067	Q61316_S429_M1_Hspa4
SNLAYDIVQLPTGLTGIK.2	-0.195259867	#N/A
LEDTENWLYEDGEDQPK.2	0.157398014	#N/A
KPVVDC[Carbamidomethyl (C)]VVSVPSFYTDAER.3	-0.028859788	#N/A
LFEELGK.2	0.313559144	#N/A
DKLSGEYEK.2	-0.177405445	#N/A
FQESEERPK.2	-0.131982146	#N/A
LM[Oxidation (M)]NETTAVALAYGIYK.2	-0.06235168	#N/A
LKETAESVLK.2	-0.126159194	#N/A
QDLPALEEKPR.3	0.329714894	#N/A
SVM[Oxidation (M)]DATQIAGLNC[Carbamidomethyl (C)]LR.2	0.26315448	#N/A
AFSDPFVEAEK.2	-0.314137485	#N/A
NKEDQYEHLDAADVTK.3	-0.145665496	#N/A
FLEM[Oxidation (M)]C[Carbamidomethyl (C)]DDLLAR.2	-0.495257717	#N/A
NAVEEYVYEMR.2	0.335981475	#N/A
WNSPAEEGLS[Phospho (STY)]DC[Carbamidomethyl (C)]EVFPK.2	0.039915162	Q61316_S415_M1_Hspa4
SQVISNAK.2	-0.156284848	#N/A
ELSTTLNADEAVTR.2	0.481313346	#N/A
TTGPGGDGIPAEVHIVR.3	0.02092235	#N/A
FAQLNLAAEDPR.2	-0.050544163	#N/A
S[Phospho (STY)]APPNLWAAQR.2	-0.390774779	Q61337_S136_M1_Bad
S[Phospho (STY)]AGTATQMR.2	0.313153867	Q61337_S170_M1_Bad
RM[Oxidation (M)]S[Phospho (STY)]DEFEGSFK.2	-0.016551078	Q61337_S155_M1_Bad
ELETPS[Phospho (STY)]EEK.2	-0.458243398	Q61361_S587_M1_Bcan

EYQWIGLNDR.2	-0.200267504	#N/A
EVGGETGS[Phospho (STY)]PELSGVPR.2	-0.343462925	Q61361_S546_M1_Bcan
SWEEAESQC[Carbamidomethyl (C)]R.2	0.249109596	#N/A
YPIITPSQR.2	0.201900097	#N/A
FNVYC[Carbamidomethyl (C)]FR.2	0.197474058	#N/A
C[Carbamidomethyl (C)]QENGLWEAPQISC[Carbamidomethyl (C)]VPR.2	0.297765816	#N/A
LTWEEAR.2	-0.242613927	#N/A
GAIYSIPIS[Phospho (STY)]EDGGGGSSTPEDPAEAPR.3	-0.175304583	Q61361_S406_M1_Bcan
ALGAHLTSIC[Carbamidomethyl (C)]TPEEQDFVNDR.3	-0.325299718	#N/A
C[Carbamidomethyl (C)]SPGWLADGSVR.2	0.513729283	#N/A
ELETPS[Phospho (STY)]EEKSGR.2	-0.323023183	Q61361_S587_M1_Bcan
DSAHPSAS[Phospho (STY)]SEASSPASDGLEAIVTVTEK.3	-0.020909515	Q61361_S361_M1_Bcan
ALGAHLTSIC[Carbamidomethyl (C)]TPEEQDFVNDRYR.4	-0.334391802	#N/A
EVEVLVAR.2	-0.374211696	#N/A
C[Carbamidomethyl (C)]EVQHGIDDSSDAVEVK.2	-0.168003879	#N/A
EAC[Carbamidomethyl (C)]S[Phospho (STY)]GDM[Oxidation (M)]DGYPGVR.2	0.134611334	Q61361_S224_M1_Bcan
NKVPELQK.2	-0.189176729	#N/A
LTSSVTAYDYSGK.2	0.551497332	#N/A
LQDVIHVQGGS[Phospho (STY)]PLQAS[Phospho (STY)]PDKVPLDVHR.4	-0.261033431	Q61410_S92_M2_Prkg2
ALS[Phospho (STY)]LEMIQLK.2	0.254198547	Q61410_S431_M1_Prkg2
SFEDIHQYR.2	-0.348054352	#N/A
QGVEDAFYTLVR.2	-0.374042451	#N/A
GAYSSHDPQGS[Phospho (STY)]RGSR.3	0.115158606	Q61423_S79_M1_Kcna4
QSSFPHC[Carbamidomethyl (C)]SDLMPS[Phospho (STY)]GSEEK.2	-0.051335042	Q61423_S111_M1_Kcna4
DTPGFIVNR.2	-0.522465779	#N/A
TFESLVDFC[Carbamidomethyl (C)]K.2	-0.128248605	#N/A
AGDEFVEK.2	-0.298946105	#N/A
FAAEHTIFASNTSSLQITNIANATTR.3	0.115088197	#N/A
SYRS[Phospho (STY)]AS[Phospho (STY)]PER.2	-0.310457475	Q61464_S560_M2_Znf638
GLS[Phospho (STY)]PAQKPK.2	0.115381044	Q61464_S606_M1_Znf638
QSSVTQVTEQS[Phospho (STY)]PK.2	-0.245442417	Q61464_S128_M1_Znf638
HTETVDKGLS[Phospho (STY)]PAQKPK.3	-0.229761974	Q61464_S606_M1_Znf638
GSTFS[Phospho (STY)]PDLK.2	0.247827784	Q61464_S1099_M1_Znf638
[Acetyl (Protein N-term)]M[Oxidation (M)]ETDAPQPGLAS[Phospho (STY)]PDSPHDPC[Carbamidomethyl (C)]K.2	-0.277724415	Q61474_S12_M1_Msi1
NNLVDIIQQNK.2	-0.385840195	#N/A
NSLQQPEGIDR.2	-0.390671834	#N/A
[Acetyl (Protein N-term)]GS[Phospho (STY)]TDTDIEELENATYK.2	0.091515677	Q61481_S3_M1_Pde1a
APFLETDQLK.2	-0.402829071	#N/A
SSNTYTLTDVR.2	-0.327687969	#N/A
EIDPGTQLYTVTSSLEYK.2	-0.189555577	#N/A
VLQPVEGEVAILFK.2	-0.222145084	#N/A
NM[Oxidation (M)]AASTTITVHYLDLSLNPSGEVTK.3	0.008157248	#N/A
KTDPSGLSK.2	-0.373283747	#N/A
SVQYDDVPEYKDR.3	-0.03030474	#N/A
DALEIYKR.2	0.038354553	#N/A
NTLFNLSNFLDK.2	-0.26780408	#N/A
GLGSDLDSSLASLVGNLGISGTTSK.2	-0.56809575	#N/A

DPLADLNIKDFL.2	0.093397579	#N/A
SSPATTVTS[Phospho (STY)]PNSTPAK.2	-0.413611613	Q61548_S313_M1_Snap91
QM[Oxidation (M)]AFDFAR.2	-0.111761242	#N/A
IAAAQYSVTGSAVAR.2	0.166323643	#N/A
ATTHEVM[Oxidation (M)]GPK.2	0.098026585	#N/A
LTGGANWQPK.2	0.170553037	#N/A
KPGNNEGSGAPS[Phospho (STY)]PLSK.2	-0.392523699	Q61548_S300_M1_Snap91
GASPVPESSLTADLLSVDAFAAPSPASTASPAK.3	-0.46580208	#N/A
KGDLQWNAGEK.2	0.002579894	#N/A
TIDTS[Phospho (STY)]PPVDIFATASAAAPVSSAK.2	0.023492741	Q61548_S325_M1_Snap91
PDAAPSIDLFGTDAFSS[Phospho (STY)]PPR.2	-0.142500041	Q61548_S594_M1_Snap91
SGSHGYDM[Oxidation (M)]STFIR.3	-0.284273703	#N/A
DALEIYK.2	-0.153554354	#N/A
VNASASS[Phospho (STY)]LKK.2	-0.118796214	Q61553_S39_M1_Fscn1
FLVVAHDDGR.2	0.227787908	#N/A
YLAPSGPSGTLK.2	-0.106898288	#N/A
GEHGFIGC[Carbamidomethyl (C)]R.2	0.217706592	#N/A
LSC[Carbamidomethyl (C)]FAQSVSPAEK.2	-0.105328627	#N/A
KVTGTLDANR.2	0.502399584	#N/A
VNASASS[Phospho (STY)]LKKK.2	-0.438005414	Q61553_S39_M1_Fscn1
YSVQTSDHR.2	0.203796865	#N/A
LVARPEPATGFTLEFR.3	-0.375993246	#N/A
WSLQSEAHR.2	0.248555746	#N/A
YFGGTEDR.2	-0.19504208	#N/A
YLTAEAFGFK.2	-0.039414936	#N/A
AC[Carbamidomethyl (C)]AETIDPASLWEY.2	0.161476858	#N/A
SSYDVFQLEFNDGAYNIK.2	-0.205622028	#N/A
VGKDELFALEQSC[Carbamidomethyl (C)]AQVVLQAANER.3	-0.504576378	#N/A
VTGTLDANR.2	0.134721408	#N/A
YLAADKDGNVTC[Carbamidomethyl (C)]ER.2	-0.175621405	#N/A
YAHLSAR.2	0.01090673	#N/A
QIWTLEQPPDEAGSAAVC[Carbamidomethyl (C)]LR.2	0.265200353	#N/A
DVPWGVDSLITLAFQDQR.2	-0.144953532	#N/A
VNASASS[Phospho (STY)]LK.2	0.119720301	Q61553_S39_M1_Fscn1
QGM[Oxidation (M)]DLSANQDEETDQETFQLEIDR.3	-0.384879756	#N/A
EIQNGTLRES[Phospho (STY)]DSEHVPR.3	-0.278998863	Q61595_S75_M1_Ktn1
DLLT[Phospho (STY)]ELQK.2	-0.141333649	Q61595_T1211_M1_Ktn1
NTNDANSC[Carbamidomethyl (C)]QIIIPQNQVNR.2	-0.228228693	#N/A
LYSESLAR.2	0.051702168	#N/A
TDDYLDQPC[Carbamidomethyl (C)]C[Carbamidomethyl (C)]ETINR.2	-0.15124566	#N/A
VIC[Carbamidomethyl (C)]ILSHPIK.2	-0.017767503	#N/A
FVSISDLFVPK.2	-0.093534034	#N/A
DLGTDSQIFISR.2	0.229978637	#N/A
M[Oxidation (M)]LLFTEVTR.2	-0.066065595	#N/A
FLM[Oxidation (M)]ANGQLVK.2	0.582000203	#N/A
SPYLYPLYGLGELPQGFAR.2	-0.309008023	#N/A
AYDATTHFETTC[Carbamidomethyl (C)]DDIKDIYKR.4	-0.50134103	#N/A

VLHM[Oxidation (M)]DQNPYYGGESASITPLEDLYK.3	-0.607653623	#N/A
FLVYVANFDEKDPR.2	-0.192943748	#N/A
VIEGSFVYK.2	-0.050826143	#N/A
FDLGQDVIDFTGHSLALYR.3	-0.273232367	#N/A
MTGSEFDFEEMKR.3	-0.1596649	#N/A
EVKSS[Phospho (STY)]PSES[Phospho (STY)]PLMEKK.2	-0.313273611	#N/A
EVKSS[Phospho (STY)]PSES[Phospho (STY)]PLM[Oxidation (M)]EK.2	0.188050447	#N/A
SS[Phospho (STY)]PSES[Phospho (STY)]PLMEKK.2	-0.339071969	#N/A
SS[Phospho (STY)]PSESPLMEK.2	0.292499499	Q61609_S269_M1_Slc20a1
IQPVTHSGQHS[Phospho (STY)]T.2	0.234311162	Q61616_S445_M1_Drd1
GGLLTSEEDS[Phospho (STY)]GFSTSPKDNSLPK.3	0.128287673	Q61624_S301_M1_Znf148
GGLLTSEEDSGFSTS[Phospho (STY)]PK.2	0.317713476	Q61624_S306_M1_Znf148
AGMTSS[Phospho (STY)]PDATTGQTFG.2	0.164398995	Q61624_S784_M1_Znf148
VPS[Phospho (STY)]KEDDKEIDLEHLHR.4	-0.261671156	Q61625_S865_M1_Grid2
TEQSVT[Phospho (STY)]PEQQK.2	-0.211003729	Q61644_T181_M1_Pacsin1
GPQYGSLER.2	-0.231219751	#N/A
GRLDS[Phospho (STY)]GQLGLYPANYVEAI.2	-0.468631372	Q61644_S427_M1_Pacsin1
EVLLDIKR.2	-0.070379209	#N/A
NSLLNEDLEK.2	0.010320103	#N/A
AYAQQLTDWAK.2	0.098215941	#N/A
LGEEDEQGWC[Carbamidomethyl (C)]R.2	-0.208353239	#N/A
GADAQEDLR.2	0.042302886	#N/A
ALYDYDGQEQDELSFK.2	-0.284769245	#N/A
EAEDGFRK.2	0.169618541	#N/A
LDS[Phospho (STY)]GQLGLYPANYVEAI.2	0.46238245	Q61644_S427_M1_Pacsin1
NSLLNEDLEKVK.2	-0.140703031	#N/A
KAEGATLSNATGAVESTSQAGDR.3	-0.347544755	#N/A
AYHLAC[Carbamidomethyl (C)]K.2	0.251006602	#N/A
LC[Carbamidomethyl (C)]NDLM[Oxidation (M)]SC[Carbamidomethyl (C)]VQER.2	0.310139783	#N/A
AYHLAC[Carbamidomethyl (C)]KEER.2	-0.174645225	#N/A
GSVSS[Phospho (STY)]YDR.2	-0.276179626	Q61644_S346_M1_Pacsin1
TEQSVT[Phospho (STY)]PEQQKK.2	-0.184156212	Q61644_T181_M1_Pacsin1
HLNLAENS[Phospho (STY)]SYM[Oxidation (M)]HVYR.3	0.021436216	Q61644_S259_M1_Pacsin1
[Acetyl (Protein N-term)]SGSYDEASEEITDSFWEVGNYKR.3	-0.231780361	#N/A
AWGAM[Oxidation (M)]M[Oxidation (M)]TEADKVSELHQEVK.3	-0.169471924	#N/A
KAEGATLSNATGAVESTS[Phospho (STY)]QAGDRGSVSSYDR.4	-0.098987568	Q61644_S336_M1_Pacsin1
AEGATLSNATGAVESTSQAGDR.2	-0.457256506	#N/A
TTPQYM[Oxidation (M)]EGM[Oxidation (M)]EQVFEQC[Carbamidomethyl (C)]QQFEEK.3	-0.402634618	#N/A
STSGPGM[Oxidation (M)]PM[Oxidation (M)]NWPQFEEWNPDLPHTTAK.3	0.17131911	#N/A
APILIATDVASR.2	-0.029928293	#N/A
QVSDLISVLR.2	0.201038758	#N/A
LIDFLEC[Carbamidomethyl (C)]GK.2	-0.272894119	#N/A
GVEIC[Carbamidomethyl (C)]IATPGR.2	0.256979363	#N/A
ELAQQVQQVAAEYC[Carbamidomethyl (C)]R.2	-0.023404323	#N/A
LLQLVEDR.2	-0.113649572	#N/A
STC[Carbamidomethyl (C)]IYGGAPK.2	-0.056638654	#N/A
NFYQEHPDLAR.2	-0.025579058	#N/A

FVINYDYPNSSEDYIHR.3	0.290419986	#N/A
DWVLNEFK.2	-0.378290766	#N/A
KSS[Phospho (STY)]FSNSADDIK.2	-0.407368724	Q61686_S93_M1_Cbx5
SS[Phospho (STY)]FSNSADDIK.2	0.356003705	Q61686_S93_M1_Cbx5
TADSSSS[Phospho (STY)]EDEEEYVVEK.2	0.126751824	Q61686_S14_M1_Cbx5
EENVNS[Phospho (STY)]PEDKR.2	-0.226023565	Q61687_S912_M1_Atrx
LTLS[Phospho (STY)]DGES[Phospho (STY)]GEEKPTKPK.2	-0.160264596	Q61687_S1335_M2_Atrx
VC[Carbamidomethyl (C)]DQTSKFS[Phospho (STY)]PK.2	-0.225412324	Q61687_S315_M1_Atrx
QNYSESSNYDS[Phospho (STY)]ELER.2	0.133364301	Q61687_S801_M1_Atrx
YVES[Phospho (STY)]DDEKPTDENVNEK.2	-0.354616492	Q61687_S92_M1_Atrx
TTSTSNPS[Phospho (STY)]S[Phospho (STY)]PAPDWYK.2	0.302489569	Q61687_S1978_M2_Atrx
EEIS[Phospho (STY)]DHENNVTILLEDSDLR.3	-0.027348828	Q61687_S626_M1_Atrx
TTQEGS[Phospho (STY)]S[Phospho (STY)]ADDTGDTEGR.2	0.392307062	Q61687_S854_M2_Atrx
AKQPVIGDQNS[Phospho (STY)]DS[Phospho (STY)]DEM[Oxidation (M)]LAVLK.3	-0.001305303	Q61687_S717_M2_Atrx
IGKQS[Phospho (STY)]EES[Phospho (STY)]PADDGELRR.3	-0.529479126	Q61687_S1287_M2_Atrx
IES[Phospho (STY)]PKLER.2	-0.34139136	Q61699_S810_M1_Hsph1
PKIES[Phospho (STY)]PKLER.2	-0.17440599	Q61699_S810_M1_Hsph1
SVLDAAQIVGLNC[Carbamidomethyl (C)]LR.2	0.295859006	#N/A
FVVQNVSAQK.2	-0.141203024	#N/A
AGGIETIANEFSDR.2	-0.203177617	#N/A
[Acetyl (Protein N-term)]SVVGLDVGSQSC[Carbamidomethyl (C)]YIAVAR.2	-0.22979671	#N/A
FIC[Carbamidomethyl (C)]EQEHEK.2	0.114932903	#N/A
LLTETEDWLYEEGEDQAK.2	-0.234500518	#N/A
NAVEEC[Carbamidomethyl (C)]VYEFR.2	0.272171527	#N/A
SQFEELC[Carbamidomethyl (C)]AELLQK.2	-0.235675623	#N/A
S[Phospho (STY)]LDQDPVVR.2	-0.193785629	Q61699_S774_M1_Hsph1
VVNVELPVEANLVWQLGR.2	0.162273734	#N/A
VLEELGQR.2	0.038163345	#N/A
QDLPNAEEKPR.3	0.038507357	#N/A
NQQITHANNTVSSFK.2	-0.184343769	#N/A
T[Phospho (STY)]PNGPNIDKKEDLEGK.3	0.232694216	Q61699_T816_M1_Hsph1
SSQALLSQLYQS[Phospho (STY)]PNR.2	-0.046694691	Q61701_S233_M1_Elavl4
ILVDQVTGVSR.2	0.051339071	#N/A
NC[Carbamidomethyl (C)]PS[Phospho (STY)]PMQTGAATDDSK.2	0.272648698	Q61701_S38_M1_Elavl4
ISVSDLINGIASLK.2	-0.188007624	#N/A
FVASNQR.2	-0.078269833	#N/A
NVEAQSTEEMFVK.2	0.194756914	#N/A
GTIQVVTQGTSLK.2	0.247505774	#N/A
GGIVDEGALLR.2	-0.040331685	#N/A
VTADVINAAEK.2	-0.092713829	#N/A
QADVNLVNAK.2	0.203817237	#N/A
NAGTC[Carbamidomethyl (C)]LSPAVIVGLLR.2	0.354118121	#N/A
VLISDSLDPC[Carbamidomethyl (C)]C[Carbamidomethyl (C)]R.2	0.387686312	#N/A
ILQDGGLQVVEK.2	-0.041020355	#N/A
AGTGVDNVDLEAATR.2	0.147589882	#N/A
ALVDHENVISC[Carbamidomethyl (C)]PHLGASTK.3	0.47182093	#N/A
RES[Phospho (STY)]GLDTGGQR.2	-0.105161761	Q61762_S535_M1_Kcna5

DYEGPAIS[Phospho (STY)]PK.2	-0.396472891	Q61771_S681_M1_Kif3b
SQNQQYLVR.2	-0.42568788	#N/A
GFSVVADTPELQR.2	0.034093299	#N/A
YHEEFEK.2	-0.196876251	#N/A
YKEEFEK.2	-0.141135374	#N/A
QSFTM[Oxidation (M)]VADTPENLR.2	-0.428972612	#N/A
TQDQISNIK.2	0.166480169	#N/A
IVYPTEK.2	0.338615658	#N/A
EAQDSSSYR.2	0.066293885	#N/A
TGDTGMLPANYVEAI.2	-0.238379549	#N/A
MGPSGGEGVEPERR.3	-0.30911239	#N/A
LKQQSELQSQVR.3	-0.045464414	#N/A
S[Phospho (STY)]PALPER.2	-0.109729833	Q61818_S1332_M1_Rai1
SGVAVPTS[Phospho (STY)]PK.2	0.091737416	Q61823_S94_M1_Pdcd4
SGVAVPTS[Phospho (STY)]PKGR.2	-0.259458149	Q61823_S94_M1_Pdcd4
TSDVNDTQPPQS[Phospho (STY)]E.2	-0.203424226	Q61879_S1975_M1_Myh10
SDLLLEGFNNYR.2	-0.29004665	#N/A
IVFQEFR.2	0.039631834	#N/A
TQLEELEDELQATEDAK.2	-0.023292053	#N/A
GGPISFS[Phospho (STY)]SSR.2	0.022067665	Q61879_S1937_M1_Myh10
EQEVAELKK.2	-0.150969625	#N/A
LQQELDDLTVDLDHQR.3	-0.097939058	#N/A
LDPHLVLDQLR.2	0.148564266	#N/A
DAAGLESQLQDTQELLQEETR.2	-0.047317156	#N/A
QLLQANPILESFGNAK.2	-0.058431185	#N/A
LVQEQGSHSK.2	-0.140936475	#N/A
DLEAQIEAANK.2	0.386721541	#N/A
QLHIEGAS[Phospho (STY)]LELSDDDTESK.3	0.191737213	Q61879_S1952_M1_Myh10
ELQAQIAELQEDFESEK.2	-0.307903443	#N/A
IAQLEEELEEEQSNM[Oxidation (M)]ELLNDR.3	-0.385564616	#N/A
SLEAEILQLQEELASSER.2	-0.177896204	#N/A
RQLHIEGASLELS[Phospho (STY)]DDDTESK.3	-0.020258597	Q61879_S1956_M1_Myh10
VIQYLAHVASSHK.3	-0.296854484	#N/A
IAEC[Carbamidomethyl (C)]SSQLAEEEEKAK.2	-0.197786961	#N/A
AVIYNPATQADWTAK.2	-0.314659196	#N/A
NKQEVMISDLEER.2	-0.012254322	#N/A
LQQLFNHTM[Oxidation (M)]FILEQEEYQR.3	0.099345859	#N/A
VIGPGYPIR.2	0.064212384	#N/A
ALVGDEAELPC[Carbamidomethyl (C)]R.2	-0.077680967	#N/A
AEVENLHR.2	0.095666046	#N/A
TFDPHFLR.2	0.156408645	#N/A
FSDEGGYTC[Carbamidomethyl (C)]FFR.2	0.163852584	#N/A
LAGQFLEELR.2	0.393772279	#N/A
LAGQFLEELRNPF.2	0.171330682	#N/A
DQDAEQAPEYR.2	-0.0172861	#N/A
LRAEVENLHR.3	-0.213194049	#N/A
DHSYQEEAAM[Oxidation (M)]ELK.2	-0.276417998	#N/A

NGKDQDAEQAPEYR.2	0.429442221	#N/A
STT[Phospho (STY)]PGTPSTIETTPR.2	0.06332051	Q61909_T565_M1_Runx1t1
QQS[Phospho (STY)]PVNPDPVALDAHR.3	-0.199505453	Q61909_S390_M1_Runx1t1
S[Phospho (STY)]LTLAAPGEVR.2	-0.060874281	Q61923_S6_M1_Kcna6
SEKS[Phospho (STY)]LTLAAPGEVR.2	-0.084473353	Q61923_S6_M1_Kcna6
MRS[Phospho (STY)]EKS[Phospho (STY)]LTLAAPGEVR.3	0.138501101	Q61923_S3_M2_Kcna6
M[Oxidation (M)]TDQEAIQDLWQWR.2	-0.452521738	#N/A
GPSSVEDIK.2	0.394873262	#N/A
VDNDENEHQLSLR.2	0.235737314	#N/A
DELHIVEAEAM[Oxidation (M)]NYEGSPIK.3	0.179852536	#N/A
MSVQPTVSLGGFEITPPVVLR.2	0.085720222	#N/A
C[Carbamidomethyl (C)]GSGPVHISGQHLVAVEEDAES[Phospho (STY)]EDEDEEDVKLLGM[Oxidation (M)]SGK.5	0.265565874	Q61937_S125_M1_Npm1
LVVPASQC[Carbamidomethyl (C)]GSLIGK.2	0.524070961	#N/A
INISEGNC[Carbamidomethyl (C)]PER.2	0.233770488	#N/A
AITIAGIPQSIIEC[Carbamidomethyl (C)]VK.2	0.042096531	#N/A
IITLAGPTNAIFK.2	0.093326238	#N/A
PSSS[Phospho (STY)]PVIFAGGQDR.2	0.068145371	Q61990_S185_M1_Pcbp2
ESTGAQVQVAGDM[Oxidation (M)]LPNSTER.2	0.198508625	#N/A
QVTITGSAASISLAQYLINVR.2	-0.506451174	#N/A
KGGEFDEFVNDDT[Phospho (STY)]DDDLPVSK.3	-0.019346451	Q62018_T925_M1_Ctr9
GGEFDEFVNDDT[Phospho (STY)]DDDLPVSK.2	-0.074500278	Q62018_T925_M1_Ctr9
NSNS[Phospho (STY)]DS[Phospho (STY)]DDDERPNR.2	-0.530533962	Q62018_S1039_M2_Ctr9
KGS[Phospho (STY)]GS[Phospho (STY)]EQEGEEEEGGER.2	-0.306065769	Q62018_S941_M2_Ctr9
RKGS[Phospho (STY)]GS[Phospho (STY)]EQEGEEEEGGER.2	0.16565397	Q62018_S941_M2_Ctr9
SNS[Phospho (STY)]TTQVNQSWTGSPR.2	0.16119528	Q62036_S78_M1_Cep131
QPS[Phospho (STY)]EEEIIK.2	-0.254772107	Q62048_S116_M1_Pea15
SAC[Carbamidomethyl (C)]KEDIPSEK.2	0.266058608	#N/A
YKDIIRQPS[Phospho (STY)]EEEIIK.3	-0.371033776	Q62048_S116_M1_Pea15
LTRIPS[Phospho (STY)]AK.2	-0.328807338	Q62048_S104_M1_Pea15
DIIRQPS[Phospho (STY)]EEEIIK.2	-0.373301502	Q62048_S116_M1_Pea15
RPDLLTM[Oxidation (M)]VVDYR.2	-0.108056196	#N/A
[Acetyl (Protein N-term)]AEYGTLLQDLTNNITLEDLEQLK.3	-0.013688423	#N/A
QPS[Phospho (STY)]EEEIIKLAPPPK.2	-0.191502965	Q62048_S116_M1_Pea15
SEEITTGSAWFSFLESHNK.2	-0.279565679	#N/A
DNLSYIEHIFEISR.2	0.001697792	#N/A
YEINSLIR.2	-0.064639408	#N/A
AQC[Carbamidomethyl (C)]GGGLLGVR.2	0.178717514	#N/A
GTVAC[Carbamidomethyl (C)]GQPPVVENAK.2	-0.204491124	#N/A
LATVGELQAAWR.2	-0.066796443	#N/A
FTFEEAEAEC[Carbamidomethyl (C)]TSR.2	0.066390737	#N/A
NGFDQC[Carbamidomethyl (C)]DYGWLSDASVR.2	-0.117975908	#N/A
VGHDYQWIGLNDK.3	0.008859335	#N/A
S[Phospho (STY)]IQDLTVTGTEPGQVSSR.2	-0.019048859	Q62073_S412_M1_Map3k7
AREGS[Phospho (STY)]FEAR.2	-0.177437028	Q62077_S1248_M1_Plcg1
S[Phospho (STY)]RS[Phospho (STY)]PPPVSK.2	-0.050291777	Q62093_S189_M2_Srsf2
TS[Phospho (STY)]PDTLRR.2	-0.486020323	Q62093_S26_M1_Srsf2
VGDVYIPR.2	-0.226899624	#N/A

S[Phospho (STY)]KS[Phospho (STY)]PPKS[Phospho (STY)]PEEEGAVSS.2	0.158767055	Q62093_S206_M3_Srsf2
VDNLTYRTS[Phospho (STY)]PDTLR.2	-0.363237738	Q62093_S26_M1_Srsf2
TS[Phospho (STY)]PDTLR.2	-0.022157963	Q62093_S26_M1_Srsf2
S[Phospho (STY)]RS[Phospho (STY)]RS[Phospho (STY)]PPPVSK.2	-0.078383989	Q62093_S187_M3_Srsf2
VDNLTYR.2	-0.055837409	#N/A
YLVLDEADR.2	-0.339102514	#N/A
GKPNYFSDRGS[Phospho (STY)]GSR.3	0.114829895	Q62095_S90_M1_Ddx3y
FS[Phospho (STY)]GGFGAR.2	0.143712903	Q62095_S592_M1_Ddx3y
HVINFDLPSDIEEYVHR.3	0.128865532	#N/A
DLLDLLVEAK.2	0.100618635	#N/A
VGNLGLATSFFNER.2	0.166633479	#N/A
RYS[Phospho (STY)]PVAK.2	-0.396532801	Q62108_S295_M1_Dlg4
IIPGGAAAQDGR.2	-0.104628494	#N/A
DYHFVSSR.2	0.125663733	#N/A
VIEDLSGPYIWVPAR.2	-0.217913113	#N/A
PIIILGPTK.2	0.318132323	#N/A
EVTHSAAVEALK.2	-0.011431858	#N/A
VHSDS[Phospho (STY)]ETDDIGFIPSKR.3	0.216946126	Q62108_S480_M1_Dlg4
REYEIDGR.2	-0.364747032	#N/A
VNDSILFVNEVDVR.2	0.075612811	#N/A
NTYDVVY[Phospho (STY)]LK.2	-0.456013458	Q62108_Y240_M1_Dlg4
AKDWGS[Phospho (STY)]SSGSQGR.2	0.124705176	Q62108_S509_M1_Dlg4
EQLM[Oxidation (M)]NSSLGS[Phospho (STY)]GTASLR.2	-0.646617007	Q62108_S418_M1_Dlg4
RVHSDS[Phospho (STY)]ETDDIGFIPSK.3	-0.261866182	Q62108_S480_M1_Dlg4
VHSDSETDDIGFIPSK.3	0.28323343	#N/A
HC[Carbamidomethyl (C)]ILDVSANAVR.2	0.711178159	#N/A
RVHS[Phospho (STY)]DS[Phospho (STY)]ETDDIGFIPSKR.3	0.022785546	Q62108_S478_M2_Dlg4
EVTHSAAVEALKEAGS[Phospho (STY)]IVR.3	-0.183510233	Q62108_S142_M1_Dlg4
DWGSSSGS[Phospho (STY)]QGR.2	0.194457646	Q62108_S513_M1_Dlg4
GNS[Phospho (STY)]GLGFSIAGGTDNPHIGDDPSIFITK.3	0.31909895	Q62108_S73_M1_Dlg4
FIEAGQYNSHLYGTSVQSVR.3	-0.438048865	#N/A
DLLGEEDIPREPR.3	-0.094742943	#N/A
EQLMNSSLGSGT[Phospho (STY)]ASLRS[Phospho (STY)]NPKR.3	0.142030992	Q62108_S425_M2_Dlg4
S[Phospho (STY)]LENVLEINKR.2	-0.125237562	Q62108_S654_M1_Dlg4
ILAVNSVGLEDVMHEDAVAALK.3	-0.233199466	#N/A
IIEGGAAHKDGR.3	0.006106991	#N/A
TNGISDVQIS[Phospho (STY)]PTLQR.2	0.056252066	Q62120_S523_M1_Jak2
NQEIHLS[Phospho (STY)]PIAR.2	-0.153144272	Q62132_S271_M1_Ptprr
TPS[Phospho (STY)]SEEISPTK.2	-0.023795373	Q62172_S29_M1_Ralbp1
[Acetyl (Protein N-term)]TEC[Carbamidomethyl (C)]FLPPSS[Phospho (STY)]SPSEHR.2	-0.211313917	Q62172_S10_M1_Ralbp1
[Acetyl (Protein N-term)]TEC[Carbamidomethyl (C)]FLPPS[Phospho (STY)]SSPSEHRR.3	0.072436787	Q62172_S9_M1_Ralbp1
TEGYAAFQEDS[Phospho (STY)]S[Phospho (STY)]GDEAESPSK.2	0.239741033	Q62172_S92_M2_Ralbp1
TPS[Phospho (STY)]SEEIS[Phospho (STY)]PTKFPGLYR.2	-0.127126301	Q62172_S29_M2_Ralbp1
TGEPS[Phospho (STY)]PPHDVLHEPPDT[Phospho (STY)]VSDDDKDHGK.4	0.15051249	Q62172_S48_M2_Ralbp1
RTEGYAAFQEDS[Phospho (STY)]S[Phospho (STY)]GDEAESPSK.3	-0.093241203	Q62172_S92_M2_Ralbp1
TSAS[Phospho (STY)]DVDADNNHLGAEVA.2	-0.18840874	Q62178_S746_M1_Sema4a
DNFTAIPEGTNGVEER.2	-0.074562905	#N/A

GAPLVVIC[Carbamidomethyl (C)]QGK.2	0.026486694	#N/A
QIGDNLIVPGGVK.2	0.146124231	#N/A
IAVGSDSDLVIWDPDALK.2	-0.047488314	#N/A
AITVASQTNC[Carbamidomethyl (C)]PLYVTK.2	0.033443731	#N/A
GM[Oxidation (M)]YDGPVFDLTTT[Phospho (STY)]PK.2	-0.042130951	Q62188_T509_M1_Dpysl3
SAADLISQAR.2	0.081602307	#N/A
GS[Phospho (STY)]PTRPNPPVR.2	-0.238748794	Q62188_S522_M1_Dpysl3
GGT[Phospho (STY)]PAGSTRGS[Phospho (STY)]PTRPNPPVR.3	-0.079356727	Q62188_S522_M2_Dpysl3
IVAPPGGRS[Phospho (STY)]NITS[Phospho (STY)]LS.2	0.113703689	Q62188_S564_M2_Dpysl3
NLHQSGFS[Phospho (STY)]LSGTQVDEGVR.3	-0.116169109	Q62188_S539_M1_Dpysl3
GM[Oxidation (M)]TTVDDFFQGTK.2	-0.034976425	#N/A
NHQSVAEYNIFEGM[Oxidation (M)]ELR.3	-0.027331567	#N/A
GM[Oxidation (M)]YDGPVFDLTTT[Phospho (STY)]PKGGT[Phospho (STY)]PAGSTR.3	-0.219094827	Q62188_T509_M2_Dpysl3
AALAGGTTM[Oxidation (M)]IIDHVVPEPESSLTEAYEK.3	-0.088935032	#N/A
MADLHAVPR.2	0.380904928	#N/A
IM[Oxidation (M)]LEDGNLHVTQGAGR.3	0.126761618	#N/A
SC[Carbamidomethyl (C)]C[Carbamidomethyl (C)]DYALHVDITHWNDSVK.3	0.024009012	#N/A
NLHQSGFS[Phospho (STY)]LSGTQVDEGVRS[Phospho (STY)]ASKR.4	0.178499599	Q62188_S539_M2_Dpysl3
S[Phospho (STY)]GS[Phospho (STY)]EES[Phospho (STY)]IRKK.2	-0.128100785	Q62205_S502_M3_Scn9a
EGC[Carbamidomethyl (C)]PS[Phospho (STY)]PPGQTSK.2	-0.246467421	Q62219_S194_M1_Tgfb1i1
IPESETESTASAPNS[Phospho (STY)]PR.2	-0.31242215	Q62245_S1082_M1_Sos1
RLS[Phospho (STY)]ESAC[Carbamidomethyl (C)]R.2	0.117996055	Q62245_S401_M1_Sos1
SAS[Phospho (STY)]VSSISLSK.2	0.128943532	Q62245_S1120_M1_Sos1
ETWLSENQR.2	-0.273721734	#N/A
DVEDEILWVGER.2	-0.149070579	#N/A
DQNTVETLQR.2	0.125762801	#N/A
SALPAQSAATLPAR.2	0.14400621	#N/A
EIGQSVDEVEK.2	-0.313575679	#N/A
VAVVNQIAR.2	-0.064656234	#N/A
FM[Oxidation (M)]ELLEPLSER.2	-0.331236114	#N/A
LLEVLSGER.2	-0.173982526	#N/A
IHC[Carbamidomethyl (C)]LENVDK.2	-0.146381507	#N/A
VLLLSQDYGK.2	-0.021894141	#N/A
EVDDLEQWIAER.2	-0.239210173	#N/A
EIEELQSQAQALSQEGK.2	-0.01056708	#N/A
ENEVLEAWK.2	-0.338059604	#N/A
SQNIITDSSSLNAEAIR.2	-0.092977102	#N/A
LVSDGNINSDR.2	0.034127862	#N/A
KLPEELGR.2	-0.151450011	#N/A
LVDTGDKFR.2	0.113469955	#N/A
LTTLELLEVR.2	-0.18233733	#N/A
ITDLYTDLR.2	-0.15471872	#N/A
VLDNAIETEK.2	0.054290999	#N/A
SLLDAC[Carbamidomethyl (C)]EGR.2	0.109322503	#N/A
VQAVVAVAR.2	-0.397973991	#N/A
IDDIFER.2	-0.167892085	#N/A
LVSQDNFGFDLPAVEAATK.2	-0.502064441	#N/A

LQALDTGWNELHK.2	0.146663419	#N/A
TQTAIASEDM[Oxidation (M)]PNTLTEAEK.2	-0.262203153	#N/A
LQAAYAGDKADDIQKR.3	-0.279202251	#N/A
TAGYPNVNIHNFTTSWR.3	0.152938439	#N/A
HLLGVEDLLQK.2	-0.29636212	#N/A
LSDGNEYLFQAK.2	0.097612336	#N/A
EQWANLEQLSAIR.2	-0.394692664	#N/A
IVSSNDVGHDEYSTQSLVK.3	-0.037167896	#N/A
EAEKLESEHPDQAQAILSR.3	-0.097056273	#N/A
LQAAYAGDK.2	-0.047764901	#N/A
QLWGLLIEETEKR.2	-0.298778228	#N/A
RPPS[Phospho (STY)]PDPNTK.2	-0.263697562	Q62261_S2102_M1_Sptbn1
TSS[Phospho (STY)]KESS[Phospho (STY)]PVPS[Phospho (STY)]PTLDRK.3	-0.333111182	#N/A
[Acetyl (Protein N-term)]TTTVATDYDNIEIQQQYSDVNNR.3	-0.257432989	#N/A
ESS[Phospho (STY)]PVPSPTLDR.2	-0.171388423	Q62261_S2164_M1_Sptbn1
FQIQDISVETEDNK.2	-0.444158997	#N/A
EIQGHQPR.2	0.170016715	#N/A
QIEAQEKPR.2	-0.27548903	#N/A
QALQDTLALYK.2	0.330411773	#N/A
DLDDFQSWLSR.2	0.014488296	#N/A
HQILEQAVEDYAETVHQLSK.3	-0.263635628	#N/A
SAATWDER.2	0.067507316	#N/A
LPEELGR.2	0.248922353	#N/A
AQTLPTSVVTITSESS[Phospho (STY)]PGKR.3	-0.169554144	Q62261_S2340_M1_Sptbn1
FATDGEGYKPC[Carbamidomethyl (C)]DPQVIR.3	0.042828238	#N/A
EAIC[Carbamidomethyl (C)]EVALDYK.2	-0.271499613	#N/A
ILSSDDYGK.2	0.190337058	#N/A
HRPDLIDFDK.3	-0.541052444	#N/A
SAASGIPYHSEVPVSLK.3	-0.082884264	#N/A
FANSLVGVQQQLQAFNTYR.2	0.082632403	#N/A
VIESTQDLGNDLAGVM[Oxidation (M)]ALQR.3	-0.295793871	#N/A
LQAAYAGDKADDIQK.2	-0.079692212	#N/A
ESS[Phospho (STY)]PVPS[Phospho (STY)]PTLDRK.2	-0.37905063	#N/A
FESLEPEM[Oxidation (M)]NNQASR.2	-0.059765446	#N/A
DDEEMNTWIQAISSAISSDKHDTSASTQS[Phospho (STY)]TPASSR.4	0.055678304	Q62261_S2318_M1_Sptbn1
INAVVETGR.2	0.196174844	#N/A
AQTLPTSVVTITSESSPGK.2	0.057000461	#N/A
STDEVDSKR.2	0.121368365	#N/A
TSS[Phospho (STY)]KESS[Phospho (STY)]PVPSPTLDR.2	0.031925136	#N/A
GDQVSQNGLPAEQGS[Phospho (STY)]PR.2	-0.356301726	Q62261_S2137_M1_Sptbn1
EGM[Oxidation (M)]QLISEKPETEAVVK.3	-0.279292601	#N/A
DLVAIEAK.2	-0.409403512	#N/A
ELEAENYHDIKR.3	0.152456696	#N/A
LILEVHQFSR.2	-0.430406491	#N/A
LISDINK.2	0.116838454	#N/A
HDTSASTQS[Phospho (STY)]T[Phospho (STY)]PASSR.2	0.008887068	Q62261_S2318_M2_Sptbn1
QNLLSQSHAYQQFLR.3	0.011574203	#N/A

DVSSVELLM[Oxidation (M)]NNHQGIK.2	-0.240304332	#N/A
VDTVNNM[Oxidation (M)]ADELINSGHSDAATIAEWK.3	-0.075033732	#N/A
EAASELLMR.2	0.307482107	#N/A
DASVAEAWLLGQEPYLSSR.2	0.179147321	#N/A
MWEVLESTTQTK.2	0.233174336	#N/A
DALLSALSIQNYHLEC[Carbamidomethyl (C)]NETK.3	0.142206563	#N/A
LAEISDVWEEMK.2	0.236152376	#N/A
WDVDDWDNENS[Phospho (STY)]SAR.2	0.245738538	Q62261_S35_M1_Sptbn1
GNLEVLLFTIQSK.2	-0.228174246	#N/A
RRPPS[Phospho (STY)]PDPNTK.2	-0.412137387	Q62261_S2102_M1_Sptbn1
VAHM[Oxidation (M)]EFC[Carbamidomethyl (C)]YQELC[Carbamidomethyl (C)]QLAAER.3	-0.686848149	#N/A
TQILAASYELHK.2	-0.036571112	#N/A
FQIQDISVETEDNKEK.3	-0.261000249	#N/A
RPPS[Phospho (STY)]PDPNTKVSEEAESQQWDTSK.3	-0.229848912	Q62261_S2102_M1_Sptbn1
TLETPAAQM[Oxidation (M)]EGFLNR.2	-0.599626437	#N/A
LLDPEDISVDHPDEK.2	0.014565792	#N/A
EVVAGSHELGQDYEHVTM[Oxidation (M)]LQER.3	-0.315348801	#N/A
KHEAIETDIAAYEER.3	0.00951394	#N/A
ITDLYTDLRDGR.3	0.445724647	#N/A
LEMNLGLQK.2	-0.386601811	#N/A
M[Oxidation (M)]HTTFEHDIQALGTQVR.3	-0.257171589	#N/A
VSEEAESQQWDTSKGDQVSQNGLPAEQGS[Phospho (STY)]PR.4	-0.063680835	Q62261_S2137_M1_Sptbn1
IQEKVDSIDDR.3	-0.074507771	#N/A
IFQEM[Oxidation (M)]LYIM[Oxidation (M)]DWM[Oxidation (M)]DEM[Oxidation (M)]K.2	-0.45466402	#N/A
QAEAFLNNQEYVLAHTEM[Oxidation (M)]PTTLEGAEAAIK.3	0.163451396	#N/A
HYASEEIKEK.3	-0.276409323	#N/A
SNAHYNLQNAFNLAEQHLGLTK.3	-0.201706424	#N/A
MLTAQDMSYDEAR.2	-0.263379076	#N/A
FFWEM[Oxidation (M)]AEEEGWIR.2	-0.012156047	#N/A
EAIC[Carbamidomethyl (C)]EVALDYKK.3	0.26834595	#N/A
AQQYYFDAAEAEAWM[Oxidation (M)]SEQELYM[Oxidation (M)]M[Oxidation (M)]SEEK.3	-0.397990386	#N/A
M[Oxidation (M)]ATDPENIIK.2	0.266711523	#N/A
LHQVY[Phospho (STY)]FDAPSC[Carbamidomethyl (C)]VK.2	-0.275718849	Q62277_Y81_M1_Syp
ETGWAAPFM[Oxidation (M)]R.2	-0.118991015	#N/A
M[Oxidation (M)]LLLADM[Oxidation (M)]DVVNQLVAGGQFR.2	-0.31704807	#N/A
AVDHVQR.2	-0.145954138	#N/A
TYTDAM[Oxidation (M)]QNYNGNDER.2	-0.06600581	#N/A
DADS[Phospho (STY)]GDSQNPPNQPSK.2	-0.022200278	Q62313_S28_M1_Tgoln1;Tgoln2
TES[Phospho (STY)]GETLAGDSDFSLKPEKGDK.3	0.043504735	Q62313_S210_M1_Tgoln1;Tgoln2
TES[Phospho (STY)]GETLAGDSDFSLKPEK.2	-0.114882732	Q62313_S210_M1_Tgoln1;Tgoln2
S[Phospho (STY)]GEGEVSGLLR.2	-0.152155522	Q62318_S473_M1_Trim28
SRS[Phospho (STY)]GEGEVSGLLR.2	-0.109642026	Q62318_S473_M1_Trim28
[Acetyl (Protein N-term)]AASAAATAAASAATAASAASGS[Phospho (STY)]PGSGEGSAGGEK.3	-0.207619072	Q62318_S23_M1_Trim28
LSPPYSSPQEFAQDVGR.2	-0.039402634	#N/A
VS[Phospho (STY)]LERLDLDLTSDSQPPVFK.3	-0.272999212	Q62318_S489_M1_Trim28
RPAASSAAAASAAASS[Phospho (STY)]PAGGGGEAQELLEHC[Carbamidomethyl (C)]GVC[Carbamidomethyl (C)]R.4	-0.144335015	Q62318_S51_M1_Trim28
[Acetyl (Protein N-term)]AASAAATAAASAATAASAASGSPGSGEGS[Phospho (STY)]AGGEKR.3	-0.037145465	Q62318_S30_M1_Trim28

YDERPGPS[Phospho (STY)]PLPHR.3	0.014717536	Q62376_S226_M1_Snrnp70
GGGGS[Phospho (STY)]GQDNGLEGLGSDGR.2	-0.54521966	Q62376_S408_M1_Snrnp70
NSPTFKS[Phospho (STY)]FEEK.2	-0.426923198	Q62393_S175_M1_Tpd52
S[Phospho (STY)]FEEKVENLK.2	-0.216212766	Q62393_S175_M1_Tpd52
NSPTFKS[Phospho (STY)]FEEKVENLK.3	-0.45730886	Q62393_S175_M1_Tpd52
ASAAFSSVGSVITK.2	-0.017140835	#N/A
PAGGDFGEVLNSTANATSTM[Oxidation (M)]TTEPPPEQMTES[Phospho (STY)]P.4	-0.207519832	Q62393_S223_M1_Tpd52
SSS[Phospho (STY)]FSQGEAEPR.2	-0.240560926	Q62407_S2135_M1_Speg
AAS[Phospho (STY)]VELPQR.2	-0.207830675	Q62407_S2042_M1_Speg
ARS[Phospho (STY)]LEQPK.2	-0.401327132	Q62407_S439_M1_Speg
AAS[Phospho (STY)]VELPQRR.2	-0.035661215	Q62407_S2042_M1_Speg
AS[Phospho (STY)]PVLAVR.2	-0.207997269	Q62407_S2451_M1_Speg
SRS[Phospho (STY)]VQDLR.2	0.305322252	Q62407_S2396_M1_Speg
SATS[Phospho (STY)]DLELR.2	0.006510291	Q62407_S493_M1_Speg
LSPS[Phospho (STY)]QDHDSSDSSSK.2	0.023761831	Q62407_S860_M1_Speg
ATS[Phospho (STY)]EGESLRR.2	-0.272448525	Q62407_S2499_M1_Speg
SEGGAAWGT[Phospho (STY)]PEASQEELR.2	0.126835654	Q62407_T453_M1_Speg
SEGGAAWGT[Phospho (STY)]PEAS[Phospho (STY)]QEELRSPR.3	0.182122454	Q62407_S457_M2_Speg
S[Phospho (STY)]TS[Phospho (STY)]REELVR.2	-0.035554338	Q62407_S509_M2_Speg
AAS[Phospho (STY)]SEAAPHHQPPPESR.3	-0.080531951	Q62407_S2114_M1_Speg
RGSS[Phospho (STY)]AESALPR.2	-0.154304487	Q62407_S2020_M1_Speg
RLS[Phospho (STY)]STLER.2	0.134712362	Q62407_S2461_M1_Speg
MPS[Phospho (STY)]IPEEPEHGDLER.3	-0.036201041	Q62407_S1177_M1_Speg
QRS[Phospho (STY)]ATS[Phospho (STY)]DLELR.2	-0.673989402	Q62407_S490_M2_Speg
SS[Phospho (STY)]ITEPEGPGGPNIQK.2	-0.398808975	Q62415_S708_M1_Ppp1r13b
VNGTSSPQS[Phospho (STY)]PLSTSGR.2	0.431304869	Q62415_S335_M1_Ppp1r13b
AGEQDPVPTPAELTS[Phospho (STY)]PGR.2	-0.193315291	Q62417_S185_M1_Sorbs1
SATVS[Phospho (STY)]PQQPQAQQR.2	0.046594553	Q62417_S1209_M1_Sorbs1
NNNPQSELAAAHGDSES[Phospho (STY)]PR.3	-0.090196336	Q62417_S969_M1_Sorbs1
S[Phospho (STY)]AESLLESTK.2	-0.507896723	Q62417_S722_M1_Sorbs1
EGS[Phospho (STY)]GGSVHGDFPK.2	-0.185619269	Q62417_S791_M1_Sorbs1
RAGEQDPVPTPAELTS[Phospho (STY)]PGR.3	-0.133230173	Q62417_S185_M1_Sorbs1
SAT[Phospho (STY)]LPLPAR.2	-0.006828489	Q62417_T286_M1_Sorbs1
S[Phospho (STY)]M[Oxidation (M)]PSLDFSGR.2	-0.218791458	Q62417_S694_M1_Sorbs1
GTPSSSPVSPQES[Phospho (STY)]PKHESK.3	-0.458036587	Q62417_S62_M1_Sorbs1
SKS[Phospho (STY)]EM[Oxidation (M)]NYIEGEK.2	-0.212039936	Q62417_S270_M1_Sorbs1
GTPSSSPVS[Phospho (STY)]PQESPK.2	-0.058631949	Q62417_S58_M1_Sorbs1
DIS[Phospho (STY)]PEEIDLK.2	0.072342761	Q62417_S345_M1_Sorbs1
SVLEGGDIPLQGLSGLK.2	0.27019463	#N/A
S[Phospho (STY)]PTPVLSR.2	-0.320805479	Q62417_S707_M1_Sorbs1
SSS[Phospho (STY)]LKSSPER.2	-0.0673735	Q62417_S295_M1_Sorbs1
RES[Phospho (STY)]DGTPGGLASLENER.2	-0.134867806	Q62417_S432_M1_Sorbs1
YS[Phospho (STY)]FSDDTKSPLSVPR.3	-0.54276067	Q62417_S254_M1_Sorbs1
AQS[Phospho (STY)]C[Carbamidomethyl (C)]ENLC[Carbamidomethyl (C)]VSLNDSK.2	-0.42430728	Q62417_S554_M1_Sorbs1
SAQDLSDVST[Phospho (STY)]DEVGIPLR.2	-0.029963734	Q62417_T213_M1_Sorbs1
DDDSDVHS[Phospho (STY)]PR.2	0.158665295	Q62417_S250_M1_Sorbs1
YS[Phospho (STY)]FSDDTK.2	-0.295720804	Q62417_S254_M1_Sorbs1

AQS[Phospho (STY)]C[Carbamidomethyl (C)]ENLC[Carbamidomethyl (C)]VSLNDSKR.3	-0.200068693	Q62417_S554_M1_Sorbs1
TPVDYIDLPYSSS[Phospho (STY)]PSR.2	0.193233844	Q62417_S1201_M1_Sorbs1
DDDSDVHSPRYS[Phospho (STY)]FSDDTK.3	-0.179886602	Q62417_S254_M1_Sorbs1
VGGS[Phospho (STY)]IENLLMR.2	-0.178822357	Q62417_S576_M1_Sorbs1
DIS[Phospho (STY)]PEEIDLKNEPWYK.3	0.465852433	Q62417_S345_M1_Sorbs1
GELGAS[Phospho (STY)]QEGSEHIPK.2	-0.294674644	Q62417_S666_M1_Sorbs1
NGPALQEAYVR.2	-0.01625999	#N/A
VAGTGEGGLEELVEELNSGK.2	-0.049775743	#N/A
ESTSFQDVGPQAPVGSVYQK.2	-0.076082637	#N/A
SPTDWALFTYEGNSNDIR.2	-0.136865476	#N/A
TRQEWESAGQQAPHPR.3	-0.157557665	#N/A
VVTEKS[Phospho (STY)]PTDWALFTYEGNSNDIR.3	-0.049236782	Q62418_S24_M1_Dbnl
VKDPNSGLPK.2	0.119412379	#N/A
FVLINWTGEGVNDVR.2	0.312128007	#N/A
AMS[Phospho (STY)]TTSVTSSQPGK.2	0.377942708	Q62418_S277_M1_Dbnl
QLT[Phospho (STY)]QPETSYGR.2	0.346487834	Q62418_T299_M1_Dbnl
AEEDVEPEC[Carbamidomethyl (C)]IMEK.2	-0.141683656	#N/A
ASGANYSFHK.2	0.089492135	#N/A
ITAS[Phospho (STY)]SSFR.2	-0.40082434	Q62419_S286_M1_Sh3gl1
TIEYLQPNPASR.2	0.062072568	#N/A
IPDEELR.2	0.123791153	#N/A
ALYDFEPENEGELGFK.2	-0.304418264	#N/A
RLDFDYK.2	-0.096709425	#N/A
KVDVTSR.2	0.139128201	#N/A
[Acetyl (Protein N-term)]S[Phospho (STY)]VAGLKK.2	-0.35327449	Q62420_S2_M1_Sh3gl2
RLDFDYKK.2	-0.24587199	#N/A
QAVQILQQVTVR.2	-0.180023484	#N/A
EIQHHLK.2	-0.068764662	#N/A
EYQPKPR.2	-0.147561521	#N/A
QNFIDPLQNLHDKDLR.3	-0.086915046	#N/A
ELGDDC[Carbamidomethyl (C)]NFGPALGEVGEAM[Oxidation (M)]R.2	-0.269844732	#N/A
GPGYPQAEALLAEAM[Oxidation (M)]LK.2	0.489887135	#N/A
VGGAEGTKLDDDFKEM[Oxidation (M)]ER.3	0.054416132	#N/A
ELSEVKDSLDM[Oxidation (M)]EVK.2	-0.04203157	#N/A
AVMEIMTK.2	0.011688666	#N/A
LDDDFKEMER.3	-0.39257119	#N/A
TLSNAEDYLDDEDS[Phospho (STY)]D.2	0.180109713	Q62422_S214_M1_Ostf1
LALFNPDVSWDR.2	0.076004825	#N/A
FYSVNVDYSK.2	0.251266688	#N/A
KNNPEPWNK.2	-0.019035402	#N/A
LGPNEQYK.2	0.067121861	#N/A
LALFNPDVSWDRK.3	0.104200206	#N/A
NNPEPWNK.2	0.073101323	#N/A
TAS[Phospho (STY)]GSSVTSLEGTR.2	0.010039991	Q62433_S330_M1_Ndrg1
LNITPNSGATGNNAGPK.2	-0.064855975	#N/A
[Acetyl (Protein N-term)]S[Phospho (STY)]RELHDVDLAEVKPLVEK.3	-0.408003475	Q62433_S2_M1_Ndrg1
SRT[Phospho (STY)]AS[Phospho (STY)]GSSVTSLEGTR.2	-0.040443729	Q62433_S330_M2_Ndrg1

DASGS[Phospho (STY)]PASTASSGSSSSLK.2	-0.158762081	Q62440_S284_M1_Tle1
DAPIS[Phospho (STY)]PASVASSSSTPSSK.2	0.382585253	Q62441_S292_M1_Tle4
SSSVS[Phospho (STY)]PSASFR.2	-0.134236377	Q62441_S208_M1_Tle4
FIC[Carbamidomethyl (C)]TSVPVDADM[Oxidation (M)]C[Carbamidomethyl (C)]AASVAAGGAEELR.3	-0.146767209	#N/A
FLQDHGSDS[Phospho (STY)]FLAEHK.3	-0.153969662	Q62446_S36_M1_Fkbp3
TQT[Phospho (STY)]PPLGQTPQLGLK.2	-0.366259802	Q62448_T507_M1_Eif4g2
LTEAVVTDYLNSGNANDAVSGVR.2	0.004683474	#N/A
FS[Phospho (STY)]PTMGR.2	0.094116607	Q62448_S394_M1_Eif4g2
VLLVPGPEKET.2	0.123837022	#N/A
SQS[Phospho (STY)]PVHLR.2	-0.376643958	Q62504_S749_M1_Spen
DLEPGEVPS[Phospho (STY)]DS[Phospho (STY)]DEDAEHR.2	-0.061065959	Q62504_S1395_M2_Spen
S[Phospho (STY)]RSPNRFDAHDPR.4	-0.650556768	Q62504_S188_M1_Spen
EAGPAAAS[Phospho (STY)]PQESESPQK.2	-0.264488729	Q62504_S2128_M1_Spen
RPQS[Phospho (STY)]PGVS[Phospho (STY)]PAHSER.3	0.392039231	Q62504_S758_M2_Spen
S[Phospho (STY)]PGGPGPLTLK.2	-0.475329884	Q62523_S336_M1_Zyx
DTQLQQIVDK.2	0.290706711	#N/A
VIDIFDTDGNGEVDFK.2	0.148027334	#N/A
IYDM[Oxidation (M)]DKDGYISNGELFQVLK.3	-0.242852052	#N/A
EFIEGVSQFSVK.2	0.413427557	#N/A
ISFEEFC[Carbamidomethyl (C)]AVVGGLDIHK.3	-0.368127683	#N/A
KLDLDNSGSLSVEEFM[Oxidation (M)]SLPELQQNPLVQR.3	-0.154656264	#N/A
LDLDNSGSLSVEEFM[Oxidation (M)]SLPELQQNPLVQR.3	-0.55289416	#N/A
TIINADKDGDGR.3	0.163523198	#N/A
[Acetyl (Protein N-term)]AAAAAAPGGGGGEPR.2	0.173759115	#N/A
IADPEHDHTGFLTEY[Phospho (STY)]VATR.3	-0.335740485	Q63844_Y205_M1_Mapk3
ELEDLLS[Phospho (STY)]PLEESVK.2	-0.231383584	Q63850_S412_M1_Nup62
TLDISNNR.2	0.02338524	#N/A
LESLPAQLPR.2	0.510704942	#N/A
LWTVPTNM[Oxidation (M)]PSK.2	-0.468529924	#N/A
AHVIGTPC[Carbamidomethyl (C)]SK.2	0.401053273	#N/A
SDTAYQWNLK.2	0.145941198	#N/A
FTFIPEQSFDQLLQLQEITLHNNR.3	-0.348549298	#N/A
S[Phospho (STY)]SPFKVS[Phospho (STY)]PLSFGR.2	-0.382188829	Q63918_S287_M2_Cavin2
LKQPST[Phospho (STY)]PTR.2	-0.248819127	Q63932_T395_M1_Map2k2
SEPVS[Phospho (STY)]PSRER.2	0.242625827	Q63943_S437_M1_Mef2d
LAPLATPPGS[Phospho (STY)]PR.2	0.042183015	Q63959_S754_M1_Kcnc3
INS[Phospho (STY)]DDKNLYLTASK.2	0.215868331	Q64008_S241_M1_Rab34
QEAVALLQGQR.2	0.113013724	#N/A
QGS[Phospho (STY)]GVILR.2	-0.184691064	Q64010_S125_M1_Crk
ALFDFNGNDEEDLPFK.2	-0.402157092	#N/A
DSS[Phospho (STY)]TSPGDYVLSVSENSR.2	0.000906436	Q64010_S41_M1_Crk
LLDQQNPDEDFS.2	-0.037934745	#N/A
IHYLDTTTLIEPVAR.3	0.556553756	#N/A
GRLS[Phospho (STY)]PVPVPR.2	-0.422450221	Q64012_S135_M1_Raly
LPAPQEDTAS[Phospho (STY)]EAGTPQGEVQTR.3	0.089790838	Q64012_S270_M1_Raly
LS[Phospho (STY)]PVPVPR.2	-0.616541678	Q64012_S135_M1_Raly
RS[Phospho (STY)]SFSMEEES.2	0.207545886	Q64092_S564_M1_Tfe3

TSS[Phospho (STY)]TGEEEESLAILR.2	0.074858154	Q64096_S621_M1_Mcf2l
QLC[Carbamidomethyl (C)]PEPAEILS[Phospho (STY)]PEPV.2	0.211352872	Q64096_S1145_M1_Mcf2l
SSS[Phospho (STY)]AQC[Carbamidomethyl (C)]LSSSGK.2	-0.281450721	Q64096_S1120_M1_Mcf2l
EALSQQLDS[Phospho (STY)]EDEQDQR.2	0.239564474	Q640N2_S328_M1_Arl13b
THTS[Phospho (STY)]PPR.2	-0.170871174	Q640R3_S379_M1_Hepacam
QNS[Phospho (STY)]LEYM[Oxidation (M)]DQNDDR.2	0.106925832	Q640R3_S280_M1_Hepacam
STTEPGPPGYSVS[Phospho (STY)]PPVPGR.2	-0.400191975	Q640R3_S345_M1_Hepacam
SPARS[Phospho (STY)]PATGR.2	-0.32801981	Q640R3_S370_M1_Hepacam
THTS[Phospho (STY)]PPRAPSS[Phospho (STY)]PGR.2	0.22788886	Q640R3_S379_M2_Hepacam
QNS[Phospho (STY)]LEYM[Oxidation (M)]DQNDDRLK.3	-0.580936105	Q640R3_S280_M1_Hepacam
DKDSS[Phospho (STY)]EPDENPATEPR.2	0.265088613	Q640R3_S321_M1_Hepacam
SEADTLPRS[Phospho (STY)]GEQER.2	0.12692389	Q640R3_S301_M1_Hepacam
S[Phospho (STY)]PGLPIR.2	-0.102505215	Q640R3_S352_M1_Hepacam
ETSKDPELR.2	0.230185102	#N/A
ALAPQLAR.2	-0.120542572	#N/A
MEADGLGC[Carbamidomethyl (C)]AVC[Carbamidomethyl (C)]VLTGASR.2	-0.461948241	#N/A
LLLINNAATLGDVSK.2	0.144389655	#N/A
DM[Oxidation (M)]LYQVLAAEEPSVR.2	-0.054256416	#N/A
LKS[Phospho (STY)]DGALVDC[Carbamidomethyl (C)]GTSAQK.2	-0.252530363	Q64105_S228_M1_Spr
SEWSDASQKS[Phospho (STY)]PVHVGETR.3	0.149141664	Q64127_S812_M1_Trim24
HEAEDC[Carbamidomethyl (C)]KFS[Phospho (STY)]DDS[Phospho (STY)]DDDFVQPR.3	-0.32560573	Q64127_S1026_M2_Trim24
EIPVDAPWQAR.2	0.391564579	#N/A
DVPALEITHTFLER.2	-0.3768319	#N/A
VLGSQEALS[Phospho (STY)]PVHYEEK.2	-0.455403785	Q64133_S388_M1_Maoa
IYFAGTETATQWSGYM[Oxidation (M)]EGAVEAGER.3	-0.187374872	#N/A
VTS[Phospho (STY)]PTTYVLDEDEPR.2	-0.050835565	Q641K1_S1160_M1_Agtpbp1
ENDFPQSGQDSVPES[Phospho (STY)]PSK.2	-0.245884214	Q641K5_S389_M1_Nuak1
SSVC[Carbamidomethyl (C)]DC[Carbamidomethyl (C)]DALPDSES[Phospho (STY)]PLLAR.2	-0.074859383	Q641K5_S326_M1_Nuak1
TAIPLPSS[Phospho (STY)]PEADM[Oxidation (M)]SGK.2	0.117194079	Q641K5_S446_M1_Nuak1
LSQELSGGR.2	0.170683225	#N/A
TLTGIVIDSGDGVTHVIPVAEGYVIGSC[Carbamidomethyl (C)]IK.3	-0.120054946	#N/A
TGDLGIPPNPEDRSPSPEPIY[Phospho (STY)]NS[Phospho (STY)]EGKR.4	-0.010927636	Q64213_S89_M2_Sf1
TGDLGIPPNPEDRSPSPEPIY[Phospho (STY)]NS[Phospho (STY)]EGK.3	0.114495989	Q64213_S89_M2_Sf1
S[Phospho (STY)]C[Carbamidomethyl (C)]TSS[Phospho (STY)]PSPLNLC[Carbamidomethyl (C)]SAR.2	-0.000326855	Q64318_S678_M2_Zeb1
GPTS[Phospho (STY)]PSTPR.2	0.25125507	Q64322_S236_M1_Npdc1
IS[Phospho (STY)]PGDQR.2	-0.072623983	Q64322_S243_M1_Npdc1
GPTS[Phospho (STY)]PST[Phospho (STY)]PRIS[Phospho (STY)]PGDQR.2	-0.025391976	Q64322_S236_M3_Npdc1
VLLVVDEPHTDWAK.2	-0.08788121	#N/A
FPLIEQTYYPNHR.3	0.111711974	#N/A
TLGGEKFPLIEQTYYPNHR.3	-0.187338432	#N/A
QLITDLVISK.2	0.095202195	#N/A
ILGDYDIK.2	-0.038279603	#N/A
SFRPDFVLIR.2	-0.148237761	#N/A
EM[Oxidation (M)]LTLPTFPVVVK.2	-0.065300919	#N/A
M[Oxidation (M)]NQLLSR.2	0.326284623	#N/A
QTAASAGLVDAPAPSAASR.2	0.024324252	#N/A
KSFASLFSD.2	-0.055328796	#N/A

TNTGSAM[Oxidation (M)]LEQIAM[Oxidation (M)]SDR.2	-0.250048332	#N/A
T[Phospho (STY)]PALS[Phospho (STY)]PQRPLTTQQPQSGTLK.3	0.034267305	Q64332_S426_M2_Syn2
VEQAEFSELNLVAHADGTYAVDM[Oxidation (M)]QVLR.3	0.012554975	#N/A
TPALS[Phospho (STY)]PQRPLTTQQPQSGTLKEPDSSK.4	-0.348188217	Q64332_S426_M1_Syn2
SS[Phospho (STY)]ANEDEAKAETIR.2	-0.169369695	Q64332_S562_M1_Syn2
LWVDAC[Carbamidomethyl (C)]SEM[Oxidation (M)]FGGLDIC[Carbamidomethyl (C)]AVK.2	-0.216122376	#N/A
QHAFGM[Oxidation (M)]AENEDFR.3	0.165592253	#N/A
TSISGNWK.2	0.331101682	#N/A
DGKDYIFEVM[Oxidation (M)]DC[Carbamidomethyl (C)]SM[Oxidation (M)]PLIGEHQVEDR.4	-0.139362426	#N/A
SQS[Phospho (STY)]LTNAFSFSESSFFR.2	-0.133644555	Q64332_S546_M1_Syn2
VENHYDFQDIASVVALTQTYATAEPFIDAK.3	-0.061206589	#N/A
S[Phospho (STY)]PPQYC[Carbamidomethyl (C)]GAK.2	0.529452947	Q64336_S549_M1_Tbr1
LTPS[Phospho (STY)]PNDSPR.2	-0.101057666	Q64336_S410_M1_Tbr1
SIDS[Phospho (STY)]SDSGIYEQAK.2	-0.019931075	Q64336_S625_M1_Tbr1
RIS[Phospho (STY)]PADTPVSESSSPLK.3	0.013812029	Q64336_S640_M1_Tbr1
SKLS[Phospho (STY)]PVLDGVS[Phospho (STY)]ELRHSFDGSAADR.4	-0.142209686	Q64336_S84_M2_Tbr1
RSS[Phospho (STY)]LNSINSSDAKR.3	-0.3411605	Q64338_S469_M1_Pde1c
[Acetyl (Protein N-term)]MES[Phospho (STY)]PTKEIEEFESNSLK.3	-0.161010893	Q64338_S3_M1_Pde1c
TLAGDVKTS[Phospho (STY)]PPR.2	-0.332998188	Q64362_S30_M1_Aktip
VSIASEVESTASS[Phospho (STY)]PSSSSLQK.2	0.239177375	Q64430_S357_M1_Atp7a
VLLPEYGGTK.2	0.077309627	#N/A
FLPLFDR.2	0.196402724	#N/A
KFLPLFDR.2	0.105491171	#N/A
SGEIEPVSVK.2	0.007250589	#N/A
VVLDDKDYFLFR.2	-0.043921729	#N/A
VLQATVVAVGSGGK.2	0.301844004	#N/A
GKSGEIEPVSVK.2	0.267662992	#N/A
GGIM[Oxidation (M)]LPEK.2	-0.214404028	#N/A
KSS[Phospho (STY)]LPNSK.2	0.001486015	Q64487_S1303_M1_Ptprd
DFLPVDTSNNNGR.2	-0.308740797	#N/A
YANVIAYDHSR.2	0.281835689	#N/A
GALQIEQSEESDQGKYEC[Carbamidomethyl (C)]VATNSAGTR.3	-0.072938261	#N/A
ASGS[Phospho (STY)]ENEGDYNPGR.2	0.314333738	Q64511_S1539_M1_Top2b
KAS[Phospho (STY)]GS[Phospho (STY)]ENEGDYNPGR.2	-0.136376688	Q64511_S1537_M2_Top2b
YFAES[Phospho (STY)]DEEEDVDFAM[Oxidation (M)]FN.2	0.188877579	Q64511_S1600_M1_Top2b
IVETINS[Phospho (STY)]DS[Phospho (STY)]DSEFGIPKK.2	0.091034022	#N/A
IVETINSDS[Phospho (STY)]DSEFGIPK.2	0.177509914	Q64511_S1511_M1_Top2b
KIVETINS[Phospho (STY)]DS[Phospho (STY)]DSEFGIPKK.3	0.049565824	#N/A
KTSFDQDS[Phospho (STY)]DVDIFPSDFTSEPPALPR.3	-0.542586827	Q64511_S1568_M1_Top2b
KIVETINS[Phospho (STY)]DS[Phospho (STY)]DSEFGIPK.2	-0.468745033	#N/A
AS[Phospho (STY)]PITNDGEDEFVPSDGLDKDEYAFSSGK.3	0.057139655	Q64511_S1387_M1_Top2b
VKAS[Phospho (STY)]PITNDGEDEFVPS[Phospho (STY)]DGLDKDEYAFSSGK.4	0.134047725	Q64511_S1387_M2_Top2b
KKDEGS[Phospho (STY)]YTLEEPK.3	0.295484047	Q64519_S418_M1_Sdc3
QAS[Phospho (STY)]VTYQKPDKQEEFYA.3	0.225076425	Q64519_S428_M1_Sdc3
IC[Carbamidomethyl (C)]VLDVDLQGVR.2	0.025762296	#N/A
NYLSSDVEVR.2	-0.096614284	#N/A
ELNWSELR.2	-0.277728077	#N/A

TVGLFLQGGK.2	0.185102851	#N/A
LAFLNVQAAEEALPR.2	0.191490748	#N/A
KQEELETATR.2	0.035003008	#N/A
FHNLNAGPSR.2	-0.336608945	#N/A
DWSPTLYIR.2	0.190681869	#N/A
TEQLTDSTEISLLPSDIDR.2	0.097721802	#N/A
LVQDYGLESEVAQHLAK.2	-0.050118408	#N/A
YGAATANYM[Oxidation (M)]EVVSLLK.2	0.175597146	#N/A
C[Carbamidomethyl (C)]VINASGPFTDSVR.2	0.296444197	#N/A
M[Oxidation (M)]NLAIALTAAR.2	0.368629776	#N/A
NTPEFDILVIGGGATGC[Carbamidomethyl (C)]GC[Carbamidomethyl (C)]ALDAVTR.3	0.028060452	#N/A
AIM[Oxidation (M)]NLDVEQYR.2	0.382927673	#N/A
TALVERDDFSSGTSSR.3	-0.198653693	#N/A
LVGAIVYYDGQHNDAR.2	-0.188501453	#N/A
EYAC[Carbamidomethyl (C)]TAVDMISR.2	-0.072721695	#N/A
M[Oxidation (M)]TIAGTTDTPTDVTHHPIPSEEDINFILNEVR.4	-0.061713578	#N/A
AGLQFPVGR.2	-0.087579616	#N/A
HLQLAIR.2	0.240176189	#N/A
VTIAQGGVLPNIQAVLLPK.2	-0.099748083	#N/A
NDEELNKLLGK.2	-0.522022263	#N/A
NDEELNK.2	0.163938576	#N/A
KGNYAER.2	0.086142958	#N/A
YQDILVFR.2	-0.221164478	#N/A
AAFVLPEFTR.2	0.230183552	#N/A
VLIIGGGDGGVLR.2	-0.07091206	#N/A
AQPAGS[Phospho (STY)]PSHQIQQR.2	-0.515540572	Q64700_S37_M1_Rbl2
GREEDSS[Phospho (STY)]PGPQSQSHR.3	0.212408458	Q64707_S418_M1_Zrsr1
ALEAPPEEDDDVS[Phospho (STY)]ANEELAER.2	0.163921499	Q64707_S50_M1_Zrsr1
GREEDSS[Phospho (STY)]PGPQSQSHRT.3	0.379913481	Q64707_S418_M1_Zrsr1
DPNAS[Phospho (STY)]PGDAGEQAIR.2	0.047544803	Q64727_S290_M1_Vcl
GWLRDPNAS[Phospho (STY)]PGDAGEQAIR.3	0.280959114	Q64727_S290_M1_Vcl
GQGAS[Phospho (STY)]PVAMQK.2	0.263471174	Q64727_S346_M1_Vcl
KGS[Phospho (STY)]DS[Phospho (STY)]DTDASNLETPR.2	-0.185584027	Q64HY3_S424_M2_Sp9
SES[Phospho (STY)]PDLILHDSGVSAAR.3	0.077497487	Q64HY3_S440_M1_Sp9
TPEELEDVS[Phospho (STY)]DLEDDHEVR.2	0.285315103	Q65CL1_S637_M1_Ctnna3
TES[Phospho (STY)]PSESC[Carbamidomethyl (C)]PVK.2	-0.379680075	Q65Z40_S226_M1_Wapl
RTES[Phospho (STY)]PSESC[Carbamidomethyl (C)]PVK.2	-0.073294682	Q65Z40_S226_M1_Wapl
[Acetyl (Protein N-term)]AAAAAAAAAAAAGDS[Phospho (STY)]DSWDADTFSM[Oxidation (M)]EDPVRK.3	-0.395409703	Q66JS6_S16_M1_Eif3j2
RVS[Phospho (STY)]VEDLR.2	-0.399284975	Q66JV4_S681_M1_Rbm12b2;Rbm12b1
S[Phospho (STY)]RS[Phospho (STY)]PLGFYVHLK.3	0.001836595	Q66JV4_S277_M2_Rbm12b2;Rbm12b1
MRS[Phospho (STY)]EEHS[Phospho (STY)]PSR.2	-0.160328333	Q66JV4_S250_M2_Rbm12b2;Rbm12b1
GSDGAS[Phospho (STY)]PPASPSIIPR.2	0.00384942	Q66L42_S502_M1_Map3k10
QWSSS[Phospho (STY)]APNLGK.2	0.132898711	Q66L42_S583_M1_Map3k10
KGSDGAS[Phospho (STY)]PPASPSIIPR.2	-0.034247559	Q66L42_S502_M1_Map3k10
HAS[Phospho (STY)]LDGASPYFK.2	-0.255898295	Q66L44_S290_M1_Cbarp
ASS[Phospho (STY)]LDTRGS[Phospho (STY)]PK.2	-0.097276402	Q66L44_S313_M2_Cbarp
RDYS[Phospho (STY)]IDEK.2	-0.185568466	Q66L44_S537_M1_Cbarp

GAGDEVSELPAPARS[Phospho (STY)]PPR.3	-0.085140917	Q66L44_S520_M1_Cbarp
LAEPVASSQVLIVAAAAPTS[Phospho (STY)]PDHSPA.3	-0.129268542	Q66L44_S692_M1_Cbarp
RHAS[Phospho (STY)]LDGASPYFK.3	-0.472764837	Q66L44_S290_M1_Cbarp
GAGDEVSELPAPARS[Phospho (STY)]PPRS[Phospho (STY)]PR.3	-0.082380398	Q66L44_S520_M2_Cbarp
ASS[Phospho (STY)]LDTRGS[Phospho (STY)]PKR.2	-0.261818905	Q66L44_S313_M2_Cbarp
LLQMDSGYAS[Phospho (STY)]IEGR.2	0.318963112	Q66L44_S501_M1_Cbarp
RGDS[Phospho (STY)]VDC[Carbamidomethyl (C)]PPEGR.2	-0.128749924	Q66L44_S610_M1_Cbarp
LEAAEAAGGAS[Phospho (STY)]PET[Phospho (STY)]PPEHGISLGPEHAQQQDPQQEQDAEHAQC[Carbamidomethyl (C)]SYR.5	0.021662877	Q66L44_S389_M2_Cbarp
RVS[Phospho (STY)]FNEAALFEQSR.3	-0.310025782	Q66L44_S129_M1_Cbarp
GHGREEEPGS[Phospho (STY)]PR.3	0.010269926	Q673H1_S146_M1_Tusc1
EAPGS[Phospho (STY)]PPLSPR.2	-0.197896032	Q67FY2_S21_M1_Bcl9l
TAMPS[Phospho (STY)]PGVSQNK.2	-0.347975782	Q67FY2_S1014_M1_Bcl9l
TELC[Carbamidomethyl (C)]ENVDPNS[Phospho (STY)]PAAK.2	-0.254989512	Q68ED2_S900_M1_Grm7
RPLS[Phospho (STY)]VDKHGR.3	-0.370675791	Q68ED7_S120_M1_Crtc1
RPLS[Phospho (STY)]VDK.2	-0.114296983	Q68ED7_S120_M1_Crtc1
TLVLSSSPTS[Phospho (STY)]PTQEPLAAAK.2	-0.537168289	Q68EF0_S218_M1_Rab3ip
AS[Phospho (STY)]PLYATYK.2	-0.165176717	Q68EF6_S473_M1_Begain
AGGDLSLS[Phospho (STY)]PSR.2	0.152671349	Q68EF6_S510_M1_Begain
VLEKPDPGS[Phospho (STY)]LSSR.2	-0.199097902	Q68EF6_S194_M1_Begain
GDIYC[Carbamidomethyl (C)]S[Phospho (STY)]DPALYC[Carbamidomethyl (C)]PDER.2	0.033458268	Q68EF6_S229_M1_Begain
YYGGGGGGGAAGGGS[Phospho (STY)]PGDKAEGR.3	-0.510724656	Q68EF6_S463_M1_Begain
KPSATYGS[Phospho (STY)]GPR.2	0.015012676	Q68EF6_S344_M1_Begain
AQNST[Phospho (STY)]DSAAEEEEEAEAAAFPEAYR.3	-0.090917088	Q68EF6_T263_M1_Begain
SADALAGYAAS[Phospho (STY)]DGDGDRLR.2	0.095822737	Q68EF6_S524_M1_Begain
LS[Phospho (STY)]PQQAFPR.2	-0.03112188	Q68EF6_S570_M1_Begain
VQLC[Carbamidomethyl (C)]GAGS[Phospho (STY)]S[Phospho (STY)]PEPEHGSR.2	0.135408616	Q68EF6_S540_M2_Begain
ADS[Phospho (STY)]FSEGDDLSQGHLAEPC[Carbamidomethyl (C)]FLR.3	0.308400228	Q68EF6_S483_M1_Begain
YYGGGGGGGAAGGGS[Phospho (STY)]PGDK.2	0.343510692	Q68EF6_S463_M1_Begain
GDIY[Phospho (STY)]C[Carbamidomethyl (C)]SDPALYC[Carbamidomethyl (C)]PDEREHAR.4	-0.158059793	Q68EF6_Y227_M1_Begain
AASTLGS[Phospho (STY)]PLEAQVAPGFAR.2	0.307547425	Q68EF6_S357_M1_Begain
RPS[Phospho (STY)]VDT[Phospho (STY)]PVTDVGFLR.2	0.123158417	Q68EF6_S246_M2_Begain
ESLEPS[Phospho (STY)]SMEAS[Phospho (STY)]PEMHPPTR.2	0.252527118	Q68EF6_S555_M2_Begain
IYIDSNNNPER.2	-0.250413218	#N/A
LLLPWLEAR.2	-0.01773567	#N/A
NNLAGAEELFAR.2	-0.161108223	#N/A
DPHLAC[Carbamidomethyl (C)]VAYER.2	0.016717743	#N/A
LLYNNVSNFGR.2	-0.045349311	#N/A
ESNC[Carbamidomethyl (C)]YDPER.2	-0.051796739	#N/A
LHIIEVGTPPTGNQPFPK.3	-0.26332248	#N/A
EVC[Carbamidomethyl (C)]FAC[Carbamidomethyl (C)]VDGK.2	-0.094363487	#N/A
LAELEEFINGPNNAHIQQVGDR.3	-0.184082031	#N/A
AHIAQLC[Carbamidomethyl (C)]EK.2	0.349001781	#N/A
GQFSTDELVAEVEKR.2	-0.196974092	#N/A
ENPYYDSR.2	0.01097161	#N/A
LTDQLPLIIVC[Carbamidomethyl (C)]DR.2	-0.021623496	#N/A
C[Carbamidomethyl (C)]NEPAVWSQLAK.2	-0.088891934	#N/A
SVDPTLALSVYLR.2	-0.110093051	#N/A

NLQNLLILTAIK.2	-0.224408291	#N/A
EHLELFWSR.2	-0.062372524	#N/A
HELIEFR.2	-0.105570586	#N/A
WLLLTGISAQQNR.2	-0.268490346	#N/A
HDVVFLITK.2	0.01856499	#N/A
LASTLVHLGEYQAAVDGAR.3	-0.162090328	#N/A
AFM[Oxidation (M)]TADLPNELIELLEK.2	-0.199362811	#N/A
VIQC[Carbamidomethyl (C)]FAETGQVQK.2	0.116156078	#N/A
LEKHELIEFR.3	-0.351528473	#N/A
IAAYLFK.2	-0.180702458	#N/A
YIEIYVQK.2	0.098413864	#N/A
SVNESLNNLFITEEDYQALR.2	-0.473972697	#N/A
ISGETIFVTAPHEATAGIIGVNR.3	-0.251805251	#N/A
YESLELC[Carbamidomethyl (C)]RPVLQQGR.3	-0.109402786	#N/A
QSVELC[Carbamidomethyl (C)]KK.2	0.326259599	#N/A
RPLIDQVVQTALSETQDPEEVSVTVK.3	-0.085600392	#N/A
EAIDSYIK.2	-0.050206572	#N/A
GQFSTDELVAEVEK.2	-0.240315015	#N/A
IVLDNSVFSEHR.2	-0.159511859	#N/A
KFNALFAQGNYSEAAK.3	0.138930202	#N/A
AYEFAER.2	-0.06205137	#N/A
RDPHLAC[Carbamidomethyl (C)]VAYER.3	0.190337954	#N/A
VGYTPDWIFLLR.2	0.173100278	#N/A
QSVELC[Carbamidomethyl (C)]K.2	-0.135387137	#N/A
VM[Oxidation (M)]EYINR.2	-0.06440282	#N/A
VANVELYYK.2	-0.334362113	#N/A
VVGAM[Oxidation (M)]QLYSVDR.2	-0.023059799	#N/A
M[Oxidation (M)]EGNAEESTLFC[Carbamidomethyl (C)]FAVR.2	-0.320908044	#N/A
LLEM[Oxidation (M)]NLM[Oxidation (M)]HAPQVADAILGNQM[Oxidation (M)]FTHYDR.4	-0.005993118	#N/A
FNALFAQGNYSEAAK.2	0.058156859	#N/A
TLQIFNIEMK.2	0.276004651	#N/A
VDKLDASESLR.2	0.296928917	#N/A
VSQPIEGHAASFAQFK.3	-0.265385223	#N/A
GQC[Carbamidomethyl (C)]DLELINVC[Carbamidomethyl (C)]NENSLFK.2	-0.311635153	#N/A
AM[Oxidation (M)]LSANIR.2	0.027202452	#N/A
ALEHFTDLYDIK.2	-0.103473534	#N/A
ESYVETELIFALAK.2	-0.14069369	#N/A
HSSLAGC[Carbamidomethyl (C)]QIINYR.3	-0.069645697	#N/A
DAMQYASESK.2	0.314442522	#N/A
IHEGC[Carbamidomethyl (C)]EEPATHNALAK.3	-0.140732704	#N/A
ALEHFTDLYDIKR.3	-0.396411608	#N/A
LPVVIGGLLDVDC[Carbamidomethyl (C)]SEDVIK.2	-0.283859568	#N/A
ADDPSSYM[Oxidation (M)]EVVQAANASGNWEELVK.3	-0.470346582	#N/A
FQSVPAQPGQTSPLLQYFGILLDQGQLNK.3	0.102367325	#N/A
GILRT[Phospho (STY)]PDTIR.2	0.167745362	Q68FD5_T394_M1_Cltc
KFDVNTSAVQVLIEHIGNLDR.3	-0.161852865	#N/A
TSIDAYDNFDNISLAQR.2	-0.197675492	#N/A

AVNYFSK.2	0.162131802	#N/A
YIQAAC[Carbamidomethyl (C)]K.2	-0.159972615	#N/A
NNRPSEGPLQTR.2	-0.403433345	#N/A
VGEQAQVVIIDM[Oxidation (M)]NDPSNPIR.2	0.004100954	#N/A
AHM[Oxidation (M)]GM[Oxidation (M)]FTELAILYSK.2	-0.045101263	#N/A
AHTM[Oxidation (M)]TDDVTFWK.2	-0.045393657	#N/A
RPIS[Phospho (STY)]ADSAIMNPASK.2	0.273205238	Q68FD5_S67_M1_Cltc
FDVNTSAVQVLIEHIGNLDR.3	0.267507358	#N/A
YGYIHLYDLETGTC[Carbamidomethyl (C)]IYM[Oxidation (M)]NR.3	-0.103127377	#N/A
EDKLEC[Carbamidomethyl (C)]SEELGDLVK.3	0.074447477	#N/A
NESSDSALGDS[Phospho (STY)]ESEDTGPDIR.2	-0.018433865	Q68FD7_S945_M1_Fnip1
NESSDSALGDS[Phospho (STY)]ESEDTGPDIRR.3	-0.446572023	Q68FD7_S945_M1_Fnip1
DHTTPSENGDVPS[Phospho (STY)]PK.2	-0.19918467	Q68FD9_S1384_M1_Kiaa1549
LITTDS[Phospho (STY)]DGTYK.2	0.147234363	Q68FD9_S1613_M1_Kiaa1549
GHYEFPVVDDLS[Phospho (STY)]SGDTKER.3	0.371300388	Q68FD9_S1543_M1_Kiaa1549
GLQGPGLGYPTSS[Phospho (STY)]TEDLQPGHSSASLIK.3	-0.402393944	Q68FD9_S1904_M1_Kiaa1549
GHYEFPVVDDLS[Phospho (STY)]SGDTK.2	0.12310596	Q68FD9_S1543_M1_Kiaa1549
RGHYEFPVVDDLS[Phospho (STY)]S[Phospho (STY)]GDTKER.4	-0.008607916	Q68FD9_S1543_M2_Kiaa1549
LEASYSDS[Phospho (STY)]PPGEEDLLVHVAEGSK.3	-0.506056758	Q68FE2_S18_M1_Atg9a
FSTYSQS[Phospho (STY)]PPDTPSLR.2	-0.167155473	Q68FE6_S347_M1_Ripor1
PLTSPAPDS[Phospho (STY)]PEQIPK.2	0.237927522	Q68FE6_S748_M1_Ripor1
HTSS[Phospho (STY)]PEVVAEDR.2	0.184275043	Q68FE6_S875_M1_Ripor1
SGS[Phospho (STY)]VNEASC[Carbamidomethyl (C)]PTEQTR.2	0.264882591	Q68FE7_S488_M1_Tmem151b
SVNS[Phospho (STY)]DAS[Phospho (STY)]DPGSVR.2	-0.407419857	Q68FE8_S904_M2_Znf280d
LQAENLQLR.2	0.232342908	#N/A
SAVPFLPVNPEYSATR.2	-0.08254604	#N/A
S[Phospho (STY)]M[Oxidation (M)]DSSDLSDGAVTLQEYLELKK.3	-0.466427661	Q68FF6_S419_M1_Git1
SLSS[Phospho (STY)]PTDNLELSAR.2	-0.164194838	Q68FF6_S371_M1_Git1
ARS[Phospho (STY)]M[Oxidation (M)]DSSDLSDGAVTLQEYLELKK.3	-0.508408474	Q68FF6_S419_M1_Git1
S[Phospho (STY)]M[Oxidation (M)]DSSDLSDGAVTLQEYLELK.2	0.232480822	Q68FF6_S419_M1_Git1
HGS[Phospho (STY)]GADSDYENTQSGDPLLGLEGK.3	0.233113511	Q68FF6_S601_M1_Git1
HGS[Phospho (STY)]GADSDYENTQSGDPLLGLEGKR.4	-0.09420143	Q68FF6_S601_M1_Git1
AEVC[Carbamidomethyl (C)]ADC[Carbamidomethyl (C)]SAPDPGWASISR.2	-0.204010753	#N/A
SQSELDDQHDYDSVASDEDT[Phospho (STY)]DQEPLPSAGATR.4	0.064432133	Q68FF6_T401_M1_Git1
ENDAVWLATQNHSTLVTER.3	-0.17484253	#N/A
S[Phospho (STY)]MPNLAR.2	0.214794492	Q68FF7_S401_M1_Slain1
SSQS[Phospho (STY)]FDSSLHGAGSGVSR.2	0.265738462	Q68FF7_S429_M1_Slain1
VGS[Phospho (STY)]PLTLTDAQTR.2	-0.429287378	Q68FH0_S313_M1_Pkp4
AQS[Phospho (STY)]PSYVTSTGVSPSR.2	-0.021590275	Q68FH0_S220_M1_Pkp4
LYLQS[Phospho (STY)]PR.2	-0.273284942	Q68FH0_S1133_M1_Pkp4
TGS[Phospho (STY)]VGIGNLQR.2	-0.337159766	Q68FH0_S446_M1_Pkp4
VAS[Phospho (STY)]PSQGQVGSSSPK.2	-0.462593661	Q68FH0_S326_M1_Pkp4
PSPIYISSYSS[Phospho (STY)]PAR.2	0.133547374	Q68FH0_S1099_M1_Pkp4
TYYS[Phospho (STY)]PVYR.2	-0.032935139	Q68FH0_S421_M1_Pkp4
S[Phospho (STY)]YEDPYC[Carbamidomethyl (C)]DDR.2	0.005185897	Q68FH0_S1136_M1_Pkp4
PAS[Phospho (STY)]PTAVR.2	-0.263660171	Q68FH0_S280_M1_Pkp4
AAS[Phospho (STY)]PYSQRPAS[Phospho (STY)]PTAVRR.3	-0.138420122	Q68FH0_S272_M2_Pkp4

TSS[Phospho (STY)]QRS[Phospho (STY)]TLTYQR.2	-0.224551226	Q68FH0_S457_M2_Pkp4
AASPYSQRPAS[Phospho (STY)]PTAVR.3	-0.058423824	Q68FH0_S280_M1_Pkp4
GLYPGSSKPSPIY[Phospho (STY)]ISSYS[Phospho (STY)]SPAR.3	-0.118123731	Q68FH0_S1098_M2_Pkp4
VASPSQGQVGSSS[Phospho (STY)]PKR.2	0.029825902	Q68FH0_S336_M1_Pkp4
LQHTGPADDGATRS[Phospho (STY)]PS[Phospho (STY)]IDSIQKDPR.4	0.27398815	Q68FH0_S509_M2_Pkp4
SAVS[Phospho (STY)]PDLHITPIYEGR.2	-0.205293495	Q68FH0_S405_M1_Pkp4
LQHTGPADDGATRS[Phospho (STY)]PS[Phospho (STY)]IDSIQK.3	0.072366394	Q68FH0_S509_M2_Pkp4
S[Phospho (STY)]PNHGTVELQGSQTALYR.3	-0.174223266	Q68FH0_S426_M1_Pkp4
AEQYPGS[Phospho (STY)]PDSWV.2	0.066081396	Q68FH0_S1185_M1_Pkp4
SSYAS[Phospho (STY)]QHS[Phospho (STY)]QLGQELR.2	0.049648309	Q68FH0_S391_M2_Pkp4
DGGEALVS[Phospho (STY)]PDGTVTEAPR.2	-0.250264082	Q68FL4_S109_M1_Ahcyl2
IVLLAEGR.2	-0.248022454	#N/A
GESEDDFWWC[Carbamidomethyl (C)]IDR.2	-0.31471076	#N/A
AQGEKPLAGAK.2	-0.041866286	#N/A
TPELTWER.2	0.063126334	#N/A
HRDGGEALVS[Phospho (STY)]PDGTVTEAPR.3	-0.326795915	Q68FL4_S109_M1_Ahcyl2
LC[Carbamidomethyl (C)]VPAM[Oxidation (M)]NVNDSVTK.2	0.08206017	#N/A
GIVEESVTGVHR.2	-0.064951311	#N/A
FDNLYC[Carbamidomethyl (C)]C[Carbamidomethyl (C)]R.2	-0.249700106	#N/A
QVVVC[Carbamidomethyl (C)]GYGEVGK.2	0.022876509	#N/A
NNSELLNNLGNFINR.2	-0.710828595	#N/A
DSTYSQLS[Phospho (STY)]PR.2	-0.169833077	Q68FM6_S750_M1_Elfn2
QLS[Phospho (STY)]ADAAVSRK.2	-0.153182792	Q68FM6_S622_M1_Elfn2
QLS[Phospho (STY)]ADAAVSR.2	-0.170680411	Q68FM6_S622_M1_Elfn2
YIEKGS[Phospho (STY)]PLNSPLDR.2	0.256129941	Q68FM6_S671_M1_Elfn2
GS[Phospho (STY)]PLNSPLDR.2	-0.335929452	Q68FM6_S671_M1_Elfn2
HYYSGYSS[Phospho (STY)]S[Phospho (STY)]PEYSSESTHK.3	-0.004679097	Q68FM6_S760_M2_Elfn2
SISELVS[Phospho (STY)]EK.2	-0.292512813	Q692V3_S1279_M1_Ttc41
ANTLS[Phospho (STY)]PVR.2	-0.135568878	Q69Z38_S282_M1_Peak1
DPSTKPVT[Phospho (STY)]SPPSK.2	0.103137973	Q69Z38_T851_M1_Peak1
SYSSTHSS[Phospho (STY)]PAK.2	-0.05992823	Q69Z38_S727_M1_Peak1
SLFTSQSSGEGEAHQTTES[Phospho (STY)]PT[Phospho (STY)]AK.3	-0.280523975	Q69Z38_S836_M2_Peak1
GTPALTSEAAQSS[Phospho (STY)]PPTR.2	0.087115223	Q69Z89_S235_M1_Radil
S[Phospho (STY)]LLLEFEPDVEDTLLQR.2	0.188893316	Q69Z89_S432_M1_Radil
TRLNS[Phospho (STY)]IK.2	0.10352479	Q69Z98_S482_M1_Brsk2
VTPHPS[Phospho (STY)]PR.2	-0.167944896	Q69Z98_S436_M1_Brsk2
S[Phospho (STY)]M[Oxidation (M)]EVLSVTDGGSPVPAR.2	-0.28455391	Q69Z98_S383_M1_Brsk2
VDS[Phospho (STY)]PMLNR.2	0.153733135	Q69Z98_S368_M1_Brsk2
S[Phospho (STY)]ISGASSGLSTS[Phospho (STY)]PLSSPR.2	0.055449674	Q69Z98_S413_M2_Brsk2
VTPHPS[Phospho (STY)]PRGS[Phospho (STY)]PLPTPK.3	0.003401473	Q69Z98_S436_M2_Brsk2
GS[Phospho (STY)]PLPTPK.2	-0.503239519	Q69Z98_S440_M1_Brsk2
KLQVPTPEEM[Oxidation (M)]SNLT[Phospho (STY)]PESS[Phospho (STY)]PELAK.3	-0.2145249	#N/A
LQVPTPEEM[Oxidation (M)]SNLT[Phospho (STY)]PESS[Phospho (STY)]PELAK.3	-0.130784074	#N/A
LQVPTPEEMSNLT[Phospho (STY)]PESS[Phospho (STY)]PELAKK.3	-0.13846391	#N/A
RVDS[Phospho (STY)]PMLNR.2	0.09729328	Q69Z98_S368_M1_Brsk2
GGDVS[Phospho (STY)]PSPYSSSSWR.2	-0.104713443	Q69ZA1_S384_M1_Cdk13
QTDPST[Phospho (STY)]PQQESSK.2	-0.210891494	Q69ZA1_T1147_M1_Cdk13

TEHAPS[Phospho (STY)]PSSGGTVK.2	-0.26799231	Q69ZA1_S526_M1_Cdk13
HSS[Phospho (STY)]IS[Phospho (STY)]PSTLTLK.2	-0.040326069	Q69ZA1_S438_M2_Cdk13
S[Phospho (STY)]LS[Phospho (STY)]PLGGRDES[Phospho (STY)]PVSHR.3	0.130414818	Q69ZA1_S316_M3_Cdk13
S[Phospho (STY)]RS[Phospho (STY)]PY[Phospho (STY)]SPVLR.2	0.119867548	Q69ZA1_S396_M3_Cdk13
S[Phospho (STY)]RS[Phospho (STY)]PYS[Phospho (STY)]PVLRR.3	0.060094807	Q69ZA1_S396_M3_Cdk13
AAAEDAWHDEKPPQS[Phospho (STY)]PR.3	-0.557009598	Q69ZB3_S41_M1_Tspyl5
NSLLHRAS[Phospho (STY)]EEETI.2	-0.383871628	Q69ZF7_S766_M1_Cnnm4
SYS[Phospho (STY)]PSFQR.2	0.055225762	Q69ZH9_S421_M1_Arhgap23
RHS[Phospho (STY)]TSDLSDATFSDIR.3	-0.166771777	Q69ZH9_S673_M1_Arhgap23
S[Phospho (STY)]AEALGPGALVSPR.2	-0.431130993	Q69ZH9_S361_M1_Arhgap23
SKS[Phospho (STY)]C[Carbamidomethyl (C)]DDGLNTFRDEGR.3	0.033397975	Q69ZH9_S619_M1_Arhgap23
HYS[Phospho (STY)]QDC[Carbamidomethyl (C)]SSIK.2	-0.024269522	Q69ZH9_S594_M1_Arhgap23
HS[Phospho (STY)]TSDLSDATFSDIRR.3	-0.102264155	Q69ZH9_S673_M1_Arhgap23
QMNLGFGDES[Phospho (STY)]PEPEAR.2	0.14335831	Q69ZH9_S515_M1_Arhgap23
ARS[Phospho (STY)]DDYLSR.2	-0.211379446	Q69ZH9_S351_M1_Arhgap23
SKS[Phospho (STY)]C[Carbamidomethyl (C)]DDGLNTFR.2	-0.222255302	Q69ZH9_S619_M1_Arhgap23
HS[Phospho (STY)]TSDLSDATFSDIR.2	0.063092087	Q69ZH9_S673_M1_Arhgap23
RS[Phospho (STY)]S[Phospho (STY)]YLLAITTER.2	-0.493589315	Q69ZH9_S606_M2_Arhgap23
TSS[Phospho (STY)]LDSAVPIAPPPR.2	-0.355618465	Q69ZI1_S805_M1_Sh3rf1
DSVLS[Phospho (STY)]YTSVR.2	-0.161119734	Q69ZK0_S1173_M1_Prex1
LVSRT[Phospho (STY)]PT[Phospho (STY)]PK.2	-0.050217608	Q69ZK6_T322_M2_Jmjd1c
RLS[Phospho (STY)]PPGGSGSGVPGGGPLLPTAGR.3	0.110000514	Q69ZK9_S714_M1_Nlgn2
AAS[Phospho (STY)]EELVEK.2	-0.286177105	Q69ZL1_S494_M1_Fgd6
S[Phospho (STY)]LDEDSERKEEVSPLGAK.3	-0.212014147	Q69ZL1_S1167_M1_Fgd6
SSS[Phospho (STY)]DNNTNTLGR.2	-0.368351142	Q69ZR2_S1576_M1_Hectd1
RLDS[Phospho (STY)]SGER.2	0.022468311	Q69ZR2_S357_M1_Hectd1
AS[Phospho (STY)]LALIR.2	-0.262345618	Q69ZR2_S545_M1_Hectd1
NNS[Phospho (STY)]RDETTEPEQQK.2	-0.287239334	Q69ZR9_S653_M1_Tasor
SSQS[Phospho (STY)]ESEIVPK.2	-0.097570871	Q69ZS7_S150_M1_Hbs1l
SSTPSDINS[Phospho (STY)]PR.2	-0.217340325	Q69ZS8_S343_M1_Kazn
VS[Phospho (STY)]PC[Carbamidomethyl (C)]HSR.2	0.035192676	Q69ZS8_S314_M1_Kazn
THSLC[Carbamidomethyl (C)]NGDS[Phospho (STY)]PGPVQK.2	0.015241253	Q69ZS8_S356_M1_Kazn
QVRVS[Phospho (STY)]PC[Carbamidomethyl (C)]HSR.3	-0.142652694	Q69ZS8_S314_M1_Kazn
S[Phospho (STY)]LDPGLFDDSDSQC[Carbamidomethyl (C)]SPTR.2	-0.133555207	Q69ZS8_S404_M1_Kazn
S[Phospho (STY)]IQEDTKR.2	-0.250154842	Q69ZW3_S1038_M1_Ehbp1
QRS[Phospho (STY)]IQEDTKR.3	-0.114288901	Q69ZW3_S1038_M1_Ehbp1
SRS[Phospho (STY)]LEC[Carbamidomethyl (C)]R.2	0.091168434	Q69ZW3_S747_M1_Ehbp1
T[Phospho (STY)]SGSDDPGLSSSTDSAQALASLGK.2	0.2735069	Q69ZW3_T673_M1_Ehbp1
QRS[Phospho (STY)]IQEDTK.2	-0.361398587	Q69ZW3_S1038_M1_Ehbp1
LDGELAITKPNVSS[Phospho (STY)]PSK.2	-0.254160233	Q69ZW3_S765_M1_Ehbp1
VGNYETDTNSS[Phospho (STY)]VDQEK.2	-0.048653647	Q69ZW3_S588_M1_Ehbp1
ITEIVNQLNALSS[Phospho (STY)]LDEDQDDC[Carbamidomethyl (C)]IK.3	0.318600116	Q69ZW3_S222_M1_Ehbp1
S[Phospho (STY)]PPLPAVIK.2	-0.237061332	Q69ZX6_S614_M1_Morc2a
ASS[Phospho (STY)]PGYIDSPTYSR.2	-0.170398429	Q69ZX8_S373_M1_Ablim3
SS[Phospho (STY)]PYHSQLDVR.2	0.322086114	Q69ZX8_S408_M1_Ablim3
SGPESGRSS[Phospho (STY)]PYHSQLDVR.3	-0.051840671	Q69ZX8_S408_M1_Ablim3
RFS[Phospho (STY)]SGGEEEDFDR.2	-0.1229385	Q69ZX8_S502_M1_Ablim3

YTS[Phospho (STY)]DEMLER.2	0.285845959	Q69ZX8_S337_M1_Ablim3
TSETSIS[Phospho (STY)]PPGSSIGSPNR.2	-0.457122643	Q69ZX8_S282_M1_Ablim3
SAS[Phospho (STY)]LPAYR.2	0.103359214	Q69ZX8_S584_M1_Ablim3
SHYLADS[Phospho (STY)]DPLISK.2	0.114019753	Q69ZX8_S575_M1_Ablim3
RAS[Phospho (STY)]SPGYIDSPTYSR.2	0.026706504	Q69ZX8_S372_M1_Ablim3
RTS[Phospho (STY)]ETSIS[Phospho (STY)]PPGSSIGSPNR.2	-0.005907067	Q69ZX8_S277_M2_Ablim3
NKFGS[Phospho (STY)]ADNIPNLK.2	-0.126591374	Q69ZZ6_S378_M1_Tmcc1
FGS[Phospho (STY)]ADNIPNLK.2	-0.047428078	Q69ZZ6_S378_M1_Tmcc1
GS[Phospho (STY)]PPDC[Carbamidomethyl (C)]TC[Carbamidomethyl (C)]VPAGDTVISEVK.2	-0.131789562	Q69ZZ9_S276_M1_Kiaa0754
KQFS[Phospho (STY)]LENVPEGELLPDGK.2	-0.112352269	Q6A037_S299_M1_N4bp1
ALLHSGS[Phospho (STY)]SSSK.2	0.073664584	Q6A065_S717_M1_Cep170
SKTS[Phospho (STY)]PVASGSTSK.2	-0.245489926	Q6A065_S1069_M1_Cep170
SRS[Phospho (STY)]ADIGAR.2	-0.073763116	Q6A065_S985_M1_Cep170
LGEAS[Phospho (STY)]DSELADADK.2	0.020278968	Q6A065_S1102_M1_Cep170
LRTS[Phospho (STY)]PALK.2	0.005095128	Q6A065_S1250_M1_Cep170
SIKS[Phospho (STY)]DVPVYLKR.3	-0.105947291	Q6A065_S358_M1_Cep170
S[Phospho (STY)]VDSRPQPAEHPDHLTITR.4	-0.19391574	Q6A065_S1384_M1_Cep170
SIKS[Phospho (STY)]DVPVYLK.2	-0.108444168	Q6A065_S358_M1_Cep170
TEEDSKS[Phospho (STY)]IKS[Phospho (STY)]DVPVYLK.2	0.268334387	Q6A065_S355_M2_Cep170
QGS[Phospho (STY)]FTIDKPSSNIPIELIPHINK.3	-0.251034744	Q6A065_S829_M1_Cep170
C[Carbamidomethyl (C)]STSS[Phospho (STY)]PSKDVTK.2	-0.237157047	Q6A065_S963_M1_Cep170
ALLHSGS[Phospho (STY)]SSSKEK.2	0.295385977	Q6A065_S717_M1_Cep170
SDS[Phospho (STY)]EATISR.2	-0.503198732	Q6A065_S1157_M1_Cep170
SDS[Phospho (STY)]LDTDSSM[Oxidation (M)]DTTLILK.2	0.043671622	Q6A065_S872_M1_Cep170
GNKHDDGTQS[Phospho (STY)]DSENAGAHR.3	0.137978936	Q6A065_S378_M1_Cep170
HDDGTQS[Phospho (STY)]DSENAGAHRR.3	-0.359946631	Q6A065_S378_M1_Cep170
RFPTDYAS[Phospho (STY)]TS[Phospho (STY)]EDEFGSNR.2	-0.123784678	Q6A065_S1229_M2_Cep170
NQATSASSEKDNDDDQSDKGTY[Phospho (STY)]TIELENPNSEEVEAR.4	0.132070332	Q6A065_Y499_M1_Cep170
GNKHDDGTQS[Phospho (STY)]DSENAGAHRR.4	-0.083979046	Q6A065_S378_M1_Cep170
SPS[Phospho (STY)]PVHLPEDPKVAEK.3	0.274313685	Q6A0A2_S603_M1_Larp4b
EQNTPPKS[Phospho (STY)]PQ.2	-0.318596964	Q6A0A2_S739_M1_Larp4b
RAS[Phospho (STY)]PPAAGKR.2	-0.282530674	Q6A0A2_S721_M1_Larp4b
KNS[Phospho (STY)]FGYR.2	0.066959919	Q6A0A2_S500_M1_Larp4b
RAS[Phospho (STY)]PPAAGK.2	-0.262597745	Q6A0A2_S721_M1_Larp4b
SPS[Phospho (STY)]PVHLPEDPK.2	-0.092104286	Q6A0A2_S603_M1_Larp4b
DSVALRHS[Phospho (STY)]NPR.2	-0.364801756	Q6A0D4_S536_M1_Rftn1
SYGSQPNS[Phospho (STY)]PTSEDLAK.2	-0.208633656	Q6AW69_S203_M1_Cgnl1
RQDS[Phospho (STY)]AGPILDGAR.2	-0.065414626	Q6AW69_S284_M1_Cgnl1
KVM[Oxidation (M)]LPESYLLVATR.2	-0.008925689	#N/A
DQGEPVS[Phospho (STY)]PSEDEPFSWPGPK.2	0.468529801	Q6DFV3_S41_M1_Arhgap21
NRS[Phospho (STY)]PTLSC[Carbamidomethyl (C)]R.2	-0.164605878	Q6DFV3_S1501_M1_Arhgap21
QLS[Phospho (STY)]HDQESVGPPSLDGQHSSK.3	-0.288112022	Q6DFV3_S850_M1_Arhgap21
VGGS[Phospho (STY)]IRPWK.2	-0.29387485	Q6DFV3_S947_M1_Arhgap21
LLSKDDTS[Phospho (STY)]PPKDK.3	-0.178249305	Q6DFV3_S1108_M1_Arhgap21
KGS[Phospho (STY)]SSEVFSDAAR.2	0.319065002	Q6DFV3_S917_M1_Arhgap21
SKS[Phospho (STY)]YDEGLDDYREDAK.3	-0.366212953	Q6DFV3_S874_M1_Arhgap21
VRTS[Phospho (STY)]ASDLSR.2	0.263734736	Q6DFV3_S1847_M1_Arhgap21

EKPQS[Phospho (STY)]GRQTPQPLR.3	-0.065446123	Q6DFV3_S736_M1_Arhgap21
T[Phospho (STY)]SQGFGFTLR.2	0.20333215	Q6DFV3_T61_M1_Arhgap21
S[Phospho (STY)]TS[Phospho (STY)]HDRVPQSVQIR.2	0.088989591	Q6DFV3_S435_M2_Arhgap21
RQLS[Phospho (STY)]HDQESVGPPSLDGQHSSK.4	-0.369388324	Q6DFV3_S850_M1_Arhgap21
TQSPHS[Phospho (STY)]PKEESER.3	-0.221985603	Q6DFV3_S1092_M1_Arhgap21
GIPS[Phospho (STY)]IVR.2	-0.352771528	Q6DFV3_S1122_M1_Arhgap21
SVS[Phospho (STY)]QERLEDSVLMK.2	0.411352855	Q6DFV3_S453_M1_Arhgap21
EVLDSLGSS[Phospho (STY)]LDK.2	-0.097354277	Q6DFV7_S612_M1_Ncoa7
HSGS[Phospho (STY)]PENLGESTLNIHEDLDKIK.4	0.160872062	Q6DFV7_S500_M1_Ncoa7
VLS[Phospho (STY)]STSEEDEPGVVK.2	-0.156062709	Q6DFV7_S211_M1_Ncoa7
YYS[Phospho (STY)]VDDNQNK.2	0.035231444	Q6DFV7_S92_M1_Ncoa7
SVRS[Phospho (STY)]PEESTER.2	-0.481261525	Q6DFV7_S353_M1_Ncoa7
[Acetyl (Protein N-term)]STIC[Carbamidomethyl (C)]PPPS[Phospho (STY)]PAVAK.2	-0.194491885	Q6DFW0_S9_M1_
EEPLS[Phospho (STY)]EEEPC[Carbamidomethyl (C)]TSTAVPS[Phospho (STY)]PEK.2	0.387764851	Q6DFW4_S509_M2_Nop58
HIKEEPLS[Phospho (STY)]EEEPC[Carbamidomethyl (C)]TSTAVPS[Phospho (STY)]PEK.3	0.144434311	Q6DFW4_S509_M2_Nop58
NTPES[Phospho (STY)]PPYSSGSYDSIK.2	-0.009365858	Q6DIB4_S189_M1_Nol4l
LEIYQSS[Phospho (STY)]QEEPIALDK.2	-0.143463753	Q6DIB4_S354_M1_Nol4l
ASEPVKEPVQTAQS[Phospho (STY)]PAPVEK.3	-0.296919591	Q6DID3_S617_M1_Scaf8
WTKPSSFS[Phospho (STY)]DSER.2	0.161617918	Q6DVA0_S507_M1_Lemd2
AS[Phospho (STY)]VRGS[Phospho (STY)]SEDDEDTRTPDR.3	-0.343710762	Q6DVA0_S145_M2_Lemd2
S[Phospho (STY)]KAIMAKY[Phospho (STY)]DPEAR.2	-0.314665674	Q6EJB6_S307_M2_Utp14b
SLS[Phospho (STY)]GGPLGR.2	0.106496354	Q6GQT5_S423_M1_Tmem151a
SQT[Phospho (STY)]PS[Phospho (STY)]PSTLSR.2	-0.322760438	Q6GYP7_S1003_M2_Ralgapa1
SSS[Phospho (STY)]TSDILEPFTVER.2	-0.205164328	Q6GYP7_S796_M1_Ralgapa1
SATTTGS[Phospho (STY)]PGTEK.2	-0.244795765	Q6GYP7_S739_M1_Ralgapa1
RGS[Phospho (STY)]SPGSLEIPK.2	0.134480635	Q6GYP7_S859_M1_Ralgapa1
GVGHEFQKVS[Phospho (STY)]VDK.3	-0.118884292	Q6GYP7_S710_M1_Ralgapa1
GWS[Phospho (STY)]RDQPGQAPMR.2	-0.195795261	Q6GYP7_S720_M1_Ralgapa1
ANS[Phospho (STY)]DTDLVTSESR.2	-0.286855792	Q6I6G8_S48_M1_Hecw2
SHS[Phospho (STY)]AGEVGEDSR.2	0.105384846	Q6I6G8_S1048_M1_Hecw2
TSSTLEIDTEDLISTSSRNS[Phospho (STY)]PPR.3	0.213887697	Q6I6G8_S406_M1_Hecw2
SNS[Phospho (STY)]IQQMEQLNR.2	0.304070551	Q6I6G8_S858_M1_Hecw2
QRSHS[Phospho (STY)]AGEVGEDSR.3	0.044181523	Q6I6G8_S1048_M1_Hecw2
DQTEDPASS[Phospho (STY)]PTSK.2	0.020846483	Q6IEE6_S1006_M1_Tmem132e
DQTEDPASS[Phospho (STY)]PTSKR.2	-0.296440266	Q6IEE6_S1006_M1_Tmem132e
KSS[Phospho (STY)]C[Carbamidomethyl (C)]DAVLSR.2	-0.130816113	Q6IFT4_S730_M1_Arhgap20
S[Phospho (STY)]APSLILDK.2	0.014132067	Q6IFT4_S46_M1_Arhgap20
SS[Phospho (STY)]EPSIDYLDTK.2	-0.212009797	Q6IFT4_S704_M1_Arhgap20
RC[Carbamidomethyl (C)]S[Phospho (STY)]EPSIDDQNYK.2	-0.000524954	Q6IFT4_S847_M1_Arhgap20
RSS[Phospho (STY)]EPSIDYLDTK.2	-0.008839933	Q6IFT4_S704_M1_Arhgap20
AAQSSVTAS[Phospho (STY)]PQTEEFFDLIASSQSR.3	-0.069710914	Q6IR34_S543_M1_Gpsm1
APS[Phospho (STY)]SDEEC[Carbamidomethyl (C)]FFDLLSK.2	-0.080005257	Q6IR34_S490_M1_Gpsm1
AS[Phospho (STY)]VGSLPGLR.2	-0.133630085	Q6IR34_S567_M1_Gpsm1
EGSHS[Phospho (STY)]PLDSADVR.2	-0.469331798	Q6IR34_S469_M1_Gpsm1
VDLAGS[Phospho (STY)]PEQEASGLPDPQQQC[Carbamidomethyl (C)]PPGAS.3	0.184601197	Q6IR34_S653_M1_Gpsm1
DLEEWNQR.2	-0.039917404	#N/A
LTQEPESIRK.2	0.067346761	#N/A

VAQLC[Carbamidomethyl (C)]DFNPK.2	0.2039613	#N/A
LTQEPESIR.2	0.468518872	#N/A
LQELDAASK.2	0.178657696	#N/A
VTEQEWR.2	-0.193434747	#N/A
KDLEEWNQR.2	0.273701327	#N/A
ESKEETPGTEWEK.2	0.252574684	#N/A
AFYQQPDADTIGYVASEEAFVK.3	0.059508757	#N/A
AITSLLGGGS[Phospho (STY)]PK.2	-0.106631566	Q6KCD5_S2652_M1_Nipbl
FPHSS[Phospho (STY)]PNVK.2	-0.172283097	Q6NS52_S95_M1_Dgkb
VLAC[Carbamidomethyl (C)]GGDGTVGWILDC[Carbamidomethyl (C)]IEK.2	0.039377752	#N/A
QDILNQTIDFEGFK.2	-0.313009806	#N/A
GGGGS[Phospho (STY)]ASGPGVR.2	0.241119447	Q6NS60_S363_M1_Fbxo41
EHHAGSAVPSTYAVS[Phospho (STY)]R.3	-0.124856794	Q6NS60_S391_M1_Fbxo41
RHS[Phospho (STY)]TEGEEGDVSDVGSR.3	-0.469291513	Q6NS60_S476_M1_Fbxo41
LSARPEGGS[Phospho (STY)]GR.2	-0.090905723	Q6NS60_S526_M1_Fbxo41
[Acetyl (Protein N-term)]AS[Phospho (STY)]LDLPYR.2	0.297029655	Q6NS60_S3_M1_Fbxo41
HGSS[Phospho (STY)]PSTGASSR.2	-0.31470385	Q6NS60_S396_M1_Fbxo41
SSQTS[Phospho (STY)]PC[Carbamidomethyl (C)]SQGYGR.2	-0.167366557	Q6NS69_S52_M1_Amer3
DGPES[Phospho (STY)]PAHS[Phospho (STY)]PQALDC[Carbamidomethyl (C)]K.2	-0.188791024	Q6NS69_S263_M2_Amer3
SAPPAGDEPLAETES[Phospho (STY)]ESEAELAGFSPVVDVK.3	0.364505936	Q6NS82_S276_M1_Retreg2
ASSC[Carbamidomethyl (C)]ES[Phospho (STY)]IPEEDSEAR.2	-0.256658556	Q6NSW3_S1070_M1_Sphkap
SNS[Phospho (STY)]LLESTDYWLQNQR.2	-0.048763299	Q6NSW3_S50_M1_Sphkap
ANS[Phospho (STY)]LDGFAQNC[Carbamidomethyl (C)]PQDSVNVQPVSR.3	-0.106315774	Q6NSW3_S1244_M1_Sphkap
QSS[Phospho (STY)]C[Carbamidomethyl (C)]ESITDEFSR.2	0.2990087	Q6NSW3_S1106_M1_Sphkap
RAS[Phospho (STY)]TDLGK.2	-0.516162914	Q6NSW3_S841_M1_Sphkap
LKAS[Phospho (STY)]S[Phospho (STY)]C[Carbamidomethyl (C)]ESIPEEDSEAR.2	0.016012112	Q6NSW3_S1066_M2_Sphkap
QSS[Phospho (STY)]TESITEEFYR.2	-0.156332175	Q6NSW3_S1171_M1_Sphkap
EILKEQS[Phospho (STY)]ES[Phospho (STY)]IKEHASGLPGR.3	-0.172231929	Q6NSW3_S1574_M2_Sphkap
ASSSGLC[Carbamidomethyl (C)]KS[Phospho (STY)]DSC[Carbamidomethyl (C)]LYR.3	-0.086512262	Q6NSW3_S1273_M1_Sphkap
RVKS[Phospho (STY)]PS[Phospho (STY)]PK.2	-0.118409953	Q6NV83_S946_M2_U2surp
RHS[Phospho (STY)]TS[Phospho (STY)]PS[Phospho (STY)]PSR.2	0.300384742	Q6NV83_S930_M3_U2surp
VKS[Phospho (STY)]PS[Phospho (STY)]PK.2	-0.169631533	Q6NV83_S946_M2_U2surp
EAENTANQAGNES[Phospho (STY)]PVQELR.2	-0.373167817	Q6NVE8_S50_M1_Wdr44
S[Phospho (STY)]NSGRELTDEEILASVMIK.3	-0.254404193	Q6NVE8_S344_M1_Wdr44
EYVSNDATQS[Phospho (STY)]DDEEK.2	-0.07622135	Q6NVE8_S405_M1_Wdr44
TKEYVSNDATQS[Phospho (STY)]DDEEK.2	-0.430831556	Q6NVE8_S405_M1_Wdr44
TKEYVSNDATQS[Phospho (STY)]DDEEKLQSQQTDTDGGR.4	0.13267449	Q6NVE8_S405_M1_Wdr44
S[Phospho (STY)]RDRHDDYYR.3	-0.255404054	Q6NVF9_S513_M1_Cpsf6
VS[Phospho (STY)]SSC[Carbamidomethyl (C)]LDLPDSTEEK.2	-0.287357305	Q6NXI6_S1086_M1_Rprd2
SFNYS[Phospho (STY)]PSSSTSEVSSTSASK.2	0.266240155	Q6NXI6_S612_M1_Rprd2
DVEDM[Oxidation (M)]ELS[Phospho (STY)]DVEDDGSK.2	0.193571355	Q6NXI6_S392_M1_Rprd2
ASVGQS[Phospho (STY)]PVLPSTTFK.2	-0.361153775	Q6NXI6_S633_M1_Rprd2
GPTSTSVDSIDGT[Phospho (STY)]PVRDER.3	-0.445015275	Q6NXI6_T742_M1_Rprd2
SDS[Phospho (STY)]DSSTLAKK.2	-0.087443754	Q6NXJ0_S1017_M1_Wwc2
LNRSDS[Phospho (STY)]DSSTLAK.2	0.161803611	Q6NXJ0_S1017_M1_Wwc2
AADKS[Phospho (STY)]PESQNLIDGTKK.3	0.098996321	Q6NXK2_S313_M1_Znf532
KAASDSC[Carbamidomethyl (C)]KEPVANS[Phospho (STY)]R.3	-0.168749418	Q6NXK2_S293_M1_Znf532

LYASAFSER.2	-0.038654573	#N/A
ATQSQPADQELGS[Phospho (STY)]NSPPQR.2	-0.166957243	Q6NXK7_S25_M1_Dpp10
QNEEPVFSR.2	-0.353642537	#N/A
ILGSAS[Phospho (STY)]PEEEQEKPILDRPTR.3	-0.449784446	Q6NXN1_S107_M1_Szrd1
KLS[Phospho (STY)]VGAYVSSVR.2	-0.268561462	Q6NZC7_S748_M1_Sec23ip
EM[Oxidation (M)]AS[Phospho (STY)]PSSESNESKR.2	-0.463887648	Q6NZC7_S735_M1_Sec23ip
KLS[Phospho (STY)]VGGDSDPPLKR.3	-0.128239513	Q6NZF1_S289_M1_Zc3h11a
KAAS[Phospho (STY)]LTEDR.2	-0.133398813	Q6NZJ6_S1211_M1_Eif4g1
SFS[Phospho (STY)]KEVEER.2	-0.26905533	Q6NZJ6_S1189_M1_Eif4g1
EATLPPVS[Phospho (STY)]PPK.2	-0.34060877	Q6NZJ6_S1231_M1_Eif4g1
AAS[Phospho (STY)]LTEDR.2	0.000345326	Q6NZJ6_S1211_M1_Eif4g1
SS[Phospho (STY)]LS[Phospho (STY)]RER.2	-0.539682681	Q6NZJ6_S1147_M2_Eif4g1
QLSYS[Phospho (STY)]DSDLKR.2	-0.069446891	Q6NZK8_S394_M1_Ptpdc1
EQSES[Phospho (STY)]LSELR.2	-0.020773021	Q6NZL0_S639_M1_Soga3
RGS[Phospho (STY)]KDLPK.2	-0.27637994	Q6NZL0_S501_M1_Soga3
EGPIGGES[Phospho (STY)]DSEEVR.2	-0.218082862	Q6NZL0_S779_M1_Soga3
MKELS[Phospho (STY)]LK.2	0.216713794	Q6NZL0_S495_M1_Soga3
AQS[Phospho (STY)]PARPR.2	-0.072592333	Q6NZL0_S41_M1_Soga3
KREGPIGGES[Phospho (STY)]DSEEVR.3	-0.170125819	Q6NZL0_S779_M1_Soga3
LIADTSTIITEAR.2	-0.250658221	#N/A
QEPIES[Phospho (STY)]EEEEAEATR.2	-0.087543936	Q6NZM9_S562_M1_Hdac4
RLNHS[Phospho (STY)]PPQSSSR.3	-0.024706143	Q6NZN0_S127_M1_Rbm26
LNHS[Phospho (STY)]PPQSSSR.2	0.269492292	Q6NZN0_S127_M1_Rbm26
AGS[Phospho (STY)]PDVLR.2	-0.180273279	Q6NZQ6_S19_M1_Znf740
LSVC[Carbamidomethyl (C)]PS[Phospho (STY)]TQDIC[Carbamidomethyl (C)]R.2	-0.195531056	Q6NZQ8_S75_M1_Marchf1
SSNISKASSPT[Phospho (STY)]T[Phospho (STY)]GTAPR.2	0.004822215	Q6NZQ8_T59_M2_Marchf1
QHFISFDSDR.3	-0.321586366	#N/A
ITFDDYIAC[Carbamidomethyl (C)]C[Carbamidomethyl (C)]VK.2	-0.319990791	#N/A
ALTDSFR.2	0.285687053	#N/A
LFEEPEDPSNR.2	-0.323892607	#N/A
VVIFQQEQENK.2	0.145128124	#N/A
NAAQFLLSTNDK.2	0.011095896	#N/A
ENIIAVATTNNLYIFQDK.2	-0.223307859	#N/A
SFFSEIISSISDVK.2	0.129867561	#N/A
INLWHLEITDR.3	0.024177051	#N/A
[Acetyl (Protein N-term)]AGAGGGNDIQWC[Carbamidomethyl (C)]FSQVK.2	0.090598032	#N/A
GEYNVYSTFQSHEPEFDYLK.3	0.205342457	#N/A
ENIIAVATTNNLYIFQDKVN.2	-0.428774525	#N/A
GAVDDDVAEADIISTVEFNHSGELLATGDK.3	-0.082069617	#N/A
KTHS[Phospho (STY)]S[Phospho (STY)]PALSLGHGPVTGSK.3	-0.163838206	Q6P1I6_S745_M2_Psd2
KPPS[Phospho (STY)]PNVSTEEK.2	-0.398021092	Q6P1Y8_S470_M1_Inpp4b
TNLSGRQS[Phospho (STY)]PSFK.2	0.100766629	Q6P2K6_S728_M1_Ppp4r3a
SEKPAQSASS[Phospho (STY)]PEATSGSAGPVEK.3	0.027513072	Q6P2L6_S561_M1_Nsd3
FFDENES[Phospho (STY)]PVDPQHGSK.2	0.029604914	Q6P2L7_S365_M1_Golm2
TWC[Carbamidomethyl (C)]GS[Phospho (STY)]PPYAAPELFEGK.2	-0.178090235	Q6P4S6_S167_M1_Sik3
QLS[Phospho (STY)]ADSAEAHSLNMNR.2	0.120928814	Q6P4S6_S856_M1_Sik3
RAS[Phospho (STY)]DGGANIQLHAQQLLK.3	-0.502363596	Q6P4S6_S493_M1_Sik3

EEAS[Phospho (STY)]DDDM[Oxidation (M)]EGDEAVVR.2	-0.07555075	Q6P4T2_S225_M1_Snrnp200
EAEQGS[Phospho (STY)]GEEKEEKEGDLK.2	-0.495392039	Q6P542_S225_M1_Abcf1
SKPAAADS[Phospho (STY)]EGEEEEEDTAKEK.3	-0.079537571	Q6P542_S194_M1_Abcf1
QLSVPAS[Phospho (STY)]DEEDEVPAPIPR.2	0.139957422	Q6P542_S107_M1_Abcf1
LKQLSVPAS[Phospho (STY)]DEEDEVPAPIPR.3	-0.021428	Q6P542_S107_M1_Abcf1
SKPAAADS[Phospho (STY)]EGEEEEEDTAK.2	0.067792326	Q6P542_S194_M1_Abcf1
GGNVFEALIQDDS[Phospho (STY)]EEEEEEEENR.3	0.345048155	Q6P542_S138_M1_Abcf1
AKGGNVFEALIQDDS[Phospho (STY)]EEEEEEEENR.3	-0.374894783	Q6P542_S138_M1_Abcf1
GDSIEEILADS[Phospho (STY)]EDEDEEEERGR.3	0.355974239	Q6P5B0_S1081_M1_Rrp12
GDSIEEILADS[Phospho (STY)]EDEDEEEER.2	-0.062626723	Q6P5B0_S1081_M1_Rrp12
TWPGSGS[Phospho (STY)]PR.2	-0.28883853	Q6P5E8_S17_M1_Dgkq
LGS[Phospho (STY)]PAGS[Phospho (STY)]PVLGISGR.2	0.072815832	Q6P5E8_S22_M2_Dgkq
YLATSQPRPDS[Phospho (STY)]SGS[Phospho (STY)]GH.2	0.317612128	Q6P5F7_S519_M2_Ttyh3
C[Carbamidomethyl (C)]ENTPLIGRES[Phospho (STY)]PPPSYTSSMR.3	0.116898641	Q6P5F7_S496_M1_Ttyh3
GYLS[Phospho (STY)]EGLVTK.2	0.24887735	Q6P5G0_S186_M1_Mapk4
VGS[Phospho (STY)]PSYLDK.2	0.110089994	Q6P5G0_S430_M1_Mapk4
AGST[Phospho (STY)]PLAEDVQVDPR.2	-0.312648587	Q6P5G0_T383_M1_Mapk4
SESLIDASEDS[Phospho (STY)]QLEAAIR.2	0.244315286	Q6P5G6_S266_M1_Ubxn7
S[Phospho (STY)]C[Carbamidomethyl (C)]PSITHGPR.2	-0.008936284	Q6P5H6_S351_M1_Frmd5
SSS[Phospho (STY)]HGDTFLPHVR.3	-0.223923336	Q6P5H6_S396_M1_Frmd5
VS[Phospho (STY)]VDC[Carbamidomethyl (C)]LWR.2	-0.18337948	Q6P5U7_S1685_M1_Nwd2
VESTTT[Phospho (STY)]PDGPC[Carbamidomethyl (C)]VVSVQETEK.2	-0.149058961	Q6P6J0_T10_M1_Adat2
LLLEQGK.2	-0.121378194	#N/A
T[Phospho (STY)]LSVAAAFNEDEDSEPEEM[Oxidation (M)]PPEAK.3	0.177074623	Q6P8I4_T106_M1_Pcnp
SLVDYENANK.2	-0.061837615	#N/A
[Acetyl (Protein N-term)]M[Oxidation (M)]EGLDDGPDFLS[Phospho (STY)]EEDR.2	-0.227339081	Q6P8X1_S13_M1_Snx6
AQPGS[Phospho (STY)]PQALGGPHGPATAK.2	-0.419550556	Q6P9K8_S791_M1_Caskin1
KVPLPGPGS[Phospho (STY)]PEVKR.3	-0.427586271	Q6P9K8_S1259_M1_Caskin1
VPLPGPGS[Phospho (STY)]PEVK.2	-0.26462196	Q6P9K8_S1259_M1_Caskin1
SGS[Phospho (STY)]LSNVAGGR.2	0.088408742	Q6P9K8_S390_M1_Caskin1
SLPQS[Phospho (STY)]PTHR.2	0.019409122	Q6P9K8_S828_M1_Caskin1
PAS[Phospho (STY)]PEPGR.2	-0.261790361	Q6P9K8_S1036_M1_Caskin1
VPLPGPGS[Phospho (STY)]PEVKR.2	-0.1397515	Q6P9K8_S1259_M1_Caskin1
IQGS[Phospho (STY)]PTPASK.2	-0.127361201	Q6P9K8_S1244_M1_Caskin1
RRAS[Phospho (STY)]DLAGSVDTGSAGSVK.3	-0.022513101	Q6P9K8_S989_M1_Caskin1
KVPLPGPGS[Phospho (STY)]PEVK.2	-0.418401116	Q6P9K8_S1259_M1_Caskin1
AAASVVSGPPVASDC[Carbamidomethyl (C)]AS[Phospho (STY)]PGDSAR.2	-0.132346614	Q6P9K8_S1363_M1_Caskin1
TPLDLAC[Carbamidomethyl (C)]EFGR.2	0.436633948	#N/A
TGSEPS[Phospho (STY)]PPQGGGSLGPSAPPEEIWVLR.3	0.036125088	Q6P9K8_S358_M1_Caskin1
IQGS[Phospho (STY)]PTPASKK.2	-0.27935435	Q6P9K8_S1244_M1_Caskin1
RPPEGHPTPRPAS[Phospho (STY)]PEPGR.4	-0.174925463	Q6P9K8_S1036_M1_Caskin1
S[Phospho (STY)]QEYLLDEGM[Oxidation (M)]APGTPPK.2	0.248772987	Q6P9K8_S728_M1_Caskin1
RAS[Phospho (STY)]DLAGSVDTGSAGSVK.2	-0.090493726	Q6P9K8_S989_M1_Caskin1
SVSESS[Phospho (STY)]PGDSPVKPPEGSSGAAR.3	-0.113529417	Q6P9K8_S428_M1_Caskin1
RGS[Phospho (STY)]GEPAEQSHFMEDGTAR.3	-0.012116047	Q6P9K8_S1083_M1_Caskin1
TPQSLEM[Oxidation (M)]M[Oxidation (M)]AIESPPPSEPAAAEC[Carbamidomethyl (C)]QS[Phospho (STY)]PK.3	-0.363037117	Q6P9K8_S650_M1_Caskin1
Y[Phospho (STY)]AASDSEPERDELLVPAAAGPYATVQR.3	0.188253744	Q6P9K8_Y888_M1_Caskin1

HISSS[Phospho (STY)]QELLGDGPPGPGSPMSR.3	0.062247945	Q6P9K8_S710_M1_Caskin1
AGDIITVLEQHPDGR.3	-0.035147627	#N/A
S[Phospho (STY)]FEQVSGAPVPR.2	-0.192971644	Q6P9N1_S453_M1_Fam126a
S[Phospho (STY)]LEGGGC[Carbamidomethyl (C)]PVR.2	-0.18908807	Q6P9Q4_S370_M1_Fhod1
S[Phospho (STY)]NSLSEQLTVNSNPDTVK.2	-0.049580908	Q6P9Q6_S342_M1_Fkbp15
VTS[Phospho (STY)]LEEELTDLR.2	-0.40622905	Q6P9Q6_S722_M1_Fkbp15
[Acetyl (Protein N-term)]MFGAGDEDDTDFLS[Phospho (STY)]PSGGAK.2	0.215924701	Q6P9Q6_S14_M1_Fkbp15
HNQDSQHC[Carbamidomethyl (C)]SLS[Phospho (STY)]GDEEDELFK.3	0.041006712	Q6P9Q6_S1159_M1_Fkbp15
TPTVPDSPGY[Phospho (STY)]VGPTR.2	-0.00565464	Q6P9S0_Y567_M1_Mtss2
GLS[Phospho (STY)]LEHQK.2	-0.243171823	Q6P9S0_S419_M1_Mtss2
GSDYSWSYQTPPS[Phospho (STY)]SPSSSNSR.2	0.073186875	Q6P9S0_S260_M1_Mtss2
AGS[Phospho (STY)]EEC[Carbamidomethyl (C)]VFYTDEVASPLAPDLAK.3	-0.079919767	Q6P9S0_S575_M1_Mtss2
ASAPRPPPAPADGADPAPVEESEARPDGEGS[Phospho (STY)]PSK.4	0.239547917	Q6PAJ1_S122_M1_Bcr
VS[Phospho (STY)]PSPTTYR.2	0.048803619	Q6PAJ1_S356_M1_Bcr
RHQDGLPYIDDS[Phospho (STY)]PSSSPHLSSK.4	-0.258265725	Q6PAJ1_S461_M1_Bcr
STLVLHDLLK.2	-0.392368562	#N/A
HQDGLPYIDDS[Phospho (STY)]PSSSPHLSSK.3	-0.020431866	Q6PAJ1_S461_M1_Bcr
ANS[Phospho (STY)]EASSSEGQSSLSSLEK.2	-0.237846214	Q6PAL7_S1180_M1_Ahdc1
AYS[Phospho (STY)]PTC[Carbamidomethyl (C)]S[Phospho (STY)]PTLGFK.2	0.009495014	Q6PAL7_S1392_M2_Ahdc1
ASTVS[Phospho (STY)]PGGYM[Oxidation (M)]VPK.2	-0.262226795	Q6PAL7_S1060_M1_Ahdc1
FNS[Phospho (STY)]YDISR.2	-0.513007581	Q6PAL8_S193_M1_Dennd5a
S[Phospho (STY)]HNDFVAILDLPEGEHQYK.3	-0.280132527	Q6PAM0_S107_M1_Prkab2
IM[Oxidation (M)]VGS[Phospho (STY)]TDDPSVFSLPDSK.2	0.23159959	Q6PAM0_S38_M1_Prkab2
DLSSS[Phospho (STY)]PPGPYGQEM[Oxidation (M)]YVFR.2	0.101276995	Q6PAM0_S183_M1_Prkab2
EQGVESPGAQPASS[Phospho (STY)]PR.2	0.276154444	Q6PAM1_S523_M1_Txlna
SRS[Phospho (STY)]SDIVSSVR.2	-0.366427495	Q6PAR5_S902_M1_Gapvd1
S[Phospho (STY)]SDIVSSVR.2	-0.222560362	Q6PAR5_S902_M1_Gapvd1
LQELESC[Carbamidomethyl (C)]SGLGS[Phospho (STY)]TSDDTDVR.2	-0.516287378	Q6PAR5_S746_M1_Gapvd1
FSLC[Carbamidomethyl (C)]S[Phospho (STY)]DNLEGISEGPSNR.2	0.364691129	Q6PAR5_S569_M1_Gapvd1
REEGEAVEAGDT[Phospho (STY)]PEELR.3	-0.300044219	Q6PB44_T744_M1_Ptpn23
ELQEAQEHDAR.2	-0.120244671	#N/A
GPS[Phospho (STY)]PPRPGK.2	0.015806425	Q6PB70_S1009_M1_Ano8
VESIGQWQK.2	-0.217046018	#N/A
S[Phospho (STY)]PGLPASTAR.2	-0.305600816	Q6PCN3_S1222_M1_Ttbk1
APPDS[Phospho (STY)]PTTPVR.2	-0.044904983	Q6PCN3_S441_M1_Ttbk1
RVNS[Phospho (STY)]PESER.2	-0.284879153	Q6PCN3_S456_M1_Ttbk1
GVGVPSS[Phospho (STY)]PVR.2	0.204106778	Q6PCN3_S433_M1_Ttbk1
KGTTISPS[Phospho (STY)]R.2	-0.118779626	Q6PCN3_S1036_M1_Ttbk1
S[Phospho (STY)]QEGAPSTLLADDQK.2	-0.212077787	Q6PCN3_S794_M1_Ttbk1
AGTDALLSTSTST[Phospho (STY)]PPQQNTR.2	-0.157065634	Q6PCN3_T325_M1_Ttbk1
SETSQPPTPGS[Phospho (STY)]PSHSPLHSGPR.3	0.0126367	Q6PCN3_S638_M1_Ttbk1
VNS[Phospho (STY)]PESER.2	0.097833184	Q6PCN3_S456_M1_Ttbk1
MDLPGS[Phospho (STY)]PSR.2	-0.000841784	Q6PCN3_S484_M1_Ttbk1
RVNS[Phospho (STY)]PES[Phospho (STY)]ERLSTAADGR.3	-0.425430613	Q6PCN3_S456_M2_Ttbk1
SETSQPPTPGSPSHS[Phospho (STY)]PLHS[Phospho (STY)]GPRPR.3	-0.070766764	Q6PCN3_S642_M2_Ttbk1
GVGVPSS[Phospho (STY)]PVRAPPDS[Phospho (STY)]PTTPVR.3	0.266906317	Q6PCN3_S433_M2_Ttbk1
SRMDLPGS[Phospho (STY)]PSR.2	0.105665442	Q6PCN3_S484_M1_Ttbk1

RRES[Phospho (STY)]DPTGPQR.2	0.002547636	Q6PCN3_S655_M1_Ttbk1
GRAS[Phospho (STY)]MADGDLEPEEGSK.2	0.077242274	Q6PCN3_S815_M1_Ttbk1
NDSIVTPS[Phospho (STY)]PPQAR.2	-0.074272352	Q6PCP5_S151_M1_Mff
ERS[Phospho (STY)]M[Oxidation (M)]SENAVR.2	-0.181548842	Q6PCP5_S129_M1_Mff
SM[Oxidation (M)]S[Phospho (STY)]ENAVR.2	0.145078665	Q6PCP5_S131_M1_Mff
GPES[Phospho (STY)]PQKT[Phospho (STY)]PPSPAPPSFEEQLR.3	-0.36321719	Q6PD24_S552_M2_Ankrd13d
SHS[Phospho (STY)]SSQFR.2	-0.026622289	Q6PD28_S46_M1_Ppp2r5b
SNDC[Carbamidomethyl (C)]LASPTTELTS[Phospho (STY)]PGLTPS.2	-0.134732874	Q6PDC0_S402_M1_Rundc3b
FEQSSDSISS[Phospho (STY)]DEEELR.2	-0.124037385	Q6PDC0_S217_M1_Rundc3b
S[Phospho (STY)]PS[Phospho (STY)]PSPTPEAK.2	-0.047757011	Q6PDG5_S302_M2_Smarcc2
TLTDEVNS[Phospho (STY)]PDSDRR.2	-0.222959037	Q6PDG5_S283_M1_Smarcc2
RS[Phospho (STY)]PS[Phospho (STY)]PSPTPEAK.2	-0.304309972	Q6PDG5_S302_M2_Smarcc2
KRS[Phospho (STY)]PS[Phospho (STY)]PSPTPEAK.2	-0.188224709	Q6PDG5_S302_M2_Smarcc2
DM[Oxidation (M)]DEPS[Phospho (STY)]PVPNVEEVTLPK.2	-0.445093828	Q6PDG5_S347_M1_Smarcc2
TLTDEVNS[Phospho (STY)]PDS[Phospho (STY)]DRRDK.3	-0.153144129	Q6PDG5_S283_M2_Smarcc2
YYEAADTVTQFDNVR.2	0.119930948	#N/A
S[Phospho (STY)]YEDLTELEDR.2	-0.02989893	Q6PDG8_S56_M1_Mon1a
S[Phospho (STY)]YEDLTELEDREASGDSPK.3	0.269604465	Q6PDG8_S56_M1_Mon1a
TLQPPES[Phospho (STY)]PR.2	-0.257501605	Q6PDH0_S445_M1_Phldb1
KLS[Phospho (STY)]SGDLR.2	-0.078683657	Q6PDH0_S565_M1_Phldb1
SS[Phospho (STY)]PPPLPAK.2	0.162838132	Q6PDH0_S951_M1_Phldb1
SQPASIPGS[Phospho (STY)]PK.2	-0.090044701	Q6PDH0_S382_M1_Phldb1
SPS[Phospho (STY)]PTLGESLAPR.2	-0.11828823	Q6PDH0_S522_M1_Phldb1
T[Phospho (STY)]RSPS[Phospho (STY)]PTLGESLAPR.2	0.258280352	Q6PDH0_S522_M2_Phldb1
KNS[Phospho (STY)]ITEISDNEDELLEYHRR.4	-0.244508283	Q6PDH0_S580_M1_Phldb1
KNS[Phospho (STY)]ITEIS[Phospho (STY)]DNEDELLEYHR.3	-0.28663325	Q6PDH0_S580_M2_Phldb1
NSITEIS[Phospho (STY)]DNEDELLEYHR.3	-0.135837808	Q6PDH0_S585_M1_Phldb1
VTAS[Phospho (STY)]PELAEAAAGR.2	-0.120740849	Q6PDI6_S99_M1_Mindy2
VS[Phospho (STY)]PPEGR.2	-0.179317329	Q6PDI6_S21_M1_Mindy2
RGS[Phospho (STY)]LPDQK.2	-0.313325471	Q6PDJ6_S488_M1_Fbxo42
ASQVEPQS[Phospho (STY)]PGLGLR.2	0.235956179	Q6PDK2_S2299_M1_Kmt2d
LC[Carbamidomethyl (C)]PQPEEPYLS[Phospho (STY)]PQPEEPR.2	0.052573615	Q6PDK2_S727_M1_Kmt2d
GGPASVPSAS[Phospho (STY)]PGTSVK.2	0.275973011	Q6PDL0_S407_M1_Dync1li2
TGSPGS[Phospho (STY)]PSAGGVQSTAK.2	-0.14962958	Q6PDL0_S446_M1_Dync1li2
EFQDYIEPEEGC[Carbamidomethyl (C)]QGS[Phospho (STY)]PQRR.3	-0.320765816	Q6PDL0_S194_M1_Dync1li2
EFQDYIEPEEGC[Carbamidomethyl (C)]QGS[Phospho (STY)]PQR.2	-0.109316001	Q6PDL0_S194_M1_Dync1li2
FAVSAESLR.2	0.214223422	#N/A
KPDSM[Oxidation (M)]VTNSS[Phospho (STY)]TENEA.2	-0.006855929	Q6PDL0_S487_M1_Dync1li2
KTGSPGS[Phospho (STY)]PSAGGVQSTAK.2	-0.322747236	Q6PDL0_S446_M1_Dync1li2
TSES[Phospho (STY)]PARGPSGSPR.2	-0.053512888	Q6PDL0_S383_M1_Dync1li2
VKVDGPRS[Phospho (STY)]PS[Phospho (STY)]YGR.3	-0.151064437	Q6PDM2_S199_M2_Srsf1
YS[Phospho (STY)]PRHS[Phospho (STY)]R.2	0.119057104	Q6PDM2_S238_M2_Srsf1
DGTGVVEFVR.2	-0.455120368	#N/A
VDGPRS[Phospho (STY)]PSYGRS[Phospho (STY)]R.2	-0.207357694	Q6PDM2_S199_M2_Srsf1
VDGPRS[Phospho (STY)]PSYGR.2	-0.13729676	Q6PDM2_S199_M1_Srsf1
GS[Phospho (STY)]PRYS[Phospho (STY)]PR.2	-0.08936987	Q6PDM2_S234_M2_Srsf1
SRGS[Phospho (STY)]PRYS[Phospho (STY)]PR.2	-0.028475794	Q6PDM2_S234_M2_Srsf1

KSS[Phospho (STY)]TGSPTSPINAEK.2	-0.03135157	Q6PDN3_S1795_M1_Mylk
MSQPGS[Phospho (STY)]PSPK.2	0.118816166	Q6PDQ2_S1528_M1_Chd4
KMS[Phospho (STY)]QPGS[Phospho (STY)]PSPK.2	0.192694705	Q6PDQ2_S1524_M2_Chd4
EM[Oxidation (M)]LHS[Phospho (STY)]PLPC[Carbamidomethyl (C)]TGGK.2	-0.011367221	Q6PDS3_S548_M1_Sarm1
S[Phospho (STY)]LTEVC[Carbamidomethyl (C)]LR.2	-0.073824486	Q6PE13_S865_M1_Prrt3
SSESHSAS[Phospho (STY)]SDTIEL.2	-0.472204695	Q6PE13_S965_M1_Prrt3
RGS[Phospho (STY)]QPDAELDGAGTSLLR.3	0.18450341	Q6PE13_S845_M1_Prrt3
C[Carbamidomethyl (C)]RS[Phospho (STY)]LTEVC[Carbamidomethyl (C)]LR.2	-0.060933341	Q6PE13_S865_M1_Prrt3
GS[Phospho (STY)]QPDAELDGAGTSLLR.2	-0.285737889	Q6PE13_S845_M1_Prrt3
GLASS[Phospho (STY)]PTGGALRPR.2	-0.191569398	Q6PE13_S833_M1_Prrt3
TSLPQHVM[Oxidation (M)]EPPVGAAAAGTSGS[Phospho (STY)]SLDSFSK.3	-0.154532077	Q6PE13_S894_M1_Prrt3
GSVGPAPSLSELDLRPPS[Phospho (STY)]PINLSR.3	-0.095983792	Q6PE13_S799_M1_Prrt3
S[Phospho (STY)]IDAALFR.2	-0.303667194	Q6PE13_S806_M1_Prrt3
EVAPLTNFSDS[Phospho (STY)]DDTVVAK.2	-0.078655832	Q6PE65_S88_M1_Gpatch2l
DNLIVPILEEEVQNSVSGESEA.2	-0.496835034	#N/A
HTLDGAAC[Carbamidomethyl (C)]LLNSNK.3	-0.474238927	#N/A
S[Phospho (STY)]QEIEELER.2	-0.24192538	Q6PEE2_S18_M1_Ctif
VNPHPEFQQFPQS[Phospho (STY)]P.2	-0.273379209	Q6PEM8_S458_M1_Slc46a1
LSNVAPPC[Carbamidomethyl (C)]ILR.2	-0.038944826	#N/A
LEHEYIHNFK.2	0.077887221	#N/A
FFDANYDGKDYNPLLAR.3	0.042783346	#N/A
QGQDVAPPPNPGDQIFNK.2	0.034718049	#N/A
FFDANYDGK.2	0.185537806	#N/A
KLIGTAVPQRT[Phospho (STY)]SPTGPK.3	0.063721663	Q6PER3_T161_M1_Mapre3
LTVDGLEK.2	0.211508639	#N/A
DYNPLLAR.2	0.242386099	#N/A
[Acetyl (Protein N-term)]AVNVYSTSVTSENLSR.2	-0.099630409	#N/A
LIGTAVPQRTS[Phospho (STY)]PTGPK.2	-0.403462814	Q6PER3_S162_M1_Mapre3
HSSS[Phospho (STY)]APPPPPPGR.2	0.199422348	Q6PEV3_S175_M1_Wipf2
DGDESS[Phospho (STY)]PILTSFELVK.2	-0.319775522	Q6PF93_S244_M1_Pik3c3
SHS[Phospho (STY)]LEAPGKR.2	-0.426710382	Q6PFD5_S185_M1_Dlgap3
S[Phospho (STY)]LDSVDR.2	-0.188770637	Q6PFD5_S930_M1_Dlgap3
SHS[Phospho (STY)]LEAPGK.2	-0.356602956	Q6PFD5_S185_M1_Dlgap3
S[Phospho (STY)]LPDS[Phospho (STY)]GRTSPC[Carbamidomethyl (C)]PR.2	-0.11248227	Q6PFD5_S779_M2_Dlgap3
LFAKS[Phospho (STY)]HS[Phospho (STY)]LEAPGKR.3	-0.000500475	Q6PFD5_S183_M2_Dlgap3
RDS[Phospho (STY)]WMER.2	0.034904086	Q6PFD5_S771_M1_Dlgap3
ERS[Phospho (STY)]LDSVDR.2	-0.440129154	Q6PFD5_S930_M1_Dlgap3
HAS[Phospho (STY)]EPQPGPR.2	0.027907769	Q6PFD5_S712_M1_Dlgap3
WRPSIGVQVETIS[Phospho (STY)]DSDTENR.3	-0.112159868	Q6PFD5_S641_M1_Dlgap3
S[Phospho (STY)]NSVTAGVQADLELEGLAGLATVATEDK.3	-0.017742542	Q6PFD5_S672_M1_Dlgap3
HSSATES[Phospho (STY)]ADSIEIYIPEAQTR.3	-0.365150645	Q6PFD5_S962_M1_Dlgap3
AMGDEESGDS[Phospho (STY)]DGS[Phospho (STY)]PKTS[Phospho (STY)]PK.2	0.303200218	Q6PFD5_S407_M3_Dlgap3
AIQAGC[Carbamidomethyl (C)]S[Phospho (STY)]QDDDC[Carbamidomethyl (C)]LPLLAAPASVSGR.3	-0.282529397	Q6PFD5_S510_M1_Dlgap3
RSSS[Phospho (STY)]VDTAR.2	0.215390701	Q6PFD5_S429_M1_Dlgap3
S[Phospho (STY)]LPDSGR.2	-0.107151637	Q6PFD5_S779_M1_Dlgap3
AMGDEESGDSDGS[Phospho (STY)]PK.2	-0.039978313	Q6PFD5_S410_M1_Dlgap3
EAEDYELPEEILEK.2	0.303646773	#N/A

EGYPLPYEPPATDGS[Phospho (STY)]PGPTPVPAPGPGSGR.3	0.042893832	Q6PFD5_S753_M1_Dlgap3
C[Carbamidomethyl (C)]S[Phospho (STY)]SADGLDGPTMGAR.2	0.199554169	Q6PFD5_S578_M1_Dlgap3
DSWMERGS[Phospho (STY)]RS[Phospho (STY)]LPDSGR.3	0.085979342	Q6PFD5_S777_M2_Dlgap3
YGLQDS[Phospho (STY)]DEEEEEHPPK.2	0.112883569	Q6PFD9_S888_M1_Nup98
GETVQEIC[Carbamidomethyl (C)]S[Phospho (STY)]PR.2	0.172561498	Q6PFD9_S1027_M1_Nup98
[Acetyl (Protein N-term)]S[Phospho (STY)]DVEENNFEGR.2	-0.307959968	Q6PFR5_S2_M1_Tra2a
S[Phospho (STY)]RS[Phospho (STY)]YT[Phospho (STY)]PEYR.2	-0.11767304	Q6PFR5_S82_M3_Tra2a
RRS[Phospho (STY)]PS[Phospho (STY)]PYYSR.2	-0.247575622	Q6PFR5_S259_M2_Tra2a
S[Phospho (STY)]PSPYYSR.2	-0.251540223	Q6PFR5_S259_M1_Tra2a
RS[Phospho (STY)]PS[Phospho (STY)]PYYSR.2	-0.138372462	Q6PFR5_S259_M2_Tra2a
[Acetyl (Protein N-term)]S[Phospho (STY)]DVEENNFEGRES[Phospho (STY)]R.2	0.082029795	Q6PFR5_S14_M2_Tra2a
S[Phospho (STY)]RS[Phospho (STY)]HS[Phospho (STY)]PMSNR.2	0.391437375	Q6PFR5_S94_M3_Tra2a
S[Phospho (STY)]HS[Phospho (STY)]PMSNR.2	-0.337470051	Q6PFR5_S96_M2_Tra2a
AHT[Phospho (STY)]PTPGIYMGR.2	0.046951324	Q6PFR5_T200_M1_Tra2a
RPPAKPR.2	0.546107677	#N/A
KYGGITSFENT[Phospho (STY)]AIEVDR.3	-0.229397024	Q6PGE7_T620_M1_Slc6a7
NEGLFENNK.2	0.165064669	#N/A
DLS[Phospho (STY)]PEGPAPLTEEELDLR.2	0.012145837	Q6PGG2_S75_M1_Gmip
S[Phospho (STY)]QGSPEDPPSQASPGSNK.2	-0.163162756	Q6PGG2_S235_M1_Gmip
VS[Phospho (STY)]PEVGSADVASIAQK.2	-0.193425594	Q6PGL7_S747_M1_Washc2
VPLLFS[Phospho (STY)]DEEDSEVPSGVKPEDLK.3	0.165253903	Q6PGL7_S723_M1_Washc2
VDNARVS[Phospho (STY)]PEVGSADVASIAQK.3	-0.415784574	Q6PGL7_S747_M1_Washc2
LAAQESS[Phospho (STY)]EAEDVTVDR.2	-0.242225607	Q6PGL7_S1049_M1_Washc2
EGLLPASDQEAGGPSDIFSSSS[Phospho (STY)]PLDK.3	0.267914243	Q6PGL7_S783_M1_Washc2
GLFS[Phospho (STY)]DEEDSEDLFSSQSSSKPK.3	0.130902758	Q6PGL7_S533_M1_Washc2
TSALLFSS[Phospho (STY)]DEEDQWNIADSHTK.3	0.153404966	Q6PGL7_S614_M1_Washc2
VASIFDDPLNAFGS[Phospho (STY)]Q.2	-0.232351666	Q6PGL7_S1333_M1_Washc2
GLFS[Phospho (STY)]DEEDSEDLFSSQSSSK.2	-0.158589436	Q6PGL7_S533_M1_Washc2
AGNS[Phospho (STY)]DS[Phospho (STY)]EEDDANER.2	-0.388952724	Q6PGL7_S157_M2_Washc2
AGNS[Phospho (STY)]DS[Phospho (STY)]EEDDANERVDLILEPK.3	-0.35950094	Q6PGL7_S157_M2_Washc2
SSSSS[Phospho (STY)]PTSPGSFR.2	-0.383485597	Q6PGN3_S340_M1_Dclk2
C[Carbamidomethyl (C)]LS[Phospho (STY)]PEGVNGNR.2	-0.225280954	Q6PGN3_S374_M1_Dclk2
SSS[Phospho (STY)]NVNGGPELDR.2	-0.332398761	Q6PGN3_S361_M1_Dclk2
S[Phospho (STY)]IELEHFEER.2	-0.381155358	Q6PGN3_S6_M1_Dclk2
YSGSRS[Phospho (STY)]PGFSR.2	-0.598002055	Q6PGN3_S307_M1_Dclk2
TISNLEGS[Phospho (STY)]PSR.2	-0.001821904	Q6PH08_S14_M1_Erc2
S[Phospho (STY)]QLQPEPAK.2	-0.126316611	Q6PH08_S364_M1_Erc2
AAILQTEVDALR.2	-0.067655224	#N/A
DLNHLLQQESGNR.3	-0.491093615	#N/A
ESELLALQTK.2	-0.127232755	#N/A
SQHSNHRPS[Phospho (STY)]PDQDDEEGIWA.3	-0.012785898	Q6PH08_S946_M1_Erc2
IAELESLTLR.2	0.17087755	#N/A
TISNLEGS[Phospho (STY)]PSRS[Phospho (STY)]PR.2	-0.387107993	Q6PH08_S14_M2_Erc2
DYDHLFK.2	-0.175790367	#N/A
QQQQQQNDVVK.2	0.140462456	#N/A
FAGQM[Oxidation (M)]GIQLFETSAK.2	-0.036653331	#N/A
WLHEINQNC[Carbamidomethyl (C)]DDVC[Carbamidomethyl (C)]R.3	0.350514096	#N/A

SGYHDDS[Phospho (STY)]DEDLLE.2	0.172448882	Q6PHU5_S819_M1_Sort1
FTDEYQLFEELGK.2	-0.326963735	#N/A
FYFENALSK.2	0.188249571	#N/A
IPTGQEYAAK.2	0.106018563	#N/A
ESTESSNTT[Phospho (STY)]IEDEDVK.2	0.127299128	Q6PHZ2_T337_M1_Camk2d
HPWIC[Carbamidomethyl (C)]QR.2	0.495524181	#N/A
IC[Carbamidomethyl (C)]DPGLTAFEPEALGNLVEGM[Oxidation (M)]DFHR.3	0.357679309	#N/A
VTEQLIEAINNGDFEAYTK.2	-0.112307717	#N/A
ITASEALK.2	0.084632922	#N/A
ASGDPGAGGAS[Phospho (STY)]PR.2	-0.100931215	Q6PHZ5_S107_M1_Rbm15b
TTHS[Phospho (STY)]PYEER.2	-0.07141754	Q6PHZ5_S606_M1_Rbm15b
QRS[Phospho (STY)]LS[Phospho (STY)]PVAAPPLREPR.3	0.340660045	Q6PHZ5_S261_M2_Rbm15b
RNS[Phospho (STY)]LEGYSR.2	-0.09609207	Q6PHZ5_S559_M1_Rbm15b
TSS[Phospho (STY)]PAIQNS[Phospho (STY)]VEDELEM[Oxidation (M)]ATVR.2	-0.186246969	Q6PHZ8_S50_M2_Kcnip4
YQLSIHETEDPNDNR.3	-0.00351879	#N/A
C[Carbamidomethyl (C)]IELSSGS[Phospho (STY)]VK.2	-0.379995639	Q6PIC6_S456_M1_Atp1a3
TDKLVNER.2	-0.005643003	#N/A
GGQDNIPVLK.2	0.047324571	#N/A
IATLASGLEVGK.2	0.144949617	#N/A
GGQDNIPVLKR.2	-0.148953823	#N/A
IISAHGC[Carbamidomethyl (C)]K.2	0.119393819	#N/A
VLGFC[Carbamidomethyl (C)]HYYLPEEQFPK.3	-0.175701739	#N/A
GVVVATGDR.2	0.07581984	#N/A
AC[Carbamidomethyl (C)]VIHGTDLK.2	0.120302606	#N/A
C[Carbamidomethyl (C)]ATILLQGK.2	0.078976185	#N/A
RDLDDLKK.2	0.008363278	#N/A
RNPGGWVEK.2	0.192775848	#N/A
EAFQNAYLELGGLGER.2	-0.100042089	#N/A
AQEILAR.2	-0.080150239	#N/A
NITFFSTNC[Carbamidomethyl (C)]VEGTAR.2	-0.211414393	#N/A
NPGGWVEKETYY.2	-0.33756484	#N/A
EQPLDEEM[Oxidation (M)]KEAFQNAYLELGGLGER.3	-0.449309704	#N/A
NPGGWVEK.2	0.22480342	#N/A
M[Oxidation (M)]QVNAEEVVVGDLVEIK.2	-0.424215676	#N/A
KYNTDC[Carbamidomethyl (C)]VQGLTHSK.3	0.029762648	#N/A
DFTSEQIDEILQNHTEIVFAR.3	-0.049271999	#N/A
S[Phospho (STY)]PDC[Carbamidomethyl (C)]THDNPLETR.2	-0.065470158	Q6PIC6_S218_M1_Atp1a3
MSVEEVC[Carbamidomethyl (C)]R.2	0.229553779	#N/A
EVAMTEHK.2	0.396406572	#N/A
GFAFDC[Carbamidomethyl (C)]DDVNFTTDNLC[Carbamidomethyl (C)]FVGLM[Oxidation (M)]SM[Oxidation (M)]IDPPR.3	-0.102861841	#N/A
SSHTWVALSHIAGLC[Carbamidomethyl (C)]NR.3	-0.384513406	#N/A
DVAGDASESALLK.2	0.224865614	#N/A
ADIGVAM[Oxidation (M)]GIAGSDVSK.2	-0.084762472	#N/A
KADIGVAM[Oxidation (M)]GIAGSDVSK.2	-0.041341561	#N/A
MSVEEVC[Carbamidomethyl (C)]RK.2	0.180778332	#N/A
TVNDLEDSYGQQWTYEQR.2	-0.442806091	#N/A
YNTDC[Carbamidomethyl (C)]VQGLTHSK.2	-0.063914499	#N/A

M[Oxidation (M)]TVAHM[Oxidation (M)]WFDNQIHEADTTEDQSGTSFDK.4	-0.586464312	#N/A
VDNSSLTGESEPQTRS[Phospho (STY)]PDC[Carbamidomethyl (C)]THDNPLETR.4	0.438557764	Q6PIC6_S218_M1_Atp1a3
AC[Carbamidomethyl (C)]VIHGTDLKDFTSEQIDEILQNHTEIVFAR.4	0.031799994	#N/A
VDNSS[Phospho (STY)]LTGESEPQTR.2	-0.177355486	Q6PIE5_S215_M1_Atp1a2
NM[Oxidation (M)]VPQQALVIR.2	-0.052465571	#N/A
LIIVEGC[Carbamidomethyl (C)]QR.2	0.046237183	#N/A
LIFDNLKK.2	-0.116275871	#N/A
AAVPDAVGK.2	-0.046536311	#N/A
C[Carbamidomethyl (C)]IELSC[Carbamidomethyl (C)]GS[Phospho (STY)]VR.2	-0.202161264	Q6PIE5_S464_M1_Atp1a2
TGTLTQNR.2	-0.099950638	#N/A
IAGLC[Carbamidomethyl (C)]NR.2	0.161621288	#N/A
DHTEIVFAR.2	0.172434228	#N/A
LSLDELGR.2	0.020914155	#N/A
FDTDELNFPTEK.2	-0.016364721	#N/A
VLGFC[Carbamidomethyl (C)]QLNLPSGK.2	0.03484055	#N/A
KYQVDLSK.2	0.085208007	#N/A
YQLSIHER.2	0.125650166	#N/A
TTNDLEDSYGQEWTYEQR.2	0.013876227	#N/A
NLEAVETLGSTSTIC[Carbamidomethyl (C)]SDKTGTLTQNR.3	-0.491315582	#N/A
LIFDNLK.2	0.302814488	#N/A
YPGGWVEK.2	-0.109377265	#N/A
ELDELKK.2	0.051948034	#N/A
C[Carbamidomethyl (C)]STILVQGK.2	0.302685248	#N/A
VIM[Oxidation (M)]VTGDHPITAK.2	-0.06992876	#N/A
NLEAVETLGSTSTIC[Carbamidomethyl (C)]SDK.2	-0.29059235	#N/A
AC[Carbamidomethyl (C)]VVHGSDLK.2	0.065274808	#N/A
DTAGDASESALLK.2	-0.339666163	#N/A
LC[Carbamidomethyl (C)]FVGLM[Oxidation (M)]SM[Oxidation (M)]IDPPR.2	-0.132559148	#N/A
AGQENISVSK.2	0.127091063	#N/A
NSVFQQGM[Oxidation (M)]K.2	0.31600664	#N/A
GFKFDTDELNFPTEK.3	0.109067176	#N/A
QGAIVAVTGDGVNDSPALK.2	0.035532025	#N/A
DM[Oxidation (M)]TSEQLDEILR.2	0.136410893	#N/A
QAADM[Oxidation (M)]ILLDDNFASIVTGVEEGR.3	-0.202689833	#N/A
DGPNALTPPPTTPEWVK.2	-0.124958088	#N/A
AIAKGVGIISEGNETVEDIAAR.3	-0.450235467	#N/A
QGAIVAVTGDGVNDSPALKK.2	0.177826992	#N/A
NSQTDKLVNER.2	-0.39578934	#N/A
IISSHGC[Carbamidomethyl (C)]K.2	0.029267772	#N/A
EIPLDKEM[Oxidation (M)]QDAFQNAYM[Oxidation (M)]ELGGLGER.3	-0.324811405	#N/A
YQVDLSK.2	0.003466364	#N/A
M[Oxidation (M)]QINAEEVVVGDLVEVK.2	-0.341137901	#N/A
LNIPVSQVNPR.2	-0.093977731	#N/A
ADIGIAM[Oxidation (M)]GISGSDVSK.2	-0.111824198	#N/A
VAEIPFNSTNK.2	-0.218170986	#N/A
SPEFTHENPLETR.2	-0.091092174	#N/A
AGQENIS[Phospho (STY)]VSKR.2	0.069907792	Q6PIE5_S439_M1_Atp1a2

GVGIISEGNETVEDIAAR.2	-0.465127819	#N/A
SPTWTALSR.2	0.028195871	#N/A
C[Carbamidomethyl (C)]IELSC[Carbamidomethyl (C)]GS[Phospho (STY)]VRK.2	-0.046806381	Q6PIE5_S464_M1_Atp1a2
EDSPQSHVLVM[Oxidation (M)]K.2	0.357994279	#N/A
NIC[Carbamidomethyl (C)]FFSTNC[Carbamidomethyl (C)]VEGTAR.2	0.187294684	#N/A
YQVDLS[Phospho (STY)]KGLTNQR.3	-0.065501473	Q6PIE5_S58_M1_Atp1a2
M[Oxidation (M)]TVAHM[Oxidation (M)]WFDNQIHEADTTEDQSGATFDKR.4	-0.647709304	#N/A
SPS[Phospho (STY)]PAVPLR.2	-0.560691502	Q6PIJ4_S228_M1_Nfrkb
QTS[Phospho (STY)]ITQTIR.2	-0.094084745	Q6PIX5_S76_M1_Rhbdf1
DSS[Phospho (STY)]LKLDVR.2	-0.147854192	Q6PNC0_S1904_M1_Dmxl1
TGS[Phospho (STY)]ESS[Phospho (STY)]LKK.2	0.185343525	Q6PNC0_S1426_M2_Dmxl1
WDS[Phospho (STY)]DNDEENEDPPISMK.2	0.2861064	Q6PNC0_S1965_M1_Dmxl1
ISEASWLPEEHY[Phospho (STY)]SSSPEK.2	-0.031370404	Q6PNC0_Y913_M1_Dmxl1
GSSFLTSKDS[Phospho (STY)]S[Phospho (STY)]LKLDVR.3	0.084646702	Q6PNC0_S1903_M2_Dmxl1
TQDGVALEIQPLNS[Phospho (STY)]QEGLDSEDKEK.3	-0.335646637	Q6Q477_S329_M1_Atp2b4
GIIDSTAGEQR.2	0.139724868	#N/A
EAGHGS[Phospho (STY)]DKEDISR.3	-0.147004149	Q6Q477_S1065_M1_Atp2b4
TQDGVALEIQPLNS[Phospho (STY)]QEGLDSEDKEKK.3	-0.245860346	Q6Q477_S329_M1_Atp2b4
DDFLC[Carbamidomethyl (C)]LEGK.2	-0.422906714	#N/A
NEKGEVEQEKLDK.3	-0.343024848	#N/A
NDS[Phospho (STY)]GEENVPLDLTR.2	0.190945194	Q6QI06_S21_M1_Rictor
GRNDS[Phospho (STY)]GEENVPLDLTR.3	0.329507392	Q6QI06_S21_M1_Rictor
[Acetyl (Protein N-term)]S[Phospho (STY)]EVLPYGDEK.2	0.070686515	Q6QWF9_S2_M1_Camk2n1
LSPYGDGGDVGQIFSC[Carbamidomethyl (C)]R.2	0.222293656	#N/A
LQDTNNFFGAGQSK.2	-0.066048308	#N/A
ISTAS[Phospho (STY)]GDGR.2	-0.161323649	Q6R0H7_S1091_M1_Gnas
ALWEDEGVR.2	-0.054269115	#N/A
VLTSGIFETK.2	-0.489345709	#N/A
EAAEM[Oxidation (M)]EGS[Phospho (STY)]PTTATAVEGK.2	-0.305955323	Q6R0H7_S367_M1_Gnas
AQLEQSVEENKER.2	0.220594791	#N/A
ERPGEQSEVAQLIQQTLEQER.3	-0.420814016	#N/A
ASS[Phospho (STY)]LNENVDHSALLK.2	-0.228056701	Q6R891_S100_M1_Ppp1r9b
TVTEGGAAHR.2	-0.284840525	#N/A
ETQAQYQALER.2	0.116991532	#N/A
KDS[Phospho (STY)]QNSSQHSVSSHR.3	-0.457545243	Q6RHR9_S733_M1_Magi1
SLHTASPSHGIQVLPEY[Phospho (STY)]LPADAPAPDQTDSSGQK.4	0.196420385	Q6RHR9_Y762_M1_Magi1
S[Phospho (STY)]PPRPSPGSLHYS[Phospho (STY)]DEDVTK.3	0.379342031	Q6V4S5_S2018_M2_Sdk2
SQDSYPGSPSLS[Phospho (STY)]PR.2	-0.207682085	Q6VN19_S369_M1_Ranbp10
LKISS[Phospho (STY)]PVR.2	-0.178375018	Q6VNB8_S3483_M1_Wdfy3
SQS[Phospho (STY)]EYC[Carbamidomethyl (C)]NVGTK.2	0.125346114	Q6VNB8_S1942_M1_Wdfy3
VFLAEC[Carbamidomethyl (C)]YNLS[Phospho (STY)]PTKDK.2	0.02142669	Q6VNS1_S561_M1_Ntrk3
VFLAEC[Carbamidomethyl (C)]YNLS[Phospho (STY)]PTK.2	-0.418967391	Q6VNS1_S561_M1_Ntrk3
GSS[Phospho (STY)]YGSLM[Oxidation (M)]TAHGK.2	-0.359733417	Q6VVW5_S523_M1_Npr2
TPPAANQAS[Phospho (STY)]DPEEK.2	-0.066499232	Q6W8Q3_S16_M1_Pcp4l1
[Acetyl (Protein N-term)]SELNTKT[Phospho (STY)]PPAANQASDPEEK.2	0.158856255	Q6W8Q3_T8_M1_Pcp4l1
TPPAANQAS[Phospho (STY)]DPEEKGKPGSIK.3	0.108897748	Q6W8Q3_S16_M1_Pcp4l1
KAEEEEEIDIDLTAPETEK.3	-0.142458873	#N/A

TSTPTGHGAS[Phospho (STY)]PTKQDDLVISAR.3	-0.012974891	Q6WQJ1_S1032_M1_Dagla
GS[Phospho (STY)]PSLHAVLER.2	0.455491309	Q6WQJ1_S808_M1_Dagla
HSQDTQPLEAALGSGGVTPERPPS[Phospho (STY)]AT[Phospho (STY)]IEEEEAAGGSEGGGVAPR.4	-0.10197168	Q6WQJ1_S874_M2_Dagla
DLQLVLPDYFPER.2	0.111191443	#N/A
EAEYFELPELVR.2	0.129882948	#N/A
YILDYLR.2	0.118494061	#N/A
S[Phospho (STY)]PSGGAAGPLLTPSQSLDGSR.2	-0.112502553	Q6WVG3_S187_M1_Kctd12
SPSGGAAGPLLT[Phospho (STY)]PS[Phospho (STY)]QSLDGSRR.3	-0.209480278	Q6WVG3_S200_M2_Kctd12
MFTQQQPQELAR.2	0.005643408	#N/A
EADEQLVAEVIEKC[Carbamidomethyl (C)]S[Phospho (STY)]PDTC[Carbamidomethyl (C)]SR.3	-0.154089157	Q6Y685_S111_M1_Tacc1
KYEAQPLDLDAC[Carbamidomethyl (C)]S[Phospho (STY)]QDEGAVISK.3	0.291662537	Q6Y685_S480_M1_Tacc1
ESC[Carbamidomethyl (C)]DSS[Phospho (STY)]PGLAEPEAKPQESR.3	-0.591859615	Q6Y685_S82_M1_Tacc1
SQS[Phospho (STY)]WEER.2	-0.105573361	Q6Y7W8_S161_M1_Gigyf2
WRPHS[Phospho (STY)]PDGPR.3	-0.084076066	Q6Y7W8_S237_M1_Gigyf2
S[Phospho (STY)]FDEVEGVFGR.2	0.260187703	Q6Y7W8_S140_M1_Gigyf2
SQS[Phospho (STY)]WEERGDR.2	-0.09159804	Q6Y7W8_S161_M1_Gigyf2
ALSSGGSITS[Phospho (STY)]PPLSPALPK.2	-0.064822535	Q6Y7W8_S26_M1_Gigyf2
QQQQQQQQQQHQQSNR.3	0.36338517	#N/A
GS[Phospho (STY)]GEGQGQILQR.2	-0.209405027	Q6ZPE2_S20_M1_Sbf1
DKGPS[Phospho (STY)]FR.2	-0.311190232	Q6ZPE2_S1047_M1_Sbf1
ALYLEPSDGVS[Phospho (STY)]PTQETGEAQSQDDER.3	-0.035338991	Q6ZPE2_S706_M1_Sbf1
C[Carbamidomethyl (C)]SGPHINS[Phospho (STY)]AR.2	0.135573415	Q6ZPE2_S559_M1_Sbf1
TSSS[Phospho (STY)]SEWPSEPSK.2	-0.086532314	Q6ZPF3_S1524_M1_Tiam2
QGS[Phospho (STY)]PTKDIEIQFQR.2	0.109914081	Q6ZPF3_S1604_M1_Tiam2
HLS[Phospho (STY)]DADR.2	-0.222059132	Q6ZPF3_S1116_M1_Tiam2
ESDILSDEDEDFHHPLK.3	0.255327938	#N/A
HQS[Phospho (STY)]LDSHPETASIDLNLVLER.3	-0.075321081	Q6ZPF3_S1672_M1_Tiam2
TAPSS[Phospho (STY)]PLT[Phospho (STY)]SPSDTR.2	-0.198845713	Q6ZPJ0_S265_M2_Tex2
SLS[Phospho (STY)]TEVEPK.2	-0.623634649	Q6ZPJ0_S195_M1_Tex2
KEEKPEVQS[Phospho (STY)]PVK.2	-0.068631169	Q6ZPJ3_S893_M1_Ube2o
LIHGEDS[Phospho (STY)]DS[Phospho (STY)]EGDDDGR.2	0.007157185	Q6ZPJ3_S82_M2_Ube2o
DSSSLEPMAAAELS[Phospho (STY)]DSGR.2	-0.100342454	Q6ZPJ3_S1175_M1_Ube2o
DHS[Phospho (STY)]M[Oxidation (M)]EDPDKKGEAR.2	0.246732538	Q6ZPJ3_S406_M1_Ube2o
S[Phospho (STY)]IPLSIK.2	-0.166583629	Q6ZPJ3_S510_M1_Ube2o
NM[Oxidation (M)]TVEQLLTGSPTS[Phospho (STY)]PTVEPEKPTR.3	-0.351402453	Q6ZPJ3_S836_M1_Ube2o
NC[Carbamidomethyl (C)]AQGEGS[Phospho (STY)]M[Oxidation (M)]AKK.2	0.110615101	Q6ZPJ3_S376_M1_Ube2o
LKSGS[Phospho (STY)]DEEER.2	0.123243031	Q6ZPK7_S394_M1_Zfyve28
TVQLTDLGVS[Phospho (STY)]DLEEDS.2	0.253786044	Q6ZPL9_S594_M1_Ddx55
KQWS[Phospho (STY)]TSSK.2	0.006265469	Q6ZPQ6_S302_M1_Pitpnm2
GAS[Phospho (STY)]PSRHS[Phospho (STY)]ISEWR.3	-0.132049841	Q6ZPQ6_S316_M2_Pitpnm2
HS[Phospho (STY)]ISEWR.2	-0.054807006	Q6ZPQ6_S321_M1_Pitpnm2
TDTGENNNVFNLS[Phospho (STY)]PVEK.2	-0.106358126	Q6ZPQ6_S129_M1_Pitpnm2
WNS[Phospho (STY)]NDLMDK.2	0.213602832	Q6ZPQ6_S365_M1_Pitpnm2
RGAS[Phospho (STY)]PSRHS[Phospho (STY)]ISEWR.3	0.160027781	Q6ZPQ6_S316_M2_Pitpnm2
S[Phospho (STY)]NIDIPR.2	0.299427926	Q6ZPQ6_S630_M1_Pitpnm2
QWSTSSKSS[Phospho (STY)]R.2	0.04279976	Q6ZPQ6_S308_M1_Pitpnm2
GGGSVGS[Phospho (STY)]DVGQR.2	-0.277425462	Q6ZPR4_S47_M1_Kcnt1

GVAPADS[Phospho (STY)]PDAPR.2	-0.266061764	Q6ZPS6_S738_M1_Ankib1
GVAPADS[Phospho (STY)]PDAPRR.2	-0.506234343	Q6ZPS6_S738_M1_Ankib1
AC[Carbamidomethyl (C)]TLTVFPPEENPGS[Phospho (STY)]PSR.2	0.154058058	Q6ZPT1_S603_M1_Klhl9
TGTGS[Phospho (STY)]PFAGNS[Phospho (STY)]PAR.2	-0.261804453	Q6ZPZ3_S1270_M2_Zc3h4
S[Phospho (STY)]PSPLQAPEK.2	-0.131515079	Q6ZQ18_S214_M1_Efr3b
SVILEAFSSPSEEVK.2	-0.100103206	#N/A
LTLIDPETLLPR.2	-0.170689236	#N/A
AVAALLTIPEAEK.2	0.173128338	#N/A
EGPAVVGQFIQDVK.2	-0.190077078	#N/A
ITSEALLVTQQLVK.2	0.114782219	#N/A
[Acetyl (Protein N-term)]ASASYHISNLLEK.2	-0.139467905	#N/A
ISGSILNELIGLVR.2	0.043688314	#N/A
DS[Phospho (STY)]SSTNLESMDTS.2	0.004802388	Q6ZQ38_S1219_M1_Cand1
NVVAEC[Carbamidomethyl (C)]LGK.2	-0.395406666	#N/A
QEFEKQDELKR.3	-0.023751443	#N/A
HTVDDGLDIRK.3	0.18845311	#N/A
DLLDSVLPHLYNETK.3	-0.077455374	#N/A
QSYYSIAK.2	-0.047850121	#N/A
HC[Carbamidomethyl (C)]EC[Carbamidomethyl (C)]AEEGTR.2	-0.095007539	#N/A
GLSAS[Phospho (STY)]LPDLDSESWIEVK.2	-0.202784715	Q6ZQ58_S525_M1_Larp1
S[Phospho (STY)]LPTTVPESPNYR.2	-0.313076407	Q6ZQ58_S743_M1_Larp1
ETESAPGS[Phospho (STY)]PR.2	-0.157944384	Q6ZQ58_S498_M1_Larp1
ESPRPPAAAEAPAGS[Phospho (STY)]DGEDGGRR.3	0.054984697	Q6ZQ58_S81_M1_Larp1
NTFTAWSEEDS[Phospho (STY)]DYEIDDRDVNK.3	-0.109188137	Q6ZQ58_S608_M1_Larp1
TASISSS[Phospho (STY)]PSEGTPAVGSYGC[Carbamidomethyl (C)]TPQSLPK.3	0.284440072	Q6ZQ58_S828_M1_Larp1
QHYQKET[Phospho (STY)]ESAPGS[Phospho (STY)]PR.2	-0.325256291	Q6ZQ58_S498_M2_Larp1
KNTFTAWS[Phospho (STY)]EEDSDY[Phospho (STY)]EIDDRDVNK.3	-0.319158807	Q6ZQ58_S604_M2_Larp1
DSQSEGTAQLDSIGFSIIR.2	-0.099023415	#N/A
NGY[Phospho (STY)]S[Phospho (STY)]C[Carbamidomethyl (C)]VPVALAEGLDIK.3	-0.024232734	Q6ZQ88_S573_M2_Kdm1a
QATPGVPAQQS[Phospho (STY)]PSM.2	-0.134511535	Q6ZQ88_S850_M1_Kdm1a
DSSIIDPGTEQDLPS[Phospho (STY)]PENSSVK.2	0.06332253	Q6ZQ93_S3443_M1_Usp34
RVS[Phospho (STY)]S[Phospho (STY)]DEEHTVDSC[Carbamidomethyl (C)]IGDIK.3	-0.412639442	Q6ZQ93_S3395_M2_Usp34
FVDKYSALLS[Phospho (STY)]PSEQEKR.3	-0.51664049	Q6ZQK5_S521_M1_Acap2
KS[Phospho (STY)]SPSTGSLDSGNESK.2	0.076357133	Q6ZQK5_S378_M1_Acap2
YSALLS[Phospho (STY)]PSEQEK.2	0.128716491	Q6ZQK5_S521_M1_Acap2
SSPSTGS[Phospho (STY)]LDSGNESK.2	-0.229900194	Q6ZQK5_S384_M1_Acap2
SSPSTGS[Phospho (STY)]LDSGNESKEK.2	-0.038039983	Q6ZQK5_S384_M1_Acap2
FVDKYSALLS[Phospho (STY)]PSEQEK.3	0.097340829	Q6ZQK5_S521_M1_Acap2
ESLPSTVS[Phospho (STY)]ANSLYEPEGER.2	-0.069971765	Q6ZQK5_S573_M1_Acap2
YSALLS[Phospho (STY)]PSEQEKR.2	-0.051943906	Q6ZQK5_S521_M1_Acap2
LIGEYGLR.2	0.081896422	#N/A
LFEGNALLR.2	-0.022367511	#N/A
IEDFLER.2	-0.288067607	#N/A
ELLTLDEKDPR.3	-0.084088022	#N/A
SISEGHPWHVPDS[Phospho (STY)]PSHSK.3	-0.111229739	Q6ZWQ0_S6448_M1_Syne2
LTSHTPGLDDEKEAS[Phospho (STY)]ENETDIEDPR.3	0.013370465	Q6ZWQ0_S6348_M1_Syne2
NPET[Phospho (STY)]PTELQFIEADLR.2	0.327096725	Q6ZWR6_T2268_M1_Syne1

YLFQTGSS[Phospho (STY)]HER.2	0.055767195	Q6ZWR6_S1360_M1_Syne1
TLPS[Phospho (STY)]EDEEGEEDKEFYLR.3	-0.124424488	Q6ZWR6_S8308_M1_Syne1
LTEGC[Carbamidomethyl (C)]SFR.2	0.436522247	#N/A
DLLHPS[Phospho (STY)]PEEEKRK.3	-0.212442641	Q6ZWU9_S11_M1_Rps27
VLTVINQTQK.2	0.171189048	#N/A
QLDDLKVELSQLR.3	-0.154882889	#N/A
ADIEVAC[Carbamidomethyl (C)]YGYEGIDAVK.2	0.110380756	#N/A
ETIEQEKR.2	0.140510743	#N/A
TET[Phospho (STY)]QEKNTLPTK.2	-0.483860174	Q6ZWY8_T23_M1_Tmsb10
NTLPTKETIEQEKR.3	-0.012462868	#N/A
[Acetyl (Protein N-term)]ADKPDM[Oxidation (M)]GEIASFDK.2	0.425374981	#N/A
[Acetyl (Protein N-term)]ADKPDMGEIAS[Phospho (STY)]FDKAK.3	-0.228717541	Q6ZWY8_S12_M1_Tmsb10
IDFSTPFQVEEVDDVSPS[Phospho (STY)]PK.2	0.113550917	Q70IV5_S780_M1_Synm
QFTQS[Phospho (STY)]PGAEEEATSFPDTK.2	-0.134920071	Q70IV5_S655_M1_Synm
S[Phospho (STY)]LGGAVGSASGGR.2	-0.242133852	Q71FD5_S75_M1_Znrf2
AYS[Phospho (STY)]GSDLPSGTGSGGGGADGAR.2	0.261710419	Q71FD5_S18_M1_Znrf2
TSS[Phospho (STY)]PEESIR.2	-0.001783184	Q71LX4_S2083_M1_Tln2
DRFGLEGDEESTM[Oxidation (M)]LEESVS[Phospho (STY)]PK.3	-0.147557574	Q71LX4_S428_M1_Tln2
VSS[Phospho (STY)]PHAR.2	-0.337549611	Q71M36_S498_M1_Cspg5
GDQDDLGVNC[Carbamidomethyl (C)]LQNNLT.2	0.066880689	#N/A
LEGGKGDQDDLGVNC[Carbamidomethyl (C)]LQNNLT.2	-0.368921452	#N/A
KFC[Carbamidomethyl (C)]DTPRVS[Phospho (STY)]S[Phospho (STY)]PHAR.3	0.125839661	Q71M36_S497_M2_Cspg5
LKEEESFNIQNSMS[Phospho (STY)]PK.2	0.34929766	Q71M36_S543_M1_Cspg5
SSS[Phospho (STY)]SDSIHSVR.2	0.084690275	Q76I79_S929_M1_Ssh1
SDS[Phospho (STY)]LPQVEELEK.2	0.21851719	Q76I79_S585_M1_Ssh1
TRAPS[Phospho (STY)]PQGR.2	-0.338672409	Q76LS9_S440_M1_Mindy1
VETAEVC[Carbamidomethyl (C)]SRPQELPQS[Phospho (STY)]PR.3	0.08813643	Q76LS9_S103_M1_Mindy1
PQELPQS[Phospho (STY)]PR.2	0.146035528	Q76LS9_S103_M1_Mindy1
LAGGDWFTSR.2	-0.004754271	#N/A
DKAVESLR.2	-0.17582162	#N/A
IGPILDNSTLQSEVKPILEK.3	-0.160064236	#N/A
VLELDNVK.2	0.159776866	#N/A
NEDVQLR.2	-0.05309734	#N/A
AVGPEITK.2	-0.012220349	#N/A
LSTIALALGVER.2	-0.522752632	#N/A
EWAHATIIPK.2	-0.157510733	#N/A
SALASVIM[Oxidation (M)]GLSPILGK.2	0.297322991	#N/A
LTQDQDVDVK.2	0.026051203	#N/A
TSAC[Carbamidomethyl (C)]GLFSVC[Carbamidomethyl (C)]YPR.2	0.083207319	#N/A
AISHEHSPSDLEAHFVPLVK.3	0.339304559	#N/A
QLSQSLLPAIVELAEDAK.2	-0.216183383	#N/A
SEIIPM[Oxidation (M)]FSNLASDEQDSVR.2	-0.347509177	#N/A
NLC[Carbamidomethyl (C)]SDDTPM[Oxidation (M)]VR.2	0.054222457	#N/A
MAGDPVANVR.2	0.651456655	#N/A
YFAQEALTVLSLA.2	-0.008653848	#N/A
LNIISNLDC[Carbamidomethyl (C)]VNEVIGIR.2	-0.012228545	#N/A
HM[Oxidation (M)]LPTVLR.2	-0.046844028	#N/A

ENVIM[Oxidation (M)]TQILPC[Carbamidomethyl (C)]IK.2	-0.200312067	#N/A
VLAM[Oxidation (M)]SGDPNYLHR.2	0.218834961	#N/A
ELVSDANQHVK.2	-0.05442616	#N/A
LNSLC[Carbamidomethyl (C)]M[Oxidation (M)]AWLVDHVYAIR.3	-0.419833315	#N/A
VKEFC[Carbamidomethyl (C)]ENLSADC[Carbamidomethyl (C)]R.2	-0.535351356	#N/A
SDGSNFANYS[Phospho (STY)]PPVNR.2	0.422645937	Q76N33_S242_M1_Stambpl1
GLS[Phospho (STY)]LDPPKEPSK.2	-0.134934048	Q78HU3_S168_M1_Mvb12a
TLS[Phospho (STY)]QDMR.2	0.036565502	Q78HU3_S161_M1_Mvb12a
YFNSYTLTGR.2	0.37336707	#N/A
AGAESDGQFQFTGIK.2	0.468907307	#N/A
VENM[Oxidation (M)]SSNQDGNDS[Phospho (STY)]DEFM[Oxidation (M)].2	-0.086198397	Q78PG9_S204_M1_Ccdc25
SSS[Phospho (STY)]LSDLTHR.2	-0.146977346	Q78T81_S252_M1_Fam102a
FYEEVHDLER.2	0.012195261	#N/A
LTDQVM[Oxidation (M)]QNPQVLAALQER.2	-0.072553001	#N/A
[Acetyl (Protein N-term)]AENSLSDGGPADSVEAAK.2	0.108806027	#N/A
FY[Phospho (STY)]EEVHDLERK.3	0.086025606	Q78ZA7_Y85_M1_Nap1l4
EFITGDVEPTDAES[Phospho (STY)]AWHSENEEEDK.3	0.022453722	Q78ZA7_S121_M1_Nap1l4
EFITGDVEPTDAESAWHS[Phospho (STY)]ENEEEDKLAGDM[Oxidation (M)]K.4	0.073880425	Q78ZA7_S125_M1_Nap1l4
LDNVSHTPSSYIETLPK.3	-0.030437082	#N/A
REFITGDVEPTDAESAWHS[Phospho (STY)]ENEEEDKLAGDM[Oxidation (M)]K.4	0.017262573	Q78ZA7_S125_M1_Nap1l4
RVS[Phospho (STY)]SGSC[Carbamidomethyl (C)]FALE.2	0.195604119	Q791T5_S381_M1_Mtch1
VLQYYQESEKPEELGSVTVQK.3	0.117937729	#N/A
TYC[Carbamidomethyl (C)]YDLR.2	-0.158515769	#N/A
EYSSSFDR.2	-0.206061199	#N/A
ATTLSNAVSSLASTGLSLTK.2	-0.09003024	#N/A
EC[Carbamidomethyl (C)]LVSSSGNDNTENGLPGSK.2	0.164937142	#N/A
IS[Phospho (STY)]ENPRRSPTHEM[Oxidation (M)]NPR.4	-0.536758561	Q7M750_S84_M1_Opalin
RS[Phospho (STY)]PTHEM[Oxidation (M)]NPR.3	0.05021852	#N/A
GS[Phospho (STY)]QEGHIYVK.2	-0.116290864	Q7M750_S100_M1_Opalin
TVSGSEEPLPDTYRPPEELER.3	0.066880204	#N/A
S[Phospho (STY)]PTHEMNPR.2	0.200685567	#N/A
LLHEDLDES[Phospho (STY)]DDDVDEK.2	0.211602891	Q7SIG6_S704_M1_Asap2
LLHEDLDES[Phospho (STY)]DDDVDEKLQPSPNRR.4	0.167404339	Q7SIG6_S704_M1_Asap2
LLHEDLDES[Phospho (STY)]DDDVDEKLQPSPNR.4	-0.017767857	Q7SIG6_S704_M1_Asap2
SGDGEST[Phospho (STY)]PVEHVR.2	-0.14707451	Q7TMB8_T1234_M1_Cyfip1
PGSSQLVHIPEETQENISTS[Phospho (STY)]PK.3	-0.275962896	Q7TME0_S549_M1_Plppr4
DASS[Phospho (STY)]LTNLKR.2	-0.284984697	Q7TME0_S386_M1_Plppr4
QQGVLQSS[Phospho (STY)]PK.2	-0.566633405	Q7TME0_S594_M1_Plppr4
DALRS[Phospho (STY)]LTDLNQDPSR.2	-0.399370423	Q7TME0_S347_M1_Plppr4
M[Oxidation (M)]SLQVM[Oxidation (M)]DTEPEGQS[Phospho (STY)]PPR.2	-0.225294898	Q7TME0_S474_M1_Plppr4
TVHTS[Phospho (STY)]PGGGLGAR.2	0.143055005	Q7TME0_S37_M1_Plppr4
S[Phospho (STY)]LTDLNQDPSR.2	-0.066668908	Q7TME0_S347_M1_Plppr4
NASIHAS[Phospho (STY)]M[Oxidation (M)]DSAR.2	-0.259149545	Q7TME0_S439_M1_Plppr4
KM[Oxidation (M)]SLQVMDTEPEGQS[Phospho (STY)]PPR.3	0.236119738	Q7TME0_S474_M1_Plppr4
DSESC[Carbamidomethyl (C)]ESLKDSFGS[Phospho (STY)]GDR.2	0.588979841	Q7TME0_S685_M1_Plppr4
SNS[Phospho (STY)]PENTR.2	-0.122884121	Q7TME0_S746_M1_Plppr4
FNVSATPEQYVPYSTTR.2	-0.210399235	#N/A

IGTLVGEDK.2	0.29351916	#N/A
[Acetyl (Protein N-term)]M[Oxidation (M)]ELVEVLKR.2	-0.106472595	#N/A
GVQQVTGHGGLR.2	-0.07323556	#N/A
WLHC[Carbamidomethyl (C)]M[Oxidation (M)]TDDPPTTNPPTAR.3	-0.154230415	#N/A
[Acetyl (Protein N-term)]M[Oxidation (M)]ELVEVLK.2	-0.0653132	#N/A
RPAS[Phospho (STY)]DDEC[Carbamidomethyl (C)]PGDSK.2	0.271708267	Q7TMI3_S668_M1_Uhrf2
NLANTVTEEILEK.2	0.366868864	#N/A
DLFEDELVPLFEK.2	0.037912971	#N/A
DSDLSHVQNK.2	-0.255109862	#N/A
LYNNHEIR.2	0.239785763	#N/A
TGYTLDVTTGQR.2	0.062397202	#N/A
GFC[Carbamidomethyl (C)]FLEYEDHK.3	-0.312615428	#N/A
VTEGLTDVILYHQPDDK.3	0.335932106	#N/A
AILVDLEPGTM[Oxidation (M)]DSVR.2	0.019770039	#N/A
ALTVPELTQQM[Oxidation (M)]FDSK.2	-0.002200436	#N/A
YLTVAAIFR.2	0.004634158	#N/A
INVYYNEAAGNK.2	0.129447427	#N/A
KESESC[Carbamidomethyl (C)]DC[Carbamidomethyl (C)]LQGFQLTHSLGGGTGSGM[Oxidation (M)]GTLLISK.4	0.153221836	#N/A
IM[Oxidation (M)]NTFSVM[Oxidation (M)]PSPK.2	0.062546782	#N/A
FWEVISDEHGIDPTGSYHGDSDLQLER.4	0.234835869	#N/A
INVYYNEAAGNKYVPR.3	-0.306214258	#N/A
ESESC[Carbamidomethyl (C)]DC[Carbamidomethyl (C)]LQGFQLTHSLGGGTGSGM[Oxidation (M)]GTLLISK.3	-0.591619951	#N/A
AES[Phospho (STY)]PEEVAC[Carbamidomethyl (C)]R.2	-0.10927752	Q7TMY8_S1395_M1_Huwe1
RAQC[Carbamidomethyl (C)]ETLS[Phospho (STY)]PDGLPEEQPQTTK.3	-0.407305171	Q7TMY8_S3663_M1_Huwe1
ESNQPPEDSS[Phospho (STY)]PPASSESSSTR.2	-0.545441522	Q7TMY8_S2918_M1_Huwe1
GSGTAS[Phospho (STY)]DDEFENLR.2	-0.039926066	Q7TMY8_S1907_M1_Huwe1
AQC[Carbamidomethyl (C)]ETLS[Phospho (STY)]PDGLPEEQPQTTK.2	-0.276728736	Q7TMY8_S3663_M1_Huwe1
DGGS[Phospho (STY)]GNSTIIVSR.2	0.475574745	Q7TMY8_S2362_M1_Huwe1
AES[Phospho (STY)]PEEVAC[Carbamidomethyl (C)]RK.2	-0.426301129	Q7TMY8_S1395_M1_Huwe1
DLS[Phospho (STY)]MSEEDQMMR.2	-0.074188861	Q7TMY8_S1368_M1_Huwe1
DLDLLASVPS[Phospho (STY)]PSSVSR.2	0.073678496	Q7TN29_S219_M1_Smap2
S[Phospho (STY)]LDINVLR.2	-0.178285557	Q7TN29_S127_M1_Smap2
KAVGSMPTAGSAGS[Phospho (STY)]VPENLNLFPEPGSK.3	-0.063421047	Q7TN29_S239_M1_Smap2
QQLSPS[Phospho (STY)]PGQEAGILPETEK.2	0.037358556	Q7TN98_S99_M1_Cpeb4
S[Phospho (STY)]PASPHPPPFTHR.3	-0.028214822	Q7TN98_S252_M1_Cpeb4
SPAS[Phospho (STY)]PSQAPYAQR.2	-0.270477166	Q7TN99_S197_M1_Cpeb3
RS[Phospho (STY)]PAS[Phospho (STY)]PSQAPYAQR.2	-0.077442473	Q7TN99_S194_M2_Cpeb3
EFAPS[Phospho (STY)]DEELDSYR.2	0.040644782	Q7TNE3_S114_M1_Spag7
GGGS[Phospho (STY)]PEKLPNR.2	-0.038025553	Q7TNF8_S1402_M1_Tspoap1
LQESFQGAPDVIS[Phospho (STY)]PR.2	-0.365664856	Q7TNM2_S627_M1_Trim46
SVS[Phospho (STY)]TDELGPPGR.2	-0.479236241	Q7TPB0_S320_M1_Plppr3
GLGLPDEAS[Phospho (STY)]PVHLR.2	-0.354671922	Q7TPB0_S426_M1_Plppr3
QKS[Phospho (STY)]LEGR.2	-0.091817182	Q7TPB0_S413_M1_Plppr3
EKTS[Phospho (STY)]LGS[Phospho (STY)]LKR.2	-0.155355324	Q7TPB0_S343_M2_Plppr3
RAS[Phospho (STY)]VDVDLLAPR.2	0.061059361	Q7TPB0_S351_M1_Plppr3
QPGM[Oxidation (M)]GPGS[Phospho (STY)]PVSDVDQEEPR.2	-0.508328791	Q7TPB0_S641_M1_Plppr3
EFDYDSPHGLDS[Phospho (STY)]D.2	0.187602605	Q7TPE5_S305_M1_Slc7a6os

SKS[Phospho (STY)]DSYTLDPDTLR.2	-0.114671572	Q7TPH6_S2943_M1_Mycbp2
LISGDAEPT[Phospho (STY)]PEQEEK.2	-0.475451169	Q7TPH6_T3992_M1_Mycbp2
ALLSS[Phospho (STY)]PEGEEK.2	0.30806912	Q7TPH6_S4003_M1_Mycbp2
SSS[Phospho (STY)]PQDKNLPQK.2	-0.062924998	Q7TPH6_S2916_M1_Mycbp2
STS[Phospho (STY)]PKPKPVPK.2	0.264445189	Q7TPH6_S2970_M1_Mycbp2
DPKDSPGS[Phospho (STY)]ENR.2	0.328861942	Q7TPH6_S2986_M1_Mycbp2
SLS[Phospho (STY)]PNHNTLQTLK.2	-0.322626814	Q7TPH6_S2861_M1_Mycbp2
QVS[Phospho (STY)]TENDSTLVHR.2	-0.202980786	Q7TPH6_S1621_M1_Mycbp2
ESGS[Phospho (STY)]PAQEFSK.2	-0.033153422	Q7TPM1_S621_M1_Prrc2b
ALS[Phospho (STY)]LSSADSTDAKR.2	0.022873798	Q7TPM1_S415_M1_Prrc2b
LKFS[Phospho (STY)]DDEDEEDVVKDGR.3	0.134006176	Q7TPM1_S387_M1_Prrc2b
LKFS[Phospho (STY)]DDEDEEDVVK.2	-0.370809592	Q7TPM1_S387_M1_Prrc2b
S[Phospho (STY)]PDEALPGGLGSHSPYALER.3	0.019645063	Q7TPM1_S793_M1_Prrc2b
QQQQQQQQQQQQQQQEQLYK.3	0.004764244	#N/A
TAS[Phospho (STY)]PVNSPAR.2	-0.043985929	Q7TPM6_S313_M1_Fsd1
RGS[Phospho (STY)]ATSSSNTSLT.2	0.203740895	Q7TPM6_S486_M1_Fsd1
LDGSTSHQNLR.2	0.016655086	#N/A
ALESSEELLETANQTLQASDSEDFSQAAK.3	-0.421045833	#N/A
IWLS[Phospho (STY)]TETLANED.2	-0.431106304	Q7TPM9_S763_M1_Mtmr10
RNS[Phospho (STY)]LVLK.2	0.210236536	Q7TPM9_S603_M1_Mtmr10
ATLPDADKER.2	-0.135631774	#N/A
TINEVENQILTR.2	-0.23045208	#N/A
LASDLLEWIR.2	-0.110376019	#N/A
ILAGDKNYITEDELR.3	-0.648418668	#N/A
EGLLLWC[Carbamidomethyl (C)]QR.2	0.132893535	#N/A
GYEEWLLNEIR.2	-0.395617138	#N/A
VGWEQLLTTIAR.2	-0.352715562	#N/A
TIPWLENR.2	0.361466904	#N/A
IC[Carbamidomethyl (C)]DQWDNLGALTQK.2	-0.142760762	#N/A
FAIQDISVEETSAK.2	-0.259787496	#N/A
LAILGIHNEVSK.2	-0.170250703	#N/A
ASFNHFDR.2	-0.222171305	#N/A
DLLLDPAWEK.2	0.305609708	#N/A
KDDPLTNLNTAFDVAER.3	-0.089920958	#N/A
LLETIDQLYLEYAK.2	-0.025300619	#N/A
RDQALTEEHAR.3	-0.086411678	#N/A
DDPLTNLNTAFDVAER.2	0.204787006	#N/A
DGLGFC[Carbamidomethyl (C)]ALIHR.2	-0.022865691	#N/A
IDQLEC[Carbamidomethyl (C)]DHQLIQEALIFDNK.3	-0.287261509	#N/A
QFGAQANVIGPWIQTK.2	0.168569245	#N/A
HRPELIDYGK.3	-0.2649062	#N/A
VLAVNQENEQLM[Oxidation (M)]EDYEK.2	-0.180049161	#N/A
GISQEQM[Oxidation (M)]NEFR.2	-0.427480064	#N/A
ALDFIASK.2	-0.169885988	#N/A
C[Carbamidomethyl (C)]QLEINFNTLQTK.2	0.100520217	#N/A
IMSIVDPNR.2	0.456444796	#N/A
NVNIQNFHISWK.2	0.036192827	#N/A

HTNYNM[Oxidation (M)]EHIR.3	-0.151065285	#N/A
MVSDINNAWGC[Carbamidomethyl (C)]LEQAEK.2	0.301574731	#N/A
AIM[Oxidation (M)]TYVSSFYHAFSGAQK.2	-0.158680081	#N/A
ASIHEAWTDGK.2	0.510451721	#N/A
KHEAFESDLAAHQDR.3	0.345882133	#N/A
VEQIAAIAQELNELDYYDSPSVNAR.3	0.234568212	#N/A
QKDYETATLSEIK.2	0.072013021	#N/A
ISIEM[Oxidation (M)]HGTLEDQLSHLR.3	-0.413233328	#N/A
MLDAEDIVGTARPDEK.3	-0.551928098	#N/A
NQTYSFS[Phospho (STY)]PSK.2	0.076613008	Q7TPS5_S295_M1_C2cd5
SQS[Phospho (STY)]ESSDEVTELDLSHGKK.3	0.081100643	Q7TPS5_S659_M1_C2cd5
SQSESS[Phospho (STY)]DEVTELDLSHGK.3	0.245035222	Q7TPS5_S662_M1_C2cd5
LSSPAAFLPAC[Carbamidomethyl (C)]S[Phospho (STY)]SPSR.2	-0.068256159	Q7TPS5_S259_M1_C2cd5
SPAPSNPTLS[Phospho (STY)]PSTPAK.2	0.029797541	Q7TPV4_S1253_M1_Mybbp1a
TVS[Phospho (STY)]QESLTPGK.2	-0.054567602	Q7TPW1_S296_M1_Nexn
EM[Oxidation (M)]LAS[Phospho (STY)]DDEEESSPK.2	-0.205754793	Q7TPW1_S16_M1_Nexn
EMLAS[Phospho (STY)]DDEEESSPKIEK.2	-0.018553478	Q7TPW1_S16_M1_Nexn
ADS[Phospho (STY)]VPNLEPSEESLVTK.2	-0.435053058	Q7TQ95_S411_M1_Lnpk
IVEYLVGDGPQNR.2	-0.023620867	#N/A
AANKT[Phospho (STY)]PPKS[Phospho (STY)]PGDPAR.2	-0.271132857	Q7TQD2_S19_M2_Tppp
AVSS[Phospho (STY)]PTVSR.2	-0.233749515	Q7TQD2_S159_M1_Tppp
VDLVDESGYVPGYK.2	-0.105541346	#N/A
APVISGVTK.2	0.130548399	#N/A
FAVHGDTR.2	0.202240645	#N/A
NVTVTDVDIVFSK.2	-0.051377722	#N/A
TITFEQFQEALEELAK.2	-0.127063629	#N/A
RLS[Phospho (STY)]LESEGANEGATAAPELSALEEAFR.3	-0.363357581	Q7TQD2_S31_M1_Tppp
LSLESEGANEGATAAPELSALEEAFR.3	0.045644252	#N/A
DC[Carbamidomethyl (C)]HVIDGK.2	0.100956951	#N/A
RLS[Phospho (STY)]LESEGANEGATAAPELSALEEAFRR.4	-0.326101375	Q7TQD2_S31_M1_Tppp
LSLESEGANEGATAAPELSALEEAFRR.3	-0.214066706	#N/A
TPPKS[Phospho (STY)]PGDPAR.2	-0.234429218	Q7TQD2_S19_M1_Tppp
DKSSEEAVR.2	-0.250629678	#N/A
[Acetyl (Protein N-term)]ADS[Phospho (STY)]KAKPAK.2	-0.167449441	Q7TQD2_S4_M1_Tppp
NASGVVNSS[Phospho (STY)]PR.2	-0.412456484	Q7TQE6_S332_M1_Maco1
EAEVGS[Phospho (STY)]DGER.2	-0.311159235	Q7TQF2_S338_M1_Fbxo10
AALPAGEGGS[Phospho (STY)]PEGAK.2	-0.092189171	Q7TQF7_S500_M1_Amph
GLFPENFTR.2	-0.11685109	#N/A
KLVDYDSAR.2	0.273530191	#N/A
ESDWLQYR.2	-0.18430244	#N/A
EIAVLC[Carbamidomethyl (C)]HK.2	0.070651278	#N/A
VGFYVNTFK.2	-0.066498989	#N/A
LVDYDSAR.2	0.123580196	#N/A
ADETKDEQFEEYVQNFKR.3	-0.292667291	#N/A
EAAEDVAAQGSAGEK.2	0.349431579	#N/A
GDVVLVVPSDSEADQDAGWLVGVK.3	-0.041976155	#N/A
ADETKDEQFEEYVQNFK.3	-0.174308746	#N/A

LVDGSLLTLDTYLGQFPDIK.2	-0.348919943	#N/A
AFSIQGAPSDS[Phospho (STY)]GPLR.2	0.050460727	Q7TQF7_S252_M1_Amph
TPS[Phospho (STY)]PPEEPSPLPSPTASPNHTLAPASPAPVRPR.4	0.102431105	Q7TQF7_S262_M1_Amph
VETLHDFEAANSDELNLQR.3	0.240369365	#N/A
LTESLHEVYEPDWYGREDVK.3	-0.153505585	#N/A
LYEVM[Oxidation (M)]TK.2	0.136227628	#N/A
C[Carbamidomethyl (C)]DVLWEDFHQK.3	-0.152387855	#N/A
TKS[Phospho (STY)]PAEEELTPSAVVR.2	-0.157955872	Q7TQG1_S901_M1_Plekha6
DPSQTS[Phospho (STY)]PLGTPR.2	-0.384651547	Q7TQG1_S864_M1_Plekha6
SHSVPRS[Phospho (STY)]PSQGSYSR.3	-0.448979352	Q7TQG1_S459_M1_Plekha6
S[Phospho (STY)]PSQGSYSR.2	-0.03657561	Q7TQG1_S459_M1_Plekha6
ANGLPSGPETAS[Phospho (STY)]EPGSPYPDGPR.2	0.11218207	Q7TQG1_S247_M1_Plekha6
QPVFYDELDAASGS[Phospho (STY)]LRR.3	-0.223216857	Q7TQG1_S443_M1_Plekha6
MVENSS[Phospho (STY)]PR.2	-0.090565994	Q7TQG1_S574_M1_Plekha6
KDPSQTS[Phospho (STY)]PLGTPR.2	-0.216327512	Q7TQG1_S864_M1_Plekha6
SISS[Phospho (STY)]PKYDYLGDR.2	-0.229812537	Q7TQG1_S506_M1_Plekha6
QDGTVWIPS[Phospho (STY)]PSR.2	-0.305557614	Q7TQG1_S426_M1_Plekha6
IYS[Phospho (STY)]PVRS[Phospho (STY)]PSAR.2	0.297143768	Q7TQG1_S472_M2_Plekha6
AYVPLES[Phospho (STY)]PPTVPPLPNESR.2	-0.24704636	Q7TQG1_S816_M1_Plekha6
GVPPPEDLRS[Phospho (STY)]PSR.2	0.13148106	Q7TQG1_S336_M1_Plekha6
S[Phospho (STY)]LQLPASPAPEPSTRPAYK.3	-0.008347429	Q7TQG1_S967_M1_Plekha6
LQPSSSPET[Phospho (STY)]GLDPFPSR.2	-0.110804186	Q7TQH0_T565_M1_Atxn2l
EVDGLLTSDPM[Oxidation (M)]GS[Phospho (STY)]PVSSK.2	0.134299638	#N/A
EKEVDGLLTSDPM[Oxidation (M)]GS[Phospho (STY)]PVSSK.3	-0.053764843	#N/A
GPPQS[Phospho (STY)]PVFEGVYNNSR.2	-0.42387935	Q7TQH0_S109_M1_Atxn2l
QGSGRES[Phospho (STY)]PSLVSR.2	-0.02093076	Q7TQH0_S337_M1_Atxn2l
ES[Phospho (STY)]PSLVSR.2	-0.152970709	Q7TQH0_S337_M1_Atxn2l
IQQEIAVQNPLVSER.2	0.028316441	#N/A
LLTSGYLQR.2	-0.24993087	#N/A
EYAEDDNIYQQK.2	-0.474416406	#N/A
FFEHFIEGGR.3	-0.437015356	#N/A
LELSVLYK.2	-0.246872411	#N/A
QEPLGSDS[Phospho (STY)]EGVNC[Carbamidomethyl (C)]LAYDEAIM[Oxidation (M)]AQQDR.3	0.243470978	Q7TQI3_S18_M1_Otub1
EFC[Carbamidomethyl (C)]QQEVEPMC[Carbamidomethyl (C)]K.2	0.166506236	#N/A
AFGFSHLEALLDDSKELQR.3	0.43838482	#N/A
GEGGTTNPHVFPEGSEPK.3	0.524906446	#N/A
SPTVDS[Phospho (STY)]GDIR.2	-0.19720527	Q7TQI7_S444_M1_Abtb2
QSELSAEES[Phospho (STY)]PEKLGTSQLHQR.3	-0.143938706	Q7TQK5_S305_M1_Ccdc93
QSELSAEES[Phospho (STY)]PEK.2	-0.151845053	Q7TQK5_S305_M1_Ccdc93
KGTS[Phospho (STY)]PGSELPPPK.2	-0.144233564	Q7TSC1_S378_M1_Prrc2a
SDS[Phospho (STY)]GGSSSEPFER.2	0.051770736	Q7TSC1_S761_M1_Prrc2a
GTS[Phospho (STY)]PGSELPPPK.2	0.083120311	Q7TSC1_S378_M1_Prrc2a
EGPEPPEEVPPPTT[Phospho (STY)]PPAPK.2	0.280642648	Q7TSC1_T609_M1_Prrc2a
S[Phospho (STY)]EGSEYEEIPKR.2	0.010585404	Q7TSC1_S1087_M1_Prrc2a
ETPPGGNLS[Phospho (STY)]PAPR.2	-0.15849935	Q7TSC1_S1002_M1_Prrc2a
KQS[Phospho (STY)]SSEISLAVER.2	0.19139111	Q7TSC1_S454_M1_Prrc2a
TASETRS[Phospho (STY)]EGSEYEEIPKR.3	-0.19664281	Q7TSC1_S1087_M1_Prrc2a

ERS[Phospho (STY)]DS[Phospho (STY)]GGSSSEPFER.2	0.288037626	Q7TSC1_S759_M2_Prrc2a
TAS[Phospho (STY)]ETRS[Phospho (STY)]EGSEYEEIPK.2	-0.49521018	Q7TSC1_S1083_M2_Prrc2a
FS[Phospho (STY)]REEFPTLQAAGDQDK.3	0.093091024	Q7TSC1_S166_M1_Prrc2a
S[Phospho (STY)]EGSEYEEIPK.2	0.245579462	Q7TSC1_S1087_M1_Prrc2a
GSETGSETHES[Phospho (STY)]DLAPSDK.2	0.385444829	Q7TSC1_S1113_M1_Prrc2a
LKFS[Phospho (STY)]DEEDGRDS[Phospho (STY)]DEEGAEGHK.4	-0.095668254	Q7TSC1_S342_M2_Prrc2a
AS[Phospho (STY)]PGAAPGPR.2	-0.105266852	Q7TSF4_S15_M1_Lrrc75a
EGTDQQDPIGS[Phospho (STY)]PVTPAR.2	-0.415177331	Q7TSF4_S322_M1_Lrrc75a
GSLAERAS[Phospho (STY)]PGAAPGPR.3	-0.337218896	Q7TSF4_S15_M1_Lrrc75a
DNS[Phospho (STY)]PAVFPDR.2	-0.472339239	Q7TSG2_S730_M1_Ctdp1
[Acetyl (Protein N-term)]ANS[Phospho (STY)]PDAAFSSPALLR.2	-0.308896282	Q7TSH2_S4_M1_Phkb
QSS[Phospho (STY)]TADAPEAQHEPGITITEWK.3	-0.167773246	Q7TSH2_S693_M1_Phkb
LSHS[Phospho (STY)]DTFIPLLTEEK.2	0.116806443	Q7TSH7_S472_M1_Kcnf1
LPS[Phospho (STY)]AETLK.2	0.021292206	Q7TSH8_S664_M1_Tmem94
VEPPHSS[Phospho (STY)]HEDLTDDLSTR.3	-0.130177191	Q7TSH8_S445_M1_Tmem94
NTVDLVNTHHLHSSS[Phospho (STY)]DDEDDRLK.4	-0.280707814	Q7TSI3_S531_M1_Ppp6r1
EQM[Oxidation (M)]IQGQDS[Phospho (STY)]PEPDQLLATLEK.3	-0.582388517	Q7TSI3_S232_M1_Ppp6r1
EADMSSIQIPSS[Phospho (STY)]PPAHGSPQLR.3	0.153053029	Q7TSI3_S739_M1_Ppp6r1
ATGPAPGPS[Phospho (STY)]VDRETVAAPGR.3	-0.362609738	Q7TSJ2_S86_M1_Map6
SEYQPSDAPFER.2	-0.342496945	#N/A
DQSFPTPAPR.2	-0.036602537	#N/A
DQVPVVPGSLK.2	0.082519169	#N/A
KDPGPVIPEPEKDR.3	0.142824842	#N/A
AVADALNR.2	0.398439682	#N/A
ADIAVPLVFTK.2	0.417734775	#N/A
NQGPVIPEPVK.2	0.09950009	#N/A
DQGPTAPDPLK.2	0.368630438	#N/A
NQGLAGPELVK.2	0.374214873	#N/A
NKDSVPLAPAK.2	0.355533008	#N/A
DQGAVLLGPVK.2	0.214249245	#N/A
NQSPVVPASTK.2	0.084740998	#N/A
GHDSVFVAPVK.2	0.02666841	#N/A
DTGTDTTAPR.2	0.018649396	#N/A
DKQVVSGQAAK.2	-0.414942489	#N/A
FWNQLDK.2	0.159141935	#N/A
DPEGAGGAGVLAAGK.2	-0.079022616	#N/A
AVAIETQPAQGESDAVAR.2	-0.079047669	#N/A
ATGPAPGPS[Phospho (STY)]VDR.2	0.110724375	Q7TSJ2_S86_M1_Map6
GQS[Phospho (STY)]PTAPGPTKDQGAVLLGPVK.3	-0.374906064	Q7TSJ2_S632_M1_Map6
NQS[Phospho (STY)]PVVLGPIK.2	-0.413769449	Q7TSJ2_S832_M1_Map6
VQDHIASELLK.2	-0.304925822	#N/A
AGPAWM[Oxidation (M)]VTR.2	0.098542497	#N/A
AQS[Phospho (STY)]PLLPEPLKNQSPVVPASTK.3	-0.408368215	Q7TSJ2_S687_M1_Map6
RPQS[Phospho (STY)]QERGPM[Oxidation (M)]QLSADAR.3	0.297235668	Q7TSJ2_S198_M1_Map6
GPM[Oxidation (M)]QLS[Phospho (STY)]ADAR.2	0.481660976	Q7TSJ2_S207_M1_Map6
S[Phospho (STY)]LYSEPFKEC[Carbamidomethyl (C)]PK.2	-0.181316523	Q7TSJ2_S491_M1_Map6
DQVPVVPGSLKGQS[Phospho (STY)]PTAPGPTK.3	-0.061177838	Q7TSJ2_S632_M1_Map6

EPHKDQGSVAPGLPK.3	0.152659428	#N/A
RSEGHEQT[Phospho (STY)]PAAHAQGTGPEGGK.3	-0.178381651	Q7TSJ2_T392_M1_Map6
AKPQYKPPDDK.3	-0.110618261	#N/A
EEVASTVSSSYR.2	0.311073504	#N/A
GQS[Phospho (STY)]PTAPGPTK.2	-0.053163758	Q7TSJ2_S632_M1_Map6
TEGHEETPLPPAQSQTQEGGPAAGK.3	-0.096124135	#N/A
TTEGPSATKPDDKEQSK.3	0.217424737	#N/A
YSEATEHPGAPPQPPAPLQPALAPPSR.3	-0.075275429	#N/A
S[Phospho (STY)]EGHEQTPAAHAQGTGPEGGK.3	-0.225804576	Q7TSJ2_S386_M1_Map6
GTQLPTVSPSPPVM[Oxidation (M)]IPTVPHTEYIEGS[Phospho (STY)]P.3	-0.300201529	Q7TSJ2_S905_M1_Map6
GPM[Oxidation (M)]QLS[Phospho (STY)]ADARDPEGAGGAGVLAAGK.3	-0.27609453	Q7TSJ2_S207_M1_Map6
M[Oxidation (M)]VHETSYSAQFK.3	0.17677906	#N/A
GEANKPSAADNK.2	-0.399408694	#N/A
SEGHEEKPLPPAQSQTQEGGPAAGK.4	0.077017063	#N/A
SGLGLGAASASTSGSGPADSVM[Oxidation (M)]R.2	-0.157572927	#N/A
RRPQS[Phospho (STY)]QER.2	0.385280843	Q7TSJ2_S198_M1_Map6
AQS[Phospho (STY)]PLLPEPLK.2	-0.094934803	Q7TSJ2_S687_M1_Map6
NEGHEEKPLPPAQSQTQEGGPAAGK.4	0.270933452	#N/A
S[Phospho (STY)]PLQLPR.2	-0.122662178	Q7TSK2_S952_M1_Sez6
EERPGAAGGGAS[Phospho (STY)]APGS[Phospho (STY)]PDETAR.2	-0.087895578	Q7TSK3_S798_M2_Pcdh8
DSGKGDSDFNDS[Phospho (STY)]DSDISGDALKK.3	-0.315486182	Q7TSK3_S926_M1_Pcdh8
[Acetyl (Protein N-term)]AAAT[Phospho (STY)]PTETPAPEGSGLGMDAR.2	-0.216426294	Q7TSV4_T5_M1_Pgm2
NKQEDLNSEALS[Phospho (STY)]PSITC[Carbamidomethyl (C)]DLSSR.3	-0.056026102	Q7TT18_S112_M1_Atf7ip
SKS[Phospho (STY)]EDMDSVESKR.2	0.136379501	Q7TT18_S593_M1_Atf7ip
SKS[Phospho (STY)]EDMDSVESK.2	0.367745264	Q7TT18_S593_M1_Atf7ip
EAFLVLS[Phospho (STY)]DEEDLSC[Carbamidomethyl (C)]GKDESEAVAQSK.3	0.032044975	Q7TT18_S533_M1_Atf7ip
S[Phospho (STY)]LDEGAPQDTPK.2	-0.350728509	Q7TT28_S487_M1_Rexo1
ALS[Phospho (STY)]HAELFGDES[Phospho (STY)]EEEDSSLGAGAPR.3	0.152631138	Q7TT28_S505_M2_Rexo1
SSGNSANDLANVAVIDGNR.2	0.060792879	#N/A
ALGNS[Phospho (STY)]NRDKEIK.3	-0.553562226	Q7TT50_S481_M1_Cdc42bpb
HSTPSNSSNPSGPPS[Phospho (STY)]PNS[Phospho (STY)]PHR.3	-0.053600183	Q7TT50_S1692_M2_Cdc42bpb
SM[Oxidation (M)]S[Phospho (STY)]DPDQDFDKEPDSDSTK.2	-0.068928137	Q7TT50_S1661_M1_Cdc42bpb
VVLLAEGR.2	0.168772179	#N/A
[Acetyl (Protein N-term)]S[Phospho (STY)]M[Oxidation (M)]PDAM[Oxidation (M)]PLPGVGEELK.2	-0.322960249	Q80SW1_S2_M1_Ahcyl1
EIEIAEQDM[Oxidation (M)]SALISLR.2	-0.444708857	#N/A
QVDVVITC[Carbamidomethyl (C)]TGNK.2	-0.307589863	#N/A
GSSNFC[Carbamidomethyl (C)]VK.2	-0.136003794	#N/A
TTDVMFGGK.2	0.207115488	#N/A
NSC[Carbamidomethyl (C)]IVC[Carbamidomethyl (C)]NM[Oxidation (M)]GHSNTEIDVTSLR.3	-0.197659045	#N/A
VAPAGSAISNAS[Phospho (STY)]GER.2	0.154026714	Q80SY4_S411_M1_Mib1
SYS[Phospho (STY)]LDLDSQNLQSFK.2	0.138106263	Q80T23_S211_M1_Sytl5
NTSGTPSIAVSGTSLS[Phospho (STY)]SER.2	-0.073851323	Q80T23_S319_M1_Sytl5
DLNS[Phospho (STY)]LEDTEDGVDLVSSR.2	-0.641227262	Q80T23_S358_M1_Sytl5
TSTS[Phospho (STY)]VTSVNQASTSR.2	-0.106113279	Q80T41_S775_M1_Gabbr2
KEDS[Phospho (STY)]KTSTSVTSVNQASTSR.3	0.197783588	Q80T41_S770_M1_Gabbr2
ITELDKDLEEVTM[Oxidation (M)]QLQDT[Phospho (STY)]PEK.3	-0.146835961	Q80T41_T818_M1_Gabbr2
TC[Carbamidomethyl (C)]KDPIEDINS[Phospho (STY)]PEHIQR.3	-0.355410581	Q80T41_S883_M1_Gabbr2

FTQFQDSR.2	0.011571222	#N/A
DPIEDINS[Phospho (STY)]PEHIQR.3	0.202441569	Q80T41_S883_M1_Gabbr2
DLEEVTMQLQDT[Phospho (STY)]PEK.2	0.337816785	Q80T41_T818_M1_Gabbr2
QLS[Phospho (STY)]LPLTQSK.2	-0.256245945	Q80TA6_S564_M1_Mtmr12
ISAEGVDIVLDC[Carbamidomethyl (C)]LC[Carbamidomethyl (C)]GDNTGK.2	0.062831533	#N/A
DSVTHLFDR.2	0.388676371	#N/A
TPLVPGFEC[Carbamidomethyl (C)]SGIVEALGDSVK.2	0.204122172	#N/A
AC[Carbamidomethyl (C)]GLNFIDLM[Oxidation (M)]VR.2	0.133408574	#N/A
TPTPLM[Oxidation (M)]ANDS[Phospho (STY)]TETSEAGEEEEDHEGDSENKER.4	0.177034994	Q80TB8_S390_M1_Vat1l
LILDVEK.2	-0.111377728	#N/A
ETSKEPAEGGDGSHR.3	0.132283021	#N/A
EYGS[Phospho (STY)]TSSIDR.2	-0.036232577	Q80TE4_S195_M1_Sipa1l2
TLS[Phospho (STY)]DESVC[Carbamidomethyl (C)]SNR.2	0.196080137	Q80TE4_S1488_M1_Sipa1l2
YTIGDIFVHS[Phospho (STY)]PQR.2	-0.187204289	Q80TE4_S149_M1_Sipa1l2
SEGS[Phospho (STY)]PPPEEPEVTEC[Carbamidomethyl (C)]PR.2	-0.220222904	Q80TE4_S1406_M1_Sipa1l2
NEFWFS[Phospho (STY)]DGSLSDK.2	0.080634088	Q80TE4_S1549_M1_Sipa1l2
TPS[Phospho (STY)]PFEDR.2	-0.33195572	Q80TE7_S850_M1_Lrrc7
GVIAISKS[Phospho (STY)]TER.2	0.129427685	Q80TE7_S928_M1_Lrrc7
S[Phospho (STY)]M[Oxidation (M)]DGYPEQFC[Carbamidomethyl (C)]VR.2	-0.287200999	Q80TE7_S1392_M1_Lrrc7
LETTPTTS[Phospho (STY)]PLPER.2	-0.087408113	Q80TE7_S869_M1_Lrrc7
VYNIPLENYASGSDHLGS[Phospho (STY)]HERPDK.3	0.222237047	Q80TE7_S977_M1_Lrrc7
TAFPSKLETT[Phospho (STY)]PTTS[Phospho (STY)]PLPERK.3	0.407196073	Q80TE7_S869_M2_Lrrc7
SQS[Phospho (STY)]IDEIDVGTYK.2	-0.152701975	Q80TE7_S949_M1_Lrrc7
KSQS[Phospho (STY)]IDEIDVGTYK.2	0.529696426	Q80TE7_S949_M1_Lrrc7
S[Phospho (STY)]VPAHGR.2	-0.096309958	Q80TE7_S801_M1_Lrrc7
FKKS[Phospho (STY)]QS[Phospho (STY)]IDEIDVGTYK.3	0.340033937	Q80TE7_S947_M2_Lrrc7
SYS[Phospho (STY)]TESYGASQTR.2	-0.521196631	Q80TE7_S1206_M1_Lrrc7
STERLS[Phospho (STY)]PLM[Oxidation (M)]K.2	-0.146836328	Q80TE7_S933_M1_Lrrc7
LETT[Phospho (STY)]PTTS[Phospho (STY)]PLPERK.2	-0.337438958	Q80TE7_S869_M2_Lrrc7
SYS[Phospho (STY)]TESYGASQTRPVSAR.2	-0.376495426	Q80TE7_S1206_M1_Lrrc7
TLAVDENFLPELPR.2	-0.106039002	#N/A
S[Phospho (STY)]REQQPYEGNINK.2	-0.131233309	Q80TE7_S1343_M1_Lrrc7
IPS[Phospho (STY)]DYNLGNYGDK.2	-0.071093397	Q80TE7_S1233_M1_Lrrc7
S[Phospho (STY)]QSVPMLDDEMLM[Oxidation (M)]YGSSK.2	0.038831799	Q80TE7_S997_M1_Lrrc7
RVS[Phospho (STY)]EPHELPPGDR.3	0.168307627	Q80TE7_S1132_M1_Lrrc7
DKES[Phospho (STY)]PSVKR.2	-0.525967746	Q80TF3_S1134_M1_Pcdh19
SKS[Phospho (STY)]PEHVR.2	0.122348796	Q80TF3_S983_M1_Pcdh19
EAGNGC[Carbamidomethyl (C)]EAIS[Phospho (STY)]PVTS[Phospho (STY)]PLHLK.2	-0.06650054	Q80TF3_S1063_M2_Pcdh19
SES[Phospho (STY)]AENHSYAK.2	-0.347105438	Q80TH2_S1013_M1_Erbin
VRQEDENFNSLLQNGVNLNNS[Phospho (STY)]PEEK.3	-0.209398209	Q80TH2_S712_M1_Erbin
STEDLS[Phospho (STY)]PQR.2	-0.32925676	Q80TH2_S854_M1_Erbin
STPAC[Carbamidomethyl (C)]S[Phospho (STY)]PILR.2	0.126522386	Q80TI0_S21_M1_Gramd1b
TESTYLAEIHRQS[Phospho (STY)]PK.3	-0.248449217	Q80TI0_S550_M1_Gramd1b
SSIETKPDAS[Phospho (STY)]PQLPKK.3	-0.334467834	Q80TI0_S274_M1_Gramd1b
S[Phospho (STY)]RSPTPQNQDGDTMVEK.3	0.037023916	Q80TI0_S28_M1_Gramd1b
SSIETKPDAS[Phospho (STY)]PQLPK.2	-0.482046432	Q80TI0_S274_M1_Gramd1b
S[Phospho (STY)]PTPQNQDGDTM[Oxidation (M)]VEK.2	0.240406281	Q80TI0_S30_M1_Gramd1b

SC[Carbamidomethyl (C)]DS[Phospho (STY)]DEDYEAGGTGR.2	-0.609772028	Q80TI1_S739_M1_Plekhh1
VILHPTPNS[Phospho (STY)]PK.2	-0.370598525	Q80TJ1_S486_M1_Cadps
DIVTPVPQEEVK.2	-0.275409196	#N/A
LDALQTFIR.2	-0.192288141	#N/A
EGDTVIFASDDEQDR.2	-0.236703961	#N/A
S[Phospho (STY)]LPEIDGLSK.2	-0.41695309	Q80TJ1_S219_M1_Cadps
SLTSNLPNVNLPNVNLPK.2	-0.223458713	#N/A
LQGVLDSTLNSK.2	0.279175131	#N/A
HSQDVLR.2	-0.312074417	#N/A
ATLSLLER.2	-0.21838376	#N/A
LQKEEEER.2	0.116618036	#N/A
EQLYEM[Oxidation (M)]FQNILGIK.2	0.078739049	#N/A
VLLENQITHFR.2	-0.071195157	#N/A
LFTESTGVLALEDKELGR.3	0.203659309	#N/A
YVDLM[Oxidation (M)]ESSIAQSIHR.2	-0.242223918	#N/A
M[Oxidation (M)]VQSGGC[Carbamidomethyl (C)]SANDSR.2	-0.179300433	#N/A
DAEYIYPSLES[Phospho (STY)]DDDDPALK.2	-0.198943892	Q80TJ7_S820_M1_Phf8
SSGSSSSGLGTVSSS[Phospho (STY)]PASQR.2	-0.516638886	Q80TJ7_S768_M1_Phf8
S[Phospho (STY)]LDSC[Carbamidomethyl (C)]PSQGLPQEGQVK.2	0.013698218	Q80TK0_S1051_M1_Kiaa1107
SSS[Phospho (STY)]DTSTPEELK.2	-0.023008313	Q80TK0_S750_M1_Kiaa1107
SAPPTLAQAQGSQGESPHS[Phospho (STY)]VK.2	0.316731798	Q80TK0_S343_M1_Kiaa1107
KQGGGDHQIHS[Phospho (STY)]AS[Phospho (STY)]DDEIPR.3	-0.062521672	#N/A
SEDYDAGGS[Phospho (STY)]QDDEGSHDR.2	0.337602651	Q80TK0_S722_M1_Kiaa1107
QGGGDHQIHS[Phospho (STY)]AS[Phospho (STY)]DDEIPR.2	-0.334472807	#N/A
SSS[Phospho (STY)]DTSTPEELKVHEGDLR.3	-0.429697109	Q80TK0_S750_M1_Kiaa1107
SSVSSRQS[Phospho (STY)]DENVTR.2	-0.573481584	Q80TK0_S353_M1_Kiaa1107
YNC[Carbamidomethyl (C)]PSFLAAALAR.2	-0.21249619	#N/A
RPWPQHQC[Carbamidomethyl (C)]S[Phospho (STY)]APADLGYEGR.3	-0.370563689	Q80TL0_S532_M1_Ppm1e
VDS[Phospho (STY)]FTDR.2	-0.161703595	Q80TL0_S545_M1_Ppm1e
AVNVS[Phospho (STY)]EESEWTENSFQGGQEDGGDDKETHGEC[Carbamidomethyl (C)]K.4	0.012339356	Q80TL0_S495_M1_Ppm1e
S[Phospho (STY)]PQHLFR.2	-0.362550879	Q80TL1_S471_M1_Adcy2
S[Phospho (STY)]PQHLFRPR.3	-0.38189548	Q80TL1_S471_M1_Adcy2
VIPDC[Carbamidomethyl (C)]SLTLEDFVR.2	-0.199712268	#N/A
ALLPTEQESR.2	-0.078522151	#N/A
GVEPEEFDR.2	-0.006546207	#N/A
QFAALDPEQR.2	0.148565924	#N/A
ALTDEQEEQAAR.2	-0.330493083	#N/A
YVDWPTFLR.2	0.124494754	#N/A
ENVIYILAAR.2	0.319683844	#N/A
KLDDDKPPDIC[Carbamidomethyl (C)]LEPR.3	-0.321850407	#N/A
VFHDGC[Carbamidomethyl (C)]LR.2	-0.066096869	#N/A
GSTISEAEC[Carbamidomethyl (C)]HHAR.3	-0.143567096	#N/A
EAEASAPVVQEESS[Phospho (STY)]INR.2	0.035998983	Q80TL4_S73_M1_Phf24
LTEAPSC[Carbamidomethyl (C)]SVSISHVGPIADSS[Phospho (STY)]PAASSSK.3	-0.140196419	Q80TL4_S348_M1_Phf24
RGT[Phospho (STY)]VEGSVQEVQEEKEAEASAPVVQEESSINR.4	-0.583214296	Q80TL4_T47_M1_Phf24
RGT[Phospho (STY)]VEGSVQEVQEEK.2	-0.088375663	Q80TL4_T47_M1_Phf24
DEQAAS[Phospho (STY)]DPM[Oxidation (M)]DQETVSR.2	-0.07437437	Q80TL7_S534_M1_Mon2

PWSS[Phospho (STY)]TDSDGSVR.2	-0.125834867	Q80TM6_S362_M1_R3hdm2
DSS[Phospho (STY)]QEYTDSTGIDLHEFLVNTLK.3	0.045742794	Q80TM6_S143_M1_R3hdm2
SKS[Phospho (STY)]IEEREEEYQR.3	-0.145088461	Q80TM6_S283_M1_R3hdm2
ASS[Phospho (STY)]FSGISILTR.2	-0.088503849	Q80TM6_S381_M1_R3hdm2
SLEPRPWSS[Phospho (STY)]TDSDGSVR.2	0.134903964	Q80TM6_S362_M1_R3hdm2
S[Phospho (STY)]IEEREEEYQR.2	-0.111011212	Q80TM6_S283_M1_R3hdm2
SAS[Phospho (STY)]TDLGTADVVLGR.2	-0.168703007	Q80TM6_S923_M1_R3hdm2
TPS[Phospho (STY)]KEDVEKEGEENGLR.3	0.210413615	Q80TM6_S37_M1_R3hdm2
SKS[Phospho (STY)]IEER.2	0.202431077	Q80TM6_S283_M1_R3hdm2
LEQEILDFINDNNNQFK.2	-0.371856496	#N/A
TPS[Phospho (STY)]PEPVDKDFYSEFGDK.3	-0.095117441	Q80TM9_S1373_M1_Nisch
AC[Carbamidomethyl (C)]SDS[Phospho (STY)]LESIPAGQVASDDLR.2	-0.120020638	Q80TM9_S545_M1_Nisch
SQS[Phospho (STY)]FNSIDK.2	0.15038376	Q80TN7_S275_M1_Nav3
S[Phospho (STY)]LPRPSK.2	-0.362114207	Q80TN7_S1140_M1_Nav3
KTS[Phospho (STY)]LDGSQNQDDVVLHVSSK.3	0.162841719	Q80TN7_S1116_M1_Nav3
WLDGAS[Phospho (STY)]FDNER.2	0.004837879	Q80TP3_S321_M1_Ubr5
SELFYTLNGSS[Phospho (STY)]VDSQQSK.2	0.109586398	Q80TQ2_S359_M1_Cyld
VTGSTS[Phospho (STY)]DPGSR.2	0.033849941	Q80TQ2_S341_M1_Cyld
YYTGTQS[Phospho (STY)]R.2	-0.135254411	Q80TR1_S1141_M1_Adgrl1
SPPGGTHGS[Phospho (STY)]LK.2	0.219383104	Q80TR1_S1123_M1_Adgrl1
PLSS[Phospho (STY)]PPGR.2	0.360127799	Q80TR1_S1369_M1_Adgrl1
QTESSFM[Oxidation (M)]AGDINST[Phospho (STY)]PTLNR.2	-0.026206638	Q80TR1_T1167_M1_Adgrl1
RPS[Phospho (STY)]HEGYLAAPSLEGPGPDGDGQM[Oxidation (M)]QLVTSL.3	0.021248453	Q80TR1_S1439_M1_Adgrl1
TDTLTEYASWEDYVAAR.2	-0.221284482	#N/A
SVYVDDDSEAAGNRVDYAFNTNANR.3	0.105815301	#N/A
GPAHLVTS[Phospho (STY)]L.2	0.039291179	Q80TS3_S1536_M1_Adgrl3
S[Phospho (STY)]MPNLGSR.2	0.117625889	Q80TS3_S1485_M1_Adgrl3
GSSDGFIVPPNKDGAS[Phospho (STY)]PEGTSK.3	-0.121392813	Q80TS3_S1522_M1_Adgrl3
STESSIGS[Phospho (STY)]GK.2	-0.313969596	Q80TS3_S1222_M1_Adgrl3
S[Phospho (STY)]PLGQPRPEPR.2	-0.286356345	Q80TS7_S138_M1_Ddn
LRPGS[Phospho (STY)]PEPPPR.2	-0.428821616	Q80TS7_S77_M1_Ddn
RS[Phospho (STY)]PLGQPRPEPR.3	-0.102598999	Q80TS7_S138_M1_Ddn
SGS[Phospho (STY)]PAPAEPVDPNR.2	-0.027287888	Q80TT2_S78_M1_Baiap3
VGLQS[Phospho (STY)]PSVETR.2	-0.074763388	Q80TT8_S1775_M1_Cul9
AVLETPSTQGQDGS[Phospho (STY)]PESLIR.2	-0.610012759	Q80TT8_S284_M1_Cul9
NSSNTGVGS[Phospho (STY)]PSNTIGR.2	-0.343968381	Q80TV8_S1088_M1_Clasp1
S[Phospho (STY)]QEDLNEPIKR.2	-0.280132551	Q80TV8_S1193_M1_Clasp1
FSFRS[Phospho (STY)]QEDLNEPIKR.3	-0.189962103	Q80TV8_S1193_M1_Clasp1
DGGAAS[Phospho (STY)]PATEGR.2	-0.221529094	Q80TV8_S1220_M1_Clasp1
SRS[Phospho (STY)]ANPAGAGSR.2	0.16008524	Q80TV8_S676_M1_Clasp1
TS[Phospho (STY)]PLTS[Phospho (STY)]PTNC[Carbamidomethyl (C)]SHGGLSPSR.2	-0.173961298	Q80TV8_S1106_M2_Clasp1
SRS[Phospho (STY)]DIDVNAAASAK.2	-0.159096891	Q80TV8_S600_M1_Clasp1
RS[Phospho (STY)]PTGSTASR.2	-0.612996535	Q80TV8_S572_M1_Clasp1
NFDDEDS[Phospho (STY)]VDGNRPSSASSSSSK.3	-0.184398431	Q80TV8_S246_M1_Clasp1
TVS[Phospho (STY)]DNSLSSSK.2	-0.240435847	Q80TY0_S296_M1_Fnbp1
TVS[Phospho (STY)]DNS[Phospho (STY)]LSSSKEGKPELR.3	-0.161276312	Q80TY0_S296_M2_Fnbp1
S[Phospho (STY)]EM[Oxidation (M)]PVPES[Phospho (STY)]APQM[Oxidation (M)]PS[Phospho (STY)]PVEK.2	0.144363394	Q80TY5_S2245_M3_Vps13b

RKEDELS[Phospho (STY)]IGS[Phospho (STY)]APLGK.3	-0.225239028	Q80TY5_S1001_M2_Vps13b
RTATSASAS[Phospho (STY)]PTLR.2	-0.249988278	Q80TZ3_S595_M1_Dnajc6
SAATS[Phospho (STY)]PTGSSHGTPTHQSK.3	-0.596801666	Q80TZ3_S683_M1_Dnajc6
VSEC[Carbamidomethyl (C)]SWPIR.2	-0.136483314	#N/A
IYSTC[Carbamidomethyl (C)]TDFER.2	0.077125365	#N/A
HLDHYTVYNLS[Phospho (STY)]PK.3	-0.437561588	Q80TZ3_S137_M1_Dnajc6
TATSASAS[Phospho (STY)]PTLR.2	-0.417462752	Q80TZ3_S595_M1_Dnajc6
GASS[Phospho (STY)]PDM[Oxidation (M)]EPSYGGGLFDMVK.2	0.261577146	Q80TZ3_S36_M1_Dnajc6
DSENKGASS[Phospho (STY)]PDM[Oxidation (M)]EPSYGGGLFDM[Oxidation (M)]VK.3	0.074140679	Q80TZ3_S36_M1_Dnajc6
AADFEDLLSSQGFNAHK.2	-0.06704193	#N/A
ATGQPYEQYAK.2	0.326302542	#N/A
SRC[Carbamidomethyl (C)]EEDHAALVNQES[Phospho (STY)]EQS[Phospho (STY)]DDELLTLSSPHGNAEGDKPHGAK.5	0.12300121	#N/A
C[Carbamidomethyl (C)]EEDHAALVNQES[Phospho (STY)]EQS[Phospho (STY)]DDELLTLSSPHGNAEGDKPHGAK.5	0.239981702	#N/A
EKVAS[Phospho (STY)]DTEDTDR.2	0.056685879	Q80TZ9_S656_M1_Rere
PHVM[Oxidation (M)]S[Phospho (STY)]PTNR.2	-0.387169274	Q80TZ9_S1258_M1_Rere
KQPTS[Phospho (STY)]PDGRAS[Phospho (STY)]PINEDIR.3	-0.021630535	Q80TZ9_S594_M2_Rere
AS[Phospho (STY)]PINEDIR.2	-0.062138045	Q80TZ9_S600_M1_Rere
VAS[Phospho (STY)]DTEDTDR.2	-0.319836885	Q80TZ9_S656_M1_Rere
EKVAS[Phospho (STY)]DTEDTDRITSK.3	-0.088629749	Q80TZ9_S656_M1_Rere
QPTS[Phospho (STY)]PDGRAS[Phospho (STY)]PINEDIR.2	-0.408700325	Q80TZ9_S594_M2_Rere
ANHHGS[Phospho (STY)]SPEQVVRPK.4	-0.119958431	Q80U04_S305_M1_Pja2
S[Phospho (STY)]IDVEELGVGR.2	-0.058695483	Q80U12_S402_M1_Sgsm2
FSSGSS[Phospho (STY)]IDSHVQR.2	-0.338051817	Q80U12_S637_M1_Sgsm2
SQS[Phospho (STY)]FAGFSGLQER.2	-0.068696631	Q80U16_S46_M1_Ripor2
ELGS[Phospho (STY)]TEDIYITSR.2	0.273148856	Q80U19_S704_M1_Daam2
LSEGPAALAGPAS[Phospho (STY)]PPR.2	-0.366339877	Q80U22_S534_M1_Rusc2
NAGPEPETSRPSPLGSYS[Phospho (STY)]PVR.3	0.174304432	Q80U22_S776_M1_Rusc2
ASVGDSSQEFS[Phospho (STY)]PIQEAQQDR.2	-0.158179379	Q80U22_S572_M1_Rusc2
AAGSGS[Phospho (STY)]PPLR.2	-0.028774969	Q80U22_S557_M1_Rusc2
RTS[Phospho (STY)]PPVSVR.2	0.076118107	Q80U23_S20_M1_Snph
EEGTGESAGGS[Phospho (STY)]PAR.2	-0.163381038	Q80U23_S204_M1_Snph
TS[Phospho (STY)]PPVSVR.2	0.016629593	Q80U23_S20_M1_Snph
AIQTDFVQYQPDLNTILEK.2	-0.113896712	#N/A
QPGDPSNTS[Phospho (STY)]AEDGADSGYVAADDTLSR.3	0.289358163	Q80U23_S236_M1_Snph
SSTYTKLS[Phospho (STY)]DPAVC[Carbamidomethyl (C)]GDR.2	-0.406142892	Q80U23_S219_M1_Snph
EEGTGESAGGS[Phospho (STY)]PARS[Phospho (STY)]LTR.2	-0.042656054	Q80U23_S204_M2_Snph
EKPAGS[Phospho (STY)]PIR.2	-0.320672084	Q80U28_S1266_M1_Madd
S[Phospho (STY)]PTENVNTPVGK.2	-0.114616805	Q80U28_S1058_M1_Madd
KRS[Phospho (STY)]PTENVNTPVGK.3	-0.173183932	Q80U28_S1058_M1_Madd
REPSS[Phospho (STY)]PQGR.2	0.396041723	Q80U28_S929_M1_Madd
LAS[Phospho (STY)]DSDAESDSR.2	-0.124680022	Q80U28_S817_M1_Madd
S[Phospho (STY)]LKEENFVASVELWNK.3	-0.444734849	Q80U28_S1109_M1_Madd
LLRPNS[Phospho (STY)]LK.2	-0.451633659	Q80U28_S812_M1_Madd
S[Phospho (STY)]PTENVNTPVGKDPGLAGR.2	0.50517385	Q80U28_S1058_M1_Madd
SSVIKHS[Phospho (STY)]PTVK.2	-0.027882625	Q80U28_S920_M1_Madd
EKPAGS[Phospho (STY)]PIRSSEDVSQR.3	0.26921754	Q80U28_S1266_M1_Madd
RNGS[Phospho (STY)]ETQLNK.2	-0.047634545	Q80U28_S1499_M1_Madd

AT[Phospho (STY)]LS[Phospho (STY)]DSEIETNSATSAIFGK.2	0.019368785	Q80U28_S1237_M2_Madd
RGS[Phospho (STY)]SDPTVQR.2	0.231567132	Q80U30_S848_M1_Clec16a
LPSLDHRDS[Phospho (STY)]PR.3	-0.327598579	Q80U35_S2005_M1_Arhgef17
SLS[Phospho (STY)]DPIPQR.2	-0.279536323	Q80U35_S728_M1_Arhgef17
RGTS[Phospho (STY)]VDDFLK.2	0.096673016	Q80U40_S737_M1_Rimbp2
DHLS[Phospho (STY)]PDFYDESETDPGAEELPAR.3	-0.159937239	Q80U40_S852_M1_Rimbp2
GS[Phospho (STY)]LDWPEEER.2	-0.076344972	Q80U49_S1032_M1_Cep170b
SQS[Phospho (STY)]FTHTPPADPK.2	0.236962659	Q80U49_S421_M1_Cep170b
HEDGTQS[Phospho (STY)]DSEDPLAK.2	-0.102830224	Q80U49_S360_M1_Cep170b
VVQTS[Phospho (STY)]PSAR.2	-0.004605882	Q80U49_S819_M1_Cep170b
AGS[Phospho (STY)]FTGPSDSETAPAR.2	0.09275542	Q80U49_S1166_M1_Cep170b
ESPLS[Phospho (STY)]PPTVPDPGGATPGSAR.2	-0.142774111	Q80U49_S981_M1_Cep170b
LGDAS[Phospho (STY)]DTEAVDGER.2	-0.004675608	Q80U49_S1122_M1_Cep170b
GASPVTPSTT[Phospho (STY)]PPPPTDPQLTK.2	0.028423448	Q80U49_T541_M1_Cep170b
DGPGDDRHS[Phospho (STY)]TKS[Phospho (STY)]DLPVHTR.3	0.040868846	Q80U49_S337_M2_Cep170b
QES[Phospho (STY)]FTKEPTSGPPAPGK.3	0.13280159	Q80U49_S862_M1_Cep170b
EIHDVAGDGDSLGS[Phospho (STY)]PGPTR.2	0.052974907	Q80U49_S1341_M1_Cep170b
GHGSRGS[Phospho (STY)]LDWPEEER.3	0.011238965	Q80U49_S1032_M1_Cep170b
GHKHEDGTQS[Phospho (STY)]DSEDPLAK.3	0.059729703	Q80U49_S360_M1_Cep170b
NGPS[Phospho (STY)]PTTPQTPGPQK.2	0.096555927	Q80U49_S965_M1_Cep170b
SNS[Phospho (STY)]LSTPRPTR.2	-0.187089253	Q80U49_S1101_M1_Cep170b
RGHGS[Phospho (STY)]RGS[Phospho (STY)]LDWPEEER.3	-0.008177942	Q80U49_S1029_M2_Cep170b
S[Phospho (STY)]PSLGNVPNTPASTISAR.2	-0.248860788	Q80U49_S1347_M1_Cep170b
SNS[Phospho (STY)]LSTPR.2	0.04009997	Q80U49_S1101_M1_Cep170b
DSLPSDS[Phospho (STY)]PPITVQPQANNR.2	-0.209307598	Q80U56_S368_M1_Avl9
TEKPYFQVEGNNNK.2	-0.041280194	#N/A
RLS[Phospho (STY)]QSS[Phospho (STY)]LESATSPSC[Carbamidomethyl (C)]S.2	0.058675828	Q80U57_S294_M2_Rims3
LSQSS[Phospho (STY)]LESATSPSC[Carbamidomethyl (C)]S.2	-0.077982142	Q80U57_S297_M1_Rims3
SSS[Phospho (STY)]ISGEIC[Carbamidomethyl (C)]GSQQAGGGAGTTTAK.2	-0.375914464	Q80U57_S22_M1_Rims3
QGSRESTDGSTNS[Phospho (STY)]NSSEGTFIFPTR.3	-0.251203333	Q80U57_S114_M1_Rims3
QAS[Phospho (STY)]PTEVVER.2	-0.330549877	Q80U58_S181_M1_Pum2
GKAS[Phospho (STY)]PFEEDQNR.2	-0.097311217	Q80U58_S136_M1_Pum2
SAQTS[Phospho (STY)]LDS[Phospho (STY)]QEEATGILPK.2	-0.118783003	Q80U59_S1194_M2_Kiaa0232
KQES[Phospho (STY)]PLLVTK.2	-0.716769811	Q80U62_S198_M1_Rubcn
ASM[Oxidation (M)]SEFLES[Phospho (STY)]EDGEVEQQR.2	-0.571187609	Q80U70_S548_M1_Suz12
NS[Phospho (STY)]LESISSIDR.2	-0.13757471	Q80U72_S1206_M1_Scrib
M[Oxidation (M)]QS[Phospho (STY)]PELPAPER.2	-0.462041889	Q80U72_S1457_M1_Scrib
VS[Phospho (STY)]PTGAAGR.2	0.021981815	Q80U72_S1126_M1_Scrib
M[Oxidation (M)]KS[Phospho (STY)]LEQDALR.2	0.012368089	Q80U72_S1490_M1_Scrib
EDGES[Phospho (STY)]EEGQPEAAWPLPSGR.2	0.214977524	Q80U72_S583_M1_Scrib
RDS[Phospho (STY)]LTGSSDLYKR.3	-0.481807655	Q80U78_S710_M1_Pum1
RDS[Phospho (STY)]LTGSSDLYK.2	0.05696725	Q80U78_S710_M1_Pum1
S[Phospho (STY)]MDELNHDFQALALEGR.3	0.084230234	Q80U78_S124_M1_Pum1
SYS[Phospho (STY)]SPDITQALQEEEK.2	-0.236804388	Q80U87_S680_M1_Usp8
SYS[Phospho (STY)]SPDITQALQEEEKR.2	0.198603133	Q80U87_S680_M1_Usp8
RSY[Phospho (STY)]SSPDITQALQEEEKR.3	0.034859616	Q80U87_Y679_M1_Usp8
S[Phospho (STY)]VENLLDSK.2	0.147384333	Q80U87_S153_M1_Usp8

S[Phospho (STY)]FEVEEIEPPNSTPPR.2	-0.169471856	Q80UG5_S30_M1_Septin9
S[Phospho (STY)]PKPSLR.2	0.003022436	Q80UG5_S85_M1_Septin9
YLQEEVNINR.2	0.327433601	#N/A
SVQPT[Phospho (STY)]SEERIPK.2	0.426846681	Q80UG5_T329_M1_Septin9
KSVQPTS[Phospho (STY)]EERIPK.3	-0.456388364	Q80UG5_S330_M1_Septin9
S[Phospho (STY)]FEVEEIEPPNSTPPRR.3	-0.046499309	Q80UG5_S30_M1_Septin9
KTS[Phospho (STY)]LSDSTTSAYPGDAGK.2	-0.156846925	Q80UJ7_S536_M1_Rab3gap1
FSVTHHYLVQES[Phospho (STY)]PDKER.3	-0.244436287	Q80UJ7_S83_M1_Rab3gap1
ITVNGDS[Phospho (STY)]SAETEELANEI.2	0.135260229	Q80UM3_S854_M1_Naa15
RPQNTLKAS[Phospho (STY)]K.2	-0.336450571	Q80UP3_S259_M1_Dgkz
ESALSRAS[Phospho (STY)]DESLR.2	0.354583287	Q80UP8_S256_M1_Slc20a2
ESALSRAS[Phospho (STY)]DESLRK.3	0.161760454	Q80UP8_S256_M1_Slc20a2
AS[Phospho (STY)]DESLRK.2	-0.312707503	Q80UP8_S256_M1_Slc20a2
ALS[Phospho (STY)]MTHGSAK.2	-0.028027346	Q80UP8_S316_M1_Slc20a2
AS[Phospho (STY)]DES[Phospho (STY)]LRKVQEAESPGFK.3	0.2007445	Q80UP8_S256_M2_Slc20a2
GLC[Carbamidomethyl (C)]SGPGAGEES[Phospho (STY)]PAATLPR.2	-0.272510465	Q80UU9_S84_M1_Pgrmc2
DFS[Phospho (STY)]LEQLR.2	-0.210025282	Q80UU9_S98_M1_Pgrmc2
LLKPGEEPSEYT[Phospho (STY)]DEEDTKDHSK.4	0.166285132	Q80UU9_T205_M1_Pgrmc2
LLKPGEEPSEYT[Phospho (STY)]DEEDTK.3	0.415051677	Q80UU9_T205_M1_Pgrmc2
VLAELPATELVQAC[Carbamidomethyl (C)]R.2	-0.142204956	#N/A
TDAGSLYELTVR.2	0.528846993	#N/A
NPC[Carbamidomethyl (C)]GEEDLEGWS[Phospho (STY)]DVEHGGDGWR.3	-0.057639973	Q80UW2_S141_M1_Fbxo2
C[Carbamidomethyl (C)]QQEGLVPEGS[Phospho (STY)]ADEERDHWQQFYFLSK.4	-0.283888114	Q80UW2_S106_M1_Fbxo2
EAEEEEEAEAVEYLAELPEPLLLR.3	0.46513156	#N/A
FEHGGQDSVYWK.3	0.179002441	#N/A
[Acetyl (Protein N-term)]S[Phospho (STY)]RHEGVSC[Carbamidomethyl (C)]DAC[Carbamidomethyl (C)]LK.2	0.293345041	Q80UY2_S2_M1_Kcmf1
IIEQPEC[Carbamidomethyl (C)]SS[Phospho (STY)]PVIETER.2	-0.13836931	Q80V94_S962_M1_Ap4e1
DLPDGHAVS[Phospho (STY)]PR.2	-0.308671546	Q80VC9_S334_M1_Camsap3
AVASS[Phospho (STY)]PAANNSEVK.2	-0.19166675	Q80VC9_S555_M1_Camsap3
RAS[Phospho (STY)]LLER.2	-0.252506508	Q80VC9_S903_M1_Camsap3
NTETVPS[Phospho (STY)]QNNSGSSSPVFNFR.2	0.278677905	Q80VC9_S343_M1_Camsap3
KFS[Phospho (STY)]PSQVPVQTR.2	-0.399652403	Q80VC9_S812_M1_Camsap3
APSPSGLM[Oxidation (M)]S[Phospho (STY)]PSR.2	-0.488885462	Q80VC9_S1083_M1_Camsap3
RS[Phospho (STY)]PGPGPSPTPR.2	0.269513696	Q80VC9_S767_M1_Camsap3
SVS[Phospho (STY)]SDSLGPPRPVSTSSR.3	-0.121326663	Q80VC9_S431_M1_Camsap3
[Acetyl (Protein N-term)]M[Oxidation (M)]DPQPPPPAQGS[Phospho (STY)]PPHR.2	-0.192742241	Q80VM4_S12_M1_Znf579
GSGTESAPSDTDAS[Phospho (STY)]LEVER.2	-0.288834938	Q80VP0_S418_M1_Tecpr1
TALPTSGS[Phospho (STY)]STGELELLAGEVPAR.2	-0.518213067	Q80VP1_S418_M1_Epn1
[Acetyl (Protein N-term)]STSS[Phospho (STY)]LRR.2	-0.357069044	Q80VP1_S5_M1_Epn1
NIVHNYSEAEIK.2	-0.131711505	#N/A
T[Phospho (STY)]PESFLGPNAALVDLDSLVSR.2	-0.11727706	Q80VP1_T469_M1_Epn1
ENMYAVQTLK.2	-0.20745932	#N/A
HIETPATSS[Phospho (STY)]PK.2	-0.168124756	Q80VY2_S281_M1_Inka2
GS[Phospho (STY)]PHLLR.2	-0.086995838	Q80W04_S189_M1_Tmcc2
NKFGS[Phospho (STY)]ADNIAHLK.3	-0.279127514	Q80W04_S435_M1_Tmcc2
ALSGSATLVSS[Phospho (STY)]PK.2	-0.327344155	Q80W04_S467_M1_Tmcc2
NKFGS[Phospho (STY)]ADNIAHLKDPMEDGPPEEAAR.4	-0.265841238	Q80W04_S435_M1_Tmcc2

C[Carbamidomethyl (C)]KS[Phospho (STY)]DELQQQQGEEDGAGMEDAAC[Carbamidomethyl (C)]LLPGADLR.3	-0.144409849	Q80W04_S6_M1_Tmcc2
IRVDILENQLM[Oxidation (M)]DNR.3	-0.126667878	#N/A
GAYVPSS[Phospho (STY)]PTR.2	-0.237916376	Q80W47_S395_M1_Wipi2
ELGGC[Carbamidomethyl (C)]SQAGNR.2	-0.438475625	#N/A
SETNWES[Phospho (STY)]PK.2	-0.31028182	Q80WJ7_S565_M1_Mtdh
SQEPISNDQKVS[Phospho (STY)]DDDKEK.3	-0.025143843	Q80WJ7_S423_M1_Mtdh
SETNWES[Phospho (STY)]PKQIK.2	-0.303244986	Q80WJ7_S565_M1_Mtdh
SETNWES[Phospho (STY)]PKQIKK.2	-0.165904271	Q80WJ7_S565_M1_Mtdh
S[Phospho (STY)]LDESTLRK.2	-0.058602792	Q80WQ4_S253_M1_Trmt9b
S[Phospho (STY)]LDESTLR.2	-0.048340135	Q80WQ4_S253_M1_Trmt9b
S[Phospho (STY)]HSM[Oxidation (M)]DYGSAVAR.2	-0.180855687	Q80WQ4_S210_M1_Trmt9b
SFS[Phospho (STY)]PGDFR.2	0.08981582	Q80WT5_S151_M1_Aftph
HLLETHSS[Phospho (STY)]PAK.2	-0.48154628	Q80WT5_S680_M1_Aftph
S[Phospho (STY)]PDPDPTGQNALDDSAASMK.2	-0.624174382	Q80WT5_S395_M1_Aftph
ARPAS[Phospho (STY)]TS[Phospho (STY)]PSPGTR.2	-0.098122831	Q80WV7_S464_M2_Srrm3
RRDS[Phospho (STY)]PSFMEPR.3	0.287423963	Q80WV7_S562_M1_Srrm3
RYPSSISSS[Phospho (STY)]PQKDLTQAK.3	-0.284103723	Q80X50_S629_M1_Ubap2l
RYPSSISSS[Phospho (STY)]PQK.2	-0.184715622	Q80X50_S629_M1_Ubap2l
YPSSISSS[Phospho (STY)]PQK.2	0.015493249	Q80X50_S629_M1_Ubap2l
TATEEWGTEDWNEDLSETK.2	-0.291882583	#N/A
YPSSISSS[Phospho (STY)]PQKDLTQAK.2	0.198514765	Q80X50_S629_M1_Ubap2l
NQDEC[Carbamidomethyl (C)]VIALHDC[Carbamidomethyl (C)]NGDVNR.3	0.081544424	#N/A
STSAPQMS[Phospho (STY)]PGSSDNQSSSPQPAQQK.3	0.113901498	Q80X50_S487_M1_Ubap2l
[Acetyl (Protein N-term)]AGRPHPYDSNSS[Phospho (STY)]DPENWDR.3	-0.485923701	Q80X66_S13_M1_Btbd10
NLGT[Phospho (STY)]PTSSTPR.2	-0.387201289	Q80X80_T417_M1_C2cd2l
VDGKLDS[Phospho (STY)]PSR.2	-0.072315159	Q80X80_S464_M1_C2cd2l
LDS[Phospho (STY)]PSRS[Phospho (STY)]PSKVEVTEK.3	0.210734566	Q80X80_S464_M2_C2cd2l
NLGT[Phospho (STY)]PTS[Phospho (STY)]STPRPSITPTKK.3	0.020395223	Q80X80_S420_M2_C2cd2l
LDS[Phospho (STY)]PSRSPSK.2	-0.229340622	Q80X80_S464_M1_C2cd2l
KAGS[Phospho (STY)]FSR.2	-0.06864897	Q80X80_S681_M1_C2cd2l
EAGLSQS[Phospho (STY)]HDDLSNTTATPSVR.3	-0.384758412	Q80X80_S662_M1_C2cd2l
S[Phospho (STY)]PSKVEVTEK.2	-0.426951961	Q80X80_S468_M1_C2cd2l
TST[Phospho (STY)]PEEAGVR.2	-0.031169305	Q80X80_T79_M1_C2cd2l
KKAGS[Phospho (STY)]FSR.2	-0.176504884	Q80X80_S681_M1_C2cd2l
NLGT[Phospho (STY)]PTSSTPRPSIT[Phospho (STY)]PTK.2	-0.076483258	Q80X80_T417_M2_C2cd2l
ESHLS[Phospho (STY)]NGLDPVAETAIR.3	0.07050228	Q80X80_S502_M1_C2cd2l
VDGKLDS[Phospho (STY)]PSRS[Phospho (STY)]PSK.2	0.082318927	Q80X80_S464_M2_C2cd2l
SLSPAATVTAELHYEQGS[Phospho (STY)]PR.3	-0.447867444	Q80X80_S411_M1_C2cd2l
QKEAGLSQS[Phospho (STY)]HDDLSNTTATPSVR.3	0.243706664	Q80X80_S662_M1_C2cd2l
SDISERPSVDDVES[Phospho (STY)]ETGSTGALETR.3	-0.000265397	Q80X80_S619_M1_C2cd2l
TSS[Phospho (STY)]PETREPESK.2	0.091336515	Q80X82_S1276_M1_Sympk
APS[Phospho (STY)]QAAESSPTK.2	0.018892396	Q80XA6_S405_M1_Reps2
ASS[Phospho (STY)]LDLNK.2	-0.198075322	Q80XA6_S354_M1_Reps2
LNS[Phospho (STY)]ELQQLK.2	-0.372233996	Q80XA6_S492_M1_Reps2
VS[Phospho (STY)]LSHGYSK.2	-0.200871379	Q80XA6_S67_M1_Reps2
SYS[Phospho (STY)]STSIEEAMK.2	0.018593271	Q80XA6_S324_M1_Reps2
SFS[Phospho (STY)]VEREPQENNSNYPDEPWR.3	-0.025708416	Q80XA6_S119_M1_Reps2

APSQAAESSPT[Phospho (STY)]KMDAPHAQPPSKPIR.4	-0.314158337	Q80XA6_T413_M1_Reps2
RGEDPPT[Phospho (STY)]PPPRPQK.3	0.359672358	Q80XA6_T340_M1_Reps2
SVGRPS[Phospho (STY)]PK.2	-0.322732872	Q80XC3_S394_M1_Usp6nl
GDNWYS[Phospho (STY)]EDEDTDTEEYK.2	0.302059997	Q80XE1_S468_M1_Ric8b
AYEAVSIEQDDS[Phospho (STY)]PSLELGR.2	-0.458399785	Q80XG9_S560_M1_Lrrtm4
TSS[Phospho (STY)]PTSLPPLAR.2	-0.429918668	Q80XI3_S267_M1_Eif4g3
NPT[Phospho (STY)]PPIGR.2	-0.277363229	Q80XI3_T168_M1_Eif4g3
GSS[Phospho (STY)]KDLLDNQSQEEQRR.3	-0.200198971	Q80XI3_S1151_M1_Eif4g3
GSS[Phospho (STY)]KDLLDNQSQEEQR.2	0.19755809	Q80XI3_S1151_M1_Eif4g3
T[Phospho (STY)]PDEVLEAEAEPK.2	0.301329319	Q80XI3_T513_M1_Eif4g3
AESES[Phospho (STY)]DGQAEETADPQSLHSGR.3	-0.133467414	Q80XI3_S472_M1_Eif4g3
FGIDDQDYQNSVTR.2	0.059917541	#N/A
DFATAPPTS[Phospho (STY)]PAK.2	-0.068083753	Q80XK6_S1571_M1_Atg2b
LS[Phospho (STY)]PSWNPK.2	-0.226228492	Q80XK6_S255_M1_Atg2b
S[Phospho (STY)]SPVC[Carbamidomethyl (C)]STAPVETEPK.2	-0.028020133	Q80XK6_S239_M1_Atg2b
S[Phospho (STY)]PGADVTC[Carbamidomethyl (C)]SLPR.2	-0.225802632	Q80XK6_S1741_M1_Atg2b
SAS[Phospho (STY)]VDESRPEFIC[Carbamidomethyl (C)]R.2	-0.404658305	Q80XK6_S496_M1_Atg2b
AFTEVFLDDSHS[Phospho (STY)]PANR.3	-0.470789323	Q80XK6_S734_M1_Atg2b
VSVVEPGNFIAATSLYSPER.2	-0.016904504	#N/A
FGIEAFSDC[Carbamidomethyl (C)]LR.2	0.028884816	#N/A
AILITGC[Carbamidomethyl (C)]DSGFGFSLAK.2	-0.563612085	#N/A
VVNISSMLGR.2	0.466445351	#N/A
SPYC[Carbamidomethyl (C)]ITK.2	-0.350459503	#N/A
VSSLS[Phospho (STY)]PEQEQGLWK.2	0.008178634	Q80XP8_S193_M1_Fam76b
EGETTVNTETSS[Phospho (STY)]PK.2	-0.516737637	Q80XP9_S1039_M1_Wnk3
S[Phospho (STY)]VEM[Oxidation (M)]TEDDKVAESSR.2	0.268981851	Q80XP9_S62_M1_Wnk3
TISSSPS[Phospho (STY)]IESLPGGR.2	0.027902366	Q80XQ2_S565_M1_Tbc1d5
EFTGS[Phospho (STY)]PPPSATKK.2	0.092993698	Q80XQ2_S578_M1_Tbc1d5
SES[Phospho (STY)]MPVQLNK.2	0.103585378	Q80XQ2_S546_M1_Tbc1d5
[Acetyl (Protein N-term)]M[Oxidation (M)]NPVYS[Phospho (STY)]PGSSGVPYANAK.2	-0.358081516	Q80XQ8_S6_M1_Fam168b
ATVTPS[Phospho (STY)]PVKGK.2	-0.239009584	Q80XU3_S181_M1_Nucks1
ATVTPS[Phospho (STY)]PVK.2	-0.047749566	Q80XU3_S181_M1_Nucks1
LKATVTPS[Phospho (STY)]PVK.2	-0.409118982	Q80XU3_S181_M1_Nucks1
TPSPKEEDEEAES[Phospho (STY)]PPEKK.3	-0.048186939	Q80XU3_S214_M1_Nucks1
EEDEEAES[Phospho (STY)]PPEKK.2	-0.57330972	Q80XU3_S214_M1_Nucks1
KVVDYSQFQES[Phospho (STY)]DDADEDYGRDSGPPAK.4	-0.192004497	#N/A
KVVDYSQFQES[Phospho (STY)]DDADEDYGR.3	0.176966635	#N/A
VVDYSQFQESDDADEDY[Phospho (STY)]GRDSGPPAK.3	-0.282218703	Q80XU3_Y26_M1_Nucks1
LKATVT[Phospho (STY)]PS[Phospho (STY)]PVKGK.2	0.048225537	Q80XU3_S181_M2_Nucks1
NSQEDS[Phospho (STY)]EDS[Phospho (STY)]EEKDVK.2	-0.363316077	Q80XU3_S58_M2_Nucks1
VVDYSQFQES[Phospho (STY)]DDADEDYGR.2	0.272684903	#N/A
VVDYSQFQES[Phospho (STY)]DDADEDYGRDSGPPAKK.4	0.02723752	#N/A
SGKNS[Phospho (STY)]QEDS[Phospho (STY)]EDS[Phospho (STY)]EEKDVK.2	-0.07683433	Q80XU3_S54_M3_Nucks1
DDSHS[Phospho (STY)]AEDS[Phospho (STY)]EDEKDDHKNVR.4	0.129588893	Q80XU3_S75_M2_Nucks1
EM[Oxidation (M)]LLEDVGS[Phospho (STY)]EEEPEEDDEAPFQEK.3	-0.09183352	Q80XU3_S113_M1_Nucks1
KDDS[Phospho (STY)]HS[Phospho (STY)]AEDS[Phospho (STY)]EDEKDDHK.4	-0.054079552	Q80XU3_S73_M3_Nucks1
SC[Carbamidomethyl (C)]S[Phospho (STY)]LDLGDTGGC[Carbamidomethyl (C)]YGYAR.2	-0.171707614	Q80XU8_S585_M1_Lrfn4

GVGGS[Phospho (STY)]AERLEESVV.2	-0.009406953	Q80XU8_S627_M1_Lrfn4
SALVDEKDQDS[Phospho (STY)]K.2	-0.163760376	Q80Y14_S151_M1_Glrx5
S[Phospho (STY)]LRQS[Phospho (STY)]FRR.2	0.058889182	Q80Y17_S658_M2_Llgl1
S[Phospho (STY)]LRQS[Phospho (STY)]FR.2	-0.26087189	Q80Y17_S658_M2_Llgl1
DIILAPES[Phospho (STY)]C[Carbamidomethyl (C)]EGS[Phospho (STY)]PSSAHSKR.3	-0.474932132	Q80Y17_S982_M2_Llgl1
DIILAPESC[Carbamidomethyl (C)]EGS[Phospho (STY)]PSSAHSK.2	-0.178396321	Q80Y17_S986_M1_Llgl1
S[Phospho (STY)]FQESLGQGSR.2	-0.14487981	Q80Y24_S732_M1_Prickle2
YVTS[Phospho (STY)]DELLHK.2	0.203854327	Q80Y24_S807_M1_Prickle2
AC[Carbamidomethyl (C)]SAGEDPNGS[Phospho (STY)]DSSDSAFQNAR.2	0.182236478	Q80Y24_S319_M1_Prickle2
S[Phospho (STY)]DNALHLASER.2	-0.492473874	Q80Y24_S696_M1_Prickle2
GHGGS[Phospho (STY)]FIQEC[Carbamidomethyl (C)]R.2	0.127002499	Q80Y24_S463_M1_Prickle2
S[Phospho (STY)]REEPFHYGNK.3	0.018180681	Q80Y24_S396_M1_Prickle2
SRS[Phospho (STY)]DNALHLASER.3	0.033840899	Q80Y24_S696_M1_Prickle2
QRS[Phospho (STY)]FQESLGQGSR.2	-0.092204266	Q80Y24_S732_M1_Prickle2
GS[Phospho (STY)]M[Oxidation (M)]ESLALSNATGLSAEGGAK.2	-0.144237296	Q80Y24_S544_M1_Prickle2
YTEDM[Oxidation (M)]T[Phospho (STY)]PTEQTPR.2	-0.125209944	Q80Y24_T535_M1_Prickle2
TVS[Phospho (STY)]DLALQNAFGER.2	0.159239357	Q80Y24_S754_M1_Prickle2
M[Oxidation (M)]HQS[Phospho (STY)]FDFDGGIASSK.2	-0.066974776	Q80Y24_S643_M1_Prickle2
LMS[Phospho (STY)]QESYSDMSSQSFNETR.2	0.404534813	Q80Y24_S479_M1_Prickle2
LPGQEGVHIQPMS[Phospho (STY)]ER.3	0.293696511	Q80Y24_S667_M1_Prickle2
ADQSISEEPGWEEEEEELEGIVPS[Phospho (STY)]PK.3	0.09105359	Q80Y55_S234_M1_Bsdc1
DSLSTHTS[Phospho (STY)]PSQS[Phospho (STY)]PNSVHGSR.3	0.234890804	Q80Y56_S214_M2_Rbsn
[Acetyl (Protein N-term)]AS[Phospho (STY)]LDDPGEVR.2	-0.052690008	Q80Y56_S3_M1_Rbsn
TS[Phospho (STY)]PNRLS[Phospho (STY)]PK.2	-0.212349635	Q80Y81_S195_M2_Elac2
AHTDEPHS[Phospho (STY)]PQSK.2	0.002308138	Q80Y81_S815_M1_Elac2
QQPS[Phospho (STY)]QSPRTS[Phospho (STY)]PNRLS[Phospho (STY)]PK.3	0.454081041	Q80Y81_S189_M3_Elac2
QQPSQS[Phospho (STY)]PR.2	0.209240079	Q80Y81_S191_M1_Elac2
TDEPDS[Phospho (STY)]PSSR.2	-0.273138727	Q80Y83_S287_M1_Dixdc1
GSES[Phospho (STY)]PNSFLDQEYR.2	-0.430566828	Q80YA9_S390_M1_Cnksr2
RENS[Phospho (STY)]LLR.2	-0.185849888	Q80YA9_S467_M1_Cnksr2
LGDS[Phospho (STY)]LQDLYR.2	0.144407069	Q80YA9_S906_M1_Cnksr2
GSES[Phospho (STY)]PNSFLDQEYRK.2	0.182737407	Q80YA9_S390_M1_Cnksr2
STYDGLHVITGTTENS[Phospho (STY)]PADR.3	0.131863536	Q80YA9_S248_M1_Cnksr2
QEVTGSSAVS[Phospho (STY)]PIRK.2	0.091225695	Q80YA9_S756_M1_Cnksr2
QEVTGSSAVS[Phospho (STY)]PIR.2	-0.357744434	Q80YA9_S756_M1_Cnksr2
IKQEQDYWS[Phospho (STY)]ES[Phospho (STY)]DKEEADTPSTPK.3	-0.253022177	Q80YA9_S685_M2_Cnksr2
EEKLGDS[Phospho (STY)]LQDLYR.2	-0.102495619	Q80YA9_S906_M1_Cnksr2
SPTSSVATPSSTIS[Phospho (STY)]T[Phospho (STY)]PTKR.2	-0.172096224	Q80YA9_S338_M2_Cnksr2
EQDLRS[Phospho (STY)]PPS[Phospho (STY)]PGHQSVQAFPR.3	0.225918219	Q80YE4_S1032_M2_Aatk
AVDSGYDTENYES[Phospho (STY)]PEFVLK.2	-0.018571379	Q80YE4_S950_M1_Aatk
SPPS[Phospho (STY)]PGHQSVQAFPR.3	0.037504333	Q80YE4_S1035_M1_Aatk
AS[Phospho (STY)]PEVGHLLSQEDPR.2	-0.402390139	Q80YE4_S718_M1_Aatk
QQS[Phospho (STY)]DGSLVR.2	-0.510322403	Q80YF9_S995_M1_Arhgap33
TPAS[Phospho (STY)]PVER.2	-0.421796509	Q80YF9_S594_M1_Arhgap33
TSAWLDDGDDLDFS[Phospho (STY)]PPR.2	0.042576667	Q80YF9_S749_M1_Arhgap33
S[Phospho (STY)]AKSEESLSSQASGAGLQR.3	-0.516402731	Q80YF9_S657_M1_Arhgap33
AQQVAEQQSQQEQGGTPPAPHS[Phospho (STY)]PFR.3	0.221392175	Q80YF9_S949_M1_Arhgap33

RQS[Phospho (STY)]LGGFLK.2	-0.510815528	Q80YN3_S601_M1_Bcas1
LGLS[Phospho (STY)]FRK.2	-0.265740665	Q80YN3_S347_M1_Bcas1
ANFTPQETR.2	-0.001370684	#N/A
AGPTS[Phospho (STY)]LPLGK.2	-0.1485699	Q80YN3_S414_M1_Bcas1
TLVSPNKTETK.2	-0.261706841	#N/A
ADS[Phospho (STY)]VC[Carbamidomethyl (C)]DGHAAGQK.2	0.276296078	Q80YN3_S316_M1_Bcas1
M[Oxidation (M)]SDAQVQTDPVSIGPVGK.2	-0.249413648	#N/A
TPS[Phospho (STY)]PPEPEPAGTAQK.2	0.077917743	Q80YN3_S525_M1_Bcas1
EPAPC[Carbamidomethyl (C)]VQPPTVEANAM[Oxidation (M)]QTGDKT[Phospho (STY)]PK.3	-0.337423431	Q80YN3_T591_M1_Bcas1
SC[Carbamidomethyl (C)]S[Phospho (STY)]PPPPPPEPTSEGR.2	0.216581181	Q80YN3_S390_M1_Bcas1
RM[Oxidation (M)]S[Phospho (STY)]DAQVQTDPVSIGPVGK.3	-0.319836297	Q80YN3_S615_M1_Bcas1
S[Phospho (STY)]VKEDTLSTGAEENAVC[Carbamidomethyl (C)]ESPVETVR.3	-0.174798096	Q80YN3_S425_M1_Bcas1
KS[Phospho (STY)]VKEDTLSTGAEENAVC[Carbamidomethyl (C)]ESPVETVR.3	-0.041178206	Q80YN3_S425_M1_Bcas1
EDTLSTGAEENAVC[Carbamidomethyl (C)]ES[Phospho (STY)]PVETVR.3	-0.117895483	Q80YN3_S443_M1_Bcas1
QS[Phospho (STY)]LGGFLK.2	0.013937902	Q80YN3_S601_M1_Bcas1
MEEEGS[Phospho (STY)]EDNGLEEDSR.2	-0.289520495	Q80YR5_S95_M1_Safb2
SGTSSPQS[Phospho (STY)]PVFR.2	-0.2952247	Q80YS6_S669_M1_Afap1
LC[Carbamidomethyl (C)]SSSS[Phospho (STY)]SDTSPR.2	-0.272296693	Q80YV2_S409_M1_Zc3hc1
SQDATVSPGSEQS[Phospho (STY)]EKSPGPIVSR.3	-0.090618954	Q80YV2_S340_M1_Zc3hc1
SQDATVS[Phospho (STY)]PGSEQSEK.2	0.286087474	Q80YV2_S334_M1_Zc3hc1
DTAATFQSVDGS[Phospho (STY)]PQAEQSPLESTSK.3	-0.202773838	Q80YV2_S62_M1_Zc3hc1
SWESSSPVDRPELEAAS[Phospho (STY)]PTTR.3	0.185518731	Q80YV2_S369_M1_Zc3hc1
VRS[Phospho (STY)]FDHPGK.2	-0.278302711	Q80YV4_S63_M1_Pank4
VIVNFAPTIQEIK.2	-0.357851958	#N/A
DQAGEYEC[Carbamidomethyl (C)]SAENDVSFPDVK.2	-0.034458635	#N/A
LFNGQQGIIIQNFSTR.2	-0.201787984	#N/A
LQQTVPADAS[Phospho (STY)]PDSK.2	-0.373920415	Q80Z37_S99_M1_Topors
APSPVVS[Phospho (STY)]PTELSK.2	-0.493786954	Q80Z38_S1338_M1_Shank2
NSPAFLS[Phospho (STY)]TDLGDEDVGLGPPAPR.3	-0.133787923	Q80Z38_S729_M1_Shank2
RNSPAFLS[Phospho (STY)]TDLGDEDVGLGPPAPR.3	-0.127237517	Q80Z38_S729_M1_Shank2
QSNVEDS[Phospho (STY)]PEK.2	0.200246367	Q80Z38_S648_M1_Shank2
TIVAAGS[Phospho (STY)]VEEAVILPFR.2	-0.076503689	Q80Z38_S1005_M1_Shank2
S[Phospho (STY)]KSM[Oxidation (M)]TAELEELGLSLVDK.3	-0.592720397	Q80Z38_S370_M1_Shank2
ELDRFS[Phospho (STY)]LDSEDVYSR.2	0.345005453	Q80Z38_S586_M1_Shank2
S[Phospho (STY)]NSDNNLNAGAPEWAVC[Carbamidomethyl (C)]SAATSHR.3	-0.164238277	Q80Z38_S41_M1_Shank2
ANVISELNSILQQM[Oxidation (M)]NR.2	-0.350236501	#N/A
QSNVEDSPEKTC[Carbamidomethyl (C)]S[Phospho (STY)]IPIPTIIVK.3	-0.229783153	Q80Z38_S654_M1_Shank2
DNEGFGFVLR.2	-0.294194932	#N/A
RAPSPVVS[Phospho (STY)]PTELSK.2	-0.085414979	Q80Z38_S1338_M1_Shank2
RELDRFS[Phospho (STY)]LDSEDVYSR.3	-0.180728241	Q80Z38_S586_M1_Shank2
IFLSGITEEER.2	-0.266734633	#N/A
SM[Oxidation (M)]T[Phospho (STY)]AELEELGLSLVDK.2	-0.439283864	Q80Z38_T374_M1_Shank2
RM[Oxidation (M)]S[Phospho (STY)]DPESVSSVFSDTPIVV.2	-0.186796105	Q80ZD8_S476_M1_Amigo1
SETGSTISM[Oxidation (M)]SS[Phospho (STY)]LER.2	-0.109515705	Q80ZF8_S1411_M1_Adgrb3
QSPS[Phospho (STY)]AGVHTFC[Carbamidomethyl (C)]DR.2	-0.387840762	Q80ZK9_S229_M1_Wdtc1
KDS[Phospho (STY)]ISEDEM[Oxidation (M)]VLR.2	-0.522883367	Q80ZK9_S511_M1_Wdtc1
GC[Carbamidomethyl (C)]IS[Phospho (STY)]PQVELPPYLER.2	0.088659557	Q80ZK9_S352_M1_Wdtc1

ESGVDPSQVLLS[Phospho (STY)]PDSSEAAR.2	0.174576716	Q80ZX8_S703_M1_Spag1
YM[Oxidation (M)]AENPTAGVVQEEEEDNLEY[Phospho (STY)]DSDGNPIAPSKK.4	0.402305669	Q810A7_Y183_M1_Ddx42
ESGAAEQVDNK.2	0.354955141	#N/A
VYDC[Carbamidomethyl (C)]GSHSLS[Phospho (STY)]D.2	-0.033345629	Q810C1_S695_M1_Slitrk1
NLILAPGEDGR.2	-0.102317345	#N/A
DLELTDLAER.2	-0.041067131	#N/A
DNILIEC[Carbamidomethyl (C)]EAK.2	0.140050868	#N/A
YVVGQTPVYVPYEIR.2	0.318267071	#N/A
EVAGDTIIFR.2	0.415549093	#N/A
EDDSLTIFGVAER.2	0.011764768	#N/A
AYLTVLADQATPTNR.2	0.163006073	#N/A
LDC[Carbamidomethyl (C)]PFFGSPIPTLR.2	0.358475149	#N/A
ITNVSEEDSGEYFC[Carbamidomethyl (C)]LASNK.2	-0.09459428	#N/A
VIAVNEVGSSHPSLPSER.2	0.116246352	#N/A
GTTVQLEC[Carbamidomethyl (C)]R.2	0.000282142	#N/A
SGTLVIDFR.2	0.173273155	#N/A
DQGSYTC[Carbamidomethyl (C)]MASTELDQDLAK.2	-0.319061384	#N/A
DKEETEGNESSEATS[Phospho (STY)]PVNAIYSLA.3	-0.140441198	Q810U3_S1231_M1_Nfasc
SVQLTWTPGDDNNSPITK.2	-0.401265764	#N/A
GFHTPEGVPSAPSSLK.3	-0.29973056	#N/A
DNGELPNDER.2	0.047610856	#N/A
ENIVIQC[Carbamidomethyl (C)]EAK.2	0.414635368	#N/A
DSTGTYTC[Carbamidomethyl (C)]VAR.2	0.550307184	#N/A
EDDGTFGEYS[Phospho (STY)]DAEDHKPLK.3	0.077753089	Q810U4_S1178_M1_Nrcam
NEVHLEIKDPTR.3	0.199218848	#N/A
ISWLTNGVPIEIALDDPSR.2	-0.400446083	#N/A
TLQITHVSEADSGNYQC[Carbamidomethyl (C)]IAK.3	-0.187113308	#N/A
HQAEVSGTQTTAQLK.3	-0.293323256	#N/A
EPAEGNESSEAPS[Phospho (STY)]PVNAMNSFV.2	-0.513153784	Q810U4_S1247_M1_Nrcam
EKEPAEGNESSEAPS[Phospho (STY)]PVNAM[Oxidation (M)]NSFV.3	-0.11781274	Q810U4_S1247_M1_Nrcam
AETYEGVYQC[Carbamidomethyl (C)]TAR.2	0.182930521	#N/A
RFST[Phospho (STY)]EEFVLL.2	0.103673227	Q811C2_T452_M1_Atg4c
DFDFTSTAAS[Phospho (STY)]EEDLFSEDERK.3	0.527466826	Q811C2_S434_M1_Atg4c
DYHFVTSR.2	-0.370644322	#N/A
AVEALKEAGS[Phospho (STY)]IVR.2	-0.401751452	Q811D0_S301_M1_Dlg1
QVTPDGESDEVGVIPSKR.3	0.047192031	#N/A
QIIEEQSGPYIWVPAK.2	0.294965382	#N/A
VNDC[Carbamidomethyl (C)]ILR.2	0.340140466	#N/A
IISVNSVDLR.2	-0.157217771	#N/A
IIEGGAAHK.2	0.196598808	#N/A
IITGGAAAQDGR.2	0.103497124	#N/A
EQM[Oxidation (M)]M[Oxidation (M)]NSSVSSGSGS[Phospho (STY)]LR.2	-0.582103701	Q811D0_S575_M1_Dlg1
ALFDYDK.2	-0.196622912	#N/A
NLSEIENVHGFVSHSHIS[Phospho (STY)]PIK.3	-0.009249517	Q811D0_S158_M1_Dlg1
T[Phospho (STY)]KDSGLPSQGLNFR.3	-0.0150333	Q811D0_T595_M1_Dlg1
HC[Carbamidomethyl (C)]ILDVSGNAIK.2	-0.154075814	#N/A
HVTSNAS[Phospho (STY)]DSESSYR.2	0.000958862	Q811D0_S688_M1_Dlg1

AASHEQAAAALK.2	0.346043982	#N/A
TRGDKGEIPDDM[Oxidation (M)]GS[Phospho (STY)]K.3	-0.385667161	Q811D0_S677_M1_Dlg1
FIEAGQYNNHLYGTSVQSVR.3	0.139885078	#N/A
NAGQAVTIVAQYRPEEYSR.3	-0.443733212	#N/A
EAGS[Phospho (STY)]IVR.2	-0.166851355	Q811D0_S301_M1_Dlg1
RGS[Phospho (STY)]GLPPEGADGSGVSELEANPFYDR.3	-0.147812874	Q811I0_S52_M1_Atpaf1
TPSPTPQPTS[Phospho (STY)]PQR.2	0.255997738	Q811L6_S1389_M1_Mast4
SDS[Phospho (STY)]LPSFR.2	0.194814934	Q811L6_S2517_M1_Mast4
KHS[Phospho (STY)]LEVTQEEVQR.3	-0.076713338	Q811L6_S1465_M1_Mast4
SLSYSAS[Phospho (STY)]FPEAQPGVR.2	0.081302146	Q811L6_S2461_M1_Mast4
AVS[Phospho (STY)]PEGDER.2	-0.437778935	Q811P8_S1823_M1_Arhgap32
DVINRS[Phospho (STY)]PTQLQLGK.2	0.078704414	Q811P8_S952_M1_Arhgap32
S[Phospho (STY)]PTQLQLGK.2	-0.185389493	Q811P8_S952_M1_Arhgap32
TQAQVSS[Phospho (STY)]PIVTENK.2	0.055489742	Q811P8_S613_M1_Arhgap32
NES[Phospho (STY)]EPSEM[Oxidation (M)]K.2	-0.050825706	Q811P8_S682_M1_Arhgap32
KLHDM[Oxidation (M)]GC[Carbamidomethyl (C)]S[Phospho (STY)]LPEHR.3	-0.432364946	Q811P8_S1876_M1_Arhgap32
LSPFFTLDLS[Phospho (STY)]PTDDK.2	0.071792065	Q811P8_S871_M1_Arhgap32
DAEAGGSQSQTPGSTASSEPVS[Phospho (STY)]PVQEK.3	-0.398172901	Q811P8_S856_M1_Arhgap32
S[Phospho (STY)]SSDALSASFNGDVLGNR.2	-0.370684909	Q811P8_S730_M1_Arhgap32
SAKS[Phospho (STY)]EESLTSLHAVDGDSK.3	-0.37225885	Q811P8_S706_M1_Arhgap32
SLLVSS[Phospho (STY)]PSTK.2	-0.115948155	Q811P8_S592_M1_Arhgap32
S[Phospho (STY)]EESLTSLHAVDGDSK.2	0.219600992	Q811P8_S706_M1_Arhgap32
LSS[Phospho (STY)]RDFAFYNPR.2	0.29761903	Q811P8_S1689_M1_Arhgap32
S[Phospho (STY)]FFNLGK.2	0.060477625	Q811P8_S662_M1_Arhgap32
AES[Phospho (STY)]FPGHSC[Carbamidomethyl (C)]GFAAPVPPTR.3	0.041595838	Q811P8_S1405_M1_Arhgap32
QS[Phospho (STY)]PVSSPTR.2	-0.27342491	Q811Q9_S319_M1_Pcyt1b
M[Oxidation (M)]LQALS[Phospho (STY)]PK.2	-0.104269659	Q811Q9_S315_M1_Pcyt1b
AASASISS[Phospho (STY)]MS[Phospho (STY)]EGDEDEK.2	-0.243341057	Q811Q9_S360_M2_Pcyt1b
MLQALS[Phospho (STY)]PKQS[Phospho (STY)]PVSSPTR.2	0.050238751	Q811Q9_S315_M2_Pcyt1b
GRIES[Phospho (STY)]PEK.2	-0.325670365	Q812A2_S908_M1_Srgap3
GRIES[Phospho (STY)]PEKR.2	-0.163419427	Q812A2_S908_M1_Srgap3
SGGDTHS[Phospho (STY)]PPR.2	-0.154671526	Q812A2_S874_M1_Srgap3
ADSEASS[Phospho (STY)]GPLLDDK.2	0.084693814	Q812A2_S821_M1_Srgap3
SSS[Phospho (STY)]SSSSGVGSPAVTPTEK.2	-0.590579623	Q812A2_S1070_M1_Srgap3
RRS[Phospho (STY)]GGDTHS[Phospho (STY)]PPR.2	0.044637362	Q812A2_S868_M2_Srgap3
IES[Phospho (STY)]PEKR.2	-0.094386936	Q812A2_S908_M1_Srgap3
NDLQS[Phospho (STY)]PTEHISDYGFGGVM[Oxidation (M)]GR.3	0.07616612	Q812A2_S837_M1_Srgap3
SGGDTHS[Phospho (STY)]PPRGLGPSIDTPPR.3	-0.094382627	Q812A2_S874_M1_Srgap3
MATFGS[Phospho (STY)]AGSINYPDKK.2	-0.220033999	Q812A2_S919_M1_Srgap3
LRS[Phospho (STY)]DGAAIPR.2	-0.223962816	Q812A2_S858_M1_Srgap3
AAAC[Carbamidomethyl (C)]PSS[Phospho (STY)]PHKIPLSR.3	0.236822751	Q812A2_S895_M1_Srgap3
HSS[Phospho (STY)]LGDHKS[Phospho (STY)]LEAEALAEDIEK.3	-0.22841335	Q812A2_S948_M2_Srgap3
SLEAEALAEDIEK.2	-0.138895658	#N/A
S[Phospho (STY)]SPTPHSGPC[Carbamidomethyl (C)]PSR.2	0.142688687	Q812A5_S310_M1_Prr5
LLDAVDTYIPVPTR.2	0.217884783	#N/A
GTVVTGTLER.2	0.442259355	#N/A
AEAGDNLGALVR.2	-0.204139198	#N/A

TVVTGIEM[Oxidation (M)]FHK.2	-0.118442662	#N/A
TIGTGLVTDVPAM[Oxidation (M)]TEEDKNIK.3	-0.325938431	#N/A
KYEEIDNAPEER.3	0.201143943	#N/A
KGDEC[Carbamidomethyl (C)]ELLGHNK.3	0.08518189	#N/A
TTLTAAITK.2	0.415142478	#N/A
YEEIDNAPEER.2	0.103942854	#N/A
ILAEGGGAK.2	0.327160046	#N/A
ELAM[Oxidation (M)]PGEDLK.2	0.360151906	#N/A
ELLTEFGYKGEETPVIVGSALC[Carbamidomethyl (C)]ALEQR.3	-0.120484974	#N/A
GEETPVIVGSALC[Carbamidomethyl (C)]ALEQR.2	-0.301996966	#N/A
GDEC[Carbamidomethyl (C)]ELLGHNK.2	-0.179762928	#N/A
NM[Oxidation (M)]ITGTAPLDGC[Carbamidomethyl (C)]ILVVAANDGPM[Oxidation (M)]PQTR.3	-0.094146564	#N/A
GITINAAHVEYSTAAR.2	0.168964065	#N/A
ADAVQDSEM[Oxidation (M)]VELVELEIR.2	-0.292153914	#N/A
DKPHVNVGTIGHVDHGK.4	-0.187679449	#N/A
HYAHTDC[Carbamidomethyl (C)]PGHADYVK.3	-0.113289703	#N/A
QIGVEHVVVYVNK.2	-0.058070518	#N/A
SEDDAAS[Phospho (STY)]GEHDIQIEGVR.3	-0.007972574	Q8BFT9_S31_M1_Svop
RTGESARS[Phospho (STY)]EDDAAS[Phospho (STY)]GEHDIQIEGVR.4	-0.007740475	Q8BFT9_S25_M2_Svop
SDESLPDGLS[Phospho (STY)]PK.2	-0.142814144	Q8BFU3_S36_M1_Rnf214
VDQDDDQDS[Phospho (STY)]SSLK.2	-0.188168187	Q8BFU3_S194_M1_Rnf214
[Acetyl (Protein N-term)]AASEVAGLGAGT[Phospho (STY)]PS[Phospho (STY)]PSESSALC[Carbamidomethyl (C)]ASK.3	-0.092996083	Q8BFU3_S15_M2_Rnf214
NLS[Phospho (STY)]PPSVSSQMITK.2	0.118904306	Q8BFU3_S48_M1_Rnf214
VC[Carbamidomethyl (C)]TQLQVLTEAFR.2	0.108724545	#N/A
VAPDEHPILLTEAPLNPK.3	-0.419330472	#N/A
IFVGGIKEDTEEYNLR.3	-0.55374381	#N/A
GFAFVTFDDHDTVDKIVVQK.3	-0.237262715	#N/A
GFAFVTFDDHDTVDK.2	-0.1964241	#N/A
EDTEEYNLR.2	0.382490955	#N/A
IETIEVM[Oxidation (M)]EDR.2	0.187223606	#N/A
EDSVKPGAHLTVK.2	0.035476184	#N/A
SSGS[Phospho (STY)]PYGGGYGSGGGSGGYGSR.2	-0.14925196	Q8BG05_S359_M1_Hnrnpa3
LFIGGLSFETTDDSLREHFEK.3	-0.12224061	#N/A
LFIGGLSFETTDDSLR.2	-0.022343856	#N/A
GEEGHDPKEPEQLRK.4	-0.414352877	#N/A
GFGFVTYSC[Carbamidomethyl (C)]VEEVDAAM[Oxidation (M)]C[Carbamidomethyl (C)]AR.2	-0.47750984	#N/A
WGTLTDC[Carbamidomethyl (C)]VVMR.2	0.048065337	#N/A
YHTINGHNC[Carbamidomethyl (C)]EVK.3	0.012605181	#N/A
YGKIETIEVMEDR.3	0.293179093	#N/A
QSGS[Phospho (STY)]PTLDTAPNGR.2	-0.444011322	Q8BG16_S701_M1_Slc6a15
IPSEMSS[Phospho (STY)]PNFGK.2	-0.384864768	Q8BG16_S687_M1_Slc6a15
KQS[Phospho (STY)]GS[Phospho (STY)]PTLDTAPNGR.2	-0.486632918	Q8BG16_S699_M2_Slc6a15
DTDAEDGS[Phospho (STY)]EADDERPAWNSK.2	0.069770247	Q8BG16_S55_M1_Slc6a15
ETSPSSNNS[Phospho (STY)]SEELSSTLQLSK.2	-0.017137941	Q8BG18_S14_M1_Necab1
AQS[Phospho (STY)]LLSTDR.2	-0.226764729	Q8BG32_S14_M1_Psmd11
LYDNLLEQNLIR.2	-0.170611697	#N/A
YQEALHLGSQLLR.2	-0.060020252	#N/A

TAYSYFYEAFEGYDSIDSPK.2	0.172836653	#N/A
DNYEGYAPSDGYYR.2	-0.107635532	#N/A
YFQDEEYKS[Phospho (STY)]K.2	-0.157243121	Q8BG39_S425_M1_Sv2b
YFQDEEYK.2	-0.088983812	#N/A
HVLFEDTFFDK.2	-0.403626303	#N/A
QVHDTNMR.2	0.154368807	#N/A
DMC[Carbamidomethyl (C)]LSS[Phospho (STY)]SKK.2	-0.501451404	Q8BG39_S144_M1_Sv2b
ADGLGGQADLMAER.2	0.429668226	#N/A
SNEQNQEEDAQS[Phospho (STY)]DVT[Phospho (STY)]EGHDEEDEIYEGEYQGIPHPDDVK.5	0.102250298	Q8BG39_S33_M2_Sv2b
[Acetyl (Protein N-term)]M[Oxidation (M)]EESAPVESQGQLPS[Phospho (STY)]PHHGS[Phospho (STY)]LRR.3	-0.34095113	#N/A
KDS[Phospho (STY)]RPESIIIYR.3	-0.236970759	Q8BG50_S47_M1_Tmem169
[Acetyl (Protein N-term)]MEESAPVESQGQLPS[Phospho (STY)]PHHGS[Phospho (STY)]LR.3	-0.227409347	#N/A
NLYFLT[Phospho (STY)]NK.2	-0.097209876	Q8BG67_T660_M1_Efr3a
LGPPSS[Phospho (STY)]PSAADKEENPAVLAESC[Carbamidomethyl (C)]FR.3	-0.413860149	Q8BG67_S220_M1_Efr3a
FLEANKIEFEEVDITM[Oxidation (M)]SEEQR.3	0.025006587	#N/A
AIHEPAES[Phospho (STY)]PR.2	-0.049987383	Q8BG89_S370_M1_Znf365
ADSLDAPC[Carbamidomethyl (C)]AS[Phospho (STY)]PGLPTQDTK.2	-0.164522213	Q8BG89_S146_M1_Znf365
MVEAVDRT[Phospho (STY)]IEKR.3	0.19195959	Q8BG89_T176_M1_Znf365
MVEAVDRT[Phospho (STY)]IEK.2	-0.081076247	Q8BG89_T176_M1_Znf365
DVDKELS[Phospho (STY)]PK.2	-0.285426826	Q8BG92_S293_M1_Clvs2
SQS[Phospho (STY)]VVDPTALK.2	-0.176141344	Q8BG92_S302_M1_Clvs2
SQS[Phospho (STY)]VVDPTALKR.2	-0.272292125	Q8BG92_S302_M1_Clvs2
SKS[Phospho (STY)]FSLR.2	-0.095738531	Q8BGB5_S30_M1_Limd2
[Acetyl (Protein N-term)]MFQAAGAAQATPSHEAK.2	0.290673588	#N/A
VLDEEGS[Phospho (STY)]ER.2	-0.096450381	Q8BGC0_S613_M1_Htatsf1
[Acetyl (Protein N-term)]SGNNLSGNDEFDEQLR.2	-0.269294218	#N/A
LFDDS[Phospho (STY)]DER.2	-0.257823559	Q8BGC0_S724_M1_Htatsf1
AEEGGES[Phospho (STY)]EGDAS[Phospho (STY)]EKDAK.2	-0.122218513	Q8BGC0_S441_M2_Htatsf1
LFDDS[Phospho (STY)]DERGTVGNVK.2	0.2429065	Q8BGC0_S724_M1_Htatsf1
VFDDDS[Phospho (STY)]DDIEEEEEADEK.2	-0.02243311	Q8BGC0_S645_M1_Htatsf1
QITAQAWDGTTDYQVEETSR.2	0.316227168	#N/A
RES[Phospho (STY)]GS[Phospho (STY)]SEASPGPR.2	0.091313147	Q8BGC9_S109_M2_Creg2
FEAGGFQSSQSTENS.2	0.139927069	#N/A
EKFEAGGFQSSQSTENS.2	0.258729606	#N/A
SQSS[Phospho (STY)]DTEQPS[Phospho (STY)]PTSGGGK.2	0.037345903	Q8BGD9_S498_M2_Eif4b
AFGS[Phospho (STY)]GYRR.2	-0.151118334	Q8BGD9_S283_M1_Eif4b
SWSS[Phospho (STY)]RDDYSR.2	0.034550101	Q8BGD9_S312_M1_Eif4b
TGS[Phospho (STY)]ESSQTGASATSGR.2	-0.353248685	Q8BGD9_S422_M1_Eif4b
ERHPS[Phospho (STY)]WR.2	-0.061392448	Q8BGD9_S406_M1_Eif4b
RAFGS[Phospho (STY)]GYR.2	-0.359530005	Q8BGD9_S283_M1_Eif4b
YAALS[Phospho (STY)]VDGEDEDEGDDC[Carbamidomethyl (C)]TE.2	0.19084912	Q8BGD9_S597_M1_Eif4b
ARPTT[Phospho (STY)]DSFDDYPPR.2	-0.055776042	Q8BGD9_T205_M1_Eif4b
SRTGS[Phospho (STY)]ESSQTGASATSGR.2	-0.272431645	Q8BGD9_S422_M1_Eif4b
RGDDSFGDKYR.3	0.140840654	#N/A
ERHPS[Phospho (STY)]WRS[Phospho (STY)]EETQER.3	0.285880518	Q8BGD9_S406_M2_Eif4b
SAPKEDDASASTSQSSR.2	-0.232774525	#N/A
FFDS[Phospho (STY)]EDEDFEILSL.2	0.173385805	Q8BGE6_S383_M1_Atg4b

VPEPESQTS[Phospho (STY)]PK.2	-0.31220918	Q8BGF7_S1188_M1_Pan2
DYLM[Oxidation (M)]ENEALPS[Phospho (STY)]P.2	-0.163624495	Q8BGF8_S421_M1_Slc35d3
KDYLMENEALPS[Phospho (STY)]P.2	0.530476961	Q8BGF8_S421_M1_Slc35d3
FYLGGPTSVR.2	0.002401629	#N/A
LELNYC[Carbamidomethyl (C)]IPM[Oxidation (M)]GVQGGDR.2	-0.113101982	#N/A
THFFLNAGNLC[Carbamidomethyl (C)]NLNYGEGPK.3	-0.160269188	#N/A
DVVVQHVHFDGLGR.3	0.144006014	#N/A
SSLSHAM[Oxidation (M)]VIDSR.2	0.175560999	#N/A
SFS[Phospho (STY)]MQDLTTIR.2	-0.084699136	Q8BGH4_S152_M1_Reep1
[Acetyl (Protein N-term)]M[Oxidation (M)]EAQELGS[Phospho (STY)]PTPTYHLLPK.2	-0.344444925	Q8BGK6_S8_M1_Slc7a6
EDAGS[Phospho (STY)]PSQGSPETMQLK.2	-0.071622878	Q8BGK6_S30_M1_Slc7a6
VDYLTPDFPSLS[Phospho (STY)]YPNYYTLM[Oxidation (M)]TGR.3	0.016994514	Q8BGN3_S71_M1_Enpp6
AFT[Phospho (STY)]FDDEDDELSQLK.2	0.098727422	Q8BGQ1_T21_M1_Vipas39
ETAGS[Phospho (STY)]SGSTPEGR.2	0.316269635	Q8BGQ1_S77_M1_Vipas39
TRPGS[Phospho (STY)]FQSLSDALSDTPAK.3	-0.439488639	Q8BGQ1_S119_M1_Vipas39
IVAVTGAEAQK.2	0.333331938	#N/A
ITC[Carbamidomethyl (C)]LC[Carbamidomethyl (C)]QVPQNAANR.2	0.182165281	#N/A
QIWQNLGLDEAR.2	0.251149033	#N/A
M[Oxidation (M)]ALELLTQEFGIPVER.2	-0.271326027	#N/A
LYVTYFGGDEAAGLEPDLEC[Carbamidomethyl (C)]R.2	-0.062784115	#N/A
MFVDEVVTGQEC[Carbamidomethyl (C)]GVVLDK.2	0.321483804	#N/A
SSS[Phospho (STY)]FSLPAR.2	-0.244349769	Q8BGR5_S168_M1_Fam53b
S[Phospho (STY)]RSQPC[Carbamidomethyl (C)]VLNDKK.3	-0.337823594	Q8BGR5_S267_M1_Fam53b
ETTGLS[Phospho (STY)]PQAAQEK.2	0.323780478	Q8BGS3_S13_M1_Zkscan1
KPS[Phospho (STY)]PSASPTVR.2	0.082745289	Q8BGT6_S647_M1_Micall1
SLHPWYGITPT[Phospho (STY)]SSPK.2	-0.125647414	Q8BGT6_T479_M1_Micall1
APS[Phospho (STY)]AS[Phospho (STY)]PLAIHASR.2	-0.580793044	Q8BGT6_S494_M2_Micall1
DLS[Phospho (STY)]SS[Phospho (STY)]FR.2	-0.111813095	Q8BGT6_S28_M2_Micall1
VHLTQLLEK.2	0.108448696	#N/A
TC[Carbamidomethyl (C)]NIS[Phospho (STY)]VGR.2	0.190550247	Q8BGT8_S372_M1_Phyhipl
SQDSGIAEM[Oxidation (M)]EELPVPHNIK.3	0.035728661	#N/A
NLSLEAIQLC[Carbamidomethyl (C)]DR.2	0.069186311	#N/A
LDHALSSPSS[Phospho (STY)]PC[Carbamidomethyl (C)]EEIK.2	-0.093681725	Q8BGT8_S15_M1_Phyhipl
GHWFLSPR.2	-0.001669062	#N/A
TEYTVAVQTASK.2	0.227068978	#N/A
ISWDMDSK.2	0.094913103	#N/A
DNSGSHGSPISGK.2	-0.555797615	#N/A
FEIAAEK.2	-0.176951025	#N/A
NLS[Phospho (STY)]LEAIQLC[Carbamidomethyl (C)]DRDGNK.3	0.317622607	Q8BGT8_S24_M1_Phyhipl
SAS[Phospho (STY)]ADNLILPR.2	-0.485900135	Q8BGU5_S326_M1_Ccny
ASTIFLSKSQT[Phospho (STY)]DVR.2	-0.539956593	Q8BGU5_T75_M1_Ccny
LESYRPDTDLS[Phospho (STY)]R.2	-0.254227659	Q8BGU5_S33_M1_Ccny
RSAS[Phospho (STY)]ADNLILPR.2	-0.086140681	Q8BGU5_S326_M1_Ccny
LESYRPDTDLS[Phospho (STY)]REDTGC[Carbamidomethyl (C)]NLQHISDR.4	0.166161014	Q8BGU5_S33_M1_Ccny
AISAPTS[Phospho (STY)]PTR.2	-0.211058781	Q8BGV8_S59_M1_Mief1
GGVS[Phospho (STY)]PDSEDKTPGR.2	-0.273506351	Q8BGW2_S238_M1_Wbp1l
SSTRPPSVADPQS[Phospho (STY)]PEVPTDR.3	-0.172144998	Q8BGW2_S183_M1_Wbp1l

[Acetyl (Protein N-term)]AESGGSSGSSQS[Phospho (STY)]PERPSSLADR.3	0.025621985	Q8BGY4_S13_M1_Klhl26
QS[Phospho (STY)]LTLSSTFTNK.2	-0.139030134	Q8BGY9_S550_M1_Slc5a7
EFIC[Carbamidomethyl (C)]ALSVTSR.2	-0.040229781	#N/A
SDPSIVLLLQC[Carbamidomethyl (C)]DM[Oxidation (M)]QK.2	0.078866888	#N/A
LNWAFEM[Oxidation (M)]YDLDGDGR.2	-0.334353055	#N/A
M[Oxidation (M)]NQDGLTPQQR.2	0.075221722	#N/A
LAPEVLEDLVQNTEFSEQELK.3	0.098904209	#N/A
LEM[Oxidation (M)]LEIIEAIYK.2	-0.077977063	#N/A
MDQDKDDQITLEEFK.3	-0.182207527	#N/A
M[Oxidation (M)]DQDKDDQITLEEFKEAAK.4	-0.191431177	#N/A
[Acetyl (Protein N-term)]M[Oxidation (M)]NPVYS[Phospho (STY)]PVQPGAPYGNPK.2	0.008898511	Q8BGZ2_S6_M1_Fam168a
YVAAAFPS[Phospho (STY)]AC[Carbamidomethyl (C)]GK.2	-0.18910067	Q8BH04_S304_M1_Pck2
[Acetyl (Protein N-term)]ANLGSEAEREPLLGPGSPGS[Phospho (STY)]R.3	-0.431662345	Q8BH31_S21_M1_Mfsd8
VLS[Phospho (STY)]PPHTK.2	-0.014795265	Q8BH48_S146_M1_Ubap1
RDS[Phospho (STY)]SESQLASTESDKPTTGR.3	-0.273410879	Q8BH50_S66_M1_
DSSESQLASTES[Phospho (STY)]DKPTTGR.2	-0.195810831	Q8BH50_S76_M1_
S[Phospho (STY)]RSESETSTMAAK.2	0.385074918	Q8BH50_S143_M1_
DTNNNVAYWDVLK.2	-0.157394775	#N/A
IDPNPVDSQST[Phospho (STY)]PSE.2	0.005306492	Q8BH58_T268_M1_Tiprl
LTVNDFVR.2	-0.033333198	#N/A
LLLADENGR.2	-0.353362421	#N/A
YLGLYNDPNSNPK.2	-0.15069566	#N/A
LTLADIER.2	-0.11799572	#N/A
IAPLAEGALPYNLAELQR.2	-0.19927285	#N/A
IVQLLAGVADQTK.2	-0.22175284	#N/A
LATATFAGIENK.2	0.225993217	#N/A
YEGFFGLYR.2	-0.348245565	#N/A
SGNGEVTFENVK.2	-0.252226156	#N/A
IYSTLAGTR.2	0.189972917	#N/A
VGGINLLTAGALAGVPAASLVTPADVIK.3	-0.16984043	#N/A
DIEVTKEEFAQSAIR.3	-0.109951045	#N/A
LQVAGEITTGPR.2	-0.067604559	#N/A
EEGPSAFWK.2	-0.018983503	#N/A
AGQTTYSGVVDC[Carbamidomethyl (C)]FR.2	-0.059452774	#N/A
FTLGSVAGAVGATAVYPIDLVK.2	-0.32335484	#N/A
SSPQFGVTLVTYELLQR.2	-0.334924221	#N/A
NSFDC[Carbamidomethyl (C)]FK.2	0.238302593	#N/A
NIFLQYASTEVDGEHYM[Oxidation (M)]TPEDFVQR.3	0.238164507	#N/A
ILIPWLLSPER.2	0.128324562	#N/A
SSDWSS[Phospho (STY)]EEEEPVR.2	0.282882155	Q8BH66_S23_M1_Atl1
SSDWSS[Phospho (STY)]EEEEPVRK.2	-0.358887625	Q8BH66_S23_M1_Atl1
DDHSFELDEAALNR.3	-0.054621553	#N/A
DRNS[Phospho (STY)]WGGFSEK.2	-0.356167957	Q8BH66_S10_M1_Atl1
VAVLLM[Oxidation (M)]DTQGTFDSQSTLR.2	0.118897003	#N/A
TS[Phospho (STY)]PLNAEVVQYAK.2	0.294398527	Q8BH70_S45_M1_Fbxl4
HPDISYILGTEGRS[Phospho (STY)]PR.3	-0.122958413	Q8BH74_S87_M1_Nup107
S[Phospho (STY)]FKLDYR.2	-0.330375574	Q8BH82_S43_M1_Napepld

QNS[Phospho (STY)]VQNSGGSVSSR.2	-0.021629411	Q8BH82_S27_M1_Napepld
AQFGQPEILLGTIPGAGGTQR.2	0.083409063	#N/A
LVEEAIQC[Carbamidomethyl (C)]AEK.2	0.344860488	#N/A
FLSHWDHITR.3	-0.004455603	#N/A
AFAAGADIK.2	-0.396646429	#N/A
LFYSTFATDDRR.2	0.039104644	#N/A
EGMTAFVEK.2	-0.511303812	#N/A
SGFVPNVVHSS[Phospho (STY)]EEVIK.2	-0.517394137	Q8BHC1_S201_M1_Rab39b
S[Phospho (STY)]LEPPSEKPWER.2	0.069817356	Q8BHE1_S181_M1_Gemin8
LGGAVEDSSS[Phospho (STY)]PPSTLNLSGAHR.3	-0.01070633	Q8BHE3_S46_M1_Atcay
IQYVHSLEELER.3	-0.154066924	#N/A
AAEATEDQETS[Phospho (STY)]MS.2	-0.384449633	Q8BHE3_S370_M1_Atcay
EIQSLETSEDQLS[Phospho (STY)]EPR.2	0.252951866	Q8BHE4_S538_M1_Tmem108
LGADES[Phospho (STY)]EEEGR.2	-0.450313567	Q8BHG1_S85_M1_Nrdc
SSS[Phospho (STY)]DALQSR.2	-0.324016299	Q8BHJ6_S339_M1_Serinc5
LVEQANS[Phospho (STY)]PK.2	0.093269475	Q8BHJ9_S215_M1_Slu7
AEEAAQENASDY[Phospho (STY)]LAIPSENK.2	0.100699094	Q8BHK2_Y198_M1_Scn3b
ASPVPGPGT[Phospho (STY)]PTR.2	0.122607136	Q8BHL3_T136_M1_Tbc1d10b
HGAPAAPS[Phospho (STY)]PPPR.2	0.004231196	Q8BHL3_S22_M1_Tbc1d10b
RHGAPAAPS[Phospho (STY)]PPPR.3	-0.381891417	Q8BHL3_S22_M1_Tbc1d10b
ELLEQNPGKFEELER.3	0.188051752	#N/A
KHS[Phospho (STY)]LEGDEGSDFITK.3	-0.066734125	Q8BHN1_S79_M1_Txlng
NPEPELLVR.2	0.227773028	#N/A
LDLLEDR.2	0.087457131	#N/A
IILTAQPFR.2	0.245306783	#N/A
M[Oxidation (M)]LDYLQGSGETPQTDIR.2	0.279290004	#N/A
DAVHYGGWEHR.3	0.054140588	#N/A
KLDAES[Phospho (STY)]DGSTEETDESET.2	-0.315682064	Q8BHR8_S319_M1_
YNDWS[Phospho (STY)]DDDDDSNESK.2	-0.245191042	Q8BHS6_S61_M1_Armcx3
ASPNSDDTVLS[Phospho (STY)]PQELQK.2	0.36464895	Q8BHS6_S119_M1_Armcx3
RAS[Phospho (STY)]PNSDDTVLSPQELQK.3	0.402325291	Q8BHS6_S110_M1_Armcx3
TPSDTGHSPIGFC[Carbamidomethyl (C)]PGS[Phospho (STY)]DEDFTR.3	0.244198252	Q8BHS8_S111_M1_Sybu
SGSS[Phospho (STY)]PPS[Phospho (STY)]PR.2	-0.585807549	Q8BHS8_S199_M2_Sybu
DAIQNPNDIQLQEK.2	0.039471196	#N/A
EQAPDSVEGLLNALR.2	0.127234866	#N/A
EIENYPHFFLDFENAQPTEGER.3	-0.297978478	#N/A
VTLPAGPDILR.2	0.067962985	#N/A
[Acetyl (Protein N-term)]SAGGAVPPPPNPAVSFPAPR.2	-0.086086081	#N/A
SAAQQENSS[Phospho (STY)]PSR.2	-0.415327645	Q8BI72_S169_M1_Cdkn2aip
EEAKPAS[Phospho (STY)]PSSVQDR.2	0.400655651	Q8BI84_S1915_M1_Mia3
DSLPSGPREEAKPAS[Phospho (STY)]PSSVQDR.3	-0.434739885	Q8BI84_S1915_M1_Mia3
DETAAQTPGS[Phospho (STY)]GEAVLSK.2	0.006731645	Q8BI84_S820_M1_Mia3
SEFGS[Phospho (STY)]LDR.2	-0.25270001	Q8BI84_S1733_M1_Mia3
HSAS[Phospho (STY)]DPGPAPVVNSSS[Phospho (STY)]R.2	0.216671478	Q8BI84_S1754_M2_Mia3
LLGSENDTGS[Phospho (STY)]PDFYTPR.2	-0.05957062	Q8BIE6_S712_M1_Frmd4a
MWSESS[Phospho (STY)]LDEPYEK.2	0.322117148	Q8BIE6_S601_M1_Frmd4a
SSS[Phospho (STY)]LESQGK.2	-0.185023012	Q8BIE6_S696_M1_Frmd4a

TSNS[Phospho (STY)]YTAGGLFK.2	0.119042825	Q8BIE6_S854_M1_Frmd4a
SVDIS[Phospho (STY)]PTR.2	-0.131437344	Q8BIE6_S679_M1_Frmd4a
IISGSSGSLLS[Phospho (STY)]SGSQESDSSQSAK.2	-0.289098201	Q8BIE6_S356_M1_Frmd4a
QETQS[Phospho (STY)]AEPPPPEPPRI.2	-0.071631687	Q8BIF0_S226_M1_Cd99l2
GENLEAVVC[Carbamidomethyl (C)]EEPQVTYSK.2	0.051779669	#N/A
KIEVNNATAR.2	-0.076937134	#N/A
VLSELGVDAQSLSAM[Oxidation (M)]EIR.2	-0.420239488	#N/A
SQLPS[Phospho (STY)]PGELR.2	-0.189438624	Q8BIJ7_S52_M1_Rufy1
GSLISTDS[Phospho (STY)]GNSLPDRNPEK.3	0.315782511	Q8BIK4_S1270_M1_Dock9
LDKLPVHVYEVDEEADKDEDAAS[Phospho (STY)]LGSQK.4	0.031405822	Q8BIK4_S178_M1_Dock9
SLGTGAPVIESPYGESIS[Phospho (STY)]PEDAPESISK.3	0.063380875	Q8BIQ5_S120_M1_Cstf2
NVSKS[Phospho (STY)]DSDLIAYPSKDK.3	0.109347144	Q8BIZ1_S738_M1_Anks1b
TLANLPWIVEPGQEAK.2	0.051373979	#N/A
NIIAEHEIR.2	-0.242014658	#N/A
LIFQSC[Carbamidomethyl (C)]DYK.2	0.572562378	#N/A
NVSKS[Phospho (STY)]DSDLIAYPSK.2	0.228625892	Q8BIZ1_S738_M1_Anks1b
NSSTGPTPDC[Carbamidomethyl (C)]S[Phospho (STY)]PPS[Phospho (STY)]PDTALK.2	0.267682249	Q8BIZ1_S507_M2_Anks1b
LS[Phospho (STY)]PEPVAHR.2	-0.064192037	Q8BIZ6_S33_M1_Snip1
QERLS[Phospho (STY)]PEPVAHR.3	0.205423798	Q8BIZ6_S33_M1_Snip1
S[Phospho (STY)]GDALTTVVVK.2	-0.049468705	Q8BIZ6_S18_M1_Snip1
RPDAPAAS[Phospho (STY)]LS[Phospho (STY)]PPAAEPGHSGHR.3	0.166847559	Q8BIZ6_S48_M2_Snip1
HRS[Phospho (STY)]GDALTTVVVK.3	-0.04816241	Q8BIZ6_S18_M1_Snip1
DLVQPDKPAS[Phospho (STY)]PK.2	-0.422823477	Q8BJ05_S515_M1_Zc3h14
WIRPQS[Phospho (STY)]SE.2	-0.03537275	Q8BJ05_S733_M1_Zc3h14
IS[Phospho (STY)]PPVKEEEAKGDNTGK.3	-0.366660433	Q8BJ05_S409_M1_Zc3h14
RFS[Phospho (STY)]HDGEEEEEDEDYGTR.3	0.237190098	Q8BJ05_S309_M1_Zc3h14
EKS[Phospho (STY)]LDLPDR.2	-0.470881968	Q8BJ42_S1012_M1_Dlgap2
SDVETATDS[Phospho (STY)]DTESR.2	-0.357453437	Q8BJ42_S745_M1_Dlgap2
IIES[Phospho (STY)]PERKEER.3	-0.499241416	Q8BJ42_S983_M1_Dlgap2
IIES[Phospho (STY)]PERK.2	-0.358325448	Q8BJ42_S983_M1_Dlgap2
S[Phospho (STY)]IDKAVLASK.2	-0.313564365	Q8BJ42_S700_M1_Dlgap2
EKS[Phospho (STY)]LDLPDRQR.3	-0.290893735	Q8BJ42_S1012_M1_Dlgap2
GLQFGSS[Phospho (STY)]FQR.2	-0.029661149	Q8BJ42_S806_M1_Dlgap2
SHS[Phospho (STY)]LEGSSK.2	-0.216138619	Q8BJ42_S248_M1_Dlgap2
C[Carbamidomethyl (C)]SS[Phospho (STY)]IGVQDSEFPDHQPYPR.3	0.272748124	Q8BJ42_S720_M1_Dlgap2
GGLYNS[Phospho (STY)]M[Oxidation (M)]DSLDSNK.2	-0.582191469	Q8BJ42_S667_M1_Dlgap2
T[Phospho (STY)]PPPVPPR.2	0.051919532	Q8BJ42_T616_M1_Dlgap2
ADS[Phospho (STY)]IEIYIPEAQTRL.2	-0.141235979	Q8BJ42_S1047_M1_Dlgap2
EAEENDLLEDILGK.2	-0.26617812	#N/A
TTSKPLISVTAQS[Phospho (STY)]STESTQDAYQDSR.3	-0.250617874	Q8BJ42_S636_M1_Dlgap2
IIES[Phospho (STY)]PER.2	0.053505807	Q8BJ42_S983_M1_Dlgap2
QNSATERADS[Phospho (STY)]IEIYIPEAQTR.3	0.069704918	Q8BJ42_S1047_M1_Dlgap2
HS[Phospho (STY)]EPSTPTQYGALR.2	-0.115004869	Q8BJ42_S811_M1_Dlgap2
SGM[Oxidation (M)]SSWWS[Phospho (STY)]SDDNLDSDSTYR.2	-0.034543086	Q8BJ42_S301_M1_Dlgap2
MRS[Phospho (STY)]GSYIK.2	-0.24170812	Q8BJ42_S438_M1_Dlgap2
AMGDEESGESDSS[Phospho (STY)]PK.2	-0.472981368	Q8BJ42_S456_M1_Dlgap2
ADSIEIYIPEAQTR.2	-0.216714276	#N/A

S[Phospho (STY)]LDLPDR.2	-0.123235945	Q8BJ42_S1012_M1_Dlgap2
DGS[Phospho (STY)]WFLK.2	-0.692746397	Q8BJ42_S873_M1_Dlgap2
ATIS[Phospho (STY)]DEEIER.2	-0.444977905	Q8BJF9_S199_M1_Chmp2b
NTT[Phospho (STY)]PPSLAR.2	-0.123492031	Q8BJH1_T243_M1_Zc2hc1a
YGSGYLLAST[Phospho (STY)]PESEL.2	-0.243359089	Q8BJI1_T722_M1_Slc6a17
DIS[Phospho (STY)]NLEENDETR.2	-0.232198905	Q8BJI1_S665_M1_Slc6a17
EHS[Phospho (STY)]NEHVTESVADLLALEEPVDYK.3	-0.475562419	Q8BJI1_S13_M1_Slc6a17
SYLGPGSTS[Phospho (STY)]PLDNSNNPNGR.2	-0.205474896	Q8BJI1_S701_M1_Slc6a17
ILGYLNSNVLSR.2	0.277341828	#N/A
VPS[Phospho (STY)]EAPSPM[Oxidation (M)]PTHR.2	-0.052557898	Q8BJI1_S682_M1_Slc6a17
ALELDPDNDTYK.2	0.235221328	#N/A
TPS[Phospho (STY)]ASHEEQQE.2	-0.077294426	Q8BJU0_S307_M1_Sgta
AIELNPANAVYFC[Carbamidomethyl (C)]NR.2	-0.204364605	#N/A
SRTPS[Phospho (STY)]ASHEEQQE.2	0.013320756	Q8BJU0_S307_M1_Sgta
APDRT[Phospho (STY)]PPSEEDSAEAER.3	0.07329362	Q8BJU0_T82_M1_Sgta
HAEAVAYYK.2	-0.220321368	#N/A
VFTAIADQPWAQR.2	0.228063857	#N/A
IVENSEAVTEILNNAELLK.2	-0.236341835	#N/A
S[Phospho (STY)]PDGDGVGSGYIEENEDASK.2	-0.032512381	Q8BK06_S126_M1_Fbxo9
TQSVLKEPEPTDTTTYK.3	-0.247344766	#N/A
LPQPSSGRES[Phospho (STY)]PRH.3	-0.330532244	Q8BK30_S101_M1_Ndufv3
LPQPSSGR.2	-0.113136739	#N/A
LPQPSSGRES[Phospho (STY)]PR.2	-0.24935717	Q8BK30_S101_M1_Ndufv3
EM[Oxidation (M)]C[Carbamidomethyl (C)]EGLLEES[Phospho (STY)]DGEDEHEAGR.3	-0.235668394	Q8BK35_S305_M1_Nop53
AAQQAASSSGQGQQAQT[Phospho (STY)]PTGK.2	0.015148422	Q8BK63_T321_M1_Csnk1a1
ETFLTSPEELYR.2	0.128355265	#N/A
VFTTQELVQAFTHAPAALEADR.3	-0.108420723	#N/A
QTFGYGAR.2	0.130362227	#N/A
ADATNVNNWHWTER.2	0.0336077	#N/A
DPT[Phospho (STY)]PPPSSRGK.2	0.060161209	Q8BKA3_T101_M1_Srrm4
SAS[Phospho (STY)]PLAATR.2	-0.536947868	Q8BKA3_S271_M1_Srrm4
GQEKGS[Phospho (STY)]PGGDLSK.2	0.420250465	Q8BKA3_S309_M1_Srrm4
SRGQEKGS[Phospho (STY)]PGGDLSK.3	0.13036747	Q8BKA3_S309_M1_Srrm4
NLIDEDGNNQWPEGLK.2	-0.236053664	#N/A
LVLEQVVTSIASVADTAEEK.3	-0.096915359	#N/A
HIVENAVQK.2	0.340640639	#N/A
S[Phospho (STY)]VENLPEC[Carbamidomethyl (C)]GITHEQR.2	-0.248272383	Q8BKC8_S428_M1_Pi4kb
RTS[Phospho (STY)]FSFQK.2	-0.390899339	Q8BKH7_S510_M1_Mapkap1
YSS[Phospho (STY)]PGLTSK.2	-0.470590853	Q8BKH7_S270_M1_Mapkap1
TS[Phospho (STY)]FSFQK.2	-0.107695857	Q8BKH7_S510_M1_Mapkap1
DDEPSGWEEPS[Phospho (STY)]PQSISR.2	-0.142488136	Q8BKI2_S913_M1_Tnrc6b
M[Oxidation (M)]GS[Phospho (STY)]PAPLLPGDLLGGGSDSI.2	-0.441428239	Q8BKI2_S1793_M1_Tnrc6b
SSSS[Phospho (STY)]AGSEVGGQSTGSNHK.2	-0.209157028	Q8BKI2_S601_M1_Tnrc6b
EGDLITLLVPEAR.2	0.037016628	#N/A
QIQNQLEETLK.2	-0.272007071	#N/A
YSDKELQYIDAISNK.2	-0.034431132	#N/A
LSDSYSNTLPVR.2	0.094395338	#N/A

SLS[Phospho (STY)]PPQSQSK.2	-0.120668836	Q8BKX1_S326_M1_Baiap2
LPLWQQAC[Carbamidomethyl (C)]ADPNKIPDR.3	-0.141217522	#N/A
SFHNELLTQLEQK.3	-0.150149485	#N/A
SSS[Phospho (STY)]M[Oxidation (M)]AAGLER.2	0.213235392	Q8BKX1_S367_M1_Baiap2
SNLVISDPIPGAKPLPVPPELAPFVGR.3	0.241218191	#N/A
TIM[Oxidation (M)]EQFNPSLR.2	-0.002049154	#N/A
AIFSHAAGDNSTLLSFK.2	-0.19957479	#N/A
LTENVYK.2	-0.056427458	#N/A
ELGDVLFQM[Oxidation (M)]AEVHR.3	-0.377777759	#N/A
VVDDELATR.2	0.105784119	#N/A
VSVNDFIIR.2	0.120484932	#N/A
DLVKDDIK.2	-0.249743295	#N/A
GIQEIADSVK.2	0.25717155	#N/A
HSLDASQGTATGPR.2	0.513812587	#N/A
LTEDEEGNPQLQQHQLITVTM[Oxidation (M)]SSDSR.3	-0.548647762	#N/A
NSSS[Phospho (STY)]PVSLDAAPPDSVNVYR.2	0.060199519	Q8BL06_S613_M1_Usp54
S[Phospho (STY)]PQHYSR.2	-0.075409135	Q8BL65_S383_M1_Ablim2
IYPYDSLIVTNR.2	-0.15477261	#N/A
TSSESIVSVPASSTSGS[Phospho (STY)]PSR.2	-0.305262032	Q8BL65_S294_M1_Ablim2
TSS[Phospho (STY)]PSSAGSVSLGHYTPTSR.3	-0.056244452	Q8BL65_S365_M1_Ablim2
S[Phospho (STY)]PQHYSRPGSESGR.3	0.075389849	Q8BL65_S383_M1_Ablim2
RLDVEDSS[Phospho (STY)]FDQDSR.2	0.019745298	Q8BL65_S453_M1_Ablim2
T[Phospho (STY)]NSPDLDSQSLSLSSGTDQEPLQR.3	-0.154534425	Q8BL65_T475_M1_Ablim2
SLGS[Phospho (STY)]ADELFK.2	-0.344351942	Q8BL66_S52_M1_Eea1
LQQQSSQAAQELAAEK.2	-0.189034925	#N/A
VSS[Phospho (STY)]PTKPK.2	-0.196950253	Q8BL74_S219_M1_Gtf3c2
LEEELDQER.2	0.117277586	#N/A
S[Phospho (STY)]RS[Phospho (STY)]AS[Phospho (STY)]LRR.2	0.125408359	Q8BL97_S200_M3_Srsf7
S[Phospho (STY)]RS[Phospho (STY)]VSLR.2	-0.159418822	Q8BL97_S192_M2_Srsf7
S[Phospho (STY)]RS[Phospho (STY)]ASLR.2	-0.432618692	Q8BL97_S200_M2_Srsf7
S[Phospho (STY)]RS[Phospho (STY)]GS[Phospho (STY)]IIGSR.2	-0.253221791	Q8BL97_S208_M3_Srsf7
SGS[Phospho (STY)]IIGSR.2	-0.231209165	Q8BL97_S212_M1_Srsf7
VLNEPGS[Phospho (STY)]PTR.2	0.127380708	Q8BLB8_S231_M1_Ankrd34c
T[Phospho (STY)]NSIDSKDPTLFPTVQEQVLK.3	-0.185635527	Q8BLB8_T299_M1_Ankrd34c
RGS[Phospho (STY)]GTLLLDR.2	-0.16868995	Q8BLB8_S446_M1_Ankrd34c
AVES[Phospho (STY)]TDEDHQTDLDAK.2	-0.301754288	Q8BLH7_S134_M1_Hirip3
LNYVFNEGEANT.2	0.062091744	#N/A
AANEVSSADVK.2	-0.10425742	#N/A
HALEYSLR.2	-0.169866089	#N/A
QGDTAILR.2	-0.180303097	#N/A
C[Carbamidomethyl (C)]EASAVPAPDFEWYR.2	0.072879753	#N/A
EFEGEEEYLEILGITR.2	-0.319717997	#N/A
C[Carbamidomethyl (C)]EASAVPAPDFEWYRDDTR.3	0.332560704	#N/A
SGIIFAGHDK.2	-0.082130165	#N/A
VDVYDEGSYTC[Carbamidomethyl (C)]SVQTQHEPK.3	-0.125337533	#N/A
IQKVDVYDEGSYTC[Carbamidomethyl (C)]SVQTQHEPK.3	-0.433195151	#N/A
VC[Carbamidomethyl (C)]LQQPSAS[Phospho (STY)]PQGGSSFESR.2	0.291103451	Q8BLK9_S448_M1_Rps6kc1

SS[Phospho (STY)]PDQFLFSSLR.2	-0.226135198	Q8BLK9_S779_M1_Rps6kc1
S[Phospho (STY)]SQESLDIK.2	-0.086353553	Q8BLK9_S422_M1_Rps6kc1
VFSGGEDLEAVSS[Phospho (STY)]PPTSESLSR.2	-0.00081768	Q8BLK9_S577_M1_Rps6kc1
GVDLLLEGVQGESS[Phospho (STY)]PTR.2	-0.01551058	Q8BLK9_S281_M1_Rps6kc1
FLNRSS[Phospho (STY)]QES[Phospho (STY)]LDIK.2	-0.033572122	Q8BLK9_S423_M2_Rps6kc1
ESEAQDSVSRGS[Phospho (STY)]DDSVPVISFK.3	0.147867654	Q8BLK9_S658_M1_Rps6kc1
ALFTLEDGDS[Phospho (STY)]PSQSLDPGESK.2	0.074264529	Q8BLK9_S634_M1_Rps6kc1
ALFTLEDGDS[Phospho (STY)]PSQSLDPGESKR.3	0.020864999	Q8BLK9_S634_M1_Rps6kc1
GRS[Phospho (STY)]PTVGNKR.2	-0.247714796	Q8BLN6_S1261_M1_Unc80
VAS[Phospho (STY)]IQS[Phospho (STY)]EPGQQNVLLQQPLGR.3	0.294585783	Q8BLN6_S3045_M2_Unc80
ASPPSS[Phospho (STY)]PC[Carbamidomethyl (C)]LAS[Phospho (STY)]PGPTAK.2	-0.290268923	Q8BLQ7_S422_M2_Slc7a4
FQKAS[Phospho (STY)]PPSS[Phospho (STY)]PC[Carbamidomethyl (C)]LAS[Phospho (STY)]PGPTAK.3	-0.036764958	Q8BLQ7_S418_M3_Slc7a4
DGAELPDPDR.2	-0.305947604	#N/A
GAEDAPDADTAIINAEGS[Phospho (STY)]QVNAEEKK.3	0.183784855	Q8BLQ9_S423_M1_Cadm2
SDDGVAVIC[Carbamidomethyl (C)]R.2	0.237861903	#N/A
TSGSKPAADIR.2	-0.000424468	#N/A
TFTVSSTLDFR.2	0.041918575	#N/A
IIPSTPFPQEGQALTLTC[Carbamidomethyl (C)]ESK.2	-0.1558265	#N/A
YLKEEDANRK.3	-0.107517165	#N/A
GAEDAPDADTAIINAEGS[Phospho (STY)]QVNAEEK.3	-0.077566283	Q8BLQ9_S423_M1_Cadm2
GAEDAPDADTAIINAEGS[Phospho (STY)]QVNAEEKKEYFI.3	-0.228994956	Q8BLQ9_S423_M1_Cadm2
QRS[Phospho (STY)]EEPSPR.2	-0.038752332	Q8BLS7_S242_M1_Sowaha
TKS[Phospho (STY)]SSEEVLER.2	-0.494203327	Q8BLV3_S694_M1_Slc9a7
VGVEELS[Phospho (STY)]EEDQNENR.2	-0.145642442	Q8BLV3_S546_M1_Slc9a7
S[Phospho (STY)]RSDVTHVDWR.3	0.058771532	Q8BM47_S150_M1_Plekhm3
LSTS[Phospho (STY)]SETVNSTAASK.2	-0.093206946	Q8BM65_S154_M1_Nyap2
RNPNTQLSTS[Phospho (STY)]FDETYIKK.3	-0.288877263	Q8BM65_S238_M1_Nyap2
FTVPASHS[Phospho (STY)]PPR.2	-0.139503396	Q8BM65_S184_M1_Nyap2
KEEDDQSEAVY[Phospho (STY)]EEM[Oxidation (M)]KYPIFDDLGHDSK.4	-0.198549016	Q8BM65_Y300_M1_Nyap2
DQTGALGPAPGASILSSS[Phospho (STY)]PPPPSTLYR.3	-0.353866783	Q8BM65_S442_M1_Nyap2
TNS[Phospho (STY)]SSSSPVVLK.2	-0.027892086	Q8BMB0_S173_M1_Emsy
VRGEEYDDLLDEFM[Oxidation (M)]QAVTDK.3	-0.190314387	#N/A
TFTPGQGNNAYVFPGVALGVIAGGIR.3	-0.167570684	#N/A
EMFAQDHPEVNSLEEVVR.3	-0.147139791	#N/A
ILVPEGTR.2	0.248524782	#N/A
DVPLGAPLC[Carbamidomethyl (C)]IIVEK.2	0.21041813	#N/A
GLETIASDVVSLASK.2	-0.166298017	#N/A
VPLPSLSPTM[Oxidation (M)]QAGTIAR.2	-0.290857253	#N/A
DIDSFVPSK.2	0.267457297	#N/A
VFVSPLAK.2	-0.008574402	#N/A
YLEKPITM[Oxidation (M)]LL.2	-0.353499346	#N/A
ISVNDFIIK.2	-0.16330081	#N/A
VAPAPAGVFTDIPISNIR.2	0.246294578	#N/A
DVPVGSIIC[Carbamidomethyl (C)]ITVEKPQDIEAFK.3	0.301324013	#N/A
LQPHEFQGGTFTISNLGM[Oxidation (M)]FGIK.3	-0.2758494	#N/A
VPEANSSWM[Oxidation (M)]DTVIR.2	0.212709878	#N/A
AAPAAAAAM[Oxidation (M)]APPGPR.2	0.032604168	#N/A

ASALAC[Carbamidomethyl (C)]LK.2	-0.230934997	#N/A
VVDGAVGAQWLAEFK.2	0.160449356	#N/A
GGFS[Phospho (STY)]PFGNTQGPSR.2	-0.347107135	Q8BMG7_S448_M1_Rab3gap2
AKS[Phospho (STY)]PRPDSFETEIK.3	0.126898344	Q8BMG7_S566_M1_Rab3gap2
TSS[Phospho (STY)]WEDDSWGAWEETEPR.2	-0.261970592	Q8BMG7_S42_M1_Rab3gap2
LAS[Phospho (STY)]ETEDNDNSLGDILQASDNLSR.3	0.243740403	Q8BMI3_S267_M1_Gga3
VGAHGS[Phospho (STY)]EEGAVFK.2	0.128874786	Q8BMK4_S367_M1_Ckap4
FVDLYGAQK.2	0.037862194	#N/A
DGPGFYTTR.2	0.178137547	#N/A
AGLEQGSDAGYLAESQK.2	-0.216792669	#N/A
SLNSEM[Oxidation (M)]DNILANLR.2	0.152101584	#N/A
DTTASAVAVGLR.2	0.062260711	#N/A
TVLGVPEVLLGILPGAGGTQR.2	0.271462016	#N/A
LPAKPEVSSDEDVQYR.3	0.200858512	#N/A
SPGAS[Phospho (STY)]PLLSR.2	0.232064176	Q8BN59_S451_M1_Larp6
YLSPGWGSAS[Phospho (STY)]EEEPSR.2	0.137443732	Q8BN59_S58_M1_Larp6
SGDLAPPESPSS[Phospho (STY)]PGQASPR.2	-0.285935112	Q8BNN1_S329_M1_Spata2l
ASAAEGSEAS[Phospho (STY)]PPSLR.2	-0.20310981	Q8BNW9_S70_M1_Kbtbd11
LLLADAYSGR.2	-0.109973896	#N/A
AC[Carbamidomethyl (C)]LLAAALGTTGER.2	0.234703076	#N/A
SGS[Phospho (STY)]RPQS[Phospho (STY)]PSGDAESR.2	-0.125569713	Q8BNW9_S316_M2_Kbtbd11
GDAAVYC[Carbamidomethyl (C)]YQAEAGEWR.2	-0.299734504	#N/A
PQS[Phospho (STY)]PSGDAESR.2	0.164347276	Q8BNW9_S320_M1_Kbtbd11
ANSKS[Phospho (STY)]PLQR.2	-0.190205985	Q8BP22_S312_M1_Fam92a
S[Phospho (STY)]SSHSFC[Carbamidomethyl (C)]SVVK.2	0.029429921	Q8BP27_S139_M1_Sfr1
ENPPS[Phospho (STY)]PHSNSSGKQPLSGTPK.3	-0.36648917	Q8BP27_S115_M1_Sfr1
ENPPS[Phospho (STY)]PPTS[Phospho (STY)]PAAPQPR.2	0.190104262	Q8BP27_S67_M2_Sfr1
ENPPS[Phospho (STY)]PHSNSSGK.2	-0.129493955	Q8BP27_S115_M1_Sfr1
ISALEGYR.2	0.026040143	#N/A
DVC[Carbamidomethyl (C)]LYPR.2	-0.330326609	#N/A
SWDSEEILEGYKR.3	0.150278188	#N/A
TTPEC[Carbamidomethyl (C)]DES[Phospho (STY)]PRKK.2	-0.100129746	Q8BP78_S243_M1_Fra10ac1
S[Phospho (STY)]RSPPSEEASK.2	0.265905535	Q8BP78_S248_M1_Fra10ac1
NAEEEDSAS[Phospho (STY)]DSELWK.2	-0.501430244	Q8BP78_S285_M1_Fra10ac1
EALLGVQEDVDEYVK.2	-0.102196392	#N/A
RDPTANEDPEWILVEK.3	0.137915724	#N/A
VIDFDENTALDDTEEGSFR.2	-0.429723868	#N/A
LSEEEILENQDLFLTSEATDYGR.3	-0.139085499	#N/A
HYAMQEAK.2	0.257340326	#N/A
SSS[Phospho (STY)]ESPSDNQVFQATR.2	0.172779545	Q8BP99_S63_M1_
ESS[Phospho (STY)]EESGEFDDLVSALR.2	-0.152941522	Q8BPM0_S1026_M1_Daam1
LDHELS[Phospho (STY)]LDR.2	-0.13661917	Q8BPN8_S451_M1_Dmxl2
KQS[Phospho (STY)]EVEADLGYPGGK.2	-0.437934557	Q8BPN8_S2636_M1_Dmxl2
NLAS[Phospho (STY)]PEGTLATLGLK.2	-0.473582409	Q8BPN8_S1856_M1_Dmxl2
ESKSS[Phospho (STY)]DDIDYR.2	0.051325679	Q8BPN8_S2708_M1_Dmxl2
YLDEYNPGIFR.2	-0.283848778	#N/A
LVYSQPLDLPEAVEVIR.2	-0.289388985	#N/A

ALLT[Phospho (STY)]PQDEEC[Carbamidomethyl (C)]ADGDTEVDVIAEQLK.3	0.301622584	Q8BPN8_T2017_M1_Dmxl2
FGNVDADS[Phospho (STY)]PVEETIQDHSALK.3	-0.035346865	Q8BPN8_S1288_M1_Dmxl2
M[Oxidation (M)]KLDHELS[Phospho (STY)]LDR.3	0.150457986	Q8BPN8_S451_M1_Dmxl2
VDPIGPLSYTGGVSELAR.2	-0.055962654	#N/A
RQS[Phospho (STY)]ESIAAPPVASEDM[Oxidation (M)]DKHR.4	-0.01058148	Q8BPN8_S2394_M1_Dmxl2
QLDHES[Phospho (STY)]DDADREDEER.3	-0.49250474	Q8BPN8_S423_M1_Dmxl2
LYQAVVDAR.2	-0.21028082	#N/A
VS[Phospho (STY)]VDSNLFVYSK.2	-0.181848432	Q8BPN8_S1141_M1_Dmxl2
RQS[Phospho (STY)]ESIAAPPVASEDMDK.2	-0.204285655	Q8BPN8_S2394_M1_Dmxl2
RKQS[Phospho (STY)]EVEADLGYPGGK.3	0.259952236	Q8BPN8_S2636_M1_Dmxl2
SS[Phospho (STY)]DDIDYR.2	-0.246212529	Q8BPN8_S2708_M1_Dmxl2
LDHELSLDRES[Phospho (STY)]EAGTGSSEHEDGEREGS[Phospho (STY)]PR.4	0.24604356	Q8BPN8_S456_M2_Dmxl2
ESEAGTGSSEHEDGEREGS[Phospho (STY)]PR.3	-0.117109718	Q8BPN8_S473_M1_Dmxl2
NIGAGVMQIAISQDNR.2	0.008769524	#N/A
VGSVLDSRVS[Phospho (STY)]VDS[Phospho (STY)]NLFVYSK.3	-0.20457859	Q8BPN8_S1141_M2_Dmxl2
QLDHES[Phospho (STY)]DDADREDEERSQDER.4	0.188638045	Q8BPN8_S423_M1_Dmxl2
C[Carbamidomethyl (C)]SSGAS[Phospho (STY)]LDSHLHR.3	-0.129136156	Q8BPQ7_S633_M1_Sgsm1
QDS[Phospho (STY)]PTKRPALC[Carbamidomethyl (C)]IQK.3	-0.183758939	Q8BPQ7_S215_M1_Sgsm1
RHS[Phospho (STY)]S[Phospho (STY)]GSM[Oxidation (M)]DDRPSISAR.3	-0.292095684	Q8BPQ7_S230_M2_Sgsm1
HSSGS[Phospho (STY)]M[Oxidation (M)]DDRPSISAR.2	-0.257941377	Q8BPQ7_S232_M1_Sgsm1
SPQGSSESTSS[Phospho (STY)]DKEDDEATDYVFR.3	-0.080222957	Q8BPQ7_S407_M1_Sgsm1
IAFDAESEPNNSSGS[Phospho (STY)]M[Oxidation (M)]EK.2	-0.565816996	Q8BPU7_S344_M1_Elmo1
SC[Carbamidomethyl (C)]LVKGS[Phospho (STY)]PER.2	-0.407097208	Q8BQ30_S510_M1_Ppp1r18
SSS[Phospho (STY)]FSEFC[Carbamidomethyl (C)]HR.2	0.013918551	Q8BQS4_S197_M1_Fam102b
C[Carbamidomethyl (C)]PVKQDS[Phospho (STY)]VESQLKR.3	-0.131188434	Q8BQS4_S267_M1_Fam102b
SAS[Phospho (STY)]VPDELGAWGHSR.2	-0.623810885	Q8BQS4_S162_M1_Fam102b
SDS[Phospho (STY)]APPTPVNR.2	-0.321127563	Q8BQZ4_S359_M1_Ralgapb
SGS[Phospho (STY)]LKGPDDPR.2	-0.133396889	Q8BR07_S548_M1_Bicd1
RVIHFVSGET[Phospho (STY)]M[Oxidation (M)]EEY[Phospho (STY)]STDEDEVDGLDKK.4	-0.204074914	Q8BR63_T60_M2_Fam177a1
EES[Phospho (STY)]LATDPAPGTQK.2	0.109304766	Q8BR92_S355_M1_Palm2
LEQEIQALESEESQISAK.2	-0.569114123	#N/A
AELVLIDEDDEKS[Phospho (STY)]LREK.3	-0.221588538	Q8BR92_S315_M1_Palm2
AELVLIDEDDEKS[Phospho (STY)]LR.2	0.023978557	Q8BR92_S315_M1_Palm2
VNLEESTGVENS[Phospho (STY)]PAGARPK.2	0.025244093	Q8BRF7_S300_M1_Scfd1
SWGS[Phospho (STY)]GGGQEPGGSQAAGGGQPR.2	0.248406575	Q8BRJ3_S195_M1_Rell2
YGLHEHRDGS[Phospho (STY)]PTDR.3	-0.221682409	Q8BRJ3_S187_M1_Rell2
GS[Phospho (STY)]EPDDAQLQPPEDDDVNEDTVER.3	-0.182876773	Q8BRJ3_S52_M1_Rell2
SC[Carbamidomethyl (C)]S[Phospho (STY)]AAGLHR.2	0.369669467	Q8BRK8_S491_M1_Prkaa2
TSC[Carbamidomethyl (C)]GS[Phospho (STY)]PNYAAPEVISGR.2	0.069283361	Q8BRK8_S176_M1_Prkaa2
MPPLIADS[Phospho (STY)]PK.2	0.411942612	Q8BRK8_S377_M1_Prkaa2
ESSRDTS[Phospho (STY)]PVR.2	-0.201610887	Q8BRT1_S547_M1_Clasp2
AQT[Phospho (STY)]GSAGADPTADVSGQS.2	-0.27258599	Q8BRT1_T1271_M1_Clasp2
RAQTGS[Phospho (STY)]AGADPTADVSGQS.2	-0.142614331	Q8BRT1_S1273_M1_Clasp2
VLTTTALSTVSSGAQR.2	0.094175533	#N/A
MVSQSQPGS[Phospho (STY)]R.2	0.327350275	Q8BRT1_S465_M1_Clasp2
SSSSS[Phospho (STY)]QESLNRPFSSK.2	-0.154776124	Q8BRT1_S333_M1_Clasp2
SRS[Phospho (STY)]DIDVNAAAGAK.2	0.199103335	Q8BRT1_S376_M1_Clasp2

IPRPSVS[Phospho (STY)]QGC[Carbamidomethyl (C)]S[Phospho (STY)]R.2	-0.142177046	Q8BRT1_S531_M2_Clasp2
S[Phospho (STY)]QEDMSEPVR.2	0.188124625	Q8BRT1_S947_M1_Clasp2
EILSDDKHDWDQR.3	-0.2007625	#N/A
IC[Carbamidomethyl (C)]DYKS[Phospho (STY)]FDDEESVDGNRPSSAASAFK.3	-0.112640729	Q8BRT1_S14_M1_Clasp2
RDES[Phospho (STY)]DEEPPRVER.2	-0.327992236	Q8BRV5_S205_M1_Kiaa1671
KNS[Phospho (STY)]AAELGASK.2	0.055223568	Q8BSD5_S80_M1_She
C[Carbamidomethyl (C)]ETS[Phospho (STY)]PPPTPR.2	0.096712726	Q8BSS9_S774_M1_Ppfia2
QAQS[Phospho (STY)]PSGVSSEVEVLK.2	-0.414382021	Q8BSS9_S163_M1_Ppfia2
S[Phospho (STY)]LGDHEWNR.2	0.147711185	Q8BSS9_S594_M1_Ppfia2
VKS[Phospho (STY)]LGDHEWNR.2	0.059003465	Q8BSS9_S594_M1_Ppfia2
RQAQS[Phospho (STY)]PSGVSSEVEVLK.3	-0.211717364	Q8BSS9_S163_M1_Ppfia2
VAS[Phospho (STY)]VS[Phospho (STY)]LEGLNLAR.2	0.171118085	Q8BSS9_S687_M2_Ppfia2
KMVSSEGS[Phospho (STY)]TES[Phospho (STY)]EHLEGMEAGQK.3	-0.682678624	Q8BSS9_S236_M2_Ppfia2
VGRPHS[Phospho (STY)]SPAR.2	0.079864446	Q8BTG3_S55_M1_Tcp11l1
LDPTTTTFLK.2	0.045820157	#N/A
TAS[Phospho (STY)]LTSASSVDGSR.2	0.101918481	Q8BTG7_S317_M1_Ndrg4
LSGGAVPSAS[Phospho (STY)]MTR.2	0.319840672	Q8BTG7_S306_M1_Ndrg4
RLS[Phospho (STY)]GGAVPS[Phospho (STY)]ASMTR.2	0.172676054	Q8BTG7_S298_M2_Ndrg4
DLDINRPGTVPNAK.2	0.216067397	#N/A
SSS[Phospho (STY)]PVTELTAR.2	-0.30860845	Q8BTI8_S1068_M1_Srrm2
S[Phospho (STY)]RT[Phospho (STY)]PPVTR.2	-0.144042411	Q8BTI8_S1878_M2_Srrm2
S[Phospho (STY)]RTS[Phospho (STY)]PVTR.2	-0.126954553	Q8BTI8_S1924_M2_Srrm2
S[Phospho (STY)]RT[Phospho (STY)]PPAIR.2	-0.192424752	Q8BTI8_S1972_M2_Srrm2
SST[Phospho (STY)]PPRQS[Phospho (STY)]PSR.2	-0.159768529	Q8BTI8_S882_M2_Srrm2
VSS[Phospho (STY)]PVLETVQQR.2	-0.352312934	Q8BTI8_S1360_M1_Srrm2
S[Phospho (STY)]RS[Phospho (STY)]PLAIR.2	-0.436125675	Q8BTI8_S1996_M2_Srrm2
S[Phospho (STY)]RTS[Phospho (STY)]PAPWK.2	-0.1973722	Q8BTI8_S1810_M2_Srrm2
AAETPAVASC[Carbamidomethyl (C)]WSGPQVS[Phospho (STY)]PEHK.3	-0.456892661	Q8BTI8_S1269_M1_Srrm2
SSS[Phospho (STY)]PVTELTARS[Phospho (STY)]PVKQDK.3	-0.173786765	Q8BTI8_S1077_M2_Srrm2
SRHS[Phospho (STY)]PRLS[Phospho (STY)]R.3	-0.058928021	Q8BTI8_S1548_M2_Srrm2
S[Phospho (STY)]PVPSAFSDQSR.2	-0.128275209	Q8BTI8_S2404_M1_Srrm2
S[Phospho (STY)]RTS[Phospho (STY)]PVSR.2	-0.251236263	Q8BTI8_S1846_M2_Srrm2
S[Phospho (STY)]RS[Phospho (STY)]PQRPGWSR.3	-0.425643648	Q8BTI8_S531_M2_Srrm2
S[Phospho (STY)]RS[Phospho (STY)]IS[Phospho (STY)]PC[Carbamidomethyl (C)]PK.2	-0.222067523	Q8BTI8_S924_M3_Srrm2
NSGPVSEVNTGFS[Phospho (STY)]PEVK.2	-0.194031888	Q8BTI8_S1305_M1_Srrm2
ERAPS[Phospho (STY)]PASR.2	-0.314034629	Q8BTI8_S2381_M1_Srrm2
ALPQT[Phospho (STY)]PR.2	-0.219668902	Q8BTI8_T1448_M1_Srrm2
DKFS[Phospho (STY)]PTQDRPESSTVLK.3	-0.50895423	Q8BTI8_S1151_M1_Srrm2
S[Phospho (STY)]RT[Phospho (STY)]PLISR.2	-0.360732989	Q8BTI8_S1834_M2_Srrm2
RVPS[Phospho (STY)]PTPVPK.2	-0.286828876	Q8BTI8_S2535_M1_Srrm2
REIS[Phospho (STY)]S[Phospho (STY)]SPTSK.2	-0.021031948	Q8BTI8_S452_M2_Srrm2
SGAGS[Phospho (STY)]PPGKR.2	-0.153935133	Q8BTI8_S1179_M1_Srrm2
T[Phospho (STY)]PPVALSSSR.2	0.102873123	Q8BTI8_T2056_M1_Srrm2
S[Phospho (STY)]RSRT[Phospho (STY)]S[Phospho (STY)]PAPWK.2	-0.07104054	Q8BTI8_S1808_M3_Srrm2
VPS[Phospho (STY)]PTPVPK.2	0.232775824	Q8BTI8_S2535_M1_Srrm2
GHS[Phospho (STY)]RS[Phospho (STY)]RS[Phospho (STY)]PQWR.3	-0.426096278	Q8BTI8_S503_M3_Srrm2
S[Phospho (STY)]RS[Phospho (STY)]RTS[Phospho (STY)]PVSR.2	-0.16662587	Q8BTI8_S1844_M3_Srrm2

S[Phospho (STY)]RS[Phospho (STY)]RS[Phospho (STY)]LVR.2	0.014141215	Q8BTI8_S700_M3_Srrm2
SSS[Phospho (STY)]PVTELTARS[Phospho (STY)]PVK.2	0.17114853	Q8BTI8_S1077_M2_Srrm2
SRS[Phospho (STY)]T[Phospho (STY)]T[Phospho (STY)]PAPK.2	-0.121791991	Q8BTI8_S250_M3_Srrm2
TS[Phospho (STY)]PAPWKR.2	-0.18738004	Q8BTI8_S1813_M1_Srrm2
ARS[Phospho (STY)]RT[Phospho (STY)]PPSAPSQSR.3	-0.22423233	Q8BTI8_S2360_M2_Srrm2
RKET[Phospho (STY)]PS[Phospho (STY)]PR.2	-0.233280843	Q8BTI8_S2691_M2_Srrm2
HSGST[Phospho (STY)]SPYLK.2	-0.259021754	Q8BTI8_T963_M1_Srrm2
GRS[Phospho (STY)]PS[Phospho (STY)]PKPR.2	-0.230471908	Q8BTI8_S1718_M2_Srrm2
NNKS[Phospho (STY)]VT[Phospho (STY)]PQR.2	-0.16209784	Q8BTI8_S1465_M2_Srrm2
VSS[Phospho (STY)]PVLETVQQRT[Phospho (STY)]PSR.3	0.276751307	Q8BTI8_S1360_M2_Srrm2
TS[Phospho (STY)]PLM[Oxidation (M)]LDR.2	0.019882407	Q8BTI8_S2351_M1_Srrm2
SLSYS[Phospho (STY)]PVER.2	-0.062053077	Q8BTI8_S2648_M1_Srrm2
S[Phospho (STY)]RTS[Phospho (STY)]PAPWKR.2	-0.021723031	Q8BTI8_S1810_M2_Srrm2
GRS[Phospho (STY)]RT[Phospho (STY)]PLTSR.2	-0.227567225	Q8BTI8_S1798_M2_Srrm2
RSS[Phospho (STY)]SELSPEVVEK.2	-0.244382248	Q8BTI8_S1339_M1_Srrm2
HAS[Phospho (STY)]S[Phospho (STY)]SPESLKPTPAPGSR.3	-0.359751915	Q8BTI8_S433_M2_Srrm2
LKSGM[Oxidation (M)]S[Phospho (STY)]PEQSK.2	0.210181896	Q8BTI8_S1097_M1_Srrm2
GRS[Phospho (STY)]EC[Carbamidomethyl (C)]DS[Phospho (STY)]S[Phospho (STY)]PEPK.2	-0.068230961	Q8BTI8_S1434_M3_Srrm2
SSSELS[Phospho (STY)]PEVVEK.2	-0.170727643	Q8BTI8_S1343_M1_Srrm2
SM[Oxidation (M)]LQT[Phospho (STY)]PPDQNLSGSK.2	-0.466007865	Q8BTI8_T973_M1_Srrm2
PSM[Oxidation (M)]S[Phospho (STY)]PTPLDR.2	-0.002921458	Q8BTI8_S2075_M1_Srrm2
S[Phospho (STY)]PVKQDKSEISTDPK.2	-0.403629669	Q8BTI8_S1077_M1_Srrm2
VSSPVLETVQQRT[Phospho (STY)]PS[Phospho (STY)]RER.3	-0.024598524	Q8BTI8_S1372_M2_Srrm2
S[Phospho (STY)]RT[Phospho (STY)]PLLPR.2	0.359440968	Q8BTI8_S1984_M2_Srrm2
S[Phospho (STY)]RAS[Phospho (STY)]PVSR.2	-0.209746576	Q8BTI8_S1866_M2_Srrm2
QSHSESS[Phospho (STY)]PDGEVK.2	0.002569407	Q8BTI8_S851_M1_Srrm2
AQS[Phospho (STY)]GTDS[Phospho (STY)]S[Phospho (STY)]PEHKIPAPR.2	-0.104580263	#N/A
NHSGS[Phospho (STY)]RT[Phospho (STY)]PPVALSSSR.3	0.296642405	Q8BTI8_S2054_M2_Srrm2
SLS[Phospho (STY)]GSS[Phospho (STY)]PC[Carbamidomethyl (C)]PK.2	-0.098373662	Q8BTI8_S775_M2_Srrm2
SQTS[Phospho (STY)]PKGS[Phospho (STY)]LSR.2	0.042413789	Q8BTI8_S1058_M2_Srrm2
EISSS[Phospho (STY)]PTSK.2	-0.001771375	Q8BTI8_S454_M1_Srrm2
SRSGS[Phospho (STY)]S[Phospho (STY)]PGLR.2	-0.301242679	Q8BTI8_S1399_M2_Srrm2
S[Phospho (STY)]VT[Phospho (STY)]PQRER.2	0.257850512	Q8BTI8_S1465_M2_Srrm2
SAVRPS[Phospho (STY)]PS[Phospho (STY)]PER.2	-0.148856142	Q8BTI8_S349_M2_Srrm2
RQPS[Phospho (STY)]PQPS[Phospho (STY)]PR.2	-0.078511632	Q8BTI8_S2656_M2_Srrm2
S[Phospho (STY)]PQRPGWSR.2	0.075700962	Q8BTI8_S533_M1_Srrm2
GGS[Phospho (STY)]GYHS[Phospho (STY)]RS[Phospho (STY)]PTRQESSR.3	-0.237877327	Q8BTI8_S1774_M3_Srrm2
S[Phospho (STY)]RS[Phospho (STY)]RT[Phospho (STY)]PLLPR.2	0.100475714	Q8BTI8_S1982_M3_Srrm2
SS[Phospho (STY)]RSS[Phospho (STY)]PELTR.2	-0.167595743	Q8BTI8_S1647_M2_Srrm2
S[Phospho (STY)]LTRS[Phospho (STY)]PPAIR.2	-0.010630586	Q8BTI8_S2019_M2_Srrm2
S[Phospho (STY)]RS[Phospho (STY)]RT[Phospho (STY)]PLISR.2	-0.239106891	Q8BTI8_S1832_M3_Srrm2
S[Phospho (STY)]RS[Phospho (STY)]RS[Phospho (STY)]PLAIR.2	-0.087046805	Q8BTI8_S1994_M3_Srrm2
KET[Phospho (STY)]PS[Phospho (STY)]PR.2	0.206190099	Q8BTI8_S2691_M2_Srrm2
SRT[Phospho (STY)]PPSAPSQSR.2	0.08296373	Q8BTI8_T2362_M1_Srrm2
RGS[Phospho (STY)]RSS[Phospho (STY)]IEPK.2	-0.49406915	Q8BTI8_S1628_M2_Srrm2
SSRRS[Phospho (STY)]S[Phospho (STY)]SELS[Phospho (STY)]PEVVEK.2	-0.028495519	Q8BTI8_S1339_M3_Srrm2
S[Phospho (STY)]RS[Phospho (STY)]RAS[Phospho (STY)]PVSR.2	-0.259579434	Q8BTI8_S1864_M3_Srrm2

SEQPLSQVLPS[Phospho (STY)]LSPEHK.2	-0.030595939	Q8BTI8_S1214_M1_Srrm2
S[Phospho (STY)]RT[Phospho (STY)]PLTSR.2	-0.068547501	Q8BTI8_S1798_M2_Srrm2
RRS[Phospho (STY)]PS[Phospho (STY)]VS[Phospho (STY)]S[Phospho (STY)]PEPTEK.2	0.075879776	Q8BTI8_S1683_M3_Srrm2
S[Phospho (STY)]SRSS[Phospho (STY)]PELTRK.2	-0.443920808	Q8BTI8_S1646_M2_Srrm2
DTPQT[Phospho (STY)]PSR.2	-0.097586265	Q8BTI8_T1428_M1_Srrm2
S[Phospho (STY)]RS[Phospho (STY)]RT[Phospho (STY)]PPVTR.2	-0.079231246	Q8BTI8_S1876_M3_Srrm2
SRS[Phospho (STY)]PQWR.2	-0.039655945	Q8BTI8_S507_M1_Srrm2
S[Phospho (STY)]RS[Phospho (STY)]RT[Phospho (STY)]PPAIR.2	-0.271854202	Q8BTI8_S1970_M3_Srrm2
S[Phospho (STY)]RS[Phospho (STY)]RTS[Phospho (STY)]PVTR.2	-0.054541817	Q8BTI8_S1922_M3_Srrm2
ELSHS[Phospho (STY)]PPRENSFESSLEFK.3	-0.09349474	Q8BTI8_S1278_M1_Srrm2
NNKS[Phospho (STY)]VT[Phospho (STY)]PQRER.2	0.055405406	Q8BTI8_S1465_M2_Srrm2
QSSS[Phospho (STY)]PYEDKDKK.2	0.028768281	Q8BTI8_S333_M1_Srrm2
ARS[Phospho (STY)]HS[Phospho (STY)]PS[Phospho (STY)]SPER.2	-0.129239494	Q8BTI8_S1453_M3_Srrm2
WGKS[Phospho (STY)]RS[Phospho (STY)]PQR.2	-0.164104285	Q8BTI8_S522_M2_Srrm2
SEC[Carbamidomethyl (C)]DS[Phospho (STY)]S[Phospho (STY)]PEPK.2	0.092253953	Q8BTI8_S1438_M2_Srrm2
SSS[Phospho (STY)]AS[Phospho (STY)]PELKDGLPR.2	-0.076327144	Q8BTI8_S1378_M2_Srrm2
SM[Oxidation (M)]LQT[Phospho (STY)]PPDQNLS[Phospho (STY)]GSKSPC[Carbamidomethyl (C)]PQK.3	-0.130528047	Q8BTI8_S980_M2_Srrm2
SGS[Phospho (STY)]S[Phospho (STY)]PEM[Oxidation (M)]KDKPR.2	-0.227274262	Q8BTI8_S1556_M2_Srrm2
RSSRS[Phospho (STY)]S[Phospho (STY)]PELTR.2	-0.145075799	Q8BTI8_S1649_M2_Srrm2
GGSGYHS[Phospho (STY)]RS[Phospho (STY)]PTR.2	0.052871261	Q8BTI8_S1778_M2_Srrm2
SGMS[Phospho (STY)]PEQSK.2	0.343776313	Q8BTI8_S1097_M1_Srrm2
SGT[Phospho (STY)]PPRPGSVTNMQADEC[Carbamidomethyl (C)]TAT[Phospho (STY)]PQR.3	-0.112896342	Q8BTI8_T823_M2_Srrm2
SGSSQELDGKPSAS[Phospho (STY)]PQER.2	-0.171787784	Q8BTI8_S1508_M1_Srrm2
MELGT[Phospho (STY)]PLR.2	0.433500821	Q8BTI8_T955_M1_Srrm2
S[Phospho (STY)]RS[Phospho (STY)]RAS[Phospho (STY)]PATHR.2	0.056904371	Q8BTI8_S1820_M3_Srrm2
SS[Phospho (STY)]PELTR.2	-0.550467069	Q8BTI8_S1650_M1_Srrm2
RGGSGYHS[Phospho (STY)]RS[Phospho (STY)]PTR.3	-0.172575097	Q8BTI8_S1778_M2_Srrm2
C[Carbamidomethyl (C)]RS[Phospho (STY)]PGMLEPLGSAR.3	-0.184534759	Q8BTI8_S2084_M1_Srrm2
SGS[Phospho (STY)]SPGLR.2	-0.257889544	Q8BTI8_S1399_M1_Srrm2
MS[Phospho (STY)]C[Carbamidomethyl (C)]FS[Phospho (STY)]RPSM[Oxidation (M)]SPTPLDR.2	-0.618198963	Q8BTI8_S2067_M2_Srrm2
TPAAAAAMNLAS[Phospho (STY)]PR.2	0.362056168	Q8BTI8_S2224_M1_Srrm2
S[Phospho (STY)]RS[Phospho (STY)]RT[Phospho (STY)]PAR.2	-0.250505401	Q8BTI8_S570_M3_Srrm2
SSS[Phospho (STY)]TEPPPPVR.2	-0.041228964	Q8BTV2_S48_M1_Cpsf7
DSSDSADGRAT[Phospho (STY)]PSENLVPSSAR.3	-0.265415902	Q8BTV2_T203_M1_Cpsf7
RES[Phospho (STY)]PPPPAR.2	-0.107559041	Q8BTW9_S104_M1_Pak4
LYSGPGIVGNVLVDPSAR.2	0.34807236	#N/A
LSTTPS[Phospho (STY)]PT[Phospho (STY)]NSLHEDGVDDFRR.3	-0.296585918	Q8BU11_S178_M2_Tox4
EDSKKEETPGT[Phospho (STY)]PK.2	0.195761787	Q8BU14_T158_M1_Sec62
SQHS[Phospho (STY)]SGNGNDFEM[Oxidation (M)]ITK.2	-0.072859679	Q8BU14_S356_M1_Sec62
EELEQQT[Phospho (STY)]DGDC[Carbamidomethyl (C)]DEEDDDKDGEVPK.3	-0.540210332	Q8BU14_T375_M1_Sec62
LNQSDSIEDPNS[Phospho (STY)]PAGR.2	-0.164435054	Q8BUK6_S238_M1_Hook3
LNQSDSIEDPNS[Phospho (STY)]PAGRR.2	-0.572515543	Q8BUK6_S238_M1_Hook3
RLS[Phospho (STY)]NPC[Carbamidomethyl (C)]IQR.2	-0.103785716	Q8BUL6_S308_M1_Plekha1
AQS[Phospho (STY)]QEDSDYINANYIR.2	0.159172612	Q8BUM3_S143_M1_Ptpn7
VTLYNADQDGSDS[Phospho (STY)]PR.2	-0.313452158	Q8BUR3_S223_M1_Foxj3
ASHS[Phospho (STY)]AVDITK.2	-0.330607684	Q8BUV3_S338_M1_Gphn
IPDSIISR.2	0.091423261	#N/A

VLAQDVYAK.2	0.422842198	#N/A
DTASLSTTPSES[Phospho (STY)]PR.2	-0.046361208	Q8BUV3_S270_M1_Gphn
DLVQDPSLLGGTISAYK.2	-0.115044929	#N/A
VGVLTVSDSC[Carbamidomethyl (C)]FR.2	0.536604916	#N/A
DTASLSTT[Phospho (STY)]PSES[Phospho (STY)]PRAQATSR.3	0.105040521	Q8BUV3_S270_M2_Gphn
LSTAS[Phospho (STY)]C[Carbamidomethyl (C)]PT[Phospho (STY)]PK.2	-0.064245396	Q8BUV3_S283_M2_Gphn
C[Carbamidomethyl (C)]SS[Phospho (STY)]KENILR.2	-0.170620695	Q8BUV3_S328_M1_Gphn
AQATSRLS[Phospho (STY)]TAS[Phospho (STY)]C[Carbamidomethyl (C)]PT[Phospho (STY)]PK.2	0.075284897	Q8BUV3_S280_M3_Gphn
EVHDELEDLPS[Phospho (STY)]PPPPLS[Phospho (STY)]PPPTTSPHK.4	0.163186993	Q8BUV3_S188_M2_Gphn
VKEVHDELEDLPS[Phospho (STY)]PPPPLS[Phospho (STY)]PPPT[Phospho (STY)]TSPHKQTEDK.5	-0.171506557	Q8BUV3_S188_M3_Gphn
VKEVHDELEDLPS[Phospho (STY)]PPPPLS[Phospho (STY)]PPPTTSPHK.4	-0.135240826	Q8BUV3_S188_M2_Gphn
DSS[Phospho (STY)]LTLTR.2	0.063073267	Q8BUV6_S220_M1_Lsm11
S[Phospho (STY)]VPSSLQASAR.2	0.258085895	Q8BUV6_S281_M1_Lsm11
KVS[Phospho (STY)]NGAVEPQGSWEGTA.2	-0.335267973	Q8BUV8_S537_M1_Gpr107
QLQIQIDNQNDPEEGQ.2	0.345560053	#N/A
QLQSEQPQTAAARS[Phospho (STY)].2	-0.266725047	Q8BVE3_S483_M1_Atp6v1h
GAVDAAVPTNIIAAK.2	-0.24538249	#N/A
LEWSPVHK.2	-0.416877945	#N/A
LLEVSDDPQVLAVAAHDVGEYVR.3	-0.301530821	#N/A
LGESVQDLSSFDEYSSELK.2	-0.378336067	#N/A
QLQSEQPQTAAAR.2	0.152547726	#N/A
YNIIPVLSDILQESVK.2	-0.202639489	#N/A
QLENLEQQKYDDEDISEDIK.3	-0.126168484	#N/A
RSS[Phospho (STY)]EDKQEM[Oxidation (M)]LQTEGSQC[Carbamidomethyl (C)]AK.3	0.360687204	Q8BVE3_S59_M1_Atp6v1h
VIEQLGGK.2	0.207589396	#N/A
SSS[Phospho (STY)]AENQEGDPGTDK.2	0.083955866	Q8BVE8_S408_M1_Nsd2
[Acetyl (Protein N-term)]C[Carbamidomethyl (C)]SGVS[Phospho (STY)]PVEQVAAGDM[Oxidation (M)]DDTAAR.2	0.107237739	Q8BVG4_S6_M1_Dpp9
EGGLLTLAGAK.2	-0.037701652	#N/A
VDAILC[Carbamidomethyl (C)]VAGGWAGGNAK.2	-0.007000189	#N/A
AALDGTPGMIGYGM[Oxidation (M)]AK.2	-0.472402382	#N/A
MTDSFTEQADQVTADVGK.2	0.214674989	#N/A
GAVHQLC[Carbamidomethyl (C)]QSLAGK.3	-0.285293216	#N/A
QSM[Oxidation (M)]WTSTISSHLATK.2	0.099473463	#N/A
S[Phospho (STY)]PPKWVDGVDEIQYDVGR.3	-0.197308951	Q8BVI5_S74_M1_Stx16
QLLAEQVSS[Phospho (STY)]HTTSSPLHSR.3	-0.18765367	Q8BVI5_S30_M1_Stx16
SIAAELDELADDR.2	-0.155375889	#N/A
VTSSVPLPS[Phospho (STY)]GGTSSPSR.2	0.298858003	Q8BVL3_S331_M1_Snx17
SPPLLES[Phospho (STY)]PDASR.2	-0.16171112	Q8BVL3_S421_M1_Snx17
VLVTVLTSDQTEDVLEAVAAK.2	-0.29712506	#N/A
SDSQQAVKS[Phospho (STY)]PPLLES[Phospho (STY)]PDASR.3	0.214491213	Q8BVL3_S415_M2_Snx17
HTS[Phospho (STY)]LNDLSLTR.2	-0.069919355	Q8BVL9_S382_M1_Jakmip1
QC[Carbamidomethyl (C)]EGITS[Phospho (STY)]PEGSK.2	0.038138991	Q8BVQ5_S243_M1_Ppme1
SIVEGIIEEEEEDEEGSESVNKR.3	-0.373816759	#N/A
SLENAIEWSVK.2	0.013994845	#N/A
FAEPIGGFQC[Carbamidomethyl (C)]VFPGC[Carbamidomethyl (C)].2	0.14140087	#N/A
YVDLGGSYVGPTQNR.2	-0.291594953	#N/A
FIGGSGQVSER.2	0.209755128	#N/A

DLKPENLLYYSQDEESK.3	0.417707642	#N/A
AAYDAFNSWK.2	0.385960576	#N/A
EVGEVLC[Carbamidomethyl (C)]TDPLVSK.2	0.278649573	#N/A
GIHDSFVTK.2	-0.069621198	#N/A
NAGQTC[Carbamidomethyl (C)]VC[Carbamidomethyl (C)]SNR.2	0.181691202	#N/A
ISFTGSTATGK.2	0.342173142	#N/A
LGTVADC[Carbamidomethyl (C)]GVPEAR.2	0.182520644	#N/A
WLPAPATFPVYDPASGAK.2	0.290085732	#N/A
DM[Oxidation (M)]LC[Carbamidomethyl (C)]ITEETFGPLAPVIK.2	0.214715548	#N/A
VAEQLEVGM[Oxidation (M)]VGVNEGLISSVEC[Carbamidomethyl (C)]PFGGVK.3	-0.349419689	#N/A
YGIDEYLEVK.2	0.024490851	#N/A
QPVGVAAIITPWNFPSAM[Oxidation (M)]ITR.2	0.12174493	#N/A
HQSGGNFFEPTLLSNVTR.2	-0.135407005	#N/A
DDKDEEDDGTGS[Phospho (STY)]PHLNNR.2	-0.108984624	#N/A
GM[Oxidation (M)]KDDKDEEDDGTGS[Phospho (STY)]PHLNNR.4	-0.424036816	#N/A
S[Phospho (STY)]LNLVDSPQPLLK.2	-0.08862259	Q8BWJ3_S729_M1_Phka2
S[Phospho (STY)]VSTS[Phospho (STY)]PSILPAYLK.2	-0.366054643	Q8BWS5_S323_M2_Gprin3
LEDTLWAGLTDQHVK.2	-0.253577914	#N/A
VGVPTETGALTLNR.2	0.570012353	#N/A
TNVSGGAIALGHPLGGSGSR.2	-0.134085146	#N/A
DFSATDLTEFAAR.2	-0.470177161	#N/A
YALQSQQR.2	0.126011991	#N/A
VPPETIDSVIVGNVM[Oxidation (M)]QSSSDAAYLAR.3	-0.154100296	#N/A
APSQPPS[Phospho (STY)]PTEER.2	0.032605475	Q8BX17_S1414_M1_Gemin5
EVST[Phospho (STY)]PQDVHTTQGVPAAR.3	0.082302659	Q8BX70_T1397_M1_Vps13c
LS[Phospho (STY)]FHNDSFR.2	-0.015949693	Q8BX70_S1956_M1_Vps13c
ILLENLGEASSQPS[Phospho (STY)]PTQYAQEAAR.3	-0.400366061	Q8BX70_S1891_M1_Vps13c
SST[Phospho (STY)]PS[Phospho (STY)]PERQVASIPVLSGGTK.3	-0.085976997	Q8BX70_S841_M2_Vps13c
TANAS[Phospho (STY)]LEEIIDK.2	-0.221077264	Q8BX70_S736_M1_Vps13c
KVS[Phospho (STY)]VEPQDSHQDAQPR.3	0.018925798	Q8BXB6_S21_M1_Slco2b1
VFQDVAQK.2	0.15172374	#N/A
ATSAC[Carbamidomethyl (C)]APISS[Phospho (STY)]PK.2	0.287975554	Q8BXK8_S422_M1_Agap1
LDPPPS[Phospho (STY)]PHANR.2	-0.085056164	Q8BXK8_S521_M1_Agap1
YLTGTYVQEES[Phospho (STY)]PEGGR.2	0.029297976	Q8BXK8_S101_M1_Agap1
GNAGGGGPQVPQQS[Phospho (STY)]PPKR.2	0.248741339	Q8BXQ8_S162_M1_Fam53c
SRS[Phospho (STY)]QPC[Carbamidomethyl (C)]DLDAR.2	0.211204158	Q8BXQ8_S273_M1_Fam53c
S[Phospho (STY)]LSVPVDLSR.2	-0.043836503	Q8BXQ8_S120_M1_Fam53c
QNSEALIANDELDC[Carbamidomethyl (C)]S[Phospho (STY)]PE.2	0.138025594	Q8BXR1_S769_M1_Slc7a14
HKQNSEALIANDELDC[Carbamidomethyl (C)]S[Phospho (STY)]PE.2	0.186106033	Q8BXR1_S769_M1_Slc7a14
ET[Phospho (STY)]C[Carbamidomethyl (C)]SPVS[Phospho (STY)]EGEEFSSPATNTC[Carbamidomethyl (C)]GAK.3	-0.113301059	Q8BXR1_S468_M2_Slc7a14
NLPS[Phospho (STY)]LGDNEM[Oxidation (M)]LIGK.2	0.109659877	Q8BXR1_S488_M1_Slc7a14
GKS[Phospho (STY)]LETLTQDHSNTVR.3	-0.379506398	Q8BXR5_S781_M1_Nalcn
TAS[Phospho (STY)]SSTEPSVSR.2	0.04105559	Q8BXR9_S44_M1_Osbpl6
[Acetyl (Protein N-term)]S[Phospho (STY)]SDEKGIS[Phospho (STY)]PAHK.2	-0.469336768	Q8BXR9_S2_M2_Osbpl6
RLS[Phospho (STY)]TEEATR.2	-0.360611316	Q8BXT1_S46_M1_Rgs8
SDS[Phospho (STY)]C[Carbamidomethyl (C)]SDFTAILPDKPNR.2	0.169026124	Q8BXT1_S26_M1_Rgs8
TPEGPDEEDVS[Phospho (STY)]D.2	-0.186468132	Q8BY35_S654_M1_Fgd2

STETSDFENIES[Phospho (STY)]PLNER.2	-0.073960344	Q8BY87_S911_M1_Usp47
LFVLLPEQS[Phospho (STY)]PGSYSK.2	-0.204696139	Q8BY87_S832_M1_Usp47
ELEQHIQTS[Phospho (STY)]DPENFQSEER.3	-0.259177234	Q8BY87_S934_M1_Usp47
PC[Carbamidomethyl (C)]HY[Phospho (STY)]LRPDAS[Phospho (STY)]LNLR.2	-0.088817939	Q8BY98_S236_M2_Sowahd
PDAS[Phospho (STY)]LNLR.2	-0.136369037	Q8BY98_S236_M1_Sowahd
YLLLASS[Phospho (STY)]PQDSR.2	-0.276284116	Q8BYH7_S176_M1_Tbc1d17
LILNYSPR.2	-0.441673444	#N/A
LYPATEYEISLNSVR.2	-0.087191642	#N/A
ASGPIDHYR.2	0.496919211	#N/A
YEVSISAVR.2	0.1365654	#N/A
YGLVGGEGGK.2	0.11766188	#N/A
SSLTSTVFTTGGR.2	0.107103839	#N/A
AAIENYVLTYK.2	0.221940633	#N/A
QSALISWQPPR.2	0.202945585	#N/A
ELIVDAEDTWIR.2	0.002707192	#N/A
LDSSVVPNTVTEFAITR.2	0.170787639	#N/A
TATSLDLEWDNSEAEAQEYK.2	-0.338757307	#N/A
DGQEAVFAYYDK.2	-0.18475121	#N/A
VATHLSTPQGLQFK.3	0.434655902	#N/A
DKEEDM[Oxidation (M)]LEVLLDATKR.3	-0.627780649	#N/A
SPPTSASVSTVIDGPTQILVR.2	0.129969632	#N/A
GHEFSIPFVEM[Oxidation (M)]K.2	-0.033290039	#N/A
LEGLSENTDYTVLLQAAQEATR.3	-0.296177439	#N/A
QSIPATM[Oxidation (M)]NAR.2	0.38149154	#N/A
KELIVDAEDTWIR.3	0.158951548	#N/A
VVYSTLAGEQYHEVLVPK.2	-0.026407458	#N/A
VGFGNLEDEFWLGLDNIHR.3	-0.105601728	#N/A
DVSDTVAFVEWTPPR.2	0.060450285	#N/A
ITFTPSSGISSEVTVPR.2	-0.011542047	#N/A
C[Carbamidomethyl (C)]LNAC[Carbamidomethyl (C)]SGR.2	-0.058759363	#N/A
DSLTVSWSSPVAPFDYYR.2	-0.200232939	#N/A
LQVYC[Carbamidomethyl (C)]DM[Oxidation (M)]TTDGGGWIVFQR.2	-0.128354589	#N/A
DGQEAVFAYYDKFAVEDSR.3	-0.300894591	#N/A
RQEPLRQPS[Phospho (STY)]PQR.3	-0.475324818	Q8BYM5_S722_M1_Nlgn3
QEPLRQPS[Phospho (STY)]PQR.2	-0.466071068	Q8BYM5_S722_M1_Nlgn3
LTALPDYTLTLR.2	0.729361502	#N/A
SENGMTGSTSS[Phospho (STY)]LNNVTQ.2	0.156782515	Q8BYN5_S501_M1_Fsd1l
PVSPSPSVLS[Phospho (STY)]EGR.2	-0.348641089	Q8BYR5_S68_M1_Cadps2
SKDAPIS[Phospho (STY)]PPAQK.2	0.029765672	Q8BYW1_S363_M1_Arhgap25
S[Phospho (STY)]SVGWDATEDPPLSR.2	-0.216226943	Q8BYW1_S379_M1_Arhgap25
DAPIS[Phospho (STY)]PPAQK.2	0.255846185	Q8BYW1_S363_M1_Arhgap25
TNS[Phospho (STY)]M[Oxidation (M)]GSATGPLPGTK.2	-0.251096993	Q8BZ47_S467_M1_Znf609
LVEPHS[Phospho (STY)]PSPSSK.2	-0.146135142	Q8BZ47_S575_M1_Znf609
AEADKIYSFTDNAPS[Phospho (STY)]PSIGGSSR.3	0.346595221	Q8BZ47_S803_M1_Znf609
APS[Phospho (STY)]LTDLVK.2	0.106088011	Q8BZ47_S1057_M1_Znf609
FC[Carbamidomethyl (C)]DS[Phospho (STY)]PTSDLEMR.2	0.001136964	Q8BZ47_S358_M1_Znf609
S[Phospho (STY)]PPPSPTTQR.2	-0.287963147	Q8BZ98_S763_M1_Dnm3

ENC[Carbamidomethyl (C)]LILAVTPANTDLANSDALK.2	0.224026272	#N/A
STIGNQVIR.2	-0.325750357	#N/A
VPVGDQPPDIEYQIR.2	0.162965021	#N/A
FLELAC[Carbamidomethyl (C)]DSQEDVDSWK.2	0.108810913	#N/A
RS[Phospho (STY)]PPPSPTTQR.2	-0.494921618	Q8BZ98_S763_M1_Dnm3
APPSVPSR.2	0.313040639	#N/A
LQGQLLSIEHEVEAFK.3	0.359020062	#N/A
RS[Phospho (STY)]PPPS[Phospho (STY)]PTTQRR.3	0.116212788	Q8BZ98_S763_M2_Dnm3
IIQGQGVDAPLSETGFR.2	0.404747221	#N/A
SLDAEPSVPSAAKPS[Phospho (STY)]SPEK.2	-0.339223243	Q8BZH4_S421_M1_Pogz
SDLAVSNISPPS[Phospho (STY)]PDSK.2	-0.072536352	Q8BZJ8_S111_M1_
DM[Oxidation (M)]SQS[Phospho (STY)]PTSSFVR.2	-0.19355094	Q8BZN6_S877_M1_Dock10
ASLASLDS[Phospho (STY)]NPSTTEK.2	-0.130636187	Q8BZN6_S1295_M1_Dock10
HATS[Phospho (STY)]VDTSFSK.2	-0.204543424	Q8BZN6_S1257_M1_Dock10
EKDM[Oxidation (M)]SQS[Phospho (STY)]PTSSFVR.2	0.011738362	Q8BZN6_S877_M1_Dock10
AEAPAGPALGLPS[Phospho (STY)]PEVESGLER.2	-0.262460626	Q8BZR9_S25_M1_Ncbp3
VAGPDPS[Phospho (STY)]PEDSPNQR.2	0.031985193	Q8BZW2_S132_M1_Sowahb
FTQGLEDQLS[Phospho (STY)]PPGNR.2	-0.506919191	Q8C008_S768_M1_Dzank1
S[Phospho (STY)]LVGTPYWM[Oxidation (M)]APEVISR.2	-0.284153082	Q8C015_S602_M1_Pak5
ESPPTPDQGAAS[Phospho (STY)]R.2	-0.149084923	Q8C015_S114_M1_Pak5
ASSSS[Phospho (STY)]PLDYSFQLTPSR.2	-0.253951226	Q8C015_S226_M1_Pak5
SNS[Phospho (STY)]LRKES[Phospho (STY)]PPT[Phospho (STY)]PDQGAASR.3	-0.369137214	Q8C015_S104_M3_Pak5
KES[Phospho (STY)]PPT[Phospho (STY)]PDQGAASR.2	-0.290223333	Q8C015_S104_M2_Pak5
SSYLHQTS[Phospho (STY)]PQPAMR.2	0.262088399	Q8C015_S277_M1_Pak5
AQMVFS[Phospho (STY)]PPLSGSDTYPR.2	0.344622447	Q8C015_S341_M1_Pak5
M[Oxidation (M)]NS[Phospho (STY)]KDNVQETQI.2	-0.41696305	Q8C031_S631_M1_Lrrc4c
NDLSSS[Phospho (STY)]SGS[Phospho (STY)]LNLSHGSSSLEHR.3	0.276400286	Q8C033_S1259_M2_Arhgef10
T[Phospho (STY)]RSLVSQDHR.3	-0.58130564	Q8C033_T353_M1_Arhgef10
TLQPSNPTEGSNPDTLFQFS[Phospho (STY)]D.2	-0.135440382	Q8C050_S862_M1_Rps6ka5
RPVVTTHDLEAPS[Phospho (STY)]RANS[Phospho (STY)]QDSLASR.4	0.046533168	Q8C052_S458_M2_Map1s
ANS[Phospho (STY)]QDSLASR.2	-0.137729253	Q8C052_S462_M1_Map1s
GGPC[Carbamidomethyl (C)]VESWGAPAPGS[Phospho (STY)]PPR.2	-0.384431484	Q8C078_S572_M1_Camkk2
S[Phospho (STY)]FGNPFEGSR.2	-0.430398347	Q8C078_S495_M1_Camkk2
SLS[Phospho (STY)]APGNLLTK.2	-0.398842361	Q8C078_S511_M1_Camkk2
DQPPEADGQELPLEAS[Phospho (STY)]DPESR.2	-0.370118614	Q8C078_S85_M1_Camkk2
C[Carbamidomethyl (C)]IC[Carbamidomethyl (C)]PSLSY[Phospho (STY)]SPASS[Phospho (STY)]PQSSPR.2	0.04844771	Q8C078_S133_M2_Camkk2
KRS[Phospho (STY)]FGNPFEGSR.3	-0.361163609	Q8C078_S495_M1_Camkk2
KMS[Phospho (STY)]LQEPSQGGPASSSNSLDMNGR.3	-0.293866634	Q8C078_S99_M1_Camkk2
AAS[Phospho (STY)]PPASASDLIEQQQKR.3	0.096954026	Q8C079_S335_M1_Strip1
AAS[Phospho (STY)]PPASASDLIEQQQK.2	-0.281858487	Q8C079_S335_M1_Strip1
SSS[Phospho (STY)]FGSVSTSSTSSK.2	-0.269579711	Q8C080_S29_M1_Snx16
EFLC[Carbamidomethyl (C)]LDDPPGPFDS[Phospho (STY)]LEESR.2	0.213823085	Q8C080_S222_M1_Snx16
GES[Phospho (STY)]PLAHAGEPALR.2	-0.165305631	Q8C0C4_S375_M1_Ccser1
SLQALGEVIEAELR.2	-0.26756415	#N/A
LQLVQAGQAEK.2	0.049085672	#N/A
ATT[Phospho (STY)]PPNQGRPDS[Phospho (STY)]PVYANLQELK.3	0.069461289	Q8C0D4_S238_M2_Arhgap12
TSFSQEQSC[Carbamidomethyl (C)]DS[Phospho (STY)]AGEGSER.2	-0.324758271	Q8C0D4_S199_M1_Arhgap12

IQQDSES[Phospho (STY)]GDELSSSSTEQM[Oxidation (M)]R.2	-0.138351653	Q8C0D4_S213_M1_Arhgap12
TPGQLSDNNSR.2	-0.116229041	#N/A
SM[Oxidation (M)]S[Phospho (STY)]HQAAIASQR.2	-0.215263772	#N/A
KSM[Oxidation (M)]S[Phospho (STY)]HQAAIASQR.3	-0.483091686	#N/A
TPGQLSDNNSRQ.2	0.278714994	#N/A
ETTGTGPNVYHENDTIAK.3	0.245811977	#N/A
AASAAGAS[Phospho (STY)]PAATPAAPESGTIPK.2	0.091720834	Q8C0I1_S57_M1_Agps
FC[Carbamidomethyl (C)]TGDS[Phospho (STY)]PPLEAK.2	0.135043878	Q8C0J6_S82_M1_Sowahc
NLVGALDEDDGDS[Phospho (STY)]PAAR.2	0.012835882	Q8C0J6_S391_M1_Sowahc
EDGAHPADTQDVVEDALR.3	0.02863331	#N/A
VKLTLEGLEEDEDDDDDKVS[Phospho (STY)]PTVLHK.4	0.019258617	Q8C0L9_S178_M1_Gpcpd1
LTLEGLEEDEDDDDDKVS[Phospho (STY)]PTVLHK.3	0.119790373	Q8C0L9_S178_M1_Gpcpd1
AVS[Phospho (STY)]PTEPTPR.2	0.307800774	Q8C0M0_S564_M1_Wdr59
ETEHISSPWGESS[Phospho (STY)]PEELR.3	0.051778088	Q8C0M0_S840_M1_Wdr59
LYSGS[Phospho (STY)]PTR.2	0.177151161	Q8C0M0_S603_M1_Wdr59
LALFHVEQGK.2	-0.105097405	#N/A
DLSAGAVSAVR.2	-0.069950655	#N/A
GNLAYATSTGGIVNK.2	0.194121944	#N/A
FAEDMGIPQVPVEK.2	0.336331155	#N/A
TVEEAAQLALDYMK.2	-0.180464649	#N/A
HTVDEYS[Phospho (STY)]PQKK.2	-0.177321681	Q8C0Q9_S445_M1_Rapgef5
TLS[Phospho (STY)]DESIYSSQR.2	-0.398271454	Q8C0T5_S1564_M1_Sipa1l1
LIDLES[Phospho (STY)]PTPESQK.2	-0.440387562	Q8C0T5_S1528_M1_Sipa1l1
QDPVVHLS[Phospho (STY)]PNK.2	-0.231294041	Q8C0T5_S1249_M1_Sipa1l1
DLQAS[Phospho (STY)]PKPTSK.2	0.177289061	Q8C0T5_S1507_M1_Sipa1l1
SIS[Phospho (STY)]SDGRPLER.2	-0.034857267	Q8C0T5_S1116_M1_Sipa1l1
KLIDLES[Phospho (STY)]PTPESQK.2	-0.098788867	Q8C0T5_S1528_M1_Sipa1l1
GSGFS[Phospho (STY)]LDVIDGPISQR.2	-0.217027402	Q8C0T5_S258_M1_Sipa1l1
EYGS[Phospho (STY)]TSSIDK.2	-0.648733535	Q8C0T5_S208_M1_Sipa1l1
RSPAS[Phospho (STY)]IDR.2	0.024641078	Q8C0T5_S1184_M1_Sipa1l1
HS[Phospho (STY)]AS[Phospho (STY)]PVVFSSAR.2	-0.07698741	Q8C0T5_S1410_M2_Sipa1l1
SKS[Phospho (STY)]ETGDSSIFR.2	-0.37269987	Q8C0T5_S288_M1_Sipa1l1
SKS[Phospho (STY)]ETGDSSIFRK.2	-0.535167973	Q8C0T5_S288_M1_Sipa1l1
SYSSKDPS[Phospho (STY)]PTLASK.2	-0.016852586	Q8C0T5_S1712_M1_Sipa1l1
QKS[Phospho (STY)]M[Oxidation (M)]PEGFGVSR.2	0.288615227	Q8C0T5_S1170_M1_Sipa1l1
SLHGEFSAS[Phospho (STY)]DSSLTDIQETR.3	-0.296474069	Q8C0T5_S1626_M1_Sipa1l1
S[Phospho (STY)]MPEGFGVSR.2	0.103923479	Q8C0T5_S1170_M1_Sipa1l1
ISDYFEYQGGNGSS[Phospho (STY)]PVR.2	-0.007490593	Q8C0V0_S159_M1_Tlk1
LSTS[Phospho (STY)]PTPGSAAAAR.2	0.069802842	Q8C0V0_S22_M1_Tlk1
HSLS[Phospho (STY)]LDDIR.2	0.074003812	Q8C0V9_S544_M1_Frmd6
LRGQS[Phospho (STY)]TDS[Phospho (STY)]LPQTIC[Carbamidomethyl (C)]R.2	-0.291847975	Q8C0V9_S522_M2_Frmd6
SQS[Phospho (STY)]FNNQAFHAK.2	-0.200221959	Q8C0Y0_S777_M1_Ppp4r4
RDS[Phospho (STY)]TPSLK.2	0.200288396	Q8C142_S198_M1_Ldlrap1
YDSQLS[Phospho (STY)]SNEEK.2	0.228653275	Q8C145_S488_M1_Slc39a6
NC[Carbamidomethyl (C)]SS[Phospho (STY)]PEFSK.2	0.199245683	Q8C166_S54_M1_Cpne1
APPT[Phospho (STY)]PGKGPAQAPQA.2	0.500934027	Q8C166_T525_M1_Cpne1
FAAQAAQQR.2	0.270293463	#N/A

DWNES[Phospho (STY)]PVR.2	0.052118738	Q8C170_S1950_M1_Myo9a
GNYPSPSS[Phospho (STY)]PVIVR.2	-0.208605373	Q8C170_S2296_M1_Myo9a
S[Phospho (STY)]LEDLHQK.2	-0.068813979	Q8C170_S1259_M1_Myo9a
S[Phospho (STY)]LEILQR.2	-0.055361733	Q8C170_S755_M1_Myo9a
RQS[Phospho (STY)]GTDLQEDVIVR.2	0.399530153	Q8C170_S1243_M1_Myo9a
AAS[Phospho (STY)]AC[Carbamidomethyl (C)]EAQGTEGPLGQAK.2	-0.105859828	Q8C170_S2458_M1_Myo9a
WDLSAQQIR.2	0.072109948	#N/A
EELGGLPEDFLSSLEK.2	0.110191951	#N/A
TSQTVATFLDELAQK.2	-0.411149959	#N/A
VDQVLHTQTDADPAEEYAR.3	0.479950341	#N/A
NLNEDTTFLPFTR.2	0.117818987	#N/A
LLEEAFNC[Carbamidomethyl (C)]R.2	-0.191575821	#N/A
SVS[Phospho (STY)]NEGLTLNNSR.2	-0.310638117	Q8C1B1_S439_M1_Camsap2
LNQSS[Phospho (STY)]PDNLTDTK.2	-0.294784498	Q8C1B1_S573_M1_Camsap2
WLKS[Phospho (STY)]PTTPVDPER.2	-0.21076992	Q8C1B1_S836_M1_Camsap2
EQQAWVIS[Phospho (STY)]PPQPSPQK.2	-0.128721802	Q8C1B1_S905_M1_Camsap2
ADVSVEKLDGES[Phospho (STY)]DKEQFDDDQK.3	-0.12959323	Q8C1B1_S1120_M1_Camsap2
SES[Phospho (STY)]VEGFLSPSR.2	-0.389393867	Q8C1B1_S1285_M1_Camsap2
SKS[Phospho (STY)]LADIK.2	-0.253215387	Q8C1B1_S818_M1_Camsap2
SSS[Phospho (STY)]MSYVDGFIGTWPK.2	0.481444193	Q8C1B1_S391_M1_Camsap2
DHIES[Phospho (STY)]PK.2	0.082847701	Q8C1B1_S1248_M1_Camsap2
LDGES[Phospho (STY)]DKEQFDDDQK.2	-0.172608053	Q8C1B1_S1120_M1_Camsap2
SYELQESNVR.2	0.051523143	#N/A
ELEEEVSNFQK.2	0.199018613	#N/A
STSQGFC[Carbamidomethyl (C)]FNILC[Carbamidomethyl (C)]VGETGIGK.2	-0.12581646	#N/A
[Acetyl (Protein N-term)]AVAVGRPS[Phospho (STY)]NEELR.2	-0.308173226	Q8C1B7_S9_M1_Septin11
FESDPATHNEPGVR.3	0.122804874	#N/A
SLFNYHDTR.2	0.060168865	#N/A
AAAQLLQSQAQQSGAQQTK.2	-0.235819442	#N/A
KKELEEEVSNFQK.3	0.071212364	#N/A
NLSLSGHVGFDSLPDQLVNK.3	-0.423132738	#N/A
LTIVDTVGFGDQINK.2	0.03995832	#N/A
EAEDS[Phospho (STY)]DS[Phospho (STY)]DDNIKR.2	-0.193530761	Q8C1D8_S458_M2_Iws1
VVC[Carbamidomethyl (C)]DADDS[Phospho (STY)]DSDVVSDK.2	-0.047519558	Q8C1D8_S366_M1_Iws1
IS[Phospho (STY)]DS[Phospho (STY)]ESEELPKPR.2	-0.072532763	Q8C1D8_S183_M2_Iws1
KAAVLS[Phospho (STY)]DS[Phospho (STY)]EDDAGNASAK.2	-0.118326845	Q8C1D8_S343_M2_Iws1
IDS[Phospho (STY)]DDDEEKEGDEEK.2	-0.172455618	Q8C1D8_S321_M1_Iws1
AAVLS[Phospho (STY)]DS[Phospho (STY)]EDDAGNASAK.2	-0.207792823	Q8C1D8_S343_M2_Iws1
ETTVAS[Phospho (STY)]DS[Phospho (STY)]EEEAGKEESSVK.2	-0.093941907	Q8C1D8_S384_M2_Iws1
GLHS[Phospho (STY)]S[Phospho (STY)]DS[Phospho (STY)]EEEEPKR.2	-0.254809488	Q8C1D8_S306_M3_Iws1
AAVLS[Phospho (STY)]DS[Phospho (STY)]EDDAGNASAKK.2	-0.204210653	Q8C1D8_S343_M2_Iws1
QKIDS[Phospho (STY)]DDDEEKEGDEEK.2	0.03920806	Q8C1D8_S321_M1_Iws1
IDS[Phospho (STY)]DDDEEKEGDEEKVAK.3	-0.147151385	Q8C1D8_S321_M1_Iws1
KGLHS[Phospho (STY)]S[Phospho (STY)]DS[Phospho (STY)]EEEEPKR.2	0.304416342	Q8C1D8_S306_M3_Iws1
GTGPPS[Phospho (STY)]PTKDR.2	0.017886747	Q8C1W1_S323_M1_Vash1
TFS[Phospho (STY)]PSKPAK.2	0.035153693	Q8C1Y8_S74_M1_Ccz1
HIEPELAGRDS[Phospho (STY)]PVR.3	-0.387446978	Q8C1Y8_S264_M1_Ccz1

TRLS[Phospho (STY)]PPR.2	-0.130075341	Q8C2Q3_S582_M1_Rbm14
DRS[Phospho (STY)]PLRR.2	-0.317847849	Q8C2Q3_S215_M1_Rbm14
S[Phospho (STY)]PTKSSLDYR.2	-0.429756943	Q8C2Q3_S627_M1_Rbm14
QPT[Phospho (STY)]PPFFGR.2	-0.2447072	Q8C2Q3_T206_M1_Rbm14
LSESQLSFR.2	-0.221024536	#N/A
RLS[Phospho (STY)]ESQLSFR.2	-0.108634585	Q8C2Q3_S618_M1_Rbm14
IFVGNVSAAC[Carbamidomethyl (C)]TSQELR.2	0.014315816	#N/A
SKS[Phospho (STY)]LQFTGK.2	-0.177430596	Q8C341_S1077_M1_Suco
KQS[Phospho (STY)]EADLAEPRPDLK.3	-0.404777372	Q8C351_S299_M1_Layn
QENS[Phospho (STY)]PNLDVYNVIR.2	0.073649245	Q8C351_S286_M1_Layn
S[Phospho (STY)]DSPVPTAPTSSGPKPSTTSVAPELATDPELEKK.4	0.306283629	Q8C3P7_S48_M1_Mettl3
EHLLS[Phospho (STY)]PPR.2	-0.110357689	Q8C3Q5_S451_M1_Shisa7
SSS[Phospho (STY)]LTPGLGGPDSM[Oxidation (M)]APR.2	0.391115931	Q8C3Q5_S250_M1_Shisa7
RVMS[Phospho (STY)]QEHLLGDGSR.3	0.006229887	Q8C3Q5_S405_M1_Shisa7
EHLLS[Phospho (STY)]PPRS[Phospho (STY)]PALPPDPTTR.3	-0.022071838	Q8C3Q5_S451_M2_Shisa7
LVSQEHLLLSS[Phospho (STY)]PEALR.2	-0.17608349	Q8C3Q5_S438_M1_Shisa7
EAIS[Phospho (STY)]DDEDEALYQK.2	0.318664887	Q8C3S2_S561_M1_Tango6
SSS[Phospho (STY)]LDALGPAR.2	-0.203629937	Q8C3W1_S176_M1_
SAS[Phospho (STY)]AHNLSSEK.2	-0.288656009	Q8C419_S881_M1_Gpr158
SLS[Phospho (STY)]VIASAK.2	-0.15653626	Q8C419_S904_M1_Gpr158
LKEDS[Phospho (STY)]EAESTESVPLVC[Carbamidomethyl (C)]K.2	-0.172474128	Q8C419_S865_M1_Gpr158
EQTVQDDLQWYQALVR.2	0.123659425	#N/A
EES[Phospho (STY)]LKNR.2	-0.334314195	Q8C419_S806_M1_Gpr158
VS[Phospho (STY)]LPTANPDVSSGITQIK.2	0.103827943	Q8C419_S976_M1_Gpr158
S[Phospho (STY)]IDKTEVC[Carbamidomethyl (C)]PWEIHSQSLLEDENR.3	0.08390814	Q8C419_S1059_M1_Gpr158
QC[Carbamidomethyl (C)]S[Phospho (STY)]KEDKEGTDHSAAK.3	0.179342798	Q8C419_S770_M1_Gpr158
KGS[Phospho (STY)]PGLTR.2	-0.480130378	Q8C437_S378_M1_Pex5l
S[Phospho (STY)]PVDSSVLEGVK.2	0.009644279	Q8C437_S388_M1_Pex5l
TGS[Phospho (STY)]KELLWSAEHR.2	-0.474520264	Q8C437_S146_M1_Pex5l
SALNSES[Phospho (STY)]ASELELVAPAQAR.2	-0.635478234	Q8C437_S172_M1_Pex5l
NKKGS[Phospho (STY)]PGLTR.2	-0.022780441	Q8C437_S378_M1_Pex5l
S[Phospho (STY)]QPELSTGK.2	-0.157791674	Q8C437_S157_M1_Pex5l
GPETS[Phospho (STY)]SLDLDIQTQLEK.2	0.27574827	Q8C437_S105_M1_Pex5l
KGS[Phospho (STY)]PGLTRR.2	-0.188982821	Q8C437_S378_M1_Pex5l
NHS[Phospho (STY)]LEEEFER.2	-0.561721833	Q8C437_S202_M1_Pex5l
M[Oxidation (M)]SKS[Phospho (STY)]PVDSSVLEGVK.2	-0.164994338	Q8C437_S388_M1_Pex5l
RM[Oxidation (M)]S[Phospho (STY)]KS[Phospho (STY)]PVDSSVLEGVK.3	0.156793782	Q8C437_S386_M2_Pex5l
VVTM[Oxidation (M)]AEPGAAS[Phospho (STY)]PPPPAR.2	-0.178598637	Q8C4U2_S511_M1_Tmem145
GGRDS[Phospho (STY)]DQDS[Phospho (STY)]AEEGTAEKPK.3	-0.205964136	Q8C4V4_S12_M2_Fbxl3
GSTTSIPS[Phospho (STY)]PQSDGGDPNQPDDR.2	-0.149681835	Q8C525_S376_M1_Mb21d2
TNSMSSSGLGS[Phospho (STY)]PNR.2	0.290533709	Q8C5L3_S165_M1_Cnot2
GGGQSS[Phospho (STY)]PQEEPTWK.2	-0.051190823	Q8C5N3_S106_M1_Cwc22
IKES[Phospho (STY)]PSEQGSR.2	-0.474529903	Q8C5W0_S317_M1_Clmn
NSHS[Phospho (STY)]DSSIYIR.2	0.203985571	Q8C5W0_S925_M1_Clmn
SNS[Phospho (STY)]LPVKK.2	-0.110398748	Q8C5W0_S419_M1_Clmn
VFVC[Carbamidomethyl (C)]DQLESPT[Phospho (STY)]GFC[Carbamidomethyl (C)]LDSAPSHK.3	-0.108444078	Q8C5W0_T360_M1_Clmn
YS[Phospho (STY)]PENFPYR.2	-0.102738945	Q8C5W3_S18_M1_Tbcel

AS[Phospho (STY)]NETLISYGIPDNDEQR.2	-0.321137193	Q8C624_S87_M1_Spata33
RPSDS[Phospho (STY)]VQPAQHSPC[Carbamidomethyl (C)]R.3	0.168391986	Q8C6B2_S108_M1_Rtkn
TFS[Phospho (STY)]LDAAPADHSLGPSR.2	-0.279441857	Q8C6B2_S520_M1_Rtkn
RLS[Phospho (STY)]QSDEDVIR.2	-0.264590865	Q8C6G8_S101_M1_Wdr26
LSQS[Phospho (STY)]DEDVIR.2	0.28402859	Q8C6G8_S103_M1_Wdr26
KRLS[Phospho (STY)]QSDEDVIR.3	0.009406259	Q8C6G8_S101_M1_Wdr26
EAQPPS[Phospho (STY)]PR.2	-0.15101978	Q8C6M1_S369_M1_Usp20
LSEQDS[Phospho (STY)]PPPSHPLK.2	0.017923128	Q8C6M1_S112_M1_Usp20
KFS[Phospho (STY)]WGQQR.2	-0.256678052	Q8C6M1_S334_M1_Usp20
AVPIAVADEGESES[Phospho (STY)]EDDDLKPR.3	0.115767315	Q8C6M1_S134_M1_Usp20
STS[Phospho (STY)]PC[Carbamidomethyl (C)]QTPEPDNEAHIR.3	0.188944075	Q8C6M1_S374_M1_Usp20
LSSSPPRAS[Phospho (STY)]PVR.2	-0.100052622	Q8C6M1_S415_M1_Usp20
TAITTAS[Phospho (STY)]IRR.2	0.055961067	Q8C729_S321_M1_Fam126b
VEVT[Phospho (STY)]PTVPR.2	-0.018455804	Q8C729_T306_M1_Fam126b
NSLPFDAPDSS[Phospho (STY)]QEGQK.2	-0.399712029	Q8C729_S294_M1_Fam126b
TNS[Phospho (STY)]LSLIR.2	0.016712427	Q8C729_S442_M1_Fam126b
YSTIS[Phospho (STY)]LQEDR.2	0.012491061	Q8C729_S491_M1_Fam126b
S[Phospho (STY)]AKDKETALAIK.3	0.061821038	Q8C729_S383_M1_Fam126b
SKS[Phospho (STY)]DLSDR.2	-0.04264479	Q8C739_S257_M1_Fam110b
ANS[Phospho (STY)]DIISLNFR.2	0.156124488	Q8C739_S297_M1_Fam110b
VYPTPGHGS[Phospho (STY)]PQESSSHVSR.3	0.263769664	Q8C739_S172_M1_Fam110b
VAAMKS[Phospho (STY)]PEADQVEPAC[Carbamidomethyl (C)]GVSR.3	0.012259473	Q8C739_S234_M1_Fam110b
QGGGQPSSFS[Phospho (STY)]PGQSQVTPQDQEK.3	-0.259552802	Q8C7E9_S579_M1_Cstf2t
NILVQTPESES[Phospho (STY)]PQSR.2	-0.085805304	Q8C7M3_S46_M1_Trim9
RAS[Phospho (STY)]GSGVSDYDYLDLDK.2	0.064048598	Q8C7M3_S53_M1_Trim9
S[Phospho (STY)]KS[Phospho (STY)]LPKR.2	-0.109160411	Q8C8K3_S187_M2_Srsf12
HC[Carbamidomethyl (C)]DSIARS[Phospho (STY)]PC[Carbamidomethyl (C)]KS[Phospho (STY)]PR.3	-0.306615226	Q8C8K3_S228_M2_Srsf12
QSTSLRQS[Phospho (STY)]RT[Phospho (STY)]PR.3	-0.24297367	Q8C8K3_S172_M2_Srsf12
VGT[Phospho (STY)]PTDIHSEK.2	-0.113931379	Q8C8R3_T2232_M1_Ank2
S[Phospho (STY)]YIETETESR.2	-0.317339914	Q8C8R3_S3362_M1_Ank2
SS[Phospho (STY)]PEDVVVTNSSNR.2	0.189029252	Q8C8R3_S2924_M1_Ank2
TEWQPS[Phospho (STY)]PTDIPLQK.2	0.127578588	Q8C8R3_S3315_M1_Ank2
QPPIS[Phospho (STY)]PTSK.2	-0.110054396	Q8C8R3_S1936_M1_Ank2
DSLESS[Phospho (STY)]PVEPK.2	0.224148357	Q8C8R3_S2431_M1_Ank2
NERHS[Phospho (STY)]PVSSLK.2	0.112721096	Q8C8R3_S1851_M1_Ank2
TQDQQEYYVTT[Phospho (STY)]PGAEVEDPQK.3	-0.231324301	Q8C8R3_T3718_M1_Ank2
LSPVS[Phospho (STY)]PSAK.2	-0.205059184	Q8C8R3_S1819_M1_Ank2
VVTEEVTTTTTTITEK.2	-0.185554724	#N/A
HTPVS[Phospho (STY)]PSSK.2	0.08286557	Q8C8R3_S1807_M1_Ank2
ATFSPIVTLEPR.2	-0.439015748	#N/A
AREEQTPLHIASR.3	0.340920356	#N/A
FWLIDC[Carbamidomethyl (C)]R.2	0.121428988	#N/A
Y[Phospho (STY)]SLEGGR.2	0.185775824	Q8C8R3_Y889_M1_Ank2
HS[Phospho (STY)]PVSSLK.2	-0.153483759	Q8C8R3_S1851_M1_Ank2
HTPVS[Phospho (STY)]PSSKNEK.2	-0.006025049	Q8C8R3_S1807_M1_Ank2
ERAEGFES[Phospho (STY)]ESEDGATKPK.3	0.047840833	Q8C8R3_S3379_M1_Ank2
SVYSGQDDES[Phospho (STY)]PESS[Phospho (STY)]PEEQK.2	0.014114727	Q8C8R3_S3226_M2_Ank2

GS[Phospho (STY)]PIVQEPEEASEPK.2	0.083585107	Q8C8R3_S3764_M1_Ank2
ETTQPETC[Carbamidomethyl (C)]DGHGC[Carbamidomethyl (C)]ETVSPSNSATPVSLGVQS[Phospho (STY)]PEHK.4	0.076198774	Q8C8R3_S2755_M1_Ank2
ATS[Phospho (STY)]PLIEETPIGSIK.2	-0.150213706	Q8C8R3_S1722_M1_Ank2
ATAVPDSLC[Carbamidomethyl (C)]K.2	-0.167320444	#N/A
HLPVS[Phospho (STY)]PGK.2	-0.36297144	Q8C8R3_S1925_M1_Ank2
ELEEEWVIVS[Phospho (STY)]DEEIQEAK.2	-0.140913567	Q8C8R3_S1562_M1_Ank2
GADSGLLTEPVIR.2	-0.15551416	#N/A
TLEQQENFSEVAR.2	-0.122766225	#N/A
KTS[Phospho (STY)]LVIVESTDDQSQVFER.3	-0.172984676	Q8C8R3_S3786_M1_Ank2
TEKHPPVS[Phospho (STY)]PGKTEK.2	-0.195075648	Q8C8R3_S1889_M1_Ank2
GAAVDFTAR.2	0.067120593	#N/A
TDADADSWSEIREDDAAFEAR.3	-0.493909196	#N/A
EGHVGLVQELLGR.3	0.001624712	#N/A
ARS[Phospho (STY)]YIETETESR.2	0.207184093	Q8C8R3_S3362_M1_Ank2
ESPC[Carbamidomethyl (C)]RDSLES[Phospho (STY)]S[Phospho (STY)]PVEPK.2	0.168913748	Q8C8R3_S2430_M2_Ank2
ATAVPDSLC[Carbamidomethyl (C)]KTPEDIS[Phospho (STY)]T[Phospho (STY)]PPEGTKPC[Carbamidomethyl (C)]LQTPVTSER.4	0.464214038	Q8C8R3_S3744_M2_Ank2
GSPIVQEPEEASEPKEESS[Phospho (STY)]PR.3	-0.129231211	Q8C8R3_S3781_M1_Ank2
TERHS[Phospho (STY)]PVFSGKPEK.3	-0.08896431	Q8C8R3_S1828_M1_Ank2
GSPIVQEPEEASEPKEESS[Phospho (STY)]PRK.3	-0.272579197	Q8C8R3_S3781_M1_Ank2
TELKKGS[Phospho (STY)]S[Phospho (STY)]EESVDEDR.2	-0.047839555	Q8C8R3_S1699_M2_Ank2
NEKLS[Phospho (STY)]PVS[Phospho (STY)]PSAK.2	-0.263976362	Q8C8R3_S1816_M2_Ank2
HLPGS[Phospho (STY)]PSIR.2	-0.197323287	Q8C8R3_S1900_M1_Ank2
NERHS[Phospho (STY)]PVSSLKTER.4	0.02627423	Q8C8R3_S1851_M1_Ank2
MDIVHLLETNTEPLQER.3	0.371718553	#N/A
KSDSNAS[Phospho (STY)]FLR.2	0.127213262	Q8C8R3_S34_M1_Ank2
S[Phospho (STY)]PLSPDTPSSEEVSYEVTPK.2	0.030969339	Q8C8R3_S2493_M1_Ank2
ETTKVGT[Phospho (STY)]PTDIHSEK.2	-0.198722839	Q8C8R3_T2232_M1_Ank2
IVDEQGDM[Oxidation (M)]DFQIS[Phospho (STY)]PDRK.2	-0.089134293	#N/A
HPPVS[Phospho (STY)]PGKTEK.2	-0.013759226	Q8C8R3_S1889_M1_Ank2
THPAVS[Phospho (STY)]PSSK.2	0.01373719	Q8C8R3_S1777_M1_Ank2
VSSSSS[Phospho (STY)]ESEPELTQLSK.2	0.281142925	Q8C8R3_S2624_M1_Ank2
HLPVS[Phospho (STY)]PGKTEK.2	0.067114714	Q8C8R3_S1925_M1_Ank2
KGS[Phospho (STY)]S[Phospho (STY)]EESVDEDR.2	0.142832865	Q8C8R3_S1699_M2_Ank2
ATS[Phospho (STY)]PLIEETPIGSIKDK.2	0.049686684	Q8C8R3_S1722_M1_Ank2
AEGFES[Phospho (STY)]ESEDGATKPK.2	-0.146866113	Q8C8R3_S3379_M1_Ank2
SDSNAS[Phospho (STY)]FLR.2	0.122750726	Q8C8R3_S34_M1_Ank2
IVDEQGDM[Oxidation (M)]DFQIS[Phospho (STY)]PDR.2	-0.118716601	#N/A
SKS[Phospho (STY)]ESDASSLDAK.2	-0.306136629	Q8C8R3_S3343_M1_Ank2
STSSSGRPGTS[Phospho (STY)]PTR.2	-0.400793775	Q8C8R3_S3411_M1_Ank2
RFT[Phospho (STY)]PEEEMFK.2	0.018236783	Q8C8R3_T2542_M1_Ank2
SHLVNEVPVLAS[Phospho (STY)]PDLLSEVSEM[Oxidation (M)]K.3	-0.013980542	Q8C8R3_S1467_M1_Ank2
ELDFTEEQIHQIR.3	-0.35192837	#N/A
HSS[Phospho (STY)]LSSSAKPER.2	-0.061815381	Q8C8R3_S1793_M1_Ank2
KGS[Phospho (STY)]S[Phospho (STY)]EESVDEDRGLVPEPLPTAK.3	0.209162085	Q8C8R3_S1699_M2_Ank2
HEGLAETPETSPESLSFS[Phospho (STY)]PK.3	-0.297680434	Q8C8R3_S2213_M1_Ank2
VGT[Phospho (STY)]PTDIHSEKELPITNDITDSSQK.3	-0.158417725	Q8C8R3_T2232_M1_Ank2
SGHDQVVELLLER.2	-0.219980461	#N/A

ESES[Phospho (STY)]DQEPEEEIGMTSEK.2	0.21273819	Q8C8R3_S1428_M1_Ank2
TPEDIST[Phospho (STY)]PPEGTKPC[Carbamidomethyl (C)]LQTPVTSER.3	-0.413216645	Q8C8R3_T3745_M1_Ank2
ASSDVM[Oxidation (M)]LNGFGGDAPTLR.2	0.174552533	#N/A
NS[Phospho (STY)]IEFFEEISDEASK.2	-0.148217704	Q8C8R3_S3427_M1_Ank2
IENPNSLQDQSHALLK.3	0.498509132	#N/A
HSPVFSGKPEKHS[Phospho (STY)]PGS[Phospho (STY)]PSTK.3	-0.230809054	Q8C8R3_S1839_M2_Ank2
KRFT[Phospho (STY)]PEEEMFK.3	-0.357289629	Q8C8R3_T2542_M1_Ank2
KS[Phospho (STY)]EEQIGEAK.2	0.111160627	Q8C8R3_S2217_M1_Ank2
TEGDSPAAALS[Phospho (STY)]PQMHQEPVQQDFSGK.3	-0.281749254	Q8C8R3_S3692_M1_Ank2
SAALLLQNDHNADVQSK.3	0.1905153	#N/A
NEKLS[Phospho (STY)]PVS[Phospho (STY)]PSAKTER.2	0.04355222	Q8C8R3_S1816_M2_Ank2
DPDGS[Phospho (STY)]AEDDSLEQTSLMESSGK.2	-0.107973463	Q8C8R3_S2475_M1_Ank2
TAEGTEPKPQGAIRS[Phospho (STY)]PQGLELPLPNR.4	-0.180564099	Q8C8R3_S2364_M1_Ank2
EIASPSS[Phospho (STY)]PVK.2	-0.468247684	Q8C8R3_S2827_M1_Ank2
LLRDPDGS[Phospho (STY)]AEDDSLEQTSLMESSGK.3	-0.111225722	Q8C8R3_S2475_M1_Ank2
HTPVSPSS[Phospho (STY)]KNEKLS[Phospho (STY)]PVS[Phospho (STY)]PSAK.3	0.24608822	Q8C8R3_S1810_M3_Ank2
S[Phospho (STY)]PQGLELPLPNR.2	-0.005338797	Q8C8R3_S2364_M1_Ank2
VVLKSDT[Phospho (STY)]QQS[Phospho (STY)]EDNNE.2	-0.177985374	Q8C8R3_S3893_M2_Ank2
FQVSAATESR.2	0.315779434	#N/A
TPTEEGT[Phospho (STY)]PTSEQNPFLFQEGK.3	0.261559258	Q8C8R3_T3050_M1_Ank2
ELGDDS[Phospho (STY)]LPSSQFLDGM[Oxidation (M)]NYLR.2	-0.17432182	Q8C8R3_S874_M1_Ank2
HPPVS[Phospho (STY)]PGK.2	0.323779098	Q8C8R3_S1889_M1_Ank2
DSEVLSPMADESLAVSHKDS[Phospho (STY)]LEAS[Phospho (STY)]PVLEDNSSHK.4	-0.459369772	Q8C8R3_S2395_M2_Ank2
PQGAIRS[Phospho (STY)]PQGLELPLPNR.3	-0.031260778	Q8C8R3_S2364_M1_Ank2
AS[Phospho (STY)]PSIWER.2	0.086570846	Q8C8T7_S773_M1_Elfn1
AVAAAAGS[Phospho (STY)]LKK.2	-0.068508209	Q8C8T7_S460_M1_Elfn1
SSS[Phospho (STY)]LGNLKK.2	0.305787466	Q8C8U0_S435_M1_Ppfibp1
ISES[Phospho (STY)]GQFSDGLEDR.2	0.249507592	Q8C996_S56_M1_Tmem163
GHAPSTAAPAPSPAPM[Oxidation (M)]SSS[Phospho (STY)]VQSDEER.3	-0.084856181	Q8C996_S42_M1_Tmem163
GHAPSTAAPAPSPAPM[Oxidation (M)]SSSVQS[Phospho (STY)]DEERQPR.4	-0.080915427	Q8C996_S45_M1_Tmem163
S[Phospho (STY)]PPGPGVPRPPPR.3	-0.023169585	Q8C996_S11_M1_Tmem163
GLS[Phospho (STY)]PSYLGGPR.2	0.019503203	Q8C9B9_S1840_M1_Dido1
AAS[Phospho (STY)]PPSYTLDLGESQLAPPPSK.2	-0.243662461	Q8C9H6_S328_M1_Strip2
QDS[Phospho (STY)]LDIYNER.2	-0.395839317	Q8C9H6_S364_M1_Strip2
QLLTKQDS[Phospho (STY)]LDIYNER.3	0.170545805	Q8C9H6_S364_M1_Strip2
DLTLLITER.2	0.316475693	#N/A
ST[Phospho (STY)]PEKLNVK.2	-0.004585859	Q8CA95_T780_M1_Pde10a
AYLSLHPQVLDEFVSESVSAETVEK.3	0.0325033	#N/A
EVDLYTGYTTR.2	-0.098073019	#N/A
IVLATDPDADRLAVAELQENGR.3	-0.081725756	#N/A
LIDALIENFLEPSK.2	0.138339353	#N/A
DITTGYDSSQPNKK.2	0.115709769	#N/A
AVAGVMITAS[Phospho (STY)]HNR.2	0.214171461	Q8CAA7_S175_M1_Pgm2l1
RDPEDS[Phospho (STY)]DVFEEDTHL.2	-0.012989597	Q8CAE9_S594_M1_Podxl2
SLS[Phospho (STY)]FSGPR.2	-0.176208313	Q8CAF4_S1482_M1_Nhsl1
KNS[Phospho (STY)]GAGAVLFSEPSAQEQR.3	-0.167893041	Q8CAF4_S1079_M1_Nhsl1
NHS[Phospho (STY)]PS[Phospho (STY)]PPVTPTSAAPNLASPK.3	-0.070857737	Q8CAF4_S1373_M2_Nhsl1

SEPSADSPDSPSSC[Carbamidomethyl (C)]S[Phospho (STY)]PNK.2	-0.340504032	Q8CAF4_S1459_M1_Nhsl1
FHGKVS[Phospho (STY)]PK.2	-0.108964695	Q8CAK3_S24_M1_Shfl
EKFHGKVS[Phospho (STY)]PK.2	0.055863228	Q8CAK3_S24_M1_Shfl
SLEDALNR.2	0.090956894	#N/A
SEIQAEQDR.2	-0.012131866	#N/A
ELDSITPDITPGWK.2	0.186664522	#N/A
LSEQELEFR.2	-0.13818801	#N/A
AVDEAADALLK.2	0.272825601	#N/A
GVYSEETLR.2	0.01281085	#N/A
TSSAEM[Oxidation (M)]PTIPLGSAVEAIR.2	-0.009836724	#N/A
EIAGATPHITAAEGR.3	0.207803526	#N/A
TSSVTLQTITAQNAAVQAVK.2	-0.230466806	#N/A
QAAAHTDHLR.2	0.088509006	#N/A
STSETTEEAFSSSVR.2	-0.072652715	#N/A
QHIELALEK.2	0.384488126	#N/A
TFDSAVAK.2	0.281259212	#N/A
VVSQYHELVVQAR.2	-0.157330566	#N/A
GIEQAVQSHAVAEEEAR.3	-0.473926988	#N/A
RIDQLNR.2	-0.344415314	#N/A
LSTDDLNSLIAHAHR.3	-0.368481218	#N/A
ISSVSEVMK.2	0.469913857	#N/A
SQEQM[Oxidation (M)]DSFTLDINTAYAR.2	-0.482542188	#N/A
YSTSSSSGLTAGK.2	-0.237666364	#N/A
VQEQELKYEFEQGLSEK.3	0.010170876	#N/A
VAVLSQNR.2	-0.052936643	#N/A
AVC[Carbamidomethyl (C)]LAAQSIAM[Oxidation (M)]GDSTIVVAGGM[Oxidation (M)]ENM[Oxidation (M)]SK.3	-0.237986639	#N/A
VNIDGGAIALGHPLGASGC[Carbamidomethyl (C)]R.3	-0.042003818	#N/A
SYS[Phospho (STY)]PEHR.2	0.1255006	Q8CB77_S125_M1_Eloa
S[Phospho (STY)]PEEDQEPIISHQKPGK.3	0.094458906	Q8CB77_S195_M1_Eloa
SHS[Phospho (STY)]LSIR.2	-0.142369298	Q8CBE3_S22_M1_Wdr37
STLLELFGQIER.2	-0.02877758	#N/A
ASHSTSQLS[Phospho (STY)]QK.2	-0.149816138	Q8CBE3_S119_M1_Wdr37
KSHS[Phospho (STY)]LSIR.2	0.173681585	Q8CBE3_S22_M1_Wdr37
DPSIHSVNVFQGHTDTVTSAVFTVGDNVVSGSDDR.4	0.162428389	#N/A
YLQDDTS[Phospho (STY)]DPTYTSSLGGK.2	0.319881203	Q8CBF3_S774_M1_Ephb1
VAILT[Phospho (STY)]DDEEEQKRK.3	-0.186069065	Q8CBH5_T11_M1_Mfsd6
[Acetyl (Protein N-term)]AADDKVAILT[Phospho (STY)]DDEEEQKR.3	0.158047906	Q8CBH5_T11_M1_Mfsd6
[Acetyl (Protein N-term)]AADDKVAILT[Phospho (STY)]DDEEEQK.2	0.183761749	Q8CBH5_T11_M1_Mfsd6
IPVPSSPVPIAT[Phospho (STY)]IDLVQQQTEDVM[Oxidation (M)]PR.3	-0.32780303	Q8CBH5_T653_M1_Mfsd6
VAILT[Phospho (STY)]DDEEEQK.2	0.315610192	Q8CBH5_T11_M1_Mfsd6
TNPPTQKPPS[Phospho (STY)]PPVSGR.2	-0.530336404	Q8CBW3_S183_M1_Abi1
NDDGWFEGVC[Carbamidomethyl (C)]NR.2	-0.195666335	#N/A
TAS[Phospho (STY)]LNQRPR.2	0.309500084	Q8CBW3_S231_M1_Abi1
HNS[Phospho (STY)]TTSSTSSGGYR.2	-0.153630229	Q8CBW3_S296_M1_Abi1
ALIESYQNLTR.2	0.153618613	#N/A
LGSQHS[Phospho (STY)]PGR.2	-0.230335899	Q8CBW3_S225_M1_Abi1
KNDDGWFEGVC[Carbamidomethyl (C)]NR.3	0.225498443	#N/A

[Acetyl (Protein N-term)]AELQM[Oxidation (M)]LLEEEIPSGKR.2	-0.031500602	#N/A
DKDDELSFK.2	0.086447566	#N/A
VVS[Phospho (STY)]EEHLR.2	0.100887818	Q8CBW7_S80_M1_Chic1
S[Phospho (STY)]SPSEVSLEAER.2	-0.223245642	Q8CBX0_S88_M1_Tmem63c
LALLIHNDS[Phospho (STY)]LTSLIYGEQSEK.3	0.239145621	Q8CBX0_S75_M1_Tmem63c
AYSS[Phospho (STY)]PSTTPEVR.2	-0.179948513	Q8CBY1_S420_M1_Samd4a
LLQPDLQPVSASQLYPR.2	-0.191925498	#N/A
IQLVAVNYIPEVR.2	-0.042720681	#N/A
LIEYYQQLAQK.2	0.259134778	#N/A
SGGNSSS[Phospho (STY)]SLGDIVPSSR.2	0.18252963	Q8CC27_S202_M1_Cacnb2
SAS[Phospho (STY)]QAEEEPC[Carbamidomethyl (C)]LEPVKK.2	0.078398718	Q8CC27_S510_M1_Cacnb2
SAS[Phospho (STY)]QAEEEPC[Carbamidomethyl (C)]LEPVK.2	-0.417396818	Q8CC27_S510_M1_Cacnb2
VASLS[Phospho (STY)]PAR.2	0.075408886	Q8CC35_S765_M1_Synpo
VRSPPSY[Phospho (STY)]STLYPSSDPKPSHLK.4	-0.314134797	Q8CC35_Y571_M1_Synpo
VTPNPDLLDLVQTADEK.2	-0.170389148	#N/A
ASPAAAEEAVPEWASC[Carbamidomethyl (C)]LK.2	-0.031726679	#N/A
YTTNAPGGFR.2	-0.133239654	#N/A
HPSPQS[Phospho (STY)]PSR.2	-0.269450457	Q8CC35_S230_M1_Synpo
VAS[Phospho (STY)]EEEEVPLVVYLK.2	-0.372048208	Q8CC35_S258_M1_Synpo
SSPGLYTAPVQDSLQPTAVS[Phospho (STY)]PTYSSDISPVSPSR.3	-0.298541971	Q8CC35_S882_M1_Synpo
LLGQRS[Phospho (STY)]PVLER.2	-0.43056725	Q8CC35_S535_M1_Synpo
PSLYALS[Phospho (STY)]PVKEPAK.2	-0.205132948	Q8CC35_S805_M1_Synpo
ASPAAAEEAVPEWASC[Carbamidomethyl (C)]LKS[Phospho (STY)]PR.2	-0.318742161	Q8CC35_S689_M1_Synpo
C[Carbamidomethyl (C)]PS[Phospho (STY)]PTM[Oxidation (M)]SLPSSWK.2	-0.332791171	Q8CC35_S740_M1_Synpo
AAS[Phospho (STY)]PAKPSSLDLVPNLPR.3	0.047562174	Q8CC35_S833_M1_Synpo
PKPNQNLSEASGK.3	0.528592781	#N/A
ENAALLTANGLHLSQSR.2	0.043076369	#N/A
DRAS[Phospho (STY)]PAAAEEAVPEWASC[Carbamidomethyl (C)]LK.3	0.141196705	Q8CC35_S672_M1_Synpo
AWAPPASSMADRS[Phospho (STY)]PQPQR.3	0.089008474	Q8CC35_S512_M1_Synpo
S[Phospho (STY)]ST[Phospho (STY)]PKGDMSAVNDESF.2	-0.30849674	Q8CCI5_S213_M2_Rybp
TVPLVDSEGGS[Phospho (STY)]GR.2	-0.095105903	Q8CCJ4_S340_M1_Amer2
IC[Carbamidomethyl (C)]LEAGS[Phospho (STY)]PTGSGDQSSR.2	0.063978542	Q8CCJ4_S280_M1_Amer2
ASAVPDPSSVDPPS[Phospho (STY)]DPSADR.2	-0.022055755	Q8CCJ4_S356_M1_Amer2
DMSS[Phospho (STY)]VKR.2	0.317223274	Q8CCJ4_S488_M1_Amer2
AAGPGNLVLPGS[Phospho (STY)]LTAS[Phospho (STY)]LEC[Carbamidomethyl (C)]VKEEPPR.3	-0.577964531	Q8CCJ4_S223_M2_Amer2
RPDS[Phospho (STY)]PGQDASR.2	-0.27141297	Q8CCJ4_S244_M1_Amer2
S[Phospho (STY)]GGTMPSIFGVK.2	0.12299878	Q8CCJ4_S71_M1_Amer2
HAAGEPAGGEQAPAS[Phospho (STY)]AESAPER.3	-0.139886012	Q8CCJ4_S266_M1_Amer2
VKS[Phospho (STY)]PPQISPSK.2	0.275963028	Q8CCN5_S565_M1_Bcas3
EGS[Phospho (STY)]IETLSNSSGSTSGSIPR.2	-0.188825994	Q8CCN5_S886_M1_Bcas3
DVVGS[Phospho (STY)]GTELQR.2	-0.384497029	Q8CCN5_S877_M1_Bcas3
LADAM[Oxidation (M)]AES[Phospho (STY)]PSR.2	0.056979563	Q8CCN5_S869_M1_Bcas3
LTSQDS[Phospho (STY)]YNNFTNNNPGNPR.2	-0.64464867	Q8CCN5_S503_M1_Bcas3
LADAM[Oxidation (M)]AES[Phospho (STY)]PSRDVVGSGTELQR.3	-0.136832102	Q8CCN5_S869_M1_Bcas3
HGSY[Phospho (STY)]DSLASDHSGQEDEEWLSQVEIVTHTGPHR.5	0.386878623	Q8CCN5_Y707_M1_Bcas3
C[Carbamidomethyl (C)]SPVPGLS[Phospho (STY)]SSPSGS[Phospho (STY)]PLHGK.2	-0.077960426	Q8CCN5_S486_M2_Bcas3
QMNMS[Phospho (STY)]PPPGNAGPVIMSLEEK.3	-0.121386966	Q8CCS6_S146_M1_Pabpn1

RPAEDYVPR.2	-0.142897061	#N/A
DAQDALVPR.2	0.07275827	#N/A
TDRGTDDS[Phospho (STY)]PKNSQEDLQDR.3	-0.436564353	Q8CCT4_S120_M1_Tceal5
GTDDS[Phospho (STY)]PKNSQEDLQDR.2	-0.374615011	Q8CCT4_S120_M1_Tceal5
M[Oxidation (M)]GGFHWVPR.2	-0.007846496	#N/A
ADEDKS[Phospho (STY)]DAEGKPAR.2	0.256699992	Q8CCT4_S35_M1_Tceal5
S[Phospho (STY)]PSADSIHTR.2	0.230548003	Q8CDA1_S941_M1_Inpp5f
GLES[Phospho (STY)]PLKK.2	-0.235171665	Q8CDA1_S936_M1_Inpp5f
IIPS[Phospho (STY)]PDDSK.2	-0.2474457	Q8CDA1_S123_M1_Inpp5f
VQKS[Phospho (STY)]PAEPEAVNEIQQNELK.3	-0.191413234	Q8CDA1_S1103_M1_Inpp5f
SQS[Phospho (STY)]ASSIDVSTHAPSEAAAGPGSELGK.3	-0.166655008	Q8CDA1_S908_M1_Inpp5f
GLES[Phospho (STY)]PLKKS[Phospho (STY)]PSADSIHTR.3	-0.26959213	Q8CDA1_S936_M2_Inpp5f
KSPS[Phospho (STY)]ADSIHTR.2	-0.369755511	Q8CDA1_S943_M1_Inpp5f
ESS[Phospho (STY)]PSHGLLK.2	-0.047493092	Q8CDG3_S997_M1_Vcpip1
DGPSS[Phospho (STY)]APATPTK.2	-0.20727969	Q8CDG3_S756_M1_Vcpip1
TVS[Phospho (STY)]PSTIR.2	-0.091156256	Q8CDG3_S746_M1_Vcpip1
APYS[Phospho (STY)]PTTSK.2	-0.048087398	Q8CDG3_S767_M1_Vcpip1
S[Phospho (STY)]RESS[Phospho (STY)]PSHGLLK.2	-0.446406233	Q8CDG3_S993_M2_Vcpip1
TS[Phospho (STY)]PDHPKNDGKTEVHK.3	-0.244921502	Q8CDM8_S552_M1_Fam160b1
QHLENDPGS[Phospho (STY)]NEDADIPK.2	-0.509020175	Q8CDN6_S113_M1_Txnl1
IKQHLENDPGS[Phospho (STY)]NEDADIPK.3	-0.154620709	Q8CDN6_S113_M1_Txnl1
FQNVNSVTLFVQSNQGEEETTR.3	-0.041547643	#N/A
DLS[Phospho (STY)]PGAPPAVAAAAPEER.2	0.136901749	Q8CDU6_S9_M1_Hectd2
RGS[Phospho (STY)]LTLEEK.2	-0.41839614	Q8CFE4_S677_M1_Scyl2
GS[Phospho (STY)]LTLEEK.2	-0.286740038	Q8CFE4_S677_M1_Scyl2
TS[Phospho (STY)]PQELSEEVSR.2	-0.351974311	Q8CFI0_S332_M1_Nedd4l
DT[Phospho (STY)]LSNPQSPQPSPYNSPKPQHK.3	-0.34591047	Q8CFI0_T502_M1_Nedd4l
SLS[Phospho (STY)]SPTVTLSAPLEGAKDS[Phospho (STY)]PIRR.3	0.163008179	Q8CFI0_S477_M2_Nedd4l
DTLS[Phospho (STY)]NPQSPQPSPYNSPK.2	0.121862353	Q8CFI0_S504_M1_Nedd4l
NS[Phospho (STY)]PGLLVSPGNLNK.2	-0.262210737	Q8CFN5_S222_M1_Mef2c
PSPDERES[Phospho (STY)]PSVKR.3	-0.368663374	Q8CFN5_S460_M1_Mef2c
S[Phospho (STY)]PPPMNLGMNNR.2	0.233707203	Q8CFN5_S240_M1_Mef2c
TVC[Carbamidomethyl (C)]TYWEDFHSC[Carbamidomethyl (C)]TVTALTDC[Carbamidomethyl (C)]QEGAK.3	-0.168593133	#N/A
ALQTTYGTNAPR.2	0.295290404	#N/A
VDLFYLHAPDHSTPVEETLR.3	-0.133769536	#N/A
IPRPLS[Phospho (STY)]PTK.2	-4.58E-06	Q8CG79_S697_M1_Tp53bp2
KNQS[Phospho (STY)]SEDILR.2	-0.135616166	Q8CG79_S479_M1_Tp53bp2
IAEELVAVAR.2	0.38650763	#N/A
GPLDGSPY[Phospho (STY)]AQVQR.2	0.194385424	Q8CGB6_Y483_M1_Tns2
APHSC[Carbamidomethyl (C)]GS[Phospho (STY)]PSEGR.2	0.205374373	Q8CGB6_S811_M1_Tns2
HLPGS[Phospho (STY)]GQQPSPPAR.2	0.093600307	Q8CGB6_S1082_M1_Tns2
SFS[Phospho (STY)]LDPLMER.2	0.211447171	Q8CGB6_S120_M1_Tns2
GYPSPGAHS[Phospho (STY)]PR.2	-0.461359606	Q8CGB6_S825_M1_Tns2
WDS[Phospho (STY)]YENFNQHHEDSVDGALAHTR.4	-0.144126388	Q8CGB6_S455_M1_Tns2
TLPDSQQVPSS[Phospho (STY)]PASK.2	-0.070437849	Q8CGE9_S850_M1_Rgs12
S[Phospho (STY)]LNEDVGEEDSEKK.2	-0.169973306	Q8CGE9_S879_M1_Rgs12
S[Phospho (STY)]LNEDVGEEDSEK.2	-0.178129297	Q8CGE9_S879_M1_Rgs12

QNSAVNSS[Phospho (STY)]PR.2	-0.491097267	Q8CGE9_S1130_M1_Rgs12
ESQGSVSS[Phospho (STY)]AGSLDLSEAC[Carbamidomethyl (C)]R.2	0.296873066	Q8CGE9_S940_M1_Rgs12
LPAGSSELALSS[Phospho (STY)]PPPVK.2	-0.102220878	Q8CGE9_S1233_M1_Rgs12
SKS[Phospho (STY)]GRS[Phospho (STY)]LNEDVGEEDSEK.2	-0.361025856	Q8CGE9_S876_M2_Rgs12
HSISS[Phospho (STY)]DHSNVSTPK.2	-0.111391804	Q8CGE9_S859_M1_Rgs12
RIS[Phospho (STY)]DLGNK.2	0.036135605	Q8CGF6_S332_M1_Wdr47
LSPYPSS[Phospho (STY)]PMR.2	0.110317068	Q8CGF6_S297_M1_Wdr47
RPQS[Phospho (STY)]ADAYM[Oxidation (M)]TR.2	-0.052407715	Q8CGF6_S304_M1_Wdr47
YQQHLEQK.2	0.134192516	#N/A
LILASS[Phospho (STY)]PQPK.2	-0.107732179	Q8CGI1_S1151_M1_Fam193a
VLEFIAVSQLR.2	0.175165952	#N/A
LALLDNHSSEFNVTR.3	-0.22213576	#N/A
VLTSGIFETR.2	0.106118359	#N/A
ESLDLFESIWNNR.2	-0.234088303	#N/A
IDSVSLVDYTPTDQDLLR.2	0.156600718	#N/A
SNEYQLIDC[Carbamidomethyl (C)]AQYFLER.2	0.305457511	#N/A
LS[Phospho (STY)]LDEDEEPK.2	-0.13303596	Q8CGM1_S1269_M1_Adgrb2
TDVLC[Carbamidomethyl (C)]TGEDEDC[Carbamidomethyl (C)]QAAS[Phospho (STY)]PLQK.2	-0.320248299	Q8CGN4_S1348_M1_Bcor
DGGS[Phospho (STY)]PPLLEK.2	0.183067962	Q8CGN4_S423_M1_Bcor
PDPS[Phospho (STY)]KSAPAPK.2	-0.130709179	Q8CGP0_S5_M1_H2bu1
ESYSIYVYK.2	0.320049301	#N/A
LPSYGLC[Carbamidomethyl (C)]ESGNTSSS[Phospho (STY)]PPGPR.2	0.224530291	Q8CGU1_S563_M1_Calcoco1
RRS[Phospho (STY)]S[Phospho (STY)]EVEIFVDC[Carbamidomethyl (C)]EC[Carbamidomethyl (C)]GK.3	0.028097761	Q8CGV2_S101_M2_Tph2
GLS[Phospho (STY)]LDSAVPEDHQLLGSLTQNK.3	-0.22207316	Q8CGV2_S19_M1_Tph2
DSGNIPEAIASYR.2	0.334031994	#N/A
AIQINPAFADAHSNLASIHK.3	-0.173748705	#N/A
SDLGNLLK.2	-0.614256137	#N/A
DIDASPS[Phospho (STY)]PLSVQDLK.2	0.193927275	Q8CH02_S409_M1_Sugp1
ALQQHQHGYDS[Phospho (STY)]DEEVDSELGTWEHQLR.4	-0.230818712	Q8CH02_S483_M1_Sugp1
GRS[Phospho (STY)]LNIADQEGTLLGK.3	-0.651685248	Q8CH09_S206_M1_Sugp2
DVQDAIAQS[Phospho (STY)]PEK.2	-0.041683406	Q8CH25_S289_M1_Sltm
S[Phospho (STY)]PGHM[Oxidation (M)]VILNQTK.2	-0.115516823	Q8CH25_S552_M1_Sltm
DVQDAIAQS[Phospho (STY)]PEKEAK.2	-0.340858635	Q8CH25_S289_M1_Sltm
ISS[Phospho (STY)]KS[Phospho (STY)]PGHM[Oxidation (M)]VILNQTK.3	-0.419061425	Q8CH25_S550_M2_Sltm
AGAGMITQHSSTAS[Phospho (STY)]PVNR.2	-0.29369751	Q8CH25_S999_M1_Sltm
EM[Oxidation (M)]DM[Oxidation (M)]S[Phospho (STY)]PEEVAPSPR.2	-0.305806885	Q8CH72_S330_M1_Trim32
EMDMSPEEVAPS[Phospho (STY)]PRAS[Phospho (STY)]PAK.2	0.111518155	Q8CH72_S337_M2_Trim32
SIGS[Phospho (STY)]PESTPK.2	0.216941534	Q8CH77_S762_M1_Nav1
S[Phospho (STY)]LTNLSFLTDSEKK.2	0.12938959	Q8CH77_S93_M1_Nav1
SS[Phospho (STY)]PLSWR.2	0.030714816	Q8CH77_S315_M1_Nav1
LYHLPPPS[Phospho (STY)]VGPHSTASPPEDR.3	-0.159258801	Q8CH77_S1816_M1_Nav1
S[Phospho (STY)]LTNLSFLTDSEK.2	0.454505846	Q8CH77_S93_M1_Nav1
RSS[Phospho (STY)]TIVLR.2	-0.114359537	Q8CH77_S455_M1_Nav1
VNSNS[Phospho (STY)]LDLPSSSDTHASK.3	-0.034762079	Q8CH77_S810_M1_Nav1
LELVES[Phospho (STY)]LDSDEVDLK.2	0.111576536	Q8CH77_S380_M1_Nav1
SVQPEVELS[Phospho (STY)]GGSGSGGDEGADESR.2	-0.27789734	Q8CH77_S16_M1_Nav1
SLAESGLNWFS[Phospho (STY)]ESEEK.2	-0.499853588	Q8CH77_S477_M1_Nav1

APEAAVS[Phospho (STY)]DDGKS[Phospho (STY)]DDELLSSK.2	-0.500293526	Q8CH77_S197_M2_Nav1
ERPES[Phospho (STY)]C[Carbamidomethyl (C)]DDASK.2	0.093955154	Q8CH77_S510_M1_Nav1
SGYMS[Phospho (STY)]DSDLMGK.2	0.005754666	Q8CH77_S394_M1_Nav1
SLAESGLNWFS[Phospho (STY)]ES[Phospho (STY)]EEKTPK.3	0.228548962	Q8CH77_S477_M2_Nav1
EFGGVGAPPS[Phospho (STY)]PGVAR.2	-0.31704748	Q8CHC4_S1147_M1_Synj1
TNC[Carbamidomethyl (C)]LDC[Carbamidomethyl (C)]LDR.2	0.098993636	#N/A
REIEAPKS[Phospho (STY)]PGTAR.3	0.081023284	Q8CHC4_S1160_M1_Synj1
IYAGTGALEGK.2	0.123407423	#N/A
GFEANAPAFDR.2	-0.09930515	#N/A
AGVISAPQSQAR.2	0.250989519	#N/A
IDLPNEEVK.2	-0.253957868	#N/A
QIVVNLLGSK.2	0.230658632	#N/A
EVIAVQGPPDGTVLVSIK.2	-0.078393774	#N/A
QQNWDSLIAGDQLINQK.2	-0.280114028	#N/A
TPGPPSSQGS[Phospho (STY)]PVDTQPAAQK.2	-0.246703545	Q8CHC4_S1084_M1_Synj1
EIEAPKS[Phospho (STY)]PGTAR.2	-0.17536511	Q8CHC4_S1160_M1_Synj1
VSEQTLQSASSK.2	0.318741483	#N/A
SAEDLDLLNASFQDESK.2	-0.061342829	#N/A
ERNEDFVEIAR.3	0.321869166	#N/A
NEDFVEIAR.2	-0.242115227	#N/A
VTSTEFISLR.2	0.234413798	#N/A
SSAQESTFFDDALIDELLR.2	-0.418468815	#N/A
NQPS[Phospho (STY)]PQAGLAGPGPAGYGAAR.2	-0.111352513	Q8CHC4_S1175_M1_Synj1
T[Phospho (STY)]SPC[Carbamidomethyl (C)]QS[Phospho (STY)]PTVPEYSAPSLPIRPSR.3	0.151873959	Q8CHC4_S1053_M2_Synj1
QEAIDVLLLGNTLNSDLADK.2	-0.28202088	#N/A
TPAWTDR.2	-0.279617389	#N/A
HLEEEMS[Phospho (STY)]LEK.2	-0.214344368	Q8CHC4_S982_M1_Synj1
IDLPNEEVKELIR.3	0.068219325	#N/A
FLDYGFFYFDGSEVQR.2	-0.388958392	#N/A
RSS[Phospho (STY)]FLNAK.2	-0.211954105	Q8CHG7_S960_M1_Rapgef2
SETS[Phospho (STY)]PVAPR.2	-0.285592111	Q8CHG7_S1022_M1_Rapgef2
HIPAALPVSGTLSSS[Phospho (STY)]NPDLLQSHHR.4	-0.287894925	Q8CHG7_S585_M1_Rapgef2
ILS[Phospho (STY)]LSEEGSLER.2	-0.005472709	Q8CHG7_S1088_M1_Rapgef2
QAEDTISNASSQLSS[Phospho (STY)]PPTS[Phospho (STY)]PQSSPR.3	0.051944994	Q8CHG7_S1115_M2_Rapgef2
SSIVSNSS[Phospho (STY)]FDSVPVSLHDERR.3	0.090067244	Q8CHG7_S1158_M1_Rapgef2
S[Phospho (STY)]LGSLSQGSANATVLDVAQTGGHK.3	-0.275543426	Q8CHG7_S930_M1_Rapgef2
SSIVSNSS[Phospho (STY)]FDSVPVSLHDER.3	0.117599689	Q8CHG7_S1158_M1_Rapgef2
NNM[Oxidation (M)]ETETLC[Carbamidomethyl (C)]S[Phospho (STY)]DEDAQELLR.2	0.221400806	Q8CHG7_S698_M1_Rapgef2
EFLSELQR.2	0.141098516	#N/A
ELEEETNAFNC[Carbamidomethyl (C)]R.2	0.022387346	#N/A
SLFDYHDTR.2	-0.122369438	#N/A
SVTQGFSFNILC[Carbamidomethyl (C)]VGETGIGK.2	-0.258358945	#N/A
LTIVDAVGFGDQINK.2	-0.127381341	#N/A
[Acetyl (Protein N-term)]AATDLERVS[Phospho (STY)]NAEPEPR.2	-0.099022074	Q8CHH9_S10_M1_Septin8
SLSLGGHVGFDSLPDQLVSK.3	-0.349903838	#N/A
S[Phospho (STY)]SPVNRPSSATNK.2	-0.191599678	Q8CHI8_S720_M1_Ep400
ISQRDPS[Phospho (STY)]PESK.2	-0.229541014	Q8CHP6_S261_M1_Phc3

KGGS[Phospho (STY)]PGLESR.2	-0.029867142	Q8CHP6_S269_M1_Phc3
M[Oxidation (M)]DRT[Phospho (STY)]PPPPTLS[Phospho (STY)]PAAVTVGR.3	0.190678716	Q8CHP6_S614_M2_Phc3
FIFDC[Carbamidomethyl (C)]VSQEYGINPER.2	0.172758935	#N/A
GETAVPGAPETLR.2	0.302941838	#N/A
FIAGTGC[Carbamidomethyl (C)]LVR.2	0.191634513	#N/A
TILTLTGVSSLEDVK.2	-0.118688	#N/A
SNQESDC[Carbamidomethyl (C)]MFK.2	-0.051438783	#N/A
GAGS[Phospho (STY)]PPPASDWGR.2	-0.134877872	Q8CHQ0_S11_M1_Fbxo4
TKFVS[Phospho (STY)]FTSR.2	-0.219847549	Q8CHT1_S606_M1_Ngef
FVS[Phospho (STY)]FTSR.2	-0.273615354	Q8CHT1_S606_M1_Ngef
MEDPQRS[Phospho (STY)]QNKDR.2	0.127509781	Q8CHT1_S696_M1_Ngef
VEELEDQGQTLANVFILR.2	0.067621111	#N/A
MEDPQRS[Phospho (STY)]QNK.2	0.251986673	Q8CHT1_S696_M1_Ngef
TTS[Phospho (STY)]PDLFESQSLTSASSK.2	-0.425967126	Q8CHU3_S443_M1_Epn2
TTS[Phospho (STY)]PDLFESQSLTSASSKPSSAR.3	0.18220354	Q8CHU3_S443_M1_Epn2
AGGS[Phospho (STY)]PASYHGSTSPR.2	-0.468966747	Q8CHU3_S192_M1_Epn2
GSS[Phospho (STY)]QPNLSTSYSEQEYGK.2	0.003589516	Q8CHU3_S173_M1_Epn2
NSDPWAASQQPASNAGK.2	0.112187953	#N/A
AGS[Phospho (STY)]PQLDDIR.2	-0.328859983	Q8CHW4_S540_M1_Eif2b5
NSS[Phospho (STY)]PSYANPK.2	-0.316342056	Q8CHX7_S228_M1_Rftn2
S[Phospho (STY)]LGLDTNASQAAGGR.2	-0.395049329	Q8CHX7_S429_M1_Rftn2
PVTWNSAAQT[Phospho (STY)]PERK.3	-0.371179467	Q8CHX7_T408_M1_Rftn2
PVTWNSAAQT[Phospho (STY)]PER.2	-0.161860597	Q8CHX7_T408_M1_Rftn2
EERPAQSDS[Phospho (STY)]FSGFSSSDSVLR.3	0.246522987	Q8CHX7_S469_M1_Rftn2
RPPS[Phospho (STY)]PDVIVLS[Phospho (STY)]DSEQPSSPR.3	0.147193201	Q8CHY6_S103_M2_Gatad2a
SHC[Carbamidomethyl (C)]HS[Phospho (STY)]PDIEGLQAR.3	-0.127003672	Q8CI03_S294_M1_Flywch1
S[Phospho (STY)]LPNLSR.2	-0.545565935	Q8CI08_S414_M1_Slain2
RTS[Phospho (STY)]SEDLR.2	0.248897075	Q8CI08_S87_M1_Slain2
ELS[Phospho (STY)]PEQSTAGKPSDGSSALDR.3	-0.062252515	Q8CI11_S505_M1_Gnl3
RGS[Phospho (STY)]QGDIK.2	-0.205458699	Q8CI51_S228_M1_Pdlim5
S[Phospho (STY)]PGNSPTPMSR.2	0.055116808	Q8CI61_S175_M1_Bag4
SVS[Phospho (STY)]RETLK.2	-0.248574747	Q8CI71_S585_M1_Vps50
KKS[Phospho (STY)]DYSLNK.2	-0.090502642	Q8CI71_S595_M1_Vps50
SAYQDYDS[Phospho (STY)]DSDVPEELKR.2	0.171678423	Q8CI71_S559_M1_Vps50
FLEQSRS[Phospho (STY)]PSVSPSK.2	-0.447801952	Q8CI71_S494_M1_Vps50
SAYQDY[Phospho (STY)]DSDSDVPEELK.2	-0.101994365	Q8CI71_Y557_M1_Vps50
S[Phospho (STY)]PSVSPSK.2	-0.070864051	Q8CI71_S494_M1_Vps50
RPGLEKAS[Phospho (STY)]DEEPED.2	-0.279770144	Q8CI75_S864_M1_Dis3l2
QKQIS[Phospho (STY)]VR.2	-0.426814007	Q8CI94_S15_M1_Pygb
[Acetyl (Protein N-term)]AKPLTDSER.2	0.174047295	#N/A
GIAGLGDVAEVR.2	0.103506529	#N/A
NLAENISR.2	0.111030309	#N/A
VIFLENYR.2	-0.128219697	#N/A
VEDVEALDQK.2	0.235705942	#N/A
DYFFALAHTVR.2	0.001442733	#N/A
DFYELEPEKFQNK.2	-0.019734838	#N/A
LLSLVDDEAFIR.2	0.031137539	#N/A

TC[Carbamidomethyl (C)]AYTNHTVLPEALER.3	-0.089093692	#N/A
YEFGIFNQK.2	0.263288564	#N/A
QAVDQISSGFFSPK.2	0.170646791	#N/A
LVTSIGDVVNHDPVVGDR.3	-0.306097276	#N/A
HLDHVAALFPGDVDR.3	-0.067161529	#N/A
IVNGWQVEEADDWLR.2	0.317568846	#N/A
LLSLVDDEAFIRDVAK.3	0.137228552	#N/A
DFNVGDYIEAVLDR.2	0.067430986	#N/A
VIPAADLSQQISTAGTEASGTGNM[Oxidation (M)]K.3	-0.4294134	#N/A
VFADYEAYIQC[Carbamidomethyl (C)]QAQVDR.2	0.084386543	#N/A
HLEIIYAINQR.2	0.040954662	#N/A
NNTVNTMR.2	-0.113598226	#N/A
WLLLC[Carbamidomethyl (C)]NPGLAEIIVER.2	0.163798525	#N/A
SFSLASSGNS[Phospho (STY)]PISQR.2	-0.437166362	Q8CI95_S186_M1_Osbpl11
SWS[Phospho (STY)]SSTTAGGLEGTVK.2	-0.133040968	Q8CI96_S608_M1_Clip4
LAS[Phospho (STY)]TDDGYIDLQFK.2	0.056358265	Q8CIB6_S24_M1_Tmem230
NLS[Phospho (STY)]PGAVESDVR.2	0.370341902	Q8CIE6_S173_M1_Copa
[Acetyl (Protein N-term)]S[Phospho (STY)]DNGELEDKPPAPPVR.2	0.01227158	Q8CIN4_S2_M1_Pak2
RS[Phospho (STY)]PTSTGDTELKEER.3	-0.557022588	Q8CIP4_S438_M1_Mark4
KAS[Phospho (STY)]C[Carbamidomethyl (C)]SAVGSGSR.2	-0.024194489	Q8CIP4_S458_M1_Mark4
GS[Phospho (STY)]PSLPDKYR.2	0.305061555	Q8CIQ7_S1763_M1_Dock3
GNVLASHSPMS[Phospho (STY)]PENIK.2	0.203789842	Q8CIQ7_S1655_M1_Dock3
GTSQVSS[Phospho (STY)]PSQPPEK.2	0.498782074	Q8CJ19_S1154_M1_Mical3
RKT[Phospho (STY)]SQSEEEEPPR.3	-0.069588368	Q8CJ19_T684_M1_Mical3
QVGVFS[Phospho (STY)]PAR.2	-0.273012467	Q8CJ19_S1167_M1_Mical3
GRS[Phospho (STY)]EEELEASK.2	-0.365942004	Q8CJ19_S977_M1_Mical3
SVFS[Phospho (STY)]GYKK.2	-0.023681182	Q8CJ19_S1736_M1_Mical3
KTS[Phospho (STY)]QSEEEEPPR.2	0.075160666	Q8CJ19_S685_M1_Mical3
S[Phospho (STY)]PGTEEAKSPLATK.2	-0.202260639	Q8CJ19_S1171_M1_Mical3
AVLS[Phospho (STY)]PIR.2	-0.04787489	Q8CJ19_S1216_M1_Mical3
SAGDQPPLLTPKS[Phospho (STY)]PS[Phospho (STY)]DKELR.3	0.066420332	Q8CJ19_S1404_M2_Mical3
QVGVFSPARS[Phospho (STY)]PGT[Phospho (STY)]EEAK.3	0.081129796	Q8CJ19_S1171_M2_Mical3
VKS[Phospho (STY)]PEEPLFPTPLLLR.3	-0.110731814	Q8CJ19_S1187_M1_Mical3
RQLS[Phospho (STY)]FSEDSDLSSDDILER.3	-0.358544428	Q8CJ19_S1783_M1_Mical3
GFSGQRT[Phospho (STY)]PT[Phospho (STY)]PPRHS[Phospho (STY)]PGR.3	0.113260139	Q8CJ40_S501_M3_Crocc
EAPAGGSGDGLS[Phospho (STY)]SPSPLEYSPR.2	-0.231932534	Q8CJ40_S1468_M1_Crocc
DLAQAALS[Phospho (STY)]DTESGVQLSSSER.2	-0.069146019	Q8CJ40_S464_M1_Crocc
TPTPPRHS[Phospho (STY)]PGR.3	-0.104835752	Q8CJ40_S501_M1_Crocc
TES[Phospho (STY)]RDVDSRPEIQR.3	-0.468242843	Q8CJ61_S194_M1_Cmtm4
SPGS[Phospho (STY)]PQLSR.2	-0.231337154	Q8JZP2_S483_M1_Syn3
LSPQGQQPVS[Phospho (STY)]PQSGSPQQQR.3	-0.181881533	Q8JZP2_S469_M1_Syn3
SQS[Phospho (STY)]LTNSLSTSDTSHR.2	-0.150887786	Q8JZP2_S540_M1_Syn3
STS[Phospho (STY)]QQGEEPQK.2	-0.30283092	Q8JZP2_S519_M1_Syn3
SAS[Phospho (STY)]PHPHLNK.2	0.289769569	Q8JZP2_S530_M1_Syn3
TGTFS[Phospho (STY)]PQR.2	-0.550366499	Q8JZP9_S292_M1_Gas2l1
GSPTPSPRPGS[Phospho (STY)]PVPGSER.3	-0.018145241	Q8JZP9_S306_M1_Gas2l1
NLETLQQELGIEGENR.2	0.557886326	#N/A

GHPSAGAEEEGGS[Phospho (STY)]DGS[Phospho (STY)]AAEAEPR.2	-0.06556306	Q8JZQ9_S120_M2_Eif3b
AKPAAQSEEETATS[Phospho (STY)]PAAS[Phospho (STY)]PTPQSAER.3	-0.267622436	Q8JZQ9_S75_M2_Eif3b
SPS[Phospho (STY)]QEPSAPGK.2	0.057730339	Q8JZQ9_S90_M1_Eif3b
KLLSS[Phospho (STY)]PGK.2	-0.013826998	Q8JZR6_S1046_M1_Slc4a8
ALS[Phospho (STY)]INSGNTK.2	-0.066635789	Q8JZR6_S1076_M1_Slc4a8
DGQTVS[Phospho (STY)]PQSATNLEVK.2	-0.086204875	Q8JZR6_S259_M1_Slc4a8
KQS[Phospho (STY)]DPHSMDR.2	-0.155659676	Q8JZR6_S246_M1_Slc4a8
EQNS[Phospho (STY)]PIYISR.2	-0.241776681	Q8JZS0_S130_M1_Lin7a
ATVAAFAASEGHSHPR.3	-0.008956251	#N/A
VLQSEFC[Carbamidomethyl (C)]TAIR.2	0.231123128	#N/A
VLEEMEAR.2	0.391143518	#N/A
QQQQLLIQQQQQQQQQPQQNHM[Oxidation (M)]S.3	-0.349476867	#N/A
LQESGEVPVHK.3	-0.315500302	#N/A
DIVQFVPFR.2	0.266542656	#N/A
[Acetyl (Protein N-term)]M[Oxidation (M)]EQPEDM[Oxidation (M)]AS[Phospho (STY)]LSEFDSLAGSIPATK.3	0.02177146	Q8JZW4_S9_M1_Cpne5
FDLYDVDSKS[Phospho (STY)]PDLSK.2	0.156050824	Q8JZW4_S103_M1_Cpne5
PRPPPAAPAQS[Phospho (STY)]PPQS[Phospho (STY)]PAHS[Phospho (STY)]PPGSPVHT[Phospho (STY)]HI.4	0.160929019	Q8JZW4_T591_M3_Cpne5
AQGIRPRPPPAAPAQS[Phospho (STY)]PPQS[Phospho (STY)]PAHS[Phospho (STY)]PPGS[Phospho (STY)]PVHTHI.5	-0.221370495	#N/A
LGNPYC[Carbamidomethyl (C)]S[Phospho (STY)]PTLVR.2	0.035777828	Q8JZW5_S237_M1_Sh2d5
RAS[Phospho (STY)]DC[Carbamidomethyl (C)]GPAPYRPR.3	0.025202254	Q8JZW5_S12_M1_Sh2d5
RPDPDS[Phospho (STY)]DEDEDYER.2	0.334767475	Q8JZX4_S155_M1_Rbm17
RPDPDS[Phospho (STY)]DEDEDYERER.3	-0.282923298	Q8JZX4_S155_M1_Rbm17
S[Phospho (STY)]ENEDIYYK.2	-0.379223967	Q8JZZ7_S1428_M1_Adgrl2
GNS[Phospho (STY)]DGYIIPINK.2	0.074957459	Q8JZZ7_S1458_M1_Adgrl2
S[Phospho (STY)]MPNLGAGR.2	0.166477029	Q8JZZ7_S1437_M1_Adgrl2
S[Phospho (STY)]VDAYDSYWESR.2	-0.230250591	Q8K004_S247_M1_Spata2
TIS[Phospho (STY)]DGTISAAK.2	-0.316894561	Q8K012_S295_M1_Fnbp1l
ES[Phospho (STY)]PEGSYTDDANQEVR.2	-0.420411331	Q8K012_S501_M1_Fnbp1l
HSSDINHLVTQGRES[Phospho (STY)]PEGSYTDDANQEVR.4	0.086557016	Q8K012_S501_M1_Fnbp1l
EVQS[Phospho (STY)]PEQVK.2	-0.086017378	Q8K019_S494_M1_Bclaf1
QKS[Phospho (STY)]PEIHR.2	-0.312792432	Q8K019_S646_M1_Bclaf1
KEVQS[Phospho (STY)]PEQVK.2	-0.223268814	Q8K019_S494_M1_Bclaf1
KAEGEPQEES[Phospho (STY)]PLKSK.2	-0.391807768	Q8K019_S177_M1_Bclaf1
KAEGEPQEES[Phospho (STY)]PLK.2	-0.479805174	Q8K019_S177_M1_Bclaf1
SATSGDIWPGLSAYDNS[Phospho (STY)]PR.2	-0.06254057	Q8K019_S221_M1_Bclaf1
AEGEPQEES[Phospho (STY)]PLKSK.2	0.05658267	Q8K019_S177_M1_Bclaf1
TIT[Phospho (STY)]PQNAPR.2	-0.203088041	Q8K019_T305_M1_Bclaf1
KEVQS[Phospho (STY)]PEQVKSEK.3	-0.261508788	Q8K019_S494_M1_Bclaf1
RIDIS[Phospho (STY)]PSALR.2	-0.058901965	Q8K019_S656_M1_Bclaf1
ELFDYS[Phospho (STY)]PPLHK.2	-0.287659758	Q8K019_S510_M1_Bclaf1
AEGEPQEES[Phospho (STY)]PLK.2	0.455650651	Q8K019_S177_M1_Bclaf1
QKS[Phospho (STY)]PEIHRR.2	-0.266822153	Q8K019_S646_M1_Bclaf1
S[Phospho (STY)]PEIHRR.2	0.364326572	Q8K019_S646_M1_Bclaf1
FHDS[Phospho (STY)]EGDDTEETEDYR.2	-0.284047774	Q8K019_S395_M1_Bclaf1
YS[Phospho (STY)]PSQNS[Phospho (STY)]PIHHIPSR.3	-0.408059004	Q8K019_S284_M2_Bclaf1
NTPSQHSHSIQHS[Phospho (STY)]PER.3	-0.403086674	Q8K019_S267_M1_Bclaf1
LKELFDYS[Phospho (STY)]PPLHK.3	-0.145796462	Q8K019_S510_M1_Bclaf1

SIFREES[Phospho (STY)]PLR.2	-0.279184211	Q8K019_S529_M1_Bclaf1
QKFHDS[Phospho (STY)]EGDDTEETEDYR.3	-0.706856658	Q8K019_S395_M1_Bclaf1
EKSIFREES[Phospho (STY)]PLR.3	-0.30593661	Q8K019_S529_M1_Bclaf1
EVQS[Phospho (STY)]PEQVKSEK.2	-0.233008239	Q8K019_S494_M1_Bclaf1
YS[Phospho (STY)]PSQNS[Phospho (STY)]PIHHIPSRR.3	0.139792802	Q8K019_S284_M2_Bclaf1
GRADGDWDDQEVLDYFS[Phospho (STY)]DKESAK.4	-0.461054663	Q8K019_S383_M1_Bclaf1
ADGDWDDQEVLDYFS[Phospho (STY)]DKESAK.3	0.007043959	Q8K019_S383_M1_Bclaf1
SQEEPKDTFEHDPSES[Phospho (STY)]IDEFNK.3	-0.220190158	Q8K019_S198_M1_Bclaf1
LRC[Carbamidomethyl (C)]DS[Phospho (STY)]ADLR.2	-0.478994033	Q8K019_S688_M1_Bclaf1
TVQTAAANAASTAATSAAQNAFK.3	0.054979462	#N/A
EMQNLS[Phospho (STY)]QHGR.2	0.189711126	Q8K021_S112_M1_Scamp1
SLS[Phospho (STY)]IESLC[Carbamidomethyl (C)]PDEGR.2	-0.546669506	Q8K031_S234_M1_Stard8
IQPQLPDEDGNHS[Phospho (STY)]DKEDEQPQVVVLKK.4	0.145098991	Q8K039_S50_M1_
KIQPQLPDEDGNHS[Phospho (STY)]DKEDEQPQVVVLK.4	0.115082522	Q8K039_S50_M1_
IQPQLPDEDGNHS[Phospho (STY)]DKEDEQPQVVVLK.4	0.02802784	Q8K039_S50_M1_
NSSLLSFDS[Phospho (STY)]EDENE.2	0.322010737	Q8K039_S150_M1_
S[Phospho (STY)]LDEDEPPPSPLAR.2	-0.215377099	Q8K070_S108_M1_Samd14
VTDGC[Carbamidomethyl (C)]GS[Phospho (STY)]PLHR.2	0.039824133	Q8K070_S77_M1_Samd14
HEGFS[Phospho (STY)]PK.2	-0.396695582	Q8K070_S267_M1_Samd14
AEPHSEDDSRDAS[Phospho (STY)]PPEPASPTIGLDKK.4	0.199425208	Q8K070_S173_M1_Samd14
GSASSGSTTSPT[Phospho (STY)]C[Carbamidomethyl (C)]SPK.2	-0.567102879	Q8K070_T258_M1_Samd14
DAS[Phospho (STY)]PPEPASPTIGLDKK.2	0.098161738	Q8K070_S173_M1_Samd14
DAS[Phospho (STY)]PPEPASPTIGLDK.2	0.321306884	Q8K070_S173_M1_Samd14
DSASSAEDGEGS[Phospho (STY)]DGPGGK.2	-0.061114975	Q8K070_S64_M1_Samd14
GSGS[Phospho (STY)]DQESQSVSR.2	-0.259191396	Q8K083_S678_M1_Znf536
RLS[Phospho (STY)]LPADIR.2	-0.588151683	Q8K0D0_S146_M1_Cdk17
RAS[Phospho (STY)]LSEIGFGK.2	-0.236517052	Q8K0D0_S180_M1_Cdk17
RIS[Phospho (STY)]M[Oxidation (M)]EDLNKR.3	-0.325873116	Q8K0D0_S137_M1_Cdk17
RIS[Phospho (STY)]MEDLNK.2	0.212166912	Q8K0D0_S137_M1_Cdk17
MGSDGESDQASGTSSDEVQS[Phospho (STY)]PTGVC[Carbamidomethyl (C)]LR.3	0.197665646	Q8K0D0_S122_M1_Cdk17
LQISS[Phospho (STY)]PPFDQPMSR.2	-0.125438216	Q8K0D0_S165_M1_Cdk17
ISWAM[Oxidation (M)]EDSDLER.2	-0.357115515	#N/A
EYFQHAR.2	0.208013417	#N/A
THC[Carbamidomethyl (C)]GNVLQPYLK.3	-0.251862444	#N/A
FLTC[Carbamidomethyl (C)]SVEDGELIFR.2	-0.057285803	#N/A
[Acetyl (Protein N-term)]M[Oxidation (M)]ELLSTPHSIEINNITC[Carbamidomethyl (C)]DSFR.3	-0.043428878	#N/A
LSSERDS[Phospho (STY)]PGAPQAGTM[Oxidation (M)]K.2	-0.15647183	Q8K0S2_S161_M1_Erich5
HQAQVDQYLGLVR.3	-0.110329038	#N/A
THINTVVAK.2	-0.221151841	#N/A
TDEGHPFK.2	0.006315053	#N/A
VSLVTAPVK.2	0.341489466	#N/A
APNQPAPVEGK.2	0.16811145	#N/A
ELC[Carbamidomethyl (C)]SGPAR.2	-0.156752396	#N/A
EQDS[Phospho (STY)]PPM[Oxidation (M)]KPGALDAIR.2	-0.185116347	#N/A
[Acetyl (Protein N-term)]AAPPDLQDEPLSLGS[Phospho (STY)]PGSQWFGGR.3	-0.247891719	Q8K0T0_S16_M1_Rtn1
REQDS[Phospho (STY)]PPM[Oxidation (M)]KPGALDAIR.3	-0.396488305	#N/A
GDGEDEATAVMGAR.2	0.131075605	#N/A

GS[Phospho (STY)]VSEDELIAAIK.2	-0.171564444	Q8K0T0_S350_M1_Rtn1
NAWLSPNDS[Phospho (STY)]DRELESDLSR.3	-0.081583979	Q8K0T7_S450_M1_Unc13c
ETAPTSTYSS[Phospho (STY)]PAR.2	-0.301429451	Q8K0U4_S24_M1_Hspa12a
LDLTGS[Phospho (STY)]GGTAVPAR.2	0.113539499	Q8K0U4_S631_M1_Hspa12a
IIIPQDVGLTILK.2	0.075538536	#N/A
TPTTILLTPER.2	-0.028034243	#N/A
[Acetyl (Protein N-term)]ADKEAGGGDAGPR.2	0.111714976	#N/A
ELSDQAGSDFENSDVR.2	-0.075085704	#N/A
GAVLFGLDPAVIK.2	0.064165256	#N/A
IFGEDFIEQFK.2	-0.033032213	#N/A
WEGGDPGVSNQK.2	0.253623967	#N/A
ATAVDITTSK.2	0.012540881	#N/A
FISADQSVALGELVK.2	0.349483335	#N/A
ATGGPYGSLGVDYEFEK.2	-0.093830841	#N/A
DFYHDLDPSEAK.3	-0.080284626	#N/A
EIQTIMQFGDTEIK.2	0.093539027	#N/A
WSS[Phospho (STY)]QGMLR.2	0.152609733	Q8K0U4_S437_M1_Hspa12a
TNPLNITLPFSFIDYYK.2	-0.205026892	#N/A
M[Oxidation (M)]SPDAM[Oxidation (M)]NALFKPTIDSIIEHLR.4	0.013588107	#N/A
STDSEVSQS[Phospho (STY)]PAK.2	-0.286700243	Q8K0V4_S299_M1_Cnot3
KGS[Phospho (STY)]SELGFPR.2	-0.153108292	Q8K0W3_S103_M1_Rnf208
GLS[Phospho (STY)]LQSNR.2	-0.164649429	Q8K0X8_S298_M1_Fez1
S[Phospho (STY)]M[Oxidation (M)]EDLVNEFDEK.2	-0.051764865	Q8K0X8_S58_M1_Fez1
SSS[Phospho (STY)]LGDLLR.2	-0.383838321	Q8K124_S395_M1_Plekho2
AEAGEGQKPSPAQLELR.3	-0.189079038	#N/A
VLSIQSHVVR.2	0.458620364	#N/A
DKSFLAM[Oxidation (M)]VVDIVR.2	-0.122358237	#N/A
VVPVADIITPNQFEAELLSGR.2	0.022479176	#N/A
VNDVNKYDYVLTGYTR.3	-0.073964981	#N/A
SQELHELYEGLK.2	-0.207163503	#N/A
DKVVPVADIITPNQFEAELLSGR.3	0.104065161	#N/A
SFLAM[Oxidation (M)]VVDIVR.2	0.452601914	#N/A
KPDGSTVTQR.2	0.357921749	#N/A
TVSAMQHVLQR.2	0.231809107	#N/A
DIEDPEIVVQATVL.2	0.405614266	#N/A
LANHDEGS[Phospho (STY)]DDEEEETPKK.3	-0.465327025	Q8K1A6_S435_M1_Cc2d1a
LANHDEGS[Phospho (STY)]DDEEEETPKKQNTPAASTTQLK.4	-0.376119728	Q8K1A6_S435_M1_Cc2d1a
SSPS[Phospho (STY)]KAPPSGPAPAGK.2	0.136389235	Q8K1A6_S461_M1_Cc2d1a
LANHDEGS[Phospho (STY)]DDEEEETPK.2	0.08329268	Q8K1A6_S435_M1_Cc2d1a
SADVDS[Phospho (STY)]LSESSPNSSPGPC[Carbamidomethyl (C)]PEK.2	-0.21529561	Q8K1D8_S43_M1_Enho
QC[Carbamidomethyl (C)]VSEDQQS[Phospho (STY)]DSESSR.2	0.169423591	Q8K1H7_S16_M1_Tcp11l2
EDGIDAVEVAADRPGS[Phospho (STY)]PR.2	0.00439526	Q8K1J5_S269_M1_Sde2
AFGESSTESDEDEEEGC[Carbamidomethyl (C)]S[Phospho (STY)]HK.3	0.209209426	Q8K1L5_S91_M1_Ppp1r11
ELPSAGS[Phospho (STY)]RDK.2	-0.26113702	Q8K1M6_S535_M1_Dnm1l
SQLDINNKK.2	0.275898212	#N/A
LYTDFDEIRQEIENETER.3	-0.393168251	#N/A
DTLQSELVGQLYK.2	-0.12436292	#N/A

VFSPNVVNLTLVDLPGM[Oxidation (M)]TK.2	-0.304700115	#N/A
ALQGASQIIAEIR.2	0.068621313	#N/A
SVTDSIRDEYAFLQK.3	0.215109337	#N/A
SQLDINNK.2	-0.220658767	#N/A
GHAVNLLDVPVPVAR.2	-0.124307111	#N/A
RLEEPSLR.2	-0.155167174	#N/A
VPSALAPASQEPPPAASAEADGK.2	-0.075883283	#N/A
DC[Carbamidomethyl (C)]EVIER.2	-0.033505799	#N/A
SKPIPIM[Oxidation (M)]PAS[Phospho (STY)]PQK.2	-0.219215318	Q8K1M6_S622_M1_Dnm1l
TLESVDPLGGLNTIDILTAIR.2	-0.028426889	#N/A
SSLLDDLLTESEDM[Oxidation (M)]AQR.2	-0.137389112	#N/A
LQDVFNTVGADIIQLPQIVVVGTQSSGK.3	-0.203346962	#N/A
YIETSELC[Carbamidomethyl (C)]GGAR.2	0.004994975	#N/A
IIQHC[Carbamidomethyl (C)]SNYSTQELLR.3	-0.235670362	#N/A
IC[Carbamidomethyl (C)]YIFHETFGR.2	-0.249111553	#N/A
FATEYC[Carbamidomethyl (C)]NTIEGTAK.2	0.035668875	#N/A
FISNPNSIILAVTAANTDM[Oxidation (M)]ATSEALK.3	-0.107934155	#N/A
RLS[Phospho (STY)]AGTTVADVQK.2	-0.088874781	Q8K1N2_S465_M1_Phldb2
DRS[Phospho (STY)]FSEHDLAQLR.3	0.28752596	Q8K1S6_S443_M1_Spire2
VYISSPHSS[Phospho (STY)]PAHNK.2	-0.169412033	Q8K1X1_S209_M1_Wdr11
YTDQS[Phospho (STY)]GEEEEDYESEEQLQHR.3	0.013421039	Q8K1Z0_S81_M1_Coq9
LVQLGQAEK.2	0.144704595	#N/A
GRDYC[Carbamidomethyl (C)]S[Phospho (STY)]EEEDIT.2	-0.358411992	Q8K207_S115_M1_
ANM[Oxidation (M)]HIS[Phospho (STY)]ESQQEFFR.3	-0.226657358	Q8K207_S93_M1_
LPISSGS[Phospho (STY)]TSSSR.2	-0.209678769	Q8K209_S681_M1_Adgrg1
NNSDS[Phospho (STY)]AKLPISSGSTSSSR.3	-0.128017787	Q8K209_S672_M1_Adgrg1
QLS[Phospho (STY)]KPLSER.2	-0.237151943	Q8K212_S517_M1_Pacs1
DVSVPVAEIK.2	0.055688877	#N/A
S[Phospho (STY)]TPLKER.2	-0.202927966	Q8K212_S508_M1_Pacs1
ADLQGSAS[Phospho (STY)]PSKVEGTHTPR.3	0.099904959	Q8K212_S493_M1_Pacs1
VAAVGSQSYLSSILR.2	0.167294413	#N/A
DTTS[Phospho (STY)]PM[Oxidation (M)]ELAALEK.2	-0.149772115	Q8K212_S428_M1_Pacs1
YSSTFLDSAWR.2	0.188434805	#N/A
TNS[Phospho (STY)]SDSERSPDLGHSTQIPR.3	-0.134244806	Q8K212_S526_M1_Pacs1
GSGVAQS[Phospho (STY)]PQQQPQQQPPQPQQPTPPK.3	-0.027466729	Q8K212_S28_M1_Pacs1
GS[Phospho (STY)]LGKDTTSPM[Oxidation (M)]ELAALEK.3	-0.513145061	Q8K212_S421_M1_Pacs1
HPSHSTS[Phospho (STY)]PSGPGDEVAR.3	0.244670865	Q8K221_S76_M1_Arfip2
GHS[Phospho (STY)]PSC[Carbamidomethyl (C)]LAR.2	0.141735111	Q8K285_S628_M1_Fcho1
RSS[Phospho (STY)]DPSLNEK.2	-0.034150573	Q8K296_S633_M1_Mtmr3
DSNLSAAS[Phospho (STY)]PKPGK.2	-0.103571027	Q8K298_S637_M1_Anln
AES[Phospho (STY)]ADS[Phospho (STY)]LGSEDR.2	0.10274934	Q8K298_S653_M2_Anln
AESADS[Phospho (STY)]LGSEDRDLLYSIDAYR.3	0.151362138	Q8K298_S656_M1_Anln
KDS[Phospho (STY)]LGPDKK.2	0.272354088	Q8K298_S887_M1_Anln
LNQVIVDIR.2	-0.256679449	#N/A
EVEMS[Phospho (STY)]VDDEDINSSR.2	-0.076193561	Q8K298_S548_M1_Anln
GEGGILINSQGER.2	-0.249806246	#N/A
NTVIATGGYGR.2	0.151459206	#N/A

ISQLYGDLK.2	-0.225628658	#N/A
AAFGLSEAGFNTAC[Carbamidomethyl (C)]LTK.2	-0.042438258	#N/A
VGSVLQEGC[Carbamidomethyl (C)]EK.2	-0.059794329	#N/A
VSDAISTQYPVVDHEFDAVVVGAGGAGLR.3	0.048433631	#N/A
LGANSLLDLVVFGR.2	-0.146913764	#N/A
WHFYDTVK.2	0.229597619	#N/A
AFGGQSLK.2	0.05725938	#N/A
SM[Oxidation (M)]TLEIR.2	-0.374158557	#N/A
SM[Oxidation (M)]QNHAAVFR.2	-0.379323811	#N/A
ANAGEESVM[Oxidation (M)]NLDK.2	0.163748719	#N/A
GVIALC[Carbamidomethyl (C)]IEDGSIHR.2	0.288198405	#N/A
VTLEYRPVIDK.2	0.210903921	#N/A
VDEYDYSKPIQGQQK.3	0.063916652	#N/A
TYFSC[Carbamidomethyl (C)]TSAHTSTGDGTAM[Oxidation (M)]VTR.3	-0.263416047	#N/A
TGHSLLHTLYGR.3	-0.189165937	#N/A
SHTVAAQGGINAALGNMEEDNWR.3	-0.047076545	#N/A
HVNGQDQIVPGLYAC[Carbamidomethyl (C)]GEAAC[Carbamidomethyl (C)]ASVHGANR.4	-0.10184105	#N/A
EGS[Phospho (STY)]PETLTNLKK.2	0.027341879	Q8K2C9_S138_M1_Hacd3
EGS[Phospho (STY)]PETLTNLK.2	-0.157166119	Q8K2C9_S138_M1_Hacd3
WLDES[Phospho (STY)]DAEM[Oxidation (M)]ELR.2	-0.208585108	Q8K2C9_S114_M1_Hacd3
SQDVAIS[Phospho (STY)]PQQQQC[Carbamidomethyl (C)]SK.2	-0.205621505	Q8K2D3_S131_M1_Edc3
HNS[Phospho (STY)]WSSSSR.2	-0.190331534	Q8K2D3_S161_M1_Edc3
SES[Phospho (STY)]PPPLSEPK.2	0.386207026	Q8K2F0_S262_M1_Brd3
SS[Phospho (STY)]PQLDPLR.2	0.116321623	Q8K2F8_S183_M1_Lsm14a
SFFDNIS[Phospho (STY)]C[Carbamidomethyl (C)]DDNRER.2	0.298635268	Q8K2F8_S373_M1_Lsm14a
SS[Phospho (STY)]PQLDPLRK.2	0.178430169	Q8K2F8_S183_M1_Lsm14a
S[Phospho (STY)]PVPARPLPPTSQK.2	-0.07225445	Q8K2F8_S216_M1_Lsm14a
DAS[Phospho (STY)]PSSSSAVASSK.2	-0.087743533	Q8K2H1_S219_M1_Pphln1
DAPFFRES[Phospho (STY)]PVGR.2	-0.365957632	Q8K2H1_S147_M1_Pphln1
DAPFFRES[Phospho (STY)]PVGRK.3	-0.209709653	Q8K2H1_S147_M1_Pphln1
[Acetyl (Protein N-term)]MEEVVAEELDDEEQLVR.2	-0.000319415	#N/A
PVPGFEEC[Carbamidomethyl (C)]EDEVTS[Phospho (STY)]DPATD.2	-0.147656481	Q8K2I1_S432_M1_Fntb
GTPSQS[Phospho (STY)]PVVGR.2	-0.355945568	Q8K2K6_S181_M1_Agfg1
SSS[Phospho (STY)]ADFGTFSTSQSHQTASTVSK.3	0.048463298	Q8K2K6_S293_M1_Agfg1
SISM[Oxidation (M)]TTFTQQEIEFLQK.2	-0.499214708	#N/A
LSSLTAPVGEKS[Phospho (STY)]PDSTSPSYSTR.3	-0.365855245	Q8K2L8_S309_M1_Trappc12
S[Phospho (STY)]LTNSHLEK.2	-0.023644056	Q8K2P7_S52_M1_Slc38a1
RS[Phospho (STY)]LTNSHLEK.2	-0.171497633	Q8K2P7_S52_M1_Slc38a1
S[Phospho (STY)]LTNSHLEKR.2	-0.146025086	Q8K2P7_S52_M1_Slc38a1
RS[Phospho (STY)]LTNS[Phospho (STY)]HLEKR.3	0.200385449	Q8K2P7_S52_M2_Slc38a1
NFEDSS[Phospho (STY)]PETLEAHK.2	-0.091563197	Q8K2V6_S343_M1_Ipo11
LKAEVDNRS[Phospho (STY)]PQK.2	0.304199599	Q8K2Y0_S210_M1_Obi1
SVEVEGPSKS[Phospho (STY)]PQGR.2	0.204354373	Q8K2Y0_S566_M1_Obi1
TAQDPGIS[Phospho (STY)]PSQSLC[Carbamidomethyl (C)]AESSR.2	-0.421600719	Q8K2Y9_S164_M1_Ccm2
GIITDS[Phospho (STY)]FGR.2	0.600769491	Q8K2Y9_S393_M1_Ccm2
RDS[Phospho (STY)]FDDR.2	-0.038171698	Q8K310_S188_M1_Matr3
S[Phospho (STY)]ATREPPYR.2	0.008120851	Q8K310_S164_M1_Matr3

ITPENLPQILLQLK.2	0.12773177	#N/A
GDTDQASNILASFGLSAR.2	0.10288665	#N/A
IGPYQPNVPVGIDYVIPK.2	-0.416360256	#N/A
KRS[Phospho (STY)]YS[Phospho (STY)]PDGK.2	-0.357215845	Q8K310_S596_M2_Matr3
S[Phospho (STY)]QESGYYDR.2	0.044560645	Q8K310_S208_M1_Matr3
TES[Phospho (STY)]PAEGKEQEEK.2	-0.095640749	Q8K310_S619_M1_Matr3
SFQQSSLGR.2	-0.091160014	#N/A
TEEGPTLSYGR.2	0.250787445	#N/A
TDAQKT[Phospho (STY)]ESPAEGK.2	-0.293308885	Q8K310_T617_M1_Matr3
RDS[Phospho (STY)]FDDRGPSLNPVLDYDHGSR.4	0.058643591	Q8K310_S188_M1_Matr3
RSYS[Phospho (STY)]PDGK.2	-0.190216235	Q8K310_S598_M1_Matr3
SYS[Phospho (STY)]PDGKES[Phospho (STY)]PSDKK.2	-0.50789315	Q8K310_S604_M2_Matr3
TDAQKTES[Phospho (STY)]PAEGKEQEEK.3	-0.129417982	Q8K310_S619_M1_Matr3
DDWEEKR.2	-0.075544897	#N/A
LC[Carbamidomethyl (C)]SLFYTNEEVAK.2	0.022850449	#N/A
SFQQSSLGRDS[Phospho (STY)]QGHGR.3	0.35040516	Q8K310_S14_M1_Matr3
S[Phospho (STY)]ATREPPYRVPR.3	-0.22888318	Q8K310_S164_M1_Matr3
RRT[Phospho (STY)]EEGPTLSYGR.3	0.565096338	Q8K310_T150_M1_Matr3
SYS[Phospho (STY)]PDGKESPSDK.2	-0.244177639	Q8K310_S598_M1_Matr3
DSFDDRGPSLNPVLDYDHGSR.4	-0.181401795	#N/A
VVHIMDFQR.2	-0.1384873	#N/A
VIHLSNLPHSGYSDSAVLK.3	-0.319963267	#N/A
GPSLNPVLDYDHGSR.3	0.167856618	#N/A
DLSAAGIGLLAAATQSLSM[Oxidation (M)]PASLGR.2	0.283652347	#N/A
AVLPAS[Phospho (STY)]PEPR.2	-0.481477332	Q8K327_S532_M1_Champ1
KPSSS[Phospho (STY)]PDLWK.2	-0.182068476	Q8K327_S434_M1_Champ1
TAS[Phospho (STY)]PEPR.2	0.055991882	Q8K327_S265_M1_Champ1
DSQENS[Phospho (STY)]DAELSSSEYIR.2	0.191141023	Q8K327_S617_M1_Champ1
SSC[Carbamidomethyl (C)]GS[Phospho (STY)]PPDLWK.2	0.110396852	Q8K327_S464_M1_Champ1
RPGPPLS[Phospho (STY)]PEIR.2	-0.017299496	Q8K327_S416_M1_Champ1
EQPSKDTESGKS[Phospho (STY)]PSPPER.3	-0.161944229	Q8K327_S108_M1_Champ1
LM[Oxidation (M)]EALES[Phospho (STY)]PLEEQQI.2	0.123158203	Q8K327_S795_M1_Champ1
GQES[Phospho (STY)]SS[Phospho (STY)]DQEQVDVESIDFSK.2	-0.184193904	Q8K327_S641_M2_Champ1
LKKDS[Phospho (STY)]QENS[Phospho (STY)]DAELSSSEYIR.3	-0.264534586	Q8K327_S613_M2_Champ1
QAS[Phospho (STY)]VDDSREEDKA.2	-0.21853349	Q8K330_S639_M1_Ssh3
S[Phospho (STY)]PPASGHSTPVGPTQDR.2	-0.183998415	Q8K330_S9_M1_Ssh3
SSS[Phospho (STY)]LGNSPDR.2	0.165333119	Q8K341_S272_M1_Atat1
LLLATDPGGS[Phospho (STY)]PAQR.2	-0.452161057	Q8K341_S315_M1_Atat1
RAT[Phospho (STY)]PPAHPPPR.3	-0.049512867	Q8K341_T261_M1_Atat1
SSS[Phospho (STY)]FKDFAK.2	-0.438922987	Q8K352_S27_M1_Sash3
FHQLDIDDPQSIR.3	-0.018634135	#N/A
EYGGLNVLVNNAGIAFR.2	-0.101412796	#N/A
S[Phospho (STY)]LEDLRAPK.2	-0.581285296	Q8K382_S592_M1_Dennd1a
RPKS[Phospho (STY)]NITVEGR.3	0.191005836	Q8K382_S510_M1_Dennd1a
TM[Oxidation (M)]PS[Phospho (STY)]PQAETQDPR.2	-0.111256757	Q8K382_S473_M1_Dennd1a
ESDSAEGDETES[Phospho (STY)]PEQLVR.2	-0.051413986	Q8K382_S546_M1_Dennd1a
RS[Phospho (STY)]LEAIPEK.2	-0.250489495	Q8K394_S1114_M1_Plcl2

S[Phospho (STY)]LEAIPEKASDENGD.2	-0.14908091	Q8K394_S1114_M1_Plcl2
LSSNC[Carbamidomethyl (C)]SGVEGDVT[Phospho (STY)]DEDEGAEM[Oxidation (M)]SQR.3	-0.020156797	Q8K394_T585_M1_Plcl2
S[Phospho (STY)]LEAIPEK.2	-0.119388616	Q8K394_S1114_M1_Plcl2
TPS[Phospho (STY)]PAAGPVPR.2	-0.27904921	Q8K394_S87_M1_Plcl2
RS[Phospho (STY)]LEAIPEKASDENGD.2	-0.008898706	Q8K394_S1114_M1_Plcl2
ALNAET[Phospho (STY)]PKSS[Phospho (STY)]PLPAK.2	0.057964802	Q8K3A9_S192_M2_Mepce
ELYPDFDLNLND.2	-0.213583565	#N/A
ERS[Phospho (STY)]PPLTPK.2	-0.096838204	Q8K3E5_S974_M1_Ahi1
GKTPTADDS[Phospho (STY)]DDSR.2	0.283241273	Q8K3E5_S26_M1_Ahi1
NLSM[Oxidation (M)]TS[Phospho (STY)]PPPGPAK.2	0.36653395	Q8K3E5_S856_M1_Ahi1
AVSNDNEDTNGDGVHEITSRDS[Phospho (STY)]PVHPK.4	0.303414159	Q8K3E5_S181_M1_Ahi1
GKTPTADDS[Phospho (STY)]DDSREK.2	-0.448327452	Q8K3E5_S26_M1_Ahi1
TPTADDS[Phospho (STY)]DDSREK.2	0.301926673	Q8K3E5_S26_M1_Ahi1
TPTADDS[Phospho (STY)]DDSR.2	-0.396120476	Q8K3E5_S26_M1_Ahi1
VKERS[Phospho (STY)]PPLT[Phospho (STY)]PK.3	-0.164545223	Q8K3E5_S974_M2_Ahi1
ERS[Phospho (STY)]PPLT[Phospho (STY)]PKEK.3	0.359550285	Q8K3E5_S974_M2_Ahi1
GSAFTYPSQQS[Phospho (STY)]PR.2	-0.296863366	Q8K3F6_S599_M1_Kcnq3
T[Phospho (STY)]PQGIGLLAK.2	0.008663791	Q8K3F6_T82_M1_Kcnq3
DLNSSFET[Phospho (STY)]SPK.2	-0.196421103	Q8K3G5_T58_M1_Vrk3
YVLLNDQADDTGGS[Phospho (STY)]PSENR.2	-0.170538773	Q8K3G9_S651_M1_Appl2
SESNLSSVC[Carbamidomethyl (C)]YIFESNNEGEK.2	-0.219731343	#N/A
VNQSALEAVTPS[Phospho (STY)]PSFQQR.2	-0.362173216	Q8K3H0_S401_M1_Appl1
EVFQIASNDHDAAINR.3	-0.191247756	#N/A
GLPS[Phospho (STY)]PGAHPR.2	-0.481326343	Q8K3I4_S540_M1_Myrip
GSLS[Phospho (STY)]SEENNQGVQEELK.2	-0.26528016	Q8K3I4_S618_M1_Myrip
SAS[Phospho (STY)]WGSADQLK.2	-0.168003073	Q8K3I9_S214_M1_Glcci1
S[Phospho (STY)]PGS[Phospho (STY)]PVC[Carbamidomethyl (C)]R.2	-0.112259364	Q8K3I9_S136_M2_Glcci1
VPC[Carbamidomethyl (C)]NVEGIS[Phospho (STY)]PELEK.2	-0.426086381	Q8K3I9_S293_M1_Glcci1
SAS[Phospho (STY)]WGSADQLKEIAK.2	-0.052744466	Q8K3I9_S214_M1_Glcci1
S[Phospho (STY)]IDTQTPSVQER.2	-0.332292123	Q8K3I9_S335_M1_Glcci1
STAGS[Phospho (STY)]PPAAAGSGTGPAGSC[Carbamidomethyl (C)]APAAGAGR.2	-0.042268693	Q8K3I9_S26_M1_Glcci1
TSS[Phospho (STY)]LDTITGPYLTGQWPR.2	-0.128494794	Q8K3I9_S163_M1_Glcci1
EPATINYPFEK.2	-0.238326931	#N/A
LC[Carbamidomethyl (C)]EAIC[Carbamidomethyl (C)]PAQAITIEAEPR.2	0.312336853	#N/A
GLGM[Oxidation (M)]TLSYLFR.2	0.297979813	#N/A
RYPSGEER.2	-0.132147341	#N/A
ILM[Oxidation (M)]WTELIR.2	-0.056772516	#N/A
YDIDMTK.2	0.2537611	#N/A
T[Phospho (STY)]SDSDQQTYLFQR.2	-0.075342686	Q8K3K8_T529_M1_Optn
SWS[Phospho (STY)]LNTEEVPDFER.2	0.031473531	Q8K3V7_S370_M1_Minar1
[Acetyl (Protein N-term)]M[Oxidation (M)]S[Phospho (STY)]PEIALNR.2	-0.421439642	Q8K3W0_S2_M1_Babam2
FGSS[Phospho (STY)]PQRDPNWIGDR.3	-0.246087777	Q8K3W3_S263_M1_Casc3
GTVTGERQS[Phospho (STY)]GDGQESTEPVENK.3	0.058114149	Q8K3W3_S145_M1_Casc3
NSSS[Phospho (STY)]PVSPASVPGQR.2	-0.031027405	Q8K3X4_S638_M1_Irf2bpl
S[Phospho (STY)]PGPPPPVGVK.2	-0.563386218	Q8K3X4_S69_M1_Irf2bpl
RKAS[Phospho (STY)]PEPPDSAESALK.3	0.037804752	Q8K3X4_S526_M1_Irf2bpl
RNS[Phospho (STY)]SS[Phospho (STY)]PVS[Phospho (STY)]PASVPGQR.2	-0.260799188	Q8K3X4_S636_M3_Irf2bpl

KAS[Phospho (STY)]PEPPDSAESALK.2	-0.380780425	Q8K3X4_S526_M1_Irf2bpl
RPGS[Phospho (STY)]VSSTDQER.2	-0.391977163	Q8K3X4_S313_M1_Irf2bpl
SGPTSS[Phospho (STY)]PFSSPASSR.2	-0.264501435	Q8K3Z9_S409_M1_Pom121
SQT[Phospho (STY)]PERPAKK.2	0.092852209	Q8K3Z9_T421_M1_Pom121
SSS[Phospho (STY)]VTS[Phospho (STY)]IDKESR.2	-0.327057826	Q8K400_S783_M2_Stxbp5
QPS[Phospho (STY)]GAGLC[Carbamidomethyl (C)]DITEGTVVPEDR.2	0.190679654	Q8K400_S693_M1_Stxbp5
RPVSVS[Phospho (STY)]PSSSQEISENQYAVIC[Carbamidomethyl (C)]SEK.3	0.048417361	Q8K400_S903_M1_Stxbp5
SAPVINNGS[Phospho (STY)]PTILGK.2	-0.260153371	Q8K4B0_S576_M1_Mta1
NNM[Oxidation (M)]S[Phospho (STY)]PHGIPAR.2	-0.260109863	Q8K4B0_S449_M1_Mta1
ATVVNGTGTPGQS[Phospho (STY)]PGAGR.2	0.144256861	Q8K4B0_S386_M1_Mta1
QSLGES[Phospho (STY)]PR.2	-0.011537336	Q8K4G5_S470_M1_Ablim1
TS[Phospho (STY)]SESIYSR.2	0.285103481	Q8K4G5_S396_M1_Ablim1
STS[Phospho (STY)]QGSINSPVYSR.2	-0.199676438	Q8K4G5_S496_M1_Ablim1
TSS[Phospho (STY)]LPGYGK.2	0.06848238	Q8K4G5_S738_M1_Ablim1
DC[Carbamidomethyl (C)]LC[Carbamidomethyl (C)]QLC[Carbamidomethyl (C)]AQPM[Oxidation (M)]SSS[Phospho (STY)]PK.2	0.092912552	Q8K4G5_S216_M1_Ablim1
RSS[Phospho (STY)]GREEDEEELLR.3	0.013377019	Q8K4G5_S671_M1_Ablim1
TLS[Phospho (STY)]PTPSAEGYQDVR.2	-0.148721312	Q8K4G5_S475_M1_Ablim1
SS[Phospho (STY)]GREEDEEELLR.3	-0.366479497	Q8K4G5_S671_M1_Ablim1
YDS[Phospho (STY)]PLHSASHAPSSK.3	-0.057185185	Q8K4G5_S723_M1_Ablim1
QERQS[Phospho (STY)]LGES[Phospho (STY)]PR.2	-0.20043818	Q8K4G5_S466_M2_Ablim1
TLS[Phospho (STY)]PTPSAEGYQDVRDR.3	-0.233551462	Q8K4G5_S475_M1_Ablim1
HSYTPTTSR.2	0.339826414	#N/A
GVS[Phospho (STY)]MPNMLEPK.2	0.071691107	Q8K4G5_S789_M1_Ablim1
KAS[Phospho (STY)]EEEYVIR.2	-0.555875215	Q8K4I3_S639_M1_Arhgef6
EIKPSERPLS[Phospho (STY)]PK.2	0.254755149	Q8K4I3_S224_M1_Arhgef6
KAS[Phospho (STY)]EEEYVIRK.2	-0.184790777	Q8K4I3_S639_M1_Arhgef6
KDS[Phospho (STY)]VPQVLLPEEEKLIIEETR.3	0.039213478	Q8K4I3_S679_M1_Arhgef6
MS[Phospho (STY)]GFMYQGK.2	0.274222561	Q8K4I3_S487_M1_Arhgef6
FGSTGST[Phospho (STY)]PPVSPTPSER.2	0.132227681	Q8K4J6_T488_M1_Mrtfa
AQQPAPASS[Phospho (STY)]PVKR.2	0.146833013	Q8K4J6_S606_M1_Mrtfa
ALPSFPTSEC[Carbamidomethyl (C)]VS[Phospho (STY)]DVEPDTR.2	-0.428315947	Q8K4L0_S74_M1_Ddx54
IDDRDS[Phospho (STY)]EEEGPSNQR.2	0.224590621	Q8K4L0_S774_M1_Ddx54
[Acetyl (Protein N-term)]ATEIGS[Phospho (STY)]PPR.2	-0.315964585	Q8K4P0_S7_M1_Wdr33
SSS[Phospho (STY)]LDGDHHDGYHR.3	-0.060235433	Q8K4P0_S1273_M1_Wdr33
DAPRPDHPPHDGHS[Phospho (STY)]PASR.4	0.088501216	Q8K4P0_S1204_M1_Wdr33
STS[Phospho (STY)]DTDLVTSDSR.2	-0.328902663	Q8K4P8_S68_M1_Hecw1
SYS[Phospho (STY)]AGEASEVSR.2	-0.146826798	Q8K4P8_S1073_M1_Hecw1
EGSLS[Phospho (STY)]PVNSQK.2	-0.192875174	Q8K4P8_S937_M1_Hecw1
GST[Phospho (STY)]TEEEDGLEEESTLK.2	0.058318648	Q8K4P8_T579_M1_Hecw1
REGSLS[Phospho (STY)]PVNSQK.2	-0.473064524	Q8K4P8_S937_M1_Hecw1
C[Carbamidomethyl (C)]SPC[Carbamidomethyl (C)]SS[Phospho (STY)]PQNSPGLQR.2	0.313618717	Q8K4P8_S1203_M1_Hecw1
APS[Phospho (STY)]PYRR.2	-0.478546622	Q8K4P8_S1219_M1_Hecw1
SGS[Phospho (STY)]VHQMEQLNR.2	0.269135783	Q8K4P8_S871_M1_Hecw1
SVSS[Phospho (STY)]YGNIR.2	0.103289045	Q8K4Q0_S722_M1_Rptor
ILDTSSLTQSAPAS[Phospho (STY)]PTNK.2	-0.321240379	Q8K4Q0_S863_M1_Rptor
KKS[Phospho (STY)]APETLTLPDPEK.3	0.185156457	Q8K4R4_S299_M1_Pitpnc1
S[Phospho (STY)]APSSAPSTPLSTDAPEFLSIPK.2	-0.199592171	Q8K4R4_S270_M1_Pitpnc1

YEDNKGS[Phospho (STY)]NDSIFDSEAK.3	0.249371737	Q8K4R4_S119_M1_Pitpnc1
S[Phospho (STY)]APETLTLPDPEKK.2	-0.079575255	Q8K4R4_S299_M1_Pitpnc1
GNPSGIQPDLLISLTAPK.2	-0.141895503	#N/A
YHYLGGR.2	0.478653747	#N/A
SPPTVLVIC[Carbamidomethyl (C)]GPGNNGGDGLVC[Carbamidomethyl (C)]AR.2	-0.136698706	#N/A
RGS[Phospho (STY)]ETMAGAAVK.2	0.374948532	Q8K4Z3_S43_M1_Naxe
GIS[Phospho (STY)]ALLLNQGNGDK.2	-0.323503272	Q8K596_S622_M1_Slc8a2
S[Phospho (STY)]GIIIGAEGDPPK.2	-0.173113951	Q8K596_S266_M1_Slc8a2
C[Carbamidomethyl (C)]QPGVLLPVWEPEDPSLGDK.2	-0.370069056	#N/A
TNLALVIGTHSWR.2	0.004864434	#N/A
GIS[Phospho (STY)]ALLLNQGNGDKK.2	0.237422565	Q8K596_S622_M1_Slc8a2
GHGHS[Phospho (STY)]DEEDEEQPR.2	0.061023439	Q8N7N5_S100_M1_Dcaf8
AHSPAEGASSESSS[Phospho (STY)]PGPK.2	-0.138605256	Q8QZS3_S73_M1_Flcn
FASEITPITISVK.2	0.119766182	#N/A
QEQDTYALSSYTR.2	0.102649715	#N/A
QATLGAGLPISTPC[Carbamidomethyl (C)]TTVNK.2	0.262770009	#N/A
LGTAAIQGAIEK.2	0.071358369	#N/A
TPIGSFLGSLASQPATK.2	0.192889791	#N/A
SKEAWDAGK.2	0.077551171	#N/A
M[Oxidation (M)]LEIDPQK.2	0.00203203	#N/A
LEDLIVK.2	0.082808557	#N/A
EVYM[Oxidation (M)]GNVIQGGEGQAPTR.2	-0.337299567	#N/A
GATPYGGVK.2	0.160394304	#N/A
ENGTITAANASTLNDGAAALVLM[Oxidation (M)]TAEAAQR.3	-0.24350511	#N/A
DGLTDVYNK.2	-0.110454858	#N/A
EEVKEVYMGNVIQGGEGQAPTR.3	-0.219559774	#N/A
IAAFADAAVDPIDFPLAPAYAVPK.3	0.326674157	#N/A
AHS[Phospho (STY)]VDVEK.2	-0.31803814	Q8QZT2_S222_M1_Ccsap
ETDGAPS[Phospho (STY)]GPGPR.2	0.045850021	Q8QZT2_S129_M1_Ccsap
GALGEPS[Phospho (STY)]APS[Phospho (STY)]AREEEQPVGER.3	0.017572254	Q8QZT2_S76_M2_Ccsap
QRAHS[Phospho (STY)]VDVEK.3	-0.216477779	Q8QZT2_S222_M1_Ccsap
QMDMGS[Phospho (STY)]QK.2	-0.131082927	Q8QZT2_S180_M1_Ccsap
GSSAAAPPSS[Phospho (STY)]PPPGR.2	-0.14604053	Q8QZV4_S18_M1_Stk32c
RGS[Phospho (STY)]SAAAPPSS[Phospho (STY)]PPPGR.2	-0.245707559	Q8QZV4_S10_M2_Stk32c
M[Oxidation (M)]S[Phospho (STY)]MSSISSGSAR.2	0.243775237	Q8QZV4_S69_M1_Stk32c
SS[Phospho (STY)]NPKPR.2	-0.01411643	Q8QZV6_S384_M1_Fam161a
SDKDLETQVIQLNEQVHSLK.3	0.041301046	#N/A
LALEGVEK.2	0.232477385	#N/A
SSPASKPGSTPSRPSS[Phospho (STY)]AK.3	0.382968341	Q8R001_S222_M1_Mapre2
EIELLC[Carbamidomethyl (C)]QEHGQENDDLVQR.3	-0.384858011	#N/A
SHHANS[Phospho (STY)]PTAGAAK.2	-0.222384822	Q8R001_S199_M1_Mapre2
NLVHSGATK.2	0.019748481	#N/A
VENSWGEDHGHK.3	-0.089835596	#N/A
ENLSAAFSR.2	0.168227956	#N/A
DTLEISDFFR.2	-0.320924051	#N/A
TTPLPDGQILDHR.2	0.087808625	#N/A
SSSGLLEWDSK.2	0.270268999	#N/A

FSTPEQAAK.2	0.021617228	#N/A
IEYAKPTR.2	0.310154078	#N/A
VFNVFC[Carbamidomethyl (C)]LYGNVEK.2	0.284785047	#N/A
YYGGGNEGGR.2	0.197613328	#N/A
NDQDTWDYTNPNLSGQGDPGSNPNKR.3	0.008843243	#N/A
ISRPGDS[Phospho (STY)]DDSR.2	-0.521259412	Q8R081_S182_M1_Hnrnpl
ASLNGADIYSGC[Carbamidomethyl (C)]C[Carbamidomethyl (C)]TLK.2	0.036367134	#N/A
LC[Carbamidomethyl (C)]FSTAQHAS.2	-0.45544903	#N/A
SKPGAAMVEMADGYAVDR.3	0.126733247	#N/A
HYQTEEKS[Phospho (STY)]D.2	-0.470411028	Q8R0A0_S248_M1_Gtf2f2
DGSIS[Phospho (STY)]PVSSEC[Carbamidomethyl (C)]SAVER.2	-0.248862002	Q8R0A2_S161_M1_Zbtb44
[Acetyl (Protein N-term)]MEEVRGENEGKLEK.3	0.218614659	#N/A
SANSWLAEK.2	0.275983064	#N/A
TVTM[Oxidation (M)]IS[Phospho (STY)]PEDEQK.2	-0.206055719	Q8R0A7_S275_M1_Kiaa0513
FWNAAFFDAVHC[Carbamidomethyl (C)]ER.2	-0.209658757	#N/A
QAVIGNLDEEQYK.2	-0.196850007	#N/A
TVTM[Oxidation (M)]IS[Phospho (STY)]PEDEQKGEK.2	-0.295006243	Q8R0A7_S275_M1_Kiaa0513
S[Phospho (STY)]SSNESFSSNQSADSAPDEETLALR.3	-0.021145252	Q8R0A7_S72_M1_Kiaa0513
IFSGGEDLDQEEK.2	-0.078013767	#N/A
IITLEEGDLILTGTPK.2	-0.014819432	#N/A
ESGADGASTS[Phospho (STY)]PR.2	0.023851124	Q8R0L9_S298_M1_Tada3
ETGTEHS[Phospho (STY)]PGVQPADVK.2	0.064248426	Q8R0P4_S46_M1_Aamdc
TDSVIIADQTPT[Phospho (STY)]PTR.2	-0.124375956	Q8R0S1_T53_M1_Atf7
SLGGQQGS[Phospho (STY)]PK.2	-0.186464623	Q8R0S2_S415_M1_Iqsec1
LQHSTS[Phospho (STY)]VLR.2	0.03396449	Q8R0S2_S88_M1_Iqsec1
SALSSS[Phospho (STY)]LRDLSEAGKR.3	0.304956489	Q8R0S2_S923_M1_Iqsec1
M[Oxidation (M)]QFS[Phospho (STY)]FEGPEK.2	-0.346876093	Q8R0S2_S179_M1_Iqsec1
SALSSS[Phospho (STY)]LR.2	-0.15828619	Q8R0S2_S923_M1_Iqsec1
SLAES[Phospho (STY)]IDDALNC[Carbamidomethyl (C)]R.2	0.314799653	Q8R0S2_S251_M1_Iqsec1
ETLIGIYER.2	0.050063346	#N/A
VLINFNAPNPQDR.2	-0.145249964	#N/A
ETRNS[Phospho (STY)]WDSPAFSNDVIR.3	-0.421493175	Q8R0S2_S510_M1_Iqsec1
LQHSTS[Phospho (STY)]VLRK.2	-0.457638218	Q8R0S2_S88_M1_Iqsec1
SALSSS[Phospho (STY)]LRDLSEAGK.2	-0.323749228	Q8R0S2_S923_M1_Iqsec1
GVDDGEDIPR.2	0.280269406	#N/A
S[Phospho (STY)]LHSEEVPASDTAR.2	-0.284032753	Q8R0S2_S260_M1_Iqsec1
STPS[Phospho (STY)]LERPEPR.2	-0.095178457	Q8R0S2_S362_M1_Iqsec1
LRSS[Phospho (STY)]MSENR.2	0.351032871	Q8R0S2_S159_M1_Iqsec1
SLS[Phospho (STY)]ESYELSSDLQDK.2	-0.11345144	Q8R0S2_S106_M1_Iqsec1
NSWDS[Phospho (STY)]PAFSNDVIR.2	0.113452188	Q8R0S2_S513_M1_Iqsec1
SRS[Phospho (STY)]LS[Phospho (STY)]ESYELSSDLQDK.2	-0.167943771	Q8R0S2_S104_M2_Iqsec1
HSNHSTENS[Phospho (STY)]PIER.2	-0.223348869	Q8R0S4_S447_M1_Cacnb4
HSNHS[Phospho (STY)]TENS[Phospho (STY)]PIERR.3	-0.514842375	Q8R0S4_S443_M2_Cacnb4
ATHTSSS[Phospho (STY)]TPM[Oxidation (M)]T[Phospho (STY)]PLLGR.2	-0.072752663	Q8R0S4_S406_M2_Cacnb4
SNTRSS[Phospho (STY)]LAEVQSEIER.3	-0.366321834	Q8R0S4_S287_M1_Cacnb4
SDGS[Phospho (STY)]TTSTS[Phospho (STY)]FILR.2	0.231102079	Q8R0S4_S38_M2_Cacnb4
NRGS[Phospho (STY)]PGGC[Carbamidomethyl (C)]SHDSR.2	-0.264709591	Q8R0S4_S507_M1_Cacnb4

NRLS[Phospho (STY)]SSSQHSR.2	0.121362318	Q8R0S4_S474_M1_Cacnb4
ILPNVPEVEDSTDFFK.2	-0.159005264	#N/A
LIAEGTAPR.2	-0.165370096	#N/A
KVS[Phospho (STY)]LELGGK.2	0.012535276	Q8R0Y6_S671_M1_Aldh1l1
KLVEYC[Carbamidomethyl (C)]QR.2	-0.083430759	#N/A
EC[Carbamidomethyl (C)]DVLPDDTVSTLYNR.2	0.224807822	#N/A
AGLILFGNDDR.2	0.061614099	#N/A
DLGEAALNEYLR.2	0.459133459	#N/A
ANATEFGLASGVFTR.2	0.339239097	#N/A
GSASSALELTEEELATAEAVR.2	-0.111100729	#N/A
LRGEDGESEC[Carbamidomethyl (C)]VINYVEK.3	-0.150447925	#N/A
IAVIGQSLFGQEVYC[Carbamidomethyl (C)]QLR.2	0.107514768	#N/A
TYSTINPTDGSVIC[Carbamidomethyl (C)]QVSLAQVSDVDK.3	0.012030676	#N/A
DTNHGPQNHEAHLR.4	-0.263534954	#N/A
LALES[Phospho (STY)]PEVQDLQLER.2	0.02982667	Q8R105_S29_M1_Vps37c
C[Carbamidomethyl (C)]LS[Phospho (STY)]PGGHPVYR.2	0.26877131	Q8R123_S468_M1_Flad1
ALDVSAS[Phospho (STY)]DEEM[Oxidation (M)]ARPK.2	-0.184037072	Q8R146_S187_M1_Apeh
VRHDT[Phospho (STY)]PDLS[Phospho (STY)]PPRR.3	-0.006802102	Q8R149_S200_M2_Bud13
HDT[Phospho (STY)]PDPS[Phospho (STY)]PPRR.2	-0.470641091	Q8R149_S187_M2_Bud13
AHHES[Phospho (STY)]PDLELHK.3	0.20310696	Q8R149_S297_M1_Bud13
HDLDAS[Phospho (STY)]PPRK.3	-0.330609245	Q8R149_S238_M1_Bud13
HDT[Phospho (STY)]PDLS[Phospho (STY)]PPRR.3	-0.073237911	Q8R149_S200_M2_Bud13
VRHDT[Phospho (STY)]PDLS[Phospho (STY)]PPR.3	0.115879678	Q8R149_S200_M2_Bud13
TDFAPQLQSLNK.2	0.198116102	#N/A
FTLVAWDPR.2	0.330242998	#N/A
TC[Carbamidomethyl (C)]EDWVDGISQFK.2	-0.276140069	#N/A
FADEFNR.2	0.150129323	#N/A
QVSLLGWSDGGITALIAAAK.2	0.373684092	#N/A
AGAAFDPVSFAR.2	0.019622385	#N/A
FQQISSVR.2	0.002835408	#N/A
TTPGPGTAQAGDAAR.2	-0.141951418	#N/A
[Acetyl (Protein N-term)]MEGASFGAGR.2	0.250836035	#N/A
SLSNS[Phospho (STY)]NPDISGTPTSPDDEVR.2	-0.374195587	Q8R1A4_S900_M1_Dock7
SNS[Phospho (STY)]WVNTGPK.2	-0.188671541	Q8R1A4_S929_M1_Dock7
SPS[Phospho (STY)]GSAFGSQENLR.2	-0.269572048	Q8R1A4_S1422_M1_Dock7
KGS[Phospho (STY)]WSER.2	0.220048467	Q8R1A4_S432_M1_Dock7
TTS[Phospho (STY)]GDDAC[Carbamidomethyl (C)]NLTSFR.2	-0.163458761	Q8R1A4_S452_M1_Dock7
RNS[Phospho (STY)]SLVGR.2	0.257479853	Q8R1A4_S439_M1_Dock7
M[Oxidation (M)]NS[Phospho (STY)]LTFKK.2	-0.127677098	Q8R1A4_S1382_M1_Dock7
SMS[Phospho (STY)]IDDTPR.2	-0.131004071	Q8R1A4_S182_M1_Dock7
SKS[Phospho (STY)]VENFFLR.2	-0.241433303	Q8R1B0_S48_M1_Stac2
QPLLLS[Phospho (STY)]EDEEDTKR.2	-0.222417491	Q8R1B4_S39_M1_Eif3c
NETDESQIQLAGGDVELPR.2	-0.046418938	#N/A
AAS[Phospho (STY)]DPNPAEPAR.2	-0.228503731	Q8R1C6_S120_M1_Mettl22
AAS[Phospho (STY)]DPNPAEPARDK.2	-0.269933956	Q8R1C6_S120_M1_Mettl22
TGS[Phospho (STY)]QEGTSM[Oxidation (M)]EGSRPAAPAEPGTLK.3	0.094022736	Q8R1F6_S593_M1_Hid1
TGS[Phospho (STY)]QEGTSMEGSR.2	0.035645506	Q8R1F6_S593_M1_Hid1

AFDQGADAIYEHINEGK.2	-0.223748604	#N/A
NVLLLGEDGAGK.2	-0.041443503	#N/A
DAVFIPAGWDNDKK.2	0.237230551	#N/A
FSLDALSLR.2	0.139705328	#N/A
KPASVSPTTPTS[Phospho (STY)]PTEGEAS.2	-0.481136988	Q8R1Q8_S516_M1_Dync1li1
DFQEYVEPGEDFPAS[Phospho (STY)]PQRR.3	-0.318095737	Q8R1Q8_S207_M1_Dync1li1
DFQEYVEPGEDFPAS[Phospho (STY)]PQR.2	-0.075557192	Q8R1Q8_S207_M1_Dync1li1
IPAVVVEK.2	-0.148176352	#N/A
SVSS[Phospho (STY)]NVASVSPIPAGSK.2	-0.110252236	Q8R1Q8_S415_M1_Dync1li1
VPGGS[Phospho (STY)]PRT[Phospho (STY)]PNR.2	-0.520465293	Q8R1Q8_S405_M2_Dync1li1
GLEYLYLNVHDEDRDDQTR.3	0.038994772	#N/A
NIDLVYK.2	-0.097214459	#N/A
ATAPS[Phospho (STY)]PERK.2	0.227634873	Q8R1R3_S356_M1_Stard7
VS[Phospho (STY)]PVNDVDVGVYQLM[Oxidation (M)]QSEQLLSR.3	-0.144738722	Q8R1T1_S232_M1_Chmp7
S[Phospho (STY)]PEQESVSTAPQR.2	-0.069309855	Q8R1X6_S126_M1_Spart
DAC[Carbamidomethyl (C)]KKS[Phospho (STY)]PEQESVSTAPQR.3	0.190628986	Q8R1X6_S126_M1_Spart
KS[Phospho (STY)]PEQESVSTAPQR.2	0.050819966	Q8R1X6_S126_M1_Spart
VASAESC[Carbamidomethyl (C)]HSEQQEPS[Phospho (STY)]PTAER.3	-0.336276859	Q8R2R3_S211_M1_Aagab
AFWM[Oxidation (M)]AIGGDRDEIEGLSS[Phospho (STY)]DDEH.3	0.415855064	Q8R2R3_S312_M1_Aagab
RIPVS[Phospho (STY)]PEQTR.2	0.308651999	Q8R2S9_S132_M1_Actr8
PGS[Phospho (STY)]DTALTVDR.2	-0.228921404	Q8R310_S4_M1_Tmcc3
ALSNLESIPGGYNALR.2	0.194646164	#N/A
EANLQALIATGGDINAAIER.2	0.023654643	#N/A
FQQQLEQLSAM[Oxidation (M)]GFLNR.2	-0.343730725	#N/A
[Acetyl (Protein N-term)]AESAESGGPPGAQDSAADGGPAEPK.2	-0.073453475	#N/A
LFVGNLPTDITEEDFK.2	-0.312114521	#N/A
FAQPGTFEFEYASR.2	0.190168376	#N/A
GQEDSLAS[Phospho (STY)]AVDATTGQEAC[Carbamidomethyl (C)]DSD.2	0.393317824	Q8R344_S152_M1_Ccdc12
TYS[Phospho (STY)]DEASQLR.2	-0.221433184	Q8R361_S307_M1_Rab11fip5
S[Phospho (STY)]PSHSSWLSTEGGR.2	-0.04011653	Q8R361_S278_M1_Rab11fip5
RTYS[Phospho (STY)]DEASQLR.2	0.029875149	Q8R361_S307_M1_Rab11fip5
GSPSLGAS[Phospho (STY)]PHHSSTGEEK.3	0.181215588	Q8R361_S486_M1_Rab11fip5
DKPRS[Phospho (STY)]PFSK.2	-0.008299057	Q8R361_S188_M1_Rab11fip5
ATVALDEVFR.2	0.393030919	#N/A
RGS[Phospho (STY)]VGEKGS[Phospho (STY)]PSLGASPHHSSTGEEK.4	-0.229601005	Q8R361_S474_M2_Rab11fip5
VLPDELQVSAAPPGPR.2	-0.021059104	#N/A
AIPEAPVGR.2	0.096429792	#N/A
LVAQLDTEGIGSLGPGYEDR.2	-0.213973293	#N/A
DSQFSYAVFGPR.2	0.166558618	#N/A
VASGDLQVQR.2	0.561210265	#N/A
ATLQEVVGLR.2	0.150944898	#N/A
HAAYSVGWEM[Oxidation (M)]APAGAPGPGR.3	-0.107468115	#N/A
LEAARPADAGTYR.3	-0.17070207	#N/A
LTVHEGQELALGC[Carbamidomethyl (C)]LAQTK.3	-0.460478328	#N/A
AVLAHVDVQTLSSQLAVTVGPGER.3	-0.296235803	#N/A
EEGVVLEAVAWLAGGTVYR.2	0.46376151	#N/A
LVKPSSSSSGS[Phospho (STY)]R.2	0.088729546	Q8R373_S293_M1_Clmp

VS[Phospho (STY)]PSTSYTPSR.2	0.122363025	Q8R3C0_S154_M1_Mcmbp
SGVTNMSS[Phospho (STY)]PHK.2	-0.137347649	Q8R3D1_S184_M1_Tbc1d13
DSS[Phospho (STY)]VAEEGAQEAVAR.2	-0.466163729	Q8R3F5_S41_M1_Mcat
RTS[Phospho (STY)]TQENVHLSQAK.3	-0.283783782	Q8R3I2_S472_M1_Mboat2
KPEPLDES[Phospho (STY)]DNDF.2	0.086510993	Q8R3P7_S409_M1_Cluap1
IRKPEPLDES[Phospho (STY)]DNDF.2	0.38840341	Q8R3P7_S409_M1_Cluap1
NVPGLAAPSS[Phospho (STY)]PTQK.2	0.188539491	Q8R3Q2_S670_M1_Ppp6r2
VGTEGLVDS[Phospho (STY)]FSQGLER.2	-0.14845343	Q8R3Q2_S289_M1_Ppp6r2
LTGSTSS[Phospho (STY)]LNK.2	0.172686406	Q8R3S6_S473_M1_Exoc1
GKVPVTYLELLS[Phospho (STY)].2	-0.403892106	Q8R3V5_S400_M1_Sh3glb2
QTEVLLQPNPSAR.2	0.163343115	#N/A
NFLEGDWK.2	-0.076670397	#N/A
VAQTEFDR.2	0.126306234	#N/A
VEEFLYEK.2	-0.207316753	#N/A
SQTTYYAQC[Carbamidomethyl (C)]YR.2	-0.171404774	#N/A
LASDAGIFFTR.2	0.227218311	#N/A
DFIHTASLSFLTPLR.2	0.217372172	#N/A
TELDAHFENLLAR.2	-0.197605906	#N/A
RLDLDAC[Carbamidomethyl (C)]K.2	-0.038505834	#N/A
LLLEGISSTHVNHLR.3	0.34092756	#N/A
AVQFTEEK.2	-0.284320921	#N/A
VPVTYLELLS[Phospho (STY)].2	0.206726751	Q8R3V5_S400_M1_Sh3glb2
NVAEALGHS[Phospho (STY)]PKDPGGGGGSVR.3	-0.359562601	Q8R3Y8_S436_M1_Irf2bp1
KAS[Phospho (STY)]PEPEGETAGK.2	-0.250812346	Q8R3Y8_S384_M1_Irf2bp1
NVAEALGHS[Phospho (STY)]PK.2	-0.128069049	Q8R3Y8_S436_M1_Irf2bp1
AGGAS[Phospho (STY)]PAASSTTQPPAQHR.2	-0.478933352	Q8R3Y8_S453_M1_Irf2bp1
RKAS[Phospho (STY)]PEPEGETAGK.2	-0.41436595	Q8R3Y8_S384_M1_Irf2bp1
S[Phospho (STY)]PGPPALKHPTSK.3	-0.170335546	Q8R3Y8_S66_M1_Irf2bp1
S[Phospho (STY)]PGPPALK.2	0.430837833	Q8R3Y8_S66_M1_Irf2bp1
QDT[Phospho (STY)]FDADTPGSR.2	0.208099772	Q8R3Z5_T499_M1_Cacnb1
SDGS[Phospho (STY)]TSSDTTSNSFVR.2	0.096268063	Q8R3Z5_S44_M1_Cacnb1
LSSSKS[Phospho (STY)]GDNSSSSLGDVVTGTR.3	0.140588045	Q8R3Z5_S186_M1_Cacnb1
S[Phospho (STY)]GDNSSSSLGDVVTGTR.2	-0.084939831	Q8R3Z5_S186_M1_Cacnb1
QGS[Phospho (STY)]WEDEEDYEEEM[Oxidation (M)]TDNR.2	0.101791527	Q8R3Z5_S547_M1_Cacnb1
ATHPPSS[Phospho (STY)]TPPNPLLNR.3	-0.038716915	Q8R3Z5_S417_M1_Cacnb1
ALS[Phospho (STY)]RQDT[Phospho (STY)]FDADTPGSR.2	-0.375695258	Q8R3Z5_S495_M2_Cacnb1
GPYPPSQEIPM[Oxidation (M)]EVFDPS[Phospho (STY)]PQGK.3	-0.12078133	Q8R3Z5_S26_M1_Cacnb1
DSWNSGIISVM[Oxidation (M)]SALSVAPSK.2	-0.046100121	#N/A
GSVAGGAVYLVYDQELLGPSDK.2	-0.159409241	#N/A
VWSVASTVR.2	-0.291384295	#N/A
GSYLTHEAS[Phospho (STY)]GLDEQGEAR.3	0.034878375	Q8R464_S361_M1_Cadm4
FQLEEFSPR.2	-0.130554423	#N/A
LHQYDGSIVVIQNPAR.3	0.18273865	#N/A
RAS[Phospho (STY)]GEEQEC[Carbamidomethyl (C)]LD.2	-0.10881777	Q8R4E6_S342_M1_Purg
DALVQLIEDYGEGDIEER.2	-0.155864522	#N/A
HSAPS[Phospho (STY)]PPVSVGS[Phospho (STY)]EEHPHSVLK.3	-0.021777304	Q8R4E6_S159_M2_Purg
QKHSAPS[Phospho (STY)]PPVS[Phospho (STY)]VGS[Phospho (STY)]EEHPHSVLK.4	0.082848483	#N/A

SEGVQDAEPQSLVGS[Phospho (STY)]PSTR.2	-0.133134263	Q8R4H2_S341_M1_Arhgef12
TGTGDIATC[Carbamidomethyl (C)]DS[Phospho (STY)]PR.2	-0.461851467	Q8R4H2_S1327_M1_Arhgef12
ITS[Phospho (STY)]PVLVGEENNVVHNQK.3	0.144805817	Q8R4H2_S190_M1_Arhgef12
HLSTPSSVS[Phospho (STY)]PEPQDPAK.2	-0.063975206	Q8R4H2_S637_M1_Arhgef12
TDWSSGDASRPSSDSADS[Phospho (STY)]PK.3	0.113617285	Q8R4H2_S309_M1_Arhgef12
VEHHDLSVAGLQS[Phospho (STY)]PDR.3	-0.158018464	Q8R4H2_S1176_M1_Arhgef12
SSS[Phospho (STY)]HDFDPTDSSSK.2	0.27640851	Q8R4H2_S41_M1_Arhgef12
SSS[Phospho (STY)]HDFDPTDSSSKK.2	0.063223312	Q8R4H2_S41_M1_Arhgef12
LLPGNELR.2	0.152766909	#N/A
GTGATADFADVAEDFENY[Phospho (STY)]HK.3	0.276727591	Q8R4I7_Y417_M1_Neto1
VDC[Carbamidomethyl (C)]AVLDC[Carbamidomethyl (C)]EDGVAENK.2	-0.079652144	#N/A
TLGT[Phospho (STY)]PTQSGSTPR.2	-0.065255364	Q8R4R6_T272_M1_Nup35
KLS[Phospho (STY)]PPQKK.2	0.059050717	Q8R4S0_S157_M1_Ppp1r14c
LAPAVS[Phospho (STY)]PEKK.2	-0.427389317	Q8R4T1_S95_M1_Cys1
GNPEDSC[Carbamidomethyl (C)]ASEAPGNS[Phospho (STY)]PK.2	0.116903659	Q8R4T1_S116_M1_Cys1
ELS[Phospho (STY)]PQHKR.2	-0.325190385	Q8R4U7_S395_M1_Luzp1
KGS[Phospho (STY)]LDYLK.2	-0.215110254	Q8R4U7_S261_M1_Luzp1
RKGS[Phospho (STY)]LDYLK.2	-0.491069228	Q8R4U7_S261_M1_Luzp1
ELS[Phospho (STY)]PQHK.2	-0.166199919	Q8R4U7_S395_M1_Luzp1
NQRPFS[Phospho (STY)]PR.2	-0.126706754	Q8R4U7_S746_M1_Luzp1
S[Phospho (STY)]QENILQGFSLPNK.2	-0.085937114	Q8R4U7_S612_M1_Luzp1
EFALS[Phospho (STY)]NEHYSLSSK.2	-0.029782146	Q8R4U7_S411_M1_Luzp1
EKPDS[Phospho (STY)]DDDLDIESFVTAK.2	0.041203885	Q8R4U7_S660_M1_Luzp1
TFS[Phospho (STY)]DSTHVSVPNDIVGK.2	0.161907794	Q8R4U7_S513_M1_Luzp1
SQS[Phospho (STY)]LKS[Phospho (STY)]IPEK.2	-0.142685903	Q8R502_S212_M2_Lrrc8c
SGT[Phospho (STY)]IQSGPEGNLVR.2	-0.518411101	Q8R502_T198_M1_Lrrc8c
S[Phospho (STY)]QSLKS[Phospho (STY)]IPEKFVVDK.2	0.01334473	Q8R502_S210_M2_Lrrc8c
RVS[Phospho (STY)]ADGQPFQR.2	-0.349111731	Q8R516_S251_M1_Mib2
HGS[Phospho (STY)]PSTPDR.2	-0.237203002	Q8R516_S44_M1_Mib2
VS[Phospho (STY)]ADGQPFQR.2	0.344078316	Q8R516_S251_M1_Mib2
S[Phospho (STY)]IEVENDFLPVEK.2	-0.147742302	Q8R550_S274_M1_Sh3kbp1
ELS[Phospho (STY)]GESDELGISQDEQLSK.2	0.013998764	Q8R550_S156_M1_Sh3kbp1
ASS[Phospho (STY)]PSLFSTEGKPK.2	-0.141384414	Q8R550_S631_M1_Sh3kbp1
AAGGAAS[Phospho (STY)]PGPGGGAR.2	-0.294645467	Q8R554_S749_M1_Otud7a
AS[Phospho (STY)]PGPTAAQPTQLVLK.2	0.26703032	Q8R554_S711_M1_Otud7a
ELLLSQPGTAANIPSHVTR.3	-0.257680159	#N/A
IELLEDALVLR.2	-0.194410331	#N/A
ELPTGGQEGEQLQLQK.2	0.036258723	#N/A
LGIAVIHGEAQDAESDLVDGRHS[Phospho (STY)]PPM[Oxidation (M)]VR.4	-0.133867226	Q8R574_S227_M1_Prpsap2
HS[Phospho (STY)]PPMVR.2	-0.05300668	Q8R574_S227_M1_Prpsap2
S[Phospho (STY)]SDTC[Carbamidomethyl (C)]GSPALPSK.2	0.171999131	Q8R5A3_S531_M1_Apbb1ip
RSSDT[Phospho (STY)]C[Carbamidomethyl (C)]GSPALPSK.2	-0.323383221	Q8R5A3_T534_M1_Apbb1ip
QGLGNHS[Phospho (STY)]PGATR.2	-0.227883352	Q8R5A3_S507_M1_Apbb1ip
SVS[Phospho (STY)]ESHTPC[Carbamidomethyl (C)]PSESTGDTVPLQR.3	-0.135742269	Q8R5A6_S144_M1_Tbc1d22a
AGFAGDQIPK.2	0.075541558	#N/A
VQYTLPDGSTLDVGPAR.2	-0.019020701	#N/A
TLFSNIVLSGGSTLFK.2	-0.125525862	#N/A

DQLQTFSEEHPVLLTEAPLNPSK.3	-0.156716417	#N/A
TTGVVLDSGDGVTHAVPIYEGFAM[Oxidation (M)]PHSIM[Oxidation (M)]R.3	-0.333661137	#N/A
AC[Carbamidomethyl (C)]YLSINPQKDEALETEK.3	-0.116410857	#N/A
KEYEEDGSR.2	-0.550306576	#N/A
S[Phospho (STY)]PGASNFSTLPK.2	0.061085912	Q8R5H1_S229_M1_Usp15
WYYFDDSSVSTASEDQIVSK.2	-0.068490221	#N/A
IENDVATILSR.2	-0.031936462	#N/A
LAQGPELAEDDADLLHK.3	-0.030346803	#N/A
SSTIQDQQLFDRK.2	-0.071193722	#N/A
AWDDFFPGSDR.2	-0.253804798	#N/A
TPMGIILDALEQQEDNINK.2	-0.325600215	#N/A
SDDSVIQLLNPNR.2	-0.00803569	#N/A
C[Carbamidomethyl (C)]EASNIVGK.2	-0.093470462	#N/A
GTYFTHEAK.2	0.069792579	#N/A
GADDAADADTAIINAEGGQNNSEEKKEYFI.3	0.089318771	#N/A
VEEEQEADEEDVS[Phospho (STY)]EEEAEDREGASK.3	-0.029066499	Q8VBT0_S245_M1_Tmx1
KVEEEQEADEEDVS[Phospho (STY)]EEEAEDR.3	-0.241011274	Q8VBT0_S245_M1_Tmx1
SANSELGGIWSVGQR.2	0.192351155	#N/A
GVLEQGWQADSTTR.2	0.178616528	#N/A
GLTTLT[Phospho (STY)]PVS[Phospho (STY)]ESSK.2	0.370141319	Q8VBW1_S628_M2_Slc6a8
LLQSVGQAPESISEK.2	0.08693175	#N/A
DNSQTPAVPAPDLEPIPSTS[Phospho (STY)]R.2	-0.184550613	Q8VBX6_S1590_M1_Mpdz
KFS[Phospho (STY)]LQER.2	0.107406954	Q8VBY2_S74_M1_Camkk1
S[Phospho (STY)]FGNPFEPQAR.2	-0.181434138	Q8VBY2_S458_M1_Camkk1
EGC[Carbamidomethyl (C)]GEGC[Carbamidomethyl (C)]KS[Phospho (STY)]PELPGVQEDEAAS.2	-0.061569555	Q8VBY2_S492_M1_Camkk1
SM[Oxidation (M)]S[Phospho (STY)]APGSLLMK.2	0.31061486	Q8VBY2_S475_M1_Camkk1
KRS[Phospho (STY)]FGNPFEPQAR.3	0.142046337	Q8VBY2_S458_M1_Camkk1
S[Phospho (STY)]M[Oxidation (M)]EKENM[Oxidation (M)]AVEEK.2	-0.293777784	Q8VC31_S60_M1_Ccdc9
LENGC[Carbamidomethyl (C)]GNVS[Phospho (STY)]PQTK.2	0.354334278	Q8VCC8_S531_M1_Rapgef3
TETS[Phospho (STY)]EDDLGDKAPK.2	0.019185259	Q8VCD6_S210_M1_Reep2
TETS[Phospho (STY)]EDDLGDKAPKR.3	0.077931528	Q8VCD6_S210_M1_Reep2
TETS[Phospho (STY)]EDDLGDK.2	-0.04360952	Q8VCD6_S210_M1_Reep2
GTLDEEDEEADS[Phospho (STY)]DTDDIDHR.3	0.041538899	Q8VCE2_S338_M1_Gpn1
GNAS[Phospho (STY)]PVLDPSDLILTR.2	-0.132342735	Q8VCE2_S314_M1_Gpn1
[Acetyl (Protein N-term)]MEAQAVPEGSGPSTAS[Phospho (STY)]PR.2	-0.020210556	Q8VCG9_S16_M1_Rfxap
AKPS[Phospho (STY)]PEHAPTISAPDASGPQK.3	-0.306306614	Q8VCI5_S35_M1_Pex19
IC[Carbamidomethyl (C)]EQFEAET[Phospho (STY)]PTDSEATQR.2	0.297514531	Q8VCI5_T236_M1_Pex19
AKPS[Phospho (STY)]PEHAPTISAPDASGPQKR.4	-0.328372915	Q8VCI5_S35_M1_Pex19
LSPWEDLSQAPTM[Oxidation (M)]DS[Phospho (STY)]PLEK.2	-0.062382919	Q8VCY8_S312_M1_Plppr2
KQES[Phospho (STY)]FC[Carbamidomethyl (C)]R.2	-0.524905751	Q8VD04_S237_M1_Gripap1
EC[Carbamidomethyl (C)]VGS[Phospho (STY)]PDPDLEPGEAN.2	-0.116727359	Q8VD04_S795_M1_Gripap1
LSKEC[Carbamidomethyl (C)]VGS[Phospho (STY)]PDPDLEPGEAN.2	-0.141153469	Q8VD04_S795_M1_Gripap1
TQTGDSSS[Phospho (STY)]VSSFSYR.2	-0.420632099	Q8VD04_S631_M1_Gripap1
KKQES[Phospho (STY)]FC[Carbamidomethyl (C)]R.2	-0.031518798	Q8VD04_S237_M1_Gripap1
TGLEELVLSEM[Oxidation (M)]NS[Phospho (STY)]PSR.2	-0.150510872	Q8VD04_S620_M1_Gripap1
QPGS[Phospho (STY)]PSPPSVPESGQGVTK.2	0.37344857	Q8VD12_S160_M1_Znf385a
SFS[Phospho (STY)]SSADEHS.2	-0.432591838	Q8VD33_S297_M1_Sgtb

SRS[Phospho (STY)]FSSSADEHS.2	0.228270871	Q8VD33_S295_M1_Sgtb
NDIRPLSNS[Phospho (STY)]VPEDVGK.3	-0.419863219	Q8VD33_S77_M1_Sgtb
TGS[Phospho (STY)]PLTVATGNDQAATEAK.2	-0.337785893	Q8VD37_S265_M1_Sgip1
AT[Phospho (STY)]PPPPPPPTYR.2	-0.275836525	Q8VD37_T409_M1_Sgip1
RST[Phospho (STY)]PT[Phospho (STY)]PELTSK.2	-0.550474528	Q8VD37_T180_M2_Sgip1
TVPAT[Phospho (STY)]PPR.2	0.218480961	Q8VD37_T259_M1_Sgip1
TVPATPPRT[Phospho (STY)]GS[Phospho (STY)]PLTVATGNDQAATEAK.3	-0.151420127	Q8VD37_S265_M2_Sgip1
RS[Phospho (STY)]PGAIKR.2	-0.257180718	Q8VD37_S160_M1_Sgip1
STPT[Phospho (STY)]PELTSK.2	-0.281488191	Q8VD37_T182_M1_Sgip1
NLS[Phospho (STY)]SEEVAR.2	0.165310029	Q8VD37_S169_M1_Sgip1
VAEQTFIKDDYLETLSS[Phospho (STY)]PK.3	-0.153710455	#N/A
S[Phospho (STY)]PGAIKR.2	-0.314860536	Q8VD37_S160_M1_Sgip1
HVPS[Phospho (STY)]PLNLEEVQKK.3	-0.536758691	Q8VD37_S371_M1_Sgip1
RS[Phospho (STY)]PGAIK.2	-0.078697638	Q8VD37_S160_M1_Sgip1
RNLS[Phospho (STY)]SEEVARPR.3	0.224811644	Q8VD37_S169_M1_Sgip1
VAEQTFIKDDYLETLSS[Phospho (STY)]PKEC[Carbamidomethyl (C)]GLGQR.4	0.040574848	#N/A
TVVSS[Phospho (STY)]PGPGSGSGTGTTSGASSPAR.2	0.086277983	Q8VD37_S424_M1_Sgip1
ITGEM[Oxidation (M)]VLSFPAGITR.2	-0.10713534	#N/A
IEKLPS[Phospho (STY)]ISDLDSIFGPVLS[Phospho (STY)]PK.3	-0.20974041	Q8VD37_S287_M2_Sgip1
YNS[Phospho (STY)]PELDEEGYSIRPEEPGSTK.3	-0.220951904	Q8VD37_S78_M1_Sgip1
KVAEQTFIKDDYLETLSS[Phospho (STY)]PK.3	0.183635478	#N/A
IPDISQK.2	-0.268800937	#N/A
NLS[Phospho (STY)]SEEVARPR.2	-0.28766961	Q8VD37_S169_M1_Sgip1
EC[Carbamidomethyl (C)]GLGQRAT[Phospho (STY)]PPPPPPPTYR.3	0.282706893	Q8VD37_T409_M1_Sgip1
DDYLETLSS[Phospho (STY)]PKEC[Carbamidomethyl (C)]GLGQR.3	0.473392658	#N/A
WVHFSDAS[Phospho (STY)]PEHVT[Phospho (STY)]PELTPR.3	-0.228935078	Q8VD37_T324_M2_Sgip1
HVPS[Phospho (STY)]PLNLEEVQK.2	-0.327242415	Q8VD37_S371_M1_Sgip1
ASIGNIALSPSPVR.2	0.090572611	#N/A
DDYLETLSS[Phospho (STY)]PK.2	0.483971793	#N/A
ASIGNIALS[Phospho (STY)]PS[Phospho (STY)]PVRK.2	-0.354313081	Q8VD37_S149_M2_Sgip1
ASIGNIALS[Phospho (STY)]PS[Phospho (STY)]PVRKS[Phospho (STY)]PR.3	0.062884215	Q8VD37_S149_M3_Sgip1
YNTDAM[Oxidation (M)]STAVALNNVQFLVPIDGGVTK.3	0.026120739	#N/A
EQVVT[Phospho (STY)]PPAASDIPADSPAPAPPGPTGSAGPPGPPGPR.4	0.058407902	Q8VD37_T335_M1_Sgip1
GPS[Phospho (STY)]PLTM[Oxidation (M)]GAQDTLPVAAAFTETVNAYFK.3	0.022858104	Q8VD37_S528_M1_Sgip1
LPS[Phospho (STY)]ISDLDSIFGPVLSPK.2	0.239276275	Q8VD37_S287_M1_Sgip1
GKHFY[Phospho (STY)]SS[Phospho (STY)]S[Phospho (STY)]ES[Phospho (STY)]EEEEESHKK.3	-0.608938734	Q8VD37_S104_M3_Sgip1
GLEDAPS[Phospho (STY)]PSKGGDAPSAPVAADDSSK.3	0.019337872	Q8VD63_S87_M1_Tspyl4
NGC[Carbamidomethyl (C)]QLEGPHS[Phospho (STY)]PAKPK.2	-0.099747294	Q8VD63_S116_M1_Tspyl4
GLEDAPS[Phospho (STY)]PSK.2	-0.154429021	Q8VD63_S87_M1_Tspyl4
VVFQEFR.2	-0.291581139	#N/A
KGTGDC[Carbamidomethyl (C)]S[Phospho (STY)]DEEVDGKADGADAK.3	-0.346771334	#N/A
GTGDC[Carbamidomethyl (C)]S[Phospho (STY)]DEEVDGK.2	-0.178457214	#N/A
GTGDC[Carbamidomethyl (C)]S[Phospho (STY)]DEEVDGKADGADAK.2	-0.652655149	#N/A
LQQELDDLLVDLDHQR.3	-0.218143673	#N/A
KGTGDC[Carbamidomethyl (C)]S[Phospho (STY)]DEEVDGK.2	0.159303854	#N/A
KQELEEIC[Carbamidomethyl (C)]HDLEAR.3	-0.002661865	#N/A
DFSALESQLQDTQELLQEENR.3	-0.009982244	#N/A

ANLQIDQINTDLNLER.2	0.468954664	#N/A
ELETQISELQEDLESER.2	0.437824853	#N/A
TAFYNEDDS[Phospho (STY)]EEEQR.2	-0.395277379	Q8VDD9_S1783_M1_Phip
VLS[Phospho (STY)]DS[Phospho (STY)]EEEEKDADVPGTSTR.2	0.05387889	Q8VDD9_S1281_M2_Phip
KVLS[Phospho (STY)]DS[Phospho (STY)]EEEEKDADVPGTSTR.3	0.354322643	Q8VDD9_S1281_M2_Phip
TLS[Phospho (STY)]PDPR.2	-0.434449451	Q8VDG3_S556_M1_Parn
ETDPGS[Phospho (STY)]PR.2	-0.147668854	Q8VDJ3_S944_M1_Hdlbp
EAKETDPGS[Phospho (STY)]PR.2	-0.171614286	Q8VDJ3_S944_M1_Hdlbp
ETDPGS[Phospho (STY)]PRR.2	0.235055843	Q8VDJ3_S944_M1_Hdlbp
VATLNS[Phospho (STY)]EEENDPPTYK.2	-0.084390688	Q8VDJ3_S31_M1_Hdlbp
GKS[Phospho (STY)]LDQC[Carbamidomethyl (C)]AEILQK.2	-0.422463831	Q8VDM1_S353_M1_Zgpat
LLSTVDQES[Phospho (STY)]PAQEDAEYLAFQK.3	0.43514344	Q8VDM1_S70_M1_Zgpat
TPVQSQQPSATT[Phospho (STY)]PSGADEK.2	-0.175068561	Q8VDM4_T20_M1_Psmd2
DKTPVQSQQPSATT[Phospho (STY)]PSGADEK.3	0.203149713	Q8VDM4_T20_M1_Psmd2
TITGFQTHTTPVLLAHGER.3	-0.490952929	#N/A
FGGS[Phospho (STY)]GSQVDSAR.2	0.003003518	Q8VDM4_S361_M1_Psmd2
LVGSQEELASWGHEYVR.3	-0.059697404	#N/A
GRS[Phospho (STY)]PQPPAEEDEDDFDDTLVAIDTYNC[Carbamidomethyl (C)]DLHFK.4	-0.120795383	Q8VDM6_S195_M1_Hnrnpul1
NYILDQTNVYGS[Phospho (STY)]AQR.2	0.298264003	Q8VDM6_S513_M1_Hnrnpul1
S[Phospho (STY)]PQPPAEEDEDDFDDTLVAIDTYNC[Carbamidomethyl (C)]DLHFK.3	0.14235726	Q8VDM6_S195_M1_Hnrnpul1
S[Phospho (STY)]PDFTNENPLETR.2	-0.280931416	Q8VDN2_S228_M1_Atp1a1
IVEIPFNSTNK.2	-0.151490635	#N/A
LNIPVNQVNPR.2	-0.239176676	#N/A
YHTEIVFAR.2	-0.263863226	#N/A
LSLDELHR.2	-0.13951401	#N/A
AVFQANQENLPILK.2	0.014835173	#N/A
TSATWFALSR.2	0.104665619	#N/A
GIVVYTGDR.2	-0.225751856	#N/A
YQLSIHK.2	0.018891946	#N/A
KYGTDLSR.2	-0.140192643	#N/A
C[Carbamidomethyl (C)]SSILLHGK.2	0.174967514	#N/A
YGTDLSR.2	-0.402117976	#N/A
NIAFFSTNC[Carbamidomethyl (C)]VEGTAR.2	-0.257277282	#N/A
IISANGC[Carbamidomethyl (C)]K.2	0.187336826	#N/A
AVAGDASESALLK.2	0.271033245	#N/A
DM[Oxidation (M)]DELKK.2	0.293818799	#N/A
EVS[Phospho (STY)]MDDHK.2	0.283441232	Q8VDN2_S40_M1_Atp1a1
WVNDVEDSYGQQWTYEQR.2	-0.447179882	#N/A
M[Oxidation (M)]SINAEDVVVGDLVEVK.2	-0.411006961	#N/A
DM[Oxidation (M)]TSEELDDILR.2	0.235843291	#N/A
ADIGVAM[Oxidation (M)]GIVGSDVSK.2	0.005702051	#N/A
EQPLDEELKDAFQNAYLELGGLGER.3	-0.079464539	#N/A
DKYEPAAVSEHGDKK.3	-0.134708151	#N/A
EVS[Phospho (STY)]M[Oxidation (M)]DDHKLSLDELHR.3	-0.125541739	Q8VDN2_S40_M1_Atp1a1
AC[Carbamidomethyl (C)]VVHGSDLKDM[Oxidation (M)]TSEELDDILR.4	-0.128107493	#N/A
M[Oxidation (M)]TVAHM[Oxidation (M)]WFDNQIHEADTTENQSGVSFDK.4	-0.528787953	#N/A
EVS[Phospho (STY)]M[Oxidation (M)]DDHKLS[Phospho (STY)]LDELHRK.3	-0.271621901	Q8VDN2_S40_M2_Atp1a1

KSLS[Phospho (STY)]APLHPEFEEVYR.3	0.335522104	Q8VDN4_S194_M1_Ccdc92
SATGQHS[Phospho (STY)]PAR.2	0.263644281	Q8VDN4_S261_M1_Ccdc92
SLS[Phospho (STY)]APLHPEFEEVYR.2	-0.075331727	Q8VDN4_S194_M1_Ccdc92
VS[Phospho (STY)]PVPSPSQPAR.2	-0.190052906	Q8VDP3_S777_M1_Mical1
EAAPDTGAEPS[Phospho (STY)]PEDSDPTYSSK.2	-0.485668119	Q8VDP4_S298_M1_Ccar2
HDLS[Phospho (STY)]PYR.2	0.302877441	Q8VDP4_S222_M1_Ccar2
RPSS[Phospho (STY)]GGEEEEKAR.2	0.033364489	Q8VDP4_S626_M1_Ccar2
S[Phospho (STY)]VAS[Phospho (STY)]NQSEM[Oxidation (M)]EYSSLQDM[Oxidation (M)]PK.2	0.102519039	Q8VDP4_S674_M2_Ccar2
VVGIC[Carbamidomethyl (C)]GTQEK.2	0.318348992	#N/A
LLDELTLEGVTR.2	0.28780314	#N/A
IFSEATPR.2	0.135021659	#N/A
NLFTQTLGLGSQK.2	0.203318832	#N/A
SPSTGLYANLEK.2	0.34036998	#N/A
ELEDLVR.2	0.037075998	#N/A
C[Carbamidomethyl (C)]EQC[Carbamidomethyl (C)]QSVVKPDIVFFGENLPSR.3	0.013167459	#N/A
VAGLEPQDLVEAHGTFYTSHC[Carbamidomethyl (C)]VNTSC[Carbamidomethyl (C)]R.4	-0.212279055	#N/A
EHANIDAQSGSQAPNPSTTISPGK.3	0.054106201	#N/A
EHANIDAQSGSQAPNPSTTISPGKS[Phospho (STY)]PPPAK.4	-0.147932517	Q8VDQ8_S372_M1_Sirt2
FFSC[Carbamidomethyl (C)]M[Oxidation (M)]QSDFSK.2	0.09586583	#N/A
YVDEDNS[Phospho (STY)]DGETVDHR.2	0.002375021	Q8VDQ9_S142_M1_Kri1
ILEGDNGLDS[Phospho (STY)]DMEEEADDGC[Carbamidomethyl (C)]K.2	0.007217908	Q8VDY9_S183_M1_Caap1
SVNEILGLAESS[Phospho (STY)]PKEPK.2	0.029166168	Q8VDY9_S307_M1_Caap1
GLGS[Phospho (STY)]PEPGTTAPYLGR.2	-0.261618983	Q8VDZ4_S621_M1_Zdhhc5
LSRGDS[Phospho (STY)]LKEPTSIADSSR.3	-0.118172134	Q8VDZ4_S380_M1_Zdhhc5
SIGSASPGPGQPPLSS[Phospho (STY)]PTR.2	0.051554082	Q8VDZ4_S694_M1_Zdhhc5
VLC[Carbamidomethyl (C)]SS[Phospho (STY)]PAPR.2	-0.424394953	Q8VDZ4_S247_M1_Zdhhc5
GDS[Phospho (STY)]LKEPTSIADSSR.2	-0.260440032	Q8VDZ4_S380_M1_Zdhhc5
SEPS[Phospho (STY)]LEPESFRS[Phospho (STY)]PTFGK.2	-0.076361432	Q8VDZ4_S401_M2_Zdhhc5
SFHFDPLSSGS[Phospho (STY)]R.2	0.141385836	Q8VDZ4_S425_M1_Zdhhc5
LYEVAIDGKEEDLGS[Phospho (STY)]PTGELTSK.3	-0.074897579	Q8VE18_S469_M1_Smg8
GFSQYGVSGSPT[Phospho (STY)]K.2	0.15149646	Q8VE19_T768_M1_Mios
QYTEDNDQKS[Phospho (STY)]PGNK.2	0.488968411	Q8VE19_S444_M1_Mios
DNIISDYDQIIDYVER.2	-0.538711105	#N/A
LDHEEEPQLSEPYLSK.3	-0.249270021	#N/A
LNLGEEVPVIIHR.3	0.032441208	#N/A
TFTGEHVVALM[Oxidation (M)]PEAGS[Phospho (STY)]PQHAR.3	-0.265493663	Q8VE33_S139_M1_Gdap1l1
KTEDSASEVTVEDSGESLEDLMAR.3	0.121593644	#N/A
IS[Phospho (STY)]PGASHVNSK.2	0.089186034	Q8VE52_S353_M1_Ogfrl1
EPGEEADKPS[Phospho (STY)]PEPGSGDPKPR.3	-0.100804976	Q8VE52_S382_M1_Ogfrl1
AS[Phospho (STY)]PVPEDHAEAAGAEQGGESTEGNAKPK.4	-0.585686505	Q8VE52_S65_M1_Ogfrl1
DSAADQSDS[Phospho (STY)]PPEK.2	-0.244122224	Q8VE52_S406_M1_Ogfrl1
DSKIS[Phospho (STY)]PGASHVNSK.3	0.08004774	Q8VE52_S353_M1_Ogfrl1
IAGTPGT[Phospho (STY)]GGRLSPENNQVLTK.3	-0.213379419	Q8VE65_T46_M1_Taf12
IPDEINDR.2	0.272752659	#N/A
AETSLGIPS[Phospho (STY)]PTEISAEVK.2	0.010153687	Q8VE88_S119_M1_Fam114a2
ADAQT[Phospho (STY)]PEEVGPSEASLR.2	-0.610448587	Q8VE99_T90_M1_Ccdc115
INSNHINSNSTGS[Phospho (STY)]S.2	-0.267626215	Q8VEB1_S589_M1_Grk5

S[Phospho (STY)]LDYLNLDK.2	-0.156443056	Q8VEB3_S167_M1_MACIR
[Acetyl (Protein N-term)]MEEGPAVGTLS[Phospho (STY)]R.2	-0.536637962	Q8VED2_S11_M1_Bloc1s4
IQTLSAIDTIK.2	0.138454848	#N/A
[Acetyl (Protein N-term)]AGS[Phospho (STY)]VADSDAVVK.2	-0.143610587	Q8VED9_S4_M1_Lgalsl
GEEQSAIPYFPFIPDQPFR.2	-0.236130013	#N/A
VFVDGHQLFDFYHR.3	-0.133322128	#N/A
LDDGHLNNS[Phospho (STY)]LGS[Phospho (STY)]PVQADVYFPR.3	0.034772159	Q8VED9_S22_M2_Lgalsl
[Acetyl (Protein N-term)]AGSVADSDAVVKLDDGHLNNSLGS[Phospho (STY)]PVQADVYFPR.4	-0.291149578	Q8VED9_S25_M1_Lgalsl
C[Carbamidomethyl (C)]AEAGANM[Oxidation (M)]IVS[Phospho (STY)]GS[Phospho (STY)]AIM[Oxidation (M)]RS[Phospho (STY)]DDPR.3	0.063245218	Q8VEE0_S196_M3_Rpe
S[Phospho (STY)]SPSSSPSLR.2	0.086930665	Q8VEF1_S11_M1_Gramd1a
EIC[Carbamidomethyl (C)]C[Carbamidomethyl (C)]YSISC[Carbamidomethyl (C)]K.2	-0.31104799	#N/A
M[Oxidation (M)]NLSAIQDR.2	0.232560656	#N/A
DLAGALDEK.2	0.154269544	#N/A
ENQSEGKGS[Phospho (STY)]DS[Phospho (STY)]DSEGDNPEK.2	-0.140764582	Q8VEJ9_S95_M2_Vps4a
EIEIDYTGTEPSS[Phospho (STY)]PC[Carbamidomethyl (C)]NK.2	0.162532963	Q8VEK0_S89_M1_Tmem30a
DLPEHAVLK.2	0.52068722	#N/A
SSGPTSLFAVTVAPPGAR.2	0.048640225	#N/A
DIDIHEVR.2	-0.249005613	#N/A
LNTLLQR.2	0.353932428	#N/A
EKPYFPIPEDC[Carbamidomethyl (C)]TFIQNVPLEDR.3	-0.315038683	#N/A
NFILDQTNVSAAAQR.2	-0.258965771	#N/A
LLEQYKEESKK.3	0.116983523	#N/A
AKS[Phospho (STY)]PQPPVEEEDEHFDDTVVC[Carbamidomethyl (C)]LDTYNC[Carbamidomethyl (C)]DLHFK.4	-0.332515913	#N/A
S[Phospho (STY)]PQPPVEEEDEHFDDTVVC[Carbamidomethyl (C)]LDTYNC[Carbamidomethyl (C)]DLHFK.4	0.233545332	#N/A
GYFEYIEENKYSR.2	-0.315029405	#N/A
[Acetyl (Protein N-term)]SSS[Phospho (STY)]PVNVKK.2	-0.194465468	Q8VEK3_S4_M1_Hnrnpu
FIEIAAR.2	0.349719971	#N/A
[Acetyl (Protein N-term)]SSS[Phospho (STY)]PVNVK.2	-0.317458877	Q8VEK3_S4_M1_Hnrnpu
NGQDLGVAFK.2	-0.311316484	#N/A
VSELKEELKK.3	-0.35053278	#N/A
PAMEPGNGS[Phospho (STY)]LDLGGDAAGR.2	0.033107479	Q8VEK3_S58_M1_Hnrnpu
LQAALDNEAGGRPAMEPGNGS[Phospho (STY)]LDLGGDAAGR.3	0.087769014	Q8VEK3_S58_M1_Hnrnpu
LSASSLTM[Oxidation (M)]ESFAFLWAGGR.2	-0.166867892	#N/A
GHAGGQRPEPSS[Phospho (STY)]PDGPAPPTRR.4	-0.678968602	Q8VEL9_S296_M1_Rem2
QAS[Phospho (STY)]PLGTPTPEADTTLLK.2	-0.426963282	Q8VEL9_S27_M1_Rem2
GHAGGQRPEPSS[Phospho (STY)]PDGPAPPTR.3	-0.339977713	Q8VEL9_S296_M1_Rem2
RGS[Phospho (STY)]M[Oxidation (M)]PVPYK.2	-0.090507059	Q8VEL9_S69_M1_Rem2
SRS[Phospho (STY)]C[Carbamidomethyl (C)]HDLSVL.2	-0.024497882	Q8VEL9_S334_M1_Rem2
RRGS[Phospho (STY)]M[Oxidation (M)]PVPYK.3	-0.569640866	Q8VEL9_S69_M1_Rem2
GS[Phospho (STY)]MPVPYK.2	-0.156427971	Q8VEL9_S69_M1_Rem2
IQTQPGYANTLR.2	0.049924333	#N/A
GSTASQVLQR.2	-0.287727279	#N/A
EEGLNAFYK.2	0.258718021	#N/A
FGFYEVFK.2	0.088530666	#N/A
GIFNGFSITLK.2	-0.03192042	#N/A
MYKEEGLNAFYK.2	0.444924777	#N/A
YYALC[Carbamidomethyl (C)]GFGGVLSC[Carbamidomethyl (C)]GLTHTAVVPLDLVK.3	-0.179579195	#N/A

YRS[Phospho (STY)]PYSGPK.2	-0.349676107	Q8VH51_S97_M1_Rbm39
DKS[Phospho (STY)]PVREPIDNLTPEER.3	-0.34144043	Q8VH51_S136_M1_Rbm39
S[Phospho (STY)]KS[Phospho (STY)]PFRK.2	-0.063339246	Q8VH51_S127_M2_Rbm39
DTTVISHS[Phospho (STY)]PNTSYDTALEAR.3	0.237229596	Q8VHG2_S693_M1_Amot
IEDLALQNQIR.2	0.248790791	#N/A
LSQSVHHGASSEGSLS[Phospho (STY)]PDTR.3	-0.058393861	Q8VHI6_S235_M1_Wasf3
SAAYNGPPAS[Phospho (STY)]PSHETGAFAHAR.3	0.544376456	Q8VHJ5_S633_M1_Mark1
RGT[Phospho (STY)]STGIISK.2	-0.187285665	Q8VHJ5_T648_M1_Mark1
SRPSS[Phospho (STY)]DLNNSTLQSPAHLK.3	-0.283479799	Q8VHJ5_S394_M1_Mark1
RDPS[Phospho (STY)]EGEASGR.2	-0.193449594	Q8VHJ5_S666_M1_Mark1
KPPEFEGGESLS[Phospho (STY)]SGSLC[Carbamidomethyl (C)]QR.3	-0.258545009	Q8VHJ5_S382_M1_Mark1
STAS[Phospho (STY)]PSNNVYSGGSMAR.2	0.243194553	Q8VHJ5_S488_M1_Mark1
RPQANS[Phospho (STY)]VESEQKEEWGK.3	-0.043654581	Q8VHJ5_S444_M1_Mark1
SIS[Phospho (STY)]ANQK.2	-0.452672257	Q8VHJ5_S414_M1_Mark1
SPS[Phospho (STY)]QES[Phospho (STY)]IGAR.2	-0.444573247	Q8VHK1_S696_M2_Caskin2
T[Phospho (STY)]SPSLTPTR.2	0.006056194	Q8VHK1_T840_M1_Caskin2
IAYVYPDQR.2	0.166593784	#N/A
VYVADSLISSAGEGLFSK.2	-0.275175445	#N/A
SGPEAPEWYQVELK.2	0.182700677	#N/A
AFQATQQK.2	-0.315788962	#N/A
AVEAEEELTVAYGYDHS[Phospho (STY)]PPGK.3	0.157316557	Q8VHL1_S340_M1_Setd7
RAS[Phospho (STY)]LSDR.2	-0.133184297	Q8VHQ3_S350_M1_Ppp1r16b
EQS[Phospho (STY)]PQTLLELKR.3	-0.468370922	Q8VHQ3_S477_M1_Ppp1r16b
[Acetyl (Protein N-term)]GTQGS[Phospho (STY)]PVKSYDYLLK.2	-0.04525422	Q8VHQ4_S6_M1_Rab40c
LQQQAALS[Phospho (STY)]PTTAPAVSSVSK.2	-0.458735541	Q8VHR5_S487_M1_Gatad2b
S[Phospho (STY)]LDPADERDDVLAKR.3	0.02161161	Q8VHR5_S17_M1_Gatad2b
GRLT[Phospho (STY)]PSPDIIVLS[Phospho (STY)]DNEASSPR.3	0.177437621	Q8VHR5_S130_M2_Gatad2b
VSS[Phospho (STY)]PLPS[Phospho (STY)]PSAM[Oxidation (M)]SDAANSQAAAK.2	-0.217875486	Q8VHR5_S335_M2_Gatad2b
LTPSPDIIVLS[Phospho (STY)]DNEASS[Phospho (STY)]PR.2	-0.126564904	Q8VHR5_S130_M2_Gatad2b
S[Phospho (STY)]LDPADERDDVLAK.2	0.051363486	Q8VHR5_S17_M1_Gatad2b
AGGGAGGS[Phospho (STY)]GGSGPSAILR.2	0.132220524	Q8VHW2_S251_M1_Cacng8
DAS[Phospho (STY)]PGGPGGPGFASTDISM[Oxidation (M)]YTLSR.3	-0.068601223	Q8VHW2_S290_M1_Cacng8
SSS[Phospho (STY)]RGS[Phospho (STY)]SEASPSR.2	-0.052232561	Q8VHW2_S277_M2_Cacng8
SGS[Phospho (STY)]PDVKGPPPVK.2	-0.167619151	Q8VI24_S20_M1_Satb2
ES[Phospho (STY)]SPPREEAPPPPPPTEDSC[Carbamidomethyl (C)]AK.3	-0.005668877	Q8VI24_S593_M1_Satb2
SES[Phospho (STY)]PC[Carbamidomethyl (C)]LRDS[Phospho (STY)]PDRR.2	-0.045290215	Q8VI24_S13_M2_Satb2
YAHQQPPS[Phospho (STY)]PLPVYSSSAK.3	-0.078677593	Q8VI36_S83_M1_Pxn
RGS[Phospho (STY)]LPDTGWK.2	-0.493658374	Q8VI56_S1887_M1_Lrp4
GS[Phospho (STY)]LPDTGWK.2	0.094112354	Q8VI56_S1887_M1_Lrp4
SQAPLDLSLSPDVSSEAS[Phospho (STY)]PAR.2	0.200882287	Q8VI59_S1980_M1_Pcnx3
SSVSS[Phospho (STY)]PANLNNSR.2	0.066585582	Q8VII8_S299_M1_Nr3c2
DAEYTYDQQNQQGS[Phospho (STY)]LSPAK.2	-0.294277029	Q8VII8_S127_M1_Nr3c2
YGEPGEVFINK.2	0.180240173	#N/A
LFVGNLPADITEDEFKR.3	0.037069859	#N/A
FATHAAALSVR.2	0.222385954	#N/A
FGQGGAGPVGGQGPR.2	0.154373636	#N/A
PVIVEPLEQLDDEDGLPEK.2	0.220471084	#N/A

LFVGNLPADITEDEFK.2	3.05E-05	#N/A
EPVETAEVNNDK.2	0.309588341	#N/A
EALS[Phospho (STY)]PEEVQSVQEHLGYHSDSLR.3	-0.020517093	Q8WTY4_S136_M1_Ciapin1
YGPLS[Phospho (STY)]EPGSAR.2	0.166434718	Q91V09_S70_M1_Wdr13
LVVHEGSPVTSISAR.3	-0.050483378	#N/A
LLWAGDDR.2	-0.244252743	#N/A
VLALSFDAPGR.2	0.252883077	#N/A
[Acetyl (Protein N-term)]AAVWQQVLAVDAR.2	0.31824569	#N/A
Y[Phospho (STY)]EAQKLER.2	-0.340471187	Q91V12_Y195_M1_Acot7
VLEVPPIVYLR.2	0.299799109	#N/A
LM[Oxidation (M)]DEVAGIVAAR.2	0.40696677	#N/A
SM[Oxidation (M)]EIEVLVDADPVVDNSQK.2	0.065966394	#N/A
AASAFFTYVSLNQEGK.2	-0.067000735	#N/A
M[Oxidation (M)]IEEAGAIISTR.2	0.261484192	#N/A
TNIVTASVDAINFHDK.2	-0.338995115	#N/A
M[Oxidation (M)]TFTSNK.2	-0.399002346	#N/A
EIQSITDESR.2	-0.159034027	#N/A
ENLWSSYLTK.2	-0.328397831	#N/A
LNEVIVNK.2	-0.063591494	#N/A
VDQDQNVVHPQLLSLTSQLK.3	-0.326986344	#N/A
LVLLNM[Oxidation (M)]PGPPR.2	-0.323963103	#N/A
GFC[Carbamidomethyl (C)]QVVISSNLR.2	-0.378677013	#N/A
NKGPS[Phospho (STY)]PVSSEGIK.2	0.108464854	Q91V14_S1044_M1_Slc12a5
DLTTFLYHLR.2	-0.075075858	#N/A
HGFDVC[Carbamidomethyl (C)]AK.2	0.254287528	#N/A
ESS[Phospho (STY)]PFINSTDTEK.2	-0.172797121	Q91V14_S49_M1_Slc12a5
EIQSITDES[Phospho (STY)]RGSIRR.3	0.10681302	Q91V14_S960_M1_Slc12a5
EREIQSITDESRGS[Phospho (STY)]IR.3	-0.21647041	Q91V14_S963_M1_Slc12a5
EIQSITDESRGS[Phospho (STY)]IR.2	-0.227126495	Q91V14_S963_M1_Slc12a5
ESSPFINS[Phospho (STY)]TDTEKGR.2	0.028511037	Q91V14_S54_M1_Slc12a5
GLTIVGSVLEGTFLDNHPQAQR.3	-0.121597717	#N/A
TLVMEQR.2	0.16979895	#N/A
ITAEVEVVEM[Oxidation (M)]HESDISAYTYEK.3	-0.314912411	#N/A
NVSMFPGNPER.2	0.180959211	#N/A
IFTVAQM[Oxidation (M)]DDNSIQM[Oxidation (M)]K.2	-0.224535351	#N/A
EDES[Phospho (STY)]EDESDILEESPC[Carbamidomethyl (C)]GR.2	0.29016601	Q91V36_S20_M1_Nrbp2
AKT[Phospho (STY)]PT[Phospho (STY)]PEPFDSETR.2	-0.25184244	Q91V36_T407_M2_Nrbp2
AKT[Phospho (STY)]PT[Phospho (STY)]PEPFDSETRK.3	-0.300323212	Q91V36_T407_M2_Nrbp2
NLTNPNTVIILIGNK.2	0.00011164	#N/A
IYQNIQDGSLDLNAAESGVQHKPSAPQGGR.4	-0.081632583	#N/A
STYNHLSSWLTDAR.3	-0.150643698	#N/A
QFAEENGLLFLEASAK.2	0.266835769	#N/A
FM[Oxidation (M)]ADC[Carbamidomethyl (C)]PHTIGVEFGTR.3	0.293123094	#N/A
TGENVEDAFLEAAKK.3	-0.349534712	#N/A
ELQVGIPVTDEAGQR.2	-0.267123494	#N/A
NLLLSGEQLEASR.2	-0.329434934	#N/A
NIVQNYR.2	-0.032127201	#N/A

HFFTVTDPR.2	-0.135892758	#N/A
[Acetyl (Protein N-term)]GDLPLNINIQEPR.2	0.322372485	#N/A
SSIHVTR.2	-0.223624012	#N/A
AGVATPGLTEDQLWR.2	0.141745977	#N/A
QGIFQVVISR.2	-0.233434779	#N/A
YVYDSAFHPDTGEK.2	-0.43094775	#N/A
AQIQAQNPSIDVVYYNK.2	-0.043130903	#N/A
SGDAPITVQQLGTAYVSATTGAVATALGLK.3	-0.120475362	#N/A
TAS[Phospho (STY)]FSESR.2	-0.265850909	Q91V92_S455_M1_Acly
TAS[Phospho (STY)]FSESRADEVAPAKK.3	-0.089122005	Q91V92_S455_M1_Acly
EAGVFVPR.2	0.153861481	#N/A
TIAIIAEGIPEALTR.2	0.115512613	#N/A
LNTEDIKR.2	-0.303774585	#N/A
DLVSSLTSGLLTIGDR.2	0.097871699	#N/A
DGVYILDLAAK.2	0.236399391	#N/A
TAS[Phospho (STY)]FSESRADEVAPAK.2	-0.41727351	Q91V92_S455_M1_Acly
LGLVGVNLSLDGVK.2	0.357953967	#N/A
SINNPDMR.2	-0.243880204	#N/A
SAYDSTM[Oxidation (M)]ETM[Oxidation (M)]NYAQIR.2	-0.192163385	#N/A
GPGGS[Phospho (STY)]PSGLQK.2	0.113463393	Q91VC7_S26_M1_Ppp1r14a
QPGFPQPSPSDDPS[Phospho (STY)]LSPRQDR.3	0.306008718	Q91VC7_S134_M1_Ppp1r14a
QPGFPQPSPSDDPSLS[Phospho (STY)]PR.2	-0.1315523	Q91VC7_S136_M1_Ppp1r14a
ARGPGGS[Phospho (STY)]PSGLQK.2	-0.162778903	Q91VC7_S26_M1_Ppp1r14a
GPGGS[Phospho (STY)]PSGLQKR.2	-0.345088037	Q91VC7_S26_M1_Ppp1r14a
VAVTPPGLAR.2	0.161279277	#N/A
FEAPLFNAR.2	0.164443334	#N/A
ILQDIASGR.2	-0.018865855	#N/A
FASEIAGVDDLGTTGR.2	-0.392144591	#N/A
GWNILTNSEK.2	0.010401757	#N/A
AVTEGAQAVEEPSIC[Carbamidomethyl (C)].2	0.324839967	#N/A
VALIGSPVDLTYR.2	-0.200597405	#N/A
SATYVNTEGR.2	-0.121108299	#N/A
GNDM[Oxidation (M)]QVGTYIEK.2	-0.293319274	#N/A
YDHLGDSPK.2	0.228932016	#N/A
ALSEIAGITLPYDTLDQVR.2	-0.063832402	#N/A
LSVAGNC[Carbamidomethyl (C)]R.2	0.162113891	#N/A
GLLTYTSWEDALSR.2	-0.084687799	#N/A
SWLHNDLK.2	0.474155893	#N/A
HSFC[Carbamidomethyl (C)]EVLK.2	0.312581138	#N/A
IASQVAALDLGYKPGVEAIR.3	-0.017696684	#N/A
M[Oxidation (M)]LFLLGADGGC[Carbamidomethyl (C)]ITR.2	0.08495755	#N/A
VTTGVAAEWK.2	0.315701887	#N/A
MC[Carbamidomethyl (C)]LVEIEK.2	0.260675494	#N/A
DFYM[Oxidation (M)]TDSISR.2	0.182138005	#N/A
LTEPMVR.2	0.217996323	#N/A
NRLEEVSPNLVR.3	0.10241701	#N/A
M[Oxidation (M)]HEDINEEWISDK.3	0.213335362	#N/A

VAGM[Oxidation (M)]LQNFEGNAVAAIAGGLVDAEALVALK.3	0.14499069	#N/A
SNYLLNTTIAGVEEADVVLLVGTNPR.3	-0.467066003	#N/A
[Acetyl (Protein N-term)]S[Phospho (STY)]LDIQC[Carbamidomethyl (C)]EQLSDAR.2	-0.115536463	Q91VI7_S2_M1_Rnh1
ELSLASNELKDEGAR.3	-0.222306793	#N/A
LLC[Carbamidomethyl (C)]EGLQDPQC[Carbamidomethyl (C)]R.2	0.237244989	#N/A
AAASGPASASAPAAEILLT[Phospho (STY)]PAR.2	-0.114164731	Q91VK4_T39_M1_Itm2c
YLLGNAPVS[Phospho (STY)]PSSQK.2	0.077772817	Q91VL8_S200_M1_Terf2ip
LST[Phospho (STY)]PKER.2	-0.371016694	Q91VL9_T203_M1_Zbtb1
DVYLS[Phospho (STY)]PR.2	0.114894916	Q91VM5_S205_M1_Rbmxl1
RDVYLS[Phospho (STY)]PR.2	0.128766485	Q91VM5_S205_M1_Rbmxl1
[Acetyl (Protein N-term)]VEADRPGK.2	0.050134386	#N/A
LFIGGLNTETNEK.2	0.186303055	#N/A
ST[Phospho (STY)]PSGPVR.2	-0.059574225	Q91VM5_T163_M1_Rbmxl1
GFAFVTFESPADAK.2	0.042026819	#N/A
VEQATKPS[Phospho (STY)]FESGR.2	-0.198256959	Q91VM5_S88_M1_Rbmxl1
DVYLS[Phospho (STY)]PRDDGYSTK.2	-0.394037981	Q91VM5_S205_M1_Rbmxl1
REPLPSR.2	0.365000703	#N/A
DSYESYGNSR.2	-0.17471992	#N/A
RST[Phospho (STY)]PSGPVR.2	-0.303516412	Q91VM5_T163_M1_Rbmxl1
DYSHS[Phospho (STY)]SSRDDYPSR.3	0.222777842	Q91VM5_S246_M1_Rbmxl1
DRDYSDHPSGGSYR.3	-0.192394553	#N/A
SDLYSSGR.2	0.273156962	#N/A
ILGTLALIDQSETDWK.2	-0.204713802	#N/A
AFALDVINSAHER.2	0.18059354	#N/A
LSES[Phospho (STY)]VVNR.2	-0.078349175	Q91VN4_S33_M1_Chchd6
KKAS[Phospho (STY)]LTHEQQQSAR.3	-0.416896731	Q91VN4_S164_M1_Chchd6
AYQHC[Carbamidomethyl (C)]VSTAR.2	0.180024296	#N/A
KAS[Phospho (STY)]LTHEQQQSAR.3	-0.244358692	Q91VN4_S164_M1_Chchd6
AS[Phospho (STY)]LTHEQQQSAR.2	-0.228123363	Q91VN4_S164_M1_Chchd6
LSSQQFHEAASK.2	-0.291516173	#N/A
HLIIGVSSDR.2	0.2187412	#N/A
ADIKAPEDK.2	-0.356230091	#N/A
NEVAALTAAGK.2	-0.107404622	#N/A
VYGTGSLALYEK.2	-0.024043051	#N/A
ELIEIISGAAALD.2	-0.096640881	#N/A
NASDM[Oxidation (M)]IDKLTLTFNR.3	-0.192875071	#N/A
EVMIVGVGEK.2	0.319872128	#N/A
THSDQFLVSFK.2	-0.071044089	#N/A
GLC[Carbamidomethyl (C)]GAIHSSVAK.2	0.376858458	#N/A
MTAMDNASK.2	0.163943551	#N/A
NASDMIDK.2	-0.393830935	#N/A
ALIVEPSR.2	0.434155319	#N/A
ELAEQTLNNVK.2	-0.426176387	#N/A
DQLSVLDNGVDIVVGTPGR.2	-0.003406471	#N/A
DNTRPGANS[Phospho (STY)]PEM[Oxidation (M)]WSEAIK.3	-0.268483744	Q91VR5_S481_M1_Ddx1
FLVLDEADGLLSQGYSDFINR.2	-0.093168446	#N/A
IPVIIER.2	0.123473669	#N/A

QLPVLDK.2	-0.260326022	#N/A
PS[Phospho (STY)]DRPFK.2	-0.155706406	Q91VR7_S3_M1_Map1lc3a
FLVPDHVNM[Oxidation (M)]SELVK.3	-0.098690641	#N/A
EIHQDWANR.2	-0.176795929	#N/A
EYIEIITSSIK.2	0.271800546	#N/A
RIEYIEAR.2	0.389935428	#N/A
ISDFLNSFDM[Oxidation (M)]SC[Carbamidomethyl (C)]R.2	0.0774942	#N/A
VTKGETLT[Phospho (STY)].2	-0.087986173	Q91VR8_T75_M1_Brk1
SRS[Phospho (STY)]WPGGPR.2	-0.356755483	Q91VU8_S36_M1_Ptov1
SQQSEVTR.2	0.179835307	#N/A
VYSTSVTGSR.2	-0.330641423	#N/A
IQYQLVDISQDNALRDEM[Oxidation (M)]R.3	0.251918832	#N/A
IQYQLVDISQDNALR.2	0.077614243	#N/A
TSS[Phospho (STY)]FTDQLDDVTPNR.2	-0.331007618	Q91VW5_S41_M1_Golga4
NS[Phospho (STY)]VEEWTTEDWTEDLSETK.2	0.013201324	Q91VX2_S254_M1_Ubap2
LLYEANLPENFR.2	0.215100829	#N/A
SS[Phospho (STY)]ADSLPGPISR.2	-0.197441791	Q91W39_S378_M1_Ncoa5
[Acetyl (Protein N-term)]M[Oxidation (M)]NTAPSRPS[Phospho (STY)]PTR.2	-0.263944405	Q91W39_S9_M1_Ncoa5
DRS[Phospho (STY)]PIRGS[Phospho (STY)]PR.2	0.042114372	Q91W39_S29_M2_Ncoa5
LLRSS[Phospho (STY)]ADS[Phospho (STY)]LPGPISR.3	-0.257061676	Q91W39_S378_M2_Ncoa5
EAFGFIER.2	-0.057873423	#N/A
ATNIEVLSNTFQFTNEAR.2	0.518336746	#N/A
LLGRNS[Phospho (STY)]NSK.2	0.126271134	Q91W50_S514_M1_Csde1
SPAAPGQS[Phospho (STY)]PTGSVC[Carbamidomethyl (C)]YER.2	0.097978085	Q91W50_S123_M1_Csde1
DM[Oxidation (M)]PGGFLFDGLS[Phospho (STY)]DDEDDFHPSTR.3	0.137648297	Q91W67_S230_M1_Ubl7
VDDS[Phospho (STY)]PSTSGGSSDGDQR.2	-0.459202301	Q91W82_S11_M1_Ube2e2
RSDS[Phospho (STY)]LLSFR.2	-0.465458794	Q91W92_S207_M1_Cdc42ep1
AS[Phospho (STY)]WGASR.2	0.17337236	Q91W92_S332_M1_Cdc42ep1
SDS[Phospho (STY)]LLSFR.2	-0.287675227	Q91W92_S207_M1_Cdc42ep1
RQS[Phospho (STY)]QQLPEEDC[Carbamidomethyl (C)]M[Oxidation (M)]QLNPSFK.3	-0.350886933	Q91WB2_S62_M1_Plpp7
S[Phospho (STY)]GTGATATVSPK.2	-0.029695904	Q91WC0_S12_M1_Setd3
LVILM[Oxidation (M)]EPFEDALR.2	0.315163131	#N/A
LPELSDLGQFFR.2	-0.091959106	#N/A
VGFFQGDIR.2	-0.024112831	#N/A
LVDAEELNYWTC[Carbamidomethyl (C)]K.2	0.113203102	#N/A
EALDSDGWLHTGDIGK.3	-0.35325501	#N/A
VLFGEITR.2	0.348063101	#N/A
IIEQC[Carbamidomethyl (C)]LNK.2	0.282309558	#N/A
TQPYDVYDQVEFDVPIGSR.2	0.134325894	#N/A
TYLQALPYFDR.2	0.004372531	#N/A
GSGIQWDLR.2	0.157983302	#N/A
APGFAHLAGLDK.2	0.013411578	#N/A
M[Oxidation (M)]FEFYER.2	-0.452108593	#N/A
TVDIGVVTAEDALNYGFSGVM[Oxidation (M)]LR.3	-0.426701072	#N/A
IDEVEEM[Oxidation (M)]LTNNR.2	0.017388609	#N/A
AVTNM[Oxidation (M)]TLNFGPQHPAAHGVLR.4	-0.012194608	#N/A
TSM[Oxidation (M)]ESLIHHFK.3	0.277386452	#N/A

LDYVSM[Oxidation (M)]M[Oxidation (M)]C[Carbamidomethyl (C)]NEQAYSIAVEK.2	0.031900509	#N/A
IYASSS[Phospho (STY)]PPDTGQR.2	0.24424046	Q91WG5_S196_M1_Prkag2
PSS[Phospho (STY)]PVS[Phospho (STY)]APVRPK.2	0.273775291	Q91WG5_S87_M2_Prkag2
SSS[Phospho (STY)]KESSPNSNPSTSPGGIR.2	-0.229210895	Q91WG5_S131_M1_Prkag2
[Acetyl (Protein N-term)]S[Phospho (STY)]EEQWVSLSSEEFDQLQK.2	-0.452227628	Q91WG7_S2_M1_Dgkg
EGASSSEPNVSDYNS[Phospho (STY)]DNAAK.2	0.143750991	Q91WG7_S107_M1_Dgkg
QET[Phospho (STY)]PDHPK.2	0.086504394	Q91WG7_T87_M1_Dgkg
DTGS[Phospho (STY)]PPASEEATGDSR.2	0.136193821	Q91WI7_S219_M1_Itfg2
SDKQYAENYTRPSS[Phospho (STY)]R.3	0.09459013	Q91WK0_S92_M1_Lrrfip2
DFS[Phospho (STY)]PEALKK.2	-0.573677948	Q91WK2_S183_M1_Eif3h
LFM[Oxidation (M)]AQALQEYNN.2	-0.333475013	#N/A
EKDFS[Phospho (STY)]PEALKK.3	0.255415846	Q91WK2_S183_M1_Eif3h
SC[Carbamidomethyl (C)]YEDGWLIK.2	0.224003737	#N/A
RLPVS[Phospho (STY)]PVAGS.2	0.026270765	Q91WM3_S470_M1_Rrp9
APWELLELR.2	0.012436565	#N/A
TSYYENVIK.2	0.11879156	#N/A
VVHAFDM[Oxidation (M)]EDLGDK.2	-0.120072387	#N/A
AM[Oxidation (M)]VNLQIQK.2	0.309155858	#N/A
HNEETGDNVGPLIIK.2	-0.598164105	#N/A
STPGVS[Phospho (STY)]DDSDQC[Carbamidomethyl (C)]DSTQDI.2	-0.216896943	Q91WZ8_S340_M1_Dtnbp1
S[Phospho (STY)]PSAAGPSGGGLR.2	0.083089897	Q91X56_S340_M1_S1pr5
S[Phospho (STY)]PSAAGPSGGGLRR.2	0.189687845	Q91X56_S340_M1_S1pr5
TLPSSSS[Phospho (STY)]PSR.2	0.013075472	Q91X58_S173_M1_Zfand2b
IYGNFFPYGDASK.2	-0.005671356	#N/A
AEM[Oxidation (M)]LEIVQAIYK.2	0.323352452	#N/A
LQEEVPS[Phospho (STY)]EEQM[Oxidation (M)]PQEK.2	-0.098989358	Q91XA2_S213_M1_Golm1
AC[Carbamidomethyl (C)]S[Phospho (STY)]PGASEITGLSK.2	-0.179202697	Q91XB0_S78_M1_Trex1
[Acetyl (Protein N-term)]SS[Phospho (STY)]PPEGKLETK.2	-0.221420576	Q91XC8_S3_M1_Dap
S[Phospho (STY)]SQIGAVVSR.2	-0.425245995	Q91XF0_S164_M1_Pnpo
FFTNYESR.2	-0.045423685	#N/A
GLATGDS[Phospho (STY)]PLGPM[Oxidation (M)]THHGEEDWVYER.4	0.169560238	Q91XF0_S241_M1_Pnpo
ITM[Oxidation (M)]PVIFNEPLSFLQR.2	0.045357842	#N/A
M[Oxidation (M)]QC[Carbamidomethyl (C)]VAAFAVSAVASQWER.2	-0.181648	#N/A
AIS[Phospho (STY)]LEGEPR.2	-0.45069369	Q91XM9_S414_M1_Dlg2
LC[Carbamidomethyl (C)]DKPAS[Phospho (STY)]PR.2	-0.161831462	Q91XM9_S360_M1_Dlg2
QPS[Phospho (STY)]VTLQR.2	-0.345115648	Q91XM9_S406_M1_Dlg2
GPELVHVS[Phospho (STY)]EK.2	-0.315894304	Q91XM9_S45_M1_Dlg2
GQEDLILSYEPVTR.2	-0.48042165	#N/A
LVIEEQSGPFIWIPSK.2	0.167976489	#N/A
AIS[Phospho (STY)]LEGEPRK.2	-0.43335899	Q91XM9_S414_M1_Dlg2
DYHFVISR.2	0.018980092	#N/A
YQDEDGPHDHS[Phospho (STY)]LPR.3	-0.444863348	Q91XM9_S28_M1_Dlg2
YGDILHVINASDDEWWQAR.3	-0.172554796	#N/A
TSLPPIS[Phospho (STY)]PGR.2	-0.053904222	Q91XM9_S323_M1_Dlg2
TSLPPIS[Phospho (STY)]PGRYS[Phospho (STY)]PIPK.2	-0.084967556	Q91XM9_S323_M2_Dlg2
HYS[Phospho (STY)]PVEC[Carbamidomethyl (C)]DK.2	-0.438215325	Q91XM9_S365_M1_Dlg2
NLSQIENVHGYVLQSHIS[Phospho (STY)]PLK.3	0.042083541	Q91XM9_S65_M1_Dlg2

FIEAGQYNDNLYGTSVQSVR.2	-0.111630221	#N/A
DSGLPSQGLSFK.2	0.033139215	#N/A
SKDS[Phospho (STY)]GLPSQGLSFK.2	-0.325104576	Q91XM9_S553_M1_Dlg2
TSS[Phospho (STY)]DPNLNNHSQEVR.2	-0.289429706	Q91XS1_S629_M1_Mtmr4
AKGPS[Phospho (STY)]PPGAK.2	-0.037331007	Q91XU0_S65_M1_Wrnip1
RPAAAAAAGSAS[Phospho (STY)]PR.2	-0.215937267	Q91XU0_S153_M1_Wrnip1
RLS[Phospho (STY)]ESSALK.2	-0.085356116	Q91XU0_S75_M1_Wrnip1
FAIDDHDYLVSLTR.3	0.286665628	#N/A
ENLPSHFK.2	0.360631745	#N/A
AEGAGTEEEGT[Phospho (STY)]PK.2	-0.029460089	Q91XV3_T36_M1_Basp1
ETPAASEAPSSAAK.2	-0.109012008	#N/A
AGEAS[Phospho (STY)]AES[Phospho (STY)]TGAADGAAPEEGEAK.2	-0.247935382	Q91XV3_S128_M2_Basp1
ESEPQAAADATEVK.2	0.365883254	#N/A
KTEAPAAAGPEAK.2	0.138409287	#N/A
APAPAAPAAAEPQAEAPAAAASS[Phospho (STY)]EQSVAVK.3	0.350081282	Q91XV3_S218_M1_Basp1
GYNVNDEK.2	-0.195767653	#N/A
TEAPAAAGPEAK.2	0.369918795	#N/A
DAADGEAKAEEK.2	0.178099188	#N/A
AAAAKEEAPK.2	-0.163328727	#N/A
SDAAPAASDSKPSSAEPAPSSK.3	0.033614338	#N/A
SDAAPAASDSKPSSAEPAPSSKET[Phospho (STY)]PAASEAPSSAAK.4	0.224737237	Q91XV3_T183_M1_Basp1
APAPAAPAAAEPQAEAPAAAASS[Phospho (STY)]EQSVAVKE.3	0.352443273	Q91XV3_S218_M1_Basp1
GYNVNDEKAK.2	-0.070042784	#N/A
ESTEEKPKDAADGEAK.2	-0.000373855	#N/A
KAEGAGTEEEGT[Phospho (STY)]PKESEPQAAADATEVK.4	-0.206642191	Q91XV3_T36_M1_Basp1
AEGAGTEEEGT[Phospho (STY)]PKESEPQAAADATEVK.3	-0.295726567	Q91XV3_T36_M1_Basp1
AEPEKS[Phospho (STY)]EGAAEEQPEPAPAPEQEAAAPGPAAGGEAPK.4	-0.151270708	Q91XV3_S92_M1_Basp1
DAADGEAKAEEKEADK.3	-0.056468231	#N/A
AGEASAESTGAADGAAPEEGEAKKTEAPAAAGPEAK.4	-0.587084684	#N/A
EADKAAAAKEEAPK.2	-0.143821291	#N/A
KAEGAGTEEEGT[Phospho (STY)]PK.2	0.198790365	Q91XV3_T36_M1_Basp1
AEEKEADKAAAAK.2	0.002688849	#N/A
ESEPQAAADATEVKESTEEKPK.3	0.13528785	#N/A
KKGYNVNDEK.2	0.207797286	#N/A
DKKAEGAGT[Phospho (STY)]EEEGTPK.2	0.038793533	Q91XV3_T31_M1_Basp1
EEAPKAEPEKS[Phospho (STY)]EGAAEEQPEPAPAPEQEAAAPGPAAGGEAPK.4	0.122050104	Q91XV3_S92_M1_Basp1
SEGAAEEQPEPAPAPEQEAAAPGPAAGGEAPK.3	-0.38686713	#N/A
TAGTSFM[Oxidation (M)]M[Oxidation (M)]T[Phospho (STY)]PY[Phospho (STY)]VVTR.2	-0.231623756	Q91Y86_T183_M2_Mapk8
GSQDS[Phospho (STY)]PENELGLDLR.2	-0.335445218	Q91YD6_S766_M1_Vill
VIY[Phospho (STY)]DFIEK.2	-0.102038835	Q91YD9_Y253_M1_Wasl
DYDS[Phospho (STY)]PERER.2	-0.087100216	Q91YE7_S59_M1_Rbm5
YDDYRDYDS[Phospho (STY)]PERER.3	0.25166842	Q91YE7_S59_M1_Rbm5
GLVAAYSGDS[Phospho (STY)]DNEEELVER.2	0.250614786	Q91YE7_S624_M1_Rbm5
AQS[Phospho (STY)]PTPSLPASWK.2	-0.443226435	Q91YE8_S895_M1_Synpo2
YFGTDAVPDGS[Phospho (STY)]DDDEAATVG.2	0.131785184	Q91YL3_S539_M1_Uckl1
NEEENIY[Phospho (STY)]SVPHDSTQGK.2	-0.228239748	Q91YM2_Y1105_M1_Arhgap35
TSFSVGS[Phospho (STY)]DDELGPIRK.2	-0.181925729	Q91YM2_S1179_M1_Arhgap35

NQKNS[Phospho (STY)]LSDLNIDR.2	-0.422579675	Q91YM2_S589_M1_Arhgap35
NIIEATHM[Oxidation (M)]YDNVAEAC[Carbamidomethyl (C)]STTEEVFNS[Phospho (STY)]PR.3	-0.490649017	Q91YM2_S970_M1_Arhgap35
TSFSVGS[Phospho (STY)]DDELGPIR.2	-0.010036203	Q91YM2_S1179_M1_Arhgap35
AQSNGSGNGS[Phospho (STY)]DSEMDTSSLER.2	0.080288715	Q91YM2_S1134_M1_Arhgap35
EAAS[Phospho (STY)]PLQAM[Oxidation (M)]SSYTAAGR.2	-0.156374715	Q91YP2_S32_M1_Nln
DLPS[Phospho (STY)]PLPTKR.2	-0.447290393	Q91YQ3_S48_M1_Csdc2
[Acetyl (Protein N-term)]TSESTLPPVVPPLHS[Phospho (STY)]PK.2	-0.234355457	Q91YQ3_S16_M1_Csdc2
DLPS[Phospho (STY)]PLPTK.2	-0.364807079	Q91YQ3_S48_M1_Csdc2
AS[Phospho (STY)]AGPVFK.2	0.169307569	Q91YQ3_S65_M1_Csdc2
VTAEVVLVHPGGGSTSR.2	0.316545791	#N/A
NIQVDSPYDISR.2	0.266613827	#N/A
SPLLEIVER.2	0.362089716	#N/A
YLLSQSS[Phospho (STY)]PAPLTAAEEELR.2	0.017528267	Q91YR1_S143_M1_Twf1
GPDWILGEM[Oxidation (M)]K.2	-0.295403967	#N/A
IFTNLYGR.2	0.346357408	#N/A
LVEGC[Carbamidomethyl (C)]LVGGR.2	0.409944122	#N/A
EAYEAGLIGK.2	-0.160864962	#N/A
NAC[Carbamidomethyl (C)]GSDYDFDVFVVR.2	-0.278779274	#N/A
YLVVNADEGEPGTC[Carbamidomethyl (C)]K.2	0.255802857	#N/A
GAGAYIC[Carbamidomethyl (C)]GEETALIESIEGK.2	-0.530564285	#N/A
HFRPELEDR.3	0.238371085	#N/A
GEFYNEASNLQVAIR.2	-0.293547504	#N/A
GDARPAEIDSLWEISK.3	0.094000924	#N/A
YLVVNADEGEPGTC[Carbamidomethyl (C)]KDR.2	-0.309607567	#N/A
KTSFGSLKDEDR.3	0.122764875	#N/A
EGVDWMNK.2	0.202835083	#N/A
HESC[Carbamidomethyl (C)]GQC[Carbamidomethyl (C)]TPC[Carbamidomethyl (C)]R.2	-0.119599179	#N/A
HAGGVTGGWDNLLAVIPGGSSTPLIPK.3	-0.24841427	#N/A
TSFGSLKDEDR.3	0.005823704	#N/A
QIEGHTIC[Carbamidomethyl (C)]ALGDGAAWPVQGLIR.3	-0.087411505	#N/A
DGLNDDDFEPYLS[Phospho (STY)]PQAR.2	0.082244745	Q91YT7_S39_M1_Ythdf2
DFGNSPLHR.2	-0.12713053	#N/A
LALDGQNIYNAC[Carbamidomethyl (C)]C[Carbamidomethyl (C)]TLR.2	0.269167502	#N/A
AAEAHPQDDHVEEEC[Carbamidomethyl (C)]FHDC[Carbamidomethyl (C)]SASFEEEQPGAHVAGSK.5	-0.542134014	#N/A
QDSSTGSYSINFVQNPNNNR.2	0.180852819	#N/A
LLDAAGANLR.2	-0.276650954	#N/A
VFVTGPLPAEGR.2	0.313008231	#N/A
NTM[Oxidation (M)]SLLAANNLLAGLR.2	-0.068562576	#N/A
VISTLSVGVDHLALDEIK.3	-0.007391326	#N/A
S[Phospho (STY)]PDSTANVR.2	-0.174421515	Q91Z67_S206_M1_Srgap2
ELEGPIYS[Phospho (STY)]R.2	-0.007726387	Q91Z67_S691_M1_Srgap2
QTPRS[Phospho (STY)]PDSTANVR.3	-0.095117101	Q91Z67_S206_M1_Srgap2
KQDS[Phospho (STY)]SQAIPLVVESC[Carbamidomethyl (C)]IR.2	0.242311286	Q91Z67_S500_M1_Srgap2
GLNNDS[Phospho (STY)]PERR.2	-0.050671696	Q91Z69_S883_M1_Srgap1
DMNS[Phospho (STY)]PTDR.2	-0.091213402	Q91Z69_S812_M1_Srgap1
IYTFNQSR.2	0.303499647	#N/A
AM[Oxidation (M)]S[Phospho (STY)]QDGASQFQEVILQELELSVK.3	-0.173947869	Q91ZJ5_S13_M1_Ugp2

NENTFLDLTVQQIEHLNK.3	-0.16083021	#N/A
ALANDGTS[Phospho (STY)]PGER.2	-0.289282115	Q91ZM2_S219_M1_Sh2b1
S[Phospho (STY)]SEDLAGPLPSSVPSSTTSSKPK.3	0.009198102	Q91ZM2_S126_M1_Sh2b1
DDFS[Phospho (STY)]DVEQLR.2	0.165801897	Q91ZP6_S195_M1_Ndfip2
TLS[Phospho (STY)]FDLQQR.2	-0.152573963	Q91ZP9_S271_M1_Necab2
VYEGGSNVDQFVTR.2	-0.070300298	#N/A
SES[Phospho (STY)]NSSITATQPTLAK.2	-0.24064764	Q91ZU6_S7186_M1_Dst
QKS[Phospho (STY)]FSEDVISHK.3	-0.007447776	Q91ZU6_S3894_M1_Dst
LLDPEDVDVSS[Phospho (STY)]PDEK.2	0.166995802	Q91ZU6_S237_M1_Dst
AM[Oxidation (M)]VESQQKS[Phospho (STY)]PVKR.3	-0.522464448	Q91ZU6_S1381_M1_Dst
AMVESQQKS[Phospho (STY)]PVK.2	0.314096141	Q91ZU6_S1381_M1_Dst
QLS[Phospho (STY)]LLDSR.2	0.261306285	Q91ZU6_S5407_M1_Dst
DNLGNSNVPDS[Phospho (STY)]PIPAEC[Carbamidomethyl (C)]EAAGR.2	0.185933828	Q91ZV0_S1271_M1_Mia2
SLS[Phospho (STY)]PPWEQDRR.2	-0.240804117	Q91ZV0_S1190_M1_Mia2
IDLETGENR.2	0.10623994	#N/A
AVTDEEPFLIFANR.2	0.340648637	#N/A
AEGSEYQVLYIADDNEIR.2	-0.532385045	#N/A
HS[Phospho (STY)]LAS[Phospho (STY)]TDEKR.2	-0.044174421	Q91ZX7_S4521_M2_Lrp1
IFFSDIHFGNIQQINDDGSGR.3	-0.22226237	#N/A
EGVVAAAEK.2	-0.188775349	#N/A
TKEQASHLGGAVFSGAGNIAAATGLVK.3	-0.421146006	#N/A
QGVTEAAEK.2	0.084797803	#N/A
TKQGVTEAAEK.2	0.206664942	#N/A
TKEGVLYVGSK.2	0.111199611	#N/A
EGVLYVGSK.2	0.086210993	#N/A
EQASHLGGAVFSGAGNIAAATGLVK.3	-0.387174145	#N/A
TSGVVQGVASVAEK.2	0.262009111	#N/A
KTDS[Phospho (STY)]C[Carbamidomethyl (C)]DSGITK.2	-0.022508104	Q920E3_S863_M1_Kcnh5
AGEARS[Phospho (STY)]PLEHS[Phospho (STY)]PSQADVK.3	0.081835468	Q920E3_S883_M2_Kcnh5
YEEDSYNR.2	-0.127772884	#N/A
TYQVPPVQPAS[Phospho (STY)]PGSHNALK.3	0.322780449	Q920I9_S1455_M1_Wdr7
GPITAVSFAPDGR.2	0.368022018	#N/A
ARDS[Phospho (STY)]PPPSSNIVQGQIK.3	-0.247028855	Q920I9_S935_M1_Wdr7
LPASC[Carbamidomethyl (C)]LPASDSFR.2	-0.083430942	#N/A
LAC[Carbamidomethyl (C)]THTGIQFYQFSVGNQQEGR.3	-0.35677395	#N/A
RQS[Phospho (STY)]REDSDPEYR.3	-0.268517496	Q920I9_S772_M1_Wdr7
RIS[Phospho (STY)]SLDSR.2	-0.483613688	Q920M7_S106_M1_Syt17
[Acetyl (Protein N-term)]M[Oxidation (M)]EANGS[Phospho (STY)]PGTSGSANDSQHDPGK.2	-0.125081144	Q920Q6_S6_M1_Msi2
NS[Phospho (STY)]PQSS[Phospho (STY)]PTSTPK.2	0.343641281	Q920Q8_S322_M2_Ivns1abp
QHKDS[Phospho (STY)]PEILSR.3	0.074083464	Q920R0_S1329_M1_Als2
QLGC[Carbamidomethyl (C)]ES[Phospho (STY)]PGQGEVWK.2	-0.077879533	Q920R0_S1302_M1_Als2
RQHKDS[Phospho (STY)]PEILSR.3	-0.072721413	Q920R0_S1329_M1_Als2
SSS[Phospho (STY)]LM[Oxidation (M)]DIREEELEGGSR.3	-0.022354675	Q920R0_S460_M1_Als2
RLS[Phospho (STY)]LPGLLSQVS[Phospho (STY)]PR.2	-0.277546133	Q920R0_S477_M2_Als2
S[Phospho (STY)]KDTTDELSVVEAK.2	0.041268455	Q921C1_S253_M1_Gjc3
KS[Phospho (STY)]SSDKESNLQVTQK.3	-0.171909137	Q921C3_S1888_M1_Brwd1
RGPEVEGS[Phospho (STY)]PVSEALR.3	-0.229981546	Q921C3_S1830_M1_Brwd1

QTS[Phospho (STY)]LDNEKDR.2	-0.069035884	Q921C5_S411_M1_Bicd2
S[Phospho (STY)]PVLLPK.2	0.391447148	Q921C5_S578_M1_Bicd2
LPNSKQS[Phospho (STY)]PDEPLR.2	-0.30189485	Q921F2_S183_M1_Tardbp
FGGNPGGFGNQGGFGNSR.2	-0.079120746	#N/A
GISVHISNAEPK.3	0.291562642	#N/A
TEEGEIVYS[Phospho (STY)]AEESENR.2	-0.001820049	Q921F4_S37_M1_Hnrnpll
LKTEEGEIVYS[Phospho (STY)]AEESENR.3	0.050445598	Q921F4_S37_M1_Hnrnpll
ITRPGNTDDPSGGNK.2	-0.392505859	#N/A
SYS[Phospho (STY)]LSELSVLQAK.2	-0.063955746	Q921I6_S245_M1_Sh3bp4
IILEAEKIDGAAS[Phospho (STY)]QGK.2	-0.085358644	Q921J2_S175_M1_Rheb
ALAESWNAAFLESSAK.2	-0.021598062	#N/A
INNVPAEGENEVNNELANR.2	-0.291266276	#N/A
DAEGILEDLQSYR.2	0.088073331	#N/A
EAIQHPADEKLQEK.3	-0.274675928	#N/A
RLS[Phospho (STY)]ADGSLGR.2	-0.021414249	Q921Q7_S446_M1_Rin1
EKPSTDPLY[Phospho (STY)]DTPDTR.2	0.109856672	Q921Q7_Y35_M1_Rin1
SM[Oxidation (M)]S[Phospho (STY)]SAFC[Carbamidomethyl (C)]SLLAPER.2	-0.197989979	Q921Q7_S340_M1_Rin1
ALPLS[Phospho (STY)]PAQELQR.2	0.150222126	Q921Q7_S590_M1_Rin1
NNAYLAQS[Phospho (STY)]PQLYK.2	-0.28869022	Q922B2_S249_M1_Dars1
FLEPTLR.2	-0.112433597	#N/A
IISAASEGGANVFTVSYFK.2	0.184151705	#N/A
C[Carbamidomethyl (C)]TAPLTPSSS[Phospho (STY)]PEQR.2	-0.064557077	Q922D4_S824_M1_Ppp6r3
IQQFDDGGS[Phospho (STY)]DEEDIWEEK.2	0.080239886	Q922D4_S588_M1_Ppp6r3
TPVPSDIAISR.2	0.199772603	#N/A
LDIDPETITWQR.2	-0.085969317	#N/A
TASESISNLSEAGS[Phospho (STY)]VK.2	-0.18572905	Q922J3_S203_M1_Clip1
TASESISNLS[Phospho (STY)]EAGS[Phospho (STY)]VKKGER.3	-0.193233095	Q922J3_S199_M2_Clip1
S[Phospho (STY)]VEELQLK.2	-0.125275589	Q922J3_S910_M1_Clip1
HVQSM[Oxidation (M)]YEEAYSDYLKDR.3	-0.162806712	#N/A
KSPSGPVKS[Phospho (STY)]PPLS[Phospho (STY)]PVGTTPVK.3	-0.222220569	Q922M7_S189_M2_
KLS[Phospho (STY)]LSEGK.2	-0.345580165	Q922P9_S130_M1_Glyr1
LQQLPADFGR.2	0.367826713	#N/A
LVNLQHLDLLNNR.2	-0.160557856	#N/A
GSFSEQGINEFLR.2	-0.204385946	#N/A
LAAVDATVNQVLASR.2	0.288392995	#N/A
VETVYTYLLDGESR.2	-0.217440702	#N/A
DIILATDLAHHLR.3	0.010174732	#N/A
RPEAVQNTS[Phospho (STY)]VDASEDQKDEK.3	0.009839412	Q922S4_S204_M1_Pde2a
GLPS[Phospho (STY)]NNSLDFLDEEYEVPDLDGTR.3	-0.160870285	Q922S4_S908_M1_Pde2a
LLHDGIQPVAAIDSNFANFTYTPR.3	-0.155469425	#N/A
SVLAALYSSEGSVM[Oxidation (M)]ER.2	0.024837688	#N/A
SQQYPAAR.2	0.005844397	#N/A
WDEQTSNTKGDDDEES[Phospho (STY)]DEEAVKK.3	0.045112881	Q922U1_S619_M1_Prpf3
GDDDEES[Phospho (STY)]DEEAVKK.2	-0.042088908	Q922U1_S619_M1_Prpf3
SS[Phospho (STY)]PPATDPGPVPSSPSQEPPTKR.3	-0.30779015	Q922Y1_S188_M1_Ubxn1
NQAIYAAVDDGDAS[Phospho (STY)]AE.2	-0.087240153	Q923B1_S548_M1_Dbr1
C[Carbamidomethyl (C)]GETVES[Phospho (STY)]GDEKDLAK.2	-0.119060349	Q923B1_S505_M1_Dbr1

RRDEDM[Oxidation (M)]LYS[Phospho (STY)]PELAQR.3	-0.077067118	Q923D5_S237_M1_Wbp11
SVS[Phospho (STY)]YHLSPEENSAPDALVR.3	-0.10490097	Q923M0_S432_M1_Ppp1r16a
RVS[Phospho (STY)]LTHR.2	-0.328965216	Q923M0_S353_M1_Ppp1r16a
KKGDDS[Phospho (STY)]DEEDLC[Carbamidomethyl (C)]ISNK.3	0.02371667	Q923Q2_S133_M1_Stard13
QET[Phospho (STY)]VEC[Carbamidomethyl (C)]LR.2	0.157073432	Q923T9_T287_M1_Camk2g
FTDDYQLFEELGK.2	-0.186389735	#N/A
FYFENLLSK.2	0.297723896	#N/A
GST[Phospho (STY)]ESC[Carbamidomethyl (C)]NTTTEDEDLK.2	0.365732346	Q923T9_T382_M1_Camk2g
SLLNKKS[Phospho (STY)]DGGVK.2	-0.628761762	Q923T9_S325_M1_Camk2g
ITEQLIEAINNGDFEAYTK.2	-0.385870192	#N/A
WLNVHYHC[Carbamidomethyl (C)]SGAPAAPLQ.3	-0.007190174	#N/A
KTS[Phospho (STY)]TQEYAAK.2	0.13993228	Q923T9_S36_M1_Camk2g
S[Phospho (STY)]SSSVHLM[Oxidation (M)]PQSNNK.2	-0.363139377	Q923T9_S334_M1_Camk2g
GSTES[Phospho (STY)]C[Carbamidomethyl (C)]NTTTEDEDLKVR.2	0.427222501	Q923T9_S384_M1_Camk2g
SAVGFSNGS[Phospho (STY)]LEQR.2	-0.024429148	Q923Z0_S355_M1_Gprc5b
KNS[Phospho (STY)]TDLDSAPEDPT[Phospho (STY)]SPK.2	-0.177869071	Q924A2_S2299_M2_Cic
NSTDLDSAPEDPTS[Phospho (STY)]PK.2	0.106884627	Q924A2_S2311_M1_Cic
TQS[Phospho (STY)]LSALPK.2	0.176820771	Q924A2_S1080_M1_Cic
S[Phospho (STY)]SSEAKPASLGLAGGHK.3	0.189800999	Q924A2_S1184_M1_Cic
SS[Phospho (STY)]PPPPLPAEERPGTK.2	0.096046854	Q924A2_S2148_M1_Cic
EGS[Phospho (STY)]TEFDWGDETSR.2	0.438364826	Q924A2_S553_M1_Cic
AQSVS[Phospho (STY)]PVQATPSGGSAQLLPGK.2	-0.049127073	Q924A2_S1809_M1_Cic
AAS[Phospho (STY)]EDMTS[Phospho (STY)]DEER.2	0.089421712	Q924A2_S1338_M2_Cic
QQSHDPVS[Phospho (STY)]PR.2	0.053671167	Q924H7_S511_M1_Wac
DAADPS[Phospho (STY)]PPNK.2	-0.622582642	Q924H7_S53_M1_Wac
QGPVSHSATQQPVTADKQQSHDPVS[Phospho (STY)]PR.4	-0.210518192	Q924H7_S511_M1_Wac
MRDAADPS[Phospho (STY)]PPNK.2	0.294196601	Q924H7_S53_M1_Wac
S[Phospho (STY)]NS[Phospho (STY)]PENKYSDSTGHNK.2	0.175110192	Q924H7_S62_M2_Wac
QGPVS[Phospho (STY)]HS[Phospho (STY)]ATQQPVT[Phospho (STY)]ADK.2	-0.197684396	Q924H7_S491_M3_Wac
HAELSGS[Phospho (STY)]PLK.2	-0.253064357	Q924K8_S516_M1_Mta3
S[Phospho (STY)]PPSLQTPATK.2	-0.00705099	Q924K8_S547_M1_Mta3
M[Oxidation (M)]PTQSDEEKS[Phospho (STY)]PS[Phospho (STY)]PTAEDPR.2	0.156755719	Q924K8_S425_M2_Mta3
ISQQVFDGNNMEDIYGK.2	-0.125585453	#N/A
IDDIPGLSDTS[Phospho (STY)]PDLSSR.2	0.085469518	Q924N4_S32_M1_Slc12a6
LTSIGS[Phospho (STY)]DEDEETETYQEK.2	-0.057396548	Q924N4_S1032_M1_Slc12a6
TISS[Phospho (STY)]PTSC[Carbamidomethyl (C)]EHR.2	-0.074607274	Q924S7_S168_M1_Spred2
HVS[Phospho (STY)]FQDEDEIVR.2	-0.063962313	Q924S8_S239_M1_Spred1
SVSS[Phospho (STY)]PGSGPAAESLGTR.2	-0.266024056	Q924W6_S767_M1_Trim66
GS[Phospho (STY)]SERGSPIEK.2	0.1700254	Q925H1_S1061_M1_Trps1
HPNYS[Phospho (STY)]PPGS[Phospho (STY)]PIEK.2	-0.030128349	Q925H1_S1081_M2_Trps1
TAGTLLGEGFR.2	0.169841502	#N/A
SQT[Phospho (STY)]PPGVATPPIPK.2	-0.015713827	Q925J9_T1051_M1_Med1
NSSQTGGKPGSS[Phospho (STY)]PITK.2	-0.224243256	Q925J9_S1158_M1_Med1
HDGGS[Phospho (STY)]PSIK.2	-0.247966764	Q925J9_S1403_M1_Med1
EAVQLLEDYK.2	0.362238152	#N/A
HALLETALTR.2	-0.127977955	#N/A
YGELEEGIDVLDADGNLVGSSK.2	-0.26428372	#N/A

HFLDIIDPR.2	0.020148617	#N/A
FQQTSFYGR.2	-0.182487906	#N/A
RQS[Phospho (STY)]IPEEFK.2	-0.164898627	Q925T6_S43_M1_Grip1
SLYSTS[Phospho (STY)]PR.2	0.032683278	Q925T6_S432_M1_Grip1
TSLS[Phospho (STY)]PVPKPR.2	-0.221231634	Q925T6_S846_M1_Grip1
QAS[Phospho (STY)]FQER.2	0.203523751	Q925T6_S952_M1_Grip1
QTDAQSASS[Phospho (STY)]PK.2	-0.18533278	Q925T6_S761_M1_Grip1
KQTDAQSASS[Phospho (STY)]PK.2	0.270300724	Q925T6_S761_M1_Grip1
SYEPQEDPGVK.2	-0.16334203	#N/A
WLHNEDQM[Oxidation (M)]AVEK.3	-0.077851113	#N/A
LSSTWEGIQAGK.2	0.009686909	#N/A
M[Oxidation (M)]ESALDQLK.2	-0.259747775	#N/A
[Acetyl (Protein N-term)]SGS[Phospho (STY)]PVKR.2	-0.31349817	Q93092_S4_M1_Taldo1
TIVM[Oxidation (M)]GAS[Phospho (STY)]FR.2	-0.466973796	Q93092_S237_M1_Taldo1
IFVGGLNPEATEEK.2	-0.056756704	#N/A
EVYQQQQYGSGGR.2	0.288572371	#N/A
EYFGQFGEIEAIELPIDPK.2	-0.018170666	#N/A
AIS[Phospho (STY)]PTSATSSGR.2	-0.108609935	Q99104_S600_M1_Myo5a
SAPEVTAPGAPAYR.2	0.291687578	#N/A
YQNLLNEFSR.2	-0.107184942	#N/A
M[Oxidation (M)]LPELFQDDEKAIS[Phospho (STY)]PTSATSSGR.3	-0.41613007	Q99104_S600_M1_Myo5a
AC[Carbamidomethyl (C)]GVLETIR.2	0.212229005	#N/A
QLELDLNDER.2	-0.330325685	#N/A
ETLEPLIQAAQLLQVK.2	0.047387982	#N/A
TSS[Phospho (STY)]IADEGTYTLDSILR.2	-0.082845391	Q99104_S1650_M1_Myo5a
VLNLYTPVNEFEER.2	-0.231734381	#N/A
SHENEAEALRGEIQSLKEENNR.4	-0.267531844	#N/A
LHIGMENK.2	-0.14132459	#N/A
YNVSQLEEWLR.2	-0.025542959	#N/A
YFATVSGSASEANVEEK.2	-0.246843016	#N/A
IGELEVGQMENIS[Phospho (STY)]PGQIIDEPIRPVNIPR.3	-0.210353223	Q99104_S1450_M1_Myo5a
VLMEQLTSVSEELDVR.2	-0.14607519	#N/A
SES[Phospho (STY)]GDEQFPTIC[Carbamidomethyl (C)]R.2	0.405473061	Q99246_S1833_M1_Cacna1d
QT[Phospho (STY)]VLSWQAAIDAAR.2	0.284585564	Q99246_T49_M1_Cacna1d
RQS[Phospho (STY)]S[Phospho (STY)]QDDVLPSPALPHR.3	0.103795233	Q99246_S1950_M2_Cacna1d
YS[Phospho (STY)]PSHSTR.2	0.272257834	Q99246_S1991_M1_Cacna1d
RIS[Phospho (STY)]ELNMK.2	-0.339872526	Q99246_S869_M1_Cacna1d
MNQQGADT[Phospho (STY)]PK.2	-0.075723025	Q99J08_T401_M1_Sec14l2
KDT[Phospho (STY)]PPPLVPPAAR.2	0.022316277	Q99J09_T5_M1_Wdr77
YEHDDIVSTVTVLSSGTQAVSGSK.3	-0.034600272	#N/A
AGATC[Carbamidomethyl (C)]VSQC[Carbamidomethyl (C)]DLS[Phospho (STY)]PVAE.2	-0.046930946	Q99J10_S416_M1_Ctu1
AGATC[Carbamidomethyl (C)]VSQC[Carbamidomethyl (C)]DLS[Phospho (STY)]PGNGEDR.2	-0.245517118	Q99J10_S378_M1_Ctu1
SELQAYIEQC[Carbamidomethyl (C)]QDS[Phospho (STY)]PTSGK.2	0.028612565	Q99J21_S547_M1_Mcoln1
[Acetyl (Protein N-term)]ATTAQQS[Phospho (STY)]PQPVAGKR.2	-0.340584382	Q99J36_S8_M1_Thumpd1
FIDKDQQPS[Phospho (STY)]GS[Phospho (STY)]EGEDDDAEAALKK.3	0.177101561	#N/A
[Acetyl (Protein N-term)]ATTAQQS[Phospho (STY)]PQPVAGK.2	-0.175943705	Q99J36_S8_M1_Thumpd1
DQQPS[Phospho (STY)]GS[Phospho (STY)]EGEDDDAEAALKK.3	-0.112585152	#N/A

FIDKDQQPS[Phospho (STY)]GS[Phospho (STY)]EGEDDDAEAALK.3	0.128862489	#N/A
[Acetyl (Protein N-term)]S[Phospho (STY)]EGESQTVVSSGSDPK.2	0.037793434	Q99J45_S2_M1_Nrbp1
TPT[Phospho (STY)]PEPAEVETR.2	0.345800793	Q99J45_T433_M1_Nrbp1
GSDHSASLEPGELAELVR.3	-0.431408275	#N/A
AELLLLQSTAEQLR.2	0.02724387	#N/A
FLC[Carbamidomethyl (C)]TPLAAAC[Carbamidomethyl (C)]PSGAEQGDAAGER.2	-0.018669296	#N/A
HIPGAAFFDIDR.2	-0.424981021	#N/A
ALVSAQWVAEALK.2	0.402809666	#N/A
AQPEHIISEGR.3	-0.144175451	#N/A
FQGTQPEPR.2	0.309881686	#N/A
THEDILENLDAR.3	0.114151726	#N/A
HWLNQNLPISSGK.2	0.292155711	#N/A
DGIEPGHIPGSVNIPFTEFLTNEGLEK.3	0.324232448	#N/A
AFGHHSVSLLDGGFR.3	0.085718322	#N/A
ILEPGLNVLIPVLDR.2	-0.167706439	#N/A
NTVILFVPQQEAWVVER.2	0.202838546	#N/A
APVPGAQNSSQSR.2	-0.010472486	#N/A
DLQQSIEAAS[Phospho (STY)]DEEDLR.2	-0.222619519	Q99JB8_S276_M1_Pacsin3
DGTAPPPQSPSSPGSGQDEDWS[Phospho (STY)]DEESPR.3	-0.030623289	Q99JB8_S354_M1_Pacsin3
GGRS[Phospho (STY)]PDEVTLTSIVPTR.3	-0.111044958	Q99JB8_S319_M1_Pacsin3
ALYDYAGQEADELS[Phospho (STY)]FR.2	-0.153431104	Q99JB8_S383_M1_Pacsin3
QSNASS[Phospho (STY)]DVEVEEK.2	-0.052558146	Q99JF8_S106_M1_Psip1
NLAKPGVTSTS[Phospho (STY)]DS[Phospho (STY)]EDEDDQEGEKK.3	-0.059124559	Q99JF8_S272_M2_Psip1
FSSQQASTK.2	-0.064541742	#N/A
QVDTEEAGM[Oxidation (M)]VTAATASNVKAS[Phospho (STY)]PK.3	0.268268959	#N/A
NLAKPGVTSTS[Phospho (STY)]DS[Phospho (STY)]EDEDDQEGEK.3	0.208499093	Q99JF8_S272_M2_Psip1
QSNASS[Phospho (STY)]DVEVEEKETNVSK.2	-0.061094983	Q99JF8_S106_M1_Psip1
PGVTSTS[Phospho (STY)]DS[Phospho (STY)]EDEDDQEGEK.2	0.060215243	Q99JF8_S272_M2_Psip1
QVDTEEAGM[Oxidation (M)]VTAATASNVKAS[Phospho (STY)]PKR.3	-0.52513609	#N/A
ETNVSKEDT[Phospho (STY)]DQEEK.2	0.117708716	Q99JF8_T122_M1_Psip1
EDTDQEEKAS[Phospho (STY)]NEDVTK.2	-0.011213059	Q99JF8_S129_M1_Psip1
NFQAAHR.2	0.045649927	#N/A
ETNVSKEDT[Phospho (STY)]DQEEKASNEDVTK.3	0.020219817	Q99JF8_T122_M1_Psip1
QSNASS[Phospho (STY)]DVEVEEKETNVSKEDTDQEEK.4	0.031034466	Q99JF8_S106_M1_Psip1
LLGDVSC[Carbamidomethyl (C)]R.2	-0.060972529	#N/A
ES[Phospho (STY)]PPLLPLGTPC[Carbamidomethyl (C)].2	0.102099978	Q99JG2_S471_M1_Gpr37l1
PRES[Phospho (STY)]PPLLPLGTPC[Carbamidomethyl (C)].2	0.123864113	Q99JG2_S471_M1_Gpr37l1
SLLHEQPRGT[Phospho (STY)]PNR.2	-0.10422603	Q99JN2_T605_M1_Klhl22
EDVEGVGTS[Phospho (STY)]DGEGAAGLSSDPK.2	-0.113695688	Q99JP4_S42_M1_Cdc26
QKEDVEGVGTS[Phospho (STY)]DGEGAAGLSSDPK.3	-0.358199059	Q99JP4_S42_M1_Cdc26
SQS[Phospho (STY)]ADTPGPTER.2	-0.175865666	Q99JP6_S159_M1_Homer3
LPSSSSEM[Oxidation (M)]GSQDGS[Phospho (STY)]PLRETR.3	-0.326486395	Q99JP7_S83_M1_Ggt7
LPSSSSEM[Oxidation (M)]GSQDGS[Phospho (STY)]PLR.2	0.193379515	Q99JP7_S83_M1_Ggt7
KDEDAFLGDPDT[Phospho (STY)]DPDSFLK.3	-0.373906649	Q99JP7_T56_M1_Ggt7
NILLTNEQLENAR.2	-0.01489789	#N/A
QAITQVVISR.2	-0.065077447	#N/A
ASHFFTVTDPR.3	-0.285017859	#N/A

ADASSLTVDVTS[Phospho (STY)]PASK.2	-0.109737545	Q99JX3_S418_M1_Gorasp2
LYVYNTDTDNC[Carbamidomethyl (C)]R.2	0.315423691	#N/A
[Acetyl (Protein N-term)]SVPAFIDISEEDQAAELR.2	-0.470086188	#N/A
AALSGLLHR.2	0.340851388	#N/A
DFIYVSQDPK.2	0.004160546	#N/A
NIVVVEGVR.2	0.345410886	#N/A
DVVDYIIFGTVIQEVK.2	-0.40321859	#N/A
EILSPVDIIDR.2	0.194344247	#N/A
NGGS[Phospho (STY)]PALNNNPR.2	-0.156718049	Q99JY8_S19_M1_Plpp3
DYEEIGPSIC[Carbamidomethyl (C)]R.2	-0.271589585	#N/A
AEPEDHYFLLTEPPLNTPENR.3	-0.397224209	#N/A
GVDDLDFFIGDEAIEKPTYATK.3	-0.068843899	#N/A
YSYVC[Carbamidomethyl (C)]PDLVK.2	0.170758755	#N/A
QYTGVNAISK.2	0.26277101	#N/A
DITYFIQQLLR.2	-0.228177942	#N/A
LPAC[Carbamidomethyl (C)]VVDC[Carbamidomethyl (C)]GTGYTK.2	0.400459002	#N/A
HNPVFGVM[Oxidation (M)]S[Phospho (STY)].2	0.103338322	Q99JY9_S418_M1_Actr3
TLTGTVIDSGDGVTHVIPVAEGYVIGSC[Carbamidomethyl (C)]IK.3	-0.181622311	#N/A
VSNQS[Phospho (STY)]FTEIER.2	0.215407694	Q99K28_S239_M1_Arfgap2
AIS[Phospho (STY)]SDM[Oxidation (M)]FFGR.2	-0.256028137	Q99K28_S431_M1_Arfgap2
HGTDLWIDSM[Oxidation (M)]NSAPSHS[Phospho (STY)]PEKK.4	-0.236656521	Q99K28_S145_M1_Arfgap2
[Acetyl (Protein N-term)]AAS[Phospho (STY)]PSKTEIQTIFK.2	-0.153664015	Q99K28_S4_M1_Arfgap2
LAYQELQIDR.2	-0.228929985	#N/A
YWGPAS[Phospho (STY)]PTHK.2	0.040704492	Q99K30_S573_M1_Eps8l2
RAEDSYTSQHTS[Phospho (STY)]PESEGAPHL.3	-0.499856014	Q99K30_S720_M1_Eps8l2
RRES[Phospho (STY)]QDEEPR.2	-0.070682234	Q99K30_S242_M1_Eps8l2
QSQTSSSDTPAS[Phospho (STY)]PVSSSTPNSDIM[Oxidation (M)]DIN.3	0.204758057	Q99K46_S906_M1_Usp11
AVVIVDDR.2	-0.198720682	#N/A
AGEVFIHK.2	-0.21492489	#N/A
VELDNMPLR.2	0.289406186	#N/A
FAC[Carbamidomethyl (C)]HSASLTVR.3	-0.267346046	#N/A
SC[Carbamidomethyl (C)]SHQTS[Phospho (STY)]APSLK.2	-0.058933724	Q99K70_S381_M1_Rragc
[Acetyl (Protein N-term)]SLQYGAEETPLAGS[Phospho (STY)]YGAADSFPK.3	0.442623769	Q99K70_S15_M1_Rragc
MSPNETLFLEST[Phospho (STY)]NK.2	0.015560806	Q99K70_T95_M1_Rragc
LLS[Phospho (STY)]SSDDDANILSSPTDR.2	-0.026791319	Q99K74_S860_M1_Med24
ELLAVPNNYK.2	-0.243959721	#N/A
ASLYNAVTTEDVEK.2	-0.161679308	#N/A
QVVNFGPGPAK.2	0.548655703	#N/A
EC[Carbamidomethyl (C)]PSVLDYK.2	0.360241153	#N/A
DDLLGFSLR.2	0.076808146	#N/A
LPHSVLLEIQK.2	-0.101469384	#N/A
SQM[Oxidation (M)]IYEIIDNSQGFYVC[Carbamidomethyl (C)]PVER.3	-0.311689262	#N/A
SADYVVTGAWSAK.2	0.097188689	#N/A
ALLEVLGR.2	0.248147252	#N/A
HVEPGNAAIQEK.2	0.181634525	#N/A
TVQQHAGETDPVTTM[Oxidation (M)]R.2	0.225371857	#N/A
VTHLSTLQVGSLSVK.3	0.305684354	#N/A

GLVAAYSGES[Phospho (STY)]DS[Phospho (STY)]EEEQER.2	0.322698502	Q99KG3_S736_M2_Rbm10
LASDDRPS[Phospho (STY)]PPR.2	0.213381403	Q99KG3_S723_M1_Rbm10
[Acetyl (Protein N-term)]AHS[Phospho (STY)]PVQSGLPGMQNLK.2	-0.174216682	Q99KH8_S4_M1_Stk24
SQFTITPGSEQIR.2	0.13659817	#N/A
NAVTQEFGPVPDTAR.2	-0.172180554	#N/A
VDVSPTSQR.2	-0.066800792	#N/A
SDFDPGQDTYQHPPK.3	0.050369468	#N/A
NTIVTSYNR.2	-0.03145044	#N/A
FNPETDFLTGK.2	-0.145586167	#N/A
EHAALEPR.2	-0.051283953	#N/A
IVYGHLDDPANQEIER.3	-0.385310315	#N/A
DFAPGKPLK.2	0.302497619	#N/A
FKLEAPDADELPR.2	-0.251932079	#N/A
LEAPDADELPR.2	0.127311901	#N/A
LTGSLSGWTSPK.2	0.104836676	#N/A
EGWPLDIR.2	0.143087592	#N/A
IHETNLK.2	0.203116593	#N/A
DVGGIVLANAC[Carbamidomethyl (C)]GPC[Carbamidomethyl (C)]IGQWDR.2	0.463722817	#N/A
QGLLPLTFADPSDYNK.2	-0.15103748	#N/A
DGYAQILR.2	-0.002240033	#N/A
QALAHGLK.2	0.184047327	#N/A
DVGGIVLANAC[Carbamidomethyl (C)]GPC[Carbamidomethyl (C)]IGQWDRK.3	0.048109682	#N/A
TDIANLAEEFK.2	0.104210576	#N/A
NDANPETHAFVTSPEIVTALAIAGTLK.3	0.190863273	#N/A
WVVIGDENYGEGSSR.2	-0.439834215	#N/A
VAGILTVK.2	-0.238192939	#N/A
AKDINQEVYNFLATAGAK.3	-0.150745847	#N/A
GHLDNISNNLLIGAINIENGK.3	-0.569642989	#N/A
C[Carbamidomethyl (C)]TTDHISAAGPWLK.2	0.14525	#N/A
VAM[Oxidation (M)]SHFEPSEYIR.3	-0.301699695	#N/A
VGLIGSC[Carbamidomethyl (C)]TNSSYEDM[Oxidation (M)]GR.2	0.034630086	#N/A
VAM[Oxidation (M)]QDATAQM[Oxidation (M)]AM[Oxidation (M)]LQFISSGLPK.3	-0.278243463	#N/A
SDFDPGQDTYQHPPKDSSGQR.4	-0.010769401	#N/A
DINQEVYNFLATAGAK.2	-0.035247305	#N/A
VAVPSTIHC[Carbamidomethyl (C)]DHLIEAQVGGEK.3	-0.018702899	#N/A
VAVPSTIHC[Carbamidomethyl (C)]DHLIEAQVGGEKDLR.4	0.070660306	#N/A
HPNGTQETILLNHTFNETQIEWFR.4	-0.406153453	#N/A
AKS[Phospho (STY)]VEVDNK.2	-0.182288942	Q99KJ0_S154_M1_Ubxn2a
C[Carbamidomethyl (C)]LS[Phospho (STY)]PTEQKK.2	0.463413149	Q99KJ0_S54_M1_Ubxn2a
YADLPGIAR.2	0.103117301	#N/A
WSPVASTLPELVQR.2	0.25036219	#N/A
LLGPDAAINLADPDGALAK.2	-0.15786943	#N/A
QQLVASHLEK.2	0.287970798	#N/A
ASVEDADTQNK.2	0.183274805	#N/A
VSALDLAVLDQVEAR.2	-0.1610889	#N/A
LLHEVQELTTEVEK.3	0.218909972	#N/A
ENLATVEGNFASIDAR.2	-0.177881256	#N/A

LTELEATVR.2	0.053826516	#N/A
DNTALLTQVQTTM[Oxidation (M)]R.2	0.157302066	#N/A
VHQLYETIQR.2	0.620280098	#N/A
GLDFSDR.2	-0.438991089	#N/A
TGYES[Phospho (STY)]GDYEM[Oxidation (M)]LGEGLGVK.2	-0.002687891	Q99KJ8_S83_M1_Dctn2
DLS[Phospho (STY)]PQQMVVR.2	0.274932737	Q99KK9_S66_M1_Hars2
TIDLGAAAHYTGDKAS[Phospho (STY)]PDQNASTHTPQSSAK.4	0.039561159	Q99KN9_S307_M1_Clint1
[Acetyl (Protein N-term)]ASPAAGGVVIVGSGLIGR.2	-0.297892292	#N/A
SYS[Phospho (STY)]FDEVRK.2	-0.495652724	Q99KQ4_S472_M1_Nampt
GLGSGDDLDSSS[Phospho (STY)]LNSSR.2	-0.077810979	Q99KR6_S163_M1_Rnf34
AS[Phospho (STY)]LSDLSSLEEVEGMSVR.2	0.194545381	Q99KR6_S258_M1_Rnf34
DQHNGS[Phospho (STY)]LTDPSSVHEK.3	0.087310116	Q99KU0_S23_M1_Vmp1
LAIQGPEDS[Phospho (STY)]PSR.2	-0.37678068	Q99KX1_S237_M1_Mlf2
DSDS[Phospho (STY)]GLEQMSIGHHIR.3	0.138774294	Q99KX1_S145_M1_Mlf2
EPETGLDNTS[Phospho (STY)]PK.2	0.010867193	Q99KY4_S809_M1_Gak
YFDNKDDDS[Phospho (STY)]DPETANDLPK.3	-0.124876864	Q99KZ6_S60_M1_Znf639
DLFSLDSEGPSPTS[Phospho (STY)]PPLR.2	-0.17211991	Q99L02_S236_M1_Pagr1a
VLPSC[Carbamidomethyl (C)]DLAR.2	0.255424267	#N/A
ATAQEAQSLGGR.2	0.066894527	#N/A
DLGLAQDSATSTK.2	0.25658851	#N/A
TPILLGSLAHQIYR.2	0.237475913	#N/A
VTQQVNPIFSNS[Phospho (STY)]Q.2	-0.384932125	Q99L27_S347_M1_Gmpr2
AETAPLPTSVDDT[Phospho (STY)]PEVLNR.2	0.167408135	Q99L43_T50_M1_Cds2
LDGETAS[Phospho (STY)]DSESR.2	-0.040033511	Q99L43_S32_M1_Cds2
ESESEAKLDGETAS[Phospho (STY)]DSESR.2	0.206653696	Q99L43_S32_M1_Cds2
EDAPPEDKES[Phospho (STY)]ESEAK.2	0.216355481	Q99L43_S20_M1_Cds2
ALS[Phospho (STY)]NLSSR.2	0.038854808	Q99L43_S59_M1_Cds2
EDAPPEDKESESEAKLDGETAS[Phospho (STY)]DSESR.4	-0.066170368	Q99L43_S32_M1_Cds2
DYTYEELLNR.2	-0.171487883	#N/A
[Acetyl (Protein N-term)]S[Phospho (STY)]GDEM[Oxidation (M)]IFDPTM[Oxidation (M)]SKK.2	-0.481982809	#N/A
[Acetyl (Protein N-term)]S[Phospho (STY)]GDEM[Oxidation (M)]IFDPTM[Oxidation (M)]SK.2	-0.512603493	#N/A
KKDAS[Phospho (STY)]DDLDDLNFFNQK.3	-0.214775332	Q99L45_S67_M1_Eif2s2
IFDIDEAEEAIKDVK.3	0.25323665	#N/A
EYVTC[Carbamidomethyl (C)]HTC[Carbamidomethyl (C)]R.2	0.04598138	#N/A
IES[Phospho (STY)]DAQEPAEPEDDLDIMLGNK.3	-0.05649452	Q99L45_S105_M1_Eif2s2
AIEINPDSAQPYK.2	0.117033202	#N/A
GAAIEALNDGELQK.2	0.006349536	#N/A
AIDLFTDAIK.2	0.274196809	#N/A
KGAAIEALNDGELQK.3	0.293046332	#N/A
QDPSVLHTEEMR.3	0.324842517	#N/A
GSPVSEIGWETPPPES[Phospho (STY)]PR.2	0.066195005	Q99L88_S218_M1_Sntb1
KGSPVSEIGWETPPPES[Phospho (STY)]PR.3	0.122167147	Q99L88_S218_M1_Sntb1
GDQVLNFS[Phospho (STY)]DAEDLIDDSK.2	-0.017316852	Q99L90_S282_M1_Mcrs1
GDQVLNFS[Phospho (STY)]DAEDLIDDSKLK.3	-0.126974093	Q99L90_S282_M1_Mcrs1
QAEEESHRGS[Phospho (STY)]PIPK.2	-0.289866079	Q99LB0_S160_M1_Dnttip1
VLITGATGLLGR.2	0.005942103	#N/A
AVLENNLGAAVLR.2	0.218994907	#N/A

LQSWLYASR.2	0.116933194	#N/A
QDDWTFHYLR.2	0.01247645	#N/A
VITVDGNIC[Carbamidomethyl (C)]SGK.2	0.031114491	#N/A
VTSAYLQDIENAYKK.3	-0.066968148	#N/A
LLQYADALEHLLSTGQGVVLER.3	-0.033172357	#N/A
VTSAYLQDIENAYK.2	0.016877395	#N/A
TEVLNYTTIPVYLPEITIGAHQGSR.3	-0.198806093	#N/A
YAPGYNAEVGDK.2	-0.292765039	#N/A
LNVAPVSDIIEIK.2	0.287775763	#N/A
GLLPEELTPLILETQK.2	0.530466858	#N/A
TIYAGNALC[Carbamidomethyl (C)]TVK.2	0.431080083	#N/A
VLVAQHDAYK.2	0.425460272	#N/A
APSSSSVGISEWLDQK.2	0.099411593	#N/A
AAVDAGFVPNDM[Oxidation (M)]QVGQTGK.2	0.013509302	#N/A
SPDTFVR.2	0.124635552	#N/A
VVPEMTEILK.2	0.268438951	#N/A
SDRPELTGAK.2	-0.033498579	#N/A
GTSFEAAATSGGSASSEK.2	-0.146224366	#N/A
LLYDLADQLHAAVGASR.2	0.200526837	#N/A
SPPREGS[Phospho (STY)]QGELTPANSQSR.3	-0.513895479	Q99LD4_S454_M1_Gps1
EGS[Phospho (STY)]QGELTPANSQSR.2	-0.027977375	Q99LD4_S454_M1_Gps1
LFLELEPQVR.2	-0.269645945	#N/A
GERDS[Phospho (STY)]QTQAILTK.2	0.142522613	Q99LD4_S220_M1_Gps1
NLDFQDVLDK.2	-0.108660206	#N/A
MLQADPNKVS[Phospho (STY)]PR.2	-0.253271392	Q99LF4_S208_M1_Rtcb
AETETS[Phospho (STY)]PGHAPDR.2	0.144046598	Q99LG1_S178_M1_Tmem51
TYSEC[Carbamidomethyl (C)]EDGTYS[Phospho (STY)]PEISWHHGK.3	0.385916477	Q99LH2_S425_M1_Ptdss1
TVAS[Phospho (STY)]DLQK.2	-0.124376927	Q99LH9_S342_M1_Sh3bp5l
RGS[Phospho (STY)]DIGVR.2	-0.423028428	Q99LH9_S377_M1_Sh3bp5l
GLSDHAS[Phospho (STY)]LDGQELGAQSR.2	-0.490674738	Q99LH9_S361_M1_Sh3bp5l
TVAS[Phospho (STY)]DLQKC[Carbamidomethyl (C)]DS[Phospho (STY)]VEHLR.3	0.08741332	Q99LH9_S342_M2_Sh3bp5l
FHSGNKS[Phospho (STY)]PEVLR.3	0.260169193	Q99LI2_S433_M1_Clcc1
KEVSPDS[Phospho (STY)]SEAEELSDC[Carbamidomethyl (C)]LHR.3	0.026845811	Q99LI2_S58_M1_Clcc1
EVSPDS[Phospho (STY)]SEAEELSDC[Carbamidomethyl (C)]LHR.3	-0.506331353	Q99LI2_S58_M1_Clcc1
RPNEDS[Phospho (STY)]DEDEEKGAVVPPVHDIYR.4	0.213962465	Q99LI7_S691_M1_Cstf3
ALQNAVSTFVNR.2	0.356107087	#N/A
ASSTTELPPEYLTSPLSQQS[Phospho (STY)]QLPPK.3	-0.215741099	Q99LI8_S246_M1_Hgs
FQSQADQDQQASGLQS[Phospho (STY)]PPSR.2	0.222228692	Q99LJ0_S481_M1_Cttnbp2nl
DLS[Phospho (STY)]PTLLDNSAAK.2	-0.166124989	Q99LJ0_S488_M1_Cttnbp2nl
GLSVSGPC[Carbamidomethyl (C)]GSRS[Phospho (STY)]PQQTPMES.2	-0.168491299	Q99LL5_S493_M1_Pwp1
GKLQEEGGS[Phospho (STY)]EEEEAGNPSEDGM[Oxidation (M)]QSGPTQAPPR.3	0.114245696	Q99LL5_S49_M1_Pwp1
DLLEVADILEK.2	-0.196831531	#N/A
VTVAGLAGKDPVQC[Carbamidomethyl (C)]SR.3	0.25994398	#N/A
VTTHPLAK.2	0.170699706	#N/A
DVMIC[Carbamidomethyl (C)]PDTSLEDAK.2	0.384322485	#N/A
VEKDGLILTSR.2	-0.376412042	#N/A
TQGPYDVVVLPGGNLGAQNLSESPM[Oxidation (M)]VK.3	0.116577735	#N/A

GAEEM[Oxidation (M)]ETVIPVDVMR.2	0.371174745	#N/A
PFLDIQK.2	0.063460618	#N/A
IEFDDFEEC[Carbamidomethyl (C)]LLR.2	0.129659987	#N/A
EC[Carbamidomethyl (C)]KIEFDDFEEC[Carbamidomethyl (C)]LLR.3	-0.030931071	#N/A
HFM[Oxidation (M)]FLSAEQPYK.2	-0.163517176	#N/A
EWIEC[Carbamidomethyl (C)]AHGIGGTR.3	0.22245737	#N/A
RGS[Phospho (STY)]FEAGR.2	-0.217547755	Q99M08_S19_M1_
RGS[Phospho (STY)]FEAGRR.2	0.008680521	Q99M08_S19_M1_
S[Phospho (STY)]PS[Phospho (STY)]PKPTK.2	0.015517152	Q99M28_S155_M2_Rnps1
RS[Phospho (STY)]PS[Phospho (STY)]PKPTK.2	-0.078345035	Q99M28_S155_M2_Rnps1
APS[Phospho (STY)]PTKR.2	-0.101634407	Q99M28_S27_M1_Rnps1
SSTRAPS[Phospho (STY)]PTK.2	-0.124982579	Q99M28_S27_M1_Rnps1
ALVSYDGLNQR.2	-0.265889984	#N/A
DC[Carbamidomethyl (C)]YPVQETFIR.2	0.075289869	#N/A
SYETWIGVYTAK.2	-0.196254002	#N/A
QVLYQQSSGHNNR.2	-0.015686565	#N/A
GGASGPATPAPPQLS[Phospho (STY)]PVQA.2	-0.351160327	Q99M85_S344_M1_Scrt1
VTIPVPAGVEDGQTVR.2	0.197142504	#N/A
NKPRS[Phospho (STY)]PVVELSK.3	-0.116953662	Q99MK8_S670_M1_Grk2
SLLEGLLQR.2	0.513479224	#N/A
GEVNAADAFDIGS[Phospho (STY)]FDEEDTK.2	-0.54187615	Q99MK8_S487_M1_Grk2
EAQQLVQR.2	0.192200811	#N/A
S[Phospho (STY)]PVVELSK.2	0.113328863	Q99MK8_S670_M1_Grk2
ETAATTETPESTEASPS[Phospho (STY)]V.2	-0.174095924	Q99MN1_S594_M1_Kars1
DTSYLFITGPEVVK.2	0.268294425	#N/A
EFFNFLPLSSQDPAPIR.2	0.164574196	#N/A
IM[Oxidation (M)]DQAITVGAPVIGLNDSGGAR.2	-0.17683978	#N/A
IQEGVESLAGYADIFLR.2	-0.392441607	#N/A
EQSESVNTAPESPS[Phospho (STY)]K.2	0.038052383	Q99MP8_S118_M1_Brap
SREQSESVNTAPES[Phospho (STY)]PSK.2	-0.438014371	Q99MP8_S116_M1_Brap
GEPGS[Phospho (STY)]PGAAGQSGLPGPK.2	-0.123716787	Q99MQ5_S265_M1_Col25a1
S[Phospho (STY)]IEEGDGAFGR.2	-0.445226164	Q99MR1_S137_M1_Gigyf1
ISS[Phospho (STY)]PPGPPGDLEDEEGLK.2	0.272340244	Q99MR1_S413_M1_Gigyf1
SQS[Phospho (STY)]WDDRGER.2	-0.107994938	Q99MR1_S157_M1_Gigyf1
STS[Phospho (STY)]PDGGPR.2	-0.151683014	Q99MR1_S228_M1_Gigyf1
GLEEEEEEEEPS[Phospho (STY)]EGVDEERPEAGGK.3	-0.209453277	Q99MR1_S343_M1_Gigyf1
[Acetyl (Protein N-term)]ASESS[Phospho (STY)]PLLAYR.2	-0.414010619	Q99MR3_S6_M1_Slc12a9
[Acetyl (Protein N-term)]GDS[Phospho (STY)]DDEYDRR.2	0.016073921	Q99MR6_S4_M1_Srrt
ERFS[Phospho (STY)]PPRHELS[Phospho (STY)]PPQK.3	0.216693453	Q99MR6_S67_M2_Srrt
HELS[Phospho (STY)]PPQKR.2	0.21242768	Q99MR6_S74_M1_Srrt
FS[Phospho (STY)]PPRHELS[Phospho (STY)]PPQKR.3	0.360846987	Q99MR6_S67_M2_Srrt
[Acetyl (Protein N-term)]GDS[Phospho (STY)]DDEYDR.2	0.085951107	Q99MR6_S4_M1_Srrt
ERFS[Phospho (STY)]PPR.2	0.155890833	Q99MR6_S67_M1_Srrt
LREC[Carbamidomethyl (C)]ELS[Phospho (STY)]PGVNR.3	-0.301772771	Q99MR6_S492_M1_Srrt
GSWVS[Phospho (STY)]PSR.2	-0.0588102	Q99MX7_S521_M1_Tmem121b
GHLENEGGPHGYVNTLAVASQN.3	-0.276207964	#N/A
GNPVPQQYVWVK.2	0.124663293	#N/A

LLLHC[Carbamidomethyl (C)]EGR.2	-0.245113689	#N/A
GTYLTHEAK.2	0.255271009	#N/A
GTYLTHEAKGS[Phospho (STY)]DDAPDADTAIINAEGGQSGGDDKK.4	-0.211157513	Q99N28_S368_M1_Cadm3
GSDDAPDADTAIINAEGGQS[Phospho (STY)]GGDDKK.3	0.433220964	Q99N28_S386_M1_Cadm3
EDDGANIVC[Carbamidomethyl (C)]SVNHESLK.2	0.299446218	#N/A
GSDDAPDADTAIINAEGGQSGGDDKKEYFI.3	-0.06111482	#N/A
LALQYVPEPS[Phospho (STY)]PGK.2	-0.449131106	Q99N50_S799_M1_Sytl2
LALQYVPEPS[Phospho (STY)]PGKK.3	0.116628549	Q99N50_S799_M1_Sytl2
SAS[Phospho (STY)]EPSLHR.2	-0.40441838	Q99N57_S621_M1_Raf1
AAHTEDINAC[Carbamidomethyl (C)]TLTTS[Phospho (STY)]PR.2	-0.161848392	Q99N57_S642_M1_Raf1
RAS[Phospho (STY)]DDGKLTDSSK.2	-0.223876114	Q99N57_S43_M1_Raf1
GGDSIGET[Phospho (STY)]PT[Phospho (STY)]PGASK.2	-0.451718944	Q99NB9_T326_M2_Sf3b1
AKGSET[Phospho (STY)]PGAT[Phospho (STY)]PGSK.2	-0.048231041	Q99NB9_T244_M2_Sf3b1
WDETPASQMGGST[Phospho (STY)]PVLTPGK.2	-0.211356055	Q99NB9_T350_M1_Sf3b1
GSET[Phospho (STY)]PGAT[Phospho (STY)]PGSK.2	-0.39197101	Q99NB9_T244_M2_Sf3b1
DT[Phospho (STY)]PGHGSGWAETPR.2	-0.332985916	Q99NB9_T303_M1_Sf3b1
TM[Oxidation (M)]IIS[Phospho (STY)]PERLDPFADGGKT[Phospho (STY)]PDPK.3	-0.478156718	Q99NB9_S129_M2_Sf3b1
KLSSWDQAET[Phospho (STY)]PGHT[Phospho (STY)]PSLR.3	-0.268927756	Q99NB9_T223_M2_Sf3b1
RWDQTADQT[Phospho (STY)]PGAT[Phospho (STY)]PK.2	0.337087344	Q99NB9_T207_M2_Sf3b1
QGS[Phospho (STY)]PTQSPPADTSFGSR.2	-0.238252853	Q99NE5_S1023_M1_Rims1
GRS[Phospho (STY)]QDYPDRLEK.3	-0.361537586	Q99NE5_S346_M1_Rims1
S[Phospho (STY)]LDEIHPTRR.3	-0.427757531	Q99NE5_S812_M1_Rims1
S[Phospho (STY)]LDEIHPTR.2	-0.279055518	Q99NE5_S812_M1_Rims1
SGS[Phospho (STY)]IEQASLVVEER.2	-0.314083279	Q99NE5_S1051_M1_Rims1
ETS[Phospho (STY)]PISSHPVTWQPSK.2	-0.196810212	Q99NE5_S413_M1_Rims1
GRS[Phospho (STY)]QDYPDR.2	-0.08811266	Q99NE5_S346_M1_Rims1
S[Phospho (STY)]QDYPDRLEK.2	-0.082967328	Q99NE5_S346_M1_Rims1
ETS[Phospho (STY)]PISSHPVTWQPSKEGDR.3	-0.050614962	Q99NE5_S413_M1_Rims1
IQIQHAS[Phospho (STY)]PENDR.2	0.117132384	Q99NE5_S927_M1_Rims1
SS[Phospho (STY)]DSDVSDVSAISR.2	0.207779396	Q99NE5_S1111_M1_Rims1
SASTNC[Carbamidomethyl (C)]LRPDTSLHS[Phospho (STY)]PER.3	-0.205861575	Q99NE5_S899_M1_Rims1
ASQSS[Phospho (STY)]LESSSGPPC[Carbamidomethyl (C)]IR.2	-0.364624376	Q99NE5_S1451_M1_Rims1
RAS[Phospho (STY)]QSSLESSSGPPC[Carbamidomethyl (C)]IR.3	-0.076496608	Q99NE5_S1448_M1_Rims1
IPESSHPPLESSSS[Phospho (STY)]SFESQK.3	0.487358753	Q99NE5_S547_M1_Rims1
SASTNC[Carbamidomethyl (C)]LRPDTSLHS[Phospho (STY)]PERER.4	-0.037325586	Q99NE5_S899_M1_Rims1
S[Phospho (STY)]AEC[Carbamidomethyl (C)]LHM[Oxidation (M)]TSELQPSLDR.3	-0.147959299	Q99NE5_S866_M1_Rims1
FKQTTGSGSS[Phospho (STY)]QELDHEQYSK.3	0.100512507	Q99NE5_S1082_M1_Rims1
S[Phospho (STY)]TETGM[Oxidation (M)]AAEMR.2	0.171313174	Q99NE5_S1240_M1_Rims1
STSQLS[Phospho (STY)]QTESGHKK.2	0.160402371	Q99NE5_S1224_M1_Rims1
SC[Carbamidomethyl (C)]DNASAKS[Phospho (STY)]SDSDVSDVSAISR.3	-0.11087153	Q99NE5_S1110_M1_Rims1
SRS[Phospho (STY)]SEHSSIR.2	-0.043109843	Q99NE5_S988_M1_Rims1
QPSRESTDGSINSY[Phospho (STY)]SSEGNLIFPGVR.3	-0.284522386	Q99NE5_Y1268_M1_Rims1
S[Phospho (STY)]VS[Phospho (STY)]PHRGDDQGRPR.3	-0.467827988	Q99NE5_S788_M2_Rims1
S[Phospho (STY)]RSVS[Phospho (STY)]PHR.2	-0.108055852	Q99NE5_S786_M2_Rims1
LQTHDESSLPLPQPS[Phospho (STY)]PFMPR.3	0.026727076	Q99NE5_S716_M1_Rims1
QGS[Phospho (STY)]KEC[Carbamidomethyl (C)]PGC[Carbamidomethyl (C)]AQLVPGPSSR.3	-0.088921111	Q99NF2_S166_M1_Nsmf
M[Oxidation (M)]YS[Phospho (STY)]VDGVSDDVPIR.2	-0.05737708	Q99NF2_S206_M1_Nsmf

SWS[Phospho (STY)]DPTPMK.2	0.193934127	Q99NF2_S294_M1_Nsmf
ERS[Phospho (STY)]FS[Phospho (STY)]RS[Phospho (STY)]WSDPTPMK.2	0.31335668	Q99NF2_S288_M3_Nsmf
LEDNDS[Phospho (STY)]AT[Phospho (STY)]SEADAEIAAK.2	0.002472788	Q99NF3_S96_M2_Cep41
GGQNLPTSC[Carbamidomethyl (C)]PASHSS[Phospho (STY)]PR.3	0.17091733	Q99NF3_S358_M1_Cep41
NS[Phospho (STY)]PLDC[Carbamidomethyl (C)]GSASPNK.2	0.182946862	Q99NH0_S2055_M1_Ankrd17
EHYPVSSPSS[Phospho (STY)]PS[Phospho (STY)]PPAQPGGVSR.3	-0.141290974	Q99NH0_S2041_M2_Ankrd17
RSSDPALTGLS[Phospho (STY)]TSVSDNNFSSEEPSR.3	0.105902094	Q99NH2_S152_M1_Pard3
NNYSPGRFS[Phospho (STY)]PDSHC[Carbamidomethyl (C)]VANR.3	0.033896706	Q99NH2_S383_M1_Pard3
LLQTC[Carbamidomethyl (C)]FSS[Phospho (STY)]PTDDSM[Oxidation (M)]DR.2	0.409852262	Q99P31_S349_M1_Hspbp1
KAQIITEKTS[Phospho (STY)]PK.2	-0.231453235	Q99P72_S489_M1_Rtn4
GSGS[Phospho (STY)]VDETLFALPAASEPVIPSSAEK.3	-0.238130688	Q99P72_S167_M1_Rtn4
LPS[Phospho (STY)]DTEKEDR.2	0.299248724	Q99P72_S953_M1_Rtn4
HQAQIDHYLGLANK.3	-0.401397413	#N/A
[Acetyl (Protein N-term)]M[Oxidation (M)]EDIDQSSLVSSSADS[Phospho (STY)]PPRPPPAFK.3	-0.357679075	Q99P72_S16_M1_Rtn4
TSNPFLVAIHDSEADYVTTDNLSK.3	-0.412025129	#N/A
LSTEPS[Phospho (STY)]PEFSNYSEIAK.2	0.349670569	Q99P72_S690_M1_Rtn4
GPLPAAPPT[Phospho (STY)]APERQPSWER.3	-0.19091307	Q99P72_T98_M1_Rtn4
EAEEKLPS[Phospho (STY)]DTEKEDR.2	-0.213157912	Q99P72_S953_M1_Rtn4
VVKEDGVM[Oxidation (M)]S[Phospho (STY)]PEK.2	-0.003357776	Q99P72_S344_M1_Rtn4
DDS[Phospho (STY)]PKEYTDLEVSNK.2	-0.081914012	Q99P72_S857_M1_Rtn4
RRGS[Phospho (STY)]GSVDETLFALPAASEPVIPSSAEK.3	-0.396494778	Q99P72_S165_M1_Rtn4
EHGYLGNLSAVASTEGTIEETLNEASR.3	-0.502287432	#N/A
RGS[Phospho (STY)]GSVDETLFALPAASEPVIPSSAEK.3	0.144471471	Q99P72_S165_M1_Rtn4
AQIITEKTS[Phospho (STY)]PK.2	0.167504593	Q99P72_S489_M1_Rtn4
AYLESEVAISEELVQK.2	-0.022480327	#N/A
ESLTEVSETVTQHK.2	0.032681663	#N/A
SKDKEDLVC[Carbamidomethyl (C)]SAALHNPQES[Phospho (STY)]PATLTK.4	0.409339464	Q99P72_S329_M1_Rtn4
EDGVMS[Phospho (STY)]PEK.2	0.180086955	Q99P72_S344_M1_Rtn4
LSAS[Phospho (STY)]PQEVGKPYLESFQPNLHITK.3	-0.52747269	Q99P72_S768_M1_Rtn4
ESAEFSVLEYSEM[Oxidation (M)]GSSFNGS[Phospho (STY)]PK.3	0.016747934	Q99P72_S291_M1_Rtn4
AAPQS[Phospho (STY)]PSVPK.2	-0.128014901	Q99P88_S992_M1_Nup155
IALNLEGC[Carbamidomethyl (C)]ALS[Phospho (STY)]PTSQEPR.2	-0.313387677	Q99PG2_S403_M1_Ogfr
S[Phospho (STY)]DSELEVKPSESLLR.2	0.171904982	Q99PI5_S243_M1_Lpin2
VTVNYPPYISEAK.2	-0.240888658	#N/A
AVGFVSEDEYLEIQGITR.2	-0.400997788	#N/A
EQSGEYEC[Carbamidomethyl (C)]SASNDVAAPVVR.2	0.180391156	#N/A
GTGVPVGQK.2	-0.285517409	#N/A
VHLIVQVSPK.2	-0.389825152	#N/A
STILYAGNDK.2	-0.345004086	#N/A
AMDNVTVR.2	0.334910938	#N/A
LASS[Phospho (STY)]PKDR.2	0.199043	Q99PL5_S135_M1_Rrbp1
GKS[Phospho (STY)]PQLALR.2	-0.219673422	Q99PL6_S36_M1_Ubxn6
EALQNDWLPFELR.2	0.184887687	#N/A
SVLRPELLAAIEQLS.2	-0.179912752	#N/A
RAS[Phospho (STY)]PS[Phospho (STY)]PPR.2	-0.069611073	Q99PP2_S101_M2_Znf318
RGS[Phospho (STY)]PS[Phospho (STY)]PPR.2	-0.365856564	Q99PP2_S79_M2_Znf318
DQVVGGNVS[Phospho (STY)]PR.2	0.038789627	Q99PP2_S2206_M1_Znf318

WAS[Phospho (STY)]PS[Phospho (STY)]PPR.2	-0.165340961	Q99PP2_S69_M2_Znf318
LGS[Phospho (STY)]PVDGLQDM[Oxidation (M)]DRDDLTDDSVFTR.3	-0.36308744	Q99PP2_S205_M1_Znf318
S[Phospho (STY)]PGLC[Carbamidomethyl (C)]SDSSLEESLR.2	0.309611015	Q99PP2_S167_M1_Znf318
RAS[Phospho (STY)]PS[Phospho (STY)]PPRGR.3	0.30835279	Q99PP2_S101_M2_Znf318
LKS[Phospho (STY)]DERPVHIK.3	-0.254016447	Q99PP7_S1134_M1_Trim33
VAVSADPNVPNVIVTR.2	-0.144288208	#N/A
[Acetyl (Protein N-term)]AEQEPTAEQLAQIAAENEEDEHSVNY[Phospho (STY)]KPPAQK.4	0.169846057	Q99PT1_Y27_M1_Arhgdia
TDYM[Oxidation (M)]VGSYGPR.2	-0.011159353	#N/A
S[Phospho (STY)]IQEIQELDKDDESLRK.3	-0.351835679	Q99PT1_S34_M1_Arhgdia
LTLVC[Carbamidomethyl (C)]STAPGPLELDLTGDLESFKK.3	-0.029196243	#N/A
YIQHTYR.2	0.16904555	#N/A
SIQEIQELDKDDESLR.3	-0.16776256	#N/A
IDKTDYM[Oxidation (M)]VGSYGPR.3	-0.334641326	#N/A
AEEYEFLTPM[Oxidation (M)]EEAPK.2	-0.093841589	#N/A
ES[Phospho (STY)]PSHGLELSAPEK.2	0.073598941	Q99PU5_S53_M1_Acsbg1
AAS[Phospho (STY)]LDGAEEALWTTR.2	0.290649625	Q99PU5_S70_M1_Acsbg1
DDQQQGASLGTSQDNSQTSSLIDGQTLSK.3	-0.121411483	#N/A
GGS[Phospho (STY)]FEM[Oxidation (M)]TDDDSAIR.2	0.025214875	Q99PU8_S249_M1_Dhx30
ELAGNSST[Phospho (STY)]PPPVS[Phospho (STY)]PGR.2	-0.166621674	Q99PV8_S381_M2_Bcl11b
FST[Phospho (STY)]PPGDLLDGGLSGR.2	-0.140097546	Q99PV8_T744_M1_Bcl11b
SDDGLSAAS[Phospho (STY)]SPEPGTSELPGDLK.2	0.086360414	Q99PV8_S495_M1_Bcl11b
VLDKSS[Phospho (STY)]PPPSSR.2	0.062069883	Q99PV8_S96_M1_Bcl11b
VSEPVEIGIQVTPDEDDHLLS[Phospho (STY)]PTK.3	-0.691670073	Q99PV8_S128_M1_Bcl11b
SPFLST[Phospho (STY)]PPLPPMPAGT[Phospho (STY)]PPPQPPAK.3	-0.148997602	Q99PV8_T406_M2_Bcl11b
TTGLVGLAVC[Carbamidomethyl (C)]DTPHER.2	0.096423792	#N/A
KYTEQITNEK.2	-0.182861408	#N/A
LEALLQGGEVEEVILQAEK.2	-0.256252259	#N/A
AGIFQSAK.2	-0.075359264	#N/A
NYDSM[Oxidation (M)]KDFEEMRK.3	0.166617859	#N/A
AYAEFYR.2	-0.18018005	#N/A
NYDSMKDFEEMR.3	-0.110841394	#N/A
[Acetyl (Protein N-term)]AAAVAAAGAGEPLS[Phospho (STY)]PEELLPK.2	-0.353258447	Q9CPQ3_S15_M1_Tomm22
LWGLTEM[Oxidation (M)]FPER.2	0.079042517	#N/A
LQM[Oxidation (M)]EQQQQLQQR.2	-0.100215319	#N/A
AEAEKAEEELEEDDDDELDETLSER.3	-0.263829	#N/A
VELVPPTPAEIPTAIQSVK.2	0.004709301	#N/A
LATFWHYAK.2	-0.258308888	#N/A
TPS[Phospho (STY)]PGEQQQVQEVK.2	0.242684637	Q9CPS8_S40_M1_
TPS[Phospho (STY)]PGEQQQVQEVKK.3	-0.040397732	Q9CPS8_S40_M1_
RFEELGVK.2	0.154863698	#N/A
GLAFIQDPDGYWIEILNPNK.2	-0.115102446	#N/A
DFLLQQTM[Oxidation (M)]LR.2	0.265534678	#N/A
FEELGVK.2	-0.018546206	#N/A
SLDFYTR.2	-0.270051501	#N/A
GFGHIGIAVPDVYSAC[Carbamidomethyl (C)]K.2	-0.474238299	#N/A
IASGLGLAWIIGR.2	0.11567313	#N/A
VLYAYGYYTGDPSK.2	0.066852171	#N/A

VTLAVSDLQK.2	0.326243936	#N/A
VAEGIFETEAPGGYK.2	0.187768483	#N/A
FQTVHFFR.2	0.107194553	#N/A
SPSQSDPVLK.2	0.142227826	#N/A
IYEQDEEKQR.2	-0.21980111	#N/A
ALLGYADNQC[Carbamidomethyl (C)]K.2	0.010828545	#N/A
FYLQDRS[Phospho (STY)]PSQSDPVLK.3	0.076032964	Q9CPV4_S131_M1_Glod4
ESHSILTPLVSLDTPGK.3	0.053757159	#N/A
DVLGM[Oxidation (M)]QVLR.2	-0.097778938	#N/A
LELQGIQGAVDHAAAFGR.3	0.275794611	#N/A
LLDDAMEADKSDEWFATR.3	0.261884005	#N/A
ALAAGGVGSIVR.2	0.092851669	#N/A
QVIDC[Carbamidomethyl (C)]QLADVNNLGK.2	-0.369881639	#N/A
ANKPGDVVR.2	0.636347559	#N/A
ADM[Oxidation (M)]GGAATIC[Carbamidomethyl (C)]SAIVSAAK.2	-0.27678912	#N/A
SAGAC[Carbamidomethyl (C)]TAAAFLR.2	0.44982364	#N/A
DKDDDLPQFTSAGESFNK.3	0.214518889	#N/A
TLIEFLLR.2	-0.098141709	#N/A
FAEIIEK.2	-0.096348951	#N/A
S[Phospho (STY)]WIEEQEMGSFLSVAK.2	0.114756425	Q9CPY7_S238_M1_Lap3
T[Phospho (STY)]TPAPS[Phospho (STY)]PGSANESFFAPSR.2	-0.220206432	Q9CQ20_S71_M2_Mid1ip1
LLLDPSST[Phospho (STY)]PTK.2	-0.013536493	Q9CQ22_T28_M1_Lamtor1
KLLLDPSST[Phospho (STY)]PTK.2	-0.166871262	Q9CQ22_T28_M1_Lamtor1
DHDMFGYIK.2	0.123945899	#N/A
TYAEILEPFHPVR.2	-0.345250656	#N/A
DLPAAAAPAGPASFAR.2	0.289943336	#N/A
LPIPDSQVLTINPALPVEDAAEDYAR.3	0.252080051	#N/A
TGALC[Carbamidomethyl (C)]WFLDEAAAR.2	-0.504969109	#N/A
VAGHPDVVINNAAGNFISPSER.3	-0.226188995	#N/A
HVISYSLSPFEQR.3	0.07069039	#N/A
EFGNLAR.2	0.333612741	#N/A
AFPSYFSK.2	0.172509646	#N/A
KNPAMYENDK.2	0.265662329	#N/A
AHPNLPILIR.2	-0.034581305	#N/A
TVSLNNLSADEVTR.2	-0.002763162	#N/A
EC[Carbamidomethyl (C)]SEVQPK.2	0.201864656	#N/A
YAFGQEK.2	0.349620739	#N/A
AM[Oxidation (M)]QNVLSGK.2	-0.216676351	#N/A
RAS[Phospho (STY)]NGEPVEK.2	0.123962339	Q9CQ86_S97_M1_Mien1
TQSSLVPALTEFVR.2	0.293992797	#N/A
GLLQTEPQNNQAK.2	-0.131072274	#N/A
[Acetyl (Protein N-term)]M[Oxidation (M)]EAVLNELVSVEDLK.2	0.28134927	#N/A
KFQSEQAAGS[Phospho (STY)]VSK.2	-0.159845605	Q9CQ92_S29_M1_Fis1
STQFEYAWC[Carbamidomethyl (C)]LVR.2	-0.022995129	#N/A
LQDPFSVYR.2	-0.071367081	#N/A
DLVPDLSNFYAQYK.2	0.167355237	#N/A
QQYLQSIEDR.2	0.067898512	#N/A

RIDTDLSK.2	-0.116869527	#N/A
WDPDKTGDKPR.2	0.181635669	#N/A
MQTYEVDLNK.2	0.417577963	#N/A
SIEPYLK.2	-0.252475851	#N/A
IKNEVDSTLTFR.2	0.090680959	#N/A
QQYLQSIEDREK.3	-0.073555685	#N/A
VVNEINIEDLC[Carbamidomethyl (C)]LTK.2	-0.008435999	#N/A
NAEEES[Phospho (STY)]ESEAEEGD.2	0.03046493	Q9CQC6_S411_M1_Bzw1
VSHIEDPALIR.2	-0.303062527	#N/A
EYLLQYNDPK.2	-0.208468173	#N/A
EYLLQYNDPKR.3	-0.337413884	#N/A
YKPAPLATLPSTLDPAEYDVSPETR.3	0.187293756	#N/A
QASPNIVIALSGNK.2	-0.431555017	#N/A
GAQAAIVVYDITNEESFAR.2	-0.630381616	#N/A
WASS[Phospho (STY)]LENLLEDPEGVQR.2	0.284645937	Q9CQE5_S41_M1_Rgs10
INEAIVAVQAIIADPK.2	0.018613927	#N/A
LPGGELNPGEDEVEGLKR.3	-0.345216992	#N/A
EADEEDS[Phospho (STY)]DEETSYPER.2	-0.140834013	Q9CQF4_S106_M1_Mtres1
NFYDGPEK.2	-0.150826901	#N/A
EFIDHSPK.2	0.357265912	#N/A
TLAILQIESEKAELR.2	-0.44419324	#N/A
NTEDLTEEWLR.2	0.214044966	#N/A
LVQTAELTK.2	0.221668442	#N/A
LVVLDEELEGVSPDELKDELPER.3	-0.44117657	#N/A
ETHNAAIIM[Oxidation (M)]K.2	-0.154525891	#N/A
[Acetyl (Protein N-term)]SESLVVC[Carbamidomethyl (C)]DVAEDLVEK.2	-0.315555091	#N/A
KAGGANYDAQS[Phospho (STY)]E.2	0.342037459	Q9CQI6_S141_M1_Cotl1
EFVISDRK.2	-0.400756203	#N/A
ELEEDFIR.2	0.077347193	#N/A
EVVQNFAK.2	0.220042367	#N/A
EFVISDR.2	-0.171137184	#N/A
YDGATIVPGDQGADYQHFIQQC[Carbamidomethyl (C)]TDDVR.3	-0.288129452	#N/A
AGGANYDAQS[Phospho (STY)]E.2	-0.217127802	Q9CQI6_S141_M1_Cotl1
FALITWIGEDVSGLQR.2	-0.136940512	#N/A
FTTGDAMSK.2	0.243585172	#N/A
LTVENS[Phospho (STY)]PK.2	-0.12731035	Q9CQJ6_S73_M1_Denr
WPEVDDDSIEDLGEVKK.3	-0.331946177	#N/A
LTVENS[Phospho (STY)]PKQETGITEGQGPVGEEEEK.3	-0.183873889	Q9CQJ6_S73_M1_Denr
HLESWC[Carbamidomethyl (C)]IHR.3	-0.13089137	#N/A
VPEWC[Carbamidomethyl (C)]LDYWHPSEK.3	0.07870315	#N/A
ARFEEHKNEK.3	-0.409045046	#N/A
EAEEEFWQNQHPQPYIFPDSPGGTSFER.3	0.297989404	#N/A
S[Phospho (STY)]PSLAFPK.2	0.031002964	Q9CQM0_S142_M1_Nicn1
SWC[Carbamidomethyl (C)]PDC[Carbamidomethyl (C)]VEAEPVIR.2	-0.077057376	#N/A
[Acetyl (Protein N-term)]ATFEEVSVLGFEEFDK.2	-0.112933953	#N/A
LVESEC[Carbamidomethyl (C)]C[Carbamidomethyl (C)]QSSLVEM[Oxidation (M)]IFSED.2	-0.499411292	#N/A
HNIQFSSFDIFSDEEVR.3	-0.262774709	#N/A

[Acetyl (Protein N-term)]AAGAAEAGEAAVAVVEVGSAQQFEELLR.3	-0.253841792	#N/A
AFLEALQNQAETSSK.2	0.09114743	#N/A
GVVDSEDIPLNLS[Phospho (STY)]R.2	0.178720417	Q9CQN1_S403_M1_Trap1
NLSDIDLM[Oxidation (M)]APQPGV.2	0.351284683	#N/A
FAMNPFYEPNS[Phospho (STY)]PIR.2	0.141959556	Q9CQP2_S119_M1_Trappc2
HYLFDVQR.2	-0.148617881	#N/A
IAQLEEVK.2	0.12766967	#N/A
AQQALVQK.2	0.144847735	#N/A
QIQDAIDMEK.2	0.299253025	#N/A
SISVQQEKETIAK.2	-0.135084571	#N/A
RKEEEHM[Oxidation (M)]IDWVEK.4	-0.209518537	#N/A
KEEEHMIDWVEK.3	0.2841748	#N/A
LDYHISVQNM[Oxidation (M)]M[Oxidation (M)]R.3	0.135690087	#N/A
TYGIC[Carbamidomethyl (C)]GAIR.2	0.401901167	#N/A
[Acetyl (Protein N-term)]M[Oxidation (M)]QNDAGEFVDLYVPR.2	-0.474113035	#N/A
DHASIQM[Oxidation (M)]NVAEVDR.2	-0.207351962	#N/A
VTLVSAAPEK.2	-0.265598167	#N/A
TLAFASVDLTNK.2	0.089183399	#N/A
[Acetyl (Protein N-term)]SSMTQNLR.2	-0.26279422	#N/A
APLDLDKYVEIAR.3	-0.291399441	#N/A
SFGSGTELGHWVTT[Phospho (STY)]PPDIPGSR.3	-0.329252883	Q9CQS4_T45_M1_Slc25a46
PGPTPSGTNVGSSGRS[Phospho (STY)]PSK.2	-0.195093404	Q9CQS8_S17_M1_Sec61b
S[Phospho (STY)]PSKAVAAR.2	-0.044892541	Q9CQS8_S17_M1_Sec61b
VDPSLM[Oxidation (M)]EDS[Phospho (STY)]DDGPSLPTK.2	0.092509652	Q9CQU3_S95_M1_Rer1
[Acetyl (Protein N-term)]S[Phospho (STY)]EGDSVGDSVHGKPSVVYR.3	0.105929107	Q9CQU3_S2_M1_Rer1
NQS[Phospho (STY)]YLQLLC[Carbamidomethyl (C)]SLQNK.2	0.061594754	Q9CQU5_S216_M1_Zwint
AM[Oxidation (M)]S[Phospho (STY)]GSWER.2	-0.442224816	Q9CQV4_S26_M1_Retreg3
GQTPLT[Phospho (STY)]EGSEDLDGHSDPEES[Phospho (STY)]FAR.3	-0.030222076	Q9CQV4_S325_M2_Retreg3
S[Phospho (STY)]FEQRVEDVR.2	-0.202025476	Q9CQV6_S12_M1_Map1lc3b
EAALILGVS[Phospho (STY)]PTANK.2	-0.184553772	Q9CQV7_S70_M1_Dnajc19
DSTLIM[Oxidation (M)]QLLR.2	0.355952833	#N/A
YLSEVASGENK.2	0.141469509	#N/A
LAEQAER.2	-0.040785285	#N/A
YLILNATQAESK.2	0.224884933	#N/A
AVTEQGHELSNEER.3	-0.12650535	#N/A
AKLAEQAER.2	-0.037872644	#N/A
QTTVSNSQQAYQEAFEISKK.3	-0.469097644	#N/A
QTTVSNSQQAYQEAFEISK.2	-0.518479476	#N/A
DNLTLWTSENQGDEGDAGEGEN.2	0.136249664	#N/A
EM[Oxidation (M)]QPTHPIR.2	0.334947156	#N/A
M[Oxidation (M)]KGDYFR.2	0.350688807	#N/A
VISS[Phospho (STY)]IEQK.2	0.126651769	Q9CQV8_S66_M1_Ywhab
[Acetyl (Protein N-term)]T[Phospho (STY)]M[Oxidation (M)]DKSELVQK.2	-0.120191502	Q9CQV8_T2_M1_Ywhab
KEM[Oxidation (M)]QPTHPIR.3	-0.12774956	#N/A
TAFDEAIAELDTLNEESYK.2	-0.142802634	#N/A
YDDM[Oxidation (M)]AAAM[Oxidation (M)]K.2	0.074875069	#N/A
NLLSVAYK.2	-0.332809773	#N/A

IEAELQDIC[Carbamidomethyl (C)]NDVLELLDK.2	-0.105201615	#N/A
DLPNALDEK.2	-0.110753569	#N/A
[Acetyl (Protein N-term)]AT[Phospho (STY)]PEASGSGEKVEGSEPSVTYYR.3	-0.410483386	Q9CQX2_T3_M1_Cyb5b
RNS[Phospho (STY)]AEETWM[Oxidation (M)]VIHGR.3	-0.681185853	Q9CQX2_S33_M1_Cyb5b
FLSEHPGGEEVLLEQAGADATESFEDVGHSPDAR.4	-0.182137826	#N/A
[Acetyl (Protein N-term)]AT[Phospho (STY)]PEASGSGEK.2	0.089763938	Q9CQX2_T3_M1_Cyb5b
NSAEETWM[Oxidation (M)]VIHGR.2	0.166887848	#N/A
LSASEALGSAALPSHSSAISQHSK.3	0.301381689	#N/A
RPPES[Phospho (STY)]PPIVEEWNSR.3	-0.039405622	Q9CQY2_S36_M1_Ramac
[Acetyl (Protein N-term)]S[Phospho (STY)]DTSEEIPNFEEMFASR.2	0.104251324	Q9CQY2_S2_M1_Ramac
TM[Oxidation (M)]QDITLVVETLLQQM[Oxidation (M)]QDK.3	-0.061594471	#N/A
NIADLM[Oxidation (M)]TQAGVEELDPENKIPTAQKS[Phospho (STY)].3	-0.278365928	Q9CQZ1_S76_M1_Hsbp1
NIADLMTQAGVEELDPENKIPTAQK.3	0.111102121	#N/A
QAAAAAASTSVKPIFSR.2	0.216858592	#N/A
NAHVTDPR.2	0.578159531	#N/A
MELQETIK.2	0.222720514	#N/A
VVDLLVIK.2	-0.111104467	#N/A
EVPNTVHLM[Oxidation (M)]QLDITVK.2	0.251071785	#N/A
MELPDYR.2	0.345144514	#N/A
[Acetyl (Protein N-term)]S[Phospho (STY)]GEDVPHR.2	-0.122385338	Q9CR00_S2_M1_Psmd9
ADVDLYQVR.2	0.081618528	#N/A
LASNS[Phospho (STY)]PVLPQAFAR.2	-0.130674833	Q9CR00_S128_M1_Psmd9
VFFDVDIGGER.2	0.114058735	#N/A
ILLISEDLK.2	0.310336247	#N/A
AIQAELLK.2	0.223355859	#N/A
IVLELFADIVPK.2	-0.430716446	#N/A
EYDQALADLKK.3	-0.176894595	#N/A
AQEIAPGDK.2	0.207450202	#N/A
[Acetyl (Protein N-term)]SHASPAAKPSNSK.3	-0.355676461	#N/A
LYDKIDPEK.2	0.246904654	#N/A
LM[Oxidation (M)]C[Carbamidomethyl (C)]PQEIVDYIADKK.3	-0.035953921	#N/A
PALPLPVGS[Phospho (STY)]PSSQPESLER.2	-0.082560377	Q9CR25_S188_M1_Dph2
YAGSALQYEDVGTAVQNLQK.2	-0.299024314	#N/A
LTPEDFAR.2	0.063149584	#N/A
DSLRDES[Phospho (STY)]QR.2	-0.196811335	Q9CR29_S186_M1_Ccdc43
AALLAQYADVT[Phospho (STY)]DEEDEADKKDDAGASTANVSSDR.4	-0.227549781	Q9CR29_T137_M1_Ccdc43
[Acetyl (Protein N-term)]ASQSQGIQQLLQAEK.2	-0.153186482	#N/A
[Acetyl (Protein N-term)]ASQSQGIQQLLQAEKR.3	0.186122494	#N/A
AAILKAS[Phospho (STY)]PK.2	-0.365814091	Q9CR57_S139_M1_Rpl14
LVAIVDVIDQNR.2	-0.188638823	#N/A
AAIAAAAAAAAAK.2	0.104901847	#N/A
DYC[Carbamidomethyl (C)]AHYLIR.2	-0.064812095	#N/A
DSFPNFLAC[Carbamidomethyl (C)]K.2	0.239435397	#N/A
VAQGQGEGEVGPEVAL.2	-0.144662917	#N/A
HEQHDWDYC[Carbamidomethyl (C)]EHLDYVK.4	0.063878561	#N/A
[Acetyl (Protein N-term)]AATASPGAGR.2	0.296661188	#N/A
NVFNALVR.2	-0.123797913	#N/A

TSFHALTSILK.2	-0.010708973	#N/A
LTGADGTPPGFLLK.2	0.498259638	#N/A
AVVVNAAQLASYSQSK.2	0.096462088	#N/A
M[Oxidation (M)]IDGKPEYK.2	0.110776692	#N/A
MQLSGEGAK.2	0.316576523	#N/A
LGIYTVLFER.2	-0.18802289	#N/A
ALIGM[Oxidation (M)]TAGATGAFVGTPAEVALIR.2	-0.094617819	#N/A
QATYTTTR.2	-0.212111797	#N/A
FLFGGLAGM[Oxidation (M)]GATVFVQPLDLVK.2	-0.396162063	#N/A
SGPFAPVLSATSR.2	0.121825643	#N/A
LSDIPEGK.2	-0.016615241	#N/A
GVAGALRPLLQGAVPAASEPPVLDVK.3	-0.185661846	#N/A
RAEVLDSTK.2	0.126590208	#N/A
VPDFSDYR.2	-0.217048995	#N/A
DPQHDLDR.2	0.228787793	#N/A
PLVATVGLNVPASVR.2	-0.005381405	#N/A
EIDQEAAVEVSQLRDPQHDLDR.3	-0.618848647	#N/A
NVVSQFVSSM[Oxidation (M)]SASADVLAM[Oxidation (M)]SK.2	-0.304142168	#N/A
GPAPLNLEVPAYEFTSDDVVVVG.2	-0.250734709	#N/A
EIDQEAAVEVSQLR.3	0.057372925	#N/A
GNVVPS[Phospho (STY)]PLPTR.2	0.089482344	Q9CR86_S42_M1_Carhsp1
TFS[Phospho (STY)]ATVR.2	-0.026355421	Q9CR86_S53_M1_Carhsp1
DRS[Phospho (STY)]PSPLR.2	-0.135936963	Q9CR86_S31_M1_Carhsp1
TRDRS[Phospho (STY)]PSPLR.2	-0.167601838	Q9CR86_S31_M1_Carhsp1
DRS[Phospho (STY)]PS[Phospho (STY)]PLRGNVVPS[Phospho (STY)]PLPTR.3	0.153429141	Q9CR86_S31_M3_Carhsp1
HETWSGHVISN.2	0.171582509	#N/A
ASGTGGLSLLPPPPGGK.2	0.123825596	#N/A
LDQPDWTGR.2	0.362156526	#N/A
LSIGNITAK.2	-0.093652657	#N/A
VSGELFAQAPVEQYPGIAVETVTDSSR.3	-0.151880429	#N/A
SAFIGIGFTDR.2	0.16685667	#N/A
ASDWKLDQPDWTGR.3	-0.222645134	#N/A
ESQEMDNRPK.2	-0.082958677	#N/A
C[Carbamidomethyl (C)]HAPLAQAQALVTSELER.3	-0.193773357	#N/A
AAGGGGGS[Phospho (STY)]GEDEAQSR.2	-0.289238975	Q9CRA5_S35_M1_Golph3
ADANLLTDTGTESS[Phospho (STY)]PR.2	-0.022785007	Q9CRA8_S20_M1_Exosc5
AESC[Carbamidomethyl (C)]GS[Phospho (STY)]FDETESEESSK.2	-0.147690275	Q9CRB0_S116_M1_Snx24
ALEELATKR.2	0.216175567	#N/A
[Acetyl (Protein N-term)]AASTDIAGLEESFRK.2	0.269364531	#N/A
AVTGTDVDIVFSK.2	-0.238184016	#N/A
LSENVIDR.2	0.092030542	#N/A
YSSVYGASVSDEDLK.2	0.135867269	#N/A
YEYHPVC[Carbamidomethyl (C)]ADLQTK.3	0.189257006	#N/A
FDSNVSGQSSFGT[Phospho (STY)]SPAADNIEK.2	0.124131429	Q9CRD0_T146_M1_Ociad1
RSS[Phospho (STY)]PPGHYTQKPK.3	0.229096958	Q9CRD0_S123_M1_Ociad1
TSS[Phospho (STY)]LSSDSSNLR.2	0.131856817	Q9CRD4_S117_M1_Dbndd2
ENGPS[Phospho (STY)]APQEK.2	0.00461105	Q9CS72_S881_M1_Filip1

ASS[Phospho (STY)]PEGEEPASPQQSK.2	-0.146215967	Q9CSB4_S338_M1_Pard3b
GPPS[Phospho (STY)]PPAPVM[Oxidation (M)]HS[Phospho (STY)]PSR.2	0.194636941	#N/A
GPPS[Phospho (STY)]PPAPVM[Oxidation (M)]HSPS[Phospho (STY)]RK.3	-0.11302986	Q9CSN1_S234_M2_Snw1
TSS[Phospho (STY)]LTHSEEK.2	-0.331369465	Q9CT10_S58_M1_Ranbp3
S[Phospho (STY)]PSESAEETHTLEEKVPQK.3	0.079827823	Q9CT10_S146_M1_Ranbp3
VLS[Phospho (STY)]PPKLNEANSDTSR.3	0.236368767	Q9CT10_S257_M1_Ranbp3
S[Phospho (STY)]PSESAEETHTLEEK.2	0.532872529	Q9CT10_S146_M1_Ranbp3
ERTSS[Phospho (STY)]LTHSEEK.2	-0.050260853	Q9CT10_S58_M1_Ranbp3
SAGSSSPEAGEDS[Phospho (STY)]DHEDGNYC[Carbamidomethyl (C)]PPVKR.4	-0.05994719	Q9CT10_S40_M1_Ranbp3
ASHTAPQVLFSHR.3	0.073175654	#N/A
NC[Carbamidomethyl (C)]FASVFEK.2	0.03139643	#N/A
DYLHYHIK.2	-0.278698104	#N/A
M[Oxidation (M)]ILLEVNNR.2	0.119187587	#N/A
IIEETLALK.2	0.259189481	#N/A
ELQAHGADELLKR.3	-0.034069133	#N/A
VFM[Oxidation (M)]QEFK.2	0.016805256	#N/A
DNTINLIHTFR.2	-0.457851716	#N/A
YFQFQEEGKEGENR.2	0.024246389	#N/A
SLQSLEASLHAM[Oxidation (M)]ESTRES[Phospho (STY)]LK.3	-0.108493792	Q9CW03_S776_M1_Smc3
GDVEGSQS[Phospho (STY)]QDEGEGSGESER.2	0.068824002	Q9CW03_S1067_M1_Smc3
KGDVEGSQS[Phospho (STY)]QDEGEGSGESER.3	0.209009017	Q9CW03_S1067_M1_Smc3
SFS[Phospho (STY)]LPADPILQAAK.2	-0.090254237	Q9CW07_S81_M1_Ppp1r3g
LLS[Phospho (STY)]PIASNR.2	0.15346595	Q9CW46_S576_M1_Raver1
ESVASM[Oxidation (M)]GADS[Phospho (STY)]GDDFASDGSSSR.2	0.076418913	Q9CW79_S41_M1_Golga1
GSIRPS[Phospho (STY)]IS[Phospho (STY)]NPR.2	-0.053820699	Q9CW79_S749_M2_Golga1
NAS[Phospho (STY)]VPNLR.2	0.026328635	Q9CWE0_S100_M1_Mtfr1l
AS[Phospho (STY)]FETLPNISDLC[Carbamidomethyl (C)]LK.2	-0.261104432	Q9CWE0_S38_M1_Mtfr1l
TTC[Carbamidomethyl (C)]SS[Phospho (STY)]S[Phospho (STY)]EEDDC[Carbamidomethyl (C)]ISLSK.2	-0.077494269	Q9CWE0_S221_M2_Mtfr1l
ASS[Phospho (STY)]FADM[Oxidation (M)]M[Oxidation (M)]GILK.2	-0.074884525	Q9CWE0_S235_M1_Mtfr1l
TTELQDELSHLR.3	-0.118764846	#N/A
INVYYNEATGNK.2	0.129332441	#N/A
GTGRPNS[Phospho (STY)]PQRLDR.3	0.293411107	Q9CWK3_S196_M1_Cd2bp2
GTGRPNS[Phospho (STY)]PQR.2	0.203808251	Q9CWK3_S196_M1_Cd2bp2
GKHSLDS[Phospho (STY)]DEEDDDEEGSSK.2	0.204792916	Q9CWK3_S49_M1_Cd2bp2
SIS[Phospho (STY)]APVIFDR.2	0.05183504	Q9CWK8_S119_M1_Snx2
ELILSSEPS[Phospho (STY)]PAVT[Phospho (STY)]PVTPTTLIAPR.3	-0.190969851	Q9CWK8_S97_M2_Snx2
EYAENIGDGRS[Phospho (STY)]PEFR.3	0.134504721	Q9CWL8_S545_M1_Ctnnbl1
LADIQIEQLNR.2	0.336273263	#N/A
S[Phospho (STY)]QGEEVDFAR.2	-0.299232153	Q9CWS0_S33_M1_Ddah1
[Acetyl (Protein N-term)]AGLGHPSAFGR.2	0.165800167	#N/A
GAEILADTFKDYAVSTVPVADSLHLK.3	-0.480187326	#N/A
TPEEYPESAK.2	-0.397256841	#N/A
GAEILADTFK.2	0.394691357	#N/A
EFFVGLSK.2	0.024767179	#N/A
LQLNIVEM[Oxidation (M)]KDENATLDGGDVLFTGR.3	-0.088346509	#N/A
SFC[Carbamidomethyl (C)]SM[Oxidation (M)]AGPNLIAIGSSESAQK.2	-0.1029945	#N/A
QHELYVGVLGSK.2	-0.167233202	#N/A

DYAVSTVPVADSLHLK.3	-0.319105105	#N/A
LLPEGEETVES[Phospho (STY)]DDDKDER.3	-0.050907467	Q9CWU4_S156_M1_
[Acetyl (Protein N-term)]AADEEPDSPS[Phospho (STY)]GALQTAAEEEETK.3	0.241044004	Q9CWX9_S11_M1_Ddx47
[Acetyl (Protein N-term)]AESSGS[Phospho (STY)]PHR.2	-0.127704945	Q9CWY9_S7_M1_Rpain
GFGS[Phospho (STY)]EEGSR.2	0.09979769	Q9CWZ3_S42_M1_Rbm8a
M[Oxidation (M)]REDYDS[Phospho (STY)]VEQDGDEPGPQR.3	-0.059860321	Q9CWZ3_S56_M1_Rbm8a
[Acetyl (Protein N-term)]ADVLDLHEAGGEDFAM[Oxidation (M)]DEDGDES[Phospho (STY)]IHK.3	0.033605836	Q9CWZ3_S24_M1_Rbm8a
LIENVDPEK.2	-0.057918312	#N/A
AVQLYQQTANVFENEER.2	-0.570345912	#N/A
INEGLEHLAK.2	0.301548769	#N/A
FDEAALSIQK.2	-0.123822146	#N/A
QAVELLGK.2	0.255590893	#N/A
LPEAVQLIEK.2	0.193610268	#N/A
TIAQVLVHLHR.3	0.194781121	#N/A
WKPDYDSAASEYGK.2	0.123492605	#N/A
YMDNDYAK.2	-0.334656775	#N/A
KS[Phospho (STY)]PAT[Phospho (STY)]PQAKPDGAAGMAAEEEEDEYSGGLC[Carbamidomethyl (C)].3	-0.39256694	Q9CWZ7_S284_M2_Napg
KKS[Phospho (STY)]PAT[Phospho (STY)]PQAK.2	0.120165927	Q9CWZ7_S284_M2_Napg
IVADQLC[Carbamidomethyl (C)]AK.2	0.189709051	#N/A
ELDSGLAESVSTLIWAAPR.2	0.012311517	#N/A
ALEQNPDDAQYYC[Carbamidomethyl (C)]QR.2	0.134782458	#N/A
[Acetyl (Protein N-term)]AAAAAGPASSQR.2	-0.022654797	#N/A
LFQQIYSDGSDEVKR.3	-0.396024315	#N/A
IEILDS[Phospho (STY)]PASK.2	-0.550412894	Q9CX66_S177_M1_D10Wsu102e
SDS[Phospho (STY)]KEEDSPEESSR.2	0.283099825	Q9CX66_S125_M1_D10Wsu102e
LQPLPSC[Carbamidomethyl (C)]EVC[Carbamidomethyl (C)]TPPS[Phospho (STY)]PK.2	-0.180428724	Q9CX84_S80_M1_Rgs19
LMHS[Phospho (STY)]PTGR.2	-0.04622919	Q9CX84_S97_M1_Rgs19
LFIGGLNVQTSESGLR.2	-0.221096478	#N/A
GDVAEGDLIEHFSQFGAVEK.3	-0.050739608	#N/A
SGGGGGGGGSWGGR.2	0.417088937	#N/A
QENSSNSS[Phospho (STY)]PAPETNPPVPSDGTEAK.3	-0.094948401	Q9CXE2_S114_M1_Bcl7a
MKLEASQQNS[Phospho (STY)]EEM[Oxidation (M)].2	0.266296878	Q9CXE2_S207_M1_Bcl7a
DDS[Phospho (STY)]PTQTLASPNAC[Carbamidomethyl (C)]R.2	0.041300722	Q9CXF4_S655_M1_Tbc1d15
SIVNTS[Phospho (STY)]PPC[Carbamidomethyl (C)]R.2	0.144319746	Q9CXK9_S792_M1_Rbm33
ANS[Phospho (STY)]GGGGDGPHVSSK.2	-0.234088121	Q9CXK9_S1032_M1_Rbm33
DIKEES[Phospho (STY)]DEEDDDDEESGR.2	0.105648946	Q9CXK9_S243_M1_Rbm33
[Acetyl (Protein N-term)]SSGASVS[Phospho (STY)]ALQR.2	-0.245490843	Q9CXP8_S8_M1_Gng10
LLLDTFEYQGLVK.2	0.079193236	#N/A
SVVTEEFNGS[Phospho (STY)]DWER.2	-0.054227304	Q9CXS4_S234_M1_Cenpv
SNPGGFGIAPHC[Carbamidomethyl (C)]LDEGTVR.2	-0.198569211	#N/A
QRLS[Phospho (STY)]FEGAAK.2	-0.409969147	Q9CXS4_S105_M1_Cenpv
WDYLTQVEK.2	0.244497411	#N/A
[Acetyl (Protein N-term)]ASVLEELQKDLEEVK.2	0.227553761	#N/A
VLEQLTGQTPVFSK.2	-0.331231348	#N/A
YDGIILPGK.2	-0.013404942	#N/A
[Acetyl (Protein N-term)]AQDQGEKENPM[Oxidation (M)]R.2	0.339077954	#N/A
VLQSALAAIR.2	-0.081224063	#N/A

NQDLAPNSAEQASILSLVTK.2	0.173421749	#N/A
EDAIAFAEK.2	0.260825143	#N/A
DTQLITVDEK.2	0.114635017	#N/A
LDITTLTGVPEEHIK.2	-0.108356498	#N/A
NNMQSGVNNTK.2	0.110600014	#N/A
DTQLITVDEKLDITTLTGVPEEHIK.3	-0.113198301	#N/A
NNMQSGVNNTKK.2	0.373469918	#N/A
S[Phospho (STY)]LKDEDVLQK.2	0.107315268	Q9CY27_S58_M1_Tecr
GHLDAELDAYM[Oxidation (M)]AQTDPETND.2	-0.429947698	#N/A
SAAQAAAQTNSNAAGK.2	0.392581967	#N/A
GGSGSHNWGTVKDELTES[Phospho (STY)]PK.3	-0.101636889	Q9CY58_S234_M1_Serbp1
EFDRHS[Phospho (STY)]GSDR.2	0.213821522	Q9CY58_S197_M1_Serbp1
TDKSSAS[Phospho (STY)]APDVDDPEAFPALA.2	0.012754218	Q9CY58_S393_M1_Serbp1
SSAS[Phospho (STY)]APDVDDPEAFPALA.2	0.295935987	Q9CY58_S393_M1_Serbp1
DELTES[Phospho (STY)]PKYIQK.2	0.382431063	Q9CY58_S234_M1_Serbp1
FDQLFDDES[Phospho (STY)]DPFEVLK.2	0.250100791	Q9CY58_S25_M1_Serbp1
GGSGSHNWGTVKDELTES[Phospho (STY)]PKYIQK.4	-0.093409331	Q9CY58_S234_M1_Serbp1
SKS[Phospho (STY)]EEAHAEDSVM[Oxidation (M)]DHHFR.4	0.035581121	Q9CY58_S329_M1_Serbp1
S[Phospho (STY)]EEAHAEDSVMDHHFR.3	0.186447794	Q9CY58_S329_M1_Serbp1
SGS[Phospho (STY)]LEEVPNVGVNK.2	-0.351473206	Q9CY64_S236_M1_Blvra
SQEVDVAYIC[Carbamidomethyl (C)]TESSSHEDYIR.3	-0.501108402	#N/A
QISLEDALR.2	-0.077496682	#N/A
DQDIFIQK.2	0.422770695	#N/A
VPS[Phospho (STY)]AGDVER.2	0.252594786	Q9CYG7_S186_M1_Tomm34
TVLQIDNSVASALEGINR.2	0.094263799	#N/A
VSDS[Phospho (STY)]VEQLR.2	0.030989604	Q9CYG7_S8_M1_Tomm34
SSLSDISSLLQIEPR.2	0.081387882	#N/A
YSESLLC[Carbamidomethyl (C)]SSLESATYSNR.2	0.001797734	#N/A
LDELGVPLYAVVK.2	0.096302963	#N/A
LTQNRGDS[Phospho (STY)]DDGR.2	-0.22499054	Q9CYI0_S55_M1_
S[Phospho (STY)]LGNDVK.2	-0.199685448	Q9CYI0_S186_M1_
RS[Phospho (STY)]EEKEAGEI.2	-0.258526378	Q9CYI4_S363_M1_Luc7l
ERS[Phospho (STY)]LREESWEYGR.3	-0.367958681	Q9CYI4_S331_M1_Luc7l
S[Phospho (STY)]LREESWEYGR.2	-0.119902421	Q9CYI4_S331_M1_Luc7l
QENTQSPYNLAYK.2	-0.081410624	#N/A
S[Phospho (STY)]GDSEVYQMGDVSQK.2	0.330780338	Q9CYR0_S67_M1_Ssbp1
THTPSPTS[Phospho (STY)]PK.2	-0.501682095	Q9CYT6_S311_M1_Cap2
IQEIQTFR.2	-0.336837716	#N/A
LEQLSAGLDGPPR.2	0.038361232	#N/A
TEGC[Carbamidomethyl (C)]HLYLSEDALDC[Carbamidomethyl (C)]EIVSAK.3	-0.359866556	#N/A
LINSM[Oxidation (M)]VAEFLK.2	-0.356787084	#N/A
SNS[Phospho (STY)]PQKHTPVLELEGK.3	-0.414245255	Q9CYT6_S316_M1_Cap2
S[Phospho (STY)]NSPQKHTPVLELEGKK.3	0.283767138	Q9CYT6_S314_M1_Cap2
HTPVLELEGKK.3	-0.224980506	#N/A
AQGQIRS[Phospho (STY)]PTKTHTPSPTS[Phospho (STY)]PK.3	0.015501268	Q9CYT6_S300_M2_Cap2
EM[Oxidation (M)]NDAATFYTNR.2	-0.188083094	#N/A
THTPS[Phospho (STY)]PTS[Phospho (STY)]PKSNSPQK.2	-0.059616754	Q9CYT6_S308_M2_Cap2

S[Phospho (STY)]PTKTHTPSPTS[Phospho (STY)]PK.2	-0.085062542	Q9CYT6_S300_M2_Cap2
VEYQEDRNDLVISETELK.3	-0.291362462	#N/A
SSEM[Oxidation (M)]NVLVPQDDDYREFPIPEQFK.3	-0.503649189	#N/A
AQGQIRS[Phospho (STY)]PTK.2	-0.243941584	Q9CYT6_S300_M1_Cap2
SALFAQLNQGEAITK.2	-0.197621136	#N/A
VLAGDVETHAEM[Oxidation (M)]VHGAFQAQR.3	0.120084789	#N/A
TIWDGSK.2	0.170212906	#N/A
DVVLHTFR.2	-0.056006388	#N/A
NSATFKS[Phospho (STY)]FEDR.2	-0.43115409	Q9CYZ2_S180_M1_Tpd52l2
S[Phospho (STY)]WHDVQVSTAYVK.2	0.06572272	Q9CYZ2_S96_M1_Tpd52l2
[Acetyl (Protein N-term)]M[Oxidation (M)]DSASQDINLNSPNKGVLS[Phospho (STY)]DFM[Oxidation (M)]TDVPVDPGVVHR.4	-0.065119563	Q9CYZ2_S19_M1_Tpd52l2
[Acetyl (Protein N-term)]MDSASQDINLNS[Phospho (STY)]PNK.2	0.327860414	Q9CYZ2_S12_M1_Tpd52l2
ENGSDNLPPS[Phospho (STY)]PGSGDQTLPDHAPF.3	-0.371942174	Q9CYZ2_S206_M1_Tpd52l2
S[Phospho (STY)]FEDRVGTIK.2	0.129346578	Q9CYZ2_S180_M1_Tpd52l2
HSIS[Phospho (STY)]M[Oxidation (M)]PVM[Oxidation (M)]R.2	-0.516212072	Q9CYZ2_S168_M1_Tpd52l2
[Acetyl (Protein N-term)]M[Oxidation (M)]ES[Phospho (STY)]PEGAGPGEIVK.2	-0.222396338	Q9CZ00_S3_M1_Dbndd1
VYEEDAVPGLTPC[Carbamidomethyl (C)]R.2	-0.136805977	#N/A
TDLTDYLNR.2	-0.546958776	#N/A
NALVSHLDGTTPVC[Carbamidomethyl (C)]EDIGR.3	0.041017099	#N/A
LC[Carbamidomethyl (C)]TSATESEVTR.2	-0.01075396	#N/A
EHTAYLIK.2	-0.226352013	#N/A
IPLAEWESR.2	-0.091720007	#N/A
RIPLAEWESR.2	-0.23748513	#N/A
VVELLADIVQNSSLEDSQIEKER.3	-0.277186322	#N/A
NRPGNALEK.2	0.319250343	#N/A
NNGAGYFLEHLAFK.2	-0.143300387	#N/A
IQEVDAQM[Oxidation (M)]LR.2	0.085905506	#N/A
SLLTYGR.2	0.199259189	#N/A
YFYDQC[Carbamidomethyl (C)]PAVAGYGPIEQLPDYNR.3	-0.238193854	#N/A
SGM[Oxidation (M)]FWLR.2	0.150550879	#N/A
FTGSEIR.2	0.076469023	#N/A
M[Oxidation (M)]VLAAAGGVEHQQLLDLAQK.3	-0.512448902	#N/A
VVELLADIVQNSSLEDSQIEK.2	-0.34001582	#N/A
VASEQSSHATC[Carbamidomethyl (C)]TVGVWIDAGSR.3	-0.348814964	#N/A
EVESIGAHLNAYSTR.2	-0.402831949	#N/A
IGIVGLPNVGK.2	-0.065225282	#N/A
IPAFLNVVDIAGLVK.2	-0.139134265	#N/A
NYIVEDGDIIFFK.2	0.150192293	#N/A
DDLLLNNVR.2	-0.277855436	#N/A
SYSPELIVHPVLDSSNAVEEVEK.3	-0.178118788	#N/A
LSQALGNITVVQK.2	0.416227999	#N/A
AILTPNHVEFSR.3	0.326061879	#N/A
STTTTDMITEVGTAFSR.2	-0.036654699	#N/A
S[Phospho (STY)]PGETSKPRPFAGGGYR.3	-0.245222254	Q9CZ44_S140_M1_Nsfl1c
EANLLNAVIVQR.2	0.325766804	#N/A
SYQDPSNAQFLESIR.2	-0.183324576	#N/A
DLIHDQDEEEEEEEGQR.3	-0.132747943	#N/A

ASSSILINEAEPTTNIQIR.2	-0.305720051	#N/A
RHS[Phospho (STY)]GQDVHVVLK.3	-0.070173686	Q9CZ44_S176_M1_Nsfl1c
S[Phospho (STY)]PNELVDDLFK.2	-0.017347411	Q9CZ44_S114_M1_Nsfl1c
RRHS[Phospho (STY)]GQDVHVVLK.3	-0.0371221	Q9CZ44_S176_M1_Nsfl1c
VTKS[Phospho (STY)]PGETSKPR.2	-0.112092755	Q9CZ44_S140_M1_Nsfl1c
TGFSLDNGDLR.2	0.431112332	#N/A
FYAGGSER.2	-0.336945964	#N/A
S[Phospho (STY)]PGETSKPR.2	-0.15134214	Q9CZ44_S140_M1_Nsfl1c
HS[Phospho (STY)]GQDVHVVLK.2	-0.455513746	Q9CZ44_S176_M1_Nsfl1c
KKS[Phospho (STY)]PNELVDDLFK.2	-0.027277252	Q9CZ44_S114_M1_Nsfl1c
EVPPPPVEES[Phospho (STY)]EEEDDDGLPK.2	0.006943293	Q9CZ52_S360_M1_Antxr1
QLM[Oxidation (M)]SQS[Phospho (STY)]QDEELSPLAAVETR.2	0.065043792	Q9CZ62_S308_M1_Cep97
HLLWDLGK.2	0.091890883	#N/A
LLQGS[Phospho (STY)]PEQGEAVTR.2	0.094005191	Q9CZB3_S172_M1_Thumpd2
DHGESQEDKLLQGS[Phospho (STY)]PEQGEAVTR.3	-0.18320836	Q9CZB3_S172_M1_Thumpd2
AVIES[Phospho (STY)]DEEQGR.2	-0.213183776	Q9CZC8_S360_M1_Scrn1
THAIVISR.2	-0.10798711	#N/A
FQEKPDR.2	0.403799109	#N/A
VEC[Carbamidomethyl (C)]TYISIDQVPR.2	0.411928429	#N/A
DKASGVC[Carbamidomethyl (C)]IDSESFLTTASIVSVLPQNR.3	-0.106983868	#N/A
AQS[Phospho (STY)]PC[Carbamidomethyl (C)]FGDDDPAK.2	-0.394275998	Q9CZC8_S322_M1_Scrn1
ASGVC[Carbamidomethyl (C)]IDSESFLTTASIVSVLPQNR.3	-0.298696226	#N/A
SSPC[Carbamidomethyl (C)]IHYFTGTPDPSR.3	-0.226471287	#N/A
PAWLWGAEM[Oxidation (M)]GANEHGVC[Carbamidomethyl (C)]IANEAINAR.3	0.098870986	#N/A
AQS[Phospho (STY)]PC[Carbamidomethyl (C)]FGDDDPAKKEPR.3	-0.118288048	Q9CZC8_S322_M1_Scrn1
EPAAETEALLGM[Oxidation (M)]DLVR.2	0.079185508	#N/A
TMLELEK.2	0.048155218	#N/A
DEVQEVVYFPAVDHDAESK.3	0.142294865	#N/A
YS[Phospho (STY)]PGQLR.2	0.111874112	Q9CZH7_S118_M1_Mxra7
VAEPEES[Phospho (STY)]EAEEPAAEGR.2	-0.256276863	Q9CZH7_S79_M1_Mxra7
SPVPS[Phospho (STY)]PPGSPR.2	0.015352887	Q9CZJ2_S25_M1_Hspa12b
RSS[Phospho (STY)]VNLVK.2	-0.3200573	Q9CZJ2_S434_M1_Hspa12b
TQESC[Carbamidomethyl (C)]GIAPLTPS[Phospho (STY)]QSPKPEAR.3	0.138226545	Q9CZJ2_S44_M1_Hspa12b
RS[Phospho (STY)]IDSS[Phospho (STY)]FR.2	-0.244984454	Q9CZJ2_S276_M2_Hspa12b
S[Phospho (STY)]IDSS[Phospho (STY)]FR.2	0.089113745	Q9CZJ2_S276_M2_Hspa12b
SLQSVAEER.2	0.11565247	#N/A
NTLQLHR.2	0.052367877	#N/A
VLNSYWVGEDSTYK.2	0.42575392	#N/A
AVGNSDGDWAVATLKS[Phospho (STY)]PK.3	-0.218104173	Q9CZN4_S296_M1_Shisa9
AFS[Phospho (STY)]PEHGPAQQNGQK.2	0.108106619	Q9CZN4_S337_M1_Shisa9
TFVEESIYR.2	-0.260151193	#N/A
LLAEQLGFDFFEASAK.2	0.027270491	#N/A
M[Oxidation (M)]SDS[Phospho (STY)]M[Oxidation (M)]DTDPSVLGASK.2	-0.127432532	Q9CZT8_S190_M1_Rab3b
LVDAIC[Carbamidomethyl (C)]DKM[Oxidation (M)]S[Phospho (STY)]DSM[Oxidation (M)]DTDPSVLGASK.3	0.09481173	Q9CZT8_S188_M1_Rab3b
VVPGYGHAVLR.2	0.186031337	#N/A
GLVYETSVLDPDEGIR.2	-0.23711427	#N/A
EGSSIGAIDSR.2	-0.16273363	#N/A

ALGFPLERPK.2	-0.051148621	#N/A
IVPNILLEQGK.2	0.134660736	#N/A
GYSIPEC[Carbamidomethyl (C)]QK.2	-0.077486185	#N/A
DYIWNTLNSGR.2	0.056619859	#N/A
LVAQLYK.2	0.023860259	#N/A
EVGKDVSDEKLR.3	-0.216720129	#N/A
EVGKDVSDEK.2	0.288296618	#N/A
ALGVLAQLIWSR.2	0.153066695	#N/A
AYAEGM[Oxidation (M)]NR.2	0.350826852	#N/A
LDWSHNFTNM[Oxidation (M)]LGYTDPQFTELM[Oxidation (M)]R.3	-0.315692104	#N/A
AALPSHVVTM[Oxidation (M)]LDNFPTNLHPM[Oxidation (M)]SQLSAAITALNSESNFAR.4	-0.108492129	#N/A
TVVGQITVDM[Oxidation (M)]M[Oxidation (M)]YGGM[Oxidation (M)]R.2	-0.571229437	#N/A
DVLSNLIPK.2	0.005881976	#N/A
YWELIYEDC[Carbamidomethyl (C)]M[Oxidation (M)]DLIAK.2	-0.246958598	#N/A
SMSTDGLMK.2	0.150010424	#N/A
NVDLSTFYQNR.2	-0.164232235	#N/A
AAAFEQLQK.2	0.070939973	#N/A
SYFSSFTDDIISQPM[Oxidation (M)]LK.2	-0.003153701	#N/A
FALAQAQK.2	-0.080456526	#N/A
YEQAIQC[Carbamidomethyl (C)]YTEAISLC[Carbamidomethyl (C)]PTEK.2	-0.489200161	#N/A
ATFYLLIGSANAAKPDLDK.2	-0.084565746	#N/A
C[Carbamidomethyl (C)]IDLEPDNATTYVHK.3	0.371111244	#N/A
GDASGLKR.2	-0.248765423	#N/A
YM[Oxidation (M)]AEALLLR.2	0.127548268	#N/A
C[Carbamidomethyl (C)]AEGYALYAQALTDQQQFGK.2	0.419216684	#N/A
AS[Phospho (STY)]PALGSGHHDGSGDSLEM[Oxidation (M)]SSLDR.3	-0.440772974	Q9CZW5_S94_M1_Tomm70
SDEDKDKEGEALEVK.3	-0.615201009	#N/A
WKEVAQDC[Carbamidomethyl (C)]TK.2	0.137574284	#N/A
GFEEIIK.2	0.40207348	#N/A
QAYTANNSSQVQAAMK.2	-0.401939385	#N/A
NSERKT[Phospho (STY)]PEGR.2	-0.598196628	Q9CZW5_T88_M1_Tomm70
EC[Carbamidomethyl (C)]LEDVTAVC[Carbamidomethyl (C)]ILEGFQNEQSM[Oxidation (M)]LLADK.3	-0.524496759	#N/A
DVNQQEFVR.2	-0.226390933	#N/A
ELAPYDENWFYTR.2	0.550777822	#N/A
IAGQVAAANK.2	-0.054940446	#N/A
GGAGVGSMTK.2	-0.229740619	#N/A
S[Phospho (STY)]DRGSGQGDSLYPVGYLDK.3	-0.236307395	Q9CZX9_S32_M1_Emc4
LPQPPEGQC[Carbamidomethyl (C)]YS[Phospho (STY)]N.2	0.176314563	Q9CZY3_S146_M1_Ube2v1
VNM[Oxidation (M)]SGVSSSNGVVDPR.2	0.285728828	#N/A
DNSNIILLGDS[Phospho (STY)]QGDLR.2	0.104873806	Q9D020_S273_M1_Nt5c3a
NS[Phospho (STY)]PNNISGISNPPGTPR.2	0.002586989	Q9D032_S347_M1_Ssbp3
VLEDNSVPQVK.2	-0.014855785	#N/A
VVSPWNSEDAK.2	-0.27039914	#N/A
EGIEC[Carbamidomethyl (C)]EVINLR.2	-0.27226925	#N/A
VFLLGEEVAQYDGAYK.2	-0.476193779	#N/A
DFLIPIGK.2	0.158432738	#N/A
TIRPM[Oxidation (M)]DIEAIEASVM[Oxidation (M)]K.3	-0.116210797	#N/A

IM[Oxidation (M)]EGPAFNFLDAPAVR.2	0.022883023	#N/A
TYYM[Oxidation (M)]SAGLQPVPIVFR.2	-0.518853168	#N/A
EAINQGM[Oxidation (M)]DEELERDEK.3	-0.216498592	#N/A
PVGHC[Carbamidomethyl (C)]LEAAAVLSK.3	0.344236294	#N/A
ASS[Phospho (STY)]QDGLETLKK.2	-0.099932994	Q9D067_S556_M1_Mdm1
VQGTHFS[Phospho (STY)]R.2	-0.375253247	Q9D067_S357_M1_Mdm1
RAS[Phospho (STY)]S[Phospho (STY)]QDGLETLKK.3	-0.352814901	Q9D067_S555_M2_Mdm1
TQSKS[Phospho (STY)]PDSTLR.2	0.136961849	Q9D074_S428_M1_Mgrn1
ESSS[Phospho (STY)]PESFGTEEGDEPSLK.2	0.101574727	Q9D074_S470_M1_Mgrn1
YKEDADS[Phospho (STY)]PTEDGEKPR.2	-0.117054451	Q9D074_S106_M1_Mgrn1
GRDS[Phospho (STY)]PYQSR.2	0.078643268	Q9D0B0_S205_M1_Srsf9
GS[Phospho (STY)]PHYFS[Phospho (STY)]PFRPY.2	0.109686867	Q9D0B0_S212_M2_Srsf9
STS[Phospho (STY)]YGYSR.2	0.294708822	Q9D0B0_S190_M1_Srsf9
EWVTYEISEEKQES.2	-0.303578019	#N/A
GADS[Phospho (STY)]GGEKEEGANR.2	-0.07160662	Q9D0B6_S184_M1_Pbdc1
GADS[Phospho (STY)]GGEKEEGANREGEK.3	-0.150333156	Q9D0B6_S184_M1_Pbdc1
AFITNIPFDVK.2	0.24936854	#N/A
INEILSNALK.2	0.615638005	#N/A
MGLS[Phospho (STY)]MDR.2	-0.134432821	Q9D0E1_S527_M1_Hnrnpm
MGLAMGGAGGAS[Phospho (STY)]FDR.2	-0.629362551	Q9D0E1_S617_M1_Hnrnpm
VGS[Phospho (STY)]EIER.2	-0.319670958	Q9D0E1_S431_M1_Hnrnpm
MGS[Phospho (STY)]GVER.2	0.268897312	Q9D0E1_S512_M1_Hnrnpm
S[Phospho (STY)]YGSLVQSSC[Carbamidomethyl (C)]SPVR.2	-0.363023303	Q9D0E3_S23_M1_Lysmd1
DLFNGLDS[Phospho (STY)]EEENDGEEEVRPSKDEIGSSSGR.4	0.153491993	Q9D0E3_S99_M1_Lysmd1
YDYEEVEAEGANK.2	-0.226203526	#N/A
ADNFEYSDPVDGSISK.2	-0.18962623	#N/A
ELLSGPNR.2	0.358617001	#N/A
TQAYPDQKPGTSGLR.3	0.064068396	#N/A
IALYETPTGWK.2	-0.012917906	#N/A
LIFADGSR.2	-0.067334153	#N/A
QQFDLENK.2	-0.444949439	#N/A
AIGGIILTAS[Phospho (STY)]HNPGGPNGDFGIK.3	-0.183371401	Q9D0F9_S117_M1_Pgm1
VDLGVLGK.2	0.087001898	#N/A
INQDPQVM[Oxidation (M)]LAPLISIALK.2	0.231256559	#N/A
QEATLVVGGDGR.2	-0.035686324	#N/A
FFGNLM[Oxidation (M)]DASK.2	0.003137441	#N/A
FNISNGGPAPEAITDK.2	-0.060810107	#N/A
DLEALMLDR.2	0.291419688	#N/A
LYIDSYEKDVAK.3	-0.435498129	#N/A
LTSS[Phospho (STY)]C[Carbamidomethyl (C)]PDLPSQSDK.2	0.075810542	Q9D0I4_S288_M1_Stx17
[Acetyl (Protein N-term)]S[Phospho (STY)]EKSVEAAAELSAK.2	0.026831092	Q9D0J8_S2_M1_Ptms
TAEEEDEADPKR.3	0.170217326	#N/A
FYTDPVEAVK.2	0.316972189	#N/A
GVFDVDKK.2	-0.110874479	#N/A
DGSVAIASKPR.2	-0.04453275	#N/A
YGDLANWM[Oxidation (M)]IPGK.2	-0.082333645	#N/A
AGGAGVPAFYTSTGYGTLVQEGGSPIK.3	-0.024270853	#N/A

GM[Oxidation (M)]GGAM[Oxidation (M)]DLVSSSK.2	-0.426880146	#N/A
C[Carbamidomethyl (C)]TLPLTGK.2	0.458057793	#N/A
QFLSGELEVELTPQGTLAER.2	-0.132111089	#N/A
DLTAVSNNAGVDNFGLGLLLR.2	-0.188217648	#N/A
ETVTVLPGASFFSSDESFAMIR.2	0.211923691	#N/A
STGC[Carbamidomethyl (C)]DFAVSPNLM[Oxidation (M)]PM[Oxidation (M)]QQIST.2	-0.364505834	#N/A
VVVTMEHSAK.2	-0.055414193	#N/A
M[Oxidation (M)]ISSYVGENAEFER.2	-0.204519953	#N/A
S[Phospho (STY)]AEDLTDGSYDDILNAEQLKK.3	0.060600283	Q9D0L7_S43_M1_Armc10
S[Phospho (STY)]AEDLTDGSYDDILNAEQLK.2	-0.062990122	Q9D0L7_S43_M1_Armc10
LLYLLESTDDPVITEK.2	-0.252668563	#N/A
ASVASDPES[Phospho (STY)]PPGGNEPAAASGQR.2	-0.336101023	Q9D0L8_S15_M1_Rnmt
GDVS[Phospho (STY)]EDEPSLGR.2	-0.33555665	Q9D0L8_S100_M1_Rnmt
EFGEDLVEQNSSYVQDS[Phospho (STY)]PSKK.3	-0.269654364	Q9D0L8_S64_M1_Rnmt
EFGEDLVEQNSSYVQDS[Phospho (STY)]PSKKR.3	-0.179273276	Q9D0L8_S64_M1_Rnmt
EFGEDLVEQNSSYVQDS[Phospho (STY)]PSK.2	-0.062865274	Q9D0L8_S64_M1_Rnmt
LPENT[Phospho (STY)]PPC[Carbamidomethyl (C)]QQVDQPK.2	0.498914447	Q9D0L8_T34_M1_Rnmt
HLVGVC[Carbamidomethyl (C)]YTEEEAK.2	0.107011111	#N/A
LSDYFPKPYPNPEAAR.3	-0.123290459	#N/A
HGGEDYVFSLLTGYC[Carbamidomethyl (C)]EPPTGVSLR.3	0.326499557	#N/A
GLLSSLDHTSIR.2	0.048603869	#N/A
DVATFLR.2	0.182455036	#N/A
ALAEEVEVQDGPNDDGEM[Oxidation (M)]FM[Oxidation (M)]RPGK.3	-0.46215637	#N/A
WASEPEHDHRK.3	0.140885901	#N/A
DIAAYIKK.2	-0.147557778	#N/A
NFGSYVTHETK.2	-0.084183621	#N/A
YNPTWHC[Carbamidomethyl (C)]IVGR.2	-0.016122118	#N/A
NADM[Oxidation (M)]SEDMQQDAVDC[Carbamidomethyl (C)]ATQAMEK.3	0.136016469	#N/A
ISQAEEDDQQLLGHLLLVAK.3	-0.487365124	#N/A
QQIQSIQQS[Phospho (STY)]IER.2	-0.090824789	Q9D0T1_S122_M1_Snu13
KLS[Phospho (STY)]GDQITLPTTVDYSSVPK.2	0.049805919	Q9D0V7_S36_M1_Ebag9
APGDEEAQVENLITTNAAEPQKAEN.3	-0.320483844	#N/A
APGDEEAQVENLITTNAAEPQK.2	-0.056284029	#N/A
SS[Phospho (STY)]PITVQTSK.2	-0.205282053	Q9D168_S127_M1_Ints12
SVS[Phospho (STY)]C[Carbamidomethyl (C)]DNVSK.2	-0.350029902	Q9D168_S377_M1_Ints12
GVTEAHVDQK.2	-0.225776492	#N/A
ITSLAQLNAANHDAAIFPGGFGAAK.3	-0.008178141	#N/A
NLSTFAVDGK.2	-0.115791043	#N/A
NVLELTGK.2	0.06675855	#N/A
VNKEVER.2	0.000336975	#N/A
NVLAESAR.2	0.127091444	#N/A
GGAEVQIFAPDVPQMHVIDHTK.3	-0.115740114	#N/A
TVFGVEPDLTR.2	0.216131269	#N/A
AVFQYIDENQDR.2	0.09956144	#N/A
TGQEIPVNLR.2	0.503427048	#N/A
LNYIEGTK.2	-0.152451228	#N/A
FAELQSPNK.2	0.205911083	#N/A

EGGSIPVTLTFQEATGK.2	0.085158986	#N/A
YEIS[Phospho (STY)]PEAYER.2	-0.074868591	Q9D1E6_S110_M1_Tbcb
AQQEAEAAQR.2	0.325856827	#N/A
AQASAISVGSR.2	0.015448718	#N/A
SSDEENGPPSS[Phospho (STY)]PDLDR.2	0.174026759	Q9D1F4_S213_M1_Akt1s1
EDEEEDEDEPTETETS[Phospho (STY)]GER.2	0.079354993	Q9D1F4_S117_M1_Akt1s1
LNT[Phospho (STY)]SDFQK.2	-0.185800002	Q9D1F4_T247_M1_Akt1s1
TEARSS[Phospho (STY)]DEENGPPSS[Phospho (STY)]PDLDR.3	0.46457429	Q9D1F4_S204_M2_Akt1s1
AATATRPPGPPPAPQPPS[Phospho (STY)]PAPS[Phospho (STY)]PPPRPALAR.4	0.102815728	Q9D1F4_S88_M2_Akt1s1
EFADSLGVPFLETSAK.2	-0.252095525	#N/A
[Acetyl (Protein N-term)]M[Oxidation (M)]NPEYDYLFK.2	-0.051264178	#N/A
GAHGIIVVYDVTDQESYANVK.3	-0.394211226	#N/A
MGPGAAS[Phospho (STY)]GGERPNLK.2	0.129162593	Q9D1G1_S179_M1_Rab1b
SLSLNNNK.2	0.400187364	#N/A
DTTINEIEDTFR.2	0.215314412	#N/A
DDIGIILINQYIAEM[Oxidation (M)]VR.2	0.287741575	#N/A
GM[Oxidation (M)]FTAEDLR.2	-0.001183028	#N/A
LIAVIGDEDTVTGFLLGGIGELNK.3	-0.025956122	#N/A
LLSVTPTS[Phospho (STY)]EGYSIK.2	-0.156089588	Q9D1K7_S99_M1_
LC[Carbamidomethyl (C)]EGFNEVLR.2	-0.165084355	#N/A
GQGSVSASITEGQQNEQ.2	0.166174183	#N/A
S[Phospho (STY)]PGRVS[Phospho (STY)]PPTPAR.2	-0.221261179	Q9D1P2_S37_M2_Kat8
AFLIEEQK.2	-0.029731773	#N/A
SAC[Carbamidomethyl (C)]GVC[Carbamidomethyl (C)]PGR.2	0.172451001	#N/A
LSYNTASNK.2	-0.433855062	#N/A
S[Phospho (STY)]DAGISSADAPR.2	-0.123401488	Q9D1T0_S596_M1_Lingo1
HNIEIEYVPRKS[Phospho (STY)]DAGISSADAPR.4	-0.221326793	Q9D1T0_S596_M1_Lingo1
FVAVPEGIPTETR.2	-0.217289356	#N/A
LTVFPDGTLEVR.2	-0.055071674	#N/A
KS[Phospho (STY)]DAGISSADAPR.2	-0.095823154	Q9D1T0_S596_M1_Lingo1
ASEQEEVGGPEGS[Phospho (STY)]EALQPR.2	0.077340159	Q9D1X0_S142_M1_Nol3
LVETLQADSGLLLDALVAR.2	-0.15865698	#N/A
IPVEGPLS[Phospho (STY)]PSR.2	-0.076321276	Q9D219_S686_M1_Bcl9
SST[Phospho (STY)]PSHGQTPAPEPISAQK.2	-0.136941142	Q9D219_T155_M1_Bcl9
FSNQGKPGGSASQSQPS[Phospho (STY)]PC[Carbamidomethyl (C)]DSK.3	0.293498427	Q9D219_S62_M1_Bcl9
AADS[Phospho (STY)]DDDVPPVPTPSDGGVNK.2	0.069648795	Q9D2E2_S5_M1_Toe1
VEGGTPLFTLR.2	0.17995027	#N/A
AKPAETPAPAHK.3	-0.000686151	#N/A
NVETM[Oxidation (M)]NYADIER.2	0.367315084	#N/A
ASAFALQEQPVVNAVIDDATK.2	-0.21699342	#N/A
TINELGEK.2	0.086989537	#N/A
NDVITVQTPAFAESVTEGDVR.2	-0.035183674	#N/A
LPQPPEGQTYNN.2	-0.093903566	#N/A
LLEELEEGQK.2	0.239144121	#N/A
VILQELR.2	0.268748564	#N/A
WTGM[Oxidation (M)]IIGPPR.2	-0.393114727	#N/A
YPEAPPSVR.2	0.170809413	#N/A

ENM[Oxidation (M)]KLPQPPEGQTYNN.2	-0.330597028	#N/A
VEC[Carbamidomethyl (C)]GS[Phospho (STY)]KYPEAPPSVR.3	-0.1556964	Q9D2M8_S71_M1_Ube2v2
GVGDGTVSWGLEDDEDM[Oxidation (M)]TLTR.2	-0.508645747	#N/A
SS[Phospho (STY)]PSHTISR.2	0.396037893	Q9D2N4_S568_M1_Dtna
HQPAAS[Phospho (STY)]PVVVR.2	-0.12639214	Q9D2P8_S85_M1_Mobp
ATS[Phospho (STY)]PQRPK.2	-0.12428187	Q9D2P8_S74_M1_Mobp
GGS[Phospho (STY)]PTRAPR.2	-0.03608329	Q9D2P8_S162_M1_Mobp
S[Phospho (STY)]PPRPAKPR.2	0.018466983	Q9D2P8_S107_M1_Mobp
RATS[Phospho (STY)]PQRPK.2	0.075487934	Q9D2P8_S74_M1_Mobp
SS[Phospho (STY)]PLRGPGTSR.2	-0.499440882	Q9D2P8_S150_M1_Mobp
S[Phospho (STY)]PLM[Oxidation (M)]PAKPR.2	0.140258488	Q9D2P8_S98_M1_Mobp
PEPSRST[Phospho (STY)]PAPK.2	-0.054316055	Q9D2U9_T8_M1_Hist3h2ba
IWDLQTGAER.2	0.076582855	#N/A
S[Phospho (STY)]LQSLLGPSSK.2	0.063958489	Q9D2V7_S459_M1_Coro7
EDPLPQDS[Phospho (STY)]FEGVDEDEWD.2	-0.118015028	Q9D2V7_S912_M1_Coro7
LGNREDPLPQDS[Phospho (STY)]FEGVDEDEWD.3	-0.452418871	Q9D2V7_S912_M1_Coro7
SPIAEAVFR.2	0.063804233	#N/A
SVLFVC[Carbamidomethyl (C)]LGNIC[Carbamidomethyl (C)]R.2	-0.128814326	#N/A
VHFVYDSSEK.2	-0.098757693	#N/A
NYVEELNR.2	-0.107321458	#N/A
TAEGQALSEAR.2	0.234599884	#N/A
GLIESALNLGR.2	0.419089876	#N/A
LQSSDLGVK.2	0.082069726	#N/A
VS[Phospho (STY)]M[Oxidation (M)]DGEWLC[Carbamidomethyl (C)]LR.2	-0.363872035	Q9D394_S34_M1_Rufy3
_[Acetyl (Protein N-term)]SALT[Phospho (STY)]PPTDM[Oxidation (M)]PT[Phospho (STY)]PTTDKITQAAM[Oxidation (M)]ETIYLC[Carbamid	-0.537067494	Q9D394_T12_M2_Rufy3
[Acetyl (Protein N-term)]SALT[Phospho (STY)]PPTDM[Oxidation (M)]PTPTTDK.2	-0.309313167	Q9D394_T5_M1_Rufy3
DGNS[Phospho (STY)]SKGSEGDGQITAILDQK.3	0.319443845	Q9D394_S247_M1_Rufy3
GEDLDSQVGVIDFSM[Oxidation (M)]YLK.2	-0.03747255	#N/A
VKEESSYLLESNR.3	0.048159285	#N/A
IEANEALVK.2	0.225254016	#N/A
IEANEALVKALE.2	0.463780946	#N/A
AQSELSGAADEAAR.2	0.254220201	#N/A
SES[Phospho (STY)]PTADSDASSLHEQPQQIAIK.3	-0.057318004	Q9D3X9_S298_M1_Mfap3l
GC[Carbamidomethyl (C)]S[Phospho (STY)]QDDEC[Carbamidomethyl (C)]VSLR.2	-0.357980903	Q9D415_S509_M1_Dlgap1
SHS[Phospho (STY)]LEGPSK.2	-0.282727822	Q9D415_S154_M1_Dlgap1
ERS[Phospho (STY)]LESSQR.2	-0.301152056	Q9D415_S947_M1_Dlgap1
SLDSLDPAGLLTS[Phospho (STY)]PK.2	-0.080090421	Q9D415_S437_M1_Dlgap1
AVSEVS[Phospho (STY)]INR.2	0.063616185	Q9D415_S421_M1_Dlgap1
AS[Phospho (STY)]PDESQTLR.2	0.047475079	Q9D415_S169_M1_Dlgap1
SRNES[Phospho (STY)]YM[Oxidation (M)]R.2	0.300413501	Q9D415_S446_M1_Dlgap1
AM[Oxidation (M)]GDEDS[Phospho (STY)]GDS[Phospho (STY)]DTS[Phospho (STY)]PKPSPK.2	-0.163334555	Q9D415_S365_M3_Dlgap1
GSVNGGKAS[Phospho (STY)]PDESQTLR.2	-0.071288869	Q9D415_S169_M1_Dlgap1
AIEKGC[Carbamidomethyl (C)]S[Phospho (STY)]QDDEC[Carbamidomethyl (C)]VSLR.3	-0.306156344	Q9D415_S509_M1_Dlgap1
GDMISQSGLSNS[Phospho (STY)]TESLDSM[Oxidation (M)]K.2	-0.38109883	Q9D415_S605_M1_Dlgap1
QNS[Phospho (STY)]ATESAESIEIYIPEAQTR.3	0.04445348	Q9D415_S973_M1_Dlgap1
C[Carbamidomethyl (C)]LS[Phospho (STY)]IGIQVDDAEEPEK.2	0.118104653	Q9D415_S666_M1_Dlgap1
RSAS[Phospho (STY)]LATK.2	-0.405745634	Q9D428_S16_M1_GOLGA7B

DYS[Phospho (STY)]DGTIC[Carbamidomethyl (C)]QFQTK.2	0.353758316	Q9D428_S28_M1_GOLGA7B
SQS[Phospho (STY)]VVEAGTLK.2	-0.02739606	Q9D4C9_S329_M1_Clvs1
HTC[Carbamidomethyl (C)]S[Phospho (STY)]NLER.2	0.331598707	Q9D4C9_S313_M1_Clvs1
SQS[Phospho (STY)]VVEAGTLKHEEK.2	-0.154044469	Q9D4C9_S329_M1_Clvs1
EC[Carbamidomethyl (C)]S[Phospho (STY)]PKPMK.2	0.24562905	Q9D4C9_S320_M1_Clvs1
EC[Carbamidomethyl (C)]S[Phospho (STY)]PKPMKR.2	0.331345556	Q9D4C9_S320_M1_Clvs1
ASDS[Phospho (STY)]PVHR.2	-0.042744135	Q9D4F2_S60_M1_Plpp6
ASNTADTLFQEVLGR.2	0.297953389	#N/A
GSS[Phospho (STY)]FQSGRDDTWR.2	-0.144205102	Q9D4H1_S432_M1_Exoc2
NSADDEELT[Phospho (STY)]NDS[Phospho (STY)]LTLSQSK.2	-0.168268228	Q9D4H9_S283_M2_Phf14
SNS[Phospho (STY)]LPHSAVSNAASK.2	-0.353107036	Q9D5R2_S432_M1_Wdr20
EYVNSTSEES[Phospho (STY)]HDEDEIRPVQQQDLHR.4	-0.247776241	Q9D5T0_S322_M1_Atad1
ESS[Phospho (STY)]PSNSPSPQGFR.2	-0.244705758	Q9D620_S340_M1_Rab11fip1
GPPGC[Carbamidomethyl (C)]SPC[Carbamidomethyl (C)]PES[Phospho (STY)]PSR.2	-0.1405582	Q9D650_S67_M1_Lratd1
C[Carbamidomethyl (C)]SLQREDS[Phospho (STY)]DGGTGGPAR.2	-0.11898542	Q9D6E4_S226_M1_Ttc9b
EDS[Phospho (STY)]DGGTGGPAR.2	0.148140947	Q9D6E4_S226_M1_Ttc9b
IREEFPDR.2	-0.030131006	#N/A
GHYTEGAELVDAVLDVVR.3	-0.078582182	#N/A
GHYTEGAELVDAVLDVVRK.3	-0.471449945	#N/A
EVDEQM[Oxidation (M)]LSVQSK.2	0.051789357	#N/A
M[Oxidation (M)]AATFIGNSTAIQELFK.2	-0.352705065	#N/A
INVYYNEATGGNYVPR.2	-0.094217909	#N/A
RGS[Phospho (STY)]WDSLPDTSSTDR.2	-0.349741177	Q9D6I9_S126_M1_Lurap1
GGDPTKEPEPVVHYDI.3	-0.062177913	#N/A
QYPYNNLYLER.2	-0.284414454	#N/A
DPWYQWDHSELR.2	0.237550362	#N/A
DSDSILETLQR.2	-0.062187252	#N/A
DIEEIIDELK.2	0.141805205	#N/A
DTPENNPDTPFDFTPENYKR.3	-0.135571961	#N/A
DTPENNPDTPFDFTPENYK.2	-0.161146004	#N/A
NYPEGHQAAAVLPVLDLAQR.3	-0.253108586	#N/A
FC[Carbamidomethyl (C)]C[Carbamidomethyl (C)]EPAGGLTSLTEPPKGPGFGVQAGL.3	-0.251459488	#N/A
VYEVATFYTM[Oxidation (M)]YNR.2	-0.127166049	#N/A
YHIQVC[Carbamidomethyl (C)]TTTPC[Carbamidomethyl (C)]M[Oxidation (M)]LR.3	-0.046201501	#N/A
LAANDFFR.2	0.062415951	#N/A
HEGPSAFLK.2	0.351753466	#N/A
NHGIAGLYK.2	8.18E-05	#N/A
GVNEDTYSGFLDC[Carbamidomethyl (C)]AR.2	-0.174614726	#N/A
SEGYFGMYR.2	0.344728551	#N/A
LINGGIAGLIGVTC[Carbamidomethyl (C)]VFPIDLAK.2	-0.179665671	#N/A
SPFYVSFLAGC[Carbamidomethyl (C)]VAGSAAAVAVNPC[Carbamidomethyl (C)]DVVK.3	0.119041098	#N/A
APIQWEER.2	0.224524741	#N/A
TPYTDVNIVTIR.2	0.177695784	#N/A
NVTAIQGPGGK.2	0.122959544	#N/A
LITEEASKR.2	0.242899318	#N/A
IAEFAFEYAR.2	-0.390617756	#N/A
SNVTAVHK.2	0.263992849	#N/A

ENTEGEYSGIEHVIVDGVVQSIK.3	-0.395833919	#N/A
C[Carbamidomethyl (C)]SDFTEEIC[Carbamidomethyl (C)]R.2	0.396930373	#N/A
IEAAC[Carbamidomethyl (C)]FATIK.2	0.27267056	#N/A
EVAENC[Carbamidomethyl (C)]KDIK.2	-0.438104924	#N/A
M[Oxidation (M)]SDGLFLQK.2	-0.105372679	#N/A
HM[Oxidation (M)]GLFDHAAK.2	-0.16023885	#N/A
TPIAAGHPSM[Oxidation (M)]NLLLR.2	-0.351399508	#N/A
C[Carbamidomethyl (C)]SDFTEEIC[Carbamidomethyl (C)]RR.2	-0.078246933	#N/A
ALVEDIISLEEVDEDM[Oxidation (M)]K.2	-0.256212406	#N/A
TLS[Phospho (STY)]GEEEAESVGVSSR.2	-0.086052811	Q9D6W8_S43_M1_Borcs6
S[Phospho (STY)]LDGLSGAC[Carbamidomethyl (C)]GGGGSSSSGETGAGGGR.2	0.03989011	Q9D6W8_S173_M1_Borcs6
S[Phospho (STY)]LDGLSGAC[Carbamidomethyl (C)]GGGGSSSSGETGAGGGRR.3	-0.172300091	Q9D6W8_S173_M1_Borcs6
RGS[Phospho (STY)]PGGVEMNVELPQQEGDDDDDEDEEAAAGR.3	-0.486787664	Q9D6W8_S130_M1_Borcs6
EEVAS[Phospho (STY)]EPEEAASPTTPK.2	-0.286521623	Q9D6Z1_S536_M1_Nop56
SSPKEEVAS[Phospho (STY)]EPEEAASPTTPK.3	-0.17058569	Q9D6Z1_S536_M1_Nop56
EELAS[Phospho (STY)]DLEEM[Oxidation (M)]ATSSAK.2	0.204325357	#N/A
KSSPKEEVAS[Phospho (STY)]EPEEAASPTTPK.3	0.012787643	Q9D6Z1_S536_M1_Nop56
APKEELAS[Phospho (STY)]DLEEM[Oxidation (M)]ATSSAK.2	-0.058533613	#N/A
KAPKEELAS[Phospho (STY)]DLEEMATSSAK.3	0.102928849	#N/A
AVDNPPRGS[Phospho (STY)]PK.2	-0.208782108	Q9D706_S429_M1_Rpap3
NGSM[Oxidation (M)]GS[Phospho (STY)]PVNQQPK.2	0.073838591	Q9D772_S44_M1_Fam219a
ENISEWR.2	0.099923329	#N/A
VYAILTHGIFSGPAISR.3	-0.309428286	#N/A
INNAC[Carbamidomethyl (C)]FEAVVVTNTIPQEDK.2	-0.216037621	#N/A
IQVIDISM[Oxidation (M)]ILAEAIR.2	0.044799037	#N/A
VAILVDDMADTC[Carbamidomethyl (C)]GTIC[Carbamidomethyl (C)]HAADK.3	-0.329663512	#N/A
IFSGSSHQDLSQK.2	-0.251749521	#N/A
ELS[Phospho (STY)]APAR.2	-0.155679304	Q9D7P6_S30_M1_Iscu
AAS[Phospho (STY)]ALLLRS[Phospho (STY)]PR.2	-0.21732831	Q9D7P6_S15_M2_Iscu
RAAS[Phospho (STY)]ALLLRS[Phospho (STY)]PR.3	0.080246349	Q9D7P6_S15_M2_Iscu
RAAS[Phospho (STY)]ALLLR.2	-0.103856437	Q9D7P6_S15_M1_Iscu
YFQISQDEDGS[Phospho (STY)]ESED.2	0.387097297	Q9D7S7_S118_M1_Rpl22l1
NKDGVLVDEFGLPQIPAS.2	-0.432920571	#N/A
[Acetyl (Protein N-term)]ADLS[Phospho (STY)]PAPALR.2	-0.249593999	Q9D7V2_S5_M1_Lysmd2
AHRPSAPS[Phospho (STY)]PPPR.3	-0.598424088	Q9D7V2_S24_M1_Lysmd2
STS[Phospho (STY)]EPEEAELSLSLAR.2	-0.218913878	Q9D7V2_S33_M1_Lysmd2
HNS[Phospho (STY)]VEDAEQGK.2	-0.515860219	Q9D7X1_S20_M1_Kctd4
HNS[Phospho (STY)]VEDAEQGKNC[Carbamidomethyl (C)]K.2	-0.46466379	Q9D7X1_S20_M1_Kctd4
VYVGNASVAQDITQLQK.2	0.256797306	#N/A
ANDTQEFNLSAYFER.2	-0.463479389	#N/A
EIGPNDGFLAQLC[Carbamidomethyl (C)]QLNDR.2	-0.195295445	#N/A
LGITHVLNAAEGR.3	-0.011968012	#N/A
GKIS[Phospho (STY)]DEM[Oxidation (M)]EDIIK.2	0.173820234	Q9D7X8_S173_M1_Ggct
AAPFTLEYR.2	0.139162131	#N/A
VIAINVDDPDAANYKDISDVER.3	-0.348038513	#N/A
HTGC[Carbamidomethyl (C)]C[Carbamidomethyl (C)]GDNDPIDVC[Carbamidomethyl (C)]EIGSK.3	0.003281124	#N/A
VLGILAM[Oxidation (M)]IDEGETDWK.2	0.274924317	#N/A

STHDYWK.2	-0.041955263	#N/A
ERDHS[Phospho (STY)]PTPSVFNSDEER.3	-0.323387826	Q9D824_S479_M1_Fip1l1
ERDHS[Phospho (STY)]PTPSVFNSDEERYR.4	-0.327281141	Q9D824_S479_M1_Fip1l1
AES[Phospho (STY)]PDLR.2	-0.057946943	Q9D824_S280_M1_Fip1l1
TTGASQLQGS[Phospho (STY)]PEITK.2	0.331019757	Q9D842_S149_M1_Aplf
RLS[Phospho (STY)]PSGNSK.2	0.109997736	Q9D842_S238_M1_Aplf
WYYNAAGFNK.2	0.110653981	#N/A
LPEDLYNDR.2	0.29805675	#N/A
DDTLHETEDVKEAIR.3	-0.07269888	#N/A
DDTLHETEDVK.2	0.415838571	#N/A
RLPEDLYNDR.2	0.024958622	#N/A
YEEDKFYLEPYLK.2	-0.364724677	#N/A
SS[Phospho (STY)]SSQQGFFNR.2	-0.04369344	Q9D882_S27_M1_Fam241b
AVIDLNNR.2	-0.299978282	#N/A
IDLPEYQGEPDEISIQK.2	0.089601034	#N/A
IVFVTGNAK.2	-0.399323236	#N/A
LQEYFSVAAGAGDH.2	-0.01776598	#N/A
ALAVGGLGSIIR.2	0.087890624	#N/A
AFHAALRNS[Phospho (STY)]PINTK.3	-0.145665317	Q9D898_S64_M1_Arpc5l
NS[Phospho (STY)]PINTK.2	0.358145306	Q9D898_S64_M1_Arpc5l
SSEIEQAVQSLDR.2	0.151125511	#N/A
GGPTPQEAIQR.2	0.218739471	#N/A
KIEQELTAAK.2	0.249724359	#N/A
QLAQIDGTLSTIEFQR.2	0.038694852	#N/A
FFESYHEVPDGTQC[Carbamidomethyl (C)]HR.3	0.265004305	#N/A
EKPDDPLNYFIGGC[Carbamidomethyl (C)]AGGLTLGAR.3	0.016259363	#N/A
ILKS[Phospho (STY)]PEIQR.2	-0.424678373	Q9D8E6_S295_M1_Rpl4
FC[Carbamidomethyl (C)]IWTESAFR.2	0.321094956	#N/A
QPYAVSELAGHQTSAESWGTGR.3	0.008608549	#N/A
SGQGAFGNM[Oxidation (M)]C[Carbamidomethyl (C)]R.2	-0.219750862	#N/A
TAGSSEDHHS[Phospho (STY)]S[Phospho (STY)]QTDNITLA.2	-0.117689923	Q9D8I2_S328_M2_P2yr13
STFVLDEFKR.2	-0.146062905	#N/A
[Acetyl (Protein N-term)]AAGTLYTYPENWR.2	-0.044964619	#N/A
TFLVGER.2	0.268291756	#N/A
QVLEPSFR.2	0.21692025	#N/A
ILGLLDTHLK.2	-0.00457682	#N/A
WFLTC[Carbamidomethyl (C)]INQPQFR.2	0.167096592	#N/A
ALIAAQYSGAQVR.2	0.242872097	#N/A
KLDPGSEETQTLVR.2	-0.259440285	#N/A
KFAESQPK.2	-0.030849823	#N/A
QATENAKEEVKR.3	0.118342426	#N/A
LTNTS[Phospho (STY)]FTEIEK.2	-0.234571921	Q9D8S3_S241_M1_Arfgap3
SSS[Phospho (STY)]FSSWDDGADSYWK.2	0.28695407	Q9D8S3_S374_M1_Arfgap3
FEGM[Oxidation (M)]S[Phospho (STY)]PLQR.2	0.005766538	Q9D8S9_S81_M1_Bola1
ENPQLDIS[Phospho (STY)]PPC[Carbamidomethyl (C)]LGGSK.2	-0.024263151	Q9D8S9_S123_M1_Bola1
AKLEQALS[Phospho (STY)]PEVLELR.3	0.159245033	Q9D8S9_S45_M1_Bola1
ALHGAQTS[Phospho (STY)]DEER.2	0.025335725	Q9D8T7_S105_M1_Slirp

[Acetyl (Protein N-term)]AAVPELLEQQEEDR.2	-0.224395599	#N/A
[Acetyl (Protein N-term)]AAVPELLEQQEEDRSK.2	-0.287806631	#N/A
FHSFEDQLR.2	0.301399283	#N/A
[Acetyl (Protein N-term)]ASVSTHGNQEK.2	0.391867214	#N/A
GAGFGPEHNR.2	0.142118893	#N/A
ADLNQGIGEPQS[Phospho (STY)]PSR.2	-0.073280202	Q9D8Y0_S74_M1_Efhd2
RADLNQGIGEPQS[Phospho (STY)]PSR.3	-0.343150495	Q9D8Y0_S74_M1_Efhd2
VQAINVSSR.2	0.083891935	#N/A
LSEIDVSTEGVK.2	0.208990697	#N/A
RADLNQGIGEPQS[Phospho (STY)]PSRR.3	-0.32013795	Q9D8Y0_S74_M1_Efhd2
ADLNQGIGEPQS[Phospho (STY)]PSRR.3	-0.428966075	Q9D8Y0_S74_M1_Efhd2
[Acetyl (Protein N-term)]ATDELASKLS[Phospho (STY)]R.2	0.034758536	Q9D8Y0_S11_M1_Efhd2
ELQSTFK.2	0.518234251	#N/A
SMIQEVDEDFDSK.2	-0.484468973	#N/A
GLLSGQTS[Phospho (STY)]PTNAK.2	-0.167799627	Q9D920_S75_M1_Borcs5
GLLSGQTS[Phospho (STY)]PTNAKLEK.2	0.112172261	Q9D920_S75_M1_Borcs5
KLS[Phospho (STY)]DEPSVQAK.2	0.134630537	Q9D9S3_S141_M1_Rwdd2a
KLS[Phospho (STY)]DEPSVQAKPVK.2	0.272390782	Q9D9S3_S141_M1_Rwdd2a
SVSAPQIS[Phospho (STY)]SPEESAEGADTIK.2	-0.154803147	Q9DAI6_S775_M1_Fam135b
NTAGFSEDLDPSSKENS[Phospho (STY)]PPR.3	-0.525216101	Q9DAI6_S805_M1_Fam135b
TASTNS[Phospho (STY)]IAQAR.2	-0.303424652	Q9DAS9_S10_M1_Gng12
SDPLLM[Oxidation (M)]GIPTSENPFK.2	-0.403694699	#N/A
LEASIER.2	-0.268416818	#N/A
ASADLM[Oxidation (M)]SYC[Carbamidomethyl (C)]EEHAR.2	-0.709333479	#N/A
VLTANSNPSS[Phospho (STY)]PSAAK.2	-0.229276873	Q9DAT2_S195_M1_Mrgbp
WS[Phospho (STY)]PPQNYK.2	0.339881983	Q9DAZ9_S69_M1_Zfyve19
VTLQDYHLPDS[Phospho (STY)]DEDEETAIQR.3	-0.112914342	Q9DAZ9_S280_M1_Zfyve19
TNS[Phospho (STY)]QGQASQSLEEEKYK.2	0.086504926	Q9DAZ9_S219_M1_Zfyve19
EHQTS[Phospho (STY)]PYHPR.2	-0.069142231	Q9DAZ9_S378_M1_Zfyve19
YEELFPAFSDSR.2	0.007079311	#N/A
TIQGDEEDLR.2	0.089905237	#N/A
VAGYAAQLEQYQK.2	0.031431959	#N/A
NSQSFFSGLFGGSSK.2	-0.463352646	#N/A
LLEAHEEQNVDSYTEAVKEYDSISR.4	-0.246246448	#N/A
AIDIYEQVGTSAM[Oxidation (M)]DSPLLK.2	0.178388976	#N/A
LLEAHEEQNVDSYTEAVK.3	-0.274916373	#N/A
AIEIYTDMGR.2	0.124687965	#N/A
HDAATC[Carbamidomethyl (C)]FVDAGNAFK.2	-0.14352815	#N/A
NYVQGINLVQAK.2	0.047952007	#N/A
LTEAKPVDK.2	0.333520202	#N/A
LVRPPVQVYGIEGR.3	-0.051066008	#N/A
VSLAVLNPYIK.2	-0.023275077	#N/A
SFLSPNQILK.2	0.065103001	#N/A
YATALYSAASK.2	0.263894531	#N/A
SLNDITKR.2	0.157690347	#N/A
EKFSPLTANLM[Oxidation (M)]NLLAENGR.3	-0.497790534	#N/A
FSPLTANLM[Oxidation (M)]NLLAENGR.2	-0.25783532	#N/A

LGNTQGIISAFSTIM[Oxidation (M)]SVHR.3	-0.165493065	#N/A
TDPSIMGGMIVR.2	0.295143792	#N/A
GEVPC[Carbamidomethyl (C)]TVTTASPLDDAVLSELK.2	-0.094682546	#N/A
YVDMSAK.2	-0.077722051	#N/A
IQLQDAGR.2	0.130421715	#N/A
GAAVNLTLVTPEK.2	0.00020879	#N/A
LAANDFLR.2	-0.14883261	#N/A
ASFTHSFVAGC[Carbamidomethyl (C)]TAGSVAAVAVTPLDVLK.3	0.326022915	#N/A
VSTTTDSPVS[Phospho (STY)]PAQAASPFIPVDELSSK.3	-0.148345554	Q9DB52_S267_M1_Pabir1
RIDFIPVS[Phospho (STY)]PAPS[Phospho (STY)]PTR.2	0.101543134	Q9DB52_S140_M2_Pabir1
RNS[Phospho (STY)]TTFPSR.2	-0.647840844	Q9DB52_S59_M1_Pabir1
SNS[Phospho (STY)]APLIHGLSDSSPVFQAEAPSAR.3	0.068637711	Q9DB52_S34_M1_Pabir1
SILDQHDVR.2	-0.263585906	#N/A
WIAQDLSNLR.2	0.383644329	#N/A
NPPPQDYES[Phospho (STY)]DDESYEVLDLTEYAR.3	0.295072288	Q9DB70_S13_M1_Fundc1
[Acetyl (Protein N-term)]S[Phospho (STY)]NEPPPPYPGGPTAPLLEEK.2	0.189260298	Q9DB75_S2_M1_Cdip1
TSAAPGGVPLQPQDLEFTK.2	0.205793936	#N/A
AVAQGNLSSADVQAAK.2	0.465936775	#N/A
GNNTTSLLSQSVAK.2	0.225178211	#N/A
AVAFQNSQTR.2	0.148299312	#N/A
NALANPLYC[Carbamidomethyl (C)]PDYR.2	0.042389268	#N/A
YRGGEIR.2	-0.287933113	#N/A
WEVAALR.2	-0.460547492	#N/A
LPNGLVIASLENYAPLSR.2	0.508844401	#N/A
YEDSNNLGTSHLLR.2	-0.265247743	#N/A
M[Oxidation (M)]ALVGLGVSHSVLK.2	-0.088929428	#N/A
ENM[Oxidation (M)]AYTVEGIR.2	0.543103135	#N/A
IIENLHDVAYK.2	-0.170083518	#N/A
SM[Oxidation (M)]AASGNLGHTPFLDEL.2	0.000264731	#N/A
QVAEQFLNM[Oxidation (M)]R.2	0.245621543	#N/A
SQLKIDK.2	-0.257876569	#N/A
LASSLTTK.2	-0.07923813	#N/A
ITSEELHYFVQNHFTSAR.3	-0.393090387	#N/A
WKEPGSSGPQNLS[Phospho (STY)]GPGGR.3	-0.505132386	Q9DB90_S32_M1_Smg9
LEQLS[Phospho (STY)]PSDLQR.2	0.029207809	Q9DBC0_S544_M1_Selenoo
HLS[Phospho (STY)]STSDDEPLSSVNHAAK.3	0.147199127	Q9DBC3_S27_M1_Cmtr1
EDEIS[Phospho (STY)]PPPPNPVVK.2	-0.246064922	Q9DBC7_S83_M1_Prkar1a
LTVADALEPVQFEDGQK.2	-0.090209809	#N/A
TDSREDEIS[Phospho (STY)]PPPPNPVVK.3	-0.165639928	Q9DBC7_S83_M1_Prkar1a
FQDEEEVFEWNNEVK.2	-0.065839618	#N/A
GEVITTYC[Carbamidomethyl (C)]PANNEPIAR.2	-0.339629729	#N/A
VNLLSFTGSTQVGK.2	0.536044608	#N/A
DIPNENELQFQIK.2	-0.015181476	#N/A
IQPGNPNYTLSLK.2	0.056825648	#N/A
LLSTDPVTAK.2	0.110806019	#N/A
LQNNNVYTIAK.2	-0.189509114	#N/A
QVFLATWK.2	0.122097159	#N/A

VNYVVQEAIVVIR.2	-0.063852241	#N/A
APEVSQYIYQVYDSILKN.3	-0.303702521	#N/A
DSDYYNM[Oxidation (M)]LLK.2	0.140445387	#N/A
KPSETQELVQQVLSLATQDSDNPDLR.3	-0.305796105	#N/A
LHDINAQM[Oxidation (M)]VEDQGFLDSLR.3	-0.399919333	#N/A
LAPPLVTLLSGEPEVQYVALR.2	0.198150008	#N/A
GLEISGTFTHR.2	0.067565745	#N/A
EC[Carbamidomethyl (C)]HLNADTVSSK.2	-0.063245337	#N/A
DLIADSNPM[Oxidation (M)]VVANAVAALSEISESHPNSNLLDLNPQNINK.4	-0.304382798	#N/A
APEVSQYIYQVYDSILK.2	-0.029821952	#N/A
LTNGIWILAELR.2	-0.148964552	#N/A
YHVPVVVVPEGSTSDTQEQAILR.3	-0.063069521	#N/A
TATAASEQS[Phospho (STY)]PGAR.2	-0.356602697	Q9DBH0_S211_M1_Wwp2
ALPFWNEEIVPQIK.2	0.167722621	#N/A
VLIAAHGNSLR.2	0.315285796	#N/A
HYGGLTGLNK.2	0.101579413	#N/A
YADLTEDQLPSC[Carbamidomethyl (C)]ESLKDTIAR.3	-0.003136057	#N/A
FSGWYDADLSPAGHEEAK.2	-0.016315329	#N/A
AM[Oxidation (M)]EAVAAQGK.2	0.244914178	#N/A
YADLTEDQLPSC[Carbamidomethyl (C)]ESLK.2	-0.335562178	#N/A
HGES[Phospho (STY)]AWNLENR.2	-0.323089624	Q9DBJ1_S14_M1_Pgam1
KAM[Oxidation (M)]EAVAAQGK.2	0.237271547	#N/A
SYDVPPPPM[Oxidation (M)]EPDHPFYSNISK.3	-0.425243024	#N/A
DAGYEFDIC[Carbamidomethyl (C)]FTSVQK.2	-0.41202347	#N/A
FSGWYDADLSPAGHEEAKR.3	0.07956342	#N/A
HLEGLSEEAIM[Oxidation (M)]ELNLPTGIPIVYELDK.3	-0.188978443	#N/A
TLWTVLDAIDQMWLPVVR.2	-0.418193645	#N/A
YAIGSLNEGR.2	-0.245186162	#N/A
IFDFQGLQHQVAQVATQLEATR.3	-0.060151966	#N/A
ENGPYFASYPPS[Phospho (STY)]PDL.2	-0.260032493	Q9DBL2_S495_M1_Gdap2
EEKHPASAQSTPSST[Phospho (STY)]PHAS[Phospho (STY)]PK.3	0.102581693	Q9DBN4_S183_M2_P33monox
HPASAQSTPSSTPHAS[Phospho (STY)]PK.3	0.030144585	Q9DBN4_S183_M1_P33monox
EGEGSLRPSS[Phospho (STY)]PQR.2	0.310859427	Q9DBN9_S150_M1_Ddx59
NQDNLQGWNK.2	0.057241479	#N/A
FLIDGFPR.2	0.282839404	#N/A
IVPVEITISLLK.2	0.121578508	#N/A
IQTYLESTKPIIDLYEEM[Oxidation (M)]GK.3	-0.019807485	#N/A
SVDEVFGEVVK.2	-0.136249566	#N/A
YGYTHLSAGELLR.3	-0.062411716	#N/A
KIDASKS[Phospho (STY)]VDEVFGEVVK.3	-0.207090269	Q9DBP5_S180_M1_Cmpk1
SDDNRESLEK.2	-0.171387948	#N/A
KNPDSQYGELIEK.3	0.26187685	#N/A
ADS[Phospho (STY)]DGDLS[Phospho (STY)]ENDDGAGDLR.2	0.166399941	Q9DBR0_S320_M2_Akap8
S[Phospho (STY)]IEDKTR.2	0.035419091	Q9DBR0_S634_M1_Akap8
SNS[Phospho (STY)]STFIR.2	0.312873886	Q9DBR0_S201_M1_Akap8
AEDS[Phospho (STY)]DS[Phospho (STY)]EPEPEDNVR.2	-0.089290104	Q9DBR1_S499_M2_Xrn2
NS[Phospho (STY)]PGC[Carbamidomethyl (C)]QVASNPR.2	-0.341448905	Q9DBR1_S448_M1_Xrn2

RKAEDS[Phospho (STY)]DS[Phospho (STY)]EPEPEDNVR.2	-0.031143752	Q9DBR1_S499_M2_Xrn2
KAEDS[Phospho (STY)]DS[Phospho (STY)]EPEPEDNVR.2	-0.246165995	Q9DBR1_S499_M2_Xrn2
AGTPAHES[Phospho (STY)]PQSSIFR.2	-0.170939249	Q9DBR2_S130_M1_Fam13c
SEAVGPEPGS[Phospho (STY)]S[Phospho (STY)]GEETSDAVVPEKR.3	0.351621348	Q9DBR2_S405_M2_Fam13c
SAS[Phospho (STY)]YSYLEDR.2	-0.357799966	Q9DBR7_S909_M1_Ppp1r12a
T[Phospho (STY)]GSYGALAEISASK.2	-0.161298218	Q9DBR7_T443_M1_Ppp1r12a
DKKS[Phospho (STY)]PLIESTANMENNQPQK.3	0.030971967	Q9DBR7_S299_M1_Ppp1r12a
IS[Phospho (STY)]PKEEER.2	-0.243407218	Q9DBR7_S422_M1_Ppp1r12a
STGVSFWTQDS[Phospho (STY)]DENEQER.2	0.187188709	Q9DBR7_S861_M1_Ppp1r12a
SPLIESTANM[Oxidation (M)]ENNQPQK.2	-0.21992372	#N/A
KTGS[Phospho (STY)]YGALAEISASK.2	-0.437810728	Q9DBR7_S445_M1_Ppp1r12a
STGVSFWTQDSDENEQERQS[Phospho (STY)]DTEDGSSKR.4	-0.000949492	Q9DBR7_S870_M1_Ppp1r12a
RIS[Phospho (STY)]EMEEELK.2	0.310698067	Q9DBR7_S994_M1_Ppp1r12a
YDFISLQC[Carbamidomethyl (C)]QQVVR.2	-0.003119516	#N/A
DTVDSAGTSPT[Phospho (STY)]AVLAAGEDAGAGRPGAGTPLR.3	0.331098721	Q9DBS2_T16_M1_Tprg1l
VSS[Phospho (STY)]PTVNTTLK.2	-0.496569234	Q9DBS5_S460_M1_Klc4
RSS[Phospho (STY)]ELLVR.2	-0.140519316	Q9DBS5_S566_M1_Klc4
S[Phospho (STY)]SELLVR.2	-0.207200614	Q9DBS5_S565_M1_Klc4
AAS[Phospho (STY)]LNYLNQPNAAPLQVSR.2	0.078668416	Q9DBS5_S590_M1_Klc4
ALVHQLS[Phospho (STY)]NESR.2	-0.133407982	Q9DBS9_S405_M1_Osbpl3
QDS[Phospho (STY)]WEVVEGLR.2	0.016726551	Q9DBS9_S33_M1_Osbpl3
S[Phospho (STY)]LPGNAPC[Carbamidomethyl (C)]LK.2	-0.18086145	Q9DBT5_S19_M1_Ampd2
QIS[Phospho (STY)]QDVKLEPDILLR.3	-0.513856644	Q9DBT5_S87_M1_Ampd2
S[Phospho (STY)]LAESELR.2	0.009178477	Q9DBT5_S53_M1_Ampd2
TDS[Phospho (STY)]DSDLQLYK.2	0.275390674	Q9DBT5_S109_M1_Ampd2
SAPYEFPEES[Phospho (STY)]PIEQLEER.2	0.385901352	Q9DBT5_S70_M1_Ampd2
QIS[Phospho (STY)]QDVK.2	-0.258517216	Q9DBT5_S87_M1_Ampd2
AASFLKDDGS[Phospho (STY)]PPVLSAD.2	-0.38712466	Q9DBU3_S512_M1_Riok3
NSATC[Carbamidomethyl (C)]HSEDS[Phospho (STY)]DLEID.2	0.029869667	Q9DBX2_S296_M1_Pdcl
ESLPLDLS[Phospho (STY)]DDQSNSK.2	0.102784018	Q9DBY8_S190_M1_Nvl
QSFTTVADTPENLR.2	-0.246886587	#N/A
GVQPHVVEMDR.3	0.000178663	#N/A
RGS[Phospho (STY)]GHPAYAEVEPVGEK.3	-0.561433283	Q9DC11_S507_M1_Plxdc2
DGEQS[Phospho (STY)]PNVSLM[Oxidation (M)]QR.2	-0.36085846	Q9DC22_S336_M1_Dcaf6
DSALQDTDDS[Phospho (STY)]DDDPVLIPGAR.2	0.193530319	Q9DC22_S673_M1_Dcaf6
GAPVNVS[Phospho (STY)]SSDLTGR.2	-0.283743303	Q9DC28_S382_M1_Csnk1d
AIQASLAK.2	-0.018792551	#N/A
S[Phospho (STY)]PLTGYVR.2	-0.145859365	Q9DC33_S104_M1_Hmg20a
DSNAPKS[Phospho (STY)]PLTGYVR.3	-0.311821734	Q9DC33_S104_M1_Hmg20a
ISQTNYIPTQQDVLR.2	-0.309195045	#N/A
EYQLNDSASYYLNDLDR.2	-0.061401411	#N/A
NFDFEDVFVNIPR.2	-0.255363107	#N/A
QPVYVADVSK.2	-0.32065611	#N/A
M[Oxidation (M)]GSQVIIPYR.2	-0.272870261	#N/A
SVFPEAIIIRPSDIFGR.3	-0.143598262	#N/A
SSVSGVVATVFGATGFLGR.2	-0.114048147	#N/A
AVQHSNVVINLIGR.3	-0.199366324	#N/A

IHISDVM[Oxidation (M)]PTDLPGLEDLGVQPTPLELK.3	0.178551752	#N/A
GIVNATKDPDAVGK.2	0.103220282	#N/A
LM[Oxidation (M)]GDLGQLTFLEWDAR.2	-0.259057304	#N/A
WLSSEIEETKPAK.3	-0.230564067	#N/A
C[Carbamidomethyl (C)]DVYDIMHLR.3	-0.127518146	#N/A
LDDLINWAR.2	0.064885086	#N/A
SRAEYVVTK.2	0.13717658	#N/A
KVYDQM[Oxidation (M)]PEPR.2	-0.645131915	#N/A
QADVM[Oxidation (M)]IVAGTLTNK.2	-0.013449599	#N/A
TES[Phospho (STY)]IEKEGE.2	-0.002406035	Q9DCB1_S93_M1_Hmgn3
AWSS[Phospho (STY)]TDSDSSNR.2	0.106598612	Q9DCB4_S362_M1_Arpp21
TGS[Phospho (STY)]ESSSSAGSSGSLSR.2	-0.110569525	Q9DCB4_S408_M1_Arpp21
SES[Phospho (STY)]LDEEEKLELQRR.3	0.190464473	Q9DCB4_S32_M1_Arpp21
SES[Phospho (STY)]LDEEEKLELQR.2	0.313932423	Q9DCB4_S32_M1_Arpp21
QSS[Phospho (STY)]SETELRWPDHQR.3	-0.454057253	Q9DCB4_S346_M1_Arpp21
TAS[Phospho (STY)]FGGITVLTR.2	-0.322071355	Q9DCB4_S381_M1_Arpp21
DNS[Phospho (STY)]S[Phospho (STY)]IDKEDNQNR.2	-0.122071947	Q9DCB4_S256_M2_Arpp21
IVS[Phospho (STY)]AQSLAEDDVE.2	0.022973956	Q9DCC8_S135_M1_Tomm20
LVPLLDTGDIIIDGGNSEYR.2	0.064510686	#N/A
VDDFLANEAK.2	0.181029399	#N/A
NPELQNLLLDDFFK.2	0.248090728	#N/A
GILFVGSGVSGGEEGAR.2	0.02965662	#N/A
KDS[Phospho (STY)]LTQAQEQGTVLS.2	-0.163396609	Q9DCD5_S527_M1_Tjap1
KLS[Phospho (STY)]EADNR.2	0.077258705	Q9DCF9_S105_M1_Ssr3
KLS[Phospho (STY)]EADNRK.2	-0.313756092	Q9DCF9_S105_M1_Ssr3
QQS[Phospho (STY)]EEDLLLQDFSR.2	-0.278174078	Q9DCF9_S11_M1_Ssr3
TC[Carbamidomethyl (C)]FS[Phospho (STY)]PNR.2	0.076825096	Q9DCH4_S262_M1_Eif3f
VIGLSSDLQQVGGASAR.2	-0.485659595	#N/A
ARPEPNPVIEGDLKPAK.3	-0.000536516	#N/A
TDRPLPENPYHSR.3	0.095361122	#N/A
FDQC[Carbamidomethyl (C)]VLDK.2	0.382601417	#N/A
PGIVELPTLEELKVEEVK.3	0.044966722	#N/A
LVNGC[Carbamidomethyl (C)]ALNFFR.2	0.029492711	#N/A
AAAHHYGAQC[Carbamidomethyl (C)]DK.3	-0.192479824	#N/A
TSAAS[Phospho (STY)]PPVVPSAEQPRPIVEEELSKK.4	0.352247579	Q9DCL8_S23_M1_Ppp1r2
TSAAS[Phospho (STY)]PPVVPSAEQPRPIVEEELSK.3	-0.361421647	Q9DCL8_S23_M1_Ppp1r2
EQESS[Phospho (STY)]GEEDNDLSPEER.2	0.064364162	Q9DCL8_S123_M1_Ppp1r2
TREQES[Phospho (STY)]S[Phospho (STY)]GEEDNDLSPEER.2	-0.201793704	Q9DCL8_S122_M2_Ppp1r2
EIVLADVIDNDSWR.2	0.255212824	#N/A
DDANNDPQWSEEQLIAAK.2	0.183224888	#N/A
S[Phospho (STY)]PSKEPHFTDFEGK.3	-0.404923544	Q9DCP2_S51_M1_Slc38a3
HLEGLLPVGVPTTDTQR.3	-0.181294834	#N/A
AYEQLGYR.2	-0.072753502	#N/A
FYQPY[Phospho (STY)]SEDTQQQIIR.2	0.345279673	Q9DCR2_Y23_M1_Ap3s1
IS[Phospho (STY)]PQSNVDFDLTLR.2	-0.449887135	Q9DCS2_S149_M1_Mettl26
DTVLLEELGQASGLVLER.2	0.16572889	#N/A
PDSWDKDVYPEPPSR.3	-0.125349012	#N/A

YLDLGAYYSAR.2	0.080341043	#N/A
AYDLVVDWPVTLVR.2	0.480108858	#N/A
RVPDITEC[Carbamidomethyl (C)]K.2	0.312731231	#N/A
EGDVLC[Carbamidomethyl (C)]IYEAEM[Oxidation (M)]QWR.2	-0.195936715	#N/A
DFKVDQEIM[Oxidation (M)]NIIQER.3	-0.431924218	#N/A
VDQEIMNIIQER.2	0.43631872	#N/A
SLADLTAVDVPTR.2	0.177002035	#N/A
VVAEPVELAQEFR.2	0.205559936	#N/A
DFPLTGYVELR.2	0.346924163	#N/A
YDDEVKR.2	0.045187628	#N/A
DHTNAQFK.2	-0.18608313	#N/A
FEIVYNLLSLR.2	-0.08128413	#N/A
LEAGDKKPETK.2	-0.023502699	#N/A
QLSAFGEYVAEILPK.2	0.375341242	#N/A
ILTDYGFEGHPFR.2	0.194058163	#N/A
FDLNSPWEAFPAYR.2	0.379864468	#N/A
VYEDSGIPLPAES[Phospho (STY)]PK.2	-0.062109311	Q9DCT6_S96_M1_Bap18
GVNTGAVGSYIYDKDPEGTVQP.2	-0.126924041	#N/A
KTS[Phospho (STY)]GPPKGPSK.2	-0.157014415	Q9DCT8_S104_M1_Crip2
TVYFAEK.2	0.201281582	#N/A
VEEILAQADYLYESGETEK.2	-0.489507705	#N/A
EIDGGLETLR.2	0.154823999	#N/A
GIHVEIPGAQAESLGPLQVAR.3	-0.065958404	#N/A
EIIAVSC[Carbamidomethyl (C)]GPSQC[Carbamidomethyl (C)]QETIR.2	-0.252482453	#N/A
AGDLGVDLTSK.2	0.155606652	#N/A
TALAMGADR.2	0.089066612	#N/A
SC[Carbamidomethyl (C)]AEFVSGSQLR.2	0.176343467	#N/A
LASLSEKPPAIDWAYYR.3	0.230644937	#N/A
ANVAKPGLVDDFEK.2	-0.115262304	#N/A
ANVAKPGLVDDFEKK.3	0.096743905	#N/A
SWNETFHAR.2	0.4111426	#N/A
IPVPEDKYTALVDQEEKEDVK.3	-0.156460149	#N/A
YTALVDQEEKEDVK.2	-0.010375907	#N/A
TIDWVSFVEVM[Oxidation (M)]PQNQK.2	0.158827628	#N/A
NIIPFDQM[Oxidation (M)]TIDDLNEIFPETK.3	0.061307915	#N/A
PGLVDDFEK.2	-0.127140151	#N/A
IDELSLYSVPEGQSK.2	-0.330113867	#N/A
LYDWGLR.2	-0.085974199	#N/A
TQLEENISQLR.2	-0.195729244	#N/A
GYIVIEDLWK.2	0.185882958	#N/A
SASS[Phospho (STY)]GAEGDVSSER.2	-0.064709909	Q9DD18_S197_M1_Dtd1
SASS[Phospho (STY)]GAEGDVSSEREP.2	-0.272684824	Q9DD18_S197_M1_Dtd1
ASVTVGGEQISAIGR.2	-0.032164904	#N/A
AKGPSESS[Phospho (STY)]KER.2	-0.558955531	Q9DD18_S182_M1_Dtd1
GGFDS[Phospho (STY)]PFYR.2	-0.152956739	Q9EP53_S502_M1_Tsc1
SPFPGS[Phospho (STY)]PEQTKK.2	-0.292223033	Q9EP82_S397_M1_Wdr4
NHLS[Phospho (STY)]PQEGGATPQVPSPC[Carbamidomethyl (C)]C[Carbamidomethyl (C)]R.3	0.085716369	Q9EP97_S163_M1_Senp3

GS[Phospho (STY)]PPVPSGPPMEEDGLR.2	-0.598073638	Q9EP97_S206_M1_Senp3
EIHPYLVM[Oxidation (M)]DLEQS[Phospho (STY)].2	-0.24787508	Q9EPB4_S193_M1_Pycard
[Acetyl (Protein N-term)]ATS[Phospho (STY)]PQKSPLVPK.2	0.026677759	Q9EPC1_S4_M1_Parva
SPTPKS[Phospho (STY)]PPSR.2	-0.024634475	Q9EPC1_S19_M1_Parva
DS[Phospho (STY)]PVLSNSGPR.2	-0.116391869	Q9EPE9_S896_M1_Atp13a1
S[Phospho (STY)]PSSDSWTC[Carbamidomethyl (C)]ADASTGR.2	0.039441575	#N/A
AAPSTADEGWDNQNW.2	0.034589916	#N/A
TLQFTAHR.2	0.10806622	#N/A
NSNSDGWESWEGASGEGR.2	0.176536611	#N/A
KAAPSTADEGWDNQNW.2	-0.278250795	#N/A
AKS[Phospho (STY)]PSSDSWTC[Carbamidomethyl (C)]ADASTGR.3	-0.192419632	#N/A
SS[Phospho (STY)]DSWDVWGSGSASNNK.2	0.248978471	Q9EPJ9_S360_M1_Arfgap1
RS[Phospho (STY)]SDSWDVWGSGSASNNK.3	0.025083613	Q9EPJ9_S359_M1_Arfgap1
AFEDWLNDDLGSYQGAQENR.2	-0.192006192	#N/A
DVTTFFSGK.2	-0.311709022	#N/A
AKS[Phospho (STY)]PSSDS[Phospho (STY)]WTC[Carbamidomethyl (C)]ADASTGRR.3	-0.112861717	#N/A
GLGVHLSFVR.2	-0.360022785	#N/A
S[Phospho (STY)]PSSDSWTC[Carbamidomethyl (C)]ADASTGRR.2	-0.12983929	#N/A
FGSQASQK.2	-0.191576205	#N/A
EWSLESSPAQNWTPPQPK.2	0.185528587	#N/A
ETENDDLTNVIQK.2	-0.051916741	#N/A
RQFS[Phospho (STY)]PGPR.2	-0.253925326	Q9EPN1_S1311_M1_Nbea
TPLENVPGNLS[Phospho (STY)]PIKDPDR.2	-0.370062626	Q9EPN1_S1519_M1_Nbea
QFS[Phospho (STY)]PGPR.2	-0.270325217	Q9EPN1_S1311_M1_Nbea
AVPNVDAGSIIS[Phospho (STY)]DTERSDDGKESGK.3	0.242600119	Q9EPN1_S1252_M1_Nbea
EIEDLSQSQS[Phospho (STY)]PES[Phospho (STY)]ETDYPVSTDTR.3	-0.318539021	Q9EPN1_S1001_M2_Nbea
SQAIVNQNS[Phospho (STY)]ETELMLEGDDDAVSLLQEK.3	-0.255131049	Q9EPN1_S2114_M1_Nbea
AGSS[Phospho (STY)]PTQGAQNEAPR.2	-0.075526754	Q9EPQ8_S588_M1_Tcf20
LSTS[Phospho (STY)]PATR.2	0.001647501	Q9EPQ8_S603_M1_Tcf20
GSQEDDPAASQRPPSNS[Phospho (STY)]GVK.3	-0.287612011	Q9EPQ8_S671_M1_Tcf20
LSTS[Phospho (STY)]PATRDEAAS[Phospho (STY)]PGAK.3	0.471495119	Q9EPQ8_S603_M2_Tcf20
SSLAETLDSTGS[Phospho (STY)]LDPQR.2	-0.312235143	Q9EPR4_S81_M1_Slc23a2
AS[Phospho (STY)]PTSTEEEQVMLR.2	0.361629848	Q9EPW0_S488_M1_Inpp4a
VNAGDQPGADLGPLITPQAK.2	0.128285922	#N/A
TLADAEGDVFR.2	-0.137695774	#N/A
EGASILLDGR.2	-0.017622242	#N/A
AISFVGSNQAGEYIFER.2	-0.127955595	#N/A
DMDLYSYR.2	0.326954765	#N/A
VDLS[Phospho (STY)]DEEAKETEHLK.3	0.000467106	Q9EQ28_S306_M1_Pold3
IPHGVSGVQDR.2	0.20904538	#N/A
ILNLYPR.2	0.100972968	#N/A
VVNDDC[Carbamidomethyl (C)]THEADVYIESGIIQQVGR.3	-0.105598829	#N/A
FVAVTSSNAAK.2	0.169856563	#N/A
AEM[Oxidation (M)]ETLVR.2	-0.114868477	#N/A
C[Carbamidomethyl (C)]HGVPLVTISR.2	-0.008104448	#N/A
EM[Oxidation (M)]GT[Phospho (STY)]PLADT[Phospho (STY)]PTRPVTR.2	-0.457228036	#N/A
KEM[Oxidation (M)]GT[Phospho (STY)]PLADT[Phospho (STY)]PTRPVTR.3	0.145647691	#N/A

DLHESSFSLSGS[Phospho (STY)]QIDDHVPKR.4	-0.13797347	Q9EQF6_S538_M1_Dpysl5
ELM[Oxidation (M)]IPGGAK.2	0.047600351	#N/A
VVYENGVFM[Oxidation (M)]C[Carbamidomethyl (C)]AEGTGK.2	0.228366214	#N/A
M[Oxidation (M)]SVVWER.2	-0.350014534	#N/A
DLHESSFSLSGS[Phospho (STY)]QIDDHVPK.3	-0.069688491	Q9EQF6_S538_M1_Dpysl5
EALDLGITGPEGIEISHPEELEAEATHR.3	-0.115996973	#N/A
LDTNTSTYLM[Oxidation (M)]SLLANDTLNIVASDHRPFTTK.4	0.157217204	#N/A
DSELYQVFHAC[Carbamidomethyl (C)]R.2	0.038514562	#N/A
TISASTQVQGGDFNLYENM[Oxidation (M)]R.2	-0.039946441	#N/A
AMGKEDFTK.2	-0.093943362	#N/A
THC[Carbamidomethyl (C)]PIYLVNVSSISAGDVIAAAK.3	0.077801242	#N/A
TPYLGDVAIVVHPGK.3	-0.32096222	#N/A
FVQKPYS[Phospho (STY)]R.2	-0.219541236	Q9EQG9_S373_M1_Cert1
SSS[Phospho (STY)]M[Oxidation (M)]SS[Phospho (STY)]IDLVSASDDVHR.2	-0.300125607	Q9EQG9_S377_M2_Cert1
RES[Phospho (STY)]PESEGPIYEGLIL.2	-0.104336384	Q9EQH3_S783_M1_Vps35
SRRES[Phospho (STY)]PESEGPIYEGLIL.3	-0.1351882	Q9EQH3_S783_M1_Vps35
IREDLPNLESSEETEQINK.3	-0.183800627	#N/A
AELAELPLR.2	-0.131592854	#N/A
SDDPDQQYLILNTAR.2	-0.207514945	#N/A
LSQLEGVNVER.2	-0.369589819	#N/A
IPVDTYNNILTVLK.2	0.078719317	#N/A
LFDIFSQQVATVIQSR.2	-0.292729216	#N/A
PTTQQS[Phospho (STY)]PQDEQEKLLDEAIQAVK.3	-0.359011322	Q9EQH3_S7_M1_Vps35
LLDEAIQAVK.2	-0.169881112	#N/A
PTTQQS[Phospho (STY)]PQDEQEK.2	0.024260981	Q9EQH3_S7_M1_Vps35
LNLEHIATSSAVSK.2	-0.216960079	#N/A
ES[Phospho (STY)]PESEGPIYEGLIL.2	0.088384079	Q9EQH3_S783_M1_Vps35
TQC[Carbamidomethyl (C)]ALAASK.2	0.145071757	#N/A
SGS[Phospho (STY)]PKLDPSEVYLK.2	0.071887863	Q9EQJ9_S702_M1_Magi3
GGPC[Carbamidomethyl (C)]S[Phospho (STY)]PTK.2	-0.149907018	Q9EQJ9_S662_M1_Magi3
QPEDESHQAFSQNGS[Phospho (STY)]PR.3	0.244591633	Q9EQJ9_S833_M1_Magi3
KQEDPSSS[Phospho (STY)]PQASTS.2	-0.247678648	Q9EQN9_S492_M1_Slc19a2
DKPVYDELFYTLS[Phospho (STY)]PINGK.3	-0.18263383	Q9EQP2_S459_M1_Ehd4
LENEGS[Phospho (STY)]DEDIETDVLYSPQM[Oxidation (M)]ALK.3	0.497627232	Q9EQQ9_S364_M1_Oga
PAVTAPEVINYADYTLETTEESAAPAS[Phospho (STY)]PQAQSDSR.4	0.021547673	Q9EQT6_S113_M1_Syt13
IDFYFDENPYFENK.2	0.046814495	#N/A
VEVTEFEDIK.2	0.097181756	#N/A
EFHLNESGDPSSK.2	0.317666876	#N/A
TGSVQTS[Phospho (STY)]PSSTPGTGR.2	-0.184886082	Q9EQZ7_S1095_M1_Rims2
TSNHS[Phospho (STY)]PPTPR.2	-0.138656526	Q9EQZ7_S472_M1_Rims2
SWS[Phospho (STY)]PSAPPPQR.2	0.083350089	Q9EQZ7_S1039_M1_Rims2
ASQSS[Phospho (STY)]LESSTGPSYSR.2	-0.22966104	Q9EQZ7_S1518_M1_Rims2
RHS[Phospho (STY)]DVSLANAELEDSR.3	0.397708592	Q9EQZ7_S409_M1_Rims2
YRS[Phospho (STY)]DPNLAR.2	0.131665103	Q9EQZ7_S375_M1_Rims2
SPS[Phospho (STY)]DYADRR.2	-0.146799212	Q9EQZ7_S311_M1_Rims2
S[Phospho (STY)]ASQLSQTEGGGKK.2	-0.175581972	Q9EQZ7_S1285_M1_Rims2
NDGSQS[Phospho (STY)]DTAVGALGTSGK.2	-0.167880949	Q9EQZ7_S1251_M1_Rims2

IPDSTHAQLESSSSSFES[Phospho (STY)]QK.3	-0.671616575	Q9EQZ7_S788_M1_Rims2
RAS[Phospho (STY)]QSS[Phospho (STY)]LESSTGPSYSR.2	-0.045157155	Q9EQZ7_S1515_M2_Rims2
RADS[Phospho (STY)]LRK.2	-0.100550877	Q9EQZ7_S493_M1_Rims2
RQLHGES[Phospho (STY)]PTR.2	-0.003685329	Q9EQZ7_S965_M1_Rims2
NDS[Phospho (STY)]LSSDQSESVRPPPPRPHK.3	-0.055261193	Q9EQZ7_S522_M1_Rims2
RS[Phospho (STY)]PIPLDRPDMR.3	-0.230487358	Q9EQZ7_S479_M1_Rims2
ELGSLPLPLSASEQR.2	0.388217606	#N/A
AGSRLS[Phospho (STY)]AEDR.2	0.080679637	Q9ER00_S142_M1_Stx12
DFNSIIQTC[Carbamidomethyl (C)]SGNIQR.2	-0.051423135	#N/A
LQENLQQLQHSTNQLAK.3	-0.111322231	#N/A
ISQATAQIK.2	0.015642601	#N/A
QLEADILDVNQIFK.2	-0.131883303	#N/A
LM[Oxidation (M)]NDFSSALNNFQVVQR.2	-0.305259525	#N/A
NPGPSGPQPR.2	0.478695052	#N/A
HVS[Phospho (STY)]DPGLPGK.2	-0.396909156	Q9ER47_S1188_M1_Kcnh7
KQS[Phospho (STY)]LPQEDPDVVVIDSSK.2	-0.301623396	Q9ER47_S174_M1_Kcnh7
SSLLGSTS[Phospho (STY)]DSNLNK.2	0.104736649	Q9ER47_S319_M1_Kcnh7
SFS[Phospho (STY)]PSSQC[Carbamidomethyl (C)]PEFLDLEK.2	0.398504418	Q9ER47_S1103_M1_Kcnh7
RLS[Phospho (STY)]FES[Phospho (STY)]EGEKDFSK.3	-0.399341704	Q9ER47_S891_M2_Kcnh7
LNTAS[Phospho (STY)]EDNLTSLLK.2	0.040740147	Q9ER47_S1135_M1_Kcnh7
SQSVNDS[Phospho (STY)]EGDTGK.2	0.015194427	Q9ER47_S878_M1_Kcnh7
ETMSGQTTFLGS[Phospho (STY)]PDSR.2	0.003788406	Q9ER64_S746_M1_Osbpl5
ENAEDS[Phospho (STY)]DAETQDHSR.2	-0.153299293	Q9ER64_S311_M1_Osbpl5
EGS[Phospho (STY)]TPEDDFPSSSGNGNK.2	-0.008533956	Q9ER69_S297_M1_Wtap
VQPQWS[Phospho (STY)]PPAGTEPC[Carbamidomethyl (C)]R.2	-0.430299758	Q9ER72_S102_M1_Cars1
ALQEGEGDLSIS[Phospho (STY)]ADR.2	0.140051898	Q9ER72_S390_M1_Cars1
TTQSLQDFPVADS[Phospho (STY)]EEEAEEEFQK.3	-0.105735935	Q9ERA6_S211_M1_Tfip11
EEATYGVWAERDS[Phospho (STY)]DEERPSFGGK.3	-0.134612394	Q9ERA6_S60_M1_Tfip11
GAAEEADS[Phospho (STY)]EDS[Phospho (STY)]DAEEKPVKQEDFPK.3	-0.039651881	Q9ERA6_S96_M2_Tfip11
EDLAGPDIGAS[Phospho (STY)]PQGGR.2	-0.203108616	Q9ERD6_S308_M1_Ralgps2
YLTVATVFR.2	0.005776814	#N/A
VREEYPDR.2	0.133722687	#N/A
M[Oxidation (M)]SSTFIGNSTAIQELFKR.3	-0.580540789	#N/A
EVDEQM[Oxidation (M)]LAIQSK.2	-0.045724462	#N/A
LHFFM[Oxidation (M)]PGFAPLTAR.3	-0.024279002	#N/A
MSSTFIGNSTAIQELFK.2	-0.43309507	#N/A
ISVYYNEASSHK.2	0.098431993	#N/A
LATPTYGDLNHLVSATM[Oxidation (M)]SGVTTSLR.3	-0.174126603	#N/A
ISVYYNEASS[Phospho (STY)]HKYVPR.3	-0.080429606	Q9ERD7_S56_M1_Tubb3
VAVC[Carbamidomethyl (C)]DIPPR.2	0.040204003	#N/A
FWEVISDEHGIDPSGNYVGDSDLQLER.3	-0.067705411	#N/A
EC[Carbamidomethyl (C)]ENC[Carbamidomethyl (C)]DC[Carbamidomethyl (C)]LQGFQLTHSLGGGTGSGM[Oxidation (M)]GTLLISK.3	-0.341115973	#N/A
[Acetyl (Protein N-term)]ASGS[Phospho (STY)]AGKPTGEAASPAPGSAVGGASSQPR.3	-0.424422711	Q9ERE8_S5_M1_Tlnrd1
MISEGSS[Phospho (STY)]PIR.2	0.315007525	Q9ERE9_S370_M1_Mapk8ip2
SSQELSS[Phospho (STY)]PGSDSEDAGGAR.2	-0.184652691	Q9ERE9_S254_M1_Mapk8ip2
ENIETVVTGSLDDLVK.2	0.051969916	#N/A
ELS[Phospho (STY)]VEEQIK.2	-0.258616842	Q9ERG0_S735_M1_Lima1

TSS[Phospho (STY)]LPESSPSK.2	-0.258043409	Q9ERG0_S367_M1_Lima1
SLLGLSNSEPNGS[Phospho (STY)]VEAK.2	-0.402997982	Q9ERG2_S214_M1_Strn3
NLEQILNGGES[Phospho (STY)]PKQK.2	0.212205591	Q9ERG2_S229_M1_Strn3
VVSHPTLPVTITAHEDR.4	0.334957977	#N/A
NLEQILNGGES[Phospho (STY)]PK.2	-0.194668574	Q9ERG2_S229_M1_Strn3
LLQTDDEEEAGLLELLK.2	0.030434139	#N/A
IVEDEPNKIC[Carbamidomethyl (C)]EADR.3	0.118060678	#N/A
VGESDC[Carbamidomethyl (C)]S[Phospho (STY)]DGEEDFYYTEIK.2	-0.008091422	Q9ERQ3_S381_M1_Znf704
S[Phospho (STY)]APSS[Phospho (STY)]PTLDC[Carbamidomethyl (C)]EK.2	-0.096839481	Q9ERR1_S194_M2_Ndel1
GTENSFPS[Phospho (STY)]PK.2	-0.311539549	Q9ERR1_S231_M1_Ndel1
KSAPS[Phospho (STY)]SPTLDC[Carbamidomethyl (C)]EK.2	-0.271980579	Q9ERR1_S197_M1_Ndel1
M[Oxidation (M)]DSAVQASLS[Phospho (STY)]LPAT[Phospho (STY)]PVGK.2	-0.280323204	Q9ERR1_S215_M2_Ndel1
ISALNIVGDLLR.2	-0.214516263	#N/A
LLIEDLEAR.2	0.040363301	#N/A
VGESVFHTTR.2	0.23235736	#N/A
IALM[Oxidation (M)]PLFQAEK.2	-0.094015431	#N/A
ENLEEEAIIM[Oxidation (M)]K.2	0.265857739	#N/A
SAS[Phospho (STY)]HPGSC[Carbamidomethyl (C)]SSER.2	0.011792824	Q9ERS4_S211_M1_Plekha3
STLSM[Oxidation (M)]S[Phospho (STY)]PR.2	-0.124118549	Q9ERT9_S67_M1_Ppp1r1a
[Acetyl (Protein N-term)]MEPDNS[Phospho (STY)]PRK.2	0.257330884	Q9ERT9_S6_M1_Ppp1r1a
[Acetyl (Protein N-term)]MEPDNS[Phospho (STY)]PR.2	0.378466834	Q9ERT9_S6_M1_Ppp1r1a
TEDSSAHMLPLDSQGASLV.2	-0.17153492	#N/A
EPDNS[Phospho (STY)]PR.2	-0.061649793	Q9ERT9_S6_M1_Ppp1r1a
SHETDGGS[Phospho (STY)]AHGDEEDDGPHFEPVVPLPDKIEVK.5	-0.303047731	Q9ERU9_S1154_M1_Ranbp2
YIASVQGSAPS[Phospho (STY)]PR.2	-0.176808186	Q9ERU9_S21_M1_Ranbp2
HSTPS[Phospho (STY)]PTKYSLS[Phospho (STY)]PSK.2	0.229471615	Q9ERU9_S781_M2_Ranbp2
SALS[Phospho (STY)]PSKSPAK.2	0.251184479	Q9ERU9_S2113_M1_Ranbp2
LNSNNSAS[Phospho (STY)]PHR.2	-0.207712901	Q9ERU9_S837_M1_Ranbp2
HSTPS[Phospho (STY)]PTK.2	-0.073457604	Q9ERU9_S781_M1_Ranbp2
NRPGYVS[Phospho (STY)]EEEEDDEDYEM[Oxidation (M)]AVK.3	-0.219598874	Q9ERU9_S2505_M1_Ranbp2
KQLS[Phospho (STY)]SEGTVR.2	0.04439205	Q9ERV1_S365_M1_Mkrn2
VNNPGGC[Carbamidomethyl (C)]SDPQTS[Phospho (STY)]PEMKPHSYLDAIR.4	0.009420541	Q9ERV1_S139_M1_Mkrn2
SC[Carbamidomethyl (C)]SSY[Phospho (STY)]ELQQQGTKR.2	-0.349508399	Q9ERZ3_Y292_M1_Chrm3
SC[Carbamidomethyl (C)]S[Phospho (STY)]SYELQQQGTK.2	0.045800069	Q9ERZ3_S290_M1_Chrm3
KEPVANQDPVS[Phospho (STY)]PSLVQGR.3	-0.079663233	Q9ERZ4_S232_M1_Chrm2
EPVANQDPVS[Phospho (STY)]PSLVQGR.2	0.405940826	Q9ERZ4_S232_M1_Chrm2
EKKEPVANQDPVS[Phospho (STY)]PSLVQGR.3	-0.484208466	Q9ERZ4_S232_M1_Chrm2
LAGGQTSQPTTPLTS[Phospho (STY)]PQR.2	-0.11545935	Q9ES00_S31_M1_Ube4b
TVEDLKNNESQWKDS[Phospho (STY)]PLATR.3	0.213719707	Q9ES00_S674_M1_Ube4b
HLLNS[Phospho (STY)]PTDPFNR.2	0.024793186	Q9ES00_S1136_M1_Ube4b
SQSSEGVSSLSSS[Phospho (STY)]PSNSLETQSQSLSR.3	0.294610144	Q9ES00_S88_M1_Ube4b
SQS[Phospho (STY)]M[Oxidation (M)]DIDGVSC[Carbamidomethyl (C)]EK.2	-0.298923503	Q9ES00_S105_M1_Ube4b
NNESQWKDS[Phospho (STY)]PLATR.2	0.272537985	Q9ES00_S674_M1_Ube4b
KPS[Phospho (STY)]DEEFAVR.2	-0.444525049	Q9ES28_S673_M1_Arhgef7
EDLSKS[Phospho (STY)]PK.2	0.142598517	Q9ES28_S655_M1_Arhgef7
EIKPSEKPVS[Phospho (STY)]PK.2	-0.279286611	Q9ES28_S228_M1_Arhgef7
M[Oxidation (M)]S[Phospho (STY)]GFIYQGK.2	-0.100149051	Q9ES28_S497_M1_Arhgef7

SFDS[Phospho (STY)]LGSQSSHSR.2	-0.058685914	Q9ES28_S135_M1_Arhgef7
LETFDANDLYQGQNFNK.2	-0.136190878	#N/A
IKS[Phospho (STY)]FDSLGSQSSHSR.3	-0.143201661	Q9ES28_S132_M1_Arhgef7
S[Phospho (STY)]LDM[Oxidation (M)]TDNTNSQLVVR.2	-0.037462918	Q9ES28_S155_M1_Arhgef7
KPS[Phospho (STY)]DEEFAVRK.3	-0.518406643	Q9ES28_S673_M1_Arhgef7
S[Phospho (STY)]TAALEEDAQILK.2	-0.115438214	Q9ES28_S682_M1_Arhgef7
SGTLKS[Phospho (STY)]PPK.2	0.037880632	Q9ES28_S236_M1_Arhgef7
SEEGHPFK.2	-0.091329352	#N/A
[Acetyl (Protein N-term)]AESSAATQSPSVSSSSSGAEPSALGGGGGS[Phospho (STY)]PGAC[Carbamidomethyl (C)]PALGAK.4	-0.402603806	Q9ES97_S31_M1_Rtn3
SALDFGQSK.2	0.345774958	#N/A
ETTGGAPTM[Oxidation (M)]S[Phospho (STY)]PDLEQEQLTIR.2	-0.266205397	Q9ES97_S673_M1_Rtn3
KLDS[Phospho (STY)]PQGTNK.2	-0.034091822	Q9ES97_S132_M1_Rtn3
TQIDHYVGIAR.2	0.095232076	#N/A
GIVDKEEGDVLEAVLEK.3	-0.286127937	#N/A
QTFAPQSGPQSSSDILEHTDVK.3	0.043144629	#N/A
KLDS[Phospho (STY)]PQGTNKDR.2	-0.159863032	Q9ES97_S132_M1_Rtn3
DQTKSIVEK.2	0.388847054	#N/A
Y[Phospho (STY)]VEDTDGSSPEDLM[Oxidation (M)]AVLTGAEEK.3	-0.022042659	Q9ES97_Y589_M1_Rtn3
S[Phospho (STY)]LEGEVASQVPNTLNEVTPEK.2	0.077721642	Q9ES97_S529_M1_Rtn3
SEMC[Carbamidomethyl (C)]ENSEQPQAQPET[Phospho (STY)]PTQK.2	-0.400983618	Q9ES97_T524_M1_Rtn3
QFSHTTAALEEVSR.3	-0.272581786	#N/A
AAPTT[Phospho (STY)]PPPPVK.2	0.162489389	Q9ESJ4_T174_M1_Nckipsd
RAAPTT[Phospho (STY)]PPPPVK.2	0.281390878	Q9ESJ4_T174_M1_Nckipsd
VSTSQAS[Phospho (STY)]PQSAASPR.2	-0.161155764	Q9ESK9_S646_M1_Rb1cc1
STELVLS[Phospho (STY)]PDM[Oxidation (M)]PR.2	-0.022921949	Q9ESK9_S243_M1_Rb1cc1
S[Phospho (STY)]MEHVAPDPTGTER.2	-0.026228653	Q9ESK9_S261_M1_Rb1cc1
[Acetyl (Protein N-term)]ASEGASIPS[Phospho (STY)]PVVR.2	-0.338887772	Q9ESN6_S10_M1_Trim2
VKS[Phospho (STY)]PGSGHVK.2	-0.181112125	Q9ESN6_S440_M1_Trim2
VIRS[Phospho (STY)]ADVS[Phospho (STY)]PTTEGVKR.3	-0.271193979	#N/A
SADVS[Phospho (STY)]PTTEGVK.2	-0.074183186	Q9ESN6_S428_M1_Trim2
VLQSQLDTLLQGQESIK.2	0.21891714	#N/A
EGDFTLSLR.2	-0.401517465	#N/A
LYDQHIR.2	0.037497877	#N/A
VIRS[Phospho (STY)]ADVS[Phospho (STY)]PTTEGVK.2	-0.425962542	#N/A
ASIVDDIHSTFDELQK.3	-0.339451973	#N/A
LNELADQDFPLHPR.2	-0.064501645	#N/A
SADVS[Phospho (STY)]PTTEGVKR.2	-0.158001604	Q9ESN6_S428_M1_Trim2
TGNAYLTAELS[Phospho (STY)]TPDGSVADGEILDNK.3	-0.014112833	Q9ESN6_S370_M1_Trim2
RVKS[Phospho (STY)]PGS[Phospho (STY)]GHVK.2	-0.018124423	Q9ESN6_S440_M2_Trim2
RPASMYS[Phospho (STY)]TGK.2	0.488820565	Q9ESN6_S459_M1_Trim2
VFNQEGEFMLK.2	-0.579377678	#N/A
LFSSSSS[Phospho (STY)]PPPAK.2	-0.673708276	Q9ESN9_S586_M1_Mapk8ip3
STHPS[Phospho (STY)]PEK.2	-0.201443612	Q9ESN9_S755_M1_Mapk8ip3
ANKTS[Phospho (STY)]PTSGEGTRPGGIIHVYGDDSSDK.4	0.221005203	Q9ESN9_S1192_M1_Mapk8ip3
QLS[Phospho (STY)]PNGGQEDTR.2	0.081190374	Q9ESN9_S677_M1_Mapk8ip3
S[Phospho (STY)]PTAAGFSQR.2	0.321251978	Q9ESN9_S603_M1_Mapk8ip3
TGSS[Phospho (STY)]PTQGIVNK.2	-0.221045944	Q9ESN9_S366_M1_Mapk8ip3

YKQLS[Phospho (STY)]PNGGQEDTR.2	-0.324965637	Q9ESN9_S677_M1_Mapk8ip3
AKET[Phospho (STY)]PEADATSSR.2	0.17974834	Q9ESN9_T764_M1_Mapk8ip3
LFSSSSS[Phospho (STY)]PPPAKR.2	-0.116250863	Q9ESN9_S586_M1_Mapk8ip3
EYNALHQR.2	0.182257903	#N/A
LMELQEAVR.2	-0.436932894	#N/A
SS[Phospho (STY)]PVESLKK.2	-0.424598324	Q9EST3_S77_M1_Eif4enif1
SSS[Phospho (STY)]PVGLAK.2	0.485461264	Q9EST3_S949_M1_Eif4enif1
SSS[Phospho (STY)]LGSTPHEELER.2	-0.446982555	Q9EST3_S352_M1_Eif4enif1
IHS[Phospho (STY)]PIIR.2	-0.477949243	Q9ESU6_S1153_M1_Brd4
MPDEPEEPVVTVSS[Phospho (STY)]PAVPPPTK.2	0.005125371	Q9ESU6_S471_M1_Brd4
AQAVS[Phospho (STY)]EEEEEEEGQSSSPK.2	-0.015638391	Q9ESV0_S80_M1_Ddx24
LASFWAQPQDASSR.2	0.117181511	#N/A
IPIGFIPLGQTSSLSHTLFAESGNK.3	0.04852868	#N/A
TVLES[Phospho (STY)]GGETGDGDNDTTK.2	-0.14734748	Q9ESX5_S481_M1_Dkc1
KRDSES[Phospho (STY)]ES[Phospho (STY)]DETPTVPQLK.3	0.160211589	Q9ESX5_S453_M2_Dkc1
VKVVEEM[Oxidation (M)]S[Phospho (STY)]E.2	0.131532274	Q9ESX5_S508_M1_Dkc1
TVLES[Phospho (STY)]GGETGDGDNDTTKK.2	0.00172514	Q9ESX5_S481_M1_Dkc1
ESTSSKS[Phospho (STY)]PPR.2	-0.422249036	Q9ESZ8_S823_M1_Gtf2i
TS[Phospho (STY)]INSLR.2	-0.332168294	Q9ET77_S163_M1_Jph3
TES[Phospho (STY)]PTTFSWTSHHR.3	-0.422769626	Q9ET77_S578_M1_Jph3
SLPVALES[Phospho (STY)]DEETGDELK.2	-0.129989227	Q9ET77_S706_M1_Jph3
S[Phospho (STY)]PLRTS[Phospho (STY)]INSLR.2	0.224500837	Q9ET77_S158_M2_Jph3
QVS[Phospho (STY)]VDEER.2	-0.15938578	Q9ET77_S506_M1_Jph3
EFS[Phospho (STY)]PSFQHR.2	-0.321466707	Q9ET77_S420_M1_Jph3
VLEKPPS[Phospho (STY)]PKES[Phospho (STY)]PHFYR.3	-0.457697361	Q9ET80_S448_M2_Jph1
QNPS[Phospho (STY)]PGAR.2	-0.25489731	Q9ET80_S490_M1_Jph1
KQNPS[Phospho (STY)]PGAR.2	-0.234366752	Q9ET80_S490_M1_Jph1
QSHS[Phospho (STY)]PQPSS[Phospho (STY)]PEPSK.2	-0.223153697	Q9ET80_S475_M2_Jph1
TS[Phospho (STY)]LASLR.2	-0.225468738	Q9ET80_S162_M1_Jph1
QS[Phospho (STY)]HSPQPSS[Phospho (STY)]PEPSKK.3	-0.26877875	Q9ET80_S473_M2_Jph1
RGS[Phospho (STY)]LLGS[Phospho (STY)]MK.2	0.017084634	Q9ET80_S216_M2_Jph1
QFLIS[Phospho (STY)]PPAS[Phospho (STY)]PPVGWK.2	-0.158012605	Q9JHG6_S161_M2_Rcan1;Rcan3
FLQENLAPK.2	0.061596144	#N/A
LYEIGAGTSEVR.2	0.184363181	#N/A
ELLLC[Carbamidomethyl (C)]NNR.2	0.523060386	#N/A
LPENVLLR.2	-0.290256248	#N/A
AQALVQYLEEPLTQVAAS.2	0.050157335	#N/A
SGPQC[Carbamidomethyl (C)]SS[Phospho (STY)]PTC[Carbamidomethyl (C)]QEETEDVR.2	-0.318350103	Q9JHU2_S385_M1_Palmd
SNGPTHTS[Phospho (STY)]PTRPTPQPR.3	-0.245898391	Q9JHU2_S321_M1_Palmd
MELS[Phospho (STY)]PSRAS[Phospho (STY)]PGK.2	0.15680423	Q9JHU2_S370_M2_Palmd
LVQT[Phospho (STY)]PLTDR.2	-0.268336233	Q9JHU4_T1880_M1_Dync1h1
VTDFGDKVEDPTFLNQLQSGVNR.3	-0.358911661	#N/A
VAAPDVVVPTLDTVR.2	-0.034188983	#N/A
EQPWVSVQPR.2	0.072011192	#N/A
EYQTQLIQR.2	-0.090676413	#N/A
SLLQALNEVK.2	0.048687095	#N/A
LLNTFLER.2	-0.27015056	#N/A

VTFVNFTVTR.2	0.087028952	#N/A
QNLDGLLNQLK.2	0.052571442	#N/A
EWTDGLFTHVLR.2	-0.296235329	#N/A
TFSEILNR.2	-0.206082862	#N/A
LNTQEIFDDWAR.2	0.04502304	#N/A
LSLSNAISTVLPLTQLR.2	-0.122657464	#N/A
FLSDPQVHTVLVER.3	0.176366485	#N/A
IQGLTVEQAEAVVR.2	0.046235096	#N/A
LQGATC[Carbamidomethyl (C)]SNNK.2	0.301243242	#N/A
SAC[Carbamidomethyl (C)]DTVDTWLDDTAK.2	-0.02366326	#N/A
EVWNTYELDLVNYQNK.2	-0.334995912	#N/A
VQVALEELQDLK.2	0.385285935	#N/A
FNYGFEYLGVQDK.2	-0.335064439	#N/A
ESPEVLLTLDILK.2	0.160599255	#N/A
LKQDGDSFR.2	0.412503215	#N/A
LQGEFQLR.2	-0.321474336	#N/A
IFTIESAR.2	-0.023393864	#N/A
ENFIPTIVNFSAEEISDAIR.2	-0.057636684	#N/A
M[Oxidation (M)]LSAVSQQVQC[Carbamidomethyl (C)]IQEALR.2	-0.275208308	#N/A
TDS[Phospho (STY)]TSDGRPAWMR.2	0.169706836	Q9JHU4_S4366_M1_Dync1h1
SPLVM[Oxidation (M)]DVLNIQGVQR.2	-0.243391514	#N/A
IAFIM[Oxidation (M)]DESNVLDSGFLER.2	-0.037299835	#N/A
DHLYGTLDPNTR.2	-0.343068965	#N/A
DVLLVAQGEM[Oxidation (M)]ALEEFLK.2	-0.299740565	#N/A
QLQNISQAAASGGAK.2	0.37171304	#N/A
IQFVGAC[Carbamidomethyl (C)]NPPTDPGR.2	0.260727776	#N/A
FTQDTQPHYIYSPR.3	-0.197929647	#N/A
LEDDAKDNQQK.2	0.197932092	#N/A
VAEVLFDAADANAIEEVNLAYENVK.3	-0.319653161	#N/A
TFSSIPVSRIC[Carbamidomethyl (C)]K.2	0.118320902	#N/A
EAIVNSC[Carbamidomethyl (C)]VFVHQTLHQANAR.3	-0.20852532	#N/A
LALESIC[Carbamidomethyl (C)]LLLGESTTDWK.2	-0.526798866	#N/A
SC[Carbamidomethyl (C)]IENIFR.2	0.112297179	#N/A
VIVLWTANTER.2	0.065381934	#N/A
HVFVGGDDFK.2	0.493954195	#N/A
AVDNQVYVATASPAR.2	0.079267687	#N/A
TLS[Phospho (STY)]PGDSFSTFDTPYC[Carbamidomethyl (C)]K.2	0.005169582	Q9JHW2_S133_M1_Nit2
FAELAQIYAQR.2	-0.034580967	#N/A
ELEEEEENS[Phospho (STY)]DEDELDSHTM[Oxidation (M)]VK.3	-0.006524305	Q9JI10_S316_M1_Stk3
LVGIFENQER.2	-0.4978747	#N/A
SESEEEVLLVSSSR.2	-0.198726065	#N/A
WIVPGGGM[Oxidation (M)]EPEEEPSVAAVR.2	-0.217968854	#N/A
LTYHGLS[Phospho (STY)]PC[Carbamidomethyl (C)]K.2	-0.316545361	Q9JI90_S349_M1_Rnf14
LTYHGLS[Phospho (STY)]PC[Carbamidomethyl (C)]KVTAEK.3	0.221306857	Q9JI90_S349_M1_Rnf14
ETADTDTAEQVIASFR.2	0.076178532	#N/A
ISSSNPYSTVTM[Oxidation (M)]DELR.2	-0.489760355	#N/A
ATLPEADGER.2	0.389763542	#N/A

AC[Carbamidomethyl (C)]LISM[Oxidation (M)]GYDLGEAEFAR.2	-0.204567933	#N/A
HTNYTMEHIR.3	-0.026638175	#N/A
VEQIAAIAQELNELDYHDAVNVNDR.3	0.250117502	#N/A
NS[Phospho (STY)]LTGEEGELVK.2	-0.081525922	Q9JI99_S101_M1_Sgpp1
RNS[Phospho (STY)]LTGEEGELVK.2	-0.309859528	Q9JI99_S101_M1_Sgpp1
DNALC[Carbamidomethyl (C)]ENAR.2	0.298076102	#N/A
FQELNVQAPR.2	0.02432727	#N/A
GLDSLTNVDLR.2	-0.192706325	#N/A
DFDC[Carbamidomethyl (C)]IITEFAK.2	0.35568119	#N/A
SLIHLSLANNNLQTLPK.3	-0.731001281	#N/A
IQDIEVLK.2	-0.189022815	#N/A
GNAFNC[Carbamidomethyl (C)]DC[Carbamidomethyl (C)]K.2	0.173878833	#N/A
KPNDIETFK.2	0.06031768	#N/A
WGGSSFQDIQR.2	0.484918788	#N/A
DTDVEYLEIARPPLALR.2	0.366645295	#N/A
C[Carbamidomethyl (C)]PAVC[Carbamidomethyl (C)]TC[Carbamidomethyl (C)]SK.2	-0.233891662	#N/A
S[Phospho (STY)]SEDVSAHAATK.2	0.07604836	Q9JID9_S106_M1_Sh2b2
ALDDFVLGSAR.2	0.151486908	#N/A
TPMGLLLEALGQEQEAGS.2	-0.297918048	#N/A
TLS[Phospho (STY)]LDDKGWR.2	0.075579055	Q9JII1_S89_M1_Inpp5e
FRGS[Phospho (STY)]QEDLTVQNGASPC[Carbamidomethyl (C)]R.3	0.277188555	Q9JII1_S103_M1_Inpp5e
LFVGGLDWSTTQETLR.2	-0.340640434	#N/A
IFVGGIPHNC[Carbamidomethyl (C)]GETELR.3	0.024107593	#N/A
SYFSQYGEVVDC[Carbamidomethyl (C)]VIM[Oxidation (M)]K.2	-0.388920255	#N/A
DAGHPLYPFNDPY.2	-0.269016567	#N/A
[Acetyl (Protein N-term)]TASSVLLHTGQK.2	0.362585016	#N/A
ALGLSNFNSR.2	0.06311715	#N/A
GLEVTAYSPLGSSDR.2	-0.225445478	#N/A
ILQNIQVFDFTFSPEEM[Oxidation (M)]K.2	-0.061608521	#N/A
HALSAGYR.2	0.133663571	#N/A
HPDEPVLLEEPVVLALAEK.3	-0.600973944	#N/A
EELFVTSK.2	-0.319887744	#N/A
YDSTHYK.2	-0.583626944	#N/A
EIITEEPS[Phospho (STY)]EEEADM[Oxidation (M)]PKPK.3	0.373760648	Q9JIK5_S118_M1_Ddx21
GGLS[Phospho (STY)]DGEGPPGGR.2	-0.201112313	Q9JIS5_S127_M1_Sv2a
HLQAVDYAAR.2	-0.0346157	#N/A
GGLS[Phospho (STY)]DGEGPPGGRGEAQR.2	-0.512215739	Q9JIS5_S127_M1_Sv2a
M[Oxidation (M)]ADGAPLAGVR.2	0.350657927	#N/A
FEEEDDDDDFPAPADGYYR.2	0.204197689	#N/A
VFSVTHIK.2	-0.026682628	#N/A
DREELAQQYETILR.3	-0.179803848	#N/A
[Acetyl (Protein N-term)]MEEGFRDR.2	-0.193848578	#N/A
GGQYFNDK.2	0.240522568	#N/A
VQDEYSR.2	0.132530307	#N/A
TIHQEDELIEIQSDTGTWYQR.3	-0.381775749	#N/A
TAQVPS[Phospho (STY)]PPR.2	-0.276980502	Q9JIX8_S1003_M1_Acin1
TAQVPS[Phospho (STY)]PPRGK.2	-0.434920623	Q9JIX8_S1003_M1_Acin1

LQPEQGS[Phospho (STY)]PK.2	-0.255004199	Q9JIX8_S729_M1_Acin1
SLS[Phospho (STY)]PLSGTTDTK.2	-0.226609247	Q9JIX8_S479_M1_Acin1
GVQAGNS[Phospho (STY)]DTEGGQPGR.2	-0.123993339	Q9JIX8_S838_M1_Acin1
SSSFSEEKGES[Phospho (STY)]DDEKPR.3	-0.391100421	Q9JIX8_S216_M1_Acin1
KS[Phospho (STY)]LS[Phospho (STY)]PGVSR.2	0.49544764	Q9JIX8_S655_M2_Acin1
S[Phospho (STY)]RST[Phospho (STY)]PVRDR.2	-0.24164001	Q9JIX8_S1326_M2_Acin1
KIS[Phospho (STY)]VVSATK.2	0.116037833	Q9JIX8_S825_M1_Acin1
SLS[Phospho (STY)]PLSGTTDTKAES[Phospho (STY)]PAGR.2	0.05475026	Q9JIX8_S479_M2_Acin1
SQS[Phospho (STY)]PSPPPLPEDLEK.2	0.073522934	Q9JIX8_S389_M1_Acin1
HLS[Phospho (STY)]HPEPEQQHVIQR.3	-0.356929873	Q9JIX8_S710_M1_Acin1
GVQAGNS[Phospho (STY)]DTEGGQPGRK.2	0.041416007	Q9JIX8_S838_M1_Acin1
AKDPPQS[Phospho (STY)]PPDSESEQLLLETER.3	-0.05109426	Q9JIY0_S341_M1_Plekho1
DLDGS[Phospho (STY)]YDQLTGHPPGPSKK.3	0.01315064	Q9JJ69_S19_M1_Kcnip2
DLDGS[Phospho (STY)]YDQLTGHPPGPSK.2	-0.294343136	Q9JJ69_S19_M1_Kcnip2
RPS[Phospho (STY)]KEVEAVVVGPY.2	-0.046719902	Q9JJ72_S98_M1_Rprm
NFTQS[Phospho (STY)]PEQQR.2	0.195430157	Q9JJA9_S93_M1_Tamalin
FIPGLNRS[Phospho (STY)]LEEEESQL.2	-0.475742491	Q9JJA9_S384_M1_Tamalin
NFTQS[Phospho (STY)]PEQQRK.2	0.023193907	Q9JJA9_S93_M1_Tamalin
S[Phospho (STY)]LEEEESQL.2	0.081467583	Q9JJA9_S384_M1_Tamalin
T[Phospho (STY)]SPQPESGIKR.2	-0.09292596	Q9JJC6_T348_M1_Rilpl1
[Acetyl (Protein N-term)]M[Oxidation (M)]EEPLGS[Phospho (STY)]PPAALSALEK.2	-0.453338566	Q9JJC6_S7_M1_Rilpl1
VQNS[Phospho (STY)]PPVGR.2	-0.238638021	Q9JJG0_S419_M1_Tacc2
FNS[Phospho (STY)]PSEELDYR.2	0.003040317	Q9JJG0_S714_M1_Tacc2
TRPPS[Phospho (STY)]LK.2	-0.168902479	Q9JJG0_S327_M1_Tacc2
KTRPPS[Phospho (STY)]LK.2	-0.056937629	Q9JJG0_S327_M1_Tacc2
LDNTPASPPRS[Phospho (STY)]PT[Phospho (STY)]EPSDTPIAK.3	-0.293153755	Q9JJG0_S514_M2_Tacc2
ETQQEPGEES[Phospho (STY)]PVPSEEHLAPETK.3	-0.144096613	Q9JJG0_S354_M1_Tacc2
SGNYPSSLSNETDR.2	-0.32531093	#N/A
SFPNPFPDYEAAASAAGLAAGSAEETGR.3	-0.466684083	#N/A
GDYDAFFEAR.2	0.007777488	#N/A
QQDVLC[Carbamidomethyl (C)]FLEANK.2	0.296361214	#N/A
SQGGEPTYNVAVGR.2	0.16313398	#N/A
DSLYVDGDC[Carbamidomethyl (C)]TM[Oxidation (M)]DIR.2	-0.448232782	#N/A
EGVHGGGLNKK.2	0.129083133	#N/A
VLVFVMGK.2	0.189463848	#N/A
_[Acetyl (Protein N-term)]AGWQSYVDNLM[Oxidation (M)]C[Carbamidomethyl (C)]DGC[Carbamidomethyl (C)]C[Carbamidomethyl (C)]Q	-0.132931054	#N/A
ASSSS[Phospho (STY)]PYAR.2	-0.299430587	Q9JJV4_S228_M1_Cacng4
STEAS[Phospho (STY)]PSRDAS[Phospho (STY)]PVGLK.2	0.119494785	Q9JJV4_S253_M2_Cacng4
DAS[Phospho (STY)]PVGLK.2	-0.180617701	Q9JJV4_S259_M1_Cacng4
SRDLS[Phospho (STY)]PISK.2	-0.369653253	Q9JJV5_S248_M1_Cacng3
S[Phospho (STY)]HSELLK.2	-0.09563141	Q9JJV5_S211_M1_Cacng3
DLS[Phospho (STY)]PISK.2	-0.15492463	Q9JJV5_S248_M1_Cacng3
DGDSGS[Phospho (STY)]LGSPSASR.2	-0.149825065	Q9JJY3_S289_M1_Smpd3
TAS[Phospho (STY)]VEYKGDGGR.2	0.362198536	Q9JJY3_S208_M1_Smpd3
IY[Phospho (STY)]IDSPTNTSISAASFSSLVSPQGGDGSR.3	-0.291239077	Q9JJY3_Y170_M1_Smpd3
DSS[Phospho (STY)]PGSLPK.2	-0.065063741	Q9JJY4_S569_M1_Ddx20
AAMHTYSSPTVATQS[Phospho (STY)]PK.2	0.326688755	Q9JJY4_S472_M1_Ddx20

ES[Phospho (STY)]IDLVLDR.2	0.164528775	Q9JJZ2_S114_M1_Tuba8
NTSMS[Phospho (STY)]PR.2	0.131860276	Q9JJZ4_S251_M1_Ube2j1
ADLES[Phospho (STY)]PQRK.2	0.140293648	Q9JJZ6_S163_M1_Klf13
SRADLES[Phospho (STY)]PQRK.3	0.152252433	Q9JJZ6_S163_M1_Klf13
TLSQFTDALVTIR.2	0.230800167	#N/A
SPSTDITAEGS[Phospho (STY)]PTK.2	0.166432719	Q9JK45_S458_M1_Kcnq5
SWS[Phospho (STY)]FNDR.2	0.073375935	Q9JK45_S467_M1_Kcnq5
RSPSTDITAEGS[Phospho (STY)]PTK.2	0.317831266	Q9JK45_S458_M1_Kcnq5
FLENYDAIR.2	0.315355133	#N/A
WLPLEANPEVTNQFLK.2	0.281487964	#N/A
FLEESVSM[Oxidation (M)]S[Phospho (STY)]PEER.2	-0.197887207	Q9JKB1_S130_M1_Uchl3
TSDETLLEDAIEVC[Carbamidomethyl (C)]KK.3	-0.148314552	#N/A
S[Phospho (STY)]RPLNAVSQDGK.2	-0.129284962	Q9JKB3_S328_M1_Ybx3
[Acetyl (Protein N-term)]S[Phospho (STY)]EAGEATTGGTTLPQAAADAPAAAPPDPAPK.3	-0.186834866	Q9JKB3_S2_M1_Ybx3
PAPTVPAAPSS[Phospho (STY)]PDATSEPK.2	0.149018757	Q9JKC6_S90_M1_Cend1
DTQAEKQEQAAAPGPAATK.3	-0.201441723	#N/A
QEQAAAPGPAATK.2	0.263053367	#N/A
DTQAEKQEQAAAPGPAATKK.3	-0.001535043	#N/A
VPQATAEAK.2	-0.051401174	#N/A
SASS[Phospho (STY)]PKPDTK.2	0.047992461	Q9JKC6_S10_M1_Cend1
SASS[Phospho (STY)]PKPDTKVPQATAEAK.3	-0.203460535	Q9JKC6_S10_M1_Cend1
GPGDGAEEDESNTGGR.2	-0.201894655	#N/A
ADPVLLNNHSNLKPAPTVPAAPSS[Phospho (STY)]PDATSEPKGPGDGAEEDESNTGGR.5	0.013334288	Q9JKC6_S90_M1_Cend1
ADPVLLNNHSNLKPAPTVPAAPSS[Phospho (STY)]PDATSEPK.4	0.187386112	Q9JKC6_S90_M1_Cend1
GKS[Phospho (STY)]ASSPKPDTK.2	-0.375611871	Q9JKC6_S7_M1_Cend1
AQEEWTTGAWK.2	-0.263849146	#N/A
NPHVQQAAQNAAM[Oxidation (M)]GAAQGAM[Oxidation (M)]NQPQTQYSATPNYTYSNEM[Oxidation (M)].4	-0.416114523	#N/A
AGPLGGSS[Phospho (STY)]YEEEEEEEGGGGGER.3	0.072092391	Q9JKF6_S434_M1_Nectin1
AGPLGGSS[Phospho (STY)]YEEEEEEEGGGGGERK.3	-0.1949852	Q9JKF6_S434_M1_Nectin1
AGIPQHHPPM[Oxidation (M)]AQNLQYPDDS[Phospho (STY)]DDEKK.4	0.035960718	Q9JKF6_S421_M1_Nectin1
[Acetyl (Protein N-term)]S[Phospho (STY)]M[Oxidation (M)]EDPFFVVK.2	-0.331072462	Q9JKK1_S2_M1_Stx6
ANDPSLQEVNLNNIK.2	-0.37613673	#N/A
SLETNTHVK.2	0.427809767	#N/A
SNDPVALAFAEM[Oxidation (M)]LK.2	-0.053528423	#N/A
NIDEDELLGK.2	0.150301928	#N/A
QLENVLDDLDPESATLPAGFR.2	0.039269178	#N/A
ENDTLTEIK.2	0.050920543	#N/A
DREDFVPFTGEK.2	-0.12155933	#N/A
SLNVESNFITGTGILALVEALR.2	-0.354643562	#N/A
VTGAALIPPPGETALASLGQASE.2	-0.219109925	#N/A
QTLPSSPT[Phospho (STY)]TTK.2	-0.278114958	Q9JKN6_T156_M1_Nova1
IKQTLPSS[Phospho (STY)]PTTTK.2	-0.227117504	Q9JKN6_S154_M1_Nova1
TLVEYQELTGAR.2	0.07422168	#N/A
REEEEDNEDDGS[Phospho (STY)]DLGEALA.2	0.237082245	Q9JKP8_S122_M1_Chrac1
DAVIYPILVEFTR.2	0.005632696	#N/A
VLQLINDNTATALSYGVFR.2	-0.165633401	#N/A
VESVFETLVEDSPEEESTLTK.2	0.150132136	#N/A

ASSEGAQGSVS[Phospho (STY)]PK.2	0.356951115	Q9JKS4_S179_M1_Ldb3
SLAASGAQQPDS[Phospho (STY)]PGGPQPPGQK.2	-0.228936986	Q9JKS5_S108_M1_Habp4
AFDS[Phospho (STY)]FDQR.2	-0.190945886	Q9JKS5_S206_M1_Habp4
KSLAASGAQQPDS[Phospho (STY)]PGGPQPPGQK.3	0.36621297	Q9JKS5_S108_M1_Habp4
S[Phospho (STY)]PAVASGHRPGAGGR.3	0.30626414	Q9JKS5_S74_M1_Habp4
RGEQQGWNDNR.3	-0.321499686	#N/A
DKKDEEEDM[Oxidation (M)]S[Phospho (STY)]LD.2	0.397141785	Q9JKV1_S405_M1_Adrm1
SQS[Phospho (STY)]AAVTPSSSTSSAR.2	-0.159011812	Q9JKV1_S213_M1_Adrm1
ESTESS[Phospho (STY)]PGKHLVTSEELISEGK.3	-0.289636269	Q9JKX6_S10_M1_Nudt5
GATADDSGGGS[Phospho (STY)]PVLAAK.2	-0.400299655	Q9JL04_S493_M1_Fmn2
SSILES[Phospho (STY)]PKK.2	-0.406762222	Q9JL04_S629_M1_Fmn2
LPEEEPS[Phospho (STY)]PKDVDTEPK.2	-0.318897388	Q9JL04_S614_M1_Fmn2
GS[Phospho (STY)]PGEEWVPEVEEASQR.2	-0.109984617	Q9JL04_S339_M1_Fmn2
SIQTS[Phospho (STY)]PTEEGR.2	-0.381220232	Q9JL04_S724_M1_Fmn2
S[Phospho (STY)]ADWTEELGVR.2	-0.280368141	Q9JL04_S459_M1_Fmn2
QLSSPNHSPS[Phospho (STY)]QSPNQSPR.3	-0.214449296	Q9JL04_S411_M1_Fmn2
SKPLDQSVEDLS[Phospho (STY)]K.2	0.172359298	Q9JL26_S189_M1_Fmnl1
EAAADTSGREEPPTPKS[Phospho (STY)]PPK.3	-0.164587641	Q9JL26_S1021_M1_Fmnl1
GPGDVVSIEILPGAAAT[Phospho (STY)]PSGDDAQAPR.3	-0.362877549	Q9JL26_T509_M1_Fmnl1
[Acetyl (Protein N-term)]ALLAEHLLKPLPADR.3	-0.023904421	#N/A
IDSILEVVQTGR.2	0.050624646	#N/A
VENNDLVIAR.2	0.156337071	#N/A
NAEFDRHEIQIYEEVAK.3	-0.112880041	#N/A
YLEHGEYEGNLYGTK.3	-0.361685205	#N/A
NLDLAS[Phospho (STY)]PEEYPK.2	0.088206236	Q9JLB2_S25_M1_Mpp5
VNVT[Phospho (STY)]NEFKSSGNTM[Oxidation (M)]K.3	0.061954079	Q9JLB4_T2533_M1_Cubn
DSAPTTPTS[Phospho (STY)]PTEFLTPGLR.2	-0.407873654	Q9JLC8_S4266_M1_Sacs
SPSPSPTS[Phospho (STY)]PGSLR.2	0.120173902	Q9JLM8_S337_M1_Dclk1
DIKPENLLVYEHQDGSK.3	-0.351317609	#N/A
SFEALLADLTR.2	-0.143496893	#N/A
S[Phospho (STY)]PASTSSVNGTPGSQLSTPR.2	-0.086205335	Q9JLM8_S307_M1_Dclk1
TLSDNVNLPQGVR.2	-0.430856597	#N/A
NVNPNWSVNVK.2	-0.285499196	#N/A
REESEEGFQIPATITER.3	-0.126148112	#N/A
TIGDGNFAVVK.2	0.137270989	#N/A
GIVYAISPDR.2	0.153639759	#N/A
SKS[Phospho (STY)]PASTSSVNGTPGSQLSTPR.3	0.026287691	Q9JLM8_S307_M1_Dclk1
SPSPSPTS[Phospho (STY)]PGSLRK.2	-0.306657901	Q9JLM8_S337_M1_Dclk1
SGKS[Phospho (STY)]PSPSPT[Phospho (STY)]SPGSLR.2	-0.056712483	Q9JLM8_T336_M2_Dclk1
DM[Oxidation (M)]ELEHFDERDK.3	-0.256341018	#N/A
AQPAPPELNSESEDYS[Phospho (STY)]PSSSETVR.3	-0.299672269	Q9JLM8_S742_M1_Dclk1
ISQHGGS[Phospho (STY)]STSLSSTK.2	-0.210342071	Q9JLM8_S352_M1_Dclk1
VC[Carbamidomethyl (C)]SS[Phospho (STY)]M[Oxidation (M)]DENDGPGEGDELGRR.3	0.004598693	#N/A
VC[Carbamidomethyl (C)]SS[Phospho (STY)]M[Oxidation (M)]DENDGPGEGDELGR.2	-0.004651937	#N/A
SGKS[Phospho (STY)]PSPSPTS[Phospho (STY)]PGSLRK.3	-0.12050368	#N/A
TLQTLSSEK.2	-0.370071054	#N/A
EES[Phospho (STY)]EEGFQIPATITER.2	0.098158435	Q9JLM8_S392_M1_Dclk1

[Acetyl (Protein N-term)]S[Phospho (STY)]FGRDMELEHFDERDK.3	-0.354729211	Q9JLM8_S2_M1_Dclk1
[Acetyl (Protein N-term)]S[Phospho (STY)]FGRDMELEHFDER.3	-0.370319035	Q9JLM8_S2_M1_Dclk1
S[Phospho (STY)]VDLDAFVAR.2	0.306765829	Q9JLQ0_S458_M1_Cd2ap
[Acetyl (Protein N-term)]STNTDLSLSSYDEGQGSK.2	-0.09972466	#N/A
SQS[Phospho (STY)]PAASDC[Carbamidomethyl (C)]SSSSSSASLPSSGR.2	0.163995273	Q9JLV1_S179_M1_Bag3
AASPFRS[Phospho (STY)]PVR.2	0.155114621	Q9JLV1_S274_M1_Bag3
FLQESFNNDR.2	0.144392606	#N/A
LAIIPGGGGTQR.2	-0.12300654	#N/A
HLEEENR.2	0.19982503	#N/A
EFLPQGPVAM[Oxidation (M)]R.2	0.058040837	#N/A
SEVPGIFC[Carbamidomethyl (C)]AGADLK.2	-0.076930372	#N/A
AVGLISHVLEQNQEGDAAYRK.3	-0.458804939	#N/A
AVGLISHVLEQNQEGDAAYR.3	-0.04147865	#N/A
QTS[Phospho (STY)]IETDR.2	0.351464721	Q9JM13_S132_M1_Rabgef1
YM[Oxidation (M)]SGQTS[Phospho (STY)]PR.2	0.208657604	Q9JM13_S390_M1_Rabgef1
LDAQSLNLSQEDFDRYM[Oxidation (M)]S[Phospho (STY)]GQTSPR.3	-0.098843054	Q9JM13_S386_M1_Rabgef1
DTEVFIC[Carbamidomethyl (C)]TT[Phospho (STY)]PLLK.2	0.262816651	Q9JM14_T102_M1_Nt5c
WVEQNLGPEFVER.2	0.14585709	#N/A
EM[Oxidation (M)]NDM[Oxidation (M)]KDTEVFIC[Carbamidomethyl (C)]TT[Phospho (STY)]PLLK.3	-0.365226974	Q9JM14_T102_M1_Nt5c
VLVDM[Oxidation (M)]DGVLADFESGLLQGFR.2	-0.18181091	#N/A
SQS[Phospho (STY)]LQDQPTR.2	-0.218839834	Q9JM52_S603_M1_Mink1
LDSS[Phospho (STY)]PVLSPGNK.2	-0.306676573	Q9JM52_S746_M1_Mink1
SDSVLPASHGHLPQAGS[Phospho (STY)]LER.3	-0.416921001	Q9JM52_S729_M1_Mink1
S[Phospho (STY)]DPGWER.2	-0.332048505	Q9JM52_S706_M1_Mink1
QQNS[Phospho (STY)]PLAK.2	0.031141779	Q9JM52_S537_M1_Mink1
GQS[Phospho (STY)]PPTKDGGSDYQSR.2	-0.235482625	Q9JM52_S903_M1_Mink1
QQQQLQQQQR.2	0.138794765	#N/A
VGASTKLDSS[Phospho (STY)]PVLS[Phospho (STY)]PGNK.2	-0.243783013	Q9JM52_S746_M2_Mink1
VAS[Phospho (STY)]PSGLR.2	-0.091822038	Q9JM63_S338_M1_Kcnj10
AYLQQLR.2	0.055177127	#N/A
DTDIVDEAIYYFK.2	0.134298483	#N/A
LIGNMALLPLR.2	0.33449274	#N/A
ETKDTDIVDEAIYYFK.3	-0.372022494	#N/A
PAYHSSLM[Oxidation (M)]DPDTK.3	-0.066018852	#N/A
VFDPQSDKPSK.3	0.400194789	#N/A
S[Phospho (STY)]AGEDNDGPVLTDEQK.2	-0.300444151	Q9JM93_S140_M1_Arl6ip4
SLSSS[Phospho (STY)]PVKK.2	-0.212038763	Q9JM96_S140_M1_Cdc42ep4
EADDES[Phospho (STY)]LDEQASASK.2	-0.231413197	Q9JM96_S64_M1_Cdc42ep4
DQWGS[Phospho (STY)]EEEEEAGGYR.2	0.19156509	Q9JM96_S223_M1_Cdc42ep4
AREADDES[Phospho (STY)]LDEQASASK.2	-0.220062008	Q9JM96_S64_M1_Cdc42ep4
DQWGS[Phospho (STY)]EEEEEAGGYRDK.3	-0.034584351	Q9JM96_S223_M1_Cdc42ep4
NAMS[Phospho (STY)]LPQLNEK.2	0.014595673	Q9JM96_S116_M1_Cdc42ep4
VNQQPNANDKNS[Phospho (STY)]PPKEIK.3	-0.26672598	Q9JMA1_S393_M1_Usp14
AS[Phospho (STY)]GEM[Oxidation (M)]ASAQYITAALR.2	-0.30433207	Q9JMA1_S143_M1_Usp14
QSPTLQR.2	0.054842114	#N/A
LEAIEDDSGRETDSSSAPAVT[Phospho (STY)]PSK.3	-0.327559435	Q9JMA1_T235_M1_Usp14
[Acetyl (Protein N-term)]AAVGS[Phospho (STY)]PGSLESAPR.2	-0.108159293	Q9JMA2_S6_M1_Qtrt1

LISQAYEVLSDPK.2	-0.053066408	#N/A
AGGAGGEGS[Phospho (STY)]DDDTSLT.2	-0.241765088	Q9JMD3_S284_M1_Stard10
AFYGS[Phospho (STY)]EEDEAK.2	0.085069365	Q9JME5_S272_M1_Ap3b2
QLIVPSEQGGALSR.2	-0.158039485	#N/A
ATGYQELPDWPEEAPDPSVR.2	-0.091898282	#N/A
AFYGS[Phospho (STY)]EEDEAKGPGSEEAATAALPAR.3	-0.237667898	Q9JME5_S272_M1_Ap3b2
TQFLS[Phospho (STY)]PTQNESLLEENPEK.2	-0.473078151	Q9JME5_S253_M1_Ap3b2
LEVLTNLANETNIPTVLR.2	0.068281677	#N/A
TTLVAGSVVM[Oxidation (M)]AFEEVC[Carbamidomethyl (C)]PER.2	0.225627819	#N/A
GGGS[Phospho (STY)]ES[Phospho (STY)]EQS[Phospho (STY)]EEEDEKKK.2	0.376927325	Q9JME5_S731_M3_Ap3b2
EEAPGAES[Phospho (STY)]PSLR.2	0.045366402	Q9JMG3_S71_M1_Tmub1
GFNEGLWEIENNPGVK.2	-0.310098161	#N/A
S[Phospho (STY)]PGDEDDKDC[Carbamidomethyl (C)]KEEENK.2	-0.348041955	Q9JMG7_S161_M1_Hdgfl3
FTGYQTIQQQSSSETEGEGGNTADAS[Phospho (STY)]S[Phospho (STY)]EEEGDRVEDGKGK.4	0.066316433	Q9JMG7_S121_M2_Hdgfl3
FTGYQTIQQQSSSETEGEGGNTADAS[Phospho (STY)]S[Phospho (STY)]EEEGDRVEDGK.4	0.297138454	Q9JMG7_S121_M2_Hdgfl3
VLVLDFVTPTPLGTR.2	0.38652278	#N/A
VTAQSTNSEETIEGEFNTVLLAVGR.3	-0.376895212	#N/A
VVYENAYGR.2	-0.527034117	#N/A
RFS[Phospho (STY)]FSQR.2	-0.283654499	Q9JMH9_S140_M1_Myo18a
S[Phospho (STY)]LAHADEAR.2	-0.477900033	Q9JMH9_S865_M1_Myo18a
FSHSYLSDS[Phospho (STY)]DTEAK.2	-0.054315592	Q9JMH9_S2039_M1_Myo18a
SSSPTSHWKPLAPDPS[Phospho (STY)]DDEHDPVDSISRPR.5	0.375958006	Q9JMH9_S2016_M1_Myo18a
NELRLS[Phospho (STY)]SDRLETR.3	-0.04643725	Q9JMH9_S1279_M1_Myo18a
VASGSDLHLTDIDSDSNR.3	0.010850581	#N/A
NKGPSKAPS[Phospho (STY)]DDGS[Phospho (STY)]LK.2	-0.129131458	Q9JMH9_S1994_M2_Myo18a
FSHSYLSDS[Phospho (STY)]DTEAKLTETSA.3	-0.179897748	Q9JMH9_S2039_M1_Myo18a
NKLEGDS[Phospho (STY)]DVDS[Phospho (STY)]ELEDRVDGVK.3	-0.362721356	Q9JMH9_S1966_M2_Myo18a
APS[Phospho (STY)]DDGSLK.2	0.243456889	Q9JMH9_S1994_M1_Myo18a
GAPANVS[Phospho (STY)]SSDLTGR.2	0.230916445	Q9JMK2_S389_M1_Csnk1e
IQQTGNTS[Phospho (STY)]PR.2	-0.379630036	Q9JMK2_S363_M1_Csnk1e
[Acetyl (Protein N-term)]AQEFVNC[Carbamidomethyl (C)]K.2	0.229270276	#N/A
LVIVGDGAC[Carbamidomethyl (C)]GK.2	0.127955418	#N/A
TC[Carbamidomethyl (C)]LLIVFSK.2	0.226339181	#N/A
M[Oxidation (M)]KQEPVKPEEGR.3	-0.164452267	#N/A
EVFEMATR.2	0.334650587	#N/A
QVELALWDTAGQEDYDR.2	-0.152143009	#N/A
AINQGGLTSVAVR.2	-0.064026645	#N/A
LLDSSTVTHLFK.2	0.338931989	#N/A
HITIFSPEGR.2	-0.200997331	#N/A
YEAANWK.2	0.514430498	#N/A
ITESIGC[Carbamidomethyl (C)]VM[Oxidation (M)]TGM[Oxidation (M)]TADSR.2	-0.248538084	#N/A
ILTEAEIDAHLVALAERD.3	-0.452039188	#N/A
PAFVGSHDHS[Phospho (STY)]EESQLPR.3	0.128608973	Q9QUN9_S295_M1_Dkk3
LLVEAEQAK.2	-0.542764435	#N/A
GGSDNDASLVITDLTLEDYGR.2	-0.018334711	#N/A
EPC[Carbamidomethyl (C)]GGQNTVPGVR.2	0.274645861	#N/A
DPTAFGSGIHK.2	-0.274190621	#N/A

YNLNFHEAR.2	-0.066623393	#N/A
C[Carbamidomethyl (C)]SPTEAAVR.2	-0.005490247	#N/A
C[Carbamidomethyl (C)]DAGWLADGSVR.2	0.47637111	#N/A
LTSDYLR.2	-0.017584753	#N/A
VFLDPNTLSDDGTVALR.2	0.522626534	#N/A
SGEEDFESLASQFSDC[Carbamidomethyl (C)]SSAK.2	-0.296641893	#N/A
TGEM[Oxidation (M)]SGPVFTDSGIHIILR.3	-0.219283676	#N/A
RNS[Phospho (STY)]LPQIPTLNLEAR.3	-0.213905344	Q9QVP9_S375_M1_Ptk2b
NLLDAVDQAK.2	0.010112633	#N/A
MSGVSEPLSRVK.2	0.0025012	#N/A
SHLSESC[Carbamidomethyl (C)]SIES[Phospho (STY)]DIYAEIPDETLR.3	-0.357402777	Q9QVP9_S399_M1_Ptk2b
NS[Phospho (STY)]LPQIPTLNLEAR.2	0.194544337	Q9QVP9_S375_M1_Ptk2b
LPPSS[Phospho (STY)]PQK.2	-0.050506152	Q9QWI6_S540_M1_Srcin1
KAES[Phospho (STY)]EELEVQKPQVK.3	-0.459046022	Q9QWI6_S1110_M1_Srcin1
AES[Phospho (STY)]EELEVQKPQVK.2	-0.35892208	Q9QWI6_S1110_M1_Srcin1
RFS[Phospho (STY)]NVGLVHTSER.3	-0.472485978	Q9QWI6_S79_M1_Srcin1
AVS[Phospho (STY)]VEAAERDWEEKR.3	-0.216730247	Q9QWI6_S974_M1_Srcin1
AVS[Phospho (STY)]VEAAER.2	-0.042284423	Q9QWI6_S974_M1_Srcin1
GVTDTLAQIR.2	0.170106101	#N/A
TLGGGGGGGS[Phospho (STY)]GGRR.2	-0.122370008	Q9QWI6_S73_M1_Srcin1
YLNDEELITQQLNDLEK.2	-0.145744695	#N/A
AAQGPAGS[Phospho (STY)]PDKGK.2	-0.202694883	Q9QWI6_S1185_M1_Srcin1
FSNVGLVHTS[Phospho (STY)]ERR.3	-0.365512834	Q9QWI6_S87_M1_Srcin1
EVSGPNETSS[Phospho (STY)]PGSEKPSGSR.3	0.308693122	Q9QWI6_S1222_M1_Srcin1
QLGETLTELK.2	0.305486043	#N/A
GEGLYADPYGLLHEGR.3	-0.026666649	#N/A
QIASLTGLVQSALLR.2	0.177562302	#N/A
DSGSSSVFAES[Phospho (STY)]PGGK.2	-0.283781043	Q9QWI6_S588_M1_Srcin1
KDS[Phospho (STY)]GSSSVFAES[Phospho (STY)]PGGK.2	-0.338737124	Q9QWI6_S579_M2_Srcin1
LSYAGGRPPSYAGS[Phospho (STY)]PVHHAAER.3	-0.263010201	Q9QWI6_S411_M1_Srcin1
HTQGAQPGLADQAAK.2	0.147350648	#N/A
S[Phospho (STY)]SPPM[Oxidation (M)]LSADDAEYPR.2	0.214038338	Q9QWI6_S46_M1_Srcin1
AALTQYSAK.2	0.410780831	#N/A
GS[Phospho (STY)]DELTVPR.2	-0.177249331	Q9QWI6_S1054_M1_Srcin1
GLQNSASDLR.2	0.292867704	#N/A
RQEDPLQR.2	0.109618118	#N/A
RGS[Phospho (STY)]DELTVPR.2	-0.424716021	Q9QWI6_S1054_M1_Srcin1
S[Phospho (STY)]PGVLFLQFGEETR.2	0.027471038	Q9QWI6_S247_M1_Srcin1
S[Phospho (STY)]FPSSHGLTTTR.2	0.164001028	Q9QWI6_S1079_M1_Srcin1
GEPDPERS[Phospho (STY)]SPPM[Oxidation (M)]LSADDAEYPR.3	-0.293841249	Q9QWI6_S46_M1_Srcin1
RFS[Phospho (STY)]NVGLVHT[Phospho (STY)]SERR.3	-0.014852862	Q9QWI6_S79_M2_Srcin1
QVDEGM[Oxidation (M)]WPPPNNLLNQS[Phospho (STY)]PK.2	-0.395461253	Q9QWI6_S911_M1_Srcin1
LKAAQGPAGS[Phospho (STY)]PDKGK.3	-0.439689518	Q9QWI6_S1185_M1_Srcin1
S[Phospho (STY)]PNTAILIKDEAR.2	-0.09524756	Q9QWI6_S293_M1_Srcin1
AAQGPAGS[Phospho (STY)]PDK.2	-0.018440049	Q9QWI6_S1185_M1_Srcin1
LGGAPTGQGVSPSPSAILER.2	0.059925091	#N/A
VEGSNGAAT[Phospho (STY)]PSAPVC[Carbamidomethyl (C)]GSGSK.2	-0.07022089	Q9QWI6_T675_M1_Srcin1

S[Phospho (STY)]S[Phospho (STY)]GATPVSGPPPPSASSTPAGQPTAVSR.3	-0.032008967	Q9QWI6_S687_M2_Srcin1
TEKPSKS[Phospho (STY)]PPPPPPR.3	-0.103094943	Q9QWI6_S1070_M1_Srcin1
S[Phospho (STY)]PNTAILIK.2	0.125862694	Q9QWI6_S293_M1_Srcin1
FRQS[Phospho (STY)]LPLSR.2	-0.249712746	Q9QWI6_S233_M1_Srcin1
DAFMDHLK.2	0.167262931	#N/A
HTVIAAQS[Phospho (STY)]LEALSGLQK.3	-0.395081138	Q9QWI6_S98_M1_Srcin1
EMVYASRES[Phospho (STY)]S[Phospho (STY)]PTR.2	0.167545496	Q9QWI6_S356_M2_Srcin1
FSNVGLVHTSER.2	-0.140691402	#N/A
KVAAETDFSK.2	0.068208583	#N/A
LADVSAPSGGPPPPHSPY[Phospho (STY)]SGPPSRGSPVR.3	0.029874441	Q9QWI6_Y561_M1_Srcin1
QVDEGMWPPPNNLLNQS[Phospho (STY)]PKK.3	0.150001387	Q9QWI6_S911_M1_Srcin1
RQVDEGMWPPPNNLLNQS[Phospho (STY)]PK.3	-0.086066449	Q9QWI6_S911_M1_Srcin1
SHTGDLS[Phospho (STY)]PNVQSR.2	-0.098281569	Q9QWY8_S1045_M1_Asap1
TLS[Phospho (STY)]DPPSPLPHGPPNK.2	-0.478630843	Q9QWY8_S854_M1_Asap1
SPRPQSFC[Carbamidomethyl (C)]HSS[Phospho (STY)]S[Phospho (STY)]ISPQDK.2	0.322395326	Q9QWY8_S758_M2_Asap1
QDEM[Oxidation (M)]DES[Phospho (STY)]DDDLDDKPSPIKK.3	0.018301327	#N/A
QDEMDES[Phospho (STY)]DDDLDDKPSPIK.2	0.319311794	#N/A
ESAQAVAVALS[Phospho (STY)]PK.2	0.014408101	Q9QX47_S1723_M1_Son
S[Phospho (STY)]RT[Phospho (STY)]PLRR.2	-0.216582072	Q9QX47_S2038_M2_Son
SAAS[Phospho (STY)]PVVISIPER.2	-0.201557996	Q9QX47_S1794_M1_Son
FS[Phospho (STY)]RS[Phospho (STY)]PIR.2	-0.048101521	Q9QX47_S2047_M2_Son
RFS[Phospho (STY)]RS[Phospho (STY)]PIR.2	0.358518085	Q9QX47_S2047_M2_Son
DVEDSLPIKESAQAVAVALS[Phospho (STY)]PK.3	-0.004567233	Q9QX47_S1723_M1_Son
RRS[Phospho (STY)]FS[Phospho (STY)]IS[Phospho (STY)]PVR.2	0.327520376	Q9QX47_S2027_M3_Son
ESTVLGDSEEPS[Phospho (STY)]E.2	-0.400972787	Q9QXG2_S664_M1_Chm
VQDNTET[Phospho (STY)]PKR.2	-0.354808873	Q9QXG2_T221_M1_Chm
GWS[Phospho (STY)]PPPEVR.2	-0.175005675	Q9QXG4_S30_M1_Acss2
VRGWS[Phospho (STY)]PPPEVR.2	-0.418140259	Q9QXG4_S30_M1_Acss2
AELGM[Oxidation (M)]NDS[Phospho (STY)]PSQSPPVK.2	-0.140519555	Q9QXG4_S263_M1_Acss2
GATDTS[Phospho (STY)]PLTR.2	-0.233225907	Q9QXL1_S1217_M1_Kif21b
AVS[Phospho (STY)]AEC[Carbamidomethyl (C)]LGPPLDSSTK.2	-0.267361569	Q9QXL1_S1150_M1_Kif21b
TVS[Phospho (STY)]LPTR.2	0.136139089	Q9QXL1_S1198_M1_Kif21b
VC[Carbamidomethyl (C)]PQTDAENATDNK.2	-0.055282534	#N/A
DQVLQNLGSVESYSEEK.2	-0.367645141	#N/A
ITQLVSEQANQVLAR.2	-0.393172783	#N/A
HSDSGASETSLS[Phospho (STY)]PPSS[Phospho (STY)]PPSRPR.3	-0.129319844	Q9QXL2_S1273_M2_Kif21a
TTASTQM[Oxidation (M)]NVQSS[Phospho (STY)]R.2	0.257695568	Q9QXL2_S224_M1_Kif21a
AHNLQDGQLSDTGDLGEDIAS[Phospho (STY)]N.2	0.054181603	Q9QXL2_S1671_M1_Kif21a
LISESS[Phospho (STY)]PM[Oxidation (M)]NEFETLTAK.2	-0.248509499	Q9QXL2_S258_M1_Kif21a
MTISNMEADMNR.2	-0.449117753	#N/A
KSQAWAEGGS[Phospho (STY)]PR.2	-0.104646691	Q9QXM1_S108_M1_Jmy
GPES[Phospho (STY)]PLRS[Phospho (STY)]PAR.2	-0.129945626	Q9QXM1_S130_M2_Jmy
IKEASPES[Phospho (STY)]EDEEEALPC[Carbamidomethyl (C)]TDWEN.3	0.252610564	Q9QXM1_S969_M1_Jmy
RGS[Phospho (STY)]GQDLAGPAPETLEQTEGATP.3	-0.141068198	Q9QXQ1_S426_M1_Pde7b
GSGQDLAGPAPETLEQTEGAT[Phospho (STY)]P.2	0.051616833	Q9QXQ1_T445_M1_Pde7b
RRGS[Phospho (STY)]GQDLAGPAPETLEQTEGATP.3	0.238936775	Q9QXQ1_S426_M1_Pde7b
AQLEPVAS[Phospho (STY)]PAK.2	-0.253224114	Q9QXS1_S1443_M1_Plec

NDESQLS[Phospho (STY)]PATR.2	-0.380794287	Q9QXS1_S728_M1_Plec
RAS[Phospho (STY)]FAEK.2	-0.287121742	Q9QXS1_S1729_M1_Plec
AQLEPVAS[Phospho (STY)]PAKKPK.2	-0.426279363	Q9QXS1_S1443_M1_Plec
LPVDVAYQR.2	-0.048982129	#N/A
ARNDESQLS[Phospho (STY)]PATR.2	0.095982281	Q9QXS1_S728_M1_Plec
SSS[Phospho (STY)]VGSSSSYPISSAGPR.2	-0.181489912	Q9QXS1_S4393_M1_Plec
WQAVLAQTDVR.2	0.336311123	#N/A
DAPDGPSVEAEPEYTFEGLR.2	0.153315797	#N/A
LTVDEAVR.2	0.064440205	#N/A
GYYSPYSVSGSGSTAGS[Phospho (STY)]R.2	-0.100293317	Q9QXS1_S4633_M1_Plec
VLALPEPSPAAPTLR.2	-0.177116686	#N/A
LLDAQLATGGIVDPR.2	-0.001974857	#N/A
FLEVQYLTGGLIEPDTPGR.2	-0.480362002	#N/A
VYHDPSTQEPVTYSQLQQR.3	-0.431332253	#N/A
LLDAQLSTGGIVDPSK.2	-0.584563408	#N/A
FLEGTSC[Carbamidomethyl (C)]IAGVFVDATK.2	0.175371909	#N/A
AQAEVEGLGK.2	-0.304582508	#N/A
TEVVETTQVYTEEETRR.3	0.270223613	#N/A
EEELQQTLQQEQNMLDR.2	0.394693803	#N/A
[Acetyl (Protein N-term)]AGVSFSGHR.2	0.115113282	#N/A
YVLINWVGEDVPDAR.2	0.21697848	#N/A
KQQS[Phospho (STY)]LEAEEAK.2	-0.294381704	Q9QXS6_S241_M1_Dbn1
EESAADWALYTYEDGSDDLK.2	0.021690852	#N/A
LSS[Phospho (STY)]PVLHR.2	-0.369438484	Q9QXS6_S142_M1_Dbn1
EGTQASEGYFSQS[Phospho (STY)]QEEEFAQSEEPC[Carbamidomethyl (C)]AK.3	-0.436094602	Q9QXS6_S658_M1_Dbn1
ASDSGPSSSSSSSSS[Phospho (STY)]PPR.2	-0.027162548	Q9QXS6_S344_M1_Dbn1
EQFWEQAK.2	-0.090655666	#N/A
LREDENAEPVGTTYQK.3	-0.09760845	#N/A
M[Oxidation (M)]APTPIPTR.2	0.03669482	#N/A
SPS[Phospho (STY)]DSSTASTPIAEQIER.2	-0.185877338	Q9QXS6_S387_M1_Dbn1
ASDSGPSSSSSSSSS[Phospho (STY)]PPRTPFPYITC[Carbamidomethyl (C)]HR.4	0.474027869	Q9QXS6_S344_M1_Dbn1
LELLAAYEEVIR.2	0.058339372	#N/A
SESEVEEAAAIIAQRPDNPR.3	0.356425216	#N/A
LAASGEGGLQELSGHFENQK.3	0.293536619	#N/A
KSESEVEEAAAIIAQRPDNPR.3	-0.352319927	#N/A
KQQS[Phospho (STY)]LEAEEAKR.3	0.07630585	Q9QXS6_S241_M1_Dbn1
EREQQIEEHR.3	0.251693216	#N/A
VAEFFQGVDVIVNASSVEDIDAGAIGQR.3	0.310480686	#N/A
NGES[Phospho (STY)]SELDLQGIR.2	-0.366007344	Q9QXT0_S115_M1_Cnpy2
SEAHLTELLEEVC[Carbamidomethyl (C)]DR.3	-0.104395159	#N/A
SQDLHC[Carbamidomethyl (C)]GAC[Carbamidomethyl (C)]R.3	0.217890212	#N/A
ILTGS[Phospho (STY)]SEPEAAPAPR.2	-0.25290206	Q9QXV0_S204_M1_Pcsk1n
ALAHLLEAER.2	0.411833591	#N/A
VLAQLLR.2	-0.322705146	#N/A
LDPAALAAQLVPAPAAAPR.2	0.292316778	#N/A
AEAQEAEDQQAR.2	0.417234044	#N/A
ASDPPLAPDDDPDAPAAQLAR.2	-0.027589172	#N/A

ARAEAQEAEDQQAR.2	-0.012705524	#N/A
VDDKPSS[Phospho (STY)]PGDSSK.2	-0.096426209	Q9QXV1_S164_M1_Cbx8
RQDS[Phospho (STY)]DLVQYGVTSPSSAEASSK.3	-0.365197511	Q9QXV1_S229_M1_Cbx8
PTPVKDPDS[Phospho (STY)]EEQP.2	-0.156748109	Q9QXW9_S527_M1_Slc7a8
NHPGSDTS[Phospho (STY)]PEAEASSGGGGVALKK.3	-0.302135411	Q9QXW9_S21_M1_Slc7a8
NHPGSDTS[Phospho (STY)]PEAEASSGGGGVALK.3	0.206302966	Q9QXW9_S21_M1_Slc7a8
SKLPNS[Phospho (STY)]VLGK.2	-0.175451426	Q9QXY6_S479_M1_Ehd3
LLPLEEYYR.2	0.2730839	#N/A
ELVNNLAEIYGR.2	-0.04811033	#N/A
IVNTPEVIR.2	0.130571996	#N/A
DKPM[Oxidation (M)]YDEIFYTLS[Phospho (STY)]PVDGK.3	-0.162076573	Q9QXY6_S456_M1_Ehd3
EHQIS[Phospho (STY)]PGDFPNLKR.3	0.315728333	Q9QXY6_S349_M1_Ehd3
LNAFGNAFLNR.2	0.257020379	#N/A
DIQS[Phospho (STY)]LPR.2	0.105271205	Q9QXY6_S284_M1_Ehd3
EHQIS[Phospho (STY)]PGDFPNLK.3	-0.148702164	Q9QXY6_S349_M1_Ehd3
IGPEPTTDSFIAVM[Oxidation (M)]QGDVEGIIPGNALVVDPK.3	0.027744085	#N/A
LLDAEDVDVPS[Phospho (STY)]PDEK.2	-0.188782522	Q9QXZ0_S280_M1_Macf1
S[Phospho (STY)]QDSVLDPAER.2	0.020400376	Q9QXZ0_S57_M1_Macf1
AFVESQQKS[Phospho (STY)]PGKR.3	-0.257934817	Q9QXZ0_S1367_M1_Macf1
DTQDIVHDLES[Phospho (STY)]PGIDPSIIK.3	-0.171763603	Q9QXZ0_S5988_M1_Macf1
RQGS[Phospho (STY)]FSEDVISHK.3	-0.343860597	Q9QXZ0_S3889_M1_Macf1
AFVESQQKS[Phospho (STY)]PGK.2	-0.268235031	Q9QXZ0_S1367_M1_Macf1
EAEEELAASGGQSPT[Phospho (STY)]GEQIPQFQQR.3	-0.048518593	Q9QXZ0_T5553_M1_Macf1
AFLAELEQNS[Phospho (STY)]PK.2	-0.028363171	Q9QXZ0_S4483_M1_Macf1
RKS[Phospho (STY)]QDSVLDPAER.3	-0.035968307	Q9QXZ0_S57_M1_Macf1
QGS[Phospho (STY)]FSEDVISHK.2	-0.258902178	Q9QXZ0_S3889_M1_Macf1
AM[Oxidation (M)]DATLS[Phospho (STY)]PTKTETVK.2	0.52831535	Q9QXZ0_S4458_M1_Macf1
LNSQES[Phospho (STY)]DEEPQISDVPHISK.3	0.296553776	Q9QXZ0_S3085_M1_Macf1
TPLPTIIGS[Phospho (STY)]PTK.2	-0.183690257	Q9QY01_S600_M1_Ulk2
AQDKPES[Phospho (STY)]PSGSTQIQR.2	-0.210674455	Q9QY06_S1264_M1_Myo9b
TNS[Phospho (STY)]LLLR.2	0.062271368	Q9QY40_S1747_M1_Plxnb3
SLTSPLDDTEVKK.2	0.02810691	#N/A
SLTS[Phospho (STY)]PLDDTEVK.2	0.357483203	Q9QY76_S159_M1_Vapb
ELAS[Phospho (STY)]PLALT[Phospho (STY)]PR.2	0.161015739	Q9QY81_S1839_M2_Nup210
FVNWQVDGEYR.2	-0.330176399	#N/A
MAIQTQQSK.2	-0.058333949	#N/A
TEEVLS[Phospho (STY)]PDGSPSK.2	-0.091997398	Q9QYB5_S673_M1_Add3
EQDHIIIIPR.3	0.051892288	#N/A
TLDNLGYR.2	0.065337519	#N/A
TEEVLSPDGSPSKS[Phospho (STY)]PSKK.3	0.083330806	Q9QYB5_S681_M1_Add3
TEEVLSPDGSPSKS[Phospho (STY)]PSK.2	0.089303897	Q9QYB5_S681_M1_Add3
GLSFSEATASTLVK.2	-0.107109931	#N/A
SPERTEEVLSPDGSPSKS[Phospho (STY)]PSK.3	-0.083856253	Q9QYB5_S681_M1_Add3
[Acetyl (Protein N-term)]SSDTSPAVVTT[Phospho (STY)]PPPPSM[Oxidation (M)]PHK.2	0.393387695	Q9QYB5_T12_M1_Add3
SDVEIPATVTAFSFEDDSAPLS[Phospho (STY)]PLK.3	-0.037085083	Q9QYB5_S423_M1_Add3
HKSDVEIPATVTAFSFEDDSAPLS[Phospho (STY)]PLK.3	-0.467101821	Q9QYB5_S423_M1_Add3
SPERTEEVLS[Phospho (STY)]PDGSPSK.2	-0.061353244	Q9QYB5_S673_M1_Add3

NMS[Phospho (STY)]PDLR.2	0.050061535	Q9QYB5_S42_M1_Add3
STPAS[Phospho (STY)]PVQS[Phospho (STY)]PSK.2	0.121757298	Q9QYB8_S602_M2_Add2
DIATEKPGS[Phospho (STY)]PVK.2	0.089126169	Q9QYB8_S594_M1_Add2
VNVADEVQR.2	0.47622457	#N/A
FSEDDPEYLR.2	-0.185440576	#N/A
WLNTPNTYLR.2	-0.337772223	#N/A
VNILGEVVEK.2	0.295345937	#N/A
GVSC[Carbamidomethyl (C)]SEVTASSLIK.2	0.049436538	#N/A
EQDHFLISPK.2	0.539538484	#N/A
SAGPQSQLLASVIAEK.2	0.143747629	#N/A
[Acetyl (Protein N-term)]SEDTVPEAAS[Phospho (STY)]PPPSQGQHYFDR.3	-0.364048583	Q9QYB8_S11_M1_Add2
TESVTSGPLSPEGS[Phospho (STY)]PSKS[Phospho (STY)]PSK.2	-0.315064716	#N/A
TESVTSGPLS[Phospho (STY)]PEGSPSK.2	-0.245251819	#N/A
LEQEQEGEKDIATEKPGS[Phospho (STY)]PVK.3	0.517240159	Q9QYB8_S594_M1_Add2
HPFVQEK.2	-0.276720371	#N/A
TESVTSGPLSPEGSPS[Phospho (STY)]KS[Phospho (STY)]PSKK.3	0.074887174	#N/A
SPAVS[Phospho (STY)]PSKTSEDTK.2	-0.257516569	Q9QYB8_S618_M1_Add2
NMGS[Phospho (STY)]PRPK.2	-0.350944864	Q9QYB8_S455_M1_Add2
AGTKS[Phospho (STY)]PAVS[Phospho (STY)]PSK.2	-0.115867266	#N/A
IENPNQFVPLYTDPQEVLDM[Oxidation (M)]R.3	-0.317831062	#N/A
AGTKS[Phospho (STY)]PAVS[Phospho (STY)]PSKTSEDTK.2	-0.469837137	#N/A
STPAS[Phospho (STY)]PVQS[Phospho (STY)]PSKAGTK.2	-0.072849815	Q9QYB8_S602_M2_Add2
SPS[Phospho (STY)]TESQLMSK.2	0.496022593	Q9QYB8_S532_M1_Add2
S[Phospho (STY)]PAVS[Phospho (STY)]PSKTSEDTKK.2	-0.269116202	#N/A
DIATEKPGS[Phospho (STY)]PVKS[Phospho (STY)]TPAS[Phospho (STY)]PVQS[Phospho (STY)]PSK.3	-0.260549917	Q9QYB8_S594_M3_Add2
DKTESVTSGPLS[Phospho (STY)]PEGS[Phospho (STY)]PSK.2	0.117268857	#N/A
DKTESVTSGPLSPEGSPS[Phospho (STY)]KSPSK.3	0.281242774	Q9QYB8_S698_M1_Add2
SRS[Phospho (STY)]PSTESQLM[Oxidation (M)]SK.2	0.186861082	Q9QYB8_S530_M1_Add2
GLGQM[Oxidation (M)]TTNADTDGDSYKDKTESVTSGPLS[Phospho (STY)]PEGSPSK.4	-0.139987325	#N/A
C[Carbamidomethyl (C)]LGPTC[Carbamidomethyl (C)]K.2	0.039444921	#N/A
NHGM[Oxidation (M)]VALGDTVEEAFYK.3	-0.356810451	#N/A
SPAVS[Phospho (STY)]PSK.2	0.126051608	Q9QYB8_S618_M1_Add2
SSSGMPIR.2	0.001315706	#N/A
LGEHEFEALMR.3	-0.124877257	#N/A
SAGPQSQLLASVIAEKS[Phospho (STY)]R.3	0.001184202	Q9QYB8_S528_M1_Add2
AAVVTS[Phospho (STY)]PPPTTAPHK.2	-0.466862283	Q9QYC0_S12_M1_Add1
TPS[Phospho (STY)]FLKK.2	-0.294194271	Q9QYC0_S724_M1_Add1
AAVVTS[Phospho (STY)]PPPTTAPHKER.3	-0.455035637	Q9QYC0_S12_M1_Add1
GS[Phospho (STY)]EENLDETR.2	-0.271318235	Q9QYC0_S586_M1_Add1
LEEDLPQEPTS[Phospho (STY)]R.2	-0.194225114	Q9QYC0_S628_M1_Add1
VNLQGDIVDR.2	0.16710278	#N/A
TSTSAVPNLFVPLNTNPK.2	-0.179106255	#N/A
TLASAGGPDNLVLLDPGK.2	0.097642327	#N/A
YFDRVDENNPEYLR.3	-0.634816272	#N/A
GSDSIAYDKGEK.2	-0.210995786	#N/A
SLVQGELVTASK.2	0.071580836	#N/A
SPGT[Phospho (STY)]PAGEGSGSPPK.2	-0.082832084	Q9QYC0_T358_M1_Add1

EKS[Phospho (STY)]PPDQSAVPNT[Phospho (STY)]PPSTPVK.3	0.054346872	Q9QYC0_S600_M2_Add1
EYQPHVIVSTTGPNPFNTLTDR.3	-0.123103879	#N/A
M[Oxidation (M)]LDNLGYR.2	0.478639838	#N/A
KQKGS[Phospho (STY)]EENLDETR.3	-0.432712654	Q9QYC0_S586_M1_Add1
SPPDQSAVPNT[Phospho (STY)]PPSTPVK.2	0.215104435	Q9QYC0_T610_M1_Add1
SRS[Phospho (STY)]PGT[Phospho (STY)]PAGEGSGSPPK.2	0.128463848	Q9QYC0_S355_M2_Add1
WQIGEQEFEALM[Oxidation (M)]R.2	-0.142303909	#N/A
YSDVEVPAS[Phospho (STY)]VTGHSFASDGDSGTC[Carbamidomethyl (C)]SPLRHSFQK.4	-0.016350225	Q9QYC0_S415_M1_Add1
GDDASEEGQNGSS[Phospho (STY)]PK.2	0.413465919	Q9QYC0_S465_M1_Add1
WLHSGRGDDASEEGQNGSS[Phospho (STY)]PK.3	-0.482171058	Q9QYC0_S465_M1_Add1
QKGS[Phospho (STY)]EENLDETR.2	-0.217403759	Q9QYC0_S586_M1_Add1
YSDVEVPASVTGHSFASDGDSGTC[Carbamidomethyl (C)]S[Phospho (STY)]PLR.3	-0.462470306	Q9QYC0_S431_M1_Add1
SPPDQSAVPNT[Phospho (STY)]PPST[Phospho (STY)]PVKLEEDLPQEPTSR.4	-0.047260736	Q9QYC0_T614_M2_Add1
NMAPDLR.2	-0.219413923	#N/A
KYSDVEVPASVTGHSFASDGDSGTC[Carbamidomethyl (C)]S[Phospho (STY)]PLR.4	-0.266641068	Q9QYC0_S431_M1_Add1
EKSPPDQS[Phospho (STY)]AVPNT[Phospho (STY)]PPSTPVKLEEDLPQEPTSR.4	0.253057602	Q9QYC0_S605_M2_Add1
IREQNLQDIK.3	0.184540398	#N/A
SSS[Phospho (STY)]LIHR.2	0.10023848	Q9QYC1_S551_M1_Pcnx1
GC[Carbamidomethyl (C)]S[Phospho (STY)]PGESASGGLSK.2	-0.134937417	Q9QYE3_S608_M1_Bcl11a
GC[Carbamidomethyl (C)]S[Phospho (STY)]PGESASGGLSKK.2	-0.330090939	Q9QYE3_S608_M1_Bcl11a
IYLESEHGS[Phospho (STY)]PLTPR.3	0.088622627	Q9QYE3_S205_M1_Bcl11a
FST[Phospho (STY)]PPGELDGGISGR.2	0.27394793	Q9QYE3_T701_M1_Bcl11a
ELAGNTSS[Phospho (STY)]PPLS[Phospho (STY)]PGRPSPMQR.3	0.184826559	Q9QYE3_S328_M2_Bcl11a
GVDKPPS[Phospho (STY)]PSPIEMK.2	-0.206102034	Q9QYE3_S86_M1_Bcl11a
LLLGS[Phospho (STY)]PSSLS[Phospho (STY)]PFSK.2	0.244054308	Q9QYE3_S625_M2_Bcl11a
LDPINTTLLK.2	0.044170354	#N/A
DLEIERPILGQNDNR.3	-0.453645225	#N/A
THSTSSS[Phospho (STY)]IGSGESPFSR.2	-0.327106582	Q9QYF9_S335_M1_Ndrg3
S[Phospho (STY)]RT[Phospho (STY)]HSTSSSIGSGESPFSR.3	-0.054635181	Q9QYF9_S327_M2_Ndrg3
HQTMEVS[Phospho (STY)]C[Carbamidomethyl (C)].2	0.527922128	Q9QYF9_S374_M1_Ndrg3
LHIAQDINQENLQLFLGSYNGR.3	0.318696499	#N/A
SVTSNQSDGTQESC[Carbamidomethyl (C)]ES[Phospho (STY)]PDVLDR.3	0.046336946	Q9QYF9_S361_M1_Ndrg3
C[Carbamidomethyl (C)]STLLVVGDNSPAVEAVVEC[Carbamidomethyl (C)]NSR.3	-0.489665165	#N/A
SC[Carbamidomethyl (C)]FQPLFR.2	0.470230504	#N/A
[Acetyl (Protein N-term)]AELQEVQITEEKPLLPGQTPETAK.3	0.150154128	#N/A
GIIQHAPNLENIELYWNSYNNR.3	-0.201834988	#N/A
LDPTQTSFLK.2	-0.018428812	#N/A
GWM[Oxidation (M)]DWAAHK.2	-0.24213659	#N/A
YALNHPDTVEGLVLINIDPNAK.3	0.28918047	#N/A
M[Oxidation (M)]ADSGGQPQLTQPGK.2	0.516451986	#N/A
TAS[Phospho (STY)]LTSAASIDGSR.2	0.179708864	Q9QYG0_S332_M1_Ndrg2
FGDM[Oxidation (M)]QEIIQNFVR.2	-0.399003008	#N/A
TASLTSAAS[Phospho (STY)]IDGS[Phospho (STY)]RSR.2	0.025428474	Q9QYG0_S338_M2_Ndrg2
SRT[Phospho (STY)]AS[Phospho (STY)]LTSAASIDGSR.2	0.040241077	Q9QYG0_T330_M2_Ndrg2
TLSQSS[Phospho (STY)]ESGTLPSGPPGHTM[Oxidation (M)]EVSC[Carbamidomethyl (C)].3	-0.117904135	Q9QYG0_S353_M1_Ndrg2
S[Phospho (STY)]RT[Phospho (STY)]LSQSSESGTLPSGPPGHTM[Oxidation (M)]EVSC[Carbamidomethyl (C)].3	0.269000355	Q9QYG0_S346_M2_Ndrg2
LSRS[Phospho (STY)]RT[Phospho (STY)]AS[Phospho (STY)]LTSAASIDGSR.3	0.321844045	#N/A

LTGLTSSIPDM[Oxidation (M)]ILGHLFSQEELSGNSELIQK.3	-0.225294868	#N/A
C[Carbamidomethyl (C)]PVMLVVGDQAPHEDAVVEC[Carbamidomethyl (C)]NSK.3	-0.015757715	#N/A
SAS[Phospho (STY)]DEDLKK.2	-0.131827668	Q9QYI4_S123_M1_Dnajb12
GEAAKLS[Phospho (STY)]PSSEEK.2	-0.173595798	Q9QYI5_S311_M1_Dnajb2
SAS[Phospho (STY)]PDDIKK.2	-0.101240052	Q9QYI5_S15_M1_Dnajb2
LS[Phospho (STY)]PSSEEK.2	0.196080231	Q9QYI5_S311_M1_Dnajb2
GEAAKLS[Phospho (STY)]PS[Phospho (STY)]SEEKASR.2	-0.323701887	Q9QYI5_S311_M2_Dnajb2
S[Phospho (STY)]PEEVFR.2	-0.344570212	Q9QYI5_S98_M1_Dnajb2
VIEPGC[Carbamidomethyl (C)]VR.2	0.331404914	#N/A
EHEVFQR.2	0.364856419	#N/A
VSLEDLYNGK.2	0.308943039	#N/A
YGEQGLR.2	0.323396527	#N/A
NVLC[Carbamidomethyl (C)]SAC[Carbamidomethyl (C)]SGQGGK.2	-0.020795959	#N/A
DGNDLHM[Oxidation (M)]TYK.2	-0.289346709	#N/A
ITFTGEADQAPGVEPGDIVLLLQEK.3	-0.493272331	#N/A
IGLVEALC[Carbamidomethyl (C)]GFQFTFK.2	-0.237954819	#N/A
GAS[Phospho (STY)]DDEIKR.2	-0.027743186	Q9QYJ3_S16_M1_Dnajb1
HSHPGLGTSQS[Phospho (STY)]PKW.3	-0.287088481	Q9QYK9_S340_M1_Pnck
KLGQS[Phospho (STY)]PEGEEASR.2	0.16852078	Q9QYK9_S316_M1_Pnck
HSHPGLGTSQS[Phospho (STY)]PK.3	0.284835106	Q9QYK9_S340_M1_Pnck
VPSAPGQES[Phospho (STY)]PVPDTKST[Phospho (STY)]PPTR.3	0.253032042	Q9QYR6_S1789_M2_Map1a
GEPVPAWEGKS[Phospho (STY)]PEQEVR.2	-0.232668883	Q9QYR6_S1648_M1_Map1a
EAELT[Phospho (STY)]PENIAAAR.2	-0.295477433	Q9QYR6_T633_M1_Map1a
EQDVVQGWR.2	-0.124368621	#N/A
VLFPGNAPQNK.2	-0.062742093	#N/A
ASYYVVSGNDPANGEPSR.2	-0.292036659	#N/A
GVEQQDGAVPEK.2	-0.124309713	#N/A
DLAAGAVPANLKPSK.2	-0.15569015	#N/A
SSRPDTLLSSEQRPGK.3	0.226067623	#N/A
GAALQQTQAPEPR.2	0.049959701	#N/A
GAAPPPAASGHR.2	0.259406505	#N/A
EVVPDFPEDK.2	0.3678218	#N/A
DTAVELGKGPEPK.2	-0.285154245	#N/A
NRAPDS[Phospho (STY)]GAEVER.2	0.024387817	Q9QYR6_S611_M1_Map1a
DLEQKDEHLELR.3	-0.084459594	#N/A
LGIQAEPLYR.2	-0.008972157	#N/A
LDM[Oxidation (M)]YVLNPVK.2	0.240958611	#N/A
EGEGGAGAPDSSS[Phospho (STY)]FSSK.2	-0.343527393	Q9QYR6_S1900_M1_Map1a
DFQEPLPQK.2	-0.053508996	#N/A
NEPTTPSWLAEIPPWVPK.2	-0.329681471	#N/A
DAEQTEPEQREPTPYPDER.3	0.317129034	#N/A
VRTES[Phospho (STY)]SEALK.2	-0.246947273	Q9QYR6_S384_M1_Map1a
AQWGENLQVTLIPTHDTEVTR.3	0.064178437	#N/A
S[Phospho (STY)]PTPGKGPVDR.2	-0.267154365	Q9QYR6_S2603_M1_Map1a
ELSSEPRT[Phospho (STY)]PPAQK.2	-0.393216419	Q9QYR6_T504_M1_Map1a
ALGLEESPEEEGK.2	-0.330893433	#N/A
ADS[Phospho (STY)]KESLK.2	-0.444594851	Q9QYR6_S319_M1_Map1a

ALGLEES[Phospho (STY)]PEEEGKAR.2	-0.40111612	Q9QYR6_S1606_M1_Map1a
ALALVPGTPTR.2	-0.269413738	#N/A
GEKELSSEPRT[Phospho (STY)]PPAQK.3	0.267896984	Q9QYR6_T504_M1_Map1a
SHWDDGTNDS[Phospho (STY)]DLEKGAR.3	-0.306759034	Q9QYR6_S2082_M1_Map1a
APEHIIPQPTQTDR.2	0.252049705	#N/A
AKPAS[Phospho (STY)]PARR.2	0.008165332	Q9QYR6_S2591_M1_Map1a
QLS[Phospho (STY)]PESLGTLQFGELSLGK.2	-0.292310148	Q9QYR6_S1205_M1_Map1a
ESTFLDEGPNEQEITPLQHTPR.3	-0.564177408	#N/A
VLVLEDR.2	-0.201074166	#N/A
EKETWEER.2	-0.192992294	#N/A
VVSNTIEPLTLFHK.2	0.14286165	#N/A
APSLDSSLPQLPSPSSPGAPLLSNLPR.3	0.009052223	#N/A
SPQAQDTLGSLAGGQTGC[Carbamidomethyl (C)]TIQLLPEQDK.3	-0.303322105	#N/A
IDSVLLTHIGADNLPGINGLLQR.3	-0.295148288	#N/A
C[Carbamidomethyl (C)]LS[Phospho (STY)]PDDSTVK.2	-0.216380436	Q9QYR6_S981_M1_Map1a
SEPQDFQEDS[Phospho (STY)]WGDTK.2	-0.054906772	Q9QYR6_S1023_M1_Map1a
AKPAS[Phospho (STY)]PAR.2	-0.014555918	Q9QYR6_S2591_M1_Map1a
YPVATQK.2	0.011514159	#N/A
TQEPAEPQKDELLGFTDQSFS[Phospho (STY)]PEDAESLSVLSVVSPDTAK.4	-0.130306714	Q9QYR6_S1133_M1_Map1a
NLISPELGVVFFNVPDK.2	-0.100166865	#N/A
ETS[Phospho (STY)]PTRGEPVPAWEGK.3	-0.413097289	Q9QYR6_S1634_M1_Map1a
ELALSS[Phospho (STY)]PEDLTQDFEELKR.3	-0.134981467	Q9QYR6_S527_M1_Map1a
EM[Oxidation (M)]TLDQKS[Phospho (STY)]PEKAK.2	-0.358980491	Q9QYR6_S1580_M1_Map1a
ETS[Phospho (STY)]PTRGEPVPAWEGKSPEQEVR.4	-0.274393763	Q9QYR6_S1634_M1_Map1a
HRADS[Phospho (STY)]KESLK.2	-0.238948701	Q9QYR6_S319_M1_Map1a
TES[Phospho (STY)]SEALKAEK.2	-0.333811187	Q9QYR6_S384_M1_Map1a
ADS[Phospho (STY)]KES[Phospho (STY)]LKAAPK.2	-0.269884468	Q9QYR6_S319_M2_Map1a
S[Phospho (STY)]PPC[Carbamidomethyl (C)]EDFSVTGESEK.2	0.300373125	Q9QYR6_S908_M1_Map1a
STPSQVTSEEKDGHS[Phospho (STY)]PM[Oxidation (M)]SK.3	-0.31189453	Q9QYR6_S2637_M1_Map1a
GFKS[Phospho (STY)]PPC[Carbamidomethyl (C)]EDFSVTGESEK.3	-0.120448355	Q9QYR6_S908_M1_Map1a
LSSFATSVAEDQSVASLTAPQTEETGK.3	-0.475775121	#N/A
TPS[Phospho (STY)]PKEAGR.2	-0.304212969	Q9QYR6_S2066_M1_Map1a
NRAPDS[Phospho (STY)]GAEVEREK.3	-0.147066126	Q9QYR6_S611_M1_Map1a
SSLLLDTVTSIPSSR.2	0.207223341	#N/A
VPSAPGQES[Phospho (STY)]PVPDTK.2	-0.44719001	Q9QYR6_S1789_M1_Map1a
SHWDDGTNDS[Phospho (STY)]DLEK.2	-0.441960385	Q9QYR6_S2082_M1_Map1a
VRTES[Phospho (STY)]SEALKAEK.3	-0.153588586	Q9QYR6_S384_M1_Map1a
APDS[Phospho (STY)]GAEVEREK.2	-0.284638048	Q9QYR6_S611_M1_Map1a
AELEEM[Oxidation (M)]EEVHPS[Phospho (STY)]DEEEEETK.3	0.012706715	Q9QYR6_S667_M1_Map1a
EM[Oxidation (M)]TLDQKS[Phospho (STY)]PEK.2	-0.101271173	Q9QYR6_S1580_M1_Map1a
S[Phospho (STY)]PC[Carbamidomethyl (C)]T[Phospho (STY)]PKEQQLHK.2	-0.476345797	#N/A
WLAES[Phospho (STY)]PVGLPPEEEDK.2	-0.076108909	Q9QYR6_S1747_M1_Map1a
TRHDEYLEVTK.3	-0.096019057	#N/A
SPFEIIS[Phospho (STY)]PPAS[Phospho (STY)]PPEMTGQR.2	-0.221995025	Q9QYR6_S1768_M2_Map1a
QLS[Phospho (STY)]PESLGTLQFGELSLGKEEK.3	-0.326825874	Q9QYR6_S1205_M1_Map1a
S[Phospho (STY)]PESLSSPAM[Oxidation (M)]EDLAM[Oxidation (M)]EWGGK.2	-0.428104584	Q9QYR6_S1310_M1_Map1a
EKEGAALDQDNR.2	-0.155477044	#N/A

GKDLYLEDQGLAEKDK.3	-0.115101545	#N/A
KVAELEEEQS[Phospho (STY)]QGSSSYSDWVK.3	-0.591698249	Q9QYR6_S114_M1_Map1a
VAELEEEQS[Phospho (STY)]QGSSSYSDWVK.2	-0.40157049	Q9QYR6_S114_M1_Map1a
M[Oxidation (M)]AS[Phospho (STY)]PPPSGPPSAAHTPFHQSPVEEK.4	-0.536339448	Q9QYR6_S991_M1_Map1a
PASPALSEGS[Phospho (STY)]S[Phospho (STY)]SEATTPVISSVAER.3	0.31118322	Q9QYR6_S2233_M2_Map1a
QEPEPGPNVEPSFT[Phospho (STY)]PPAVPPR.2	-0.021400658	Q9QYR6_T2031_M1_Map1a
VPEVTESHTTR.2	-0.141188227	#N/A
APDS[Phospho (STY)]GAEVER.2	-0.054655348	Q9QYR6_S611_M1_Map1a
DEALDEENKPGGQQDK.2	-0.124234725	#N/A
GKDLYLEDQGLAEK.3	0.21010701	#N/A
S[Phospho (STY)]APC[Carbamidomethyl (C)]GSLAFSGDR.2	-0.273703244	Q9QYR6_S2162_M1_Map1a
EES[Phospho (STY)]EPEVKEDVIEK.2	-0.274907954	Q9QYR6_S644_M1_Map1a
QEATPRS[Phospho (STY)]PC[Carbamidomethyl (C)]T[Phospho (STY)]PKEQQLHK.3	0.041877292	#N/A
WLAESPVGLPPEEEDKLTR.3	-0.125751122	#N/A
EKEVVPDFPEDK.2	-0.406800335	#N/A
HMQEALK.2	0.202738273	#N/A
TEATQGLDYVPSAGTIS[Phospho (STY)]PTSSLEEDK.3	-0.087051125	Q9QYR6_S895_M1_Map1a
APISLSQDPSPPLNGSTTSC[Carbamidomethyl (C)]GPDRRT[Phospho (STY)]PS[Phospho (STY)]PK.4	-0.065234825	Q9QYR6_S2066_M2_Map1a
RS[Phospho (STY)]PT[Phospho (STY)]PGKGPVDR.2	0.202218297	Q9QYR6_S2603_M2_Map1a
EAELTPENIAAAREES[Phospho (STY)]EPEVKEDVIEK.4	0.106655725	Q9QYR6_S644_M1_Map1a
KHS[Phospho (STY)]PGEITGPGGHFM[Oxidation (M)]TSDSSLTK.4	-0.185818516	Q9QYR6_S1289_M1_Map1a
S[Phospho (STY)]IEEAC[Carbamidomethyl (C)]LTLQHLNR.2	0.036813778	Q9QYR6_S155_M1_Map1a
EAELTPENIAAAREES[Phospho (STY)]EPEVK.3	0.051619824	Q9QYR6_S644_M1_Map1a
GDSALFAVNGFNILVDGGSDR.2	0.2393576	#N/A
QEATPRS[Phospho (STY)]PC[Carbamidomethyl (C)]T[Phospho (STY)]PK.2	-0.197789933	#N/A
DQILSEK.2	0.030066499	#N/A
PRYS[Phospho (STY)]EER.2	0.32206749	Q9QYR6_S1694_M1_Map1a
ELALS[Phospho (STY)]S[Phospho (STY)]PEDLTQDFEELK.2	-0.415040577	Q9QYR6_S526_M2_Map1a
SPEQEVR.2	-0.232718175	#N/A
APDS[Phospho (STY)]GAEVEREKETWEER.3	-0.122989584	Q9QYR6_S611_M1_Map1a
AELEEM[Oxidation (M)]EEVHPS[Phospho (STY)]DEEEEETKAESFYQK.4	0.028389635	Q9QYR6_S667_M1_Map1a
DSVMHQKDEALDEENKPGGQQDK.4	0.359676564	#N/A
TEATQGLDYVPSAGTIS[Phospho (STY)]PTSSLEEDKGFK.4	-0.232675321	Q9QYR6_S895_M1_Map1a
DEAS[Phospho (STY)]EEKEQVLEQK.2	-0.333003117	Q9QYR6_S1516_M1_Map1a
FPTSTYDLSGPEGPGPFEASQSAESAVPASSSK.3	-0.29703115	#N/A
SSFLEDK.2	0.102404403	#N/A
DRDITLQQDAYWK.3	-0.41165165	#N/A
EQKDEAS[Phospho (STY)]EEKEQVLEQK.3	-0.15418701	Q9QYR6_S1516_M1_Map1a
DKT[Phospho (STY)]PEEKDK.2	0.284227541	Q9QYR6_T1472_M1_Map1a
EHKENPELVDDLS[Phospho (STY)]PR.3	-0.313789202	Q9QYX7_S1439_M1_Pclo
ENPELVDDLS[Phospho (STY)]PR.2	-0.200006431	Q9QYX7_S1439_M1_Pclo
TQPQKVS[Phospho (STY)]PEQPQDQQK.2	-0.290339927	Q9QYX7_S1378_M1_Pclo
SPVPSQQAS[Phospho (STY)]PK.2	0.028848794	Q9QYX7_S625_M1_Pclo
SHSSQNSQQS[Phospho (STY)]PKPSVIK.3	-0.403472981	Q9QYX7_S4774_M1_Pclo
AKS[Phospho (STY)]PGVDPK.2	-0.324817893	Q9QYX7_S4544_M1_Pclo
SLVSVLPEKKPS[Phospho (STY)]EEEK.3	-0.448201021	Q9QYX7_S1149_M1_Pclo
SPVPSQQAS[Phospho (STY)]PKKELPSK.3	-0.237110669	Q9QYX7_S625_M1_Pclo

S[Phospho (STY)]QEVTDFLAPLQTSSR.2	0.165061729	Q9QYX7_S4016_M1_Pclo
KSS[Phospho (STY)]TS[Phospho (STY)]FDDDAGR.2	-0.526953221	Q9QYX7_S1551_M2_Pclo
SEGITKPSLQQPS[Phospho (STY)]PK.2	-0.321923444	Q9QYX7_S212_M1_Pclo
SQPTT[Phospho (STY)]PQETVTGK.2	-0.025029703	Q9QYX7_T860_M1_Pclo
ITQHFSTEEFEDEYVTDYTR.3	-0.100264346	#N/A
ELPSKQDS[Phospho (STY)]PKAPESK.2	-0.105497572	Q9QYX7_S636_M1_Pclo
YNS[Phospho (STY)]LDLR.2	-0.062505899	Q9QYX7_S3936_M1_Pclo
SSTS[Phospho (STY)]FDDDAGRR.2	0.232861967	Q9QYX7_S1553_M1_Pclo
KTQPQKVS[Phospho (STY)]PEQPQDQQK.3	-0.176641001	Q9QYX7_S1378_M1_Pclo
DSFSQESSPSS[Phospho (STY)]PSDLAK.2	-0.275685925	Q9QYX7_S1341_M1_Pclo
KIVDSGVQT[Phospho (STY)]DDEETADR.2	-0.297927858	Q9QYX7_T3376_M1_Pclo
FS[Phospho (STY)]PIQESR.2	-0.053035259	Q9QYX7_S4132_M1_Pclo
VYKLPTAVSLYS[Phospho (STY)]PTDEQSVM[Oxidation (M)]QK.3	0.019499092	Q9QYX7_S1829_M1_Pclo
SSTS[Phospho (STY)]FDDDAGR.2	-0.343695178	Q9QYX7_S1553_M1_Pclo
ASYDSVEDSSESENS[Phospho (STY)]PVAR.2	0.31988531	Q9QYX7_S1457_M1_Pclo
TQT[Phospho (STY)]PSETR.2	0.062450116	Q9QYX7_T1390_M1_Pclo
AMPRPAS[Phospho (STY)]DSK.2	-0.015566312	Q9QYX7_S769_M1_Pclo
DQFGSSHS[Phospho (STY)]LPEVQQHM[Oxidation (M)]R.3	0.064696106	Q9QYX7_S4325_M1_Pclo
LPAQQPGPQTAAK.2	-0.086841131	#N/A
SHGPLLPTIEDSS[Phospho (STY)]EEEELREEEELLKEQEK.4	-0.078844192	Q9QYX7_S1709_M1_Pclo
SSS[Phospho (STY)]EGHLR.2	-0.236701331	Q9QYX7_S4814_M1_Pclo
DQGKSEGITKPSLQQPS[Phospho (STY)]PK.3	-0.251549534	Q9QYX7_S212_M1_Pclo
SM[Oxidation (M)]S[Phospho (STY)]DPKPLSPTADESSR.2	-0.358199272	Q9QYX7_S3610_M1_Pclo
SPVPSQQAS[Phospho (STY)]PKK.2	0.023219731	Q9QYX7_S625_M1_Pclo
KDS[Phospho (STY)]FSQESSPSS[Phospho (STY)]PSDLAK.2	0.140575076	Q9QYX7_S1332_M2_Pclo
GSVPAAAAES[Phospho (STY)]PSM[Oxidation (M)]HR.2	-0.502908907	Q9QYX7_S75_M1_Pclo
DISISEEEIKESQEK.2	-0.029657156	#N/A
TPPSNLSPIEDAS[Phospho (STY)]PTEELR.2	0.110968733	Q9QYX7_S1772_M1_Pclo
M[Oxidation (M)]SKFS[Phospho (STY)]PIQESR.2	0.138119387	Q9QYX7_S4132_M1_Pclo
HSWHDEDDETFDES[Phospho (STY)]PELK.3	0.269284903	Q9QYX7_S1575_M1_Pclo
ADPTVQLAPS[Phospho (STY)]PPKS[Phospho (STY)]PK.2	-0.417039453	Q9QYX7_S3545_M2_Pclo
IVDSGVQT[Phospho (STY)]DDEETADR.2	-0.532449533	Q9QYX7_T3376_M1_Pclo
LPTAVSLY[Phospho (STY)]SPTDEQSVM[Oxidation (M)]QK.2	0.105667032	Q9QYX7_Y1828_M1_Pclo
ERPKT[Phospho (STY)]PPS[Phospho (STY)]NLSPIEDAS[Phospho (STY)]PTEELR.3	0.078968928	Q9QYX7_S1763_M3_Pclo
KSS[Phospho (STY)]TS[Phospho (STY)]FDDDAGRR.2	-0.159756327	Q9QYX7_S1551_M2_Pclo
T[Phospho (STY)]PPAVATDQK.2	0.26773055	Q9QYX7_T1120_M1_Pclo
GAHAHSGPTSAGSSSVPS[Phospho (STY)]PGQPGS[Phospho (STY)]PSVSK.3	-0.322772289	Q9QYX7_S4592_M2_Pclo
TSQS[Phospho (STY)]FPPTGDTITPLDSK.2	-0.178903683	Q9QYX7_S748_M1_Pclo
ETGDGIILEVLDAYK.2	0.112071936	#N/A
AYLQGVAEDRDY[Phospho (STY)]M[Oxidation (M)]SDSEVSSTRPSR.3	-0.056152953	Q9QYX7_Y3761_M1_Pclo
S[Phospho (STY)]RSHGIFPDPSK.3	-0.083728161	Q9QYX7_S4782_M1_Pclo
GSVPAAAAES[Phospho (STY)]PSMHRK.2	0.31476634	Q9QYX7_S75_M1_Pclo
S[Phospho (STY)]RTDFKEEYK.2	0.196870231	Q9QYX7_S137_M1_Pclo
KTTLYFDEEPELEM[Oxidation (M)]ESLTDS[Phospho (STY)]PEDR.3	-0.008561389	Q9QYX7_S1642_M1_Pclo
QAELDEEEKEIDAK.2	0.196333428	#N/A
RASYDSVEDSSESENS[Phospho (STY)]PVAR.3	-0.082645153	Q9QYX7_S1457_M1_Pclo
SIS[Phospho (STY)]M[Oxidation (M)]EQLMK.2	0.052107993	Q9QYX7_S4706_M1_Pclo

DYMS[Phospho (STY)]DSEVSSTRPSR.2	0.215022327	Q9QYX7_S3763_M1_Pclo
DIAFIMDDFQHAM[Oxidation (M)]S[Phospho (STY)]DSEAYHLR.3	-0.089866138	Q9QYX7_S4358_M1_Pclo
AEEDS[Phospho (STY)]MEDPYELK.2	0.177507711	Q9QYX7_S4042_M1_Pclo
RHS[Phospho (STY)]WHDEDDETFDESPELK.4	-0.45562663	Q9QYX7_S1563_M1_Pclo
SPSTIS[Phospho (STY)]LK.2	0.491906789	Q9QYX7_S131_M1_Pclo
TSVAQTHLEDAGAAIAAAEAAVQQLR.3	-0.355184453	#N/A
TTLYFDEEPELEM[Oxidation (M)]ESLTDS[Phospho (STY)]PEDR.3	0.14571048	Q9QYX7_S1642_M1_Pclo
SPS[Phospho (STY)]TIS[Phospho (STY)]LKESK.2	-0.136405393	Q9QYX7_S128_M2_Pclo
GGSLGC[Carbamidomethyl (C)]QTETDPDTQS[Phospho (STY)]PPYMGATSPPK.3	-0.03327654	Q9QYX7_S3506_M1_Pclo
KSVDTSVQT[Phospho (STY)]DDEDQDEWDM[Oxidation (M)]PSR.3	0.176336347	Q9QYX7_T3403_M1_Pclo
LPS[Phospho (STY)]PT[Phospho (STY)]SPLSPHSNK.2	0.168905381	Q9QYX7_S2489_M2_Pclo
LPSPTSPLSPHSNKS[Phospho (STY)]S[Phospho (STY)]PR.3	-0.215530195	Q9QYX7_S2501_M2_Pclo
TPPSNLS[Phospho (STY)]PIEDAS[Phospho (STY)]PTEELRQAAEM[Oxidation (M)]EELHR.4	0.234404454	Q9QYX7_S1766_M2_Pclo
KQIIEM[Oxidation (M)]SADEDAS[Phospho (STY)]GSEDEEFIR.3	-0.06054454	Q9QYX7_S1505_M1_Pclo
SSSEGHLRS[Phospho (STY)]HGPS[Phospho (STY)]R.3	-0.176384159	Q9QYX7_S4820_M2_Pclo
SYS[Phospho (STY)]HDVLPK.2	0.291404188	Q9QYY0_S266_M1_Gab1
SSS[Phospho (STY)]LEGFHSQYK.2	-0.510433619	Q9QYY0_S420_M1_Gab1
SVLTAGGVSGEELDENYVPM[Oxidation (M)]NPNS[Phospho (STY)]PPR.3	0.018497835	Q9QYY0_S455_M1_Gab1
SVS[Phospho (STY)]PQTLEAYIR.2	0.093212788	Q9QYY8_S595_M1_Spast
SSGTAPAPAS[Phospho (STY)]PS[Phospho (STY)]PPEPGPGGEAESVR.3	0.224369705	Q9QYY8_S101_M2_Spast
GPVYIGELPQDFLR.2	-0.230286912	#N/A
LSITVVQAK.2	0.162884438	#N/A
QGQVEDEWYSLSGR.2	-0.081353667	#N/A
[Acetyl (Protein N-term)]AALYGGVEGGGTR.2	-0.219762717	#N/A
LLIEELR.2	-0.104007885	#N/A
SLGLSLS[Phospho (STY)]GGEQEDAVR.2	-0.054540387	Q9QZ08_S76_M1_Nagk
IRPTVQEDGGDVIYR.3	-0.226382283	#N/A
GFEDGIVR.2	-0.278031781	#N/A
IQHILC[Carbamidomethyl (C)]TGNLC[Carbamidomethyl (C)]TK.3	-0.163437763	#N/A
VVTVGQFK.2	0.123438788	#N/A
TLAGDVHIVR.2	0.169605554	#N/A
FEAFEHENK.2	-0.087116944	#N/A
ARAS[Phospho (STY)]PSASPADPGLPEGSER.3	-0.177428141	Q9QZB1_S13_M1_Rgs20
NQEDQRPQRAS[Phospho (STY)]HEIR.3	0.168011133	Q9QZB1_S86_M1_Rgs20
RAFS[Phospho (STY)]TEK.2	-0.606978208	Q9QZB7_S414_M1_Actr10
DSVVEILFEQDNEEK.2	-0.152722977	#N/A
KQS[Phospho (STY)]QQLELLESELRK.3	-0.166044941	Q9QZC2_S984_M1_Plxnc1
TSS[Phospho (STY)]PEVQETKK.2	-0.331543441	Q9QZD4_S363_M1_Ercc4
S[Phospho (STY)]SPLEASATPADSLDSASLPK.2	0.195313256	Q9QZD5_S122_M1_Chml
LVVQDS[Phospho (STY)]IEQPKR.2	-0.175635085	Q9QZD5_S207_M1_Chml
LTLTS[Phospho (STY)]DESTLIEDGNGR.2	0.161790785	Q9QZI8_S348_M1_Serinc1
SDGS[Phospho (STY)]LDDGDGIHR.2	-0.188174022	Q9QZI8_S364_M1_Serinc1
GGAREDS[Phospho (STY)]LEDGVHIPQHAK.4	0.083639142	Q9QZL9_S200_M1_Dkkl1
GPAAAPGAAS[Phospho (STY)]PPAEPK.2	-0.27311073	Q9QZM0_S25_M1_Ubqln2
EKEEFAVPENSTVQQFK.3	0.150022366	#N/A
SQTDQLVLIFAGK.2	0.716470679	#N/A
NQDLALSNLESIPGGYNALR.2	-0.001283578	#N/A

NPEISHLLNNPDIM[Oxidation (M)]R.3	0.137380493	#N/A
YVLSSQHRPDIS[Phospho (STY)]PTER.3	0.183118998	Q9QZQ1_S655_M1_Afdn
S[Phospho (STY)]QEELREEK.2	-0.210794356	Q9QZQ1_S1275_M1_Afdn
S[Phospho (STY)]QDADLPGSSGAPENLTFK.2	0.006566218	Q9QZQ1_S1770_M1_Afdn
TSS[Phospho (STY)]VVTLEVAK.2	0.020885491	Q9QZQ1_S1083_M1_Afdn
TQVLS[Phospho (STY)]PDSLFTAK.2	0.173086946	Q9QZQ1_S1719_M1_Afdn
SSPNVANQPPS[Phospho (STY)]PGGKGPYTSGTAAK.3	0.25507722	Q9QZQ1_S1182_M1_Afdn
ADHRS[Phospho (STY)]SPNVANQPPS[Phospho (STY)]PGGK.3	-0.08688183	Q9QZQ1_S1172_M2_Afdn
SSPNVANQPPS[Phospho (STY)]PGGK.2	-0.342473407	Q9QZQ1_S1182_M1_Afdn
VTRS[Phospho (STY)]QEELREEK.3	-0.23503882	Q9QZQ1_S1275_M1_Afdn
LPYLVELS[Phospho (STY)]PGR.2	0.148992818	Q9QZQ1_S391_M1_Afdn
TIS[Phospho (STY)]NPEVVMK.2	0.085809136	Q9QZQ1_S216_M1_Afdn
EYFTFPASKS[Phospho (STY)]QDR.2	-0.164272874	Q9QZQ1_S1238_M1_Afdn
EFVEAVLELR.2	0.374879087	#N/A
AASADSTTEGT[Phospho (STY)]PTDGFTVLSTK.2	0.068724586	Q9QZQ8_T178_M1_Macroh2a1
AVC[Carbamidomethyl (C)]STY[Phospho (STY)]LQSR.2	0.049614911	Q9QZR5_Y361_M1_Hipk2
NVSTC[Carbamidomethyl (C)]EVTSSQAISS[Phospho (STY)]PQR.2	0.050693232	Q9QZR5_S827_M1_Hipk2
LRQDHADSC[Carbamidomethyl (C)]VVS[Phospho (STY)]S[Phospho (STY)]DDEELSR.3	-0.119779162	Q9QZS2_S98_M2_Rnf4
TNPS[Phospho (STY)]PTNPFSSDLQK.2	-0.327193486	Q9QZS3_S636_M1_Numb
RTPS[Phospho (STY)]EADR.2	0.414752616	Q9QZS3_S440_M1_Numb
TASPAS[Phospho (STY)]PSGSK.2	0.220447258	Q9QZS8_S341_M1_Sh2d3c
VHAPSATPSTSAQPAS[Phospho (STY)]PVAR.3	-0.169060126	Q9QZS8_S435_M1_Sh2d3c
S[Phospho (STY)]ITMTDGLTTDK.2	-0.026536067	Q9QZS8_S354_M1_Sh2d3c
RAS[Phospho (STY)]DSLSAR.2	-0.095118243	Q9QZW0_S1105_M1_Atp11c
GLIPDT[Phospho (STY)]PEEKPK.2	-0.23915494	Q9QZX7_T71_M1_Srr
DLVDDVFTVTEDEIK.2	-0.242684807	#N/A
VYAAEPSNADDC[Carbamidomethyl (C)]YQSK.2	-0.051569577	#N/A
LAIQAYGASIVYC[Carbamidomethyl (C)]DPSDESR.2	-0.312769959	#N/A
S[Phospho (STY)]RPSS[Phospho (STY)]PAVR.2	0.241939554	Q9R020_S290_M2_Zranb2
YNLDAS[Phospho (STY)]EEEDSNK.2	-0.313226818	Q9R020_S188_M1_Zranb2
EES[Phospho (STY)]DGEYDEFGR.2	0.148259123	Q9R020_S120_M1_Zranb2
YNLDAS[Phospho (STY)]EEEDSNKKK.2	-0.38966927	Q9R020_S188_M1_Zranb2
S[Phospho (STY)]RPSS[Phospho (STY)]PAVRK.2	0.086554447	Q9R020_S290_M2_Zranb2
ENVEYIEREES[Phospho (STY)]DGEYDEFGR.3	0.190568497	Q9R020_S120_M1_Zranb2
SRS[Phospho (STY)]RPSS[Phospho (STY)]PAVR.2	-0.371685682	Q9R020_S290_M2_Zranb2
ENVEYIEREES[Phospho (STY)]DGEYDEFGRK.3	0.325317479	Q9R020_S120_M1_Zranb2
YNLDAS[Phospho (STY)]EEEDSNKK.2	-0.004115846	Q9R020_S188_M1_Zranb2
PS[Phospho (STY)]S[Phospho (STY)]PAVRK.2	0.081344194	Q9R020_S293_M2_Zranb2
S[Phospho (STY)]QNNFVAILDLPEGEHQYK.3	-0.365565714	Q9R078_S108_M1_Prkab1
[Acetyl (Protein N-term)]ASSEQAEQPNQPSS[Phospho (STY)]PPGSENVVPR.3	0.194045115	Q9R0A0_S15_M1_Pex14
AYLQSITPQGYSDS[Phospho (STY)]EER.2	-0.06946685	Q9R0G7_S1124_M1_Zeb2
SNT[Phospho (STY)]PS[Phospho (STY)]PLNLSSTSSK.2	-0.079877602	Q9R0G7_S784_M2_Zeb2
S[Phospho (STY)]DHEEDNMEDGME.2	0.096355286	Q9R0G7_S1203_M1_Zeb2
VYQYSNSRS[Phospho (STY)]PSLER.3	0.342599243	Q9R0G7_S705_M1_Zeb2
TSPVEGLPGTAPDLEK.2	-0.311603682	#N/A
EIPDPSSINAK.2	-0.313834841	#N/A
ILSGAGEAR.2	0.131810026	#N/A

GIIDSTHTEQR.2	0.001701649	#N/A
GASEIVLKK.2	-0.334622279	#N/A
YGDLLPADGLFIQGNDLK.2	-0.464510007	#N/A
NNSAIDS[Phospho (STY)]GINLTTDTSK.2	-0.266499464	Q9R0K7_S1170_M1_Atp2b2
AFRSS[Phospho (STY)]LYEGLEKPESR.3	0.062447022	Q9R0K7_S1105_M1_Atp2b2
AGQVVQIPVAEIVVGDIAQIK.2	-0.210853249	#N/A
IDESSLTGESDQVR.2	0.168061631	#N/A
MPDESFR.2	0.313336187	#N/A
EASDIILTDDNFSSIVK.2	-0.400605516	#N/A
NES[Phospho (STY)]SHGGEFGC[Carbamidomethyl (C)]TM[Oxidation (M)]EELR.3	0.183449076	Q9R0K7_S18_M1_Atp2b2
T[Phospho (STY)]SIHNFM[Oxidation (M)]AHPEFR.3	-0.472628192	Q9R0K7_T1117_M1_Atp2b2
QNSS[Phospho (STY)]PPSSLNK.2	-0.104448971	Q9R0K7_S1156_M1_Atp2b2
IDESSLTGESDQVRK.2	-0.074149061	#N/A
TLELLVNAIAINSAYTTK.2	0.20495074	#N/A
VIEPMAC[Carbamidomethyl (C)]DGLR.2	0.262844576	#N/A
IEDSQPHIPLIDDTDLEEDAALKQNSS[Phospho (STY)]PPSSLNK.4	-0.382319597	Q9R0K7_S1156_M1_Atp2b2
TEC[Carbamidomethyl (C)]GLLGFVLDLR.2	-0.324660043	#N/A
EEIPEEELNEDVEEIDHAER.3	-0.051721366	#N/A
LTQKEEIPEEELNEDVEEIDHAER.4	-0.01572615	#N/A
M[Oxidation (M)]TVVQAYVGDVHYK.3	0.02271429	#N/A
SNRPVS[Phospho (STY)]ADGNYRPLAK.3	-0.004568181	Q9R0L6_S957_M1_Pcm1
VTNAIS[Phospho (STY)]PESSPGVGR.2	-0.506383818	Q9R0L6_S65_M1_Pcm1
ILEGDHGS[Phospho (STY)]PAGEIDDEDKDKDETETVK.4	-0.237197511	Q9R0L6_S1729_M1_Pcm1
RS[Phospho (STY)]S[Phospho (STY)]LVDEAPEDEEFEQK.2	0.167613835	Q9R0L6_S644_M2_Pcm1
HIS[Phospho (STY)]ESDER.2	-0.357418269	Q9R0L6_S1432_M1_Pcm1
NVRS[Phospho (STY)]DVS[Phospho (STY)]DQEEDEESER.2	-0.556516666	Q9R0L6_S1766_M2_Pcm1
KAS[Phospho (STY)]AQASLASK.2	-0.694272617	Q9R0L6_S1280_M1_Pcm1
STLTSSYNC[Carbamidomethyl (C)]PS[Phospho (STY)]PYADGR.2	-0.155200552	Q9R0M0_S2648_M1_Celsr2
DYGEELRS[Phospho (STY)]PM[Oxidation (M)]TSLT[Phospho (STY)]PGESK.3	-0.154491592	Q9R0N3_S133_M2_Syt11
NSLETVGT[Phospho (STY)]PDSGR.2	-0.173621547	Q9R0N7_T58_M1_Syt7
NSLETVGT[Phospho (STY)]PDS[Phospho (STY)]GRGRGEK.3	-0.022028763	Q9R0N7_S61_M2_Syt7
YKNSLETVGT[Phospho (STY)]PDS[Phospho (STY)]GRGR.3	-0.317436209	Q9R0N7_S61_M2_Syt7
YKNSLETVGT[Phospho (STY)]PDSGR.2	0.278157206	Q9R0N7_T58_M1_Syt7
VLYLQVLDYDR.2	-0.012764563	#N/A
NSLETVGT[Phospho (STY)]PDS[Phospho (STY)]GRGR.2	-0.275363587	Q9R0N7_S61_M2_Syt7
S[Phospho (STY)]VDGDDAKSEAAK.2	-0.037127695	Q9R0N8_S217_M1_Syt6
EASSPSSANPASETLQS[Phospho (STY)]PSSR.2	0.27250522	Q9R0N8_S108_M1_Syt6
QKS[Phospho (STY)]VDGDDAKSEAAK.2	-0.150374199	Q9R0N8_S217_M1_Syt6
QKS[Phospho (STY)]VDGDDAK.2	-0.435047589	Q9R0N8_S217_M1_Syt6
AFSGYLGPDESK.2	0.220913068	#N/A
M[Oxidation (M)]YSYVTEELPQLINANFPVDPQR.3	-0.382372357	#N/A
AYDATC[Carbamidomethyl (C)]LVK.2	-0.339104262	#N/A
SAS[Phospho (STY)]PDDDLGSSNWEAADLGNEERK.3	0.293918943	Q9R0P4_S17_M1_Smap
SAS[Phospho (STY)]PDDDLGSSNWEAADLGNEER.3	-0.029777448	Q9R0P4_S17_M1_Smap
[Acetyl (Protein N-term)]AS[Phospho (STY)]GVQVADEVC[Carbamidomethyl (C)]R.2	-0.389703284	Q9R0P5_S3_M1_Dstn
YALYDASFETK.2	0.308629699	#N/A
HEYQANGPEDLNR.2	-0.39746254	#N/A

EILVGDVGATITDPFK.2	0.196997936	#N/A
HFVGM[Oxidation (M)]LPEKDC[Carbamidomethyl (C)]R.3	0.11946748	#N/A
C[Carbamidomethyl (C)]IVVEEGKEILVGDVGATITDPFK.3	-0.124244804	#N/A
M[Oxidation (M)]PFPVNHGASSEDSLLQDAAK.3	-0.128635977	#N/A
LGVAGQWR.2	-0.029005588	#N/A
QFLSETEK.2	0.300442	#N/A
NEAIQAAHDSVAQEGQC[Carbamidomethyl (C)]R.3	0.167815081	#N/A
FSAVALC[Carbamidomethyl (C)]K.2	0.265397807	#N/A
QFLSETEKLS[Phospho (STY)]PEDR.2	-0.16826974	Q9R0P9_S125_M1_Uchl1
QTIGNSC[Carbamidomethyl (C)]GTIGLIHAVANNQDKLEFEDGSVLK.4	-0.209039095	#N/A
C[Carbamidomethyl (C)]FEKNEAIQAAHDSVAQEGQC[Carbamidomethyl (C)]R.4	0.02969114	#N/A
QIEELKGQEVSPK.2	0.277170237	#N/A
TLESSIQGLR.2	0.135373367	#N/A
WSPLENK.2	0.081529257	#N/A
[Acetyl (Protein N-term)]SLHQFLLEPITC[Carbamidomethyl (C)]HAWNR.3	0.411827225	#N/A
FC[Carbamidomethyl (C)]TTGIDGAM[Oxidation (M)]TIWDFK.2	-0.540723793	#N/A
NAYVWSQK.2	0.210488342	#N/A
IVTC[Carbamidomethyl (C)]GADR.2	0.294005282	#N/A
EVDEKPASTPWGSK.2	-0.342717605	#N/A
SVQVSTLR.2	0.079430252	#N/A
NM[Oxidation (M)]S[Phospho (STY)]AMER.2	0.201881468	Q9R0Q6_S298_M1_Arpc1a
LAWVSHDSTVSVADASK.2	-0.206245082	#N/A
EHNGHITGIDWAPK.3	-0.050533054	#N/A
LISVC[Carbamidomethyl (C)]YFESENDWWVSK.2	-0.193352206	#N/A
HLNEIDLFHC[Carbamidomethyl (C)]IDPNDSK.3	-0.104641285	#N/A
DWEDDSDEDM[Oxidation (M)]SNFDR.2	0.121071861	#N/A
SILC[Carbamidomethyl (C)]C[Carbamidomethyl (C)]LR.2	-0.089539549	#N/A
LTFSC[Carbamidomethyl (C)]LGGSDNFK.2	0.18870547	#N/A
KGESGQSWPR.2	-0.034224237	#N/A
S[Phospho (STY)]RS[Phospho (STY)]FDYNYRR.3	-0.092216439	Q9R0U0_S131_M2_Srsf10
S[Phospho (STY)]RS[Phospho (STY)]FDYNYR.2	-0.321444415	Q9R0U0_S131_M2_Srsf10
S[Phospho (STY)]FDYNYR.2	0.110154385	Q9R0U0_S133_M1_Srsf10
SRS[Phospho (STY)]WT[Phospho (STY)]SPK.2	0.070286709	Q9R0U0_S253_M2_Srsf10
S[Phospho (STY)]RS[Phospho (STY)]RS[Phospho (STY)]FDYNYR.2	-0.093277638	Q9R0U0_S129_M3_Srsf10
S[Phospho (STY)]RS[Phospho (STY)]RS[Phospho (STY)]YER.2	-0.473196104	Q9R0U0_S119_M3_Srsf10
S[Phospho (STY)]RS[Phospho (STY)]HSDNDRFK.3	0.047035074	Q9R0U0_S156_M2_Srsf10
SS[Phospho (STY)]SSEILGGNDPTSK.2	0.005925205	Q9R0X5_S913_M1_Rpgr
IIFVVGGPGSGK.2	0.054043464	#N/A
VDSSNGFLIDGYPR.2	0.159923569	#N/A
GELVPLDTVLDM[Oxidation (M)]LR.2	-0.014708928	#N/A
RLETYYNATEPVISFYDKR.3	-0.359987066	#N/A
VDDNEETIKK.2	-0.37418235	#N/A
LETYYNATEPVISFYDKR.3	0.562656727	#N/A
YGYTHLSTGDLLR.3	-0.19673199	#N/A
AEVSSGSER.2	0.058482559	#N/A
SEDS[Phospho (STY)]DEDEPC[Carbamidomethyl (C)]AISGK.2	0.054642911	Q9R0Z9_S89_M1_Dlc1
QDPIPGS[Phospho (STY)]PDNSR.2	0.066045966	Q9R0Z9_S129_M1_Dlc1

FSDEGDSDSALDSVSPC[Carbamidomethyl (C)]PSS[Phospho (STY)]PK.2	-0.136676577	Q9R0Z9_S510_M1_Dlc1
RSEDS[Phospho (STY)]DEDEPC[Carbamidomethyl (C)]AISGK.2	0.212754319	Q9R0Z9_S89_M1_Dlc1
KRSEDS[Phospho (STY)]DEDEPC[Carbamidomethyl (C)]AISGK.3	0.233096477	Q9R0Z9_S89_M1_Dlc1
YTFPTEQR.2	0.125356065	#N/A
TPPLALVFR.2	0.150685185	#N/A
DHLLGVSDSGK.2	-0.35298051	#N/A
STDVAEEVYTR.2	0.131656856	#N/A
IVFLEESSQQEK.2	0.117115569	#N/A
NIEEVYVGGK.2	0.072979108	#N/A
EFDALLINPR.2	0.156763596	#N/A
FLYLGDDR.2	0.058339236	#N/A
ETTEESVKETER.2	0.093674767	#N/A
GTFVHSTWTC[Carbamidomethyl (C)]PM[Oxidation (M)]EVLR.3	0.102184151	#N/A
FVSEM[Oxidation (M)]LQK.2	0.153480633	#N/A
NYTDVYDKNNLLTNK.2	0.206205918	#N/A
TVM[Oxidation (M)]AHGC[Carbamidomethyl (C)]YLSEEELNIFSER.3	-0.438841391	#N/A
FTLSC[Carbamidomethyl (C)]TETLM[Oxidation (M)]SELGNIAK.2	-0.455166516	#N/A
AVM[Oxidation (M)]VSNVLLINK.2	-0.450534494	#N/A
ASDSPIDLFYGDFVGDISEAVIQK.3	-0.024827458	#N/A
IGLGTDVAGGYSYSM[Oxidation (M)]LDAIR.2	-0.539910333	#N/A
THDLYIQSHISENREEIEAVK.4	-0.467873637	#N/A
ASLQSGQEGPGDS[Phospho (STY)]PGSQFSK.2	-0.173597949	Q9R1E0_S284_M1_Foxo1
M[Oxidation (M)]S[Phospho (STY)]PKPELTEDQKQEIR.3	-0.22514996	Q9R1K9_S20_M1_Cetn2
SRS[Phospho (STY)]LS[Phospho (STY)]PGR.2	-0.191449884	Q9R1L5_S161_M2_Mast1
HFGS[Phospho (STY)]TESITDEDGGRR.3	-0.30863651	Q9R1L5_S139_M1_Mast1
LEEQDS[Phospho (STY)]GGSNTPEQDDTSEGR.2	0.009819901	Q9R1L5_S346_M1_Mast1
GGS[Phospho (STY)]PEIKR.2	0.021623215	Q9R1L5_S772_M1_Mast1
DSS[Phospho (STY)]PSRDYSPAVSGLR.3	0.050303092	Q9R1L5_S954_M1_Mast1
AALS[Phospho (STY)]PVQEHETGRR.3	-0.463869877	Q9R1L5_S1414_M1_Mast1
AALS[Phospho (STY)]PVQEHETGR.2	0.05706355	Q9R1L5_S1414_M1_Mast1
HFGS[Phospho (STY)]TESITDEDGGR.2	-0.33636269	Q9R1L5_S139_M1_Mast1
S[Phospho (STY)]PITIQR.2	-0.218546538	Q9R1L5_S968_M1_Mast1
FSASEASFLEGEAS[Phospho (STY)]PPLGAR.2	-0.048149699	Q9R1L5_S791_M1_Mast1
TTIFSPEGR.2	-0.181585752	#N/A
LLDEVFFSEK.2	-0.241005949	#N/A
QAYTQFGGK.2	-0.079325233	#N/A
LYIGLAGLATDVQTVAQR.3	-0.305402559	#N/A
DAVSGM[Oxidation (M)]GVIVHVIEK.3	-0.331264738	#N/A
FILNLPTFSVR.2	0.132437136	#N/A
YYTPTISR.2	0.459553003	#N/A
[Acetyl (Protein N-term)]M[Oxidation (M)]EYLIGIQGPDYVLVASDR.2	0.180698021	#N/A
NQYDNDVTVWSPQGR.2	-0.092657504	#N/A
ETLPAEQDLTTK.2	-0.400271764	#N/A
TQIPTQR.2	-0.335165148	#N/A
AQPS[Phospho (STY)]QAAEEPAEK.2	0.115680691	Q9R1P4_S247_M1_Psma1
AQPS[Phospho (STY)]QAAEEPAEKADEPMEH.3	0.155059136	Q9R1P4_S247_M1_Psma1
IHQIEYAMEAVK.2	0.119153462	#N/A

GFS[Phospho (STY)]EEQLR.2	-0.214717013	Q9R1Q8_S163_M1_Tagln3
LINSLYPPGQEPIPK.2	0.324198311	#N/A
RGFS[Phospho (STY)]EEQLR.2	-0.386615029	Q9R1Q8_S163_M1_Tagln3
GEPSWFHR.2	0.172580658	#N/A
TTDIFQTVDLWEGK.2	0.09233901	#N/A
GPSYGLSR.2	0.254918349	#N/A
TLM[Oxidation (M)]ALGSVAVTK.2	0.093536623	#N/A
AAEVYGVR.2	-0.014912906	#N/A
WLM[Oxidation (M)]DGTVLC[Carbamidomethyl (C)]K.2	0.115501009	#N/A
LVDWIILQC[Carbamidomethyl (C)]AEDIEHPPPGR.3	0.164633972	#N/A
QM[Oxidation (M)]EQISQFLK.2	0.242981742	#N/A
QGQNVIGLQMGSNK.2	0.195575247	#N/A
FY[Phospho (STY)]PVSAR.2	-0.420297734	Q9R1Q9_Y326_M1_Atp6ap1
Y[Phospho (STY)]WFAMER.2	-0.153298467	Q9R1Q9_Y332_M1_Atp6ap1
S[Phospho (STY)]PGGPGSHVR.2	-0.248872872	Q9R1R2_S437_M1_Trim3
TGS[Phospho (STY)]AELC[Carbamidomethyl (C)]AEITGPDGVR.2	0.158956428	Q9R1R2_S359_M1_Trim3
EDS[Phospho (STY)]PGPEVQPMDK.2	0.208063052	Q9R1R2_S7_M1_Trim3
ALRPGDLPPS[Phospho (STY)]PDDVKR.3	0.191496972	Q9R1R2_S427_M1_Trim3
VKS[Phospho (STY)]PGGPGSHVR.2	-0.170755846	Q9R1R2_S437_M1_Trim3
REDS[Phospho (STY)]PGPEVQPMDK.2	0.239827955	Q9R1R2_S7_M1_Trim3
ALRPGDLPPS[Phospho (STY)]PDDVK.2	0.061130859	Q9R1R2_S427_M1_Trim3
RNEFLGELQK.2	-0.056837339	#N/A
SDAISKS[Phospho (STY)]ELAK.2	-0.047762418	Q9R1T4_S192_M1_Septin6
EAELKEAEKELHEK.3	-0.150330929	#N/A
NEFLGELQK.2	0.147172321	#N/A
AAAELLQSQGSQAGGS[Phospho (STY)]QTLK.2	-0.030904673	Q9R1T4_S416_M1_Septin6
STLM[Oxidation (M)]DTLFNTK.2	0.020527107	#N/A
VNMEDLR.2	0.240445724	#N/A
SVSQGFC[Carbamidomethyl (C)]FNILC[Carbamidomethyl (C)]VGETGLGK.2	-0.266016232	#N/A
TVPLAGHVGFDSLPDQLVNK.3	-0.306086873	#N/A
DTDPDSKPFSLQETYEAK.3	0.34270857	#N/A
QVGEDC[Carbamidomethyl (C)]R.2	-0.149455153	#N/A
SNS[Phospho (STY)]TETLSPAK.2	-0.253179341	Q9R1V6_S855_M1_Adam22
SPSSS[Phospho (STY)]TGSIASSR.2	-0.11759859	Q9R1V6_S868_M1_Adam22
FAISENPLITLR.2	0.220618271	#N/A
FQPLGTVC[Carbamidomethyl (C)]R.2	0.093594757	#N/A
KLAS[Phospho (STY)]GEC[Carbamidomethyl (C)]K.2	-0.191399776	Q9R1V6_S387_M1_Adam22
S[Phospho (STY)]NSTETLSPAKS[Phospho (STY)]PSSSTGSIASSR.3	-0.119330966	Q9R1V6_S853_M2_Adam22
DVLC[Carbamidomethyl (C)]GYLLC[Carbamidomethyl (C)]TNIGNIPR.2	-0.605668103	#N/A
LSVVYTNTYAK.2	0.474909351	#N/A
SNS[Phospho (STY)]WQGNMGGNK.2	0.303903059	Q9R1V6_S832_M1_Adam22
SNGLSHS[Phospho (STY)]WSERIPDTK.3	-0.158580546	Q9R1V6_S808_M1_Adam22
SNS[Phospho (STY)]WQGNM[Oxidation (M)]GGNKK.2	-0.182763251	Q9R1V6_S832_M1_Adam22
ADAVHLFSGSQFESSR.3	-0.107278792	#N/A
SNGLSHS[Phospho (STY)]WSER.2	-0.343058937	Q9R1V6_S808_M1_Adam22
DNQC[Carbamidomethyl (C)]QYIWGTK.2	0.070786137	#N/A
QDGYSC[Carbamidomethyl (C)]NQNQGR.2	-0.016342497	#N/A

S[Phospho (STY)]LEC[Carbamidomethyl (C)]QDALETAAR.2	0.04660867	Q9R1X5_S43_M1_Abcc5
NATLAWDSSHSSIQNS[Phospho (STY)]PKLT[Phospho (STY)]PK.3	-0.008760443	Q9R1X5_S509_M2_Abcc5
T[Phospho (STY)]RSLEC[Carbamidomethyl (C)]QDALETAAR.3	0.240599272	Q9R1X5_T41_M1_Abcc5
LHS[Phospho (STY)]PSLSDEENLR.3	0.229363387	Q9R1Z7_S27_M1_Pts
VFETDNNIVVYKGE.2	0.210367365	#N/A
RS[Phospho (STY)]SFSM[Oxidation (M)]EEGDVL.2	-0.146417762	Q9R210_S465_M1_Tfeb
FLIPDYNDEIR.2	-0.325939725	#N/A
FATVEVTDKPVDEALR.3	0.2326216	#N/A
IPNQFQGS[Phospho (STY)]PPAPSDESVK.2	0.282400432	Q9R257_S111_M1_Hebp1
IPNQFQGS[Phospho (STY)]PPAPSDESVKIEER.3	0.137651774	Q9R257_S111_M1_Hebp1
EADYVAHATQLR.2	0.012561262	#N/A
HLGY[Phospho (STY)]KPEEY[Phospho (STY)]K.2	-0.144404689	Q9WTI7_Y703_M2_Myo1c
DAES[Phospho (STY)]PSWR.2	-0.239520869	Q9WTI7_S408_M1_Myo1c
IDS[Phospho (STY)]TEVIYQPR.2	-0.310039622	Q9WTK7_S31_M1_Stk11
DLAILQC[Carbamidomethyl (C)]HGELDPM[Oxidation (M)]VPVR.3	-0.107077219	#N/A
GPSS[Phospho (STY)]PEPDFSVFR.2	-0.039770702	Q9WTP5_S724_M1_Cdh22
TVGIDDLTGEPLIQR.2	0.427343284	#N/A
HLSSGDLLR.2	0.500380888	#N/A
EDDKPETVIKR.3	0.133962778	#N/A
TLTQC[Carbamidomethyl (C)]SWLLDGFPR.2	-0.081584562	#N/A
GVLETFSGTETNK.2	0.492216392	#N/A
EGITPWAS[Phospho (STY)]FK.2	0.065114858	Q9WTQ5_S598_M1_Akap12
GPSEAPQEAEAEEGAT[Phospho (STY)]SDGEKKR.3	-0.138271268	Q9WTQ5_T583_M1_Akap12
GPSEAPQEAEAEEGATS[Phospho (STY)]DGEK.2	-0.088900897	Q9WTQ5_S584_M1_Akap12
AEADASGNLTKES[Phospho (STY)]PDTNGPK.2	0.231612715	Q9WTQ5_S1645_M1_Akap12
EGITPWAS[Phospho (STY)]FKK.2	-0.348066686	Q9WTQ5_S598_M1_Akap12
EVC[Carbamidomethyl (C)]VSGGDHTQLTDLS[Phospho (STY)]PEEK.3	0.025094711	Q9WTQ5_S467_M1_Akap12
EKEPTKPLES[Phospho (STY)]PTS[Phospho (STY)]PVSNETTSSFK.3	0.111068165	#N/A
VELPLEDQVGDLEALS[Phospho (STY)]EK.2	0.221792876	Q9WTQ5_S371_M1_Akap12
EPTKPLES[Phospho (STY)]PTS[Phospho (STY)]PVSNETTSSFKK.3	-0.219416694	#N/A
SATLSSTESTAS[Phospho (STY)]GM[Oxidation (M)]QDEVR.2	0.193866047	Q9WTQ5_S637_M1_Akap12
EPT[Phospho (STY)]KPLES[Phospho (STY)]PTSPVSNETTSSFK.3	-0.285979902	Q9WTQ5_T265_M2_Akap12
LS[Phospho (STY)]ADYEKVELPLEDQVGDLEALSEK.3	0.278085889	Q9WTQ5_S350_M1_Akap12
C[Carbamidomethyl (C)]QETESNEEQSIS[Phospho (STY)]PEKR.2	-0.09572591	Q9WTQ5_S1292_M1_Akap12
TKEVC[Carbamidomethyl (C)]VSGGDHTQLTDLS[Phospho (STY)]PEEK.3	0.023733032	Q9WTQ5_S467_M1_Akap12
AEDSGAEQLASEIEPSREESWVS[Phospho (STY)]IKK.4	-0.026454083	Q9WTQ5_S786_M1_Akap12
HPEGIVSEVEM[Oxidation (M)]LSS[Phospho (STY)]QER.3	-0.146624405	Q9WTQ5_S489_M1_Akap12
RPS[Phospho (STY)]ES[Phospho (STY)]DKEEELDK.2	0.433016201	Q9WTQ5_S613_M2_Akap12
LETS[Phospho (STY)]DGDEEGSAEVNK.2	0.356166752	Q9WTR1_S15_M1_Trpv2
NFS[Phospho (STY)]PQIK.2	-0.073716386	Q9WTR1_S47_M1_Trpv2
NSAS[Phospho (STY)]EEDHLPLQVLQSH.3	-0.071944974	Q9WTR1_S743_M1_Trpv2
INENTGSVSVTR.2	0.020789655	#N/A
ETIATYQLYVETTDASGK.2	0.185830733	#N/A
ANS[Phospho (STY)]ALS[Phospho (STY)]LTDTDHER.2	-0.206542869	Q9WTS4_S142_M2_Tenm1
[Acetyl (Protein N-term)]ASQTQGIQQLLQAEK.2	0.010508573	#N/A
[Acetyl (Protein N-term)]ASQTQGIQQLLQAEKR.3	-0.173453933	#N/A
EREQEFQSK.2	-0.016163824	#N/A

VLAQLLGM[Oxidation (M)]VC[Carbamidomethyl (C)]EVRPQVHPNYR.3	-0.216536037	#N/A
QVQGM[Oxidation (M)]QSSQQR.2	0.233083346	#N/A
EEAQM[Oxidation (M)]EVEQYRR.3	0.016887987	#N/A
QQAAM[Oxidation (M)]GS[Phospho (STY)]QGNLSAEVEQATR.2	-0.249155737	Q9WTT4_S65_M1_Atp6v1g2
QQAAM[Oxidation (M)]GSQGNLSAEVEQATRR.3	0.189613179	#N/A
EEAQMEVEQYR.2	0.311519178	#N/A
RPSASS[Phospho (STY)]PNNTAAK.2	0.348153969	Q9WTU0_S1060_M1_Phf2
DSDYVYPSLES[Phospho (STY)]DEDNPVFK.2	0.076913228	Q9WTU0_S876_M1_Phf2
RKGS[Phospho (STY)]DDAPYSPTAR.3	-0.475799055	Q9WTU0_S893_M1_Phf2
ELS[Phospho (STY)]EGEEKGDPEK.2	0.105841712	Q9WTU3_S504_M1_Scn8a
VFKS[Phospho (STY)]ES[Phospho (STY)]EDGMR.2	0.397690857	Q9WTU3_S518_M2_Scn8a
FRDPGSENEFADDEHS[Phospho (STY)]T[Phospho (STY)]VEESEGRR.4	0.275961284	Q9WTU3_S589_M2_Scn8a
VFKS[Phospho (STY)]ES[Phospho (STY)]EDGMRR.3	-0.109074261	Q9WTU3_S518_M2_Scn8a
NYVENRPK.2	0.027863821	#N/A
TAC[Carbamidomethyl (C)]TNFM[Oxidation (M)]M[Oxidation (M)]T[Phospho (STY)]PY[Phospho (STY)]VVTR.2	-0.065591599	#N/A
DYIDQWNK.2	-0.05413329	#N/A
EDS[Phospho (STY)]GTFSLGK.2	0.080980315	Q9WTX2_S18_M1_Prkra
AEAPPLQREDS[Phospho (STY)]GTFSLGK.3	-0.566892716	Q9WTX2_S18_M1_Prkra
HRAEAPPLQREDS[Phospho (STY)]GTFSLGK.4	0.111060952	Q9WTX2_S18_M1_Prkra
GKT[Phospho (STY)]PEEIRK.2	-0.180181744	Q9WTX5_T131_M1_Skp1
GKT[Phospho (STY)]PEEIR.2	-0.075061031	Q9WTX5_T131_M1_Skp1
TDDIPVWDQEFLK.2	0.144999549	#N/A
T[Phospho (STY)]PEEIRK.2	0.14457441	Q9WTX5_T131_M1_Skp1
RTDDIPVWDQEFLK.3	0.162850084	#N/A
LQSSDGEIFEVDVEIAK.2	-0.565665792	#N/A
S[Phospho (STY)]PSKPLAR.2	0.02014577	Q9WU22_S358_M1_Ptpn4
NSS[Phospho (STY)]FTQEATR.2	0.168132872	Q9WU22_S403_M1_Ptpn4
IQPALQEK.2	0.316197862	#N/A
NQLDQEVEFLSTSIAQLK.3	0.324221319	#N/A
VLLGFSSDES[Phospho (STY)]DVEASPR.2	-0.121676931	Q9WU40_S140_M1_Lemd3
EGT[Phospho (STY)]PPPPPPPR.2	-0.207205104	Q9WU42_T1350_M1_Ncor2
S[Phospho (STY)]PGNTSQPPAFFSK.2	-0.174268483	Q9WU42_S2215_M1_Ncor2
SDHALTS[Phospho (STY)]PGGGGK.2	-0.360588762	Q9WU42_S2351_M1_Ncor2
TDDT[Phospho (STY)]S[Phospho (STY)]GEDNDEKEAVASK.2	0.031284679	Q9WU42_S550_M2_Ncor2
PASHTHQHS[Phospho (STY)]PIS[Phospho (STY)]PR.3	-0.310533775	Q9WU42_S1816_M2_Ncor2
LEPVS[Phospho (STY)]PPS[Phospho (STY)]PPHADPELELAPSR.3	-0.057371877	Q9WU42_S149_M2_Ncor2
EKTDDT[Phospho (STY)]S[Phospho (STY)]GEDNDEKEAVASK.2	-0.129613078	Q9WU42_S550_M2_Ncor2
LLDEEEATDNDLR.2	0.141815989	#N/A
STSVVEQGGIQTVDQLIK.2	-0.079131643	#N/A
LANQAADYFGDAFK.2	-0.267409702	#N/A
FYNELTEILVR.2	0.238402172	#N/A
NIQVSHQEFSK.2	0.090767625	#N/A
[Acetyl (Protein N-term)]S[Phospho (STY)]DLDSSSSSAYPK.2	-0.286205929	Q9WUA3_S2_M1_Pfkp
DLLFQPVAELK.2	0.259812417	#N/A
YLEHLSGDGK.2	-0.200008094	#N/A
NVLGHM[Oxidation (M)]QQGGAPSPFDR.3	-0.394992668	#N/A
EIGWADVGGWTGQGGSILGTK.2	-0.040456928	#N/A

FVSDDSIC[Carbamidomethyl (C)]VLGIC[Carbamidomethyl (C)]K.2	0.026839981	#N/A
LNIIIVSEGAIDM[Oxidation (M)]QNKPITSEK.3	0.042808243	#N/A
KVTDFEHR.2	0.225055662	#N/A
IIEVVDAIM[Oxidation (M)]TTAQSHQR.2	-0.448059997	#N/A
LGITNLC[Carbamidomethyl (C)]VIGGDGSLTGANLFR.2	-0.191553543	#N/A
WDC[Carbamidomethyl (C)]VSSILQVGGTIIGSAR.2	-0.24197231	#N/A
ALLS[Phospho (STY)]PEDYQR.2	-0.001602305	Q9WUB0_S344_M1_Rbck1
LITAIGDVVNHDPAVGDR.3	-0.20684757	#N/A
IGEDYISDLDQLRK.3	0.002880343	#N/A
[Acetyl (Protein N-term)]SRPLSDQDKR.3	-0.009099727	#N/A
QC[Carbamidomethyl (C)]QPTDTVC[Carbamidomethyl (C)]ASVR.2	0.521626324	#N/A
WNS[Phospho (STY)]IEER.2	-0.337835688	Q9WUD1_S150_M1_Stub1
LGTGGGGS[Phospho (STY)]PDKSPSAQELK.2	-0.428838283	Q9WUD1_S20_M1_Stub1
KNS[Phospho (STY)]PGVSHSSQK.2	-0.22968501	Q9WUF3_S1818_M1_Casp8ap2
TDS[Phospho (STY)]PLPLEEASTPPGK.2	-0.148572548	Q9WUH1_S320_M1_Tmem115
EALTYDGALLGDR.2	0.165526481	#N/A
[Acetyl (Protein N-term)]ADFDTYDDR.2	-0.05706682	#N/A
TVATPLNQVANPNSAIFGGAR.2	0.097685606	#N/A
GSNM[Oxidation (M)]DFREPTEEER.3	-0.179397027	#N/A
DKDTDKFK.2	0.177718259	#N/A
AYSSFGGGR.2	-0.441022043	#N/A
TVAS[Phospho (STY)]PC[Carbamidomethyl (C)]SSTESLSQR.2	-0.47070692	Q9WUK6_S214_M1_Zbtb18
ILLLGLDNAGK.2	0.166432021	#N/A
LNVWDIGGQR.2	0.011838401	#N/A
QDLLTAAPASEIAEGLNLHTIR.3	0.032531027	#N/A
LEEQLGR.2	0.218459896	#N/A
VTWDSTFC[Carbamidomethyl (C)]AVNPK.2	-0.290091903	#N/A
NDQC[Carbamidomethyl (C)]YDDIR.2	0.28685418	#N/A
VTWDSSFC[Carbamidomethyl (C)]AVNPR.2	0.113043083	#N/A
LIGPNC[Carbamidomethyl (C)]PGVINPGEC[Carbamidomethyl (C)]K.2	-0.247340838	#N/A
HLGLPVFNTVK.2	0.24611167	#N/A
LVGGTTPGK.2	0.076037264	#N/A
IIC[Carbamidomethyl (C)]QGFTGK.2	0.308386284	#N/A
MGHAGAIIAGGK.2	-0.066835411	#N/A
ISALQSAGVVVSM[Oxidation (M)]SPAQLGTTIYK.3	0.561120742	#N/A
QGTFHSQQALEYGTK.3	-0.440812011	#N/A
ASQQDFENALNQVK.2	0.002188271	#N/A
IAQNFGLQHLSSGHLLR.3	0.317325244	#N/A
YLQDQLS[Phospho (STY)]PLTR.2	0.058123379	Q9WUU8_S416_M1_Tnip1
HPPT[Phospho (STY)]PPDPSGGLPR.2	0.006012774	Q9WV18_T929_M1_Gabbr1
RHPPT[Phospho (STY)]PPDPSGGLPR.3	-0.16910216	Q9WV18_T929_M1_Gabbr1
RSS[Phospho (STY)]PIFQTTK.2	0.165400288	Q9WV30_S618_M1_Nfat5
TLVLIGAQGVGR.2	0.226099661	#N/A
VEGGELVIAR.2	0.034818956	#N/A
DNNLELVQEILR.2	0.500067943	#N/A
GIM[Oxidation (M)]ES[Phospho (STY)]PIVR.2	-0.302863639	Q9WV34_S42_M1_Mpp2
DLAELAEQSS[Phospho (STY)]TAAELAR.2	0.25853607	Q9WV34_S86_M1_Mpp2

EGQGYSFVSR.2	0.049620501	#N/A
DLELT[Phospho (STY)]PTSGTLC[Carbamidomethyl (C)]GSLSGK.2	0.211126244	Q9WV34_T303_M1_Mpp2
ALQELLR.2	-0.010072565	#N/A
TYET[Phospho (STY)]PPPS[Phospho (STY)]PGLDPTFSNQPVPPDAVR.3	0.40139426	Q9WV34_S121_M2_Mpp2
ILPSYQEPHLPR.3	-0.029131453	#N/A
NAEFDRHELLIYEEVAR.4	0.084349033	#N/A
YLEHGEYEGNLYGTR.3	0.220593828	#N/A
VAHSDKPGS[Phospho (STY)]TSAVSFR.3	0.133444515	Q9WV55_S214_M1_Vapa
IQAAAS[Phospho (STY)]PPANATAASDTNAGDR.2	-0.258868828	Q9WV60_S389_M1_Gsk3b
GQTNNAASASASNST.2	0.009430331	#N/A
VTTVVATPGQGPDRPQEVSYTDTK.3	0.198345098	#N/A
GNS[Phospho (STY)]LPC[Carbamidomethyl (C)]VLEQK.2	-0.308018099	#N/A
ESVGGS[Phospho (STY)]PQSK.2	0.085833754	Q9WV69_S156_M1_Dmtn
HLS[Phospho (STY)]AEDFSR.2	-0.547150174	Q9WV69_S372_M1_Dmtn
GAEEEEEEEDDDS[Phospho (STY)]EEEIK.2	-0.235833716	Q9WV69_S226_M1_Dmtn
QPLTS[Phospho (STY)]PGSVSSSR.2	-0.349823625	Q9WV69_S11_M1_Dmtn
SREC[Carbamidomethyl (C)]SLS[Phospho (STY)]PK.2	-0.32427839	Q9WV69_S87_M1_Dmtn
TLGIISQASTPR.2	-0.11821587	#N/A
DSSVPGS[Phospho (STY)]PSSIVAK.2	-0.110216584	Q9WV69_S26_M1_Dmtn
SSS[Phospho (STY)]LPSYGR.2	0.005479313	Q9WV69_S289_M1_Dmtn
M[Oxidation (M)]DRGNS[Phospho (STY)]LPC[Carbamidomethyl (C)]VLEQK.3	-0.468272658	#N/A
EC[Carbamidomethyl (C)]SLS[Phospho (STY)]PK.2	-0.013808626	Q9WV69_S87_M1_Dmtn
STS[Phospho (STY)]PPPSPEVWAESR.2	-0.377421981	Q9WV69_S92_M1_Dmtn
KGAEEEEEEEDDDS[Phospho (STY)]EEEIK.2	-0.290270158	Q9WV69_S226_M1_Dmtn
GAEEEEEEEDDDS[Phospho (STY)]EEEIKAIR.3	0.008154246	Q9WV69_S226_M1_Dmtn
QRESVGGS[Phospho (STY)]PQSK.2	0.095041737	Q9WV69_S156_M1_Dmtn
TRS[Phospho (STY)]LPDR.2	-0.241300143	Q9WV69_S269_M1_Dmtn
S[Phospho (STY)]LPDRTPFHTSLHSGTSK.3	-0.033149287	Q9WV69_S269_M1_Dmtn
VFAM[Oxidation (M)]S[Phospho (STY)]PEEFGK.2	0.120440533	Q9WV69_S383_M1_Dmtn
MDNQVLGYK.2	-0.291842024	#N/A
LQS[Phospho (STY)]TEFSPSGSEAGSPGLQNGEGQR.3	-0.121190637	Q9WV69_S303_M1_Dmtn
KGAEEEEEEEDDDS[Phospho (STY)]EEEIKAIR.3	-0.006731659	Q9WV69_S226_M1_Dmtn
S[Phospho (STY)]VDVADNR.2	-0.444486764	Q9WV72_S35_M1_Asb3
GRS[Phospho (STY)]VDVADNR.2	-0.087524082	Q9WV72_S35_M1_Asb3
SSS[Phospho (STY)]PLDRDPAFR.2	-0.14546271	Q9WV89_S12_M1_Stxbp4
VESTSVGSIS[Phospho (STY)]PGGAK.2	-0.186153736	Q9WV92_S804_M1_Epb41l3
ETEIT[Phospho (STY)]PEDGED.2	0.26878719	Q9WV92_T923_M1_Epb41l3
DSVS[Phospho (STY)]AAEVGTGQYATTK.2	-0.046344173	Q9WV92_S470_M1_Epb41l3
KKAEEAT[Phospho (STY)]PVTALR.3	-0.082127992	Q9WV92_T518_M1_Epb41l3
RLS[Phospho (STY)]TSPVR.2	-0.430205592	Q9WV92_S599_M1_Epb41l3
EVPVVHTETK.2	0.119002038	#N/A
AEEAT[Phospho (STY)]PVTALR.2	0.143774286	Q9WV92_T518_M1_Epb41l3
ASALIDRPAPYFER.3	-0.049012038	#N/A
SHFESSTVR.2	0.174743739	#N/A
LM[Oxidation (M)]DGSEILSLLESAR.2	-0.264349999	#N/A
KAEEAT[Phospho (STY)]PVTALR.2	-0.273363525	Q9WV92_T518_M1_Epb41l3
DEVDAAEST[Phospho (STY)]PTDR.2	0.005367122	Q9WV92_T725_M1_Epb41l3

TEPVEAEVESTPHPQPLS[Phospho (STY)]TEK.3	0.010080366	Q9WV92_S686_M1_Epb41l3
QLEYQQFEDDKLS[Phospho (STY)]QR.3	-0.23947651	Q9WV92_S96_M1_Epb41l3
TFLETSTETALTNEWEK.2	0.114732043	#N/A
VLQETILVEER.2	0.207861837	#N/A
ELEDKVIELHK.2	-0.448638973	#N/A
LSTS[Phospho (STY)]PVR.2	-0.312713982	Q9WV92_S601_M1_Epb41l3
EIGDKDRDFAAAAAK.3	0.185294343	#N/A
VVVHKETEIT[Phospho (STY)]PEDGED.2	-0.394329649	Q9WV92_T923_M1_Epb41l3
S[Phospho (STY)]SSS[Phospho (STY)]KLSR.2	0.151635949	Q9WV92_S102_M2_Epb41l3
FAPNHTK.2	0.231388836	#N/A
GKEGSS[Phospho (STY)]VTEAAK.2	-0.151862732	Q9WV92_S740_M1_Epb41l3
QQPALEQFPEAAAHST[Phospho (STY)]PVKR.4	0.007470209	Q9WV92_T64_M1_Epb41l3
TITYESSQVDPGADLEPGVLM[Oxidation (M)]SAQTITSETTSTTTTTHITK.4	-0.156729749	#N/A
GIS[Phospho (STY)]QTNLITTVT[Phospho (STY)]PEKK.2	-0.282675448	Q9WV92_S486_M2_Epb41l3
SSSSKLS[Phospho (STY)]RS[Phospho (STY)]PLK.2	0.072778744	Q9WV92_S105_M2_Epb41l3
VTLLDGSEYGC[Carbamidomethyl (C)]DVDKR.3	0.065828877	#N/A
LSRS[Phospho (STY)]PLK.2	0.19039672	Q9WV92_S107_M1_Epb41l3
GISQTNLITTVT[Phospho (STY)]PEK.2	-0.065039829	Q9WV92_T495_M1_Epb41l3
QLEYQQFEDDKLSQRS[Phospho (STY)]S[Phospho (STY)]SSK.3	0.064913119	Q9WV92_S100_M2_Epb41l3
DEVDAAEST[Phospho (STY)]PTDRR.2	0.096363318	Q9WV92_T725_M1_Epb41l3
NWLDPAK.2	-0.230515999	#N/A
EQHPDMSVTK.2	0.011994189	#N/A
IVITGDADIDHDQALAQAIK.3	-0.251279768	#N/A
S[Phospho (STY)]LDGASVSENHEIYM[Oxidation (M)]K.2	-0.067206293	Q9WV92_S451_M1_Epb41l3
AEEERVEEEDRR.3	0.07455885	#N/A
TFLETSTETALTNEWEKR.3	-0.245039321	#N/A
VC[Carbamidomethyl (C)]VEHHTFFR.3	-0.334100335	#N/A
LTETC[Carbamidomethyl (C)]FLDC[Carbamidomethyl (C)]VK.2	0.388153404	#N/A
EFLGTYNK.2	-0.080309938	#N/A
EVKPEEVTC[Carbamidomethyl (C)]SEHC[Carbamidomethyl (C)]LQK.3	0.180857492	#N/A
FQEYHIQQNEALAAK.2	0.228545888	#N/A
FIDTSQFILNR.2	-0.003004328	#N/A
SKPVFSESLS[Phospho (STY)]D.2	0.150577952	Q9WVA2_S96_M1_Timm8a1
LDS[Phospho (STY)]RAEAC[Carbamidomethyl (C)]FVNC[Carbamidomethyl (C)]VER.3	-0.169175461	Q9WVA2_S57_M1_Timm8a1
VAVEYLDPS[Phospho (STY)]PEVQK.2	0.114497779	Q9WVA3_S211_M1_Bub3
NFS[Phospho (STY)]DNQLQEGK.2	0.485839444	Q9WVA4_S163_M1_Tagln2
LDSGQVGLYPANYVEAIQ.2	-0.133707919	#N/A
AYAQQLTEWAR.2	0.265591256	#N/A
SSS[Phospho (STY)]PIPLTPSK.2	-0.249675742	Q9WVG9_S404_M1_Msl3
WPGS[Phospho (STY)]PTSR.2	-0.081684514	Q9WVH4_S293_M1_Foxo3
PHPSISEEDEGFDC[Carbamidomethyl (C)]LSS[Phospho (STY)]PER.3	0.245748835	Q9WVI9_S337_M1_Mapk8ip1
GSIDEVDKR.2	-0.27426567	#N/A
DKPTYDEIFYTLS[Phospho (STY)]PVNGK.3	-0.043303376	Q9WVK4_S456_M1_Ehd1
S[Phospho (STY)]SFPDSTEAFDPR.2	-0.126435785	Q9WVQ1_S725_M1_Magi2
GGFFS[Phospho (STY)]PWK.2	-0.190598781	Q9WVQ1_S685_M1_Magi2
IIPQEELNS[Phospho (STY)]PTSAPSSEK.2	0.426656944	Q9WVQ1_S1013_M1_Magi2
M[Oxidation (M)]DS[Phospho (STY)]SGPDYKELDVHLR.3	-0.10084755	Q9WVQ1_S768_M1_Magi2

GAQGFGFTIADS[Phospho (STY)]PTGQR.2	0.17686087	Q9WVQ1_S621_M1_Magi2
SPGSVSTHHSS[Phospho (STY)]PR.2	0.106426307	Q9WVQ1_S884_M1_Magi2
NKS[Phospho (STY)]VTNMEK.2	0.13790977	Q9WVQ1_S219_M1_Magi2
AGPGDRDPPS[Phospho (STY)]RGEESR.3	0.003363978	Q9WVR4_S477_M1_Fxr2
TDGS[Phospho (STY)]ISGDRQPVTVADYISR.3	0.000425185	Q9WVR4_S602_M1_Fxr2
EAEPDLLAVLR.2	-0.031959597	#N/A
IFDAVGFTFPNR.2	-0.05919806	#N/A
IPILSTFLTAR.2	-0.152708544	#N/A
GLSEDFQR.2	0.161124192	#N/A
EVC[Carbamidomethyl (C)]QLLPFLVR.2	-0.06236649	#N/A
EGAPSLLC[Carbamidomethyl (C)]K.2	-0.088535215	#N/A
DLQAGLAR.2	0.160273344	#N/A
TLYEEAEEASDISQQVANLAISPTT[Phospho (STY)]PGPSWPGDALR.4	-0.255325465	Q9Z0E0_T451_M1_Ncdn
NDSEQFAALLLVTK.2	0.10080789	#N/A
EAPDGC[Carbamidomethyl (C)]PDHVLR.2	0.20565907	#N/A
SM[Oxidation (M)]IDDTYQC[Carbamidomethyl (C)]LTAVAGTPR.2	-0.482796107	#N/A
LQAGEETASHYR.2	0.477660197	#N/A
LLSTS[Phospho (STY)]PALQGTPASR.2	0.054458309	Q9Z0E0_S583_M1_Ncdn
EAIGAVIHYLLQVGPEK.2	0.133963545	#N/A
DGEES[Phospho (STY)]PTVLK.2	0.1054996	Q9Z0E8_S548_M1_Slc22a5
S[Phospho (STY)]FEDLTDHPVTR.2	0.163940655	Q9Z0F8_S794_M1_Adam17
ASENQS[Phospho (STY)]PSSSNYFSPR.2	0.017668882	Q9Z0G7_S203_M1_Zbtb22
ELFEPYGAVYQINVLR.2	0.126510178	#N/A
SWS[Phospho (STY)]EKELK.2	-0.466666312	Q9Z0H4_S52_M1_Celf2
VM[Oxidation (M)]FSPFGQIEEC[Carbamidomethyl (C)]R.2	-0.174746393	#N/A
IGFPSTS[Phospho (STY)]PAK.2	-0.440065692	Q9Z0H8_S295_M1_Clip2
QAS[Phospho (STY)]GPSSSGAATTVSEKPGPK.3	0.028783848	Q9Z0H8_S50_M1_Clip2
YSLIDPAS[Phospho (STY)]PPELLK.2	-0.427688841	Q9Z0H8_S980_M1_Clip2
TGNESGSNLS[Phospho (STY)]DSGSVK.2	-0.05321373	Q9Z0H8_S208_M1_Clip2
EGS[Phospho (STY)]PLHK.2	-0.386309221	Q9Z0H8_S43_M1_Clip2
VLLLEANRHS[Phospho (STY)]PGPER.3	-0.346325367	Q9Z0H8_S924_M1_Clip2
QQHIEQLLAER.3	0.139266246	#N/A
TGNESGSNLS[Phospho (STY)]DSGS[Phospho (STY)]VKR.2	0.001648627	Q9Z0H8_S208_M2_Clip2
VTQPLGT[Phospho (STY)]PTK.2	-0.058089102	Q9Z0J4_T128_M1_Nos1
KSS[Phospho (STY)]GDGPDLR.2	-0.024113037	Q9Z0J4_S858_M1_Nos1
GNVPVVLNNPYSENEQSPASGKQS[Phospho (STY)]PTK.3	-0.239082389	Q9Z0J4_S287_M1_Nos1
SETLVNAQQT[Phospho (STY)]PLGT[Phospho (STY)]PKENR.2	-0.325649507	#N/A
EPAPLNGS[Phospho (STY)]AAELPATK.2	-0.148000513	Q9Z0P4_S345_M1_Palm
VLGLQDTIK.2	0.422334443	#N/A
GLAEDVTR.2	0.049476892	#N/A
GLEESITR.2	0.042822357	#N/A
ADEVTLS[Phospho (STY)]EAGSTAGPAEPR.2	-0.016223221	Q9Z0P4_S244_M1_Palm
SETLVNAQQT[Phospho (STY)]PLGT[Phospho (STY)]PK.2	-0.139208962	#N/A
EIDVLEFGESAPAASK.2	-0.226951247	#N/A
EENQTGPTTT[Phospho (STY)]PS[Phospho (STY)]DTQDLDM[Oxidation (M)]K.2	-0.16322925	Q9Z0P4_S365_M2_Palm
[Acetyl (Protein N-term)]M[Oxidation (M)]EVLATDTAS[Phospho (STY)]QQER.2	-0.296255606	Q9Z0P4_S10_M1_Palm
AELVVIEDAATPR.2	0.198192925	#N/A

EENQTGPTTTPS[Phospho (STY)]DT[Phospho (STY)]QDLDM[Oxidation (M)]KKPR.3	0.117350567	Q9Z0P4_S365_M2_Palm
ENSAAPSPGRPQSAS[Phospho (STY)]PAKEEQK.3	0.011939872	Q9Z0P4_S124_M1_Palm
TST[Phospho (STY)]PVRS[Phospho (STY)]PGGSTMM[Oxidation (M)]K.2	0.304378442	Q9Z0P4_S157_M2_Palm
WLLEGTPSSASEGDEDMRK.3	0.156136791	#N/A
PQSAS[Phospho (STY)]PAKEEQK.2	-0.150917343	Q9Z0P4_S124_M1_Palm
EEQKSETLVNAQQT[Phospho (STY)]PLGT[Phospho (STY)]PKENR.3	-0.138787601	#N/A
ENSAAPS[Phospho (STY)]PGRPQSASPAK.2	-0.6897282	Q9Z0P4_S116_M1_Palm
S[Phospho (STY)]PGGSTMMK.2	0.352010448	#N/A
ETIELVHTEPTNVAQLPSR.3	0.106358507	#N/A
VIIEDEQLVLGASQEPVGR.2	0.194196497	#N/A
AVLPLLDAQEPC[Carbamidomethyl (C)]YLLFR.2	-0.314050316	#N/A
KDS[Phospho (STY)]LGSDSSTAIIPHELIR.3	-0.309438154	Q9Z0P7_S346_M1_Sufu
RLS[Phospho (STY)]GKDTEQIR.3	-0.256193732	Q9Z0P7_S301_M1_Sufu
LPEEPSS[Phospho (STY)]EDEQQPEK.2	-0.164338637	Q9Z0R4_S335_M1_Itsn1
STS[Phospho (STY)]IDTGPTESPASLK.2	-0.03505518	Q9Z0R4_S971_M1_Itsn1
QRS[Phospho (STY)]LEAAR.2	-0.313060335	Q9Z0R4_S623_M1_Itsn1
QVQQNS[Phospho (STY)]LHR.2	-0.024027514	Q9Z0R4_S558_M1_Itsn1
STS[Phospho (STY)]IDTGPTESPASLKR.2	-0.388170698	Q9Z0R4_S971_M1_Itsn1
KSTS[Phospho (STY)]IDTGPTESPASLKR.3	-0.318616348	Q9Z0R4_S971_M1_Itsn1
SAFTPATATGSSPS[Phospho (STY)]PVLGQGEK.2	0.133519836	Q9Z0R4_S897_M1_Itsn1
LLS[Phospho (STY)]PGTSKIT[Phospho (STY)]PTELPK.2	0.181443057	Q9Z0R4_S1130_M2_Itsn1
LPEEPSS[Phospho (STY)]EDEQQPEKK.2	-0.443862941	Q9Z0R4_S335_M1_Itsn1
SGS[Phospho (STY)]GM[Oxidation (M)]SVISSSSVDQR.2	0.44117126	Q9Z0R4_S315_M1_Itsn1
LLS[Phospho (STY)]PGTSK.2	-0.337424935	Q9Z0R4_S1130_M1_Itsn1
SNPYC[Carbamidomethyl (C)]EVTM[Oxidation (M)]GS[Phospho (STY)]QC[Carbamidomethyl (C)]HITK.2	-0.160531712	Q9Z0R4_S1620_M1_Itsn1
DQFS[Phospho (STY)]PDDFLGR.2	-0.717507081	Q9Z0R4_S1663_M1_Itsn1
AQS[Phospho (STY)]FDVASAPPAAEWAVPQSSR.2	-0.161613548	Q9Z0R4_S203_M1_Itsn1
NYEYYASHVPESVK.2	-0.062780281	#N/A
IIQLIEGK.2	-0.02995071	#N/A
LTDIHGNALQYNK.3	0.146330544	#N/A
SHSNQLVTDC[Carbamidomethyl (C)]ISAMNPDTVLR.3	-0.088948633	#N/A
LVQM[Oxidation (M)]SIC[Carbamidomethyl (C)]SSLAR.2	-0.096282186	#N/A
[Acetyl (Protein N-term)]ASSHTVLMR.2	0.112387122	#N/A
VLIEDT[Phospho (STY)]DDEANT.2	0.301212115	Q9Z0U0_T689_M1_Xpr1
GSYGS[Phospho (STY)]DPEEEEYR.2	0.123628778	Q9Z0U1_S1136_M1_Tjp2
AAS[Phospho (STY)]RDQLR.2	-0.203430308	Q9Z0U1_S960_M1_Tjp2
SNLPATAGSEIPGGST[Phospho (STY)]K.2	-0.236697466	Q9Z0U1_T1009_M1_Tjp2
S[Phospho (STY)]IDRDYDR.2	-0.121605245	Q9Z0U1_S213_M1_Tjp2
S[Phospho (STY)]IDRDYDRDYER.3	-0.072084946	Q9Z0U1_S213_M1_Tjp2
S[Phospho (STY)]QNREDSFDYSK.2	0.15354961	Q9Z0U1_S982_M1_Tjp2
S[Phospho (STY)]RGRS[Phospho (STY)]LER.2	-0.241059942	Q9Z0U1_S169_M2_Tjp2
GRS[Phospho (STY)]IDRDYDR.3	0.302963918	Q9Z0U1_S213_M1_Tjp2
KVQVAPLQGS[Phospho (STY)]PPLSHDDR.3	-0.321257474	Q9Z0U1_S107_M1_Tjp2
EHLRS[Phospho (STY)]RS[Phospho (STY)]PS[Phospho (STY)]PESR.3	-0.178940865	Q9Z0U1_S263_M3_Tjp2
DAS[Phospho (STY)]PPPAFKPEPPK.2	-0.291227607	Q9Z0U1_S968_M1_Tjp2
SRS[Phospho (STY)]PS[Phospho (STY)]PESR.2	0.01355562	Q9Z0U1_S265_M2_Tjp2
RQQYS[Phospho (STY)]DQDYHS[Phospho (STY)]S[Phospho (STY)]TEKLK.3	-0.321013697	Q9Z0U1_S404_M3_Tjp2

VQVAPLQGS[Phospho (STY)]PPLSHDDR.3	0.104052786	Q9Z0U1_S107_M1_Tjp2
GSYGS[Phospho (STY)]DPEEEEYRQQLAAHSK.3	-0.315093321	Q9Z0U1_S1136_M1_Tjp2
M[Oxidation (M)]SYLTAM[Oxidation (M)]GADYLS[Phospho (STY)]C[Carbamidomethyl (C)]DSR.2	-0.48698449	#N/A
SYHEAYEPDYGGGYS[Phospho (STY)]PSYDR.3	0.145657413	Q9Z0U1_S239_M1_Tjp2
ERPS[Phospho (STY)]SREETSGR.3	-0.391134466	Q9Z0U1_S420_M1_Tjp2
HSSHDMLSHS[Phospho (STY)]WEGNRER.4	0.325567618	Q9Z0U1_S147_M1_Tjp2
HSSHDMLSHS[Phospho (STY)]WEGNR.3	-0.072667874	Q9Z0U1_S147_M1_Tjp2
RQQYS[Phospho (STY)]DQDYHSSTEK.3	-0.279112001	Q9Z0U1_S404_M1_Tjp2
SRHS[Phospho (STY)]S[Phospho (STY)]HDMLSHSWEGNR.4	-0.349196694	Q9Z0U1_S139_M2_Tjp2
LAGGNDVGIFVAGIQEGTSAEQEGLQEGDQILK.3	-0.043724323	#N/A
GS[Phospho (STY)]VQELSTIQIR.2	0.031089573	Q9Z0V2_S552_M1_Kcnd2
TPLSNS[Phospho (STY)]RSS[Phospho (STY)]LNAK.2	-0.25588926	Q9Z0V2_S572_M2_Kcnd2
IPNANVS[Phospho (STY)]GSHR.2	0.036491562	Q9Z0V2_S546_M1_Kcnd2
ADIVESLC[Carbamidomethyl (C)]SESTATS[Phospho (STY)]PV.2	-0.029355521	Q9Z0W1_S425_M1_Ngfr
AIASATEGGSVPQIR.2	0.269185427	#N/A
SITVIGGGFLGSELAC[Carbamidomethyl (C)]ALGR.2	0.235798374	#N/A
[Acetyl (Protein N-term)]AALTDVQR.2	0.276121522	#N/A
VQVALGNIASK.2	-0.033226192	#N/A
LAQIHIQQQDQC[Carbamidomethyl (C)]VAITEESK.3	0.049349001	#N/A
LTGPSSVGPGPTDPDS[Phospho (STY)]DDDSSDLVIENR.3	0.090652508	Q9Z101_S278_M1_Pard6a
LM[Oxidation (M)]HDAS[Phospho (STY)]DS[Phospho (STY)]EVDQDDVVEWK.3	0.239199153	Q9Z103_S959_M2_Adnp
VFVGGLS[Phospho (STY)]PDTSEEQIK.2	0.441593306	Q9Z130_S122_M1_Hnrnpdl
DLTEYLSR.2	-0.229117751	#N/A
YHQIGSGK.2	-0.244350631	#N/A
[Acetyl (Protein N-term)]MEDM[Oxidation (M)]NEYSNIEEFAEGSK.2	0.02957936	#N/A
NNLNPSWEPFR.2	0.037164206	#N/A
LLDGDDGPLR.2	0.218114353	#N/A
SC[Carbamidomethyl (C)]SS[Phospho (STY)]PVFSR.2	-0.232129692	Q9Z140_S68_M1_Cpne6
C[Carbamidomethyl (C)]VLAEVPR.2	0.002122187	#N/A
PSTPAM[Oxidation (M)]TPSPS[Phospho (STY)]P.2	-0.248478226	Q9Z140_S556_M1_Cpne6
SSQSLHC[Carbamidomethyl (C)]LS[Phospho (STY)]PR.2	-0.349439426	Q9Z140_S330_M1_Cpne6
[Acetyl (Protein N-term)]S[Phospho (STY)]DPEM[Oxidation (M)]GWVPEPPAM[Oxidation (M)]TLGASR.2	-0.283924285	#N/A
LYSDEQWVEVER.2	-0.144897869	#N/A
YSVLLVLTDGVVSDM[Oxidation (M)]AETR.2	-0.263436579	#N/A
TEVLRSC[Carbamidomethyl (C)]S[Phospho (STY)]S[Phospho (STY)]PVFSR.2	-0.280507809	Q9Z140_S67_M2_Cpne6
VLAIEYFFEEK.2	-0.179939452	#N/A
VAGQDGSVVQFK.2	0.027940941	#N/A
[Acetyl (Protein N-term)]S[Phospho (STY)]EEKPKEGVK.2	-0.449962099	Q9Z172_S2_M1_Sumo3
NNPS[Phospho (STY)]PPPDSDLER.2	0.060889006	Q9Z191_S338_M1_Eya4
NDSIPQEDFTPDVYR.2	-0.325725398	#N/A
YNEIQNDYLR.2	0.075622349	#N/A
WDDDSTIVTPIILR.2	0.196421683	#N/A
SYIQEVVQYIK.2	0.348727522	#N/A
SPVEFVEYNK.2	0.248254569	#N/A
EVIEAIAEC[Carbamidomethyl (C)]AFK.2	0.151876098	#N/A
LQVTPEGR.2	0.18413658	#N/A
LIFGDALLM[Oxidation (M)]EPLEK.2	0.396687273	#N/A

EADPGETS[Phospho (STY)]SEAPSETR.2	-0.069731486	Q9Z1B3_S848_M1_Plcb1
VGTYVEVDM[Oxidation (M)]FGLPVDTR.2	0.055871775	#N/A
TSPFPILLSFENHVDSPK.2	0.062768981	#N/A
SEPSS[Phospho (STY)]PDHGSSAIEQDLAALDAEM[Oxidation (M)]TQK.3	-0.359842942	Q9Z1B3_S982_M1_Plcb1
VNLKS[Phospho (STY)]PSSEEIERENPGR.3	-0.382015829	Q9Z1B3_S1197_M1_Plcb1
YVNLM[Oxidation (M)]EQR.2	-0.011534835	#N/A
SPS[Phospho (STY)]SEEIER.2	-0.040898314	Q9Z1B3_S1199_M1_Plcb1
ELLDVGNIGHLEQR.2	-0.278253619	#N/A
KSEPSSPDHGSS[Phospho (STY)]AIEQDLAALDAEM[Oxidation (M)]TQK.4	-0.429321312	Q9Z1B3_S988_M1_Plcb1
FESFEISK.2	-0.456954923	#N/A
C[Carbamidomethyl (C)]VELDC[Carbamidomethyl (C)]WK.2	-0.263433963	#N/A
VNLKS[Phospho (STY)]PSSEEIER.2	-0.45034858	Q9Z1B3_S1197_M1_Plcb1
LTDVAEEC[Carbamidomethyl (C)]QNNQLKK.2	-0.111594598	#N/A
KKSEPSSPDHGS[Phospho (STY)]S[Phospho (STY)]AIEQDLAALDAEM[Oxidation (M)]TQK.4	0.014248891	Q9Z1B3_S987_M2_Plcb1
SPS[Phospho (STY)]SEEIERENPGR.2	-0.489200758	Q9Z1B3_S1199_M1_Plcb1
DYVPDTYADVIEALSNPIR.2	-0.083958561	#N/A
SPS[Phospho (STY)]SEEIERENPGREFDTPL.3	0.21107058	Q9Z1B3_S1199_M1_Plcb1
VFLNNLC[Carbamidomethyl (C)]PRPEIDNIFSEFGAK.3	-0.077044101	#N/A
GIPLPTGDTS[Phospho (STY)]PEPELLPGDPLPPPK.3	-0.071883524	Q9Z1D1_S42_M1_Eif3g
HSS[Phospho (STY)]PHQS[Phospho (STY)]EDEEEPR.2	-0.06354663	Q9Z1E4_S653_M2_Gys1
GVTQIDNDLK.2	-0.156497946	#N/A
[Acetyl (Protein N-term)]TEFWLISAPGEK.2	-0.111828844	#N/A
GNLQNLER.2	-0.168096915	#N/A
VGTLDVLVGLSDELAK.2	-0.1731409	#N/A
IDC[Carbamidomethyl (C)]NLLEFK.2	-0.035846575	#N/A
SSNVLSEDQDSYLC[Carbamidomethyl (C)]NVTLFR.2	-0.364652701	#N/A
ASAYNNLK.2	0.30480772	#N/A
NNNLAVSSK.2	-0.238113015	#N/A
KAVDDFR.2	0.109508422	#N/A
VQENLLASGVDLVTYITR.3	-0.507034884	#N/A
FNIPDLK.2	-0.005769825	#N/A
NISEIIAK.2	0.278449201	#N/A
TC[Carbamidomethyl (C)]QQTWEK.2	-0.099410569	#N/A
SRAS[Phospho (STY)]AYNNLK.2	0.469792609	Q9Z1G3_S141_M1_Atp6v1c1
LDAFVEGVVK.2	-0.2789594	#N/A
VAQYMADVLEDSKDK.3	-0.026336556	#N/A
DLNPDVNVFQR.2	-0.243693993	#N/A
FLPFSFEHIR.2	-0.111897627	#N/A
HLGTLNFGGIR.2	-0.255112315	#N/A
LGFVAGVINR.2	-0.066740999	#N/A
NFLELTELK.2	0.129567114	#N/A
FYTGTGFK.2	-0.168753758	#N/A
GTEHSGSTVPSILNR.2	0.175452129	#N/A
KANIPIMDTGENPEVPFPR.3	0.503878273	#N/A
ANIPIM[Oxidation (M)]DTGENPEVPFPR.2	-0.116365261	#N/A
LHWVEFQNK.2	0.187794785	#N/A
IDDLQM[Oxidation (M)]VLNQTEDHR.3	-0.132145482	#N/A

QAEIENPLEDPVTGDYVHK.3	-0.095114996	#N/A
FTHGFQNIVDAYGIGTYR.3	-0.356791768	#N/A
AIYHTLNLC[Carbamidomethyl (C)]NIDVTQK.2	-0.494270311	#N/A
ASLYPC[Carbamidomethyl (C)]PETPQER.2	-0.107586189	#N/A
EINTNQEALKR.2	0.34067153	#N/A
DM[Oxidation (M)]IDLEANFEKIENELK.3	-0.121419843	#N/A
M[Oxidation (M)]QTNQTPPTYNK.2	0.141392956	#N/A
DMIDLEANFEK.2	0.249218468	#N/A
ERIPTFER.2	0.244611189	#N/A
SVFIIFFQGDQLK.2	0.313207786	#N/A
SPAS[Phospho (STY)]LEGHT[Phospho (STY)]GK.2	-0.020283426	Q9Z1J2_S343_M2_Nek4
RSSDGGDGEGS[Phospho (STY)]ELVKPLYPSNK.3	0.195945862	Q9Z1J2_S438_M1_Nek4
VSS[Phospho (STY)]PEDSPAGTPQR.2	-0.102584644	Q9Z1K7_S2143_M1_Apc2
RGS[Phospho (STY)]DGEARPLPR.3	-0.381873771	Q9Z1K7_S2099_M1_Apc2
APS[Phospho (STY)]PEQQR.2	0.099965001	Q9Z1K7_S1805_M1_Apc2
FHAPALGPEPAART[Phospho (STY)]PEGS[Phospho (STY)]PVHGSGPSK.4	0.121222488	Q9Z1K7_S108_M2_Apc2
GLAITFVSDENDAK.2	0.337024547	#N/A
DVQEIFR.2	0.005441723	#N/A
NWASGQDLQAK.2	-0.133449693	#N/A
LSNNYYC[Carbamidomethyl (C)]TR.2	0.363749396	#N/A
AAESSAM[Oxidation (M)]AATEK.2	0.139390377	#N/A
YQEIAKR.2	0.370453959	#N/A
DINLDVNR.2	-0.427125328	#N/A
[Acetyl (Protein N-term)]SILYVSPHPDAFPSLR.2	-0.016971931	#N/A
LSATVTEAFVR.2	-0.20649935	#N/A
PLTS[Phospho (STY)]PESLSR.2	-0.046887847	Q9Z1R2_S1103_M1_Bag6
LQEDPNYS[Phospho (STY)]PQR.2	0.195565346	Q9Z1R2_S1139_M1_Bag6
ESFSLVQVQPGVDIIR.2	0.152839493	#N/A
TSPEPQRENAS[Phospho (STY)]PAPGTTAEEAM[Oxidation (M)]SR.3	0.067387361	Q9Z1R2_S995_M1_Bag6
LQEDPNYS[Phospho (STY)]PQRFPNAHR.3	-0.07464173	Q9Z1R2_S1139_M1_Bag6
AAGARPLTS[Phospho (STY)]PESLSR.2	0.275716638	Q9Z1R2_S1103_M1_Bag6
ENAS[Phospho (STY)]PAPGTTAEEAMSR.2	0.206466604	Q9Z1R2_S995_M1_Bag6
SNHVLAQMDHGDS[Phospho (STY)]A.2	0.294026996	Q9Z1S3_S794_M1_Rasgrp1
VVNIIPVIAK.2	0.290750085	#N/A
STLVNTLFK.2	0.158662814	#N/A
KASS[Phospho (STY)]WNR.2	-0.050641637	Q9Z1S5_S92_M1_Septin3
THLQDLK.2	0.183742699	#N/A
YINEQYEK.2	0.140464221	#N/A
EVTHNIHYETYR.3	0.270014812	#N/A
LTVIDTPGFGDQINNENC[Carbamidomethyl (C)]WEPIEK.3	-0.365365636	#N/A
ADTM[Oxidation (M)]TLEEK.2	0.069909264	#N/A
AIGHVIEEGGVK.2	0.606425704	#N/A
ASSWNREEK.2	-0.186203007	#N/A
ASS[Phospho (STY)]C[Carbamidomethyl (C)]ETYEYPAR.2	0.146558337	Q9Z1S8_S402_M1_Gab2
SAS[Phospho (STY)]FSQGTR.2	-0.098893956	Q9Z1S8_S211_M1_Gab2
SSLTGS[Phospho (STY)]ETDNEDVYTFK.2	-0.013825608	Q9Z1S8_S282_M1_Gab2
EDGGLEDNEKNFYES[Phospho (STY)]EEEEEEKEK.4	-0.434254661	Q9Z1T1_S276_M1_Ap3b1

NFYES[Phospho (STY)]EEEEEEKEK.2	0.090047643	Q9Z1T1_S276_M1_Ap3b1
SAS[Phospho (STY)]ITNLSLDR.2	-0.243724093	Q9Z1T6_S307_M1_Pikfyve
[Acetyl (Protein N-term)]ATDDKSS[Phospho (STY)]PTLDSANDLPR.2	-0.584091965	Q9Z1T6_S8_M1_Pikfyve
SGSPMVPSYETSVS[Phospho (STY)]PQANR.2	-0.037065698	Q9Z1T6_S329_M1_Pikfyve
T[Phospho (STY)]PDIAQR.2	-0.202724167	Q9Z1W9_T366_M1_Stk39
RVPGSS[Phospho (STY)]GHLHK.3	-0.511448049	Q9Z1W9_S383_M1_Stk39
TEDGDWEWS[Phospho (STY)]DDEM[Oxidation (M)]DEKSEEGK.3	-0.037072121	Q9Z1W9_S397_M1_Stk39
TEDGDWEWS[Phospho (STY)]DDEM[Oxidation (M)]DEK.2	0.231343229	Q9Z1W9_S397_M1_Stk39
LLTRT[Phospho (STY)]PDIAQR.2	-0.12842719	Q9Z1W9_T366_M1_Stk39
VAGGSGSES[Phospho (STY)]PLLK.2	-0.190473674	Q9Z1X2_S16_M1_Ptdss2
RVAGGSGSES[Phospho (STY)]PLLK.2	0.208692606	Q9Z1X2_S16_M1_Ptdss2
RPM[Oxidation (M)]EEDGEEKS[Phospho (STY)]PSK.3	0.057783706	Q9Z1X4_S382_M1_Ilf3
TVDFTQDSNYLLTGGQDK.2	0.106619856	#N/A
C[Carbamidomethyl (C)]VLPEEDS[Phospho (STY)]GELAKPK.2	-0.003221238	Q9Z1Z2_S312_M1_Strap
GFAFVQYVNER.2	-0.012294941	#N/A
VFIGNLNTLVVK.2	-0.431317206	#N/A
M[Oxidation (M)]IAGQVLDINLAAEPK.2	-0.073807176	#N/A
SEEEQSSASVK.2	0.430403036	#N/A
QADLSFSS[Phospho (STY)]PVEM[Oxidation (M)]K.2	-0.079094307	Q9Z204_S232_M1_Hnrnpc
NEKS[Phospho (STY)]EEEQSSASVKK.3	-0.507626582	Q9Z204_S241_M1_Hnrnpc
NEKS[Phospho (STY)]EEEQSSASVK.2	-0.26308884	Q9Z204_S241_M1_Hnrnpc
SEEEQSSAS[Phospho (STY)]VKKDETNVK.3	0.343757255	Q9Z204_S249_M1_Hnrnpc
SDVEAIFSK.2	0.102256051	#N/A
KHEDESEAWLHR.3	-0.109108232	#N/A
IYFLSTEDLPR.2	-0.060258465	#N/A
HLYSANTVDDFNR.2	-0.541247191	#N/A
TGEPDEEEGTFRSS[Phospho (STY)]IR.3	0.138953794	Q9Z239_S83_M1_Fxyd1
[Acetyl (Protein N-term)]AAAGSAAVSGAGT[Phospho (STY)]PVAGPTGR.2	0.140654605	Q9Z266_T14_M1_Snapin
AMLDSGVYPPGS[Phospho (STY)]PSK.2	0.110274242	Q9Z266_S133_M1_Snapin
VVLVNNILQNAQER.2	-0.003568786	#N/A
RIS[Phospho (STY)]FKGT[Phospho (STY)]PTEEQVR.3	-0.089265598	Q9Z268_S396_M2_Rasal1
LLEDSS[Phospho (STY)]LDTEVDPGR.2	-0.369070154	Q9Z268_S749_M1_Rasal1
LADDEGDS[Phospho (STY)]DSESVGQSR.2	-0.158837425	Q9Z277_S1464_M1_Baz1b
EENVLGKVDR.2	-0.255208364	#N/A
ANS[Phospho (STY)]FVGTAQYVSPELLTEK.2	-0.397447948	Q9Z2A0_S244_M1_Pdpk1
KEGS[Phospho (STY)]KEFIHPQSIEEK.3	-0.131326841	Q9Z2A5_S166_M1_Ate1
EFIHPQS[Phospho (STY)]IEEK.2	-0.17250063	Q9Z2A5_S174_M1_Ate1
EGS[Phospho (STY)]KEFIHPQSIEEK.3	-0.415507098	Q9Z2A5_S166_M1_Ate1
LEPVYSPAGS[Phospho (STY)]PPPGDPR.2	0.390868905	Q9Z2B9_S347_M1_Rps6ka4
RGS[Phospho (STY)]PVPASSGR.2	0.300004343	Q9Z2B9_S745_M1_Rps6ka4
GSS[Phospho (STY)]PTHSATPVHTSV.2	-0.335567088	Q9Z2C4_S657_M1_Mtmr1
HPSIETLDS[Phospho (STY)]PTGSHVEWC[Carbamidomethyl (C)]K.3	-0.147921559	Q9Z2C4_S53_M1_Mtmr1
SPS[Phospho (STY)]QGDEDSALILTQDNLK.2	-0.17236965	Q9Z2C9_S595_M1_Mtmr7
S[Phospho (STY)]PSGGEHAPSEDSGKDR.3	0.307266114	Q9Z2C9_S634_M1_Mtmr7
S[Phospho (STY)]PSGGEHAPSEDSGK.2	-0.062289945	Q9Z2C9_S634_M1_Mtmr7
AS[Phospho (STY)]SPAQC[Carbamidomethyl (C)]VTPVQTVV.2	0.194360973	Q9Z2D1_S630_M1_Mtmr2
SSS[Phospho (STY)]C[Carbamidomethyl (C)]ESLGAQLPAAR.2	0.124024357	Q9Z2D1_S6_M1_Mtmr2

SASAISSDSISTSADNFS[Phospho (STY)]PDLR.2	0.004191106	Q9Z2D1_S58_M1_Mtmr2
AETSESSGSAPAVPEASAS[Phospho (STY)]PK.2	-0.191772662	Q9Z2D6_S80_M1_Mecp2
VLEKS[Phospho (STY)]PGK.2	0.169009119	Q9Z2D6_S216_M1_Mecp2
GGS[Phospho (STY)]LESDGC[Carbamidomethyl (C)]PKEPAK.2	-0.193245459	Q9Z2D6_S421_M1_Mecp2
EGKHEPLQPSAHHSAEPAEAGKAETSESSGSAPAVPEASAS[Phospho (STY)]PK.5	-0.07449111	Q9Z2D6_S80_M1_Mecp2
VGDTSLDPNDFDFTVTGR.2	-0.053409053	#N/A
MPFQAS[Phospho (STY)]PGGK.2	-0.027212165	Q9Z2D6_S229_M1_Mecp2
VRYDS[Phospho (STY)]SNQVK.2	-0.095777335	Q9Z2D8_S85_M1_Mbd3
YLGGS[Phospho (STY)]M[Oxidation (M)]DLSTFDFR.2	-0.205191068	Q9Z2D8_S56_M1_Mbd3
AADTEEVDIDM[Oxidation (M)]DS[Phospho (STY)]GDEA.2	0.184388217	Q9Z2E1_S410_M1_Mbd2
ILLDAQHES[Phospho (STY)]GQSSSR.2	-0.043033269	Q9Z2F7_S81_M1_Bnip3l
DHSSQS[Phospho (STY)]EEEVVEGEKEVEALKK.4	0.003259868	Q9Z2F7_S119_M1_Bnip3l
SVYGVTDETQSQS[Phospho (STY)]PVHIPSQPIR.3	-0.147705646	Q9Z2H2_S244_M1_Rgs6
KSVYGVTDETQSQS[Phospho (STY)]PVHIPSQPIRK.4	-0.165693689	Q9Z2H2_S244_M1_Rgs6
SVYGVTDETQSQS[Phospho (STY)]PVHIPSQPIRK.3	-0.572235664	Q9Z2H2_S244_M1_Rgs6
KSVYGVTDETQSQS[Phospho (STY)]PVHIPSQPIR.3	-0.203530985	Q9Z2H2_S244_M1_Rgs6
ETDPS[Phospho (STY)]PEER.2	-0.356951289	Q9Z2H5_S868_M1_Epb41l1
LVS[Phospho (STY)]PEPPPK.2	-0.260744456	Q9Z2H5_S378_M1_Epb41l1
S[Phospho (STY)]LPELDR.2	-0.227387654	Q9Z2H5_S639_M1_Epb41l1
S[Phospho (STY)]LDGAEFSR.2	-0.15149977	Q9Z2H5_S430_M1_Epb41l1
IKELKPEQETT[Phospho (STY)]PR.2	-0.046051703	Q9Z2H5_T489_M1_Epb41l1
S[Phospho (STY)]LPELDRDK.2	-0.32543135	Q9Z2H5_S639_M1_Epb41l1
AGLREGS[Phospho (STY)]EEK.2	-0.37782463	Q9Z2H5_S564_M1_Epb41l1
VTLLDASEYEC[Carbamidomethyl (C)]EVEK.2	-0.332195775	#N/A
ETDPS[Phospho (STY)]PEERDK.2	0.182888029	Q9Z2H5_S868_M1_Epb41l1
DNHLAIER.2	0.065284136	#N/A
FAPNQTR.2	-0.06564317	#N/A
KAQEETPQQPEAAAAVTT[Phospho (STY)]PVT[Phospho (STY)]PAGHSHPETNSNEK.4	-0.546849048	Q9Z2H5_T30_M2_Epb41l1
HQAS[Phospho (STY)]INELKR.2	-0.286407248	Q9Z2H5_S510_M1_Epb41l1
S[Phospho (STY)]EAEEGEVR.2	0.294210707	Q9Z2H5_S466_M1_Epb41l1
S[Phospho (STY)]EAEEGEVRT[Phospho (STY)]PTK.2	-0.155654802	Q9Z2H5_S466_M2_Epb41l1
AQEETPQQPEAAAAVT[Phospho (STY)]T[Phospho (STY)]PVTPAGHSHPETNSNEK.4	-0.113544443	Q9Z2H5_T29_M2_Epb41l1
RLPSSPAS[Phospho (STY)]PS[Phospho (STY)]PKGT[Phospho (STY)]PEK.2	-0.105493577	Q9Z2H5_S544_M3_Epb41l1
GAC[Carbamidomethyl (C)]ST[Phospho (STY)]PEM[Oxidation (M)]PQFESVK.2	-0.251210308	Q9Z2H5_T685_M1_Epb41l1
GAAAM[Oxidation (M)]IPGPQTVATEIR.2	-0.474257566	#N/A
LPSSPAS[Phospho (STY)]PS[Phospho (STY)]PK.2	0.129834929	#N/A
IIITGDEDVDQDQALALAIK.2	-0.390907198	#N/A
GPSSQEDESGGLEDS[Phospho (STY)]PDR.2	0.011441479	Q9Z2H5_S677_M1_Epb41l1
SLS[Phospho (STY)]PIIGK.2	-0.488018935	Q9Z2H5_S782_M1_Epb41l1
RLPSSPAS[Phospho (STY)]PS[Phospho (STY)]PK.2	-0.118503261	#N/A
EDDAES[Phospho (STY)]GGRRS[Phospho (STY)]EAEEGEVR.3	0.208087262	Q9Z2H5_S461_M2_Epb41l1
DVLTSTYGATAETLSTSTTTHVTK.3	0.446984372	#N/A
RS[Phospho (STY)]EAEEGEVR.2	0.410332562	Q9Z2H5_S466_M1_Epb41l1
SLDGAEFSRPAS[Phospho (STY)]VSENHDAGPEGDKR.4	0.231365744	Q9Z2H5_S441_M1_Epb41l1
LPSSPASPS[Phospho (STY)]PKGT[Phospho (STY)]PEK.2	-0.012079358	#N/A
RSEAEEGEVRT[Phospho (STY)]PTK.3	0.023018374	Q9Z2H5_T475_M1_Epb41l1
DYSEADGLS[Phospho (STY)]ERTT[Phospho (STY)]PSK.2	0.394406093	Q9Z2H5_S75_M2_Epb41l1

ELKPEQETT[Phospho (STY)]PR.2	-0.34301668	Q9Z2H5_T489_M1_Epb41l1
AETMTVSSLAIR.2	0.205149457	#N/A
LPSSPASPS[Phospho (STY)]PKGT[Phospho (STY)]PEKASER.3	0.357015156	#N/A
SLPELDRDKS[Phospho (STY)]DSETEGLVFAQDLK.3	-0.025246608	Q9Z2H5_S648_M1_Epb41l1
DKS[Phospho (STY)]DS[Phospho (STY)]ETEGLVFAQDLK.2	0.254557766	Q9Z2H5_S648_M2_Epb41l1
APES[Phospho (STY)]DMGDEDQDQER.2	0.067919399	Q9Z2H5_S578_M1_Epb41l1
VSAADSTQVDGGTPMVK.2	0.094248748	#N/A
PASVSENHDAGPEGDKREDDAES[Phospho (STY)]GGR.4	-0.196257623	Q9Z2H5_S461_M1_Epb41l1
LLELQSIGTNNFLR.2	-0.445043337	#N/A
VAEVEGEKVDNK.2	-0.146624443	#N/A
LISLTSALDDNKDGNINIDDLVK.3	0.459479036	#N/A
FLQDTIEEMALK.2	0.149589968	#N/A
INFDS[Phospho (STY)]NSAYR.2	0.09259634	Q9Z2I9_S279_M1_Sucla2
EQAVTLAQK.2	0.452200059	#N/A
IVFSPEEAK.2	-0.172640866	#N/A
ILAC[Carbamidomethyl (C)]DDLDEAAK.2	0.254901953	#N/A
IC[Carbamidomethyl (C)]NQVLVC[Carbamidomethyl (C)]ER.2	0.025655922	#N/A
LSEIVTLAK.2	0.28541478	#N/A
LHGGTPANFLDVGGGATVQQVTEAFK.3	-0.311937812	#N/A
SSDEAYAIAK.2	0.112173775	#N/A
IFDLQDWSQEDER.2	0.08072975	#N/A
VQAILVNIFGGIM[Oxidation (M)]R.2	-0.134183955	#N/A
AVSSQM[Oxidation (M)]IGQK.2	0.301894777	#N/A
C[Carbamidomethyl (C)]DVIAQGIVM[Oxidation (M)]AVK.2	0.144527748	#N/A
YDATMVEINPM[Oxidation (M)]VEDSDGK.2	0.229892185	#N/A
VLC[Carbamidomethyl (C)]MDAK.2	0.059487141	#N/A
EFLFS[Phospho (STY)]PNDSASDIAK.2	-0.142243477	Q9Z2M6_S27_M1_Ubl3
EGS[Phospho (STY)]PANWK.2	-0.370683211	Q9Z2N8_S233_M1_Actl6a
EGS[Phospho (STY)]PANWKR.2	0.148563522	Q9Z2N8_S233_M1_Actl6a
ATS[Phospho (STY)]PSNNVDEVQIPEISLSK.2	0.181049259	Q9Z2Q2_S57_M1_Knop1
ADC[Carbamidomethyl (C)]LVPSEIR.2	0.249264578	#N/A
IREEIDK.2	0.119226111	#N/A
VNIVPLIAK.2	0.274780677	#N/A
KLLSAEER.2	0.010077569	#N/A
INQTVEILK.2	-0.282436087	#N/A
HTVDIEEK.2	-0.309341125	#N/A
DVTC[Carbamidomethyl (C)]DVHYENYR.2	0.347217967	#N/A
M[Oxidation (M)]KDEELR.2	-0.260923509	#N/A
LATPEDKQDIDKQYVGFATLPNQVHR.4	-0.383404797	#N/A
QYVGFATLPNQVHR.2	0.008353761	#N/A
SKLAT[Phospho (STY)]PEDKQDIDK.2	0.136188718	Q9Z2Q6_T13_M1_Septin5
STLVHSLFLTDLYKDR.3	0.004027826	#N/A
M[Oxidation (M)]ES[Phospho (STY)]PIPILPLPTPDAETEK.2	-0.522967257	Q9Z2Q6_S327_M1_Septin5
STLVHSLFLTDLYK.2	0.207430641	#N/A
LYPWGIVEVENQAHC[Carbamidomethyl (C)]DFVK.2	-0.392635373	#N/A
FGIHVYQFPEC[Carbamidomethyl (C)]DSDEDEDFKQQDR.4	0.161645524	#N/A
ESAPFAVIGSNTVVEAK.2	-0.003626989	#N/A

LAT[Phospho (STY)]PEDKQDIDK.2	-0.243809459	Q9Z2Q6_T13_M1_Septin5
AHC[Carbamidomethyl (C)]IQQM[Oxidation (M)]TSK.2	0.406245671	#N/A
ADC[Carbamidomethyl (C)]LVPSEIRK.3	-0.317892049	#N/A
THMHDLK.2	0.211529748	#N/A
THM[Oxidation (M)]HDLKDVTC[Carbamidomethyl (C)]DVHYENYR.4	-0.181637627	#N/A
FGIHVYQFPEC[Carbamidomethyl (C)]DS[Phospho (STY)]DEDEDFK.3	-0.034989983	Q9Z2Q6_S225_M1_Septin5
MKDEELRR.2	-0.293204258	#N/A
LSPEEPAPEAPET[Phospho (STY)]PETPEAPGGSAV.3	0.022170944	Q9Z2S7_T125_M1_Tsc22d3
ALLEVVQSGGK.2	0.619409331	#N/A
ILNPEEIEK.2	0.235900647	#N/A
NYTDDAIETDDLTIK.2	0.139398084	#N/A
YVAEIEKEKEENEK.3	-0.030755861	#N/A
AITVFSPDGHLFQVEYAQEAVKK.3	-0.038549293	#N/A
GPQLFHM[Oxidation (M)]DPSGTFVQC[Carbamidomethyl (C)]DAR.3	-0.288391249	#N/A
GVNTFS[Phospho (STY)]PEGR.2	0.309612314	Q9Z2U1_S16_M1_Psma5
ITS[Phospho (STY)]PLM[Oxidation (M)]EPSSIEK.2	-0.239644333	Q9Z2U1_S56_M1_Psma5
LFQVEYAIEAIK.2	0.208792437	#N/A
EELEEVIKDI.2	0.049808348	#N/A
AIGSASEGAQSSLQEVYHK.3	-0.265183399	#N/A
PFGVALLFGGVDEK.2	-0.452154475	#N/A
AVLAQGQS[Phospho (STY)]SEQAAK.2	-0.33559432	Q9Z2V5_S909_M1_Hdac6
HNPQS[Phospho (STY)]PLQESSATLK.2	-0.057438582	Q9Z2V5_S21_M1_Hdac6
KTAS[Phospho (STY)]EPNLK.2	-0.307699011	Q9Z2V6_S250_M1_Hdac5
DEDGES[Phospho (STY)]GPDEGPDLEESSAGYKK.3	-0.101180703	Q9Z2V6_S600_M1_Hdac5
DEDGES[Phospho (STY)]GPDEGPDLEESSAGYK.2	0.355725717	Q9Z2V6_S600_M1_Hdac5
RSS[Phospho (STY)]PLLR.2	0.141475548	Q9Z2V6_S270_M1_Hdac5
VAERRS[Phospho (STY)]S[Phospho (STY)]PLLR.3	-0.26810498	Q9Z2V6_S269_M2_Hdac5
TQS[Phospho (STY)]SPAAPGSMK.2	-0.504384613	Q9Z2V6_S650_M1_Hdac5
ETAC[Carbamidomethyl (C)]TTGVLQTLTLFK.2	-0.134325777	#N/A
SPSPFHVVAEC[Carbamidomethyl (C)]R.3	-0.530354358	#N/A
ITGEAFVQFASQELAEK.2	-0.093945084	#N/A
LTAALLESTANVK.2	-0.254389642	#N/A
LQEVEIR.2	-0.208892833	#N/A
FGQWADSR.2	-0.173716126	#N/A
IFELTELR.2	0.266210824	#N/A
TELNQTVQELEETLK.2	0.332532364	#N/A
[Acetyl (Protein N-term)]GEQPIFSTR.2	0.348699439	#N/A
TLLEILDGK.2	-0.137262575	#N/A
AIINSTITPNM[Oxidation (M)]TFTK.2	-0.200677215	#N/A
TQALSHASS[Phospho (STY)]AISK.2	0.309844139	Q9Z2Y3_S189_M1_Homer1
IISLDGSK.2	0.35279005	#N/A
NKDLEGQLSDLEQR.3	0.14631551	#N/A
AEPTQNALPFPHSAGDR.3	0.426245323	#N/A
HWEAELATLK.2	-0.524578062	#N/A
DLEGQLS[Phospho (STY)]DLEQR.2	-0.053165461	Q9Z2Y3_S318_M1_Homer1
NKDLEGQLS[Phospho (STY)]DLEQRLEK.3	-0.217005523	Q9Z2Y3_S318_M1_Homer1
ANTVYGLGFSSEHHLSK.3	0.130401354	#N/A

HAVTVSYFYDSTR.2	-0.104314729	#N/A
IGS[Phospho (STY)]TIFGER.2	0.063920974	Q9Z2Y8_S244_M1_Plpbp
KGPTEPLK.2	0.212583325	#N/A
GKGS[Phospho (STY)]PQAQTVR.2	-0.225300931	Q9Z351_S429_M1_Kcnq2
RSPSADQSLDDS[Phospho (STY)]PSKVPK.3	-0.239481521	Q9Z351_S448_M1_Kcnq2
SPS[Phospho (STY)]ADQSLDDSPSK.2	0.053155083	Q9Z351_S440_M1_Kcnq2
SWS[Phospho (STY)]FGDR.2	0.099815377	Q9Z351_S457_M1_Kcnq2
YPQT[Phospho (STY)]WNGR.2	-0.264521947	Q9Z351_T287_M1_Kcnq2
S[Phospho (STY)]PSADQSLDDSPSKVPK.2	0.005339074	Q9Z351_S438_M1_Kcnq2
QNS[Phospho (STY)]EEASLPGEDIVEDNK.2	-0.176428043	Q9Z351_S479_M1_Kcnq2
EPEPAPPYHS[Phospho (STY)]PEDSR.2	0.202333782	Q9Z351_S681_M1_Kcnq2
EPEPAPPYHS[Phospho (STY)]PEDSRDHADK.3	0.248427789	Q9Z351_S681_M1_Kcnq2
RS[Phospho (STY)]PSADQSLDDSPSK.2	-0.281530683	Q9Z351_S438_M1_Kcnq2
SSPGSVAAS[Phospho (STY)]PSGGGGR.2	-0.02942375	S4R1M9_S66_M1_Osbpl10
ATSAGSS[Phospho (STY)]PSC[Carbamidomethyl (C)]SLAGR.2	0.052734575	S4R1M9_S32_M1_Osbpl10
