# Supplementary material for: The Chronic Effects of a Single Low-Intensity Blast Exposure on Phosphoproteome Networks and Cognitive Function Influenced by Mutant Tau Overexpression
Source: Int J Mol Sci. 2024 Mar 15;25(6):3338. doi: 10.3390/ijms25063338 (PMC10970429; doi:10.3390/ijms25063338)
Supplement: Supplementary file 1 [file ijms-25-03338-s001.zip › Supplementary Table S5- Phospep module assigment.pdf]

Supplementary Table S5. Phospep module assigment

PhospepName	Module (color)	Phos site
MSS[Phospho (STY)]VEEDSDTKR.2	black	D3YXK2_S406_M1_Safb
APS[Phospho (STY)]ESS[Phospho (STY)]DDQGQPAAK.2	black	E9PZ19_S794_M2_Igsf9b
ES[Phospho (STY)]RTPEELTILGETQEEEDEILPR.3	black	O35454_S23_M1_Clcn6
TNS[Phospho (STY)]PSIS[Phospho (STY)]PSMLSNAEHKR.3	black	O70305_S832_M2_Atxn2
VSVC[Carbamidomethyl (C)]AETFNPDEEEEDNDPR.2	black	#N/A
IVVQGEPGDDFYIITEGTASVLQR.3	black	#N/A
C[Carbamidomethyl (C)]PQIVIAFYEER.2	black	#N/A
TLDRS[Phospho (STY)]GDLGDMEPLK.3	black	P30999_S920_M1_Ctnnd1
S[Phospho (STY)]HTGEAAAVR.2	black	P59017_S387_M1_Bcl2l13
QWC[Carbamidomethyl (C)]NC[Carbamidomethyl (C)]AFLESSAK.2	black	#N/A
S[Phospho (STY)]PPTRKDDAAPNLHSEAR.4	black	Q5NBX1_S982_M1_Cobl
LNS[Phospho (STY)]DEEGESSGKR.2	black	Q5XJE5_S631_M1_Leo1
ASDPHLGTLS[Phospho (STY)]PEDSELETHDMFPDTIHSR.4	black	Q61161_S298_M1_Map4k2
SLS[Phospho (STY)]TEVEPK.2	black	Q6ZPJ0_S195_M1_Tex2
S[Phospho (STY)]PDPDPTGQNALDDSAASMK.2	black	Q80WT5_S395_M1_Aftph
DNSGSHGSPISGK.2	black	#N/A
T[Phospho (STY)]RSLVSQDHR.3	black	Q8C033_T353_M1_Arhgef10
SDEDKDKEGEALEVK.3	black	#N/A
MGLAMGGAGGAS[Phospho (STY)]FDR.2	black	Q9D0E1_S617_M1_Hnrnpm
RNS[Phospho (STY)]TTFPSR.2	black	Q9DB52_S59_M1_Pabir1
ISALQSAGVVVSM[Oxidation (M)]SPAQLGTTIYK.3	black	#N/A
RKPGAGGS[Phospho (STY)]PALAR.3	blue	A2AJI0_S401_M1_Map7d1
SNS[Phospho (STY)]QENVEASHPSQDGKR.3	blue	B2RUJ5_S573_M1_Apba1
M[Oxidation (M)]TDIM[Oxidation (M)]TEGITIVEDINKR.3	blue	#N/A
DSLGDFIEHYAQLGPSS[Phospho (STY)]PEQLAAGAEEGGGPR.4	blue	O35295_S113_M1_Purb
HTGPNS[Phospho (STY)]PDTANDGFVR.2	blue	O35737_S104_M1_Hnrnph1
SPS[Phospho (STY)]IDSIQKDPR.2	blue	O35927_S531_M1_Ctnnd2
EIAVGDSEGQIVIYDVGEQIAVPR.3	blue	#N/A
AS[Phospho (STY)]PQAKPLR.2	blue	O88737_S600_M1_Bsn
GGVVGIKVDK.2	blue	#N/A
RPWFAGDK.2	blue	#N/A
DDKHGS[Phospho (STY)]YENAVHSGALDD.3	blue	P11983_S544_M1_Tcp1
M[Oxidation (M)]LVDDIGDVTITNDGATILK.2	blue	#N/A
ASLSPM[Oxidation (M)]DEPVPDSES[Phospho (STY)]PVEK.2	blue	P14873_S1384_M1_Map1b
SAPTS[Phospho (STY)]PC[Carbamidomethyl (C)]DQELK.2	blue	P16054_S350_M1_Prkce
VAEDLESEGLM[Oxidation (M)]AEEVQAVQQQEVYGAM[Oxidation (M)]PR.3	blue	#N/A
AQALQEEC[Carbamidomethyl (C)]GYLR.2	blue	#N/A
GAKEEHGGLIRS[Phospho (STY)]PR.3	blue	P26369_S79_M1_U2af2
TTEQLIEAVNNGDFEAYAK.2	blue	#N/A
DSNGAILVYDVTDEDSFQK.2	blue	#N/A
NFRPDDPAR.2	blue	#N/A
LQSQLLSIEKEVDEYK.3	blue	#N/A
TVQIEASTVEIEER.2	blue	#N/A
SNVASTAAC[Carbamidomethyl (C)]SSASSLGLGLAYR.2	blue	#N/A
ALDEYYDKHFTEFVPLR.3	blue	#N/A
AGDVLEDS[Phospho (STY)]PKRPK.2	blue	P51859_S165_M1_Hdgf
VSEQGLIEILEK.2	blue	#N/A
C[Carbamidomethyl (C)]PEALFQPSFLGM[Oxidation (M)]ESC[Carbamidomethyl (C)]GIHETTFNSIM[Oxidation (M)]K.3	blue	#N/A
RADQLADESLESTRR.3	blue	#N/A

QHSAILAAPNPDEK.2	blue	#N/A
VLSVPESTPFTAVLK.2	blue	#N/A
LAPDYDALDVANK.2	blue	#N/A
LVDIIC[Carbamidomethyl (C)]DK.2	blue	#N/A
HWPFM[Oxidation (M)]VVNDAGRPK.3	blue	#N/A
M[Oxidation (M)]SATFIGNSTAIQELFKR.3	blue	#N/A
NS[Phospho (STY)]FTPLSSSNTIR.2	blue	P70265_S469_M1_Pfkfb2
NDQWNWDNIR.2	blue	#N/A
TLTSDVANLANEKEELNNK.3	blue	#N/A
VQDDEVGDGTTSVTVLAAELLR.2	blue	#N/A
IDTIAADESFTQVDIGDR.2	blue	#N/A
S[Phospho (STY)]GQLGLPDEENRR.2	blue	Q03517_S287_M1_Scg2
FKAFS[Phospho (STY)]GEGQK.2	blue	Q0KL01_S216_M1_Ubxn2b
AGS[Phospho (STY)]RDAAQERPSGPGGR.3	blue	Q14BB9_S82_M1_Map6d1
TASAVAGKT[Phospho (STY)]PDAS[Phospho (STY)]PEPKDQTLK.3	blue	Q3TXS7_S315_M2_Psmd1
QQSSSSKGDS[Phospho (STY)]PELKPR.3	blue	Q3UHC7_S978_M1_Dab2ip
RGS[Phospho (STY)]LPDNSILHR.3	blue	Q3UTJ2_S829_M1_Sorbs2
DSGLEDGRES[Phospho (STY)]PSFDTPSQR.3	blue	Q5DTL9_S89_M1_Slc4a10
TLTSGS[Phospho (STY)]RESNISSK.2	blue	Q5DTY9_S372_M1_Kctd16
IQPNDGPVFFK.2	blue	#N/A
HALGGS[Phospho (STY)]LEHLPR.3	blue	Q5SP85_S342_M1_Ccdc85a
DPQALSEHLK.2	blue	#N/A
[Acetyl (Protein N-term)]C[Carbamidomethyl (C)]NT[Phospho (STY)]PTYC[Carbamidomethyl (C)]DLGK.2	blue	Q60931_T4_M1_Vdac3
LTLDTIFVPNTGKK.2	blue	#N/A
GLGSDLDSSLASLVGNLGISGTTSK.2	blue	#N/A
VGKDELFALEQSC[Carbamidomethyl (C)]AQVVLQAANER.3	blue	#N/A
LVSQDNFGFDLPAVEAATK.2	blue	#N/A
TLETPAAQM[Oxidation (M)]EGFLNR.2	blue	#N/A
GVAPADS[Phospho (STY)]PDAPRR.2	blue	Q6ZPS6_S738_M1_Ankib1
ALGNS[Phospho (STY)]NRDKEIK.3	blue	Q7TT50_S481_M1_Cdc42bpb
SGFVPNVVHSS[Phospho (STY)]EEVIK.2	blue	Q8BHC1_S201_M1_Rab39b
GGLYNS[Phospho (STY)]M[Oxidation (M)]DSLDSNK.2	blue	Q8BJ42_S667_M1_Dlgap2
LEQEIQALESEESQISAK.2	blue	#N/A
LNQSDSIEDPNS[Phospho (STY)]PAGRR.2	blue	Q8BUK6_S238_M1_Hook3
NLETLQQELGIEGENR.2	blue	#N/A
QKFHDS[Phospho (STY)]EGDDTEETEDYR.3	blue	Q8K019_S395_M1_Bclaf1
RLS[Phospho (STY)]LPADIR.2	blue	Q8K0D0_S146_M1_Cdk17
SYS[Phospho (STY)]PDGKES[Phospho (STY)]PSDKK.2	blue	Q8K310_S604_M2_Matr3
S[Phospho (STY)]LEDLRAPK.2	blue	Q8K382_S592_M1_Dennd1a
RST[Phospho (STY)]PT[Phospho (STY)]PELTSK.2	blue	Q8VD37_T180_M2_Sgip1
HVPS[Phospho (STY)]PLNLEEVQKK.3	blue	Q8VD37_S371_M1_Sgip1
GTGDC[Carbamidomethyl (C)]S[Phospho (STY)]DEEVDGKADGADAK.2	blue	#N/A
M[Oxidation (M)]TVAHM[Oxidation (M)]WFDNQIHEADTTENQSGVSFDK.4	blue	#N/A
AM[Oxidation (M)]VESQQKS[Phospho (STY)]PVKR.3	blue	Q91ZU6_S1381_M1_Dst
SLLNKKS[Phospho (STY)]DGGVK.2	blue	Q923T9_S325_M1_Camk2g
GHLDNISNNLLIGAINIENGK.3	blue	#N/A
VHQLYETIQR.2	blue	#N/A
SPPREGS[Phospho (STY)]QGELTPANSQSR.3	blue	Q99LD4_S454_M1_Gps1
EIDQEAAVEVSQLRDPQHDLDR.3	blue	#N/A
S[Phospho (STY)]PSESAEETHTLEEK.2	blue	Q9CT10_S146_M1_Ranbp3
AVQLYQQTANVFENEER.2	blue	#N/A
SQEVDVAYIC[Carbamidomethyl (C)]TESSSHEDYIR.3	blue	#N/A

TDLTDYLNR.2	blue	#N/A
M[Oxidation (M)]VLAAAGGVEHQQLLDLAQK.3	blue	#N/A
INEILSNALK.2	blue	#N/A
GVGDGTVSWGLEDDEDM[Oxidation (M)]TLTR.2	blue	#N/A
LPNGLVIASLENYAPLSR.2	blue	#N/A
VNLLSFTGSTQVGK.2	blue	#N/A
QIS[Phospho (STY)]QDVKLEPDILLR.3	blue	Q9DBT5_S87_M1_Ampd2
M[Oxidation (M)]SSTFIGNSTAIQELFKR.3	blue	#N/A
GGLS[Phospho (STY)]DGEGPPGGRGEAQR.2	blue	Q9JIS5_S127_M1_Sv2a
VFLDPNTLSDDGTVALR.2	blue	#N/A
LEQEQEGEKDIATEKPGS[Phospho (STY)]PVK.3	blue	Q9QYB8_S594_M1_Add2
YFDRVDENNPEYLR.3	blue	#N/A
ESTFLDEGPNEQEITPLQHTPR.3	blue	#N/A
KVAELEEEQS[Phospho (STY)]QGSSSYSDWVK.3	blue	Q9QYR6_S114_M1_Map1a
M[Oxidation (M)]AS[Phospho (STY)]PPPSGPPSAAHTPFHQSPVEEK.4	blue	Q9QYR6_S991_M1_Map1a
IVDSGVQT[Phospho (STY)]DDEETADR.2	blue	Q9QYX7_T3376_M1_Pclo
DVLC[Carbamidomethyl (C)]GYLLC[Carbamidomethyl (C)]TNIGNIPR.2	blue	#N/A
LQSSDGEIFEVDVEIAK.2	blue	#N/A
HLS[Phospho (STY)]AEDFSR.2	blue	Q9WV69_S372_M1_Dmtn
VQENLLASGVDLVTYITR.3	blue	#N/A
M[Oxidation (M)]ES[Phospho (STY)]PIPILPLPTPDAETEK.2	blue	Q9Z2Q6_S327_M1_Septin5
LDQPVSAPPS[Phospho (STY)]PR.2	brown	D3YZP9_S237_M1_Ccdc6
HPS[Phospho (STY)]SQLAGPGVEGGEGTQKPR.3	brown	E9PUL5_S254_M1_Prrt2
VKEYEEEIHS[Phospho (STY)]LK.3	brown	F6SEU4_S1201_M1_Syngap1
SSS[Phospho (STY)]EITLSEC[Carbamidomethyl (C)]DVEEPGDPR.2	brown	G3X9J0_S166_M1_Sipa1l3
AQGLTEEDIVFLPQPDK.2	brown	#N/A
GAADMVAQAK.2	brown	#N/A
SGLS[Phospho (STY)]PANDTGAKK.2	brown	O70310_S47_M1_Nmt1
NM[Oxidation (M)]GGPYGGGNYGPGGSGGS[Phospho (STY)]GGYGGR.3	brown	O88569_S344_M1_Hnrnpa2b1
GENHC[Carbamidomethyl (C)]GIESEIVAGIPR.3	brown	#N/A
[Acetyl (Protein N-term)]S[Phospho (STY)]EAAPAAPAAAPPAEKAPAK.2	brown	P15864_S2_M1_H1-2
YTEHSTVGLAQQWDQLDQLGM[Oxidation (M)]R.3	brown	#N/A
YTVPLPSPVQDSENLSGESGSFYEGTDDKVR.3	brown	#N/A
QLLTLSNELSQAR.2	brown	#N/A
VGS[Phospho (STY)]TENIK.2	brown	P27546_S914_M1_Map4
NAYHAVYK.2	brown	#N/A
PAQYIAVHVVPDQLM[Oxidation (M)]TFSGTNDPC[Carbamidomethyl (C)]ALC[Carbamidomethyl (C)]SLHSIGK.4	brown	#N/A
LLEAGDFIC[Carbamidomethyl (C)]QALNR.2	brown	#N/A
SAS[Phospho (STY)]LENKK.2	brown	P39447_S1277_M1_Tjp1
VYC[Carbamidomethyl (C)]M[Oxidation (M)]LGDGEVSEGSVWEAM[Oxidation (M)]AFAGIYK.3	brown	#N/A
YLMEEDEDAYKK.3	brown	#N/A
AGVLSQADYLNLVQC[Carbamidomethyl (C)]ETLEDLK.3	brown	#N/A
SSDVPLPLELLET[Phospho (STY)]GGSPSK.2	brown	P51954_T615_M1_Nek1
VNEVDVSEVVHSR.3	brown	#N/A
S[Phospho (STY)]IDDSEMESPVDDVFYPGTGR.2	brown	P70257_S280_M1_Nfix
C[Carbamidomethyl (C)]AADLGLK.2	brown	#N/A
NVLLDPQLVPGGGASEM[Oxidation (M)]AVAHALTEK.3	brown	#N/A
LNNS[Phospho (STY)]LLSR.2	brown	P97292_S321_M1_Hrh2
RM[Oxidation (M)]S[Phospho (STY)]QEHPSQASEAELAQR.3	brown	P97412_S2614_M1_Lyst
SAGS[Phospho (STY)]FKR.2	brown	Q04690_S2831_M1_Nf1
EGPEGFAR.2	brown	#N/A
SFISSSPSS[Phospho (STY)]PSR.2	brown	Q3UTJ2_S382_M1_Sorbs2

S[Phospho (STY)]KDPGSPQLNQEAMADGVEGTPWSAEKPR.4	brown	Q3UZA1_S211_M1_Rcsd1
FANNPSQK.2	brown	#N/A
[Acetyl (Protein N-term)]SQGDSNPAAIPHAAEDIQGDDRWM[Oxidation (M)]SQHNR.4	brown	#N/A
YLEIC[Carbamidomethyl (C)]AADGQDAVFLR.2	brown	#N/A
C[Carbamidomethyl (C)]SPGWLADGSVR.2	brown	#N/A
FLM[Oxidation (M)]ANGQLVK.2	brown	#N/A
AYDATTHFETTC[Carbamidomethyl (C)]DDIKDIYKR.4	brown	#N/A
HC[Carbamidomethyl (C)]ILDVSANAVR.2	brown	#N/A
YS[Phospho (STY)]FSDDTKSPLSVPR.3	brown	Q62417_S254_M1_Sorbs1
QNS[Phospho (STY)]LEYM[Oxidation (M)]DQNDDRLK.3	brown	Q640R3_S280_M1_Hepacam
KTSFDQDS[Phospho (STY)]DVDIFPSDFTSEPPALPR.3	brown	Q64511_S1568_M1_Top2b
NDEELNKLLGK.2	brown	#N/A
NNSELLNNLGNFINR.2	brown	#N/A
RPPAKPR.2	brown	#N/A
ESC[Carbamidomethyl (C)]DSS[Phospho (STY)]PGLAEPEAKPQESR.3	brown	Q6Y685_S82_M1_Tacc1
FVDKYSALLS[Phospho (STY)]PSEQEKR.3	brown	Q6ZQK5_S521_M1_Acap2
ESESC[Carbamidomethyl (C)]DC[Carbamidomethyl (C)]LQGFQLTHSLGGGTGSGM[Oxidation (M)]GTLLISK.3	brown	#N/A
QPGM[Oxidation (M)]GPGS[Phospho (STY)]PVSDVDQEEPR.2	brown	Q7TPB0_S641_M1_Plppr3
EQM[Oxidation (M)]IQGQDS[Phospho (STY)]PEPDQLLATLEK.3	brown	Q7TSI3_S232_M1_Ppp6r1
SSVSSRQS[Phospho (STY)]DENVTR.2	brown	Q80TK0_S353_M1_Kiaa1107
S[Phospho (STY)]PTENVNTPVGKDPGLAGR.2	brown	Q80U28_S1058_M1_Madd
EPAEGNESSEAPS[Phospho (STY)]PVNAMNSFV.2	brown	Q810U4_S1247_M1_Nrcam
DMC[Carbamidomethyl (C)]LSS[Phospho (STY)]SKK.2	brown	Q8BG39_S144_M1_Sv2b
SGSS[Phospho (STY)]PPS[Phospho (STY)]PR.2	brown	Q8BHS8_S199_M2_Sybu
LIFQSC[Carbamidomethyl (C)]DYK.2	brown	#N/A
LTEDEEGNPQLQQHQLITVTM[Oxidation (M)]SSDSR.3	brown	#N/A
SS[Phospho (STY)]PELTR.2	brown	Q8BTI8_S1650_M1_Srrm2
APPT[Phospho (STY)]PGKGPAQAPQA.2	brown	Q8C166_T525_M1_Cpne1
SALNSES[Phospho (STY)]ASELELVAPAQAR.2	brown	Q8C437_S172_M1_Pex5l
LTSQDS[Phospho (STY)]YNNFTNNNPGNPR.2	brown	Q8CCN5_S503_M1_Bcas3
GSASSGSTTSPT[Phospho (STY)]C[Carbamidomethyl (C)]SPK.2	brown	Q8K070_T258_M1_Samd14
DNIISDYDQIIDYVER.2	brown	#N/A
RRGS[Phospho (STY)]M[Oxidation (M)]PVPYK.3	brown	Q8VEL9_S69_M1_Rem2
SAAYNGPPAS[Phospho (STY)]PSHETGAFAHAR.3	brown	Q8VHJ5_S633_M1_Mark1
QVDTEEAGM[Oxidation (M)]VTAATASNVKAS[Phospho (STY)]PKR.3	brown	#N/A
SSEM[Oxidation (M)]NVLVPQDDDYREFPIPEQFK.3	brown	#N/A
ELQSTFK.2	brown	#N/A
NTAGFSEDLDPSSKENS[Phospho (STY)]PPR.3	brown	Q9DAI6_S805_M1_Fam135b
ASADLM[Oxidation (M)]SYC[Carbamidomethyl (C)]EEHAR.2	brown	#N/A
AKGPSESS[Phospho (STY)]KER.2	brown	Q9DD18_S182_M1_Dtd1
LALESIC[Carbamidomethyl (C)]LLLGESTTDWK.2	brown	#N/A
YEAANWK.2	brown	#N/A
M[Oxidation (M)]ADSGGQPQLTQPGK.2	brown	#N/A
LETYYNATEPVISFYDKR.3	brown	#N/A
ENSAAPS[Phospho (STY)]PGRPQSASPAK.2	brown	Q9Z0P4_S116_M1_Palm
HLYSANTVDDFNR.2	brown	#N/A
SVYGVTDETQSQS[Phospho (STY)]PVHIPSQPIRK.3	brown	Q9Z2H2_S244_M1_Rgs6
TQS[Phospho (STY)]SPAAPGSMK.2	brown	Q9Z2V6_S650_M1_Hdac5
SPSPFHVVAEC[Carbamidomethyl (C)]R.3	brown	#N/A
SYDYQSVC[Carbamidomethyl (C)]DPGAAPK.2	green	#N/A
STM[Oxidation (M)]VGTPYWM[Oxidation (M)]APEVVTR.2	green	#N/A
QIAAVM[Oxidation (M)]QR.2	green	#N/A

DNSTM[Oxidation (M)]GYMAAK.2	green	#N/A
RTLS[Phospho (STY)]IDKGF.2	green	P12382_S775_M1_Pfkl
VNEVSQFAAK.2	green	#N/A
LVPHQMAH.2	green	#N/A
S[Phospho (STY)]TVASMMHR.2	green	P28652_S276_M1_Camk2b
LAEM[Oxidation (M)]PADSGYPAYLGAR.2	green	#N/A
GKLDGNQDLIR.2	green	#N/A
RWS[Phospho (STY)]APETR.2	green	P54254_S751_M1_Atxn1
[Acetyl (Protein N-term)]AAAMDVDTPSGTNSGAGK.2	green	#N/A
YDDM[Oxidation (M)]AAC[Carbamidomethyl (C)]M[Oxidation (M)]K.2	green	#N/A
IVS[Phospho (STY)]PKQEHEDR.2	green	Q50H33_S331_M1_Kctd8
TSFDEM[Oxidation (M)]LPGTHFQR.3	green	#N/A
HRPDLIDFDK.3	green	#N/A
M[Oxidation (M)]TVAHM[Oxidation (M)]WFDNQIHEADTTEDQSGTSFDK.4	green	#N/A
KDS[Phospho (STY)]ISEDEM[Oxidation (M)]VLR.2	green	Q80ZK9_S511_M1_Wdtc1
KDYLMENEALPS[Phospho (STY)]P.2	green	Q8BGF8_S421_M1_Slc35d3
RS[Phospho (STY)]PTSTGDTELKEER.3	green	Q8CIP4_S438_M1_Mark4
DFS[Phospho (STY)]PEALKK.2	green	Q91WK2_S183_M1_Eif3h
[Acetyl (Protein N-term)]S[Phospho (STY)]GDEM[Oxidation (M)]IFDPTM[Oxidation (M)]SK.2	green	#N/A
THTPSPTS[Phospho (STY)]PK.2	green	Q9CYT6_S311_M1_Cap2
TVVGQITVDM[Oxidation (M)]M[Oxidation (M)]YGGM[Oxidation (M)]R.2	green	#N/A
AM[Oxidation (M)]DATLS[Phospho (STY)]PTKTETVK.2	green	Q9QXZ0_S4458_M1_Macf1
HQTMEVS[Phospho (STY)]C[Carbamidomethyl (C)].2	green	Q9QYF9_S374_M1_Ndrg3
FC[Carbamidomethyl (C)]TTGIDGAM[Oxidation (M)]TIWDFK.2	green	#N/A
DNNLELVQEILR.2	green	#N/A
KANIPIMDTGENPEVPFPR.3	green	#N/A
IVHSES[Phospho (STY)]QPEKESR.2	grey	A2ALU4_S1089_M1_Shroom2
VPLHLES[Phospho (STY)]PHEER.3	grey	D3YXJ0_S1067_M1_Dgkh
DLQQYQSQAK.2	grey	#N/A
LC[Carbamidomethyl (C)]DFNPK.2	grey	#N/A
M[Oxidation (M)]TDIM[Oxidation (M)]TEGITIVEDINK.2	grey	#N/A
SVLWVSADGLR.2	grey	#N/A
DLVLANLASPSS[Phospho (STY)]PAHK.2	grey	O35144_S367_M1_Terf2
EVIC[Carbamidomethyl (C)]QVLYLGNVIDYK.2	grey	#N/A
KGS[Phospho (STY)]ITEYTATEEK.2	grey	O54940_S114_M1_Bnip2
MSDEEILEK.2	grey	#N/A
LTDFGFC[Carbamidomethyl (C)]AQITPEQSK.2	grey	#N/A
EYLPIGGLAEFC[Carbamidomethyl (C)]K.2	grey	#N/A
SGS[Phospho (STY)]GYEGSTSWK.2	grey	P0C7M9_S47_M1_Clec2l
M[Oxidation (M)]DSTEPPYSQK.2	grey	#N/A
S[Phospho (STY)]TVASC[Carbamidomethyl (C)]M[Oxidation (M)]HR.2	grey	P11798_S275_M1_Camk2a
AAHFVFR.2	grey	#N/A
RSES[Phospho (STY)]PFEGK.2	grey	P14873_S1422_M1_Map1b
ITGTNAEVM[Oxidation (M)]PAQWEFQIGPC[Carbamidomethyl (C)]EGIR.3	grey	#N/A
DC[Carbamidomethyl (C)]EQAENWM[Oxidation (M)]AAR.2	grey	#N/A
AKFEELNM[Oxidation (M)]DLFR.3	grey	#N/A
ELVDDSITNVKK.3	grey	#N/A
ELVSC[Carbamidomethyl (C)]SNC[Carbamidomethyl (C)]TDYQAR.2	grey	#N/A
QAVTNPNNTFYATK.2	grey	#N/A
VYSPHVLNLTLVDLPGM[Oxidation (M)]TK.3	grey	#N/A
AVPVS[Phospho (STY)]PSAVEEDEDEDGHTVVATAR.3	grey	P39447_S1614_M1_Tjp1
NEFNLESK.2	grey	#N/A

TIGISVDPR.2	grey	#N/A
EAINVEQAFQTIAR.2	grey	#N/A
FT[Phospho (STY)]DTRKDEQER.2	grey	P58252_T57_M1_Eef2
RDS[Phospho (STY)]LGSFSTR.2	grey	P58871_S866_M1_Tnks1bp1
INVNEIFYDLVR.2	grey	#N/A
VIVWDSFTTNK.2	grey	#N/A
VC[Carbamidomethyl (C)]NPIITK.2	grey	#N/A
IHIDPESQDC[Carbamidomethyl (C)]S[Phospho (STY)]PATSR.2	grey	P70271_S111_M1_Pdlim4
TC[Carbamidomethyl (C)]TIILR.2	grey	#N/A
VEEVLEEEEEEYVVEK.2	grey	#N/A
LINSELGS[Phospho (STY)]PSR.2	grey	Q3UDW8_S238_M1_Hgsnat
KTDPEFSGKLT[Phospho (STY)]PGSSGK.3	grey	Q3UNH4_T256_M1_Gprin1
VS[Phospho (STY)]VS[Phospho (STY)]PGR.2	grey	Q52KI8_S427_M2_Srrm1
KSS[Phospho (STY)]LGNDETDKEK.2	grey	Q5NBX1_S235_M1_Cobl
ETAQTDS[Phospho (STY)]PQPQER.2	grey	Q5PR69_S487_M1_Crad
VAVFGAGGVGK.2	grey	#N/A
FQVDISSSEGPEPS[Phospho (STY)]PR.2	grey	Q5RJI5_S676_M1_Brsk1
SSM[Oxidation (M)]S[Phospho (STY)]GLHLVK.2	grey	Q5SWU9_S79_M1_Acaca
T[Phospho (STY)]RYGSQEHPIGDK.3	grey	Q60790_T805_M1_Rasa3
LTSSVTAYDYSGK.2	grey	#N/A
VLHM[Oxidation (M)]DQNPYYGGESASITPLEDLYK.3	grey	#N/A
EQLM[Oxidation (M)]NSSLGS[Phospho (STY)]GTASLR.2	grey	Q62108_S418_M1_Dlg4
VAHM[Oxidation (M)]EFC[Carbamidomethyl (C)]YQELC[Carbamidomethyl (C)]QLAAER.3	grey	#N/A
LS[Phospho (STY)]PVPVPR.2	grey	Q64012_S135_M1_Raly
TLVLSSSPTS[Phospho (STY)]PTQEPLAAAK.2	grey	Q68EF0_S218_M1_Rab3ip
YYGGGGGGGAAGGGS[Phospho (STY)]PGDKAEGR.3	grey	Q68EF6_S463_M1_Begain
ARS[Phospho (STY)]M[Oxidation (M)]DSSDLSDGAVTLQEYLELKK.3	grey	Q68FF6_S419_M1_Git1
AAAEDAWHDEKPPQS[Phospho (STY)]PR.3	grey	Q69ZB3_S41_M1_Tspyl5
SDS[Phospho (STY)]EATISR.2	grey	Q6A065_S1157_M1_Cep170
RAS[Phospho (STY)]TDLGK.2	grey	Q6NSW3_S841_M1_Sphkap
FNS[Phospho (STY)]YDISR.2	grey	Q6PAL8_S193_M1_Dennd5a
LSTIALALGVER.2	grey	#N/A
MAGDPVANVR.2	grey	#N/A
QQGVLQSS[Phospho (STY)]PK.2	grey	Q7TME0_S594_M1_Plppr4
DSESC[Carbamidomethyl (C)]ESLKDSFGS[Phospho (STY)]GDR.2	grey	Q7TME0_S685_M1_Plppr4
ILAGDKNYITEDELR.3	grey	#N/A
ASIHEAWTDGK.2	grey	#N/A
KSQS[Phospho (STY)]IDEIDVGTYK.2	grey	Q80TE7_S949_M1_Lrrc7
AVLETPSTQGQDGS[Phospho (STY)]PESLIR.2	grey	Q80TT8_S284_M1_Cul9
SAATS[Phospho (STY)]PTGSSHGTPTHQSK.3	grey	Q80TZ3_S683_M1_Dnajc6
TALPTSGS[Phospho (STY)]STGELELLAGEVPAR.2	grey	Q80VP1_S418_M1_Epn1
AILITGC[Carbamidomethyl (C)]DSGFGFSLAK.2	grey	#N/A
EGETTVNTETSS[Phospho (STY)]PK.2	grey	Q80XP9_S1039_M1_Wnk3
EQM[Oxidation (M)]M[Oxidation (M)]NSSVSSGSGS[Phospho (STY)]LR.2	grey	Q811D0_S575_M1_Dlg1
APS[Phospho (STY)]AS[Phospho (STY)]PLAIHASR.2	grey	Q8BGT6_S494_M2_Micall1
SAS[Phospho (STY)]PLAATR.2	grey	Q8BKA3_S271_M1_Srrm4
QHLENDPGS[Phospho (STY)]NEDADIPK.2	grey	Q8CDN6_S113_M1_Txnl1
SDLGNLLK.2	grey	#N/A
TGTFS[Phospho (STY)]PQR.2	grey	Q8JZP9_S292_M1_Gas2l1
SLS[Phospho (STY)]IESLC[Carbamidomethyl (C)]PDEGR.2	grey	Q8K031_S234_M1_Stard8
S[Phospho (STY)]PGPPPPVGVK.2	grey	Q8K3X4_S69_M1_Irf2bpl
KAS[Phospho (STY)]EEEYVIR.2	grey	Q8K4I3_S639_M1_Arhgef6

HSNHS[Phospho (STY)]TENS[Phospho (STY)]PIERR.3	grey	Q8R0S4_S443_M2_Cacnb4
SGT[Phospho (STY)]IQSGPEGNLVR.2	grey	Q8R502_T198_M1_Lrrc8c
KQES[Phospho (STY)]FC[Carbamidomethyl (C)]R.2	grey	Q8VD04_S237_M1_Gripap1
GHAGGQRPEPSS[Phospho (STY)]PDGPAPPTRR.4	grey	Q8VEL9_S296_M1_Rem2
HNEETGDNVGPLIIK.2	grey	#N/A
AGEASAESTGAADGAAPEEGEAKKTEAPAAAGPEAK.4	grey	#N/A
QVVNFGPGPAK.2	grey	#N/A
EVSPDS[Phospho (STY)]SEAEELSDC[Carbamidomethyl (C)]LHR.3	grey	Q99LI2_S58_M1_Clcc1
SLLEGLLQR.2	grey	#N/A
EHGYLGNLSAVASTEGTIEETLNEASR.3	grey	#N/A
ANKPGDVVR.2	grey	#N/A
QTTVSNSQQAYQEAFEISK.2	grey	#N/A
NAHVTDPR.2	grey	#N/A
IEILDS[Phospho (STY)]PASK.2	grey	Q9CX66_S177_M1_D10Wsu102e
HSIS[Phospho (STY)]M[Oxidation (M)]PVM[Oxidation (M)]R.2	grey	Q9CYZ2_S168_M1_Tpd52l2
TGQEIPVNLR.2	grey	#N/A
[Acetyl (Protein N-term)]SALT[Phospho (STY)]PPTDM[Oxidation (M)]PT[Phospho (STY)]PTTDKITQAAM[Oxidation (M)]ETIYLC[Carbamidomethyl (C)]K.3	grey	Q9D394_T12_M2_Rufy3
AHRPSAPS[Phospho (STY)]PPPR.3	grey	Q9D7V2_S24_M1_Lysmd2
HNS[Phospho (STY)]VEDAEQGK.2	grey	Q9D7X1_S20_M1_Kctd4
ENM[Oxidation (M)]AYTVEGIR.2	grey	#N/A
SLIHLSLANNNLQTLPK.3	grey	#N/A
LLVEAEQAK.2	grey	#N/A
EQDHFLISPK.2	grey	#N/A
KSS[Phospho (STY)]TS[Phospho (STY)]FDDDAGR.2	grey	Q9QYX7_S1551_M2_Pclo
GSVPAAAAES[Phospho (STY)]PSM[Oxidation (M)]HR.2	grey	Q9QYX7_S75_M1_Pclo
RAFS[Phospho (STY)]TEK.2	grey	Q9QZB7_S414_M1_Actr10
SQTDQLVLIFAGK.2	grey	#N/A
VTNAIS[Phospho (STY)]PESSPGVGR.2	grey	Q9R0L6_S65_M1_Pcm1
NVRS[Phospho (STY)]DVS[Phospho (STY)]DQEEDEESER.2	grey	Q9R0L6_S1766_M2_Pcm1
HLSSGDLLR.2	grey	#N/A
RVPGSS[Phospho (STY)]GHLHK.3	grey	Q9Z1W9_S383_M1_Stk39
HWEAELATLK.2	grey	#N/A
PhospepName	Module (color)	Phos site
SSAIQM[Oxidation (M)]GT[Phospho (STY)]PELEKETK.2	pink	D3YVF0_T220_M1_Akap5
TKEQVTNVGGAVVTGVTAVAQK.3	pink	#N/A
VVTVFSVADGYSENNVFYGHHAK.3	pink	#N/A
VKVDNQHDFQDIASVVALTK.3	pink	#N/A
TVPPAVPGVTFLSGGQSEEEASLNLNAINR.3	pink	#N/A
TFTTQETITNAETAKEWFLEAAK.3	pink	#N/A
SM[Oxidation (M)]S[Phospho (STY)]ADEDLQEPSR.2	pink	P19426_S115_M1_Nelfe
DDFLQQNGYTPYDR.2	pink	#N/A
HGGYQPSDEHKTDLNPDNLQGGDDLDPNYVLSSR.5	pink	#N/A
WNTDNTLGTEITVEDQLAR.2	pink	#N/A
IFVGGIKEDTEEYNLR.3	pink	#N/A
DKEEDM[Oxidation (M)]LEVLLDATKR.3	pink	#N/A
NHS[Phospho (STY)]LEEEFER.2	pink	Q8C437_S202_M1_Pex5l
S[Phospho (STY)]LPNLSR.2	pink	Q8CI08_S414_M1_Slain2
ISRPGDS[Phospho (STY)]DDSR.2	pink	Q8R081_S182_M1_Hnrnpl
GAGAYIC[Carbamidomethyl (C)]GEETALIESIEGK.2	pink	#N/A
DAADPS[Phospho (STY)]PPNK.2	pink	Q924H7_S53_M1_Wac
TGALC[Carbamidomethyl (C)]WFLDEAAAR.2	pink	#N/A
TYYM[Oxidation (M)]SAGLQPVPIVFR.2	pink	#N/A

KPS[Phospho (STY)]DEEFAVRK.3	pink	Q9ES28_S673_M1_Arhgef7
ITVQVEPVC[Carbamidomethyl (C)]GVGVVPK.2	red	#N/A
SC[Carbamidomethyl (C)]GQVVEEWKEFVWNPR.3	red	#N/A
SST[Phospho (STY)]PEEVKKR.2	red	P18760_T25_M1_Cfl1
NM[Oxidation (M)]APGAGC[Carbamidomethyl (C)]S[Phospho (STY)]PGEPR.2	red	P58871_S1290_M1_Tnks1bp1
QGANINEIR.2	red	#N/A
RRDPS[Phospho (STY)]LEEIQK.3	red	P63042_S90_M1_Stmn4
EAFEISK.2	red	#N/A
NSRPSS[Phospho (STY)]PVNTPSSQPPAAK.3	red	Q03173_S355_M1_Enah
DRT[Phospho (STY)]PPLLYR.2	red	Q0VBL3_T567_M1_Rbm15
ERDSGLEDGRES[Phospho (STY)]PSFDTPSQR.4	red	Q5DTL9_S89_M1_Slc4a10
EM[Oxidation (M)]TTS[Phospho (STY)]ADQIAEVK.2	red	Q5SW75_S487_M1_Ssh2
[Acetyl (Protein N-term)]ADKEAAFDDAVEER.2	red	#N/A
S[Phospho (STY)]KS[Phospho (STY)]PVDLRDK.2	red	Q61136_S292_M2_Prpf4b
DTPGFIVNR.2	red	#N/A
GGGGS[Phospho (STY)]GQDNGLEGLGSDGR.2	red	Q62376_S408_M1_Snrnp70
S[Phospho (STY)]AESLLESTK.2	red	Q62417_S722_M1_Sorbs1
LEASYSDS[Phospho (STY)]PPGEEDLLVHVAEGSK.3	red	Q68FE2_S18_M1_Atg9a
IS[Phospho (STY)]ENPRRSPTHEM[Oxidation (M)]NPR.4	red	Q7M750_S84_M1_Opalin
SYS[Phospho (STY)]TESYGASQTR.2	red	Q80TE7_S1206_M1_Lrrc7
RQS[Phospho (STY)]LGGFLK.2	red	Q80YN3_S601_M1_Bcas1
DKFS[Phospho (STY)]PTQDRPESSTVLK.3	red	Q8BTI8_S1151_M1_Srrm2
FTQGLEDQLS[Phospho (STY)]PPGNR.2	red	Q8C008_S768_M1_Dzank1
TNPPTQKPPS[Phospho (STY)]PPVSGR.2	red	Q8CBW3_S183_M1_Abi1
GRS[Phospho (STY)]LNIADQEGTLLGK.3	red	Q8CH09_S206_M1_Sugp2
ADAQT[Phospho (STY)]PEEVGPSEASLR.2	red	Q8VE99_T90_M1_Ccdc115
RNS[Phospho (STY)]AEETWM[Oxidation (M)]VIHGR.3	red	Q9CQX2_S33_M1_Cyb5b
HPDEPVLLEEPVVLALAEK.3	red	#N/A
[Acetyl (Protein N-term)]ATDDKSS[Phospho (STY)]PTLDSANDLPR.2	red	Q9Z1T6_S8_M1_Pikfyve
TDPGS[Phospho (STY)]IENLC[Carbamidomethyl (C)]PGK.2	turquoise	A2ARP1_S939_M1_Ppip5k1
TGGEGS[Phospho (STY)]LDEEAEGSKK.2	turquoise	A2TJV2_S565_M1_Palm3
KIVEPEVVGES[Phospho (STY)]DS[Phospho (STY)]EVEGDAWR.3	turquoise	C0HKD8_S116_M2_Mfap1a;Mfap1b
YAIAVNDLGTEYVHR.2	turquoise	#N/A
KLS[Phospho (STY)]SQPSTDVSTDKER.3	turquoise	E9Q6J5_S236_M1_Bod1l
DDSLDLS[Phospho (STY)]PQGR.2	turquoise	G5E870_S1024_M1_Trip12
HIAEVSQEVTR.2	turquoise	#N/A
IGGVQQDTILAEGLHFR.3	turquoise	#N/A
FFPLEAWQIGK.2	turquoise	#N/A
EGEEPTVYS[Phospho (STY)]DDEEPKDETARK.4	turquoise	O55022_S181_M1_Pgrmc1
FLHGVIVAADSR.3	turquoise	#N/A
ALEENNNFSR.2	turquoise	#N/A
[Acetyl (Protein N-term)]S[Phospho (STY)]YTPGIGGDSAQLAQR.2	turquoise	O70439_S2_M1_Stx7
S[Phospho (STY)]AAEELELTVLK.2	turquoise	O88327_S373_M1_Ctnnal1
RFS[Phospho (STY)]GNEYILATK.2	turquoise	O88502_S452_M1_Pde8a
M[Oxidation (M)]FASFPTTK.2	turquoise	#N/A
VDPVNFK.2	turquoise	#N/A
EGDGSATTDAAPATS[Phospho (STY)]PKAEEPSKAGDAPSEEK.4	turquoise	#N/A
QLFALSC[Carbamidomethyl (C)]AAEEQGM[Oxidation (M)]LPEDLSGVIR.3	turquoise	#N/A
NGDTITIK.2	turquoise	#N/A
TNVLGHLQQGGAPTPFDR.3	turquoise	#N/A
ERLDQLFEM[Oxidation (M)]ADQYQYQVVLVEPK.3	turquoise	#N/A
PPYTIVYFPVR.2	turquoise	#N/A

LESIISGAALM[Oxidation (M)]ADSSC[Carbamidomethyl (C)]TR.2	turquoise	#N/A
DDGQSDS[Phospho (STY)]QAVDGDGK.2	turquoise	P26339_S282_M1_Chga
LVVVGAGGVGK.2	turquoise	#N/A
DKGS[Phospho (STY)]PHSEGSDRYR.3	turquoise	P35436_S1198_M1_Grin2a
EVSVFGAVSELFTR.2	turquoise	#N/A
TQSIY[Phospho (STY)]DDKNHRESNSNQC[Carbamidomethyl (C)]VYAAHNSVVIDEC[Carbamidomethyl (C)]K.5	turquoise	P43006_Y522_M1_Slc1a2
ELPVNAQNYVR.2	turquoise	#N/A
LLC[Carbamidomethyl (C)]GGGAAADR.2	turquoise	#N/A
QAWTGSS[Phospho (STY)]QR.2	turquoise	P54285_S418_M1_Cacnb3
[Acetyl (Protein N-term)]ATNFLAHEK.2	turquoise	#N/A
GLTLS[Phospho (STY)]DDLER.2	turquoise	P59325_S227_M1_Eif5
NYLSQPR.2	turquoise	#N/A
QTFTGHESDINAIC[Carbamidomethyl (C)]FFPNGNAFATGSDDATC[Carbamidomethyl (C)]R.3	turquoise	#N/A
IPS[Phospho (STY)]S[Phospho (STY)]PDLK.2	turquoise	P63141_S440_M2_Kcna2
EFTEAVEAK.2	turquoise	#N/A
EWHHFLVVNM[Oxidation (M)]K.2	turquoise	#N/A
ATISNDGATILK.2	turquoise	#N/A
ALIAGGGAPEIELALR.2	turquoise	#N/A
GPGTGGDEASAAGGS[Phospho (STY)]PPQPPR.2	turquoise	P81122_S66_M1_Irs2
QHEVEIPSPT[Phospho (STY)]QKEK.3	turquoise	Q01063_T289_M1_Pde4d
NAEPPS[Phospho (STY)]PTPAAHTR.2	turquoise	Q01341_S53_M1_Adcy6
KTS[Phospho (STY)]FDENDS[Phospho (STY)]EELEDKDSK.3	turquoise	Q05D44_S108_M2_Eif5b
SESS[Phospho (STY)]DFEVVPK.2	turquoise	Q3TLH4_S1222_M1_Prrc2c
SAT[Phospho (STY)]PATDGR.2	turquoise	Q3UHL1_T438_M1_Camkv
S[Phospho (STY)]TQSLSLQR.2	turquoise	Q3UPL5_S217_M1_Ag2
AKESDLSDTLSPS[Phospho (STY)]KEK.2	turquoise	Q3URD3_S471_M1_Slmap
KATGVS[Phospho (STY)]AEDADPR.2	turquoise	Q4KMM3_S308_M1_Oxr1
KQHRES[Phospho (STY)]GEGEEEVADSAR.3	turquoise	Q505F5_S293_M1_Lrrc47
VNLS[Phospho (STY)]PVTPAK.2	turquoise	Q5PR69_S552_M1_Crad
QLS[Phospho (STY)]LEGAGLAMEEFK.2	turquoise	Q5SV85_S744_M1_Synrg
ELGYPQEDDEEDEDEEEDEEEDSQAEVLK.3	turquoise	#N/A
LM[Oxidation (M)]DVGLIAIR.2	turquoise	#N/A
VLATAFDTTLGGR.2	turquoise	#N/A
KVTGTLDANR.2	turquoise	#N/A
LVVPASQC[Carbamidomethyl (C)]GSLIGK.2	turquoise	#N/A
QRS[Phospho (STY)]ATS[Phospho (STY)]DLELR.2	turquoise	Q62407_S490_M2_Speg
S[Phospho (STY)]RSPNRFDAHDPR.4	turquoise	Q62504_S188_M1_Spen
LESLPAQLPR.2	turquoise	#N/A
IHYLDTTTLIEPVAR.3	turquoise	#N/A
S[Phospho (STY)]PPQYC[Carbamidomethyl (C)]GAK.2	turquoise	Q64336_S549_M1_Tbr1
SS[Phospho (STY)]LS[Phospho (STY)]RER.2	turquoise	Q6NZJ6_S1147_M2_Eif4g1
YSGSRS[Phospho (STY)]PGFSR.2	turquoise	Q6PGN3_S307_M1_Dclk2
M[Oxidation (M)]TVAHM[Oxidation (M)]WFDNQIHEADTTEDQSGATFDKR.4	turquoise	#N/A
SPS[Phospho (STY)]PAVPLR.2	turquoise	Q6PIJ4_S228_M1_Nfrkb
VKEFC[Carbamidomethyl (C)]ENLSADC[Carbamidomethyl (C)]R.2	turquoise	#N/A
ESNQPPEDSS[Phospho (STY)]PPASSESSSTR.2	turquoise	Q7TMY8_S2918_M1_Huwe1
GEGGTTNPHVFPEGSEPK.3	turquoise	#N/A
DLNS[Phospho (STY)]LEDTEDGVDLVSSR.2	turquoise	Q80T23_S358_M1_Sytl5
SC[Carbamidomethyl (C)]DS[Phospho (STY)]DEDYEAGGTGR.2	turquoise	Q80TI1_S739_M1_Plekhh1
RGT[Phospho (STY)]VEGSVQEVQEEKEAEASAPVVQEESSINR.4	turquoise	Q80TL4_T47_M1_Phf24
KQES[Phospho (STY)]PLLVTK.2	turquoise	Q80U62_S198_M1_Rubcn
ASM[Oxidation (M)]SEFLES[Phospho (STY)]EDGEVEQQR.2	turquoise	Q80U70_S548_M1_Suz12

TDAGSLYELTVR.2	turquoise	#N/A
EEDEEAES[Phospho (STY)]PPEKK.2	turquoise	Q80XU3_S214_M1_Nucks1
QQS[Phospho (STY)]DGSLVR.2	turquoise	Q80YF9_S995_M1_Arhgap33
S[Phospho (STY)]AKSEESLSSQASGAGLQR.3	turquoise	Q80YF9_S657_M1_Arhgap33
S[Phospho (STY)]KSM[Oxidation (M)]TAELEELGLSLVDK.3	turquoise	Q80Z38_S370_M1_Shank2
DSTGTYTC[Carbamidomethyl (C)]VAR.2	turquoise	#N/A
DFDFTSTAAS[Phospho (STY)]EEDLFSEDERK.3	turquoise	Q811C2_S434_M1_Atg4c
EGMTAFVEK.2	turquoise	#N/A
DGS[Phospho (STY)]WFLK.2	turquoise	Q8BJ42_S873_M1_Dlgap2
HSLDASQGTATGPR.2	turquoise	#N/A
IAFDAESEPNNSSGS[Phospho (STY)]M[Oxidation (M)]EK.2	turquoise	Q8BPU7_S344_M1_Elmo1
SAS[Phospho (STY)]VPDELGAWGHSR.2	turquoise	Q8BQS4_S162_M1_Fam102b
EELEQQT[Phospho (STY)]DGDC[Carbamidomethyl (C)]DEEDDDKDGEVPK.3	turquoise	Q8BU14_T375_M1_Sec62
VGVLTVSDSC[Carbamidomethyl (C)]FR.2	turquoise	#N/A
VGVPTETGALTLNR.2	turquoise	#N/A
LTALPDYTLTLR.2	turquoise	#N/A
EYGS[Phospho (STY)]TSSIDK.2	turquoise	Q8C0T5_S208_M1_Sipa1l1
SKS[Phospho (STY)]ETGDSSIFRK.2	turquoise	Q8C0T5_S288_M1_Sipa1l1
GIITDS[Phospho (STY)]FGR.2	turquoise	Q8K2Y9_S393_M1_Ccm2
RRT[Phospho (STY)]EEGPTLSYGR.3	turquoise	Q8K310_T150_M1_Matr3
VPGGS[Phospho (STY)]PRT[Phospho (STY)]PNR.2	turquoise	Q8R1Q8_S405_M2_Dync1li1
VASGDLQVQR.2	turquoise	#N/A
GKHFY[Phospho (STY)]SS[Phospho (STY)]S[Phospho (STY)]ES[Phospho (STY)]EEEEESHKK.3	turquoise	Q8VD37_S104_M3_Sgip1
AS[Phospho (STY)]PVPEDHAEAAGAEQGGESTEGNAKPK.4	turquoise	Q8VE52_S65_M1_Ogfrl1
[Acetyl (Protein N-term)]MEEGPAVGTLS[Phospho (STY)]R.2	turquoise	Q8VED2_S11_M1_Bloc1s4
DLPEHAVLK.2	turquoise	#N/A
ATNIEVLSNTFQFTNEAR.2	turquoise	#N/A
AAEAHPQDDHVEEEC[Carbamidomethyl (C)]FHDC[Carbamidomethyl (C)]SASFEEEQPGAHVAGSK.5	turquoise	#N/A
AEGSEYQVLYIADDNEIR.2	turquoise	#N/A
GLLPEELTPLILETQK.2	turquoise	#N/A
GEVNAADAFDIGS[Phospho (STY)]FDEEDTK.2	turquoise	Q99MK8_S487_M1_Grk2
LSAS[Phospho (STY)]PQEVGKPYLESFQPNLHITK.3	turquoise	Q99P72_S768_M1_Rtn4
VSEPVEIGIQVTPDEDDHLLS[Phospho (STY)]PTK.3	turquoise	Q99PV8_S128_M1_Bcl11b
GAQAAIVVYDITNEESFAR.2	turquoise	#N/A
NSERKT[Phospho (STY)]PEGR.2	turquoise	Q9CZW5_T88_M1_Tomm70
ELAPYDENWFYTR.2	turquoise	#N/A
WKEPGSSGPQNLS[Phospho (STY)]GPGGR.3	turquoise	Q9DB90_S32_M1_Smg9
RGS[Phospho (STY)]GHPAYAEVEPVGEK.3	turquoise	Q9DC11_S507_M1_Plxdc2
KVYDQM[Oxidation (M)]PEPR.2	turquoise	#N/A
GS[Phospho (STY)]PPVPSGPPMEEDGLR.2	turquoise	Q9EP97_S206_M1_Senp3
IPDSTHAQLESSSSSFES[Phospho (STY)]QK.3	turquoise	Q9EQZ7_S788_M1_Rims2
LFSSSSS[Phospho (STY)]PPPAK.2	turquoise	Q9ESN9_S586_M1_Mapk8ip3
ELLLC[Carbamidomethyl (C)]NNR.2	turquoise	#N/A
YDSTHYK.2	turquoise	#N/A
LLDAQLSTGGIVDPSK.2	turquoise	#N/A
SSS[Phospho (STY)]LEGFHSQYK.2	turquoise	Q9QYY0_S420_M1_Gab1
KAS[Phospho (STY)]AQASLASK.2	turquoise	Q9R0L6_S1280_M1_Pcm1
AEAPPLQREDS[Phospho (STY)]GTFSLGK.3	turquoise	Q9WTX2_S18_M1_Prkra
QC[Carbamidomethyl (C)]QPTDTVC[Carbamidomethyl (C)]ASVR.2	turquoise	#N/A
DQFS[Phospho (STY)]PDDFLGR.2	turquoise	Q9Z0R4_S1663_M1_Itsn1
AIGHVIEEGGVK.2	turquoise	#N/A
NEKS[Phospho (STY)]EEEQSSASVKK.3	turquoise	Q9Z204_S241_M1_Hnrnpc

KAQEETPQQPEAAAAVTT[Phospho (STY)]PVT[Phospho (STY)]PAGHSHPETNSNEK.4	turquoise	Q9Z2H5_T30_M2_Epb41l1
ALLEVVQSGGK.2	turquoise	#N/A
S[Phospho (STY)]MDSLC[Carbamidomethyl (C)]SVPVEGK.2	yellow	A6X8Z5_S343_M1_Arhgap31
IVNDDQSFYADIYM[Oxidation (M)]EDGLIK.2	yellow	#N/A
ALIFVSHGAGEHC[Carbamidomethyl (C)]GR.3	yellow	#N/A
IVEFLQSFDEITAM[Oxidation (M)]TGDGVNDAPALKK.3	yellow	#N/A
IM[Oxidation (M)]DYSLLLGVHNIDQQER.3	yellow	#N/A
GDEELDSLIK.2	yellow	#N/A
DS[Phospho (STY)]SDDWEIPDGQITVGQR.2	yellow	P28028_S431_M1_Braf
YAPTTTPS[Phospho (STY)]PEDGFEVQPLHPDK.3	yellow	P28571_S659_M1_Slc6a9
TC[Carbamidomethyl (C)]DEGSS[Phospho (STY)]PSKPK.2	yellow	P30415_S1060_M1_Nktr
SPS[Phospho (STY)]PGRREEDGDELAR.3	yellow	P36552_S101_M1_Cpox
QTSAYNISNS[Phospho (STY)]STFTK.2	yellow	P70206_S1611_M1_Plxna1
NLLLLANSTEEQQK.2	yellow	#N/A
VDGDREKS[Phospho (STY)]PR.2	yellow	P97868_S1053_M1_Rbbp6
FDGGVVIAADM[Oxidation (M)]LGSYGSLAR.2	yellow	#N/A
TPVAS[Phospho (STY)]THS[Phospho (STY)]ISSATTPDR.2	yellow	Q03141_S540_M2_Mark3
ATSNVFAM[Oxidation (M)]FDQSQIQEFK.2	yellow	#N/A
C[Carbamidomethyl (C)]GFVGHDQLAGS[Phospho (STY)]DES[Phospho (STY)]EMEDEAEPPGAPPAPPPSYGATHSTVQPPRPPPPVR.5	yellow	Q3TXX4_S519_M2_Slc17a7
RRT[Phospho (STY)]AS[Phospho (STY)]PPPPPK.2	yellow	Q52KI8_S635_M2_Srrm1
APQASIC[Carbamidomethyl (C)]SDVDGFPTSQAT[Phospho (STY)]SPEPGKKDEEAK.4	yellow	Q5DTX6_T1164_M1_Jcad
TKEGS[Phospho (STY)]AELVPR.2	yellow	Q5GH64_S154_M1_Xkr7
IGKQS[Phospho (STY)]EES[Phospho (STY)]PADDGELRR.3	yellow	Q61687_S1287_M2_Atrx
QVTITGSAASISLAQYLINVR.2	yellow	#N/A
NSNS[Phospho (STY)]DS[Phospho (STY)]DDDERPNR.2	yellow	Q62018_S1039_M2_Ctr9
LDLDNSGSLSVEEFM[Oxidation (M)]SLPELQQNPLVQR.3	yellow	#N/A
AQPAGS[Phospho (STY)]PSHQIQQR.2	yellow	Q64700_S37_M1_Rbl2
GS[Phospho (STY)]PLPTPK.2	yellow	Q69Z98_S440_M1_Brsk2
RAS[Phospho (STY)]DGGANIQLHAQQLLK.3	yellow	Q6P4S6_S493_M1_Sik3
LQELESC[Carbamidomethyl (C)]SGLGS[Phospho (STY)]TSDDTDVR.2	yellow	Q6PAR5_S746_M1_Gapvd1
MLDAEDIVGTARPDEK.3	yellow	#N/A
DKES[Phospho (STY)]PSVKR.2	yellow	Q80TF3_S1134_M1_Pcdh19
SSGSSSSGLGTVSSS[Phospho (STY)]PASQR.2	yellow	Q80TJ7_S768_M1_Phf8
RS[Phospho (STY)]PTGSTASR.2	yellow	Q80TV8_S572_M1_Clasp1
SSS[Phospho (STY)]SSSSGVGSPAVTPTEK.2	yellow	Q812A2_S1070_M1_Srgap3
ASTIFLSKSQT[Phospho (STY)]DVR.2	yellow	Q8BGU5_T75_M1_Ccny
NAEEEDSAS[Phospho (STY)]DSELWK.2	yellow	Q8BP78_S285_M1_Fra10ac1
KMVSSEGS[Phospho (STY)]TES[Phospho (STY)]EHLEGMEAGQK.3	yellow	Q8BSS9_S236_M2_Ppfia2
MS[Phospho (STY)]C[Carbamidomethyl (C)]FS[Phospho (STY)]RPSM[Oxidation (M)]SPTPLDR.2	yellow	Q8BTI8_S2067_M2_Srrm2
PKPNQNLSEASGK.3	yellow	#N/A
AAGPGNLVLPGS[Phospho (STY)]LTAS[Phospho (STY)]LEC[Carbamidomethyl (C)]VKEEPPR.3	yellow	Q8CCJ4_S223_M2_Amer2
APEAAVS[Phospho (STY)]DDGKS[Phospho (STY)]DDELLSSK.2	yellow	Q8CH77_S197_M2_Nav1
GS[Phospho (STY)]LGKDTTSPM[Oxidation (M)]ELAALEK.3	yellow	Q8K212_S421_M1_Pacs1
KEYEEDGSR.2	yellow	#N/A
EC[Carbamidomethyl (C)]LEDVTAVC[Carbamidomethyl (C)]ILEGFQNEQSM[Oxidation (M)]LLADK.3	yellow	#N/A
VFNQEGEFMLK.2	yellow	#N/A
VVYENAYGR.2	yellow	#N/A
IGLGTDVAGGYSYSM[Oxidation (M)]LDAIR.2	yellow	#N/A
