# Supplementary material for: The Chronic Effects of a Single Low-Intensity Blast Exposure on Phosphoproteome Networks and Cognitive Function Influenced by Mutant Tau Overexpression
Source: Int J Mol Sci. 2024 Mar 15;25(6):3338. doi: 10.3390/ijms25063338 (PMC10970429; doi:10.3390/ijms25063338)
Supplement: Supplementary file 1 [file ijms-25-03338-s001.zip › Supplementary Table S6- Module eigenpeptides for each animal.pdf]

Supplementary Table S6. Module eigenpeptides for each animal

Group	MEblack	MEblue	MEbrown	MEgreen	MEgrey	MEpink
Blast NC 1	0.14236454	0.465605	0.11698	0.142551	0.354514	0.243125
Blast NC 2	0.27099925	0.031235	0.320044	0.102538	0.071733	0.019664
Blast NC 3	0.25775529	0.064419	0.273287	0.001449	0.030882	0.028504
Blast NC 4	0.10680082	0.007188	0.026704	0.103323	0.097757	0.01042
Blast NC 5	0.16863997	0.474705	0.299861	0.246551	0.247745	0.425627
Blast NC 6	0.14776993	0.265158	0.070764	0.173421	0.009145	0.376104
Blast rTg4510 1	0.18707839	0.322887	0.298467	0.632364	0.255881	0.486014
Blast rTg4510 2	0.24743609	0.07482	0.261329	0.454802	0.294503	0.168188
Blast rTg4510 3	0.30486868	0.235942	0.309988	0.257521	0.326909	0.10077
Blast rTg4510 4	0.03669218	0.224841	0.150341	0.030119	0.193568	0.059894
Blast rTg4510 5	0.01581643	0.297603	0.204685	0.019188	0.335537	0.191285
Sham NC 1	0.30038764	0.013339	0.051806	0.168581	0.160183	0.001878
Sham NC 2	0.22024769	0.010613	0.12033	0.082023	0.196421	0.180184
Sham NC 3	0.33496403	0.051659	0.14398	0.129326	0.008419	0.063179
Sham NC 4	0.24986357	0.03391	0.23551	0.070075	0.086414	0.266119
Sham rTg4510 1	0.16100741	0.139891	0.15984	0.07883	0.033633	0.107124
Sham rTg4510 2	0.45466143	0.254441	0.368559	0.264546	0.497061	0.304843
Sham rTg4510 3	0.10576707	0.210932	0.08493	0.117763	0.222694	0.211342
Sham rTg4510 4	0.03716639	0.073802	0.080803	0.148297	0.078895	0.207736
Sham rTg4510 5	0.16977903	0.198445	0.356528	0.156402	0.110803	0.051338

MEred	MEturquoi	MEyellow
0.05374	0.20106	0.071054
0.020737	0.261683	0.104263
0.019243	0.298837	0.264721
0.016728	0.143155	0.22095
0.189671	0.217829	0.189293
0.224661	0.087951	0.028228
0.432442	0.243183	0.02348
0.011568	0.201923	0.252487
0.111323	0.311866	0.250383
0.165845	0.051437	0.334639
0.779569	0.109275	0.411708
0.069478	0.00259	0.181141
0.069307	0.184726	0.14981
0.085633	0.025689	0.080035
0.078242	0.202527	0.315628
0.034409	0.062754	0.118712
0.201878	0.633402	0.253652
0.064223	0.017701	0.25433
0.057124	0.022497	0.080064
0.049055	0.180537	0.330558
