# Supplementary material for: The Chronic Effects of a Single Low-Intensity Blast Exposure on Phosphoproteome Networks and Cognitive Function Influenced by Mutant Tau Overexpression
Source: Int J Mol Sci. 2024 Mar 15;25(6):3338. doi: 10.3390/ijms25063338 (PMC10970429; doi:10.3390/ijms25063338)
Supplement: Supplementary file 1 [file ijms-25-03338-s001.zip › Supplementary Table S7- Network Screening.pdf]

Supplementary Table S7. Network Screening

PG.Genes	Phospep Name	Phos site	p.Weighted	q.Weighted	cor.Weighted	Z.Weighted	p.Standard	q.Standard	cor.Standard	Z.Standard
Atp6v1a	_ALDEYYDKHFTEFVPLR_.3	#N/A	7.63E-06	NA	-8.25E-01	-6.19E+00	3.56E-04	NA	-7.19E-01	-4.39E+00
Rubcn	_KQES[Phospho (STY)]PLLVTK_.2	Q80U62_S198_M1_Rubcn	2.07E-05	NA	-8.03E-01	-5.71E+00	3.77E-04	NA	-7.17E-01	-4.36E+00
Itsn1	_DQFS[Phospho (STY)]PDDFLGR_.2	Q9Z0R4_S1663_M1_Itsn1	3.38E-05	NA	-7.90E-01	-5.47E+00	3.69E-04	NA	-7.18E-01	-4.37E+00
Pcm1	_KAS[Phospho (STY)]AQASLASK_.2	Q9R0L6_S1280_M1_Pcm1	3.74E-05	NA	-7.88E-01	-5.42E+00	6.83E-04	NA	-6.94E-01	-4.09E+00
Dlgap2	_DGS[Phospho (STY)]WFLK_.2	Q8BJ42_S873_M1_Dlgap2	3.76E-05	NA	-7.88E-01	-5.42E+00	7.10E-04	NA	-6.93E-01	-4.08E+00
Bcl11b	_VSEPVEIGIQVTPDEDDHLLS[Phospho (STY)]PTK_.3	Q99PV8_S128_M1_Bcl11b	3.77E-05	NA	-7.87E-01	-5.42E+00	7.30E-04	NA	-6.92E-01	-4.06E+00
Gng12	_ASADLM[Oxidation (M)]SYC[Carbamidomethyl (C)]EEHAR_.2	#N/A	3.84E-05	NA	-7.87E-01	-5.41E+00	4.61E-04	NA	-7.09E-01	-4.27E+00
Eif5b	_KTS[Phospho (STY)]FDENDS[Phospho (STY)]EELEDKDSK_.3	Q05D44_S108_M2_Eif5b	4.50E-05	NA	-7.83E-01	-5.34E+00	1.78E-04	NA	-7.42E-01	-4.70E+00
Speg	_QRS[Phospho (STY)]ATS[Phospho (STY)]DLELR_.2	Q62407_S490_M2_Speg	4.54E-05	NA	-7.83E-01	-5.33E+00	1.12E-03	NA	-6.74E-01	-3.87E+00
Rims2	_IPDSTHAQLESSSSSFES[Phospho (STY)]QK_.3	Q9EQZ7_S788_M1_Rims2	4.72E-05	NA	-7.82E-01	-5.32E+00	1.18E-03	NA	-6.72E-01	-3.85E+00
Palm3	_TGGEGS[Phospho (STY)]LDEEAEGSKK_.2	A2TJV2_S565_M1_Palm3	5.14E-05	NA	-7.79E-01	-5.27E+00	6.29E-04	NA	-6.98E-01	-4.13E+00
Pgrmc1	_EGEEPTVYS[Phospho (STY)]DDEEPKDETARK_.4	O55022_S181_M1_Pgrmc1	5.44E-05	NA	-7.78E-01	-5.25E+00	1.43E-03	NA	-6.63E-01	-3.76E+00
Cct2	_VQDDEVGDGTTSVTVLAAELLR_.2	#N/A	6.23E-05	NA	-7.74E-01	-5.19E+00	2.64E-03	NA	-6.35E-01	-3.49E+00
Map6d1	_AGS[Phospho (STY)]RDAAQERPSGPGGR_.3	Q14BB9_S82_M1_Map6d1	7.26E-05	NA	-7.70E-01	-5.11E+00	6.28E-04	NA	-6.98E-01	-4.13E+00
Cyb5b	_RNS[Phospho (STY)]AEETWM[Oxidation (M)]VIHGR_.3	Q9CQX2_S33_M1_Cyb5b	7.42E-05	NA	-7.69E-01	-5.10E+00	9.44E-04	NA	-6.81E-01	-3.95E+00
Lgi1	_SLIHLSLANNNLQTLPK_.3	#N/A	8.33E-05	NA	-7.66E-01	-5.05E+00	2.51E-04	NA	-7.31E-01	-4.54E+00
Sytl5	_DLNS[Phospho (STY)]LEDTEDGVDLVSSR_.2	Q80T23_S358_M1_Sytl5	8.63E-05	NA	-7.65E-01	-5.03E+00	2.31E-03	NA	-6.41E-01	-3.55E+00
Nlgn3	_LTALPDYTLTLR_.2	#N/A	9.06E-05	NA	7.63E-01	5.01E+00	2.63E-04	NA	7.29E-01	4.52E+00
Atp1a2	_M[Oxidation (M)]TVAHM[Oxidation (M)]WFDNQIHEADTTEDQSGATFDKR_.4	#N/A	1.05E-04	NA	-7.59E-01	-4.94E+00	2.02E-03	NA	-6.48E-01	-3.61E+00
Ubqln2	_SQTDQLVLIFAGK_.2	#N/A	1.06E-04	NA	7.59E-01	4.94E+00	3.80E-04	NA	7.16E-01	4.36E+00
Atxn2	_TNS[Phospho (STY)]PSIS[Phospho (STY)]PSMLSNAEHKR_.3	O70305_S832_M2_Atxn2	1.13E-04	NA	-7.57E-01	-4.91E+00	7.00E-04	NA	-6.93E-01	-4.08E+00
Prkar2a	_VSVC[Carbamidomethyl (C)]AETFNPDEEEEDNDPR_.2	#N/A	1.18E-04	NA	-7.55E-01	-4.89E+00	5.19E-04	NA	-7.05E-01	-4.22E+00
Bclaf1	_QKFHDS[Phospho (STY)]EGDDTEETEDYR_.3	Q8K019_S395_M1_Bclaf1	1.40E-04	NA	-7.50E-01	-4.81E+00	4.93E-04	NA	-7.07E-01	-4.24E+00
Eif5	_GLTLS[Phospho (STY)]DDLER_.2	P59325_S227_M1_Eif5	1.45E-04	NA	-7.49E-01	-4.80E+00	1.94E-04	NA	-7.40E-01	-4.66E+00
Camkv	_SAT[Phospho (STY)]PATDGR_.2	Q3UHL1_T438_M1_Camkv	1.53E-04	NA	-7.47E-01	-4.77E+00	1.13E-03	NA	-6.74E-01	-3.87E+00
Sptbn1	_VAHM[Oxidation (M)]EFC[Carbamidomethyl (C)]YQELC[Carbamidomethyl (C)]QLAAER_.3	#N/A	1.61E-04	NA	-7.46E-01	-4.75E+00	8.22E-04	NA	-6.87E-01	-4.01E+00
Actb	_C[Carbamidomethyl (C)]PEALFQPSFLGM[Oxidation (M)]ESC[Carbamidomethyl (C)]GIHETTFNSIM[Oxidation (M)	#N/A	1.63E-04	NA	-7.45E-01	-4.74E+00	2.60E-03	NA	-6.35E-01	-3.49E+00
Ctnna1	_LESIISGAALM[Oxidation (M)]ADSSC[Carbamidomethyl (C)]TR_.2	#N/A	1.80E-04	NA	-7.42E-01	-4.70E+00	1.91E-03	NA	-6.50E-01	-3.63E+00
Cobl	_S[Phospho (STY)]PPTRKDDAAPNLHSEAR_.4	Q5NBX1_S982_M1_Cobl	1.95E-04	NA	-7.39E-01	-4.66E+00	1.47E-03	NA	-6.62E-01	-3.75E+00
Hspa8	_VC[Carbamidomethyl (C)]NPIITK_.2	#N/A	2.16E-04	NA	7.36E-01	4.61E+00	1.72E-03	NA	6.55E-01	3.68E+00
Ppp2r1a	_MAGDPVANVR_.2	#N/A	2.16E-04	NA	7.36E-01	4.61E+00	1.86E-03	NA	6.51E-01	3.64E+00
Slmap	_AKESDLSDTLSPS[Phospho (STY)]KEK_.2	Q3URD3_S471_M1_Slmap	2.18E-04	NA	-7.36E-01	-4.61E+00	3.87E-03	NA	-6.16E-01	-3.31E+00
Pabir1	_RNS[Phospho (STY)]TTFPSR_.2	Q9DB52_S59_M1_Pabir1	2.77E-04	NA	-7.28E-01	-4.50E+00	2.01E-03	NA	-6.48E-01	-3.61E+00
Dennd1a	_S[Phospho (STY)]LEDLRAPK_.2	Q8K382_S592_M1_Dennd1a	2.78E-04	NA	-7.27E-01	-4.50E+00	7.19E-03	NA	-5.81E-01	-3.03E+00
Dlg4	_HC[Carbamidomethyl (C)]ILDVSANAVR_.2	#N/A	3.12E-04	NA	7.23E-01	4.45E+00	4.39E-04	NA	7.11E-01	4.29E+00
Slc4a10	_ERDSGLEDGRES[Phospho (STY)]PSFDTPSQR_.4	Q5DTL9_S89_M1_Slc4a10	3.15E-04	NA	-7.23E-01	-4.44E+00	4.83E-04	NA	-7.08E-01	-4.25E+00
Nefh	_AQALQEEC[Carbamidomethyl (C)]GYLR_.2	#N/A	3.28E-04	NA	7.22E-01	4.42E+00	3.05E-03	NA	6.28E-01	3.42E+00
Mars1	_NNSELLNNLGNFINR_.2	#N/A	3.30E-04	NA	-7.22E-01	-4.42E+00	4.43E-04	NA	-7.11E-01	-4.29E+00
Gpi	_TFTTQETITNAETAKEWFLEAAK_.3	#N/A	3.70E-04	NA	-7.17E-01	-4.37E+00	4.06E-04	NA	-7.14E-01	-4.33E+00
Sugp2	_GRS[Phospho (STY)]LNIADQEGTLLGK_.3	Q8CH09_S206_M1_Sugp2	3.91E-04	NA	-7.15E-01	-4.34E+00	1.85E-03	NA	-6.52E-01	-3.65E+00
Sptan1	_VAEDLESEGLM[Oxidation (M)]AEEVQAVQQQEVYGAM[Oxidation (M)]PR_.3	#N/A	4.03E-04	NA	-7.14E-01	-4.33E+00	4.97E-03	NA	-6.02E-01	-3.20E+00
Palm	_ENSAAPS[Phospho (STY)]PGRPQSASPAK_.2	Q9Z0P4_S116_M1_Palm	4.05E-04	NA	-7.14E-01	-4.33E+00	7.66E-04	NA	-6.90E-01	-4.04E+00
Igsf9b	_APS[Phospho (STY)]ESS[Phospho (STY)]DDQGQPAAK_.2	E9PZ19_S794_M2_Igsf9b	4.07E-04	NA	-7.14E-01	-4.33E+00	2.07E-03	NA	-6.46E-01	-3.59E+00
Aftph	_S[Phospho (STY)]PDPDPTGQNALDDSAASMK_.2	Q80WT5_S395_M1_Aftph	4.22E-04	NA	-7.13E-01	-4.31E+00	3.27E-03	NA	-6.24E-01	-3.39E+00
Hnrnpm	_MGLAMGGAGGAS[Phospho (STY)]FDR_.2	Q9D0E1_S617_M1_Hnrnpm	4.30E-04	NA	-7.12E-01	-4.30E+00	2.95E-03	NA	-6.29E-01	-3.44E+00
Ctnnd1	_TLDRS[Phospho (STY)]GDLGDMEPLK_.3	P30999_S920_M1_Ctnnd1	4.30E-04	NA	-7.12E-01	-4.30E+00	3.05E-03	NA	-6.28E-01	-3.42E+00
Ppfia2	_KMVSSEGS[Phospho (STY)]TES[Phospho (STY)]EHLEGMEAGQK_.3	Q8BSS9_S236_M2_Ppfia2	4.35E-04	NA	-7.11E-01	-4.30E+00	9.11E-04	NA	-6.83E-01	-3.96E+00
Aldh2	_LLC[Carbamidomethyl (C)]GGGAAADR_.2	#N/A	4.39E-04	NA	7.11E-01	4.29E+00	1.19E-03	NA	6.71E-01	3.84E+00
Map4k2	_ASDPHLGTLS[Phospho (STY)]PEDSELETHDMFPDTIHSR_.4	Q61161_S298_M1_Map4k2	4.61E-04	NA	-7.09E-01	-4.27E+00	2.51E-03	NA	-6.37E-01	-3.51E+00
Hint1	_C[Carbamidomethyl (C)]AADLGLK_.2	#N/A	4.66E-04	NA	-7.09E-01	-4.27E+00	6.05E-03	NA	-5.91E-01	-3.11E+00
Hook3	_LNQSDSIEDPNS[Phospho (STY)]PAGRR_.2	Q8BUK6_S238_M1_Hook3	4.66E-04	NA	-7.09E-01	-4.26E+00	8.34E-03	NA	-5.73E-01	-2.96E+00
Tkt	_VYC[Carbamidomethyl (C)]M[Oxidation (M)]LGDGEVSEGSVWEAM[Oxidation (M)]AFAGIYK_.3	#N/A	4.66E-04	NA	-7.09E-01	-4.26E+00	7.40E-03	NA	-5.80E-01	-3.02E+00
Sybu	_SGSS[Phospho (STY)]PPS[Phospho (STY)]PR_.2	Q8BHS8_S199_M2_Sybu	4.79E-04	NA	-7.08E-01	-4.25E+00	6.65E-03	NA	-5.86E-01	-3.07E+00
Clcn6	_ES[Phospho (STY)]RTPEELTILGETQEEEDEILPR_.3	O35454_S23_M1_Clcn6	4.96E-04	NA	-7.07E-01	-4.24E+00	3.19E-03	NA	-6.25E-01	-3.40E+00

Leo1	_LNS[Phospho (STY)]DEEGESSGKR_.2	Q5XJE5_S631_M1_Leo1	5.21E-04	NA	-7.05E-01	-4.21E+00	1.24E-03	NA	-6.70E-01	-3.83E+00
Tnr	_DKEEDM[Oxidation (M)]LEVLLDATKR_.3	#N/A	5.28E-04	NA	-7.04E-01	-4.21E+00	3.04E-03	NA	-6.28E-01	-3.42E+00
Kiaa1107	_SSVSSRQS[Phospho (STY)]DENVTR_.2	Q80TK0_S353_M1_Kiaa1107	5.66E-04	NA	-7.02E-01	-4.18E+00	8.20E-03	NA	-5.73E-01	-2.97E+00
Sptbn1	_TLETPAAQM[Oxidation (M)]EGFLNR_.2	#N/A	5.79E-04	NA	-7.01E-01	-4.17E+00	5.20E-03	NA	-6.00E-01	-3.18E+00
Cacnb3	_QAWTGSS[Phospho (STY)]QR_.2	P54285_S418_M1_Cacnb3	5.91E-04	NA	-7.00E-01	-4.16E+00	3.83E-03	NA	-6.16E-01	-3.32E+00
Adam22	_DVLC[Carbamidomethyl (C)]GYLLC[Carbamidomethyl (C)]TNIGNIPR_.2	#N/A	6.03E-04	NA	-6.99E-01	-4.15E+00	4.65E-03	NA	-6.06E-01	-3.23E+00
Tacc1	_ESC[Carbamidomethyl (C)]DSS[Phospho (STY)]PGLAEPEAKPQESR_.3	Q6Y685_S82_M1_Tacc1	6.31E-04	NA	-6.97E-01	-4.13E+00	5.98E-03	NA	-5.92E-01	-3.12E+00
Sec22b	_DLQQYQSQAK_.2	#N/A	7.73E-04	NA	6.89E-01	4.04E+00	1.89E-03	NA	6.51E-01	3.64E+00
Rem2	_RRGS[Phospho (STY)]M[Oxidation (M)]PVPYK_.3	Q8VEL9_S69_M1_Rem2	7.91E-04	NA	-6.88E-01	-4.03E+00	8.74E-03	NA	-5.70E-01	-2.94E+00
Add1	_YFDRVDENNPEYLR_.3	#N/A	8.07E-04	NA	-6.88E-01	-4.02E+00	2.64E-03	NA	-6.35E-01	-3.49E+00
Dtd1	_AKGPSESS[Phospho (STY)]KER_.2	Q9DD18_S182_M1_Dtd1	8.08E-04	NA	-6.88E-01	-4.02E+00	1.04E-02	NA	-5.59E-01	-2.86E+00
Dlg3	_VNEVDVSEVVHSR_.3	#N/A	8.63E-04	NA	-6.85E-01	-3.99E+00	1.12E-02	NA	-5.54E-01	-2.83E+00
Pde4d	_QHEVEIPSPT[Phospho (STY)]QKEK_.3	Q01063_T289_M1_Pde4d	8.82E-04	NA	-6.84E-01	-3.98E+00	4.49E-03	NA	-6.08E-01	-3.25E+00
Sgip1	_GKHFY[Phospho (STY)]SS[Phospho (STY)]S[Phospho (STY)]ES[Phospho (STY)]EEEEESHKK_.3	Q8VD37_S104_M3_Sgip1	8.96E-04	NA	-6.83E-01	-3.97E+00	4.38E-03	NA	-6.09E-01	-3.26E+00
Srrm2	_SS[Phospho (STY)]PELTR_.2	Q8BTI8_S1650_M1_Srrm2	9.20E-04	NA	-6.82E-01	-3.96E+00	1.19E-02	NA	-5.50E-01	-2.80E+00
Tex2	_SLS[Phospho (STY)]TEVEPK_.2	Q6ZPJ0_S195_M1_Tex2	9.33E-04	NA	-6.82E-01	-3.95E+00	3.30E-03	NA	-6.24E-01	-3.38E+00
Mapk8ip3	_LFSSSSS[Phospho (STY)]PPPAK_.2	Q9ESN9_S586_M1_Mapk8ip3	9.39E-04	NA	-6.81E-01	-3.95E+00	1.13E-03	NA	-6.74E-01	-3.87E+00
Sipa1l3	_SSS[Phospho (STY)]EITLSEC[Carbamidomethyl (C)]DVEEPGDPR_.2	G3X9J0_S166_M1_Sipa1l3	9.45E-04	NA	-6.81E-01	-3.95E+00	1.22E-02	NA	-5.49E-01	-2.79E+00
Snca	_TKEQVTNVGGAVVTGVTAVAQK_.3	#N/A	9.80E-04	NA	-6.80E-01	-3.93E+00	3.14E-03	NA	-6.26E-01	-3.41E+00
Snta1	_YLEIC[Carbamidomethyl (C)]AADGQDAVFLR_.2	#N/A	9.94E-04	NA	-6.79E-01	-3.92E+00	1.26E-02	NA	-5.47E-01	-2.77E+00
Shank2	_S[Phospho (STY)]KSM[Oxidation (M)]TAELEELGLSLVDK_.3	Q80Z38_S370_M1_Shank2	1.00E-03	NA	-6.79E-01	-3.92E+00	5.89E-03	NA	-5.93E-01	-3.12E+00
Atp1a3	_M[Oxidation (M)]TVAHM[Oxidation (M)]WFDNQIHEADTTEDQSGTSFDK_.4	#N/A	1.02E-03	NA	-6.78E-01	-3.91E+00	6.57E-03	NA	-5.86E-01	-3.07E+00
Dlg1	_EQM[Oxidation (M)]M[Oxidation (M)]NSSVSSGSGS[Phospho (STY)]LR_.2	Q811D0_S575_M1_Dlg1	1.06E-03	NA	-6.76E-01	-3.89E+00	7.09E-03	NA	-5.82E-01	-3.04E+00
Sorbs1	_YS[Phospho (STY)]FSDDTKSPLSVPR_.3	Q62417_S254_M1_Sorbs1	1.07E-03	NA	-6.76E-01	-3.89E+00	1.34E-02	NA	-5.43E-01	-2.74E+00
Ctnnal1	_S[Phospho (STY)]AAEELELTVLK_.2	O88327_S373_M1_Ctnnal1	1.09E-03	NA	-6.75E-01	-3.88E+00	2.79E-03	NA	-6.32E-01	-3.46E+00
Sipa1l1	_EYGS[Phospho (STY)]TSSIDK_.2	Q8C0T5_S208_M1_Sipa1l1	1.13E-03	NA	-6.73E-01	-3.86E+00	1.97E-03	NA	-6.49E-01	-3.62E+00
Tomm70	_SDEDKDKEGEALEVK_.3	#N/A	1.14E-03	NA	-6.73E-01	-3.86E+00	3.89E-03	NA	-6.15E-01	-3.31E+00
Rbx1	_[Acetyl (Protein N-term)]AAAMDVDTPSGTNSGAGK_.2	#N/A	1.19E-03	NA	6.71E-01	3.84E+00	4.88E-03	NA	6.03E-01	3.21E+00
Fam102b	_SAS[Phospho (STY)]VPDELGAWGHSR_.2	Q8BQS4_S162_M1_Fam102b	1.21E-03	NA	-6.71E-01	-3.84E+00	3.29E-03	NA	-6.24E-01	-3.39E+00
Map4	_VGS[Phospho (STY)]TENIK_.2	P27546_S914_M1_Map4	1.23E-03	NA	-6.70E-01	-3.83E+00	1.44E-02	NA	-5.38E-01	-2.71E+00
Kcna2	_IPS[Phospho (STY)]S[Phospho (STY)]PDLK_.2	P63141_S440_M2_Kcna2	1.25E-03	NA	-6.69E-01	-3.82E+00	4.26E-03	NA	-6.10E-01	-3.27E+00
Tcp1	_DDKHGS[Phospho (STY)]YENAVHSGALDD_.3	P11983_S544_M1_Tcp1	1.26E-03	NA	-6.69E-01	-3.82E+00	2.69E-03	NA	-6.34E-01	-3.48E+00
Sptan1	_DC[Carbamidomethyl (C)]EQAENWM[Oxidation (M)]AAR_.2	#N/A	1.26E-03	NA	6.69E-01	3.82E+00	7.63E-03	NA	5.78E-01	3.00E+00
Actn1	_ILAGDKNYITEDELR_.3	#N/A	1.26E-03	NA	-6.69E-01	-3.82E+00	1.99E-03	NA	-6.48E-01	-3.61E+00
Kctd16	_TLTSGS[Phospho (STY)]RESNISSK_.2	Q5DTY9_S372_M1_Kctd16	1.32E-03	NA	-6.67E-01	-3.80E+00	1.49E-02	NA	-5.36E-01	-2.69E+00
Cnp	_ERLDQLFEM[Oxidation (M)]ADQYQYQVVLVEPK_.3	#N/A	1.32E-03	NA	-6.67E-01	-3.80E+00	4.72E-03	NA	-6.05E-01	-3.22E+00
Ctnnd2	_SPS[Phospho (STY)]IDSIQKDPR_.2	O35927_S531_M1_Ctnnd2	1.37E-03	NA	-6.65E-01	-3.78E+00	6.66E-03	NA	-5.86E-01	-3.07E+00
Myh9	_GTGDC[Carbamidomethyl (C)]S[Phospho (STY)]DEEVDGKADGADAK_.2	#N/A	1.40E-03	NA	-6.64E-01	-3.77E+00	1.81E-03	NA	-6.53E-01	-3.65E+00
Camk2g	_SLLNKKS[Phospho (STY)]DGGVK_.2	Q923T9_S325_M1_Camk2g	1.41E-03	NA	-6.64E-01	-3.77E+00	2.98E-03	NA	-6.29E-01	-3.43E+00
Cd200	_EVIC[Carbamidomethyl (C)]QVLYLGNVIDYK_.2	#N/A	1.42E-03	NA	-6.64E-01	-3.76E+00	1.01E-03	NA	-6.78E-01	-3.92E+00
Ndufs7	_KVYDQM[Oxidation (M)]PEPR_.2	#N/A	1.47E-03	NA	-6.62E-01	-3.75E+00	2.13E-03	NA	-6.45E-01	-3.58E+00
Map1a	_KVAELEEEQS[Phospho (STY)]QGSSSYSDWVK_.3	Q9QYR6_S114_M1_Map1a	1.53E-03	NA	-6.60E-01	-3.73E+00	5.99E-03	NA	-5.92E-01	-3.11E+00
Syn1	_VKVDNQHDFQDIASVVALTK_.3	#N/A	1.54E-03	NA	-6.60E-01	-3.73E+00	5.54E-03	NA	-5.96E-01	-3.15E+00
Septin5	_M[Oxidation (M)]ES[Phospho (STY)]PIPILPLPTPDAETEK_.2	Q9Z2Q6_S327_M1_Septin5	1.54E-03	NA	-6.60E-01	-3.73E+00	1.80E-02	NA	-5.23E-01	-2.60E+00
Dnm1	_VYSPHVLNLTLVDLPGM[Oxidation (M)]TK_.3	#N/A	1.56E-03	NA	-6.60E-01	-3.72E+00	7.24E-03	NA	-5.81E-01	-3.03E+00
Pex5l	_NHS[Phospho (STY)]LEEEFER_.2	Q8C437_S202_M1_Pex5l	1.56E-03	NA	-6.60E-01	-3.72E+00	9.95E-03	NA	-5.62E-01	-2.88E+00
Cbx3	_C[Carbamidomethyl (C)]PQIVIAFYEER_.2	#N/A	1.60E-03	NA	-6.58E-01	-3.71E+00	8.54E-03	NA	-5.71E-01	-2.95E+00
Dync1h1	_LALESIC[Carbamidomethyl (C)]LLLGESTTDWK_.2	#N/A	1.66E-03	NA	-6.57E-01	-3.69E+00	1.70E-02	NA	-5.27E-01	-2.63E+00
Snap91	_GLGSDLDSSLASLVGNLGISGTTSK_.2	#N/A	1.71E-03	NA	-6.55E-01	-3.68E+00	8.97E-03	NA	-5.68E-01	-2.93E+00
Tubb2a	_ESESC[Carbamidomethyl (C)]DC[Carbamidomethyl (C)]LQGFQLTHSLGGGTGSGM[Oxidation (M)]GTLLISK_.3	#N/A	1.71E-03	NA	-6.55E-01	-3.68E+00	6.00E-03	NA	-5.92E-01	-3.11E+00
Stxbp1	_M[Oxidation (M)]TDIM[Oxidation (M)]TEGITIVEDINKR_.3	#N/A	1.72E-03	NA	-6.55E-01	-3.68E+00	1.13E-02	NA	-5.54E-01	-2.82E+00
Dmtn	_HLS[Phospho (STY)]AEDFSR_.2	Q9WV69_S372_M1_Dmtn	1.76E-03	NA	-6.54E-01	-3.67E+00	1.25E-02	NA	-5.47E-01	-2.77E+00
Dclk2	_YSGSRS[Phospho (STY)]PGFSR_.2	Q6PGN3_S307_M1_Dclk2	1.76E-03	NA	-6.54E-01	-3.67E+00	5.35E-03	NA	-5.98E-01	-3.17E+00
Akr1a1	_YDSTHYK_.2	#N/A	1.80E-03	NA	-6.53E-01	-3.66E+00	6.90E-03	NA	-5.84E-01	-3.05E+00
Akap5	_SSAIQM[Oxidation (M)]GT[Phospho (STY)]PELEKETK_.2	D3YVF0_T220_M1_Akap5	1.80E-03	NA	-6.53E-01	-3.66E+00	1.75E-02	NA	-5.25E-01	-2.62E+00
Rock2	_TLTSDVANLANEKEELNNK_.3	#N/A	1.83E-03	NA	-6.52E-01	-3.65E+00	1.93E-02	NA	-5.18E-01	-2.57E+00
Ssh2	_EM[Oxidation (M)]TTS[Phospho (STY)]ADQIAEVK_.2	Q5SW75_S487_M1_Ssh2	1.88E-03	NA	-6.51E-01	-3.64E+00	3.33E-03	NA	-6.23E-01	-3.38E+00

Cdk17	_RLS[Phospho (STY)]LPADIR_.2	Q8K0D0_S146_M1_Cdk17	1.91E-03	NA	-6.50E-01	-3.63E+00	6.38E-03	NA	-5.88E-01	-3.09E+00
Ndufv1	_GAGAYIC[Carbamidomethyl (C)]GEETALIESIEGK_.2	#N/A	1.93E-03	NA	-6.50E-01	-3.63E+00	1.61E-02	NA	-5.31E-01	-2.66E+00
Stip1	_DPQALSEHLK_.2	#N/A	2.03E-03	NA	6.47E-01	3.60E+00	7.06E-03	NA	5.82E-01	3.04E+00
Pc	_EGPEGFAR_.2	#N/A	2.11E-03	NA	6.46E-01	3.59E+00	1.44E-02	NA	5.38E-01	2.71E+00
Atp6v0d1	_AGVLSQADYLNLVQC[Carbamidomethyl (C)]ETLEDLK_.3	#N/A	2.14E-03	NA	-6.45E-01	-3.58E+00	1.44E-02	NA	-5.38E-01	-2.71E+00
Ankib1	_GVAPADS[Phospho (STY)]PDAPRR_.2	Q6ZPS6_S738_M1_Ankib1	2.16E-03	NA	-6.45E-01	-3.58E+00	2.28E-02	NA	-5.06E-01	-2.49E+00
Ccdc115	_ADAQT[Phospho (STY)]PEEVGPSEASLR_.2	Q8VE99_T90_M1_Ccdc115	2.17E-03	NA	-6.44E-01	-3.57E+00	4.26E-03	NA	-6.10E-01	-3.27E+00
Top2b	_KTSFDQDS[Phospho (STY)]DVDIFPSDFTSEPPALPR_.3	Q64511_S1568_M1_Top2b	2.18E-03	NA	-6.44E-01	-3.57E+00	1.34E-02	NA	-5.43E-01	-2.74E+00
Sgip1	_RST[Phospho (STY)]PT[Phospho (STY)]PELTSK_.2	Q8VD37_T180_M2_Sgip1	2.22E-03	NA	-6.43E-01	-3.56E+00	1.19E-02	NA	-5.50E-01	-2.80E+00
Ppp3r1	_LDLDNSGSLSVEEFM[Oxidation (M)]SLPELQQNPLVQR_.3	#N/A	2.24E-03	NA	-6.43E-01	-3.56E+00	1.15E-02	NA	-5.53E-01	-2.82E+00
Sgip1	_HVPS[Phospho (STY)]PLNLEEVQKK_.3	Q8VD37_S371_M1_Sgip1	2.26E-03	NA	-6.42E-01	-3.56E+00	1.47E-02	NA	-5.37E-01	-2.70E+00
Slc17a7	_C[Carbamidomethyl (C)]GFVGHDQLAGS[Phospho (STY)]DES[Phospho (STY)]EMEDEAEPPGAPPAPPPSYGATHST	Q3TXX4_S519_M2_Slc17a7	2.29E-03	NA	-6.42E-01	-3.55E+00	1.16E-02	NA	-5.52E-01	-2.81E+00
Myl12b	_ATSNVFAM[Oxidation (M)]FDQSQIQEFK_.2	#N/A	2.30E-03	NA	-6.42E-01	-3.55E+00	4.51E-03	NA	-6.07E-01	-3.24E+00
Actn1	_MLDAEDIVGTARPDEK_.3	#N/A	2.31E-03	NA	-6.41E-01	-3.55E+00	1.16E-02	NA	-5.52E-01	-2.81E+00
Prrt2	_HPS[Phospho (STY)]SQLAGPGVEGGEGTQKPR_.3	E9PUL5_S254_M1_Prrt2	2.32E-03	NA	-6.41E-01	-3.54E+00	3.78E-03	NA	-6.17E-01	-3.32E+00
Ccm2	_GIITDS[Phospho (STY)]FGR_.2	Q8K2Y9_S393_M1_Ccm2	2.39E-03	NA	6.40E-01	3.53E+00	5.09E-03	NA	6.01E-01	3.19E+00
H1-2	_[Acetyl (Protein N-term)]S[Phospho (STY)]EAAPAAPAAAPPAEKAPAK_.2	P15864_S2_M1_H1-2	2.44E-03	NA	-6.39E-01	-3.52E+00	1.41E-02	NA	-5.40E-01	-2.72E+00
Rab5a	_GAQAAIVVYDITNEESFAR_.2	#N/A	2.47E-03	NA	-6.38E-01	-3.52E+00	2.89E-03	NA	-6.30E-01	-3.45E+00
Clasp1	_RS[Phospho (STY)]PTGSTASR_.2	Q80TV8_S572_M1_Clasp1	2.48E-03	NA	-6.38E-01	-3.51E+00	4.05E-03	NA	-6.13E-01	-3.29E+00
Nktr	_TC[Carbamidomethyl (C)]DEGSS[Phospho (STY)]PSKPK_.2	P30415_S1060_M1_Nktr	2.49E-03	NA	-6.38E-01	-3.51E+00	1.10E-02	NA	-5.56E-01	-2.83E+00
Camk2b	_TTEQLIEAVNNGDFEAYAK_.2	#N/A	2.51E-03	NA	-6.37E-01	-3.51E+00	1.88E-02	NA	-5.20E-01	-2.58E+00
Dctn2	_VHQLYETIQR_.2	#N/A	2.57E-03	NA	6.36E-01	3.50E+00	3.53E-03	NA	6.20E-01	3.36E+00
Actr1b	_KEYEEDGSR_.2	#N/A	2.58E-03	NA	-6.36E-01	-3.50E+00	1.19E-02	NA	-5.50E-01	-2.80E+00
Senp3	_GS[Phospho (STY)]PPVPSGPPMEEDGLR_.2	Q9EP97_S206_M1_Senp3	2.60E-03	NA	-6.36E-01	-3.49E+00	5.35E-03	NA	-5.98E-01	-3.17E+00
Tomm70	_NSERKT[Phospho (STY)]PEGR_.2	Q9CZW5_T88_M1_Tomm70	2.61E-03	NA	-6.35E-01	-3.49E+00	5.33E-03	NA	-5.98E-01	-3.17E+00
Apba1	_SNS[Phospho (STY)]QENVEASHPSQDGKR_.3	B2RUJ5_S573_M1_Apba1	2.66E-03	NA	-6.34E-01	-3.48E+00	6.96E-03	NA	-5.83E-01	-3.05E+00
Hnrnpm	_INEILSNALK_.2	#N/A	2.67E-03	NA	6.34E-01	3.48E+00	3.86E-03	NA	6.16E-01	3.31E+00
Pdcd5	_VSEQGLIEILEK_.2	#N/A	2.70E-03	NA	6.34E-01	3.47E+00	4.70E-03	NA	6.05E-01	3.22E+00
Plec	_LLDAQLSTGGIVDPSK_.2	#N/A	2.74E-03	NA	-6.33E-01	-3.47E+00	6.79E-03	NA	-5.85E-01	-3.06E+00
Cbx1	_VEEVLEEEEEEYVVEK_.2	#N/A	2.75E-03	NA	-6.33E-01	-3.47E+00	7.89E-03	NA	-5.76E-01	-2.99E+00
Rem2	_GHAGGQRPEPSS[Phospho (STY)]PDGPAPPTRR_.4	Q8VEL9_S296_M1_Rem2	2.80E-03	NA	-6.32E-01	-3.46E+00	9.96E-04	NA	-6.79E-01	-3.92E+00
Pex5l	_SALNSES[Phospho (STY)]ASELELVAPAQAR_.2	Q8C437_S172_M1_Pex5l	2.82E-03	NA	-6.32E-01	-3.46E+00	2.60E-03	NA	-6.35E-01	-3.49E+00
Gnb1	_QTFTGHESDINAIC[Carbamidomethyl (C)]FFPNGNAFATGSDDATC[Carbamidomethyl (C)]R_.3	#N/A	2.86E-03	NA	-6.31E-01	-3.45E+00	1.53E-02	NA	-5.34E-01	-2.68E+00
Hepacam	_QNS[Phospho (STY)]LEYM[Oxidation (M)]DQNDDRLK_.3	Q640R3_S280_M1_Hepacam	2.89E-03	NA	-6.30E-01	-3.44E+00	7.23E-03	NA	-5.81E-01	-3.03E+00
Ppp2r1a	_VKEFC[Carbamidomethyl (C)]ENLSADC[Carbamidomethyl (C)]R_.2	#N/A	2.92E-03	NA	-6.30E-01	-3.44E+00	1.50E-02	NA	-5.35E-01	-2.69E+00
Dnpep	_SPSPFHVVAEC[Carbamidomethyl (C)]R_.3	#N/A	2.92E-03	NA	-6.30E-01	-3.44E+00	1.61E-02	NA	-5.30E-01	-2.65E+00
Cs	_TVVGQITVDM[Oxidation (M)]M[Oxidation (M)]YGGM[Oxidation (M)]R_.2	#N/A	2.93E-03	NA	-6.30E-01	-3.44E+00	8.52E-03	NA	-5.71E-01	-2.95E+00
Psmd1	_TASAVAGKT[Phospho (STY)]PDAS[Phospho (STY)]PEPKDQTLK_.3	Q3TXS7_S315_M2_Psmd1	2.95E-03	NA	-6.29E-01	-3.44E+00	1.39E-02	NA	-5.40E-01	-2.72E+00
Arhgef7	_KPS[Phospho (STY)]DEEFAVRK_.3	Q9ES28_S673_M1_Arhgef7	2.95E-03	NA	-6.29E-01	-3.43E+00	1.92E-02	NA	-5.18E-01	-2.57E+00
Dlgap2	_GGLYNS[Phospho (STY)]M[Oxidation (M)]DSLDSNK_.2	Q8BJ42_S667_M1_Dlgap2	2.97E-03	NA	-6.29E-01	-3.43E+00	7.08E-03	NA	-5.82E-01	-3.04E+00
Pclo	_IVDSGVQT[Phospho (STY)]DDEETADR_.2	Q9QYX7_T3376_M1_Pclo	2.98E-03	NA	-6.29E-01	-3.43E+00	1.57E-02	NA	-5.32E-01	-2.67E+00
Synrg	_QLS[Phospho (STY)]LEGAGLAMEEFK_.2	Q5SV85_S744_M1_Synrg	2.98E-03	NA	-6.29E-01	-3.43E+00	1.48E-02	NA	-5.36E-01	-2.70E+00
Dlg4	_EQLM[Oxidation (M)]NSSLGS[Phospho (STY)]GTASLR_.2	Q62108_S418_M1_Dlg4	3.00E-03	NA	-6.29E-01	-3.43E+00	2.06E-03	NA	-6.47E-01	-3.60E+00
Spen	_S[Phospho (STY)]RSPNRFDAHDPR_.4	Q62504_S188_M1_Spen	3.00E-03	NA	-6.28E-01	-3.43E+00	1.90E-03	NA	-6.51E-01	-3.63E+00
Slc6a9	_YAPTTTPS[Phospho (STY)]PEDGFEVQPLHPDK_.3	P28571_S659_M1_Slc6a9	3.02E-03	NA	-6.28E-01	-3.43E+00	1.02E-02	NA	-5.60E-01	-2.87E+00
Wac	_DAADPS[Phospho (STY)]PPNK_.2	Q924H7_S53_M1_Wac	3.02E-03	NA	-6.28E-01	-3.43E+00	3.37E-03	NA	-6.23E-01	-3.38E+00
Cisd1	_HNEETGDNVGPLIIK_.2	#N/A	3.03E-03	NA	-6.28E-01	-3.42E+00	5.34E-03	NA	-5.98E-01	-3.17E+00
Map1b	_ASLSPM[Oxidation (M)]DEPVPDSES[Phospho (STY)]PVEK_.2	P14873_S1384_M1_Map1b	3.09E-03	NA	-6.27E-01	-3.42E+00	1.78E-02	NA	-5.24E-01	-2.61E+00
Pdhx	_LTEDEEGNPQLQQHQLITVTM[Oxidation (M)]SSDSR_.3	#N/A	3.14E-03	NA	-6.26E-01	-3.41E+00	1.22E-02	NA	-5.49E-01	-2.78E+00
Plekhh1	_SC[Carbamidomethyl (C)]DS[Phospho (STY)]DEDYEAGGTGR_.2	Q80TI1_S739_M1_Plekhh1	3.15E-03	NA	-6.26E-01	-3.41E+00	4.31E-03	NA	-6.10E-01	-3.26E+00
Epha4	_IDTIAADESFTQVDIGDR_.2	#N/A	3.16E-03	NA	-6.26E-01	-3.40E+00	1.07E-02	NA	-5.57E-01	-2.85E+00
Slc4a10	_DSGLEDGRES[Phospho (STY)]PSFDTPSQR_.3	Q5DTL9_S89_M1_Slc4a10	3.22E-03	NA	-6.25E-01	-3.40E+00	9.39E-03	NA	-5.65E-01	-2.91E+00
Stip1	_LM[Oxidation (M)]DVGLIAIR_.2	#N/A	3.32E-03	NA	6.23E-01	3.38E+00	3.76E-03	NA	6.17E-01	3.33E+00
Uqcrc1	_TDLTDYLNR_.2	#N/A	3.34E-03	NA	-6.23E-01	-3.38E+00	1.26E-02	NA	-5.47E-01	-2.77E+00
Septin2	_TVQIEASTVEIEER_.2	#N/A	3.36E-03	NA	-6.23E-01	-3.38E+00	1.44E-02	NA	-5.38E-01	-2.71E+00
Ubxn2b	_FKAFS[Phospho (STY)]GEGQK_.2	Q0KL01_S216_M1_Ubxn2b	3.45E-03	NA	-6.21E-01	-3.37E+00	1.38E-02	NA	-5.41E-01	-2.73E+00
Lap3	_ANKPGDVVR_.2	#N/A	3.49E-03	NA	6.21E-01	3.36E+00	2.56E-03	NA	6.36E-01	3.50E+00

Epb41l1	_KAQEETPQQPEAAAAVTT[Phospho (STY)]PVT[Phospho (STY)]PAGHSHPETNSNEK_.4	Q9Z2H5_T30_M2_Epb41l1	3.55E-03	NA	-6.20E-01	-3.35E+00	1.26E-02	NA	-5.47E-01	-2.77E+00
Chga	_DDGQSDS[Phospho (STY)]QAVDGDGK_.2	P26339_S282_M1_Chga	3.56E-03	NA	-6.20E-01	-3.35E+00	1.40E-02	NA	-5.40E-01	-2.72E+00
Map7d1	_RKPGAGGS[Phospho (STY)]PALAR_.3	A2AJI0_S401_M1_Map7d1	3.60E-03	NA	-6.19E-01	-3.35E+00	8.29E-03	NA	-5.73E-01	-2.97E+00
Rbbp6	_VDGDREKS[Phospho (STY)]PR_.2	P97868_S1053_M1_Rbbp6	3.64E-03	NA	-6.19E-01	-3.34E+00	4.90E-03	NA	-6.03E-01	-3.21E+00
Safb	_MSS[Phospho (STY)]VEEDSDTKR_.2	D3YXK2_S406_M1_Safb	3.72E-03	NA	-6.18E-01	-3.33E+00	5.76E-03	NA	-5.94E-01	-3.13E+00
Rap1a	_QWC[Carbamidomethyl (C)]NC[Carbamidomethyl (C)]AFLESSAK_.2	#N/A	3.72E-03	NA	-6.17E-01	-3.33E+00	6.91E-03	NA	-5.84E-01	-3.05E+00
Cpox	_SPS[Phospho (STY)]PGRREEDGDELAR_.3	P36552_S101_M1_Cpox	3.78E-03	NA	-6.17E-01	-3.32E+00	6.40E-03	NA	-5.88E-01	-3.08E+00
Palm2	_LEQEIQALESEESQISAK_.2	#N/A	3.93E-03	NA	-6.15E-01	-3.31E+00	8.82E-03	NA	-5.69E-01	-2.94E+00
Phyhipl	_DNSGSHGSPISGK_.2	#N/A	4.09E-03	NA	-6.13E-01	-3.29E+00	1.09E-02	NA	-5.56E-01	-2.84E+00
Rbm15	_DRT[Phospho (STY)]PPLLYR_.2	Q0VBL3_T567_M1_Rbm15	4.09E-03	NA	-6.13E-01	-3.29E+00	5.47E-03	NA	-5.97E-01	-3.16E+00
Sorbs2	_RGS[Phospho (STY)]LPDNSILHR_.3	Q3UTJ2_S829_M1_Sorbs2	4.13E-03	NA	-6.12E-01	-3.28E+00	1.39E-02	NA	-5.40E-01	-2.72E+00
Rgs6	_SVYGVTDETQSQS[Phospho (STY)]PVHIPSQPIRK_.3	Q9Z2H2_S244_M1_Rgs6	4.16E-03	NA	-6.12E-01	-3.28E+00	8.38E-03	NA	-5.72E-01	-2.96E+00
U2af2	_GAKEEHGGLIRS[Phospho (STY)]PR_.3	P26369_S79_M1_U2af2	4.18E-03	NA	-6.11E-01	-3.28E+00	1.89E-02	NA	-5.20E-01	-2.58E+00
Sec62	_EELEQQT[Phospho (STY)]DGDC[Carbamidomethyl (C)]DEEDDDKDGEVPK_.3	Q8BU14_T375_M1_Sec62	4.25E-03	NA	-6.10E-01	-3.27E+00	1.39E-02	NA	-5.40E-01	-2.72E+00
Nelfe	_SM[Oxidation (M)]S[Phospho (STY)]ADEDLQEPSR_.2	P19426_S115_M1_Nelfe	4.53E-03	NA	-6.07E-01	-3.24E+00	1.60E-02	NA	-5.31E-01	-2.66E+00
Sv2a	_GGLS[Phospho (STY)]DGEGPPGGRGEAQR_.2	Q9JIS5_S127_M1_Sv2a	4.63E-03	NA	-6.06E-01	-3.23E+00	2.09E-02	NA	-5.12E-01	-2.53E+00
Atp2a2	_IVEFLQSFDEITAM[Oxidation (M)]TGDGVNDAPALKK_.3	#N/A	4.67E-03	NA	-6.05E-01	-3.23E+00	6.61E-03	NA	-5.86E-01	-3.07E+00
Ogdh	_TSFDEM[Oxidation (M)]LPGTHFQR_.3	#N/A	4.73E-03	NA	-6.05E-01	-3.22E+00	7.80E-03	NA	-5.76E-01	-2.99E+00
Arhgef10	_T[Phospho (STY)]RSLVSQDHR_.3	Q8C033_T353_M1_Arhgef10	4.87E-03	NA	-6.03E-01	-3.21E+00	7.18E-03	NA	-5.81E-01	-3.03E+00
Uqcrfs1	_EIDQEAAVEVSQLRDPQHDLDR_.3	#N/A	4.94E-03	NA	-6.02E-01	-3.20E+00	3.62E-03	NA	-6.19E-01	-3.34E+00
Blvra	_SQEVDVAYIC[Carbamidomethyl (C)]TESSSHEDYIR_.3	#N/A	4.95E-03	NA	-6.02E-01	-3.20E+00	2.44E-02	NA	-5.01E-01	-2.46E+00
Skp1	_LQSSDGEIFEVDVEIAK_.2	#N/A	5.12E-03	NA	-6.00E-01	-3.19E+00	9.34E-03	NA	-5.66E-01	-2.91E+00
Ccdc85a	_HALGGS[Phospho (STY)]LEHLPR_.3	Q5SP85_S342_M1_Ccdc85a	5.12E-03	NA	-6.00E-01	-3.19E+00	1.29E-02	NA	-5.45E-01	-2.76E+00
Srrm2	_MS[Phospho (STY)]C[Carbamidomethyl (C)]FS[Phospho (STY)]RPSM[Oxidation (M)]SPTPLDR_.2	Q8BTI8_S2067_M2_Srrm2	5.28E-03	NA	-5.99E-01	-3.17E+00	3.67E-03	NA	-6.18E-01	-3.34E+00
Stx1b	_QHSAILAAPNPDEK_.2	#N/A	5.29E-03	NA	5.99E-01	3.17E+00	1.53E-02	NA	5.34E-01	2.68E+00
Stmn3	_ALEENNNFSR_.2	#N/A	5.31E-03	NA	-5.98E-01	-3.17E+00	6.79E-03	NA	-5.85E-01	-3.06E+00
Phb2	_IGGVQQDTILAEGLHFR_.3	#N/A	5.44E-03	NA	-5.97E-01	-3.16E+00	2.23E-02	NA	-5.08E-01	-2.50E+00
Dync1i2	_EIAVGDSEGQIVIYDVGEQIAVPR_.3	#N/A	5.47E-03	NA	-5.97E-01	-3.16E+00	1.99E-02	NA	-5.16E-01	-2.55E+00
Gps1	_SPPREGS[Phospho (STY)]QGELTPANSQSR_.3	Q99LD4_S454_M1_Gps1	5.51E-03	NA	-5.96E-01	-3.15E+00	2.05E-02	NA	-5.14E-01	-2.54E+00
Lrp1	_AEGSEYQVLYIADDNEIR_.2	#N/A	5.51E-03	NA	-5.96E-01	-3.15E+00	1.57E-02	NA	-5.32E-01	-2.67E+00
Hdgf	_AGDVLEDS[Phospho (STY)]PKRPK_.2	P51859_S165_M1_Hdgf	5.52E-03	NA	-5.96E-01	-3.15E+00	2.14E-02	NA	-5.11E-01	-2.52E+00
Uqcrc2	_LPNGLVIASLENYAPLSR_.2	#N/A	5.53E-03	NA	5.96E-01	3.15E+00	2.19E-02	NA	5.09E-01	2.51E+00
Gdap1l1	_DNIISDYDQIIDYVER_.2	#N/A	5.57E-03	NA	-5.96E-01	-3.15E+00	1.43E-02	NA	-5.39E-01	-2.71E+00
Stmn4	_RRDPS[Phospho (STY)]LEEIQK_.3	P63042_S90_M1_Stmn4	5.63E-03	NA	-5.95E-01	-3.14E+00	6.06E-03	NA	-5.91E-01	-3.11E+00
Nfix	_S[Phospho (STY)]IDDSEMESPVDDVFYPGTGR_.2	P70257_S280_M1_Nfix	5.69E-03	NA	-5.95E-01	-3.14E+00	4.96E-03	NA	-6.02E-01	-3.20E+00
Dpysl2	_IVNDDQSFYADIYM[Oxidation (M)]EDGLIK_.2	#N/A	5.81E-03	NA	-5.93E-01	-3.13E+00	2.10E-02	NA	-5.12E-01	-2.53E+00
Tjp1	_SAS[Phospho (STY)]LENKK_.2	P39447_S1277_M1_Tjp1	5.82E-03	NA	-5.93E-01	-3.13E+00	7.79E-03	NA	-5.77E-01	-2.99E+00
Tubb3	_M[Oxidation (M)]SSTFIGNSTAIQELFKR_.3	#N/A	5.87E-03	NA	-5.93E-01	-3.12E+00	7.28E-03	NA	-5.81E-01	-3.02E+00
Lysmd2	_AHRPSAPS[Phospho (STY)]PPPR_.3	Q9D7V2_S24_M1_Lysmd2	5.90E-03	NA	-5.93E-01	-3.12E+00	5.31E-03	NA	-5.98E-01	-3.17E+00
Hdac5	_TQS[Phospho (STY)]SPAAPGSMK_.2	Q9Z2V6_S650_M1_Hdac5	6.06E-03	NA	-5.91E-01	-3.11E+00	2.33E-02	NA	-5.04E-01	-2.48E+00
Adss2	_ELPVNAQNYVR_.2	#N/A	6.07E-03	NA	5.91E-01	3.11E+00	4.43E-03	NA	6.08E-01	3.25E+00
Txnrd1	_VVYENAYGR_.2	#N/A	6.15E-03	NA	-5.90E-01	-3.10E+00	1.70E-02	NA	-5.27E-01	-2.63E+00
Map2	_YTVPLPSPVQDSENLSGESGSFYEGTDDKVR_.3	#N/A	6.15E-03	NA	-5.90E-01	-3.10E+00	2.26E-02	NA	-5.07E-01	-2.49E+00
Trim2	_VFNQEGEFMLK_.2	#N/A	6.18E-03	NA	-5.90E-01	-3.10E+00	7.43E-03	NA	-5.79E-01	-3.02E+00
Map1b	_RSES[Phospho (STY)]PFEGK_.2	P14873_S1422_M1_Map1b	6.19E-03	NA	-5.90E-01	-3.10E+00	8.51E-03	NA	-5.71E-01	-2.95E+00
Plxna1	_QTSAYNISNS[Phospho (STY)]STFTK_.2	P70206_S1611_M1_Plxna1	6.26E-03	NA	-5.89E-01	-3.09E+00	8.58E-03	NA	-5.71E-01	-2.95E+00
Slc1a2	_TQSIY[Phospho (STY)]DDKNHRESNSNQC[Carbamidomethyl (C)]VYAAHNSVVIDEC[Carbamidomethyl (C)]K_.5	P43006_Y522_M1_Slc1a2	6.34E-03	NA	-5.88E-01	-3.09E+00	3.24E-03	NA	-6.25E-01	-3.39E+00
Mark3	_TPVAS[Phospho (STY)]THS[Phospho (STY)]ISSATTPDR_.2	Q03141_S540_M2_Mark3	6.41E-03	NA	-5.88E-01	-3.08E+00	7.90E-03	NA	-5.76E-01	-2.99E+00
Rab21	_DSNGAILVYDVTDEDSFQK_.2	#N/A	6.45E-03	NA	-5.88E-01	-3.08E+00	2.20E-02	NA	-5.09E-01	-2.51E+00
Vdac3	_LTLDTIFVPNTGKK_.2	#N/A	6.51E-03	NA	-5.87E-01	-3.08E+00	1.43E-02	NA	-5.38E-01	-2.71E+00
Ctsb	_GENHC[Carbamidomethyl (C)]GIESEIVAGIPR_.3	#N/A	6.73E-03	NA	5.85E-01	3.06E+00	1.51E-02	NA	5.35E-01	2.69E+00
Arhgap31	_S[Phospho (STY)]MDSLC[Carbamidomethyl (C)]SVPVEGK_.2	A6X8Z5_S343_M1_Arhgap31	6.73E-03	NA	-5.85E-01	-3.06E+00	7.83E-03	NA	-5.76E-01	-2.99E+00
Ccny	_ASTIFLSKSQT[Phospho (STY)]DVR_.2	Q8BGU5_T75_M1_Ccny	6.79E-03	NA	-5.85E-01	-3.06E+00	1.40E-02	NA	-5.40E-01	-2.72E+00
Hnrnph1	_HTGPNS[Phospho (STY)]PDTANDGFVR_.2	O35737_S104_M1_Hnrnph1	6.81E-03	NA	-5.84E-01	-3.06E+00	1.69E-02	NA	-5.27E-01	-2.63E+00
Wdtc1	_KDS[Phospho (STY)]ISEDEM[Oxidation (M)]VLR_.2	Q80ZK9_S511_M1_Wdtc1	6.84E-03	NA	-5.84E-01	-3.05E+00	1.80E-02	NA	-5.23E-01	-2.60E+00
Bdh1	_AILITGC[Carbamidomethyl (C)]DSGFGFSLAK_.2	#N/A	6.84E-03	NA	-5.84E-01	-3.05E+00	9.65E-03	NA	-5.64E-01	-2.89E+00
Braf	_DS[Phospho (STY)]SDDWEIPDGQITVGQR_.2	P28028_S431_M1_Braf	6.96E-03	NA	-5.83E-01	-3.05E+00	1.85E-02	NA	-5.21E-01	-2.59E+00

Hspa8	_HWPFM[Oxidation (M)]VVNDAGRPK_.3	#N/A	6.96E-03	NA	-5.83E-01	-3.05E+00	1.52E-02	NA	-5.34E-01	-2.68E+00
Trip12	_DDSLDLS[Phospho (STY)]PQGR_.2	G5E870_S1024_M1_Trip12	6.97E-03	NA	5.83E-01	3.04E+00	4.20E-03	NA	6.11E-01	3.28E+00
H2az1;H2az2	_GDEELDSLIK_.2	#N/A	7.13E-03	NA	-5.82E-01	-3.03E+00	7.83E-03	NA	-5.76E-01	-2.99E+00
Sipa1l1	_SKS[Phospho (STY)]ETGDSSIFRK_.2	Q8C0T5_S288_M1_Sipa1l1	7.14E-03	NA	-5.82E-01	-3.03E+00	1.50E-02	NA	-5.35E-01	-2.69E+00
Napg	_AVQLYQQTANVFENEER_.2	#N/A	7.31E-03	NA	-5.80E-01	-3.02E+00	8.64E-03	NA	-5.70E-01	-2.95E+00
Bloc1s4	_[Acetyl (Protein N-term)]MEEGPAVGTLS[Phospho (STY)]R_.2	Q8VED2_S11_M1_Bloc1s4	7.41E-03	NA	-5.79E-01	-3.02E+00	1.47E-02	NA	-5.37E-01	-2.70E+00
Slain2	_S[Phospho (STY)]LPNLSR_.2	Q8CI08_S414_M1_Slain2	7.50E-03	NA	-5.79E-01	-3.01E+00	1.28E-02	NA	-5.46E-01	-2.76E+00
Pip5k1c	_IM[Oxidation (M)]DYSLLLGVHNIDQQER_.3	#N/A	7.84E-03	NA	-5.76E-01	-2.99E+00	1.04E-02	NA	-5.59E-01	-2.86E+00
Wdr1	_VVTVFSVADGYSENNVFYGHHAK_.3	#N/A	7.86E-03	NA	-5.76E-01	-2.99E+00	5.48E-03	NA	-5.97E-01	-3.16E+00
Efhd2	_ELQSTFK_.2	#N/A	7.94E-03	NA	5.75E-01	2.99E+00	1.92E-02	NA	5.18E-01	2.57E+00
Fra10ac1	_NAEEEDSAS[Phospho (STY)]DSELWK_.2	Q8BP78_S285_M1_Fra10ac1	7.95E-03	NA	-5.75E-01	-2.98E+00	2.43E-02	NA	-5.01E-01	-2.46E+00
Eif2s2	_[Acetyl (Protein N-term)]S[Phospho (STY)]GDEM[Oxidation (M)]IFDPTM[Oxidation (M)]SK_.2	#N/A	8.02E-03	NA	-5.75E-01	-2.98E+00	2.08E-02	NA	-5.13E-01	-2.53E+00
Prkar1b	_IVVQGEPGDDFYIITEGTASVLQR_.3	#N/A	8.04E-03	NA	5.75E-01	2.98E+00	1.38E-02	NA	5.41E-01	2.73E+00
Ogfrl1	_AS[Phospho (STY)]PVPEDHAEAAGAEQGGESTEGNAKPK_.4	Q8VE52_S65_M1_Ogfrl1	8.13E-03	NA	-5.74E-01	-2.97E+00	6.66E-03	NA	-5.86E-01	-3.07E+00
Gstp1	_PPYTIVYFPVR_.2	#N/A	8.14E-03	NA	5.74E-01	2.97E+00	2.60E-03	NA	6.36E-01	3.49E+00
Mfap1a;Mfap1b	_KIVEPEVVGES[Phospho (STY)]DS[Phospho (STY)]EVEGDAWR_.3	C0HKD8_S116_M2_Mfap1a;Mf	8.19E-03	NA	-5.74E-01	-2.97E+00	1.53E-02	NA	-5.34E-01	-2.68E+00
Tubb4b	_M[Oxidation (M)]SATFIGNSTAIQELFKR_.3	#N/A	8.35E-03	NA	-5.72E-01	-2.96E+00	1.80E-02	NA	-5.23E-01	-2.60E+00
Rbl2	_AQPAGS[Phospho (STY)]PSHQIQQR_.2	Q64700_S37_M1_Rbl2	8.46E-03	NA	-5.72E-01	-2.96E+00	2.00E-02	NA	-5.16E-01	-2.55E+00
Sv2b	_DMC[Carbamidomethyl (C)]LSS[Phospho (STY)]SKK_.2	Q8BG39_S144_M1_Sv2b	8.49E-03	NA	-5.71E-01	-2.95E+00	2.43E-02	NA	-5.01E-01	-2.46E+00
Pfkfb2	_NS[Phospho (STY)]FTPLSSSNTIR_.2	P70265_S469_M1_Pfkfb2	8.49E-03	NA	-5.71E-01	-2.95E+00	1.06E-02	NA	-5.58E-01	-2.85E+00
Mif	_PAQYIAVHVVPDQLM[Oxidation (M)]TFSGTNDPC[Carbamidomethyl (C)]ALC[Carbamidomethyl (C)]SLHSIGK_.4	#N/A	8.52E-03	NA	-5.71E-01	-2.95E+00	1.63E-02	NA	-5.30E-01	-2.65E+00
Ppp1r1b	_ELGYPQEDDEEDEDEEEDEEEDSQAEVLK_.3	#N/A	8.54E-03	NA	-5.71E-01	-2.95E+00	1.78E-02	NA	-5.24E-01	-2.61E+00
Pcm1	_VTNAIS[Phospho (STY)]PESSPGVGR_.2	Q9R0L6_S65_M1_Pcm1	8.62E-03	NA	-5.71E-01	-2.95E+00	2.27E-02	NA	-5.06E-01	-2.49E+00
Ndufa6	_NAHVTDPR_.2	#N/A	8.71E-03	NA	5.70E-01	2.94E+00	7.58E-03	NA	5.78E-01	3.01E+00
Abi1	_TNPPTQKPPS[Phospho (STY)]PPVSGR_.2	Q8CBW3_S183_M1_Abi1	8.82E-03	NA	-5.69E-01	-2.94E+00	1.62E-02	NA	-5.30E-01	-2.65E+00
Aco2	_GHLDNISNNLLIGAINIENGK_.3	#N/A	8.93E-03	NA	-5.68E-01	-2.93E+00	8.74E-03	NA	-5.70E-01	-2.94E+00
Actr10	_RAFS[Phospho (STY)]TEK_.2	Q9QZB7_S414_M1_Actr10	9.01E-03	NA	-5.68E-01	-2.93E+00	4.54E-03	NA	-6.07E-01	-3.24E+00
Atrx	_IGKQS[Phospho (STY)]EES[Phospho (STY)]PADDGELRR_.3	Q61687_S1287_M2_Atrx	9.05E-03	NA	-5.68E-01	-2.92E+00	1.64E-02	NA	-5.29E-01	-2.65E+00
Ywhaz	_YDDM[Oxidation (M)]AAC[Carbamidomethyl (C)]M[Oxidation (M)]K_.2	#N/A	9.13E-03	NA	5.67E-01	2.92E+00	1.35E-02	NA	5.43E-01	2.74E+00
Dab2ip	_QQSSSSKGDS[Phospho (STY)]PELKPR_.3	Q3UHC7_S978_M1_Dab2ip	9.45E-03	NA	-5.65E-01	-2.90E+00	9.79E-03	NA	-5.63E-01	-2.89E+00
Bcan	_C[Carbamidomethyl (C)]SPGWLADGSVR_.2	#N/A	9.51E-03	NA	5.65E-01	2.90E+00	2.05E-02	NA	5.14E-01	2.54E+00
Ckb	_HGGYQPSDEHKTDLNPDNLQGGDDLDPNYVLSSR_.5	#N/A	9.54E-03	NA	-5.64E-01	-2.90E+00	1.61E-02	NA	-5.31E-01	-2.66E+00
Cap2	_SSEM[Oxidation (M)]NVLVPQDDDYREFPIPEQFK_.3	#N/A	9.84E-03	NA	-5.62E-01	-2.89E+00	2.36E-02	NA	-5.04E-01	-2.47E+00
Rock2	_NLLLLANSTEEQQK_.2	#N/A	9.96E-03	NA	5.62E-01	2.88E+00	7.85E-03	NA	5.76E-01	2.99E+00
Rock2	_NDQWNWDNIR_.2	#N/A	1.00E-02	NA	-5.61E-01	-2.88E+00	2.31E-02	NA	-5.05E-01	-2.48E+00
Hnrnpa2b1	_NM[Oxidation (M)]GGPYGGGNYGPGGSGGS[Phospho (STY)]GGYGGR_.3	O88569_S344_M1_Hnrnpa2b1	1.00E-02	NA	-5.61E-01	-2.88E+00	1.49E-02	NA	-5.36E-01	-2.69E+00
Jcad	_APQASIC[Carbamidomethyl (C)]SDVDGFPTSQAT[Phospho (STY)]SPEPGKKDEEAK_.4	Q5DTX6_T1164_M1_Jcad	1.01E-02	NA	-5.61E-01	-2.88E+00	1.48E-02	NA	-5.36E-01	-2.70E+00
Gdi2	_VLHM[Oxidation (M)]DQNPYYGGESASITPLEDLYK_.3	#N/A	1.02E-02	NA	-5.60E-01	-2.87E+00	4.48E-03	NA	-6.08E-01	-3.25E+00
Arhgap33	_S[Phospho (STY)]AKSEESLSSQASGAGLQR_.3	Q80YF9_S657_M1_Arhgap33	1.08E-02	NA	-5.57E-01	-2.84E+00	1.97E-02	NA	-5.16E-01	-2.56E+00
Bcas3	_LTSQDS[Phospho (STY)]YNNFTNNNPGNPR_.2	Q8CCN5_S503_M1_Bcas3	1.08E-02	NA	-5.57E-01	-2.84E+00	2.15E-03	NA	-6.45E-01	-3.58E+00
Dzank1	_FTQGLEDQLS[Phospho (STY)]PPGNR_.2	Q8C008_S768_M1_Dzank1	1.09E-02	NA	-5.56E-01	-2.84E+00	2.25E-02	NA	-5.07E-01	-2.50E+00
Add2	_LEQEQEGEKDIATEKPGS[Phospho (STY)]PVK_.3	Q9QYB8_S594_M1_Add2	1.09E-02	NA	5.56E-01	2.84E+00	1.95E-02	NA	5.17E-01	2.56E+00
Mgll	_ALIFVSHGAGEHC[Carbamidomethyl (C)]GR_.3	#N/A	1.10E-02	NA	5.55E-01	2.83E+00	7.08E-03	NA	5.82E-01	3.04E+00
Cox5b	_LVPHQMAH_.2	#N/A	1.11E-02	NA	5.55E-01	2.83E+00	2.01E-02	NA	5.15E-01	2.55E+00
Atp1a1	_M[Oxidation (M)]TVAHM[Oxidation (M)]WFDNQIHEADTTENQSGVSFDK_.4	#N/A	1.12E-02	NA	-5.54E-01	-2.83E+00	1.65E-02	NA	-5.29E-01	-2.64E+00
Map1a	_ESTFLDEGPNEQEITPLQHTPR_.3	#N/A	1.12E-02	NA	-5.54E-01	-2.82E+00	9.57E-03	NA	-5.64E-01	-2.90E+00
Amer2	_AAGPGNLVLPGS[Phospho (STY)]LTAS[Phospho (STY)]LEC[Carbamidomethyl (C)]VKEEPPR_.3	Q8CCJ4_S223_M2_Amer2	1.14E-02	NA	-5.53E-01	-2.82E+00	7.61E-03	NA	-5.78E-01	-3.00E+00
Dnajc6	_SAATS[Phospho (STY)]PTGSSHGTPTHQSK_.3	Q80TZ3_S683_M1_Dnajc6	1.16E-02	NA	-5.52E-01	-2.81E+00	5.47E-03	NA	-5.97E-01	-3.16E+00
Snrnp70	_GGGGS[Phospho (STY)]GQDNGLEGLGSDGR_.2	Q62376_S408_M1_Snrnp70	1.17E-02	NA	-5.52E-01	-2.81E+00	1.29E-02	NA	-5.45E-01	-2.76E+00
Eif4g1	_SS[Phospho (STY)]LS[Phospho (STY)]RER_.2	Q6NZJ6_S1147_M2_Eif4g1	1.19E-02	NA	-5.51E-01	-2.80E+00	1.40E-02	NA	-5.40E-01	-2.72E+00
Lyst	_RM[Oxidation (M)]S[Phospho (STY)]QEHPSQASEAELAQR_.3	P97412_S2614_M1_Lyst	1.21E-02	NA	-5.49E-01	-2.79E+00	2.21E-02	NA	-5.08E-01	-2.50E+00
Rab7a	_EAINVEQAFQTIAR_.2	#N/A	1.22E-02	NA	-5.49E-01	-2.79E+00	2.42E-02	NA	-5.02E-01	-2.46E+00
Matr3	_SYS[Phospho (STY)]PDGKES[Phospho (STY)]PSDKK_.2	Q8K310_S604_M2_Matr3	1.22E-02	NA	-5.49E-01	-2.79E+00	2.22E-02	NA	-5.08E-01	-2.50E+00
Rpl5	_YLMEEDEDAYKK_.3	#N/A	1.22E-02	NA	-5.49E-01	-2.78E+00	2.05E-02	NA	-5.14E-01	-2.54E+00
Samd14	_GSASSGSTTSPT[Phospho (STY)]C[Carbamidomethyl (C)]SPK_.2	Q8K070_T258_M1_Samd14	1.23E-02	NA	-5.49E-01	-2.78E+00	9.12E-03	NA	-5.67E-01	-2.92E+00
Prpf4b	_S[Phospho (STY)]KS[Phospho (STY)]PVDLRDK_.2	Q61136_S292_M2_Prpf4b	1.23E-02	NA	-5.48E-01	-2.78E+00	5.93E-03	NA	-5.92E-01	-3.12E+00
Nek1	_SSDVPLPLELLET[Phospho (STY)]GGSPSK_.2	P51954_T615_M1_Nek1	1.24E-02	NA	5.48E-01	2.78E+00	3.60E-03	NA	6.19E-01	3.35E+00

Pebp1	_EWHHFLVVNM[Oxidation (M)]K_.2	#N/A	1.25E-02	NA	-5.47E-01	-2.77E+00	6.20E-03	NA	-5.90E-01	-3.10E+00
Gda	_IGLGTDVAGGYSYSM[Oxidation (M)]LDAIR_.2	#N/A	1.26E-02	NA	-5.47E-01	-2.77E+00	1.40E-02	NA	-5.40E-01	-2.72E+00
Micall1	_APS[Phospho (STY)]AS[Phospho (STY)]PLAIHASR_.2	Q8BGT6_S494_M2_Micall1	1.28E-02	NA	-5.46E-01	-2.76E+00	7.25E-03	NA	-5.81E-01	-3.03E+00
Brsk2	_GS[Phospho (STY)]PLPTPK_.2	Q69Z98_S440_M1_Brsk2	1.29E-02	NA	-5.45E-01	-2.76E+00	2.37E-02	NA	-5.03E-01	-2.47E+00
Vdac1	_WNTDNTLGTEITVEDQLAR_.2	#N/A	1.30E-02	NA	-5.45E-01	-2.75E+00	6.30E-03	NA	-5.89E-01	-3.09E+00
Nrcam	_EPAEGNESSEAPS[Phospho (STY)]PVNAMNSFV_.2	Q810U4_S1247_M1_Nrcam	1.31E-02	NA	-5.44E-01	-2.75E+00	2.07E-02	NA	-5.13E-01	-2.54E+00
Phb	_EFTEAVEAK_.2	#N/A	1.31E-02	NA	5.44E-01	2.75E+00	2.45E-02	NA	5.01E-01	2.46E+00
Psmb5	_FLHGVIVAADSR_.3	#N/A	1.33E-02	NA	5.43E-01	2.75E+00	1.14E-02	NA	5.53E-01	2.82E+00
H2ac20;Hist2h2aa1	_NDEELNKLLGK_.2	#N/A	1.33E-02	NA	-5.43E-01	-2.75E+00	1.82E-02	NA	-5.22E-01	-2.60E+00
Shroom2	_IVHSES[Phospho (STY)]QPEKESR_.2	A2ALU4_S1089_M1_Shroom2	1.35E-02	NA	-5.42E-01	-2.74E+00	1.30E-02	NA	-5.45E-01	-2.76E+00
Dpysl4	_GAADMVAQAK_.2	#N/A	1.36E-02	NA	5.42E-01	2.74E+00	1.56E-02	NA	5.33E-01	2.67E+00
Nyap1	_RPPAKPR_.2	#N/A	1.37E-02	NA	5.41E-01	2.73E+00	1.27E-02	NA	5.46E-01	2.77E+00
Fam135b	_NTAGFSEDLDPSSKENS[Phospho (STY)]PPR_.3	Q9DAI6_S805_M1_Fam135b	1.38E-02	NA	-5.41E-01	-2.73E+00	1.74E-02	NA	-5.25E-01	-2.62E+00
Aldoa	_GGVVGIKVDK_.2	#N/A	1.38E-02	NA	-5.41E-01	-2.73E+00	1.17E-02	NA	-5.51E-01	-2.80E+00
Kctd8	_IVS[Phospho (STY)]PKQEHEDR_.2	Q50H33_S331_M1_Kctd8	1.39E-02	NA	-5.40E-01	-2.72E+00	1.99E-02	NA	-5.16E-01	-2.55E+00
Psip1	_QVDTEEAGM[Oxidation (M)]VTAATASNVKAS[Phospho (STY)]PKR_.3	#N/A	1.43E-02	NA	-5.38E-01	-2.71E+00	1.74E-02	NA	-5.25E-01	-2.62E+00
Dync1li1	_VPGGS[Phospho (STY)]PRT[Phospho (STY)]PNR_.2	Q8R1Q8_S405_M2_Dync1li1	1.44E-02	NA	-5.38E-01	-2.71E+00	1.86E-02	NA	-5.20E-01	-2.59E+00
Clta	_LC[Carbamidomethyl (C)]DFNPK_.2	#N/A	1.45E-02	NA	5.38E-01	2.71E+00	9.52E-03	NA	5.64E-01	2.90E+00
Ak1	_LETYYNATEPVISFYDKR_.3	#N/A	1.45E-02	NA	5.38E-01	2.70E+00	9.80E-03	NA	5.63E-01	2.89E+00
Cdc42bpb	_ALGNS[Phospho (STY)]NRDKEIK_.3	Q7TT50_S481_M1_Cdc42bpb	1.46E-02	NA	-5.37E-01	-2.70E+00	1.13E-02	NA	-5.54E-01	-2.82E+00
Rab3c	_LVDIIC[Carbamidomethyl (C)]DK_.2	#N/A	1.46E-02	NA	5.37E-01	2.70E+00	1.06E-02	NA	5.58E-01	2.85E+00
Ptgds	_AQGLTEEDIVFLPQPDK_.2	#N/A	1.47E-02	NA	5.37E-01	2.70E+00	1.18E-02	NA	5.51E-01	2.80E+00
Fscn1	_KVTGTLDANR_.2	#N/A	1.49E-02	NA	5.36E-01	2.69E+00	2.40E-02	NA	5.02E-01	2.47E+00
Mark1	_SAAYNGPPAS[Phospho (STY)]PSHETGAFAHAR_.3	Q8VHJ5_S633_M1_Mark1	1.53E-02	NA	5.34E-01	2.68E+00	1.31E-02	NA	5.44E-01	2.75E+00
Nav1	_APEAAVS[Phospho (STY)]DDGKS[Phospho (STY)]DDELLSSK_.2	Q8CH77_S197_M2_Nav1	1.54E-02	NA	-5.34E-01	-2.68E+00	2.47E-02	NA	-5.00E-01	-2.45E+00
Aldoc	_TVPPAVPGVTFLSGGQSEEEASLNLNAINR_.3	#N/A	1.54E-02	NA	-5.34E-01	-2.68E+00	2.38E-02	NA	-5.03E-01	-2.47E+00
Sptan1	_YTEHSTVGLAQQWDQLDQLGM[Oxidation (M)]R_.3	#N/A	1.54E-02	NA	-5.34E-01	-2.68E+00	8.90E-03	NA	-5.69E-01	-2.93E+00
Acaca	_SSM[Oxidation (M)]S[Phospho (STY)]GLHLVK_.2	Q5SWU9_S79_M1_Acaca	1.56E-02	NA	-5.33E-01	-2.67E+00	1.45E-02	NA	-5.38E-01	-2.71E+00
Pcm1	_NVRS[Phospho (STY)]DVS[Phospho (STY)]DQEEDEESER_.2	Q9R0L6_S1766_M2_Pcm1	1.58E-02	NA	-5.32E-01	-2.66E+00	1.08E-02	NA	-5.57E-01	-2.84E+00
Gdi2	_FLM[Oxidation (M)]ANGQLVK_.2	#N/A	1.58E-02	NA	5.32E-01	2.66E+00	7.10E-03	NA	5.82E-01	3.04E+00
Acaa2	_VGVPTETGALTLNR_.2	#N/A	1.59E-02	NA	5.31E-01	2.66E+00	8.69E-03	NA	5.70E-01	2.94E+00
Phf8	_SSGSSSSGLGTVSSS[Phospho (STY)]PASQR_.2	Q80TJ7_S768_M1_Phf8	1.60E-02	NA	-5.31E-01	-2.66E+00	1.97E-02	NA	-5.17E-01	-2.56E+00
Ampd2	_QIS[Phospho (STY)]QDVKLEPDILLR_.3	Q9DBT5_S87_M1_Ampd2	1.63E-02	NA	-5.30E-01	-2.65E+00	2.05E-02	NA	-5.14E-01	-2.54E+00
Lrrc47	_KQHRES[Phospho (STY)]GEGEEEVADSAR_.3	Q505F5_S293_M1_Lrrc47	1.63E-02	NA	-5.30E-01	-2.65E+00	1.29E-02	NA	-5.45E-01	-2.76E+00
Bcas1	_RQS[Phospho (STY)]LGGFLK_.2	Q80YN3_S601_M1_Bcas1	1.65E-02	NA	-5.29E-01	-2.64E+00	2.14E-02	NA	-5.11E-01	-2.52E+00
Nucks1	_EEDEEAES[Phospho (STY)]PPEKK_.2	Q80XU3_S214_M1_Nucks1	1.65E-02	NA	-5.29E-01	-2.64E+00	8.23E-03	NA	-5.73E-01	-2.97E+00
Tomm70	_EC[Carbamidomethyl (C)]LEDVTAVC[Carbamidomethyl (C)]ILEGFQNEQSM[Oxidation (M)]LLADK_.3	#N/A	1.66E-02	NA	-5.29E-01	-2.64E+00	1.76E-02	NA	-5.24E-01	-2.61E+00
Mark4	_RS[Phospho (STY)]PTSTGDTELKEER_.3	Q8CIP4_S438_M1_Mark4	1.66E-02	NA	-5.29E-01	-2.64E+00	1.07E-02	NA	-5.57E-01	-2.85E+00
Got1	_QIAAVM[Oxidation (M)]QR_.2	#N/A	1.67E-02	NA	5.28E-01	2.64E+00	1.90E-02	NA	5.19E-01	2.58E+00
Gapvd1	_LQELESC[Carbamidomethyl (C)]SGLGS[Phospho (STY)]TSDDTDVR_.2	Q6PAR5_S746_M1_Gapvd1	1.68E-02	NA	-5.27E-01	-2.63E+00	1.98E-02	NA	-5.16E-01	-2.56E+00
Pde8a	_RFS[Phospho (STY)]GNEYILATK_.2	O88502_S452_M1_Pde8a	1.70E-02	NA	5.27E-01	2.63E+00	2.20E-02	NA	5.09E-01	2.51E+00
Dnm1	_NFRPDDPAR_.2	#N/A	1.71E-02	NA	5.27E-01	2.63E+00	1.53E-02	NA	5.34E-01	2.68E+00
Srgap3	_SSS[Phospho (STY)]SSSSGVGSPAVTPTEK_.2	Q812A2_S1070_M1_Srgap3	1.71E-02	NA	-5.27E-01	-2.63E+00	6.11E-03	NA	-5.91E-01	-3.10E+00
Atg4c	_DFDFTSTAAS[Phospho (STY)]EEDLFSEDERK_.3	Q811C2_S434_M1_Atg4c	1.72E-02	NA	5.26E-01	2.62E+00	1.68E-02	NA	5.27E-01	2.63E+00
Tcp1	_M[Oxidation (M)]LVDDIGDVTITNDGATILK_.2	#N/A	1.73E-02	NA	-5.26E-01	-2.62E+00	1.73E-02	NA	-5.26E-01	-2.62E+00
Ctr9	_NSNS[Phospho (STY)]DS[Phospho (STY)]DDDERPNR_.2	Q62018_S1039_M2_Ctr9	1.73E-02	NA	-5.26E-01	-2.62E+00	1.61E-02	NA	-5.31E-01	-2.66E+00
Got2	_EYLPIGGLAEFC[Carbamidomethyl (C)]K_.2	#N/A	1.73E-02	NA	5.26E-01	2.62E+00	1.28E-02	NA	5.46E-01	2.76E+00
Eef1a1	_M[Oxidation (M)]DSTEPPYSQK_.2	#N/A	1.78E-02	NA	5.24E-01	2.61E+00	1.14E-02	NA	5.53E-01	2.82E+00
Pafah1b2	_[Acetyl (Protein N-term)]SQGDSNPAAIPHAAEDIQGDDRWM[Oxidation (M)]SQHNR_.4	#N/A	1.79E-02	NA	-5.23E-01	-2.60E+00	1.40E-02	NA	-5.40E-01	-2.72E+00
Pfkl	_TNVLGHLQQGGAPTPFDR_.3	#N/A	1.80E-02	NA	-5.23E-01	-2.60E+00	1.30E-02	NA	-5.45E-01	-2.76E+00
Rpl23a	_LAPDYDALDVANK_.2	#N/A	1.81E-02	NA	-5.23E-01	-2.60E+00	1.91E-02	NA	-5.19E-01	-2.57E+00
Nf1	_SAGS[Phospho (STY)]FKR_.2	Q04690_S2831_M1_Nf1	1.81E-02	NA	-5.22E-01	-2.60E+00	1.34E-02	NA	-5.43E-01	-2.74E+00
Dnm1	_LQSQLLSIEKEVDEYK_.3	#N/A	1.82E-02	NA	-5.22E-01	-2.60E+00	1.70E-02	NA	-5.27E-01	-2.63E+00
Pak1	_STM[Oxidation (M)]VGTPYWM[Oxidation (M)]APEVVTR_.2	#N/A	1.82E-02	NA	-5.22E-01	-2.60E+00	1.46E-02	NA	-5.37E-01	-2.70E+00
Huwe1	_ESNQPPEDSS[Phospho (STY)]PPASSESSSTR_.2	Q7TMY8_S2918_M1_Huwe1	1.83E-02	NA	-5.22E-01	-2.59E+00	1.29E-02	NA	-5.45E-01	-2.76E+00
Acap2	_FVDKYSALLS[Phospho (STY)]PSEQEKR_.3	Q6ZQK5_S521_M1_Acap2	1.83E-02	NA	-5.22E-01	-2.59E+00	1.97E-02	NA	-5.17E-01	-2.56E+00
Arpc1a	_FC[Carbamidomethyl (C)]TTGIDGAM[Oxidation (M)]TIWDFK_.2	#N/A	1.94E-02	NA	-5.18E-01	-2.57E+00	1.38E-02	NA	-5.41E-01	-2.73E+00

Fscn1	_VGKDELFALEQSC[Carbamidomethyl (C)]AQVVLQAANER_.3	#N/A	1.96E-02	NA	-5.17E-01	-2.56E+00	2.33E-02	NA	-5.05E-01	-2.48E+00
Prep	_VFLDPNTLSDDGTVALR_.2	#N/A	1.97E-02	NA	5.17E-01	2.56E+00	1.81E-02	NA	5.23E-01	2.60E+00
Nfrkb	_SPS[Phospho (STY)]PAVPLR_.2	Q6PIJ4_S228_M1_Nfrkb	1.97E-02	NA	-5.17E-01	-2.56E+00	1.01E-02	NA	-5.61E-01	-2.87E+00
Snap25	_RADQLADESLESTRR_.3	#N/A	1.97E-02	NA	-5.17E-01	-2.56E+00	8.87E-03	NA	-5.69E-01	-2.93E+00
Syngap1	_VKEYEEEIHS[Phospho (STY)]LK_.3	F6SEU4_S1201_M1_Syngap1	2.02E-02	NA	-5.15E-01	-2.55E+00	4.33E-03	NA	-6.09E-01	-3.26E+00
Idh2	_GKLDGNQDLIR_.2	#N/A	2.02E-02	NA	-5.15E-01	-2.55E+00	1.16E-02	NA	-5.52E-01	-2.81E+00
Fabp3	_NGDTITIK_.2	#N/A	2.02E-02	NA	5.15E-01	2.55E+00	7.46E-03	NA	5.79E-01	3.01E+00
Suz12	_ASM[Oxidation (M)]SEFLES[Phospho (STY)]EDGEVEQQR_.2	Q80U70_S548_M1_Suz12	2.02E-02	NA	-5.15E-01	-2.55E+00	8.52E-03	NA	-5.71E-01	-2.95E+00
Matr3	_RRT[Phospho (STY)]EEGPTLSYGR_.3	Q8K310_T150_M1_Matr3	2.07E-02	NA	5.13E-01	2.54E+00	9.42E-03	NA	5.65E-01	2.91E+00
Eif3h	_DFS[Phospho (STY)]PEALKK_.2	Q91WK2_S183_M1_Eif3h	2.07E-02	NA	-5.13E-01	-2.54E+00	8.18E-03	NA	-5.74E-01	-2.97E+00
Vdac3	_[Acetyl (Protein N-term)]C[Carbamidomethyl (C)]NT[Phospho (STY)]PTYC[Carbamidomethyl (C)]DLGK_.2	Q60931_T4_M1_Vdac3	2.08E-02	NA	-5.13E-01	-2.53E+00	2.06E-02	NA	-5.13E-01	-2.54E+00
Hnrnpc	_NEKS[Phospho (STY)]EEEQSSASVKK_.3	Q9Z204_S241_M1_Hnrnpc	2.09E-02	NA	-5.13E-01	-2.53E+00	2.23E-02	NA	-5.08E-01	-2.50E+00
Rab39b	_SGFVPNVVHSS[Phospho (STY)]EEVIK_.2	Q8BHC1_S201_M1_Rab39b	2.09E-02	NA	-5.12E-01	-2.53E+00	1.95E-02	NA	-5.17E-01	-2.57E+00
Hnrnpu	_DLPEHAVLK_.2	#N/A	2.12E-02	NA	5.11E-01	2.52E+00	1.86E-02	NA	5.21E-01	2.59E+00
Pip5k1c	_ITVQVEPVC[Carbamidomethyl (C)]GVGVVPK_.2	#N/A	2.13E-02	NA	-5.11E-01	-2.52E+00	5.90E-03	NA	-5.93E-01	-3.12E+00
Kras	_LVVVGAGGVGK_.2	#N/A	2.16E-02	NA	5.10E-01	2.51E+00	1.04E-02	NA	5.59E-01	2.86E+00
Rps19	_ELAPYDENWFYTR_.2	#N/A	2.19E-02	NA	5.09E-01	2.51E+00	1.18E-02	NA	5.51E-01	2.80E+00
Fbxo2	_TDAGSLYELTVR_.2	#N/A	2.21E-02	NA	5.08E-01	2.51E+00	1.65E-02	NA	5.29E-01	2.64E+00
Rtn4	_LSAS[Phospho (STY)]PQEVGKPYLESFQPNLHITK_.3	Q99P72_S768_M1_Rtn4	2.23E-02	NA	-5.08E-01	-2.50E+00	1.68E-02	NA	-5.27E-01	-2.63E+00
Map1a	_M[Oxidation (M)]AS[Phospho (STY)]PPPSGPPSAAHTPFHQSPVEEK_.4	Q9QYR6_S991_M1_Map1a	2.24E-02	NA	-5.07E-01	-2.50E+00	1.48E-02	NA	-5.36E-01	-2.70E+00
Irs2	_GPGTGGDEASAAGGS[Phospho (STY)]PPQPPR_.2	P81122_S66_M1_Irs2	2.25E-02	NA	-5.07E-01	-2.50E+00	2.17E-02	NA	-5.10E-01	-2.51E+00
Hba	_VDPVNFK_.2	#N/A	2.27E-02	NA	5.07E-01	2.49E+00	9.25E-03	NA	5.66E-01	2.91E+00
Rcsd1	_S[Phospho (STY)]KDPGSPQLNQEAMADGVEGTPWSAEKPR_.4	Q3UZA1_S211_M1_Rcsd1	2.29E-02	NA	-5.06E-01	-2.49E+00	1.69E-02	NA	-5.27E-01	-2.63E+00
Afg3l2	_NLETLQQELGIEGENR_.2	#N/A	2.32E-02	NA	5.05E-01	2.48E+00	1.06E-02	NA	5.58E-01	2.85E+00
Prkce	_SAPTS[Phospho (STY)]PC[Carbamidomethyl (C)]DQELK_.2	P16054_S350_M1_Prkce	2.33E-02	NA	5.04E-01	2.48E+00	4.37E-03	NA	6.09E-01	3.26E+00
Echs1	_EGMTAFVEK_.2	#N/A	2.34E-02	NA	-5.04E-01	-2.48E+00	2.12E-02	NA	-5.11E-01	-2.52E+00
Uqcrc1	_M[Oxidation (M)]VLAAAGGVEHQQLLDLAQK_.3	#N/A	2.35E-02	NA	-5.04E-01	-2.47E+00	2.09E-02	NA	-5.12E-01	-2.53E+00
Rbbp4	_[Acetyl (Protein N-term)]ADKEAAFDDAVEER_.2	#N/A	2.43E-02	NA	-5.01E-01	-2.46E+00	3.68E-03	NA	-6.18E-01	-3.34E+00
Clec2l	_SGS[Phospho (STY)]GYEGSTSWK_.2	P0C7M9_S47_M1_Clec2l	2.43E-02	NA	-5.01E-01	-2.46E+00	8.81E-03	NA	-5.69E-01	-2.94E+00
Dpp6	_HLYSANTVDDFNR_.2	#N/A	2.45E-02	NA	-5.01E-01	-2.45E+00	1.37E-02	NA	-5.41E-01	-2.73E+00
Stxbp1	_M[Oxidation (M)]TDIM[Oxidation (M)]TEGITIVEDINK_.2	#N/A	2.46E-02	NA	-5.01E-01	-2.45E+00	1.50E-02	NA	-5.35E-01	-2.69E+00
Suclg1	_ISALQSAGVVVSM[Oxidation (M)]SPAQLGTTIYK_.3	#N/A	2.47E-02	NA	5.00E-01	2.45E+00	1.01E-02	NA	5.61E-01	2.88E+00
Ak3	_HLSSGDLLR_.2	#N/A	2.48E-02	NA	5.00E-01	2.45E+00	2.46E-02	NA	5.00E-01	2.45E+00
Sptbn1	_HRPDLIDFDK_.3	#N/A	2.49E-02	NA	-4.99E-01	-2.45E+00	1.38E-02	NA	-5.41E-01	-2.73E+00
Septin3	_AIGHVIEEGGVK_.2	#N/A	2.59E-02	NA	4.97E-01	2.43E+00	4.59E-03	NA	6.06E-01	3.24E+00
Bsn	_AS[Phospho (STY)]PQAKPLR_.2	O88737_S600_M1_Bsn	2.60E-02	NA	-4.96E-01	-2.43E+00	1.84E-02	NA	-5.21E-01	-2.59E+00
Ndrg2	_M[Oxidation (M)]ADSGGQPQLTQPGK_.2	#N/A	2.60E-02	NA	4.96E-01	2.43E+00	1.97E-02	NA	5.16E-01	2.56E+00
Pfkl	_RTLS[Phospho (STY)]IDKGF_.2	P12382_S775_M1_Pfkl	2.61E-02	NA	-4.96E-01	-2.42E+00	1.80E-02	NA	-5.23E-01	-2.60E+00
Scg2	_S[Phospho (STY)]GQLGLPDEENRR_.2	Q03517_S287_M1_Scg2	2.64E-02	NA	-4.95E-01	-2.42E+00	2.40E-02	NA	-5.02E-01	-2.46E+00
Pacs1	_GS[Phospho (STY)]LGKDTTSPM[Oxidation (M)]ELAALEK_.3	Q8K212_S421_M1_Pacs1	2.67E-02	NA	-4.95E-01	-2.41E+00	2.07E-02	NA	-5.13E-01	-2.54E+00
Hadh	_DTPGFIVNR_.2	#N/A	2.68E-02	NA	-4.94E-01	-2.41E+00	1.81E-02	NA	-5.22E-01	-2.60E+00
Akr1a1	_HPDEPVLLEEPVVLALAEK_.3	#N/A	2.69E-02	NA	-4.94E-01	-2.41E+00	5.07E-03	NA	-6.01E-01	-3.19E+00
Psma7	_ALLEVVQSGGK_.2	#N/A	2.70E-02	NA	4.94E-01	2.41E+00	3.59E-03	NA	6.19E-01	3.35E+00
Cacna2d1	_SYDYQSVC[Carbamidomethyl (C)]DPGAAPK_.2	#N/A	2.79E-02	NA	4.91E-01	2.39E+00	7.92E-03	NA	5.76E-01	2.99E+00
Elavl2	_FANNPSQK_.2	#N/A	2.80E-02	NA	4.91E-01	2.39E+00	6.65E-03	NA	5.86E-01	3.07E+00
Grk2	_GEVNAADAFDIGS[Phospho (STY)]FDEEDTK_.2	Q99MK8_S487_M1_Grk2	2.84E-02	NA	-4.90E-01	-2.38E+00	1.36E-02	NA	-5.42E-01	-2.74E+00
Lrrc7	_KSQS[Phospho (STY)]IDEIDVGTYK_.2	Q80TE7_S949_M1_Lrrc7	2.85E-02	NA	4.89E-01	2.38E+00	1.63E-02	NA	5.30E-01	2.65E+00
Raly	_LS[Phospho (STY)]PVPVPR_.2	Q64012_S135_M1_Raly	2.89E-02	NA	-4.88E-01	-2.37E+00	3.79E-03	NA	-6.17E-01	-3.32E+00
Pcbp1	_QGANINEIR_.2	#N/A	2.95E-02	NA	-4.87E-01	-2.36E+00	8.22E-03	NA	-5.73E-01	-2.97E+00
Psmb4	_FDGGVVIAADM[Oxidation (M)]LGSYGSLAR_.2	#N/A	2.95E-02	NA	-4.87E-01	-2.36E+00	1.36E-02	NA	-5.42E-01	-2.73E+00
Camk2b	_S[Phospho (STY)]TVASMMHR_.2	P28652_S276_M1_Camk2b	2.96E-02	NA	4.86E-01	2.36E+00	2.26E-02	NA	5.07E-01	2.49E+00
Dgkh	_VPLHLES[Phospho (STY)]PHEER_.3	D3YXJ0_S1067_M1_Dgkh	2.99E-02	NA	-4.86E-01	-2.36E+00	1.85E-02	NA	-5.21E-01	-2.59E+00
Brsk1	_FQVDISSSEGPEPS[Phospho (STY)]PR_.2	Q5RJI5_S676_M1_Brsk1	3.00E-02	NA	-4.86E-01	-2.36E+00	2.23E-02	NA	-5.08E-01	-2.50E+00
Sik3	_RAS[Phospho (STY)]DGGANIQLHAQQLLK_.3	Q6P4S6_S493_M1_Sik3	3.00E-02	NA	-4.85E-01	-2.36E+00	2.40E-02	NA	-5.02E-01	-2.46E+00
Gab1	_SSS[Phospho (STY)]LEGFHSQYK_.2	Q9QYY0_S420_M1_Gab1	3.04E-02	NA	-4.85E-01	-2.35E+00	2.15E-02	NA	-5.10E-01	-2.52E+00
Dst	_AM[Oxidation (M)]VESQQKS[Phospho (STY)]PVKR_.3	Q91ZU6_S1381_M1_Dst	3.04E-02	NA	-4.85E-01	-2.35E+00	1.81E-02	NA	-5.22E-01	-2.60E+00
Gls	_YAIAVNDLGTEYVHR_.2	#N/A	3.07E-02	NA	-4.84E-01	-2.35E+00	2.11E-02	NA	-5.12E-01	-2.53E+00

Sptbn1	_LVSQDNFGFDLPAVEAATK_.2	#N/A	3.07E-02	NA	-4.84E-01	-2.35E+00	2.41E-02	NA	-5.02E-01	-2.46E+00
Numbl	_SVLWVSADGLR_.2	#N/A	3.07E-02	NA	4.84E-01	2.34E+00	1.85E-02	NA	5.21E-01	2.59E+00
Slc35d3	_KDYLMENEALPS[Phospho (STY)]P_.2	Q8BGF8_S421_M1_Slc35d3	3.07E-02	NA	4.84E-01	2.34E+00	1.61E-02	NA	5.30E-01	2.65E+00
Rufy3	_[Acetyl (Protein N-term)]SALT[Phospho (STY)]PPTDM[Oxidation (M)]PT[Phospho (STY)]PTTDKITQAAM[Oxidati	Q9D394_T12_M2_Rufy3	3.08E-02	NA	-4.83E-01	-2.34E+00	1.46E-02	NA	-5.37E-01	-2.70E+00
Tnks1bp1	_NM[Oxidation (M)]APGAGC[Carbamidomethyl (C)]S[Phospho (STY)]PGEPR_.2	P58871_S1290_M1_Tnks1bp1	3.12E-02	NA	-4.82E-01	-2.34E+00	2.09E-02	NA	-5.13E-01	-2.53E+00
Gphn	_VGVLTVSDSC[Carbamidomethyl (C)]FR_.2	#N/A	3.12E-02	NA	4.82E-01	2.34E+00	1.47E-02	NA	5.37E-01	2.70E+00
Hnrnpa3	_IFVGGIKEDTEEYNLR_.3	#N/A	3.14E-02	NA	-4.82E-01	-2.33E+00	1.13E-02	NA	-5.54E-01	-2.82E+00
Cul9	_AVLETPSTQGQDGS[Phospho (STY)]PESLIR_.2	Q80TT8_S284_M1_Cul9	3.29E-02	NA	-4.78E-01	-2.31E+00	4.29E-03	NA	-6.10E-01	-3.27E+00
Cfl1	_SST[Phospho (STY)]PEEVKKR_.2	P18760_T25_M1_Cfl1	3.40E-02	NA	4.76E-01	2.29E+00	9.76E-03	NA	5.63E-01	2.89E+00
Cndp2	_TGQEIPVNLR_.2	#N/A	3.40E-02	NA	4.76E-01	2.29E+00	2.36E-02	NA	5.03E-01	2.47E+00
Diras2	_VAVFGAGGVGK_.2	#N/A	3.44E-02	NA	-4.75E-01	-2.29E+00	1.25E-02	NA	-5.47E-01	-2.78E+00
Phf24	_RGT[Phospho (STY)]VEGSVQEVQEEKEAEASAPVVQEESSINR_.4	Q80TL4_T47_M1_Phf24	3.49E-02	NA	-4.74E-01	-2.28E+00	6.95E-03	NA	-5.83E-01	-3.05E+00
Psat1	_QVVNFGPGPAK_.2	#N/A	3.54E-02	NA	4.72E-01	2.27E+00	1.22E-02	NA	5.49E-01	2.78E+00
Psma6	_YEAANWK_.2	#N/A	3.55E-02	NA	4.72E-01	2.27E+00	2.03E-02	NA	5.14E-01	2.55E+00
Pcdh19	_DKES[Phospho (STY)]PSVKR_.2	Q80TF3_S1134_M1_Pcdh19	3.60E-02	NA	-4.71E-01	-2.27E+00	1.72E-02	NA	-5.26E-01	-2.62E+00
Hgsnat	_LINSELGS[Phospho (STY)]PSR_.2	Q3UDW8_S238_M1_Hgsnat	3.68E-02	NA	-4.69E-01	-2.26E+00	1.43E-02	NA	-5.38E-01	-2.71E+00
Kctd4	_HNS[Phospho (STY)]VEDAEQGK_.2	Q9D7X1_S20_M1_Kctd4	3.69E-02	NA	-4.69E-01	-2.25E+00	1.99E-02	NA	-5.16E-01	-2.55E+00
Camk2a	_S[Phospho (STY)]TVASC[Carbamidomethyl (C)]M[Oxidation (M)]HR_.2	P11798_S275_M1_Camk2a	3.75E-02	NA	4.68E-01	2.25E+00	2.41E-02	NA	5.02E-01	2.46E+00
Uqcrc2	_ENM[Oxidation (M)]AYTVEGIR_.2	#N/A	3.78E-02	NA	4.67E-01	2.24E+00	1.33E-02	NA	5.43E-01	2.74E+00
Anks1b	_LIFQSC[Carbamidomethyl (C)]DYK_.2	#N/A	3.79E-02	NA	4.67E-01	2.24E+00	8.33E-03	NA	5.73E-01	2.96E+00
Atp6v1b2	_NYLSQPR_.2	#N/A	3.79E-02	NA	4.67E-01	2.24E+00	1.30E-02	NA	5.45E-01	2.76E+00
Atxn1	_RWS[Phospho (STY)]APETR_.2	P54254_S751_M1_Atxn1	3.80E-02	NA	-4.67E-01	-2.24E+00	2.02E-02	NA	-5.15E-01	-2.55E+00
Atg9a	_LEASYSDS[Phospho (STY)]PPGEEDLLVHVAEGSK_.3	Q68FE2_S18_M1_Atg9a	3.87E-02	NA	-4.65E-01	-2.23E+00	2.28E-02	NA	-5.06E-01	-2.49E+00
Ag2	_S[Phospho (STY)]TQSLSLQR_.2	Q3UPL5_S217_M1_Ag2	3.91E-02	NA	-4.64E-01	-2.23E+00	6.22E-03	NA	-5.90E-01	-3.10E+00
Homer1	_HWEAELATLK_.2	#N/A	3.92E-02	NA	-4.64E-01	-2.22E+00	1.76E-02	NA	-5.25E-01	-2.61E+00
Hsp90aa1	_DNSTM[Oxidation (M)]GYMAAK_.2	#N/A	3.99E-02	NA	4.63E-01	2.21E+00	1.20E-02	NA	5.50E-01	2.79E+00
Prrc2c	_SESS[Phospho (STY)]DFEVVPK_.2	Q3TLH4_S1222_M1_Prrc2c	4.03E-02	NA	-4.62E-01	-2.21E+00	1.23E-02	NA	-5.48E-01	-2.78E+00
Add2	_EQDHFLISPK_.2	#N/A	4.03E-02	NA	4.62E-01	2.21E+00	1.41E-02	NA	5.40E-01	2.72E+00
Bcl2l13	_S[Phospho (STY)]HTGEAAAVR_.2	P59017_S387_M1_Bcl2l13	4.04E-02	NA	-4.62E-01	-2.21E+00	1.96E-02	NA	-5.17E-01	-2.56E+00
Aldh7a1	_VNLLSFTGSTQVGK_.2	#N/A	4.06E-02	NA	4.61E-01	2.21E+00	1.48E-02	NA	5.36E-01	2.69E+00
D10Wsu102e	_IEILDS[Phospho (STY)]PASK_.2	Q9CX66_S177_M1_D10Wsu10	4.06E-02	NA	-4.61E-01	-2.21E+00	1.19E-02	NA	-5.50E-01	-2.80E+00
Atp6v1a	_LAEM[Oxidation (M)]PADSGYPAYLGAR_.2	#N/A	4.08E-02	NA	4.61E-01	2.20E+00	2.13E-02	NA	5.11E-01	2.52E+00
Hrh2	_LNNS[Phospho (STY)]LLSR_.2	P97292_S321_M1_Hrh2	4.16E-02	NA	-4.59E-01	-2.19E+00	1.85E-02	NA	-5.21E-01	-2.59E+00
Crad	_VNLS[Phospho (STY)]PVTPAK_.2	Q5PR69_S552_M1_Crad	4.18E-02	NA	-4.59E-01	-2.19E+00	2.39E-02	NA	-5.03E-01	-2.47E+00
Ogt	_SDLGNLLK_.2	#N/A	4.30E-02	NA	-4.57E-01	-2.18E+00	3.96E-03	NA	-6.14E-01	-3.30E+00
Opalin	_IS[Phospho (STY)]ENPRRSPTHEM[Oxidation (M)]NPR_.4	Q7M750_S84_M1_Opalin	4.31E-02	NA	-4.56E-01	-2.18E+00	1.47E-02	NA	-5.37E-01	-2.70E+00
Crk	_IHYLDTTTLIEPVAR_.3	#N/A	4.31E-02	NA	4.56E-01	2.18E+00	1.08E-02	NA	5.57E-01	2.84E+00
Omg	_LESLPAQLPR_.2	#N/A	4.31E-02	NA	4.56E-01	2.18E+00	2.14E-02	NA	5.11E-01	2.52E+00
Bnip2	_KGS[Phospho (STY)]ITEYTATEEK_.2	O54940_S114_M1_Bnip2	4.31E-02	NA	-4.56E-01	-2.18E+00	1.54E-02	NA	-5.34E-01	-2.68E+00
Srrm2	_DKFS[Phospho (STY)]PTQDRPESSTVLK_.3	Q8BTI8_S1151_M1_Srrm2	4.32E-02	NA	-4.56E-01	-2.17E+00	2.19E-02	NA	-5.09E-01	-2.51E+00
Elmo1	_IAFDAESEPNNSSGS[Phospho (STY)]M[Oxidation (M)]EK_.2	Q8BPU7_S344_M1_Elmo1	4.39E-02	NA	-4.55E-01	-2.17E+00	9.31E-03	NA	-5.66E-01	-2.91E+00
Cap2	_THTPSPTS[Phospho (STY)]PK_.2	Q9CYT6_S311_M1_Cap2	4.43E-02	NA	-4.54E-01	-2.16E+00	2.42E-02	NA	-5.02E-01	-2.46E+00
Ufm1	_VLSVPESTPFTAVLK_.2	#N/A	4.49E-02	NA	4.53E-01	2.16E+00	5.29E-03	NA	5.99E-01	3.17E+00
Eef1d	_[Acetyl (Protein N-term)]ATNFLAHEK_.2	#N/A	4.50E-02	NA	4.53E-01	2.15E+00	1.26E-02	NA	5.47E-01	2.77E+00
Hmgcl	_EVSVFGAVSELFTR_.2	#N/A	4.51E-02	NA	-4.53E-01	-2.15E+00	2.41E-02	NA	-5.02E-01	-2.46E+00
Cox7a2l	_LTSSVTAYDYSGK_.2	#N/A	4.52E-02	NA	4.52E-01	2.15E+00	1.17E-02	NA	5.51E-01	2.80E+00
Nrcam	_DSTGTYTC[Carbamidomethyl (C)]VAR_.2	#N/A	4.61E-02	NA	4.51E-01	2.14E+00	1.19E-02	NA	5.50E-01	2.80E+00
Nckap1	_NAYHAVYK_.2	#N/A	4.62E-02	NA	-4.51E-01	-2.14E+00	2.16E-02	NA	-5.10E-01	-2.52E+00
Atp6v1c1	_VQENLLASGVDLVTYITR_.3	#N/A	4.67E-02	NA	-4.50E-01	-2.14E+00	2.25E-02	NA	-5.07E-01	-2.50E+00
Ube2v2	_GVGDGTVSWGLEDDEDM[Oxidation (M)]TLTR_.2	#N/A	4.68E-02	NA	-4.50E-01	-2.14E+00	2.20E-02	NA	-5.09E-01	-2.51E+00
Pak1	_LTDFGFC[Carbamidomethyl (C)]AQITPEQSK_.2	#N/A	4.71E-02	NA	4.49E-01	2.13E+00	1.40E-02	NA	5.40E-01	2.72E+00
Lrrc7	_SYS[Phospho (STY)]TESYGASQTR_.2	Q80TE7_S1206_M1_Lrrc7	4.80E-02	NA	-4.47E-01	-2.12E+00	1.84E-02	NA	-5.21E-01	-2.59E+00
Gdi2	_AYDATTHFETTC[Carbamidomethyl (C)]DDIKDIYKR_.4	#N/A	4.95E-02	NA	-4.45E-01	-2.11E+00	2.43E-02	NA	-5.01E-01	-2.46E+00
Etfa	_GLLPEELTPLILETQK_.2	#N/A	4.99E-02	NA	4.44E-01	2.10E+00	1.61E-02	NA	5.30E-01	2.65E+00
Arhgap33	_QQS[Phospho (STY)]DGSLVR_.2	Q80YF9_S995_M1_Arhgap33	5.04E-02	NA	-4.43E-01	-2.10E+00	2.15E-02	NA	-5.10E-01	-2.52E+00
Macf1	_AM[Oxidation (M)]DATLS[Phospho (STY)]PTKTETVK_.2	Q9QXZ0_S4458_M1_Macf1	5.26E-02	NA	4.39E-01	2.08E+00	1.66E-02	NA	5.28E-01	2.64E+00
Xkr7	_TKEGS[Phospho (STY)]AELVPR_.2	Q5GH64_S154_M1_Xkr7	5.27E-02	NA	4.39E-01	2.07E+00	8.08E-03	NA	5.74E-01	2.98E+00

Adcy6	_NAEPPS[Phospho (STY)]PTPAAHTR_.2	Q01341_S53_M1_Adcy6	5.31E-02	NA	-4.38E-01	-2.07E+00	2.10E-02	NA	-5.12E-01	-2.53E+00
Ndrg3	_HQTMEVS[Phospho (STY)]C[Carbamidomethyl (C)]_.2	Q9QYF9_S374_M1_Ndrg3	5.48E-02	NA	4.36E-01	2.05E+00	1.67E-02	NA	5.28E-01	2.64E+00
Sorbs1	_S[Phospho (STY)]AESLLESTK_.2	Q62417_S722_M1_Sorbs1	5.50E-02	NA	-4.35E-01	-2.05E+00	2.22E-02	NA	-5.08E-01	-2.50E+00
Enah	_NSRPSS[Phospho (STY)]PVNTPSSQPPAAK_.3	Q03173_S355_M1_Enah	5.52E-02	NA	-4.35E-01	-2.05E+00	1.98E-02	NA	-5.16E-01	-2.56E+00
Wnk3	_EGETTVNTETSS[Phospho (STY)]PK_.2	Q80XP9_S1039_M1_Wnk3	5.62E-02	NA	-4.33E-01	-2.04E+00	1.97E-02	NA	-5.17E-01	-2.56E+00
Pgls	_TGALC[Carbamidomethyl (C)]WFLDEAAAR_.2	#N/A	5.67E-02	NA	-4.33E-01	-2.04E+00	2.32E-02	NA	-5.05E-01	-2.48E+00
Stx7	_[Acetyl (Protein N-term)]S[Phospho (STY)]YTPGIGGDSAQLAQR_.2	O70439_S2_M1_Stx7	5.70E-02	NA	-4.32E-01	-2.03E+00	8.64E-03	NA	-5.70E-01	-2.95E+00
Cep170	_SDS[Phospho (STY)]EATISR_.2	Q6A065_S1157_M1_Cep170	5.73E-02	NA	-4.32E-01	-2.03E+00	2.37E-02	NA	-5.03E-01	-2.47E+00
Igsf8	_VASGDLQVQR_.2	#N/A	5.77E-02	NA	4.31E-01	2.03E+00	1.00E-02	NA	5.61E-01	2.88E+00
Ly6h	_QC[Carbamidomethyl (C)]QPTDTVC[Carbamidomethyl (C)]ASVR_.2	#N/A	5.77E-02	NA	4.31E-01	2.03E+00	1.83E-02	NA	5.22E-01	2.59E+00
Hnrnpl	_ISRPGDS[Phospho (STY)]DDSR_.2	Q8R081_S182_M1_Hnrnpl	5.86E-02	NA	-4.30E-01	-2.02E+00	1.84E-02	NA	-5.21E-01	-2.59E+00
Atp1b2	_SC[Carbamidomethyl (C)]GQVVEEWKEFVWNPR_.3	#N/A	5.90E-02	NA	-4.29E-01	-2.02E+00	1.49E-02	NA	-5.36E-01	-2.69E+00
Cpne1	_APPT[Phospho (STY)]PGKGPAQAPQA_.2	Q8C166_T525_M1_Cpne1	5.92E-02	NA	4.29E-01	2.01E+00	2.45E-02	NA	5.01E-01	2.46E+00
Irf2bpl	_S[Phospho (STY)]PGPPPPVGVK_.2	Q8K3X4_S69_M1_Irf2bpl	5.94E-02	NA	-4.29E-01	-2.01E+00	9.69E-03	NA	-5.63E-01	-2.89E+00
Plppr4	_DSESC[Carbamidomethyl (C)]ESLKDSFGS[Phospho (STY)]GDR_.2	Q7TME0_S685_M1_Plppr4	5.98E-02	NA	4.28E-01	2.01E+00	6.29E-03	NA	5.89E-01	3.09E+00
Purb	_DSLGDFIEHYAQLGPSS[Phospho (STY)]PEQLAAGAEEGGGPR_.4	O35295_S113_M1_Purb	6.00E-02	NA	-4.28E-01	-2.01E+00	1.87E-02	NA	-5.20E-01	-2.59E+00
Sptan1	_VNEVSQFAAK_.2	#N/A	6.02E-02	NA	4.27E-01	2.01E+00	6.21E-03	NA	5.90E-01	3.10E+00
Nmt1	_SGLS[Phospho (STY)]PANDTGAKK_.2	O70310_S47_M1_Nmt1	6.07E-02	NA	-4.27E-01	-2.00E+00	1.34E-02	NA	-5.43E-01	-2.74E+00
Ppp6r1	_EQM[Oxidation (M)]IQGQDS[Phospho (STY)]PEPDQLLATLEK_.3	Q7TSI3_S232_M1_Ppp6r1	6.09E-02	NA	-4.26E-01	-2.00E+00	7.05E-03	NA	-5.82E-01	-3.04E+00
Pak1	_MSDEEILEK_.2	#N/A	6.14E-02	NA	4.26E-01	2.00E+00	2.14E-02	NA	5.11E-01	2.52E+00
Plxdc2	_RGS[Phospho (STY)]GHPAYAEVEPVGEK_.3	Q9DC11_S507_M1_Plxdc2	6.14E-02	NA	-4.26E-01	-1.99E+00	1.00E-02	NA	-5.61E-01	-2.88E+00
Ppip5k1	_TDPGS[Phospho (STY)]IENLC[Carbamidomethyl (C)]PGK_.2	A2ARP1_S939_M1_Ppip5k1	6.19E-02	NA	-4.25E-01	-1.99E+00	2.15E-02	NA	-5.10E-01	-2.52E+00
Stard8	_SLS[Phospho (STY)]IESLC[Carbamidomethyl (C)]PDEGR_.2	Q8K031_S234_M1_Stard8	6.29E-02	NA	-4.23E-01	-1.98E+00	1.26E-02	NA	-5.47E-01	-2.77E+00
Smg9	_WKEPGSSGPQNLS[Phospho (STY)]GPGGR_.3	Q9DB90_S32_M1_Smg9	6.41E-02	NA	-4.22E-01	-1.97E+00	2.31E-02	NA	-5.05E-01	-2.48E+00
Rasa3	_T[Phospho (STY)]RYGSQEHPIGDK_.3	Q60790_T805_M1_Rasa3	6.60E-02	NA	-4.19E-01	-1.96E+00	2.14E-02	NA	-5.11E-01	-2.52E+00
Hspa9	_QAVTNPNNTFYATK_.2	#N/A	6.67E-02	NA	4.18E-01	1.95E+00	1.24E-02	NA	5.48E-01	2.78E+00
Srrm1	_VS[Phospho (STY)]VS[Phospho (STY)]PGR_.2	Q52KI8_S427_M2_Srrm1	6.77E-02	NA	-4.16E-01	-1.94E+00	1.40E-02	NA	-5.40E-01	-2.72E+00
Cobl	_KSS[Phospho (STY)]LGNDETDKEK_.2	Q5NBX1_S235_M1_Cobl	6.86E-02	NA	-4.15E-01	-1.94E+00	1.79E-02	NA	-5.23E-01	-2.61E+00
Epn1	_TALPTSGS[Phospho (STY)]STGELELLAGEVPAR_.2	Q80VP1_S418_M1_Epn1	6.94E-02	NA	-4.14E-01	-1.93E+00	1.92E-02	NA	-5.18E-01	-2.57E+00
Tpd52l2	_HSIS[Phospho (STY)]M[Oxidation (M)]PVM[Oxidation (M)]R_.2	Q9CYZ2_S168_M1_Tpd52l2	6.96E-02	NA	-4.14E-01	-1.93E+00	1.98E-02	NA	-5.16E-01	-2.56E+00
Gnb5	_VIVWDSFTTNK_.2	#N/A	7.06E-02	NA	-4.13E-01	-1.92E+00	1.85E-02	NA	-5.21E-01	-2.59E+00
Txnl1	_QHLENDPGS[Phospho (STY)]NEDADIPK_.2	Q8CDN6_S113_M1_Txnl1	7.09E-02	NA	-4.12E-01	-1.92E+00	2.19E-02	NA	-5.09E-01	-2.51E+00
Ina	_SNVASTAAC[Carbamidomethyl (C)]SSASSLGLGLAYR_.2	#N/A	7.20E-02	NA	-4.11E-01	-1.91E+00	2.33E-02	NA	-5.05E-01	-2.48E+00
Pdhb	_TYYM[Oxidation (M)]SAGLQPVPIVFR_.2	#N/A	7.20E-02	NA	-4.11E-01	-1.91E+00	1.91E-02	NA	-5.19E-01	-2.58E+00
Cct3	_NVLLDPQLVPGGGASEM[Oxidation (M)]AVAHALTEK_.3	#N/A	7.26E-02	NA	-4.10E-01	-1.91E+00	2.07E-02	NA	-5.13E-01	-2.54E+00
Stxbp1	_HIAEVSQEVTR_.2	#N/A	7.32E-02	NA	4.09E-01	1.90E+00	2.18E-02	NA	5.09E-01	2.51E+00
Rab3ip	_TLVLSSSPTS[Phospho (STY)]PTQEPLAAAK_.2	Q68EF0_S218_M1_Rab3ip	7.45E-02	NA	-4.07E-01	-1.89E+00	1.46E-02	NA	-5.37E-01	-2.70E+00
Rabggta	_ELLLC[Carbamidomethyl (C)]NNR_.2	#N/A	7.53E-02	NA	4.07E-01	1.89E+00	1.80E-02	NA	5.23E-01	2.60E+00
Terf2	_DLVLANLASPSS[Phospho (STY)]PAHK_.2	O35144_S367_M1_Terf2	7.55E-02	NA	-4.06E-01	-1.89E+00	2.36E-02	NA	-5.04E-01	-2.47E+00
Gap43	_EGDGSATTDAAPATS[Phospho (STY)]PKAEEPSKAGDAPSEEK_.4	#N/A	7.57E-02	NA	-4.06E-01	-1.88E+00	1.71E-02	NA	-5.27E-01	-2.63E+00
Ezr	_QLLTLSNELSQAR_.2	#N/A	7.61E-02	NA	4.06E-01	1.88E+00	1.74E-02	NA	5.25E-01	2.62E+00
Crad	_ETAQTDS[Phospho (STY)]PQPQER_.2	Q5PR69_S487_M1_Crad	7.67E-02	NA	4.05E-01	1.88E+00	1.31E-02	NA	5.44E-01	2.75E+00
Pdlim4	_IHIDPESQDC[Carbamidomethyl (C)]S[Phospho (STY)]PATSR_.2	P70271_S111_M1_Pdlim4	7.70E-02	NA	-4.04E-01	-1.88E+00	1.14E-02	NA	-5.53E-01	-2.82E+00
Ranbp3	_S[Phospho (STY)]PSESAEETHTLEEK_.2	Q9CT10_S146_M1_Ranbp3	7.75E-02	NA	4.04E-01	1.87E+00	1.56E-02	NA	5.33E-01	2.67E+00
Ywhab	_QTTVSNSQQAYQEAFEISK_.2	#N/A	7.77E-02	NA	-4.03E-01	-1.87E+00	1.92E-02	NA	-5.18E-01	-2.57E+00
Pikfyve	_[Acetyl (Protein N-term)]ATDDKSS[Phospho (STY)]PTLDSANDLPR_.2	Q9Z1T6_S8_M1_Pikfyve	7.84E-02	NA	-4.03E-01	-1.87E+00	6.85E-03	NA	-5.84E-01	-3.05E+00
Srrm1	_RRT[Phospho (STY)]AS[Phospho (STY)]PPPPPK_.2	Q52KI8_S635_M2_Srrm1	7.92E-02	NA	-4.02E-01	-1.86E+00	1.63E-02	NA	-5.30E-01	-2.65E+00
Tspyl5	_AAAEDAWHDEKPPQS[Phospho (STY)]PR_.3	Q69ZB3_S41_M1_Tspyl5	7.97E-02	NA	-4.01E-01	-1.86E+00	1.07E-02	NA	-5.57E-01	-2.85E+00
Hspa4	_VLATAFDTTLGGR_.2	#N/A	8.06E-02	NA	4.00E-01	1.85E+00	2.21E-02	NA	5.08E-01	2.50E+00
Hspa5	_AKFEELNM[Oxidation (M)]DLFR_.3	#N/A	8.08E-02	NA	-4.00E-01	-1.85E+00	2.09E-02	NA	-5.12E-01	-2.53E+00
Ttc1	_AAEAHPQDDHVEEEC[Carbamidomethyl (C)]FHDC[Carbamidomethyl (C)]SASFEEEQPGAHVAGSK_.5	#N/A	8.26E-02	NA	-3.98E-01	-1.84E+00	1.35E-02	NA	-5.42E-01	-2.74E+00
Ccdc6	_LDQPVSAPPS[Phospho (STY)]PR_.2	D3YZP9_S237_M1_Ccdc6	8.32E-02	NA	-3.97E-01	-1.83E+00	2.15E-02	NA	-5.10E-01	-2.52E+00
Gas2l1	_TGTFS[Phospho (STY)]PQR_.2	Q8JZP9_S292_M1_Gas2l1	8.38E-02	NA	-3.96E-01	-1.83E+00	1.19E-02	NA	-5.50E-01	-2.80E+00
Grin2a	_DKGS[Phospho (STY)]PHSEGSDRYR_.3	P35436_S1198_M1_Grin2a	8.42E-02	NA	-3.96E-01	-1.83E+00	2.45E-02	NA	-5.01E-01	-2.45E+00
Atp6v0a1	_KANIPIMDTGENPEVPFPR_.3	#N/A	8.48E-02	NA	3.95E-01	1.82E+00	2.35E-02	NA	5.04E-01	2.47E+00
Pcbp2	_LVVPASQC[Carbamidomethyl (C)]GSLIGK_.2	#N/A	8.50E-02	NA	3.95E-01	1.82E+00	1.77E-02	NA	5.24E-01	2.61E+00
Pclo	_KSS[Phospho (STY)]TS[Phospho (STY)]FDDDAGR_.2	Q9QYX7_S1551_M2_Pclo	8.61E-02	NA	-3.93E-01	-1.82E+00	1.70E-02	NA	-5.27E-01	-2.63E+00

Tbr1	_S[Phospho (STY)]PPQYC[Carbamidomethyl (C)]GAK_.2	Q64336_S549_M1_Tbr1	8.64E-02	NA	3.93E-01	1.81E+00	1.64E-02	NA	5.29E-01	2.65E+00
Cacnb4	_HSNHS[Phospho (STY)]TENS[Phospho (STY)]PIERR_.3	Q8R0S4_S443_M2_Cacnb4	8.75E-02	NA	-3.92E-01	-1.81E+00	2.02E-02	NA	-5.15E-01	-2.55E+00
Csde1	_ATNIEVLSNTFQFTNEAR_.2	#N/A	8.87E-02	NA	3.91E-01	1.80E+00	1.92E-02	NA	5.18E-01	2.57E+00
Gstm1	_RPWFAGDK_.2	#N/A	9.05E-02	NA	3.88E-01	1.79E+00	2.00E-02	NA	5.16E-01	2.55E+00
Mpp2	_DNNLELVQEILR_.2	#N/A	9.07E-02	NA	3.88E-01	1.79E+00	2.47E-02	NA	5.00E-01	2.45E+00
Oxr1	_KATGVS[Phospho (STY)]AEDADPR_.2	Q4KMM3_S308_M1_Oxr1	9.08E-02	NA	-3.88E-01	-1.79E+00	9.72E-03	NA	-5.63E-01	-2.89E+00
Cct7	_ATISNDGATILK_.2	#N/A	9.11E-02	NA	3.88E-01	1.79E+00	2.17E-02	NA	5.10E-01	2.51E+00
Rab11b	_NEFNLESK_.2	#N/A	9.33E-02	NA	-3.85E-01	-1.77E+00	1.76E-02	NA	-5.24E-01	-2.61E+00
Atp6v1a	_DDFLQQNGYTPYDR_.2	#N/A	9.35E-02	NA	3.85E-01	1.77E+00	2.30E-02	NA	5.05E-01	2.48E+00
Cnrip1	_IQPNDGPVFFK_.2	#N/A	9.42E-02	NA	-3.84E-01	-1.77E+00	2.36E-02	NA	-5.03E-01	-2.47E+00
Gprin1	_KTDPEFSGKLT[Phospho (STY)]PGSSGK_.3	Q3UNH4_T256_M1_Gprin1	9.47E-02	NA	3.84E-01	1.76E+00	1.62E-02	NA	5.30E-01	2.65E+00
Git1	_ARS[Phospho (STY)]M[Oxidation (M)]DSSDLSDGAVTLQEYLELKK_.3	Q68FF6_S419_M1_Git1	9.50E-02	NA	-3.84E-01	-1.76E+00	2.21E-02	NA	-5.08E-01	-2.50E+00
Grk2	_SLLEGLLQR_.2	#N/A	9.57E-02	NA	3.83E-01	1.76E+00	2.06E-02	NA	5.13E-01	2.54E+00
Ppp2r1a	_LSTIALALGVER_.2	#N/A	9.61E-02	NA	-3.82E-01	-1.76E+00	1.80E-02	NA	-5.23E-01	-2.60E+00
Rap1a	_INVNEIFYDLVR_.2	#N/A	9.76E-02	NA	3.81E-01	1.75E+00	1.88E-02	NA	5.20E-01	2.58E+00
Hba	_M[Oxidation (M)]FASFPTTK_.2	#N/A	9.78E-02	NA	3.81E-01	1.75E+00	1.70E-02	NA	5.27E-01	2.63E+00
Pdhx	_HSLDASQGTATGPR_.2	#N/A	1.01E-01	NA	3.78E-01	1.73E+00	2.05E-02	NA	5.14E-01	2.54E+00
Prkra	_AEAPPLQREDS[Phospho (STY)]GTFSLGK_.3	Q9WTX2_S18_M1_Prkra	1.03E-01	NA	-3.75E-01	-1.72E+00	9.15E-03	NA	-5.67E-01	-2.92E+00
Sars1	_ELVSC[Carbamidomethyl (C)]SNC[Carbamidomethyl (C)]TDYQAR_.2	#N/A	1.03E-01	NA	3.75E-01	1.72E+00	2.31E-02	NA	5.05E-01	2.48E+00
Cct4	_ALIAGGGAPEIELALR_.2	#N/A	1.06E-01	NA	3.73E-01	1.70E+00	1.27E-02	NA	5.46E-01	2.77E+00
Eef2	_FT[Phospho (STY)]DTRKDEQER_.2	P58252_T57_M1_Eef2	1.06E-01	NA	3.72E-01	1.70E+00	1.50E-02	NA	5.35E-01	2.69E+00
Plppr4	_QQGVLQSS[Phospho (STY)]PK_.2	Q7TME0_S594_M1_Plppr4	1.07E-01	NA	-3.71E-01	-1.70E+00	9.19E-03	NA	-5.67E-01	-2.92E+00
Sphkap	_RAS[Phospho (STY)]TDLGK_.2	Q6NSW3_S841_M1_Sphkap	1.15E-01	NA	-3.64E-01	-1.66E+00	1.98E-02	NA	-5.16E-01	-2.56E+00
Ddt	_FFPLEAWQIGK_.2	#N/A	1.18E-01	NA	3.61E-01	1.64E+00	2.04E-02	NA	5.14E-01	2.54E+00
Sorbs2	_SFISSSPSS[Phospho (STY)]PSR_.2	Q3UTJ2_S382_M1_Sorbs2	1.22E-01	NA	-3.58E-01	-1.62E+00	9.89E-03	NA	-5.62E-01	-2.88E+00
Rtn4	_EHGYLGNLSAVASTEGTIEETLNEASR_.3	#N/A	1.22E-01	NA	-3.57E-01	-1.62E+00	2.40E-02	NA	-5.02E-01	-2.46E+00
Glul	_ITGTNAEVM[Oxidation (M)]PAQWEFQIGPC[Carbamidomethyl (C)]EGIR_.3	#N/A	1.24E-01	NA	-3.56E-01	-1.62E+00	2.02E-02	NA	-5.15E-01	-2.55E+00
Gripap1	_KQES[Phospho (STY)]FC[Carbamidomethyl (C)]R_.2	Q8VD04_S237_M1_Gripap1	1.27E-01	NA	-3.53E-01	-1.60E+00	1.75E-02	NA	-5.25E-01	-2.62E+00
Pcbp2	_QVTITGSAASISLAQYLINVR_.2	#N/A	1.28E-01	NA	-3.52E-01	-1.59E+00	2.27E-02	NA	-5.06E-01	-2.49E+00
Srrm4	_SAS[Phospho (STY)]PLAATR_.2	Q8BKA3_S271_M1_Srrm4	1.30E-01	NA	-3.50E-01	-1.59E+00	1.46E-02	NA	-5.37E-01	-2.70E+00
Lrrc8c	_SGT[Phospho (STY)]IQSGPEGNLVR_.2	Q8R502_T198_M1_Lrrc8c	1.35E-01	NA	-3.46E-01	-1.57E+00	1.92E-02	NA	-5.18E-01	-2.57E+00
Arhgef6	_KAS[Phospho (STY)]EEEYVIR_.2	Q8K4I3_S639_M1_Arhgef6	1.40E-01	NA	-3.42E-01	-1.54E+00	1.09E-02	NA	-5.56E-01	-2.84E+00
Dennd5a	_FNS[Phospho (STY)]YDISR_.2	Q6PAL8_S193_M1_Dennd5a	1.41E-01	NA	-3.41E-01	-1.54E+00	2.07E-02	NA	-5.13E-01	-2.54E+00
Hapln1	_LLVEAEQAK_.2	#N/A	1.44E-01	NA	-3.39E-01	-1.53E+00	1.34E-02	NA	-5.43E-01	-2.74E+00
Basp1	_AGEASAESTGAADGAAPEEGEAKKTEAPAAAGPEAK_.4	#N/A	1.44E-01	NA	-3.38E-01	-1.53E+00	6.50E-03	NA	-5.87E-01	-3.08E+00
Actn1	_ASIHEAWTDGK_.2	#N/A	1.45E-01	NA	3.38E-01	1.52E+00	2.15E-02	NA	5.10E-01	2.52E+00
Synpo	_PKPNQNLSEASGK_.3	#N/A	1.52E-01	NA	3.32E-01	1.49E+00	1.66E-02	NA	5.29E-01	2.64E+00
Ncam1	_AAHFVFR_.2	#N/A	1.55E-01	NA	3.30E-01	1.48E+00	2.15E-02	NA	5.10E-01	2.52E+00
Hmgcl	_LLEAGDFIC[Carbamidomethyl (C)]QALNR_.2	#N/A	1.58E-01	NA	3.28E-01	1.47E+00	2.14E-02	NA	5.11E-01	2.52E+00
Begain	_YYGGGGGGGAAGGGS[Phospho (STY)]PGDKAEGR_.3	Q68EF6_S463_M1_Begain	1.58E-01	NA	-3.28E-01	-1.47E+00	2.14E-02	NA	-5.11E-01	-2.52E+00
Pclo	_GSVPAAAAES[Phospho (STY)]PSM[Oxidation (M)]HR_.2	Q9QYX7_S75_M1_Pclo	1.70E-01	NA	-3.19E-01	-1.43E+00	2.38E-02	NA	-5.03E-01	-2.47E+00
Tnks1bp1	_RDS[Phospho (STY)]LGSFSTR_.2	P58871_S866_M1_Tnks1bp1	1.74E-01	NA	-3.17E-01	-1.42E+00	1.16E-02	NA	-5.52E-01	-2.81E+00
Tjp1	_AVPVS[Phospho (STY)]PSAVEEDEDEDGHTVVATAR_.3	P39447_S1614_M1_Tjp1	1.80E-01	NA	-3.12E-01	-1.39E+00	2.10E-02	NA	-5.12E-01	-2.53E+00
Ywhah	_EAFEISK_.2	#N/A	1.99E-01	NA	-3.00E-01	-1.33E+00	2.17E-02	NA	-5.10E-01	-2.51E+00
Bod1l	_KLS[Phospho (STY)]SQPSTDVSTDKER_.3	E9Q6J5_S236_M1_Bod1l	2.05E-01	NA	-2.96E-01	-1.31E+00	2.33E-02	NA	-5.04E-01	-2.48E+00
Plppr3	_QPGM[Oxidation (M)]GPGS[Phospho (STY)]PVSDVDQEEPR_.2	Q7TPB0_S641_M1_Plppr3	2.05E-01	NA	-2.96E-01	-1.31E+00	2.21E-02	NA	-5.08E-01	-2.50E+00
Pcmt1	_ELVDDSITNVKK_.3	#N/A	2.26E-01	NA	2.83E-01	1.25E+00	1.84E-02	NA	5.21E-01	2.59E+00
Madd	_S[Phospho (STY)]PTENVNTPVGKDPGLAGR_.2	Q80U28_S1058_M1_Madd	2.37E-01	NA	2.77E-01	1.22E+00	2.31E-02	NA	5.05E-01	2.48E+00
Otub1	_GEGGTTNPHVFPEGSEPK_.3	#N/A	2.37E-01	NA	2.77E-01	1.22E+00	1.75E-02	NA	5.25E-01	2.62E+00
Cct7	_TC[Carbamidomethyl (C)]TIILR_.2	#N/A	2.54E-01	NA	-2.67E-01	-1.18E+00	1.90E-02	NA	-5.19E-01	-2.58E+00
Clcc1	_EVSPDS[Phospho (STY)]SEAEELSDC[Carbamidomethyl (C)]LHR_.3	Q99LI2_S58_M1_Clcc1	2.63E-01	NA	-2.63E-01	-1.16E+00	2.27E-02	NA	-5.06E-01	-2.49E+00
Gnai2	_QLFALSC[Carbamidomethyl (C)]AAEEQGM[Oxidation (M)]LPEDLSGVIR_.3	#N/A	2.73E-01	NA	2.57E-01	1.13E+00	2.44E-02	NA	5.01E-01	2.46E+00
Rpl13	_TIGISVDPR_.2	#N/A	2.80E-01	NA	2.54E-01	1.11E+00	1.94E-02	NA	5.18E-01	2.57E+00
Stk39	_RVPGSS[Phospho (STY)]GHLHK_.3	Q9Z1W9_S383_M1_Stk39	3.18E-01	NA	-2.35E-01	-1.03E+00	2.12E-02	NA	-5.11E-01	-2.53E+00
