# Supplementary material for: The Chronic Effects of a Single Low-Intensity Blast Exposure on Phosphoproteome Networks and Cognitive Function Influenced by Mutant Tau Overexpression
Source: Int J Mol Sci. 2024 Mar 15;25(6):3338. doi: 10.3390/ijms25063338 (PMC10970429; doi:10.3390/ijms25063338)
Supplement: Supplementary file 1 [file ijms-25-03338-s001.zip › Supplementary Table S8- Black and turquoise module GO.pdf]

Supplementary Table S8. Black and turquoise module GO

Module	GO:ID	ONTOLOGY	Description	GeneRatio	BgRatio	pvalue	p.adjust	qvalue	geneID	Count
black	GO:0017038	BP	protein import	02/18'	10/28943	1.6E-05	0.008359	0.005521	28185/76936	2
black	GO:0098686	CC	hippocampal mossy fiber to CA3 synapse	02/18'	49/28804	0.00043	0.046037	0.025576	19085/12388	2
turquoise	GO:0043209	CC	myelin sheath	13/121	215/28804	7.5E-12	2.4E-09	1.62E-09	20910/12799/11966/14688/18673/23980/20867/14086/18377/98660/140559/68585/18810	13
turquoise	GO:0097060	CC	synaptic membrane	15/121	469/28804	1.3E-09	2.15E-07	1.45E-07	20910/14811/20511/16490/18673/210274/319504/268566/245537/68585/75406/116838/16443/24050/13821	15
turquoise	GO:0099572	CC	postsynaptic specialization	14/121	438/28804	5E-09	5.31E-07	3.59E-07	12034/14432/14811/18673/18521/210274/319504/244310/268566/245537/217692/68585/20085/13821	14
turquoise	GO:0043197	CC	dendritic spine	10/121	200/28804	1.3E-08	1.08E-06	7.31E-07	14811/20511/19049/98660/230904/210274/244310/217692/110355/16443	10
turquoise	GO:0044309	CC	neuron spine	10/121	205/28804	1.7E-08	1.09E-06	7.39E-07	14811/20511/19049/98660/230904/210274/244310/217692/110355/16443	10
turquoise	GO:0014069	CC	postsynaptic density	12/121	398/28804	1.2E-07	6.64E-06	4.49E-06	12034/14432/14811/18673/18521/210274/319504/268566/217692/68585/20085/13821	12
turquoise	GO:0032279	CC	asymmetric synapse	12/121	406/28804	1.5E-07	7.05E-06	4.76E-06	12034/14432/14811/18673/18521/210274/319504/268566/217692/68585/20085/13821	12
turquoise	GO:0098984	CC	neuron to neuron synapse	12/121	437/28804	3.4E-07	1.35E-05	9.14E-06	12034/14432/14811/18673/18521/210274/319504/268566/217692/68585/20085/13821	12
turquoise	GO:0008306	BP	associative learning	8/122	120/28943	4.5E-08	5.6E-05	3.94E-05	53328/16653/14811/19049/21375/98660/210274/245537	8
turquoise	GO:0010563	BP	negative regulation of phosphorus metabolic process	13/122	453/28943	6.7E-08	5.6E-05	3.94E-05	665775/14678/14870/23980/384783/238871/19049/208643/100502698/51810/16971/110355/18810	13
turquoise	GO:0045936	BP	negative regulation of phosphate metabolic process	13/122	453/28943	6.7E-08	5.6E-05	3.94E-05	665775/14678/14870/23980/384783/238871/19049/208643/100502698/51810/16971/110355/18810	13
turquoise	GO:0042734	CC	presynaptic membrane	8/121	201/28804	2.2E-06	8E-05	5.4E-05	20910/14811/20511/16490/18673/116838/16443/24050	8
turquoise	GO:0010001	BP	glial cell differentiation	10/122	261/28943	1.7E-07	0.000107	7.54E-05	14432/12799/16653/18377/52615/245537/16971/110355/68585/18810	10
turquoise	GO:0098982	CC	GABA-ergic synapse	6/121	109/28804	6.9E-06	0.000222	0.00015	12034/14432/18673/268566/245537/116838	6
turquoise	GO:2000463	BP	positive regulation of excitatory postsynaptic potential	5/122	41/28943	8.1E-07	0.000367	0.000258	14811/12297/245537/110355/116838	5
turquoise	GO:0007632	BP	visual behavior	6/122	77/28943	9.2E-07	0.000367	0.000258	16653/14811/20511/19049/98660/245537	6
turquoise	GO:0007409	BP	axonogenesis	12/122	493/28943	1.2E-06	0.000367	0.000258	20910/14432/12799/18377/21375/319504/245537/217692/16971/68585/58208/30957	12
turquoise	GO:0050808	BP	synapse organization	12/122	496/28943	1.3E-06	0.000367	0.000258	53328/14432/14811/12297/235604/208643/210274/319504/268566/245537/217692/16443	12
turquoise	GO:0007612	BP	learning	8/122	187/28943	1.3E-06	0.000367	0.000258	53328/16653/14811/19049/21375/98660/210274/245537	8
turquoise	GO:0030534	BP	adult behavior	8/122	189/28943	1.5E-06	0.000367	0.000258	12799/20511/11669/170719/19049/98660/210274/245537	8
turquoise	GO:0050821	BP	protein stabilization	8/122	193/28943	1.7E-06	0.00039	0.000274	20910/12034/18673/12468/12464/68585/30957/23992	8
turquoise	GO:0016601	BP	Rac protein signal transduction	5/122	49/28943	2E-06	0.000423	0.000298	20262/16653/12928/140580/68585	5
turquoise	GO:0005741	CC	mitochondrial outer membrane	7/121	188/28804	1.5E-05	0.000444	0.0003	12034/53328/12799/18673/23980/28185/18810	7
turquoise	GO:0097110	MF	scaffold protein binding	6/121	84/28404	1.6E-06	0.000633	0.000506	14811/238871/11512/12928/245537/110355	6
turquoise	GO:0042063	BP	gliogenesis	10/122	363/28943	3.4E-06	0.000655	0.000461	14432/12799/16653/18377/52615/245537/16971/110355/68585/18810	10
turquoise	GO:0019867	CC	outer membrane	7/121	209/28804	3E-05	0.000742	0.000501	12034/53328/12799/18673/23980/28185/18810	7
turquoise	GO:0031968	CC	organelle outer membrane	7/121	209/28804	3E-05	0.000742	0.000501	12034/53328/12799/18673/23980/28185/18810	7
turquoise	GO:0035020	BP	regulation of Rac protein signal transduction	4/122	26/28943	4.2E-06	0.000753	0.000529	20262/16653/12928/68585	4
turquoise	GO:0098815	BP	modulation of excitatory postsynaptic potential	5/122	59/28943	5.1E-06	0.000859	0.000604	14811/12297/245537/110355/116838	5
turquoise	GO:0045121	CC	membrane raft	9/121	394/28804	4.5E-05	0.00098	0.000662	14678/12385/16653/20511/11512/12928/98660/110355/271457	9
turquoise	GO:0098857	CC	membrane microdomain	9/121	395/28804	4.6E-05	0.00098	0.000662	14678/12385/16653/20511/11512/12928/98660/110355/271457	9
turquoise	GO:0031102	BP	neuron projection regeneration	5/122	62/28943	6.5E-06	0.001029	0.000724	14432/18377/16971/68585/30957	5
turquoise	GO:0042326	BP	negative regulation of phosphorylation	10/122	394/28943	6.9E-06	0.001029	0.000724	14870/23980/384783/238871/208643/100502698/51810/16971/110355/18810	10
turquoise	GO:0031647	BP	regulation of protein stability	9/122	312/28943	7.4E-06	0.00103	0.000725	20910/12034/16653/18673/12468/12464/68585/30957/23992	9
turquoise	GO:0002026	BP	regulation of the force of heart contraction	4/122	31/28943	8.7E-06	0.001109	0.00078	12652/23980/98660/110355	4
turquoise	GO:0030900	BP	forebrain development	10/122	407/28943	9.2E-06	0.001109	0.00078	16653/20511/12928/21375/70762/98660/68585/58208/30957/18536	10
turquoise	GO:0007611	BP	learning or memory	9/122	321/28943	9.2E-06	0.001109	0.00078	53328/16653/14811/238871/19049/21375/98660/210274/245537	9
turquoise	GO:0048786	CC	presynaptic active zone	5/121	99/28804	6.3E-05	0.00126	0.000851	20910/12034/18673/210274/116838	5
turquoise	GO:0099240	CC	intrinsic component of synaptic membrane	7/121	247/28804	8.6E-05	0.001628	0.0011	14811/20511/16490/319504/268566/245537/68585	7
turquoise	GO:0008542	BP	visual learning	5/122	73/28943	1.5E-05	0.001677	0.001179	16653/14811/19049/98660/245537	5
turquoise	GO:0050807	BP	regulation of synapse organization	8/122	264/28943	1.7E-05	0.001855	0.001305	53328/235604/208643/210274/319504/245537/217692/16443	8
turquoise	GO:0031644	BP	regulation of nervous system process	7/122	194/28943	1.9E-05	0.002002	0.001409	14811/12297/244310/245537/110355/68585/116838	7
turquoise	GO:0002087	BP	regulation of respiratory gaseous exchange by nervous system process	3/122	13/28943	2E-05	0.002033	0.00143	14660/98660/245537	3
turquoise	GO:0050803	BP	regulation of synapse structure or activity	8/122	272/28943	2.1E-05	0.002033	0.00143	53328/235604/208643/210274/319504/245537/217692/16443	8
turquoise	GO:0050890	BP	cognition	9/122	358/28943	2.2E-05	0.002046	0.001439	53328/16653/14811/238871/19049/21375/98660/210274/245537	9
turquoise	GO:0035591	MF	signaling adaptor activity	5/121	74/28404	1.6E-05	0.002213	0.00177	14688/12928/210274/30957/14388	5
turquoise	GO:0060090	MF	molecular adaptor activity	10/121	433/28404	1.7E-05	0.002213	0.00177	14688/14086/12928/210274/244310/268566/30957/14388/16443/24050	10
turquoise	GO:0030159	MF	signaling receptor complex adaptor activity	4/121	39/28404	2.3E-05	0.002222	0.001777	14688/12928/210274/30957	4
turquoise	GO:0051968	BP	positive regulation of synaptic transmission, glutamatergic	4/122	41/28943	2.7E-05	0.002351	0.001654	20910/14811/210274/245537	4
turquoise	GO:0008277	BP	regulation of G protein-coupled receptor signaling pathway	6/122	138/28943	2.7E-05	0.002351	0.001654	14678/12652/18673/238871/230085/110355	6
turquoise	GO:0099173	BP	postsynapse organization	7/122	208/28943	3E-05	0.002504	0.001761	14432/14811/210274/319504/268566/245537/217692	7
turquoise	GO:0019897	CC	extrinsic component of plasma membrane	6/121	190/28804	0.00016	0.002783	0.00188	14678/12385/16653/14688/18673/268566	6
turquoise	GO:0099084	BP	postsynaptic specialization organization	4/122	44/28943	3.6E-05	0.00291	0.002047	14432/210274/245537/217692	4
turquoise	GO:0044065	BP	regulation of respiratory system process	3/122	16/28943	3.9E-05	0.003096	0.002178	14660/98660/245537	3
turquoise	GO:0060079	BP	excitatory postsynaptic potential	5/122	91/28943	4.3E-05	0.003203	0.002253	14811/12297/245537/110355/116838	5
turquoise	GO:0019233	BP	sensory perception of pain	6/122	150/28943	4.3E-05	0.003203	0.002253	14811/12297/16490/230085/110355/68585	6
turquoise	GO:1903829	BP	positive regulation of protein localization	10/122	493/28943	4.7E-05	0.003397	0.00239	53328/12297/12468/12464/384783/245537/16971/68585/28185/18536	10
turquoise	GO:0150034	CC	distal axon	8/121	379/28804	0.00021	0.003453	0.002332	14811/16490/23980/14086/210274/16971/271457/16443	8
turquoise	GO:0060077	CC	inhibitory synapse	3/121	28/28804	0.00022	0.003453	0.002332	268566/245537/116838	3
turquoise	GO:0045177	CC	apical part of cell	9/121	488/28804	0.00023	0.003453	0.002332	12297/11966/23980/210274/16971/110355/58810/18810/18536	9
turquoise	GO:0046395	BP	carboxylic acid catabolic process	7/122	226/28943	5E-05	0.003534	0.002486	14660/15356/384783/93747/52538/110842/58810	7
turquoise	GO:0016054	BP	organic acid catabolic process	7/122	228/28943	5.3E-05	0.003635	0.002557	14660/15356/384783/93747/52538/110842/58810	7
turquoise	GO:1901077	BP	regulation of relaxation of muscle	3/122	18/28943	5.7E-05	0.003775	0.002656	12652/238871/110355	3
turquoise	GO:0006979	BP	response to oxidative stress	9/122	407/28943	5.9E-05	0.00383	0.002694	19173/18584/14870/11669/170719/12928/110355/14388/23992	9
turquoise	GO:0044306	CC	neuron projection terminus	6/121	215/28804	0.0003	0.004432	0.002994	14811/20511/16490/23980/271457/16443	6
turquoise	GO:0099565	BP	chemical synaptic transmission, postsynaptic	5/122	102/28943	7.4E-05	0.00461	0.003243	14811/12297/245537/110355/116838	5

turquoise	GO:0031103	BP	axon regeneration	4/122	53/28943	7.5E-05	0.00461	0.003243	14432/18377/16971/30957	4
turquoise	GO:0031252	CC	cell leading edge	8/121	409/28804	0.00034	0.004798	0.003241	12385/20511/11966/16490/14086/271457/30957/16443	8
turquoise	GO:0009150	BP	purine ribonucleotide metabolic process	9/122	425/28943	8.2E-05	0.004947	0.00348	18641/15356/11566/11966/11512/98660/27402/52538/75406	9
turquoise	GO:0016574	BP	histone ubiquitination	4/122	55/28943	8.7E-05	0.005086	0.003578	14897/59026/107260/52615	4
turquoise	GO:0101031	CC	chaperone complex	3/121	34/28804	0.00039	0.005173	0.003495	12468/12464/20867	3
turquoise	GO:0099699	CC	integral component of synaptic membrane	6/121	227/28804	0.00041	0.005173	0.003495	14811/20511/16490/319504/245537/68585	6
turquoise	GO:0031253	CC	cell projection membrane	7/121	320/28804	0.00042	0.005173	0.003495	14432/20511/14688/11512/14086/210274/30957	7
turquoise	GO:0044304	CC	main axon	4/121	84/28804	0.00044	0.005244	0.003542	20511/16490/319504/30957	4
turquoise	GO:0009314	BP	response to radiation	9/122	432/28943	9.3E-05	0.005345	0.00376	16653/14811/20511/66656/14688/19049/98660/98415/245537	9
turquoise	GO:0007264	BP	small GTPase mediated signal transduction	9/122	434/28943	9.7E-05	0.005412	0.003807	20262/54366/16653/12928/233071/140580/217692/68585/16443	9
turquoise	GO:0009259	BP	ribonucleotide metabolic process	9/122	445/28943	0.00012	0.006259	0.004402	18641/15356/11566/11966/11512/98660/27402/52538/75406	9
turquoise	GO:0034329	BP	cell junction assembly	9/122	445/28943	0.00012	0.006259	0.004402	14432/12385/14086/208643/210274/245537/217692/16971/18810	9
turquoise	GO:0044282	BP	small molecule catabolic process	8/122	350/28943	0.00012	0.006421	0.004517	14660/18641/15356/384783/93747/52538/110842/58810	8
turquoise	GO:0006163	BP	purine nucleotide metabolic process	9/122	449/28943	0.00012	0.006421	0.004517	18641/15356/11566/11966/11512/98660/27402/52538/75406	9
turquoise	GO:0032515	BP	negative regulation of phosphoprotein phosphatase activity	3/122	24/28943	0.00014	0.006698	0.004711	665775/14678/19049	3
turquoise	GO:0019693	BP	ribose phosphate metabolic process	9/122	456/28943	0.00014	0.006698	0.004711	18641/15356/11566/11966/11512/98660/27402/52538/75406	9
turquoise	GO:0051952	BP	regulation of amine transport	5/122	117/28943	0.00014	0.006698	0.004711	20910/12652/16490/110355/68585	5
turquoise	GO:0060078	BP	regulation of postsynaptic membrane potential	5/122	117/28943	0.00014	0.006698	0.004711	14811/12297/245537/110355/116838	5
turquoise	GO:0021537	BP	telencephalon development	7/122	268/28943	0.00015	0.006717	0.004725	20511/12928/21375/70762/98660/68585/58208	7
turquoise	GO:0019395	BP	fatty acid oxidation	5/122	118/28943	0.00015	0.006717	0.004725	14077/384783/93747/52538/110842	5
turquoise	GO:0048709	BP	oligodendrocyte differentiation	5/122	119/28943	0.00015	0.006864	0.004828	12799/18377/52615/245537/110355	5
turquoise	GO:0015837	BP	amine transport	5/122	122/28943	0.00017	0.007577	0.00533	20910/12652/16490/110355/68585	5
turquoise	GO:0043576	BP	regulation of respiratory gaseous exchange	3/122	26/28943	0.00018	0.007689	0.005409	14660/98660/245537	3
turquoise	GO:0050433	BP	regulation of catecholamine secretion	4/122	68/28943	0.0002	0.008368	0.005886	12652/16490/110355/68585	4
turquoise	GO:0034440	BP	lipid oxidation	5/122	126/28943	0.0002	0.008368	0.005886	14077/384783/93747/52538/110842	5
turquoise	GO:1990778	BP	protein localization to cell periphery	8/122	377/28943	0.0002	0.008424	0.005925	20910/53328/14811/12297/11512/83997/268566/16971	8
turquoise	GO:0048167	BP	regulation of synaptic plasticity	8/122	378/28943	0.00021	0.008438	0.005935	20910/16653/14811/210274/245537/217692/271457/116838	8
turquoise	GO:0072329	BP	monocarboxylic acid catabolic process	5/122	128/28943	0.00021	0.008462	0.005952	384783/93747/52538/110842/58810	5
turquoise	GO:0072521	BP	purine-containing compound metabolic process	9/122	483/28943	0.00021	0.008462	0.005952	18641/15356/11566/11966/11512/98660/27402/52538/75406	9
turquoise	GO:0045822	BP	negative regulation of heart contraction	3/122	28/28943	0.00022	0.008462	0.005952	238871/98660/110355	3
turquoise	GO:0098698	BP	postsynaptic specialization assembly	3/122	28/28943	0.00022	0.008462	0.005952	14432/245537/217692	3
turquoise	GO:0019079	BP	viral genome replication	5/122	130/28943	0.00023	0.008666	0.006096	18673/18521/98415/110355/271457	5
turquoise	GO:0005832	CC	chaperonin-containing T-complex	2/121	10/28804	0.00077	0.008832	0.005966	12468/12464	2
turquoise	GO:0007585	BP	respiratory gaseous exchange by respiratory system	4/122	72/28943	0.00025	0.009048	0.006364	14660/98660/245537/30957	4
turquoise	GO:0048678	BP	response to axon injury	4/122	72/28943	0.00025	0.009048	0.006364	14432/18377/16971/30957	4
turquoise	GO:0032412	BP	regulation of ion transmembrane transporter activity	7/122	293/28943	0.00025	0.009069	0.00638	12034/14811/12297/238871/83997/98660/245537	7
turquoise	GO:1903523	BP	negative regulation of blood circulation	3/122	30/28943	0.00027	0.009635	0.006777	238871/98660/110355	3
turquoise	GO:0050432	BP	catecholamine secretion	4/122	74/28943	0.00028	0.009635	0.006777	12652/16490/110355/68585	4
turquoise	GO:0099569	CC	presynaptic cytoskeleton	2/121	11/28804	0.00094	0.010394	0.007021	116838/24050	2
turquoise	GO:0005902	CC	microvillus	4/121	105/28804	0.00102	0.010575	0.007143	12799/11966/11512/14086	4
turquoise	GO:0031234	CC	extrinsic component of cytoplasmic side of plasma membrane	4/121	105/28804	0.00102	0.010575	0.007143	14678/16653/14688/268566	4
turquoise	GO:0022898	BP	regulation of transmembrane transporter activity	7/122	303/28943	0.00031	0.010652	0.007493	12034/14811/12297/238871/83997/98660/245537	7
turquoise	GO:0039694	BP	viral RNA genome replication	3/122	32/28943	0.00033	0.011137	0.007834	18673/18521/271457	3
turquoise	GO:0071875	BP	adrenergic receptor signaling pathway	3/122	32/28943	0.00033	0.011137	0.007834	14678/12652/238871	3
turquoise	GO:0006635	BP	fatty acid beta-oxidation	4/122	78/28943	0.00034	0.01117	0.007857	384783/93747/52538/110842	4
turquoise	GO:0099060	CC	integral component of postsynaptic specialization membrane	4/121	108/28804	0.00113	0.011374	0.007684	14811/319504/245537/68585	4
turquoise	GO:0099068	BP	postsynapse assembly	3/122	33/28943	0.00036	0.011896	0.008368	14432/245537/217692	3
turquoise	GO:0060326	BP	cell chemotaxis	7/122	314/28943	0.00038	0.01235	0.008687	14870/12652/238871/12928/20085/18810/14388	7
turquoise	GO:0035308	BP	negative regulation of protein dephosphorylation	3/122	34/28943	0.0004	0.012519	0.008806	665775/14678/19049	3
turquoise	GO:0099563	BP	modification of synaptic structure	3/122	34/28943	0.0004	0.012519	0.008806	53328/235604/16443	3
turquoise	GO:0032409	BP	regulation of transporter activity	7/122	317/28943	0.0004	0.012588	0.008855	12034/14811/12297/238871/83997/98660/245537	7
turquoise	GO:0001508	BP	action potential	5/122	148/28943	0.00042	0.012745	0.008965	14811/12297/16490/83997/319504	5
turquoise	GO:0051047	BP	positive regulation of secretion	8/122	420/28943	0.00042	0.012745	0.008965	20910/14678/384783/16971/110355/68585/271457/16443	8
turquoise	GO:0098948	CC	intrinsic component of postsynaptic specialization membrane	4/121	114/28804	0.00138	0.013141	0.008877	14811/319504/245537/68585	4
turquoise	GO:0043679	CC	axon terminus	5/121	194/28804	0.00139	0.013141	0.008877	14811/16490/23980/271457/16443	5
turquoise	GO:0009416	BP	response to light stimulus	7/122	322/28943	0.00044	0.013169	0.009264	16653/14811/20511/14688/19049/98660/245537	7
turquoise	GO:1901653	BP	cellular response to peptide	7/122	322/28943	0.00044	0.013169	0.009264	14678/384783/11512/12928/98415/16971/110355	7
turquoise	GO:0098798	CC	mitochondrial protein-containing complex	6/121	291/28804	0.00147	0.013487	0.009111	12034/18673/27402/110842/28185/75406	6
turquoise	GO:0051966	BP	regulation of synaptic transmission, glutamatergic	4/122	85/28943	0.00047	0.013652	0.009603	20910/14811/210274/245537	4
turquoise	GO:0048169	BP	regulation of long-term neuronal synaptic plasticity	3/122	36/28943	0.00047	0.013652	0.009603	16653/14811/271457	3
turquoise	GO:0004112	MF	cyclic-nucleotide phosphodiesterase activity	3/121	26/28404	0.00018	0.01368	0.01094	18584/12799/238871	3
turquoise	GO:0019001	MF	guanyl nucleotide binding	8/121	384/28404	0.00025	0.01368	0.01094	14678/16653/11566/217869/226982/235661/271457/24050	8
turquoise	GO:0032561	MF	guanyl ribonucleotide binding	8/121	384/28404	0.00025	0.01368	0.01094	14678/16653/11566/217869/226982/235661/271457/24050	8
turquoise	GO:0003018	BP	vascular process in circulatory system	6/122	235/28943	0.0005	0.01416	0.00996	12652/11512/98660/16971/110355/18810	6
turquoise	GO:0006942	BP	regulation of striated muscle contraction	4/122	87/28943	0.00051	0.01416	0.00996	12652/238871/98660/110355	4
turquoise	GO:0010923	BP	negative regulation of phosphatase activity	3/122	37/28943	0.00051	0.01416	0.00996	665775/14678/19049	3
turquoise	GO:0090075	BP	relaxation of muscle	3/122	37/28943	0.00051	0.01416	0.00996	12652/238871/110355	3
turquoise	GO:0031099	BP	regeneration	5/122	155/28943	0.00052	0.014167	0.009966	14432/18377/16971/68585/30957	5
turquoise	GO:0035418	BP	protein localization to synapse	4/122	89/28943	0.00056	0.015078	0.010606	14811/268566/245537/30957	4
turquoise	GO:0050805	BP	negative regulation of synaptic transmission	4/122	90/28943	0.00058	0.015274	0.010744	20910/14678/210274/245537	4
turquoise	GO:1902600	BP	proton transmembrane transport	4/122	90/28943	0.00058	0.015274	0.010744	12034/11966/98660/75406	4

turquoise	GO:1904951	BP	positive regulation of establishment of protein localization	7/122	337/28943	0.00058	0.015274	0.010744	12297/12468/12464/384783/16971/28185/18536	7
turquoise	GO:0007613	BP	memory	5/122	160/28943	0.0006	0.01537	0.010812	53328/14811/238871/19049/210274	5
turquoise	GO:0070570	BP	regulation of neuron projection regeneration	3/122	39/28943	0.0006	0.01537	0.010812	18377/16971/68585	3
turquoise	GO:0051937	BP	catecholamine transport	4/122	91/28943	0.00061	0.015404	0.010836	12652/16490/110355/68585	4
turquoise	GO:0098562	CC	cytoplasmic side of membrane	5/121	207/28804	0.00185	0.016496	0.011143	14678/16653/14688/268566/271457	5
turquoise	GO:0001933	BP	negative regulation of protein phosphorylation	7/122	346/28943	0.00068	0.017062	0.012002	14870/23980/238871/208643/16971/110355/18810	7
turquoise	GO:0032204	BP	regulation of telomere maintenance	4/122	94/28943	0.00068	0.017062	0.012002	12468/12464/235134/51810	4
turquoise	GO:0030139	CC	endocytic vesicle	5/121	211/28804	0.00201	0.017446	0.011785	20910/245537/73094/271457/16443	5
turquoise	GO:0051341	BP	regulation of oxidoreductase activity	4/122	95/28943	0.00071	0.01758	0.012366	12034/14678/14870/16653	4
turquoise	GO:0003015	BP	heart process	6/122	254/28943	0.00074	0.018175	0.012785	14678/12652/23980/238871/98660/110355	6
turquoise	GO:0001558	BP	regulation of cell growth	8/122	459/28943	0.00075	0.018175	0.012785	18673/18377/12928/208643/16971/68585/28185/116838	8
turquoise	GO:0001505	BP	regulation of neurotransmitter levels	6/122	255/28943	0.00076	0.018249	0.012836	20910/20511/23980/98660/271457/116838	6
turquoise	GO:0110095	BP	cellular detoxification of aldehyde	2/122	10/28943	0.00078	0.018268	0.01285	11669/58810	2
turquoise	GO:1904851	BP	positive regulation of establishment of protein localization to telomere	2/122	10/28943	0.00078	0.018268	0.01285	12468/12464	2
turquoise	GO:0032228	BP	regulation of synaptic transmission, GABAergic	3/122	43/28943	0.0008	0.018604	0.013086	20910/16653/230085	3
turquoise	GO:0007411	BP	axon guidance	6/122	259/28943	0.00082	0.019056	0.013405	14432/21375/319504/16971/58208/30957	6
turquoise	GO:0097485	BP	neuron projection guidance	6/122	260/28943	0.00084	0.019264	0.013551	14432/21375/319504/16971/58208/30957	6
turquoise	GO:0042391	BP	regulation of membrane potential	8/122	469/28943	0.00086	0.019404	0.013649	14811/12297/16490/83997/319504/245537/110355/116838	8
turquoise	GO:0014910	BP	regulation of smooth muscle cell migration	4/122	100/28943	0.00086	0.019404	0.013649	14870/238871/12928/16971	4
turquoise	GO:1903532	BP	positive regulation of secretion by cell	7/122	365/28943	0.00093	0.020551	0.014456	20910/384783/16971/110355/68585/271457/16443	7
turquoise	GO:0070203	BP	regulation of establishment of protein localization to telomere	2/122	11/28943	0.00095	0.020551	0.014456	12468/12464	2
turquoise	GO:1902915	BP	negative regulation of protein polyubiquitination	2/122	11/28943	0.00095	0.020551	0.014456	14897/107260	2
turquoise	GO:0007626	BP	locomotory behavior	6/122	266/28943	0.00095	0.020551	0.014456	12799/14811/170719/19049/98660/210274	6
turquoise	GO:0009062	BP	fatty acid catabolic process	4/122	103/28943	0.00096	0.020597	0.014489	384783/93747/52538/110842	4
turquoise	GO:1901215	BP	negative regulation of neuron death	6/122	267/28943	0.00096	0.020597	0.014489	20910/12652/16653/170719/16971/16443	6
turquoise	GO:0055013	BP	cardiac muscle cell development	4/122	104/28943	0.001	0.02114	0.01487	11790/51810/28185/18810	4
turquoise	GO:0008022	MF	protein C-terminus binding	6/121	233/28404	0.0005	0.021232	0.016979	12034/20262/18673/226562/20867/210274	6
turquoise	GO:0008081	MF	phosphoric diester hydrolase activity	4/121	86/28404	0.00051	0.021232	0.016979	18584/12799/14688/238871	4
turquoise	GO:0031369	MF	translation initiation factor binding	3/121	38/28404	0.00057	0.021232	0.016979	217869/208643/20085	3
turquoise	GO:0008135	MF	translation factor activity, RNA binding	4/121	90/28404	0.0006	0.021232	0.016979	66656/217869/226982/208643	4
turquoise	GO:0003016	BP	respiratory system process	3/122	47/28943	0.00103	0.021731	0.015286	14660/98660/245537	3
turquoise	GO:0045211	CC	postsynaptic membrane	6/121	326/28804	0.0026	0.021941	0.014821	14811/210274/319504/268566/245537/68585	6
turquoise	GO:0099634	CC	postsynaptic specialization membrane	4/121	138/28804	0.00278	0.022421	0.015146	14811/319504/245537/68585	4
turquoise	GO:0005839	CC	proteasome core complex	2/121	19/28804	0.00286	0.022421	0.015146	19173/26444	2
turquoise	GO:0098858	CC	actin-based cell projection	5/121	229/28804	0.00286	0.022421	0.015146	14432/12799/11966/11512/14086	5
turquoise	GO:0048588	BP	developmental cell growth	6/122	273/28943	0.00108	0.02245	0.015792	18377/245537/16971/68585/28185/116838	6
turquoise	GO:0043949	BP	regulation of cAMP-mediated signaling	3/122	48/28943	0.0011	0.02245	0.015792	12652/23980/238871	3
turquoise	GO:0006898	BP	receptor-mediated endocytosis	6/122	274/28943	0.0011	0.02245	0.015792	245537/73094/16971/110355/271457/16443	6
turquoise	GO:0021761	BP	limbic system development	4/122	107/28943	0.00111	0.02245	0.015792	12928/21375/70762/98660	4
turquoise	GO:0033182	BP	regulation of histone ubiquitination	2/122	12/28943	0.00113	0.02245	0.015792	14897/107260	2
turquoise	GO:0070202	BP	regulation of establishment of protein localization to chromosome	2/122	12/28943	0.00113	0.02245	0.015792	12468/12464	2
turquoise	GO:0075522	BP	IRES-dependent viral translational initiation	2/122	12/28943	0.00113	0.02245	0.015792	18521/229663	2
turquoise	GO:0048660	BP	regulation of smooth muscle cell proliferation	5/122	185/28943	0.00115	0.022556	0.015866	14678/14870/18673/238871/110355	5
turquoise	GO:0032210	BP	regulation of telomere maintenance via telomerase	3/122	49/28943	0.00117	0.022689	0.01596	12468/12464/51810	3
turquoise	GO:0071375	BP	cellular response to peptide hormone stimulus	6/122	278/28943	0.00119	0.022689	0.01596	14678/384783/11512/12928/98415/110355	6
turquoise	GO:0015844	BP	monoamine transport	4/122	109/28943	0.00119	0.022689	0.01596	12652/16490/110355/68585	4
turquoise	GO:0048661	BP	positive regulation of smooth muscle cell proliferation	4/122	109/28943	0.00119	0.022689	0.01596	14678/18673/238871/110355	4
turquoise	GO:0055006	BP	cardiac cell development	4/122	110/28943	0.00123	0.023293	0.016385	11790/51810/28185/18810	4
turquoise	GO:0035305	BP	negative regulation of dephosphorylation	3/122	50/28943	0.00124	0.023308	0.016396	665775/14678/19049	3
turquoise	GO:0019898	CC	extrinsic component of membrane	6/121	337/28804	0.00306	0.023378	0.015792	14678/12385/16653/14688/18673/268566	6
turquoise	GO:0030673	CC	axolemma	2/121	20/28804	0.00316	0.023526	0.015892	20511/30957	2
turquoise	GO:0005905	CC	clathrin-coated pit	3/121	70/28804	0.00322	0.023526	0.015892	73094/16971/16443	3
turquoise	GO:0008344	BP	adult locomotory behavior	4/122	111/28943	0.00127	0.023554	0.016569	12799/170719/98660/210274	4
turquoise	GO:0014909	BP	smooth muscle cell migration	4/122	111/28943	0.00127	0.023554	0.016569	14870/238871/12928/16971	4
turquoise	GO:0008038	BP	neuron recognition	3/122	51/28943	0.00131	0.023663	0.016645	14432/319504/68585	3
turquoise	GO:0021879	BP	forebrain neuron differentiation	3/122	51/28943	0.00131	0.023663	0.016645	21375/70762/58208	3
turquoise	GO:0070934	BP	CRD-mediated mRNA stabilization	2/122	13/28943	0.00133	0.023663	0.016645	51810/229663	2
turquoise	GO:0099159	BP	regulation of modification of postsynaptic structure	2/122	13/28943	0.00133	0.023663	0.016645	235604/16443	2
turquoise	GO:1904816	BP	positive regulation of protein localization to chromosome, telomeric region	2/122	13/28943	0.00133	0.023663	0.016645	12468/12464	2
turquoise	GO:1905214	BP	regulation of RNA binding	2/122	13/28943	0.00133	0.023663	0.016645	208643/98415	2
turquoise	GO:0048659	BP	smooth muscle cell proliferation	5/122	192/28943	0.00135	0.023797	0.01674	14678/14870/18673/238871/110355	5
turquoise	GO:0007416	BP	synapse assembly	5/122	194/28943	0.00141	0.024737	0.017401	14432/208643/210274/245537/217692	5
turquoise	GO:0005697	CC	telomerase holoenzyme complex	2/121	21/28804	0.00349	0.024882	0.016809	51810/15381	2
turquoise	GO:0043524	BP	negative regulation of neuron apoptotic process	5/122	195/28943	0.00145	0.025129	0.017677	20910/16653/170719/16971/16443	5
turquoise	GO:0051954	BP	positive regulation of amine transport	3/122	53/28943	0.00147	0.025335	0.017821	20910/110355/68585	3
turquoise	GO:2001257	BP	regulation of cation channel activity	5/122	196/28943	0.00148	0.025352	0.017833	14811/12297/238871/83997/245537	5
turquoise	GO:0030072	BP	peptide hormone secretion	6/122	291/28943	0.0015	0.02549	0.01793	18641/12652/384783/16971/116838/16443	6
turquoise	GO:0035249	BP	synaptic transmission, glutamatergic	4/122	117/28943	0.00154	0.02572	0.018092	20910/14811/210274/245537	4
turquoise	GO:0106072	BP	negative regulation of adenylate cyclase-activating G protein-coupled receptor signaling pathway	2/122	14/28943	0.00155	0.02572	0.018092	14678/110355	2
turquoise	GO:1900152	BP	negative regulation of nuclear-transcribed mRNA catabolic process, deadenylation-dependent decay	2/122	14/28943	0.00155	0.02572	0.018092	51810/229663	2
turquoise	GO:2001241	BP	positive regulation of extrinsic apoptotic signaling pathway in absence of ligand	2/122	14/28943	0.00155	0.02572	0.018092	12385/51792	2

turquoise	GO:0008016	BP	regulation of heart contraction	5/122	199/28943	0.00158	0.026039	0.018316	12652/23980/238871/98660/110355	5
turquoise	GO:0099061	CC	integral component of postsynaptic density membrane	3/121	74/28804	0.00377	0.026326	0.017784	14811/319504/68585	3
turquoise	GO:0035097	CC	histone methyltransferase complex	3/121	76/28804	0.00407	0.027774	0.018762	665775/52615/80886	3
turquoise	GO:0002790	BP	peptide secretion	6/122	299/28943	0.00172	0.028093	0.019762	18641/12652/384783/16971/116838/16443	6
turquoise	GO:0110096	BP	cellular response to aldehyde	2/122	15/28943	0.00179	0.028524	0.020064	11669/58810	2
turquoise	GO:1900044	BP	regulation of protein K63-linked ubiquitination	2/122	15/28943	0.00179	0.028524	0.020064	14897/107260	2
turquoise	GO:1904814	BP	regulation of protein localization to chromosome, telomeric region	2/122	15/28943	0.00179	0.028524	0.020064	12468/12464	2
turquoise	GO:0021782	BP	glial cell development	4/122	122/28943	0.0018	0.028524	0.020064	16653/18377/16971/18810	4
turquoise	GO:0031646	BP	positive regulation of nervous system process	3/122	57/28943	0.00181	0.028524	0.020064	245537/110355/116838	3
turquoise	GO:0051489	BP	regulation of filopodium assembly	3/122	57/28943	0.00181	0.028524	0.020064	14432/14086/271457	3
turquoise	GO:1902414	BP	protein localization to cell junction	4/122	123/28943	0.00185	0.029016	0.020411	14811/268566/245537/30957	4
turquoise	GO:0043666	BP	regulation of phosphoprotein phosphatase activity	3/122	58/28943	0.0019	0.029307	0.020616	665775/14678/19049	3
turquoise	GO:1904356	BP	regulation of telomere maintenance via telomere lengthening	3/122	58/28943	0.0019	0.029307	0.020616	12468/12464/51810	3
turquoise	GO:1904062	BP	regulation of cation transmembrane transport	7/122	414/28943	0.00191	0.029307	0.020616	12034/14811/12297/238871/83997/98660/245537	7
turquoise	GO:0048511	BP	rhythmic process	6/122	306/28943	0.00193	0.029454	0.020719	14811/16490/59026/245537/51810/110355	6
turquoise	GO:0072659	BP	protein localization to plasma membrane	6/122	308/28943	0.00199	0.029808	0.020968	20910/53328/12297/11512/83997/16971	6
turquoise	GO:0014812	BP	muscle cell migration	4/122	126/28943	0.00202	0.029808	0.020968	14870/238871/12928/16971	4
turquoise	GO:0023035	BP	CD40 signaling pathway	2/122	16/28943	0.00203	0.029808	0.020968	12034/18673	2
turquoise	GO:0042428	BP	serotonin metabolic process	2/122	16/28943	0.00203	0.029808	0.020968	14811/11669	2
turquoise	GO:0046185	BP	aldehyde catabolic process	2/122	16/28943	0.00203	0.029808	0.020968	11669/58810	2
turquoise	GO:0055119	BP	relaxation of cardiac muscle	2/122	16/28943	0.00203	0.029808	0.020968	12652/238871	2
turquoise	GO:0086103	BP	G protein-coupled receptor signaling pathway involved in heart process	2/122	16/28943	0.00203	0.029808	0.020968	14678/12652	2
turquoise	GO:0005525	MF	GTP binding	7/121	361/28404	0.00092	0.029819	0.023846	14678/16653/11566/217869/226982/271457/24050	7
turquoise	GO:0006278	BP	RNA-templated DNA biosynthetic process	3/122	60/28943	0.0021	0.030395	0.021381	12468/12464/51810	3
turquoise	GO:0007004	BP	telomere maintenance via telomerase	3/122	60/28943	0.0021	0.030395	0.021381	12468/12464/51810	3
turquoise	GO:0006887	BP	exocytosis	7/122	422/28943	0.00212	0.030581	0.021512	20910/14678/12652/236643/271457/116838/16443	7
turquoise	GO:0006446	BP	regulation of translational initiation	3/122	61/28943	0.0022	0.031329	0.022038	217869/226982/208643	3
turquoise	GO:0051931	BP	regulation of sensory perception	3/122	61/28943	0.0022	0.031329	0.022038	12297/110355/68585	3
turquoise	GO:0099146	CC	intrinsic component of postsynaptic density membrane	3/121	80/28804	0.0047	0.031399	0.021211	14811/319504/68585	3
turquoise	GO:0016324	CC	apical plasma membrane	6/121	370/28804	0.00481	0.031534	0.021302	12297/11966/210274/110355/58810/18810	6
turquoise	GO:0003334	BP	keratinocyte development	2/122	17/28943	0.0023	0.03192	0.022453	58208/18810	2
turquoise	GO:0140374	BP	antiviral innate immune response	2/122	17/28943	0.0023	0.03192	0.022453	12034/18673	2
turquoise	GO:2000767	BP	positive regulation of cytoplasmic translation	2/122	17/28943	0.0023	0.03192	0.022453	51810/229663	2
turquoise	GO:0021872	BP	forebrain generation of neurons	3/122	62/28943	0.00231	0.03192	0.022453	21375/70762/58208	3
turquoise	GO:0035176	BP	social behavior	3/122	62/28943	0.00231	0.03192	0.022453	210274/245537/18536	3
turquoise	GO:1902495	CC	transmembrane transporter complex	6/121	373/28804	0.005	0.032123	0.021699	14811/12297/16490/238871/98660/75406	6
turquoise	GO:1901652	BP	response to peptide	7/122	430/28943	0.00236	0.03248	0.022847	14678/384783/11512/12928/98415/16971/110355	7
turquoise	GO:0060048	BP	cardiac muscle contraction	4/122	132/28943	0.0024	0.032695	0.022999	12652/238871/98660/110355	4
turquoise	GO:0015833	BP	peptide transport	6/122	320/28943	0.00241	0.032695	0.022999	18641/12652/384783/16971/116838/16443	6
turquoise	GO:0010517	BP	regulation of phospholipase activity	3/122	63/28943	0.00241	0.032695	0.022999	12034/18673/16971	3
turquoise	GO:0030258	BP	lipid modification	5/122	221/28943	0.00249	0.03351	0.023572	14077/384783/93747/52538/110842	5
turquoise	GO:0014911	BP	positive regulation of smooth muscle cell migration	3/122	64/28943	0.00252	0.03351	0.023572	238871/12928/16971	3
turquoise	GO:0009895	BP	negative regulation of catabolic process	6/122	324/28943	0.00256	0.03351	0.023572	14811/18673/208643/100502698/51810/229663	6
turquoise	GO:0060382	BP	regulation of DNA strand elongation	2/122	18/28943	0.00258	0.03351	0.023572	235134/98415	2
turquoise	GO:0070200	BP	establishment of protein localization to telomere	2/122	18/28943	0.00258	0.03351	0.023572	12468/12464	2
turquoise	GO:0099150	BP	regulation of postsynaptic specialization assembly	2/122	18/28943	0.00258	0.03351	0.023572	14432/217692	2
turquoise	GO:1901160	BP	primary amino compound metabolic process	2/122	18/28943	0.00258	0.03351	0.023572	14811/11669	2
turquoise	GO:1905244	BP	regulation of modification of synaptic structure	2/122	18/28943	0.00258	0.03351	0.023572	235604/16443	2
turquoise	GO:0051703	BP	biological process involved in intraspecies interaction between organisms	3/122	65/28943	0.00264	0.033653	0.023672	210274/245537/18536	3
turquoise	GO:0051932	BP	synaptic transmission, GABAergic	3/122	65/28943	0.00264	0.033653	0.023672	20910/16653/230085	3
turquoise	GO:0070372	BP	regulation of ERK1 and ERK2 cascade	6/122	326/28943	0.00264	0.033653	0.023672	12034/18584/14678/14870/18673/16971	6
turquoise	GO:0035264	BP	multicellular organism growth	5/122	224/28943	0.00264	0.033653	0.023672	20511/238871/216527/73094/18810	5
turquoise	GO:0042383	CC	sarcolemma	4/121	166/28804	0.00536	0.033735	0.022789	12297/11512/98660/18810	4
turquoise	GO:0033673	BP	negative regulation of kinase activity	5/122	226/28943	0.00275	0.034788	0.024471	14870/384783/100502698/51810/18810	5
turquoise	GO:0070374	BP	positive regulation of ERK1 and ERK2 cascade	5/122	227/28943	0.0028	0.035174	0.024742	12034/18584/14678/18673/16971	5
turquoise	GO:0044242	BP	cellular lipid catabolic process	5/122	228/28943	0.00285	0.035174	0.024742	384783/93747/52538/16971/110842	5
turquoise	GO:0019081	BP	viral translation	2/122	19/28943	0.00288	0.035174	0.024742	18521/229663	2
turquoise	GO:0033605	BP	positive regulation of catecholamine secretion	2/122	19/28943	0.00288	0.035174	0.024742	110355/68585	2
turquoise	GO:0044539	BP	long-chain fatty acid import into cell	2/122	19/28943	0.00288	0.035174	0.024742	14077/384783	2
turquoise	GO:0044827	BP	modulation by host of viral genome replication	2/122	19/28943	0.00288	0.035174	0.024742	18673/98415	2
turquoise	GO:0106070	BP	regulation of adenylate cyclase-activating G protein-coupled receptor signaling pathway	2/122	19/28943	0.00288	0.035174	0.024742	14678/110355	2
turquoise	GO:0043194	CC	axon initial segment	2/121	27/28804	0.00574	0.035283	0.023835	16490/319504	2
turquoise	GO:0099055	CC	integral component of postsynaptic membrane	4/121	170/28804	0.00583	0.035283	0.023835	14811/319504/245537/68585	4
turquoise	GO:0090079	MF	translation regulator activity, nucleic acid binding	4/121	108/28404	0.00119	0.035529	0.028412	66656/217869/226982/208643	4
turquoise	GO:0099504	BP	synaptic vesicle cycle	5/122	230/28943	0.00296	0.036057	0.025363	20910/245537/271457/116838/16443	5
turquoise	GO:0019933	BP	cAMP-mediated signaling	3/122	68/28943	0.003	0.036339	0.025562	12652/23980/238871	3
turquoise	GO:0007188	BP	adenylate cyclase-modulating G protein-coupled receptor signaling pathway	5/122	231/28943	0.00302	0.036381	0.025591	14678/12652/11512/110355/116838	5
turquoise	GO:0016661	MF	oxidoreductase activity, acting on other nitrogenous compounds as donors	2/121	13/28404	0.00136	0.037477	0.029969	11669/268566	2
turquoise	GO:0042043	MF	neurexin family protein binding	2/121	14/28404	0.00158	0.037477	0.029969	236643/245537	2
turquoise	GO:0003924	MF	GTPase activity	6/121	296/28404	0.00172	0.037477	0.029969	14678/16653/14688/226982/271457/24050	6
turquoise	GO:0004115	MF	3',5'-cyclic-AMP phosphodiesterase activity	2/121	15/28404	0.00182	0.037477	0.029969	18584/238871	2

turquoise	GO:0106222	MF	lncRNA binding	2/121	15/28404	0.00182	0.037477	0.029969	52615/51810	2
turquoise	GO:0003697	MF	single-stranded DNA binding	4/121	124/28404	0.00198	0.037477	0.029969	18521/56381/98415/51810	4
turquoise	GO:0008432	MF	JUN kinase binding	2/121	16/28404	0.00208	0.037477	0.029969	14870/30957	2
turquoise	GO:0050839	MF	cell adhesion molecule binding	6/121	308/28404	0.0021	0.037477	0.029969	54366/12385/14811/319504/245537/68585	6
turquoise	GO:0003743	MF	translation initiation factor activity	3/121	61/28404	0.00227	0.037477	0.029969	217869/226982/208643	3
turquoise	GO:0031625	MF	ubiquitin protein ligase binding	6/121	315/28404	0.00235	0.037477	0.029969	18521/12928/107260/217692/110355/68585	6
turquoise	GO:0070034	MF	telomerase RNA binding	2/121	17/28404	0.00235	0.037477	0.029969	51810/15381	2
turquoise	GO:0017124	MF	SH3 domain binding	4/121	131/28404	0.00242	0.037477	0.029969	12928/210274/140580/73094	4
turquoise	GO:0046324	BP	regulation of glucose import	3/122	69/28943	0.00313	0.037514	0.026388	20511/384783/110355	3
turquoise	GO:0010801	BP	negative regulation of peptidyl-threonine phosphorylation	2/122	20/28943	0.00319	0.037641	0.026477	208643/110355	2
turquoise	GO:0033599	BP	regulation of mammary gland epithelial cell proliferation	2/122	20/28943	0.00319	0.037641	0.026477	12034/68585	2
turquoise	GO:0050966	BP	detection of mechanical stimulus involved in sensory perception of pain	2/122	20/28943	0.00319	0.037641	0.026477	12297/230085	2
turquoise	GO:0030073	BP	insulin secretion	5/122	235/28943	0.00325	0.037641	0.026477	18641/12652/384783/16971/116838	5
turquoise	GO:0090276	BP	regulation of peptide hormone secretion	5/122	235/28943	0.00325	0.037641	0.026477	18641/12652/384783/16971/16443	5
turquoise	GO:0032206	BP	positive regulation of telomere maintenance	3/122	70/28943	0.00326	0.037641	0.026477	12468/12464/235134	3
turquoise	GO:0032507	BP	maintenance of protein location in cell	3/122	70/28943	0.00326	0.037641	0.026477	11512/51810/110355	3
turquoise	GO:0099601	BP	regulation of neurotransmitter receptor activity	3/122	70/28943	0.00326	0.037641	0.026477	14811/244310/245537	3
turquoise	GO:0000723	BP	telomere maintenance	4/122	144/28943	0.00328	0.037726	0.026538	12468/12464/235134/51810	4
turquoise	GO:0030027	CC	lamellipodium	4/121	175/28804	0.00644	0.037819	0.025548	12385/16490/14086/16443	4
turquoise	GO:1990351	CC	transporter complex	6/121	395/28804	0.00657	0.037819	0.025548	14811/12297/16490/238871/98660/75406	6
turquoise	GO:0044305	CC	calyx of Held	2/121	29/28804	0.0066	0.037819	0.025548	16490/16443	2
turquoise	GO:0051056	BP	regulation of small GTPase mediated signal transduction	5/122	236/28943	0.00331	0.03787	0.026639	20262/16653/12928/217692/68585	5
turquoise	GO:0055117	BP	regulation of cardiac muscle contraction	3/122	71/28943	0.00339	0.03865	0.027187	12652/238871/98660	3
turquoise	GO:0007265	BP	Ras protein signal transduction	6/122	344/28943	0.00344	0.038923	0.027379	20262/54366/16653/12928/140580/68585	6
turquoise	GO:0002181	BP	cytoplasmic translation	4/122	146/28943	0.00344	0.038923	0.027379	217869/51810/229663/20085	4
turquoise	GO:1904705	BP	regulation of vascular associated smooth muscle cell proliferation	3/122	72/28943	0.00353	0.039529	0.027805	14678/14870/110355	3
turquoise	GO:0010469	BP	regulation of signaling receptor activity	4/122	147/28943	0.00353	0.039529	0.027805	14811/238871/244310/245537	4
turquoise	GO:0002791	BP	regulation of peptide secretion	5/122	240/28943	0.00355	0.039597	0.027853	18641/12652/384783/16971/16443	5
turquoise	GO:0051015	MF	actin filament binding	5/121	222/28404	0.00266	0.039598	0.031666	54366/12385/14086/217692/18810	5
turquoise	GO:0033044	BP	regulation of chromosome organization	5/122	241/28943	0.00361	0.039715	0.027936	12468/12464/235134/235661/51810	5
turquoise	GO:0006091	BP	generation of precursor metabolites and energy	7/122	465/28943	0.00363	0.039715	0.027936	12034/18641/15356/384783/110842/75406/18810	7
turquoise	GO:0017156	BP	calcium-ion regulated exocytosis	3/122	73/28943	0.00367	0.039715	0.027936	20910/14678/116838	3
turquoise	GO:0019080	BP	viral gene expression	3/122	73/28943	0.00367	0.039715	0.027936	18521/98415/229663	3
turquoise	GO:0048168	BP	regulation of neuronal synaptic plasticity	3/122	73/28943	0.00367	0.039715	0.027936	16653/14811/271457	3
turquoise	GO:0060047	BP	heart contraction	5/122	242/28943	0.00368	0.039715	0.027936	12652/23980/238871/98660/110355	5
turquoise	GO:0090087	BP	regulation of peptide transport	5/122	242/28943	0.00368	0.039715	0.027936	18641/12652/384783/16971/16443	5
turquoise	GO:0032200	BP	telomere organization	4/122	149/28943	0.0037	0.039715	0.027936	12468/12464/235134/51810	4
turquoise	GO:0034763	BP	negative regulation of transmembrane transport	4/122	149/28943	0.0037	0.039715	0.027936	12297/384783/98660/110355	4
turquoise	GO:0006836	BP	neurotransmitter transport	5/122	243/28943	0.00374	0.039786	0.027986	20910/20511/98660/271457/116838	5
turquoise	GO:0070371	BP	ERK1 and ERK2 cascade	6/122	350/28943	0.00374	0.039786	0.027986	12034/18584/14678/14870/18673/16971	6
turquoise	GO:0055007	BP	cardiac muscle cell differentiation	4/122	150/28943	0.00379	0.039786	0.027986	11790/51810/28185/18810	4
turquoise	GO:0010833	BP	telomere maintenance via telomere lengthening	3/122	74/28943	0.00381	0.039786	0.027986	12468/12464/51810	3
turquoise	GO:0098586	BP	cellular response to virus	3/122	74/28943	0.00381	0.039786	0.027986	12034/18673/28185	3
turquoise	GO:0001975	BP	response to amphetamine	2/122	22/28943	0.00385	0.039786	0.027986	14811/19049	2
turquoise	GO:0048791	BP	calcium ion-regulated exocytosis of neurotransmitter	2/122	22/28943	0.00385	0.039786	0.027986	20910/116838	2
turquoise	GO:0060080	BP	inhibitory postsynaptic potential	2/122	22/28943	0.00385	0.039786	0.027986	245537/116838	2
turquoise	GO:0071625	BP	vocalization behavior	2/122	22/28943	0.00385	0.039786	0.027986	210274/245537	2
turquoise	GO:0098936	CC	intrinsic component of postsynaptic membrane	4/121	180/28804	0.00711	0.040019	0.027034	14811/319504/245537/68585	4
turquoise	GO:0042886	BP	amide transport	6/122	353/28943	0.0039	0.040138	0.028234	18641/12652/384783/16971/116838/16443	6
turquoise	GO:1990874	BP	vascular associated smooth muscle cell proliferation	3/122	75/28943	0.00396	0.040524	0.028506	14678/14870/110355	3
turquoise	GO:0009898	CC	cytoplasmic side of plasma membrane	4/121	182/28804	0.00738	0.040861	0.027602	14678/16653/14688/268566	4
turquoise	GO:0016032	BP	viral process	6/122	355/28943	0.00401	0.040918	0.028783	18673/18521/98415/229663/110355/271457	6
turquoise	GO:0007189	BP	adenylate cyclase-activating G protein-coupled receptor signaling pathway	4/122	153/28943	0.00407	0.04135	0.029087	14678/12652/11512/110355	4
turquoise	GO:0046847	BP	filopodium assembly	3/122	76/28943	0.00411	0.041555	0.029231	14432/14086/271457	3
turquoise	GO:0044389	MF	ubiquitin-like protein ligase binding	6/121	332/28404	0.00304	0.041633	0.033293	18521/12928/107260/217692/110355/68585	6
turquoise	GO:0003779	MF	actin binding	7/121	447/28404	0.0031	0.041633	0.033293	54366/12385/14086/217692/51810/18810/13821	7
turquoise	GO:0045296	MF	cadherin binding	3/121	69/28404	0.00322	0.041633	0.033293	54366/12385/68585	3
turquoise	GO:0030674	MF	protein-macromolecule adaptor activity	6/121	336/28404	0.00323	0.041633	0.033293	14688/14086/12928/210274/30957/14388	6
turquoise	GO:0045056	BP	transcytosis	2/122	23/28943	0.00421	0.042076	0.029597	16971/271457	2
turquoise	GO:0048670	BP	regulation of collateral sprouting	2/122	23/28943	0.00421	0.042076	0.029597	18377/16971	2
turquoise	GO:0140354	BP	lipid import into cell	2/122	23/28943	0.00421	0.042076	0.029597	14077/384783	2
turquoise	GO:0005901	CC	caveola	3/121	96/28804	0.00778	0.042323	0.02859	12385/98660/110355	3
turquoise	GO:0030125	CC	clathrin vesicle coat	2/121	32/28804	0.00799	0.04277	0.028892	217030/73094	2
turquoise	GO:0098839	CC	postsynaptic density membrane	3/121	99/28804	0.00846	0.044531	0.030082	14811/319504/68585	3
turquoise	GO:0014075	BP	response to amine	2/122	24/28943	0.00458	0.045236	0.03182	14811/19049	2
turquoise	GO:0032930	BP	positive regulation of superoxide anion generation	2/122	24/28943	0.00458	0.045236	0.03182	14678/14870	2
turquoise	GO:0051481	BP	negative regulation of cytosolic calcium ion concentration	2/122	24/28943	0.00458	0.045236	0.03182	98660/16971	2
turquoise	GO:0010977	BP	negative regulation of neuron projection development	4/122	160/28943	0.00477	0.046925	0.033008	11512/245537/16971/68585	4
turquoise	GO:0090257	BP	regulation of muscle system process	5/122	258/28943	0.00482	0.047201	0.033202	12652/238871/98660/110355/28185	5
turquoise	GO:0043434	BP	response to peptide hormone	6/122	369/28943	0.00483	0.047201	0.033202	14678/384783/11512/12928/98415/110355	6
turquoise	GO:0007202	BP	activation of phospholipase C activity	2/122	25/28943	0.00496	0.047855	0.033662	12034/18673	2

turquoise	GO:0046628	BP	positive regulation of insulin receptor signaling pathway	2/122	25/28943	0.00496	0.047855	0.033662	14678/98415	2
turquoise	GO:0051457	BP	maintenance of protein location in nucleus	2/122	25/28943	0.00496	0.047855	0.033662	51810/110355	2
turquoise	GO:0099003	BP	vesicle-mediated transport in synapse	5/122	260/28943	0.00498	0.047855	0.033662	20910/245537/271457/116838/16443	5
turquoise	GO:0019058	BP	viral life cycle	5/122	261/28943	0.00506	0.048443	0.034076	18673/18521/98415/110355/271457	5
turquoise	GO:0035637	BP	multicellular organismal signaling	4/122	163/28943	0.00509	0.048577	0.03417	16490/238871/319504/18810	4
turquoise	GO:0051348	BP	negative regulation of transferase activity	5/122	262/28943	0.00514	0.048851	0.034363	14870/384783/100502698/51810/18810	5
turquoise	GO:0005834	CC	heterotrimeric G-protein complex	2/121	35/28804	0.00951	0.049247	0.033267	14678/14688	2
turquoise	GO:0046879	BP	hormone secretion	6/122	375/28943	0.00522	0.049454	0.034787	18641/12652/384783/16971/116838/16443	6
turquoise	GO:0016082	BP	synaptic vesicle priming	2/122	26/28943	0.00536	0.049847	0.035064	20910/116838	2
turquoise	GO:0039529	BP	RIG-I signaling pathway	2/122	26/28943	0.00536	0.049847	0.035064	12034/18673	2
turquoise	GO:0071880	BP	adenylate cyclase-activating adrenergic receptor signaling pathway	2/122	26/28943	0.00536	0.049847	0.035064	14678/12652	2
turquoise	GO:0097164	BP	ammonium ion metabolic process	2/122	26/28943	0.00536	0.049847	0.035064	14811/11669	2
turquoise	GO:0099010	BP	modification of postsynaptic structure	2/122	26/28943	0.00536	0.049847	0.035064	235604/16443	2
turquoise	GO:0008088	BP	axo-dendritic transport	3/122	84/28943	0.00543	0.049952	0.035137	117197/51810/30957	3
turquoise	GO:0060191	BP	regulation of lipase activity	3/122	84/28943	0.00543	0.049952	0.035137	12034/18673/16971	3
turquoise	GO:1900076	BP	regulation of cellular response to insulin stimulus	3/122	84/28943	0.00543	0.049952	0.035137	14678/98415/110355	3
